# Supplementary material for: Cross-cohort transcriptomic meta-analysis identifies conserved melanogenesis signatures and developmental background in black-boned chicken breast muscle
Source: Anim Biosci. 2026 Jun 24;39(10):260430. doi: 10.5713/ab.260430 (PMC13662945; doi:10.5713/ab.260430)
Supplement: Supplementary file 14 [file ab-260430-Supplementary-6.pdf]

Supplement 6. Leave-one-cohort-out meta-analysis results for genes detected in all three pigmentation -related cohorts.

gene_id	symbol	k	meta_log2FC	meta_se	meta_z	meta_pvalue	tau2	Q	I2	direction_consistency	cohorts	dropped_cohort	meta_fdr_leave_one
ENSGALG00010000007	ND1	2	0.554916396	0.697008026	0.796140612	0.425950311	0.88516254	10.36109687	90.3485122	0.5	PRJNA827465;Yang2025_Silkie_Wenchang	CRA004775	0.84125685
ENSGALG00010000007	ND1	2	0.589327243	0.692369456	0.851174526	0.394672411	0.827359314	7.176686385	86.06599277	0.5	CRA004775;Yang2025_Silkie_Wenchang	PRJNA827465	0.830643015
ENSGALG00010000007	ND1	2	-0.080602985	0.072384054	-1.113546148	0.26547394	0	0.001941588	0	1	CRA004775;PRJNA827465	Yang2025_Silkie_Wenchang	0.769520043
ENSGALG00010000011	ND2	2	0.406337675	0.800504559	0.507601949	0.611732523	1.142242998	8.306802499	87.96167358	0.5	PRJNA827465;Yang2025_Silkie_Wenchang	CRA004775	0.897777844
ENSGALG00010000011	ND2	2	0.620318982	0.585788914	1.058946265	0.289624252	0.506124933	3.572400471	72.00761762	1	CRA004775;Yang2025_Silkie_Wenchang	PRJNA827465	0.784014188
ENSGALG00010000011	ND2	2	-0.189329355	0.19063814	-0.993134719	0.320644306	0.042328577	1.87501152	46.66699434	0.5	CRA004775;PRJNA827465	Yang2025_Silkie_Wenchang	0.798348216
ENSGALG00010000017	COX1	2	0.395239445	0.724003183	0.545908436	0.585128879	0.764849388	2.758348218	63.7464192	0.5	PRJNA827465;Yang2025_Silkie_Wenchang	CRA004775	0.891086887
ENSGALG00010000017	COX1	2	0.496064703	0.557827133	0.889280341	0.373852434	0.350324602	1.75365433	42.97621926	1	CRA004775;Yang2025_Silkie_Wenchang	PRJNA827465	0.822579781
ENSGALG00010000017	COX1	2	-0.073456091	0.087735579	-0.837244045	0.402455404	0	0.961894048	0	0.5	CRA004775;PRJNA827465	Yang2025_Silkie_Wenchang	0.832905052
ENSGALG00010000020	COX2	2	0.3358062	0.527100729	0.637081646	0.524071663	0.483905554	6.916015744	85.54080793	0.5	PRJNA827465;Yang2025_Silkie_Wenchang	CRA004775	0.869740342
ENSGALG00010000020	COX2	2	0.472167913	0.416039002	1.134912617	0.256411933	0.233394532	2.989653961	66.55131286	1	CRA004775;Yang2025_Silkie_Wenchang	PRJNA827465	0.764677554
ENSGALG00010000020	COX2	2	-0.107789289	0.077252871	-1.395278747	0.162931798	0	0.609387611	0	0.5	CRA004775;PRJNA827465	Yang2025_Silkie_Wenchang	0.660959169
ENSGALG00010000022	ATP8	2	0.149572292	0.457491309	0.326940182	0.743713129	0.32809815	3.870569162	74.16400642	0.5	PRJNA827465;Yang2025_Silkie_Wenchang	CRA004775	0.935821071
ENSGALG00010000022	ATP8	2	0.274225172	0.344422798	0.796187633	0.425922984	0.108517258	1.735547983	42.38131069	1	CRA004775;Yang2025_Silkie_Wenchang	PRJNA827465	0.84125685
ENSGALG00010000022	ATP8	2	-0.182385586	0.100837474	-1.808708396	0.070496318	0	0.563559129	0	0.5	CRA004775;PRJNA827465	Yang2025_Silkie_Wenchang	0.458021454
ENSGALG00010000023	ATP6	2	0.265962744	0.504027889	0.527674657	0.597725166	0.432416495	5.911913458	83.08500273	0.5	PRJNA827465;Yang2025_Silkie_Wenchang	CRA004775	0.894645663
ENSGALG00010000023	ATP6	2	0.474034352	0.265131516	1.787921552	0.073788665	0.046959161	1.416154191	29.38622034	1	CRA004775;Yang2025_Silkie_Wenchang	PRJNA827465	0.469350366
ENSGALG00010000023	ATP6	2	0.007387646	0.220823856	0.033454928	0.973311808	0.070845304	3.171295455	68.46714492	0.5	CRA004775;PRJNA827465	Yang2025_Silkie_Wenchang	0.992392211
ENSGALG00010000024	COX3	2	0.418669003	0.517282719	0.809362053	0.418306923	0.506581269	18.19248988	94.50322629	0.5	PRJNA827465;Yang2025_Silkie_Wenchang	CRA004775	0.838112595
ENSGALG00010000024	COX3	2	0.540627836	0.423933433	1.275265864	0.202215156	0.302187609	6.268643145	84.04758451	1	CRA004775;Yang2025_Silkie_Wenchang	PRJNA827465	0.715232385
ENSGALG00010000024	COX3	2	-0.057744385	0.082869592	-0.696810296	0.485921522	0	0.493520221	0	0.5	CRA004775;PRJNA827465	Yang2025_Silkie_Wenchang	0.857391223
ENSGALG00010000026	ND3	2	0.184578527	0.644735645	0.286285593	0.774659392	0.756364602	10.27076326	90.26362526	0.5	PRJNA827465;Yang2025_Silkie_Wenchang	CRA004775	0.943713409
ENSGALG00010000026	ND3	2	0.546175348	0.278829483	1.958814909	0.050134467	0.0395785	1.322586812	24.39059642	1	CRA004775;Yang2025_Silkie_Wenchang	PRJNA827465	0.387575719
ENSGALG00010000026	ND3	2	-0.099599358	0.357977966	-0.278227621	0.780837633	0.214305067	5.425737204	81.56932482	0.5	CRA004775;PRJNA827465	Yang2025_Silkie_Wenchang	0.945235709
ENSGALG00010000028	ND4L	2	1.134618837	1.472285912	0.770651154	0.440913732	4.109836506	18.30084395	94.53577112	0.5	PRJNA827465;Yang2025_Silkie_Wenchang	CRA004775	0.844888742
ENSGALG00010000028	ND4L	2	1.455514196	1.151324928	1.26420801	0.206155377	2.374518506	9.228623484	89.16414781	1	CRA004775;Yang2025_Silkie_Wenchang	PRJNA827465	0.718403416
ENSGALG00010000028	ND4L	2	-0.021018058	0.310372918	-0.067718723	0.946009545	0.146466139	3.48787939	71.32928384	0.5	CRA004775;PRJNA827465	Yang2025_Silkie_Wenchang	0.986321197
ENSGALG00010000029	ND4	2	0.521285173	0.84360396	0.617926418	0.536623844	1.286906063	9.545107856	89.52342902	0.5	PRJNA827465;Yang2025_Silkie_Wenchang	CRA004775	0.874599474
ENSGALG00010000029	ND4	2	0.847843159	0.503365571	1.684348727	0.092114293	0.326569186	2.652261515	62.29632733	1	CRA004775;Yang2025_Silkie_Wenchang	PRJNA827465	0.519672986
ENSGALG00010000029	ND4	2	0.023449673	0.328440418	0.071397038	0.943081767	0.170643139	4.107312292	75.65317831	0.5	CRA004775;PRJNA827465	Yang2025_Silkie_Wenchang	0.985827325
ENSGALG00010000033	ND5	2	0.474457583	0.762089635	0.622574512	0.533564165	1.029278432	7.892290535	87.32940715	0.5	PRJNA827465;Yang2025_Silkie_Wenchang	CRA004775	0.873379507
ENSGALG00010000033	ND5	2	0.779900373	0.438440987	1.778803526	0.075271979	0.208657084	2.056909613	51.3833766	1	CRA004775;Yang2025_Silkie_Wenchang	PRJNA827465	0.473509649
ENSGALG00010000033	ND5	2	0.040375521	0.307864349	0.131147113	0.895658932	0.14541696	3.613370525	72.3250081	0.5	CRA004775;PRJNA827465	Yang2025_Silkie_Wenchang	0.974927462
ENSGALG00010000034	CYTB	2	0.509325607	0.702510619	0.725007699	0.468447307	0.907175037	11.57718525	91.36232186	0.5	PRJNA827465;Yang2025_Silkie_Wenchang	CRA004775	0.853198008
ENSGALG00010000034	CYTB	2	0.773233153	0.457535731	1.689995122	0.091028888	0.292070442	3.260469792	69.32957323	1	CRA004775;Yang2025_Silkie_Wenchang	PRJNA827465	0.517594064
ENSGALG00010000034	CYTB	2	0.016265451	0.229188372	0.070969793	0.943421797	0.068021525	2.29149275	56.3603245	0.5	CRA004775;PRJNA827465	Yang2025_Silkie_Wenchang	0.985827325
ENSGALG00010000037	ND6	2	0.489839217	0.655926163	0.746790179	0.455190225	0.773354805	9.140579512	89.05977462	0.5	PRJNA827465;Yang2025_Silkie_Wenchang	CRA004775	0.849072665
ENSGALG00010000037	ND6	2	0.790220649	0.374052958	2.112590298	0.03463585	0.139703609	1.969458263	49.22461577	1	CRA004775;Yang2025_Silkie_Wenchang	PRJNA827465	0.320530447
ENSGALG00010000037	ND6	2	0.088895399	0.268475951	0.331111217	0.74056048	0.097892925	2.594271815	61.45353798	0.5	CRA004775;PRJNA827465	Yang2025_Silkie_Wenchang	0.935128797
ENSGALG00010000039	PDIA3	2	0.070052726	0.041538851	1.686438703	0.091711332	0	0.761916094	0	1	PRJNA827465;Yang2025_Silkie_Wenchang	CRA004775	0.518914432
ENSGALG00010000039	PDIA3	2	0.054086389	0.150871245	0.358493688	0.719973891	0.021923903	1.607515144	37.79218791	0.5	CRA004775;Yang2025_Silkie_Wenchang	PRJNA827465	0.928564173
ENSGALG00010000039	PDIA3	2	0.044512464	0.045342064	0.981703529	0.326245926	0	0.943151082	0	0.5	CRA004775;PRJNA827465	Yang2025_Silkie_Wenchang	0.800023788
ENSGALG00010000042	CKMT1B	2	0.351456391	0.357670075	0.98262733	0.325790888	0.111722254	1.770865266	43.53043005	1	PRJNA827465;Yang2025_Silkie_Wenchang	CRA004775	0.800023788
ENSGALG00010000042	CKMT1B	2	0.921855289	0.346357336	2.661572869	0.00777765	0	0.940629992	0	1	CRA004775;Yang2025_Silkie_Wenchang	PRJNA827465	0.136710641
ENSGALG00010000042	CKMT1B	2	0.642145162	0.73835833	0.869693123	0.384468131	0.810123415	3.625922596	72.42081226	1	CRA004775;PRJNA827465	Yang2025_Silkie_Wenchang	0.826099934
ENSGALG00010000043	PPIP5K1	2	-0.058133525	0.070900922	-0.819926223	0.412258167	0	0.933811786	0	1	PRJNA827465;Yang2025_Silkie_Wenchang	CRA004775	0.835611629
ENSGALG00010000043	PPIP5K1	2	-0.138162422	0.102410882	-1.349099039	0.177305157	0	0.009181495	0	1	CRA004775;Yang2025_Silkie_Wenchang	PRJNA827465	0.683332476
ENSGALG00010000043	PPIP5K1	2	-0.016339615	0.085596546	-0.190891059	0.848610945	0	0.188326553	0	1	CRA004775;PRJNA827465	Yang2025_Silkie_Wenchang	0.961364896
ENSGALG00010000044	MAP1A	2	-0.066921482	0.107148555	-0.624567284	0.532255098	0.005650498	1.240789035	19.4061221	0.5	PRJNA827465;Yang2025_Silkie_Wenchang	CRA004775	0.873104119
ENSGALG00010000044	MAP1A	2	0.235567717	0.224247049	1.050483018	0.293496095	0.029397609	1.272028746	21.38542444	1	CRA004775;Yang2025_Silkie_Wenchang	PRJNA827465	0.786979185
ENSGALG00010000044	MAP1A	2	0.14022997	0.366535463	0.382582272	0.702029517	0.198434535	3.095835985	67.69854718	0.5	CRA004775;PRJNA827465	Yang2025_Silkie_Wenchang	0.923051857
ENSGALG00010000045	TP53BP1	2	0.106124693	0.143269756	0.740733401	0.458855103	0.034642329	6.145621446	83.72825257	0.5	PRJNA827465;Yang2025_Silkie_Wenchang	CRA004775	0.850089963
ENSGALG00010000045	TP53BP1	2	0.063618902	0.262178039	0.242655343	0.808272405	0.097966608	2.95097396	66.11288294	0.5	CRA004775;Yang2025_Silkie_Wenchang	PRJNA827465	0.952496413
ENSGALG00010000045	TP53BP1	2	-0.0335056	0.054579281	-0.613888642	0.539288916	0	0.707754193	0	1	CRA004775;PRJNA827465	Yang2025_Silkie_Wenchang	0.874983893
ENSGALG00010000046	TUBGCP4	2	0.049527749	0.058287449	0.849715505	0.395483276	0	0.022235196	0	1	PRJNA827465;Yang2025_Silkie_Wenchang	CRA004775	0.830946177
ENSGALG00010000046	TUBGCP4	2	-0.117170566	0.202173079	-0.579555728	0.562214255	0.059480539	3.572598282	72.00916752	0.5	CRA004775;Yang2025_Silkie_Wenchang	PRJNA827465	0.881955027
ENSGALG00010000046	TUBGCP4	2	-0.113022272	0.189686657	-0.5958367	0.551284324	0.056462285	4.142611584	75.86063816	0.5	CRA004775;PRJNA827465	Yang2025_Silkie_Wenchang	0.878879109
ENSGALG00010000047	IQGAP1	2	0.091502111	0.115235394	0.794045201	0.427169117	0.018245056	3.076060279	67.49088414	0.5	PRJNA827465;Yang2025_Silkie_Wenchang	CRA004775	0.84125685
ENSGALG00010000047	IQGAP1	2	0.210438045	0.107059319	1.965620986	0.049342408	0	0.178261854	0	1	CRA004775;Yang2025_Silkie_Wenchang	PRJNA827465	0.384401321
ENSGALG00010000047	IQGAP1	2	-0.007430037	0.070970219	-0.104692324	0.916619953	0	0.021879678	0	0.5	CRA004775;PRJNA827465	Yang2025_Silkie_Wenchang	0.980643874
ENSGALG00010000048	MAN2A2	2	0.271773757	0.216256134	1.256721612	0.20885446	0.08643354	13.17388798	92.40922648	1	PRJNA827465;Yang2025_Silkie_Wenchang	CRA004775	0.721011841
ENSGALG00010000048	MAN2A2	2	0.144281818	0.44448249	0.324606304	0.745479056	0.308654355	3.729327913	73.18551698	0.5	CRA004775;Yang2025_Silkie_Wenchang	PRJNA827465	0.936178015
ENSGALG00010000048	MAN2A2	2	0.030798362	0.106985874	0.287873165	0.773443829	0.005007804	1.044300169	4.242091486	0.5	CRA004775;PRJNA827465	Yang2025_Silkie_Wenchang	0.943248868
ENSGALG00010000049	FES	2	0.188444142	0.17989474	1.04752447	0.294857752	0.034772422	2.139943509	53.26979447	1	PRJNA827465;Yang2025_Silkie_Wenchang	CRA004775	0.78756211
ENSGALG00010000049	FES	2	-0.060470848	0.505743665	-0.119568177	0.90482523	0.418352832	5.130925582	80.51033904	0.5	CRA004775;Yang2025_Silkie_Wenchang	PRJNA827465	0.977793401
ENSGALG00010000049	FES	2	-0.197130298	0.31099217	-0.633875438	0.526162117	0.121287571	2.273070908	56.00665178	0.5	CRA004775;PRJNA827465	Yang2025_Silkie_Wenchang	0.870525481
ENSGALG00010000050	CIB1	2	-0.185023724	0.078476394	-2.357699097	0.018388592	0	0.193949241	0	1	PRJNA827465;Yang2025_Silkie_Wenchang	CRA004775	0.227043528
ENSGALG00010000050	CIB1	2	-0.232859632	0.129468981	-1.798574679	0.072085986	0	0.011924183	0	1	CRA004775;Yang2025_Silkie_Wenchang	PRJNA827465	0.463470554
ENSGALG00010000050	CIB1	2	-0.168680725	0.086911095	-1.940842249	0.052277415	0	0.026205572	0	1	CRA004775;PRJNA827465	Yang2025_Silkie_Wenchang	0.396958892
ENSGALG00010000051	GDPGP1	2	0.088407984	0.126601265	0.69831833	0.484978138	0	0.189481071	0	1	PRJNA827465;Yang2025_Silkie_Wenchang	CRA004775	0.857004733
ENSGALG00010000051	GDPGP1	2	-0.079638684	0.247393229	-0.321911333	0.747519867	0.075756496	2.618726503	61.81349985	0.5	CRA004775;Yang2025_Silkie_Wenchang	PRJNA827465	0.93680487
ENSGALG00010000051	GDPGP1	2	-0.111902856	0.187767618	-0.595964613	0.551198867	0.034318766	1.893600407	47.1905479	0.5	CRA004775;PRJNA827465	Yang2025_Silkie_Wenchang	0.878879109
ENSGALG00010000052	TTLL13	2	0.005432685	0.09973747	0.054469854	0.956560826	0	0.059876398	0	0.5	PRJNA827465;Yang2025_Silkie_Wenchang	CRA004775	0.988540618
ENSGALG00010000052	TTLL13	2	0.029761022	0.170112182	0.174949387	0.861119404	0	0.02932696	0	0.5	CRA004775;Yang2025_Silkie_Wenchang	PRJNA827465	0.965343309
ENSGALG00010000052	TTLL13	2	-0.009275194	0.107784706	-0.08605297	0.93142431	0	0.000472834	0	1	CRA004775;PRJNA827465	Yang2025_Silkie_Wenchang	0.984076328
ENSGALG00010000053	HDDC3	2	0.448347744	0.360547864	1.243517958	0.213677028	0.213796314	5.550947098	81.9850562	1	PRJNA827465;Yang2025_Silkie_Wenchang	CRA004775	0.726416385
ENSGALG00010000053	HDDC3	2	0.35628947	0.45680612	0.779957742	0.435415749	0.371079241	8.934313105	88.80719773	0.5	CRA004775;Yang2025_Silkie_Wenchang	PRJNA827465	0.842762754
ENSGALG00010000053	HDDC3	2	0.010460328	0.127393127	0.082110616	0.934558751	0	0.567732735	0	0.5	CRA004775;PRJNA827465	Yang2025_Silkie_Wenchang	0.984397102
ENSGALG00010000054	VPS33B	2	0.021313506	0.056420874	0.377759222	0.705609469	0	0.027757726	0	0.5	PRJNA827465;Yang2025_Silkie_Wenchang	CRA004775	0.924358822
ENSGALG00010000054	VPS33B	2	-0.053051341	0.129670192	-0.409125181	0.682447798	0	0.748910786	0	1	CRA004775;Yang2025_Silkie_Wenchang	PRJNA827465	0.918625039
ENSGALG00010000054	VPS33B	2	0.007418567	0.073403542	0.101065522	0.919498447	0.001940522	1.039783662	3.826148024	0.5	CRA004775;PRJNA827465	Yang2025_Silkie_Wenchang	0.981192162
ENSGALG00010000055	PRC1	2	0.14666188	0.192996328	0.759920573	0.447302064	0.05965738	4.908050849	79.62531296	0.5	PRJNA827465;Yang2025_Silkie_Wenchang	CRA004775	0.84650962
ENSGALG00010000055	PRC1	2	0.322141225	0.127028812	2.535969755	0.011213642	0	0.22317647	0	1	CRA004775;Yang2025_Silkie_Wenchang	PRJNA827465	0.168281697
ENSGALG00010000055	PRC1	2	-0.002984954	0.091947491	-0.032463684	0.974102277	0	0.74454816	0	0.5	CRA004775;PRJNA827465	Yang2025_Silkie_Wenchang	0.992492038
ENSGALG00010000056	UNC45A	2	0.283165752	0.210716412	1.343823907	0.179005337	0.078114858	8.070411233	87.60905769	1	PRJNA827465;Yang2025_Silkie_Wenchang	CRA004775	0.685713211
ENSGALG00010000056	UNC45A	2	0.141703541	0.372500319	0.380411864	0.703639709	0.256015874	12.86222776	92.22529706	0.5	CRA004775;Yang2025_Silkie_Wenchang	PRJNA827465	0.923639641
ENSGALG00010000056	UNC45A	2	-0.043340797	0.158081531	-0.274167367	0.783956005	0.036193683	3.27412121	69.45745328	0.5	CRA004775;PRJNA827465	Yang2025_Silkie_Wenchang	0.945989465
ENSGALG00010000058	FURIN	2	0.119621648	0.10313765	1.159825225	0.246119972	0.017389798	5.474114532	81.73220538	1	PRJNA827465;Yang2025_Silkie_Wenchang	CRA004775	0.756727496
ENSGALG00010000058	FURIN	2	0.079498932	0.213148114	0.372975066	0.709167001	0.061994809	2.392509487	58.20288257	0.5	CRA004775;Yang2025_Silkie_Wenchang	PRJNA827465	0.925298042
ENSGALG00010000058	FURIN	2	0.00387611	0.062420842	0.062096406	0.950486059	0	0.728613037	0	0.5	CRA004775;PRJNA827465	Yang2025_Silkie_Wenchang	0.987277827
ENSGALG00010000059	MFAP1	2	0.22608185	0.271642159	0.832278212	0.405251926	0.13236431	9.029651807	88.92537585	0.5	PRJNA827465;Yang2025_Silkie_Wenchang	CRA004775	0.833112102
ENSGALG00010000059	MFAP1	2	0.133920285	0.389739307	0.343615034	0.731135817	0.273894686	10.1606226	90.15808342	0.5	CRA004775;Yang2025_Silkie_Wenchang	PRJNA827465	0.932769533
ENSGALG00010000059	MFAP1	2	-0.081098755	0.101894918	-0.795905791	0.426086795	0.011194847	1.69313736	40.93804652	1	CRA004775;PRJNA827465	Yang2025_Silkie_Wenchang	0.84125685
ENSGALG00010000060	HYPK	2	0.322378899	0.376289693	0.856730612	0.391593781	0.265038985	14.9591048	93.315108	0.5	PRJNA827465;Yang2025_Silkie_Wenchang	CRA004775	0.829251057
ENSGALG00010000060	HYPK	2	0.516401079	0.251809395	2.050761764	0.040290153	0.070321894	2.127090913	52.98743492	1	CRA004775;Yang2025_Silkie_Wenchang	PRJNA827465	0.347442284
ENSGALG00010000060	HYPK	2	-0.024960102	0.058521787	-0.426509566	0.669736579	0	0.605549266	0	0.5	CRA004775;PRJNA827465	Yang2025_Silkie_Wenchang	0.914377769
ENSGALG00010000061	SERINC4	2	-0.01167521	0.174948548	-0.066735105	0.946792587	0	0.724100033	0	0.5	PRJNA827465;Yang2025_Silkie_Wenchang	CRA004775	0.986445196
ENSGALG00010000061	SERINC4	2	0.056114394	0.239375388	0.234420067	0.814658881	0	0.574332087	0	0.5	CRA004775;Yang2025_Silkie_Wenchang	PRJNA827465	0.953869515
ENSGALG00010000061	SERINC4	2	-0.122420069	0.182830243	-0.669583255	0.503123491	0	0.011389847	0	1	CRA004775;PRJNA827465	Yang2025_Silkie_Wenchang	0.863052662
ENSGALG00010000063	SERF2	2	-0.099882435	0.091535239	-1.091191062	0.27518882	0	0.514422515	0	1	PRJNA827465;Yang2025_Silkie_Wenchang	CRA004775	0.775068889
ENSGALG00010000063	SERF2	2	-0.152809429	0.202449629	-0.754802219	0.450367662	0	0.465899857	0	1	CRA004775;Yang2025_Silkie_Wenchang	PRJNA827465	0.847577105
ENSGALG00010000063	SERF2	2	-0.073778904	0.09043145	-0.815854478	0.414583371	0	0.02903093	0	1	CRA004775;PRJNA827465	Yang2025_Silkie_Wenchang	0.837197258
ENSGALG00010000069	ENSGALG00010000069	2	0.455387122	0.583995185	0.779778898	0.435521029	0.643991204	17.88357755	94.40827767	0.5	PRJNA827465;Yang2025_Silkie_Wenchang	CRA004775	0.842762754
ENSGALG00010000069	ENSGALG00010000069	2	0.980427784	0.190968977	5.133963638	2.83703E-07	0	0.616120998	0	1	CRA004775;Yang2025_Silkie_Wenchang	PRJNA827465	8.08275E-05
ENSGALG00010000069	ENSGALG00010000069	2	0.110102394	0.347282221	0.317040112	0.751213166	0.142294649	2.031543008	50.7763313	0.5	CRA004775;PRJNA827465	Yang2025_Silkie_Wenchang	0.937684688
ENSGALG00010000075	ENSGALG00010000075	2	0.308125407	0.354576429	0.868995741	0.38484946	0.046626573	1.223163866	18.24480532	0.5	PRJNA827465;Yang2025_Silkie_Wenchang	CRA004775	0.826444827
ENSGALG00010000075	ENSGALG00010000075	2	0.133048203	0.645285171	0.206185124	0.836646308	0.508991603	2.524060061	60.38129141	0.5	CRA004775;Yang2025_Silkie_Wenchang	PRJNA827465	0.958399882
ENSGALG00010000075	ENSGALG00010000075	2	-0.174775471	0.353347425	-0.494627833	0.620862852	0	0.564537085	0	1	CRA004775;PRJNA827465	Yang2025_Silkie_Wenchang	0.899538197
ENSGALG00010000076	ENSGALG00010000076	2	-0.106543239	0.230456881	-0.462313119	0.643856793	0.085523377	5.056365396	80.22294827	0.5	PRJNA827465;Yang2025_Silkie_Wenchang	CRA004775	0.906747296
ENSGALG00010000076	ENSGALG00010000076	2	0.103081047	0.150393612	0.685408415	0.493086235	0	0.253008545	0	0.5	CRA004775;Yang2025_Silkie_Wenchang	PRJNA827465	0.859667262
ENSGALG00010000076	ENSGALG00010000076	2	-0.296282753	0.114680207	-2.583556138	0.009778755	0	0.489919257	0	1	CRA004775;PRJNA827465	Yang2025_Silkie_Wenchang	0.155979876
ENSGALG00010000078	ENSGALG00010000078	2	1.689325186	0.450313104	3.751445762	0.000175818	0.201418121	1.959953707	48.97838676	1	PRJNA827465;Yang2025_Silkie_Wenchang	CRA004775	0.01153893
ENSGALG00010000078	ENSGALG00010000078	2	0.332162566	0.89217869	0.372304976	0.709665794	1.261104015	4.769906458	79.03522828	0.5	CRA004775;Yang2025_Silkie_Wenchang	PRJNA827465	0.925298042
ENSGALG00010000078	ENSGALG00010000078	2	0.789153289	1.345859458	0.586356387	0.557636046	3.34075314	12.67240724	92.10883946	0.5	CRA004775;PRJNA827465	Yang2025_Silkie_Wenchang	0.880678789
ENSGALG00010000081	ENSGALG00010000081	2	-0.439666286	0.322285146	-1.364215176	0.172499853	0.155249293	3.895622298	74.33016028	1	PRJNA827465;Yang2025_Silkie_Wenchang	CRA004775	0.675388277
ENSGALG00010000081	ENSGALG00010000081	2	-0.077241565	0.245274956	-0.314918271	0.752823708	0	0.027283867	0	0.5	CRA004775;Yang2025_Silkie_Wenchang	PRJNA827465	0.938087082
ENSGALG00010000081	ENSGALG00010000081	2	-0.636999817	0.260648003	-2.443908292	0.014529119	0.040096098	1.157502918	13.60712921	0.5	CRA004775;PRJNA827465	Yang2025_Silkie_Wenchang	0.198277074
ENSGALG00010000084	ENSGALG00010000084	2	0.30233221	0.330910823	0.913636511	0.360907875	0.159919951	3.697063405	72.95150528	0.5	PRJNA827465;Yang2025_Silkie_Wenchang	CRA004775	0.815139251
ENSGALG00010000084	ENSGALG00010000084	2	-0.206754999	0.896197455	-0.230702507	0.817545926	1.460280001	10.70400039	90.6576984	0.5	CRA004775;Yang2025_Silkie_Wenchang	PRJNA827465	0.954888783
ENSGALG00010000084	ENSGALG00010000084	2	-0.504344918	0.560610113	-0.899635784	0.368314107	0.497833424	4.464499305	77.60107166	1	CRA004775;PRJNA827465	Yang2025_Silkie_Wenchang	0.820213684
ENSGALG00010000085	ENSGALG00010000085	2	0.113706784	0.075382466	1.508398301	0.131452618	0	0.306054083	0	1	PRJNA827465;Yang2025_Silkie_Wenchang	CRA004775	0.60724991
ENSGALG00010000085	ENSGALG00010000085	2	0.032333727	0.110517313	0.292567075	0.769853083	0	0.102168342	0	0.5	CRA004775;Yang2025_Silkie_Wenchang	PRJNA827465	0.941986107
ENSGALG00010000085	ENSGALG00010000085	2	0.115506133	0.084282129	1.370470043	0.17054022	0	0.541396799	0	0.5	CRA004775;PRJNA827465	Yang2025_Silkie_Wenchang	0.672201941
ENSGALG00010000089	ENSGALG00010000089	2	0.227758983	0.14375941	1.58430661	0.113123962	0	0.196196416	0	1	PRJNA827465;Yang2025_Silkie_Wenchang	CRA004775	0.567482422
ENSGALG00010000089	ENSGALG00010000089	2	0.301881745	0.235359186	1.282642711	0.199617279	0	0.053996038	0	1	CRA004775;Yang2025_Silkie_Wenchang	PRJNA827465	0.711489865
ENSGALG00010000089	ENSGALG00010000089	2	0.200613711	0.149084398	1.345638542	0.178419116	0	0.010793882	0	1	CRA004775;PRJNA827465	Yang2025_Silkie_Wenchang	0.684796269
ENSGALG00010000092	INPPL1	2	0.108266696	0.262334693	0.412704452	0.679823162	0.121407777	8.158705221	87.7431532	0.5	PRJNA827465;Yang2025_Silkie_Wenchang	CRA004775	0.917701868
ENSGALG00010000092	INPPL1	2	-0.06496248	0.507652284	-0.127966489	0.898175493	0.438046813	6.246377856	83.99072193	0.5	CRA004775;Yang2025_Silkie_Wenchang	PRJNA827465	0.975750151
ENSGALG00010000092	INPPL1	2	-0.248963467	0.208018771	-1.196831736	0.231372146	0.049183987	1.669563118	40.10409137	1	CRA004775;PRJNA827465	Yang2025_Silkie_Wenchang	0.743891332
ENSGALG00010000093	ENSGALG00010000093	2	0.025046105	0.216913112	0.11546607	0.908075713	0	0.241025057	0	0.5	PRJNA827465;Yang2025_Silkie_Wenchang	CRA004775	0.978196186
ENSGALG00010000093	ENSGALG00010000093	2	0.186710469	0.306381094	0.609405973	0.54225538	0	0.022836465	0	1	CRA004775;Yang2025_Silkie_Wenchang	PRJNA827465	0.876721948
ENSGALG00010000093	ENSGALG00010000093	2	-0.005283292	0.252351655	-0.020936231	0.983296525	0	0.229543606	0	0.5	CRA004775;PRJNA827465	Yang2025_Silkie_Wenchang	0.995363468
ENSGALG00010000098	ENSGALG00010000098	2	0.31985303	0.403542681	0.792612641	0.428003541	0.30341028	14.39486004	93.05307591	0.5	PRJNA827465;Yang2025_Silkie_Wenchang	CRA004775	0.84125685
ENSGALG00010000098	ENSGALG00010000098	2	0.18151799	0.579705858	0.313120847	0.754188851	0.611223951	10.8426579	90.77716913	0.5	CRA004775;Yang2025_Silkie_Wenchang	PRJNA827465	0.938385954
ENSGALG00010000098	ENSGALG00010000098	2	-0.135025423	0.136534148	-0.988949836	0.322687686	0.011129235	1.217608325	17.87178359	1	CRA004775;PRJNA827465	Yang2025_Silkie_Wenchang	0.798652022
ENSGALG00010000105	CACNA1F	2	-0.693171236	0.236015828	-2.936969281	0.003314369	0	0.083297359	0	1	PRJNA827465;Yang2025_Silkie_Wenchang	CRA004775	0.080463346
ENSGALG00010000105	CACNA1F	2	-0.366893543	0.366577736	-1.000861501	0.316893771	0.148511293	2.184384033	54.22050405	1	CRA004775;Yang2025_Silkie_Wenchang	PRJNA827465	0.796959041
ENSGALG00010000105	CACNA1F	2	-0.348500173	0.298255526	-1.168461746	0.242620561	0.091678725	2.062960203	51.52596746	1	CRA004775;PRJNA827465	Yang2025_Silkie_Wenchang	0.7550024
ENSGALG00010000107	ENSGALG00010000107	2	-0.493381374	0.403208826	-1.223637337	0.221089068	0.171530182	2.090810672	52.17166176	1	PRJNA827465;Yang2025_Silkie_Wenchang	CRA004775	0.734279503
ENSGALG00010000107	ENSGALG00010000107	2	-0.189544317	0.325614703	-0.582112279	0.560491055	0	0.208708421	0	1	CRA004775;Yang2025_Silkie_Wenchang	PRJNA827465	0.881203589
ENSGALG00010000107	ENSGALG00010000107	2	-0.866939693	0.39229858	-2.209897604	0.02711227	0	0.146410027	0	1	CRA004775;PRJNA827465	Yang2025_Silkie_Wenchang	0.280945645
ENSGALG00010000121	ENSGALG00010000121	2	-0.099752364	0.099882711	-0.998695	0.317942463	0	0.583812628	0	0.5	PRJNA827465;Yang2025_Silkie_Wenchang	CRA004775	0.797439961
ENSGALG00010000121	ENSGALG00010000121	2	-0.090768059	0.198745198	-0.45670567	0.647882615	0	0.897674728	0	0.5	CRA004775;Yang2025_Silkie_Wenchang	PRJNA827465	0.907506697
ENSGALG00010000121	ENSGALG00010000121	2	-0.146603267	0.099299714	-1.476371488	0.139844192	0	0.233878139	0	1	CRA004775;PRJNA827465	Yang2025_Silkie_Wenchang	0.623665962
ENSGALG00010000123	ENSGALG00010000123	2	0.068113274	0.115709492	0.588657617	0.556090975	0	0.038200309	0	1	PRJNA827465;Yang2025_Silkie_Wenchang	CRA004775	0.880324407
ENSGALG00010000123	ENSGALG00010000123	2	-0.496385013	0.625679951	-0.793352916	0.427572235	0.708727164	10.04202819	90.0418523	0.5	CRA004775;Yang2025_Silkie_Wenchang	PRJNA827465	0.84125685
ENSGALG00010000123	ENSGALG00010000123	2	-0.524373854	0.602490541	-0.870343712	0.384112598	0.643360705	8.480903149	88.20880297	0.5	CRA004775;PRJNA827465	Yang2025_Silkie_Wenchang	0.825767461
ENSGALG00010000124	ENSGALG00010000124	2	-0.051815079	0.164350557	-0.315271695	0.752555374	0	0.410550481	0	0.5	PRJNA827465;Yang2025_Silkie_Wenchang	CRA004775	0.938087082
ENSGALG00010000124	ENSGALG00010000124	2	-0.103465465	0.21908555	-0.472260564	0.636740815	0	0.283464462	0	0.5	CRA004775;Yang2025_Silkie_Wenchang	PRJNA827465	0.904765881
ENSGALG00010000124	ENSGALG00010000124	2	0.013503642	0.164124869	0.082276636	0.934426734	0	1.16516E-05	0	1	CRA004775;PRJNA827465	Yang2025_Silkie_Wenchang	0.984353495
ENSGALG00010000126	ENSGALG00010000126	2	0.63748124	0.772618892	0.825091447	0.409319675	1.109971012	13.97021916	92.84191616	0.5	PRJNA827465;Yang2025_Silkie_Wenchang	CRA004775	0.834355341
ENSGALG00010000126	ENSGALG00010000126	2	0.520243931	0.934860039	0.556493924	0.577873247	1.588799608	10.95362703	90.87060389	0.5	CRA004775;Yang2025_Silkie_Wenchang	PRJNA827465	0.88750546
ENSGALG00010000126	ENSGALG00010000126	2	-0.164372245	0.183369553	-0.896398787	0.370039815	0	0.455701181	0	1	CRA004775;PRJNA827465	Yang2025_Silkie_Wenchang	0.820855964
ENSGALG00010000127	ENSGALG00010000127	2	0.128529492	0.204077282	0.629807937	0.528820252	0.064177264	4.041290453	75.25542864	0.5	PRJNA827465;Yang2025_Silkie_Wenchang	CRA004775	0.871809618
ENSGALG00010000127	ENSGALG00010000127	2	-0.011081267	0.446000511	-0.024845863	0.980177909	0.307389571	4.026329745	75.16348478	0.5	CRA004775;Yang2025_Silkie_Wenchang	PRJNA827465	0.994676009
ENSGALG00010000127	ENSGALG00010000127	2	-0.128413507	0.183176564	-0.70103677	0.483280069	0.032174974	1.363297513	26.64843951	1	CRA004775;PRJNA827465	Yang2025_Silkie_Wenchang	0.85691916
ENSGALG00010000133	USP39	2	0.193227405	0.199269706	0.969677776	0.332207132	0.067030773	6.030288811	83.41704633	1	PRJNA827465;Yang2025_Silkie_Wenchang	CRA004775	0.802178578
ENSGALG00010000133	USP39	2	0.013285887	0.415039196	0.032011163	0.974463149	0.31189499	10.46052505	90.44025042	0.5	CRA004775;Yang2025_Silkie_Wenchang	PRJNA827465	0.992492038
ENSGALG00010000133	USP39	2	-0.1546269	0.209881471	-0.736734402	0.461283882	0.068001638	3.800742577	73.68935202	0.5	CRA004775;PRJNA827465	Yang2025_Silkie_Wenchang	0.85014477
ENSGALG00010000134	ENSGALG00010000134	2	0.530729504	0.464292599	1.14309275	0.253000084	0.42180529	46.00790031	97.82646025	1	PRJNA827465;Yang2025_Silkie_Wenchang	CRA004775	0.762731469
ENSGALG00010000134	ENSGALG00010000134	2	0.442392183	0.579364676	0.763581559	0.445116642	0.632545568	16.89281247	94.08032261	0.5	CRA004775;Yang2025_Silkie_Wenchang	PRJNA827465	0.846013379
ENSGALG00010000134	ENSGALG00010000134	2	0.054346324	0.068950888	0.788188887	0.430586228	0	0.750318052	0	0.5	CRA004775;PRJNA827465	Yang2025_Silkie_Wenchang	0.842407512
ENSGALG00010000137	ENSGALG00010000137	2	-0.997787929	0.350579033	-2.846114099	0.004425633	0.12099421	1.915045614	47.78192266	1	PRJNA827465;Yang2025_Silkie_Wenchang	CRA004775	0.097602941
ENSGALG00010000137	ENSGALG00010000137	2	-0.573553524	0.35695622	-1.606789552	0.108100541	0	0.001181196	0	1	CRA004775;Yang2025_Silkie_Wenchang	PRJNA827465	0.556254984
ENSGALG00010000137	ENSGALG00010000137	2	-1.174046159	0.273188226	-4.29757233	1.72679E-05	0	0.986779726	0	1	CRA004775;PRJNA827465	Yang2025_Silkie_Wenchang	0.002196375
ENSGALG00010000138	ENSGALG00010000138	2	0.638734402	1.003074563	0.636776592	0.524270375	1.666070892	5.52810035	81.91060334	0.5	PRJNA827465;Yang2025_Silkie_Wenchang	CRA004775	0.869790047
ENSGALG00010000138	ENSGALG00010000138	2	0.948112367	0.607255855	1.561306257	0.118451508	0.435948882	2.208320905	54.71672629	1	CRA004775;Yang2025_Silkie_Wenchang	PRJNA827465	0.578338927
ENSGALG00010000138	ENSGALG00010000138	2	0.121195802	0.377183184	0.321318148	0.747969305	0.133986928	1.888937762	47.0601933	0.5	CRA004775;PRJNA827465	Yang2025_Silkie_Wenchang	0.936938957
ENSGALG00010000140	SEPHS3	2	0.165235747	0.18574518	0.889582956	0.373689862	0.060688018	8.093964962	87.64511578	0.5	PRJNA827465;Yang2025_Silkie_Wenchang	CRA004775	0.822578137
ENSGALG00010000140	SEPHS3	2	0.031593295	0.362801129	0.087081578	0.930606668	0.228335097	7.140672693	85.99571717	0.5	CRA004775;Yang2025_Silkie_Wenchang	PRJNA827465	0.983903639
ENSGALG00010000140	SEPHS3	2	-0.107876962	0.159193645	-0.67764616	0.497996071	0.030472628	1.920498086	47.9301746	1	CRA004775;PRJNA827465	Yang2025_Silkie_Wenchang	0.861203659
ENSGALG00010000141	ENSGALG00010000141	2	-0.008425375	0.07621942	-0.110541051	0.911980295	0.005201056	1.72719072	42.1025143	0.5	PRJNA827465;Yang2025_Silkie_Wenchang	CRA004775	0.978933642
ENSGALG00010000141	ENSGALG00010000141	2	-0.010052654	0.134808728	-0.074569757	0.940557037	0.017885849	1.815018388	44.90413945	0.5	CRA004775;Yang2025_Silkie_Wenchang	PRJNA827465	0.985559481
ENSGALG00010000141	ENSGALG00010000141	2	-0.081526435	0.060723666	-1.342580909	0.179407716	0	0.413045736	0	1	CRA004775;PRJNA827465	Yang2025_Silkie_Wenchang	0.686254155
ENSGALG00010000142	ENSGALG00010000142	2	-1.097969648	0.29586116	-3.711097621	0.000206362	0	0.559839551	0	1	PRJNA827465;Yang2025_Silkie_Wenchang	CRA004775	0.013022445
ENSGALG00010000142	ENSGALG00010000142	2	-1.279987585	0.466911277	-2.741393595	0.006117917	0	0.342390343	0	1	CRA004775;Yang2025_Silkie_Wenchang	PRJNA827465	0.118495743
ENSGALG00010000142	ENSGALG00010000142	2	-0.92900235	0.334976693	-2.773334289	0.005548509	0	0.027985902	0	1	CRA004775;PRJNA827465	Yang2025_Silkie_Wenchang	0.111717753
ENSGALG00010000143	SCARF2	2	0.054885593	0.06091462	0.901024958	0.36757505	0	0.000127863	0	1	PRJNA827465;Yang2025_Silkie_Wenchang	CRA004775	0.819975483
ENSGALG00010000143	SCARF2	2	0.010961279	0.131138123	0.083585756	0.933385792	0	0.618382913	0	0.5	CRA004775;Yang2025_Silkie_Wenchang	PRJNA827465	0.984260547
ENSGALG00010000143	SCARF2	2	0.043170865	0.065905869	0.655038246	0.51244313	0	0.698895882	0	0.5	CRA004775;PRJNA827465	Yang2025_Silkie_Wenchang	0.865809047
ENSGALG00010000145	ENSGALG00010000145	2	0.202562183	0.082053916	2.468647345	0.01356248	0	0.01768214	0	1	PRJNA827465;Yang2025_Silkie_Wenchang	CRA004775	0.190509709
ENSGALG00010000145	ENSGALG00010000145	2	-0.490928706	0.764240783	-0.642374389	0.520630124	1.064569404	10.73861283	90.68781028	0.5	CRA004775;Yang2025_Silkie_Wenchang	PRJNA827465	0.869331539
ENSGALG00010000145	ENSGALG00010000145	2	-0.496632447	0.751055102	-0.661246353	0.508454339	1.035270251	11.32846374	91.17267775	0.5	CRA004775;PRJNA827465	Yang2025_Silkie_Wenchang	0.864902608
ENSGALG00010000146	ACADM	2	-0.234557069	0.289771189	-0.809456142	0.41825282	0.151640137	10.14502062	90.14294758	0.5	PRJNA827465;Yang2025_Silkie_Wenchang	CRA004775	0.838112595
ENSGALG00010000146	ACADM	2	-0.045571141	0.21681415	-0.210185272	0.833523074	0.036248774	1.349718265	25.91046396	0.5	CRA004775;Yang2025_Silkie_Wenchang	PRJNA827465	0.957968425

ENSGALG00010000146	ACADM	2	-0.510049654	0.09566437	-5.331657497	9.73204E-08	0	0.013488884	0	1	CRA004775;PRJNA827465	Yang2025_Silkie_Wenchang	3.48334E-05
ENSGALG00010000147	ENSGALG00010000147	2	0.332326102	0.336204549	0.988446025	0.322925445	0.200490662	8.230597217	87.85021337	1	PRJNA827465;Yang2025_Silkie_Wenchang	CRA004775	0.79874391
ENSGALG00010000147	ENSGALG00010000147	2	0.180247233	0.516585674	0.348920308	0.727149138	0.490167179	12.23521258	91.82686861	0.5	CRA004775;Yang2025_Silkie_Wenchang	PRJNA827465	0.931522817
ENSGALG00010000147	ENSGALG00010000147	2	-0.107037436	0.173205966	-0.617977764	0.536589996	0.04248584	2.979064418	66.43241436	0.5	CRA004775;PRJNA827465	Yang2025_Silkie_Wenchang	0.874599474
ENSGALG00010000148	ENSGALG00010000148	2	0.204282918	0.385848839	0.529437691	0.596501857	0.27012501	10.62162872	90.58524802	0.5	PRJNA827465;Yang2025_Silkie_Wenchang	CRA004775	0.894064255
ENSGALG00010000148	ENSGALG00010000148	2	0.080572939	0.556695233	0.14473438	0.884920594	0.54567135	8.149545056	87.72937639	0.5	CRA004775;Yang2025_Silkie_Wenchang	PRJNA827465	0.972188235
ENSGALG00010000148	ENSGALG00010000148	2	-0.210921683	0.120331227	-1.752842454	0.079629054	0	0.907425558	0	1	CRA004775;PRJNA827465	Yang2025_Silkie_Wenchang	0.48542321
ENSGALG00010000149	ENSGALG00010000149	2	0.261576543	0.272879353	0.95857946	0.337770644	0.13644677	11.91673705	91.60844117	0.5	PRJNA827465;Yang2025_Silkie_Wenchang	CRA004775	0.80415385
ENSGALG00010000149	ENSGALG00010000149	2	0.251174262	0.294118557	0.853989848	0.393110615	0.154339774	9.212291319	89.14493729	0.5	CRA004775;Yang2025_Silkie_Wenchang	PRJNA827465	0.830282579
ENSGALG00010000149	ENSGALG00010000149	2	-0.021683445	0.085960391	-0.252249265	0.800848401	0	0.053200781	0	1	CRA004775;PRJNA827465	Yang2025_Silkie_Wenchang	0.950158029
ENSGALG00010000150	B3GAT3	2	0.142546509	0.106679774	1.336209325	0.181480864	0	0.217533351	0	1	PRJNA827465;Yang2025_Silkie_Wenchang	CRA004775	0.689004635
ENSGALG00010000150	B3GAT3	2	0.156177811	0.190194826	0.821146475	0.41156284	0	0.367077095	0	0.5	CRA004775;Yang2025_Silkie_Wenchang	PRJNA827465	0.835128673
ENSGALG00010000150	B3GAT3	2	0.102931581	0.112123988	0.918015694	0.35861065	0	0.118899068	0	0.5	CRA004775;PRJNA827465	Yang2025_Silkie_Wenchang	0.814191806
ENSGALG00010000151	TBX5	2	0.379212946	0.314439446	1.205996737	0.227881749	0.142089392	3.267684959	69.39729465	1	PRJNA827465;Yang2025_Silkie_Wenchang	CRA004775	0.740853612
ENSGALG00010000151	TBX5	2	0.598750473	0.27201514	2.201165987	0.027724275	0	0.959965346	0	1	CRA004775;Yang2025_Silkie_Wenchang	PRJNA827465	0.284073056
ENSGALG00010000151	TBX5	2	0.121294065	0.150919787	0.803698892	0.421570894	0	0.004075285	0	1	CRA004775;PRJNA827465	Yang2025_Silkie_Wenchang	0.839352211
ENSGALG00010000153	NF2	2	0.100821652	0.096010894	1.05010637	0.29366921	0.012826498	3.01139592	66.79280883	1	PRJNA827465;Yang2025_Silkie_Wenchang	CRA004775	0.786979185
ENSGALG00010000153	NF2	2	0.181133825	0.106124829	1.706799694	0.087859289	0.002435627	1.04412487	4.226014683	0.5	CRA004775;Yang2025_Silkie_Wenchang	PRJNA827465	0.509491082
ENSGALG00010000153	NF2	2	0.018872259	0.04975712	0.3792876	0.704474307	0	0.200567762	0	0.5	CRA004775;PRJNA827465	Yang2025_Silkie_Wenchang	0.924152671
ENSGALG00010000154	ENSGALG00010000154	2	-5.703190192	5.506022081	-1.035809539	0.300291007	54.16840449	9.281455268	89.22582751	1	PRJNA827465;Yang2025_Silkie_Wenchang	CRA004775	0.790442127
ENSGALG00010000154	ENSGALG00010000154	2	-5.250506988	5.828498378	-0.900833568	0.367676817	63.19394853	13.75444903	92.72962517	0.5	CRA004775;Yang2025_Silkie_Wenchang	PRJNA827465	0.819975483
ENSGALG00010000154	ENSGALG00010000154	2	0.124200197	0.973759234	0.127550713	0.898488709	0	0.076875024	0	0.5	CRA004775;PRJNA827465	Yang2025_Silkie_Wenchang	0.97575249
ENSGALG00010000155	MAZ	2	-0.028437191	0.059897417	-0.474764897	0.634954556	0	0.298092229	0	0.5	PRJNA827465;Yang2025_Silkie_Wenchang	CRA004775	0.904036852
ENSGALG00010000155	MAZ	2	-0.235008879	0.32957053	-0.713076133	0.475798647	0.157976405	2.880183928	65.27999513	0.5	CRA004775;Yang2025_Silkie_Wenchang	PRJNA827465	0.854637093
ENSGALG00010000155	MAZ	2	-0.257225348	0.290491039	-0.885484623	0.37589531	0.113838001	2.344912231	57.35448062	1	CRA004775;PRJNA827465	Yang2025_Silkie_Wenchang	0.825974013
ENSGALG00010000156	ENSGALG00010000156	2	0.660420528	0.419679187	1.573631832	0.11557259	0.097227229	1.371828703	27.10460151	1	PRJNA827465;Yang2025_Silkie_Wenchang	CRA004775	0.572716099
ENSGALG00010000156	ENSGALG00010000156	2	0.59024632	0.723731318	0.815560008	0.414751831	0.51721636	1.846806676	45.85248077	0.5	CRA004775;Yang2025_Silkie_Wenchang	PRJNA827465	0.837298284
ENSGALG00010000156	ENSGALG00010000156	2	0.169510982	0.415840535	0.407634581	0.683541972	0	0.381220805	0	0.5	CRA004775;PRJNA827465	Yang2025_Silkie_Wenchang	0.918800744
ENSGALG00010000157	ENSGALG00010000157	2	-3.141556393	2.655467933	-1.183051904	0.236785578	9.149998583	2.78473938	64.69099861	1	PRJNA827465;Yang2025_Silkie_Wenchang	CRA004775	0.749020867
ENSGALG00010000157	ENSGALG00010000157	2	-2.372050075	3.410553675	-0.695502931	0.486740175	18.88805764	5.1334746	80.5200166	0.5	CRA004775;Yang2025_Silkie_Wenchang	PRJNA827465	0.857603818
ENSGALG00010000157	ENSGALG00010000157	2	0.128533312	1.202230562	0.106912365	0.914858504	0	0.378664617	0	0.5	CRA004775;PRJNA827465	Yang2025_Silkie_Wenchang	0.979879651
ENSGALG00010000161	CDC42EP2	2	0.073253596	0.104053596	0.703971085	0.481450781	0	0.417184238	0	1	PRJNA827465;Yang2025_Silkie_Wenchang	CRA004775	0.85686777
ENSGALG00010000161	CDC42EP2	2	0.019848186	0.169052867	0.11740816	0.906536618	0.019205443	1.482335191	32.53887474	0.5	CRA004775;Yang2025_Silkie_Wenchang	PRJNA827465	0.978196186
ENSGALG00010000161	CDC42EP2	2	-0.030148178	0.113134142	-0.266481697	0.789868254	0	0.618622427	0	0.5	CRA004775;PRJNA827465	Yang2025_Silkie_Wenchang	0.947390191
ENSGALG00010000166	PFDN6	2	0.560375744	0.545221159	1.0277953	0.304046128	0.57276947	26.94271262	96.28842124	1	PRJNA827465;Yang2025_Silkie_Wenchang	CRA004775	0.79225794
ENSGALG00010000166	PFDN6	2	0.589595828	0.554532083	1.063231228	0.287677104	0.551257749	9.466853066	89.43012863	1	CRA004775;Yang2025_Silkie_Wenchang	PRJNA827465	0.782840067
ENSGALG00010000166	PFDN6	2	0.025974154	0.087731658	0.296063641	0.76718148	0	0.003730771	0	1	CRA004775;PRJNA827465	Yang2025_Silkie_Wenchang	0.941432527
ENSGALG00010000171	DPF2	2	-0.048777042	0.12593853	-0.387308332	0.698527958	0.024646567	4.416875069	77.35955886	0.5	PRJNA827465;Yang2025_Silkie_Wenchang	CRA004775	0.921716388
ENSGALG00010000171	DPF2	2	-0.029989867	0.136875596	-0.2191031	0.826569736	0.022818606	2.452154655	59.21953788	0.5	CRA004775;Yang2025_Silkie_Wenchang	PRJNA827465	0.956393839
ENSGALG00010000171	DPF2	2	-0.171389028	0.064430685	-2.66005284	0.007812839	0	0.023286287	0	1	CRA004775;PRJNA827465	Yang2025_Silkie_Wenchang	0.13708394
ENSGALG00010000172	ENSGALG00010000172	2	0.020712726	0.651809025	0.031777292	0.974649656	0	0.171738416	0	0.5	PRJNA827465;Yang2025_Silkie_Wenchang	CRA004775	0.99250325
ENSGALG00010000172	ENSGALG00010000172	2	-0.123258207	0.652849272	-0.188800405	0.850249254	0	0.416245764	0	0.5	CRA004775;Yang2025_Silkie_Wenchang	PRJNA827465	0.961982453
ENSGALG00010000172	ENSGALG00010000172	2	-0.410872815	0.665969992	-0.616953946	0.537265103	0	0.455781041	0	1	CRA004775;PRJNA827465	Yang2025_Silkie_Wenchang	0.87481821
ENSGALG00010000173	NIPSNAP1	2	0.154119009	0.120694885	1.276930735	0.201626702	0	0.051309133	0	1	PRJNA827465;Yang2025_Silkie_Wenchang	CRA004775	0.714501332
ENSGALG00010000173	NIPSNAP1	2	-0.138652174	0.225271076	-0.615490354	0.538230938	0.034366664	1.5096297	33.75858993	0.5	CRA004775;Yang2025_Silkie_Wenchang	PRJNA827465	0.87481821
ENSGALG00010000173	NIPSNAP1	2	-0.050056396	0.256402558	-0.195225806	0.845216177	0.094991803	3.373601334	70.35808618	0.5	CRA004775;PRJNA827465	Yang2025_Silkie_Wenchang	0.960863849
ENSGALG00010000174	ENSGALG00010000174	2	-0.051923759	0.151280364	-0.343228676	0.731426433	0	0.000425272	0	1	PRJNA827465;Yang2025_Silkie_Wenchang	CRA004775	0.932851968
ENSGALG00010000174	ENSGALG00010000174	2	-0.207328686	0.181816199	-1.140320213	0.25415292	0	0.671374419	0	1	CRA004775;Yang2025_Silkie_Wenchang	PRJNA827465	0.763894203
ENSGALG00010000174	ENSGALG00010000174	2	-0.15555148	0.1505542	-1.033192564	0.301513792	0	0.928045349	0	1	CRA004775;PRJNA827465	Yang2025_Silkie_Wenchang	0.791273005
ENSGALG00010000176	ENSGALG00010000176	2	1.06688867	0.777269574	1.372610874	0.16987335	1.021859838	6.349330627	84.25030739	1	PRJNA827465;Yang2025_Silkie_Wenchang	CRA004775	0.671232541
ENSGALG00010000176	ENSGALG00010000176	2	0.631265825	1.275570729	0.494888924	0.62067853	2.954773719	10.85762024	90.78987865	0.5	CRA004775;Yang2025_Silkie_Wenchang	PRJNA827465	0.895993593
ENSGALG00010000176	ENSGALG00010000176	2	-0.051449575	0.484704069	-0.106146364	0.915466226	0.268677079	2.193404174	54.40876735	0.5	CRA004775;PRJNA827465	Yang2025_Silkie_Wenchang	0.980165711
ENSGALG00010000177	ENSGALG00010000177	2	-0.023311791	0.141478274	-0.164772937	0.869122706	0.021079141	1.930086251	48.18884393	0.5	PRJNA827465;Yang2025_Silkie_Wenchang	CRA004775	0.968461055
ENSGALG00010000177	ENSGALG00010000177	2	-0.106960214	0.295076204	-0.360932808	0.718149677	0.126052287	3.628516473	72.43779752	1	CRA004775;Yang2025_Silkie_Wenchang	PRJNA827465	0.927579712
ENSGALG00010000177	ENSGALG00010000177	2	-0.189639982	0.120090243	-1.579145617	0.11430266	0.00828182	1.233184083	18.90910577	1	CRA004775;PRJNA827465	Yang2025_Silkie_Wenchang	0.57019364
ENSGALG00010000184	ENSGALG00010000184	2	1.33129463	2.095189855	0.635405248	0.525164146	0	0.001738759	0	1	PRJNA827465;Yang2025_Silkie_Wenchang	CRA004775	0.870173964
ENSGALG00010000184	ENSGALG00010000184	2	0.087439908	1.889716765	0.046271436	0.963093906	0	0.251233116	0	0.5	CRA004775;Yang2025_Silkie_Wenchang	PRJNA827465	0.989998461
ENSGALG00010000184	ENSGALG00010000184	2	0.169718423	1.880664418	0.090241843	0.928095034	0	0.302986391	0	0.5	CRA004775;PRJNA827465	Yang2025_Silkie_Wenchang	0.983019892
ENSGALG00010000186	THOC5	2	-0.083924962	0.039285672	-2.136274049	0.032657083	0	0.662554377	0	1	PRJNA827465;Yang2025_Silkie_Wenchang	CRA004775	0.310329141
ENSGALG00010000186	THOC5	2	-0.055370015	0.07535549	-0.734784093	0.462470997	0	0.966103733	0	1	CRA004775;Yang2025_Silkie_Wenchang	PRJNA827465	0.850823289
ENSGALG00010000186	THOC5	2	-0.108126621	0.043903897	-2.462802322	0.01378559	0	0.455400978	0	1	CRA004775;PRJNA827465	Yang2025_Silkie_Wenchang	0.192701185
ENSGALG00010000188	ENSGALG00010000188	2	-1.054760723	1.121521704	-0.940472859	0.346975065	0	5.70712E-05	0	1	PRJNA827465;Yang2025_Silkie_Wenchang	CRA004775	0.80855929
ENSGALG00010000188	ENSGALG00010000188	2	0.12048429	1.239750492	0.097184305	0.922580032	0	0.397529808	0	0.5	CRA004775;Yang2025_Silkie_Wenchang	PRJNA827465	0.981804675
ENSGALG00010000188	ENSGALG00010000188	2	-0.324951115	0.980730152	-0.331335907	0.740390771	0	0.742388963	0	0.5	CRA004775;PRJNA827465	Yang2025_Silkie_Wenchang	0.935128797
ENSGALG00010000189	ENSGALG00010000189	2	0.803283651	0.377609924	2.127284269	0.033396471	0	0.135595177	0	1	PRJNA827465;Yang2025_Silkie_Wenchang	CRA004775	0.314014713
ENSGALG00010000189	ENSGALG00010000189	2	0.519912717	0.582310454	0.892844553	0.371940423	0.003952868	1.005828515	0.579473988	0.5	CRA004775;Yang2025_Silkie_Wenchang	PRJNA827465	0.821597118
ENSGALG00010000189	ENSGALG00010000189	2	0.558967549	0.38389681	1.456035931	0.145382687	0	0.764745486	0	0.5	CRA004775;PRJNA827465	Yang2025_Silkie_Wenchang	0.633907279
ENSGALG00010000191	NEFH	2	-0.203483991	0.636038337	-0.319924097	0.74902587	0.673748711	5.978130186	83.27236161	0.5	PRJNA827465;Yang2025_Silkie_Wenchang	CRA004775	0.937357674
ENSGALG00010000191	NEFH	2	0.268142928	0.267218047	1.003461144	0.315638415	0	0.425013505	0	1	CRA004775;Yang2025_Silkie_Wenchang	PRJNA827465	0.796883862
ENSGALG00010000191	NEFH	2	-0.387384101	0.461414777	-0.839557206	0.401156702	0.281744918	2.95366927	66.14380594	0.5	CRA004775;PRJNA827465	Yang2025_Silkie_Wenchang	0.832453107
ENSGALG00010000197	ENSGALG00010000197	2	-0.168375648	0.430603901	-0.391022115	0.695780889	0	0.093473621	0	0.5	PRJNA827465;Yang2025_Silkie_Wenchang	CRA004775	0.921038437
ENSGALG00010000197	ENSGALG00010000197	2	-0.306263027	0.590937646	-0.518266232	0.60427253	0	0.595729783	0	0.5	CRA004775;Yang2025_Silkie_Wenchang	PRJNA827465	0.89612415
ENSGALG00010000197	ENSGALG00010000197	2	-0.415150218	0.478709261	-0.867228298	0.385816934	0	0.358436347	0	1	CRA004775;PRJNA827465	Yang2025_Silkie_Wenchang	0.826543277
ENSGALG00010000200	GAL3ST1	2	0.338252532	0.15711502	2.152897494	0.031326735	0	0.31920942	0	1	PRJNA827465;Yang2025_Silkie_Wenchang	CRA004775	0.302812269
ENSGALG00010000200	GAL3ST1	2	0.591692117	0.259395111	2.281045751	0.02254574	0	0.545318343	0	1	CRA004775;Yang2025_Silkie_Wenchang	PRJNA827465	0.255291042
ENSGALG00010000200	GAL3ST1	2	0.419412461	0.26248592	1.597847461	0.110076929	0.053288504	1.347855094	25.80804834	1	CRA004775;PRJNA827465	Yang2025_Silkie_Wenchang	0.561937076
ENSGALG00010000202	ENSGALG00010000202	2	0.529236556	0.318689967	1.660662745	0.0967812	0.095650779	1.880893553	46.83378023	1	PRJNA827465;Yang2025_Silkie_Wenchang	CRA004775	0.531627042
ENSGALG00010000202	ENSGALG00010000202	2	0.155593136	0.233881651	0.6652644	0.505881397	0	0.155129951	0	1	CRA004775;Yang2025_Silkie_Wenchang	PRJNA827465	0.863927652
ENSGALG00010000202	ENSGALG00010000202	2	0.464212218	0.413000093	1.124000275	0.261012909	0.213503798	2.67214523	62.57688434	1	CRA004775;PRJNA827465	Yang2025_Silkie_Wenchang	0.767929536
ENSGALG00010000205	RNF215	2	0.314629162	0.268949018	1.169846854	0.242062604	0.12883321	8.735875672	88.55295064	1	PRJNA827465;Yang2025_Silkie_Wenchang	CRA004775	0.754284194
ENSGALG00010000205	RNF215	2	0.18968487	0.415779599	0.456214952	0.648235415	0.317440917	12.21462737	91.8130945	0.5	CRA004775;Yang2025_Silkie_Wenchang	PRJNA827465	0.90762535
ENSGALG00010000205	RNF215	2	-0.041017407	0.140519517	-0.29189829	0.770364391	0.025289889	2.454848222	59.264284	0.5	CRA004775;PRJNA827465	Yang2025_Silkie_Wenchang	0.942377032
ENSGALG00010000210	CCDC157	2	-0.728930214	0.302619793	-2.408732772	0.016008014	0	0.360421654	0	1	PRJNA827465;Yang2025_Silkie_Wenchang	CRA004775	0.209024249
ENSGALG00010000210	CCDC157	2	-0.323342994	0.272829184	-1.185148117	0.235958889	0	0.616056757	0	1	CRA004775;Yang2025_Silkie_Wenchang	PRJNA827465	0.748669015
ENSGALG00010000210	CCDC157	2	-0.487496207	0.395673823	-1.232065854	0.217924485	0.149056948	1.888887218	47.0587767	1	CRA004775;PRJNA827465	Yang2025_Silkie_Wenchang	0.73141715
ENSGALG00010000215	SF3A1	2	-0.06075334	0.046371884	-1.310133084	0.190150818	0	0.478815026	0	0.5	PRJNA827465;Yang2025_Silkie_Wenchang	CRA004775	0.699403589
ENSGALG00010000215	SF3A1	2	-0.16982939	0.191872429	-0.885116176	0.376093976	0.057243988	4.491229797	77.73438356	0.5	CRA004775;Yang2025_Silkie_Wenchang	PRJNA827465	0.822974013
ENSGALG00010000215	SF3A1	2	-0.193349474	0.141464402	-1.366771228	0.171697024	0.032075462	4.568750584	78.1121779	1	CRA004775;PRJNA827465	Yang2025_Silkie_Wenchang	0.674249474
ENSGALG00010000221	TBC1D10A	2	0.11854979	0.05420902	2.186901553	0.028749712	0	0.000827482	0	1	PRJNA827465;Yang2025_Silkie_Wenchang	CRA004775	0.288915856
ENSGALG00010000221	TBC1D10A	2	0.068798372	0.121541203	0.56604979	0.571359934	0	0.333705374	0	0.5	CRA004775;Yang2025_Silkie_Wenchang	PRJNA827465	0.885354083
ENSGALG00010000221	TBC1D10A	2	0.107027352	0.055661004	1.922842639	0.054499815	0	0.45778907	0	0.5	CRA004775;PRJNA827465	Yang2025_Silkie_Wenchang	0.404097297
ENSGALG00010000223	CASTOR1	2	0.084024862	0.095165761	0.882931652	0.377273201	0.000204294	1.010453428	1.034528465	1	PRJNA827465;Yang2025_Silkie_Wenchang	CRA004775	0.823181514
ENSGALG00010000223	CASTOR1	2	0.256058859	0.145256593	1.762803701	0.077933603	0	0.471000645	0	1	CRA004775;Yang2025_Silkie_Wenchang	PRJNA827465	0.480724545
ENSGALG00010000223	CASTOR1	2	0.124419609	0.199247421	0.624447777	0.532333557	0.038197245	1.534673673	34.83956768	1	CRA004775;PRJNA827465	Yang2025_Silkie_Wenchang	0.873104119
ENSGALG00010000224	MYBPC2	2	-0.707825103	0.170792605	-4.144354523	3.40772E-05	0	0.17829853	0	1	PRJNA827465;Yang2025_Silkie_Wenchang	CRA004775	0.003661746
ENSGALG00010000224	MYBPC2	2	-0.586403742	0.222789471	-2.632098089	0.008485936	0	0.598139322	0	1	CRA004775;Yang2025_Silkie_Wenchang	PRJNA827465	0.14369705
ENSGALG00010000224	MYBPC2	2	-0.70895327	0.237300171	-2.987580105	0.002811956	0	0.907570237	0	1	CRA004775;PRJNA827465	Yang2025_Silkie_Wenchang	0.07222107
ENSGALG00010000225	LIF	2	0.176843203	0.316953826	0.557946266	0.576881077	0.038507319	1.23106936	18.76980839	0.5	PRJNA827465;Yang2025_Silkie_Wenchang	CRA004775	0.887113945
ENSGALG00010000225	LIF	2	-0.076810057	0.311132757	-0.246872294	0.805007059	0	0.127756422	0	0.5	CRA004775;Yang2025_Silkie_Wenchang	PRJNA827465	0.951863117
ENSGALG00010000225	LIF	2	0.276411437	0.282596248	0.978114318	0.328017787	0	0.532485766	0	1	CRA004775;PRJNA827465	Yang2025_Silkie_Wenchang	0.800530298
ENSGALG00010000226	ENSGALG00010000226	2	0.115189124	0.215614277	0.534236997	0.593177575	0	0.885434401	0	0.5	PRJNA827465;Yang2025_Silkie_Wenchang	CRA004775	0.892776493
ENSGALG00010000226	ENSGALG00010000226	2	-0.08861857	0.464166714	-0.190919701	0.848588505	0.316949443	3.748150987	73.32017831	0.5	CRA004775;Yang2025_Silkie_Wenchang	PRJNA827465	0.961364896
ENSGALG00010000226	ENSGALG00010000226	2	-0.256377898	0.248695113	-1.030892386	0.302591287	0.044318301	1.55200628	35.56727101	1	CRA004775;PRJNA827465	Yang2025_Silkie_Wenchang	0.791852471
ENSGALG00010000227	ENSGALG00010000227	2	0.002364689	0.197510142	0.011972496	0.990447558	0	0.872792021	0	0.5	PRJNA827465;Yang2025_Silkie_Wenchang	CRA004775	0.997468525
ENSGALG00010000227	ENSGALG00010000227	2	-0.039961797	0.334472707	-0.119477004	0.904897458	0.086944528	1.635560175	38.85886836	0.5	CRA004775;Yang2025_Silkie_Wenchang	PRJNA827465	0.977844568
ENSGALG00010000227	ENSGALG00010000227	2	-0.188213422	0.197389754	-0.953511609	0.340330917	0	0.353845258	0	1	CRA004775;PRJNA827465	Yang2025_Silkie_Wenchang	0.805432415
ENSGALG00010000231	ENSGALG00010000231	2	0.349269767	0.206456434	1.691735926	0.090696335	0	0.010396567	0	1	PRJNA827465;Yang2025_Silkie_Wenchang	CRA004775	0.516759185
ENSGALG00010000231	ENSGALG00010000231	2	-0.001298021	0.431749126	-0.003006425	0.997601224	0.247432641	2.679707602	62.68249569	0.5	CRA004775;Yang2025_Silkie_Wenchang	PRJNA827465	0.999290321
ENSGALG00010000231	ENSGALG00010000231	2	-0.058130707	0.469371183	-0.123848053	0.901435584	0.222166264	2.012418497	50.30854658	0.5	CRA004775;PRJNA827465	Yang2025_Silkie_Wenchang	0.976655087
ENSGALG00010000232	ENSGALG00010000232	2	0.160235481	0.135074842	1.186271833	0.235514967	0.011872933	1.180237767	15.27130992	0.5	PRJNA827465;Yang2025_Silkie_Wenchang	CRA004775	0.748604097
ENSGALG00010000232	ENSGALG00010000232	2	-0.020223627	0.240362827	-0.084137914	0.93294678	0	0.397089306	0	0.5	CRA004775;Yang2025_Silkie_Wenchang	PRJNA827465	0.984224753
ENSGALG00010000232	ENSGALG00010000232	2	0.206690888	0.092297424	2.239400398	0.025129875	0	0.069694679	0	1	CRA004775;PRJNA827465	Yang2025_Silkie_Wenchang	0.269931537
ENSGALG00010000234	ENSGALG00010000234	2	-5.111401491	5.109355512	-1.000400438	0.317116758	46.32214639	8.687258125	88.48888814	1	PRJNA827465;Yang2025_Silkie_Wenchang	CRA004775	0.797007794
ENSGALG00010000234	ENSGALG00010000234	2	-5.092391302	5.025577898	-1.013294671	0.310919432	45.67789067	9.970733404	89.9706475	1	CRA004775;Yang2025_Silkie_Wenchang	PRJNA827465	0.795218958
ENSGALG00010000234	ENSGALG00010000234	2	-0.364802116	1.013174749	-0.360058436	0.718803434	0	0.005002587	0	1	CRA004775;PRJNA827465	Yang2025_Silkie_Wenchang	0.928449671
ENSGALG00010000239	ENSGALG00010000239	2	-6.548857391	6.33885391	-1.033129566	0.301543269	73.87029198	12.2793235	91.85622889	1	PRJNA827465;Yang2025_Silkie_Wenchang	CRA004775	0.791273005
ENSGALG00010000239	ENSGALG00010000239	2	-6.404602435	6.36028655	-1.006967592	0.313950348	76.04159962	16.11568611	93.79486549	1	CRA004775;Yang2025_Silkie_Wenchang	PRJNA827465	0.796531448
ENSGALG00010000239	ENSGALG00010000239	2	-0.337842871	1.020706687	-0.330989182	0.740652657	0	0.000457549	0	1	CRA004775;PRJNA827465	Yang2025_Silkie_Wenchang	0.935166776
ENSGALG00010000241	ENSGALG00010000241	2	0.070473023	0.367106093	0.191969091	0.847766417	0	0.025265202	0	1	PRJNA827465;Yang2025_Silkie_Wenchang	CRA004775	0.96130416
ENSGALG00010000241	ENSGALG00010000241	2	-0.851601503	0.957671031	-0.889242209	0.373872923	1.359325785	3.73530518	73.22842574	0.5	CRA004775;Yang2025_Silkie_Wenchang	PRJNA827465	0.822579781
ENSGALG00010000241	ENSGALG00010000241	2	-0.787658071	1.0159992	-0.775254618	0.43818922	1.615158553	4.409822867	77.3233522	0.5	CRA004775;PRJNA827465	Yang2025_Silkie_Wenchang	0.843979791
ENSGALG00010000242	ENSGALG00010000242	2	-0.049285126	0.541098012	-0.091083547	0.927426206	0.508851442	7.485375341	86.64061648	0.5	PRJNA827465;Yang2025_Silkie_Wenchang	CRA004775	0.982993195
ENSGALG00010000242	ENSGALG00010000242	2	0.318106463	0.328506978	0.968340049	0.33287457	0.039113386	1.170398041	14.55898207	0.5	CRA004775;Yang2025_Silkie_Wenchang	PRJNA827465	0.802178578
ENSGALG00010000242	ENSGALG00010000242	2	-0.520418672	0.202565448	-2.569138402	0.010195173	0	0.311388722	0	1	CRA004775;PRJNA827465	Yang2025_Silkie_Wenchang	0.160364698
ENSGALG00010000246	ENSGALG00010000246	2	-3.145785821	2.728599182	-1.152894072	0.248953866	10.46004636	3.177698966	68.53068807	1	PRJNA827465;Yang2025_Silkie_Wenchang	CRA004775	0.759411487
ENSGALG00010000246	ENSGALG00010000246	2	-2.428858175	3.529441951	-0.688170597	0.491345357	20.56587752	5.45942355	81.68304784	0.5	CRA004775;Yang2025_Silkie_Wenchang	PRJNA827465	0.85895707
ENSGALG00010000246	ENSGALG00010000246	2	0.038108144	1.05117077	0.036253047	0.971080588	0	0.554311973	0	0.5	CRA004775;PRJNA827465	Yang2025_Silkie_Wenchang	0.991916113
ENSGALG00010000247	ENSGALG00010000247	2	0.501019832	0.186359578	2.688457643	0.007178294	0	0.336865117	0	1	PRJNA827465;Yang2025_Silkie_Wenchang	CRA004775	0.130253231
ENSGALG00010000247	ENSGALG00010000247	2	0.53389967	0.232701456	2.294354658	0.021770135	0	0.557638282	0	1	CRA004775;Yang2025_Silkie_Wenchang	PRJNA827465	0.249990082
ENSGALG00010000247	ENSGALG00010000247	2	0.328574154	0.263945782	1.244854725	0.213185156	0	0.198875832	0	1	CRA004775;PRJNA827465	Yang2025_Silkie_Wenchang	0.72579791
ENSGALG00010000249	ENSGALG00010000249	2	-6.077676947	5.911907733	-1.028039885	0.303931066	63.6014464	10.97380721	90.8873923	1	PRJNA827465;Yang2025_Silkie_Wenchang	CRA004775	0.792208658
ENSGALG00010000249	ENSGALG00010000249	2	-5.818463872	6.067949006	-0.958884768	0.337616799	68.70627926	14.44993761	93.0795549	1	CRA004775;Yang2025_Silkie_Wenchang	PRJNA827465	0.80415385
ENSGALG00010000249	ENSGALG00010000249	2	-0.125928856	1.051153087	-0.119800681	0.904641042	0	0.018363786	0	1	CRA004775;PRJNA827465	Yang2025_Silkie_Wenchang	0.977683364
ENSGALG00010000255	ENSGALG00010000255	2	1.508660447	1.615912271	0.933627694	0.350495975	5.180544226	124.6403226	99.19769142	0.5	PRJNA827465;Yang2025_Silkie_Wenchang	CRA004775	0.810111037
ENSGALG00010000255	ENSGALG00010000255	2	1.52009094	1.623633888	0.93622765	0.34915599	5.168586547	50.658875	98.02601222	0.5	CRA004775;Yang2025_Silkie_Wenchang	PRJNA827465	0.809731578
ENSGALG00010000255	ENSGALG00010000255	2	-0.102039727	0.129876767	-0.785665749	0.432063334	0	0.001478204	0	1	CRA004775;PRJNA827465	Yang2025_Silkie_Wenchang	0.842503929
ENSGALG00010000256	ENSGALG00010000256	2	-0.232253137	0.208111623	-1.116002717	0.264420971	0	0.404018026	0	1	PRJNA827465;Yang2025_Silkie_Wenchang	CRA004775	0.768790489
ENSGALG00010000256	ENSGALG00010000256	2	-0.075913893	0.313850194	-0.241879388	0.808873622	0	0.022852869	0	1	CRA004775;Yang2025_Silkie_Wenchang	PRJNA827465	0.952595795
ENSGALG00010000256	ENSGALG00010000256	2	-0.309791022	0.241848213	-1.280931618	0.200217686	0	0.046682125	0	1	CRA004775;PRJNA827465	Yang2025_Silkie_Wenchang	0.712517205
ENSGALG00010000262	HNRNPKL	2	-5.606524807	5.614459108	-0.99858681	0.317994893	57.53986803	11.18209215	91.05712968	1	PRJNA827465;Yang2025_Silkie_Wenchang	CRA004775	0.797439961
ENSGALG00010000262	HNRNPKL	2	-5.407290434	5.759160158	-0.938902598	0.347780755	61.57302406	13.38585931	92.52942992	0.5	CRA004775;Yang2025_Silkie_Wenchang	PRJNA827465	0.808847403
ENSGALG00010000262	HNRNPKL	2	-0.05717566	0.91848097	-0.062250239	0.950363555	0	0.022492416	0	0.5	CRA004775;PRJNA827465	Yang2025_Silkie_Wenchang	0.987277827
ENSGALG00010000263	MTMR3	2	-0.111889511	0.052970231	-2.112309294	0.034659929	0	0.719426313	0	1	PRJNA827465;Yang2025_Silkie_Wenchang	CRA004775	0.320651825
ENSGALG00010000263	MTMR3	2	-0.155812451	0.074052272	-2.104087368	0.035370827	0	0.04342093	0	1	CRA004775;Yang2025_Silkie_Wenchang	PRJNA827465	0.324684241
ENSGALG00010000263	MTMR3	2	-0.075446611	0.067811902	-1.112586571	0.265886031	0	0.035550913	0	1	CRA004775;PRJNA827465	Yang2025_Silkie_Wenchang	0.770065639
ENSGALG00010000267	ENSGALG00010000267	2	0.381731006	0.372770654	1.024037172	0.305817718	0.260078341	15.30088091	93.46442858	1	PRJNA827465;Yang2025_Silkie_Wenchang	CRA004775	0.79225794
ENSGALG00010000267	ENSGALG00010000267	2	0.302646368	0.484643092	0.624472675	0.53231721	0.423339703	9.969324705	89.96923032	0.5	CRA004775;Yang2025_Silkie_Wenchang	PRJNA827465	0.873104119
ENSGALG00010000267	ENSGALG00010000267	2	-0.003081026	0.08606212	-0.035800025	0.971441813	0	0.672200251	0	0.5	CRA004775;PRJNA827465	Yang2025_Silkie_Wenchang	0.991955348
ENSGALG00010000271	RTN4R	2	0.034326788	0.195058521	0.175981996	0.860308087	0	0.093530981	0	0.5	PRJNA827465;Yang2025_Silkie_Wenchang	CRA004775	0.964999892
ENSGALG00010000271	RTN4R	2	0.291345957	0.329660466	0.883775845	0.376817228	0	0.708236986	0	1	CRA004775;Yang2025_Silkie_Wenchang	PRJNA827465	0.823054769
ENSGALG00010000271	RTN4R	2	0.137139139	0.298442871	0.459515547	0.64586399	0.056534961	1.22740252	18.52713484	0.5	CRA004775;PRJNA827465	Yang2025_Silkie_Wenchang	0.907098003
ENSGALG00010000272	ENSGALG00010000272	2	-0.342581516	0.385889632	-0.887770721	0.374664095	0	0.969254648	0	0.5	PRJNA827465;Yang2025_Silkie_Wenchang	CRA004775	0.822805106
ENSGALG00010000272	ENSGALG00010000272	2	0.742986523	0.49123469	1.512487896	0.13040979	0.128517427	1.353817861	26.13482	1	CRA004775;Yang2025_Silkie_Wenchang	PRJNA827465	0.605614597
ENSGALG00010000272	ENSGALG00010000272	2	0.257019456	0.894940735	0.287191594	0.773965623	1.340578942	6.116888677	83.65181953	0.5	CRA004775;PRJNA827465	Yang2025_Silkie_Wenchang	0.943248868
ENSGALG00010000279	ENSGALG00010000279	2	-0.221553034	0.100722341	-2.199641422	0.027832346	0	0.783758466	0	1	PRJNA827465;Yang2025_Silkie_Wenchang	CRA004775	0.284423997
ENSGALG00010000279	ENSGALG00010000279	2	-0.36448522	0.1436745	-2.536881762	0.011184472	0	0.055501413	0	1	CRA004775;Yang2025_Silkie_Wenchang	PRJNA827465	0.167908064
ENSGALG00010000279	ENSGALG00010000279	2	-0.202799895	0.11325661	-1.790623041	0.073353807	0	0.783088536	0	1	CRA004775;PRJNA827465	Yang2025_Silkie_Wenchang	0.467957919
ENSGALG00010000281	OSBP2	2	-0.047938627	0.119773029	-0.400245594	0.688975636	0.016788897	2.40821982	58.47555146	0.5	PRJNA827465;Yang2025_Silkie_Wenchang	CRA004775	0.919854035
ENSGALG00010000281	OSBP2	2	0.090139091	0.108070717	0.834075074	0.40423868	0	0.229185844	0	1	CRA004775;Yang2025_Silkie_Wenchang	PRJNA827465	0.833112102
ENSGALG00010000281	OSBP2	2	-0.107705621	0.146058774	-0.737412876	0.460871307	0.0134029	1.16788698	14.3752763	0.5	CRA004775;PRJNA827465	Yang2025_Silkie_Wenchang	0.85014477
ENSGALG00010000282	ENSGALG00010000282	2	-6.299050654	5.728755057	-1.099549656	0.271528387	59.4005823	10.39502533	90.38001382	1	PRJNA827465;Yang2025_Silkie_Wenchang	CRA004775	0.772805786
ENSGALG00010000282	ENSGALG00010000282	2	-5.452568388	6.523398634	-0.835847799	0.403240532	79.90892651	15.98285979	93.74329743	0.5	CRA004775;Yang2025_Silkie_Wenchang	PRJNA827465	0.83306585
ENSGALG00010000282	ENSGALG00010000282	2	0.288443726	1.146054468	0.251684134	0.801285222	0	0.428433982	0	0.5	CRA004775;PRJNA827465	Yang2025_Silkie_Wenchang	0.950158029
ENSGALG00010000283	ENSGALG00010000283	2	-0.643896849	0.358706444	-1.795052359	0.072645358	0	0.578801109	0	1	PRJNA827465;Yang2025_Silkie_Wenchang	CRA004775	0.465519917
ENSGALG00010000283	ENSGALG00010000283	2	-0.117354184	0.347346623	-0.337859003	0.735469444	0	0.195181273	0	0.5	CRA004775;Yang2025_Silkie_Wenchang	PRJNA827465	0.933592889
ENSGALG00010000283	ENSGALG00010000283	2	-0.420725662	0.435820232	-0.965365147	0.334361952	0.174696971	1.850136618	45.94993742	0.5	CRA004775;PRJNA827465	Yang2025_Silkie_Wenchang	0.803094614
ENSGALG00010000284	ENSGALG00010000284	2	0.072371316	0.241635929	0.299505607	0.764554294	0.077014258	2.573679456	61.1451225	0.5	PRJNA827465;Yang2025_Silkie_Wenchang	CRA004775	0.940627228
ENSGALG00010000284	ENSGALG00010000284	2	-0.193877925	0.571973795	-0.338962951	0.73463764	0.58637166	9.589607386	89.5720444	0.5	CRA004775;Yang2025_Silkie_Wenchang	PRJNA827465	0.933520234
ENSGALG00010000284	ENSGALG00010000284	2	-0.401918645	0.319753645	-1.256963451	0.208766871	0.172028922	6.020338498	83.3896383	1	CRA004775;PRJNA827465	Yang2025_Silkie_Wenchang	0.721011841
ENSGALG00010000285	MYLPF	2	-0.252908348	0.115516292	-2.189373836	0.02856968	0	0.0292682	0	1	PRJNA827465;Yang2025_Silkie_Wenchang	CRA004775	0.287842636
ENSGALG00010000285	MYLPF	2	-0.227588841	0.289344412	-0.786567258	0.431535232	0	0.046289633	0	1	CRA004775;Yang2025_Silkie_Wenchang	PRJNA827465	0.842503929
ENSGALG00010000285	MYLPF	2	-0.263839544	0.123036562	-2.144399514	0.032000889	0	0.024918901	0	1	CRA004775;PRJNA827465	Yang2025_Silkie_Wenchang	0.306763646
ENSGALG00010000286	TCN2	2	0.016234658	0.179435837	0.090476119	0.927908871	0.043156519	2.824845648	64.59983572	0.5	PRJNA827465;Yang2025_Silkie_Wenchang	CRA004775	0.982993195
ENSGALG00010000286	TCN2	2	0.184656349	0.146338579	1.261843258	0.207005193	0	0.161288902	0	1	CRA004775;Yang2025_Silkie_Wenchang	PRJNA827465	0.718911823
ENSGALG00010000286	TCN2	2	-0.089074754	0.095153027	-0.936121075	0.349210853	0	0.959667387	0	0.5	CRA004775;PRJNA827465	Yang2025_Silkie_Wenchang	0.809731578
ENSGALG00010000287	IQCD	2	-0.053990109	0.152237686	-0.354643521	0.722856668	0	0.518771268	0	0.5	PRJNA827465;Yang2025_Silkie_Wenchang	CRA004775	0.930032096
ENSGALG00010000287	IQCD	2	-0.16796656	0.223615359	-0.751140534	0.452568084	0	0.072347909	0	1	CRA004775;Yang2025_Silkie_Wenchang	PRJNA827465	0.848321192
ENSGALG00010000287	IQCD	2	0.016479933	0.17650639	0.093367347	0.925611731	0	0.027264504	0	0.5	CRA004775;PRJNA827465	Yang2025_Silkie_Wenchang	0.982594411
ENSGALG00010000288	VARS2	2	-0.096165465	0.065017768	-1.47906438	0.139123109	0.001447579	1.160834215	13.85505463	0.5	PRJNA827465;Yang2025_Silkie_Wenchang	CRA004775	0.622860674
ENSGALG00010000288	VARS2	2	-0.151988819	0.201732892	-0.753416148	0.451199882	0.052626947	2.580520345	61.24812572	0.5	CRA004775;Yang2025_Silkie_Wenchang	PRJNA827465	0.847847413
ENSGALG00010000288	VARS2	2	-0.181876903	0.100625304	-1.807466878	0.070689515	0.007714101	1.268470071	21.1648723	1	CRA004775;PRJNA827465	Yang2025_Silkie_Wenchang	0.458542662
ENSGALG00010000289	QPRT	2	0.65954037	0.581134761	1.13491812	0.256409627	0.495842843	3.73932306	73.25719164	1	PRJNA827465;Yang2025_Silkie_Wenchang	CRA004775	0.764677554
ENSGALG00010000289	QPRT	2	-0.028381671	1.330893994	-0.021325268	0.982986188	3.239650039	11.60900633	91.38599833	0.5	CRA004775;Yang2025_Silkie_Wenchang	PRJNA827465	0.995363468
ENSGALG00010000289	QPRT	2	-0.562250464	0.745008594	-0.754689903	0.450435066	0.849436722	4.095616557	75.58365179	0.5	CRA004775;PRJNA827465	Yang2025_Silkie_Wenchang	0.847577105
ENSGALG00010000292	ABHD16A	2	0.17461601	0.12311146	1.41835707	0.156086543	0.006233099	1.151359015	13.14611803	1	PRJNA827465;Yang2025_Silkie_Wenchang	CRA004775	0.649132948
ENSGALG00010000292	ABHD16A	2	-0.069507055	0.472598304	-0.147074279	0.883073392	0.394345272	8.443975979	88.15723775	0.5	CRA004775;Yang2025_Silkie_Wenchang	PRJNA827465	0.972001076
ENSGALG00010000292	ABHD16A	2	-0.187353262	0.318420178	-0.58838376	0.556274736	0.178966519	8.304765718	87.95872112	0.5	CRA004775;PRJNA827465	Yang2025_Silkie_Wenchang	0.880324407

ENSGALG00010000293	ENSGALG00010000293	2	0.039900759	0.295980637	0.134808681	0.892763142	0.118324022	3.068675143	67.41264704	0.5	PRJNA827465;Yang2025_Silkie_Wenchang	CRA004775	0.974381724
ENSGALG00010000293	ENSGALG00010000293	2	0.203601812	0.269847895	0.754505838	0.450545541	0.029248959	1.16240678	13.97159607	0.5	CRA004775;Yang2025_Silkie_Wenchang	PRJNA827465	0.84760941
ENSGALG00010000293	ENSGALG00010000293	2	-0.251702826	0.206421619	-1.219362713	0.222706556	0	0.008623615	0	1	CRA004775;PRJNA827465	Yang2025_Silkie_Wenchang	0.736291702
ENSGALG00010000294	DUSP21	2	-0.150144796	0.208145381	-0.721345796	0.470696787	0	0.321021836	0	1	PRJNA827465;Yang2025_Silkie_Wenchang	CRA004775	0.853744788
ENSGALG00010000294	DUSP21	2	0.126949286	0.282534007	0.449323913	0.65319801	0	0.54804904	0	0.5	CRA004775;Yang2025_Silkie_Wenchang	PRJNA827465	0.909597225
ENSGALG00010000294	DUSP21	2	-0.042596007	0.319794784	-0.133197941	0.894036839	0.071934741	1.402414766	28.69441878	0.5	CRA004775;PRJNA827465	Yang2025_Silkie_Wenchang	0.974381724
ENSGALG00010000298	SLC35E4	2	0.576271587	0.327949999	1.757193441	0.078884843	0.165007052	4.277771384	76.62334168	1	PRJNA827465;Yang2025_Silkie_Wenchang	CRA004775	0.482993365
ENSGALG00010000298	SLC35E4	2	0.133432181	0.825694662	0.161599908	0.87162093	1.235778868	10.3770926	90.36338946	0.5	CRA004775;Yang2025_Silkie_Wenchang	PRJNA827465	0.969425739
ENSGALG00010000298	SLC35E4	2	-0.157906333	0.49204649	-0.320917508	0.748272906	0.373041287	3.99606726	74.97539618	0.5	CRA004775;PRJNA827465	Yang2025_Silkie_Wenchang	0.937171219
ENSGALG00010000301	UQCR10	2	-0.043821654	0.229848277	-0.190654698	0.848796133	0.07554822	2.850477687	64.91816075	0.5	PRJNA827465;Yang2025_Silkie_Wenchang	CRA004775	0.961364896
ENSGALG00010000301	UQCR10	2	0.245161706	0.226228392	1.083691149	0.278501761	0	0.025908873	0	1	CRA004775;Yang2025_Silkie_Wenchang	PRJNA827465	0.776763074
ENSGALG00010000301	UQCR10	2	-0.195421018	0.079885585	-2.446261352	0.014434632	0.001136024	1.014055795	1.386096782	0.5	CRA004775;PRJNA827465	Yang2025_Silkie_Wenchang	0.197673991
ENSGALG00010000302	ENSGALG00010000302	2	-0.093119249	0.326491961	-0.285211459	0.775482141	0.195378213	11.88570081	91.58652892	0.5	PRJNA827465;Yang2025_Silkie_Wenchang	CRA004775	0.943815265
ENSGALG00010000302	ENSGALG00010000302	2	-0.390885471	0.140844796	-2.775292249	0.005515212	0	0.398927576	0	1	CRA004775;Yang2025_Silkie_Wenchang	PRJNA827465	0.111469898
ENSGALG00010000302	ENSGALG00010000302	2	0.176105484	0.131810475	1.336050747	0.181532687	0.007163586	1.097214607	8.86012695	0.5	CRA004775;PRJNA827465	Yang2025_Silkie_Wenchang	0.689004635
ENSGALG00010000303	ENSGALG00010000303	2	-0.055384292	0.054224852	-1.021382091	0.307073447	0	0.010408731	0	1	PRJNA827465;Yang2025_Silkie_Wenchang	CRA004775	0.79266003
ENSGALG00010000303	ENSGALG00010000303	2	-0.217858906	0.193356023	-1.126724178	0.259859117	0.056221827	3.861622485	74.10414913	1	CRA004775;Yang2025_Silkie_Wenchang	PRJNA827465	0.767047318
ENSGALG00010000303	ENSGALG00010000303	2	-0.214709176	0.185741682	-1.155955812	0.247699232	0.054719535	4.332162651	76.91684084	1	CRA004775;PRJNA827465	Yang2025_Silkie_Wenchang	0.7580874
ENSGALG00010000306	ENSGALG00010000306	2	0.286034251	0.465349063	0.614666007	0.538775313	0.422387982	40.40732234	97.52520102	0.5	PRJNA827465;Yang2025_Silkie_Wenchang	CRA004775	0.87481821
ENSGALG00010000306	ENSGALG00010000306	2	0.121640778	0.652234463	0.186498544	0.852053821	0.811890112	21.39533009	95.32608286	0.5	CRA004775;Yang2025_Silkie_Wenchang	PRJNA827465	0.962361955
ENSGALG00010000306	ENSGALG00010000306	2	-0.286978601	0.170338615	-1.684753638	0.092036113	0.032034352	1.841527207	45.6972454	1	CRA004775;PRJNA827465	Yang2025_Silkie_Wenchang	0.51957704
ENSGALG00010000307	ZMAT5	2	0.207477139	0.07438738	2.789144339	0.005284751	0	0.063373943	0	1	PRJNA827465;Yang2025_Silkie_Wenchang	CRA004775	0.108631224
ENSGALG00010000307	ZMAT5	2	0.158519563	0.179084384	0.885166868	0.376066639	0	0.540500943	0	0.5	CRA004775;Yang2025_Silkie_Wenchang	PRJNA827465	0.822974013
ENSGALG00010000307	ZMAT5	2	0.186588297	0.075977928	2.455822405	0.01405626	0	0.489833869	0	0.5	CRA004775;PRJNA827465	Yang2025_Silkie_Wenchang	0.194183902
ENSGALG00010000308	ENSGALG00010000308	2	0.805804485	0.711439943	1.132638802	0.257365967	0.938216385	13.52009875	92.60360432	1	PRJNA827465;Yang2025_Silkie_Wenchang	CRA004775	0.764987374
ENSGALG00010000308	ENSGALG00010000308	2	0.443288078	1.089361675	0.406924613	0.684063358	2.281264151	25.73810699	96.11471038	0.5	CRA004775;Yang2025_Silkie_Wenchang	PRJNA827465	0.918806497
ENSGALG00010000308	ENSGALG00010000308	2	-0.239960792	0.376940491	-0.63660126	0.524384604	0.222510046	4.548514848	78.0148019	0.5	CRA004775;PRJNA827465	Yang2025_Silkie_Wenchang	0.869790047
ENSGALG00010000309	ENSGALG00010000309	2	-6.276195021	6.302636278	-0.995804731	0.319345031	73.2045033	12.59507657	92.06038967	1	PRJNA827465;Yang2025_Silkie_Wenchang	CRA004775	0.798079048
ENSGALG00010000309	ENSGALG00010000309	2	-5.843739015	6.645979123	-0.879289403	0.37924438	83.45451107	17.57445951	94.30992459	0.5	CRA004775;Yang2025_Silkie_Wenchang	PRJNA827465	0.824128991
ENSGALG00010000309	ENSGALG00010000309	2	0.353372077	1.008688366	0.350328297	0.726092331	0	0.090418172	0	0.5	CRA004775;PRJNA827465	Yang2025_Silkie_Wenchang	0.931029023
ENSGALG00010000312	CABP7	2	1.050404563	0.668755118	1.570686393	0.116255511	0.711902616	4.795178943	79.1457209	1	PRJNA827465;Yang2025_Silkie_Wenchang	CRA004775	0.57401889
ENSGALG00010000312	CABP7	2	0.478526323	1.291764997	0.370443791	0.71105185	3.08468823	13.21045809	92.43023979	0.5	CRA004775;Yang2025_Silkie_Wenchang	PRJNA827465	0.925588272
ENSGALG00010000312	CABP7	2	-0.138917506	0.618926562	-0.224449094	0.822407874	0.586170527	4.160752057	75.96588342	0.5	CRA004775;PRJNA827465	Yang2025_Silkie_Wenchang	0.955795631
ENSGALG00010000313	ENSGALG00010000313	2	-3.814144337	3.911619835	-0.975080529	0.329520316	26.43440676	7.182195804	86.07668146	1	PRJNA827465;Yang2025_Silkie_Wenchang	CRA004775	0.801177491
ENSGALG00010000313	ENSGALG00010000313	2	-3.409510517	4.313887107	-0.790356918	0.429319355	33.26687264	9.186121628	89.11401307	0.5	CRA004775;Yang2025_Silkie_Wenchang	PRJNA827465	0.842008086
ENSGALG00010000313	ENSGALG00010000313	2	0.311104473	1.052643909	0.295545788	0.76757698	0	0.144371317	0	0.5	CRA004775;PRJNA827465	Yang2025_Silkie_Wenchang	0.941444269
ENSGALG00010000318	ENSGALG00010000318	2	-6.049035072	6.366305053	-0.950164188	0.342028832	75.08830887	13.39636431	92.53528811	0.5	PRJNA827465;Yang2025_Silkie_Wenchang	CRA004775	0.805904173
ENSGALG00010000318	ENSGALG00010000318	2	-5.895782729	6.442027644	-0.915206059	0.360083465	78.09753763	16.43141805	93.914098	0.5	CRA004775;Yang2025_Silkie_Wenchang	PRJNA827465	0.814880744
ENSGALG00010000318	ENSGALG00010000318	2	0.224220705	1.002615586	0.223635766	0.823040731	0	0.00509398	0	1	CRA004775;PRJNA827465	Yang2025_Silkie_Wenchang	0.955822987
ENSGALG00010000323	AP1B1	2	-0.105725546	0.04619013	-2.288920717	0.022083959	0.000106567	1.016688086	1.641416521	1	PRJNA827465;Yang2025_Silkie_Wenchang	CRA004775	0.252169045
ENSGALG00010000323	AP1B1	2	-0.218740242	0.091900749	-2.380179092	0.017304226	0	0.295493442	0	1	CRA004775;Yang2025_Silkie_Wenchang	PRJNA827465	0.218613945
ENSGALG00010000323	AP1B1	2	-0.110510428	0.078735303	-1.403568957	0.160447245	0.004421208	1.170051171	14.53365247	1	CRA004775;PRJNA827465	Yang2025_Silkie_Wenchang	0.65630165
ENSGALG00010000324	ENSGALG00010000324	2	-0.234494596	0.108931103	-2.152687253	0.031343266	0	0.003114215	0	1	PRJNA827465;Yang2025_Silkie_Wenchang	CRA004775	0.302897562
ENSGALG00010000324	ENSGALG00010000324	2	-0.235008831	0.145642299	-1.613602867	0.106613595	0	0.007040618	0	1	CRA004775;Yang2025_Silkie_Wenchang	PRJNA827465	0.553677869
ENSGALG00010000324	ENSGALG00010000324	2	-0.226004725	0.13237553	-1.707299877	0.087766329	0	0.002573128	0	1	CRA004775;PRJNA827465	Yang2025_Silkie_Wenchang	0.509353637
ENSGALG00010000326	ENSGALG00010000326	2	-2.784544781	2.767909322	-1.006010117	0.314410703	13.594486	8.860605989	88.7140902	1	PRJNA827465;Yang2025_Silkie_Wenchang	CRA004775	0.796531448
ENSGALG00010000326	ENSGALG00010000326	2	-2.63350106	2.914354637	-0.903630954	0.366191122	15.29329005	10.02031625	90.02027506	0.5	CRA004775;Yang2025_Silkie_Wenchang	PRJNA827465	0.819159863
ENSGALG00010000326	ENSGALG00010000326	2	0.107800722	0.881546435	0.122285926	0.902672578	0	0.027597811	0	0.5	CRA004775;PRJNA827465	Yang2025_Silkie_Wenchang	0.976923607
ENSGALG00010000331	ENSGALG00010000331	2	0.153614457	0.255362523	0.601554431	0.547470769	0.111050516	6.613341992	84.87905205	0.5	PRJNA827465;Yang2025_Silkie_Wenchang	CRA004775	0.878281266
ENSGALG00010000331	ENSGALG00010000331	2	0.222661005	0.273908388	0.812903198	0.416273561	0.088327707	2.154464809	53.58476054	0.5	CRA004775;Yang2025_Silkie_Wenchang	PRJNA827465	0.837742614
ENSGALG00010000331	ENSGALG00010000331	2	-0.093396463	0.106344303	-0.878246037	0.379810214	0	0.028369319	0	1	CRA004775;PRJNA827465	Yang2025_Silkie_Wenchang	0.824369385
ENSGALG00010000338	ENSGALG00010000338	2	0.379482672	0.571584161	0.663913905	0.506745416	0.538425186	4.908371962	79.62664591	0.5	PRJNA827465;Yang2025_Silkie_Wenchang	CRA004775	0.863929781
ENSGALG00010000338	ENSGALG00010000338	2	0.424136735	0.554290192	0.76518896	0.444159032	0.459490111	3.722527903	73.13653447	0.5	CRA004775;Yang2025_Silkie_Wenchang	PRJNA827465	0.846013379
ENSGALG00010000338	ENSGALG00010000338	2	-0.088852048	0.107630771	-0.825526447	0.409072774	0	0.019705719	0	1	CRA004775;PRJNA827465	Yang2025_Silkie_Wenchang	0.834316328
ENSGALG00010000340	NAA10	2	-0.034493451	0.066901336	-0.515586875	0.606142979	0	0.000480399	0	1	PRJNA827465;Yang2025_Silkie_Wenchang	CRA004775	0.896709756
ENSGALG00010000340	NAA10	2	-0.074461807	0.131617333	-0.565744692	0.571567349	0	0.337886194	0	1	CRA004775;Yang2025_Silkie_Wenchang	PRJNA827465	0.885429878
ENSGALG00010000340	NAA10	2	-0.047772994	0.071916678	-0.664282547	0.50650949	0	0.39571433	0	1	CRA004775;PRJNA827465	Yang2025_Silkie_Wenchang	0.863929781
ENSGALG00010000341	EWSR1	2	0.410227876	0.473891845	0.865657176	0.386678191	0.432948184	27.12247731	96.31302116	0.5	PRJNA827465;Yang2025_Silkie_Wenchang	CRA004775	0.827062786
ENSGALG00010000341	EWSR1	2	0.282689798	0.615870143	0.459008773	0.646227866	0.728358447	25.09053013	96.01443256	0.5	CRA004775;Yang2025_Silkie_Wenchang	PRJNA827465	0.907220702
ENSGALG00010000341	EWSR1	2	-0.143774863	0.133345402	-1.078213875	0.280938321	0.022952871	2.330547789	57.09163293	1	CRA004775;PRJNA827465	Yang2025_Silkie_Wenchang	0.778593637
ENSGALG00010000346	ENSGALG00010000346	2	-0.138605455	0.057894751	-2.394093636	0.016661495	0	0.191099057	0	1	PRJNA827465;Yang2025_Silkie_Wenchang	CRA004775	0.214072156
ENSGALG00010000346	ENSGALG00010000346	2	-0.179637733	0.108334419	-1.658177842	0.097281576	0	0.015408628	0	1	CRA004775;Yang2025_Silkie_Wenchang	PRJNA827465	0.532942995
ENSGALG00010000346	ENSGALG00010000346	2	-0.128336472	0.062215355	-2.062778099	0.039133716	0	0.020718935	0	1	CRA004775;PRJNA827465	Yang2025_Silkie_Wenchang	0.341717936
ENSGALG00010000349	CHIR-IG1-5	2	0.596362668	0.613628979	0.971861969	0.331119222	0.647691356	7.132305363	85.97928791	0.5	PRJNA827465;Yang2025_Silkie_Wenchang	CRA004775	0.801725552
ENSGALG00010000349	CHIR-IG1-5	2	-0.087353336	0.295277277	-0.295834942	0.767356137	0	0.11121499	0	1	CRA004775;Yang2025_Silkie_Wenchang	PRJNA827465	0.941444269
ENSGALG00010000349	CHIR-IG1-5	2	0.555353422	0.72060179	0.770680048	0.440896601	0.841556846	5.004629096	80.01849926	0.5	CRA004775;PRJNA827465	Yang2025_Silkie_Wenchang	0.844888742
ENSGALG00010000350	ENSGALG00010000350	2	-0.170939247	0.056733281	-3.013032972	0.002586508	0	0.000200011	0	1	PRJNA827465;Yang2025_Silkie_Wenchang	CRA004775	0.068641141
ENSGALG00010000350	ENSGALG00010000350	2	-0.183449682	0.113320095	-1.618862754	0.105476796	0	0.013288644	0	1	CRA004775;Yang2025_Silkie_Wenchang	PRJNA827465	0.551018754
ENSGALG00010000350	ENSGALG00010000350	2	-0.173639192	0.058251132	-2.980872418	0.002874285	0	0.02318868	0	1	CRA004775;PRJNA827465	Yang2025_Silkie_Wenchang	0.073213239
ENSGALG00010000353	ENSGALG00010000353	2	-0.585676047	0.440771182	-1.328753038	0.18392946	0.333532988	6.398899726	84.37231332	1	PRJNA827465;Yang2025_Silkie_Wenchang	CRA004775	0.692470323
ENSGALG00010000353	ENSGALG00010000353	2	-0.531349496	0.578628336	-0.918291524	0.358466263	0.531634706	4.825879109	79.27838685	0.5	CRA004775;Yang2025_Silkie_Wenchang	PRJNA827465	0.814005058
ENSGALG00010000353	ENSGALG00010000353	2	-0.183619846	0.097604728	-1.881259739	0.059936595	0	0.414248634	0	0.5	CRA004775;PRJNA827465	Yang2025_Silkie_Wenchang	0.423882851
ENSGALG00010000356	ENSGALG00010000356	2	0.237505884	0.383180674	0.619827408	0.535371422	0.270466929	12.65601608	92.0986194	0.5	PRJNA827465;Yang2025_Silkie_Wenchang	CRA004775	0.874151593
ENSGALG00010000356	ENSGALG00010000356	2	0.33221516	0.393529571	0.844193638	0.398561209	0.215429351	2.69356873	62.87453189	0.5	CRA004775;Yang2025_Silkie_Wenchang	PRJNA827465	0.831980453
ENSGALG00010000356	ENSGALG00010000356	2	-0.15455064	0.151298777	-1.021492987	0.30702093	0	0.014581748	0	1	CRA004775;PRJNA827465	Yang2025_Silkie_Wenchang	0.79266003
ENSGALG00010000366	EMID1	2	0.230248739	0.178397045	1.290653323	0.196823917	0	0.191570476	0	1	PRJNA827465;Yang2025_Silkie_Wenchang	CRA004775	0.707994211
ENSGALG00010000366	EMID1	2	0.202787947	0.247525899	0.81925951	0.412638368	0	0.433877873	0	1	CRA004775;Yang2025_Silkie_Wenchang	PRJNA827465	0.835704442
ENSGALG00010000366	EMID1	2	0.144099143	0.180833586	0.796860509	0.425532048	0	0.132292551	0	1	CRA004775;PRJNA827465	Yang2025_Silkie_Wenchang	0.84125685
ENSGALG00010000367	HSD17B10	2	-0.291666004	0.219276183	-1.330130799	0.183475179	0.067961009	2.707543667	63.06615431	1	PRJNA827465;Yang2025_Silkie_Wenchang	CRA004775	0.691847354
ENSGALG00010000367	HSD17B10	2	-0.552458867	0.20257212	-2.727220638	0.006387032	0	0.066073202	0	1	CRA004775;Yang2025_Silkie_Wenchang	PRJNA827465	0.121505083
ENSGALG00010000367	HSD17B10	2	-0.196042656	0.134399612	-1.458654926	0.144660108	0.0172567	1.363906788	26.6812066	1	CRA004775;PRJNA827465	Yang2025_Silkie_Wenchang	0.63222242
ENSGALG00010000376	ASCC2	2	0.109515629	0.125746676	0.870922661	0.383796383	0.022820329	3.205245103	68.80113789	1	PRJNA827465;Yang2025_Silkie_Wenchang	CRA004775	0.825601288
ENSGALG00010000376	ASCC2	2	0.00946594	0.281036403	0.033682257	0.973130527	0.127328168	4.986011446	79.9438888	0.5	CRA004775;Yang2025_Silkie_Wenchang	PRJNA827465	0.992392211
ENSGALG00010000376	ASCC2	2	-0.074516033	0.136322177	-0.546617101	0.584641818	0.022287193	1.899137772	47.34452578	0.5	CRA004775;PRJNA827465	Yang2025_Silkie_Wenchang	0.891016154
ENSGALG00010000377	ENSGALG00010000377	2	0.401403416	0.575106699	0.697963382	0.485200095	0.607286032	11.6425539	91.41081924	0.5	PRJNA827465;Yang2025_Silkie_Wenchang	CRA004775	0.857056537
ENSGALG00010000377	ENSGALG00010000377	2	0.345282479	0.673838405	0.512411399	0.608363115	0.802675177	8.610650779	88.38647594	0.5	CRA004775;Yang2025_Silkie_Wenchang	PRJNA827465	0.897204334
ENSGALG00010000377	ENSGALG00010000377	2	-0.158493312	0.109777318	-1.443789395	0.14885451	0	0.306997251	0	1	CRA004775;PRJNA827465	Yang2025_Silkie_Wenchang	0.863896865
ENSGALG00010000381	ENSGALG00010000381	2	0.432994201	0.180829077	2.394494339	0.0166433	0.01515549	1.277991572	21.75222264	1	PRJNA827465;Yang2025_Silkie_Wenchang	CRA004775	0.213908317
ENSGALG00010000381	ENSGALG00010000381	2	0.253559745	0.476102671	0.532573665	0.594328736	0.32489977	3.356161347	70.20405468	0.5	CRA004775;Yang2025_Silkie_Wenchang	PRJNA827465	0.893334186
ENSGALG00010000381	ENSGALG00010000381	2	0.139797291	0.258096232	0.541647918	0.588061075	0.052697253	1.436831146	30.40239936	0.5	CRA004775;PRJNA827465	Yang2025_Silkie_Wenchang	0.891761457
ENSGALG00010000382	ENSGALG00010000382	2	1.382167061	1.460068192	0.946635511	0.343662468	4.171560182	46.23688132	97.87318695	0.5	PRJNA827465;Yang2025_Silkie_Wenchang	CRA004775	0.806937247
ENSGALG00010000382	ENSGALG00010000382	2	1.345896502	1.535072799	0.876763958	0.38061486	4.505483867	22.5468647	95.56479354	0.5	CRA004775;Yang2025_Silkie_Wenchang	PRJNA827465	0.824392515
ENSGALG00010000382	ENSGALG00010000382	2	-0.094782376	0.230370783	-0.411434009	0.680734321	0	0.062416479	0	1	CRA004775;PRJNA827465	Yang2025_Silkie_Wenchang	0.917851751
ENSGALG00010000384	ENSGALG00010000384	2	0.190333581	0.098657162	1.929242167	0.053700776	0	0.081243306	0	1	PRJNA827465;Yang2025_Silkie_Wenchang	CRA004775	0.460942791
ENSGALG00010000384	ENSGALG00010000384	2	-0.135381639	0.376168691	-0.359896085	0.718924845	0.193705118	2.741072056	63.51792365	0.5	CRA004775;Yang2025_Silkie_Wenchang	PRJNA827465	0.928449671
ENSGALG00010000384	ENSGALG00010000384	2	-0.104092021	0.406742257	-0.255916416	0.798015379	0.249613699	3.404455547	70.62672764	0.5	CRA004775;PRJNA827465	Yang2025_Silkie_Wenchang	0.949922217
ENSGALG00010000386	ENSGALG00010000386	2	-0.949049681	0.823575187	-1.152353417	0.249157875	1.317690963	34.74718739	97.12206922	1	PRJNA827465;Yang2025_Silkie_Wenchang	CRA004775	0.759411487
ENSGALG00010000386	ENSGALG00010000386	2	-0.930712071	0.876387438	-1.062009121	0.288233814	1.438339874	15.52303325	93.55796007	1	CRA004775;Yang2025_Silkie_Wenchang	PRJNA827465	0.782882265
ENSGALG00010000386	ENSGALG00010000386	2	-0.12020955	0.141136626	-0.850519454	0.395036349	0	0.067819256	0	1	CRA004775;PRJNA827465	Yang2025_Silkie_Wenchang	0.830643015
ENSGALG00010000387	DTX1	2	-0.162318579	0.267456792	-0.606896456	0.54391963	0	0.207703972	0	0.5	PRJNA827465;Yang2025_Silkie_Wenchang	CRA004775	0.876968972
ENSGALG00010000387	DTX1	2	0.137196204	0.348745005	0.393405991	0.727280754	0	0.030906471	0	0.5	CRA004775;Yang2025_Silkie_Wenchang	PRJNA827465	0.931522817
ENSGALG00010000387	DTX1	2	-0.099992868	0.255484882	-0.391384677	0.695512917	0	0.571728424	0	0.5	CRA004775;PRJNA827465	Yang2025_Silkie_Wenchang	0.921038437
ENSGALG00010000388	ENSGALG00010000388	2	0.079622598	0.15033807	0.52962365	0.596372893	0.039951998	8.586327469	88.3535772	0.5	PRJNA827465;Yang2025_Silkie_Wenchang	CRA004775	0.894064255
ENSGALG00010000388	ENSGALG00010000388	2	-0.023046248	0.291075439	-0.079176202	0.936892473	0.14405842	6.101875873	83.61159714	0.5	CRA004775;Yang2025_Silkie_Wenchang	PRJNA827465	0.985038972
ENSGALG00010000388	ENSGALG00010000388	2	-0.130892148	0.11854622	-1.104144421	0.269530495	0.013575035	1.495328182	33.12504825	1	CRA004775;PRJNA827465	Yang2025_Silkie_Wenchang	0.77189992
ENSGALG00010000402	PFKFB1	2	0.103762823	0.188091111	0.551662557	0.581179568	0	0.251591851	0	0.5	PRJNA827465;Yang2025_Silkie_Wenchang	CRA004775	0.88890219
ENSGALG00010000402	PFKFB1	2	0.148042509	0.213290806	0.694087623	0.48762726	0	0.060458007	0	0.5	CRA004775;Yang2025_Silkie_Wenchang	PRJNA827465	0.85768885
ENSGALG00010000402	PFKFB1	2	-0.034354732	0.306605818	-0.112048531	0.910784926	0	0.001602083	0	1	CRA004775;PRJNA827465	Yang2025_Silkie_Wenchang	0.97880407
ENSGALG00010000405	ENSGALG00010000405	2	0.228687065	0.899976541	0.254103362	0.799415706	1.543588698	21.19526452	95.28196499	0.5	PRJNA827465;Yang2025_Silkie_Wenchang	CRA004775	0.949922217
ENSGALG00010000405	ENSGALG00010000405	2	0.546657544	0.675575322	0.809173345	0.418415445	0.714150119	4.30949604	76.79543058	0.5	CRA004775;Yang2025_Silkie_Wenchang	PRJNA827465	0.838158675
ENSGALG00010000405	ENSGALG00010000405	2	-0.59589104	0.231126527	-2.578202724	0.009931571	0	0.469511564	0	1	CRA004775;PRJNA827465	Yang2025_Silkie_Wenchang	0.157756967
ENSGALG00010000406	NSMCE3	2	-0.020785213	0.09829569	-0.211455998	0.832531464	0.000486027	1.01712874	1.684028706	0.5	PRJNA827465;Yang2025_Silkie_Wenchang	CRA004775	0.957861248
ENSGALG00010000406	NSMCE3	2	-0.294834863	0.170278657	-1.731484541	0.08336538	0	0.450997961	0	1	CRA004775;Yang2025_Silkie_Wenchang	PRJNA827465	0.497345101
ENSGALG00010000406	NSMCE3	2	-0.136431394	0.228247782	-0.597733712	0.550017628	0.068107878	2.458104509	59.31824719	0.5	CRA004775;PRJNA827465	Yang2025_Silkie_Wenchang	0.878652984
ENSGALG00010000407	WSB2	2	-0.157241412	0.162628842	-0.966872846	0.333607603	0.034468066	2.236840093	55.29407743	1	PRJNA827465;Yang2025_Silkie_Wenchang	CRA004775	0.802691373
ENSGALG00010000407	WSB2	2	-0.397611629	0.193664959	-2.053090195	0.040063831	0	0.001625754	0	1	CRA004775;Yang2025_Silkie_Wenchang	PRJNA827465	0.346798223
ENSGALG00010000407	WSB2	2	-0.05822272	0.059290852	-0.981984868	0.326107303	0	0.820585924	0	1	CRA004775;PRJNA827465	Yang2025_Silkie_Wenchang	0.800023788
ENSGALG00010000408	ENSGALG00010000408	2	0.285107624	0.723988942	0.393801076	0.69372791	1.012440039	29.1495735	96.56941807	0.5	PRJNA827465;Yang2025_Silkie_Wenchang	CRA004775	0.920721644
ENSGALG00010000408	ENSGALG00010000408	2	0.601836509	0.582514934	1.033169235	0.301524707	0.473451629	2.553869205	60.84372692	0.5	CRA004775;Yang2025_Silkie_Wenchang	PRJNA827465	0.791273005
ENSGALG00010000408	ENSGALG00010000408	2	-0.430972646	0.205419002	-2.098017433	0.035903605	0	0.063985289	0	1	CRA004775;PRJNA827465	Yang2025_Silkie_Wenchang	0.326969036
ENSGALG00010000412	RFC5	2	0.028829526	0.069440691	0.415167616	0.678019204	0	0.433557788	0	1	PRJNA827465;Yang2025_Silkie_Wenchang	CRA004775	0.917534751
ENSGALG00010000412	RFC5	2	-0.02481989	0.230268414	-0.107786778	0.914164831	0.044638354	1.644724052	39.19952718	0.5	CRA004775;Yang2025_Silkie_Wenchang	PRJNA827465	0.979510383
ENSGALG00010000412	RFC5	2	-0.018561166	0.096533566	-0.192276806	0.847525386	0.004742668	1.090693239	8.315192222	0.5	CRA004775;PRJNA827465	Yang2025_Silkie_Wenchang	0.961252712
ENSGALG00010000422	ITIH6	2	0.945395588	0.493284908	1.916530534	0.0552976	0.233826139	1.92424204	48.03148567	1	PRJNA827465;Yang2025_Silkie_Wenchang	CRA004775	0.407301647
ENSGALG00010000422	ITIH6	2	0.466084285	0.942784639	0.494368889	0.621045681	1.590565566	9.370572758	89.32829374	0.5	CRA004775;Yang2025_Silkie_Wenchang	PRJNA827465	0.899579438
ENSGALG00010000422	ITIH6	2	-0.059197741	0.444721462	-0.13311195	0.894104845	0.224102234	2.236523355	55.28774615	0.5	CRA004775;PRJNA827465	Yang2025_Silkie_Wenchang	0.974381724
ENSGALG00010000423	ENSGALG00010000423	2	0.274279589	0.488740409	0.561196873	0.574663334	0.435777721	11.38479915	91.216358	0.5	PRJNA827465;Yang2025_Silkie_Wenchang	CRA004775	0.88613451
ENSGALG00010000423	ENSGALG00010000423	2	-0.210390072	0.179604175	-1.171409692	0.241434138	0	0.00065739	0	1	CRA004775;Yang2025_Silkie_Wenchang	PRJNA827465	0.75393977
ENSGALG00010000423	ENSGALG00010000423	2	0.34359322	0.479001039	0.717312056	0.47318157	0.363156036	4.513725232	77.8453506	0.5	CRA004775;PRJNA827465	Yang2025_Silkie_Wenchang	0.854341771
ENSGALG00010000428	GNL3L	2	0.029727469	0.075510878	0.39368459	0.69381392	0	0.655716914	0	1	PRJNA827465;Yang2025_Silkie_Wenchang	CRA004775	0.92072583
ENSGALG00010000428	GNL3L	2	-0.046084404	0.209582085	-0.219887134	0.825959057	0.054761046	2.650514631	62.27147784	0.5	CRA004775;Yang2025_Silkie_Wenchang	PRJNA827465	0.956393839
ENSGALG00010000428	GNL3L	2	-0.075958772	0.119285205	-0.636782846	0.524266301	0.013028558	1.605837635	37.72720366	0.5	CRA004775;PRJNA827465	Yang2025_Silkie_Wenchang	0.869790047
ENSGALG00010000432	ENSGALG00010000432	2	-0.006386159	0.150625963	-0.042397463	0.966181851	0.036184705	4.930314473	79.71731813	0.5	PRJNA827465;Yang2025_Silkie_Wenchang	CRA004775	0.990863074
ENSGALG00010000432	ENSGALG00010000432	2	0.132417281	0.085699955	1.545126602	0.122315644	0	0.079966242	0	1	CRA004775;Yang2025_Silkie_Wenchang	PRJNA827465	0.586622543
ENSGALG00010000432	ENSGALG00010000432	2	-0.126791108	0.092613193	-1.369039366	0.17098697	0	0.748887802	0	0.5	CRA004775;PRJNA827465	Yang2025_Silkie_Wenchang	0.673107059
ENSGALG00010000434	TP53	2	-0.220230718	0.109431211	-2.012503716	0.044166865	0.009078524	1.315684232	23.99392077	0.5	PRJNA827465;Yang2025_Silkie_Wenchang	CRA004775	0.363282309
ENSGALG00010000434	TP53	2	-0.237455491	0.213021548	-1.114701744	0.264978253	0.050387076	2.214213973	54.83724643	0.5	CRA004775;Yang2025_Silkie_Wenchang	PRJNA827465	0.769274038
ENSGALG00010000434	TP53	2	-0.298577959	0.07013872	-4.256963352	2.07222E-05	0	0.635008587	0	1	CRA004775;PRJNA827465	Yang2025_Silkie_Wenchang	0.002521504
ENSGALG00010000435	P2RX6	2	0.482600133	0.216558554	2.228497203	0.025847378	0.006981806	1.065893007	6.181953208	1	PRJNA827465;Yang2025_Silkie_Wenchang	CRA004775	0.274043562
ENSGALG00010000435	P2RX6	2	0.637638859	0.264819169	2.40782743	0.016047765	0	0.395438979	0	1	CRA004775;Yang2025_Silkie_Wenchang	PRJNA827465	0.209265194
ENSGALG00010000435	P2RX6	2	0.387126839	0.201955109	1.916895492	0.05525121	0	0.105212016	0	1	CRA004775;PRJNA827465	Yang2025_Silkie_Wenchang	0.407036233
ENSGALG00010000436	ENSGALG00010000436	2	0.153203666	0.161627625	0.947880454	0.343190316	0	0.131744555	0	1	PRJNA827465;Yang2025_Silkie_Wenchang	CRA004775	0.806218116
ENSGALG00010000436	ENSGALG00010000436	2	-0.290639384	0.596161044	-0.487518242	0.625891119	0.556061623	4.204387341	76.21532178	0.5	CRA004775;Yang2025_Silkie_Wenchang	PRJNA827465	0.901504244
ENSGALG00010000436	ENSGALG00010000436	2	-0.335397418	0.533739176	-0.628391981	0.529747189	0.424794574	3.504862086	71.46820686	0.5	CRA004775;PRJNA827465	Yang2025_Silkie_Wenchang	0.871944741
ENSGALG00010000440	ENSGALG00010000440	2	1.160014318	0.299130634	3.877952259	0.000105339	0	0.008273282	0	1	PRJNA827465;Yang2025_Silkie_Wenchang	CRA004775	0.008070475
ENSGALG00010000440	ENSGALG00010000440	2	1.000998571	0.353277172	2.833465191	0.004604632	0	0.389777007	0	1	CRA004775;Yang2025_Silkie_Wenchang	PRJNA827465	0.099711218
ENSGALG00010000440	ENSGALG00010000440	2	1.003686256	0.31524623	3.183816838	0.00145347	0	0.344955234	0	1	CRA004775;PRJNA827465	Yang2025_Silkie_Wenchang	0.047250382
ENSGALG00010000441	SLC7A4	2	-0.015863053	0.135125596	-0.11739488	0.906547141	0	0.774111478	0	0.5	PRJNA827465;Yang2025_Silkie_Wenchang	CRA004775	0.978196186
ENSGALG00010000441	SLC7A4	2	-0.27170651	0.21831605	-1.244555817	0.21329507	0	0.809221997	0	1	CRA004775;Yang2025_Silkie_Wenchang	PRJNA827465	0.725875498
ENSGALG00010000441	SLC7A4	2	-0.155882244	0.35744328	-0.436103439	0.662761683	0.150175881	1.896691848	47.27662267	0.5	CRA004775;PRJNA827465	Yang2025_Silkie_Wenchang	0.912798206
ENSGALG00010000442	DGCR6	2	-0.052803362	0.090226639	-0.585230289	0.558392881	0.006487868	1.35025932	25.940152	0.5	PRJNA827465;Yang2025_Silkie_Wenchang	CRA004775	0.880678789
ENSGALG00010000442	DGCR6	2	-0.107667665	0.243656543	-0.441882922	0.65857393	0.080245571	3.07137225	67.44126343	0.5	CRA004775;Yang2025_Silkie_Wenchang	PRJNA827465	0.911105434
ENSGALG00010000442	DGCR6	2	-0.154118078	0.107652431	-1.431626544	0.152250729	0.010927887	1.456004111	31.31887522	1	CRA004775;PRJNA827465	Yang2025_Silkie_Wenchang	0.643777343
ENSGALG00010000443	TBX3	2	0.119321872	0.285230373	0.418335085	0.675702141	0.141711267	7.487650221	86.64467529	0.5	PRJNA827465;Yang2025_Silkie_Wenchang	CRA004775	0.916522765
ENSGALG00010000443	TBX3	2	0.39782771	0.161903046	2.457197189	0.01400258	0	0.106285852	0	1	CRA004775;Yang2025_Silkie_Wenchang	PRJNA827465	0.193842584
ENSGALG00010000443	TBX3	2	-0.047044108	0.18496804	-0.254336412	0.799235672	0.033921443	1.523375499	34.35630281	0.5	CRA004775;PRJNA827465	Yang2025_Silkie_Wenchang	0.949922217
ENSGALG00010000444	RBM19	2	0.111743618	0.061754906	1.809469487	0.070378096	0.002358152	1.380589108	27.56715273	1	PRJNA827465;Yang2025_Silkie_Wenchang	CRA004775	0.457882855
ENSGALG00010000444	RBM19	2	0.166820706	0.088308474	1.889067936	0.058882726	0	0.835979022	0	0.5	CRA004775;Yang2025_Silkie_Wenchang	PRJNA827465	0.42013793
ENSGALG00010000444	RBM19	2	0.06204049	0.056514224	1.097785404	0.272298207	0	0.182402506	0	0.5	CRA004775;PRJNA827465	Yang2025_Silkie_Wenchang	0.773331629
ENSGALG00010000449	PLBD2	2	-0.081158916	0.056509149	-1.436208427	0.150943042	0	0.373981104	0	1	PRJNA827465;Yang2025_Silkie_Wenchang	CRA004775	0.642092467
ENSGALG00010000449	PLBD2	2	-0.188757754	0.096182125	-1.962503463	0.049703898	0	0.818242327	0	1	CRA004775;Yang2025_Silkie_Wenchang	PRJNA827465	0.386162809
ENSGALG00010000449	PLBD2	2	-0.141546546	0.127319299	-1.11174462	0.266247969	0.018437751	1.873378222	46.62049616	1	CRA004775;PRJNA827465	Yang2025_Silkie_Wenchang	0.77034334
ENSGALG00010000450	ENSGALG00010000450	2	-0.334993923	0.2111184	-1.586758535	0.112567343	0	0.603415542	0	1	PRJNA827465;Yang2025_Silkie_Wenchang	CRA004775	0.566223663
ENSGALG00010000450	ENSGALG00010000450	2	-0.457802603	0.213839379	-2.140871365	0.032284413	0	0.02777107	0	1	CRA004775;Yang2025_Silkie_Wenchang	PRJNA827465	0.30850335
ENSGALG00010000450	ENSGALG00010000450	2	-0.287246182	0.219740326	-1.307207404	0.191142282	0	0.341544592	0	1	CRA004775;PRJNA827465	Yang2025_Silkie_Wenchang	0.700443419
ENSGALG00010000452	SLC8B1	2	0.179308788	0.110908559	1.616726341	0.105937365	0	0.008097782	0	1	PRJNA827465;Yang2025_Silkie_Wenchang	CRA004775	0.552209054
ENSGALG00010000452	SLC8B1	2	-0.192338767	0.413126592	-0.465568596	0.641524326	0.291701821	6.63174718	84.92101745	0.5	CRA004775;Yang2025_Silkie_Wenchang	PRJNA827465	0.906634523
ENSGALG00010000452	SLC8B1	2	-0.201491645	0.403045845	-0.499922397	0.617129721	0.275371306	6.31798384	84.17216591	0.5	CRA004775;PRJNA827465	Yang2025_Silkie_Wenchang	0.899121942
ENSGALG00010000453	ACHE	2	0.073982013	0.132726511	0.557401931	0.577252847	0	0.371434167	0	1	PRJNA827465;Yang2025_Silkie_Wenchang	CRA004775	0.887145528
ENSGALG00010000453	ACHE	2	0.11609694	0.203072999	0.571700525	0.567524879	0	0.551697204	0	0.5	CRA004775;Yang2025_Silkie_Wenchang	PRJNA827465	0.883717772
ENSGALG00010000453	ACHE	2	-0.009866351	0.157450312	-0.062663267	0.950034649	0	0.205835978	0	0.5	CRA004775;PRJNA827465	Yang2025_Silkie_Wenchang	0.987277827
ENSGALG00010000457	TPCN1	2	0.082680012	0.049819339	1.659596728	0.096995607	0	0.472146021	0	1	PRJNA827465;Yang2025_Silkie_Wenchang	CRA004775	0.532209736
ENSGALG00010000457	TPCN1	2	-0.120815643	0.189207397	-0.638535514	0.523125144	0.04368832	2.247389169	55.50392367	0.5	CRA004775;Yang2025_Silkie_Wenchang	PRJNA827465	0.869569689
ENSGALG00010000457	TPCN1	2	-0.0811596	0.232720286	-0.348743125	0.727282165	0.084084404	3.71846404	73.10717573	0.5	CRA004775;PRJNA827465	Yang2025_Silkie_Wenchang	0.931522817
ENSGALG00010000458	ENSGALG00010000458	2	0.295713173	0.463839199	0.637533811	0.523777193	0.420040085	41.96361427	97.61698315	0.5	PRJNA827465;Yang2025_Silkie_Wenchang	CRA004775	0.869661505
ENSGALG00010000458	ENSGALG00010000458	2	0.152979125	0.624817491	0.244838096	0.806581795	0.747937755	23.27398609	95.7033574	0.5	CRA004775;Yang2025_Silkie_Wenchang	PRJNA827465	0.952270941
ENSGALG00010000458	ENSGALG00010000458	2	-0.257554858	0.144143159	-1.786799035	0.073969975	0.017990721	1.528773035	34.58806658	1	CRA004775;PRJNA827465	Yang2025_Silkie_Wenchang	0.469669846
ENSGALG00010000462	ENSGALG00010000462	2	-0.057206862	0.209223334	-0.273424865	0.784526641	0.020934943	1.229307505	18.65338849	0.5	PRJNA827465;Yang2025_Silkie_Wenchang	CRA004775	0.946117405
ENSGALG00010000462	ENSGALG00010000462	2	0.129336251	0.263686917	0.490491729	0.623785981	0	0.35029835	0	0.5	CRA004775;Yang2025_Silkie_Wenchang	PRJNA827465	0.900549196
ENSGALG00010000462	ENSGALG00010000462	2	-0.146953898	0.173297014	-0.847988631	0.396444302	0	0.150544495	0	1	CRA004775;PRJNA827465	Yang2025_Silkie_Wenchang	0.831365249
ENSGALG00010000465	ENSGALG00010000465	2	-0.074805659	0.129972754	-0.575548778	0.564920212	0	0.019837337	0	1	PRJNA827465;Yang2025_Silkie_Wenchang	CRA004775	0.883416369
ENSGALG00010000465	ENSGALG00010000465	2	0.071333216	0.22142763	0.322151379	0.747338016	0	0.62414948	0	0.5	CRA004775;Yang2025_Silkie_Wenchang	PRJNA827465	0.93680487
ENSGALG00010000465	ENSGALG00010000465	2	-0.034506741	0.140133516	-0.246241885	0.805494994	0	0.966799847	0	0.5	CRA004775;PRJNA827465	Yang2025_Silkie_Wenchang	0.951922776
ENSGALG00010000466	FBXO21	2	-0.08112639	0.048845389	-1.660881232	0.096737302	0	0.05815325	0	1	PRJNA827465;Yang2025_Silkie_Wenchang	CRA004775	0.531460189
ENSGALG00010000466	FBXO21	2	-0.198885914	0.162408631	-1.224601875	0.220725261	0.035634728	2.933682232	65.9131453	1	CRA004775;Yang2025_Silkie_Wenchang	PRJNA827465	0.734053249
ENSGALG00010000466	FBXO21	2	-0.198937909	0.145352467	-1.368658637	0.171106006	0.030365748	3.025977772	66.95283062	1	CRA004775;PRJNA827465	Yang2025_Silkie_Wenchang	0.673238496
ENSGALG00010000467	RNASEK	2	-0.09289664	0.122147206	-0.760530207	0.44693772	0	0.567746455	0	1	PRJNA827465;Yang2025_Silkie_Wenchang	CRA004775	0.84650962

ENSGALG00010000467	RNASEK	2	0.003825119	0.274645867	0.013927459	0.988887855	0.08215277	2.091025954	52.17658594	0.5	CRA004775;Yang2025_Silkie_Wenchang	PRJNA827465	0.996891127
ENSGALG00010000467	RNASEK	2	0.042797229	0.143333208	0.298585581	0.765256269	0.002441583	1.037466052	3.611303936	0.5	CRA004775;PRJNA827465	Yang2025_Silkie_Wenchang	0.940779391
ENSGALG00010000468	TESC	2	0.136892933	0.215782767	0.634401599	0.525818766	0	0.026995187	0	1	PRJNA827465;Yang2025_Silkie_Wenchang	CRA004775	0.870414822
ENSGALG00010000468	TESC	2	-0.017441847	0.250065569	-0.069749093	0.944393367	0	0.22826213	0	0.5	CRA004775;Yang2025_Silkie_Wenchang	PRJNA827465	0.985935636
ENSGALG00010000468	TESC	2	0.052018474	0.222007211	0.234309842	0.814744446	0	0.457610868	0	0.5	CRA004775;PRJNA827465	Yang2025_Silkie_Wenchang	0.953884449
ENSGALG00010000469	ENSGALG00010000469	2	-0.063107641	0.141093253	-0.447276105	0.654675717	0	0.314821617	0	0.5	PRJNA827465;Yang2025_Silkie_Wenchang	CRA004775	0.910222014
ENSGALG00010000469	ENSGALG00010000469	2	-0.132542752	0.183608074	-0.721742512	0.4704528	0	0.005197696	0	1	CRA004775;Yang2025_Silkie_Wenchang	PRJNA827465	0.853744788
ENSGALG00010000469	ENSGALG00010000469	2	0.010190206	0.196033583	0.051981942	0.958543082	0	0.031123125	0	0.5	CRA004775;PRJNA827465	Yang2025_Silkie_Wenchang	0.989070013
ENSGALG00010000470	FBXW8	2	0.066113619	0.089861409	0.735728716	0.46189581	0.008887326	2.115433636	52.72836817	0.5	PRJNA827465;Yang2025_Silkie_Wenchang	CRA004775	0.850747865
ENSGALG00010000470	FBXW8	2	-0.083458882	0.30389969	-0.274626413	0.783603272	0.148616632	4.661114272	78.54590251	0.5	CRA004775;Yang2025_Silkie_Wenchang	PRJNA827465	0.945815798
ENSGALG00010000470	FBXW8	2	-0.147093437	0.202108863	-0.727821914	0.466722608	0.055973581	2.506825449	60.10890984	1	CRA004775;PRJNA827465	Yang2025_Silkie_Wenchang	0.852842649
ENSGALG00010000471	ENSGALG00010000471	2	0.459753962	0.355986852	1.29149141	0.196533332	0.225618988	9.061918768	88.96480949	1	PRJNA827465;Yang2025_Silkie_Wenchang	CRA004775	0.707876607
ENSGALG00010000471	ENSGALG00010000471	2	0.229159581	0.611735627	0.374605582	0.707953821	0.694526421	13.75211277	92.72839006	0.5	CRA004775;Yang2025_Silkie_Wenchang	PRJNA827465	0.925061251
ENSGALG00010000471	ENSGALG00010000471	2	-0.091761708	0.250580569	-0.366196422	0.714218512	0.082669997	2.712813526	63.13790128	0.5	CRA004775;PRJNA827465	Yang2025_Silkie_Wenchang	0.92653974
ENSGALG00010000475	RNFT2	2	0.029235723	0.065955155	0.443266681	0.657572853	0	0.459686825	0	1	PRJNA827465;Yang2025_Silkie_Wenchang	CRA004775	0.910774485
ENSGALG00010000475	RNFT2	2	-0.275362254	0.414015724	-0.665100956	0.505985923	0.302379401	8.214936624	87.82705156	0.5	CRA004775;Yang2025_Silkie_Wenchang	PRJNA827465	0.863929781
ENSGALG00010000475	RNFT2	2	-0.315402517	0.358993913	-0.878573441	0.379632601	0.226052351	7.456221201	86.58838072	0.5	CRA004775;PRJNA827465	Yang2025_Silkie_Wenchang	0.824239628
ENSGALG00010000476	ENSGALG00010000476	2	-0.514167195	0.242645964	-2.119001635	0.034090329	0.067772921	2.286940045	56.27344923	1	PRJNA827465;Yang2025_Silkie_Wenchang	CRA004775	0.3174253
ENSGALG00010000476	ENSGALG00010000476	2	-0.215643757	0.248103524	-0.869168455	0.384754999	0	0.030068356	0	1	CRA004775;Yang2025_Silkie_Wenchang	PRJNA827465	0.826430523
ENSGALG00010000476	ENSGALG00010000476	2	-0.681124411	0.179863052	-3.786905662	0.000152535	0	0.746632329	0	1	CRA004775;PRJNA827465	Yang2025_Silkie_Wenchang	0.010517688
ENSGALG00010000479	C12orf49	2	-0.098663564	0.317480478	-0.310770492	0.755975104	0.181325513	9.445505551	89.41295419	0.5	PRJNA827465;Yang2025_Silkie_Wenchang	CRA004775	0.938501783
ENSGALG00010000479	C12orf49	2	-0.220204077	0.264787342	-0.831626146	0.405619999	0.082038028	2.30820142	56.67622455	0.5	CRA004775;Yang2025_Silkie_Wenchang	PRJNA827465	0.833257341
ENSGALG00010000479	C12orf49	2	0.189082321	0.073887759	2.559047993	0.010495925	0	0.103736491	0	1	CRA004775;PRJNA827465	Yang2025_Silkie_Wenchang	0.1632418
ENSGALG00010000483	ENSGALG00010000483	2	0.13962047	0.30849624	0.452584024	0.650848305	0.162292516	6.753588623	85.19305726	0.5	PRJNA827465;Yang2025_Silkie_Wenchang	CRA004775	0.908769703
ENSGALG00010000483	ENSGALG00010000483	2	0.438530774	0.168887024	2.596592459	0.009415356	0	0.066804955	0	1	CRA004775;Yang2025_Silkie_Wenchang	PRJNA827465	0.157226616
ENSGALG00010000483	ENSGALG00010000483	2	-0.075324339	0.186755048	-0.403332277	0.6867038	0.019369391	1.186610245	15.72633016	0.5	CRA004775;PRJNA827465	Yang2025_Silkie_Wenchang	0.919235539
ENSGALG00010000485	MED13L	2	-0.158744995	0.062722677	-2.530902744	0.011376939	0	0.377938815	0	1	PRJNA827465;Yang2025_Silkie_Wenchang	CRA004775	0.170082857
ENSGALG00010000485	MED13L	2	0.104082083	0.22408255	0.464480983	0.642303182	0.041910816	1.586771563	36.97895631	0.5	CRA004775;Yang2025_Silkie_Wenchang	PRJNA827465	0.906634523
ENSGALG00010000485	MED13L	2	0.036433421	0.284267777	0.128165848	0.898017726	0.120160652	3.105448966	67.798537	0.5	CRA004775;PRJNA827465	Yang2025_Silkie_Wenchang	0.975733636
ENSGALG00010000486	ENSGALG00010000486	2	0.531305458	0.52834384	1.005605475	0.314605389	0.461327472	5.527822412	81.90969381	1	PRJNA827465;Yang2025_Silkie_Wenchang	CRA004775	0.796531448
ENSGALG00010000486	ENSGALG00010000486	2	0.733113462	0.457750131	1.60155817	0.109253347	0.198236902	1.839346411	45.63286209	1	CRA004775;Yang2025_Silkie_Wenchang	PRJNA827465	0.559388543
ENSGALG00010000486	ENSGALG00010000486	2	0.067574225	0.206491253	0.32724982	0.743478943	0	0.045489551	0	1	CRA004775;PRJNA827465	Yang2025_Silkie_Wenchang	0.935816088
ENSGALG00010000491	ENSGALG00010000491	2	0.094744947	0.133478773	0.709812841	0.477820205	0.01530048	1.440258109	30.56800071	1	PRJNA827465;Yang2025_Silkie_Wenchang	CRA004775	0.853366076
ENSGALG00010000491	ENSGALG00010000491	2	0.250952716	0.179108723	1.401119451	0.161178356	0	0.255670104	0	1	CRA004775;Yang2025_Silkie_Wenchang	PRJNA827465	0.657426695
ENSGALG00010000491	ENSGALG00010000491	2	0.035705263	0.083995826	0.42508378	0.670775602	0	0.244051536	0	1	CRA004775;PRJNA827465	Yang2025_Silkie_Wenchang	0.914509972
ENSGALG00010000495	ENSGALG00010000495	2	0.114847224	0.299452183	0.389945549	0.696576812	0	0.747851564	0	0.5	PRJNA827465;Yang2025_Silkie_Wenchang	CRA004775	0.921081523
ENSGALG00010000495	ENSGALG00010000495	2	-0.389600843	0.385247944	-1.011298955	0.311873366	0	0.386610913	0	1	CRA004775;Yang2025_Silkie_Wenchang	PRJNA827465	0.795993742
ENSGALG00010000495	ENSGALG00010000495	2	-0.063659665	0.494707626	-0.12868139	0.897609762	0.247402966	1.88604105	46.97888468	0.5	CRA004775;PRJNA827465	Yang2025_Silkie_Wenchang	0.975608116
ENSGALG00010000501	ENSGALG00010000501	2	0.245199306	0.197211037	1.243334599	0.21374456	0.068225125	7.977869566	87.46532528	1	PRJNA827465;Yang2025_Silkie_Wenchang	CRA004775	0.726416385
ENSGALG00010000501	ENSGALG00010000501	2	0.189944304	0.301643186	0.629698639	0.528891773	0.146352994	4.749722774	78.94613965	0.5	CRA004775;Yang2025_Silkie_Wenchang	PRJNA827465	0.871809618
ENSGALG00010000501	ENSGALG00010000501	2	0.040832081	0.072076884	0.566507306	0.571048968	0	0.655574463	0	0.5	CRA004775;PRJNA827465	Yang2025_Silkie_Wenchang	0.885354083
ENSGALG00010000507	TRPT1	2	0.22269152	0.296471882	0.751138753	0.452569155	0.107929609	2.502225029	60.0355688	0.5	PRJNA827465;Yang2025_Silkie_Wenchang	CRA004775	0.848321192
ENSGALG00010000507	TRPT1	2	0.204199344	0.404764395	0.504489039	0.613937515	0.196949488	2.462875529	59.39705486	0.5	CRA004775;Yang2025_Silkie_Wenchang	PRJNA827465	0.893925604
ENSGALG00010000507	TRPT1	2	-0.077354257	0.18883977	-0.409629057	0.682078079	0	0.213714824	0	1	CRA004775;PRJNA827465	Yang2025_Silkie_Wenchang	0.918515969
ENSGALG00010000511	FERMT3	2	0.496936078	0.13003149	3.821659505	0.000132557	0.008646754	1.254915106	20.31333473	1	PRJNA827465;Yang2025_Silkie_Wenchang	CRA004775	0.009541893
ENSGALG00010000511	FERMT3	2	0.217226554	0.573539059	0.378747621	0.704875287	0.517538995	4.316401566	76.83255405	0.5	CRA004775;Yang2025_Silkie_Wenchang	PRJNA827465	0.924152671
ENSGALG00010000511	FERMT3	2	0.123300734	0.411086878	0.299938384	0.764224155	0.240744939	2.760415816	63.77357374	0.5	CRA004775;PRJNA827465	Yang2025_Silkie_Wenchang	0.940483053
ENSGALG00010000526	POLD1	2	-0.037091779	0.153787602	-0.241188354	0.809409137	0.031887169	2.943203971	66.02342175	0.5	PRJNA827465;Yang2025_Silkie_Wenchang	CRA004775	0.952770549
ENSGALG00010000526	POLD1	2	0.09564861	0.139925622	0.683567517	0.494248302	0	0.537164189	0	0.5	CRA004775;Yang2025_Silkie_Wenchang	PRJNA827465	0.860175061
ENSGALG00010000526	POLD1	2	-0.167866556	0.093753258	-1.79051437	0.073371259	0	0.007794914	0	1	CRA004775;PRJNA827465	Yang2025_Silkie_Wenchang	0.467957919
ENSGALG00010000529	STIP1	2	0.134968157	0.184508767	0.731519792	0.46446172	0.063969885	16.37263358	93.89224711	0.5	PRJNA827465;Yang2025_Silkie_Wenchang	CRA004775	0.852159649
ENSGALG00010000529	STIP1	2	0.178093579	0.209387023	0.85054716	0.395020952	0.059289744	2.477016289	59.62884845	0.5	CRA004775;Yang2025_Silkie_Wenchang	PRJNA827465	0.830643015
ENSGALG00010000529	STIP1	2	-0.047066291	0.04777723	-0.985119711	0.324563273	0	0.07693539	0	1	CRA004775;PRJNA827465	Yang2025_Silkie_Wenchang	0.799859262
ENSGALG00010000532	ENSGALG00010000532	2	-0.057000116	0.315791518	-0.180499198	0.856760684	0	0.801370368	0	0.5	PRJNA827465;Yang2025_Silkie_Wenchang	CRA004775	0.965662684
ENSGALG00010000532	ENSGALG00010000532	2	-0.364082162	0.371152595	-0.980950066	0.326617366	0	0.01493971	0	1	CRA004775;Yang2025_Silkie_Wenchang	PRJNA827465	0.800028239
ENSGALG00010000532	ENSGALG00010000532	2	0.001481943	0.327560643	0.00452418	0.996390239	0	0.502927897	0	0.5	CRA004775;PRJNA827465	Yang2025_Silkie_Wenchang	0.998855419
ENSGALG00010000555	OTUB1	2	0.022741868	0.060748145	0.374363164	0.708134144	0	0.02192764	0	0.5	PRJNA827465;Yang2025_Silkie_Wenchang	CRA004775	0.925061251
ENSGALG00010000555	OTUB1	2	-0.12675424	0.17319068	-0.731876797	0.46441377	0.005517066	1.088548323	8.134533054	1	CRA004775;Yang2025_Silkie_Wenchang	PRJNA827465	0.852159649
ENSGALG00010000555	OTUB1	2	-0.072099004	0.17163062	-0.42008241	0.674425252	0.034936641	1.781339504	43.86246992	0.5	CRA004775;PRJNA827465	Yang2025_Silkie_Wenchang	0.915949608
ENSGALG00010000561	ENSGALG00010000561	2	-0.048353347	0.111902966	-0.432100675	0.665668244	0.009913962	1.362300852	26.5947754	0.5	PRJNA827465;Yang2025_Silkie_Wenchang	CRA004775	0.913532078
ENSGALG00010000561	ENSGALG00010000561	2	-0.153273023	0.16329987	-0.938596565	0.34793689	0	0.546848744	0	0.5	CRA004775;Yang2025_Silkie_Wenchang	PRJNA827465	0.805847403
ENSGALG00010000561	ENSGALG00010000561	2	0.007144685	0.073843316	0.096754661	0.92292123	0	0.014272151	0	0.5	CRA004775;PRJNA827465	Yang2025_Silkie_Wenchang	0.981827844
ENSGALG00010000564	NAA40	2	0.004013375	0.054583732	0.073526935	0.941386811	0	0.03398806	0	1	PRJNA827465;Yang2025_Silkie_Wenchang	CRA004775	0.985827325
ENSGALG00010000564	NAA40	2	-0.164431025	0.234733412	-0.700501151	0.483614386	0.059483511	2.118374048	52.79398362	0.5	CRA004775;Yang2025_Silkie_Wenchang	PRJNA827465	0.85691916
ENSGALG00010000564	NAA40	2	-0.14188967	0.205335939	-0.691013252	0.489557779	0.059339913	2.623108548	61.87729247	0.5	CRA004775;PRJNA827465	Yang2025_Silkie_Wenchang	0.885819004
ENSGALG00010000569	SIPA1	2	0.325203905	0.29756494	1.092883809	0.274444818	0.15284399	7.178706901	86.06991463	1	PRJNA827465;Yang2025_Silkie_Wenchang	CRA004775	0.774526342
ENSGALG00010000569	SIPA1	2	0.117803348	0.570939234	0.206332549	0.836531155	0.563389147	6.992274915	85.69850282	0.5	CRA004775;Yang2025_Silkie_Wenchang	PRJNA827465	0.958399882
ENSGALG00010000569	SIPA1	2	-0.106641004	0.244928611	-0.435396273	0.663274815	0.066957409	1.790733471	44.15696046	0.5	CRA004775;PRJNA827465	Yang2025_Silkie_Wenchang	0.91293776
ENSGALG00010000574	ENSGALG00010000574	2	0.158051298	0.283467703	0.55756369	0.577142356	0.138937425	7.049070237	85.81373193	0.5	PRJNA827465;Yang2025_Silkie_Wenchang	CRA004775	0.857145528
ENSGALG00010000574	ENSGALG00010000574	2	0.354674788	0.175186446	2.0245561	0.042912966	0.006422781	1.094624737	8.644490986	1	CRA004775;Yang2025_Silkie_Wenchang	PRJNA827465	0.358166978
ENSGALG00010000574	ENSGALG00010000574	2	-0.086615224	0.091016478	-0.951643132	0.341277898	0	0.312275681	0	0.5	CRA004775;PRJNA827465	Yang2025_Silkie_Wenchang	0.805665554
ENSGALG00010000575	ENSGALG00010000575	2	-0.330234447	0.275797114	-1.184401495	0.23623043	0.121070202	4.381197853	77.17516591	1	PRJNA827465;Yang2025_Silkie_Wenchang	CRA004775	0.748669015
ENSGALG00010000575	ENSGALG00010000575	2	-0.466347743	0.261569906	-1.782879961	0.074605846	0.046784903	1.401236692	28.63446943	1	CRA004775;Yang2025_Silkie_Wenchang	PRJNA827465	0.471679921
ENSGALG00010000575	ENSGALG00010000575	2	-0.075634498	0.136861139	-0.55263677	0.580512158	0	0.000650753	0	1	CRA004775;PRJNA827465	Yang2025_Silkie_Wenchang	0.888433837
ENSGALG00010000576	NOS1	2	-0.263599902	0.228688563	-1.152658879	0.249050426	0	0.086010868	0	1	PRJNA827465;Yang2025_Silkie_Wenchang	CRA004775	0.759411487
ENSGALG00010000576	NOS1	2	-0.211880181	0.266852119	-0.793998493	0.427196309	0	0.000467134	0	1	CRA004775;Yang2025_Silkie_Wenchang	PRJNA827465	0.84125685
ENSGALG00010000576	NOS1	2	-0.322821946	0.344213771	-0.937853083	0.348319917	0	0.033633144	0	1	CRA004775;PRJNA827465	Yang2025_Silkie_Wenchang	0.809042603
ENSGALG00010000577	ENSGALG00010000577	2	0.704904665	0.411168914	1.714391923	0.086456771	0.264948321	4.242032039	76.42639209	1	PRJNA827465;Yang2025_Silkie_Wenchang	CRA004775	0.506036629
ENSGALG00010000577	ENSGALG00010000577	2	0.81565606	0.408186873	1.998241769	0.045690455	0.168067382	1.999092177	49.97729412	1	CRA004775;Yang2025_Silkie_Wenchang	PRJNA827465	0.368613377
ENSGALG00010000577	ENSGALG00010000577	2	0.355460053	0.154938006	2.294234076	0.021777057	0	0.000693862	0	1	CRA004775;PRJNA827465	Yang2025_Silkie_Wenchang	0.230990082
ENSGALG00010000579	ENSGALG00010000579	2	1.045965347	0.517991329	2.019271923	0.043458964	0.237746351	1.781608965	43.87096048	1	PRJNA827465;Yang2025_Silkie_Wenchang	CRA004775	0.360199848
ENSGALG00010000579	ENSGALG00010000579	2	-1.293157228	2.977344364	-0.434332435	0.664047054	16.98135772	23.43900389	95.73360709	0.5	CRA004775;Yang2025_Silkie_Wenchang	PRJNA827465	0.91312035
ENSGALG00010000579	ENSGALG00010000579	2	-1.788610149	2.455833191	-0.728310927	0.466423275	11.37596534	17.1768073	94.17819632	0.5	CRA004775;PRJNA827465	Yang2025_Silkie_Wenchang	0.852763198
ENSGALG00010000581	ENSGALG00010000581	2	0.29271185	0.263005124	1.112951131	0.265729418	0.135450439	47.49506883	97.89451826	1	PRJNA827465;Yang2025_Silkie_Wenchang	CRA004775	0.769919462
ENSGALG00010000581	ENSGALG00010000581	2	0.187939354	0.40119895	0.468444282	0.639466907	0.293999156	10.83269829	90.76868964	0.5	CRA004775;Yang2025_Silkie_Wenchang	PRJNA827465	0.906064918
ENSGALG00010000581	ENSGALG00010000581	2	-0.00974124	0.100324182	-0.097097626	0.922648866	0.010642223	1.377043498	27.38065271	0.5	CRA004775;PRJNA827465	Yang2025_Silkie_Wenchang	0.981804675
ENSGALG00010000583	CCS	2	0.749570022	0.740726915	1.011938418	0.311567499	1.07449813	47.66259745	97.90191879	1	PRJNA827465;Yang2025_Silkie_Wenchang	CRA004775	0.795778238
ENSGALG00010000583	CCS	2	0.621307236	0.890221281	0.697931549	0.485220003	1.527162836	27.29452865	96.33626207	0.5	CRA004775;Yang2025_Silkie_Wenchang	PRJNA827465	0.857056537
ENSGALG00010000583	CCS	2	-0.0142136	0.092794005	-0.153173685	0.878261309	0.000380325	1.008580427	0.850742922	0.5	CRA004775;PRJNA827465	Yang2025_Silkie_Wenchang	0.971037793
ENSGALG00010000584	RBM14	2	0.027630725	0.151731649	0.182102581	0.855502223	0.037637565	5.149545516	80.58081054	0.5	PRJNA827465;Yang2025_Silkie_Wenchang	CRA004775	0.963463555
ENSGALG00010000584	RBM14	2	0.010545851	0.216900958	0.048620584	0.961221666	0.067796878	3.402743184	70.61194613	0.5	CRA004775;Yang2025_Silkie_Wenchang	PRJNA827465	0.989727676
ENSGALG00010000584	RBM14	2	-0.117243155	0.056874429	-2.061438819	0.039261195	0	0.387919315	0	1	CRA004775;PRJNA827465	Yang2025_Silkie_Wenchang	0.342602739
ENSGALG00010000585	SPTBN2	2	0.736861263	0.952577763	0.773544473	0.43920022	1.622373739	9.056287774	88.95794806	0.5	PRJNA827465;Yang2025_Silkie_Wenchang	CRA004775	0.844102016
ENSGALG00010000585	SPTBN2	2	0.325823498	1.442867697	0.225816614	0.821344054	3.805783933	11.62613626	91.39869018	0.5	CRA004775;Yang2025_Silkie_Wenchang	PRJNA827465	0.955598716
ENSGALG00010000585	SPTBN2	2	-0.478530835	0.460783398	-1.038515791	0.299029988	0.2458192	2.066782015	51.61560374	1	CRA004775;PRJNA827465	Yang2025_Silkie_Wenchang	0.789748017
ENSGALG00010000586	ENSGALG00010000586	2	0.347148504	0.649064933	0.534844029	0.592757714	0.674568892	4.277827326	76.62364738	0.5	PRJNA827465;Yang2025_Silkie_Wenchang	CRA004775	0.892776493
ENSGALG00010000586	ENSGALG00010000586	2	0.240448471	0.865976138	0.277661774	0.781272007	1.185502296	4.732217946	78.86825984	0.5	CRA004775;Yang2025_Silkie_Wenchang	PRJNA827465	0.945235709
ENSGALG00010000586	ENSGALG00010000586	2	-0.214031194	0.146161804	-1.464344234	0.143099917	0	0.612294362	0	1	CRA004775;PRJNA827465	Yang2025_Silkie_Wenchang	0.630019027
ENSGALG00010000589	ENSGALG00010000589	2	0.605400479	0.588706567	1.028356931	0.30378196	0.630181746	10.55629021	90.5269751	1	PRJNA827465;Yang2025_Silkie_Wenchang	CRA004775	0.792208658
ENSGALG00010000589	ENSGALG00010000589	2	0.718055727	0.532437415	1.348619963	0.177459065	0.438171084	4.386855512	77.20462875	1	CRA004775;Yang2025_Silkie_Wenchang	PRJNA827465	0.683574458
ENSGALG00010000589	ENSGALG00010000589	2	0.067954493	0.133961936	0.507267176	0.611967366	0	0.078204919	0	1	CRA004775;PRJNA827465	Yang2025_Silkie_Wenchang	0.897777844
ENSGALG00010000591	SBK2	2	-0.054434437	0.260165107	-0.209230354	0.834268418	0.05559638	1.654317339	39.55210549	0.5	PRJNA827465;Yang2025_Silkie_Wenchang	CRA004775	0.957990393
ENSGALG00010000591	SBK2	2	-0.265923186	0.310210335	-0.857235097	0.391314969	0	0.694092052	0	0.5	CRA004775;Yang2025_Silkie_Wenchang	PRJNA827465	0.829251057
ENSGALG00010000591	SBK2	2	0.176359447	0.231136316	0.763010548	0.445457105	0	0.06118573	0	1	CRA004775;PRJNA827465	Yang2025_Silkie_Wenchang	0.846013379
ENSGALG00010000592	CCT5	2	0.027828072	0.06867121	0.405236367	0.685303776	0.00379524	1.549233631	35.45195639	0.5	PRJNA827465;Yang2025_Silkie_Wenchang	CRA004775	0.919235539
ENSGALG00010000592	CCT5	2	0.088850025	0.089454474	0.993242932	0.320591581	0	0.53716794	0	0.5	CRA004775;Yang2025_Silkie_Wenchang	PRJNA827465	0.798348216
ENSGALG00010000592	CCT5	2	-0.020640188	0.054115261	-0.38141159	0.702897863	0	0.001023403	0	1	CRA004775;PRJNA827465	Yang2025_Silkie_Wenchang	0.923347492
ENSGALG00010000593	ATPSCKMT	2	-0.154473866	0.277167953	-0.557329463	0.577302349	0.109718494	3.407000369	70.64866769	0.5	PRJNA827465;Yang2025_Silkie_Wenchang	CRA004775	0.887148664
ENSGALG00010000593	ATPSCKMT	2	0.10319392	0.578341949	0.178430632	0.858384794	0.59220653	8.679974303	88.47922857	0.5	CRA004775;Yang2025_Silkie_Wenchang	PRJNA827465	0.964330358
ENSGALG00010000593	ATPSCKMT	2	0.342661428	0.295192658	1.160806066	0.245720776	0.118738719	2.944408348	66.0373195	1	CRA004775;PRJNA827465	Yang2025_Silkie_Wenchang	0.75661406
ENSGALG00010000595	MYO10	2	-0.078217933	0.119326342	-0.655495941	0.512148499	0	0.404463692	0	0.5	PRJNA827465;Yang2025_Silkie_Wenchang	CRA004775	0.865694231
ENSGALG00010000595	MYO10	2	0.089210223	0.185284875	0.48147601	0.630178226	0	0.245150084	0	1	CRA004775;Yang2025_Silkie_Wenchang	PRJNA827465	0.902620074
ENSGALG00010000595	MYO10	2	-0.079049223	0.133606465	-0.591657169	0.554080183	0	0.906779911	0	0.5	CRA004775;PRJNA827465	Yang2025_Silkie_Wenchang	0.880092687
ENSGALG00010000596	ENSGALG00010000596	2	0.579085077	0.713976689	0.811070006	0.417325469	0.357579926	1.49815546	33.25125286	1	PRJNA827465;Yang2025_Silkie_Wenchang	CRA004775	0.838112595
ENSGALG00010000596	ENSGALG00010000596	2	-0.460028977	2.123237306	-0.216663948	0.828470246	7.30695359	5.097267414	80.38164533	0.5	CRA004775;Yang2025_Silkie_Wenchang	PRJNA827465	0.956777964
ENSGALG00010000596	ENSGALG00010000596	2	-1.004804776	1.345791633	-0.746627302	0.455288564	2.374241414	2.543446147	60.68326427	0.5	CRA004775;PRJNA827465	Yang2025_Silkie_Wenchang	0.849072665
ENSGALG00010000598	ENSGALG00010000598	2	1.415440161	0.337775398	4.190477365	2.78368E-05	0	0.929004339	0	1	PRJNA827465;Yang2025_Silkie_Wenchang	CRA004775	0.00309935
ENSGALG00010000598	ENSGALG00010000598	2	0.541023595	1.476878445	0.366329129	0.714119496	3.741744595	6.931828108	85.57379115	0.5	CRA004775;Yang2025_Silkie_Wenchang	PRJNA827465	0.926457784
ENSGALG00010000598	ENSGALG00010000598	2	0.257714379	1.102038673	0.233852391	0.815099575	1.97706768	4.959566215	79.83694629	0.5	CRA004775;PRJNA827465	Yang2025_Silkie_Wenchang	0.953933933
ENSGALG00010000599	RETREG1	2	-0.409607653	0.106224609	-3.856052345	0.000115233	0	0.141027234	0	1	PRJNA827465;Yang2025_Silkie_Wenchang	CRA004775	0.008571592
ENSGALG00010000599	RETREG1	2	0.136917804	0.731793849	0.187098872	0.851583113	0.855188627	4.149914352	75.90311715	0.5	CRA004775;Yang2025_Silkie_Wenchang	PRJNA827465	0.96214473
ENSGALG00010000599	RETREG1	2	0.168611803	0.688667951	0.244837592	0.806582185	0.736785177	3.698341205	72.9608507	0.5	CRA004775;PRJNA827465	Yang2025_Silkie_Wenchang	0.952270941
ENSGALG00010000601	ENSGALG00010000601	2	0.054887853	0.204440952	0.268477781	0.788331575	0	0.114088015	0	0.5	PRJNA827465;Yang2025_Silkie_Wenchang	CRA004775	0.947024357
ENSGALG00010000601	ENSGALG00010000601	2	0.159049742	0.289650256	0.549109621	0.582930223	0	0.030291227	0	1	CRA004775;Yang2025_Silkie_Wenchang	PRJNA827465	0.890230846
ENSGALG00010000601	ENSGALG00010000601	2	0.030578599	0.250549836	0.122045973	0.902862609	0	0.131075012	0	0.5	CRA004775;PRJNA827465	Yang2025_Silkie_Wenchang	0.97693511
ENSGALG00010000603	ZNF622	2	0.110249151	0.063304722	1.741562825	0.081584971	0.00012058	1.010112897	1.00116504	1	PRJNA827465;Yang2025_Silkie_Wenchang	CRA004775	0.492101614
ENSGALG00010000603	ZNF622	2	-0.018466948	0.264888444	-0.069715945	0.94441975	0.114378386	5.340937726	81.27669613	0.5	CRA004775;Yang2025_Silkie_Wenchang	PRJNA827465	0.985935636
ENSGALG00010000603	ZNF622	2	-0.071693983	0.183681654	-0.390316512	0.696302515	0.051049641	3.633769076	72.48036463	0.5	CRA004775;PRJNA827465	Yang2025_Silkie_Wenchang	0.921038437
ENSGALG00010000604	ENSGALG00010000604	2	0.008177348	0.116758947	0.070036162	0.944164877	0.005310332	1.235569338	19.06565099	0.5	PRJNA827465;Yang2025_Silkie_Wenchang	CRA004775	0.985827325
ENSGALG00010000604	ENSGALG00010000604	2	-0.381206126	0.269505831	-1.414463371	0.157225895	0.110679785	4.154890654	75.93197792	1	CRA004775;Yang2025_Silkie_Wenchang	PRJNA827465	0.650687662
ENSGALG00010000604	ENSGALG00010000604	2	-0.262767353	0.38763822	-0.677867507	0.497855704	0.27001255	9.699831381	89.69054244	0.5	CRA004775;PRJNA827465	Yang2025_Silkie_Wenchang	0.861098503
ENSGALG00010000606	UBE2QL1	2	-0.221048915	0.080300012	-2.752788085	0.005909013	0	0.78732135	0	1	PRJNA827465;Yang2025_Silkie_Wenchang	CRA004775	0.116113758
ENSGALG00010000606	UBE2QL1	2	-0.152633209	0.09924848	-1.537889633	0.124075602	0	0.071585163	0	1	CRA004775;Yang2025_Silkie_Wenchang	PRJNA827465	0.590534404
ENSGALG00010000606	UBE2QL1	2	-0.274160778	0.116272392	-2.357918096	0.018377747	0	0.477988023	0	1	CRA004775;PRJNA827465	Yang2025_Silkie_Wenchang	0.227043528
ENSGALG00010000607	ENSGALG00010000607	2	-0.078860237	0.169210492	-0.466048152	0.641181034	0	0.100639992	0	1	PRJNA827465;Yang2025_Silkie_Wenchang	CRA004775	0.906429868
ENSGALG00010000607	ENSGALG00010000607	2	-0.754747635	0.799452892	-0.944080186	0.345128674	1.117137881	7.614046348	86.86637887	1	CRA004775;Yang2025_Silkie_Wenchang	PRJNA827465	0.806961063
ENSGALG00010000607	ENSGALG00010000607	2	-0.798042423	0.743804997	-1.072918879	0.283307515	0.958293613	7.026083899	85.76732054	1	CRA004775;PRJNA827465	Yang2025_Silkie_Wenchang	0.779568117
ENSGALG00010000610	ENSGALG00010000610	2	-0.328658481	0.23456741	-1.40112593	0.161176419	0.090100851	4.900813063	79.59522253	1	PRJNA827465;Yang2025_Silkie_Wenchang	CRA004775	0.657426695
ENSGALG00010000610	ENSGALG00010000610	2	-0.490445578	0.161725174	-3.032586483	0.002424676	0	0.847042181	0	1	CRA004775;Yang2025_Silkie_Wenchang	PRJNA827465	0.06593704
ENSGALG00010000610	ENSGALG00010000610	2	-0.140105537	0.062926159	-2.226507064	0.025980236	0	0.387101983	0	1	CRA004775;PRJNA827465	Yang2025_Silkie_Wenchang	0.274758802
ENSGALG00010000612	ENSGALG00010000612	2	0.098169965	0.171615109	0.572035677	0.567297804	0	0.000350584	0	1	PRJNA827465;Yang2025_Silkie_Wenchang	CRA004775	0.883696313
ENSGALG00010000612	ENSGALG00010000612	2	-0.349545063	0.458611074	-0.762181906	0.445951444	0.289156521	3.198184757	68.73226295	0.5	CRA004775;Yang2025_Silkie_Wenchang	PRJNA827465	0.846013379
ENSGALG00010000612	ENSGALG00010000612	2	-0.30464781	0.451642629	-0.674532895	0.49997258	0.32646864	4.740534484	78.90533223	0.5	CRA004775;PRJNA827465	Yang2025_Silkie_Wenchang	0.862100651
ENSGALG00010000613	ENSGALG00010000613	2	0.014747766	0.191725215	0.076921367	0.9386861	0.057171929	4.401270708	77.27928895	0.5	PRJNA827465;Yang2025_Silkie_Wenchang	CRA004775	0.985349045
ENSGALG00010000613	ENSGALG00010000613	2	-0.037056996	0.313587976	-0.118170972	0.90593219	0.142939444	3.346728821	70.12007685	0.5	CRA004775;Yang2025_Silkie_Wenchang	PRJNA827465	0.978196186
ENSGALG00010000613	ENSGALG00010000613	2	-0.187601988	0.099307839	-1.889095456	0.058879039	0	0.585282592	0	1	CRA004775;PRJNA827465	Yang2025_Silkie_Wenchang	0.42013793
ENSGALG00010000614	KAT8	2	-0.354370283	0.10908263	-3.248640814	0.001159578	0	0.079933664	0	1	PRJNA827465;Yang2025_Silkie_Wenchang	CRA004775	0.041469421
ENSGALG00010000614	KAT8	2	-0.410162767	0.159854301	-2.565853811	0.01029222	0	0.04558194	0	1	CRA004775;Yang2025_Silkie_Wenchang	PRJNA827465	0.161161396
ENSGALG00010000614	KAT8	2	-0.348796522	0.129675181	-2.689770844	0.00715011	0	0.146209047	0	1	CRA004775;PRJNA827465	Yang2025_Silkie_Wenchang	0.130102208
ENSGALG00010000616	PSENEN	2	-0.509993133	0.215638523	-2.365037219	0.018028259	0	0.158079661	0	1	PRJNA827465;Yang2025_Silkie_Wenchang	CRA004775	0.223945849
ENSGALG00010000616	PSENEN	2	-0.304816829	0.269133425	-1.132586295	0.257388027	0	0.954934478	0	0.5	CRA004775;Yang2025_Silkie_Wenchang	PRJNA827465	0.764987374
ENSGALG00010000616	PSENEN	2	-0.349542375	0.389054402	-0.898440869	0.368950556	0.116188595	1.466878263	31.82801703	0.5	CRA004775;PRJNA827465	Yang2025_Silkie_Wenchang	0.820553374
ENSGALG00010000619	ENSGALG00010000619	2	0.702997614	0.717181879	0.98022222	0.326976439	0.965789004	16.35077862	93.8840833	0.5	PRJNA827465;Yang2025_Silkie_Wenchang	CRA004775	0.800028239
ENSGALG00010000619	ENSGALG00010000619	2	0.505941335	0.934839903	0.541206396	0.588365329	1.647401949	17.27842934	94.2124369	0.5	CRA004775;Yang2025_Silkie_Wenchang	PRJNA827465	0.891874519
ENSGALG00010000619	ENSGALG00010000619	2	-0.146535779	0.20542838	-0.713318088	0.475648947	0	0.95707421	0	1	CRA004775;PRJNA827465	Yang2025_Silkie_Wenchang	0.854555922
ENSGALG00010000621	UPP1	2	-0.248473494	0.130909126	-1.898060907	0.057688059	0	0.838167325	0	1	PRJNA827465;Yang2025_Silkie_Wenchang	CRA004775	0.416173603
ENSGALG00010000621	UPP1	2	-0.351766609	0.230960912	-1.523056896	0.127744471	0	0.667657186	0	1	CRA004775;Yang2025_Silkie_Wenchang	PRJNA827465	0.600026204
ENSGALG00010000621	UPP1	2	-0.166423453	0.142638225	-1.166752127	0.243310485	0	0.100065276	0	1	CRA004775;PRJNA827465	Yang2025_Silkie_Wenchang	0.755238542
ENSGALG00010000623	ENSGALG00010000623	2	-0.123497851	0.08575691	-1.440092135	0.149841334	0	0.004184943	0	1	PRJNA827465;Yang2025_Silkie_Wenchang	CRA004775	0.639784242
ENSGALG00010000623	ENSGALG00010000623	2	-0.123602793	0.134453072	-0.919300624	0.357938352	0	0.008948718	0	1	CRA004775;Yang2025_Silkie_Wenchang	PRJNA827465	0.813641308
ENSGALG00010000623	ENSGALG00010000623	2	-0.117837262	0.092256384	-1.277280298	0.201503307	0	0.003271069	0	1	CRA004775;PRJNA827465	Yang2025_Silkie_Wenchang	0.714377024
ENSGALG00010000625	ENSGALG00010000625	2	0.312733264	0.318447536	0.982055844	0.326072337	0.141489988	3.305967776	69.75167129	0.5	PRJNA827465;Yang2025_Silkie_Wenchang	CRA004775	0.800023788
ENSGALG00010000625	ENSGALG00010000625	2	0.19313918	0.479899626	0.402457451	0.687347396	0.359928059	4.417606113	77.3633055	0.5	CRA004775;Yang2025_Silkie_Wenchang	PRJNA827465	0.919465461
ENSGALG00010000625	ENSGALG00010000625	2	-0.106674963	0.211989631	-0.503208399	0.614817761	0	0.497994513	0	1	CRA004775;PRJNA827465	Yang2025_Silkie_Wenchang	0.898764504
ENSGALG00010000627	ENSGALG00010000627	2	0.505093279	0.632849732	0.798125138	0.424797875	0.715230653	8.838075984	88.68532018	0.5	PRJNA827465;Yang2025_Silkie_Wenchang	CRA004775	0.841099792
ENSGALG00010000627	ENSGALG00010000627	2	0.558024496	0.660803795	0.844463213	0.398410612	0.681427952	4.524445732	77.89784519	0.5	CRA004775;Yang2025_Silkie_Wenchang	PRJNA827465	0.831980453
ENSGALG00010000627	ENSGALG00010000627	2	-0.082694557	0.14944407	-0.553347864	0.580025232	0	0.010938825	0	1	CRA004775;PRJNA827465	Yang2025_Silkie_Wenchang	0.888203646
ENSGALG00010000630	ENSGALG00010000630	2	0.026708411	0.609265267	0.043837082	0.965034269	0.633498922	6.727401187	85.13541898	0.5	PRJNA827465;Yang2025_Silkie_Wenchang	CRA004775	0.990532586
ENSGALG00010000630	ENSGALG00010000630	2	-0.053138411	0.716830387	-0.074129685	0.940907195	0.884577639	7.180542714	86.07347606	0.5	CRA004775;Yang2025_Silkie_Wenchang	PRJNA827465	0.98556663
ENSGALG00010000630	ENSGALG00010000630	2	-0.628674204	0.221063577	-2.843861536	0.004457041	0	0.212361958	0	1	CRA004775;PRJNA827465	Yang2025_Silkie_Wenchang	0.097699437
ENSGALG00010000634	ENSGALG00010000634	2	0.351595148	0.449816262	0.7816417	0.434425173	0.342337565	6.020369164	83.38972291	0.5	PRJNA827465;Yang2025_Silkie_Wenchang	CRA004775	0.842762754
ENSGALG00010000634	ENSGALG00010000634	2	0.19341237	0.65890237	0.293537221	0.769111554	0.759631392	7.98473431	87.47610176	0.5	CRA004775;Yang2025_Silkie_Wenchang	PRJNA827465	0.941790377
ENSGALG00010000634	ENSGALG00010000634	2	-0.154696358	0.180768457	-0.85577075	0.392124597	0.025051021	1.419497621	29.55254131	1	CRA004775;PRJNA827465	Yang2025_Silkie_Wenchang	0.829253325
ENSGALG00010000635	ENSGALG00010000635	2	0.086605329	0.12567088	0.689143971	0.490732672	0	0.526736735	0	1	PRJNA827465;Yang2025_Silkie_Wenchang	CRA004775	0.858798485
ENSGALG00010000635	ENSGALG00010000635	2	0.120703237	0.165516766	0.729250822	0.465848248	0	0.488587244	0	0.5	CRA004775;Yang2025_Silkie_Wenchang	PRJNA827465	0.852511753
ENSGALG00010000635	ENSGALG00010000635	2	-0.000532542	0.140886148	-0.003779943	0.996984049	0	0.03303206	0	0.5	CRA004775;PRJNA827465	Yang2025_Silkie_Wenchang	0.999065103
ENSGALG00010000636	ENSGALG00010000636	2	0.314278966	0.334671569	0.939066822	0.347696437	0.204613892	11.54003212	91.33451286	0.5	PRJNA827465;Yang2025_Silkie_Wenchang	CRA004775	0.808847403
ENSGALG00010000636	ENSGALG00010000636	2	0.408501384	0.297947291	1.371052521	0.170358584	0.126643668	3.1732875	68.48693981	1	CRA004775;Yang2025_Silkie_Wenchang	PRJNA827465	0.672114378
ENSGALG00010000636	ENSGALG00010000636	2	-0.008011165	0.121504224	-0.06593322	0.947430992	0	0.033275518	0	0.5	CRA004775;PRJNA827465	Yang2025_Silkie_Wenchang	0.986538092
ENSGALG00010000639	SCN1B	2	-0.21561984	0.218569914	-0.986502834	0.323886425	0.064819535	3.068733197	67.41326352	0.5	PRJNA827465;Yang2025_Silkie_Wenchang	CRA004775	0.799600434
ENSGALG00010000639	SCN1B	2	0.202265173	0.250779265	0.806546635	0.41992773	0.056752711	1.685551346	40.67223153	1	CRA004775;Yang2025_Silkie_Wenchang	PRJNA827465	0.838596594
ENSGALG00010000639	SCN1B	2	0.012718822	0.480461672	0.026472085	0.978880799	0.392206008	6.219314221	83.92105682	0.5	CRA004775;PRJNA827465	Yang2025_Silkie_Wenchang	0.994259589
ENSGALG00010000641	ENSGALG00010000641	2	-0.330122618	0.667041081	-0.494905978	0.620666491	0.799992048	9.873471664	89.87185021	0.5	PRJNA827465;Yang2025_Silkie_Wenchang	CRA004775	0.899395593
ENSGALG00010000641	ENSGALG00010000641	2	0.325077545	0.296640222	1.095864689	0.273137994	0	0.031355874	0	1	CRA004775;Yang2025_Silkie_Wenchang	PRJNA827465	0.773904763
ENSGALG00010000641	ENSGALG00010000641	2	-0.581844402	0.564487935	-1.030747276	0.302659348	0.406931362	2.33704566	57.21093443	0.5	CRA004775;PRJNA827465	Yang2025_Silkie_Wenchang	0.791852471
ENSGALG00010000643	VKORC1	2	-0.249205759	0.144895644	-1.719898212	0.085450946	0	0.036873298	0	1	PRJNA827465;Yang2025_Silkie_Wenchang	CRA004775	0.50266296
ENSGALG00010000643	VKORC1	2	-0.495203594	0.383793367	-1.290287006	0.196951027	0.150771268	1.911576225	47.68715016	1	CRA004775;Yang2025_Silkie_Wenchang	PRJNA827465	0.707994211

ENSGALG00010000643	VKORC1	2	-0.480707134	0.333282527	-1.442341244	0.149206131	0.129299957	1.936587367	48.36277376	1	CRA004775;PRJNA827465	Yang2025_Silkie_Wenchang	0.639246608
ENSGALG00010000646	ENSGALG00010000646	2	-0.316134279	0.706812931	-0.447267255	0.654682107	0.720144771	3.572939771	72.01184279	0.5	PRJNA827465;Yang2025_Silkie_Wenchang	CRA004775	0.910222014
ENSGALG00010000646	ENSGALG00010000646	2	-1.595825107	0.692988352	-2.302816638	0.021289162	0.475307781	1.886890931	47.00276612	1	CRA004775;Yang2025_Silkie_Wenchang	PRJNA827465	0.246749612
ENSGALG00010000646	ENSGALG00010000646	2	-0.957760766	1.415311382	-0.676713816	0.498587549	3.516038038	7.942226606	87.40907242	0.5	CRA004775;PRJNA827465	Yang2025_Silkie_Wenchang	0.861478809
ENSGALG00010000649	PAPD7	2	-0.177464052	0.059307498	-2.99227006	0.002769112	0	0.063157362	0	1	PRJNA827465;Yang2025_Silkie_Wenchang	CRA004775	0.071743179
ENSGALG00010000649	PAPD7	2	0.156008205	0.367082406	0.424995049	0.670840284	0.227607774	5.772499047	82.67648047	0.5	CRA004775;Yang2025_Silkie_Wenchang	PRJNA827465	0.914552186
ENSGALG00010000649	PAPD7	2	0.142303068	0.382377257	0.372153587	0.709778501	0.250904139	6.332419174	84.20824692	0.5	CRA004775;PRJNA827465	Yang2025_Silkie_Wenchang	0.925298042
ENSGALG00010000650	BCKDK	2	-0.009962067	0.343993799	-0.02896002	0.976896477	0.208747846	8.136740014	87.71006572	0.5	PRJNA827465;Yang2025_Silkie_Wenchang	CRA004775	0.993272855
ENSGALG00010000650	BCKDK	2	-0.005735545	0.429183887	-0.01336384	0.989337516	0.271498483	3.557955034	71.89396745	0.5	CRA004775;Yang2025_Silkie_Wenchang	PRJNA827465	0.997119019
ENSGALG00010000650	BCKDK	2	-0.340689105	0.103397229	-3.294953909	0.000984378	0	0.181678566	0	1	CRA004775;PRJNA827465	Yang2025_Silkie_Wenchang	0.037419142
ENSGALG00010000651	ENSGALG00010000651	2	0.26835278	0.423558968	0.633566518	0.526363758	0.276846707	4.324449253	76.87566806	0.5	PRJNA827465;Yang2025_Silkie_Wenchang	CRA004775	0.870546826
ENSGALG00010000651	ENSGALG00010000651	2	0.630021422	0.269436931	2.338289036	0.019372259	0	0.240364075	0	1	CRA004775;Yang2025_Silkie_Wenchang	PRJNA827465	0.23313687
ENSGALG00010000651	ENSGALG00010000651	2	-0.002980366	0.235476539	-0.012656743	0.98990165	0.010174568	1.068653609	6.424308942	0.5	CRA004775;PRJNA827465	Yang2025_Silkie_Wenchang	0.997311131
ENSGALG00010000652	SRD5A1	2	0.260775532	0.106422287	2.450384593	0.01427037	0	0.129777646	0	1	PRJNA827465;Yang2025_Silkie_Wenchang	CRA004775	0.195970774
ENSGALG00010000652	SRD5A1	2	0.001363415	0.44268095	0.003079904	0.997542596	0.261693288	2.394318147	58.23445596	0.5	CRA004775;Yang2025_Silkie_Wenchang	PRJNA827465	0.999290321
ENSGALG00010000652	SRD5A1	2	-0.01803864	0.392016048	-0.046015055	0.963298251	0.194809727	2.059159199	51.43648921	0.5	CRA004775;PRJNA827465	Yang2025_Silkie_Wenchang	0.989998461
ENSGALG00010000653	NSUN2	2	0.181384319	0.126659686	1.432060386	0.15212654	0.027920838	7.513546174	86.69070534	1	PRJNA827465;Yang2025_Silkie_Wenchang	CRA004775	0.643667715
ENSGALG00010000653	NSUN2	2	0.247859551	0.123472089	2.007413603	0.044705642	0.01363235	1.471731473	32.05282223	1	CRA004775;Yang2025_Silkie_Wenchang	PRJNA827465	0.364399881
ENSGALG00010000653	NSUN2	2	0.060676347	0.04695954	1.292098421	0.196323063	0	0.026616942	0	1	CRA004775;PRJNA827465	Yang2025_Silkie_Wenchang	0.707702956
ENSGALG00010000654	ENSGALG00010000654	2	0.75385637	0.78340943	0.962276355	0.335910803	1.21364359	88.56046308	98.87082795	0.5	PRJNA827465;Yang2025_Silkie_Wenchang	CRA004775	0.803504387
ENSGALG00010000654	ENSGALG00010000654	2	0.545053067	1.004844	0.542425557	0.587525377	1.982335113	54.26673455	98.15725046	0.5	CRA004775;Yang2025_Silkie_Wenchang	PRJNA827465	0.89167336
ENSGALG00010000654	ENSGALG00010000654	2	-0.193622134	0.215082873	-0.900221071	0.368002615	0.06817895	3.27997621	69.51197399	1	CRA004775;PRJNA827465	Yang2025_Silkie_Wenchang	0.82021134
ENSGALG00010000656	ENSGALG00010000656	2	0.150450186	0.607096695	0.247819148	0.804274339	0.699919388	19.77856668	94.9440219	0.5	PRJNA827465;Yang2025_Silkie_Wenchang	CRA004775	0.95154709
ENSGALG00010000656	ENSGALG00010000656	2	0.309826094	0.48473381	0.639167494	0.522713976	0.396470696	6.24453588	83.98599961	0.5	CRA004775;Yang2025_Silkie_Wenchang	PRJNA827465	0.869568211
ENSGALG00010000656	ENSGALG00010000656	2	-0.397315212	0.154478766	-2.571970982	0.010112078	0	0.426331862	0	1	CRA004775;PRJNA827465	Yang2025_Silkie_Wenchang	0.159642386
ENSGALG00010000659	ENSGALG00010000659	2	0.028609893	0.310527828	0.092133106	0.926592285	0	0.000397374	0	1	PRJNA827465;Yang2025_Silkie_Wenchang	CRA004775	0.982834319
ENSGALG00010000659	ENSGALG00010000659	2	-0.582081369	0.651231249	-0.893816705	0.37141997	0.554250319	2.854172393	64.96357394	0.5	CRA004775;Yang2025_Silkie_Wenchang	PRJNA827465	0.821526338
ENSGALG00010000659	ENSGALG00010000659	2	-0.54983546	0.655052441	-0.839376248	0.401258208	0.605617363	3.293495088	69.63711882	0.5	CRA004775;PRJNA827465	Yang2025_Silkie_Wenchang	0.83248071
ENSGALG00010000660	ENSGALG00010000660	2	0.038594128	0.211617964	0.18237737	0.855286584	0.06743404	4.014531728	75.09049454	0.5	PRJNA827465;Yang2025_Silkie_Wenchang	CRA004775	0.953406774
ENSGALG00010000660	ENSGALG00010000660	2	0.1967592	0.1495193	1.315945165	0.188192448	0	0.899548582	0	0.5	CRA004775;Yang2025_Silkie_Wenchang	PRJNA827465	0.697483156
ENSGALG00010000660	ENSGALG00010000660	2	-0.156575846	0.124908989	-1.253519433	0.210016731	0	0.015733384	0	1	CRA004775;PRJNA827465	Yang2025_Silkie_Wenchang	0.721835135
ENSGALG00010000674	GRB10	2	-0.170998641	0.114957639	-1.487492623	0.136888475	0.018349456	3.176256524	68.51639682	1	PRJNA827465;Yang2025_Silkie_Wenchang	CRA004775	0.619029034
ENSGALG00010000674	GRB10	2	0.204765686	0.563822333	0.363174131	0.716474811	0.561144011	7.80381735	87.18575852	0.5	CRA004775;Yang2025_Silkie_Wenchang	PRJNA827465	0.927487786
ENSGALG00010000674	GRB10	2	0.301058678	0.444060046	0.677968397	0.497791731	0.328411109	5.13560467	80.52809622	0.5	CRA004775;PRJNA827465	Yang2025_Silkie_Wenchang	0.861072323
ENSGALG00010000684	COBL	2	-0.213418673	0.109934882	-1.941318982	0.052219599	0	0.099302634	0	1	PRJNA827465;Yang2025_Silkie_Wenchang	CRA004775	0.396826543
ENSGALG00010000684	COBL	2	-0.221388726	0.165443467	-1.338153323	0.180864461	0	0.286315595	0	1	CRA004775;Yang2025_Silkie_Wenchang	PRJNA827465	0.68848481
ENSGALG00010000684	COBL	2	-0.16965685	0.13263521	-1.279123779	0.200853471	0	0.155400988	0	1	CRA004775;PRJNA827465	Yang2025_Silkie_Wenchang	0.71349819
ENSGALG00010000685	ENSGALG00010000685	2	0.019697036	0.277994719	0.070853994	0.943513959	0	0.016623904	0	0.5	PRJNA827465;Yang2025_Silkie_Wenchang	CRA004775	0.985827325
ENSGALG00010000685	ENSGALG00010000685	2	-0.146076357	0.382015492	-0.382383332	0.702177052	0	0.147046565	0	1	CRA004775;Yang2025_Silkie_Wenchang	PRJNA827465	0.923083392
ENSGALG00010000685	ENSGALG00010000685	2	-0.042339823	0.297649992	-0.142247017	0.886884895	0	0.286216689	0	0.5	CRA004775;PRJNA827465	Yang2025_Silkie_Wenchang	0.972850601
ENSGALG00010000686	ENSGALG00010000686	2	0.198541256	0.098752465	2.010494184	0.044378911	0	0.781021287	0	1	PRJNA827465;Yang2025_Silkie_Wenchang	CRA004775	0.363683141
ENSGALG00010000686	ENSGALG00010000686	2	-0.023296439	0.369250179	-0.063091205	0.949693878	0.224506507	5.491915355	81.79141638	0.5	CRA004775;Yang2025_Silkie_Wenchang	PRJNA827465	0.98723759
ENSGALG00010000686	ENSGALG00010000686	2	-0.093477924	0.274300049	-0.34078712	0.733263848	0.112122066	3.571447269	72.00014659	0.5	CRA004775;PRJNA827465	Yang2025_Silkie_Wenchang	0.933121871
ENSGALG00010000687	IKZF1	2	-0.181353503	0.195518915	-0.92754966	0.35364122	0.001393414	1.017621489	1.731634918	1	PRJNA827465;Yang2025_Silkie_Wenchang	CRA004775	0.811318534
ENSGALG00010000687	IKZF1	2	-0.446776483	0.264419127	-1.689652666	0.091094424	0	0.014813028	0	1	CRA004775;Yang2025_Silkie_Wenchang	PRJNA827465	0.517711803
ENSGALG00010000687	IKZF1	2	-0.118404867	0.221603535	-0.534309468	0.593127443	0	0.718490943	0	1	CRA004775;PRJNA827465	Yang2025_Silkie_Wenchang	0.892776493
ENSGALG00010000688	ENSGALG00010000688	2	0.142088861	0.619545116	0.229343948	0.818601676	0.675929766	8.101312002	87.65632036	0.5	PRJNA827465;Yang2025_Silkie_Wenchang	CRA004775	0.955256039
ENSGALG00010000688	ENSGALG00010000688	2	0.281164284	0.462519039	0.607897752	0.543255291	0.344204702	4.958307421	79.83182737	0.5	CRA004775;Yang2025_Silkie_Wenchang	PRJNA827465	0.876968972
ENSGALG00010000688	ENSGALG00010000688	2	-0.276474522	0.167322059	-1.652349507	0.098463322	0	0.849205191	0	1	CRA004775;PRJNA827465	Yang2025_Silkie_Wenchang	0.535930498
ENSGALG00010000695	FIGNL1	2	-0.031088685	0.106986234	-0.290588539	0.771368092	0	0.553770914	0	0.5	PRJNA827465;Yang2025_Silkie_Wenchang	CRA004775	0.94283287
ENSGALG00010000695	FIGNL1	2	-0.214175401	0.184204552	-1.162704169	0.244949549	0	0.677878198	0	1	CRA004775;Yang2025_Silkie_Wenchang	PRJNA827465	0.756475101
ENSGALG00010000695	FIGNL1	2	-0.098339836	0.244274873	-0.402578598	0.687258256	0.057521217	1.42282199	29.7171391	0.5	CRA004775;PRJNA827465	Yang2025_Silkie_Wenchang	0.919451777
ENSGALG00010000697	ENSGALG00010000697	2	0.412620727	0.45692478	0.90303863	0.366503393	0.364777269	7.720276578	87.04709618	0.5	PRJNA827465;Yang2025_Silkie_Wenchang	CRA004775	0.819290267
ENSGALG00010000697	ENSGALG00010000697	2	0.181792871	0.734861749	0.247384612	0.804612795	0.972258519	10.05026674	90.05003533	0.5	CRA004775;Yang2025_Silkie_Wenchang	PRJNA827465	0.951665484
ENSGALG00010000697	ENSGALG00010000697	2	-0.199134587	0.260230588	-0.765223598	0.44413841	0.071380173	1.865278541	46.38870399	1	CRA004775;PRJNA827465	Yang2025_Silkie_Wenchang	0.846013379
ENSGALG00010000702	DDC	2	0.202774693	0.309338745	0.65551017	0.512139341	0.019570823	1.106930546	9.660095308	0.5	PRJNA827465;Yang2025_Silkie_Wenchang	CRA004775	0.865694231
ENSGALG00010000702	DDC	2	0.847127015	0.497849994	1.701570804	0.088835854	0.273262222	2.110667632	52.62162622	1	CRA004775;Yang2025_Silkie_Wenchang	PRJNA827465	0.511771357
ENSGALG00010000702	DDC	2	0.599395358	0.826953899	0.724823184	0.468560511	1.076904552	4.66671057	78.57163017	0.5	CRA004775;PRJNA827465	Yang2025_Silkie_Wenchang	0.853198008
ENSGALG00010000704	ENSGALG00010000704	2	-0.030461928	0.068964142	-0.441706769	0.658701411	0	0.145438754	0	1	PRJNA827465;Yang2025_Silkie_Wenchang	CRA004775	0.911229508
ENSGALG00010000704	ENSGALG00010000704	2	-0.21847179	0.163751658	-1.334163564	0.182149668	0.030470164	2.272489282	55.99539202	1	CRA004775;Yang2025_Silkie_Wenchang	PRJNA827465	0.690033956
ENSGALG00010000704	ENSGALG00010000704	2	-0.178010124	0.192004881	-0.927112493	0.353868131	0.057111219	4.084522558	75.51733318	1	CRA004775;PRJNA827465	Yang2025_Silkie_Wenchang	0.813456203
ENSGALG00010000710	ENSGALG00010000710	2	-0.15913747	0.21275781	-0.74797475	0.454475388	0	0.503452453	0	0.5	PRJNA827465;Yang2025_Silkie_Wenchang	CRA004775	0.849072665
ENSGALG00010000710	ENSGALG00010000710	2	-0.554456654	0.713805845	-0.776761157	0.437299698	0.746851358	3.534695847	71.70902269	0.5	CRA004775;Yang2025_Silkie_Wenchang	PRJNA827465	0.843708704
ENSGALG00010000710	ENSGALG00010000710	2	-0.65211225	0.533244312	-1.22291459	0.221361957	0.369576283	2.437678109	58.97735651	1	CRA004775;PRJNA827465	Yang2025_Silkie_Wenchang	0.734689158
ENSGALG00010000711	SPRY2	2	-0.320738577	0.309243537	-1.037171481	0.299655947	0.183190581	23.37791814	95.72172712	1	PRJNA827465;Yang2025_Silkie_Wenchang	CRA004775	0.79004225
ENSGALG00010000711	SPRY2	2	0.197182971	0.865106809	0.227929047	0.819701401	1.432494991	22.63135608	95.58135184	0.5	CRA004775;Yang2025_Silkie_Wenchang	PRJNA827465	0.955256039
ENSGALG00010000711	SPRY2	2	0.485169159	0.553868005	0.875965311	0.381048892	0.557451901	10.05998528	90.0596276	0.5	CRA004775;PRJNA827465	Yang2025_Silkie_Wenchang	0.824392515
ENSGALG00010000714	FBXL7	2	-0.14226446	0.053664372	-2.651003905	0.008025291	0	0.06502287	0	1	PRJNA827465;Yang2025_Silkie_Wenchang	CRA004775	0.139297234
ENSGALG00010000714	FBXL7	2	-0.112964515	0.132119196	-0.855019699	0.392540243	0.009603197	1.14451743	12.62693137	0.5	CRA004775;Yang2025_Silkie_Wenchang	PRJNA827465	0.825981969
ENSGALG00010000714	FBXL7	2	-0.118979126	0.071590984	-1.661928918	0.096527028	0.001380218	1.021846827	2.137974762	0.5	CRA004775;PRJNA827465	Yang2025_Silkie_Wenchang	0.530898652
ENSGALG00010000717	NDFIP2	2	-0.104013597	0.053684612	-1.937493677	0.052685026	0	0.003977221	0	1	PRJNA827465;Yang2025_Silkie_Wenchang	CRA004775	0.398821179
ENSGALG00010000717	NDFIP2	2	0.00137846	0.115367264	0.01194845	0.990466313	0	0.664430119	0	0.5	CRA004775;Yang2025_Silkie_Wenchang	PRJNA827465	0.997468525
ENSGALG00010000717	NDFIP2	2	-0.063343768	0.081098487	-0.781072132	0.434760071	0.005101945	1.346930675	25.75712927	0.5	CRA004775;PRJNA827465	Yang2025_Silkie_Wenchang	0.842762754
ENSGALG00010000718	EEF1G	2	-0.062914478	0.056399169	-1.115521376	0.264627064	0	0.078971877	0	1	PRJNA827465;Yang2025_Silkie_Wenchang	CRA004775	0.768855521
ENSGALG00010000718	EEF1G	2	-0.10419976	0.110912771	-0.939474863	0.347486992	0	0.042882846	0	1	CRA004775;Yang2025_Silkie_Wenchang	PRJNA827465	0.808705451
ENSGALG00010000718	EEF1G	2	-0.060176444	0.061747901	-0.974550439	0.329783308	0	0.134010216	0	1	CRA004775;PRJNA827465	Yang2025_Silkie_Wenchang	0.801177491
ENSGALG00010000720	RBM26	2	0.106407049	0.162242268	0.655852822	0.511918826	0.042429795	4.634208409	78.42134165	0.5	PRJNA827465;Yang2025_Silkie_Wenchang	CRA004775	0.865694231
ENSGALG00010000720	RBM26	2	0.313847123	0.110488237	2.840547845	0.004503612	0	0.03043924	0	1	CRA004775;Yang2025_Silkie_Wenchang	PRJNA827465	0.098172739
ENSGALG00010000720	RBM26	2	0.116033926	0.180341319	0.643412872	0.519956234	0.051369524	4.129130701	75.7818274	0.5	CRA004775;PRJNA827465	Yang2025_Silkie_Wenchang	0.869253622
ENSGALG00010000721	ENSGALG00010000721	2	0.364625838	0.111046721	3.283562576	0.001025039	0	0.154568124	0	1	PRJNA827465;Yang2025_Silkie_Wenchang	CRA004775	0.038424195
ENSGALG00010000721	ENSGALG00010000721	2	-0.100439987	0.543608024	-0.184765462	0.853412999	0.533479164	10.0596601	90.05930628	0.5	CRA004775;Yang2025_Silkie_Wenchang	PRJNA827465	0.962956221
ENSGALG00010000721	ENSGALG00010000721	2	-0.137629117	0.498599975	-0.276031135	0.782524153	0.445547089	9.270885579	89.21354393	0.5	CRA004775;PRJNA827465	Yang2025_Silkie_Wenchang	0.945432561
ENSGALG00010000722	ANKH	2	0.046601073	0.256465881	0.181704766	0.855814426	0.117198198	9.039124125	88.93698121	0.5	PRJNA827465;Yang2025_Silkie_Wenchang	CRA004775	0.965486168
ENSGALG00010000722	ANKH	2	-0.19248663	0.121713726	-1.581470202	0.113770567	0	0.152506318	0	1	CRA004775;Yang2025_Silkie_Wenchang	PRJNA827465	0.568978529
ENSGALG00010000722	ANKH	2	0.148818981	0.193869803	0.767623316	0.442711006	0.049160233	2.49338208	59.89383223	0.5	CRA004775;PRJNA827465	Yang2025_Silkie_Wenchang	0.845623604
ENSGALG00010000724	OBI1	2	0.020357954	0.068308908	0.298027809	0.765681939	0	0.116012328	0	0.5	PRJNA827465;Yang2025_Silkie_Wenchang	CRA004775	0.940926806
ENSGALG00010000724	OBI1	2	-0.11586389	0.255064963	-0.454252473	0.649664712	0.079660319	2.081204136	51.9508931	0.5	CRA004775;Yang2025_Silkie_Wenchang	PRJNA827465	0.908200019
ENSGALG00010000724	OBI1	2	-0.123053459	0.21891537	-0.562105162	0.574044371	0.057046956	1.812944084	44.84110078	0.5	CRA004775;PRJNA827465	Yang2025_Silkie_Wenchang	0.886081433
ENSGALG00010000727	ENSGALG00010000727	2	-0.024096442	0.150359778	-0.160258561	0.8726774	0.016799591	1.512912133	33.90230811	0.5	PRJNA827465;Yang2025_Silkie_Wenchang	CRA004775	0.969629321
ENSGALG00010000727	ENSGALG00010000727	2	0.016012357	0.193514357	0.082745062	0.934054253	0.016885938	1.280960793	21.93359819	0.5	CRA004775;Yang2025_Silkie_Wenchang	PRJNA827465	0.98432801
ENSGALG00010000727	ENSGALG00010000727	2	-0.150381828	0.120380668	-1.249219808	0.211564951	0	0.066230522	0	1	CRA004775;PRJNA827465	Yang2025_Silkie_Wenchang	0.724013667
ENSGALG00010000729	EDNRB	2	-0.31906678	0.253697673	-1.257665376	0.208512799	0.106357089	5.734822385	82.56266833	1	PRJNA827465;Yang2025_Silkie_Wenchang	CRA004775	0.721011841
ENSGALG00010000729	EDNRB	2	0.143053846	0.755631669	0.189316902	0.849844449	1.054729884	12.48156945	91.98818703	0.5	CRA004775;Yang2025_Silkie_Wenchang	PRJNA827465	0.961856718
ENSGALG00010000729	EDNRB	2	0.3760466	0.498629723	0.754160016	0.450753144	0.411409654	5.314384619	81.1831459	0.5	CRA004775;PRJNA827465	Yang2025_Silkie_Wenchang	0.847631399
ENSGALG00010000730	INTS5	2	-0.304737737	0.087593894	-3.478983784	0.000503319	0	0.043809923	0	1	PRJNA827465;Yang2025_Silkie_Wenchang	CRA004775	0.023568345
ENSGALG00010000730	INTS5	2	-0.264704469	0.13971126	-1.8946538	0.058138282	0	0.054095458	0	1	CRA004775;Yang2025_Silkie_Wenchang	PRJNA827465	0.417658363
ENSGALG00010000730	INTS5	2	-0.306827853	0.102520864	-2.992833277	0.002764007	0	0.121026338	0	1	CRA004775;PRJNA827465	Yang2025_Silkie_Wenchang	0.071732358
ENSGALG00010000731	ENSGALG00010000731	2	0.519747848	0.327350528	1.587740979	0.112344922	0.047004431	1.214504761	17.6619119	1	PRJNA827465;Yang2025_Silkie_Wenchang	CRA004775	0.56602666
ENSGALG00010000731	ENSGALG00010000731	2	0.32378826	0.838186829	0.386296048	0.699277427	0.886853353	2.612598347	61.72392894	0.5	CRA004775;Yang2025_Silkie_Wenchang	PRJNA827465	0.921931076
ENSGALG00010000731	ENSGALG00010000731	2	0.175730148	0.340175347	0.51658696	0.605444521	0.03367708	1.07786245	7.223783525	0.5	CRA004775;PRJNA827465	Yang2025_Silkie_Wenchang	0.896474603
ENSGALG00010000732	ENSGALG00010000732	2	-0.069718074	0.11100512	-0.628061793	0.529963461	0	0.009135297	0	1	PRJNA827465;Yang2025_Silkie_Wenchang	CRA004775	0.87207153
ENSGALG00010000732	ENSGALG00010000732	2	-0.105756167	0.221234426	-0.478027626	0.632630542	0	0.00195711	0	1	CRA004775;Yang2025_Silkie_Wenchang	PRJNA827465	0.903058174
ENSGALG00010000732	ENSGALG00010000732	2	-0.071581765	0.111983299	-0.639218223	0.522680979	0	0.021538549	0	1	CRA004775;PRJNA827465	Yang2025_Silkie_Wenchang	0.869568211
ENSGALG00010000735	ENSGALG00010000735	2	-0.679939128	0.885029599	-0.768267105	0.442328513	1.037511082	2.786343097	64.1106653	0.5	PRJNA827465;Yang2025_Silkie_Wenchang	CRA004775	0.845442667
ENSGALG00010000735	ENSGALG00010000735	2	-0.843123039	0.845029747	-0.99774362	0.318403696	0.648857289	1.82717251	45.2706302	1	CRA004775;Yang2025_Silkie_Wenchang	PRJNA827465	0.797723439
ENSGALG00010000735	ENSGALG00010000735	2	0.027533981	0.453459525	0.060719822	0.951582345	0	0.011342205	0	0.5	CRA004775;PRJNA827465	Yang2025_Silkie_Wenchang	0.987713746
ENSGALG00010000741	FAM105A	2	0.235912585	0.210326222	1.121650847	0.262010923	0.06472621	3.705629656	73.0140329	1	PRJNA827465;Yang2025_Silkie_Wenchang	CRA004775	0.768363052
ENSGALG00010000741	FAM105A	2	0.014013554	0.136850855	0.102400197	0.918439027	0	0.340876187	0	0.5	CRA004775;Yang2025_Silkie_Wenchang	PRJNA827465	0.981033557
ENSGALG00010000741	FAM105A	2	0.260497895	0.329135533	0.791460869	0.428675102	0.118612013	1.729324487	42.17395245	0.5	CRA004775;PRJNA827465	Yang2025_Silkie_Wenchang	0.841695436
ENSGALG00010000753	TRIO	2	0.034409941	0.058877686	0.584430936	0.558930418	0	0.244414952	0	1	PRJNA827465;Yang2025_Silkie_Wenchang	CRA004775	0.880926437
ENSGALG00010000753	TRIO	2	0.200463048	0.166791745	1.201876314	0.229411453	0	0.517962424	0	1	CRA004775;Yang2025_Silkie_Wenchang	PRJNA827465	0.742409084
ENSGALG00010000753	TRIO	2	0.07871965	0.130239386	0.604422761	0.545562607	0.016012066	1.304055772	23.31616321	1	CRA004775;PRJNA827465	Yang2025_Silkie_Wenchang	0.87777334
ENSGALG00010000768	ENSGALG00010000768	2	0.204267393	0.493038557	0.41430308	0.678652159	0.333834783	3.140560575	68.1585508	0.5	PRJNA827465;Yang2025_Silkie_Wenchang	CRA004775	0.917701868
ENSGALG00010000768	ENSGALG00010000768	2	-0.21988356	0.341866744	-0.643184995	0.520104069	0	0.157989177	0	1	CRA004775;Yang2025_Silkie_Wenchang	PRJNA827465	0.869331539
ENSGALG00010000768	ENSGALG00010000768	2	0.419919283	0.335920596	1.250055185	0.211279389	0.055000073	1.275687543	21.61089872	0.5	CRA004775;PRJNA827465	Yang2025_Silkie_Wenchang	0.723589563
ENSGALG00010000777	MYCBP2	2	-0.094495131	0.060895358	-1.551762453	0.120719073	0	0.254361296	0	1	PRJNA827465;Yang2025_Silkie_Wenchang	CRA004775	0.582519549
ENSGALG00010000777	MYCBP2	2	0.005902631	0.151986327	0.03883659	0.969020672	0	0.091678991	0	0.5	CRA004775;Yang2025_Silkie_Wenchang	PRJNA827465	0.991497956
ENSGALG00010000777	MYCBP2	2	-0.099682595	0.064291266	-1.55048425	0.121025331	0	0.334155677	0	0.5	CRA004775;PRJNA827465	Yang2025_Silkie_Wenchang	0.583253909
ENSGALG00010000783	DAP	2	0.088744127	0.21273176	0.417164449	0.676558127	0.07018581	4.102546719	75.62489672	0.5	PRJNA827465;Yang2025_Silkie_Wenchang	CRA004775	0.916786558
ENSGALG00010000783	DAP	2	0.002158674	0.395006909	0.005464901	0.995639661	0.231632451	3.61142996	72.31013723	0.5	CRA004775;Yang2025_Silkie_Wenchang	PRJNA827465	0.998484999
ENSGALG00010000783	DAP	2	-0.112768633	0.087084613	-1.294931782	0.195343764	0	0.921735655	0	1	CRA004775;PRJNA827465	Yang2025_Silkie_Wenchang	0.70643959
ENSGALG00010000784	FBXL3	2	-0.155947323	0.122369455	-1.274397462	0.202522591	0.02210695	3.736462523	73.23671805	1	PRJNA827465;Yang2025_Silkie_Wenchang	CRA004775	0.715289846
ENSGALG00010000784	FBXL3	2	-0.027384848	0.290071916	-0.0944071	0.924785776	0.142190461	6.114228039	83.64470554	0.5	CRA004775;Yang2025_Silkie_Wenchang	PRJNA827465	0.982493782
ENSGALG00010000784	FBXL3	2	0.066244517	0.158234209	0.418648517	0.675473026	0.031784068	2.264887334	55.84769339	0.5	CRA004775;PRJNA827465	Yang2025_Silkie_Wenchang	0.916463558
ENSGALG00010000785	CLN5	2	-0.095435954	0.189492819	-0.503638892	0.614515157	0.060752256	6.396860555	84.36733158	0.5	PRJNA827465;Yang2025_Silkie_Wenchang	CRA004775	0.898614239
ENSGALG00010000785	CLN5	2	-0.257866618	0.111882055	-2.304807669	0.021177347	0	0.557461028	0	1	CRA004775;Yang2025_Silkie_Wenchang	PRJNA827465	0.246205433
ENSGALG00010000785	CLN5	2	0.070499784	0.082697866	0.852498229	0.393937622	0	0.287080003	0	0.5	CRA004775;PRJNA827465	Yang2025_Silkie_Wenchang	0.830486298
ENSGALG00010000786	ANKRD33B	2	0.174076436	0.22055247	0.789274482	0.429951598	0	0.024476129	0	1	PRJNA827465;Yang2025_Silkie_Wenchang	CRA004775	0.842012722
ENSGALG00010000786	ANKRD33B	2	0.123482852	0.299418264	0.412409217	0.680039509	0	0.380887026	0	0.5	CRA004775;Yang2025_Silkie_Wenchang	PRJNA827465	0.917701868
ENSGALG00010000786	ANKRD33B	2	0.081134694	0.270687981	0.299735119	0.764379208	0	0.288578351	0	0.5	CRA004775;PRJNA827465	Yang2025_Silkie_Wenchang	0.940559031
ENSGALG00010000787	SNRNP48	2	-0.079738556	0.103242307	-0.772343801	0.439910829	0	0.873395458	0	0.5	PRJNA827465;Yang2025_Silkie_Wenchang	CRA004775	0.844353254
ENSGALG00010000787	SNRNP48	2	-0.058382663	0.139579551	-0.418275187	0.675745929	0.000331807	1.008397243	0.832731682	0.5	CRA004775;Yang2025_Silkie_Wenchang	PRJNA827465	0.916522765
ENSGALG00010000787	SNRNP48	2	-0.164700227	0.107798191	-1.52785706	0.126548031	0	0.092121302	0	1	CRA004775;PRJNA827465	Yang2025_Silkie_Wenchang	0.597084907
ENSGALG00010000788	GATD3AL1	2	-0.161287851	0.081771822	-1.97241356	0.048562415	0.002404544	1.05257231	4.99465066	1	PRJNA827465;Yang2025_Silkie_Wenchang	CRA004775	0.381043841
ENSGALG00010000788	GATD3AL1	2	-0.366435486	0.212488222	-1.724497867	0.084618004	0	0.161440912	0	1	CRA004775;Yang2025_Silkie_Wenchang	PRJNA827465	0.5001477
ENSGALG00010000788	GATD3AL1	2	-0.14477601	0.067749484	-2.136931537	0.03260356	0	0.196064336	0	1	CRA004775;PRJNA827465	Yang2025_Silkie_Wenchang	0.310045424
ENSGALG00010000789	ROPN1L	2	1.793607257	0.275963942	6.499426145	8.0627E-11	0	0.393733342	0	1	PRJNA827465;Yang2025_Silkie_Wenchang	CRA004775	5.7616E-08
ENSGALG00010000789	ROPN1L	2	1.085340615	1.019706378	1.06436582	0.287163011	1.651472225	4.417552247	77.36302948	0.5	CRA004775;Yang2025_Silkie_Wenchang	PRJNA827465	0.782742686
ENSGALG00010000789	ROPN1L	2	0.937438546	0.837624252	1.119163567	0.263070365	0.980457903	2.975084405	66.38750826	0.5	CRA004775;PRJNA827465	Yang2025_Silkie_Wenchang	0.768643821
ENSGALG00010000790	MARCHF6	2	-0.071643338	0.143958413	-0.497666906	0.618718838	0.037576651	10.65779267	90.61719409	0.5	PRJNA827465;Yang2025_Silkie_Wenchang	CRA004775	0.899168321
ENSGALG00010000790	MARCHF6	2	-0.07503093	0.183645295	-0.408564399	0.682859361	0.049593015	3.25490856	69.27717072	0.5	CRA004775;Yang2025_Silkie_Wenchang	PRJNA827465	0.918793207
ENSGALG00010000790	MARCHF6	2	0.075647026	0.052736938	1.434422046	0.15145186	0	0.193677916	0	1	CRA004775;PRJNA827465	Yang2025_Silkie_Wenchang	0.643006634
ENSGALG00010000791	ACOD1	2	0.982997577	1.121105011	0.876811331	0.380589124	2.396034807	21.33139446	95.31207394	0.5	PRJNA827465;Yang2025_Silkie_Wenchang	CRA004775	0.824392515
ENSGALG00010000791	ACOD1	2	1.043756259	1.118173408	0.933447577	0.350588925	2.255941275	10.02895241	90.02886882	0.5	CRA004775;Yang2025_Silkie_Wenchang	PRJNA827465	0.810111037
ENSGALG00010000791	ACOD1	2	-0.12926908	0.279740582	-0.46210342	0.644007158	0	2.81079E-05	0	1	CRA004775;PRJNA827465	Yang2025_Silkie_Wenchang	0.906826466
ENSGALG00010000795	ENSGALG00010000795	2	0.347164283	0.635119312	0.546612701	0.584644842	0.75467249	15.44521138	93.52550137	0.5	PRJNA827465;Yang2025_Silkie_Wenchang	CRA004775	0.891016154
ENSGALG00010000795	ENSGALG00010000795	2	0.218182337	0.829649424	0.262981364	0.792564943	1.222435073	8.590478754	88.35920525	0.5	CRA004775;Yang2025_Silkie_Wenchang	PRJNA827465	0.948435155
ENSGALG00010000795	ENSGALG00010000795	2	-0.332552621	0.187649847	-1.772197662	0.076361752	0	0.516438834	0	1	CRA004775;PRJNA827465	Yang2025_Silkie_Wenchang	0.476766627
ENSGALG00010000796	ENSGALG00010000796	2	-0.137212382	0.13461892	-1.019265214	0.308077074	0.004583323	1.115472146	10.35186283	0.5	PRJNA827465;Yang2025_Silkie_Wenchang	CRA004775	0.793314108
ENSGALG00010000796	ENSGALG00010000796	2	-0.15152189	0.328076036	-0.461849916	0.644188952	0.100371374	1.688177278	40.76451491	0.5	CRA004775;Yang2025_Silkie_Wenchang	PRJNA827465	0.906873169
ENSGALG00010000796	ENSGALG00010000796	2	-0.255787662	0.138297893	-1.849541275	0.064379693	0	0.643149559	0	1	CRA004775;PRJNA827465	Yang2025_Silkie_Wenchang	0.437453135
ENSGALG00010000797	ENSGALG00010000797	2	-0.128703629	1.023995424	-0.125687699	0.899979139	1.928362186	12.36198739	91.91068581	0.5	PRJNA827465;Yang2025_Silkie_Wenchang	CRA004775	0.976407823
ENSGALG00010000797	ENSGALG00010000797	2	-0.444864054	0.799154596	-0.55666833	0.577754059	0.956983854	3.892493097	74.30952413	0.5	CRA004775;Yang2025_Silkie_Wenchang	PRJNA827465	0.887391761
ENSGALG00010000797	ENSGALG00010000797	2	0.776808052	0.312657222	2.484535768	0.012972048	0	0.343705132	0	1	CRA004775;PRJNA827465	Yang2025_Silkie_Wenchang	0.185261768
ENSGALG00010000799	ENSGALG00010000799	2	-0.216660817	0.094499456	-2.292720249	0.021864116	0	0.002116502	0	1	PRJNA827465;Yang2025_Silkie_Wenchang	CRA004775	0.25067834
ENSGALG00010000799	ENSGALG00010000799	2	-0.104744859	0.264241458	-0.396398278	0.691811236	0	0.795838863	0	0.5	CRA004775;Yang2025_Silkie_Wenchang	PRJNA827465	0.920348122
ENSGALG00010000799	ENSGALG00010000799	2	-0.198432222	0.098085144	-2.023060917	0.043066867	0	0.938990096	0	0.5	CRA004775;PRJNA827465	Yang2025_Silkie_Wenchang	0.358841867
ENSGALG00010000800	LMO7	2	0.147119793	0.277850792	0.529492076	0.59646414	0.138183223	9.257429802	89.1978657	0.5	PRJNA827465;Yang2025_Silkie_Wenchang	CRA004775	0.894064255
ENSGALG00010000800	LMO7	2	0.334323718	0.103392207	3.233548503	0.001222626	0	0.766595156	0	1	CRA004775;Yang2025_Silkie_Wenchang	PRJNA827465	0.042624337
ENSGALG00010000800	LMO7	2	0.057255031	0.185963003	0.307883988	0.75817061	0.056504296	5.327203451	81.22842484	0.5	CRA004775;PRJNA827465	Yang2025_Silkie_Wenchang	0.938954369
ENSGALG00010000802	SEMA5A	2	0.014808578	0.079161402	0.187068163	0.851607189	0	4.89388E-06	0	1	PRJNA827465;Yang2025_Silkie_Wenchang	CRA004775	0.96214473
ENSGALG00010000802	SEMA5A	2	0.033704422	0.149629571	0.225252416	0.821782915	0	0.192855884	0	1	CRA004775;Yang2025_Silkie_Wenchang	PRJNA827465	0.95563486
ENSGALG00010000802	SEMA5A	2	0.021551155	0.0907071	0.237590611	0.812198633	0	0.203275744	0	1	CRA004775;PRJNA827465	Yang2025_Silkie_Wenchang	0.953371387
ENSGALG00010000803	MTRR	2	-0.047937144	0.141014293	-0.339945282	0.733897735	0.03069864	4.376302906	77.14966213	0.5	PRJNA827465;Yang2025_Silkie_Wenchang	CRA004775	0.933391141
ENSGALG00010000803	MTRR	2	-0.205142189	0.090330131	-2.271027256	0.023145328	0	0.090365587	0	1	CRA004775;Yang2025_Silkie_Wenchang	PRJNA827465	0.259577892
ENSGALG00010000803	MTRR	2	-0.034992773	0.168448482	-0.207735756	0.835435297	0.035788226	2.365910692	57.73297769	0.5	CRA004775;PRJNA827465	Yang2025_Silkie_Wenchang	0.958339923
ENSGALG00010000805	FASTKD3	2	0.006277168	0.067686462	0.092738898	0.926110994	0	0.948019295	0	0.5	PRJNA827465;Yang2025_Silkie_Wenchang	CRA004775	0.98280842
ENSGALG00010000805	FASTKD3	2	-0.052449805	0.10760661	-0.487421776	0.625959465	0	0.460332043	0	0.5	CRA004775;Yang2025_Silkie_Wenchang	PRJNA827465	0.901504244
ENSGALG00010000805	FASTKD3	2	0.049062919	0.073931164	0.663629741	0.506927317	0	0.003725221	0	1	CRA004775;PRJNA827465	Yang2025_Silkie_Wenchang	0.863929781
ENSGALG00010000806	CFAP90	2	0.440721343	0.390763788	1.127845919	0.259384997	0.234972166	4.334694141	76.93032155	1	PRJNA827465;Yang2025_Silkie_Wenchang	CRA004775	0.766684856
ENSGALG00010000806	CFAP90	2	0.426086201	0.416205049	1.023741067	0.305957593	0.267289949	4.373136391	77.13311659	1	CRA004775;Yang2025_Silkie_Wenchang	PRJNA827465	0.792366985
ENSGALG00010000806	CFAP90	2	0.032027273	0.193163892	0.165803622	0.868311495	0	0.017139559	0	1	CRA004775;PRJNA827465	Yang2025_Silkie_Wenchang	0.968121378
ENSGALG00010000808	ADCY2	2	0.179492038	0.144709996	1.240356873	0.214843425	0.02194954	2.096962066	52.3119652	1	PRJNA827465;Yang2025_Silkie_Wenchang	CRA004775	0.728055797
ENSGALG00010000808	ADCY2	2	0.310109724	0.140988851	2.199533667	0.027839998	0	0.216393679	0	1	CRA004775;Yang2025_Silkie_Wenchang	PRJNA827465	0.28442824
ENSGALG00010000808	ADCY2	2	0.045252089	0.129796985	0.348637444	0.727361513	0	0.014179571	0	1	CRA004775;PRJNA827465	Yang2025_Silkie_Wenchang	0.931522817
ENSGALG00010000810	UCHL3	2	-0.047790498	0.066563375	-0.717969872	0.472775864	0	0.089239857	0	1	PRJNA827465;Yang2025_Silkie_Wenchang	CRA004775	0.854086678
ENSGALG00010000810	UCHL3	2	-0.089873027	0.120284089	-0.747173029	0.454959122	0	0.693997514	0	1	CRA004775;Yang2025_Silkie_Wenchang	PRJNA827465	0.849072665
ENSGALG00010000810	UCHL3	2	-0.077416068	0.068324475	-1.133064943	0.257186982	0	0.57101883	0	1	CRA004775;PRJNA827465	Yang2025_Silkie_Wenchang	0.764870705
ENSGALG00010000811	ENSGALG00010000811	2	-0.470964186	0.300546101	-1.5670281	0.117108124	0	0.744019811	0	1	PRJNA827465;Yang2025_Silkie_Wenchang	CRA004775	0.576418357
ENSGALG00010000811	ENSGALG00010000811	2	-0.767277447	0.44756055	-1.71435451	0.086463638	0	0.016250805	0	1	CRA004775;Yang2025_Silkie_Wenchang	PRJNA827465	0.506036629
ENSGALG00010000811	ENSGALG00010000811	2	-0.284619776	0.37361435	-0.761800975	0.446178799	0	0.059864097	0	1	CRA004775;PRJNA827465	Yang2025_Silkie_Wenchang	0.846013379
ENSGALG00010000812	ENSGALG00010000812	2	-0.32419733	0.281532877	-1.151543412	0.249508746	0.147756105	14.33728265	93.02517761	1	PRJNA827465;Yang2025_Silkie_Wenchang	CRA004775	0.759622031
ENSGALG00010000812	ENSGALG00010000812	2	-0.219844297	0.409967108	-0.536248624	0.591786733	0.308884483	12.22239909	91.81830022	0.5	CRA004775;Yang2025_Silkie_Wenchang	PRJNA827465	0.892776493
ENSGALG00010000812	ENSGALG00010000812	2	0.00933769	0.111169272	0.083995244	0.933060213	0.012457266	1.608475468	37.82932847	0.5	CRA004775;PRJNA827465	Yang2025_Silkie_Wenchang	0.984247176
ENSGALG00010000813	ENSGALG00010000813	2	-0.396675937	0.17721094	-2.23843932	0.02519242	0	0.116327909	0	1	PRJNA827465;Yang2025_Silkie_Wenchang	CRA004775	0.270285761
ENSGALG00010000813	ENSGALG00010000813	2	-0.548086944	0.235721399	-2.325147167	0.020064092	0	0.325505574	0	1	CRA004775;Yang2025_Silkie_Wenchang	PRJNA827465	0.238261187
ENSGALG00010000813	ENSGALG00010000813	2	-0.432225811	0.207365777	-2.08436424	0.037127048	0	0.689493956	0	1	CRA004775;PRJNA827465	Yang2025_Silkie_Wenchang	0.332241432

ENSGALG00010000815	ENSGALG00010000815	2	0.269900235	0.239475027	1.127049607	0.259721508	0.097225495	6.423758912	84.43279062	1	PRJNA827465;Yang2025_Silkie_Wenchang	CRA004775	0.767047318
ENSGALG00010000815	ENSGALG00010000815	2	-0.096851296	0.634994673	-0.152523005	0.878774447	0.764666381	19.15154278	94.77848855	0.5	CRA004775;Yang2025_Silkie_Wenchang	PRJNA827465	0.971109264
ENSGALG00010000815	ENSGALG00010000815	2	-0.319535871	0.393997761	-0.811009357	0.417360297	0.277453333	8.952168837	88.82952256	0.5	CRA004775;PRJNA827465	Yang2025_Silkie_Wenchang	0.838112595
ENSGALG00010000816	TBC1D4	2	-0.090388313	0.089726785	-1.007372688	0.313755711	0.004354357	1.313516419	23.86848113	0.5	PRJNA827465;Yang2025_Silkie_Wenchang	CRA004775	0.796531448
ENSGALG00010000816	TBC1D4	2	0.014323115	0.128308828	0.111630005	0.91111678	0	0.150451102	0	0.5	CRA004775;Yang2025_Silkie_Wenchang	PRJNA827465	0.978845851
ENSGALG00010000816	TBC1D4	2	-0.148320132	0.083917916	-1.767442984	0.07715407	0	0.039507146	0	1	CRA004775;PRJNA827465	Yang2025_Silkie_Wenchang	0.478533748
ENSGALG00010000817	ENSGALG00010000817	2	-0.163589586	0.076540035	-2.137307425	0.032572995	0	0.011322886	0	1	PRJNA827465;Yang2025_Silkie_Wenchang	CRA004775	0.310037591
ENSGALG00010000817	ENSGALG00010000817	2	-0.114812456	0.212627846	-0.53996905	0.589218377	0	0.014442908	0	1	CRA004775;Yang2025_Silkie_Wenchang	PRJNA827465	0.892171138
ENSGALG00010000817	ENSGALG00010000817	2	-0.161584538	0.077572282	-2.083019013	0.037249491	0	0.056843497	0	1	CRA004775;PRJNA827465	Yang2025_Silkie_Wenchang	0.333009775
ENSGALG00010000818	KLF12	2	-0.052991187	0.088645043	-0.597790759	0.549979558	0	0.16484614	0	0.5	PRJNA827465;Yang2025_Silkie_Wenchang	CRA004775	0.878652984
ENSGALG00010000818	KLF12	2	0.0999548	0.190532216	0.524608397	0.599855448	0	0.344034365	0	1	CRA004775;Yang2025_Silkie_Wenchang	PRJNA827465	0.895092498
ENSGALG00010000818	KLF12	2	-0.045283675	0.093241012	-0.485662631	0.627206383	0	0.868263732	0	0.5	CRA004775;PRJNA827465	Yang2025_Silkie_Wenchang	0.901583369
ENSGALG00010000820	KLF5	2	-0.208213331	0.13151504	-1.583190261	0.113378106	0.011541474	1.379512588	27.51062887	1	PRJNA827465;Yang2025_Silkie_Wenchang	CRA004775	0.568380061
ENSGALG00010000820	KLF5	2	0.553260761	0.614649603	0.90012384	0.36805435	0.638921209	6.140250877	83.71402049	0.5	CRA004775;Yang2025_Silkie_Wenchang	PRJNA827465	0.820213684
ENSGALG00010000820	KLF5	2	0.408638086	0.760089035	0.53761871	0.590840311	1.056324285	10.89759885	90.82366663	0.5	CRA004775;PRJNA827465	Yang2025_Silkie_Wenchang	0.892752146
ENSGALG00010000821	ENSGALG00010000821	2	-0.138509351	0.121109428	-1.143671082	0.252760071	0	0.817469541	0	1	PRJNA827465;Yang2025_Silkie_Wenchang	CRA004775	0.762731469
ENSGALG00010000821	ENSGALG00010000821	2	-0.2638646	0.161847977	-1.630323744	0.103033092	0	0.167897579	0	1	CRA004775;Yang2025_Silkie_Wenchang	PRJNA827465	0.547231029
ENSGALG00010000821	ENSGALG00010000821	2	-0.064131006	0.166713976	-0.384676846	0.700476852	0	0.595072511	0	1	CRA004775;PRJNA827465	Yang2025_Silkie_Wenchang	0.922303014
ENSGALG00010000822	BASP1	2	-0.019471971	0.15209294	-0.128026792	0.89812777	0.030837889	2.629457328	61.9693391	0.5	PRJNA827465;Yang2025_Silkie_Wenchang	CRA004775	0.975733636
ENSGALG00010000822	BASP1	2	0.170891308	0.16493545	1.036110236	0.300150717	0	0.017217532	0	1	CRA004775;Yang2025_Silkie_Wenchang	PRJNA827465	0.790301041
ENSGALG00010000822	BASP1	2	-0.12740966	0.074508272	-1.71000691	0.087264595	0	0.355459413	0	0.5	CRA004775;PRJNA827465	Yang2025_Silkie_Wenchang	0.507886922
ENSGALG00010000823	PIBF1	2	-0.417570025	0.246963413	-1.690817357	0.090871691	0.109495528	9.660334417	89.64839149	1	PRJNA827465;Yang2025_Silkie_Wenchang	CRA004775	0.517213717
ENSGALG00010000823	PIBF1	2	-0.289712511	0.415237177	-0.697703693	0.485362518	0.302732663	7.841837535	87.24788679	0.5	CRA004775;Yang2025_Silkie_Wenchang	PRJNA827465	0.857076964
ENSGALG00010000823	PIBF1	2	-0.10319164	0.141401467	-0.729777713	0.465526069	0.017523916	1.439547261	30.53371523	0.5	CRA004775;PRJNA827465	Yang2025_Silkie_Wenchang	0.852424459
ENSGALG00010000843	DIS3	2	0.01837701	0.076344618	0.240711274	0.809778901	0.005440206	1.801527962	44.49156376	0.5	PRJNA827465;Yang2025_Silkie_Wenchang	CRA004775	0.952818762
ENSGALG00010000843	DIS3	2	0.117235503	0.086051295	1.362390926	0.173074546	0	0.007055806	0	1	CRA004775;Yang2025_Silkie_Wenchang	PRJNA827465	0.676043418
ENSGALG00010000843	DIS3	2	-0.024258242	0.060838415	-0.398732306	0.690090461	0	0.783288398	0	0.5	CRA004775;PRJNA827465	Yang2025_Silkie_Wenchang	0.919979266
ENSGALG00010000853	ENSGALG00010000853	2	-0.01746209	0.109563766	-0.159378327	0.873370814	0	0.039835305	0	0.5	PRJNA827465;Yang2025_Silkie_Wenchang	CRA004775	0.969910234
ENSGALG00010000853	ENSGALG00010000853	2	-0.277323694	0.37521292	-0.739110194	0.459840087	0.183337873	2.604571621	61.60597037	0.5	CRA004775;Yang2025_Silkie_Wenchang	PRJNA827465	0.850098539
ENSGALG00010000853	ENSGALG00010000853	2	-0.275961345	0.341964702	-0.806987808	0.419673508	0.160789806	2.610554209	61.69395768	1	CRA004775;PRJNA827465	Yang2025_Silkie_Wenchang	0.838527387
ENSGALG00010000857	ENSGALG00010000857	2	-0.647681361	0.528874441	-1.22464107	0.220710487	0.382838916	3.159955884	68.35398858	1	PRJNA827465;Yang2025_Silkie_Wenchang	CRA004775	0.734053249
ENSGALG00010000857	ENSGALG00010000857	2	-1.11182665	0.388778153	-2.859797142	0.004239121	0	0.152781298	0	1	CRA004775;Yang2025_Silkie_Wenchang	PRJNA827465	0.095151436
ENSGALG00010000857	ENSGALG00010000857	2	-0.272450252	0.356670978	-0.763869977	0.44494473	0	0.599663931	0	1	CRA004775;PRJNA827465	Yang2025_Silkie_Wenchang	0.846013379
ENSGALG00010000859	BORA	2	0.061057613	0.064935819	0.940276324	0.347075841	0	0.602181761	0	0.5	PRJNA827465;Yang2025_Silkie_Wenchang	CRA004775	0.808602357
ENSGALG00010000859	BORA	2	0.007764783	0.113380018	0.068484584	0.945399891	0	0.591900015	0	0.5	CRA004775;Yang2025_Silkie_Wenchang	PRJNA827465	0.986321197
ENSGALG00010000859	BORA	2	0.101851465	0.07695514	1.323517383	0.185663379	0	0.300588817	0	1	CRA004775;PRJNA827465	Yang2025_Silkie_Wenchang	0.694265174
ENSGALG00010000864	MZT1	2	-0.436653832	0.518518904	-0.842117479	0.399722198	0.5279068	54.56008437	98.16715826	0.5	PRJNA827465;Yang2025_Silkie_Wenchang	CRA004775	0.832246851
ENSGALG00010000864	MZT1	2	-0.654375802	0.348786208	-1.8761516	0.060634466	0.200371757	5.237874113	80.90828495	1	CRA004775;Yang2025_Silkie_Wenchang	PRJNA827465	0.426470368
ENSGALG00010000864	MZT1	2	0.023082434	0.123138883	0.187450408	0.851307503	0.012867278	1.301511805	23.16627506	0.5	CRA004775;PRJNA827465	Yang2025_Silkie_Wenchang	0.962071965
ENSGALG00010000870	DACH1	2	0.108514388	0.104518946	1.038226962	0.299164403	0	0.159102921	0	1	PRJNA827465;Yang2025_Silkie_Wenchang	CRA004775	0.789767499
ENSGALG00010000870	DACH1	2	0.148909833	0.2180139	0.683029075	0.494588471	0.016664336	1.131614015	11.63064552	1	CRA004775;Yang2025_Silkie_Wenchang	PRJNA827465	0.860238557
ENSGALG00010000870	DACH1	2	0.161219124	0.1167879	1.380443729	0.167450064	0	0.859829871	0	1	CRA004775;PRJNA827465	Yang2025_Silkie_Wenchang	0.667202947
ENSGALG00010001066	SLC33A1	2	-0.165678093	0.232385012	-0.712946552	0.475878831	0.09376195	7.119182708	85.9534438	0.5	PRJNA827465;Yang2025_Silkie_Wenchang	CRA004775	0.854742058
ENSGALG00010001066	SLC33A1	2	-0.293881857	0.158228382	-1.857327077	0.063264634	0.01539384	1.417542697	29.4553877	1	CRA004775;Yang2025_Silkie_Wenchang	PRJNA827465	0.433102794
ENSGALG00010001066	SLC33A1	2	0.032276659	0.063945447	0.504753037	0.613732304	0	0.397296592	0	0.5	CRA004775;PRJNA827465	Yang2025_Silkie_Wenchang	0.898256044
ENSGALG00010001070	C3orf33	2	0.059149648	0.337135619	0.175447637	0.860727912	0.202521381	9.142872646	89.06251855	0.5	PRJNA827465;Yang2025_Silkie_Wenchang	CRA004775	0.965141911
ENSGALG00010001070	C3orf33	2	0.054598805	0.414140732	0.131836356	0.89511373	0.263517628	3.9248258	74.52116219	0.5	CRA004775;Yang2025_Silkie_Wenchang	PRJNA827465	0.974641371
ENSGALG00010001070	C3orf33	2	-0.292226453	0.13533408	-2.159296846	0.030827145	0	0.161233869	0	1	CRA004775;PRJNA827465	Yang2025_Silkie_Wenchang	0.299826595
ENSGALG00010001082	P2RY1	2	-0.001291501	0.103490928	-0.012479361	0.990043169	0	0.543883089	0	0.5	PRJNA827465;Yang2025_Silkie_Wenchang	CRA004775	0.997368295
ENSGALG00010001082	P2RY1	2	0.046066348	0.141258157	0.326114607	0.744337646	0	0.41941838	0	0.5	CRA004775;Yang2025_Silkie_Wenchang	PRJNA827465	0.935821071
ENSGALG00010001082	P2RY1	2	-0.088110612	0.135276035	-0.651339409	0.514827414	0	0.093528264	0	1	CRA004775;PRJNA827465	Yang2025_Silkie_Wenchang	0.866930857
ENSGALG00010001104	MBNL1	2	-0.060127263	0.056595533	-1.062402996	0.288052774	0	0.832693978	0	0.5	PRJNA827465;Yang2025_Silkie_Wenchang	CRA004775	0.782840067
ENSGALG00010001104	MBNL1	2	0.077926432	0.116852662	0.666877682	0.504850268	0.003824612	1.080465439	7.447294106	1	CRA004775;Yang2025_Silkie_Wenchang	PRJNA827465	0.863602458
ENSGALG00010001104	MBNL1	2	0.039245428	0.200184966	0.19604583	0.844574295	0.052619851	2.209754938	54.74611311	0.5	CRA004775;PRJNA827465	Yang2025_Silkie_Wenchang	0.960769014
ENSGALG00010001111	SUCNR1	2	0.622154187	0.270136007	2.303114617	0.021272395	0	0.207257042	0	1	PRJNA827465;Yang2025_Silkie_Wenchang	CRA004775	0.246628007
ENSGALG00010001111	SUCNR1	2	0.609975006	0.340008662	1.793998433	0.072813419	0	0.467692488	0	1	CRA004775;Yang2025_Silkie_Wenchang	PRJNA827465	0.466088893
ENSGALG00010001111	SUCNR1	2	0.445310108	0.315065714	1.413388027	0.157541664	0	0.132391819	0	1	CRA004775;PRJNA827465	Yang2025_Silkie_Wenchang	0.650796198
ENSGALG00010001112	TSC22D2	2	-0.044342004	0.073752653	-0.60122588	0.547689549	0.002833644	1.284690224	22.16022341	0.5	PRJNA827465;Yang2025_Silkie_Wenchang	CRA004775	0.878281266
ENSGALG00010001112	TSC22D2	2	-0.144532568	0.103094525	-1.401942234	0.160932497	0	0.02663277	0	1	CRA004775;Yang2025_Silkie_Wenchang	PRJNA827465	0.657013704
ENSGALG00010001112	TSC22D2	2	-0.009872578	0.066461601	-0.148545587	0.881912212	0	0.357347789	0	0.5	CRA004775;PRJNA827465	Yang2025_Silkie_Wenchang	0.971928118
ENSGALG00010001113	IGSF10	2	0.535624083	0.513387215	1.043314029	0.296802876	0.42613756	5.071444232	80.28175103	1	PRJNA827465;Yang2025_Silkie_Wenchang	CRA004775	0.788458494
ENSGALG00010001113	IGSF10	2	0.641590654	0.510155606	1.257637172	0.208523003	0.315798818	2.487021737	59.79126417	1	CRA004775;Yang2025_Silkie_Wenchang	PRJNA827465	0.721011841
ENSGALG00010001113	IGSF10	2	0.063748306	0.222802772	0.286119895	0.774786294	0	1.94671E-05	0	1	CRA004775;PRJNA827465	Yang2025_Silkie_Wenchang	0.943713409
ENSGALG00010001114	MED12L	2	0.409691444	0.387331604	1.057727899	0.290179512	0.288200318	25.17998054	96.02859105	1	PRJNA827465;Yang2025_Silkie_Wenchang	CRA004775	0.784195688
ENSGALG00010001114	MED12L	2	0.388089997	0.432033748	0.8982863	0.369032935	0.342281494	11.81662493	91.5373467	0.5	CRA004775;Yang2025_Silkie_Wenchang	PRJNA827465	0.820553374
ENSGALG00010001114	MED12L	2	0.015842679	0.079989549	0.198059356	0.842998624	0	0.149362815	0	0.5	CRA004775;PRJNA827465	Yang2025_Silkie_Wenchang	0.960416186
ENSGALG00010001115	PFN2	2	-0.103277024	0.138267703	-0.746935267	0.455102637	0.026599487	2.664919617	62.47541601	1	PRJNA827465;Yang2025_Silkie_Wenchang	CRA004775	0.849072665
ENSGALG00010001115	PFN2	2	-0.243921074	0.135613063	-1.798654707	0.072073318	0	0.240427524	0	1	CRA004775;Yang2025_Silkie_Wenchang	PRJNA827465	0.463464923
ENSGALG00010001115	PFN2	2	-0.011179651	0.047663109	-0.234555644	0.81455364	0	0.468834272	0	1	CRA004775;PRJNA827465	Yang2025_Silkie_Wenchang	0.953869515
ENSGALG00010001116	RNF13	2	-0.196296	0.183795068	-1.068015598	0.285513476	0.065340649	30.14988637	96.68323791	1	PRJNA827465;Yang2025_Silkie_Wenchang	CRA004775	0.781226409
ENSGALG00010001116	RNF13	2	-0.164306744	0.234981498	-0.699232685	0.484406628	0.099767387	9.89940348	89.89838123	0.5	CRA004775;Yang2025_Silkie_Wenchang	PRJNA827465	0.857004733
ENSGALG00010001116	RNF13	2	-0.010276087	0.031572932	-0.325471428	0.744824304	0	0.534225096	0	0.5	CRA004775;PRJNA827465	Yang2025_Silkie_Wenchang	0.935893304
ENSGALG00010001117	P2RY13	2	-0.067229869	0.178670247	-0.376279038	0.706709461	0	0.198974225	0	1	PRJNA827465;Yang2025_Silkie_Wenchang	CRA004775	0.924553846
ENSGALG00010001117	P2RY13	2	-0.088339841	0.251087849	-0.35182842	0.724966943	0	0.337453216	0	0.5	CRA004775;Yang2025_Silkie_Wenchang	PRJNA827465	0.930608654
ENSGALG00010001117	P2RY13	2	0.026518499	0.211000111	0.125680023	0.899985216	0	0.105194935	0	0.5	CRA004775;PRJNA827465	Yang2025_Silkie_Wenchang	0.976407823
ENSGALG00010001118	P2RY12	2	-0.314253393	0.182730901	-1.719760541	0.085475978	0	0.27279227	0	1	PRJNA827465;Yang2025_Silkie_Wenchang	CRA004775	0.502675386
ENSGALG00010001118	P2RY12	2	-0.118206599	0.292077523	-0.404709675	0.68569093	0	0.131007329	0	0.5	CRA004775;Yang2025_Silkie_Wenchang	PRJNA827465	0.919235539
ENSGALG00010001118	P2RY12	2	-0.329317373	0.208236092	-1.581461553	0.113772543	0	0.473015866	0	0.5	CRA004775;PRJNA827465	Yang2025_Silkie_Wenchang	0.568978529
ENSGALG00010001119	COMMD2	2	-0.096500094	0.165474149	-0.583173229	0.559776691	0.0499895	11.29527578	91.14674117	0.5	PRJNA827465;Yang2025_Silkie_Wenchang	CRA004775	0.881203589
ENSGALG00010001119	COMMD2	2	-0.261607346	0.076195056	-3.433390038	0.000596084	0	0.040591081	0	1	CRA004775;Yang2025_Silkie_Wenchang	PRJNA827465	0.026382755
ENSGALG00010001119	COMMD2	2	-0.027235338	0.134162718	-0.203002281	0.83913325	0.023350241	2.247392786	55.50399528	0.5	CRA004775;PRJNA827465	Yang2025_Silkie_Wenchang	0.959403482
ENSGALG00010001121	WWTR1	2	0.029709299	0.053018621	0.560355926	0.575236688	0	0.045031839	0	1	PRJNA827465;Yang2025_Silkie_Wenchang	CRA004775	0.88644908
ENSGALG00010001121	WWTR1	2	0.103352092	0.135039215	0.765348735	0.444063911	0	0.482471588	0	1	CRA004775;Yang2025_Silkie_Wenchang	PRJNA827465	0.846013379
ENSGALG00010001121	WWTR1	2	0.034009845	0.055838146	0.609079053	0.542472042	0	0.740282251	0	1	CRA004775;PRJNA827465	Yang2025_Silkie_Wenchang	0.876817086
ENSGALG00010001122	P2RY14	2	-0.534334315	0.731247734	-0.730715857	0.464952728	0.989801424	13.42758716	92.55264562	0.5	PRJNA827465;Yang2025_Silkie_Wenchang	CRA004775	0.852233226
ENSGALG00010001122	P2RY14	2	0.078638792	0.233199835	0.337216327	0.73595383	0	0.564538957	0	0.5	CRA004775;Yang2025_Silkie_Wenchang	PRJNA827465	0.933592889
ENSGALG00010001122	P2RY14	2	-0.76751569	0.540295998	-1.420546686	0.155448587	0.460128594	4.608903985	78.30286759	1	CRA004775;PRJNA827465	Yang2025_Silkie_Wenchang	0.648167852
ENSGALG00010001123	CP	2	0.364978285	0.335594314	1.08755801	0.276790276	0.207683394	12.60735303	92.0681209	1	PRJNA827465;Yang2025_Silkie_Wenchang	CRA004775	0.775940673
ENSGALG00010001123	CP	2	0.67333489	0.151535601	4.443410557	8.8544E-06	0	0.458803348	0	1	CRA004775;Yang2025_Silkie_Wenchang	PRJNA827465	0.001270089
ENSGALG00010001123	CP	2	0.06091089	0.093840465	0.649089814	0.516280325	0	0.755424109	0	1	CRA004775;PRJNA827465	Yang2025_Silkie_Wenchang	0.867685411
ENSGALG00010001125	CLRN1	2	-0.14383342	0.149201016	-0.9640244	0.335033693	0	0.030819478	0	1	PRJNA827465;Yang2025_Silkie_Wenchang	CRA004775	0.803101658
ENSGALG00010001125	CLRN1	2	-0.178844509	0.212562211	-0.8413749	0.400137942	0	0.299772517	0	1	CRA004775;Yang2025_Silkie_Wenchang	PRJNA827465	0.832246851
ENSGALG00010001125	CLRN1	2	-0.199105856	0.170285133	-1.169249785	0.242303008	0	0.2130691	0	1	CRA004775;PRJNA827465	Yang2025_Silkie_Wenchang	0.754674311
ENSGALG00010001127	SIAH2	2	-0.118249928	0.065288071	-1.811202675	0.070109484	0	0.042216409	0	1	PRJNA827465;Yang2025_Silkie_Wenchang	CRA004775	0.456815825
ENSGALG00010001127	SIAH2	2	0.004528948	0.153541309	0.029496609	0.976468524	0.033078414	3.258428665	69.31036083	0.5	CRA004775;Yang2025_Silkie_Wenchang	PRJNA827465	0.993126926
ENSGALG00010001127	SIAH2	2	0.013522184	0.139361674	0.097029432	0.922703021	0.025922546	2.873003043	65.19321473	0.5	CRA004775;PRJNA827465	Yang2025_Silkie_Wenchang	0.981804675
ENSGALG00010001131	HPS3	2	0.127103986	0.111411429	1.140852311	0.253931388	0.015618943	2.680273652	62.69037681	1	PRJNA827465;Yang2025_Silkie_Wenchang	CRA004775	0.763717886
ENSGALG00010001131	HPS3	2	0.105185168	0.171712893	0.61256418	0.540164549	0.038358088	2.662880403	62.44667996	0.5	CRA004775;Yang2025_Silkie_Wenchang	PRJNA827465	0.87529516
ENSGALG00010001131	HPS3	2	-0.000838135	0.079949581	-0.010483293	0.991635696	0	0.375424592	0	0.5	CRA004775;PRJNA827465	Yang2025_Silkie_Wenchang	0.997575757

ENSGALG00010001169	ENSGALG00010001169	2	0.019840689	0.184856571	0.10733018	0.914527042	0.051989635	4.175090122	76.04842121	0.5	CRA004775;Yang2025_Silkie_Wenchang	PRJNA827465	0.979711523
ENSGALG00010001169	ENSGALG00010001169	2	-0.056271112	0.254745039	-0.220891884	0.825176617	0.117130197	10.12891427	90.12727353	0.5	CRA004775;PRJNA827465	Yang2025_Silkie_Wenchang	0.956355172
ENSGALG00010001170	DCAF10	2	-0.043071327	0.097910291	-0.43990603	0.660005168	0	0.07275046	0	1	PRJNA827465;Yang2025_Silkie_Wenchang	CRA004775	0.911785463
ENSGALG00010001170	DCAF10	2	-0.050566978	0.129202137	-0.391378806	0.695517256	0	0.237717268	0	1	CRA004775;Yang2025_Silkie_Wenchang	PRJNA827465	0.921038437
ENSGALG00010001170	DCAF10	2	-0.084024528	0.113746269	-0.738701396	0.460088338	0	0.099329113	0	1	CRA004775;PRJNA827465	Yang2025_Silkie_Wenchang	0.850104868
ENSGALG00010001171	POLR1E	2	0.088619117	0.122693078	0.722282935	0.470120542	0	0.522689487	0	1	PRJNA827465;Yang2025_Silkie_Wenchang	CRA004775	0.853648049
ENSGALG00010001171	POLR1E	2	-0.00512749	0.216100553	-0.023727335	0.981070102	0.050009049	2.10963384	52.59840922	0.5	CRA004775;Yang2025_Silkie_Wenchang	PRJNA827465	0.990928779
ENSGALG00010001171	POLR1E	2	-0.078470339	0.137450287	-0.570899783	0.568067578	0	0.777100012	0	0.5	CRA004775;PRJNA827465	Yang2025_Silkie_Wenchang	0.883948057
ENSGALG00010001172	TMEM200C	2	-0.143192414	0.126089115	-1.135644536	0.256105362	0	0.423072714	0	1	PRJNA827465;Yang2025_Silkie_Wenchang	CRA004775	0.764570254
ENSGALG00010001172	TMEM200C	2	-0.376511517	0.235974375	-1.595561029	0.110586834	0	0.431557121	0	1	CRA004775;Yang2025_Silkie_Wenchang	PRJNA827465	0.562832796
ENSGALG00010001172	TMEM200C	2	-0.177698046	0.197476756	-0.899842847	0.368203889	0.024679593	1.175766949	14.94913164	1	CRA004775;PRJNA827465	Yang2025_Silkie_Wenchang	0.820213684
ENSGALG00010001173	IFT80	2	-0.187378856	0.102387593	-1.830093374	0.067235978	0.010886342	2.066589465	51.61109562	1	PRJNA827465;Yang2025_Silkie_Wenchang	CRA004775	0.446758347
ENSGALG00010001173	IFT80	2	-0.274796294	0.094057486	-2.921578139	0.003482629	0	0.180312984	0	1	CRA004775;Yang2025_Silkie_Wenchang	PRJNA827465	0.083407175
ENSGALG00010001173	IFT80	2	-0.114975064	0.082979353	-1.385586414	0.165873229	0	0.295737662	0	1	CRA004775;PRJNA827465	Yang2025_Silkie_Wenchang	0.69529196
ENSGALG00010001174	ENSGALG00010001174	2	-0.057504473	0.049564496	-1.160194848	0.245969484	0	0.752736111	0	1	PRJNA827465;Yang2025_Silkie_Wenchang	CRA004775	0.75661406
ENSGALG00010001174	ENSGALG00010001174	2	-0.121591273	0.078845806	-1.542140032	0.123039569	0	0.331542576	0	1	CRA004775;Yang2025_Silkie_Wenchang	PRJNA827465	0.58801364
ENSGALG00010001174	ENSGALG00010001174	2	-0.031676218	0.06217064	-0.509504463	0.610398671	0	0.638648365	0	1	CRA004775;PRJNA827465	Yang2025_Silkie_Wenchang	0.897438028
ENSGALG00010001177	SEH1L	2	0.063107586	0.047226778	1.336267017	0.181462013	0	0.33718131	0	1	PRJNA827465;Yang2025_Silkie_Wenchang	CRA004775	0.689004635
ENSGALG00010001177	SEH1L	2	-0.056846576	0.184906767	-0.307433724	0.758513262	0.052497114	3.950087596	74.68410571	0.5	CRA004775;Yang2025_Silkie_Wenchang	PRJNA827465	0.938954369
ENSGALG00010001177	SEH1L	2	-0.075579266	0.153582402	-0.492108895	0.622642364	0.034063167	3.07273785	67.45573333	0.5	CRA004775;PRJNA827465	Yang2025_Silkie_Wenchang	0.900255255
ENSGALG00010001178	ZBTB5	2	-0.056407501	0.094073173	-0.599613034	0.548764158	0	0.803342457	0	0.5	PRJNA827465;Yang2025_Silkie_Wenchang	CRA004775	0.878281266
ENSGALG00010001178	ZBTB5	2	-0.153720583	0.12183242	-1.261737905	0.207043113	0	0.040758104	0	1	CRA004775;Yang2025_Silkie_Wenchang	PRJNA827465	0.718979983
ENSGALG00010001178	ZBTB5	2	-0.01366301	0.119969723	-0.113887152	0.90932725	0	0.536818117	0	0.5	CRA004775;PRJNA827465	Yang2025_Silkie_Wenchang	0.978607662
ENSGALG00010001179	GRHPR	2	-0.057839424	0.486157982	-0.118972487	0.905297153	0.397031731	6.21947943	83.92148392	0.5	PRJNA827465;Yang2025_Silkie_Wenchang	CRA004775	0.977980705
ENSGALG00010001179	GRHPR	2	-0.773572824	0.28901232	-2.676608471	0.007437149	0.040030588	1.28197397	21.99529602	1	CRA004775;Yang2025_Silkie_Wenchang	PRJNA827465	0.132925083
ENSGALG00010001179	GRHPR	2	-0.331825688	0.786912359	-0.421680616	0.673258155	1.113747321	9.686807071	89.67668095	0.5	CRA004775;PRJNA827465	Yang2025_Silkie_Wenchang	0.915223605
ENSGALG00010001180	SPIRE1	2	0.396287694	0.260402378	1.521828246	0.128052122	0.094745649	3.068419352	67.40993048	1	PRJNA827465;Yang2025_Silkie_Wenchang	CRA004775	0.600542912
ENSGALG00010001180	SPIRE1	2	0.293356914	0.437934196	0.669865282	0.502943673	0.294113916	4.256398274	76.50595796	0.5	CRA004775;Yang2025_Silkie_Wenchang	PRJNA827465	0.863050365
ENSGALG00010001180	SPIRE1	2	0.128100138	0.128497472	0.99690784	0.318809246	0	0.939015219	0	0.5	CRA004775;PRJNA827465	Yang2025_Silkie_Wenchang	0.797974202
ENSGALG00010001181	MFSD1	2	-0.043703462	0.123814473	-0.352975389	0.724106891	0.026348034	6.954440063	85.62069712	0.5	PRJNA827465;Yang2025_Silkie_Wenchang	CRA004775	0.930355448
ENSGALG00010001181	MFSD1	2	-0.177472491	0.070301341	-2.524453863	0.011587821	0	0.003634847	0	1	CRA004775;Yang2025_Silkie_Wenchang	PRJNA827465	0.172187579
ENSGALG00010001181	MFSD1	2	-0.016684602	0.122958326	-0.135693145	0.892063867	0.020569867	2.604557879	61.6057678	0.5	CRA004775;PRJNA827465	Yang2025_Silkie_Wenchang	0.974077808
ENSGALG00010001182	ZCCHC7	2	0.255867397	0.108039971	2.36826606	0.017871679	0	0.245773755	0	1	PRJNA827465;Yang2025_Silkie_Wenchang	CRA004775	0.223129158
ENSGALG00010001182	ZCCHC7	2	0.205856012	0.13535276	1.520885213	0.128288645	0.006846958	1.203154003	16.88512051	1	CRA004775;Yang2025_Silkie_Wenchang	PRJNA827465	0.601040485
ENSGALG00010001182	ZCCHC7	2	0.132464397	0.129200906	1.025259042	0.305240976	0	0.432983572	0	1	CRA004775;PRJNA827465	Yang2025_Silkie_Wenchang	0.79225794
ENSGALG00010001183	RARRES1	2	0.01965383	0.108604179	0.180967533	0.85639306	0	0.045344169	0	0.5	PRJNA827465;Yang2025_Silkie_Wenchang	CRA004775	0.96353738
ENSGALG00010001183	RARRES1	2	0.101586518	0.149342257	0.680226211	0.496361239	0	0.684353888	0	1	CRA004775;Yang2025_Silkie_Wenchang	PRJNA827465	0.860939846
ENSGALG00010001183	RARRES1	2	0.05098146	0.134668166	0.378570979	0.705006476	0	0.925715138	0	0.5	CRA004775;PRJNA827465	Yang2025_Silkie_Wenchang	0.924152671
ENSGALG00010001185	PTPN2	2	-0.064432999	0.039942333	-1.613150603	0.106711792	0	0.015484021	0	1	PRJNA827465;Yang2025_Silkie_Wenchang	CRA004775	0.553895846
ENSGALG00010001185	PTPN2	2	-0.060659273	0.100230324	-0.605198807	0.545046909	0	0.190012554	0	0.5	CRA004775;Yang2025_Silkie_Wenchang	PRJNA827465	0.877354247
ENSGALG00010001185	PTPN2	2	-0.059830261	0.04259869	-1.404509411	0.160167211	0	0.168601401	0	0.5	CRA004775;PRJNA827465	Yang2025_Silkie_Wenchang	0.655558469
ENSGALG00010001187	GFM1	2	0.169325354	0.236365914	0.716369595	0.473763164	0.103016984	12.75737373	92.16139606	0.5	PRJNA827465;Yang2025_Silkie_Wenchang	CRA004775	0.85444425
ENSGALG00010001187	GFM1	2	0.374713063	0.097644894	3.837508011	0.000124289	0	0.920790595	0	1	CRA004775;Yang2025_Silkie_Wenchang	PRJNA827465	0.009096718
ENSGALG00010001187	GFM1	2	-0.047250119	0.078002632	-0.605750323	0.544680562	0	0.440125022	0	0.5	CRA004775;PRJNA827465	Yang2025_Silkie_Wenchang	0.877345822
ENSGALG00010001188	PSMG2	2	0.156502683	0.211318818	0.740599841	0.458936105	0.078374899	8.012048906	87.5187981	0.5	PRJNA827465;Yang2025_Silkie_Wenchang	CRA004775	0.850089963
ENSGALG00010001188	PSMG2	2	0.105692626	0.324694321	0.325514242	0.744791906	0.164345508	4.112197996	75.68210478	0.5	CRA004775;Yang2025_Silkie_Wenchang	PRJNA827465	0.935893304
ENSGALG00010001188	PSMG2	2	-0.060122826	0.078041135	-0.770399176	0.441063142	0	0.577274347	0	1	CRA004775;PRJNA827465	Yang2025_Silkie_Wenchang	0.844888742
ENSGALG00010001190	LXN	2	0.450802549	0.225807786	1.996399488	0.045890456	0	0.283517423	0	1	PRJNA827465;Yang2025_Silkie_Wenchang	CRA004775	0.369489827
ENSGALG00010001190	LXN	2	0.055505161	0.352448918	0.15748427	0.874863204	0.019108213	1.067158406	6.293199359	0.5	CRA004775;Yang2025_Silkie_Wenchang	PRJNA827465	0.970159044
ENSGALG00010001190	LXN	2	0.180609401	0.491184203	0.367701973	0.713095469	0.290856084	2.187297737	54.28148701	0.5	CRA004775;PRJNA827465	Yang2025_Silkie_Wenchang	0.926168706
ENSGALG00010001191	TOMM5	2	-0.009349594	0.157071649	-0.059524392	0.952534438	0	0.626503142	0	0.5	PRJNA827465;Yang2025_Silkie_Wenchang	CRA004775	0.987758913
ENSGALG00010001191	TOMM5	2	-0.305885871	0.242581878	-1.260959283	0.207323518	0	0.148674524	0	1	CRA004775;Yang2025_Silkie_Wenchang	PRJNA827465	0.719377239
ENSGALG00010001191	TOMM5	2	-0.063530815	0.210337956	-0.302041612	0.762620342	0.027193191	1.311164458	23.73191676	0.5	CRA004775;PRJNA827465	Yang2025_Silkie_Wenchang	0.940082621
ENSGALG00010001192	ENSGALG00010001192	2	0.13722851	0.110180654	1.245486437	0.212952998	0.009804453	1.610045917	37.88997014	1	PRJNA827465;Yang2025_Silkie_Wenchang	CRA004775	0.725779715
ENSGALG00010001192	ENSGALG00010001192	2	0.285382221	0.140207138	2.035432899	0.041807334	0	0.013330906	0	1	CRA004775;Yang2025_Silkie_Wenchang	PRJNA827465	0.354116889
ENSGALG00010001192	ENSGALG00010001192	2	0.067851065	0.095297143	0.711994745	0.47646803	0	0.461543683	0	1	CRA004775;PRJNA827465	Yang2025_Silkie_Wenchang	0.854986476
ENSGALG00010001193	PRELID3A	2	0.259921492	0.142000121	1.830431475	0.067185445	0	0.898036184	0	1	PRJNA827465;Yang2025_Silkie_Wenchang	CRA004775	0.446498062
ENSGALG00010001193	PRELID3A	2	0.157022187	0.196335461	0.799764783	0.423847091	0	0.324298535	0	1	CRA004775;Yang2025_Silkie_Wenchang	PRJNA827465	0.84030043
ENSGALG00010001193	PRELID3A	2	0.365396605	0.165498778	2.207850766	0.027254678	0	0.001441793	0	1	CRA004775;PRJNA827465	Yang2025_Silkie_Wenchang	0.281669893
ENSGALG00010001194	POT1	2	-0.286885898	0.192341468	-1.491544702	0.135818544	0.063764434	6.917350115	85.54359714	1	PRJNA827465;Yang2025_Silkie_Wenchang	CRA004775	0.617261359
ENSGALG00010001194	POT1	2	-0.302731782	0.246892473	-1.226168534	0.22013526	0.081332481	2.69900445	62.94930155	0.5	CRA004775;Yang2025_Silkie_Wenchang	PRJNA827465	0.733715749
ENSGALG00010001194	POT1	2	-0.103964942	0.064369553	-1.615126045	0.106283402	0	0.180128844	0	0.5	CRA004775;PRJNA827465	Yang2025_Silkie_Wenchang	0.553132487
ENSGALG00010001195	ZNF608	2	0.589386876	0.403722503	1.459881158	0.144322738	0.287656182	8.197188949	87.80069599	1	PRJNA827465;Yang2025_Silkie_Wenchang	CRA004775	0.631870428
ENSGALG00010001195	ZNF608	2	0.581361925	0.475033792	1.223832779	0.221015316	0.357341457	4.706444595	78.75253857	1	CRA004775;Yang2025_Silkie_Wenchang	PRJNA827465	0.734231106
ENSGALG00010001195	ZNF608	2	0.196880829	0.121909653	1.614973247	0.106316488	0	0.13992961	0	1	CRA004775;PRJNA827465	Yang2025_Silkie_Wenchang	0.553168483
ENSGALG00010001196	LSMEM1	2	-0.173164346	0.109985054	-1.574435242	0.115386862	0	0.00820933	0	1	PRJNA827465;Yang2025_Silkie_Wenchang	CRA004775	0.57246274
ENSGALG00010001196	LSMEM1	2	-0.111218303	0.207625361	-0.535668199	0.592187886	0.018621673	1.094701812	8.650923074	0.5	CRA004775;Yang2025_Silkie_Wenchang	PRJNA827465	0.892776493
ENSGALG00010001196	LSMEM1	2	-0.116795584	0.168249045	-0.694182747	0.487567611	0.010345024	1.053939144	5.117861337	0.5	CRA004775;PRJNA827465	Yang2025_Silkie_Wenchang	0.85768885
ENSGALG00010001197	CEP76	2	-0.141447068	0.232783012	-0.607634837	0.54342969	0.092575077	6.658538708	84.98168977	0.5	PRJNA827465;Yang2025_Silkie_Wenchang	CRA004775	0.876968972
ENSGALG00010001197	CEP76	2	-0.371845397	0.132729984	-2.80151768	0.005086285	0	0.05931236	0	1	CRA004775;Yang2025_Silkie_Wenchang	PRJNA827465	0.106389715
ENSGALG00010001197	CEP76	2	-0.05018007	0.18353831	-0.273403794	0.784542837	0.040855683	2.110856059	52.62585549	0.5	CRA004775;PRJNA827465	Yang2025_Silkie_Wenchang	0.946117405
ENSGALG00010001198	IFRD1	2	0.096124464	0.067399316	1.42619347	0.153812504	0	0.524965982	0	1	PRJNA827465;Yang2025_Silkie_Wenchang	CRA004775	0.646370532
ENSGALG00010001198	IFRD1	2	0.234847417	0.158890028	1.478050071	0.139394377	0	0.12561428	0	1	CRA004775;Yang2025_Silkie_Wenchang	PRJNA827465	0.623009874
ENSGALG00010001198	IFRD1	2	0.084482866	0.072079964	1.17207142	0.241168383	0	0.497249239	0	1	CRA004775;PRJNA827465	Yang2025_Silkie_Wenchang	0.753758453
ENSGALG00010001199	AFG3L2	2	0.067776128	0.121772348	0.556580607	0.577814007	0.026749946	9.94262199	89.94229087	0.5	PRJNA827465;Yang2025_Silkie_Wenchang	CRA004775	0.887449156
ENSGALG00010001199	AFG3L2	2	0.198400648	0.064918693	3.056140529	0.002242061	0	0.013340279	0	1	CRA004775;Yang2025_Silkie_Wenchang	PRJNA827465	0.062585044
ENSGALG00010001199	AFG3L2	2	-0.007860092	0.096101216	-0.08178972	0.934813931	0.009412442	1.342120366	25.49103451	0.5	CRA004775;PRJNA827465	Yang2025_Silkie_Wenchang	0.984432558
ENSGALG00010001200	CTTNBP2	2	0.078761367	0.123785462	0.636273159	0.524598396	0.018530363	2.255652615	55.66693235	0.5	PRJNA827465;Yang2025_Silkie_Wenchang	CRA004775	0.869811145
ENSGALG00010001200	CTTNBP2	2	0.298380742	0.141265543	2.112197605	0.034669504	0	0.704358295	0	1	CRA004775;Yang2025_Silkie_Wenchang	PRJNA827465	0.320651825
ENSGALG00010001200	CTTNBP2	2	0.175628608	0.26900409	0.652884527	0.513830725	0.10290625	2.706463703	63.05141654	0.5	CRA004775;PRJNA827465	Yang2025_Silkie_Wenchang	0.866620841
ENSGALG00010001201	TUBB6	2	-0.028163138	0.072151198	-0.390335009	0.696288839	0.000520224	1.046755383	4.466696172	0.5	PRJNA827465;Yang2025_Silkie_Wenchang	CRA004775	0.921038437
ENSGALG00010001201	TUBB6	2	-0.13498729	0.111281699	-1.213023268	0.225120925	0	0.012643703	0	1	CRA004775;Yang2025_Silkie_Wenchang	PRJNA827465	0.738126946
ENSGALG00010001201	TUBB6	2	0.006293344	0.081627522	0.077098314	0.938545334	0	0.43110302	0	0.5	CRA004775;PRJNA827465	Yang2025_Silkie_Wenchang	0.985349045
ENSGALG00010001202	CFTR	2	-0.405483158	0.121811809	-3.328767228	0.000872313	0	0.141438199	0	1	PRJNA827465;Yang2025_Silkie_Wenchang	CRA004775	0.034665833
ENSGALG00010001202	CFTR	2	0.171581474	0.665508274	0.257820196	0.796545677	0.700898418	4.071691384	75.44018184	0.5	CRA004775;Yang2025_Silkie_Wenchang	PRJNA827465	0.949491531
ENSGALG00010001202	CFTR	2	0.136427418	0.714618251	0.190909507	0.848596492	0.828174393	4.59528614	78.23856949	0.5	CRA004775;PRJNA827465	Yang2025_Silkie_Wenchang	0.961364896
ENSGALG00010001203	TMX3	2	-0.179169039	0.22429709	-0.798802333	0.424405037	0.096330174	23.38363789	95.72350545	0.5	PRJNA827465;Yang2025_Silkie_Wenchang	CRA004775	0.840628469
ENSGALG00010001203	TMX3	2	-0.04085949	0.37565118	-0.108769762	0.913385108	0.269081607	21.13757628	95.26908863	0.5	CRA004775;Yang2025_Silkie_Wenchang	PRJNA827465	0.979193041
ENSGALG00010001203	TMX3	2	0.163950721	0.148500517	1.104041417	0.269575172	0.033775697	3.787432207	73.59688715	1	CRA004775;PRJNA827465	Yang2025_Silkie_Wenchang	0.77189992
ENSGALG00010001204	SNX2	2	-0.069059933	0.086481778	-0.798548941	0.424552004	0	0.020508773	0	1	PRJNA827465;Yang2025_Silkie_Wenchang	CRA004775	0.840782395
ENSGALG00010001204	SNX2	2	0.112136597	0.179275382	0.625499141	0.531643512	0.042650448	2.942621112	66.01669185	0.5	CRA004775;Yang2025_Silkie_Wenchang	PRJNA827465	0.873042777
ENSGALG00010001204	SNX2	2	0.0949654	0.191442842	0.496050932	0.619858477	0.054066954	3.724890441	73.15357282	0.5	CRA004775;PRJNA827465	Yang2025_Silkie_Wenchang	0.899395593
ENSGALG00010001205	CEP192	2	-0.034189611	0.108826359	-0.314166636	0.753394481	0.017158601	3.480831344	71.27123088	0.5	PRJNA827465;Yang2025_Silkie_Wenchang	CRA004775	0.938087082
ENSGALG00010001205	CEP192	2	0.070931986	0.095841142	0.740099553	0.45923959	0	0.434827142	0	0.5	CRA004775;Yang2025_Silkie_Wenchang	PRJNA827465	0.850098539
ENSGALG00010001205	CEP192	2	-0.130433625	0.061099439	-2.134776155	0.032779299	0	0.001776266	0	1	CRA004775;PRJNA827465	Yang2025_Silkie_Wenchang	0.31117452
ENSGALG00010001206	RSRC1	2	0.079320135	0.230077391	0.344754146	0.730279208	0.098274791	13.80079235	92.75403923	0.5	PRJNA827465;Yang2025_Silkie_Wenchang	CRA004775	0.932615145
ENSGALG00010001206	RSRC1	2	0.19693933	0.166955331	1.179592942	0.238162151	0.031408454	2.012763157	50.31705562	0.5	CRA004775;Yang2025_Silkie_Wenchang	PRJNA827465	0.750039025
ENSGALG00010001206	RSRC1	2	-0.135438293	0.065947876	-2.0537173	0.040003061	0	0.20381897	0	1	CRA004775;PRJNA827465	Yang2025_Silkie_Wenchang	0.346537428
ENSGALG00010001209	CDH7	2	0.052943907	0.109816593	0.482112089	0.629726323	0	0.282610932	0	1	PRJNA827465;Yang2025_Silkie_Wenchang	CRA004775	0.902457014
ENSGALG00010001209	CDH7	2	-0.113105955	0.434549087	-0.260283495	0.794645103	0.215124302	1.674960654	40.29710502	0.5	CRA004775;Yang2025_Silkie_Wenchang	PRJNA827465	0.948905846
ENSGALG00010001209	CDH7	2	-0.130886384	0.325261259	-0.402403852	0.687386835	0.104092734	1.331691003	24.90750498	0.5	CRA004775;PRJNA827465	Yang2025_Silkie_Wenchang	0.919486924
ENSGALG00010001213	PPIC	2	0.160502947	0.143944922	1.115030283	0.264837445	0	0.002657299	0	1	PRJNA827465;Yang2025_Silkie_Wenchang	CRA004775	0.769263623
ENSGALG00010001213	PPIC	2	0.092258161	0.18214166	0.506518721	0.61249255	0	0.715575848	0	0.5	CRA004775;Yang2025_Silkie_Wenchang	PRJNA827465	0.897938231
ENSGALG00010001213	PPIC	2	0.079513673	0.183121101	0.434213601	0.664133337	0	0.660374222	0	0.5	CRA004775;PRJNA827465	Yang2025_Silkie_Wenchang	0.91312035
ENSGALG00010001214	TES	2	-0.18918313	0.061315513	-3.085404023	0.002032758	0	0.210201545	0	1	PRJNA827465;Yang2025_Silkie_Wenchang	CRA004775	0.058631999
ENSGALG00010001214	TES	2	-0.215212338	0.088549928	-2.430406679	0.015081889	0	0.051168664	0	1	CRA004775;Yang2025_Silkie_Wenchang	PRJNA827465	0.203100811
ENSGALG00010001214	TES	2	-0.162202389	0.080886614	-2.0053057	0.044930376	0	0.006309166	0	1	CRA004775;PRJNA827465	Yang2025_Silkie_Wenchang	0.365383522
ENSGALG00010001218	ENSGALG00010001218	2	0.169111288	0.093570049	1.80732285	0.070711955	0.00393612	1.283088317	22.06304218	1	PRJNA827465;Yang2025_Silkie_Wenchang	CRA004775	0.4585765
ENSGALG00010001218	ENSGALG00010001218	2	-0.024504129	0.335712961	-0.072991312	0.941813031	0.186914777	5.536321532	81.93746526	0.5	CRA004775;Yang2025_Silkie_Wenchang	PRJNA827465	0.985827325
ENSGALG00010001218	ENSGALG00010001218	2	-0.099669822	0.236721711	-0.421042167	0.673724291	0.079591462	3.052434917	67.23926875	0.5	CRA004775;PRJNA827465	Yang2025_Silkie_Wenchang	0.915497675
ENSGALG00010001220	PLXNB2	2	0.012823906	0.113279071	0.113206314	0.90986699	0.015629661	2.073887322	51.78137262	0.5	PRJNA827465;Yang2025_Silkie_Wenchang	CRA004775	0.978730675
ENSGALG00010001220	PLXNB2	2	0.267815096	0.138982902	1.926964334	0.053984075	0.000239902	1.004924226	0.490009687	1	CRA004775;Yang2025_Silkie_Wenchang	PRJNA827465	0.402825653
ENSGALG00010001220	PLXNB2	2	0.155661105	0.272529908	0.571170724	0.567883921	0.118987177	4.212501997	76.26113885	0.5	CRA004775;PRJNA827465	Yang2025_Silkie_Wenchang	0.88383817
ENSGALG00010001221	GRAMD2B	2	0.019988104	0.167556649	0.11929162	0.905044322	0.026983115	1.921267374	47.9510237	0.5	PRJNA827465;Yang2025_Silkie_Wenchang	CRA004775	0.977931125
ENSGALG00010001221	GRAMD2B	2	-0.259089306	0.157148264	-1.648693403	0.099210462	0.003508387	1.0637966	5.997067467	1	CRA004775;Yang2025_Silkie_Wenchang	PRJNA827465	0.537385447
ENSGALG00010001221	GRAMD2B	2	-0.120905989	0.336636062	-0.359159349	0.719475885	0.17715069	4.382316615	77.1810189	0.5	CRA004775;PRJNA827465	Yang2025_Silkie_Wenchang	0.928449671
ENSGALG00010001222	ZNF800	2	0.025344673	0.074833083	0.338682736	0.734848747	0	0.482072196	0	0.5	PRJNA827465;Yang2025_Silkie_Wenchang	CRA004775	0.933537081
ENSGALG00010001222	ZNF800	2	-0.051966135	0.14914557	-0.3484256	0.727520579	0	0.123790091	0	0.5	CRA004775;Yang2025_Silkie_Wenchang	PRJNA827465	0.931630234
ENSGALG00010001222	ZNF800	2	0.052460265	0.081817462	0.641186658	0.521401417	0	0.00072904	0	1	CRA004775;PRJNA827465	Yang2025_Silkie_Wenchang	0.869518515
ENSGALG00010001224	DNAJB9	2	-0.295146941	0.395606995	-0.746060976	0.455630581	0.297916776	20.29917941	95.07369249	0.5	PRJNA827465;Yang2025_Silkie_Wenchang	CRA004775	0.84907427
ENSGALG00010001224	DNAJB9	2	-0.009488229	0.711211313	-0.013340942	0.989355784	0.962465542	20.34870194	95.08568162	0.5	CRA004775;Yang2025_Silkie_Wenchang	PRJNA827465	0.997119019
ENSGALG00010001224	DNAJB9	2	0.348799647	0.310870054	1.122011088	0.261857726	0.159824361	5.060101169	80.23754928	1	CRA004775;PRJNA827465	Yang2025_Silkie_Wenchang	0.768363052
ENSGALG00010001225	SOCS6	2	-0.155575477	0.163217284	-0.953180157	0.340498798	0.046128245	7.372047284	86.43524707	0.5	PRJNA827465;Yang2025_Silkie_Wenchang	CRA004775	0.805524125
ENSGALG00010001225	SOCS6	2	-0.012441274	0.344067375	-0.036159411	0.97115525	0.207015458	7.497486309	86.66219638	0.5	CRA004775;Yang2025_Silkie_Wenchang	PRJNA827465	0.991925437
ENSGALG00010001225	SOCS6	2	0.115852788	0.169265572	0.684443899	0.493694903	0.036729566	2.236759797	55.29247255	1	CRA004775;PRJNA827465	Yang2025_Silkie_Wenchang	0.860054554
ENSGALG00010001228	THAP5	2	-0.124154232	0.139030748	-0.892998376	0.371858043	0.028023025	3.22407696	68.98337067	1	PRJNA827465;Yang2025_Silkie_Wenchang	CRA004775	0.821597118
ENSGALG00010001228	THAP5	2	-0.005430935	0.319931652	-0.016975298	0.986456322	0.16642582	5.168304023	80.65129304	0.5	CRA004775;Yang2025_Silkie_Wenchang	PRJNA827465	0.99618774
ENSGALG00010001228	THAP5	2	0.08925827	0.16150651	0.552660506	0.580495902	0.032866982	2.060400501	51.46574661	0.5	CRA004775;PRJNA827465	Yang2025_Silkie_Wenchang	0.888433837
ENSGALG00010001229	ZNF407	2	-0.003221823	0.136051666	-0.023680877	0.98110716	0.028265067	3.800319304	73.68642159	0.5	PRJNA827465;Yang2025_Silkie_Wenchang	CRA004775	0.994928779
ENSGALG00010001229	ZNF407	2	0.227548688	0.111244381	2.045484785	0.040807088	0	0.903138456	0	1	CRA004775;Yang2025_Silkie_Wenchang	PRJNA827465	0.349602411
ENSGALG00010001229	ZNF407	2	0.100124614	0.252715103	0.396195607	0.691960732	0.107578116	5.610783981	82.17717874	0.5	CRA004775;PRJNA827465	Yang2025_Silkie_Wenchang	0.920348122
ENSGALG00010001230	PTX3	2	-0.337773074	0.331308618	-1.019511887	0.307960013	0.163704376	3.880682224	74.23133505	1	PRJNA827465;Yang2025_Silkie_Wenchang	CRA004775	0.793278812
ENSGALG00010001230	PTX3	2	-0.93064516	0.391156942	-2.37921167	0.01734971	0.138157966	1.554464022	35.66914475	1	CRA004775;Yang2025_Silkie_Wenchang	PRJNA827465	0.219047753
ENSGALG00010001230	PTX3	2	-0.675676271	0.762026598	-0.886683315	0.375249424	0.958086982	5.088982742	80.34970738	1	CRA004775;PRJNA827465	Yang2025_Silkie_Wenchang	0.822941424
ENSGALG00010001232	TSPAN12	2	0.074329913	0.069934374	1.062852344	0.287848918	0	0.615723369	0	1	PRJNA827465;Yang2025_Silkie_Wenchang	CRA004775	0.782840067
ENSGALG00010001232	TSPAN12	2	0.006324933	0.09776195	0.064697283	0.948415026	0	0.022749268	0	0.5	CRA004775;Yang2025_Silkie_Wenchang	PRJNA827465	0.986778445
ENSGALG00010001232	TSPAN12	2	0.105383349	0.087874169	1.199252871	0.230429635	0	0.306855344	0	0.5	CRA004775;PRJNA827465	Yang2025_Silkie_Wenchang	0.743256129
ENSGALG00010001233	CNDP2	2	0.027473159	0.068694425	0.39993288	0.689205954	0	0.257610491	0	0.5	PRJNA827465;Yang2025_Silkie_Wenchang	CRA004775	0.919854035
ENSGALG00010001233	CNDP2	2	-0.039446693	0.102506982	-0.384819576	0.700371094	0	0.105315341	0	1	CRA004775;Yang2025_Silkie_Wenchang	PRJNA827465	0.922303014
ENSGALG00010001233	CNDP2	2	0.03143913	0.078561716	0.400183851	0.689021108	0	0.447684723	0	0.5	CRA004775;PRJNA827465	Yang2025_Silkie_Wenchang	0.919854035
ENSGALG00010001234	AASS	2	-0.234778434	0.096872478	-2.423582419	0.015368266	0.009979936	2.082425838	51.9790822	1	PRJNA827465;Yang2025_Silkie_Wenchang	CRA004775	0.205049211
ENSGALG00010001234	AASS	2	0.135120234	0.318149005	0.424707391	0.671049995	0.155289985	3.751878182	73.34668261	0.5	CRA004775;Yang2025_Silkie_Wenchang	PRJNA827465	0.914582784
ENSGALG00010001234	AASS	2	0.049509758	0.41965291	0.117977874	0.906085189	0.304882666	6.693132777	85.05931328	0.5	CRA004775;PRJNA827465	Yang2025_Silkie_Wenchang	0.978196186
ENSGALG00010001237	MELK	2	0.187196062	0.124881749	1.498986548	0.133877122	0.00965181	1.416645013	29.41068577	1	PRJNA827465;Yang2025_Silkie_Wenchang	CRA004775	0.613104446
ENSGALG00010001237	MELK	2	0.372035776	0.168710473	2.205172975	0.027441959	0	0.39672997	0	1	CRA004775;Yang2025_Silkie_Wenchang	PRJNA827465	0.282861607
ENSGALG00010001237	MELK	2	0.125958974	0.157398592	0.800254771	0.423563202	0.010384775	1.046387114	4.433073929	1	CRA004775;PRJNA827465	Yang2025_Silkie_Wenchang	0.84030043
ENSGALG00010001238	GOLIM4	2	0.392900259	0.387304944	1.014446794	0.310369605	0.258988513	6.616646495	84.8866038	1	PRJNA827465;Yang2025_Silkie_Wenchang	CRA004775	0.794899969
ENSGALG00010001238	GOLIM4	2	0.469755851	0.392463029	1.196942939	0.231328796	0.201127712	2.851062817	64.92536068	1	CRA004775;Yang2025_Silkie_Wenchang	PRJNA827465	0.743891332
ENSGALG00010001238	GOLIM4	2	0.052478727	0.079886455	0.656916461	0.511234634	0	0.000257581	0	1	CRA004775;PRJNA827465	Yang2025_Silkie_Wenchang	0.865658376
ENSGALG00010001239	ENSGALG00010001239	2	0.009066641	0.228821633	0.039623183	0.968393545	0	0.145104984	0	0.5	PRJNA827465;Yang2025_Silkie_Wenchang	CRA004775	0.991215679
ENSGALG00010001239	ENSGALG00010001239	2	-0.340694305	0.567778627	-0.600047782	0.548474392	0.405422179	2.45740664	59.30669414	0.5	CRA004775;Yang2025_Silkie_Wenchang	PRJNA827465	0.878281266
ENSGALG00010001239	ENSGALG00010001239	2	-0.394017539	0.465816617	-0.845864068	0.397628579	0.225663388	1.840226051	45.65884992	1	CRA004775;PRJNA827465	Yang2025_Silkie_Wenchang	0.831689959
ENSGALG00010001244	ENSGALG00010001244	2	-0.212553225	0.121163693	-1.754264998	0.07938511	0	0.195471301	0	1	PRJNA827465;Yang2025_Silkie_Wenchang	CRA004775	0.484805397
ENSGALG00010001244	ENSGALG00010001244	2	-0.175551475	0.197148522	-0.890452908	0.373222744	0	0.404917611	0	1	CRA004775;Yang2025_Silkie_Wenchang	PRJNA827465	0.822187352
ENSGALG00010001244	ENSGALG00010001244	2	-0.168898021	0.114010822	-1.481420958	0.138494433	0	0.146591142	0	1	CRA004775;PRJNA827465	Yang2025_Silkie_Wenchang	0.622343774
ENSGALG00010001245	SERPINI1	2	-0.153039268	0.163893076	-0.933775067	0.350419933	0.014401537	1.309818961	23.65357126	1	PRJNA827465;Yang2025_Silkie_Wenchang	CRA004775	0.810111037
ENSGALG00010001245	SERPINI1	2	-0.310187837	0.24466465	-1.267808145	0.204866473	0	0.793428628	0	0.5	CRA004775;Yang2025_Silkie_Wenchang	PRJNA827465	0.717411187
ENSGALG00010001245	SERPINI1	2	-0.022777663	0.156317655	-0.145713949	0.884147209	0	0.209813169	0	0.5	CRA004775;PRJNA827465	Yang2025_Silkie_Wenchang	0.972001076
ENSGALG00010001248	PTPRZ1	2	0.318672813	0.226030512	1.409866349	0.158579151	0.045086224	1.659650142	39.74633722	1	PRJNA827465;Yang2025_Silkie_Wenchang	CRA004775	0.652563749
ENSGALG00010001248	PTPRZ1	2	1.175324965	0.606028233	1.939389784	0.052453894	0.558560553	4.023645338	75.1469149	1	CRA004775;Yang2025_Silkie_Wenchang	PRJNA827465	0.39754556
ENSGALG00010001248	PTPRZ1	2	0.934678874	0.844998584	1.106130699	0.268669953	1.288468658	9.647484838	89.63460408	1	CRA004775;PRJNA827465	Yang2025_Silkie_Wenchang	0.771398294
ENSGALG00010001249	PDCD10	2	0.037505232	0.055688356	0.673484267	0.500639254	0	0.036800992	0	1	PRJNA827465;Yang2025_Silkie_Wenchang	CRA004775	0.862400704
ENSGALG00010001249	PDCD10	2	0.057109727	0.112813052	0.506233331	0.612692859	0	0.000687587	0	1	CRA004775;Yang2025_Silkie_Wenchang	PRJNA827465	0.898034557
ENSGALG00010001249	PDCD10	2	0.032787942	0.061240124	0.535399662	0.592373524	0	0.003489529	0	1	CRA004775;PRJNA827465	Yang2025_Silkie_Wenchang	0.892776493
ENSGALG00010001251	ZNF277	2	-0.048009122	0.067058814	-0.715925597	0.474037292	0	0.913212396	0	0.5	PRJNA827465;Yang2025_Silkie_Wenchang	CRA004775	0.85444425
ENSGALG00010001251	ZNF277	2	0.045106981	0.097476896	0.462745352	0.643546906	0	0.009988725	0	1	CRA004775;Yang2025_Silkie_Wenchang	PRJNA827465	0.906643868
ENSGALG00010001251	ZNF277	2	-0.070071972	0.076257003	-0.918892287	0.358151915	0	0.564810668	0	0.5	CRA004775;PRJNA827465	Yang2025_Silkie_Wenchang	0.813853337
ENSGALG00010001252	SNCAIP	2	0.098293171	0.224290415	0.438240622	0.661211864	0.05939451	2.439849104	59.01385876	0.5	PRJNA827465;Yang2025_Silkie_Wenchang	CRA004775	0.91217832
ENSGALG00010001252	SNCAIP	2	0.227430813	0.180054547	1.26312174	0.206545432	0	0.925122328	0	0.5	CRA004775;Yang2025_Silkie_Wenchang	PRJNA827465	0.71859917
ENSGALG00010001252	SNCAIP	2	-0.111089332	0.174130669	-0.637965345	0.523496239	0	0.013043142	0	1	CRA004775;PRJNA827465	Yang2025_Silkie_Wenchang	0.869661505
ENSGALG00010001254	NETO1	2	-0.053037147	0.226137727	-0.234534714	0.814569887	0	0.143759262	0	0.5	PRJNA827465;Yang2025_Silkie_Wenchang	CRA004775	0.953869515
ENSGALG00010001254	NETO1	2	0.055102069	0.348763349	0.157992717	0.874462538	0	0.000177831	0	1	CRA004775;Yang2025_Silkie_Wenchang	PRJNA827465	0.970135804
ENSGALG00010001254	NETO1	2	-0.110078427	0.277998235	-0.39596808	0.692128577	0	0.019530496	0	0.5	CRA004775;PRJNA827465	Yang2025_Silkie_Wenchang	0.920348122
ENSGALG00010001258	ENSGALG00010001258	2	-0.001319066	0.093687021	-0.014079493	0.988766561	0	0.002515074	0	0.5	PRJNA827465;Yang2025_Silkie_Wenchang	CRA004775	0.996891127
ENSGALG00010001258	ENSGALG00010001258	2	-0.133133165	0.210597101	-0.63216998	0.527275811	0.038705072	1.58009666	36.71273251	0.5	CRA004775;Yang2025_Silkie_Wenchang	PRJNA827465	0.871138813
ENSGALG00010001258	ENSGALG00010001258	2	-0.123666308	0.198203763	-0.623935217	0.532670131	0.039806756	1.650869776	39.42587026	1	CRA004775;PRJNA827465	Yang2025_Silkie_Wenchang	0.873183244
ENSGALG00010001259	ENSGALG00010001259	2	0.184493464	0.395200469	0.466835134	0.640617837	0.213167955	3.148516519	68.23901053	0.5	PRJNA827465;Yang2025_Silkie_Wenchang	CRA004775	0.906257309
ENSGALG00010001259	ENSGALG00010001259	2	0.561748994	0.261667604	2.146803755	0.031808908	0	0.01321876	0	1	CRA004775;Yang2025_Silkie_Wenchang	PRJNA827465	0.305444789
ENSGALG00010001259	ENSGALG00010001259	2	0.077348778	0.354637768	0.218106431	0.827346187	0.110176307	1.717625483	41.78009061	0.5	CRA004775;PRJNA827465	Yang2025_Silkie_Wenchang	0.956550752
ENSGALG00010001262	ENSGALG00010001262	2	0.559552488	0.571423174	0.979226103	0.327468274	0.64123093	55.24330083	98.18982576	0.5	PRJNA827465;Yang2025_Silkie_Wenchang	CRA004775	0.800077397
ENSGALG00010001262	ENSGALG00010001262	2	0.539624493	0.627541516	0.859902459	0.389842813	0.731738835	13.51768999	92.60228633	0.5	CRA004775;Yang2025_Silkie_Wenchang	PRJNA827465	0.829112125
ENSGALG00010001262	ENSGALG00010001262	2	-0.020921606	0.096899319	-0.215910768	0.829057303	0	0.114653449	0	1	CRA004775;PRJNA827465	Yang2025_Silkie_Wenchang	0.956860127
ENSGALG00010001264	FAM3C	2	-0.095084498	0.058075306	-1.637262117	0.101575713	0.001314671	1.189943888	15.96242396	1	PRJNA827465;Yang2025_Silkie_Wenchang	CRA004775	0.543686036
ENSGALG00010001264	FAM3C	2	0.220387603	0.436551114	0.504838027	0.613672605	0.345754916	10.27406683	90.26675594	0.5	CRA004775;Yang2025_Silkie_Wenchang	PRJNA827465	0.898256044
ENSGALG00010001264	FAM3C	2	0.27459584	0.371237656	0.739676687	0.459496197	0.245161165	8.257979454	87.89050027	0.5	CRA004775;PRJNA827465	Yang2025_Silkie_Wenchang	0.850098539
ENSGALG00010001265	OPN1LW	2	0.687994382	0.969874281	0.709364497	0.478098313	1.737893469	13.11290662	92.37392571	0.5	PRJNA827465;Yang2025_Silkie_Wenchang	CRA004775	0.855366076
ENSGALG00010001265	OPN1LW	2	0.519802623	1.190300423	0.436698679	0.662329887	2.570371418	10.59636754	90.56280376	0.5	CRA004775;Yang2025_Silkie_Wenchang	PRJNA827465	0.91272639

ENSGALG00010001265	OPN1LW	2	-0.39110914	0.314292092	-1.24441292	0.21334763	0	0.38047874	0	1	CRA004775;PRJNA827465	Yang2025_Silkie_Wenchang	0.725928657
ENSGALG00010001267	BCHE	2	-0.277129214	0.067988802	-4.076100848	4.57972E-05	0	0.667328692	0	1	PRJNA827465;Yang2025_Silkie_Wenchang	CRA004775	0.004568441
ENSGALG00010001267	BCHE	2	-0.23100108	0.098082063	-2.355181694	0.018513651	0	0.745743254	0	1	CRA004775;Yang2025_Silkie_Wenchang	PRJNA827465	0.227601507
ENSGALG00010001267	BCHE	2	-0.338600907	0.092412336	-3.664022828	0.000248285	0	0.483897211	0	1	CRA004775;PRJNA827465	Yang2025_Silkie_Wenchang	0.01478535
ENSGALG00010001268	CBLN2	2	-0.562805152	0.156846173	-3.58826192	0.00033289	0	0.132259548	0	1	PRJNA827465;Yang2025_Silkie_Wenchang	CRA004775	0.018046299
ENSGALG00010001268	CBLN2	2	-0.376795874	0.304649905	-1.236815991	0.216155404	0.011131429	1.018991491	1.863753613	0.5	CRA004775;Yang2025_Silkie_Wenchang	PRJNA827465	0.729796053
ENSGALG00010001268	CBLN2	2	-0.416380631	0.434205907	-0.958947413	0.337585238	0.187602285	1.336272251	25.16495055	0.5	CRA004775;PRJNA827465	Yang2025_Silkie_Wenchang	0.80415385
ENSGALG00010001270	DRAP1	2	0.100651516	0.144120777	0.698383109	0.484937637	0.02892637	2.712600753	63.13500987	0.5	PRJNA827465;Yang2025_Silkie_Wenchang	CRA004775	0.857004733
ENSGALG00010001270	DRAP1	2	0.127364605	0.222283731	0.572982127	0.566656796	0.047722813	1.821494261	45.10001918	0.5	CRA004775;Yang2025_Silkie_Wenchang	PRJNA827465	0.883487517
ENSGALG00010001270	DRAP1	2	-0.009633978	0.052914248	-0.182067754	0.855529555	0	0.28063762	0	1	CRA004775;PRJNA827465	Yang2025_Silkie_Wenchang	0.963463555
ENSGALG00010001271	ENSGALG00010001271	2	0.145363365	0.341176574	0.426064905	0.670060552	0.185965197	4.489041136	77.72352782	0.5	PRJNA827465;Yang2025_Silkie_Wenchang	CRA004775	0.914428878
ENSGALG00010001271	ENSGALG00010001271	2	0.171044941	0.378103893	0.45237551	0.650998487	0.192500789	3.060449708	67.32506346	0.5	CRA004775;Yang2025_Silkie_Wenchang	PRJNA827465	0.908769703
ENSGALG00010001271	ENSGALG00010001271	2	-0.151999594	0.110330892	-1.377670305	0.168305104	0	0.038388748	0	1	CRA004775;PRJNA827465	Yang2025_Silkie_Wenchang	0.668962553
ENSGALG00010001273	ENSGALG00010001273	2	0.682707262	0.29424066	2.320234269	0.020328208	0	0.839092966	0	1	PRJNA827465;Yang2025_Silkie_Wenchang	CRA004775	0.240144138
ENSGALG00010001273	ENSGALG00010001273	2	1.410698352	0.593654112	2.376296775	0.017487391	0.266912014	1.455001432	31.27154528	1	CRA004775;Yang2025_Silkie_Wenchang	PRJNA827465	0.220149566
ENSGALG00010001273	ENSGALG00010001273	2	1.189283783	0.906179601	1.312415091	0.189380118	1.194699592	3.209230811	68.83988535	1	CRA004775;PRJNA827465	Yang2025_Silkie_Wenchang	0.698499134
ENSGALG00010001276	ENSGALG00010001276	2	-0.390667218	0.212654776	-1.837095904	0.066195739	0	0.192114214	0	1	PRJNA827465;Yang2025_Silkie_Wenchang	CRA004775	0.442991847
ENSGALG00010001276	ENSGALG00010001276	2	0.406042617	0.738184189	0.550055966	0.582280988	0.929245613	6.648141903	84.95820314	0.5	CRA004775;Yang2025_Silkie_Wenchang	PRJNA827465	0.889547544
ENSGALG00010001276	ENSGALG00010001276	2	0.312793533	0.831901567	0.37599825	0.706918197	1.231277355	8.856253376	88.70854347	0.5	CRA004775;PRJNA827465	Yang2025_Silkie_Wenchang	0.924623312
ENSGALG00010001277	ENSGALG00010001277	2	0.04905046	0.103116029	0.475682209	0.634300797	0.010532191	1.84931538	45.92593503	0.5	PRJNA827465;Yang2025_Silkie_Wenchang	CRA004775	0.903815945
ENSGALG00010001277	ENSGALG00010001277	2	0.060995752	0.1874276	0.325436341	0.744850855	0.031923062	1.625709719	38.48840367	0.5	CRA004775;Yang2025_Silkie_Wenchang	PRJNA827465	0.935893304
ENSGALG00010001277	ENSGALG00010001277	2	-0.042550134	0.076068488	-0.559366102	0.575911891	0	0.415933754	0	1	CRA004775;PRJNA827465	Yang2025_Silkie_Wenchang	0.886924804
ENSGALG00010001279	LPIN2	2	0.077655684	0.106614615	0.728377477	0.466382547	0.008896074	1.640500791	39.04300409	0.5	PRJNA827465;Yang2025_Silkie_Wenchang	CRA004775	0.852763198
ENSGALG00010001279	LPIN2	2	-0.177544155	0.30015376	-0.591510682	0.5541783	0.100872162	1.582699731	36.81682128	1	CRA004775;Yang2025_Silkie_Wenchang	PRJNA827465	0.880141825
ENSGALG00010001279	LPIN2	2	-0.120422406	0.446681337	-0.269593547	0.787472965	0.280593232	2.610748422	61.69680726	0.5	CRA004775;PRJNA827465	Yang2025_Silkie_Wenchang	0.949919116
ENSGALG00010001281	LRRN3	2	0.311533217	0.241408547	1.290481306	0.196883598	0.095132687	5.230168088	80.88015561	1	PRJNA827465;Yang2025_Silkie_Wenchang	CRA004775	0.707994211
ENSGALG00010001281	LRRN3	2	0.509723344	0.161597295	3.154281414	0.001608939	0	0.600063197	0	1	CRA004775;Yang2025_Silkie_Wenchang	PRJNA827465	0.050669962
ENSGALG00010001281	LRRN3	2	0.10850764	0.101454728	1.069517832	0.284836398	0	0.268864703	0	1	CRA004775;PRJNA827465	Yang2025_Silkie_Wenchang	0.780351663
ENSGALG00010001283	ENSGALG00010001283	2	0.053080198	0.164499814	0.322676339	0.746940373	0.04184456	4.257466994	76.51185549	0.5	PRJNA827465;Yang2025_Silkie_Wenchang	CRA004775	0.936729365
ENSGALG00010001283	ENSGALG00010001283	2	-0.009745092	0.258603066	-0.037683589	0.969939961	0.108506046	5.233250371	80.89141682	0.5	CRA004775;Yang2025_Silkie_Wenchang	PRJNA827465	0.991572914
ENSGALG00010001283	ENSGALG00010001283	2	-0.129314516	0.077118892	-1.676820203	0.093577646	0	0.87420143	0	1	CRA004775;PRJNA827465	Yang2025_Silkie_Wenchang	0.52406339
ENSGALG00010001285	ENSGALG00010001285	2	0.035944443	0.098388006	0.36177241	0.717519248	0	0.305965101	0	0.5	PRJNA827465;Yang2025_Silkie_Wenchang	CRA004775	0.9277944
ENSGALG00010001285	ENSGALG00010001285	2	0.037952967	0.123619861	0.307013506	0.758833092	0	0.514489725	0	0.5	CRA004775;Yang2025_Silkie_Wenchang	PRJNA827465	0.938996879
ENSGALG00010001285	ENSGALG00010001285	2	-0.033607022	0.112958661	-0.297516112	0.766072509	0	0.101231679	0	1	CRA004775;PRJNA827465	Yang2025_Silkie_Wenchang	0.941081072
ENSGALG00010001286	SRFBP1	2	0.396037771	0.336030575	1.178576596	0.238560816	0.173894757	4.324533995	76.87612119	1	PRJNA827465;Yang2025_Silkie_Wenchang	CRA004775	0.750652917
ENSGALG00010001286	SRFBP1	2	0.391993547	0.377435737	1.038570302	0.299006624	0.206282224	3.584526802	72.10231489	0.5	CRA004775;Yang2025_Silkie_Wenchang	PRJNA827465	0.789748017
ENSGALG00010001286	SRFBP1	2	0.047698331	0.173182118	0.275422958	0.782991308	0	0.04970219	0	0.5	CRA004775;PRJNA827465	Yang2025_Silkie_Wenchang	0.945580193
ENSGALG00010001287	PLPP4	2	0.124106582	0.253298538	0.489961698	0.624161002	0	0.214983266	0	0.5	PRJNA827465;Yang2025_Silkie_Wenchang	CRA004775	0.900829901
ENSGALG00010001287	PLPP4	2	0.048464291	0.36464601	0.132907779	0.894266315	0	0.140741205	0	0.5	CRA004775;Yang2025_Silkie_Wenchang	PRJNA827465	0.974381724
ENSGALG00010001287	PLPP4	2	0.219713241	0.285352955	0.769970093	0.441317634	0	0.005854784	0	1	CRA004775;PRJNA827465	Yang2025_Silkie_Wenchang	0.844907543
ENSGALG00010001288	TGIF1	2	-0.26027497	0.23974589	-1.085628496	0.277643387	0.099483655	7.233203417	86.1748669	1	PRJNA827465;Yang2025_Silkie_Wenchang	CRA004775	0.776763074
ENSGALG00010001288	TGIF1	2	-0.145380795	0.418779483	-0.347153577	0.728475945	0.289378539	5.344940023	81.29071616	0.5	CRA004775;Yang2025_Silkie_Wenchang	PRJNA827465	0.932184127
ENSGALG00010001288	TGIF1	2	0.006593498	0.116437783	0.056626794	0.95484249	0.006884783	1.11649887	10.43450251	0.5	CRA004775;PRJNA827465	Yang2025_Silkie_Wenchang	0.988802548
ENSGALG00010001289	ENSGALG00010001289	2	1.035536328	0.629403947	1.64526507	0.09991516	0.679864267	6.766302849	85.22088026	1	PRJNA827465;Yang2025_Silkie_Wenchang	CRA004775	0.540011761
ENSGALG00010001289	ENSGALG00010001289	2	0.749067786	0.9496474	0.78878517	0.430237579	1.659419717	12.43810813	91.9601921	0.5	CRA004775;Yang2025_Silkie_Wenchang	PRJNA827465	0.842040417
ENSGALG00010001289	ENSGALG00010001289	2	0.180647559	0.315419904	0.572720861	0.56683371	0.123596338	2.563961637	60.99785638	0.5	CRA004775;PRJNA827465	Yang2025_Silkie_Wenchang	0.883487517
ENSGALG00010001291	ENSGALG00010001291	2	0.058591067	0.080653405	0.726454971	0.467559902	0	0.954617193	0	1	PRJNA827465;Yang2025_Silkie_Wenchang	CRA004775	0.852885838
ENSGALG00010001291	ENSGALG00010001291	2	0.214812919	0.184044197	1.167181155	0.24313722	0	0.109821436	0	1	CRA004775;Yang2025_Silkie_Wenchang	PRJNA827465	0.7550024
ENSGALG00010001291	ENSGALG00010001291	2	0.029998889	0.085052909	0.35266153	0.724342203	0	0.042204445	0	1	CRA004775;PRJNA827465	Yang2025_Silkie_Wenchang	0.930387861
ENSGALG00010001292	LOX	2	-0.078043603	0.126259381	-0.618121224	0.536495433	0.000312645	1.00965759	0.95652151	0.5	PRJNA827465;Yang2025_Silkie_Wenchang	CRA004775	0.874599474
ENSGALG00010001292	LOX	2	0.045321588	0.176368499	0.257063688	0.797129608	0	0.093675415	0	0.5	CRA004775;Yang2025_Silkie_Wenchang	PRJNA827465	0.949622804
ENSGALG00010001292	LOX	2	-0.17120014	0.153941554	-1.112111288	0.266090304	0	0.060398665	0	1	CRA004775;PRJNA827465	Yang2025_Silkie_Wenchang	0.770092601
ENSGALG00010001295	ENSGALG00010001295	2	0.180420235	0.236439485	0.763071511	0.445420749	0.106897496	22.70814103	95.59629298	0.5	PRJNA827465;Yang2025_Silkie_Wenchang	CRA004775	0.846013379
ENSGALG00010001295	ENSGALG00010001295	2	0.098437714	0.338863208	0.290493958	0.771438373	0.211755708	12.40636663	91.93962237	0.5	CRA004775;Yang2025_Silkie_Wenchang	PRJNA827465	0.94283287
ENSGALG00010001295	ENSGALG00010001295	2	-0.089954776	0.078706683	-1.142911542	0.25307532	0.003886327	1.224778688	18.35259627	1	CRA004775;PRJNA827465	Yang2025_Silkie_Wenchang	0.762731469
ENSGALG00010001297	MYL12A	2	0.291456966	0.17289404	1.685754847	0.091843028	0.040311116	2.619199148	61.82039076	1	PRJNA827465;Yang2025_Silkie_Wenchang	CRA004775	0.519097275
ENSGALG00010001297	MYL12A	2	0.109351771	0.404693864	0.270208622	0.7869976	0.292085199	9.189473554	89.11798381	0.5	CRA004775;Yang2025_Silkie_Wenchang	PRJNA827465	0.946784559
ENSGALG00010001297	MYL12A	2	-0.037276891	0.222961866	-0.167189537	0.867220917	0.083714562	5.964469003	83.23404817	0.5	CRA004775;PRJNA827465	Yang2025_Silkie_Wenchang	0.967702313
ENSGALG00010001298	DOCK4	2	0.112771272	0.117618005	0.958792595	0.33766324	0.011670712	1.601131756	37.54417797	1	PRJNA827465;Yang2025_Silkie_Wenchang	CRA004775	0.80415385
ENSGALG00010001298	DOCK4	2	0.282330623	0.155285161	1.818142963	0.069042285	0	0.002251752	0	1	CRA004775;Yang2025_Silkie_Wenchang	PRJNA827465	0.452607154
ENSGALG00010001298	DOCK4	2	0.047277083	0.092206295	0.512733619	0.608139068	0	0.525387953	0	1	CRA004775;PRJNA827465	Yang2025_Silkie_Wenchang	0.897204334
ENSGALG00010001300	MRPL49	2	0.211781063	0.189747638	1.116119627	0.264370932	0.034403333	1.806182105	44.63459707	1	PRJNA827465;Yang2025_Silkie_Wenchang	CRA004775	0.768790489
ENSGALG00010001300	MRPL49	2	0.230698548	0.213746126	1.079311012	0.280449105	0.03511211	1.620018589	38.27231324	1	CRA004775;Yang2025_Silkie_Wenchang	PRJNA827465	0.778536485
ENSGALG00010001300	MRPL49	2	0.054713496	0.125658022	0.435415859	0.663260601	0	0.017311677	0	1	CRA004775;PRJNA827465	Yang2025_Silkie_Wenchang	0.91293776
ENSGALG00010001301	GMPS	2	-0.138335036	0.180375662	-0.766927139	0.443124688	0.061574754	18.43197935	94.5046467	0.5	PRJNA827465;Yang2025_Silkie_Wenchang	CRA004775	0.845730569
ENSGALG00010001301	GMPS	2	-0.307809901	0.06853197	-4.491478979	7.07303E-06	0	0.620912893	0	1	CRA004775;Yang2025_Silkie_Wenchang	PRJNA827465	0.001061035
ENSGALG00010001301	GMPS	2	0.033170866	0.043987723	0.754093724	0.450792946	0	0.35952574	0	0.5	CRA004775;PRJNA827465	Yang2025_Silkie_Wenchang	0.847631399
ENSGALG00010001304	SYVN1	2	0.24078687	0.363289253	0.662796568	0.507460852	0.242097058	11.50730412	91.30986728	0.5	PRJNA827465;Yang2025_Silkie_Wenchang	CRA004775	0.864156579
ENSGALG00010001304	SYVN1	2	0.167353765	0.465503824	0.359511041	0.719212819	0.389245182	9.812475215	89.80889146	0.5	CRA004775;Yang2025_Silkie_Wenchang	PRJNA827465	0.92849671
ENSGALG00010001304	SYVN1	2	-0.119532556	0.069379838	-1.722871653	0.084911738	0	0.77674969	0	1	CRA004775;PRJNA827465	Yang2025_Silkie_Wenchang	0.501162525
ENSGALG00010001305	WNT16	2	-0.034810171	0.244356384	-0.142456566	0.886719384	0.029525155	1.291081913	22.54558056	0.5	PRJNA827465;Yang2025_Silkie_Wenchang	CRA004775	0.972831955
ENSGALG00010001305	WNT16	2	0.420552266	0.844914097	0.49774519	0.618664337	1.199265449	5.93793723	83.15931488	0.5	CRA004775;Yang2025_Silkie_Wenchang	PRJNA827465	0.918968321
ENSGALG00010001305	WNT16	2	0.617665742	0.579121523	1.066556358	0.286172217	0.496114017	3.446539216	70.98538745	1	CRA004775;PRJNA827465	Yang2025_Silkie_Wenchang	0.781941993
ENSGALG00010001306	CEP120	2	0.077210416	0.097017523	0.795839897	0.426125009	0	0.115093778	0	1	PRJNA827465;Yang2025_Silkie_Wenchang	CRA004775	0.84125685
ENSGALG00010001306	CEP120	2	0.147866484	0.123097776	1.201211661	0.229669107	0	0.293207077	0	1	CRA004775;Yang2025_Silkie_Wenchang	PRJNA827465	0.742686486
ENSGALG00010001306	CEP120	2	0.093799989	0.119122336	0.787425701	0.431032706	0	0.598417839	0	0.5	CRA004775;PRJNA827465	Yang2025_Silkie_Wenchang	0.842503929
ENSGALG00010001307	CALU	2	0.251322456	0.174624267	1.439218387	0.150088658	0.042470189	2.879472549	65.27141749	1	PRJNA827465;Yang2025_Silkie_Wenchang	CRA004775	0.64035329
ENSGALG00010001307	CALU	2	0.083863044	0.385190188	0.217718537	0.82764842	0.263926843	9.013434917	88.90545048	0.5	CRA004775;Yang2025_Silkie_Wenchang	PRJNA827465	0.956550752
ENSGALG00010001307	CALU	2	-0.063551114	0.203338013	-0.312539271	0.754630723	0.067323083	5.045223542	80.1792727	0.5	CRA004775;PRJNA827465	Yang2025_Silkie_Wenchang	0.93838723
ENSGALG00010001308	MGMT	2	-0.071484002	0.192462425	-0.371417962	0.710326249	0.059476311	4.860829948	79.42738152	0.5	PRJNA827465;Yang2025_Silkie_Wenchang	CRA004775	0.925364369
ENSGALG00010001308	MGMT	2	-0.21865459	0.127800215	-1.71090941	0.087097837	0	0.646254555	0	1	CRA004775;Yang2025_Silkie_Wenchang	PRJNA827465	0.507587624
ENSGALG00010001308	MGMT	2	0.082436095	0.082122247	1.003821712	0.315465557	0	0.412416408	0	0.5	CRA004775;PRJNA827465	Yang2025_Silkie_Wenchang	0.796883862
ENSGALG00010001313	EMILIN2	2	-0.147287352	0.143747109	-1.024628273	0.305535619	0.022861442	2.215757282	54.86870298	1	PRJNA827465;Yang2025_Silkie_Wenchang	CRA004775	0.79525794
ENSGALG00010001313	EMILIN2	2	0.093248277	0.449380665	0.207503981	0.835616284	0.337700035	5.677579728	82.38686116	0.5	CRA004775;Yang2025_Silkie_Wenchang	PRJNA827465	0.958339923
ENSGALG00010001313	EMILIN2	2	0.205831545	0.295975387	0.695434669	0.48678294	0.121755309	2.779118922	64.01737285	0.5	CRA004775;PRJNA827465	Yang2025_Silkie_Wenchang	0.857603818
ENSGALG00010001320	CPED1	2	0.11906268	0.130173022	0.914649433	0.360375698	0	0.185932668	0	1	PRJNA827465;Yang2025_Silkie_Wenchang	CRA004775	0.814880744
ENSGALG00010001320	CPED1	2	0.404390522	0.42103545	0.960466682	0.336820392	0.259059807	3.250354998	69.23412979	1	CRA004775;Yang2025_Silkie_Wenchang	PRJNA827465	0.803904536
ENSGALG00010001320	CPED1	2	0.453262807	0.360179888	1.258434526	0.208234652	0.161943202	2.344817426	57.35275639	1	CRA004775;PRJNA827465	Yang2025_Silkie_Wenchang	0.720917211
ENSGALG00010001330	ENSGALG00010001330	2	0.340368241	0.410209256	0.829742956	0.406684129	0.312944258	13.91603144	92.81404326	0.5	PRJNA827465;Yang2025_Silkie_Wenchang	CRA004775	0.833618122
ENSGALG00010001330	ENSGALG00010001330	2	0.263401196	0.508321271	0.518178583	0.604333676	0.474923668	12.34129751	91.89712428	0.5	CRA004775;Yang2025_Silkie_Wenchang	PRJNA827465	0.89612415
ENSGALG00010001330	ENSGALG00010001330	2	-0.087034605	0.089142747	-0.97635094	0.328890584	0	0.69983173	0	1	CRA004775;PRJNA827465	Yang2025_Silkie_Wenchang	0.800761292
ENSGALG00010001333	PLCH1	2	-0.050127541	0.162918856	-0.307684098	0.758322721	0	0.150468926	0	0.5	PRJNA827465;Yang2025_Silkie_Wenchang	CRA004775	0.938954369
ENSGALG00010001333	PLCH1	2	0.332850371	0.43522073	0.764785196	0.444399464	0.196640516	1.807312119	44.66921406	1	CRA004775;Yang2025_Silkie_Wenchang	PRJNA827465	0.846013379
ENSGALG00010001333	PLCH1	2	0.269471901	0.507880794	0.530581003	0.595709163	0.35085807	2.566760713	61.04038858	0.5	CRA004775;PRJNA827465	Yang2025_Silkie_Wenchang	0.893802528
ENSGALG00010001334	EBF3	2	0.151322453	0.245464107	0.61647487	0.537581153	0.091741737	3.776008597	73.51700945	0.5	PRJNA827465;Yang2025_Silkie_Wenchang	CRA004775	0.87481821
ENSGALG00010001334	EBF3	2	0.319442571	0.197050272	1.621122201	0.104991433	0	0.944075318	0	1	CRA004775;Yang2025_Silkie_Wenchang	PRJNA827465	0.550168852
ENSGALG00010001334	EBF3	2	-0.045955104	0.095912393	-0.479136246	0.631841706	0	0.053418516	0	0.5	CRA004775;PRJNA827465	Yang2025_Silkie_Wenchang	0.903058174
ENSGALG00010001343	ENSGALG00010001343	2	-0.327233382	0.246589961	-1.327034488	0.184497274	0	0.243530352	0	1	PRJNA827465;Yang2025_Silkie_Wenchang	CRA004775	0.692816808
ENSGALG00010001343	ENSGALG00010001343	2	-0.183631537	0.335622416	-0.547137283	0.58428442	0	0.00727307	0	1	CRA004775;Yang2025_Silkie_Wenchang	PRJNA827465	0.890842213
ENSGALG00010001343	ENSGALG00010001343	2	-0.373993065	0.270427614	-1.382969218	0.166674305	0	0.089418891	0	1	CRA004775;PRJNA827465	Yang2025_Silkie_Wenchang	0.665626918
ENSGALG00010001347	MYOM1	2	-0.067794482	0.137833943	-0.491856221	0.622820989	0.031931254	6.080305749	83.55345864	0.5	PRJNA827465;Yang2025_Silkie_Wenchang	CRA004775	0.900255255
ENSGALG00010001347	MYOM1	2	0.063457373	0.057961406	1.094821154	0.273594997	0	0.491161764	0	1	CRA004775;Yang2025_Silkie_Wenchang	PRJNA827465	0.774328808
ENSGALG00010001347	MYOM1	2	-0.137469771	0.230287588	-0.596948245	0.550541935	0.050095204	1.216769421	17.81516015	0.5	CRA004775;PRJNA827465	Yang2025_Silkie_Wenchang	0.878805526
ENSGALG00010001349	ING3	2	0.079250438	0.136156072	0.582055849	0.560529063	0.025438159	2.91476306	65.69189401	0.5	PRJNA827465;Yang2025_Silkie_Wenchang	CRA004775	0.881203589
ENSGALG00010001349	ING3	2	0.241154444	0.127672692	1.888849053	0.058912058	0	0.007086678	0	1	CRA004775;Yang2025_Silkie_Wenchang	PRJNA827465	0.42013793
ENSGALG00010001349	ING3	2	-0.013346305	0.07018666	-0.190154432	0.849188117	0	0.852009961	0	0.5	CRA004775;PRJNA827465	Yang2025_Silkie_Wenchang	0.961473816
ENSGALG00010001351	CAPN1	2	-0.32362224	0.063402727	-5.104232185	3.3214E-07	0	0.009090633	0	1	PRJNA827465;Yang2025_Silkie_Wenchang	CRA004775	9.12875E-05
ENSGALG00010001351	CAPN1	2	-0.310881262	0.098742767	-3.148395283	0.001641695	0	0.015139129	0	1	CRA004775;Yang2025_Silkie_Wenchang	PRJNA827465	0.051226717
ENSGALG00010001351	CAPN1	2	-0.324411685	0.07612019	-4.261834924	2.02755E-05	0	0.029122013	0	1	CRA004775;PRJNA827465	Yang2025_Silkie_Wenchang	0.00247481
ENSGALG00010001353	GLRX3	2	-0.127562367	0.139159698	-0.916661711	0.359319936	0.026072614	2.548719432	60.76461036	1	PRJNA827465;Yang2025_Silkie_Wenchang	CRA004775	0.814335519
ENSGALG00010001353	GLRX3	2	-0.211012969	0.158432222	-1.331881646	0.182899083	0.006698614	1.131437531	11.61686152	0.5	CRA004775;Yang2025_Silkie_Wenchang	PRJNA827465	0.691347415
ENSGALG00010001353	GLRX3	2	-0.024649464	0.057356136	-0.429761583	0.667369081	0	0.028961915	0	0.5	CRA004775;PRJNA827465	Yang2025_Silkie_Wenchang	0.913859905
ENSGALG00010001363	HIBADH	2	0.21011822	0.225737174	0.930809119	0.351952312	0.093237692	11.54290064	91.33666631	0.5	PRJNA827465;Yang2025_Silkie_Wenchang	CRA004775	0.810678834
ENSGALG00010001363	HIBADH	2	0.419702602	0.108567207	3.865832168	0.000110711	0	0.548287963	0	1	CRA004775;Yang2025_Silkie_Wenchang	PRJNA827465	0.008378897
ENSGALG00010001363	HIBADH	2	-0.000590625	0.067462251	-0.008754895	0.993014694	0	0.222534918	0	0.5	CRA004775;PRJNA827465	Yang2025_Silkie_Wenchang	0.99794341
ENSGALG00010001364	SMCHD1	2	0.028194372	0.052257846	0.539524179	0.589525218	0	0.589765648	0	1	PRJNA827465;Yang2025_Silkie_Wenchang	CRA004775	0.892320726
ENSGALG00010001364	SMCHD1	2	0.168996526	0.131695479	1.283237109	0.199409019	0	0.297310279	0	1	CRA004775;Yang2025_Silkie_Wenchang	PRJNA827465	0.711195341
ENSGALG00010001364	SMCHD1	2	0.023190837	0.054957815	0.421975242	0.673043089	0	0.991723591	0	1	CRA004775;PRJNA827465	Yang2025_Silkie_Wenchang	0.915153756
ENSGALG00010001365	ENSGALG00010001365	2	-0.628983231	0.121460455	-5.178502168	2.23674E-07	0.012111317	1.643069699	39.13830919	1	PRJNA827465;Yang2025_Silkie_Wenchang	CRA004775	6.71075E-05
ENSGALG00010001365	ENSGALG00010001365	2	-0.73781857	0.150467535	-4.903506733	9.41407E-07	0	0.626391052	0	1	CRA004775;Yang2025_Silkie_Wenchang	PRJNA827465	0.000211429
ENSGALG00010001365	ENSGALG00010001365	2	-0.524123627	0.107082975	-4.89455608	9.85279E-07	0	0.07698749	0	1	CRA004775;PRJNA827465	Yang2025_Silkie_Wenchang	0.000218782
ENSGALG00010001367	ENSGALG00010001367	2	0.075368291	0.512631908	0.14702224	0.883114466	0.334673858	2.722702583	63.27178715	0.5	PRJNA827465;Yang2025_Silkie_Wenchang	CRA004775	0.972001076
ENSGALG00010001367	ENSGALG00010001367	2	-0.260099672	0.895428509	-0.290475084	0.771452809	1.303740869	5.118825006	80.46426672	0.5	CRA004775;Yang2025_Silkie_Wenchang	PRJNA827465	0.94283287
ENSGALG00010001367	ENSGALG00010001367	2	-0.734222269	0.398146877	-1.844099029	0.065168709	0	0.829894902	0	1	CRA004775;PRJNA827465	Yang2025_Silkie_Wenchang	0.439917301
ENSGALG00010001368	WIPF3	2	-0.094887265	0.096810917	-0.980129811	0.327022046	0	0.001258538	0	1	PRJNA827465;Yang2025_Silkie_Wenchang	CRA004775	0.800028239
ENSGALG00010001368	WIPF3	2	-0.044055701	0.215704433	-0.204241055	0.83816513	0	0.27563375	0	0.5	CRA004775;Yang2025_Silkie_Wenchang	PRJNA827465	0.95916157
ENSGALG00010001368	WIPF3	2	-0.085917681	0.104997535	-0.818282834	0.413195705	0	0.323052323	0	0.5	CRA004775;PRJNA827465	Yang2025_Silkie_Wenchang	0.836055586
ENSGALG00010001369	PPP2R2D	2	-0.040275554	0.09313403	-0.432447233	0.665416396	0.013188985	4.060642597	75.37335591	0.5	PRJNA827465;Yang2025_Silkie_Wenchang	CRA004775	0.91350776
ENSGALG00010001369	PPP2R2D	2	0.154281424	0.325610378	0.473822195	0.635626706	0.188159303	8.317099249	87.97657729	0.5	CRA004775;Yang2025_Silkie_Wenchang	PRJNA827465	0.904261643
ENSGALG00010001369	PPP2R2D	2	0.232226133	0.228931248	1.014392466	0.310395518	0.084928698	4.525002264	77.90056354	1	CRA004775;PRJNA827465	Yang2025_Silkie_Wenchang	0.794899969
ENSGALG00010001372	ENSGALG00010001372	2	-0.028729139	0.288334461	-0.099638244	0.920631531	0.109261653	2.915396584	65.69934926	0.5	PRJNA827465;Yang2025_Silkie_Wenchang	CRA004775	0.981469977
ENSGALG00010001372	ENSGALG00010001372	2	-0.075037872	0.383681892	-0.19557314	0.844944286	0.202559092	3.099651028	67.73830373	0.5	CRA004775;Yang2025_Silkie_Wenchang	PRJNA827465	0.960779519
ENSGALG00010001372	ENSGALG00010001372	2	-0.370236834	0.197048038	-1.878916621	0.060255879	0	0.205276678	0	1	CRA004775;PRJNA827465	Yang2025_Silkie_Wenchang	0.425316544
ENSGALG00010001373	IMPA2	2	-0.068404835	0.113806214	-0.601064145	0.547797263	0.019465335	3.774169361	73.5041037	0.5	PRJNA827465;Yang2025_Silkie_Wenchang	CRA004775	0.878281266
ENSGALG00010001373	IMPA2	2	0.077508046	0.098407594	0.787622615	0.430917483	0	0.153739494	0	1	CRA004775;Yang2025_Silkie_Wenchang	PRJNA827465	0.842503929
ENSGALG00010001373	IMPA2	2	-0.107112983	0.13169722	-0.813327597	0.416030259	0.017764081	1.403438769	28.74644609	0.5	CRA004775;PRJNA827465	Yang2025_Silkie_Wenchang	0.837742614
ENSGALG00010001378	POLA2	2	0.312680093	0.200426851	1.560070873	0.118743134	0.072342226	9.727897995	89.72028695	1	PRJNA827465;Yang2025_Silkie_Wenchang	CRA004775	0.578883823
ENSGALG00010001378	POLA2	2	-0.039450397	0.592803965	-0.066548807	0.946940901	0.657455978	14.98615214	93.32717304	0.5	CRA004775;Yang2025_Silkie_Wenchang	PRJNA827465	0.986445196
ENSGALG00010001378	POLA2	2	-0.219492344	0.389597609	-0.563382163	0.573174681	0.266252781	7.312241155	86.32430224	0.5	CRA004775;PRJNA827465	Yang2025_Silkie_Wenchang	0.88575805
ENSGALG00010001380	SCRN1	2	0.072504721	0.097965013	0.740108321	0.45923427	0	0.2471191	0	0.5	PRJNA827465;Yang2025_Silkie_Wenchang	CRA004775	0.850098539
ENSGALG00010001380	SCRN1	2	0.104736237	0.175907604	0.595404832	0.551572896	0.010237269	1.149761258	13.02542221	0.5	CRA004775;Yang2025_Silkie_Wenchang	PRJNA827465	0.878987534
ENSGALG00010001380	SCRN1	2	0.137859392	0.110012808	1.253121298	0.210161566	0	0.711395416	0	1	CRA004775;PRJNA827465	Yang2025_Silkie_Wenchang	0.721895543
ENSGALG00010001381	MDFIC	2	-0.328322256	0.389635793	-0.842638849	0.399430457	0.297208769	47.08548778	97.87620338	0.5	PRJNA827465;Yang2025_Silkie_Wenchang	CRA004775	0.832246851
ENSGALG00010001381	MDFIC	2	-0.231616749	0.539394639	-0.429401281	0.667631222	0.521748179	8.960581967	88.84001057	0.5	CRA004775;Yang2025_Silkie_Wenchang	PRJNA827465	0.913995707
ENSGALG00010001381	MDFIC	2	0.066606189	0.059555755	1.118383761	0.263403127	0	0.738739246	0	1	CRA004775;PRJNA827465	Yang2025_Silkie_Wenchang	0.768643821
ENSGALG00010001382	BNIP3	2	-0.199642665	0.092454918	-2.159351488	0.030822908	0	0.187574603	0	1	PRJNA827465;Yang2025_Silkie_Wenchang	CRA004775	0.299826595
ENSGALG00010001382	BNIP3	2	-0.298843381	0.171737455	-1.740117675	0.081838357	0	0.119514556	0	1	CRA004775;Yang2025_Silkie_Wenchang	PRJNA827465	0.493100254
ENSGALG00010001382	BNIP3	2	-0.193041431	0.102522725	-1.882913579	0.059712081	0	0.350844771	0	1	CRA004775;PRJNA827465	Yang2025_Silkie_Wenchang	0.423249156
ENSGALG00010001385	MME	2	0.322438804	0.192113845	1.678373596	0.093274189	0.038706806	2.058730998	51.42638833	1	PRJNA827465;Yang2025_Silkie_Wenchang	CRA004775	0.523343073
ENSGALG00010001385	MME	2	0.475545232	0.205499162	2.314098158	0.020662338	0	0.962275309	0	0.5	CRA004775;Yang2025_Silkie_Wenchang	PRJNA827465	0.242632772
ENSGALG00010001385	MME	2	0.138877938	0.156732221	0.886084162	0.375572177	0	0.176354054	0	0.5	CRA004775;PRJNA827465	Yang2025_Silkie_Wenchang	0.822974013
ENSGALG00010001386	FKBP14	2	0.042538182	0.312959769	0.135922205	0.891882782	0.181489175	13.6015542	92.64789902	0.5	PRJNA827465;Yang2025_Silkie_Wenchang	CRA004775	0.974015927
ENSGALG00010001386	FKBP14	2	-0.273848707	0.116758428	-2.345429886	0.019005153	0	0.002649677	0	1	CRA004775;Yang2025_Silkie_Wenchang	PRJNA827465	0.230995614
ENSGALG00010001386	FKBP14	2	0.120507243	0.310183967	0.388502489	0.697644213	0.137246975	2.931618693	65.88915187	0.5	CRA004775;PRJNA827465	Yang2025_Silkie_Wenchang	0.921416442
ENSGALG00010001388	MPPE1	2	0.24193861	0.220159466	1.098924404	0.271801041	0.086930435	9.660066866	89.64810478	1	PRJNA827465;Yang2025_Silkie_Wenchang	CRA004775	0.773148753
ENSGALG00010001388	MPPE1	2	-0.005118457	0.51351019	-0.009967585	0.992047149	0.473843035	9.242126999	89.17997989	0.5	CRA004775;Yang2025_Silkie_Wenchang	PRJNA827465	0.997699848
ENSGALG00010001388	MPPE1	2	-0.189376516	0.284089467	-0.66660872	0.505022098	0.118491364	3.122029048	67.96954851	0.5	CRA004775;PRJNA827465	Yang2025_Silkie_Wenchang	0.863821199
ENSGALG00010001395	GNAL	2	-0.058253362	0.060601987	-0.961245084	0.336428953	0	0.421593168	0	0.5	PRJNA827465;Yang2025_Silkie_Wenchang	CRA004775	0.803806405
ENSGALG00010001395	GNAL	2	0.011532515	0.165517664	0.069675436	0.944451994	0	0.239651705	0	0.5	CRA004775;Yang2025_Silkie_Wenchang	PRJNA827465	0.985935636
ENSGALG00010001395	GNAL	2	-0.072964785	0.062452641	-1.168321834	0.242676972	0	0.021467294	0	1	CRA004775;PRJNA827465	Yang2025_Silkie_Wenchang	0.7550024
ENSGALG00010001397	KLHL7	2	0.030126067	0.041097005	0.733047758	0.463529302	0	0.002218081	0	1	PRJNA827465;Yang2025_Silkie_Wenchang	CRA004775	0.852069227
ENSGALG00010001397	KLHL7	2	0.032029661	0.070122768	0.456765504	0.647839603	0	0.001268332	0	1	CRA004775;Yang2025_Silkie_Wenchang	PRJNA827465	0.907478791
ENSGALG00010001397	KLHL7	2	0.028759909	0.0462535	0.621788827	0.534080734	0	0.000143653	0	1	CRA004775;PRJNA827465	Yang2025_Silkie_Wenchang	0.873408008
ENSGALG00010001398	WDR11	2	-0.069056006	0.063632622	-1.085229621	0.277819968	0.004603407	2.315340364	56.80980578	1	PRJNA827465;Yang2025_Silkie_Wenchang	CRA004775	0.776763074
ENSGALG00010001398	WDR11	2	-0.126493484	0.059276301	-2.133963866	0.032845739	0	0.324980383	0	0.5	CRA004775;Yang2025_Silkie_Wenchang	PRJNA827465	0.311420523
ENSGALG00010001398	WDR11	2	-0.005756253	0.056123339	-0.102564328	0.918308755	0	0.010035487	0	0.5	CRA004775;PRJNA827465	Yang2025_Silkie_Wenchang	0.981033557
ENSGALG00010001400	SNX24	2	0.008407617	0.115983963	0.072489479	0.942212378	0	0.933659933	0	0.5	PRJNA827465;Yang2025_Silkie_Wenchang	CRA004775	0.985827325
ENSGALG00010001400	SNX24	2	-0.102608256	0.159674067	-0.642610651	0.52047677	0	0.022911378	0	1	CRA004775;Yang2025_Silkie_Wenchang	PRJNA827465	0.869331539
ENSGALG00010001400	SNX24	2	0.09812155	0.150543045	0.651784011	0.514540518	0	0.089595023	0	0.5	CRA004775;PRJNA827465	Yang2025_Silkie_Wenchang	0.866869697
ENSGALG00010001402	NUP42	2	0.091291204	0.095138394	0.959562176	0.337275613	0	0.882512507	0	0.5	PRJNA827465;Yang2025_Silkie_Wenchang	CRA004775	0.804086136
ENSGALG00010001402	NUP42	2	-0.189616268	0.157278807	-1.205605967	0.227969457	0.010892503	1.275441698	21.59578903	1	CRA004775;Yang2025_Silkie_Wenchang	PRJNA827465	0.740853612
ENSGALG00010001402	NUP42	2	-0.078962717	0.256464517	-0.307889442	0.75816646	0.104179629	4.528138376	77.91586924	0.5	CRA004775;PRJNA827465	Yang2025_Silkie_Wenchang	0.938954369
ENSGALG00010001406	EML3	2	0.21235364	0.170199095	1.247677846	0.212149057	0.045507614	4.211897723	76.25773307	1	PRJNA827465;Yang2025_Silkie_Wenchang	CRA004775	0.72461062
ENSGALG00010001406	EML3	2	-0.002482411	0.421186726	-0.00589385	0.995297415	0.326988581	12.74439261	92.15341185	0.5	CRA004775;Yang2025_Silkie_Wenchang	PRJNA827465	0.998269135
ENSGALG00010001406	EML3	2	-0.151836832	0.24694187	-0.614868721	0.538641421	0.105824467	6.995607185	85.70531516	0.5	CRA004775;PRJNA827465	Yang2025_Silkie_Wenchang	0.87481821
ENSGALG00010001407	FOXP2	2	0.158014942	0.263530313	0.59960822	0.548767367	0.116688622	5.689388539	82.42341874	0.5	PRJNA827465;Yang2025_Silkie_Wenchang	CRA004775	0.878281266
ENSGALG00010001407	FOXP2	2	0.467345111	0.177490343	2.633073452	0.008461605	0	0.003491843	0	1	CRA004775;Yang2025_Silkie_Wenchang	PRJNA827465	0.143515254
ENSGALG00010001407	FOXP2	2	0.113082874	0.261552426	0.432352608	0.665485157	0.097046955	2.699699575	62.95884145	0.5	CRA004775;PRJNA827465	Yang2025_Silkie_Wenchang	0.91350776
ENSGALG00010001409	GPNMB	2	6.618976911	0.620995987	10.65864683	1.58892E-26	0	0.384402558	0	1	PRJNA827465;Yang2025_Silkie_Wenchang	CRA004775	2.31294E-23
ENSGALG00010001409	GPNMB	2	6.367700534	0.481725063	13.21853692	6.85851E-40	0	0.70691146	0	1	CRA004775;Yang2025_Silkie_Wenchang	PRJNA827465	1.3478E-36
ENSGALG00010001409	GPNMB	2	6.200942796	0.451158399	13.74449155	5.49578E-43	0	0.030955381	0	1	CRA004775;PRJNA827465	Yang2025_Silkie_Wenchang	1.13685E-39
ENSGALG00010001411	FGFR2	2	0.08094042	0.185245236	0.43693658	0.662157342	0	0.737557528	0	0.5	PRJNA827465;Yang2025_Silkie_Wenchang	CRA004775	0.912691648
ENSGALG00010001411	FGFR2	2	0.279690504	0.255424292	1.095003542	0.273515085	0	0.001799244	0	1	CRA004775;Yang2025_Silkie_Wenchang	PRJNA827465	0.774252172
ENSGALG00010001411	FGFR2	2	0.008794451	0.218927028	0.040170697	0.967957039	0	0.358538285	0	0.5	CRA004775;PRJNA827465	Yang2025_Silkie_Wenchang	0.991128245
ENSGALG00010001412	ENSGALG00010001412	2	0.230948861	0.316751845	0.729116071	0.465930665	0.173000811	7.13972904	85.99386623	0.5	PRJNA827465;Yang2025_Silkie_Wenchang	CRA004775	0.852511753
ENSGALG00010001412	ENSGALG00010001412	2	-0.089217545	0.742285138	-0.120193091	0.904330191	0.939520848	6.205316318	83.88478606	0.5	CRA004775;Yang2025_Silkie_Wenchang	PRJNA827465	0.977586231
ENSGALG00010001412	ENSGALG00010001412	2	-0.324719381	0.393341583	-0.825540434	0.409064837	0.201511702	2.183152682	54.19468331	1	CRA004775;PRJNA827465	Yang2025_Silkie_Wenchang	0.834316328

ENSGALG00010001414	CCDC126	2	-0.026770761	0.057062128	-0.469151127	0.638961616	0	0.208094416	0	1	PRJNA827465;Yang2025_Silkie_Wenchang	CRA004775	0.905858255
ENSGALG00010001414	CCDC126	2	-0.144848661	0.13383982	-1.082253852	0.279139745	0.013969688	1.43200423	30.16780406	1	CRA004775;Yang2025_Silkie_Wenchang	PRJNA827465	0.777370468
ENSGALG00010001414	CCDC126	2	-0.120403164	0.166489186	-0.723189098	0.469563716	0.035351362	2.19578849	54.45827298	1	CRA004775;PRJNA827465	Yang2025_Silkie_Wenchang	0.853485658
ENSGALG00010001415	MC5R	2	-0.217409469	0.290672682	-0.747953495	0.454488209	0.126225091	3.92151767	74.49966864	0.5	PRJNA827465;Yang2025_Silkie_Wenchang	CRA004775	0.849072665
ENSGALG00010001415	MC5R	2	-0.089208036	0.535803148	-0.166494049	0.867768166	0.423726138	3.405111016	70.63238187	0.5	CRA004775;Yang2025_Silkie_Wenchang	PRJNA827465	0.967946362
ENSGALG00010001415	MC5R	2	0.111504853	0.175872897	0.634008167	0.526075492	0	0.786531256	0	1	CRA004775;PRJNA827465	Yang2025_Silkie_Wenchang	0.870509645
ENSGALG00010001416	AP4M1	2	0.246257109	0.250506105	0.983038356	0.325588561	0.095604862	3.488230522	71.33216989	1	PRJNA827465;Yang2025_Silkie_Wenchang	CRA004775	0.799987949
ENSGALG00010001416	AP4M1	2	0.125238538	0.439397652	0.285023229	0.775626346	0.319022021	5.748470535	82.60406844	0.5	CRA004775;Yang2025_Silkie_Wenchang	PRJNA827465	0.943815265
ENSGALG00010001416	AP4M1	2	-0.048262305	0.162051291	-0.297821169	0.765839657	0.031817397	1.952908161	48.79431505	0.5	CRA004775;PRJNA827465	Yang2025_Silkie_Wenchang	0.941000908
ENSGALG00010001418	RNMT	2	-0.154380503	0.11329246	-1.362672357	0.172985794	0.020997624	5.459701156	81.68397919	1	PRJNA827465;Yang2025_Silkie_Wenchang	CRA004775	0.676043418
ENSGALG00010001418	RNMT	2	-0.130862989	0.200723501	-0.651959649	0.514429241	0.054536182	2.495245691	59.92378612	0.5	CRA004775;Yang2025_Silkie_Wenchang	PRJNA827465	0.866869697
ENSGALG00010001418	RNMT	2	-0.034667559	0.059615187	-0.581522266	0.560888516	0	0.562517457	0	0.5	CRA004775;PRJNA827465	Yang2025_Silkie_Wenchang	0.881354866
ENSGALG00010001422	AP1S1	2	0.216882784	0.152914828	1.418324086	0.156096168	0.019869357	1.496762399	33.18912871	1	PRJNA827465;Yang2025_Silkie_Wenchang	CRA004775	0.649132948
ENSGALG00010001422	AP1S1	2	0.122745563	0.340309326	0.360688215	0.718332535	0.168970733	3.691970998	72.91419676	0.5	CRA004775;Yang2025_Silkie_Wenchang	PRJNA827465	0.928033122
ENSGALG00010001422	AP1S1	2	0.025441999	0.15293927	0.166353605	0.867878683	0.021271667	1.611913564	37.96193406	0.5	CRA004775;PRJNA827465	Yang2025_Silkie_Wenchang	0.967946362
ENSGALG00010001423	FAM210A	2	-0.041873561	0.060088992	-0.696859089	0.485890983	0	0.568269756	0	0.5	PRJNA827465;Yang2025_Silkie_Wenchang	CRA004775	0.857391223
ENSGALG00010001423	FAM210A	2	0.254188518	0.27633477	0.919857163	0.357647407	0.099916159	2.491163356	59.85811217	1	CRA004775;Yang2025_Silkie_Wenchang	PRJNA827465	0.813508654
ENSGALG00010001423	FAM210A	2	0.201133052	0.336359412	0.597970637	0.549859526	0.179103823	3.980398027	74.87688434	0.5	CRA004775;PRJNA827465	Yang2025_Silkie_Wenchang	0.878608324
ENSGALG00010001426	ATE1	2	-0.129128886	0.143315331	-0.901012372	0.367581742	0.035855903	7.320290516	86.33933998	1	PRJNA827465;Yang2025_Silkie_Wenchang	CRA004775	0.819975483
ENSGALG00010001426	ATE1	2	-0.203620041	0.119374363	-1.705726721	0.088058972	0.011411671	1.55397438	35.64887474	1	CRA004775;Yang2025_Silkie_Wenchang	PRJNA827465	0.509839669
ENSGALG00010001426	ATE1	2	-0.001494299	0.035945336	-0.041571423	0.966840355	0	0.038004714	0	1	CRA004775;PRJNA827465	Yang2025_Silkie_Wenchang	0.990893423
ENSGALG00010001427	ASB11	2	-0.646187246	0.681726656	-0.947868534	0.343196385	0.82615052	8.988495955	88.87466819	0.5	PRJNA827465;Yang2025_Silkie_Wenchang	CRA004775	0.806218116
ENSGALG00010001427	ASB11	2	0.072921054	0.283134441	0.257549217	0.796754826	0	0.120187437	0	1	CRA004775;Yang2025_Silkie_Wenchang	PRJNA827465	0.949622804
ENSGALG00010001427	ASB11	2	-0.636651887	0.806506992	-0.789394131	0.429881685	1.025054421	4.374715653	77.14137148	0.5	CRA004775;PRJNA827465	Yang2025_Silkie_Wenchang	0.842012722
ENSGALG00010001432	NSMCE4A	2	0.052396726	0.068708442	0.762595163	0.44570487	0	0.745641268	0	0.5	PRJNA827465;Yang2025_Silkie_Wenchang	CRA004775	0.846013379
ENSGALG00010001432	NSMCE4A	2	-0.01651815	0.132464121	-0.124699053	0.900761807	0	0.38675691	0	0.5	CRA004775;Yang2025_Silkie_Wenchang	PRJNA827465	0.976497577
ENSGALG00010001432	NSMCE4A	2	0.083152338	0.072554981	1.146059679	0.25177046	0	0.010924266	0	1	CRA004775;PRJNA827465	Yang2025_Silkie_Wenchang	0.761871849
ENSGALG00010001440	CDCA7L	2	-0.13734381	0.052622969	-2.609959349	0.009055298	0	0.000309005	0	1	PRJNA827465;Yang2025_Silkie_Wenchang	CRA004775	0.149037013
ENSGALG00010001440	CDCA7L	2	-0.094072883	0.119423878	-0.78772563	0.430859005	0	0.72309598	0	0.5	CRA004775;Yang2025_Silkie_Wenchang	PRJNA827465	0.842503929
ENSGALG00010001440	CDCA7L	2	-0.128383837	0.056720867	-2.263432229	0.023609058	0	0.829217819	0	0.5	CRA004775;PRJNA827465	Yang2025_Silkie_Wenchang	0.26228978
ENSGALG00010001441	HMGCLL1	2	0.288068651	0.14554612	1.979225904	0.047790578	0	0.072023049	0	1	PRJNA827465;Yang2025_Silkie_Wenchang	CRA004775	0.378006253
ENSGALG00010001441	HMGCLL1	2	0.222853082	0.224794935	0.991361672	0.321509009	0	0.88449645	0	0.5	CRA004775;Yang2025_Silkie_Wenchang	PRJNA827465	0.798513866
ENSGALG00010001441	HMGCLL1	2	0.207907282	0.16344235	1.272052695	0.203354397	0	0.701191863	0	0.5	CRA004775;PRJNA827465	Yang2025_Silkie_Wenchang	0.717242017
ENSGALG00010001442	LDLRAD4	2	0.166217663	0.214444936	0.775106494	0.438276735	0.0743508	5.092571207	80.3635539	0.5	PRJNA827465;Yang2025_Silkie_Wenchang	CRA004775	0.843979791
ENSGALG00010001442	LDLRAD4	2	0.204184429	0.263672928	0.774385258	0.438702999	0.081744095	2.151466259	53.52007053	0.5	CRA004775;Yang2025_Silkie_Wenchang	PRJNA827465	0.844002503
ENSGALG00010001442	LDLRAD4	2	-0.042970164	0.100544048	-0.427376502	0.669105119	0	0.117503754	0	1	CRA004775;PRJNA827465	Yang2025_Silkie_Wenchang	0.914377769
ENSGALG00010001443	ANKRD26	2	0.657597005	0.588897929	1.116657018	0.264141002	0.670536841	29.68209532	96.63096561	1	PRJNA827465;Yang2025_Silkie_Wenchang	CRA004775	0.768790489
ENSGALG00010001443	ANKRD26	2	0.891429856	0.406068174	2.195271419	0.028144134	0.261980116	4.686813153	78.66354029	1	CRA004775;Yang2025_Silkie_Wenchang	PRJNA827465	0.285752749
ENSGALG00010001443	ANKRD26	2	0.130309045	0.124064562	1.050332524	0.293565257	0.009186809	1.163151376	14.02666753	1	CRA004775;PRJNA827465	Yang2025_Silkie_Wenchang	0.786979185
ENSGALG00010001448	GPR37	2	0.13100519	0.362954974	0.36094061	0.718143845	0.216112432	5.557447727	82.00612855	0.5	PRJNA827465;Yang2025_Silkie_Wenchang	CRA004775	0.927975712
ENSGALG00010001448	GPR37	2	-0.214503168	0.216636871	-0.990150786	0.322100423	0	0.118364006	0	0.5	CRA004775;Yang2025_Silkie_Wenchang	PRJNA827465	0.798513866
ENSGALG00010001448	GPR37	2	0.454360035	0.200594447	2.265067865	0.023508515	0	0.362559307	0	1	CRA004775;PRJNA827465	Yang2025_Silkie_Wenchang	0.261669548
ENSGALG00010001453	TAX1BP1	2	0.148936183	0.231449949	0.643491965	0.519904927	0.099837041	13.94413803	92.82852767	0.5	PRJNA827465;Yang2025_Silkie_Wenchang	CRA004775	0.869253622
ENSGALG00010001453	TAX1BP1	2	0.370306141	0.109455782	3.383157403	0.000716576	0	0.259156506	0	1	CRA004775;Yang2025_Silkie_Wenchang	PRJNA827465	0.029993149
ENSGALG00010001453	TAX1BP1	2	-0.024661661	0.10895891	-0.22633909	0.820937698	0.012745482	1.348420021	25.83913141	0.5	CRA004775;PRJNA827465	Yang2025_Silkie_Wenchang	0.955548641
ENSGALG00010001454	VEGFD	2	-0.150098612	0.288676788	-0.519953866	0.60309573	0.138103918	5.651125793	82.3044109	0.5	PRJNA827465;Yang2025_Silkie_Wenchang	CRA004775	0.895538966
ENSGALG00010001454	VEGFD	2	-0.443187103	0.196848099	-2.251416732	0.024359155	0	0.087764705	0	1	CRA004775;Yang2025_Silkie_Wenchang	PRJNA827465	0.266904902
ENSGALG00010001454	VEGFD	2	0.099126923	0.123517568	0.802532988	0.422244714	0	0.409819938	0	0.5	CRA004775;PRJNA827465	Yang2025_Silkie_Wenchang	0.839649223
ENSGALG00010001456	LSM8	2	-0.075154925	0.255763672	-0.293845192	0.7688762	0.090737699	2.97076282	66.33861198	0.5	PRJNA827465;Yang2025_Silkie_Wenchang	CRA004775	0.941670291
ENSGALG00010001456	LSM8	2	-0.254689935	0.198567006	-1.282639748	0.199618318	0	0.530394196	0	1	CRA004775;Yang2025_Silkie_Wenchang	PRJNA827465	0.711489865
ENSGALG00010001456	LSM8	2	0.09142984	0.120346815	0.759719642	0.447422185	0	0.538021291	0	0.5	CRA004775;PRJNA827465	Yang2025_Silkie_Wenchang	0.84650962
ENSGALG00010001457	NDC80	2	-0.087612898	0.09876904	-0.887048189	0.375052957	0.004970291	1.335966355	25.14781558	0.5	PRJNA827465;Yang2025_Silkie_Wenchang	CRA004775	0.822916144
ENSGALG00010001457	NDC80	2	0.019974921	0.398749474	0.050093911	0.960047552	0.189604933	1.733283814	42.30604407	0.5	CRA004775;Yang2025_Silkie_Wenchang	PRJNA827465	0.989338243
ENSGALG00010001457	NDC80	2	0.048574038	0.183365824	0.264903255	0.791084684	0.023375899	1.091194344	8.357296248	1	CRA004775;PRJNA827465	Yang2025_Silkie_Wenchang	0.947840178
ENSGALG00010001458	JAZF1	2	-0.025901986	0.084288142	-0.307302845	0.75861287	0.004375974	1.286986044	22.29907972	0.5	PRJNA827465;Yang2025_Silkie_Wenchang	CRA004775	0.938954369
ENSGALG00010001458	JAZF1	2	-0.212107238	0.14812683	-1.431929905	0.152163883	0	0.341117751	0	1	CRA004775;Yang2025_Silkie_Wenchang	PRJNA827465	0.643687268
ENSGALG00010001458	JAZF1	2	-0.033840486	0.146669393	-0.2307263	0.81752744	0.019592979	1.244822009	19.66723011	0.5	CRA004775;PRJNA827465	Yang2025_Silkie_Wenchang	0.954888783
ENSGALG00010001460	MALSU1	2	-0.046726838	0.054713204	-0.854032149	0.393087149	0.001590768	1.2217296	18.14882772	0.5	PRJNA827465;Yang2025_Silkie_Wenchang	CRA004775	0.838302579
ENSGALG00010001460	MALSU1	2	0.033017685	0.101094885	0.326600949	0.743969725	0	0.298040801	0	0.5	CRA004775;Yang2025_Silkie_Wenchang	PRJNA827465	0.935821071
ENSGALG00010001460	MALSU1	2	-0.076309487	0.046537623	-1.639737515	0.101059755	0	0.005731484	0	1	CRA004775;PRJNA827465	Yang2025_Silkie_Wenchang	0.542487465
ENSGALG00010001461	ASB9	2	0.281202802	0.208995144	1.345490215	0.178464075	0.037912663	1.753465031	42.96941264	1	PRJNA827465;Yang2025_Silkie_Wenchang	CRA004775	0.684796269
ENSGALG00010001461	ASB9	2	0.085369139	0.182334131	0.468201637	0.639640402	0	0.005410024	0	1	CRA004775;Yang2025_Silkie_Wenchang	PRJNA827465	0.906064918
ENSGALG00010001461	ASB9	2	0.393322018	0.208852369	1.883253806	0.05966598	0	0.91202344	0	1	CRA004775;PRJNA827465	Yang2025_Silkie_Wenchang	0.423235714
ENSGALG00010001462	DHX36	2	0.127543974	0.10774271	1.183782864	0.236499027	0.017153909	3.392493629	70.52315762	1	PRJNA827465;Yang2025_Silkie_Wenchang	CRA004775	0.7487803
ENSGALG00010001462	DHX36	2	0.032869901	0.230968776	0.142313179	0.886832637	0.093191013	7.895547068	87.33463316	0.5	CRA004775;Yang2025_Silkie_Wenchang	PRJNA827465	0.972850601
ENSGALG00010001462	DHX36	2	-0.053630804	0.117805004	-0.455250645	0.648928929	0.020811592	3.499580065	71.42514298	0.5	CRA004775;PRJNA827465	Yang2025_Silkie_Wenchang	0.907783669
ENSGALG00010001463	DSEL	2	-0.166452918	0.114681015	-1.451442661	0.146656638	0.021463868	5.234423519	80.89569947	1	PRJNA827465;Yang2025_Silkie_Wenchang	CRA004775	0.635558525
ENSGALG00010001463	DSEL	2	-0.071105268	0.301094148	-0.23615626	0.813311419	0.132067088	2.970474582	66.33534567	0.5	CRA004775;Yang2025_Silkie_Wenchang	PRJNA827465	0.953587297
ENSGALG00010001463	DSEL	2	-0.016284508	0.127358709	-0.127863323	0.898257137	0.01570602	1.243291327	19.56832815	0.5	CRA004775;PRJNA827465	Yang2025_Silkie_Wenchang	0.97575249
ENSGALG00010001465	TACC2	2	-0.034492536	0.275737243	-0.125092046	0.90045068	0.131879437	7.068053728	85.85183364	0.5	PRJNA827465;Yang2025_Silkie_Wenchang	CRA004775	0.976475201
ENSGALG00010001465	TACC2	2	0.305061067	0.165835915	1.839535589	0.065836448	0	0.123621022	0	1	CRA004775;Yang2025_Silkie_Wenchang	PRJNA827465	0.442023131
ENSGALG00010001465	TACC2	2	-0.012545004	0.335893622	-0.037348147	0.970207416	0.180097399	4.214714206	76.27359885	0.5	CRA004775;PRJNA827465	Yang2025_Silkie_Wenchang	0.991681174
ENSGALG00010001466	PRKX	2	-0.140886076	0.061795826	-2.279863962	0.022615758	0	0.727674984	0	1	PRJNA827465;Yang2025_Silkie_Wenchang	CRA004775	0.255732111
ENSGALG00010001466	PRKX	2	-0.213758451	0.116350531	-1.837193606	0.066181319	0	0.249604888	0	0.5	CRA004775;Yang2025_Silkie_Wenchang	PRJNA827465	0.442974406
ENSGALG00010001466	PRKX	2	-0.106465883	0.071191704	-1.495481608	0.134788796	0	0.065113596	0	0.5	CRA004775;PRJNA827465	Yang2025_Silkie_Wenchang	0.615292791
ENSGALG00010001468	ANOS1	2	-0.063084184	0.098932018	-0.637651846	0.523700338	0	0.001756915	0	1	PRJNA827465;Yang2025_Silkie_Wenchang	CRA004775	0.869661505
ENSGALG00010001468	ANOS1	2	0.062130938	0.223604227	0.277861194	0.781118914	0.031377257	1.274864736	21.5603058	0.5	CRA004775;Yang2025_Silkie_Wenchang	PRJNA827465	0.945235709
ENSGALG00010001468	ANOS1	2	0.042966	0.218705715	0.196455772	0.844253447	0.045701007	1.436245255	30.37400843	0.5	CRA004775;PRJNA827465	Yang2025_Silkie_Wenchang	0.960723102
ENSGALG00010001469	ARHGEF26	2	0.130849117	0.078359036	1.669866341	0.094945811	0	0.015882281	0	1	PRJNA827465;Yang2025_Silkie_Wenchang	CRA004775	0.527442434
ENSGALG00010001469	ARHGEF26	2	0.127754729	0.159206453	0.802446925	0.422294478	0	0.052685997	0	1	CRA004775;Yang2025_Silkie_Wenchang	PRJNA827465	0.839696443
ENSGALG00010001469	ARHGEF26	2	0.139368267	0.085461345	1.630775491	0.102937701	0	0.033622077	0	1	CRA004775;PRJNA827465	Yang2025_Silkie_Wenchang	0.546946121
ENSGALG00010001470	GPR85	2	-0.192979954	0.280703675	-0.68748638	0.491776281	0.068888537	1.734471896	42.34556339	0.5	PRJNA827465;Yang2025_Silkie_Wenchang	CRA004775	0.858983177
ENSGALG00010001470	GPR85	2	0.419155794	0.434970628	0.963641604	0.335225641	0.143058791	1.449139295	30.99352124	1	CRA004775;Yang2025_Silkie_Wenchang	PRJNA827465	0.803198384
ENSGALG00010001470	GPR85	2	0.191123643	0.751409171	0.25435362	0.799222379	0.882575639	4.046157482	75.28519331	0.5	CRA004775;PRJNA827465	Yang2025_Silkie_Wenchang	0.949922217
ENSGALG00010001471	BMP5	2	0.438783798	0.287800076	1.524613207	0.127355601	0.109668689	2.799780267	64.28291134	1	PRJNA827465;Yang2025_Silkie_Wenchang	CRA004775	0.599191336
ENSGALG00010001471	BMP5	2	0.570438602	0.360040017	1.584375556	0.113108281	0.083804843	1.324606566	24.50588534	0.5	CRA004775;Yang2025_Silkie_Wenchang	PRJNA827465	0.567482422
ENSGALG00010001471	BMP5	2	0.179544199	0.172246873	1.042365503	0.297242254	0	0.128737855	0	0.5	CRA004775;PRJNA827465	Yang2025_Silkie_Wenchang	0.788466926
ENSGALG00010001472	ENSGALG00010001472	2	0.18405595	0.282187304	0.652247453	0.514241552	0.132721278	5.893304235	83.03159043	0.5	PRJNA827465;Yang2025_Silkie_Wenchang	CRA004775	0.866866841
ENSGALG00010001472	ENSGALG00010001472	2	0.454029586	0.177721941	2.55471881	0.010627361	0	0.170909707	0	1	CRA004775;Yang2025_Silkie_Wenchang	PRJNA827465	0.164355008
ENSGALG00010001472	ENSGALG00010001472	2	-0.055822172	0.127229161	-0.438752971	0.66084054	0	0.522700908	0	0.5	CRA004775;PRJNA827465	Yang2025_Silkie_Wenchang	0.91192683
ENSGALG00010001475	BMT2	2	0.011932509	0.129327157	0.092266072	0.926486644	0.023500615	3.33195108	69.98755456	0.5	PRJNA827465;Yang2025_Silkie_Wenchang	CRA004775	0.98280842
ENSGALG00010001475	BMT2	2	0.056109763	0.228813002	0.245221044	0.806285283	0.073235234	2.972233399	66.35526671	0.5	CRA004775;Yang2025_Silkie_Wenchang	PRJNA827465	0.952174506
ENSGALG00010001475	BMT2	2	0.157593081	0.084235389	1.870865464	0.061363731	0	0.632894961	0	1	CRA004775;PRJNA827465	Yang2025_Silkie_Wenchang	0.427938152
ENSGALG00010001476	IGF2BP3	2	-0.184712208	0.061374503	-3.009591895	0.002615989	0	0.245916151	0	1	PRJNA827465;Yang2025_Silkie_Wenchang	CRA004775	0.069143394
ENSGALG00010001476	IGF2BP3	2	-0.212308989	0.113352028	-1.873005644	0.061067606	0	0.722823989	0	0.5	CRA004775;Yang2025_Silkie_Wenchang	PRJNA827465	0.42722323
ENSGALG00010001476	IGF2BP3	2	-0.158518457	0.071635119	-2.212859564	0.026907329	0	0.539974285	0	0.5	CRA004775;PRJNA827465	Yang2025_Silkie_Wenchang	0.279402579
ENSGALG00010001477	NLGN4	2	0.061483294	0.188363915	0.326406966	0.744116468	0	0.339133401	0	0.5	PRJNA827465;Yang2025_Silkie_Wenchang	CRA004775	0.935821071
ENSGALG00010001477	NLGN4	2	0.340085349	0.297877016	1.141697178	0.253579914	0	0.330094224	0	1	CRA004775;Yang2025_Silkie_Wenchang	PRJNA827465	0.763346521
ENSGALG00010001477	NLGN4	2	0.088237391	0.225695495	0.390957696	0.695828505	0.010403138	1.056471367	5.345281375	0.5	CRA004775;PRJNA827465	Yang2025_Silkie_Wenchang	0.921038437
ENSGALG00010001479	TMEM168	2	-0.303004076	0.233485415	-1.297743056	0.194375643	0.103822729	20.804629	95.1933774	1	PRJNA827465;Yang2025_Silkie_Wenchang	CRA004775	0.705148332
ENSGALG00010001479	TMEM168	2	-0.468621313	0.13798287	-3.396228187	0.000683214	0.017055413	1.43175173	30.15548862	1	CRA004775;Yang2025_Silkie_Wenchang	PRJNA827465	0.028935723
ENSGALG00010001479	TMEM168	2	-0.079376954	0.056260064	-1.41089341	0.158276046	0	0.227388757	0	1	CRA004775;PRJNA827465	Yang2025_Silkie_Wenchang	0.652026484
ENSGALG00010001482	TRA2A	2	0.094819506	0.127474133	0.743833307	0.456977327	0.024877613	3.662687832	72.69764594	0.5	PRJNA827465;Yang2025_Silkie_Wenchang	CRA004775	0.849541335
ENSGALG00010001482	TRA2A	2	0.129959076	0.158805412	0.818354198	0.413154967	0.023827144	1.806172879	44.63431427	0.5	CRA004775;Yang2025_Silkie_Wenchang	PRJNA827465	0.836055586
ENSGALG00010001482	TRA2A	2	-0.009132512	0.040055713	-0.227995251	0.819649932	0	0.09695535	0	1	CRA004775;PRJNA827465	Yang2025_Silkie_Wenchang	0.955256039
ENSGALG00010001486	WNT2	2	-0.266481938	0.186793324	-1.426613822	0.153691238	0	0.020151377	0	1	PRJNA827465;Yang2025_Silkie_Wenchang	CRA004775	0.646153969
ENSGALG00010001486	WNT2	2	0.236760994	0.625098302	0.378758018	0.704867565	0.529636462	2.679719025	62.68265476	0.5	CRA004775;Yang2025_Silkie_Wenchang	PRJNA827465	0.924152671
ENSGALG00010001486	WNT2	2	0.208094347	0.652531199	0.318903291	0.749799849	0.612985021	3.020840654	66.89663194	0.5	CRA004775;PRJNA827465	Yang2025_Silkie_Wenchang	0.937362187
ENSGALG00010001487	CDH19	2	0.417111339	0.112239932	3.716247241	0.000202204	0	0.211977112	0	1	PRJNA827465;Yang2025_Silkie_Wenchang	CRA004775	0.01288041
ENSGALG00010001487	CDH19	2	0.631506162	0.295495868	2.137106572	0.032589325	0.081598913	1.459692305	31.49241132	1	CRA004775;Yang2025_Silkie_Wenchang	PRJNA827465	0.310037591
ENSGALG00010001487	CDH19	2	0.600689596	0.368797477	1.62877903	0.103359806	0.166092667	1.956200033	48.88048342	1	CRA004775;PRJNA827465	Yang2025_Silkie_Wenchang	0.547576788
ENSGALG00010001491	PHF3	2	0.171506094	0.241646807	0.709738713	0.477866181	0.09551615	4.878095663	79.50019702	0.5	PRJNA827465;Yang2025_Silkie_Wenchang	CRA004775	0.855366076
ENSGALG00010001491	PHF3	2	0.442006116	0.168759789	2.619143573	0.008815084	0	0.014950753	0	1	CRA004775;Yang2025_Silkie_Wenchang	PRJNA827465	0.147241496
ENSGALG00010001491	PHF3	2	0.10844436	0.207834206	0.52178302	0.601821414	0.058551329	2.411497564	58.53199211	0.5	CRA004775;PRJNA827465	Yang2025_Silkie_Wenchang	0.895283385
ENSGALG00010001492	ENSGALG00010001492	2	0.550952636	0.602025569	0.915164845	0.360105097	0.610729044	6.337289228	84.2203825	0.5	PRJNA827465;Yang2025_Silkie_Wenchang	CRA004775	0.814880744
ENSGALG00010001492	ENSGALG00010001492	2	0.405059962	0.79649668	0.508551978	0.611066297	1.083173867	6.748314615	85.1814852	0.5	CRA004775;Yang2025_Silkie_Wenchang	PRJNA827465	0.897559444
ENSGALG00010001492	ENSGALG00010001492	2	-0.151432154	0.266975982	-0.567212648	0.570569716	0	0.438031702	0	1	CRA004775;PRJNA827465	Yang2025_Silkie_Wenchang	0.885152854
ENSGALG00010001495	CCNL1	2	0.382755995	0.334865597	1.14301379	0.253032866	0.205683416	11.68339389	91.44084322	1	PRJNA827465;Yang2025_Silkie_Wenchang	CRA004775	0.762731469
ENSGALG00010001495	CCNL1	2	0.495734609	0.263614417	1.880529203	0.06003599	0.09590392	3.167775419	68.43210557	1	CRA004775;Yang2025_Silkie_Wenchang	PRJNA827465	0.424082409
ENSGALG00010001495	CCNL1	2	0.081124638	0.079017631	1.026665022	0.304578225	0	0.31196946	0	1	CRA004775;PRJNA827465	Yang2025_Silkie_Wenchang	0.79225794
ENSGALG00010001496	PTP4A1	2	-0.290234206	0.25135404	-1.154682876	0.248220314	0.111202071	8.112999416	87.6741024	1	PRJNA827465;Yang2025_Silkie_Wenchang	CRA004775	0.758364066
ENSGALG00010001496	PTP4A1	2	-0.171200189	0.405252611	-0.422453019	0.672694385	0.290054887	8.407541723	88.10591689	0.5	CRA004775;Yang2025_Silkie_Wenchang	PRJNA827465	0.914956406
ENSGALG00010001496	PTP4A1	2	0.023593047	0.133565942	0.176639696	0.859791411	0.015992339	1.509792174	33.76571839	0.5	CRA004775;PRJNA827465	Yang2025_Silkie_Wenchang	0.964858345
ENSGALG00010001499	TIPARP	2	-0.133700326	0.077111895	-1.733848278	0.082945007	0	0.147502564	0	1	PRJNA827465;Yang2025_Silkie_Wenchang	CRA004775	0.495859127
ENSGALG00010001499	TIPARP	2	0.209927704	0.492835569	0.425958915	0.670137784	0.371963449	3.505854262	71.47628152	0.5	CRA004775;Yang2025_Silkie_Wenchang	PRJNA827465	0.914452132
ENSGALG00010001499	TIPARP	2	0.229649402	0.45864239	0.500715607	0.616571287	0.31450829	3.147490075	68.22865279	0.5	CRA004775;PRJNA827465	Yang2025_Silkie_Wenchang	0.899111936
ENSGALG00010001501	CAPZA2	2	-0.22475229	0.181422017	-1.238836904	0.215405904	0.058513803	8.576069424	88.33964663	1	PRJNA827465;Yang2025_Silkie_Wenchang	CRA004775	0.728768034
ENSGALG00010001501	CAPZA2	2	-0.067536475	0.408241255	-0.165432755	0.868603374	0.278698264	5.525908034	81.90342666	0.5	CRA004775;Yang2025_Silkie_Wenchang	PRJNA827465	0.968254536
ENSGALG00010001501	CAPZA2	2	0.056592983	0.19869277	0.284826584	0.775777005	0.049883126	1.88490912	46.94704431	0.5	CRA004775;PRJNA827465	Yang2025_Silkie_Wenchang	0.943820051
ENSGALG00010001504	RBMS3	2	0.048891289	0.247817693	0.197287323	0.843602699	0.095331763	3.650640504	72.60754657	0.5	PRJNA827465;Yang2025_Silkie_Wenchang	CRA004775	0.96057435
ENSGALG00010001504	RBMS3	2	0.298879617	0.200325264	1.491971661	0.135706575	0	0.174240625	0	1	CRA004775;Yang2025_Silkie_Wenchang	PRJNA827465	0.617196137
ENSGALG00010001504	RBMS3	2	-0.105810035	0.105707707	-1.000968026	0.316842267	0.009884723	1.20187213	16.79647317	0.5	CRA004775;PRJNA827465	Yang2025_Silkie_Wenchang	0.796959041
ENSGALG00010001505	SSR3	2	-0.119912544	0.15680867	-0.764706085	0.444446582	0.038706942	4.160731662	75.96576561	0.5	PRJNA827465;Yang2025_Silkie_Wenchang	CRA004775	0.846013379
ENSGALG00010001505	SSR3	2	-0.385679878	0.132497196	-2.910853139	0.003604434	0.001629589	1.035997958	3.474713176	1	CRA004775;Yang2025_Silkie_Wenchang	PRJNA827465	0.085443345
ENSGALG00010001505	SSR3	2	-0.250041919	0.308256929	-0.811147766	0.417280818	0.159861029	5.470605616	81.72048818	0.5	CRA004775;PRJNA827465	Yang2025_Silkie_Wenchang	0.838112595
ENSGALG00010001506	DPYSL4	2	0.07642444	0.159033881	0.480554457	0.63083319	0	0.436682843	0	0.5	PRJNA827465;Yang2025_Silkie_Wenchang	CRA004775	0.902834349
ENSGALG00010001506	DPYSL4	2	0.504036615	0.603304619	0.835459565	0.403459004	0.599115012	5.308621993	81.16271979	0.5	CRA004775;Yang2025_Silkie_Wenchang	PRJNA827465	0.83306585
ENSGALG00010001506	DPYSL4	2	0.592640594	0.494609847	1.198198132	0.230839893	0.367307594	3.672151924	72.76801122	1	CRA004775;PRJNA827465	Yang2025_Silkie_Wenchang	0.743374303
ENSGALG00010001507	METTL4	2	-0.011131229	0.090682491	-0.122749488	0.902305474	0	0.235999912	0	0.5	PRJNA827465;Yang2025_Silkie_Wenchang	CRA004775	0.976923607
ENSGALG00010001507	METTL4	2	-0.151756597	0.166404044	-0.911976618	0.361781026	0	0.611142231	0	1	CRA004775;Yang2025_Silkie_Wenchang	PRJNA827465	0.815545459
ENSGALG00010001507	METTL4	2	-0.047595747	0.151605469	-0.313944793	0.753562969	0.01590443	1.200722626	16.71681884	0.5	CRA004775;PRJNA827465	Yang2025_Silkie_Wenchang	0.938087082
ENSGALG00010001508	CD109	2	0.412837935	0.284567749	1.450754474	0.146848241	0.084149981	2.02164132	50.53524133	1	PRJNA827465;Yang2025_Silkie_Wenchang	CRA004775	0.635558525
ENSGALG00010001508	CD109	2	0.611817811	0.2994792	2.042939248	0.041058453	0	0.931820213	0	1	CRA004775;Yang2025_Silkie_Wenchang	PRJNA827465	0.35111409
ENSGALG00010001508	CD109	2	0.154965848	0.221967092	0.69814785	0.485084736	0	0.052597726	0	1	CRA004775;PRJNA827465	Yang2025_Silkie_Wenchang	0.857004733
ENSGALG00010001510	AP1S2	2	-0.015981225	0.048060129	-0.332525639	0.739492383	0	0.999130728	0	0.5	PRJNA827465;Yang2025_Silkie_Wenchang	CRA004775	0.934873078
ENSGALG00010001510	AP1S2	2	-0.064435652	0.072641268	-0.887039185	0.375057804	0	0.225914598	0	0.5	CRA004775;Yang2025_Silkie_Wenchang	PRJNA827465	0.822916144
ENSGALG00010001510	AP1S2	2	0.025524991	0.060755227	0.420128319	0.674391716	0	0.016268086	0	1	CRA004775;PRJNA827465	Yang2025_Silkie_Wenchang	0.915945041
ENSGALG00010001512	PLCL2	2	-0.028692807	0.082008878	-0.349874399	0.726432961	0	0.169286435	0	0.5	PRJNA827465;Yang2025_Silkie_Wenchang	CRA004775	0.93109166
ENSGALG00010001512	PLCL2	2	0.077899671	0.122811393	0.634303297	0.525882905	0	0.770976648	0	1	CRA004775;Yang2025_Silkie_Wenchang	PRJNA827465	0.870414822
ENSGALG00010001512	PLCL2	2	0.026815887	0.140054635	0.191467333	0.848159472	0.015772905	1.423546945	29.75293133	0.5	CRA004775;PRJNA827465	Yang2025_Silkie_Wenchang	0.961363811
ENSGALG00010001513	ZRSR2	2	0.284514871	0.287554046	0.989430946	0.32245234	0.151847615	11.5099298	91.31184971	1	PRJNA827465;Yang2025_Silkie_Wenchang	CRA004775	0.798652022
ENSGALG00010001513	ZRSR2	2	0.450223348	0.155803074	2.889694897	0.003856159	0.01821705	1.594773447	37.29516867	1	CRA004775;Yang2025_Silkie_Wenchang	PRJNA827465	0.089204598
ENSGALG00010001513	ZRSR2	2	0.088441031	0.117012095	0.755828117	0.449752256	0.01663493	1.91135151	47.68099982	1	CRA004775;PRJNA827465	Yang2025_Silkie_Wenchang	0.847341953
ENSGALG00010001514	MET	2	-0.154780535	0.127331729	-1.215569254	0.224149052	0.018712892	2.330454902	57.0899227	1	PRJNA827465;Yang2025_Silkie_Wenchang	CRA004775	0.737452676
ENSGALG00010001514	MET	2	0.399972482	0.737923091	0.542024619	0.587801546	1.011521441	13.44716284	92.56348709	0.5	CRA004775;Yang2025_Silkie_Wenchang	PRJNA827465	0.891742161
ENSGALG00010001514	MET	2	0.516175401	0.608687102	0.848014356	0.396429976	0.669172537	9.593191507	89.5759404	0.5	CRA004775;PRJNA827465	Yang2025_Silkie_Wenchang	0.831365249
ENSGALG00010001517	CAV2	2	-0.232335198	0.155803754	-1.49120411	0.135907915	0.034582181	3.178774302	68.54133371	1	PRJNA827465;Yang2025_Silkie_Wenchang	CRA004775	0.617467165
ENSGALG00010001517	CAV2	2	0.101493097	0.541299956	0.187498808	0.851269558	0.541403747	12.96171192	92.28496972	0.5	CRA004775;Yang2025_Silkie_Wenchang	PRJNA827465	0.962071965
ENSGALG00010001517	CAV2	2	0.240015192	0.380840967	0.630224196	0.528547911	0.257569397	8.264099618	87.89946823	0.5	CRA004775;PRJNA827465	Yang2025_Silkie_Wenchang	0.871701504
ENSGALG00010001518	SLC17A5	2	-0.292833857	0.456663425	-0.641246576	0.521362493	0.404781492	33.66717197	97.02974755	0.5	PRJNA827465;Yang2025_Silkie_Wenchang	CRA004775	0.869518515
ENSGALG00010001518	SLC17A5	2	-0.344663032	0.492281105	-0.700134594	0.483843254	0.39323107	4.669699205	78.58534445	0.5	CRA004775;Yang2025_Silkie_Wenchang	PRJNA827465	0.85691916
ENSGALG00010001518	SLC17A5	2	0.159886439	0.077988307	2.050133478	0.040351407	0	0.037422124	0	1	CRA004775;PRJNA827465	Yang2025_Silkie_Wenchang	0.347442284
ENSGALG00010001520	KCNAB1	2	0.136486004	0.094635054	1.442235183	0.149236039	0	0.090692927	0	1	PRJNA827465;Yang2025_Silkie_Wenchang	CRA004775	0.639246608
ENSGALG00010001520	KCNAB1	2	0.050960213	0.157242955	0.324085827	0.745873056	0	0.449546919	0	0.5	CRA004775;Yang2025_Silkie_Wenchang	PRJNA827465	0.936373614
ENSGALG00010001520	KCNAB1	2	0.130272467	0.109913083	1.185231673	0.23592586	0	0.707805221	0	0.5	CRA004775;PRJNA827465	Yang2025_Silkie_Wenchang	0.748669015
ENSGALG00010001521	TBC1D5	2	-0.157801941	0.064928083	-2.430411204	0.015081701	0	0.733827874	0	1	PRJNA827465;Yang2025_Silkie_Wenchang	CRA004775	0.203100811
ENSGALG00010001521	TBC1D5	2	-0.035025336	0.134831515	-0.259771138	0.795040315	0	0.017469603	0	0.5	CRA004775;Yang2025_Silkie_Wenchang	PRJNA827465	0.948905846
ENSGALG00010001521	TBC1D5	2	-0.175734692	0.070418507	-2.495575387	0.012575303	0	0.328106761	0	0.5	CRA004775;PRJNA827465	Yang2025_Silkie_Wenchang	0.181574995
ENSGALG00010001525	EEF1A1	2	-0.313093702	0.369287873	-0.847830987	0.396532103	0.252288685	12.64145599	92.0895188	0.5	PRJNA827465;Yang2025_Silkie_Wenchang	CRA004775	0.831365249
ENSGALG00010001525	EEF1A1	2	-0.522119125	0.178677968	-2.922123697	0.003476534	0.026512271	1.708228966	41.45983825	1	CRA004775;Yang2025_Silkie_Wenchang	PRJNA827465	0.083315994
ENSGALG00010001525	EEF1A1	2	-0.117711312	0.186807665	-0.630120353	0.528615845	0.053985474	3.811204915	73.76157876	0.5	CRA004775;PRJNA827465	Yang2025_Silkie_Wenchang	0.871776961
ENSGALG00010001526	PPP1R3A	2	-0.10336016	0.135623667	-0.762110051	0.445994325	0.021923647	2.446382816	59.12332307	0.5	PRJNA827465;Yang2025_Silkie_Wenchang	CRA004775	0.846013379
ENSGALG00010001526	PPP1R3A	2	0.069355162	0.133223526	0.52059245	0.60265071	0	0.842141775	0	1	CRA004775;Yang2025_Silkie_Wenchang	PRJNA827465	0.895527108
ENSGALG00010001526	PPP1R3A	2	-0.02096132	0.380698755	-0.055060122	0.956090566	0.175693571	1.741407262	42.57517917	0.5	CRA004775;PRJNA827465	Yang2025_Silkie_Wenchang	0.988513657
ENSGALG00010001527	STK32C	2	-0.314614487	0.351122773	-0.896024157	0.370239861	0.223408993	10.46675603	90.44594144	0.5	PRJNA827465;Yang2025_Silkie_Wenchang	CRA004775	0.821045695
ENSGALG00010001527	STK32C	2	-0.293295392	0.442155611	-0.663330703	0.507118777	0.311690318	4.617361721	78.34261077	0.5	CRA004775;Yang2025_Silkie_Wenchang	PRJNA827465	0.864023291
ENSGALG00010001527	STK32C	2	0.038613842	0.109951421	0.351190025	0.725445793	0	0.235994949	0	1	CRA004775;PRJNA827465	Yang2025_Silkie_Wenchang	0.930910808
ENSGALG00010001528	NR1D2	2	0.008824576	0.099969537	0.088272648	0.929659978	0	0.11059151	0	0.5	PRJNA827465;Yang2025_Silkie_Wenchang	CRA004775	0.983767889
ENSGALG00010001528	NR1D2	2	0.127373462	0.156729895	0.812694105	0.416393462	0	0.415592389	0	1	CRA004775;Yang2025_Silkie_Wenchang	PRJNA827465	0.837839156
ENSGALG00010001528	NR1D2	2	0.030597507	0.109324896	0.279876845	0.779571992	0	0.900573938	0	0.5	CRA004775;PRJNA827465	Yang2025_Silkie_Wenchang	0.944807046
ENSGALG00010001532	ENSGALG00010001532	2	0.255061954	0.110357342	2.311236845	0.020819776	0	0.16756247	0	1	PRJNA827465;Yang2025_Silkie_Wenchang	CRA004775	0.243971278
ENSGALG00010001532	ENSGALG00010001532	2	0.263690775	0.148013826	1.781528	0.074826235	0	0.344237443	0	1	CRA004775;Yang2025_Silkie_Wenchang	PRJNA827465	0.472257613
ENSGALG00010001532	ENSGALG00010001532	2	0.192716276	0.137435505	1.402230639	0.160846385	0	0.126959558	0	1	CRA004775;PRJNA827465	Yang2025_Silkie_Wenchang	0.656872971
ENSGALG00010001533	ENSGALG00010001533	2	0.421004647	0.178038939	2.364677355	0.018045785	0.043650972	3.173462877	68.48868134	1	PRJNA827465;Yang2025_Silkie_Wenchang	CRA004775	0.224092724
ENSGALG00010001533	ENSGALG00010001533	2	0.209449034	0.450617602	0.464804378	0.642071552	0.34080379	5.84263618	82.8844383	0.5	CRA004775;Yang2025_Silkie_Wenchang	PRJNA827465	0.906634523
ENSGALG00010001533	ENSGALG00010001533	2	0.072072358	0.25924168	0.278012232	0.781002969	0.085418061	2.299559421	56.51340901	0.5	CRA004775;PRJNA827465	Yang2025_Silkie_Wenchang	0.945235709
ENSGALG00010001534	SLITRK3	2	0.498005673	0.761846893	0.653682095	0.513316641	0.996009905	6.884104082	85.47378151	0.5	PRJNA827465;Yang2025_Silkie_Wenchang	CRA004775	0.866301448

ENSGALG00010001534	SLITRK3	2	1.100338998	0.406544876	2.706562211	0.006798384	0	0.569727487	0	1	CRA004775;Yang2025_Silkie_Wenchang	PRJNA827465	0.126544334
ENSGALG00010001534	SLITRK3	2	-0.031590489	0.356017172	-0.088733049	0.929294067	0.064345627	1.207063597	17.15432373	0.5	CRA004775;PRJNA827465	Yang2025_Silkie_Wenchang	0.983653628
ENSGALG00010001535	RAP2B	2	-0.084292161	0.105990399	-0.795281103	0.426450004	0.016052153	3.073367295	67.4623986	1	PRJNA827465;Yang2025_Silkie_Wenchang	CRA004775	0.84125685
ENSGALG00010001535	RAP2B	2	-0.220150798	0.106819545	-2.060959883	0.039306867	0	0.000547561	0	1	CRA004775;Yang2025_Silkie_Wenchang	PRJNA827465	0.342849048
ENSGALG00010001535	RAP2B	2	-0.006926576	0.045477744	-0.152306946	0.878944847	0	0.631521677	0	1	CRA004775;PRJNA827465	Yang2025_Silkie_Wenchang	0.971127951
ENSGALG00010001540	TRABD	2	-0.077220966	0.080521892	-0.95900586	0.337555793	0.00359385	1.352458044	26.06055292	1	PRJNA827465;Yang2025_Silkie_Wenchang	CRA004775	0.80415385
ENSGALG00010001540	TRABD	2	-0.184622496	0.109152342	-1.691420381	0.090756542	0	0.010087506	0	1	CRA004775;Yang2025_Silkie_Wenchang	PRJNA827465	0.516920505
ENSGALG00010001540	TRABD	2	-0.027481004	0.079892456	-0.343974959	0.730865118	0	0.380444186	0	1	CRA004775;PRJNA827465	Yang2025_Silkie_Wenchang	0.932769533
ENSGALG00010001542	MTO1	2	-0.089727213	0.044616954	-2.011056433	0.044319496	0	0.566462877	0	1	PRJNA827465;Yang2025_Silkie_Wenchang	CRA004775	0.363642137
ENSGALG00010001542	MTO1	2	-0.1473183	0.104962032	-1.403538949	0.160456186	0	0.280620156	0	0.5	CRA004775;Yang2025_Silkie_Wenchang	PRJNA827465	0.65630165
ENSGALG00010001542	MTO1	2	-0.072949228	0.048224825	-1.512690363	0.130358329	0	0.078111999	0	0.5	CRA004775;PRJNA827465	Yang2025_Silkie_Wenchang	0.605608015
ENSGALG00010001545	SELENOO	2	-0.066524506	0.119991928	-0.554408173	0.579299534	0.003636481	1.142445213	12.46845025	0.5	PRJNA827465;Yang2025_Silkie_Wenchang	CRA004775	0.887888423
ENSGALG00010001545	SELENOO	2	-0.213779761	0.148050353	-1.443966569	0.148748379	0	0.024171331	0	1	CRA004775;Yang2025_Silkie_Wenchang	PRJNA827465	0.638795624
ENSGALG00010001545	SELENOO	2	-0.01707959	0.133793433	-0.127656414	0.898420885	0	0.766673272	0	0.5	CRA004775;PRJNA827465	Yang2025_Silkie_Wenchang	0.97575249
ENSGALG00010001546	CGAS	2	-0.069813576	0.070764446	-0.986562876	0.323856977	0	0.784006649	0	0.5	PRJNA827465;Yang2025_Silkie_Wenchang	CRA004775	0.799600434
ENSGALG00010001546	CGAS	2	-0.007088374	0.119088077	-0.059522113	0.952536253	0	0.870386018	0	0.5	CRA004775;Yang2025_Silkie_Wenchang	PRJNA827465	0.987758913
ENSGALG00010001546	CGAS	2	-0.124085099	0.085679922	-1.44824009	0.147549916	0	0.488618834	0	1	CRA004775;PRJNA827465	Yang2025_Silkie_Wenchang	0.636640065
ENSGALG00010001547	PWWP2B	2	-0.186376507	0.06110026	-3.050339011	0.002285832	0	0.000927596	0	1	PRJNA827465;Yang2025_Silkie_Wenchang	CRA004775	0.063536104
ENSGALG00010001547	PWWP2B	2	-0.124178119	0.115736639	-1.072936978	0.283299394	0.008756631	1.280271966	21.89159599	0.5	CRA004775;Yang2025_Silkie_Wenchang	PRJNA827465	0.779568117
ENSGALG00010001547	PWWP2B	2	-0.127502838	0.11699818	-1.089784794	0.275807954	0.010542069	1.34538773	25.67198451	0.5	CRA004775;PRJNA827465	Yang2025_Silkie_Wenchang	0.7754953
ENSGALG00010001548	AKAP17A	2	0.255126678	0.163715119	1.558357463	0.119148534	0.045088093	5.920340611	83.10907994	1	PRJNA827465;Yang2025_Silkie_Wenchang	CRA004775	0.579471011
ENSGALG00010001548	AKAP17A	2	0.274502834	0.191567414	1.43293073	0.151877634	0.048279791	2.787950109	64.13135239	1	CRA004775;Yang2025_Silkie_Wenchang	PRJNA827465	0.643294424
ENSGALG00010001548	AKAP17A	2	0.103897767	0.054702949	1.899308321	0.057523949	0	0.081527574	0	1	CRA004775;PRJNA827465	Yang2025_Silkie_Wenchang	0.415417952
ENSGALG00010001551	FHIP1B	2	0.06502132	0.15730239	0.413352394	0.679348448	0.042141013	6.614682509	84.88211643	0.5	PRJNA827465;Yang2025_Silkie_Wenchang	CRA004775	0.917701868
ENSGALG00010001551	FHIP1B	2	0.063525409	0.264886287	0.239821434	0.81046869	0.088061955	2.054978158	51.33768229	0.5	CRA004775;Yang2025_Silkie_Wenchang	PRJNA827465	0.952997426
ENSGALG00010001551	FHIP1B	2	-0.091517347	0.067557147	-1.354665654	0.175524105	0	0.453906075	0	1	CRA004775;PRJNA827465	Yang2025_Silkie_Wenchang	0.680297576
ENSGALG00010001552	TUBGCP6	2	-0.136512811	0.090945435	-1.501040829	0.133345002	0.010327499	2.576506039	61.18774865	1	PRJNA827465;Yang2025_Silkie_Wenchang	CRA004775	0.611963872
ENSGALG00010001552	TUBGCP6	2	-0.023573467	0.087189929	-0.27036915	0.786876271	0	0.049393489	0	0.5	CRA004775;Yang2025_Silkie_Wenchang	PRJNA827465	0.946784559
ENSGALG00010001552	TUBGCP6	2	-0.194705118	0.067100734	-2.901683863	0.003711629	0.000762642	1.027637338	2.689405773	0.5	CRA004775;PRJNA827465	Yang2025_Silkie_Wenchang	0.087190443
ENSGALG00010001553	CNGA4	2	0.390836817	0.397151788	0.984099352	0.325066665	0.223811299	3.417001669	70.73457677	1	PRJNA827465;Yang2025_Silkie_Wenchang	CRA004775	0.799914637
ENSGALG00010001553	CNGA4	2	0.493976352	0.38611467	1.279351421	0.200773332	0.139258577	1.814928548	44.90141218	1	CRA004775;Yang2025_Silkie_Wenchang	PRJNA827465	0.713340819
ENSGALG00010001553	CNGA4	2	0.016832536	0.238350724	0.070620872	0.943699498	0	0.000204143	0	1	CRA004775;PRJNA827465	Yang2025_Silkie_Wenchang	0.985827325
ENSGALG00010001554	ENSGALG00010001554	2	0.329380905	0.234398412	1.40521816	0.159956416	0.022958035	1.258795414	20.55897335	1	PRJNA827465;Yang2025_Silkie_Wenchang	CRA004775	0.655404348
ENSGALG00010001554	ENSGALG00010001554	2	0.05470713	0.491753392	0.111249116	0.911418804	0.398682973	5.67982749	82.39383147	0.5	CRA004775;Yang2025_Silkie_Wenchang	PRJNA827465	0.978845851
ENSGALG00010001554	ENSGALG00010001554	2	-0.199241324	0.255485129	-0.779854878	0.4354763	0.03133408	1.315530485	23.98503789	0.5	CRA004775;PRJNA827465	Yang2025_Silkie_Wenchang	0.842762754
ENSGALG00010001556	EPB41L3	2	-0.05612151	0.089582401	-0.626479185	0.531000685	0.007615505	1.565290664	36.1141018	0.5	PRJNA827465;Yang2025_Silkie_Wenchang	CRA004775	0.872840748
ENSGALG00010001556	EPB41L3	2	0.208423513	0.180961911	1.151753493	0.249422384	0.02144048	1.354180862	26.15462025	1	CRA004775;Yang2025_Silkie_Wenchang	PRJNA827465	0.759622031
ENSGALG00010001556	EPB41L3	2	0.119400883	0.295231092	0.404431939	0.685895118	0.13598848	3.707375175	73.02673852	0.5	CRA004775;PRJNA827465	Yang2025_Silkie_Wenchang	0.919235539
ENSGALG00010001558	CCKBR	2	0.389738949	0.34200318	1.139576972	0.254462584	0.099355601	1.725317879	42.0396663	1	PRJNA827465;Yang2025_Silkie_Wenchang	CRA004775	0.764087626
ENSGALG00010001558	CCKBR	2	0.369281123	0.425044826	0.868805126	0.384953729	0.178955047	1.973481514	49.32812937	0.5	CRA004775;Yang2025_Silkie_Wenchang	PRJNA827465	0.826444827
ENSGALG00010001558	CCKBR	2	0.022570829	0.270433813	0.083461564	0.933484538	0	0.084354326	0	0.5	CRA004775;PRJNA827465	Yang2025_Silkie_Wenchang	0.984260547
ENSGALG00010001559	HDAC10	2	-0.145154165	0.091224186	-1.59118071	0.111568913	0	0.750745181	0	1	PRJNA827465;Yang2025_Silkie_Wenchang	CRA004775	0.564762672
ENSGALG00010001559	HDAC10	2	-0.005090541	0.146106426	-0.034841319	0.972206273	0	0.085525131	0	0.5	CRA004775;Yang2025_Silkie_Wenchang	PRJNA827465	0.99196841
ENSGALG00010001559	HDAC10	2	-0.169049347	0.103833019	-1.628088532	0.103506115	0	0.664740581	0	0.5	CRA004775;PRJNA827465	Yang2025_Silkie_Wenchang	0.547892368
ENSGALG00010001567	INPP5A	2	-0.190074939	0.148512523	-1.279857994	0.200595083	0.039816937	10.22214169	90.21731423	1	PRJNA827465;Yang2025_Silkie_Wenchang	CRA004775	0.712970384
ENSGALG00010001567	INPP5A	2	-0.139108211	0.24539218	-0.56688119	0.570794904	0.096060325	4.336890074	76.94200261	0.5	CRA004775;Yang2025_Silkie_Wenchang	PRJNA827465	0.885224727
ENSGALG00010001567	INPP5A	2	-0.032778683	0.05587394	-0.586654219	0.557435961	0	0.738473725	0	0.5	CRA004775;PRJNA827465	Yang2025_Silkie_Wenchang	0.880678789
ENSGALG00010001570	SMPD1	2	0.092249653	0.066112651	1.395340405	0.162913213	0	0.086800264	0	1	PRJNA827465;Yang2025_Silkie_Wenchang	CRA004775	0.660959169
ENSGALG00010001570	SMPD1	2	0.081305502	0.182524442	0.44544994	0.655994629	0	0.444440372	0	0.5	CRA004775;Yang2025_Silkie_Wenchang	PRJNA827465	0.910411938
ENSGALG00010001570	SMPD1	2	0.078267831	0.068417304	1.143977129	0.252633122	0	0.336609229	0	0.5	CRA004775;PRJNA827465	Yang2025_Silkie_Wenchang	0.762687335
ENSGALG00010001585	ENSGALG00010001585	2	0.038469542	0.109356212	0.351781959	0.72500179	0.012099458	1.920563463	47.93194709	0.5	PRJNA827465;Yang2025_Silkie_Wenchang	CRA004775	0.93062299
ENSGALG00010001585	ENSGALG00010001585	2	0.034297024	0.163446914	0.209835861	0.833795783	0.025720626	1.861399801	46.27699007	0.5	CRA004775;Yang2025_Silkie_Wenchang	PRJNA827465	0.957968425
ENSGALG00010001585	ENSGALG00010001585	2	0.14168786	0.078958596	1.7944577	0.072740144	0	0.245462738	0	1	CRA004775;PRJNA827465	Yang2025_Silkie_Wenchang	0.465709132
ENSGALG00010001586	ZBTB14	2	-0.013079757	0.068123252	-0.19200136	0.84774114	0	0.912428799	0	0.5	PRJNA827465;Yang2025_Silkie_Wenchang	CRA004775	0.961304071
ENSGALG00010001586	ZBTB14	2	0.064655587	0.215087085	0.300601903	0.76371808	0.063854608	3.117864202	67.9267622	0.5	CRA004775;Yang2025_Silkie_Wenchang	PRJNA827465	0.940361611
ENSGALG00010001586	ZBTB14	2	0.103778361	0.127226404	0.815698298	0.414672712	0.016216981	1.673495908	40.2448494	1	CRA004775;PRJNA827465	Yang2025_Silkie_Wenchang	0.837298284
ENSGALG00010001588	ENSGALG00010001588	2	0.656227902	0.383151289	1.712712239	0.086765492	0.081687489	1.361755719	26.56539013	1	PRJNA827465;Yang2025_Silkie_Wenchang	CRA004775	0.506632618
ENSGALG00010001588	ENSGALG00010001588	2	0.152724671	0.440277347	0.346882874	0.728679314	0	0.008097852	0	1	CRA004775;Yang2025_Silkie_Wenchang	PRJNA827465	0.932236477
ENSGALG00010001588	ENSGALG00010001588	2	0.772514083	0.360653822	2.141982247	0.032194911	0.006662045	1.017994458	1.767638156	1	CRA004775;PRJNA827465	Yang2025_Silkie_Wenchang	0.307722902
ENSGALG00010001595	AKAIN1	2	0.171816659	0.220712857	0.778462392	0.436296469	0	0.460706649	0	0.5	PRJNA827465;Yang2025_Silkie_Wenchang	CRA004775	0.843122822
ENSGALG00010001595	AKAIN1	2	0.605610667	0.413434727	1.464827766	0.142967914	0.181713271	1.950146179	48.72179272	1	CRA004775;Yang2025_Silkie_Wenchang	PRJNA827465	0.62972856
ENSGALG00010001595	AKAIN1	2	0.500409046	0.578524615	0.86497451	0.387052779	0.47364143	3.278175217	69.49522421	0.5	CRA004775;PRJNA827465	Yang2025_Silkie_Wenchang	0.827206927
ENSGALG00010001599	TBLX1	2	-0.130143163	0.038203944	-3.406537335	0.000657926	0	0.497650783	0	1	PRJNA827465;Yang2025_Silkie_Wenchang	CRA004775	0.028283199
ENSGALG00010001599	TBLX1	2	-0.098890209	0.105881549	-0.933970178	0.350319277	0.011679438	1.727631844	42.11729754	0.5	CRA004775;Yang2025_Silkie_Wenchang	PRJNA827465	0.810111037
ENSGALG00010001599	TBLX1	2	-0.091422826	0.053898505	-1.696203361	0.089847374	0.000901296	1.059080372	5.578459704	0.5	CRA004775;PRJNA827465	Yang2025_Silkie_Wenchang	0.514237855
ENSGALG00010001604	ENSGALG00010001604	2	-0.364972329	0.168013734	-2.172276753	0.029834794	0	0.002348265	0	1	PRJNA827465;Yang2025_Silkie_Wenchang	CRA004775	0.29494131
ENSGALG00010001604	ENSGALG00010001604	2	-0.418097876	0.300446888	-1.39158664	0.164047607	0	0.060162599	0	1	CRA004775;Yang2025_Silkie_Wenchang	PRJNA827465	0.662784035
ENSGALG00010001604	ENSGALG00010001604	2	-0.376649469	0.18518359	-2.033924652	0.041959193	0	0.086253683	0	1	CRA004775;PRJNA827465	Yang2025_Silkie_Wenchang	0.354529664
ENSGALG00010001605	APBB1	2	-0.002106229	0.07177981	-0.029342911	0.976591104	0	0.071072359	0	0.5	PRJNA827465;Yang2025_Silkie_Wenchang	CRA004775	0.99314221
ENSGALG00010001605	APBB1	2	-0.042275451	0.160608141	-0.263221099	0.79238017	0	0.654256663	0	0.5	CRA004775;Yang2025_Silkie_Wenchang	PRJNA827465	0.948368502
ENSGALG00010001605	APBB1	2	-0.024956508	0.074575323	-0.334648344	0.737890372	0	0.571278241	0	1	CRA004775;PRJNA827465	Yang2025_Silkie_Wenchang	0.934161348
ENSGALG00010001606	PIGA	2	-0.151481879	0.234434965	-0.646157364	0.518177448	0.099512375	10.39052039	90.37584295	0.5	PRJNA827465;Yang2025_Silkie_Wenchang	CRA004775	0.868371989
ENSGALG00010001606	PIGA	2	-0.222689581	0.241559717	-0.92188211	0.356590071	0.073170249	2.284987732	56.23608889	0.5	CRA004775;Yang2025_Silkie_Wenchang	PRJNA827465	0.812655664
ENSGALG00010001606	PIGA	2	0.075758497	0.075346081	1.005473625	0.314668843	0	0.01593075	0	1	CRA004775;PRJNA827465	Yang2025_Silkie_Wenchang	0.796531448
ENSGALG00010001609	ENSGALG00010001609	2	-0.576146455	0.372274921	-1.547637037	0.121709709	0	0.343119403	0	1	PRJNA827465;Yang2025_Silkie_Wenchang	CRA004775	0.5849268
ENSGALG00010001609	ENSGALG00010001609	2	-0.398110813	0.485942	-0.819255823	0.412640472	0	0.05844964	0	1	CRA004775;Yang2025_Silkie_Wenchang	PRJNA827465	0.835704442
ENSGALG00010001609	ENSGALG00010001609	2	-0.734846553	0.443088025	-1.658466289	0.097223386	0	0.02355753	0	1	CRA004775;PRJNA827465	Yang2025_Silkie_Wenchang	0.53286442
ENSGALG00010001611	MAPK12	2	-0.113887557	0.09603588	-1.185885485	0.235667526	0	0.432430322	0	1	PRJNA827465;Yang2025_Silkie_Wenchang	CRA004775	0.748626601
ENSGALG00010001611	MAPK12	2	-0.216343257	0.134100984	-1.61328612	0.106682361	0	0.409173296	0	1	CRA004775;Yang2025_Silkie_Wenchang	PRJNA827465	0.553888616
ENSGALG00010001611	MAPK12	2	-0.097198215	0.122406833	-0.794058739	0.427161237	0	0.92990944	0	1	CRA004775;PRJNA827465	Yang2025_Silkie_Wenchang	0.84125685
ENSGALG00010001612	HPX	2	0.832322136	0.397172721	2.095617576	0.036116129	0	0.751671401	0	1	PRJNA827465;Yang2025_Silkie_Wenchang	CRA004775	0.327822681
ENSGALG00010001612	HPX	2	0.411054437	0.405069228	1.014775767	0.310212728	0	0.14951899	0	1	CRA004775;Yang2025_Silkie_Wenchang	PRJNA827465	0.794813537
ENSGALG00010001612	HPX	2	0.745706465	0.508325583	1.466985904	0.14237989	0.124262531	1.316016005	24.01308221	1	CRA004775;PRJNA827465	Yang2025_Silkie_Wenchang	0.628759193
ENSGALG00010001617	TRIM3	2	0.229592919	0.231731822	0.990769921	0.32179794	0.099615002	13.30807545	92.48576548	1	PRJNA827465;Yang2025_Silkie_Wenchang	CRA004775	0.798513866
ENSGALG00010001617	TRIM3	2	0.211839318	0.291657407	0.726329293	0.467636926	0.139976068	5.363255022	81.35460656	0.5	CRA004775;Yang2025_Silkie_Wenchang	PRJNA827465	0.852885838
ENSGALG00010001617	TRIM3	2	0.00455519	0.04711781	0.096676609	0.922983216	0	0.285097747	0	0.5	CRA004775;PRJNA827465	Yang2025_Silkie_Wenchang	0.981836718
ENSGALG00010001620	MOSPD2	2	-0.079599483	0.103372067	-0.770028944	0.441282723	0.006813514	1.402745941	28.71125333	1	PRJNA827465;Yang2025_Silkie_Wenchang	CRA004775	0.844907543
ENSGALG00010001620	MOSPD2	2	-0.184149514	0.140640439	-1.309363901	0.190411113	0	0.324567189	0	1	CRA004775;Yang2025_Silkie_Wenchang	PRJNA827465	0.699413831
ENSGALG00010001620	MOSPD2	2	-0.006125299	0.093021405	-0.065848275	0.947498622	0	0.001567521	0	1	CRA004775;PRJNA827465	Yang2025_Silkie_Wenchang	0.986560458
ENSGALG00010001627	ARFIP2	2	0.056387754	0.127299243	0.442954357	0.657798749	0.026745079	5.471218433	81.72253563	0.5	PRJNA827465;Yang2025_Silkie_Wenchang	CRA004775	0.910774485
ENSGALG00010001627	ARFIP2	2	0.131420146	0.103018787	1.275691058	0.202064751	0.005698777	1.282158295	22.00651015	0.5	CRA004775;Yang2025_Silkie_Wenchang	PRJNA827465	0.71497298
ENSGALG00010001627	ARFIP2	2	-0.056652247	0.050382567	-1.12444146	0.260825792	0	0.023519454	0	1	CRA004775;PRJNA827465	Yang2025_Silkie_Wenchang	0.76792161
ENSGALG00010001628	MAPK11	2	-0.1543273	0.236932455	-0.65135568	0.514816913	0.095431068	6.542355212	84.71498463	0.5	PRJNA827465;Yang2025_Silkie_Wenchang	CRA004775	0.866930857
ENSGALG00010001628	MAPK11	2	-0.343393431	0.140623435	-2.441936012	0.014608736	0	0.972542333	0	1	CRA004775;Yang2025_Silkie_Wenchang	PRJNA827465	0.19908708
ENSGALG00010001628	MAPK11	2	0.060833472	0.09913465	0.613644903	0.539450004	0	0.080837513	0	0.5	CRA004775;PRJNA827465	Yang2025_Silkie_Wenchang	0.874993194
ENSGALG00010001632	FANCB	2	-0.565066795	0.362578174	-1.558468863	0.119122144	0.226894801	7.146451643	86.00704168	1	PRJNA827465;Yang2025_Silkie_Wenchang	CRA004775	0.579471011
ENSGALG00010001632	FANCB	2	-0.970282991	0.216533108	-4.480991382	7.42971E-06	0	0.083330726	0	1	CRA004775;Yang2025_Silkie_Wenchang	PRJNA827465	0.001106098
ENSGALG00010001632	FANCB	2	-0.453718035	0.402688773	-1.126721344	0.259860315	0.193788972	1.801486188	44.4902766	1	CRA004775;PRJNA827465	Yang2025_Silkie_Wenchang	0.767047318
ENSGALG00010001633	B3GAT2	2	-0.122184572	0.23516287	-0.519574249	0.60336035	0	0.199466645	0	0.5	PRJNA827465;Yang2025_Silkie_Wenchang	CRA004775	0.895538966
ENSGALG00010001633	B3GAT2	2	0.255091529	0.360589391	0.707429379	0.479299685	0	0.53076509	0	1	CRA004775;Yang2025_Silkie_Wenchang	PRJNA827465	0.856035423
ENSGALG00010001633	B3GAT2	2	0.030290051	0.349091955	0.086768114	0.930855833	0.093038132	1.439650163	30.53868048	0.5	CRA004775;PRJNA827465	Yang2025_Silkie_Wenchang	0.983943003
ENSGALG00010001635	TIMM10B	2	0.125158725	0.090198655	1.387589702	0.165262019	0	0.569668591	0	1	PRJNA827465;Yang2025_Silkie_Wenchang	CRA004775	0.664874006
ENSGALG00010001635	TIMM10B	2	-0.054222567	0.386494499	-0.140293245	0.888428301	0.215839438	3.589473069	72.14075769	0.5	CRA004775;Yang2025_Silkie_Wenchang	PRJNA827465	0.973135755
ENSGALG00010001635	TIMM10B	2	-0.10156472	0.258527861	-0.392857927	0.694424417	0.100315855	3.482148041	71.28209404	0.5	CRA004775;PRJNA827465	Yang2025_Silkie_Wenchang	0.920728119
ENSGALG00010001639	ENSGALG00010001639	2	0.341372366	0.334791413	1.019656875	0.307891221	0.194642935	7.063383884	85.8424798	1	PRJNA827465;Yang2025_Silkie_Wenchang	CRA004775	0.793251305
ENSGALG00010001639	ENSGALG00010001639	2	0.6266913	0.213245593	2.938824157	0.003294599	0	0.70603499	0	1	CRA004775;Yang2025_Silkie_Wenchang	PRJNA827465	0.080078935
ENSGALG00010001639	ENSGALG00010001639	2	0.047608937	0.087673263	0.543026857	0.587111311	0	0.194258153	0	1	CRA004775;PRJNA827465	Yang2025_Silkie_Wenchang	0.891596903
ENSGALG00010001642	ENSGALG00010001642	2	0.347661556	0.481814462	0.721567291	0.470560555	0.423228247	10.8167554	90.75508354	0.5	PRJNA827465;Yang2025_Silkie_Wenchang	CRA004775	0.853744788
ENSGALG00010001642	ENSGALG00010001642	2	0.782487294	0.217717436	3.594049739	0.000325578	0	0.240708098	0	1	CRA004775;Yang2025_Silkie_Wenchang	PRJNA827465	0.017894809
ENSGALG00010001642	ENSGALG00010001642	2	0.187960594	0.362783852	0.518106285	0.604384115	0.204195331	3.837227138	73.93951507	0.5	CRA004775;PRJNA827465	Yang2025_Silkie_Wenchang	0.89614475
ENSGALG00010001647	ARL8BL	2	-0.061934151	0.167883469	-0.368911549	0.712193655	0.02547899	1.802607064	44.524793	0.5	PRJNA827465;Yang2025_Silkie_Wenchang	CRA004775	0.92597752
ENSGALG00010001647	ARL8BL	2	0.003103945	0.160065098	0.019391765	0.98452858	0.002453605	1.047156383	4.503279923	0.5	CRA004775;Yang2025_Silkie_Wenchang	PRJNA827465	0.995703389
ENSGALG00010001647	ARL8BL	2	-0.208162225	0.133979797	-1.553683683	0.120259887	0	0.000745907	0	1	CRA004775;PRJNA827465	Yang2025_Silkie_Wenchang	0.581589066
ENSGALG00010001649	UBE2E2	2	-0.024014148	0.153670271	-0.156270615	0.875819718	0.009060169	1.23022836	18.71427835	0.5	PRJNA827465;Yang2025_Silkie_Wenchang	CRA004775	0.970347777
ENSGALG00010001649	UBE2E2	2	0.09512273	0.19711565	0.4825732	0.629398812	0	0.457781801	0	0.5	CRA004775;Yang2025_Silkie_Wenchang	PRJNA827465	0.902390168
ENSGALG00010001649	UBE2E2	2	-0.160478665	0.166920586	-0.961407271	0.33634743	0	0.006929171	0	1	CRA004775;PRJNA827465	Yang2025_Silkie_Wenchang	0.803806405
ENSGALG00010001654	RPS11	2	-0.118127546	0.124131945	-0.951628901	0.341285219	0.014269474	1.334350184	25.05715425	1	PRJNA827465;Yang2025_Silkie_Wenchang	CRA004775	0.805665554
ENSGALG00010001654	RPS11	2	-0.377904903	0.18698569	-2.021036491	0.043275988	0	0.011695182	0	1	CRA004775;Yang2025_Silkie_Wenchang	PRJNA827465	0.359703576
ENSGALG00010001654	RPS11	2	-0.115480003	0.112610975	-1.025477343	0.305138011	0.011260715	1.343968599	25.59349969	1	CRA004775;PRJNA827465	Yang2025_Silkie_Wenchang	0.79225794
ENSGALG00010001657	SMAP1	2	0.107505327	0.090985077	1.181570988	0.237375971	0.012030607	3.371886882	70.34301461	1	PRJNA827465;Yang2025_Silkie_Wenchang	CRA004775	0.749366344
ENSGALG00010001657	SMAP1	2	0.168959553	0.094484171	1.788231324	0.073738695	0.003059239	1.124228996	11.05015051	0.5	CRA004775;Yang2025_Silkie_Wenchang	PRJNA827465	0.469294822
ENSGALG00010001657	SMAP1	2	0.029204209	0.043835683	0.666220008	0.505270486	0	0.058792482	0	0.5	CRA004775;PRJNA827465	Yang2025_Silkie_Wenchang	0.863862536
ENSGALG00010001661	TCTN3	2	0.015598372	0.083945618	0.185815198	0.852589686	0.009289461	2.708763354	63.08278468	0.5	PRJNA827465;Yang2025_Silkie_Wenchang	CRA004775	0.962607579
ENSGALG00010001661	TCTN3	2	-0.176203454	0.155973664	-1.129700035	0.258602642	0.023622699	1.554243413	35.66001365	1	CRA004775;Yang2025_Silkie_Wenchang	PRJNA827465	0.765798911
ENSGALG00010001661	TCTN3	2	-0.114747584	0.258646457	-0.443646455	0.657298214	0.103865455	3.624812977	72.41236979	0.5	CRA004775;PRJNA827465	Yang2025_Silkie_Wenchang	0.910711339
ENSGALG00010001666	PIEZO2	2	-0.120737831	0.085918992	-1.405251953	0.15994637	0	0.100202656	0	1	PRJNA827465;Yang2025_Silkie_Wenchang	CRA004775	0.655404348
ENSGALG00010001666	PIEZO2	2	-0.170649446	0.179582674	-0.950255626	0.34198238	0	0.004901966	0	1	CRA004775;Yang2025_Silkie_Wenchang	PRJNA827465	0.805904173
ENSGALG00010001666	PIEZO2	2	-0.108567766	0.093947464	-1.155622098	0.247835765	0	0.00449496	0	1	CRA004775;PRJNA827465	Yang2025_Silkie_Wenchang	0.7580874
ENSGALG00010001668	UBE2E1	2	-0.18815268	0.085438907	-2.202189677	0.027651913	0.007721858	1.981834114	49.54169004	1	PRJNA827465;Yang2025_Silkie_Wenchang	CRA004775	0.28376061
ENSGALG00010001668	UBE2E1	2	-0.346012964	0.095751612	-3.613651569	0.000301915	0	0.856793914	0	1	CRA004775;Yang2025_Silkie_Wenchang	PRJNA827465	0.017073604
ENSGALG00010001668	UBE2E1	2	-0.269088672	0.188910718	-1.424422467	0.154324214	0.053354032	3.360149844	70.23942245	1	CRA004775;PRJNA827465	Yang2025_Silkie_Wenchang	0.646989113
ENSGALG00010001670	SHOX	2	0.320700769	0.169179133	1.895628397	0.0580092	0	0.04735481	0	1	PRJNA827465;Yang2025_Silkie_Wenchang	CRA004775	0.417494153
ENSGALG00010001670	SHOX	2	0.394473808	0.21813004	1.808434125	0.070538961	0	0.112945107	0	1	CRA004775;Yang2025_Silkie_Wenchang	PRJNA827465	0.458155954
ENSGALG00010001670	SHOX	2	0.332713526	0.213010193	1.561960592	0.118297273	0	0.222762649	0	1	CRA004775;PRJNA827465	Yang2025_Silkie_Wenchang	0.578072574
ENSGALG00010001674	ENSGALG00010001674	2	0.082737436	0.093432451	0.885532125	0.375869701	0.011594655	2.648094936	62.23700343	1	PRJNA827465;Yang2025_Silkie_Wenchang	CRA004775	0.822974013
ENSGALG00010001674	ENSGALG00010001674	2	0.094062463	0.150650796	0.624374153	0.532381895	0.022954936	1.84241256	45.72334006	0.5	CRA004775;Yang2025_Silkie_Wenchang	PRJNA827465	0.873115194
ENSGALG00010001674	ENSGALG00010001674	2	0.003929859	0.047343354	0.083007626	0.933845476	0	0.339637721	0	0.5	CRA004775;PRJNA827465	Yang2025_Silkie_Wenchang	0.984309396
ENSGALG00010001677	SDHAF4	2	-0.105205938	0.21491687	-0.489519219	0.624474152	0.068480059	3.476458518	71.23509471	0.5	PRJNA827465;Yang2025_Silkie_Wenchang	CRA004775	0.900892482
ENSGALG00010001677	SDHAF4	2	-0.304399096	0.176218699	-1.727393847	0.084096955	0	0.257492623	0	1	CRA004775;Yang2025_Silkie_Wenchang	PRJNA827465	0.499714459
ENSGALG00010001677	SDHAF4	2	0.049489595	0.088982029	0.556175169	0.578091112	0	0.610292643	0	0.5	CRA004775;PRJNA827465	Yang2025_Silkie_Wenchang	0.887735993
ENSGALG00010001678	SYAP1	2	-0.140723285	0.104902627	-1.341465785	0.179769271	0.018585309	6.312001207	84.15716399	1	PRJNA827465;Yang2025_Silkie_Wenchang	CRA004775	0.68659821
ENSGALG00010001678	SYAP1	2	-0.086543217	0.198762118	-0.435411022	0.663264112	0.062072446	4.160304035	75.96329519	0.5	CRA004775;Yang2025_Silkie_Wenchang	PRJNA827465	0.91293776
ENSGALG00010001678	SYAP1	2	-0.027879532	0.050020197	-0.557365494	0.577277736	0.000482375	1.026280391	2.560741813	0.5	CRA004775;PRJNA827465	Yang2025_Silkie_Wenchang	0.887145528
ENSGALG00010001681	NAPG	2	-0.143464087	0.154294142	-0.929809032	0.352469969	0.043410949	10.87440243	90.80409239	0.5	PRJNA827465;Yang2025_Silkie_Wenchang	CRA004775	0.810930586
ENSGALG00010001681	NAPG	2	-0.198001719	0.144363024	-1.371554247	0.170202245	0.025481416	2.315167562	56.8065821	1	CRA004775;Yang2025_Silkie_Wenchang	PRJNA827465	0.671969748
ENSGALG00010001681	NAPG	2	0.000896442	0.035996647	0.024903492	0.980131942	0	0.002877769	0	0.5	CRA004775;PRJNA827465	Yang2025_Silkie_Wenchang	0.994658414
ENSGALG00010001686	WASL	2	-0.06764791	0.039231564	-1.724323576	0.084649446	0	0.424362248	0	0.5	PRJNA827465;Yang2025_Silkie_Wenchang	CRA004775	0.500222096
ENSGALG00010001686	WASL	2	0.080140389	0.13709567	0.584558134	0.558844865	0	0.050057217	0	1	CRA004775;Yang2025_Silkie_Wenchang	PRJNA827465	0.880896757
ENSGALG00010001686	WASL	2	-0.066008419	0.039348275	-1.67754287	0.093436374	0	0.823293942	0	0.5	CRA004775;PRJNA827465	Yang2025_Silkie_Wenchang	0.52381273
ENSGALG00010001688	ENSGALG00010001688	2	0.213186541	0.203536985	1.04740935	0.294910821	0.073242283	8.596648416	88.36755964	1	PRJNA827465;Yang2025_Silkie_Wenchang	CRA004775	0.787569357
ENSGALG00010001688	ENSGALG00010001688	2	0.269612894	0.18811543	1.433231148	0.151791791	0.04698236	2.722531584	63.26948029	1	CRA004775;Yang2025_Silkie_Wenchang	PRJNA827465	0.643294424
ENSGALG00010001688	ENSGALG00010001688	2	0.016849109	0.082566803	0.204066383	0.838301624	0	0.009211256	0	1	CRA004775;PRJNA827465	Yang2025_Silkie_Wenchang	0.959197438
ENSGALG00010001689	ILK	2	0.144104101	0.151270463	0.952625501	0.340779852	0.039965045	7.616111863	86.86994075	1	PRJNA827465;Yang2025_Silkie_Wenchang	CRA004775	0.80558586
ENSGALG00010001689	ILK	2	0.066473103	0.245660515	0.270589284	0.786706939	0.108707209	10.02079088	90.02074775	0.5	CRA004775;Yang2025_Silkie_Wenchang	PRJNA827465	0.946722706
ENSGALG00010001689	ILK	2	-0.05870997	0.088461469	-0.663678445	0.506896138	0.00896535	2.02729079	50.67308524	0.5	CRA004775;PRJNA827465	Yang2025_Silkie_Wenchang	0.863929781
ENSGALG00010001691	CTPS2	2	-0.080899274	0.072673769	-1.113183972	0.265629425	0	0.002362637	0	1	PRJNA827465;Yang2025_Silkie_Wenchang	CRA004775	0.769794389
ENSGALG00010001691	CTPS2	2	-0.053612997	0.103960101	-0.515707436	0.60605876	0	0.222891166	0	0.5	CRA004775;Yang2025_Silkie_Wenchang	PRJNA827465	0.896709756
ENSGALG00010001691	CTPS2	2	-0.066527737	0.087686073	-0.758703575	0.448029898	0	0.264450256	0	0.5	CRA004775;PRJNA827465	Yang2025_Silkie_Wenchang	0.846601815
ENSGALG00010001692	ALDH18A1	2	0.159478812	0.088494912	1.802124072	0.071525887	0	0.6120542	0	1	PRJNA827465;Yang2025_Silkie_Wenchang	CRA004775	0.46165999
ENSGALG00010001692	ALDH18A1	2	-0.239432547	0.426352174	-0.561583972	0.574399503	0.27423506	3.399836119	70.58681757	0.5	CRA004775;Yang2025_Silkie_Wenchang	PRJNA827465	0.886083039
ENSGALG00010001692	ALDH18A1	2	-0.185220582	0.50073039	-0.369900821	0.71145639	0.406027031	4.557630956	78.05877638	0.5	CRA004775;PRJNA827465	Yang2025_Silkie_Wenchang	0.92587565
ENSGALG00010001693	NKIRAS1	2	-0.15959967	0.163418341	-0.976632545	0.328751101	0.017312601	1.472989419	32.11084976	1	PRJNA827465;Yang2025_Silkie_Wenchang	CRA004775	0.800761292
ENSGALG00010001693	NKIRAS1	2	-0.323726792	0.191182744	-1.693284576	0.090401313	0	0.048233533	0	1	CRA004775;Yang2025_Silkie_Wenchang	PRJNA827465	0.5160164
ENSGALG00010001693	NKIRAS1	2	-0.030977068	0.168306142	-0.184051914	0.853972729	0	0.126589752	0	1	CRA004775;PRJNA827465	Yang2025_Silkie_Wenchang	0.962975689
ENSGALG00010001694	ENSGALG00010001694	2	0.037375873	0.109434975	0.341534992	0.732700867	0.011107522	1.864519583	46.36688136	0.5	PRJNA827465;Yang2025_Silkie_Wenchang	CRA004775	0.932985777
ENSGALG00010001694	ENSGALG00010001694	2	0.154072442	0.103652183	1.486437018	0.137163564	0	0.014462089	0	1	CRA004775;Yang2025_Silkie_Wenchang	PRJNA827465	0.619615281
ENSGALG00010001694	ENSGALG00010001694	2	-0.026531697	0.101700457	-0.260880806	0.794184427	0	0.883988385	0	0.5	CRA004775;PRJNA827465	Yang2025_Silkie_Wenchang	0.948905846
ENSGALG00010001695	LMOD2	2	0.246004323	0.53255737	0.461930182	0.644131389	0.522989418	12.56910008	92.04398092	0.5	PRJNA827465;Yang2025_Silkie_Wenchang	CRA004775	0.906873169
ENSGALG00010001695	LMOD2	2	0.013690782	0.977362978	0.014007879	0.988823695	1.506411739	4.044012456	75.27208408	0.5	CRA004775;Yang2025_Silkie_Wenchang	PRJNA827465	0.996891127
ENSGALG00010001695	LMOD2	2	-0.286740514	0.146079541	-1.962906729	0.049657013	0	0.92629818	0	1	CRA004775;PRJNA827465	Yang2025_Silkie_Wenchang	0.386010596
ENSGALG00010001696	FAM135A	2	-0.190674946	0.089530989	-2.129708916	0.033195652	0.010750878	2.795146648	64.2237018	1	PRJNA827465;Yang2025_Silkie_Wenchang	CRA004775	0.313175403
ENSGALG00010001696	FAM135A	2	-0.066903922	0.282374827	-0.236933026	0.812708748	0.1250947	4.123103856	75.74642708	0.5	CRA004775;Yang2025_Silkie_Wenchang	PRJNA827465	0.95348724
ENSGALG00010001696	FAM135A	2	-0.009093256	0.175509266	-0.051810688	0.958679539	0.040119324	2.095808091	52.28570762	0.5	CRA004775;PRJNA827465	Yang2025_Silkie_Wenchang	0.989079457
ENSGALG00010001697	TAF10	2	0.026345251	0.071683242	0.367523145	0.71322883	0	0.741109487	0	0.5	PRJNA827465;Yang2025_Silkie_Wenchang	CRA004775	0.926216079
ENSGALG00010001697	TAF10	2	-0.274766171	0.201982405	-1.360347062	0.173720122	0	0.054584291	0	1	CRA004775;Yang2025_Silkie_Wenchang	PRJNA827465	0.677447326
ENSGALG00010001697	TAF10	2	-0.041350193	0.151501849	-0.272935237	0.784902999	0.024380742	1.605016346	37.6953386	0.5	CRA004775;PRJNA827465	Yang2025_Silkie_Wenchang	0.946260623
ENSGALG00010001698	RPL15	2	-0.499115696	0.514424729	-0.97024048	0.331926638	0.476844596	9.236270263	89.17311889	1	PRJNA827465;Yang2025_Silkie_Wenchang	CRA004775	0.802002658
ENSGALG00010001698	RPL15	2	-0.705771562	0.334256109	-2.11146945	0.034731981	0.131306672	2.402166044	58.37090436	1	CRA004775;Yang2025_Silkie_Wenchang	PRJNA827465	0.32104211
ENSGALG00010001698	RPL15	2	-0.117686631	0.152770521	-0.770349087	0.441092845	0.026493695	1.670066792	40.12215532	1	CRA004775;PRJNA827465	Yang2025_Silkie_Wenchang	0.844888742
ENSGALG00010001699	ASB15	2	-0.502510909	0.117493692	-4.276918207	1.89498E-05	0	0.092467945	0	1	PRJNA827465;Yang2025_Silkie_Wenchang	CRA004775	0.002357128
ENSGALG00010001699	ASB15	2	-0.331416808	0.385263177	-0.860234842	0.389659602	0.157347336	1.646334811	39.25901381	0.5	CRA004775;Yang2025_Silkie_Wenchang	PRJNA827465	0.829068422
ENSGALG00010001699	ASB15	2	-0.325803092	0.317484468	-1.026201674	0.304796533	0.102162009	1.435832073	30.35397254	0.5	CRA004775;PRJNA827465	Yang2025_Silkie_Wenchang	0.79225794
ENSGALG00010001700	ENSGALG00010001700	2	-0.044652208	0.246372668	-0.181238482	0.856180391	0.0714618	2.392115879	58.19600511	0.5	PRJNA827465;Yang2025_Silkie_Wenchang	CRA004775	0.963499334
ENSGALG00010001700	ENSGALG00010001700	2	0.35503445	0.220132128	1.61282432	0.106782681	0	0.986064659	0	1	CRA004775;Yang2025_Silkie_Wenchang	PRJNA827465	0.553925396
ENSGALG00010001700	ENSGALG00010001700	2	0.156415314	0.494736422	0.316158882	0.751881916	0.384973258	4.293836828	76.71080574	0.5	CRA004775;PRJNA827465	Yang2025_Silkie_Wenchang	0.937865846
ENSGALG00010001701	NDUFA5	2	-0.247128546	0.114033501	-2.167157412	0.030222853	0.006757225	1.199414624	16.62599571	1	PRJNA827465;Yang2025_Silkie_Wenchang	CRA004775	0.295973612
ENSGALG00010001701	NDUFA5	2	-0.047836286	0.203471963	-0.235100137	0.814131013	0	0.049169125	0	1	CRA004775;Yang2025_Silkie_Wenchang	PRJNA827465	0.953869515

ENSGALG00010001701	NDUFA5	2	-0.292577092	0.094366612	-3.100430197	0.001932398	0	0.225983349	0	1	CRA004775;PRJNA827465	Yang2025_Silkie_Wenchang	0.056763095
ENSGALG00010001702	PLEKHA1	2	0.090779007	0.051465696	1.763874085	0.077753181	0	0.829614301	0	1	PRJNA827465;Yang2025_Silkie_Wenchang	CRA004775	0.480392844
ENSGALG00010001702	PLEKHA1	2	0.097676153	0.104753578	0.932437387	0.351110531	0.008368696	1.514421818	33.96819906	1	CRA004775;Yang2025_Silkie_Wenchang	PRJNA827465	0.810161472
ENSGALG00010001702	PLEKHA1	2	0.14028561	0.058176703	2.411370932	0.015892676	0	0.52492766	0	1	CRA004775;PRJNA827465	Yang2025_Silkie_Wenchang	0.208471156
ENSGALG00010001704	ENSGALG00010001704	2	-0.068106856	0.09224211	-0.738348848	0.46030249	0	0.164511425	0	1	PRJNA827465;Yang2025_Silkie_Wenchang	CRA004775	0.85014477
ENSGALG00010001704	ENSGALG00010001704	2	-0.053260399	0.145325472	-0.366490459	0.71399913	0	0.452481409	0	1	CRA004775;Yang2025_Silkie_Wenchang	PRJNA827465	0.926399535
ENSGALG00010001704	ENSGALG00010001704	2	-0.111308025	0.110975626	-1.002995242	0.315863157	0	0.263995546	0	1	CRA004775;PRJNA827465	Yang2025_Silkie_Wenchang	0.796883862
ENSGALG00010001705	HTRA1	2	-0.157839704	0.09746393	-1.619467883	0.105346631	0.0004023	1.021515896	2.106271311	1	PRJNA827465;Yang2025_Silkie_Wenchang	CRA004775	0.550809984
ENSGALG00010001705	HTRA1	2	0.106500654	0.395947197	0.268976912	0.78794745	0.272987638	7.484621036	86.63927011	0.5	CRA004775;Yang2025_Silkie_Wenchang	PRJNA827465	0.946919116
ENSGALG00010001705	HTRA1	2	0.192841513	0.296625488	0.650117811	0.515616125	0.138327652	4.398368878	77.26429893	0.5	CRA004775;PRJNA827465	Yang2025_Silkie_Wenchang	0.867300757
ENSGALG00010001706	IQUB	2	0.499509825	0.167178974	2.987874689	0.002809247	0	0.772673573	0	1	PRJNA827465;Yang2025_Silkie_Wenchang	CRA004775	0.07222107
ENSGALG00010001706	IQUB	2	0.644915737	0.218320138	2.953991064	0.003136931	0	0.020763409	0	1	CRA004775;Yang2025_Silkie_Wenchang	PRJNA827465	0.07754138
ENSGALG00010001706	IQUB	2	0.411023595	0.201479132	2.040030601	0.041347278	0	0.192558237	0	1	CRA004775;PRJNA827465	Yang2025_Silkie_Wenchang	0.352537164
ENSGALG00010001708	AADAC	2	0.087326533	0.287044059	0.304226929	0.76095501	0	0.086404392	0	1	PRJNA827465;Yang2025_Silkie_Wenchang	CRA004775	0.939493404
ENSGALG00010001708	AADAC	2	-0.109461765	0.249539642	-0.438654814	0.660911673	0	0.291755502	0	0.5	CRA004775;Yang2025_Silkie_Wenchang	PRJNA827465	0.911975967
ENSGALG00010001708	AADAC	2	-0.091194296	0.285654616	-0.31924671	0.749539439	0	0.55016847	0	0.5	CRA004775;PRJNA827465	Yang2025_Silkie_Wenchang	0.937362187
ENSGALG00010001710	LCMT2	2	0.10503131	0.070746612	1.484612573	0.137646483	0	0.06910939	0	1	PRJNA827465;Yang2025_Silkie_Wenchang	CRA004775	0.620631442
ENSGALG00010001710	LCMT2	2	0.042095598	0.109215454	0.385436278	0.699914209	0	0.65306478	0	0.5	CRA004775;Yang2025_Silkie_Wenchang	PRJNA827465	0.922198312
ENSGALG00010001710	LCMT2	2	0.092908805	0.083920448	1.107105677	0.268248241	0	0.944149568	0	0.5	CRA004775;PRJNA827465	Yang2025_Silkie_Wenchang	0.771398294
ENSGALG00010001711	CREB5	2	0.219229133	0.374271403	0.585749086	0.558044142	0.217210237	3.89126004	74.30138337	0.5	PRJNA827465;Yang2025_Silkie_Wenchang	CRA004775	0.880678789
ENSGALG00010001711	CREB5	2	0.859913046	0.306230191	2.808060963	0.004984079	0	0.792811903	0	1	CRA004775;Yang2025_Silkie_Wenchang	PRJNA827465	0.105203684
ENSGALG00010001711	CREB5	2	0.485735954	0.670005016	0.724973608	0.468468222	0.759252275	5.692019918	82.43154426	0.5	CRA004775;PRJNA827465	Yang2025_Silkie_Wenchang	0.853198008
ENSGALG00010001712	ATP6AP1	2	-0.206347344	0.160220841	-1.287893276	0.197783117	0.039334694	4.143501775	75.86582426	1	PRJNA827465;Yang2025_Silkie_Wenchang	CRA004775	0.709650373
ENSGALG00010001712	ATP6AP1	2	-0.127838192	0.320527468	-0.398836932	0.690013362	0.15034507	3.281265498	69.52395347	0.5	CRA004775;Yang2025_Silkie_Wenchang	PRJNA827465	0.919979266
ENSGALG00010001712	ATP6AP1	2	-0.040846254	0.083309113	-0.490297547	0.623932162	0	0.926661402	0	0.5	CRA004775;PRJNA827465	Yang2025_Silkie_Wenchang	0.900619212
ENSGALG00010001713	PNPLA4	2	0.138018196	0.228612468	0.603721211	0.54602901	0.066625115	2.725141636	63.30465959	0.5	PRJNA827465;Yang2025_Silkie_Wenchang	CRA004775	0.877958239
ENSGALG00010001713	PNPLA4	2	-0.101754663	0.188938306	-0.53856026	0.590190315	0	0.00632203	0	1	CRA004775;Yang2025_Silkie_Wenchang	PRJNA827465	0.892451983
ENSGALG00010001713	PNPLA4	2	0.27275059	0.16236573	1.679853192	0.092985883	0.002167907	1.024727506	2.413081081	0.5	CRA004775;PRJNA827465	Yang2025_Silkie_Wenchang	0.52216376
ENSGALG00010001716	TRIL	2	-0.228220283	0.121511085	-1.878184883	0.060358877	0.022636236	4.273276606	76.97076427	1	PRJNA827465;Yang2025_Silkie_Wenchang	CRA004775	0.425577153
ENSGALG00010001716	TRIL	2	0.035312386	0.49823791	0.070874547	0.943497601	0.391370258	3.866080814	74.13401199	0.5	CRA004775;Yang2025_Silkie_Wenchang	PRJNA827465	0.985827325
ENSGALG00010001716	TRIL	2	0.116986736	0.355698672	0.32889281	0.742236709	0.171916777	2.266425897	55.87766618	0.5	CRA004775;PRJNA827465	Yang2025_Silkie_Wenchang	0.935497348
ENSGALG00010001717	STARD10	2	1.17177288	0.858176618	1.365421587	0.172120583	1.266826942	7.91222925	87.36133688	1	PRJNA827465;Yang2025_Silkie_Wenchang	CRA004775	0.674880614
ENSGALG00010001717	STARD10	2	0.908437249	1.153769946	0.787364285	0.431068648	2.400005713	10.04629801	90.04608465	0.5	CRA004775;Yang2025_Silkie_Wenchang	PRJNA827465	0.842503929
ENSGALG00010001717	STARD10	2	0.097261341	0.354864628	0.274080123	0.784023049	0	0.638915181	0	0.5	CRA004775;PRJNA827465	Yang2025_Silkie_Wenchang	0.946012276
ENSGALG00010001721	TM4SF1	2	-0.52106806	0.525738977	-0.991115521	0.321629175	0.521501629	16.90215716	94.08359542	1	PRJNA827465;Yang2025_Silkie_Wenchang	CRA004775	0.798513866
ENSGALG00010001721	TM4SF1	2	-0.77433293	0.346055593	-2.237596923	0.025243752	0.148496051	2.549818636	60.78152438	1	CRA004775;Yang2025_Silkie_Wenchang	PRJNA827465	0.270438551
ENSGALG00010001721	TM4SF1	2	-0.032567861	0.062492547	-0.521147922	0.602263729	0	0.933915079	0	1	CRA004775;PRJNA827465	Yang2025_Silkie_Wenchang	0.895349419
ENSGALG00010001725	MTA2	2	-0.050241206	0.054763027	-0.91742931	0.35891772	0	0.687523315	0	0.5	PRJNA827465;Yang2025_Silkie_Wenchang	CRA004775	0.814241185
ENSGALG00010001725	MTA2	2	-0.131322049	0.178087105	-0.737403468	0.460877027	0.04430822	3.145695747	68.21053019	0.5	CRA004775;Yang2025_Silkie_Wenchang	PRJNA827465	0.85014477
ENSGALG00010001725	MTA2	2	-0.158673153	0.120055092	-1.321669496	0.186278231	0.016406629	1.924086276	48.02727859	1	CRA004775;PRJNA827465	Yang2025_Silkie_Wenchang	0.695534555
ENSGALG00010001727	COL9A1	2	1.196488863	1.045956042	1.143918879	0.252657281	1.301836283	2.452064417	59.21803713	1	PRJNA827465;Yang2025_Silkie_Wenchang	CRA004775	0.762687335
ENSGALG00010001727	COL9A1	2	-0.806747028	3.200124499	-0.252098638	0.800964823	18.9499721	13.27611376	92.46767527	0.5	CRA004775;Yang2025_Silkie_Wenchang	PRJNA827465	0.950158029
ENSGALG00010001727	COL9A1	2	-1.782402648	2.147387785	-0.830032964	0.406520146	7.87478785	6.655308625	84.96988406	0.5	CRA004775;PRJNA827465	Yang2025_Silkie_Wenchang	0.833618122
ENSGALG00010001728	STS	2	-0.217759972	0.096754563	-2.250642923	0.024408163	0	0.189651964	0	1	PRJNA827465;Yang2025_Silkie_Wenchang	CRA004775	0.267184364
ENSGALG00010001728	STS	2	-0.115443018	0.139797829	-0.825785483	0.408925791	0.000174154	1.001962793	0.195889816	0.5	CRA004775;Yang2025_Silkie_Wenchang	PRJNA827465	0.834316328
ENSGALG00010001728	STS	2	-0.137440578	0.213177365	-0.644724068	0.519106016	0.042778468	1.498937419	33.28607402	0.5	CRA004775;PRJNA827465	Yang2025_Silkie_Wenchang	0.868939943
ENSGALG00010001729	ENSGALG00010001729	2	-0.065751027	0.437088989	-0.150429382	0.880425061	0.330980947	7.45709495	86.58995216	0.5	PRJNA827465;Yang2025_Silkie_Wenchang	CRA004775	0.971652891
ENSGALG00010001729	ENSGALG00010001729	2	-0.017606678	0.498725256	-0.035303361	0.971837843	0.43225136	7.528415858	86.71699307	0.5	CRA004775;Yang2025_Silkie_Wenchang	PRJNA827465	0.991955348
ENSGALG00010001729	ENSGALG00010001729	2	0.429425506	0.187940691	2.284899049	0.022318748	0	0.104541666	0	1	CRA004775;PRJNA827465	Yang2025_Silkie_Wenchang	0.253377742
ENSGALG00010001731	ENSGALG00010001731	2	-0.021975669	0.100109257	-0.219516854	0.826247452	0	0.002568724	0	1	PRJNA827465;Yang2025_Silkie_Wenchang	CRA004775	0.956393839
ENSGALG00010001731	ENSGALG00010001731	2	-0.13528517	0.240722595	-0.561993809	0.574120237	0	0.238630595	0	1	CRA004775;Yang2025_Silkie_Wenchang	PRJNA827465	0.886081433
ENSGALG00010001731	ENSGALG00010001731	2	-0.039027475	0.101685542	-0.383805552	0.701122573	0	0.430026715	0	1	CRA004775;PRJNA827465	Yang2025_Silkie_Wenchang	0.922547052
ENSGALG00010001738	PUDP	2	-0.103837954	0.084511082	-1.228690386	0.219187912	0.001147773	1.061141484	5.76185971	0.5	PRJNA827465;Yang2025_Silkie_Wenchang	CRA004775	0.73285529
ENSGALG00010001738	PUDP	2	-0.021004474	0.133005665	-0.15792647	0.87451854	0	0.420221994	0	0.5	CRA004775;Yang2025_Silkie_Wenchang	PRJNA827465	0.970143165
ENSGALG00010001738	PUDP	2	-0.146916439	0.083109841	-1.76773817	0.077104686	0	0.010421389	0	1	CRA004775;PRJNA827465	Yang2025_Silkie_Wenchang	0.478533748
ENSGALG00010001741	SATB1	2	0.04373262	0.155364812	0.281483431	0.778339635	0.000858354	1.017341398	1.704580021	0.5	PRJNA827465;Yang2025_Silkie_Wenchang	CRA004775	0.944577369
ENSGALG00010001741	SATB1	2	0.332407028	0.182406715	1.822339858	0.068403434	0	0.390468913	0	1	CRA004775;Yang2025_Silkie_Wenchang	PRJNA827465	0.450776288
ENSGALG00010001741	SATB1	2	0.155350471	0.271176831	0.572875323	0.566729222	0.09127131	2.571603484	61.11175622	0.5	CRA004775;PRJNA827465	Yang2025_Silkie_Wenchang	0.883487517
ENSGALG00010001745	CADPS2	2	-0.236236464	0.119928593	-1.969809352	0.048860224	0.007363314	1.342919437	25.53536925	1	PRJNA827465;Yang2025_Silkie_Wenchang	CRA004775	0.382007837
ENSGALG00010001745	CADPS2	2	-0.179883062	0.273783428	-0.657026846	0.511163655	0.085313556	1.939988378	48.45329945	0.5	CRA004775;Yang2025_Silkie_Wenchang	PRJNA827465	0.865649044
ENSGALG00010001745	CADPS2	2	-0.082433916	0.133421771	-0.617844565	0.536677016	0	0.698463731	0	0.5	CRA004775;PRJNA827465	Yang2025_Silkie_Wenchang	0.874599474
ENSGALG00010001749	VAPA	2	-0.085027323	0.052205632	-1.628700202	0.1033765	0	0.002688742	0	1	PRJNA827465;Yang2025_Silkie_Wenchang	CRA004775	0.547576788
ENSGALG00010001749	VAPA	2	0.000915269	0.147485339	0.006205829	0.995048497	0.018930828	1.530166283	34.64762549	0.5	CRA004775;Yang2025_Silkie_Wenchang	PRJNA827465	0.998242335
ENSGALG00010001749	VAPA	2	-0.00883636	0.136507147	-0.064731851	0.948387502	0.020586995	1.643436068	39.15187699	0.5	CRA004775;PRJNA827465	Yang2025_Silkie_Wenchang	0.986778445
ENSGALG00010001752	ARRB1	2	0.146904965	0.301984918	0.329982207	0.741413407	0.149677516	4.895787492	79.57432763	0.5	PRJNA827465;Yang2025_Silkie_Wenchang	CRA004775	0.93543545
ENSGALG00010001752	ARRB1	2	0.053692457	0.397489186	0.135079039	0.892549383	0.251690456	4.905295935	79.61387013	0.5	CRA004775;Yang2025_Silkie_Wenchang	PRJNA827465	0.974359592
ENSGALG00010001752	ARRB1	2	-0.169822508	0.071755991	-2.366666607	0.017949093	0	0.521681462	0	1	CRA004775;PRJNA827465	Yang2025_Silkie_Wenchang	0.223669379
ENSGALG00010001753	PNPLA8	2	-0.1362241	0.054296337	-2.508900384	0.012110763	0	0.037681867	0	1	PRJNA827465;Yang2025_Silkie_Wenchang	CRA004775	0.177409365
ENSGALG00010001753	PNPLA8	2	-0.113263335	0.131617658	-0.860548169	0.389486943	0.010627514	1.098065691	8.262409477	0.5	CRA004775;Yang2025_Silkie_Wenchang	PRJNA827465	0.828911149
ENSGALG00010001753	PNPLA8	2	-0.115600226	0.077217054	-1.497081548	0.13437204	0.001470643	1.012645601	1.248768673	0.5	CRA004775;PRJNA827465	Yang2025_Silkie_Wenchang	0.61409585
ENSGALG00010001754	APCDD1	2	-0.214683396	0.234108585	-0.917024872	0.359129607	0.069489018	2.674303115	62.60708092	0.5	PRJNA827465;Yang2025_Silkie_Wenchang	CRA004775	0.814241185
ENSGALG00010001754	APCDD1	2	-0.457500431	0.16491213	-2.774205281	0.005533641	0	0.579119552	0	1	CRA004775;Yang2025_Silkie_Wenchang	PRJNA827465	0.11704511
ENSGALG00010001754	APCDD1	2	-0.258150563	0.452121494	-0.57097609	0.56801585	0.24374751	2.053260342	51.2990005	0.5	CRA004775;PRJNA827465	Yang2025_Silkie_Wenchang	0.88390256
ENSGALG00010001756	ENSGALG00010001756	2	0.606194127	0.36559763	1.658090963	0.097299108	0.146984853	2.215385152	54.86112204	1	PRJNA827465;Yang2025_Silkie_Wenchang	CRA004775	0.532942995
ENSGALG00010001756	ENSGALG00010001756	2	0.490214382	0.507155671	0.966595487	0.333746292	0.364867648	3.377139215	70.38913896	0.5	CRA004775;Yang2025_Silkie_Wenchang	PRJNA827465	0.802691373
ENSGALG00010001756	ENSGALG00010001756	2	0.107838455	0.282643487	0.381521466	0.702816546	0	0.246455543	0	0.5	CRA004775;PRJNA827465	Yang2025_Silkie_Wenchang	0.923345061
ENSGALG00010001758	COL19A1	2	-0.09099253	0.108706579	-0.837047135	0.402566074	0	0.725268055	0	0.5	PRJNA827465;Yang2025_Silkie_Wenchang	CRA004775	0.832937979
ENSGALG00010001758	COL19A1	2	-0.769390134	0.955154506	-0.805513797	0.420523248	1.531277205	5.447939354	81.6444359	0.5	CRA004775;Yang2025_Silkie_Wenchang	PRJNA827465	0.839061082
ENSGALG00010001758	COL19A1	2	-0.844456969	0.850951132	-0.992368348	0.321017873	1.179370795	4.515155893	77.85237047	1	CRA004775;PRJNA827465	Yang2025_Silkie_Wenchang	0.798513866
ENSGALG00010001759	C10orf88	2	0.016086257	0.052926572	0.303936508	0.761176587	0.001672345	1.309922737	23.6287195	0.5	PRJNA827465;Yang2025_Silkie_Wenchang	CRA004775	0.93984592
ENSGALG00010001759	C10orf88	2	0.100783132	0.087388936	1.153271078	0.248799137	0	0.003801294	0	1	CRA004775;Yang2025_Silkie_Wenchang	PRJNA827465	0.759204386
ENSGALG00010001759	C10orf88	2	-0.012985197	0.045877223	-0.283042353	0.77714437	0	0.153052912	0	0.5	CRA004775;PRJNA827465	Yang2025_Silkie_Wenchang	0.944218792
ENSGALG00010001760	RAB5A	2	-0.084048427	0.120113093	-0.699734089	0.484087117	0.019348812	2.606652251	61.5481078	0.5	PRJNA827465;Yang2025_Silkie_Wenchang	CRA004775	0.853704733
ENSGALG00010001760	RAB5A	2	-0.261328238	0.131462514	-1.987853648	0.046827878	0	0.066849352	0	1	CRA004775;Yang2025_Silkie_Wenchang	PRJNA827465	0.37376485
ENSGALG00010001760	RAB5A	2	-0.027766529	0.100710028	-0.275707685	0.782772593	0.008164259	1.165556904	14.2041031	0.5	CRA004775;PRJNA827465	Yang2025_Silkie_Wenchang	0.945490372
ENSGALG00010001761	ELOB	2	0.251488102	0.286973596	0.876345789	0.38084208	0.142350409	6.961994671	85.63630041	0.5	PRJNA827465;Yang2025_Silkie_Wenchang	CRA004775	0.824392515
ENSGALG00010001761	ELOB	2	0.369221733	0.246664927	1.496855423	0.134430882	0.060328286	1.896436919	47.26953532	1	CRA004775;Yang2025_Silkie_Wenchang	PRJNA827465	0.614221918
ENSGALG00010001761	ELOB	2	-0.003703537	0.085823529	-0.043152938	0.96557962	0	0.049532076	0	0.5	CRA004775;PRJNA827465	Yang2025_Silkie_Wenchang	0.990668014
ENSGALG00010001763	ENSGALG00010001763	2	0.341634469	0.303427539	1.125917804	0.260200313	0.017930089	1.10768442	9.721579378	1	PRJNA827465;Yang2025_Silkie_Wenchang	CRA004775	0.767536242
ENSGALG00010001763	ENSGALG00010001763	2	0.486922444	0.264425939	1.841432216	0.065558245	0	0.239586388	0	1	CRA004775;Yang2025_Silkie_Wenchang	PRJNA827465	0.441431509
ENSGALG00010001763	ENSGALG00010001763	2	0.227543095	0.269536108	0.844202642	0.398556178	0	0.402129543	0	1	CRA004775;PRJNA827465	Yang2025_Silkie_Wenchang	0.831980453
ENSGALG00010001767	ENSGALG00010001767	2	0.326575798	0.484680507	0.673796023	0.500441004	0.425945326	10.25249021	90.24627209	0.5	PRJNA827465;Yang2025_Silkie_Wenchang	CRA004775	0.862400704
ENSGALG00010001767	ENSGALG00010001767	2	0.392105912	0.483560206	0.810872993	0.417438611	0.361189455	4.330144255	76.90608116	0.5	CRA004775;Yang2025_Silkie_Wenchang	PRJNA827465	0.838112595
ENSGALG00010001767	ENSGALG00010001767	2	-0.126198066	0.113418444	-1.112676753	0.265847283	0	3.76661E-05	0	1	CRA004775;PRJNA827465	Yang2025_Silkie_Wenchang	0.770065639
ENSGALG00010001771	ENSGALG00010001771	2	0.130119536	0.226589054	0.574253405	0.565796335	0.079263905	4.184130073	76.10016939	0.5	PRJNA827465;Yang2025_Silkie_Wenchang	CRA004775	0.883487517
ENSGALG00010001771	ENSGALG00010001771	2	0.0697889	0.338239037	0.206330117	0.836533055	0.176776151	4.327354862	76.89119493	0.5	CRA004775;Yang2025_Silkie_Wenchang	PRJNA827465	0.958399882
ENSGALG00010001771	ENSGALG00010001771	2	-0.104989772	0.102907616	-1.02023325	0.307617852	0	0.603021303	0	1	CRA004775;PRJNA827465	Yang2025_Silkie_Wenchang	0.792968388
ENSGALG00010001773	NRCAM	2	0.302094443	0.434985361	0.694493355	0.487372866	0.348514922	12.28093056	91.85729457	0.5	PRJNA827465;Yang2025_Silkie_Wenchang	CRA004775	0.85768885
ENSGALG00010001773	NRCAM	2	1.208948934	0.596327804	2.027322768	0.042629415	0.50709068	2.92930105	65.86216361	1	CRA004775;Yang2025_Silkie_Wenchang	PRJNA827465	0.357019514
ENSGALG00010001773	NRCAM	2	0.834915499	1.050279187	0.794946249	0.42664477	1.986784853	9.158978217	89.08175152	0.5	CRA004775;PRJNA827465	Yang2025_Silkie_Wenchang	0.84125685
ENSGALG00010001776	LMBRD1	2	-0.342915735	0.323962421	-1.058504667	0.289825424	0.187137039	8.601770078	88.37448582	1	PRJNA827465;Yang2025_Silkie_Wenchang	CRA004775	0.784072731
ENSGALG00010001776	LMBRD1	2	-0.487510746	0.270427077	-1.802743832	0.071428455	0.07332651	1.893681453	47.19280804	1	CRA004775;Yang2025_Silkie_Wenchang	PRJNA827465	0.461553385
ENSGALG00010001776	LMBRD1	2	-0.051563823	0.068055737	-0.757670482	0.448648274	0	0.069953847	0	1	CRA004775;PRJNA827465	Yang2025_Silkie_Wenchang	0.846878812
ENSGALG00010001778	KAT2B	2	0.042297925	0.055473424	0.762489903	0.445767667	0	0.044655011	0	1	PRJNA827465;Yang2025_Silkie_Wenchang	CRA004775	0.846013379
ENSGALG00010001778	KAT2B	2	0.100886707	0.111900492	0.901575188	0.367282576	0	0.415789796	0	1	CRA004775;Yang2025_Silkie_Wenchang	PRJNA827465	0.819739734
ENSGALG00010001778	KAT2B	2	0.048242155	0.060236369	0.800880853	0.423200625	0	0.653288903	0	1	CRA004775;PRJNA827465	Yang2025_Silkie_Wenchang	0.840095671
ENSGALG00010001780	ENSGALG00010001780	2	0.35686263	0.10445089	3.416559024	0.000634179	0	0.541284712	0	1	PRJNA827465;Yang2025_Silkie_Wenchang	CRA004775	0.027633196
ENSGALG00010001780	ENSGALG00010001780	2	0.263161594	0.157907644	1.666553866	0.095603149	0	0.001296105	0	1	CRA004775;Yang2025_Silkie_Wenchang	PRJNA827465	0.529447732
ENSGALG00010001780	ENSGALG00010001780	2	0.412836464	0.132874567	3.106963757	0.001890196	0	0.078311977	0	1	CRA004775;PRJNA827465	Yang2025_Silkie_Wenchang	0.055941538
ENSGALG00010001783	SGO1	2	0.028827333	0.123687335	0.233066165	0.815710028	0.013126396	1.688181509	40.76466337	0.5	PRJNA827465;Yang2025_Silkie_Wenchang	CRA004775	0.954210034
ENSGALG00010001783	SGO1	2	-0.09232488	0.156739185	-0.589035092	0.555837733	0	0.685110379	0	0.5	CRA004775;Yang2025_Silkie_Wenchang	PRJNA827465	0.880324407
ENSGALG00010001783	SGO1	2	0.135704798	0.107374521	1.263845433	0.206285511	0	0.191819017	0	1	CRA004775;PRJNA827465	Yang2025_Silkie_Wenchang	0.718451597
ENSGALG00010001785	CNTN1	2	-0.192092439	0.154721028	-1.241540612	0.214406105	0	0.167500368	0	1	PRJNA827465;Yang2025_Silkie_Wenchang	CRA004775	0.727205473
ENSGALG00010001785	CNTN1	2	-0.263197504	0.222750191	-1.181581495	0.2373718	0	0.004592954	0	1	CRA004775;Yang2025_Silkie_Wenchang	PRJNA827465	0.749366344
ENSGALG00010001785	CNTN1	2	-0.152011569	0.186306631	-0.815921411	0.414545086	0	0.025500465	0	1	CRA004775;PRJNA827465	Yang2025_Silkie_Wenchang	0.837162959
ENSGALG00010001786	P2RY8	2	-0.258063614	0.362745028	-0.711418748	0.476824785	0.22656743	7.077590473	85.87089768	0.5	PRJNA827465;Yang2025_Silkie_Wenchang	CRA004775	0.854986476
ENSGALG00010001786	P2RY8	2	-0.649865217	0.213140255	-3.049002713	0.002296024	0	0.019761269	0	1	CRA004775;Yang2025_Silkie_Wenchang	PRJNA827465	0.063663608
ENSGALG00010001786	P2RY8	2	-0.073562708	0.326385852	-0.225385712	0.821679226	0.108323141	1.467127141	31.83958145	0.5	CRA004775;PRJNA827465	Yang2025_Silkie_Wenchang	0.955598716
ENSGALG00010001788	HOXA1	2	0.374959665	0.199001775	1.884202616	0.059537574	0	0.679782601	0	1	PRJNA827465;Yang2025_Silkie_Wenchang	CRA004775	0.422736519
ENSGALG00010001788	HOXA1	2	0.243242976	0.379404656	0.641117531	0.521446325	0.154842762	2.101286763	52.41011281	0.5	CRA004775;Yang2025_Silkie_Wenchang	PRJNA827465	0.869518515
ENSGALG00010001788	HOXA1	2	0.117124612	0.224358689	0.52204179	0.601641234	0	0.743217059	0	0.5	CRA004775;PRJNA827465	Yang2025_Silkie_Wenchang	0.895230519
ENSGALG00010001790	HOXA2	2	0.239795763	0.402383943	0.595937702	0.551216845	0.291071529	9.706828923	89.69797441	0.5	PRJNA827465;Yang2025_Silkie_Wenchang	CRA004775	0.878879109
ENSGALG00010001790	HOXA2	2	0.562556243	0.174657652	3.220908081	0.001277851	0	0.54622192	0	1	CRA004775;Yang2025_Silkie_Wenchang	PRJNA827465	0.043386199
ENSGALG00010001790	HOXA2	2	0.0645061	0.262209705	0.246009583	0.805674815	0.093870251	2.84805294	64.88829312	0.5	CRA004775;PRJNA827465	Yang2025_Silkie_Wenchang	0.951984168
ENSGALG00010001793	IKZF5	2	-0.188056207	0.191993411	-0.979493024	0.327336434	0.064483795	7.643209945	86.9164918	1	PRJNA827465;Yang2025_Silkie_Wenchang	CRA004775	0.800028239
ENSGALG00010001793	IKZF5	2	-0.018499059	0.395632521	-0.046758187	0.962705954	0.283396003	10.33829202	90.32722235	0.5	CRA004775;Yang2025_Silkie_Wenchang	PRJNA827465	0.989980504
ENSGALG00010001793	IKZF5	2	0.142001496	0.197045531	0.720653221	0.471122901	0.058341534	3.385383202	70.46124647	0.5	CRA004775;PRJNA827465	Yang2025_Silkie_Wenchang	0.853923969
ENSGALG00010001795	ENSGALG00010001795	2	0.710475527	0.353122174	2.011982199	0.044221814	0	0.657528546	0	1	PRJNA827465;Yang2025_Silkie_Wenchang	CRA004775	0.36338071
ENSGALG00010001795	ENSGALG00010001795	2	0.322683345	0.722758601	0.446460747	0.655264461	0.74905758	3.533134183	71.69651793	0.5	CRA004775;Yang2025_Silkie_Wenchang	PRJNA827465	0.910411938
ENSGALG00010001795	ENSGALG00010001795	2	0.078642438	0.43049208	0.182680336	0.855048846	0.118374773	1.461303513	31.5679466	0.5	CRA004775;PRJNA827465	Yang2025_Silkie_Wenchang	0.963317625
ENSGALG00010001796	SLCO2B1	2	-0.229106644	0.162357105	-1.411127924	0.158206898	0.043118895	5.490256496	81.78591473	1	PRJNA827465;Yang2025_Silkie_Wenchang	CRA004775	0.65200722
ENSGALG00010001796	SLCO2B1	2	-0.380032615	0.092936273	-4.089174242	4.32912E-05	0	0.169938427	0	1	CRA004775;Yang2025_Silkie_Wenchang	PRJNA827465	0.00440796
ENSGALG00010001796	SLCO2B1	2	-0.07850868	0.095905992	-0.818600363	0.41301446	0	0.223184163	0	1	CRA004775;PRJNA827465	Yang2025_Silkie_Wenchang	0.835888542
ENSGALG00010001798	PSTK	2	0.188257493	0.087690402	2.146842627	0.031805812	0	0.00034907	0	1	PRJNA827465;Yang2025_Silkie_Wenchang	CRA004775	0.305444789
ENSGALG00010001798	PSTK	2	0.217083725	0.180908636	1.199963304	0.230153592	0	0.094407609	0	1	CRA004775;Yang2025_Silkie_Wenchang	PRJNA827465	0.742845187
ENSGALG00010001798	PSTK	2	0.194726068	0.095299759	2.043300736	0.041022677	0	0.114976587	0	1	CRA004775;PRJNA827465	Yang2025_Silkie_Wenchang	0.350884497
ENSGALG00010001800	ENSGALG00010001800	2	0.072944454	0.105602094	0.690748174	0.48972381	0.009619249	1.733830189	42.32422494	0.5	PRJNA827465;Yang2025_Silkie_Wenchang	CRA004775	0.858425426
ENSGALG00010001800	ENSGALG00010001800	2	-0.097865161	0.3546576	-0.275942657	0.78259211	0.194815909	3.934600678	74.58446023	0.5	CRA004775;Yang2025_Silkie_Wenchang	PRJNA827465	0.945446706
ENSGALG00010001800	ENSGALG00010001800	2	-0.169330758	0.232346174	-0.728786513	0.466132263	0.066550439	2.051651223	51.25877202	1	CRA004775;PRJNA827465	Yang2025_Silkie_Wenchang	0.852631007
ENSGALG00010001801	HOXA3	2	0.324242295	0.33321784	0.973064031	0.330521479	0.186831064	5.598361376	82.13763041	1	PRJNA827465;Yang2025_Silkie_Wenchang	CRA004775	0.80156474
ENSGALG00010001801	HOXA3	2	0.436989901	0.280839765	1.55601149	0.11970536	0.079624851	2.018783144	50.46520955	1	CRA004775;Yang2025_Silkie_Wenchang	PRJNA827465	0.580639051
ENSGALG00010001801	HOXA3	2	0.046255651	0.072402979	0.638863928	0.522911456	0	0.147307661	0	1	CRA004775;PRJNA827465	Yang2025_Silkie_Wenchang	0.869568211
ENSGALG00010001803	PPP4R1	2	-0.038339247	0.03786766	-1.012453562	0.311321239	0	0.784585727	0	1	PRJNA827465;Yang2025_Silkie_Wenchang	CRA004775	0.795445189
ENSGALG00010001803	PPP4R1	2	-0.063984841	0.054021373	-1.184435652	0.236240654	0	0.566303528	0	0.5	CRA004775;Yang2025_Silkie_Wenchang	PRJNA827465	0.748669015
ENSGALG00010001803	PPP4R1	2	-0.00198074	0.05003081	-0.039590395	0.968419685	0	0.199594886	0	0.5	CRA004775;PRJNA827465	Yang2025_Silkie_Wenchang	0.991215679
ENSGALG00010001805	ENSGALG00010001805	2	-0.176873056	0.085522856	-2.068137862	0.038627061	0	0.768236244	0	1	PRJNA827465;Yang2025_Silkie_Wenchang	CRA004775	0.338965227
ENSGALG00010001805	ENSGALG00010001805	2	-0.324149789	0.141664494	-2.288151257	0.022128714	0	0.358370649	0	1	CRA004775;Yang2025_Silkie_Wenchang	PRJNA827465	0.252386783
ENSGALG00010001805	ENSGALG00010001805	2	-0.171684039	0.126522669	-1.356942921	0.174799348	0.007436979	1.102362107	9.285706222	1	CRA004775;PRJNA827465	Yang2025_Silkie_Wenchang	0.679506889
ENSGALG00010001807	ASMTL	2	-0.103603466	0.141748476	-0.730896508	0.464842369	0.031971959	4.874051231	79.48318652	0.5	PRJNA827465;Yang2025_Silkie_Wenchang	CRA004775	0.85217126
ENSGALG00010001807	ASMTL	2	-0.222199744	0.092413889	-2.404397715	0.016199139	0	0.786166473	0	0.5	CRA004775;Yang2025_Silkie_Wenchang	PRJNA827465	0.210262467
ENSGALG00010001807	ASMTL	2	0.032739229	0.080318425	0.407617922	0.683554204	0	0.00195794	0	1	CRA004775;PRJNA827465	Yang2025_Silkie_Wenchang	0.918800744
ENSGALG00010001808	HOXA4	2	0.137837945	0.191642402	0.719245553	0.471989637	0.051779192	3.357113871	70.21250877	0.5	PRJNA827465;Yang2025_Silkie_Wenchang	CRA004775	0.853998373
ENSGALG00010001808	HOXA4	2	0.351393239	0.139629167	2.516617748	0.01184873	0	0.012500088	0	1	CRA004775;Yang2025_Silkie_Wenchang	PRJNA827465	0.174649902
ENSGALG00010001808	HOXA4	2	0.101297126	0.198908164	0.509265805	0.610565923	0.042802861	1.9488406	48.68744011	0.5	CRA004775;PRJNA827465	Yang2025_Silkie_Wenchang	0.897438028
ENSGALG00010001810	OPN2SW	2	1.075356834	0.67608265	1.590570081	0.111706362	0.749664889	5.522465383	81.89214543	1	PRJNA827465;Yang2025_Silkie_Wenchang	CRA004775	0.565091513
ENSGALG00010001810	OPN2SW	2	0.392843057	1.430314165	0.274655084	0.783581242	3.761885814	12.25886192	91.84263591	0.5	CRA004775;Yang2025_Silkie_Wenchang	PRJNA827465	0.945815798
ENSGALG00010001810	OPN2SW	2	-0.219122414	0.746245828	-0.293633018	0.769038343	0.838414928	3.79243858	73.63174172	0.5	CRA004775;PRJNA827465	Yang2025_Silkie_Wenchang	0.941780831
ENSGALG00010001811	ACADSB	2	-0.517682773	0.181795473	-2.847610908	0.004404874	0.040902648	2.538097996	60.60041805	1	PRJNA827465;Yang2025_Silkie_Wenchang	CRA004775	0.097319196
ENSGALG00010001811	ACADSB	2	-0.147961568	0.598349038	-0.247283038	0.804689185	0.657947362	12.18917547	91.79599963	0.5	CRA004775;Yang2025_Silkie_Wenchang	PRJNA827465	0.951665484
ENSGALG00010001811	ACADSB	2	0.015725274	0.413119116	0.038064746	0.96963606	0.295253931	7.014100889	85.74300519	0.5	CRA004775;PRJNA827465	Yang2025_Silkie_Wenchang	0.991572914
ENSGALG00010001813	HOXA5	2	0.258319637	0.274762124	0.940157373	0.347136843	0.130243804	7.178530675	86.06957266	0.5	PRJNA827465;Yang2025_Silkie_Wenchang	CRA004775	0.808616989
ENSGALG00010001813	HOXA5	2	0.513487795	0.150708173	3.40716622	0.000656411	0	0.196499513	0	1	CRA004775;Yang2025_Silkie_Wenchang	PRJNA827465	0.028276881
ENSGALG00010001813	HOXA5	2	0.052284656	0.135076087	0.387075588	0.698700249	0.007435026	1.11385031	10.22132947	0.5	CRA004775;PRJNA827465	Yang2025_Silkie_Wenchang	0.921748813
ENSGALG00010001817	SLC25A6	2	0.162099434	0.045016626	3.60087928	0.000317143	0	0.103620723	0	1	PRJNA827465;Yang2025_Silkie_Wenchang	CRA004775	0.017593592
ENSGALG00010001817	SLC25A6	2	0.067847643	0.1312191	0.517056152	0.605116962	0	0.702987356	0	0.5	CRA004775;Yang2025_Silkie_Wenchang	PRJNA827465	0.896474603
ENSGALG00010001817	SLC25A6	2	0.140399542	0.091140293	1.540477194	0.123444078	0.006761466	1.132148354	11.67235318	0.5	CRA004775;PRJNA827465	Yang2025_Silkie_Wenchang	0.589015732
ENSGALG00010001819	HOXA6	2	0.147484915	0.091839867	1.605892076	0.108297625	0.01019539	2.48674228	59.78674557	1	PRJNA827465;Yang2025_Silkie_Wenchang	CRA004775	0.556717702
ENSGALG00010001819	HOXA6	2	0.235563755	0.090031066	2.616471891	0.008884368	0	0.367036714	0	1	CRA004775;Yang2025_Silkie_Wenchang	PRJNA827465	0.147645798
ENSGALG00010001819	HOXA6	2	0.063120931	0.070006389	0.901645284	0.367245327	0	0.013948584	0	1	CRA004775;PRJNA827465	Yang2025_Silkie_Wenchang	0.819739734
ENSGALG00010001820	PGM2L1	2	-0.125636726	0.124354323	-1.010312493	0.312345597	0	0.026404639	0	1	PRJNA827465;Yang2025_Silkie_Wenchang	CRA004775	0.796054997
ENSGALG00010001820	PGM2L1	2	-0.077843934	0.180274124	-0.431808692	0.665880462	0	0.044631121	0	1	CRA004775;Yang2025_Silkie_Wenchang	PRJNA827465	0.913546323
ENSGALG00010001820	PGM2L1	2	-0.121229445	0.143829044	-0.842871801	0.399300146	0	0.106802363	0	1	CRA004775;PRJNA827465	Yang2025_Silkie_Wenchang	0.832152064
ENSGALG00010001822	KCNE3	2	-0.270777953	0.402279049	-0.673109757	0.500877467	0.220761323	3.040291152	67.10841331	0.5	PRJNA827465;Yang2025_Silkie_Wenchang	CRA004775	0.862421093
ENSGALG00010001822	KCNE3	2	-0.197928243	0.53401153	-0.370644138	0.710902602	0.416002655	3.694880246	72.93552339	0.5	CRA004775;Yang2025_Silkie_Wenchang	PRJNA827465	0.925563881
ENSGALG00010001822	KCNE3	2	0.159069779	0.216403852	0.735059833	0.462303055	0	0.293151262	0	1	CRA004775;PRJNA827465	Yang2025_Silkie_Wenchang	0.850823289
ENSGALG00010001823	RALBP1	2	0.055980981	0.101742861	0.550220235	0.582168326	0.010934126	1.648727729	39.34717161	0.5	PRJNA827465;Yang2025_Silkie_Wenchang	CRA004775	0.889444576
ENSGALG00010001823	RALBP1	2	0.244368066	0.119747021	2.040702665	0.04128039	0	0.007300208	0	1	CRA004775;Yang2025_Silkie_Wenchang	PRJNA827465	0.352245589
ENSGALG00010001823	RALBP1	2	0.079045425	0.119313024	0.662504576	0.507647903	0.018041845	2.219548262	54.94578708	0.5	CRA004775;PRJNA827465	Yang2025_Silkie_Wenchang	0.864325313
ENSGALG00010001825	LIPT2	2	0.138855269	0.432077038	0.321366925	0.747932345	0.338501557	10.39699949	90.38184045	0.5	PRJNA827465;Yang2025_Silkie_Wenchang	CRA004775	0.936938957
ENSGALG00010001825	LIPT2	2	0.272865564	0.40785435	0.669026979	0.503478268	0.213725754	2.553450838	60.83731141	0.5	CRA004775;Yang2025_Silkie_Wenchang	PRJNA827465	0.863108263
ENSGALG00010001825	LIPT2	2	-0.268191548	0.119703104	-2.240472789	0.025060245	0	0.003177317	0	1	CRA004775;PRJNA827465	Yang2025_Silkie_Wenchang	0.269672828
ENSGALG00010001826	HOXA7	2	0.027259894	0.055032328	0.495343278	0.620357827	0	0.802902777	0	1	PRJNA827465;Yang2025_Silkie_Wenchang	CRA004775	0.899395593
ENSGALG00010001826	HOXA7	2	0.115135914	0.12523051	0.91939188	0.357890635	0	0.19427042	0	0.5	CRA004775;Yang2025_Silkie_Wenchang	PRJNA827465	0.813641308
ENSGALG00010001826	HOXA7	2	0.00528643	0.058895329	0.089759743	0.928478139	0	0.001496102	0	0.5	CRA004775;PRJNA827465	Yang2025_Silkie_Wenchang	0.983311758
ENSGALG00010001827	PDZRN4	2	0.277583997	0.232711756	1.192823266	0.232938596	0.053863582	1.87244958	46.59402258	1	PRJNA827465;Yang2025_Silkie_Wenchang	CRA004775	0.746005924
ENSGALG00010001827	PDZRN4	2	0.555126414	0.267370492	2.076244128	0.037871378	0	0.028051844	0	1	CRA004775;Yang2025_Silkie_Wenchang	PRJNA827465	0.335620339
ENSGALG00010001827	PDZRN4	2	0.13044728	0.172645598	0.755578373	0.449902027	0	0.37356682	0	1	CRA004775;PRJNA827465	Yang2025_Silkie_Wenchang	0.84744374

ENSGALG00010001829	POLD3	2	-0.064776005	0.056491929	-1.146641758	0.251529711	0	0.020150863	0	1	PRJNA827465;Yang2025_Silkie_Wenchang	CRA004775	0.761706068
ENSGALG00010001829	POLD3	2	-0.116075056	0.093628458	-1.239741195	0.215071135	0	0.289613046	0	1	CRA004775;Yang2025_Silkie_Wenchang	PRJNA827465	0.728473987
ENSGALG00010001829	POLD3	2	-0.078094183	0.059874887	-1.304289431	0.192134918	0	0.529728772	0	1	CRA004775;PRJNA827465	Yang2025_Silkie_Wenchang	0.701849591
ENSGALG00010001831	CSF2RA	2	0.052184521	0.134225283	0.388783098	0.697436605	0.006168668	1.178848448	15.17145384	0.5	PRJNA827465;Yang2025_Silkie_Wenchang	CRA004775	0.921328007
ENSGALG00010001831	CSF2RA	2	-0.343185909	0.349078386	-0.983119902	0.32554843	0.115546606	1.565128601	36.10748667	1	CRA004775;Yang2025_Silkie_Wenchang	PRJNA827465	0.799987949
ENSGALG00010001831	CSF2RA	2	-0.239289247	0.519488639	-0.460624602	0.645067958	0.398057282	3.086857639	67.6045961	0.5	CRA004775;PRJNA827465	Yang2025_Silkie_Wenchang	0.906966303
ENSGALG00010001832	BUB3	2	0.007264207	0.121877788	0.059602385	0.952472319	0.021495168	3.139251259	68.1452704	0.5	PRJNA827465;Yang2025_Silkie_Wenchang	CRA004775	0.987758913
ENSGALG00010001832	BUB3	2	-0.223324602	0.108461913	-2.05901403	0.039492894	0	0.947534942	0	1	CRA004775;Yang2025_Silkie_Wenchang	PRJNA827465	0.343708864
ENSGALG00010001832	BUB3	2	-0.100441682	0.235013657	-0.427386575	0.669097784	0.094000966	5.987526917	83.2986137	0.5	CRA004775;PRJNA827465	Yang2025_Silkie_Wenchang	0.914377769
ENSGALG00010001834	ZCRB1	2	-0.022760441	0.08380158	-0.271599182	0.785930228	0.004104508	1.264952569	20.94565249	0.5	PRJNA827465;Yang2025_Silkie_Wenchang	CRA004775	0.946639719
ENSGALG00010001834	ZCRB1	2	0.12816477	0.132932973	0.964130771	0.334980367	0	6.6491E-05	0	1	CRA004775;Yang2025_Silkie_Wenchang	PRJNA827465	0.803101658
ENSGALG00010001834	ZCRB1	2	-0.052717531	0.06811218	-0.773980965	0.438942048	0	0.631758421	0	0.5	CRA004775;PRJNA827465	Yang2025_Silkie_Wenchang	0.844002503
ENSGALG00010001835	MECP2	2	-0.089790143	0.081639328	-1.099839318	0.271402138	0	0.051559799	0	1	PRJNA827465;Yang2025_Silkie_Wenchang	CRA004775	0.772743863
ENSGALG00010001835	MECP2	2	-0.256656441	0.162212036	-1.582228101	0.113597507	0	0.563245291	0	1	CRA004775;Yang2025_Silkie_Wenchang	PRJNA827465	0.568794964
ENSGALG00010001835	MECP2	2	-0.154561272	0.127722578	-1.210132731	0.226227966	0.013828379	1.449113667	30.99230083	1	CRA004775;PRJNA827465	Yang2025_Silkie_Wenchang	0.739677001
ENSGALG00010001836	TWSG1	2	-0.168884778	0.27788282	-0.607755376	0.543349729	0.14703392	20.73645924	95.17757594	0.5	PRJNA827465;Yang2025_Silkie_Wenchang	CRA004775	0.876968972
ENSGALG00010001836	TWSG1	2	-0.182034168	0.306646672	-0.593628382	0.552760694	0.156584871	5.523049875	81.89406175	0.5	CRA004775;Yang2025_Silkie_Wenchang	PRJNA827465	0.87928478
ENSGALG00010001836	TWSG1	2	0.10892113	0.066516812	1.637495736	0.101526512	0	0.06121784	0	1	CRA004775;PRJNA827465	Yang2025_Silkie_Wenchang	0.543637124
ENSGALG00010001837	TAB3	2	0.236577383	0.342899081	0.689932973	0.490236339	0.200880724	5.991088519	83.3085424	0.5	PRJNA827465;Yang2025_Silkie_Wenchang	CRA004775	0.858529056
ENSGALG00010001837	TAB3	2	0.622393681	0.200112541	3.110218268	0.001869491	0	0.006743999	0	1	CRA004775;Yang2025_Silkie_Wenchang	PRJNA827465	0.055579895
ENSGALG00010001837	TAB3	2	0.214474194	0.325243472	0.659426591	0.509621871	0.176638337	5.185803828	80.71658641	0.5	CRA004775;PRJNA827465	Yang2025_Silkie_Wenchang	0.865257661
ENSGALG00010001838	PPHLN1	2	0.010740741	0.050644765	0.21207998	0.832044638	0	0.481160713	0	0.5	PRJNA827465;Yang2025_Silkie_Wenchang	CRA004775	0.957762335
ENSGALG00010001838	PPHLN1	2	0.060773276	0.084977521	0.715168853	0.474504713	0	0.001711737	0	1	CRA004775;Yang2025_Silkie_Wenchang	PRJNA827465	0.854462864
ENSGALG00010001838	PPHLN1	2	-0.010249576	0.059895986	-0.171122926	0.86412711	0	0.052453092	0	0.5	CRA004775;PRJNA827465	Yang2025_Silkie_Wenchang	0.96655438
ENSGALG00010001840	HOXA9	2	0.252817321	0.123660423	2.047760516	0.040583316	0.013079236	1.730426253	42.21077044	1	PRJNA827465;Yang2025_Silkie_Wenchang	CRA004775	0.314856948
ENSGALG00010001840	HOXA9	2	0.368607747	0.136677461	2.696916842	0.006998474	0	0.189717149	0	1	CRA004775;Yang2025_Silkie_Wenchang	PRJNA827465	0.12854434
ENSGALG00010001840	HOXA9	2	0.151964456	0.112263112	1.353645499	0.175849504	0	0.044533615	0	1	CRA004775;PRJNA827465	Yang2025_Silkie_Wenchang	0.680860313
ENSGALG00010001843	PRICKLE1	2	0.063442748	0.06264032	1.012810097	0.311150877	0	0.482328615	0	1	PRJNA827465;Yang2025_Silkie_Wenchang	CRA004775	0.79534098
ENSGALG00010001843	PRICKLE1	2	0.028587788	0.101766986	0.280914164	0.778776237	0	0.496956458	0	1	CRA004775;Yang2025_Silkie_Wenchang	PRJNA827465	0.944673731
ENSGALG00010001843	PRICKLE1	2	0.102369605	0.074142778	1.380709053	0.167368437	0	0.177069587	0	1	CRA004775;PRJNA827465	Yang2025_Silkie_Wenchang	0.666945317
ENSGALG00010001844	HOXA10	2	0.224855316	0.101122588	2.223591394	0.026175948	0	0.088794349	0	1	PRJNA827465;Yang2025_Silkie_Wenchang	CRA004775	0.276012526
ENSGALG00010001844	HOXA10	2	0.292040783	0.149555008	1.95273155	0.050851413	0.011270834	1.267478769	21.10321495	1	CRA004775;Yang2025_Silkie_Wenchang	PRJNA827465	0.390049394
ENSGALG00010001844	HOXA10	2	0.319183878	0.124047426	2.573079402	0.010079808	0	0.846341959	0	1	CRA004775;PRJNA827465	Yang2025_Silkie_Wenchang	0.159487396
ENSGALG00010001845	ANKRD12	2	0.24097702	0.410010641	0.587733576	0.556711135	0.296456788	8.011199315	87.51747447	0.5	PRJNA827465;Yang2025_Silkie_Wenchang	CRA004775	0.880358001
ENSGALG00010001845	ANKRD12	2	0.721736222	0.19307056	3.738199253	0.000185343	0	0.033165376	0	1	CRA004775;Yang2025_Silkie_Wenchang	PRJNA827465	0.012060492
ENSGALG00010001845	ANKRD12	2	0.275417607	0.445263906	0.618549141	0.536213415	0.353695601	8.781242533	88.61208996	0.5	CRA004775;PRJNA827465	Yang2025_Silkie_Wenchang	0.874599474
ENSGALG00010001848	HOXA11	2	0.822056952	0.191210133	4.299233192	1.7139E-05	0	0.920986153	0	1	PRJNA827465;Yang2025_Silkie_Wenchang	CRA004775	0.00218706
ENSGALG00010001848	HOXA11	2	0.224749401	0.781768871	0.287488297	0.773738461	1.135585682	14.05243965	92.88379794	0.5	CRA004775;Yang2025_Silkie_Wenchang	PRJNA827465	0.943248868
ENSGALG00010001848	HOXA11	2	0.040878054	0.597930662	0.068365877	0.945494385	0.624020459	7.836228344	87.2387588	0.5	CRA004775;PRJNA827465	Yang2025_Silkie_Wenchang	0.986321197
ENSGALG00010001849	NDUFV2	2	-0.101504887	0.117847681	-0.861322741	0.389060314	0.015172716	1.740693389	42.55162878	1	PRJNA827465;Yang2025_Silkie_Wenchang	CRA004775	0.820615121
ENSGALG00010001849	NDUFV2	2	-0.211336767	0.16545138	-1.277334573	0.201484153	0	0.757179766	0	0.5	CRA004775;Yang2025_Silkie_Wenchang	PRJNA827465	0.714377024
ENSGALG00010001849	NDUFV2	2	-0.028698038	0.059929439	-0.478863776	0.632035542	0	0.03268462	0	0.5	CRA004775;PRJNA827465	Yang2025_Silkie_Wenchang	0.903058174
ENSGALG00010001850	MYOM2	2	-0.058989902	0.136149104	-0.433274257	0.664815538	0.026680152	3.494884293	71.38674943	0.5	PRJNA827465;Yang2025_Silkie_Wenchang	CRA004775	0.913393112
ENSGALG00010001850	MYOM2	2	0.071242987	0.085066035	0.837502143	0.402310373	0	0.323309592	0	1	CRA004775;Yang2025_Silkie_Wenchang	PRJNA827465	0.832879175
ENSGALG00010001850	MYOM2	2	-0.189681743	0.1169255	-1.622244441	0.10475102	0	0.888089694	0	0.5	CRA004775;PRJNA827465	Yang2025_Silkie_Wenchang	0.549890386
ENSGALG00010001852	CPXM2	2	0.352079227	0.209547278	1.680189929	0.092920368	0.032805296	1.44252188	30.6769614	1	PRJNA827465;Yang2025_Silkie_Wenchang	CRA004775	0.522019616
ENSGALG00010001852	CPXM2	2	0.617072519	0.271035521	2.276721946	0.022802835	0	0.089289014	0	1	CRA004775;Yang2025_Silkie_Wenchang	PRJNA827465	0.227312612
ENSGALG00010001852	CPXM2	2	0.257524212	0.158480426	1.624959116	0.104171271	0	0.398254765	0	1	CRA004775;PRJNA827465	Yang2025_Silkie_Wenchang	0.548605581
ENSGALG00010001853	ENSGALG00010001853	2	-0.017056223	0.200584253	-0.085032713	0.932235384	0.061914211	4.336613254	76.94053074	0.5	PRJNA827465;Yang2025_Silkie_Wenchang	CRA004775	0.984202262
ENSGALG00010001853	ENSGALG00010001853	2	-0.133406029	0.359526376	-0.371060478	0.710592487	0.21139331	5.141281937	80.54959809	0.5	CRA004775;Yang2025_Silkie_Wenchang	PRJNA827465	0.925404854
ENSGALG00010001853	ENSGALG00010001853	2	-0.277776563	0.126626292	-2.193672091	0.028258991	0.001634354	1.032261713	3.125342394	1	CRA004775;PRJNA827465	Yang2025_Silkie_Wenchang	0.286481335
ENSGALG00010001854	CHRDL2	2	0.064292088	0.17541714	0.366509724	0.713984758	0	0.009086367	0	1	PRJNA827465;Yang2025_Silkie_Wenchang	CRA004775	0.926399535
ENSGALG00010001854	CHRDL2	2	0.311653687	0.300627133	1.036678506	0.299883712	0.01959124	1.108917809	9.821991144	1	CRA004775;Yang2025_Silkie_Wenchang	PRJNA827465	0.790115099
ENSGALG00010001854	CHRDL2	2	0.240410997	0.299972032	0.801443904	0.422874707	0.083052035	1.587998658	37.0276546	1	CRA004775;PRJNA827465	Yang2025_Silkie_Wenchang	0.830998514
ENSGALG00010001856	CHST15	2	0.043423448	0.123629592	0.351238299	0.72540958	0.020445111	2.98542018	66.50387752	0.5	PRJNA827465;Yang2025_Silkie_Wenchang	CRA004775	0.930910808
ENSGALG00010001856	CHST15	2	-0.060759101	0.105416589	-0.576371346	0.564364208	0	0.566112728	0	0.5	CRA004775;Yang2025_Silkie_Wenchang	PRJNA827465	0.883094571
ENSGALG00010001856	CHST15	2	0.157846202	0.085764018	1.84047115	0.065699096	0	8.15107E-05	0	1	CRA004775;PRJNA827465	Yang2025_Silkie_Wenchang	0.441718274
ENSGALG00010001857	HNRNPA2B1	2	0.190474312	0.243234055	0.783090641	0.433573887	0.11083515	15.24102593	93.4387619	0.5	PRJNA827465;Yang2025_Silkie_Wenchang	CRA004775	0.842762754
ENSGALG00010001857	HNRNPA2B1	2	0.235085199	0.244041316	0.963300819	0.335396582	0.090411365	3.894326175	74.32161676	0.5	CRA004775;Yang2025_Silkie_Wenchang	PRJNA827465	0.803198384
ENSGALG00010001857	HNRNPA2B1	2	-0.041208587	0.042700023	-0.964890748	0.334590536	0	0.000754169	0	1	CRA004775;PRJNA827465	Yang2025_Silkie_Wenchang	0.803101658
ENSGALG00010001858	ADAMTS20	2	0.023873165	0.214674202	0.111206495	0.911457602	0.070935466	4.1658077	75.99505133	0.5	PRJNA827465;Yang2025_Silkie_Wenchang	CRA004775	0.979845851
ENSGALG00010001858	ADAMTS20	2	0.288482555	0.174260236	1.655469786	0.09782924	0	0.237434775	0	1	CRA004775;Yang2025_Silkie_Wenchang	PRJNA827465	0.534130658
ENSGALG00010001858	ADAMTS20	2	-0.049121179	0.274376383	-0.179028453	0.85791536	0.077456665	1.376803752	27.36800733	0.5	CRA004775;PRJNA827465	Yang2025_Silkie_Wenchang	0.964106119
ENSGALG00010001860	RNF169	2	0.125586778	0.069286613	1.812569159	0.069898298	0	0.530738592	0	1	PRJNA827465;Yang2025_Silkie_Wenchang	CRA004775	0.455893265
ENSGALG00010001860	RNF169	2	-0.018347233	0.312920995	-0.058632158	0.953245097	0.138422327	3.180809251	68.56234916	0.5	CRA004775;Yang2025_Silkie_Wenchang	PRJNA827465	0.987758913
ENSGALG00010001860	RNF169	2	-0.048034411	0.224051536	-0.214390011	0.830242931	0.068504243	2.472595837	59.55667379	0.5	CRA004775;PRJNA827465	Yang2025_Silkie_Wenchang	0.957231
ENSGALG00010001861	CBX3	2	-0.038645191	0.044086167	-0.876583155	0.380713092	0.001025868	1.292847975	22.65138523	1	PRJNA827465;Yang2025_Silkie_Wenchang	CRA004775	0.824392515
ENSGALG00010001861	CBX3	2	-0.078486157	0.064064955	-1.225102817	0.220536484	0	0.596561224	0	0.5	CRA004775;Yang2025_Silkie_Wenchang	PRJNA827465	0.734053249
ENSGALG00010001861	CBX3	2	-0.006770598	0.040564245	-0.166914026	0.867440484	0	0.03718402	0	0.5	CRA004775;PRJNA827465	Yang2025_Silkie_Wenchang	0.967862205
ENSGALG00010001864	ENSGALG00010001864	2	-0.110935524	0.275816855	-0.402207197	0.687531546	0.117383781	4.351869321	77.02136883	0.5	PRJNA827465;Yang2025_Silkie_Wenchang	CRA004775	0.91949273
ENSGALG00010001864	ENSGALG00010001864	2	0.003885554	0.214482139	0.01811598	0.98554633	0.034513762	1.557339092	35.78790867	0.5	CRA004775;Yang2025_Silkie_Wenchang	PRJNA827465	0.995939127
ENSGALG00010001864	ENSGALG00010001864	2	-0.346520672	0.146123298	-2.371426578	0.017719567	0	0.11444769	0	1	CRA004775;PRJNA827465	Yang2025_Silkie_Wenchang	0.2722112
ENSGALG00010001865	CYBB	2	-0.014985734	0.283927447	-0.052780153	0.957907075	0.121077655	3.890960525	74.29940516	0.5	PRJNA827465;Yang2025_Silkie_Wenchang	CRA004775	0.988864712
ENSGALG00010001865	CYBB	2	-0.336811245	0.232398048	-1.44928603	0.147257721	0	0.010430558	0	1	CRA004775;Yang2025_Silkie_Wenchang	PRJNA827465	0.636077613
ENSGALG00010001865	CYBB	2	0.217608223	0.155563795	1.398835908	0.161862196	0	0.615406584	0	0.5	CRA004775;PRJNA827465	Yang2025_Silkie_Wenchang	0.659099502
ENSGALG00010001866	PUS7L	2	-0.00296188	0.066091054	-0.044815136	0.96425466	0	0.10156999	0	0.5	PRJNA827465;Yang2025_Silkie_Wenchang	CRA004775	0.990266302
ENSGALG00010001866	PUS7L	2	0.069619839	0.100295005	0.694150607	0.487587764	0	0.987810674	0	1	CRA004775;Yang2025_Silkie_Wenchang	PRJNA827465	0.85768885
ENSGALG00010001866	PUS7L	2	0.050903682	0.12975665	0.392301142	0.69483572	0.014854093	1.442496951	30.67576336	0.5	CRA004775;PRJNA827465	Yang2025_Silkie_Wenchang	0.92087205
ENSGALG00010001868	ENSGALG00010001868	2	0.124764561	0.113427554	1.099949319	0.271354205	0.010236279	1.534987054	34.85287077	1	PRJNA827465;Yang2025_Silkie_Wenchang	CRA004775	0.772743863
ENSGALG00010001868	ENSGALG00010001868	2	0.048844126	0.265243295	0.184148389	0.853897046	0.098365	3.246584019	69.19839456	0.5	CRA004775;Yang2025_Silkie_Wenchang	PRJNA827465	0.962975689
ENSGALG00010001868	ENSGALG00010001868	2	-0.014423485	0.118160944	-0.122066437	0.902846402	0.008844967	1.26444709	20.9140495	0.5	CRA004775;PRJNA827465	Yang2025_Silkie_Wenchang	0.97693511
ENSGALG00010001870	PLP2	2	0.064410295	0.17626793	0.365411307	0.714804402	0.025681688	1.608790501	37.84150269	0.5	PRJNA827465;Yang2025_Silkie_Wenchang	CRA004775	0.926755613
ENSGALG00010001870	PLP2	2	0.03266352	0.290470868	0.112450245	0.910466418	0.095085877	2.274375991	56.03189605	0.5	CRA004775;Yang2025_Silkie_Wenchang	PRJNA827465	0.97880407
ENSGALG00010001870	PLP2	2	-0.110124145	0.133690215	-0.823726292	0.410095097	0	0.417701376	0	1	CRA004775;PRJNA827465	Yang2025_Silkie_Wenchang	0.834652129
ENSGALG00010001871	ENSGALG00010001871	2	0.019590812	0.18404797	0.106444052	0.915230043	0.034868164	1.999725087	49.99312624	0.5	PRJNA827465;Yang2025_Silkie_Wenchang	CRA004775	0.980117272
ENSGALG00010001871	ENSGALG00010001871	2	-0.043245374	0.305225931	-0.141683157	0.88733028	0.125546926	3.023806704	66.92910302	0.5	CRA004775;Yang2025_Silkie_Wenchang	PRJNA827465	0.972987228
ENSGALG00010001871	ENSGALG00010001871	2	-0.190434455	0.133313967	-1.428465898	0.153157803	0	0.56870774	0	1	CRA004775;PRJNA827465	Yang2025_Silkie_Wenchang	0.645321735
ENSGALG00010001873	ENSGALG00010001873	2	0.204181126	0.368782574	0.553662621	0.579809761	0.216225839	4.72658048	78.84305569	0.5	PRJNA827465;Yang2025_Silkie_Wenchang	CRA004775	0.888015862
ENSGALG00010001873	ENSGALG00010001873	2	0.018587744	0.590166299	0.031495774	0.974874162	0.612860318	8.319192165	87.9796021	0.5	CRA004775;Yang2025_Silkie_Wenchang	PRJNA827465	0.99260328
ENSGALG00010001873	ENSGALG00010001873	2	-0.293995442	0.211946776	-1.387119195	0.16540542	0.03777245	1.64121275	39.06944727	1	CRA004775;PRJNA827465	Yang2025_Silkie_Wenchang	0.664874006
ENSGALG00010001877	OAT	2	-0.263238588	0.179459141	-1.46684413	0.142418462	0.05806357	9.947459054	89.94718154	1	PRJNA827465;Yang2025_Silkie_Wenchang	CRA004775	0.628788228
ENSGALG00010001877	OAT	2	-0.402861384	0.087000944	-4.630540385	3.64713E-06	0.000402102	1.019574319	1.919852071	1	CRA004775;Yang2025_Silkie_Wenchang	PRJNA827465	0.00065654
ENSGALG00010001877	OAT	2	-0.107292582	0.055869947	-1.920398837	0.05480754	0	0.682632293	0	1	CRA004775;PRJNA827465	Yang2025_Silkie_Wenchang	0.405341722
ENSGALG00010001878	XK	2	-0.088724452	0.219063581	-0.405016899	0.685465089	0.005135708	1.048601237	4.634863616	0.5	PRJNA827465;Yang2025_Silkie_Wenchang	CRA004775	0.919235539
ENSGALG00010001878	XK	2	0.143882884	0.302544408	0.475576081	0.634376419	0	0.144480932	0	0.5	CRA004775;Yang2025_Silkie_Wenchang	PRJNA827465	0.903815945
ENSGALG00010001878	XK	2	-0.189010323	0.226816857	-0.833316913	0.40466602	0	0.17529512	0	1	CRA004775;PRJNA827465	Yang2025_Silkie_Wenchang	0.833112102
ENSGALG00010001879	IRAK4	2	0.376199192	0.138586516	2.714543971	0.006636709	0.00511192	1.151670135	13.16958131	1	PRJNA827465;Yang2025_Silkie_Wenchang	CRA004775	0.124602584
ENSGALG00010001879	IRAK4	2	0.160577351	0.372470711	0.431114035	0.666385456	0.225084042	5.12300996	80.48022534	0.5	CRA004775;Yang2025_Silkie_Wenchang	PRJNA827465	0.913579507
ENSGALG00010001879	IRAK4	2	0.0325543	0.230375868	0.141309506	0.88762544	0.051558029	1.880572633	46.82470742	0.5	CRA004775;PRJNA827465	Yang2025_Silkie_Wenchang	0.973038314
ENSGALG00010001880	MTCL1	2	0.137123762	0.233739931	0.586650993	0.557438128	0.086737696	4.322786708	76.86677443	0.5	PRJNA827465;Yang2025_Silkie_Wenchang	CRA004775	0.880678789
ENSGALG00010001880	MTCL1	2	0.435455514	0.195269125	2.230027473	0.025745619	0	0.042659835	0	1	CRA004775;Yang2025_Silkie_Wenchang	PRJNA827465	0.273555033
ENSGALG00010001880	MTCL1	2	0.049648847	0.225932513	0.219750786	0.82606525	0.056639248	1.504139229	33.51679282	0.5	CRA004775;PRJNA827465	Yang2025_Silkie_Wenchang	0.956393839
ENSGALG00010001883	KBTBD11	2	-0.296578086	0.274893227	-1.07888466	0.280639148	0.137178584	10.65543923	90.61512174	1	PRJNA827465;Yang2025_Silkie_Wenchang	CRA004775	0.778536485
ENSGALG00010001883	KBTBD11	2	-0.195898257	0.431477045	-0.454017796	0.649816018	0.315586489	6.159169381	83.7640445	0.5	CRA004775;Yang2025_Silkie_Wenchang	PRJNA827465	0.908240362
ENSGALG00010001883	KBTBD11	2	-0.009547978	0.086195503	-0.11077119	0.911797792	0	0.918571584	0	0.5	CRA004775;PRJNA827465	Yang2025_Silkie_Wenchang	0.978895589
ENSGALG00010001885	SMPX	2	-0.191240115	0.14252763	-1.341775729	0.179668724	0	0.008569645	0	1	PRJNA827465;Yang2025_Silkie_Wenchang	CRA004775	0.68659821
ENSGALG00010001885	SMPX	2	-0.238896804	0.223464293	-1.0690603	0.285042499	0	0.666198303	0	1	CRA004775;Yang2025_Silkie_Wenchang	PRJNA827465	0.780501722
ENSGALG00010001885	SMPX	2	-0.238010458	0.172152658	-1.382554652	0.166801462	0	0.635481864	0	1	CRA004775;PRJNA827465	Yang2025_Silkie_Wenchang	0.665766006
ENSGALG00010001886	NEU3	2	0.042051831	0.040179721	1.0465934	0.295287147	0	0.197983977	0	0.5	PRJNA827465;Yang2025_Silkie_Wenchang	CRA004775	0.787628307
ENSGALG00010001886	NEU3	2	-0.281362812	0.291445961	-0.965403021	0.334342989	0.144195131	6.445736366	84.48586875	1	CRA004775;Yang2025_Silkie_Wenchang	PRJNA827465	0.803094614
ENSGALG00010001886	NEU3	2	-0.244265997	0.319593619	-0.764301859	0.444687378	0.185921023	10.3152582	90.30562318	0.5	CRA004775;PRJNA827465	Yang2025_Silkie_Wenchang	0.846013379
ENSGALG00010001888	SNX10	2	0.138973492	0.250545723	0.554683155	0.5791114	0.096410778	4.209150963	76.24223961	0.5	PRJNA827465;Yang2025_Silkie_Wenchang	CRA004775	0.887888423
ENSGALG00010001888	SNX10	2	-0.202030168	0.156977173	-1.287003476	0.198093077	0	0.289233411	0	1	CRA004775;Yang2025_Silkie_Wenchang	PRJNA827465	0.710234722
ENSGALG00010001888	SNX10	2	0.063168337	0.336282935	0.187842825	0.850999864	0.187364046	5.588414193	82.10583601	0.5	CRA004775;PRJNA827465	Yang2025_Silkie_Wenchang	0.962056252
ENSGALG00010001890	KLHL34	2	0.343910643	0.436963529	0.787046562	0.43125461	0.350364141	12.11716989	91.74724784	0.5	PRJNA827465;Yang2025_Silkie_Wenchang	CRA004775	0.842503929
ENSGALG00010001890	KLHL34	2	0.76973979	0.174758662	4.40458734	1.05985E-05	0	0.067588296	0	1	CRA004775;Yang2025_Silkie_Wenchang	PRJNA827465	0.001477144
ENSGALG00010001890	KLHL34	2	-0.017104458	0.215572134	-0.079344476	0.936758631	0.021921946	1.098338326	8.953372938	0.5	CRA004775;PRJNA827465	Yang2025_Silkie_Wenchang	0.985038972
ENSGALG00010001892	RAB12	2	-0.203307603	0.160446596	-1.267135655	0.205106788	0.047744923	13.73648895	92.72011936	1	PRJNA827465;Yang2025_Silkie_Wenchang	CRA004775	0.717838399
ENSGALG00010001892	RAB12	2	-0.017466923	0.395146266	-0.04420369	0.964742041	0.273032168	7.167872032	86.04885808	0.5	CRA004775;Yang2025_Silkie_Wenchang	PRJNA827465	0.990394578
ENSGALG00010001892	RAB12	2	0.107904587	0.222076129	0.485890075	0.627045106	0.069494872	2.592072337	61.42082975	0.5	CRA004775;PRJNA827465	Yang2025_Silkie_Wenchang	0.901573209
ENSGALG00010001895	P4HA3	2	0.008793921	0.128889953	0.068228132	0.945604033	0.010110244	1.335311036	25.11108099	0.5	PRJNA827465;Yang2025_Silkie_Wenchang	CRA004775	0.986321197
ENSGALG00010001895	P4HA3	2	-0.24863219	0.201810356	-1.232009075	0.217945694	0	0.505293739	0	1	CRA004775;Yang2025_Silkie_Wenchang	PRJNA827465	0.73141715
ENSGALG00010001895	P4HA3	2	-0.054468853	0.285815411	-0.190573534	0.848859727	0.089637165	1.561691972	35.96688606	0.5	CRA004775;PRJNA827465	Yang2025_Silkie_Wenchang	0.961364896
ENSGALG00010001896	ARHGEF10	2	-0.252164194	0.163442235	-1.542833732	0.122871123	0.037333658	3.275379259	69.46918446	1	PRJNA827465;Yang2025_Silkie_Wenchang	CRA004775	0.587708865
ENSGALG00010001896	ARHGEF10	2	-0.192925119	0.302327226	-0.638133462	0.523386806	0.126438503	2.861332914	65.05125303	0.5	CRA004775;Yang2025_Silkie_Wenchang	PRJNA827465	0.869661505
ENSGALG00010001896	ARHGEF10	2	-0.072302184	0.105410376	-0.685911452	0.492768948	0	0.684794901	0	0.5	CRA004775;PRJNA827465	Yang2025_Silkie_Wenchang	0.859474364
ENSGALG00010001897	TLR7	2	0.449845459	0.150721254	2.984618611	0.002839321	0	0.181649607	0	1	PRJNA827465;Yang2025_Silkie_Wenchang	CRA004775	0.072753867
ENSGALG00010001897	TLR7	2	-0.060378899	0.590754645	-0.102206389	0.918592856	0.45692668	2.401135638	58.35303995	0.5	CRA004775;Yang2025_Silkie_Wenchang	PRJNA827465	0.981033557
ENSGALG00010001897	TLR7	2	-0.000288581	0.664907355	-0.000434018	0.999653704	0.653631154	3.114320344	67.8902653	0.5	CRA004775;PRJNA827465	Yang2025_Silkie_Wenchang	0.999889665
ENSGALG00010001898	LHPP	2	0.056884563	0.056726562	1.002785319	0.315964454	0	0.291089428	0	1	PRJNA827465;Yang2025_Silkie_Wenchang	CRA004775	0.796883862
ENSGALG00010001898	LHPP	2	-0.190889557	0.339303682	-0.562592059	0.573712701	0.189163806	4.986574734	79.94615436	0.5	CRA004775;Yang2025_Silkie_Wenchang	PRJNA827465	0.886016235
ENSGALG00010001898	LHPP	2	-0.214006793	0.305465206	-0.700593024	0.483557033	0.149370268	4.269768315	76.57952548	0.5	CRA004775;PRJNA827465	Yang2025_Silkie_Wenchang	0.85691916
ENSGALG00010001899	TWF1	2	0.012167667	0.122488129	0.099337518	0.92087029	0.024150845	4.812388485	79.22029772	0.5	PRJNA827465;Yang2025_Silkie_Wenchang	CRA004775	0.981469977
ENSGALG00010001899	TWF1	2	-0.00309873	0.182541469	-0.016975483	0.986456175	0.038459801	1.991064347	49.77560611	0.5	CRA004775;Yang2025_Silkie_Wenchang	PRJNA827465	0.99618774
ENSGALG00010001899	TWF1	2	0.125405108	0.049226242	2.547525512	0.010848992	0	0.305405734	0	1	CRA004775;PRJNA827465	Yang2025_Silkie_Wenchang	0.166012234
ENSGALG00010001901	PRPS2	2	-0.245511616	0.569554054	-0.431059377	0.666425197	0.622634728	24.69346368	95.95034535	0.5	PRJNA827465;Yang2025_Silkie_Wenchang	CRA004775	0.913579507
ENSGALG00010001901	PRPS2	2	-0.006377273	0.860686782	-0.007409517	0.994088115	1.364783421	11.9595309	91.63846803	0.5	CRA004775;Yang2025_Silkie_Wenchang	PRJNA827465	0.99818842
ENSGALG00010001901	PRPS2	2	0.458002376	0.240810876	1.901917318	0.057181965	0.038369113	1.286960944	22.29756429	1	CRA004775;PRJNA827465	Yang2025_Silkie_Wenchang	0.414005744
ENSGALG00010001902	WDR45	2	0.045226093	0.05395086	0.838283084	0.401871734	0	0.205410679	0	1	PRJNA827465;Yang2025_Silkie_Wenchang	CRA004775	0.832806663
ENSGALG00010001902	WDR45	2	-0.136038708	0.275936902	-0.493006577	0.62200794	0.114559596	3.607121496	72.27706355	0.5	CRA004775;Yang2025_Silkie_Wenchang	PRJNA827465	0.899936613
ENSGALG00010001902	WDR45	2	-0.14993966	0.242416192	-0.618521638	0.536231539	0.08800041	3.224333111	68.98583473	0.5	CRA004775;PRJNA827465	Yang2025_Silkie_Wenchang	0.874599474
ENSGALG00010001903	NARS2	2	-0.038616166	0.200612104	-0.192491705	0.847357065	0.06389275	4.611589354	78.31550203	0.5	PRJNA827465;Yang2025_Silkie_Wenchang	CRA004775	0.961252712
ENSGALG00010001903	NARS2	2	-0.251795062	0.119292445	-2.1107377	0.034794864	0	0.011481143	0	1	CRA004775;Yang2025_Silkie_Wenchang	PRJNA827465	0.321263903
ENSGALG00010001903	NARS2	2	-0.020552804	0.186990917	-0.109913383	0.912478069	0.052487589	3.750213242	73.33484964	0.5	CRA004775;PRJNA827465	Yang2025_Silkie_Wenchang	0.978967245
ENSGALG00010001906	SKAP2	2	-0.089720559	0.106028841	-0.846190137	0.397446683	0.012734941	2.226904153	55.09460977	0.5	PRJNA827465;Yang2025_Silkie_Wenchang	CRA004775	0.831571329
ENSGALG00010001906	SKAP2	2	-0.217423722	0.112717152	-1.928931994	0.053739305	0	0.004002128	0	1	CRA004775;Yang2025_Silkie_Wenchang	PRJNA827465	0.401964436
ENSGALG00010001906	SKAP2	2	-0.012237067	0.078895306	-0.155105132	0.876738437	0	0.44538663	0	0.5	CRA004775;PRJNA827465	Yang2025_Silkie_Wenchang	0.97046577
ENSGALG00010001910	FAM53B	2	0.019869199	0.046972799	0.422993718	0.672299844	0	0.827146143	0	0.5	PRJNA827465;Yang2025_Silkie_Wenchang	CRA004775	0.914742762
ENSGALG00010001910	FAM53B	2	-0.080989836	0.112484326	-0.720009966	0.47151886	0	0.000138534	0	1	CRA004775;Yang2025_Silkie_Wenchang	PRJNA827465	0.853998373
ENSGALG00010001910	FAM53B	2	0.035083823	0.050428925	0.695708326	0.48661151	0	0.139680478	0	0.5	CRA004775;PRJNA827465	Yang2025_Silkie_Wenchang	0.857603818
ENSGALG00010001911	USP35	2	-0.326765062	0.404477756	-0.807869055	0.419165967	0	0.849186434	0	0.5	PRJNA827465;Yang2025_Silkie_Wenchang	CRA004775	0.838481271
ENSGALG00010001911	USP35	2	-0.705993915	0.89737967	-0.786728225	0.431440978	1.186443701	3.683305468	72.85047334	0.5	CRA004775;Yang2025_Silkie_Wenchang	PRJNA827465	0.842503929
ENSGALG00010001911	USP35	2	-1.007681095	0.480317938	-2.097945995	0.035909916	0.215671279	1.873264986	46.61726944	1	CRA004775;PRJNA827465	Yang2025_Silkie_Wenchang	0.326969036
ENSGALG00010001912	TMEM117	2	-0.195870647	0.168506213	-1.162394208	0.245075375	0.03988777	3.333647298	70.00282542	1	PRJNA827465;Yang2025_Silkie_Wenchang	CRA004775	0.756475101
ENSGALG00010001912	TMEM117	2	0.066398127	0.495331431	0.134047878	0.893364715	0.409035082	5.321375308	81.2078656	0.5	CRA004775;Yang2025_Silkie_Wenchang	PRJNA827465	0.974381724
ENSGALG00010001912	TMEM117	2	0.199315881	0.312032813	0.638765774	0.522975317	0.123888327	2.264714366	55.84432125	0.5	CRA004775;PRJNA827465	Yang2025_Silkie_Wenchang	0.869568211
ENSGALG00010001914	ENSGALG00010001914	2	-0.044050705	0.307824451	-0.143103334	0.886208572	0.146304475	4.378805738	77.1627229	0.5	PRJNA827465;Yang2025_Silkie_Wenchang	CRA004775	0.972624487
ENSGALG00010001914	ENSGALG00010001914	2	0.199208676	0.187910765	1.060123806	0.289088279	0	0.418815447	0	0.5	CRA004775;Yang2025_Silkie_Wenchang	PRJNA827465	0.783530627
ENSGALG00010001914	ENSGALG00010001914	2	-0.270438201	0.174473657	-1.550023115	0.121135968	0	0.644566766	0	1	CRA004775;PRJNA827465	Yang2025_Silkie_Wenchang	0.583385241
ENSGALG00010001915	EEF1AKMT2	2	-0.035034039	0.457236437	-0.076621275	0.938924834	0.395004273	18.02708877	94.45279261	0.5	PRJNA827465;Yang2025_Silkie_Wenchang	CRA004775	0.985349045
ENSGALG00010001915	EEF1AKMT2	2	0.452659823	0.141050621	3.209201211	0.001331043	0	0.067545824	0	1	CRA004775;Yang2025_Silkie_Wenchang	PRJNA827465	0.044565154
ENSGALG00010001915	EEF1AKMT2	2	-0.012976815	0.496451014	-0.026139164	0.979146339	0.45279606	11.97552603	91.64963612	0.5	CRA004775;PRJNA827465	Yang2025_Silkie_Wenchang	0.994324552
ENSGALG00010001918	CNKSR2	2	0.004571207	0.205120913	0.022285424	0.982220276	0.061057452	3.436145263	70.89762151	0.5	PRJNA827465;Yang2025_Silkie_Wenchang	CRA004775	0.995107581
ENSGALG00010001918	CNKSR2	2	0.331334547	0.173801799	1.906393087	0.056599225	0	0.978815661	0	1	CRA004775;Yang2025_Silkie_Wenchang	PRJNA827465	0.411574294
ENSGALG00010001918	CNKSR2	2	0.168416671	0.412060153	0.408718654	0.682746143	0.273332654	4.461064771	77.58382693	0.5	CRA004775;PRJNA827465	Yang2025_Silkie_Wenchang	0.918751383
ENSGALG00010001921	ENSGALG00010001921	2	0.112381977	0.636967316	0.176432879	0.859953876	0	0.810875531	0	0.5	PRJNA827465;Yang2025_Silkie_Wenchang	CRA004775	0.964879591
ENSGALG00010001921	ENSGALG00010001921	2	0.241438753	1.139101316	0.211955469	0.832141776	0.890448668	1.508505615	33.70922918	0.5	CRA004775;Yang2025_Silkie_Wenchang	PRJNA827465	0.957762335
ENSGALG00010001921	ENSGALG00010001921	2	0.616231135	0.608275222	1.013079461	0.311022208	0	0.375387156	0	1	CRA004775;PRJNA827465	Yang2025_Silkie_Wenchang	0.795218958
ENSGALG00010001923	RAPGEF5	2	-0.028952914	0.127464402	-0.227145096	0.820310919	0.024287144	3.806730747	73.73073993	0.5	PRJNA827465;Yang2025_Silkie_Wenchang	CRA004775	0.955308792
ENSGALG00010001923	RAPGEF5	2	0.276987859	0.493867754	0.560854312	0.574896858	0.433208682	8.342879388	88.01373059	0.5	CRA004775;Yang2025_Silkie_Wenchang	PRJNA827465	0.886211989
ENSGALG00010001923	RAPGEF5	2	0.383753591	0.361325293	1.062072316	0.288202857	0.215317993	4.903213798	79.60521321	1	CRA004775;PRJNA827465	Yang2025_Silkie_Wenchang	0.782882265
ENSGALG00010001928	KCTD21	2	-0.248834732	0.349661395	-0.711644854	0.476684724	0.227643391	14.2780472	92.99624111	0.5	PRJNA827465;Yang2025_Silkie_Wenchang	CRA004775	0.854986476
ENSGALG00010001928	KCTD21	2	-0.564477811	0.148789311	-3.793806211	0.000148355	0	0.692607025	0	1	CRA004775;Yang2025_Silkie_Wenchang	PRJNA827465	0.010301794
ENSGALG00010001928	KCTD21	2	0.074604377	0.093340471	0.799271484	0.42413301	0	0.520972905	0	0.5	CRA004775;PRJNA827465	Yang2025_Silkie_Wenchang	0.840447358
ENSGALG00010001929	POLR2I	2	0.177813253	0.071416658	2.489800808	0.012781471	0	0.305086255	0	1	PRJNA827465;Yang2025_Silkie_Wenchang	CRA004775	0.183473076
ENSGALG00010001929	POLR2I	2	0.067527355	0.245262355	0.275327025	0.783065003	0.068437424	2.208224244	54.71474409	0.5	CRA004775;Yang2025_Silkie_Wenchang	PRJNA827465	0.945640135
ENSGALG00010001929	POLR2I	2	0.060307344	0.171133584	0.352399236	0.724538875	0.033306034	1.764451273	43.32515636	0.5	CRA004775;PRJNA827465	Yang2025_Silkie_Wenchang	0.930388018
ENSGALG00010001935	ABRAXAS2	2	0.02597834	0.121181372	0.214375691	0.830254097	0.016881357	2.113511967	52.68538737	0.5	PRJNA827465;Yang2025_Silkie_Wenchang	CRA004775	0.957231
ENSGALG00010001935	ABRAXAS2	2	0.193913494	0.135296094	1.433252714	0.15178563	0	0.003788132	0	1	CRA004775;Yang2025_Silkie_Wenchang	PRJNA827465	0.643294424
ENSGALG00010001935	ABRAXAS2	2	-0.043693823	0.073934064	-0.590983652	0.554531375	0	0.938262106	0	0.5	CRA004775;PRJNA827465	Yang2025_Silkie_Wenchang	0.880223402
ENSGALG00010001938	ALG8	2	-0.176491426	0.310102082	-0.569139766	0.569261293	0.179193504	14.33221227	93.02271009	0.5	PRJNA827465;Yang2025_Silkie_Wenchang	CRA004775	0.884658821
ENSGALG00010001938	ALG8	2	-0.526971812	0.1290701	-4.082834141	4.44898E-05	0	0.171696722	0	1	CRA004775;Yang2025_Silkie_Wenchang	PRJNA827465	0.004483543
ENSGALG00010001938	ALG8	2	-0.205844523	0.372648116	-0.552383104	0.580685904	0.240249902	6.695113874	85.06373426	0.5	CRA004775;PRJNA827465	Yang2025_Silkie_Wenchang	0.888561342
ENSGALG00010001940	PTPRM	2	-0.056945029	0.16329399	-0.348727033	0.727294247	0.042262267	4.627568067	78.39037729	0.5	PRJNA827465;Yang2025_Silkie_Wenchang	CRA004775	0.931522817
ENSGALG00010001940	PTPRM	2	0.121422199	0.117810604	1.030655938	0.302702194	0	0.002466605	0	1	CRA004775;Yang2025_Silkie_Wenchang	PRJNA827465	0.791852471
ENSGALG00010001940	PTPRM	2	-0.150713253	0.117832606	-1.279045399	0.200881069	0.010685014	1.277582567	21.72717241	0.5	CRA004775;PRJNA827465	Yang2025_Silkie_Wenchang	0.71352535
ENSGALG00010001947	TINAG	2	-0.034069097	0.263541888	-0.129273936	0.897140894	0.050211708	1.5651818	36.10965832	0.5	PRJNA827465;Yang2025_Silkie_Wenchang	CRA004775	0.975346564

ENSGALG00010001947	TINAG	2	-0.300457489	0.265022385	-1.133706077	0.25691786	0	0.008911016	0	1	CRA004775;Yang2025_Silkie_Wenchang	PRJNA827465	0.764799109
ENSGALG00010001947	TINAG	2	0.129981082	0.276312578	0.470413193	0.63805984	0	0.688683225	0	0.5	CRA004775;PRJNA827465	Yang2025_Silkie_Wenchang	0.905365679
ENSGALG00010001948	EMX2	2	-0.108009277	0.181603968	-0.594751744	0.552009428	0	0.035261303	0	1	PRJNA827465;Yang2025_Silkie_Wenchang	CRA004775	0.879061525
ENSGALG00010001948	EMX2	2	-0.040073361	0.287200268	-0.13953107	0.889030506	0	0.656869517	0	0.5	CRA004775;Yang2025_Silkie_Wenchang	PRJNA827465	0.973135755
ENSGALG00010001948	EMX2	2	-0.030803954	0.207860766	-0.148195132	0.882188773	0	0.56204757	0	0.5	CRA004775;PRJNA827465	Yang2025_Silkie_Wenchang	0.971942333
ENSGALG00010001952	ENSGALG00010001952	2	0.082258601	0.171697145	0.479091257	0.631873709	0.046658678	4.096001624	75.58594718	0.5	PRJNA827465;Yang2025_Silkie_Wenchang	CRA004775	0.903058174
ENSGALG00010001952	ENSGALG00010001952	2	0.086548672	0.217225041	0.398428611	0.690314271	0.062557218	2.956685175	66.17834025	0.5	CRA004775;Yang2025_Silkie_Wenchang	PRJNA827465	0.919979266
ENSGALG00010001952	ENSGALG00010001952	2	-0.060618531	0.043475917	-1.394301397	0.163226609	0	0.184223562	0	1	CRA004775;PRJNA827465	Yang2025_Silkie_Wenchang	0.661668506
ENSGALG00010001957	NDUFC2	2	-0.117934455	0.089903587	-1.311788095	0.189591643	0	0.107104287	0	1	PRJNA827465;Yang2025_Silkie_Wenchang	CRA004775	0.698754721
ENSGALG00010001957	NDUFC2	2	-0.135615061	0.297292353	-0.456167336	0.648269652	0	0.138981893	0	1	CRA004775;Yang2025_Silkie_Wenchang	PRJNA827465	0.90762535
ENSGALG00010001957	NDUFC2	2	-0.108262061	0.089320084	-1.212068513	0.225486155	0	0.032078819	0	1	CRA004775;PRJNA827465	Yang2025_Silkie_Wenchang	0.73884362
ENSGALG00010001958	RAB11FIP2	2	-0.130688353	0.077709552	-1.681754039	0.092616544	0.003696637	1.280813835	21.924641	1	PRJNA827465;Yang2025_Silkie_Wenchang	CRA004775	0.52105755
ENSGALG00010001958	RAB11FIP2	2	-0.212413115	0.140734622	-1.509316699	0.131217869	0.00114237	1.014882448	1.466420848	0.5	CRA004775;Yang2025_Silkie_Wenchang	PRJNA827465	0.606878893
ENSGALG00010001958	RAB11FIP2	2	-0.081260354	0.063675511	-1.276163358	0.201897779	0	0.328695354	0	0.5	CRA004775;PRJNA827465	Yang2025_Silkie_Wenchang	0.714969076
ENSGALG00010001961	ENSGALG00010001961	2	0.709412403	0.511592504	1.386674742	0.165540966	0.457642111	7.949001391	87.41980343	1	PRJNA827465;Yang2025_Silkie_Wenchang	CRA004775	0.664874006
ENSGALG00010001961	ENSGALG00010001961	2	0.138350529	1.194952027	0.115779149	0.907827576	2.539716953	8.435677387	88.14558744	0.5	CRA004775;Yang2025_Silkie_Wenchang	PRJNA827465	0.978196186
ENSGALG00010001961	ENSGALG00010001961	2	-0.285904648	0.658915841	-0.433901616	0.664359886	0.611806518	2.817210205	64.50389119	0.5	CRA004775;PRJNA827465	Yang2025_Silkie_Wenchang	0.913219726
ENSGALG00010001966	ARHGAP6	2	0.143751777	0.218373644	0.658283547	0.510355949	0.070391658	3.277557467	69.4894747	0.5	PRJNA827465;Yang2025_Silkie_Wenchang	CRA004775	0.865365179
ENSGALG00010001966	ARHGAP6	2	0.26981149	0.19226841	1.403306394	0.160525492	0.005100752	1.070195624	6.559139518	1	CRA004775;Yang2025_Silkie_Wenchang	PRJNA827465	0.65630165
ENSGALG00010001966	ARHGAP6	2	-0.023168609	0.07735	-0.29952953	0.764536044	0	0.032243278	0	0.5	CRA004775;PRJNA827465	Yang2025_Silkie_Wenchang	0.940627228
ENSGALG00010001967	LAMA1	2	-0.209754639	0.141599449	-1.481323836	0.1385203	0.004103019	1.113101187	10.16090794	1	PRJNA827465;Yang2025_Silkie_Wenchang	CRA004775	0.622343774
ENSGALG00010001967	LAMA1	2	0.109608513	0.580753052	0.188735147	0.850300403	0.521630198	3.873004607	74.18025277	0.5	CRA004775;Yang2025_Silkie_Wenchang	PRJNA827465	0.961985357
ENSGALG00010001967	LAMA1	2	0.21025773	0.421092001	0.499315422	0.617557192	0.228353237	2.28142708	56.16778599	0.5	CRA004775;PRJNA827465	Yang2025_Silkie_Wenchang	0.899121942
ENSGALG00010001969	PLEKHA8	2	0.161623522	0.114032898	1.417341173	0.156383203	0.008360276	1.457556782	31.39203823	1	PRJNA827465;Yang2025_Silkie_Wenchang	CRA004775	0.649484056
ENSGALG00010001969	PLEKHA8	2	-0.070749982	0.257222088	-0.27505407	0.783274698	0.053673589	1.224219836	18.3153245	0.5	CRA004775;Yang2025_Silkie_Wenchang	PRJNA827465	0.945748071
ENSGALG00010001969	PLEKHA8	2	-0.01692501	0.446026476	-0.0379462	0.969730577	0.261071258	2.109524903	52.59596136	0.5	CRA004775;PRJNA827465	Yang2025_Silkie_Wenchang	0.991572914
ENSGALG00010001971	MLIP	2	-0.049013201	0.293161039	-0.167188658	0.867221608	0.136900839	4.642696312	78.46079233	0.5	PRJNA827465;Yang2025_Silkie_Wenchang	CRA004775	0.967702313
ENSGALG00010001971	MLIP	2	0.284950887	0.224120453	1.271418486	0.203579809	0	0.000763102	0	1	CRA004775;Yang2025_Silkie_Wenchang	PRJNA827465	0.717254253
ENSGALG00010001971	MLIP	2	-0.272282221	0.14365466	-1.895394282	0.058040186	0.005639433	1.031446625	3.048788361	0.5	CRA004775;PRJNA827465	Yang2025_Silkie_Wenchang	0.417532916
ENSGALG00010001972	FAM204A	2	-0.188249145	0.09639005	-1.952993549	0.05082036	0.006252603	1.408240366	28.98939527	1	PRJNA827465;Yang2025_Silkie_Wenchang	CRA004775	0.390049394
ENSGALG00010001972	FAM204A	2	-0.041138819	0.130865408	-0.314359768	0.753247809	0	0.003434128	0	1	CRA004775;Yang2025_Silkie_Wenchang	PRJNA827465	0.938087082
ENSGALG00010001972	FAM204A	2	-0.230540103	0.080918698	-2.849033776	0.004385222	0	0.707347197	0	1	CRA004775;PRJNA827465	Yang2025_Silkie_Wenchang	0.097063291
ENSGALG00010001974	ENSGALG00010001974	2	0.184430253	0.256185814	0.719908139	0.471581556	0.076027103	2.349101319	57.4305292	0.5	PRJNA827465;Yang2025_Silkie_Wenchang	CRA004775	0.853998373
ENSGALG00010001974	ENSGALG00010001974	2	0.008275025	0.588787611	0.014054347	0.988786623	0.487070318	2.93526053	65.93147389	0.5	CRA004775;Yang2025_Silkie_Wenchang	PRJNA827465	0.996891127
ENSGALG00010001974	ENSGALG00010001974	2	-0.121031381	0.212591653	-0.56931389	0.569143142	0.006170937	1.025764529	2.511739081	1	CRA004775;PRJNA827465	Yang2025_Silkie_Wenchang	0.884646991
ENSGALG00010001976	THRSP	2	-1.561486239	0.697831378	-2.237626866	0.025245398	0	0.366993807	0	1	PRJNA827465;Yang2025_Silkie_Wenchang	CRA004775	0.270438551
ENSGALG00010001976	THRSP	2	-1.8562849	0.55741542	-3.330164242	0.000867948	0	0.018861552	0	1	CRA004775;Yang2025_Silkie_Wenchang	PRJNA827465	0.034597311
ENSGALG00010001976	THRSP	2	-1.587672324	0.566210369	-2.804032585	0.00504678	0	0.314152745	0	1	CRA004775;PRJNA827465	Yang2025_Silkie_Wenchang	0.105958126
ENSGALG00010001982	DMD	2	-0.097284216	0.252405199	-0.385428732	0.699919799	0.106683019	5.744495791	82.59203181	0.5	PRJNA827465;Yang2025_Silkie_Wenchang	CRA004775	0.922198312
ENSGALG00010001982	DMD	2	0.379399644	0.29840878	1.27140912	0.20358314	0.08875012	1.70771258	41.44213659	1	CRA004775;Yang2025_Silkie_Wenchang	PRJNA827465	0.717254253
ENSGALG00010001982	DMD	2	0.171023411	0.574582968	0.297647894	0.765971916	0.565599778	6.124984085	83.67342697	0.5	CRA004775;PRJNA827465	Yang2025_Silkie_Wenchang	0.941045738
ENSGALG00010001991	TOMM7	2	0.282385781	0.299909363	0.941570409	0.346412626	0.133829567	3.250176963	69.23244453	1	PRJNA827465;Yang2025_Silkie_Wenchang	CRA004775	0.80782339
ENSGALG00010001991	TOMM7	2	0.3680882	0.307426336	1.197321624	0.231181219	0.078414688	1.708945782	41.48439284	1	CRA004775;Yang2025_Silkie_Wenchang	PRJNA827465	0.743891332
ENSGALG00010001991	TOMM7	2	0.053887108	0.089116299	0.60468297	0.545389666	0	0.00040186	0	1	CRA004775;PRJNA827465	Yang2025_Silkie_Wenchang	0.877636727
ENSGALG00010001995	LRRC1	2	-0.022661684	0.095896443	-0.236314128	0.813188925	0.002832102	1.156419287	13.52617416	0.5	PRJNA827465;Yang2025_Silkie_Wenchang	CRA004775	0.953526116
ENSGALG00010001995	LRRC1	2	-0.147946178	0.151593638	-0.975939228	0.329094581	0	0.066593271	0	1	CRA004775;Yang2025_Silkie_Wenchang	PRJNA827465	0.800761292
ENSGALG00010001995	LRRC1	2	0.040906235	0.101689717	0.402265212	0.687488854	0	0.020342635	0	0.5	CRA004775;PRJNA827465	Yang2025_Silkie_Wenchang	0.91949273
ENSGALG00010001997	INTS4	2	-0.208678823	0.129849678	-1.607080013	0.108036817	0.030031312	8.675318976	88.47304632	1	PRJNA827465;Yang2025_Silkie_Wenchang	CRA004775	0.556189254
ENSGALG00010001997	INTS4	2	-0.335174897	0.076263361	-4.394966262	1.1079E-05	0	0.243354603	0	1	CRA004775;Yang2025_Silkie_Wenchang	PRJNA827465	0.00152251
ENSGALG00010001997	INTS4	2	-0.092590147	0.033731649	-2.744904284	0.006052855	0	0.460970212	0	1	CRA004775;PRJNA827465	Yang2025_Silkie_Wenchang	0.117595331
ENSGALG00010002005	COLQ	2	-0.14714727	0.23651639	-0.622144072	0.533847138	0.090314979	5.176099836	80.68043447	0.5	PRJNA827465;Yang2025_Silkie_Wenchang	CRA004775	0.873408008
ENSGALG00010002005	COLQ	2	0.063819501	0.149909149	0.425721187	0.670311022	0	0.545654776	0	0.5	CRA004775;Yang2025_Silkie_Wenchang	PRJNA827465	0.914482883
ENSGALG00010002005	COLQ	2	-0.375343277	0.133755026	-2.806199425	0.005012966	0	0.007242028	0	1	CRA004775;PRJNA827465	Yang2025_Silkie_Wenchang	0.105530043
ENSGALG00010002006	ENSGALG00010002006	2	0.084529463	0.349492369	0.241863545	0.808885899	0.130703375	2.140355531	53.2787901	0.5	PRJNA827465;Yang2025_Silkie_Wenchang	CRA004775	0.952595795
ENSGALG00010002006	ENSGALG00010002006	2	0.542686728	0.308445457	1.759425259	0.078505305	0.013479152	1.037922377	3.653681401	1	CRA004775;Yang2025_Silkie_Wenchang	PRJNA827465	0.481915278
ENSGALG00010002006	ENSGALG00010002006	2	0.33689437	0.764269924	0.440805479	0.659353826	0.844579164	3.274436567	69.4603948	0.5	CRA004775;PRJNA827465	Yang2025_Silkie_Wenchang	0.911449691
ENSGALG00010002012	MID1	2	-0.309004327	0.180169307	-1.715077513	0.086331017	0.044392286	3.160494135	68.35937808	1	PRJNA827465;Yang2025_Silkie_Wenchang	CRA004775	0.505748687
ENSGALG00010002012	MID1	2	-0.483223285	0.1419292	-3.404678424	0.00066242	0	0.054292871	0	1	CRA004775;Yang2025_Silkie_Wenchang	PRJNA827465	0.028422595
ENSGALG00010002012	MID1	2	-0.14594109	0.135181116	-1.079596724	0.280321801	0	0.166617064	0	1	CRA004775;PRJNA827465	Yang2025_Silkie_Wenchang	0.778461864
ENSGALG00010002017	ARHGAP28	2	-0.070906923	0.060021977	-1.181349348	0.237463971	0	0.376355916	0	1	PRJNA827465;Yang2025_Silkie_Wenchang	CRA004775	0.749366344
ENSGALG00010002017	ARHGAP28	2	-0.114296325	0.109232746	-1.046355873	0.295396758	0	0.155768185	0	1	CRA004775;Yang2025_Silkie_Wenchang	PRJNA827465	0.787628307
ENSGALG00010002017	ARHGAP28	2	-0.049238593	0.065719841	-0.74921959	0.453724863	0	0.004554706	0	1	CRA004775;PRJNA827465	Yang2025_Silkie_Wenchang	0.849044791
ENSGALG00010002023	CLCN4	2	-0.160000095	0.188952643	-0.846773519	0.397121371	0.030446589	1.706005349	41.38353667	1	PRJNA827465;Yang2025_Silkie_Wenchang	CRA004775	0.831438925
ENSGALG00010002023	CLCN4	2	-0.679624728	0.393955957	-1.725128701	0.084504281	0.186433269	2.236917759	55.29562962	1	CRA004775;Yang2025_Silkie_Wenchang	PRJNA827465	0.500072565
ENSGALG00010002023	CLCN4	2	-0.514177352	0.595722099	-0.863116129	0.388073621	0.587620973	5.254985312	80.97045109	1	CRA004775;PRJNA827465	Yang2025_Silkie_Wenchang	0.828154121
ENSGALG00010002024	KLHL31	2	-0.339078205	0.176786923	-1.918005013	0.055110376	0.050508922	5.190423782	80.73375042	1	PRJNA827465;Yang2025_Silkie_Wenchang	CRA004775	0.406379567
ENSGALG00010002024	KLHL31	2	0.150552222	0.786725128	0.191365722	0.848239074	1.056266753	5.964434523	83.23395125	0.5	CRA004775;Yang2025_Silkie_Wenchang	PRJNA827465	0.961363811
ENSGALG00010002024	KLHL31	2	0.29029451	0.598166585	0.485307132	0.627458497	0.557148086	3.641638869	72.53983616	0.5	CRA004775;PRJNA827465	Yang2025_Silkie_Wenchang	0.90174789
ENSGALG00010002027	ENSGALG00010002027	2	-0.141215752	0.38777759	-0.364166871	0.715733404	0.127382902	1.701989927	41.24524571	0.5	PRJNA827465;Yang2025_Silkie_Wenchang	CRA004775	0.927131632
ENSGALG00010002027	ENSGALG00010002027	2	-0.356457029	0.378766918	-0.941098633	0.346654316	0	0.661914928	0	0.5	CRA004775;Yang2025_Silkie_Wenchang	PRJNA827465	0.808232887
ENSGALG00010002027	ENSGALG00010002027	2	0.149065154	0.312225188	0.477428341	0.633057137	0	0.04489256	0	1	CRA004775;PRJNA827465	Yang2025_Silkie_Wenchang	0.903225139
ENSGALG00010002034	WWC3	2	0.098959141	0.16750223	0.590792974	0.554659143	0.047447664	5.994104859	83.31694184	0.5	PRJNA827465;Yang2025_Silkie_Wenchang	CRA004775	0.880223402
ENSGALG00010002034	WWC3	2	0.265555516	0.117325856	2.263401475	0.023610952	0	0.193822239	0	1	CRA004775;Yang2025_Silkie_Wenchang	PRJNA827465	0.26228978
ENSGALG00010002034	WWC3	2	-0.043559811	0.050304538	-0.865922092	0.386532887	0	0.437464785	0	0.5	CRA004775;PRJNA827465	Yang2025_Silkie_Wenchang	0.826995213
ENSGALG00010002039	AAMDC	2	0.145693632	0.084087449	1.732644206	0.083158927	0	0.229974653	0	1	PRJNA827465;Yang2025_Silkie_Wenchang	CRA004775	0.496641135
ENSGALG00010002039	AAMDC	2	0.194600212	0.136772966	1.422797341	0.154794912	0	0.027088892	0	1	CRA004775;Yang2025_Silkie_Wenchang	PRJNA827465	0.647505492
ENSGALG00010002039	AAMDC	2	0.118751488	0.099083162	1.198503223	0.230721169	0	0.00230428	0	1	CRA004775;PRJNA827465	Yang2025_Silkie_Wenchang	0.743374303
ENSGALG00010002041	GCLC	2	0.243795374	0.158132623	1.541714601	0.123142962	0.031535654	2.635738295	62.0599662	1	PRJNA827465;Yang2025_Silkie_Wenchang	CRA004775	0.588293162
ENSGALG00010002041	GCLC	2	0.41269719	0.146364762	2.81964856	0.004807627	0	0.026136012	0	1	CRA004775;Yang2025_Silkie_Wenchang	PRJNA827465	0.102692483
ENSGALG00010002041	GCLC	2	0.127522145	0.108281154	1.17769474	0.238918325	0	0.456848601	0	1	CRA004775;PRJNA827465	Yang2025_Silkie_Wenchang	0.751036304
ENSGALG00010002046	FAM126A	2	0.029352296	0.052990444	0.553916779	0.579635802	0	7.62582E-09	0	1	PRJNA827465;Yang2025_Silkie_Wenchang	CRA004775	0.887983094
ENSGALG00010002046	FAM126A	2	0.014700698	0.079297796	0.185385957	0.852926322	0	0.800715273	0	0.5	CRA004775;Yang2025_Silkie_Wenchang	PRJNA827465	0.962708451
ENSGALG00010002046	FAM126A	2	0.018255067	0.068988063	0.264611969	0.791308399	0	0.808979984	0	0.5	CRA004775;PRJNA827465	Yang2025_Silkie_Wenchang	0.947963729
ENSGALG00010002050	ENSGALG00010002050	2	0.799741548	1.226450726	0.65207801	0.514350849	2.551261478	6.001050288	83.33625029	0.5	PRJNA827465;Yang2025_Silkie_Wenchang	CRA004775	0.866869697
ENSGALG00010002050	ENSGALG00010002050	2	1.413881438	0.459994412	3.073692638	0.002114271	0	0.880493627	0	1	CRA004775;Yang2025_Silkie_Wenchang	PRJNA827465	0.060171765
ENSGALG00010002050	ENSGALG00010002050	2	0.393146393	0.723405306	0.543466283	0.586808799	0.862055469	5.506459689	81.83951111	0.5	CRA004775;PRJNA827465	Yang2025_Silkie_Wenchang	0.891596903
ENSGALG00010002054	RSF1	2	-0.062743669	0.057399666	-1.093101651	0.27449971	0	0.23109901	0	1	PRJNA827465;Yang2025_Silkie_Wenchang	CRA004775	0.774526342
ENSGALG00010002054	RSF1	2	0.048119443	0.13606194	0.353658365	0.723594928	0	0.311572769	0	1	CRA004775;Yang2025_Silkie_Wenchang	PRJNA827465	0.930032096
ENSGALG00010002054	RSF1	2	-0.061899334	0.060365332	-1.025411962	0.305168846	0	0.807518577	0	0.5	CRA004775;PRJNA827465	Yang2025_Silkie_Wenchang	0.79225794
ENSGALG00010002062	GSTA4	2	0.22635958	0.189947865	1.191693205	0.233381564	0	0.607188249	0	1	PRJNA827465;Yang2025_Silkie_Wenchang	CRA004775	0.746672784
ENSGALG00010002062	GSTA4	2	0.092721543	0.295067615	0.314238291	0.753384062	0	0.258313281	0	1	CRA004775;Yang2025_Silkie_Wenchang	PRJNA827465	0.938087082
ENSGALG00010002062	GSTA4	2	0.327302851	0.212018377	1.543747555	0.122649501	0	0.001027984	0	1	CRA004775;PRJNA827465	Yang2025_Silkie_Wenchang	0.58762464
ENSGALG00010002065	CLNS1A	2	0.002782304	0.056571996	0.049181651	0.960774534	0	0.034307047	0	0.5	PRJNA827465;Yang2025_Silkie_Wenchang	CRA004775	0.98952228
ENSGALG00010002065	CLNS1A	2	-0.016236802	0.097838109	-0.1659558	0.868191734	0	0.001004163	0	1	CRA004775;Yang2025_Silkie_Wenchang	PRJNA827465	0.968042774
ENSGALG00010002065	CLNS1A	2	0.006382158	0.063101833	0.101140613	0.919438838	0	0.019434552	0	0.5	CRA004775;PRJNA827465	Yang2025_Silkie_Wenchang	0.981192162
ENSGALG00010002072	NEURL4	2	0.233013302	0.266026439	0.875902797	0.381082879	0.134251043	19.41378909	94.84902202	0.5	PRJNA827465;Yang2025_Silkie_Wenchang	CRA004775	0.824392515
ENSGALG00010002072	NEURL4	2	0.252779061	0.274609621	0.920503296	0.357309812	0.128729179	6.41616622	84.41436887	0.5	CRA004775;Yang2025_Silkie_Wenchang	PRJNA827465	0.813292764
ENSGALG00010002072	NEURL4	2	-0.035046046	0.076203922	-0.459879821	0.645602485	0	0.008770108	0	1	CRA004775;PRJNA827465	Yang2025_Silkie_Wenchang	0.907052464
ENSGALG00010002079	ARX	2	-0.112738362	0.176789922	-0.63769677	0.523671089	0.024815266	1.556062096	35.73521245	0.5	PRJNA827465;Yang2025_Silkie_Wenchang	CRA004775	0.869661505
ENSGALG00010002079	ARX	2	0.107431186	0.238944497	0.449607282	0.652993637	0	0.066194337	0	0.5	CRA004775;Yang2025_Silkie_Wenchang	PRJNA827465	0.909572411
ENSGALG00010002079	ARX	2	-0.22967053	0.146531063	-1.567384586	0.117024825	0	0.091280244	0	1	CRA004775;PRJNA827465	Yang2025_Silkie_Wenchang	0.57615266
ENSGALG00010002084	HACL1	2	0.040567796	0.077762736	0.521686844	0.601888386	0	0.05133241	0	1	PRJNA827465;Yang2025_Silkie_Wenchang	CRA004775	0.895304733
ENSGALG00010002084	HACL1	2	0.018816116	0.095526132	0.196973496	0.843848279	0	0.69034937	0	0.5	CRA004775;Yang2025_Silkie_Wenchang	PRJNA827465	0.96057435
ENSGALG00010002084	HACL1	2	-0.014096585	0.101668913	-0.138651874	0.88972525	0	0.45709518	0	0.5	CRA004775;PRJNA827465	Yang2025_Silkie_Wenchang	0.9731958
ENSGALG00010002087	POLA1	2	-0.027977208	0.047830751	-0.584920932	0.558600883	0	0.241396707	0	1	PRJNA827465;Yang2025_Silkie_Wenchang	CRA004775	0.880830111
ENSGALG00010002087	POLA1	2	-0.010890388	0.069923765	-0.155746589	0.876232773	0	0.369364009	0	1	CRA004775;Yang2025_Silkie_Wenchang	PRJNA827465	0.970347777
ENSGALG00010002087	POLA1	2	-0.054843064	0.063223283	-0.867450425	0.385695264	0	0.223296465	0	1	CRA004775;PRJNA827465	Yang2025_Silkie_Wenchang	0.82648313
ENSGALG00010002088	OZFL1	2	0.201719003	0.192800549	1.04625741	0.295442203	0.051778035	3.264983732	69.37197603	1	PRJNA827465;Yang2025_Silkie_Wenchang	CRA004775	0.787628307
ENSGALG00010002088	OZFL1	2	0.204323611	0.253248404	0.806811048	0.419775353	0.077678022	2.36147592	57.65360165	0.5	CRA004775;Yang2025_Silkie_Wenchang	PRJNA827465	0.838527387
ENSGALG00010002088	OZFL1	2	-0.002368218	0.122559496	-0.01932301	0.984583428	0	0.168220968	0	0.5	CRA004775;PRJNA827465	Yang2025_Silkie_Wenchang	0.995703389
ENSGALG00010002090	AQP11	2	0.329377309	0.150777879	2.184520114	0.028924051	0	0.215588553	0	1	PRJNA827465;Yang2025_Silkie_Wenchang	CRA004775	0.289667055
ENSGALG00010002090	AQP11	2	0.35423058	0.188335164	1.880852058	0.059992046	0	0.179180191	0	1	CRA004775;Yang2025_Silkie_Wenchang	PRJNA827465	0.42407687
ENSGALG00010002090	AQP11	2	0.260489339	0.171189079	1.521646942	0.128097569	0	0.005596087	0	1	CRA004775;PRJNA827465	Yang2025_Silkie_Wenchang	0.600574823
ENSGALG00010002097	PCYT1B	2	0.090599839	0.876548842	0.103359716	0.917677483	1.376507717	9.531102346	89.50803418	0.5	PRJNA827465;Yang2025_Silkie_Wenchang	CRA004775	0.980974192
ENSGALG00010002097	PCYT1B	2	-0.124430579	1.206197401	-0.103159383	0.917836476	2.496896881	6.800787961	85.29582152	0.5	CRA004775;Yang2025_Silkie_Wenchang	PRJNA827465	0.981033557
ENSGALG00010002097	PCYT1B	2	-0.863860737	0.31705446	-2.724644646	0.006437073	0	0.571701277	0	1	CRA004775;PRJNA827465	Yang2025_Silkie_Wenchang	0.12222042
ENSGALG00010002098	ENSGALG00010002098	2	0.799778762	0.853724977	0.936810779	0.348855899	1.24450396	6.832348913	85.36374514	0.5	PRJNA827465;Yang2025_Silkie_Wenchang	CRA004775	0.809343216
ENSGALG00010002098	ENSGALG00010002098	2	0.812776245	0.819220031	0.992134242	0.321132045	1.165007933	7.563080853	86.77787506	1	CRA004775;Yang2025_Silkie_Wenchang	PRJNA827465	0.798513866
ENSGALG00010002098	ENSGALG00010002098	2	-0.022924512	0.306237676	-0.074858563	0.940327246	0	0.012242424	0	0.5	CRA004775;PRJNA827465	Yang2025_Silkie_Wenchang	0.985511899
ENSGALG00010002100	BTD	2	-0.282670202	0.07988266	-3.538567708	0.000402304	0	0.343145251	0	1	PRJNA827465;Yang2025_Silkie_Wenchang	CRA004775	0.020297507
ENSGALG00010002100	BTD	2	-0.350816554	0.1238206	-2.833264843	0.004607519	0	0.008819103	0	1	CRA004775;Yang2025_Silkie_Wenchang	PRJNA827465	0.099718798
ENSGALG00010002100	BTD	2	-0.259559532	0.094879995	-2.735661317	0.006225506	0	0.151972411	0	1	CRA004775;PRJNA827465	Yang2025_Silkie_Wenchang	0.119648449
ENSGALG00010002102	PAK1	2	-0.055094345	0.158616914	-0.347342182	0.728334264	0	0.509420347	0	0.5	PRJNA827465;Yang2025_Silkie_Wenchang	CRA004775	0.932099951
ENSGALG00010002102	PAK1	2	0.017251291	0.209526821	0.082334522	0.934380703	0	0.245770702	0	0.5	CRA004775;Yang2025_Silkie_Wenchang	PRJNA827465	0.984345577
ENSGALG00010002102	PAK1	2	-0.125338204	0.165293192	-0.758278079	0.448284527	0	0.007235967	0	1	CRA004775;PRJNA827465	Yang2025_Silkie_Wenchang	0.846800937
ENSGALG00010002113	GDPD4	2	0.084374893	0.161481199	0.522505988	0.601318079	0	0.80306855	0	0.5	PRJNA827465;Yang2025_Silkie_Wenchang	CRA004775	0.895230519
ENSGALG00010002113	GDPD4	2	0.501059594	0.774019628	0.6473474	0.517407131	0.932215945	4.118313378	75.718215	0.5	CRA004775;Yang2025_Silkie_Wenchang	PRJNA827465	0.867952013
ENSGALG00010002113	GDPD4	2	0.605826436	0.59226475	1.022898012	0.306356069	0.503569549	2.879133933	65.26733305	1	CRA004775;PRJNA827465	Yang2025_Silkie_Wenchang	0.792485409
ENSGALG00010002118	ENSGALG00010002118	2	-0.169232869	0.273154963	-0.619548944	0.53555479	0	0.003486245	0	1	PRJNA827465;Yang2025_Silkie_Wenchang	CRA004775	0.874171355
ENSGALG00010002118	ENSGALG00010002118	2	-0.370177776	0.333462596	-1.110102843	0.266954712	0	0.266477791	0	1	CRA004775;Yang2025_Silkie_Wenchang	PRJNA827465	0.771241774
ENSGALG00010002118	ENSGALG00010002118	2	-0.286463391	0.270290841	-1.05983388	0.289220181	0	0.440271594	0	1	CRA004775;PRJNA827465	Yang2025_Silkie_Wenchang	0.783530627
ENSGALG00010002122	ENSGALG00010002122	2	-0.85082532	0.498209325	-1.70776675	0.087679631	0	0.241976921	0	1	PRJNA827465;Yang2025_Silkie_Wenchang	CRA004775	0.509096253
ENSGALG00010002122	ENSGALG00010002122	2	-1.324325218	0.548129816	-2.416079511	0.015688635	0	0.171230592	0	1	CRA004775;Yang2025_Silkie_Wenchang	PRJNA827465	0.207264008
ENSGALG00010002122	ENSGALG00010002122	2	-1.011265262	0.520277656	-1.943703039	0.051931272	0	0.800390255	0	1	CRA004775;PRJNA827465	Yang2025_Silkie_Wenchang	0.395399997
ENSGALG00010002126	ENSGALG00010002126	2	0.018688349	0.20915937	0.089349804	0.928803914	0.055828483	2.677507279	62.65182889	0.5	PRJNA827465;Yang2025_Silkie_Wenchang	CRA004775	0.983452686
ENSGALG00010002126	ENSGALG00010002126	2	0.215735404	0.204410017	1.055405245	0.291240025	0	0.5161921	0	0.5	CRA004775;Yang2025_Silkie_Wenchang	PRJNA827465	0.78467882
ENSGALG00010002126	ENSGALG00010002126	2	-0.168840199	0.143123594	-1.179681106	0.238127071	0	0.029307059	0	1	CRA004775;PRJNA827465	Yang2025_Silkie_Wenchang	0.750031946
ENSGALG00010002134	ANKRD28	2	0.256392257	0.229069822	1.119275574	0.263022593	0.097239377	13.14147908	92.39050647	1	PRJNA827465;Yang2025_Silkie_Wenchang	CRA004775	0.768643821
ENSGALG00010002134	ANKRD28	2	0.449144226	0.100994395	4.447219315	8.6989E-06	0	0.694412539	0	1	CRA004775;Yang2025_Silkie_Wenchang	PRJNA827465	0.001256959
ENSGALG00010002134	ANKRD28	2	0.100204853	0.111537129	0.898399073	0.36897283	0.013783036	1.645117569	39.21407086	1	CRA004775;PRJNA827465	Yang2025_Silkie_Wenchang	0.820553374
ENSGALG00010002135	ELOVL5	2	-0.330206408	0.262853925	-1.256235407	0.209030633	0.130971386	19.02438535	94.74358839	1	PRJNA827465;Yang2025_Silkie_Wenchang	CRA004775	0.721011841
ENSGALG00010002135	ELOVL5	2	-0.286122867	0.372589001	-0.767931599	0.442527823	0.226280056	4.76175701	78.9993484	0.5	CRA004775;Yang2025_Silkie_Wenchang	PRJNA827465	0.845535782
ENSGALG00010002135	ELOVL5	2	-0.063313616	0.066107486	-0.957737469	0.338195158	0	0.464798403	0	0.5	CRA004775;PRJNA827465	Yang2025_Silkie_Wenchang	0.80415385
ENSGALG00010002136	ACAP1	2	0.160641757	0.204842189	0.78422203	0.43290984	0.022106175	1.335731274	25.13464204	0.5	PRJNA827465;Yang2025_Silkie_Wenchang	CRA004775	0.842762754
ENSGALG00010002136	ACAP1	2	-0.130486086	0.610774348	-0.213640416	0.83082748	0.586185526	4.460207251	77.57951719	0.5	CRA004775;Yang2025_Silkie_Wenchang	PRJNA827465	0.957336479
ENSGALG00010002136	ACAP1	2	-0.281247331	0.384228955	-0.731978491	0.464181696	0.177385991	2.178017453	54.08668565	1	CRA004775;PRJNA827465	Yang2025_Silkie_Wenchang	0.852159649
ENSGALG00010002137	PDK3	2	-0.352951744	0.087761696	-4.021706049	5.77781E-05	0.005256168	1.501260783	33.38932106	1	PRJNA827465;Yang2025_Silkie_Wenchang	CRA004775	0.005336024
ENSGALG00010002137	PDK3	2	-0.113631407	0.390118147	-0.291274342	0.770841509	0.255705667	5.716496176	82.50676692	0.5	CRA004775;Yang2025_Silkie_Wenchang	PRJNA827465	0.942491331
ENSGALG00010002137	PDK3	2	-0.044001882	0.296381008	-0.148463906	0.881976669	0.134057127	3.582538159	72.08682907	0.5	CRA004775;PRJNA827465	Yang2025_Silkie_Wenchang	0.971928118
ENSGALG00010002138	MYO7A	2	-0.21742722	0.342886322	-0.634108758	0.526009847	0.198845618	6.273541107	84.06003909	0.5	PRJNA827465;Yang2025_Silkie_Wenchang	CRA004775	0.870474317
ENSGALG00010002138	MYO7A	2	-0.613382493	0.220578331	-2.780792154	0.005422644	0	0.088857282	0	1	CRA004775;Yang2025_Silkie_Wenchang	PRJNA827465	0.110428077
ENSGALG00010002138	MYO7A	2	-0.141670757	0.393628347	-0.359909946	0.718914479	0.193872402	1.989679448	49.74064786	0.5	CRA004775;PRJNA827465	Yang2025_Silkie_Wenchang	0.928449671
ENSGALG00010002142	CLDN34	2	1.157529376	0.342654888	3.378120137	0.000729832	0	0.610329424	0	1	PRJNA827465;Yang2025_Silkie_Wenchang	CRA004775	0.030276194
ENSGALG00010002142	CLDN34	2	1.793771646	0.531758789	3.373280673	0.000742782	0.151705031	1.291493661	22.57027425	1	CRA004775;Yang2025_Silkie_Wenchang	PRJNA827465	0.030569172
ENSGALG00010002142	CLDN34	2	1.588592779	0.831344494	1.910871834	0.056021053	0.93480439	2.855869816	64.9843983	1	CRA004775;PRJNA827465	Yang2025_Silkie_Wenchang	0.409427048
ENSGALG00010002145	ENSGALG00010002145	2	-0.269170981	0.276715524	-0.972735384	0.330684835	0	0.000889858	0	1	PRJNA827465;Yang2025_Silkie_Wenchang	CRA004775	0.801575303
ENSGALG00010002145	ENSGALG00010002145	2	-0.172905161	0.341737022	-0.505959699	0.612884941	0	0.264092625	0	0.5	CRA004775;Yang2025_Silkie_Wenchang	PRJNA827465	0.898076834
ENSGALG00010002145	ENSGALG00010002145	2	-0.165539133	0.333447419	-0.496447484	0.61957873	0	0.246825762	0	0.5	CRA004775;PRJNA827465	Yang2025_Silkie_Wenchang	0.899301701
ENSGALG00010002146	CACUL1	2	-0.177995072	0.203751433	-0.873589302	0.382341954	0.076079064	11.8993335	91.59616797	0.5	PRJNA827465;Yang2025_Silkie_Wenchang	CRA004775	0.825080207
ENSGALG00010002146	CACUL1	2	-0.356643582	0.087475422	-4.077071861	4.56064E-05	0	0.929948005	0	1	CRA004775;Yang2025_Silkie_Wenchang	PRJNA827465	0.004560988
ENSGALG00010002146	CACUL1	2	0.012665185	0.070908756	0.17861242	0.858242041	0	0.228355659	0	0.5	CRA004775;PRJNA827465	Yang2025_Silkie_Wenchang	0.964291891
ENSGALG00010002148	POLR2A	2	0.105407412	0.475238943	0.221798768	0.824470539	0.367799711	4.768039826	79.02702082	0.5	PRJNA827465;Yang2025_Silkie_Wenchang	CRA004775	0.95619211
ENSGALG00010002148	POLR2A	2	0.121785591	0.499970418	0.243585594	0.807551786	0.373673154	3.86073398	74.09818949	0.5	CRA004775;Yang2025_Silkie_Wenchang	PRJNA827465	0.952471502
ENSGALG00010002148	POLR2A	2	-0.301108317	0.121000806	-2.488481918	0.012828976	0	0.015367136	0	1	CRA004775;PRJNA827465	Yang2025_Silkie_Wenchang	0.183819631
ENSGALG00010002150	EIF3A	2	0.030696466	0.202923859	0.151270858	0.879762057	0.071195298	7.023706303	85.76250263	0.5	PRJNA827465;Yang2025_Silkie_Wenchang	CRA004775	0.971407853
ENSGALG00010002150	EIF3A	2	0.154909445	0.135206651	1.145723554	0.251909555	0.00860722	1.269300241	21.21643345	0.5	CRA004775;Yang2025_Silkie_Wenchang	PRJNA827465	0.761871849
ENSGALG00010002150	EIF3A	2	-0.143847707	0.063466083	-2.26652883	0.023419024	0	0.305990941	0	1	CRA004775;PRJNA827465	Yang2025_Silkie_Wenchang	0.261142791
ENSGALG00010002159	GALNT15	2	-0.299875907	0.097142169	-3.086979715	0.002022014	0	0.470545403	0	1	PRJNA827465;Yang2025_Silkie_Wenchang	CRA004775	0.058563901
ENSGALG00010002159	GALNT15	2	-0.170565137	0.172478425	-0.988907088	0.322708602	0	0.097911225	0	1	CRA004775;Yang2025_Silkie_Wenchang	PRJNA827465	0.798653498
ENSGALG00010002159	GALNT15	2	-0.323983083	0.110912497	-2.921069222	0.003488323	0	0.401054899	0	1	CRA004775;PRJNA827465	Yang2025_Silkie_Wenchang	0.083445869
ENSGALG00010002160	CAPN5	2	0.014928279	0.065631949	0.227454456	0.82007038	0	0.132657789	0	1	PRJNA827465;Yang2025_Silkie_Wenchang	CRA004775	0.955308792
ENSGALG00010002160	CAPN5	2	0.083453488	0.124566363	0.669952031	0.50288837	0	0.423613158	0	1	CRA004775;Yang2025_Silkie_Wenchang	PRJNA827465	0.863050365
ENSGALG00010002160	CAPN5	2	0.012780448	0.074401426	0.17177692	0.863612911	0	0.658998682	0	1	CRA004775;PRJNA827465	Yang2025_Silkie_Wenchang	0.966309237
ENSGALG00010002161	CDKL5	2	-0.051102493	0.106777833	-0.478587095	0.632232401	0	0.900353199	0	0.5	PRJNA827465;Yang2025_Silkie_Wenchang	CRA004775	0.903058174
ENSGALG00010002161	CDKL5	2	-0.142743502	0.378012889	-0.377615437	0.705716295	0.17153262	2.259014649	55.73291212	0.5	CRA004775;Yang2025_Silkie_Wenchang	PRJNA827465	0.924374044
ENSGALG00010002161	CDKL5	2	-0.181483194	0.189030241	-0.960074923	0.337017509	0.027354001	1.234834978	19.01751909	1	CRA004775;PRJNA827465	Yang2025_Silkie_Wenchang	0.804050409
ENSGALG00010002174	ENSGALG00010002174	2	-0.403505427	0.308965715	-1.305987711	0.191556737	0	0.015273324	0	1	PRJNA827465;Yang2025_Silkie_Wenchang	CRA004775	0.701184773
ENSGALG00010002174	ENSGALG00010002174	2	0.060268902	0.630277824	0.095622755	0.923820192	0.372728183	1.67190535	40.18800168	0.5	CRA004775;Yang2025_Silkie_Wenchang	PRJNA827465	0.982219519
ENSGALG00010002174	ENSGALG00010002174	2	0.027447094	0.673492679	0.040753366	0.967492517	0.49177498	1.905956917	47.53291689	0.5	CRA004775;PRJNA827465	Yang2025_Silkie_Wenchang	0.99104377
ENSGALG00010002177	FBXO9	2	-0.297619129	0.136816115	-2.175322172	0.029605982	0.024786757	2.677157813	62.64695361	1	PRJNA827465;Yang2025_Silkie_Wenchang	CRA004775	0.293468832
ENSGALG00010002177	FBXO9	2	-0.323639652	0.216931736	-1.49189629	0.135726335	0.044787173	1.69783992	41.10163224	1	CRA004775;Yang2025_Silkie_Wenchang	PRJNA827465	0.617196137
ENSGALG00010002177	FBXO9	2	-0.180141856	0.072916751	-2.470514019	0.013491903	0	0.312907029	0	1	CRA004775;PRJNA827465	Yang2025_Silkie_Wenchang	0.190061744
ENSGALG00010002181	FIS1	2	0.765203197	0.559209081	1.368366899	0.17119726	0.601764734	26.42655767	96.21592788	1	PRJNA827465;Yang2025_Silkie_Wenchang	CRA004775	0.673243267
ENSGALG00010002181	FIS1	2	0.439081394	0.914540254	0.48011161	0.631148033	1.59716947	21.67716418	95.38685046	0.5	CRA004775;Yang2025_Silkie_Wenchang	PRJNA827465	0.903058174
ENSGALG00010002181	FIS1	2	-0.07351243	0.347721256	-0.211411954	0.83256583	0.177011358	3.324593083	69.92113095	0.5	CRA004775;PRJNA827465	Yang2025_Silkie_Wenchang	0.957861248
ENSGALG00010002185	ENSGALG00010002185	2	0.734772543	0.362085914	2.029276795	0.042430105	0	0.007346854	0	1	PRJNA827465;Yang2025_Silkie_Wenchang	CRA004775	0.356451428
ENSGALG00010002185	ENSGALG00010002185	2	0.053003181	0.603169595	0.087874424	0.929976485	0.505873371	3.051477132	67.22898594	0.5	CRA004775;Yang2025_Silkie_Wenchang	PRJNA827465	0.983813577

ENSGALG00010002185	ENSGALG00010002185	2	0.093246997	0.579389903	0.160939976	0.872140676	0.519772076	4.362590049	77.07783705	0.5	CRA004775;PRJNA827465	Yang2025_Silkie_Wenchang	0.969585183
ENSGALG00010002186	CILK1	2	0.064941412	0.043118517	1.50611423	0.132037852	0	0.165085558	0	1	PRJNA827465;Yang2025_Silkie_Wenchang	CRA004775	0.608737092
ENSGALG00010002186	CILK1	2	0.078638151	0.075752787	1.038089214	0.299228523	0	0.410582455	0	0.5	CRA004775;Yang2025_Silkie_Wenchang	PRJNA827465	0.78978392
ENSGALG00010002186	CILK1	2	0.048270258	0.050859885	0.957930954	0.338097577	0	0.276383571	0	0.5	CRA004775;PRJNA827465	Yang2025_Silkie_Wenchang	0.80415385
ENSGALG00010002192	ENSGALG00010002192	2	-0.465957519	0.480943815	-0.96883982	0.332625115	0.441554524	21.83174453	95.41951401	0.5	PRJNA827465;Yang2025_Silkie_Wenchang	CRA004775	0.802178578
ENSGALG00010002192	ENSGALG00010002192	2	-0.496044938	0.51878325	-0.956169918	0.338986395	0.450846124	5.722006601	82.52361331	0.5	CRA004775;Yang2025_Silkie_Wenchang	PRJNA827465	0.804767595
ENSGALG00010002192	ENSGALG00010002192	2	0.013083069	0.110621559	0.118268708	0.90585475	0	0.039611332	0	1	CRA004775;PRJNA827465	Yang2025_Silkie_Wenchang	0.978196186
ENSGALG00010002194	SHROOM2	2	0.000581883	0.105449129	0.005518141	0.995597183	0	0.319864807	0	0.5	PRJNA827465;Yang2025_Silkie_Wenchang	CRA004775	0.998484999
ENSGALG00010002194	SHROOM2	2	0.126585646	0.17360092	0.729176125	0.465893934	0	0.078812566	0	1	CRA004775;Yang2025_Silkie_Wenchang	PRJNA827465	0.852511753
ENSGALG00010002194	SHROOM2	2	-0.007539873	0.11630654	-0.064827591	0.948311273	0	0.455842734	0	0.5	CRA004775;PRJNA827465	Yang2025_Silkie_Wenchang	0.986763555
ENSGALG00010002196	ENSGALG00010002196	2	-0.225920314	0.267876195	-0.843375851	0.399018274	0.138523463	28.74957777	96.52168805	0.5	PRJNA827465;Yang2025_Silkie_Wenchang	CRA004775	0.832054308
ENSGALG00010002196	ENSGALG00010002196	2	-0.350710608	0.197489116	-1.775847781	0.07575801	0.054439047	2.70752891	63.065953	1	CRA004775;Yang2025_Silkie_Wenchang	PRJNA827465	0.475110429
ENSGALG00010002196	ENSGALG00010002196	2	0.03251579	0.067223898	0.48369391	0.628603116	0	0.227462804	0	0.5	CRA004775;PRJNA827465	Yang2025_Silkie_Wenchang	0.901974673
ENSGALG00010002197	GSTA2	2	0.564589383	0.193441718	2.918653679	0.003515465	0.018067417	1.31126711	23.73788737	1	PRJNA827465;Yang2025_Silkie_Wenchang	CRA004775	0.083890916
ENSGALG00010002197	GSTA2	2	0.256166506	0.224781921	1.139622369	0.254443662	0	0.447261634	0	0.5	CRA004775;Yang2025_Silkie_Wenchang	PRJNA827465	0.764087626
ENSGALG00010002197	GSTA2	2	0.468729821	0.35302478	1.327753312	0.184259615	0.147027363	2.196375109	54.4704365	0.5	CRA004775;PRJNA827465	Yang2025_Silkie_Wenchang	0.692546205
ENSGALG00010002202	SFXN4	2	-0.107957442	0.126943416	-0.850437507	0.395081891	0.017239856	1.87628838	46.70328877	1	PRJNA827465;Yang2025_Silkie_Wenchang	CRA004775	0.830643015
ENSGALG00010002202	SFXN4	2	-0.226598735	0.151019575	-1.500459159	0.133495453	0	0.429761413	0	1	CRA004775;Yang2025_Silkie_Wenchang	PRJNA827465	0.612368324
ENSGALG00010002202	SFXN4	2	-0.025050593	0.077461148	-0.323395583	0.746395674	0	0.039578303	0	1	CRA004775;PRJNA827465	Yang2025_Silkie_Wenchang	0.936640779
ENSGALG00010002206	DPH3P1	2	0.095457229	0.159216877	0.599542154	0.548811408	0.033936563	2.805701232	64.35828631	0.5	PRJNA827465;Yang2025_Silkie_Wenchang	CRA004775	0.878281266
ENSGALG00010002206	DPH3P1	2	0.179722876	0.163725144	1.097710907	0.272330745	0.009757943	1.184801803	15.59769765	0.5	CRA004775;Yang2025_Silkie_Wenchang	PRJNA827465	0.773337076
ENSGALG00010002206	DPH3P1	2	-0.037934965	0.087212884	-0.434969729	0.6635844	0	0.009887909	0	1	CRA004775;PRJNA827465	Yang2025_Silkie_Wenchang	0.913119244
ENSGALG00010002211	ACER3	2	-0.105851304	0.26233052	-0.403503578	0.686577803	0.128066641	14.11522625	92.91545185	0.5	PRJNA827465;Yang2025_Silkie_Wenchang	CRA004775	0.919235539
ENSGALG00010002211	ACER3	2	-0.364526774	0.115450351	-3.157433225	0.001591647	0	0.115212413	0	1	CRA004775;Yang2025_Silkie_Wenchang	PRJNA827465	0.050489505
ENSGALG00010002211	ACER3	2	0.093090079	0.136260678	0.683176398	0.494495385	0.016889151	1.26814814	21.14485932	0.5	CRA004775;PRJNA827465	Yang2025_Silkie_Wenchang	0.894190852
ENSGALG00010002215	YLEC18	2	-0.539237033	0.290218064	-1.858040902	0.063163206	0.008876969	1.05535436	5.245096976	1	PRJNA827465;Yang2025_Silkie_Wenchang	CRA004775	0.432793495
ENSGALG00010002215	YLEC18	2	-0.700495757	0.354852236	-1.974049155	0.048376154	0	0.485869457	0	1	CRA004775;Yang2025_Silkie_Wenchang	PRJNA827465	0.380196722
ENSGALG00010002215	YLEC18	2	-0.273762257	0.336230779	-0.814209389	0.415525008	0	0.000860606	0	1	CRA004775;PRJNA827465	Yang2025_Silkie_Wenchang	0.837678466
ENSGALG00010002225	PRDX3	2	-0.425267246	0.215152696	-1.9765834	0.04808874	0.074835566	4.583671158	78.18342622	1	PRJNA827465;Yang2025_Silkie_Wenchang	CRA004775	0.379219854
ENSGALG00010002225	PRDX3	2	-0.388444971	0.352886255	-1.100765375	0.270998785	0.173456909	3.074276788	67.47202451	0.5	CRA004775;Yang2025_Silkie_Wenchang	PRJNA827465	0.772743863
ENSGALG00010002225	PRDX3	2	-0.238606979	0.058821703	-4.05644459	4.98254E-05	0	0.589244061	0	0.5	CRA004775;PRJNA827465	Yang2025_Silkie_Wenchang	0.004835276
ENSGALG00010002226	OXNAD1	2	0.188158473	0.095314503	1.974080194	0.048372625	0.000273564	1.101208138	1.108390788	1	PRJNA827465;Yang2025_Silkie_Wenchang	CRA004775	0.380196722
ENSGALG00010002226	OXNAD1	2	-0.080810295	0.146631235	-0.551112421	0.581556611	0	0.652273395	0	0.5	CRA004775;Yang2025_Silkie_Wenchang	PRJNA827465	0.889045686
ENSGALG00010002226	OXNAD1	2	0.053807003	0.227075176	0.236956781	0.81269032	0.075319976	3.395102289	70.54580643	0.5	CRA004775;PRJNA827465	Yang2025_Silkie_Wenchang	0.95348724
ENSGALG00010002231	TMEM14A	2	-0.374047536	0.363302733	-1.029575344	0.303209395	0.254017129	26.00365512	96.15438678	1	PRJNA827465;Yang2025_Silkie_Wenchang	CRA004775	0.792084776
ENSGALG00010002231	TMEM14A	2	-0.565098737	0.241120777	-2.343635525	0.019096925	0.073989578	2.433857755	58.91129645	1	CRA004775;Yang2025_Silkie_Wenchang	PRJNA827465	0.231442015
ENSGALG00010002231	TMEM14A	2	-0.027991619	0.053107774	-0.527071979	0.598143604	0	0.574173219	0	1	CRA004775;PRJNA827465	Yang2025_Silkie_Wenchang	0.894645663
ENSGALG00010002235	MXRA5	2	-0.142058895	0.137676807	-1.031828805	0.302152323	0	0.140162138	0	1	PRJNA827465;Yang2025_Silkie_Wenchang	CRA004775	0.791603969
ENSGALG00010002235	MXRA5	2	0.010694433	0.252428908	0.042366119	0.966206837	0	0.223296417	0	0.5	CRA004775;Yang2025_Silkie_Wenchang	PRJNA827465	0.990863074
ENSGALG00010002235	MXRA5	2	-0.140111594	0.151714323	-0.92352252	0.35573497	0	0.515588151	0	0.5	CRA004775;PRJNA827465	Yang2025_Silkie_Wenchang	0.812301302
ENSGALG00010002238	ENSGALG00010002238	2	0.332097675	0.324462062	1.023533147	0.306055836	0	0.648652959	0	1	PRJNA827465;Yang2025_Silkie_Wenchang	CRA004775	0.792366985
ENSGALG00010002238	ENSGALG00010002238	2	-0.12482614	0.88087049	-0.141707711	0.887310884	1.150116758	3.838243613	73.94641662	0.5	CRA004775;Yang2025_Silkie_Wenchang	PRJNA827465	0.972987228
ENSGALG00010002238	ENSGALG00010002238	2	-0.312262068	0.585793474	-0.533056298	0.59399923	0.40626728	2.283771195	56.27277638	0.5	CRA004775;PRJNA827465	Yang2025_Silkie_Wenchang	0.893224663
ENSGALG00010002239	AGPAT5	2	-0.232870097	0.059987677	-3.881965596	0.000103616	0	0.805004488	0	1	PRJNA827465;Yang2025_Silkie_Wenchang	CRA004775	0.008000789
ENSGALG00010002239	AGPAT5	2	-0.012110094	0.340377363	-0.035578436	0.971618503	0.19722869	6.333774058	84.211625	0.5	CRA004775;Yang2025_Silkie_Wenchang	PRJNA827465	0.991955348
ENSGALG00010002239	AGPAT5	2	0.033368083	0.277907581	0.120068992	0.904428495	0.126336301	4.820161631	79.25380772	0.5	CRA004775;PRJNA827465	Yang2025_Silkie_Wenchang	0.977606588
ENSGALG00010002243	ARSH	2	0.367624328	0.119336842	3.080560219	0.002066116	0	0.83871667	0	1	PRJNA827465;Yang2025_Silkie_Wenchang	CRA004775	0.059057851
ENSGALG00010002243	ARSH	2	0.140059153	0.416370507	0.336381062	0.736583529	0.26135662	3.663976538	72.70724881	0.5	CRA004775;Yang2025_Silkie_Wenchang	PRJNA827465	0.933688397
ENSGALG00010002243	ARSH	2	0.061269475	0.29436329	0.208142379	0.835117798	0.101469722	2.054096127	51.31678665	0.5	CRA004775;PRJNA827465	Yang2025_Silkie_Wenchang	0.958269147
ENSGALG00010002247	RFTN1	2	-0.352081946	0.183566019	-1.918012654	0.055109407	0.053323714	4.785934551	79.1054393	1	PRJNA827465;Yang2025_Silkie_Wenchang	CRA004775	0.406379567
ENSGALG00010002247	RFTN1	2	-0.510463465	0.117002459	-4.362843895	1.28383E-05	0	0.553341132	0	1	CRA004775;Yang2025_Silkie_Wenchang	PRJNA827465	0.00172802
ENSGALG00010002247	RFTN1	2	-0.176895108	0.109509404	-1.615341702	0.106236717	0	0.029475885	0	1	CRA004775;PRJNA827465	Yang2025_Silkie_Wenchang	0.553035988
ENSGALG00010002251	GRK5	2	0.008312765	0.129095235	0.064392504	0.948657699	0	0.190594095	0	0.5	PRJNA827465;Yang2025_Silkie_Wenchang	CRA004775	0.986942018
ENSGALG00010002251	GRK5	2	0.095274731	0.182530913	0.521964905	0.601694766	0	0.067155913	0	1	CRA004775;Yang2025_Silkie_Wenchang	PRJNA827465	0.895230519
ENSGALG00010002251	GRK5	2	-0.010049268	0.158829581	-0.06327076	0.949550899	0	0.250193727	0	0.5	CRA004775;PRJNA827465	Yang2025_Silkie_Wenchang	0.98723759
ENSGALG00010002253	ENSGALG00010002253	2	0.274998287	0.162863252	1.688522638	0.091310947	0	0.019116496	0	1	PRJNA827465;Yang2025_Silkie_Wenchang	CRA004775	0.518013013
ENSGALG00010002253	ENSGALG00010002253	2	0.285443901	0.199408879	1.431450304	0.1523012	0	0.012330617	0	1	CRA004775;Yang2025_Silkie_Wenchang	PRJNA827465	0.643791086
ENSGALG00010002253	ENSGALG00010002253	2	0.245757808	0.225015544	1.092181469	0.274753346	0	0.000925037	0	1	CRA004775;PRJNA827465	Yang2025_Silkie_Wenchang	0.774706274
ENSGALG00010002254	ENSGALG00010002254	2	-0.240572374	0.852584246	-0.282168449	0.77781435	1.199884195	5.723838528	82.52920667	0.5	PRJNA827465;Yang2025_Silkie_Wenchang	CRA004775	0.944406469
ENSGALG00010002254	ENSGALG00010002254	2	-0.512071629	0.582058829	-0.879759234	0.378989753	0.426176592	2.694351645	62.88531967	0.5	CRA004775;Yang2025_Silkie_Wenchang	PRJNA827465	0.823912132
ENSGALG00010002254	ENSGALG00010002254	2	0.34571874	0.362615895	0.953402057	0.340386399	0	0.556422207	0	1	CRA004775;PRJNA827465	Yang2025_Silkie_Wenchang	0.805432415
ENSGALG00010002261	NR0B1	2	-0.608927682	0.332450982	-1.831631473	0.067006344	0	0.049136003	0	1	PRJNA827465;Yang2025_Silkie_Wenchang	CRA004775	0.445965231
ENSGALG00010002261	NR0B1	2	0.483719852	1.236642673	0.391155717	0.695682138	2.654973382	7.473233291	86.61891097	0.5	CRA004775;Yang2025_Silkie_Wenchang	PRJNA827465	0.921038437
ENSGALG00010002261	NR0B1	2	0.537583751	1.160461961	0.463249783	0.643185336	2.335733397	7.315335832	86.3300876	0.5	CRA004775;PRJNA827465	Yang2025_Silkie_Wenchang	0.906634523
ENSGALG00010002269	MCPH1	2	0.006796262	0.081427507	0.083463954	0.933482638	0	0.67638186	0	0.5	PRJNA827465;Yang2025_Silkie_Wenchang	CRA004775	0.984260547
ENSGALG00010002269	MCPH1	2	-0.004474978	0.247180116	-0.018104117	0.985555793	0.052276952	1.300784784	23.12333201	0.5	CRA004775;Yang2025_Silkie_Wenchang	PRJNA827465	0.995939127
ENSGALG00010002269	MCPH1	2	-0.054798496	0.097488511	-0.562102096	0.574046461	0	0.831327742	0	1	CRA004775;PRJNA827465	Yang2025_Silkie_Wenchang	0.886081433
ENSGALG00010002270	IMMP2L	2	-0.549365509	0.170740146	-3.21755324	0.00129289	0	0.653560502	0	1	PRJNA827465;Yang2025_Silkie_Wenchang	CRA004775	0.043633159
ENSGALG00010002270	IMMP2L	2	-0.420924631	0.364550693	-1.154639501	0.248238084	0.150740885	2.290967237	56.35031422	1	CRA004775;Yang2025_Silkie_Wenchang	PRJNA827465	0.758364066
ENSGALG00010002270	IMMP2L	2	-0.353221221	0.18498251	-1.909484417	0.05619963	0.003162531	1.03510886	3.391803623	1	CRA004775;PRJNA827465	Yang2025_Silkie_Wenchang	0.410231561
ENSGALG00010002274	ENSGALG00010002274	2	1.505359972	0.756273885	1.990495776	0.046536349	0	0.440689832	0	1	PRJNA827465;Yang2025_Silkie_Wenchang	CRA004775	0.372346812
ENSGALG00010002274	ENSGALG00010002274	2	0.565858235	1.372699169	0.412223047	0.680175946	2.878322692	3.977776528	74.8603273	0.5	CRA004775;Yang2025_Silkie_Wenchang	PRJNA827465	0.917743664
ENSGALG00010002274	ENSGALG00010002274	2	0.101041079	0.857345365	0.117853415	0.906183805	0.837748838	2.244596366	55.44856014	0.5	CRA004775;PRJNA827465	Yang2025_Silkie_Wenchang	0.978196186
ENSGALG00010002277	RGS10	2	-0.237611304	0.135623277	-1.751995004	0.079774667	0.027849643	4.095832986	75.58494198	1	PRJNA827465;Yang2025_Silkie_Wenchang	CRA004775	0.485804731
ENSGALG00010002277	RGS10	2	-0.385128972	0.098253344	-3.919754327	8.86393E-05	0	0.050757856	0	1	CRA004775;Yang2025_Silkie_Wenchang	PRJNA827465	0.007095294
ENSGALG00010002277	RGS10	2	-0.127371931	0.084291663	-1.511085743	0.130766607	0	0.945895015	0	1	CRA004775;PRJNA827465	Yang2025_Silkie_Wenchang	0.606504595
ENSGALG00010002280	ST7	2	0.1095838	0.260375493	0.420868334	0.67385123	0.111486361	5.613774262	82.1866724	0.5	PRJNA827465;Yang2025_Silkie_Wenchang	CRA004775	0.915590641
ENSGALG00010002280	ST7	2	0.209864069	0.234606681	0.894535772	0.371035299	0.055462126	1.809382932	44.73253935	0.5	CRA004775;Yang2025_Silkie_Wenchang	PRJNA827465	0.8214684
ENSGALG00010002280	ST7	2	-0.141683152	0.133656087	-1.060057604	0.289118394	0	0.003990733	0	1	CRA004775;PRJNA827465	Yang2025_Silkie_Wenchang	0.783530627
ENSGALG00010002281	TGFBR2	2	-0.114435036	0.064656215	-1.769900014	0.076743799	0	0.368752824	0	1	PRJNA827465;Yang2025_Silkie_Wenchang	CRA004775	0.477904868
ENSGALG00010002281	TGFBR2	2	-0.063938806	0.124609003	-0.513115459	0.607870559	0	0.171172395	0	1	CRA004775;Yang2025_Silkie_Wenchang	PRJNA827465	0.897203929
ENSGALG00010002281	TGFBR2	2	-0.13841678	0.07183468	-1.92687961	0.053994635	0	0.024479301	0	1	CRA004775;PRJNA827465	Yang2025_Silkie_Wenchang	0.402825653
ENSGALG00010002282	TSKU	2	-0.066560956	0.173472364	-0.383697754	0.701202478	0.055646339	13.25663336	92.45660664	0.5	PRJNA827465;Yang2025_Silkie_Wenchang	CRA004775	0.9226126
ENSGALG00010002282	TSKU	2	-0.238610155	0.068437237	-3.486554479	0.000489286	0	0.040768482	0	1	CRA004775;Yang2025_Silkie_Wenchang	PRJNA827465	0.023197101
ENSGALG00010002282	TSKU	2	0.100626677	0.064718676	1.554832132	0.119986054	0	0.332364894	0	0.5	CRA004775;PRJNA827465	Yang2025_Silkie_Wenchang	0.580979659
ENSGALG00010002285	CAV1	2	-0.264482192	0.104137157	-2.539748542	0.011093219	0.013859013	2.763174537	63.80974178	1	PRJNA827465;Yang2025_Silkie_Wenchang	CRA004775	0.167433488
ENSGALG00010002285	CAV1	2	-0.013393411	0.415074244	-0.032267508	0.974258721	0.292629152	5.942912395	83.17323337	0.5	CRA004775;Yang2025_Silkie_Wenchang	PRJNA827465	0.992492038
ENSGALG00010002285	CAV1	2	0.069616624	0.304511061	0.228617719	0.819166052	0.140326564	3.403175599	70.61568024	0.5	CRA004775;PRJNA827465	Yang2025_Silkie_Wenchang	0.955256039
ENSGALG00010002288	ANGPT2	2	0.027402519	0.224173305	0.122238101	0.902710453	0.081533878	5.056224024	80.2223953	0.5	PRJNA827465;Yang2025_Silkie_Wenchang	CRA004775	0.97693171
ENSGALG00010002288	ANGPT2	2	0.192833423	0.172369095	1.118723879	0.263257955	0.005917779	1.065576317	6.154070435	0.5	CRA004775;Yang2025_Silkie_Wenchang	PRJNA827465	0.768643821
ENSGALG00010002288	ANGPT2	2	-0.173554409	0.094027878	-1.845776087	0.064924724	0	0.000952659	0	1	CRA004775;PRJNA827465	Yang2025_Silkie_Wenchang	0.439424217
ENSGALG00010002289	ENSGALG00010002289	2	0.771300421	0.665864923	1.158343674	0.246723815	0.79495842	9.173782938	89.09937147	1	PRJNA827465;Yang2025_Silkie_Wenchang	CRA004775	0.757157859
ENSGALG00010002289	ENSGALG00010002289	2	1.06232856	0.363309168	2.924034552	0.003455264	0.149088942	2.219151738	54.93773666	1	CRA004775;Yang2025_Silkie_Wenchang	PRJNA827465	0.083008703
ENSGALG00010002289	ENSGALG00010002289	2	0.413423651	0.296624855	1.393759306	0.1633903	0.126992422	3.42020823	70.7620141	1	CRA004775;PRJNA827465	Yang2025_Silkie_Wenchang	0.661692837
ENSGALG00010002291	RNASEH1	2	0.055586766	0.185006621	0.300458253	0.763827636	0.05985423	7.675082932	86.97082483	0.5	PRJNA827465;Yang2025_Silkie_Wenchang	CRA004775	0.940361611
ENSGALG00010002291	RNASEH1	2	0.10315925	0.179765644	0.573854091	0.566066542	0.041268795	2.632554985	62.01408875	0.5	CRA004775;Yang2025_Silkie_Wenchang	PRJNA827465	0.883487517
ENSGALG00010002291	RNASEH1	2	-0.115781553	0.059686794	-1.939818609	0.052401738	0	0.000942527	0	1	CRA004775;PRJNA827465	Yang2025_Silkie_Wenchang	0.397401935
ENSGALG00010002293	GYG2	2	-0.364013673	0.233317128	-1.560166949	0.118720434	0.060668862	2.163564919	53.7799864	1	PRJNA827465;Yang2025_Silkie_Wenchang	CRA004775	0.578883823
ENSGALG00010002293	GYG2	2	-0.43677956	0.309589193	-1.410835938	0.158292995	0.066353231	1.424217529	29.78600672	0.5	CRA004775;Yang2025_Silkie_Wenchang	PRJNA827465	0.652026484
ENSGALG00010002293	GYG2	2	-0.148782538	0.16584203	-0.897134084	0.369647373	0	0.136882507	0	0.5	CRA004775;PRJNA827465	Yang2025_Silkie_Wenchang	0.820606801
ENSGALG00010002294	DAZL	2	-0.99784793	0.503256881	-1.982780501	0.047391953	0	0.043642724	0	1	PRJNA827465;Yang2025_Silkie_Wenchang	CRA004775	0.375988278
ENSGALG00010002294	DAZL	2	0.374286756	1.373066507	0.272591862	0.785166968	2.686895902	3.379477272	70.40962494	0.5	CRA004775;Yang2025_Silkie_Wenchang	PRJNA827465	0.946325593
ENSGALG00010002294	DAZL	2	0.221956021	1.478569252	0.150115404	0.880673567	3.477727536	4.587794912	78.20303612	0.5	CRA004775;PRJNA827465	Yang2025_Silkie_Wenchang	0.971623434
ENSGALG00010002295	YAF2	2	0.05406317	0.164759892	0.328133078	0.742811045	0.044008521	5.145030398	80.56376887	0.5	PRJNA827465;Yang2025_Silkie_Wenchang	CRA004775	0.935661872
ENSGALG00010002295	YAF2	2	0.205613475	0.104142266	1.97435184	0.04834175	0	0.17551578	0	1	CRA004775;Yang2025_Silkie_Wenchang	PRJNA827465	0.38014722
ENSGALG00010002295	YAF2	2	-0.054321774	0.09102188	-0.596799074	0.550641536	0.003871796	1.16731045	14.33298654	0.5	CRA004775;PRJNA827465	Yang2025_Silkie_Wenchang	0.878857433
ENSGALG00010002308	GXYLT1	2	-0.349074497	0.319679962	-1.091949882	0.274855131	0.194434375	20.32376006	95.07965063	1	PRJNA827465;Yang2025_Silkie_Wenchang	CRA004775	0.774719194
ENSGALG00010002308	GXYLT1	2	-0.403673371	0.317862573	-1.269961946	0.204098185	0.16054535	4.462115416	77.58910502	1	CRA004775;Yang2025_Silkie_Wenchang	PRJNA827465	0.717254253
ENSGALG00010002308	GXYLT1	2	-0.036338604	0.071138099	-0.510817754	0.609478676	0	0.000170746	0	1	CRA004775;PRJNA827465	Yang2025_Silkie_Wenchang	0.897238578
ENSGALG00010002309	GADL1	2	-0.049923869	0.174603303	-0.285927402	0.774933726	0.026961284	1.771419701	43.54810443	0.5	PRJNA827465;Yang2025_Silkie_Wenchang	CRA004775	0.943736877
ENSGALG00010002309	GADL1	2	-0.240964486	0.199411397	-1.208378706	0.226901627	0	0.015356493	0	1	CRA004775;Yang2025_Silkie_Wenchang	PRJNA827465	0.740444589
ENSGALG00010002309	GADL1	2	0.093405386	0.161680905	0.577714392	0.563456961	0	0.101089491	0	0.5	CRA004775;PRJNA827465	Yang2025_Silkie_Wenchang	0.882407972
ENSGALG00010002310	PRRG1	2	-0.068435574	0.233995029	-0.29246593	0.769930406	0.099894721	11.19402401	91.06666201	0.5	PRJNA827465;Yang2025_Silkie_Wenchang	CRA004775	0.941995757
ENSGALG00010002310	PRRG1	2	0.088049325	0.417528951	0.210881963	0.832979379	0.320278747	12.0446442	91.69755467	0.5	CRA004775;Yang2025_Silkie_Wenchang	PRJNA827465	0.957916437
ENSGALG00010002310	PRRG1	2	0.287544154	0.176114379	1.632712535	0.10252947	0.043021024	2.757904344	63.74058432	1	CRA004775;PRJNA827465	Yang2025_Silkie_Wenchang	0.545514519
ENSGALG00010002312	ENSGALG00010002312	2	-0.096039377	0.102312147	-0.938689878	0.347889992	0	0.730152573	0	1	PRJNA827465;Yang2025_Silkie_Wenchang	CRA004775	0.808847403
ENSGALG00010002312	ENSGALG00010002312	2	0.109812717	0.354188914	0.310039962	0.756530567	0.195845132	4.275624823	76.61160552	0.5	CRA004775;Yang2025_Silkie_Wenchang	PRJNA827465	0.938659623
ENSGALG00010002312	ENSGALG00010002312	2	0.175884754	0.259318327	0.678258094	0.497608064	0.088334202	2.569207354	61.07748959	0.5	CRA004775;PRJNA827465	Yang2025_Silkie_Wenchang	0.86098656
ENSGALG00010002315	ENSGALG00010002315	2	-0.061743322	0.160875103	-0.383796629	0.701129187	0.033064787	2.575751597	61.17638047	0.5	PRJNA827465;Yang2025_Silkie_Wenchang	CRA004775	0.922547052
ENSGALG00010002315	ENSGALG00010002315	2	-0.061027308	0.239054981	-0.255285656	0.798502478	0.064615618	2.205887333	54.666769	0.5	CRA004775;Yang2025_Silkie_Wenchang	PRJNA827465	0.949922217
ENSGALG00010002315	ENSGALG00010002315	2	0.087121306	0.093381921	0.932956885	0.35084223	0	0.291801145	0	1	CRA004775;PRJNA827465	Yang2025_Silkie_Wenchang	0.810146221
ENSGALG00010002328	ENSGALG00010002328	2	-0.060145117	0.211433495	-0.284463523	0.776055184	0	0.63857008	0	0.5	PRJNA827465;Yang2025_Silkie_Wenchang	CRA004775	0.943991115
ENSGALG00010002328	ENSGALG00010002328	2	0.010432852	0.31531301	0.033087288	0.97360498	0.000242209	1.000746042	0.074548555	0.5	CRA004775;Yang2025_Silkie_Wenchang	PRJNA827465	0.992442268
ENSGALG00010002328	ENSGALG00010002328	2	-0.24209282	0.249307791	-0.971059984	0.331518409	0	0.34689964	0	1	CRA004775;PRJNA827465	Yang2025_Silkie_Wenchang	0.801798188
ENSGALG00010002329	STT3B	2	-0.061174854	0.077341617	-0.790969412	0.428961841	0.009205356	4.005654236	75.03528909	0.5	PRJNA827465;Yang2025_Silkie_Wenchang	CRA004775	0.841898328
ENSGALG00010002329	STT3B	2	-0.081084929	0.093872076	-0.863781142	0.387708129	0.008821888	1.860737253	46.25786104	0.5	CRA004775;Yang2025_Silkie_Wenchang	PRJNA827465	0.828154121
ENSGALG00010002329	STT3B	2	0.007430188	0.032448654	0.228982932	0.818882182	0	0.089862517	0	1	CRA004775;PRJNA827465	Yang2025_Silkie_Wenchang	0.955256039
ENSGALG00010002330	KHDRBS2	2	0.29198418	0.178012793	1.640242675	0.100954719	0	0.935809893	0	1	PRJNA827465;Yang2025_Silkie_Wenchang	CRA004775	0.542348734
ENSGALG00010002330	KHDRBS2	2	0.485641827	0.300463344	1.616309734	0.106027363	0	0.396352767	0	0.5	CRA004775;Yang2025_Silkie_Wenchang	PRJNA827465	0.552458367
ENSGALG00010002330	KHDRBS2	2	0.15990299	0.212039648	0.754118355	0.450778157	0	0.092673513	0	0.5	CRA004775;PRJNA827465	Yang2025_Silkie_Wenchang	0.847631399
ENSGALG00010002333	ENSGALG00010002333	2	0.15908866	0.527270703	0.301721031	0.762864734	0	0.612830472	0	0.5	PRJNA827465;Yang2025_Silkie_Wenchang	CRA004775	0.940082621
ENSGALG00010002333	ENSGALG00010002333	2	-0.027903161	0.619072078	-0.045072557	0.964049476	0.173305269	1.28975674	22.46599927	0.5	CRA004775;Yang2025_Silkie_Wenchang	PRJNA827465	0.990214803
ENSGALG00010002333	ENSGALG00010002333	2	-0.385120386	0.503490708	-0.764900682	0.444330688	0	0.162219859	0	1	CRA004775;PRJNA827465	Yang2025_Silkie_Wenchang	0.846013379
ENSGALG00010002334	OSBPL10	2	-0.126500121	0.069790499	-1.812569381	0.069898264	0	0.125331913	0	1	PRJNA827465;Yang2025_Silkie_Wenchang	CRA004775	0.455893265
ENSGALG00010002334	OSBPL10	2	-0.054597954	0.146724177	-0.372112863	0.70980882	0	0.062364023	0	0.5	CRA004775;Yang2025_Silkie_Wenchang	PRJNA827465	0.925298042
ENSGALG00010002334	OSBPL10	2	-0.129560878	0.074866182	-1.730566123	0.083529178	0	0.220284092	0	0.5	CRA004775;PRJNA827465	Yang2025_Silkie_Wenchang	0.497929561
ENSGALG00010002335	TMEM47	2	-0.176352133	0.106420947	-1.65711863	0.097495493	0	0.077149103	0	1	PRJNA827465;Yang2025_Silkie_Wenchang	CRA004775	0.533219726
ENSGALG00010002335	TMEM47	2	0.184529213	0.434339896	0.424849788	0.67094618	0.318413503	6.076603439	83.5434382	0.5	CRA004775;Yang2025_Silkie_Wenchang	PRJNA827465	0.914552186
ENSGALG00010002335	TMEM47	2	0.208870518	0.403925868	0.517101117	0.605085574	0.270127819	5.428171104	81.57758883	0.5	CRA004775;PRJNA827465	Yang2025_Silkie_Wenchang	0.896474603
ENSGALG00010002336	PRIM2	2	-0.284747143	0.244522452	-1.164503057	0.244220205	0.106516967	8.961292748	88.84089575	1	PRJNA827465;Yang2025_Silkie_Wenchang	CRA004775	0.755675227
ENSGALG00010002336	PRIM2	2	-0.508982836	0.12974824	-3.92284963	8.75078E-05	0	0.409794389	0	1	CRA004775;Yang2025_Silkie_Wenchang	PRJNA827465	0.00703337
ENSGALG00010002336	PRIM2	2	-0.066286704	0.082708783	-0.801446975	0.422872929	0	0.474950702	0	1	CRA004775;PRJNA827465	Yang2025_Silkie_Wenchang	0.839988514
ENSGALG00010002338	CD99	2	0.043234646	0.0943798	0.458092156	0.646886234	0.009782001	2.206391705	54.67713201	0.5	PRJNA827465;Yang2025_Silkie_Wenchang	CRA004775	0.907350807
ENSGALG00010002338	CD99	2	0.151426878	0.090086663	1.680902278	0.092781897	0	0.048391543	0	1	CRA004775;Yang2025_Silkie_Wenchang	PRJNA827465	0.52167685
ENSGALG00010002338	CD99	2	-0.021204366	0.079233892	-0.267617374	0.788993856	0	0.860864291	0	0.5	CRA004775;PRJNA827465	Yang2025_Silkie_Wenchang	0.94717823
ENSGALG00010002339	ZNFY4	2	0.189912032	0.742794834	0.255672257	0.798203919	0.770595556	3.314518437	69.82970471	0.5	PRJNA827465;Yang2025_Silkie_Wenchang	CRA004775	0.949922217
ENSGALG00010002339	ZNFY4	2	0.247274765	0.616972615	0.400787262	0.688576758	0.517020841	3.036060088	67.06257547	0.5	CRA004775;Yang2025_Silkie_Wenchang	PRJNA827465	0.919798626
ENSGALG00010002339	ZNFY4	2	-0.387050689	0.332730449	-1.163255991	0.244725656	0	0.120542688	0	1	CRA004775;PRJNA827465	Yang2025_Silkie_Wenchang	0.756114592
ENSGALG00010002340	RAB23	2	-0.008074575	0.155501302	-0.051926095	0.958587582	0.040190503	5.538853656	81.94572267	0.5	PRJNA827465;Yang2025_Silkie_Wenchang	CRA004775	0.989070013
ENSGALG00010002340	RAB23	2	-0.17247411	0.110866335	-1.555694169	0.119780834	0	0.048522216	0	1	CRA004775;Yang2025_Silkie_Wenchang	PRJNA827465	0.580639051
ENSGALG00010002340	RAB23	2	0.120524314	0.054267323	2.220937177	0.026355217	0	0.765641828	0	0.5	CRA004775;PRJNA827465	Yang2025_Silkie_Wenchang	0.276862168
ENSGALG00010002341	FAM49A	2	0.068058423	0.06654723	1.022708577	0.306445655	0	0.171646063	0	1	PRJNA827465;Yang2025_Silkie_Wenchang	CRA004775	0.792485409
ENSGALG00010002341	FAM49A	2	0.139808191	0.135005154	1.035576687	0.300399673	0	0.10739722	0	1	CRA004775;Yang2025_Silkie_Wenchang	PRJNA827465	0.790532865
ENSGALG00010002341	FAM49A	2	0.062050386	0.073215	0.847509191	0.396711366	0	0.283012295	0	1	CRA004775;PRJNA827465	Yang2025_Silkie_Wenchang	0.831375233
ENSGALG00010002342	BAG2	2	-0.160494999	0.073115379	-2.195092223	0.028156983	0	0.017650642	0	1	PRJNA827465;Yang2025_Silkie_Wenchang	CRA004775	0.285809376
ENSGALG00010002342	BAG2	2	-0.045793321	0.197201117	-0.232216335	0.816369991	0.026701852	1.277615859	21.72921204	0.5	CRA004775;Yang2025_Silkie_Wenchang	PRJNA827465	0.954546835
ENSGALG00010002342	BAG2	2	-0.064659122	0.20559499	-0.314497556	0.753143173	0.045458062	1.511556124	33.84301222	0.5	CRA004775;PRJNA827465	Yang2025_Silkie_Wenchang	0.938087082
ENSGALG00010002343	GPD1L	2	-0.209893924	0.279256625	-0.751616631	0.452281639	0.14361017	12.07763467	91.72023308	0.5	PRJNA827465;Yang2025_Silkie_Wenchang	CRA004775	0.848321192
ENSGALG00010002343	GPD1L	2	-0.50025606	0.137095266	-3.648966692	0.000263297	0	0.011967382	0	1	CRA004775;Yang2025_Silkie_Wenchang	PRJNA827465	0.015514799
ENSGALG00010002343	GPD1L	2	-0.105484959	0.239106428	-0.441163211	0.659094845	0.078905859	2.403446587	58.39308411	0.5	CRA004775;PRJNA827465	Yang2025_Silkie_Wenchang	0.911449691
ENSGALG00010002345	SCML2	2	-0.256416069	0.093946319	-2.729389203	0.006345177	0	0.031494892	0	1	PRJNA827465;Yang2025_Silkie_Wenchang	CRA004775	0.121060428
ENSGALG00010002345	SCML2	2	-0.1371982	0.200564064	-0.684061724	0.49393619	0.036444135	1.668593374	40.06928136	0.5	CRA004775;Yang2025_Silkie_Wenchang	PRJNA827465	0.860175061
ENSGALG00010002345	SCML2	2	-0.138462913	0.17364405	-0.797395087	0.425221612	0.027094734	1.544562461	35.25674583	0.5	CRA004775;PRJNA827465	Yang2025_Silkie_Wenchang	0.84125685
ENSGALG00010002346	FBXO15	2	-0.805552495	0.513368445	-1.569150778	0.11661281	0.417260627	4.617005554	78.34094007	1	PRJNA827465;Yang2025_Silkie_Wenchang	CRA004775	0.57477217
ENSGALG00010002346	FBXO15	2	-1.412533693	0.351962089	-4.01331205	5.98727E-05	0	0.032168056	0	1	CRA004775;Yang2025_Silkie_Wenchang	PRJNA827465	0.005459805
ENSGALG00010002346	FBXO15	2	-0.759675757	0.562157421	-1.351357696	0.176580878	0.429312882	2.649707965	62.25999193	1	CRA004775;PRJNA827465	Yang2025_Silkie_Wenchang	0.682016071
ENSGALG00010002347	ZNF451	2	-0.086819628	0.098489797	-0.881508856	0.378042463	0.015052311	4.335195749	76.93299085	0.5	PRJNA827465;Yang2025_Silkie_Wenchang	CRA004775	0.82325159
ENSGALG00010002347	ZNF451	2	-0.045381834	0.20709719	-0.219133025	0.826546426	0.058908205	2.621197397	61.84949668	0.5	CRA004775;Yang2025_Silkie_Wenchang	PRJNA827465	0.956393839
ENSGALG00010002347	ZNF451	2	0.012388999	0.051084167	0.242521312	0.808376245	0	0.824369638	0	1	CRA004775;PRJNA827465	Yang2025_Silkie_Wenchang	0.952496413
ENSGALG00010002348	THAP12	2	0.003971453	0.050333424	0.078902897	0.937109859	0	0.517556337	0	0.5	PRJNA827465;Yang2025_Silkie_Wenchang	CRA004775	0.985108291
ENSGALG00010002348	THAP12	2	-0.030905532	0.073519924	-0.420369476	0.674215563	0	0.094008592	0	0.5	CRA004775;Yang2025_Silkie_Wenchang	PRJNA827465	0.915800735
ENSGALG00010002348	THAP12	2	0.034528048	0.063224062	0.546121956	0.584982109	0	2.65975E-05	0	1	CRA004775;PRJNA827465	Yang2025_Silkie_Wenchang	0.891072617
ENSGALG00010002349	ZBED1	2	-0.197791481	0.189187818	-1.045476833	0.295802643	0.067043087	15.55451193	93.57099725	1	PRJNA827465;Yang2025_Silkie_Wenchang	CRA004775	0.787878237
ENSGALG00010002349	ZBED1	2	-0.121297292	0.309057277	-0.39247512	0.694707191	0.162450212	6.106872234	83.62500538	0.5	CRA004775;Yang2025_Silkie_Wenchang	PRJNA827465	0.92082578
ENSGALG00010002349	ZBED1	2	-0.002993432	0.051235127	-0.058425388	0.953409793	0.000309238	1.010431041	1.032335772	0.5	CRA004775;PRJNA827465	Yang2025_Silkie_Wenchang	0.987758913
ENSGALG00010002350	BEND6	2	0.470359809	0.10120096	4.647780108	3.35526E-06	0	0.234096297	0	1	PRJNA827465;Yang2025_Silkie_Wenchang	CRA004775	0.000622037
ENSGALG00010002350	BEND6	2	0.679297416	0.190825684	3.55977981	0.000371166	0.020436985	1.330997509	24.86837931	1	CRA004775;Yang2025_Silkie_Wenchang	PRJNA827465	0.01934739
ENSGALG00010002350	BEND6	2	0.621268246	0.245443149	2.531210382	0.011366965	0.079094255	2.519310775	60.3066041	1	CRA004775;PRJNA827465	Yang2025_Silkie_Wenchang	0.169998406
ENSGALG00010002352	ENSGALG00010002352	2	-0.384266582	0.078383831	-4.902370539	9.4687E-07	0	0.304230595	0	1	PRJNA827465;Yang2025_Silkie_Wenchang	CRA004775	0.000211448
ENSGALG00010002352	ENSGALG00010002352	2	-0.420950543	0.141260697	-2.979955164	0.002882906	0	0.21847834	0	1	CRA004775;Yang2025_Silkie_Wenchang	PRJNA827465	0.073385263
ENSGALG00010002352	ENSGALG00010002352	2	-0.361004118	0.080599272	-4.478999731	7.49936E-06	0	0.008553447	0	1	CRA004775;PRJNA827465	Yang2025_Silkie_Wenchang	0.001108073
ENSGALG00010002353	CMTM8	2	0.097759096	0.108085438	0.904461296	0.365750848	0	0.123042603	0	1	PRJNA827465;Yang2025_Silkie_Wenchang	CRA004775	0.818968857
ENSGALG00010002353	CMTM8	2	0.113060252	0.18055879	0.626168643	0.531204332	0	0.633421737	0	1	CRA004775;Yang2025_Silkie_Wenchang	PRJNA827465	0.872895889
ENSGALG00010002353	CMTM8	2	0.147242175	0.120940873	1.217472403	0.22342453	0	0.419014755	0	1	CRA004775;PRJNA827465	Yang2025_Silkie_Wenchang	0.736744216
ENSGALG00010002355	UFM1	2	-0.301724678	0.388828522	-0.775983914	0.437758484	0.288201349	20.8299966	95.19923109	0.5	PRJNA827465;Yang2025_Silkie_Wenchang	CRA004775	0.843899392
ENSGALG00010002355	UFM1	2	-0.363195091	0.359485825	-1.010318255	0.312342838	0.220542301	6.707796022	85.09197363	0.5	CRA004775;Yang2025_Silkie_Wenchang	PRJNA827465	0.796054997
ENSGALG00010002355	UFM1	2	0.069699435	0.061647445	1.130613518	0.258217796	0	0.059938434	0	1	CRA004775;PRJNA827465	Yang2025_Silkie_Wenchang	0.765480013

ENSGALG00010002356	WNT11	2	-0.349836475	0.35616388	-0.982234568	0.325984301	0.20665099	5.350805566	81.31122524	1	PRJNA827465;Yang2025_Silkie_Wenchang	CRA004775	0.800023788
ENSGALG00010002356	WNT11	2	-0.789784579	0.223482039	-3.533995772	0.000409328	0	0.576577001	0	1	CRA004775;Yang2025_Silkie_Wenchang	PRJNA827465	0.020366982
ENSGALG00010002356	WNT11	2	-0.476266612	0.585846027	-0.812955265	0.416243707	0.52537445	3.677085068	72.80454541	1	CRA004775;PRJNA827465	Yang2025_Silkie_Wenchang	0.837742614
ENSGALG00010002357	RAB31	2	-0.06309128	0.12071291	-0.522655611	0.601213934	0.017011254	2.328737438	57.05827615	0.5	PRJNA827465;Yang2025_Silkie_Wenchang	CRA004775	0.895230519
ENSGALG00010002357	RAB31	2	-0.179058657	0.122841333	-1.457641765	0.144939311	0	0.273448799	0	1	CRA004775;Yang2025_Silkie_Wenchang	PRJNA827465	0.632809348
ENSGALG00010002357	RAB31	2	0.03737012	0.088247189	0.423470937	0.671951699	0	0.020290425	0	0.5	CRA004775;PRJNA827465	Yang2025_Silkie_Wenchang	0.914714609
ENSGALG00010002358	FREM2	2	-0.042554833	0.122991209	-0.345989983	0.729343479	0	0.215017915	0	0.5	PRJNA827465;Yang2025_Silkie_Wenchang	CRA004775	0.9323457
ENSGALG00010002358	FREM2	2	0.094408815	0.343911924	0.274514515	0.783689251	0.12298442	1.687765863	40.75007548	0.5	CRA004775;Yang2025_Silkie_Wenchang	PRJNA827465	0.945815798
ENSGALG00010002358	FREM2	2	0.110570008	0.243496446	0.45409323	0.649761726	0.045866448	1.2666104	21.04912448	1	CRA004775;PRJNA827465	Yang2025_Silkie_Wenchang	0.90820125
ENSGALG00010002359	CMTM6	2	-0.276411729	0.518397785	-0.533203916	0.593892431	0.51576449	24.68659823	95.94921912	0.5	PRJNA827465;Yang2025_Silkie_Wenchang	CRA004775	0.893216744
ENSGALG00010002359	CMTM6	2	-0.283291753	0.548243637	-0.516726022	0.605347428	0.540433688	9.614185355	89.59870272	0.5	CRA004775;Yang2025_Silkie_Wenchang	PRJNA827465	0.896474603
ENSGALG00010002359	CMTM6	2	0.244806764	0.116610926	2.099346714	0.035786348	0	0.033293018	0	1	CRA004775;PRJNA827465	Yang2025_Silkie_Wenchang	0.326412357
ENSGALG00010002360	DHRSX	2	-0.222906614	0.192917363	-1.15545128	0.247905673	0.056208344	3.540696492	71.75696922	1	PRJNA827465;Yang2025_Silkie_Wenchang	CRA004775	0.75810588
ENSGALG00010002360	DHRSX	2	-0.335680564	0.163101289	-2.058111041	0.039579475	0.003718008	1.070937004	6.62326005	1	CRA004775;Yang2025_Silkie_Wenchang	PRJNA827465	0.344081425
ENSGALG00010002360	DHRSX	2	-0.073190133	0.065888537	-1.1108174	0.266646955	0	0.052816008	0	1	CRA004775;PRJNA827465	Yang2025_Silkie_Wenchang	0.770816805
ENSGALG00010002361	ENSGALG00010002361	2	0.18173195	0.143690687	1.264744107	0.205963073	0.035005961	6.322266073	84.18288651	1	PRJNA827465;Yang2025_Silkie_Wenchang	CRA004775	0.718403416
ENSGALG00010002361	ENSGALG00010002361	2	0.307431183	0.091345454	3.365588215	0.000763806	0	0.547521056	0	1	CRA004775;Yang2025_Silkie_Wenchang	PRJNA827465	0.031270711
ENSGALG00010002361	ENSGALG00010002361	2	0.055985224	0.054081903	1.035193288	0.300578652	0	0.223385078	0	1	CRA004775;PRJNA827465	Yang2025_Silkie_Wenchang	0.790579721
ENSGALG00010002362	TRPC4	2	-0.279675553	0.242289777	-1.154301909	0.248376414	0	0.550364412	0	0.5	PRJNA827465;Yang2025_Silkie_Wenchang	CRA004775	0.75856854
ENSGALG00010002362	TRPC4	2	0.639657998	0.680106721	0.940525919	0.346947861	0.664378301	3.509767697	71.50808582	1	CRA004775;Yang2025_Silkie_Wenchang	PRJNA827465	0.80855929
ENSGALG00010002362	TRPC4	2	0.416868514	0.877295336	0.475174661	0.634662486	1.344751408	7.588900761	86.82286102	0.5	CRA004775;PRJNA827465	Yang2025_Silkie_Wenchang	0.903872221
ENSGALG00010002364	VSNL1	2	-0.054857065	0.19009964	-0.288570062	0.772910408	0	0.814242686	0	0.5	PRJNA827465;Yang2025_Silkie_Wenchang	CRA004775	0.943096979
ENSGALG00010002364	VSNL1	2	0.057707147	0.319459268	0.180640078	0.856650096	0	0.880580177	0	0.5	CRA004775;Yang2025_Silkie_Wenchang	PRJNA827465	0.963625561
ENSGALG00010002364	VSNL1	2	-0.190906002	0.20972499	-0.910268262	0.362681052	0	0.203265522	0	1	CRA004775;PRJNA827465	Yang2025_Silkie_Wenchang	0.815941879
ENSGALG00010002365	RAI2	2	0.040393592	0.274750946	0.147018938	0.883117072	0.126659455	5.657097543	82.32509073	0.5	PRJNA827465;Yang2025_Silkie_Wenchang	CRA004775	0.972001076
ENSGALG00010002365	RAI2	2	0.344149686	0.177908674	1.934417689	0.053061793	0	0.008042089	0	1	CRA004775;Yang2025_Silkie_Wenchang	PRJNA827465	0.399631766
ENSGALG00010002365	RAI2	2	-0.020834384	0.247199717	-0.084281586	0.932832552	0.086710797	2.766753858	63.85656074	0.5	CRA004775;PRJNA827465	Yang2025_Silkie_Wenchang	0.984224753
ENSGALG00010002366	ENSGALG00010002366	2	1.082771223	0.971809515	1.114180512	0.265201754	1.83740873	36.43061368	97.25505585	1	PRJNA827465;Yang2025_Silkie_Wenchang	CRA004775	0.769274038
ENSGALG00010002366	ENSGALG00010002366	2	1.427352771	0.641933032	2.223532859	0.026180564	0.743767	10.25208057	90.24558236	1	CRA004775;Yang2025_Silkie_Wenchang	PRJNA827465	0.325012526
ENSGALG00010002366	ENSGALG00010002366	2	0.412452594	0.327146091	1.260759656	0.207395453	0.166249404	4.225819797	76.3359526	1	CRA004775;PRJNA827465	Yang2025_Silkie_Wenchang	0.719377239
ENSGALG00010002368	UVRAG	2	-0.153393133	0.100018865	-1.533642007	0.125117748	0.011285788	2.157666378	53.65363199	1	PRJNA827465;Yang2025_Silkie_Wenchang	CRA004775	0.593686207
ENSGALG00010002368	UVRAG	2	-0.058439418	0.231418108	-0.252527419	0.800633423	0.087631638	5.438553917	81.61275929	0.5	CRA004775;Yang2025_Silkie_Wenchang	PRJNA827465	0.950150676
ENSGALG00010002368	UVRAG	2	0.016959088	0.123219515	0.137633138	0.890530364	0.01826491	2.209958376	54.75027897	0.5	CRA004775;PRJNA827465	Yang2025_Silkie_Wenchang	0.973453342
ENSGALG00010002369	GEMIN8	2	0.165182478	0.143044103	1.15476608	0.248186231	0.029687578	3.599485614	72.21825262	1	PRJNA827465;Yang2025_Silkie_Wenchang	CRA004775	0.758364066
ENSGALG00010002369	GEMIN8	2	0.022606945	0.340160477	0.066459648	0.947011883	0.188885923	5.007399292	80.02955344	0.5	CRA004775;Yang2025_Silkie_Wenchang	PRJNA827465	0.986445196
ENSGALG00010002369	GEMIN8	2	-0.079514884	0.179177668	-0.443776754	0.657201996	0.035750053	1.805939805	44.61655787	0.5	CRA004775;PRJNA827465	Yang2025_Silkie_Wenchang	0.910711339
ENSGALG00010002370	ENSGALG00010002370	2	-0.063241247	0.092657251	-0.68252885	0.494904607	0	0.050768544	0	1	PRJNA827465;Yang2025_Silkie_Wenchang	CRA004775	0.860445712
ENSGALG00010002370	ENSGALG00010002370	2	-0.114708676	0.149051128	-0.769592805	0.441541475	0	0.017187254	0	1	CRA004775;Yang2025_Silkie_Wenchang	PRJNA827465	0.845053556
ENSGALG00010002370	ENSGALG00010002370	2	-0.065391777	0.097880412	-0.668078275	0.504083629	0	0.11006142	0	1	CRA004775;PRJNA827465	Yang2025_Silkie_Wenchang	0.863448292
ENSGALG00010002371	BAG3	2	0.11137524	0.258893321	0.439379429	0.660386629	0.108894413	4.8314147	79.30212863	0.5	PRJNA827465;Yang2025_Silkie_Wenchang	CRA004775	0.911923994
ENSGALG00010002371	BAG3	2	0.451680563	0.20080669	2.249330257	0.024491492	0	0.13595789	0	1	CRA004775;Yang2025_Silkie_Wenchang	PRJNA827465	0.267263775
ENSGALG00010002371	BAG3	2	0.103121346	0.325771762	0.316544765	0.751589053	0.14339668	2.286223926	56.25975266	0.5	CRA004775;PRJNA827465	Yang2025_Silkie_Wenchang	0.937716403
ENSGALG00010002372	SMC6	2	0.013023542	0.072641448	0.179285278	0.857713706	0	0.758870327	0	0.5	PRJNA827465;Yang2025_Silkie_Wenchang	CRA004775	0.964072462
ENSGALG00010002372	SMC6	2	0.150792375	0.124001445	1.216053372	0.223964591	0.005774448	1.111092114	9.998461203	1	CRA004775;Yang2025_Silkie_Wenchang	PRJNA827465	0.737452676
ENSGALG00010002372	SMC6	2	0.109419824	0.227377748	0.481224855	0.630356697	0.06748564	2.341597581	57.29411372	0.5	CRA004775;PRJNA827465	Yang2025_Silkie_Wenchang	0.902650914
ENSGALG00010002373	POSTN	2	-0.201876298	0.09451713	-2.135869956	0.032690015	0	0.790704943	0	1	PRJNA827465;Yang2025_Silkie_Wenchang	CRA004775	0.310566996
ENSGALG00010002373	POSTN	2	-0.296549413	0.175428074	-1.690433047	0.090945137	0	0.689793414	0	0.5	CRA004775;Yang2025_Silkie_Wenchang	PRJNA827465	0.517431489
ENSGALG00010002373	POSTN	2	-0.140582247	0.108330046	-1.297721658	0.194382999	0	0.288507454	0	0.5	CRA004775;PRJNA827465	Yang2025_Silkie_Wenchang	0.705148332
ENSGALG00010002374	DST	2	0.263192549	0.502881991	0.523368411	0.600717902	0.478230682	17.87738494	94.40634073	0.5	PRJNA827465;Yang2025_Silkie_Wenchang	CRA004775	0.895230519
ENSGALG00010002374	DST	2	0.704773364	0.187211452	3.764584679	0.000166826	0	0.501925276	0	1	CRA004775;Yang2025_Silkie_Wenchang	PRJNA827465	0.011248673
ENSGALG00010002374	DST	2	0.05013062	0.344462674	0.145532808	0.884290215	0.183379774	3.692218947	72.9160157	0.5	CRA004775;PRJNA827465	Yang2025_Silkie_Wenchang	0.972001076
ENSGALG00010002375	NPY	2	0.43375016	0.286916797	1.51176287	0.130594198	0	0.3887517	0	1	PRJNA827465;Yang2025_Silkie_Wenchang	CRA004775	0.606134125
ENSGALG00010002375	NPY	2	0.074826486	0.394045451	0.189893033	0.849392952	0	0.386848398	0	0.5	CRA004775;Yang2025_Silkie_Wenchang	PRJNA827465	0.961622629
ENSGALG00010002375	NPY	2	0.377420193	0.384953593	0.980430369	0.326873725	0.051952041	1.135021294	11.89592604	0.5	CRA004775;PRJNA827465	Yang2025_Silkie_Wenchang	0.808002539
ENSGALG00010002376	GEN1	2	0.012329457	0.085494855	0.144212852	0.885332393	0	0.149745998	0	0.5	PRJNA827465;Yang2025_Silkie_Wenchang	CRA004775	0.972280795
ENSGALG00010002376	GEN1	2	-0.030085362	0.141926618	-0.211977996	0.832124201	0	0.010504607	0	0.5	CRA004775;Yang2025_Silkie_Wenchang	PRJNA827465	0.957762335
ENSGALG00010002376	GEN1	2	0.035200679	0.102725854	0.342666204	0.73184959	0	0.000861039	0	1	CRA004775;PRJNA827465	Yang2025_Silkie_Wenchang	0.932851968
ENSGALG00010002377	ART1A	2	0.627637436	0.179638403	3.494092824	0.000475674	0	0.242369821	0	1	PRJNA827465;Yang2025_Silkie_Wenchang	CRA004775	0.022808588
ENSGALG00010002377	ART1A	2	0.554911118	0.28187592	1.968636123	0.048994891	0.042542903	1.240025189	19.35647688	1	CRA004775;Yang2025_Silkie_Wenchang	PRJNA827465	0.382604056
ENSGALG00010002377	ART1A	2	0.443517949	0.23465523	1.890083374	0.05874681	0	0.654565543	0	1	CRA004775;PRJNA827465	Yang2025_Silkie_Wenchang	0.419728393
ENSGALG00010002378	ENSGALG00010002378	2	0.106374484	0.11765397	0.904130002	0.36592541	0	0.678265914	0	1	PRJNA827465;Yang2025_Silkie_Wenchang	CRA004775	0.81896857
ENSGALG00010002378	ENSGALG00010002378	2	0.167721778	0.163715137	1.024473248	0.3056118	0	0.432532963	0	0.5	CRA004775;Yang2025_Silkie_Wenchang	PRJNA827465	0.79225794
ENSGALG00010002378	ENSGALG00010002378	2	0.015099475	0.140923949	0.107146264	0.914672945	0	0.030978896	0	0.5	CRA004775;PRJNA827465	Yang2025_Silkie_Wenchang	0.97982748
ENSGALG00010002379	NHS	2	0.348709726	0.306520409	1.137639503	0.255271045	0.146067945	4.12126067	75.73557996	1	PRJNA827465;Yang2025_Silkie_Wenchang	CRA004775	0.764467569
ENSGALG00010002379	NHS	2	0.264220494	0.482093142	0.548069389	0.583644258	0.352551174	4.057816437	75.35620412	0.5	CRA004775;Yang2025_Silkie_Wenchang	PRJNA827465	0.890558522
ENSGALG00010002379	NHS	2	0.05537129	0.119942146	0.461649984	0.644332344	0	0.710385384	0	0.5	CRA004775;PRJNA827465	Yang2025_Silkie_Wenchang	0.90692956
ENSGALG00010002380	CMPK2	2	0.218945959	0.22422236	0.976468001	0.328832597	0	0.281361916	0	1	PRJNA827465;Yang2025_Silkie_Wenchang	CRA004775	0.800761292
ENSGALG00010002380	CMPK2	2	0.154847978	0.295519592	0.523985488	0.600288633	0	0.907342025	0	0.5	CRA004775;Yang2025_Silkie_Wenchang	PRJNA827465	0.895230519
ENSGALG00010002380	CMPK2	2	0.047383721	0.239529493	0.197819986	0.843185909	0	0.356803684	0	0.5	CRA004775;PRJNA827465	Yang2025_Silkie_Wenchang	0.96054421
ENSGALG00010002381	TDRD3	2	0.188384864	0.301660955	0.624492036	0.532304499	0.158352911	7.131839377	85.97837182	0.5	PRJNA827465;Yang2025_Silkie_Wenchang	CRA004775	0.873104119
ENSGALG00010002381	TDRD3	2	0.514600178	0.169882513	3.029153326	0.002452402	0	0.006184984	0	1	CRA004775;Yang2025_Silkie_Wenchang	PRJNA827465	0.066199691
ENSGALG00010002381	TDRD3	2	0.145352374	0.282870591	0.513847598	0.607358547	0.125944511	4.011093603	75.06914326	0.5	CRA004775;PRJNA827465	Yang2025_Silkie_Wenchang	0.896998866
ENSGALG00010002383	INPP5F	2	0.046765575	0.083482394	0.560184879	0.57535334	0.003642868	1.332772777	24.96845542	0.5	PRJNA827465;Yang2025_Silkie_Wenchang	CRA004775	0.886545353
ENSGALG00010002383	INPP5F	2	-0.06365723	0.108796395	-0.585104226	0.558477638	0	0.022136077	0	1	CRA004775;Yang2025_Silkie_Wenchang	PRJNA827465	0.880777119
ENSGALG00010002383	INPP5F	2	0.09386623	0.084407427	1.112061269	0.266111808	0	0.555010948	0	0.5	CRA004775;PRJNA827465	Yang2025_Silkie_Wenchang	0.770092601
ENSGALG00010002385	PALS2	2	-0.041124097	0.078248758	-0.525555913	0.599196795	0	0.003394046	0	1	PRJNA827465;Yang2025_Silkie_Wenchang	CRA004775	0.894908299
ENSGALG00010002385	PALS2	2	-0.169736121	0.242055605	-0.701227807	0.483160859	0.054724723	1.432534361	30.1936465	1	CRA004775;Yang2025_Silkie_Wenchang	PRJNA827465	0.85691916
ENSGALG00010002385	PALS2	2	-0.159810349	0.244342192	-0.654043199	0.513083974	0.065464399	1.537993001	34.98019827	1	CRA004775;PRJNA827465	Yang2025_Silkie_Wenchang	0.866225921
ENSGALG00010002386	MHCY2B2	2	-0.087138877	0.197841106	-0.440448797	0.659612089	0.027507708	1.503034216	33.46791516	0.5	PRJNA827465;Yang2025_Silkie_Wenchang	CRA004775	0.911617103
ENSGALG00010002386	MHCY2B2	2	0.127610582	0.243305959	0.524486052	0.599940519	0	0.077226262	0	0.5	CRA004775;Yang2025_Silkie_Wenchang	PRJNA827465	0.895092498
ENSGALG00010002386	MHCY2B2	2	-0.221861693	0.181595533	-1.221735411	0.2218077	0	0.149616193	0	1	CRA004775;PRJNA827465	Yang2025_Silkie_Wenchang	0.735224622
ENSGALG00010002387	MCMBP	2	0.119886395	0.046260481	2.591550962	0.009554439	0	0.371024261	0	1	PRJNA827465;Yang2025_Silkie_Wenchang	CRA004775	0.153870904
ENSGALG00010002387	MCMBP	2	0.142146142	0.085054261	1.671240698	0.094674145	0	0.466944212	0	1	CRA004775;Yang2025_Silkie_Wenchang	PRJNA827465	0.526919621
ENSGALG00010002387	MCMBP	2	0.098375163	0.051270103	1.918762743	0.055014367	0	0.167097129	0	1	CRA004775;PRJNA827465	Yang2025_Silkie_Wenchang	0.405976279
ENSGALG00010002388	ENSGALG00010002388	2	0.115623965	0.591030332	0.195631187	0.844898848	0.442834713	2.667847135	62.51659299	0.5	PRJNA827465;Yang2025_Silkie_Wenchang	CRA004775	0.960769014
ENSGALG00010002388	ENSGALG00010002388	2	-0.21832782	0.412826549	-0.528860899	0.596901948	0	0.614879271	0	0.5	CRA004775;Yang2025_Silkie_Wenchang	PRJNA827465	0.894172266
ENSGALG00010002388	ENSGALG00010002388	2	0.449108139	0.343492873	1.307474404	0.191051642	0	0.57056811	0	1	CRA004775;PRJNA827465	Yang2025_Silkie_Wenchang	0.700443419
ENSGALG00010002389	COL21A1	2	0.112464692	0.129502273	0.868437975	0.385154614	0	0.169250572	0	1	PRJNA827465;Yang2025_Silkie_Wenchang	CRA004775	0.826444827
ENSGALG00010002389	COL21A1	2	0.23090244	0.211232276	1.093121019	0.274340668	0	0.100443053	0	1	CRA004775;Yang2025_Silkie_Wenchang	PRJNA827465	0.774526342
ENSGALG00010002389	COL21A1	2	0.107437351	0.145684253	0.737467153	0.460838311	0	0.343511916	0	1	CRA004775;PRJNA827465	Yang2025_Silkie_Wenchang	0.85014477
ENSGALG00010002391	REPS2	2	0.116959961	0.132308535	0.883994076	0.376699411	0.024545809	3.341788344	70.07590257	0.5	PRJNA827465;Yang2025_Silkie_Wenchang	CRA004775	0.822974013
ENSGALG00010002391	REPS2	2	-0.042212794	0.101958613	-0.41401891	0.678860258	0	0.681571678	0	1	CRA004775;Yang2025_Silkie_Wenchang	PRJNA827465	0.917701868
ENSGALG00010002391	REPS2	2	0.064600299	0.270492606	0.238824639	0.811241564	0.096506998	2.301645084	56.55281489	0.5	CRA004775;PRJNA827465	Yang2025_Silkie_Wenchang	0.953179464
ENSGALG00010002392	DFNA5	2	0.104456628	0.094209153	1.10877367	0.267527832	0	0.252620188	0	1	PRJNA827465;Yang2025_Silkie_Wenchang	CRA004775	0.771398294
ENSGALG00010002392	DFNA5	2	0.048045208	0.140222543	0.342635406	0.731872762	0	0.019400777	0	1	CRA004775;Yang2025_Silkie_Wenchang	PRJNA827465	0.932851968
ENSGALG00010002392	DFNA5	2	0.127629383	0.104726994	1.21868659	0.22296317	0	0.041889277	0	1	CRA004775;PRJNA827465	Yang2025_Silkie_Wenchang	0.736458649
ENSGALG00010002393	GPM6B	2	-0.029857676	0.049469683	-0.603555026	0.546139521	0	0.06187613	0	1	PRJNA827465;Yang2025_Silkie_Wenchang	CRA004775	0.878012469
ENSGALG00010002393	GPM6B	2	0.160483991	0.24577881	0.652961055	0.513781386	0.075041912	2.139394918	53.25781175	0.5	CRA004775;Yang2025_Silkie_Wenchang	PRJNA827465	0.866584405
ENSGALG00010002393	GPM6B	2	0.141898061	0.261360311	0.54292123	0.587184038	0.096881173	2.586942078	61.34432198	0.5	CRA004775;PRJNA827465	Yang2025_Silkie_Wenchang	0.891596903
ENSGALG00010002395	CLN8	2	-0.211747418	0.245470965	-0.862616962	0.388348101	0.115729234	25.10268408	96.01636225	0.5	PRJNA827465;Yang2025_Silkie_Wenchang	CRA004775	0.828154121
ENSGALG00010002395	CLN8	2	-0.219918605	0.292008032	-0.753125191	0.451374688	0.136746766	4.390890268	77.22557525	0.5	CRA004775;Yang2025_Silkie_Wenchang	PRJNA827465	0.847847413
ENSGALG00010002395	CLN8	2	0.035103212	0.056196177	0.624654808	0.532197639	0	0.139626628	0	1	CRA004775;PRJNA827465	Yang2025_Silkie_Wenchang	0.873104119
ENSGALG00010002396	SEC23IP	2	-0.002337712	0.07063684	-0.033094798	0.973598991	0.003346212	1.354536745	26.17402198	0.5	PRJNA827465;Yang2025_Silkie_Wenchang	CRA004775	0.992442268
ENSGALG00010002396	SEC23IP	2	-0.160975449	0.099930588	-1.610872635	0.107207486	0	0.312493184	0	1	CRA004775;Yang2025_Silkie_Wenchang	PRJNA827465	0.554473355
ENSGALG00010002396	SEC23IP	2	-0.053334817	0.130919131	-0.407387496	0.683723409	0.022431841	2.412404122	58.54757539	0.5	CRA004775;PRJNA827465	Yang2025_Silkie_Wenchang	0.918800744
ENSGALG00010002397	ENSGALG00010002397	2	-0.325881113	0.769137689	-0.423696717	0.67178701	1.002911759	6.304088968	84.13727971	0.5	PRJNA827465;Yang2025_Silkie_Wenchang	CRA004775	0.914674175
ENSGALG00010002397	ENSGALG00010002397	2	-0.669925355	0.450889299	-1.48578677	0.137335531	0.156026972	1.614162928	38.04838518	1	CRA004775;Yang2025_Silkie_Wenchang	PRJNA827465	0.619926307
ENSGALG00010002397	ENSGALG00010002397	2	0.162176488	0.302541626	0.536046858	0.591926166	0.052429291	1.346421837	25.72907147	0.5	CRA004775;PRJNA827465	Yang2025_Silkie_Wenchang	0.892776493
ENSGALG00010002400	RBBP7	2	-0.093866032	0.073681031	-1.273951125	0.202680738	0	0.167574231	0	1	PRJNA827465;Yang2025_Silkie_Wenchang	CRA004775	0.715655471
ENSGALG00010002400	RBBP7	2	-0.015691747	0.188300031	-0.083333746	0.933586168	0	0.001323398	0	1	CRA004775;Yang2025_Silkie_Wenchang	PRJNA827465	0.984262625
ENSGALG00010002400	RBBP7	2	-0.102197735	0.077082098	-1.325829711	0.184896109	0	0.03454137	0	1	CRA004775;PRJNA827465	Yang2025_Silkie_Wenchang	0.693265167
ENSGALG00010002401	ZRANB1	2	-0.053314467	0.100458089	-0.530713527	0.595617311	0.014150897	3.12762312	68.02683822	0.5	PRJNA827465;Yang2025_Silkie_Wenchang	CRA004775	0.893802528
ENSGALG00010002401	ZRANB1	2	0.063582526	0.098483362	0.645616927	0.518527473	0	0.013247101	0	1	CRA004775;Yang2025_Silkie_Wenchang	PRJNA827465	0.868525042
ENSGALG00010002401	ZRANB1	2	-0.135213443	0.05490601	-2.462634659	0.013792037	0	0.125149261	0	0.5	CRA004775;PRJNA827465	Yang2025_Silkie_Wenchang	0.192701185
ENSGALG00010002402	TXLNG	2	-0.061839942	0.083065492	-0.74447211	0.456590908	0.003612598	1.324382525	24.49311424	0.5	PRJNA827465;Yang2025_Silkie_Wenchang	CRA004775	0.849515887
ENSGALG00010002402	TXLNG	2	0.158091831	0.350749624	0.450725589	0.652187337	0.215466864	7.790051134	87.16311379	0.5	CRA004775;Yang2025_Silkie_Wenchang	PRJNA827465	0.909376064
ENSGALG00010002402	TXLNG	2	0.229177696	0.262849144	0.871898202	0.383263915	0.113193789	5.044067887	80.17473154	1	CRA004775;PRJNA827465	Yang2025_Silkie_Wenchang	0.825476201
ENSGALG00010002403	ZNFY3	2	-0.449394342	0.661028957	-0.679840628	0.496605378	0.795355932	11.0400128	90.94203949	0.5	PRJNA827465;Yang2025_Silkie_Wenchang	CRA004775	0.860939846
ENSGALG00010002403	ZNFY3	2	-0.794451544	0.398217764	-1.995017839	0.046040934	0.167183376	2.046406609	51.13385601	1	CRA004775;Yang2025_Silkie_Wenchang	PRJNA827465	0.370050478
ENSGALG00010002403	ZNFY3	2	0.089212242	0.209814117	0.425196568	0.670693386	0	0.972278682	0	0.5	CRA004775;PRJNA827465	Yang2025_Silkie_Wenchang	0.914482883
ENSGALG00010002404	OSBPL3	2	0.499998954	0.125589614	3.981212597	6.85646E-05	0	0.327312286	0	1	PRJNA827465;Yang2025_Silkie_Wenchang	CRA004775	0.006001768
ENSGALG00010002404	OSBPL3	2	0.405427824	0.278976221	1.453270182	0.146148753	0.073372543	1.601322237	37.55160722	0.5	CRA004775;Yang2025_Silkie_Wenchang	PRJNA827465	0.634916196
ENSGALG00010002404	OSBPL3	2	0.373774939	0.160819883	2.324183621	0.020115655	0	0.965808435	0	0.5	CRA004775;PRJNA827465	Yang2025_Silkie_Wenchang	0.238628722
ENSGALG00010002405	KCNQ5	2	0.124197547	0.366441677	0.338928552	0.734663555	0.209067723	4.435234217	77.4532764	0.5	PRJNA827465;Yang2025_Silkie_Wenchang	CRA004775	0.933520234
ENSGALG00010002405	KCNQ5	2	0.400668347	0.238100234	1.682771744	0.092419287	0	0.585919004	0	1	CRA004775;Yang2025_Silkie_Wenchang	PRJNA827465	0.520543885
ENSGALG00010002405	KCNQ5	2	-0.158036581	0.185162551	-0.853501855	0.393381059	0	0.473004279	0	0.5	CRA004775;PRJNA827465	Yang2025_Silkie_Wenchang	0.830346712
ENSGALG00010002406	DIAPH3	2	-0.007021622	0.097618675	-0.071929083	0.942658345	0	0.567211277	0	0.5	PRJNA827465;Yang2025_Silkie_Wenchang	CRA004775	0.985827325
ENSGALG00010002406	DIAPH3	2	0.147555327	0.179365312	0.822652526	0.41070562	0	0.571826806	0	1	CRA004775;Yang2025_Silkie_Wenchang	PRJNA827465	0.835028074
ENSGALG00010002406	DIAPH3	2	-0.032341415	0.118346346	-0.273277683	0.784639769	0.001233931	1.004860995	0.483748018	0.5	CRA004775;PRJNA827465	Yang2025_Silkie_Wenchang	0.946117405
ENSGALG00010002408	OFD1	2	0.105321025	0.151013192	0.697429301	0.48553417	0.024380942	1.943722505	48.55232692	0.5	PRJNA827465;Yang2025_Silkie_Wenchang	CRA004775	0.857198341
ENSGALG00010002408	OFD1	2	0.180749209	0.14598906	1.238101053	0.215678594	0	0.869988919	0	1	CRA004775;Yang2025_Silkie_Wenchang	PRJNA827465	0.729213578
ENSGALG00010002408	OFD1	2	0.003433751	0.092585726	0.037087259	0.970415431	0	0.035818315	0	0.5	CRA004775;PRJNA827465	Yang2025_Silkie_Wenchang	0.991711632
ENSGALG00010002409	CYCS	2	-0.100131978	0.250239733	-0.400144202	0.689050309	0.100664929	4.57796642	78.15623995	0.5	PRJNA827465;Yang2025_Silkie_Wenchang	CRA004775	0.919854035
ENSGALG00010002409	CYCS	2	-0.082779351	0.383773722	-0.215698331	0.829222902	0.195899748	2.76560991	63.84161062	0.5	CRA004775;Yang2025_Silkie_Wenchang	PRJNA827465	0.956860127
ENSGALG00010002409	CYCS	2	0.121805206	0.080435308	1.514325106	0.129943404	0	0.424913401	0	1	CRA004775;PRJNA827465	Yang2025_Silkie_Wenchang	0.604756141
ENSGALG00010002410	RNF121	2	-0.00850242	0.063149303	-0.134639967	0.89289654	0	0.097553299	0	1	PRJNA827465;Yang2025_Silkie_Wenchang	CRA004775	0.974381724
ENSGALG00010002410	RNF121	2	-0.081720198	0.180914312	-0.451706654	0.651480324	0	0.000118599	0	1	CRA004775;Yang2025_Silkie_Wenchang	PRJNA827465	0.908927523
ENSGALG00010002410	RNF121	2	-0.007722892	0.06338095	-0.121848786	0.903018776	0	0.078801691	0	1	CRA004775;PRJNA827465	Yang2025_Silkie_Wenchang	0.977050157
ENSGALG00010002411	ENSGALG00010002411	2	0.223594202	0.110948548	2.015296332	0.043873609	0	0.560031138	0	1	PRJNA827465;Yang2025_Silkie_Wenchang	CRA004775	0.361804689
ENSGALG00010002411	ENSGALG00010002411	2	0.026078604	0.396831485	0.065717073	0.94760308	0.23064959	3.727071558	73.16928359	0.5	CRA004775;Yang2025_Silkie_Wenchang	PRJNA827465	0.986560458
ENSGALG00010002411	ENSGALG00010002411	2	-0.028381932	0.276164374	-0.102771881	0.918144023	0.107883174	2.986105722	66.51156747	0.5	CRA004775;PRJNA827465	Yang2025_Silkie_Wenchang	0.981033557
ENSGALG00010002413	TSSC1	2	-0.088499038	0.07138905	-1.239672444	0.215096573	0	0.076021017	0	1	PRJNA827465;Yang2025_Silkie_Wenchang	CRA004775	0.728473987
ENSGALG00010002413	TSSC1	2	-0.13968937	0.113441613	-1.231376803	0.218181969	0	0.269604334	0	1	CRA004775;Yang2025_Silkie_Wenchang	PRJNA827465	0.731735294
ENSGALG00010002413	TSSC1	2	-0.090761658	0.084420771	-1.075110505	0.282325257	0	0.448254593	0	1	CRA004775;PRJNA827465	Yang2025_Silkie_Wenchang	0.779519046
ENSGALG00010002414	ENSGALG00010002414	2	0.835279968	0.56157731	1.487381973	0.136913955	0.349932378	2.175125626	54.02564394	1	PRJNA827465;Yang2025_Silkie_Wenchang	CRA004775	0.619089874
ENSGALG00010002414	ENSGALG00010002414	2	0.672546511	0.766261588	0.877698323	0.380107455	0.863407041	3.722779158	73.13834752	0.5	CRA004775;Yang2025_Silkie_Wenchang	PRJNA827465	0.824392515
ENSGALG00010002414	ENSGALG00010002414	2	0.177266778	0.324408987	0.546429924	0.584770445	0	0.373905912	0	0.5	CRA004775;PRJNA827465	Yang2025_Silkie_Wenchang	0.891016154
ENSGALG00010002416	SH3BGRL2	2	-0.040933164	0.628372389	-0.06514157	0.948061282	0.717546052	10.91210156	90.83586242	0.5	PRJNA827465;Yang2025_Silkie_Wenchang	CRA004775	0.986697717
ENSGALG00010002416	SH3BGRL2	2	-0.537346794	0.252128842	-2.131238892	0.033069466	0	0.911169261	0	1	CRA004775;Yang2025_Silkie_Wenchang	PRJNA827465	0.312461555
ENSGALG00010002416	SH3BGRL2	2	0.353100113	0.329326726	1.072187847	0.283635668	0.099650584	1.65814511	39.69164739	0.5	CRA004775;PRJNA827465	Yang2025_Silkie_Wenchang	0.779713546
ENSGALG00010002418	TRAPPC2	2	-0.197144935	0.302679783	-0.651331689	0.514832396	0.17567086	24.08511829	95.84805859	0.5	PRJNA827465;Yang2025_Silkie_Wenchang	CRA004775	0.866930857
ENSGALG00010002418	TRAPPC2	2	-0.410339705	0.143289236	-2.863716182	0.00418703	0.018820638	1.632438089	38.74193413	1	CRA004775;Yang2025_Silkie_Wenchang	PRJNA827465	0.094197388
ENSGALG00010002418	TRAPPC2	2	0.029087919	0.126035517	0.230791447	0.817476826	0.016423688	1.614710345	38.0693879	0.5	CRA004775;PRJNA827465	Yang2025_Silkie_Wenchang	0.954888783
ENSGALG00010002419	CTBP2	2	0.083667169	0.134363553	0.622692443	0.53348665	0.027588448	4.007576933	75.0472663	0.5	PRJNA827465;Yang2025_Silkie_Wenchang	CRA004775	0.873379507
ENSGALG00010002419	CTBP2	2	0.195269599	0.104473595	1.869080877	0.061611563	0	0.559366568	0	1	CRA004775;Yang2025_Silkie_Wenchang	PRJNA827465	0.428734062
ENSGALG00010002419	CTBP2	2	-0.027042047	0.062640544	-0.43170198	0.665958028	0	0.133296303	0	0.5	CRA004775;PRJNA827465	Yang2025_Silkie_Wenchang	0.913546323
ENSGALG00010002421	ENSGALG00010002421	2	-0.343333356	0.419296577	-0.818831764	0.412882406	0.311913718	8.7165114	88.52752031	0.5	PRJNA827465;Yang2025_Silkie_Wenchang	CRA004775	0.835870654
ENSGALG00010002421	ENSGALG00010002421	2	-0.701324854	0.214381729	-3.271383516	0.001070227	0	0.628747382	0	1	CRA004775;Yang2025_Silkie_Wenchang	PRJNA827465	0.039495886
ENSGALG00010002421	ENSGALG00010002421	2	0.021687355	0.149484175	0.145081278	0.8846467	0	0.584020043	0	0.5	CRA004775;PRJNA827465	Yang2025_Silkie_Wenchang	0.972062774
ENSGALG00010002422	TRAPPC12	2	0.012604923	0.049615427	0.254052485	0.799455011	0	0.339241511	0	0.5	PRJNA827465;Yang2025_Silkie_Wenchang	CRA004775	0.949922217
ENSGALG00010002422	TRAPPC12	2	-0.036187321	0.104665026	-0.345744155	0.729534998	0	0.074759434	0	0.5	CRA004775;Yang2025_Silkie_Wenchang	PRJNA827465	0.9323457
ENSGALG00010002422	TRAPPC12	2	0.022828378	0.051438517	0.443799299	0.657187696	0	0.013188409	0	1	CRA004775;PRJNA827465	Yang2025_Silkie_Wenchang	0.910711339
ENSGALG00010002423	ENSGALG00010002423	2	-0.561840277	0.340253734	-1.651239119	0.098689758	0	0.072342666	0	1	PRJNA827465;Yang2025_Silkie_Wenchang	CRA004775	0.536091342
ENSGALG00010002423	ENSGALG00010002423	2	-0.844932352	0.451948488	-1.869532424	0.061548777	0.057298327	1.160451359	13.82663369	1	CRA004775;Yang2025_Silkie_Wenchang	PRJNA827465	0.428529953
ENSGALG00010002423	ENSGALG00010002423	2	-0.859074842	0.356969052	-2.406580728	0.016102645	0	0.879097405	0	1	CRA004775;PRJNA827465	Yang2025_Silkie_Wenchang	0.209649548
ENSGALG00010002424	CASK	2	0.051886065	0.042676711	1.215793415	0.224063628	0	0.107790774	0	1	PRJNA827465;Yang2025_Silkie_Wenchang	CRA004775	0.737452676
ENSGALG00010002424	CASK	2	0.218891575	0.166380774	1.315606189	0.188306255	0.032360769	2.248142903	55.51884185	1	CRA004775;Yang2025_Silkie_Wenchang	PRJNA827465	0.697615303
ENSGALG00010002424	CASK	2	0.18872775	0.184607328	1.02231993	0.306629503	0.052273038	3.54364797	71.78049263	1	CRA004775;PRJNA827465	Yang2025_Silkie_Wenchang	0.792500392
ENSGALG00010002425	FDX2	2	0.019540499	0.09423229	0.207365218	0.835724644	0	0.065169027	0	0.5	PRJNA827465;Yang2025_Silkie_Wenchang	CRA004775	0.958339923
ENSGALG00010002425	FDX2	2	-0.044975712	0.204948833	-0.219448489	0.826300702	0	0.001588701	0	1	CRA004775;Yang2025_Silkie_Wenchang	PRJNA827465	0.956393839
ENSGALG00010002425	FDX2	2	0.023007273	0.097965142	0.234851628	0.814323895	0	0.052909864	0	0.5	CRA004775;PRJNA827465	Yang2025_Silkie_Wenchang	0.953869515
ENSGALG00010002427	CYB5R4	2	0.019199865	0.079182288	0.24247677	0.808410754	0.005088132	1.620704715	38.29844569	0.5	PRJNA827465;Yang2025_Silkie_Wenchang	CRA004775	0.952496413
ENSGALG00010002427	CYB5R4	2	0.109970725	0.096316785	1.141760743	0.253553484	0	0.048767093	0	1	CRA004775;Yang2025_Silkie_Wenchang	PRJNA827465	0.763346521
ENSGALG00010002427	CYB5R4	2	-0.033250812	0.06777722	-0.490589795	0.623716606	0	0.208189052	0	0.5	CRA004775;PRJNA827465	Yang2025_Silkie_Wenchang	0.900519204
ENSGALG00010002428	IBSP	2	0.165531239	0.593507402	0.278903411	0.780318951	0	0.197330545	0	0.5	PRJNA827465;Yang2025_Silkie_Wenchang	CRA004775	0.945041817
ENSGALG00010002428	IBSP	2	0.854842539	0.492580067	1.735438757	0.08266312	0	0.334221206	0	1	CRA004775;Yang2025_Silkie_Wenchang	PRJNA827465	0.495638233
ENSGALG00010002428	IBSP	2	0.61971338	0.551705473	1.123268503	0.261323477	0.132170097	1.257534785	20.47933686	0.5	CRA004775;PRJNA827465	Yang2025_Silkie_Wenchang	0.768082306
ENSGALG00010002429	ENSGALG00010002429	2	-0.070292957	0.319545976	-0.219977599	0.825888601	0	0.33681506	0	0.5	PRJNA827465;Yang2025_Silkie_Wenchang	CRA004775	0.956393839
ENSGALG00010002429	ENSGALG00010002429	2	-0.168745744	0.394678006	-0.427552947	0.668976629	0.010029724	1.031816969	3.083586563	0.5	CRA004775;Yang2025_Silkie_Wenchang	PRJNA827465	0.914377769
ENSGALG00010002429	ENSGALG00010002429	2	-0.359322985	0.345327676	-1.04052762	0.298094839	0	0.332342304	0	1	CRA004775;PRJNA827465	Yang2025_Silkie_Wenchang	0.789110621
ENSGALG00010002430	NUMA1	2	0.359536482	0.424344378	0.847275233	0.396841728	0.347322248	27.7352155	96.39447546	0.5	PRJNA827465;Yang2025_Silkie_Wenchang	CRA004775	0.831375233
ENSGALG00010002430	NUMA1	2	0.32583237	0.487126034	0.668887202	0.503567435	0.434927554	11.7512415	91.49026084	0.5	CRA004775;Yang2025_Silkie_Wenchang	PRJNA827465	0.863108263
ENSGALG00010002430	NUMA1	2	-0.065221608	0.067947108	-0.959887917	0.337111628	0	0.245183952	0	1	CRA004775;PRJNA827465	Yang2025_Silkie_Wenchang	0.804086136
ENSGALG00010002431	BCKDHB	2	-0.177354801	0.160129801	-1.107568983	0.268048005	0.039848998	4.344055419	76.98003585	1	PRJNA827465;Yang2025_Silkie_Wenchang	CRA004775	0.771398294
ENSGALG00010002431	BCKDHB	2	-0.059290003	0.350461565	-0.169176906	0.865657495	0.192625512	4.181847547	76.08712444	0.5	CRA004775;Yang2025_Silkie_Wenchang	PRJNA827465	0.967088846
ENSGALG00010002431	BCKDHB	2	0.03816556	0.149760795	0.254843462	0.798844005	0.020431879	1.367860595	26.89313491	0.5	CRA004775;PRJNA827465	Yang2025_Silkie_Wenchang	0.949922217
ENSGALG00010002432	RAVER1	2	0.439481745	0.409611023	1.072924604	0.283304946	0.321660722	23.93803263	95.82254726	1	PRJNA827465;Yang2025_Silkie_Wenchang	CRA004775	0.779568117

ENSGALG00010002432	RAVER1	2	0.150507444	0.709492077	0.212134074	0.832002438	0.982043896	40.70627521	97.54337631	0.5	CRA004775;Yang2025_Silkie_Wenchang	PRJNA827465	0.957762335
ENSGALG00010002432	RAVER1	2	-0.244366201	0.299281065	-0.816510732	0.414208088	0.161426583	9.797219243	89.79302213	0.5	CRA004775;PRJNA827465	Yang2025_Silkie_Wenchang	0.836914812
ENSGALG00010002433	GRHL1	2	-0.03372634	0.30844935	-0.109341581	0.912931567	0	9.26774E-05	0	1	PRJNA827465;Yang2025_Silkie_Wenchang	CRA004775	0.979071811
ENSGALG00010002433	GRHL1	2	-0.08730484	0.240733201	-0.362662233	0.716857216	0	0.013081345	0	1	CRA004775;Yang2025_Silkie_Wenchang	PRJNA827465	0.927632716
ENSGALG00010002433	GRHL1	2	-0.079135417	0.225352716	-0.351162473	0.725466462	0	0.021991368	0	1	CRA004775;PRJNA827465	Yang2025_Silkie_Wenchang	0.930910808
ENSGALG00010002434	GPR34	2	-0.294172151	0.271319812	-1.084226576	0.278264351	0.126271608	7.02418295	85.76346876	1	PRJNA827465;Yang2025_Silkie_Wenchang	CRA004775	0.776763074
ENSGALG00010002434	GPR34	2	-0.519806105	0.138447753	-3.754529016	0.000173668	0	0.879839326	0	1	CRA004775;Yang2025_Silkie_Wenchang	PRJNA827465	0.011507636
ENSGALG00010002434	GPR34	2	-0.039102786	0.134293202	-0.29117472	0.770917696	0	0.100980625	0	1	CRA004775;PRJNA827465	Yang2025_Silkie_Wenchang	0.942531226
ENSGALG00010002436	ENSGALG00010002436	2	0.583430344	0.499438388	1.168172808	0.242737066	0	0.021373439	0	1	PRJNA827465;Yang2025_Silkie_Wenchang	CRA004775	0.7550024
ENSGALG00010002436	ENSGALG00010002436	2	-0.00776631	0.491758871	-0.015792923	0.987399594	0.021479796	1.041372846	3.972913871	0.5	CRA004775;Yang2025_Silkie_Wenchang	PRJNA827465	0.996552983
ENSGALG00010002436	ENSGALG00010002436	2	0.059300001	0.443085855	0.133834109	0.893533754	0.027570981	1.075249254	6.998307932	0.5	CRA004775;PRJNA827465	Yang2025_Silkie_Wenchang	0.974381724
ENSGALG00010002437	TTK	2	-0.18487178	0.20964182	-0.881845902	0.377860145	0.071696095	5.179344743	80.69253835	0.5	PRJNA827465;Yang2025_Silkie_Wenchang	CRA004775	0.82325159
ENSGALG00010002437	TTK	2	-0.396425777	0.15317594	-2.588042066	0.009652319	0	0.153220189	0	1	CRA004775;Yang2025_Silkie_Wenchang	PRJNA827465	0.154906126
ENSGALG00010002437	TTK	2	-0.004138702	0.08760472	-0.047242915	0.962319624	0	0.237317798	0	0.5	CRA004775;PRJNA827465	Yang2025_Silkie_Wenchang	0.989950484
ENSGALG00010002438	RARB	2	0.145008755	0.062365235	2.325153677	0.020063744	0	0.005100553	0	1	PRJNA827465;Yang2025_Silkie_Wenchang	CRA004775	0.238261187
ENSGALG00010002438	RARB	2	0.197217878	0.13031245	1.513423152	0.130172208	0	0.637299449	0	1	CRA004775;Yang2025_Silkie_Wenchang	PRJNA827465	0.605390875
ENSGALG00010002438	RARB	2	0.162169929	0.066059569	2.454904447	0.014092204	0	0.726951278	0	1	CRA004775;PRJNA827465	Yang2025_Silkie_Wenchang	0.194475385
ENSGALG00010002440	LRRC51	2	-0.517799821	0.169316782	-3.058171872	0.002226918	0	0.05823526	0	1	PRJNA827465;Yang2025_Silkie_Wenchang	CRA004775	0.062383865
ENSGALG00010002440	LRRC51	2	-0.562123745	0.287344994	-1.956267753	0.050433621	0	0.021899558	0	1	CRA004775;Yang2025_Silkie_Wenchang	PRJNA827465	0.38897029
ENSGALG00010002440	LRRC51	2	-0.495715114	0.181026463	-2.738357475	0.006174692	0	8.51891E-05	0	1	CRA004775;PRJNA827465	Yang2025_Silkie_Wenchang	0.11894719
ENSGALG00010002441	USO1	2	0.173136634	0.195421362	0.885965755	0.375635981	0.073426818	25.75707733	96.11123083	0.5	PRJNA827465;Yang2025_Silkie_Wenchang	CRA004775	0.822974013
ENSGALG00010002441	USO1	2	0.122471015	0.269989077	0.453614703	0.650106169	0.130813252	9.219169024	89.1530354	0.5	CRA004775;Yang2025_Silkie_Wenchang	PRJNA827465	0.908335059
ENSGALG00010002441	USO1	2	-0.028340414	0.040836871	-0.693990838	0.487687954	0	0.772749868	0	1	CRA004775;PRJNA827465	Yang2025_Silkie_Wenchang	0.85768885
ENSGALG00010002442	ENSGALG00010002442	2	0.753999781	0.523219419	1.441077593	0.149562763	0.401020815	3.736581693	73.2375716	1	PRJNA827465;Yang2025_Silkie_Wenchang	CRA004775	0.639428399
ENSGALG00010002442	ENSGALG00010002442	2	-0.313122384	1.654899747	-0.189209077	0.849928797	5.111364403	14.59270782	93.14726223	0.5	CRA004775;Yang2025_Silkie_Wenchang	PRJNA827465	0.961869125
ENSGALG00010002442	ENSGALG00010002442	2	-0.805864905	1.128637747	-0.714015553	0.475217562	2.187182687	6.741058933	85.16553542	0.5	CRA004775;PRJNA827465	Yang2025_Silkie_Wenchang	0.854462864
ENSGALG00010002443	TYK2	2	0.06811356	0.142050756	0.479501568	0.631581853	0.035086215	7.580810421	86.80879821	0.5	PRJNA827465;Yang2025_Silkie_Wenchang	CRA004775	0.903058174
ENSGALG00010002443	TYK2	2	-0.322271387	0.551306153	-0.584559749	0.558843779	0.586550444	28.04758666	96.43463086	0.5	CRA004775;Yang2025_Silkie_Wenchang	PRJNA827465	0.880896757
ENSGALG00010002443	TYK2	2	-0.458576175	0.408823229	-1.119956791	0.262740699	0.314955086	16.65693973	93.99649626	1	CRA004775;PRJNA827465	Yang2025_Silkie_Wenchang	0.765969343
ENSGALG00010002444	KLF11	2	-0.062327275	0.126885734	-0.491207901	0.623279411	0.018372724	2.095632482	52.28170928	0.5	PRJNA827465;Yang2025_Silkie_Wenchang	CRA004775	0.900348922
ENSGALG00010002444	KLF11	2	0.139022962	0.149170938	0.93197083	0.351351601	0	0.197826511	0	1	CRA004775;Yang2025_Silkie_Wenchang	PRJNA827465	0.810492544
ENSGALG00010002444	KLF11	2	-0.074298136	0.171559421	-0.433075217	0.664960117	0.029593291	1.439889495	30.55025603	0.5	CRA004775;PRJNA827465	Yang2025_Silkie_Wenchang	0.901399315
ENSGALG00010002445	EDRF1	2	-0.009712183	0.041965911	-0.231430302	0.816980526	0	0.528465932	0	0.5	PRJNA827465;Yang2025_Silkie_Wenchang	CRA004775	0.954817505
ENSGALG00010002445	EDRF1	2	0.07845928	0.10019142	0.783093803	0.43357203	0.00744332	1.287263436	22.31582345	1	CRA004775;Yang2025_Silkie_Wenchang	PRJNA827465	0.842762754
ENSGALG00010002445	EDRF1	2	0.059519302	0.145487907	0.409101371	0.68246527	0.026908722	2.082505969	51.98092995	0.5	CRA004775;PRJNA827465	Yang2025_Silkie_Wenchang	0.918625039
ENSGALG00010002446	RIMS1	2	-0.207134173	0.318965363	-0.649393938	0.516063781	0.104434981	1.84646084	45.84233913	0.5	PRJNA827465;Yang2025_Silkie_Wenchang	CRA004775	0.867498525
ENSGALG00010002446	RIMS1	2	0.423436938	0.403190268	1.050216166	0.293618738	0	0.946940687	0	1	CRA004775;Yang2025_Silkie_Wenchang	PRJNA827465	0.786979185
ENSGALG00010002446	RIMS1	2	0.161927978	0.811257191	0.199601285	0.841792423	0.964773826	3.008478804	66.76061009	0.5	CRA004775;PRJNA827465	Yang2025_Silkie_Wenchang	0.960031145
ENSGALG00010002447	YLEC12	2	0.250477842	0.382066643	0.655586784	0.512090032	0	0.656938972	0	0.5	PRJNA827465;Yang2025_Silkie_Wenchang	CRA004775	0.865694231
ENSGALG00010002447	YLEC12	2	-0.176270847	0.439834329	-0.40076646	0.688592074	0	0.257096316	0	1	CRA004775;Yang2025_Silkie_Wenchang	PRJNA827465	0.919798626
ENSGALG00010002447	YLEC12	2	0.190374048	0.533573203	0.356790872	0.72124837	0.123935702	1.248785071	19.92216892	0.5	CRA004775;PRJNA827465	Yang2025_Silkie_Wenchang	0.928950734
ENSGALG00010002448	RAB9A	2	-0.173266777	0.16631233	-1.041815585	0.297497187	0.042636412	4.156831789	75.94321708	1	PRJNA827465;Yang2025_Silkie_Wenchang	CRA004775	0.788562929
ENSGALG00010002448	RAB9A	2	-0.280779584	0.164171912	-1.710277846	0.087214506	0.013099981	1.178512091	15.1472431	0.5	CRA004775;Yang2025_Silkie_Wenchang	PRJNA827465	0.507822786
ENSGALG00010002448	RAB9A	2	-0.021935552	0.079818744	-0.274817054	0.783456796	0	0.0490297	0	0.5	CRA004775;PRJNA827465	Yang2025_Silkie_Wenchang	0.945815798
ENSGALG00010002449	ALG11	2	-0.2481692	0.255533931	-0.971179049	0.331459125	0.114512509	7.748649066	87.09452459	1	PRJNA827465;Yang2025_Silkie_Wenchang	CRA004775	0.801798188
ENSGALG00010002449	ALG11	2	-0.381409019	0.200543621	-1.901875596	0.05718742	0.033253151	1.591122165	37.15127461	1	CRA004775;Yang2025_Silkie_Wenchang	PRJNA827465	0.414005744
ENSGALG00010002449	ALG11	2	-0.018906662	0.075882333	-0.249157625	0.803238856	0	0.08848118	0	1	CRA004775;PRJNA827465	Yang2025_Silkie_Wenchang	0.950951274
ENSGALG00010002450	ENSGALG00010002450	2	0.227486378	0.372989789	0.609899747	0.54192822	0.17553407	2.389530427	58.15077353	0.5	PRJNA827465;Yang2025_Silkie_Wenchang	CRA004775	0.876554789
ENSGALG00010002450	ENSGALG00010002450	2	-0.083576459	0.785280206	-0.106428836	0.915242115	1.059137076	7.044735336	85.80500257	0.5	CRA004775;Yang2025_Silkie_Wenchang	PRJNA827465	0.980117272
ENSGALG00010002450	ENSGALG00010002450	2	-0.387893778	0.392308519	-0.988746762	0.322787057	0.224608691	3.474154983	71.21602218	1	CRA004775;PRJNA827465	Yang2025_Silkie_Wenchang	0.79874391
ENSGALG00010002453	TOP2B	2	0.046635771	0.098471531	0.473596481	0.635787685	0.014400492	3.380573864	70.41922348	0.5	PRJNA827465;Yang2025_Silkie_Wenchang	CRA004775	0.904387873
ENSGALG00010002453	TOP2B	2	0.172232956	0.091959869	1.872914327	0.061080216	0	0.001000201	0	1	CRA004775;Yang2025_Silkie_Wenchang	PRJNA827465	0.427235406
ENSGALG00010002453	TOP2B	2	-0.019880762	0.047935663	-0.414733429	0.678333397	0.001031513	1.04938034	4.705666612	0.5	CRA004775;PRJNA827465	Yang2025_Silkie_Wenchang	0.917670684
ENSGALG00010002455	NYX	2	0.395412794	0.119701528	3.303322865	0.000955463	0	0.000753128	0	1	PRJNA827465;Yang2025_Silkie_Wenchang	CRA004775	0.016708276
ENSGALG00010002455	NYX	2	0.346584451	0.156341298	2.216845167	0.026633674	0	0.594459272	0	1	CRA004775;Yang2025_Silkie_Wenchang	PRJNA827465	0.278694271
ENSGALG00010002455	NYX	2	0.350221072	0.159855939	2.190854308	0.028462337	0	0.612100694	0	1	CRA004775;PRJNA827465	Yang2025_Silkie_Wenchang	0.287202885
ENSGALG00010002457	ENSGALG00010002457	2	-0.194786258	0.32445363	-0.600351607	0.548271932	0	0.066021612	0	1	PRJNA827465;Yang2025_Silkie_Wenchang	CRA004775	0.878281266
ENSGALG00010002457	ENSGALG00010002457	2	-0.093748644	0.369461177	-0.253744177	0.799691356	0	0.010301326	0	1	CRA004775;Yang2025_Silkie_Wenchang	PRJNA827465	0.94992217
ENSGALG00010002457	ENSGALG00010002457	2	-0.202283713	0.391348186	-0.516889359	0.605233396	0	0.09025763	0	1	CRA004775;PRJNA827465	Yang2025_Silkie_Wenchang	0.896474603
ENSGALG00010002458	ENSGALG00010002458	2	0.168628034	0.072738434	2.318279696	0.020434125	0	0.272775682	0	1	PRJNA827465;Yang2025_Silkie_Wenchang	CRA004775	0.240888548
ENSGALG00010002458	ENSGALG00010002458	2	0.238076338	0.116295608	2.04716535	0.040641852	0	0.240992336	0	1	CRA004775;Yang2025_Silkie_Wenchang	PRJNA827465	0.348841826
ENSGALG00010002458	ENSGALG00010002458	2	0.155140602	0.088127242	1.76041595	0.078337307	0	0.495063738	0	1	CRA004775;PRJNA827465	Yang2025_Silkie_Wenchang	0.481707034
ENSGALG00010002459	G3BP2	2	-0.003656299	0.037036303	-0.098722025	0.921358981	0	0.369231187	0	0.5	PRJNA827465;Yang2025_Silkie_Wenchang	CRA004775	0.981519273
ENSGALG00010002459	G3BP2	2	-0.038838131	0.070869488	-0.5480233	0.583675904	0	0.045121032	0	1	CRA004775;Yang2025_Silkie_Wenchang	PRJNA827465	0.890558522
ENSGALG00010002459	G3BP2	2	0.00687072	0.040328838	0.170367423	0.86472119	0	0.01286656	0	0.5	CRA004775;PRJNA827465	Yang2025_Silkie_Wenchang	0.966723659
ENSGALG00010002460	LAMTOR1	2	-0.045749707	0.246540097	-0.185567004	0.852784331	0.092430307	3.568543748	71.9773647	0.5	PRJNA827465;Yang2025_Silkie_Wenchang	CRA004775	0.962680849
ENSGALG00010002460	LAMTOR1	2	0.090953369	0.228104215	0.39873603	0.690087716	0.017085669	1.181018663	15.327333	0.5	CRA004775;Yang2025_Silkie_Wenchang	PRJNA827465	0.919979266
ENSGALG00010002460	LAMTOR1	2	-0.238920644	0.077549873	-3.080864424	0.002064006	0	0.009256976	0	1	CRA004775;PRJNA827465	Yang2025_Silkie_Wenchang	0.059057851
ENSGALG00010002461	TOMT	2	0.345641471	0.433977296	0.796450584	0.425770187	0.35182234	15.00205988	93.33424871	0.5	PRJNA827465;Yang2025_Silkie_Wenchang	CRA004775	0.84125685
ENSGALG00010002461	TOMT	2	0.249450912	0.550619067	0.453037184	0.650521967	0.561700546	13.5350653	92.61178296	0.5	CRA004775;Yang2025_Silkie_Wenchang	PRJNA827465	0.908618725
ENSGALG00010002461	TOMT	2	-0.12699993	0.108190304	-1.173856858	0.240452365	0	0.785052021	0	1	CRA004775;PRJNA827465	Yang2025_Silkie_Wenchang	0.753027834
ENSGALG00010002462	RRM2	2	-0.027192984	0.107489928	-0.252981697	0.800282357	0.004107132	1.18896908	15.89358208	0.5	PRJNA827465;Yang2025_Silkie_Wenchang	CRA004775	0.950064546
ENSGALG00010002462	RRM2	2	0.103065202	0.164502327	0.626527316	0.530969126	0	0.134948458	0	0.5	CRA004775;Yang2025_Silkie_Wenchang	PRJNA827465	0.872840748
ENSGALG00010002462	RRM2	2	-0.102564497	0.112657165	-0.910412553	0.36260498	0	0.002769787	0	1	CRA004775;PRJNA827465	Yang2025_Silkie_Wenchang	0.815907913
ENSGALG00010002463	ASRGL1	2	0.029182998	0.088595038	0.329397659	0.741855137	0	0.162096856	0	1	PRJNA827465;Yang2025_Silkie_Wenchang	CRA004775	0.93543545
ENSGALG00010002463	ASRGL1	2	-0.154617361	0.267617029	-0.577756064	0.563428822	0.101576998	3.35190903	70.16625478	0.5	CRA004775;Yang2025_Silkie_Wenchang	PRJNA827465	0.882407972
ENSGALG00010002463	ASRGL1	2	-0.170615985	0.223339474	-0.763931171	0.444908261	0.070601484	3.048689239	67.19901828	0.5	CRA004775;PRJNA827465	Yang2025_Silkie_Wenchang	0.846013379
ENSGALG00010002464	NGLY1	2	0.127936589	0.143703008	0.890284696	0.373313036	0.030984765	3.763584501	73.42958556	1	PRJNA827465;Yang2025_Silkie_Wenchang	CRA004775	0.822187352
ENSGALG00010002464	NGLY1	2	0.234554051	0.106676344	2.198744751	0.027896077	0	0.597999317	0	1	CRA004775;Yang2025_Silkie_Wenchang	PRJNA827465	0.284557362
ENSGALG00010002464	NGLY1	2	0.01938238	0.066269215	0.292479387	0.769920118	0	0.358649315	0	1	CRA004775;PRJNA827465	Yang2025_Silkie_Wenchang	0.941995757
ENSGALG00010002466	UROS	2	0.068541756	0.077343776	0.886196146	0.37551184	0	0.428440122	0	1	PRJNA827465;Yang2025_Silkie_Wenchang	CRA004775	0.822974013
ENSGALG00010002466	UROS	2	-0.208762713	0.374813898	-0.556976979	0.577543158	0.243825485	7.294724184	86.2914625	0.5	CRA004775;Yang2025_Silkie_Wenchang	PRJNA827465	0.88731056
ENSGALG00010002466	UROS	2	-0.250929517	0.320056657	-0.784015927	0.433030765	0.173478192	6.027997916	83.4107441	0.5	CRA004775;PRJNA827465	Yang2025_Silkie_Wenchang	0.842762754
ENSGALG00010002467	NEK3	2	0.424321647	0.31184706	1.360672267	0.173617282	0.161978069	5.95201808	83.19897577	1	PRJNA827465;Yang2025_Silkie_Wenchang	CRA004775	0.677355574
ENSGALG00010002467	NEK3	2	0.177498722	0.18084993	0.981469674	0.326361182	0	0.92183804	0	1	CRA004775;Yang2025_Silkie_Wenchang	PRJNA827465	0.800028239
ENSGALG00010002467	NEK3	2	0.70994151	0.153638538	4.620855668	3.82161E-06	0	0.091606526	0	1	CRA004775;PRJNA827465	Yang2025_Silkie_Wenchang	0.000673545
ENSGALG00010002468	ENSGALG00010002468	2	-0.209442096	0.330174652	-0.634337296	0.525860722	0	0.596924863	0	0.5	PRJNA827465;Yang2025_Silkie_Wenchang	CRA004775	0.870414822
ENSGALG00010002468	ENSGALG00010002468	2	0.052275525	0.358754716	0.145713832	0.884147301	0	0.040887191	0	1	CRA004775;Yang2025_Silkie_Wenchang	PRJNA827465	0.972001076
ENSGALG00010002468	ENSGALG00010002468	2	-0.253308118	0.403026329	-0.628515063	0.529666582	0	0.666214124	0	0.5	CRA004775;PRJNA827465	Yang2025_Silkie_Wenchang	0.871944741
ENSGALG00010002470	OXSM	2	-0.012756927	0.098184529	-0.129928081	0.896623328	0	0.002152187	0	1	PRJNA827465;Yang2025_Silkie_Wenchang	CRA004775	0.975115773
ENSGALG00010002470	OXSM	2	-0.039537781	0.127077893	-0.311130281	0.755701582	0	0.056733853	0	1	CRA004775;Yang2025_Silkie_Wenchang	PRJNA827465	0.938501783
ENSGALG00010002470	OXSM	2	-0.027231023	0.110039196	-0.247466573	0.804547158	0	0.083416372	0	1	CRA004775;PRJNA827465	Yang2025_Silkie_Wenchang	0.951665484
ENSGALG00010002471	OGFRL1	2	-0.040889308	0.208342035	-0.196260481	0.844406291	0.070793866	5.257554281	80.97974939	0.5	PRJNA827465;Yang2025_Silkie_Wenchang	CRA004775	0.960752464
ENSGALG00010002471	OGFRL1	2	-0.161790591	0.18249436	-0.886551182	0.375320587	0.020535742	1.301724491	23.17882877	0.5	CRA004775;Yang2025_Silkie_Wenchang	PRJNA827465	0.822941424
ENSGALG00010002471	OGFRL1	2	0.150948925	0.092819117	1.626269786	0.103892276	0	6.45725E-05	0	1	CRA004775;PRJNA827465	Yang2025_Silkie_Wenchang	0.548175896
ENSGALG00010002472	CDC37	2	0.25361721	0.290277791	0.873705182	0.382278828	0.161341482	23.32514853	95.71278186	0.5	PRJNA827465;Yang2025_Silkie_Wenchang	CRA004775	0.825080207
ENSGALG00010002472	CDC37	2	0.146312784	0.430119588	0.340167683	0.733730254	0.336931117	10.70508343	90.65864357	0.5	CRA004775;Yang2025_Silkie_Wenchang	PRJNA827465	0.933382967
ENSGALG00010002472	CDC37	2	-0.075009211	0.10137219	-0.739938746	0.459337163	0.007691525	1.240865738	19.41110392	1	CRA004775;PRJNA827465	Yang2025_Silkie_Wenchang	0.850098539
ENSGALG00010002473	ENSGALG00010002473	2	0.787655707	0.752088537	1.0472912	0.294965294	0.348664611	1.369029478	26.95555383	1	PRJNA827465;Yang2025_Silkie_Wenchang	CRA004775	0.787569357
ENSGALG00010002473	ENSGALG00010002473	2	0.656477906	0.964175942	0.680869412	0.495954125	1.015572058	2.070482874	51.70208783	0.5	CRA004775;Yang2025_Silkie_Wenchang	PRJNA827465	0.860911322
ENSGALG00010002473	ENSGALG00010002473	2	0.082893117	0.49528998	0.167362798	0.867084595	0	0.142277271	0	0.5	CRA004775;PRJNA827465	Yang2025_Silkie_Wenchang	0.967699
ENSGALG00010002474	HPCAL1	2	0.060310346	0.073542608	0.820073519	0.412174197	0	0.684460714	0	0.5	PRJNA827465;Yang2025_Silkie_Wenchang	CRA004775	0.835532508
ENSGALG00010002474	HPCAL1	2	-0.14092182	0.211841273	-0.665223628	0.50590747	0.047918162	1.717489106	41.77546765	1	CRA004775;Yang2025_Silkie_Wenchang	PRJNA827465	0.863927652
ENSGALG00010002474	HPCAL1	2	-0.098487753	0.286957698	-0.343213489	0.731437858	0.116990442	2.8081489	64.38935272	0.5	CRA004775;PRJNA827465	Yang2025_Silkie_Wenchang	0.932851968
ENSGALG00010002475	ENSGALG00010002475	2	0.204990057	0.163150769	1.256445546	0.208954477	0	0.001315138	0	1	PRJNA827465;Yang2025_Silkie_Wenchang	CRA004775	0.721011841
ENSGALG00010002475	ENSGALG00010002475	2	0.210108621	0.215437948	0.975262822	0.329429907	0	0.000215402	0	1	CRA004775;Yang2025_Silkie_Wenchang	PRJNA827465	0.801013588
ENSGALG00010002475	ENSGALG00010002475	2	0.201199136	0.187241351	1.074544352	0.282578777	0	0.000125716	0	1	CRA004775;PRJNA827465	Yang2025_Silkie_Wenchang	0.779532565
ENSGALG00010002476	DDX3X	2	0.076467944	0.064744309	1.181075917	0.237572566	0.003886638	1.709919701	41.5177216	1	PRJNA827465;Yang2025_Silkie_Wenchang	CRA004775	0.749400455
ENSGALG00010002476	DDX3X	2	0.432087948	0.340333064	1.269603204	0.204226008	0.177601224	3.580842837	72.0736138	1	CRA004775;Yang2025_Silkie_Wenchang	PRJNA827465	0.717271785
ENSGALG00010002476	DDX3X	2	0.374080169	0.412573087	0.906700367	0.364565269	0.286046371	5.347883135	81.30101248	1	CRA004775;PRJNA827465	Yang2025_Silkie_Wenchang	0.817496251
ENSGALG00010002478	PPME1	2	0.074666244	0.087072526	0.85751784	0.39115876	0.009453345	2.170207284	53.92145224	1	PRJNA827465;Yang2025_Silkie_Wenchang	CRA004775	0.829251057
ENSGALG00010002478	PPME1	2	-0.109707932	0.319203058	-0.343693236	0.731076999	0.184970187	10.78407643	90.72706869	0.5	CRA004775;Yang2025_Silkie_Wenchang	PRJNA827465	0.932769533
ENSGALG00010002478	PPME1	2	-0.186207727	0.224371839	-0.829906853	0.40659145	0.089375114	8.172154797	87.76332528	0.5	CRA004775;PRJNA827465	Yang2025_Silkie_Wenchang	0.833618122
ENSGALG00010002481	CKAP2	2	0.024555022	0.078902405	0.31120752	0.755642867	0	0.018530861	0	1	PRJNA827465;Yang2025_Silkie_Wenchang	CRA004775	0.938501783
ENSGALG00010002481	CKAP2	2	0.01433075	0.125518162	0.114172721	0.909100876	0	0.015875582	0	1	CRA004775;Yang2025_Silkie_Wenchang	PRJNA827465	0.978513889
ENSGALG00010002481	CKAP2	2	0.034426821	0.098288898	0.350261547	0.726142421	0	0.007804073	0	1	CRA004775;PRJNA827465	Yang2025_Silkie_Wenchang	0.931029023
ENSGALG00010002483	COQ2	2	-0.205647701	0.087371633	-2.353712466	0.018586982	0	0.163511863	0	1	PRJNA827465;Yang2025_Silkie_Wenchang	CRA004775	0.228074982
ENSGALG00010002483	COQ2	2	-0.372481053	0.155497189	-2.39541986	0.016601343	0	0.228055235	0	1	CRA004775;Yang2025_Silkie_Wenchang	PRJNA827465	0.213438855
ENSGALG00010002483	COQ2	2	-0.244473462	0.102305324	-2.389645549	0.01686464	0.004389131	1.164087238	14.09578536	1	CRA004775;PRJNA827465	Yang2025_Silkie_Wenchang	0.215695069
ENSGALG00010002487	BCCIP	2	-0.026575801	0.052696762	-0.504315645	0.614039585	0	0.788486903	0	0.5	PRJNA827465;Yang2025_Silkie_Wenchang	CRA004775	0.898282836
ENSGALG00010002487	BCCIP	2	0.05303742	0.104570212	0.507194339	0.612018466	0	0.059480234	0	1	CRA004775;Yang2025_Silkie_Wenchang	PRJNA827465	0.897777844
ENSGALG00010002487	BCCIP	2	-0.04746239	0.057401901	-0.826843518	0.40832576	0	0.042431948	0	0.5	CRA004775;PRJNA827465	Yang2025_Silkie_Wenchang	0.834134074
ENSGALG00010002492	RPL8	2	0.04465496	0.147001058	0.303773054	0.761300797	0.030290847	2.856745905	64.99513666	0.5	PRJNA827465;Yang2025_Silkie_Wenchang	CRA004775	0.939618592
ENSGALG00010002492	RPL8	2	-0.049403147	0.324406102	-0.152287972	0.878959811	0.15922439	3.933605226	74.57802849	0.5	CRA004775;Yang2025_Silkie_Wenchang	PRJNA827465	0.971127951
ENSGALG00010002492	RPL8	2	-0.131885196	0.135693205	-0.971936626	0.331082077	0.0188907	1.452865607	31.17050917	1	CRA004775;PRJNA827465	Yang2025_Silkie_Wenchang	0.801722786
ENSGALG00010002500	OSR1	2	0.219878673	0.249422854	0.881549822	0.3780203	0.083164668	2.378413364	57.95516393	1	PRJNA827465;Yang2025_Silkie_Wenchang	CRA004775	0.82325159
ENSGALG00010002500	OSR1	2	0.609796672	0.273133623	2.232594673	0.025575686	0	0.01564448	0	1	CRA004775;Yang2025_Silkie_Wenchang	PRJNA827465	0.272875365
ENSGALG00010002500	OSR1	2	0.178597831	0.248509825	0.71867513	0.47234111	0.070362086	1.614825418	38.0738011	1	CRA004775;PRJNA827465	Yang2025_Silkie_Wenchang	0.853998373
ENSGALG00010002501	TCEANC	2	-0.094652481	0.099735401	-0.949035952	0.342602327	0	0.358239802	0	1	PRJNA827465;Yang2025_Silkie_Wenchang	CRA004775	0.806011702
ENSGALG00010002501	TCEANC	2	-0.105823672	0.159580596	-0.663136213	0.50724332	0	0.704260831	0	0.5	CRA004775;Yang2025_Silkie_Wenchang	PRJNA827465	0.864048203
ENSGALG00010002501	TCEANC	2	-0.035646314	0.109261815	-0.326246767	0.74423766	0	0.258950421	0	0.5	CRA004775;PRJNA827465	Yang2025_Silkie_Wenchang	0.935821071
ENSGALG00010002504	ENSGALG00010002504	2	-0.061669165	0.039953302	-1.543531124	0.122701962	0.000546607	1.062619939	5.89297613	0.5	PRJNA827465;Yang2025_Silkie_Wenchang	CRA004775	0.58762464
ENSGALG00010002504	ENSGALG00010002504	2	0.194289422	0.180181544	1.07829813	0.280900731	0.033836083	1.905699747	47.52583656	1	CRA004775;Yang2025_Silkie_Wenchang	PRJNA827465	0.778581075
ENSGALG00010002504	ENSGALG00010002504	2	0.126404512	0.250649406	0.504308045	0.614044925	0.102018778	4.418003016	77.36533913	0.5	CRA004775;PRJNA827465	Yang2025_Silkie_Wenchang	0.898282836
ENSGALG00010002507	C2CD3	2	-0.024318219	0.064579341	-0.376563441	0.706498061	0	0.074168452	0	1	PRJNA827465;Yang2025_Silkie_Wenchang	CRA004775	0.924548793
ENSGALG00010002507	C2CD3	2	-0.075320038	0.122694131	-0.613884601	0.539291587	0	0.262487108	0	1	CRA004775;Yang2025_Silkie_Wenchang	PRJNA827465	0.874983893
ENSGALG00010002507	C2CD3	2	-0.022892499	0.073285792	-0.312372945	0.754757109	0	0.397855556	0	1	CRA004775;PRJNA827465	Yang2025_Silkie_Wenchang	0.93838723
ENSGALG00010002511	VPS36	2	0.015765204	0.054656772	0.28844009	0.773009884	0.001549477	1.305445561	23.3978015	0.5	PRJNA827465;Yang2025_Silkie_Wenchang	CRA004775	0.943100418
ENSGALG00010002511	VPS36	2	0.069888304	0.074737215	0.935120529	0.349726188	0	0.294309114	0	0.5	CRA004775;Yang2025_Silkie_Wenchang	PRJNA827465	0.809903518
ENSGALG00010002511	VPS36	2	-0.022009665	0.051836219	-0.424600125	0.671128202	0	0.009985895	0	1	CRA004775;PRJNA827465	Yang2025_Silkie_Wenchang	0.914621672
ENSGALG00010002512	DAXX	2	0.192518849	0.34142453	0.563869411	0.572843009	0.22010144	17.70991978	94.35344704	0.5	PRJNA827465;Yang2025_Silkie_Wenchang	CRA004775	0.88573298
ENSGALG00010002512	DAXX	2	0.402478075	0.200751859	2.004853543	0.044978706	0.041093449	1.812362756	44.82340818	1	CRA004775;Yang2025_Silkie_Wenchang	PRJNA827465	0.365625254
ENSGALG00010002512	DAXX	2	-0.119309574	0.083540826	-1.428158903	0.153246127	0	0.720990993	0	0.5	CRA004775;PRJNA827465	Yang2025_Silkie_Wenchang	0.645602272
ENSGALG00010002518	MAST1	2	0.18696768	0.780028354	0.239693441	0.81056792	1.127357169	13.55789532	92.62422392	0.5	PRJNA827465;Yang2025_Silkie_Wenchang	CRA004775	0.952997426
ENSGALG00010002518	MAST1	2	0.498842549	0.4866108	1.025136617	0.30529873	0.359524257	4.149112569	75.89846062	1	CRA004775;Yang2025_Silkie_Wenchang	PRJNA827465	0.79225794
ENSGALG00010002518	MAST1	2	-0.330277833	0.290479077	-1.137010749	0.255533792	0.075188823	1.774960142	43.66070673	0.5	CRA004775;PRJNA827465	Yang2025_Silkie_Wenchang	0.764467569
ENSGALG00010002520	TTC32	2	0.44109316	0.236753131	1.863093252	0.062449147	0.061316769	2.108542035	52.57386463	1	PRJNA827465;Yang2025_Silkie_Wenchang	CRA004775	0.430841401
ENSGALG00010002520	TTC32	2	-0.039522558	0.23845552	-0.165743943	0.868358463	0.01526947	1.146831465	12.80322954	0.5	CRA004775;Yang2025_Silkie_Wenchang	PRJNA827465	0.96814628
ENSGALG00010002520	TTC32	2	0.199444175	0.483087449	0.412853149	0.679714208	0.390751408	5.822978956	82.82665956	0.5	CRA004775;PRJNA827465	Yang2025_Silkie_Wenchang	0.917701868
ENSGALG00010002522	HPSE	2	-0.152659621	0.186623974	-0.818006485	0.413353484	0	0.400021895	0	1	PRJNA827465;Yang2025_Silkie_Wenchang	CRA004775	0.836088312
ENSGALG00010002522	HPSE	2	-0.362575395	0.254756849	-1.423221381	0.154671989	0	0.175185361	0	1	CRA004775;Yang2025_Silkie_Wenchang	PRJNA827465	0.647505492
ENSGALG00010002522	HPSE	2	-0.148970081	0.21598607	-0.689720781	0.490369795	0	0.769859136	0	1	CRA004775;PRJNA827465	Yang2025_Silkie_Wenchang	0.858529056
ENSGALG00010002523	DNAJB13	2	0.421692721	0.201316869	2.094671565	0.036200199	0.012750347	1.184310385	15.56267573	1	PRJNA827465;Yang2025_Silkie_Wenchang	CRA004775	0.328206784
ENSGALG00010002523	DNAJB13	2	0.575254345	0.262548971	2.191036376	0.02844916	0	0.55871096	0	1	CRA004775;Yang2025_Silkie_Wenchang	PRJNA827465	0.287193179
ENSGALG00010002523	DNAJB13	2	0.220648593	0.235095466	0.938548909	0.347962395	0	0.069894767	0	1	CRA004775;PRJNA827465	Yang2025_Silkie_Wenchang	0.808847403
ENSGALG00010002526	ZBTB22	2	-0.03192719	0.256181261	-0.124627344	0.90081858	0.099335247	3.819235218	73.81674752	0.5	PRJNA827465;Yang2025_Silkie_Wenchang	CRA004775	0.976497577
ENSGALG00010002526	ZBTB22	2	0.184478022	0.187035065	0.986328538	0.32397192	0	0.327778421	0	1	CRA004775;Yang2025_Silkie_Wenchang	PRJNA827465	0.799600434
ENSGALG00010002526	ZBTB22	2	-0.211053888	0.110667808	-1.907093775	0.056508445	0	0.835240805	0	0.5	CRA004775;PRJNA827465	Yang2025_Silkie_Wenchang	0.411523553
ENSGALG00010002527	LRRC3B	2	-0.22682163	0.136696393	-1.659309541	0.097053434	0	0.297411387	0	1	PRJNA827465;Yang2025_Silkie_Wenchang	CRA004775	0.532304089
ENSGALG00010002527	LRRC3B	2	0.149552348	0.617148157	0.242328113	0.808525932	0.559495872	3.318242676	69.86356642	0.5	CRA004775;Yang2025_Silkie_Wenchang	PRJNA827465	0.952496413
ENSGALG00010002527	LRRC3B	2	0.193544067	0.521110032	0.371407295	0.710334193	0.384138629	2.727953667	63.34248591	0.5	CRA004775;PRJNA827465	Yang2025_Silkie_Wenchang	0.925364369
ENSGALG00010002528	MED14	2	0.127527495	0.192126139	0.663769623	0.506837771	0.060410755	4.931324684	79.72147315	0.5	PRJNA827465;Yang2025_Silkie_Wenchang	CRA004775	0.863929781
ENSGALG00010002528	MED14	2	0.116005175	0.252697543	0.459067284	0.64618585	0.092692758	3.609819766	72.2977859	0.5	CRA004775;Yang2025_Silkie_Wenchang	PRJNA827465	0.907220702
ENSGALG00010002528	MED14	2	-0.043908074	0.054622137	-0.803851257	0.421482883	0	0.293474242	0	1	CRA004775;PRJNA827465	Yang2025_Silkie_Wenchang	0.839352211
ENSGALG00010002530	STAG3	2	0.107284374	0.106133695	1.010841788	0.312092159	0.010365538	1.847942835	45.88577196	1	PRJNA827465;Yang2025_Silkie_Wenchang	CRA004775	0.796054997
ENSGALG00010002530	STAG3	2	0.056676462	0.205223536	0.276169405	0.782417955	0.055095693	2.628483266	61.95524572	0.5	CRA004775;Yang2025_Silkie_Wenchang	PRJNA827465	0.945410671
ENSGALG00010002530	STAG3	2	-0.028849128	0.095811703	-0.30110234	0.763336457	0	0.669901202	0	0.5	CRA004775;PRJNA827465	Yang2025_Silkie_Wenchang	0.940276829
ENSGALG00010002533	LPAR2	2	-0.190135588	0.193179172	-0.984244763	0.324995181	0.053576337	3.479859288	71.26320586	1	PRJNA827465;Yang2025_Silkie_Wenchang	CRA004775	0.799914637
ENSGALG00010002533	LPAR2	2	-0.240543149	0.214241856	-1.122764499	0.261537527	0.043488832	1.771526668	43.55151304	0.5	CRA004775;Yang2025_Silkie_Wenchang	PRJNA827465	0.768310285
ENSGALG00010002533	LPAR2	2	-0.003757086	0.113545124	-0.033088928	0.973603672	0	0.034981145	0	0.5	CRA004775;PRJNA827465	Yang2025_Silkie_Wenchang	0.992442268
ENSGALG00010002536	RGL2	2	-0.075881393	0.163865478	-0.46307126	0.64331329	0.016979145	1.406045742	28.8785585	0.5	PRJNA827465;Yang2025_Silkie_Wenchang	CRA004775	0.906634523
ENSGALG00010002536	RGL2	2	-0.080545289	0.222437935	-0.362102305	0.717275581	0.041697577	1.727791507	42.12264639	0.5	CRA004775;Yang2025_Silkie_Wenchang	PRJNA827465	0.9277944
ENSGALG00010002536	RGL2	2	-0.228158176	0.130387772	-1.749843359	0.080145347	0	0.130171747	0	1	CRA004775;PRJNA827465	Yang2025_Silkie_Wenchang	0.487141092
ENSGALG00010002539	SNAP91	2	0.741710127	0.267424049	2.77353562	0.005545077	0.042763283	1.423297557	29.74062277	1	PRJNA827465;Yang2025_Silkie_Wenchang	CRA004775	0.111717753
ENSGALG00010002539	SNAP91	2	0.277645087	0.798424891	0.347741021	0.728034687	1.090993251	6.783904214	85.25922583	0.5	CRA004775;Yang2025_Silkie_Wenchang	PRJNA827465	0.931898625
ENSGALG00010002539	SNAP91	2	0.04364494	0.525332306	0.083080632	0.933787426	0.387319743	3.173532857	68.48937619	0.5	CRA004775;PRJNA827465	Yang2025_Silkie_Wenchang	0.984301003
ENSGALG00010002543	UCP3	2	-0.213627362	0.220450557	-0.969048864	0.33252081	0	0.002560612	0	1	PRJNA827465;Yang2025_Silkie_Wenchang	CRA004775	0.802178578
ENSGALG00010002543	UCP3	2	-0.173488937	0.351555248	-0.493489822	0.621666529	0	0.034552341	0	1	CRA004775;Yang2025_Silkie_Wenchang	PRJNA827465	0.899796991
ENSGALG00010002543	UCP3	2	-0.206275928	0.262494377	-0.785829892	0.431967152	0	0.046004523	0	1	CRA004775;PRJNA827465	Yang2025_Silkie_Wenchang	0.842503929
ENSGALG00010002544	THSD1	2	-0.212812213	0.283058757	-0.751830521	0.452152985	0.148648482	13.55055923	92.62023077	0.5	PRJNA827465;Yang2025_Silkie_Wenchang	CRA004775	0.848321192
ENSGALG00010002544	THSD1	2	-0.117632223	0.433772073	-0.271184409	0.786249204	0.321787875	6.464441599	84.53075978	0.5	CRA004775;Yang2025_Silkie_Wenchang	PRJNA827465	0.946639719
ENSGALG00010002544	THSD1	2	0.075440099	0.072418741	1.041720663	0.297541206	0	0.890416529	0	1	CRA004775;PRJNA827465	Yang2025_Silkie_Wenchang	0.788562929
ENSGALG00010002546	ATP6AP2	2	-0.170363448	0.204545333	-0.832888461	0.404907638	0.079246402	18.53487534	94.60476544	0.5	PRJNA827465;Yang2025_Silkie_Wenchang	CRA004775	0.833112102
ENSGALG00010002546	ATP6AP2	2	-0.375218059	0.083289105	-4.505007693	6.63705E-06	0	0.138170665	0	1	CRA004775;Yang2025_Silkie_Wenchang	PRJNA827465	0.001018969
ENSGALG00010002546	ATP6AP2	2	0.024944462	0.042219096	0.590833636	0.554631896	0	0.381395659	0	0.5	CRA004775;PRJNA827465	Yang2025_Silkie_Wenchang	0.880223402
ENSGALG00010002547	PBX4	2	-0.012225681	0.060417288	-0.202354024	0.839639968	0.00119258	1.119910655	10.70716259	0.5	PRJNA827465;Yang2025_Silkie_Wenchang	CRA004775	0.959493354
ENSGALG00010002547	PBX4	2	0.016512058	0.133506717	0.123679608	0.901568958	0.009385374	1.291412491	22.56540751	0.5	CRA004775;Yang2025_Silkie_Wenchang	PRJNA827465	0.976674416

ENSGALG00010002547	PBX4	2	-0.052284884	0.056404437	-0.926964024	0.353945214	0	0.377240618	0	1	CRA004775;PRJNA827465	Yang2025_Silkie_Wenchang	0.811521918
ENSGALG00010002551	WDR35	2	-0.025919241	0.231344416	-0.11203746	0.910793705	0.093675971	8.009083616	87.51417705	0.5	PRJNA827465;Yang2025_Silkie_Wenchang	CRA004775	0.97880407
ENSGALG00010002551	WDR35	2	-0.251414953	0.110442104	-2.276441178	0.022819617	0	0.030438515	0	1	CRA004775;Yang2025_Silkie_Wenchang	PRJNA827465	0.257428078
ENSGALG00010002551	WDR35	2	0.176907002	0.112099826	1.578120221	0.114537994	0	0.828857509	0	0.5	CRA004775;PRJNA827465	Yang2025_Silkie_Wenchang	0.570628313
ENSGALG00010002553	ENSGALG00010002553	2	0.559789049	0.456500006	1.226262962	0.220099734	0.340976991	5.483244969	81.76262404	1	PRJNA827465;Yang2025_Silkie_Wenchang	CRA004775	0.733691086
ENSGALG00010002553	ENSGALG00010002553	2	0.702694494	0.52160043	1.347189254	0.177919288	0.291377418	1.75854496	43.13480618	0.5	CRA004775;Yang2025_Silkie_Wenchang	PRJNA827465	0.683951879
ENSGALG00010002553	ENSGALG00010002553	2	0.092357593	0.245104739	0.376808682	0.706315788	0	0.082937943	0	0.5	CRA004775;PRJNA827465	Yang2025_Silkie_Wenchang	0.924548793
ENSGALG00010002554	EGFL6	2	-0.226850964	0.139270349	-1.628853271	0.103344084	0.019736821	2.021323316	50.5274593	1	PRJNA827465;Yang2025_Silkie_Wenchang	CRA004775	0.547576788
ENSGALG00010002554	EGFL6	2	0.25146885	0.73070901	0.344143629	0.730738274	0.907855855	5.938298909	83.16016059	0.5	CRA004775;Yang2025_Silkie_Wenchang	PRJNA827465	0.932769533
ENSGALG00010002554	EGFL6	2	0.363053915	0.583113883	0.622612367	0.533539282	0.537154322	3.97431643	74.83844033	0.5	CRA004775;PRJNA827465	Yang2025_Silkie_Wenchang	0.873379507
ENSGALG00010002556	ENSGALG00010002556	2	-0.362568605	0.113833943	-3.18506586	0.001447211	0	0.15884834	0	1	PRJNA827465;Yang2025_Silkie_Wenchang	CRA004775	0.047151552
ENSGALG00010002556	ENSGALG00010002556	2	-0.126600771	0.377481965	-0.335382304	0.737336715	0.194189159	2.811150448	64.42737525	0.5	CRA004775;Yang2025_Silkie_Wenchang	PRJNA827465	0.933968189
ENSGALG00010002556	ENSGALG00010002556	2	-0.104112414	0.320467188	-0.324876986	0.745274176	0.133365621	2.350151558	57.4495527	0.5	CRA004775;PRJNA827465	Yang2025_Silkie_Wenchang	0.935980538
ENSGALG00010002557	ENSGALG00010002557	2	0.120134276	0.168966432	0.710994928	0.477085379	0	0.446633862	0	1	PRJNA827465;Yang2025_Silkie_Wenchang	CRA004775	0.854986476
ENSGALG00010002557	ENSGALG00010002557	2	-0.22021831	0.328184948	-0.671018921	0.502208476	0.11106199	1.866135398	46.41332022	0.5	CRA004775;Yang2025_Silkie_Wenchang	PRJNA827465	0.862860181
ENSGALG00010002557	ENSGALG00010002557	2	-0.139162364	0.452725746	-0.307387784	0.758548226	0.288674658	3.168816074	68.44247262	0.5	CRA004775;PRJNA827465	Yang2025_Silkie_Wenchang	0.938954369
ENSGALG00010002561	PAAF1	2	-0.086640653	0.073351066	-1.18117783	0.237532087	0.003597082	1.385207824	27.8086665	0.5	PRJNA827465;Yang2025_Silkie_Wenchang	CRA004775	0.749400455
ENSGALG00010002561	PAAF1	2	-0.22359774	0.275118222	-0.8127333	0.416370985	0.123724067	5.263279932	81.00765747	0.5	CRA004775;Yang2025_Silkie_Wenchang	PRJNA827465	0.837836822
ENSGALG00010002561	PAAF1	2	-0.279815151	0.189382793	-1.477510951	0.139538725	0.053067157	3.238751563	69.12390529	1	CRA004775;PRJNA827465	Yang2025_Silkie_Wenchang	0.623285658
ENSGALG00010002565	MRPS18C	2	0.004400955	0.075061859	0.058631046	0.953245982	0	0.601248099	0	0.5	PRJNA827465;Yang2025_Silkie_Wenchang	CRA004775	0.987758913
ENSGALG00010002565	MRPS18C	2	-0.089076042	0.171094057	-0.52062616	0.602627222	0	0.232168434	0	0.5	CRA004775;Yang2025_Silkie_Wenchang	PRJNA827465	0.895527108
ENSGALG00010002565	MRPS18C	2	0.027652545	0.077986051	0.354583221	0.722901848	0	0.000490947	0	1	CRA004775;PRJNA827465	Yang2025_Silkie_Wenchang	0.930032096
ENSGALG00010002566	TMSB4X	2	-0.010629307	0.087759031	-0.121119238	0.903596591	0.004674036	1.148216283	12.90839409	0.5	PRJNA827465;Yang2025_Silkie_Wenchang	CRA004775	0.97732491
ENSGALG00010002566	TMSB4X	2	-0.300904628	0.186944355	-1.609594619	0.107486384	0	0.120100513	0	1	CRA004775;Yang2025_Silkie_Wenchang	PRJNA827465	0.554966053
ENSGALG00010002566	TMSB4X	2	-0.070695448	0.17267708	-0.444155346	0.656930274	0.035456516	1.789209649	44.10940045	0.5	CRA004775;PRJNA827465	Yang2025_Silkie_Wenchang	0.910670519
ENSGALG00010002567	COA4	2	-0.12354439	0.325977444	-0.378996745	0.704690281	0.148895928	3.230704087	69.04699493	0.5	PRJNA827465;Yang2025_Silkie_Wenchang	CRA004775	0.924152671
ENSGALG00010002567	COA4	2	-0.505990567	0.239437388	-2.113247937	0.034579552	0	0.006007372	0	1	CRA004775;Yang2025_Silkie_Wenchang	PRJNA827465	0.320322559
ENSGALG00010002567	COA4	2	-0.103392179	0.338420592	-0.305513854	0.75997482	0.145927109	2.527442568	60.43431361	0.5	CRA004775;PRJNA827465	Yang2025_Silkie_Wenchang	0.939215731
ENSGALG00010002569	GTPBP6	2	-0.081507925	0.109194562	-0.746446979	0.455397449	0.009535148	1.629995885	38.65015188	0.5	PRJNA827465;Yang2025_Silkie_Wenchang	CRA004775	0.84907427
ENSGALG00010002569	GTPBP6	2	-0.203633945	0.128568419	-1.58385663	0.113226347	0	0.027366462	0	1	CRA004775;Yang2025_Silkie_Wenchang	PRJNA827465	0.567909023
ENSGALG00010002569	GTPBP6	2	-0.005889676	0.09820992	-0.059970273	0.952179311	0	0.233680574	0	0.5	CRA004775;PRJNA827465	Yang2025_Silkie_Wenchang	0.987758913
ENSGALG00010002570	ADAM12	2	0.051147772	0.09922361	0.515478949	0.606217747	0	0.298628891	0	0.5	PRJNA827465;Yang2025_Silkie_Wenchang	CRA004775	0.896709756
ENSGALG00010002570	ADAM12	2	0.130470634	0.30827617	0.423226466	0.672130039	0.083436383	1.501830485	33.41458907	0.5	CRA004775;Yang2025_Silkie_Wenchang	PRJNA827465	0.914742762
ENSGALG00010002570	ADAM12	2	0.129199192	0.160805832	0.803448422	0.421715597	0.015599913	1.103670127	9.393216712	1	CRA004775;PRJNA827465	Yang2025_Silkie_Wenchang	0.839352211
ENSGALG00010002571	BCOR	2	0.132416859	0.140384002	0.943247501	0.345554322	0.028395783	3.012439448	66.80431201	1	PRJNA827465;Yang2025_Silkie_Wenchang	CRA004775	0.807354745
ENSGALG00010002571	BCOR	2	0.208358673	0.161833609	1.287487034	0.197923586	0.012552627	1.258936133	20.56785297	0.5	CRA004775;Yang2025_Silkie_Wenchang	PRJNA827465	0.710035882
ENSGALG00010002571	BCOR	2	0.023880771	0.050533135	0.472576477	0.636515368	0	0.044792652	0	1	CRA004775;PRJNA827465	Yang2025_Silkie_Wenchang	0.904566695
ENSGALG00010002573	MVB12A	2	0.063860379	0.056879484	1.122731334	0.261551616	0	0.827785051	0	1	PRJNA827465;Yang2025_Silkie_Wenchang	CRA004775	0.768310285
ENSGALG00010002573	MVB12A	2	-0.006037663	0.237547095	-0.0254167	0.97972259	0.071392385	2.464731825	59.42763469	0.5	CRA004775;Yang2025_Silkie_Wenchang	PRJNA827465	0.994474095
ENSGALG00010002573	MVB12A	2	-0.029739817	0.140710743	-0.211354273	0.832610836	0.02084774	1.507256774	33.65430382	0.5	CRA004775;PRJNA827465	Yang2025_Silkie_Wenchang	0.957861248
ENSGALG00010002575	BGV	2	0.290118657	0.226352468	1.281711922	0.199943721	0	0.200032485	0	1	PRJNA827465;Yang2025_Silkie_Wenchang	CRA004775	0.712003993
ENSGALG00010002575	BGV	2	0.194836675	0.278516073	0.699552714	0.484206682	0.012554965	1.077911018	7.227963764	0.5	CRA004775;Yang2025_Silkie_Wenchang	PRJNA827465	0.857004733
ENSGALG00010002575	BGV	2	0.068477685	0.262384552	0.260982153	0.79410627	0	0.46990187	0	0.5	CRA004775;PRJNA827465	Yang2025_Silkie_Wenchang	0.948905846
ENSGALG00010002576	HELQ	2	0.163515528	0.076768222	2.129989788	0.033172457	0	0.352325008	0	1	PRJNA827465;Yang2025_Silkie_Wenchang	CRA004775	0.313106882
ENSGALG00010002576	HELQ	2	0.026674603	0.130868254	0.203827916	0.838487977	0	0.672870982	0	0.5	CRA004775;Yang2025_Silkie_Wenchang	PRJNA827465	0.959197438
ENSGALG00010002576	HELQ	2	0.102415482	0.153448037	0.667427775	0.50448893	0.02334823	1.588019988	37.02850041	0.5	CRA004775;PRJNA827465	Yang2025_Silkie_Wenchang	0.863512504
ENSGALG00010002577	MRPL48	2	-0.004981017	0.06621639	-0.075223233	0.940037022	0	0.633279043	0	0.5	PRJNA827465;Yang2025_Silkie_Wenchang	CRA004775	0.985444231
ENSGALG00010002577	MRPL48	2	0.066629381	0.141948084	0.469392608	0.638789031	0	0.357885092	0	0.5	CRA004775;Yang2025_Silkie_Wenchang	PRJNA827465	0.905768875
ENSGALG00010002577	MRPL48	2	-0.03243299	0.070731008	-0.458539904	0.646564601	0	0.044541179	0	1	CRA004775;PRJNA827465	Yang2025_Silkie_Wenchang	0.907309644
ENSGALG00010002579	MATN3	2	0.353279025	0.815054961	0.433123125	0.664925324	0.591206077	1.773731029	43.62166622	0.5	PRJNA827465;Yang2025_Silkie_Wenchang	CRA004775	0.913399315
ENSGALG00010002579	MATN3	2	-0.509231476	1.730772637	-0.294222652	0.768588231	5.277213245	8.305561271	87.95987451	0.5	CRA004775;Yang2025_Silkie_Wenchang	PRJNA827465	0.941493634
ENSGALG00010002579	MATN3	2	-1.290504818	0.908502221	-1.420475139	0.155469401	1.112481883	3.062728018	67.34936977	1	CRA004775;PRJNA827465	Yang2025_Silkie_Wenchang	0.648167852
ENSGALG00010002581	ATP13A1	2	-0.030861351	0.047981318	-0.643195158	0.520097475	0	0.59388412	0	1	PRJNA827465;Yang2025_Silkie_Wenchang	CRA004775	0.869331539
ENSGALG00010002581	ATP13A1	2	-0.27841514	0.18662376	-1.491852589	0.135737794	0.045978872	2.826172327	64.61645749	1	CRA004775;Yang2025_Silkie_Wenchang	PRJNA827465	0.617196137
ENSGALG00010002581	ATP13A1	2	-0.220529012	0.237248674	-0.929526849	0.352616117	0.095795672	6.011177245	83.36432351	1	CRA004775;PRJNA827465	Yang2025_Silkie_Wenchang	0.811035834
ENSGALG00010002584	ENSGALG00010002584	2	0.512388472	0.245326049	2.088601982	0.036743567	0	0.893440564	0	1	PRJNA827465;Yang2025_Silkie_Wenchang	CRA004775	0.330692908
ENSGALG00010002584	ENSGALG00010002584	2	-0.058223971	0.901367795	-0.064595132	0.948496361	1.426605312	8.177360319	87.77111487	0.5	CRA004775;Yang2025_Silkie_Wenchang	PRJNA827465	0.986836946
ENSGALG00010002584	ENSGALG00010002584	2	-0.265683046	0.656467514	-0.404716213	0.685668123	0.707916427	5.450013249	81.65142075	0.5	CRA004775;PRJNA827465	Yang2025_Silkie_Wenchang	0.919235539
ENSGALG00010002586	GPR63	2	0.231498944	0.079242376	2.921403381	0.003484583	0	0.445713176	0	1	PRJNA827465;Yang2025_Silkie_Wenchang	CRA004775	0.083407175
ENSGALG00010002586	GPR63	2	0.201591262	0.174887489	1.152691156	0.249037173	0	0.757286061	0	1	CRA004775;Yang2025_Silkie_Wenchang	PRJNA827465	0.759411487
ENSGALG00010002586	GPR63	2	0.263569209	0.082769025	3.206141549	0.001345278	0	0.302041049	0	1	CRA004775;PRJNA827465	Yang2025_Silkie_Wenchang	0.044378793
ENSGALG00010002587	DOCK1	2	-0.039253704	0.056717109	-0.692096354	0.488876821	0	0.074316374	0	0.5	PRJNA827465;Yang2025_Silkie_Wenchang	CRA004775	0.858203837
ENSGALG00010002587	DOCK1	2	0.135630005	0.166065429	0.816726306	0.414084854	0.011910797	1.25366807	20.23406963	1	CRA004775;Yang2025_Silkie_Wenchang	PRJNA827465	0.836792485
ENSGALG00010002587	DOCK1	2	0.07924519	0.183060359	0.432891044	0.665093927	0.045226765	2.385080314	58.07269071	0.5	CRA004775;PRJNA827465	Yang2025_Silkie_Wenchang	0.913399315
ENSGALG00010002588	FAM175A	2	-0.012520945	0.05587752	-0.22407839	0.822696308	0	0.621325864	0	0.5	PRJNA827465;Yang2025_Silkie_Wenchang	CRA004775	0.955822987
ENSGALG00010002588	FAM175A	2	0.035787181	0.112345933	0.318544518	0.750071931	0	0.958978914	0	0.5	CRA004775;Yang2025_Silkie_Wenchang	PRJNA827465	0.937374353
ENSGALG00010002588	FAM175A	2	-0.04396697	0.062732267	-0.700866908	0.483386078	0	0.553340742	0	1	CRA004775;PRJNA827465	Yang2025_Silkie_Wenchang	0.85691916
ENSGALG00010002589	SLC4A7	2	0.173989653	0.202736154	0.858207329	0.39077799	0.076141755	13.38254048	92.52757724	0.5	PRJNA827465;Yang2025_Silkie_Wenchang	CRA004775	0.829251057
ENSGALG00010002589	SLC4A7	2	0.383796576	0.091424533	4.197960493	2.6933E-05	0	0.000291824	0	1	CRA004775;Yang2025_Silkie_Wenchang	PRJNA827465	0.003025068
ENSGALG00010002589	SLC4A7	2	-0.003462097	0.086040467	-0.040236321	0.967904721	0.004283181	1.052967146	5.030275301	0.5	CRA004775;PRJNA827465	Yang2025_Silkie_Wenchang	0.991128245
ENSGALG00010002590	ATP6V1C2	2	0.359516646	0.128912317	2.788846366	0.005289615	0	0.85604286	0	1	PRJNA827465;Yang2025_Silkie_Wenchang	CRA004775	0.108631224
ENSGALG00010002590	ATP6V1C2	2	0.583145929	0.247688993	2.354347366	0.018555262	0	0.07340371	0	1	CRA004775;Yang2025_Silkie_Wenchang	PRJNA827465	0.227943938
ENSGALG00010002590	ATP6V1C2	2	0.303387492	0.147249068	2.060369521	0.039363228	0	0.319033803	0	1	CRA004775;PRJNA827465	Yang2025_Silkie_Wenchang	0.34316319
ENSGALG00010002591	ENSGALG00010002591	2	-0.469218413	0.165055027	-2.842799895	0.004471914	0.026729653	1.769339775	43.48174308	1	PRJNA827465;Yang2025_Silkie_Wenchang	CRA004775	0.097752851
ENSGALG00010002591	ENSGALG00010002591	2	-0.199241428	0.516031345	-0.386103345	0.699420132	0.465240753	7.880963557	87.31119624	0.5	CRA004775;Yang2025_Silkie_Wenchang	PRJNA827465	0.922020923
ENSGALG00010002591	ENSGALG00010002591	2	-0.060375476	0.337419086	-0.178933049	0.857990156	0.185464919	4.955480556	79.82032239	0.5	CRA004775;PRJNA827465	Yang2025_Silkie_Wenchang	0.964162605
ENSGALG00010002592	RPS6KA3	2	-0.149320711	0.127823114	-1.168182395	0.2427332	0.018348169	2.038716163	50.94952312	0.5	PRJNA827465;Yang2025_Silkie_Wenchang	CRA004775	0.7550024
ENSGALG00010002592	RPS6KA3	2	0.072469352	0.151804282	0.477386745	0.633086751	0	0.392069813	0	1	CRA004775;Yang2025_Silkie_Wenchang	PRJNA827465	0.903231036
ENSGALG00010002592	RPS6KA3	2	-0.098210853	0.229271434	-0.428360616	0.668388594	0.0667295	2.06816926	51.64805806	0.5	CRA004775;PRJNA827465	Yang2025_Silkie_Wenchang	0.914248324
ENSGALG00010002597	ENSGALG00010002597	2	0.249497732	0.232633748	1.072557825	0.283461476	0.088091796	5.122291475	80.47748737	1	PRJNA827465;Yang2025_Silkie_Wenchang	CRA004775	0.779568117
ENSGALG00010002597	ENSGALG00010002597	2	0.019896107	0.482129904	0.041267109	0.967082954	0.438017704	17.24697735	94.20188257	0.5	CRA004775;Yang2025_Silkie_Wenchang	PRJNA827465	0.990893423
ENSGALG00010002597	ENSGALG00010002597	2	-0.196497768	0.247969455	-0.792427308	0.428111562	0.107928804	8.053017912	87.5822951	0.5	CRA004775;PRJNA827465	Yang2025_Silkie_Wenchang	0.84125685
ENSGALG00010002598	NMT2	2	-0.166548464	0.115013578	-1.448076547	0.147595644	0.022090718	5.926632285	83.12701123	1	PRJNA827465;Yang2025_Silkie_Wenchang	CRA004775	0.636767464
ENSGALG00010002598	NMT2	2	-0.005467306	0.306360925	-0.017845965	0.985761736	0.168281495	9.205574066	89.13701641	0.5	CRA004775;Yang2025_Silkie_Wenchang	PRJNA827465	0.996013375
ENSGALG00010002598	NMT2	2	0.091257424	0.187374644	0.487031876	0.626235742	0.054968185	3.93409444	74.58118977	0.5	CRA004775;PRJNA827465	Yang2025_Silkie_Wenchang	0.901504244
ENSGALG00010002599	KIFC1	2	-0.234620252	0.218465531	-1.073946313	0.282846745	0	0.007899277	0	1	PRJNA827465;Yang2025_Silkie_Wenchang	CRA004775	0.779532565
ENSGALG00010002599	KIFC1	2	-0.365383962	0.257234466	-1.420433591	0.155482071	0.003145866	1.018075254	1.775433958	1	CRA004775;Yang2025_Silkie_Wenchang	PRJNA827465	0.648167852
ENSGALG00010002599	KIFC1	2	-0.419509932	0.277637194	-1.511000476	0.13078833	0	0.831045834	0	1	CRA004775;PRJNA827465	Yang2025_Silkie_Wenchang	0.606533772
ENSGALG00010002601	PLCXD1	2	-0.321014814	0.261419478	-1.227968235	0.219458893	0.11628384	6.670868782	85.0094488	1	PRJNA827465;Yang2025_Silkie_Wenchang	CRA004775	0.732888275
ENSGALG00010002601	PLCXD1	2	-0.562088365	0.116919885	-4.807465928	1.52855E-06	0	0.057423533	0	1	CRA004775;Yang2025_Silkie_Wenchang	PRJNA827465	0.000311279
ENSGALG00010002601	PLCXD1	2	-0.219101872	0.217358072	-1.008022706	0.313445362	0.050764272	2.006664675	50.15861211	1	CRA004775;PRJNA827465	Yang2025_Silkie_Wenchang	0.796531448
ENSGALG00010002603	INSYN2A	2	0.135868915	0.09244283	1.46976153	0.141626352	0	0.02418064	0	1	PRJNA827465;Yang2025_Silkie_Wenchang	CRA004775	0.627088401
ENSGALG00010002603	INSYN2A	2	0.278533569	0.247927632	1.123447057	0.261247674	0.063066688	1.758453108	43.13183585	1	CRA004775;Yang2025_Silkie_Wenchang	PRJNA827465	0.768039422
ENSGALG00010002603	INSYN2A	2	0.275739693	0.222103454	1.241492145	0.214423998	0.053050068	1.686576611	40.70829669	1	CRA004775;PRJNA827465	Yang2025_Silkie_Wenchang	0.727205473
ENSGALG00010002604	BLEC3	2	-0.620689786	0.441884673	-1.404642035	0.16012775	0.082080866	1.237355598	19.18248874	1	PRJNA827465;Yang2025_Silkie_Wenchang	CRA004775	0.655509678
ENSGALG00010002604	BLEC3	2	-0.337798988	0.8114427	-0.416294322	0.677194647	0.964323506	3.665618413	72.71947357	0.5	CRA004775;Yang2025_Silkie_Wenchang	PRJNA827465	0.917144807
ENSGALG00010002604	BLEC3	2	0.034217717	0.35099884	0.097486696	0.922339899	0.012119334	1.051503876	4.898115663	0.5	CRA004775;PRJNA827465	Yang2025_Silkie_Wenchang	0.981804675
ENSGALG00010002606	ENSGALG00010002606	2	0.219599495	0.302323443	0.726372997	0.467610324	0.125354948	3.065938356	67.37398088	0.5	PRJNA827465;Yang2025_Silkie_Wenchang	CRA004775	0.843288838
ENSGALG00010002606	ENSGALG00010002606	2	-0.119368903	0.21558064	-0.553708825	0.579778135	0	0.001056968	0	0.5	CRA004775;Yang2025_Silkie_Wenchang	PRJNA827465	0.888013685
ENSGALG00010002606	ENSGALG00010002606	2	0.241630775	0.292781342	0.825294268	0.409204522	0.108501195	2.57086329	61.10256024	0.5	CRA004775;PRJNA827465	Yang2025_Silkie_Wenchang	0.834316328
ENSGALG00010002608	RPP38	2	-0.080718619	0.147036059	-0.548971587	0.583024949	0.02346345	2.1123925	52.66336159	0.5	PRJNA827465;Yang2025_Silkie_Wenchang	CRA004775	0.890269423
ENSGALG00010002608	RPP38	2	-0.355615006	0.167519779	-2.122823989	0.033768604	0.010523509	1.174041058	14.82410323	1	CRA004775;Yang2025_Silkie_Wenchang	PRJNA827465	0.315701103
ENSGALG00010002608	RPP38	2	-0.234243129	0.332208512	-0.705108751	0.480742561	0.174213502	4.263170456	76.54327899	0.5	CRA004775;PRJNA827465	Yang2025_Silkie_Wenchang	0.856722567
ENSGALG00010002609	PDIA6	2	0.081965867	0.066918129	1.224867878	0.220625005	0.004546605	1.827539304	45.28161458	1	PRJNA827465;Yang2025_Silkie_Wenchang	CRA004775	0.734053249
ENSGALG00010002609	PDIA6	2	0.178750778	0.077900927	2.294591153	0.021756566	0	0.002561678	0	1	CRA004775;Yang2025_Silkie_Wenchang	PRJNA827465	0.249955657
ENSGALG00010002609	PDIA6	2	0.051654574	0.047901938	1.078339959	0.280882071	0.000398331	1.036588088	3.529665062	1	CRA004775;PRJNA827465	Yang2025_Silkie_Wenchang	0.778581075
ENSGALG00010002611	NPS	2	0.089481898	0.362878811	0.246588932	0.805226371	0	0.129661499	0	0.5	PRJNA827465;Yang2025_Silkie_Wenchang	CRA004775	0.951864391
ENSGALG00010002611	NPS	2	-0.259501562	0.41436112	-0.626269092	0.531138456	0	0.376494	0	1	CRA004775;Yang2025_Silkie_Wenchang	PRJNA827465	0.872848946
ENSGALG00010002611	NPS	2	-0.072869437	0.394459107	-0.18473255	0.853483814	0	0.90614393	0	0.5	CRA004775;PRJNA827465	Yang2025_Silkie_Wenchang	0.963577704
ENSGALG00010002612	JPH1	2	-0.120506117	0.100408481	-1.200158747	0.230077693	0.006586682	1.401286046	28.63698295	0.5	PRJNA827465;Yang2025_Silkie_Wenchang	CRA004775	0.742845187
ENSGALG00010002612	JPH1	2	0.179340435	0.288451127	0.621735999	0.534115509	0.082720257	1.527998199	34.55489669	1	CRA004775;Yang2025_Silkie_Wenchang	PRJNA827465	0.873408008
ENSGALG00010002612	JPH1	2	0.108389756	0.425974648	0.254451191	0.799147007	0.262204428	2.764617785	63.82863463	0.5	CRA004775;PRJNA827465	Yang2025_Silkie_Wenchang	0.949922217
ENSGALG00010002613	ENSGALG00010002613	2	0.001382988	0.059301066	0.023321475	0.981393842	0.000484228	1.054646354	5.181486095	0.5	PRJNA827465;Yang2025_Silkie_Wenchang	CRA004775	0.994962782
ENSGALG00010002613	ENSGALG00010002613	2	0.149589678	0.111504742	1.341554409	0.179740517	0.001025903	1.020839874	2.041443966	1	CRA004775;Yang2025_Silkie_Wenchang	PRJNA827465	0.68659821
ENSGALG00010002613	ENSGALG00010002613	2	0.104934639	0.208701135	0.502798604	0.615105876	0.058361981	2.309166586	56.69433265	0.5	CRA004775;PRJNA827465	Yang2025_Silkie_Wenchang	0.898817943
ENSGALG00010002614	GPAT3	2	-0.201576787	0.188819612	-1.067562764	0.285717788	0.051751652	3.577506097	72.04090621	1	PRJNA827465;Yang2025_Silkie_Wenchang	CRA004775	0.781404649
ENSGALG00010002614	GPAT3	2	-0.281635209	0.179147533	-1.572085331	0.115930763	0.021674797	1.425343017	29.84144952	1	CRA004775;Yang2025_Silkie_Wenchang	PRJNA827465	0.573234866
ENSGALG00010002614	GPAT3	2	-0.027875452	0.108563951	-0.256765271	0.797359981	0	5.02298E-05	0	1	CRA004775;PRJNA827465	Yang2025_Silkie_Wenchang	0.949715736
ENSGALG00010002616	ZNFB3	2	0.031029378	0.106756839	0.290654712	0.771315411	0	0.569844749	0	0.5	PRJNA827465;Yang2025_Silkie_Wenchang	CRA004775	0.94283287
ENSGALG00010002616	ZNFB3	2	-0.276862002	0.217559825	-1.272578714	0.203165576	0.053582154	2.275740486	56.07755886	1	CRA004775;Yang2025_Silkie_Wenchang	PRJNA827465	0.716895255
ENSGALG00010002616	ZNFB3	2	-0.185517904	0.301903333	-0.61449439	0.538888679	0.149514437	5.356417515	81.33080558	0.5	CRA004775;PRJNA827465	Yang2025_Silkie_Wenchang	0.87481821
ENSGALG00010002619	RAB6A	2	0.125231633	0.280542822	0.446390438	0.655315239	0.147394418	15.23234719	93.43502359	0.5	PRJNA827465;Yang2025_Silkie_Wenchang	CRA004775	0.910411938
ENSGALG00010002619	RAB6A	2	0.207114961	0.238799828	0.867316208	0.385768779	0.084284869	3.685605048	72.86741289	0.5	CRA004775;Yang2025_Silkie_Wenchang	PRJNA827465	0.820485163
ENSGALG00010002619	RAB6A	2	-0.137483099	0.053113277	-2.588488353	0.009639821	0	0.135533038	0	1	CRA004775;PRJNA827465	Yang2025_Silkie_Wenchang	0.154831987
ENSGALG00010002621	SLC25A15	2	0.318907768	0.120302025	2.650892777	0.008027932	0	0.577360577	0	1	PRJNA827465;Yang2025_Silkie_Wenchang	CRA004775	0.139297234
ENSGALG00010002621	SLC25A15	2	0.099000423	0.187888501	0.526930755	0.598241676	0.010024888	1.116987402	10.4734755	0.5	CRA004775;Yang2025_Silkie_Wenchang	PRJNA827465	0.894645663
ENSGALG00010002621	SLC25A15	2	0.169163798	0.299406375	0.565008338	0.572068093	0.115712499	2.458891432	59.33126648	0.5	CRA004775;PRJNA827465	Yang2025_Silkie_Wenchang	0.883538639
ENSGALG00010002622	AZI2	2	0.048657646	0.040214178	1.209962468	0.226293295	0	0.396433935	0	1	PRJNA827465;Yang2025_Silkie_Wenchang	CRA004775	0.739727883
ENSGALG00010002622	AZI2	2	0.093473649	0.075258667	1.242031686	0.214224871	0	0.00196344	0	1	CRA004775;Yang2025_Silkie_Wenchang	PRJNA827465	0.72702531
ENSGALG00010002622	AZI2	2	0.038415018	0.044200439	0.86910943	0.384787728	0	0.087915206	0	1	CRA004775;PRJNA827465	Yang2025_Silkie_Wenchang	0.826444827
ENSGALG00010002623	MID1IP1	2	0.06702185	0.05662661	1.183575174	0.236581273	0	0.149949187	0	1	PRJNA827465;Yang2025_Silkie_Wenchang	CRA004775	0.7487803
ENSGALG00010002623	MID1IP1	2	0.021857296	0.131650008	0.166025788	0.868136656	0	0.007027542	0	0.5	CRA004775;Yang2025_Silkie_Wenchang	PRJNA827465	0.968042774
ENSGALG00010002623	MID1IP1	2	0.076637892	0.061611472	1.243889964	0.213540064	0	0.001437575	0	1	CRA004775;PRJNA827465	Yang2025_Silkie_Wenchang	0.726080554
ENSGALG00010002625	FHL5	2	-0.284431458	0.24507212	-1.160603083	0.245803351	0.112365711	15.47338436	93.53728973	1	PRJNA827465;Yang2025_Silkie_Wenchang	CRA004775	0.75661406
ENSGALG00010002625	FHL5	2	-0.444636621	0.158384967	-2.807315809	0.004995624	0.025196087	1.51196467	33.86088842	1	CRA004775;Yang2025_Silkie_Wenchang	PRJNA827465	0.105390772
ENSGALG00010002625	FHL5	2	-0.046397786	0.089522728	-0.518279396	0.604263346	0	0.108464167	0	1	CRA004775;PRJNA827465	Yang2025_Silkie_Wenchang	0.89612415
ENSGALG00010002626	CHD7	2	0.151858414	0.244721161	0.620536504	0.534904629	0.098816413	5.033107957	80.13156069	0.5	PRJNA827465;Yang2025_Silkie_Wenchang	CRA004775	0.873896294
ENSGALG00010002626	CHD7	2	0.509817667	0.169385019	3.009815574	0.002614064	0	0.273303296	0	1	CRA004775;Yang2025_Silkie_Wenchang	PRJNA827465	0.069138993
ENSGALG00010002626	CHD7	2	0.231086159	0.335700636	0.68836974	0.491219975	0.190579105	5.672701175	82.37171377	0.5	CRA004775;PRJNA827465	Yang2025_Silkie_Wenchang	0.85895707
ENSGALG00010002627	MRPS31	2	-0.112558476	0.058185188	-1.934486779	0.053053306	0	0.027810882	0	1	PRJNA827465;Yang2025_Silkie_Wenchang	CRA004775	0.399631766
ENSGALG00010002627	MRPS31	2	-0.050337009	0.158881138	-0.316821809	0.751378815	0	0.135361953	0	0.5	CRA004775;Yang2025_Silkie_Wenchang	PRJNA827465	0.937684688
ENSGALG00010002627	MRPS31	2	-0.109884744	0.060282262	-1.822837111	0.068328065	0	0.264583377	0	0.5	CRA004775;PRJNA827465	Yang2025_Silkie_Wenchang	0.450487609
ENSGALG00010002628	TRIM7.2	2	-0.06975609	0.051998482	-1.341502445	0.179757377	0	0.668503781	0	1	PRJNA827465;Yang2025_Silkie_Wenchang	CRA004775	0.68659821
ENSGALG00010002628	TRIM7.2	2	-0.200787243	0.094342281	-2.128284812	0.033313477	0	0.643193329	0	1	CRA004775;Yang2025_Silkie_Wenchang	PRJNA827465	0.313584825
ENSGALG00010002628	TRIM7.2	2	-0.133194717	0.12534309	-1.062641085	0.287944748	0.01959615	2.178208877	54.09072057	1	CRA004775;PRJNA827465	Yang2025_Silkie_Wenchang	0.782840067
ENSGALG00010002632	HPGD	2	0.092077952	0.216116881	0.426056268	0.670066845	0.049387498	2.047026554	51.14865521	0.5	PRJNA827465;Yang2025_Silkie_Wenchang	CRA004775	0.914428878
ENSGALG00010002632	HPGD	2	0.655445396	0.406338868	1.613051193	0.106733386	0.192933378	2.163071955	53.76945285	1	CRA004775;Yang2025_Silkie_Wenchang	PRJNA827465	0.553925396
ENSGALG00010002632	HPGD	2	0.463020232	0.63663741	0.72729033	0.467048121	0.679182272	5.574855921	82.06231669	0.5	CRA004775;PRJNA827465	Yang2025_Silkie_Wenchang	0.852885838
ENSGALG00010002633	ENSGALG00010002633	2	0.349727846	0.233347346	1.498743619	0.133940156	0.092170711	6.185392573	83.83287741	1	PRJNA827465;Yang2025_Silkie_Wenchang	CRA004775	0.61312019
ENSGALG00010002633	ENSGALG00010002633	2	0.161116138	0.456817748	0.352692378	0.724319074	0.379039255	10.82755756	90.76430677	0.5	CRA004775;Yang2025_Silkie_Wenchang	PRJNA827465	0.930387861
ENSGALG00010002633	ENSGALG00010002633	2	-0.037647289	0.21752503	-0.173071069	0.862595565	0.071145477	3.554019223	71.86284212	0.5	CRA004775;PRJNA827465	Yang2025_Silkie_Wenchang	0.966099097
ENSGALG00010002634	RGN	2	0.112163011	0.15252954	0.735352716	0.462124711	0	0.64251314	0	0.5	PRJNA827465;Yang2025_Silkie_Wenchang	CRA004775	0.850747865
ENSGALG00010002634	RGN	2	-0.142878251	0.226096525	-0.631934748	0.527429517	0	0.453511779	0	1	CRA004775;Yang2025_Silkie_Wenchang	PRJNA827465	0.871149047
ENSGALG00010002634	RGN	2	0.037308317	0.270821851	0.137759628	0.890430393	0.064579552	1.525953126	34.46718755	0.5	CRA004775;PRJNA827465	Yang2025_Silkie_Wenchang	0.973417023
ENSGALG00010002638	FABP9	2	0.564331775	0.268491439	2.10186134	0.035565424	0	0.039497081	0	1	PRJNA827465;Yang2025_Silkie_Wenchang	CRA004775	0.325417923
ENSGALG00010002638	FABP9	2	1.100849563	0.759129552	1.450147159	0.147017487	0.752140986	2.631310068	61.99611699	1	CRA004775;Yang2025_Silkie_Wenchang	PRJNA827465	0.635668678
ENSGALG00010002638	FABP9	2	1.117187762	0.691897584	1.614672154	0.106381711	0.623712233	2.47004982	59.51498663	1	CRA004775;PRJNA827465	Yang2025_Silkie_Wenchang	0.553237654
ENSGALG00010002646	FOXO1	2	-0.239463954	0.307667163	-0.778321458	0.436379529	0.1848077	41.89096892	97.61285063	0.5	PRJNA827465;Yang2025_Silkie_Wenchang	CRA004775	0.843122822
ENSGALG00010002646	FOXO1	2	-0.005552698	0.604285496	-0.009188865	0.99266845	0.654956728	8.839733165	88.68744134	0.5	CRA004775;Yang2025_Silkie_Wenchang	PRJNA827465	0.997852036
ENSGALG00010002646	FOXO1	2	0.234354485	0.269523056	0.869515538	0.384565213	0.097748215	2.188664067	54.31002797	1	CRA004775;PRJNA827465	Yang2025_Silkie_Wenchang	0.826203486
ENSGALG00010002647	FAM168A	2	-0.006008213	0.072848664	-0.082475274	0.93426878	0.00448249	1.44778539	30.92898944	0.5	PRJNA827465;Yang2025_Silkie_Wenchang	CRA004775	0.98432801
ENSGALG00010002647	FAM168A	2	0.159037991	0.108976193	1.459382886	0.144459754	0	0.228574154	0	1	CRA004775;Yang2025_Silkie_Wenchang	PRJNA827465	0.631870428
ENSGALG00010002647	FAM168A	2	0.032753643	0.126981789	0.257939688	0.796453454	0.019987576	2.0138687	50.3443298	0.5	CRA004775;PRJNA827465	Yang2025_Silkie_Wenchang	0.949467989
ENSGALG00010002649	UFL1	2	0.042317838	0.074229114	0.570097576	0.568611519	0.006737442	2.291293438	56.35652844	0.5	PRJNA827465;Yang2025_Silkie_Wenchang	CRA004775	0.884199349
ENSGALG00010002649	UFL1	2	0.15168613	0.082727931	1.83355402	0.066720223	0	0.063359549	0	1	CRA004775;Yang2025_Silkie_Wenchang	PRJNA827465	0.444986411
ENSGALG00010002649	UFL1	2	0.011914457	0.067718579	0.175940735	0.860340503	0.003351241	1.186211108	15.69797375	0.5	CRA004775;PRJNA827465	Yang2025_Silkie_Wenchang	0.964999892
ENSGALG00010002653	EIF1AX	2	-0.123109599	0.111055626	-1.108539954	0.267628694	0.019943058	4.873898393	79.48254314	1	PRJNA827465;Yang2025_Silkie_Wenchang	CRA004775	0.771398294
ENSGALG00010002653	EIF1AX	2	-0.11615256	0.170905093	-0.679631941	0.49673754	0.038627821	2.649387917	62.2554329	0.5	CRA004775;Yang2025_Silkie_Wenchang	PRJNA827465	0.860939846
ENSGALG00010002653	EIF1AX	2	-0.019673383	0.042387017	-0.464137004	0.642549592	0	0.408057691	0	0.5	CRA004775;PRJNA827465	Yang2025_Silkie_Wenchang	0.906634523
ENSGALG00010002655	FABP4	2	-1.009846427	0.531179502	-1.901139677	0.05728372	0.043897369	1.082962959	7.660738399	1	PRJNA827465;Yang2025_Silkie_Wenchang	CRA004775	0.414473866
ENSGALG00010002655	FABP4	2	-0.845381389	0.620977687	-1.361371603	0.173396285	0.328760481	1.742484282	42.61067315	1	CRA004775;Yang2025_Silkie_Wenchang	PRJNA827465	0.676868485
ENSGALG00010002655	FABP4	2	-0.30829847	0.49946515	-0.617257219	0.53706508	0	0.028702532	0	1	CRA004775;PRJNA827465	Yang2025_Silkie_Wenchang	0.87481821
ENSGALG00010002658	FBXL12	2	0.103398434	0.276963886	0.373328217	0.708904178	0.125113958	4.90369537	79.6072161	0.5	PRJNA827465;Yang2025_Silkie_Wenchang	CRA004775	0.925298042
ENSGALG00010002658	FBXL12	2	0.068930064	0.344491895	0.200091975	0.84140865	0.19057588	5.043755633	80.17350418	0.5	CRA004775;Yang2025_Silkie_Wenchang	PRJNA827465	0.95989279
ENSGALG00010002658	FBXL12	2	-0.155586045	0.080375637	-1.93573638	0.052899996	0	0.363058228	0	1	CRA004775;PRJNA827465	Yang2025_Silkie_Wenchang	0.399377178
ENSGALG00010002661	ACBD7	2	-0.00671724	0.154934825	-0.04335526	0.965418342	0	0.037745811	0	0.5	PRJNA827465;Yang2025_Silkie_Wenchang	CRA004775	0.990668014
ENSGALG00010002661	ACBD7	2	0.544180514	0.530875123	1.025063127	0.305333403	0.470360668	5.988139048	83.30032099	1	CRA004775;Yang2025_Silkie_Wenchang	PRJNA827465	0.79225794
ENSGALG00010002661	ACBD7	2	0.498516273	0.561941929	0.887131299	0.375008215	0.556965077	8.21256454	87.82353557	0.5	CRA004775;PRJNA827465	Yang2025_Silkie_Wenchang	0.822916144
ENSGALG00010002664	ZNFB1	2	0.12108513	0.102872236	1.177043825	0.239178015	0	0.034407692	0	1	PRJNA827465;Yang2025_Silkie_Wenchang	CRA004775	0.751401241
ENSGALG00010002664	ZNFB1	2	-0.024033214	0.240905226	-0.099762113	0.920533187	0.044062065	1.53702293	34.93916191	0.5	CRA004775;Yang2025_Silkie_Wenchang	PRJNA827465	0.98144788
ENSGALG00010002664	ZNFB1	2	-0.003858223	0.19799517	-0.019486451	0.984453046	0.038626972	1.588216543	37.03629368	0.5	CRA004775;PRJNA827465	Yang2025_Silkie_Wenchang	0.995703389
ENSGALG00010002667	ENSGALG00010002667	2	-0.658892863	1.696373725	-0.388412561	0.69771075	4.773909111	5.828358276	82.8425098	0.5	PRJNA827465;Yang2025_Silkie_Wenchang	CRA004775	0.921422004
ENSGALG00010002667	ENSGALG00010002667	2	0.098650765	2.362706257	0.041753292	0.96669537	10.53294254	17.61762749	94.32386682	0.5	CRA004775;Yang2025_Silkie_Wenchang	PRJNA827465	0.990893423
ENSGALG00010002667	ENSGALG00010002667	2	2.030932904	0.609723888	3.330905913	0.000865639	0.047271674	1.056380132	5.337106465	1	CRA004775;PRJNA827465	Yang2025_Silkie_Wenchang	0.034540296
ENSGALG00010002669	IL4I1	2	-0.579962896	0.406383579	-1.427131717	0.153541932	0	0.019524317	0	1	PRJNA827465;Yang2025_Silkie_Wenchang	CRA004775	0.645911732
ENSGALG00010002669	IL4I1	2	-0.977268289	0.362564977	-2.69542937	0.007029798	0	0.478940014	0	1	CRA004775;Yang2025_Silkie_Wenchang	PRJNA827465	0.12862763
ENSGALG00010002669	IL4I1	2	-0.90722133	0.349433666	-2.596261948	0.009424419	0	0.800773678	0	1	CRA004775;PRJNA827465	Yang2025_Silkie_Wenchang	0.152808549

ENSGALG00010002673	SLC66A3	2	-0.228904218	0.140823837	-1.625465007	0.104063514	0.020490432	1.990625526	49.76453446	1	PRJNA827465;Yang2025_Silkie_Wenchang	CRA004775	0.548383482
ENSGALG00010002673	SLC66A3	2	-0.138338867	0.329232293	-0.420186203	0.674349433	0.148224625	2.847004185	64.87535897	0.5	CRA004775;Yang2025_Silkie_Wenchang	PRJNA827465	0.915919265
ENSGALG00010002673	SLC66A3	2	-0.071531397	0.119945663	-0.596365019	0.550931404	0.003305838	1.045685393	4.368942413	0.5	CRA004775;PRJNA827465	Yang2025_Silkie_Wenchang	0.878879109
ENSGALG00010002678	CCNI	2	-0.061263266	0.076246932	-0.803484998	0.421694465	0.000819431	1.058353646	5.51362453	0.5	PRJNA827465;Yang2025_Silkie_Wenchang	CRA004775	0.839352211
ENSGALG00010002678	CCNI	2	0.076601305	0.1331272	0.575399358	0.565021238	0	0.040263824	0	1	CRA004775;Yang2025_Silkie_Wenchang	PRJNA827465	0.883427937
ENSGALG00010002678	CCNI	2	-0.092830631	0.081808168	-1.134735485	0.256486165	0	0.499372594	0	0.5	CRA004775;PRJNA827465	Yang2025_Silkie_Wenchang	0.764677554
ENSGALG00010002681	PLEKHB1	2	0.16445511	0.087151738	1.88699747	0.059160666	0	0.850790126	0	1	PRJNA827465;Yang2025_Silkie_Wenchang	CRA004775	0.421306696
ENSGALG00010002681	PLEKHB1	2	0.075381488	0.205498767	0.366822095	0.713751725	0.057106225	2.967045072	66.2964338	0.5	CRA004775;Yang2025_Silkie_Wenchang	PRJNA827465	0.926324888
ENSGALG00010002681	PLEKHB1	2	0.01949968	0.114624652	0.170117682	0.864917588	0.004706228	1.171825877	14.66308946	0.5	CRA004775;PRJNA827465	Yang2025_Silkie_Wenchang	0.966805721
ENSGALG00010002682	UBL5	2	0.269238659	0.320226182	0.840776533	0.400473136	0.188317904	12.01678648	91.67830766	0.5	PRJNA827465;Yang2025_Silkie_Wenchang	CRA004775	0.832251175
ENSGALG00010002682	UBL5	2	0.551236981	0.145270167	3.794564231	0.000147903	0	0.634972464	0	1	CRA004775;Yang2025_Silkie_Wenchang	PRJNA827465	0.010288551
ENSGALG00010002682	UBL5	2	-0.017269723	0.09153808	-0.18866162	0.850358034	0	0.778712926	0	0.5	CRA004775;PRJNA827465	Yang2025_Silkie_Wenchang	0.961985357
ENSGALG00010002685	CA8	2	-0.302146988	0.314945973	-0.95936133	0.337376749	0.175367076	8.590290602	88.35895028	0.5	PRJNA827465;Yang2025_Silkie_Wenchang	CRA004775	0.80415385
ENSGALG00010002685	CA8	2	-0.329429902	0.400419401	-0.822712137	0.410671712	0.219702446	2.661104897	62.42162415	0.5	CRA004775;Yang2025_Silkie_Wenchang	PRJNA827465	0.835028074
ENSGALG00010002685	CA8	2	0.021605956	0.132290457	0.163322109	0.870264828	0	0.170512029	0	1	CRA004775;PRJNA827465	Yang2025_Silkie_Wenchang	0.96881514
ENSGALG00010002689	COG6	2	-0.186938581	0.050005942	-3.738327396	0.000185249	0	0.165410887	0	1	PRJNA827465;Yang2025_Silkie_Wenchang	CRA004775	0.012060492
ENSGALG00010002689	COG6	2	-0.188345583	0.095548482	-1.971204345	0.048700507	0	0.478593059	0	1	CRA004775;Yang2025_Silkie_Wenchang	PRJNA827465	0.381461853
ENSGALG00010002689	COG6	2	-0.168477168	0.053720292	-3.136192343	0.001711569	0	0.262687585	0	1	CRA004775;PRJNA827465	Yang2025_Silkie_Wenchang	0.052390811
ENSGALG00010002693	MSC	2	-0.367339344	0.338888465	-1.08395352	0.278385408	0.2074888	10.16090558	90.15835752	1	PRJNA827465;Yang2025_Silkie_Wenchang	CRA004775	0.776763074
ENSGALG00010002693	MSC	2	0.111692093	0.908080792	0.122997969	0.902108707	1.486047026	9.471509803	89.44202117	0.5	CRA004775;Yang2025_Silkie_Wenchang	PRJNA827465	0.976899171
ENSGALG00010002693	MSC	2	0.395663919	0.556439619	0.71106353	0.477044869	0.489616743	3.962526732	74.76357719	0.5	CRA004775;PRJNA827465	Yang2025_Silkie_Wenchang	0.854986476
ENSGALG00010002700	LHFPL6	2	-0.309182923	0.280532971	-1.102126862	0.270406523	0.144802576	12.14047335	91.76308888	1	PRJNA827465;Yang2025_Silkie_Wenchang	CRA004775	0.772529292
ENSGALG00010002700	LHFPL6	2	-0.451997545	0.233914169	-1.932322212	0.053319748	0.056930409	1.769975737	43.50205039	1	CRA004775;Yang2025_Silkie_Wenchang	PRJNA827465	0.400004977
ENSGALG00010002700	LHFPL6	2	-0.044396389	0.069515324	-0.63865614	0.523046651	0	0.019087735	0	1	CRA004775;PRJNA827465	Yang2025_Silkie_Wenchang	0.869568211
ENSGALG00010002703	PIN1	2	0.005314485	0.154006126	0.034508269	0.972471849	0.037804769	4.588682959	78.20725448	0.5	PRJNA827465;Yang2025_Silkie_Wenchang	CRA004775	0.992162113
ENSGALG00010002703	PIN1	2	0.057333449	0.177420454	0.323150164	0.746581521	0.029885349	1.72488028	42.02496188	0.5	CRA004775;Yang2025_Silkie_Wenchang	PRJNA827465	0.936668296
ENSGALG00010002703	PIN1	2	-0.132651191	0.06085552	-2.179772516	0.029274328	0	0.063290399	0	1	CRA004775;PRJNA827465	Yang2025_Silkie_Wenchang	0.291357032
ENSGALG00010002704	KCNB2	2	-0.005953509	0.188423407	-0.03159644	0.974793882	0	0.605360181	0	0.5	PRJNA827465;Yang2025_Silkie_Wenchang	CRA004775	0.992572967
ENSGALG00010002704	KCNB2	2	0.134595935	0.247805807	0.543150853	0.587025942	0	0.001042074	0	1	CRA004775;Yang2025_Silkie_Wenchang	PRJNA827465	0.891596903
ENSGALG00010002704	KCNB2	2	-0.131427009	0.26162276	-0.502353117	0.615419152	0	0.12870902	0	0.5	CRA004775;PRJNA827465	Yang2025_Silkie_Wenchang	0.898864379
ENSGALG00010002705	JADE3	2	0.098459547	0.039541303	2.490043057	0.012772762	0	0.002058957	0	1	PRJNA827465;Yang2025_Silkie_Wenchang	CRA004775	0.183473076
ENSGALG00010002705	JADE3	2	0.103748695	0.082637624	1.255465612	0.209309784	0	0.008580155	0	1	CRA004775;Yang2025_Silkie_Wenchang	PRJNA827465	0.721187439
ENSGALG00010002705	JADE3	2	0.098125958	0.044295262	2.215269837	0.026741549	0	0.011450649	0	1	CRA004775;PRJNA827465	Yang2025_Silkie_Wenchang	0.278911632
ENSGALG00010002706	ROCK2	2	0.236388514	0.283946783	0.832509922	0.40512118	0.137509263	6.105858399	83.62228642	0.5	PRJNA827465;Yang2025_Silkie_Wenchang	CRA004775	0.833112102
ENSGALG00010002706	ROCK2	2	0.64526423	0.198897716	3.244201304	0.001177805	0	0.657774655	0	1	CRA004775;Yang2025_Silkie_Wenchang	PRJNA827465	0.041666301
ENSGALG00010002706	ROCK2	2	0.379082381	0.476423872	0.79568301	0.426216305	0.374660585	4.824229563	79.27130152	0.5	CRA004775;PRJNA827465	Yang2025_Silkie_Wenchang	0.84125685
ENSGALG00010002707	RELT	2	0.032535581	0.126514759	0.257168263	0.797048883	0	0.165260565	0	0.5	PRJNA827465;Yang2025_Silkie_Wenchang	CRA004775	0.949622804
ENSGALG00010002707	RELT	2	0.080578371	0.204681711	0.393676457	0.693819925	0	0.100852786	0	0.5	CRA004775;Yang2025_Silkie_Wenchang	PRJNA827465	0.92072583
ENSGALG00010002707	RELT	2	-0.00979461	0.14983513	-0.065369251	0.947880005	0	0.021450076	0	1	CRA004775;PRJNA827465	Yang2025_Silkie_Wenchang	0.986644937
ENSGALG00010002712	PTPRE	2	0.002680921	0.087186998	0.030749094	0.975469638	0.008278581	2.188197257	54.30028089	0.5	PRJNA827465;Yang2025_Silkie_Wenchang	CRA004775	0.992754467
ENSGALG00010002712	PTPRE	2	0.144977968	0.312964086	0.463241549	0.643191238	0.145036028	3.140058718	68.15346177	0.5	CRA004775;Yang2025_Silkie_Wenchang	PRJNA827465	0.906634523
ENSGALG00010002712	PTPRE	2	0.196985454	0.20216339	0.97438737	0.329864238	0.047169333	1.704394536	41.32813858	1	CRA004775;PRJNA827465	Yang2025_Silkie_Wenchang	0.801177491
ENSGALG00010002713	OLFM2	2	0.151546317	0.110621051	1.369959118	0.170699664	0.009199494	1.528424824	34.57316418	1	PRJNA827465;Yang2025_Silkie_Wenchang	CRA004775	0.672386723
ENSGALG00010002713	OLFM2	2	0.015785975	0.382159305	0.041307316	0.9670509	0.201628284	2.721623624	63.25722663	0.5	CRA004775;Yang2025_Silkie_Wenchang	PRJNA827465	0.990893423
ENSGALG00010002713	OLFM2	2	-0.039377544	0.223023707	-0.176562145	0.859852331	0.051585082	1.470759437	32.00791543	0.5	CRA004775;PRJNA827465	Yang2025_Silkie_Wenchang	0.964858345
ENSGALG00010002716	NHLRC3	2	-0.110471565	0.257440505	-0.429114932	0.667839586	0.118604901	9.194972103	89.1244912	0.5	PRJNA827465;Yang2025_Silkie_Wenchang	CRA004775	0.914120722
ENSGALG00010002716	NHLRC3	2	-0.381688888	0.143180232	-2.66579319	0.007680693	0	0.003738814	0	1	CRA004775;Yang2025_Silkie_Wenchang	PRJNA827465	0.135612875
ENSGALG00010002716	NHLRC3	2	0.086632774	0.140504632	0.616583047	0.53750978	0.015630697	1.151788291	13.1784888	0.5	CRA004775;PRJNA827465	Yang2025_Silkie_Wenchang	0.87481821
ENSGALG00010002722	TRIM7.1	2	0.110521521	0.297749007	0.371190224	0.710495854	0.159934741	10.09751512	90.09657338	0.5	PRJNA827465;Yang2025_Silkie_Wenchang	CRA004775	0.925404854
ENSGALG00010002722	TRIM7.1	2	0.379771026	0.148267811	2.56138553	0.010425559	0	0.917887949	0	0.5	CRA004775;Yang2025_Silkie_Wenchang	PRJNA827465	0.162472542
ENSGALG00010002722	TRIM7.1	2	-0.175946546	0.105495905	-1.66780451	0.09535454	0	0.004220395	0	1	CRA004775;PRJNA827465	Yang2025_Silkie_Wenchang	0.528666878
ENSGALG00010002725	TSPAN7	2	0.14969145	0.167361444	0.894420164	0.371097128	0.039081954	3.19196422	68.67132803	1	PRJNA827465;Yang2025_Silkie_Wenchang	CRA004775	0.8214684
ENSGALG00010002725	TSPAN7	2	0.042326058	0.398191758	0.106295667	0.91534777	0.221913292	2.802148319	64.31309531	0.5	CRA004775;Yang2025_Silkie_Wenchang	PRJNA827465	0.980163067
ENSGALG00010002725	TSPAN7	2	-0.031096052	0.123628283	-0.251528629	0.801405431	0.0056822	1.048978729	4.669182263	0.5	CRA004775;PRJNA827465	Yang2025_Silkie_Wenchang	0.95021231
ENSGALG00010002728	RP2	2	-0.429031308	0.352014188	-1.218789818	0.222923977	0.240684579	34.47368154	97.09923642	1	PRJNA827465;Yang2025_Silkie_Wenchang	CRA004775	0.736458649
ENSGALG00010002728	RP2	2	-0.580878026	0.271713571	-2.137832219	0.032530363	0.105271673	2.935105515	65.92967459	1	CRA004775;Yang2025_Silkie_Wenchang	PRJNA827465	0.310024457
ENSGALG00010002728	RP2	2	-0.086978688	0.060346297	-1.441326022	0.149492599	0	0.189417366	0	1	CRA004775;PRJNA827465	Yang2025_Silkie_Wenchang	0.639246608
ENSGALG00010002730	E2F6	2	-0.107083752	0.062467858	-1.714221609	0.086488034	0	0.05792998	0	1	PRJNA827465;Yang2025_Silkie_Wenchang	CRA004775	0.506036629
ENSGALG00010002730	E2F6	2	0.047986161	0.167117564	0.287140139	0.774005019	0.031659241	2.152003624	53.53167677	0.5	CRA004775;Yang2025_Silkie_Wenchang	PRJNA827465	0.943248868
ENSGALG00010002730	E2F6	2	0.024264118	0.182441022	0.13299705	0.894195714	0.047659631	3.032356255	67.02234448	0.5	CRA004775;PRJNA827465	Yang2025_Silkie_Wenchang	0.974381724
ENSGALG00010002733	RDH8	2	-0.127116626	0.171720473	-0.740253178	0.459146386	0	0.589617393	0	0.5	PRJNA827465;Yang2025_Silkie_Wenchang	CRA004775	0.850098539
ENSGALG00010002733	RDH8	2	-0.011884231	0.250998747	-0.047347769	0.962236057	0	0.197240112	0	0.5	CRA004775;Yang2025_Silkie_Wenchang	PRJNA827465	0.989942249
ENSGALG00010002733	RDH8	2	-0.217772417	0.193851686	-1.123397078	0.26126889	0	0.002609016	0	1	CRA004775;PRJNA827465	Yang2025_Silkie_Wenchang	0.768039422
ENSGALG00010002741	PDK4	2	-0.27551096	0.189536012	-1.453607453	0.146055169	0	0.077782166	0	1	PRJNA827465;Yang2025_Silkie_Wenchang	CRA004775	0.634897819
ENSGALG00010002741	PDK4	2	0.11014983	0.521304472	0.211296538	0.832655884	0.323573939	1.991668745	49.79084736	0.5	CRA004775;Yang2025_Silkie_Wenchang	PRJNA827465	0.957861248
ENSGALG00010002741	PDK4	2	0.084362718	0.589369289	0.143140676	0.886179081	0.441128369	2.312724002	56.76094514	0.5	CRA004775;PRJNA827465	Yang2025_Silkie_Wenchang	0.97261928
ENSGALG00010002742	PROSER1	2	0.611078209	0.621104477	0.983857356	0.325185654	0.723154192	15.11577189	93.38439342	1	PRJNA827465;Yang2025_Silkie_Wenchang	CRA004775	0.799914637
ENSGALG00010002742	PROSER1	2	0.666469165	0.57667601	1.15570815	0.247800553	0.599896313	9.994199642	89.99419628	1	CRA004775;Yang2025_Silkie_Wenchang	PRJNA827465	0.7580874
ENSGALG00010002742	PROSER1	2	0.036323881	0.061972876	0.586125468	0.557791203	0	0.204122414	0	1	CRA004775;PRJNA827465	Yang2025_Silkie_Wenchang	0.880678789
ENSGALG00010002750	ENSGALG00010002750	2	-0.039646921	0.090563066	-0.437782458	0.661543988	0	0.715198202	0	0.5	PRJNA827465;Yang2025_Silkie_Wenchang	CRA004775	0.912368004
ENSGALG00010002750	ENSGALG00010002750	2	0.040923963	0.127796494	0.320227589	0.74879581	0	0.004748087	0	1	CRA004775;Yang2025_Silkie_Wenchang	PRJNA827465	0.937249951
ENSGALG00010002750	ENSGALG00010002750	2	-0.107477374	0.124151841	-0.865692955	0.386658564	0	0.08233481	0	0.5	CRA004775;PRJNA827465	Yang2025_Silkie_Wenchang	0.827062786
ENSGALG00010002752	NDUFA4	2	0.012722564	0.14889549	0.085446265	0.931906613	0.030870354	2.883474131	65.31961258	0.5	PRJNA827465;Yang2025_Silkie_Wenchang	CRA004775	0.98412915
ENSGALG00010002752	NDUFA4	2	0.178430719	0.15505573	1.150752179	0.249834204	0	0.167917087	0	1	CRA004775;Yang2025_Silkie_Wenchang	PRJNA827465	0.759798915
ENSGALG00010002752	NDUFA4	2	-0.099304271	0.06718926	-1.477978334	0.139413578	0	0.0954335	0	0.5	CRA004775;PRJNA827465	Yang2025_Silkie_Wenchang	0.623009874
ENSGALG00010002753	SLC9A7	2	0.11817612	0.083328494	1.418195789	0.156133611	0	0.034453634	0	1	PRJNA827465;Yang2025_Silkie_Wenchang	CRA004775	0.649132948
ENSGALG00010002753	SLC9A7	2	0.04333697	0.150650672	0.287665291	0.773602961	0	0.69698954	0	0.5	CRA004775;Yang2025_Silkie_Wenchang	PRJNA827465	0.943248868
ENSGALG00010002753	SLC9A7	2	0.106257952	0.094654379	1.122588866	0.261612146	0	0.911757906	0	0.5	CRA004775;PRJNA827465	Yang2025_Silkie_Wenchang	0.768310285
ENSGALG00010002760	RAB2A	2	0.147755081	0.177793215	0.83105017	0.405945288	0.058023147	11.86970247	91.57518899	0.5	PRJNA827465;Yang2025_Silkie_Wenchang	CRA004775	0.833416425
ENSGALG00010002760	RAB2A	2	0.201180729	0.173814337	1.15744611	0.247090145	0.039564818	2.563500633	60.99084248	0.5	CRA004775;Yang2025_Silkie_Wenchang	PRJNA827465	0.757459165
ENSGALG00010002760	RAB2A	2	-0.022080541	0.047240027	-0.467411692	0.640205359	0	0.000397694	0	1	CRA004775;PRJNA827465	Yang2025_Silkie_Wenchang	0.906137307
ENSGALG00010002762	PPAN	2	0.383355121	0.425122701	0.901751705	0.36718878	0.352804971	41.76595863	97.60570562	0.5	PRJNA827465;Yang2025_Silkie_Wenchang	CRA004775	0.819739734
ENSGALG00010002762	PPAN	2	0.244303639	0.57966701	0.421455136	0.673422765	0.645823098	25.2251791	96.03570704	0.5	CRA004775;Yang2025_Silkie_Wenchang	PRJNA827465	0.915261599
ENSGALG00010002762	PPAN	2	-0.137718114	0.14338692	-0.960464975	0.336821251	0.021750282	1.832114037	45.41824472	1	CRA004775;PRJNA827465	Yang2025_Silkie_Wenchang	0.803904536
ENSGALG00010002763	SEPTIN11	2	0.104188568	0.079134869	1.316594934	0.187974438	0.004822448	1.32596091	24.58299541	1	PRJNA827465;Yang2025_Silkie_Wenchang	CRA004775	0.697306213
ENSGALG00010002763	SEPTIN11	2	0.212204901	0.125636613	1.689037108	0.091212319	0	0.236684143	0	1	CRA004775;Yang2025_Silkie_Wenchang	PRJNA827465	0.517862098
ENSGALG00010002763	SEPTIN11	2	0.069978146	0.05276563	1.326206969	0.184771152	0	0.129566191	0	1	CRA004775;PRJNA827465	Yang2025_Silkie_Wenchang	0.69308359
ENSGALG00010002765	MAP7D2	2	0.331078773	0.359905271	0.91990532	0.357622239	0	0.00761114	0	1	PRJNA827465;Yang2025_Silkie_Wenchang	CRA004775	0.813498486
ENSGALG00010002765	MAP7D2	2	0.896771417	0.592292994	1.514067237	0.130008787	0.216178655	1.434589409	30.29364402	1	CRA004775;Yang2025_Silkie_Wenchang	PRJNA827465	0.604845569
ENSGALG00010002765	MAP7D2	2	0.782264019	0.610818316	1.280681995	0.200305386	0.406107442	2.040792891	50.99943731	1	CRA004775;PRJNA827465	Yang2025_Silkie_Wenchang	0.712517205
ENSGALG00010002766	NANOS1	2	0.644641891	0.210481231	3.062704873	0.002193463	0	0.699115271	0	1	PRJNA827465;Yang2025_Silkie_Wenchang	CRA004775	0.061754774
ENSGALG00010002766	NANOS1	2	0.057988659	0.936766501	0.061903003	0.950640076	1.454196673	5.565617727	82.0325425	0.5	CRA004775;Yang2025_Silkie_Wenchang	PRJNA827465	0.98727979
ENSGALG00010002766	NANOS1	2	-0.082422951	0.737871415	-0.111703678	0.911058363	0.850073486	4.036904903	75.22854702	0.5	CRA004775;PRJNA827465	Yang2025_Silkie_Wenchang	0.978845851
ENSGALG00010002768	TRIM39.2	2	0.648378972	0.350705959	1.848782307	0.064489253	0	0.411727694	0	1	PRJNA827465;Yang2025_Silkie_Wenchang	CRA004775	0.437650344
ENSGALG00010002768	TRIM39.2	2	0.470349528	0.520749276	0.903216864	0.366410809	0.172629978	1.450971059	31.08063776	0.5	CRA004775;Yang2025_Silkie_Wenchang	PRJNA827465	0.819255965
ENSGALG00010002768	TRIM39.2	2	0.274828611	0.38049804	0.722286535	0.47011833	0	0.525930615	0	0.5	CRA004775;PRJNA827465	Yang2025_Silkie_Wenchang	0.853648049
ENSGALG00010002774	TBX18	2	0.15140485	0.084090072	1.800508027	0.071780457	0	0.933515073	0	1	PRJNA827465;Yang2025_Silkie_Wenchang	CRA004775	0.46218665
ENSGALG00010002774	TBX18	2	0.286770686	0.136458623	2.101521187	0.03559524	0	0.801242022	0	1	CRA004775;Yang2025_Silkie_Wenchang	PRJNA827465	0.325575919
ENSGALG00010002774	TBX18	2	0.205996705	0.269010333	0.765757593	0.443820551	0.074684939	1.373656352	27.20158879	1	CRA004775;PRJNA827465	Yang2025_Silkie_Wenchang	0.845949521
ENSGALG00010002779	FBXO8	2	-0.102880014	0.102830719	-1.000479388	0.317078568	0.012727101	2.391533773	58.1858299	1	PRJNA827465;Yang2025_Silkie_Wenchang	CRA004775	0.797007794
ENSGALG00010002779	FBXO8	2	-0.088785401	0.203079521	-0.437195246	0.661969757	0.047077536	1.970884245	49.26135299	0.5	CRA004775;Yang2025_Silkie_Wenchang	PRJNA827465	0.912556664
ENSGALG00010002779	FBXO8	2	-0.004110436	0.069746667	-0.058933793	0.953004842	0	0.584156558	0	0.5	CRA004775;PRJNA827465	Yang2025_Silkie_Wenchang	0.987758913
ENSGALG00010002781	EIF3G	2	-0.046032636	0.055815184	-0.824733209	0.409523073	0	0.121558363	0	0.5	PRJNA827465;Yang2025_Silkie_Wenchang	CRA004775	0.83448182
ENSGALG00010002781	EIF3G	2	-0.047426542	0.144708361	-0.327738783	0.743109177	0	0.3845016	0	0.5	CRA004775;Yang2025_Silkie_Wenchang	PRJNA827465	0.93573824
ENSGALG00010002781	EIF3G	2	-0.05892791	0.057580202	-1.023405743	0.306116045	0	0.238303378	0	1	CRA004775;PRJNA827465	Yang2025_Silkie_Wenchang	0.792417764
ENSGALG00010002782	GSTA3	2	0.310654401	0.206002428	1.508013297	0.131551124	0.064250299	4.009887244	75.06164291	1	PRJNA827465;Yang2025_Silkie_Wenchang	CRA004775	0.607490755
ENSGALG00010002782	GSTA3	2	0.08280193	0.106871275	0.774781905	0.438468544	0	0.776192389	0	0.5	CRA004775;Yang2025_Silkie_Wenchang	PRJNA827465	0.844002503
ENSGALG00010002782	GSTA3	2	0.232525389	0.336042343	0.691952647	0.488967067	0.176138555	4.388353799	77.21241163	0.5	CRA004775;PRJNA827465	Yang2025_Silkie_Wenchang	0.858232954
ENSGALG00010002784	TRIM27.2	2	1.184342717	0.280810963	4.21758005	2.46938E-05	0	0.533318695	0	1	PRJNA827465;Yang2025_Silkie_Wenchang	CRA004775	0.002862953
ENSGALG00010002784	TRIM27.2	2	0.319546889	1.229953409	0.259804059	0.79501492	2.632115642	7.57723012	86.80256526	0.5	CRA004775;Yang2025_Silkie_Wenchang	PRJNA827465	0.948905846
ENSGALG00010002784	TRIM27.2	2	0.145511655	1.003282053	0.14503564	0.884682732	1.707414564	6.199616372	83.86996969	0.5	CRA004775;PRJNA827465	Yang2025_Silkie_Wenchang	0.972062774
ENSGALG00010002787	CEP44	2	-0.106260622	0.112527878	-0.944304855	0.345013886	0.016158725	2.7627075	63.8036238	0.5	PRJNA827465;Yang2025_Silkie_Wenchang	CRA004775	0.806961063
ENSGALG00010002787	CEP44	2	-0.093030589	0.194540904	-0.478205805	0.632503731	0.044479627	1.948314732	48.67359037	0.5	CRA004775;Yang2025_Silkie_Wenchang	PRJNA827465	0.903058174
ENSGALG00010002787	CEP44	2	0.026856576	0.091532991	0.293408704	0.769209773	0	0.435722182	0	1	CRA004775;PRJNA827465	Yang2025_Silkie_Wenchang	0.941790377
ENSGALG00010002788	ENSGALG00010002788	2	-0.72157621	0.382908884	-1.884459308	0.059502874	0.123762829	1.730208711	42.2035045	1	PRJNA827465;Yang2025_Silkie_Wenchang	CRA004775	0.422595132
ENSGALG00010002788	ENSGALG00010002788	2	-1.216549655	0.325924279	-3.732614391	0.000189503	0	0.183310982	0	1	CRA004775;Yang2025_Silkie_Wenchang	PRJNA827465	0.012270948
ENSGALG00010002788	ENSGALG00010002788	2	-0.814949749	0.523942621	-1.555417936	0.119846565	0.331391687	2.484380072	59.74850985	1	CRA004775;PRJNA827465	Yang2025_Silkie_Wenchang	0.580639051
ENSGALG00010002790	BCLAF3	2	-0.079868955	0.065247874	-1.224085175	0.220920098	4.501E-06	1.000464998	0.046478208	0.5	PRJNA827465;Yang2025_Silkie_Wenchang	CRA004775	0.73419654
ENSGALG00010002790	BCLAF3	2	0.166409531	0.212399431	0.783474468	0.433348541	0.055328308	2.225024799	55.0566807	1	CRA004775;Yang2025_Silkie_Wenchang	PRJNA827465	0.842762754
ENSGALG00010002790	BCLAF3	2	0.108068464	0.287465244	0.375935756	0.706964658	0.130769754	4.12848921	75.77806435	0.5	CRA004775;PRJNA827465	Yang2025_Silkie_Wenchang	0.924653309
ENSGALG00010002792	ENSGALG00010002792	2	-0.011715097	0.243413443	-0.04812839	0.96161392	0.088935647	3.667069886	72.73027155	0.5	PRJNA827465;Yang2025_Silkie_Wenchang	CRA004775	0.98973612
ENSGALG00010002792	ENSGALG00010002792	2	-0.319640584	0.204777844	-1.560913902	0.118544067	0	0.022847225	0	1	CRA004775;Yang2025_Silkie_Wenchang	PRJNA827465	0.578415575
ENSGALG00010002792	ENSGALG00010002792	2	0.054805914	0.243010177	0.225529295	0.821567538	0.066052548	1.695529413	41.02137114	0.5	CRA004775;PRJNA827465	Yang2025_Silkie_Wenchang	0.955598716
ENSGALG00010002796	PRDM8	2	0.0824817	0.201202788	0.409943128	0.681847668	0.018938298	1.301489901	23.16498198	0.5	PRJNA827465;Yang2025_Silkie_Wenchang	CRA004775	0.918430638
ENSGALG00010002796	PRDM8	2	0.185009181	0.2177135	0.849782769	0.395445871	0	0.86204917	0	0.5	CRA004775;Yang2025_Silkie_Wenchang	PRJNA827465	0.830946177
ENSGALG00010002796	PRDM8	2	-0.171007025	0.242826881	-0.704234323	0.481286859	0	0.08323447	0	1	CRA004775;PRJNA827465	Yang2025_Silkie_Wenchang	0.85682839
ENSGALG00010002800	TUBGCP5	2	-0.022745541	0.050791377	-0.447822886	0.654281025	0	0.058066286	0	1	PRJNA827465;Yang2025_Silkie_Wenchang	CRA004775	0.910015116
ENSGALG00010002800	TUBGCP5	2	-0.034101786	0.088246438	-0.386438105	0.699172234	0	0.049832625	0	0.5	CRA004775;Yang2025_Silkie_Wenchang	PRJNA827465	0.92185597
ENSGALG00010002800	TUBGCP5	2	-0.013244285	0.058077015	-0.228046929	0.819609757	0	0.01445331	0	0.5	CRA004775;PRJNA827465	Yang2025_Silkie_Wenchang	0.955256039
ENSGALG00010002805	FAM171A1	2	-0.067137308	0.034860832	-1.92586651	0.054121044	0	0.197643038	0	1	PRJNA827465;Yang2025_Silkie_Wenchang	CRA004775	0.403227287
ENSGALG00010002805	FAM171A1	2	-0.03950704	0.108414324	-0.364407938	0.715553409	0	0.262294725	0	1	CRA004775;Yang2025_Silkie_Wenchang	PRJNA827465	0.927131632
ENSGALG00010002805	FAM171A1	2	-0.073626624	0.036263352	-2.030331462	0.042322858	0	0.133009193	0	1	CRA004775;PRJNA827465	Yang2025_Silkie_Wenchang	0.356191713
ENSGALG00010002807	P2RY6	2	0.126490355	0.173580718	0.728712015	0.466177842	0.033779937	2.141502879	53.30382182	0.5	PRJNA827465;Yang2025_Silkie_Wenchang	CRA004775	0.852631007
ENSGALG00010002807	P2RY6	2	-0.271289089	0.24197983	-1.121122734	0.262235623	0.047436615	1.592253886	37.19594539	1	CRA004775;Yang2025_Silkie_Wenchang	PRJNA827465	0.768363052
ENSGALG00010002807	P2RY6	2	-0.106822016	0.426511306	-0.250455296	0.802235271	0.304652036	5.612311058	82.18202823	0.5	CRA004775;PRJNA827465	Yang2025_Silkie_Wenchang	0.950637102
ENSGALG00010002809	SH3KBP1	2	-0.118079707	0.057947867	-2.037688578	0.041581087	0	0.308846235	0	1	PRJNA827465;Yang2025_Silkie_Wenchang	CRA004775	0.353355943
ENSGALG00010002809	SH3KBP1	2	0.01207986	0.133046776	0.090794082	0.927656213	0	0.805492127	0	0.5	CRA004775;Yang2025_Silkie_Wenchang	PRJNA827465	0.982993195
ENSGALG00010002809	SH3KBP1	2	-0.05077367	0.16132185	-0.314735233	0.75296269	0.028632374	1.54961186	35.46771124	0.5	CRA004775;PRJNA827465	Yang2025_Silkie_Wenchang	0.938087082
ENSGALG00010002810	TRIM27.1	2	-0.105055796	0.311365237	-0.337403743	0.735812564	0.118904643	2.520553437	60.3261734	0.5	PRJNA827465;Yang2025_Silkie_Wenchang	CRA004775	0.933592889
ENSGALG00010002810	TRIM27.1	2	-0.547893073	0.26180344	-2.092764987	0.036370139	0	0.226969814	0	1	CRA004775;Yang2025_Silkie_Wenchang	PRJNA827465	0.32890494
ENSGALG00010002810	TRIM27.1	2	-0.19267807	0.43783349	-0.440071566	0.659885275	0.269329851	3.099125968	67.7328379	0.5	CRA004775;PRJNA827465	Yang2025_Silkie_Wenchang	0.911773501
ENSGALG00010002812	TRIM13	2	-0.280912524	0.198968316	-1.411845513	0.157995454	0.071848656	10.80092092	90.74153021	1	PRJNA827465;Yang2025_Silkie_Wenchang	CRA004775	0.651594473
ENSGALG00010002812	TRIM13	2	-0.461096431	0.079087058	-5.830238742	5.53481E-09	0	0.505165669	0	1	CRA004775;Yang2025_Silkie_Wenchang	PRJNA827465	2.93966E-06
ENSGALG00010002812	TRIM13	2	-0.100965562	0.083723177	-1.205945181	0.227838628	0	0.520885292	0	1	CRA004775;PRJNA827465	Yang2025_Silkie_Wenchang	0.740853612
ENSGALG00010002814	TERF1	2	-0.151387627	0.087749092	-1.725232971	0.084485496	0	0.414145636	0	1	PRJNA827465;Yang2025_Silkie_Wenchang	CRA004775	0.500072565
ENSGALG00010002814	TERF1	2	-0.211196786	0.136441285	-1.547895017	0.121647574	0	0.086608709	0	1	CRA004775;Yang2025_Silkie_Wenchang	PRJNA827465	0.584917374
ENSGALG00010002814	TERF1	2	-0.108465173	0.105746238	-1.025711889	0.305027409	0	0.000127718	0	1	CRA004775;PRJNA827465	Yang2025_Silkie_Wenchang	0.79225794
ENSGALG00010002817	SHFM1	2	-0.218467807	0.122161669	-1.788349892	0.073719575	0.015884287	1.749544541	42.84226685	1	PRJNA827465;Yang2025_Silkie_Wenchang	CRA004775	0.469290648
ENSGALG00010002817	SHFM1	2	-0.247340975	0.215077455	-1.150008845	0.250140228	0.036718537	1.594820617	37.2970233	0.5	CRA004775;Yang2025_Silkie_Wenchang	PRJNA827465	0.75993411
ENSGALG00010002817	SHFM1	2	-0.13332651	0.067557932	-1.973513788	0.048437055	0	0.323925148	0	0.5	CRA004775;PRJNA827465	Yang2025_Silkie_Wenchang	0.380432851
ENSGALG00010002819	BMP3	2	-0.274687609	0.110960199	-2.475550803	0.013303083	0	0.392033094	0	1	PRJNA827465;Yang2025_Silkie_Wenchang	CRA004775	0.188414806
ENSGALG00010002819	BMP3	2	-0.359965399	0.17694857	-2.034293915	0.04192197	0	0.01848866	0	1	CRA004775;Yang2025_Silkie_Wenchang	PRJNA827465	0.35448778
ENSGALG00010002819	BMP3	2	-0.225309025	0.135350273	-1.664636655	0.095985268	0	0.008338529	0	1	CRA004775;PRJNA827465	Yang2025_Silkie_Wenchang	0.5301492
ENSGALG00010002825	TRIM41	2	-0.057155123	0.185751775	-0.307696239	0.758313481	0.060406938	7.911402244	87.36001572	0.5	PRJNA827465;Yang2025_Silkie_Wenchang	CRA004775	0.938954369
ENSGALG00010002825	TRIM41	2	-0.224626527	0.363443933	-0.618050011	0.536542373	0.251119661	20.21495864	95.05316821	0.5	CRA004775;Yang2025_Silkie_Wenchang	PRJNA827465	0.874599474
ENSGALG00010002825	TRIM41	2	-0.400052723	0.177088866	-2.259050673	0.023880233	0.053057118	6.326398214	84.19321759	1	CRA004775;PRJNA827465	Yang2025_Silkie_Wenchang	0.264093469
ENSGALG00010002827	SBSPON	2	-0.61381258	0.644194626	-0.952837164	0.340672581	0.686161988	5.325231336	81.22147308	1	PRJNA827465;Yang2025_Silkie_Wenchang	CRA004775	0.805526077
ENSGALG00010002827	SBSPON	2	-0.350222575	1.000399638	-0.350082669	0.726276657	1.729477644	7.355513098	86.40475536	0.5	CRA004775;Yang2025_Silkie_Wenchang	PRJNA827465	0.931029023
ENSGALG00010002827	SBSPON	2	0.138580841	0.310842268	0.445823671	0.655724621	0.085475079	1.533582259	34.79319455	0.5	CRA004775;PRJNA827465	Yang2025_Silkie_Wenchang	0.910411938
ENSGALG00010002832	LPIN1	2	-0.266139381	0.133853169	-1.988293469	0.046779243	0	0.189423085	0	1	PRJNA827465;Yang2025_Silkie_Wenchang	CRA004775	0.373540141
ENSGALG00010002832	LPIN1	2	-0.150053002	0.188981074	-0.794010737	0.427189181	0	0.411901324	0	0.5	CRA004775;Yang2025_Silkie_Wenchang	PRJNA827465	0.84125685
ENSGALG00010002832	LPIN1	2	-0.265296512	0.167341713	-1.585357926	0.112885035	0	0.763044176	0	0.5	CRA004775;PRJNA827465	Yang2025_Silkie_Wenchang	0.566980581
ENSGALG00010002834	MRPL53	2	-0.035009509	0.074492864	-0.469971304	0.63837552	0	0.361920021	0	1	PRJNA827465;Yang2025_Silkie_Wenchang	CRA004775	0.905582655
ENSGALG00010002834	MRPL53	2	-0.224291592	0.182869399	-1.226512433	0.2200059	0	0.121144848	0	1	CRA004775;Yang2025_Silkie_Wenchang	PRJNA827465	0.733691086
ENSGALG00010002834	MRPL53	2	-0.03994431	0.075620652	-0.528219586	0.597346937	0	0.897101187	0	1	CRA004775;PRJNA827465	Yang2025_Silkie_Wenchang	0.894395424
ENSGALG00010002835	CYFIP1	2	-0.121051732	0.159741114	-0.757799473	0.448571038	0.047750199	15.31067349	93.46860867	0.5	PRJNA827465;Yang2025_Silkie_Wenchang	CRA004775	0.846878812
ENSGALG00010002835	CYFIP1	2	-0.268945697	0.067885285	-3.961767235	7.43971E-05	0	0.796453728	0	1	CRA004775;Yang2025_Silkie_Wenchang	PRJNA827465	0.006301783
ENSGALG00010002835	CYFIP1	2	0.031349545	0.04068381	0.770565633	0.440964438	0	0.118217091	0	0.5	CRA004775;PRJNA827465	Yang2025_Silkie_Wenchang	0.844888742
ENSGALG00010002837	DNMT1	2	-0.01823798	0.045783366	-0.398353849	0.690369372	0	0.644496056	0	0.5	PRJNA827465;Yang2025_Silkie_Wenchang	CRA004775	0.919979266
ENSGALG00010002837	DNMT1	2	-0.169609428	0.2213068	-0.76639953	0.443438613	0.084039129	6.749184802	85.18339578	0.5	CRA004775;Yang2025_Silkie_Wenchang	PRJNA827465	0.845733906
ENSGALG00010002837	DNMT1	2	-0.200309398	0.181109177	-1.10601462	0.268720191	0.054017139	5.140563847	80.54688105	1	CRA004775;PRJNA827465	Yang2025_Silkie_Wenchang	0.771398294
ENSGALG00010002838	ENSGALG00010002838	2	0.105613435	0.260126634	0.406007771	0.684736891	0.089736347	2.856027106	64.98632671	0.5	PRJNA827465;Yang2025_Silkie_Wenchang	CRA004775	0.919003112
ENSGALG00010002838	ENSGALG00010002838	2	0.407996076	0.217693358	1.874177878	0.060905912	0	0.000310556	0	1	CRA004775;Yang2025_Silkie_Wenchang	PRJNA827465	0.427127227
ENSGALG00010002838	ENSGALG00010002838	2	0.026712285	0.238353481	0.112070042	0.910767871	0.049499128	1.53216919	34.73305649	0.5	CRA004775;PRJNA827465	Yang2025_Silkie_Wenchang	0.97880407
ENSGALG00010002840	SRPX	2	-0.214870668	0.157653961	-1.362925903	0.172905864	0.036647376	3.650853141	72.60914199	1	PRJNA827465;Yang2025_Silkie_Wenchang	CRA004775	0.676043418
ENSGALG00010002840	SRPX	2	-0.300520885	0.193545579	-1.552713761	0.120491533	0.026030981	1.267492748	21.10408511	0.5	CRA004775;Yang2025_Silkie_Wenchang	PRJNA827465	0.581921692
ENSGALG00010002840	SRPX	2	-0.068054175	0.08545221	-0.79640041	0.42579934	0	0.175762998	0	0.5	CRA004775;PRJNA827465	Yang2025_Silkie_Wenchang	0.84125685
ENSGALG00010002848	TRIB2	2	-0.151715523	0.32885909	-0.461338997	0.644555411	0.204836747	18.65117364	94.63840711	0.5	PRJNA827465;Yang2025_Silkie_Wenchang	CRA004775	0.906953008
ENSGALG00010002848	TRIB2	2	0.013601292	0.516233104	0.02634719	0.978980416	0.502096816	17.05824815	94.1377333	0.5	CRA004775;Yang2025_Silkie_Wenchang	PRJNA827465	0.994259988
ENSGALG00010002848	TRIB2	2	0.303737531	0.179955915	1.687844109	0.091441158	0.044602197	2.737664435	63.47251375	1	CRA004775;PRJNA827465	Yang2025_Silkie_Wenchang	0.51833799
ENSGALG00010002853	CEP162	2	0.597527783	0.534837038	1.117214668	0.26390255	0.537796972	16.59080854	93.97256621	1	PRJNA827465;Yang2025_Silkie_Wenchang	CRA004775	0.768643821
ENSGALG00010002853	CEP162	2	0.516619291	0.649613791	0.795271433	0.426455627	0.768428481	11.00985115	90.91722507	0.5	CRA004775;Yang2025_Silkie_Wenchang	PRJNA827465	0.84125685
ENSGALG00010002853	CEP162	2	0.030582687	0.140351377	0.21790087	0.82750635	0	0.399766652	0	0.5	CRA004775;PRJNA827465	Yang2025_Silkie_Wenchang	0.956550752
ENSGALG00010002855	RACK1	2	-0.199584208	0.159810405	-1.248881181	0.211708536	0.036552291	2.982461422	66.47064761	1	PRJNA827465;Yang2025_Silkie_Wenchang	CRA004775	0.724049825
ENSGALG00010002855	RACK1	2	-0.434939063	0.146368922	-2.971526031	0.002963238	0	0.054869042	0	1	CRA004775;Yang2025_Silkie_Wenchang	PRJNA827465	0.074704377
ENSGALG00010002855	RACK1	2	-0.209093386	0.188935385	-1.106692565	0.268426871	0.049112431	2.523037959	60.36524158	1	CRA004775;PRJNA827465	Yang2025_Silkie_Wenchang	0.771398294
ENSGALG00010002857	DCAF15	2	0.243945106	0.282478032	0.863589653	0.38781335	0.137945135	7.102326504	85.92010661	0.5	PRJNA827465;Yang2025_Silkie_Wenchang	CRA004775	0.828154121

ENSGALG00010002857	DCAF15	2	0.061814865	0.494179258	0.125085915	0.900455534	0.446733508	11.69099177	91.44640575	0.5	CRA004775;Yang2025_Silkie_Wenchang	PRJNA827465	0.976475201
ENSGALG00010002857	DCAF15	2	-0.182866728	0.20593667	-0.887975548	0.374553905	0.05960248	3.030535605	67.00253254	1	CRA004775;PRJNA827465	Yang2025_Silkie_Wenchang	0.822805106
ENSGALG00010002860	NIPA2	2	-0.479794083	0.37820145	-1.268620422	0.204576477	0.273139638	21.54921092	95.35945885	1	PRJNA827465;Yang2025_Silkie_Wenchang	CRA004775	0.71729696
ENSGALG00010002860	NIPA2	2	-0.522812978	0.357297789	-1.463241569	0.14340129	0.225902991	8.640165137	88.42614714	1	CRA004775;Yang2025_Silkie_Wenchang	PRJNA827465	0.630506868
ENSGALG00010002860	NIPA2	2	-0.118165616	0.053819961	-2.195572311	0.02812257	0	0.045561341	0	1	CRA004775;PRJNA827465	Yang2025_Silkie_Wenchang	0.285731308
ENSGALG00010002864	RNASEH2B	2	-0.274334846	0.11107757	-2.469759154	0.013520405	0.013499072	2.151392852	53.5184846	1	PRJNA827465;Yang2025_Silkie_Wenchang	CRA004775	0.190294637
ENSGALG00010002864	RNASEH2B	2	-0.408634624	0.115158863	-3.548442692	0.000387516	0	0.013721833	0	1	CRA004775;Yang2025_Silkie_Wenchang	PRJNA827465	0.01983145
ENSGALG00010002864	RNASEH2B	2	-0.199232859	0.085918566	-2.318856885	0.020402798	0	0.67943147	0	1	CRA004775;PRJNA827465	Yang2025_Silkie_Wenchang	0.240591405
ENSGALG00010002866	ENSGALG00010002866	2	0.703310191	0.504464859	1.39417083	0.163266024	0.493382152	32.59637532	96.93217424	1	PRJNA827465;Yang2025_Silkie_Wenchang	CRA004775	0.661668506
ENSGALG00010002866	ENSGALG00010002866	2	0.302981513	1.003724075	0.301857374	0.762760792	1.813517452	9.188206995	89.11648377	0.5	CRA004775;Yang2025_Silkie_Wenchang	PRJNA827465	0.940082621
ENSGALG00010002866	ENSGALG00010002866	2	-0.097542448	0.461062948	-0.211559936	0.832450368	0.290077835	2.335538777	57.18332703	0.5	CRA004775;PRJNA827465	Yang2025_Silkie_Wenchang	0.957861248
ENSGALG00010002868	PPP1R9A	2	-0.05777678	0.060741308	-0.951194199	0.341505803	0	0.673017271	0	0.5	PRJNA827465;Yang2025_Silkie_Wenchang	CRA004775	0.805758959
ENSGALG00010002868	PPP1R9A	2	0.054681019	0.127889694	0.42756392	0.668968639	0	0.015431618	0	1	CRA004775;Yang2025_Silkie_Wenchang	PRJNA827465	0.914377769
ENSGALG00010002868	PPP1R9A	2	-0.073235546	0.065596837	-1.116449344	0.264229842	0	0.30787109	0	0.5	CRA004775;PRJNA827465	Yang2025_Silkie_Wenchang	0.768790489
ENSGALG00010002870	FABP5	2	-0.051708854	0.095505183	-0.541424584	0.588214966	0	0.052187837	0	1	PRJNA827465;Yang2025_Silkie_Wenchang	CRA004775	0.891852974
ENSGALG00010002870	FABP5	2	0.179613199	0.374218314	0.479969025	0.631249418	0.177539366	2.310839789	56.72568886	0.5	CRA004775;Yang2025_Silkie_Wenchang	PRJNA827465	0.903058174
ENSGALG00010002870	FABP5	2	0.18123158	0.338738731	0.535018774	0.592636876	0.150551004	2.207384441	54.69751524	0.5	CRA004775;PRJNA827465	Yang2025_Silkie_Wenchang	0.892776493
ENSGALG00010002878	P2RY2	2	-0.24805009	0.130073831	-1.906994569	0.056521291	0	0.025408832	0	1	PRJNA827465;Yang2025_Silkie_Wenchang	CRA004775	0.411523553
ENSGALG00010002878	P2RY2	2	-0.28615156	0.179221886	-1.596632901	0.110347561	0	0.064302036	0	1	CRA004775;Yang2025_Silkie_Wenchang	PRJNA827465	0.562436811
ENSGALG00010002878	P2RY2	2	-0.246993204	0.169388033	-1.45815026	0.14479913	0	0.107860131	0	1	CRA004775;PRJNA827465	Yang2025_Silkie_Wenchang	0.63257278
ENSGALG00010002880	RASGEF1B	2	0.095993496	0.225124597	0.426401632	0.669815213	0.07158909	3.380459085	70.41821909	0.5	PRJNA827465;Yang2025_Silkie_Wenchang	CRA004775	0.914377769
ENSGALG00010002880	RASGEF1B	2	0.236089922	0.163782024	1.441488607	0.149446694	0.007622719	1.085458685	7.873048117	0.5	CRA004775;Yang2025_Silkie_Wenchang	PRJNA827465	0.639246608
ENSGALG00010002880	RASGEF1B	2	-0.139681076	0.17003316	-0.821493146	0.411365427	0	0.000623663	0	1	CRA004775;PRJNA827465	Yang2025_Silkie_Wenchang	0.835128673
ENSGALG00010002881	ENSGALG00010002881	2	0.509504414	0.542085745	0.939893652	0.347270729	0.491421413	6.094166766	83.59086585	0.5	PRJNA827465;Yang2025_Silkie_Wenchang	CRA004775	0.808705451
ENSGALG00010002881	ENSGALG00010002881	2	0.290873967	0.844925129	0.34426005	0.730650725	1.192240423	5.670395164	82.36454478	0.5	CRA004775;Yang2025_Silkie_Wenchang	PRJNA827465	0.932769533
ENSGALG00010002881	ENSGALG00010002881	2	-0.165959392	0.291888759	-0.568570685	0.569647524	0	0.717489776	0	1	CRA004775;PRJNA827465	Yang2025_Silkie_Wenchang	0.884865095
ENSGALG00010002883	MAP3K15	2	0.299382826	0.255608345	1.171256073	0.241495862	0.07216229	2.225207126	55.06036323	1	PRJNA827465;Yang2025_Silkie_Wenchang	CRA004775	0.753952806
ENSGALG00010002883	MAP3K15	2	0.61874704	0.233432395	2.650647701	0.008033759	0	0.211558921	0	1	CRA004775;Yang2025_Silkie_Wenchang	PRJNA827465	0.139297234
ENSGALG00010002883	MAP3K15	2	0.291155344	0.362114896	0.804041334	0.421373104	0.134038778	1.737765379	42.45483238	1	CRA004775;PRJNA827465	Yang2025_Silkie_Wenchang	0.839352211
ENSGALG00010002884	ADHFE1	2	0.164896904	0.133548409	1.234735063	0.216929122	0	0.518239662	0	1	PRJNA827465;Yang2025_Silkie_Wenchang	CRA004775	0.730505165
ENSGALG00010002884	ADHFE1	2	0.189358787	0.161997815	1.168897169	0.242445064	0	0.730923129	0	0.5	CRA004775;Yang2025_Silkie_Wenchang	PRJNA827465	0.754698112
ENSGALG00010002884	ADHFE1	2	0.014109738	0.185364962	0.076118691	0.93932467	0	0.175205766	0	0.5	CRA004775;PRJNA827465	Yang2025_Silkie_Wenchang	0.985393654
ENSGALG00010002886	HERC2	2	0.049772263	0.126838448	0.392406747	0.694757702	0.024103333	3.804259918	73.71367831	0.5	PRJNA827465;Yang2025_Silkie_Wenchang	CRA004775	0.92082578
ENSGALG00010002886	HERC2	2	0.204609579	0.105307425	1.942973904	0.052019311	0	0.071253755	0	1	CRA004775;Yang2025_Silkie_Wenchang	PRJNA827465	0.395759125
ENSGALG00010002886	HERC2	2	-0.012291561	0.119978736	-0.102447828	0.918401222	0.01239716	1.273771905	21.49300858	0.5	CRA004775;PRJNA827465	Yang2025_Silkie_Wenchang	0.981033557
ENSGALG00010002890	VXN	2	0.735088819	0.229208888	3.207069436	0.001340946	0.059397289	2.144248051	53.36360458	1	PRJNA827465;Yang2025_Silkie_Wenchang	CRA004775	0.044739568
ENSGALG00010002890	VXN	2	0.764941561	0.286566903	2.66932976	0.007600279	0.07245407	1.778045683	43.75847542	1	CRA004775;Yang2025_Silkie_Wenchang	PRJNA827465	0.134538115
ENSGALG00010002890	VXN	2	0.535165667	0.143785756	3.721965807	0.000197678	0	0.080420538	0	1	CRA004775;PRJNA827465	Yang2025_Silkie_Wenchang	0.012694984
ENSGALG00010002894	FCHSD2	2	0.04312448	0.043408576	0.993455319	0.320488114	0	0.06811297	0	1	PRJNA827465;Yang2025_Silkie_Wenchang	CRA004775	0.798348216
ENSGALG00010002894	FCHSD2	2	0.051175051	0.082592419	0.61960954	0.535514884	0	0.524251509	0	1	CRA004775;Yang2025_Silkie_Wenchang	PRJNA827465	0.874171355
ENSGALG00010002894	FCHSD2	2	0.057166821	0.047644501	1.199861879	0.230192987	0	0.409160769	0	1	CRA004775;PRJNA827465	Yang2025_Silkie_Wenchang	0.742845187
ENSGALG00010002896	OCA2	2	3.466960998	0.229228245	15.12449303	1.11657E-51	0	0.348722993	0	1	PRJNA827465;Yang2025_Silkie_Wenchang	CRA004775	2.74279E-48
ENSGALG00010002896	OCA2	2	3.056956141	0.525640868	5.815674406	6.03899E-09	0.266573115	1.527235315	34.52220557	1	CRA004775;Yang2025_Silkie_Wenchang	PRJNA827465	3.12267E-06
ENSGALG00010002896	OCA2	2	3.125238749	0.7105003	4.398645222	1.08929E-05	0.629854778	2.21500102	54.85329393	1	CRA004775;PRJNA827465	Yang2025_Silkie_Wenchang	0.001507474
ENSGALG00010002904	PON1	2	-0.007403497	0.12881619	-0.057473342	0.954168141	0.022062043	2.98307294	66.47752099	0.5	PRJNA827465;Yang2025_Silkie_Wenchang	CRA004775	0.987925986
ENSGALG00010002904	PON1	2	-0.141645971	0.099390498	-1.425146001	0.154115002	0	0.023889514	0	1	CRA004775;Yang2025_Silkie_Wenchang	PRJNA827465	0.646788679
ENSGALG00010002904	PON1	2	0.087193065	0.099431179	0.876918745	0.380530774	0	0.942592344	0	0.5	CRA004775;PRJNA827465	Yang2025_Silkie_Wenchang	0.824392515
ENSGALG00010002908	S1PR2	2	-0.044710564	0.110573112	-0.40435295	0.685953193	0	0.409816471	0	0.5	PRJNA827465;Yang2025_Silkie_Wenchang	CRA004775	0.919235539
ENSGALG00010002908	S1PR2	2	-0.029285199	0.188208388	-0.155599862	0.876348434	0	0.884482432	0	0.5	CRA004775;Yang2025_Silkie_Wenchang	PRJNA827465	0.970347777
ENSGALG00010002908	S1PR2	2	-0.112047916	0.120897299	-0.926802477	0.354029099	0	0.379614268	0	1	CRA004775;PRJNA827465	Yang2025_Silkie_Wenchang	0.811619556
ENSGALG00010002911	MYBL1	2	-0.145439315	0.110105232	-1.320911937	0.186530729	0	0.014798652	0	1	PRJNA827465;Yang2025_Silkie_Wenchang	CRA004775	0.695457578
ENSGALG00010002911	MYBL1	2	0.142930153	0.453090506	0.315456077	0.752415395	0.251336693	1.916521066	47.82212323	0.5	CRA004775;Yang2025_Silkie_Wenchang	PRJNA827465	0.93806821
ENSGALG00010002911	MYBL1	2	0.132116302	0.469740299	0.281253924	0.778515648	0.283091217	2.046143023	51.12756103	0.5	CRA004775;PRJNA827465	Yang2025_Silkie_Wenchang	0.944673731
ENSGALG00010002912	RPS3	2	-0.541293641	0.478203999	-1.131930395	0.257663699	0.426591216	14.21873267	92.96702439	1	PRJNA827465;Yang2025_Silkie_Wenchang	CRA004775	0.7650601
ENSGALG00010002912	RPS3	2	-0.675758396	0.354419622	-1.906661919	0.056564381	0.205414905	5.427238373	81.57442273	1	CRA004775;Yang2025_Silkie_Wenchang	PRJNA827465	0.411552605
ENSGALG00010002912	RPS3	2	-0.157001971	0.110356963	-1.422673897	0.154830711	0.010709341	1.5322571	34.73680102	1	CRA004775;PRJNA827465	Yang2025_Silkie_Wenchang	0.647505492
ENSGALG00010002913	NXNL1	2	-0.155897023	0.253449905	-0.615099947	0.538488717	0	0.04331529	0	1	PRJNA827465;Yang2025_Silkie_Wenchang	CRA004775	0.87481821
ENSGALG00010002913	NXNL1	2	0.396348294	0.604434221	0.655734372	0.511995049	0.428927505	2.268091751	55.91007289	0.5	CRA004775;Yang2025_Silkie_Wenchang	PRJNA827465	0.865694231
ENSGALG00010002913	NXNL1	2	0.327575168	0.657028055	0.498571051	0.618081603	0.60377039	2.984519699	66.49377116	0.5	CRA004775;PRJNA827465	Yang2025_Silkie_Wenchang	0.899121942
ENSGALG00010002914	SLC27A1	2	-0.100357863	0.099398316	-1.009653556	0.312661301	0	0.354503037	0	1	PRJNA827465;Yang2025_Silkie_Wenchang	CRA004775	0.796054997
ENSGALG00010002914	SLC27A1	2	0.017643422	0.180284585	0.097864283	0.922040062	0	0.126045807	0	0.5	CRA004775;Yang2025_Silkie_Wenchang	PRJNA827465	0.981720024
ENSGALG00010002914	SLC27A1	2	-0.122633193	0.114202354	-1.073823686	0.282901712	0	0.355616591	0	0.5	CRA004775;PRJNA827465	Yang2025_Silkie_Wenchang	0.779532565
ENSGALG00010002916	SDCBP	2	-0.333744061	0.38762652	-0.860993879	0.389241414	0.279899889	13.76680485	92.73615039	0.5	PRJNA827465;Yang2025_Silkie_Wenchang	CRA004775	0.828814078
ENSGALG00010002916	SDCBP	2	-0.332392958	0.424083927	-0.783790511	0.433163042	0.310613606	7.309148122	86.31851505	0.5	CRA004775;Yang2025_Silkie_Wenchang	PRJNA827465	0.842762754
ENSGALG00010002916	SDCBP	2	0.031520889	0.043947018	0.717247505	0.473221392	0	0.086969637	0	1	CRA004775;PRJNA827465	Yang2025_Silkie_Wenchang	0.854341771
ENSGALG00010002917	PGLS	2	0.134218063	0.113323249	1.184382408	0.236261721	0.017786326	2.898100611	65.49464169	1	PRJNA827465;Yang2025_Silkie_Wenchang	CRA004775	0.748669015
ENSGALG00010002917	PGLS	2	0.029246986	0.258759766	0.113027564	0.910008702	0.113080314	6.372885159	84.30852	0.5	CRA004775;Yang2025_Silkie_Wenchang	PRJNA827465	0.978730675
ENSGALG00010002917	PGLS	2	-0.056627638	0.136415417	-0.415111722	0.678060119	0.025855335	2.756164834	63.71769976	0.5	CRA004775;PRJNA827465	Yang2025_Silkie_Wenchang	0.917534751
ENSGALG00010002918	LRATD1	2	0.444772296	0.18442577	2.411660231	0.015880072	0	0.702137746	0	1	PRJNA827465;Yang2025_Silkie_Wenchang	CRA004775	0.208471156
ENSGALG00010002918	LRATD1	2	0.400453228	0.235616989	1.699594032	0.089207314	0.028024982	1.281817183	21.98575484	1	CRA004775;Yang2025_Silkie_Wenchang	PRJNA827465	0.512814839
ENSGALG00010002918	LRATD1	2	0.169657299	0.238000524	0.712844224	0.475942156	0	0.142430684	0	1	CRA004775;PRJNA827465	Yang2025_Silkie_Wenchang	0.854800041
ENSGALG00010002919	ENSGALG00010002919	2	0.33273755	0.555405756	0.599089127	0.549113452	0.568883469	12.06901739	91.71432133	0.5	PRJNA827465;Yang2025_Silkie_Wenchang	CRA004775	0.878431377
ENSGALG00010002919	ENSGALG00010002919	2	0.571375386	0.325678333	1.754416331	0.079359195	0.138162019	2.818029449	64.51421044	1	CRA004775;Yang2025_Silkie_Wenchang	PRJNA827465	0.484805397
ENSGALG00010002919	ENSGALG00010002919	2	-0.007265251	0.223340406	-0.032529946	0.974049436	0.074927844	3.450960718	71.02256207	0.5	CRA004775;PRJNA827465	Yang2025_Silkie_Wenchang	0.992492038
ENSGALG00010002920	VCPIP1	2	-0.065318223	0.077458524	-0.843267084	0.399079087	0.005472835	1.636710359	38.90183472	1	PRJNA827465;Yang2025_Silkie_Wenchang	CRA004775	0.832054308
ENSGALG00010002920	VCPIP1	2	-0.161626438	0.111952974	-1.443699359	0.148823562	0	0.267031179	0	0.5	CRA004775;Yang2025_Silkie_Wenchang	PRJNA827465	0.63890906
ENSGALG00010002920	VCPIP1	2	-0.013047983	0.055837953	-0.233675881	0.815236613	0	0.003802304	0	0.5	CRA004775;PRJNA827465	Yang2025_Silkie_Wenchang	0.953933933
ENSGALG00010002922	TMEM106B	2	-0.049310821	0.117458418	-0.419815133	0.674620509	0.018229797	2.932641374	65.90104713	0.5	PRJNA827465;Yang2025_Silkie_Wenchang	CRA004775	0.915970608
ENSGALG00010002922	TMEM106B	2	-0.054849584	0.193539223	-0.283402933	0.77686798	0.040553379	1.747125883	42.76313974	0.5	CRA004775;Yang2025_Silkie_Wenchang	PRJNA827465	0.944200431
ENSGALG00010002922	TMEM106B	2	0.077639427	0.086224966	0.900428619	0.367892196	0	0.379229085	0	1	CRA004775;PRJNA827465	Yang2025_Silkie_Wenchang	0.820152313
ENSGALG00010002923	SERPINH1	2	-0.120993542	0.195731431	-0.618161025	0.536469199	0.067577196	8.209241147	87.8186061	0.5	PRJNA827465;Yang2025_Silkie_Wenchang	CRA004775	0.874599474
ENSGALG00010002923	SERPINH1	2	-0.318999948	0.115213698	-2.76876754	0.005626877	0	0.106310687	0	1	CRA004775;Yang2025_Silkie_Wenchang	PRJNA827465	0.112546123
ENSGALG00010002923	SERPINH1	2	0.055988704	0.065046112	0.860754039	0.389373524	0	0.369130617	0	0.5	CRA004775;PRJNA827465	Yang2025_Silkie_Wenchang	0.828911149
ENSGALG00010002925	INTS6	2	0.160391816	0.172909843	0.927603735	0.35361316	0.04668199	4.425378662	77.40306364	1	PRJNA827465;Yang2025_Silkie_Wenchang	CRA004775	0.811318534
ENSGALG00010002925	INTS6	2	0.286785915	0.112457767	2.550165475	0.01076718	0	0.61147662	0	1	CRA004775;Yang2025_Silkie_Wenchang	PRJNA827465	0.165305655
ENSGALG00010002925	INTS6	2	0.031684872	0.081351756	0.389479881	0.696921191	0	0.580576237	0	1	CRA004775;PRJNA827465	Yang2025_Silkie_Wenchang	0.921086208
ENSGALG00010002928	ASB4	2	-0.665752865	0.397044512	-1.676771356	0.093587201	0.201844614	2.771879603	63.923397	1	PRJNA827465;Yang2025_Silkie_Wenchang	CRA004775	0.52406339
ENSGALG00010002928	ASB4	2	-0.595441535	0.591967203	-1.00586913	0.314478528	0.457603359	2.6783499	62.6635788	0.5	CRA004775;Yang2025_Silkie_Wenchang	PRJNA827465	0.796531448
ENSGALG00010002928	ASB4	2	-0.202957366	0.286099482	-0.709394385	0.478079772	0	0.328772329	0	0.5	CRA004775;PRJNA827465	Yang2025_Silkie_Wenchang	0.855366076
ENSGALG00010002929	PDHA2	2	0.002458215	0.076844223	0.031989593	0.974480351	0	0.778216246	0	0.5	PRJNA827465;Yang2025_Silkie_Wenchang	CRA004775	0.992492038
ENSGALG00010002929	PDHA2	2	0.114146739	0.1410354	0.809348141	0.418314923	0	0.04765012	0	1	CRA004775;Yang2025_Silkie_Wenchang	PRJNA827465	0.838112595
ENSGALG00010002929	PDHA2	2	-0.022700032	0.082059209	-0.27662991	0.782064296	0	0.129372832	0	0.5	CRA004775;PRJNA827465	Yang2025_Silkie_Wenchang	0.945372683
ENSGALG00010002930	SERAF	2	0.227069829	0.134450629	1.688871448	0.091244068	0	0.341441713	0	1	PRJNA827465;Yang2025_Silkie_Wenchang	CRA004775	0.51792097
ENSGALG00010002930	SERAF	2	0.248581894	0.317326665	0.783362765	0.433414115	0.058558446	1.201428107	16.76572288	1	CRA004775;Yang2025_Silkie_Wenchang	PRJNA827465	0.842762754
ENSGALG00010002930	SERAF	2	0.307453103	0.1552521	1.98034747	0.047664498	0	0.806048124	0	1	CRA004775;PRJNA827465	Yang2025_Silkie_Wenchang	0.377236765
ENSGALG00010002937	GALNT7	2	0.00369385	0.062378152	0.059217048	0.952779231	0.002904124	1.53363309	34.7953558	0.5	PRJNA827465;Yang2025_Silkie_Wenchang	CRA004775	0.987758913
ENSGALG00010002937	GALNT7	2	-0.068560568	0.080609813	-0.850523845	0.395033909	0	0.074999069	0	1	CRA004775;Yang2025_Silkie_Wenchang	PRJNA827465	0.830643015
ENSGALG00010002937	GALNT7	2	0.046915015	0.055203807	0.849851076	0.395407889	0	0.093479839	0	0.5	CRA004775;PRJNA827465	Yang2025_Silkie_Wenchang	0.830946177
ENSGALG00010002939	HMGB2	2	-0.168404752	0.116394232	-1.446847923	0.147939521	0.016433802	2.523392547	60.37081106	1	PRJNA827465;Yang2025_Silkie_Wenchang	CRA004775	0.637411423
ENSGALG00010002939	HMGB2	2	-0.005626226	0.329212475	-0.017089953	0.986364854	0.178331094	5.200270192	80.77022995	0.5	CRA004775;Yang2025_Silkie_Wenchang	PRJNA827465	0.99618774
ENSGALG00010002939	HMGB2	2	0.082459776	0.202694126	0.406818774	0.684141097	0.052355434	2.289429326	56.3209928	0.5	CRA004775;PRJNA827465	Yang2025_Silkie_Wenchang	0.918806497
ENSGALG00010002940	BG1	2	-0.372936608	0.234651651	-1.589320195	0.111988122	0	0.27787009	0	1	PRJNA827465;Yang2025_Silkie_Wenchang	CRA004775	0.56547057
ENSGALG00010002940	BG1	2	0.05633595	0.386032751	0.145935674	0.88397217	0	0.242608997	0	0.5	CRA004775;Yang2025_Silkie_Wenchang	PRJNA827465	0.972001076
ENSGALG00010002940	BG1	2	-0.267544076	0.295938851	-0.904051885	0.36596789	0.046804424	1.242501095	19.51717357	0.5	CRA004775;PRJNA827465	Yang2025_Silkie_Wenchang	0.81897375
ENSGALG00010002943	SAP30	2	-0.291165871	0.307339087	-0.947376638	0.343446891	0.176648885	15.31366885	93.46988622	0.5	PRJNA827465;Yang2025_Silkie_Wenchang	CRA004775	0.806276913
ENSGALG00010002943	SAP30	2	-0.169965403	0.464733001	-0.365726993	0.714568802	0.388861673	9.63821284	89.62463253	0.5	CRA004775;Yang2025_Silkie_Wenchang	PRJNA827465	0.926596201
ENSGALG00010002943	SAP30	2	0.065292999	0.120155655	0.543403462	0.586852043	0.008921651	1.216343563	17.78638615	1	CRA004775;PRJNA827465	Yang2025_Silkie_Wenchang	0.891596903
ENSGALG00010002946	GDPD5	2	-0.096693715	0.12357179	-0.782490206	0.433926539	0.026698238	7.940969416	87.40707906	0.5	PRJNA827465;Yang2025_Silkie_Wenchang	CRA004775	0.842762754
ENSGALG00010002946	GDPD5	2	-0.223872332	0.064073642	-3.493984837	0.000475868	0	0.034939239	0	1	CRA004775;Yang2025_Silkie_Wenchang	PRJNA827465	0.022808588
ENSGALG00010002946	GDPD5	2	0.017804742	0.058173891	0.30606071	0.759558422	0	0.694094543	0	0.5	CRA004775;PRJNA827465	Yang2025_Silkie_Wenchang	0.939163539
ENSGALG00010002949	ENSGALG00010002949	2	-0.742833982	0.431916866	-1.719854077	0.08545897	0	0.024159226	0	1	PRJNA827465;Yang2025_Silkie_Wenchang	CRA004775	0.50266296
ENSGALG00010002949	ENSGALG00010002949	2	1.741245017	2.52220149	0.690367135	0.489963339	11.80966435	13.59896649	92.64650001	0.5	CRA004775;Yang2025_Silkie_Wenchang	PRJNA827465	0.858512413
ENSGALG00010002949	ENSGALG00010002949	2	1.674867403	2.589457471	0.646802437	0.517759815	12.50755856	14.49695783	93.10200104	0.5	CRA004775;PRJNA827465	Yang2025_Silkie_Wenchang	0.867973554
ENSGALG00010002952	NBAS	2	-0.113105743	0.105430278	-1.072801336	0.283360261	0.017614373	4.622951648	78.3687982	1	PRJNA827465;Yang2025_Silkie_Wenchang	CRA004775	0.779568117
ENSGALG00010002952	NBAS	2	-0.155254401	0.133152364	-1.165990567	0.243618259	0.016135203	1.498111782	33.24930677	0.5	CRA004775;Yang2025_Silkie_Wenchang	PRJNA827465	0.755365463
ENSGALG00010002952	NBAS	2	-0.014003305	0.049083799	-0.285293831	0.775419039	0	0.164814558	0	0.5	CRA004775;PRJNA827465	Yang2025_Silkie_Wenchang	0.943815265
ENSGALG00010002953	WDFY2	2	-0.284342958	0.083356417	-3.411170565	0.000646846	0	0.005850957	0	1	PRJNA827465;Yang2025_Silkie_Wenchang	CRA004775	0.0280607
ENSGALG00010002953	WDFY2	2	-0.295207041	0.106737453	-2.765730601	0.005679543	0	0.011094542	0	1	CRA004775;Yang2025_Silkie_Wenchang	PRJNA827465	0.113196293
ENSGALG00010002953	WDFY2	2	-0.284786495	0.110130677	-2.585896162	0.009712619	0	0.021654651	0	1	CRA004775;PRJNA827465	Yang2025_Silkie_Wenchang	0.155303112
ENSGALG00010002954	CHST7	2	0.01869907	0.118802568	0.157396173	0.87493263	0	0.004324624	0	1	PRJNA827465;Yang2025_Silkie_Wenchang	CRA004775	0.970159044
ENSGALG00010002954	CHST7	2	0.034419801	0.22280996	0.154480531	0.877230861	0	0.047186435	0	1	CRA004775;Yang2025_Silkie_Wenchang	PRJNA827465	0.970575794
ENSGALG00010002954	CHST7	2	0.030835689	0.126386476	0.243979342	0.807246819	0	0.04035165	0	1	CRA004775;PRJNA827465	Yang2025_Silkie_Wenchang	0.95234753
ENSGALG00010002955	BLEC1	2	-0.634883478	0.311010165	-2.041359252	0.041215132	0	0.579319219	0	1	PRJNA827465;Yang2025_Silkie_Wenchang	CRA004775	0.351928866
ENSGALG00010002955	BLEC1	2	-0.26067453	0.314829082	-0.827987453	0.407677605	0	0.010984702	0	1	CRA004775;Yang2025_Silkie_Wenchang	PRJNA827465	0.833982683
ENSGALG00010002955	BLEC1	2	-0.525707875	0.28426641	-1.849349263	0.064407397	0.012126255	1.081092247	7.500955336	1	CRA004775;PRJNA827465	Yang2025_Silkie_Wenchang	0.437453135
ENSGALG00010002956	SCRG1	2	0.393975969	0.169321536	2.326791845	0.019976347	0	0.397460468	0	1	PRJNA827465;Yang2025_Silkie_Wenchang	CRA004775	0.237630252
ENSGALG00010002956	SCRG1	2	0.617547457	0.26097028	2.366351668	0.017964371	0	0.140804595	0	1	CRA004775;Yang2025_Silkie_Wenchang	PRJNA827465	0.223717898
ENSGALG00010002956	SCRG1	2	0.389165017	0.187386071	2.076808668	0.037819222	0	0.716862667	0	1	CRA004775;PRJNA827465	Yang2025_Silkie_Wenchang	0.335608236
ENSGALG00010002957	ADGRG2	2	-0.027764372	0.156617081	-0.177275502	0.859291992	0	0.006483476	0	1	PRJNA827465;Yang2025_Silkie_Wenchang	CRA004775	0.964605083
ENSGALG00010002957	ADGRG2	2	0.370704327	0.474106864	0.781900357	0.434273135	0.295676473	2.680544479	62.69414636	0.5	CRA004775;Yang2025_Silkie_Wenchang	PRJNA827465	0.842762754
ENSGALG00010002957	ADGRG2	2	0.341479678	0.484134372	0.705340703	0.480598236	0.340661327	3.165311433	68.40753205	0.5	CRA004775;PRJNA827465	Yang2025_Silkie_Wenchang	0.856719542
ENSGALG00010002958	ENSGALG00010002958	2	-0.052408679	0.140471821	-0.373090334	0.709081212	0	0.728689495	0	0.5	PRJNA827465;Yang2025_Silkie_Wenchang	CRA004775	0.925298042
ENSGALG00010002958	ENSGALG00010002958	2	-0.382936747	0.576149636	-0.664648075	0.506275613	0.512923101	3.974263411	74.83810466	0.5	CRA004775;Yang2025_Silkie_Wenchang	PRJNA827465	0.863929781
ENSGALG00010002958	ENSGALG00010002958	2	-0.468214059	0.4400467	-1.064009933	0.2873242	0.26437072	2.622277104	61.86520492	1	CRA004775;PRJNA827465	Yang2025_Silkie_Wenchang	0.782840067
ENSGALG00010002960	BLEC2	2	-1.748038566	2.12204782	-0.823750789	0.410081175	7.976947718	8.747782124	88.56853102	0.5	PRJNA827465;Yang2025_Silkie_Wenchang	CRA004775	0.834652129
ENSGALG00010002960	BLEC2	2	-0.200310047	0.557980657	-0.358991024	0.719601804	0	0.475620095	0	0.5	CRA004775;Yang2025_Silkie_Wenchang	PRJNA827465	0.928449671
ENSGALG00010002960	BLEC2	2	-2.081959729	1.707116099	-1.219577117	0.222625226	5.073528043	7.573327608	86.79576466	1	CRA004775;PRJNA827465	Yang2025_Silkie_Wenchang	0.736208602
ENSGALG00010002965	PRSS35	2	-0.095580122	0.086964083	-1.099075834	0.27173499	0	0.003773148	0	1	PRJNA827465;Yang2025_Silkie_Wenchang	CRA004775	0.773086433
ENSGALG00010002965	PRSS35	2	0.250576515	0.401691148	0.623803927	0.53275636	0.261488292	4.942850411	79.76875857	0.5	CRA004775;Yang2025_Silkie_Wenchang	PRJNA827465	0.873183244
ENSGALG00010002965	PRSS35	2	0.246480043	0.394405279	0.624941035	0.532009759	0.259435312	5.398931376	81.47781606	0.5	CRA004775;PRJNA827465	Yang2025_Silkie_Wenchang	0.873104119
ENSGALG00010002972	ME1	2	0.023913942	0.093523052	0.255701045	0.798181688	0	0.963487902	0	0.5	PRJNA827465;Yang2025_Silkie_Wenchang	CRA004775	0.949922217
ENSGALG00010002972	ME1	2	0.139509192	0.135245901	1.031522515	0.302295855	0	0.025884439	0	1	CRA004775;Yang2025_Silkie_Wenchang	PRJNA827465	0.791706138
ENSGALG00010002972	ME1	2	-0.031745334	0.11730367	-0.27062524	0.786679281	0	0.379546872	0	0.5	CRA004775;PRJNA827465	Yang2025_Silkie_Wenchang	0.946722706
ENSGALG00010002978	DYNLT3	2	0.042143642	0.063943981	0.659071289	0.509849992	0	0.596456914	0	1	PRJNA827465;Yang2025_Silkie_Wenchang	CRA004775	0.865257661
ENSGALG00010002978	DYNLT3	2	0.116728386	0.102674434	1.136878784	0.255588963	0	0.004481803	0	1	CRA004775;Yang2025_Silkie_Wenchang	PRJNA827465	0.764467569
ENSGALG00010002978	DYNLT3	2	0.018885409	0.074235716	0.254397884	0.799188185	0	0.222810674	0	1	CRA004775;PRJNA827465	Yang2025_Silkie_Wenchang	0.949922217
ENSGALG00010002982	HAND2	2	-0.120672609	0.164481213	-0.733655879	0.463158495	0	0.443041045	0	1	PRJNA827465;Yang2025_Silkie_Wenchang	CRA004775	0.851507079
ENSGALG00010002982	HAND2	2	-0.043709677	0.281192685	-0.155443864	0.876471406	0.056232223	1.485007411	32.66026874	0.5	CRA004775;Yang2025_Silkie_Wenchang	PRJNA827465	0.970347777
ENSGALG00010002982	HAND2	2	0.048236637	0.188825575	0.25545606	0.798370877	0	0.628503207	0	0.5	CRA004775;PRJNA827465	Yang2025_Silkie_Wenchang	0.949922217
ENSGALG00010002983	NSMAF	2	-0.002536707	0.061505359	-0.041243682	0.96710163	0.00390083	2.0316708	50.77942746	0.5	PRJNA827465;Yang2025_Silkie_Wenchang	CRA004775	0.990893423
ENSGALG00010002983	NSMAF	2	-0.076707679	0.063586426	-1.206353054	0.227681389	0	0.030703782	0	1	CRA004775;Yang2025_Silkie_Wenchang	PRJNA827465	0.740853612
ENSGALG00010002983	NSMAF	2	0.037504111	0.050752537	0.738960305	0.459931102	0	0.785516072	0	0.5	CRA004775;PRJNA827465	Yang2025_Silkie_Wenchang	0.850104868
ENSGALG00010002985	ENSGALG00010002985	2	0.054807317	0.100251726	0.546696989	0.584586923	0	0.000751047	0	1	PRJNA827465;Yang2025_Silkie_Wenchang	CRA004775	0.891016154
ENSGALG00010002985	ENSGALG00010002985	2	0.04387298	0.189067949	0.232048746	0.816500153	0	0.0007391	0	1	CRA004775;Yang2025_Silkie_Wenchang	PRJNA827465	0.95460349
ENSGALG00010002985	ENSGALG00010002985	2	0.05369573	0.101945139	0.526712021	0.598393586	0	0.003449703	0	1	CRA004775;PRJNA827465	Yang2025_Silkie_Wenchang	0.894645663
ENSGALG00010002987	PCDH8	2	-0.426444284	0.198109106	-2.152572858	0.031352264	0	0.002213075	0	1	PRJNA827465;Yang2025_Silkie_Wenchang	CRA004775	0.302910033
ENSGALG00010002987	PCDH8	2	0.192888861	0.630327649	0.306013644	0.759594257	0.662615413	5.978244705	83.27268205	0.5	CRA004775;Yang2025_Silkie_Wenchang	PRJNA827465	0.939163539
ENSGALG00010002987	PCDH8	2	0.167838917	0.639190078	0.262580605	0.792873851	0.70726548	7.265949023	86.23717292	0.5	CRA004775;PRJNA827465	Yang2025_Silkie_Wenchang	0.948451453
ENSGALG00010002991	BLB1	2	-0.674191946	0.288141801	-2.339792231	0.01929447	0.09484787	2.259506539	55.74254898	1	PRJNA827465;Yang2025_Silkie_Wenchang	CRA004775	0.232624497
ENSGALG00010002991	BLB1	2	-0.654647743	0.369235329	-1.772982407	0.076231622	0.161710397	2.45299535	59.23351423	1	CRA004775;Yang2025_Silkie_Wenchang	PRJNA827465	0.476484008
ENSGALG00010002991	BLB1	2	-0.382999609	0.186567861	-2.052870235	0.040085164	0	0.142567683	0	1	CRA004775;PRJNA827465	Yang2025_Silkie_Wenchang	0.346798223
ENSGALG00010002992	ENSGALG00010002992	2	0.405764683	0.416512289	0.974196185	0.329959138	0.268668232	4.412875199	77.33903737	1	PRJNA827465;Yang2025_Silkie_Wenchang	CRA004775	0.801209935
ENSGALG00010002992	ENSGALG00010002992	2	0.170301131	0.693682387	0.245503035	0.806066958	0.834957829	7.475780568	86.62347041	0.5	CRA004775;Yang2025_Silkie_Wenchang	PRJNA827465	0.952063038
ENSGALG00010002992	ENSGALG00010002992	2	-0.183388899	0.262285636	-0.699195357	0.484429952	0.037182474	1.318393354	24.15010307	0.5	CRA004775;PRJNA827465	Yang2025_Silkie_Wenchang	0.857004733
ENSGALG00010002994	DUSP4	2	0.044605663	0.112353541	0.397011634	0.691358881	0	0.183606748	0	0.5	PRJNA827465;Yang2025_Silkie_Wenchang	CRA004775	0.920268407
ENSGALG00010002994	DUSP4	2	-0.270543662	0.333994379	-0.810024598	0.417926039	0.10801168	1.719472549	41.84263072	1	CRA004775;Yang2025_Silkie_Wenchang	PRJNA827465	0.838112595
ENSGALG00010002994	DUSP4	2	-0.208286214	0.393214286	-0.529701542	0.596318879	0.216165956	2.652618134	62.30139622	0.5	CRA004775;PRJNA827465	Yang2025_Silkie_Wenchang	0.894064255
ENSGALG00010002995	RWDD2A	2	-0.308287471	0.275592731	-1.118634264	0.2632962	0.132599535	7.838741957	87.24285089	1	PRJNA827465;Yang2025_Silkie_Wenchang	CRA004775	0.768643821
ENSGALG00010002995	RWDD2A	2	-0.525671311	0.126797663	-4.145749221	3.38704E-05	0	0.606672131	0	1	CRA004775;Yang2025_Silkie_Wenchang	PRJNA827465	0.003661746
ENSGALG00010002995	RWDD2A	2	-0.151306601	0.158467771	-0.954809927	0.339673826	0.021915342	1.634052344	38.80245001	1	CRA004775;PRJNA827465	Yang2025_Silkie_Wenchang	0.805053392
ENSGALG00010002998	LECT1	2	0.393088827	0.21937356	1.791869662	0.073153847	0	0.252096539	0	1	PRJNA827465;Yang2025_Silkie_Wenchang	CRA004775	0.467506607
ENSGALG00010002998	LECT1	2	-0.047695337	0.650573518	-0.073312755	0.941557242	0.626895111	3.49872926	71.41819428	0.5	CRA004775;Yang2025_Silkie_Wenchang	PRJNA827465	0.985827325
ENSGALG00010002998	LECT1	2	-0.145147751	0.533662492	-0.271984172	0.785634189	0.344441476	2.306511013	56.64447322	0.5	CRA004775;PRJNA827465	Yang2025_Silkie_Wenchang	0.946557017
ENSGALG00010002999	CMTM7	2	0.117603974	0.158032738	0.744174753	0.45677076	0	0.015326397	0	1	PRJNA827465;Yang2025_Silkie_Wenchang	CRA004775	0.849515887
ENSGALG00010002999	CMTM7	2	-0.177958396	0.411119346	-0.432863102	0.665114227	0.203945571	2.315512264	56.81301215	0.5	CRA004775;Yang2025_Silkie_Wenchang	PRJNA827465	0.913399315
ENSGALG00010002999	CMTM7	2	-0.175160074	0.383348416	-0.456921346	0.64772758	0.184834017	2.315131898	56.80591673	0.5	CRA004775;PRJNA827465	Yang2025_Silkie_Wenchang	0.90745124
ENSGALG00010003001	MAP6	2	-0.147359319	0.352133792	-0.418475372	0.675599589	0.101760248	1.597487439	37.40169873	0.5	PRJNA827465;Yang2025_Silkie_Wenchang	CRA004775	0.916508721
ENSGALG00010003001	MAP6	2	0.598426705	0.385326647	1.553037429	0.120414193	0	0.694857655	0	1	CRA004775;Yang2025_Silkie_Wenchang	PRJNA827465	0.581775014
ENSGALG00010003001	MAP6	2	0.19066242	0.688493288	0.276927058	0.781836116	0.741213858	4.277132589	76.61985035	0.5	CRA004775;PRJNA827465	Yang2025_Silkie_Wenchang	0.945273413
ENSGALG00010003002	PHKA2	2	-0.016729533	0.128798409	-0.129889281	0.896654025	0.00697557	1.248913752	19.93041968	0.5	PRJNA827465;Yang2025_Silkie_Wenchang	CRA004775	0.975115773
ENSGALG00010003002	PHKA2	2	-0.129585941	0.338468559	-0.382859611	0.70182386	0.140731104	2.012875739	50.31983443	0.5	CRA004775;Yang2025_Silkie_Wenchang	PRJNA827465	0.922904951
ENSGALG00010003002	PHKA2	2	-0.238421319	0.177753069	-1.341306344	0.179821011	0	0.794951449	0	1	CRA004775;PRJNA827465	Yang2025_Silkie_Wenchang	0.68659821
ENSGALG00010003006	CYP7B1	2	-0.228096231	0.139149375	-1.639218506	0.101167761	0.003186167	1.085231275	7.853743005	1	PRJNA827465;Yang2025_Silkie_Wenchang	CRA004775	0.542825462
ENSGALG00010003006	CYP7B1	2	-0.402547433	0.190594445	-2.112062775	0.034681065	0	0.000286038	0	1	CRA004775;Yang2025_Silkie_Wenchang	PRJNA827465	0.320651825
ENSGALG00010003006	CYP7B1	2	-0.157878018	0.1569303	-1.006039098	0.314396763	0	0.426307125	0	1	CRA004775;PRJNA827465	Yang2025_Silkie_Wenchang	0.796531448
ENSGALG00010003009	TNKS	2	-0.108643158	0.041045998	-2.646863582	0.008124212	0	0.045446183	0	1	PRJNA827465;Yang2025_Silkie_Wenchang	CRA004775	0.139923707
ENSGALG00010003009	TNKS	2	0.083368277	0.227722105	0.36609655	0.714293032	0.087558481	6.169926496	83.79235149	0.5	CRA004775;Yang2025_Silkie_Wenchang	PRJNA827465	0.92653974

ENSGALG00010003009	TNKS	2	0.086467445	0.216170047	0.399997347	0.68915847	0.080901024	6.757193573	85.20095674	0.5	CRA004775;PRJNA827465	Yang2025_Silkie_Wenchang	0.919854035
ENSGALG00010003017	PGM3	2	0.337330949	0.140090808	2.407944911	0.016042602	0.0222252	2.186619388	54.26730388	1	PRJNA827465;Yang2025_Silkie_Wenchang	CRA004775	0.209265194
ENSGALG00010003017	PGM3	2	0.283242311	0.299192742	0.946688443	0.343797651	0.113679533	2.427387596	58.80344772	0.5	CRA004775;Yang2025_Silkie_Wenchang	PRJNA827465	0.806388726
ENSGALG00010003017	PGM3	2	0.198998873	0.098084011	2.028861483	0.042472401	0	0.789983315	0	0.5	CRA004775;PRJNA827465	Yang2025_Silkie_Wenchang	0.356610291
ENSGALG00010003018	MOGAT2	2	-0.603749484	0.317819111	-1.899663878	0.057477243	0	0.013454557	0	1	PRJNA827465;Yang2025_Silkie_Wenchang	CRA004775	0.415229176
ENSGALG00010003018	MOGAT2	2	0.997351717	1.544828116	0.645606917	0.518533957	4.351544133	11.32284388	91.16829649	0.5	CRA004775;Yang2025_Silkie_Wenchang	PRJNA827465	0.868525042
ENSGALG00010003018	MOGAT2	2	0.913493724	1.586511652	0.575787592	0.564758761	4.749390958	17.41313712	94.25720941	0.5	CRA004775;PRJNA827465	Yang2025_Silkie_Wenchang	0.883345814
ENSGALG00010003019	TAPBP	2	-0.153002879	0.12286379	-1.245304898	0.213019696	0	0.875504733	0	1	PRJNA827465;Yang2025_Silkie_Wenchang	CRA004775	0.72579791
ENSGALG00010003019	TAPBP	2	-0.365235681	0.163494816	-2.233928211	0.025487797	0	0.307579652	0	1	CRA004775;Yang2025_Silkie_Wenchang	PRJNA827465	0.272213827
ENSGALG00010003019	TAPBP	2	-0.21349944	0.204715117	-1.042909985	0.296989985	0.040140885	1.769993028	43.50260232	1	CRA004775;PRJNA827465	Yang2025_Silkie_Wenchang	0.788466926
ENSGALG00010003022	BLB2	2	-0.258571579	0.41445662	-0.623880924	0.532705789	0.243394133	3.429360114	70.84004109	0.5	PRJNA827465;Yang2025_Silkie_Wenchang	CRA004775	0.873183244
ENSGALG00010003022	BLB2	2	-0.237536204	0.392321339	-0.605463379	0.54487115	0.208367201	3.093736693	67.67662865	0.5	CRA004775;Yang2025_Silkie_Wenchang	PRJNA827465	0.877345822
ENSGALG00010003022	BLB2	2	-0.646642508	0.219191929	-2.95012006	0.003176505	0	0.010184661	0	1	CRA004775;PRJNA827465	Yang2025_Silkie_Wenchang	0.078092601
ENSGALG00010003024	SUGT1	2	-0.143913175	0.20583278	-0.6991752	0.484442547	0.076110458	7.755362302	87.10569589	0.5	PRJNA827465;Yang2025_Silkie_Wenchang	CRA004775	0.857004733
ENSGALG00010003024	SUGT1	2	-0.18073917	0.235663256	-0.766938268	0.443118219	0.072345721	2.630225103	61.98044043	0.5	CRA004775;Yang2025_Silkie_Wenchang	PRJNA827465	0.845730569
ENSGALG00010003024	SUGT1	2	0.047335952	0.056388215	0.839465337	0.401208233	0	0.066430614	0	1	CRA004775;PRJNA827465	Yang2025_Silkie_Wenchang	0.832472134
ENSGALG00010003025	MFHAS1	2	-0.166254465	0.063377489	-2.623241608	0.008709748	0	0.634703896	0	1	PRJNA827465;Yang2025_Silkie_Wenchang	CRA004775	0.146236539
ENSGALG00010003025	MFHAS1	2	-0.216357298	0.09537538	-2.268481633	0.023299867	0	0.156752731	0	1	CRA004775;Yang2025_Silkie_Wenchang	PRJNA827465	0.260268986
ENSGALG00010003025	MFHAS1	2	-0.121906184	0.080414719	-1.515968552	0.129527305	0	0.014586496	0	1	CRA004775;PRJNA827465	Yang2025_Silkie_Wenchang	0.60360584
ENSGALG00010003027	SCIN	2	-0.155998331	0.166188219	-0.938684654	0.347892675	0	0.303990309	0	1	PRJNA827465;Yang2025_Silkie_Wenchang	CRA004775	0.808847403
ENSGALG00010003027	SCIN	2	0.073854113	0.364868995	0.202412684	0.839594113	0.177557338	2.717947234	63.20752708	0.5	CRA004775;Yang2025_Silkie_Wenchang	PRJNA827465	0.959493354
ENSGALG00010003027	SCIN	2	0.19242701	0.265933705	0.723590153	0.469317389	0.020033608	1.153508185	13.30794067	0.5	CRA004775;PRJNA827465	Yang2025_Silkie_Wenchang	0.853485658
ENSGALG00010003030	PPEF1	2	-0.161922578	0.364396643	-0.44435804	0.656783745	0	0.530617348	0	0.5	PRJNA827465;Yang2025_Silkie_Wenchang	CRA004775	0.910563743
ENSGALG00010003030	PPEF1	2	-0.049296529	0.432981728	-0.113853602	0.909353847	0	0.299734028	0	0.5	CRA004775;Yang2025_Silkie_Wenchang	PRJNA827465	0.978607662
ENSGALG00010003030	PPEF1	2	-0.415223894	0.425713499	-0.975359943	0.329381746	0	0.000422394	0	1	CRA004775;PRJNA827465	Yang2025_Silkie_Wenchang	0.800993593
ENSGALG00010003034	ERI1	2	-0.147981529	0.123421516	-1.19899297	0.230530679	0.026915175	8.266179144	87.90251236	1	PRJNA827465;Yang2025_Silkie_Wenchang	CRA004775	0.743335644
ENSGALG00010003034	ERI1	2	-0.275067903	0.071280494	-3.858950582	0.000113875	0	0.03695064	0	1	CRA004775;Yang2025_Silkie_Wenchang	PRJNA827465	0.008492652
ENSGALG00010003034	ERI1	2	-0.049535346	0.056150819	-0.882183854	0.377637392	0.001890442	1.096419299	8.794016932	1	CRA004775;PRJNA827465	Yang2025_Silkie_Wenchang	0.82325159
ENSGALG00010003035	DGAT2	2	-0.791077847	0.335551444	-2.357545651	0.018396193	0	0.808972818	0	1	PRJNA827465;Yang2025_Silkie_Wenchang	CRA004775	0.227043528
ENSGALG00010003035	DGAT2	2	-0.609426109	0.399858682	-1.52410373	0.127482801	0.159435126	1.972683286	49.30762545	1	CRA004775;Yang2025_Silkie_Wenchang	PRJNA827465	0.599452995
ENSGALG00010003035	DGAT2	2	-0.314300848	0.29455548	-1.067034459	0.285956276	0	0.096968483	0	1	CRA004775;PRJNA827465	Yang2025_Silkie_Wenchang	0.781767418
ENSGALG00010003042	TOX	2	0.328364527	0.15564597	2.10968859	0.034885188	0	0.596772791	0	1	PRJNA827465;Yang2025_Silkie_Wenchang	CRA004775	0.321626212
ENSGALG00010003042	TOX	2	-0.101865576	0.287065863	-0.354850888	0.722701303	0	0.978271462	0	0.5	CRA004775;Yang2025_Silkie_Wenchang	PRJNA827465	0.929978368
ENSGALG00010003042	TOX	2	0.06086055	0.437687604	0.139050202	0.889410479	0.268808494	2.827443005	64.63235516	0.5	CRA004775;PRJNA827465	Yang2025_Silkie_Wenchang	0.973135755
ENSGALG00010003052	ENSGALG00010003052	2	-0.022063013	0.063995169	-0.344760606	0.730274351	0.003224531	1.249065679	19.9401587	0.5	PRJNA827465;Yang2025_Silkie_Wenchang	CRA004775	0.932615145
ENSGALG00010003052	ENSGALG00010003052	2	0.199994437	0.120181617	1.664101729	0.096092102	0	0.482305114	0	1	CRA004775;Yang2025_Silkie_Wenchang	PRJNA827465	0.330362009
ENSGALG00010003052	ENSGALG00010003052	2	0.07748108	0.168015672	0.461153886	0.644688204	0.042593445	3.291078597	69.61482473	0.5	CRA004775;PRJNA827465	Yang2025_Silkie_Wenchang	0.906953008
ENSGALG00010003057	DDX1	2	0.013382601	0.022694985	0.589672177	0.555410452	0	0.977608397	0	1	PRJNA827465;Yang2025_Silkie_Wenchang	CRA004775	0.880255849
ENSGALG00010003057	DDX1	2	0.066646246	0.057697693	1.155093784	0.248052023	0	3.29178E-05	0	1	CRA004775;Yang2025_Silkie_Wenchang	PRJNA827465	0.758257812
ENSGALG00010003057	DDX1	2	0.00424152	0.024561502	0.17268975	0.862895301	0	0.030312365	0	1	CRA004775;PRJNA827465	Yang2025_Silkie_Wenchang	0.966099097
ENSGALG00010003061	ENSGALG00010003061	2	-0.267610094	0.286902565	-0.932756018	0.350945955	0	0.015061112	0	1	PRJNA827465;Yang2025_Silkie_Wenchang	CRA004775	0.810146221
ENSGALG00010003061	ENSGALG00010003061	2	0.850764459	1.178693237	0.721786155	0.470425963	2.36918079	6.494936378	84.60339037	0.5	CRA004775;Yang2025_Silkie_Wenchang	PRJNA827465	0.853744788
ENSGALG00010003061	ENSGALG00010003061	2	0.810967719	1.213861981	0.668088903	0.504076845	2.55738087	7.190615632	86.09298492	0.5	CRA004775;PRJNA827465	Yang2025_Silkie_Wenchang	0.863348292
ENSGALG00010003063	GGH	2	-0.31403041	0.357747672	-0.877798613	0.380053017	0.250615668	47.80474123	97.90815728	0.5	PRJNA827465;Yang2025_Silkie_Wenchang	CRA004775	0.824392515
ENSGALG00010003063	GGH	2	-0.199548829	0.504852777	-0.395261426	0.692649964	0.472823307	13.11633232	92.37591748	0.5	CRA004775;Yang2025_Silkie_Wenchang	PRJNA827465	0.920574244
ENSGALG00010003063	GGH	2	0.076920253	0.095031159	0.809421397	0.418272799	0.005794978	1.152118271	13.20335551	1	CRA004775;PRJNA827465	Yang2025_Silkie_Wenchang	0.838112595
ENSGALG00010003064	VAV1	2	0.334823265	0.1779827	1.881212416	0.059943029	0	0.457630011	0	1	PRJNA827465;Yang2025_Silkie_Wenchang	CRA004775	0.243882851
ENSGALG00010003064	VAV1	2	-0.051512548	0.58576965	-0.08793994	0.929924412	0.55013247	4.891639569	79.5569566	0.5	CRA004775;Yang2025_Silkie_Wenchang	PRJNA827465	0.983813577
ENSGALG00010003064	VAV1	2	-0.145611882	0.45756867	-0.318229573	0.750310802	0.304932935	3.405441348	70.63523057	0.5	CRA004775;PRJNA827465	Yang2025_Silkie_Wenchang	0.937393444
ENSGALG00010003068	BRD2	2	0.12178727	0.186674758	0.652403526	0.51414089	0.055310475	4.279390332	76.63218537	0.5	PRJNA827465;Yang2025_Silkie_Wenchang	CRA004775	0.866885965
ENSGALG00010003068	BRD2	2	0.277041032	0.124565867	2.224052539	0.02614491	0	0.332026215	0	1	CRA004775;Yang2025_Silkie_Wenchang	PRJNA827465	0.275965687
ENSGALG00010003068	BRD2	2	0.025177905	0.101782476	0.24736974	0.804622091	0.010256621	1.58042904	36.72604243	0.5	CRA004775;PRJNA827465	Yang2025_Silkie_Wenchang	0.951665484
ENSGALG00010003072	ELF1	2	0.018677093	0.103286223	0.180828506	0.856502187	0.00905866	1.330796276	24.85701849	0.5	PRJNA827465;Yang2025_Silkie_Wenchang	CRA004775	0.96353738
ENSGALG00010003072	ELF1	2	0.043379875	0.271491448	0.159783578	0.873035561	0.061745455	1.609765348	37.87918345	0.5	CRA004775;Yang2025_Silkie_Wenchang	PRJNA827465	0.9697477
ENSGALG00010003072	ELF1	2	-0.036983976	0.061821708	-0.59823607	0.549682427	0	0.582293059	0	1	CRA004775;PRJNA827465	Yang2025_Silkie_Wenchang	0.878554604
ENSGALG00010003074	TTPA	2	-0.1335814	0.179134789	-0.745703279	0.455846678	0	0.215317049	0	1	PRJNA827465;Yang2025_Silkie_Wenchang	CRA004775	0.84907427
ENSGALG00010003074	TTPA	2	-0.049743832	0.229792609	-0.216472723	0.828619285	0	0.016885875	0	0.5	CRA004775;Yang2025_Silkie_Wenchang	PRJNA827465	0.956818279
ENSGALG00010003074	TTPA	2	-0.197032636	0.253495135	-0.777263972	0.437000446	0	0.110666649	0	0.5	CRA004775;PRJNA827465	Yang2025_Silkie_Wenchang	0.84342618
ENSGALG00010003077	EIF2S3	2	-0.014631777	0.049879718	-0.293341205	0.769261361	0	0.093078771	0	1	PRJNA827465;Yang2025_Silkie_Wenchang	CRA004775	0.941790377
ENSGALG00010003077	EIF2S3	2	-0.046086781	0.105044456	-0.43873597	0.66086286	0	0.003997102	0	1	CRA004775;Yang2025_Silkie_Wenchang	PRJNA827465	0.91192683
ENSGALG00010003077	EIF2S3	2	-0.010526777	0.051884285	-0.202889514	0.83922139	0	0.02236616	0	1	CRA004775;PRJNA827465	Yang2025_Silkie_Wenchang	0.959420527
ENSGALG00010003081	DOP1A	2	0.395553521	0.176758489	2.237819093	0.025232855	0.049414701	4.592768135	78.22663869	1	PRJNA827465;Yang2025_Silkie_Wenchang	CRA004775	0.270438551
ENSGALG00010003081	DOP1A	2	0.287325245	0.34372843	0.83590771	0.403206825	0.19049803	4.863663694	79.43936787	0.5	CRA004775;Yang2025_Silkie_Wenchang	PRJNA827465	0.83306585
ENSGALG00010003081	DOP1A	2	0.173207729	0.131476733	1.317402139	0.187703864	0.014073237	1.329246504	24.76940908	0.5	CRA004775;PRJNA827465	Yang2025_Silkie_Wenchang	0.696930043
ENSGALG00010003087	TWISTNB	2	0.079703628	0.214823837	0.371018549	0.710623716	0.074779502	5.022663761	80.09024598	0.5	PRJNA827465;Yang2025_Silkie_Wenchang	CRA004775	0.925404854
ENSGALG00010003087	TWISTNB	2	0.354994577	0.142892091	2.484354275	0.012978662	0	0.159440525	0	1	CRA004775;Yang2025_Silkie_Wenchang	PRJNA827465	0.185288899
ENSGALG00010003087	TWISTNB	2	0.109702485	0.273481869	0.401132569	0.688322523	0.115897884	3.895813012	74.33141691	0.5	CRA004775;PRJNA827465	Yang2025_Silkie_Wenchang	0.919768134
ENSGALG00010003090	DMA	2	0.097811169	0.152437958	0.641645756	0.521103217	0	0.108761389	0	1	PRJNA827465;Yang2025_Silkie_Wenchang	CRA004775	0.869518515
ENSGALG00010003090	DMA	2	-0.310610198	0.330421517	-0.940042286	0.347195871	0.144893687	2.971929121	66.35182202	0.5	CRA004775;Yang2025_Silkie_Wenchang	PRJNA827465	0.808660951
ENSGALG00010003090	DMA	2	-0.228729321	0.384054214	-0.59556519	0.551465737	0.243580163	5.627524416	82.23019704	0.5	CRA004775;PRJNA827465	Yang2025_Silkie_Wenchang	0.878888037
ENSGALG00010003097	TFPI2	2	-0.129681667	0.178698426	-0.72570123	0.468021948	0.047489351	3.718421844	73.10687055	0.5	PRJNA827465;Yang2025_Silkie_Wenchang	CRA004775	0.853145337
ENSGALG00010003097	TFPI2	2	-0.347021152	0.143346791	-2.420850503	0.015484244	0	0.035350043	0	1	CRA004775;Yang2025_Silkie_Wenchang	PRJNA827465	0.205809006
ENSGALG00010003097	TFPI2	2	-0.080394367	0.185370371	-0.433695884	0.664509296	0.036886419	1.679428895	40.45594886	0.5	CRA004775;PRJNA827465	Yang2025_Silkie_Wenchang	0.913255304
ENSGALG00010003101	KLHL15	2	0.033199016	0.058474267	0.567754281	0.570201828	0.001608461	1.20004839	16.67002692	0.5	PRJNA827465;Yang2025_Silkie_Wenchang	CRA004775	0.885061508
ENSGALG00010003101	KLHL15	2	-0.075817849	0.108390635	-0.699487081	0.484247684	0	0.002867246	0	1	CRA004775;Yang2025_Silkie_Wenchang	PRJNA827465	0.857004733
ENSGALG00010003101	KLHL15	2	0.06099463	0.052514349	1.161485029	0.245444708	0	0.231816986	0	0.5	CRA004775;PRJNA827465	Yang2025_Silkie_Wenchang	0.75661406
ENSGALG00010003103	YTHDF3	2	-0.196651882	0.056886279	-3.456929938	0.000546367	0	0.427090208	0	1	PRJNA827465;Yang2025_Silkie_Wenchang	CRA004775	0.024894026
ENSGALG00010003103	YTHDF3	2	-0.047592573	0.242120386	-0.196565741	0.844167382	0.089476734	3.807726759	73.73761136	0.5	CRA004775;Yang2025_Silkie_Wenchang	PRJNA827465	0.960723102
ENSGALG00010003103	YTHDF3	2	-0.022540164	0.198295964	-0.113669305	0.909499946	0.056193015	2.902753633	65.54995268	0.5	CRA004775;PRJNA827465	Yang2025_Silkie_Wenchang	0.978607662
ENSGALG00010003107	GNG11	2	-0.101041108	0.216406053	-0.466905186	0.640567715	0.048893692	1.723924683	41.99282546	0.5	PRJNA827465;Yang2025_Silkie_Wenchang	CRA004775	0.90623926
ENSGALG00010003107	GNG11	2	-0.273140869	0.226496307	-1.205939616	0.227840774	0	0.456742032	0	1	CRA004775;Yang2025_Silkie_Wenchang	PRJNA827465	0.740853612
ENSGALG00010003107	GNG11	2	0.007326378	0.116723349	0.062767032	0.949952019	0	0.28329831	0	0.5	CRA004775;PRJNA827465	Yang2025_Silkie_Wenchang	0.987277827
ENSGALG00010003108	PPP1R3B	2	-0.157669229	0.158834471	-0.992663796	0.320873824	0.015512161	1.433578944	30.24451116	1	PRJNA827465;Yang2025_Silkie_Wenchang	CRA004775	0.798436461
ENSGALG00010003108	PPP1R3B	2	-0.358307867	0.174953482	-2.048017923	0.040558243	0	0.034518419	0	1	CRA004775;Yang2025_Silkie_Wenchang	PRJNA827465	0.34850473
ENSGALG00010003108	PPP1R3B	2	-0.110794219	0.166933288	-0.663703569	0.506880055	0.007247293	1.103597547	9.387257786	1	CRA004775;PRJNA827465	Yang2025_Silkie_Wenchang	0.863929781
ENSGALG00010003110	BF1	2	-0.263355456	0.210370085	-1.251867422	0.210618178	0.052281305	2.441998746	59.04993802	1	PRJNA827465;Yang2025_Silkie_Wenchang	CRA004775	0.722132818
ENSGALG00010003110	BF1	2	-0.244559389	0.187791911	-1.302289264	0.19281752	0.035871706	2.034960771	50.85900357	1	CRA004775;Yang2025_Silkie_Wenchang	PRJNA827465	0.702807541
ENSGALG00010003110	BF1	2	-0.453776305	0.134379041	-3.376838386	0.000733241	0	0.028253125	0	1	CRA004775;PRJNA827465	Yang2025_Silkie_Wenchang	0.030276194
ENSGALG00010003113	WBP4	2	0.176017939	0.149248803	1.179359135	0.2382552	0.022681205	1.740697023	42.55174871	1	PRJNA827465;Yang2025_Silkie_Wenchang	CRA004775	0.750271944
ENSGALG00010003113	WBP4	2	0.233273565	0.149024616	1.56533579	0.117504195	0	0.987443178	0	1	CRA004775;Yang2025_Silkie_Wenchang	PRJNA827465	0.577067024
ENSGALG00010003113	WBP4	2	0.08402282	0.085525268	0.9824327	0.325886723	0	0.015259418	0	1	CRA004775;PRJNA827465	Yang2025_Silkie_Wenchang	0.800023788
ENSGALG00010003115	UBE3D	2	-0.061919547	0.25529989	-0.242536521	0.808364462	0.099429476	4.077189394	75.47330027	0.5	PRJNA827465;Yang2025_Silkie_Wenchang	CRA004775	0.952496413
ENSGALG00010003115	UBE3D	2	-0.35438182	0.181755859	-1.949768339	0.051203737	0	0.007047452	0	1	CRA004775;Yang2025_Silkie_Wenchang	PRJNA827465	0.391986846
ENSGALG00010003115	UBE3D	2	-0.014267691	0.258096686	-0.05528041	0.955915069	0.081184209	2.183481841	54.20158843	0.5	CRA004775;PRJNA827465	Yang2025_Silkie_Wenchang	0.988406776
ENSGALG00010003117	ID2	2	0.212052772	0.145469989	1.457708034	0.144921037	0.032761479	4.267490141	76.56702261	1	PRJNA827465;Yang2025_Silkie_Wenchang	CRA004775	0.632799856
ENSGALG00010003117	ID2	2	0.207210787	0.270142684	0.767042008	0.44305654	0.086164571	1.889486788	47.07557594	0.5	CRA004775;Yang2025_Silkie_Wenchang	PRJNA827465	0.84572371
ENSGALG00010003117	ID2	2	0.071621199	0.073475194	0.974767073	0.329675814	0	0.528396656	0	0.5	CRA004775;PRJNA827465	Yang2025_Silkie_Wenchang	0.801177491
ENSGALG00010003121	ARMC1	2	-0.010130085	0.055291638	-0.183211881	0.854631772	0.00221168	1.409576501	29.05670611	0.5	PRJNA827465;Yang2025_Silkie_Wenchang	CRA004775	0.963225297
ENSGALG00010003121	ARMC1	2	-0.075596501	0.08816366	-0.857456474	0.39119266	0	0.554645789	0	0.5	CRA004775;Yang2025_Silkie_Wenchang	PRJNA827465	0.829251057
ENSGALG00010003121	ARMC1	2	0.026598318	0.044334373	0.599947992	0.548540897	0	0.098340017	0	1	CRA004775;PRJNA827465	Yang2025_Silkie_Wenchang	0.878281266
ENSGALG00010003125	APOO	2	0.090682196	0.105145811	0.862442303	0.38844417	0.008282113	1.309582586	23.639791	1	PRJNA827465;Yang2025_Silkie_Wenchang	CRA004775	0.828154121
ENSGALG00010003125	APOO	2	0.196512125	0.183116985	1.073150726	0.283203495	0	0.788279991	0	0.5	CRA004775;Yang2025_Silkie_Wenchang	PRJNA827465	0.779568117
ENSGALG00010003125	APOO	2	0.034512655	0.072229779	0.477817532	0.632780081	0	0.068644354	0	0.5	CRA004775;PRJNA827465	Yang2025_Silkie_Wenchang	0.903112631
ENSGALG00010003126	ENSGALG00010003126	2	0.796546062	0.348089067	2.288339791	0.022117741	0.125136836	2.066666667	51.61290324	1	PRJNA827465;Yang2025_Silkie_Wenchang	CRA004775	0.252379025
ENSGALG00010003126	ENSGALG00010003126	2	1.511187787	0.567060601	2.664949362	0.007699992	0.327499725	1.711638867	41.57646107	1	CRA004775;Yang2025_Silkie_Wenchang	PRJNA827465	0.135831585
ENSGALG00010003126	ENSGALG00010003126	2	1.256183634	0.959802159	1.308794341	0.190604024	1.449387841	4.186889309	76.11591981	1	CRA004775;PRJNA827465	Yang2025_Silkie_Wenchang	0.69979542
ENSGALG00010003131	TAP1	2	0.405038043	0.366851081	1.104093908	0.269552403	0.16616496	2.611256984	61.70426709	1	PRJNA827465;Yang2025_Silkie_Wenchang	CRA004775	0.77189992
ENSGALG00010003131	TAP1	2	0.069678103	0.711372428	0.097948838	0.92197292	0.901688275	9.166366977	89.09055242	0.5	CRA004775;Yang2025_Silkie_Wenchang	PRJNA827465	0.981720024
ENSGALG00010003131	TAP1	2	-0.28759946	0.344268771	-0.83539224	0.403496898	0.132837024	2.272034741	55.9865885	0.5	CRA004775;PRJNA827465	Yang2025_Silkie_Wenchang	0.83306585
ENSGALG00010003132	TPBG	2	-0.069811138	0.316366875	-0.220665133	0.825353182	0.171640251	6.981102943	85.6756159	0.5	PRJNA827465;Yang2025_Silkie_Wenchang	CRA004775	0.956381808
ENSGALG00010003132	TPBG	2	-0.092645731	0.418764519	-0.221235865	0.824908785	0.232573166	2.486432039	59.78172802	0.5	CRA004775;Yang2025_Silkie_Wenchang	PRJNA827465	0.956260685
ENSGALG00010003132	TPBG	2	0.256709983	0.145793498	1.76077799	0.078275987	0	0.205762142	0	1	CRA004775;PRJNA827465	Yang2025_Silkie_Wenchang	0.481707034
ENSGALG00010003133	SAT1	2	-0.101192366	0.115923263	-0.87292544	0.382703719	0	0.51946921	0	1	PRJNA827465;Yang2025_Silkie_Wenchang	CRA004775	0.825121999
ENSGALG00010003133	SAT1	2	-0.229957202	0.170430403	-1.349273362	0.177249178	0	0.035979314	0	1	CRA004775;Yang2025_Silkie_Wenchang	PRJNA827465	0.683250732
ENSGALG00010003133	SAT1	2	-0.093876713	0.112842008	-0.831930543	0.405448151	0	0.294701565	0	1	CRA004775;PRJNA827465	Yang2025_Silkie_Wenchang	0.833112102
ENSGALG00010003136	NUP98	2	-0.161567094	0.095949105	-1.683883279	0.092204228	0.014468499	4.639609151	78.44646031	1	PRJNA827465;Yang2025_Silkie_Wenchang	CRA004775	0.519928664
ENSGALG00010003136	NUP98	2	-0.256077447	0.06435558	-3.979102468	6.91759E-05	0	0.050539676	0	1	CRA004775;Yang2025_Silkie_Wenchang	PRJNA827465	0.006028429
ENSGALG00010003136	NUP98	2	-0.082655651	0.054129129	-1.527008695	0.126758849	0	0.607377584	0	1	CRA004775;PRJNA827465	Yang2025_Silkie_Wenchang	0.597577432
ENSGALG00010003152	ENSGALG00010003152	2	0.169294086	0.139694476	1.211888191	0.225555183	0.009972167	1.343189951	25.55036616	1	PRJNA827465;Yang2025_Silkie_Wenchang	CRA004775	0.73884362
ENSGALG00010003152	ENSGALG00010003152	2	0.175032108	0.184356154	0.949423731	0.342405145	0.022879435	1.436517582	30.38720774	0.5	CRA004775;Yang2025_Silkie_Wenchang	PRJNA827465	0.806005903
ENSGALG00010003152	ENSGALG00010003152	2	-0.000998396	0.1451671	-0.006877563	0.994512542	0	0.112388095	0	0.5	CRA004775;PRJNA827465	Yang2025_Silkie_Wenchang	0.99823774
ENSGALG00010003154	MTFR1	2	0.061244193	0.050109236	1.222213667	0.221626837	0	0.194540589	0	1	PRJNA827465;Yang2025_Silkie_Wenchang	CRA004775	0.734944791
ENSGALG00010003154	MTFR1	2	0.266656861	0.198389345	1.344108786	0.178913212	0.055395526	3.122985427	67.97935746	1	CRA004775;Yang2025_Silkie_Wenchang	PRJNA827465	0.685601971
ENSGALG00010003154	MTFR1	2	0.236017068	0.224432818	1.051615669	0.292975916	0.081996922	4.695961331	78.70510574	1	CRA004775;PRJNA827465	Yang2025_Silkie_Wenchang	0.786774237
ENSGALG00010003159	TAP2	2	0.162635022	0.33737503	0.482060043	0.629763293	0.163159428	3.501772884	71.44303663	0.5	PRJNA827465;Yang2025_Silkie_Wenchang	CRA004775	0.902457014
ENSGALG00010003159	TAP2	2	-0.147580034	0.678925862	-0.217372827	0.827917806	0.828044308	9.798460451	89.79431509	0.5	CRA004775;Yang2025_Silkie_Wenchang	PRJNA827465	0.956550752
ENSGALG00010003159	TAP2	2	-0.45489237	0.338469962	-1.343966734	0.178959144	0.151538648	2.866861479	65.11864952	1	CRA004775;PRJNA827465	Yang2025_Silkie_Wenchang	0.685672767
ENSGALG00010003160	KIDINS220	2	-0.022624927	0.051237132	-0.441572871	0.658798319	0	0.011186046	0	1	PRJNA827465;Yang2025_Silkie_Wenchang	CRA004775	0.911311264
ENSGALG00010003160	KIDINS220	2	-0.006732347	0.090232278	-0.074611296	0.940523986	0	0.085572036	0	0.5	CRA004775;Yang2025_Silkie_Wenchang	PRJNA827465	0.985559481
ENSGALG00010003160	KIDINS220	2	-0.022394179	0.060108082	-0.372565192	0.709472083	0	0.112057817	0	0.5	CRA004775;PRJNA827465	Yang2025_Silkie_Wenchang	0.925298042
ENSGALG00010003164	C3	2	-0.287358031	0.575845333	-0.499019468	0.61776567	0.540335468	5.0706257	80.27856799	0.5	PRJNA827465;Yang2025_Silkie_Wenchang	CRA004775	0.899121942
ENSGALG00010003164	C3	2	-0.808057006	0.210085994	-3.846315458	0.000119907	0	0.016728234	0	1	CRA004775;Yang2025_Silkie_Wenchang	PRJNA827465	0.008808821
ENSGALG00010003164	C3	2	-0.213752057	0.618052755	-0.345847592	0.729457257	0.500397479	2.872515756	65.18731019	0.5	CRA004775;PRJNA827465	Yang2025_Silkie_Wenchang	0.9323457
ENSGALG00010003165	BMP2K	2	-0.050191812	0.054870708	-0.914728705	0.360334071	0	0.034502202	0	1	PRJNA827465;Yang2025_Silkie_Wenchang	CRA004775	0.814880744
ENSGALG00010003165	BMP2K	2	-0.0625722	0.129942717	-0.481536797	0.630135034	0	0.969154209	0	1	CRA004775;Yang2025_Silkie_Wenchang	PRJNA827465	0.902591101
ENSGALG00010003165	BMP2K	2	-0.061748873	0.059626072	-1.035601895	0.300387907	0	0.917597068	0	1	CRA004775;PRJNA827465	Yang2025_Silkie_Wenchang	0.790532865
ENSGALG00010003166	PGAP2	2	-0.008147146	0.053022283	-0.153655144	0.877881654	0	0.969609111	0	0.5	PRJNA827465;Yang2025_Silkie_Wenchang	CRA004775	0.970941655
ENSGALG00010003166	PGAP2	2	0.060962087	0.097553473	0.624909449	0.53203049	0	0.31664829	0	0.5	CRA004775;Yang2025_Silkie_Wenchang	PRJNA827465	0.873104119
ENSGALG00010003166	PGAP2	2	-0.042602249	0.061061085	-0.697698863	0.485365539	0	0.055432668	0	1	CRA004775;PRJNA827465	Yang2025_Silkie_Wenchang	0.857076964
ENSGALG00010003169	MTRF1	2	-0.010926954	0.120285551	-0.090841786	0.927618307	0.015373071	2.088932515	52.12865935	0.5	PRJNA827465;Yang2025_Silkie_Wenchang	CRA004775	0.982993195
ENSGALG00010003169	MTRF1	2	0.003947673	0.157983543	0.024987873	0.980064636	0.021977191	1.706805261	41.41100788	0.5	CRA004775;Yang2025_Silkie_Wenchang	PRJNA827465	0.994658414
ENSGALG00010003169	MTRF1	2	-0.131169485	0.090557829	-1.448461018	0.147488161	0	0.127116108	0	1	CRA004775;PRJNA827465	Yang2025_Silkie_Wenchang	0.63651336
ENSGALG00010003170	C4	2	0.427265445	0.267448623	1.59756083	0.11014075	0	0.401863562	0	1	PRJNA827465;Yang2025_Silkie_Wenchang	CRA004775	0.562189855
ENSGALG00010003170	C4	2	-0.048908641	0.315252781	-0.15514103	0.876710137	0.043337312	1.27814709	21.76174341	0.5	CRA004775;Yang2025_Silkie_Wenchang	PRJNA827465	0.97046577
ENSGALG00010003170	C4	2	0.120016599	0.484984533	0.247464797	0.804548532	0.318939171	3.100769903	67.74994497	0.5	CRA004775;PRJNA827465	Yang2025_Silkie_Wenchang	0.951665484
ENSGALG00010003171	PAQR3	2	0.105939447	0.087298812	1.213526786	0.22492848	0	0.661320738	0	1	PRJNA827465;Yang2025_Silkie_Wenchang	CRA004775	0.737917946
ENSGALG00010003171	PAQR3	2	-0.114092786	0.325101881	-0.350944713	0.725629826	0.176248375	5.728622394	82.5437962	0.5	CRA004775;Yang2025_Silkie_Wenchang	PRJNA827465	0.930973071
ENSGALG00010003171	PAQR3	2	-0.174817915	0.252041319	-0.693608159	0.487927976	0.094115154	3.568819045	71.97952635	0.5	CRA004775;PRJNA827465	Yang2025_Silkie_Wenchang	0.85768885
ENSGALG00010003176	PRAG1	2	-0.431802321	0.083444966	-5.174695859	2.28283E-07	0	0.20829243	0	1	PRJNA827465;Yang2025_Silkie_Wenchang	CRA004775	6.79711E-05
ENSGALG00010003176	PRAG1	2	-0.292095003	0.197949029	-1.475607158	0.140049382	0.030689499	1.304629529	23.34988763	0.5	CRA004775;Yang2025_Silkie_Wenchang	PRJNA827465	0.623792706
ENSGALG00010003176	PRAG1	2	-0.314179882	0.256420155	-1.225254243	0.220479443	0.076262505	1.778414735	43.77014651	0.5	CRA004775;PRJNA827465	Yang2025_Silkie_Wenchang	0.734053249
ENSGALG00010003178	CENPA	2	0.225932773	0.659978661	0.342333451	0.732099964	0.676186988	4.429745122	77.42533775	0.5	PRJNA827465;Yang2025_Silkie_Wenchang	CRA004775	0.93287651
ENSGALG00010003178	CENPA	2	0.58133207	0.315725219	1.841259532	0.065583535	0	0.811321913	0	1	CRA004775;Yang2025_Silkie_Wenchang	PRJNA827465	0.441526152
ENSGALG00010003178	CENPA	2	-0.040840034	0.371923575	-0.109807597	0.912561966	0.115221334	1.712633892	41.6104046	0.5	CRA004775;PRJNA827465	Yang2025_Silkie_Wenchang	0.978967245
ENSGALG00010003180	IBTK	2	-0.029338205	0.038998718	-0.752286408	0.45187884	0	0.230197672	0	1	PRJNA827465;Yang2025_Silkie_Wenchang	CRA004775	0.848106301
ENSGALG00010003180	IBTK	2	-0.070195247	0.099907173	-0.702604676	0.482302148	0	0.037943692	0	1	CRA004775;Yang2025_Silkie_Wenchang	PRJNA827465	0.85691916
ENSGALG00010003180	IBTK	2	-0.023190989	0.040573784	-0.571575704	0.567609459	0	0.00463315	0	1	CRA004775;PRJNA827465	Yang2025_Silkie_Wenchang	0.883717772
ENSGALG00010003183	NAA16	2	-0.109550118	0.056502744	-1.938845981	0.052520095	0.001640798	1.244994297	19.67834691	1	PRJNA827465;Yang2025_Silkie_Wenchang	CRA004775	0.39795591
ENSGALG00010003183	NAA16	2	0.095001251	0.163221994	0.582037069	0.560541713	0.027731966	1.796052307	44.32233426	0.5	CRA004775;Yang2025_Silkie_Wenchang	PRJNA827465	0.881203589
ENSGALG00010003183	NAA16	2	0.0402987	0.234304202	0.171993075	0.863442973	0.085787107	3.78515853	73.58102727	0.5	CRA004775;PRJNA827465	Yang2025_Silkie_Wenchang	0.966256632
ENSGALG00010003184	BET1	2	-0.396908177	0.416495775	-0.952970477	0.340605029	0.332734719	23.83701933	95.80484462	0.5	PRJNA827465;Yang2025_Silkie_Wenchang	CRA004775	0.805524125
ENSGALG00010003184	BET1	2	-0.489836219	0.356869224	-1.372593056	0.169878892	0.215289589	6.351548674	84.25580829	1	CRA004775;Yang2025_Silkie_Wenchang	PRJNA827465	0.671232541
ENSGALG00010003184	BET1	2	-0.000455269	0.055597463	-0.00818866	0.993466467	0	0.242901508	0	0.5	CRA004775;PRJNA827465	Yang2025_Silkie_Wenchang	0.997979742
ENSGALG00010003186	CYP21A1	2	-0.274933436	0.287982523	-0.954687919	0.339735541	0.066064463	1.475224909	32.21372591	0.5	PRJNA827465;Yang2025_Silkie_Wenchang	CRA004775	0.805078728
ENSGALG00010003186	CYP21A1	2	0.047317635	0.370281467	0.12778829	0.898316518	0	0.207915897	0	0.5	CRA004775;Yang2025_Silkie_Wenchang	PRJNA827465	0.97575249
ENSGALG00010003186	CYP21A1	2	-0.417861138	0.207665319	-2.012185477	0.044200389	0	0.223473691	0	1	CRA004775;PRJNA827465	Yang2025_Silkie_Wenchang	0.363282309
ENSGALG00010003187	RHOG	2	0.279261476	0.329905427	0.846489487	0.397279736	0.185495249	6.349962654	84.25187589	0.5	PRJNA827465;Yang2025_Silkie_Wenchang	CRA004775	0.831504091
ENSGALG00010003187	RHOG	2	0.290267104	0.382849136	0.758176203	0.448345506	0.215272138	3.710647489	73.05052547	0.5	CRA004775;Yang2025_Silkie_Wenchang	PRJNA827465	0.846853297
ENSGALG00010003187	RHOG	2	-0.027684957	0.100586681	-0.275234813	0.783135842	0	0.096831227	0	1	CRA004775;PRJNA827465	Yang2025_Silkie_Wenchang	0.945669422
ENSGALG00010003189	ANTXR2	2	-0.129325006	0.060513316	-2.13713303	0.032587173	0	0.001424127	0	1	PRJNA827465;Yang2025_Silkie_Wenchang	CRA004775	0.310037591
ENSGALG00010003189	ANTXR2	2	-0.150501629	0.096849523	-1.553973882	0.120190647	0	0.152897693	0	1	CRA004775;Yang2025_Silkie_Wenchang	PRJNA827465	0.581468857
ENSGALG00010003189	ANTXR2	2	-0.137492792	0.069807124	-1.969609748	0.048883114	0	0.186503287	0	1	CRA004775;PRJNA827465	Yang2025_Silkie_Wenchang	0.382067319
ENSGALG00010003191	CC2D1A	2	0.196809331	0.432500789	0.455049646	0.649073523	0.344137582	12.32155423	91.88414074	0.5	PRJNA827465;Yang2025_Silkie_Wenchang	CRA004775	0.907783669
ENSGALG00010003191	CC2D1A	2	0.208205098	0.444769828	0.468118754	0.639699668	0.344837385	7.766086687	87.12350196	0.5	CRA004775;Yang2025_Silkie_Wenchang	PRJNA827465	0.906064918
ENSGALG00010003191	CC2D1A	2	-0.227066023	0.11705767	-1.939779116	0.05240654	0	0.007644038	0	1	CRA004775;PRJNA827465	Yang2025_Silkie_Wenchang	0.397401935
ENSGALG00010003192	RGCC	2	-0.152482309	0.208887736	-0.729972532	0.465406974	0.05794054	2.815827351	64.48645903	0.5	PRJNA827465;Yang2025_Silkie_Wenchang	CRA004775	0.852411124
ENSGALG00010003192	RGCC	2	-0.324154935	0.534041268	-0.606984806	0.543860996	0.414085622	3.177413292	68.52785873	0.5	CRA004775;Yang2025_Silkie_Wenchang	PRJNA827465	0.876968972
ENSGALG00010003192	RGCC	2	-0.431900816	0.243188971	-1.775988501	0.075734812	0.053149524	1.303807146	23.30154017	1	CRA004775;PRJNA827465	Yang2025_Silkie_Wenchang	0.475110429
ENSGALG00010003193	ACOT9	2	-0.431931877	0.068970252	-6.262582283	3.78654E-10	0	0.059479537	0	1	PRJNA827465;Yang2025_Silkie_Wenchang	CRA004775	2.48037E-07
ENSGALG00010003193	ACOT9	2	-0.227518261	0.192138225	-1.184138453	0.236358261	0.040864167	2.198273173	54.50974827	1	CRA004775;Yang2025_Silkie_Wenchang	PRJNA827465	0.748669015
ENSGALG00010003193	ACOT9	2	-0.268642924	0.210543268	-1.275951145	0.20197279	0.067847974	3.758180212	73.39137711	1	CRA004775;PRJNA827465	Yang2025_Silkie_Wenchang	0.714969076
ENSGALG00010003198	PRKG2	2	0.173694841	0.204805522	0.84809647	0.396384248	0	0.229700676	0	1	PRJNA827465;Yang2025_Silkie_Wenchang	CRA004775	0.831365249
ENSGALG00010003198	PRKG2	2	-0.071373941	0.455344535	-0.156747111	0.875444156	0.227997917	2.15056025	53.50048901	0.5	CRA004775;Yang2025_Silkie_Wenchang	PRJNA827465	0.970347777
ENSGALG00010003198	PRKG2	2	-0.107652062	0.326625884	-0.329588277	0.741711082	0.089669619	1.542698187	35.17850682	0.5	CRA004775;PRJNA827465	Yang2025_Silkie_Wenchang	0.93543545
ENSGALG00010003201	MBOAT2	2	-0.434999069	0.191651996	-2.269734089	0.023223722	0.052607295	3.247423717	69.20635903	1	PRJNA827465;Yang2025_Silkie_Wenchang	CRA004775	0.259824064
ENSGALG00010003201	MBOAT2	2	0.000895076	0.724859922	0.001234826	0.999014752	0.932416487	8.410372465	88.10992017	0.5	CRA004775;Yang2025_Silkie_Wenchang	PRJNA827465	0.999828799
ENSGALG00010003201	MBOAT2	2	0.160496093	0.523693815	0.306469331	0.759247327	0.455118053	5.067477559	80.26631616	0.5	CRA004775;PRJNA827465	Yang2025_Silkie_Wenchang	0.939145717

ENSGALG00010003202	FAM46A	2	0.270322847	0.22877472	1.181611531	0.237359876	0.089004737	6.53589482	84.69987618	1	PRJNA827465;Yang2025_Silkie_Wenchang	CRA004775	0.749366344
ENSGALG00010003202	FAM46A	2	0.101179334	0.505646637	0.200098896	0.841403237	0.403320907	4.115301198	75.70044203	0.5	CRA004775;Yang2025_Silkie_Wenchang	PRJNA827465	0.95989279
ENSGALG00010003202	FAM46A	2	-0.033951441	0.208025859	-0.163207791	0.870354832	0.041712123	1.339324907	25.33551831	0.5	CRA004775;PRJNA827465	Yang2025_Silkie_Wenchang	0.968860451
ENSGALG00010003204	PKD2	2	0.066656884	0.156412026	0.426162138	0.669989704	0.034290732	3.242133327	69.15611114	0.5	PRJNA827465;Yang2025_Silkie_Wenchang	CRA004775	0.914428878
ENSGALG00010003204	PKD2	2	0.070665594	0.226135563	0.312492173	0.75466651	0.059571885	2.174742836	54.01755172	0.5	CRA004775;Yang2025_Silkie_Wenchang	PRJNA827465	0.93838723
ENSGALG00010003204	PKD2	2	-0.089191737	0.09258467	-0.963353189	0.335370309	0	0.265956241	0	1	CRA004775;PRJNA827465	Yang2025_Silkie_Wenchang	0.803198384
ENSGALG00010003205	ENSGALG00010003205	2	-0.380173965	0.146390869	-2.59697868	0.009404776	0	0.121843337	0	1	PRJNA827465;Yang2025_Silkie_Wenchang	CRA004775	0.152724616
ENSGALG00010003205	ENSGALG00010003205	2	-0.27607716	0.188250136	-1.466544279	0.142500068	0	0.47872455	0	0.5	CRA004775;Yang2025_Silkie_Wenchang	PRJNA827465	0.628893678
ENSGALG00010003205	ENSGALG00010003205	2	-0.359538422	0.192996135	-1.86293068	0.06247202	0	0.77664807	0	0.5	CRA004775;PRJNA827465	Yang2025_Silkie_Wenchang	0.430912213
ENSGALG00010003210	EYA1	2	0.209329896	0.451177373	0.463963638	0.642673798	0.360572078	8.094801224	87.64639214	0.5	PRJNA827465;Yang2025_Silkie_Wenchang	CRA004775	0.906634523
ENSGALG00010003210	EYA1	2	0.561200058	0.252519469	2.22240313	0.026256074	0	0.754702112	0	1	CRA004775;Yang2025_Silkie_Wenchang	PRJNA827465	0.276215866
ENSGALG00010003210	EYA1	2	-0.179865819	0.09234531	-1.94775262	0.05144457	0	0.883460873	0	0.5	CRA004775;PRJNA827465	Yang2025_Silkie_Wenchang	0.393141348
ENSGALG00010003214	PRDX4	2	-0.020258125	0.167669236	-0.120821958	0.903832058	0.041894693	3.46219376	71.11657899	0.5	PRJNA827465;Yang2025_Silkie_Wenchang	CRA004775	0.977333734
ENSGALG00010003214	PRDX4	2	-0.22687526	0.141685687	-1.601257436	0.109319912	0	0.000282742	0	1	CRA004775;Yang2025_Silkie_Wenchang	PRJNA827465	0.559453191
ENSGALG00010003214	PRDX4	2	0.031405704	0.145717516	0.21552456	0.829358366	0.023736107	1.709897873	41.51697504	0.5	CRA004775;PRJNA827465	Yang2025_Silkie_Wenchang	0.95688454
ENSGALG00010003218	MAP3K7	2	-0.172060193	0.108063127	-1.592219275	0.111335444	0.020077361	6.816793933	85.33034723	1	PRJNA827465;Yang2025_Silkie_Wenchang	CRA004775	0.564466729
ENSGALG00010003218	MAP3K7	2	-0.038078989	0.268919112	-0.141600158	0.887395842	0.130229756	9.66203583	89.65021433	0.5	CRA004775;Yang2025_Silkie_Wenchang	PRJNA827465	0.973030878
ENSGALG00010003218	MAP3K7	2	0.052160908	0.156972331	0.332293645	0.739667538	0.038854402	3.985836515	74.91116366	0.5	CRA004775;PRJNA827465	Yang2025_Silkie_Wenchang	0.934914078
ENSGALG00010003220	SPP1	2	-0.502363778	0.674192603	-0.745133922	0.456190764	0	0.06575225	0	1	PRJNA827465;Yang2025_Silkie_Wenchang	CRA004775	0.849223966
ENSGALG00010003220	SPP1	2	-0.135525776	0.769233014	-0.176182995	0.86015018	0	0.078599954	0	0.5	CRA004775;Yang2025_Silkie_Wenchang	PRJNA827465	0.964955275
ENSGALG00010003220	SPP1	2	-0.374241543	0.729042855	-0.513332708	0.607718608	0	0.269415196	0	0.5	CRA004775;PRJNA827465	Yang2025_Silkie_Wenchang	0.897203929
ENSGALG00010003222	ITGA8	2	0.22625951	0.086324186	2.621044229	0.008766089	0.005933032	1.469096103	31.9309337	1	PRJNA827465;Yang2025_Silkie_Wenchang	CRA004775	0.146797439
ENSGALG00010003222	ITGA8	2	0.39336431	0.132553955	2.967578834	0.003001553	0	0.231839133	0	1	CRA004775;Yang2025_Silkie_Wenchang	PRJNA827465	0.075380216
ENSGALG00010003222	ITGA8	2	0.21240969	0.11392685	1.864439252	0.062260045	0.010170145	1.177954245	15.10705921	1	CRA004775;PRJNA827465	Yang2025_Silkie_Wenchang	0.430280737
ENSGALG00010003229	STIM1	2	0.081101596	0.184180907	0.440336608	0.659693331	0.056946995	6.118489055	83.65609563	0.5	PRJNA827465;Yang2025_Silkie_Wenchang	CRA004775	0.911617103
ENSGALG00010003229	STIM1	2	0.024045507	0.264915584	0.090766678	0.927677988	0.118729663	6.404142587	84.38510719	0.5	CRA004775;Yang2025_Silkie_Wenchang	PRJNA827465	0.982993195
ENSGALG00010003229	STIM1	2	-0.124914641	0.075874187	-1.646339111	0.099693961	0	0.723046009	0	1	CRA004775;PRJNA827465	Yang2025_Silkie_Wenchang	0.539319724
ENSGALG00010003239	RRM1	2	-0.011908438	0.04371619	-0.272403377	0.785311876	0.000160909	1.027509014	2.677252844	0.5	PRJNA827465;Yang2025_Silkie_Wenchang	CRA004775	0.946376178
ENSGALG00010003239	RRM1	2	-0.304173388	0.265659596	-1.144974218	0.252219838	0.102866515	3.133351699	68.08529344	1	CRA004775;Yang2025_Silkie_Wenchang	PRJNA827465	0.762479523
ENSGALG00010003239	RRM1	2	-0.255574134	0.32334519	-0.790406483	0.429290417	0.172770987	4.8744925	79.48504383	0.5	CRA004775;PRJNA827465	Yang2025_Silkie_Wenchang	0.842008086
ENSGALG00010003242	ASAP2	2	0.18047657	0.195497494	0.923165648	0.355920888	0.050567804	2.67270413	62.58471004	1	PRJNA827465;Yang2025_Silkie_Wenchang	CRA004775	0.81231505
ENSGALG00010003242	ASAP2	2	0.506619092	0.194567629	2.603820044	0.009219115	0	0.552272992	0	1	CRA004775;Yang2025_Silkie_Wenchang	PRJNA827465	0.150534672
ENSGALG00010003242	ASAP2	2	0.295893394	0.358768785	0.824746761	0.409515378	0.190003044	3.135958988	68.11182787	1	CRA004775;PRJNA827465	Yang2025_Silkie_Wenchang	0.83448182
ENSGALG00010003243	VPS50	2	-0.122068773	0.060909483	-2.004101293	0.045059211	0.00128802	1.204570064	16.98282813	1	PRJNA827465;Yang2025_Silkie_Wenchang	CRA004775	0.3659545
ENSGALG00010003243	VPS50	2	-0.179441554	0.083138564	-2.1583432	0.030901158	0	0.351882078	0	0.5	CRA004775;Yang2025_Silkie_Wenchang	PRJNA827465	0.300270431
ENSGALG00010003243	VPS50	2	-0.066018347	0.070216672	-0.940208995	0.347110369	0	0.050066095	0	0.5	CRA004775;PRJNA827465	Yang2025_Silkie_Wenchang	0.808616989
ENSGALG00010003245	ENSGALG00010003245	2	1.347888426	1.057354493	1.274774388	0.202389108	2.072875842	13.14342486	92.391633	1	PRJNA827465;Yang2025_Silkie_Wenchang	CRA004775	0.715289846
ENSGALG00010003245	ENSGALG00010003245	2	1.224634882	1.215270632	1.007705485	0.313595871	2.723212104	12.72983911	92.14444117	1	CRA004775;Yang2025_Silkie_Wenchang	PRJNA827465	0.796531448
ENSGALG00010003245	ENSGALG00010003245	2	0.288333055	0.179790139	1.603720073	0.108775769	0	0.481067645	0	1	CRA004775;PRJNA827465	Yang2025_Silkie_Wenchang	0.558486487
ENSGALG00010003246	VWA8	2	0.057533664	0.069843519	0.823750935	0.410081091	0	0.073107634	0	1	PRJNA827465;Yang2025_Silkie_Wenchang	CRA004775	0.834652129
ENSGALG00010003246	VWA8	2	0.035845866	0.088495613	0.405058113	0.685434795	0	0.022130409	0	0.5	CRA004775;Yang2025_Silkie_Wenchang	PRJNA827465	0.919235539
ENSGALG00010003246	VWA8	2	0.06771485	0.09707052	0.697584088	0.485437336	0	0.078879934	0	0.5	CRA004775;PRJNA827465	Yang2025_Silkie_Wenchang	0.857076964
ENSGALG00010003247	ENSGALG00010003247	2	0.017987552	0.153200448	0.117411876	0.906533674	0	0.735446614	0	0.5	PRJNA827465;Yang2025_Silkie_Wenchang	CRA004775	0.978196186
ENSGALG00010003247	ENSGALG00010003247	2	0.108306227	0.224690494	0.482024073	0.629788845	0	0.470197328	0	0.5	CRA004775;Yang2025_Silkie_Wenchang	PRJNA827465	0.902457014
ENSGALG00010003247	ENSGALG00010003247	2	-0.089428286	0.174271873	-0.513153871	0.607843691	0	0.025416616	0	1	CRA004775;PRJNA827465	Yang2025_Silkie_Wenchang	0.897203929
ENSGALG00010003248	MRI1	2	0.61960549	0.552302464	1.121859001	0.261922395	0.534567289	7.718847677	87.04469836	1	PRJNA827465;Yang2025_Silkie_Wenchang	CRA004775	0.768363052
ENSGALG00010003248	MRI1	2	0.515677931	0.669703006	0.77000988	0.441294032	0.813369632	10.48443424	90.46205091	0.5	CRA004775;Yang2025_Silkie_Wenchang	PRJNA827465	0.844907543
ENSGALG00010003248	MRI1	2	0.014233245	0.131896915	0.107911889	0.914065586	0	0.755067819	0	0.5	CRA004775;PRJNA827465	Yang2025_Silkie_Wenchang	0.979467654
ENSGALG00010003249	YJU2B	2	0.772654653	0.773151576	0.999357277	0.317621648	1.14827942	25.09001723	96.01435108	1	PRJNA827465;Yang2025_Silkie_Wenchang	CRA004775	0.797215846
ENSGALG00010003249	YJU2B	2	0.540080995	1.027658229	0.525545341	0.599204142	2.027112131	24.80389865	95.96837572	0.5	CRA004775;Yang2025_Silkie_Wenchang	PRJNA827465	0.894908299
ENSGALG00010003249	YJU2B	2	-0.166868499	0.243491299	-0.685316067	0.493144495	0.06665983	2.061862057	51.50015024	0.5	CRA004775;PRJNA827465	Yang2025_Silkie_Wenchang	0.859667262
ENSGALG00010003251	ELOF1	2	0.312331706	0.160755793	1.942895493	0.052028787	0.020813193	1.648428785	39.33617216	1	PRJNA827465;Yang2025_Silkie_Wenchang	CRA004775	0.395759125
ENSGALG00010003251	ELOF1	2	0.207219299	0.302171844	0.685766405	0.492860424	0.136726018	3.967019939	74.79216098	0.5	CRA004775;Yang2025_Silkie_Wenchang	PRJNA827465	0.859553304
ENSGALG00010003251	ELOF1	2	0.084819644	0.130401452	0.650450153	0.51540149	0.000993569	1.02612796	2.546267235	0.5	CRA004775;PRJNA827465	Yang2025_Silkie_Wenchang	0.867199306
ENSGALG00010003252	PHEX	2	-0.252661035	0.135471651	-1.865047287	0.062174776	0	0.69126781	0	1	PRJNA827465;Yang2025_Silkie_Wenchang	CRA004775	0.430069559
ENSGALG00010003252	PHEX	2	-0.353300072	0.195240722	-1.809561384	0.070363833	0	0.181020914	0	1	CRA004775;Yang2025_Silkie_Wenchang	PRJNA827465	0.457865848
ENSGALG00010003252	PHEX	2	-0.154146865	0.169672674	-0.908495523	0.363616478	0	0.001748364	0	1	CRA004775;PRJNA827465	Yang2025_Silkie_Wenchang	0.817042253
ENSGALG00010003253	ITGB1BP1	2	0.379681711	0.362230454	1.048177221	0.294556963	0.249059764	19.56539921	94.88893639	1	PRJNA827465;Yang2025_Silkie_Wenchang	CRA004775	0.787408474
ENSGALG00010003253	ITGB1BP1	2	0.280425177	0.477511965	0.587263142	0.557026991	0.428657247	16.52085947	93.94704615	0.5	CRA004775;Yang2025_Silkie_Wenchang	PRJNA827465	0.880601733
ENSGALG00010003253	ITGB1BP1	2	-0.031243814	0.097578282	-0.320192294	0.748822564	0.002740219	1.114747366	10.29357588	0.5	CRA004775;PRJNA827465	Yang2025_Silkie_Wenchang	0.937249951
ENSGALG00010003254	DGKH	2	0.403986691	0.606649184	0.665931319	0.505455001	0.694334121	17.2891284	94.21601843	0.5	PRJNA827465;Yang2025_Silkie_Wenchang	CRA004775	0.863922501
ENSGALG00010003254	DGKH	2	0.739506179	0.427662983	1.72917977	0.083776926	0.200102675	1.906792947	47.55592098	1	CRA004775;Yang2025_Silkie_Wenchang	PRJNA827465	0.498685546
ENSGALG00010003254	DGKH	2	-0.168960736	0.126364273	-1.337092608	0.18119241	0	0.226024075	0	0.5	CRA004775;PRJNA827465	Yang2025_Silkie_Wenchang	0.688657314
ENSGALG00010003255	CALCR	2	-0.098957651	0.195320888	-0.506641414	0.612406445	0	0.868319451	0	0.5	PRJNA827465;Yang2025_Silkie_Wenchang	CRA004775	0.897938231
ENSGALG00010003255	CALCR	2	-0.081230277	0.31338693	-0.259201227	0.795479984	0.059784525	1.421945312	29.67380732	0.5	CRA004775;Yang2025_Silkie_Wenchang	PRJNA827465	0.948998325
ENSGALG00010003255	CALCR	2	0.097489996	0.20912955	0.466170352	0.641093569	0	0.274855182	0	1	CRA004775;PRJNA827465	Yang2025_Silkie_Wenchang	0.906429868
ENSGALG00010003259	VEGFC	2	-0.073915022	0.164070149	-0.450508655	0.652343715	0.042600204	4.763218103	79.00579024	0.5	PRJNA827465;Yang2025_Silkie_Wenchang	CRA004775	0.909377929
ENSGALG00010003259	VEGFC	2	-0.133354313	0.203857659	-0.654154051	0.513012561	0.040490111	1.525050203	34.42838813	0.5	CRA004775;Yang2025_Silkie_Wenchang	PRJNA827465	0.866156104
ENSGALG00010003259	VEGFC	2	0.093481742	0.092991376	1.005273242	0.314765296	0	0.163463114	0	1	CRA004775;PRJNA827465	Yang2025_Silkie_Wenchang	0.796531448
ENSGALG00010003260	ACP5	2	0.232087034	0.154203896	1.505065957	0.13230712	0.001336233	1.028907282	2.809512795	1	PRJNA827465;Yang2025_Silkie_Wenchang	CRA004775	0.60919245
ENSGALG00010003260	ACP5	2	0.10028526	0.206225892	0.486288404	0.6267627	0	0.135226325	0	1	CRA004775;Yang2025_Silkie_Wenchang	PRJNA827465	0.901506107
ENSGALG00010003260	ACP5	2	0.386492508	0.20787906	1.859218083	0.062996233	0	0.000351111	0	1	CRA004775;PRJNA827465	Yang2025_Silkie_Wenchang	0.432176808
ENSGALG00010003261	PEX2	2	-0.299165028	0.311283431	-0.961069552	0.336517198	0.188499768	36.38518096	97.25162834	0.5	PRJNA827465;Yang2025_Silkie_Wenchang	CRA004775	0.803806405
ENSGALG00010003261	PEX2	2	-0.300904108	0.35263697	-0.853297114	0.39349456	0.214861532	6.762290575	85.21211136	0.5	CRA004775;Yang2025_Silkie_Wenchang	PRJNA827465	0.830407898
ENSGALG00010003261	PEX2	2	0.011700626	0.052128356	0.22445799	0.822400952	0	0.110078366	0	1	CRA004775;PRJNA827465	Yang2025_Silkie_Wenchang	0.955795631
ENSGALG00010003262	ENSGALG00010003262	2	-0.10346765	0.931090936	-0.111125182	0.91151708	1.553601548	9.351225436	89.30621439	0.5	PRJNA827465;Yang2025_Silkie_Wenchang	CRA004775	0.978851978
ENSGALG00010003262	ENSGALG00010003262	2	-1.119036576	2.103042321	-0.532103689	0.594654183	7.926027284	8.79825636	88.63411159	0.5	CRA004775;Yang2025_Silkie_Wenchang	PRJNA827465	0.893379204
ENSGALG00010003262	ENSGALG00010003262	2	-1.920435952	1.128470962	-1.701803606	0.08879219	1.677089117	2.501954484	60.03124732	1	CRA004775;PRJNA827465	Yang2025_Silkie_Wenchang	0.5117008
ENSGALG00010003263	ENSGALG00010003263	2	1.000886922	2.029095605	0.493267503	0.621823587	7.643762613	13.80135108	92.75433257	0.5	PRJNA827465;Yang2025_Silkie_Wenchang	CRA004775	0.89980238
ENSGALG00010003263	ENSGALG00010003263	2	1.785398031	1.298326879	1.375152945	0.169084033	2.553878188	4.124186064	75.75279135	1	CRA004775;Yang2025_Silkie_Wenchang	PRJNA827465	0.67030648
ENSGALG00010003263	ENSGALG00010003263	2	-0.391432633	0.715883333	-0.546782716	0.584528019	0.482731616	1.821518818	45.10075933	0.5	CRA004775;PRJNA827465	Yang2025_Silkie_Wenchang	0.891016154
ENSGALG00010003264	ENSGALG00010003264	2	-0.699779534	0.581150399	-1.204128114	0.228540065	0.475522883	3.056796413	67.28601238	1	PRJNA827465;Yang2025_Silkie_Wenchang	CRA004775	0.741270737
ENSGALG00010003264	ENSGALG00010003264	2	-0.259367628	0.265046363	-0.97857456	0.327790234	0	0.068296779	0	1	CRA004775;Yang2025_Silkie_Wenchang	PRJNA827465	0.800344138
ENSGALG00010003264	ENSGALG00010003264	2	-0.967298218	0.495224286	-1.953252789	0.050789649	0.069675932	1.163967464	14.08694564	1	CRA004775;PRJNA827465	Yang2025_Silkie_Wenchang	0.390032349
ENSGALG00010003268	SMS	2	-0.176737888	0.138599294	-1.275171628	0.202248501	0.033259029	7.427331944	86.53621506	1	PRJNA827465;Yang2025_Silkie_Wenchang	CRA004775	0.715285957
ENSGALG00010003268	SMS	2	-0.00885237	0.335175917	-0.026411116	0.978929428	0.202016194	9.368995291	89.32649693	0.5	CRA004775;Yang2025_Silkie_Wenchang	PRJNA827465	0.994259589
ENSGALG00010003268	SMS	2	0.108899561	0.19173416	0.567971614	0.570054243	0.054725306	3.338502617	70.04645151	0.5	CRA004775;PRJNA827465	Yang2025_Silkie_Wenchang	0.884966131
ENSGALG00010003269	ENSGALG00010003269	2	0.58647084	0.554456018	1.057740959	0.290173557	0.532589775	7.451547982	86.57996966	1	PRJNA827465;Yang2025_Silkie_Wenchang	CRA004775	0.784195688
ENSGALG00010003269	ENSGALG00010003269	2	-0.102791721	1.324027813	-0.077635621	0.938117907	3.20096102	10.89416556	90.82077471	0.5	CRA004775;Yang2025_Silkie_Wenchang	PRJNA827465	0.985349045
ENSGALG00010003269	ENSGALG00010003269	2	-0.597366841	0.757020026	-0.789103089	0.430051757	0.857675184	3.544398788	71.78647044	0.5	CRA004775;PRJNA827465	Yang2025_Silkie_Wenchang	0.842012722
ENSGALG00010003271	AKAP11	2	0.001841423	0.074970956	0.024561819	0.980404474	0	0.656754466	0	0.5	PRJNA827465;Yang2025_Silkie_Wenchang	CRA004775	0.994703832
ENSGALG00010003271	AKAP11	2	0.111437479	0.141738269	0.786220125	0.431738538	0	0.00020424	0	1	CRA004775;Yang2025_Silkie_Wenchang	PRJNA827465	0.842503929
ENSGALG00010003271	AKAP11	2	-0.024374028	0.083611613	-0.291514863	0.770657578	0	0.155498064	0	0.5	CRA004775;PRJNA827465	Yang2025_Silkie_Wenchang	0.942491331
ENSGALG00010003272	CPSF3	2	0.224391684	0.137156217	1.636029983	0.101833312	0.033292665	8.36905986	88.05122658	1	PRJNA827465;Yang2025_Silkie_Wenchang	CRA004775	0.54379819
ENSGALG00010003272	CPSF3	2	0.154729973	0.235108398	0.658121845	0.51045984	0.095112893	6.883832855	85.47320917	0.5	CRA004775;Yang2025_Silkie_Wenchang	PRJNA827465	0.865365179
ENSGALG00010003272	CPSF3	2	0.057373063	0.078583085	0.730094309	0.46533254	0.005851606	1.433236577	30.22784821	0.5	CRA004775;PRJNA827465	Yang2025_Silkie_Wenchang	0.852411124
ENSGALG00010003273	NFIX	2	0.01245445	0.079932519	0.155812052	0.876181171	0	0.029279109	0	0.5	PRJNA827465;Yang2025_Silkie_Wenchang	CRA004775	0.970347777
ENSGALG00010003273	NFIX	2	-0.238027335	0.299117359	-0.795765703	0.42616823	0.126889222	2.999398336	66.65998018	1	CRA004775;Yang2025_Silkie_Wenchang	PRJNA827465	0.84125685
ENSGALG00010003273	NFIX	2	-0.222139735	0.313199334	-0.709259921	0.478163195	0.147967217	3.458803488	71.08826785	0.5	CRA004775;PRJNA827465	Yang2025_Silkie_Wenchang	0.855366076
ENSGALG00010003274	BACH2	2	-0.050545627	0.069910677	-0.723002969	0.46967806	0	0.618299387	0	0.5	PRJNA827465;Yang2025_Silkie_Wenchang	CRA004775	0.853485658
ENSGALG00010003274	BACH2	2	0.586451424	0.575945377	1.018241395	0.308563252	0.579797086	7.461028109	86.59702141	1	CRA004775;Yang2025_Silkie_Wenchang	PRJNA827465	0.793561833
ENSGALG00010003274	BACH2	2	0.514684476	0.646218468	0.796455845	0.42576713	0.76181266	10.539339	90.51173892	0.5	CRA004775;PRJNA827465	Yang2025_Silkie_Wenchang	0.84125685
ENSGALG00010003275	MBTPS2	2	-0.28907458	0.432200422	-0.668843817	0.503595112	0.357328997	22.85534703	95.62465624	0.5	PRJNA827465;Yang2025_Silkie_Wenchang	CRA004775	0.863108263
ENSGALG00010003275	MBTPS2	2	-0.664045937	0.13250793	-5.011367512	5.40446E-07	0	0.923307228	0	1	CRA004775;Yang2025_Silkie_Wenchang	PRJNA827465	0.000136161
ENSGALG00010003275	MBTPS2	2	-0.066292441	0.264061679	-0.251049078	0.801776165	0.100190462	3.03865983	67.09075527	0.5	CRA004775;PRJNA827465	Yang2025_Silkie_Wenchang	0.950422506
ENSGALG00010003276	TNFSF11	2	-0.139776432	0.325767451	-0.429068132	0.667873643	0	0.491137584	0	0.5	PRJNA827465;Yang2025_Silkie_Wenchang	CRA004775	0.914120722
ENSGALG00010003276	TNFSF11	2	0.33040775	0.752731887	0.438944803	0.660701532	0.879289746	4.403764129	77.29215347	0.5	CRA004775;Yang2025_Silkie_Wenchang	PRJNA827465	0.91192683
ENSGALG00010003276	TNFSF11	2	0.512540427	0.509747377	1.005479284	0.31466612	0.348072948	3.022850854	66.91864574	1	CRA004775;PRJNA827465	Yang2025_Silkie_Wenchang	0.796531448
ENSGALG00010003280	ENSGALG00010003280	2	-0.059930867	0.18475241	-0.324384765	0.745646753	0.059755269	7.762258559	87.11715163	0.5	PRJNA827465;Yang2025_Silkie_Wenchang	CRA004775	0.936239037
ENSGALG00010003280	ENSGALG00010003280	2	-0.148574099	0.180739252	-0.822035598	0.411056637	0.031054368	1.594326886	37.27760544	0.5	CRA004775;Yang2025_Silkie_Wenchang	PRJNA827465	0.835128673
ENSGALG00010003280	ENSGALG00010003280	2	0.114150585	0.062748026	1.819190066	0.068882438	0	0.01502878	0	1	CRA004775;PRJNA827465	Yang2025_Silkie_Wenchang	0.452044654
ENSGALG00010003292	GADD45GIP1	2	0.197806281	0.15598394	1.268119534	0.204755267	0.036356094	3.513001212	71.53431099	1	PRJNA827465;Yang2025_Silkie_Wenchang	CRA004775	0.717318293
ENSGALG00010003292	GADD45GIP1	2	0.332966286	0.140884442	2.363399974	0.018108114	0	0.639891304	0	1	CRA004775;Yang2025_Silkie_Wenchang	PRJNA827465	0.224653792
ENSGALG00010003292	GADD45GIP1	2	0.072821408	0.061881234	1.176793093	0.2392781	0	0.015323268	0	1	CRA004775;PRJNA827465	Yang2025_Silkie_Wenchang	0.751446039
ENSGALG00010003295	IAH1	2	0.174650661	0.153238832	1.139728483	0.254399437	0.024082544	1.67200455	40.19155033	1	PRJNA827465;Yang2025_Silkie_Wenchang	CRA004775	0.764087626
ENSGALG00010003295	IAH1	2	0.049977634	0.407732075	0.122574692	0.902443896	0.245616413	3.767457597	73.45690099	0.5	CRA004775;Yang2025_Silkie_Wenchang	PRJNA827465	0.976923607
ENSGALG00010003295	IAH1	2	-0.0415413	0.207383666	-0.200311339	0.841237095	0.050306276	1.826181252	45.24092289	0.5	CRA004775;PRJNA827465	Yang2025_Silkie_Wenchang	0.95989279
ENSGALG00010003299	RAD23A	2	0.106711608	0.246792641	0.432393802	0.665455222	0.106219241	7.371823042	86.43483445	0.5	PRJNA827465;Yang2025_Silkie_Wenchang	CRA004775	0.91350776
ENSGALG00010003299	RAD23A	2	0.312619534	0.154388947	2.024882863	0.042879394	0	0.97988103	0	0.5	CRA004775;Yang2025_Silkie_Wenchang	PRJNA827465	0.358114919
ENSGALG00010003299	RAD23A	2	-0.115640541	0.070733011	-1.634887853	0.102072559	0	0.032557534	0	1	CRA004775;PRJNA827465	Yang2025_Silkie_Wenchang	0.544451388
ENSGALG00010003300	CASP8AP2	2	0.053721331	0.069249833	0.775761162	0.437890019	0	0.592200202	0	1	PRJNA827465;Yang2025_Silkie_Wenchang	CRA004775	0.843936225
ENSGALG00010003300	CASP8AP2	2	0.228439992	0.168149077	1.358556325	0.174287223	0	0.259508849	0	1	CRA004775;Yang2025_Silkie_Wenchang	PRJNA827465	0.678487591
ENSGALG00010003300	CASP8AP2	2	0.04580302	0.073224033	0.625518951	0.531630514	0	0.898261948	0	1	CRA004775;PRJNA827465	Yang2025_Silkie_Wenchang	0.873042777
ENSGALG00010003303	MTHFD2L	2	-0.143213452	0.111590733	-1.283381229	0.199358547	0	0.164944652	0	1	PRJNA827465;Yang2025_Silkie_Wenchang	CRA004775	0.711181159
ENSGALG00010003303	MTHFD2L	2	-0.037639317	0.175442413	-0.21453944	0.830126414	0	0.143629392	0	0.5	CRA004775;Yang2025_Silkie_Wenchang	PRJNA827465	0.957212554
ENSGALG00010003303	MTHFD2L	2	-0.142071113	0.125504757	-1.131997829	0.257635347	0	0.441992705	0	0.5	CRA004775;PRJNA827465	Yang2025_Silkie_Wenchang	0.7650601
ENSGALG00010003305	CALR	2	0.123541727	0.037759964	3.271764956	0.001068784	0	0.036384131	0	1	PRJNA827465;Yang2025_Silkie_Wenchang	CRA004775	0.039479712
ENSGALG00010003305	CALR	2	0.017108741	0.136549906	0.125292951	0.900291632	0.023471218	2.259556839	55.74353418	0.5	CRA004775;Yang2025_Silkie_Wenchang	PRJNA827465	0.976475201
ENSGALG00010003305	CALR	2	0.026074669	0.144260917	0.18074659	0.856566488	0.029198538	2.66936664	62.53792998	0.5	CRA004775;PRJNA827465	Yang2025_Silkie_Wenchang	0.963582136
ENSGALG00010003307	COL1A2	2	-0.222807266	0.103443775	-2.153897283	0.031248228	0	0.310933913	0	1	PRJNA827465;Yang2025_Silkie_Wenchang	CRA004775	0.302425291
ENSGALG00010003307	COL1A2	2	-0.397972544	0.256644158	-1.550678367	0.120978781	0.041214984	1.143872058	12.57763547	1	CRA004775;Yang2025_Silkie_Wenchang	PRJNA827465	0.583128409
ENSGALG00010003307	COL1A2	2	-0.368448628	0.372359778	-0.989496315	0.322420372	0.157255921	1.565987757	36.14254036	1	CRA004775;PRJNA827465	Yang2025_Silkie_Wenchang	0.798652022
ENSGALG00010003308	MDN1	2	0.148060346	0.2756055	0.5372184	0.591116763	0.134793214	8.320259906	87.98114468	0.5	PRJNA827465;Yang2025_Silkie_Wenchang	CRA004775	0.892776493
ENSGALG00010003308	MDN1	2	0.323244133	0.189510419	1.705680006	0.088067674	0.019567235	1.303827871	23.30275931	1	CRA004775;Yang2025_Silkie_Wenchang	PRJNA827465	0.509839669
ENSGALG00010003308	MDN1	2	-0.097097946	0.064546344	-1.504313629	0.132500632	0	0.205211198	0	0.5	CRA004775;PRJNA827465	Yang2025_Silkie_Wenchang	0.609713444
ENSGALG00010003317	LYRM2	2	0.095701752	0.224863653	0.425599027	0.67040005	0.066217594	2.716190276	63.18372801	0.5	PRJNA827465;Yang2025_Silkie_Wenchang	CRA004775	0.914482883
ENSGALG00010003317	LYRM2	2	0.202229519	0.224925537	0.899095414	0.368601841	0.019690543	1.215384644	17.7215209	0.5	CRA004775;Yang2025_Silkie_Wenchang	PRJNA827465	0.82033738
ENSGALG00010003317	LYRM2	2	-0.092141439	0.128285079	-0.718255306	0.472599882	0	0.001284205	0	1	CRA004775;PRJNA827465	Yang2025_Silkie_Wenchang	0.853998373
ENSGALG00010003320	OR51E2	2	-0.150317489	0.326478089	-0.460421371	0.645213798	0.13717934	2.757309199	63.732758	0.5	PRJNA827465;Yang2025_Silkie_Wenchang	CRA004775	0.906966303
ENSGALG00010003320	OR51E2	2	0.092828557	0.798910512	0.116193936	0.907498843	0.90312855	2.894586084	65.45274624	0.5	CRA004775;Yang2025_Silkie_Wenchang	PRJNA827465	0.978196186
ENSGALG00010003320	OR51E2	2	0.251549649	0.308648652	0.815003232	0.41507046	0.049512553	1.108674664	9.802214105	1	CRA004775;PRJNA827465	Yang2025_Silkie_Wenchang	0.837489728
ENSGALG00010003323	STMN2	2	0.682367555	0.691980441	0.986108153	0.324080043	0.436032828	1.824760145	45.198277	1	PRJNA827465;Yang2025_Silkie_Wenchang	CRA004775	0.799600434
ENSGALG00010003323	STMN2	2	1.440811065	0.424964808	3.390424424	0.000697845	0	4.93047E-05	0	1	CRA004775;Yang2025_Silkie_Wenchang	PRJNA827465	0.02942854
ENSGALG00010003323	STMN2	2	0.813000867	0.694462466	1.170690868	0.241723057	0.622495632	2.771871933	63.92329718	1	CRA004775;PRJNA827465	Yang2025_Silkie_Wenchang	0.75406671
ENSGALG00010003324	ENSGALG00010003324	2	0.567425047	0.732747172	0.774380398	0.438705872	0	0.749047665	0	0.5	PRJNA827465;Yang2025_Silkie_Wenchang	CRA004775	0.844002503
ENSGALG00010003324	ENSGALG00010003324	2	0.233032463	0.453774875	0.513542014	0.607572229	0	0.977237476	0	0.5	CRA004775;Yang2025_Silkie_Wenchang	PRJNA827465	0.897160522
ENSGALG00010003324	ENSGALG00010003324	2	-0.101520806	0.500549876	-0.202818562	0.839276849	0	0.08223277	0	1	CRA004775;PRJNA827465	Yang2025_Silkie_Wenchang	0.959428114
ENSGALG00010003330	HEY1	2	-0.0336118	0.067230192	-0.499950966	0.617109605	0	0.043918795	0	1	PRJNA827465;Yang2025_Silkie_Wenchang	CRA004775	0.899121942
ENSGALG00010003330	HEY1	2	0.203943716	0.286909286	0.71082996	0.477189613	0.131972615	4.896648871	79.57786996	0.5	CRA004775;Yang2025_Silkie_Wenchang	PRJNA827465	0.854986476
ENSGALG00010003330	HEY1	2	0.206412556	0.268400624	0.769046481	0.441865718	0.120157519	5.460179491	81.68558375	0.5	CRA004775;PRJNA827465	Yang2025_Silkie_Wenchang	0.845135451
ENSGALG00010003331	EPSTI1	2	-0.444133576	0.329924339	-1.346167963	0.178248354	0.075879433	1.517922991	34.12050505	1	PRJNA827465;Yang2025_Silkie_Wenchang	CRA004775	0.684689586
ENSGALG00010003331	EPSTI1	2	-0.262358858	0.296477835	-0.884918963	0.37620034	0	0.440477144	0	1	CRA004775;Yang2025_Silkie_Wenchang	PRJNA827465	0.822974013
ENSGALG00010003331	EPSTI1	2	-0.62045147	0.262266982	-2.365724669	0.017994821	0	0.260180508	0	1	CRA004775;PRJNA827465	Yang2025_Silkie_Wenchang	0.22387506
ENSGALG00010003333	ENSGALG00010003333	2	0.83930669	0.989881672	0.847885877	0.396501531	1.27945256	2.514294077	60.22740501	1	PRJNA827465;Yang2025_Silkie_Wenchang	CRA004775	0.831365249
ENSGALG00010003333	ENSGALG00010003333	2	0.92657074	0.729030358	1.270963176	0.203741749	0.608740009	1.787661361	44.06099379	1	CRA004775;Yang2025_Silkie_Wenchang	PRJNA827465	0.717254253
ENSGALG00010003333	ENSGALG00010003333	2	0.374316968	0.276422758	1.354146711	0.175689576	0	0.438629832	0	1	CRA004775;PRJNA827465	Yang2025_Silkie_Wenchang	0.6803934
ENSGALG00010003344	ZFAND1	2	0.176526578	0.099403386	1.77586081	0.075755862	0	0.099975923	0	1	PRJNA827465;Yang2025_Silkie_Wenchang	CRA004775	0.475110429
ENSGALG00010003344	ZFAND1	2	0.046077955	0.229208457	0.201030779	0.840674507	0.041787448	1.626917931	38.53408453	0.5	CRA004775;Yang2025_Silkie_Wenchang	PRJNA827465	0.959769655
ENSGALG00010003344	ZFAND1	2	0.060696102	0.169630019	0.357814627	0.720482043	0.026246855	1.534312862	34.82424445	0.5	CRA004775;PRJNA827465	Yang2025_Silkie_Wenchang	0.928764162
ENSGALG00010003348	ADAM17	2	0.04512044	0.06839083	0.659744008	0.50941812	0.004519048	1.744043007	42.6619644	0.5	PRJNA827465;Yang2025_Silkie_Wenchang	CRA004775	0.865257661
ENSGALG00010003348	ADAM17	2	0.010243076	0.200625836	0.051055619	0.959281201	0.048120898	2.023201064	50.57337514	0.5	CRA004775;Yang2025_Silkie_Wenchang	PRJNA827465	0.989206828
ENSGALG00010003348	ADAM17	2	-0.010985762	0.04881569	-0.225045712	0.821943713	0	0.973278651	0	1	CRA004775;PRJNA827465	Yang2025_Silkie_Wenchang	0.95563486
ENSGALG00010003352	FARSA	2	0.110715495	0.109571124	1.010444095	0.312282571	0.015537892	2.500037326	60.00059721	1	PRJNA827465;Yang2025_Silkie_Wenchang	CRA004775	0.796054997
ENSGALG00010003352	FARSA	2	-0.054121006	0.333563709	-0.162250881	0.871108293	0.189601148	6.544300751	84.71952867	0.5	CRA004775;Yang2025_Silkie_Wenchang	PRJNA827465	0.969259951
ENSGALG00010003352	FARSA	2	-0.139099207	0.214015755	-0.649948444	0.515725525	0.070226406	3.580756198	72.0729381	0.5	CRA004775;PRJNA827465	Yang2025_Silkie_Wenchang	0.86733249
ENSGALG00010003356	ENSGALG00010003356	2	0.627744784	0.524146461	1.197651478	0.231052726	0.453759678	5.340663378	81.27573432	1	PRJNA827465;Yang2025_Silkie_Wenchang	CRA004775	0.743739992
ENSGALG00010003356	ENSGALG00010003356	2	0.94612881	0.225070083	4.203707563	2.62578E-05	0	0.615650198	0	1	CRA004775;Yang2025_Silkie_Wenchang	PRJNA827465	0.002991336
ENSGALG00010003356	ENSGALG00010003356	2	0.471847824	0.332563327	1.418820976	0.155951216	0.169923875	4.217499415	76.28926761	1	CRA004775;PRJNA827465	Yang2025_Silkie_Wenchang	0.649132948
ENSGALG00010003358	DNAJC15	2	-0.051384258	0.181476676	-0.283145244	0.7770655	0.046120197	2.999196485	66.65773633	0.5	PRJNA827465;Yang2025_Silkie_Wenchang	CRA004775	0.944218792
ENSGALG00010003358	DNAJC15	2	0.119429137	0.16130888	0.740375461	0.459072204	0	0.256286233	0	1	CRA004775;Yang2025_Silkie_Wenchang	PRJNA827465	0.850098539
ENSGALG00010003358	DNAJC15	2	-0.179209216	0.084905185	-2.110698143	0.034798266	0	0.433481406	0	0.5	CRA004775;PRJNA827465	Yang2025_Silkie_Wenchang	0.321263903
ENSGALG00010003363	HBE1	2	-1.014168718	0.361551468	-2.805046608	0.00503093	0.109366804	1.705879236	41.37920323	1	PRJNA827465;Yang2025_Silkie_Wenchang	CRA004775	0.105738321
ENSGALG00010003363	HBE1	2	-0.446694714	1.082717158	-0.412568241	0.679922973	1.84257986	4.123521789	75.74888527	0.5	CRA004775;Yang2025_Silkie_Wenchang	PRJNA827465	0.917701868
ENSGALG00010003363	HBE1	2	-0.167660133	0.678260171	-0.247191477	0.804760041	0.47572703	1.770505757	43.51896365	0.5	CRA004775;PRJNA827465	Yang2025_Silkie_Wenchang	0.951720645
ENSGALG00010003364	ANKRD6	2	-0.073949194	0.087989936	-0.840427862	0.400668533	0	0.379136758	0	1	PRJNA827465;Yang2025_Silkie_Wenchang	CRA004775	0.832353691
ENSGALG00010003364	ANKRD6	2	-0.157981103	0.131797089	-1.198669136	0.230656624	0	0.075471439	0	1	CRA004775;Yang2025_Silkie_Wenchang	PRJNA827465	0.743374303
ENSGALG00010003364	ANKRD6	2	-0.051783365	0.10638469	-0.4867558	0.626431397	0	0.348200122	0	1	CRA004775;PRJNA827465	Yang2025_Silkie_Wenchang	0.901504244
ENSGALG00010003366	CASD1	2	-0.224436312	0.21057133	-1.065844589	0.286493901	0.081184502	11.78840143	91.51708562	1	PRJNA827465;Yang2025_Silkie_Wenchang	CRA004775	0.782018115
ENSGALG00010003366	CASD1	2	-0.261926935	0.22008054	-1.19014128	0.233990868	0.070607719	3.281287994	69.52416241	0.5	CRA004775;Yang2025_Silkie_Wenchang	PRJNA827465	0.747279664
ENSGALG00010003366	CASD1	2	-0.014672408	0.07294986	-0.201130034	0.840596898	0	0.014801545	0	0.5	CRA004775;PRJNA827465	Yang2025_Silkie_Wenchang	0.959736808
ENSGALG00010003371	ENSGALG00010003371	2	0.048722363	0.483930114	0.100680578	0.91980403	0.36639859	4.581803558	78.17453352	0.5	PRJNA827465;Yang2025_Silkie_Wenchang	CRA004775	0.981237238
ENSGALG00010003371	ENSGALG00010003371	2	-0.404805573	0.30441493	-1.32978226	0.183590023	0	0.085368436	0	1	CRA004775;Yang2025_Silkie_Wenchang	PRJNA827465	0.692102743
ENSGALG00010003371	ENSGALG00010003371	2	0.40433528	0.275744787	1.466338799	0.14255601	0	0.953893051	0	0.5	CRA004775;PRJNA827465	Yang2025_Silkie_Wenchang	0.628893678
ENSGALG00010003372	ENOX1	2	0.12393719	0.228401719	0.542628094	0.587385892	0.095147645	11.17911441	91.0547476	0.5	PRJNA827465;Yang2025_Silkie_Wenchang	CRA004775	0.891660017
ENSGALG00010003372	ENOX1	2	0.339442136	0.108812356	3.119518314	0.00181147	0	0.303199827	0	1	CRA004775;Yang2025_Silkie_Wenchang	PRJNA827465	0.05435515
ENSGALG00010003372	ENOX1	2	-0.082032054	0.070646941	-1.161155074	0.245578841	0	0.707197415	0	0.5	CRA004775;PRJNA827465	Yang2025_Silkie_Wenchang	0.75661406
ENSGALG00010003374	SYCE2	2	0.294776432	0.330609393	0.891615419	0.372599103	0.099985251	1.816723636	44.95585459	1	PRJNA827465;Yang2025_Silkie_Wenchang	CRA004775	0.822016521

ENSGALG00010003374	SYCE2	2	-0.013888992	0.760950098	-0.018252172	0.985437682	0.897782496	4.289843921	76.6891286	0.5	CRA004775;Yang2025_Silkie_Wenchang	PRJNA827465	0.99592834
ENSGALG00010003374	SYCE2	2	-0.252375792	0.394833176	-0.639196014	0.522695425	0.128746439	1.528051453	34.5571775	0.5	CRA004775;PRJNA827465	Yang2025_Silkie_Wenchang	0.869568211
ENSGALG00010003375	YWHAQ	2	0.004976692	0.078055486	0.063758387	0.949162613	0.008419538	2.871381608	65.17355975	0.5	PRJNA827465;Yang2025_Silkie_Wenchang	CRA004775	0.99723759
ENSGALG00010003375	YWHAQ	2	-0.106883792	0.084017428	-1.272162146	0.203315514	0	0.29402485	0	1	CRA004775;Yang2025_Silkie_Wenchang	PRJNA827465	0.717188086
ENSGALG00010003375	YWHAQ	2	0.044758877	0.082803472	0.540543477	0.588822286	0.005894866	1.112649593	10.12444473	0.5	CRA004775;PRJNA827465	Yang2025_Silkie_Wenchang	0.892171138
ENSGALG00010003382	ASNS	2	-0.051332347	0.130764227	-0.392556502	0.694647072	0.007545167	1.263499213	20.85471918	0.5	PRJNA827465;Yang2025_Silkie_Wenchang	CRA004775	0.92082578
ENSGALG00010003382	ASNS	2	-0.586439518	0.461653735	-1.270301687	0.203977187	0.312284735	3.302267353	69.7177759	1	CRA004775;Yang2025_Silkie_Wenchang	PRJNA827465	0.717254253
ENSGALG00010003382	ASNS	2	-0.468016605	0.601336083	-0.778294565	0.436395379	0.611534915	5.808567172	82.78405034	0.5	CRA004775;PRJNA827465	Yang2025_Silkie_Wenchang	0.843122822
ENSGALG00010003383	UBE2J1	2	-0.010104588	0.054602218	-0.185058196	0.85318339	0	0.382742374	0	0.5	PRJNA827465;Yang2025_Silkie_Wenchang	CRA004775	0.962780062
ENSGALG00010003383	UBE2J1	2	-0.001120791	0.099820042	-0.011228115	0.991041449	0.004318537	1.188172617	15.83714476	0.5	CRA004775;Yang2025_Silkie_Wenchang	PRJNA827465	0.99755571
ENSGALG00010003383	UBE2J1	2	0.03164283	0.063403051	0.499074256	0.617727073	0	0.631981614	0	1	CRA004775;PRJNA827465	Yang2025_Silkie_Wenchang	0.899121942
ENSGALG00010003388	PXDN	2	-0.231233468	0.200899181	-1.150992585	0.249735286	0.066253813	5.546083409	81.96925783	1	PRJNA827465;Yang2025_Silkie_Wenchang	CRA004775	0.759757983
ENSGALG00010003388	PXDN	2	-0.109392181	0.436381295	-0.25068027	0.802061316	0.281934894	3.180113203	68.55457853	0.5	CRA004775;Yang2025_Silkie_Wenchang	PRJNA827465	0.950588502
ENSGALG00010003388	PXDN	2	-0.012359566	0.109829653	-0.112533962	0.910400043	0.001057363	1.008366213	0.829680005	0.5	CRA004775;PRJNA827465	Yang2025_Silkie_Wenchang	0.97880407
ENSGALG00010003397	SPATA4	2	-0.214726576	0.257086716	-0.83523015	0.403588137	0.021799751	1.188026579	15.82679902	0.5	PRJNA827465;Yang2025_Silkie_Wenchang	CRA004775	0.83306585
ENSGALG00010003397	SPATA4	2	0.107735427	0.32861921	0.327842754	0.743030559	0	0.001514132	0	1	CRA004775;Yang2025_Silkie_Wenchang	PRJNA827465	0.935735651
ENSGALG00010003397	SPATA4	2	-0.331017136	0.270934762	-1.221759564	0.221798564	0	0.586914419	0	0.5	CRA004775;PRJNA827465	Yang2025_Silkie_Wenchang	0.735224622
ENSGALG00010003400	PKIA	2	-0.203646552	0.114623867	-1.776650513	0.075625759	0.016208501	2.530302062	60.47902679	1	PRJNA827465;Yang2025_Silkie_Wenchang	CRA004775	0.474784631
ENSGALG00010003400	PKIA	2	0.081534235	0.489015993	0.166731225	0.867581536	0.398781726	5.34913663	81.30539432	0.5	CRA004775;Yang2025_Silkie_Wenchang	PRJNA827465	0.96788231
ENSGALG00010003400	PKIA	2	0.168533132	0.364096539	0.462880345	0.643450136	0.200024036	3.274331058	69.45941072	0.5	CRA004775;PRJNA827465	Yang2025_Silkie_Wenchang	0.906634523
ENSGALG00010003401	ENSGALG00010003401	2	0.115813114	0.66309116	0.174656399	0.861349629	0	0.307338267	0	0.5	PRJNA827465;Yang2025_Silkie_Wenchang	CRA004775	0.965343309
ENSGALG00010003401	ENSGALG00010003401	2	0.372527119	0.511931761	0.727689015	0.466803977	0	0.08072294	0	1	CRA004775;Yang2025_Silkie_Wenchang	PRJNA827465	0.852842649
ENSGALG00010003401	ENSGALG00010003401	2	0.162046067	0.457913992	0.353878829	0.723429694	0	0.193164611	0	0.5	CRA004775;PRJNA827465	Yang2025_Silkie_Wenchang	0.930032096
ENSGALG00010003403	GCDH	2	0.239806336	0.214951857	1.115628117	0.264581352	0.066676631	3.202651039	68.77586762	1	PRJNA827465;Yang2025_Silkie_Wenchang	CRA004775	0.768855521
ENSGALG00010003403	GCDH	2	0.064259892	0.46472111	0.138276248	0.890022096	0.361279953	6.045168013	83.45786258	0.5	CRA004775;Yang2025_Silkie_Wenchang	PRJNA827465	0.973310644
ENSGALG00010003403	GCDH	2	-0.093449974	0.230098274	-0.406130704	0.684646567	0.069485265	2.372370069	57.84806033	0.5	CRA004775;PRJNA827465	Yang2025_Silkie_Wenchang	0.919003112
ENSGALG00010003406	C1GALT1	2	-0.112493098	0.205894002	-0.546364132	0.584815661	0.079454554	15.593657	93.5871361	0.5	PRJNA827465;Yang2025_Silkie_Wenchang	CRA004775	0.891016154
ENSGALG00010003406	C1GALT1	2	-0.285094187	0.094009512	-3.032610003	0.002424487	0.001958161	1.063247146	5.948489568	1	CRA004775;Yang2025_Silkie_Wenchang	PRJNA827465	0.06593704
ENSGALG00010003406	C1GALT1	2	0.079476898	0.047238854	1.682447543	0.092482069	0	0.434071741	0	0.5	CRA004775;PRJNA827465	Yang2025_Silkie_Wenchang	0.520673653
ENSGALG00010003407	CHMP4C	2	-0.097289933	0.275408707	-0.353256562	0.723896107	0.120596206	4.859500332	79.42175262	0.5	PRJNA827465;Yang2025_Silkie_Wenchang	CRA004775	0.93026709
ENSGALG00010003407	CHMP4C	2	-0.191330307	0.261686065	-0.731144422	0.464690943	0.068656275	1.828693524	45.31615128	0.5	CRA004775;Yang2025_Silkie_Wenchang	PRJNA827465	0.85217126
ENSGALG00010003407	CHMP4C	2	0.169691968	0.148553094	1.142298445	0.253329987	0	2.40404E-06	0	1	CRA004775;PRJNA827465	Yang2025_Silkie_Wenchang	0.763018507
ENSGALG00010003408	ENSGALG00010003408	2	0.647314778	0.610390452	1.060492962	0.28892039	0.625366548	5.709424466	82.48509975	1	PRJNA827465;Yang2025_Silkie_Wenchang	CRA004775	0.783530627
ENSGALG00010003408	ENSGALG00010003408	2	0.910111252	0.348468858	2.611743433	0.009008183	0.077297264	1.43588241	30.35641408	1	CRA004775;Yang2025_Silkie_Wenchang	PRJNA827465	0.14870051
ENSGALG00010003408	ENSGALG00010003408	2	0.285102129	0.244165598	1.167658881	0.242944389	0.055994915	1.717494131	41.775638	1	CRA004775;PRJNA827465	Yang2025_Silkie_Wenchang	0.7550024
ENSGALG00010003412	ASB5	2	-0.12699889	0.066435172	-1.911621292	0.055923786	0	0.000144494	0	1	PRJNA827465;Yang2025_Silkie_Wenchang	CRA004775	0.409250851
ENSGALG00010003412	ASB5	2	-0.109624074	0.120172271	-0.912374152	0.361571791	0	0.272619282	0	0.5	CRA004775;Yang2025_Silkie_Wenchang	PRJNA827465	0.81550034
ENSGALG00010003412	ASB5	2	-0.120739899	0.077643695	-1.55505092	0.119933943	0	0.286920238	0	0.5	CRA004775;PRJNA827465	Yang2025_Silkie_Wenchang	0.580870455
ENSGALG00010003420	ENSGALG00010003420	2	-0.228115213	0.559752417	-0.407528768	0.68361967	0.312248951	1.791738043	44.18826993	0.5	PRJNA827465;Yang2025_Silkie_Wenchang	CRA004775	0.918800744
ENSGALG00010003420	ENSGALG00010003420	2	0.030931955	0.317887701	0.097304661	0.922484455	0	0.329669758	0	0.5	CRA004775;Yang2025_Silkie_Wenchang	PRJNA827465	0.981804675
ENSGALG00010003420	ENSGALG00010003420	2	-0.465894179	0.443078281	-1.051494057	0.293031737	0	0.710895119	0	1	CRA004775;PRJNA827465	Yang2025_Silkie_Wenchang	0.786774237
ENSGALG00010003428	SNX16	2	0.058241318	0.067431858	0.863706267	0.38774927	0	0.885224822	0	0.5	PRJNA827465;Yang2025_Silkie_Wenchang	CRA004775	0.828154121
ENSGALG00010003428	SNX16	2	-0.048484193	0.12815738	-0.378317603	0.70519467	0.000061649	1.037070765	3.574564677	1	CRA004775;Yang2025_Silkie_Wenchang	PRJNA827465	0.924345236
ENSGALG00010003428	SNX16	2	-0.028805255	0.282374437	-0.102010844	0.918748068	0.091511073	1.565898147	36.13888606	0.5	CRA004775;PRJNA827465	Yang2025_Silkie_Wenchang	0.981033557
ENSGALG00010003431	PM20D2	2	-0.385330884	0.13837237	-2.784738638	0.005357088	0	0.70490233	0	1	PRJNA827465;Yang2025_Silkie_Wenchang	CRA004775	0.109433716
ENSGALG00010003431	PM20D2	2	-0.225172492	0.199149623	-1.13066994	0.258194038	0	0.161833662	0	0.5	CRA004775;Yang2025_Silkie_Wenchang	PRJNA827465	0.765467321
ENSGALG00010003431	PM20D2	2	-0.446606911	0.176165256	-2.535158864	0.011239634	0	0.593009586	0	0.5	CRA004775;PRJNA827465	Yang2025_Silkie_Wenchang	0.168607382
ENSGALG00010003432	LACC1	2	-0.389610512	0.225665719	-1.726494013	0.084258576	0.008799247	1.091467963	8.380270108	1	PRJNA827465;Yang2025_Silkie_Wenchang	CRA004775	0.49990641
ENSGALG00010003432	LACC1	2	-0.435626409	0.25047705	-1.73918692	0.082001889	0	0.96110551	0	1	CRA004775;Yang2025_Silkie_Wenchang	PRJNA827465	0.493597945
ENSGALG00010003432	LACC1	2	-0.187514319	0.223524311	-0.838898992	0.401525993	0	0.005705588	0	1	CRA004775;PRJNA827465	Yang2025_Silkie_Wenchang	0.832623399
ENSGALG00010003437	PNRC1	2	0.125410802	0.173125752	0.724391376	0.468825493	0.046591372	4.456209242	77.55940205	0.5	PRJNA827465;Yang2025_Silkie_Wenchang	CRA004775	0.85338312
ENSGALG00010003437	PNRC1	2	0.306992237	0.118297079	2.595095659	0.00945646	0	4.28189E-05	0	1	CRA004775;Yang2025_Silkie_Wenchang	PRJNA827465	0.152962732
ENSGALG00010003437	PNRC1	2	0.004845973	0.116692142	0.041527842	0.966857097	0.004901901	1.087612676	8.055503397	0.5	CRA004775;PRJNA827465	Yang2025_Silkie_Wenchang	0.990893423
ENSGALG00010003438	KCNS3	2	-0.254136336	0.202745487	-1.253474689	0.210033005	0.037207763	1.804287724	44.5764671	1	PRJNA827465;Yang2025_Silkie_Wenchang	CRA004775	0.721835135
ENSGALG00010003438	KCNS3	2	0.437149155	0.538232602	0.812193749	0.416680466	0.446554381	4.048440885	75.29913297	0.5	CRA004775;Yang2025_Silkie_Wenchang	PRJNA827465	0.837984974
ENSGALG00010003438	KCNS3	2	0.248556842	0.745830967	0.333261628	0.738936802	0.985244523	8.23168406	87.85181753	0.5	CRA004775;PRJNA827465	Yang2025_Silkie_Wenchang	0.934613258
ENSGALG00010003439	GMIP	2	-0.001114672	0.156986448	-0.007100433	0.994334722	0	0.021945416	0	0.5	PRJNA827465;Yang2025_Silkie_Wenchang	CRA004775	0.99823774
ENSGALG00010003439	GMIP	2	-0.220573044	0.365948901	-0.602741243	0.546679842	0.142627391	1.827262691	45.2733127	0.5	CRA004775;Yang2025_Silkie_Wenchang	PRJNA827465	0.878259987
ENSGALG00010003439	GMIP	2	-0.232728659	0.335349053	-0.693989313	0.487688911	0.107932041	1.630235923	38.65918509	1	CRA004775;PRJNA827465	Yang2025_Silkie_Wenchang	0.85768885
ENSGALG00010003442	SPCS3	2	-0.038732103	0.139990686	-0.276676285	0.782028683	0.031462997	4.538344919	77.9655355	0.5	PRJNA827465;Yang2025_Silkie_Wenchang	CRA004775	0.945372683
ENSGALG00010003442	SPCS3	2	-0.199855682	0.109107944	-1.83174394	0.066993492	0	0.006652073	0	1	CRA004775;Yang2025_Silkie_Wenchang	PRJNA827465	0.446966231
ENSGALG00010003442	SPCS3	2	0.036379674	0.097239653	0.374123855	0.708312171	0.009242257	1.362756275	26.61930691	0.5	CRA004775;PRJNA827465	Yang2025_Silkie_Wenchang	0.925061251
ENSGALG00010003447	ZC2HC1A	2	0.063167898	0.095532315	0.661220213	0.5084711	0.005490864	1.302425218	23.22015986	1	PRJNA827465;Yang2025_Silkie_Wenchang	CRA004775	0.864902608
ENSGALG00010003447	ZC2HC1A	2	0.357139265	0.202918724	1.760011387	0.078405876	0.02949539	1.43202442	30.16878861	1	CRA004775;Yang2025_Silkie_Wenchang	PRJNA827465	0.481856686
ENSGALG00010003447	ZC2HC1A	2	0.260225897	0.320964113	0.810763067	0.417501748	0.160601113	3.818626784	73.81257566	1	CRA004775;PRJNA827465	Yang2025_Silkie_Wenchang	0.838312595
ENSGALG00010003449	BORCS6	2	0.120044668	0.155487246	0.772054755	0.440081998	0.031916345	2.644182575	62.18112889	0.5	PRJNA827465;Yang2025_Silkie_Wenchang	CRA004775	0.844353254
ENSGALG00010003449	BORCS6	2	0.231903624	0.161389285	1.436920822	0.150740493	0.001830357	1.025107963	2.449299429	0.5	CRA004775;Yang2025_Silkie_Wenchang	PRJNA827465	0.642051813
ENSGALG00010003449	BORCS6	2	-0.00544058	0.081502265	-0.066753727	0.946777762	0	0.036194127	0	1	CRA004775;PRJNA827465	Yang2025_Silkie_Wenchang	0.986445196
ENSGALG00010003454	BPNT2	2	-0.059343407	0.182505191	-0.325160102	0.745059903	0.059238401	9.024684968	88.9192808	0.5	PRJNA827465;Yang2025_Silkie_Wenchang	CRA004775	0.93597621
ENSGALG00010003454	BPNT2	2	-0.118200088	0.152606127	-0.774543533	0.438609435	0.031029794	2.782003285	64.05467939	0.5	CRA004775;Yang2025_Silkie_Wenchang	PRJNA827465	0.844002503
ENSGALG00010003454	BPNT2	2	0.110815895	0.073926184	1.499007374	0.133871719	0	0.085559776	0	1	CRA004775;PRJNA827465	Yang2025_Silkie_Wenchang	0.613104446
ENSGALG00010003456	CDS1	2	0.162982181	0.274610787	0.593502472	0.552844929	0	0.133318101	0	1	PRJNA827465;Yang2025_Silkie_Wenchang	CRA004775	0.87928478
ENSGALG00010003456	CDS1	2	0.329583513	0.348719072	0.945126149	0.344594477	0.055090599	1.256496178	20.4136059	1	CRA004775;Yang2025_Silkie_Wenchang	PRJNA827465	0.806886906
ENSGALG00010003456	CDS1	2	0.480126981	0.335135711	1.432634498	0.151962318	0	0.589414909	0	1	CRA004775;PRJNA827465	Yang2025_Silkie_Wenchang	0.643294424
ENSGALG00010003466	TSC22D1	2	-0.167633286	0.113092074	-1.482272632	0.138267767	0.017581259	3.006590601	66.73973504	1	PRJNA827465;Yang2025_Silkie_Wenchang	CRA004775	0.621841044
ENSGALG00010003466	TSC22D1	2	-0.144001458	0.224865998	-0.640387874	0.521920464	0.062262325	2.196768199	54.47858357	0.5	CRA004775;Yang2025_Silkie_Wenchang	PRJNA827465	0.86956507
ENSGALG00010003466	TSC22D1	2	-0.061221211	0.063913557	-0.957875199	0.338125694	0	0.649783327	0	0.5	CRA004775;PRJNA827465	Yang2025_Silkie_Wenchang	0.80415385
ENSGALG00010003468	ILVBL	2	-0.114432491	0.095943382	-1.192708535	0.232983542	0	0.108544818	0	1	PRJNA827465;Yang2025_Silkie_Wenchang	CRA004775	0.746005924
ENSGALG00010003468	ILVBL	2	-0.315170356	0.303865325	-1.037204086	0.299640754	0.109202642	2.408545892	58.48117308	1	CRA004775;Yang2025_Silkie_Wenchang	PRJNA827465	0.79004225
ENSGALG00010003468	ILVBL	2	-0.308353382	0.249138058	-1.237680765	0.215834454	0.083588593	2.546489819	60.7302573	1	CRA004775;PRJNA827465	Yang2025_Silkie_Wenchang	0.729213578
ENSGALG00010003470	METTL3	2	-0.079373861	0.074109527	-1.071034523	0.284153907	0	0.990704403	0	0.5	PRJNA827465;Yang2025_Silkie_Wenchang	CRA004775	0.780058742
ENSGALG00010003470	METTL3	2	0.093656342	0.164229873	0.570275919	0.568490571	0	0.000630363	0	1	CRA004775;Yang2025_Silkie_Wenchang	PRJNA827465	0.884151198
ENSGALG00010003470	METTL3	2	-0.098708483	0.077974118	-1.265913427	0.205544079	0	0.355991092	0	0.5	CRA004775;PRJNA827465	Yang2025_Silkie_Wenchang	0.71825498
ENSGALG00010003471	ADI1	2	0.001110244	0.086670466	0.012809951	0.989779417	0.00687929	1.646887078	39.2793827	0.5	PRJNA827465;Yang2025_Silkie_Wenchang	CRA004775	0.997311131
ENSGALG00010003471	ADI1	2	-0.024824515	0.156216468	-0.158911001	0.873738993	0.017759595	1.443466857	30.72234424	0.5	CRA004775;Yang2025_Silkie_Wenchang	PRJNA827465	0.9699681
ENSGALG00010003471	ADI1	2	0.068613691	0.06154337	1.114883554	0.264900325	0	0.349357936	0	1	CRA004775;PRJNA827465	Yang2025_Silkie_Wenchang	0.769274038
ENSGALG00010003472	FAM110B	2	-0.204983918	0.339667306	-0.603484391	0.546186496	0.223484622	31.61856331	96.83730095	0.5	PRJNA827465;Yang2025_Silkie_Wenchang	CRA004775	0.878012469
ENSGALG00010003472	FAM110B	2	-0.537755885	0.098319903	-5.469450953	4.51432E-08	0	0.244610958	0	1	CRA004775;Yang2025_Silkie_Wenchang	PRJNA827465	1.82224E-05
ENSGALG00010003472	FAM110B	2	0.109361362	0.09585414	1.140914334	0.253905574	0.005211428	1.052704738	5.006602125	0.5	CRA004775;PRJNA827465	Yang2025_Silkie_Wenchang	0.763698689
ENSGALG00010003473	ENSGALG00010003473	2	0.285588172	0.60788779	0.469804093	0.63849499	0.582329686	3.918571816	74.48049833	0.5	PRJNA827465;Yang2025_Silkie_Wenchang	CRA004775	0.905621386
ENSGALG00010003473	ENSGALG00010003473	2	0.43036617	0.463664096	0.928185239	0.35331149	0.225346149	1.920245006	47.92331203	1	CRA004775;Yang2025_Silkie_Wenchang	PRJNA827465	0.811157428
ENSGALG00010003473	ENSGALG00010003473	2	-0.155764153	0.119884654	-1.2992835	0.193846655	0	0.636062837	0	0.5	CRA004775;PRJNA827465	Yang2025_Silkie_Wenchang	0.704071257
ENSGALG00010003474	RPS7	2	-0.356815291	0.33806394	-1.055466879	0.29121185	0.195166163	6.031336887	83.41992797	1	PRJNA827465;Yang2025_Silkie_Wenchang	CRA004775	0.78467882
ENSGALG00010003474	RPS7	2	-0.459785845	0.258413899	-1.779261283	0.075196935	0.077140503	2.323182925	56.95560651	1	CRA004775;Yang2025_Silkie_Wenchang	PRJNA827465	0.473260364
ENSGALG00010003474	RPS7	2	-0.078520831	0.055376849	-1.417936054	0.156209435	0	0.586420192	0	1	CRA004775;PRJNA827465	Yang2025_Silkie_Wenchang	0.649132948
ENSGALG00010003476	ENSGALG00010003476	2	-0.895841924	1.497053617	-0.598403366	0.54957082	4.233042448	17.74785512	94.36551632	0.5	PRJNA827465;Yang2025_Silkie_Wenchang	CRA004775	0.878554604
ENSGALG00010003476	ENSGALG00010003476	2	-1.98347489	0.472194644	-4.200545087	2.66273E-05	0.049634629	1.124963827	11.10825287	1	CRA004775;Yang2025_Silkie_Wenchang	PRJNA827465	0.00300728
ENSGALG00010003476	ENSGALG00010003476	2	-0.394275852	1.022539457	-0.385584976	0.699804062	1.828398741	7.704682809	87.02088036	0.5	CRA004775;PRJNA827465	Yang2025_Silkie_Wenchang	0.92216184
ENSGALG00010003478	COLEC11	2	-0.144494679	0.226516094	-0.637900275	0.523538598	0.084941354	5.760398173	82.64008893	0.5	PRJNA827465;Yang2025_Silkie_Wenchang	CRA004775	0.869661505
ENSGALG00010003478	COLEC11	2	0.122783865	0.549084016	0.223615807	0.823056263	0.522882129	6.843749883	85.3881276	0.5	CRA004775;Yang2025_Silkie_Wenchang	PRJNA827465	0.955822987
ENSGALG00010003478	COLEC11	2	0.308286017	0.307680455	1.001968153	0.316358974	0.119779597	2.279657551	56.1337623	1	CRA004775;PRJNA827465	Yang2025_Silkie_Wenchang	0.796883862
ENSGALG00010003479	SYDE1	2	0.008083386	0.058292636	0.138669078	0.889711654	0	0.01113289	0	1	PRJNA827465;Yang2025_Silkie_Wenchang	CRA004775	0.9731958
ENSGALG00010003479	SYDE1	2	-0.010873567	0.091376398	-0.118997547	0.905277299	0	0.630454046	0	0.5	CRA004775;Yang2025_Silkie_Wenchang	PRJNA827465	0.977980705
ENSGALG00010003479	SYDE1	2	-0.011540301	0.070002212	-0.164856235	0.86905714	0	0.591719897	0	0.5	CRA004775;PRJNA827465	Yang2025_Silkie_Wenchang	0.968461055
ENSGALG00010003485	RNGTT	2	-0.039014853	0.049012973	-0.796010738	0.426025794	0	0.513276402	0	1	PRJNA827465;Yang2025_Silkie_Wenchang	CRA004775	0.84125685
ENSGALG00010003485	RNGTT	2	-0.119663519	0.099307655	-1.204977794	0.228211874	0	0.203519702	0	1	CRA004775;Yang2025_Silkie_Wenchang	PRJNA827465	0.741040754
ENSGALG00010003485	RNGTT	2	-0.027235845	0.054693361	-0.497973506	0.618502716	0	0.529491109	0	1	CRA004775;PRJNA827465	Yang2025_Silkie_Wenchang	0.899168321
ENSGALG00010003489	SGCE	2	0.026352394	0.055532595	0.474539208	0.635115445	0.001312833	1.178351593	15.13568564	0.5	PRJNA827465;Yang2025_Silkie_Wenchang	CRA004775	0.904051292
ENSGALG00010003489	SGCE	2	-0.040166924	0.102748582	-0.390924359	0.695853148	0	0.82298448	0	0.5	CRA004775;Yang2025_Silkie_Wenchang	PRJNA827465	0.921038437
ENSGALG00010003489	SGCE	2	0.06143896	0.051157659	1.200972877	0.229761723	0	0.262756815	0	1	CRA004775;PRJNA827465	Yang2025_Silkie_Wenchang	0.742686486
ENSGALG00010003495	ENSGALG00010003495	2	-0.361681679	0.172069348	-2.101952975	0.035557395	0	0.491811285	0	1	PRJNA827465;Yang2025_Silkie_Wenchang	CRA004775	0.325417923
ENSGALG00010003495	ENSGALG00010003495	2	-0.420268912	0.334964571	-1.254666756	0.209599759	0	0.986348936	0	0.5	CRA004775;Yang2025_Silkie_Wenchang	PRJNA827465	0.721420379
ENSGALG00010003495	ENSGALG00010003495	2	-0.265952783	0.186075268	-1.429275289	0.152925126	0	0.461488242	0	0.5	CRA004775;PRJNA827465	Yang2025_Silkie_Wenchang	0.645068129
ENSGALG00010003503	NUFIP1	2	-0.087051488	0.043527674	-1.999911331	0.045509839	0	0.482195956	0	1	PRJNA827465;Yang2025_Silkie_Wenchang	CRA004775	0.367822707
ENSGALG00010003503	NUFIP1	2	0.0320702	0.215658759	0.148708081	0.881783985	0.067012938	2.976495777	66.40344637	0.5	CRA004775;Yang2025_Silkie_Wenchang	PRJNA827465	0.971900875
ENSGALG00010003503	NUFIP1	2	0.049988608	0.176544037	0.28315093	0.777061142	0.041690682	2.283041624	56.19878371	0.5	CRA004775;PRJNA827465	Yang2025_Silkie_Wenchang	0.944218792
ENSGALG00010003506	DYNC1I1	2	0.312082635	0.180907085	1.725099016	0.08450963	0.017128589	1.339370898	25.33808212	1	PRJNA827465;Yang2025_Silkie_Wenchang	CRA004775	0.500072565
ENSGALG00010003506	DYNC1I1	2	0.530981672	0.208345616	2.548561769	0.010816813	0	0.000276554	0	1	CRA004775;Yang2025_Silkie_Wenchang	PRJNA827465	0.165679345
ENSGALG00010003506	DYNC1I1	2	0.240891483	0.173911151	1.385141099	0.166009327	0	0.780186422	0	1	CRA004775;PRJNA827465	Yang2025_Silkie_Wenchang	0.66529196
ENSGALG00010003507	WDFY3	2	0.151089471	0.235872808	0.640554848	0.521811943	0.092755892	5.267398134	81.01529494	0.5	PRJNA827465;Yang2025_Silkie_Wenchang	CRA004775	0.86956507
ENSGALG00010003507	WDFY3	2	0.292456846	0.184597597	1.584293894	0.113126855	0.012189028	1.196943665	16.45387921	1	CRA004775;Yang2025_Silkie_Wenchang	PRJNA827465	0.567482422
ENSGALG00010003507	WDFY3	2	-0.045267743	0.049602564	-0.912608938	0.361448252	0	0.102624715	0	0.5	CRA004775;PRJNA827465	Yang2025_Silkie_Wenchang	0.81550034
ENSGALG00010003509	GPALPP1	2	-0.009197826	0.057303937	-0.160509492	0.872479745	0	0.516219941	0	0.5	PRJNA827465;Yang2025_Silkie_Wenchang	CRA004775	0.969629321
ENSGALG00010003509	GPALPP1	2	-0.085085627	0.193878835	-0.438859801	0.660763126	0.045815394	2.172820871	53.976878	0.5	CRA004775;Yang2025_Silkie_Wenchang	PRJNA827465	0.91192683
ENSGALG00010003509	GPALPP1	2	-0.101687606	0.129222273	-0.786920118	0.43132863	0.016047502	1.442285021	30.66557683	1	CRA004775;PRJNA827465	Yang2025_Silkie_Wenchang	0.842503929
ENSGALG00010003511	UBXN2B	2	-0.204763991	0.068245456	-3.000404768	0.00269621	0	0.609794815	0	1	PRJNA827465;Yang2025_Silkie_Wenchang	CRA004775	0.070599041
ENSGALG00010003511	UBXN2B	2	-0.258452951	0.107487135	-2.404501242	0.016194551	0	0.241169259	0	1	CRA004775;Yang2025_Silkie_Wenchang	PRJNA827465	0.210262467
ENSGALG00010003511	UBXN2B	2	-0.159894721	0.083739567	-1.909428572	0.056206828	0	0.044366023	0	1	CRA004775;PRJNA827465	Yang2025_Silkie_Wenchang	0.410231561
ENSGALG00010003512	ENSGALG00010003512	2	-0.982988856	0.285497817	-3.443069609	0.000575151	0	0.113969913	0	1	PRJNA827465;Yang2025_Silkie_Wenchang	CRA004775	0.02583448
ENSGALG00010003512	ENSGALG00010003512	2	-1.195054043	0.351748941	-3.397463087	0.000680138	0	0.052596938	0	1	CRA004775;Yang2025_Silkie_Wenchang	PRJNA827465	0.028867656
ENSGALG00010003512	ENSGALG00010003512	2	-1.03244928	0.288759379	-3.575465791	0.000349605	0	0.35040308	0	1	CRA004775;PRJNA827465	Yang2025_Silkie_Wenchang	0.018545851
ENSGALG00010003517	GTF2F2	2	0.139331589	0.457112095	0.304808363	0.760512112	0.394502496	17.52015816	94.29228897	0.5	PRJNA827465;Yang2025_Silkie_Wenchang	CRA004775	0.93932961
ENSGALG00010003517	GTF2F2	2	0.109410252	0.512167283	0.213622103	0.830841761	0.477058866	10.9904959	90.90122949	0.5	CRA004775;Yang2025_Silkie_Wenchang	PRJNA827465	0.957336479
ENSGALG00010003517	GTF2F2	2	-0.318430486	0.091156002	-3.493247598	0.000477184	0	0.179355633	0	1	CRA004775;PRJNA827465	Yang2025_Silkie_Wenchang	0.022813698
ENSGALG00010003522	ENSGALG00010003522	2	-0.770965813	0.379127942	-2.033524116	0.0419996	0	0.035113632	0	1	PRJNA827465;Yang2025_Silkie_Wenchang	CRA004775	0.354529664
ENSGALG00010003522	ENSGALG00010003522	2	-0.955231274	0.371505258	-2.571245639	0.010133342	0	0.385304644	0	1	CRA004775;Yang2025_Silkie_Wenchang	PRJNA827465	0.159819725
ENSGALG00010003522	ENSGALG00010003522	2	-1.002856343	0.356235271	-2.815151737	0.004875421	0	0.204382401	0	1	CRA004775;PRJNA827465	Yang2025_Silkie_Wenchang	0.103802093
ENSGALG00010003525	NOL10	2	-0.126822425	0.051069741	-2.483318329	0.013016471	0	0.949899052	0	1	PRJNA827465;Yang2025_Silkie_Wenchang	CRA004775	0.185626401
ENSGALG00010003525	NOL10	2	-0.18252146	0.082424684	-2.214402903	0.026801075	0	0.208788162	0	1	CRA004775;Yang2025_Silkie_Wenchang	PRJNA827465	0.279174307
ENSGALG00010003525	NOL10	2	-0.092419091	0.059617818	-1.550192459	0.121095329	0	6.16219E-05	0	1	CRA004775;PRJNA827465	Yang2025_Silkie_Wenchang	0.583289761
ENSGALG00010003528	GDAP1	2	0.066343235	0.380533994	0.174342465	0.861596328	0.235218391	5.144595901	80.56212734	0.5	PRJNA827465;Yang2025_Silkie_Wenchang	CRA004775	0.965482137
ENSGALG00010003528	GDAP1	2	0.06267792	0.483738492	0.129569843	0.896906764	0.327481297	3.184657744	68.59945149	0.5	CRA004775;Yang2025_Silkie_Wenchang	PRJNA827465	0.975244266
ENSGALG00010003528	GDAP1	2	-0.307662525	0.163537108	-1.881301008	0.059930984	0	0.183378126	0	1	CRA004775;PRJNA827465	Yang2025_Silkie_Wenchang	0.423882851
ENSGALG00010003531	PPM1K	2	-0.08954032	0.088304129	-1.013999249	0.310583111	0	0.000166968	0	1	PRJNA827465;Yang2025_Silkie_Wenchang	CRA004775	0.794899969
ENSGALG00010003531	PPM1K	2	-0.10901387	0.143418955	-0.760107824	0.447190136	0	0.161844564	0	1	CRA004775;Yang2025_Silkie_Wenchang	PRJNA827465	0.84650962
ENSGALG00010003531	PPM1K	2	-0.101450782	0.104781764	-0.968210292	0.332939356	0	0.167305028	0	1	CRA004775;PRJNA827465	Yang2025_Silkie_Wenchang	0.802178578
ENSGALG00010003539	ENSGALG00010003539	2	0.247044231	0.206628005	1.195598977	0.231853091	0.051243854	2.443491856	59.07496079	1	PRJNA827465;Yang2025_Silkie_Wenchang	CRA004775	0.744183098
ENSGALG00010003539	ENSGALG00010003539	2	-0.062340922	0.587660202	-0.106083281	0.915516276	0.594574056	6.927546612	85.56487519	0.5	CRA004775;Yang2025_Silkie_Wenchang	PRJNA827465	0.980165711
ENSGALG00010003539	ENSGALG00010003539	2	-0.231373987	0.371581859	-0.622672988	0.533499437	0.2011275	3.247953193	69.21137897	0.5	CRA004775;PRJNA827465	Yang2025_Silkie_Wenchang	0.873379507
ENSGALG00010003541	CNR1	2	-0.062570879	0.160516728	-0.389809084	0.696677726	0.038583071	3.957465614	74.73130287	0.5	PRJNA827465;Yang2025_Silkie_Wenchang	CRA004775	0.92106851
ENSGALG00010003541	CNR1	2	-0.225430294	0.120458941	-1.871428496	0.061285712	0	0.029466588	0	1	CRA004775;Yang2025_Silkie_Wenchang	PRJNA827465	0.427873874
ENSGALG00010003541	CNR1	2	0.082754011	0.10199178	0.811379224	0.417147926	0	0.175936577	0	0.5	CRA004775;PRJNA827465	Yang2025_Silkie_Wenchang	0.838112595
ENSGALG00010003543	SOX11	2	0.212209294	0.197195389	1.076137203	0.2818659	0.069585205	9.499200472	89.47279823	1	PRJNA827465;Yang2025_Silkie_Wenchang	CRA004775	0.779420863
ENSGALG00010003543	SOX11	2	0.430249531	0.088527602	4.860060824	1.1735E-06	0	0.780075541	0	1	CRA004775;Yang2025_Silkie_Wenchang	PRJNA827465	0.000252033
ENSGALG00010003543	SOX11	2	0.292270143	0.34816209	0.839465731	0.401208012	0.189827056	3.89418273	74.32067088	1	CRA004775;PRJNA827465	Yang2025_Silkie_Wenchang	0.832472134
ENSGALG00010003552	PI15	2	-0.571820429	0.413531288	-1.38277428	0.166734088	0.311637663	11.11090868	90.99983603	1	PRJNA827465;Yang2025_Silkie_Wenchang	CRA004775	0.665699904
ENSGALG00010003552	PI15	2	0.354887095	1.389125474	0.255475191	0.798356103	3.72362221	27.97311589	96.42513904	0.5	CRA004775;Yang2025_Silkie_Wenchang	PRJNA827465	0.949922217
ENSGALG00010003552	PI15	2	0.74788985	0.974197654	0.767698267	0.442666466	1.778432654	15.14511434	93.39721063	0.5	CRA004775;PRJNA827465	Yang2025_Silkie_Wenchang	0.845623604
ENSGALG00010003558	TPT1	2	-0.209870882	0.283379489	-0.740600115	0.458935938	0.136613769	6.111353477	83.63701259	0.5	PRJNA827465;Yang2025_Silkie_Wenchang	CRA004775	0.850089963
ENSGALG00010003558	TPT1	2	-0.384162784	0.232568872	-1.651823739	0.098570488	0.028270448	1.273918726	21.50205667	1	CRA004775;Yang2025_Silkie_Wenchang	PRJNA827465	0.53598695
ENSGALG00010003558	TPT1	2	0.036936928	0.073529568	0.502341154	0.615427565	0	0.021040156	0	0.5	CRA004775;PRJNA827465	Yang2025_Silkie_Wenchang	0.898864379
ENSGALG00010003560	ADGRL3	2	-0.133566193	0.078474048	-1.70204287	0.088747332	0	0.735447331	0	0.5	PRJNA827465;Yang2025_Silkie_Wenchang	CRA004775	0.511667361
ENSGALG00010003560	ADGRL3	2	0.020343361	0.174635822	0.116490198	0.907264054	0	0.000289931	0	1	CRA004775;Yang2025_Silkie_Wenchang	PRJNA827465	0.978196186
ENSGALG00010003560	ADGRL3	2	-0.154857696	0.083855296	-1.846725288	0.064786965	0	0.216960193	0	0.5	CRA004775;PRJNA827465	Yang2025_Silkie_Wenchang	0.438869713
ENSGALG00010003571	RPL7	2	-0.394596849	0.385329495	-1.024050464	0.30581144	0.262222627	7.803339344	87.18497356	1	PRJNA827465;Yang2025_Silkie_Wenchang	CRA004775	0.79225794
ENSGALG00010003571	RPL7	2	-0.553091145	0.242500214	-2.280786213	0.022561101	0.060912713	2.043890324	51.07369568	1	CRA004775;Yang2025_Silkie_Wenchang	PRJNA827465	0.255317863
ENSGALG00010003571	RPL7	2	-0.125062864	0.125945825	-0.992989359	0.32071514	0.016731078	1.682673166	40.57075254	1	CRA004775;PRJNA827465	Yang2025_Silkie_Wenchang	0.798348216
ENSGALG00010003582	RDH10	2	-0.034727188	0.116083374	-0.299157294	0.764820032	0.017280383	2.785702637	64.10241399	0.5	PRJNA827465;Yang2025_Silkie_Wenchang	CRA004775	0.940711384
ENSGALG00010003582	RDH10	2	0.031721518	0.23707681	0.133802703	0.89355859	0.07961065	2.927995543	65.84694255	0.5	CRA004775;Yang2025_Silkie_Wenchang	PRJNA827465	0.974381724
ENSGALG00010003582	RDH10	2	0.114638761	0.094236049	1.216506446	0.223792057	0	0.807327573	0	1	CRA004775;PRJNA827465	Yang2025_Silkie_Wenchang	0.737337515
ENSGALG00010003583	RSAD2	2	-0.005196394	0.31164229	-0.016674225	0.98669651	0	0.513802733	0	0.5	PRJNA827465;Yang2025_Silkie_Wenchang	CRA004775	0.996259695
ENSGALG00010003583	RSAD2	2	-0.279338985	0.384289934	-0.726896442	0.467289397	0	0.010153086	0	1	CRA004775;Yang2025_Silkie_Wenchang	PRJNA827465	0.852885838
ENSGALG00010003583	RSAD2	2	0.026742092	0.314997725	0.084896142	0.932343959	0	0.34376319	0	0.5	CRA004775;PRJNA827465	Yang2025_Silkie_Wenchang	0.984224753
ENSGALG00010003585	PNPLA6	2	0.184083834	0.142413048	1.292605113	0.19614767	0.033658708	5.85578562	82.9228721	1	PRJNA827465;Yang2025_Silkie_Wenchang	CRA004775	0.707524953
ENSGALG00010003585	PNPLA6	2	-0.155051844	0.498587102	-0.310982461	0.755813955	0.476118433	23.22506536	95.69430706	0.5	CRA004775;Yang2025_Silkie_Wenchang	PRJNA827465	0.938501783
ENSGALG00010003585	PNPLA6	2	-0.290647163	0.355727072	-0.817051008	0.413899279	0.233543037	12.46647094	91.97848369	0.5	CRA004775;PRJNA827465	Yang2025_Silkie_Wenchang	0.836666158
ENSGALG00010003589	SLC25A30	2	0.349318226	0.100740337	3.467511002	0.000525302	0	0.516891818	0	1	PRJNA827465;Yang2025_Silkie_Wenchang	CRA004775	0.024203932
ENSGALG00010003589	SLC25A30	2	0.424750178	0.15867015	2.676938149	0.007429835	0	0.221203772	0	1	CRA004775;Yang2025_Silkie_Wenchang	PRJNA827465	0.132915249
ENSGALG00010003589	SLC25A30	2	0.285944423	0.126663298	2.257516001	0.023975851	0	0.078270063	0	1	CRA004775;PRJNA827465	Yang2025_Silkie_Wenchang	0.264463641
ENSGALG00010003595	SNX13	2	-0.016642417	0.050154036	-0.331826083	0.740020588	0	0.037401124	0	0.5	PRJNA827465;Yang2025_Silkie_Wenchang	CRA004775	0.935072094
ENSGALG00010003595	SNX13	2	0.085928487	0.122515713	0.701367069	0.483073968	0	0.831277196	0	1	CRA004775;Yang2025_Silkie_Wenchang	PRJNA827465	0.85691916
ENSGALG00010003595	SNX13	2	0.032535059	0.107238418	0.303389962	0.761592695	0.011432484	1.452062955	31.1324625	0.5	CRA004775;PRJNA827465	Yang2025_Silkie_Wenchang	0.939738254
ENSGALG00010003596	CRISPLD1	2	0.107175924	0.139086641	0.7705695	0.440962145	0.026363912	2.899845396	65.51540295	0.5	PRJNA827465;Yang2025_Silkie_Wenchang	CRA004775	0.844888742
ENSGALG00010003596	CRISPLD1	2	0.238418462	0.136619212	1.745131292	0.080962018	0	0.475129693	0	1	CRA004775;Yang2025_Silkie_Wenchang	PRJNA827465	0.489847627
ENSGALG00010003596	CRISPLD1	2	-0.006187859	0.075605051	-0.081844524	0.934770349	0	0.001558214	0	0.5	CRA004775;PRJNA827465	Yang2025_Silkie_Wenchang	0.984413039
ENSGALG00010003597	RNF144A	2	-0.380612262	0.296548645	-1.283473277	0.199326316	0.158905228	10.28049605	90.27284291	1	PRJNA827465;Yang2025_Silkie_Wenchang	CRA004775	0.711181159
ENSGALG00010003597	RNF144A	2	-0.682556687	0.145483482	-4.691643885	2.71019E-06	0	0.010417004	0	1	CRA004775;Yang2025_Silkie_Wenchang	PRJNA827465	0.00051708

ENSGALG00010003597	RNF144A	2	-0.10578703	0.108415036	-0.975759765	0.329183528	0	0.576735293	0	1	CRA004775;PRJNA827465	Yang2025_Silkie_Wenchang	0.800761292
ENSGALG00010003604	ODC	2	0.119874166	0.458026242	0.261718991	0.793538098	0.385660157	11.90264745	91.59850777	0.5	PRJNA827465;Yang2025_Silkie_Wenchang	CRA004775	0.948743674
ENSGALG00010003604	ODC	2	0.067210223	0.557623214	0.120529816	0.904063462	0.540758921	7.603654203	86.84842875	0.5	CRA004775;Yang2025_Silkie_Wenchang	PRJNA827465	0.977427069
ENSGALG00010003604	ODC	2	-0.330360139	0.098079742	-3.368281063	0.000756384	0	0.344170194	0	1	CRA004775;PRJNA827465	Yang2025_Silkie_Wenchang	0.031031501
ENSGALG00010003617	COG3	2	0.087298714	0.05054548	1.727131966	0.084143966	0	0.000374332	0	1	PRJNA827465;Yang2025_Silkie_Wenchang	CRA004775	0.499714459
ENSGALG00010003617	COG3	2	0.060245027	0.081495018	0.739247973	0.459756435	0	0.512846567	0	0.5	CRA004775;Yang2025_Silkie_Wenchang	PRJNA827465	0.850098539
ENSGALG00010003617	COG3	2	0.072483601	0.057844611	1.253074404	0.21017863	0	0.557310067	0	0.5	CRA004775;PRJNA827465	Yang2025_Silkie_Wenchang	0.721895543
ENSGALG00010003619	HNF4G	2	0.355877152	0.283090274	1.257115432	0.20871184	0	0.098335804	0	1	PRJNA827465;Yang2025_Silkie_Wenchang	CRA004775	0.721011841
ENSGALG00010003619	HNF4G	2	1.271736289	1.149596128	1.106246149	0.268619993	2.165565792	5.302126828	81.13964391	1	CRA004775;Yang2025_Silkie_Wenchang	PRJNA827465	0.771398294
ENSGALG00010003619	HNF4G	2	1.327372366	1.050012761	1.264148795	0.206176626	1.808439331	5.083090374	80.32692857	1	CRA004775;PRJNA827465	Yang2025_Silkie_Wenchang	0.718403416
ENSGALG00010003627	MCOLN1	2	0.058663184	0.098767082	0.593954814	0.552542336	0.011586207	2.211951979	54.79106195	0.5	PRJNA827465;Yang2025_Silkie_Wenchang	CRA004775	0.879228216
ENSGALG00010003627	MCOLN1	2	-0.10239756	0.295299642	-0.346758158	0.728773014	0.157309729	10.19403657	90.19034321	0.5	CRA004775;Yang2025_Silkie_Wenchang	PRJNA827465	0.932236477
ENSGALG00010003627	MCOLN1	2	-0.188435027	0.191485971	-0.984067008	0.325082567	0.062875301	6.60002065	84.84853225	1	CRA004775;PRJNA827465	Yang2025_Silkie_Wenchang	0.799914637
ENSGALG00010003634	CYP4F11	2	0.503047405	0.208900319	2.408073892	0.016036935	0	0.155959282	0	1	PRJNA827465;Yang2025_Silkie_Wenchang	CRA004775	0.209265194
ENSGALG00010003634	CYP4F11	2	0.636541446	0.289112613	2.201707626	0.027685968	0.02765903	1.189076226	15.90110223	1	CRA004775;Yang2025_Silkie_Wenchang	PRJNA827465	0.283891176
ENSGALG00010003634	CYP4F11	2	0.688297658	0.226336021	3.041043373	0.002357599	0	0.70187748	0	1	CRA004775;PRJNA827465	Yang2025_Silkie_Wenchang	0.064662038
ENSGALG00010003639	ENSGALG00010003639	2	-0.841307808	0.502035744	-1.675792645	0.093778815	0	0.398885688	0	1	PRJNA827465;Yang2025_Silkie_Wenchang	CRA004775	0.524592766
ENSGALG00010003639	ENSGALG00010003639	2	-0.168554556	0.635675258	-0.265158277	0.790887535	0.035918781	1.033770704	3.266749943	0.5	CRA004775;Yang2025_Silkie_Wenchang	PRJNA827465	0.947777321
ENSGALG00010003639	ENSGALG00010003639	2	-0.368220385	1.025047448	-0.359222771	0.719428443	1.170930212	2.094156188	52.24806986	0.5	CRA004775;PRJNA827465	Yang2025_Silkie_Wenchang	0.928449671
ENSGALG00010003652	MYCN	2	-0.119309057	0.205164095	-0.581529908	0.560883367	0.073657846	7.839698048	87.24440669	0.5	PRJNA827465;Yang2025_Silkie_Wenchang	CRA004775	0.881354866
ENSGALG00010003652	MYCN	2	-0.059975718	0.378897263	-0.158290185	0.874228142	0.204336703	2.812530291	64.44482738	0.5	CRA004775;Yang2025_Silkie_Wenchang	PRJNA827465	0.970094827
ENSGALG00010003652	MYCN	2	0.08626295	0.077787557	1.107657004	0.268009975	0	0.686355787	0	1	CRA004775;PRJNA827465	Yang2025_Silkie_Wenchang	0.771398294
ENSGALG00010003660	IGFBP7	2	-0.088337987	0.083807854	-1.054053801	0.291858286	0	0.942235975	0	1	PRJNA827465;Yang2025_Silkie_Wenchang	CRA004775	0.785409532
ENSGALG00010003660	IGFBP7	2	-0.021159612	0.297186169	-0.071199855	0.943238697	0.108337269	2.471901332	59.54531084	0.5	CRA004775;Yang2025_Silkie_Wenchang	PRJNA827465	0.985827325
ENSGALG00010003660	IGFBP7	2	0.014597639	0.145554698	0.100289711	0.920114326	0.027230684	1.304768314	23.35804073	0.5	CRA004775;PRJNA827465	Yang2025_Silkie_Wenchang	0.981364549
ENSGALG00010003665	RDH14	2	0.049352635	0.220580084	0.223740212	0.822959455	0.09010772	13.37893159	92.5255616	0.5	PRJNA827465;Yang2025_Silkie_Wenchang	CRA004775	0.955822987
ENSGALG00010003665	RDH14	2	0.290911743	0.095479289	3.04685705	0.002312477	0	0.247540707	0	1	CRA004775;Yang2025_Silkie_Wenchang	PRJNA827465	0.063780538
ENSGALG00010003665	RDH14	2	0.057975095	0.293487876	0.197538298	0.843406314	0.131360902	3.424755097	70.80083184	0.5	CRA004775;PRJNA827465	Yang2025_Silkie_Wenchang	0.96057435
ENSGALG00010003666	SLC25A13	2	0.013579571	0.119289874	0.113836748	0.909367207	0.015918413	2.117370993	52.77162089	0.5	PRJNA827465;Yang2025_Silkie_Wenchang	CRA004775	0.978607662
ENSGALG00010003666	SLC25A13	2	-0.149313075	0.136496495	-1.093896769	0.274000256	0	0.042560246	0	1	CRA004775;Yang2025_Silkie_Wenchang	PRJNA827465	0.774526342
ENSGALG00010003666	SLC25A13	2	0.091693759	0.08211343	1.116671881	0.264134645	0	0.721539529	0	0.5	CRA004775;PRJNA827465	Yang2025_Silkie_Wenchang	0.768790489
ENSGALG00010003671	ENSGALG00010003671	2	0.700805134	0.38242053	1.832551022	0.066869368	0.049068339	1.201558322	16.7747431	1	PRJNA827465;Yang2025_Silkie_Wenchang	CRA004775	0.445476026
ENSGALG00010003671	ENSGALG00010003671	2	0.910011136	0.427489063	2.128735482	0.033276151	0	0.492891615	0	1	CRA004775;Yang2025_Silkie_Wenchang	PRJNA827465	0.313333153
ENSGALG00010003671	ENSGALG00010003671	2	0.341493039	0.42416249	0.805099571	0.420762224	0	0.006419504	0	1	CRA004775;PRJNA827465	Yang2025_Silkie_Wenchang	0.839211146
ENSGALG00010003674	AKIRIN2	2	-0.078167113	0.066973663	-1.167132119	0.243157019	0.003814876	1.473685214	32.14290337	1	PRJNA827465;Yang2025_Silkie_Wenchang	CRA004775	0.7550024
ENSGALG00010003674	AKIRIN2	2	0.070908005	0.284694975	0.249066586	0.803309276	0.136920156	6.247709914	83.99413523	0.5	CRA004775;Yang2025_Silkie_Wenchang	PRJNA827465	0.950977845
ENSGALG00010003674	AKIRIN2	2	0.125997525	0.203918983	0.617880314	0.536654236	0.066682431	4.296678202	76.72620678	0.5	CRA004775;PRJNA827465	Yang2025_Silkie_Wenchang	0.874599474
ENSGALG00010003676	POLR2B	2	-0.134091987	0.095773554	-1.40009409	0.161485145	0.01420383	4.215920086	76.28038531	1	PRJNA827465;Yang2025_Silkie_Wenchang	CRA004775	0.657909261
ENSGALG00010003676	POLR2B	2	-0.199296626	0.073193339	-2.722879293	0.00647157	0.000526692	1.038985912	3.752304193	1	CRA004775;Yang2025_Silkie_Wenchang	PRJNA827465	0.122697593
ENSGALG00010003676	POLR2B	2	-0.051770315	0.044116017	-1.173503835	0.24059382	0	0.028752736	0	1	CRA004775;PRJNA827465	Yang2025_Silkie_Wenchang	0.753158164
ENSGALG00010003678	ZNFY1	2	0.412476418	0.230017623	1.793238332	0.072934823	0	0.772238236	0	1	PRJNA827465;Yang2025_Silkie_Wenchang	CRA004775	0.466486145
ENSGALG00010003678	ZNFY1	2	0.426665888	0.329721736	1.294018083	0.195659175	0.030975257	1.1449143	12.65721808	0.5	CRA004775;Yang2025_Silkie_Wenchang	PRJNA827465	0.706714537
ENSGALG00010003678	ZNFY1	2	0.181121513	0.260724811	0.694684606	0.487252977	0	0.214831657	0	0.5	CRA004775;PRJNA827465	Yang2025_Silkie_Wenchang	0.85768885
ENSGALG00010003680	TRAPPC5	2	-0.056980329	0.065776164	-0.866276253	0.386338685	0	0.459436947	0	1	PRJNA827465;Yang2025_Silkie_Wenchang	CRA004775	0.826894808
ENSGALG00010003680	TRAPPC5	2	-0.451940443	0.207053881	-2.182719014	0.029056509	0	0.967080582	0	1	CRA004775;Yang2025_Silkie_Wenchang	PRJNA827465	0.290070603
ENSGALG00010003680	TRAPPC5	2	-0.284810356	0.29769312	-0.956724683	0.338706236	0.140775858	4.097861552	75.59702817	1	CRA004775;PRJNA827465	Yang2025_Silkie_Wenchang	0.804450436
ENSGALG00010003681	NT5C1B	2	-0.306188709	0.192108265	-1.593834129	0.110973191	0.05881946	4.815963488	79.23572298	1	PRJNA827465;Yang2025_Silkie_Wenchang	CRA004775	0.563777127
ENSGALG00010003681	NT5C1B	2	-0.336779636	0.195274638	-1.724646064	0.084591277	0.048305668	2.68124537	62.70389829	1	CRA004775;Yang2025_Silkie_Wenchang	PRJNA827465	0.500108811
ENSGALG00010003681	NT5C1B	2	-0.12653373	0.087935614	-1.438936119	0.150168623	0	0.0011237	0	1	CRA004775;PRJNA827465	Yang2025_Silkie_Wenchang	0.640555393
ENSGALG00010003691	ERICH1	2	0.420247713	0.282181929	1.489279325	0.13641383	0.132694348	5.985268209	83.29231097	1	PRJNA827465;Yang2025_Silkie_Wenchang	CRA004775	0.61829693
ENSGALG00010003691	ERICH1	2	0.266647349	0.496986717	0.536528121	0.591593606	0.412018404	5.629432707	82.23622073	0.5	CRA004775;Yang2025_Silkie_Wenchang	PRJNA827465	0.892776493
ENSGALG00010003691	ERICH1	2	0.068039404	0.164997069	0.41236735	0.68007019	0.009337529	1.108638333	9.799258242	0.5	CRA004775;PRJNA827465	Yang2025_Silkie_Wenchang	0.917701868
ENSGALG00010003692	ZNFY2	2	0.166171909	0.278309252	0.597076479	0.550456321	0.099870851	2.814241974	64.4664528	0.5	PRJNA827465;Yang2025_Silkie_Wenchang	CRA004775	0.878740242
ENSGALG00010003692	ZNFY2	2	0.004024418	0.468101896	0.008597312	0.993140422	0.357474616	5.326451373	81.22577435	0.5	CRA004775;Yang2025_Silkie_Wenchang	PRJNA827465	0.99794341
ENSGALG00010003692	ZNFY2	2	-0.241916149	0.193104999	-1.252769994	0.210289425	0	0.870022679	0	1	CRA004775;PRJNA827465	Yang2025_Silkie_Wenchang	0.721897568
ENSGALG00010003695	NOA1	2	-0.116054196	0.046001979	-2.52280878	0.011642169	0	0.136922655	0	1	PRJNA827465;Yang2025_Silkie_Wenchang	CRA004775	0.172635381
ENSGALG00010003695	NOA1	2	-0.169959767	0.126264477	-1.34606163	0.178282642	0	0.000814663	0	1	CRA004775;Yang2025_Silkie_Wenchang	PRJNA827465	0.684689586
ENSGALG00010003695	NOA1	2	-0.112906902	0.047512147	-2.376379712	0.017483461	0	0.068510178	0	1	CRA004775;PRJNA827465	Yang2025_Silkie_Wenchang	0.220149566
ENSGALG00010003700	REST	2	0.249201251	0.251665092	0.990209841	0.322071564	0.109702215	6.694813844	85.06306488	1	PRJNA827465;Yang2025_Silkie_Wenchang	CRA004775	0.798513866
ENSGALG00010003700	REST	2	0.473052725	0.166482598	2.841454489	0.004490826	0	0.467435785	0	1	CRA004775;Yang2025_Silkie_Wenchang	PRJNA827465	0.098057189
ENSGALG00010003700	REST	2	0.033369608	0.04497881	0.7418962	0.458150229	0	0.495501334	0	1	CRA004775;PRJNA827465	Yang2025_Silkie_Wenchang	0.850026426
ENSGALG00010003701	SARAF	2	-0.226515007	0.178973978	-1.265630957	0.205645237	0.052413461	5.264096054	81.00338615	1	PRJNA827465;Yang2025_Silkie_Wenchang	CRA004775	0.71825498
ENSGALG00010003701	SARAF	2	-0.293442727	0.151833221	-1.93266483	0.0532775	0.021440027	1.834454053	45.48786882	1	CRA004775;Yang2025_Silkie_Wenchang	PRJNA827465	0.400004977
ENSGALG00010003701	SARAF	2	-0.072567054	0.070420301	-1.030484855	0.302782458	0	0.07216244	0	1	CRA004775;PRJNA827465	Yang2025_Silkie_Wenchang	0.791977836
ENSGALG00010003703	TDRP	2	-0.268479409	0.181783421	-1.476919115	0.13969732	0.035529627	2.162124577	53.74919602	1	PRJNA827465;Yang2025_Silkie_Wenchang	CRA004775	0.623568857
ENSGALG00010003703	TDRP	2	-0.411135078	0.156432875	-2.628188466	0.008584094	0	0.258473562	0	1	CRA004775;Yang2025_Silkie_Wenchang	PRJNA827465	0.144612369
ENSGALG00010003703	TDRP	2	-0.112633812	0.160248438	-0.702869951	0.482136799	0	0.139506222	0	1	CRA004775;PRJNA827465	Yang2025_Silkie_Wenchang	0.85691916
ENSGALG00010003713	SPERT	2	0.275952154	0.267480665	1.031671408	0.302226076	0.026297012	1.224240049	18.31667322	1	PRJNA827465;Yang2025_Silkie_Wenchang	CRA004775	0.791647454
ENSGALG00010003713	SPERT	2	1.003142493	1.085466071	0.924158314	0.355403896	2.029001599	6.758684723	85.20422181	1	CRA004775;Yang2025_Silkie_Wenchang	PRJNA827465	0.812301302
ENSGALG00010003713	SPERT	2	1.239805391	0.810778412	1.529154418	0.126226168	0.99487571	3.75885188	73.3961318	1	CRA004775;PRJNA827465	Yang2025_Silkie_Wenchang	0.596627648
ENSGALG00010003714	ORC3	2	-0.070428027	0.210108654	-0.335198125	0.737475637	0.074774581	6.238673414	83.9709513	0.5	PRJNA827465;Yang2025_Silkie_Wenchang	CRA004775	0.933968189
ENSGALG00010003714	ORC3	2	-0.346873539	0.128654337	-2.696166696	0.007014256	0	0.356524306	0	1	CRA004775;Yang2025_Silkie_Wenchang	PRJNA827465	0.12854434
ENSGALG00010003714	ORC3	2	-0.129514771	0.294352268	-0.43999923	0.659937665	0.143400042	5.168197768	80.65089525	0.5	CRA004775;PRJNA827465	Yang2025_Silkie_Wenchang	0.911773501
ENSGALG00010003716	YLEC8	2	-0.078597764	0.397843684	-0.197559412	0.843389793	0	0.161497997	0	0.5	PRJNA827465;Yang2025_Silkie_Wenchang	CRA004775	0.96057435
ENSGALG00010003716	YLEC8	2	-0.291785371	0.456102269	-0.639736724	0.522343776	0	0.028732081	0	1	CRA004775;Yang2025_Silkie_Wenchang	PRJNA827465	0.869568211
ENSGALG00010003716	YLEC8	2	-0.081706541	0.45929773	-0.177894501	0.858805829	0	0.248863928	0	0.5	CRA004775;PRJNA827465	Yang2025_Silkie_Wenchang	0.964472541
ENSGALG00010003717	LEPROTL1	2	-0.462072321	0.453956366	-1.017878271	0.308735809	0.397573574	27.82194093	96.40571446	1	PRJNA827465;Yang2025_Silkie_Wenchang	CRA004775	0.793670558
ENSGALG00010003717	LEPROTL1	2	-0.633654628	0.302528533	-2.094528481	0.036212929	0.152846862	6.033379389	83.42554089	1	CRA004775;Yang2025_Silkie_Wenchang	PRJNA827465	0.328246479
ENSGALG00010003717	LEPROTL1	2	-0.11911469	0.142460536	-0.836124115	0.403085084	0.025513108	2.253988253	55.63419647	1	CRA004775;PRJNA827465	Yang2025_Silkie_Wenchang	0.833046091
ENSGALG00010003719	RPGR	2	0.280912038	0.410514313	0.68429292	0.493790217	0.311579593	12.62658763	92.08020386	0.5	PRJNA827465;Yang2025_Silkie_Wenchang	CRA004775	0.860144347
ENSGALG00010003719	RPGR	2	0.594636613	0.16141983	3.683789111	0.000229792	0	0.692766007	0	1	CRA004775;Yang2025_Silkie_Wenchang	PRJNA827465	0.0140037
ENSGALG00010003719	RPGR	2	0.125449675	0.272558388	0.46026716	0.64532447	0.12165421	4.894391067	79.56844914	0.5	CRA004775;PRJNA827465	Yang2025_Silkie_Wenchang	0.906966303
ENSGALG00010003722	MHCY11	2	0.183062816	0.580090235	0.315576448	0.752324017	0.450732313	3.027622556	66.97078379	0.5	PRJNA827465;Yang2025_Silkie_Wenchang	CRA004775	0.93806821
ENSGALG00010003722	MHCY11	2	-0.691076943	0.357057715	-1.935476851	0.052931807	0	0.860606504	0	1	CRA004775;Yang2025_Silkie_Wenchang	PRJNA827465	0.399458294
ENSGALG00010003722	MHCY11	2	-0.133803793	0.913899909	-0.14640968	0.883597986	1.415523739	6.535417988	84.69875987	0.5	CRA004775;PRJNA827465	Yang2025_Silkie_Wenchang	0.972001076
ENSGALG00010003724	ENSGALG00010003724	2	0.284167079	0.128264934	2.215469739	0.026727839	0	0.0369672	0	1	PRJNA827465;Yang2025_Silkie_Wenchang	CRA004775	0.278911632
ENSGALG00010003724	ENSGALG00010003724	2	0.117504471	0.155298899	0.75663428	0.449268998	0.010843852	1.28687772	22.2925392	0.5	CRA004775;Yang2025_Silkie_Wenchang	PRJNA827465	0.847263197
ENSGALG00010003724	ENSGALG00010003724	2	0.141423426	0.180109662	0.785207323	0.432332023	0.027881427	1.746209995	42.73311899	0.5	CRA004775;PRJNA827465	Yang2025_Silkie_Wenchang	0.842762754
ENSGALG00010003726	ENPEP	2	0.022486143	0.288340411	0.077984708	0.937840217	0	0.444932224	0	0.5	PRJNA827465;Yang2025_Silkie_Wenchang	CRA004775	0.985348964
ENSGALG00010003726	ENPEP	2	0.188186608	0.724079786	0.259897614	0.794942752	0.466566517	1.535139713	34.85934918	0.5	CRA004775;Yang2025_Silkie_Wenchang	PRJNA827465	0.948905846
ENSGALG00010003726	ENPEP	2	0.253311027	0.339400408	0.746348624	0.455456846	0	0.931551278	0	1	CRA004775;PRJNA827465	Yang2025_Silkie_Wenchang	0.84907427
ENSGALG00010003727	FBXO25	2	-0.019984352	0.116970451	-0.170849576	0.864342046	0.017961822	2.726676377	63.32531398	0.5	PRJNA827465;Yang2025_Silkie_Wenchang	CRA004775	0.966643645
ENSGALG00010003727	FBXO25	2	-0.203960334	0.114059161	-1.788197734	0.073744112	0	0.664971672	0	1	CRA004775;Yang2025_Silkie_Wenchang	PRJNA827465	0.469294822
ENSGALG00010003727	FBXO25	2	-0.092857421	0.230966474	-0.402038526	0.687655674	0.077033196	2.918083332	65.73093068	0.5	CRA004775;PRJNA827465	Yang2025_Silkie_Wenchang	0.919564865
ENSGALG00010003729	RARS2	2	0.090920858	0.090738606	1.002008534	0.316339471	0.004442021	1.354258682	26.15886365	1	PRJNA827465;Yang2025_Silkie_Wenchang	CRA004775	0.796883862
ENSGALG00010003729	RARS2	2	0.070526181	0.184767852	0.38170158	0.702682728	0.035175984	1.828024999	45.29615291	0.5	CRA004775;Yang2025_Silkie_Wenchang	PRJNA827465	0.923272306
ENSGALG00010003729	RARS2	2	-0.010630374	0.091774995	-0.115830833	0.907786614	0	0.556847691	0	0.5	CRA004775;PRJNA827465	Yang2025_Silkie_Wenchang	0.978196186
ENSGALG00010003731	ENSGALG00010003731	2	0.106203393	0.460101973	0.230825771	0.81745016	0	0.369746543	0	0.5	PRJNA827465;Yang2025_Silkie_Wenchang	CRA004775	0.954888783
ENSGALG00010003731	ENSGALG00010003731	2	-0.19631811	0.444683271	-0.441478515	0.658866612	0.007039702	1.013893757	1.370336606	0.5	CRA004775;Yang2025_Silkie_Wenchang	PRJNA827465	0.911329736
ENSGALG00010003731	ENSGALG00010003731	2	-0.276437442	0.371677562	-0.743756067	0.457024063	0	0.275977789	0	1	CRA004775;PRJNA827465	Yang2025_Silkie_Wenchang	0.849541335
ENSGALG00010003733	STAU2	2	0.167858573	0.226017307	0.742680173	0.457675337	0.093488539	11.15316759	91.03393729	0.5	PRJNA827465;Yang2025_Silkie_Wenchang	CRA004775	0.849573219
ENSGALG00010003733	STAU2	2	0.209288697	0.223653802	0.935770798	0.34939121	0.074018721	3.728993816	73.18311455	0.5	CRA004775;Yang2025_Silkie_Wenchang	PRJNA827465	0.809903518
ENSGALG00010003733	STAU2	2	-0.042323475	0.041875601	-1.010695333	0.312162272	0	0.000300081	0	1	CRA004775;PRJNA827465	Yang2025_Silkie_Wenchang	0.796054997
ENSGALG00010003734	ZC3H13	2	0.275475488	0.373667536	0.737220822	0.460988073	0.251967346	9.378797799	89.33765264	0.5	PRJNA827465;Yang2025_Silkie_Wenchang	CRA004775	0.85014477
ENSGALG00010003734	ZC3H13	2	0.407694922	0.262483823	1.553219228	0.120370769	0.089549032	2.832675661	64.697688	1	CRA004775;Yang2025_Silkie_Wenchang	PRJNA827465	0.581775014
ENSGALG00010003734	ZC3H13	2	-0.033827146	0.075275974	-0.449375076	0.653161108	0.004454198	1.213748314	17.61059619	0.5	CRA004775;PRJNA827465	Yang2025_Silkie_Wenchang	0.909597225
ENSGALG00010003737	SRP72	2	-0.031387886	0.039986834	-0.78495551	0.432479654	0	0.011955431	0	1	PRJNA827465;Yang2025_Silkie_Wenchang	CRA004775	0.842762754
ENSGALG00010003737	SRP72	2	-0.077794203	0.069784124	-1.114783682	0.264943131	0	0.733755914	0	1	CRA004775;Yang2025_Silkie_Wenchang	PRJNA827465	0.769274038
ENSGALG00010003737	SRP72	2	-0.048504075	0.048842398	-0.993073174	0.320674296	0.000684068	1.07308917	6.811099422	1	CRA004775;PRJNA827465	Yang2025_Silkie_Wenchang	0.798348216
ENSGALG00010003740	PITX2	2	-0.013107655	0.176211272	-0.07438602	0.940703232	0.048338422	4.498959395	77.7726378	0.5	PRJNA827465;Yang2025_Silkie_Wenchang	CRA004775	0.985565295
ENSGALG00010003740	PITX2	2	0.197005561	0.121192466	1.625559467	0.104043404	0	0.878753577	0	1	CRA004775;Yang2025_Silkie_Wenchang	PRJNA827465	0.548383482
ENSGALG00010003740	PITX2	2	0.091140361	0.391190099	0.23298228	0.815775166	0.216870205	2.68821072	62.80053522	0.5	CRA004775;PRJNA827465	Yang2025_Silkie_Wenchang	0.954210034
ENSGALG00010003742	ZFHX4	2	0.128370969	0.315175486	0.40729998	0.683787678	0.150876382	3.528235936	71.65722423	0.5	PRJNA827465;Yang2025_Silkie_Wenchang	CRA004775	0.918800744
ENSGALG00010003742	ZFHX4	2	0.275810895	0.295538444	0.933248789	0.35069153	0.042216119	1.309305267	23.62361743	0.5	CRA004775;Yang2025_Silkie_Wenchang	PRJNA827465	0.810111037
ENSGALG00010003742	ZFHX4	2	-0.116161645	0.095497005	-1.216390453	0.223836219	0	0.015094424	0	1	CRA004775;PRJNA827465	Yang2025_Silkie_Wenchang	0.7374212
ENSGALG00010003743	ARL9	2	0.354960961	0.351919284	1.00864311	0.313145824	0.216411877	7.749089968	87.09525887	1	PRJNA827465;Yang2025_Silkie_Wenchang	CRA004775	0.796531448
ENSGALG00010003743	ARL9	2	0.228860218	0.526477071	0.434701207	0.663779322	0.479528596	7.268414025	86.24184043	0.5	CRA004775;Yang2025_Silkie_Wenchang	PRJNA827465	0.913119244
ENSGALG00010003743	ARL9	2	-0.028359033	0.124475969	-0.22782737	0.819780448	0	0.976893889	0	0.5	CRA004775;PRJNA827465	Yang2025_Silkie_Wenchang	0.955256039
ENSGALG00010003746	HOPX	2	-0.230529557	0.344819665	-0.668551072	0.503781893	0.170099234	3.466630822	71.15354789	0.5	PRJNA827465;Yang2025_Silkie_Wenchang	CRA004775	0.863240168
ENSGALG00010003746	HOPX	2	-0.10911534	0.319669355	-0.34133813	0.732849046	0.078084757	1.561167149	35.94535982	0.5	CRA004775;Yang2025_Silkie_Wenchang	PRJNA827465	0.932985777
ENSGALG00010003746	HOPX	2	-0.544329782	0.20169329	-2.698799651	0.006959006	0	0.004288701	0	1	CRA004775;PRJNA827465	Yang2025_Silkie_Wenchang	0.128156372
ENSGALG00010003749	MHCY15	2	0.624051379	0.77756845	0.802567774	0.422224601	0	0.143983033	0	1	PRJNA827465;Yang2025_Silkie_Wenchang	CRA004775	0.839649223
ENSGALG00010003749	MHCY15	2	-0.460554922	1.189900306	-0.387053369	0.698716698	2.091858487	3.581039894	72.07515053	0.5	CRA004775;Yang2025_Silkie_Wenchang	PRJNA827465	0.921748813
ENSGALG00010003749	MHCY15	2	-0.788457029	0.882192384	-0.893747263	0.371457131	0.894987062	2.179545529	54.11887539	0.5	CRA004775;PRJNA827465	Yang2025_Silkie_Wenchang	0.821526338
ENSGALG00010003753	SLCO5A1	2	0.083895866	0.116162095	0.722231002	0.470152466	0	0.218201923	0	1	PRJNA827465;Yang2025_Silkie_Wenchang	CRA004775	0.853663603
ENSGALG00010003753	SLCO5A1	2	0.072710413	0.165141456	0.440291704	0.659725849	0	0.668724056	0	1	CRA004775;Yang2025_Silkie_Wenchang	PRJNA827465	0.911619908
ENSGALG00010003753	SLCO5A1	2	0.160693501	0.146562798	1.09641398	0.272897649	0	0.369638694	0	1	CRA004775;PRJNA827465	Yang2025_Silkie_Wenchang	0.773799118
ENSGALG00010003754	FAM110C	2	-0.034010366	0.484344234	-0.070219409	0.944019026	0.428069655	11.404788	91.23175284	0.5	PRJNA827465;Yang2025_Silkie_Wenchang	CRA004775	0.985827325
ENSGALG00010003754	FAM110C	2	-0.214884931	0.379405377	-0.56637292	0.571140299	0.196387314	2.819462734	64.53224978	0.5	CRA004775;Yang2025_Silkie_Wenchang	PRJNA827465	0.885354083
ENSGALG00010003754	FAM110C	2	0.416410735	0.189679558	2.195337964	0.028139364	0	0.15894883	0	1	CRA004775;PRJNA827465	Yang2025_Silkie_Wenchang	0.285752749
ENSGALG00010003755	SLC35A1	2	0.077894638	0.092598558	0.841207898	0.400231477	0.006308314	1.487274008	32.7628941	0.5	PRJNA827465;Yang2025_Silkie_Wenchang	CRA004775	0.832246851
ENSGALG00010003755	SLC35A1	2	-0.020112896	0.121560181	-0.165456286	0.868584854	0	0.233656025	0	0.5	CRA004775;Yang2025_Silkie_Wenchang	PRJNA827465	0.968254536
ENSGALG00010003755	SLC35A1	2	0.138111314	0.0771764	1.789553719	0.073525684	0	0.047790101	0	1	CRA004775;PRJNA827465	Yang2025_Silkie_Wenchang	0.468547845
ENSGALG00010003756	MHCY7	2	-0.616717052	0.369729375	-1.668022665	0.09531188	0	0.002268115	0	1	PRJNA827465;Yang2025_Silkie_Wenchang	CRA004775	0.52850665
ENSGALG00010003756	MHCY7	2	-0.749393798	0.401278902	-1.867513577	0.061829903	0	0.160180506	0	1	CRA004775;Yang2025_Silkie_Wenchang	PRJNA827465	0.429075295
ENSGALG00010003756	MHCY7	2	-0.752995248	0.379540107	-1.983962823	0.047259985	0	0.1403695	0	1	CRA004775;PRJNA827465	Yang2025_Silkie_Wenchang	0.375333084
ENSGALG00010003761	UGT8	2	0.104499473	0.1632955	0.639940922	0.522211008	0	0.796028457	0	0.5	PRJNA827465;Yang2025_Silkie_Wenchang	CRA004775	0.869568211
ENSGALG00010003761	UGT8	2	0.58453686	0.441463024	1.324090019	0.185473093	0.209228459	1.834411526	45.47768846	1	CRA004775;Yang2025_Silkie_Wenchang	PRJNA827465	0.693919245
ENSGALG00010003761	UGT8	2	0.467631431	0.610953293	0.765412735	0.444025812	0.557242005	3.354380792	70.18823855	0.5	CRA004775;PRJNA827465	Yang2025_Silkie_Wenchang	0.846013379
ENSGALG00010003762	EVI5L	2	0.224020017	0.328151997	0.682671502	0.49481444	0.208963297	33.60642846	97.07243883	0.5	PRJNA827465;Yang2025_Silkie_Wenchang	CRA004775	0.860403132
ENSGALG00010003762	EVI5L	2	0.054672745	0.521898872	0.104758959	0.916567076	0.513979757	17.13396209	94.16363831	0.5	CRA004775;Yang2025_Silkie_Wenchang	PRJNA827465	0.980604962
ENSGALG00010003762	EVI5L	2	-0.231397892	0.182884699	-1.265266553	0.205775792	0.044600039	2.444571176	59.09302991	1	CRA004775;PRJNA827465	Yang2025_Silkie_Wenchang	0.71832154
ENSGALG00010003764	HDAC9	2	-0.361916475	0.130595865	-2.77127056	0.005583801	0.020982555	2.510266437	60.16359119	1	PRJNA827465;Yang2025_Silkie_Wenchang	CRA004775	0.112129505
ENSGALG00010003764	HDAC9	2	-0.346086624	0.244067587	-1.417995023	0.156192218	0.068546896	2.003295862	50.082261	0.5	CRA004775;Yang2025_Silkie_Wenchang	PRJNA827465	0.649132948
ENSGALG00010003764	HDAC9	2	-0.231523609	0.090133834	-2.568664832	0.010209915	0	0.531461899	0	1	CRA004775;PRJNA827465	Yang2025_Silkie_Wenchang	0.16043518
ENSGALG00010003765	ENSGALG00010003765	2	0.185537598	0.632864949	0.293170918	0.769391512	0.470754691	2.33171837	57.113174	0.5	PRJNA827465;Yang2025_Silkie_Wenchang	CRA004775	0.941790377
ENSGALG00010003765	ENSGALG00010003765	2	-0.949029479	0.504378751	-1.881581008	0.059892927	0	0.623880878	0	1	CRA004775;Yang2025_Silkie_Wenchang	PRJNA827465	0.423882851
ENSGALG00010003765	ENSGALG00010003765	2	-0.245909027	1.037896039	-0.236930307	0.812710858	1.794599854	5.836146987	82.86540757	0.5	CRA004775;PRJNA827465	Yang2025_Silkie_Wenchang	0.95348724
ENSGALG00010003767	ENSGALG00010003767	2	-1.560597171	1.232072159	-1.266644295	0.205282507	0	0.175131243	0	1	PRJNA827465;Yang2025_Silkie_Wenchang	CRA004775	0.718123692
ENSGALG00010003767	ENSGALG00010003767	2	-1.109738447	1.405167728	-0.789755148	0.429670777	0	0.017543409	0	1	CRA004775;Yang2025_Silkie_Wenchang	PRJNA827465	0.842012722
ENSGALG00010003767	ENSGALG00010003767	2	-1.725525009	1.254683687	-1.375266951	0.169048698	0	0.068979868	0	1	CRA004775;PRJNA827465	Yang2025_Silkie_Wenchang	0.67030648
ENSGALG00010003768	ARSJ	2	-0.16096328	0.056566571	-2.845554845	0.004431423	0	0.271585332	0	1	PRJNA827465;Yang2025_Silkie_Wenchang	CRA004775	0.099760941
ENSGALG00010003768	ARSJ	2	-0.118730259	0.113732624	-1.043941965	0.296512241	0	0.116671686	0	1	CRA004775;Yang2025_Silkie_Wenchang	PRJNA827465	0.788355067
ENSGALG00010003768	ARSJ	2	-0.178585373	0.06321816	-2.824906223	0.004729446	0	0.026601314	0	1	CRA004775;PRJNA827465	Yang2025_Silkie_Wenchang	0.101352998
ENSGALG00010003771	SPARCL1	2	0.192889375	0.603354208	0.319693506	0.749199485	0.613691748	6.259074126	84.02319609	0.5	PRJNA827465;Yang2025_Silkie_Wenchang	CRA004775	0.937362187
ENSGALG00010003771	SPARCL1	2	0.728131433	0.333859919	2.18094408	0.029187179	0	0.229440739	0	1	CRA004775;Yang2025_Silkie_Wenchang	PRJNA827465	0.290710666
ENSGALG00010003771	SPARCL1	2	-0.125979127	0.390381467	-0.322707755	0.746916578	0.140017679	1.615033746	38.08178917	0.5	CRA004775;PRJNA827465	Yang2025_Silkie_Wenchang	0.936729365
ENSGALG00010003772	ENSGALG00010003772	2	0.211230315	0.738609086	0.285983911	0.774890444	0.748437597	3.048422816	67.19615157	0.5	PRJNA827465;Yang2025_Silkie_Wenchang	CRA004775	0.943713409
ENSGALG00010003772	ENSGALG00010003772	2	0.22857108	0.7621318	0.299910173	0.764245674	0.754461581	2.808946918	64.39946966	0.5	CRA004775;Yang2025_Silkie_Wenchang	PRJNA827465	0.940483053
ENSGALG00010003772	ENSGALG00010003772	2	-0.443706103	0.351867671	-1.261002754	0.207307854	0	0.002826214	0	1	CRA004775;PRJNA827465	Yang2025_Silkie_Wenchang	0.717877239
ENSGALG00010003775	NCOA2	2	-0.044300313	0.100811917	-0.439435283	0.660346165	0.008954186	1.507066588	33.64593126	0.5	PRJNA827465;Yang2025_Silkie_Wenchang	CRA004775	0.911923994
ENSGALG00010003775	NCOA2	2	0.147926896	0.159178902	0.929312202	0.352727313	0	0.082371337	0	1	CRA004775;Yang2025_Silkie_Wenchang	PRJNA827465	0.811035834
ENSGALG00010003775	NCOA2	2	-0.092669043	0.067655296	-1.369721935	0.170773264	0	0.813195441	0	1	CRA004775;PRJNA827465	Yang2025_Silkie_Wenchang	0.672602625
ENSGALG00010003777	ENSGALG00010003777	2	0.412215685	0.850319302	0.484777523	0.627834168	1.02900529	3.433041338	70.8713091	0.5	PRJNA827465;Yang2025_Silkie_Wenchang	CRA004775	0.901878949
ENSGALG00010003777	ENSGALG00010003777	2	1.152352544	0.462650188	2.49076424	0.012746867	0	0.028641417	0	1	CRA004775;Yang2025_Silkie_Wenchang	PRJNA827465	0.183311423
ENSGALG00010003777	ENSGALG00010003777	2	0.246525203	0.769541179	0.320353491	0.748700378	0.625339634	2.115064366	52.720115	0.5	CRA004775;PRJNA827465	Yang2025_Silkie_Wenchang	0.937249951
ENSGALG00010003778	LCA5	2	0.23341144	0.355208869	0.657125196	0.511100419	0.227821284	10.17761839	90.17451861	0.5	PRJNA827465;Yang2025_Silkie_Wenchang	CRA004775	0.865628707
ENSGALG00010003778	LCA5	2	0.359271075	0.286760782	1.25285866	0.21025671	0.106988842	2.722845669	63.27371723	1	CRA004775;Yang2025_Silkie_Wenchang	PRJNA827465	0.721897568
ENSGALG00010003778	LCA5	2	-0.089925105	0.115059064	-0.781556022	0.434475541	0	0.158471089	0	0.5	CRA004775;PRJNA827465	Yang2025_Silkie_Wenchang	0.842762754
ENSGALG00010003786	ENSGALG00010003786	2	-0.491244491	0.451402447	-1.09022991	0.275307166	0	0.002159548	0	1	PRJNA827465;Yang2025_Silkie_Wenchang	CRA004775	0.775135547
ENSGALG00010003786	ENSGALG00010003786	2	-0.127087303	0.477801362	-0.265983551	0.790251877	0	0.655454252	0	0.5	CRA004775;Yang2025_Silkie_Wenchang	PRJNA827465	0.947390191
ENSGALG00010003786	ENSGALG00010003786	2	-0.152280635	0.477361793	-0.319004657	0.749722981	0	0.732374145	0	0.5	CRA004775;PRJNA827465	Yang2025_Silkie_Wenchang	0.937362187
ENSGALG00010003787	SMIM8	2	-0.280255969	0.154075692	-1.818949936	0.068919068	0	0.006732354	0	1	PRJNA827465;Yang2025_Silkie_Wenchang	CRA004775	0.452044654
ENSGALG00010003787	SMIM8	2	-0.152768409	0.234805722	-0.65061621	0.515294264	0	0.775623815	0	0.5	CRA004775;Yang2025_Silkie_Wenchang	PRJNA827465	0.867163796
ENSGALG00010003787	SMIM8	2	-0.203130479	0.166164567	-1.222465674	0.221531577	0	0.852091584	0	1	CRA004775;PRJNA827465	Yang2025_Silkie_Wenchang	0.734941806
ENSGALG00010003788	NUDT9	2	0.29374025	0.382485236	0.767978009	0.44250025	0.271193021	13.63864156	92.66789148	0.5	PRJNA827465;Yang2025_Silkie_Wenchang	CRA004775	0.845535782
ENSGALG00010003788	NUDT9	2	0.296366054	0.411006519	0.721073854	0.470864077	0.29179355	7.148337175	86.01073263	0.5	CRA004775;Yang2025_Silkie_Wenchang	PRJNA827465	0.853762555
ENSGALG00010003788	NUDT9	2	-0.094979472	0.117509404	-0.808271239	0.418934455	0	0.039787233	0	1	CRA004775;PRJNA827465	Yang2025_Silkie_Wenchang	0.838264828
ENSGALG00010003790	ELOVL6	2	0.493368402	0.383416313	1.28676946	0.198174654	0.277119163	17.29960425	94.21952095	1	PRJNA827465;Yang2025_Silkie_Wenchang	CRA004775	0.710234722
ENSGALG00010003790	ELOVL6	2	0.426888189	0.527115891	0.809856422	0.4180227	0.464331759	5.521233517	81.88810531	0.5	CRA004775;Yang2025_Silkie_Wenchang	PRJNA827465	0.838112595
ENSGALG00010003790	ELOVL6	2	0.099551444	0.103823532	0.958852405	0.337633105	0	0.456893698	0	0.5	CRA004775;PRJNA827465	Yang2025_Silkie_Wenchang	0.80415385
ENSGALG00010003791	ITGB8	2	0.137940143	0.208896	0.660329271	0.50904255	0.051755996	2.277101011	56.08451294	0.5	PRJNA827465;Yang2025_Silkie_Wenchang	CRA004775	0.865193604
ENSGALG00010003791	ITGB8	2	0.638895233	0.303903015	2.102299753	0.035527026	0.086085504	1.778019693	43.75765332	1	CRA004775;Yang2025_Silkie_Wenchang	PRJNA827465	0.325417923
ENSGALG00010003791	ITGB8	2	0.435262267	0.524525052	0.830248598	0.40639977	0.469343521	6.264574135	84.03722299	0.5	CRA004775;PRJNA827465	Yang2025_Silkie_Wenchang	0.833618122
ENSGALG00010003793	TRAM1L1	2	-0.213645762	0.212075856	-1.00740257	0.313741357	0.087164578	32.18975658	96.89342168	1	PRJNA827465;Yang2025_Silkie_Wenchang	CRA004775	0.796531448
ENSGALG00010003793	TRAM1L1	2	-0.221620068	0.22473134	-0.986155993	0.324056767	0.089344117	8.185408999	87.78313949	0.5	CRA004775;Yang2025_Silkie_Wenchang	PRJNA827465	0.799600434
ENSGALG00010003793	TRAM1L1	2	-0.001091616	0.042826964	-0.025488984	0.979664936	0	0.031306877	0	0.5	CRA004775;PRJNA827465	Yang2025_Silkie_Wenchang	0.994472942

ENSGALG00010003795	LCP1	2	-0.046356905	0.211831627	-0.218838452	0.826775893	0.075078255	6.08589759	83.56857004	0.5	PRJNA827465;Yang2025_Silkie_Wenchang	CRA004775	0.956501588
ENSGALG00010003795	LCP1	2	-0.296692809	0.128730009	-2.304768035	0.021179568	0	0.891898502	0	1	CRA004775;Yang2025_Silkie_Wenchang	PRJNA827465	0.246205433
ENSGALG00010003795	LCP1	2	-0.167560285	0.441830894	-0.379240762	0.704509084	0.289816118	3.105742592	67.80158142	0.5	CRA004775;PRJNA827465	Yang2025_Silkie_Wenchang	0.924152671
ENSGALG00010003796	ENSGALG00010003796	2	-0.044590174	0.408801965	-0.109075243	0.913142811	0.157993452	1.862641177	46.3127943	0.5	PRJNA827465;Yang2025_Silkie_Wenchang	CRA004775	0.979129568
ENSGALG00010003796	ENSGALG00010003796	2	-1.177126904	0.667112548	-1.764510212	0.077646118	0.649966391	3.706015553	73.01684287	1	CRA004775;Yang2025_Silkie_Wenchang	PRJNA827465	0.480057473
ENSGALG00010003796	ENSGALG00010003796	2	-0.744091337	1.079824006	-0.689085752	0.490769306	2.141779792	12.17521787	91.78659462	0.5	CRA004775;PRJNA827465	Yang2025_Silkie_Wenchang	0.858802583
ENSGALG00010003797	ENSGALG00010003797	2	0.213547512	0.246598648	0.865971951	0.386505544	0.090942574	3.964142693	74.77386468	0.5	PRJNA827465;Yang2025_Silkie_Wenchang	CRA004775	0.826981729
ENSGALG00010003797	ENSGALG00010003797	2	0.320374985	0.21447859	1.493738771	0.135243911	0.035053884	1.46450825	31.71769431	0.5	CRA004775;Yang2025_Silkie_Wenchang	PRJNA827465	0.616533955
ENSGALG00010003797	ENSGALG00010003797	2	-0.029244627	0.157321777	-0.185890521	0.852530616	0	0.003500783	0	1	CRA004775;PRJNA827465	Yang2025_Silkie_Wenchang	0.962607579
ENSGALG00010003803	LACTB2	2	-0.210900063	0.064433776	-3.273129046	0.001063639	0.000144241	1.016357361	1.609410387	1	PRJNA827465;Yang2025_Silkie_Wenchang	CRA004775	0.039400759
ENSGALG00010003803	LACTB2	2	-0.294447435	0.095649309	-3.078406305	0.00208111	0	0.001138181	0	1	CRA004775;Yang2025_Silkie_Wenchang	PRJNA827465	0.059400037
ENSGALG00010003803	LACTB2	2	-0.176591753	0.075175919	-2.349046831	0.018821539	0	0.284745618	0	1	CRA004775;PRJNA827465	Yang2025_Silkie_Wenchang	0.229831303
ENSGALG00010003804	PSME1	2	-0.056383365	0.254007836	-0.221974903	0.82433342	0.103587807	5.049411824	80.19571319	0.5	PRJNA827465;Yang2025_Silkie_Wenchang	CRA004775	0.95619211
ENSGALG00010003804	PSME1	2	-0.180679213	0.416070527	-0.43425141	0.664105884	0.292802632	6.293094447	84.10956631	0.5	CRA004775;Yang2025_Silkie_Wenchang	PRJNA827465	0.91312035
ENSGALG00010003804	PSME1	2	-0.37438581	0.135198515	-2.769156232	0.005620168	0.00194506	1.037960497	3.657219809	1	CRA004775;PRJNA827465	Yang2025_Silkie_Wenchang	0.112499196
ENSGALG00010003806	EGF	2	-0.157443353	0.164243787	-0.958595485	0.337762568	0.016022274	1.421647236	29.65906204	0.5	PRJNA827465;Yang2025_Silkie_Wenchang	CRA004775	0.80415385
ENSGALG00010003806	EGF	2	0.03443378	0.190675743	0.180588151	0.856690857	0	0.130764355	0	1	CRA004775;Yang2025_Silkie_Wenchang	PRJNA827465	0.963627293
ENSGALG00010003806	EGF	2	-0.269358444	0.18198353	-1.480125393	0.138839786	0	0.735744791	0	0.5	CRA004775;PRJNA827465	Yang2025_Silkie_Wenchang	0.622497285
ENSGALG00010003808	TWIST1	2	-0.173324957	0.141993613	-1.220653191	0.222217358	0.012374061	1.429331363	30.03721699	1	PRJNA827465;Yang2025_Silkie_Wenchang	CRA004775	0.73559121
ENSGALG00010003808	TWIST1	2	-0.219521613	0.130649096	-1.680238277	0.092910965	8.34948E-05	1.00186233	0.185886813	0.5	CRA004775;Yang2025_Silkie_Wenchang	PRJNA827465	0.522019616
ENSGALG00010003808	TWIST1	2	-0.004587982	0.151295482	-0.030324646	0.975808141	0	0.001582724	0	0.5	CRA004775;PRJNA827465	Yang2025_Silkie_Wenchang	0.992834072
ENSGALG00010003810	HMGN2P46	2	0.031434223	0.192410816	0.163370355	0.870226843	0.064530261	7.378628553	86.44734597	0.5	PRJNA827465;Yang2025_Silkie_Wenchang	CRA004775	0.96881514
ENSGALG00010003810	HMGN2P46	2	0.253142816	0.119401951	2.120089449	0.033998503	0	0.048400755	0	1	CRA004775;Yang2025_Silkie_Wenchang	PRJNA827465	0.317170413
ENSGALG00010003810	HMGN2P46	2	-0.012594995	0.207797248	-0.060611942	0.951668262	0.057143446	2.180932373	54.14805097	0.5	CRA004775;PRJNA827465	Yang2025_Silkie_Wenchang	0.987713746
ENSGALG00010003812	BG8	2	-0.392612625	0.250760286	-1.565689014	0.117421439	0	0.992998346	0	1	PRJNA827465;Yang2025_Silkie_Wenchang	CRA004775	0.5768585
ENSGALG00010003812	BG8	2	-0.468265273	0.282963628	-1.654860296	0.09795284	0	0.660553306	0	1	CRA004775;Yang2025_Silkie_Wenchang	PRJNA827465	0.534130658
ENSGALG00010003812	BG8	2	-0.12789056	0.296076292	-0.431951369	0.665776758	0	0.000162962	0	1	CRA004775;PRJNA827465	Yang2025_Silkie_Wenchang	0.913532078
ENSGALG00010003814	TPP1	2	0.162235001	0.059193096	2.740775742	0.006129433	0	0.057206945	0	1	PRJNA827465;Yang2025_Silkie_Wenchang	CRA004775	0.118495743
ENSGALG00010003814	TPP1	2	-0.147058045	0.257468155	-0.57116984	0.56788452	0.08882352	3.02917214	66.98768626	0.5	CRA004775;Yang2025_Silkie_Wenchang	PRJNA827465	0.88383817
ENSGALG00010003814	TPP1	2	-0.083774906	0.281950871	-0.297125898	0.766370393	0.138253681	7.002438556	85.71926063	0.5	CRA004775;PRJNA827465	Yang2025_Silkie_Wenchang	0.941152991
ENSGALG00010003816	KLHL8	2	-0.16858815	0.080286279	-2.099837642	0.035743126	0	0.360349303	0	1	PRJNA827465;Yang2025_Silkie_Wenchang	CRA004775	0.326169508
ENSGALG00010003816	KLHL8	2	-0.243775772	0.132876174	-1.834608606	0.066563703	0	0.001493421	0	1	CRA004775;Yang2025_Silkie_Wenchang	PRJNA827465	0.444449088
ENSGALG00010003816	KLHL8	2	-0.149383175	0.089008368	-1.678304838	0.093287604	0	0.113491092	0	1	CRA004775;PRJNA827465	Yang2025_Silkie_Wenchang	0.523343073
ENSGALG00010003817	BF2	2	0.055194968	0.367021182	0.150386328	0.880459827	0.182512696	3.018502941	66.87099467	0.5	PRJNA827465;Yang2025_Silkie_Wenchang	CRA004775	0.971623434
ENSGALG00010003817	BF2	2	0.021564199	0.405843486	0.053134274	0.957624923	0.241318581	3.654647387	72.63757912	0.5	CRA004775;Yang2025_Silkie_Wenchang	PRJNA827465	0.988770061
ENSGALG00010003817	BF2	2	-0.310091327	0.173045729	-1.791961747	0.073139094	0	0.04861106	0	1	CRA004775;PRJNA827465	Yang2025_Silkie_Wenchang	0.46748834
ENSGALG00010003818	GAB2	2	-0.007897775	0.133283899	-0.059254984	0.952749015	0	0.937186461	0	0.5	PRJNA827465;Yang2025_Silkie_Wenchang	CRA004775	0.987758913
ENSGALG00010003818	GAB2	2	0.16000515	0.240641174	0.664911776	0.506106923	0	0.245528445	0	0.5	CRA004775;Yang2025_Silkie_Wenchang	PRJNA827465	0.863929781
ENSGALG00010003818	GAB2	2	-0.072369076	0.143429039	-0.504563628	0.613865361	0	0.008514045	0	1	CRA004775;PRJNA827465	Yang2025_Silkie_Wenchang	0.898256044
ENSGALG00010003819	PHIP	2	0.215455548	0.253968015	0.848357017	0.396239173	0.108865081	5.791809816	82.7342397	0.5	PRJNA827465;Yang2025_Silkie_Wenchang	CRA004775	0.831365249
ENSGALG00010003819	PHIP	2	0.568390771	0.17109145	3.322145965	0.00089328	0	0.310877533	0	1	CRA004775;Yang2025_Silkie_Wenchang	PRJNA827465	0.035143708
ENSGALG00010003819	PHIP	2	0.28934639	0.355533995	0.813836072	0.415738868	0.208824043	4.936799866	79.74396315	0.5	CRA004775;PRJNA827465	Yang2025_Silkie_Wenchang	0.837701508
ENSGALG00010003820	SH3YL1	2	-0.438457227	0.192083705	-2.282630241	0.022451804	0	0.009290098	0	1	PRJNA827465;Yang2025_Silkie_Wenchang	CRA004775	0.225447309
ENSGALG00010003820	SH3YL1	2	-0.428750028	0.249837457	-1.716115881	0.086140835	0	0.035766055	0	1	CRA004775;Yang2025_Silkie_Wenchang	PRJNA827465	0.504895124
ENSGALG00010003820	SH3YL1	2	-0.407971538	0.21692212	-1.880728154	0.060008908	0	0.015731951	0	1	CRA004775;PRJNA827465	Yang2025_Silkie_Wenchang	0.424082409
ENSGALG00010003821	RUBCNL	2	0.017977324	0.14665284	0.122584223	0.902436349	0.023990921	2.043407772	51.0621417	0.5	PRJNA827465;Yang2025_Silkie_Wenchang	CRA004775	0.976923607
ENSGALG00010003821	RUBCNL	2	-0.235724822	0.165695257	-1.422640731	0.154840033	0	0.339603502	0	1	CRA004775;Yang2025_Silkie_Wenchang	PRJNA827465	0.647505492
ENSGALG00010003821	RUBCNL	2	-0.043380255	0.247166439	-0.175510297	0.86067868	0.080681359	2.358624903	57.60241492	0.5	CRA004775;PRJNA827465	Yang2025_Silkie_Wenchang	0.965114242
ENSGALG00010003822	ENSGALG00010003822	2	0.449831251	0.398670895	1.128327293	0.259181721	0.266893062	5.511447087	81.85594483	1	PRJNA827465;Yang2025_Silkie_Wenchang	CRA004775	0.766314541
ENSGALG00010003822	ENSGALG00010003822	2	0.224667235	0.682597545	0.329135721	0.742053106	0.835210848	9.621476005	89.60658428	0.5	CRA004775;Yang2025_Silkie_Wenchang	PRJNA827465	0.935481151
ENSGALG00010003822	ENSGALG00010003822	2	-0.101293601	0.270588906	-0.374344987	0.708147666	0.110333339	3.438477681	70.91736248	0.5	CRA004775;PRJNA827465	Yang2025_Silkie_Wenchang	0.925061251
ENSGALG00010003823	PTPN13	2	-0.001242855	0.078902477	-0.015751791	0.987432409	0.004016535	1.300652957	23.11554019	0.5	PRJNA827465;Yang2025_Silkie_Wenchang	CRA004775	0.99656051
ENSGALG00010003823	PTPN13	2	0.156161891	0.137116104	1.138902624	0.254743771	0	0.055758709	0	1	CRA004775;Yang2025_Silkie_Wenchang	PRJNA827465	0.764394153
ENSGALG00010003823	PTPN13	2	-0.035138159	0.062952288	-0.558171277	0.576727432	0	0.643755757	0	0.5	CRA004775;PRJNA827465	Yang2025_Silkie_Wenchang	0.838711945
ENSGALG00010003824	SP4	2	-0.078156045	0.067319106	-1.160978656	0.245650579	0	0.004090968	0	1	PRJNA827465;Yang2025_Silkie_Wenchang	CRA004775	0.75661406
ENSGALG00010003824	SP4	2	0.039086517	0.20019099	0.195246137	0.845200262	0.028268091	1.405897716	28.87107018	0.5	CRA004775;Yang2025_Silkie_Wenchang	PRJNA827465	0.960863849
ENSGALG00010003824	SP4	2	0.016218869	0.176893916	0.091686978	0.926946744	0.034435051	1.58988863	37.10251264	0.5	CRA004775;PRJNA827465	Yang2025_Silkie_Wenchang	0.982896295
ENSGALG00010003826	ENSGALG00010003826	2	-0.004753437	0.113246047	-0.041974418	0.966519091	0.013447983	1.711484347	41.57118634	0.5	PRJNA827465;Yang2025_Silkie_Wenchang	CRA004775	0.990881287
ENSGALG00010003826	ENSGALG00010003826	2	0.427677609	0.29547577	1.447420237	0.147779261	0.114875726	2.758993335	63.75489613	1	CRA004775;Yang2025_Silkie_Wenchang	PRJNA827465	0.637081344
ENSGALG00010003826	ENSGALG00010003826	2	0.298980599	0.423868723	0.705361313	0.480583413	0.31464027	7.219956762	86.14950154	0.5	CRA004775;PRJNA827465	Yang2025_Silkie_Wenchang	0.856719542
ENSGALG00010003829	ENSGALG00010003829	2	0.276343136	0.317545713	0.870243011	0.384167364	0.171298685	6.099951183	83.60642618	0.5	PRJNA827465;Yang2025_Silkie_Wenchang	CRA004775	0.832567461
ENSGALG00010003829	ENSGALG00010003829	2	0.34863935	0.331814104	1.050706844	0.293393252	0.133712279	2.469970718	59.51369007	0.5	CRA004775;Yang2025_Silkie_Wenchang	PRJNA827465	0.786979185
ENSGALG00010003829	ENSGALG00010003829	2	-0.005454831	0.086240857	-0.063251117	0.949566541	0	0.006984144	0	1	CRA004775;PRJNA827465	Yang2025_Silkie_Wenchang	0.98723759
ENSGALG00010003830	DMB2	2	-0.832376188	0.28688867	-2.901390944	0.003715101	0	0.608606769	0	1	PRJNA827465;Yang2025_Silkie_Wenchang	CRA004775	0.087190443
ENSGALG00010003830	DMB2	2	-0.54760685	0.246859258	-2.218293574	0.026534672	0	0.000206438	0	1	CRA004775;Yang2025_Silkie_Wenchang	PRJNA827465	0.277582281
ENSGALG00010003830	DMB2	2	-0.726358597	0.229216311	-3.168878314	0.001530284	0	0.985634999	0	1	CRA004775;PRJNA827465	Yang2025_Silkie_Wenchang	0.048927262
ENSGALG00010003831	IRAK1BP1	2	0.011309915	0.10902926	0.103732845	0.917381361	0	0.066364184	0	0.5	PRJNA827465;Yang2025_Silkie_Wenchang	CRA004775	0.980871069
ENSGALG00010003831	IRAK1BP1	2	-0.313480592	0.271474559	-1.154732853	0.248199842	0.075104075	2.037099911	50.91060608	1	CRA004775;Yang2025_Silkie_Wenchang	PRJNA827465	0.758364066
ENSGALG00010003831	IRAK1BP1	2	-0.233895129	0.304436822	-0.768287905	0.442316158	0.144906713	4.181107418	76.08328145	0.5	CRA004775;PRJNA827465	Yang2025_Silkie_Wenchang	0.894265867
ENSGALG00010003833	ACP1	2	-0.075791891	0.117887563	-0.642916763	0.520278112	0.014415838	1.541649145	35.13439789	1	PRJNA827465;Yang2025_Silkie_Wenchang	CRA004775	0.869331539
ENSGALG00010003833	ACP1	2	-0.431818601	0.166155093	-2.598888751	0.009352608	0	0.77441245	0	1	CRA004775;Yang2025_Silkie_Wenchang	PRJNA827465	0.15202049
ENSGALG00010003833	ACP1	2	-0.252843137	0.285879096	-0.8879363	0.374575017	0.135984146	5.178984794	80.69119644	1	CRA004775;PRJNA827465	Yang2025_Silkie_Wenchang	0.822805106
ENSGALG00010003835	ENSGALG00010003835	2	0.231808261	0.16822203	1.377989914	0.168206402	0.032328512	1.825790797	45.22921236	1	PRJNA827465;Yang2025_Silkie_Wenchang	CRA004775	0.668792739
ENSGALG00010003835	ENSGALG00010003835	2	0.161578137	0.403352065	0.400588348	0.688723226	0.210323881	2.711145068	63.11521608	0.5	CRA004775;Yang2025_Silkie_Wenchang	PRJNA827465	0.919799842
ENSGALG00010003835	ENSGALG00010003835	2	0.100993415	0.107223644	0.941895003	0.346246399	0.00574595	1.063630496	5.982387347	0.5	CRA004775;PRJNA827465	Yang2025_Silkie_Wenchang	0.807744676
ENSGALG00010003836	CD1F	2	0.433883382	0.250304919	1.733419323	0.083021166	0.053054306	1.686537488	40.70059241	1	PRJNA827465;Yang2025_Silkie_Wenchang	CRA004775	0.496604523
ENSGALG00010003836	CD1F	2	0.370870951	0.366793609	1.011116173	0.311960831	0.167414727	2.64670507	62.21717291	1	CRA004775;Yang2025_Silkie_Wenchang	PRJNA827465	0.795993742
ENSGALG00010003836	CD1F	2	0.153066458	0.184667385	0.82887651	0.407174288	0	0.327104027	0	1	CRA004775;PRJNA827465	Yang2025_Silkie_Wenchang	0.833863243
ENSGALG00010003837	ALKAL2	2	-0.169190199	0.228805431	-0.739450102	0.459633729	0	0.104247571	0	1	PRJNA827465;Yang2025_Silkie_Wenchang	CRA004775	0.850098539
ENSGALG00010003837	ALKAL2	2	-0.112259328	0.314496144	-0.356945385	0.721200819	0	0.034581938	0	1	CRA004775;Yang2025_Silkie_Wenchang	PRJNA827465	0.929950734
ENSGALG00010003837	ALKAL2	2	-0.232019757	0.277033427	-0.837515385	0.402302933	0	1.77068E-05	0	1	CRA004775;PRJNA827465	Yang2025_Silkie_Wenchang	0.832879175
ENSGALG00010003838	GAR1	2	0.13839794	0.159913055	0.86545742	0.386787777	0.040711249	4.877934839	79.49952115	0.5	PRJNA827465;Yang2025_Silkie_Wenchang	CRA004775	0.827124608
ENSGALG00010003838	GAR1	2	0.119660437	0.247020529	0.484414947	0.628091411	0.082719586	2.653465102	62.31342936	0.5	CRA004775;Yang2025_Silkie_Wenchang	PRJNA827465	0.901904489
ENSGALG00010003838	GAR1	2	-0.033477169	0.088840826	-0.376821903	0.706305602	0	0.39536988	0	1	CRA004775;PRJNA827465	Yang2025_Silkie_Wenchang	0.924548793
ENSGALG00010003841	UBE2W	2	-0.072866487	0.120704088	-0.603678702	0.546057277	0.006890754	1.295121664	22.78717687	0.5	PRJNA827465;Yang2025_Silkie_Wenchang	CRA004775	0.877958239
ENSGALG00010003841	UBE2W	2	-0.175161429	0.155614938	-1.125608065	0.260331453	0	0.397217151	0	1	CRA004775;Yang2025_Silkie_Wenchang	PRJNA827465	0.767635013
ENSGALG00010003841	UBE2W	2	0.028399893	0.125160537	0.226907723	0.820495496	0	0.001623249	0	1	CRA004775;PRJNA827465	Yang2025_Silkie_Wenchang	0.955381125
ENSGALG00010003842	ENSGALG00010003842	2	-0.046781365	0.076769167	-0.609377002	0.542274578	0.005325284	1.736394319	42.40939461	0.5	PRJNA827465;Yang2025_Silkie_Wenchang	CRA004775	0.876721948
ENSGALG00010003842	ENSGALG00010003842	2	0.033794808	0.090569895	0.373135118	0.709047883	0	0.156304438	0	0.5	CRA004775;Yang2025_Silkie_Wenchang	PRJNA827465	0.925298042
ENSGALG00010003842	ENSGALG00010003842	2	-0.099805277	0.06110998	-1.633207494	0.102425364	0	0.11203922	0	0.5	CRA004775;PRJNA827465	Yang2025_Silkie_Wenchang	0.545320547
ENSGALG00010003843	LRCH1	2	0.292877659	0.314495459	0.931261963	0.351718073	0.170353096	6.609324016	84.86985965	1	PRJNA827465;Yang2025_Silkie_Wenchang	CRA004775	0.810678834
ENSGALG00010003843	LRCH1	2	0.532729254	0.189654957	2.808939251	0.004970502	0	0.713509257	0	1	CRA004775;Yang2025_Silkie_Wenchang	PRJNA827465	0.104973487
ENSGALG00010003843	LRCH1	2	0.030611311	0.076585219	0.399702605	0.689375573	0	0.780462317	0	1	CRA004775;PRJNA827465	Yang2025_Silkie_Wenchang	0.91986176
ENSGALG00010003844	TMEM18	2	-0.032246614	0.080048595	-0.402837978	0.68706742	0	0.405201707	0	0.5	PRJNA827465;Yang2025_Silkie_Wenchang	CRA004775	0.919403862
ENSGALG00010003844	TMEM18	2	0.04658279	0.149618052	0.311344715	0.755538578	0	0.099781169	0	1	CRA004775;Yang2025_Silkie_Wenchang	PRJNA827465	0.938501652
ENSGALG00010003844	TMEM18	2	-0.044395012	0.079456247	-0.558735332	0.576342363	0	0.058719092	0	0.5	CRA004775;PRJNA827465	Yang2025_Silkie_Wenchang	0.887048016
ENSGALG00010003848	ESD	2	0.075415157	0.292280203	0.258023487	0.796388781	0.141046445	5.51378711	81.86364508	0.5	PRJNA827465;Yang2025_Silkie_Wenchang	CRA004775	0.949419687
ENSGALG00010003848	ESD	2	-0.163664511	0.148697662	-1.100652889	0.271047757	0	0.265320998	0	1	CRA004775;Yang2025_Silkie_Wenchang	PRJNA827465	0.772743863
ENSGALG00010003848	ESD	2	0.155599271	0.214656503	0.724875643	0.468528325	0.065357743	3.282771576	69.53793535	0.5	CRA004775;PRJNA827465	Yang2025_Silkie_Wenchang	0.853198008
ENSGALG00010003850	PLA2G12A	2	-0.14624927	0.151902405	-0.962784427	0.335655717	0.039099181	6.315767821	84.16661238	1	PRJNA827465;Yang2025_Silkie_Wenchang	CRA004775	0.803317552
ENSGALG00010003850	PLA2G12A	2	-0.300196138	0.087750358	-3.421024643	0.000623857	0	0.034211433	0	1	CRA004775;Yang2025_Silkie_Wenchang	PRJNA827465	0.027274131
ENSGALG00010003850	PLA2G12A	2	-0.101299413	0.128835684	-0.786268288	0.431710327	0.02205448	2.542223248	60.66435153	1	CRA004775;PRJNA827465	Yang2025_Silkie_Wenchang	0.842503929
ENSGALG00010003851	PHF14	2	0.264100829	0.256615421	1.029169755	0.303399912	0.11909836	9.757709115	89.75169286	1	PRJNA827465;Yang2025_Silkie_Wenchang	CRA004775	0.792088386
ENSGALG00010003851	PHF14	2	0.350959459	0.203980381	1.720554974	0.08533161	0.053778682	2.811268751	64.42887221	1	CRA004775;Yang2025_Silkie_Wenchang	PRJNA827465	0.502590779
ENSGALG00010003851	PHF14	2	0.034998277	0.044589104	0.784906479	0.432508403	0	0.312042398	0	1	CRA004775;PRJNA827465	Yang2025_Silkie_Wenchang	0.842762754
ENSGALG00010003852	TCEB1	2	-0.114103438	0.115003321	-0.992175154	0.321112091	0.020000091	3.893139712	74.31379108	1	PRJNA827465;Yang2025_Silkie_Wenchang	CRA004775	0.798513866
ENSGALG00010003852	TCEB1	2	-0.160088473	0.144269157	-1.109651406	0.26714927	0.017636742	1.481342782	32.4936799	0.5	CRA004775;Yang2025_Silkie_Wenchang	PRJNA827465	0.771247817
ENSGALG00010003852	TCEB1	2	-0.008304687	0.056703692	-0.146457603	0.883560157	0	0.143445987	0	0.5	CRA004775;PRJNA827465	Yang2025_Silkie_Wenchang	0.972001076
ENSGALG00010003853	ENSGALG00010003853	2	-0.073719348	0.123696913	-0.595967562	0.551196897	0.017726185	2.286022155	56.25589201	0.5	PRJNA827465;Yang2025_Silkie_Wenchang	CRA004775	0.878879109
ENSGALG00010003853	ENSGALG00010003853	2	-0.204758258	0.132699481	-1.543022298	0.122825366	0	0.163587268	0	1	CRA004775;Yang2025_Silkie_Wenchang	PRJNA827465	0.587706885
ENSGALG00010003853	ENSGALG00010003853	2	0.028551621	0.090137091	0.316757736	0.751427436	0	0.012140678	0	0.5	CRA004775;PRJNA827465	Yang2025_Silkie_Wenchang	0.937685817
ENSGALG00010003858	SUCLA2	2	0.063477939	0.127714456	0.497030182	0.619167768	0.022678251	3.217549171	68.92044388	0.5	PRJNA827465;Yang2025_Silkie_Wenchang	CRA004775	0.899168321
ENSGALG00010003858	SUCLA2	2	0.215569985	0.107960001	1.996757909	0.045851488	0	0.087684537	0	1	CRA004775;Yang2025_Silkie_Wenchang	PRJNA827465	0.369434406
ENSGALG00010003858	SUCLA2	2	-0.000332396	0.125901222	-0.002640132	0.997893482	0.011438705	1.223120306	18.24189372	0.5	CRA004775;PRJNA827465	Yang2025_Silkie_Wenchang	0.999290321
ENSGALG00010003860	TMEM70	2	-0.318482461	0.188150722	-1.692698584	0.090512855	0.064114117	10.12176905	90.12030412	1	PRJNA827465;Yang2025_Silkie_Wenchang	CRA004775	0.516242452
ENSGALG00010003860	TMEM70	2	-0.161835866	0.385297797	-0.420028008	0.674464993	0.264887644	8.886172247	88.7465607	0.5	CRA004775;Yang2025_Silkie_Wenchang	PRJNA827465	0.915949608
ENSGALG00010003860	TMEM70	2	-0.012404018	0.185950073	-0.066706175	0.946815618	0.049517954	2.727747384	63.33971371	0.5	CRA004775;PRJNA827465	Yang2025_Silkie_Wenchang	0.986445196
ENSGALG00010003861	NUDT15	2	-0.190178104	0.150168602	-1.266430539	0.205358983	0.033567696	3.737421823	73.24358749	1	PRJNA827465;Yang2025_Silkie_Wenchang	CRA004775	0.71825498
ENSGALG00010003861	NUDT15	2	-0.3049596	0.105400996	-2.893327492	0.003811836	0	0.447279348	0	1	CRA004775;Yang2025_Silkie_Wenchang	PRJNA827465	0.08859645
ENSGALG00010003861	NUDT15	2	-0.08680158	0.072767273	-1.192865653	0.232921993	0	0.703897296	0	1	CRA004775;PRJNA827465	Yang2025_Silkie_Wenchang	0.746005924
ENSGALG00010003862	PCK2	2	0.085929377	0.334288597	0.257051475	0.797139036	0.17716267	4.611208998	78.31371338	0.5	PRJNA827465;Yang2025_Silkie_Wenchang	CRA004775	0.949622804
ENSGALG00010003862	PCK2	2	-0.108839274	0.124922662	-0.871253238	0.383615897	0.002321357	1.077690916	7.2090165	0.5	CRA004775;Yang2025_Silkie_Wenchang	PRJNA827465	0.825509017
ENSGALG00010003862	PCK2	2	0.203932404	0.204365412	0.997881205	0.318336967	0.03302034	1.603623805	37.64123501	1	CRA004775;PRJNA827465	Yang2025_Silkie_Wenchang	0.797679172
ENSGALG00010003863	HTR1B	2	-0.021357181	0.214133658	-0.099737618	0.920552635	0.050552453	2.071945227	51.73617589	0.5	PRJNA827465;Yang2025_Silkie_Wenchang	CRA004775	0.98144788
ENSGALG00010003863	HTR1B	2	0.351159254	0.228672667	1.535641571	0.124626313	0	0.489611737	0	1	CRA004775;Yang2025_Silkie_Wenchang	PRJNA827465	0.592140711
ENSGALG00010003863	HTR1B	2	0.106803734	0.387162304	0.275862948	0.782653334	0.213629101	2.955929997	66.16969952	0.5	CRA004775;PRJNA827465	Yang2025_Silkie_Wenchang	0.945490372
ENSGALG00010003865	AKAP8L	2	0.273229406	0.331392437	0.824488961	0.409661786	0.192315851	7.360585962	86.41412511	0.5	PRJNA827465;Yang2025_Silkie_Wenchang	CRA004775	0.834634657
ENSGALG00010003865	AKAP8L	2	0.250064616	0.39089084	0.639730048	0.522348117	0.253048856	5.813189753	82.79774027	0.5	CRA004775;Yang2025_Silkie_Wenchang	PRJNA827465	0.869568211
ENSGALG00010003865	AKAP8L	2	-0.032330856	0.066079242	-0.489274021	0.624647711	0	0.242323056	0	1	CRA004775;PRJNA827465	Yang2025_Silkie_Wenchang	0.90097623
ENSGALG00010003868	HBBA	2	-0.119341221	0.508120833	-0.234867797	0.814311345	0.474853194	12.39046334	91.92927679	0.5	PRJNA827465;Yang2025_Silkie_Wenchang	CRA004775	0.953869515
ENSGALG00010003868	HBBA	2	-0.442162735	0.223120816	-1.981718889	0.047510712	0.036622288	1.56495048	36.1002145	1	CRA004775;Yang2025_Silkie_Wenchang	PRJNA827465	0.376702342
ENSGALG00010003868	HBBA	2	0.137580453	0.280017871	0.491327402	0.623194902	0.10466171	2.885790858	65.34745416	0.5	CRA004775;PRJNA827465	Yang2025_Silkie_Wenchang	0.900348922
ENSGALG00010003869	LY96	2	-0.428919951	0.178680552	-2.400484805	0.016373371	0	0.76507877	0	1	PRJNA827465;Yang2025_Silkie_Wenchang	CRA004775	0.21161545
ENSGALG00010003869	LY96	2	-0.536415015	0.261443867	-2.051740665	0.040194872	0	0.464528981	0	1	CRA004775;Yang2025_Silkie_Wenchang	PRJNA827465	0.347204191
ENSGALG00010003869	LY96	2	-0.310786297	0.19789829	-1.570434475	0.116314067	0	0.010899002	0	1	CRA004775;PRJNA827465	Yang2025_Silkie_Wenchang	0.574235873
ENSGALG00010003870	MED4	2	0.004649321	0.086838895	0.053539617	0.957301966	0.006279111	1.707263089	41.42671937	0.5	PRJNA827465;Yang2025_Silkie_Wenchang	CRA004775	0.988566452
ENSGALG00010003870	MED4	2	-0.111048139	0.090230946	-1.230710127	0.218431299	0	0.280959073	0	1	CRA004775;Yang2025_Silkie_Wenchang	PRJNA827465	0.732175117
ENSGALG00010003870	MED4	2	-0.001377664	0.135540537	-0.010164223	0.991890263	0.018121141	1.687702298	40.7478439	0.5	CRA004775;PRJNA827465	Yang2025_Silkie_Wenchang	0.997575757
ENSGALG00010003871	ENSGALG00010003871	2	0.74410374	0.699741073	1.063398691	0.287601186	0.9558451	41.53757767	97.59254137	1	PRJNA827465;Yang2025_Silkie_Wenchang	CRA004775	0.782840067
ENSGALG00010003871	ENSGALG00010003871	2	0.512974995	0.950006912	0.539969751	0.589217894	1.749618914	32.45209734	96.91853507	0.5	CRA004775;Yang2025_Silkie_Wenchang	PRJNA827465	0.892171138
ENSGALG00010003871	ENSGALG00010003871	2	-0.133295829	0.241941632	-0.550942091	0.581673373	0.081731088	2.877163618	65.24354772	0.5	CRA004775;PRJNA827465	Yang2025_Silkie_Wenchang	0.889045686
ENSGALG00010003874	YES1	2	0.060326849	0.05302703	1.137662222	0.255261554	0	0.000385517	0	1	PRJNA827465;Yang2025_Silkie_Wenchang	CRA004775	0.764467569
ENSGALG00010003874	YES1	2	0.349659665	0.312100368	1.120343647	0.262567349	0.165385258	6.37368371	84.31048597	1	CRA004775;Yang2025_Silkie_Wenchang	PRJNA827465	0.768418419
ENSGALG00010003874	YES1	2	0.33968913	0.312584366	1.086711835	0.277164182	0.172550006	7.82946754	87.22773937	1	CRA004775;PRJNA827465	Yang2025_Silkie_Wenchang	0.776194032
ENSGALG00010003875	AKAP8	2	0.30895807	0.238345092	1.29626361	0.194884681	0.106409613	15.31265386	93.46945338	1	PRJNA827465;Yang2025_Silkie_Wenchang	CRA004775	0.706054964
ENSGALG00010003875	AKAP8	2	0.237633289	0.343613239	0.691571982	0.489206162	0.208313767	8.191749982	87.79259618	0.5	CRA004775;Yang2025_Silkie_Wenchang	PRJNA827465	0.858337006
ENSGALG00010003875	AKAP8	2	0.070640973	0.047225947	1.495808496	0.134703567	0	0.938648792	0	0.5	CRA004775;PRJNA827465	Yang2025_Silkie_Wenchang	0.615110292
ENSGALG00010003878	ARHGAP24	2	0.085475922	0.067058774	1.274641897	0.202436021	0	0.015600609	0	1	PRJNA827465;Yang2025_Silkie_Wenchang	CRA004775	0.715289846
ENSGALG00010003878	ARHGAP24	2	0.083557029	0.098742804	0.846208789	0.39743628	0	0.068911036	0	1	CRA004775;Yang2025_Silkie_Wenchang	PRJNA827465	0.831571329
ENSGALG00010003878	ARHGAP24	2	0.073021044	0.078999766	0.924319753	0.355319861	0	0.040387189	0	1	CRA004775;PRJNA827465	Yang2025_Silkie_Wenchang	0.812301302
ENSGALG00010003879	CASP6	2	-0.227787979	0.291330835	-0.781887639	0.43428061	0.15369108	10.54897859	90.52040923	0.5	PRJNA827465;Yang2025_Silkie_Wenchang	CRA004775	0.842762754
ENSGALG00010003879	CASP6	2	-0.224398842	0.341889687	-0.65634867	0.511599809	0.187722269	4.735975526	78.88502602	0.5	CRA004775;Yang2025_Silkie_Wenchang	PRJNA827465	0.865694231
ENSGALG00010003879	CASP6	2	0.074373325	0.107746168	0.690264229	0.490028039	0	0.120407379	0	1	CRA004775;PRJNA827465	Yang2025_Silkie_Wenchang	0.858529056
ENSGALG00010003882	ENSGALG00010003882	2	-0.118258095	0.093258962	-1.26806146	0.204776003	0.006541248	1.255648067	20.35985029	1	PRJNA827465;Yang2025_Silkie_Wenchang	CRA004775	0.717318293
ENSGALG00010003882	ENSGALG00010003882	2	-0.186408925	0.127410633	-1.463056265	0.143451984	0	0.670456617	0	1	CRA004775;Yang2025_Silkie_Wenchang	PRJNA827465	0.630564924
ENSGALG00010003882	ENSGALG00010003882	2	-0.081868167	0.057583515	-1.421729257	0.155104863	0	0.03981112	0	1	CRA004775;PRJNA827465	Yang2025_Silkie_Wenchang	0.647765412
ENSGALG00010003884	TYMS	2	-0.112449334	0.101920644	-1.103302818	0.269895683	0.008972936	1.504692316	33.54123037	1	PRJNA827465;Yang2025_Silkie_Wenchang	CRA004775	0.772187652
ENSGALG00010003884	TYMS	2	-0.232804006	0.164264609	-1.417249937	0.156409866	0	0.725600103	0	0.5	CRA004775;Yang2025_Silkie_Wenchang	PRJNA827465	0.649484056
ENSGALG00010003884	TYMS	2	-0.047024083	0.070898177	-0.663262237	0.507162618	0	0.181815582	0	0.5	CRA004775;PRJNA827465	Yang2025_Silkie_Wenchang	0.864048203
ENSGALG00010003887	GIPC1	2	0.165174006	0.139490119	1.184126932	0.236362821	0.025730749	2.848894184	64.89866118	1	PRJNA827465;Yang2025_Silkie_Wenchang	CRA004775	0.748669015
ENSGALG00010003887	GIPC1	2	0.228692663	0.138188136	1.654937033	0.097937271	0.009086013	1.265121867	20.9562315	1	CRA004775;Yang2025_Silkie_Wenchang	PRJNA827465	0.534130658
ENSGALG00010003887	GIPC1	2	0.041614655	0.08525583	0.488115066	0.62546834	0	0.002871416	0	1	CRA004775;PRJNA827465	Yang2025_Silkie_Wenchang	0.901367193
ENSGALG00010003889	ITM2B	2	-0.126128184	0.074898764	-1.683982182	0.092185112	0.006101	2.025666893	50.63354181	1	PRJNA827465;Yang2025_Silkie_Wenchang	CRA004775	0.519895462
ENSGALG00010003889	ITM2B	2	-0.206820535	0.08429893	-2.453418278	0.014150569	0	0.149254398	0	1	CRA004775;Yang2025_Silkie_Wenchang	PRJNA827465	0.194938594
ENSGALG00010003889	ITM2B	2	-0.074960138	0.050648461	-1.480008207	0.138871057	0	0.167249057	0	1	CRA004775;PRJNA827465	Yang2025_Silkie_Wenchang	0.622497285
ENSGALG00010003894	COPS4	2	-0.087607408	0.056453644	-1.551846805	0.120698884	0	0.155716016	0	1	PRJNA827465;Yang2025_Silkie_Wenchang	CRA004775	0.582493642
ENSGALG00010003894	COPS4	2	-0.091885129	0.102480903	-0.896607332	0.369928484	0	0.882306953	0	1	CRA004775;Yang2025_Silkie_Wenchang	PRJNA827465	0.820855964
ENSGALG00010003894	COPS4	2	-0.113625685	0.063301165	-1.795001469	0.072653466	0	0.638512698	0	1	CRA004775;PRJNA827465	Yang2025_Silkie_Wenchang	0.465519917
ENSGALG00010003895	THSD7A	2	0.084749142	0.277901163	0.304961452	0.760395512	0.108011653	3.307798435	69.76841184	0.5	PRJNA827465;Yang2025_Silkie_Wenchang	CRA004775	0.939327609
ENSGALG00010003895	THSD7A	2	0.41457671	0.186416071	2.223932237	0.026153004	0	0.91256533	0	1	CRA004775;Yang2025_Silkie_Wenchang	PRJNA827465	0.275965687
ENSGALG00010003895	THSD7A	2	0.239068357	0.544011861	0.439454311	0.660332381	0.440536086	3.516088651	71.5593064	0.5	CRA004775;PRJNA827465	Yang2025_Silkie_Wenchang	0.911923994
ENSGALG00010003896	MCUB	2	-0.014184702	0.255528767	-0.055511174	0.955731228	0.115810257	8.649688576	88.43889013	0.5	PRJNA827465;Yang2025_Silkie_Wenchang	CRA004775	0.9883727
ENSGALG00010003896	MCUB	2	-0.259473363	0.139785851	-1.856220498	0.063422135	0	0.23405191	0	1	CRA004775;Yang2025_Silkie_Wenchang	PRJNA827465	0.433586349
ENSGALG00010003896	MCUB	2	0.214841738	0.088898131	2.416718287	0.015661133	0	0.482768683	0	0.5	CRA004775;PRJNA827465	Yang2025_Silkie_Wenchang	0.207039858
ENSGALG00010003899	LIN54	2	0.098829598	0.252980761	0.390660529	0.696048178	0.106341255	5.355712024	81.32834634	0.5	PRJNA827465;Yang2025_Silkie_Wenchang	CRA004775	0.921038437
ENSGALG00010003899	LIN54	2	0.41120274	0.179772546	2.287350031	0.0221754	0	0.034503968	0	1	CRA004775;Yang2025_Silkie_Wenchang	PRJNA827465	0.252609681
ENSGALG00010003899	LIN54	2	0.07123403	0.275341284	0.258711768	0.795857639	0.106853587	2.619106112	61.81903454	0.5	CRA004775;PRJNA827465	Yang2025_Silkie_Wenchang	0.949107318
ENSGALG00010003900	RB1	2	0.007975433	0.055336375	0.144126405	0.885400654	0	0.000124833	0	1	PRJNA827465;Yang2025_Silkie_Wenchang	CRA004775	0.972280795
ENSGALG00010003900	RB1	2	0.002777795	0.115744576	0.023999355	0.980853123	0	0.009664139	0	0.5	CRA004775;Yang2025_Silkie_Wenchang	PRJNA827465	0.994928779
ENSGALG00010003900	RB1	2	0.006107012	0.058809666	0.103843675	0.917293406	0	0.010589333	0	0.5	CRA004775;PRJNA827465	Yang2025_Silkie_Wenchang	0.98082192
ENSGALG00010003902	ENSGALG00010003902	2	0.248593893	0.340748783	0.729551814	0.465664186	0.201902779	7.090029214	85.89568576	0.5	PRJNA827465;Yang2025_Silkie_Wenchang	CRA004775	0.852424459
ENSGALG00010003902	ENSGALG00010003902	2	0.206672287	0.446740607	0.462622567	0.64363493	0.319028459	4.920245864	79.6758124	0.5	CRA004775;Yang2025_Silkie_Wenchang	PRJNA827465	0.906643868
ENSGALG00010003902	ENSGALG00010003902	2	-0.07037837	0.082757068	-0.850421258	0.395090922	0	0.393992981	0	1	CRA004775;PRJNA827465	Yang2025_Silkie_Wenchang	0.830643015
ENSGALG00010003903	COL28A1	2	0.029681048	0.157654613	0.188266283	0.850667915	0	0.003429109	0	1	PRJNA827465;Yang2025_Silkie_Wenchang	CRA004775	0.961985357
ENSGALG00010003903	COL28A1	2	0.10018076	0.235965706	0.424556437	0.671160055	0	0.675631745	0	1	CRA004775;Yang2025_Silkie_Wenchang	PRJNA827465	0.914621672
ENSGALG00010003903	COL28A1	2	0.088273237	0.189803468	0.465077052	0.641876279	0	0.668765507	0	1	CRA004775;PRJNA827465	Yang2025_Silkie_Wenchang	0.906634523
ENSGALG00010003909	DNAJB1	2	-0.069450086	0.130466354	-0.532321809	0.59450313	0.02325072	2.92690837	65.83425671	0.5	PRJNA827465;Yang2025_Silkie_Wenchang	CRA004775	0.89334442

ENSGALG00010003909	DNAJB1	2	-0.344943189	0.228482548	-1.509713507	0.131116543	0.047205067	1.426011729	29.87434959	1	CRA004775;Yang2025_Silkie_Wenchang	PRJNA827465	0.606767161
ENSGALG00010003909	DNAJB1	2	-0.256508258	0.396413943	-0.647071735	0.517585517	0.238635926	3.292395967	69.62698259	0.5	CRA004775;PRJNA827465	Yang2025_Silkie_Wenchang	0.867962259
ENSGALG00010003910	THAP9	2	-0.019351917	0.11204518	-0.172715305	0.862875213	0.015091775	2.410333608	58.51196711	0.5	PRJNA827465;Yang2025_Silkie_Wenchang	CRA004775	0.966099097
ENSGALG00010003910	THAP9	2	-0.177579164	0.327829761	-0.541681034	0.588038258	0.176836966	5.281465971	81.06586305	0.5	CRA004775;Yang2025_Silkie_Wenchang	PRJNA827465	0.891761457
ENSGALG00010003910	THAP9	2	-0.260500123	0.204868336	-1.271548975	0.203533416	0.056812357	2.531269932	60.49413825	1	CRA004775;PRJNA827465	Yang2025_Silkie_Wenchang	0.717254253
ENSGALG00010003913	PSMB5	2	0.066458893	0.076474619	0.869032023	0.384829615	0	0.58127286	0	1	PRJNA827465;Yang2025_Silkie_Wenchang	CRA004775	0.826444827
ENSGALG00010003913	PSMB5	2	0.046126395	0.253912989	0.181662211	0.855847824	0.03604236	1.382491104	27.66680401	0.5	CRA004775;Yang2025_Silkie_Wenchang	PRJNA827465	0.963486168
ENSGALG00010003913	PSMB5	2	0.034397203	0.077159747	0.445792063	0.655747455	0	0.714796774	0	0.5	CRA004775;PRJNA827465	Yang2025_Silkie_Wenchang	0.910411938
ENSGALG00010003914	CAMK2D	2	-0.128456217	0.139105328	-0.923445699	0.355774986	0.02636536	2.671754768	62.57141517	0.5	PRJNA827465;Yang2025_Silkie_Wenchang	CRA004775	0.812301302
ENSGALG00010003914	CAMK2D	2	0.05567492	0.143634682	0.387614741	0.698301158	0	0.000847599	0	1	CRA004775;Yang2025_Silkie_Wenchang	PRJNA827465	0.921624968
ENSGALG00010003914	CAMK2D	2	-0.219618789	0.059765108	-3.674699085	0.00023813	0	0.941483964	0	0.5	CRA004775;PRJNA827465	Yang2025_Silkie_Wenchang	0.014332649
ENSGALG00010003915	MIOS	2	0.004670561	0.079731479	0.058578636	0.953287727	0.005055698	1.437369269	30.42845555	0.5	PRJNA827465;Yang2025_Silkie_Wenchang	CRA004775	0.987758913
ENSGALG00010003915	MIOS	2	-0.009647939	0.171648918	-0.056207397	0.955176589	0.032241803	2.159661129	53.69643937	0.5	CRA004775;Yang2025_Silkie_Wenchang	PRJNA827465	0.988131974
ENSGALG00010003915	MIOS	2	-0.056899899	0.056542837	-1.00631491	0.31426411	0	0.682894296	0	1	CRA004775;PRJNA827465	Yang2025_Silkie_Wenchang	0.796531448
ENSGALG00010003920	LPAR6	2	-0.136833818	0.219217062	-0.624193283	0.532500658	0.071573012	3.449489396	71.01020223	0.5	PRJNA827465;Yang2025_Silkie_Wenchang	CRA004775	0.873183244
ENSGALG00010003920	LPAR6	2	-0.107459388	0.354305786	-0.303295607	0.761664594	0.165942932	2.813229229	64.45366095	0.5	CRA004775;Yang2025_Silkie_Wenchang	PRJNA827465	0.939778474
ENSGALG00010003920	LPAR6	2	0.053712691	0.084243568	0.637588039	0.523741883	0	0.516810382	0	1	CRA004775;PRJNA827465	Yang2025_Silkie_Wenchang	0.869661505
ENSGALG00010003921	COLEC12	2	-0.03830901	0.089484669	-0.428106963	0.668573247	0	0.002350887	0	1	PRJNA827465;Yang2025_Silkie_Wenchang	CRA004775	0.914377769
ENSGALG00010003921	COLEC12	2	0.008822314	0.161784497	0.054531271	0.956511895	0	0.342019455	0	0.5	CRA004775;Yang2025_Silkie_Wenchang	PRJNA827465	0.988540618
ENSGALG00010003921	COLEC12	2	-0.024808044	0.100927685	-0.245800188	0.805836911	0	0.407864864	0	0.5	CRA004775;PRJNA827465	Yang2025_Silkie_Wenchang	0.952057827
ENSGALG00010003923	ENSGALG00010003923	2	-0.264257218	0.275714423	-0.958445391	0.337838216	0.127203862	5.373237771	81.38924718	1	PRJNA827465;Yang2025_Silkie_Wenchang	CRA004775	0.80415385
ENSGALG00010003923	ENSGALG00010003923	2	-0.470569445	0.17854269	-2.635613057	0.008398544	0	0.619502163	0	1	CRA004775;Yang2025_Silkie_Wenchang	PRJNA827465	0.142771619
ENSGALG00010003923	ENSGALG00010003923	2	-0.0422425	0.056258449	-0.750864999	0.452733907	0	0.947767413	0	1	CRA004775;PRJNA827465	Yang2025_Silkie_Wenchang	0.848446539
ENSGALG00010003925	SEC31A	2	0.032558267	0.043903276	0.741590837	0.458335277	0	0.245073758	0	1	PRJNA827465;Yang2025_Silkie_Wenchang	CRA004775	0.850026426
ENSGALG00010003925	SEC31A	2	-0.052557747	0.170813846	-0.307690202	0.758318076	0.036400697	2.304643083	56.60933325	0.5	CRA004775;Yang2025_Silkie_Wenchang	PRJNA827465	0.938954369
ENSGALG00010003925	SEC31A	2	-0.059675	0.134946943	-0.442210838	0.658336644	0.022156126	1.88691838	47.00353707	0.5	CRA004775;PRJNA827465	Yang2025_Silkie_Wenchang	0.911045566
ENSGALG00010003928	ENSGALG00010003928	2	-0.073071856	0.110268573	-0.662671635	0.50754088	0.004847222	1.079083558	7.328770562	1	PRJNA827465;Yang2025_Silkie_Wenchang	CRA004775	0.864180532
ENSGALG00010003928	ENSGALG00010003928	2	-0.582296064	0.219892575	-2.648093343	0.008094717	0	0.509730828	0	1	CRA004775;Yang2025_Silkie_Wenchang	PRJNA827465	0.139660521
ENSGALG00010003928	ENSGALG00010003928	2	-0.321205527	0.336146276	-0.955552835	0.3392982	0.185736627	5.00603092	80.02409462	1	CRA004775;PRJNA827465	Yang2025_Silkie_Wenchang	0.804824608
ENSGALG00010003929	RCBTB2	2	-0.017931922	0.134532809	-0.133290328	0.893963777	0.025588207	3.37701535	70.38805287	0.5	PRJNA827465;Yang2025_Silkie_Wenchang	CRA004775	0.974381724
ENSGALG00010003929	RCBTB2	2	-0.038725722	0.227263102	-0.170400395	0.864695261	0.05359718	1.570160265	36.31223372	0.5	CRA004775;Yang2025_Silkie_Wenchang	PRJNA827465	0.966723659
ENSGALG00010003929	RCBTB2	2	0.120620296	0.089485046	1.347938021	0.177678318	0	0.408721885	0	1	CRA004775;PRJNA827465	Yang2025_Silkie_Wenchang	0.683951879
ENSGALG00010003933	TECR	2	-0.198091958	0.187207039	-1.058143744	0.289989914	0.048728223	3.044844023	67.15759518	0.5	PRJNA827465;Yang2025_Silkie_Wenchang	CRA004775	0.784195688
ENSGALG00010003933	TECR	2	0.044109044	0.178690426	0.246846152	0.805027291	0	0.052399613	0	1	CRA004775;Yang2025_Silkie_Wenchang	PRJNA827465	0.951863117
ENSGALG00010003933	TECR	2	-0.319927184	0.124819693	-2.563114654	0.010373778	0.006248149	1.053012746	5.034387908	0.5	CRA004775;PRJNA827465	Yang2025_Silkie_Wenchang	0.162122532
ENSGALG00010003937	ENSGALG00010003937	2	0.230413611	0.372567868	0.618447351	0.536280493	0.211343575	3.782421111	73.56190729	0.5	PRJNA827465;Yang2025_Silkie_Wenchang	CRA004775	0.874599474
ENSGALG00010003937	ENSGALG00010003937	2	0.334511309	0.290178617	1.152777254	0.249001823	0.074178993	1.747490026	42.7750668	1	CRA004775;Yang2025_Silkie_Wenchang	PRJNA827465	0.759411487
ENSGALG00010003937	ENSGALG00010003937	2	-0.036800577	0.131953761	-0.278889947	0.780329284	0	0.307576625	0	0.5	CRA004775;PRJNA827465	Yang2025_Silkie_Wenchang	0.945041817
ENSGALG00010003948	SCD5	2	0.234586107	0.089084036	2.633312515	0.00845565	0	0.107724037	0	1	PRJNA827465;Yang2025_Silkie_Wenchang	CRA004775	0.143515254
ENSGALG00010003948	SCD5	2	0.354458979	0.149112052	2.377131646	0.01744786	0	0.395516495	0	1	CRA004775;Yang2025_Silkie_Wenchang	PRJNA827465	0.219933687
ENSGALG00010003948	SCD5	2	0.254874468	0.094673098	2.692153043	0.007099236	0	0.938934597	0	1	CRA004775;PRJNA827465	Yang2025_Silkie_Wenchang	0.129476228
ENSGALG00010003958	CYSLTR2	2	-0.06636834	0.260236213	-0.255031149	0.798699041	0.036029069	1.348540467	25.84575515	0.5	PRJNA827465;Yang2025_Silkie_Wenchang	CRA004775	0.949922217
ENSGALG00010003958	CYSLTR2	2	-0.029986603	0.387226204	-0.077439498	0.93827392	0.142019625	1.877131363	46.72722327	0.5	CRA004775;Yang2025_Silkie_Wenchang	PRJNA827465	0.985349045
ENSGALG00010003958	CYSLTR2	2	0.225785714	0.238409	0.947051977	0.343612294	0	0.228713849	0	1	CRA004775;PRJNA827465	Yang2025_Silkie_Wenchang	0.806314104
ENSGALG00010003960	ASPH	2	-0.084201413	0.268176749	-0.313977307	0.753538274	0.128042186	9.035550674	88.93260592	0.5	PRJNA827465;Yang2025_Silkie_Wenchang	CRA004775	0.938087082
ENSGALG00010003960	ASPH	2	0.199381687	0.133070718	1.498313757	0.134051751	0	0.017808044	0	1	CRA004775;Yang2025_Silkie_Wenchang	PRJNA827465	0.61327389
ENSGALG00010003960	ASPH	2	-0.153479847	0.275599564	-0.556894375	0.577599598	0.100857911	2.3890708	58.14272226	0.5	CRA004775;PRJNA827465	Yang2025_Silkie_Wenchang	0.887312588
ENSGALG00010003962	UMAD1	2	-0.423034763	0.199865565	-2.116596532	0.034294105	0.065030858	5.24546178	80.93590151	1	PRJNA827465;Yang2025_Silkie_Wenchang	CRA004775	0.318385282
ENSGALG00010003962	UMAD1	2	-0.597777331	0.132689794	-4.505073921	6.63498E-06	0	0.442024015	0	1	CRA004775;Yang2025_Silkie_Wenchang	PRJNA827465	0.001018969
ENSGALG00010003962	UMAD1	2	-0.252062422	0.092076998	-2.737517802	0.006190477	0	0.262457518	0	1	CRA004775;PRJNA827465	Yang2025_Silkie_Wenchang	0.119150008
ENSGALG00010003968	DSN1	2	0.019208455	0.071080426	0.270235508	0.786979077	0	0.8917811	0	0.5	PRJNA827465;Yang2025_Silkie_Wenchang	CRA004775	0.946784559
ENSGALG00010003968	DSN1	2	-0.167652917	0.182503729	-0.918627351	0.358290521	0	0.03718377	0	1	CRA004775;Yang2025_Silkie_Wenchang	PRJNA827465	0.813964918
ENSGALG00010003968	DSN1	2	0.028971358	0.071905289	0.402909964	0.687014461	0	0.330909068	0	0.5	CRA004775;PRJNA827465	Yang2025_Silkie_Wenchang	0.919369765
ENSGALG00010003972	PENK	2	0.114357898	0.099505758	1.1492591	0.250449158	0	0.261087154	0	1	PRJNA827465;Yang2025_Silkie_Wenchang	CRA004775	0.759943035
ENSGALG00010003972	PENK	2	0.067056732	0.141739234	0.473099295	0.63614234	0	0.048466079	0	1	CRA004775;Yang2025_Silkie_Wenchang	PRJNA827465	0.904466477
ENSGALG00010003972	PENK	2	0.164649886	0.134663188	1.222679248	0.221450868	0	0.006480467	0	1	CRA004775;PRJNA827465	Yang2025_Silkie_Wenchang	0.734798098
ENSGALG00010003977	MYO6	2	0.123562521	0.089822499	1.375629958	0.168936227	0	0.316102956	0	1	PRJNA827465;Yang2025_Silkie_Wenchang	CRA004775	0.670135297
ENSGALG00010003977	MYO6	2	0.068809725	0.286056177	0.240546194	0.809906857	0.06789667	1.385650139	27.83171079	0.5	CRA004775;Yang2025_Silkie_Wenchang	PRJNA827465	0.952818762
ENSGALG00010003977	MYO6	2	0.072539225	0.115069624	0.630394216	0.528436694	0.003423111	1.020974358	2.054347186	0.5	CRA004775;PRJNA827465	Yang2025_Silkie_Wenchang	0.871701504
ENSGALG00010003978	ANK2	2	0.065556528	0.210388574	0.311597377	0.755346528	0.074299437	5.847886589	82.89980517	0.5	PRJNA827465;Yang2025_Silkie_Wenchang	CRA004775	0.938464456
ENSGALG00010003978	ANK2	2	0.296255433	0.153163293	1.934245644	0.053082933	0	0.010963731	0	1	CRA004775;Yang2025_Silkie_Wenchang	PRJNA827465	0.399631766
ENSGALG00010003978	ANK2	2	-0.116892568	0.072905456	-1.603344584	0.108858599	0	0.387570429	0	0.5	CRA004775;PRJNA827465	Yang2025_Silkie_Wenchang	0.558759109
ENSGALG00010003980	CHCHD7	2	0.233439596	0.308007112	0.757903267	0.448508895	0.166219865	8.002425697	87.503789	0.5	PRJNA827465;Yang2025_Silkie_Wenchang	CRA004775	0.846878812
ENSGALG00010003980	CHCHD7	2	0.185532132	0.399288826	0.464656458	0.642177495	0.263613226	5.578006115	82.07244705	0.5	CRA004775;Yang2025_Silkie_Wenchang	PRJNA827465	0.906634523
ENSGALG00010003980	CHCHD7	2	-0.095511168	0.119785557	-0.797351288	0.425247042	0	0.335258461	0	1	CRA004775;PRJNA827465	Yang2025_Silkie_Wenchang	0.84125685
ENSGALG00010003981	TMEM150C	2	-0.124255129	0.226553568	-0.548458055	0.583377423	0.069251527	2.860118391	65.03641237	0.5	PRJNA827465;Yang2025_Silkie_Wenchang	CRA004775	0.890479822
ENSGALG00010003981	TMEM150C	2	-0.3551377	0.162535114	-2.184990625	0.028889534	0	0.061336667	0	1	CRA004775;Yang2025_Silkie_Wenchang	PRJNA827465	0.289654427
ENSGALG00010003981	TMEM150C	2	-0.085741512	0.185692164	-0.461740068	0.644267734	0.039486625	2.197124839	54.48597265	0.5	CRA004775;PRJNA827465	Yang2025_Silkie_Wenchang	0.906890059
ENSGALG00010003982	GLCCI1	2	0.192767286	0.144916317	1.330197247	0.183453291	0.031697409	3.876375295	74.20270423	1	PRJNA827465;Yang2025_Silkie_Wenchang	CRA004775	0.691847354
ENSGALG00010003982	GLCCI1	2	0.106068546	0.33333584	0.318203245	0.750330771	0.157947958	2.92041515	65.75829297	0.5	CRA004775;Yang2025_Silkie_Wenchang	PRJNA827465	0.937393444
ENSGALG00010003982	GLCCI1	2	0.042843081	0.092639209	0.462472445	0.643742558	0.003607093	1.047018198	4.490676308	0.5	CRA004775;PRJNA827465	Yang2025_Silkie_Wenchang	0.906651393
ENSGALG00010003985	PRMT5	2	0.048335266	0.090541983	0.533843691	0.593449683	0.00764831	1.734152235	42.33493578	0.5	PRJNA827465;Yang2025_Silkie_Wenchang	CRA004775	0.89290073
ENSGALG00010003985	PRMT5	2	-0.052203241	0.28483802	-0.18327343	0.854583481	0.115613276	3.06347308	67.35731068	0.5	CRA004775;Yang2025_Silkie_Wenchang	PRJNA827465	0.963225297
ENSGALG00010003985	PRMT5	2	-0.100485131	0.160903541	-0.624505408	0.53229572	0.027801362	1.550141691	35.48976809	1	CRA004775;PRJNA827465	Yang2025_Silkie_Wenchang	0.873104119
ENSGALG00010003986	FNDC3A	2	-0.091959437	0.049157219	-1.870720897	0.061383777	0	0.250954714	0	1	PRJNA827465;Yang2025_Silkie_Wenchang	CRA004775	0.427938152
ENSGALG00010003986	FNDC3A	2	0.033310562	0.211669025	0.157370979	0.874952484	0.063331368	3.04441487	67.15296559	0.5	CRA004775;Yang2025_Silkie_Wenchang	PRJNA827465	0.970159044
ENSGALG00010003986	FNDC3A	2	0.045693864	0.176980551	0.258185793	0.796263522	0.043424155	2.572502423	61.12734468	0.5	CRA004775;PRJNA827465	Yang2025_Silkie_Wenchang	0.949325935
ENSGALG00010003988	ENOPH1	2	0.020780003	0.054947768	0.378177371	0.705298835	0	0.117852016	0	0.5	PRJNA827465;Yang2025_Silkie_Wenchang	CRA004775	0.924281103
ENSGALG00010003988	ENOPH1	2	-0.228502614	0.2299632	-0.993648608	0.320393971	0.084749999	4.925636548	79.69805546	1	CRA004775;Yang2025_Silkie_Wenchang	PRJNA827465	0.798348216
ENSGALG00010003988	ENOPH1	2	-0.197434704	0.252134845	-0.783052036	0.433596556	0.111783168	7.722024985	87.05002895	0.5	CRA004775;PRJNA827465	Yang2025_Silkie_Wenchang	0.842762754
ENSGALG00010003990	ICA1	2	0.256841536	0.269076372	0.954530246	0.339815307	0.119670084	5.62530816	82.22319611	1	PRJNA827465;Yang2025_Silkie_Wenchang	CRA004775	0.805097414
ENSGALG00010003990	ICA1	2	0.494660488	0.175137444	2.824413079	0.004736729	0	0.470015552	0	1	CRA004775;Yang2025_Silkie_Wenchang	PRJNA827465	0.101435303
ENSGALG00010003990	ICA1	2	0.021187395	0.119942116	0.176646832	0.859785806	0	0.198377739	0	1	CRA004775;PRJNA827465	Yang2025_Silkie_Wenchang	0.964858345
ENSGALG00010003993	ENSGALG00010003993	2	-0.005500346	0.058524839	-0.093983096	0.925122585	0	0.495695407	0	0.5	PRJNA827465;Yang2025_Silkie_Wenchang	CRA004775	0.982594411
ENSGALG00010003993	ENSGALG00010003993	2	-0.152214138	0.212217006	-0.717257025	0.473215519	0	0.077715059	0	1	CRA004775;Yang2025_Silkie_Wenchang	PRJNA827465	0.854341771
ENSGALG00010003993	ENSGALG00010003993	2	-0.000667685	0.058589668	-0.011395957	0.990907539	0	0.091960941	0	0.5	CRA004775;PRJNA827465	Yang2025_Silkie_Wenchang	0.997555603
ENSGALG00010003994	SDR16C5	2	-0.286087181	0.377695378	-0.757454811	0.448777429	0.270505171	19.26057513	94.80804704	0.5	PRJNA827465;Yang2025_Silkie_Wenchang	CRA004775	0.847017831
ENSGALG00010003994	SDR16C5	2	-0.240855847	0.434486985	-0.554345366	0.579342509	0.353058182	15.30997213	93.46830947	0.5	CRA004775;Yang2025_Silkie_Wenchang	PRJNA827465	0.887888423
ENSGALG00010003994	SDR16C5	2	0.121629092	0.096535831	1.259937279	0.207691989	0	0.282778431	0	1	CRA004775;PRJNA827465	Yang2025_Silkie_Wenchang	0.720024543
ENSGALG00010003995	MLNR	2	-0.391980609	0.14716703	-2.66350832	0.00773305	0	0.417241928	0	1	PRJNA827465;Yang2025_Silkie_Wenchang	CRA004775	0.136109298
ENSGALG00010003995	MLNR	2	-0.479944064	0.218909199	-2.192434428	0.028348153	0	0.152945073	0	1	CRA004775;Yang2025_Silkie_Wenchang	PRJNA827465	0.286858918
ENSGALG00010003995	MLNR	2	-0.303877289	0.187880781	-1.617394219	0.105793213	0	0.027083199	0	1	CRA004775;PRJNA827465	Yang2025_Silkie_Wenchang	0.55189682
ENSGALG00010004000	ENSGALG00010004000	2	0.280160702	0.172006154	1.628783014	0.103358962	0.041644942	3.1062422	67.8067602	1	PRJNA827465;Yang2025_Silkie_Wenchang	CRA004775	0.547576788
ENSGALG00010004000	ENSGALG00010004000	2	0.231080479	0.295816557	0.78116141	0.434707567	0.122465261	3.191652952	68.66827268	0.5	CRA004775;Yang2025_Silkie_Wenchang	PRJNA827465	0.842762754
ENSGALG00010004000	ENSGALG00010004000	2	0.115977223	0.08543833	1.357437855	0.174642127	0	0.689777667	0	0.5	CRA004775;PRJNA827465	Yang2025_Silkie_Wenchang	0.679129269
ENSGALG00010004001	LARP7	2	0.167560232	0.286096229	0.585677877	0.558092003	0.145803096	8.541746288	88.29279206	0.5	PRJNA827465;Yang2025_Silkie_Wenchang	CRA004775	0.880678789
ENSGALG00010004001	LARP7	2	0.372057204	0.141943485	2.621164363	0.008763	0	0.824686044	0	1	CRA004775;Yang2025_Silkie_Wenchang	PRJNA827465	0.146797439
ENSGALG00010004001	LARP7	2	-0.006532745	0.140115533	-0.046623986	0.962812915	0.023165448	1.891827238	47.14105074	0.5	CRA004775;PRJNA827465	Yang2025_Silkie_Wenchang	0.989998461
ENSGALG00010004002	CDADC1	2	-0.054834756	0.081753282	-0.670734617	0.502389606	0	0.318600014	0	1	PRJNA827465;Yang2025_Silkie_Wenchang	CRA004775	0.8629188
ENSGALG00010004002	CDADC1	2	-0.221018838	0.174573042	-1.266053654	0.205493874	0.029537768	1.703146024	41.28512846	1	CRA004775;Yang2025_Silkie_Wenchang	PRJNA827465	0.71825498
ENSGALG00010004002	CDADC1	2	-0.186857043	0.227468925	-0.821461846	0.411383248	0.069866387	2.704992051	63.03131466	1	CRA004775;PRJNA827465	Yang2025_Silkie_Wenchang	0.835128673
ENSGALG00010004003	HNRNPDL	2	0.212770639	0.148090909	1.436756921	0.150787075	0.037750342	6.752335563	85.19030948	1	PRJNA827465;Yang2025_Silkie_Wenchang	CRA004775	0.642051813
ENSGALG00010004003	HNRNPDL	2	0.30642062	0.133023052	2.303515183	0.021249874	0.011592192	1.287505023	22.3304001	1	CRA004775;Yang2025_Silkie_Wenchang	PRJNA827465	0.246512336
ENSGALG00010004003	HNRNPDL	2	0.078223547	0.045061483	1.735929256	0.082576344	0	0.008383404	0	1	CRA004775;PRJNA827465	Yang2025_Silkie_Wenchang	0.495344636
ENSGALG00010004006	NKAIN3	2	-0.456621936	0.197201118	-2.315513934	0.020584823	0	0.046877778	0	1	PRJNA827465;Yang2025_Silkie_Wenchang	CRA004775	0.242156628
ENSGALG00010004006	NKAIN3	2	-0.354636679	0.260699893	-1.360325372	0.173726983	0	0.055313799	0	1	CRA004775;Yang2025_Silkie_Wenchang	PRJNA827465	0.677447326
ENSGALG00010004006	NKAIN3	2	-0.436110609	0.215339675	-2.02522182	0.042844592	0	0.186786521	0	1	CRA004775;PRJNA827465	Yang2025_Silkie_Wenchang	0.357943462
ENSGALG00010004007	ENSGALG00010004007	2	-0.027276176	0.087405324	-0.312065389	0.75499083	0	0.363615922	0	0.5	PRJNA827465;Yang2025_Silkie_Wenchang	CRA004775	0.93838723
ENSGALG00010004007	ENSGALG00010004007	2	0.023430347	0.122315397	0.191556806	0.84808938	0	0.027839731	0	0.5	CRA004775;Yang2025_Silkie_Wenchang	PRJNA827465	0.961363811
ENSGALG00010004007	ENSGALG00010004007	2	-0.070992777	0.112126827	-0.63314711	0.52663758	0	0.0124089	0	1	CRA004775;PRJNA827465	Yang2025_Silkie_Wenchang	0.87066367
ENSGALG00010004016	ENSGALG00010004016	2	0.470320226	0.654836557	0.718225366	0.47261834	0.677659124	4.762743298	79.0036973	0.5	PRJNA827465;Yang2025_Silkie_Wenchang	CRA004775	0.853998373
ENSGALG00010004016	ENSGALG00010004016	2	0.404806151	0.716668363	0.564844455	0.572179567	0.851488546	5.844095807	82.8887131	0.5	CRA004775;Yang2025_Silkie_Wenchang	PRJNA827465	0.885543355
ENSGALG00010004016	ENSGALG00010004016	2	-0.255989912	0.305298972	-0.83848927	0.401755972	0	0.040946422	0	1	CRA004775;PRJNA827465	Yang2025_Silkie_Wenchang	0.832689704
ENSGALG00010004018	HNRNPD	2	-0.022006054	0.098762466	-0.222817993	0.823677163	0.012242802	2.357326133	57.57905595	0.5	PRJNA827465;Yang2025_Silkie_Wenchang	CRA004775	0.955934625
ENSGALG00010004018	HNRNPD	2	-0.062562247	0.206787786	-0.302543243	0.762237974	0.057660574	2.886675442	65.35807297	0.5	CRA004775;Yang2025_Silkie_Wenchang	PRJNA827465	0.94006317
ENSGALG00010004018	HNRNPD	2	-0.108226942	0.052331074	-2.068120037	0.038628737	0	0.934943193	0	1	CRA004775;PRJNA827465	Yang2025_Silkie_Wenchang	0.338965227
ENSGALG00010004029	ARL4A	2	0.172004484	0.254043201	0.677067853	0.498362904	0.118992326	12.22408981	91.81943183	0.5	PRJNA827465;Yang2025_Silkie_Wenchang	CRA004775	0.861478809
ENSGALG00010004029	ARL4A	2	0.422554538	0.126907771	3.329619094	0.000869649	0	0.150183991	0	1	CRA004775;Yang2025_Silkie_Wenchang	PRJNA827465	0.03462999
ENSGALG00010004029	ARL4A	2	-0.031561293	0.107915615	-0.292462703	0.769932873	0.010454671	1.181841075	15.38625448	0.5	CRA004775;PRJNA827465	Yang2025_Silkie_Wenchang	0.941995757
ENSGALG00010004034	NEDD8	2	-0.254555284	0.116660273	-2.182022024	0.029107907	0	0.362141053	0	1	PRJNA827465;Yang2025_Silkie_Wenchang	CRA004775	0.290337858
ENSGALG00010004034	NEDD8	2	0.123171473	0.499783394	0.24644971	0.805334129	0.43516837	7.610985374	86.86109681	0.5	CRA004775;Yang2025_Silkie_Wenchang	PRJNA827465	0.951870339
ENSGALG00010004034	NEDD8	2	0.181577071	0.424741955	0.42749973	0.669015382	0.307618605	6.423605778	84.43241951	0.5	CRA004775;PRJNA827465	Yang2025_Silkie_Wenchang	0.914377769
ENSGALG00010004038	SENP6	2	0.053794873	0.139704495	0.385061861	0.700191584	0.027828763	2.901055287	65.52978482	0.5	PRJNA827465;Yang2025_Silkie_Wenchang	CRA004775	0.922299095
ENSGALG00010004038	SENP6	2	0.327268367	0.134471433	2.433738966	0.014943768	0	0.863563074	0	1	CRA004775;Yang2025_Silkie_Wenchang	PRJNA827465	0.201836433
ENSGALG00010004038	SENP6	2	0.181030051	0.275204372	0.657802235	0.510665218	0.126776162	5.331490031	81.2435174	0.5	CRA004775;PRJNA827465	Yang2025_Silkie_Wenchang	0.865386785
ENSGALG00010004039	ALPK1	2	0.22637153	0.201210013	1.125051016	0.260567417	0	0.357804236	0	1	PRJNA827465;Yang2025_Silkie_Wenchang	CRA004775	0.767694434
ENSGALG00010004039	ALPK1	2	0.221782563	0.276944048	0.800820832	0.423235377	0	0.949283813	0	0.5	CRA004775;Yang2025_Silkie_Wenchang	PRJNA827465	0.840122224
ENSGALG00010004039	ALPK1	2	0.06041991	0.236221325	0.255776697	0.798123269	0	0.385283513	0	0.5	CRA004775;PRJNA827465	Yang2025_Silkie_Wenchang	0.949922217
ENSGALG00010004040	LGALS1B	2	0.214069045	0.130387415	1.641792239	0.100633068	0	0.308571835	0	1	PRJNA827465;Yang2025_Silkie_Wenchang	CRA004775	0.541412814
ENSGALG00010004040	LGALS1B	2	0.310363507	0.200313006	1.549392689	0.12128735	0	0.005084678	0	1	CRA004775;Yang2025_Silkie_Wenchang	PRJNA827465	0.583828135
ENSGALG00010004040	LGALS1B	2	0.178921797	0.148458647	1.205196202	0.228127568	0	0.072845386	0	1	CRA004775;PRJNA827465	Yang2025_Silkie_Wenchang	0.740938585
ENSGALG00010004042	PLAG1	2	0.100798422	0.069327532	1.453945062	0.145961538	0	0.704342569	0	1	PRJNA827465;Yang2025_Silkie_Wenchang	CRA004775	0.634891538
ENSGALG00010004042	PLAG1	2	0.269164383	0.188937809	1.424618947	0.15426738	0	0.011764889	0	1	CRA004775;Yang2025_Silkie_Wenchang	PRJNA827465	0.646945245
ENSGALG00010004042	PLAG1	2	0.08454383	0.073007125	1.158021628	0.24685521	0	0.215985852	0	1	CRA004775;PRJNA827465	Yang2025_Silkie_Wenchang	0.757388785
ENSGALG00010004043	CAB39L	2	-0.234724287	0.190615955	-1.231398952	0.218173689	0.059409387	5.436087033	81.6044152	1	PRJNA827465;Yang2025_Silkie_Wenchang	CRA004775	0.731735294
ENSGALG00010004043	CAB39L	2	-0.420262398	0.122952879	-3.418076913	0.000630653	0	0.129729676	0	1	CRA004775;Yang2025_Silkie_Wenchang	PRJNA827465	0.027540609
ENSGALG00010004043	CAB39L	2	-0.064056661	0.0986302	-0.649462955	0.516039184	0	0.258305145	0	1	CRA004775;PRJNA827465	Yang2025_Silkie_Wenchang	0.867498525
ENSGALG00010004044	RNF138	2	-0.297809802	0.227243287	-1.310532891	0.190015625	0.091632788	8.839385432	88.68699631	1	PRJNA827465;Yang2025_Silkie_Wenchang	CRA004775	0.699333655
ENSGALG00010004044	RNF138	2	-0.263261052	0.293462807	-0.897084898	0.369673617	0.142693827	5.383023137	81.42307817	0.5	CRA004775;Yang2025_Silkie_Wenchang	PRJNA827465	0.820606801
ENSGALG00010004044	RNF138	2	-0.038568293	0.105086731	-0.367013921	0.713608634	0	0.272599918	0	0.5	CRA004775;PRJNA827465	Yang2025_Silkie_Wenchang	0.926266259
ENSGALG00010004046	ENSGALG00010004046	2	0.116736287	0.288235562	0.405003066	0.685475257	0.130737566	4.200146154	76.1913047	0.5	PRJNA827465;Yang2025_Silkie_Wenchang	CRA004775	0.919235539
ENSGALG00010004046	ENSGALG00010004046	2	0.206908529	0.233848896	0.884795833	0.376266757	0.048251221	1.779121048	43.79246983	0.5	CRA004775;Yang2025_Silkie_Wenchang	PRJNA827465	0.822974013
ENSGALG00010004046	ENSGALG00010004046	2	-0.104924835	0.092882246	-1.129654372	0.25862189	0	0.215700273	0	1	CRA004775;PRJNA827465	Yang2025_Silkie_Wenchang	0.765798911
ENSGALG00010004047	PDE7A	2	0.012431329	0.074705837	0.166403723	0.867839244	0.002042383	1.119190189	10.64968133	0.5	PRJNA827465;Yang2025_Silkie_Wenchang	CRA004775	0.967946362
ENSGALG00010004047	PDE7A	2	0.218825412	0.150368031	1.455265527	0.145595766	0	0.294588731	0	1	CRA004775;Yang2025_Silkie_Wenchang	PRJNA827465	0.634195988
ENSGALG00010004047	PDE7A	2	0.054028148	0.154270928	0.350216004	0.726176597	0.024870945	1.491588501	32.9573807	0.5	CRA004775;PRJNA827465	Yang2025_Silkie_Wenchang	0.931029023
ENSGALG00010004049	SETDB2	2	0.118320975	0.079515876	1.488017002	0.13674641	0.00786837	2.51766033	60.2805832	1	PRJNA827465;Yang2025_Silkie_Wenchang	CRA004775	0.618582531
ENSGALG00010004049	SETDB2	2	-0.013133082	0.278658178	-0.04712972	0.962409841	0.121467697	3.992145369	74.95081197	0.5	CRA004775;Yang2025_Silkie_Wenchang	PRJNA827465	0.98997945
ENSGALG00010004049	SETDB2	2	-0.065225246	0.184451967	-0.353620236	0.723632924	0.044839641	2.17106557	53.93965458	0.5	CRA004775;PRJNA827465	Yang2025_Silkie_Wenchang	0.930055658
ENSGALG00010004055	FILIP1	2	0.260808305	0.550879366	0.473439959	0.635899327	0.568084414	14.91421679	93.29498817	0.5	PRJNA827465;Yang2025_Silkie_Wenchang	CRA004775	0.904387873
ENSGALG00010004055	FILIP1	2	0.778023778	0.233954905	3.325528822	0.00088251	0	0.209579767	0	1	CRA004775;Yang2025_Silkie_Wenchang	PRJNA827465	0.034801769
ENSGALG00010004055	FILIP1	2	0.060735406	0.417197437	0.145579528	0.884254933	0.27048583	3.638171342	72.51366398	0.5	CRA004775;PRJNA827465	Yang2025_Silkie_Wenchang	0.972001076
ENSGALG00010004056	SHROOM3	2	0.120469378	0.095418882	1.262531855	0.206757471	0	4.72812E-05	0	1	PRJNA827465;Yang2025_Silkie_Wenchang	CRA004775	0.71868063
ENSGALG00010004056	SHROOM3	2	0.222399944	0.202658783	1.097410836	0.272461839	0.030944106	1.324337362	24.49053929	1	CRA004775;Yang2025_Silkie_Wenchang	PRJNA827465	0.773461007
ENSGALG00010004056	SHROOM3	2	0.218287279	0.198233785	1.101160825	0.270826668	0.032477412	1.348431355	25.83975477	1	CRA004775;PRJNA827465	Yang2025_Silkie_Wenchang	0.772743863
ENSGALG00010004057	ENSGALG00010004057	2	0.104423649	0.57891672	0.17418977	0.859261065	0.607088178	7.870926038	87.29501465	0.5	PRJNA827465;Yang2025_Silkie_Wenchang	CRA004775	0.964605083
ENSGALG00010004057	ENSGALG00010004057	2	0.223302483	0.509557398	0.438228321	0.66122078	0.386715686	3.913369188	74.44657143	0.5	CRA004775;Yang2025_Silkie_Wenchang	PRJNA827465	0.91217832
ENSGALG00010004057	ENSGALG00010004057	2	-0.411707833	0.196396663	-2.096307676	0.036054906	0	0.131603414	0	1	CRA004775;PRJNA827465	Yang2025_Silkie_Wenchang	0.327683285
ENSGALG00010004058	TMEM30A	2	-0.213031254	0.282040173	-0.819143071	0.412704791	0.156654444	65.0857629	98.46356568	0.5	PRJNA827465;Yang2025_Silkie_Wenchang	CRA004775	0.835722417
ENSGALG00010004058	TMEM30A	2	-0.252014707	0.299165004	-0.842393675	0.399592611	0.155141159	6.76861926	85.22593809	0.5	CRA004775;Yang2025_Silkie_Wenchang	PRJNA827465	0.832246851
ENSGALG00010004058	TMEM30A	2	0.050481723	0.038198447	1.321564804	0.18631311	0	0.029744587	0	1	CRA004775;PRJNA827465	Yang2025_Silkie_Wenchang	0.695347453
ENSGALG00010004060	ETV1	2	-0.095772785	0.076442969	-1.25286585	0.210254532	0.005458929	1.784621387	43.96570571	1	PRJNA827465;Yang2025_Silkie_Wenchang	CRA004775	0.721897568
ENSGALG00010004060	ETV1	2	-0.145027875	0.104686013	-1.385360765	0.165942232	0.003357714	1.09825166	8.946188122	0.5	CRA004775;Yang2025_Silkie_Wenchang	PRJNA827465	0.665529196
ENSGALG00010004060	ETV1	2	-0.028703524	0.060812492	-0.47200045	0.636926469	0	0.217354493	0	0.5	CRA004775;PRJNA827465	Yang2025_Silkie_Wenchang	0.904841694
ENSGALG00010004063	ENSGALG00010004063	2	1.014143441	0.807356788	1.256127967	0.209069578	1.150322914	8.429754643	88.13725853	1	PRJNA827465;Yang2025_Silkie_Wenchang	CRA004775	0.721011841
ENSGALG00010004063	ENSGALG00010004063	2	1.305936058	0.530723177	2.460672746	0.013867679	0.377896373	3.034411078	67.04467608	1	CRA004775;Yang2025_Silkie_Wenchang	PRJNA827465	0.193277094
ENSGALG00010004063	ENSGALG00010004063	2	0.454036524	0.271263416	1.67647746	0.093640012	0.011888074	1.08400623	7.749607708	1	CRA004775;PRJNA827465	Yang2025_Silkie_Wenchang	0.524074161
ENSGALG00010004065	TIFA	2	-0.350799446	0.241842357	-1.450529384	0.146910951	0	0.015048629	0	1	PRJNA827465;Yang2025_Silkie_Wenchang	CRA004775	0.6356276
ENSGALG00010004065	TIFA	2	-0.161361584	0.266501456	-0.60548106	0.544859405	0	0.694226857	0	0.5	CRA004775;Yang2025_Silkie_Wenchang	PRJNA827465	0.877345822
ENSGALG00010004065	TIFA	2	-0.161781388	0.240388253	-0.698376008	0.484942077	0	0.617660062	0	0.5	CRA004775;PRJNA827465	Yang2025_Silkie_Wenchang	0.857004733
ENSGALG00010004070	ARL11	2	-0.110451953	0.175192318	-0.630461168	0.528392901	0	0.602602819	0	0.5	PRJNA827465;Yang2025_Silkie_Wenchang	CRA004775	0.871701504
ENSGALG00010004070	ARL11	2	-0.199134947	0.28578425	-0.696801683	0.485926913	0.07062289	1.719043801	41.82812563	0.5	CRA004775;Yang2025_Silkie_Wenchang	PRJNA827465	0.857391223
ENSGALG00010004070	ARL11	2	-0.327696432	0.197664956	-1.65783778	0.097350214	0	0.513031016	0	1	CRA004775;PRJNA827465	Yang2025_Silkie_Wenchang	0.532942995
ENSGALG00010004072	SGK3	2	-0.077515769	0.319889768	-0.242320252	0.808532023	0.186411433	11.20149897	91.07262338	0.5	PRJNA827465;Yang2025_Silkie_Wenchang	CRA004775	0.952496413
ENSGALG00010004072	SGK3	2	-0.400023205	0.133650855	-2.99304635	0.002762078	0	0.000349046	0	1	CRA004775;Yang2025_Silkie_Wenchang	PRJNA827465	0.071732358
ENSGALG00010004072	SGK3	2	0.017777492	0.301550824	0.05895355	0.952989105	0.123805078	2.625342889	61.90973743	0.5	CRA004775;PRJNA827465	Yang2025_Silkie_Wenchang	0.987758913
ENSGALG00010004076	COX7A2	2	-0.230932831	0.471202557	-0.490092483	0.624068457	0.345437905	3.654010241	72.63280796	0.5	PRJNA827465;Yang2025_Silkie_Wenchang	CRA004775	0.900762489
ENSGALG00010004076	COX7A2	2	-0.640861417	0.302653569	-2.117475165	0.034219541	0	0.256630406	0	1	CRA004775;Yang2025_Silkie_Wenchang	PRJNA827465	0.318100902
ENSGALG00010004076	COX7A2	2	-0.093427616	0.311402365	-0.30002218	0.764160238	0.139708377	2.845469665	64.85641677	0.5	CRA004775;PRJNA827465	Yang2025_Silkie_Wenchang	0.940483053
ENSGALG00010004079	AP1AR	2	-0.083919672	0.095747858	-0.876465273	0.380777147	0.000313889	1.017386688	1.708955707	0.5	PRJNA827465;Yang2025_Silkie_Wenchang	CRA004775	0.824392515
ENSGALG00010004079	AP1AR	2	0.133268465	0.327548669	0.406866146	0.684106302	0.185495051	7.22267364	86.15471154	0.5	CRA004775;Yang2025_Silkie_Wenchang	PRJNA827465	0.918806497
ENSGALG00010004079	AP1AR	2	0.222876139	0.230890045	0.965291243	0.334398957	0.077325388	3.528964946	71.66307925	1	CRA004775;PRJNA827465	Yang2025_Silkie_Wenchang	0.803094614
ENSGALG00010004081	RPS20	2	-0.294149673	0.287552862	-1.022941214	0.306335642	0.131968296	4.343980051	76.97963646	1	PRJNA827465;Yang2025_Silkie_Wenchang	CRA004775	0.792485409
ENSGALG00010004081	RPS20	2	-0.5966401	0.197519439	-3.020665219	0.002522201	0	0.062864275	0	1	CRA004775;Yang2025_Silkie_Wenchang	PRJNA827465	0.067541822
ENSGALG00010004081	RPS20	2	-0.218881834	0.229305156	-0.954543885	0.339808406	0.071740799	2.545761051	60.71901566	1	CRA004775;PRJNA827465	Yang2025_Silkie_Wenchang	0.805097414
ENSGALG00010004082	FAM241A	2	-0.280770313	0.145245296	-1.9330768	0.053226738	0.025423997	2.501755551	60.02806911	1	PRJNA827465;Yang2025_Silkie_Wenchang	CRA004775	0.399994353
ENSGALG00010004082	FAM241A	2	-0.224023741	0.237640114	-0.942701707	0.3458335	0.081555257	3.474115527	71.21569527	0.5	CRA004775;Yang2025_Silkie_Wenchang	PRJNA827465	0.807479003

ENSGALG00010004082	FAM241A	2	-0.099991858	0.104596172	-0.955980091	0.339082293	0	0.579367681	0	0.5	CRA004775;PRJNA827465	Yang2025_Silkie_Wenchang	0.804767595
ENSGALG00010004083	PHF11	2	0.127823586	0.085133348	1.501451411	0.133238845	0	0.277897769	0	1	PRJNA827465;Yang2025_Silkie_Wenchang	CRA004775	0.611633901
ENSGALG00010004083	PHF11	2	0.005630804	0.203339133	0.027691688	0.977908053	0.053340952	2.802624471	64.31915833	0.5	CRA004775;Yang2025_Silkie_Wenchang	PRJNA827465	0.993759894
ENSGALG00010004083	PHF11	2	-0.016124345	0.149150095	-0.108108177	0.913909882	0.025378409	2.147919778	53.44332641	0.5	CRA004775;PRJNA827465	Yang2025_Silkie_Wenchang	0.979374752
ENSGALG00010004085	RB1CC1	2	0.013982437	0.123221794	0.113473734	0.909654986	0.019763825	2.62643501	61.92557606	0.5	PRJNA827465;Yang2025_Silkie_Wenchang	CRA004775	0.978659044
ENSGALG00010004085	RB1CC1	2	0.381258124	0.300190917	1.270052164	0.204066049	0.116357509	2.35525936	57.54183098	1	CRA004775;Yang2025_Silkie_Wenchang	PRJNA827465	0.717254253
ENSGALG00010004085	RB1CC1	2	0.27683317	0.436485841	0.63423173	0.525929603	0.31597273	5.008439048	80.03369931	0.5	CRA004775;PRJNA827465	Yang2025_Silkie_Wenchang	0.870414822
ENSGALG00010004086	UTP3	2	0.244463421	0.25847091	0.945806324	0.344247379	0.123937443	13.61189274	92.65348311	0.5	PRJNA827465;Yang2025_Silkie_Wenchang	CRA004775	0.806633216
ENSGALG00010004086	UTP3	2	0.292804446	0.258154704	1.134220846	0.256701922	0.100269981	3.734422607	73.22209869	0.5	CRA004775;Yang2025_Silkie_Wenchang	PRJNA827465	0.764677554
ENSGALG00010004086	UTP3	2	-0.005997721	0.070020274	-0.085656918	0.931739151	0	0.000578461	0	1	CRA004775;PRJNA827465	Yang2025_Silkie_Wenchang	0.98412915
ENSGALG00010004087	NCOA7	2	0.189273957	0.129430551	1.462359203	0.143642803	0.018049764	2.03936507	50.96513053	1	PRJNA827465;Yang2025_Silkie_Wenchang	CRA004775	0.630564924
ENSGALG00010004087	NCOA7	2	0.342623142	0.149729994	2.288273261	0.022121613	0	0.035635296	0	1	CRA004775;Yang2025_Silkie_Wenchang	PRJNA827465	0.252379025
ENSGALG00010004087	NCOA7	2	0.095798994	0.093267042	1.027147336	0.304351092	0	0.171383308	0	1	CRA004775;PRJNA827465	Yang2025_Silkie_Wenchang	0.79225794
ENSGALG00010004095	ANKRD50	2	0.086450337	0.063146105	1.369052562	0.170982845	0	0.000776448	0	1	PRJNA827465;Yang2025_Silkie_Wenchang	CRA004775	0.673107059
ENSGALG00010004095	ANKRD50	2	-0.075723803	0.230692942	-0.328244992	0.742726432	0.061190958	2.007972308	50.19851639	0.5	CRA004775;Yang2025_Silkie_Wenchang	PRJNA827465	0.935621057
ENSGALG00010004095	ANKRD50	2	-0.062208018	0.228287377	-0.272498718	0.785238577	0.069319627	2.268432808	55.91670177	0.5	CRA004775;PRJNA827465	Yang2025_Silkie_Wenchang	0.946325593
ENSGALG00010004096	COL12A1	2	0.205899286	0.378995316	0.543276597	0.586939375	0.229195655	4.80648409	79.19477145	0.5	PRJNA827465;Yang2025_Silkie_Wenchang	CRA004775	0.891596903
ENSGALG00010004096	COL12A1	2	0.152652971	0.563102678	0.271092603	0.786319811	0.44946523	3.165702098	68.41143073	0.5	CRA004775;Yang2025_Silkie_Wenchang	PRJNA827465	0.946639719
ENSGALG00010004096	COL12A1	2	-0.17916068	0.178730875	-1.002404759	0.316148145	0	0.40672298	0	1	CRA004775;PRJNA827465	Yang2025_Silkie_Wenchang	0.796883862
ENSGALG00010004097	ENSGALG00010004097	2	0.387695108	0.223497964	1.734669527	0.082799357	0.081862775	5.487072428	81.77534536	1	PRJNA827465;Yang2025_Silkie_Wenchang	CRA004775	0.495704284
ENSGALG00010004097	ENSGALG00010004097	2	0.080981445	0.579456341	0.139754179	0.888854218	0.605193042	9.695763327	89.68621689	0.5	CRA004775;Yang2025_Silkie_Wenchang	PRJNA827465	0.973135755
ENSGALG00010004097	ENSGALG00010004097	2	-0.111160934	0.349818899	-0.31776709	0.750661616	0.190009782	3.919873019	74.48896954	0.5	CRA004775;PRJNA827465	Yang2025_Silkie_Wenchang	0.937473087
ENSGALG00010004101	ARID1B	2	0.002483382	0.151785373	0.016361144	0.986946278	0.034700676	3.515722233	71.55634223	0.5	PRJNA827465;Yang2025_Silkie_Wenchang	CRA004775	0.996351319
ENSGALG00010004101	ARID1B	2	0.153448311	0.136701668	1.122505033	0.261647769	0	0.263177268	0	1	CRA004775;Yang2025_Silkie_Wenchang	PRJNA827465	0.768310285
ENSGALG00010004101	ARID1B	2	-0.113036487	0.05346631	-2.114162871	0.034501359	0	0.269961351	0	0.5	CRA004775;PRJNA827465	Yang2025_Silkie_Wenchang	0.319895202
ENSGALG00010004102	PCMTD1	2	-0.038349791	0.087926787	-0.436155946	0.662723589	0.002504413	1.140617115	12.32816107	0.5	PRJNA827465;Yang2025_Silkie_Wenchang	CRA004775	0.912778072
ENSGALG00010004102	PCMTD1	2	0.183553106	0.411861353	0.445667225	0.655837643	0.280232347	5.479024835	81.74857698	0.5	CRA004775;Yang2025_Silkie_Wenchang	PRJNA827465	0.910411938
ENSGALG00010004102	PCMTD1	2	0.25495374	0.30496335	0.83601436	0.403146824	0.143576655	3.727670865	73.17359724	1	CRA004775;PRJNA827465	Yang2025_Silkie_Wenchang	0.833064124
ENSGALG00010004104	SCARB2	2	-0.202048757	0.224670687	-0.899310719	0.368487179	0.090306132	9.480433326	89.45195894	0.5	PRJNA827465;Yang2025_Silkie_Wenchang	CRA004775	0.820279089
ENSGALG00010004104	SCARB2	2	-0.274068759	0.197583661	-1.387102344	0.165410558	0.051588739	2.632290873	62.01027742	1	CRA004775;Yang2025_Silkie_Wenchang	PRJNA827465	0.664874006
ENSGALG00010004104	SCARB2	2	0.015057683	0.092835586	0.162197317	0.871150473	0	0.027448309	0	0.5	CRA004775;PRJNA827465	Yang2025_Silkie_Wenchang	0.969278071
ENSGALG00010004108	PERPB	2	-0.039708387	0.389229369	-0.102017962	0.918742418	0.23779342	4.585849218	78.19378806	0.5	PRJNA827465;Yang2025_Silkie_Wenchang	CRA004775	0.981033557
ENSGALG00010004108	PERPB	2	0.36434161	0.849766546	0.428754947	0.668101569	1.305792131	10.28897108	90.28085518	0.5	CRA004775;Yang2025_Silkie_Wenchang	PRJNA827465	0.914120722
ENSGALG00010004108	PERPB	2	0.706510959	0.453345351	1.558438743	0.119129279	0.301837062	3.4697927	71.17983446	1	CRA004775;PRJNA827465	Yang2025_Silkie_Wenchang	0.579471011
ENSGALG00010004109	AGMO	2	-0.221319229	0.489222579	-0.452389644	0.650988306	0.302433838	2.715723504	63.17740011	0.5	PRJNA827465;Yang2025_Silkie_Wenchang	CRA004775	0.908769703
ENSGALG00010004109	AGMO	2	0.765560467	0.620968424	1.232849268	0.217632006	0.464413011	2.355235878	57.54663494	1	CRA004775;Yang2025_Silkie_Wenchang	PRJNA827465	0.731013651
ENSGALG00010004109	AGMO	2	0.336183307	1.122026409	0.299621563	0.764465834	2.182541306	7.302712318	86.3064577	0.5	CRA004775;PRJNA827465	Yang2025_Silkie_Wenchang	0.940606727
ENSGALG00010004113	ECHDC1	2	-0.038190357	0.130514837	-0.292613149	0.769817862	0.001625722	1.049057126	4.676306484	0.5	PRJNA827465;Yang2025_Silkie_Wenchang	CRA004775	0.941972336
ENSGALG00010004113	ECHDC1	2	-0.250880956	0.17720464	-1.41576968	0.15684295	0	0.580575462	0	1	CRA004775;Yang2025_Silkie_Wenchang	PRJNA827465	0.650212649
ENSGALG00010004113	ECHDC1	2	-0.112385166	0.284403509	-0.39516097	0.692724095	0.087776086	1.845309904	45.80856051	0.5	CRA004775;PRJNA827465	Yang2025_Silkie_Wenchang	0.920579378
ENSGALG00010004116	SPRY1	2	-0.210273699	0.231568951	-0.908039258	0.363857481	0.097451099	10.78311815	90.72624462	0.5	PRJNA827465;Yang2025_Silkie_Wenchang	CRA004775	0.817042253
ENSGALG00010004116	SPRY1	2	0.088301952	0.560577403	0.157519641	0.874835329	0.591014298	16.38513514	93.89690722	0.5	CRA004775;Yang2025_Silkie_Wenchang	PRJNA827465	0.970159044
ENSGALG00010004116	SPRY1	2	0.298458378	0.326400671	0.914392063	0.360505556	0.182590015	6.33593322	84.21700537	1	CRA004775;PRJNA827465	Yang2025_Silkie_Wenchang	0.814880744
ENSGALG00010004117	STBD1	2	0.064292373	0.123944135	0.518720575	0.603955612	0	0.056449358	0	1	PRJNA827465;Yang2025_Silkie_Wenchang	CRA004775	0.895982615
ENSGALG00010004117	STBD1	2	0.000581074	0.197352953	0.002944338	0.997650762	0	0.132409053	0	0.5	CRA004775;Yang2025_Silkie_Wenchang	PRJNA827465	0.999290321
ENSGALG00010004117	STBD1	2	0.068745353	0.149773316	0.458996001	0.646235708	0	0.219350607	0	0.5	CRA004775;PRJNA827465	Yang2025_Silkie_Wenchang	0.907220702
ENSGALG00010004122	TRIM55	2	0.275002852	0.308983925	0.890023168	0.373453446	0.173635896	10.830724886	90.76700763	0.5	PRJNA827465;Yang2025_Silkie_Wenchang	CRA004775	0.822254022
ENSGALG00010004122	TRIM55	2	0.570555866	0.155881409	3.660191857	0.000252026	0	0.452274749	0	1	CRA004775;Yang2025_Silkie_Wenchang	PRJNA827465	0.014940266
ENSGALG00010004122	TRIM55	2	-0.016540533	0.095833564	-0.172596448	0.862968644	0	0.086371296	0	0.5	CRA004775;PRJNA827465	Yang2025_Silkie_Wenchang	0.966099097
ENSGALG00010004123	NDUFAF4	2	-0.046997702	0.242943338	-0.19345129	0.84660555	0.104874911	8.526745809	88.27219642	0.5	PRJNA827465;Yang2025_Silkie_Wenchang	CRA004775	0.961252712
ENSGALG00010004123	NDUFAF4	2	-0.083097248	0.253786317	-0.327429978	0.743342696	0.092554432	3.44561759	70.97762668	0.5	CRA004775;Yang2025_Silkie_Wenchang	PRJNA827465	0.935767527
ENSGALG00010004123	NDUFAF4	2	0.178970217	0.063677995	2.810550424	0.004945684	0	0.009527387	0	1	CRA004775;PRJNA827465	Yang2025_Silkie_Wenchang	0.104831857
ENSGALG00010004125	MANEA	2	-0.068314127	0.091640619	-0.74545685	0.455995587	0.007392128	1.657422163	39.66534163	1	PRJNA827465;Yang2025_Silkie_Wenchang	CRA004775	0.84907427
ENSGALG00010004125	MANEA	2	-0.084848889	0.17092314	-0.496415457	0.619601322	0.024286917	1.527144777	34.51832369	0.5	CRA004775;Yang2025_Silkie_Wenchang	PRJNA827465	0.899301701
ENSGALG00010004125	MANEA	2	0.010078737	0.070901325	0.142151597	0.886960263	0	0.412114086	0	0.5	CRA004775;PRJNA827465	Yang2025_Silkie_Wenchang	0.972906121
ENSGALG00010004127	EPHA7	2	-0.311637828	0.13417474	-2.322626664	0.020199217	0	0.546074146	0	1	PRJNA827465;Yang2025_Silkie_Wenchang	CRA004775	0.239129045
ENSGALG00010004127	EPHA7	2	-0.138863009	0.22325084	-0.622004421	0.533938961	0	0.255644502	0	0.5	CRA004775;Yang2025_Silkie_Wenchang	PRJNA827465	0.873408008
ENSGALG00010004127	EPHA7	2	-0.356397771	0.161019808	-2.213378438	0.026871566	0	0.595357848	0	0.5	CRA004775;PRJNA827465	Yang2025_Silkie_Wenchang	0.27936077
ENSGALG00010004131	NUDT6	2	0.608775773	0.107316202	5.672729379	1.4054E-08	0	0.521687037	0	1	PRJNA827465;Yang2025_Silkie_Wenchang	CRA004775	6.73616E-06
ENSGALG00010004131	NUDT6	2	0.39086139	0.329363289	1.186718142	0.235338817	0.173521551	4.805785028	79.19174507	1	CRA004775;Yang2025_Silkie_Wenchang	PRJNA827465	0.748403715
ENSGALG00010004131	NUDT6	2	0.324928936	0.249506914	1.302284297	0.192819218	0.083666646	2.867843698	65.13059618	1	CRA004775;PRJNA827465	Yang2025_Silkie_Wenchang	0.702807541
ENSGALG00010004135	NUP54	2	-0.076106603	0.049754351	-1.529647199	0.12610408	0	0.793151656	0	1	PRJNA827465;Yang2025_Silkie_Wenchang	CRA004775	0.596422219
ENSGALG00010004135	NUP54	2	-0.178258101	0.100989579	-1.765113819	0.077544639	0	0.001172068	0	1	CRA004775;Yang2025_Silkie_Wenchang	PRJNA827465	0.479609428
ENSGALG00010004135	NUP54	2	-0.067259149	0.052064366	-1.291846116	0.196410441	0	0.46362198	0	1	CRA004775;PRJNA827465	Yang2025_Silkie_Wenchang	0.707832911
ENSGALG00010004137	CROT	2	-0.002770195	0.065702898	-0.042162442	0.966369203	0.002930493	1.380922148	27.58462153	0.5	PRJNA827465;Yang2025_Silkie_Wenchang	CRA004775	0.990881287
ENSGALG00010004137	CROT	2	-0.145056201	0.100321554	-1.44591262	0.148201712	0	0.643790127	0	1	CRA004775;Yang2025_Silkie_Wenchang	PRJNA827465	0.637841861
ENSGALG00010004137	CROT	2	-0.06487243	0.161235724	-0.402345266	0.687429946	0.033601805	2.145797224	53.39727403	0.5	CRA004775;PRJNA827465	Yang2025_Silkie_Wenchang	0.91949273
ENSGALG00010004141	KPNA3	2	-0.186596654	0.079614024	-2.343766138	0.019090137	0.007477753	2.248283878	55.52163097	1	PRJNA827465;Yang2025_Silkie_Wenchang	CRA004775	0.231442015
ENSGALG00010004141	KPNA3	2	0.006791932	0.351320153	0.019332601	0.984575777	0.201397696	4.802454873	79.17731605	0.5	CRA004775;Yang2025_Silkie_Wenchang	PRJNA827465	0.996703389
ENSGALG00010004141	KPNA3	2	0.064591873	0.260897117	0.247576033	0.804462456	0.10106988	3.035582022	67.03566962	0.5	CRA004775;PRJNA827465	Yang2025_Silkie_Wenchang	0.951655066
ENSGALG00010004142	RBBP8	2	0.063078043	0.173758481	0.363021378	0.716589914	0.051777471	6.863650122	85.43049278	0.5	PRJNA827465;Yang2025_Silkie_Wenchang	CRA004775	0.927487786
ENSGALG00010004142	RBBP8	2	0.238998133	0.106193788	2.25058488	0.024411842	0	0.061782148	0	1	CRA004775;Yang2025_Silkie_Wenchang	PRJNA827465	0.267184364
ENSGALG00010004142	RBBP8	2	-0.089228366	0.07018658	-1.27130238	0.203621096	0	0.564807099	0	0.5	CRA004775;PRJNA827465	Yang2025_Silkie_Wenchang	0.717254253
ENSGALG00010004143	TCF24	2	0.231267405	0.216665032	1.067396078	0.285793019	0	0.965051662	0	1	PRJNA827465;Yang2025_Silkie_Wenchang	CRA004775	0.781501636
ENSGALG00010004143	TCF24	2	-0.115946194	0.687809153	-0.168573206	0.866132358	0.696676533	3.435041869	70.88827332	0.5	CRA004775;Yang2025_Silkie_Wenchang	PRJNA827465	0.967318736
ENSGALG00010004143	TCF24	2	-0.267326896	0.44661829	-0.598557878	0.549464752	0.19277864	1.679622405	40.46280897	0.5	CRA004775;PRJNA827465	Yang2025_Silkie_Wenchang	0.878554604
ENSGALG00010004146	NAAA	2	-0.347401631	0.117185953	-2.964533053	0.003031427	0	0.026034281	0	1	PRJNA827465;Yang2025_Silkie_Wenchang	CRA004775	0.075885251
ENSGALG00010004146	NAAA	2	-0.243752484	0.124768817	-1.953633045	0.05074463	0	0.494024168	0	1	CRA004775;Yang2025_Silkie_Wenchang	PRJNA827465	0.38992636
ENSGALG00010004146	NAAA	2	-0.271391668	0.11992689	-2.262975958	0.023637171	0	0.780538301	0	1	CRA004775;PRJNA827465	Yang2025_Silkie_Wenchang	0.262358584
ENSGALG00010004147	SPRYD7	2	0.011595695	0.08418667	0.137737849	0.890447606	0.006875459	1.748133791	42.79614035	0.5	PRJNA827465;Yang2025_Silkie_Wenchang	CRA004775	0.973417023
ENSGALG00010004147	SPRYD7	2	-0.067254281	0.111245264	-0.604558625	0.545472305	0	0.741214541	0	0.5	CRA004775;Yang2025_Silkie_Wenchang	PRJNA827465	0.877672787
ENSGALG00010004147	SPRYD7	2	0.074210673	0.059273762	1.25199871	0.210570334	0	0.084489299	0	1	CRA004775;PRJNA827465	Yang2025_Silkie_Wenchang	0.722116705
ENSGALG00010004148	ENSGALG00010004148	2	0.229605089	0.165936398	1.383693345	0.166452372	0.035988217	2.849136854	64.90165088	0.5	PRJNA827465;Yang2025_Silkie_Wenchang	CRA004775	0.665350942
ENSGALG00010004148	ENSGALG00010004148	2	0.171546205	0.294358411	0.582780035	0.560041387	0.119407532	2.934762126	65.92568811	0.5	CRA004775;Yang2025_Silkie_Wenchang	PRJNA827465	0.881203589
ENSGALG00010004148	ENSGALG00010004148	2	0.041902313	0.113724141	0.368455745	0.712533438	0	0.632690286	0	0.5	CRA004775;PRJNA827465	Yang2025_Silkie_Wenchang	0.92597752
ENSGALG00010004149	SDAD1	2	-0.054266584	0.14772967	-0.367337073	0.713367604	0.035901726	5.553517158	81.99339317	0.5	PRJNA827465;Yang2025_Silkie_Wenchang	CRA004775	0.926216079
ENSGALG00010004149	SDAD1	2	0.045623676	0.098423667	0.463553909	0.642967383	0.002523148	1.107298992	9.690155303	0.5	CRA004775;Yang2025_Silkie_Wenchang	PRJNA827465	0.906634523
ENSGALG00010004149	SDAD1	2	-0.185196875	0.068753369	-2.693640719	0.00706763	0	0.108906046	0	1	CRA004775;PRJNA827465	Yang2025_Silkie_Wenchang	0.129079497
ENSGALG00010004154	COPS5	2	-0.012147268	0.058948967	-0.206064136	0.836740813	0	0.177037379	0	1	PRJNA827465;Yang2025_Silkie_Wenchang	CRA004775	0.958399882
ENSGALG00010004154	COPS5	2	-0.038403923	0.142714132	-0.269096843	0.787855159	0	0.276598862	0	0.5	CRA004775;Yang2025_Silkie_Wenchang	PRJNA827465	0.946919116
ENSGALG00010004154	COPS5	2	0.002125566	0.062445429	0.034038757	0.972846232	0	0.130644222	0	0.5	CRA004775;PRJNA827465	Yang2025_Silkie_Wenchang	0.992213869
ENSGALG00010004158	JCHAIN	2	-0.519900612	0.249704999	-2.082059289	0.037337054	0	0.666201348	0	1	PRJNA827465;Yang2025_Silkie_Wenchang	CRA004775	0.333285999
ENSGALG00010004158	JCHAIN	2	0.355851589	1.185805612	0.300092684	0.76410646	2.541992667	10.27032907	90.26321364	0.5	CRA004775;Yang2025_Silkie_Wenchang	PRJNA827465	0.940483053
ENSGALG00010004158	JCHAIN	2	0.539458885	0.972783137	0.554551052	0.579201093	1.663848787	7.958518827	87.43464784	0.5	CRA004775;PRJNA827465	Yang2025_Silkie_Wenchang	0.887888423
ENSGALG00010004172	ST8SIA6	2	-0.038988448	0.109637999	-0.355610725	0.722132111	0	0.000504721	0	1	PRJNA827465;Yang2025_Silkie_Wenchang	CRA004775	0.929404251
ENSGALG00010004172	ST8SIA6	2	0.035663122	0.170083893	0.209679595	0.833971753	0.011551426	1.115568557	10.35961044	0.5	CRA004775;Yang2025_Silkie_Wenchang	PRJNA827465	0.957968425
ENSGALG00010004172	ST8SIA6	2	0.038090435	0.177987927	0.21400572	0.830542596	0.012367132	1.12186704	10.86287728	0.5	CRA004775;PRJNA827465	Yang2025_Silkie_Wenchang	0.957295395
ENSGALG00010004177	CSPP1	2	0.51274104	0.369764185	1.386670373	0.165542299	0.252068983	12.31829118	91.88199089	0.5	PRJNA827465;Yang2025_Silkie_Wenchang	CRA004775	0.664874006
ENSGALG00010004177	CSPP1	2	0.60756075	0.298633262	2.034471128	0.041904117	0.139282508	4.563191613	78.08551372	1	CRA004775;Yang2025_Silkie_Wenchang	PRJNA827465	0.35448778
ENSGALG00010004177	CSPP1	2	0.183793652	0.075578282	2.431831563	0.015022692	0	0.438308739	0	1	CRA004775;PRJNA827465	Yang2025_Silkie_Wenchang	0.202690302
ENSGALG00010004181	BBS12	2	0.011641753	0.076371263	0.152436306	0.878842824	0	0.452994926	0	0.5	PRJNA827465;Yang2025_Silkie_Wenchang	CRA004775	0.971127951
ENSGALG00010004181	BBS12	2	-0.135661299	0.130094668	-1.042789075	0.297045992	0.00022911	1.004324873	0.430624944	1	CRA004775;Yang2025_Silkie_Wenchang	PRJNA827465	0.788466926
ENSGALG00010004181	BBS12	2	-0.089434491	0.202353049	-0.441972547	0.658509076	0.050562514	2.085527457	52.05049943	0.5	CRA004775;PRJNA827465	Yang2025_Silkie_Wenchang	0.911105434
ENSGALG00010004187	ARFGEF1	2	-0.038811173	0.071857272	-0.540114758	0.589117894	0.004709465	1.734069333	42.33217895	0.5	PRJNA827465;Yang2025_Silkie_Wenchang	CRA004775	0.892171138
ENSGALG00010004187	ARFGEF1	2	0.047012119	0.088812578	0.529340972	0.596569072	0	0.050610592	0	1	CRA004775;Yang2025_Silkie_Wenchang	PRJNA827465	0.894064255
ENSGALG00010004187	ARFGEF1	2	-0.087228628	0.056668945	-1.539266834	0.123739167	0	0.170627138	0	0.5	CRA004775;PRJNA827465	Yang2025_Silkie_Wenchang	0.590002486
ENSGALG00010004188	BMI1	2	-0.036704614	0.051993124	-0.705951317	0.480218413	0	0.42852379	0	0.5	PRJNA827465;Yang2025_Silkie_Wenchang	CRA004775	0.856469768
ENSGALG00010004188	BMI1	2	0.123276795	0.097411548	1.265525476	0.205683022	0	0.873973464	0	1	CRA004775;Yang2025_Silkie_Wenchang	PRJNA827465	0.71825498
ENSGALG00010004188	BMI1	2	0.054015129	0.134317725	0.402144458	0.687577716	0.025955018	3.114082402	67.88781185	0.5	CRA004775;PRJNA827465	Yang2025_Silkie_Wenchang	0.919521188
ENSGALG00010004191	CABLES1	2	-0.421436296	0.298031566	-1.414065971	0.157342533	0.167473166	17.27285419	94.21056886	1	PRJNA827465;Yang2025_Silkie_Wenchang	CRA004775	0.650687662
ENSGALG00010004191	CABLES1	2	-0.50089419	0.274508009	-1.824697907	0.068046629	0.111714407	3.501332889	71.43944801	1	CRA004775;Yang2025_Silkie_Wenchang	PRJNA827465	0.449787533
ENSGALG00010004191	CABLES1	2	-0.13365099	0.072705496	-1.838251549	0.066025347	0	0.01710503	0	1	CRA004775;PRJNA827465	Yang2025_Silkie_Wenchang	0.442444044
ENSGALG00010004200	ABCB1	2	0.148669969	0.168104174	0.884391895	0.376484697	0.015708459	1.320524437	24.27251082	1	PRJNA827465;Yang2025_Silkie_Wenchang	CRA004775	0.822974013
ENSGALG00010004200	ABCB1	2	0.45513855	0.472150864	0.963968478	0.33506173	0.375312098	5.931251891	83.1401529	1	CRA004775;Yang2025_Silkie_Wenchang	PRJNA827465	0.803101658
ENSGALG00010004200	ABCB1	2	0.64188674	0.291614865	2.201145475	0.027725727	0.075148171	1.757969595	43.11619479	1	CRA004775;PRJNA827465	Yang2025_Silkie_Wenchang	0.284073056
ENSGALG00010004210	SYNCRIP	2	0.160568083	0.23163809	0.693185142	0.488193371	0.097263941	10.00830166	90.00829477	0.5	PRJNA827465;Yang2025_Silkie_Wenchang	CRA004775	0.85768885
ENSGALG00010004210	SYNCRIP	2	0.241483057	0.172775914	1.397666213	0.162213325	0.039258913	2.919619055	65.74895624	1	CRA004775;Yang2025_Silkie_Wenchang	PRJNA827465	0.659645143
ENSGALG00010004210	SYNCRIP	2	-0.04314253	0.040752233	-1.058654372	0.289757215	0	0.621308646	0	0.5	CRA004775;PRJNA827465	Yang2025_Silkie_Wenchang	0.784067533
ENSGALG00010004211	RNF146	2	0.059820432	0.041369192	1.446014033	0.148173266	0	0.036730063	0	1	PRJNA827465;Yang2025_Silkie_Wenchang	CRA004775	0.637789275
ENSGALG00010004211	RNF146	2	0.0465577	0.094027814	0.49514817	0.620495534	0	0.279629672	0	0.5	CRA004775;Yang2025_Silkie_Wenchang	PRJNA827465	0.899395593
ENSGALG00010004211	RNF146	2	0.051241848	0.042961221	1.192746539	0.232968653	0	0.23270382	0	0.5	CRA004775;PRJNA827465	Yang2025_Silkie_Wenchang	0.746005924
ENSGALG00010004212	RSPO3	2	0.180230957	0.30632248	0.588369997	0.556283972	0.141617147	4.05871486	75.36165918	0.5	PRJNA827465;Yang2025_Silkie_Wenchang	CRA004775	0.880324407
ENSGALG00010004212	RSPO3	2	-0.038674966	0.653131822	-0.059214641	0.952781148	0.65159275	3.593280592	72.17027798	0.5	CRA004775;Yang2025_Silkie_Wenchang	PRJNA827465	0.987758913
ENSGALG00010004212	RSPO3	2	-0.222332005	0.234110861	-0.949686843	0.342271397	0.008858628	1.034320721	3.318189453	1	CRA004775;PRJNA827465	Yang2025_Silkie_Wenchang	0.806005903
ENSGALG00010004214	REPS1	2	-0.072201388	0.12461416	-0.57939955	0.562319608	0.018923228	1.899029444	47.34152211	0.5	PRJNA827465;Yang2025_Silkie_Wenchang	CRA004775	0.881955027
ENSGALG00010004214	REPS1	2	0.182383275	0.130299595	1.399722506	0.161596432	0	0.094030125	0	1	CRA004775;Yang2025_Silkie_Wenchang	PRJNA827465	0.658226195
ENSGALG00010004214	REPS1	2	-0.002667751	0.177841847	-0.015000694	0.988031627	0.050232897	4.168107079	76.00829391	0.5	CRA004775;PRJNA827465	Yang2025_Silkie_Wenchang	0.996832504
ENSGALG00010004216	SNX14	2	0.119851264	0.045246722	2.648838591	0.00807689	0	0.886637799	0	1	PRJNA827465;Yang2025_Silkie_Wenchang	CRA004775	0.139633915
ENSGALG00010004216	SNX14	2	0.197075047	0.108852089	1.810484759	0.070220646	0	0.289660715	0	1	CRA004775;Yang2025_Silkie_Wenchang	PRJNA827465	0.457187499
ENSGALG00010004216	SNX14	2	0.106427978	0.046164102	2.305427223	0.021142658	0	0.009860294	0	1	CRA004775;PRJNA827465	Yang2025_Silkie_Wenchang	0.246043752
ENSGALG00010004217	CCDC28A	2	0.059910921	0.086229962	0.694780788	0.48719269	0	0.102401054	0	1	PRJNA827465;Yang2025_Silkie_Wenchang	CRA004775	0.85768885
ENSGALG00010004217	CCDC28A	2	-0.024629198	0.120612435	-0.204201152	0.838196311	0	0.768159347	0	0.5	CRA004775;Yang2025_Silkie_Wenchang	PRJNA827465	0.959165366
ENSGALG00010004217	CCDC28A	2	0.017979134	0.129831463	0.138480562	0.889860631	0.008428624	1.191108539	16.04459484	0.5	CRA004775;PRJNA827465	Yang2025_Silkie_Wenchang	0.973208458
ENSGALG00010004219	FGF2	2	-0.114071456	0.196649912	-0.58007377	0.561864871	0.065651878	6.582728857	84.80873173	0.5	PRJNA827465;Yang2025_Silkie_Wenchang	CRA004775	0.881802301
ENSGALG00010004219	FGF2	2	-0.123391119	0.272043972	-0.453570495	0.650137993	0.09751814	2.281935116	56.17754452	0.5	CRA004775;Yang2025_Silkie_Wenchang	PRJNA827465	0.908335059
ENSGALG00010004219	FGF2	2	0.106128922	0.113192127	0.937599858	0.348450084	0	0.244395229	0	1	CRA004775;PRJNA827465	Yang2025_Silkie_Wenchang	0.809147894
ENSGALG00010004222	SPAG6	2	-0.355493971	0.356075966	-0.998365532	0.318102141	0.201581092	4.480221403	77.6796745	1	PRJNA827465;Yang2025_Silkie_Wenchang	CRA004775	0.797501246
ENSGALG00010004222	SPAG6	2	-0.417833717	0.414213885	-1.008739042	0.313099803	0.198554626	2.292099222	56.37187124	0.5	CRA004775;Yang2025_Silkie_Wenchang	PRJNA827465	0.796531448
ENSGALG00010004222	SPAG6	2	-0.039981679	0.131099523	-0.30497196	0.760387509	0	0.061745706	0	0.5	CRA004775;PRJNA827465	Yang2025_Silkie_Wenchang	0.939327609
ENSGALG00010004224	NT5E	2	-0.277822046	0.135406874	-2.051757326	0.040193252	0	0.000668693	0	1	PRJNA827465;Yang2025_Silkie_Wenchang	CRA004775	0.347204191
ENSGALG00010004224	NT5E	2	-0.080544956	0.250110177	-0.322037898	0.747423984	0.054997751	1.723863026	41.99075071	0.5	CRA004775;Yang2025_Silkie_Wenchang	PRJNA827465	0.93680487
ENSGALG00010004224	NT5E	2	-0.096233749	0.241370417	-0.398697366	0.690116209	0.060677456	1.910633335	47.66133398	0.5	CRA004775;PRJNA827465	Yang2025_Silkie_Wenchang	0.919979266
ENSGALG00010004225	CETN4	2	-0.154941346	0.167164399	-0.926880043	0.35398882	0.047971744	6.964547841	85.64156607	0.5	PRJNA827465;Yang2025_Silkie_Wenchang	CRA004775	0.811574555
ENSGALG00010004225	CETN4	2	-0.309632357	0.092845682	-3.334913924	0.000853258	0	0.203822861	0	1	CRA004775;Yang2025_Silkie_Wenchang	PRJNA827465	0.034080896
ENSGALG00010004225	CETN4	2	-0.025100613	0.076752883	-0.327031529	0.743644038	0.001385509	1.05584891	5.289479316	0.5	CRA004775;PRJNA827465	Yang2025_Silkie_Wenchang	0.935821071
ENSGALG00010004226	RUNDC3B	2	-0.309976442	0.121939797	-2.54204492	0.011020601	0	0.456362494	0	1	PRJNA827465;Yang2025_Silkie_Wenchang	CRA004775	0.166914327
ENSGALG00010004226	RUNDC3B	2	-0.211886069	0.280745509	-0.754726476	0.450413117	0.076783141	1.915019864	47.78122052	0.5	CRA004775;Yang2025_Silkie_Wenchang	PRJNA827465	0.847577105
ENSGALG00010004226	RUNDC3B	2	-0.192007134	0.147187443	-1.304507572	0.19206058	0.007199261	1.114564266	10.27883893	0.5	CRA004775;PRJNA827465	Yang2025_Silkie_Wenchang	0.701846421
ENSGALG00010004230	TAF1B	2	0.020162846	0.070098467	0.287636043	0.773625352	0	0.047374308	0	0.5	PRJNA827465;Yang2025_Silkie_Wenchang	CRA004775	0.943248868
ENSGALG00010004230	TAF1B	2	-0.16861356	0.213994493	-0.787934108	0.430735249	0.050987135	2.059800086	51.45159927	1	CRA004775;Yang2025_Silkie_Wenchang	PRJNA827465	0.842503929
ENSGALG00010004230	TAF1B	2	-0.140258489	0.229641086	-0.610772628	0.541350117	0.074831023	2.829131108	64.65345855	0.5	CRA004775;PRJNA827465	Yang2025_Silkie_Wenchang	0.876088431
ENSGALG00010004233	NHSL1	2	0.108626933	0.055152516	1.969573472	0.048887274	0	0.025432124	0	1	PRJNA827465;Yang2025_Silkie_Wenchang	CRA004775	0.382067319
ENSGALG00010004233	NHSL1	2	0.074567674	0.195014555	0.382369785	0.702187099	0	0.000208403	0	1	CRA004775;Yang2025_Silkie_Wenchang	PRJNA827465	0.923083392
ENSGALG00010004233	NHSL1	2	0.110396644	0.056705614	1.946837998	0.05155416	0	0.007713021	0	1	CRA004775;PRJNA827465	Yang2025_Silkie_Wenchang	0.393596182
ENSGALG00010004236	DBF4	2	0.208763203	0.092786336	2.249934754	0.024453087	0.003883029	1.272995619	21.44513422	1	PRJNA827465;Yang2025_Silkie_Wenchang	CRA004775	0.267184364
ENSGALG00010004236	DBF4	2	0.300733143	0.127126417	2.365622738	0.017999776	0	0.283757286	0	1	CRA004775;Yang2025_Silkie_Wenchang	PRJNA827465	0.22387506
ENSGALG00010004236	DBF4	2	0.133143304	0.097615935	1.363950505	0.172583144	0	0.003482966	0	1	CRA004775;PRJNA827465	Yang2025_Silkie_Wenchang	0.675533841
ENSGALG00010004237	RUFY3	2	0.157647089	0.060302494	2.614271464	0.008941796	0	0.003868074	0	1	PRJNA827465;Yang2025_Silkie_Wenchang	CRA004775	0.148099201
ENSGALG00010004237	RUFY3	2	0.171921455	0.087200944	1.971554987	0.04866043	0	0.24893516	0	1	CRA004775;Yang2025_Silkie_Wenchang	PRJNA827465	0.381461853
ENSGALG00010004237	RUFY3	2	0.173260215	0.073515947	2.356770508	0.018434636	0	0.229874302	0	1	CRA004775;PRJNA827465	Yang2025_Silkie_Wenchang	0.227269913
ENSGALG00010004238	CCNC	2	-0.19149495	0.161524079	-1.185550483	0.235799867	0.044960121	7.013028942	85.74082599	1	PRJNA827465;Yang2025_Silkie_Wenchang	CRA004775	0.748669015
ENSGALG00010004238	CCNC	2	-0.330948728	0.099680739	-3.320087035	0.000899894	0	0.942116033	0	1	CRA004775;Yang2025_Silkie_Wenchang	PRJNA827465	0.035297933
ENSGALG00010004238	CCNC	2	-0.041438794	0.060436852	-0.685654406	0.492931064	0	0.0018159	0	1	CRA004775;PRJNA827465	Yang2025_Silkie_Wenchang	0.859562076
ENSGALG00010004240	ENSGALG00010004240	2	-0.184125122	0.221978984	-0.829470965	0.40683796	0.08080148	5.347458985	81.29952931	0.5	PRJNA827465;Yang2025_Silkie_Wenchang	CRA004775	0.833618122
ENSGALG00010004240	ENSGALG00010004240	2	-0.359808603	0.146906591	-2.449233891	0.014316045	0	0.747750188	0	1	CRA004775;Yang2025_Silkie_Wenchang	PRJNA827465	0.196460728
ENSGALG00010004240	ENSGALG00010004240	2	0.005968578	0.092861721	0.064273826	0.948752194	0	0.182610498	0	0.5	CRA004775;PRJNA827465	Yang2025_Silkie_Wenchang	0.986972485
ENSGALG00010004241	PREX2	2	0.006427032	0.073381532	0.087583779	0.930207495	0	0.774563804	0	0.5	PRJNA827465;Yang2025_Silkie_Wenchang	CRA004775	0.983813577
ENSGALG00010004241	PREX2	2	0.171107618	0.156624	1.092473815	0.274624894	0	0.233525593	0	1	CRA004775;Yang2025_Silkie_Wenchang	PRJNA827465	0.774575583
ENSGALG00010004241	PREX2	2	-0.01063049	0.079948134	-0.132967334	0.894219215	0	0.81100337	0	0.5	CRA004775;PRJNA827465	Yang2025_Silkie_Wenchang	0.974381724
ENSGALG00010004242	ADAM22	2	0.103525058	0.108480318	0.954321109	0.339921126	0	0.168465849	0	1	PRJNA827465;Yang2025_Silkie_Wenchang	CRA004775	0.805202509
ENSGALG00010004242	ADAM22	2	0.177646065	0.218204667	0.814125872	0.415572846	0	0.01978028	0	1	CRA004775;Yang2025_Silkie_Wenchang	PRJNA827465	0.837689773
ENSGALG00010004242	ADAM22	2	0.083060363	0.117907391	0.704454252	0.481149931	0	0.004075164	0	1	CRA004775;PRJNA827465	Yang2025_Silkie_Wenchang	0.85681099
ENSGALG00010004243	BLTP1	2	-0.015828759	0.044783921	-0.353447378	0.723753072	0	0.017499021	0	0.5	PRJNA827465;Yang2025_Silkie_Wenchang	CRA004775	0.93017452
ENSGALG00010004243	BLTP1	2	0.044184669	0.131774751	0.335304514	0.737395389	0	0.329455505	0	1	CRA004775;Yang2025_Silkie_Wenchang	PRJNA827465	0.933968189
ENSGALG00010004243	BLTP1	2	-0.012035715	0.046261932	-0.260164567	0.794736835	0	0.515766757	0	0.5	CRA004775;PRJNA827465	Yang2025_Silkie_Wenchang	0.948905846
ENSGALG00010004244	TMEM241	2	-0.043404217	0.05806548	-0.747504662	0.45475899	0	0.029914459	0	1	PRJNA827465;Yang2025_Silkie_Wenchang	CRA004775	0.849072665
ENSGALG00010004244	TMEM241	2	-0.111751797	0.097544713	-1.14564689	0.251941287	0	0.598653468	0	1	CRA004775;Yang2025_Silkie_Wenchang	PRJNA827465	0.761871849
ENSGALG00010004244	TMEM241	2	-0.061407855	0.062399842	-0.984102733	0.325065003	0	0.991058186	0	1	CRA004775;PRJNA827465	Yang2025_Silkie_Wenchang	0.799914637
ENSGALG00010004245	ARFGEF3	2	-0.176276194	0.16192914	-1.088600813	0.276329958	0	0.029714986	0	1	PRJNA827465;Yang2025_Silkie_Wenchang	CRA004775	0.77582325
ENSGALG00010004245	ARFGEF3	2	-0.138406509	0.270224608	-0.512190618	0.608517609	0	0.001751715	0	1	CRA004775;Yang2025_Silkie_Wenchang	PRJNA827465	0.897208691
ENSGALG00010004245	ARFGEF3	2	-0.191712301	0.184691335	-1.038014591	0.299263262	0	0.002234462	0	1	CRA004775;PRJNA827465	Yang2025_Silkie_Wenchang	0.78978392
ENSGALG00010004247	MEOX2	2	0.231821144	0.181294399	1.278699978	0.201002728	0.045421974	2.756660457	63.724223	1	PRJNA827465;Yang2025_Silkie_Wenchang	CRA004775	0.713576931
ENSGALG00010004247	MEOX2	2	0.410322269	0.19027085	2.156516713	0.03104334	0	0.415466214	0	1	CRA004775;Yang2025_Silkie_Wenchang	PRJNA827465	0.301035381
ENSGALG00010004247	MEOX2	2	0.098201574	0.076024847	1.291703669	0.196459786	0	0.031832846	0	1	CRA004775;PRJNA827465	Yang2025_Silkie_Wenchang	0.707837424
ENSGALG00010004248	GRSF1	2	-0.023765534	0.132582457	-0.179250974	0.85774064	0.027771417	4.341323334	76.96554891	0.5	PRJNA827465;Yang2025_Silkie_Wenchang	CRA004775	0.964075166
ENSGALG00010004248	GRSF1	2	0.095885985	0.106810985	0.897716514	0.369336703	0	0.487770716	0	0.5	CRA004775;Yang2025_Silkie_Wenchang	PRJNA827465	0.820606801
ENSGALG00010004248	GRSF1	2	-0.133369507	0.049258939	-2.707518872	0.006778822	0	0.099226183	0	1	CRA004775;PRJNA827465	Yang2025_Silkie_Wenchang	0.126389007

ENSGALG00010004249	USP45	2	-0.037090142	0.10487485	-0.353660984	0.723592966	0.014549218	2.830861039	64.67505871	0.5	PRJNA827465;Yang2025_Silkie_Wenchang	CRA004775	0.930032096
ENSGALG00010004249	USP45	2	0.097404063	0.099052878	0.983354189	0.325433149	0	0.107890968	0	1	CRA004775;Yang2025_Silkie_Wenchang	PRJNA827465	0.799987949
ENSGALG00010004249	USP45	2	-0.086780153	0.1042571	-0.832366839	0.405201914	0.007918072	1.19971115	16.64660282	0.5	CRA004775;PRJNA827465	Yang2025_Silkie_Wenchang	0.833112102
ENSGALG00010004250	MOB1B	2	-0.096457103	0.077385823	-1.246444108	0.212601396	0.00729448	2.333665846	57.14896364	1	PRJNA827465;Yang2025_Silkie_Wenchang	CRA004775	0.725336171
ENSGALG00010004250	MOB1B	2	-0.048605207	0.214328217	-0.226779321	0.820595345	0.059076096	2.278798399	56.11722387	0.5	CRA004775;Yang2025_Silkie_Wenchang	PRJNA827465	0.955383907
ENSGALG00010004250	MOB1B	2	-0.023041527	0.061790215	-0.372899279	0.709223409	0.001696217	1.039421745	3.792661161	0.5	CRA004775;PRJNA827465	Yang2025_Silkie_Wenchang	0.925298042
ENSGALG00010004251	CCN2	2	0.316021826	0.143087097	2.208597641	0.02720264	0	0.217757919	0	1	PRJNA827465;Yang2025_Silkie_Wenchang	CRA004775	0.281498612
ENSGALG00010004251	CCN2	2	0.312476507	0.187553988	1.666061653	0.095701135	0	0.598192487	0	1	CRA004775;Yang2025_Silkie_Wenchang	PRJNA827465	0.529841065
ENSGALG00010004251	CCN2	2	0.213963197	0.174572105	1.225643681	0.220332793	0	0.237216304	0	1	CRA004775;PRJNA827465	Yang2025_Silkie_Wenchang	0.734031787
ENSGALG00010004255	RPS12	2	-0.136880786	0.077426123	-1.767888944	0.077079471	0	0.013542453	0	1	PRJNA827465;Yang2025_Silkie_Wenchang	CRA004775	0.478533748
ENSGALG00010004255	RPS12	2	-0.225457887	0.18201985	-1.238644505	0.215477179	0	0.076449864	0	1	CRA004775;Yang2025_Silkie_Wenchang	PRJNA827465	0.728883686
ENSGALG00010004255	RPS12	2	-0.147801449	0.07649706	-1.932119342	0.053344778	0	0.281459584	0	1	CRA004775;PRJNA827465	Yang2025_Silkie_Wenchang	0.400116372
ENSGALG00010004257	ENSGALG00010004257	2	-0.213843792	0.742069757	-0.288172089	0.773215013	0.841363662	4.206171057	76.22540818	0.5	PRJNA827465;Yang2025_Silkie_Wenchang	CRA004775	0.943222001
ENSGALG00010004257	ENSGALG00010004257	2	0.104841536	1.188752333	0.08831946	0.92972201	2.3011896	5.233798308	80.89341734	0.5	CRA004775;Yang2025_Silkie_Wenchang	PRJNA827465	0.983767889
ENSGALG00010004257	ENSGALG00010004257	2	0.686593315	0.406679036	1.688292863	0.091355025	0	0.822962993	0	1	CRA004775;PRJNA827465	Yang2025_Silkie_Wenchang	0.518188272
ENSGALG00010004265	PIP4K2A	2	-0.275240984	0.076556187	-3.595280729	0.000324042	0.00373315	1.467226862	31.84421399	1	PRJNA827465;Yang2025_Silkie_Wenchang	CRA004775	0.017837285
ENSGALG00010004265	PIP4K2A	2	-0.156985612	0.207988115	-0.75478165	0.450380006	0.073020616	6.245711615	83.9890142	0.5	CRA004775;Yang2025_Silkie_Wenchang	PRJNA827465	0.847577105
ENSGALG00010004265	PIP4K2A	2	-0.088362536	0.130006307	-0.679678841	0.496707836	0.020762186	2.477608918	59.63850498	0.5	CRA004775;PRJNA827465	Yang2025_Silkie_Wenchang	0.860939846
ENSGALG00010004281	VIM	2	-0.020595788	0.083482402	-0.246708143	0.805134104	0.008244804	2.433782891	58.91170065	0.5	PRJNA827465;Yang2025_Silkie_Wenchang	CRA004775	0.951863117
ENSGALG00010004281	VIM	2	0.075723381	0.077993989	0.970887389	0.331604359	0	0.0969565	0	1	CRA004775;Yang2025_Silkie_Wenchang	PRJNA827465	0.801846521
ENSGALG00010004281	VIM	2	-0.082914921	0.067492677	-1.228502469	0.219258403	0	0.839829596	0	0.5	CRA004775;PRJNA827465	Yang2025_Silkie_Wenchang	0.73285529
ENSGALG00010004283	DCK	2	-0.259227336	0.347531384	-0.74591058	0.455721434	0.221007112	11.33279567	91.17605197	0.5	PRJNA827465;Yang2025_Silkie_Wenchang	CRA004775	0.84907427
ENSGALG00010004283	DCK	2	-0.105200698	0.54165698	-0.194220147	0.846003508	0.530902455	10.37479647	90.3612567	0.5	CRA004775;Yang2025_Silkie_Wenchang	PRJNA827465	0.96122283
ENSGALG00010004283	DCK	2	0.171390778	0.171240305	1.000878725	0.316885443	0.032806616	1.774425507	43.64373166	1	CRA004775;PRJNA827465	Yang2025_Silkie_Wenchang	0.796959041
ENSGALG00010004285	SLC18B1	2	-0.463553331	0.448146277	-1.034379522	0.300989374	0.380429467	18.73812291	94.66328615	1	PRJNA827465;Yang2025_Silkie_Wenchang	CRA004775	0.791009438
ENSGALG00010004285	SLC18B1	2	-0.640046513	0.287141211	-2.229030487	0.025811876	0.131379187	4.912842302	79.64518422	1	CRA004775;Yang2025_Silkie_Wenchang	PRJNA827465	0.273814893
ENSGALG00010004285	SLC18B1	2	-0.148867797	0.156609256	-0.950568317	0.341823559	0.027417072	2.120380336	52.83864961	1	CRA004775;PRJNA827465	Yang2025_Silkie_Wenchang	0.805823616
ENSGALG00010004297	SULF1	2	0.065269446	0.106683322	0.611805523	0.540664635	0	0.453543217	0	1	PRJNA827465;Yang2025_Silkie_Wenchang	CRA004775	0.875624671
ENSGALG00010004297	SULF1	2	0.201838441	0.16492235	1.223839231	0.221012882	0	0.368225009	0	1	CRA004775;Yang2025_Silkie_Wenchang	PRJNA827465	0.734231106
ENSGALG00010004297	SULF1	2	0.048417013	0.127517769	0.379688366	0.704176757	0	0.908809504	0	1	CRA004775;PRJNA827465	Yang2025_Silkie_Wenchang	0.924020402
ENSGALG00010004300	VNN2	2	-0.127872256	0.151111536	-0.846211079	0.397435003	0	0.13092055	0	1	PRJNA827465;Yang2025_Silkie_Wenchang	CRA004775	0.831571329
ENSGALG00010004300	VNN2	2	-0.199318378	0.221359659	-0.900427744	0.367892662	0	0.012018887	0	1	CRA004775;Yang2025_Silkie_Wenchang	PRJNA827465	0.820152313
ENSGALG00010004300	VNN2	2	-0.094349307	0.192113318	-0.491112786	0.623346679	0	0.065754518	0	1	CRA004775;PRJNA827465	Yang2025_Silkie_Wenchang	0.900348922
ENSGALG00010004302	CRPPA	2	-0.207605657	0.103012763	-2.015339187	0.043869122	0	0.577828338	0	1	PRJNA827465;Yang2025_Silkie_Wenchang	CRA004775	0.361804689
ENSGALG00010004302	CRPPA	2	0.049948913	0.388799399	0.128469624	0.897777335	0.247860474	5.271990678	81.03183293	0.5	CRA004775;Yang2025_Silkie_Wenchang	PRJNA827465	0.975696709
ENSGALG00010004302	CRPPA	2	0.113142217	0.306358284	0.369313394	0.711894143	0.139709279	3.556150095	71.87976537	0.5	CRA004775;PRJNA827465	Yang2025_Silkie_Wenchang	0.925597752
ENSGALG00010004306	PNISR	2	0.80213326	0.78018681	1.028129737	0.303888804	1.183534361	35.33983963	97.17033238	1	PRJNA827465;Yang2025_Silkie_Wenchang	CRA004775	0.792208658
ENSGALG00010004306	PNISR	2	0.810280686	0.781296547	1.037097487	0.299690426	1.172331173	25.09208936	96.01468022	1	CRA004775;Yang2025_Silkie_Wenchang	PRJNA827465	0.79004225
ENSGALG00010004306	PNISR	2	0.038991921	0.076713272	0.508281289	0.61125609	0	8.63496E-05	0	1	CRA004775;PRJNA827465	Yang2025_Silkie_Wenchang	0.897609796
ENSGALG00010004307	NPC2	2	-0.135590285	0.34141997	-0.394207417	0.69342791	0.206920561	8.892442234	88.7544954	0.5	PRJNA827465;Yang2025_Silkie_Wenchang	CRA004775	0.920721644
ENSGALG00010004307	NPC2	2	-0.106356932	0.418370616	-0.254217023	0.7993279	0.28553134	5.099702469	80.39101289	0.5	CRA004775;Yang2025_Silkie_Wenchang	PRJNA827465	0.949922217
ENSGALG00010004307	NPC2	2	0.235135164	0.143900924	1.63400733	0.102257311	0	0.185948584	0	1	CRA004775;PRJNA827465	Yang2025_Silkie_Wenchang	0.54496402
ENSGALG00010004308	DSG2	2	0.016368083	0.338395532	0.048369677	0.961421625	0	0.412857548	0	0.5	PRJNA827465;Yang2025_Silkie_Wenchang	CRA004775	0.98973612
ENSGALG00010004308	DSG2	2	0.744051136	0.993203668	0.749142558	0.453771287	1.660662471	6.273284808	84.05938786	0.5	CRA004775;Yang2025_Silkie_Wenchang	PRJNA827465	0.849044791
ENSGALG00010004308	DSG2	2	0.943483337	0.774176796	1.218692348	0.222960983	0.902321609	3.983814403	74.89842902	1	CRA004775;PRJNA827465	Yang2025_Silkie_Wenchang	0.736458649
ENSGALG00010004309	MRPS28	2	0.038146753	0.069464412	0.54915535	0.582898843	0	0.141798557	0	0.5	PRJNA827465;Yang2025_Silkie_Wenchang	CRA004775	0.890230846
ENSGALG00010004309	MRPS28	2	-0.089879081	0.165417472	-0.543346967	0.586890932	0	0.25131291	0	1	CRA004775;Yang2025_Silkie_Wenchang	PRJNA827465	0.891596903
ENSGALG00010004309	MRPS28	2	0.032173491	0.071760644	0.448344513	0.65390458	0	0.735528439	0	0.5	CRA004775;PRJNA827465	Yang2025_Silkie_Wenchang	0.909861322
ENSGALG00010004312	PDLIM5	2	-0.056093112	0.075327923	-0.744652313	0.456481934	0	0.558187446	0	1	PRJNA827465;Yang2025_Silkie_Wenchang	CRA004775	0.849515887
ENSGALG00010004312	PDLIM5	2	0.007296078	0.100749424	0.072418066	0.942269208	0	0.390218909	0	0.5	CRA004775;Yang2025_Silkie_Wenchang	PRJNA827465	0.985827325
ENSGALG00010004312	PDLIM5	2	-0.091366238	0.099331583	-0.911352793	0.36210958	0	0.674168569	0	0.5	CRA004775;PRJNA827465	Yang2025_Silkie_Wenchang	0.815579273
ENSGALG00010004323	TRDMT1	2	0.154215707	0.079687948	1.935245056	0.052960231	0	0.552642898	0	1	PRJNA827465;Yang2025_Silkie_Wenchang	CRA004775	0.399596077
ENSGALG00010004323	TRDMT1	2	-0.337324675	0.450646822	-0.74853446	0.454137847	0.350946969	6.930304315	85.5706192	0.5	CRA004775;Yang2025_Silkie_Wenchang	PRJNA827465	0.849072665
ENSGALG00010004323	TRDMT1	2	-0.275532384	0.513681782	-0.536387299	0.591690907	0.477015004	9.748534335	89.74204772	0.5	CRA004775;PRJNA827465	Yang2025_Silkie_Wenchang	0.892776493
ENSGALG00010004325	LTBP2	2	-0.223456657	0.107092511	-2.086575952	0.036926483	0	0.540776648	0	1	PRJNA827465;Yang2025_Silkie_Wenchang	CRA004775	0.331631487
ENSGALG00010004325	LTBP2	2	-0.244761141	0.111579351	-2.193606059	0.028263742	0	0.131956552	0	1	CRA004775;Yang2025_Silkie_Wenchang	PRJNA827465	0.286481335
ENSGALG00010004325	LTBP2	2	-0.056957011	0.228947104	-0.248778035	0.803532482	0	0.019371469	0	1	CRA004775;PRJNA827465	Yang2025_Silkie_Wenchang	0.951064358
ENSGALG00010004326	ENSGALG00010004326	2	-0.741491469	0.69419034	-1.068120259	0.285458015	0.607274735	2.662053136	62.43500979	1	PRJNA827465;Yang2025_Silkie_Wenchang	CRA004775	0.785183426
ENSGALG00010004326	ENSGALG00010004326	2	-1.356825893	0.515529432	-2.63190772	0.008490692	0	0.114714664	0	1	CRA004775;Yang2025_Silkie_Wenchang	PRJNA827465	0.14369705
ENSGALG00010004326	ENSGALG00010004326	2	-0.45805121	0.490861225	-0.933158267	0.350738259	0.089042321	1.196667893	16.43462602	1	CRA004775;PRJNA827465	Yang2025_Silkie_Wenchang	0.810146221
ENSGALG00010004328	ENSGALG00010004328	2	-0.219616451	0.621881784	-0.353149829	0.723977418	0.557696535	3.507754361	71.4917324	0.5	PRJNA827465;Yang2025_Silkie_Wenchang	CRA004775	0.930341034
ENSGALG00010004328	ENSGALG00010004328	2	-0.650306689	0.376879455	-1.725503156	0.084436836	0	0.415146678	0	1	CRA004775;Yang2025_Silkie_Wenchang	PRJNA827465	0.500072565
ENSGALG00010004328	ENSGALG00010004328	2	0.047140281	0.372802295	0.126448474	0.899376933	0.078577594	1.369607128	26.98636132	0.5	CRA004775;PRJNA827465	Yang2025_Silkie_Wenchang	0.976081354
ENSGALG00010004329	COQ3	2	-0.286679979	0.086829826	-3.301630225	0.000961247	0	0.152230849	0	1	PRJNA827465;Yang2025_Silkie_Wenchang	CRA004775	0.036786652
ENSGALG00010004329	COQ3	2	-0.234981742	0.181569363	-1.294170663	0.195606478	0	0.817192562	0	1	CRA004775;Yang2025_Silkie_Wenchang	PRJNA827465	0.706675375
ENSGALG00010004329	COQ3	2	-0.250320431	0.088604819	-2.825133366	0.004726094	0	0.618492766	0	1	CRA004775;PRJNA827465	Yang2025_Silkie_Wenchang	0.101336428
ENSGALG00010004330	STX7	2	-0.168793882	0.248542959	-0.679133628	0.497053197	0.114700395	13.6151657	92.65524914	0.5	PRJNA827465;Yang2025_Silkie_Wenchang	CRA004775	0.86098489
ENSGALG00010004330	STX7	2	-0.149116044	0.292777854	-0.50931463	0.610531705	0.149260158	7.620680619	86.87781249	0.5	CRA004775;Yang2025_Silkie_Wenchang	PRJNA827465	0.897438028
ENSGALG00010004330	STX7	2	0.078229291	0.056601681	1.382101901	0.166940415	0	0.230457349	0	1	CRA004775;PRJNA827465	Yang2025_Silkie_Wenchang	0.666022689
ENSGALG00010004332	ENSGALG00010004332	2	-0.654434941	0.472159839	-1.386045332	0.165733062	0	0.544709231	0	1	PRJNA827465;Yang2025_Silkie_Wenchang	CRA004775	0.66529196
ENSGALG00010004332	ENSGALG00010004332	2	-1.182633576	0.492845656	-2.399602316	0.016412892	0	0.061478487	0	1	CRA004775;Yang2025_Silkie_Wenchang	PRJNA827465	0.211917183
ENSGALG00010004332	ENSGALG00010004332	2	-0.788902176	0.472750914	-1.668748072	0.095167318	0.023042034	1.054036829	5.126654771	1	CRA004775;PRJNA827465	Yang2025_Silkie_Wenchang	0.528150394
ENSGALG00010004333	DIO2	2	-0.050868445	0.286576486	-0.177503904	0.859112599	0	0.444595224	0	0.5	PRJNA827465;Yang2025_Silkie_Wenchang	CRA004775	0.964605083
ENSGALG00010004333	DIO2	2	0.107917051	0.483315169	0.223285049	0.823313663	0.17368406	1.574880289	36.50311028	0.5	CRA004775;Yang2025_Silkie_Wenchang	PRJNA827465	0.955918024
ENSGALG00010004333	DIO2	2	0.234080268	0.305605833	0.765954845	0.443703171	0	0.669186074	0	1	CRA004775;PRJNA827465	Yang2025_Silkie_Wenchang	0.845840387
ENSGALG00010004334	ENSGALG00010004334	2	0.315449565	0.15494913	2.035826627	0.041767768	0.021258354	1.793667165	44.24829646	1	PRJNA827465;Yang2025_Silkie_Wenchang	CRA004775	0.354014899
ENSGALG00010004334	ENSGALG00010004334	2	0.484458599	0.158371051	3.059009806	0.002220699	0	0.039380499	0	1	CRA004775;Yang2025_Silkie_Wenchang	PRJNA827465	0.062298448
ENSGALG00010004334	ENSGALG00010004334	2	0.202617606	0.152637194	1.327445823	0.18436125	0	0.623398767	0	1	CRA004775;PRJNA827465	Yang2025_Silkie_Wenchang	0.692608786
ENSGALG00010004337	BMPR1B	2	0.06048099	0.232543598	0.26008452	0.794798578	0.093701054	7.408265677	86.50156402	0.5	PRJNA827465;Yang2025_Silkie_Wenchang	CRA004775	0.948905846
ENSGALG00010004337	BMPR1B	2	0.182396771	0.259938433	0.701692199	0.482871139	0.063062571	1.370611609	27.03987084	0.5	CRA004775;Yang2025_Silkie_Wenchang	PRJNA827465	0.85691916
ENSGALG00010004337	BMPR1B	2	-0.16980248	0.100050068	-1.697175062	0.089663568	0	0.142618813	0	1	CRA004775;PRJNA827465	Yang2025_Silkie_Wenchang	0.513709509
ENSGALG00010004338	ENSGALG00010004338	2	0.284862817	0.141605395	2.011666416	0.044255113	0.005524312	1.158764215	13.70116652	1	PRJNA827465;Yang2025_Silkie_Wenchang	CRA004775	0.363578326
ENSGALG00010004338	ENSGALG00010004338	2	0.327289513	0.13742176	2.381642561	0.017235618	0	0.640925627	0	1	CRA004775;Yang2025_Silkie_Wenchang	PRJNA827465	0.218097712
ENSGALG00010004338	ENSGALG00010004338	2	0.182503893	0.131463916	1.388243237	0.16506299	0	0.058703821	0	1	CRA004775;PRJNA827465	Yang2025_Silkie_Wenchang	0.664377375
ENSGALG00010004339	B4GALT6	2	0.479157887	0.116330969	4.118919399	3.80653E-05	0	0.889939929	0	1	PRJNA827465;Yang2025_Silkie_Wenchang	CRA004775	0.004000873
ENSGALG00010004339	B4GALT6	2	0.333205279	0.184336893	1.80758867	0.070670543	0	0.02087402	0	1	CRA004775;Yang2025_Silkie_Wenchang	PRJNA827465	0.458542662
ENSGALG00010004339	B4GALT6	2	0.555046003	0.145642946	3.811005049	0.000138403	0	0.162933103	0	1	CRA004775;PRJNA827465	Yang2025_Silkie_Wenchang	0.00985444
ENSGALG00010004340	SLC4A4	2	0.147428622	0.198388873	0.743129493	0.457403285	0.059316237	3.865839281	74.13239591	0.5	PRJNA827465;Yang2025_Silkie_Wenchang	CRA004775	0.849573219
ENSGALG00010004340	SLC4A4	2	0.329642732	0.163823393	2.012183516	0.044200595	0	0.462494579	0	1	CRA004775;Yang2025_Silkie_Wenchang	PRJNA827465	0.363282309
ENSGALG00010004340	SLC4A4	2	-0.023702599	0.099975091	-0.237085049	0.812590811	0	0.022722803	0	0.5	CRA004775;PRJNA827465	Yang2025_Silkie_Wenchang	0.95348724
ENSGALG00010004341	ENSGALG00010004341	2	0.091308529	0.123622795	0.738605927	0.460146324	0	0.217269881	0	1	PRJNA827465;Yang2025_Silkie_Wenchang	CRA004775	0.850104868
ENSGALG00010004341	ENSGALG00010004341	2	0.109455821	0.184393635	0.593598694	0.552780554	0	0.264176604	0	0.5	CRA004775;Yang2025_Silkie_Wenchang	PRJNA827465	0.87928478
ENSGALG00010004341	ENSGALG00010004341	2	0.043057434	0.130440053	0.330093654	0.741329198	0	0.039533377	0	0.5	CRA004775;PRJNA827465	Yang2025_Silkie_Wenchang	0.935419978
ENSGALG00010004342	ZNF438	2	0.115433784	0.130508654	0.884491418	0.376430993	0.021175258	2.353777717	57.51510464	1	PRJNA827465;Yang2025_Silkie_Wenchang	CRA004775	0.822974013
ENSGALG00010004342	ZNF438	2	0.177131416	0.158219473	1.119529812	0.26291418	0.011663611	1.259561479	20.60728939	0.5	CRA004775;Yang2025_Silkie_Wenchang	PRJNA827465	0.768643821
ENSGALG00010004342	ZNF438	2	0.011644881	0.071625879	0.16257924	0.870849734	0	0.06075893	0	0.5	CRA004775;PRJNA827465	Yang2025_Silkie_Wenchang	0.969164319
ENSGALG00010004345	FAXC	2	-0.086683423	0.100865008	-0.859400349	0.390119677	0	0.480661854	0	1	PRJNA827465;Yang2025_Silkie_Wenchang	CRA004775	0.829204867
ENSGALG00010004345	FAXC	2	-0.190117721	0.192195449	-0.989189505	0.322570432	0	0.080950517	0	1	CRA004775;Yang2025_Silkie_Wenchang	PRJNA827465	0.798652022
ENSGALG00010004345	FAXC	2	-0.047515632	0.113017684	-0.42042652	0.674173898	0	8.28829E-06	0	1	CRA004775;PRJNA827465	Yang2025_Silkie_Wenchang	0.915800735
ENSGALG00010004346	CEP128	2	0.194644497	0.255577896	0.761585804	0.44630725	0.101335363	4.383658583	77.18800447	0.5	PRJNA827465;Yang2025_Silkie_Wenchang	CRA004775	0.846093664
ENSGALG00010004346	CEP128	2	0.420362595	0.186819623	2.250098726	0.024442679	0	0.553190908	0	1	CRA004775;Yang2025_Silkie_Wenchang	PRJNA827465	0.267184364
ENSGALG00010004346	CEP128	2	-0.03966954	0.139640922	-0.284082487	0.776347168	0	0.012437641	0	0.5	CRA004775;PRJNA827465	Yang2025_Silkie_Wenchang	0.944109792
ENSGALG00010004350	MOXD1	2	-0.2816391	0.111752798	-2.520197306	0.011728907	0	0.596876979	0	1	PRJNA827465;Yang2025_Silkie_Wenchang	CRA004775	0.173431612
ENSGALG00010004350	MOXD1	2	-0.220391934	0.255719664	-0.861849772	0.388770192	0.061478767	1.735506922	42.37994747	0.5	CRA004775;Yang2025_Silkie_Wenchang	PRJNA827465	0.828400391
ENSGALG00010004350	MOXD1	2	-0.176674688	0.127461341	-1.386104105	0.165715117	0	0.852092293	0	0.5	CRA004775;PRJNA827465	Yang2025_Silkie_Wenchang	0.66529196
ENSGALG00010004352	CHMP2A	2	-0.194436729	0.159070482	-1.222330672	0.221582605	0.038565037	3.625643489	72.41868918	1	PRJNA827465;Yang2025_Silkie_Wenchang	CRA004775	0.734944791
ENSGALG00010004352	CHMP2A	2	-0.371958107	0.130765854	-2.84445898	0.004448691	0	0.051300769	0	1	CRA004775;Yang2025_Silkie_Wenchang	PRJNA827465	0.097660808
ENSGALG00010004352	CHMP2A	2	-0.114448356	0.100949784	-1.133715709	0.256913818	0.009565129	1.376353958	27.34427112	1	CRA004775;PRJNA827465	Yang2025_Silkie_Wenchang	0.764799109
ENSGALG00010004355	ANKMY2	2	-0.141145666	0.146872761	-0.961006417	0.336548941	0.034982344	5.089377254	80.35123061	1	PRJNA827465;Yang2025_Silkie_Wenchang	CRA004775	0.803806405
ENSGALG00010004355	ANKMY2	2	0.027304218	0.348315858	0.078389248	0.937518426	0.217542653	9.427834479	89.39311035	0.5	CRA004775;Yang2025_Silkie_Wenchang	PRJNA827465	0.985139256
ENSGALG00010004355	ANKMY2	2	0.150779258	0.196663563	0.76668629	0.443268057	0.059355826	3.738701257	73.2527439	0.5	CRA004775;PRJNA827465	Yang2025_Silkie_Wenchang	0.845733906
ENSGALG00010004356	TRAPPC8	2	0.017949014	0.03931275	0.456569802	0.647980289	0.000371052	1.105236821	9.521653528	0.5	PRJNA827465;Yang2025_Silkie_Wenchang	CRA004775	0.907553901
ENSGALG00010004356	TRAPPC8	2	-0.032293223	0.068765046	-0.469616825	0.638628802	0	0.34288522	0	0.5	CRA004775;Yang2025_Silkie_Wenchang	PRJNA827465	0.905713124
ENSGALG00010004356	TRAPPC8	2	0.043615721	0.041066439	1.062077025	0.288200719	0	0.038054801	0	1	CRA004775;PRJNA827465	Yang2025_Silkie_Wenchang	0.782882265
ENSGALG00010004358	GC	2	-3.611902843	3.817998144	-0.946020063	0.344138353	25.32986733	7.546964408	86.74963938	0.5	PRJNA827465;Yang2025_Silkie_Wenchang	CRA004775	0.806491545
ENSGALG00010004358	GC	2	-2.661019298	4.683394613	-0.56818174	0.569911569	41.14159846	15.51505024	93.55464543	0.5	CRA004775;Yang2025_Silkie_Wenchang	PRJNA827465	0.884923673
ENSGALG00010004358	GC	2	1.458359167	0.728431702	2.002053403	0.045278988	0	0.890343685	0	1	CRA004775;PRJNA827465	Yang2025_Silkie_Wenchang	0.366947722
ENSGALG00010004362	FBXL4	2	-0.248402625	0.087005822	-2.855011529	0.004303527	0	0.401534263	0	1	PRJNA827465;Yang2025_Silkie_Wenchang	CRA004775	0.096212462
ENSGALG00010004362	FBXL4	2	0.051196206	0.407565868	0.125614556	0.90003704	0.28600794	6.941292266	85.59346067	0.5	CRA004775;Yang2025_Silkie_Wenchang	PRJNA827465	0.976431373
ENSGALG00010004362	FBXL4	2	0.09636905	0.34741169	0.277391499	0.781479508	0.202900599	5.782205659	82.70556153	0.5	CRA004775;PRJNA827465	Yang2025_Silkie_Wenchang	0.945235709
ENSGALG00010004365	TPD52	2	0.219625957	0.090148313	2.436273612	0.014839455	0	0.436672172	0	1	PRJNA827465;Yang2025_Silkie_Wenchang	CRA004775	0.201254349
ENSGALG00010004365	TPD52	2	0.009709645	0.402516013	0.024122381	0.980754991	0.227548123	2.939017304	65.97502169	0.5	CRA004775;Yang2025_Silkie_Wenchang	PRJNA827465	0.994928779
ENSGALG00010004365	TPD52	2	-0.023156903	0.317772397	-0.072872608	0.941907492	0.133577932	2.254187202	55.63811208	0.5	CRA004775;PRJNA827465	Yang2025_Silkie_Wenchang	0.985827325
ENSGALG00010004366	UNC5C	2	0.187763178	0.242097098	0.77556972	0.438003085	0	0.038493706	0	1	PRJNA827465;Yang2025_Silkie_Wenchang	CRA004775	0.843947213
ENSGALG00010004366	UNC5C	2	0.277882312	0.355898035	0.780791926	0.434924884	0	0.032920202	0	1	CRA004775;Yang2025_Silkie_Wenchang	PRJNA827465	0.842762754
ENSGALG00010004366	UNC5C	2	0.181387958	0.289602433	0.626334372	0.531095646	0	0.087465473	0	1	CRA004775;PRJNA827465	Yang2025_Silkie_Wenchang	0.872848946
ENSGALG00010004367	TSPAN13	2	-0.884435803	0.536177594	-1.649520256	0.099041097	0.543696587	18.38241724	94.56001903	1	PRJNA827465;Yang2025_Silkie_Wenchang	CRA004775	0.536947311
ENSGALG00010004367	TSPAN13	2	-0.803991435	0.632414833	-1.271303886	0.20362056	0.749067614	15.60935573	93.59358569	1	CRA004775;Yang2025_Silkie_Wenchang	PRJNA827465	0.717254253
ENSGALG00010004367	TSPAN13	2	-0.291377234	0.145167623	-2.007177828	0.044730732	0	0.370892873	0	1	CRA004775;PRJNA827465	Yang2025_Silkie_Wenchang	0.364438629
ENSGALG00010004369	UBE2M	2	-0.404969609	0.257111125	-1.575076179	0.115238862	0.121312669	11.91616518	91.60803845	1	PRJNA827465;Yang2025_Silkie_Wenchang	CRA004775	0.572203827
ENSGALG00010004369	UBE2M	2	-0.448184861	0.305709146	-1.466049892	0.142634695	0.127398936	2.634477206	62.04180481	1	CRA004775;Yang2025_Silkie_Wenchang	PRJNA827465	0.628905865
ENSGALG00010004369	UBE2M	2	-0.152947904	0.074594177	-2.05040005	0.040325408	0	0.109665412	0	1	CRA004775;PRJNA827465	Yang2025_Silkie_Wenchang	0.347442284
ENSGALG00010004370	ATP6V1H	2	0.026249607	0.145089391	0.180920234	0.856430187	0.030010044	3.127397606	68.02453266	0.5	PRJNA827465;Yang2025_Silkie_Wenchang	CRA004775	0.96353738
ENSGALG00010004370	ATP6V1H	2	-0.103632824	0.12718797	-0.814800522	0.415186502	0	0.344932376	0	0.5	CRA004775;Yang2025_Silkie_Wenchang	PRJNA827465	0.837489728
ENSGALG00010004370	ATP6V1H	2	0.131762287	0.065505047	2.011482985	0.044274466	0	0.327316772	0	1	CRA004775;PRJNA827465	Yang2025_Silkie_Wenchang	0.363642137
ENSGALG00010004372	SYNJ2	2	-0.117020115	0.250545529	-0.467061278	0.640456037	0.093358611	3.829363908	73.88600238	0.5	PRJNA827465;Yang2025_Silkie_Wenchang	CRA004775	0.906211744
ENSGALG00010004372	SYNJ2	2	0.018029075	0.215651781	0.083602719	0.933372305	0.022008313	1.239441901	19.31852564	0.5	CRA004775;Yang2025_Silkie_Wenchang	PRJNA827465	0.984260547
ENSGALG00010004372	SYNJ2	2	-0.344712683	0.140304898	-2.456882747	0.014014842	0	0.003933825	0	1	CRA004775;PRJNA827465	Yang2025_Silkie_Wenchang	0.193842584
ENSGALG00010004373	MMS22L	2	-0.184820093	0.176152864	-1.049202881	0.294084753	0.045249122	3.673290169	72.77644961	1	PRJNA827465;Yang2025_Silkie_Wenchang	CRA004775	0.787324026
ENSGALG00010004373	MMS22L	2	-0.357505316	0.136024427	-2.62824351	0.008582705	0	0.114129793	0	1	CRA004775;Yang2025_Silkie_Wenchang	PRJNA827465	0.144612369
ENSGALG00010004373	MMS22L	2	-0.022510566	0.116830333	-0.192677414	0.847211613	0	0.068971983	0	1	CRA004775;PRJNA827465	Yang2025_Silkie_Wenchang	0.961252712
ENSGALG00010004375	ENSGALG00010004375	2	1.228095859	0.812431687	1.511629691	0.130628094	1.220991361	13.21855752	92.43487802	1	PRJNA827465;Yang2025_Silkie_Wenchang	CRA004775	0.606219859
ENSGALG00010004375	ENSGALG00010004375	2	1.452246749	0.780301527	1.861135341	0.062725066	0.846597841	2.891440904	65.41516728	1	CRA004775;Yang2025_Silkie_Wenchang	PRJNA827465	0.431521668
ENSGALG00010004375	ENSGALG00010004375	2	0.437114106	0.247850617	1.763619196	0.077796114	0	0.000329565	0	1	CRA004775;PRJNA827465	Yang2025_Silkie_Wenchang	0.480531299
ENSGALG00010004378	TRIM28	2	-0.070092626	0.052406356	-1.337483302	0.181064929	0	0.216590792	0	1	PRJNA827465;Yang2025_Silkie_Wenchang	CRA004775	0.688594651
ENSGALG00010004378	TRIM28	2	-0.131372403	0.075358143	-1.743307342	0.081279944	0	0.3618049	0	1	CRA004775;Yang2025_Silkie_Wenchang	PRJNA827465	0.491015312
ENSGALG00010004378	TRIM28	2	-0.07822159	0.059189071	-1.321554603	0.186316509	0	0.939159016	0	1	CRA004775;PRJNA827465	Yang2025_Silkie_Wenchang	0.695347453
ENSGALG00010004379	ZBTB10	2	-0.039130337	0.041146212	-0.951007039	0.341600803	0	0.640974138	0	1	PRJNA827465;Yang2025_Silkie_Wenchang	CRA004775	0.805823616
ENSGALG00010004379	ZBTB10	2	0.072993965	0.2108691	0.346157712	0.729224193	0.068149586	3.981761008	74.88548413	0.5	CRA004775;Yang2025_Silkie_Wenchang	PRJNA827465	0.9323457
ENSGALG00010004379	ZBTB10	2	0.097080708	0.163332995	0.594372915	0.55226272	0.039114481	3.022919851	66.9194008	0.5	CRA004775;PRJNA827465	Yang2025_Silkie_Wenchang	0.879195629
ENSGALG00010004380	ISCA2	2	-0.404110953	0.354689564	-1.139337026	0.254562611	0.224406972	8.464085993	88.18537523	1	PRJNA827465;Yang2025_Silkie_Wenchang	CRA004775	0.764286735
ENSGALG00010004380	ISCA2	2	-0.562065386	0.256007481	-2.195503751	0.028127482	0.064455153	1.955702141	48.86746918	1	CRA004775;Yang2025_Silkie_Wenchang	PRJNA827465	0.285731308
ENSGALG00010004380	ISCA2	2	-0.09283615	0.054393429	-1.706753038	0.087867965	0	0.493370505	0	1	CRA004775;PRJNA827465	Yang2025_Silkie_Wenchang	0.509491082
ENSGALG00010004381	ENSGALG00010004381	2	0.126296466	0.603272506	0.209352266	0.834173253	0.634032412	7.746204519	87.09045188	0.5	PRJNA827465;Yang2025_Silkie_Wenchang	CRA004775	0.957968425
ENSGALG00010004381	ENSGALG00010004381	2	0.235236087	0.514555648	0.457163552	0.647553494	0.413450298	4.552928487	78.0361145	0.5	CRA004775;Yang2025_Silkie_Wenchang	PRJNA827465	0.907350807
ENSGALG00010004381	ENSGALG00010004381	2	-0.398688045	0.225318878	-1.769439149	0.076820617	0	0.14788302	0	1	CRA004775;PRJNA827465	Yang2025_Silkie_Wenchang	0.477904868
ENSGALG00010004385	ADAMTS3	2	0.559365395	0.268697574	2.081765706	0.037363876	0.09541156	2.892755964	65.4308897	1	PRJNA827465;Yang2025_Silkie_Wenchang	CRA004775	0.333449683
ENSGALG00010004385	ADAMTS3	2	0.757366133	0.213812142	3.542203568	0.000396799	0	0.509982012	0	1	CRA004775;Yang2025_Silkie_Wenchang	PRJNA827465	0.020084189
ENSGALG00010004385	ADAMTS3	2	0.354662414	0.170938063	2.074800709	0.038005009	0	0.220138465	0	1	CRA004775;PRJNA827465	Yang2025_Silkie_Wenchang	0.336269892
ENSGALG00010004386	CFAP44	2	0.282744076	0.078783326	3.58888222	0.000332099	0	0.042034813	0	1	PRJNA827465;Yang2025_Silkie_Wenchang	CRA004775	0.018046299
ENSGALG00010004386	CFAP44	2	0.288252667	0.152606281	1.888865031	0.058909916	0	0.336524099	0	1	CRA004775;Yang2025_Silkie_Wenchang	PRJNA827465	0.42013793
ENSGALG00010004386	CFAP44	2	0.301452763	0.087774138	3.434414376	0.000593836	0	0.269734424	0	1	CRA004775;PRJNA827465	Yang2025_Silkie_Wenchang	0.026342576
ENSGALG00010004389	ENSGALG00010004389	2	-0.065989683	0.066083442	-0.998581209	0.317997607	0.000966225	1.112597674	10.1202507	1	PRJNA827465;Yang2025_Silkie_Wenchang	CRA004775	0.797439961
ENSGALG00010004389	ENSGALG00010004389	2	-0.199247811	0.095143649	-2.094178777	0.036244058	0	0.836023408	0	1	CRA004775;Yang2025_Silkie_Wenchang	PRJNA827465	0.328411494
ENSGALG00010004389	ENSGALG00010004389	2	-0.139602016	0.16848017	-0.828596123	0.407332983	0.03760656	2.497250383	59.9559577	1	CRA004775;PRJNA827465	Yang2025_Silkie_Wenchang	0.833863243
ENSGALG00010004393	ZBTB45	2	0.315146396	0.295356186	1.067004558	0.285969778	0.160402127	12.39685346	91.93343696	1	PRJNA827465;Yang2025_Silkie_Wenchang	CRA004775	0.781767418
ENSGALG00010004393	ZBTB45	2	0.297169679	0.379099552	0.783882959	0.43310879	0.227887065	4.278691255	76.6283674	0.5	CRA004775;Yang2025_Silkie_Wenchang	PRJNA827465	0.842762754
ENSGALG00010004393	ZBTB45	2	0.004275247	0.108720866	0.039323146	0.968632753	0	0.237822265	0	0.5	CRA004775;PRJNA827465	Yang2025_Silkie_Wenchang	0.991255873
ENSGALG00010004394	CCNY	2	-0.03164752	0.057375755	-0.551583496	0.581233747	0	0.58035355	0	1	PRJNA827465;Yang2025_Silkie_Wenchang	CRA004775	0.88890219
ENSGALG00010004394	CCNY	2	0.049636827	0.213039798	0.232993212	0.815766677	0.056531483	2.246698261	55.49024017	0.5	CRA004775;Yang2025_Silkie_Wenchang	PRJNA827465	0.954210034
ENSGALG00010004394	CCNY	2	0.067727682	0.142563399	0.475070616	0.634736642	0.020935072	1.501085273	33.38153282	0.5	CRA004775;PRJNA827465	Yang2025_Silkie_Wenchang	0.903935677
ENSGALG00010004395	BOC	2	0.215711886	0.076850921	2.806887463	0.005002271	0	0.788821962	0	1	PRJNA827465;Yang2025_Silkie_Wenchang	CRA004775	0.105474395
ENSGALG00010004395	BOC	2	0.104770782	0.129990752	0.805986433	0.420250672	0	0.034476257	0	1	CRA004775;Yang2025_Silkie_Wenchang	PRJNA827465	0.838687527
ENSGALG00010004395	BOC	2	0.248873782	0.089809238	2.771137884	0.005586077	0	0.324614741	0	1	CRA004775;PRJNA827465	Yang2025_Silkie_Wenchang	0.112129505
ENSGALG00010004397	ENSGALG00010004397	2	0.172390011	0.083735169	2.058752772	0.039517928	0	0.497010237	0	1	PRJNA827465;Yang2025_Silkie_Wenchang	CRA004775	0.343774486
ENSGALG00010004397	ENSGALG00010004397	2	0.159164347	0.151251793	1.052313782	0.292655611	0.0134301	1.245261944	19.69561064	0.5	CRA004775;Yang2025_Silkie_Wenchang	PRJNA827465	0.786263139
ENSGALG00010004397	ENSGALG00010004397	2	0.089321355	0.105785285	0.844364647	0.398465671	0	0.589195876	0	0.5	CRA004775;PRJNA827465	Yang2025_Silkie_Wenchang	0.831980453
ENSGALG00010004398	COX18	2	-0.062996183	0.242785162	-0.259472954	0.795270347	0.091417042	3.9969296	74.98079526	0.5	PRJNA827465;Yang2025_Silkie_Wenchang	CRA004775	0.948935068
ENSGALG00010004398	COX18	2	-0.369843466	0.162927433	-2.269988918	0.023208255	0	0.008668543	0	1	CRA004775;Yang2025_Silkie_Wenchang	PRJNA827465	0.259724961
ENSGALG00010004398	COX18	2	-0.079962381	0.258502881	-0.309328779	0.757071441	0.106438519	4.441921756	77.48722164	0.5	CRA004775;PRJNA827465	Yang2025_Silkie_Wenchang	0.938795135
ENSGALG00010004404	RGS20	2	0.069307827	0.224411996	0.308841898	0.757441796	0	0.185237081	0	1	PRJNA827465;Yang2025_Silkie_Wenchang	CRA004775	0.938842663
ENSGALG00010004404	RGS20	2	0.438879063	0.389728231	1.126115658	0.260116567	0	0.936633591	0	1	CRA004775;Yang2025_Silkie_Wenchang	PRJNA827465	0.76744615
ENSGALG00010004404	RGS20	2	0.305687558	0.492412513	0.620795675	0.534734069	0.260650661	1.710056417	41.52239716	1	CRA004775;PRJNA827465	Yang2025_Silkie_Wenchang	0.873800646
ENSGALG00010004406	ENSGALG00010004406	2	0.305790418	0.183266713	1.668554061	0.09520579	0.045514835	2.812151852	64.44004262	1	PRJNA827465;Yang2025_Silkie_Wenchang	CRA004775	0.528214731
ENSGALG00010004406	ENSGALG00010004406	2	0.240132935	0.335523189	0.715696985	0.474178473	0.158952346	3.273413866	69.45085342	0.5	CRA004775;Yang2025_Silkie_Wenchang	PRJNA827465	0.85444425
ENSGALG00010004406	ENSGALG00010004406	2	0.133168132	0.093054911	1.43107044	0.152410028	0	0.832846347	0	0.5	CRA004775;PRJNA827465	Yang2025_Silkie_Wenchang	0.643965957
ENSGALG00010004408	SERAC1	2	-0.38746273	0.159499752	-2.429237197	0.01513063	0.028492608	2.148926186	53.46513033	1	PRJNA827465;Yang2025_Silkie_Wenchang	CRA004775	0.203234917

ENSGALG00010004408	SERAC1	2	-0.186246904	0.405058047	-0.459802996	0.645657632	0.287277879	8.017667854	87.52754519	0.5	CRA004775;Yang2025_Silkie_Wenchang	PRJNA827465	0.907052464
ENSGALG00010004408	SERAC1	2	-0.050445545	0.239553816	-0.210581263	0.833214034	0.087398029	3.952661076	74.70058827	0.5	CRA004775;PRJNA827465	Yang2025_Silkie_Wenchang	0.957968425
ENSGALG00010004416	STXBP6	2	0.022067633	0.138284042	0.159581924	0.873210419	0	0.067871362	0	0.5	PRJNA827465;Yang2025_Silkie_Wenchang	CRA004775	0.969837713
ENSGALG00010004416	STXBP6	2	0.058736657	0.216673654	0.27108352	0.786326796	0	0.599648951	0	0.5	CRA004775;Yang2025_Silkie_Wenchang	PRJNA827465	0.946639719
ENSGALG00010004416	STXBP6	2	0.084739169	0.157254658	0.538865877	0.589979404	0	0.444620951	0	1	CRA004775;PRJNA827465	Yang2025_Silkie_Wenchang	0.892320726
ENSGALG00010004420	ENSGALG00010004420	2	-0.023952779	0.229067693	-0.104566377	0.916719896	0	0.111749948	0	0.5	PRJNA827465;Yang2025_Silkie_Wenchang	CRA004775	0.980670715
ENSGALG00010004420	ENSGALG00010004420	2	-0.293192525	0.315160013	-0.930297351	0.352217149	0	0.416726951	0	1	CRA004775;Yang2025_Silkie_Wenchang	PRJNA827465	0.810728586
ENSGALG00010004420	ENSGALG00010004420	2	-0.104495041	0.245058216	-0.426409052	0.669809807	0	0.999919816	0	0.5	CRA004775;PRJNA827465	Yang2025_Silkie_Wenchang	0.914377769
ENSGALG00010004421	RAP1GDS1	2	0.141597163	0.143977189	0.983469421	0.325376458	0.032988297	4.626769444	78.38664727	1	PRJNA827465;Yang2025_Silkie_Wenchang	CRA004775	0.799987949
ENSGALG00010004421	RAP1GDS1	2	0.207941657	0.120203261	1.729916941	0.083645116	0.009515548	1.457191213	31.37482637	1	CRA004775;Yang2025_Silkie_Wenchang	PRJNA827465	0.498257654
ENSGALG00010004421	RAP1GDS1	2	0.019761403	0.058862282	0.335722685	0.737079998	0	0.061246344	0	1	CRA004775;PRJNA827465	Yang2025_Silkie_Wenchang	0.93387966
ENSGALG00010004424	MRPL18	2	0.131322658	0.076176834	1.723918555	0.084722547	0	0.75147677	0	1	PRJNA827465;Yang2025_Silkie_Wenchang	CRA004775	0.500353159
ENSGALG00010004424	MRPL18	2	0.212444384	0.185254793	1.146768623	0.25147726	0	0.670968337	0	0.5	CRA004775;Yang2025_Silkie_Wenchang	PRJNA827465	0.761706068
ENSGALG00010004424	MRPL18	2	0.100173233	0.078928474	1.269164708	0.204382325	0	0.13403726	0	0.5	CRA004775;PRJNA827465	Yang2025_Silkie_Wenchang	0.717281769
ENSGALG00010004425	CREM	2	-0.099754043	0.172967411	-0.576721603	0.564127537	0.047969144	4.705319509	78.74745811	0.5	PRJNA827465;Yang2025_Silkie_Wenchang	CRA004775	0.88288554
ENSGALG00010004425	CREM	2	0.022236687	0.414772578	0.053611757	0.957244489	0.253218204	3.208035969	68.82827969	0.5	CRA004775;Yang2025_Silkie_Wenchang	PRJNA827465	0.988541306
ENSGALG00010004425	CREM	2	0.105932776	0.157215049	0.673808118	0.500433313	0.02283991	1.214317656	17.64922504	1	CRA004775;PRJNA827465	Yang2025_Silkie_Wenchang	0.862400704
ENSGALG00010004429	ENSGALG00010004429	2	0.880877633	0.458146338	1.922699276	0.054517828	0.259140427	2.432245632	58.8857315	1	PRJNA827465;Yang2025_Silkie_Wenchang	CRA004775	0.404097297
ENSGALG00010004429	ENSGALG00010004429	2	0.659258778	0.744165638	0.885903278	0.37566965	0.904683945	5.368782589	81.3738034	0.5	CRA004775;Yang2025_Silkie_Wenchang	PRJNA827465	0.822974013
ENSGALG00010004429	ENSGALG00010004429	2	0.283275446	0.272421277	1.039843322	0.298412698	0.040282787	1.357703418	26.34621177	0.5	CRA004775;PRJNA827465	Yang2025_Silkie_Wenchang	0.789324547
ENSGALG00010004430	TCEA1	2	-0.145814018	0.112516552	-1.295933932	0.194998248	0.019520566	4.212641006	76.26192219	1	PRJNA827465;Yang2025_Silkie_Wenchang	CRA004775	0.706113528
ENSGALG00010004430	TCEA1	2	-0.126216475	0.194282399	-0.649658708	0.515915286	0.049885538	2.538737467	60.61034223	0.5	CRA004775;Yang2025_Silkie_Wenchang	PRJNA827465	0.867498525
ENSGALG00010004430	TCEA1	2	-0.034152579	0.056732127	-0.601997154	0.547176032	0	0.541145206	0	0.5	CRA004775;PRJNA827465	Yang2025_Silkie_Wenchang	0.878281266
ENSGALG00010004433	AHR1A	2	-0.045549198	0.138921391	-0.327877495	0.74300429	0.026600205	3.205463302	68.80326162	0.5	PRJNA827465;Yang2025_Silkie_Wenchang	CRA004775	0.935735651
ENSGALG00010004433	AHR1A	2	-0.211236729	0.112446371	-1.878555322	0.060305237	0	0.453264593	0	1	CRA004775;Yang2025_Silkie_Wenchang	PRJNA827465	0.423432635
ENSGALG00010004433	AHR1A	2	-0.052796795	0.246173434	-0.214469912	0.830180628	0.069888951	1.748270679	42.80061938	0.5	CRA004775;PRJNA827465	Yang2025_Silkie_Wenchang	0.957215045
ENSGALG00010004434	TCP1	2	0.028446938	0.057780672	0.492326195	0.622488765	0	0.00077334	0	1	PRJNA827465;Yang2025_Silkie_Wenchang	CRA004775	0.900102128
ENSGALG00010004434	TCP1	2	-0.014630001	0.109562877	-0.133530636	0.893773737	0	0.421069625	0	0.5	CRA004775;Yang2025_Silkie_Wenchang	PRJNA827465	0.974381724
ENSGALG00010004434	TCP1	2	0.013516211	0.061325631	0.220400687	0.825559111	0	0.51590716	0	0.5	CRA004775;PRJNA827465	Yang2025_Silkie_Wenchang	0.956386623
ENSGALG00010004436	NOVA1	2	-0.058664129	0.050652547	-1.158167408	0.246795726	0	0.016642865	0	1	PRJNA827465;Yang2025_Silkie_Wenchang	CRA004775	0.757265392
ENSGALG00010004436	NOVA1	2	-0.03776224	0.134778583	-0.280179828	0.779339542	0	0.533503657	0	0.5	CRA004775;Yang2025_Silkie_Wenchang	PRJNA827465	0.944727706
ENSGALG00010004436	NOVA1	2	-0.050585625	0.053510487	-0.94534039	0.344485124	0	0.522200428	0	0.5	CRA004775;PRJNA827465	Yang2025_Silkie_Wenchang	0.806768255
ENSGALG00010004440	GTF2H5	2	-0.177591595	0.194977271	-0.910832295	0.362383744	0.064418469	6.381089386	84.32869469	0.5	PRJNA827465;Yang2025_Silkie_Wenchang	CRA004775	0.815644888
ENSGALG00010004440	GTF2H5	2	-0.325034949	0.112897087	-2.879037528	0.003988909	0	0.845892294	0	1	CRA004775;Yang2025_Silkie_Wenchang	PRJNA827465	0.091148882
ENSGALG00010004440	GTF2H5	2	-0.014056077	0.077025647	-0.18248568	0.855201591	0	0.422495962	0	0.5	CRA004775;PRJNA827465	Yang2025_Silkie_Wenchang	0.963353229
ENSGALG00010004443	ANKRD17	2	0.112050615	0.193120219	0.580211724	0.561771849	0.060859257	4.787778131	79.11348495	0.5	PRJNA827465;Yang2025_Silkie_Wenchang	CRA004775	0.881802301
ENSGALG00010004443	ANKRD17	2	0.25732125	0.144956647	1.775160053	0.075871464	0	0.854576195	0	1	CRA004775;Yang2025_Silkie_Wenchang	PRJNA827465	0.47539798
ENSGALG00010004443	ANKRD17	2	-0.046525982	0.050689839	-0.917856182	0.358694164	0	0.121523509	0	0.5	CRA004775;PRJNA827465	Yang2025_Silkie_Wenchang	0.814232788
ENSGALG00010004446	ACAT2	2	0.160508636	0.09189905	1.746575575	0.080710987	0	0.034591538	0	1	PRJNA827465;Yang2025_Silkie_Wenchang	CRA004775	0.489081703
ENSGALG00010004446	ACAT2	2	0.141746367	0.13347066	1.062003945	0.288233894	0	0.007130377	0	1	CRA004775;Yang2025_Silkie_Wenchang	PRJNA827465	0.782882265
ENSGALG00010004446	ACAT2	2	0.1667433	0.093613587	1.781186943	0.074881916	0	0.005518432	0	1	CRA004775;PRJNA827465	Yang2025_Silkie_Wenchang	0.472257613
ENSGALG00010004451	CLDND1	2	-0.385090899	0.381256821	-1.010056417	0.312468261	0.277686691	21.63083006	95.3769689	1	PRJNA827465;Yang2025_Silkie_Wenchang	CRA004775	0.796054997
ENSGALG00010004451	CLDND1	2	-0.519876337	0.257759749	-2.016902714	0.043705667	0.110118644	5.83217041	82.8537246	1	CRA004775;Yang2025_Silkie_Wenchang	PRJNA827465	0.361330211
ENSGALG00010004451	CLDND1	2	-0.10432707	0.117875289	-0.885063116	0.376122591	0.019069092	2.649390901	62.25547541	1	CRA004775;PRJNA827465	Yang2025_Silkie_Wenchang	0.822974013
ENSGALG00010004454	SRI	2	-0.112259401	0.310176481	-0.361921061	0.717411021	0.17926419	14.03135671	92.87310543	0.5	PRJNA827465;Yang2025_Silkie_Wenchang	CRA004775	0.9277944
ENSGALG00010004454	SRI	2	-0.403955155	0.134988407	-2.992517385	0.002766869	0	0.208904341	0	1	CRA004775;Yang2025_Silkie_Wenchang	PRJNA827465	0.071732358
ENSGALG00010004454	SRI	2	0.011625409	0.229455296	0.050665244	0.959592272	0.078376995	3.123639599	67.98606343	0.5	CRA004775;PRJNA827465	Yang2025_Silkie_Wenchang	0.989259322
ENSGALG00010004457	AGA	2	0.193597131	0.101618067	1.905144781	0.056761254	0.00912357	1.691297893	40.87381033	1	PRJNA827465;Yang2025_Silkie_Wenchang	CRA004775	0.412257771
ENSGALG00010004457	AGA	2	0.282293595	0.128402207	2.198510459	0.02791275	0	0.746544999	0	1	CRA004775;Yang2025_Silkie_Wenchang	PRJNA827465	0.284609505
ENSGALG00010004457	AGA	2	0.110754054	0.081704354	1.355546531	0.175243494	0	0.0884282	0	1	CRA004775;PRJNA827465	Yang2025_Silkie_Wenchang	0.680258136
ENSGALG00010004468	LYPLA1	2	-0.17448887	0.25837721	-0.675171273	0.499566956	0.122634025	11.66966665	91.38648826	0.5	PRJNA827465;Yang2025_Silkie_Wenchang	CRA004775	0.865078151
ENSGALG00010004468	LYPLA1	2	-0.160025357	0.312270148	-0.512458068	0.60833046	0.16159818	5.708219133	82.48140135	0.5	CRA004775;Yang2025_Silkie_Wenchang	PRJNA827465	0.897204334
ENSGALG00010004468	LYPLA1	2	0.070645379	0.044229768	1.597236012	0.110213108	0	0.234744853	0	1	CRA004775;PRJNA827465	Yang2025_Silkie_Wenchang	0.562335482
ENSGALG00010004471	C1H3ORF52	2	0.219475359	0.206679673	1.061910712	0.288276222	0	0.0049749	0	1	PRJNA827465;Yang2025_Silkie_Wenchang	CRA004775	0.782899416
ENSGALG00010004471	C1H3ORF52	2	0.371955902	0.310808301	1.196737346	0.231408946	0	0.190438847	0	1	CRA004775;Yang2025_Silkie_Wenchang	PRJNA827465	0.74391181
ENSGALG00010004471	C1H3ORF52	2	0.276298986	0.210000734	1.315704858	0.188273123	0	0.355288096	0	1	CRA004775;PRJNA827465	Yang2025_Silkie_Wenchang	0.697615303
ENSGALG00010004472	NEIL3	2	-0.219238162	0.136811219	-1.602486729	0.10904802	0	0.20635461	0	1	PRJNA827465;Yang2025_Silkie_Wenchang	CRA004775	0.559060907
ENSGALG00010004472	NEIL3	2	-0.167674301	0.205401383	-0.816325084	0.414314232	0	0.155159706	0	1	CRA004775;Yang2025_Silkie_Wenchang	PRJNA827465	0.836962991
ENSGALG00010004472	NEIL3	2	-0.280782333	0.173480061	-1.618527984	0.105548861	0	0.055533788	0	1	CRA004775;PRJNA827465	Yang2025_Silkie_Wenchang	0.5512074
ENSGALG00010004473	JDP2	2	0.129422828	0.269938224	0.479453504	0.631616038	0.135054278	13.54797662	92.61882399	0.5	PRJNA827465;Yang2025_Silkie_Wenchang	CRA004775	0.903058174
ENSGALG00010004473	JDP2	2	0.225078095	0.258751192	0.869863025	0.384375164	0.085392848	2.285180316	56.23977711	0.5	CRA004775;Yang2025_Silkie_Wenchang	PRJNA827465	0.820066827
ENSGALG00010004473	JDP2	2	-0.134748276	0.082727212	-1.628826507	0.103349752	0	0.000967187	0	1	CRA004775;PRJNA827465	Yang2025_Silkie_Wenchang	0.547576788
ENSGALG00010004477	TULP4	2	-0.075053707	0.071374074	-1.051554196	0.293004132	0	0.567677001	0	0.5	PRJNA827465;Yang2025_Silkie_Wenchang	CRA004775	0.786774237
ENSGALG00010004477	TULP4	2	0.10467755	0.147062737	0.711788401	0.476595816	0	0.132429008	0	1	CRA004775;Yang2025_Silkie_Wenchang	PRJNA827465	0.854986476
ENSGALG00010004477	TULP4	2	-0.051345524	0.099595234	-0.51554198	0.606174343	0.006054231	1.208963014	17.28448362	0.5	CRA004775;PRJNA827465	Yang2025_Silkie_Wenchang	0.896709756
ENSGALG00010004481	ZNF704	2	-0.041801519	0.061934932	-0.674926365	0.499722549	0	0.003369698	0	1	PRJNA827465;Yang2025_Silkie_Wenchang	CRA004775	0.862078151
ENSGALG00010004481	ZNF704	2	0.011913598	0.11889458	0.100203035	0.920183137	0.001823664	1.043647501	4.182207231	0.5	CRA004775;Yang2025_Silkie_Wenchang	PRJNA827465	0.981364549
ENSGALG00010004481	ZNF704	2	-0.005393215	0.093180085	-0.057879486	0.953844623	0.004811511	1.132379816	11.69040756	0.5	CRA004775;PRJNA827465	Yang2025_Silkie_Wenchang	0.987838152
ENSGALG00010004487	ABHD10	2	-0.212830625	0.157635233	-1.350146289	0.176969063	0.035425025	3.42459061	70.79942937	1	PRJNA827465;Yang2025_Silkie_Wenchang	CRA004775	0.682639618
ENSGALG00010004487	ABHD10	2	-0.263816433	0.157301531	-1.677138364	0.093515428	0.020464929	1.625943832	38.49726045	1	CRA004775;Yang2025_Silkie_Wenchang	PRJNA827465	0.523998859
ENSGALG00010004487	ABHD10	2	-0.065874546	0.092575348	-0.711577628	0.476726365	0	0.001654452	0	1	CRA004775;PRJNA827465	Yang2025_Silkie_Wenchang	0.854986476
ENSGALG00010004489	LIG1	2	-0.095756097	0.085989873	-1.113574119	0.265461935	0	0.331955693	0	1	PRJNA827465;Yang2025_Silkie_Wenchang	CRA004775	0.769520043
ENSGALG00010004489	LIG1	2	-0.264852859	0.295845992	-0.895238961	0.370659362	0.122932713	3.179626343	68.54976365	1	CRA004775;Yang2025_Silkie_Wenchang	PRJNA827465	0.821427961
ENSGALG00010004489	LIG1	2	-0.291415189	0.230495517	-1.264298731	0.206122825	0.070875083	2.506693575	60.10681122	1	CRA004775;PRJNA827465	Yang2025_Silkie_Wenchang	0.718403416
ENSGALG00010004494	WTAP	2	-0.207716745	0.208269375	-0.997346564	0.318596319	0.084892992	46.23947199	97.83734555	1	PRJNA827465;Yang2025_Silkie_Wenchang	CRA004775	0.797754958
ENSGALG00010004494	WTAP	2	-0.147719955	0.287243385	-0.514267561	0.607064938	0.152971126	13.14981136	92.39532817	0.5	CRA004775;Yang2025_Silkie_Wenchang	PRJNA827465	0.89694739
ENSGALG00010004494	WTAP	2	0.009617378	0.041948443	0.229266616	0.818661699	0.001250174	1.109983407	9.908563202	0.5	CRA004775;PRJNA827465	Yang2025_Silkie_Wenchang	0.955256039
ENSGALG00010004495	MRPL15	2	-0.327657147	0.147375973	-2.223273847	0.02619734	0	0.006443555	0	1	PRJNA827465;Yang2025_Silkie_Wenchang	CRA004775	0.27602655
ENSGALG00010004495	MRPL15	2	-0.274123155	0.190809706	-1.436631086	0.150822846	0	0.123979332	0	1	CRA004775;Yang2025_Silkie_Wenchang	PRJNA827465	0.642051813
ENSGALG00010004495	MRPL15	2	-0.28708862	0.143996339	-1.993721667	0.04618248	0	0.12041536	0	1	CRA004775;PRJNA827465	Yang2025_Silkie_Wenchang	0.370491759
ENSGALG00010004501	TAGLN3	2	0.485217107	0.457769015	1.059960573	0.289162537	0.279110291	2.957017166	66.18213748	1	PRJNA827465;Yang2025_Silkie_Wenchang	CRA004775	0.783530627
ENSGALG00010004501	TAGLN3	2	0.716435598	0.454846166	1.575116273	0.115229609	0.100798159	1.225221907	18.38213191	0.5	CRA004775;Yang2025_Silkie_Wenchang	PRJNA827465	0.572203827
ENSGALG00010004501	TAGLN3	2	0.045247026	0.308081055	0.146867278	0.88323678	0	0.02003215	0	0.5	CRA004775;PRJNA827465	Yang2025_Silkie_Wenchang	0.972001076
ENSGALG00010004503	ENSGALG00010004503	2	0.02843539	0.11772812	0.241534396	0.809140961	0	0.138233067	0	0.5	PRJNA827465;Yang2025_Silkie_Wenchang	CRA004775	0.952716213
ENSGALG00010004503	ENSGALG00010004503	2	0.130607158	0.262248887	0.4980275	0.618464659	0.005800355	1.042468425	4.073833201	0.5	CRA004775;Yang2025_Silkie_Wenchang	PRJNA827465	0.899168321
ENSGALG00010004503	ENSGALG00010004503	2	0.080649962	0.120185066	0.671048119	0.502189876	0	0.902508601	0	1	CRA004775;PRJNA827465	Yang2025_Silkie_Wenchang	0.862860181
ENSGALG00010004505	ENSGALG00010004505	2	-0.094051371	0.07628722	-1.232858809	0.217628446	0	0.018809331	0	1	PRJNA827465;Yang2025_Silkie_Wenchang	CRA004775	0.731013651
ENSGALG00010004505	ENSGALG00010004505	2	-0.042378058	0.191141765	-0.221710092	0.824539573	0	0.042344043	0	0.5	CRA004775;Yang2025_Silkie_Wenchang	PRJNA827465	0.95619211
ENSGALG00010004505	ENSGALG00010004505	2	-0.092456812	0.079425965	-1.164062817	0.244398556	0	0.100668917	0	0.5	CRA004775;PRJNA827465	Yang2025_Silkie_Wenchang	0.755929523
ENSGALG00010004507	CUL2	2	0.012935911	0.111618008	0.115894476	0.907736174	0.019214136	3.788255419	73.60262471	0.5	PRJNA827465;Yang2025_Silkie_Wenchang	CRA004775	0.978196186
ENSGALG00010004507	CUL2	2	0.174101651	0.099800538	1.74449612	0.081072617	0	0.203303774	0	1	CRA004775;Yang2025_Silkie_Wenchang	PRJNA827465	0.490290365
ENSGALG00010004507	CUL2	2	0.022611755	0.154991821	0.14588999	0.884008233	0.032610879	2.279782785	56.13617197	0.5	CRA004775;PRJNA827465	Yang2025_Silkie_Wenchang	0.972001076
ENSGALG00010004515	ENSGALG00010004515	2	-0.278883977	0.409148099	-0.6816211	0.495478569	0.300529918	9.75278231	89.74651573	0.5	PRJNA827465;Yang2025_Silkie_Wenchang	CRA004775	0.860770265
ENSGALG00010004515	ENSGALG00010004515	2	-0.1563224	0.600918548	-0.260139084	0.794756491	0.610906086	6.074325346	83.53726639	0.5	CRA004775;Yang2025_Silkie_Wenchang	PRJNA827465	0.948905846
ENSGALG00010004515	ENSGALG00010004515	2	0.175090292	0.161187414	1.086252873	0.277367131	0	0.659710635	0	1	CRA004775;PRJNA827465	Yang2025_Silkie_Wenchang	0.776450166
ENSGALG00010004516	ENSGALG00010004516	2	0.242929151	0.283006392	0.858387506	0.390678524	0.128797482	4.720950508	78.81782496	0.5	PRJNA827465;Yang2025_Silkie_Wenchang	CRA004775	0.829251057
ENSGALG00010004516	ENSGALG00010004516	2	0.299414996	0.385601551	0.77648805	0.437460874	0.163754529	1.944218574	48.56545383	0.5	CRA004775;Yang2025_Silkie_Wenchang	PRJNA827465	0.843803603
ENSGALG00010004516	ENSGALG00010004516	2	-0.012410166	0.105837009	-0.117257337	0.906656132	0	0.206879023	0	1	CRA004775;PRJNA827465	Yang2025_Silkie_Wenchang	0.978196186
ENSGALG00010004522	TSPAN5	2	-0.366199681	0.341762541	-1.071503271	0.283943201	0.225668516	29.1141279	96.56524144	1	PRJNA827465;Yang2025_Silkie_Wenchang	CRA004775	0.77978499
ENSGALG00010004522	TSPAN5	2	-0.368220515	0.36075974	-1.020680732	0.307405726	0.237609562	11.28460782	91.1383717	0.5	CRA004775;Yang2025_Silkie_Wenchang	PRJNA827465	0.792675978
ENSGALG00010004522	TSPAN5	2	-0.027630827	0.055465162	-0.498165448	0.618367434	0	0.040011577	0	0.5	CRA004775;PRJNA827465	Yang2025_Silkie_Wenchang	0.899168321
ENSGALG00010004533	PAG1	2	-0.003407904	0.078860266	-0.043214467	0.965530573	0.001512586	1.063646099	5.98376652	0.5	PRJNA827465;Yang2025_Silkie_Wenchang	CRA004775	0.990668014
ENSGALG00010004533	PAG1	2	0.085132584	0.346682746	0.245563372	0.806020246	0.157418823	2.680227799	62.68973852	0.5	CRA004775;Yang2025_Silkie_Wenchang	PRJNA827465	0.952063038
ENSGALG00010004533	PAG1	2	0.126279927	0.193996978	0.650937597	0.51508677	0.041370115	1.546696431	35.3460718	1	CRA004775;PRJNA827465	Yang2025_Silkie_Wenchang	0.867111635
ENSGALG00010004534	CUBN	2	-0.272428362	0.141912724	-1.919689463	0.054897136	0	0.122494766	0	1	PRJNA827465;Yang2025_Silkie_Wenchang	CRA004775	0.405568071
ENSGALG00010004534	CUBN	2	-0.103662098	0.230692763	-0.449351321	0.653178241	0.025718491	1.188359708	15.85039499	0.5	CRA004775;Yang2025_Silkie_Wenchang	PRJNA827465	0.909597225
ENSGALG00010004534	CUBN	2	-0.130124108	0.302013501	-0.430855269	0.66657361	0.088327743	1.651983203	39.46669686	0.5	CRA004775;PRJNA827465	Yang2025_Silkie_Wenchang	0.913579507
ENSGALG00010004542	ERMARD	2	-0.312906149	0.25941448	-1.206201555	0.227739784	0.111014394	5.547973145	81.97539941	1	PRJNA827465;Yang2025_Silkie_Wenchang	CRA004775	0.740853612
ENSGALG00010004542	ERMARD	2	-0.449886851	0.169835801	-2.648951803	0.008074185	0.014448764	1.321981131	24.35595515	1	CRA004775;Yang2025_Silkie_Wenchang	PRJNA827465	0.139633915
ENSGALG00010004542	ERMARD	2	-0.108517132	0.104958789	-1.033902285	0.301181847	0	0.451993402	0	1	CRA004775;PRJNA827465	Yang2025_Silkie_Wenchang	0.791222412
ENSGALG00010004553	BATF	2	0.625978861	0.792724548	0.789654947	0.429729309	1.152712842	11.9911237	91.66049801	0.5	PRJNA827465;Yang2025_Silkie_Wenchang	CRA004775	0.842012722
ENSGALG00010004553	BATF	2	0.535519314	0.923993493	0.579570438	0.562204333	1.533248837	9.752293652	89.74600196	0.5	CRA004775;Yang2025_Silkie_Wenchang	PRJNA827465	0.881955027
ENSGALG00010004553	BATF	2	-0.214680583	0.23438847	-0.915917846	0.359709986	0	0.239067922	0	1	CRA004775;PRJNA827465	Yang2025_Silkie_Wenchang	0.814879426
ENSGALG00010004565	ENSGALG00010004565	2	0.184749689	0.307533004	0.60074752	0.548008163	0	0.669866451	0	0.5	PRJNA827465;Yang2025_Silkie_Wenchang	CRA004775	0.878281266
ENSGALG00010004565	ENSGALG00010004565	2	0.672409025	0.442429907	1.519809159	0.128558947	0.043200772	1.075659933	7.033815273	1	CRA004775;Yang2025_Silkie_Wenchang	PRJNA827465	0.601638797
ENSGALG00010004565	ENSGALG00010004565	2	0.517589778	0.770889777	0.671418656	0.501953863	0.740609272	2.323310261	56.95796567	0.5	CRA004775;PRJNA827465	Yang2025_Silkie_Wenchang	0.862795569
ENSGALG00010004568	RSU1	2	-0.107258544	0.056715407	-1.8911712	0.058601494	0	0.109787296	0	1	PRJNA827465;Yang2025_Silkie_Wenchang	CRA004775	0.419299934
ENSGALG00010004568	RSU1	2	-0.040097891	0.092893946	-0.431652362	0.665994096	0	0.861940984	0	0.5	CRA004775;Yang2025_Silkie_Wenchang	PRJNA827465	0.913546323
ENSGALG00010004568	RSU1	2	-0.069965616	0.104792092	-0.667661222	0.504349869	0.008811288	1.318556311	24.15947718	0.5	CRA004775;PRJNA827465	Yang2025_Silkie_Wenchang	0.863512504
ENSGALG00010004576	STEAP2	2	-0.240289184	0.089435622	-2.686727934	0.00721557	0.001291658	1.08038858	7.440709902	1	PRJNA827465;Yang2025_Silkie_Wenchang	CRA004775	0.130628071
ENSGALG00010004576	STEAP2	2	-0.250803696	0.203145438	-1.234601668	0.216978788	0.032163639	1.36959122	26.98551322	0.5	CRA004775;Yang2025_Silkie_Wenchang	PRJNA827465	0.730505165
ENSGALG00010004576	STEAP2	2	-0.15263189	0.102182873	-1.49371305	0.135250636	0	0.554687439	0	0.5	CRA004775;PRJNA827465	Yang2025_Silkie_Wenchang	0.616533955
ENSGALG00010004577	ZPLD1	2	-0.710693994	0.394411143	-1.801911549	0.071559323	0	0.067929689	0	1	PRJNA827465;Yang2025_Silkie_Wenchang	CRA004775	0.461684844
ENSGALG00010004577	ZPLD1	2	-0.834866446	0.43802617	-1.905973896	0.056653592	0	0.282385121	0	1	CRA004775;Yang2025_Silkie_Wenchang	PRJNA827465	0.411670741
ENSGALG00010004577	ZPLD1	2	-0.892617835	0.376892981	-2.368358875	0.017867195	0	0.110438088	0	1	CRA004775;PRJNA827465	Yang2025_Silkie_Wenchang	0.223129158
ENSGALG00010004583	XKR4	2	-0.156747132	0.125383118	-1.250145432	0.211246426	0	0.367120436	0	1	PRJNA827465;Yang2025_Silkie_Wenchang	CRA004775	0.72353972
ENSGALG00010004583	XKR4	2	0.080348123	0.231548333	0.347003676	0.728588557	0	0.865028568	0	0.5	CRA004775;Yang2025_Silkie_Wenchang	PRJNA827465	0.932187391
ENSGALG00010004583	XKR4	2	-0.017280922	0.317430416	-0.054440032	0.956584585	0.113253146	1.720678557	41.88339269	0.5	CRA004775;PRJNA827465	Yang2025_Silkie_Wenchang	0.988540618
ENSGALG00010004584	EIF4E	2	-0.271422621	0.205563064	-1.320386139	0.186706129	0.081491913	27.92123328	96.41849631	1	PRJNA827465;Yang2025_Silkie_Wenchang	CRA004775	0.695457578
ENSGALG00010004584	EIF4E	2	-0.201410418	0.31775334	-0.633857752	0.526173659	0.174144271	6.521821154	84.66685951	0.5	CRA004775;Yang2025_Silkie_Wenchang	PRJNA827465	0.870525481
ENSGALG00010004584	EIF4E	2	-0.059148366	0.046850092	-1.262502679	0.206767963	0	0.860802063	0	0.5	CRA004775;PRJNA827465	Yang2025_Silkie_Wenchang	0.71868063
ENSGALG00010004588	MINDY3	2	-0.156835645	0.138006354	-1.136437853	0.255773361	0.029118475	4.167133973	76.00269138	1	PRJNA827465;Yang2025_Silkie_Wenchang	CRA004775	0.764478331
ENSGALG00010004588	MINDY3	2	-0.279378919	0.101193471	-2.760839365	0.005765303	0	0.373308811	0	1	CRA004775;Yang2025_Silkie_Wenchang	PRJNA827465	0.114441255
ENSGALG00010004588	MINDY3	2	-0.038045399	0.075777165	-0.502069436	0.615618679	0	0.152841375	0	1	CRA004775;PRJNA827465	Yang2025_Silkie_Wenchang	0.898886043
ENSGALG00010004591	RRS1	2	0.363265142	0.415665766	0.873935674	0.382153286	0.319590297	12.76930955	92.16872302	0.5	PRJNA827465;Yang2025_Silkie_Wenchang	CRA004775	0.825035462
ENSGALG00010004591	RRS1	2	0.363374668	0.50655432	0.717345906	0.473160688	0.403313009	4.36330101	77.08157201	0.5	CRA004775;Yang2025_Silkie_Wenchang	PRJNA827465	0.854341771
ENSGALG00010004591	RRS1	2	-0.035276247	0.081339106	-0.43369356	0.664510984	0	0.181592249	0	1	CRA004775;PRJNA827465	Yang2025_Silkie_Wenchang	0.913255304
ENSGALG00010004602	DYNLT2	2	-0.026544865	0.26445272	-0.100376601	0.920045346	0	0.003398922	0	1	PRJNA827465;Yang2025_Silkie_Wenchang	CRA004775	0.981364549
ENSGALG00010004602	DYNLT2	2	0.2012158	0.338208068	0.594946776	0.551879049	0	0.616393582	0	0.5	CRA004775;Yang2025_Silkie_Wenchang	PRJNA827465	0.879061525
ENSGALG00010004602	DYNLT2	2	0.119058636	0.286224343	0.415962649	0.677437335	0	0.810303075	0	0.5	CRA004775;PRJNA827465	Yang2025_Silkie_Wenchang	0.917144807
ENSGALG00010004610	TGS1	2	0.04865807	0.045528735	1.068733187	0.285189913	0	0.638241695	0	1	PRJNA827465;Yang2025_Silkie_Wenchang	CRA004775	0.780667164
ENSGALG00010004610	TGS1	2	0.092721708	0.091066028	1.018181085	0.308591907	0	0.443955276	0	0.5	CRA004775;Yang2025_Silkie_Wenchang	PRJNA827465	0.793561833
ENSGALG00010004610	TGS1	2	0.026239114	0.0499698	0.525099449	0.599514059	0	0.106488274	0	0.5	CRA004775;PRJNA827465	Yang2025_Silkie_Wenchang	0.895010988
ENSGALG00010004622	ENSGALG00010004622	2	0.367163073	0.591286685	0.620956099	0.534628509	0.638653498	10.86989863	90.80028219	0.5	PRJNA827465;Yang2025_Silkie_Wenchang	CRA004775	0.873700802
ENSGALG00010004622	ENSGALG00010004622	2	0.373713034	0.663058663	0.563619865	0.573012865	0.720360019	5.474939386	81.73495761	0.5	CRA004775;Yang2025_Silkie_Wenchang	PRJNA827465	0.88573298
ENSGALG00010004622	ENSGALG00010004622	2	-0.187655177	0.101581603	-1.847334281	0.064698708	0	0.093577585	0	1	CRA004775;PRJNA827465	Yang2025_Silkie_Wenchang	0.438551994
ENSGALG00010004623	MBIP	2	0.058919316	0.062495794	0.942772504	0.345797278	0	0.165970335	0	1	PRJNA827465;Yang2025_Silkie_Wenchang	CRA004775	0.807479003
ENSGALG00010004623	MBIP	2	0.052278123	0.105207923	0.496902911	0.619257519	0	0.684155481	0	0.5	CRA004775;Yang2025_Silkie_Wenchang	PRJNA827465	0.899168321
ENSGALG00010004623	MBIP	2	0.028342175	0.069224907	0.409421639	0.682230262	0	0.416327708	0	0.5	CRA004775;PRJNA827465	Yang2025_Silkie_Wenchang	0.918550276
ENSGALG00010004624	PHF10	2	-0.102124282	0.098226743	-1.039679	0.29848906	0.015442088	4.876841315	79.49492437	1	PRJNA827465;Yang2025_Silkie_Wenchang	CRA004775	0.789416293
ENSGALG00010004624	PHF10	2	-0.1218658	0.129048554	-0.944340682	0.344995583	0.018629504	1.922713033	47.99015856	0.5	CRA004775;Yang2025_Silkie_Wenchang	PRJNA827465	0.806961063
ENSGALG00010004624	PHF10	2	-0.00631269	0.047427262	-0.133102553	0.894112276	0	0.178586205	0	0.5	CRA004775;PRJNA827465	Yang2025_Silkie_Wenchang	0.974381724
ENSGALG00010004630	ENSGALG00010004630	2	0.324019544	0.140775848	2.301669974	0.021353791	0	0.237717547	0	1	PRJNA827465;Yang2025_Silkie_Wenchang	CRA004775	0.247158031
ENSGALG00010004630	ENSGALG00010004630	2	0.197544631	0.28997557	0.68124577	0.495715991	0.062642411	1.573228133	36.43642782	0.5	CRA004775;Yang2025_Silkie_Wenchang	PRJNA827465	0.860911322
ENSGALG00010004630	ENSGALG00010004630	2	0.201420051	0.169517617	1.188195387	0.234756441	0.00991596	1.123469561	10.99002278	0.5	CRA004775;PRJNA827465	Yang2025_Silkie_Wenchang	0.747791642
ENSGALG00010004639	HBS1L	2	-0.036373701	0.035422455	-1.026854333	0.304489061	0	0.120550154	0	1	PRJNA827465;Yang2025_Silkie_Wenchang	CRA004775	0.79225794
ENSGALG00010004639	HBS1L	2	-0.005073787	0.071217357	-0.07124368	0.943203818	0	0.288170008	0	0.5	CRA004775;Yang2025_Silkie_Wenchang	PRJNA827465	0.985827325
ENSGALG00010004639	HBS1L	2	-0.039806809	0.040135562	-0.99180895	0.321290731	0	0.409935106	0	0.5	CRA004775;PRJNA827465	Yang2025_Silkie_Wenchang	0.798513866
ENSGALG00010004641	METAP1	2	-0.096223883	0.215824899	-0.445842362	0.655711119	0.088125622	18.33620826	94.54630976	0.5	PRJNA827465;Yang2025_Silkie_Wenchang	CRA004775	0.910411938
ENSGALG00010004641	METAP1	2	-0.281685178	0.076466653	-3.683764948	0.000229814	0	0.920897398	0	1	CRA004775;Yang2025_Silkie_Wenchang	PRJNA827465	0.0140037
ENSGALG00010004641	METAP1	2	0.050343059	0.107553493	0.468074607	0.639731237	0.012751582	1.749229309	42.83196634	0.5	CRA004775;PRJNA827465	Yang2025_Silkie_Wenchang	0.906064918
ENSGALG00010004650	GTPBP10	2	-0.119275907	0.361162344	-0.330255657	0.741206795	0.247388769	19.3348118	94.82798172	0.5	PRJNA827465;Yang2025_Silkie_Wenchang	CRA004775	0.93538565
ENSGALG00010004650	GTPBP10	2	0.225463715	0.111768514	2.017238187	0.043670663	0	0.150601888	0	1	CRA004775;Yang2025_Silkie_Wenchang	PRJNA827465	0.361192773
ENSGALG00010004650	GTPBP10	2	-0.23925353	0.293833976	-0.814247328	0.415503277	0.131404478	3.705676045	73.01437072	0.5	CRA004775;PRJNA827465	Yang2025_Silkie_Wenchang	0.837678466
ENSGALG00010004666	C6orf120	2	-0.055773368	0.062920545	-0.886409488	0.375396908	0	0.340618276	0	1	PRJNA827465;Yang2025_Silkie_Wenchang	CRA004775	0.822974013
ENSGALG00010004666	C6orf120	2	0.112571294	0.227680607	0.49442636	0.621005102	0.086273917	5.693663159	82.43661467	0.5	CRA004775;Yang2025_Silkie_Wenchang	PRJNA827465	0.899578487
ENSGALG00010004666	C6orf120	2	0.144226878	0.19005951	0.758851151	0.447941603	0.055677628	4.084886364	75.51951484	0.5	CRA004775;PRJNA827465	Yang2025_Silkie_Wenchang	0.846601815
ENSGALG00010004673	RAB18	2	-0.022081674	0.069880181	-0.315993363	0.752007546	0.005732199	2.182967377	54.19079505	0.5	PRJNA827465;Yang2025_Silkie_Wenchang	CRA004775	0.937903487
ENSGALG00010004673	RAB18	2	-0.042967072	0.135191564	-0.317823618	0.750618734	0.015682832	1.41910004	29.53280447	0.5	CRA004775;Yang2025_Silkie_Wenchang	PRJNA827465	0.937473087
ENSGALG00010004673	RAB18	2	0.035514193	0.043160923	0.822832097	0.410603481	0	0.472007054	0	1	CRA004775;PRJNA827465	Yang2025_Silkie_Wenchang	0.835020871
ENSGALG00010004689	BBX	2	0.06815121	0.062257692	1.094663288	0.273664178	0	0.107407832	0	1	PRJNA827465;Yang2025_Silkie_Wenchang	CRA004775	0.774413073
ENSGALG00010004689	BBX	2	0.134969953	0.13366615	1.009754172	0.312613081	0	0.192395028	0	1	CRA004775;Yang2025_Silkie_Wenchang	PRJNA827465	0.796054997
ENSGALG00010004689	BBX	2	0.066530787	0.067699752	0.982733104	0.325738813	0	0.374182283	0	1	CRA004775;PRJNA827465	Yang2025_Silkie_Wenchang	0.800023788
ENSGALG00010004705	ENSGALG00010004705	2	0.652932354	0.398183863	1.639776031	0.101051744	0.26259581	5.238420636	80.91027679	1	PRJNA827465;Yang2025_Silkie_Wenchang	CRA004775	0.542487465
ENSGALG00010004705	ENSGALG00010004705	2	0.635001097	0.503759603	1.260524054	0.207480376	0.374776309	3.801442543	73.69419664	1	CRA004775;Yang2025_Silkie_Wenchang	PRJNA827465	0.719608298
ENSGALG00010004705	ENSGALG00010004705	2	0.295813517	0.108696474	2.721463784	0.006499351	0	0.24318105	0	1	CRA004775;PRJNA827465	Yang2025_Silkie_Wenchang	0.123006701
ENSGALG00010004707	OTUD1	2	-0.282787572	0.286089408	-0.988458725	0.322928039	0.133612724	5.421937432	81.55640834	1	PRJNA827465;Yang2025_Silkie_Wenchang	CRA004775	0.79874391
ENSGALG00010004707	OTUD1	2	0.235817452	0.92681435	0.254438714	0.799156645	1.485057637	6.655861298	84.97564845	0.5	CRA004775;Yang2025_Silkie_Wenchang	PRJNA827465	0.949922217
ENSGALG00010004707	OTUD1	2	0.464670946	0.623346481	0.745445688	0.456002333	0.583415579	3.262362065	69.34736304	0.5	CRA004775;PRJNA827465	Yang2025_Silkie_Wenchang	0.84907427
ENSGALG00010004716	ADH6	2	0.923116641	0.880788785	1.04805676	0.294612456	1.123930717	3.591304435	72.15496436	1	PRJNA827465;Yang2025_Silkie_Wenchang	CRA004775	0.787429675
ENSGALG00010004716	ADH6	2	1.099857082	1.040648189	1.056896167	0.290558978	1.754782599	5.23978563	80.91524977	1	CRA004775;Yang2025_Silkie_Wenchang	PRJNA827465	0.784499619
ENSGALG00010004716	ADH6	2	2.025751845	0.505206841	4.009747455	6.07837E-05	0	0.09865152	0	1	CRA004775;PRJNA827465	Yang2025_Silkie_Wenchang	0.005517281
ENSGALG00010004717	ALKBH8	2	-0.17756619	0.10761722	-1.649979338	0.098947162	0	0.035800795	0	1	PRJNA827465;Yang2025_Silkie_Wenchang	CRA004775	0.536907794
ENSGALG00010004717	ALKBH8	2	-0.168817529	0.138657714	-1.217512712	0.223409203	0	0.159764671	0	1	CRA004775;Yang2025_Silkie_Wenchang	PRJNA827465	0.736744216
ENSGALG00010004717	ALKBH8	2	-0.14007063	0.130323731	-1.074789905	0.282468801	0	0.078121728	0	1	CRA004775;PRJNA827465	Yang2025_Silkie_Wenchang	0.779532565
ENSGALG00010004721	LAMA2	2	-0.113684063	0.164085793	-0.692833065	0.488414319	0.031655095	2.347322626	57.39827201	0.5	PRJNA827465;Yang2025_Silkie_Wenchang	CRA004775	0.857837717
ENSGALG00010004721	LAMA2	2	0.411680077	0.442369211	0.930625521	0.352047308	0.275143024	2.857738822	65.00729905	1	CRA004775;Yang2025_Silkie_Wenchang	PRJNA827465	0.810678834

ENSGALG00010004721	LAMA2	2	0.272337044	0.61812421	0.440586276	0.65951254	0.64432835	5.614413067	82.18869919	0.5	CRA004775;PRJNA827465	Yang2025_Silkie_Wenchang	0.91158155
ENSGALG00010004728	ENSGALG00010004728	2	0.176110541	0.303512136	0.580242172	0.561751318	0.164306462	8.79266489	88.62688374	0.5	PRJNA827465;Yang2025_Silkie_Wenchang	CRA004775	0.881802301
ENSGALG00010004728	ENSGALG00010004728	2	0.387010269	0.222748551	1.737431141	0.082311099	0.027738455	1.235361353	19.05202495	0.5	CRA004775;Yang2025_Silkie_Wenchang	PRJNA827465	0.494583872
ENSGALG00010004728	ENSGALG00010004728	2	-0.102177311	0.079170661	-1.290595659	0.196843922	0	0.023260133	0	1	CRA004775;PRJNA827465	Yang2025_Silkie_Wenchang	0.707994211
ENSGALG00010004729	ENSGALG00010004729	2	0.32622822	0.332200894	0.982020897	0.326089553	0	0.702229365	0	1	PRJNA827465;Yang2025_Silkie_Wenchang	CRA004775	0.800023788
ENSGALG00010004729	ENSGALG00010004729	2	0.663304309	0.456038188	1.454492905	0.145809699	0	0.034978089	0	1	CRA004775;Yang2025_Silkie_Wenchang	PRJNA827465	0.634635505
ENSGALG00010004729	ENSGALG00010004729	2	0.166105446	0.423517418	0.39220452	0.694907105	0	0.378939144	0	1	CRA004775;PRJNA827465	Yang2025_Silkie_Wenchang	0.92087205
ENSGALG00010004730	CLDN12	2	-0.247170451	0.272191164	-0.908076688	0.363837706	0.13994745	17.76210272	94.37003594	0.5	PRJNA827465;Yang2025_Silkie_Wenchang	CRA004775	0.817042253
ENSGALG00010004730	CLDN12	2	-0.298116357	0.277808561	-1.073099967	0.283226266	0.117096524	3.719253337	73.1128829	0.5	CRA004775;Yang2025_Silkie_Wenchang	PRJNA827465	0.779568117
ENSGALG00010004730	CLDN12	2	0.018505468	0.062483357	0.296166362	0.767103036	0	0.005875213	0	1	CRA004775;PRJNA827465	Yang2025_Silkie_Wenchang	0.941432527
ENSGALG00010004735	ENSGALG00010004735	2	-0.641297176	0.490919347	-1.306318808	0.191444164	0.453551612	16.44272624	93.91828347	1	PRJNA827465;Yang2025_Silkie_Wenchang	CRA004775	0.700915693
ENSGALG00010004735	ENSGALG00010004735	2	-0.571348932	0.592613619	-0.964117113	0.334987214	0.640373773	11.29447329	91.14611213	0.5	CRA004775;Yang2025_Silkie_Wenchang	PRJNA827465	0.803101658
ENSGALG00010004735	ENSGALG00010004735	2	-0.150861709	0.08646062	-1.744860351	0.08100918	0	0.498832069	0	0.5	CRA004775;PRJNA827465	Yang2025_Silkie_Wenchang	0.48998212
ENSGALG00010004736	CD47	2	0.115071216	0.051966352	2.214340854	0.02680534	0	0.9784586	0	1	PRJNA827465;Yang2025_Silkie_Wenchang	CRA004775	0.279174307
ENSGALG00010004736	CD47	2	0.023022325	0.09233472	0.249335521	0.803101257	0	0.098592426	0	0.5	CRA004775;Yang2025_Silkie_Wenchang	PRJNA827465	0.950932085
ENSGALG00010004736	CD47	2	0.137845378	0.05992857	2.300161299	0.021439084	0	0.522188427	0	0.5	CRA004775;PRJNA827465	Yang2025_Silkie_Wenchang	0.247729473
ENSGALG00010004746	LYN	2	-0.120647296	0.172681089	-0.698671153	0.484757565	0.045787728	4.261195669	76.53240833	0.5	PRJNA827465;Yang2025_Silkie_Wenchang	CRA004775	0.857004733
ENSGALG00010004746	LYN	2	-0.309297754	0.12827295	-2.411246908	0.015898082	0	0.036680995	0	1	CRA004775;Yang2025_Silkie_Wenchang	PRJNA827465	0.208471156
ENSGALG00010004746	LYN	2	0.026890304	0.101473827	0.264997432	0.79101144	0	0.665985102	0	0.5	CRA004775;PRJNA827465	Yang2025_Silkie_Wenchang	0.947823629
ENSGALG00010004751	PTER	2	-0.253500483	0.257270152	-0.985347432	0.324453441	0.106984223	5.203954494	80.78384426	0.5	PRJNA827465;Yang2025_Silkie_Wenchang	CRA004775	0.799859262
ENSGALG00010004751	PTER	2	-0.300397166	0.307854441	-0.975776619	0.329175174	0.114134523	2.052537782	51.27982496	0.5	CRA004775;Yang2025_Silkie_Wenchang	PRJNA827465	0.800761292
ENSGALG00010004751	PTER	2	0.028495325	0.156900144	0.181614396	0.85588535	0	0.104642763	0	1	CRA004775;PRJNA827465	Yang2025_Silkie_Wenchang	0.963486168
ENSGALG00010004757	TRMT10A	2	0.050411822	0.081006014	0.622321971	0.533730177	0	0.552073137	0	0.5	PRJNA827465;Yang2025_Silkie_Wenchang	CRA004775	0.873408008
ENSGALG00010004757	TRMT10A	2	0.058166552	0.097088087	0.599111109	0.549098794	0	0.948649269	0	0.5	CRA004775;Yang2025_Silkie_Wenchang	PRJNA827465	0.878431377
ENSGALG00010004757	TRMT10A	2	-0.046932601	0.108557579	-0.432329102	0.665502239	0	0.227864792	0	1	CRA004775;PRJNA827465	Yang2025_Silkie_Wenchang	0.91350776
ENSGALG00010004760	THBS2	2	-0.028575033	0.133686244	-0.213746992	0.830744364	0	0.410721485	0	0.5	PRJNA827465;Yang2025_Silkie_Wenchang	CRA004775	0.95733143
ENSGALG00010004760	THBS2	2	-0.192903134	0.212713485	-0.906868384	0.364476402	0	0.395268652	0	1	CRA004775;Yang2025_Silkie_Wenchang	PRJNA827465	0.817496251
ENSGALG00010004760	THBS2	2	-0.004305423	0.160461673	-0.026831474	0.97859415	0	0.846341453	0	0.5	CRA004775;PRJNA827465	Yang2025_Silkie_Wenchang	0.994183692
ENSGALG00010004762	IFT57	2	[illegible]	0.26446096	1.07137724	0.283999842	0.122242319	7.864491665	87.28462	1	PRJNA827465;Yang2025_Silkie_Wenchang	CRA004775	0.779853685
ENSGALG00010004762	IFT57	2	0.496930155	0.116597911	4.261913005	2.02684E-05	0	0.835020635	0	1	CRA004775;Yang2025_Silkie_Wenchang	PRJNA827465	0.00247481
ENSGALG00010004762	IFT57	2	0.06451009	0.124275992	0.519087305	0.603699861	0	0.620788971	0	1	CRA004775;PRJNA827465	Yang2025_Silkie_Wenchang	0.895856405
ENSGALG00010004765	ENSGALG00010004765	2	0.096110486	0.385433465	0.249355879	0.803084737	0.255495832	6.593580992	84.83373449	0.5	PRJNA827465;Yang2025_Silkie_Wenchang	CRA004775	0.950932085
ENSGALG00010004765	ENSGALG00010004765	2	-0.849976757	0.686041215	-1.238958737	0.21536078	0.794400242	5.697072109	82.44712405	1	CRA004775;Yang2025_Silkie_Wenchang	PRJNA827465	0.728757551
ENSGALG00010004765	ENSGALG00010004765	2	-0.51156727	1.079104972	-0.474066271	0.63545265	2.143853094	12.18657436	91.79424857	0.5	CRA004775;PRJNA827465	Yang2025_Silkie_Wenchang	0.90421144
ENSGALG00010004769	HTR2A	2	-0.346449729	0.48235972	-0.718239343	0.472609724	0.407459877	7.974853569	87.46058481	0.5	PRJNA827465;Yang2025_Silkie_Wenchang	CRA004775	0.853998373
ENSGALG00010004769	HTR2A	2	-0.856229089	0.253981501	-3.371226191	0.000748344	0	0.011917437	0	1	CRA004775;Yang2025_Silkie_Wenchang	PRJNA827465	0.030765857
ENSGALG00010004769	HTR2A	2	-0.189144967	0.477666291	-0.395977214	0.692121838	0.281009168	2.050193973	51.22412742	0.5	CRA004775;PRJNA827465	Yang2025_Silkie_Wenchang	0.920348122
ENSGALG00010004777	CDK14	2	-0.181636038	0.150499879	-1.206884947	0.227476455	0.033320023	3.531093567	71.68016137	1	PRJNA827465;Yang2025_Silkie_Wenchang	CRA004775	0.740853612
ENSGALG00010004777	CDK14	2	-0.208637792	0.224214933	-0.930525855	0.352098884	0.052124309	1.793471386	44.24221052	0.5	CRA004775;Yang2025_Silkie_Wenchang	PRJNA827465	0.810678834
ENSGALG00010004777	CDK14	2	-0.04461249	0.073909833	-0.603606966	0.546104981	0	0.279583126	0	0.5	CRA004775;PRJNA827465	Yang2025_Silkie_Wenchang	0.87799902
ENSGALG00010004778	ENSGALG00010004778	2	-0.698065441	1.208220337	-0.577763359	0.563423896	2.677227783	12.03046612	91.68777011	0.5	PRJNA827465;Yang2025_Silkie_Wenchang	CRA004775	0.882407972
ENSGALG00010004778	ENSGALG00010004778	2	-0.453603024	1.63355002	-0.277679299	0.781258553	4.542884314	6.301597484	84.13100801	0.5	CRA004775;Yang2025_Silkie_Wenchang	PRJNA827465	0.945235709
ENSGALG00010004778	ENSGALG00010004778	2	0.608186144	0.428866139	1.418145141	0.15614927	0	0.458363103	0	1	CRA004775;PRJNA827465	Yang2025_Silkie_Wenchang	0.649132948
ENSGALG00010004780	ENSGALG00010004780	2	0.33390288	0.230365401	1.449448917	0.147212256	0.04938413	1.870123029	46.52758216	1	PRJNA827465;Yang2025_Silkie_Wenchang	CRA004775	0.63602103
ENSGALG00010004780	ENSGALG00010004780	2	0.556556195	0.21356268	2.606055491	0.009159162	0	0.006834189	0	1	CRA004775;Yang2025_Silkie_Wenchang	PRJNA827465	0.150243135
ENSGALG00010004780	ENSGALG00010004780	2	0.188622418	0.211866523	0.89028892	0.373310769	0	0.622273046	0	1	CRA004775;PRJNA827465	Yang2025_Silkie_Wenchang	0.822187352
ENSGALG00010004781	ENSGALG00010004781	2	0.006335196	0.159845669	0.039633201	0.968385558	0.034372915	2.709135082	63.08785019	0.5	PRJNA827465;Yang2025_Silkie_Wenchang	CRA004775	0.999125679
ENSGALG00010004781	ENSGALG00010004781	2	-0.162595906	0.154665695	-1.051273238	0.293133115	0	0.108711495	0	1	CRA004775;Yang2025_Silkie_Wenchang	PRJNA827465	0.786774237
ENSGALG00010004781	ENSGALG00010004781	2	0.123787225	0.093397493	1.325380599	0.185044949	0	0.297271462	0	0.5	CRA004775;PRJNA827465	Yang2025_Silkie_Wenchang	0.693265167
ENSGALG00010004787	RIOK3	2	-0.099913876	0.15234868	-0.655823704	0.511937563	0.041508832	9.178502612	89.10497668	0.5	PRJNA827465;Yang2025_Silkie_Wenchang	CRA004775	0.865694231
ENSGALG00010004787	RIOK3	2	-0.069471023	0.240084297	-0.289360696	0.772305167	0.08670943	3.521016069	71.59910718	0.5	CRA004775;Yang2025_Silkie_Wenchang	PRJNA827465	0.943066211
ENSGALG00010004787	RIOK3	2	0.050479855	0.047194132	1.069621427	0.284789746	0	0.552240852	0	1	CRA004775;PRJNA827465	Yang2025_Silkie_Wenchang	0.780278244
ENSGALG00010004791	FZD1	2	-0.075349268	0.170336626	-0.442355056	0.658232297	0.051690303	9.044596407	88.94367471	0.5	PRJNA827465;Yang2025_Silkie_Wenchang	CRA004775	0.910991163
ENSGALG00010004791	FZD1	2	-0.240373967	0.089893624	-2.673982398	0.007495639	0	0.245536601	0	1	CRA004775;Yang2025_Silkie_Wenchang	PRJNA827465	0.133545368
ENSGALG00010004791	FZD1	2	0.082972548	0.063558379	1.305454125	0.191738258	0	0.217864923	0	0.5	CRA004775;PRJNA827465	Yang2025_Silkie_Wenchang	0.701469679
ENSGALG00010004798	PTPRK	2	-0.030446291	0.11139103	-0.273328036	0.784601066	0.008742853	1.441915827	30.64782416	0.5	PRJNA827465;Yang2025_Silkie_Wenchang	CRA004775	0.946117405
ENSGALG00010004798	PTPRK	2	0.149976003	0.157675422	0.951169188	0.341518497	0	0.061605965	0	1	CRA004775;Yang2025_Silkie_Wenchang	PRJNA827465	0.805758959
ENSGALG00010004798	PTPRK	2	-0.085555454	0.094370142	-0.906594526	0.364621257	0	0.765802533	0	0.5	CRA004775;PRJNA827465	Yang2025_Silkie_Wenchang	0.817496251
ENSGALG00010004800	ZC3H12C	2	-0.046679947	0.141731874	-0.329353909	0.741888202	0	0.396337533	0	0.5	PRJNA827465;Yang2025_Silkie_Wenchang	CRA004775	0.93543545
ENSGALG00010004800	ZC3H12C	2	-0.218607561	0.198229333	-1.102801275	0.270113474	0	0.582261719	0	1	CRA004775;Yang2025_Silkie_Wenchang	PRJNA827465	0.772373218
ENSGALG00010004800	ZC3H12C	2	-0.092777648	0.238096849	-0.389664486	0.6965837	0.036837205	1.275032239	21.57005699	0.5	CRA004775;PRJNA827465	Yang2025_Silkie_Wenchang	0.937086208
ENSGALG00010004803	RMC1	2	-0.113282836	0.249986453	-0.4531559	0.650436485	0.114647591	11.7784022	91.50988408	0.5	PRJNA827465;Yang2025_Silkie_Wenchang	CRA004775	0.908618725
ENSGALG00010004803	RMC1	2	-0.34248248	0.117718193	-2.909341973	0.003621904	0	0.383635241	0	1	CRA004775;Yang2025_Silkie_Wenchang	PRJNA827465	0.085650848
ENSGALG00010004803	RMC1	2	0.094237356	0.092008337	1.02422627	0.305728414	0.004872059	1.114822492	10.29962107	0.5	CRA004775;PRJNA827465	Yang2025_Silkie_Wenchang	0.79225794
ENSGALG00010004824	ATM	2	0.046815892	0.104286011	0.448918237	0.65349064	0.014316809	2.70262212	62.99889679	0.5	PRJNA827465;Yang2025_Silkie_Wenchang	CRA004775	0.909648135
ENSGALG00010004824	ATM	2	0.152462927	0.105560175	1.444322412	0.148648304	0	0.268842539	0	1	CRA004775;Yang2025_Silkie_Wenchang	PRJNA827465	0.638795624
ENSGALG00010004824	ATM	2	-0.034851538	0.059387806	-0.586846696	0.557306672	0	0.04632	0	0.5	CRA004775;PRJNA827465	Yang2025_Silkie_Wenchang	0.880678789
ENSGALG00010004835	HHLA2	2	0.996320709	0.330012504	3.019039266	0.002535777	0.152346071	3.282428491	69.53475139	1	PRJNA827465;Yang2025_Silkie_Wenchang	CRA004775	0.067660305
ENSGALG00010004835	HHLA2	2	0.709983588	0.233200343	3.04452206	0.002330503	0	0.167998921	0	1	CRA004775;Yang2025_Silkie_Wenchang	PRJNA827465	0.064063502
ENSGALG00010004835	HHLA2	2	1.186252736	0.200027644	5.930443983	3.02117E-09	0.003394573	1.032946639	3.18957798	1	CRA004775;PRJNA827465	Yang2025_Silkie_Wenchang	1.64918E-06
ENSGALG00010004837	CENPC	2	0.07982631	0.068778043	1.16063654	0.245789739	0	0.004679604	0	1	PRJNA827465;Yang2025_Silkie_Wenchang	CRA004775	0.75661406
ENSGALG00010004837	CENPC	2	0.079518587	0.111977531	0.710129844	0.477623621	0	0.035737882	0	1	CRA004775;Yang2025_Silkie_Wenchang	PRJNA827465	0.855218676
ENSGALG00010004837	CENPC	2	0.086236623	0.083335551	1.034811938	0.300756746	0	0.028406415	0	1	CRA004775;PRJNA827465	Yang2025_Silkie_Wenchang	0.790767266
ENSGALG00010004841	AKAP9	2	0.539938049	0.61476803	0.878279323	0.379792154	0.726871518	25.54891191	96.08593899	0.5	PRJNA827465;Yang2025_Silkie_Wenchang	CRA004775	0.824369385
ENSGALG00010004841	AKAP9	2	0.88495154	0.340301361	2.60049368	0.009308973	0.147432651	2.622459905	61.86786314	1	CRA004775;Yang2025_Silkie_Wenchang	PRJNA827465	0.151478671
ENSGALG00010004841	AKAP9	2	0.098575116	0.244789249	0.402693812	0.687173487	0.076071579	2.093856479	52.24123477	0.5	CRA004775;PRJNA827465	Yang2025_Silkie_Wenchang	0.919451777
ENSGALG00010004846	TTLL2	2	-0.07601047	0.202014765	-0.376261952	0.706722163	0	0.011152951	0	1	PRJNA827465;Yang2025_Silkie_Wenchang	CRA004775	0.924553846
ENSGALG00010004846	TTLL2	2	0.003946254	0.283853769	0.013902419	0.988907832	0	0.827312736	0	0.5	CRA004775;Yang2025_Silkie_Wenchang	PRJNA827465	0.996891127
ENSGALG00010004846	TTLL2	2	0.021005904	0.25341102	0.082892623	0.93393692	0	0.758651202	0	0.5	CRA004775;PRJNA827465	Yang2025_Silkie_Wenchang	0.98432801
ENSGALG00010004852	ENSGALG00010004852	2	0.013686786	0.265093018	0.051630127	0.958823413	0.117765088	6.16636298	83.78298515	0.5	PRJNA827465;Yang2025_Silkie_Wenchang	CRA004775	0.989102387
ENSGALG00010004852	ENSGALG00010004852	2	-0.762242246	0.607717849	-1.254269968	0.209743896	0.603826293	4.808513813	79.20355355	1	CRA004775;Yang2025_Silkie_Wenchang	PRJNA827465	0.721625118
ENSGALG00010004852	ENSGALG00010004852	2	-0.529154456	0.879148405	-0.601894348	0.547244467	1.400335036	9.902325186	89.90136174	0.5	CRA004775;PRJNA827465	Yang2025_Silkie_Wenchang	0.878281266
ENSGALG00010004855	ZEB1	2	0.166137932	0.239986102	0.692281471	0.488760584	0.086301104	3.387913343	70.48330643	0.5	PRJNA827465;Yang2025_Silkie_Wenchang	CRA004775	0.85803811
ENSGALG00010004855	ZEB1	2	0.367400149	0.169188846	2.171538825	0.029890466	0	0.303318066	0	1	CRA004775;Yang2025_Silkie_Wenchang	PRJNA827465	0.295167651
ENSGALG00010004855	ZEB1	2	0.060274562	0.135435818	0.44504152	0.656289749	0.018622671	1.654397262	39.5550257	0.5	CRA004775;PRJNA827465	Yang2025_Silkie_Wenchang	0.910456956
ENSGALG00010004859	NPC1	2	-0.221987158	0.127673746	-1.738706394	0.082086421	0.023832696	3.49896384	71.42011048	1	PRJNA827465;Yang2025_Silkie_Wenchang	CRA004775	0.493815353
ENSGALG00010004859	NPC1	2	-0.122288692	0.329813612	-0.370781217	0.710800493	0.155913779	2.959699217	66.2127829	0.5	CRA004775;Yang2025_Silkie_Wenchang	PRJNA827465	0.925543061
ENSGALG00010004859	NPC1	2	-0.062099182	0.137844162	-0.450502807	0.652347931	0.017060436	1.229012003	18.63382968	0.5	CRA004775;PRJNA827465	Yang2025_Silkie_Wenchang	0.909377929
ENSGALG00010004866	SLC25A21	2	0.151286523	0.171245998	0.883445597	0.376995564	0.030501583	2.049970254	51.21880436	0.5	PRJNA827465;Yang2025_Silkie_Wenchang	CRA004775	0.823064945
ENSGALG00010004866	SLC25A21	2	-0.650480164	0.739387186	-0.879755798	0.378991615	0.879961613	4.439684443	77.47587666	1	CRA004775;Yang2025_Silkie_Wenchang	PRJNA827465	0.823912132
ENSGALG00010004866	SLC25A21	2	-0.501097227	0.918016012	-0.545848024	0.585170409	1.466984562	6.903343407	85.51426546	0.5	CRA004775;PRJNA827465	Yang2025_Silkie_Wenchang	0.891086887
ENSGALG00010004868	NPAT	2	0.026544892	0.158214768	0.167777585	0.866758257	0.035322893	2.715204764	63.17036515	0.5	PRJNA827465;Yang2025_Silkie_Wenchang	CRA004775	0.967598306
ENSGALG00010004868	NPAT	2	0.258469194	0.137701135	1.877030233	0.06051395	0	0.005717431	0	1	CRA004775;Yang2025_Silkie_Wenchang	PRJNA827465	0.426049285
ENSGALG00010004868	NPAT	2	0.042233724	0.170780213	0.247298693	0.80467707	0.043252141	3.179287285	68.54640961	0.5	CRA004775;PRJNA827465	Yang2025_Silkie_Wenchang	0.951665484
ENSGALG00010004871	C11orf87	2	-0.349119455	0.175860222	-1.985209907	0.047121122	0.019214277	1.430228959	30.08112485	1	PRJNA827465;Yang2025_Silkie_Wenchang	CRA004775	0.374478176
ENSGALG00010004871	C11orf87	2	-0.451629946	0.206132555	-2.190968554	0.028454068	0	0.861772606	0	1	CRA004775;Yang2025_Silkie_Wenchang	PRJNA827465	0.287193179
ENSGALG00010004871	C11orf87	2	-0.187930199	0.167326759	-1.123132968	0.261381026	0	0.038416406	0	1	CRA004775;PRJNA827465	Yang2025_Silkie_Wenchang	0.76815058
ENSGALG00010004872	KIAA0408	2	0.097822572	0.355036379	0.275528306	0.782910382	0.218312663	7.306446957	86.31345706	0.5	PRJNA827465;Yang2025_Silkie_Wenchang	CRA004775	0.945555411
ENSGALG00010004872	KIAA0408	2	0.290748287	0.306782909	0.947733	0.343265397	0.078529018	1.477312509	32.30951517	0.5	CRA004775;Yang2025_Silkie_Wenchang	PRJNA827465	0.806218116
ENSGALG00010004872	KIAA0408	2	-0.236503518	0.137078549	-1.725313847	0.084470928	0	0.000655588	0	1	CRA004775;PRJNA827465	Yang2025_Silkie_Wenchang	0.500072565
ENSGALG00010004881	MIPOL1	2	-0.064378678	0.080208782	-0.802638763	0.422183557	0.000799943	1.050676307	4.823208346	1	PRJNA827465;Yang2025_Silkie_Wenchang	CRA004775	0.839649223
ENSGALG00010004881	MIPOL1	2	-0.156808088	0.144231339	-1.087198453	0.276949114	0	0.614764518	0	0.5	CRA004775;Yang2025_Silkie_Wenchang	PRJNA827465	0.776084088
ENSGALG00010004881	MIPOL1	2	-0.009175376	0.087093662	-0.105350671	0.916097557	0	0.151811236	0	0.5	CRA004775;PRJNA827465	Yang2025_Silkie_Wenchang	0.980406784
ENSGALG00010004883	KRIT1	2	-0.070264787	0.09002906	-0.78046786	0.435115538	0.012317261	4.080994996	75.49617187	0.5	PRJNA827465;Yang2025_Silkie_Wenchang	CRA004775	0.842762754
ENSGALG00010004883	KRIT1	2	0.119799791	0.331949851	0.360897259	0.718176253	0.187609477	6.025412354	83.40362549	0.5	CRA004775;Yang2025_Silkie_Wenchang	PRJNA827465	0.927975712
ENSGALG00010004883	KRIT1	2	0.189853377	0.235419067	0.8064486	0.419984234	0.084070946	3.333638476	70.00274603	1	CRA004775;PRJNA827465	Yang2025_Silkie_Wenchang	0.838603917
ENSGALG00010004887	MYH15	2	0.437691808	0.398915911	1.097203187	0.27255258	0.254374506	4.979345844	79.91704068	1	PRJNA827465;Yang2025_Silkie_Wenchang	CRA004775	0.773495131
ENSGALG00010004887	MYH15	2	0.545558055	0.531893369	1.02569065	0.305037423	0.302882011	1.62644669	38.51627561	0.5	CRA004775;Yang2025_Silkie_Wenchang	PRJNA827465	0.79225794
ENSGALG00010004887	MYH15	2	0.013777972	0.238671115	0.057727857	0.953965404	0	0.216639362	0	0.5	CRA004775;PRJNA827465	Yang2025_Silkie_Wenchang	0.987846193
ENSGALG00010004890	ACAT1	2	0.091713179	0.120994601	0.757993979	0.448454587	0.013402832	1.816726199	44.95593224	0.5	PRJNA827465;Yang2025_Silkie_Wenchang	CRA004775	0.846878812
ENSGALG00010004890	ACAT1	2	0.186746596	0.129116809	1.44633838	0.148082316	0	0.505074616	0	0.5	CRA004775;Yang2025_Silkie_Wenchang	PRJNA827465	0.63772401
ENSGALG00010004890	ACAT1	2	-0.013230509	0.104556529	-0.126539295	0.899305046	0	0.000441495	0	1	CRA004775;PRJNA827465	Yang2025_Silkie_Wenchang	0.976081354
ENSGALG00010004891	ANKIB1	2	0.020952536	0.047866878	0.437725141	0.661585542	0	0.068457967	0	1	PRJNA827465;Yang2025_Silkie_Wenchang	CRA004775	0.912393297
ENSGALG00010004891	ANKIB1	2	0.174813874	0.20267637	0.862527161	0.388397493	0.048567774	1.969729216	49.23160037	1	CRA004775;Yang2025_Silkie_Wenchang	PRJNA827465	0.828154121
ENSGALG00010004891	ANKIB1	2	0.158588258	0.218144477	0.726987269	0.467233755	0.065494766	2.402152079	58.37066234	1	CRA004775;PRJNA827465	Yang2025_Silkie_Wenchang	0.852885838
ENSGALG00010004892	HEY2	2	0.281315827	0.369967425	0.76037999	0.447027481	0.218306848	4.79588907	79.1488088	0.5	PRJNA827465;Yang2025_Silkie_Wenchang	CRA004775	0.84650962
ENSGALG00010004892	HEY2	2	0.600343174	0.236140861	2.542309584	0.011012259	0	0.278520294	0	1	CRA004775;Yang2025_Silkie_Wenchang	PRJNA827465	0.166852275
ENSGALG00010004892	HEY2	2	0.02807339	0.182741882	0.153623184	0.877906856	0.007148797	1.067839817	6.352995667	0.5	CRA004775;PRJNA827465	Yang2025_Silkie_Wenchang	0.970942205
ENSGALG00010004896	UBA6	2	-0.12602563	0.098581701	-1.278387658	0.201112774	0.012247108	2.662495225	62.44124719	1	PRJNA827465;Yang2025_Silkie_Wenchang	CRA004775	0.713804636
ENSGALG00010004896	UBA6	2	-0.214189468	0.090094119	-2.377396781	0.017435322	0	0.369203326	0	1	CRA004775;Yang2025_Silkie_Wenchang	PRJNA827465	0.21990233
ENSGALG00010004896	UBA6	2	-0.038673077	0.07132224	-0.542230265	0.587659889	0	0.012352505	0	1	CRA004775;PRJNA827465	Yang2025_Silkie_Wenchang	0.891742161
ENSGALG00010004899	GATAD1	2	-0.242662627	0.074973724	-3.236635617	0.001209477	0	0.004576918	0	1	PRJNA827465;Yang2025_Silkie_Wenchang	CRA004775	0.042380792
ENSGALG00010004899	GATAD1	2	-0.263833424	0.168853558	-1.562498459	0.118170609	0	0.10825731	0	1	CRA004775;Yang2025_Silkie_Wenchang	PRJNA827465	0.578003524
ENSGALG00010004899	GATAD1	2	-0.251187869	0.078679033	-3.192564267	0.001410156	0	0.108982988	0	1	CRA004775;PRJNA827465	Yang2025_Silkie_Wenchang	0.046301876
ENSGALG00010004901	CUL5	2	-0.112169947	0.055859001	-2.008090827	0.044633641	0.002138066	1.352000445	26.0355273	1	PRJNA827465;Yang2025_Silkie_Wenchang	CRA004775	0.364257243
ENSGALG00010004901	CUL5	2	0.013157941	0.096673027	0.13610768	0.891736157	0.00013888	1.002367569	0.236197731	0.5	CRA004775;Yang2025_Silkie_Wenchang	PRJNA827465	0.973985832
ENSGALG00010004901	CUL5	2	-0.020937373	0.207409381	-0.100947086	0.919592465	0.056322946	2.03088308	50.76033623	0.5	CRA004775;PRJNA827465	Yang2025_Silkie_Wenchang	0.981192162
ENSGALG00010004902	ANKRD29	2	0.216684037	0.207433902	1.044593172	0.296211037	0	0.122560787	0	1	PRJNA827465;Yang2025_Silkie_Wenchang	CRA004775	0.788267543
ENSGALG00010004902	ANKRD29	2	0.188094949	0.253938098	0.740711811	0.458868196	0	0.36717888	0	0.5	CRA004775;Yang2025_Silkie_Wenchang	PRJNA827465	0.850089963
ENSGALG00010004902	ANKRD29	2	0.100760807	0.23389412	0.430796664	0.666616225	0	0.119594516	0	0.5	CRA004775;PRJNA827465	Yang2025_Silkie_Wenchang	0.913579507
ENSGALG00010004907	ENSGALG00010004907	2	-0.259901508	0.239839096	-1.083649465	0.27852025	0.101080724	8.236308996	87.8586391	1	PRJNA827465;Yang2025_Silkie_Wenchang	CRA004775	0.776763074
ENSGALG00010004907	ENSGALG00010004907	2	-0.23578268	0.350451001	-0.672797849	0.501075906	0.178590519	3.063202821	67.35443069	0.5	CRA004775;Yang2025_Silkie_Wenchang	PRJNA827465	0.86247641
ENSGALG00010004907	ENSGALG00010004907	2	-0.002153328	0.110837648	-0.019427767	0.98449986	0	0.359498708	0	0.5	CRA004775;PRJNA827465	Yang2025_Silkie_Wenchang	0.995703389
ENSGALG00010004917	ENSGALG00010004917	2	-0.177232767	0.066005339	-2.685127748	0.007250209	0	0.010935268	0	1	PRJNA827465;Yang2025_Silkie_Wenchang	CRA004775	0.130953566
ENSGALG00010004917	ENSGALG00010004917	2	-0.312954155	0.185427598	-1.687743127	0.091460549	0.037653692	1.900591064	47.38478892	1	CRA004775;Yang2025_Silkie_Wenchang	PRJNA827465	0.51833799
ENSGALG00010004917	ENSGALG00010004917	2	-0.3009879	0.191368463	-1.572818716	0.115760801	0.046556065	2.199759463	54.54048422	1	CRA004775;PRJNA827465	Yang2025_Silkie_Wenchang	0.572799541
ENSGALG00010004921	RRAGD	2	-0.258320569	0.142529026	-1.812406759	0.069923369	0.030095129	3.829154067	73.8845713	1	PRJNA827465;Yang2025_Silkie_Wenchang	CRA004775	0.455981115
ENSGALG00010004921	RRAGD	2	-0.159521507	0.29292117	-0.544588521	0.586036551	0.134966064	4.269886241	76.58017231	0.5	CRA004775;Yang2025_Silkie_Wenchang	PRJNA827465	0.891402106
ENSGALG00010004921	RRAGD	2	-0.068048828	0.117823623	-0.57754826	0.563569147	0.008297398	1.211552911	17.46130188	0.5	CRA004775;PRJNA827465	Yang2025_Silkie_Wenchang	0.882532874
ENSGALG00010004924	ENSGALG00010004924	2	0.737201122	0.349495844	2.109327292	0.034916341	0.184975506	4.118206498	75.71758481	1	PRJNA827465;Yang2025_Silkie_Wenchang	CRA004775	0.321837931
ENSGALG00010004924	ENSGALG00010004924	2	0.349286919	0.230807961	1.513322664	0.130197719	0	0.207995795	0	1	CRA004775;Yang2025_Silkie_Wenchang	PRJNA827465	0.605437876
ENSGALG00010004924	ENSGALG00010004924	2	0.770914308	0.475301604	1.621947624	0.104814563	0.266377385	1.999224227	49.98059816	1	CRA004775;PRJNA827465	Yang2025_Silkie_Wenchang	0.550003575
ENSGALG00010004928	HDDC2	2	0.129239894	0.079458057	1.626517169	0.103839684	0	0.476783395	0	1	PRJNA827465;Yang2025_Silkie_Wenchang	CRA004775	0.548175896
ENSGALG00010004928	HDDC2	2	0.185267483	0.150851719	1.228143	0.219393292	0	0.307843897	0	1	CRA004775;Yang2025_Silkie_Wenchang	PRJNA827465	0.732888275
ENSGALG00010004928	HDDC2	2	0.099356035	0.083445457	1.19067039	0.233783007	0	0.017509285	0	1	CRA004775;PRJNA827465	Yang2025_Silkie_Wenchang	0.747082975
ENSGALG00010004929	ENSGALG00010004929	2	0.536590766	0.112236786	4.780881414	1.74528E-06	0	0.131467844	0	1	PRJNA827465;Yang2025_Silkie_Wenchang	CRA004775	0.000349974
ENSGALG00010004929	ENSGALG00010004929	2	0.44954929	0.173827379	2.586182289	0.009704559	0	0.486457628	0	1	CRA004775;Yang2025_Silkie_Wenchang	PRJNA827465	0.155237398
ENSGALG00010004929	ENSGALG00010004929	2	0.541671389	0.139327218	3.887764344	0.000101172	0	0.705359237	0	1	CRA004775;PRJNA827465	Yang2025_Silkie_Wenchang	0.007873969
ENSGALG00010004933	SPTLC2	2	-0.141264774	0.186882455	-0.755901744	0.449708108	0.061257131	7.960242386	87.43756846	0.5	PRJNA827465;Yang2025_Silkie_Wenchang	CRA004775	0.847309577
ENSGALG00010004933	SPTLC2	2	-0.320675045	0.104838752	-3.058745362	0.00222266	0	0.197956435	0	1	CRA004775;Yang2025_Silkie_Wenchang	PRJNA827465	0.062308987
ENSGALG00010004933	SPTLC2	2	0.022705654	0.068615261	0.330912599	0.740710505	0	0.613027113	0	0.5	CRA004775;PRJNA827465	Yang2025_Silkie_Wenchang	0.935170497
ENSGALG00010004934	CIP2A	2	-0.210262588	0.253094711	-0.830766423	0.406105595	0.114043218	9.084613469	88.99237702	0.5	PRJNA827465;Yang2025_Silkie_Wenchang	CRA004775	0.833477131
ENSGALG00010004934	CIP2A	2	-0.469912829	0.125185052	-3.753745537	0.000174212	0	0.009319326	0	1	CRA004775;Yang2025_Silkie_Wenchang	PRJNA827465	0.011507636
ENSGALG00010004934	CIP2A	2	0.023727724	0.107977778	0.219746367	0.826068692	0	0.712963379	0	0.5	CRA004775;PRJNA827465	Yang2025_Silkie_Wenchang	0.956393839
ENSGALG00010004935	SVIL	2	0.125246882	0.224844517	0.557037739	0.577501645	0.09271625	11.59636853	91.37661072	0.5	PRJNA827465;Yang2025_Silkie_Wenchang	CRA004775	0.88731056
ENSGALG00010004935	SVIL	2	0.300363956	0.110065558	2.728955	0.006353537	0.001382516	1.044647964	4.273972196	1	CRA004775;Yang2025_Silkie_Wenchang	PRJNA827465	0.1211211
ENSGALG00010004935	SVIL	2	-0.075884811	0.050888675	-1.49119251	0.13591096	0	0.770712027	0	0.5	CRA004775;PRJNA827465	Yang2025_Silkie_Wenchang	0.617467165
ENSGALG00010004939	DDX10	2	0.183758699	0.252034193	0.729102259	0.465939113	0.113681851	9.023364821	88.91765966	0.5	PRJNA827465;Yang2025_Silkie_Wenchang	CRA004775	0.852511753
ENSGALG00010004939	DDX10	2	0.445835887	0.134061256	3.325613241	0.000882243	0	0.018539239	0	1	CRA004775;Yang2025_Silkie_Wenchang	PRJNA827465	0.034801769
ENSGALG00010004939	DDX10	2	0.113344566	0.221335158	0.51209472	0.60858472	0.071332776	2.961046999	66.22816186	0.5	CRA004775;PRJNA827465	Yang2025_Silkie_Wenchang	0.897208691
ENSGALG00010004944	ALKBH1	2	0.016310653	0.058140447	0.280538834	0.779064136	0	0.56605592	0	0.5	PRJNA827465;Yang2025_Silkie_Wenchang	CRA004775	0.944714533
ENSGALG00010004944	ALKBH1	2	0.058085783	0.099111652	0.586064124	0.557832424	0	0.318656268	0	0.5	CRA004775;Yang2025_Silkie_Wenchang	PRJNA827465	0.880678789
ENSGALG00010004944	ALKBH1	2	-0.012362084	0.063775091	-0.193838751	0.846302143	0	0.018521891	0	1	CRA004775;PRJNA827465	Yang2025_Silkie_Wenchang	0.96122283
ENSGALG00010004946	SH2D1B	2	1.12277358	0.81273737	1.381471581	0.16713401	0.556848965	1.693864618	40.96340468	1	PRJNA827465;Yang2025_Silkie_Wenchang	CRA004775	0.666416556
ENSGALG00010004946	SH2D1B	2	0.553058865	1.370317054	0.403599198	0.686507475	3.195707617	6.26448189	84.03698793	0.5	CRA004775;Yang2025_Silkie_Wenchang	PRJNA827465	0.919235539
ENSGALG00010004946	SH2D1B	2	-0.294044932	0.525694344	-0.559345816	0.575925733	0.236006373	1.622932987	38.38316135	0.5	CRA004775;PRJNA827465	Yang2025_Silkie_Wenchang	0.886924804
ENSGALG00010004949	RAB39A	2	-0.428587497	0.124925608	-3.430741722	0.000601933	0	0.238652635	0	1	PRJNA827465;Yang2025_Silkie_Wenchang	CRA004775	0.026551952
ENSGALG00010004949	RAB39A	2	-0.199350169	0.392875411	-0.507413197	0.611864928	0.20772325	2.712362142	63.13176679	0.5	CRA004775;Yang2025_Silkie_Wenchang	PRJNA827465	0.897777844
ENSGALG00010004949	RAB39A	2	-0.165268328	0.317947522	-0.519797503	0.60320472	0.121104593	2.058217276	51.41426458	0.5	CRA004775;PRJNA827465	Yang2025_Silkie_Wenchang	0.895538966
ENSGALG00010004950	SLIRP	2	-0.038894475	0.200474074	-0.194012495	0.846166098	0.048479965	2.072810338	51.75631935	0.5	PRJNA827465;Yang2025_Silkie_Wenchang	CRA004775	0.96122283
ENSGALG00010004950	SLIRP	2	-0.145785619	0.205633544	-0.708958353	0.478350322	0	0.955712109	0	0.5	CRA004775;Yang2025_Silkie_Wenchang	PRJNA827465	0.855464436
ENSGALG00010004950	SLIRP	2	0.0929759	0.094423474	0.984669348	0.324786514	0	0.009240355	0	1	CRA004775;PRJNA827465	Yang2025_Silkie_Wenchang	0.799914637
ENSGALG00010004951	AFDN	2	0.008411285	0.101015368	0.08326738	0.933638937	0.00864135	1.411503026	29.1535348	0.5	PRJNA827465;Yang2025_Silkie_Wenchang	CRA004775	0.984262625
ENSGALG00010004951	AFDN	2	0.136662999	0.169866139	0.804533498	0.421088931	0	0.434377145	0	0.5	CRA004775;Yang2025_Silkie_Wenchang	PRJNA827465	0.839269089
ENSGALG00010004951	AFDN	2	-0.046143007	0.065596732	-0.70343454	0.481784988	0	0.003741776	0	1	CRA004775;PRJNA827465	Yang2025_Silkie_Wenchang	0.85691916
ENSGALG00010004952	PEX1	2	0.088245699	0.056101878	1.572954451	0.115729366	0	0.38648688	0	1	PRJNA827465;Yang2025_Silkie_Wenchang	CRA004775	0.572716099
ENSGALG00010004952	PEX1	2	0.191642801	0.198122378	0.967295075	0.333396545	0.052595512	2.54325763	60.68034996	1	CRA004775;Yang2025_Silkie_Wenchang	PRJNA827465	0.802565346
ENSGALG00010004952	PEX1	2	0.210968117	0.153283709	1.376324454	0.168721205	0.026695651	1.807312892	44.66923771	1	CRA004775;PRJNA827465	Yang2025_Silkie_Wenchang	0.669861707
ENSGALG00010004957	ENSGALG00010004957	2	0.521877493	0.566622585	0.921031931	0.357033757	0.596780038	14.15996159	92.93783395	0.5	PRJNA827465;Yang2025_Silkie_Wenchang	CRA004775	0.813148156
ENSGALG00010004957	ENSGALG00010004957	2	1.035473083	0.195438119	5.298214547	1.16941E-07	0	0.329467075	0	1	CRA004775;Yang2025_Silkie_Wenchang	PRJNA827465	3.99662E-05
ENSGALG00010004957	ENSGALG00010004957	2	0.289860997	0.414351204	0.699553893	0.484205946	0.239100955	2.976205666	66.40017148	0.5	CRA004775;PRJNA827465	Yang2025_Silkie_Wenchang	0.857004733
ENSGALG00010004958	TPD52L1	2	-0.323420386	0.232819233	-1.38914806	0.164787732	0	0.846696587	0	1	PRJNA827465;Yang2025_Silkie_Wenchang	CRA004775	0.663999614
ENSGALG00010004958	TPD52L1	2	0.470050016	1.192560804	0.394151824	0.693468952	2.484403314	7.642588374	86.91542772	0.5	CRA004775;Yang2025_Silkie_Wenchang	PRJNA827465	0.920721644
ENSGALG00010004958	TPD52L1	2	0.656563159	0.955652176	0.687031512	0.492062871	1.533086265	5.69624977	82.44459003	0.5	CRA004775;PRJNA827465	Yang2025_Silkie_Wenchang	0.859158569
ENSGALG00010004962	ENSGALG00010004962	2	-0.011461882	0.056851588	-0.20161059	0.840221166	0	0.604375809	0	0.5	PRJNA827465;Yang2025_Silkie_Wenchang	CRA004775	0.959662809
ENSGALG00010004962	ENSGALG00010004962	2	-0.291845809	0.189064206	-1.543633321	0.122677188	0.043051312	2.4826043	59.71971853	1	CRA004775;Yang2025_Silkie_Wenchang	PRJNA827465	0.58762464
ENSGALG00010004962	ENSGALG00010004962	2	-0.214696696	0.250858711	-0.855847082	0.392082369	0.108151164	6.492601946	84.59785448	0.5	CRA004775;PRJNA827465	Yang2025_Silkie_Wenchang	0.829251057

ENSGALG00010004963	ENSGALG00010004963	2	0.654390011	0.182794806	3.579915779	0.000343705	0.001570961	1.023935562	2.337604314	1	PRJNA827465;Yang2025_Silkie_Wenchang	CRA004775	0.018390471
ENSGALG00010004963	ENSGALG00010004963	2	0.454361066	0.470589048	0.965515599	0.334286627	0.297471245	2.731769644	63.39369236	0.5	CRA004775;Yang2025_Silkie_Wenchang	PRJNA827465	0.803094614
ENSGALG00010004963	ENSGALG00010004963	2	0.332572104	0.244072387	1.362596189	0.173009811	0.00448617	1.025374292	2.474637004	0.5	CRA004775;PRJNA827465	Yang2025_Silkie_Wenchang	0.676043418
ENSGALG00010004965	JCAD	2	0.029152785	0.058168005	0.501182483	0.616242701	0	0.849898434	0	0.5	PRJNA827465;Yang2025_Silkie_Wenchang	CRA004775	0.899111936
ENSGALG00010004965	JCAD	2	-0.044076181	0.125410546	-0.351455143	0.725246919	0	0.642257913	0	0.5	CRA004775;Yang2025_Silkie_Wenchang	PRJNA827465	0.930868485
ENSGALG00010004965	JCAD	2	0.060301028	0.063492866	0.949729189	0.342249874	0	0.212062535	0	1	CRA004775;PRJNA827465	Yang2025_Silkie_Wenchang	0.806005903
ENSGALG00010004966	LAMA3	2	0.271697721	0.360145437	0.754411115	0.4506024	0.219377903	6.478739385	84.5648985	0.5	PRJNA827465;Yang2025_Silkie_Wenchang	CRA004775	0.84760941
ENSGALG00010004966	LAMA3	2	0.144655897	0.601923801	0.240322607	0.810080171	0.559062728	3.79630801	73.65861786	0.5	CRA004775;Yang2025_Silkie_Wenchang	PRJNA827465	0.952908565
ENSGALG00010004966	LAMA3	2	-0.143594234	0.193274822	-0.742953647	0.457509744	0	0.649715844	0	1	CRA004775;PRJNA827465	Yang2025_Silkie_Wenchang	0.849573219
ENSGALG00010004967	SLC35F2	2	-0.050558212	0.15497976	-0.326224612	0.744254422	0	0.330585816	0	1	PRJNA827465;Yang2025_Silkie_Wenchang	CRA004775	0.935821071
ENSGALG00010004967	SLC35F2	2	-0.086257916	0.28062411	-0.307378848	0.758555026	0	0.569971768	0	0.5	CRA004775;Yang2025_Silkie_Wenchang	PRJNA827465	0.938954369
ENSGALG00010004967	SLC35F2	2	0.021993025	0.166504839	0.132086399	0.894915953	0	0.210718249	0	0.5	CRA004775;PRJNA827465	Yang2025_Silkie_Wenchang	0.974641371
ENSGALG00010004969	YTHDC1	2	0.140436145	0.24271452	0.578606277	0.562854869	0.107462938	10.87065364	90.80092115	0.5	PRJNA827465;Yang2025_Silkie_Wenchang	CRA004775	0.882070607
ENSGALG00010004969	YTHDC1	2	0.261020744	0.15612886	1.671828926	0.094558061	0.024879207	2.013855701	50.34400929	1	CRA004775;Yang2025_Silkie_Wenchang	PRJNA827465	0.526888901
ENSGALG00010004969	YTHDC1	2	-0.071167627	0.052021122	-1.3680525	0.171295643	0	0.891200981	0	0.5	CRA004775;PRJNA827465	Yang2025_Silkie_Wenchang	0.673243267
ENSGALG00010004970	RBM48	2	0.033396548	0.082969523	0.402515845	0.68730443	0	0.152177708	0	1	PRJNA827465;Yang2025_Silkie_Wenchang	CRA004775	0.919465461
ENSGALG00010004970	RBM48	2	0.046222712	0.136888834	0.337666052	0.735614861	0	0.257269181	0	0.5	CRA004775;Yang2025_Silkie_Wenchang	PRJNA827465	0.933592889
ENSGALG00010004970	RBM48	2	0.003437452	0.092133615	0.037309423	0.970238292	0	0.092807577	0	0.5	CRA004775;PRJNA827465	Yang2025_Silkie_Wenchang	0.991681174
ENSGALG00010004973	SLN	2	-0.668660858	0.161275899	-4.146068098	3.38233E-05	0	0.129954507	0	1	PRJNA827465;Yang2025_Silkie_Wenchang	CRA004775	0.003661746
ENSGALG00010004973	SLN	2	-0.557833709	0.225375002	-2.475135677	0.013318557	0	0.418456366	0	1	CRA004775;Yang2025_Silkie_Wenchang	PRJNA827465	0.188498106
ENSGALG00010004973	SLN	2	-0.670731451	0.210256723	-3.190059481	0.001422435	0	0.650259231	0	1	CRA004775;PRJNA827465	Yang2025_Silkie_Wenchang	0.046588307
ENSGALG00010004974	MTPAP	2	-0.065373638	0.041810636	-1.563564791	0.117919809	0	0.749862591	0	1	PRJNA827465;Yang2025_Silkie_Wenchang	CRA004775	0.577693705
ENSGALG00010004974	MTPAP	2	-0.104218286	0.085699282	-1.21609287	0.223949546	0	0.764395247	0	0.5	CRA004775;Yang2025_Silkie_Wenchang	PRJNA827465	0.737452676
ENSGALG00010004974	MTPAP	2	-0.042232586	0.045498131	-0.928226833	0.353289918	0	0.256401448	0	0.5	CRA004775;PRJNA827465	Yang2025_Silkie_Wenchang	0.811157428
ENSGALG00010004975	SNW1	2	0.228233748	0.255614653	0.89288821	0.37192034	0.119033195	10.49717164	90.47362438	0.5	PRJNA827465;Yang2025_Silkie_Wenchang	CRA004775	0.821597118
ENSGALG00010004975	SNW1	2	0.248305443	0.253134563	0.980922718	0.326630854	0.107031873	6.058284175	83.49367624	0.5	CRA004775;Yang2025_Silkie_Wenchang	PRJNA827465	0.800028239
ENSGALG00010004975	SNW1	2	-0.006615312	0.039694685	-0.166654866	0.867641621	0	0.002119781	0	1	CRA004775;PRJNA827465	Yang2025_Silkie_Wenchang	0.967896191
ENSGALG00010004976	ENSGALG00010004976	2	0.116796328	0.187524999	0.622830706	0.533395778	0.045783557	2.801344391	64.3028539	0.5	PRJNA827465;Yang2025_Silkie_Wenchang	CRA004775	0.873379507
ENSGALG00010004976	ENSGALG00010004976	2	-0.01040383	0.432754725	-0.024040939	0.980805953	0.26737635	3.037302613	67.07305523	0.5	CRA004775;Yang2025_Silkie_Wenchang	PRJNA827465	0.993928779
ENSGALG00010004976	ENSGALG00010004976	2	-0.103510256	0.155872457	-0.664070218	0.506645371	0.009496982	1.076964742	7.146449512	1	CRA004775;PRJNA827465	Yang2025_Silkie_Wenchang	0.863929781
ENSGALG00010004977	RNF217	2	-0.360247649	0.112690473	-3.196788853	0.001389666	0	0.014312843	0	1	PRJNA827465;Yang2025_Silkie_Wenchang	CRA004775	0.045782101
ENSGALG00010004977	RNF217	2	-0.087324906	0.27494852	-0.317604567	0.750784909	0.100469483	2.942056881	66.0101745	0.5	CRA004775;Yang2025_Silkie_Wenchang	PRJNA827465	0.937538899
ENSGALG00010004977	RNF217	2	-0.118353789	0.287639728	-0.411465378	0.680731323	0.127086098	4.066631376	75.40962267	0.5	CRA004775;PRJNA827465	Yang2025_Silkie_Wenchang	0.917851751
ENSGALG00010004980	FNDC1	2	0.317792587	0.491429827	0.646669309	0.51784599	0.412909033	6.696864787	85.06763938	0.5	PRJNA827465;Yang2025_Silkie_Wenchang	CRA004775	0.868075618
ENSGALG00010004980	FNDC1	2	0.272512689	0.681711509	0.39974782	0.689342267	0.685925666	3.519979366	71.59074256	0.5	CRA004775;Yang2025_Silkie_Wenchang	PRJNA827465	0.91986176
ENSGALG00010004980	FNDC1	2	-0.172557551	0.188058925	-0.91757172	0.358843129	0	0.334196383	0	1	CRA004775;PRJNA827465	Yang2025_Silkie_Wenchang	0.814241185
ENSGALG00010004981	ENSGALG00010004981	2	0.135477307	0.149936348	0.903565471	0.366225857	0.001889473	1.043867258	4.202378932	0.5	PRJNA827465;Yang2025_Silkie_Wenchang	CRA004775	0.819159863
ENSGALG00010004981	ENSGALG00010004981	2	0.417954534	0.531897385	0.785780388	0.431996158	0.426657197	3.602404012	72.24075932	0.5	CRA004775;Yang2025_Silkie_Wenchang	PRJNA827465	0.842503929
ENSGALG00010004981	ENSGALG00010004981	2	0.525593103	0.362263639	1.450858015	0.146819401	0.145617971	1.887719862	47.02603813	1	CRA004775;PRJNA827465	Yang2025_Silkie_Wenchang	0.635558525
ENSGALG00010004983	CLXN	2	0.008388864	0.259197818	0.032364717	0.974181199	0.079896212	2.41455766	58.5845467	0.5	PRJNA827465;Yang2025_Silkie_Wenchang	CRA004775	0.992492038
ENSGALG00010004983	CLXN	2	-0.520125461	0.377321875	-1.378466223	0.16805939	0.124875455	1.548340091	35.414706	1	CRA004775;Yang2025_Silkie_Wenchang	PRJNA827465	0.668614049
ENSGALG00010004983	CLXN	2	-0.317471132	0.669308832	-0.474326824	0.635266866	0.716391552	4.392414131	77.23347639	0.5	CRA004775;PRJNA827465	Yang2025_Silkie_Wenchang	0.904141515
ENSGALG00010004987	MAP3K8	2	0.08961702	0.27023606	0.331624951	0.740172477	0.116391689	3.492181491	79.1326768	0.5	PRJNA827465;Yang2025_Silkie_Wenchang	CRA004775	0.93508234
ENSGALG00010004987	MAP3K8	2	0.125767211	0.368157004	0.341612979	0.732642168	0.158963591	2.073498198	51.77232366	0.5	CRA004775;Yang2025_Silkie_Wenchang	PRJNA827465	0.932985777
ENSGALG00010004987	MAP3K8	2	-0.175222931	0.130934338	-1.338250404	0.180814823	0	0.214353646	0	1	CRA004775;PRJNA827465	Yang2025_Silkie_Wenchang	0.688484814
ENSGALG00010004988	HJURP	2	-0.104573389	0.123172598	-0.84899881	0.395881955	0.0214867	3.424404558	70.79784286	0.5	PRJNA827465;Yang2025_Silkie_Wenchang	CRA004775	0.831296327
ENSGALG00010004988	HJURP	2	0.006125877	0.402770974	0.01520933	0.98786578	0.207671266	1.919990237	47.91396027	0.5	CRA004775;Yang2025_Silkie_Wenchang	PRJNA827465	0.996800355
ENSGALG00010004988	HJURP	2	0.045959948	0.129794506	0.354093632	0.723268715	0.008196739	1.036237556	3.497031678	1	CRA004775;PRJNA827465	Yang2025_Silkie_Wenchang	0.930032096
ENSGALG00010004991	FAM133B	2	-0.005276877	0.049862497	-0.105828576	0.915718364	0.000503078	1.076389897	7.096861199	0.5	PRJNA827465;Yang2025_Silkie_Wenchang	CRA004775	0.980265984
ENSGALG00010004991	FAM133B	2	0.222045747	0.155079092	1.431817971	0.152195923	0.031886008	2.862678036	65.06767483	1	CRA004775;Yang2025_Silkie_Wenchang	PRJNA827465	0.643706371
ENSGALG00010004991	FAM133B	2	0.159409592	0.214687204	0.742520228	0.457772201	0.080124127	7.049283937	85.81416199	0.5	CRA004775;PRJNA827465	Yang2025_Silkie_Wenchang	0.849712894
ENSGALG00010004993	SULT1B	2	0.024817781	0.204279615	0.121489268	0.903303515	0.023821035	1.284730298	22.16265147	0.5	PRJNA827465;Yang2025_Silkie_Wenchang	CRA004775	0.977253688
ENSGALG00010004993	SULT1B	2	-0.117066477	0.211659995	-0.553087404	0.580203561	0	0.443975408	0	1	CRA004775;Yang2025_Silkie_Wenchang	PRJNA827465	0.888376663
ENSGALG00010004993	SULT1B	2	0.093048121	0.14853077	0.626458667	0.53101532	0	0.270264183	0	0.5	CRA004775;PRJNA827465	Yang2025_Silkie_Wenchang	0.93728407
ENSGALG00010004995	PIT54	2	-1.519940391	1.170892069	-1.298104609	0.194251391	0.945735492	1.519276902	34.17921389	1	PRJNA827465;Yang2025_Silkie_Wenchang	CRA004775	0.704982807
ENSGALG00010004995	PIT54	2	-0.385070602	2.249480508	-0.171182013	0.86408065	8.846388038	7.652055431	86.93161584	0.5	CRA004775;Yang2025_Silkie_Wenchang	PRJNA827465	0.96655438
ENSGALG00010004995	PIT54	2	0.851517776	1.055200092	0.806972804	0.419682152	1.263711555	2.184592791	54.22487869	0.5	CRA004775;PRJNA827465	Yang2025_Silkie_Wenchang	0.838527387
ENSGALG00010004996	GPR158	2	0.367062745	0.20351889	1.803569103	0.071379062	0	0.068894151	0	1	PRJNA827465;Yang2025_Silkie_Wenchang	CRA004775	0.461027938
ENSGALG00010004996	GPR158	2	0.245950422	0.272561048	0.902368197	0.36686131	0	0.136017319	0	1	CRA004775;Yang2025_Silkie_Wenchang	PRJNA827465	0.819577857
ENSGALG00010004996	GPR158	2	0.346509373	0.239593458	1.446238874	0.148110214	0	0.314806678	0	1	CRA004775;PRJNA827465	Yang2025_Silkie_Wenchang	0.63772401
ENSGALG00010004999	ADCK1	2	0.175318521	0.178497424	0.994515872	0.319977182	0.039047619	2.556193889	60.87933689	1	PRJNA827465;Yang2025_Silkie_Wenchang	CRA004775	0.796348216
ENSGALG00010004999	ADCK1	2	-0.07963461	0.470601366	-0.169218825	0.865624523	0.394305374	8.972825205	88.85523815	0.5	CRA004775;Yang2025_Silkie_Wenchang	PRJNA827465	0.967088846
ENSGALG00010004999	ADCK1	2	-0.237364004	0.288965183	-0.821427696	0.411402693	0.12619741	3.84957818	74.0231279	0.5	CRA004775;PRJNA827465	Yang2025_Silkie_Wenchang	0.835128673
ENSGALG00010005003	EXPH5	2	0.528872179	0.492696117	1.073424696	0.283080609	0.056061432	1.12922677	11.44382803	0.5	PRJNA827465;Yang2025_Silkie_Wenchang	CRA004775	0.779568117
ENSGALG00010005003	EXPH5	2	0.63514919	0.524111556	1.21185878	0.225566443	0.214448314	1.595604438	37.32782536	0.5	CRA004775;Yang2025_Silkie_Wenchang	PRJNA827465	0.73884362
ENSGALG00010005003	EXPH5	2	1.027658186	0.384940048	2.669657758	0.00759286	0	0.010738556	0	1	CRA004775;PRJNA827465	Yang2025_Silkie_Wenchang	0.134484974
ENSGALG00010005004	CDK6	2	-0.010356775	0.080707799	-0.12832434	0.897892304	0	0.647765575	0	0.5	PRJNA827465;Yang2025_Silkie_Wenchang	CRA004775	0.975696709
ENSGALG00010005004	CDK6	2	0.050770903	0.162510434	0.31241627	0.754724187	0	0.790043244	0	0.5	CRA004775;Yang2025_Silkie_Wenchang	PRJNA827465	0.93838723
ENSGALG00010005004	CDK6	2	-0.054333698	0.08790667	-0.618083908	0.536520029	0	0.29501723	0	1	CRA004775;PRJNA827465	Yang2025_Silkie_Wenchang	0.874599474
ENSGALG00010005010	NKAIN2	2	-0.686686675	0.194295467	-3.534239305	0.000408951	0	0.26060618	0	1	PRJNA827465;Yang2025_Silkie_Wenchang	CRA004775	0.020366982
ENSGALG00010005010	NKAIN2	2	-0.278927926	0.713084526	-0.391156891	0.695681271	0.695389506	2.820821933	64.54933974	0.5	CRA004775;Yang2025_Silkie_Wenchang	PRJNA827465	0.921038437
ENSGALG00010005010	NKAIN2	2	-0.229840629	0.580363909	-0.396028485	0.692084015	0.43702914	2.265748058	55.86446621	0.5	CRA004775;PRJNA827465	Yang2025_Silkie_Wenchang	0.920348122
ENSGALG00010005014	SULT1B1	2	0.201270842	0.106367888	1.892214339	0.058462429	0	0.479268769	0	1	PRJNA827465;Yang2025_Silkie_Wenchang	CRA004775	0.418915011
ENSGALG00010005014	SULT1B1	2	0.102879205	0.263838988	0.389931777	0.696586995	0.075372505	2.122353812	52.8825027	0.5	CRA004775;Yang2025_Silkie_Wenchang	PRJNA827465	0.921041503
ENSGALG00010005014	SULT1B1	2	0.078553199	0.150848438	0.520754966	0.602537478	0.014141475	1.272139989	21.39229889	0.5	CRA004775;PRJNA827465	Yang2025_Silkie_Wenchang	0.895518685
ENSGALG00010005016	STX11	2	0.120965991	0.103478999	1.168990737	0.24240736	0	0.340292652	0	1	PRJNA827465;Yang2025_Silkie_Wenchang	CRA004775	0.754698112
ENSGALG00010005016	STX11	2	-0.202734946	0.449004948	-0.451520516	0.651614442	0.323373776	4.461244474	77.58472987	0.5	CRA004775;Yang2025_Silkie_Wenchang	PRJNA827465	0.908943868
ENSGALG00010005016	STX11	2	-0.256299072	0.384717274	-0.666201103	0.505282568	0.214909079	3.206786423	68.81613341	0.5	CRA004775;PRJNA827465	Yang2025_Silkie_Wenchang	0.863862536
ENSGALG00010005017	ENSGALG00010005017	2	-0.129152427	0.141733665	-0.911233237	0.362172495	0.007649523	1.203032371	16.87671718	1	PRJNA827465;Yang2025_Silkie_Wenchang	CRA004775	0.815579273
ENSGALG00010005017	ENSGALG00010005017	2	-0.18205622	0.155547997	-1.170383822	0.241832682	0	0.765559547	0	1	CRA004775;Yang2025_Silkie_Wenchang	PRJNA827465	0.75406671
ENSGALG00010005017	ENSGALG00010005017	2	-0.03867453	0.12208753	-0.316777074	0.751412761	0	0.01160225	0	1	CRA004775;PRJNA827465	Yang2025_Silkie_Wenchang	0.937685817
ENSGALG00010005023	POGLUT3	2	-0.40922652	0.267424662	-1.530249744	0.125954923	0.128502531	9.834771528	89.83199562	1	PRJNA827465;Yang2025_Silkie_Wenchang	CRA004775	0.596003652
ENSGALG00010005023	POGLUT3	2	-0.266558948	0.273124406	-1.74941916	0.080218559	0.099187501	2.53448016	60.54417655	1	CRA004775;Yang2025_Silkie_Wenchang	PRJNA827465	0.863175395
ENSGALG00010005023	POGLUT3	2	-0.140713392	0.108156645	-1.301014768	0.1932534	0	0.011154678	0	1	CRA004775;PRJNA827465	Yang2025_Silkie_Wenchang	0.703216219
ENSGALG00010005025	THOC1	2	0.225669984	0.213758773	1.055722678	0.291094933	0.08381509	11.54975041	91.34180425	1	PRJNA827465;Yang2025_Silkie_Wenchang	CRA004775	0.78467882
ENSGALG00010005025	THOC1	2	0.178310488	0.302069913	0.590295424	0.554992606	0.153195486	5.960808044	83.223751	0.5	CRA004775;Yang2025_Silkie_Wenchang	PRJNA827465	0.880223402
ENSGALG00010005025	THOC1	2	0.01772145	0.044699695	0.396455741	0.691768859	0	0.635734618	0	0.5	CRA004775;PRJNA827465	Yang2025_Silkie_Wenchang	0.920348122
ENSGALG00010005029	FAT4	2	-0.180519633	0.104704774	-1.724082155	0.084693013	0	0.004568949	0	1	PRJNA827465;Yang2025_Silkie_Wenchang	CRA004775	0.500353159
ENSGALG00010005029	FAT4	2	-0.079660015	0.186829592	-0.426377934	0.669832478	0	0.42781301	0	0.5	CRA004775;Yang2025_Silkie_Wenchang	PRJNA827465	0.914377769
ENSGALG00010005029	FAT4	2	-0.151626074	0.110527186	-1.371844157	0.170111958	0	0.648245598	0	0.5	CRA004775;PRJNA827465	Yang2025_Silkie_Wenchang	0.671748244
ENSGALG00010005033	USP14	2	-0.072469875	0.062099913	-1.183091437	0.236772991	0	0.501400887	0	1	PRJNA827465;Yang2025_Silkie_Wenchang	CRA004775	0.749020867
ENSGALG00010005033	USP14	2	-0.154381817	0.113982224	-1.354437654	0.175596792	0	0.007329556	0	1	CRA004775;Yang2025_Silkie_Wenchang	PRJNA827465	0.680297576
ENSGALG00010005033	USP14	2	-0.056039417	0.069390312	-0.807597137	0.419322535	0	0.195391887	0	1	CRA004775;PRJNA827465	Yang2025_Silkie_Wenchang	0.838527387
ENSGALG00010005035	NECTIN3	2	-0.079801802	0.060312355	-1.323149376	0.185780201	0	0.534984858	0	1	PRJNA827465;Yang2025_Silkie_Wenchang	CRA004775	0.69450577
ENSGALG00010005035	NECTIN3	2	-0.114035876	0.091465642	-1.246761888	0.212484819	0	0.377937704	0	0.5	CRA004775;Yang2025_Silkie_Wenchang	PRJNA827465	0.725190243
ENSGALG00010005035	NECTIN3	2	-0.039583616	0.072589611	-0.545306907	0.585542454	0	0.074348982	0	0.5	CRA004775;PRJNA827465	Yang2025_Silkie_Wenchang	0.891148209
ENSGALG00010005037	THNSL1	2	0.113953566	0.176465652	0.645754937	0.518438076	0.036399355	2.391050878	58.17738513	0.5	PRJNA827465;Yang2025_Silkie_Wenchang	CRA004775	0.868525042
ENSGALG00010005037	THNSL1	2	-0.097020516	0.4268576	-0.227290121	0.820198154	0.311651706	6.736479553	85.15545112	0.5	CRA004775;Yang2025_Silkie_Wenchang	PRJNA827465	0.955308792
ENSGALG00010005037	THNSL1	2	-0.245743513	0.244546206	-1.004896035	0.314946914	0.075805936	2.513134216	60.20904917	1	CRA004775;PRJNA827465	Yang2025_Silkie_Wenchang	0.796572019
ENSGALG00010005042	SLC46A3	2	-0.174943637	0.326337302	-0.536082256	0.591901703	0.156379988	3.582001744	72.08264899	0.5	PRJNA827465;Yang2025_Silkie_Wenchang	CRA004775	0.892776493
ENSGALG00010005042	SLC46A3	2	0.00148486	0.623320774	0.002382176	0.9980993	0.601441798	4.203194339	76.20857093	0.5	CRA004775;Yang2025_Silkie_Wenchang	PRJNA827465	0.9980993
ENSGALG00010005042	SLC46A3	2	0.195938654	0.209754948	0.934131262	0.350236188	0.02011178	1.12690632	11.2614791	1	CRA004775;PRJNA827465	Yang2025_Silkie_Wenchang	0.810111037
ENSGALG00010005045	UTRN	2	-0.100189874	0.067259039	-1.489612036	0.136326275	0	0.350763504	0	1	PRJNA827465;Yang2025_Silkie_Wenchang	CRA004775	0.618139317
ENSGALG00010005045	UTRN	2	0.137313728	0.351587962	0.390552985	0.696127683	0.203681063	5.578883616	82.07526687	0.5	CRA004775;Yang2025_Silkie_Wenchang	PRJNA827465	0.921038437
ENSGALG00010005045	UTRN	2	0.168284272	0.29363133	0.573114156	0.566567403	0.144086915	5.428573036	81.57895282	0.5	CRA004775;PRJNA827465	Yang2025_Silkie_Wenchang	0.883487517
ENSGALG00010005048	ROCK1	2	0.221054216	0.346095187	0.638709303	0.523012059	0.213914456	8.646220211	88.43425248	0.5	PRJNA827465;Yang2025_Silkie_Wenchang	CRA004775	0.869568211
ENSGALG00010005048	ROCK1	2	0.546089494	0.185972185	2.936404143	0.003320414	0	0.188803875	0	1	CRA004775;Yang2025_Silkie_Wenchang	PRJNA827465	0.080507243
ENSGALG00010005048	ROCK1	2	0.073650848	0.243822024	0.30206807	0.762600172	0.082840251	2.523945613	60.37949491	0.5	CRA004775;PRJNA827465	Yang2025_Silkie_Wenchang	0.940082621
ENSGALG00010005057	FLVCR2	2	0.047640259	0.108518613	0.439005416	0.660657612	0	0.576893352	0	0.5	PRJNA827465;Yang2025_Silkie_Wenchang	CRA004775	0.91192683
ENSGALG00010005057	FLVCR2	2	0.012468571	0.134313947	0.092831546	0.926037389	0	0.56593638	0	0.5	CRA004775;Yang2025_Silkie_Wenchang	PRJNA827465	0.982800408
ENSGALG00010005057	FLVCR2	2	0.168774833	0.156510736	1.078359465	0.280873369	0	0.134720666	0	1	CRA004775;PRJNA827465	Yang2025_Silkie_Wenchang	0.778581075
ENSGALG00010005060	POMP	2	-0.211007769	0.246187353	-0.857102391	0.391383	0.106256545	7.367072286	86.42608677	0.5	PRJNA827465;Yang2025_Silkie_Wenchang	CRA004775	0.829251057
ENSGALG00010005060	POMP	2	-0.390294932	0.146507965	-2.663984387	0.007722115	0	0.980772312	0	0.5	CRA004775;Yang2025_Silkie_Wenchang	PRJNA827465	0.136027818
ENSGALG00010005060	POMP	2	0.002054573	0.045247379	0.045407567	0.96378245	0	0.475086056	0	0.5	CRA004775;PRJNA827465	Yang2025_Silkie_Wenchang	0.990167386
ENSGALG00010005068	PLCXD2	2	-0.465918323	0.12507379	-3.725147564	0.000195201	0.001091438	1.036107426	3.484911391	1	PRJNA827465;Yang2025_Silkie_Wenchang	CRA004775	0.01255644
ENSGALG00010005068	PLCXD2	2	-0.567832769	0.159649404	-3.556748442	0.000375473	0	0.129110107	0	1	CRA004775;Yang2025_Silkie_Wenchang	PRJNA827465	0.019391893
ENSGALG00010005068	PLCXD2	2	-0.362574243	0.155361832	-2.333740775	0.0196093	0	0.06220261	0	1	CRA004775;PRJNA827465	Yang2025_Silkie_Wenchang	0.234756108
ENSGALG00010005070	ALB	2	-1.368465003	2.426703372	-0.563919356	0.572809016	10.59116038	9.922377322	89.92177008	0.5	PRJNA827465;Yang2025_Silkie_Wenchang	CRA004775	0.88573298
ENSGALG00010005070	ALB	2	-0.891722611	2.838970325	-0.314100716	0.753444546	15.28943226	19.31053057	94.82147838	0.5	CRA004775;Yang2025_Silkie_Wenchang	PRJNA827465	0.938087082
ENSGALG00010005070	ALB	2	1.64587493	0.619681659	2.656000718	0.007907345	0	0.376346496	0	1	CRA004775;PRJNA827465	Yang2025_Silkie_Wenchang	0.138064133
ENSGALG00010005075	TRDN	2	0.158702238	0.554129313	0.286399284	0.774572323	0.569902117	13.64303214	92.67025109	0.5	PRJNA827465;Yang2025_Silkie_Wenchang	CRA004775	0.943713409
ENSGALG00010005075	TRDN	2	0.702444368	0.242858015	2.892493351	0.003821973	0	0.109157569	0	1	CRA004775;Yang2025_Silkie_Wenchang	PRJNA827465	0.088674731
ENSGALG00010005075	TRDN	2	-0.178551826	0.364675846	-0.489617913	0.624404299	0.14953141	1.645300598	39.22083287	0.5	CRA004775;PRJNA827465	Yang2025_Silkie_Wenchang	0.900886179
ENSGALG00010005077	ERG28	2	0.06808994	0.136119461	0.500221929	0.616918819	0.018840166	1.855693808	46.11179951	0.5	PRJNA827465;Yang2025_Silkie_Wenchang	CRA004775	0.899111936
ENSGALG00010005077	ERG28	2	-0.045690488	0.306282616	-0.149177543	0.88141354	0.149511691	4.916034663	79.65840217	0.5	CRA004775;Yang2025_Silkie_Wenchang	PRJNA827465	0.971790895
ENSGALG00010005077	ERG28	2	-0.14520873	0.155750864	-0.932314128	0.351174209	0.027689377	2.076647722	51.84546761	1	CRA004775;PRJNA827465	Yang2025_Silkie_Wenchang	0.810178441
ENSGALG00010005081	GREB1L	2	0.245078383	0.115455854	2.122702101	0.033778823	0	0.516199482	0	1	PRJNA827465;Yang2025_Silkie_Wenchang	CRA004775	0.315721539
ENSGALG00010005081	GREB1L	2	0.047280637	0.356484249	0.132630367	0.894485715	0.186390467	3.601295336	72.2322135	0.5	CRA004775;Yang2025_Silkie_Wenchang	PRJNA827465	0.974394857
ENSGALG00010005081	GREB1L	2	-0.007968807	0.26010394	-0.03063701	0.975559026	0.085457428	2.386264471	58.09349666	0.5	CRA004775;PRJNA827465	Yang2025_Silkie_Wenchang	0.992754467
ENSGALG00010005082	ENSGALG00010005082	2	0.033207665	0.049024693	0.677366096	0.498173704	0	0.860245821	0	0.5	PRJNA827465;Yang2025_Silkie_Wenchang	CRA004775	0.86140436
ENSGALG00010005082	ENSGALG00010005082	2	0.173472702	0.119382776	1.453079812	0.146201596	0	0.012459003	0	1	CRA004775;Yang2025_Silkie_Wenchang	PRJNA827465	0.634916196
ENSGALG00010005082	ENSGALG00010005082	2	0.028343506	0.050254643	0.56399777	0.57275565	0	0.70993816	0	0.5	CRA004775;PRJNA827465	Yang2025_Silkie_Wenchang	0.88573298
ENSGALG00010005087	ENPP1	2	-0.134926005	0.221386461	-0.609459155	0.542220138	0.068110874	3.064994624	67.37351537	0.5	PRJNA827465;Yang2025_Silkie_Wenchang	CRA004775	0.876721948
ENSGALG00010005087	ENPP1	2	0.231768189	0.159488479	1.453197066	0.146169046	0	0.41899379	0	1	CRA004775;Yang2025_Silkie_Wenchang	PRJNA827465	0.634916196
ENSGALG00010005087	ENPP1	2	-0.019773311	0.326929873	-0.060481811	0.951771902	0.18199719	6.472590615	84.55023561	0.5	CRA004775;PRJNA827465	Yang2025_Silkie_Wenchang	0.987713746
ENSGALG00010005092	ESCO1	2	-0.013065067	0.046746905	-0.279485181	0.779872509	0	0.086933811	0	0.5	PRJNA827465;Yang2025_Silkie_Wenchang	CRA004775	0.944978771
ENSGALG00010005092	ESCO1	2	0.379762463	0.416383711	0.912049277	0.361742778	0.298887859	6.608144063	84.867158	1	CRA004775;Yang2025_Silkie_Wenchang	PRJNA827465	0.815545459
ENSGALG00010005092	ESCO1	2	0.361461055	0.432683456	0.835393751	0.403496047	0.33032975	7.615282747	86.86851121	0.5	CRA004775;PRJNA827465	Yang2025_Silkie_Wenchang	0.83306585
ENSGALG00010005104	ENPP3	2	-0.430401909	0.161255303	-2.669071349	0.007606129	0	0.094170263	0	1	PRJNA827465;Yang2025_Silkie_Wenchang	CRA004775	0.134538115
ENSGALG00010005104	ENPP3	2	-0.055814846	0.462849388	-0.12058965	0.904016067	0.341239335	4.794810236	79.14411727	0.5	CRA004775;Yang2025_Silkie_Wenchang	PRJNA827465	0.977427069
ENSGALG00010005104	ENPP3	2	-0.014879602	0.412213057	-0.036096873	0.971205116	0.257582333	3.981837276	74.88596518	0.5	CRA004775;PRJNA827465	Yang2025_Silkie_Wenchang	0.991925437
ENSGALG00010005107	TTLL5	2	0.118145771	0.139165054	0.848961485	0.395902724	0.03093179	4.621774158	78.36328722	0.5	PRJNA827465;Yang2025_Silkie_Wenchang	CRA004775	0.831296327
ENSGALG00010005107	TTLL5	2	0.043957046	0.274387687	0.160200506	0.872723131	0.116053463	4.022503158	75.13985793	0.5	CRA004775;Yang2025_Silkie_Wenchang	PRJNA827465	0.969629321
ENSGALG00010005107	TTLL5	2	-0.031892993	0.084133966	-0.379073932	0.704632964	0.004764667	1.144394896	12.61757602	1	CRA004775;PRJNA827465	Yang2025_Silkie_Wenchang	0.924152671
ENSGALG00010005109	ABHD3	2	0.342775256	0.18997238	1.804342592	0.071177617	0	0.960293543	0	1	PRJNA827465;Yang2025_Silkie_Wenchang	CRA004775	0.460681013
ENSGALG00010005109	ABHD3	2	0.725093014	0.321517377	2.255221848	0.024119407	0	0.124394685	0	1	CRA004775;Yang2025_Silkie_Wenchang	PRJNA827465	0.265075657
ENSGALG00010005109	ABHD3	2	0.318927301	0.226868608	1.405779776	0.159789529	0.010204951	1.045626127	4.36352206	1	CRA004775;PRJNA827465	Yang2025_Silkie_Wenchang	0.655404348
ENSGALG00010005111	RASSF6	2	-0.098558039	0.271951377	-0.362410517	0.717045281	0.036653933	1.329374383	24.77664587	0.5	PRJNA827465;Yang2025_Silkie_Wenchang	CRA004775	0.927742393
ENSGALG00010005111	RASSF6	2	-0.26167205	0.270652791	-0.966818222	0.333634913	0	0.30745117	0	1	CRA004775;Yang2025_Silkie_Wenchang	PRJNA827465	0.802691373
ENSGALG00010005111	RASSF6	2	0.100690521	0.273610965	0.368006162	0.712868639	0	0.151310746	0	0.5	CRA004775;PRJNA827465	Yang2025_Silkie_Wenchang	0.926097326
ENSGALG00010005114	PHLDB2	2	-0.111856241	0.059771789	-1.871388528	0.061291248	0	0.104721194	0	1	PRJNA827465;Yang2025_Silkie_Wenchang	CRA004775	0.427873874
ENSGALG00010005114	PHLDB2	2	0.266554347	0.398399952	0.669062197	0.503455803	0.26154094	5.156294911	80.60622953	0.5	CRA004775;Yang2025_Silkie_Wenchang	PRJNA827465	0.863108263
ENSGALG00010005114	PHLDB2	2	0.240744576	0.421007964	0.571829032	0.567437807	0.30458244	6.281860235	84.08114854	0.5	CRA004775;PRJNA827465	Yang2025_Silkie_Wenchang	0.883717772
ENSGALG00010005118	PRTFDC1	2	-0.421579019	0.289595128	-1.455753149	0.145460872	0.154701897	12.72065932	92.13877225	1	PRJNA827465;Yang2025_Silkie_Wenchang	CRA004775	0.633959706
ENSGALG00010005118	PRTFDC1	2	-0.710100781	0.126284141	-5.623040054	1.87626E-08	0	0.042006305	0	1	CRA004775;Yang2025_Silkie_Wenchang	PRJNA827465	8.6756E-06
ENSGALG00010005118	PRTFDC1	2	-0.288591246	0.22961882	-1.256827493	0.208816108	0.066637123	2.095390415	52.27619671	1	CRA004775;PRJNA827465	Yang2025_Silkie_Wenchang	0.721011841
ENSGALG00010005119	MIB1	2	-0.024635109	0.08238079	-0.299039489	0.764909916	0.008836878	2.607798071	61.65347267	0.5	PRJNA827465;Yang2025_Silkie_Wenchang	CRA004775	0.940711384
ENSGALG00010005119	MIB1	2	-0.131536092	0.088909294	-1.479441415	0.139022378	0	0.008495288	0	1	CRA004775;Yang2025_Silkie_Wenchang	PRJNA827465	0.622849816
ENSGALG00010005119	MIB1	2	0.03514644	0.046214073	0.760513791	0.446947529	0	0.481435977	0	0.5	CRA004775;PRJNA827465	Yang2025_Silkie_Wenchang	0.84650962
ENSGALG00010005123	MTIF3	2	0.034118029	0.100390351	0.339853664	0.733966732	0.010202724	1.855664395	46.11094534	0.5	PRJNA827465;Yang2025_Silkie_Wenchang	CRA004775	0.933391141
ENSGALG00010005123	MTIF3	2	0.176063558	0.128231349	1.37301494	0.169747702	0	0.01389079	0	1	CRA004775;Yang2025_Silkie_Wenchang	PRJNA827465	0.671232541
ENSGALG00010005123	MTIF3	2	-0.030614489	0.071656708	-0.427238285	0.669205779	0	0.439824601	0	0.5	CRA004775;PRJNA827465	Yang2025_Silkie_Wenchang	0.914377769
ENSGALG00010005126	MED23	2	-0.122213862	0.12847322	-0.951278883	0.341462824	0.030245225	11.87439102	91.57851549	0.5	PRJNA827465;Yang2025_Silkie_Wenchang	CRA004775	0.805758959
ENSGALG00010005126	MED23	2	-0.24028768	0.057079258	-4.209719765	2.55688E-05	0	0.712897657	0	1	CRA004775;Yang2025_Silkie_Wenchang	PRJNA827465	0.002938389
ENSGALG00010005126	MED23	2	0.000574563	0.044398248	0.012941128	0.989674762	0	0.096429148	0	0.5	CRA004775;PRJNA827465	Yang2025_Silkie_Wenchang	0.997287059
ENSGALG00010005127	GATA6	2	-0.21242486	0.128589191	-1.651965131	0.098541659	0	0.402419085	0	1	PRJNA827465;Yang2025_Silkie_Wenchang	CRA004775	0.53598695
ENSGALG00010005127	GATA6	2	0.365544166	0.714502152	0.511606809	0.608926219	0.911257743	8.811945828	88.65176864	0.5	CRA004775;Yang2025_Silkie_Wenchang	PRJNA827465	0.897208691
ENSGALG00010005127	GATA6	2	0.440009915	0.63190915	0.696318316	0.486229505	0.690093644	6.889370783	85.48488633	0.5	CRA004775;PRJNA827465	Yang2025_Silkie_Wenchang	0.857603818
ENSGALG00010005132	LNX2	2	-0.031723201	0.044130437	-0.718850817	0.472232843	0	0.00081152	0	1	PRJNA827465;Yang2025_Silkie_Wenchang	CRA004775	0.853998373
ENSGALG00010005132	LNX2	2	0.14263961	0.178018129	0.801264516	0.422978529	0.035635424	2.278226136	56.10620104	0.5	CRA004775;Yang2025_Silkie_Wenchang	PRJNA827465	0.840012882
ENSGALG00010005132	LNX2	2	0.108752983	0.176128787	0.617462851	0.536929477	0.048745526	3.963158449	74.76759981	0.5	CRA004775;PRJNA827465	Yang2025_Silkie_Wenchang	0.874713452
ENSGALG00010005140	DCT	2	7.730883437	0.461120153	16.76544256	4.3675E-63	0	0.053956938	0	1	PRJNA827465;Yang2025_Silkie_Wenchang	CRA004775	1.56051E-59
ENSGALG00010005140	DCT	2	7.881556372	0.519860329	15.16091136	6.41727E-52	0	0.004119905	0	1	CRA004775;Yang2025_Silkie_Wenchang	PRJNA827465	1.68145E-48
ENSGALG00010005140	DCT	2	7.744346843	0.48389202	16.00428717	1.19271E-57	0	0.080803594	0	1	CRA004775;PRJNA827465	Yang2025_Silkie_Wenchang	3.90644E-54
ENSGALG00010005143	GTF3A	2	0.090715729	0.067489992	1.344136007	0.178904411	0	0.624698457	0	1	PRJNA827465;Yang2025_Silkie_Wenchang	CRA004775	0.685601971
ENSGALG00010005143	GTF3A	2	0.215975539	0.125844026	1.716216061	0.086122505	0	0.05451718	0	1	CRA004775;Yang2025_Silkie_Wenchang	PRJNA827465	0.504895124
ENSGALG00010005143	GTF3A	2	0.083036199	0.071779345	1.156825809	0.247343535	0	0.661212606	0	1	CRA004775;PRJNA827465	Yang2025_Silkie_Wenchang	0.757947282
ENSGALG00010005145	AKAP7	2	-0.427657169	0.085150737	-5.022354298	5.10419E-07	0	0.781493112	0	1	PRJNA827465;Yang2025_Silkie_Wenchang	CRA004775	0.000131118
ENSGALG00010005145	AKAP7	2	-0.486942426	0.136191344	-3.575428586	0.000349655	0	0.787237555	0	1	CRA004775;Yang2025_Silkie_Wenchang	PRJNA827465	0.018545851
ENSGALG00010005145	AKAP7	2	-0.358918397	0.101544341	-3.534597736	0.000408396	0	0.274457348	0	1	CRA004775;PRJNA827465	Yang2025_Silkie_Wenchang	0.020366982
ENSGALG00010005146	TGFB3	2	0.044086449	0.062865873	0.701277923	0.483129589	0	0.909337663	0	0.5	PRJNA827465;Yang2025_Silkie_Wenchang	CRA004775	0.85691916
ENSGALG00010005146	TGFB3	2	-0.374393026	0.561313599	-0.666994398	0.504775711	0.552065484	7.331852941	86.36088301	0.5	CRA004775;Yang2025_Silkie_Wenchang	PRJNA827465	0.863512504
ENSGALG00010005146	TGFB3	2	-0.426035505	0.496915271	-0.85736046	0.391245704	0.421221437	5.964335183	83.233672	1	CRA004775;PRJNA827465	Yang2025_Silkie_Wenchang	0.829251057
ENSGALG00010005148	TTC39C	2	-0.115155595	0.169358033	-0.679953548	0.496533874	0.049589633	7.21157864	86.13341059	0.5	PRJNA827465;Yang2025_Silkie_Wenchang	CRA004775	0.860939846
ENSGALG00010005148	TTC39C	2	-0.283603035	0.103385922	-2.743149451	0.006085298	0	0.171687402	0	1	CRA004775;Yang2025_Silkie_Wenchang	PRJNA827465	0.118095213
ENSGALG00010005148	TTC39C	2	0.039716507	0.066280196	0.599221321	0.549025307	0	0.169661503	0	0.5	CRA004775;PRJNA827465	Yang2025_Silkie_Wenchang	0.878431377
ENSGALG00010005150	ALCAM	2	0.113171973	0.1985768	0.569915384	0.56873509	0.053092147	2.801172784	64.300667	0.5	PRJNA827465;Yang2025_Silkie_Wenchang	CRA004775	0.884293565

ENSGALG00010005150	ALCAM	2	0.154662287	0.27084258	0.571041255	0.567971678	0.068305482	1.757260427	43.09323849	0.5	CRA004775;Yang2025_Silkie_Wenchang	PRJNA827465	0.88390256
ENSGALG00010005150	ALCAM	2	-0.059900868	0.104889827	-0.571083674	0.567942925	0	0.168774687	0	1	CRA004775;PRJNA827465	Yang2025_Silkie_Wenchang	0.883894067
ENSGALG00010005154	CWF19L2	2	0.218985443	0.353440024	0.619583035	0.535532339	0.233510927	14.82309807	93.25377195	0.5	PRJNA827465;Yang2025_Silkie_Wenchang	CRA004775	0.874171355
ENSGALG00010005154	CWF19L2	2	0.086629254	0.517931317	0.16726012	0.867165382	0.492472993	12.06863449	91.71405845	0.5	CRA004775;Yang2025_Silkie_Wenchang	PRJNA827465	0.967699
ENSGALG00010005154	CWF19L2	2	-0.19838967	0.141229646	-1.404731063	0.160101265	0.021390141	1.655970564	39.61245315	1	CRA004775;PRJNA827465	Yang2025_Silkie_Wenchang	0.655509678
ENSGALG00010005157	IMPA1	2	-0.192664503	0.189525615	-1.016561813	0.309361928	0.063280767	8.346315584	88.01866536	1	PRJNA827465;Yang2025_Silkie_Wenchang	CRA004775	0.794043289
ENSGALG00010005157	IMPA1	2	-0.28090817	0.16793849	-1.67268486	0.094389351	0.028965679	1.725505237	42.40595971	1	CRA004775;Yang2025_Silkie_Wenchang	PRJNA827465	0.526210592
ENSGALG00010005157	IMPA1	2	-0.009000435	0.077781644	-0.115714125	0.907879111	0	0.000844931	0	1	CRA004775;PRJNA827465	Yang2025_Silkie_Wenchang	0.978196186
ENSGALG00010005158	IFT43	2	-0.028958613	0.132507248	-0.218543615	0.82700558	0	0.798633489	0	0.5	PRJNA827465;Yang2025_Silkie_Wenchang	CRA004775	0.956540044
ENSGALG00010005158	IFT43	2	-0.336547782	0.234509129	-1.435115907	0.151254071	0	0.07125117	0	1	CRA004775;Yang2025_Silkie_Wenchang	PRJNA827465	0.642835272
ENSGALG00010005158	IFT43	2	-0.06995729	0.180570814	-0.38742302	0.698443064	0.020503543	1.277725852	21.73595	0.5	CRA004775;PRJNA827465	Yang2025_Silkie_Wenchang	0.921666253
ENSGALG00010005159	ARHGAP21	2	0.200442987	0.184645001	1.085558698	0.277674281	0.055277805	4.605650645	78.28754117	1	PRJNA827465;Yang2025_Silkie_Wenchang	CRA004775	0.776763074
ENSGALG00010005159	ARHGAP21	2	0.4080982	0.14093324	2.895684514	0.003783325	0	0.026449712	0	1	CRA004775;Yang2025_Silkie_Wenchang	PRJNA827465	0.088142287
ENSGALG00010005159	ARHGAP21	2	0.112364002	0.130218854	0.862885814	0.388200251	0.018928874	1.554337078	35.66389078	1	CRA004775;PRJNA827465	Yang2025_Silkie_Wenchang	0.828154121
ENSGALG00010005165	METTL21C	2	0.305644317	0.48831448	0.625916965	0.531369406	0	0.160067852	0	1	PRJNA827465;Yang2025_Silkie_Wenchang	CRA004775	0.87298465
ENSGALG00010005165	METTL21C	2	0.0517012	0.504087443	0.10256395	0.918309055	0	0.004137857	0	1	CRA004775;Yang2025_Silkie_Wenchang	PRJNA827465	0.981033557
ENSGALG00010005165	METTL21C	2	0.263680509	0.47549201	0.554542461	0.579207655	0	0.231521584	0	1	CRA004775;PRJNA827465	Yang2025_Silkie_Wenchang	0.887888423
ENSGALG00010005166	CBLB	2	-0.019927349	0.055484329	-0.359152734	0.719480834	0	0.158682194	0	0.5	PRJNA827465;Yang2025_Silkie_Wenchang	CRA004775	0.928449671
ENSGALG00010005166	CBLB	2	-0.004055266	0.189506006	-0.021399142	0.982927258	0	0.255618455	0	0.5	CRA004775;Yang2025_Silkie_Wenchang	PRJNA827465	0.995363468
ENSGALG00010005166	CBLB	2	-0.028381498	0.055916235	-0.507571688	0.61175375	0	0.09713635	0	1	CRA004775;PRJNA827465	Yang2025_Silkie_Wenchang	0.897777844
ENSGALG00010005167	GUCY1A2	2	-0.174015514	0.09954821	-1.748052662	0.080454909	0	0.240209205	0	1	PRJNA827465;Yang2025_Silkie_Wenchang	CRA004775	0.488261768
ENSGALG00010005167	GUCY1A2	2	-0.193014152	0.15194795	-1.270264931	0.203990275	0	0.716348235	0	0.5	CRA004775;Yang2025_Silkie_Wenchang	PRJNA827465	0.717254253
ENSGALG00010005167	GUCY1A2	2	-0.113888623	0.123012442	-0.925830111	0.354534276	0	0.438860066	0	0.5	CRA004775;PRJNA827465	Yang2025_Silkie_Wenchang	0.81193713
ENSGALG00010005170	GPATCH2L	2	-0.046607223	0.07793028	-0.598063078	0.549797845	0.00241923	1.183022472	15.47075195	0.5	PRJNA827465;Yang2025_Silkie_Wenchang	CRA004775	0.878603751
ENSGALG00010005170	GPATCH2L	2	-0.16931309	0.129251875	-1.309946874	0.190213808	0	0.014411967	0	1	CRA004775;Yang2025_Silkie_Wenchang	PRJNA827465	0.699403589
ENSGALG00010005170	GPATCH2L	2	-0.00846757	0.073947831	-0.114507352	0.908835619	0	0.20019183	0	0.5	CRA004775;PRJNA827465	Yang2025_Silkie_Wenchang	0.978387968
ENSGALG00010005172	OSBPL1A	2	0.144360306	0.301112304	0.479423471	0.631637399	0.165302151	11.16011202	91.03951646	0.5	PRJNA827465;Yang2025_Silkie_Wenchang	CRA004775	0.903058174
ENSGALG00010005172	OSBPL1A	2	0.103174104	0.372432573	0.277027605	0.781758909	0.240747434	7.431100016	86.54304211	0.5	CRA004775;Yang2025_Silkie_Wenchang	PRJNA827465	0.945265125
ENSGALG00010005172	OSBPL1A	2	-0.1686984	0.09059334	-1.86213013	0.062581932	0	0.332123345	0	1	CRA004775;PRJNA827465	Yang2025_Silkie_Wenchang	0.431065134
ENSGALG00010005174	TPP2	2	-0.087352448	0.07302248	-1.196240506	0.231602718	0.006125917	2.116205489	52.74560975	1	PRJNA827465;Yang2025_Silkie_Wenchang	CRA004775	0.744047869
ENSGALG00010005174	TPP2	2	-0.175755399	0.091625573	-1.918191534	0.055086729	0	0.222290962	0	0.5	CRA004775;Yang2025_Silkie_Wenchang	PRJNA827465	0.406379567
ENSGALG00010005174	TPP2	2	-0.032317118	0.046275202	-0.698367959	0.484947109	0	0.014820276	0	0.5	CRA004775;PRJNA827465	Yang2025_Silkie_Wenchang	0.857004733
ENSGALG00010005176	BAMBI	2	0.268849076	0.304351031	0.883351946	0.377046145	0	0.272605026	0	1	PRJNA827465;Yang2025_Silkie_Wenchang	CRA004775	0.823064945
ENSGALG00010005176	BAMBI	2	0.508361445	0.334717217	1.518778894	0.12881816	0.091805158	1.676682521	40.35841685	1	CRA004775;Yang2025_Silkie_Wenchang	PRJNA827465	0.602157487
ENSGALG00010005176	BAMBI	2	0.685688864	0.267722595	2.561191613	0.010431381	0	0.389348943	0	1	CRA004775;PRJNA827465	Yang2025_Silkie_Wenchang	0.162498831
ENSGALG00010005177	AASDHPPT	2	-0.010211516	0.114684953	-0.089039719	0.929050345	0	0.085183982	0	0.5	PRJNA827465;Yang2025_Silkie_Wenchang	CRA004775	0.983542314
ENSGALG00010005177	AASDHPPT	2	-0.040245581	0.160254625	-0.251135223	0.801709564	0	0.018672852	0	1	CRA004775;Yang2025_Silkie_Wenchang	PRJNA827465	0.950372222
ENSGALG00010005177	AASDHPPT	2	0.011933397	0.130950719	0.091128915	0.927390158	0	0.003489388	0	0.5	CRA004775;PRJNA827465	Yang2025_Silkie_Wenchang	0.982993195
ENSGALG00010005178	EPHA5	2	0.04618881	0.088376436	0.52263717	0.601226769	0	0.005017015	0	1	PRJNA827465;Yang2025_Silkie_Wenchang	CRA004775	0.895230519
ENSGALG00010005178	EPHA5	2	-0.015170558	0.15768076	-0.096210583	0.923353325	0	0.99212986	0	0.5	CRA004775;Yang2025_Silkie_Wenchang	PRJNA827465	0.981993607
ENSGALG00010005178	EPHA5	2	0.003220075	0.152868621	0.021064332	0.983194338	0.014664961	1.109525914	9.871415576	0.5	CRA004775;PRJNA827465	Yang2025_Silkie_Wenchang	0.995363468
ENSGALG00010005180	WAC	2	0.079796431	0.220004933	0.362702915	0.716826822	0.07939255	4.743453728	78.91831443	0.5	PRJNA827465;Yang2025_Silkie_Wenchang	CRA004775	0.927632716
ENSGALG00010005180	WAC	2	0.39116031	0.152683036	2.561958301	0.010408617	0	0.108617984	0	1	CRA004775;Yang2025_Silkie_Wenchang	PRJNA827465	0.162401701
ENSGALG00010005180	WAC	2	0.126230719	0.270911529	0.465948125	0.641252632	0.1233217	5.393407053	81.45884429	0.5	CRA004775;PRJNA827465	Yang2025_Silkie_Wenchang	0.906490386
ENSGALG00010005181	IMPACT	2	-0.027947897	0.0899995	-0.310533917	0.756154971	0	0.007052275	0	1	PRJNA827465;Yang2025_Silkie_Wenchang	CRA004775	0.93854559
ENSGALG00010005181	IMPACT	2	0.013411117	0.170130901	0.078828225	0.937169254	0	0.12275493	0	0.5	CRA004775;Yang2025_Silkie_Wenchang	PRJNA827465	0.985114195
ENSGALG00010005181	IMPACT	2	-0.021587537	0.099467455	-0.217031153	0.828184067	0	0.173964153	0	0.5	CRA004775;PRJNA827465	Yang2025_Silkie_Wenchang	0.956681118
ENSGALG00010005182	NEPRO	2	0.104240132	0.119150023	0.87486456	0.381647604	0.011140629	1.49496078	33.10861305	0.5	PRJNA827465;Yang2025_Silkie_Wenchang	CRA004775	0.824699008
ENSGALG00010005182	NEPRO	2	-0.118774826	0.142511064	-0.833442839	0.404595023	0	0.114079348	0	1	CRA004775;Yang2025_Silkie_Wenchang	PRJNA827465	0.833112102
ENSGALG00010005182	NEPRO	2	0.057292657	0.170258383	0.336504175	0.736490703	0.035501781	2.264835367	55.84668031	0.5	CRA004775;PRJNA827465	Yang2025_Silkie_Wenchang	0.933661344
ENSGALG00010005186	ESR1	2	-0.156485509	0.170235271	-0.919230829	0.357974849	0.029409952	1.438021005	30.45998655	0.5	PRJNA827465;Yang2025_Silkie_Wenchang	CRA004775	0.813641308
ENSGALG00010005186	ESR1	2	0.015134356	0.224222793	0.067496957	0.946186085	0	0.277808092	0	0.5	CRA004775;Yang2025_Silkie_Wenchang	PRJNA827465	0.986370359
ENSGALG00010005186	ESR1	2	-0.27510577	0.150931327	-1.822721472	0.068345586	0	0.016217008	0	1	CRA004775;PRJNA827465	Yang2025_Silkie_Wenchang	0.450487609
ENSGALG00010005187	KBTBD3	2	-0.101577847	0.160848392	-0.631512979	0.527705166	0	0.105342605	0	1	PRJNA827465;Yang2025_Silkie_Wenchang	CRA004775	0.871796643
ENSGALG00010005187	KBTBD3	2	-0.253261435	0.354455566	-0.714508276	0.47491294	0.115993295	1.522911442	34.33630004	1	CRA004775;Yang2025_Silkie_Wenchang	PRJNA827465	0.854462864
ENSGALG00010005187	KBTBD3	2	-0.276481014	0.269339535	-1.026514781	0.304649	0.034691844	1.155750627	13.47614469	1	CRA004775;PRJNA827465	Yang2025_Silkie_Wenchang	0.79225794
ENSGALG00010005190	ENSGALG00010005190	2	-0.570453821	0.224675869	-2.539007965	0.011116729	0.047423242	1.856626584	46.13887313	1	PRJNA827465;Yang2025_Silkie_Wenchang	CRA004775	0.167530985
ENSGALG00010005190	ENSGALG00010005190	2	-0.756808962	0.195843121	-3.864363127	0.000111379	0	0.031029744	0	1	CRA004775;Yang2025_Silkie_Wenchang	PRJNA827465	0.008402194
ENSGALG00010005190	ENSGALG00010005190	2	-0.354310399	0.245799226	-1.441462632	0.149454027	0	0.192323808	0	1	CRA004775;PRJNA827465	Yang2025_Silkie_Wenchang	0.639246608
ENSGALG00010005191	MSANTD4	2	0.288538753	0.227214699	1.269894748	0.204122124	0.096488906	15.17221543	93.40900474	1	PRJNA827465;Yang2025_Silkie_Wenchang	CRA004775	0.717254253
ENSGALG00010005191	MSANTD4	2	0.24904119	0.307164072	0.810775779	0.417490446	0.159096191	5.895484012	83.0376341	0.5	CRA004775;Yang2025_Silkie_Wenchang	PRJNA827465	0.838112595
ENSGALG00010005191	MSANTD4	2	0.053319786	0.065669942	0.81193594	0.416828391	0	0.445600939	0	0.5	CRA004775;PRJNA827465	Yang2025_Silkie_Wenchang	0.837984974
ENSGALG00010005193	EPB41L2	2	-0.037809277	0.065117089	-0.580635248	0.561486311	0	0.690052585	0	0.5	PRJNA827465;Yang2025_Silkie_Wenchang	CRA004775	0.881701086
ENSGALG00010005193	EPB41L2	2	0.142813835	0.142626557	1.001313066	0.316675478	0.010032842	1.235420541	19.0559031	1	CRA004775;Yang2025_Silkie_Wenchang	PRJNA827465	0.796935817
ENSGALG00010005193	EPB41L2	2	0.088112905	0.214603103	0.41058542	0.681356557	0.064949355	2.771916741	63.92388035	0.5	CRA004775;PRJNA827465	Yang2025_Silkie_Wenchang	0.918242009
ENSGALG00010005198	TRPC3	2	0.17278172	0.192375439	0.898148812	0.369106219	0	3.10975E-05	0	1	PRJNA827465;Yang2025_Silkie_Wenchang	CRA004775	0.820577053
ENSGALG00010005198	TRPC3	2	-0.244667904	0.571188321	-0.428348926	0.668397103	0.382177947	2.129763633	53.04643273	0.5	CRA004775;Yang2025_Silkie_Wenchang	PRJNA827465	0.914248324
ENSGALG00010005198	TRPC3	2	-0.217716766	0.563441731	-0.386405114	0.699196663	0.416401696	2.357110712	57.57517902	0.5	CRA004775;PRJNA827465	Yang2025_Silkie_Wenchang	0.92185597
ENSGALG00010005203	ENSGALG00010005203	2	-0.083992489	0.066440423	-1.264177516	0.206166319	0	0.146229708	0	1	PRJNA827465;Yang2025_Silkie_Wenchang	CRA004775	0.718403416
ENSGALG00010005203	ENSGALG00010005203	2	-0.099665254	0.104968712	-0.949475828	0.34237866	0	0.13818142	0	1	CRA004775;Yang2025_Silkie_Wenchang	PRJNA827465	0.806005903
ENSGALG00010005203	ENSGALG00010005203	2	-0.063518907	0.073374821	-0.865677167	0.386662725	0	0.0213061	0	1	CRA004775;PRJNA827465	Yang2025_Silkie_Wenchang	0.827062786
ENSGALG00010005204	ITGBL1	2	0.426013911	0.288982536	1.474185666	0.140431608	0.054737458	1.451995514	31.12926382	1	PRJNA827465;Yang2025_Silkie_Wenchang	CRA004775	0.624550824
ENSGALG00010005204	ITGBL1	2	0.109361865	0.760587105	0.143786115	0.885669368	0.879820543	4.014043536	75.08746502	0.5	CRA004775;Yang2025_Silkie_Wenchang	PRJNA827465	0.972376043
ENSGALG00010005204	ITGBL1	2	-0.090873298	0.432087706	-0.210312159	0.833424046	0.187212443	1.74134348	42.57307582	0.5	CRA004775;PRJNA827465	Yang2025_Silkie_Wenchang	0.957968425
ENSGALG00010005208	ENSGALG00010005208	2	-0.331458472	0.374175589	-0.885836709	0.375705526	0.257790365	12.24091379	91.83067525	0.5	PRJNA827465;Yang2025_Silkie_Wenchang	CRA004775	0.822974013
ENSGALG00010005208	ENSGALG00010005208	2	-0.362906353	0.385371177	-0.94170601	0.346343177	0.242534998	5.358362497	81.33758213	0.5	CRA004775;Yang2025_Silkie_Wenchang	PRJNA827465	0.807757292
ENSGALG00010005208	ENSGALG00010005208	2	0.026802544	0.088820623	0.301760364	0.762834748	0	0.006339327	0	1	CRA004775;PRJNA827465	Yang2025_Silkie_Wenchang	0.940082621
ENSGALG00010005211	ESRRB	2	0.284628137	0.294828747	0.965401576	0.334343712	0.141475209	5.353703643	81.32134188	0.5	PRJNA827465;Yang2025_Silkie_Wenchang	CRA004775	0.803094614
ENSGALG00010005211	ESRRB	2	-0.106010222	0.747208567	-0.141875009	0.887178735	1.002011125	9.289381965	89.23502119	0.5	CRA004775;Yang2025_Silkie_Wenchang	PRJNA827465	0.972982834
ENSGALG00010005211	ESRRB	2	-0.359578087	0.443831806	-0.81016746	0.417843937	0.296632049	3.55144084	71.84241424	1	CRA004775;PRJNA827465	Yang2025_Silkie_Wenchang	0.838112595
ENSGALG00010005212	TAF13	2	-0.133349099	0.139040184	-0.95906878	0.337524097	0.022516431	1.98118717	49.5252132	1	PRJNA827465;Yang2025_Silkie_Wenchang	CRA004775	0.80415385
ENSGALG00010005212	TAF13	2	-0.327139796	0.163623375	-1.999346339	0.045570894	0	0.016996415	0	1	CRA004775;Yang2025_Silkie_Wenchang	PRJNA827465	0.367927862
ENSGALG00010005212	TAF13	2	-0.057223887	0.07087471	-0.80739501	0.419438941	0	0.781048889	0	1	CRA004775;PRJNA827465	Yang2025_Silkie_Wenchang	0.838527387
ENSGALG00010005213	ENSGALG00010005213	2	-0.215905314	0.119421891	-1.80792074	0.070618837	0	0.311780795	0	1	PRJNA827465;Yang2025_Silkie_Wenchang	CRA004775	0.458507662
ENSGALG00010005213	ENSGALG00010005213	2	-0.343538065	0.235527154	-1.458592183	0.144677386	0	0.000421137	0	1	CRA004775;Yang2025_Silkie_Wenchang	PRJNA827465	0.632227632
ENSGALG00010005213	ENSGALG00010005213	2	-0.190495936	0.130273976	-1.462271599	0.143666798	0	0.074192114	0	1	CRA004775;PRJNA827465	Yang2025_Silkie_Wenchang	0.630564924
ENSGALG00010005214	BBS7	2	-0.037718084	0.12508891	-0.301530201	0.763010224	0.024273073	4.29362099	76.70963501	0.5	PRJNA827465;Yang2025_Silkie_Wenchang	CRA004775	0.940139746
ENSGALG00010005214	BBS7	2	0.08200526	0.093815805	0.874109221	0.382058776	0	0.196386049	0	0.5	CRA004775;Yang2025_Silkie_Wenchang	PRJNA827465	0.825001735
ENSGALG00010005214	BBS7	2	-0.139588785	0.061199428	-2.28088381	0.022555323	0	0.37717608	0	1	CRA004775;PRJNA827465	Yang2025_Silkie_Wenchang	0.255317863
ENSGALG00010005215	VASH1	2	-0.242235952	0.277545912	-0.872777949	0.382784121	0.145105325	16.90374374	94.08415073	0.5	PRJNA827465;Yang2025_Silkie_Wenchang	CRA004775	0.825121999
ENSGALG00010005215	VASH1	2	-0.397836604	0.200075178	-1.988435582	0.046763537	0.043062376	1.826224982	45.24223412	1	CRA004775;Yang2025_Silkie_Wenchang	PRJNA827465	0.373490609
ENSGALG00010005215	VASH1	2	0.021208413	0.062377875	0.339998961	0.73385731	0	0.150661673	0	0.5	CRA004775;PRJNA827465	Yang2025_Silkie_Wenchang	0.933391141
ENSGALG00010005216	ENSGALG00010005216	2	0.129279369	0.251007292	0.515042284	0.606523473	0.085092992	2.925913474	65.82263936	0.5	PRJNA827465;Yang2025_Silkie_Wenchang	CRA004775	0.896799223
ENSGALG00010005216	ENSGALG00010005216	2	0.352361451	0.229206822	1.537307869	0.124217936	0	0.419971321	0	1	CRA004775;Yang2025_Silkie_Wenchang	PRJNA827465	0.590771723
ENSGALG00010005216	ENSGALG00010005216	2	-0.074492772	0.147600554	-0.504691683	0.613775404	0	0.054720264	0	0.5	CRA004775;PRJNA827465	Yang2025_Silkie_Wenchang	0.898256044
ENSGALG00010005217	CCNA2	2	-0.065496732	0.076750715	-0.853369657	0.393454343	0	0.226087978	0	1	PRJNA827465;Yang2025_Silkie_Wenchang	CRA004775	0.830367612
ENSGALG00010005217	CCNA2	2	0.066091945	0.206065917	0.320732054	0.748413454	0.058899129	3.065062953	67.3742427	0.5	CRA004775;Yang2025_Silkie_Wenchang	PRJNA827465	0.937249951
ENSGALG00010005217	CCNA2	2	0.090393857	0.165401545	0.54651156	0.584714343	0.032506083	2.213087253	54.81425332	0.5	CRA004775;PRJNA827465	Yang2025_Silkie_Wenchang	0.891016154
ENSGALG00010005218	GTPBP8	2	-0.092961152	0.083032747	-1.119572159	0.262896125	0	0.249776395	0	1	PRJNA827465;Yang2025_Silkie_Wenchang	CRA004775	0.768643821
ENSGALG00010005218	GTPBP8	2	-0.226191839	0.156576076	-1.444613028	0.148566611	0	0.211190079	0	1	CRA004775;Yang2025_Silkie_Wenchang	PRJNA827465	0.638659263
ENSGALG00010005218	GTPBP8	2	-0.098201033	0.0877974	-1.118495915	0.26335525	0	0.778500631	0	1	CRA004775;PRJNA827465	Yang2025_Silkie_Wenchang	0.768643821
ENSGALG00010005219	NALCN	2	-0.187390273	0.171712753	-1.091300844	0.275140526	0.017548541	1.373869583	27.21288742	0.5	PRJNA827465;Yang2025_Silkie_Wenchang	CRA004775	0.775068889
ENSGALG00010005219	NALCN	2	-0.047171231	0.233972916	-0.201609794	0.840221788	0	0.763372213	0	0.5	CRA004775;Yang2025_Silkie_Wenchang	PRJNA827465	0.959662809
ENSGALG00010005219	NALCN	2	-0.329326495	0.160781384	-2.048287477	0.040531839	0	0.099501202	0	1	CRA004775;PRJNA827465	Yang2025_Silkie_Wenchang	0.348354009
ENSGALG00010005220	ANGEL1	2	-0.27057586	0.228351649	-1.184908717	0.23605354	0.097611895	15.40737287	93.50960084	1	PRJNA827465;Yang2025_Silkie_Wenchang	CRA004775	0.748669015
ENSGALG00010005220	ANGEL1	2	-0.33285269	0.194311988	-1.712980721	0.086716086	0.057775343	4.082357718	75.50435143	1	CRA004775;Yang2025_Silkie_Wenchang	PRJNA827465	0.506494627
ENSGALG00010005220	ANGEL1	2	-0.056677649	0.05625146	-1.007576493	0.313657819	0	0.13891653	0	1	CRA004775;PRJNA827465	Yang2025_Silkie_Wenchang	0.796531448
ENSGALG00010005221	ENSGALG00010005221	2	-0.556854351	0.290387839	-1.917622831	0.055158853	0	0.222689347	0	1	PRJNA827465;Yang2025_Silkie_Wenchang	CRA004775	0.406584471
ENSGALG00010005221	ENSGALG00010005221	2	-0.029819122	0.39966709	-0.074609902	0.940525096	0.10866063	1.511836457	33.85527944	0.5	CRA004775;Yang2025_Silkie_Wenchang	PRJNA827465	0.985559481
ENSGALG00010005221	ENSGALG00010005221	2	-0.178921887	0.537162765	-0.333086913	0.739068678	0.387298406	3.009378208	66.77054425	0.5	CRA004775;PRJNA827465	Yang2025_Silkie_Wenchang	0.934652713
ENSGALG00010005226	MYCT1	2	-0.015951329	0.066208761	-0.24092474	0.809613447	0	0.070285784	0	1	PRJNA827465;Yang2025_Silkie_Wenchang	CRA004775	0.952787295
ENSGALG00010005226	MYCT1	2	0.022721378	0.162192331	0.1400891	0.888589592	0	0.80825394	0	0.5	CRA004775;Yang2025_Silkie_Wenchang	PRJNA827465	0.973135755
ENSGALG00010005226	MYCT1	2	0.003672197	0.069090346	0.05315065	0.957611875	0	0.731601971	0	0.5	CRA004775;PRJNA827465	Yang2025_Silkie_Wenchang	0.988770061
ENSGALG00010005227	EXOSC9	2	0.057390853	0.077683478	0.73877811	0.460041746	0.004636123	1.577497705	36.60846562	0.5	PRJNA827465;Yang2025_Silkie_Wenchang	CRA004775	0.850104868
ENSGALG00010005227	EXOSC9	2	0.05576653	0.141882677	0.393046784	0.694284928	0.019049256	1.709845097	41.51516988	0.5	CRA004775;Yang2025_Silkie_Wenchang	PRJNA827465	0.920728119
ENSGALG00010005227	EXOSC9	2	-0.020708249	0.068588409	-0.301920538	0.762712639	0	0.429088296	0	1	CRA004775;PRJNA827465	Yang2025_Silkie_Wenchang	0.940082621
ENSGALG00010005228	ZNF521	2	-0.009860386	0.071586238	-0.137741365	0.890444826	0	0.047292421	0	0.5	PRJNA827465;Yang2025_Silkie_Wenchang	CRA004775	0.973417023
ENSGALG00010005228	ZNF521	2	0.004414115	0.131002117	0.033694993	0.973120372	0	0.043396309	0	0.5	CRA004775;Yang2025_Silkie_Wenchang	PRJNA827465	0.992392211
ENSGALG00010005228	ZNF521	2	-0.02094	0.079203251	-0.26438309	0.791484741	0	0.011189063	0	1	CRA004775;PRJNA827465	Yang2025_Silkie_Wenchang	0.948059392
ENSGALG00010005229	CIPC	2	0.064456692	0.063404182	1.016600016	0.309343747	0	0.032210334	0	1	PRJNA827465;Yang2025_Silkie_Wenchang	CRA004775	0.794043289
ENSGALG00010005229	CIPC	2	0.068242937	0.124224467	0.549351819	0.582764032	0	0.096757434	0	0.5	CRA004775;Yang2025_Silkie_Wenchang	PRJNA827465	0.890112496
ENSGALG00010005229	CIPC	2	0.054431726	0.069486269	0.783345061	0.433424508	0	0.058444465	0	0.5	CRA004775;PRJNA827465	Yang2025_Silkie_Wenchang	0.842762754
ENSGALG00010005231	ENSGALG00010005231	2	-0.621632031	0.366464286	-1.696296351	0.089829771	0.134589115	1.991167707	49.77821322	1	PRJNA827465;Yang2025_Silkie_Wenchang	CRA004775	0.514211987
ENSGALG00010005231	ENSGALG00010005231	2	-0.033130716	0.299365622	-0.110669743	0.91187824	0	0.535064044	0	0.5	CRA004775;Yang2025_Silkie_Wenchang	PRJNA827465	0.978928739
ENSGALG00010005231	ENSGALG00010005231	2	-0.410493758	0.587373472	-0.698863292	0.48463747	0.532525921	4.314739517	76.82362988	0.5	CRA004775;PRJNA827465	Yang2025_Silkie_Wenchang	0.857004733
ENSGALG00010005232	GATA3	2	0.178444071	0.407379059	0.438029561	0.661364854	0	0.124880634	0	1	PRJNA827465;Yang2025_Silkie_Wenchang	CRA004775	0.912313031
ENSGALG00010005232	GATA3	2	-0.37458065	0.684502703	-0.547230345	0.584220491	0.47829864	2.000681091	50.01702147	0.5	CRA004775;Yang2025_Silkie_Wenchang	PRJNA827465	0.890842213
ENSGALG00010005232	GATA3	2	-0.384360992	0.5266502	-0.729822171	0.465498891	0.27560082	1.96695401	49.15997043	0.5	CRA004775;PRJNA827465	Yang2025_Silkie_Wenchang	0.852424459
ENSGALG00010005236	TMTC4	2	0.080442136	0.082381438	0.976459482	0.328836817	0	7.43667E-05	0	1	PRJNA827465;Yang2025_Silkie_Wenchang	CRA004775	0.800761292
ENSGALG00010005236	TMTC4	2	0.036703714	0.142551556	0.257476768	0.796810748	0	0.423735351	0	0.5	CRA004775;Yang2025_Silkie_Wenchang	PRJNA827465	0.949622804
ENSGALG00010005236	TMTC4	2	0.061219759	0.092524625	0.661659083	0.508189733	0	0.474681192	0	0.5	CRA004775;PRJNA827465	Yang2025_Silkie_Wenchang	0.864714223
ENSGALG00010005237	RAP2A	2	-0.028039785	0.067543898	-0.415134241	0.678043635	0	0.331313384	0	1	PRJNA827465;Yang2025_Silkie_Wenchang	CRA004775	0.917534751
ENSGALG00010005237	RAP2A	2	-0.146223555	0.14123727	-1.035304314	0.300526816	0	0.265723039	0	1	CRA004775;Yang2025_Silkie_Wenchang	PRJNA827465	0.790579721
ENSGALG00010005237	RAP2A	2	-0.02448291	0.072758201	-0.336496912	0.73649618	0	0.753770603	0	1	CRA004775;PRJNA827465	Yang2025_Silkie_Wenchang	0.933661344
ENSGALG00010005238	TMEM63C	2	-0.117196762	0.070476233	-1.662926028	0.096327244	0	0.070280223	0	1	PRJNA827465;Yang2025_Silkie_Wenchang	CRA004775	0.530839828
ENSGALG00010005238	TMEM63C	2	-0.539389578	0.464012835	-1.162445383	0.245054598	0.365060228	5.885457503	83.00896745	1	CRA004775;Yang2025_Silkie_Wenchang	PRJNA827465	0.756475101
ENSGALG00010005238	TMEM63C	2	-0.522004625	0.483210069	-1.080285074	0.280015254	0.402205192	6.467303934	84.53760624	1	CRA004775;PRJNA827465	Yang2025_Silkie_Wenchang	0.778098827
ENSGALG00010005239	ENSGALG00010005239	2	0.088481595	0.589327701	0.150139888	0.880654251	0.637084294	12.06557949	91.71196045	0.5	PRJNA827465;Yang2025_Silkie_Wenchang	CRA004775	0.971623434
ENSGALG00010005239	ENSGALG00010005239	2	0.64992152	0.230126317	2.824194682	0.004739958	0	0.104891049	0	1	CRA004775;Yang2025_Silkie_Wenchang	PRJNA827465	0.101435303
ENSGALG00010005239	ENSGALG00010005239	2	-0.150976118	0.4664903	-0.323642567	0.746208657	0.284717673	2.489429549	59.83015465	0.5	CRA004775;PRJNA827465	Yang2025_Silkie_Wenchang	0.936550805
ENSGALG00010005240	VIP	2	0.305748882	0.137085208	2.230356481	0.025723786	0	0.688080615	0	1	PRJNA827465;Yang2025_Silkie_Wenchang	CRA004775	0.273470915
ENSGALG00010005240	VIP	2	0.463581549	0.239688036	1.93410383	0.053100363	0	0.049634313	0	1	CRA004775;Yang2025_Silkie_Wenchang	PRJNA827465	0.399631766
ENSGALG00010005240	VIP	2	0.234058349	0.160343917	1.459727028	0.144365111	0	0.005479421	0	1	CRA004775;PRJNA827465	Yang2025_Silkie_Wenchang	0.631870428
ENSGALG00010005241	C4orf54	2	-0.118250842	0.09838224	-1.201953143	0.229381683	0	0.850720288	0	1	PRJNA827465;Yang2025_Silkie_Wenchang	CRA004775	0.742373871
ENSGALG00010005241	C4orf54	2	-0.213108917	0.181508457	-1.174099105	0.240355332	0	0.826517111	0	0.5	CRA004775;Yang2025_Silkie_Wenchang	PRJNA827465	0.752843928
ENSGALG00010005241	C4orf54	2	-0.050763769	0.112142484	-0.452672057	0.650784903	0	0.332655759	0	0.5	CRA004775;PRJNA827465	Yang2025_Silkie_Wenchang	0.908769703
ENSGALG00010005242	CCDC80	2	-0.049168851	0.092828875	-0.529671946	0.596339402	0	0.058646567	0	1	PRJNA827465;Yang2025_Silkie_Wenchang	CRA004775	0.894064255
ENSGALG00010005242	CCDC80	2	-0.083465642	0.173309694	-0.481598232	0.630091383	0	0.005368727	0	1	CRA004775;Yang2025_Silkie_Wenchang	PRJNA827465	0.90256717
ENSGALG00010005242	CCDC80	2	-0.037405275	0.103670048	-0.360810817	0.718240876	0	0.001457859	0	1	CRA004775;PRJNA827465	Yang2025_Silkie_Wenchang	0.927975712
ENSGALG00010005244	FBXO5	2	-0.148064602	0.106926496	-1.384732572	0.166134255	0.010499264	1.762400009	43.25919229	1	PRJNA827465;Yang2025_Silkie_Wenchang	CRA004775	0.66529196
ENSGALG00010005244	FBXO5	2	0.037756236	0.384487132	0.09819896	0.921774308	0.232212808	4.194634482	76.16002051	0.5	CRA004775;Yang2025_Silkie_Wenchang	PRJNA827465	0.981649741
ENSGALG00010005244	FBXO5	2	0.110788331	0.259837894	0.426374805	0.669834757	0.090272897	2.350363171	57.45338371	0.5	CRA004775;PRJNA827465	Yang2025_Silkie_Wenchang	0.914377769
ENSGALG00010005245	KIAA1217	2	0.126310263	0.275692565	0.458156217	0.646840213	0.128514481	5.699711697	82.45525295	0.5	PRJNA827465;Yang2025_Silkie_Wenchang	CRA004775	0.907350807
ENSGALG00010005245	KIAA1217	2	0.550486084	0.203617591	2.703529106	0.006860744	0	0.986694376	0	1	CRA004775;Yang2025_Silkie_Wenchang	PRJNA827465	0.127252392
ENSGALG00010005245	KIAA1217	2	0.326907969	0.52319546	0.624829522	0.532082953	0.460883138	5.388110263	81.44061737	0.5	CRA004775;PRJNA827465	Yang2025_Silkie_Wenchang	0.873104119
ENSGALG00010005246	ENSGALG00010005246	2	0.152954179	0.117745871	1.29901947	0.193937248	0	0.0012647	0	1	PRJNA827465;Yang2025_Silkie_Wenchang	CRA004775	0.70433521
ENSGALG00010005246	ENSGALG00010005246	2	0.151002699	0.179217632	0.842566087	0.399471165	0	0.014336368	0	1	CRA004775;Yang2025_Silkie_Wenchang	PRJNA827465	0.832246851
ENSGALG00010005246	ENSGALG00010005246	2	0.145449342	0.145379125	1.000482992	0.317076825	0	0.011506345	0	1	CRA004775;PRJNA827465	Yang2025_Silkie_Wenchang	0.797007794
ENSGALG00010005247	THAP7	2	-0.152451283	0.055033764	-2.770140936	0.005603204	0	0.672901305	0	1	PRJNA827465;Yang2025_Silkie_Wenchang	CRA004775	0.112358537
ENSGALG00010005247	THAP7	2	-0.03198464	0.123100244	-0.259825963	0.794998023	0	0.206847677	0	0.5	CRA004775;Yang2025_Silkie_Wenchang	PRJNA827465	0.948905846
ENSGALG00010005247	THAP7	2	-0.164293375	0.059582776	-2.75739713	0.005826354	0	0.685431801	0	0.5	CRA004775;PRJNA827465	Yang2025_Silkie_Wenchang	0.115022409
ENSGALG00010005248	TMEM200A	2	-0.242272928	0.247865467	-0.977437199	0.328352754	0.110654758	9.97682718	89.97677336	1	PRJNA827465;Yang2025_Silkie_Wenchang	CRA004775	0.800761292
ENSGALG00010005248	TMEM200A	2	0.063390213	0.611915852	0.103593022	0.917492325	0.673150162	9.268609878	89.21089556	0.5	CRA004775;Yang2025_Silkie_Wenchang	PRJNA827465	0.980936342
ENSGALG00010005248	TMEM200A	2	0.270337907	0.353907717	0.763865532	0.44494738	0.19050544	3.449139206	71.0072589	0.5	CRA004775;PRJNA827465	Yang2025_Silkie_Wenchang	0.846013379
ENSGALG00010005249	PDGFD	2	-0.134254059	0.10093062	-1.330161833	0.183464956	0	0.295128548	0	1	PRJNA827465;Yang2025_Silkie_Wenchang	CRA004775	0.691847354
ENSGALG00010005249	PDGFD	2	-0.186222809	0.166563512	-1.118028835	0.263554678	0	0.17959659	0	1	CRA004775;Yang2025_Silkie_Wenchang	PRJNA827465	0.768643821
ENSGALG00010005249	PDGFD	2	-0.091248429	0.117363697	-0.777484276	0.436873108	0	0.032772176	0	1	CRA004775;PRJNA827465	Yang2025_Silkie_Wenchang	0.843387647
ENSGALG00010005250	SLC35A5	2	0.042715884	0.140562309	0.303892871	0.761209509	0.030478697	4.033838915	75.20971905	0.5	PRJNA827465;Yang2025_Silkie_Wenchang	CRA004775	0.939618592
ENSGALG00010005250	SLC35A5	2	-0.140367607	0.119150653	-1.1780683	0.238769378	0	0.148692729	0	1	CRA004775;Yang2025_Silkie_Wenchang	PRJNA827465	0.750806421
ENSGALG00010005250	SLC35A5	2	0.073200106	0.171524033	0.426762973	0.669551978	0.033761785	1.627442776	38.5539071	0.5	CRA004775;PRJNA827465	Yang2025_Silkie_Wenchang	0.914377769
ENSGALG00010005251	MTRF1L	2	-0.013567723	0.104494618	-0.129841358	0.896691941	0.012415853	2.314233839	56.78915488	0.5	PRJNA827465;Yang2025_Silkie_Wenchang	CRA004775	0.975115773
ENSGALG00010005251	MTRF1L	2	0.084143789	0.092257758	0.912051092	0.361741822	0	0.064243192	0	1	CRA004775;Yang2025_Silkie_Wenchang	PRJNA827465	0.815545459
ENSGALG00010005251	MTRF1L	2	-0.093594421	0.086355808	-1.083823129	0.278443228	0	0.352034734	0	0.5	CRA004775;PRJNA827465	Yang2025_Silkie_Wenchang	0.776763074
ENSGALG00010005252	KLHL14	2	-0.109577485	0.104232757	-1.05127686	0.293131452	0	0.235483324	0	1	PRJNA827465;Yang2025_Silkie_Wenchang	CRA004775	0.786774237
ENSGALG00010005252	KLHL14	2	-0.150161122	0.147412539	-1.018645513	0.308371289	0	0.162618049	0	0.5	CRA004775;Yang2025_Silkie_Wenchang	PRJNA827465	0.793457188
ENSGALG00010005252	KLHL14	2	-0.053493541	0.141923458	-0.376918251	0.706234358	0	0.072969019	0	0.5	CRA004775;PRJNA827465	Yang2025_Silkie_Wenchang	0.924548793
ENSGALG00010005253	POMT2	2	-0.229264268	0.215461876	-1.064059554	0.287301722	0.081009051	7.472461089	86.61752817	1	PRJNA827465;Yang2025_Silkie_Wenchang	CRA004775	0.782840067
ENSGALG00010005253	POMT2	2	-0.37895245	0.14038258	-2.69942646	0.006945911	0.006450451	1.147379555	12.84488244	1	CRA004775;Yang2025_Silkie_Wenchang	PRJNA827465	0.12805992
ENSGALG00010005253	POMT2	2	-0.038774829	0.065320041	-0.593613049	0.552770951	0	0.186519122	0	1	CRA004775;PRJNA827465	Yang2025_Silkie_Wenchang	0.87928478
ENSGALG00010005254	ENSGALG00010005254	2	0.801168632	0.372411905	2.151297046	0.031452761	0	0.503574796	0	1	PRJNA827465;Yang2025_Silkie_Wenchang	CRA004775	0.303358988
ENSGALG00010005254	ENSGALG00010005254	2	0.586283911	0.570133362	1.028327669	0.30379572	0.278220808	1.69896384	41.14059543	0.5	CRA004775;Yang2025_Silkie_Wenchang	PRJNA827465	0.792208658
ENSGALG00010005254	ENSGALG00010005254	2	0.299926424	0.433550141	0.691791782	0.489068098	0	0.488779442	0	0.5	CRA004775;PRJNA827465	Yang2025_Silkie_Wenchang	0.858232954
ENSGALG00010005255	BZW2	2	-0.149403667	0.083878673	-1.781187771	0.074881781	0.007198461	1.955661754	48.86641323	1	PRJNA827465;Yang2025_Silkie_Wenchang	CRA004775	0.472257613
ENSGALG00010005255	BZW2	2	-0.094328557	0.224784642	-0.419639687	0.674748691	0.065177986	2.345060802	57.35718241	0.5	CRA004775;Yang2025_Silkie_Wenchang	PRJNA827465	0.915970608
ENSGALG00010005255	BZW2	2	-0.063342619	0.07262863	-0.872143927	0.383129865	0.001198239	1.026427806	2.574735997	0.5	CRA004775;PRJNA827465	Yang2025_Silkie_Wenchang	0.825405345
ENSGALG00010005258	ITIH5	2	-0.401113717	0.105594409	-3.798626476	0.0001455	0	0.22779602	0	1	PRJNA827465;Yang2025_Silkie_Wenchang	CRA004775	0.010211774
ENSGALG00010005258	ITIH5	2	-0.13645052	0.432568502	-0.31544258	0.752425642	0.259761525	2.534113187	60.53846351	0.5	CRA004775;Yang2025_Silkie_Wenchang	PRJNA827465	0.93806821

ENSGALG00010005258	ITIH5	2	-0.090641251	0.368797618	-0.245775044	0.805856377	0.153146585	1.850729875	45.96726331	0.5	CRA004775;PRJNA827465	Yang2025_Silkie_Wenchang	0.952057827
ENSGALG00010005259	SLC27A5	2	-0.053465329	1.059325851	-0.050471089	0.959746988	1.609951852	3.489528057	71.34282964	0.5	PRJNA827465;Yang2025_Silkie_Wenchang	CRA004775	0.989302378
ENSGALG00010005259	SLC27A5	2	0.456987384	1.4606199	0.312872215	0.754377748	3.886125886	11.14899211	91.03057936	0.5	CRA004775;Yang2025_Silkie_Wenchang	PRJNA827465	0.93838723
ENSGALG00010005259	SLC27A5	2	1.677377977	0.459825233	3.647859785	0.000264434	0	0.578779828	0	1	CRA004775;PRJNA827465	Yang2025_Silkie_Wenchang	0.015535194
ENSGALG00010005260	ANXA5	2	-0.187133642	0.140440214	-1.332479048	0.182702822	0.032210685	5.171943822	80.66490986	1	PRJNA827465;Yang2025_Silkie_Wenchang	CRA004775	0.6911231
ENSGALG00010005260	ANXA5	2	-0.305896417	0.103240222	-2.962957755	0.003046984	0	0.993696424	0	1	CRA004775;Yang2025_Silkie_Wenchang	PRJNA827465	0.075987059
ENSGALG00010005260	ANXA5	2	-0.060186855	0.056583554	-1.063681058	0.287473208	0	0.01221176	0	1	CRA004775;PRJNA827465	Yang2025_Silkie_Wenchang	0.782840067
ENSGALG00010005261	ATG3	2	0.173794573	0.248483196	0.699421835	0.484288446	0.116591056	17.56212835	94.30592933	0.5	PRJNA827465;Yang2025_Silkie_Wenchang	CRA004775	0.857004733
ENSGALG00010005261	ATG3	2	0.284157643	0.199432794	1.424829068	0.154206618	0.050056542	2.344703519	57.35068456	1	CRA004775;Yang2025_Silkie_Wenchang	PRJNA827465	0.646830576
ENSGALG00010005261	ATG3	2	-0.062967781	0.052104894	-1.208481131	0.226862249	0	0.095568273	0	0.5	CRA004775;PRJNA827465	Yang2025_Silkie_Wenchang	0.740444589
ENSGALG00010005262	ENSGALG00010005262	2	-0.049092074	0.073770945	-0.665466249	0.505752324	0	0.773942653	0	1	PRJNA827465;Yang2025_Silkie_Wenchang	CRA004775	0.863927652
ENSGALG00010005262	ENSGALG00010005262	2	-0.151883747	0.120015493	-1.265534494	0.205679791	0	0.006694774	0	1	CRA004775;Yang2025_Silkie_Wenchang	PRJNA827465	0.71825498
ENSGALG00010005262	ENSGALG00010005262	2	-0.02185666	0.084178211	-0.259647476	0.795135711	0	0.333599786	0	1	CRA004775;PRJNA827465	Yang2025_Silkie_Wenchang	0.948933254
ENSGALG00010005263	ENSGALG00010005263	2	-0.847642944	0.263026326	-3.222654385	0.001270087	0	0.089609856	0	1	PRJNA827465;Yang2025_Silkie_Wenchang	CRA004775	0.043294215
ENSGALG00010005263	ENSGALG00010005263	2	-0.520713495	0.499055904	-1.043397124	0.296764405	0.26572864	2.064627704	51.56511762	0.5	CRA004775;Yang2025_Silkie_Wenchang	PRJNA827465	0.788458494
ENSGALG00010005263	ENSGALG00010005263	2	-0.476586182	0.408865175	-1.165631636	0.243763411	0.132803843	1.566973229	36.18270041	0.5	CRA004775;PRJNA827465	Yang2025_Silkie_Wenchang	0.755365463
ENSGALG00010005264	ITIH2	2	-0.478625011	1.39209173	-0.34381715	0.730983802	3.426147166	8.444458985	88.15791513	0.5	PRJNA827465;Yang2025_Silkie_Wenchang	CRA004775	0.932769533
ENSGALG00010005264	ITIH2	2	-0.612400641	1.153350532	-0.530975297	0.595435898	2.482798686	14.79958153	93.24305219	0.5	CRA004775;Yang2025_Silkie_Wenchang	PRJNA827465	0.893794893
ENSGALG00010005264	ITIH2	2	0.57171654	0.297263909	1.923262538	0.054447087	0	0.30170006	0	1	CRA004775;PRJNA827465	Yang2025_Silkie_Wenchang	0.404097297
ENSGALG00010005265	DYNC2H1	2	-0.176964146	0.063164683	-2.801631205	0.005084496	0	0.177769232	0	1	PRJNA827465;Yang2025_Silkie_Wenchang	CRA004775	0.106389715
ENSGALG00010005265	DYNC2H1	2	-0.116637583	0.201114908	-0.579954931	0.561945011	0.032274047	1.429083957	30.02510487	0.5	CRA004775;Yang2025_Silkie_Wenchang	PRJNA827465	0.881836663
ENSGALG00010005265	DYNC2H1	2	-0.123681559	0.120004797	-1.030638454	0.302710396	0.011168192	1.167226143	14.32679894	0.5	CRA004775;PRJNA827465	Yang2025_Silkie_Wenchang	0.791852471
ENSGALG00010005266	SLC19A2	2	0.081350024	0.1430505	0.568680459	0.569573011	0	0.25893114	0	0.5	PRJNA827465;Yang2025_Silkie_Wenchang	CRA004775	0.88485924
ENSGALG00010005266	SLC19A2	2	-0.159994476	0.220427653	-0.725836679	0.467938898	0	0.708379882	0	1	CRA004775;Yang2025_Silkie_Wenchang	PRJNA827465	0.853115491
ENSGALG00010005266	SLC19A2	2	-0.041125861	0.256678073	-0.160223507	0.872705013	0.065628287	1.723131106	41.96611061	0.5	CRA004775;PRJNA827465	Yang2025_Silkie_Wenchang	0.969629321
ENSGALG00010005267	CYP51A1	2	0.211710275	0.109804265	1.928069689	0.053846459	0	0.39371186	0	1	PRJNA827465;Yang2025_Silkie_Wenchang	CRA004775	0.402267128
ENSGALG00010005267	CYP51A1	2	0.030464083	0.145613918	0.209211344	0.834283257	0	0.227618895	0	0.5	CRA004775;Yang2025_Silkie_Wenchang	PRJNA827465	0.957990393
ENSGALG00010005267	CYP51A1	2	0.151079367	0.139871991	1.08012595	0.280086098	0.013551129	1.463722479	31.68103827	0.5	CRA004775;PRJNA827465	Yang2025_Silkie_Wenchang	0.778131328
ENSGALG00010005268	MTTP	2	0.144528828	0.286988573	0.503604817	0.614539107	0	0.336583992	0	1	PRJNA827465;Yang2025_Silkie_Wenchang	CRA004775	0.898614239
ENSGALG00010005268	MTTP	2	1.035179494	1.121266116	0.923223738	0.355890621	2.107153283	5.627343356	82.2296253	1	CRA004775;Yang2025_Silkie_Wenchang	PRJNA827465	0.81231505
ENSGALG00010005268	MTTP	2	1.281591907	0.921311203	1.391052125	0.164209621	1.125220482	2.882698281	65.31027869	1	CRA004775;PRJNA827465	Yang2025_Silkie_Wenchang	0.663029662
ENSGALG00010005269	KIN	2	-0.029328889	0.058681837	-0.499795001	0.617219431	0	0.226338551	0	1	PRJNA827465;Yang2025_Silkie_Wenchang	CRA004775	0.899121942
ENSGALG00010005269	KIN	2	-0.067803325	0.11862292	-0.571587055	0.567601767	0	0.087065478	0	1	CRA004775;Yang2025_Silkie_Wenchang	PRJNA827465	0.883717772
ENSGALG00010005269	KIN	2	-0.016985622	0.06133934	-0.276912371	0.781847393	0	8.57774E-06	0	1	CRA004775;PRJNA827465	Yang2025_Silkie_Wenchang	0.945273413
ENSGALG00010005270	GSTZ1	2	-0.0112715	0.111757694	-0.100856595	0.9196643	0	0.819618049	0	0.5	PRJNA827465;Yang2025_Silkie_Wenchang	CRA004775	0.981229904
ENSGALG00010005270	GSTZ1	2	0.145541965	0.333878173	0.435913386	0.662899573	0.15920392	3.262725322	69.35077577	0.5	CRA004775;Yang2025_Silkie_Wenchang	PRJNA827465	0.91282818
ENSGALG00010005270	GSTZ1	2	0.212343314	0.215830338	0.98384368	0.325192379	0.045827558	1.704676047	41.33782768	1	CRA004775;PRJNA827465	Yang2025_Silkie_Wenchang	0.799914637
ENSGALG00010005272	MBNL2	2	0.135821299	0.322570126	0.421059757	0.673711447	0.18484204	8.420868917	88.12474093	0.5	PRJNA827465;Yang2025_Silkie_Wenchang	CRA004775	0.915497675
ENSGALG00010005272	MBNL2	2	0.522304395	0.181391346	2.879433918	0.003983898	0	0.108711364	0	1	CRA004775;Yang2025_Silkie_Wenchang	PRJNA827465	0.091140358
ENSGALG00010005272	MBNL2	2	0.152383662	0.384914366	0.395889775	0.692186344	0.239000485	4.380397103	77.17101951	0.5	CRA004775;PRJNA827465	Yang2025_Silkie_Wenchang	0.920393799
ENSGALG00010005273	ENSGALG00010005273	2	-0.509564379	0.433808213	-1.174630548	0.240142556	0.197845186	2.08663828	52.07602537	1	PRJNA827465;Yang2025_Silkie_Wenchang	CRA004775	0.75252297
ENSGALG00010005273	ENSGALG00010005273	2	-0.478648333	0.471435948	-1.015298758	0.309963436	0.203314034	1.838573248	45.60999942	1	CRA004775;Yang2025_Silkie_Wenchang	PRJNA827465	0.794395438
ENSGALG00010005273	ENSGALG00010005273	2	-0.92897645	0.306658655	-3.029350172	0.002450804	0	0.012779951	0	1	CRA004775;PRJNA827465	Yang2025_Silkie_Wenchang	0.066199691
ENSGALG00010005275	L3MBTL3	2	0.047430168	0.068952446	0.687867803	0.491536033	0.002128464	1.255999016	20.38210324	0.5	PRJNA827465;Yang2025_Silkie_Wenchang	CRA004775	0.85895707
ENSGALG00010005275	L3MBTL3	2	0.138832084	0.103182242	1.34550366	0.17846264	0	0.00769503	0	1	CRA004775;Yang2025_Silkie_Wenchang	PRJNA827465	0.684796269
ENSGALG00010005275	L3MBTL3	2	0.000329653	0.070866726	0.004651732	0.996288468	0	0.074414838	0	0.5	CRA004775;PRJNA827465	Yang2025_Silkie_Wenchang	0.99878373
ENSGALG00010005277	ENSGALG00010005277	2	0.062665277	0.154374418	0.40593045	0.684793704	0	0.095922247	0	1	PRJNA827465;Yang2025_Silkie_Wenchang	CRA004775	0.919003112
ENSGALG00010005277	ENSGALG00010005277	2	-0.277048364	0.462499056	-0.599024713	0.549156405	0.307913974	3.431179709	70.85550496	0.5	CRA004775;Yang2025_Silkie_Wenchang	PRJNA827465	0.878449091
ENSGALG00010005277	ENSGALG00010005277	2	-0.298559265	0.405928294	-0.735497548	0.462036533	0.236694751	3.18815837	68.63392956	0.5	CRA004775;PRJNA827465	Yang2025_Silkie_Wenchang	0.850747865
ENSGALG00010005278	DAPP1	2	-0.09963205	0.092977513	-1.071571467	0.283912555	0	0.048489328	0	1	PRJNA827465;Yang2025_Silkie_Wenchang	CRA004775	0.77978499
ENSGALG00010005278	DAPP1	2	-0.088886349	0.157223265	-0.56535112	0.571834965	0	0.121676101	0	1	CRA004775;Yang2025_Silkie_Wenchang	PRJNA827465	0.885470687
ENSGALG00010005278	DAPP1	2	-0.120369476	0.108721339	-1.107137537	0.268234468	0	0.071460929	0	1	CRA004775;PRJNA827465	Yang2025_Silkie_Wenchang	0.771398294
ENSGALG00010005281	TMED8	2	-0.11004436	0.187626655	-0.586507071	0.557534811	0.065154198	13.05875416	92.34230167	0.5	PRJNA827465;Yang2025_Silkie_Wenchang	CRA004775	0.880678789
ENSGALG00010005281	TMED8	2	-0.299271273	0.089187781	-3.355518775	0.000792163	0	0.075235193	0	1	CRA004775;Yang2025_Silkie_Wenchang	PRJNA827465	0.032196871
ENSGALG00010005281	TMED8	2	0.063013508	0.045897959	1.372904356	0.169782082	0	0.812144386	0	0.5	CRA004775;PRJNA827465	Yang2025_Silkie_Wenchang	0.671232541
ENSGALG00010005282	FLT1	2	-0.018536877	0.075141296	-0.246693601	0.80514536	0	0.994977749	0	0.5	PRJNA827465;Yang2025_Silkie_Wenchang	CRA004775	0.951863117
ENSGALG00010005282	FLT1	2	0.201650064	0.21467208	0.939339963	0.347556227	0.046772812	1.61961633	38.25698214	1	CRA004775;Yang2025_Silkie_Wenchang	PRJNA827465	0.808762722
ENSGALG00010005282	FLT1	2	0.149244423	0.309308476	0.482509969	0.629443718	0.137877026	2.898377696	65.49794041	0.5	CRA004775;PRJNA827465	Yang2025_Silkie_Wenchang	0.902390168
ENSGALG00010005283	ATP5C1	2	-0.055197751	0.069019366	-0.79974294	0.423859749	0	0.47248505	0	0.5	PRJNA827465;Yang2025_Silkie_Wenchang	CRA004775	0.84030043
ENSGALG00010005283	ATP5C1	2	0.063197961	0.176637404	0.35778357	0.720505287	0	0.012563509	0	1	CRA004775;Yang2025_Silkie_Wenchang	PRJNA827465	0.928764162
ENSGALG00010005283	ATP5C1	2	-0.069081536	0.072196812	-0.956850221	0.338642859	0	0.065178404	0	0.5	CRA004775;PRJNA827465	Yang2025_Silkie_Wenchang	0.804450436
ENSGALG00010005285	MPP7	2	-0.100150266	0.083045206	-1.205972883	0.227827946	0	0.233048431	0	1	PRJNA827465;Yang2025_Silkie_Wenchang	CRA004775	0.740853612
ENSGALG00010005285	MPP7	2	0.102113893	0.307082523	0.33252916	0.739489725	0.139953087	3.441615237	70.94387573	0.5	CRA004775;Yang2025_Silkie_Wenchang	PRJNA827465	0.934873078
ENSGALG00010005285	MPP7	2	0.129358664	0.261925678	0.493875459	0.621394137	0.094299143	2.701657785	62.98568954	0.5	CRA004775;PRJNA827465	Yang2025_Silkie_Wenchang	0.899693435
ENSGALG00010005286	ENSGALG00010005286	2	0.226982313	1.204384627	0.18846331	0.850513474	2.564334173	8.597449325	88.36864328	0.5	PRJNA827465;Yang2025_Silkie_Wenchang	CRA004775	0.961985357
ENSGALG00010005286	ENSGALG00010005286	2	-0.512749266	0.470503	-1.089789577	0.275805847	0.071160254	1.191308205	16.05866595	1	CRA004775;Yang2025_Silkie_Wenchang	PRJNA827465	0.7754953
ENSGALG00010005286	ENSGALG00010005286	2	0.70146636	0.733465015	0.956373305	0.338883667	0.752505303	3.318697922	69.86770042	0.5	CRA004775;PRJNA827465	Yang2025_Silkie_Wenchang	0.804646102
ENSGALG00010005288	ENSGALG00010005288	2	0.090885348	0.064171994	1.416277443	0.156694289	0.003597939	1.775705204	43.68434594	1	PRJNA827465;Yang2025_Silkie_Wenchang	CRA004775	0.649910897
ENSGALG00010005288	ENSGALG00010005288	2	0.089801566	0.114679252	0.783067245	0.433587625	0.012492749	1.515853944	34.03058361	1	CRA004775;Yang2025_Silkie_Wenchang	PRJNA827465	0.842762754
ENSGALG00010005288	ENSGALG00010005288	2	0.168382432	0.064552668	2.608450398	0.009095319	0	0.420505626	0	1	CRA004775;PRJNA827465	Yang2025_Silkie_Wenchang	0.149507876
ENSGALG00010005291	IFNGR1	2	-0.364998078	0.252112305	-1.447759869	0.14768422	0.113154287	8.975054891	88.85800686	1	PRJNA827465;Yang2025_Silkie_Wenchang	CRA004775	0.636995621
ENSGALG00010005291	IFNGR1	2	-0.418577381	0.267522557	-1.564643314	0.117666567	0.095865261	2.709566819	63.0937317	1	CRA004775;Yang2025_Silkie_Wenchang	PRJNA827465	0.577281149
ENSGALG00010005291	IFNGR1	2	-0.119118521	0.090002501	-1.323502345	0.185668377	0	0.021380709	0	1	CRA004775;PRJNA827465	Yang2025_Silkie_Wenchang	0.694265174
ENSGALG00010005292	TAF3	2	-0.086602723	0.047525774	-1.822226456	0.068420632	0	0.469501456	0	1	PRJNA827465;Yang2025_Silkie_Wenchang	CRA004775	0.450776288
ENSGALG00010005292	TAF3	2	0.289110915	0.301714461	0.95822691	0.337948351	0.16150052	8.814067412	88.65450021	0.5	CRA004775;Yang2025_Silkie_Wenchang	PRJNA827465	0.80415385
ENSGALG00010005292	TAF3	2	0.233182084	0.348546739	0.669012381	0.50348758	0.229651883	17.60049291	94.31834094	0.5	CRA004775;PRJNA827465	Yang2025_Silkie_Wenchang	0.863108263
ENSGALG00010005293	BLZF1	2	-0.141195215	0.116637014	-1.210552379	0.226067005	0.017649314	2.779442616	64.02156338	1	PRJNA827465;Yang2025_Silkie_Wenchang	CRA004775	0.73950158
ENSGALG00010005293	BLZF1	2	-0.259916515	0.101700539	-2.55570441	0.01059731	0	0.053663326	0	1	CRA004775;Yang2025_Silkie_Wenchang	PRJNA827465	0.164107985
ENSGALG00010005293	BLZF1	2	-0.0584209	0.07718824	-0.756862708	0.449132119	0	0.554259132	0	1	CRA004775;PRJNA827465	Yang2025_Silkie_Wenchang	0.847239725
ENSGALG00010005295	ENSGALG00010005295	2	0.424776977	0.299853996	1.416612691	0.156596196	0	0.006153483	0	1	PRJNA827465;Yang2025_Silkie_Wenchang	CRA004775	0.64980313
ENSGALG00010005295	ENSGALG00010005295	2	0.619051428	0.41971909	1.474918446	0.140234471	0	0.145383347	0	1	CRA004775;Yang2025_Silkie_Wenchang	PRJNA827465	0.624052921
ENSGALG00010005295	ENSGALG00010005295	2	0.504525548	0.30235824	1.66863502	0.095189734	0	0.287151819	0	1	CRA004775;PRJNA827465	Yang2025_Silkie_Wenchang	0.528200215
ENSGALG00010005296	PAN3	2	-0.016362327	0.055773055	-0.293373325	0.769236813	0	0.00153635	0	1	PRJNA827465;Yang2025_Silkie_Wenchang	CRA004775	0.941790377
ENSGALG00010005296	PAN3	2	-0.012580956	0.122918667	-0.102351877	0.91847738	0	0.041849532	0	0.5	CRA004775;Yang2025_Silkie_Wenchang	PRJNA827465	0.981033557
ENSGALG00010005296	PAN3	2	-0.013395721	0.06103021	-0.21949328	0.826265814	0	0.039466326	0	0.5	CRA004775;PRJNA827465	Yang2025_Silkie_Wenchang	0.956393839
ENSGALG00010005301	ENSGALG00010005301	2	-0.393520226	0.357470694	-1.100846117	0.270963636	0.055998639	1.274269163	21.52364439	1	PRJNA827465;Yang2025_Silkie_Wenchang	CRA004775	0.772743863
ENSGALG00010005301	ENSGALG00010005301	2	-0.101691144	0.664158522	-0.15311276	0.878309354	0.692992463	4.614547795	78.32940421	0.5	CRA004775;Yang2025_Silkie_Wenchang	PRJNA827465	0.971037793
ENSGALG00010005301	ENSGALG00010005301	2	0.24406035	0.303737377	0.803524256	0.421671783	0.028228533	1.179661513	15.22992073	0.5	CRA004775;PRJNA827465	Yang2025_Silkie_Wenchang	0.839352211
ENSGALG00010005302	PSPC1	2	-0.08973411	0.057215595	-1.568350558	0.116799343	0.002262374	1.400621184	28.60310757	1	PRJNA827465;Yang2025_Silkie_Wenchang	CRA004775	0.57540293
ENSGALG00010005302	PSPC1	2	-0.122006198	0.176574266	-0.690962512	0.4895891	0.036952218	2.066911828	51.61864253	1	CRA004775;Yang2025_Silkie_Wenchang	PRJNA827465	0.858419004
ENSGALG00010005302	PSPC1	2	-0.138074432	0.052770464	-2.616509705	0.008883384	0.000553551	1.017655879	1.734955745	1	CRA004775;PRJNA827465	Yang2025_Silkie_Wenchang	0.147645798
ENSGALG00010005303	ENSGALG00010005303	2	-0.230251996	0.302799204	-0.760411495	0.447008654	0.073654681	1.663904138	39.90038384	0.5	PRJNA827465;Yang2025_Silkie_Wenchang	CRA004775	0.84650962
ENSGALG00010005303	ENSGALG00010005303	2	-0.040899517	0.274543968	-0.148972559	0.881575286	0	0.382883262	0	0.5	CRA004775;Yang2025_Silkie_Wenchang	PRJNA827465	0.971802788
ENSGALG00010005303	ENSGALG00010005303	2	-0.421041511	0.251953058	-1.671110939	0.094699767	0	0.240325276	0	1	CRA004775;PRJNA827465	Yang2025_Silkie_Wenchang	0.526919621
ENSGALG00010005304	ENSGALG00010005304	2	-0.019721247	0.284252611	-0.0693793	0.944687705	0.128981358	4.871547115	79.47264028	0.5	PRJNA827465;Yang2025_Silkie_Wenchang	CRA004775	0.986014725
ENSGALG00010005304	ENSGALG00010005304	2	-0.167312879	0.211980868	-0.78928292	0.429946667	0.025301149	1.345130947	25.65779548	0.5	CRA004775;Yang2025_Silkie_Wenchang	PRJNA827465	0.842012722
ENSGALG00010005304	ENSGALG00010005304	2	0.223740163	0.136284353	1.641715711	0.100648934	0	0.125934078	0	1	CRA004775;PRJNA827465	Yang2025_Silkie_Wenchang	0.541412814
ENSGALG00010005305	SCAF8	2	-0.123358388	0.043385772	-2.843291279	0.004465024	0	0.171126243	0	1	PRJNA827465;Yang2025_Silkie_Wenchang	CRA004775	0.097752851
ENSGALG00010005305	SCAF8	2	-0.013836518	0.18383415	-0.075266312	0.940002825	0.043320413	2.670829348	62.55844647	0.5	CRA004775;Yang2025_Silkie_Wenchang	PRJNA827465	0.985444231
ENSGALG00010005305	SCAF8	2	-0.012088818	0.149252608	-0.08099569	0.93544538	0.031053768	2.601105091	61.55480209	0.5	CRA004775;PRJNA827465	Yang2025_Silkie_Wenchang	0.984701764
ENSGALG00010005307	TIAM2	2	0.113277602	0.070507575	1.60660187	0.108141732	0	0.029588873	0	1	PRJNA827465;Yang2025_Silkie_Wenchang	CRA004775	0.556321269
ENSGALG00010005307	TIAM2	2	0.168043633	0.104567092	1.607041278	0.108045314	0	0.695097558	0	1	CRA004775;Yang2025_Silkie_Wenchang	PRJNA827465	0.556189254
ENSGALG00010005307	TIAM2	2	0.132088909	0.084371819	1.565557207	0.117452315	0	0.912480998	0	1	CRA004775;PRJNA827465	Yang2025_Silkie_Wenchang	0.57688585
ENSGALG00010005309	ASXL3	2	0.029899803	0.068548139	0.436186936	0.662701106	0	0.408398887	0	1	PRJNA827465;Yang2025_Silkie_Wenchang	CRA004775	0.912778072
ENSGALG00010005309	ASXL3	2	0.161593794	0.191893254	0.842102528	0.399730567	0	0.003558851	0	1	CRA004775;Yang2025_Silkie_Wenchang	PRJNA827465	0.832246851
ENSGALG00010005309	ASXL3	2	0.018671928	0.071704603	0.260400687	0.794554713	0	0.128910549	0	1	CRA004775;PRJNA827465	Yang2025_Silkie_Wenchang	0.948905846
ENSGALG00010005310	LAMTOR3	2	-0.189033709	0.166351944	-1.136348064	0.255810923	0.039281135	2.95597291	66.17019065	1	PRJNA827465;Yang2025_Silkie_Wenchang	CRA004775	0.764478331
ENSGALG00010005310	LAMTOR3	2	-0.306526407	0.144050713	-2.127906205	0.033344861	0	0.671541889	0	1	CRA004775;Yang2025_Silkie_Wenchang	PRJNA827465	0.313679529
ENSGALG00010005310	LAMTOR3	2	-0.070507974	0.065146943	-1.082291367	0.279123081	0	0.202431896	0	1	CRA004775;PRJNA827465	Yang2025_Silkie_Wenchang	0.777370468
ENSGALG00010005311	YME1L1	2	-0.146901148	0.146524492	-1.0025706	0.316068087	0.040695128	19.00517684	94.73827574	1	PRJNA827465;Yang2025_Silkie_Wenchang	CRA004775	0.796883862
ENSGALG00010005311	YME1L1	2	-0.291737672	0.054721264	-5.331340175	9.74906E-08	0	0.196936713	0	1	CRA004775;Yang2025_Silkie_Wenchang	PRJNA827465	3.48334E-05
ENSGALG00010005311	YME1L1	2	-0.005785002	0.037368713	-0.154808699	0.876972133	0	0.289263078	0	1	CRA004775;PRJNA827465	Yang2025_Silkie_Wenchang	0.970493387
ENSGALG00010005313	MPHOSPH8	2	0.441214948	0.505884913	0.872164668	0.383118551	0.478006758	14.31963657	93.01658254	0.5	PRJNA827465;Yang2025_Silkie_Wenchang	CRA004775	0.825405345
ENSGALG00010005313	MPHOSPH8	2	0.56271579	0.409437627	1.37436267	0.169329119	0.273522007	5.42002991	81.54991732	1	CRA004775;Yang2025_Silkie_Wenchang	PRJNA827465	0.670729184
ENSGALG00010005313	MPHOSPH8	2	-0.022394478	0.0565203	-0.39622008	0.691942679	0	0.645484217	0	0.5	CRA004775;PRJNA827465	Yang2025_Silkie_Wenchang	0.920348122
ENSGALG00010005314	IL20RA	2	-0.125313852	0.791328619	-0.158358802	0.874174075	1.068698964	6.803593298	85.30188452	0.5	PRJNA827465;Yang2025_Silkie_Wenchang	CRA004775	0.970092404
ENSGALG00010005314	IL20RA	2	-0.786971146	0.340072128	-2.314130095	0.020660587	0	0.293789355	0	1	CRA004775;Yang2025_Silkie_Wenchang	PRJNA827465	0.242632772
ENSGALG00010005314	IL20RA	2	0.1823689	0.579631894	0.314628823	0.753043493	0.376956263	2.214708139	54.84732357	0.5	CRA004775;PRJNA827465	Yang2025_Silkie_Wenchang	0.938087082
ENSGALG00010005315	ENSGALG00010005315	2	-0.168211603	0.215666427	-0.779961931	0.435413283	0	0.842546878	0	0.5	PRJNA827465;Yang2025_Silkie_Wenchang	CRA004775	0.842762754
ENSGALG00010005315	ENSGALG00010005315	2	0.20810244	0.320108384	0.650099935	0.515627671	0	0.005173049	0	1	CRA004775;Yang2025_Silkie_Wenchang	PRJNA827465	0.867300757
ENSGALG00010005315	ENSGALG00010005315	2	-0.165917151	0.218053505	-0.760901096	0.446716144	0	0.945298988	0	0.5	CRA004775;PRJNA827465	Yang2025_Silkie_Wenchang	0.84648396
ENSGALG00010005316	PARP4	2	-0.097935434	0.055307893	-1.770731588	0.076605347	0	0.580496881	0	1	PRJNA827465;Yang2025_Silkie_Wenchang	CRA004775	0.47762877
ENSGALG00010005316	PARP4	2	0.033155723	0.117482365	0.282218726	0.777775801	0.008218129	1.283892354	22.11185018	0.5	CRA004775;Yang2025_Silkie_Wenchang	PRJNA827465	0.944388838
ENSGALG00010005316	PARP4	2	-0.003123931	0.172151887	-0.018146363	0.985522092	0.040276867	2.543523753	60.68446388	0.5	CRA004775;PRJNA827465	Yang2025_Silkie_Wenchang	0.995939127
ENSGALG00010005317	CHCHD3	2	-0.096394393	0.053408787	-1.804841459	0.071099495	0	0.005086317	0	1	PRJNA827465;Yang2025_Silkie_Wenchang	CRA004775	0.460518035
ENSGALG00010005317	CHCHD3	2	0.031150164	0.174774475	0.178230627	0.858541858	0.022565354	1.539122023	35.0278935	0.5	CRA004775;Yang2025_Silkie_Wenchang	PRJNA827465	0.964420815
ENSGALG00010005317	CHCHD3	2	0.001236227	0.1552403	0.00796331	0.993646265	0.030191943	2.011686586	50.29046737	0.5	CRA004775;PRJNA827465	Yang2025_Silkie_Wenchang	0.997979742
ENSGALG00010005320	ENSGALG00010005320	2	1.125108162	0.372511011	3.020335315	0.00252495	0.054885555	1.221890963	18.15963695	1	PRJNA827465;Yang2025_Silkie_Wenchang	CRA004775	0.067541822
ENSGALG00010005320	ENSGALG00010005320	2	0.315395932	1.330894027	0.2369805	0.812671918	3.152576052	9.064858859	88.96838864	0.5	CRA004775;Yang2025_Silkie_Wenchang	PRJNA827465	0.95348724
ENSGALG00010005320	ENSGALG00010005320	2	-0.018637555	0.939242615	-0.019843174	0.984168477	1.471473677	5.832701103	82.85528467	0.5	CRA004775;PRJNA827465	Yang2025_Silkie_Wenchang	0.995669738
ENSGALG00010005321	HS6ST3	2	0.053742673	0.239496656	0.224398427	0.822447295	0	0.997240184	0	0.5	PRJNA827465;Yang2025_Silkie_Wenchang	CRA004775	0.955813183
ENSGALG00010005321	HS6ST3	2	0.427144087	0.367689403	1.161698116	0.245358111	0	0.066809543	0	1	CRA004775;Yang2025_Silkie_Wenchang	PRJNA827465	0.75661406
ENSGALG00010005321	HS6ST3	2	0.019844309	0.237638189	0.083506396	0.933448892	0	0.487966925	0	0.5	CRA004775;PRJNA827465	Yang2025_Silkie_Wenchang	0.984260547
ENSGALG00010005322	TFB1M	2	0.254200057	0.174513546	1.456620779	0.145221089	0.046378283	4.191530603	76.14236672	1	PRJNA827465;Yang2025_Silkie_Wenchang	CRA004775	0.633476631
ENSGALG00010005322	TFB1M	2	0.225162875	0.256916652	0.876404365	0.380810246	0.092909192	3.00319634	66.70214376	0.5	CRA004775;Yang2025_Silkie_Wenchang	PRJNA827465	0.824392515
ENSGALG00010005322	TFB1M	2	0.051724777	0.110667178	0.467390407	0.640220584	0	0.344642925	0	0.5	CRA004775;PRJNA827465	Yang2025_Silkie_Wenchang	0.906137307
ENSGALG00010005323	DNAJB14	2	0.099114638	0.16052318	0.617447513	0.536939591	0.04047005	4.207268943	76.23161216	0.5	PRJNA827465;Yang2025_Silkie_Wenchang	CRA004775	0.874713452
ENSGALG00010005323	DNAJB14	2	0.198412998	0.121254317	1.636337592	0.101768953	0.001375157	1.045198011	4.324349097	1	CRA004775;Yang2025_Silkie_Wenchang	PRJNA827465	0.543700483
ENSGALG00010005323	DNAJB14	2	-0.029839132	0.056967101	-0.52379586	0.600420533	0	0.127079957	0	0.5	CRA004775;PRJNA827465	Yang2025_Silkie_Wenchang	0.895230519
ENSGALG00010005326	MASTL	2	-0.232711665	0.077438698	-3.005108168	0.002654864	0	0.533639622	0	1	PRJNA827465;Yang2025_Silkie_Wenchang	CRA004775	0.069655625
ENSGALG00010005326	MASTL	2	-0.399038893	0.234185172	-1.70394602	0.088391172	0.044632366	1.209945967	17.3516812	1	CRA004775;Yang2025_Silkie_Wenchang	PRJNA827465	0.510512601
ENSGALG00010005326	MASTL	2	-0.372824261	0.344185775	-1.083206477	0.278716786	0.141983074	1.681882278	40.54280654	1	CRA004775;PRJNA827465	Yang2025_Silkie_Wenchang	0.776992195
ENSGALG00010005329	VIPAS39	2	0.17903432	0.210183202	0.851801275	0.394324399	0.079548681	9.756759947	89.75069587	0.5	PRJNA827465;Yang2025_Silkie_Wenchang	CRA004775	0.830486298
ENSGALG00010005329	VIPAS39	2	0.053778397	0.354548752	0.151681246	0.879438349	0.232460725	13.20397295	92.4265219	0.5	CRA004775;Yang2025_Silkie_Wenchang	PRJNA827465	0.971291607
ENSGALG00010005329	VIPAS39	2	-0.12920235	0.13989545	-0.923563628	0.355713558	0.027456503	2.950685175	66.1095664	1	CRA004775;PRJNA827465	Yang2025_Silkie_Wenchang	0.812301302
ENSGALG00010005330	ENSGALG00010005330	2	0.039557766	0.322511012	0.12265555	0.902379864	0	0.090681464	0	0.5	PRJNA827465;Yang2025_Silkie_Wenchang	CRA004775	0.976923607
ENSGALG00010005330	ENSGALG00010005330	2	0.001577166	0.406536307	0.003879521	0.996904598	0	0.22854062	0	0.5	CRA004775;Yang2025_Silkie_Wenchang	PRJNA827465	0.999065103
ENSGALG00010005330	ENSGALG00010005330	2	-0.0900901	0.339101795	-0.265672732	0.790491266	0	0.066139593	0	1	CRA004775;PRJNA827465	Yang2025_Silkie_Wenchang	0.947530415
ENSGALG00010005332	ENSGALG00010005332	2	-0.361648896	0.613456068	-0.589526968	0.555507827	0.576268371	4.266510985	76.56164478	0.5	PRJNA827465;Yang2025_Silkie_Wenchang	CRA004775	0.880255849
ENSGALG00010005332	ENSGALG00010005332	2	-0.46883633	0.55890074	-0.838854375	0.401551034	0.391474059	2.641389209	62.14113404	0.5	CRA004775;Yang2025_Silkie_Wenchang	PRJNA827465	0.832623399
ENSGALG00010005332	ENSGALG00010005332	2	0.209662734	0.329670525	0.635976583	0.524791685	0	0.023331141	0	1	CRA004775;PRJNA827465	Yang2025_Silkie_Wenchang	0.869876873
ENSGALG00010005333	PLXNA4	2	-0.23551489	0.117268513	-2.008338664	0.044607316	0	0.311150354	0	1	PRJNA827465;Yang2025_Silkie_Wenchang	CRA004775	0.364257243
ENSGALG00010005333	PLXNA4	2	-0.327107299	0.158310189	-2.06624287	0.038805553	0	0.563106857	0	1	CRA004775;Yang2025_Silkie_Wenchang	PRJNA827465	0.339979907
ENSGALG00010005333	PLXNA4	2	-0.20596506	0.163995003	-1.255922775	0.20914397	0	0.877006005	0	1	CRA004775;PRJNA827465	Yang2025_Silkie_Wenchang	0.721051356
ENSGALG00010005334	NME7	2	0.000211871	0.210886928	0.001004668	0.999198391	0.064594934	3.391712822	70.51637174	0.5	PRJNA827465;Yang2025_Silkie_Wenchang	CRA004775	0.999889665
ENSGALG00010005334	NME7	2	-0.025795856	0.252785333	-0.10204649	0.918719774	0.071670915	2.23687494	55.29477388	0.5	CRA004775;Yang2025_Silkie_Wenchang	PRJNA827465	0.981033557
ENSGALG00010005334	NME7	2	0.188710072	0.099359855	1.89925873	0.057530466	0	0.077703723	0	1	CRA004775;PRJNA827465	Yang2025_Silkie_Wenchang	0.415417952
ENSGALG00010005335	AHSA1	2	0.10416684	0.122736249	0.848704767	0.396045593	0.018063338	2.23230509	55.20325584	1	PRJNA827465;Yang2025_Silkie_Wenchang	CRA004775	0.831365249
ENSGALG00010005335	AHSA1	2	0.19831032	0.132669856	1.49476547	0.134975661	0	0.776655058	0	1	CRA004775;Yang2025_Silkie_Wenchang	PRJNA827465	0.61578043
ENSGALG00010005335	AHSA1	2	0.011523272	0.070974231	0.162358529	0.871023526	0	0.001346001	0	1	CRA004775;PRJNA827465	Yang2025_Silkie_Wenchang	0.969230271
ENSGALG00010005336	PEX7	2	-0.202245154	0.346912193	-0.582986583	0.559902332	0.22567804	15.91822913	93.71789417	0.5	PRJNA827465;Yang2025_Silkie_Wenchang	CRA004775	0.881203589
ENSGALG00010005336	PEX7	2	-0.434920758	0.166946019	-2.605158002	0.00918319	0.02242176	1.573842962	36.46125922	1	CRA004775;Yang2025_Silkie_Wenchang	PRJNA827465	0.150281327
ENSGALG00010005336	PEX7	2	0.03545621	0.156829022	0.226081944	0.821137688	0.024985483	1.736245052	42.4044435	0.5	CRA004775;PRJNA827465	Yang2025_Silkie_Wenchang	0.955598716
ENSGALG00010005337	DCTN1	2	0.969549482	0.817695946	1.185709048	0.23573722	0.849297857	2.723032842	63.27624168	1	PRJNA827465;Yang2025_Silkie_Wenchang	CRA004775	0.748669015
ENSGALG00010005337	DCTN1	2	0.614595072	1.099676983	0.558886911	0.576238903	2.132845957	7.802142856	87.18300833	0.5	CRA004775;Yang2025_Silkie_Wenchang	PRJNA827465	0.887048016
ENSGALG00010005337	DCTN1	2	-0.308350428	0.216072948	-1.427066327	0.153560777	0	0.708909066	0	0.5	CRA004775;PRJNA827465	Yang2025_Silkie_Wenchang	0.645911732
ENSGALG00010005338	PCGF1	2	0.212284881	0.180177929	1.17819581	0.238718553	0.044389058	2.892945565	65.43315532	1	PRJNA827465;Yang2025_Silkie_Wenchang	CRA004775	0.75079325
ENSGALG00010005338	PCGF1	2	0.026360418	0.427436491	0.061670959	0.950824868	0.311864534	6.742195765	85.16803672	0.5	CRA004775;Yang2025_Silkie_Wenchang	PRJNA827465	0.987351575
ENSGALG00010005338	PCGF1	2	-0.114139998	0.235347623	-0.484984708	0.627687193	0.078535142	2.950735614	66.11014571	0.5	CRA004775;PRJNA827465	Yang2025_Silkie_Wenchang	0.901856848
ENSGALG00010005341	UGGT2	2	-0.185079466	0.091336661	-2.026343686	0.042729577	0.005285016	1.439517835	30.53229521	1	PRJNA827465;Yang2025_Silkie_Wenchang	CRA004775	0.357546035
ENSGALG00010005341	UGGT2	2	0.142248855	0.455472464	0.312310549	0.754804524	0.384016259	13.18067656	92.41313604	0.5	CRA004775;Yang2025_Silkie_Wenchang	PRJNA827465	0.93838723
ENSGALG00010005341	UGGT2	2	0.22618686	0.361791361	0.625185908	0.53184905	0.234992844	9.321867228	89.27253547	0.5	CRA004775;PRJNA827465	Yang2025_Silkie_Wenchang	0.873042777
ENSGALG00010005342	MAP3K5	2	0.03346336	0.068746679	0.486763296	0.626426084	0	0.480161773	0	0.5	PRJNA827465;Yang2025_Silkie_Wenchang	CRA004775	0.901504244
ENSGALG00010005342	MAP3K5	2	0.11059611	0.088747661	1.246186198	0.212696045	0	0.760974101	0	1	CRA004775;Yang2025_Silkie_Wenchang	PRJNA827465	0.725541376
ENSGALG00010005342	MAP3K5	2	0.0702581	0.141677732	0.495900794	0.619964406	0.017698077	1.531835398	34.71883459	0.5	CRA004775;PRJNA827465	Yang2025_Silkie_Wenchang	0.899395593
ENSGALG00010005346	FBXO30	2	-0.287592045	0.282859396	-1.016731452	0.309281199	0.143518407	9.360870279	89.31723258	1	PRJNA827465;Yang2025_Silkie_Wenchang	CRA004775	0.794043289
ENSGALG00010005346	FBXO30	2	-0.197698746	0.505931288	-0.390762047	0.695973131	0.381794509	3.321089845	69.88940238	0.5	CRA004775;Yang2025_Silkie_Wenchang	PRJNA827465	0.921038437
ENSGALG00010005346	FBXO30	2	-0.011785498	0.081713286	-0.144229881	0.885318946	0	0.742236	0	0.5	CRA004775;PRJNA827465	Yang2025_Silkie_Wenchang	0.972280795
ENSGALG00010005347	DOK1	2	0.068455594	0.147665381	0.46358594	0.64294443	0.023878076	1.999033094	49.97581567	0.5	PRJNA827465;Yang2025_Silkie_Wenchang	CRA004775	0.906634523
ENSGALG00010005347	DOK1	2	-0.145781464	0.172182127	-0.846670129	0.397179013	0	0.023479741	0	1	CRA004775;Yang2025_Silkie_Wenchang	PRJNA827465	0.831438925
ENSGALG00010005347	DOK1	2	0.152104076	0.095521396	1.592356081	0.111304719	0	0.962044035	0	0.5	CRA004775;PRJNA827465	Yang2025_Silkie_Wenchang	0.564465723
ENSGALG00010005349	ACBD5	2	0.067703269	0.192373225	0.351937067	0.72488546	0.063714857	7.133879958	85.98238258	0.5	PRJNA827465;Yang2025_Silkie_Wenchang	CRA004775	0.930564843
ENSGALG00010005349	ACBD5	2	0.284337323	0.103095321	2.75800414	0.005815546	0	0.133634714	0	1	CRA004775;Yang2025_Silkie_Wenchang	PRJNA827465	0.114916244
ENSGALG00010005349	ACBD5	2	0.069945191	0.235722737	0.296726535	0.766675297	0.083111089	3.461069517	71.10719692	0.5	CRA004775;PRJNA827465	Yang2025_Silkie_Wenchang	0.941271762
ENSGALG00010005350	ARHGAP20	2	-0.553523516	0.157397193	-3.516730542	0.000436897	0	0.429631613	0	1	PRJNA827465;Yang2025_Silkie_Wenchang	CRA004775	0.02125169
ENSGALG00010005350	ARHGAP20	2	-0.590372667	0.244924449	-2.410427661	0.015933831	0	0.866880498	0	1	CRA004775;Yang2025_Silkie_Wenchang	PRJNA827465	0.208471156
ENSGALG00010005350	ARHGAP20	2	-0.44182896	0.183287349	-2.410580781	0.015927144	0	0.378753005	0	1	CRA004775;PRJNA827465	Yang2025_Silkie_Wenchang	0.208471156

ENSGALG00010005351	DTNA	2	-0.137076703	0.31168104	-0.439798018	0.660083404	0.17042481	7.905444634	87.35049012	0.5	PRJNA827465;Yang2025_Silkie_Wenchang	CRA004775	0.911825461
ENSGALG00010005351	DTNA	2	0.174452581	0.177873145	0.980769644	0.326706351	0	0.077982549	0	1	CRA004775;Yang2025_Silkie_Wenchang	PRJNA827465	0.800028239
ENSGALG00010005351	DTNA	2	-0.388649855	0.134460721	-2.890434116	0.003847102	0.007652324	1.069254012	6.476853129	0.5	CRA004775;PRJNA827465	Yang2025_Silkie_Wenchang	0.089058443
ENSGALG00010005353	ENSGALG00010005353	2	-0.487369758	0.469155453	-1.038823602	0.298886782	0.430535787	45.0197098	97.77875068	1	PRJNA827465;Yang2025_Silkie_Wenchang	CRA004775	0.78974322
ENSGALG00010005353	ENSGALG00010005353	2	-0.489241465	0.50232754	-0.973949118	0.330081804	0.460982145	11.17071858	91.04802442	0.5	CRA004775;Yang2025_Silkie_Wenchang	PRJNA827465	0.801237299
ENSGALG00010005353	ENSGALG00010005353	2	-0.0226348	0.054194402	-0.41765938	0.676196175	0	0.059658272	0	0.5	CRA004775;PRJNA827465	Yang2025_Silkie_Wenchang	0.916685233
ENSGALG00010005354	M1AP	2	0.290963123	0.16644864	1.748065485	0.080452689	0	0.582895419	0	1	PRJNA827465;Yang2025_Silkie_Wenchang	CRA004775	0.488261768
ENSGALG00010005354	M1AP	2	0.555031225	0.2067703	2.684288922	0.007268426	0	0.486889374	0	1	CRA004775;Yang2025_Silkie_Wenchang	PRJNA827465	0.131077397
ENSGALG00010005354	M1AP	2	0.402801624	0.271784514	1.482062457	0.138323677	0.074295074	1.9174551	47.84754021	1	CRA004775;PRJNA827465	Yang2025_Silkie_Wenchang	0.621886923
ENSGALG00010005355	FDX1	2	-0.0861933	0.065927	-1.30740517	0.191075142	0	0.432459567	0	1	PRJNA827465;Yang2025_Silkie_Wenchang	CRA004775	0.700443419
ENSGALG00010005355	FDX1	2	-0.024883787	0.144065012	-0.172726096	0.862866731	0.023029905	2.057842748	51.40542196	0.5	CRA004775;Yang2025_Silkie_Wenchang	PRJNA827465	0.966099097
ENSGALG00010005355	FDX1	2	-0.00199383	0.086652944	-0.023009379	0.981642791	0.001599035	1.076442521	7.101402925	0.5	CRA004775;PRJNA827465	Yang2025_Silkie_Wenchang	0.99503081
ENSGALG00010005356	DNAJC3	2	0.042503932	0.035078383	1.211684475	0.225633184	0	0.454227278	0	1	PRJNA827465;Yang2025_Silkie_Wenchang	CRA004775	0.73884362
ENSGALG00010005356	DNAJC3	2	0.078580576	0.071512627	1.098834973	0.271840054	0	0.127008183	0	1	CRA004775;Yang2025_Silkie_Wenchang	PRJNA827465	0.773184343
ENSGALG00010005356	DNAJC3	2	0.029693896	0.038670568	0.767868116	0.442565542	0	0.007306973	0	1	CRA004775;PRJNA827465	Yang2025_Silkie_Wenchang	0.845566744
ENSGALG00010005358	SHPRH	2	-0.054008901	0.060906952	-0.886744434	0.37521651	0	0.027135372	0	1	PRJNA827465;Yang2025_Silkie_Wenchang	CRA004775	0.822941424
ENSGALG00010005358	SHPRH	2	-0.113597157	0.132623947	-0.856535793	0.391701483	0	0.179578224	0	1	CRA004775;Yang2025_Silkie_Wenchang	PRJNA827465	0.829251057
ENSGALG00010005358	SHPRH	2	-0.059937971	0.063830962	-0.939010924	0.347725136	0	0.353839642	0	1	CRA004775;PRJNA827465	Yang2025_Silkie_Wenchang	0.808847403
ENSGALG00010005359	MEIS2	2	-0.074032368	0.181040952	-0.408926086	0.682593905	0.040325396	2.500118441	60.00189496	0.5	PRJNA827465;Yang2025_Silkie_Wenchang	CRA004775	0.918716001
ENSGALG00010005359	MEIS2	2	0.344325341	0.259200988	1.328410603	0.184042498	0.072306788	2.044973798	51.09961795	1	CRA004775;Yang2025_Silkie_Wenchang	PRJNA827465	0.692524875
ENSGALG00010005359	MEIS2	2	0.173638264	0.446828383	0.388601689	0.697570818	0.345098034	6.884255402	85.4741008	0.5	CRA004775;PRJNA827465	Yang2025_Silkie_Wenchang	0.921350467
ENSGALG00010005360	CAMK1D	2	0.008050499	0.1186182	0.067869003	0.945889914	0	0.005105583	0	0.5	PRJNA827465;Yang2025_Silkie_Wenchang	CRA004775	0.986321197
ENSGALG00010005360	CAMK1D	2	-0.192324866	0.269525559	-0.713568193	0.475494232	0.078296468	1.922749163	47.99113586	1	CRA004775;Yang2025_Silkie_Wenchang	PRJNA827465	0.854497479
ENSGALG00010005360	CAMK1D	2	-0.182283827	0.278001976	-0.65569256	0.512021957	0.090671748	2.101161133	52.40726738	0.5	CRA004775;PRJNA827465	Yang2025_Silkie_Wenchang	0.865694231
ENSGALG00010005361	ENSGALG00010005361	2	0.848824861	0.342779013	2.476303472	0.013275069	0	0.207280613	0	1	PRJNA827465;Yang2025_Silkie_Wenchang	CRA004775	0.188244411
ENSGALG00010005361	ENSGALG00010005361	2	0.147749815	0.921634263	0.16031285	0.872634637	1.350424984	4.800042513	79.16685118	0.5	CRA004775;Yang2025_Silkie_Wenchang	PRJNA827465	0.969629321
ENSGALG00010005361	ENSGALG00010005361	2	0.010578975	0.763309083	0.01385936	0.988942185	0.832907435	3.41717137	70.73603013	0.5	CRA004775;PRJNA827465	Yang2025_Silkie_Wenchang	0.996891127
ENSGALG00010005362	ATP1B1	2	-0.188587999	0.080852454	-2.33249566	0.019674631	0.007332221	2.27575446	56.05852839	1	PRJNA827465;Yang2025_Silkie_Wenchang	CRA004775	0.235251604
ENSGALG00010005362	ATP1B1	2	0.082038079	0.253009605	0.325608505	0.744720577	0.093521862	3.035962381	67.06151545	0.5	CRA004775;Yang2025_Silkie_Wenchang	PRJNA827465	0.935893304
ENSGALG00010005362	ATP1B1	2	0.018853105	0.339975503	0.055454305	0.955776534	0.192283275	5.210217912	80.8069448	0.5	CRA004775;PRJNA827465	Yang2025_Silkie_Wenchang	0.988393546
ENSGALG00010005363	RDX	2	-0.078210248	0.045419596	-1.721949437	0.085078678	0	0.076156243	0	1	PRJNA827465;Yang2025_Silkie_Wenchang	CRA004775	0.501658247
ENSGALG00010005363	RDX	2	0.136915213	0.195244848	0.701248789	0.483147767	0.052351997	3.146542445	68.21908436	0.5	CRA004775;Yang2025_Silkie_Wenchang	PRJNA827465	0.85691916
ENSGALG00010005363	RDX	2	0.100201492	0.213148203	0.470102449	0.638281825	0.076555606	5.652507673	82.30873697	0.5	CRA004775;PRJNA827465	Yang2025_Silkie_Wenchang	0.905547795
ENSGALG00010005364	MAP7	2	1.13451251	0.579125021	1.959011386	0.050111453	0.507639205	4.109873646	75.66835173	1	PRJNA827465;Yang2025_Silkie_Wenchang	CRA004775	0.387474022
ENSGALG00010005364	MAP7	2	1.714686616	0.370350126	4.629906935	3.6583E-06	0	0.001099671	0	1	CRA004775;Yang2025_Silkie_Wenchang	PRJNA827465	0.00065654
ENSGALG00010005364	MAP7	2	0.891440731	0.512573471	1.739143729	0.082008869	0.215986315	1.514479357	33.97070779	1	CRA004775;PRJNA827465	Yang2025_Silkie_Wenchang	0.49359745
ENSGALG00010005366	AUP1	2	-0.057093503	0.162184963	-0.352027105	0.724817935	0.032129053	1.839850002	45.64774308	0.5	PRJNA827465;Yang2025_Silkie_Wenchang	CRA004775	0.930538946
ENSGALG00010005366	AUP1	2	-0.458185119	0.193257808	-2.370849205	0.01774727	0	0.391752816	0	1	CRA004775;Yang2025_Silkie_Wenchang	PRJNA827465	0.2222112
ENSGALG00010005366	AUP1	2	-0.213270471	0.302861843	-0.704184022	0.48131818	0.151089852	4.840582743	79.34133031	0.5	CRA004775;PRJNA827465	Yang2025_Silkie_Wenchang	0.85682839
ENSGALG00010005367	H2AZ1	2	-0.102331992	0.051581134	-1.983903512	0.047266597	0.000145823	1.006562374	0.65195901	1	PRJNA827465;Yang2025_Silkie_Wenchang	CRA004775	0.375333084
ENSGALG00010005367	H2AZ1	2	-0.124864511	0.194425445	-0.642223093	0.520728341	0.026868087	1.542497522	35.17007413	0.5	CRA004775;Yang2025_Silkie_Wenchang	PRJNA827465	0.869353339
ENSGALG00010005367	H2AZ1	2	-0.080841811	0.050736153	-1.593376844	0.111075677	0	0.53626722	0	0.5	CRA004775;PRJNA827465	Yang2025_Silkie_Wenchang	0.56392513
ENSGALG00010005368	CCDC3	2	-0.118251397	0.118900398	-0.994541638	0.319959245	0	0.247232779	0	1	PRJNA827465;Yang2025_Silkie_Wenchang	CRA004775	0.798348216
ENSGALG00010005368	CCDC3	2	-0.124325156	0.184217472	-0.674882545	0.499750391	0	0.727998263	0	0.5	CRA004775;Yang2025_Silkie_Wenchang	PRJNA827465	0.862078151
ENSGALG00010005368	CCDC3	2	-0.046678297	0.137765453	-0.338824402	0.734742018	0	0.378010491	0	0.5	CRA004775;PRJNA827465	Yang2025_Silkie_Wenchang	0.933533809
ENSGALG00010005369	DPT	2	-0.831412556	0.443408691	-1.875047947	0.060786128	0.369003802	16.2010869	93.82757462	1	PRJNA827465;Yang2025_Silkie_Wenchang	CRA004775	0.42696726
ENSGALG00010005369	DPT	2	-0.668224935	0.708905271	-0.942615725	0.345857725	0.844568844	5.492529304	81.79345171	0.5	CRA004775;Yang2025_Silkie_Wenchang	PRJNA827465	0.807492544
ENSGALG00010005369	DPT	2	-0.341315771	0.163688185	-2.085158263	0.037054937	0	0.785431703	0	0.5	CRA004775;PRJNA827465	Yang2025_Silkie_Wenchang	0.332064293
ENSGALG00010005372	CDIN1	2	-0.298586728	0.143236036	-2.084578262	0.037107599	0.028681265	3.291720188	69.62074712	1	PRJNA827465;Yang2025_Silkie_Wenchang	CRA004775	0.332143015
ENSGALG00010005372	CDIN1	2	-0.209011275	0.279760941	-0.747106707	0.454999152	0.121785392	4.201850741	76.2009633	0.5	CRA004775;Yang2025_Silkie_Wenchang	PRJNA827465	0.849072665
ENSGALG00010005372	CDIN1	2	-0.116631465	0.104685094	-1.114117209	0.265228907	0.003291073	1.09285762	8.496771953	0.5	CRA004775;PRJNA827465	Yang2025_Silkie_Wenchang	0.769274038
ENSGALG00010005376	HTRA2	2	-0.015857127	0.056769874	-0.279322911	0.779997025	0	0.405578407	0	1	PRJNA827465;Yang2025_Silkie_Wenchang	CRA004775	0.945041817
ENSGALG00010005376	HTRA2	2	-0.221337851	0.212428931	-1.041938355	0.29740026	0	0.022315487	0	1	CRA004775;Yang2025_Silkie_Wenchang	PRJNA827465	0.788562929
ENSGALG00010005376	HTRA2	2	-0.016380281	0.057010621	-0.287319816	0.773867451	0	0.584689757	0	1	CRA004775;PRJNA827465	Yang2025_Silkie_Wenchang	0.943288868
ENSGALG00010005377	EMCN	2	-0.081922277	0.084025162	-0.97497316	0.329573574	0.008376621	2.406348249	58.44325524	1	PRJNA827465;Yang2025_Silkie_Wenchang	CRA004775	0.801177491
ENSGALG00010005377	EMCN	2	-0.159270456	0.085328122	-1.866564647	0.061962408	0	0.737887123	0	0.5	CRA004775;Yang2025_Silkie_Wenchang	PRJNA827465	0.429507678
ENSGALG00010005377	EMCN	2	-0.002880863	0.063893293	-0.045088659	0.964036641	0	0.170871652	0	0.5	CRA004775;PRJNA827465	Yang2025_Silkie_Wenchang	0.990214803
ENSGALG00010005379	DZIP1	2	0.249421097	0.177258312	1.407101232	0.159396187	0.049234043	4.400180502	77.27165958	1	PRJNA827465;Yang2025_Silkie_Wenchang	CRA004775	0.654972506
ENSGALG00010005379	DZIP1	2	0.239753815	0.283446918	0.845850845	0.397635957	0.102103994	2.380837941	57.99798119	0.5	CRA004775;Yang2025_Silkie_Wenchang	PRJNA827465	0.831689959
ENSGALG00010005379	DZIP1	2	0.079341992	0.081557045	0.972840446	0.330632607	0	0.420295856	0	0.5	CRA004775;PRJNA827465	Yang2025_Silkie_Wenchang	0.801575303
ENSGALG00010005381	FGF9	2	0.875853115	0.338305952	2.588937941	0.009627244	0.172298984	3.920384059	74.49929501	1	PRJNA827465;Yang2025_Silkie_Wenchang	CRA004775	0.154693206
ENSGALG00010005381	FGF9	2	0.485001196	0.941944746	0.514893468	0.606627466	1.390670869	4.014256214	75.0887849	0.5	CRA004775;Yang2025_Silkie_Wenchang	PRJNA827465	0.896799223
ENSGALG00010005381	FGF9	2	0.271394389	0.531637725	0.510487454	0.609710002	0.336465104	1.768377172	43.45097778	0.5	CRA004775;PRJNA827465	Yang2025_Silkie_Wenchang	0.897238578
ENSGALG00010005382	RAB32	2	-0.185634865	0.276457296	-0.671477541	0.501916362	0.134800268	8.310832897	87.96751165	0.5	PRJNA827465;Yang2025_Silkie_Wenchang	CRA004775	0.862795569
ENSGALG00010005382	RAB32	2	-0.432348035	0.147515358	-2.93086796	0.003380164	0	0.445794423	0	1	CRA004775;Yang2025_Silkie_Wenchang	PRJNA827465	0.081503432
ENSGALG00010005382	RAB32	2	0.055426661	0.09986701	0.555004714	0.578891436	0	0.58665176	0	0.5	CRA004775;PRJNA827465	Yang2025_Silkie_Wenchang	0.887888423
ENSGALG00010005383	OPTN	2	0.143278011	0.178828417	0.801203822	0.423013659	0.056748957	8.512923891	88.2531547	0.5	PRJNA827465;Yang2025_Silkie_Wenchang	CRA004775	0.840021515
ENSGALG00010005383	OPTN	2	0.311136157	0.106441648	2.923067824	0.00346601	0	0.653818604	0	1	CRA004775;Yang2025_Silkie_Wenchang	PRJNA827465	0.083153429
ENSGALG00010005383	OPTN	2	-0.022327127	0.05233597	-0.421776102	0.673188451	0	0.006813042	0	0.5	CRA004775;PRJNA827465	Yang2025_Silkie_Wenchang	0.915210237
ENSGALG00010005384	MAPRE2	2	0.046790713	0.058508647	0.799723034	0.423871284	0	0.736098416	0	1	PRJNA827465;Yang2025_Silkie_Wenchang	CRA004775	0.84030043
ENSGALG00010005384	MAPRE2	2	0.157698461	0.170581546	0.924475511	0.355238796	0	0.26442369	0	1	CRA004775;Yang2025_Silkie_Wenchang	PRJNA827465	0.812301302
ENSGALG00010005384	MAPRE2	2	0.034355802	0.059040734	0.58183789	0.56065588	0	0.006643441	0	1	CRA004775;PRJNA827465	Yang2025_Silkie_Wenchang	0.88830295
ENSGALG00010005385	PDSS1	2	-0.062635329	0.067101964	-0.933435108	0.350595361	0	0.531687551	0	1	PRJNA827465;Yang2025_Silkie_Wenchang	CRA004775	0.810111037
ENSGALG00010005385	PDSS1	2	-0.133753086	0.099167784	-1.348755416	0.177415539	0	0.046962327	0	1	CRA004775;Yang2025_Silkie_Wenchang	PRJNA827465	0.683556803
ENSGALG00010005385	PDSS1	2	-0.040714755	0.080947483	-0.502977412	0.614980153	0	0.366004929	0	1	CRA004775;PRJNA827465	Yang2025_Silkie_Wenchang	0.896781283
ENSGALG00010005386	CPOX	2	0.187387699	0.105688129	1.773025041	0.076224558	0.012421513	1.984949042	49.62087295	1	PRJNA827465;Yang2025_Silkie_Wenchang	CRA004775	0.476484008
ENSGALG00010005386	CPOX	2	-0.005141584	0.135112096	-0.038054208	0.969644461	0	0.752938498	0	0.5	CRA004775;Yang2025_Silkie_Wenchang	PRJNA827465	0.991572914
ENSGALG00010005386	CPOX	2	0.096173543	0.262465297	0.366423845	0.71404883	0.092175086	2.208789879	54.72634089	0.5	CRA004775;PRJNA827465	Yang2025_Silkie_Wenchang	0.926427265
ENSGALG00010005388	ENSGALG00010005388	2	-0.390456949	0.292182442	-1.336346378	0.181436084	0	0.155329599	0	1	PRJNA827465;Yang2025_Silkie_Wenchang	CRA004775	0.689004635
ENSGALG00010005388	ENSGALG00010005388	2	-0.252832659	0.278574078	-0.907599493	0.364117646	0	0.768944872	0	1	CRA004775;Yang2025_Silkie_Wenchang	PRJNA827465	0.815579273
ENSGALG00010005388	ENSGALG00010005388	2	-0.118048388	0.296524823	-0.39810626	0.69055186	0	0.184584993	0	1	CRA004775;PRJNA827465	Yang2025_Silkie_Wenchang	0.919979266
ENSGALG00010005389	MCM10	2	0.056109949	0.096942377	0.578796922	0.562726208	0	0.185955507	0	0.5	PRJNA827465;Yang2025_Silkie_Wenchang	CRA004775	0.882059323
ENSGALG00010005389	MCM10	2	0.100929266	0.221221014	0.456232747	0.648219384	0.028835076	1.230191885	18.71186827	0.5	CRA004775;Yang2025_Silkie_Wenchang	PRJNA827465	0.90762535
ENSGALG00010005389	MCM10	2	0.10950255	0.111861468	0.978921251	0.327623388	0	0.926388952	0	1	CRA004775;PRJNA827465	Yang2025_Silkie_Wenchang	0.80013317
ENSGALG00010005392	ENSGALG00010005392	2	-0.108340416	0.09740839	-1.1122288	0.266039788	0.003883726	1.251498484	20.09578817	1	PRJNA827465;Yang2025_Silkie_Wenchang	CRA004775	0.770081145
ENSGALG00010005392	ENSGALG00010005392	2	-0.035618632	0.120575825	-0.295404425	0.767684953	0	0.429757693	0	0.5	CRA004775;Yang2025_Silkie_Wenchang	PRJNA827465	0.941444269
ENSGALG00010005392	ENSGALG00010005392	2	-0.193901623	0.104708987	-1.851814519	0.064052462	0	0.000120319	0	1	CRA004775;PRJNA827465	Yang2025_Silkie_Wenchang	0.436074984
ENSGALG00010005394	PHYH	2	-0.127037803	0.078806128	-1.612029504	0.10695552	0	0.83714497	0	1	PRJNA827465;Yang2025_Silkie_Wenchang	CRA004775	0.553983328
ENSGALG00010005394	PHYH	2	-0.222074944	0.131468425	-1.689188432	0.091183325	0	0.049092351	0	1	CRA004775;Yang2025_Silkie_Wenchang	PRJNA827465	0.517862098
ENSGALG00010005394	PHYH	2	-0.081284206	0.092734528	-0.876525796	0.380744259	0	0.024477484	0	1	CRA004775;PRJNA827465	Yang2025_Silkie_Wenchang	0.824392515
ENSGALG00010005396	MICU2	2	-0.105512508	0.060004987	-1.758395665	0.07868021	0	0.808171173	0	1	PRJNA827465;Yang2025_Silkie_Wenchang	CRA004775	0.482554155
ENSGALG00010005396	MICU2	2	0.146881133	0.209162886	0.702233249	0.482533713	0.060406868	2.948329223	66.08248522	0.5	CRA004775;Yang2025_Silkie_Wenchang	PRJNA827465	0.85691916
ENSGALG00010005396	MICU2	2	0.09108046	0.26966485	0.33775429	0.735548359	0.121286166	5.406810848	81.50480888	0.5	CRA004775;PRJNA827465	Yang2025_Silkie_Wenchang	0.933592889
ENSGALG00010005398	SEPHS1	2	-0.322766163	0.138759905	-2.36135669	0.018208206	0.026283282	3.023132666	66.91297384	1	PRJNA827465;Yang2025_Silkie_Wenchang	CRA004775	0.231297543
ENSGALG00010005398	SEPHS1	2	-0.127007349	0.381323695	-0.333069648	0.73908171	0.256819491	8.354471115	88.03036139	0.5	CRA004775;Yang2025_Silkie_Wenchang	PRJNA827465	0.934652713
ENSGALG00010005398	SEPHS1	2	-0.010705744	0.237498147	-0.045077167	0.964045801	0.087505195	3.974708334	74.84092125	0.5	CRA004775;PRJNA827465	Yang2025_Silkie_Wenchang	0.990214803
ENSGALG00010005399	NDNF	2	0.180633223	0.221114062	0.81692327	0.413972279	0	0.007024243	0	1	PRJNA827465;Yang2025_Silkie_Wenchang	CRA004775	0.836724075
ENSGALG00010005399	NDNF	2	0.333303321	0.277439034	1.201356985	0.229612754	0	0.674034865	0	1	CRA004775;Yang2025_Silkie_Wenchang	PRJNA827465	0.742632495
ENSGALG00010005399	NDNF	2	0.294638884	0.266522859	1.105491981	0.268946467	0	0.793502042	0	1	CRA004775;PRJNA827465	Yang2025_Silkie_Wenchang	0.771398294
ENSGALG00010005401	BCLAF1	2	0.209924426	0.24987129	0.840130238	0.400835368	0.111504769	8.567674593	88.32822151	0.5	PRJNA827465;Yang2025_Silkie_Wenchang	CRA004775	0.832353691
ENSGALG00010005401	BCLAF1	2	0.280827714	0.237133325	1.184260853	0.23630982	0.070993224	2.626824092	61.93121561	1	CRA004775;Yang2025_Silkie_Wenchang	PRJNA827465	0.748669015
ENSGALG00010005401	BCLAF1	2	-0.01454201	0.039399516	-0.369091077	0.712059839	0	0.009174348	0	0.5	CRA004775;PRJNA827465	Yang2025_Silkie_Wenchang	0.92597752
ENSGALG00010005402	ABCC4	2	0.193064219	0.110149343	1.752749621	0.079644994	0	0.025154236	0	1	PRJNA827465;Yang2025_Silkie_Wenchang	CRA004775	0.48542321
ENSGALG00010005402	ABCC4	2	0.212314594	0.195378575	1.086683094	0.277176888	0	0.010931295	0	1	CRA004775;Yang2025_Silkie_Wenchang	PRJNA827465	0.776194032
ENSGALG00010005402	ABCC4	2	0.183849765	0.116732672	1.57496408	0.115264736	0	6.26258E-06	0	1	CRA004775;PRJNA827465	Yang2025_Silkie_Wenchang	0.572216963
ENSGALG00010005403	ENSGALG00010005403	2	0.583105379	0.460701138	1.265691205	0.205623658	0.399643295	17.06238304	94.13915396	1	PRJNA827465;Yang2025_Silkie_Wenchang	CRA004775	0.71825498
ENSGALG00010005403	ENSGALG00010005403	2	0.287298625	0.81150776	0.354030657	0.723315908	1.208411839	11.57989873	91.36434589	0.5	CRA004775;Yang2025_Silkie_Wenchang	PRJNA827465	0.930032096
ENSGALG00010005403	ENSGALG00010005403	2	-0.099329049	0.328927695	-0.301978369	0.762668553	0.138023833	2.249314184	55.54200445	0.5	CRA004775;PRJNA827465	Yang2025_Silkie_Wenchang	0.940082621
ENSGALG00010005404	BEND7	2	0.008951478	0.151545461	0.05906794	0.952897994	0.032855619	3.1722692	68.4768241	0.5	PRJNA827465;Yang2025_Silkie_Wenchang	CRA004775	0.987758913
ENSGALG00010005404	BEND7	2	0.243541957	0.133263303	1.827524551	0.067620938	0	0.31250371	0	1	CRA004775;Yang2025_Silkie_Wenchang	PRJNA827465	0.448330927
ENSGALG00010005404	BEND7	2	0.064025864	0.229460794	0.279027467	0.780223746	0.079110503	3.386761243	70.47326551	0.5	CRA004775;PRJNA827465	Yang2025_Silkie_Wenchang	0.945041817
ENSGALG00010005405	CEBPD	2	-0.144177365	0.141725868	-1.017297457	0.309011946	0	0.004793073	0	1	PRJNA827465;Yang2025_Silkie_Wenchang	CRA004775	0.794014496
ENSGALG00010005405	CEBPD	2	-0.052115161	0.246670558	-0.211274345	0.8326732	0	0.622029992	0	0.5	CRA004775;Yang2025_Silkie_Wenchang	PRJNA827465	0.957861248
ENSGALG00010005405	CEBPD	2	-0.115796208	0.162741161	-0.711536079	0.476752102	0	0.728856332	0	0.5	CRA004775;PRJNA827465	Yang2025_Silkie_Wenchang	0.854986476
ENSGALG00010005407	DPH6	2	0.103065474	0.102098396	1.009472019	0.312748314	0	0.078045017	0	1	PRJNA827465;Yang2025_Silkie_Wenchang	CRA004775	0.796054997
ENSGALG00010005407	DPH6	2	-0.011607553	0.20350671	-0.057037692	0.95451517	0.026773709	1.446301059	30.8581022	0.5	CRA004775;Yang2025_Silkie_Wenchang	PRJNA827465	0.987987175
ENSGALG00010005407	DPH6	2	0.003154971	0.14933032	0.021127468	0.983143974	0.015335242	1.331987899	24.92424288	0.5	CRA004775;PRJNA827465	Yang2025_Silkie_Wenchang	0.995363468
ENSGALG00010005408	APBB1IP	2	-0.06031031	0.135126635	-0.446324366	0.655362958	0	0.54896225	0	1	PRJNA827465;Yang2025_Silkie_Wenchang	CRA004775	0.910411938
ENSGALG00010005408	APBB1IP	2	-0.355924214	0.268100015	-1.327579985	0.1843169	0.012158065	1.049569562	4.72284672	1	CRA004775;Yang2025_Silkie_Wenchang	PRJNA827465	0.692608786
ENSGALG00010005408	APBB1IP	2	-0.271725167	0.427813665	-0.635148405	0.52533163	0.230957786	2.048480209	51.18332138	1	CRA004775;PRJNA827465	Yang2025_Silkie_Wenchang	0.870194674
ENSGALG00010005409	PRKCQ	2	0.010606614	0.119572599	0.088704388	0.929316845	0	0.369959714	0	0.5	PRJNA827465;Yang2025_Silkie_Wenchang	CRA004775	0.983653628
ENSGALG00010005409	PRKCQ	2	0.207207395	0.207002662	1.000989037	0.316832108	0.022210177	1.223667572	18.27845872	1	CRA004775;Yang2025_Silkie_Wenchang	PRJNA827465	0.796959041
ENSGALG00010005409	PRKCQ	2	0.15797369	0.300616561	0.525498958	0.599236377	0.109600996	2.151881258	53.52903435	0.5	CRA004775;PRJNA827465	Yang2025_Silkie_Wenchang	0.894908299
ENSGALG00010005411	STXBP5	2	0.016427562	0.065028987	0.252619072	0.800562591	0	0.181354736	0	0.5	PRJNA827465;Yang2025_Silkie_Wenchang	CRA004775	0.950156876
ENSGALG00010005411	STXBP5	2	0.030224825	0.115801833	0.261004717	0.794088869	0	0.640686057	0	0.5	CRA004775;Yang2025_Silkie_Wenchang	PRJNA827465	0.948905846
ENSGALG00010005411	STXBP5	2	-0.011323461	0.075363727	-0.150250815	0.880566736	0	0.441452645	0	1	CRA004775;PRJNA827465	Yang2025_Silkie_Wenchang	0.971623434
ENSGALG00010005412	MTFR2	2	-0.106740316	0.083756531	-1.274411865	0.20251749	0	0.742642314	0	0.5	PRJNA827465;Yang2025_Silkie_Wenchang	CRA004775	0.715289846
ENSGALG00010005412	MTFR2	2	-0.192003092	0.368957594	-0.520393387	0.602789418	0.153496303	1.80778392	44.68365446	0.5	CRA004775;Yang2025_Silkie_Wenchang	PRJNA827465	0.895538966
ENSGALG00010005412	MTFR2	2	-0.210361575	0.19723867	-1.066533123	0.286182714	0.034459233	1.19394731	16.24421015	1	CRA004775;PRJNA827465	Yang2025_Silkie_Wenchang	0.781941993
ENSGALG00010005414	ENSGALG00010005414	2	0.014802671	0.471246609	0.03141173	0.974941186	0.412313971	13.95211503	92.8326279	0.5	PRJNA827465;Yang2025_Silkie_Wenchang	CRA004775	0.992609821
ENSGALG00010005414	ENSGALG00010005414	2	0.136712784	0.46797935	0.292134223	0.770184001	0.309039226	2.850963825	64.9241428	0.5	CRA004775;Yang2025_Silkie_Wenchang	PRJNA827465	0.942185688
ENSGALG00010005414	ENSGALG00010005414	2	-0.458866622	0.168103968	-2.729659664	0.006339974	0	0.003316038	0	1	CRA004775;PRJNA827465	Yang2025_Silkie_Wenchang	0.121049048
ENSGALG00010005415	PRKDC	2	0.080095159	0.166063868	0.482315387	0.629581918	0.044629481	4.890395716	79.55175699	0.5	PRJNA827465;Yang2025_Silkie_Wenchang	CRA004775	0.902457014
ENSGALG00010005415	PRKDC	2	0.210936421	0.132800581	1.58836971	0.112202763	0.00228676	1.036498555	3.521331998	0.5	CRA004775;Yang2025_Silkie_Wenchang	PRJNA827465	0.5658803
ENSGALG00010005415	PRKDC	2	-0.066591076	0.063447871	-1.04953996	0.293929674	0	0.005836921	0	1	CRA004775;PRJNA827465	Yang2025_Silkie_Wenchang	0.787318065
ENSGALG00010005416	PPP3CA	2	-0.094700145	0.139629527	-0.678224345	0.497629459	0.031580567	5.244372605	80.93194219	0.5	PRJNA827465;Yang2025_Silkie_Wenchang	CRA004775	0.86098656
ENSGALG00010005416	PPP3CA	2	0.051567314	0.088492149	0.582733209	0.560072913	0	0.012057607	0	1	CRA004775;Yang2025_Silkie_Wenchang	PRJNA827465	0.881203589
ENSGALG00010005416	PPP3CA	2	-0.211764913	0.077508195	-2.732161579	0.006292028	0	0.931282845	0	0.5	CRA004775;PRJNA827465	Yang2025_Silkie_Wenchang	0.120477095
ENSGALG00010005417	LOXL3	2	0.431972862	0.249616453	1.730546432	0.083532693	0.098434081	4.750134303	78.94796365	1	PRJNA827465;Yang2025_Silkie_Wenchang	CRA004775	0.497929561
ENSGALG00010005417	LOXL3	2	0.356902916	0.339040379	1.052685575	0.292485122	0.195055908	6.574388134	84.78945904	1	CRA004775;Yang2025_Silkie_Wenchang	PRJNA827465	0.786073766
ENSGALG00010005417	LOXL3	2	0.122886926	0.121973701	1.007487063	0.313700771	0	0.498210129	0	1	CRA004775;PRJNA827465	Yang2025_Silkie_Wenchang	0.796531448
ENSGALG00010005420	DHRS4	2	0.203020311	0.165281524	1.22833034	0.219322986	0.043640078	4.535665421	77.95251838	1	PRJNA827465;Yang2025_Silkie_Wenchang	CRA004775	0.732888275
ENSGALG00010005420	DHRS4	2	0.088428451	0.339251391	0.260657593	0.794356572	0.188836409	5.338568106	81.26838545	0.5	CRA004775;Yang2025_Silkie_Wenchang	PRJNA827465	0.948905846
ENSGALG00010005420	DHRS4	2	-0.022043139	0.148483909	-0.148454733	0.881983908	0.025395334	1.728883037	42.15918726	0.5	CRA004775;PRJNA827465	Yang2025_Silkie_Wenchang	0.971928118
ENSGALG00010005421	ENSGALG00010005421	2	-0.646366197	0.36588947	-1.766561351	0.077301718	0.209683097	4.587482429	78.20155139	1	PRJNA827465;Yang2025_Silkie_Wenchang	CRA004775	0.478832063
ENSGALG00010005421	ENSGALG00010005421	2	-0.91784066	0.233446494	-3.931696057	8.43487E-05	0	0.836809569	0	1	CRA004775;Yang2025_Silkie_Wenchang	PRJNA827465	0.006879732
ENSGALG00010005421	ENSGALG00010005421	2	-0.325343178	0.202225442	-1.608814274	0.107656959	0	0.131814845	0	1	CRA004775;PRJNA827465	Yang2025_Silkie_Wenchang	0.55523359
ENSGALG00010005423	ST3GAL6	2	-0.104705044	0.076402118	-1.370446878	0.170547447	0	0.031109562	0	1	PRJNA827465;Yang2025_Silkie_Wenchang	CRA004775	0.672201941
ENSGALG00010005423	ST3GAL6	2	-0.104640584	0.106062825	-0.986590586	0.323843387	0	0.108890683	0	1	CRA004775;Yang2025_Silkie_Wenchang	PRJNA827465	0.799600434
ENSGALG00010005423	ST3GAL6	2	-0.125312475	0.094990348	-1.319212711	0.18709801	0	0.057844385	0	1	CRA004775;PRJNA827465	Yang2025_Silkie_Wenchang	0.695933761
ENSGALG00010005424	ENSGALG00010005424	2	0.025052651	0.112643125	0.222407281	0.823996844	0	0.303994402	0	0.5	PRJNA827465;Yang2025_Silkie_Wenchang	CRA004775	0.956170356
ENSGALG00010005424	ENSGALG00010005424	2	-0.145253849	0.228854807	-0.634698704	0.525624939	0	0.030271588	0	1	CRA004775;Yang2025_Silkie_Wenchang	PRJNA827465	0.870277065
ENSGALG00010005424	ENSGALG00010005424	2	0.02978503	0.117382721	0.253742887	0.799694199	0	0.376138633	0	0.5	CRA004775;PRJNA827465	Yang2025_Silkie_Wenchang	0.949922217
ENSGALG00010005425	ELAPOR2	2	0.018140305	0.140193382	0.129394873	0.897045205	0	0.007208719	0	0.5	PRJNA827465;Yang2025_Silkie_Wenchang	CRA004775	0.975312393
ENSGALG00010005425	ELAPOR2	2	-0.076436235	0.325754437	-0.234643726	0.814485268	0	0.117786371	0	1	CRA004775;Yang2025_Silkie_Wenchang	PRJNA827465	0.953869515
ENSGALG00010005425	ELAPOR2	2	0.007907487	0.147112974	0.053751122	0.957133452	0	0.192028707	0	0.5	CRA004775;PRJNA827465	Yang2025_Silkie_Wenchang	0.988541306
ENSGALG00010005427	ENSGALG00010005427	2	0.14346091	0.130318826	1.10084563	0.270963849	0.017874833	1.988202199	49.70330479	1	PRJNA827465;Yang2025_Silkie_Wenchang	CRA004775	0.772743863
ENSGALG00010005427	ENSGALG00010005427	2	0.083067699	0.305146299	0.272222534	0.785450916	0.117667133	2.371171438	57.82675246	0.5	CRA004775;Yang2025_Silkie_Wenchang	PRJNA827465	0.946456675
ENSGALG00010005427	ENSGALG00010005427	2	0.017985266	0.09495275	0.189412796	0.849769296	0	0.883839856	0	0.5	CRA004775;PRJNA827465	Yang2025_Silkie_Wenchang	0.961856718
ENSGALG00010005428	ZNF770	2	0.053702304	0.048479902	1.107723039	0.267981446	0	0.025756735	0	1	PRJNA827465;Yang2025_Silkie_Wenchang	CRA004775	0.771398294
ENSGALG00010005428	ZNF770	2	0.047905159	0.071165976	0.673146946	0.50085381	0	0.025345413	0	1	CRA004775;Yang2025_Silkie_Wenchang	PRJNA827465	0.862421093
ENSGALG00010005428	ZNF770	2	0.062029023	0.062465674	0.993009754	0.320705201	0	0.010650505	0	1	CRA004775;PRJNA827465	Yang2025_Silkie_Wenchang	0.798348216
ENSGALG00010005429	ZDHHC20	2	-0.140119535	0.052800508	-2.653753564	0.007960194	0	0.970810409	0	1	PRJNA827465;Yang2025_Silkie_Wenchang	CRA004775	0.138706114
ENSGALG00010005429	ZDHHC20	2	-0.186871925	0.072068551	-2.592974656	0.009514978	0	0.062466197	0	1	CRA004775;Yang2025_Silkie_Wenchang	PRJNA827465	0.153474704
ENSGALG00010005429	ZDHHC20	2	-0.085635354	0.07517234	-1.13918702	0.254625158	0	0.00012714	0	1	CRA004775;PRJNA827465	Yang2025_Silkie_Wenchang	0.764296259
ENSGALG00010005430	MCM4	2	-0.11384192	0.088296642	-1.289312	0.197289641	0.008360169	2.059375279	51.44158473	1	PRJNA827465;Yang2025_Silkie_Wenchang	CRA004775	0.708846764
ENSGALG00010005430	MCM4	2	-0.234154378	0.101982767	-2.296019084	0.02167479	0	0.188887856	0	1	CRA004775;Yang2025_Silkie_Wenchang	PRJNA827465	0.249411453
ENSGALG00010005430	MCM4	2	-0.052348663	0.066950156	-0.78190502	0.434270395	0	0.808611866	0	1	CRA004775;PRJNA827465	Yang2025_Silkie_Wenchang	0.842762754
ENSGALG00010005434	PRDM5	2	-0.035340256	0.150493939	-0.234828432	0.814341899	0.031129414	2.976859418	66.40755039	0.5	PRJNA827465;Yang2025_Silkie_Wenchang	CRA004775	0.953869515
ENSGALG00010005434	PRDM5	2	-0.205877921	0.151004235	-1.363391702	0.172759095	0	0.073345896	0	1	CRA004775;Yang2025_Silkie_Wenchang	PRJNA827465	0.675885995
ENSGALG00010005434	PRDM5	2	0.086944112	0.083682341	1.038978009	0.298814964	0	0.052578003	0	0.5	CRA004775;PRJNA827465	Yang2025_Silkie_Wenchang	0.78974322
ENSGALG00010005435	MAD2L1	2	-0.220947058	0.283394999	-0.779643459	0.435600768	0.135593622	5.784187875	82.71148826	0.5	PRJNA827465;Yang2025_Silkie_Wenchang	CRA004775	0.842762754
ENSGALG00010005435	MAD2L1	2	-0.574567512	0.208244765	-2.759097025	0.005796132	0	0.085784437	0	1	CRA004775;Yang2025_Silkie_Wenchang	PRJNA827465	0.114590236
ENSGALG00010005435	MAD2L1	2	-0.16499269	0.322752515	-0.511204971	0.609207538	0.135094254	1.979728066	49.48801217	0.5	CRA004775;PRJNA827465	Yang2025_Silkie_Wenchang	0.897238578
ENSGALG00010005436	AQR	2	-0.197791355	0.141249012	-1.400302567	0.161422733	0.033318153	5.977898277	83.27171267	1	PRJNA827465;Yang2025_Silkie_Wenchang	CRA004775	0.657858553
ENSGALG00010005436	AQR	2	-0.312142203	0.086759169	-3.597800757	0.000320919	0	0.948423421	0	1	CRA004775;Yang2025_Silkie_Wenchang	PRJNA827465	0.01770607
ENSGALG00010005436	AQR	2	-0.067041149	0.065090911	-1.029961759	0.303027957	0	0.029716084	0	1	CRA004775;PRJNA827465	Yang2025_Silkie_Wenchang	0.792084776
ENSGALG00010005437	PDE7B	2	-0.060718529	0.239784756	-0.253220972	0.800097461	0.102711174	9.35021897	89.3050633	0.5	PRJNA827465;Yang2025_Silkie_Wenchang	CRA004775	0.94999675
ENSGALG00010005437	PDE7B	2	0.439048329	0.379604153	1.156595167	0.247437798	0.196379234	2.423226173	58.7327006	1	CRA004775;Yang2025_Silkie_Wenchang	PRJNA827465	0.757947282
ENSGALG00010005437	PDE7B	2	0.254295015	0.641406315	0.396464783	0.691762183	0.705035832	6.163673338	83.77590853	0.5	CRA004775;PRJNA827465	Yang2025_Silkie_Wenchang	0.920348122
ENSGALG00010005438	GPR180	2	0.008612063	0.072642151	0.118554626	0.905628215	0	0.032083119	0	0.5	PRJNA827465;Yang2025_Silkie_Wenchang	CRA004775	0.978182365
ENSGALG00010005438	GPR180	2	0.054403402	0.105733463	0.514533434	0.606879091	0	0.351790917	0	1	CRA004775;Yang2025_Silkie_Wenchang	PRJNA827465	0.896799223
ENSGALG00010005438	GPR180	2	0.021416087	0.086935904	0.246343405	0.805416412	0	0.51040304	0	0.5	CRA004775;PRJNA827465	Yang2025_Silkie_Wenchang	0.951870339
ENSGALG00010005441	UBE2V2	2	-0.025294954	0.136365625	-0.185493625	0.85284188	0.026329564	2.826819377	64.62455267	0.5	PRJNA827465;Yang2025_Silkie_Wenchang	CRA004775	0.962698731
ENSGALG00010005441	UBE2V2	2	0.113546459	0.139808397	0.812157651	0.416701176	0	0.307215895	0	0.5	CRA004775;Yang2025_Silkie_Wenchang	PRJNA827465	0.837984974
ENSGALG00010005441	UBE2V2	2	-0.122525879	0.048071105	-2.54884673	0.010807979	0	0.153492043	0	1	CRA004775;PRJNA827465	Yang2025_Silkie_Wenchang	0.165608575
ENSGALG00010005442	SASH1	2	-0.046725881	0.055795973	-0.837441809	0.402344273	0	0.638748332	0	1	PRJNA827465;Yang2025_Silkie_Wenchang	CRA004775	0.832879175

ENSGALG00010005442	SASH1	2	-0.11951061	0.108817684	-1.098264597	0.272088966	0	0.037360732	0	1	CRA004775;Yang2025_Silkie_Wenchang	PRJNA827465	0.773184343
ENSGALG00010005442	SASH1	2	-0.022360948	0.063154296	-0.354068517	0.723287536	0	0.005263295	0	1	CRA004775;PRJNA827465	Yang2025_Silkie_Wenchang	0.930032096
ENSGALG00010005443	BANK1	2	-0.385617878	0.588892714	-0.654818559	0.51258458	0.431069134	2.594624808	61.45878214	0.5	PRJNA827465;Yang2025_Silkie_Wenchang	CRA004775	0.865859727
ENSGALG00010005443	BANK1	2	-0.389889209	0.585231202	-0.666213981	0.505274338	0.421195666	2.551594568	60.80882079	0.5	CRA004775;Yang2025_Silkie_Wenchang	PRJNA827465	0.863862536
ENSGALG00010005443	BANK1	2	0.140424584	0.314211975	0.446910351	0.65493979	0	0.000138747	0	1	CRA004775;PRJNA827465	Yang2025_Silkie_Wenchang	0.910254909
ENSGALG00010005444	ACTC1	2	-0.065790287	0.123120066	-0.534358765	0.593093342	0	0.855611531	0	0.5	PRJNA827465;Yang2025_Silkie_Wenchang	CRA004775	0.892776493
ENSGALG00010005444	ACTC1	2	-0.457020445	0.432857147	-1.055822802	0.291049179	0.203091112	1.63910975	38.99127255	1	CRA004775;Yang2025_Silkie_Wenchang	PRJNA827465	0.78467882
ENSGALG00010005444	ACTC1	2	-0.382257723	0.586577956	-0.651674204	0.514611367	0.482113385	2.561707824	60.96354196	0.5	CRA004775;PRJNA827465	Yang2025_Silkie_Wenchang	0.866869697
ENSGALG00010005445	SFT2D2	2	-0.100049712	0.220286219	-0.454180533	0.649698893	0.085483706	8.324582145	87.98738504	0.5	PRJNA827465;Yang2025_Silkie_Wenchang	CRA004775	0.90820125
ENSGALG00010005445	SFT2D2	2	-0.289552708	0.111153992	-2.604969046	0.009188256	0	0.58620174	0	1	CRA004775;Yang2025_Silkie_Wenchang	PRJNA827465	0.150281327
ENSGALG00010005445	SFT2D2	2	0.091308219	0.087397412	1.044747401	0.296139731	0	0.546006794	0	0.5	CRA004775;PRJNA827465	Yang2025_Silkie_Wenchang	0.788241896
ENSGALG00010005446	ENSGALG00010005446	2	-0.330889831	0.302797683	-1.092775306	0.274492467	0	0.323594867	0	1	PRJNA827465;Yang2025_Silkie_Wenchang	CRA004775	0.774526342
ENSGALG00010005446	ENSGALG00010005446	2	-0.133269338	0.352009032	-0.37859636	0.704987626	0	0.003818307	0	1	CRA004775;Yang2025_Silkie_Wenchang	PRJNA827465	0.924152671
ENSGALG00010005446	ENSGALG00010005446	2	-0.352697309	0.306825083	-1.149506114	0.250347347	0	0.238230774	0	1	CRA004775;PRJNA827465	Yang2025_Silkie_Wenchang	0.759943035
ENSGALG00010005448	ENSGALG00010005448	2	0.515572978	0.540510428	0.953863147	0.34015292	0.574318545	57.89017985	98.27259131	0.5	PRJNA827465;Yang2025_Silkie_Wenchang	CRA004775	0.805377063
ENSGALG00010005448	ENSGALG00010005448	2	0.495670091	0.579074189	0.855969926	0.392014414	0.6397037	21.50031537	95.34890543	0.5	CRA004775;Yang2025_Silkie_Wenchang	PRJNA827465	0.829251057
ENSGALG00010005448	ENSGALG00010005448	2	-0.020969392	0.044278553	-0.473578984	0.635800165	0	0.128811527	0	1	CRA004775;PRJNA827465	Yang2025_Silkie_Wenchang	0.904387873
ENSGALG00010005449	TIPRL	2	-0.052110676	0.047394173	-1.099516522	0.271542832	0	4.56453E-05	0	1	PRJNA827465;Yang2025_Silkie_Wenchang	CRA004775	0.772805786
ENSGALG00010005449	TIPRL	2	0.022848633	0.124103405	0.184109636	0.853927448	0.01233038	1.461361976	31.57068429	0.5	CRA004775;Yang2025_Silkie_Wenchang	PRJNA827465	0.962975689
ENSGALG00010005449	TIPRL	2	0.01272728	0.117651101	0.108178159	0.91385437	0.015002089	1.629030018	38.61377699	0.5	CRA004775;PRJNA827465	Yang2025_Silkie_Wenchang	0.979364626
ENSGALG00010005450	ENSGALG00010005450	2	-0.038350949	0.381355243	-0.100564893	0.919895867	0.213718837	3.587285512	72.12376889	0.5	PRJNA827465;Yang2025_Silkie_Wenchang	CRA004775	0.981237238
ENSGALG00010005450	ENSGALG00010005450	2	-0.18257055	0.657860535	-0.277521664	0.781379574	0.647960946	3.806384258	73.72834868	0.5	CRA004775;Yang2025_Silkie_Wenchang	PRJNA827465	0.945235709
ENSGALG00010005450	ENSGALG00010005450	2	-0.432817157	0.194097785	-2.229892308	0.025754593	0	0.810507011	0	1	CRA004775;PRJNA827465	Yang2025_Silkie_Wenchang	0.273576426
ENSGALG00010005453	UST	2	-0.298043765	0.20542232	-1.450883068	0.146812423	0.078874503	14.99747353	93.33221027	1	PRJNA827465;Yang2025_Silkie_Wenchang	CRA004775	0.635558525
ENSGALG00010005453	UST	2	-0.253333574	0.294391426	-0.860533125	0.389495232	0.144394107	5.510356363	81.85235339	0.5	CRA004775;Yang2025_Silkie_Wenchang	PRJNA827465	0.828911149
ENSGALG00010005453	UST	2	-0.091938968	0.049353929	-1.862850031	0.062483369	0	0.577994316	0	0.5	CRA004775;PRJNA827465	Yang2025_Silkie_Wenchang	0.430914869
ENSGALG00010005454	GPR161	2	-0.110001468	0.150285136	-0.731951749	0.464198019	0.03894001	7.109924252	85.93515255	0.5	PRJNA827465;Yang2025_Silkie_Wenchang	CRA004775	0.852159649
ENSGALG00010005454	GPR161	2	-0.225136114	0.105553486	-2.13291027	0.032932088	0.004151194	1.10304814	9.342125366	0.5	CRA004775;Yang2025_Silkie_Wenchang	PRJNA827465	0.311661412
ENSGALG00010005454	GPR161	2	0.03194448	0.060345675	0.52935823	0.596556969	0	0.000113785	0	1	CRA004775;PRJNA827465	Yang2025_Silkie_Wenchang	0.894064255
ENSGALG00010005455	ENSGALG00010005455	2	-0.616214634	0.329724609	-1.868876687	0.061639972	0	0.714211693	0	1	PRJNA827465;Yang2025_Silkie_Wenchang	CRA004775	0.428734062
ENSGALG00010005455	ENSGALG00010005455	2	-0.442023736	0.404410815	-1.093006712	0.274390853	0	0.238296892	0	1	CRA004775;Yang2025_Silkie_Wenchang	PRJNA827465	0.774526342
ENSGALG00010005455	ENSGALG00010005455	2	-0.817848249	0.369617415	-2.21268862	0.026919121	0	0.032640555	0	1	CRA004775;PRJNA827465	Yang2025_Silkie_Wenchang	0.279419618
ENSGALG00010005456	RPL7L1	2	0.038190442	0.050339959	0.758650622	0.448061582	0	0.666881275	0	0.5	PRJNA827465;Yang2025_Silkie_Wenchang	CRA004775	0.846601815
ENSGALG00010005456	RPL7L1	2	-0.022728459	0.099618559	-0.228154866	0.819525847	0	0.179725163	0	0.5	CRA004775;Yang2025_Silkie_Wenchang	PRJNA827465	0.955256039
ENSGALG00010005456	RPL7L1	2	0.054917271	0.052606873	1.04391819	0.296523241	0	0.012232028	0	1	CRA004775;PRJNA827465	Yang2025_Silkie_Wenchang	0.788355067
ENSGALG00010005457	SEMA3D	2	-0.308681774	0.185523353	-1.663843225	0.096143763	0.050986926	3.743534519	73.28727717	1	PRJNA827465;Yang2025_Silkie_Wenchang	CRA004775	0.530364903
ENSGALG00010005457	SEMA3D	2	-0.369153942	0.245662065	-1.502690053	0.132918989	0.055701365	1.548359334	35.41550865	0.5	CRA004775;Yang2025_Silkie_Wenchang	PRJNA827465	0.610864713
ENSGALG00010005457	SEMA3D	2	-0.129944858	0.103671773	-1.253425635	0.210050847	0	0.186811171	0	0.5	CRA004775;PRJNA827465	Yang2025_Silkie_Wenchang	0.721835135
ENSGALG00010005458	PRKD1	2	0.004121615	0.056559917	0.072871654	0.94190825	0	0.253023238	0	0.5	PRJNA827465;Yang2025_Silkie_Wenchang	CRA004775	0.985827325
ENSGALG00010005458	PRKD1	2	0.181223392	0.252386587	0.71803892	0.47273329	0.101045514	4.40683275	77.30796568	0.5	CRA004775;Yang2025_Silkie_Wenchang	PRJNA827465	0.854086678
ENSGALG00010005458	PRKD1	2	0.201242179	0.220357422	0.913253465	0.361109251	0.074441297	3.684757063	72.86116878	1	CRA004775;PRJNA827465	Yang2025_Silkie_Wenchang	0.815240214
ENSGALG00010005459	ZC3H12D	2	0.396093166	0.252681197	1.567560905	0.116983642	0	0.444694381	0	1	PRJNA827465;Yang2025_Silkie_Wenchang	CRA004775	0.57615266
ENSGALG00010005459	ZC3H12D	2	-0.070500133	0.288929868	-0.244004308	0.807227483	0	0.906882735	0	0.5	CRA004775;Yang2025_Silkie_Wenchang	PRJNA827465	0.95234753
ENSGALG00010005459	ZC3H12D	2	0.125720563	0.445656369	0.282102023	0.777865282	0.258751525	2.829058295	64.65254881	0.5	CRA004775;PRJNA827465	Yang2025_Silkie_Wenchang	0.944432731
ENSGALG00010005460	TGDS	2	-0.368390334	0.412987796	-0.892012641	0.372386157	0.329474411	29.1151323	96.56535993	0.5	PRJNA827465;Yang2025_Silkie_Wenchang	CRA004775	0.821871806
ENSGALG00010005460	TGDS	2	-0.441422974	0.376200863	-1.173370444	0.240647284	0.244550086	7.042442673	85.8003814	1	CRA004775;Yang2025_Silkie_Wenchang	PRJNA827465	0.753158164
ENSGALG00010005460	TGDS	2	0.031833439	0.077618759	0.410125586	0.681713825	0	0.071304175	0	0.5	CRA004775;PRJNA827465	Yang2025_Silkie_Wenchang	0.918368413
ENSGALG00010005462	AHI1	2	0.006256549	0.208010918	0.030077985	0.976004858	0.067811006	4.46647844	77.61099682	0.5	PRJNA827465;Yang2025_Silkie_Wenchang	CRA004775	0.992880004
ENSGALG00010005462	AHI1	2	0.23900999	0.154391983	1.548072544	0.121604832	0	0.002545418	0	1	CRA004775;Yang2025_Silkie_Wenchang	PRJNA827465	0.584814231
ENSGALG00010005462	AHI1	2	-0.12830571	0.14433191	-0.888962882	0.374023029	0.012732395	1.151672007	13.16972244	0.5	CRA004775;PRJNA827465	Yang2025_Silkie_Wenchang	0.822579781
ENSGALG00010005463	SKA3	2	-0.071308425	0.102717323	-0.694220049	0.487544221	0.002359987	1.114389618	10.26477782	0.5	PRJNA827465;Yang2025_Silkie_Wenchang	CRA004775	0.85768885
ENSGALG00010005463	SKA3	2	-0.166887999	0.158200168	-1.054916698	0.291463425	0	0.818118402	0	0.5	CRA004775;Yang2025_Silkie_Wenchang	PRJNA827465	0.784884343
ENSGALG00010005463	SKA3	2	0.0186367	0.115899461	0.160800578	0.87225047	0	0.298372625	0	1	CRA004775;PRJNA827465	Yang2025_Silkie_Wenchang	0.969624964
ENSGALG00010005465	ENSGALG00010005465	2	0.133697119	0.056982462	2.346285426	0.018961581	0	0.764086469	0	1	PRJNA827465;Yang2025_Silkie_Wenchang	CRA004775	0.230726626
ENSGALG00010005465	ENSGALG00010005465	2	-0.128547952	0.276731213	-0.464522777	0.642273245	0.113698837	3.205770775	68.80625378	0.5	CRA004775;Yang2025_Silkie_Wenchang	PRJNA827465	0.906634523
ENSGALG00010005465	ENSGALG00010005465	2	-0.089648094	0.330650875	-0.271126137	0.78629402	0.176372261	4.444815177	77.50187668	0.5	CRA004775;PRJNA827465	Yang2025_Silkie_Wenchang	0.946639719
ENSGALG00010005466	LATS2	2	0.050384126	0.101773225	0.495062685	0.620555873	0.010837826	1.770402838	43.51568025	0.5	PRJNA827465;Yang2025_Silkie_Wenchang	CRA004775	0.899395593
ENSGALG00010005466	LATS2	2	0.257011318	0.136939412	1.876825042	0.060542077	0	0.438812283	0	1	CRA004775;Yang2025_Silkie_Wenchang	PRJNA827465	0.426049285
ENSGALG00010005466	LATS2	2	0.115015511	0.200782693	0.572835783	0.566755888	0.05347914	2.241009799	55.37725893	0.5	CRA004775;PRJNA827465	Yang2025_Silkie_Wenchang	0.883487517
ENSGALG00010005467	ENSGALG00010005467	2	0.05004498	0.235634648	0.21238379	0.831807631	0.073115292	2.917794458	65.72753789	0.5	PRJNA827465;Yang2025_Silkie_Wenchang	CRA004775	0.957762335
ENSGALG00010005467	ENSGALG00010005467	2	-0.489728294	0.830450975	-0.589713672	0.555382628	1.263788351	11.54917836	91.34137539	0.5	CRA004775;Yang2025_Silkie_Wenchang	PRJNA827465	0.880255849
ENSGALG00010005467	ENSGALG00010005467	2	-0.703353	0.591948949	-1.188198748	0.234755117	0.595028891	6.166478152	83.78328804	1	CRA004775;PRJNA827465	Yang2025_Silkie_Wenchang	0.747791642
ENSGALG00010005468	PPIL4	2	0.342809907	0.20649917	1.660103073	0.096893719	0.07700026	10.17420967	90.17122674	1	PRJNA827465;Yang2025_Silkie_Wenchang	CRA004775	0.532106661
ENSGALG00010005468	PPIL4	2	0.307571338	0.285912678	1.07575271	0.282037867	0.132430629	4.877255793	79.49666693	0.5	CRA004775;Yang2025_Silkie_Wenchang	PRJNA827465	0.779519046
ENSGALG00010005468	PPIL4	2	0.129518103	0.069834026	1.854656116	0.063645349	0	0.432177987	0	0.5	CRA004775;PRJNA827465	Yang2025_Silkie_Wenchang	0.434657369
ENSGALG00010005470	SEMA3A	2	-0.18162252	0.143813068	-1.262906928	0.20662263	0.028259588	2.976361605	66.40193187	1	PRJNA827465;Yang2025_Silkie_Wenchang	CRA004775	0.71859917
ENSGALG00010005470	SEMA3A	2	-0.30728401	0.139212208	-2.207306486	0.027292654	0	0.973669471	0	0.5	CRA004775;Yang2025_Silkie_Wenchang	PRJNA827465	0.281782062
ENSGALG00010005470	SEMA3A	2	-0.054188375	0.083220111	-0.651145194	0.514952765	0	0.146742969	0	0.5	CRA004775;PRJNA827465	Yang2025_Silkie_Wenchang	0.866997453
ENSGALG00010005472	PFKP	2	0.039014313	0.041098831	0.949280369	0.342478035	0	0.958003116	0	1	PRJNA827465;Yang2025_Silkie_Wenchang	CRA004775	0.806005903
ENSGALG00010005472	PFKP	2	0.079672781	0.065247044	1.221094103	0.222050392	0	0.720330932	0	0.5	CRA004775;Yang2025_Silkie_Wenchang	PRJNA827465	0.735359501
ENSGALG00010005472	PFKP	2	0.002535101	0.051776756	0.048962153	0.960949457	0	0.388750415	0	0.5	CRA004775;PRJNA827465	Yang2025_Silkie_Wenchang	0.989599947
ENSGALG00010005474	COL8A1	2	0.520990175	0.322745879	1.614242688	0.106474796	0.13904081	2.923062377	65.78930345	1	PRJNA827465;Yang2025_Silkie_Wenchang	CRA004775	0.553422082
ENSGALG00010005474	COL8A1	2	0.274899332	0.664916885	0.413434127	0.679288576	0.71664782	5.114538062	80.44789211	0.5	CRA004775;Yang2025_Silkie_Wenchang	PRJNA827465	0.917701868
ENSGALG00010005474	COL8A1	2	0.04120858	0.308289409	0.133668492	0.893664721	0.085425037	1.573549136	36.44939475	0.5	CRA004775;PRJNA827465	Yang2025_Silkie_Wenchang	0.974381724
ENSGALG00010005475	TMEM181	2	-0.091934509	0.232474438	-0.395460722	0.692502904	0.099388533	12.38896658	91.92830174	0.5	PRJNA827465;Yang2025_Silkie_Wenchang	CRA004775	0.920559211
ENSGALG00010005475	TMEM181	2	-0.236619251	0.149568248	-1.582015263	0.113646086	0.020315066	1.561954492	35.97764818	0.5	CRA004775;Yang2025_Silkie_Wenchang	PRJNA827465	0.568794964
ENSGALG00010005475	TMEM181	2	0.123765622	0.079467132	1.557444181	0.119365064	0	0.241300899	0	1	CRA004775;PRJNA827465	Yang2025_Silkie_Wenchang	0.579860319
ENSGALG00010005476	NFKB1	2	0.011608775	0.127054005	0.091368823	0.927199533	0.025658692	4.533174936	77.94040569	0.5	PRJNA827465;Yang2025_Silkie_Wenchang	CRA004775	0.982993195
ENSGALG00010005476	NFKB1	2	0.134453089	0.097849406	1.374081808	0.169416286	0	0.239618799	0	1	CRA004775;Yang2025_Silkie_Wenchang	PRJNA827465	0.670820905
ENSGALG00010005476	NFKB1	2	-0.092002364	0.050511984	-1.821396781	0.068546564	0	0.343986878	0	0.5	CRA004775;PRJNA827465	Yang2025_Silkie_Wenchang	0.451043966
ENSGALG00010005477	TMEM161B	2	0.054955538	0.105353169	0.521631563	0.601926883	0	0.012400682	0	1	PRJNA827465;Yang2025_Silkie_Wenchang	CRA004775	0.895304733
ENSGALG00010005477	TMEM161B	2	0.191566289	0.176775765	1.083668276	0.278511906	0.025191226	1.558942417	35.85394885	1	CRA004775;Yang2025_Silkie_Wenchang	PRJNA827465	0.776763074
ENSGALG00010005477	TMEM161B	2	0.184737133	0.190718252	0.968638984	0.332725346	0.033772477	1.74125048	42.57000865	1	CRA004775;PRJNA827465	Yang2025_Silkie_Wenchang	0.802178578
ENSGALG00010005481	ENSGALG00010005481	2	-0.5156489	1.208424555	-0.426711703	0.669589326	2.525579143	7.184619258	86.08137795	0.5	PRJNA827465;Yang2025_Silkie_Wenchang	CRA004775	0.914377769
ENSGALG00010005481	ENSGALG00010005481	2	-0.984148038	0.990842769	-0.993243396	0.320591355	1.055077632	2.081474773	51.95714054	0.5	CRA004775;Yang2025_Silkie_Wenchang	PRJNA827465	0.798348216
ENSGALG00010005481	ENSGALG00010005481	2	0.558729923	0.426497221	1.310043524	0.190181112	0	0.106566079	0	1	CRA004775;PRJNA827465	Yang2025_Silkie_Wenchang	0.699403589
ENSGALG00010005482	XPO4	2	-0.112723514	0.06195166	-1.819539866	0.068829106	0.001555735	1.224521373	18.33543931	1	PRJNA827465;Yang2025_Silkie_Wenchang	CRA004775	0.451995049
ENSGALG00010005482	XPO4	2	-0.187072293	0.090183703	-2.074346998	0.038047096	0	0.083849236	0	1	CRA004775;Yang2025_Silkie_Wenchang	PRJNA827465	0.336310939
ENSGALG00010005482	XPO4	2	-0.071835466	0.063210207	-1.136453581	0.255766782	0	0.054492136	0	1	CRA004775;PRJNA827465	Yang2025_Silkie_Wenchang	0.764478331
ENSGALG00010005484	GINM1	2	-0.371090472	0.331002066	-1.121112254	0.262240083	0.208528103	20.57689448	95.14018016	1	PRJNA827465;Yang2025_Silkie_Wenchang	CRA004775	0.768363052
ENSGALG00010005484	GINM1	2	-0.450673688	0.286209457	-1.574628918	0.115342124	0.133688497	5.166126266	80.64313668	1	CRA004775;Yang2025_Silkie_Wenchang	PRJNA827465	0.57238529
ENSGALG00010005484	GINM1	2	-0.056115277	0.079222987	-0.708320646	0.478746159	0	0.130076915	0	1	CRA004775;PRJNA827465	Yang2025_Silkie_Wenchang	0.855746783
ENSGALG00010005485	PITRM1	2	0.03596574	0.061060458	0.589018518	0.555848851	0.000363162	1.051193019	4.869992287	0.5	PRJNA827465;Yang2025_Silkie_Wenchang	CRA004775	0.880324407
ENSGALG00010005485	PITRM1	2	0.014998478	0.135809504	0.110437616	0.912062322	0.018097566	1.629298039	38.62387507	0.5	CRA004775;Yang2025_Silkie_Wenchang	PRJNA827465	0.978967245
ENSGALG00010005485	PITRM1	2	-0.047224435	0.078594905	-0.600858731	0.547934082	0	0.589722622	0	1	CRA004775;PRJNA827465	Yang2025_Silkie_Wenchang	0.878281266
ENSGALG00010005486	IRF5	2	0.125326807	0.228678962	0.548046947	0.583659667	0.06446981	2.564624203	61.00793251	0.5	PRJNA827465;Yang2025_Silkie_Wenchang	CRA004775	0.890558522
ENSGALG00010005486	IRF5	2	0.188896203	0.271551376	0.695618656	0.486667679	0.057769298	1.527206886	34.52098669	0.5	CRA004775;Yang2025_Silkie_Wenchang	PRJNA827465	0.857603818
ENSGALG00010005486	IRF5	2	-0.099550137	0.160587905	-0.619910555	0.535316676	0	0.07137693	0	1	CRA004775;PRJNA827465	Yang2025_Silkie_Wenchang	0.874112994
ENSGALG00010005487	ENSGALG00010005487	2	0.358881164	0.315829255	1.136313874	0.255825226	0.172699136	6.896591438	85.5000835	1	PRJNA827465;Yang2025_Silkie_Wenchang	CRA004775	0.764478331
ENSGALG00010005487	ENSGALG00010005487	2	0.300996192	0.442706589	0.67989996	0.496567807	0.313167497	4.867044873	79.45365152	0.5	CRA004775;Yang2025_Silkie_Wenchang	PRJNA827465	0.860939846
ENSGALG00010005487	ENSGALG00010005487	2	0.061604194	0.080349339	0.766704431	0.443257269	0	0.54345624	0	0.5	CRA004775;PRJNA827465	Yang2025_Silkie_Wenchang	0.845733906
ENSGALG00010005489	G2E3	2	-0.089389991	0.161138799	-0.554739094	0.579073132	0.043553029	6.1167201	83.65136898	0.5	PRJNA827465;Yang2025_Silkie_Wenchang	CRA004775	0.887888423
ENSGALG00010005489	G2E3	2	-0.236773917	0.101725375	-2.327579698	0.019934433	0	0.612029325	0	0.5	CRA004775;Yang2025_Silkie_Wenchang	PRJNA827465	0.237275288
ENSGALG00010005489	G2E3	2	0.063221485	0.075135242	0.841435838	0.400103816	0	0.001382417	0	1	CRA004775;PRJNA827465	Yang2025_Silkie_Wenchang	0.832246851
ENSGALG00010005490	DCBLD2	2	-0.303171194	0.277225205	-1.093591737	0.274134075	0.146796003	22.06669667	95.46828411	1	PRJNA827465;Yang2025_Silkie_Wenchang	CRA004775	0.774526342
ENSGALG00010005490	DCBLD2	2	-0.10471515	0.495861802	-0.211178093	0.832748305	0.467370345	19.81524381	94.95338029	0.5	CRA004775;Yang2025_Silkie_Wenchang	PRJNA827465	0.957861248
ENSGALG00010005490	DCBLD2	2	0.146764233	0.214969048	0.682722629	0.494782128	0.073867132	4.374201604	77.13868517	0.5	CRA004775;PRJNA827465	Yang2025_Silkie_Wenchang	0.860385009
ENSGALG00010005491	GGACT	2	0.237640331	0.093285428	2.547453923	0.010851218	0	0.645020754	0	1	PRJNA827465;Yang2025_Silkie_Wenchang	CRA004775	0.166012234
ENSGALG00010005491	GGACT	2	-0.095354035	0.310124568	-0.30747011	0.758485571	0.144576476	3.645629732	72.56989674	0.5	CRA004775;Yang2025_Silkie_Wenchang	PRJNA827465	0.938954369
ENSGALG00010005491	GGACT	2	-0.031355078	0.387620399	-0.080891196	0.935528481	0.250643949	5.607312398	82.16614433	0.5	CRA004775;PRJNA827465	Yang2025_Silkie_Wenchang	0.984736493
ENSGALG00010005492	TNPO3	2	-0.016952243	0.045685275	-0.371065811	0.710588515	0	0.002439664	0	1	PRJNA827465;Yang2025_Silkie_Wenchang	CRA004775	0.925404854
ENSGALG00010005492	TNPO3	2	-0.079396937	0.133812656	-0.593344004	0.552950955	0.016182125	1.392868916	28.20573506	1	CRA004775;Yang2025_Silkie_Wenchang	PRJNA827465	0.87928478
ENSGALG00010005492	TNPO3	2	-0.076065321	0.126062683	-0.603392842	0.546247383	0.015999567	1.402227925	28.68491759	1	CRA004775;PRJNA827465	Yang2025_Silkie_Wenchang	0.878012469
ENSGALG00010005493	KLF6	2	-0.007456148	0.052488797	-0.142052188	0.887038784	0	0.011606389	0	1	PRJNA827465;Yang2025_Silkie_Wenchang	CRA004775	0.972937943
ENSGALG00010005493	KLF6	2	0.540953854	0.63109429	0.857168038	0.391352024	0.69836555	7.349669688	86.39394636	0.5	CRA004775;Yang2025_Silkie_Wenchang	PRJNA827465	0.829251057
ENSGALG00010005493	KLF6	2	0.543414255	0.623701954	0.87127233	0.383605475	0.685589525	7.493731173	86.65551276	0.5	CRA004775;PRJNA827465	Yang2025_Silkie_Wenchang	0.825509017
ENSGALG00010005494	PPDPFL	2	-0.344568807	0.523287517	-0.65846938	0.510236567	0.33840396	2.602864175	61.58078437	0.5	PRJNA827465;Yang2025_Silkie_Wenchang	CRA004775	0.865365179
ENSGALG00010005494	PPDPFL	2	-1.695779822	0.949559934	-1.785858649	0.074122148	1.458063532	5.005226964	80.02088602	1	CRA004775;Yang2025_Silkie_Wenchang	PRJNA827465	0.470243057
ENSGALG00010005494	PPDPFL	2	-1.216783653	1.477765618	-0.82339421	0.410283855	3.976842907	11.04033186	90.94230126	0.5	CRA004775;PRJNA827465	Yang2025_Silkie_Wenchang	0.834737228
ENSGALG00010005495	TXN	2	0.009091714	0.146157034	0.062205107	0.950399496	0	0.128705822	0	0.5	PRJNA827465;Yang2025_Silkie_Wenchang	CRA004775	0.987277827
ENSGALG00010005495	TXN	2	-0.147853441	0.190478752	-0.776220125	0.437619025	0	0.350072595	0	1	CRA004775;Yang2025_Silkie_Wenchang	PRJNA827465	0.843827727
ENSGALG00010005495	TXN	2	-0.039797641	0.157898724	-0.252045364	0.801006	0	0.898602723	0	0.5	CRA004775;PRJNA827465	Yang2025_Silkie_Wenchang	0.950158029
ENSGALG00010005497	MKX	2	0.334570779	0.15435247	2.167576446	0.030190927	0	0.435791542	0	1	PRJNA827465;Yang2025_Silkie_Wenchang	CRA004775	0.295973612
ENSGALG00010005497	MKX	2	0.386559214	0.214892959	1.798845414	0.072043137	0	0.323371449	0	1	CRA004775;Yang2025_Silkie_Wenchang	PRJNA827465	0.463464923
ENSGALG00010005497	MKX	2	0.261816911	0.161334415	1.622821216	0.104627629	0	0.004488561	0	1	CRA004775;PRJNA827465	Yang2025_Silkie_Wenchang	0.549756644
ENSGALG00010005498	SMO	2	-0.295873211	0.154175612	-1.919066236	0.054975952	0.040792155	6.978721759	85.67072833	1	PRJNA827465;Yang2025_Silkie_Wenchang	CRA004775	0.405768983
ENSGALG00010005498	SMO	2	-0.452919159	0.090908017	-4.982169606	6.28753E-07	0	0.025418975	0	1	CRA004775;Yang2025_Silkie_Wenchang	PRJNA827465	0.000152542
ENSGALG00010005498	SMO	2	-0.154896228	0.069203863	-2.238259853	0.025204115	0	0.35444081	0	1	CRA004775;PRJNA827465	Yang2025_Silkie_Wenchang	0.270285761
ENSGALG00010005499	PDE5A	2	-0.06231619	0.100487135	-0.62014098	0.535164974	0.011721402	2.337942953	57.22735669	0.5	PRJNA827465;Yang2025_Silkie_Wenchang	CRA004775	0.87403237
ENSGALG00010005499	PDE5A	2	0.11451449	0.347658065	0.329388275	0.741862229	0.193784225	4.461474267	77.58588439	0.5	CRA004775;Yang2025_Silkie_Wenchang	PRJNA827465	0.93543545
ENSGALG00010005499	PDE5A	2	0.185472532	0.234167857	0.792049491	0.428331819	0.073640924	2.374941935	57.89370741	1	CRA004775;PRJNA827465	Yang2025_Silkie_Wenchang	0.841329286
ENSGALG00010005500	KATNA1	2	0.038257609	0.063177973	0.605552964	0.544811644	0	0.417397552	0	0.5	PRJNA827465;Yang2025_Silkie_Wenchang	CRA004775	0.877345822
ENSGALG00010005500	KATNA1	2	0.063019274	0.072541256	0.86873702	0.384990988	0	0.029284882	0	1	CRA004775;Yang2025_Silkie_Wenchang	PRJNA827465	0.826444827
ENSGALG00010005500	KATNA1	2	-0.006120074	0.093932815	-0.065153742	0.948051592	0	0.050382524	0	0.5	CRA004775;PRJNA827465	Yang2025_Silkie_Wenchang	0.986697717
ENSGALG00010005502	SCFD1	2	0.062890672	0.077643812	0.809989494	0.417946214	0.008811483	3.58964281	72.14207505	0.5	PRJNA827465;Yang2025_Silkie_Wenchang	CRA004775	0.838112595
ENSGALG00010005502	SCFD1	2	-0.032983785	0.064345171	-0.512607005	0.608226252	0	0.121593297	0	1	CRA004775;Yang2025_Silkie_Wenchang	PRJNA827465	0.897204334
ENSGALG00010005502	SCFD1	2	0.085703832	0.090301677	0.949083506	0.342578142	0.008296351	1.50803859	33.68869958	0.5	CRA004775;PRJNA827465	Yang2025_Silkie_Wenchang	0.806011702
ENSGALG00010005503	DYNLT1	2	-0.431873761	0.461484992	-0.935834899	0.3493582	0.380799428	8.867352146	88.72267636	1	PRJNA827465;Yang2025_Silkie_Wenchang	CRA004775	0.809903518
ENSGALG00010005503	DYNLT1	2	-0.67046088	0.238607728	-2.809887528	0.004955882	0.041357114	1.560590453	35.92168925	1	CRA004775;Yang2025_Silkie_Wenchang	PRJNA827465	0.104831857
ENSGALG00010005503	DYNLT1	2	-0.175545046	0.215976557	-0.812796759	0.416334594	0.063406445	2.744860773	63.56827968	1	CRA004775;PRJNA827465	Yang2025_Silkie_Wenchang	0.83780649
ENSGALG00010005504	LATS1	2	0.126172368	0.173862079	0.72570378	0.468020384	0.04399754	3.000691193	66.67434482	0.5	PRJNA827465;Yang2025_Silkie_Wenchang	CRA004775	0.853145337
ENSGALG00010005504	LATS1	2	0.305586118	0.1586998	1.92556083	0.054159234	0	0.188893436	0	1	CRA004775;Yang2025_Silkie_Wenchang	PRJNA827465	0.40335757
ENSGALG00010005504	LATS1	2	0.00653575	0.052548389	0.124375839	0.901017703	0	0.723464057	0	0.5	CRA004775;PRJNA827465	Yang2025_Silkie_Wenchang	0.976551823
ENSGALG00010005507	USP53	2	0.016626862	0.079944076	0.207981171	0.835243669	0	0.127219637	0	1	PRJNA827465;Yang2025_Silkie_Wenchang	CRA004775	0.958309459
ENSGALG00010005507	USP53	2	0.474655768	0.494655761	0.959567856	0.337272753	0.384580822	4.118708576	75.72054489	1	CRA004775;Yang2025_Silkie_Wenchang	PRJNA827465	0.804086136
ENSGALG00010005507	USP53	2	0.439049131	0.528050872	0.831452335	0.405718145	0.462742646	5.045700979	80.18114819	1	CRA004775;PRJNA827465	Yang2025_Silkie_Wenchang	0.833357445
ENSGALG00010005508	SS18	2	0.002273282	0.048962535	0.046429004	0.96296832	0	0.931148756	0	0.5	PRJNA827465;Yang2025_Silkie_Wenchang	CRA004775	0.989998461
ENSGALG00010005508	SS18	2	0.077481081	0.090459001	0.856532573	0.391703264	0	0.028418479	0	1	CRA004775;Yang2025_Silkie_Wenchang	PRJNA827465	0.829251057
ENSGALG00010005508	SS18	2	-0.021842136	0.055146676	-0.396073487	0.692050817	0	0.067198941	0	0.5	CRA004775;PRJNA827465	Yang2025_Silkie_Wenchang	0.920348122
ENSGALG00010005509	COCH	2	0.376350423	0.152862408	2.462020774	0.013815666	0.001168043	1.025633411	2.499276135	1	PRJNA827465;Yang2025_Silkie_Wenchang	CRA004775	0.192817251
ENSGALG00010005509	COCH	2	0.497495576	0.198938606	2.500749273	0.012393088	0	0.16628663	0	1	CRA004775;Yang2025_Silkie_Wenchang	PRJNA827465	0.180135188
ENSGALG00010005509	COCH	2	0.230518136	0.199929189	1.152998904	0.248910835	0	0.01060723	0	1	CRA004775;PRJNA827465	Yang2025_Silkie_Wenchang	0.759368356
ENSGALG00010005510	MYOZ2	2	0.27542263	0.262745112	1.048250253	0.294523323	0.110216956	4.696408088	78.70713147	1	PRJNA827465;Yang2025_Silkie_Wenchang	CRA004775	0.787408474
ENSGALG00010005510	MYOZ2	2	-0.106282943	0.828026624	-0.128356914	0.897866526	1.098438157	4.34242259	76.97137993	0.5	CRA004775;Yang2025_Silkie_Wenchang	PRJNA827465	0.975696709
ENSGALG00010005510	MYOZ2	2	-0.280393521	0.519793357	-0.539432674	0.589588341	0.360990018	2.156833171	53.6357279	0.5	CRA004775;PRJNA827465	Yang2025_Silkie_Wenchang	0.892320726
ENSGALG00010005511	AHCYL2	2	-0.052166815	0.047507117	-1.098084209	0.27216772	0	0.156817325	0	1	PRJNA827465;Yang2025_Silkie_Wenchang	CRA004775	0.773286758
ENSGALG00010005511	AHCYL2	2	-0.017187443	0.085029107	-0.202135995	0.839810409	0	0.053250089	0	0.5	CRA004775;Yang2025_Silkie_Wenchang	PRJNA827465	0.959507805
ENSGALG00010005511	AHCYL2	2	-0.060544548	0.055476357	-1.091357684	0.275115525	0	0.133632427	0	0.5	CRA004775;PRJNA827465	Yang2025_Silkie_Wenchang	0.775068889
ENSGALG00010005512	SMARCAD1	2	-0.108416574	0.070834321	-1.530565576	0.125876795	0	0.354762722	0	1	PRJNA827465;Yang2025_Silkie_Wenchang	CRA004775	0.595777417
ENSGALG00010005512	SMARCAD1	2	-0.075166404	0.092018708	-0.816860027	0.414008424	0	0.041465429	0	1	CRA004775;Yang2025_Silkie_Wenchang	PRJNA827465	0.836724075
ENSGALG00010005512	SMARCAD1	2	-0.150789324	0.097420619	-1.547817349	0.121666278	0	0.005521384	0	1	CRA004775;PRJNA827465	Yang2025_Silkie_Wenchang	0.5849268
ENSGALG00010005513	TMEM30CP	2	0.053563413	0.145065075	0.369237134	0.711950979	0	0.08685669	0	1	PRJNA827465;Yang2025_Silkie_Wenchang	CRA004775	0.92597752
ENSGALG00010005513	TMEM30CP	2	0.234840343	0.206492051	1.137285147	0.255419102	0	0.732506725	0	1	CRA004775;Yang2025_Silkie_Wenchang	PRJNA827465	0.764467569
ENSGALG00010005513	TMEM30CP	2	0.149602975	0.208265627	0.718327727	0.472555238	0.027679224	1.33537572	25.11470852	1	CRA004775;PRJNA827465	Yang2025_Silkie_Wenchang	0.853998373
ENSGALG00010005514	EEF1AKMT1	2	-0.302211678	0.227306116	-1.329536062	0.183671176	0.089571912	7.442589368	86.56381602	1	PRJNA827465;Yang2025_Silkie_Wenchang	CRA004775	0.692148694
ENSGALG00010005514	EEF1AKMT1	2	-0.307647961	0.310742129	-0.99004265	0.322153273	0.131930409	2.669609687	62.5413406	0.5	CRA004775;Yang2025_Silkie_Wenchang	PRJNA827465	0.798513866
ENSGALG00010005514	EEF1AKMT1	2	-0.07000469	0.097122518	-0.720787427	0.471040312	0	0.251745719	0	0.5	CRA004775;PRJNA827465	Yang2025_Silkie_Wenchang	0.853855613
ENSGALG00010005515	PCCA	2	-0.139192424	0.058289134	-2.387965216	0.016941945	0.000271418	1.039885144	3.83553355	1	PRJNA827465;Yang2025_Silkie_Wenchang	CRA004775	0.216191322
ENSGALG00010005515	PCCA	2	0.037655959	0.158187777	0.238045948	0.811845457	0.027940063	1.907920705	47.58692029	0.5	CRA004775;Yang2025_Silkie_Wenchang	PRJNA827465	0.953371387
ENSGALG00010005515	PCCA	2	-0.007917969	0.224905992	-0.035205682	0.971915732	0.077193714	3.628929799	72.44366644	0.5	CRA004775;PRJNA827465	Yang2025_Silkie_Wenchang	0.991955348
ENSGALG00010005516	LARP4B	2	0.083394006	0.2993064	0.278624199	0.78053324	0.158731198	8.656401729	88.44785592	0.5	PRJNA827465;Yang2025_Silkie_Wenchang	CRA004775	0.945167388
ENSGALG00010005516	LARP4B	2	0.418610084	0.133297925	3.140409598	0.001687118	0	0.05411828	0	1	CRA004775;Yang2025_Silkie_Wenchang	PRJNA827465	0.052006891
ENSGALG00010005516	LARP4B	2	0.101227687	0.330690104	0.306110421	0.759520573	0.189072373	7.090537879	85.89669758	0.5	CRA004775;PRJNA827465	Yang2025_Silkie_Wenchang	0.939163539
ENSGALG00010005518	HPGDS	2	3.356667138	0.65808033	5.100695137	3.38408E-07	0.497351367	2.337816553	57.22504409	1	PRJNA827465;Yang2025_Silkie_Wenchang	CRA004775	9.23643E-05
ENSGALG00010005518	HPGDS	2	1.83068263	0.770476833	2.376038513	0.017499636	0.856940128	3.546344804	71.80195228	1	CRA004775;Yang2025_Silkie_Wenchang	PRJNA827465	0.220220599
ENSGALG00010005518	HPGDS	2	2.533305345	1.431645514	1.769506013	0.076809468	3.813835723	14.34980628	93.03126481	1	CRA004775;PRJNA827465	Yang2025_Silkie_Wenchang	0.477904868
ENSGALG00010005519	PTBP3	2	0.583390276	0.140542011	4.151002765	3.31022E-05	0	0.008982807	0	1	PRJNA827465;Yang2025_Silkie_Wenchang	CRA004775	0.00361393
ENSGALG00010005519	PTBP3	2	0.586420715	0.191611797	3.060462483	0.002209954	0	0.024331885	0	1	CRA004775;Yang2025_Silkie_Wenchang	PRJNA827465	0.062174546
ENSGALG00010005519	PTBP3	2	0.563627076	0.182882261	3.081912221	0.002056755	0	0.012431577	0	1	CRA004775;PRJNA827465	Yang2025_Silkie_Wenchang	0.059004842
ENSGALG00010005522	SYNPO2	2	0.041426947	0.295813949	0.140043928	0.888625283	0.138864192	4.276326657	76.61544404	0.5	PRJNA827465;Yang2025_Silkie_Wenchang	CRA004775	0.973135755
ENSGALG00010005522	SYNPO2	2	0.343610927	0.248222473	1.384286132	0.166270859	0	0.207889403	0	1	CRA004775;Yang2025_Silkie_Wenchang	PRJNA827465	0.66529196
ENSGALG00010005522	SYNPO2	2	-0.195557248	0.092376991	-2.116947583	0.034264297	0	0.293523644	0	0.5	CRA004775;PRJNA827465	Yang2025_Silkie_Wenchang	0.318366348
ENSGALG00010005524	STRIP2	2	-0.089032479	0.215146552	-0.413822474	0.679004124	0.063951486	3.234127547	69.07975998	0.5	PRJNA827465;Yang2025_Silkie_Wenchang	CRA004775	0.917701868
ENSGALG00010005524	STRIP2	2	-0.07516644	0.292362158	-0.25710044	0.797101238	0.097374108	1.985986735	49.64719641	0.5	CRA004775;Yang2025_Silkie_Wenchang	PRJNA827465	0.949622804
ENSGALG00010005524	STRIP2	2	-0.333020433	0.156793085	-2.123948471	0.033674453	0	0.194246465	0	1	CRA004775;PRJNA827465	Yang2025_Silkie_Wenchang	0.315120717
ENSGALG00010005526	STRN3	2	-0.138658297	0.084620646	-1.638587074	0.101299286	0.010787201	3.608863925	72.29044872	1	PRJNA827465;Yang2025_Silkie_Wenchang	CRA004775	0.543134617
ENSGALG00010005526	STRN3	2	0.045510009	0.316594841	0.143748422	0.885699133	0.175976281	7.689468808	86.99520051	0.5	CRA004775;Yang2025_Silkie_Wenchang	PRJNA827465	0.972376043

ENSGALG00010005526	STRN3	2	0.11418225	0.226653061	0.503775461	0.614419174	0.084011111	4.603697174	78.278328	0.5	CRA004775;PRJNA827465	Yang2025_Silkie_Wenchang	0.898549462
ENSGALG00010005527	NUP43	2	-0.119260467	0.100062732	-1.191856993	0.233317324	0.014261027	3.361338206	70.24994396	1	PRJNA827465;Yang2025_Silkie_Wenchang	CRA004775	0.746566049
ENSGALG00010005527	NUP43	2	-0.218307984	0.084881721	-2.571908066	0.010113975	0	0.150559519	0	1	CRA004775;Yang2025_Silkie_Wenchang	PRJNA827465	0.159642386
ENSGALG00010005527	NUP43	2	-0.037522831	0.058532368	-0.641061219	0.521482909	0	0.21250155	0	1	CRA004775;PRJNA827465	Yang2025_Silkie_Wenchang	0.869518515
ENSGALG00010005529	ENSGALG00010005529	2	-0.050774129	0.382842361	-0.132624114	0.894490661	0.191755851	2.791692504	64.17943601	0.5	PRJNA827465;Yang2025_Silkie_Wenchang	CRA004775	0.974394857
ENSGALG00010005529	ENSGALG00010005529	2	-0.340029135	0.304530696	-1.116567686	0.264179215	0	0.408549113	0	1	CRA004775;Yang2025_Silkie_Wenchang	PRJNA827465	0.768790489
ENSGALG00010005529	ENSGALG00010005529	2	0.204764283	0.22826978	0.897027559	0.369704211	0	0.437506641	0	0.5	CRA004775;PRJNA827465	Yang2025_Silkie_Wenchang	0.820606801
ENSGALG00010005530	IL17D	2	-0.27197131	0.102541223	-2.652311943	0.007994265	0	0.617026455	0	1	PRJNA827465;Yang2025_Silkie_Wenchang	CRA004775	0.139087465
ENSGALG00010005530	IL17D	2	-0.380476638	0.160499148	-2.370583541	0.01776003	0	0.004128099	0	1	CRA004775;Yang2025_Silkie_Wenchang	PRJNA827465	0.22222937
ENSGALG00010005530	IL17D	2	-0.22192365	0.125383727	-1.769955766	0.07673451	0	0.1410182	0	1	CRA004775;PRJNA827465	Yang2025_Silkie_Wenchang	0.477904868
ENSGALG00010005531	SUSD1	2	0.251063723	0.110388913	2.274356324	0.022944571	0	0.26919547	0	1	PRJNA827465;Yang2025_Silkie_Wenchang	CRA004775	0.258318672
ENSGALG00010005531	SUSD1	2	0.353377246	0.233598873	1.512752363	0.130342573	0.056264439	1.76270025	43.26885698	1	CRA004775;Yang2025_Silkie_Wenchang	PRJNA827465	0.605608015
ENSGALG00010005531	SUSD1	2	0.389112542	0.153221249	2.539546867	0.011099617	0.00092017	1.011966674	1.182516604	1	CRA004775;PRJNA827465	Yang2025_Silkie_Wenchang	0.167461782
ENSGALG00010005532	GTPBP4	2	-0.018943179	0.055123424	-0.34365026	0.731109322	0	0.051514623	0	0.5	PRJNA827465;Yang2025_Silkie_Wenchang	CRA004775	0.932769533
ENSGALG00010005532	GTPBP4	2	0.005467461	0.101313116	0.053965976	0.956962272	0	0.000404319	0	1	CRA004775;Yang2025_Silkie_Wenchang	PRJNA827465	0.988540618
ENSGALG00010005532	GTPBP4	2	-0.022735283	0.05988757	-0.379632745	0.70421805	0	0.026053719	0	0.5	CRA004775;PRJNA827465	Yang2025_Silkie_Wenchang	0.92402786
ENSGALG00010005534	ENSGALG00010005534	2	0.247203021	0.117405393	2.105550821	0.035243388	0	0.228801078	0	1	PRJNA827465;Yang2025_Silkie_Wenchang	CRA004775	0.323934696
ENSGALG00010005534	ENSGALG00010005534	2	0.122667325	0.14809395	0.828307466	0.407496396	0	0.088747605	0	1	CRA004775;Yang2025_Silkie_Wenchang	PRJNA827465	0.833925651
ENSGALG00010005534	ENSGALG00010005534	2	0.221006446	0.11832518	1.867788802	0.061791515	0	0.665426875	0	1	CRA004775;PRJNA827465	Yang2025_Silkie_Wenchang	0.429075295
ENSGALG00010005535	AP4S1	2	0.049727981	0.151323459	0.328620438	0.742442598	0.026426561	2.244843274	55.45346031	0.5	PRJNA827465;Yang2025_Silkie_Wenchang	CRA004775	0.935510459
ENSGALG00010005535	AP4S1	2	0.209809622	0.152376174	1.376918826	0.168537346	0	0.082616662	0	1	CRA004775;Yang2025_Silkie_Wenchang	PRJNA827465	0.669363714
ENSGALG00010005535	AP4S1	2	-0.051494562	0.1025786	-0.502001019	0.615666804	0	0.395901917	0	0.5	CRA004775;PRJNA827465	Yang2025_Silkie_Wenchang	0.898902352
ENSGALG00010005536	TAF4B	2	-0.015476278	0.130556528	-0.11854082	0.905639153	0	0.87250218	0	0.5	PRJNA827465;Yang2025_Silkie_Wenchang	CRA004775	0.978182365
ENSGALG00010005536	TAF4B	2	0.159458138	0.225456979	0.707266365	0.479400963	0	0.050798147	0	1	CRA004775;Yang2025_Silkie_Wenchang	PRJNA827465	0.85611011
ENSGALG00010005536	TAF4B	2	-0.078567302	0.146803473	-0.535186942	0.592520595	0	0.07064473	0	0.5	CRA004775;PRJNA827465	Yang2025_Silkie_Wenchang	0.892776493
ENSGALG00010005537	GRIN3A	2	-0.358365655	0.196543234	-1.823342616	0.068251516	0	0.323332558	0	1	PRJNA827465;Yang2025_Silkie_Wenchang	CRA004775	0.450415709
ENSGALG00010005537	GRIN3A	2	-0.208509831	0.282490061	-0.738113866	0.460445258	0	0.018183234	0	1	CRA004775;Yang2025_Silkie_Wenchang	PRJNA827465	0.85014477
ENSGALG00010005537	GRIN3A	2	-0.414902318	0.241544363	-1.71770648	0.085850167	0	0.187246818	0	1	CRA004775;PRJNA827465	Yang2025_Silkie_Wenchang	0.503983436
ENSGALG00010005538	ENSGALG00010005538	2	0.576361291	0.452637791	1.273338866	0.202897821	0.112923149	1.372329393	27.13119712	1	PRJNA827465;Yang2025_Silkie_Wenchang	CRA004775	0.716164621
ENSGALG00010005538	ENSGALG00010005538	2	-0.249879787	0.466931313	-0.535153202	0.592543924	0	0.715939573	0	0.5	CRA004775;Yang2025_Silkie_Wenchang	PRJNA827465	0.892776493
ENSGALG00010005538	ENSGALG00010005538	2	0.204681788	0.8571519	0.2387929	0.811266176	1.077794154	3.640733396	72.53300665	0.5	CRA004775;PRJNA827465	Yang2025_Silkie_Wenchang	0.953179464
ENSGALG00010005539	IFT88	2	0.055616878	0.091608746	0.607113188	0.543775799	0.001573213	1.100083726	9.097828068	0.5	PRJNA827465;Yang2025_Silkie_Wenchang	CRA004775	0.876968972
ENSGALG00010005539	IFT88	2	0.108018661	0.114951267	0.939690911	0.347376128	0	0.584106334	0	0.5	CRA004775;Yang2025_Silkie_Wenchang	PRJNA827465	0.808705451
ENSGALG00010005539	IFT88	2	-0.02299924	0.0997263	-0.230623619	0.817607217	0	0.000873726	0	1	CRA004775;PRJNA827465	Yang2025_Silkie_Wenchang	0.954931991
ENSGALG00010005540	PCMT1	2	-0.123562536	0.04986451	-2.477965519	0.013213391	0	0.005852308	0	1	PRJNA827465;Yang2025_Silkie_Wenchang	CRA004775	0.187685552
ENSGALG00010005540	PCMT1	2	-0.076732062	0.132481637	-0.579190168	0.562460865	0.012809414	1.204736268	16.99428114	0.5	CRA004775;Yang2025_Silkie_Wenchang	PRJNA827465	0.881955027
ENSGALG00010005540	PCMT1	2	-0.080628878	0.116955266	-0.689399301	0.490572023	0.011570675	1.190639712	16.01153646	0.5	CRA004775;PRJNA827465	Yang2025_Silkie_Wenchang	0.858798485
ENSGALG00010005542	KCTD1	2	0.075911012	0.104199936	0.728513034	0.466299593	0	0.918288063	0	0.5	PRJNA827465;Yang2025_Silkie_Wenchang	CRA004775	0.85273464
ENSGALG00010005542	KCTD1	2	0.149090909	0.124554568	1.196992702	0.231309399	0	0.169289243	0	1	CRA004775;Yang2025_Silkie_Wenchang	PRJNA827465	0.743891332
ENSGALG00010005542	KCTD1	2	0.017335402	0.115188388	0.150496093	0.880373233	0	0.147127299	0	0.5	CRA004775;PRJNA827465	Yang2025_Silkie_Wenchang	0.971592092
ENSGALG00010005543	SEC24D	2	0.060234383	0.070744328	0.851437635	0.394526293	0	0.314792777	0	1	PRJNA827465;Yang2025_Silkie_Wenchang	CRA004775	0.830643015
ENSGALG00010005543	SEC24D	2	0.092327281	0.164663901	0.560701411	0.575001105	0	0.842772616	0	0.5	CRA004775;Yang2025_Silkie_Wenchang	PRJNA827465	0.886281107
ENSGALG00010005543	SEC24D	2	0.033369456	0.076116357	0.438400589	0.66109592	0	0.54236575	0	0.5	CRA004775;PRJNA827465	Yang2025_Silkie_Wenchang	0.912102115
ENSGALG00010005544	AQP4	2	-0.512736779	0.133200845	-3.849350805	0.000118431	0	0.321336993	0	1	PRJNA827465;Yang2025_Silkie_Wenchang	CRA004775	0.00873303
ENSGALG00010005544	AQP4	2	0.129993409	0.727994318	0.178563769	0.858280246	0.881938463	5.177975185	80.68743159	0.5	CRA004775;Yang2025_Silkie_Wenchang	PRJNA827465	0.964291891
ENSGALG00010005544	AQP4	2	0.069233057	0.839956274	0.082424596	0.934309077	1.166645686	5.510190972	81.85180868	0.5	CRA004775;PRJNA827465	Yang2025_Silkie_Wenchang	0.98432801
ENSGALG00010005545	RPL34	2	-0.259566767	0.252558679	-1.027748354	0.304068216	0.085998482	2.322896455	56.95029807	1	PRJNA827465;Yang2025_Silkie_Wenchang	CRA004775	0.79225794
ENSGALG00010005545	RPL34	2	-0.36234448	0.21495951	-1.68564061	0.091865042	0.000250709	1.002537057	0.253063656	1	CRA004775;Yang2025_Silkie_Wenchang	PRJNA827465	0.519097275
ENSGALG00010005545	RPL34	2	-0.100363049	0.07164475	-1.400843051	0.161261012	0	0.138631565	0	1	CRA004775;PRJNA827465	Yang2025_Silkie_Wenchang	0.657549988
ENSGALG00010005546	NRF1	2	0.095696738	0.194658904	0.491612438	0.622993349	0.06273551	5.530275809	81.91771921	0.5	PRJNA827465;Yang2025_Silkie_Wenchang	CRA004775	0.900334887
ENSGALG00010005546	NRF1	2	0.431639532	0.142785705	3.022988419	0.002502918	0.013179682	1.457489155	31.38885482	1	CRA004775;Yang2025_Silkie_Wenchang	PRJNA827465	0.067332098
ENSGALG00010005546	NRF1	2	0.238619107	0.33986768	0.702094142	0.482620455	0.211000477	11.05510381	90.95440425	0.5	CRA004775;PRJNA827465	Yang2025_Silkie_Wenchang	0.85691916
ENSGALG00010005547	LRP11	2	-0.000881152	0.090870034	-0.009696833	0.992263168	0.00251169	1.171658897	14.65092766	0.5	PRJNA827465;Yang2025_Silkie_Wenchang	CRA004775	0.997797603
ENSGALG00010005547	LRP11	2	-0.371956131	0.411243864	-0.904466093	0.365748306	0.245520987	2.882062964	65.30263174	1	CRA004775;Yang2025_Silkie_Wenchang	PRJNA827465	0.818968857
ENSGALG00010005547	LRP11	2	-0.306661052	0.514518263	-0.596015873	0.551164623	0.419949359	4.140125781	75.84614446	0.5	CRA004775;PRJNA827465	Yang2025_Silkie_Wenchang	0.878879109
ENSGALG00010005548	EZR	2	-0.010679508	0.117885687	-0.090592068	0.927816735	0	0.760643541	0	0.5	PRJNA827465;Yang2025_Silkie_Wenchang	CRA004775	0.982993195
ENSGALG00010005548	EZR	2	0.098532584	0.147876602	0.666316256	0.505208977	0	0.092709569	0	1	CRA004775;Yang2025_Silkie_Wenchang	PRJNA827465	0.863862536
ENSGALG00010005548	EZR	2	-0.073065364	0.162618654	-0.449304937	0.653211697	0	0.574641131	0	0.5	CRA004775;PRJNA827465	Yang2025_Silkie_Wenchang	0.909597225
ENSGALG00010005549	RNF20	2	0.28968264	0.326069401	0.888407926	0.374321363	0.17636808	5.771426102	82.67325991	0.5	PRJNA827465;Yang2025_Silkie_Wenchang	CRA004775	0.822805106
ENSGALG00010005549	RNF20	2	0.240272885	0.388758433	0.61805189	0.536541134	0.257729555	6.778278007	85.24699048	0.5	CRA004775;Yang2025_Silkie_Wenchang	PRJNA827465	0.874599474
ENSGALG00010005549	RNF20	2	-0.065926934	0.123860317	-0.532268406	0.594540111	0	0.238261328	0	1	CRA004775;PRJNA827465	Yang2025_Silkie_Wenchang	0.89334442
ENSGALG00010005550	ENSGALG00010005550	2	0.749834861	0.254279926	2.94885591	0.003189526	0	0.854015426	0	1	PRJNA827465;Yang2025_Silkie_Wenchang	CRA004775	0.078299787
ENSGALG00010005550	ENSGALG00010005550	2	-0.027958931	1.178421765	-0.023725742	0.981071373	2.489224599	9.601163404	89.58459555	0.5	CRA004775;Yang2025_Silkie_Wenchang	PRJNA827465	0.994928779
ENSGALG00010005550	ENSGALG00010005550	2	-0.243956901	0.914649049	-0.266721866	0.789683317	1.463689512	7.670218372	86.96256154	0.5	CRA004775;PRJNA827465	Yang2025_Silkie_Wenchang	0.947390191
ENSGALG00010005551	METTL14	2	0.035763949	0.046389409	0.770950728	0.440736137	0	0.907450709	0	0.5	PRJNA827465;Yang2025_Silkie_Wenchang	CRA004775	0.844848889
ENSGALG00010005551	METTL14	2	-0.114972061	0.106927633	-1.075232447	0.282270672	0	0.663030847	0	1	CRA004775;Yang2025_Silkie_Wenchang	PRJNA827465	0.779519046
ENSGALG00010005551	METTL14	2	-0.038031208	0.150513821	-0.252675855	0.800518707	0.028638135	2.007624968	50.1899002	0.5	CRA004775;PRJNA827465	Yang2025_Silkie_Wenchang	0.950155719
ENSGALG00010005552	WDR37	2	-0.041645569	0.084655774	-0.491940085	0.6227617	0	0.089053001	0	0.5	PRJNA827465;Yang2025_Silkie_Wenchang	CRA004775	0.900255255
ENSGALG00010005552	WDR37	2	0.043308727	0.137357683	0.315298905	0.752534716	0	0.196685753	0	1	CRA004775;Yang2025_Silkie_Wenchang	PRJNA827465	0.938087082
ENSGALG00010005552	WDR37	2	-0.029877348	0.091011559	-0.328280812	0.742699351	0	0.51927253	0	0.5	CRA004775;PRJNA827465	Yang2025_Silkie_Wenchang	0.935621057
ENSGALG00010005553	PPP1R14C	2	-0.354012441	0.157019219	-2.254580317	0.024159684	0.034910678	3.395770151	70.55159932	1	PRJNA827465;Yang2025_Silkie_Wenchang	CRA004775	0.265236889
ENSGALG00010005553	PPP1R14C	2	-0.505024879	0.123811103	-4.078995058	4.52308E-05	0	0.111993584	0	1	CRA004775;Yang2025_Silkie_Wenchang	PRJNA827465	0.004546563
ENSGALG00010005553	PPP1R14C	2	-0.222219608	0.10319844	-2.153323319	0.031293277	0	0.262676324	0	1	CRA004775;PRJNA827465	Yang2025_Silkie_Wenchang	0.302637717
ENSGALG00010005554	CRYL1	2	-0.267611943	0.244999648	-1.092295213	0.274703364	0.107204926	9.299540218	89.2467802	1	PRJNA827465;Yang2025_Silkie_Wenchang	CRA004775	0.774706274
ENSGALG00010005554	CRYL1	2	-0.074243483	0.508973225	-0.145869133	0.884024699	0.440728452	6.068315092	83.52096118	0.5	CRA004775;Yang2025_Silkie_Wenchang	PRJNA827465	0.972001076
ENSGALG00010005554	CRYL1	2	0.102198913	0.230517413	0.443345739	0.657515677	0.060262083	1.717305423	41.76923997	0.5	CRA004775;PRJNA827465	Yang2025_Silkie_Wenchang	0.910743213
ENSGALG00010005555	PRSS12	2	0.053824033	0.123456522	0.435975611	0.662854425	0.008029241	1.333288035	24.99745191	0.5	PRJNA827465;Yang2025_Silkie_Wenchang	CRA004775	0.91282818
ENSGALG00010005555	PRSS12	2	0.254709412	0.169324917	1.504264208	0.132513351	0	0.776406154	0	1	CRA004775;Yang2025_Silkie_Wenchang	PRJNA827465	0.609713444
ENSGALG00010005555	PRSS12	2	0.146790326	0.337658322	0.434730367	0.663758154	0.133686795	1.757053723	43.08654386	0.5	CRA004775;PRJNA827465	Yang2025_Silkie_Wenchang	0.913119244
ENSGALG00010005557	CMSS1	2	-0.192174336	0.076018772	-2.527986441	0.011471878	0	0.522549031	0	1	PRJNA827465;Yang2025_Silkie_Wenchang	CRA004775	0.171041171
ENSGALG00010005557	CMSS1	2	-0.134432438	0.285091531	-0.471541325	0.637254216	0.07364455	1.733760242	42.32189806	0.5	CRA004775;Yang2025_Silkie_Wenchang	PRJNA827465	0.905135429
ENSGALG00010005557	CMSS1	2	-0.137739561	0.115610504	-1.191410435	0.233492498	0.008195668	1.108220752	9.765270272	0.5	CRA004775;PRJNA827465	Yang2025_Silkie_Wenchang	0.746761792
ENSGALG00010005558	TAGAP	2	-0.335340109	0.331661957	-1.011090064	0.311973325	0	0.047715426	0	1	PRJNA827465;Yang2025_Silkie_Wenchang	CRA004775	0.975993742
ENSGALG00010005558	TAGAP	2	-0.537743716	0.352510665	-1.525467936	0.127142425	0	0.062758092	0	1	CRA004775;Yang2025_Silkie_Wenchang	PRJNA827465	0.598452543
ENSGALG00010005558	TAGAP	2	-0.429653552	0.30774669	-1.396127291	0.162676167	0	0.285335736	0	1	CRA004775;PRJNA827465	Yang2025_Silkie_Wenchang	0.660479266
ENSGALG00010005559	FILIP1L	2	0.212653196	0.422923503	0.502817164	0.615092825	0.338172575	17.79131557	94.37928018	0.5	PRJNA827465;Yang2025_Silkie_Wenchang	CRA004775	0.898817943
ENSGALG00010005559	FILIP1L	2	0.603495988	0.166995829	3.614736906	0.000300653	0	0.36289794	0	1	CRA004775;Yang2025_Silkie_Wenchang	PRJNA827465	0.017051308
ENSGALG00010005559	FILIP1L	2	-0.014002854	0.272176614	-0.051447674	0.958968797	0.100633053	2.347215503	57.39632774	0.5	CRA004775;PRJNA827465	Yang2025_Silkie_Wenchang	0.989102387
ENSGALG00010005560	RSPH3	2	-0.26518414	0.19151536	-1.384662515	0.166155686	0	0.236177927	0	1	PRJNA827465;Yang2025_Silkie_Wenchang	CRA004775	0.66529196
ENSGALG00010005560	RSPH3	2	-0.103796582	0.335736893	-0.309160489	0.757199448	0	0.000237042	0	1	CRA004775;Yang2025_Silkie_Wenchang	PRJNA827465	0.938795135
ENSGALG00010005560	RSPH3	2	-0.297672836	0.208962917	-1.4245246	0.154284669	0	0.085599609	0	1	CRA004775;PRJNA827465	Yang2025_Silkie_Wenchang	0.646989113
ENSGALG00010005561	CLYBL	2	-0.689608325	0.22122204	-3.117267724	0.001825357	0.058331274	2.351516125	57.47424441	1	PRJNA827465;Yang2025_Silkie_Wenchang	CRA004775	0.054556666
ENSGALG00010005561	CLYBL	2	-0.367135738	0.662649192	-0.554042383	0.57954984	0.725181181	5.399354393	81.4792672	0.5	CRA004775;Yang2025_Silkie_Wenchang	PRJNA827465	0.887969027
ENSGALG00010005561	CLYBL	2	-0.202719791	0.419816474	-0.482877177	0.629182953	0.244726675	2.679768916	62.68334952	0.5	CRA004775;PRJNA827465	Yang2025_Silkie_Wenchang	0.902246702
ENSGALG00010005562	HECTD1	2	0.115600371	0.201525781	0.573625717	0.566221105	0.075506407	2.13779047	92.74260428	0.5	PRJNA827465;Yang2025_Silkie_Wenchang	CRA004775	0.883487517
ENSGALG00010005562	HECTD1	2	0.336200832	0.095398534	3.524171897	0.000424809	0	0.112187991	0	1	CRA004775;Yang2025_Silkie_Wenchang	PRJNA827465	0.020876453
ENSGALG00010005562	HECTD1	2	0.072292358	0.236501798	0.305673607	0.75985317	0.076070355	2.233875684	55.23475155	0.5	CRA004775;PRJNA827465	Yang2025_Silkie_Wenchang	0.939165042
ENSGALG00010005564	TM9SF2	2	-0.348013087	0.35593368	-0.977746997	0.328199472	0.24692061	38.49255979	97.40209535	1	PRJNA827465;Yang2025_Silkie_Wenchang	CRA004775	0.830761292
ENSGALG00010005564	TM9SF2	2	-0.407650908	0.325656672	-1.251781226	0.210649593	0.186418207	7.994299811	87.4910871	1	CRA004775;Yang2025_Silkie_Wenchang	PRJNA827465	0.722132818
ENSGALG00010005564	TM9SF2	2	-0.001247318	0.029180421	-0.042745041	0.965904775	0	0.083845825	0	1	CRA004775;PRJNA827465	Yang2025_Silkie_Wenchang	0.990745532
ENSGALG00010005565	PGAP4	2	-0.068411335	0.131274514	-0.521131883	0.602274902	0	0.657209328	0	0.5	PRJNA827465;Yang2025_Silkie_Wenchang	CRA004775	0.893349419
ENSGALG00010005565	PGAP4	2	-0.096322606	0.161103844	-0.597891417	0.549912387	0	0.772217365	0	0.5	CRA004775;Yang2025_Silkie_Wenchang	PRJNA827465	0.878621348
ENSGALG00010005565	PGAP4	2	0.072853168	0.166519421	0.43750553	0.661744767	0	0.110479918	0	1	CRA004775;PRJNA827465	Yang2025_Silkie_Wenchang	0.912545506
ENSGALG00010005567	NDST3	2	-0.243688402	0.153061045	-1.592099432	0.111362365	0.024145243	1.968217693	49.19261199	1	PRJNA827465;Yang2025_Silkie_Wenchang	CRA004775	0.564466729
ENSGALG00010005567	NDST3	2	-0.033365254	0.181954532	-0.183371383	0.854506628	0	0.180030245	0	0.5	CRA004775;Yang2025_Silkie_Wenchang	PRJNA827465	0.965822236
ENSGALG00010005567	NDST3	2	-0.349728099	0.118672255	-2.947008117	0.003208648	0	0.747004229	0	0.5	CRA004775;PRJNA827465	Yang2025_Silkie_Wenchang	0.07867092
ENSGALG00010005568	PLEKHG1	2	0.170379885	0.214079499	0.795872025	0.426106423	0.076126088	5.883321831	83.00279963	0.5	PRJNA827465;Yang2025_Silkie_Wenchang	CRA004775	0.84125685
ENSGALG00010005568	PLEKHG1	2	0.201240483	0.287478472	0.700019315	0.483915242	0.100351368	2.043747575	51.07027833	0.5	CRA004775;Yang2025_Silkie_Wenchang	PRJNA827465	0.85691916
ENSGALG00010005568	PLEKHG1	2	-0.052629261	0.111331321	-0.472726459	0.636408347	0	0.210032557	0	1	CRA004775;PRJNA827465	Yang2025_Silkie_Wenchang	0.904566695
ENSGALG00010005572	ENSGALG00010005572	2	-0.103894947	0.083511742	-1.244075921	0.213471622	0	0.065042811	0	1	PRJNA827465;Yang2025_Silkie_Wenchang	CRA004775	0.726080554
ENSGALG00010005572	ENSGALG00010005572	2	-0.432797463	0.270746103	-1.598536263	0.109923682	0	0.821450542	0	1	CRA004775;Yang2025_Silkie_Wenchang	PRJNA827465	0.561519426
ENSGALG00010005572	ENSGALG00010005572	2	-0.270279723	0.267583838	-1.010079915	0.312439399	0.09358871	2.16839138	53.88711726	1	CRA004775;PRJNA827465	Yang2025_Silkie_Wenchang	0.796054997
ENSGALG00010005573	CHST9	2	-0.105691427	0.126273856	-0.837001665	0.402591632	0	0.536096744	0	0.5	PRJNA827465;Yang2025_Silkie_Wenchang	CRA004775	0.832937979
ENSGALG00010005573	CHST9	2	0.487513031	0.52678242	0.925454253	0.354729669	0.419552257	3.77635788	73.51945891	1	CRA004775;Yang2025_Silkie_Wenchang	PRJNA827465	0.812016118
ENSGALG00010005573	CHST9	2	0.385566481	0.630839366	0.611199974	0.541069948	0.677363928	6.057171646	83.49064451	0.5	CRA004775;PRJNA827465	Yang2025_Silkie_Wenchang	0.875829389
ENSGALG00010005575	ENSGALG00010005575	2	1.514926171	0.354211421	4.276898155	1.89515E-05	0	0.003176434	0	1	PRJNA827465;Yang2025_Silkie_Wenchang	CRA004775	0.002357128
ENSGALG00010005575	ENSGALG00010005575	2	0.050382188	1.472701292	0.034210731	0.972709109	3.983970335	12.20681059	91.80785192	0.5	CRA004775;Yang2025_Silkie_Wenchang	PRJNA827465	0.992213869
ENSGALG00010005575	ENSGALG00010005575	2	0.075335799	1.492602051	0.050472796	0.959745627	4.118601895	13.1304774	92.38413068	0.5	CRA004775;PRJNA827465	Yang2025_Silkie_Wenchang	0.989302378
ENSGALG00010005578	HEATR5A	2	0.041842582	0.060826884	0.6878962	0.491518149	0	0.434514214	0	1	PRJNA827465;Yang2025_Silkie_Wenchang	CRA004775	0.85895707
ENSGALG00010005578	HEATR5A	2	0.115549076	0.093485526	1.236465231	0.216265622	0	0.425405622	0	1	CRA004775;Yang2025_Silkie_Wenchang	PRJNA827465	0.72998072
ENSGALG00010005578	HEATR5A	2	0.030573886	0.074093789	0.412637632	0.679872125	0	0.915128793	0	1	CRA004775;PRJNA827465	Yang2025_Silkie_Wenchang	0.917701868
ENSGALG00010005583	ALDOB	2	-0.868440139	1.476492429	-0.588177848	0.556412925	3.703507579	6.633227133	84.92438175	0.5	PRJNA827465;Yang2025_Silkie_Wenchang	CRA004775	0.880324407
ENSGALG00010005583	ALDOB	2	-0.22980666	2.066365911	-0.111212955	0.911447979	8.066038258	17.76070958	94.36959432	0.5	CRA004775;Yang2025_Silkie_Wenchang	PRJNA827465	0.978845831
ENSGALG00010005583	ALDOB	2	1.368803586	0.570616967	2.398813327	0.016448298	0.152861179	1.281448831	21.96332966	1	CRA004775;PRJNA827465	Yang2025_Silkie_Wenchang	0.212234878
ENSGALG00010005587	LEF1	2	0.19394932	0.240321219	0.807042011	0.41964228	0.04927272	1.743657461	42.64928621	0.5	PRJNA827465;Yang2025_Silkie_Wenchang	CRA004775	0.838527387
ENSGALG00010005587	LEF1	2	0.429657726	0.241544708	1.778791719	0.075273915	0	0.006591828	0	1	CRA004775;Yang2025_Silkie_Wenchang	PRJNA827465	0.473509649
ENSGALG00010005587	LEF1	2	0.013816241	0.236644974	0.058383634	0.953442891	0	0.373350134	0	0.5	CRA004775;PRJNA827465	Yang2025_Silkie_Wenchang	0.987758913
ENSGALG00010005588	PLAGL1	2	-0.203725134	0.062160082	-3.277427042	0.001047578	0	0.107032705	0	1	PRJNA827465;Yang2025_Silkie_Wenchang	CRA004775	0.039063527
ENSGALG00010005588	PLAGL1	2	0.029503533	0.282794611	0.104328483	0.916908675	0.091881586	1.951825707	48.76591714	0.5	CRA004775;Yang2025_Silkie_Wenchang	PRJNA827465	0.980728718
ENSGALG00010005588	PLAGL1	2	-0.002033301	0.310613419	-0.006546081	0.994777017	0.13626842	2.562695289	60.97858359	0.5	CRA004775;PRJNA827465	Yang2025_Silkie_Wenchang	0.999822355
ENSGALG00010005590	TBC1D23	2	0.028518882	0.041888446	0.68082931	0.495979502	0	0.019460131	0	1	PRJNA827465;Yang2025_Silkie_Wenchang	CRA004775	0.860911322
ENSGALG00010005590	TBC1D23	2	0.044878558	0.095057873	0.472118265	0.636842376	0	0.220870738	0	1	CRA004775;Yang2025_Silkie_Wenchang	PRJNA827465	0.904812056
ENSGALG00010005590	TBC1D23	2	0.036118062	0.043325587	0.83364278	0.404482311	0	0.205314107	0	1	CRA004775;PRJNA827465	Yang2025_Silkie_Wenchang	0.833112102
ENSGALG00010005594	MRPL50	2	0.088031945	0.161982514	0.543465731	0.58680917	0	0.052345957	0	1	PRJNA827465;Yang2025_Silkie_Wenchang	CRA004775	0.891596903
ENSGALG00010005594	MRPL50	2	-0.048544138	0.226269052	-0.214541658	0.830124685	0	0.206252701	0	0.5	CRA004775;Yang2025_Silkie_Wenchang	PRJNA827465	0.957212554
ENSGALG00010005594	MRPL50	2	0.047832934	0.172216481	0.277748877	0.781205137	0	0.496580662	0	0.5	CRA004775;PRJNA827465	Yang2025_Silkie_Wenchang	0.945235709
ENSGALG00010005596	PARD3	2	0.025421141	0.113551822	0.223872597	0.822856439	0.017272638	2.423223483	58.7326548	0.5	PRJNA827465;Yang2025_Silkie_Wenchang	CRA004775	0.955822987
ENSGALG00010005596	PARD3	2	0.105845713	0.117068513	0.904146726	0.365917605	0	0.794598238	0	0.5	CRA004775;Yang2025_Silkie_Wenchang	PRJNA827465	0.818968857
ENSGALG00010005596	PARD3	2	-0.051889422	0.041566723	-1.248340459	0.211906404	0	0.022022143	0	1	CRA004775;PRJNA827465	Yang2025_Silkie_Wenchang	0.724222381
ENSGALG00010005601	TMEM215	2	0.397685674	0.254216979	1.564355287	0.117734155	0	0.030734836	0	1	PRJNA827465;Yang2025_Silkie_Wenchang	CRA004775	0.577330692
ENSGALG00010005601	TMEM215	2	-0.492185498	1.04362856	-0.47160984	0.637205302	1.820407182	5.791365332	82.72591456	0.5	CRA004775;Yang2025_Silkie_Wenchang	PRJNA827465	0.903096662
ENSGALG00010005601	TMEM215	2	-0.520060459	0.995984405	-0.522157231	0.601560862	1.659683109	5.684659416	82.40879661	0.5	CRA004775;PRJNA827465	Yang2025_Silkie_Wenchang	0.895230519
ENSGALG00010005604	ENSGALG00010005604	2	0.612566191	0.373729342	1.639063679	0.101199998	0	0.611090088	0	1	PRJNA827465;Yang2025_Silkie_Wenchang	CRA004775	0.542850216
ENSGALG00010005604	ENSGALG00010005604	2	0.401123161	0.578562261	0.693310276	0.488114855	0.308832935	1.856036624	46.12175281	0.5	CRA004775;Yang2025_Silkie_Wenchang	PRJNA827465	0.85768885
ENSGALG00010005604	ENSGALG00010005604	2	0.16913091	0.369970208	0.457147377	0.647565119	0	0.53419276	0	0.5	CRA004775;PRJNA827465	Yang2025_Silkie_Wenchang	0.907350807
ENSGALG00010005609	GJB6	2	-0.05819301	0.372762889	-0.156112669	0.875944212	0.024523054	1.08996007	8.253519807	0.5	PRJNA827465;Yang2025_Silkie_Wenchang	CRA004775	0.970347777
ENSGALG00010005609	GJB6	2	-0.290517955	0.43959544	-0.660875724	0.508692015	0	0.407110905	0	0.5	CRA004775;Yang2025_Silkie_Wenchang	PRJNA827465	0.865144635
ENSGALG00010005609	GJB6	2	0.164778433	0.368158464	0.447574753	0.654460128	0	0.067526378	0	1	CRA004775;PRJNA827465	Yang2025_Silkie_Wenchang	0.910177199
ENSGALG00010005610	MTHFD1L	2	-0.091589357	0.06592032	-1.389394913	0.164712696	0	0.152139773	0	1	PRJNA827465;Yang2025_Silkie_Wenchang	CRA004775	0.663901457
ENSGALG00010005610	MTHFD1L	2	-0.14496949	0.10260669	-1.412865866	0.157695167	0	0.317977762	0	1	CRA004775;Yang2025_Silkie_Wenchang	PRJNA827465	0.651135279
ENSGALG00010005610	MTHFD1L	2	-0.087450469	0.080310944	-1.08889854	0.27619863	0	0.546265592	0	1	CRA004775;PRJNA827465	Yang2025_Silkie_Wenchang	0.77570997
ENSGALG00010005617	DTD2	2	-0.609220379	0.208635501	-2.920022598	0.00350006	0.04064452	1.826980616	45.26488178	1	PRJNA827465;Yang2025_Silkie_Wenchang	CRA004775	0.083675706
ENSGALG00010005617	DTD2	2	-0.124656	0.312941852	-0.398335982	0.690382541	0.088666173	1.718735522	41.81769171	0.5	CRA004775;Yang2025_Silkie_Wenchang	PRJNA827465	0.919979266
ENSGALG00010005617	DTD2	2	-0.315680337	0.533034658	-0.592232292	0.553695048	0.470092043	5.365118786	81.3610837	0.5	CRA004775;PRJNA827465	Yang2025_Silkie_Wenchang	0.879765382
ENSGALG00010005618	PHACTR2	2	0.032838752	0.061258445	0.53606898	0.591910878	0	0.012479697	0	1	PRJNA827465;Yang2025_Silkie_Wenchang	CRA004775	0.892776493
ENSGALG00010005618	PHACTR2	2	0.111152853	0.10818445	1.027438353	0.3042141	0	0.514200754	0	1	CRA004775;Yang2025_Silkie_Wenchang	PRJNA827465	0.79225794
ENSGALG00010005618	PHACTR2	2	0.053835236	0.0631825	0.852059289	0.394181186	0	0.920060617	0	1	CRA004775;PRJNA827465	Yang2025_Silkie_Wenchang	0.830486298
ENSGALG00010005619	CELF2	2	-0.053739361	0.052648466	-1.020720348	0.307386951	0	0.008406848	0	1	PRJNA827465;Yang2025_Silkie_Wenchang	CRA004775	0.792675978
ENSGALG00010005619	CELF2	2	-0.012344352	0.122302919	-0.100932604	0.919603961	0	0.375185286	0	0.5	CRA004775;Yang2025_Silkie_Wenchang	PRJNA827465	0.981192162
ENSGALG00010005619	CELF2	2	-0.042256816	0.054227746	-0.779247136	0.435834149	0	0.438539367	0	0.5	CRA004775;PRJNA827465	Yang2025_Silkie_Wenchang	0.842834885
ENSGALG00010005621	CDH2	2	-0.198862473	0.068316486	-2.910900202	0.003603891	0.002313988	1.207656144	17.19497266	1	PRJNA827465;Yang2025_Silkie_Wenchang	CRA004775	0.085443345
ENSGALG00010005621	CDH2	2	0.070947469	0.483409507	0.146764737	0.883317719	0.372894187	4.308130894	76.7880776	0.5	CRA004775;Yang2025_Silkie_Wenchang	PRJNA827465	0.972001076
ENSGALG00010005621	CDH2	2	0.124832759	0.391670073	0.318719167	0.749939479	0.232120616	3.204968176	68.79844214	0.5	CRA004775;PRJNA827465	Yang2025_Silkie_Wenchang	0.937362187
ENSGALG00010005629	NRP1	2	-0.139836728	0.072759259	-1.92190974	0.054617116	0.000635466	1.059750294	5.638148387	1	PRJNA827465;Yang2025_Silkie_Wenchang	CRA004775	0.404444428
ENSGALG00010005629	NRP1	2	-0.217645765	0.11275707	-1.930218344	0.05357979	0	0.356098233	0	0.5	CRA004775;Yang2025_Silkie_Wenchang	PRJNA827465	0.401444454
ENSGALG00010005629	NRP1	2	-0.078484338	0.087272568	-0.899301355	0.368492166	0	0.092847368	0	0.5	CRA004775;PRJNA827465	Yang2025_Silkie_Wenchang	0.820279089
ENSGALG00010005630	NIT2	2	-0.202979035	0.07809184	-2.599234878	0.009343182	0	0.328220555	0	1	PRJNA827465;Yang2025_Silkie_Wenchang	CRA004775	0.151930112
ENSGALG00010005630	NIT2	2	-0.155959112	0.103191364	-1.511358181	0.130697218	0	0.014419689	0	1	CRA004775;Yang2025_Silkie_Wenchang	PRJNA827465	0.606397445
ENSGALG00010005630	NIT2	2	-0.23626273	0.106851947	-2.211122376	0.027027364	0	0.137673803	0	1	CRA004775;PRJNA827465	Yang2025_Silkie_Wenchang	0.280280365
ENSGALG00010005632	ITGB1	2	0.024408031	0.043172798	0.56535671	0.571831163	0	0.048440376	0	1	PRJNA827465;Yang2025_Silkie_Wenchang	CRA004775	0.885470687
ENSGALG00010005632	ITGB1	2	0.09288374	0.095651225	0.971066915	0.331514958	0	0.773003667	0	1	CRA004775;Yang2025_Silkie_Wenchang	PRJNA827465	0.801798188
ENSGALG00010005632	ITGB1	2	0.050596105	0.076785356	0.65892909	0.509941305	0.004603731	1.22226889	18.18494209	1	CRA004775;PRJNA827465	Yang2025_Silkie_Wenchang	0.865268882
ENSGALG00010005634	HADH	2	-0.251304702	0.064515611	-3.895254134	9.80958E-05	0	0.355036159	0	1	PRJNA827465;Yang2025_Silkie_Wenchang	CRA004775	0.00771092
ENSGALG00010005634	HADH	2	-0.140178765	0.275205304	-0.509360697	0.61049942	0.08778765	2.185565506	54.24525154	0.5	CRA004775;Yang2025_Silkie_Wenchang	PRJNA827465	0.897438028
ENSGALG00010005634	HADH	2	-0.131912143	0.191029346	-0.690533398	0.489858814	0.042717331	1.713430069	41.63753641	0.5	CRA004775;PRJNA827465	Yang2025_Silkie_Wenchang	0.858512413
ENSGALG00010005637	TOPORS	2	0.076209398	0.109385102	0.696707287	0.485985997	0	0.347001393	0	1	PRJNA827465;Yang2025_Silkie_Wenchang	CRA004775	0.857418308
ENSGALG00010005637	TOPORS	2	0.153290698	0.124337259	1.232862127	0.217627208	0	0.001926846	0	1	CRA004775;Yang2025_Silkie_Wenchang	PRJNA827465	0.731013651
ENSGALG00010005637	TOPORS	2	0.071320989	0.115989718	0.614890618	0.538626959	0	0.344426101	0	1	CRA004775;PRJNA827465	Yang2025_Silkie_Wenchang	0.87481821
ENSGALG00010005645	FUCA2	2	0.028475369	0.101625024	0.280200365	0.779323787	0.000136538	1.005936148	0.590111857	0.5	PRJNA827465;Yang2025_Silkie_Wenchang	CRA004775	0.944727706
ENSGALG00010005645	FUCA2	2	-0.155368971	0.165478992	-0.938904502	0.347779778	0	0.527294317	0	1	CRA004775;Yang2025_Silkie_Wenchang	PRJNA827465	0.808847403
ENSGALG00010005645	FUCA2	2	-0.000342814	0.226391849	-0.001514249	0.998791804	0.046061369	1.332111928	24.93123295	0.5	CRA004775;PRJNA827465	Yang2025_Silkie_Wenchang	0.999703568
ENSGALG00010005649	TOMM70	2	-0.109619519	0.043503238	-2.519801396	0.011742107	0.000635576	1.184631589	15.58557031	1	PRJNA827465;Yang2025_Silkie_Wenchang	CRA004775	0.173482822
ENSGALG00010005649	TOMM70	2	-0.132536872	0.072129157	-1.837493713	0.066137044	0.0018465	1.119728096	10.69260442	0.5	CRA004775;Yang2025_Silkie_Wenchang	PRJNA827465	0.442825253
ENSGALG00010005649	TOMM70	2	-0.068734501	0.046441994	-1.480007534	0.138871236	0	0.31845557	0	0.5	CRA004775;PRJNA827465	Yang2025_Silkie_Wenchang	0.622497285
ENSGALG00010005651	USP6NL	2	0.365381856	0.300698146	1.215111769	0.224323466	0.152045347	5.866419021	82.9538259	1	PRJNA827465;Yang2025_Silkie_Wenchang	CRA004775	0.737471949
ENSGALG00010005651	USP6NL	2	0.439645737	0.265701765	1.654658703	0.097993748	0.087576056	2.633002091	62.02053908	1	CRA004775;Yang2025_Silkie_Wenchang	PRJNA827465	0.534130658
ENSGALG00010005651	USP6NL	2	0.109126736	0.092162069	1.184074286	0.236383658	0	0.084490729	0	1	CRA004775;PRJNA827465	Yang2025_Silkie_Wenchang	0.748669015

ENSGALG00010005656	CYP2U1	2	0.110257207	0.494720334	0.222867748	0.823638439	0.456696937	14.75443866	93.22237854	0.5	PRJNA827465;Yang2025_Silkie_Wenchang	CRA004775	0.955934625
ENSGALG00010005656	CYP2U1	2	-0.194258524	0.207798644	-0.934840194	0.349870662	0.05509152	2.637622711	62.08707198	0.5	CRA004775;Yang2025_Silkie_Wenchang	PRJNA827465	0.809953147
ENSGALG00010005656	CYP2U1	2	0.335874774	0.284290285	1.181450059	0.237423982	0.113931419	3.387913709	70.48330962	1	CRA004775;PRJNA827465	Yang2025_Silkie_Wenchang	0.749366344
ENSGALG00010005660	UBAC2	2	-0.164543604	0.208609877	-0.788762288	0.430250955	0.071744996	5.021840944	80.08698381	0.5	PRJNA827465;Yang2025_Silkie_Wenchang	CRA004775	0.842040417
ENSGALG00010005660	UBAC2	2	-0.202363398	0.229111033	-0.883254709	0.377098668	0.063441034	2.496798764	59.94871456	0.5	CRA004775;Yang2025_Silkie_Wenchang	PRJNA827465	0.823064945
ENSGALG00010005660	UBAC2	2	0.013247765	0.050938826	0.260072059	0.79480819	0	0.024787848	0	1	CRA004775;PRJNA827465	Yang2025_Silkie_Wenchang	0.948905846
ENSGALG00010005670	SGMS2	2	-0.014245973	0.156855269	-0.090822404	0.927633708	0	0.842133225	0	0.5	PRJNA827465;Yang2025_Silkie_Wenchang	CRA004775	0.982993195
ENSGALG00010005670	SGMS2	2	0.082177439	0.376881863	0.218045618	0.827393569	0.153872611	1.920276122	47.92415587	0.5	CRA004775;Yang2025_Silkie_Wenchang	PRJNA827465	0.956550752
ENSGALG00010005670	SGMS2	2	0.177070043	0.192139025	0.921572506	0.356751605	0	0.815696596	0	1	CRA004775;PRJNA827465	Yang2025_Silkie_Wenchang	0.812929518
ENSGALG00010005675	GJA3	2	-0.068481481	0.252712658	-0.270985558	0.78640214	0.02384012	1.202892663	16.86706296	0.5	PRJNA827465;Yang2025_Silkie_Wenchang	CRA004775	0.946651901
ENSGALG00010005675	GJA3	2	-0.419391354	0.33198323	-1.263290782	0.206484698	0	0.000128734	0	1	CRA004775;Yang2025_Silkie_Wenchang	PRJNA827465	0.718564554
ENSGALG00010005675	GJA3	2	0.020512489	0.246506494	0.083212775	0.933682355	0	0.691555887	0	0.5	CRA004775;PRJNA827465	Yang2025_Silkie_Wenchang	0.984262625
ENSGALG00010005679	TMEM45A	2	-0.066454014	0.220538259	-0.301326464	0.763165563	0.045975627	1.785410814	43.99048151	0.5	PRJNA827465;Yang2025_Silkie_Wenchang	CRA004775	0.940184187
ENSGALG00010005679	TMEM45A	2	-0.317771884	0.258489189	-1.229343034	0.218943219	0	0.072906852	0	1	CRA004775;Yang2025_Silkie_Wenchang	PRJNA827465	0.73259614
ENSGALG00010005679	TMEM45A	2	0.074751605	0.16767465	0.445813398	0.655732043	0	0.254156906	0	0.5	CRA004775;PRJNA827465	Yang2025_Silkie_Wenchang	0.910411938
ENSGALG00010005680	ENSGALG00010005680	2	-0.14227485	0.365560334	-0.38919663	0.697130696	0.014137921	1.047782579	4.560352494	0.5	PRJNA827465;Yang2025_Silkie_Wenchang	CRA004775	0.921116491
ENSGALG00010005680	ENSGALG00010005680	2	0.307979432	0.425886877	0.723148444	0.469588689	0	0.035008138	0	1	CRA004775;Yang2025_Silkie_Wenchang	PRJNA827465	0.853485658
ENSGALG00010005680	ENSGALG00010005680	2	-0.165561621	0.340750073	-0.485874058	0.627056463	0	0.777701957	0	0.5	CRA004775;PRJNA827465	Yang2025_Silkie_Wenchang	0.901573209
ENSGALG00010005681	EPC1	2	0.053740024	0.114240547	0.470411126	0.638061317	0.019879774	3.814623636	73.78509401	0.5	PRJNA827465;Yang2025_Silkie_Wenchang	CRA004775	0.905365679
ENSGALG00010005681	EPC1	2	0.144849182	0.097154494	1.49091593	0.135983568	0	0.866930699	0	0.5	CRA004775;Yang2025_Silkie_Wenchang	PRJNA827465	0.617575642
ENSGALG00010005681	EPC1	2	-0.041507049	0.046849834	-0.885959366	0.375639424	0	0.00090837	0	1	CRA004775;PRJNA827465	Yang2025_Silkie_Wenchang	0.822974013
ENSGALG00010005692	SEC24B	2	0.141684157	0.164868446	0.859377041	0.390132532	0.046168634	5.988318195	83.30082057	0.5	PRJNA827465;Yang2025_Silkie_Wenchang	CRA004775	0.829204867
ENSGALG00010005692	SEC24B	2	0.314330414	0.113894443	2.759839774	0.005782972	0	0.07188676	0	1	CRA004775;Yang2025_Silkie_Wenchang	PRJNA827465	0.114502839
ENSGALG00010005692	SEC24B	2	0.025126732	0.07928969	0.316897844	0.751321118	0.005421748	1.189741062	15.94809728	0.5	CRA004775;PRJNA827465	Yang2025_Silkie_Wenchang	0.937684688
ENSGALG00010005694	ENSGALG00010005694	2	0.001059521	0.098119844	0.010798237	0.991384421	0.012680975	2.65526169	62.33892863	0.5	PRJNA827465;Yang2025_Silkie_Wenchang	CRA004775	0.99755571
ENSGALG00010005694	ENSGALG00010005694	2	0.086114084	0.102998955	0.836067551	0.403116902	0	0.87180043	0	0.5	CRA004775;Yang2025_Silkie_Wenchang	PRJNA827465	0.833046091
ENSGALG00010005694	ENSGALG00010005694	2	-0.079292075	0.053348883	-1.486293064	0.13720162	0	0.070252162	0	1	CRA004775;PRJNA827465	Yang2025_Silkie_Wenchang	0.619615281
ENSGALG00010005695	KIF5B	2	0.068929387	0.038665698	1.782701238	0.07463495	0	0.728631384	0	1	PRJNA827465;Yang2025_Silkie_Wenchang	CRA004775	0.471679921
ENSGALG00010005695	KIF5B	2	0.282308014	0.159064122	1.774806351	0.075929867	0.022956357	1.682017718	40.54759417	1	CRA004775;Yang2025_Silkie_Wenchang	PRJNA827465	0.47539798
ENSGALG00010005695	KIF5B	2	0.225321342	0.216454242	1.04096524	0.297891681	0.073180203	3.722056167	73.13312978	1	CRA004775;PRJNA827465	Yang2025_Silkie_Wenchang	0.788927563
ENSGALG00010005696	NUBPL	2	-0.21196483	0.077179136	-2.746400639	0.006025313	0	0.555283904	0	1	PRJNA827465;Yang2025_Silkie_Wenchang	CRA004775	0.117234105
ENSGALG00010005696	NUBPL	2	-0.198345214	0.136453253	-1.453576291	0.146063814	0.005221135	1.098971963	9.005867914	1	CRA004775;Yang2025_Silkie_Wenchang	PRJNA827465	0.634897819
ENSGALG00010005696	NUBPL	2	-0.272396078	0.089797666	-3.033442733	0.002417806	0	0.494133083	0	1	CRA004775;PRJNA827465	Yang2025_Silkie_Wenchang	0.06593704
ENSGALG00010005699	PRPF18	2	-0.018115126	0.074999291	-0.241537302	0.809138709	0.006935591	2.41996746	58.67713031	0.5	PRJNA827465;Yang2025_Silkie_Wenchang	CRA004775	0.952716213
ENSGALG00010005699	PRPF18	2	0.041212047	0.078741908	0.523381364	0.600708891	0	0.926282541	0	0.5	CRA004775;Yang2025_Silkie_Wenchang	PRJNA827465	0.895230519
ENSGALG00010005699	PRPF18	2	-0.080927582	0.04639445	-1.744337574	0.081100243	0	0.058505766	0	1	CRA004775;PRJNA827465	Yang2025_Silkie_Wenchang	0.49038198
ENSGALG00010005704	AKAP12	2	-0.063012072	0.095741775	-0.658146058	0.510444283	0	0.635632067	0	0.5	PRJNA827465;Yang2025_Silkie_Wenchang	CRA004775	0.865365179
ENSGALG00010005704	AKAP12	2	0.33348106	0.309200639	1.078526426	0.280798895	0.091058275	1.758940966	43.14760875	1	CRA004775;Yang2025_Silkie_Wenchang	PRJNA827465	0.778581075
ENSGALG00010005704	AKAP12	2	0.228848488	0.415221163	0.551148419	0.581531936	0.26762384	3.735630155	73.23075469	0.5	CRA004775;PRJNA827465	Yang2025_Silkie_Wenchang	0.889045686
ENSGALG00010005705	ECHDC3	2	-0.136819881	0.103970236	-1.315952386	0.188190024	0.006451106	1.300375513	23.09913638	1	PRJNA827465;Yang2025_Silkie_Wenchang	CRA004775	0.697483156
ENSGALG00010005705	ECHDC3	2	-0.321654006	0.163178222	-1.971182194	0.04870304	0	0.013580996	0	1	CRA004775;Yang2025_Silkie_Wenchang	PRJNA827465	0.381461853
ENSGALG00010005705	ECHDC3	2	-0.093708015	0.087478629	-1.071210381	0.284074845	0	0.64785713	0	1	CRA004775;PRJNA827465	Yang2025_Silkie_Wenchang	0.779896175
ENSGALG00010005712	ENSGALG00010005712	2	0.048635636	0.20731031	0.234603074	0.814516824	0.068514576	4.86022485	79.42482023	0.5	PRJNA827465;Yang2025_Silkie_Wenchang	CRA004775	0.953869515
ENSGALG00010005712	ENSGALG00010005712	2	0.23524198	0.139493411	1.686402094	0.091718378	0	0.325353991	0	1	CRA004775;Yang2025_Silkie_Wenchang	PRJNA827465	0.518914432
ENSGALG00010005712	ENSGALG00010005712	2	-0.129515408	0.107117939	-1.20909167	0.226627629	0	0.262112797	0	0.5	CRA004775;PRJNA827465	Yang2025_Silkie_Wenchang	0.740165008
ENSGALG00010005714	ARHGAP5	2	-0.127020779	0.065157611	-1.949438854	0.051243038	0	0.401558624	0	1	PRJNA827465;Yang2025_Silkie_Wenchang	CRA004775	0.392134956
ENSGALG00010005714	ARHGAP5	2	0.251621092	0.492451596	0.510955988	0.609381876	0.42786841	7.993806196	87.49031468	0.5	CRA004775;Yang2025_Silkie_Wenchang	PRJNA827465	0.897238578
ENSGALG00010005714	ARHGAP5	2	0.288135864	0.4434939	0.649695213	0.515889116	0.343989425	7.183955921	86.08009276	0.5	CRA004775;PRJNA827465	Yang2025_Silkie_Wenchang	0.867498525
ENSGALG00010005716	ZBTB2	2	0.059128813	0.103500579	0.571289681	0.567803296	0.010463284	1.598026826	37.42282771	0.5	PRJNA827465;Yang2025_Silkie_Wenchang	CRA004775	0.883781749
ENSGALG00010005716	ZBTB2	2	0.287026865	0.13094682	2.191934599	0.028384229	0	0.198773205	0	1	CRA004775;Yang2025_Silkie_Wenchang	PRJNA827465	0.286930393
ENSGALG00010005716	ZBTB2	2	0.12643888	0.170152715	0.743090582	0.457426841	0.04145539	2.958669014	66.20101825	0.5	CRA004775;PRJNA827465	Yang2025_Silkie_Wenchang	0.849573219
ENSGALG00010005720	COL25A1	2	0.259841844	0.335726629	0.773968526	0.438949404	0.192011558	6.667385387	85.00161695	0.5	PRJNA827465;Yang2025_Silkie_Wenchang	CRA004775	0.844002503
ENSGALG00010005720	COL25A1	2	0.382809854	0.475786939	0.804582518	0.421060634	0.234383062	1.460207883	31.51660036	0.5	CRA004775;Yang2025_Silkie_Wenchang	PRJNA827465	0.839269089
ENSGALG00010005720	COL25A1	2	-0.073902959	0.151827557	-0.4867559	0.626431325	0	0.300259295	0	1	CRA004775;PRJNA827465	Yang2025_Silkie_Wenchang	0.901504244
ENSGALG00010005724	PROSER2	2	-0.142161904	0.151462842	-0.938592607	0.34793995	0.03394116	3.774206172	73.50436212	1	PRJNA827465;Yang2025_Silkie_Wenchang	CRA004775	0.808847403
ENSGALG00010005724	PROSER2	2	0.322709848	0.690632351	0.467267205	0.640308717	0.856617826	9.103785734	89.0155587	0.5	CRA004775;Yang2025_Silkie_Wenchang	PRJNA827465	0.906161493
ENSGALG00010005724	PROSER2	2	0.453103649	0.534797209	0.847243854	0.396859215	0.484879453	5.752643876	82.61668858	0.5	CRA004775;PRJNA827465	Yang2025_Silkie_Wenchang	0.831375233
ENSGALG00010005740	ENSGALG00010005740	2	0.032934295	0.233544851	0.141019143	0.88785482	0	0.714255283	0	0.5	PRJNA827465;Yang2025_Silkie_Wenchang	CRA004775	0.973099776
ENSGALG00010005740	ENSGALG00010005740	2	0.073210297	0.306044287	0.239214715	0.810939093	0	0.925557977	0	0.5	CRA004775;Yang2025_Silkie_Wenchang	PRJNA827465	0.953118313
ENSGALG00010005740	ENSGALG00010005740	2	-0.178247932	0.260954676	-0.683060885	0.494568371	0	0.131808246	0	1	CRA004775;PRJNA827465	Yang2025_Silkie_Wenchang	0.860238557
ENSGALG00010005742	GPR183	2	-0.117873185	0.113267448	-1.0406625	0.298032213	0.017216261	3.038584357	67.08993786	1	PRJNA827465;Yang2025_Silkie_Wenchang	CRA004775	0.789057601
ENSGALG00010005742	GPR183	2	-0.23659482	0.09147145	-2.586542808	0.009694413	0	0.057535454	0	1	CRA004775;Yang2025_Silkie_Wenchang	PRJNA827465	0.155237398
ENSGALG00010005742	GPR183	2	-0.017884361	0.088899137	-0.20117587	0.840561059	0	0.507833102	0	1	CRA004775;PRJNA827465	Yang2025_Silkie_Wenchang	0.959723769
ENSGALG00010005743	ETNPPL	2	0.04615206	0.122313571	0.377325751	0.705931538	0	0.157660147	0	1	PRJNA827465;Yang2025_Silkie_Wenchang	CRA004775	0.924548793
ENSGALG00010005743	ETNPPL	2	0.211257241	0.375951706	0.56192654	0.574166071	0.158666453	1.627196991	38.54462579	1	CRA004775;Yang2025_Silkie_Wenchang	PRJNA827465	0.886081433
ENSGALG00010005743	ETNPPL	2	0.243750318	0.295683342	0.8243627	0.409733502	0.059826417	1.226719695	18.48178488	1	CRA004775;PRJNA827465	Yang2025_Silkie_Wenchang	0.834652129
ENSGALG00010005748	AIG1	2	-0.260277871	0.160066291	-1.626062978	0.103936259	0.037471235	3.675528416	72.79302765	1	PRJNA827465;Yang2025_Silkie_Wenchang	CRA004775	0.548175896
ENSGALG00010005748	AIG1	2	-0.429152888	0.126864847	-3.382756524	0.000717622	0	0.004075267	0	1	CRA004775;Yang2025_Silkie_Wenchang	PRJNA827465	0.030005007
ENSGALG00010005748	AIG1	2	-0.124965767	0.100083185	-1.248619006	0.211804858	0	0.394881713	0	1	CRA004775;PRJNA827465	Yang2025_Silkie_Wenchang	0.724118735
ENSGALG00010005752	RMND1	2	0.005913425	0.067681404	0.08737149	0.93037623	0	0.157356485	0	0.5	PRJNA827465;Yang2025_Silkie_Wenchang	CRA004775	0.983813577
ENSGALG00010005752	RMND1	2	0.103669584	0.196657611	0.527157752	0.598084044	0.038957492	1.885877471	46.97428569	0.5	CRA004775;Yang2025_Silkie_Wenchang	PRJNA827465	0.894645663
ENSGALG00010005752	RMND1	2	0.102134898	0.146012277	0.699495277	0.484242564	0.022392447	1.628532532	38.59502463	1	CRA004775;PRJNA827465	Yang2025_Silkie_Wenchang	0.857004733
ENSGALG00010005755	ZMYM2	2	0.154679244	0.231301198	0.668735163	0.503664433	0.094421691	8.060934603	87.5944906	0.5	PRJNA827465;Yang2025_Silkie_Wenchang	CRA004775	0.863151792
ENSGALG00010005755	ZMYM2	2	0.158630379	0.256397902	0.618688287	0.536121728	0.10468496	4.886396911	79.53502308	0.5	CRA004775;Yang2025_Silkie_Wenchang	PRJNA827465	0.874599474
ENSGALG00010005755	ZMYM2	2	-0.063241342	0.060354454	-1.047832225	0.294715912	0	0.067202155	0	1	CRA004775;PRJNA827465	Yang2025_Silkie_Wenchang	0.787482391
ENSGALG00010005756	TET2	2	0.272596796	0.247297759	1.102301927	0.270330432	0.096736662	4.210589532	76.25035657	1	PRJNA827465;Yang2025_Silkie_Wenchang	CRA004775	0.772430311
ENSGALG00010005756	TET2	2	0.632890514	0.218162171	2.901009432	0.003719627	0	0.56064303	0	1	CRA004775;Yang2025_Silkie_Wenchang	PRJNA827465	0.087190443
ENSGALG00010005756	TET2	2	0.378160938	0.463691282	0.815544638	0.414760625	0.304833209	2.530879115	60.48803777	1	CRA004775;PRJNA827465	Yang2025_Silkie_Wenchang	0.837298284
ENSGALG00010005757	ARHGAP12	2	0.097295805	0.097268031	1.000285532	0.317172347	0.008027634	1.619104642	38.23746938	1	PRJNA827465;Yang2025_Silkie_Wenchang	CRA004775	0.797047618
ENSGALG00010005757	ARHGAP12	2	0.486774844	0.28004198	1.738220979	0.082171884	0.123803274	4.594425028	78.23449084	1	CRA004775;Yang2025_Silkie_Wenchang	PRJNA827465	0.986179568
ENSGALG00010005757	ARHGAP12	2	0.382788332	0.382722085	1.000173096	0.317226747	0.266670819	10.57605555	90.54467901	1	CRA004775;PRJNA827465	Yang2025_Silkie_Wenchang	0.797082395
ENSGALG00010005758	HIVEP2	2	0.023269602	0.078514576	0.296373022	0.766945226	0	0.845863528	0	0.5	PRJNA827465;Yang2025_Silkie_Wenchang	CRA004775	0.941375309
ENSGALG00010005758	HIVEP2	2	0.295655754	0.158065956	1.870458136	0.061420025	0	0.800833762	0	1	CRA004775;Yang2025_Silkie_Wenchang	PRJNA827465	0.622958152
ENSGALG00010005758	HIVEP2	2	0.175133823	0.238141316	0.735419731	0.462083909	0.082938876	3.155499025	68.30929143	0.5	CRA004775;PRJNA827465	Yang2025_Silkie_Wenchang	0.850747865
ENSGALG00010005760	GPR18	2	0.069637646	0.221945399	0.313760261	0.753703129	0	0.294820305	0	0.5	PRJNA827465;Yang2025_Silkie_Wenchang	CRA004775	0.938112996
ENSGALG00010005760	GPR18	2	0.307956691	0.514053791	0.599074837	0.549122981	0.28708433	2.035584066	50.87405052	0.5	CRA004775;Yang2025_Silkie_Wenchang	PRJNA827465	0.878431377
ENSGALG00010005760	GPR18	2	0.356449518	0.347173593	1.026718405	0.30455308	0.081221204	1.326918258	24.63740745	1	CRA004775;PRJNA827465	Yang2025_Silkie_Wenchang	0.79225794
ENSGALG00010005763	AKAP6	2	-0.1243047	0.115725705	-1.074132149	0.282763457	0.008314174	1.436272447	30.37532662	0.5	PRJNA827465;Yang2025_Silkie_Wenchang	CRA004775	0.779532565
ENSGALG00010005763	AKAP6	2	0.15259286	0.267771418	0.569862387	0.568771038	0.069024957	1.496747026	33.18844249	1	CRA004775;Yang2025_Silkie_Wenchang	PRJNA827465	0.884307452
ENSGALG00010005763	AKAP6	2	0.078881595	0.417751632	0.188824145	0.850230647	0.251303244	2.862237318	65.06229607	0.5	CRA004775;PRJNA827465	Yang2025_Silkie_Wenchang	0.961982453
ENSGALG00010005769	TFG	2	0.109313815	0.215516151	0.507218669	0.612001397	0.085063624	11.54500716	91.33824704	0.5	PRJNA827465;Yang2025_Silkie_Wenchang	CRA004775	0.897777844
ENSGALG00010005769	TFG	2	0.216662455	0.158138499	1.370080382	0.170661811	0.026486653	1.959405467	48.96411096	1	CRA004775;Yang2025_Silkie_Wenchang	PRJNA827465	0.672386723
ENSGALG00010005769	TFG	2	-0.088046717	0.055948324	-1.573715002	0.115553352	0	0.229909376	0	0.5	CRA004775;PRJNA827465	Yang2025_Silkie_Wenchang	0.572716099
ENSGALG00010005772	SVEP1	2	-0.197432643	0.174210609	-1.133298621	0.257088871	0.04172816	3.155966424	68.31398482	1	PRJNA827465;Yang2025_Silkie_Wenchang	CRA004775	0.764870705
ENSGALG00010005772	SVEP1	2	0.005158583	0.14260081	0.036174993	0.971142826	0	0.050501627	0	0.5	CRA004775;Yang2025_Silkie_Wenchang	PRJNA827465	0.991925437
ENSGALG00010005772	SVEP1	2	-0.268930026	0.176285884	-1.525533523	0.127126079	0.024559456	1.346263394	25.7203305	0.5	CRA004775;PRJNA827465	Yang2025_Silkie_Wenchang	0.598452543
ENSGALG00010005777	ENSGALG00010005777	2	-0.468470464	1.447611512	-0.32361615	0.74622866	2.864725721	3.158316583	68.33756295	0.5	PRJNA827465;Yang2025_Silkie_Wenchang	CRA004775	0.936550805
ENSGALG00010005777	ENSGALG00010005777	2	0.468820122	0.783093614	0.598676982	0.54938831	0	0.366722255	0	1	CRA004775;Yang2025_Silkie_Wenchang	PRJNA827465	0.878554604
ENSGALG00010005777	ENSGALG00010005777	2	-0.912573792	0.971949028	-0.938911163	0.347776357	0.606688748	1.471063428	32.02196581	0.5	CRA004775;PRJNA827465	Yang2025_Silkie_Wenchang	0.808847403
ENSGALG00010005779	ARMT1	2	-0.137344705	0.185093766	-0.742027717	0.458070542	0.061931305	10.23179865	90.22654731	0.5	PRJNA827465;Yang2025_Silkie_Wenchang	CRA004775	0.850025804
ENSGALG00010005779	ARMT1	2	-0.307135113	0.090120544	-3.408047718	0.000654295	0	0.369105956	0	1	CRA004775;Yang2025_Silkie_Wenchang	PRJNA827465	0.028276881
ENSGALG00010005779	ARMT1	2	0.025501029	0.059804245	0.426408333	0.66981033	0	0.83392724	0	0.5	CRA004775;PRJNA827465	Yang2025_Silkie_Wenchang	0.914377769
ENSGALG00010005784	RASL11A	2	0.048985543	0.286578682	0.170932263	0.864277028	0	0.361932265	0	0.5	PRJNA827465;Yang2025_Silkie_Wenchang	CRA004775	0.966639539
ENSGALG00010005784	RASL11A	2	-0.379246878	0.672922813	-0.563581543	0.573038951	0.657821713	3.649823473	72.60141463	0.5	CRA004775;Yang2025_Silkie_Wenchang	PRJNA827465	0.88573298
ENSGALG00010005784	RASL11A	2	-0.502700397	0.488291262	-1.029509302	0.303240411	0.288888364	2.459141824	59.33540758	1	CRA004775;PRJNA827465	Yang2025_Silkie_Wenchang	0.792084776
ENSGALG00010005785	RPL21	2	0.130258008	0.20175008	0.645640427	0.518512249	0.069255387	6.210287538	83.89768599	0.5	PRJNA827465;Yang2025_Silkie_Wenchang	CRA004775	0.868525042
ENSGALG00010005785	RPL21	2	0.159862662	0.235995962	0.677395751	0.498154894	0.071679524	2.655789767	62.34641716	0.5	CRA004775;Yang2025_Silkie_Wenchang	PRJNA827465	0.86140436
ENSGALG00010005785	RPL21	2	-0.052921747	0.058262972	-0.908325557	0.363706244	0	0.079776265	0	1	CRA004775;PRJNA827465	Yang2025_Silkie_Wenchang	0.817042253
ENSGALG00010005789	RNASET2	2	0.071610531	0.070629382	1.013891512	0.310634522	0	0.509219662	0	1	PRJNA827465;Yang2025_Silkie_Wenchang	CRA004775	0.794899969
ENSGALG00010005789	RNASET2	2	0.129311881	0.117864572	1.097122564	0.272587818	0	0.182492712	0	0.5	CRA004775;Yang2025_Silkie_Wenchang	PRJNA827465	0.773539278
ENSGALG00010005789	RNASET2	2	0.032416324	0.08524858	0.38025647	0.703755045	0	0.044087657	0	0.5	CRA004775;PRJNA827465	Yang2025_Silkie_Wenchang	0.923682903
ENSGALG00010005790	MUSK	2	0.176906826	0.355976024	0.496962756	0.619215316	0.227224286	9.667148245	89.65568775	0.5	PRJNA827465;Yang2025_Silkie_Wenchang	CRA004775	0.899168321
ENSGALG00010005790	MUSK	2	0.518231132	0.159210582	3.255004321	0.001133906	0	0.14357293	0	1	CRA004775;Yang2025_Silkie_Wenchang	PRJNA827465	0.040858726
ENSGALG00010005790	MUSK	2	-0.149307946	0.154199886	-0.968275333	0.33290688	0	0.526081382	0	0.5	CRA004775;PRJNA827465	Yang2025_Silkie_Wenchang	0.802178578
ENSGALG00010005792	ADGRG6	2	0.073979375	0.107593991	0.687579052	0.491717904	0	0.969804083	0	0.5	PRJNA827465;Yang2025_Silkie_Wenchang	CRA004775	0.85895707
ENSGALG00010005792	ADGRG6	2	0.260990019	0.16439213	1.587606527	0.112375341	0	0.239058034	0	1	CRA004775;Yang2025_Silkie_Wenchang	PRJNA827465	0.56602666
ENSGALG00010005792	ADGRG6	2	0.071232665	0.160733908	0.443171361	0.657641792	0.013664156	1.179160141	15.19387698	0.5	CRA004775;PRJNA827465	Yang2025_Silkie_Wenchang	0.910774485
ENSGALG00010005799	UFSP2	2	-0.046725155	0.106081962	-0.440462777	0.659601966	0.010650482	1.892256725	47.15304818	0.5	PRJNA827465;Yang2025_Silkie_Wenchang	CRA004775	0.911617103
ENSGALG00010005799	UFSP2	2	-0.08739618	0.186225158	-0.469303829	0.638852479	0.044890703	2.654546238	62.32877824	0.5	CRA004775;Yang2025_Silkie_Wenchang	PRJNA827465	0.905768875
ENSGALG00010005799	UFSP2	2	-0.18095192	0.091734982	-1.972550884	0.048546754	0	0.540734516	0	1	CRA004775;PRJNA827465	Yang2025_Silkie_Wenchang	0.380997016
ENSGALG00010005800	FRMD4A	2	0.019227868	0.052228531	0.36814873	0.712762337	0	0.105681657	0	1	PRJNA827465;Yang2025_Silkie_Wenchang	CRA004775	0.926097326
ENSGALG00010005800	FRMD4A	2	0.298536022	0.274572909	1.087274135	0.276915676	0.09364301	2.474227475	59.58334429	1	CRA004775;Yang2025_Silkie_Wenchang	PRJNA827465	0.776084088
ENSGALG00010005800	FRMD4A	2	0.250487614	0.30166771	0.830342809	0.406344991	0.143931646	3.932143192	74.56857619	1	CRA004775;PRJNA827465	Yang2025_Silkie_Wenchang	0.833580937
ENSGALG00010005814	UPF2	2	0.078125391	0.154752076	0.504842281	0.613669617	0.036234302	3.397716747	70.5684707	0.5	PRJNA827465;Yang2025_Silkie_Wenchang	CRA004775	0.898256044
ENSGALG00010005814	UPF2	2	0.195923536	0.126647917	1.546993748	0.121864753	0	0.569369348	0	1	CRA004775;Yang2025_Silkie_Wenchang	PRJNA827465	0.585419059
ENSGALG00010005814	UPF2	2	-0.034608945	0.041454714	-0.834861493	0.403795698	0	0.422410534	0	0.5	CRA004775;PRJNA827465	Yang2025_Silkie_Wenchang	0.833112102
ENSGALG00010005817	VTA1	2	-0.248627854	0.203017684	-1.224661066	0.220702949	0.078678043	21.57696639	95.3654282	1	PRJNA827465;Yang2025_Silkie_Wenchang	CRA004775	0.734053249
ENSGALG00010005817	VTA1	2	-0.308418723	0.187028399	-1.649047551	0.099137894	0.051025216	3.260941148	69.33400652	1	CRA004775;Yang2025_Silkie_Wenchang	PRJNA827465	0.537214481
ENSGALG00010005817	VTA1	2	-0.052211369	0.037174722	-1.404485806	0.160174235	0	0.012608392	0	1	CRA004775;PRJNA827465	Yang2025_Silkie_Wenchang	0.655558469
ENSGALG00010005821	DOCK9	2	-0.060308818	0.064486438	-0.935217083	0.349676437	0	0.069796278	0	1	PRJNA827465;Yang2025_Silkie_Wenchang	CRA004775	0.809903518
ENSGALG00010005821	DOCK9	2	0.32170724	0.424607267	0.757658347	0.448655541	0.276796343	3.789423456	73.61076133	0.5	CRA004775;Yang2025_Silkie_Wenchang	PRJNA827465	0.846878812
ENSGALG00010005821	DOCK9	2	0.293988321	0.4460638	0.659072359	0.509849305	0.325156051	4.602583136	78.27307035	0.5	CRA004775;PRJNA827465	Yang2025_Silkie_Wenchang	0.865257661
ENSGALG00010005826	LRP2BP	2	0.046393582	0.182955876	0.253577985	0.799821606	0.00663103	1.107494323	9.706083438	0.5	PRJNA827465;Yang2025_Silkie_Wenchang	CRA004775	0.949922217
ENSGALG00010005826	LRP2BP	2	-0.460459787	0.379467828	-1.213435642	0.224963306	0.160142921	2.0963275	52.29752985	1	CRA004775;Yang2025_Silkie_Wenchang	PRJNA827465	0.737917946
ENSGALG00010005826	LRP2BP	2	-0.29785652	0.571437542	-0.521240728	0.602199085	0.527420378	4.867042924	79.4536433	0.5	CRA004775;PRJNA827465	Yang2025_Silkie_Wenchang	0.895349419
ENSGALG00010005828	LPAR1	2	-0.017753901	0.076069597	-0.233390232	0.815458397	0	0.559869923	0	0.5	PRJNA827465;Yang2025_Silkie_Wenchang	CRA004775	0.954009864
ENSGALG00010005828	LPAR1	2	-0.126814614	0.125067491	-1.013969442	0.310597334	0	0.750857411	0	1	CRA004775;Yang2025_Silkie_Wenchang	PRJNA827465	0.794899969
ENSGALG00010005828	LPAR1	2	-0.053147275	0.187875596	-0.282885461	0.77726464	0.032344969	1.288167839	22.37036437	0.5	CRA004775;PRJNA827465	Yang2025_Silkie_Wenchang	0.944218792
ENSGALG00010005829	NPAS3	2	-0.197756266	0.206271027	-0.958720518	0.337699559	0.057365119	2.609348911	61.67626353	0.5	PRJNA827465;Yang2025_Silkie_Wenchang	CRA004775	0.80415385
ENSGALG00010005829	NPAS3	2	-0.190707284	0.34782485	-0.548285391	0.583495957	0.131922108	2.053733989	51.30820227	0.5	CRA004775;Yang2025_Silkie_Wenchang	PRJNA827465	0.890558522
ENSGALG00010005829	NPAS3	2	-0.362905112	0.090241911	-4.021469729	5.78361E-05	0	0.416476822	0	1	CRA004775;PRJNA827465	Yang2025_Silkie_Wenchang	0.005336024
ENSGALG00010005830	USP12	2	-0.184091746	0.162012033	-1.136284402	0.255837557	0	0.129760182	0	1	PRJNA827465;Yang2025_Silkie_Wenchang	CRA004775	0.764478331
ENSGALG00010005830	USP12	2	-0.201639769	0.212755412	-0.947753886	0.343254761	0	0.149197472	0	1	CRA004775;Yang2025_Silkie_Wenchang	PRJNA827465	0.806218116
ENSGALG00010005830	USP12	2	-0.120825604	0.187272466	-0.645186168	0.518806549	0	0.02000079	0	1	CRA004775;PRJNA827465	Yang2025_Silkie_Wenchang	0.868833516
ENSGALG00010005831	SEC61A2	2	-0.289940967	0.247820312	-1.169964495	0.242015257	0.11417671	13.86353558	92.78683281	1	PRJNA827465;Yang2025_Silkie_Wenchang	CRA004775	0.75421685
ENSGALG00010005831	SEC61A2	2	-0.443167432	0.16809899	-2.636347973	0.008380374	0.025248114	1.567175572	36.19094006	1	CRA004775;Yang2025_Silkie_Wenchang	PRJNA827465	0.142771619
ENSGALG00010005831	SEC61A2	2	-0.058129728	0.058909251	-0.986767394	0.323756682	0	0.191201026	0	1	CRA004775;PRJNA827465	Yang2025_Silkie_Wenchang	0.799600434
ENSGALG00010005836	NEBL	2	0.010985741	0.146001021	0.075244277	0.940020356	0	0.000146301	0	1	PRJNA827465;Yang2025_Silkie_Wenchang	CRA004775	0.985444231
ENSGALG00010005836	NEBL	2	0.014007552	0.203061414	0.068981848	0.945004068	0	0.00012563	0	1	CRA004775;Yang2025_Silkie_Wenchang	PRJNA827465	0.986179568
ENSGALG00010005836	NEBL	2	0.010696958	0.180988862	0.059102851	0.952870188	0	0.000324964	0	1	CRA004775;PRJNA827465	Yang2025_Silkie_Wenchang	0.987758913
ENSGALG00010005839	RPS6KA2	2	0.061372029	0.083258481	0.737126452	0.461045454	0	0.06773815	0	1	PRJNA827465;Yang2025_Silkie_Wenchang	CRA004775	0.85014477
ENSGALG00010005839	RPS6KA2	2	-0.225641222	0.334638036	-0.674284443	0.500130493	0.166328357	3.353510422	70.1805012	0.5	CRA004775;Yang2025_Silkie_Wenchang	PRJNA827465	0.862205775
ENSGALG00010005839	RPS6KA2	2	-0.207147226	0.357683324	-0.579135822	0.562497531	0.198343981	3.852277957	74.04133317	0.5	CRA004775;PRJNA827465	Yang2025_Silkie_Wenchang	0.881955027
ENSGALG00010005841	ENSGALG00010005841	2	-0.490370418	0.20326726	-2.412441719	0.015846071	0	0.664984997	0	1	PRJNA827465;Yang2025_Silkie_Wenchang	CRA004775	0.208293683
ENSGALG00010005841	ENSGALG00010005841	2	-0.289642086	0.545500241	-0.530966008	0.595442334	0.36646733	2.48306166	59.72713782	0.5	CRA004775;Yang2025_Silkie_Wenchang	PRJNA827465	0.893794893
ENSGALG00010005841	ENSGALG00010005841	2	-0.20993519	0.314606483	-0.667294544	0.504584011	0.07062877	1.342925096	25.53568303	0.5	CRA004775;PRJNA827465	Yang2025_Silkie_Wenchang	0.863512504
ENSGALG00010005847	ENSGALG00010005847	2	-0.306006135	0.643130714	-0.475807062	0.634211838	0.481095617	2.189295907	54.32321429	0.5	PRJNA827465;Yang2025_Silkie_Wenchang	CRA004775	0.903815945
ENSGALG00010005847	ENSGALG00010005847	2	-1.364672687	0.521281385	-2.617919471	0.008846768	0	0.137294246	0	1	CRA004775;Yang2025_Silkie_Wenchang	PRJNA827465	0.147549629
ENSGALG00010005847	ENSGALG00010005847	2	-0.584888154	0.856096384	-0.683203627	0.494478181	1.154015713	4.529749991	77.92372643	0.5	CRA004775;PRJNA827465	Yang2025_Silkie_Wenchang	0.860190852
ENSGALG00010005848	WASF3	2	-0.156539399	0.107909369	-1.450656235	0.146875608	0	0.038153816	0	1	PRJNA827465;Yang2025_Silkie_Wenchang	CRA004775	0.635558525
ENSGALG00010005848	WASF3	2	-0.109789036	0.187786487	-0.584648224	0.558784275	0	0.010500184	0	1	CRA004775;Yang2025_Silkie_Wenchang	PRJNA827465	0.880871906
ENSGALG00010005848	WASF3	2	-0.160064492	0.118937552	-1.345786003	0.178371541	0	0.052792642	0	1	CRA004775;PRJNA827465	Yang2025_Silkie_Wenchang	0.684689586
ENSGALG00010005849	SNX25	2	0.062481409	0.055699842	1.121751987	0.261967906	0	0.081434571	0	1	PRJNA827465;Yang2025_Silkie_Wenchang	CRA004775	0.768363052
ENSGALG00010005849	SNX25	2	0.143429058	0.154532975	0.928145322	0.353332193	0.018268507	1.55025344	35.49441824	1	CRA004775;Yang2025_Silkie_Wenchang	PRJNA827465	0.811157428
ENSGALG00010005849	SNX25	2	0.130102676	0.114700772	1.134278992	0.256677538	0.013145128	1.51846297	34.14393239	1	CRA004775;PRJNA827465	Yang2025_Silkie_Wenchang	0.764677554
ENSGALG00010005853	COMMD3	2	0.310444836	0.27898974	1.112746429	0.265817349	0.13815587	8.642417657	88.42916369	1	PRJNA827465;Yang2025_Silkie_Wenchang	CRA004775	0.770061124
ENSGALG00010005853	COMMD3	2	0.109682634	0.520424969	0.210755902	0.833077751	0.490427455	10.3586778	90.34625828	0.5	CRA004775;Yang2025_Silkie_Wenchang	PRJNA827465	0.95792089
ENSGALG00010005853	COMMD3	2	-0.122681624	0.230785689	-0.53158246	0.595015216	0.073600733	2.710600353	63.10780382	0.5	CRA004775;PRJNA827465	Yang2025_Silkie_Wenchang	0.893412691
ENSGALG00010005858	CFAP97	2	-0.022158717	0.129274729	-0.171407956	0.863902999	0.022263943	2.949645071	66.09761596	0.5	PRJNA827465;Yang2025_Silkie_Wenchang	CRA004775	0.966447685
ENSGALG00010005858	CFAP97	2	-0.132395512	0.11146576	-1.187768441	0.234924651	0	0.490229986	0	0.5	CRA004775;Yang2025_Silkie_Wenchang	PRJNA827465	0.747791642
ENSGALG00010005858	CFAP97	2	0.093543209	0.088014977	1.062810123	0.287868068	0	0.010046673	0	1	CRA004775;PRJNA827465	Yang2025_Silkie_Wenchang	0.782840067
ENSGALG00010005859	ENSGALG00010005859	2	-0.498388477	0.452362267	-1.101746351	0.27057196	0	0.0774536	0	1	PRJNA827465;Yang2025_Silkie_Wenchang	CRA004775	0.772536273
ENSGALG00010005859	ENSGALG00010005859	2	-0.693881784	0.543786775	-1.27601813	0.20194911	0	0.001315054	0	1	CRA004775;Yang2025_Silkie_Wenchang	PRJNA827465	0.714969076
ENSGALG00010005859	ENSGALG00010005859	2	-0.514210757	0.449645094	-1.1435925	0.252792674	0	0.104526201	0	1	CRA004775;PRJNA827465	Yang2025_Silkie_Wenchang	0.762731469
ENSGALG00010005860	ABI3BP	2	0.174916206	0.405707475	0.43113873	0.666367501	0.272107265	5.443602462	81.62981211	0.5	PRJNA827465;Yang2025_Silkie_Wenchang	CRA004775	0.913579507
ENSGALG00010005860	ABI3BP	2	0.564486186	0.277159848	2.036680967	0.041682023	0	0.190956727	0	1	CRA004775;Yang2025_Silkie_Wenchang	PRJNA827465	0.353826658
ENSGALG00010005860	ABI3BP	2	-0.154120975	0.150043948	-1.027172217	0.304339378	0	0.733461709	0	0.5	CRA004775;PRJNA827465	Yang2025_Silkie_Wenchang	0.79225794
ENSGALG00010005861	MPC1L	2	-0.506093574	0.368454562	-1.373557629	0.169579059	0.251358122	13.22205681	92.43688018	1	PRJNA827465;Yang2025_Silkie_Wenchang	CRA004775	0.671059781
ENSGALG00010005861	MPC1L	2	-0.300120901	0.614995504	-0.488005032	0.625546277	0.694013499	11.87084862	91.57600243	0.5	CRA004775;Yang2025_Silkie_Wenchang	PRJNA827465	0.901405877
ENSGALG00010005861	MPC1L	2	0.007560848	0.230751287	0.032766223	0.973861014	0.068533061	2.285134019	56.23889051	0.5	CRA004775;PRJNA827465	Yang2025_Silkie_Wenchang	0.992472082
ENSGALG00010005866	DNAJC1	2	0.514075444	0.422188654	1.21764391	0.223359321	0.340304015	21.64675793	95.38037057	1	PRJNA827465;Yang2025_Silkie_Wenchang	CRA004775	0.736744216
ENSGALG00010005866	DNAJC1	2	0.538765386	0.419598091	1.284003426	0.199140757	0.317272046	10.05328753	90.05300508	1	CRA004775;Yang2025_Silkie_Wenchang	PRJNA827465	0.71096127
ENSGALG00010005866	DNAJC1	2	0.104610322	0.073336277	1.426447133	0.153739317	0	0.000570103	0	1	CRA004775;PRJNA827465	Yang2025_Silkie_Wenchang	0.64624774
ENSGALG00010005871	EGLN3	2	-0.144522185	0.108837556	-1.327870547	0.184220876	0.012008301	1.999050571	49.97625301	1	PRJNA827465;Yang2025_Silkie_Wenchang	CRA004775	0.692545576
ENSGALG00010005871	EGLN3	2	0.100573805	0.456554597	0.220288671	0.825646343	0.328767046	4.045175253	75.27919219	0.5	CRA004775;Yang2025_Silkie_Wenchang	PRJNA827465	0.956388903
ENSGALG00010005871	EGLN3	2	0.175680181	0.331591378	0.529809256	0.596244187	0.150761631	2.434205603	58.91883583	0.5	CRA004775;PRJNA827465	Yang2025_Silkie_Wenchang	0.894064255
ENSGALG00010005872	STK24	2	0.039888987	0.0577717	0.690458946	0.489905619	0	0.676219605	0	0.5	PRJNA827465;Yang2025_Silkie_Wenchang	CRA004775	0.858512413

ENSGALG00010005872	STK24	2	0.106969712	0.18749631	0.570516356	0.568327532	0.04383087	2.397142637	58.2836672	0.5	CRA004775;Yang2025_Silkie_Wenchang	PRJNA827465	0.884142534
ENSGALG00010005872	STK24	2	0.128007092	0.114481164	1.118149812	0.263503015	0.011984677	1.434620326	30.29514626	1	CRA004775;PRJNA827465	Yang2025_Silkie_Wenchang	0.768643821
ENSGALG00010005875	CDK8	2	0.197647304	0.180721742	1.093655375	0.274106153	0.051061389	4.40472045	77.29708363	1	PRJNA827465;Yang2025_Silkie_Wenchang	CRA004775	0.774526342
ENSGALG00010005875	CDK8	2	0.221301066	0.246933318	0.896197681	0.370147194	0.070430248	2.076834893	51.84980745	0.5	CRA004775;Yang2025_Silkie_Wenchang	PRJNA827465	0.821033646
ENSGALG00010005875	CDK8	2	0.024295396	0.086403325	0.28118589	0.778567827	0	0.212947732	0	0.5	CRA004775;PRJNA827465	Yang2025_Silkie_Wenchang	0.944673731
ENSGALG00010005879	FAM107B	2	0.041201905	0.066189483	0.62248417	0.533623549	0.000962898	1.08003726	7.410601784	0.5	PRJNA827465;Yang2025_Silkie_Wenchang	CRA004775	0.873408008
ENSGALG00010005879	FAM107B	2	-0.00912369	0.135220599	-0.067472634	0.946205447	0.004656747	1.102865267	9.327092843	0.5	CRA004775;Yang2025_Silkie_Wenchang	PRJNA827465	0.986370359
ENSGALG00010005879	FAM107B	2	0.085244375	0.065835412	1.294810374	0.195385652	0	0.316475276	0	1	CRA004775;PRJNA827465	Yang2025_Silkie_Wenchang	0.70643959
ENSGALG00010005880	SLC25A4	2	-0.144310053	0.107146192	-1.346851902	0.178027935	0	0.75169824	0	1	PRJNA827465;Yang2025_Silkie_Wenchang	CRA004775	0.684191679
ENSGALG00010005880	SLC25A4	2	-0.281636761	0.232445452	-1.211625174	0.225655894	0	0.398544796	0	0.5	CRA004775;Yang2025_Silkie_Wenchang	PRJNA827465	0.73884362
ENSGALG00010005880	SLC25A4	2	-0.093948858	0.116440863	-0.806837526	0.419760096	0	0.083755109	0	0.5	CRA004775;PRJNA827465	Yang2025_Silkie_Wenchang	0.838527387
ENSGALG00010005881	CORO2A	2	0.832139929	0.265951642	3.128914422	0.001754534	0	0.429131388	0	1	PRJNA827465;Yang2025_Silkie_Wenchang	CRA004775	0.053126695
ENSGALG00010005881	CORO2A	2	1.254226834	0.374500154	3.34906894	0.000810836	0.092073026	1.423312025	29.74133693	1	CRA004775;Yang2025_Silkie_Wenchang	PRJNA827465	0.032752615
ENSGALG00010005881	CORO2A	2	1.134756496	0.566168602	2.004273094	0.045040814	0.409461118	2.707812954	63.06982731	1	CRA004775;PRJNA827465	Yang2025_Silkie_Wenchang	0.3659545
ENSGALG00010005884	MYB	2	-0.106784079	0.163879917	-0.651599544	0.514659542	0	0.107467204	0	1	PRJNA827465;Yang2025_Silkie_Wenchang	CRA004775	0.866869697
ENSGALG00010005884	MYB	2	0.319096408	0.479061964	0.666085876	0.505356212	0.265431232	2.092887635	52.21912619	0.5	CRA004775;Yang2025_Silkie_Wenchang	PRJNA827465	0.863862536
ENSGALG00010005884	MYB	2	0.257402225	0.538272919	0.478200214	0.63250771	0.411051832	2.866165698	65.11018185	0.5	CRA004775;PRJNA827465	Yang2025_Silkie_Wenchang	0.903058174
ENSGALG00010005887	CCNH	2	0.219626626	0.165822087	1.324471483	0.185346508	0.019056469	1.520898422	34.24938933	1	PRJNA827465;Yang2025_Silkie_Wenchang	CRA004775	0.693795574
ENSGALG00010005887	CCNH	2	0.117737749	0.314084379	0.374860252	0.707764401	0.138242138	3.2771889	69.48604336	0.5	CRA004775;Yang2025_Silkie_Wenchang	PRJNA827465	0.925061251
ENSGALG00010005887	CCNH	2	-0.01226836	0.148447677	-0.082644339	0.934134345	0	0.807259143	0	0.5	CRA004775;PRJNA827465	Yang2025_Silkie_Wenchang	0.98432801
ENSGALG00010005888	IMPG2	2	0.647205618	0.362484263	1.78547232	0.074184737	0.214066836	5.347863942	81.30094537	1	PRJNA827465;Yang2025_Silkie_Wenchang	CRA004775	0.470322999
ENSGALG00010005888	IMPG2	2	0.254815608	0.804861716	0.316595513	0.751550541	1.181749425	11.16843633	91.0461951	0.5	CRA004775;Yang2025_Silkie_Wenchang	PRJNA827465	0.937716403
ENSGALG00010005888	IMPG2	2	-0.06674558	0.43624844	-0.152999011	0.878399055	0.287463416	3.748071321	73.31961122	0.5	CRA004775;PRJNA827465	Yang2025_Silkie_Wenchang	0.971037793
ENSGALG00010005890	SNX32	2	-0.215954138	0.153695286	-1.405079776	0.159997557	0.043164956	11.55463125	91.34546159	1	PRJNA827465;Yang2025_Silkie_Wenchang	CRA004775	0.655404348
ENSGALG00010005890	SNX32	2	-0.144668499	0.238258457	-0.607191457	0.543723861	0.103627421	11.19850411	91.0702359	0.5	CRA004775;Yang2025_Silkie_Wenchang	PRJNA827465	0.885696972
ENSGALG00010005890	SNX32	2	-0.015473682	0.076890794	-0.201242325	0.840509098	0.004917911	1.519386617	34.18396684	0.5	CRA004775;PRJNA827465	Yang2025_Silkie_Wenchang	0.959692321
ENSGALG00010005891	RNF6	2	0.359623569	0.296013351	1.214889691	0.224408166	0.164759585	16.61711537	93.98210834	1	PRJNA827465;Yang2025_Silkie_Wenchang	CRA004775	0.737471949
ENSGALG00010005891	RNF6	2	0.487059856	0.210714655	2.311466447	0.020807104	0.060901592	2.954617991	66.15467709	1	CRA004775;Yang2025_Silkie_Wenchang	PRJNA827465	0.243895504
ENSGALG00010005891	RNF6	2	0.090351765	0.079466117	1.136983913	0.255545011	0	0.476919763	0	1	CRA004775;PRJNA827465	Yang2025_Silkie_Wenchang	0.764467569
ENSGALG00010005892	ENSGALG00010005892	2	-1.998097551	0.519821212	-3.843816886	0.000121135	0	0.455432627	0	1	PRJNA827465;Yang2025_Silkie_Wenchang	CRA004775	0.008882434
ENSGALG00010005892	ENSGALG00010005892	2	-1.449099346	0.935448063	-1.549096526	0.121358518	1.155389753	2.9392564	65.97778948	1	CRA004775;Yang2025_Silkie_Wenchang	PRJNA827465	0.583884665
ENSGALG00010005892	ENSGALG00010005892	2	-1.153392696	0.579552872	-1.99014232	0.04657526	0.163822443	1.320851107	24.2912396	1	CRA004775;PRJNA827465	Yang2025_Silkie_Wenchang	0.372516778
ENSGALG00010005893	SFT2D1	2	-0.02797594	0.050497493	-0.554006512	0.579574389	0	0.460518954	0	1	PRJNA827465;Yang2025_Silkie_Wenchang	CRA004775	0.887969027
ENSGALG00010005893	SFT2D1	2	-0.147147208	0.112247654	-1.310915663	0.189886258	0	0.075481487	0	1	CRA004775;Yang2025_Silkie_Wenchang	PRJNA827465	0.699271855
ENSGALG00010005893	SFT2D1	2	-0.029987431	0.051412762	-0.583268223	0.559712751	0	0.850053915	0	1	CRA004775;PRJNA827465	Yang2025_Silkie_Wenchang	0.881205589
ENSGALG00010005895	MLLT10	2	0.281871165	0.286699788	0.983164458	0.325526506	0.145364023	7.83363996	87.23454224	1	PRJNA827465;Yang2025_Silkie_Wenchang	CRA004775	0.325967949
ENSGALG00010005895	MLLT10	2	0.44824596	0.17082893	2.623946428	0.008691746	0.012836672	1.278897002	21.80762029	1	CRA004775;Yang2025_Silkie_Wenchang	PRJNA827465	0.14605031
ENSGALG00010005895	MLLT10	2	0.038108359	0.050365625	0.756634282	0.449268997	0.00073396	1.027502437	2.676629809	1	CRA004775;PRJNA827465	Yang2025_Silkie_Wenchang	0.847263197
ENSGALG00010005899	ENSGALG00010005899	2	0.165481799	0.129071752	1.282091519	0.199810544	0	0.382619733	0	1	PRJNA827465;Yang2025_Silkie_Wenchang	CRA004775	0.711723202
ENSGALG00010005899	ENSGALG00010005899	2	0.262152696	0.131454576	1.994245486	0.046125233	0	0.005103126	0	1	CRA004775;Yang2025_Silkie_Wenchang	PRJNA827465	0.370377506
ENSGALG00010005899	ENSGALG00010005899	2	0.178500557	0.126109169	1.415444723	0.156938145	0	0.503316286	0	1	CRA004775;PRJNA827465	Yang2025_Silkie_Wenchang	0.65037325
ENSGALG00010005900	SPTSSA	2	-0.600052785	0.53941555	-1.112412843	0.265960685	0.562650742	29.47374656	96.60715003	1	PRJNA827465;Yang2025_Silkie_Wenchang	CRA004775	0.770078617
ENSGALG00010005900	SPTSSA	2	-0.485988924	0.669299386	-0.732092296	0.464112236	0.853930419	21.31771855	95.3090665	0.5	CRA004775;Yang2025_Silkie_Wenchang	PRJNA827465	0.852359649
ENSGALG00010005900	SPTSSA	2	-0.031976069	0.097182139	-0.329032364	0.742131226	0.008198186	1.322323237	24.37552541	0.5	CRA004775;PRJNA827465	Yang2025_Silkie_Wenchang	0.935481151
ENSGALG00010005902	EAPP	2	0.182552142	0.259805374	0.702649598	0.482274145	0.121146646	9.265262276	89.20699738	0.5	PRJNA827465;Yang2025_Silkie_Wenchang	CRA004775	0.85691916
ENSGALG00010005902	EAPP	2	0.349584936	0.141561944	2.469483857	0.013530812	0.007829112	1.228796815	18.6159807	1	CRA004775;Yang2025_Silkie_Wenchang	PRJNA827465	0.190294637
ENSGALG00010005902	EAPP	2	-0.026984676	0.078735109	-0.342727366	0.731803573	0.002964907	1.124814979	11.09948974	0.5	CRA004775;PRJNA827465	Yang2025_Silkie_Wenchang	0.932851968
ENSGALG00010005903	PRR18	2	0.28758542	0.193041389	1.489760416	0.136287243	0	0.174896155	0	1	PRJNA827465;Yang2025_Silkie_Wenchang	CRA004775	0.618139317
ENSGALG00010005903	PRR18	2	-0.037026942	0.51342393	-0.072117679	0.942508257	0.33813928	2.709251065	63.0894304	0.5	CRA004775;Yang2025_Silkie_Wenchang	PRJNA827465	0.985827325
ENSGALG00010005903	PRR18	2	-0.067493112	0.409962597	-0.164632366	0.869233355	0.207957131	2.305027763	56.6165746	0.5	CRA004775;PRJNA827465	Yang2025_Silkie_Wenchang	0.96851728
ENSGALG00010005905	CITED2	2	0.226852769	0.141873014	1.598984637	0.109826416	0.014504309	1.445814178	30.83481851	1	PRJNA827465;Yang2025_Silkie_Wenchang	CRA004775	0.561156177
ENSGALG00010005905	CITED2	2	0.471911411	0.164869071	2.862340446	0.004205249	0	0.048234021	0	1	CRA004775;Yang2025_Silkie_Wenchang	PRJNA827465	0.094553154
ENSGALG00010005905	CITED2	2	0.262975215	0.180456558	1.457277133	0.145039896	0.035118008	1.953246047	48.80317298	1	CRA004775;PRJNA827465	Yang2025_Silkie_Wenchang	0.632983695
ENSGALG00010005909	BRMS1L	2	-0.033907369	0.048948773	-0.692711319	0.488490734	0	0.311472079	0	1	PRJNA827465;Yang2025_Silkie_Wenchang	CRA004775	0.85783717
ENSGALG00010005909	BRMS1L	2	-0.016026626	0.114988793	-0.139375547	0.889153394	0.007991469	1.282752167	22.04261854	0.5	CRA004775;Yang2025_Silkie_Wenchang	PRJNA827465	0.973135755
ENSGALG00010005909	BRMS1L	2	-0.004739971	0.054861103	-0.086399493	0.93114885	0	0.847797709	0	0.5	CRA004775;PRJNA827465	Yang2025_Silkie_Wenchang	0.983943003
ENSGALG00010005910	ENSGALG00010005910	2	0.188931508	0.351485129	0.537523475	0.590906074	0	0.415767026	0	0.5	PRJNA827465;Yang2025_Silkie_Wenchang	CRA004775	0.892752146
ENSGALG00010005910	ENSGALG00010005910	2	-0.310175875	0.402630403	-0.770373703	0.441078248	0	0.31003959	0	1	CRA004775;Yang2025_Silkie_Wenchang	PRJNA827465	0.844888742
ENSGALG00010005910	ENSGALG00010005910	2	-0.013264599	0.450637427	-0.029435191	0.976517507	0.147436949	1.548031638	35.40183705	0.5	CRA004775;PRJNA827465	Yang2025_Silkie_Wenchang	0.993126926
ENSGALG00010005911	TBXT	2	0.090089171	0.474856321	0.1897188	0.849529488	0.378991261	6.138195669	83.70856757	0.5	PRJNA827465;Yang2025_Silkie_Wenchang	CRA004775	0.961721801
ENSGALG00010005911	TBXT	2	0.345876069	0.46483637	0.74408134	0.456827267	0.176366042	1.396283011	28.38128145	0.5	CRA004775;Yang2025_Silkie_Wenchang	PRJNA827465	0.849322638
ENSGALG00010005911	TBXT	2	-0.364815866	0.207232484	-1.760418342	0.078336092	0	0.031975604	0	1	CRA004775;PRJNA827465	Yang2025_Silkie_Wenchang	0.481707034
ENSGALG00010005913	NFKBIA	2	-0.13095723	0.103381842	-1.266733369	0.205250644	0.010557236	1.965243639	49.11572386	1	PRJNA827465;Yang2025_Silkie_Wenchang	CRA004775	0.718084927
ENSGALG00010005913	NFKBIA	2	0.008743752	0.104371653	0.083775163	0.933235196	0	0.487986777	0	0.5	CRA004775;Yang2025_Silkie_Wenchang	PRJNA827465	0.984260547
ENSGALG00010005913	NFKBIA	2	-0.102343216	0.192930656	-0.530466328	0.595788649	0.043401771	1.938983523	48.42658599	0.5	CRA004775;PRJNA827465	Yang2025_Silkie_Wenchang	0.893853544
ENSGALG00010005915	PAPSS1	2	0.11658619	0.07452416	1.564407976	0.117721789	0	0.064395543	0	1	PRJNA827465;Yang2025_Silkie_Wenchang	CRA004775	0.577330692
ENSGALG00010005915	PAPSS1	2	0.085344917	0.09827818	0.868401481	0.385174584	0	0.032440205	0	1	CRA004775;Yang2025_Silkie_Wenchang	PRJNA827465	0.826444827
ENSGALG00010005915	PAPSS1	2	0.117741378	0.089249665	1.319236076	0.187090201	0	0.125453745	0	1	CRA004775;PRJNA827465	Yang2025_Silkie_Wenchang	0.695933761
ENSGALG00010005926	NUDT5	2	0.307773472	0.153254443	2.008251544	0.044616568	0	0.554380342	0	1	PRJNA827465;Yang2025_Silkie_Wenchang	CRA004775	0.364257243
ENSGALG00010005926	NUDT5	2	0.059913252	0.270152013	0.221776069	0.82448821	0.080972808	1.982304044	49.54677983	0.5	CRA004775;Yang2025_Silkie_Wenchang	PRJNA827465	0.95619211
ENSGALG00010005926	NUDT5	2	0.119102357	0.430930778	0.276383964	0.782253172	0.242095952	2.870637689	65.16453456	0.5	CRA004775;PRJNA827465	Yang2025_Silkie_Wenchang	0.945372683
ENSGALG00010005935	ATP8A2	2	-0.309085593	0.228198574	-1.354453745	0.175590354	0.012249107	1.130955588	11.5790417	1	PRJNA827465;Yang2025_Silkie_Wenchang	CRA004775	0.680397576
ENSGALG00010005935	ATP8A2	2	0.273663491	0.997636189	0.274311912	0.783844931	1.596446531	4.528395651	77.91712392	0.5	CRA004775;Yang2025_Silkie_Wenchang	PRJNA827465	0.945884474
ENSGALG00010005935	ATP8A2	2	0.44501322	0.747757824	0.595130142	0.551756482	0.784272999	2.78529873	64.09720834	0.5	CRA004775;PRJNA827465	Yang2025_Silkie_Wenchang	0.879061525
ENSGALG00010005941	TXLNB	2	-0.063420631	0.332312101	-0.190846588	0.848645787	0.19310918	7.948603349	87.41917346	0.5	PRJNA827465;Yang2025_Silkie_Wenchang	CRA004775	0.961364896
ENSGALG00010005941	TXLNB	2	0.246202853	0.151319587	1.627038894	0.103725838	0	0.167695285	0	1	CRA004775;Yang2025_Silkie_Wenchang	PRJNA827465	0.543796435
ENSGALG00010005941	TXLNB	2	-0.357426844	0.166368281	-2.14840738	0.031681407	0	0.608034057	0	0.5	CRA004775;PRJNA827465	Yang2025_Silkie_Wenchang	0.30481624
ENSGALG00010005949	SLITRK1	2	-0.329552406	0.235599292	-1.398783519	0.16187791	0	0.634342279	0	1	PRJNA827465;Yang2025_Silkie_Wenchang	CRA004775	0.659099502
ENSGALG00010005949	SLITRK1	2	0.018602822	0.32789452	0.056734166	0.954756957	0	0.822984416	0	0.5	CRA004775;Yang2025_Silkie_Wenchang	PRJNA827465	0.987987175
ENSGALG00010005949	SLITRK1	2	-0.137200725	0.541221152	-0.422627445	0.799880202	0.321853621	1.861597195	46.28268657	0.5	CRA004775;PRJNA827465	Yang2025_Silkie_Wenchang	0.949802217
ENSGALG00010005956	TXNL4B	2	0.10322688	0.072953652	1.414965229	0.157078691	0	0.097917606	0	1	PRJNA827465;Yang2025_Silkie_Wenchang	CRA004775	0.650687662
ENSGALG00010005956	TXNL4B	2	0.083590567	0.114233634	0.731750922	0.464320609	0	0.775477001	0	0.5	CRA004775;Yang2025_Silkie_Wenchang	PRJNA827465	0.852159649
ENSGALG00010005956	TXNL4B	2	0.065141896	0.083181812	0.783126669	0.433557732	0	0.560014854	0	0.5	CRA004775;PRJNA827465	Yang2025_Silkie_Wenchang	0.842762754
ENSGALG00010005963	UGCG	2	0.029246628	0.075065703	0.389613723	0.696822203	0	0.771081012	0	0.5	PRJNA827465;Yang2025_Silkie_Wenchang	CRA004775	0.921086208
ENSGALG00010005963	UGCG	2	0.388557359	0.476389793	0.815629061	0.414712323	0.408062352	9.443787448	89.41102809	0.5	CRA004775;Yang2025_Silkie_Wenchang	PRJNA827465	0.837298284
ENSGALG00010005963	UGCG	2	0.447120579	0.407594654	1.09697361	0.272652928	0.290590445	7.368883457	86.42942305	1	CRA004775;PRJNA827465	Yang2025_Silkie_Wenchang	0.773556489
ENSGALG00010005964	HSPA14	2	0.034470479	0.063194709	0.545464645	0.58543399	0	0.838589194	0	0.5	PRJNA827465;Yang2025_Silkie_Wenchang	CRA004775	0.891353566
ENSGALG00010005964	HSPA14	2	-0.136884772	0.124688163	-1.097816892	0.272284454	0	0.52844348	0	1	CRA004775;Yang2025_Silkie_Wenchang	PRJNA827465	0.773331629
ENSGALG00010005964	HSPA14	2	-0.03542764	0.158939709	-0.222899864	0.823613442	0.029945049	1.899863533	47.36464054	0.5	CRA004775;PRJNA827465	Yang2025_Silkie_Wenchang	0.955934625
ENSGALG00010005966	PLXDC2	2	-0.060050173	0.089072525	-0.674171666	0.500202181	0	0.599764709	0	1	PRJNA827465;Yang2025_Silkie_Wenchang	CRA004775	0.862256418
ENSGALG00010005966	PLXDC2	2	0.013954089	0.266640571	0.052332954	0.958263396	0.076669778	1.994139227	49.85807898	0.5	CRA004775;Yang2025_Silkie_Wenchang	PRJNA827465	0.989861643
ENSGALG00010005966	PLXDC2	2	0.032547011	0.135922474	0.239452753	0.810754529	0.011252304	1.173679183	14.79784134	0.5	CRA004775;PRJNA827465	Yang2025_Silkie_Wenchang	0.953020781
ENSGALG00010005967	GJD2	2	0.495700461	0.166405838	2.978864599	0.002893186	0.021363699	1.599748261	37.4901649	1	PRJNA827465;Yang2025_Silkie_Wenchang	CRA004775	0.073504129
ENSGALG00010005967	GJD2	2	0.264550789	0.208281603	1.270159178	0.204027935	0	0.644725713	0	0.5	CRA004775;Yang2025_Silkie_Wenchang	PRJNA827465	0.717254253
ENSGALG00010005967	GJD2	2	0.485548246	0.396302604	1.225195701	0.220501494	0.153684108	1.275307818	21.58755824	0.5	CRA004775;PRJNA827465	Yang2025_Silkie_Wenchang	0.734053249
ENSGALG00010005969	HECA	2	-0.293134491	0.264398977	-1.108682394	0.26756722	0.132566894	18.98314964	94.73217027	1	PRJNA827465;Yang2025_Silkie_Wenchang	CRA004775	0.771398294
ENSGALG00010005969	HECA	2	-0.165699298	0.41754374	-0.396842971	0.69148326	0.322674961	13.1014441	92.36725362	0.5	CRA004775;Yang2025_Silkie_Wenchang	PRJNA827465	0.920268407
ENSGALG00010005969	HECA	2	0.054019674	0.139941689	0.386015593	0.699485121	0.024591965	2.093848939	52.24106279	0.5	CRA004775;PRJNA827465	Yang2025_Silkie_Wenchang	0.922022134
ENSGALG00010005970	SHISA2	2	-0.00851745	0.104965932	-0.0811449	0.935326718	0	7.47429E-05	0	1	PRJNA827465;Yang2025_Silkie_Wenchang	CRA004775	0.984629597
ENSGALG00010005970	SHISA2	2	-0.105816868	0.178325872	-0.593390446	0.552919882	0.015894688	1.211661302	17.46868546	1	CRA004775;Yang2025_Silkie_Wenchang	PRJNA827465	0.87928478
ENSGALG00010005970	SHISA2	2	-0.098889529	0.175350191	-0.563954498	0.5727851	0.019466718	1.269231032	21.21213755	1	CRA004775;PRJNA827465	Yang2025_Silkie_Wenchang	0.88573298
ENSGALG00010005975	ABRACL	2	-0.22147085	0.269164109	-0.82280974	0.410616197	0.124116753	6.446269206	84.48715112	0.5	PRJNA827465;Yang2025_Silkie_Wenchang	CRA004775	0.835020871
ENSGALG00010005975	ABRACL	2	-0.511653389	0.179537279	-2.84984484	0.004374056	0	0.020285995	0	1	CRA004775;Yang2025_Silkie_Wenchang	PRJNA827465	0.097063291
ENSGALG00010005975	ABRACL	2	-0.052064555	0.168786542	-0.308463901	0.757729365	0.027763181	1.322285675	24.37337719	0.5	CRA004775;PRJNA827465	Yang2025_Silkie_Wenchang	0.938954369
ENSGALG00010005978	VPS16	2	-0.201599343	0.060953467	-3.307430303	0.000941561	0	0.228293228	0	1	PRJNA827465;Yang2025_Silkie_Wenchang	CRA004775	0.036351854
ENSGALG00010005978	VPS16	2	-0.277314065	0.113783535	-2.437207328	0.01480119	0	0.433077637	0	1	CRA004775;Yang2025_Silkie_Wenchang	PRJNA827465	0.200897639
ENSGALG00010005978	VPS16	2	-0.196836647	0.069236679	-2.842953312	0.004469762	0	0.759716904	0	1	CRA004775;PRJNA827465	Yang2025_Silkie_Wenchang	0.097752851
ENSGALG00010005979	SUV39H2	2	-0.341974031	0.316926874	-1.079031344	0.280573755	0.193544292	27.24911218	96.33015566	1	PRJNA827465;Yang2025_Silkie_Wenchang	CRA004775	0.778536485
ENSGALG00010005979	SUV39H2	2	-0.269731698	0.408767483	-0.65986584	0.509339927	0.311787489	14.61688738	93.15859818	0.5	CRA004775;Yang2025_Silkie_Wenchang	PRJNA827465	0.865257661
ENSGALG00010005979	SUV39H2	2	-0.007207457	0.065203406	-0.110538043	0.91198268	0	0.834187534	0	0.5	CRA004775;PRJNA827465	Yang2025_Silkie_Wenchang	0.978933642
ENSGALG00010005981	AREL1	2	0.004486298	0.098123744	0.045720819	0.96353277	0.010016712	2.004530665	50.11301062	0.5	PRJNA827465;Yang2025_Silkie_Wenchang	CRA004775	0.990084145
ENSGALG00010005981	AREL1	2	-0.155526535	0.107892279	-1.441498291	0.14944396	0	0.776767357	0	1	CRA004775;Yang2025_Silkie_Wenchang	PRJNA827465	0.639246608
ENSGALG00010005981	AREL1	2	-0.07158099	0.218744145	-0.32723614	0.743489288	0.066322738	2.644409154	62.1843693	0.5	CRA004775;PRJNA827465	Yang2025_Silkie_Wenchang	0.935816088
ENSGALG00010005984	IDH3B	2	0.119643166	0.11909367	1.00461398	0.315082763	0.014286883	1.72856307	42.14848059	1	PRJNA827465;Yang2025_Silkie_Wenchang	CRA004775	0.796635323
ENSGALG00010005984	IDH3B	2	0.039214334	0.347304453	0.112910542	0.910101478	0.156592488	2.510365041	60.16515591	0.5	CRA004775;Yang2025_Silkie_Wenchang	PRJNA827465	0.978730675
ENSGALG00010005984	IDH3B	2	-0.00620025	0.141129071	-0.043933188	0.964957661	0.016326029	1.18142558	15.35649665	0.5	CRA004775;PRJNA827465	Yang2025_Silkie_Wenchang	0.990512444
ENSGALG00010005985	ENSGALG00010005985	2	0.150439849	0.140727097	1.069018353	0.2850614	0	0.996659393	0	0.5	PRJNA827465;Yang2025_Silkie_Wenchang	CRA004775	0.780501722
ENSGALG00010005985	ENSGALG00010005985	2	1.092133034	1.199179617	0.910733487	0.362435815	2.665268387	13.11141162	92.37305617	0.5	CRA004775;Yang2025_Silkie_Wenchang	PRJNA827465	0.815644888
ENSGALG00010005985	ENSGALG00010005985	2	1.223689403	1.050526387	1.164834524	0.244085981	2.014211561	10.72894769	90.67942142	1	CRA004775;PRJNA827465	Yang2025_Silkie_Wenchang	0.755564606
ENSGALG00010005988	NUP58	2	0.015964245	0.218053803	0.073212411	0.94163709	0.091465746	26.03329496	96.15876514	0.5	PRJNA827465;Yang2025_Silkie_Wenchang	CRA004775	0.985827325
ENSGALG00010005988	NUP58	2	0.226735889	0.067527079	3.357703187	0.00078593	0	0.18774512	0	1	CRA004775;Yang2025_Silkie_Wenchang	PRJNA827465	0.03200973
ENSGALG00010005988	NUP58	2	-0.077216036	0.165197924	-0.467415292	0.640202784	0.039902761	2.992669829	66.58502082	0.5	CRA004775;PRJNA827465	Yang2025_Silkie_Wenchang	0.906137307
ENSGALG00010005989	FCF1	2	-0.109280355	0.130483836	-0.837501094	0.402310962	0.02057517	2.136056729	53.18476394	1	PRJNA827465;Yang2025_Silkie_Wenchang	CRA004775	0.832879175
ENSGALG00010005989	FCF1	2	-0.251884281	0.138548978	-1.818016164	0.069061662	0	0.161742625	0	1	CRA004775;Yang2025_Silkie_Wenchang	PRJNA827465	0.452607154
ENSGALG00010005989	FCF1	2	-0.032708514	0.06563305	-0.49835432	0.618234328	0	0.499039362	0	1	CRA004775;PRJNA827465	Yang2025_Silkie_Wenchang	0.899168321
ENSGALG00010005990	SLC2A12	2	-0.325510667	0.146028945	-2.229083194	0.02580837	0.030142252	3.261937601	69.34337433	1	PRJNA827465;Yang2025_Silkie_Wenchang	CRA004775	0.273814893
ENSGALG00010005990	SLC2A12	2	-0.497508802	0.131811995	-3.774381855	0.000160405	0	0.009792893	0	1	CRA004775;Yang2025_Silkie_Wenchang	PRJNA827465	0.010945128
ENSGALG00010005990	SLC2A12	2	-0.212003744	0.084030305	-2.522943876	0.011637697	0	0.629139366	0	1	CRA004775;PRJNA827465	Yang2025_Silkie_Wenchang	0.172635381
ENSGALG00010005991	FARP1	2	-0.076837388	0.040838424	-1.881497372	0.059904292	0	0.000479744	0	1	PRJNA827465;Yang2025_Silkie_Wenchang	CRA004775	0.423882851
ENSGALG00010005991	FARP1	2	-0.067342209	0.095820779	-0.702793374	0.482184527	0	0.023649268	0	1	CRA004775;Yang2025_Silkie_Wenchang	PRJNA827465	0.85691916
ENSGALG00010005991	FARP1	2	-0.075609045	0.043279034	-1.747013254	0.080635039	0	0.03232316	0	1	CRA004775;PRJNA827465	Yang2025_Silkie_Wenchang	0.488841242
ENSGALG00010005997	ENSGALG00010005997	2	-0.126311327	0.156534886	-0.806921253	0.419711853	0	0.435972331	0	0.5	PRJNA827465;Yang2025_Silkie_Wenchang	CRA004775	0.838527387
ENSGALG00010005997	ENSGALG00010005997	2	-0.318903628	0.498561942	-0.639646955	0.522402149	0.289652715	2.076007666	51.83062104	0.5	CRA004775;Yang2025_Silkie_Wenchang	PRJNA827465	0.869568211
ENSGALG00010005997	ENSGALG00010005997	2	-0.354980218	0.329056474	-1.078782051	0.280684898	0.099853089	1.407903969	28.97242835	1	CRA004775;PRJNA827465	Yang2025_Silkie_Wenchang	0.778536485
ENSGALG00010005998	YLPM1	2	0.026801151	0.125940531	0.212807991	0.831476731	0.020669735	2.353907932	57.51745485	0.5	PRJNA827465;Yang2025_Silkie_Wenchang	CRA004775	0.957641903
ENSGALG00010005998	YLPM1	2	0.173753072	0.131726437	1.319044804	0.187154134	0	0.106528418	0	1	CRA004775;Yang2025_Silkie_Wenchang	PRJNA827465	0.695933761
ENSGALG00010005998	YLPM1	2	-0.050688909	0.052764537	-0.960662451	0.336721917	0	0.644346537	0	0.5	CRA004775;PRJNA827465	Yang2025_Silkie_Wenchang	0.803887433
ENSGALG00010005999	PDE10A	2	0.031304583	0.084254466	0.371548058	0.710229369	0	0.026028267	0	1	PRJNA827465;Yang2025_Silkie_Wenchang	CRA004775	0.925364369
ENSGALG00010005999	PDE10A	2	0.583656184	0.676494017	0.862766217	0.388266017	0.69735502	3.507964125	71.49343709	1	CRA004775;Yang2025_Silkie_Wenchang	PRJNA827465	0.828154121
ENSGALG00010005999	PDE10A	2	0.561279793	0.692744809	0.81022591	0.417810349	0.759119803	3.8696288	74.157728	1	CRA004775;PRJNA827465	Yang2025_Silkie_Wenchang	0.838112595
ENSGALG00010006001	NOP56	2	0.170011904	0.165898372	1.024795494	0.305459693	0.046361327	5.982545511	83.28470718	1	PRJNA827465;Yang2025_Silkie_Wenchang	CRA004775	0.79225794
ENSGALG00010006001	NOP56	2	-0.001031765	0.36996659	-0.002788806	0.997774858	0.248072029	10.49957515	90.47580511	0.5	CRA004775;Yang2025_Silkie_Wenchang	PRJNA827465	0.999290321
ENSGALG00010006001	NOP56	2	-0.141305303	0.199188813	-0.709403811	0.478073923	0.062534243	4.090862306	75.555276	0.5	CRA004775;PRJNA827465	Yang2025_Silkie_Wenchang	0.855366076
ENSGALG00010006004	DCLRE1C	2	0.045112549	0.101096386	0.446233051	0.655428911	0	0.747897996	0	0.5	PRJNA827465;Yang2025_Silkie_Wenchang	CRA004775	0.910411938
ENSGALG00010006004	DCLRE1C	2	0.090575327	0.167712157	0.54006417	0.589152779	0.004027134	1.041868757	4.01862105	0.5	CRA004775;Yang2025_Silkie_Wenchang	PRJNA827465	0.892171138
ENSGALG00010006004	DCLRE1C	2	-0.04103833	0.119137433	-0.344462093	0.730498799	0	0.389800754	0	1	CRA004775;PRJNA827465	Yang2025_Silkie_Wenchang	0.932653142
ENSGALG00010006010	IPO5	2	-0.099212173	0.190022708	-0.522106933	0.60159588	0.064207127	8.59539788	88.36586725	0.5	PRJNA827465;Yang2025_Silkie_Wenchang	CRA004775	0.895230519
ENSGALG00010006010	IPO5	2	-0.176443034	0.157700038	-1.1188522	0.263203198	0.02719163	2.100840265	52.39999838	0.5	CRA004775;Yang2025_Silkie_Wenchang	PRJNA827465	0.768643821
ENSGALG00010006010	IPO5	2	0.072193631	0.050746688	1.422627445	0.154844184	0	0.091648821	0	1	CRA004775;PRJNA827465	Yang2025_Silkie_Wenchang	0.647505492
ENSGALG00010006013	TRMT10C	2	-0.122854748	0.123964398	-0.991048644	0.321661828	0.019105739	2.278206424	56.10582125	1	PRJNA827465;Yang2025_Silkie_Wenchang	CRA004775	0.798513866
ENSGALG00010006013	TRMT10C	2	-0.151077279	0.186116857	-0.811733453	0.416944594	0.029509993	1.660280115	39.76919972	0.5	CRA004775;Yang2025_Silkie_Wenchang	PRJNA827465	0.838063117
ENSGALG00010006013	TRMT10C	2	-0.025230399	0.063585298	-0.396796114	0.691517816	0	0.226664922	0	0.5	CRA004775;PRJNA827465	Yang2025_Silkie_Wenchang	0.920271344
ENSGALG00010006015	CDC123	2	-0.227533441	0.311093	-0.731400066	0.464534825	0.185557239	23.95857019	95.82612822	0.5	PRJNA827465;Yang2025_Silkie_Wenchang	CRA004775	0.85217126
ENSGALG00010006015	CDC123	2	-0.506602103	0.100266058	-5.052578213	4.35886E-07	0	0.737328213	0	1	CRA004775;Yang2025_Silkie_Wenchang	PRJNA827465	0.000114211
ENSGALG00010006015	CDC123	2	-0.046472658	0.181915071	-0.255463486	0.798365142	0.044292993	2.374065493	57.87816288	0.5	CRA004775;PRJNA827465	Yang2025_Silkie_Wenchang	0.949922217
ENSGALG00010006016	MTMR6	2	-0.15802547	0.470669741	-0.335745973	0.737062435	0.43557307	58.85176465	98.30081561	0.5	PRJNA827465;Yang2025_Silkie_Wenchang	CRA004775	0.93387966
ENSGALG00010006016	MTMR6	2	-0.314750904	0.339372066	-0.927450829	0.35369251	0.204800924	8.737170036	88.55464646	0.5	CRA004775;Yang2025_Silkie_Wenchang	PRJNA827465	0.811318534
ENSGALG00010006016	MTMR6	2	0.249925496	0.108682253	2.299598054	0.021471002	0.01198733	1.54127699	35.11873553	1	CRA004775;PRJNA827465	Yang2025_Silkie_Wenchang	0.247834011
ENSGALG00010006021	ENSGALG00010006021	2	0.291283383	0.304963663	0.955141278	0.339506257	0.177147687	20.8441658	95.2024945	0.5	PRJNA827465;Yang2025_Silkie_Wenchang	CRA004775	0.80490319
ENSGALG00010006021	ENSGALG00010006021	2	0.332198972	0.303261097	1.095422312	0.273331664	0.152345571	5.469401261	81.71646306	0.5	CRA004775;Yang2025_Silkie_Wenchang	PRJNA827465	0.774113093
ENSGALG00010006021	ENSGALG00010006021	2	-0.007916282	0.069832941	-0.113360289	0.909744921	0	5.61726E-06	0	1	CRA004775;PRJNA827465	Yang2025_Silkie_Wenchang	0.978696684
ENSGALG00010006022	AFG2A	2	0.138580766	0.060607271	2.28653699	0.022222863	0.002712526	1.564053987	36.06358805	1	PRJNA827465;Yang2025_Silkie_Wenchang	CRA004775	0.252865604
ENSGALG00010006022	AFG2A	2	0.059108117	0.195030002	0.30307192	0.761835053	0.054626116	3.040640143	67.11218846	0.5	CRA004775;Yang2025_Silkie_Wenchang	PRJNA827465	0.939811773
ENSGALG00010006022	AFG2A	2	0.024398287	0.116620238	0.20921143	0.83428319	0.013809043	1.538958126	35.02097403	0.5	CRA004775;PRJNA827465	Yang2025_Silkie_Wenchang	0.957990393
ENSGALG00010006024	AMER2	2	0.724695043	0.494231315	1.466307415	0.142564556	0.259436516	2.051323136	51.25097638	1	PRJNA827465;Yang2025_Silkie_Wenchang	CRA004775	0.628893678
ENSGALG00010006024	AMER2	2	0.621660235	0.739257218	0.840925486	0.40038968	0.695336048	2.734442476	63.42947388	0.5	CRA004775;Yang2025_Silkie_Wenchang	PRJNA827465	0.832251175
ENSGALG00010006024	AMER2	2	0.197748541	0.338188725	0.584728367	0.558730377	0	0.367549369	0	0.5	CRA004775;PRJNA827465	Yang2025_Silkie_Wenchang	0.880871906
ENSGALG00010006025	TNFAIP3	2	-0.163295117	0.18779603	-0.869534445	0.384554877	0.059797821	6.474845163	84.55561523	0.5	PRJNA827465;Yang2025_Silkie_Wenchang	CRA004775	0.826203486
ENSGALG00010006025	TNFAIP3	2	-0.181628571	0.260371742	-0.697574054	0.485443612	0.085412047	2.221699363	54.98940961	0.5	CRA004775;Yang2025_Silkie_Wenchang	PRJNA827465	0.857076964
ENSGALG00010006025	TNFAIP3	2	0.025101838	0.083061102	0.302209306	0.762492511	0	0.247846029	0	1	CRA004775;PRJNA827465	Yang2025_Silkie_Wenchang	0.940082621
ENSGALG00010006028	ARL5B	2	-0.307877163	0.130106024	-2.366355939	0.017964164	0.029952594	8.628783383	88.4108807	1	PRJNA827465;Yang2025_Silkie_Wenchang	CRA004775	0.223717898
ENSGALG00010006028	ARL5B	2	-0.008662031	0.473256219	-0.018303047	0.985397097	0.406472793	10.02170826	90.02166124	0.5	CRA004775;Yang2025_Silkie_Wenchang	PRJNA827465	0.99592834
ENSGALG00010006028	ARL5B	2	0.104218904	0.33980612	0.306701081	0.759070907	0.193883651	5.390588966	81.44915136	0.5	CRA004775;PRJNA827465	Yang2025_Silkie_Wenchang	0.939081587
ENSGALG00010006029	HSDL2	2	0.094483792	0.241493533	0.391247711	0.695614145	0.087144122	3.944274739	74.64679653	0.5	PRJNA827465;Yang2025_Silkie_Wenchang	CRA004775	0.921038437
ENSGALG00010006029	HSDL2	2	0.169496474	0.236139514	0.717781074	0.472892285	0.052887265	1.761499008	43.23016955	0.5	CRA004775;Yang2025_Silkie_Wenchang	PRJNA827465	0.854257732
ENSGALG00010006029	HSDL2	2	-0.142182619	0.144735494	-0.982361791	0.325921643	0	0.000993138	0	1	CRA004775;PRJNA827465	Yang2025_Silkie_Wenchang	0.800023788
ENSGALG00010006031	ERCC5	2	0.320520086	0.298216968	1.074788224	0.282469554	0.16670827	15.68591424	93.62485358	1	PRJNA827465;Yang2025_Silkie_Wenchang	CRA004775	0.779532565
ENSGALG00010006031	ERCC5	2	0.611109065	0.117275937	5.210864926	1.87962E-07	0	0.089640643	0	1	CRA004775;Yang2025_Silkie_Wenchang	PRJNA827465	5.91663E-05
ENSGALG00010006031	ERCC5	2	0.224772179	0.246969711	0.910120426	0.362759003	0.092680073	3.508933097	71.50130902	1	CRA004775;PRJNA827465	Yang2025_Silkie_Wenchang	0.816067604
ENSGALG00010006033	NSUN6	2	0.142798598	0.103254067	1.382982794	0.166670142	0	0.048823339	0	1	PRJNA827465;Yang2025_Silkie_Wenchang	CRA004775	0.665626918
ENSGALG00010006033	NSUN6	2	0.036243171	0.209630355	0.172890855	0.86273722	0.033660174	1.470798309	32.00971241	0.5	CRA004775;Yang2025_Silkie_Wenchang	PRJNA827465	0.966099097
ENSGALG00010006033	NSUN6	2	0.037854885	0.168322794	0.224894587	0.82206128	0.019089082	1.291425023	22.56615891	0.5	CRA004775;PRJNA827465	Yang2025_Silkie_Wenchang	0.955636543
ENSGALG00010006035	ADH1C	2	0.949703865	0.677817626	1.401120048	0.161178178	0.676399455	3.521280227	71.60123775	1	PRJNA827465;Yang2025_Silkie_Wenchang	CRA004775	0.657426695
ENSGALG00010006035	ADH1C	2	0.84678388	0.599562706	1.41233581	0.157851107	0.468943293	2.601128553	61.55514887	1	CRA004775;Yang2025_Silkie_Wenchang	PRJNA827465	0.651369486
ENSGALG00010006035	ADH1C	2	1.684309371	0.473105912	3.560110599	0.000370699	0	0.021880089	0	1	CRA004775;PRJNA827465	Yang2025_Silkie_Wenchang	0.01934739
ENSGALG00010006037	METTL21EP	2	0.099171861	0.644129753	0.153962552	0.877639263	0.470071946	2.247681632	55.50971339	0.5	PRJNA827465;Yang2025_Silkie_Wenchang	CRA004775	0.970782842
ENSGALG00010006037	METTL21EP	2	-0.390399625	0.593731619	-0.657535514	0.510836643	0	0.444937651	0	0.5	CRA004775;Yang2025_Silkie_Wenchang	PRJNA827465	0.865436876

ENSGALG00010006037	METTL21EP	2	0.58272451	0.460306698	1.26594836	0.205531571	0	0.125171219	0	1	CRA004775;PRJNA827465	Yang2025_Silkie_Wenchang	0.71825498
ENSGALG00010006038	ENSGALG00010006038	2	0.140800322	0.129757547	1.085103141	0.277875977	0	0.006578495	0	1	PRJNA827465;Yang2025_Silkie_Wenchang	CRA004775	0.776763074
ENSGALG00010006038	ENSGALG00010006038	2	0.045260617	0.248018142	0.182489137	0.855198879	0	0.264568886	0	0.5	CRA004775;Yang2025_Silkie_Wenchang	PRJNA827465	0.963353229
ENSGALG00010006038	ENSGALG00010006038	2	0.106787629	0.130673914	0.817206941	0.413810176	0	0.340522476	0	0.5	CRA004775;PRJNA827465	Yang2025_Silkie_Wenchang	0.836538492
ENSGALG00010006039	PCNP	2	-0.013195004	0.032616724	-0.404547195	0.68581038	0	0.301766987	0	1	PRJNA827465;Yang2025_Silkie_Wenchang	CRA004775	0.919235539
ENSGALG00010006039	PCNP	2	-0.049284679	0.066057333	-0.746089446	0.455613385	0	0.013629434	0	1	CRA004775;Yang2025_Silkie_Wenchang	PRJNA827465	0.84907427
ENSGALG00010006039	PCNP	2	-0.005616358	0.036433355	-0.154154287	0.877488085	0	0.100562746	0	1	CRA004775;PRJNA827465	Yang2025_Silkie_Wenchang	0.970697577
ENSGALG00010006041	QKI	2	-0.036031054	0.068061229	-0.529391763	0.596533711	0.002809576	1.17025185	14.54830854	0.5	PRJNA827465;Yang2025_Silkie_Wenchang	CRA004775	0.894064255
ENSGALG00010006041	QKI	2	0.244214677	0.13403501	1.822021549	0.068451716	0	0.99013863	0	1	CRA004775;Yang2025_Silkie_Wenchang	PRJNA827465	0.450797217
ENSGALG00010006041	QKI	2	0.126322186	0.229668333	0.55002004	0.582305629	0.086176118	4.712290097	78.77889562	0.5	CRA004775;PRJNA827465	Yang2025_Silkie_Wenchang	0.889550611
ENSGALG00010006047	ZBTB11	2	0.162332292	0.082515023	1.9673059	0.049147955	0	0.451044157	0	1	PRJNA827465;Yang2025_Silkie_Wenchang	CRA004775	0.383342347
ENSGALG00010006047	ZBTB11	2	0.120297728	0.100262818	1.199823928	0.230207729	0	0.038606781	0	1	CRA004775;Yang2025_Silkie_Wenchang	PRJNA827465	0.742845187
ENSGALG00010006047	ZBTB11	2	0.205318176	0.104364689	1.96731459	0.049146954	0	0.068267815	0	1	CRA004775;PRJNA827465	Yang2025_Silkie_Wenchang	0.383342347
ENSGALG00010006049	AIMP1	2	0.068286143	0.059679277	1.144218678	0.252532959	0	0.111781251	0	1	PRJNA827465;Yang2025_Silkie_Wenchang	CRA004775	0.762655223
ENSGALG00010006049	AIMP1	2	0.112623453	0.127912297	0.880474009	0.378602579	0	0.000961507	0	1	CRA004775;Yang2025_Silkie_Wenchang	PRJNA827465	0.823771464
ENSGALG00010006049	AIMP1	2	0.063104581	0.062516795	1.00940205	0.312781856	0	0.03670759	0	1	CRA004775;PRJNA827465	Yang2025_Silkie_Wenchang	0.796054997
ENSGALG00010006052	RALGAPA1	2	0.201453059	0.255568762	0.788253847	0.430548238	0.121649617	14.02939914	92.87211099	0.5	PRJNA827465;Yang2025_Silkie_Wenchang	CRA004775	0.842385375
ENSGALG00010006052	RALGAPA1	2	0.407131891	0.129760743	3.137558254	0.001703614	0.004719359	1.095882746	8.749361755	1	CRA004775;Yang2025_Silkie_Wenchang	PRJNA827465	0.052228664
ENSGALG00010006052	RALGAPA1	2	-0.035696536	0.04938895	-0.722763607	0.469825129	0	0.396597323	0	0.5	CRA004775;PRJNA827465	Yang2025_Silkie_Wenchang	0.853485658
ENSGALG00010006053	TMEM243	2	-0.411513432	0.16183605	-2.54277975	0.010997453	0	0.007352262	0	1	PRJNA827465;Yang2025_Silkie_Wenchang	CRA004775	0.166811819
ENSGALG00010006053	TMEM243	2	-0.30087937	0.26087888	-1.153329739	0.248775068	0	0.390102138	0	1	CRA004775;Yang2025_Silkie_Wenchang	PRJNA827465	0.759196439
ENSGALG00010006053	TMEM243	2	-0.373789066	0.181763676	-2.056456354	0.039738549	0	0.523873211	0	1	CRA004775;PRJNA827465	Yang2025_Silkie_Wenchang	0.344755224
ENSGALG00010006059	BIVM	2	-0.142205801	0.189480268	-0.750504539	0.452950891	0.068818765	23.976057	95.82917241	0.5	PRJNA827465;Yang2025_Silkie_Wenchang	CRA004775	0.848618976
ENSGALG00010006059	BIVM	2	-0.243450618	0.122543171	-1.986651855	0.046960991	0.019396834	2.434245676	58.91951212	1	CRA004775;Yang2025_Silkie_Wenchang	PRJNA827465	0.374003609
ENSGALG00010006059	BIVM	2	0.034589836	0.044979782	0.76900854	0.441888241	0	0.587635812	0	0.5	CRA004775;PRJNA827465	Yang2025_Silkie_Wenchang	0.845135451
ENSGALG00010006061	RPL24	2	-0.031001802	0.096863288	-0.320057297	0.748924896	0.008168865	1.394183003	28.27340473	0.5	PRJNA827465;Yang2025_Silkie_Wenchang	CRA004775	0.937330676
ENSGALG00010006061	RPL24	2	-0.024238045	0.208674506	-0.116152399	0.907531761	0.039788648	1.818391947	45.00635567	0.5	CRA004775;Yang2025_Silkie_Wenchang	PRJNA827465	0.978196186
ENSGALG00010006061	RPL24	2	-0.090373204	0.058396545	-1.547577936	0.121723947	0	0.514696419	0	1	CRA004775;PRJNA827465	Yang2025_Silkie_Wenchang	0.5849268
ENSGALG00010006062	ENSGALG00010006062	2	-1.442408572	0.78212863	-1.84420889	0.065152703	0.689859232	2.277122228	56.08492212	1	PRJNA827465;Yang2025_Silkie_Wenchang	CRA004775	0.439906665
ENSGALG00010006062	ENSGALG00010006062	2	-0.978075673	1.281373413	-0.763302612	0.445282945	2.717192265	5.785271305	82.71472594	0.5	CRA004775;Yang2025_Silkie_Wenchang	PRJNA827465	0.846013379
ENSGALG00010006062	ENSGALG00010006062	2	-0.245948083	0.496598349	-0.495265608	0.620412644	0.016877382	1.035316571	3.411185678	0.5	CRA004775;PRJNA827465	Yang2025_Silkie_Wenchang	0.899395593
ENSGALG00010006063	POGLUT2	2	-0.103879237	0.056279222	-1.845783115	0.064923703	0.001804801	1.392629951	28.19341568	1	PRJNA827465;Yang2025_Silkie_Wenchang	CRA004775	0.439424217
ENSGALG00010006063	POGLUT2	2	-0.049406889	0.155329085	-0.318078802	0.750425163	0.032524972	2.646682944	62.21685705	0.5	CRA004775;Yang2025_Silkie_Wenchang	PRJNA827465	0.937393444
ENSGALG00010006063	POGLUT2	2	-0.020084114	0.076468065	-0.262647084	0.792822606	0.002993507	1.156745268	13.55054329	0.5	CRA004775;PRJNA827465	Yang2025_Silkie_Wenchang	0.948451453
ENSGALG00010006064	DMTF1	2	-0.002441994	0.066935652	-0.036482704	0.97089747	0	0.720197186	0	0.5	PRJNA827465;Yang2025_Silkie_Wenchang	CRA004775	0.991868977
ENSGALG00010006064	DMTF1	2	0.154578593	0.111046847	1.392012448	0.163918628	0	0.284142521	0	1	CRA004775;Yang2025_Silkie_Wenchang	PRJNA827465	0.662467234
ENSGALG00010006064	DMTF1	2	0.049211773	0.12012753	0.409662737	0.682053369	0.015354244	1.849318593	45.92602898	0.5	CRA004775;PRJNA827465	Yang2025_Silkie_Wenchang	0.918515969
ENSGALG00010006065	WDR54	2	0.054483838	0.123411999	0.441479257	0.658866075	0	0.041826307	0	1	PRJNA827465;Yang2025_Silkie_Wenchang	CRA004775	0.911329736
ENSGALG00010006065	WDR54	2	-0.097061943	0.200478731	-0.484150825	0.628278834	0.024349533	1.433065237	30.21950612	0.5	CRA004775;Yang2025_Silkie_Wenchang	PRJNA827465	0.901904489
ENSGALG00010006065	WDR54	2	-0.078737238	0.163658418	-0.481107166	0.630440335	0.018993074	1.465231681	31.75140743	0.5	CRA004775;PRJNA827465	Yang2025_Silkie_Wenchang	0.902666539
ENSGALG00010006066	ARHGAP18	2	-0.24474335	0.330746155	-0.739973379	0.459316147	0.199479473	11.32520592	91.17013847	0.5	PRJNA827465;Yang2025_Silkie_Wenchang	CRA004775	0.850098539
ENSGALG00010006066	ARHGAP18	2	-0.262494916	0.340880614	-0.770049411	0.441270583	0.194767443	5.991658081	83.31012907	0.5	CRA004775;Yang2025_Silkie_Wenchang	PRJNA827465	0.844907543
ENSGALG00010006066	ARHGAP18	2	0.088714769	0.116038128	0.764531202	0.44455075	0	0.006649698	0	1	CRA004775;PRJNA827465	Yang2025_Silkie_Wenchang	0.846013379
ENSGALG00010006068	PACRG	2	0.017537139	0.156517411	0.112045932	0.910786987	0	0.000509373	0	1	PRJNA827465;Yang2025_Silkie_Wenchang	CRA004775	0.97880407
ENSGALG00010006068	PACRG	2	0.049082757	0.241304808	0.203405632	0.838817998	0	0.072327946	0	1	CRA004775;Yang2025_Silkie_Wenchang	PRJNA827465	0.959283372
ENSGALG00010006068	PACRG	2	0.031612278	0.186794517	0.169235577	0.865611347	0	0.083597019	0	1	CRA004775;PRJNA827465	Yang2025_Silkie_Wenchang	0.967088846
ENSGALG00010006069	TTR	2	0.057023821	2.487613044	0.022923107	0.981711608	10.40799297	6.250916995	84.00234716	0.5	PRJNA827465;Yang2025_Silkie_Wenchang	CRA004775	0.99503081
ENSGALG00010006069	TTR	2	0.439760362	2.876068026	0.152903324	0.878474514	14.60659997	8.494808202	88.22810385	0.5	CRA004775;Yang2025_Silkie_Wenchang	PRJNA827465	0.971050845
ENSGALG00010006069	TTR	2	2.859805255	0.876473786	3.262853151	0.001102967	0	0.195997412	0	1	CRA004775;PRJNA827465	Yang2025_Silkie_Wenchang	0.04010166
ENSGALG00010006070	TMEM242	2	-0.175388805	0.079173689	-2.215241036	0.026743525	0	0.012042216	0	1	PRJNA827465;Yang2025_Silkie_Wenchang	CRA004775	0.278911632
ENSGALG00010006070	TMEM242	2	-0.130894859	0.112421974	-1.164317386	0.244295413	0.000758161	1.018908469	1.855757353	0.5	CRA004775;Yang2025_Silkie_Wenchang	PRJNA827465	0.755788935
ENSGALG00010006070	TMEM242	2	-0.129801299	0.096410422	-1.346340952	0.178192585	0	0.944849568	0	0.5	CRA004775;PRJNA827465	Yang2025_Silkie_Wenchang	0.684604414
ENSGALG00010006071	TEX30	2	-0.078017304	0.145687816	-0.53551015	0.592297141	0	0.369762834	0	1	PRJNA827465;Yang2025_Silkie_Wenchang	CRA004775	0.892776493
ENSGALG00010006071	TEX30	2	-0.511275503	0.352317886	-1.451176689	0.146730666	0.139719448	2.20333431	54.61424101	1	CRA004775;Yang2025_Silkie_Wenchang	PRJNA827465	0.635558525
ENSGALG00010006071	TEX30	2	-0.40414838	0.449185118	-0.899736798	0.368260335	0.318648307	4.350486649	77.01406577	1	CRA004775;PRJNA827465	Yang2025_Silkie_Wenchang	0.820213684
ENSGALG00010006072	ENSGALG00010006072	2	-0.014679507	0.138964857	-0.105634671	0.915872215	0.028205949	3.593430384	72.17143806	0.5	PRJNA827465;Yang2025_Silkie_Wenchang	CRA004775	0.980324237
ENSGALG00010006072	ENSGALG00010006072	2	0.120617412	0.118498796	1.017878798	0.308735559	0	0.360370507	0	0.5	CRA004775;Yang2025_Silkie_Wenchang	PRJNA827465	0.793670558
ENSGALG00010006072	ENSGALG00010006072	2	-0.139235196	0.080146057	-1.737268195	0.082339843	0	2.76934E-05	0	1	CRA004775;PRJNA827465	Yang2025_Silkie_Wenchang	0.494680959
ENSGALG00010006073	CEP97	2	-0.26632864	0.240868039	-1.105703528	0.268854863	0.095574149	5.557405511	82.00599186	1	PRJNA827465;Yang2025_Silkie_Wenchang	CRA004775	0.771398294
ENSGALG00010006073	CEP97	2	-0.346591316	0.206881578	-1.675312612	0.093872912	0.045483791	2.077835341	51.87299109	1	CRA004775;Yang2025_Silkie_Wenchang	PRJNA827465	0.524867361
ENSGALG00010006073	CEP97	2	-0.053215285	0.103486029	-0.514226753	0.607093465	0	0.059437622	0	1	CRA004775;PRJNA827465	Yang2025_Silkie_Wenchang	0.89694739
ENSGALG00010006075	SOX21	2	-0.556258949	0.332822785	-1.671336742	0.094655183	0	0.481616565	0	1	PRJNA827465;Yang2025_Silkie_Wenchang	CRA004775	0.526919621
ENSGALG00010006075	SOX21	2	-0.129489195	0.37549441	-0.34484986	0.730207247	0	0.147636081	0	0.5	CRA004775;Yang2025_Silkie_Wenchang	PRJNA827465	0.932614156
ENSGALG00010006075	SOX21	2	-0.43048287	0.374343506	-1.14996751	0.250157253	0.054480947	1.232118776	18.83899352	0.5	CRA004775;PRJNA827465	Yang2025_Silkie_Wenchang	0.75993411
ENSGALG00010006076	PODXL	2	0.261946482	0.506146674	0.517530778	0.604785689	0.435609312	5.847641736	82.89908915	0.5	PRJNA827465;Yang2025_Silkie_Wenchang	CRA004775	0.896344437
ENSGALG00010006076	PODXL	2	0.59829838	0.290388056	2.060340868	0.039365966	0	0.747200965	0	1	CRA004775;Yang2025_Silkie_Wenchang	PRJNA827465	0.34316319
ENSGALG00010006076	PODXL	2	-0.059052804	0.213593775	-0.276472493	0.782185184	0.050973469	1.594235386	37.27400552	0.5	CRA004775;PRJNA827465	Yang2025_Silkie_Wenchang	0.945372683
ENSGALG00010006077	TBCK	2	0.034429647	0.120006931	0.286897151	0.774191072	0.020835042	3.53051738	71.67553952	0.5	PRJNA827465;Yang2025_Silkie_Wenchang	CRA004775	0.94338785
ENSGALG00010006077	TBCK	2	-0.094469567	0.100512566	-0.93987818	0.347280051	0	0.011107793	0	1	CRA004775;Yang2025_Silkie_Wenchang	PRJNA827465	0.808705451
ENSGALG00010006077	TBCK	2	0.134838163	0.072279446	1.86551185	0.062109693	0	0.320545803	0	0.5	CRA004775;PRJNA827465	Yang2025_Silkie_Wenchang	0.429956168
ENSGALG00010006078	SPATA13	2	0.059406223	0.136444456	0.435387589	0.663281117	0	0.017366652	0	1	PRJNA827465;Yang2025_Silkie_Wenchang	CRA004775	0.91293776
ENSGALG00010006078	SPATA13	2	0.017545526	0.200141249	0.087665718	0.930142368	0.001377583	1.005562577	0.553180562	0.5	CRA004775;Yang2025_Silkie_Wenchang	PRJNA827465	0.983813577
ENSGALG00010006078	SPATA13	2	-0.002057896	0.175619163	-0.011717946	0.990650646	0	0.922538753	0	0.5	CRA004775;PRJNA827465	Yang2025_Silkie_Wenchang	0.997528754
ENSGALG00010006079	ENSGALG00010006079	2	0.357815084	0.514110673	0.695988438	0.486436071	0.154530219	1.409623085	29.05905056	0.5	PRJNA827465;Yang2025_Silkie_Wenchang	CRA004775	0.857603818
ENSGALG00010006079	ENSGALG00010006079	2	0.235879261	0.719810232	0.327696454	0.743141184	0.287769932	1.277047889	21.69440094	0.5	CRA004775;Yang2025_Silkie_Wenchang	PRJNA827465	0.93573824
ENSGALG00010006079	ENSGALG00010006079	2	0.93439447	0.52876327	1.767131953	0.077206132	0	0.178910671	0	1	CRA004775;PRJNA827465	Yang2025_Silkie_Wenchang	0.478692636
ENSGALG00010006080	ENSGALG00010006080	2	0.488821688	0.431596032	1.132590782	0.257386142	0.351867387	17.86417954	94.40220583	1	PRJNA827465;Yang2025_Silkie_Wenchang	CRA004775	0.764987374
ENSGALG00010006080	ENSGALG00010006080	2	0.433504575	0.520588591	0.832720084	0.405002615	0.488480747	9.921042629	89.92041424	0.5	CRA004775;Yang2025_Silkie_Wenchang	PRJNA827465	0.833112102
ENSGALG00010006080	ENSGALG00010006080	2	0.042366083	0.104480384	0.405493181	0.68511503	0	0.344123894	0	0.5	CRA004775;PRJNA827465	Yang2025_Silkie_Wenchang	0.919196979
ENSGALG00010006081	ZDHHC14	2	-0.060930676	0.065438867	-0.931108359	0.351797515	0.002182252	1.312653429	23.81842931	1	PRJNA827465;Yang2025_Silkie_Wenchang	CRA004775	0.810678834
ENSGALG00010006081	ZDHHC14	2	-0.118248217	0.09000459	-1.313802072	0.18891282	0	0.627325706	0	0.5	CRA004775;Yang2025_Silkie_Wenchang	PRJNA827465	0.698162702
ENSGALG00010006081	ZDHHC14	2	-0.005231368	0.065701147	-0.079623693	0.936536551	0	0.08691108	0	0.5	CRA004775;PRJNA827465	Yang2025_Silkie_Wenchang	0.985038972
ENSGALG00010006083	CACNB2	2	0.168043043	0.227654009	0.73815104	0.460422671	0.07368498	3.269279478	69.41222043	0.5	PRJNA827465;Yang2025_Silkie_Wenchang	CRA004775	0.85014477
ENSGALG00010006083	CACNB2	2	0.470535717	0.209116405	2.250113841	0.02444172	0	0.267613058	0	1	CRA004775;Yang2025_Silkie_Wenchang	PRJNA827465	0.267184364
ENSGALG00010006083	CACNB2	2	0.131807867	0.323061409	0.407996323	0.683276374	0.11340784	1.516401877	34.05442084	0.5	CRA004775;PRJNA827465	Yang2025_Silkie_Wenchang	0.918800744
ENSGALG00010006084	TMEM68	2	-0.126552475	0.072630007	-1.742426866	0.081433778	0	0.134275172	0	1	PRJNA827465;Yang2025_Silkie_Wenchang	CRA004775	0.491466018
ENSGALG00010006084	TMEM68	2	-0.20224688	0.130779543	-1.546471836	0.121990657	0	0.702315568	0	1	CRA004775;Yang2025_Silkie_Wenchang	PRJNA827465	0.585707157
ENSGALG00010006084	TMEM68	2	-0.125724643	0.084224969	-1.492724115	0.135509418	0	0.978785909	0	1	CRA004775;PRJNA827465	Yang2025_Silkie_Wenchang	0.616997992
ENSGALG00010006085	DLST	2	0.014109828	0.058087865	0.242904912	0.80807906	0	0.109274747	0	1	PRJNA827465;Yang2025_Silkie_Wenchang	CRA004775	0.952496413
ENSGALG00010006085	DLST	2	-0.007940601	0.106134355	-0.074816501	0.940360713	0.001285588	1.039056142	3.75880962	0.5	CRA004775;Yang2025_Silkie_Wenchang	PRJNA827465	0.985520695
ENSGALG00010006085	DLST	2	-0.014600762	0.064825282	-0.22523253	0.821798384	0	0.82561326	0	0.5	CRA004775;PRJNA827465	Yang2025_Silkie_Wenchang	0.95563486
ENSGALG00010006086	ENSGALG00010006086	2	0.483562453	0.296305407	1.631973097	0.102685154	0	0.026005337	0	1	PRJNA827465;Yang2025_Silkie_Wenchang	CRA004775	0.546039025
ENSGALG00010006086	ENSGALG00010006086	2	0.493252104	0.388139888	1.270810138	0.203796201	0	0.045700431	0	1	CRA004775;Yang2025_Silkie_Wenchang	PRJNA827465	0.717254253
ENSGALG00010006086	ENSGALG00010006086	2	0.530999986	0.292911404	1.812834801	0.069857305	0	0.008639094	0	1	CRA004775;PRJNA827465	Yang2025_Silkie_Wenchang	0.455777165
ENSGALG00010006087	ENSGALG00010006087	2	-0.149544122	0.269587406	-0.554714792	0.579089757	0	0.113571053	0	1	PRJNA827465;Yang2025_Silkie_Wenchang	CRA004775	0.887888423
ENSGALG00010006087	ENSGALG00010006087	2	1.054151	1.422030761	0.741299717	0.458511733	3.580818165	8.480900575	88.20879939	0.5	CRA004775;Yang2025_Silkie_Wenchang	PRJNA827465	0.850089963
ENSGALG00010006087	ENSGALG00010006087	2	1.122044243	1.321731537	0.848919929	0.395925849	3.104964001	8.458187881	88.17713659	0.5	CRA004775;PRJNA827465	Yang2025_Silkie_Wenchang	0.831296327
ENSGALG00010006091	RTKN	2	0.040712524	0.212706039	0.191402765	0.848210054	0.070336627	4.060726904	75.3738672	0.5	PRJNA827465;Yang2025_Silkie_Wenchang	CRA004775	0.961363811
ENSGALG00010006091	RTKN	2	0.175959584	0.182658708	0.963324365	0.33538477	0.00814671	1.113232769	10.17152679	0.5	CRA004775;Yang2025_Silkie_Wenchang	PRJNA827465	0.803198384
ENSGALG00010006091	RTKN	2	-0.135761203	0.079529189	-1.707061341	0.087810652	0	0.009065585	0	1	CRA004775;PRJNA827465	Yang2025_Silkie_Wenchang	0.509480667
ENSGALG00010006096	EDIL3	2	-0.468021185	0.08598787	-5.442874528	5.24276E-08	0	0.054383118	0	1	PRJNA827465;Yang2025_Silkie_Wenchang	CRA004775	2.06056E-05
ENSGALG00010006096	EDIL3	2	-0.290519038	0.265887535	-1.092638802	0.274552419	0.055162136	1.356482343	26.2799103	0.5	CRA004775;Yang2025_Silkie_Wenchang	PRJNA827465	0.774528691
ENSGALG00010006096	EDIL3	2	-0.320261522	0.291925025	-1.09706772	0.27261179	0.100851176	1.710105589	41.5240786	0.5	CRA004775;PRJNA827465	Yang2025_Silkie_Wenchang	0.773551453
ENSGALG00010006100	RPS6KL1	2	-0.153763067	0.133684311	-1.150195302	0.250063441	0	0.03039782	0	1	PRJNA827465;Yang2025_Silkie_Wenchang	CRA004775	0.75993411
ENSGALG00010006100	RPS6KL1	2	0.014425513	0.190376157	0.075773738	0.93959911	0	0.789798021	0	0.5	CRA004775;Yang2025_Silkie_Wenchang	PRJNA827465	0.985425793
ENSGALG00010006100	RPS6KL1	2	-0.056505507	0.179580544	-0.31465272	0.753025346	0.019241697	1.319072162	24.18913623	0.5	CRA004775;PRJNA827465	Yang2025_Silkie_Wenchang	0.938087082
ENSGALG00010006101	ENSGALG00010006101	2	0.063281908	0.088668648	0.713689786	0.475419024	0	0.005658741	0	1	PRJNA827465;Yang2025_Silkie_Wenchang	CRA004775	0.854462864
ENSGALG00010006101	ENSGALG00010006101	2	0.072153545	0.190691249	0.378378898	0.705149142	0	0.003201858	0	1	CRA004775;Yang2025_Silkie_Wenchang	PRJNA827465	0.924245236
ENSGALG00010006101	ENSGALG00010006101	2	0.059982096	0.093579391	0.640975488	0.521538608	0	0.000265945	0	1	CRA004775;PRJNA827465	Yang2025_Silkie_Wenchang	0.869518515
ENSGALG00010006103	NXPE3	2	0.302629327	0.142552196	2.122936968	0.033759134	0.022436379	2.223483399	55.02552434	1	PRJNA827465;Yang2025_Silkie_Wenchang	CRA004775	0.315687663
ENSGALG00010006103	NXPE3	2	0.092662623	0.448017048	0.206828341	0.836143922	0.310294877	3.815321501	73.78988901	0.5	CRA004775;Yang2025_Silkie_Wenchang	PRJNA827465	0.958399882
ENSGALG00010006103	NXPE3	2	-0.002284729	0.280235625	-0.008152886	0.993495011	0.091504878	1.848096135	45.89026073	0.5	CRA004775;PRJNA827465	Yang2025_Silkie_Wenchang	0.997979742
ENSGALG00010006107	PGF	2	0.211113976	0.235717168	0.895624097	0.370453561	0.083007695	3.80409315	73.71252594	1	PRJNA827465;Yang2025_Silkie_Wenchang	CRA004775	0.821221934
ENSGALG00010006107	PGF	2	0.385346769	0.184681873	2.086543546	0.036929415	0	0.967772669	0	1	CRA004775;Yang2025_Silkie_Wenchang	PRJNA827465	0.331631487
ENSGALG00010006107	PGF	2	0.002729081	0.123286566	0.022136079	0.982339407	0	0.001859473	0	1	CRA004775;PRJNA827465	Yang2025_Silkie_Wenchang	0.99513702
ENSGALG00010006108	MKLN1	2	-0.187946378	0.158495651	-1.185814098	0.235695723	0.046238023	12.50976302	92.00624346	1	PRJNA827465;Yang2025_Silkie_Wenchang	CRA004775	0.74863011
ENSGALG00010006108	MKLN1	2	-0.293706827	0.102521414	-2.864833938	0.00417228	0.008732483	1.409337208	29.04466057	1	CRA004775;Yang2025_Silkie_Wenchang	PRJNA827465	0.09397313
ENSGALG00010006108	MKLN1	2	-0.037622264	0.054251381	-0.693480306	0.48800818	0	0.125751153	0	1	CRA004775;PRJNA827465	Yang2025_Silkie_Wenchang	0.85768885
ENSGALG00010006111	WBP1	2	0.022494998	0.05845529	0.384823997	0.700367819	0	0.486988091	0	1	PRJNA827465;Yang2025_Silkie_Wenchang	CRA004775	0.922303014
ENSGALG00010006111	WBP1	2	0.126970207	0.196907999	0.644819958	0.519043867	0	0.178288183	0	1	CRA004775;Yang2025_Silkie_Wenchang	PRJNA827465	0.868939943
ENSGALG00010006111	WBP1	2	0.012497106	0.059237351	0.21096665	0.832913295	0	2.00213E-05	0	1	CRA004775;PRJNA827465	Yang2025_Silkie_Wenchang	0.957905818
ENSGALG00010006113	EIF2B2	2	-0.165471648	0.117464638	-1.408693297	0.158925878	0.020808682	3.590708963	72.15034663	1	PRJNA827465;Yang2025_Silkie_Wenchang	CRA004775	0.653716774
ENSGALG00010006113	EIF2B2	2	-0.332760219	0.104244885	-3.192101153	0.001412419	0	0.156896193	0	1	CRA004775;Yang2025_Silkie_Wenchang	PRJNA827465	0.046337469
ENSGALG00010006113	EIF2B2	2	-0.17166747	0.155199801	-1.106106252	0.268680533	0.032315004	2.284511371	56.22696333	1	CRA004775;PRJNA827465	Yang2025_Silkie_Wenchang	0.771398294
ENSGALG00010006118	NPNT	2	-0.128108041	0.132040477	-0.970217947	0.331937867	0	0.861346925	0	1	PRJNA827465;Yang2025_Silkie_Wenchang	CRA004775	0.802002658
ENSGALG00010006118	NPNT	2	0.428627533	0.793361994	0.540267288	0.589012714	1.152396247	11.7153493	91.46418964	0.5	CRA004775;Yang2025_Silkie_Wenchang	PRJNA827465	0.892171138
ENSGALG00010006118	NPNT	2	0.547361102	0.650098004	0.841967056	0.399806394	0.765659582	10.12135503	90.11989999	0.5	CRA004775;PRJNA827465	Yang2025_Silkie_Wenchang	0.832246851
ENSGALG00010006120	MOGS	2	0.391829068	0.292349328	1.340276957	0.18015532	0.151575774	8.408271284	88.10694891	1	PRJNA827465;Yang2025_Silkie_Wenchang	CRA004775	0.687225928
ENSGALG00010006120	MOGS	2	0.2646179	0.470234617	0.562735899	0.573614735	0.383996378	7.431067667	86.54298353	0.5	CRA004775;Yang2025_Silkie_Wenchang	PRJNA827465	0.885985221
ENSGALG00010006120	MOGS	2	0.054688586	0.13925877	0.392711968	0.694532231	0.017217873	1.374676048	27.2555886	0.5	CRA004775;PRJNA827465	Yang2025_Silkie_Wenchang	0.920728119
ENSGALG00010006121	ENSGALG00010006121	2	0.278385291	0.171167567	1.626390414	0.103866629	0	0.110281284	0	1	PRJNA827465;Yang2025_Silkie_Wenchang	CRA004775	0.548175896
ENSGALG00010006121	ENSGALG00010006121	2	0.290770313	0.26336806	1.10404547	0.269573414	0	0.271444828	0	1	CRA004775;Yang2025_Silkie_Wenchang	PRJNA827465	0.77189992
ENSGALG00010006121	ENSGALG00010006121	2	0.212387089	0.199249022	1.065937925	0.286451704	0	0.129135311	0	1	CRA004775;PRJNA827465	Yang2025_Silkie_Wenchang	0.782018115
ENSGALG00010006123	MLH3	2	0.443728924	0.081145638	5.468302843	4.54365E-08	0	0.469843732	0	1	PRJNA827465;Yang2025_Silkie_Wenchang	CRA004775	1.82224E-05
ENSGALG00010006123	MLH3	2	0.475953821	0.105691678	4.503228901	6.69287E-06	0	0.256243419	0	1	CRA004775;Yang2025_Silkie_Wenchang	PRJNA827465	0.001019574
ENSGALG00010006123	MLH3	2	0.386473761	0.101548486	3.805805237	0.000141344	0	0.008250259	0	1	CRA004775;PRJNA827465	Yang2025_Silkie_Wenchang	0.010009417
ENSGALG00010006126	ENSGALG00010006126	2	0.194641314	0.165482769	1.176202908	0.239513803	0	2.54504E-06	0	1	PRJNA827465;Yang2025_Silkie_Wenchang	CRA004775	0.751810791
ENSGALG00010006126	ENSGALG00010006126	2	-0.467645372	0.745112178	-0.627617405	0.530254604	0.921116163	5.501550163	81.82330488	0.5	CRA004775;Yang2025_Silkie_Wenchang	PRJNA827465	0.872246963
ENSGALG00010006126	ENSGALG00010006126	2	-0.458091933	0.743615839	-0.61603305	0.537872707	0.934988756	5.922780402	83.11603787	0.5	CRA004775;PRJNA827465	Yang2025_Silkie_Wenchang	0.87481821
ENSGALG00010006128	ACYP1	2	-0.176874999	0.108944288	-1.623536236	0.104474824	0.008317072	1.382293606	27.65646926	0.5	PRJNA827465;Yang2025_Silkie_Wenchang	CRA004775	0.549394434
ENSGALG00010006128	ACYP1	2	-0.097005204	0.161407251	-0.600996566	0.547842273	0.000301669	1.004622866	0.460159343	0.5	CRA004775;Yang2025_Silkie_Wenchang	PRJNA827465	0.878281266
ENSGALG00010006128	ACYP1	2	-0.252247946	0.085664531	-2.944601971	0.003233704	0	0.132687794	0	1	CRA004775;PRJNA827465	Yang2025_Silkie_Wenchang	0.079016811
ENSGALG00010006131	ENSGALG00010006131	2	0.138815743	0.106835277	1.299343695	0.193826005	0.006185014	1.183322254	15.49216654	1	PRJNA827465;Yang2025_Silkie_Wenchang	CRA004775	0.704061321
ENSGALG00010006131	ENSGALG00010006131	2	0.044221706	0.353619402	0.125054523	0.900480386	0.172987016	3.204069856	68.78969421	0.5	CRA004775;Yang2025_Silkie_Wenchang	PRJNA827465	0.976475201
ENSGALG00010006131	ENSGALG00010006131	2	-0.018433649	0.185737097	-0.099245918	0.920943017	0.038639649	1.737961235	42.46131732	0.5	CRA004775;PRJNA827465	Yang2025_Silkie_Wenchang	0.981469977
ENSGALG00010006132	MIPEP	2	-0.137702697	0.073505544	-1.873364767	0.061018032	0.003699008	1.495010829	33.11085242	1	PRJNA827465;Yang2025_Silkie_Wenchang	CRA004775	0.42722323
ENSGALG00010006132	MIPEP	2	-0.248184638	0.089104275	-2.785328077	0.005347359	0	0.317970513	0	1	CRA004775;Yang2025_Silkie_Wenchang	PRJNA827465	0.109348202
ENSGALG00010006132	MIPEP	2	-0.143654094	0.121042304	-1.186808993	0.235302971	0.013711296	1.508141396	33.69321982	1	CRA004775;PRJNA827465	Yang2025_Silkie_Wenchang	0.748350272
ENSGALG00010006135	ZC2HC1C	2	0.181038965	0.096769077	1.870834883	0.061367971	0	0.760131821	0	1	PRJNA827465;Yang2025_Silkie_Wenchang	CRA004775	0.427938152
ENSGALG00010006135	ZC2HC1C	2	0.076726855	0.144979313	0.52922623	0.596648523	0	0.009960567	0	1	CRA004775;Yang2025_Silkie_Wenchang	PRJNA827465	0.894070734
ENSGALG00010006135	ZC2HC1C	2	0.229809502	0.114547955	2.006229629	0.044831754	0	0.142524327	0	1	CRA004775;PRJNA827465	Yang2025_Silkie_Wenchang	0.36505868
ENSGALG00010006139	GAREM1	2	-0.212832353	0.091323136	-2.330541444	0.019777552	0	0.206028348	0	1	PRJNA827465;Yang2025_Silkie_Wenchang	CRA004775	0.236123067
ENSGALG00010006139	GAREM1	2	0.331497938	0.529807279	0.625695326	0.531514799	0.473963127	6.028261635	83.41146983	0.5	CRA004775;Yang2025_Silkie_Wenchang	PRJNA827465	0.873042777
ENSGALG00010006139	GAREM1	2	0.280151807	0.576827921	0.485676571	0.627196497	0.589373163	8.022658741	87.53530429	0.5	CRA004775;PRJNA827465	Yang2025_Silkie_Wenchang	0.901583369
ENSGALG00010006146	CCDC142	2	0.323451254	0.276682856	1.169032511	0.242390533	0.106415336	3.056746515	67.28547836	1	PRJNA827465;Yang2025_Silkie_Wenchang	CRA004775	0.754698112
ENSGALG00010006146	CCDC142	2	0.180290743	0.476554233	0.378321564	0.705191728	0.372361193	5.549073751	81.97897442	0.5	CRA004775;Yang2025_Silkie_Wenchang	PRJNA827465	0.924245236
ENSGALG00010006146	CCDC142	2	-0.025788381	0.179501976	-0.14366628	0.885764	0.022725626	1.423998096	29.77518699	0.5	CRA004775;PRJNA827465	Yang2025_Silkie_Wenchang	0.972380942
ENSGALG00010006147	ENSGALG00010006147	2	0.087488767	0.284996355	0.306982056	0.75885703	0.09171512	2.248730377	55.53046243	0.5	PRJNA827465;Yang2025_Silkie_Wenchang	CRA004775	0.938996879
ENSGALG00010006147	ENSGALG00010006147	2	-0.36048975	0.273334168	-1.318860909	0.187215617	0	0.769090635	0	1	CRA004775;Yang2025_Silkie_Wenchang	PRJNA827465	0.696068053
ENSGALG00010006147	ENSGALG00010006147	2	-0.113211995	0.566632949	-0.199797762	0.841638752	0.472404232	3.319887226	69.8784949	0.5	CRA004775;PRJNA827465	Yang2025_Silkie_Wenchang	0.959922457
ENSGALG00010006151	ENSGALG00010006151	2	0.303520826	0.297907239	1.018843407	0.308277315	0.147703736	5.638017191	82.26326799	1	PRJNA827465;Yang2025_Silkie_Wenchang	CRA004775	0.793457188
ENSGALG00010006151	ENSGALG00010006151	2	0.554787443	0.182077671	3.046982315	0.002311513	0	0.361243953	0	1	CRA004775;Yang2025_Silkie_Wenchang	PRJNA827465	0.063780538
ENSGALG00010006151	ENSGALG00010006151	2	0.110122308	0.148114687	0.743493501	0.457182955	0.014793374	1.275916414	21.62496002	1	CRA004775;PRJNA827465	Yang2025_Silkie_Wenchang	0.849541335
ENSGALG00010006155	TNFRSF19	2	0.008474857	0.078264967	0.108284172	0.913770278	0.003523003	1.38504576	27.80021938	0.5	PRJNA827465;Yang2025_Silkie_Wenchang	CRA004775	0.979364626
ENSGALG00010006155	TNFRSF19	2	-0.233007395	0.313826284	-0.74247253	0.45780109	0.108740361	1.48556573	32.68557696	1	CRA004775;Yang2025_Silkie_Wenchang	PRJNA827465	0.849726386
ENSGALG00010006155	TNFRSF19	2	-0.193573443	0.434818096	-0.445182584	0.656187812	0.253586108	2.14416702	53.36184213	0.5	CRA004775;PRJNA827465	Yang2025_Silkie_Wenchang	0.910446833
ENSGALG00010006156	NFKBIZ	2	0.174082882	0.172741218	1.007766901	0.313566379	0	0.505009234	0	1	PRJNA827465;Yang2025_Silkie_Wenchang	CRA004775	0.796531448
ENSGALG00010006156	NFKBIZ	2	0.076702111	0.324915632	0.236067778	0.813380076	0.090088543	1.696276528	41.04734791	0.5	CRA004775;Yang2025_Silkie_Wenchang	PRJNA827465	0.953587297
ENSGALG00010006156	NFKBIZ	2	0.000560362	0.189741838	0.002953289	0.99764362	0	0.750582684	0	0.5	CRA004775;PRJNA827465	Yang2025_Silkie_Wenchang	0.999290321
ENSGALG00010006157	SNX9	2	0.066273082	0.043250133	1.532320881	0.125443271	0	0.888822621	0	1	PRJNA827465;Yang2025_Silkie_Wenchang	CRA004775	0.594653364
ENSGALG00010006157	SNX9	2	0.143570606	0.079900322	1.796871437	0.072356032	0	0.013718946	0	1	CRA004775;Yang2025_Silkie_Wenchang	PRJNA827465	0.46459878
ENSGALG00010006157	SNX9	2	0.05603856	0.045264137	1.238034424	0.215703297	0	0.347449037	0	1	CRA004775;PRJNA827465	Yang2025_Silkie_Wenchang	0.729213578
ENSGALG00010006163	ENSGALG00010006163	2	0.501863217	0.347872902	1.442662578	0.149115546	0	0.003554618	0	1	PRJNA827465;Yang2025_Silkie_Wenchang	CRA004775	0.639246608
ENSGALG00010006163	ENSGALG00010006163	2	0.855668068	0.394071298	2.171353439	0.029904466	0	0.828462978	0	1	CRA004775;Yang2025_Silkie_Wenchang	PRJNA827465	0.295167651
ENSGALG00010006163	ENSGALG00010006163	2	0.784234414	0.339945663	2.30694049	0.021058138	0	0.943633716	0	1	CRA004775;PRJNA827465	Yang2025_Silkie_Wenchang	0.245414483
ENSGALG00010006172	ENSGALG00010006172	2	0.248006367	0.323135263	0.767500162	0.442784197	0.196277392	16.41568613	93.90826559	0.5	PRJNA827465;Yang2025_Silkie_Wenchang	CRA004775	0.845624071
ENSGALG00010006172	ENSGALG00010006172	2	0.098325281	0.49152725	0.200040345	0.841449028	0.455455433	17.31638475	94.22512254	0.5	CRA004775;Yang2025_Silkie_Wenchang	PRJNA827465	0.95989279
ENSGALG00010006172	ENSGALG00010006172	2	-0.18911123	0.162190831	-1.16597978	0.243622621	0.03484508	2.595969621	61.47874799	1	CRA004775;PRJNA827465	Yang2025_Silkie_Wenchang	0.755365463
ENSGALG00010006173	TBPL1	2	-0.234402125	0.187669334	-1.249016661	0.21165898	0.066488661	17.77787162	94.37502969	1	PRJNA827465;Yang2025_Silkie_Wenchang	CRA004775	0.724013667
ENSGALG00010006173	TBPL1	2	-0.238577607	0.24524052	-0.972831108	0.330637249	0.089841437	3.252070245	69.25035671	0.5	CRA004775;Yang2025_Silkie_Wenchang	PRJNA827465	0.801575303
ENSGALG00010006173	TBPL1	2	-0.043930782	0.054085752	-0.812243148	0.416652126	0	0.23039229	0	0.5	CRA004775;PRJNA827465	Yang2025_Silkie_Wenchang	0.837984974
ENSGALG00010006175	IL2RA	2	0.26222958	0.336286963	0.779779202	0.43552085	0	0.031471672	0	1	PRJNA827465;Yang2025_Silkie_Wenchang	CRA004775	0.842762754
ENSGALG00010006175	IL2RA	2	0.109573058	0.35643297	0.307415607	0.75852705	0	0.040747995	0	1	CRA004775;Yang2025_Silkie_Wenchang	PRJNA827465	0.938954369
ENSGALG00010006175	IL2RA	2	0.191180309	0.322131682	0.593484963	0.552856643	0	0.16924961	0	1	CRA004775;PRJNA827465	Yang2025_Silkie_Wenchang	0.87928478
ENSGALG00010006176	MMR1L3	2	0.062860317	0.40583885	0.154889847	0.876908158	0.254012387	4.167243335	76.00332115	0.5	PRJNA827465;Yang2025_Silkie_Wenchang	CRA004775	0.97046577
ENSGALG00010006176	MMR1L3	2	-0.324803215	0.295663625	-1.098556558	0.271961535	0	0.149713339	0	1	CRA004775;Yang2025_Silkie_Wenchang	PRJNA827465	0.773184343
ENSGALG00010006176	MMR1L3	2	0.360157286	0.188856252	1.907044547	0.056514819	0	0.873084251	0	0.5	CRA004775;PRJNA827465	Yang2025_Silkie_Wenchang	0.411523553

ENSGALG00010006177	NEK9	2	-0.1889646	0.155456402	-1.215547235	0.224157445	0.037239258	4.007250603	75.04523427	1	PRJNA827465;Yang2025_Silkie_Wenchang	CRA004775	0.737452676
ENSGALG00010006177	NEK9	2	-0.358755169	0.130007055	-2.75950539	0.005788893	0	0.049419611	0	1	CRA004775;Yang2025_Silkie_Wenchang	PRJNA827465	0.114529757
ENSGALG00010006177	NEK9	2	-0.066939588	0.064802509	-1.032978333	0.301614038	0	0.548829538	0	1	CRA004775;PRJNA827465	Yang2025_Silkie_Wenchang	0.791273005
ENSGALG00010006178	RPS23	2	0.03057453	0.166212848	0.183948054	0.854054206	0	0.021172096	0	0.5	PRJNA827465;Yang2025_Silkie_Wenchang	CRA004775	0.962975689
ENSGALG00010006178	RPS23	2	-0.228915479	0.21687196	-1.055532855	0.291181691	0	0.945335221	0	1	CRA004775;Yang2025_Silkie_Wenchang	PRJNA827465	0.78467882
ENSGALG00010006178	RPS23	2	-0.13817162	0.231760142	-0.596183705	0.55105251	0.049124692	1.765549171	43.36039935	0.5	CRA004775;PRJNA827465	Yang2025_Silkie_Wenchang	0.878879109
ENSGALG00010006180	CD200L	2	0.82032842	0.503974749	1.627717305	0.103584842	0	0.103073162	0	1	PRJNA827465;Yang2025_Silkie_Wenchang	CRA004775	0.547964315
ENSGALG00010006180	CD200L	2	0.094150915	0.947596493	0.099357602	0.920854345	1.193108142	2.94395039	66.0320363	0.5	CRA004775;Yang2025_Silkie_Wenchang	PRJNA827465	0.981469977
ENSGALG00010006180	CD200L	2	-0.076898464	0.785530937	-0.097893617	0.922016769	0.590971666	1.906848232	47.55744148	0.5	CRA004775;PRJNA827465	Yang2025_Silkie_Wenchang	0.981720024
ENSGALG00010006183	PRKN	2	-0.083905707	0.10052116	-0.834706908	0.403882751	0	0.352913136	0	1	PRJNA827465;Yang2025_Silkie_Wenchang	CRA004775	0.833112102
ENSGALG00010006183	PRKN	2	0.009999465	0.139710231	0.071572887	0.942941818	0	0.157083594	0	0.5	CRA004775;Yang2025_Silkie_Wenchang	PRJNA827465	0.985827325
ENSGALG00010006183	PRKN	2	-0.099859234	0.123861923	-0.806214141	0.420119386	0	0.542526198	0	0.5	CRA004775;PRJNA827465	Yang2025_Silkie_Wenchang	0.838638439
ENSGALG00010006184	LBX2	2	0.12065286	0.528218886	0.228414513	0.819324008	0.485903694	7.736780993	87.07472784	0.5	PRJNA827465;Yang2025_Silkie_Wenchang	CRA004775	0.955256039
ENSGALG00010006184	LBX2	2	0.270602501	0.486695224	0.555999911	0.578210916	0.307717886	2.565922262	61.02765797	0.5	CRA004775;Yang2025_Silkie_Wenchang	PRJNA827465	0.88778445
ENSGALG00010006184	LBX2	2	-0.397632639	0.242086157	-1.642525304	0.100481187	0	0.006952798	0	1	CRA004775;PRJNA827465	Yang2025_Silkie_Wenchang	0.54128455
ENSGALG00010006186	CHIR1AB3	2	-0.238931171	0.251165128	-0.951291179	0.341456583	0	0.194970169	0	1	PRJNA827465;Yang2025_Silkie_Wenchang	CRA004775	0.805758959
ENSGALG00010006186	CHIR1AB3	2	-0.509196066	0.351958206	-1.446751512	0.147966531	0	0.361882031	0	1	CRA004775;Yang2025_Silkie_Wenchang	PRJNA827465	0.637457918
ENSGALG00010006186	CHIR1AB3	2	-0.278763833	0.288085155	-0.967643667	0.333222261	0	0.883508923	0	1	CRA004775;PRJNA827465	Yang2025_Silkie_Wenchang	0.802342371
ENSGALG00010006187	TCF21	2	-0.398740061	0.384789292	-1.036255605	0.300082911	0.01474954	1.052255391	4.96603686	1	PRJNA827465;Yang2025_Silkie_Wenchang	CRA004775	0.790228387
ENSGALG00010006187	TCF21	2	0.596565665	1.439345843	0.414469995	0.678529937	3.737962263	10.15302518	90.1507188	0.5	CRA004775;Yang2025_Silkie_Wenchang	PRJNA827465	0.917701868
ENSGALG00010006187	TCF21	2	0.960299286	1.052792425	0.912144943	0.361692227	1.831702949	5.670400035	82.36455993	0.5	CRA004775;PRJNA827465	Yang2025_Silkie_Wenchang	0.815545459
ENSGALG00010006188	TMED10	2	-0.121224357	0.141598644	-0.856112414	0.391935602	0.035690455	8.630251759	88.41285251	0.5	PRJNA827465;Yang2025_Silkie_Wenchang	CRA004775	0.829251057
ENSGALG00010006188	TMED10	2	-0.278342545	0.073871774	-3.767914734	0.000164617	0	0.00483242	0	1	CRA004775;Yang2025_Silkie_Wenchang	PRJNA827465	0.011193665
ENSGALG00010006188	TMED10	2	-0.111314305	0.145107177	-0.767117845	0.443011453	0.034207994	4.65360725	78.51129358	0.5	CRA004775;PRJNA827465	Yang2025_Silkie_Wenchang	0.845720426
ENSGALG00010006193	RBM17	2	0.155466072	0.232828179	0.667738765	0.504306745	0.098441179	10.13575028	90.13393214	0.5	PRJNA827465;Yang2025_Silkie_Wenchang	CRA004775	0.863512504
ENSGALG00010006193	RBM17	2	0.200913691	0.209726313	0.957980371	0.338072657	0.0672257	4.239929071	76.41469979	0.5	CRA004775;Yang2025_Silkie_Wenchang	PRJNA827465	0.80415385
ENSGALG00010006193	RBM17	2	-0.055220572	0.036682696	-1.50535751	0.132232187	0	0.100566168	0	1	CRA004775;PRJNA827465	Yang2025_Silkie_Wenchang	0.608990115
ENSGALG00010006197	ENSGALG00010006197	2	-0.422031485	0.285691345	-1.477228805	0.139614315	0	0.749518103	0	1	PRJNA827465;Yang2025_Silkie_Wenchang	CRA004775	0.623410749
ENSGALG00010006197	ENSGALG00010006197	2	-0.390604301	0.397334013	-0.983062834	0.325576515	0.042540531	1.10142213	9.208288752	1	CRA004775;Yang2025_Silkie_Wenchang	PRJNA827465	0.799987949
ENSGALG00010006197	ENSGALG00010006197	2	-0.767287496	0.366241407	-2.095032077	0.036168141	0	0.257002616	0	1	CRA004775;PRJNA827465	Yang2025_Silkie_Wenchang	0.32816796
ENSGALG00010006198	ENSGALG00010006198	2	-0.143423808	0.792704037	-0.180929831	0.856422653	1.077455538	6.901480967	85.51035633	0.5	PRJNA827465;Yang2025_Silkie_Wenchang	CRA004775	0.96353738
ENSGALG00010006198	ENSGALG00010006198	2	-0.578311007	0.415015278	-1.393469198	0.163477953	0.084901385	1.326432361	24.60980072	1	CRA004775;Yang2025_Silkie_Wenchang	PRJNA827465	0.661791656
ENSGALG00010006198	ENSGALG00010006198	2	0.333889001	0.364284713	0.916560561	0.359372959	0.090487209	1.461904895	31.59609745	0.5	CRA004775;PRJNA827465	Yang2025_Silkie_Wenchang	0.814335519
ENSGALG00010006200	GSTCD	2	-0.079588268	0.193876962	-0.410509156	0.681432489	0.059255823	4.580422614	78.16795339	0.5	PRJNA827465;Yang2025_Silkie_Wenchang	CRA004775	0.918242009
ENSGALG00010006200	GSTCD	2	-0.30578465	0.130787061	-2.338034421	0.019385463	0	0.029865721	0	1	CRA004775;Yang2025_Silkie_Wenchang	PRJNA827465	0.23321299
ENSGALG00010006200	GSTCD	2	-0.061960833	0.211556598	-0.292880645	0.769613383	0.06049614	2.661575791	62.42827262	0.5	CRA004775;PRJNA827465	Yang2025_Silkie_Wenchang	0.941843401
ENSGALG00010006202	SACS	2	0.011833037	0.083380119	0.141916771	0.887145747	0	0.068042712	0	0.5	PRJNA827465;Yang2025_Silkie_Wenchang	CRA004775	0.972973806
ENSGALG00010006202	SACS	2	0.070290253	0.131143831	0.535978338	0.591973521	0	0.354869658	0	1	CRA004775;Yang2025_Silkie_Wenchang	PRJNA827465	0.892776493
ENSGALG00010006202	SACS	2	0.015888976	0.100054479	0.158803245	0.873823892	0	0.532169944	0	0.5	CRA004775;PRJNA827465	Yang2025_Silkie_Wenchang	0.969974876
ENSGALG00010006203	MMR1L4	2	-0.355335246	0.34513445	-1.029556004	0.303218477	0.21909205	12.36657464	91.91368646	1	PRJNA827465;Yang2025_Silkie_Wenchang	CRA004775	0.792084776
ENSGALG00010006203	MMR1L4	2	-0.716642607	0.15254688	-4.697851616	2.62912E-06	0	0.039536127	0	1	CRA004775;Yang2025_Silkie_Wenchang	PRJNA827465	0.000510017
ENSGALG00010006203	MMR1L4	2	-0.212031861	0.346677067	-0.61161202	0.540794484	0.144074699	1.769813188	43.49686131	1	CRA004775;PRJNA827465	Yang2025_Silkie_Wenchang	0.875657957
ENSGALG00010006205	CGGBP1	2	-0.203976813	0.164881603	-1.237110804	0.216045949	0.051070447	16.26275713	93.85098116	1	PRJNA827465;Yang2025_Silkie_Wenchang	CRA004775	0.729551847
ENSGALG00010006205	CGGBP1	2	0.001944064	0.393525666	0.00494012	0.996058371	0.291029156	16.02485975	93.75969578	0.5	CRA004775;Yang2025_Silkie_Wenchang	PRJNA827465	0.99872652
ENSGALG00010006205	CGGBP1	2	0.150266546	0.226316181	0.66396731	0.506711234	0.087074828	5.888768762	83.01852152	0.5	CRA004775;PRJNA827465	Yang2025_Silkie_Wenchang	0.863929781
ENSGALG00010006207	ENSGALG00010006207	2	-0.034670942	0.147946194	-0.234348323	0.814714574	0	0.063953642	0	0.5	PRJNA827465;Yang2025_Silkie_Wenchang	CRA004775	0.953884449
ENSGALG00010006207	ENSGALG00010006207	2	0.284480654	0.300933323	0.945327858	0.34449152	0	0.626422535	0	1	CRA004775;Yang2025_Silkie_Wenchang	PRJNA827465	0.806768255
ENSGALG00010006207	ENSGALG00010006207	2	0.101459768	0.256931036	0.394891055	0.692923292	0.063539948	1.577944277	36.62640598	0.5	CRA004775;PRJNA827465	Yang2025_Silkie_Wenchang	0.92062328
ENSGALG00010006212	FOS	2	-0.054562764	0.152677381	-0.357372938	0.720812634	0	0.005943345	0	1	PRJNA827465;Yang2025_Silkie_Wenchang	CRA004775	0.928918486
ENSGALG00010006212	FOS	2	-0.027364305	0.283297446	-0.096592134	0.923050303	0	0.001971765	0	1	CRA004775;Yang2025_Silkie_Wenchang	PRJNA827465	0.981836718
ENSGALG00010006212	FOS	2	-0.057128129	0.168798996	-0.338438798	0.735032541	0	0.007822013	0	1	CRA004775;PRJNA827465	Yang2025_Silkie_Wenchang	0.93359216
ENSGALG00010006219	CLEC14A	2	-0.033647811	0.124898258	-0.269401766	0.787620528	0	0.798510211	0	0.5	PRJNA827465;Yang2025_Silkie_Wenchang	CRA004775	0.946919116
ENSGALG00010006219	CLEC14A	2	-0.184181598	0.241623732	-0.762266176	0.445901157	0	0.269132176	0	0.5	CRA004775;Yang2025_Silkie_Wenchang	PRJNA827465	0.846013379
ENSGALG00010006219	CLEC14A	2	0.019847129	0.132661442	0.149607369	0.881074394	0	0.000238267	0	1	CRA004775;PRJNA827465	Yang2025_Silkie_Wenchang	0.971685305
ENSGALG00010006220	SGCG	2	-0.19133976	0.072646559	-2.633844784	0.008442407	0	0.006721045	0	1	PRJNA827465;Yang2025_Silkie_Wenchang	CRA004775	0.143393228
ENSGALG00010006220	SGCG	2	-0.133124468	0.172867058	-0.770097377	0.441242132	0.01050756	1.091975049	8.422816021	0.5	CRA004775;Yang2025_Silkie_Wenchang	PRJNA827465	0.844907543
ENSGALG00010006220	SGCG	2	-0.149265318	0.13178915	-1.132607033	0.257379314	0.012134836	1.11500505	10.31430752	0.5	CRA004775;PRJNA827465	Yang2025_Silkie_Wenchang	0.764987374
ENSGALG00010006223	ATG10	2	0.041831517	0.131673163	0.317692051	0.750718542	0	0.000600499	0	1	PRJNA827465;Yang2025_Silkie_Wenchang	CRA004775	0.937485808
ENSGALG00010006223	ATG10	2	0.072718437	0.16065487	0.452637614	0.650809709	0	0.085847451	0	1	CRA004775;Yang2025_Silkie_Wenchang	PRJNA827465	0.908769703
ENSGALG00010006223	ATG10	2	0.067866043	0.160503836	0.422831284	0.672418361	0	0.096498734	0	1	CRA004775;PRJNA827465	Yang2025_Silkie_Wenchang	0.914742762
ENSGALG00010006227	AGPAT4	2	0.118615903	0.066469668	1.784511736	0.07434055	0	0.000557291	0	1	PRJNA827465;Yang2025_Silkie_Wenchang	CRA004775	0.470803516
ENSGALG00010006227	AGPAT4	2	-0.028369842	0.272267878	-0.104198269	0.917012008	0.080810499	1.634083554	38.80361885	0.5	CRA004775;Yang2025_Silkie_Wenchang	PRJNA827465	0.980728718
ENSGALG00010006227	AGPAT4	2	-0.02176254	0.264660399	-0.082228168	0.934465274	0.083322428	1.67913973	40.44569478	0.5	CRA004775;PRJNA827465	Yang2025_Silkie_Wenchang	0.984355517
ENSGALG00010006229	INTS12	2	-0.113189477	0.182539818	-0.620081021	0.535204447	0.05282768	4.555906408	78.05047096	0.5	PRJNA827465;Yang2025_Silkie_Wenchang	CRA004775	0.874060516
ENSGALG00010006229	INTS12	2	-0.170832018	0.185864219	-0.919122674	0.358031411	0.033535025	1.860834758	46.26067706	0.5	CRA004775;Yang2025_Silkie_Wenchang	PRJNA827465	0.813691224
ENSGALG00010006229	INTS12	2	0.049299135	0.075989755	0.648760278	0.516493336	0	0.002698644	0	1	CRA004775;PRJNA827465	Yang2025_Silkie_Wenchang	0.867769743
ENSGALG00010006232	ZNF654	2	-0.067743728	0.111367176	-0.608291688	0.542994033	0.016644734	2.802585246	64.31865894	0.5	PRJNA827465;Yang2025_Silkie_Wenchang	CRA004775	0.876968972
ENSGALG00010006232	ZNF654	2	0.067112383	0.302311992	0.221997093	0.824316146	0.150748745	5.418556152	81.5448992	0.5	CRA004775;Yang2025_Silkie_Wenchang	PRJNA827465	0.95619211
ENSGALG00010006232	ZNF654	2	0.147731739	0.178689216	0.826752403	0.408377413	0.043563586	2.51015612	60.16184045	1	CRA004775;PRJNA827465	Yang2025_Silkie_Wenchang	0.834136652
ENSGALG00010006236	C3orf38	2	0.162840925	0.066579603	2.445807985	0.014452795	0.002455954	1.346124741	25.71267954	1	PRJNA827465;Yang2025_Silkie_Wenchang	CRA004775	0.197724547
ENSGALG00010006236	C3orf38	2	0.132400045	0.150832373	0.877795941	0.380054468	0.027034928	2.272205461	55.9898954	0.5	CRA004775;Yang2025_Silkie_Wenchang	PRJNA827465	0.824392515
ENSGALG00010006236	C3orf38	2	0.091314718	0.063132244	1.446403808	0.148063974	0	0.79641645	0	0.5	CRA004775;PRJNA827465	Yang2025_Silkie_Wenchang	0.63772401
ENSGALG00010006237	ENSGALG00010006237	2	0.42882223	0.223364918	1.919828029	0.054879625	0	0.194252905	0	1	PRJNA827465;Yang2025_Silkie_Wenchang	CRA004775	0.405555409
ENSGALG00010006237	ENSGALG00010006237	2	0.187729267	0.454289743	0.413236861	0.679433084	0.216661267	1.971694521	49.28220426	0.5	CRA004775;Yang2025_Silkie_Wenchang	PRJNA827465	0.917701868
ENSGALG00010006237	ENSGALG00010006237	2	0.13700664	0.331000022	0.413917314	0.678934664	0.063093979	1.300285869	23.09383469	0.5	CRA004775;PRJNA827465	Yang2025_Silkie_Wenchang	0.917701868
ENSGALG00010006239	EYA4	2	0.253472921	0.224081941	1.131161752	0.257987017	0.078723756	4.280622255	76.63891041	1	PRJNA827465;Yang2025_Silkie_Wenchang	CRA004775	0.765157824
ENSGALG00010006239	EYA4	2	0.548873522	0.174852823	3.139060101	0.001694907	0	0.138766274	0	1	CRA004775;Yang2025_Silkie_Wenchang	PRJNA827465	0.052083598
ENSGALG00010006239	EYA4	2	0.26026012	0.286090985	0.909711013	0.362974934	0.113021672	2.551233183	60.80326932	1	CRA004775;PRJNA827465	Yang2025_Silkie_Wenchang	0.816366458
ENSGALG00010006241	MRC1	2	-0.140281076	0.336226688	-0.417221718	0.676516241	0.166282186	3.674726218	72.78708832	0.5	PRJNA827465;Yang2025_Silkie_Wenchang	CRA004775	0.916786558
ENSGALG00010006241	MRC1	2	-0.453281496	0.184276782	-2.459786263	0.013901978	0	0.017332923	0	1	CRA004775;Yang2025_Silkie_Wenchang	PRJNA827465	0.19354185
ENSGALG00010006241	MRC1	2	-0.023148392	0.35473786	-0.065254923	0.947971032	0.105581765	1.60527957	37.70555493	0.5	CRA004775;PRJNA827465	Yang2025_Silkie_Wenchang	0.986697717
ENSGALG00010006242	ENSGALG00010006242	2	-0.389623702	0.375234702	-1.038346664	0.299108691	0.023727407	1.075676902	7.035281871	0.5	PRJNA827465;Yang2025_Silkie_Wenchang	CRA004775	0.789748017
ENSGALG00010006242	ENSGALG00010006242	2	0.647516205	0.477593443	1.35578956	0.175166134	0	0.949840463	0	1	CRA004775;Yang2025_Silkie_Wenchang	PRJNA827465	0.680194504
ENSGALG00010006242	ENSGALG00010006242	2	0.161247233	0.872351303	0.184842084	0.853352898	1.216012021	4.808961286	79.20548866	0.5	CRA004775;PRJNA827465	Yang2025_Silkie_Wenchang	0.962916051
ENSGALG00010006244	EPHA3	2	-0.395067972	0.194639487	-2.029742157	0.042382755	0.05825028	4.216888309	76.28583148	1	PRJNA827465;Yang2025_Silkie_Wenchang	CRA004775	0.356238117
ENSGALG00010006244	EPHA3	2	-0.587087015	0.147901844	-3.969436746	7.20427E-05	0	0.143507183	0	1	CRA004775;Yang2025_Silkie_Wenchang	PRJNA827465	0.006128778
ENSGALG00010006244	EPHA3	2	-0.229469831	0.10363023	-2.214313632	0.026807212	0	0.24092541	0	1	CRA004775;PRJNA827465	Yang2025_Silkie_Wenchang	0.279174307
ENSGALG00010006245	PFKFB3	2	-0.364219903	0.159214326	-2.287607608	0.022160382	0	0.184048379	0	1	PRJNA827465;Yang2025_Silkie_Wenchang	CRA004775	0.252601367
ENSGALG00010006245	PFKFB3	2	-0.173117493	0.255205502	-0.678345459	0.497552682	0	0.864715486	0	0.5	CRA004775;Yang2025_Silkie_Wenchang	PRJNA827465	0.86098656
ENSGALG00010006245	PFKFB3	2	-0.256841742	0.324823252	-0.790712304	0.429111895	0.0916714	1.359525975	26.44495077	0.5	CRA004775;PRJNA827465	Yang2025_Silkie_Wenchang	0.841964195
ENSGALG00010006246	SUPT20H	2	-0.054054345	0.036257783	-1.490834256	0.136005016	0	0.183499582	0	1	PRJNA827465;Yang2025_Silkie_Wenchang	CRA004775	0.617575642
ENSGALG00010006246	SUPT20H	2	0.007362571	0.092566728	0.07953799	0.936604716	0	0.072815045	0	0.5	CRA004775;Yang2025_Silkie_Wenchang	PRJNA827465	0.985038972
ENSGALG00010006246	SUPT20H	2	-0.05423701	0.037400841	-1.450154823	0.14701535	0	0.368706139	0	0.5	CRA004775;PRJNA827465	Yang2025_Silkie_Wenchang	0.635668678
ENSGALG00010006249	C9orf72	2	-0.208310709	0.12820582	-1.624814767	0.104202034	0	0.620459883	0	1	PRJNA827465;Yang2025_Silkie_Wenchang	CRA004775	0.548669102
ENSGALG00010006249	C9orf72	2	-0.299492499	0.148506194	-2.016700387	0.043726789	0	0.037310945	0	1	CRA004775;Yang2025_Silkie_Wenchang	PRJNA827465	0.361428813
ENSGALG00010006249	C9orf72	2	-0.171770092	0.136739742	-1.256182651	0.209049755	0	0.259880881	0	1	CRA004775;PRJNA827465	Yang2025_Silkie_Wenchang	0.721011841
ENSGALG00010006251	SEMA3E	2	-0.033830549	0.225575714	-0.149974254	0.880784928	0	0.029378468	0	1	PRJNA827465;Yang2025_Silkie_Wenchang	CRA004775	0.971676583
ENSGALG00010006251	SEMA3E	2	0.926446383	1.148309011	0.806791877	0.4197864	2.197266354	5.56993658	82.04647422	0.5	CRA004775;Yang2025_Silkie_Wenchang	PRJNA827465	0.838527387
ENSGALG00010006251	SEMA3E	2	0.945691253	1.104144192	0.856492531	0.391725403	2.04811776	5.610029006	82.17478022	0.5	CRA004775;PRJNA827465	Yang2025_Silkie_Wenchang	0.829251057
ENSGALG00010006252	COX7C	2	-0.465082325	0.462963297	-1.004577099	0.315100529	0.359686105	5.985018492	83.29161386	1	PRJNA827465;Yang2025_Silkie_Wenchang	CRA004775	0.796635323
ENSGALG00010006252	COX7C	2	-0.526090225	0.466582212	-1.127540252	0.259514132	0.30521742	3.324492151	69.92021775	1	CRA004775;Yang2025_Silkie_Wenchang	PRJNA827465	0.766893528
ENSGALG00010006252	COX7C	2	-0.039282826	0.169127711	-0.232267237	0.816330458	0	0.00013389	0	1	CRA004775;PRJNA827465	Yang2025_Silkie_Wenchang	0.954546835
ENSGALG00010006254	PROS1	2	-0.21265866	0.222106057	-0.957464475	0.338332869	0.088173315	9.278183153	89.22202781	0.5	PRJNA827465;Yang2025_Silkie_Wenchang	CRA004775	0.804154375
ENSGALG00010006254	PROS1	2	-0.397342691	0.116752639	-3.403286588	0.000665804	0.001146775	1.020360287	1.995401764	1	CRA004775;Yang2025_Silkie_Wenchang	PRJNA827465	0.028470581
ENSGALG00010006254	PROS1	2	-0.006084019	0.078886736	-0.077123472	0.938525321	0	0.106465787	0	0.5	CRA004775;PRJNA827465	Yang2025_Silkie_Wenchang	0.985349045
ENSGALG00010006259	PPA2	2	0.052423767	0.07356256	0.712641963	0.476067338	0	0.107196574	0	1	PRJNA827465;Yang2025_Silkie_Wenchang	CRA004775	0.854810196
ENSGALG00010006259	PPA2	2	0.10200536	0.137002628	0.744550391	0.456543568	0	0.000472018	0	1	CRA004775;Yang2025_Silkie_Wenchang	PRJNA827465	0.849515887
ENSGALG00010006259	PPA2	2	0.046641992	0.078353971	0.595272851	0.5516611	0	0.062418866	0	1	CRA004775;PRJNA827465	Yang2025_Silkie_Wenchang	0.879021172
ENSGALG00010006260	MAP3K4	2	0.10637164	0.097438068	1.09168462	0.274971748	0.012920897	2.894868521	65.45611682	1	PRJNA827465;Yang2025_Silkie_Wenchang	CRA004775	0.774932927
ENSGALG00010006260	MAP3K4	2	0.998189096	0.818730086	1.219191908	0.222771362	1.257311791	15.32958428	93.47666589	1	CRA004775;Yang2025_Silkie_Wenchang	PRJNA827465	0.736382074
ENSGALG00010006260	MAP3K4	2	0.901824842	0.918371017	0.981983126	0.326108161	1.606792999	20.15334139	95.03804366	1	CRA004775;PRJNA827465	Yang2025_Silkie_Wenchang	0.800023788
ENSGALG00010006263	EXOSC8	2	-0.416951154	0.34366485	-1.213249343	0.225034504	0.214044389	10.02335914	90.0233047	1	PRJNA827465;Yang2025_Silkie_Wenchang	CRA004775	0.738087485
ENSGALG00010006263	EXOSC8	2	-0.421214395	0.393912808	-1.069308708	0.284930588	0.247946747	4.887513146	79.53969698	1	CRA004775;Yang2025_Silkie_Wenchang	PRJNA827465	0.780497609
ENSGALG00010006263	EXOSC8	2	-0.094990545	0.06533988	-1.453791237	0.146004194	0	0.112227887	0	1	CRA004775;PRJNA827465	Yang2025_Silkie_Wenchang	0.634897819
ENSGALG00010006266	CENPW	2	-0.261623925	0.249854291	-1.047105992	0.295050696	0.108170894	7.24015481	86.18814064	1	PRJNA827465;Yang2025_Silkie_Wenchang	CRA004775	0.787628307
ENSGALG00010006266	CENPW	2	-0.394496067	0.31082147	-1.26920469	0.204368069	0.088643209	1.327479569	24.66927376	0.5	CRA004775;Yang2025_Silkie_Wenchang	PRJNA827465	0.717281769
ENSGALG00010006266	CENPW	2	-0.023667792	0.090347632	-0.261963607	0.7933495	0	0.229735864	0	0.5	CRA004775;PRJNA827465	Yang2025_Silkie_Wenchang	0.948684977
ENSGALG00010006270	STAM	2	-0.134158556	0.055793877	-2.404539033	0.016192877	0	0.002213445	0	1	PRJNA827465;Yang2025_Silkie_Wenchang	CRA004775	0.210262467
ENSGALG00010006270	STAM	2	0.001138181	0.189981683	0.005991006	0.995219898	0.032257657	1.629380821	38.62699334	0.5	CRA004775;Yang2025_Silkie_Wenchang	PRJNA827465	0.998242335
ENSGALG00010006270	STAM	2	-0.02142459	0.185468078	-0.115516319	0.908035886	0.043688727	2.019860349	50.49162678	0.5	CRA004775;PRJNA827465	Yang2025_Silkie_Wenchang	0.978196186
ENSGALG00010006273	ARL13B	2	0.104698603	0.249448243	0.419720748	0.674689466	0.102693707	5.109068296	80.42695963	0.5	PRJNA827465;Yang2025_Silkie_Wenchang	CRA004775	0.915970608
ENSGALG00010006273	ARL13B	2	0.436133926	0.145075801	3.006248625	0.002644926	0	0.06117088	0	1	CRA004775;Yang2025_Silkie_Wenchang	PRJNA827465	0.069534142
ENSGALG00010006273	ARL13B	2	0.15090421	0.287081098	0.525650107	0.599131336	0.144651811	7.581401247	86.80982621	0.5	CRA004775;PRJNA827465	Yang2025_Silkie_Wenchang	0.894908299
ENSGALG00010006277	GNG10	2	0.149427974	0.106500083	1.403078477	0.160593438	0	0.905688229	0	1	PRJNA827465;Yang2025_Silkie_Wenchang	CRA004775	0.65630165
ENSGALG00010006277	GNG10	2	0.001442537	0.160738398	0.008974441	0.992839528	0	0.003511688	0	0.5	CRA004775;Yang2025_Silkie_Wenchang	PRJNA827465	0.997880823
ENSGALG00010006277	GNG10	2	0.189431688	0.121501625	1.559087687	0.118975628	0	0.444470463	0	0.5	CRA004775;PRJNA827465	Yang2025_Silkie_Wenchang	0.579372036
ENSGALG00010006279	HACD1	2	-0.158063132	0.064928131	-2.434432186	0.014915174	0	0.354594492	0	1	PRJNA827465;Yang2025_Silkie_Wenchang	CRA004775	0.201655005
ENSGALG00010006279	HACD1	2	-0.026049828	0.298774822	-0.087188832	0.930521416	0.11001133	1.84584968	45.82440754	0.5	CRA004775;Yang2025_Silkie_Wenchang	PRJNA827465	0.983892906
ENSGALG00010006279	HACD1	2	-0.009551384	0.236011641	-0.040469972	0.967718447	0.059498423	1.457753625	31.40130245	0.5	CRA004775;PRJNA827465	Yang2025_Silkie_Wenchang	0.991065147
ENSGALG00010006281	TRMT11	2	0.012848096	0.066753305	0.192471302	0.847373045	0	0.810232456	0	0.5	PRJNA827465;Yang2025_Silkie_Wenchang	CRA004775	0.961252712
ENSGALG00010006281	TRMT11	2	0.112802595	0.108962301	1.035244239	0.300554863	0	0.006950709	0	1	CRA004775;Yang2025_Silkie_Wenchang	PRJNA827465	0.790579721
ENSGALG00010006281	TRMT11	2	-0.002644928	0.070594619	-0.037466423	0.970113112	0	0.379916572	0	0.5	CRA004775;PRJNA827465	Yang2025_Silkie_Wenchang	0.991660528
ENSGALG00010006283	DNAJC25	2	-0.026253467	0.135631552	-0.193564601	0.846516819	0.028323893	4.197182634	76.17449401	0.5	PRJNA827465;Yang2025_Silkie_Wenchang	CRA004775	0.96122283
ENSGALG00010006283	DNAJC25	2	0.061892094	0.267683623	0.231213598	0.817148866	0.115854674	4.943933024	79.77318877	0.5	CRA004775;Yang2025_Silkie_Wenchang	PRJNA827465	0.954833525
ENSGALG00010006283	DNAJC25	2	0.152175839	0.108550385	1.401891288	0.160947712	0.009880446	1.386526881	27.8773449	1	CRA004775;PRJNA827465	Yang2025_Silkie_Wenchang	0.657013704
ENSGALG00010006285	FZD8	2	0.063249883	0.072428375	0.873274917	0.382513249	0	0.73398417	0	0.5	PRJNA827465;Yang2025_Silkie_Wenchang	CRA004775	0.825121999
ENSGALG00010006285	FZD8	2	0.113351478	0.09492466	1.194120459	0.232430851	0	0.076054964	0	1	CRA004775;Yang2025_Silkie_Wenchang	PRJNA827465	0.745124776
ENSGALG00010006285	FZD8	2	-0.000431564	0.101013838	-0.004272327	0.996591187	0	0.007163285	0	0.5	CRA004775;PRJNA827465	Yang2025_Silkie_Wenchang	0.99895145
ENSGALG00010006287	ECPAS	2	0.071455218	0.172914006	0.413241354	0.679429793	0.054332649	10.94022709	90.85942191	0.5	PRJNA827465;Yang2025_Silkie_Wenchang	CRA004775	0.917701868
ENSGALG00010006287	ECPAS	2	0.250766278	0.071985583	3.483562504	0.000494788	0	0.109044902	0	1	CRA004775;Yang2025_Silkie_Wenchang	PRJNA827465	0.023284834
ENSGALG00010006287	ECPAS	2	0.034607969	0.204337786	0.169366467	0.865508398	0.054816804	2.263436582	55.81939393	0.5	CRA004775;PRJNA827465	Yang2025_Silkie_Wenchang	0.967088846
ENSGALG00010006288	ARF5	2	0.178888868	0.095562645	1.871953912	0.06121298	0.000528157	1.010097813	0.999686684	0.5	PRJNA827465;Yang2025_Silkie_Wenchang	CRA004775	0.427707334
ENSGALG00010006288	ARF5	2	-0.171388912	0.247186485	-0.693358749	0.488084443	0	0.093326307	0	1	CRA004775;Yang2025_Silkie_Wenchang	PRJNA827465	0.85768885
ENSGALG00010006288	ARF5	2	0.135958747	0.173892019	0.781857313	0.434298434	0.026144367	1.289408451	22.44505618	0.5	CRA004775;PRJNA827465	Yang2025_Silkie_Wenchang	0.842762754
ENSGALG00010006289	SKIDA1	2	0.142535842	0.097006088	1.469349449	0.141738031	0	0.330007605	0	1	PRJNA827465;Yang2025_Silkie_Wenchang	CRA004775	0.627129189
ENSGALG00010006289	SKIDA1	2	0.152368241	0.143215299	1.063910361	0.287369309	0	0.870028817	0	0.5	CRA004775;Yang2025_Silkie_Wenchang	PRJNA827465	0.782840067
ENSGALG00010006289	SKIDA1	2	0.071975676	0.115770474	0.621710125	0.534132492	0	0.422978284	0	0.5	CRA004775;PRJNA827465	Yang2025_Silkie_Wenchang	0.873408008
ENSGALG00010006291	ENSGALG00010006291	2	0.858869486	0.240452101	3.571894287	0.000354408	0.085805698	3.835962841	73.93092578	1	PRJNA827465;Yang2025_Silkie_Wenchang	CRA004775	0.018772663
ENSGALG00010006291	ENSGALG00010006291	2	-0.069441233	1.213960401	-0.057202223	0.954384108	2.837848187	26.4712102	96.22231098	0.5	CRA004775;Yang2025_Silkie_Wenchang	PRJNA827465	0.987987175
ENSGALG00010006291	ENSGALG00010006291	2	-0.299167553	0.972554702	-0.307610001	0.758379109	1.790831103	18.15855299	94.49295326	0.5	CRA004775;PRJNA827465	Yang2025_Silkie_Wenchang	0.938954369
ENSGALG00010006293	SLC22A3	2	0.292225619	0.260543669	1.121599386	0.262032813	0	0.044151449	0	1	PRJNA827465;Yang2025_Silkie_Wenchang	CRA004775	0.768363052
ENSGALG00010006293	SLC22A3	2	0.216935829	0.343287763	0.631935805	0.527428826	0	0.551212127	0	0.5	CRA004775;Yang2025_Silkie_Wenchang	PRJNA827465	0.871149047
ENSGALG00010006293	SLC22A3	2	0.148232381	0.316106132	0.468932316	0.639118016	0	0.387251281	0	0.5	CRA004775;PRJNA827465	Yang2025_Silkie_Wenchang	0.905968982
ENSGALG00010006300	GCC1	2	0.240208766	0.134844477	1.781376378	0.074850985	0.027216417	3.960807273	74.7526216	1	PRJNA827465;Yang2025_Silkie_Wenchang	CRA004775	0.472257613
ENSGALG00010006300	GCC1	2	0.158191904	0.236512071	0.668853405	0.503588996	0.094586962	6.323700694	84.18647485	0.5	CRA004775;Yang2025_Silkie_Wenchang	PRJNA827465	0.863108263
ENSGALG00010006300	GCC1	2	0.049801229	0.093056385	0.535172616	0.5925305	0.004324154	1.261720206	20.74312554	0.5	CRA004775;PRJNA827465	Yang2025_Silkie_Wenchang	0.892776493
ENSGALG00010006301	ENSGALG00010006301	2	0.072981853	0.151019029	0.483262627	0.628909273	0.03612494	4.753404379	78.96244628	0.5	PRJNA827465;Yang2025_Silkie_Wenchang	CRA004775	0.902084638
ENSGALG00010006301	ENSGALG00010006301	2	-0.034726814	0.102220593	-0.339724245	0.734064201	0.001011264	1.032477217	3.145562589	0.5	CRA004775;Yang2025_Silkie_Wenchang	PRJNA827465	0.933415035
ENSGALG00010006301	ENSGALG00010006301	2	0.209890441	0.079009791	2.656511778	0.007895369	0	0.041772253	0	1	CRA004775;PRJNA827465	Yang2025_Silkie_Wenchang	0.137977634
ENSGALG00010006310	ALG5	2	0.350435336	0.250906125	1.396679081	0.162510098	0.115952703	12.55482421	92.03493427	1	PRJNA827465;Yang2025_Silkie_Wenchang	CRA004775	0.660237168
ENSGALG00010006310	ALG5	2	0.271809046	0.391427858	0.694403938	0.487428925	0.252859016	5.159477055	80.61819077	0.5	CRA004775;Yang2025_Silkie_Wenchang	PRJNA827465	0.85768885
ENSGALG00010006310	ALG5	2	0.088501166	0.080329974	1.101720339	0.270583272	0	0.73007103	0	0.5	CRA004775;PRJNA827465	Yang2025_Silkie_Wenchang	0.772536273
ENSGALG00010006311	ABCD4	2	0.362674977	0.093691558	3.870946178	0.000108414	0	0.026809775	0	1	PRJNA827465;Yang2025_Silkie_Wenchang	CRA004775	0.008228823
ENSGALG00010006311	ABCD4	2	0.054190566	0.384880245	0.140798512	0.888029119	0.233244454	4.291721713	76.69932799	0.5	CRA004775;Yang2025_Silkie_Wenchang	PRJNA827465	0.973135755
ENSGALG00010006311	ABCD4	2	0.046120289	0.367627393	0.125453897	0.900164222	0.213022527	4.173190815	76.03752035	0.5	CRA004775;PRJNA827465	Yang2025_Silkie_Wenchang	0.976466979
ENSGALG00010006323	ANAPC10	2	-0.087512342	0.062270688	-1.405353706	0.159916126	0	0.463427956	0	1	PRJNA827465;Yang2025_Silkie_Wenchang	CRA004775	0.655404348
ENSGALG00010006323	ANAPC10	2	0.030433779	0.113089408	0.269112554	0.78784307	0	0.607834727	0	0.5	CRA004775;Yang2025_Silkie_Wenchang	PRJNA827465	0.946919116
ENSGALG00010006323	ANAPC10	2	-0.045148058	0.130145491	-0.34690451	0.728663058	0.01622421	1.458297046	31.42686516	0.5	CRA004775;PRJNA827465	Yang2025_Silkie_Wenchang	0.932236477
ENSGALG00010006327	DENND6B	2	0.031442315	0.090943635	0.345734096	0.729542558	0.011855417	3.471041388	71.19020236	0.5	PRJNA827465;Yang2025_Silkie_Wenchang	CRA004775	0.9323457
ENSGALG00010006327	DENND6B	2	0.116456636	0.073238312	1.590105407	0.111811047	0	0.235600969	0	1	CRA004775;Yang2025_Silkie_Wenchang	PRJNA827465	0.565344663
ENSGALG00010006327	DENND6B	2	-0.04647296	0.056313784	-0.825250163	0.40922958	0	0.130775888	0	0.5	CRA004775;PRJNA827465	Yang2025_Silkie_Wenchang	0.834316328
ENSGALG00010006328	SYNDIG1L	2	0.217765967	0.195219092	1.115495235	0.264638259	0.026474945	1.501475907	33.39886472	1	PRJNA827465;Yang2025_Silkie_Wenchang	CRA004775	0.768855521
ENSGALG00010006328	SYNDIG1L	2	0.439805758	0.236691933	1.858135815	0.06314973	0	0.021003722	0	1	CRA004775;Yang2025_Silkie_Wenchang	PRJNA827465	0.432776606
ENSGALG00010006328	SYNDIG1L	2	0.090774649	0.182385357	0.497707986	0.618689879	0	0.274124405	0	1	CRA004775;PRJNA827465	Yang2025_Silkie_Wenchang	0.899168321
ENSGALG00010006330	STX19	2	-0.059231505	0.366842921	-0.161462854	0.871728866	0.119369918	1.754980297	43.01930332	0.5	PRJNA827465;Yang2025_Silkie_Wenchang	CRA004775	0.969462602
ENSGALG00010006330	STX19	2	0.962733913	0.677343017	1.421338802	0.155218288	0.594755464	2.735438384	63.44278834	1	CRA004775;Yang2025_Silkie_Wenchang	PRJNA827465	0.647874911
ENSGALG00010006330	STX19	2	0.602642375	1.05350737	0.572034323	0.567298722	1.940375106	7.585851952	86.81756504	0.5	CRA004775;PRJNA827465	Yang2025_Silkie_Wenchang	0.883696313
ENSGALG00010006331	SMAD9	2	0.272546187	0.230181537	1.184048859	0.236393722	0.076144109	3.280529184	69.51711312	1	PRJNA827465;Yang2025_Silkie_Wenchang	CRA004775	0.748669015
ENSGALG00010006331	SMAD9	2	0.420843309	0.250335327	1.681118339	0.092739931	0.02588716	1.156976958	13.56785517	0.5	CRA004775;Yang2025_Silkie_Wenchang	PRJNA827465	0.52160239
ENSGALG00010006331	SMAD9	2	0.073722266	0.113957303	0.646928844	0.517677997	0	0.077186737	0	0.5	CRA004775;PRJNA827465	Yang2025_Silkie_Wenchang	0.867962259
ENSGALG00010006341	HHIP	2	0.09264784	0.113476882	0.816446823	0.414244626	0	0.010773897	0	1	PRJNA827465;Yang2025_Silkie_Wenchang	CRA004775	0.836914812
ENSGALG00010006341	HHIP	2	0.488286997	0.444309481	1.098979468	0.271777022	0.313690387	4.479013612	77.67365571	1	CRA004775;Yang2025_Silkie_Wenchang	PRJNA827465	0.773136384
ENSGALG00010006341	HHIP	2	0.474304373	0.456014388	1.040108351	0.298289564	0.33887031	4.911461959	79.6394636	1	CRA004775;PRJNA827465	Yang2025_Silkie_Wenchang	0.789207321
ENSGALG00010006342	RFXAP	2	-0.441664274	0.420933253	-1.04925014	0.294063007	0.343250476	31.58057366	96.83349641	1	PRJNA827465;Yang2025_Silkie_Wenchang	CRA004775	0.787324026
ENSGALG00010006342	RFXAP	2	-0.325501281	0.565402032	-0.575698817	0.564818775	0.598733253	15.41314423	93.51203113	0.5	CRA004775;Yang2025_Silkie_Wenchang	PRJNA827465	0.883351845
ENSGALG00010006342	RFXAP	2	0.01220946	0.100065705	0.122014431	0.90288759	0.00650993	1.183738758	15.52190102	0.5	CRA004775;PRJNA827465	Yang2025_Silkie_Wenchang	0.97693511
ENSGALG00010006343	SEC23A	2	-0.087050015	0.154080277	-0.564965328	0.572097347	0.044958621	18.67017666	94.64386429	0.5	PRJNA827465;Yang2025_Silkie_Wenchang	CRA004775	0.885538639

ENSGALG00010006343	SEC23A	2	-0.08116592	0.202211357	-0.401391498	0.688131908	0.063994167	3.965074265	74.77979142	0.5	CRA004775;Yang2025_Silkie_Wenchang	PRJNA827465	0.919679555
ENSGALG00010006343	SEC23A	2	0.067720988	0.038431811	1.762107623	0.078051116	0	0.270394028	0	1	CRA004775;PRJNA827465	Yang2025_Silkie_Wenchang	0.480897161
ENSGALG00010006344	HINT3	2	-0.182902969	0.08314518	-2.199802428	0.027820916	0.001331114	1.10004379	9.094527915	1	PRJNA827465;Yang2025_Silkie_Wenchang	CRA004775	0.284423997
ENSGALG00010006344	HINT3	2	-0.121310934	0.211274924	-0.574185195	0.565842487	0.055865946	2.456049721	59.28421191	0.5	CRA004775;Yang2025_Silkie_Wenchang	PRJNA827465	0.883487517
ENSGALG00010006344	HINT3	2	-0.080346204	0.092029343	-0.873049845	0.38263591	0	0.990534463	0	0.5	CRA004775;PRJNA827465	Yang2025_Silkie_Wenchang	0.825121999
ENSGALG00010006345	SMC2	2	0.263967708	0.287450104	0.918307922	0.358457681	0.120251327	3.671958402	72.76657602	0.5	PRJNA827465;Yang2025_Silkie_Wenchang	CRA004775	0.814005058
ENSGALG00010006345	SMC2	2	0.53155354	0.202781761	2.621308437	0.008759297	0	0.105846996	0	1	CRA004775;Yang2025_Silkie_Wenchang	PRJNA827465	0.146797439
ENSGALG00010006345	SMC2	2	0.001608141	0.204594672	0.007860129	0.993728589	0	0.186591891	0	0.5	CRA004775;PRJNA827465	Yang2025_Silkie_Wenchang	0.998020001
ENSGALG00010006346	NSUN3	2	-0.120460843	0.127110394	-0.947686797	0.343288924	0	0.149865323	0	1	PRJNA827465;Yang2025_Silkie_Wenchang	CRA004775	0.806218116
ENSGALG00010006346	NSUN3	2	-0.247191184	0.191462874	-1.291065883	0.196680833	0	0.074418702	0	1	CRA004775;Yang2025_Silkie_Wenchang	PRJNA827465	0.707994211
ENSGALG00010006346	NSUN3	2	-0.13425244	0.132411114	-1.013906131	0.310627546	0	0.429635563	0	1	CRA004775;PRJNA827465	Yang2025_Silkie_Wenchang	0.794899969
ENSGALG00010006347	PPP6R2	2	0.020125213	0.189552208	0.1061724	0.915445569	0.06247554	7.408600178	86.50217347	0.5	PRJNA827465;Yang2025_Silkie_Wenchang	CRA004775	0.980165711
ENSGALG00010006347	PPP6R2	2	0.257827149	0.108475271	2.376828821	0.01746219	0	0.375530689	0	1	CRA004775;Yang2025_Silkie_Wenchang	PRJNA827465	0.220043745
ENSGALG00010006347	PPP6R2	2	0.067829739	0.267579915	0.253493387	0.799886971	0.117263568	4.850805823	79.38486848	0.5	CRA004775;PRJNA827465	Yang2025_Silkie_Wenchang	0.949922217
ENSGALG00010006351	IGF2R	2	-0.091007375	0.04787968	-1.900751509	0.057334568	0.000566663	1.081512272	7.536879079	0.5	PRJNA827465;Yang2025_Silkie_Wenchang	CRA004775	0.414689094
ENSGALG00010006351	IGF2R	2	-0.006489574	0.104644341	-0.06201553	0.950550465	0	0.32646268	0	0.5	CRA004775;Yang2025_Silkie_Wenchang	PRJNA827465	0.987277827
ENSGALG00010006351	IGF2R	2	-0.1155976	0.047201179	-2.449040493	0.014323734	0	0.090370864	0	1	CRA004775;PRJNA827465	Yang2025_Silkie_Wenchang	0.196497638
ENSGALG00010006352	GEMIN2	2	-0.103340262	0.068448747	-1.509746579	0.131108101	0	0.259497299	0	1	PRJNA827465;Yang2025_Silkie_Wenchang	CRA004775	0.606767161
ENSGALG00010006352	GEMIN2	2	-0.164350833	0.147355374	-1.115336536	0.264706234	0	0.042994881	0	1	CRA004775;Yang2025_Silkie_Wenchang	PRJNA827465	0.768996166
ENSGALG00010006352	GEMIN2	2	-0.088070724	0.07345744	-1.198935385	0.230553071	0	0.001888529	0	1	CRA004775;PRJNA827465	Yang2025_Silkie_Wenchang	0.743335644
ENSGALG00010006353	ABCE1	2	-0.135138123	0.165817177	-0.814982657	0.415082238	0.047730207	7.088031841	85.89171123	0.5	PRJNA827465;Yang2025_Silkie_Wenchang	CRA004775	0.837489728
ENSGALG00010006353	ABCE1	2	-0.203373373	0.146887061	-1.384556072	0.166188252	0.021395556	1.880380263	46.81926737	1	CRA004775;Yang2025_Silkie_Wenchang	PRJNA827465	0.66529196
ENSGALG00010006353	ABCE1	2	0.012722931	0.045157758	0.281744088	0.778139746	0	0.025893199	0	0.5	CRA004775;PRJNA827465	Yang2025_Silkie_Wenchang	0.944509772
ENSGALG00010006355	PTGR1	2	-0.033210458	0.078277776	-0.424264205	0.671373141	0.003525044	1.391024132	28.11052111	0.5	PRJNA827465;Yang2025_Silkie_Wenchang	CRA004775	0.914674175
ENSGALG00010006355	PTGR1	2	0.165864796	0.324191418	0.511626114	0.608912705	0.178409386	6.189267376	83.84299887	0.5	CRA004775;Yang2025_Silkie_Wenchang	PRJNA827465	0.897208691
ENSGALG00010006355	PTGR1	2	0.229684472	0.241603055	0.950668741	0.341772561	0.089527624	3.755580548	73.37295826	1	CRA004775;PRJNA827465	Yang2025_Silkie_Wenchang	0.805823616
ENSGALG00010006359	SGK1	2	0.129234066	0.071842498	1.798852631	0.072041995	0.001961566	1.20135543	16.76068757	1	PRJNA827465;Yang2025_Silkie_Wenchang	CRA004775	0.463464923
ENSGALG00010006359	SGK1	2	0.052045035	0.110230473	0.472147434	0.636821557	0	0.406262176	0	1	CRA004775;Yang2025_Silkie_Wenchang	PRJNA827465	0.904812056
ENSGALG00010006359	SGK1	2	0.182880807	0.073641634	2.483388755	0.013013898	0	0.039236539	0	1	CRA004775;PRJNA827465	Yang2025_Silkie_Wenchang	0.185626401
ENSGALG00010006361	SERTM1	2	-0.508654779	0.306148644	-1.661463442	0.096620405	0.163110042	7.697485698	87.00874494	1	PRJNA827465;Yang2025_Silkie_Wenchang	CRA004775	0.531114935
ENSGALG00010006361	SERTM1	2	-0.533467207	0.391033502	-1.364249366	0.172489096	0.207602069	2.586137644	61.3322979	0.5	CRA004775;Yang2025_Silkie_Wenchang	PRJNA827465	0.675388277
ENSGALG00010006361	SERTM1	2	-0.186055056	0.144728759	-1.285543088	0.19860257	0	0.17101201	0	0.5	CRA004775;PRJNA827465	Yang2025_Silkie_Wenchang	0.710809025
ENSGALG00010006363	TRAPPC6A	2	0.152669201	0.153096066	0.997211782	0.318661723	0.033797961	3.329420741	69.96474529	1	PRJNA827465;Yang2025_Silkie_Wenchang	CRA004775	0.797754958
ENSGALG00010006363	TRAPPC6A	2	0.072890003	0.304802171	0.239138728	0.810998012	0.138693261	3.697444208	72.95429102	0.5	CRA004775;Yang2025_Silkie_Wenchang	PRJNA827465	0.953118313
ENSGALG00010006363	TRAPPC6A	2	-0.008203369	0.093860773	-0.087399336	0.930354097	0.003649889	1.08467589	7.806561475	0.5	CRA004775;PRJNA827465	Yang2025_Silkie_Wenchang	0.983813577
ENSGALG00010006372	SBF1	2	0.051975295	0.071366931	0.728282613	0.466440604	0.00620139	2.522088478	60.35032043	0.5	PRJNA827465;Yang2025_Silkie_Wenchang	CRA004775	0.852763198
ENSGALG00010006372	SBF1	2	-0.114462998	0.27301069	-0.419261962	0.675024693	0.129427586	7.041456199	85.7983921	0.5	CRA004775;Yang2025_Silkie_Wenchang	PRJNA827465	0.916045007
ENSGALG00010006372	SBF1	2	-0.173243905	0.19840695	-0.873174578	0.382567929	0.061918109	4.025415056	75.15784121	1	CRA004775;PRJNA827465	Yang2025_Silkie_Wenchang	0.825121999
ENSGALG00010006378	CAAP1	2	0.218455111	0.165619017	1.319021901	0.187161791	0.018017065	1.488853493	32.83422412	1	PRJNA827465;Yang2025_Silkie_Wenchang	CRA004775	0.695933761
ENSGALG00010006378	CAAP1	2	0.040421089	0.359339776	0.112487098	0.910437199	0.211312681	5.468251929	81.71262018	0.5	CRA004775;Yang2025_Silkie_Wenchang	PRJNA827465	0.97880407
ENSGALG00010006378	CAAP1	2	-0.111627944	0.192071728	-0.58117842	0.56112021	0.028799605	1.620263465	38.28164236	0.5	CRA004775;PRJNA827465	Yang2025_Silkie_Wenchang	0.881478381
ENSGALG00010006381	SPART	2	-0.158879503	0.073438361	-2.163440192	0.030507339	0	0.081685988	0	1	PRJNA827465;Yang2025_Silkie_Wenchang	CRA004775	0.29774769
ENSGALG00010006381	SPART	2	-0.111755868	0.106812368	-1.04628209	0.295430812	0	0.671199749	0	0.5	CRA004775;Yang2025_Silkie_Wenchang	PRJNA827465	0.787628307
ENSGALG00010006381	SPART	2	-0.156452574	0.09554168	-1.637532171	0.101519323	0	0.855547794	0	0.5	CRA004775;PRJNA827465	Yang2025_Silkie_Wenchang	0.543637124
ENSGALG00010006382	PNN	2	0.751206801	0.738584676	1.017089612	0.309110802	1.058856213	33.10564493	96.97936711	1	PRJNA827465;Yang2025_Silkie_Wenchang	CRA004775	0.794043289
ENSGALG00010006382	PNN	2	0.828587727	0.670658123	1.235484516	0.216650236	0.85163384	18.67863414	94.64628948	1	CRA004775;Yang2025_Silkie_Wenchang	PRJNA827465	0.730172415
ENSGALG00010006575	SSBP2	2	-0.059707874	0.140760425	-0.424180831	0.671433939	0	0.101256049	0	0.5	CRA004775;Yang2025_Silkie_Wenchang	PRJNA827465	0.914674175
ENSGALG00010006575	SSBP2	2	-0.076937821	0.12952364	-0.594006018	0.552508088	0	0.135892538	0	0.5	CRA004775;PRJNA827465	Yang2025_Silkie_Wenchang	0.879228216
ENSGALG00010006576	CCDC191	2	0.120693361	0.109811354	1.099097281	0.271725636	0.010347705	1.396513827	28.39311861	1	PRJNA827465;Yang2025_Silkie_Wenchang	CRA004775	0.773086433
ENSGALG00010006576	CCDC191	2	-0.031193489	0.373184576	-0.083587294	0.933384569	0.225480804	5.240201259	80.91676349	0.5	CRA004775;Yang2025_Silkie_Wenchang	PRJNA827465	0.984260547
ENSGALG00010006576	CCDC191	2	-0.117240621	0.23140317	-0.506650884	0.612399799	0.081571873	3.550628136	71.83596925	0.5	CRA004775;PRJNA827465	Yang2025_Silkie_Wenchang	0.897938231
ENSGALG00010006580	ALDH6A1	2	-0.323830246	0.108819277	-2.975853684	0.002921742	0.017235296	3.666096079	72.72302802	1	PRJNA827465;Yang2025_Silkie_Wenchang	CRA004775	0.074041237
ENSGALG00010006580	ALDH6A1	2	-0.291729782	0.221691992	-1.315923859	0.1881996	0.062745403	2.138386361	53.23576608	0.5	CRA004775;Yang2025_Silkie_Wenchang	PRJNA827465	0.697483156
ENSGALG00010006580	ALDH6A1	2	-0.203515109	0.074323349	-2.7382392	0.006176913	0	0.658084598	0	0.5	CRA004775;PRJNA827465	Yang2025_Silkie_Wenchang	0.11894719
ENSGALG00010006581	CEP41	2	0.02983275	0.138817258	0.214906639	0.829840109	0.018500616	1.82973697	45.34733591	0.5	PRJNA827465;Yang2025_Silkie_Wenchang	CRA004775	0.957079054
ENSGALG00010006581	CEP41	2	-0.070616942	0.301360032	-0.234327496	0.814730742	0.135580736	3.862576075	74.11054227	0.5	CRA004775;Yang2025_Silkie_Wenchang	PRJNA827465	0.953884449
ENSGALG00010006581	CEP41	2	-0.160497603	0.138971214	-1.154898184	0.248132123	0.013189344	1.349228399	25.88356423	1	CRA004775;PRJNA827465	Yang2025_Silkie_Wenchang	0.758346566
ENSGALG00010006585	TTC29	2	0.323724206	0.14664188	2.207583579	0.027273315	0	0.053202202	0	1	PRJNA827465;Yang2025_Silkie_Wenchang	CRA004775	0.281782062
ENSGALG00010006585	TTC29	2	0.27038802	0.1968672	1.37345388	0.169611289	0	0.92447966	0	0.5	CRA004775;Yang2025_Silkie_Wenchang	PRJNA827465	0.671099233
ENSGALG00010006585	TTC29	2	0.226718375	0.181594962	1.24848384	0.211853923	0	0.707349259	0	0.5	CRA004775;PRJNA827465	Yang2025_Silkie_Wenchang	0.724118735
ENSGALG00010006592	STARD13	2	0.10163264	0.045475231	2.234901004	0.025423849	0	0.520285296	0	1	PRJNA827465;Yang2025_Silkie_Wenchang	CRA004775	0.271950723
ENSGALG00010006592	STARD13	2	0.091893703	0.108442746	0.847393725	0.396775701	0.005736302	1.148336725	12.91752864	1	CRA004775;Yang2025_Silkie_Wenchang	PRJNA827465	0.831375233
ENSGALG00010006592	STARD13	2	0.129209453	0.051550594	2.506459045	0.012194718	0	0.688257073	0	1	CRA004775;PRJNA827465	Yang2025_Silkie_Wenchang	0.178269287
ENSGALG00010006598	ENSGALG00010006598	2	0.591622724	0.355683182	1.663341857	0.096244025	0.220902216	7.437733457	86.55504387	1	PRJNA827465;Yang2025_Silkie_Wenchang	CRA004775	0.530678858
ENSGALG00010006598	ENSGALG00010006598	2	0.927713999	0.181271394	5.117817978	3.09091E-07	0	0.117187801	0	1	CRA004775;Yang2025_Silkie_Wenchang	PRJNA827465	8.55506E-05
ENSGALG00010006598	ENSGALG00010006598	2	0.506814092	0.289367628	1.751454013	0.079867736	0.13034566	4.014289647	75.08899237	1	CRA004775;PRJNA827465	Yang2025_Silkie_Wenchang	0.486052267
ENSGALG00010006602	EDNRA	2	-0.052919478	0.111528227	-0.474494035	0.635147651	0.018981752	4.145411754	75.87694397	0.5	PRJNA827465;Yang2025_Silkie_Wenchang	CRA004775	0.904051292
ENSGALG00010006602	EDNRA	2	-0.15806611	0.084742694	-1.865247646	0.0621467	0	0.313886616	0	1	CRA004775;Yang2025_Silkie_Wenchang	PRJNA827465	0.429956168
ENSGALG00010006602	EDNRA	2	0.047616419	0.062910786	0.756888	0.449116966	0	0.041694122	0	0.5	CRA004775;PRJNA827465	Yang2025_Silkie_Wenchang	0.847239725
ENSGALG00010006603	MEST	2	-0.309672023	0.116877488	-2.649543797	0.008060052	0.007255778	1.358347133	26.38111603	1	PRJNA827465;Yang2025_Silkie_Wenchang	CRA004775	0.13949108
ENSGALG00010006603	MEST	2	-0.454253692	0.138456111	-3.280849699	0.001034949	0	0.094845084	0	1	CRA004775;Yang2025_Silkie_Wenchang	PRJNA827465	0.038656163
ENSGALG00010006603	MEST	2	-0.245173449	0.125744327	-1.949777417	0.051202654	0	0.851222098	0	1	CRA004775;PRJNA827465	Yang2025_Silkie_Wenchang	0.391986846
ENSGALG00010006608	KL	2	0.412716951	0.199822099	2.065421956	0.038883093	0	0.010116416	0	1	PRJNA827465;Yang2025_Silkie_Wenchang	CRA004775	0.340209751
ENSGALG00010006608	KL	2	0.466696345	0.290044111	1.609052991	0.107604755	0	0.594537152	0	1	CRA004775;Yang2025_Silkie_Wenchang	PRJNA827465	0.555230364
ENSGALG00010006608	KL	2	0.485267334	0.252367328	1.92286116	0.054497489	0	0.544988103	0	1	CRA004775;PRJNA827465	Yang2025_Silkie_Wenchang	0.404097297
ENSGALG00010006614	TMEM184C	2	-0.023599557	0.041047666	-0.574930534	0.565338278	0	0.891168954	0	0.5	PRJNA827465;Yang2025_Silkie_Wenchang	CRA004775	0.883427937
ENSGALG00010006614	TMEM184C	2	0.007638425	0.131661345	0.058015697	0.953736125	0.020060375	2.071234886	51.71962354	0.5	CRA004775;Yang2025_Silkie_Wenchang	PRJNA827465	0.987838152

ENSGALG00010006614	TMEM184C	2	0.020671791	0.05902678	0.350210378	0.726180819	0.001566921	1.093011361	8.509642658	1	CRA004775;PRJNA827465	Yang2025_Silkie_Wenchang	0.931029023
ENSGALG00010006615	GRAMD1C	2	0.019301345	0.07416139	0.26026137	0.794662168	0	0.249442484	0	0.5	PRJNA827465;Yang2025_Silkie_Wenchang	CRA004775	0.948905846
ENSGALG00010006615	GRAMD1C	2	0.003571343	0.096260683	0.037100749	0.970404674	0.001185936	1.046916395	4.481388906	0.5	CRA004775;Yang2025_Silkie_Wenchang	PRJNA827465	0.991711632
ENSGALG00010006615	GRAMD1C	2	-0.052783032	0.093539566	-0.564285615	0.57255977	0	0.477127515	0	1	CRA004775;PRJNA827465	Yang2025_Silkie_Wenchang	0.885678394
ENSGALG00010006616	COPG2	2	-0.051680923	0.049737597	-1.039071586	0.298771444	0	0.938168481	0	1	PRJNA827465;Yang2025_Silkie_Wenchang	CRA004775	0.78974322
ENSGALG00010006616	COPG2	2	-0.22342528	0.103690571	-2.154730917	0.031182897	0	0.288020404	0	1	CRA004775;Yang2025_Silkie_Wenchang	PRJNA827465	0.302017199
ENSGALG00010006616	COPG2	2	-0.116621946	0.119717509	-0.974142771	0.329985655	0.018490029	2.320378209	56.90357735	1	CRA004775;PRJNA827465	Yang2025_Silkie_Wenchang	0.801212404
ENSGALG00010006619	LIN52	2	-0.365931154	0.226422882	-1.616140339	0.106063974	0.096260711	16.3098521	93.86873655	1	PRJNA827465;Yang2025_Silkie_Wenchang	CRA004775	0.552502635
ENSGALG00010006619	LIN52	2	-0.578340195	0.08407839	-6.878583154	6.04508E-12	0	0.695251912	0	1	CRA004775;Yang2025_Silkie_Wenchang	PRJNA827465	5.27977E-09
ENSGALG00010006619	LIN52	2	-0.147317522	0.070131859	-2.100579162	0.035677925	0	0.143839719	0	1	CRA004775;PRJNA827465	Yang2025_Silkie_Wenchang	0.325801459
ENSGALG00010006621	DACT2	2	-0.062571286	0.107152305	-0.583947175	0.559255853	0.005068651	1.247548069	19.842768	0.5	PRJNA827465;Yang2025_Silkie_Wenchang	CRA004775	0.881014886
ENSGALG00010006621	DACT2	2	0.094922461	0.464244093	0.204466708	0.837988806	0.280826815	2.226397142	55.08438359	0.5	CRA004775;Yang2025_Silkie_Wenchang	PRJNA827465	0.959032758
ENSGALG00010006621	DACT2	2	0.140343047	0.287716765	0.487781959	0.625704292	0.086256447	1.390537834	28.08537994	1	CRA004775;PRJNA827465	Yang2025_Silkie_Wenchang	0.901501367
ENSGALG00010006623	ENSGALG00010006623	2	0.449792049	0.409004267	1.099724586	0.271452139	0.282913794	6.212649157	83.90380698	1	PRJNA827465;Yang2025_Silkie_Wenchang	CRA004775	0.772805786
ENSGALG00010006623	ENSGALG00010006623	2	0.818241196	0.252756783	3.237267008	0.001206804	0	0.305239812	0	1	CRA004775;Yang2025_Silkie_Wenchang	PRJNA827465	0.042380792
ENSGALG00010006623	ENSGALG00010006623	2	0.116999378	0.149956004	0.7802247	0.435258626	0	0.837203906	0	1	CRA004775;PRJNA827465	Yang2025_Silkie_Wenchang	0.842762754
ENSGALG00010006627	SF3B5	2	-0.06048536	0.060374455	-1.001836954	0.316422346	0	0.021006487	0	1	PRJNA827465;Yang2025_Silkie_Wenchang	CRA004775	0.796883862
ENSGALG00010006627	SF3B5	2	-0.051402554	0.132683839	-0.387406291	0.698455447	0	0.151235144	0	0.5	CRA004775;Yang2025_Silkie_Wenchang	PRJNA827465	0.921666253
ENSGALG00010006627	SF3B5	2	-0.05127908	0.06383483	-0.803308798	0.421796274	0	0.120680239	0	0.5	CRA004775;PRJNA827465	Yang2025_Silkie_Wenchang	0.839445682
ENSGALG00010006633	FBXO18	2	-0.00264872	0.081797519	-0.032381424	0.974167877	0.00663677	1.876916446	46.72112325	0.5	PRJNA827465;Yang2025_Silkie_Wenchang	CRA004775	0.992492038
ENSGALG00010006633	FBXO18	2	-0.093759812	0.086765144	-1.080616098	0.279867919	0	0.05232794	0	1	CRA004775;Yang2025_Silkie_Wenchang	PRJNA827465	0.778040488
ENSGALG00010006633	FBXO18	2	0.042733464	0.05945519	0.718750776	0.472294492	0	0.564273458	0	0.5	CRA004775;PRJNA827465	Yang2025_Silkie_Wenchang	0.853998373
ENSGALG00010006634	PRMT9	2	-0.075796367	0.163236779	-0.464333884	0.642408551	0.040617844	3.750487979	73.33680295	0.5	PRJNA827465;Yang2025_Silkie_Wenchang	CRA004775	0.906634523
ENSGALG00010006634	PRMT9	2	-0.080919232	0.214897032	-0.376548859	0.7065089	0.058142138	2.650579006	62.27239415	0.5	CRA004775;Yang2025_Silkie_Wenchang	PRJNA827465	0.924548793
ENSGALG00010006634	PRMT9	2	0.066388858	0.060612815	1.095290088	0.273387817	0	0.201596283	0	1	CRA004775;PRJNA827465	Yang2025_Silkie_Wenchang	0.77418844
ENSGALG00010006635	ENSGALG00010006635	2	0.037612512	0.816645913	0.046057307	0.963264574	1.123325236	6.336549253	84.21853978	0.5	PRJNA827465;Yang2025_Silkie_Wenchang	CRA004775	0.989998461
ENSGALG00010006635	ENSGALG00010006635	2	-0.692434546	0.387751562	-1.785768553	0.07413674	0	0.124399777	0	1	CRA004775;Yang2025_Silkie_Wenchang	PRJNA827465	0.470243057
ENSGALG00010006635	ENSGALG00010006635	2	0.304557234	0.655488342	0.464626469	0.642198974	0.521655335	2.429571004	58.84047026	0.5	CRA004775;PRJNA827465	Yang2025_Silkie_Wenchang	0.906634523
ENSGALG00010006651	ENSGALG00010006651	2	0.090274894	0.179998416	0.501531599	0.615997045	0.048902964	3.499790729	71.426863	0.5	PRJNA827465;Yang2025_Silkie_Wenchang	CRA004775	0.899050535
ENSGALG00010006651	ENSGALG00010006651	2	0.058110557	0.291835793	0.199120733	0.842168303	0.117362872	3.124002842	67.98978585	0.5	CRA004775;Yang2025_Silkie_Wenchang	PRJNA827465	0.960064308
ENSGALG00010006651	ENSGALG00010006651	2	-0.06218811	0.059519781	-1.044830958	0.296101104	0	0.60031329	0	1	CRA004775;PRJNA827465	Yang2025_Silkie_Wenchang	0.788203784
ENSGALG00010006653	CFL2	2	-0.03643495	0.062413383	-0.583768228	0.559376257	0	0.562621864	0	0.5	PRJNA827465;Yang2025_Silkie_Wenchang	CRA004775	0.881050436
ENSGALG00010006653	CFL2	2	0.063753325	0.12594981	0.5061804	0.612730013	0	0.045971575	0	1	CRA004775;Yang2025_Silkie_Wenchang	PRJNA827465	0.898034557
ENSGALG00010006653	CFL2	2	-0.05328595	0.069008744	-0.772162298	0.440018308	0	0.297044279	0	0.5	CRA004775;PRJNA827465	Yang2025_Silkie_Wenchang	0.844353254
ENSGALG00010006657	PDS5B	2	-0.050258313	0.097807975	-0.513846777	0.607359121	0.007171758	1.379223196	27.49541897	0.5	PRJNA827465;Yang2025_Silkie_Wenchang	CRA004775	0.896998866
ENSGALG00010006657	PDS5B	2	0.231785158	0.14602447	1.587303536	0.112443915	0.000804038	1.017624429	1.731918782	1	CRA004775;Yang2025_Silkie_Wenchang	PRJNA827465	0.566223663
ENSGALG00010006657	PDS5B	2	0.110949632	0.261805826	0.423785954	0.671721922	0.109948629	4.419229488	77.37162094	0.5	CRA004775;PRJNA827465	Yang2025_Silkie_Wenchang	0.914674175
ENSGALG00010006670	ANKRD16	2	0.089864289	0.219790916	0.408862616	0.682640485	0.059406748	2.562516056	60.97585428	0.5	PRJNA827465;Yang2025_Silkie_Wenchang	CRA004775	0.918716001
ENSGALG00010006670	ANKRD16	2	-0.158567833	0.497278716	-0.318871145	0.749824226	0.439769527	9.005570503	88.8957618	0.5	CRA004775;Yang2025_Silkie_Wenchang	PRJNA827465	0.937362187
ENSGALG00010006670	ANKRD16	2	-0.35677867	0.274938974	-1.297664952	0.194402492	0.107005548	3.323868291	69.91457204	1	CRA004775;PRJNA827465	Yang2025_Silkie_Wenchang	0.705148332
ENSGALG00010006673	BAZ1A	2	0.191658328	0.187096649	1.024381403	0.305655162	0.058309915	5.721388782	82.52172614	1	PRJNA827465;Yang2025_Silkie_Wenchang	CRA004775	0.79225794
ENSGALG00010006673	BAZ1A	2	0.311001567	0.184371313	1.686821894	0.091637604	0.022373951	1.242319469	19.50540704	0.5	CRA004775;Yang2025_Silkie_Wenchang	PRJNA827465	0.518914432
ENSGALG00010006673	BAZ1A	2	0.018363106	0.073434993	0.250059343	0.802541457	0	0.062053902	0	0.5	CRA004775;PRJNA827465	Yang2025_Silkie_Wenchang	0.950688375
ENSGALG00010006678	ENSGALG00010006678	2	0.341381251	0.458756764	0.744144343	0.456789155	0.125880282	1.348803711	25.86022772	1	PRJNA827465;Yang2025_Silkie_Wenchang	CRA004775	0.849515887
ENSGALG00010006678	ENSGALG00010006678	2	-0.33664304	1.722290372	-0.195462418	0.845030957	4.256488637	3.098098582	67.72213751	0.5	CRA004775;Yang2025_Silkie_Wenchang	PRJNA827465	0.960808045
ENSGALG00010006678	ENSGALG00010006678	2	-0.598091809	1.100913877	-0.543268481	0.586944963	1.424036829	1.769877927	43.4989281	0.5	CRA004775;PRJNA827465	Yang2025_Silkie_Wenchang	0.891596903
ENSGALG00010006681	CRISPLD2	2	0.054861905	0.234894671	0.233559599	0.815326896	0.042510231	1.574396197	36.48358641	0.5	PRJNA827465;Yang2025_Silkie_Wenchang	CRA004775	0.953933933
ENSGALG00010006681	CRISPLD2	2	0.144434408	0.248988457	0.580084755	0.561857464	0.004164598	1.033577567	3.248674091	0.5	CRA004775;Yang2025_Silkie_Wenchang	PRJNA827465	0.881802301
ENSGALG00010006681	CRISPLD2	2	-0.136705891	0.189057632	-0.723091098	0.469623918	0	0.002866237	0	1	CRA004775;PRJNA827465	Yang2025_Silkie_Wenchang	0.853485658
ENSGALG00010006682	ARHGAP10	2	0.131175492	0.078913353	1.662272448	0.096458159	0	0.321335403	0	1	PRJNA827465;Yang2025_Silkie_Wenchang	CRA004775	0.530885356
ENSGALG00010006682	ARHGAP10	2	0.227835548	0.124391358	1.831602705	0.067010634	0	0.220834922	0	1	CRA004775;Yang2025_Silkie_Wenchang	PRJNA827465	0.445965231
ENSGALG00010006682	ARHGAP10	2	0.127720301	0.089819905	1.421959881	0.155037898	0	0.704471906	0	1	CRA004775;PRJNA827465	Yang2025_Silkie_Wenchang	0.647688616
ENSGALG00010006685	ENSGALG00010006685	2	0.081691971	0.088201792	0.92619401	0.354345165	0	0.010837894	0	1	PRJNA827465;Yang2025_Silkie_Wenchang	CRA004775	0.81193713
ENSGALG00010006685	ENSGALG00010006685	2	0.00637859	0.148018521	0.043093188	0.965627249	0	0.79090341	0	0.5	CRA004775;Yang2025_Silkie_Wenchang	PRJNA827465	0.990668014
ENSGALG00010006685	ENSGALG00010006685	2	0.042264723	0.094187958	0.448727453	0.653628278	0	0.868908328	0	0.5	CRA004775;PRJNA827465	Yang2025_Silkie_Wenchang	0.909751123
ENSGALG00010006715	IRF8	2	-0.13744928	0.12572885	-1.09321989	0.274297266	0.018665991	2.002819152	50.07037959	1	PRJNA827465;Yang2025_Silkie_Wenchang	CRA004775	0.774526342
ENSGALG00010006715	IRF8	2	-0.540255606	0.261206982	-2.068304609	0.038611389	0.080480008	2.303938158	56.59605721	1	CRA004775;Yang2025_Silkie_Wenchang	PRJNA827465	0.338965227
ENSGALG00010006715	IRF8	2	-0.403852629	0.399613972	-1.010606878	0.312204623	0.277908897	6.889190732	85.48450698	1	CRA004775;PRJNA827465	Yang2025_Silkie_Wenchang	0.796054997
ENSGALG00010006716	ENSGALG00010006716	2	0.029654027	0.059518116	0.498235307	0.6183182	0	0.736212924	0	0.5	PRJNA827465;Yang2025_Silkie_Wenchang	CRA004775	0.899168321
ENSGALG00010006716	ENSGALG00010006716	2	0.132798654	0.101151693	1.312866344	0.189227989	0	0.730178952	0	1	CRA004775;Yang2025_Silkie_Wenchang	PRJNA827465	0.698184834
ENSGALG00010006716	ENSGALG00010006716	2	0.070291786	0.158122379	0.444540405	0.656651923	0.025492276	1.44240327	30.6712609	0.5	CRA004775;PRJNA827465	Yang2025_Silkie_Wenchang	0.910509456
ENSGALG00010006720	SRP54	2	-0.034465895	0.046585906	-0.73983523	0.45939998	0	0.457517325	0	1	PRJNA827465;Yang2025_Silkie_Wenchang	CRA004775	0.850098539
ENSGALG00010006720	SRP54	2	-0.061393302	0.075723917	-0.8107518	0.41750822	0	0.426427101	0	0.5	CRA004775;Yang2025_Silkie_Wenchang	PRJNA827465	0.838112595
ENSGALG00010006720	SRP54	2	-0.006584225	0.055548579	-0.118530938	0.905646983	0	0.152313997	0	0.5	CRA004775;PRJNA827465	Yang2025_Silkie_Wenchang	0.978182365
ENSGALG00010006728	ENSGALG00010006728	2	0.027054488	0.177097723	0.152765869	0.878582914	0.049491246	4.734848078	78.87999819	0.5	PRJNA827465;Yang2025_Silkie_Wenchang	CRA004775	0.971079508
ENSGALG00010006728	ENSGALG00010006728	2	-0.154411454	0.107704575	-1.433657334	0.151670073	0	0.075078973	0	1	CRA004775;Yang2025_Silkie_Wenchang	PRJNA827465	0.643081249
ENSGALG00010006728	ENSGALG00010006728	2	0.168925136	0.13018208	1.297606677	0.194422527	0.004162845	1.036869563	3.555853515	0.5	CRA004775;PRJNA827465	Yang2025_Silkie_Wenchang	0.705148332
ENSGALG00010006730	HAPLN1	2	1.515783608	0.918397554	1.650465641	0.098847735	1.049535958	2.640939222	62.13468331	1	PRJNA827465;Yang2025_Silkie_Wenchang	CRA004775	0.536752215
ENSGALG00010006730	HAPLN1	2	-0.45726331	2.995874871	-0.152630978	0.878689295	16.83227586	15.91647395	93.71720142	0.5	CRA004775;Yang2025_Silkie_Wenchang	PRJNA827465	0.971097077
ENSGALG00010006730	HAPLN1	2	-1.329966644	2.074388832	-0.64113662	0.521433924	7.566690822	8.090460572	87.63976425	0.5	CRA004775;PRJNA827465	Yang2025_Silkie_Wenchang	0.869518515
ENSGALG00010006735	NR3C2	2	-0.08058903	0.073015471	-1.103725394	0.269712277	0	0.00940461	0	1	PRJNA827465;Yang2025_Silkie_Wenchang	CRA004775	0.77189992
ENSGALG00010006735	NR3C2	2	0.178811326	0.310607731	0.575682148	0.564830044	0.149796404	4.206105393	76.22503702	0.5	CRA004775;Yang2025_Silkie_Wenchang	PRJNA827465	0.883351845
ENSGALG00010006735	NR3C2	2	0.17508205	0.300614859	0.582413161	0.560288419	0.146355293	4.639595297	78.44639594	0.5	CRA004775;PRJNA827465	Yang2025_Silkie_Wenchang	0.881203589
ENSGALG00010006739	VCAN	2	0.051816619	0.09631894	0.537969154	0.590598344	0	1.34103E-05	0	1	PRJNA827465;Yang2025_Silkie_Wenchang	CRA004775	0.892642928
ENSGALG00010006739	VCAN	2	0.079666281	0.15751636	0.505765124	0.613021544	0	0.202878893	0	1	CRA004775;Yang2025_Silkie_Wenchang	PRJNA827465	0.898130964
ENSGALG00010006739	VCAN	2	0.066550985	0.112800884	0.589986378	0.555199782	0	0.217071785	0	1	CRA004775;PRJNA827465	Yang2025_Silkie_Wenchang	0.880255849
ENSGALG00010006740	BRCA2	2	0.096575722	0.184003247	0.524858791	0.599681358	0.05336221	4.442227902	77.48877316	0.5	PRJNA827465;Yang2025_Silkie_Wenchang	CRA004775	0.895092498
ENSGALG00010006740	BRCA2	2	0.344273005	0.149247798	2.306720836	0.021070388	0	0.684680871	0	1	CRA004775;Yang2025_Silkie_Wenchang	PRJNA827465	0.245444415
ENSGALG00010006740	BRCA2	2	0.185582689	0.374059405	0.496131596	0.619801568	0.1961111	2.495938626	59.93491228	0.5	CRA004775;PRJNA827465	Yang2025_Silkie_Wenchang	0.899359856
ENSGALG00010006748	FAM177A1	2	-0.180648354	0.155008933	-1.165406088	0.243854654	0.039452656	5.148294578	80.57609205	1	PRJNA827465;Yang2025_Silkie_Wenchang	CRA004775	0.755365463
ENSGALG00010006748	FAM177A1	2	-0.064364661	0.307116515	-0.209577335	0.833997571	0.164610764	7.749948494	87.09668844	0.5	CRA004775;Yang2025_Silkie_Wenchang	PRJNA827465	0.957968425
ENSGALG00010006748	FAM177A1	2	0.056175731	0.142669787	0.393746512	0.693768198	0.027845793	2.571399931	61.11067796	0.5	CRA004775;PRJNA827465	Yang2025_Silkie_Wenchang	0.920721644
ENSGALG00010006760	TASOR2	2	-0.211117903	0.109467377	-1.928591953	0.053781539	0.019210571	4.829007797	79.29181227	1	PRJNA827465;Yang2025_Silkie_Wenchang	CRA004775	0.401964436
ENSGALG00010006760	TASOR2	2	-0.270286529	0.129500492	-2.087146734	0.036874873	0.01312116	1.313980623	23.89537696	1	CRA004775;Yang2025_Silkie_Wenchang	PRJNA827465	0.331459091
ENSGALG00010006760	TASOR2	2	-0.108947198	0.049199747	-2.214385334	0.026802283	0	0.157622218	0	1	CRA004775;PRJNA827465	Yang2025_Silkie_Wenchang	0.279174307
ENSGALG00010006761	PPP2R3C	2	-0.145777531	0.135443456	-1.076298078	0.281793968	0.025115203	2.907234971	65.60305549	1	PRJNA827465;Yang2025_Silkie_Wenchang	CRA004775	0.779295548
ENSGALG00010006761	PPP2R3C	2	-0.250799709	0.124093072	-2.021061322	0.043273418	0	0.679105759	0	1	CRA004775;Yang2025_Silkie_Wenchang	PRJNA827465	0.359703576
ENSGALG00010006761	PPP2R3C	2	-0.039486951	0.070025029	-0.56389768	0.572823769	0	0.032403786	0	1	CRA004775;PRJNA827465	Yang2025_Silkie_Wenchang	0.88573298
ENSGALG00010006767	ENSGALG00010006767	2	-0.261693589	0.221827071	-1.179718903	0.238112033	0	0.73625422	0	1	PRJNA827465;Yang2025_Silkie_Wenchang	CRA004775	0.750031946
ENSGALG00010006767	ENSGALG00010006767	2	-0.397984549	0.276386513	-1.439956475	0.149879714	0	0.116672787	0	1	CRA004775;Yang2025_Silkie_Wenchang	PRJNA827465	0.639822112
ENSGALG00010006767	ENSGALG00010006767	2	-0.090410182	0.286981032	-0.315038875	0.752732138	0	0.037983397	0	1	CRA004775;PRJNA827465	Yang2025_Silkie_Wenchang	0.938087082
ENSGALG00010006794	PERPA	2	0.204632285	0.875732195	0.233669935	0.81524123	0.942004999	2.586804865	61.34227155	0.5	PRJNA827465;Yang2025_Silkie_Wenchang	CRA004775	0.953933933
ENSGALG00010006794	PERPA	2	0.232017684	0.978433223	0.237131854	0.812554501	1.182834324	2.607172607	61.6442733	0.5	CRA004775;Yang2025_Silkie_Wenchang	PRJNA827465	0.95348724
ENSGALG00010006794	PERPA	2	1.132273007	0.571466538	1.981346117	0.047552472	0	0.031215707	0	1	CRA004775;PRJNA827465	Yang2025_Silkie_Wenchang	0.376812254
ENSGALG00010006803	COX4I1	2	-0.108780747	0.069290379	-1.569925713	0.116432395	0	0.206868164	0	0.5	PRJNA827465;Yang2025_Silkie_Wenchang	CRA004775	0.574631355
ENSGALG00010006803	COX4I1	2	0.076339746	0.206484233	0.369712228	0.71159692	0	0.192524577	0	1	CRA004775;Yang2025_Silkie_Wenchang	PRJNA827465	0.925966552
ENSGALG00010006803	COX4I1	2	-0.103901937	0.070273554	-1.478535391	0.139264531	0	0.813655891	0	0.5	CRA004775;PRJNA827465	Yang2025_Silkie_Wenchang	0.62298132
ENSGALG00010006804	PSMA6	2	-0.041995148	0.057983568	-0.724259465	0.468906458	0	0.767316793	0	1	PRJNA827465;Yang2025_Silkie_Wenchang	CRA004775	0.85345437
ENSGALG00010006804	PSMA6	2	-0.179731164	0.167700551	-1.071738662	0.283837431	0	0.126938438	0	1	CRA004775;Yang2025_Silkie_Wenchang	PRJNA827465	0.77978499
ENSGALG00010006804	PSMA6	2	-0.033277325	0.058303936	-0.570756069	0.568165006	0	0.111972999	0	1	CRA004775;PRJNA827465	Yang2025_Silkie_Wenchang	0.883994665
ENSGALG00010006816	ENSGALG00010006816	2	0.282707945	0.262804909	1.075733122	0.28204663	0	0.166943104	0	1	PRJNA827465;Yang2025_Silkie_Wenchang	CRA004775	0.779519046
ENSGALG00010006816	ENSGALG00010006816	2	0.178548871	0.323873627	0.55129179	0.581433666	0.036797192	1.13906851	12.20896804	0.5	CRA004775;Yang2025_Silkie_Wenchang	PRJNA827465	0.889045686
ENSGALG00010006816	ENSGALG00010006816	2	-0.071310408	0.388034484	-0.183773378	0.854191242	0	0.443109643	0	0.5	CRA004775;PRJNA827465	Yang2025_Silkie_Wenchang	0.963077783
ENSGALG00010006819	EMC8	2	-0.146375966	0.226820001	-0.645339765	0.518707029	0.087872732	6.13172328	83.69137102	0.5	PRJNA827465;Yang2025_Silkie_Wenchang	CRA004775	0.868703867
ENSGALG00010006819	EMC8	2	-0.363397779	0.13711513	-2.650311312	0.008041763	0	0.1504016	0	1	CRA004775;Yang2025_Silkie_Wenchang	PRJNA827465	0.139297234
ENSGALG00010006819	EMC8	2	-0.062876537	0.163802481	-0.383855831	0.701085305	0.037378646	2.560921234	60.95155186	0.5	CRA004775;PRJNA827465	Yang2025_Silkie_Wenchang	0.922547052
ENSGALG00010006821	XRCC4	2	0.251638411	0.225207781	1.11736109	0.263839965	0.052905229	2.088187772	52.11158626	1	PRJNA827465;Yang2025_Silkie_Wenchang	CRA004775	0.768643821
ENSGALG00010006821	XRCC4	2	0.185021421	0.329060544	0.56227167	0.573930937	0.145796437	3.001787436	66.68651524	0.5	CRA004775;Yang2025_Silkie_Wenchang	PRJNA827465	0.886081433
ENSGALG00010006821	XRCC4	2	-0.035938817	0.175341255	-0.204964983	0.837599484	0	0.317494166	0	0.5	CRA004775;PRJNA827465	Yang2025_Silkie_Wenchang	0.958988945
ENSGALG00010006828	GTF2A1	2	0.003960125	0.035527597	0.111466161	0.911246698	0	0.047685783	0	1	PRJNA827465;Yang2025_Silkie_Wenchang	CRA004775	0.978845851
ENSGALG00010006828	GTF2A1	2	0.171252594	0.166961436	1.02570149	0.305032312	0.020026732	1.518632953	34.15130378	1	CRA004775;Yang2025_Silkie_Wenchang	PRJNA827465	0.79225794
ENSGALG00010006828	GTF2A1	2	0.124856958	0.177079256	0.705090822	0.480753718	0.045075766	2.702322836	62.9947989	1	CRA004775;PRJNA827465	Yang2025_Silkie_Wenchang	0.856722567
ENSGALG00010006833	LTV1	2	-0.088592911	0.06341464	-1.39704194	0.16240096	0	0.176956222	0	1	PRJNA827465;Yang2025_Silkie_Wenchang	CRA004775	0.659930202
ENSGALG00010006833	LTV1	2	-0.065795846	0.093751309	-0.701812562	0.482796064	0	0.071263116	0	1	CRA004775;Yang2025_Silkie_Wenchang	PRJNA827465	0.85691916
ENSGALG00010006833	LTV1	2	-0.110909845	0.076119714	-1.457044956	0.145103971	0	0.002585665	0	1	CRA004775;PRJNA827465	Yang2025_Silkie_Wenchang	0.63310628
ENSGALG00010006835	FRY	2	-0.13764255	0.148700991	-0.925633033	0.35463672	0.023536405	1.930130649	48.19003571	0.5	PRJNA827465;Yang2025_Silkie_Wenchang	CRA004775	0.812016118
ENSGALG00010006835	FRY	2	0.294215723	0.271382244	1.084137704	0.278303748	0.085102909	2.273729382	56.01939229	1	CRA004775;Yang2025_Silkie_Wenchang	PRJNA827465	0.776763074
ENSGALG00010006835	FRY	2	0.137951795	0.42919375	0.321420791	0.747891529	0.321235928	7.201749948	86.11448596	0.5	CRA004775;PRJNA827465	Yang2025_Silkie_Wenchang	0.936938957
ENSGALG00010006838	ASB13	2	-0.044869804	0.078208411	-0.573720952	0.566156648	0	0.0165752	0	1	PRJNA827465;Yang2025_Silkie_Wenchang	CRA004775	0.883487517
ENSGALG00010006838	ASB13	2	-0.244454236	0.251212498	-0.973097429	0.330504882	0.074997183	2.170080727	53.91876498	1	CRA004775;Yang2025_Silkie_Wenchang	PRJNA827465	0.80156474
ENSGALG00010006838	ASB13	2	-0.224457231	0.260100578	-0.862963217	0.388157691	0.093697929	2.630607536	61.98596764	1	CRA004775;PRJNA827465	Yang2025_Silkie_Wenchang	0.828154121
ENSGALG00010006840	ENSGALG00010006840	2	-0.105501159	0.096273016	-1.095853885	0.273142723	0	0.041710767	0	1	PRJNA827465;Yang2025_Silkie_Wenchang	CRA004775	0.773904763
ENSGALG00010006840	ENSGALG00010006840	2	-0.129735278	0.220292544	-0.588922693	0.555913134	0	0.043503198	0	1	CRA004775;Yang2025_Silkie_Wenchang	PRJNA827465	0.880324407
ENSGALG00010006840	ENSGALG00010006840	2	-0.094449431	0.102832278	-0.918480394	0.358367419	0	0.01546159	0	1	CRA004775;PRJNA827465	Yang2025_Silkie_Wenchang	0.814005058
ENSGALG00010006843	STON2	2	-0.084806162	0.122423613	-0.692727163	0.488480789	0.005543396	1.22086993	18.09119254	0.5	PRJNA827465;Yang2025_Silkie_Wenchang	CRA004775	0.857837717
ENSGALG00010006843	STON2	2	0.12683585	0.150831111	0.840913051	0.400396647	0	0.705728506	0	1	CRA004775;Yang2025_Silkie_Wenchang	PRJNA827465	0.832251175
ENSGALG00010006843	STON2	2	0.012793805	0.264599686	0.04835155	0.961436071	0.0906929	2.528044609	60.44373598	0.5	CRA004775;PRJNA827465	Yang2025_Silkie_Wenchang	0.98973612
ENSGALG00010006847	SEL1L	2	0.034563995	0.104362983	0.33119018	0.740500838	0.01661995	3.91123606	74.43263498	0.5	PRJNA827465;Yang2025_Silkie_Wenchang	CRA004775	0.935128797
ENSGALG00010006847	SEL1L	2	-0.084988438	0.091489161	-0.92894543	0.352917368	0	0.008317709	0	1	CRA004775;Yang2025_Silkie_Wenchang	PRJNA827465	0.811035834
ENSGALG00010006847	SEL1L	2	0.119459628	0.046549303	2.566303282	0.010278891	0	0.34897993	0	0.5	CRA004775;PRJNA827465	Yang2025_Silkie_Wenchang	0.161081043
ENSGALG00010006850	IRF2BPL	2	-0.016058457	0.061080708	-0.262905554	0.792623375	0.000820026	1.10546186	9.540072239	0.5	PRJNA827465;Yang2025_Silkie_Wenchang	CRA004775	0.948435155
ENSGALG00010006850	IRF2BPL	2	0.062884882	0.099252451	0.633585181	0.526351575	0	0.103853505	0	0.5	CRA004775;Yang2025_Silkie_Wenchang	PRJNA827465	0.870546826
ENSGALG00010006850	IRF2BPL	2	-0.056826725	0.066721006	-0.851706657	0.394376925	0	0.004743202	0	1	CRA004775;PRJNA827465	Yang2025_Silkie_Wenchang	0.830486298
ENSGALG00010006851	TMEM167A	2	-0.307917605	0.243568801	-1.264191487	0.206161305	0.102631851	7.406226146	86.49784681	1	PRJNA827465;Yang2025_Silkie_Wenchang	CRA004775	0.718403416
ENSGALG00010006851	TMEM167A	2	-0.382486866	0.20179713	-1.895402903	0.058039044	0.053366955	2.73328742	63.41401959	1	CRA004775;Yang2025_Silkie_Wenchang	PRJNA827465	0.417532916
ENSGALG00010006851	TMEM167A	2	-0.083853904	0.10982185	-0.763544818	0.445138544	0	0.094937635	0	1	CRA004775;PRJNA827465	Yang2025_Silkie_Wenchang	0.846013379
ENSGALG00010006853	NET1	2	0.00491864	0.068448238	0.071859265	0.942713908	0	0.243020988	0	0.5	PRJNA827465;Yang2025_Silkie_Wenchang	CRA004775	0.985827325
ENSGALG00010006853	NET1	2	0.025538364	0.090270478	0.282909366	0.777246315	0	0.142198272	0	0.5	CRA004775;Yang2025_Silkie_Wenchang	PRJNA827465	0.944218792
ENSGALG00010006853	NET1	2	-0.033370167	0.090531666	-0.368602157	0.712424288	0	0.016307631	0	1	CRA004775;PRJNA827465	Yang2025_Silkie_Wenchang	0.92597752
ENSGALG00010006855	DCLK2	2	0.087574565	0.154218462	0.567860448	0.57012973	0.033307615	3.32657333	69.93903633	0.5	PRJNA827465;Yang2025_Silkie_Wenchang	CRA004775	0.885019503
ENSGALG00010006855	DCLK2	2	0.150557509	0.195667438	0.769456127	0.44162258	0.031665803	1.352957638	26.08785582	0.5	CRA004775;Yang2025_Silkie_Wenchang	PRJNA827465	0.845079715
ENSGALG00010006855	DCLK2	2	-0.074631564	0.108424881	-0.688325069	0.491248098	0	0.192401784	0	1	CRA004775;PRJNA827465	Yang2025_Silkie_Wenchang	0.85895707
ENSGALG00010006877	GSE1	2	0.24060146	0.34390521	0.699615628	0.48416738	0.212700673	9.421615799	89.38610933	0.5	PRJNA827465;Yang2025_Silkie_Wenchang	CRA004775	0.857004733
ENSGALG00010006877	GSE1	2	0.344674873	0.300782146	1.145928634	0.251824682	0.118396752	2.820131698	64.54066309	1	CRA004775;Yang2025_Silkie_Wenchang	PRJNA827465	0.761871849
ENSGALG00010006877	GSE1	2	-0.071506701	0.079954597	-0.894341331	0.371139293	0	0.075825579	0	0.5	CRA004775;PRJNA827465	Yang2025_Silkie_Wenchang	0.8214684
ENSGALG00010006883	LRBA	2	-0.058449994	0.09857284	-0.592962463	0.553206274	0.00493467	1.317766188	24.11400373	0.5	PRJNA827465;Yang2025_Silkie_Wenchang	CRA004775	0.87934426
ENSGALG00010006883	LRBA	2	-0.15656275	0.133147306	-1.175861192	0.239650349	0	0.276828849	0	0.5	CRA004775;Yang2025_Silkie_Wenchang	PRJNA827465	0.751887674
ENSGALG00010006883	LRBA	2	0.020964736	0.10093998	0.207695063	0.835467073	0	0.001240284	0	1	CRA004775;PRJNA827465	Yang2025_Silkie_Wenchang	0.958339923
ENSGALG00010006885	RHOU	2	-0.059144638	0.148998056	-0.396949056	0.691405028	0.030476404	3.183428429	68.58732583	0.5	PRJNA827465;Yang2025_Silkie_Wenchang	CRA004775	0.920268407
ENSGALG00010006885	RHOU	2	-0.050665016	0.254952323	-0.198723491	0.842479048	0.075945951	1.915242514	47.78729103	0.5	CRA004775;Yang2025_Silkie_Wenchang	PRJNA827465	0.960252241
ENSGALG00010006885	RHOU	2	0.106503207	0.108369446	0.982778921	0.325716258	0	0.431658456	0	1	CRA004775;PRJNA827465	Yang2025_Silkie_Wenchang	0.800023788
ENSGALG00010006901	TUBAL3	2	0.049023858	0.074505216	0.657992289	0.510543086	0	0.002144018	0	1	PRJNA827465;Yang2025_Silkie_Wenchang	CRA004775	0.865365179
ENSGALG00010006901	TUBAL3	2	0.040703955	0.122140404	0.333255451	0.738941464	0	0.028021479	0	0.5	CRA004775;Yang2025_Silkie_Wenchang	PRJNA827465	0.934613258
ENSGALG00010006901	TUBAL3	2	0.049355856	0.092286665	0.534810267	0.592781063	0	0.032048904	0	0.5	CRA004775;PRJNA827465	Yang2025_Silkie_Wenchang	0.892776493
ENSGALG00010006903	B3GLCT	2	-0.375054879	0.417131819	-0.899127955	0.368584509	0.341388891	52.49746832	98.09514624	0.5	PRJNA827465;Yang2025_Silkie_Wenchang	CRA004775	0.82033738
ENSGALG00010006903	B3GLCT	2	-0.393897784	0.446766877	-0.881662909	0.377959125	0.351505405	7.724377855	87.05397355	0.5	CRA004775;Yang2025_Silkie_Wenchang	PRJNA827465	0.82325159
ENSGALG00010006903	B3GLCT	2	0.041488227	0.061784055	0.671503796	0.501899642	0	0.041731102	0	1	CRA004775;PRJNA827465	Yang2025_Silkie_Wenchang	0.862795569
ENSGALG00010006912	ENSGALG00010006912	2	0.174257649	0.102366869	1.70228562	0.088701839	0	0.041128329	0	1	PRJNA827465;Yang2025_Silkie_Wenchang	CRA004775	0.51163023
ENSGALG00010006912	ENSGALG00010006912	2	-0.104064682	0.332503201	-0.312973472	0.754300816	0.167814703	4.105710661	75.64368065	0.5	CRA004775;Yang2025_Silkie_Wenchang	PRJNA827465	0.93838723
ENSGALG00010006912	ENSGALG00010006912	2	-0.104411382	0.30624688	-0.340938598	0.733149807	0.15079939	4.731633296	78.86564876	0.5	CRA004775;PRJNA827465	Yang2025_Silkie_Wenchang	0.933089653
ENSGALG00010006913	HSPH1	2	0.183333628	0.094702804	1.935883837	0.05288193	0	0.000682723	0	1	PRJNA827465;Yang2025_Silkie_Wenchang	CRA004775	0.399377178
ENSGALG00010006913	HSPH1	2	0.357973782	0.245643309	1.457290991	0.145036073	0.061589014	1.857467117	46.16324613	1	CRA004775;Yang2025_Silkie_Wenchang	PRJNA827465	0.632983695
ENSGALG00010006913	HSPH1	2	0.342310937	0.235950263	1.450775825	0.146842293	0.067756613	2.077802949	51.8722408	1	CRA004775;PRJNA827465	Yang2025_Silkie_Wenchang	0.635558525
ENSGALG00010006922	SMYD3	2	-0.218681614	0.157986088	-1.384182732	0.16630251	0.027866236	2.252386244	55.60264131	1	PRJNA827465;Yang2025_Silkie_Wenchang	CRA004775	0.665303687
ENSGALG00010006922	SMYD3	2	-0.225423442	0.207679273	-1.085440249	0.277726714	0.043814319	1.912413299	47.71004779	0.5	CRA004775;Yang2025_Silkie_Wenchang	PRJNA827465	0.776763074
ENSGALG00010006922	SMYD3	2	-0.046487229	0.12262746	-0.379093144	0.704618697	0	0.139693965	0	0.5	CRA004775;PRJNA827465	Yang2025_Silkie_Wenchang	0.924152671
ENSGALG00010006926	ID4	2	0.253461281	0.358256613	0.70748528	0.479264957	0.198836324	4.197282514	76.17506097	0.5	PRJNA827465;Yang2025_Silkie_Wenchang	CRA004775	0.856035423
ENSGALG00010006926	ID4	2	0.259947144	0.423101271	0.614385164	0.538960837	0.244255477	3.128105485	68.03176859	0.5	CRA004775;Yang2025_Silkie_Wenchang	PRJNA827465	0.87481821
ENSGALG00010006926	ID4	2	-0.083252161	0.153432684	-0.542597301	0.587407097	0	0.096040374	0	1	CRA004775;PRJNA827465	Yang2025_Silkie_Wenchang	0.891660017
ENSGALG00010006939	FBXO31	2	-0.041125628	0.068859314	-0.597241323	0.550346274	0	0.734819252	0	0.5	PRJNA827465;Yang2025_Silkie_Wenchang	CRA004775	0.878702974
ENSGALG00010006939	FBXO31	2	-0.137661327	0.114134053	-1.206137195	0.227764595	0	0.150608961	0	1	CRA004775;Yang2025_Silkie_Wenchang	PRJNA827465	0.740853612
ENSGALG00010006939	FBXO31	2	-0.011582433	0.082564029	-0.140284252	0.888435406	0	0.494315555	0	0.5	CRA004775;PRJNA827465	Yang2025_Silkie_Wenchang	0.973135755
ENSGALG00010006942	ALOX5AP	2	-0.163835977	0.321540756	-0.50953409	0.610377909	0.171871073	5.879169033	82.99079352	0.5	PRJNA827465;Yang2025_Silkie_Wenchang	CRA004775	0.897438028
ENSGALG00010006942	ALOX5AP	2	-0.773504105	0.338598101	-2.284431312	0.022346195	0.147042517	2.594149573	61.45172158	1	CRA004775;Yang2025_Silkie_Wenchang	PRJNA827465	0.253542872
ENSGALG00010006942	ALOX5AP	2	-0.479673958	0.666313845	-0.719891927	0.471591539	0.807491261	10.60124536	90.56714597	0.5	CRA004775;PRJNA827465	Yang2025_Silkie_Wenchang	0.853998373
ENSGALG00010006944	MAB21L2	2	0.873365226	0.457188771	1.910294566	0.056095297	0	0.587346204	0	1	PRJNA827465;Yang2025_Silkie_Wenchang	CRA004775	0.409721884
ENSGALG00010006944	MAB21L2	2	1.492038177	0.325845754	4.57897075	4.6727E-06	0	0.367104419	0	1	CRA004775;Yang2025_Silkie_Wenchang	PRJNA827465	0.000768414
ENSGALG00010006944	MAB21L2	2	1.190693637	0.552901977	2.153534782	0.031276674	0.338774677	2.076566598	51.84358638	1	CRA004775;PRJNA827465	Yang2025_Silkie_Wenchang	0.30260177
ENSGALG00010006953	MAP1LC3B	2	-0.064822598	0.18093716	-0.358260279	0.720148541	0.057482908	7.926762227	87.38450869	0.5	PRJNA827465;Yang2025_Silkie_Wenchang	CRA004775	0.928564173
ENSGALG00010006953	MAP1LC3B	2	-0.23642731	0.104951768	-2.25272346	0.024276591	0	0.277053626	0	1	CRA004775;Yang2025_Silkie_Wenchang	PRJNA827465	0.266297194
ENSGALG00010006953	MAP1LC3B	2	0.094936081	0.060370431	1.572559268	0.115820906	0	0.490551923	0	0.5	CRA004775;PRJNA827465	Yang2025_Silkie_Wenchang	0.572808488
ENSGALG00010006957	PALM2AKAP2	2	-0.055400034	0.079976181	-0.692706673	0.48849365	0.003630347	1.310285514	23.68075589	0.5	PRJNA827465;Yang2025_Silkie_Wenchang	CRA004775	0.857837717
ENSGALG00010006957	PALM2AKAP2	2	0.182831973	0.189725619	0.963665181	0.335213816	0.031296493	1.51939396	34.1842849	1	CRA004775;Yang2025_Silkie_Wenchang	PRJNA827465	0.803198384
ENSGALG00010006957	PALM2AKAP2	2	0.113654069	0.29055323	0.391164364	0.695675747	0.127791942	3.365722717	70.28869921	0.5	CRA004775;PRJNA827465	Yang2025_Silkie_Wenchang	0.921038437
ENSGALG00010006958	KIF26B	2	0.086922367	0.157512564	0.551844022	0.581055223	0	0.005704993	0	1	PRJNA827465;Yang2025_Silkie_Wenchang	CRA004775	0.888894576
ENSGALG00010006958	KIF26B	2	-0.068458432	0.304011412	-0.225183756	0.821836326	0	0.732710478	0	0.5	CRA004775;Yang2025_Silkie_Wenchang	PRJNA827465	0.95563486
ENSGALG00010006958	KIF26B	2	0.048963398	0.171643779	0.28526171	0.775443646	0	0.941755574	0	0.5	CRA004775;PRJNA827465	Yang2025_Silkie_Wenchang	0.943815265

ENSGALG00010006961	ZCCHC14	2	0.028540762	0.142530316	0.200243447	0.84129019	0.023164146	1.851767579	45.99754248	0.5	PRJNA827465;Yang2025_Silkie_Wenchang	CRA004775	0.95989279
ENSGALG00010006961	ZCCHC14	2	0.277169905	0.192822997	1.437431789	0.150595341	0	0.033579974	0	1	CRA004775;Yang2025_Silkie_Wenchang	PRJNA827465	0.641696412
ENSGALG00010006961	ZCCHC14	2	-0.043362592	0.080691084	-0.537390135	0.590998156	0.001741281	1.022321836	2.183445144	0.5	CRA004775;PRJNA827465	Yang2025_Silkie_Wenchang	0.892766566
ENSGALG00010006963	ENSGALG00010006963	2	-0.228736033	0.354132145	-0.645905875	0.518340314	0	0.40644683	0	1	PRJNA827465;Yang2025_Silkie_Wenchang	CRA004775	0.868496797
ENSGALG00010006963	ENSGALG00010006963	2	-0.472541874	0.450157948	-1.049724606	0.293844747	0	0.066252541	0	1	CRA004775;Yang2025_Silkie_Wenchang	PRJNA827465	0.787195888
ENSGALG00010006963	ENSGALG00010006963	2	-0.185265346	0.338373159	-0.547517855	0.584023007	0	0.149430777	0	1	CRA004775;PRJNA827465	Yang2025_Silkie_Wenchang	0.890763231
ENSGALG00010006983	GTPBP2	2	-0.091908221	0.188733787	-0.486972801	0.626277606	0.054960426	3.940882009	74.6249698	0.5	PRJNA827465;Yang2025_Silkie_Wenchang	CRA004775	0.901504244
ENSGALG00010006983	GTPBP2	2	-0.218622439	0.117703335	-1.857402247	0.063253947	0	0.549613064	0	1	CRA004775;Yang2025_Silkie_Wenchang	PRJNA827465	0.433102794
ENSGALG00010006983	GTPBP2	2	0.007331843	0.093153247	0.07870733	0.937265415	0.006665322	1.447342804	30.90786807	0.5	CRA004775;PRJNA827465	Yang2025_Silkie_Wenchang	0.985114195
ENSGALG00010006988	USPL1	2	0.276894465	0.135667258	2.040982246	0.041252592	0.02574324	3.287634057	69.58298939	1	PRJNA827465;Yang2025_Silkie_Wenchang	CRA004775	0.352161298
ENSGALG00010006988	USPL1	2	0.279162165	0.1656154	1.685605113	0.091871884	0.034110738	2.557850498	60.90467364	1	CRA004775;Yang2025_Silkie_Wenchang	PRJNA827465	0.519097275
ENSGALG00010006988	USPL1	2	0.136474863	0.081861387	1.667145735	0.09548543	0	0.101857936	0	1	CRA004775;PRJNA827465	Yang2025_Silkie_Wenchang	0.529041803
ENSGALG00010006992	SLC35B4	2	-0.17149775	0.216606676	-0.791747294	0.42850804	0.086096695	12.02778743	91.68591891	0.5	PRJNA827465;Yang2025_Silkie_Wenchang	CRA004775	0.841538794
ENSGALG00010006992	SLC35B4	2	-0.402390357	0.089817809	-4.480073161	7.46175E-06	0	0.031514333	0	1	CRA004775;Yang2025_Silkie_Wenchang	PRJNA827465	0.001106675
ENSGALG00010006992	SLC35B4	2	-0.163008314	0.233590075	-0.697839213	0.485277753	0.090233888	5.26617527	81.01088649	0.5	CRA004775;PRJNA827465	Yang2025_Silkie_Wenchang	0.857076964
ENSGALG00010006998	POLH	2	0.143649796	0.119361818	1.203481971	0.228789864	0.020196523	3.189316457	68.64531904	1	PRJNA827465;Yang2025_Silkie_Wenchang	CRA004775	0.741553101
ENSGALG00010006998	POLH	2	0.249481711	0.110502274	2.257706578	0.023963959	0	0.611539395	0	1	CRA004775;Yang2025_Silkie_Wenchang	PRJNA827465	0.264417596
ENSGALG00010006998	POLH	2	0.044070038	0.061717868	0.714056389	0.475192312	0	0.000164578	0	1	CRA004775;PRJNA827465	Yang2025_Silkie_Wenchang	0.854462864
ENSGALG00010007001	SNX30	2	0.269548915	0.205590764	1.311094474	0.189825847	0.075819234	9.525524965	89.50189093	1	PRJNA827465;Yang2025_Silkie_Wenchang	CRA004775	0.699160501
ENSGALG00010007001	SNX30	2	0.365630527	0.180261985	2.02832853	0.042526729	0.033919972	1.791316421	44.17513355	1	CRA004775;Yang2025_Silkie_Wenchang	PRJNA827465	0.356625761
ENSGALG00010007001	SNX30	2	0.074428057	0.068517914	1.08625689	0.277365354	0	0.004992968	0	1	CRA004775;PRJNA827465	Yang2025_Silkie_Wenchang	0.776450166
ENSGALG00010007002	EXOC4	2	-0.05538	0.052981943	-1.045261782	0.295901996	0	0.123738508	0	1	PRJNA827465;Yang2025_Silkie_Wenchang	CRA004775	0.788089459
ENSGALG00010007002	EXOC4	2	-0.056371623	0.110346188	-0.510861533	0.609448019	0	0.536249173	0	1	CRA004775;Yang2025_Silkie_Wenchang	PRJNA827465	0.897238578
ENSGALG00010007002	EXOC4	2	-0.072461436	0.057111031	-1.268781796	0.2045189	0	0.368907515	0	1	CRA004775;PRJNA827465	Yang2025_Silkie_Wenchang	0.71729696
ENSGALG00010007007	ACSL1	2	-0.008338993	0.169659534	-0.049151338	0.960798691	0	0.025491824	0	0.5	PRJNA827465;Yang2025_Silkie_Wenchang	CRA004775	0.98952228
ENSGALG00010007007	ACSL1	2	0.103846587	0.183441661	0.566101431	0.57132483	0	0.438689394	0	1	CRA004775;Yang2025_Silkie_Wenchang	PRJNA827465	0.885354083
ENSGALG00010007007	ACSL1	2	0.085648303	0.19790894	0.432766217	0.665184619	0	0.590234807	0	0.5	CRA004775;PRJNA827465	Yang2025_Silkie_Wenchang	0.913399315
ENSGALG00010007011	HMGB1	2	-0.148195077	0.11706528	-1.265918268	0.205542345	0.022788711	5.644694067	82.28424804	1	PRJNA827465;Yang2025_Silkie_Wenchang	CRA004775	0.71825498
ENSGALG00010007011	HMGB1	2	-0.274013367	0.084896027	-3.227634737	0.001248182	0	0.028376218	0	1	CRA004775;Yang2025_Silkie_Wenchang	PRJNA827465	0.042993261
ENSGALG00010007011	HMGB1	2	-0.045873572	0.045272534	-1.01327601	0.310928343	0	0.288473696	0	1	CRA004775;PRJNA827465	Yang2025_Silkie_Wenchang	0.795218958
ENSGALG00010007014	ENSGALG00010007014	2	1.366711506	0.303888193	4.497415627	6.87844E-06	0	0.30584866	0	1	PRJNA827465;Yang2025_Silkie_Wenchang	CRA004775	0.001035799
ENSGALG00010007014	ENSGALG00010007014	2	1.664055542	0.425272763	3.912913518	9.11892E-05	0	0.155788621	0	1	CRA004775;Yang2025_Silkie_Wenchang	PRJNA827465	0.007245791
ENSGALG00010007014	ENSGALG00010007014	2	1.349155698	0.358755205	3.760658186	0.000169467	0	0.578972037	0	1	CRA004775;PRJNA827465	Yang2025_Silkie_Wenchang	0.011330586
ENSGALG00010007017	SRD5A2	2	-0.014864725	0.277205241	-0.053623534	0.957235106	0	0.05526742	0	0.5	PRJNA827465;Yang2025_Silkie_Wenchang	CRA004775	0.988541306
ENSGALG00010007017	SRD5A2	2	-0.094201056	0.371042463	-0.253882144	0.79958661	0	0.271628864	0	0.5	CRA004775;Yang2025_Silkie_Wenchang	PRJNA827465	0.949922217
ENSGALG00010007017	SRD5A2	2	-0.123770582	0.28355933	-0.436489191	0.66248184	0	0.1478698	0	1	CRA004775;PRJNA827465	Yang2025_Silkie_Wenchang	0.912778072
ENSGALG00010007021	SULT6B1	2	-0.195829189	0.232121878	-0.84364813	0.39886606	0.040780141	1.544381153	35.24914505	1	PRJNA827465;Yang2025_Silkie_Wenchang	CRA004775	0.832054308
ENSGALG00010007021	SULT6B1	2	0.061914591	0.632295593	0.097920327	0.921995559	0.609568992	4.025923619	75.16097932	0.5	CRA004775;Yang2025_Silkie_Wenchang	PRJNA827465	0.981720024
ENSGALG00010007021	SULT6B1	2	0.218343089	0.362700733	0.601992412	0.547179188	0.142923494	1.835029807	45.5049724	0.5	CRA004775;PRJNA827465	Yang2025_Silkie_Wenchang	0.878281266
ENSGALG00010007028	KATNAL1	2	-0.189433178	0.220784596	-0.857999976	0.390892477	0.077017461	4.564765341	78.09306886	0.5	PRJNA827465;Yang2025_Silkie_Wenchang	CRA004775	0.829251057
ENSGALG00010007028	KATNAL1	2	-0.292246171	0.165559538	-1.765202867	0.077529677	0.015700671	1.389587376	28.0361914	1	CRA004775;Yang2025_Silkie_Wenchang	PRJNA827465	0.479609428
ENSGALG00010007028	KATNAL1	2	-0.011475015	0.094833062	-0.121002265	0.903689241	0	0.201421559	0	0.5	CRA004775;PRJNA827465	Yang2025_Silkie_Wenchang	0.977333734
ENSGALG00010007033	CEBPZOS	2	-0.361262074	0.277973959	-1.299625604	0.19372932	0.130720718	6.347457573	84.24566075	1	PRJNA827465;Yang2025_Silkie_Wenchang	CRA004775	0.703970364
ENSGALG00010007033	CEBPZOS	2	-0.389121588	0.324206687	-1.200226902	0.23005123	0.142830882	2.904795567	65.57416944	0.5	CRA004775;Yang2025_Silkie_Wenchang	PRJNA827465	0.742845187
ENSGALG00010007033	CEBPZOS	2	-0.088905886	0.11379301	-0.781294795	0.434629131	0	0.081375952	0	0.5	CRA004775;PRJNA827465	Yang2025_Silkie_Wenchang	0.842762754
ENSGALG00010007038	CENPU	2	-0.055824223	0.095039211	-0.58738096	0.556947878	0	0.729169622	0	0.5	PRJNA827465;Yang2025_Silkie_Wenchang	CRA004775	0.880521579
ENSGALG00010007038	CENPU	2	-0.166702708	0.138683474	-1.2020373	0.229349077	0	0.367666524	0	1	CRA004775;Yang2025_Silkie_Wenchang	PRJNA827465	0.742329471
ENSGALG00010007038	CENPU	2	-0.006651622	0.124668059	-0.053354662	0.957449328	0	0.769981714	0	0.5	CRA004775;PRJNA827465	Yang2025_Silkie_Wenchang	0.98869265
ENSGALG00010007041	NAE1	2	-0.211487881	0.219942992	-0.961557713	0.336271822	0.087150276	9.798875929	89.79474781	1	PRJNA827465;Yang2025_Silkie_Wenchang	CRA004775	0.803727281
ENSGALG00010007041	NAE1	2	-0.390032506	0.108638838	-3.590175587	0.000330455	0	0.825678577	0	1	CRA004775;Yang2025_Silkie_Wenchang	PRJNA827465	0.017988759
ENSGALG00010007041	NAE1	2	-0.02012224	0.064064627	-0.314092827	0.753450537	0	0.766243031	0	1	CRA004775;PRJNA827465	Yang2025_Silkie_Wenchang	0.938087082
ENSGALG00010007051	E2F3	2	0.053472962	0.106315604	0.502964382	0.614989315	0	0.089222912	0	1	PRJNA827465;Yang2025_Silkie_Wenchang	CRA004775	0.898781283
ENSGALG00010007051	E2F3	2	-0.061731911	0.166608503	-0.370520775	0.7109945	0	0.37946724	0	0.5	CRA004775;Yang2025_Silkie_Wenchang	PRJNA827465	0.925563881
ENSGALG00010007051	E2F3	2	0.031612271	0.116819339	0.270608199	0.78669239	0	0.785011698	0	0.5	CRA004775;PRJNA827465	Yang2025_Silkie_Wenchang	0.946722706
ENSGALG00010007066	INIP	2	-0.103721132	0.13521483	-0.767084	0.443031574	0	0.010086361	0	1	PRJNA827465;Yang2025_Silkie_Wenchang	CRA004775	0.845720426
ENSGALG00010007066	INIP	2	-0.21475178	0.171458561	-1.252499608	0.210387871	0	0.448284013	0	1	CRA004775;Yang2025_Silkie_Wenchang	PRJNA827465	0.721968882
ENSGALG00010007066	INIP	2	-0.167699088	0.144642177	-1.159406557	0.246290505	0	0.669437588	0	1	CRA004775;PRJNA827465	Yang2025_Silkie_Wenchang	0.756778652
ENSGALG00010007067	CASP3	2	-0.192113435	0.207079488	-0.92772798	0.353548691	0.078695737	11.98770059	91.65811665	0.5	PRJNA827465;Yang2025_Silkie_Wenchang	CRA004775	0.811318534
ENSGALG00010007067	CASP3	2	-0.39802133	0.096662498	-4.117639611	3.82773E-05	0	0.100397641	0	1	CRA004775;Yang2025_Silkie_Wenchang	PRJNA827465	0.004008225
ENSGALG00010007067	CASP3	2	-0.000400752	0.064747005	-0.006189502	0.995061524	0	0.580863051	0	0.5	CRA004775;PRJNA827465	Yang2025_Silkie_Wenchang	0.998242335
ENSGALG00010007070	UBL3	2	-0.076921437	0.1488115	-0.516905189	0.605222345	0.040274967	10.93094171	90.85165738	0.5	PRJNA827465;Yang2025_Silkie_Wenchang	CRA004775	0.896474603
ENSGALG00010007070	UBL3	2	0.049473186	0.303935356	0.162775356	0.870695313	0.165258638	8.958994669	88.83803332	0.5	CRA004775;Yang2025_Silkie_Wenchang	PRJNA827465	0.969047343
ENSGALG00010007070	UBL3	2	0.170023679	0.146636155	1.159493569	0.246255056	0.029313027	2.509440794	60.15048443	1	CRA004775;PRJNA827465	Yang2025_Silkie_Wenchang	0.756728888
ENSGALG00010007085	PRIMPOL	2	-0.188594286	0.088355046	-2.134504979	0.032801466	0	0.349005379	0	1	PRJNA827465;Yang2025_Silkie_Wenchang	CRA004775	0.31117452
ENSGALG00010007085	PRIMPOL	2	-0.231815833	0.133594713	-1.735217118	0.082702356	0	0.242793473	0	1	CRA004775;Yang2025_Silkie_Wenchang	PRJNA827465	0.495704284
ENSGALG00010007085	PRIMPOL	2	-0.138725737	0.110029521	-1.260804704	0.207379219	0	0.069655289	0	1	CRA004775;PRJNA827465	Yang2025_Silkie_Wenchang	0.719377239
ENSGALG00010007090	CEBPZ	2	0.092595671	0.170460588	0.54320868	0.586986131	0.051540852	8.519044425	88.26159426	0.5	PRJNA827465;Yang2025_Silkie_Wenchang	CRA004775	0.891596903
ENSGALG00010007090	CEBPZ	2	0.257637131	0.09344126	2.757209517	0.005829698	0	0.143728082	0	1	CRA004775;Yang2025_Silkie_Wenchang	PRJNA827465	0.115022409
ENSGALG00010007090	CEBPZ	2	-0.020578604	0.097475419	-0.211115832	0.832796887	0.008804362	1.37882416	27.47443592	0.5	CRA004775;PRJNA827465	Yang2025_Silkie_Wenchang	0.957861248
ENSGALG00010007092	DYNC1LI2	2	0.017454824	0.070169083	0.248753771	0.803551253	0.005426351	1.714079531	41.65964987	0.5	PRJNA827465;Yang2025_Silkie_Wenchang	CRA004775	0.951064358
ENSGALG00010007092	DYNC1LI2	2	0.156337882	0.099775142	1.566902122	0.117137572	0	0.082294736	0	1	CRA004775;Yang2025_Silkie_Wenchang	PRJNA827465	0.57646425
ENSGALG00010007092	DYNC1LI2	2	0.015116241	0.084610014	0.178657824	0.858206387	0.007475964	1.422100023	29.68145813	0.5	CRA004775;PRJNA827465	Yang2025_Silkie_Wenchang	0.964291891
ENSGALG00010007097	ZCCHC9	2	-0.0032322	0.143111893	-0.022585125	0.981981209	0	0.328595705	0	0.5	PRJNA827465;Yang2025_Silkie_Wenchang	CRA004775	0.995091431
ENSGALG00010007097	ZCCHC9	2	-0.276148426	0.176462883	-1.564909404	0.117604153	0	0.913208299	0	1	CRA004775;Yang2025_Silkie_Wenchang	PRJNA827465	0.577197307
ENSGALG00010007097	ZCCHC9	2	-0.164982081	0.252858258	-0.652468628	0.514098904	0.081646542	2.683526591	62.73560309	0.5	CRA004775;PRJNA827465	Yang2025_Silkie_Wenchang	0.866855965
ENSGALG00010007098	NDUFAF7	2	0.09437851	0.069807618	1.351980096	0.176381683	0	0.979804766	0	1	PRJNA827465;Yang2025_Silkie_Wenchang	CRA004775	0.681691731
ENSGALG00010007098	NDUFAF7	2	0.130446356	0.109717651	1.188927713	0.234468114	0.000516461	1.018146305	1.78228858	0.5	CRA004775;Yang2025_Silkie_Wenchang	PRJNA827465	0.747509756
ENSGALG00010007098	NDUFAF7	2	0.036702501	0.076803683	0.477874238	0.632739718	0	0.168092292	0	0.5	CRA004775;PRJNA827465	Yang2025_Silkie_Wenchang	0.903112631
ENSGALG00010007102	CMTM3	2	-0.213281968	0.265266663	-0.804028539	0.421380493	0.134819728	23.70734315	95.78189764	0.5	PRJNA827465;Yang2025_Silkie_Wenchang	CRA004775	0.839352211
ENSGALG00010007102	CMTM3	2	-0.418171029	0.129532616	-3.228306842	0.001245253	0.013076397	1.32496839	24.52650133	1	CRA004775;Yang2025_Silkie_Wenchang	PRJNA827465	0.042969436
ENSGALG00010007102	CMTM3	2	0.03765673	0.062239211	0.605032254	0.545157566	0	0.542154695	0	0.5	CRA004775;PRJNA827465	Yang2025_Silkie_Wenchang	0.877408247
ENSGALG00010007106	SLC7A1	2	-0.323709547	0.151371306	-2.138513274	0.032475108	0.035752007	4.46328698	77.59498763	1	PRJNA827465;Yang2025_Silkie_Wenchang	CRA004775	0.309572925
ENSGALG00010007106	SLC7A1	2	-0.579619115	0.197074258	-2.941120377	0.003270274	0.033775777	1.349552786	25.90137929	1	CRA004775;Yang2025_Silkie_Wenchang	PRJNA827465	0.079684802
ENSGALG00010007106	SLC7A1	2	-0.484212956	0.393087049	-1.231821191	0.218015886	0.238261408	3.555322491	71.8731563	1	CRA004775;PRJNA827465	Yang2025_Silkie_Wenchang	0.731552834
ENSGALG00010007108	MBOAT1	2	0.143681607	0.107390985	1.337929874	0.180919298	0	0.715363426	0	1	PRJNA827465;Yang2025_Silkie_Wenchang	CRA004775	0.688484814
ENSGALG00010007108	MBOAT1	2	-0.118360431	0.264708659	-0.447134716	0.654777795	0.064873551	1.528792825	34.58891332	0.5	CRA004775;Yang2025_Silkie_Wenchang	PRJNA827465	0.910222014
ENSGALG00010007108	MBOAT1	2	-0.055989686	0.378965431	-0.147743518	0.882545186	0.199714262	2.68954435	62.81898084	0.5	CRA004775;PRJNA827465	Yang2025_Silkie_Wenchang	0.972001076
ENSGALG00010007110	ARL6	2	-0.113711492	0.160302108	-0.709357436	0.478102694	0.044557004	7.079540018	85.87478851	0.5	PRJNA827465;Yang2025_Silkie_Wenchang	CRA004775	0.855366076
ENSGALG00010007110	ARL6	2	-0.249255987	0.103184474	-2.415634618	0.015707815	0	0.977999853	0	0.5	CRA004775;Yang2025_Silkie_Wenchang	PRJNA827465	0.207334357
ENSGALG00010007110	ARL6	2	0.030842219	0.046988058	0.6563842	0.511576954	0	0.010229493	0	1	CRA004775;PRJNA827465	Yang2025_Silkie_Wenchang	0.865694231
ENSGALG00010007114	CKLF	2	-0.031272014	0.227134625	-0.137680525	0.890492911	0.064420761	2.626080364	61.92043421	0.5	PRJNA827465;Yang2025_Silkie_Wenchang	CRA004775	0.973439475
ENSGALG00010007114	CKLF	2	-0.347164733	0.190866518	-1.81888755	0.068928588	0	0.356466331	0	1	CRA004775;Yang2025_Silkie_Wenchang	PRJNA827465	0.452044654
ENSGALG00010007114	CKLF	2	-0.10464412	0.349874026	-0.29909085	0.764870727	0.171055994	2.99306716	66.58945668	0.5	CRA004775;PRJNA827465	Yang2025_Silkie_Wenchang	0.940711384
ENSGALG00010007115	ENSGALG00010007115	2	0.138343782	0.291679078	0.474301356	0.635285025	0.107228341	2.396208284	58.26740076	0.5	PRJNA827465;Yang2025_Silkie_Wenchang	CRA004775	0.904141515
ENSGALG00010007115	ENSGALG00010007115	2	-0.074463468	0.601768281	-0.123741098	0.90152027	0.594118427	5.565133351	82.03097865	0.5	CRA004775;Yang2025_Silkie_Wenchang	PRJNA827465	0.976665597
ENSGALG00010007115	ENSGALG00010007115	2	-0.290767223	0.284738585	-1.021172537	0.307172701	0.098514352	2.237427817	55.30582071	1	CRA004775;PRJNA827465	Yang2025_Silkie_Wenchang	0.79266003
ENSGALG00010007118	ENSGALG00010007118	2	0.059826218	0.107163016	0.558272995	0.576657982	0	0.216408721	0	0.5	PRJNA827465;Yang2025_Silkie_Wenchang	CRA004775	0.887113945
ENSGALG00010007118	ENSGALG00010007118	2	0.15331487	0.209167823	0.732975407	0.463573429	0.034899218	1.613166128	38.01010433	0.5	CRA004775;Yang2025_Silkie_Wenchang	PRJNA827465	0.85211049
ENSGALG00010007118	ENSGALG00010007118	2	0.159847293	0.130093844	1.228707594	0.219181458	0.004709106	1.100766168	9.154184696	1	CRA004775;PRJNA827465	Yang2025_Silkie_Wenchang	0.73285529
ENSGALG00010007123	ENSGALG00010007123	2	0.338306498	0.20018318	1.689984631	0.091030895	0	0.818577914	0	1	PRJNA827465;Yang2025_Silkie_Wenchang	CRA004775	0.517594064
ENSGALG00010007123	ENSGALG00010007123	2	0.011972637	0.526448368	0.022742281	0.981855849	0.443309435	4.882730316	79.51965529	0.5	CRA004775;Yang2025_Silkie_Wenchang	PRJNA827465	0.995055866
ENSGALG00010007123	ENSGALG00010007123	2	-0.162514398	0.342368205	-0.47467725	0.635017036	0.115645328	1.937603164	48.3898448	0.5	CRA004775;PRJNA827465	Yang2025_Silkie_Wenchang	0.904051292
ENSGALG00010007126	ENSGALG00010007126	2	1.574688623	0.697376286	2.258018597	0.0239445	0	0.256871903	0	1	PRJNA827465;Yang2025_Silkie_Wenchang	CRA004775	0.264351316
ENSGALG00010007126	ENSGALG00010007126	2	0.540780794	0.680132072	0.795111443	0.426548679	0.090175077	1.10770344	9.72312948	0.5	CRA004775;Yang2025_Silkie_Wenchang	PRJNA827465	0.84125685
ENSGALG00010007126	ENSGALG00010007126	2	0.859530486	1.033862919	0.831377613	0.405760342	1.210710853	2.29226019	56.37493491	0.5	CRA004775;PRJNA827465	Yang2025_Silkie_Wenchang	0.833357445
ENSGALG00010007130	IRF2	2	-0.070034386	0.041604294	-1.683345154	0.092308294	0	0.02329897	0	1	PRJNA827465;Yang2025_Silkie_Wenchang	CRA004775	0.520440808
ENSGALG00010007130	IRF2	2	-0.045511529	0.09982307	-0.455921958	0.648446099	0	0.565646048	0	0.5	CRA004775;Yang2025_Silkie_Wenchang	PRJNA827465	0.907649027
ENSGALG00010007130	IRF2	2	-0.060541991	0.043732621	-1.384366843	0.166246156	0	0.561844265	0	0.5	CRA004775;PRJNA827465	Yang2025_Silkie_Wenchang	0.66529196
ENSGALG00010007133	BLVRA	2	0.031361588	0.232168984	0.135080869	0.892547936	0.070610658	2.795793328	64.23197702	0.5	PRJNA827465;Yang2025_Silkie_Wenchang	CRA004775	0.974359592
ENSGALG00010007133	BLVRA	2	-0.266470253	0.213914904	-1.245683442	0.212880635	0	0.10710088	0	1	CRA004775;Yang2025_Silkie_Wenchang	PRJNA827465	0.725659838
ENSGALG00010007133	BLVRA	2	0.099849985	0.264731295	0.377174845	0.706043673	0.063833514	1.416581179	29.40750488	0.5	CRA004775;PRJNA827465	Yang2025_Silkie_Wenchang	0.924548793
ENSGALG00010007135	TK2	2	-0.109547771	0.170341154	-0.643108068	0.52015398	0.04914468	6.529417343	84.68469777	0.5	PRJNA827465;Yang2025_Silkie_Wenchang	CRA004775	0.869331539
ENSGALG00010007135	TK2	2	-0.253240165	0.088709727	-2.854705711	0.004307672	0	0.63351209	0	1	CRA004775;Yang2025_Silkie_Wenchang	PRJNA827465	0.096250398
ENSGALG00010007135	TK2	2	0.044278921	0.087526748	0.505890158	0.612933762	0	0.238143117	0	0.5	CRA004775;PRJNA827465	Yang2025_Silkie_Wenchang	0.898114888
ENSGALG00010007137	ENSGALG00010007137	2	0.652844414	0.448549489	1.455456824	0.145542835	0.279160961	3.187109516	68.62360722	1	PRJNA827465;Yang2025_Silkie_Wenchang	CRA004775	0.63417628
ENSGALG00010007137	ENSGALG00010007137	2	-0.459385556	0.590086901	-0.77850492	0.436271407	0.555575013	4.913713635	79.64879368	0.5	CRA004775;Yang2025_Silkie_Wenchang	PRJNA827465	0.843122822
ENSGALG00010007137	ENSGALG00010007137	2	0.022212525	1.041540025	0.021326617	0.982985112	2.07009035	21.78083363	95.4088075	0.5	CRA004775;PRJNA827465	Yang2025_Silkie_Wenchang	0.995363468
ENSGALG00010007141	PRKD3	2	0.005089633	0.105585498	0.048203898	0.961553744	0.015891053	3.4086087	70.66251694	0.5	PRJNA827465;Yang2025_Silkie_Wenchang	CRA004775	0.98973612
ENSGALG00010007141	PRKD3	2	-0.111164585	0.089892754	-1.236635662	0.216222374	0	0.000289807	0	1	CRA004775;Yang2025_Silkie_Wenchang	PRJNA827465	0.72989044
ENSGALG00010007141	PRKD3	2	0.093269344	0.065972194	1.413767508	0.157430176	0	0.389027491	0	0.5	CRA004775;PRJNA827465	Yang2025_Silkie_Wenchang	0.650765484
ENSGALG00010007143	CKMT2	2	-0.207479191	0.258523901	-0.802553227	0.422233012	0	0.704406668	0	0.5	PRJNA827465;Yang2025_Silkie_Wenchang	CRA004775	0.839649223
ENSGALG00010007143	CKMT2	2	-0.001582415	0.336553038	-0.004701831	0.996248496	0	0.001274237	0	0.5	CRA004775;Yang2025_Silkie_Wenchang	PRJNA827465	0.99878373
ENSGALG00010007143	CKMT2	2	-0.377916119	0.345574993	-1.093586419	0.274136408	0	0.153724973	0	1	CRA004775;PRJNA827465	Yang2025_Silkie_Wenchang	0.774526342
ENSGALG00010007146	ENSGALG00010007146	2	-0.124574327	0.161954287	-0.769194378	0.441777927	0.045352815	7.165876448	86.0449729	0.5	PRJNA827465;Yang2025_Silkie_Wenchang	CRA004775	0.845135451
ENSGALG00010007146	ENSGALG00010007146	2	-0.080127201	0.246156895	-0.325512722	0.744793056	0.097117082	4.753863423	78.96447771	0.5	CRA004775;Yang2025_Silkie_Wenchang	PRJNA827465	0.935893304
ENSGALG00010007146	ENSGALG00010007146	2	0.040935026	0.05843053	0.700575982	0.483567672	0	0.65949978	0	1	CRA004775;PRJNA827465	Yang2025_Silkie_Wenchang	0.85691916
ENSGALG00010007160	ENSGALG00010007160	2	0.21339956	0.112982931	1.888776987	0.058921718	0	0.324202395	0	1	PRJNA827465;Yang2025_Silkie_Wenchang	CRA004775	0.42013793
ENSGALG00010007160	ENSGALG00010007160	2	-0.19027897	0.573360549	-0.331866171	0.739990316	0.548921658	5.910802021	83.08182212	0.5	CRA004775;Yang2025_Silkie_Wenchang	PRJNA827465	0.935072094
ENSGALG00010007160	ENSGALG00010007160	2	-0.2397132	0.488196553	-0.491017805	0.623413855	0.400168972	5.607209879	82.16581827	0.5	CRA004775;PRJNA827465	Yang2025_Silkie_Wenchang	0.900357342
ENSGALG00010007162	ENSGALG00010007162	2	-0.116509441	0.141770151	-0.821819264	0.411179768	0	0.037176834	0	1	PRJNA827465;Yang2025_Silkie_Wenchang	CRA004775	0.835128673
ENSGALG00010007162	ENSGALG00010007162	2	-0.204456166	0.211885778	-0.964935766	0.334576986	0	0.836602673	0	1	CRA004775;Yang2025_Silkie_Wenchang	PRJNA827465	0.803101658
ENSGALG00010007162	ENSGALG00010007162	2	-0.136583244	0.180049505	-0.758587165	0.448099553	0	0.990046829	0	1	CRA004775;PRJNA827465	Yang2025_Silkie_Wenchang	0.846602312
ENSGALG00010007165	ENSGALG00010007165	2	-0.362289694	0.193853927	-1.868879831	0.061639535	0	0.002116199	0	1	PRJNA827465;Yang2025_Silkie_Wenchang	CRA004775	0.428734062
ENSGALG00010007165	ENSGALG00010007165	2	-0.378869677	0.21397386	-1.770635335	0.076621362	0	0.030467076	0	1	CRA004775;Yang2025_Silkie_Wenchang	PRJNA827465	0.47762877
ENSGALG00010007165	ENSGALG00010007165	2	-0.396021279	0.237797095	-1.665374759	0.095838012	0	0.016680247	0	1	CRA004775;PRJNA827465	Yang2025_Silkie_Wenchang	0.5301492
ENSGALG00010007166	RASGRF2	2	0.205614226	0.259226687	0.793183096	0.427671154	0.093128362	3.073148915	67.46008645	0.5	PRJNA827465;Yang2025_Silkie_Wenchang	CRA004775	0.84125685
ENSGALG00010007166	RASGRF2	2	0.524591854	0.227315873	2.30776605	0.021012152	0	0.011633773	0	1	CRA004775;Yang2025_Silkie_Wenchang	PRJNA827465	0.245277621
ENSGALG00010007166	RASGRF2	2	0.106609267	0.236538725	0.450705343	0.652201931	0.048122173	1.394183853	28.27344845	0.5	CRA004775;PRJNA827465	Yang2025_Silkie_Wenchang	0.909376064
ENSGALG00010007168	MINA	2	0.147682837	0.267295052	0.552508683	0.580599887	0.130141937	10.76664858	90.7120587	0.5	PRJNA827465;Yang2025_Silkie_Wenchang	CRA004775	0.888464311
ENSGALG00010007168	MINA	2	0.179297536	0.278308112	0.644241143	0.519419075	0.11914441	4.206195688	76.2255474	0.5	CRA004775;Yang2025_Silkie_Wenchang	PRJNA827465	0.869086345
ENSGALG00010007168	MINA	2	-0.104985707	0.06269196	-1.674627918	0.094007258	0	0.009791691	0	1	CRA004775;PRJNA827465	Yang2025_Silkie_Wenchang	0.525272571
ENSGALG00010007169	QPCT	2	0.035526997	0.066705708	0.53259306	0.594315307	0	0.00449018	0	1	PRJNA827465;Yang2025_Silkie_Wenchang	CRA004775	0.893334186
ENSGALG00010007169	QPCT	2	0.034127152	0.111517389	0.306025385	0.759585318	0	0.01371702	0	1	CRA004775;Yang2025_Silkie_Wenchang	PRJNA827465	0.939163539
ENSGALG00010007169	QPCT	2	0.040396202	0.07733876	0.522328021	0.601441962	0	0.008176913	0	1	CRA004775;PRJNA827465	Yang2025_Silkie_Wenchang	0.895230519
ENSGALG00010007175	FOXC2	2	0.938553151	0.621223661	1.510813592	0.130835951	0.607722624	4.647102072	78.48121293	1	PRJNA827465;Yang2025_Silkie_Wenchang	CRA004775	0.606578636
ENSGALG00010007175	FOXC2	2	0.485602457	1.185780815	0.409521263	0.682157166	2.428862931	7.143540721	86.00133968	0.5	CRA004775;Yang2025_Silkie_Wenchang	PRJNA827465	0.918524893
ENSGALG00010007175	FOXC2	2	-0.012276259	0.528811139	-0.023214826	0.981478912	0.289698349	1.824863087	45.20136841	0.5	CRA004775;PRJNA827465	Yang2025_Silkie_Wenchang	0.995004677
ENSGALG00010007182	ENSGALG00010007182	2	0.090865351	0.435031816	0.208870587	0.834549266	0.293228712	4.395127677	77.24753242	0.5	PRJNA827465;Yang2025_Silkie_Wenchang	CRA004775	0.958120284
ENSGALG00010007182	ENSGALG00010007182	2	-0.277557293	0.240148836	-1.155771971	0.247774441	0	0.167326528	0	1	CRA004775;Yang2025_Silkie_Wenchang	PRJNA827465	0.7580874
ENSGALG00010007182	ENSGALG00010007182	2	0.210170214	0.334204103	0.628867845	0.529435579	0.131522357	2.373566202	57.86930235	0.5	CRA004775;PRJNA827465	Yang2025_Silkie_Wenchang	0.871944741
ENSGALG00010007183	VOPP1	2	0.164691248	0.215443144	0.764430208	0.444610913	0.010120134	1.078840859	7.307922974	1	PRJNA827465;Yang2025_Silkie_Wenchang	CRA004775	0.846013379
ENSGALG00010007183	VOPP1	2	0.171647851	0.353549404	0.485498912	0.627322484	0.082986885	1.482366207	32.54028623	0.5	CRA004775;Yang2025_Silkie_Wenchang	PRJNA827465	0.901651329
ENSGALG00010007183	VOPP1	2	0.003031091	0.189622072	0.015984907	0.987246433	0	0.190797959	0	0.5	CRA004775;PRJNA827465	Yang2025_Silkie_Wenchang	0.996492495
ENSGALG00010007184	VGLL3	2	-0.034513748	0.092561294	-0.372874518	0.709241839	0.005710211	1.280290603	21.89273295	0.5	PRJNA827465;Yang2025_Silkie_Wenchang	CRA004775	0.925298042
ENSGALG00010007184	VGLL3	2	0.139233909	0.171436991	0.812157913	0.416701026	0	0.008601686	0	1	CRA004775;Yang2025_Silkie_Wenchang	PRJNA827465	0.837984974
ENSGALG00010007184	VGLL3	2	-0.076314164	0.070323081	-1.085193697	0.277835875	0	0.201849568	0	0.5	CRA004775;PRJNA827465	Yang2025_Silkie_Wenchang	0.776763074
ENSGALG00010007187	ENPP6	2	-0.216116437	0.130330554	-1.658217746	0.097273524	0.017560323	1.953077253	48.79874831	1	PRJNA827465;Yang2025_Silkie_Wenchang	CRA004775	0.532942995
ENSGALG00010007187	ENPP6	2	-0.154279211	0.258897538	-0.595908373	0.55123644	0.091939005	3.07353243	67.46414678	0.5	CRA004775;Yang2025_Silkie_Wenchang	PRJNA827465	0.878879109
ENSGALG00010007187	ENPP6	2	-0.078593248	0.092423355	-0.850361341	0.395124222	0	0.898914107	0	0.5	CRA004775;PRJNA827465	Yang2025_Silkie_Wenchang	0.830643015
ENSGALG00010007188	MAB21L1	2	-0.110672904	0.101353751	-1.0919468	0.274856485	0	0.051775537	0	1	PRJNA827465;Yang2025_Silkie_Wenchang	CRA004775	0.774719194
ENSGALG00010007188	MAB21L1	2	-0.092623214	0.173239576	-0.534653894	0.592889209	0	0.508625344	0	0.5	CRA004775;Yang2025_Silkie_Wenchang	PRJNA827465	0.892776493
ENSGALG00010007188	MAB21L1	2	-0.07551575	0.11557932	-0.653367311	0.513519508	0	0.404861154	0	0.5	CRA004775;PRJNA827465	Yang2025_Silkie_Wenchang	0.866402971
ENSGALG00010007190	ENSGALG00010007190	2	0.027997805	0.248539017	0.112649536	0.910308411	0.085887368	3.135132175	68.10341816	0.5	PRJNA827465;Yang2025_Silkie_Wenchang	CRA004775	0.97880407
ENSGALG00010007190	ENSGALG00010007190	2	0.258290579	0.222533169	1.160683508	0.245770631	0	0.368743908	0	0.5	CRA004775;Yang2025_Silkie_Wenchang	PRJNA827465	0.75661406
ENSGALG00010007190	ENSGALG00010007190	2	-0.179513169	0.144310854	-1.243933936	0.213523878	0	0.043018497	0	1	CRA004775;PRJNA827465	Yang2025_Silkie_Wenchang	0.726080554
ENSGALG00010007201	ENSGALG00010007201	2	-0.234436067	0.326161875	-0.718772134	0.47228133	0.062137894	1.411492915	29.15302732	0.5	PRJNA827465;Yang2025_Silkie_Wenchang	CRA004775	0.853998373
ENSGALG00010007201	ENSGALG00010007201	2	0.043079069	0.346560442	0.124304634	0.901074078	0	0.098616109	0	0.5	CRA004775;Yang2025_Silkie_Wenchang	PRJNA827465	0.976585994
ENSGALG00010007201	ENSGALG00010007201	2	-0.455686903	0.331150277	-1.376072842	0.168799083	0	0.258370271	0	1	CRA004775;PRJNA827465	Yang2025_Silkie_Wenchang	0.669861707
ENSGALG00010007203	SLC30A6	2	-0.009336198	0.140584511	-0.066409861	0.94705152	0.032502221	5.454231682	81.66561198	0.5	PRJNA827465;Yang2025_Silkie_Wenchang	CRA004775	0.986445196
ENSGALG00010007203	SLC30A6	2	-0.246220674	0.132198479	-1.862507618	0.062531573	0.013979957	1.48894243	32.83823604	1	CRA004775;Yang2025_Silkie_Wenchang	PRJNA827465	0.431065134
ENSGALG00010007203	SLC30A6	2	-0.129772688	0.284554479	-0.456055686	0.648349935	0.139388764	6.507862066	84.63397058	0.5	CRA004775;PRJNA827465	Yang2025_Silkie_Wenchang	0.90762535
ENSGALG00010007204	LANCL2	2	-0.015483583	0.04933449	-0.313849053	0.753635687	0	0.295707967	0	1	PRJNA827465;Yang2025_Silkie_Wenchang	CRA004775	0.938110356
ENSGALG00010007204	LANCL2	2	-0.200071285	0.136696445	-1.463617318	0.143298538	0	0.914637481	0	1	CRA004775;Yang2025_Silkie_Wenchang	PRJNA827465	0.630352362
ENSGALG00010007204	LANCL2	2	-0.124295931	0.172130206	-0.722104122	0.470230464	0.040650195	2.456749926	59.29581641	1	CRA004775;PRJNA827465	Yang2025_Silkie_Wenchang	0.853726346
ENSGALG00010007207	EPM2A	2	-0.052649789	0.065835523	-0.799717037	0.42387476	0	0.092713213	0	1	PRJNA827465;Yang2025_Silkie_Wenchang	CRA004775	0.84030043
ENSGALG00010007207	EPM2A	2	-0.063569599	0.095169399	-0.667962601	0.504157466	0	0.250849751	0	0.5	CRA004775;Yang2025_Silkie_Wenchang	PRJNA827465	0.863469622
ENSGALG00010007207	EPM2A	2	-0.026812919	0.087175098	-0.307575439	0.758405411	0	0.16766416	0	0.5	CRA004775;PRJNA827465	Yang2025_Silkie_Wenchang	0.938954369
ENSGALG00010007214	MTHFSD	2	0.301056492	0.111431164	2.701726175	0.006898055	0.011923103	1.749962454	42.85591685	1	PRJNA827465;Yang2025_Silkie_Wenchang	CRA004775	0.127631755
ENSGALG00010007214	MTHFSD	2	0.466334449	0.134455074	3.468329127	0.000523705	0	0.010873268	0	1	CRA004775;Yang2025_Silkie_Wenchang	PRJNA827465	0.024184895
ENSGALG00010007214	MTHFSD	2	0.246939032	0.078373483	3.15079824	0.001628249	0	0.992033912	0	1	CRA004775;PRJNA827465	Yang2025_Silkie_Wenchang	0.050992096
ENSGALG00010007217	MSH3	2	0.150367385	0.112884838	1.332042349	0.182846273	0	0.363483338	0	1	PRJNA827465;Yang2025_Silkie_Wenchang	CRA004775	0.691266551
ENSGALG00010007217	MSH3	2	0.163385856	0.141907367	1.151355702	0.24958593	0	0.591670081	0	0.5	CRA004775;Yang2025_Silkie_Wenchang	PRJNA827465	0.759622031
ENSGALG00010007217	MSH3	2	0.053070873	0.143980004	0.368598913	0.712426706	0	0.150003552	0	0.5	CRA004775;PRJNA827465	Yang2025_Silkie_Wenchang	0.92597752
ENSGALG00010007223	FOXF1	2	0.142616904	0.286728167	0.49739412	0.618911151	0	0.862232718	0	0.5	PRJNA827465;Yang2025_Silkie_Wenchang	CRA004775	0.899168321
ENSGALG00010007223	FOXF1	2	1.112020109	0.736560635	1.509746864	0.131108028	0.868626111	4.965591035	79.8614104	1	CRA004775;Yang2025_Silkie_Wenchang	PRJNA827465	0.606767161
ENSGALG00010007223	FOXF1	2	0.854784904	1.003334573	0.851944034	0.394245155	1.796985279	9.248531074	89.18747213	0.5	CRA004775;PRJNA827465	Yang2025_Silkie_Wenchang	0.830486298
ENSGALG00010007224	DHFR	2	0.213554525	0.157454218	1.356295996	0.175005009	0	0.130653194	0	1	PRJNA827465;Yang2025_Silkie_Wenchang	CRA004775	0.679934941

ENSGALG00010007224	DHFR	2	0.11168831	0.279377317	0.399775868	0.689321607	0.05130058	1.331316241	24.88636663	0.5	CRA004775;Yang2025_Silkie_Wenchang	PRJNA827465	0.91986176
ENSGALG00010007224	DHFR	2	0.079601094	0.198158015	0.401705142	0.68790104	0	0.92074728	0	0.5	CRA004775;PRJNA827465	Yang2025_Silkie_Wenchang	0.919631238
ENSGALG00010007226	ENSGALG00010007226	2	-0.463347709	2.070403207	-0.22379588	0.822916136	7.250707736	6.482327555	84.57344231	0.5	PRJNA827465;Yang2025_Silkie_Wenchang	CRA004775	0.955822987
ENSGALG00010007226	ENSGALG00010007226	2	-0.251783174	2.169105006	-0.116076987	0.907591527	8.593366563	11.17712089	91.05315215	0.5	CRA004775;Yang2025_Silkie_Wenchang	PRJNA827465	0.978196186
ENSGALG00010007226	ENSGALG00010007226	2	1.760978433	0.514408146	3.423309771	0.000618635	0	0.025591851	0	1	CRA004775;PRJNA827465	Yang2025_Silkie_Wenchang	0.027106159
ENSGALG00010007230	ENSGALG00010007230	2	0.227671176	0.283555175	0.802916667	0.422022902	0.065236852	1.646765292	39.27489211	0.5	PRJNA827465;Yang2025_Silkie_Wenchang	CRA004775	0.839649223
ENSGALG00010007230	ENSGALG00010007230	2	-0.279249428	0.225090232	-1.240611049	0.214749468	0	0.350370452	0	1	CRA004775;Yang2025_Silkie_Wenchang	PRJNA827465	0.727862915
ENSGALG00010007230	ENSGALG00010007230	2	0.046339832	0.424830835	0.109078317	0.913140373	0.28404781	4.688555127	78.67146759	0.5	CRA004775;PRJNA827465	Yang2025_Silkie_Wenchang	0.979129568
ENSGALG00010007243	BEAN1	2	0.190889391	0.360920319	0.528896215	0.596877448	0.233054799	9.196580127	89.12639279	0.5	PRJNA827465;Yang2025_Silkie_Wenchang	CRA004775	0.894172266
ENSGALG00010007243	BEAN1	2	0.156820043	0.48776738	0.321505804	0.747827115	0.368000652	4.08174091	75.50064979	0.5	CRA004775;Yang2025_Silkie_Wenchang	PRJNA827465	0.936938957
ENSGALG00010007243	BEAN1	2	-0.164821086	0.108704651	-1.516228468	0.129461592	0	0.336912939	0	1	CRA004775;PRJNA827465	Yang2025_Silkie_Wenchang	0.603529077
ENSGALG00010007251	SPAST	2	-0.2571699	0.248136818	-1.036403635	0.300013875	0.119662263	35.35708274	97.17171236	1	PRJNA827465;Yang2025_Silkie_Wenchang	CRA004775	0.790152472
ENSGALG00010007251	SPAST	2	-0.189977329	0.323765067	-0.586775253	0.557354659	0.200887628	23.62698129	95.76755072	0.5	CRA004775;Yang2025_Silkie_Wenchang	PRJNA827465	0.880678789
ENSGALG00010007251	SPAST	2	0.030221578	0.066812386	0.452334959	0.651027695	0.003003664	1.354925544	26.19520648	0.5	CRA004775;PRJNA827465	Yang2025_Silkie_Wenchang	0.908769703
ENSGALG00010007252	NFATC1	2	0.137807733	0.235452228	0.585289571	0.558353026	0.080218784	3.407043944	70.64904309	0.5	PRJNA827465;Yang2025_Silkie_Wenchang	CRA004775	0.880678789
ENSGALG00010007252	NFATC1	2	0.38540658	0.19871581	1.939486248	0.052442157	0	0.065804776	0	1	CRA004775;Yang2025_Silkie_Wenchang	PRJNA827465	0.39754556
ENSGALG00010007252	NFATC1	2	-0.034898783	0.120869918	-0.2887301	0.772787927	0	0.645322959	0	0.5	CRA004775;PRJNA827465	Yang2025_Silkie_Wenchang	0.943066211
ENSGALG00010007259	ANKRD34B	2	0.087519781	0.101872235	0.859112176	0.390273631	0	0.177014085	0	1	PRJNA827465;Yang2025_Silkie_Wenchang	CRA004775	0.82923632
ENSGALG00010007259	ANKRD34B	2	0.145922014	0.131657727	1.108343709	0.267713405	0.004863959	1.155716669	13.47360239	1	CRA004775;Yang2025_Silkie_Wenchang	PRJNA827465	0.771398294
ENSGALG00010007259	ANKRD34B	2	0.183544513	0.111253583	1.649785184	0.09898688	0	0.58541057	0	1	CRA004775;PRJNA827465	Yang2025_Silkie_Wenchang	0.536907794
ENSGALG00010007265	CD200R1A	2	-0.426498583	0.298475307	-1.428924181	0.153026027	0.123297476	3.208196437	68.82983883	1	PRJNA827465;Yang2025_Silkie_Wenchang	CRA004775	0.645187993
ENSGALG00010007265	CD200R1A	2	-0.74094419	0.229735987	-3.225198624	0.001258853	0	0.002799107	0	1	CRA004775;Yang2025_Silkie_Wenchang	PRJNA827465	0.04309817
ENSGALG00010007265	CD200R1A	2	-0.27401549	0.235745511	-1.162335984	0.245099015	0.028444645	1.212387475	17.51811855	1	CRA004775;PRJNA827465	Yang2025_Silkie_Wenchang	0.75648866
ENSGALG00010007270	CDH5	2	-0.259946948	0.241496746	-1.076399383	0.281748678	0.10947308	16.12906098	93.80001104	1	PRJNA827465;Yang2025_Silkie_Wenchang	CRA004775	0.779295548
ENSGALG00010007270	CDH5	2	-0.058261575	0.512226634	-0.113745791	0.909442483	0.453992812	6.682106322	85.03465896	0.5	CRA004775;Yang2025_Silkie_Wenchang	PRJNA827465	0.978607662
ENSGALG00010007270	CDH5	2	0.118838702	0.241328286	0.49243586	0.622411254	0.074272873	1.963476643	49.06993147	0.5	CRA004775;PRJNA827465	Yang2025_Silkie_Wenchang	0.900093208
ENSGALG00010007275	DPY30	2	0.000217467	0.112752918	0.001928707	0.998461116	0.013276386	1.772990655	43.59812346	0.5	PRJNA827465;Yang2025_Silkie_Wenchang	CRA004775	0.999554692
ENSGALG00010007275	DPY30	2	0.028443064	0.172659832	0.164734691	0.86915281	0.021375886	1.533861552	34.80506771	0.5	CRA004775;Yang2025_Silkie_Wenchang	PRJNA827465	0.968461055
ENSGALG00010007275	DPY30	2	-0.082052094	0.066239541	-1.238717731	0.21545905	0	0.194276876	0	1	CRA004775;PRJNA827465	Yang2025_Silkie_Wenchang	0.728854649
ENSGALG00010007277	FAM151B	2	0.568480571	0.247155633	2.300091503	0.021443037	0	0.521188645	0	1	PRJNA827465;Yang2025_Silkie_Wenchang	CRA004775	0.247729473
ENSGALG00010007277	FAM151B	2	-0.168583407	0.906579384	-0.185955483	0.852479672	1.502554984	11.57145647	91.35804553	0.5	CRA004775;Yang2025_Silkie_Wenchang	PRJNA827465	0.962607579
ENSGALG00010007277	FAM151B	2	-0.360597201	0.725275208	-0.497186719	0.619057386	0.887095981	6.370214775	84.30194216	0.5	CRA004775;PRJNA827465	Yang2025_Silkie_Wenchang	0.909168321
ENSGALG00010007283	STOX2	2	0.031942461	0.143849918	0.222054075	0.824271788	0.023173942	1.970154873	49.24256902	0.5	PRJNA827465;Yang2025_Silkie_Wenchang	CRA004775	0.95619211
ENSGALG00010007283	STOX2	2	0.526566458	0.336835646	1.563274148	0.117988126	0.156149073	3.044083827	67.14939348	1	CRA004775;Yang2025_Silkie_Wenchang	PRJNA827465	0.577760513
ENSGALG00010007283	STOX2	2	0.373094529	0.491519917	0.75906289	0.447814937	0.42877485	8.140512773	87.71576155	0.5	CRA004775;PRJNA827465	Yang2025_Silkie_Wenchang	0.846601815
ENSGALG00010007284	ENSGALG00010007284	2	0.189853768	0.112472505	1.687996364	0.091411927	0.009422588	1.589917385	37.10365019	1	PRJNA827465;Yang2025_Silkie_Wenchang	CRA004775	0.718828636
ENSGALG00010007284	ENSGALG00010007284	2	0.254666477	0.116564219	2.184774016	0.02890542	0	0.761396146	0	1	CRA004775;Yang2025_Silkie_Wenchang	PRJNA827465	0.289667055
ENSGALG00010007284	ENSGALG00010007284	2	0.08053832	0.10790007	0.746415833	0.455416258	0	0.00447363	0	1	CRA004775;PRJNA827465	Yang2025_Silkie_Wenchang	0.84907427
ENSGALG00010007287	ENSGALG00010007287	2	0.552103467	0.919110233	0.600693418	0.548044204	1.306708081	4.173981336	76.04205866	0.5	PRJNA827465;Yang2025_Silkie_Wenchang	CRA004775	0.878281266
ENSGALG00010007287	ENSGALG00010007287	2	0.158573127	1.43024033	0.110941584	0.91166267	3.486230148	6.760894157	85.20905702	0.5	CRA004775;Yang2025_Silkie_Wenchang	PRJNA827465	0.978895589
ENSGALG00010007287	ENSGALG00010007287	2	-0.565843754	0.465216304	-1.2163025	0.223869709	0.124311294	1.325079053	24.53280447	1	CRA004775;PRJNA827465	Yang2025_Silkie_Wenchang	0.737452676
ENSGALG00010007294	TRAPPC11	2	-0.074340559	0.248367418	-0.299316873	0.764698282	0.116817106	18.3949974	94.56373938	0.5	PRJNA827465;Yang2025_Silkie_Wenchang	CRA004775	0.940711384
ENSGALG00010007294	TRAPPC11	2	-0.191461022	0.167287401	-1.144503535	0.252414873	0.036522945	2.723561866	63.28337516	0.5	CRA004775;Yang2025_Silkie_Wenchang	PRJNA827465	0.762655223
ENSGALG00010007294	TRAPPC11	2	0.154579186	0.0460103	3.359664834	0.000780371	0	0.738902161	0	1	CRA004775;PRJNA827465	Yang2025_Silkie_Wenchang	0.03184934
ENSGALG00010007300	CCDC125	2	0.422327521	0.451445861	0.935499819	0.349530776	0.297506471	3.700863786	72.97928111	0.5	PRJNA827465;Yang2025_Silkie_Wenchang	CRA004775	0.809903518
ENSGALG00010007300	CCDC125	2	-0.021035085	0.997778218	-0.021081924	0.983180304	1.690693886	6.255315114	84.01359513	0.5	CRA004775;Yang2025_Silkie_Wenchang	PRJNA827465	0.995363468
ENSGALG00010007300	CCDC125	2	-0.383905091	0.517217924	-0.742250168	0.457935778	0.291469755	1.919163052	47.8939531	1	CRA004775;PRJNA827465	Yang2025_Silkie_Wenchang	0.845955976
ENSGALG00010007302	CDH11	2	-0.006403432	0.130353392	-0.049123632	0.960820771	0.019313161	2.236952954	55.29633298	0.5	PRJNA827465;Yang2025_Silkie_Wenchang	CRA004775	0.98952228
ENSGALG00010007302	CDH11	2	0.133917135	0.135625054	0.987407055	0.323443127	0	0.061978498	0	1	CRA004775;Yang2025_Silkie_Wenchang	PRJNA827465	0.799456433
ENSGALG00010007302	CDH11	2	-0.105314743	0.095875309	-1.098455312	0.272005721	0	0.194411786	0	0.5	CRA004775;PRJNA827465	Yang2025_Silkie_Wenchang	0.773184343
ENSGALG00010007304	F5	2	0.530967334	0.357116098	1.486819937	0.137062374	0.151883557	2.236483852	55.2869564	1	PRJNA827465;Yang2025_Silkie_Wenchang	CRA004775	0.619333468
ENSGALG00010007304	F5	2	0.520064232	0.301513719	1.724844341	0.084555528	0.10946095	2.43541158	58.93917856	1	CRA004775;Yang2025_Silkie_Wenchang	PRJNA827465	0.500108811
ENSGALG00010007304	F5	2	0.914332477	0.260048821	3.516003168	0.000438096	0	0.057990975	0	1	CRA004775;PRJNA827465	Yang2025_Silkie_Wenchang	0.021283656
ENSGALG00010007318	RMDN2	2	0.158123033	0.165288294	0.956649923	0.338741982	0.036919898	2.984865905	66.48866758	1	PRJNA827465;Yang2025_Silkie_Wenchang	CRA004775	0.804450436
ENSGALG00010007318	RMDN2	2	0.347420028	0.141745019	2.451021069	0.014245162	0	0.00046478	0	1	CRA004775;Yang2025_Silkie_Wenchang	PRJNA827465	0.195761393
ENSGALG00010007318	RMDN2	2	0.063425965	0.121409722	0.522412574	0.601383103	0.005739663	1.109765028	9.890834996	1	CRA004775;PRJNA827465	Yang2025_Silkie_Wenchang	0.895230519
ENSGALG00010007319	ATP9B	2	-0.013966144	0.057441885	-0.243135187	0.807900673	0	0.040333339	0	1	PRJNA827465;Yang2025_Silkie_Wenchang	CRA004775	0.952496413
ENSGALG00010007319	ATP9B	2	0.051816399	0.135867414	0.381374731	0.702925308	0.016345388	1.515238774	34.00308076	0.5	CRA004775;Yang2025_Silkie_Wenchang	PRJNA827465	0.923347492
ENSGALG00010007319	ATP9B	2	0.051884944	0.11413086	0.454609245	0.649390383	0.010879802	1.361225543	26.53678849	0.5	CRA004775;PRJNA827465	Yang2025_Silkie_Wenchang	0.908043243
ENSGALG00010007320	ENSGALG00010007320	2	-0.429752355	0.107389233	-4.001819752	6.28572E-05	0	0.575374075	0	1	PRJNA827465;Yang2025_Silkie_Wenchang	CRA004775	0.005666228
ENSGALG00010007320	ENSGALG00010007320	2	-0.380397765	0.195323105	-1.93756922	0.054305014	0	0.054779964	0	1	CRA004775;Yang2025_Silkie_Wenchang	PRJNA827465	0.550247672
ENSGALG00010007320	ENSGALG00010007320	2	-0.470189179	0.118712589	-3.960735624	7.47192E-05	0	0.027840191	0	1	CRA004775;PRJNA827465	Yang2025_Silkie_Wenchang	0.006315462
ENSGALG00010007321	CD200	2	0.032029665	0.091981916	0.348216983	0.727677235	0	0.767708521	0	0.5	PRJNA827465;Yang2025_Silkie_Wenchang	CRA004775	0.931639802
ENSGALG00010007321	CD200	2	-0.053220281	0.115630222	-0.460262728	0.645327651	0	0.365032866	0	1	CRA004775;Yang2025_Silkie_Wenchang	PRJNA827465	0.906966303
ENSGALG00010007321	CD200	2	0.088338397	0.137299214	0.643400603	0.519964193	0	0.881635418	0	0.5	CRA004775;PRJNA827465	Yang2025_Silkie_Wenchang	0.869253622
ENSGALG00010007322	AK6	2	0.118928617	0.151031681	0.787441526	0.431023446	0	0.418309952	0	1	PRJNA827465;Yang2025_Silkie_Wenchang	CRA004775	0.842503929
ENSGALG00010007322	AK6	2	0.059724212	0.19147285	0.311920004	0.75510132	0.014426382	1.237995557	19.22426587	0.5	CRA004775;Yang2025_Silkie_Wenchang	PRJNA827465	0.93838723
ENSGALG00010007322	AK6	2	-0.042870972	0.16344661	-0.262293428	0.793095228	0	0.320428655	0	0.5	CRA004775;PRJNA827465	Yang2025_Silkie_Wenchang	0.948571413
ENSGALG00010007324	ENSGALG00010007324	2	-0.032402911	0.131189143	-0.246993845	0.804912988	0	0.777883171	0	0.5	PRJNA827465;Yang2025_Silkie_Wenchang	CRA004775	0.95184424
ENSGALG00010007324	ENSGALG00010007324	2	-0.010191398	0.242279568	-0.042064622	0.966447183	0.042861424	1.428880082	30.01512077	0.5	CRA004775;Yang2025_Silkie_Wenchang	PRJNA827465	0.990881287
ENSGALG00010007324	ENSGALG00010007324	2	0.116761144	0.159788661	0.730722337	0.464948769	0	0.47525114	0	1	CRA004775;PRJNA827465	Yang2025_Silkie_Wenchang	0.852233226
ENSGALG00010007326	RWDD4	2	0.131221969	0.213239758	0.615372905	0.538308481	0.075456687	5.370786022	81.38075142	0.5	PRJNA827465;Yang2025_Silkie_Wenchang	CRA004775	0.87481821
ENSGALG00010007326	RWDD4	2	0.35906137	0.148069562	2.424950583	0.015310471	0	0.03655827	0	1	CRA004775;Yang2025_Silkie_Wenchang	PRJNA827465	0.204675996
ENSGALG00010007326	RWDD4	2	0.025306248	0.151234719	0.167330941	0.86710966	0.024781509	1.582305629	36.80108432	0.5	CRA004775;PRJNA827465	Yang2025_Silkie_Wenchang	0.967699
ENSGALG00010007328	NOVA1	2	0.034063709	0.103885118	0.32789787	0.742988884	0.003415223	1.159490742	13.75524063	0.5	PRJNA827465;Yang2025_Silkie_Wenchang	CRA004775	0.935735651
ENSGALG00010007328	NOVA1	2	0.150510798	0.159971384	0.940860761	0.346776218	0	0.258975226	0	0.5	CRA004775;Yang2025_Silkie_Wenchang	PRJNA827465	0.80837881
ENSGALG00010007328	NOVA1	2	-0.037110457	0.107479656	-0.345278894	0.729884712	0	1.064E-06	0	1	CRA004775;PRJNA827465	Yang2025_Silkie_Wenchang	0.932444623
ENSGALG00010007330	MMP13	2	-0.231257369	0.515877339	-0.448279758	0.653951308	0	0.092020975	0	1	PRJNA827465;Yang2025_Silkie_Wenchang	CRA004775	0.909861322
ENSGALG00010007330	MMP13	2	-0.464680594	0.549262734	-0.846007867	0.397548356	0	0.024435562	0	1	CRA004775;Yang2025_Silkie_Wenchang	PRJNA827465	0.831683772
ENSGALG00010007330	MMP13	2	-0.286801422	0.546890489	-0.524422032	0.599985036	0	0.194018898	0	1	CRA004775;PRJNA827465	Yang2025_Silkie_Wenchang	0.895092498
ENSGALG00010007333	QTRT2	2	0.131712963	0.053568213	2.458789567	0.01394063	0	0.457693571	0	1	PRJNA827465;Yang2025_Silkie_Wenchang	CRA004775	0.193650332
ENSGALG00010007333	QTRT2	2	-0.142522079	0.375956159	-0.379092285	0.704619335	0.245277685	7.13183219	85.97835769	0.5	CRA004775;Yang2025_Silkie_Wenchang	PRJNA827465	0.924152671
ENSGALG00010007333	QTRT2	2	-0.174484837	0.330827038	-0.527420122	0.597901873	0.187958195	6.302559376	84.13342992	0.5	CRA004775;PRJNA827465	Yang2025_Silkie_Wenchang	0.894645663
ENSGALG00010007337	PRORP	2	0.007914246	0.150201636	0.052690813	0.957978259	0.036700217	5.238085641	80.90905593	0.5	PRJNA827465;Yang2025_Silkie_Wenchang	CRA004775	0.988864712
ENSGALG00010007337	PRORP	2	-0.207865342	0.102009062	-2.037714477	0.041578496	0.001398773	1.048447659	4.62089436	1	CRA004775;Yang2025_Silkie_Wenchang	PRJNA827465	0.353355943
ENSGALG00010007337	PRORP	2	-0.089103564	0.271119963	-0.328649957	0.742420283	0.124159534	5.853619514	82.91655278	0.5	CRA004775;PRJNA827465	Yang2025_Silkie_Wenchang	0.935510459
ENSGALG00010007339	ENSGALG00010007339	2	-0.013936771	0.150683941	-0.092490087	0.926308667	0.005984314	1.148053273	12.89602812	0.5	PRJNA827465;Yang2025_Silkie_Wenchang	CRA004775	0.98280842
ENSGALG00010007339	ENSGALG00010007339	2	-0.18803209	0.193827195	-0.970101692	0.331995806	0	2.69559E-05	0	1	CRA004775;Yang2025_Silkie_Wenchang	PRJNA827465	0.802002658
ENSGALG00010007339	ENSGALG00010007339	2	0.069409329	0.168624764	0.411620022	0.680617954	0	0.419499836	0	0.5	CRA004775;PRJNA827465	Yang2025_Silkie_Wenchang	0.917851751
ENSGALG00010007344	RAD17	2	0.062807474	0.077008602	0.815590369	0.414734459	0	0.019821201	0	1	PRJNA827465;Yang2025_Silkie_Wenchang	CRA004775	0.837298284
ENSGALG00010007344	RAD17	2	0.072832257	0.102923127	0.707637429	0.479170443	0	0.0032429	0	1	CRA004775;Yang2025_Silkie_Wenchang	PRJNA827465	0.856035423
ENSGALG00010007344	RAD17	2	0.057000529	0.084488933	0.674650824	0.499897635	0	0.002635573	0	1	CRA004775;PRJNA827465	Yang2025_Silkie_Wenchang	0.862078151
ENSGALG00010007353	ING2	2	-0.259163268	0.092686721	-2.796120775	0.005172007	0	0.69167902	0	1	PRJNA827465;Yang2025_Silkie_Wenchang	CRA004775	0.107382666
ENSGALG00010007353	ING2	2	-0.300403044	0.144537065	-2.078380685	0.03767431	0	0.688811907	0	1	CRA004775;Yang2025_Silkie_Wenchang	PRJNA827465	0.33492726
ENSGALG00010007353	ING2	2	-0.198146909	0.100267454	-1.976183703	0.048133975	0	0.09329512	0	1	CRA004775;PRJNA827465	Yang2025_Silkie_Wenchang	0.379424312
ENSGALG00010007357	NOX4	2	0.078696384	0.098054402	0.802578794	0.422218229	0	0.036274906	0	1	PRJNA827465;Yang2025_Silkie_Wenchang	CRA004775	0.839649223
ENSGALG00010007357	NOX4	2	0.038403938	0.148290212	0.258978236	0.795652033	0	0.160470592	0	0.5	CRA004775;Yang2025_Silkie_Wenchang	PRJNA827465	0.94908992
ENSGALG00010007357	NOX4	2	0.079799111	0.12326449	0.647381185	0.51738527	0	0.227124887	0	0.5	CRA004775;PRJNA827465	Yang2025_Silkie_Wenchang	0.867952013
ENSGALG00010007360	ROBO1	2	0.075999786	0.094276328	0.806138583	0.420162947	0	0.015143675	0	1	PRJNA827465;Yang2025_Silkie_Wenchang	CRA004775	0.838662794
ENSGALG00010007360	ROBO1	2	-0.033105993	0.225878814	-0.146565286	0.883475156	0.027756034	1.149306239	12.99098833	0.5	CRA004775;Yang2025_Silkie_Wenchang	PRJNA827465	0.972001076
ENSGALG00010007360	ROBO1	2	-0.015132335	0.239126183	-0.063281799	0.949542109	0.0544804	1.3089247	23.60141115	0.5	CRA004775;PRJNA827465	Yang2025_Silkie_Wenchang	0.98723759
ENSGALG00010007365	MEMO1	2	-0.056545785	0.061332444	-0.921955512	0.356551782	0	0.996334914	0	1	PRJNA827465;Yang2025_Silkie_Wenchang	CRA004775	0.812615522
ENSGALG00010007365	MEMO1	2	-0.10089225	0.153344451	-0.657945232	0.510573324	0.011261743	1.168568793	14.42523486	0.5	CRA004775;Yang2025_Silkie_Wenchang	PRJNA827465	0.865369933
ENSGALG00010007365	MEMO1	2	-0.014866362	0.067794421	-0.219285915	0.826427333	0	0.500326715	0	0.5	CRA004775;PRJNA827465	Yang2025_Silkie_Wenchang	0.956393839
ENSGALG00010007369	ENSGALG00010007369	2	0.234737787	0.2075991	1.130726421	0.258170257	0	0.016416182	0	1	PRJNA827465;Yang2025_Silkie_Wenchang	CRA004775	0.765454558
ENSGALG00010007369	ENSGALG00010007369	2	0.067799427	0.240048187	0.282440903	0.777605455	0	0.798238842	0	0.5	CRA004775;Yang2025_Silkie_Wenchang	PRJNA827465	0.944388838
ENSGALG00010007369	ENSGALG00010007369	2	0.075153545	0.213468504	0.352059175	0.724793885	0	0.719751335	0	0.5	CRA004775;PRJNA827465	Yang2025_Silkie_Wenchang	0.930538466
ENSGALG00010007374	CMIP	2	-0.033402821	0.085531585	-0.390532001	0.696143196	0	0.967250506	0	0.5	PRJNA827465;Yang2025_Silkie_Wenchang	CRA004775	0.921038437
ENSGALG00010007374	CMIP	2	0.052295841	0.151279	0.345691342	0.729574692	0	0.590472756	0	0.5	CRA004775;Yang2025_Silkie_Wenchang	PRJNA827465	0.9323457
ENSGALG00010007374	CMIP	2	-0.090744408	0.095333714	-0.951860617	0.341167675	0	0.080231048	0	1	CRA004775;PRJNA827465	Yang2025_Silkie_Wenchang	0.805646109
ENSGALG00010007375	CHORDC1	2	0.078246674	0.107053197	0.730913941	0.46483172	0.015018562	2.480460146	59.68489952	0.5	PRJNA827465;Yang2025_Silkie_Wenchang	CRA004775	0.85217126
ENSGALG00010007375	CHORDC1	2	7.77609E-05	0.119167699	0.000652533	0.999479354	0.001098712	1.029282135	2.844908481	0.5	CRA004775;Yang2025_Silkie_Wenchang	PRJNA827465	0.999889665
ENSGALG00010007375	CHORDC1	2	0.159281246	0.050174334	3.174556266	0.001500658	0	0.047843423	0	1	CRA004775;PRJNA827465	Yang2025_Silkie_Wenchang	0.048147232
ENSGALG00010007376	CDKN2AIP	2	-0.108507486	0.049145901	-2.207864393	0.027253728	0.000567867	1.087878844	8.077999212	1	PRJNA827465;Yang2025_Silkie_Wenchang	CRA004775	0.281669893
ENSGALG00010007376	CDKN2AIP	2	-0.062035316	0.16647726	-0.372635376	0.70941984	0.036619265	2.773732144	63.94749211	0.5	CRA004775;Yang2025_Silkie_Wenchang	PRJNA827465	0.925298042
ENSGALG00010007376	CDKN2AIP	2	-0.036970992	0.089001262	-0.415398517	0.677850192	0.00745322	1.445941584	30.84091289	0.5	CRA004775;PRJNA827465	Yang2025_Silkie_Wenchang	0.917408612
ENSGALG00010007381	EGFR	2	-0.129579069	0.060417536	-2.144726152	0.031974749	0	0.056562143	0	1	PRJNA827465;Yang2025_Silkie_Wenchang	CRA004775	0.306656545
ENSGALG00010007381	EGFR	2	-0.043812926	0.181800682	-0.24099429	0.809559542	0.024045148	1.439878083	30.54967559	0.5	CRA004775;Yang2025_Silkie_Wenchang	PRJNA827465	0.952770549
ENSGALG00010007381	EGFR	2	-0.059207695	0.137143776	-0.431719884	0.665945014	0.018591434	1.410884548	29.12247839	0.5	CRA004775;PRJNA827465	Yang2025_Silkie_Wenchang	0.913546323
ENSGALG00010007382	EIF2AK2	2	-0.060434988	0.076111749	-0.79402968	0.427178153	0	0.15419475	0	1	PRJNA827465;Yang2025_Silkie_Wenchang	CRA004775	0.84125685
ENSGALG00010007382	EIF2AK2	2	-0.151307935	0.119145743	-1.269939925	0.20410603	0	0.820002664	0	1	CRA004775;Yang2025_Silkie_Wenchang	PRJNA827465	0.717254253
ENSGALG00010007382	EIF2AK2	2	-0.107821635	0.138931032	-0.776080283	0.437701584	0.015275547	1.333520459	25.01052435	1	CRA004775;PRJNA827465	Yang2025_Silkie_Wenchang	0.843889118
ENSGALG00010007387	ENSGALG00010007387	2	0.235887498	0.309357481	0.762507818	0.445756979	0.007491437	1.037789762	3.641369688	0.5	PRJNA827465;Yang2025_Silkie_Wenchang	CRA004775	0.846013379
ENSGALG00010007387	ENSGALG00010007387	2	-0.626379471	0.646626404	-0.968688361	0.332700701	0.374354162	1.660371289	39.77250711	1	CRA004775;Yang2025_Silkie_Wenchang	PRJNA827465	0.802178578
ENSGALG00010007387	ENSGALG00010007387	2	-0.351679888	0.989955461	-0.355248192	0.722403664	1.514472718	3.954532413	74.71256028	0.5	CRA004775;PRJNA827465	Yang2025_Silkie_Wenchang	0.929656239
ENSGALG00010007388	CYP1B1	2	0.293803366	0.156803837	1.873700105	0.060971772	0.028832989	2.377335743	57.93610545	1	PRJNA827465;Yang2025_Silkie_Wenchang	CRA004775	0.427185264
ENSGALG00010007388	CYP1B1	2	0.60719159	0.291604262	2.082245256	0.037320073	0.099403378	1.817617977	44.98293851	1	CRA004775;Yang2025_Silkie_Wenchang	PRJNA827465	0.333247689
ENSGALG00010007388	CYP1B1	2	0.50633298	0.479272439	1.05646171	0.290757325	0.353480282	3.78320765	73.56740384	1	CRA004775;PRJNA827465	Yang2025_Silkie_Wenchang	0.784646441
ENSGALG00010007391	NANP	2	-0.129071841	0.130117108	-0.991966718	0.321213761	0.027702582	5.241112207	80.92008031	1	PRJNA827465;Yang2025_Silkie_Wenchang	CRA004775	0.798513866
ENSGALG00010007391	NANP	2	-0.233614754	0.089967593	-2.596654481	0.009413656	0	0.792759005	0	1	CRA004775;Yang2025_Silkie_Wenchang	PRJNA827465	0.152724616
ENSGALG00010007391	NANP	2	-0.016051023	0.052490679	-0.30578807	0.759766012	0	0.085711519	0	1	CRA004775;PRJNA827465	Yang2025_Silkie_Wenchang	0.939165042
ENSGALG00010007392	NAALAD2	2	-0.052922728	0.105435601	-0.501943634	0.615707171	0	0.098872378	0	1	PRJNA827465;Yang2025_Silkie_Wenchang	CRA004775	0.898927895
ENSGALG00010007392	NAALAD2	2	-0.056238554	0.151495912	-0.371221591	0.710472493	0	0.412067339	0	0.5	CRA004775;Yang2025_Silkie_Wenchang	PRJNA827465	0.925404854
ENSGALG00010007392	NAALAD2	2	-0.003091619	0.133223865	-0.023206194	0.981485798	0	0.258602699	0	0.5	CRA004775;PRJNA827465	Yang2025_Silkie_Wenchang	0.995004677
ENSGALG00010007393	PLCG2	2	0.217781645	0.098197702	2.217787599	0.026569318	0	0.136239535	0	1	PRJNA827465;Yang2025_Silkie_Wenchang	CRA004775	0.278170993
ENSGALG00010007393	PLCG2	2	0.266422946	0.161956337	1.645029464	0.099963735	0	0.001295878	0	1	CRA004775;Yang2025_Silkie_Wenchang	PRJNA827465	0.540125747
ENSGALG00010007393	PLCG2	2	0.193584934	0.118850341	1.628812608	0.103352695	0	0.00712929	0	1	CRA004775;PRJNA827465	Yang2025_Silkie_Wenchang	0.547576788
ENSGALG00010007399	WWC2	2	0.321364369	0.236099284	1.361140799	0.173469199	0.093640819	6.142029289	83.71873606	1	PRJNA827465;Yang2025_Silkie_Wenchang	CRA004775	0.676912223
ENSGALG00010007399	WWC2	2	0.560407007	0.144133162	3.888119848	0.000101024	0	0.031388648	0	1	CRA004775;Yang2025_Silkie_Wenchang	PRJNA827465	0.007873969
ENSGALG00010007399	WWC2	2	0.148755304	0.133169591	1.117036578	0.263978686	0.00845623	1.116521405	10.43611028	1	CRA004775;PRJNA827465	Yang2025_Silkie_Wenchang	0.768643821
ENSGALG00010007401	SEC61G	2	-0.771752006	0.849157003	-0.908844894	0.363432005	1.396731861	31.09190856	96.78372912	0.5	PRJNA827465;Yang2025_Silkie_Wenchang	CRA004775	0.817042253
ENSGALG00010007401	SEC61G	2	-0.881487226	0.761566576	-1.157465747	0.247082126	1.076322209	13.86759755	92.78894562	1	CRA004775;Yang2025_Silkie_Wenchang	PRJNA827465	0.757459165
ENSGALG00010007401	SEC61G	2	0.039348872	0.086354008	0.455669312	0.648627793	0	0.340076942	0	0.5	CRA004775;PRJNA827465	Yang2025_Silkie_Wenchang	0.907730933
ENSGALG00010007403	SDR42E1	2	0.127134907	0.133787222	0.9502769	0.341971573	0	0.135236796	0	1	PRJNA827465;Yang2025_Silkie_Wenchang	CRA004775	0.805904173
ENSGALG00010007403	SDR42E1	2	0.046245383	0.202616049	0.228241461	0.819458531	0	0.048046487	0	0.5	CRA004775;Yang2025_Silkie_Wenchang	PRJNA827465	0.955256039
ENSGALG00010007403	SDR42E1	2	0.145324812	0.161945792	0.897367018	0.369523106	0	0.161477776	0	0.5	CRA004775;PRJNA827465	Yang2025_Silkie_Wenchang	0.820606801
ENSGALG00010007404	FOLH1	2	-0.512287125	0.126619146	-4.045889919	5.21247E-05	0	0.179413564	0	1	PRJNA827465;Yang2025_Silkie_Wenchang	CRA004775	0.004985845
ENSGALG00010007404	FOLH1	2	-0.180239519	0.454158032	-0.396865202	0.691466866	0.324209091	4.368733286	77.1100698	0.5	CRA004775;Yang2025_Silkie_Wenchang	PRJNA827465	0.920268407
ENSGALG00010007404	FOLH1	2	-0.141137343	0.396454521	-0.355998824	0.721841446	0.235873211	3.599721102	72.22007007	0.5	CRA004775;PRJNA827465	Yang2025_Silkie_Wenchang	0.929372448
ENSGALG00010007408	HCLS1	2	0.237441329	0.227209811	1.045031144	0.296008576	0.079256632	4.299358972	76.74071864	1	PRJNA827465;Yang2025_Silkie_Wenchang	CRA004775	0.788124569
ENSGALG00010007408	HCLS1	2	-0.09542452	0.604600577	-0.15783068	0.874590223	0.657828988	9.565694998	89.54597653	0.5	CRA004775;Yang2025_Silkie_Wenchang	PRJNA827465	0.970159044
ENSGALG00010007408	HCLS1	2	-0.295099993	0.372361749	-0.792508882	0.428064015	0.211969267	3.824679045	73.85401524	0.5	CRA004775;PRJNA827465	Yang2025_Silkie_Wenchang	0.84125685
ENSGALG00010007413	GPATCH11	2	0.274987213	0.302744279	0.908315143	0.363711744	0.17522277	22.65305854	95.58558506	0.5	PRJNA827465;Yang2025_Silkie_Wenchang	CRA004775	0.817042253
ENSGALG00010007413	GPATCH11	2	0.568817062	0.09124787	6.233757149	4.55379E-10	0	0.130742781	0	1	CRA004775;Yang2025_Silkie_Wenchang	PRJNA827465	2.88673E-07
ENSGALG00010007413	GPATCH11	2	0.140442187	0.233346303	0.601861636	0.547266243	0.075152907	2.609160263	61.67349264	0.5	CRA004775;PRJNA827465	Yang2025_Silkie_Wenchang	0.878281266
ENSGALG00010007416	DCTD	2	-0.441479158	0.365329064	-1.208442476	0.22687711	0.250670768	16.02376003	93.7592675	1	PRJNA827465;Yang2025_Silkie_Wenchang	CRA004775	0.740444589
ENSGALG00010007416	DCTD	2	-0.799993348	0.157735393	-5.071742833	3.94189E-07	0	0.190754858	0	1	CRA004775;Yang2025_Silkie_Wenchang	PRJNA827465	0.000105393
ENSGALG00010007416	DCTD	2	-0.100919275	0.076783997	-1.314326932	0.188736208	0	0.853686525	0	1	CRA004775;PRJNA827465	Yang2025_Silkie_Wenchang	0.698162702
ENSGALG00010007418	NME6	2	-0.045255608	0.182971046	-0.247337539	0.804647009	0.052472713	4.569427506	78.1154204	0.5	PRJNA827465;Yang2025_Silkie_Wenchang	CRA004775	0.951665484
ENSGALG00010007418	NME6	2	-0.199821186	0.114911654	-1.738911408	0.082050347	0	0.288020695	0	1	CRA004775;Yang2025_Silkie_Wenchang	PRJNA827465	0.493696386
ENSGALG00010007418	NME6	2	0.083591015	0.093835627	0.890823851	0.373023677	0	0.930571209	0	0.5	CRA004775;PRJNA827465	Yang2025_Silkie_Wenchang	0.822162024
ENSGALG00010007419	RABL2A	2	-0.059543222	0.106383107	-0.559705611	0.575680254	0	0.16456557	0	1	PRJNA827465;Yang2025_Silkie_Wenchang	CRA004775	0.88677096
ENSGALG00010007419	RABL2A	2	-0.217343595	0.187966572	-1.156288554	0.247563149	0	0.211329541	0	1	CRA004775;Yang2025_Silkie_Wenchang	PRJNA827465	0.757947282
ENSGALG00010007419	RABL2A	2	-0.077156245	0.110159271	-0.700406281	0.483673615	0	0.789784769	0	1	CRA004775;PRJNA827465	Yang2025_Silkie_Wenchang	0.85691916
ENSGALG00010007423	HEATR5B	2	-0.021055907	0.048133674	-0.437446488	0.661787576	0	0.006086301	0	1	PRJNA827465;Yang2025_Silkie_Wenchang	CRA004775	0.912545506
ENSGALG00010007423	HEATR5B	2	-0.009359111	0.112732601	-0.083020449	0.93383528	0	0.003609948	0	0.5	CRA004775;Yang2025_Silkie_Wenchang	PRJNA827465	0.984309396
ENSGALG00010007423	HEATR5B	2	-0.021718372	0.051482751	-0.421857251	0.673129214	0	0.009999247	0	0.5	CRA004775;PRJNA827465	Yang2025_Silkie_Wenchang	0.915210237
ENSGALG00010007425	ARSA	2	-0.09596688	0.050184468	-1.912282487	0.055839971	0	0.196904411	0	1	PRJNA827465;Yang2025_Silkie_Wenchang	CRA004775	0.408997088
ENSGALG00010007425	ARSA	2	-0.078180149	0.088005778	-0.888352453	0.374351193	0	0.295310262	0	1	CRA004775;Yang2025_Silkie_Wenchang	PRJNA827465	0.822805106
ENSGALG00010007425	ARSA	2	-0.115146643	0.057960044	-1.986655555	0.04696058	0	0.143065569	0	1	CRA004775;PRJNA827465	Yang2025_Silkie_Wenchang	0.374003609
ENSGALG00010007429	C21orf91	2	-0.091329255	0.079578158	-1.147667359	0.251105911	0	0.107187782	0	1	PRJNA827465;Yang2025_Silkie_Wenchang	CRA004775	0.761043771
ENSGALG00010007429	C21orf91	2	0.233567853	0.322327837	0.724628239	0.46868013	0.162285217	4.318743055	76.84511472	0.5	CRA004775;Yang2025_Silkie_Wenchang	PRJNA827465	0.853198008
ENSGALG00010007429	C21orf91	2	0.198736993	0.350607028	0.566836878	0.570825013	0.208034864	5.921262421	83.11170948	0.5	CRA004775;PRJNA827465	Yang2025_Silkie_Wenchang	0.885224727
ENSGALG00010007430	MEF2C	2	0.455769079	0.450847528	1.01091622	0.31205653	0.362061346	8.770957857	88.59873669	1	PRJNA827465;Yang2025_Silkie_Wenchang	CRA004775	0.796054997
ENSGALG00010007430	MEF2C	2	0.61547888	0.378245416	1.627194551	0.103695785	0.172574917	2.435449877	58.93982424	1	CRA004775;Yang2025_Silkie_Wenchang	PRJNA827465	0.547964315
ENSGALG00010007430	MEF2C	2	0.049438091	0.124508334	0.397066521	0.691318408	0	0.106157538	0	1	CRA004775;PRJNA827465	Yang2025_Silkie_Wenchang	0.920268407
ENSGALG00010007432	NAT	2	-0.33474158	0.473688826	-0.706669783	0.479771712	0.379636981	6.416351486	84.41481889	0.5	PRJNA827465;Yang2025_Silkie_Wenchang	CRA004775	0.856274132
ENSGALG00010007432	NAT	2	-0.618919853	0.275922908	-2.243089774	0.024891026	0.03014411	1.226736945	18.48293114	1	CRA004775;Yang2025_Silkie_Wenchang	PRJNA827465	0.269034231
ENSGALG00010007432	NAT	2	0.034611456	0.196042549	0.176550736	0.859861293	0	0.634809056	0	0.5	CRA004775;PRJNA827465	Yang2025_Silkie_Wenchang	0.964858345
ENSGALG00010007433	RNF144B	2	0.081201637	0.168768284	0.481142757	0.630415041	0	0.775529975	0	0.5	PRJNA827465;Yang2025_Silkie_Wenchang	CRA004775	0.902665749
ENSGALG00010007433	RNF144B	2	0.457561669	0.268904847	1.701574643	0.088835134	0.061097305	1.684165492	40.62341233	1	CRA004775;Yang2025_Silkie_Wenchang	PRJNA827465	0.511771357
ENSGALG00010007433	RNF144B	2	0.324457313	0.421302953	0.770128268	0.441223809	0.271312209	4.133716575	75.80869463	0.5	CRA004775;PRJNA827465	Yang2025_Silkie_Wenchang	0.844907543
ENSGALG00010007434	ENSGALG00010007434	2	-0.058128358	0.214202417	-0.271371159	0.786105582	0.021341816	1.274430423	21.53357439	0.5	PRJNA827465;Yang2025_Silkie_Wenchang	CRA004775	0.946639719
ENSGALG00010007434	ENSGALG00010007434	2	0.0626737	0.442355045	0.141681894	0.887331277	0.254154132	2.809847285	64.41087723	0.5	CRA004775;Yang2025_Silkie_Wenchang	PRJNA827465	0.972987228
ENSGALG00010007434	ENSGALG00010007434	2	0.2030727	0.19854505	1.022804146	0.306400458	0	0.865433821	0	1	CRA004775;PRJNA827465	Yang2025_Silkie_Wenchang	0.792485409
ENSGALG00010007436	PNAT3	2	-0.229701917	0.125943085	-1.823854931	0.068174007	0.011452992	1.494218691	33.0753921	1	PRJNA827465;Yang2025_Silkie_Wenchang	CRA004775	0.450274056
ENSGALG00010007436	PNAT3	2	-0.421124392	0.16844471	-2.500074895	0.012416705	0	0.097898354	0	1	CRA004775;Yang2025_Silkie_Wenchang	PRJNA827465	0.18021188

ENSGALG00010007436	PNAT3	2	-0.162271146	0.110982068	-1.462138428	0.14370328	0	0.817618508	0	1	CRA004775;PRJNA827465	Yang2025_Silkie_Wenchang	0.630564924
ENSGALG00010007437	TUSC1	2	0.267344675	0.167806234	1.593174866	0.111120968	0	0.541797272	0	1	PRJNA827465;Yang2025_Silkie_Wenchang	CRA004775	0.563970481
ENSGALG00010007437	TUSC1	2	0.094595927	0.373247662	0.253440107	0.799928138	0.17508151	2.650384118	62.26961997	0.5	CRA004775;Yang2025_Silkie_Wenchang	PRJNA827465	0.949922217
ENSGALG00010007437	TUSC1	2	0.020545697	0.228539269	0.089900074	0.928366623	0.032674849	1.370361195	27.02653841	0.5	CRA004775;PRJNA827465	Yang2025_Silkie_Wenchang	0.983228062
ENSGALG00010007441	STRN	2	0.196802364	0.26785743	0.734728038	0.462505141	0.123509663	6.93845232	85.587564	0.5	PRJNA827465;Yang2025_Silkie_Wenchang	CRA004775	0.850823289
ENSGALG00010007441	STRN	2	0.28330299	0.236789314	1.196434864	0.231526903	0.065355393	2.317923936	56.85794583	1	CRA004775;Yang2025_Silkie_Wenchang	PRJNA827465	0.743924694
ENSGALG00010007441	STRN	2	-0.044226167	0.093201692	-0.47452108	0.635128369	0	0.043039035	0	0.5	CRA004775;PRJNA827465	Yang2025_Silkie_Wenchang	0.904051292
ENSGALG00010007455	TRPC6	2	0.053905153	0.128124292	0.420725471	0.67395556	0	0.012535879	0	1	PRJNA827465;Yang2025_Silkie_Wenchang	CRA004775	0.915650814
ENSGALG00010007455	TRPC6	2	0.066195676	0.194188379	0.340883818	0.733191048	0	0.140530262	0	1	CRA004775;Yang2025_Silkie_Wenchang	PRJNA827465	0.933089653
ENSGALG00010007455	TRPC6	2	0.081359398	0.153255986	0.53087256	0.595507094	0	0.109140674	0	1	CRA004775;PRJNA827465	Yang2025_Silkie_Wenchang	0.893794893
ENSGALG00010007457	SHANK3	2	-0.174985053	0.073545678	-2.379270381	0.017346947	0	0.369178346	0	1	PRJNA827465;Yang2025_Silkie_Wenchang	CRA004775	0.219047753
ENSGALG00010007457	SHANK3	2	-0.099219481	0.119479672	-0.830429811	0.406295817	0	0.037701844	0	1	CRA004775;Yang2025_Silkie_Wenchang	PRJNA827465	0.833567076
ENSGALG00010007457	SHANK3	2	-0.192302929	0.08524944	-2.255767646	0.024085187	0	0.26366801	0	1	CRA004775;PRJNA827465	Yang2025_Silkie_Wenchang	0.265011806
ENSGALG00010007465	PNAT10	2	-0.128382203	0.252551657	-0.508340368	0.611214665	0.069483195	2.195757943	54.4576394	0.5	PRJNA827465;Yang2025_Silkie_Wenchang	CRA004775	0.897609796
ENSGALG00010007465	PNAT10	2	-0.22433547	0.21513904	-1.042746448	0.297065739	0.012021563	1.13536775	11.92281088	0.5	CRA004775;Yang2025_Silkie_Wenchang	PRJNA827465	0.788466926
ENSGALG00010007465	PNAT10	2	0.10240298	0.195100066	0.524874144	0.599670685	0	0.018090964	0	1	CRA004775;PRJNA827465	Yang2025_Silkie_Wenchang	0.895092498
ENSGALG00010007467	CHODL	2	-0.109657931	0.135091506	-0.811730759	0.41694614	0	0.042328703	0	1	PRJNA827465;Yang2025_Silkie_Wenchang	CRA004775	0.838063117
ENSGALG00010007467	CHODL	2	-0.125474826	0.235293233	-0.533270017	0.59384668	0	0.096485239	0	0.5	CRA004775;Yang2025_Silkie_Wenchang	PRJNA827465	0.893190313
ENSGALG00010007467	CHODL	2	-0.083211763	0.155542788	-0.534976672	0.592665989	0	0.05411924	0	0.5	CRA004775;PRJNA827465	Yang2025_Silkie_Wenchang	0.892776493
ENSGALG00010007479	NFX1	2	-0.00544326	0.056152237	-0.09693755	0.922775989	0	0.048514584	0	0.5	PRJNA827465;Yang2025_Silkie_Wenchang	CRA004775	0.981804675
ENSGALG00010007479	NFX1	2	0.011921712	0.104845714	0.113707196	0.909469908	0	0.012186255	0	0.5	CRA004775;Yang2025_Silkie_Wenchang	PRJNA827465	0.978607662
ENSGALG00010007479	NFX1	2	-0.013393452	0.064785214	-0.206736245	0.836215849	0	0.002027631	0	1	CRA004775;PRJNA827465	Yang2025_Silkie_Wenchang	0.958399882
ENSGALG00010007480	TENM3	2	-0.086875733	0.071471598	-1.215528072	0.224164749	0	0.782960219	0	0.5	PRJNA827465;Yang2025_Silkie_Wenchang	CRA004775	0.737452676
ENSGALG00010007480	TENM3	2	0.125312993	0.141775526	0.883883108	0.376759317	0	0.520260175	0	1	CRA004775;Yang2025_Silkie_Wenchang	PRJNA827465	0.822974013
ENSGALG00010007480	TENM3	2	0.006979644	0.179482036	0.038887702	0.968979921	0.041052072	2.23383736	55.23398356	0.5	CRA004775;PRJNA827465	Yang2025_Silkie_Wenchang	0.991482072
ENSGALG00010007485	CTSC	2	-0.367972019	0.265422301	-1.386364365	0.165635672	0.12890867	11.46859063	91.28053279	1	PRJNA827465;Yang2025_Silkie_Wenchang	CRA004775	0.665165916
ENSGALG00010007485	CTSC	2	-0.445182114	0.317041217	-1.404177406	0.160266028	0.121935424	1.986919868	49.67084399	0.5	CRA004775;Yang2025_Silkie_Wenchang	PRJNA827465	0.655729305
ENSGALG00010007485	CTSC	2	-0.110965283	0.073569261	-1.508310418	0.131475098	0	0.122630812	0	0.5	CRA004775;PRJNA827465	Yang2025_Silkie_Wenchang	0.607282381
ENSGALG00010007500	MPHOSPH6	2	0.19503809	0.22272751	0.87559905	0.381252092	0.078856196	4.650144848	78.49529353	0.5	PRJNA827465;Yang2025_Silkie_Wenchang	CRA004775	0.824329525
ENSGALG00010007500	MPHOSPH6	2	0.30630083	0.165983656	1.845367409	0.064984111	0.013749341	1.317651481	24.10739757	1	CRA004775;Yang2025_Silkie_Wenchang	PRJNA827465	0.439528572
ENSGALG00010007500	MPHOSPH6	2	0.013780893	0.093433618	0.147493945	0.882742159	0	0.190184231	0	0.5	CRA004775;PRJNA827465	Yang2025_Silkie_Wenchang	0.972001076
ENSGALG00010007506	RAB38	2	4.432512926	0.280022876	15.82911005	1.95973E-56	0	0.091491446	0	1	PRJNA827465;Yang2025_Silkie_Wenchang	CRA004775	5.92488E-53
ENSGALG00010007506	RAB38	2	2.754889555	1.804682628	1.526523009	0.126879665	6.228008304	22.6756575	95.58998455	1	CRA004775;Yang2025_Silkie_Wenchang	PRJNA827465	0.597716824
ENSGALG00010007506	RAB38	2	2.677858309	1.718747346	1.558029059	0.11922636	5.648761725	22.54924586	95.56526189	1	CRA004775;PRJNA827465	Yang2025_Silkie_Wenchang	0.5795861
ENSGALG00010007513	GALNT12	2	-0.128479299	0.100339811	-1.280441905	0.200389764	0.010333427	2.012748114	50.31668429	1	PRJNA827465;Yang2025_Silkie_Wenchang	CRA004775	0.71262386
ENSGALG00010007513	GALNT12	2	-0.034658421	0.260665897	-0.132966121	0.894220122	0.098679245	3.250371926	69.23429002	0.5	CRA004775;Yang2025_Silkie_Wenchang	PRJNA827465	0.974381724
ENSGALG00010007513	GALNT12	2	0.022744509	0.130814649	0.173868215	0.861969033	0.013879114	1.338665109	25.29871784	0.5	CRA004775;PRJNA827465	Yang2025_Silkie_Wenchang	0.96567952
ENSGALG00010007514	VIT	2	1.097861757	0.466137405	2.355232053	0.018511142	0	0.880719371	0	1	PRJNA827465;Yang2025_Silkie_Wenchang	CRA004775	0.227601507
ENSGALG00010007514	VIT	2	0.96578595	0.753359825	1.281971667	0.199852585	0.473318601	1.706875121	41.41340584	1	CRA004775;Yang2025_Silkie_Wenchang	PRJNA827465	0.711808443
ENSGALG00010007514	VIT	2	0.565746923	0.489568336	1.155603582	0.247843342	0	0.333825597	0	1	CRA004775;PRJNA827465	Yang2025_Silkie_Wenchang	0.7580874
ENSGALG00010007521	SLC12A7	2	-0.04284769	0.0927722	-0.461859155	0.644182327	0.006687773	1.572943262	36.42491599	0.5	PRJNA827465;Yang2025_Silkie_Wenchang	CRA004775	0.906873169
ENSGALG00010007521	SLC12A7	2	0.076558556	0.121455671	0.630341549	0.528471144	0	8.38259E-05	0	1	CRA004775;Yang2025_Silkie_Wenchang	PRJNA827465	0.871701504
ENSGALG00010007521	SLC12A7	2	-0.106694587	0.082347036	-1.295670027	0.195089192	0	0.212570534	0	0.5	CRA004775;PRJNA827465	Yang2025_Silkie_Wenchang	0.706113528
ENSGALG00010007529	CDH13	2	-0.012270974	0.088795052	-0.138194346	0.890086824	0	0.591644923	0	0.5	PRJNA827465;Yang2025_Silkie_Wenchang	CRA004775	0.973347498
ENSGALG00010007529	CDH13	2	0.182857009	0.16478739	1.109654139	0.267148092	0	0.377565291	0	1	CRA004775;Yang2025_Silkie_Wenchang	PRJNA827465	0.771247817
ENSGALG00010007529	CDH13	2	0.037696529	0.162187901	0.232425039	0.816207903	0.023580224	1.436372038	30.38015407	0.5	CRA004775;PRJNA827465	Yang2025_Silkie_Wenchang	0.95448249
ENSGALG00010007532	DCUN1D5	2	-0.046771643	0.055184671	-0.847547729	0.396689896	0	0.788321551	0	1	PRJNA827465;Yang2025_Silkie_Wenchang	CRA004775	0.831375233
ENSGALG00010007532	DCUN1D5	2	-0.318210126	0.16142857	-1.971213191	0.048699496	0	0.437370947	0	1	CRA004775;Yang2025_Silkie_Wenchang	PRJNA827465	0.381461853
ENSGALG00010007532	DCUN1D5	2	-0.166919024	0.192419722	-0.867473577	0.385682583	0.051551952	2.567402517	61.05012776	1	CRA004775;PRJNA827465	Yang2025_Silkie_Wenchang	0.82648313
ENSGALG00010007536	GABBR2	2	0.189859686	0.329096805	0.576911361	0.563999335	0.169689336	4.348338414	77.00270989	0.5	PRJNA827465;Yang2025_Silkie_Wenchang	CRA004775	0.882790358
ENSGALG00010007536	GABBR2	2	0.264059584	0.471013841	0.560619585	0.575056897	0.233051244	1.76261921	43.26624865	0.5	CRA004775;Yang2025_Silkie_Wenchang	PRJNA827465	0.886297057
ENSGALG00010007536	GABBR2	2	-0.113524762	0.145026598	-0.782785807	0.433752904	0	0.25271573	0	1	CRA004775;PRJNA827465	Yang2025_Silkie_Wenchang	0.842762754
ENSGALG00010007539	RPS3A	2	0.140749352	0.236925358	0.594066221	0.552467823	0.093208399	5.390073822	81.4473784	0.5	PRJNA827465;Yang2025_Silkie_Wenchang	CRA004775	0.879228216
ENSGALG00010007539	RPS3A	2	0.022791341	0.398155617	0.057242293	0.954352188	0.27372542	7.298039249	86.29768948	0.5	CRA004775;Yang2025_Silkie_Wenchang	PRJNA827465	0.985798175
ENSGALG00010007539	RPS3A	2	-0.154635387	0.142849137	-1.082508377	0.279026695	0.022888559	1.826501879	45.25053538	1	CRA004775;PRJNA827465	Yang2025_Silkie_Wenchang	0.777322971
ENSGALG00010007542	MRPL36	2	-0.155550959	0.095577822	-1.627479647	0.103635268	0	0.963607334	0	1	PRJNA827465;Yang2025_Silkie_Wenchang	CRA004775	0.547964315
ENSGALG00010007542	MRPL36	2	-0.308861325	0.187306929	-1.64895835	0.099156168	0	0.101618417	0	1	CRA004775;Yang2025_Silkie_Wenchang	PRJNA827465	0.537239434
ENSGALG00010007542	MRPL36	2	-0.112584327	0.103893299	-1.086549083	0.27727972	0	0.038276313	0	1	CRA004775;PRJNA827465	Yang2025_Silkie_Wenchang	0.776259364
ENSGALG00010007544	MLYCD	2	-0.010365313	0.083159504	-0.124643755	0.900805587	0	0.077729122	0	0.5	PRJNA827465;Yang2025_Silkie_Wenchang	CRA004775	0.976497577
ENSGALG00010007544	MLYCD	2	0.134266463	0.146414748	0.917028273	0.359127825	0	0.475425676	0	1	CRA004775;Yang2025_Silkie_Wenchang	PRJNA827465	0.814241185
ENSGALG00010007544	MLYCD	2	0.03916471	0.110649713	0.353952207	0.723374701	0.007497789	1.274823203	21.55775033	0.5	CRA004775;PRJNA827465	Yang2025_Silkie_Wenchang	0.930032096
ENSGALG00010007548	HSBP1	2	-0.194393809	0.153049573	-1.270136234	0.204036107	0.040906872	7.531691729	86.72277045	1	PRJNA827465;Yang2025_Silkie_Wenchang	CRA004775	0.717254253
ENSGALG00010007548	HSBP1	2	-0.335934824	0.091281795	-3.680195209	0.000233055	0	0.334739716	0	1	CRA004775;Yang2025_Silkie_Wenchang	PRJNA827465	0.014132351
ENSGALG00010007548	HSBP1	2	-0.061734024	0.047479866	-1.300214786	0.193527364	0	0.589494162	0	1	CRA004775;PRJNA827465	Yang2025_Silkie_Wenchang	0.703540661
ENSGALG00010007549	SH3D19	2	-0.147861283	0.074484026	-1.997223983	0.045809057	0	0.273111493	0	1	PRJNA827465;Yang2025_Silkie_Wenchang	CRA004775	0.369177826
ENSGALG00010007549	SH3D19	2	0.062435896	0.167708011	0.372289288	0.709677474	0	0.391430606	0	0.5	CRA004775;Yang2025_Silkie_Wenchang	PRJNA827465	0.925298042
ENSGALG00010007549	SH3D19	2	-0.074082434	0.14807485	-0.50030396	0.616861066	0.023476208	1.704278778	41.32415348	0.5	CRA004775;PRJNA827465	Yang2025_Silkie_Wenchang	0.899111936
ENSGALG00010007551	FEZ2	2	0.008486246	0.047881025	0.177236097	0.859322943	0	0.264370526	0	0.5	PRJNA827465;Yang2025_Silkie_Wenchang	CRA004775	0.964606195
ENSGALG00010007551	FEZ2	2	0.160582619	0.160870063	0.998213191	0.318175991	0.011030995	1.159494489	13.75551937	1	CRA004775;Yang2025_Silkie_Wenchang	PRJNA827465	0.797512752
ENSGALG00010007551	FEZ2	2	0.120924183	0.208428436	0.580171235	0.56179915	0.055507041	1.929127429	48.16309255	0.5	CRA004775;PRJNA827465	Yang2025_Silkie_Wenchang	0.881802301
ENSGALG00010007555	NDUFS6	2	-0.028966941	0.087351583	-0.331613232	0.740181326	0	0.37858968	0	0.5	PRJNA827465;Yang2025_Silkie_Wenchang	CRA004775	0.93508234
ENSGALG00010007555	NDUFS6	2	0.193504224	0.242279333	0.798682335	0.424474632	0	0.089722951	0	1	CRA004775;Yang2025_Silkie_Wenchang	PRJNA827465	0.840671527
ENSGALG00010007555	NDUFS6	2	-0.03061875	0.089458049	-0.34226937	0.732148185	0	0.620193604	0	0.5	CRA004775;PRJNA827465	Yang2025_Silkie_Wenchang	0.93287651
ENSGALG00010007566	FHIP1A	2	-0.036890729	0.128946359	-0.286093609	0.774806427	0	0.143730222	0	0.5	PRJNA827465;Yang2025_Silkie_Wenchang	CRA004775	0.943713409
ENSGALG00010007566	FHIP1A	2	0.035801173	0.170447417	0.210042333	0.833634633	0	0.03827426	0	1	CRA004775;Yang2025_Silkie_Wenchang	PRJNA827465	0.957968425
ENSGALG00010007566	FHIP1A	2	-0.047070191	0.155808178	-0.302103472	0.762573186	0	0.199704615	0	0.5	CRA004775;PRJNA827465	Yang2025_Silkie_Wenchang	0.940082621
ENSGALG00010007567	TRIP13	2	-0.044245754	0.072147765	-0.613265753	0.539700634	0	0.359195677	0	0.5	PRJNA827465;Yang2025_Silkie_Wenchang	CRA004775	0.875184801
ENSGALG00010007567	TRIP13	2	-0.138949674	0.280526249	-0.495317904	0.620375734	0.08724759	1.691935318	40.89608574	0.5	CRA004775;Yang2025_Silkie_Wenchang	PRJNA827465	0.899395593
ENSGALG00010007567	TRIP13	2	-0.149456409	0.192024639	-0.778318917	0.436381026	0.035158746	1.289515489	22.4514937	1	CRA004775;PRJNA827465	Yang2025_Silkie_Wenchang	0.843122822
ENSGALG00010007569	GPR89A	2	0.162413553	0.191954426	0.84610476	0.397494306	0.063179131	6.84281278	85.38612633	0.5	PRJNA827465;Yang2025_Silkie_Wenchang	CRA004775	0.830486298
ENSGALG00010007569	GPR89A	2	-0.056405811	0.443576713	-0.127161343	0.898812701	0.360505315	11.64406737	91.41193564	0.5	CRA004775;Yang2025_Silkie_Wenchang	PRJNA827465	0.975884294
ENSGALG00010007569	GPR89A	2	-0.22361369	0.247512472	-0.903444129	0.366290028	0.097556293	4.345153609	76.9858539	1	CRA004775;PRJNA827465	Yang2025_Silkie_Wenchang	0.819159863
ENSGALG00010007572	PDZK1	2	-0.26178927	0.387179253	-0.676148583	0.498948064	0.195012848	2.624371669	61.89564109	0.5	PRJNA827465;Yang2025_Silkie_Wenchang	CRA004775	0.861571318
ENSGALG00010007572	PDZK1	2	0.62283616	1.405654816	0.443093249	0.657698288	3.660258646	13.43911996	92.55903658	0.5	CRA004775;Yang2025_Silkie_Wenchang	PRJNA827465	0.910774485
ENSGALG00010007572	PDZK1	2	0.970732325	1.005547754	0.965376653	0.334356191	1.812214971	9.056664593	88.95840748	1	CRA004775;PRJNA827465	Yang2025_Silkie_Wenchang	0.803094614
ENSGALG00010007574	ENSGALG00010007574	2	0.669046745	0.598195083	1.118442402	0.263378093	0.655383211	11.85834506	91.56712008	1	PRJNA827465;Yang2025_Silkie_Wenchang	CRA004775	0.768643821
ENSGALG00010007574	ENSGALG00010007574	2	0.873852626	0.488490615	1.788883142	0.073633637	0.330721286	2.989247086	66.54676006	1	CRA004775;Yang2025_Silkie_Wenchang	PRJNA827465	0.308996768
ENSGALG00010007574	ENSGALG00010007574	2	0.110039175	0.210969995	0.521586851	0.601958021	0	0.12482846	0	1	CRA004775;PRJNA827465	Yang2025_Silkie_Wenchang	0.895317165
ENSGALG00010007575	MMP27	2	-0.055932343	0.280477501	-0.199418287	0.841935557	0.105302752	2.964398471	66.26634342	0.5	PRJNA827465;Yang2025_Silkie_Wenchang	CRA004775	0.960041272
ENSGALG00010007575	MMP27	2	-0.362115712	0.254604782	-1.42226595	0.15494906	0	0.00304274	0	1	CRA004775;Yang2025_Silkie_Wenchang	PRJNA827465	0.647628882
ENSGALG00010007575	MMP27	2	0.179058021	0.190764616	0.938633301	0.347919049	0	0.252859267	0	0.5	CRA004775;PRJNA827465	Yang2025_Silkie_Wenchang	0.808847403
ENSGALG00010007577	WFDC1	2	0.041084477	0.291245667	0.141064681	0.887818845	0.160172667	17.90306401	94.41436394	0.5	PRJNA827465;Yang2025_Silkie_Wenchang	CRA004775	0.973090153
ENSGALG00010007577	WFDC1	2	-0.252577933	0.091365334	-2.764483227	0.005701304	0	0.010471606	0	1	CRA004775;Yang2025_Silkie_Wenchang	PRJNA827465	0.113457389
ENSGALG00010007577	WFDC1	2	0.092799618	0.300084634	0.309244818	0.757135304	0.137896229	3.686562023	72.8744561	0.5	CRA004775;PRJNA827465	Yang2025_Silkie_Wenchang	0.938795135
ENSGALG00010007579	GATB	2	-0.173492127	0.070078854	-2.475670162	0.013298637	0	0.250086748	0	1	PRJNA827465;Yang2025_Silkie_Wenchang	CRA004775	0.188414806
ENSGALG00010007579	GATB	2	-0.164837277	0.093225152	-1.768163136	0.077033635	0	0.506299389	0	1	CRA004775;Yang2025_Silkie_Wenchang	PRJNA827465	0.478453375
ENSGALG00010007579	GATB	2	-0.223430311	0.090221405	-2.476466777	0.013268997	0	0.198981772	0	1	CRA004775;PRJNA827465	Yang2025_Silkie_Wenchang	0.188244411
ENSGALG00010007580	BRD9	2	0.236248501	0.235818467	1.001823582	0.316428806	0.102828041	13.04334162	92.33325302	1	PRJNA827465;Yang2025_Silkie_Wenchang	CRA004775	0.796883862
ENSGALG00010007580	BRD9	2	0.417467127	0.111960569	3.728697785	0.000192472	0.00397633	1.131758454	11.64192356	1	CRA004775;Yang2025_Silkie_Wenchang	PRJNA827465	0.01240118
ENSGALG00010007580	BRD9	2	0.026972993	0.064213337	0.420052813	0.674446873	0	0.84778505	0	1	CRA004775;PRJNA827465	Yang2025_Silkie_Wenchang	0.915949608
ENSGALG00010007581	LPCAT1	2	-0.127102803	0.086780403	-1.464648686	0.143016792	0.006148312	1.667281036	40.02210915	1	PRJNA827465;Yang2025_Silkie_Wenchang	CRA004775	0.629873259
ENSGALG00010007581	LPCAT1	2	-0.215824393	0.102437159	-2.10689553	0.035126636	0	0.143751302	0	1	CRA004775;Yang2025_Silkie_Wenchang	PRJNA827465	0.323167978
ENSGALG00010007581	LPCAT1	2	-0.053150794	0.08196837	-0.648430538	0.516706524	0	0.005908397	0	1	CRA004775;PRJNA827465	Yang2025_Silkie_Wenchang	0.867952013
ENSGALG00010007584	ELP2	2	-0.020352781	0.098715892	-0.206175324	0.836653962	0.008697261	1.688256935	40.7673098	0.5	PRJNA827465;Yang2025_Silkie_Wenchang	CRA004775	0.958399882
ENSGALG00010007584	ELP2	2	-0.133247614	0.109794729	-1.213606664	0.224897961	0	0.06283217	0	1	CRA004775;Yang2025_Silkie_Wenchang	PRJNA827465	0.737888351
ENSGALG00010007584	ELP2	2	0.028578204	0.073087845	0.391011722	0.695788571	0	0.562687957	0	0.5	CRA004775;PRJNA827465	Yang2025_Silkie_Wenchang	0.921038437
ENSGALG00010007588	TTC27	2	-0.076515926	0.058559429	-1.306637154	0.191335972	0	0.055629774	0	1	PRJNA827465;Yang2025_Silkie_Wenchang	CRA004775	0.700715403
ENSGALG00010007588	TTC27	2	-0.112293164	0.089415138	-1.255863015	0.20916564	0	0.189690704	0	1	CRA004775;Yang2025_Silkie_Wenchang	PRJNA827465	0.721062814
ENSGALG00010007588	TTC27	2	-0.079463941	0.06938174	-1.145314907	0.252078733	0	0.332217092	0	1	CRA004775;PRJNA827465	Yang2025_Silkie_Wenchang	0.762170202
ENSGALG00010007590	FBXW7	2	-0.04782407	0.194134363	-0.24634521	0.805415015	0.064154367	6.534193212	84.69589179	0.5	PRJNA827465;Yang2025_Silkie_Wenchang	CRA004775	0.951870339
ENSGALG00010007590	FBXW7	2	0.512476941	0.386294154	1.326649487	0.184624658	0.256294733	6.730364644	85.14196403	1	CRA004775;Yang2025_Silkie_Wenchang	PRJNA827465	0.692938714
ENSGALG00010007590	FBXW7	2	0.325304348	0.581872799	0.559064367	0.576117792	0.639340587	17.21511084	94.1911498	0.5	CRA004775;PRJNA827465	Yang2025_Silkie_Wenchang	0.887027759
ENSGALG00010007593	SEC61B	2	-0.195957593	0.363571005	-0.538980254	0.589900479	0.212205879	4.357488367	77.05100012	0.5	PRJNA827465;Yang2025_Silkie_Wenchang	CRA004775	0.892320726
ENSGALG00010007593	SEC61B	2	-0.632264428	0.239227066	-2.642946875	0.008218793	0	0.001349587	0	1	CRA004775;Yang2025_Silkie_Wenchang	PRJNA827465	0.140904587
ENSGALG00010007593	SEC61B	2	-0.190559044	0.355285924	-0.536354048	0.591713884	0.203074841	4.416620414	77.35825346	0.5	CRA004775;PRJNA827465	Yang2025_Silkie_Wenchang	0.892776493
ENSGALG00010007595	ATP2C2	2	0.109480034	0.142811953	0.766602739	0.443317746	0	0.416900115	0	1	PRJNA827465;Yang2025_Silkie_Wenchang	CRA004775	0.845733906
ENSGALG00010007595	ATP2C2	2	-0.198849162	0.310254239	-0.640923271	0.521572535	0.094169604	1.715843241	41.71961774	0.5	CRA004775;Yang2025_Silkie_Wenchang	PRJNA827465	0.869518515
ENSGALG00010007595	ATP2C2	2	-0.129613005	0.41537721	-0.312036872	0.755012502	0.239605284	2.876010572	65.22961321	0.5	CRA004775;PRJNA827465	Yang2025_Silkie_Wenchang	0.93838723
ENSGALG00010007596	ENSGALG00010007596	2	-0.284098916	0.628505003	-0.452023317	0.651252185	0.537197295	3.123838523	67.98810205	0.5	PRJNA827465;Yang2025_Silkie_Wenchang	CRA004775	0.908887045
ENSGALG00010007596	ENSGALG00010007596	2	0.467894054	1.512759186	0.309298438	0.757094519	3.93094883	6.707715149	85.09179389	0.5	CRA004775;Yang2025_Silkie_Wenchang	PRJNA827465	0.938795135
ENSGALG00010007596	ENSGALG00010007596	2	0.998217434	0.857085277	1.164665245	0.244154523	0.891247032	2.277826059	56.09849154	1	CRA004775;PRJNA827465	Yang2025_Silkie_Wenchang	0.755585822
ENSGALG00010007601	TMEM154	2	0.280398494	0.252946484	1.108528925	0.267633454	0	0.132576991	0	1	PRJNA827465;Yang2025_Silkie_Wenchang	CRA004775	0.771398294
ENSGALG00010007601	TMEM154	2	-0.168167026	0.604708151	-0.27809618	0.780938528	0.527808792	3.502496045	71.44893279	0.5	CRA004775;Yang2025_Silkie_Wenchang	PRJNA827465	0.945235709
ENSGALG00010007601	TMEM154	2	-0.241297571	0.509213212	-0.473863531	0.635597226	0.329009362	2.62587419	61.91744434	0.5	CRA004775;PRJNA827465	Yang2025_Silkie_Wenchang	0.904252436
ENSGALG00010007603	ALG2	2	-0.017775018	0.2569044	-0.069189233	0.944838993	0.113704653	6.905673346	85.51915288	0.5	PRJNA827465;Yang2025_Silkie_Wenchang	CRA004775	0.986080619
ENSGALG00010007603	ALG2	2	-0.167741463	0.131119604	-1.279301171	0.20079102	0.003488146	1.112883898	10.14336701	1	CRA004775;Yang2025_Silkie_Wenchang	PRJNA827465	0.713340819
ENSGALG00010007603	ALG2	2	0.137420536	0.118079742	1.16379434	0.244507366	0.012498033	1.637261264	38.92239301	0.5	CRA004775;PRJNA827465	Yang2025_Silkie_Wenchang	0.756028087
ENSGALG00010007604	ENSGALG00010007604	2	-1.020790278	0.302124936	-3.378702502	0.000728288	0	0.002308738	0	1	PRJNA827465;Yang2025_Silkie_Wenchang	CRA004775	0.030257815
ENSGALG00010007604	ENSGALG00010007604	2	-0.941589961	0.382804491	-2.459715033	0.013904737	0	0.347397394	0	1	CRA004775;Yang2025_Silkie_Wenchang	PRJNA827465	0.19354185
ENSGALG00010007604	ENSGALG00010007604	2	-0.906282015	0.404695005	-2.239419818	0.025128613	0	0.310293849	0	1	CRA004775;PRJNA827465	Yang2025_Silkie_Wenchang	0.269931537
ENSGALG00010007610	TLDC1	2	-0.164381649	0.182199028	-0.90220925	0.366945722	0.061081308	12.35780617	91.90794882	0.5	PRJNA827465;Yang2025_Silkie_Wenchang	CRA004775	0.819578942
ENSGALG00010007610	TLDC1	2	-0.33700372	0.081819229	-4.11888163	3.80716E-05	0	0.304250422	0	1	CRA004775;Yang2025_Silkie_Wenchang	PRJNA827465	0.004000873
ENSGALG00010007610	TLDC1	2	0.001572298	0.055854805	0.028149733	0.977542728	0	0.700395616	0	0.5	CRA004775;PRJNA827465	Yang2025_Silkie_Wenchang	0.99364718
ENSGALG00010007614	BIRC6	2	-0.178745622	0.060085514	-2.974853847	0.002931282	0	0.312322681	0	1	PRJNA827465;Yang2025_Silkie_Wenchang	CRA004775	0.074041237
ENSGALG00010007614	BIRC6	2	0.054274944	0.212765847	0.25509237	0.798651757	0.050787082	2.079406195	51.90934784	0.5	CRA004775;Yang2025_Silkie_Wenchang	PRJNA827465	0.949922217
ENSGALG00010007614	BIRC6	2	0.00532598	0.256906096	0.020731234	0.983460053	0.101481522	3.596607942	72.19602425	0.5	CRA004775;PRJNA827465	Yang2025_Silkie_Wenchang	0.995439878
ENSGALG00010007624	TPPP	2	0.333085285	0.27494329	1.211469043	0.225715692	0.110958624	3.43929951	70.92431185	1	PRJNA827465;Yang2025_Silkie_Wenchang	CRA004775	0.738905868
ENSGALG00010007624	TPPP	2	0.589219389	0.22893334	2.57375963	0.010060014	0	0.257309816	0	1	CRA004775;Yang2025_Silkie_Wenchang	PRJNA827465	0.159238308
ENSGALG00010007624	TPPP	2	0.13084479	0.12393402	1.055761685	0.291077107	0	0.473839652	0	1	CRA004775;PRJNA827465	Yang2025_Silkie_Wenchang	0.78467882
ENSGALG00010007626	DLK2	2	-0.059884292	0.09666351	-0.619512909	0.535578521	0	0.816727831	0	0.5	PRJNA827465;Yang2025_Silkie_Wenchang	CRA004775	0.874171355
ENSGALG00010007626	DLK2	2	-0.761889933	0.741418648	-1.027610966	0.304132864	0.877700003	4.164780965	75.98913344	1	CRA004775;Yang2025_Silkie_Wenchang	PRJNA827465	0.79225794
ENSGALG00010007626	DLK2	2	-0.687483487	0.835765499	-0.822579405	0.410747214	1.16994717	5.284089578	81.07526405	0.5	CRA004775;PRJNA827465	Yang2025_Silkie_Wenchang	0.835028074
ENSGALG00010007633	ENSGALG00010007633	2	0.317650698	0.325657653	0.975412969	0.329355453	0.195816718	12.93393894	92.26840327	0.5	PRJNA827465;Yang2025_Silkie_Wenchang	CRA004775	0.800981213
ENSGALG00010007633	ENSGALG00010007633	2	0.083689015	0.574304035	0.145722491	0.884140466	0.630366504	22.45615485	95.54687787	0.5	CRA004775;Yang2025_Silkie_Wenchang	PRJNA827465	0.972001076
ENSGALG00010007633	ENSGALG00010007633	2	-0.222883544	0.247223734	-0.901545899	0.367298141	0.099252659	5.076471976	80.30128001	1	CRA004775;PRJNA827465	Yang2025_Silkie_Wenchang	0.819739734
ENSGALG00010007640	TMEM123	2	-0.025543747	0.468858171	-0.054480755	0.956552141	0.382152075	7.255781098	86.21788631	0.5	PRJNA827465;Yang2025_Silkie_Wenchang	CRA004775	0.988540618
ENSGALG00010007640	TMEM123	2	-0.422272937	0.138896105	-3.040207197	0.002364154	0	0.518348814	0	1	CRA004775;Yang2025_Silkie_Wenchang	PRJNA827465	0.064796626
ENSGALG00010007640	TMEM123	2	0.283108979	0.282358914	1.002656424	0.316026662	0.020579635	1.131850669	11.64912233	0.5	CRA004775;PRJNA827465	Yang2025_Silkie_Wenchang	0.796883862
ENSGALG00010007642	ZNF318	2	0.241555473	0.283422723	0.852279841	0.394058792	0.142283625	7.936683467	87.40027867	0.5	PRJNA827465;Yang2025_Silkie_Wenchang	CRA004775	0.830486298
ENSGALG00010007642	ZNF318	2	0.623589552	0.149732861	4.164680675	3.11789E-05	0	0.24477543	0	1	CRA004775;Yang2025_Silkie_Wenchang	PRJNA827465	0.003422969
ENSGALG00010007642	ZNF318	2	0.314018386	0.358456354	0.876029627	0.381013928	0.232305765	9.559260227	89.53893945	0.5	CRA004775;PRJNA827465	Yang2025_Silkie_Wenchang	0.824392515
ENSGALG00010007643	COTL1	2	-0.331306839	0.394772351	-0.839235164	0.401337358	0.303387896	37.3900131	97.32548904	0.5	PRJNA827465;Yang2025_Silkie_Wenchang	CRA004775	0.832528277
ENSGALG00010007643	COTL1	2	-0.63358	0.180070044	-3.518519711	0.000433962	0.03015115	1.477154065	32.30225447	1	CRA004775;Yang2025_Silkie_Wenchang	PRJNA827465	0.021187568
ENSGALG00010007643	COTL1	2	0.033991099	0.091742162	0.370506845	0.711004877	0.0040055	1.066863614	6.267306628	0.5	CRA004775;PRJNA827465	Yang2025_Silkie_Wenchang	0.925563881
ENSGALG00010007650	KLHL36	2	-0.037101428	0.061413771	-0.604122294	0.545762338	0	0.281855656	0	1	PRJNA827465;Yang2025_Silkie_Wenchang	CRA004775	0.877885919
ENSGALG00010007650	KLHL36	2	-0.099581349	0.091924571	-1.083294142	0.278677885	0	0.265927003	0	1	CRA004775;Yang2025_Silkie_Wenchang	PRJNA827465	0.776992195
ENSGALG00010007650	KLHL36	2	-0.031770709	0.073712571	-0.431008022	0.666462537	0	0.652827651	0	1	CRA004775;PRJNA827465	Yang2025_Silkie_Wenchang	0.913579507
ENSGALG00010007651	CTNNAL1	2	0.210551031	0.154107351	1.366262082	0.171856718	0.036863601	4.341756979	76.96784954	1	PRJNA827465;Yang2025_Silkie_Wenchang	CRA004775	0.674565888
ENSGALG00010007651	CTNNAL1	2	0.492899493	0.186121339	2.648269648	0.008090496	0.032137242	1.578948205	36.66670021	1	CRA004775;Yang2025_Silkie_Wenchang	PRJNA827465	0.139660521
ENSGALG00010007651	CTNNAL1	2	0.372892702	0.358929776	1.038901554	0.298850523	0.213972044	5.183138429	80.70667003	1	CRA004775;PRJNA827465	Yang2025_Silkie_Wenchang	0.78974322
ENSGALG00010007658	BIRC2	2	0.066368606	0.098930412	0.670861518	0.502308753	0.013343633	3.138058247	68.13316002	0.5	PRJNA827465;Yang2025_Silkie_Wenchang	CRA004775	0.862860181
ENSGALG00010007658	BIRC2	2	-0.033151255	0.071551439	-0.463320585	0.643134593	0	0.001827819	0	1	CRA004775;Yang2025_Silkie_Wenchang	PRJNA827465	0.906634523
ENSGALG00010007658	BIRC2	2	0.109781789	0.08556772	1.28298136	0.199498607	0.00436688	1.316101269	24.01800501	0.5	CRA004775;PRJNA827465	Yang2025_Silkie_Wenchang	0.711321213
ENSGALG00010007660	FAM98A	2	0.018447964	0.054773621	0.336803816	0.736264797	0	0.000416274	0	1	PRJNA827465;Yang2025_Silkie_Wenchang	CRA004775	0.933661344
ENSGALG00010007660	FAM98A	2	0.021253742	0.086547251	0.245573855	0.80601213	0	0.002190955	0	1	CRA004775;Yang2025_Silkie_Wenchang	PRJNA827465	0.952063038
ENSGALG00010007660	FAM98A	2	0.018521407	0.066590576	0.278138554	0.780906001	0	0.003125822	0	1	CRA004775;PRJNA827465	Yang2025_Silkie_Wenchang	0.945235709
ENSGALG00010007663	ARFIP1	2	-0.213555657	0.192104054	-1.111666585	0.266281532	0.069631204	17.48736526	94.28158568	1	PRJNA827465;Yang2025_Silkie_Wenchang	CRA004775	0.770383736
ENSGALG00010007663	ARFIP1	2	-0.078013277	0.352966535	-0.221021739	0.825075506	0.229085447	11.92314511	91.61295119	0.5	CRA004775;Yang2025_Silkie_Wenchang	PRJNA827465	0.956315185
ENSGALG00010007663	ARFIP1	2	0.083335323	0.152866802	0.545149911	0.585650418	0.033001291	2.726207526	63.3190067	0.5	CRA004775;PRJNA827465	Yang2025_Silkie_Wenchang	0.891161809
ENSGALG00010007671	FAM206A	2	-0.182488336	0.2259871	-0.807516607	0.419368911	0.076843036	3.686466657	72.87375438	0.5	PRJNA827465;Yang2025_Silkie_Wenchang	CRA004775	0.838527387
ENSGALG00010007671	FAM206A	2	-0.332857168	0.164353628	-2.0252499	0.04284171	0	0.70951856	0	1	CRA004775;Yang2025_Silkie_Wenchang	PRJNA827465	0.357943462
ENSGALG00010007671	FAM206A	2	-0.0179676	0.092625045	-0.193982095	0.846189902	0	0.441606602	0	0.5	CRA004775;PRJNA827465	Yang2025_Silkie_Wenchang	0.96122283
ENSGALG00010007673	FHDC1	2	0.061912088	0.16954282	0.365170806	0.714983909	0.024511992	1.721846482	41.92281306	0.5	PRJNA827465;Yang2025_Silkie_Wenchang	CRA004775	0.926823015
ENSGALG00010007673	FHDC1	2	-0.049636463	0.15726811	-0.315616832	0.752293361	0	0.840534028	0	0.5	CRA004775;Yang2025_Silkie_Wenchang	PRJNA827465	0.93806821
ENSGALG00010007673	FHDC1	2	0.279103553	0.19382922	1.439945702	0.149882762	0	0.14651003	0	1	CRA004775;PRJNA827465	Yang2025_Silkie_Wenchang	0.639822112
ENSGALG00010007678	CDH8	2	-0.112717959	0.271494181	-0.415176333	0.678012823	0.047676783	1.463237965	31.65841619	0.5	PRJNA827465;Yang2025_Silkie_Wenchang	CRA004775	0.917534751
ENSGALG00010007678	CDH8	2	0.290421575	0.318843489	0.910859354	0.362369484	0	0.341238883	0	1	CRA004775;Yang2025_Silkie_Wenchang	PRJNA827465	0.815644888
ENSGALG00010007678	CDH8	2	-0.060462846	0.461074085	-0.131134774	0.895668693	0.213798975	1.679470619	40.45742816	0.5	CRA004775;PRJNA827465	Yang2025_Silkie_Wenchang	0.974927462
ENSGALG00010007679	TMEM135	2	-0.475543239	0.079520126	-5.980162039	2.22916E-09	0	0.400497986	0	1	PRJNA827465;Yang2025_Silkie_Wenchang	CRA004775	1.25161E-06
ENSGALG00010007679	TMEM135	2	-0.153366721	0.431390821	-0.355516885	0.722202398	0.315092293	5.960466901	83.22279082	0.5	CRA004775;Yang2025_Silkie_Wenchang	PRJNA827465	0.929458097
ENSGALG00010007679	TMEM135	2	-0.112042056	0.378754804	-0.295816856	0.767369949	0.23351546	4.7605753	78.99413544	0.5	CRA004775;PRJNA827465	Yang2025_Silkie_Wenchang	0.941444269
ENSGALG00010007680	CHMP2B	2	-0.073897666	0.048713676	-1.516979877	0.129271765	0	0.310392193	0	1	PRJNA827465;Yang2025_Silkie_Wenchang	CRA004775	0.603386934
ENSGALG00010007680	CHMP2B	2	-0.118187134	0.092532679	-1.277247517	0.201514876	0	0.024554855	0	1	CRA004775;Yang2025_Silkie_Wenchang	PRJNA827465	0.714377024
ENSGALG00010007680	CHMP2B	2	-0.062284265	0.052737094	-1.181033314	0.237589489	0	0.026340016	0	1	CRA004775;PRJNA827465	Yang2025_Silkie_Wenchang	0.749400455
ENSGALG00010007695	RASGRP3	2	0.216626462	0.065261459	3.319362817	0.000902231	0	0.195146011	0	1	PRJNA827465;Yang2025_Silkie_Wenchang	CRA004775	0.035319117
ENSGALG00010007695	RASGRP3	2	0.255971847	0.087766277	2.916517088	0.003539633	0	0.124132971	0	1	CRA004775;Yang2025_Silkie_Wenchang	PRJNA827465	0.084314066
ENSGALG00010007695	RASGRP3	2	0.203960866	0.087322445	2.335720978	0.019505788	0	0.302966058	0	1	CRA004775;PRJNA827465	Yang2025_Silkie_Wenchang	0.234012699

ENSGALG00010007698	ADAMTS18	2	-0.330053896	0.128033118	-2.577879074	0.009940878	0	0.268865706	0	1	PRJNA827465;Yang2025_Silkie_Wenchang	CRA004775	0.157756967
ENSGALG00010007698	ADAMTS18	2	-1.11989517	1.082296627	-1.034739592	0.300790541	1.934772454	5.083313996	80.32779402	1	CRA004775;Yang2025_Silkie_Wenchang	PRJNA827465	0.790767266
ENSGALG00010007698	ADAMTS18	2	-1.172626921	0.995117623	-1.178380218	0.238645061	1.621571259	4.631500711	78.40872619	1	CRA004775;PRJNA827465	Yang2025_Silkie_Wenchang	0.750694914
ENSGALG00010007699	SLC39A6	2	-0.210689488	0.058064759	-3.628525998	0.000285044	0.000628201	1.089234925	8.192440666	1	PRJNA827465;Yang2025_Silkie_Wenchang	CRA004775	0.016499391
ENSGALG00010007699	SLC39A6	2	-0.103514413	0.288306759	-0.359042616	0.719563209	0.109746251	2.26683745	55.88567675	0.5	CRA004775;Yang2025_Silkie_Wenchang	PRJNA827465	0.928449671
ENSGALG00010007699	SLC39A6	2	-0.077120514	0.195361986	-0.394757012	0.693022223	0.04246769	1.50576759	33.58868883	0.5	CRA004775;PRJNA827465	Yang2025_Silkie_Wenchang	0.920631027
ENSGALG00010007713	SCLT1	2	0.388241851	0.397412317	0.976924554	0.328606505	0.28948968	11.66544654	91.42767492	1	PRJNA827465;Yang2025_Silkie_Wenchang	CRA004775	0.800761292
ENSGALG00010007713	SCLT1	2	0.555232855	0.274532944	2.022463487	0.043128492	0.095411767	2.685527598	62.76336908	1	CRA004775;Yang2025_Silkie_Wenchang	PRJNA827465	0.35927917
ENSGALG00010007713	SCLT1	2	0.044433776	0.099947446	0.444571398	0.656629521	0	0.72655578	0	1	CRA004775;PRJNA827465	Yang2025_Silkie_Wenchang	0.910509456
ENSGALG00010007719	LTBP1	2	0.180994645	0.178049	1.016544009	0.309370401	0.039880552	2.588510715	61.36774733	1	PRJNA827465;Yang2025_Silkie_Wenchang	CRA004775	0.794043289
ENSGALG00010007719	LTBP1	2	0.311748736	0.171310754	1.819784968	0.068791758	0.000126243	1.001217696	0.121621493	0.5	CRA004775;Yang2025_Silkie_Wenchang	PRJNA827465	0.451825277
ENSGALG00010007719	LTBP1	2	0.021264602	0.117325761	0.181244101	0.85617598	0	0.048362904	0	0.5	CRA004775;PRJNA827465	Yang2025_Silkie_Wenchang	0.963499334
ENSGALG00010007724	RBM11	2	0.050312148	0.222560514	0.226060533	0.82115434	0	0.605994726	0	0.5	PRJNA827465;Yang2025_Silkie_Wenchang	CRA004775	0.955598716
ENSGALG00010007724	RBM11	2	-0.503687678	0.258271154	-1.95022816	0.051148931	0	0.609533267	0	1	CRA004775;Yang2025_Silkie_Wenchang	PRJNA827465	0.391850688
ENSGALG00010007724	RBM11	2	-0.227400332	0.40469889	-0.561900062	0.574184111	0.242502082	3.816896822	73.80070653	0.5	CRA004775;PRJNA827465	Yang2025_Silkie_Wenchang	0.886081433
ENSGALG00010007730	COG2	2	-0.218332143	0.283449459	-0.770268335	0.441140736	0.151229013	16.50597624	93.94158827	0.5	PRJNA827465;Yang2025_Silkie_Wenchang	CRA004775	0.844898121
ENSGALG00010007730	COG2	2	-0.457691698	0.104546721	-4.377867557	1.19846E-05	0	0.558715737	0	1	CRA004775;Yang2025_Silkie_Wenchang	PRJNA827465	0.001635525
ENSGALG00010007730	COG2	2	-0.112201924	0.198173919	-0.566179064	0.571272061	0.063778568	4.674844976	78.60891634	0.5	CRA004775;PRJNA827465	Yang2025_Silkie_Wenchang	0.885354083
ENSGALG00010007731	ENSGALG00010007731	2	-0.126410364	0.19626918	-0.644066296	0.519532445	0.046102834	2.488478721	59.81480606	0.5	PRJNA827465;Yang2025_Silkie_Wenchang	CRA004775	0.869086345
ENSGALG00010007731	ENSGALG00010007731	2	-0.312998757	0.157993398	-1.981087571	0.047581454	0	0.007281801	0	1	CRA004775;Yang2025_Silkie_Wenchang	PRJNA827465	0.376883088
ENSGALG00010007731	ENSGALG00010007731	2	0.015709422	0.164982427	0.09521876	0.924141069	0	0.634777951	0	0.5	CRA004775;PRJNA827465	Yang2025_Silkie_Wenchang	0.98230395
ENSGALG00010007741	ENSGALG00010007741	2	0.133391305	0.110710076	1.204870502	0.228253298	0	0.028829427	0	1	PRJNA827465;Yang2025_Silkie_Wenchang	CRA004775	0.741040754
ENSGALG00010007741	ENSGALG00010007741	2	0.049294065	0.160073188	0.307947043	0.758122629	0	0.894899912	0	0.5	CRA004775;Yang2025_Silkie_Wenchang	PRJNA827465	0.938954369
ENSGALG00010007741	ENSGALG00010007741	2	0.082201766	0.164641771	0.499276494	0.617584612	0.010788188	1.107128214	9.676224733	0.5	CRA004775;PRJNA827465	Yang2025_Silkie_Wenchang	0.899121942
ENSGALG00010007748	CSGALNACT1	2	0.608630267	0.395725894	1.538009707	0.124046241	0.243135041	4.423691771	77.39444673	1	PRJNA827465;Yang2025_Silkie_Wenchang	CRA004775	0.590534404
ENSGALG00010007748	CSGALNACT1	2	0.481034958	0.617838419	0.778577284	0.436228765	0.591249073	4.213089464	76.26444896	0.5	CRA004775;Yang2025_Silkie_Wenchang	PRJNA827465	0.843122822
ENSGALG00010007748	CSGALNACT1	2	0.161219221	0.212280194	0.759464263	0.447574884	0	0.614218066	0	0.5	CRA004775;PRJNA827465	Yang2025_Silkie_Wenchang	0.846601815
ENSGALG00010007751	HSPA13	2	-0.040319078	0.218615743	-0.184428978	0.853676938	0.083766107	8.072770304	87.61267864	0.5	PRJNA827465;Yang2025_Silkie_Wenchang	CRA004775	0.962975689
ENSGALG00010007751	HSPA13	2	0.102069974	0.376278617	0.27126169	0.786189769	0.261082418	12.66802829	92.10611173	0.5	CRA004775;Yang2025_Silkie_Wenchang	PRJNA827465	0.946639719
ENSGALG00010007751	HSPA13	2	0.29932724	0.15425876	1.940422966	0.052328308	0.028953587	2.387684687	58.11842303	1	CRA004775;PRJNA827465	Yang2025_Silkie_Wenchang	0.397191866
ENSGALG00010007753	PRSS23	2	-0.258533858	0.171802262	-1.504833844	0.1323668	0.043375281	3.759805168	73.40287713	1	PRJNA827465;Yang2025_Silkie_Wenchang	CRA004775	0.609324473
ENSGALG00010007753	PRSS23	2	-0.141141733	0.402859398	-0.350349857	0.726076153	0.231579214	2.841738652	64.81027559	0.5	CRA004775;Yang2025_Silkie_Wenchang	PRJNA827465	0.931029023
ENSGALG00010007753	PRSS23	2	-0.06068253	0.121579583	-0.499117767	0.617696422	0.001936871	1.0156381	1.539731519	0.5	CRA004775;PRJNA827465	Yang2025_Silkie_Wenchang	0.899121942
ENSGALG00010007758	DEK	2	-0.001120403	0.121419278	-0.009227557	0.99263758	0.019695322	2.480130167	59.67953565	0.5	PRJNA827465;Yang2025_Silkie_Wenchang	CRA004775	0.997852036
ENSGALG00010007758	DEK	2	0.156862278	0.128099083	1.224538646	0.220749097	0	0.020062779	0	1	CRA004775;Yang2025_Silkie_Wenchang	PRJNA827465	0.734053249
ENSGALG00010007758	DEK	2	-0.07742684	0.04914249	-1.57557943	0.115127715	0	0.87898714	0	0.5	CRA004775;PRJNA827465	Yang2025_Silkie_Wenchang	0.572141484
ENSGALG00010007759	AGT	2	-0.706539685	1.805834404	-0.391253862	0.695609599	5.037382773	4.392805867	77.23550664	0.5	PRJNA827465;Yang2025_Silkie_Wenchang	CRA004775	0.921038437
ENSGALG00010007759	AGT	2	-0.475391671	1.908175756	-0.249134111	0.803257044	6.307582031	7.259396461	86.22475015	0.5	CRA004775;Yang2025_Silkie_Wenchang	PRJNA827465	0.950951274
ENSGALG00010007759	AGT	2	1.25319452	0.618154621	2.027315623	0.042630145	0	0.022904518	0	1	CRA004775;PRJNA827465	Yang2025_Silkie_Wenchang	0.357019514
ENSGALG00010007761	ENSGALG00010007761	2	0.011135241	0.076011505	0.146494155	0.883531303	0	0.66895395	0	0.5	PRJNA827465;Yang2025_Silkie_Wenchang	CRA004775	0.972001076
ENSGALG00010007761	ENSGALG00010007761	2	-0.152371459	0.156837522	-0.971524272	0.331287273	0	0.040875858	0	1	CRA004775;Yang2025_Silkie_Wenchang	PRJNA827465	0.801798188
ENSGALG00010007761	ENSGALG00010007761	2	0.019103641	0.079543206	0.240166844	0.810200916	0	0.66395294	0	0.5	CRA004775;PRJNA827465	Yang2025_Silkie_Wenchang	0.952960588
ENSGALG00010007762	SH2D4A	2	0.582873083	0.268882308	2.167767872	0.030176733	0	0.437860182	0	1	PRJNA827465;Yang2025_Silkie_Wenchang	CRA004775	0.259937612
ENSGALG00010007762	SH2D4A	2	0.18962917	0.692860195	0.27369038	0.784322571	0.674996972	3.276111701	69.47601024	0.5	CRA004775;Yang2025_Silkie_Wenchang	PRJNA827465	0.946083234
ENSGALG00010007762	SH2D4A	2	0.069636431	0.494343124	0.140866592	0.887975335	0.268297674	2.035322374	50.86773415	0.5	CRA004775;PRJNA827465	Yang2025_Silkie_Wenchang	0.973135755
ENSGALG00010007763	FZD4	2	-0.393253824	0.332979776	-1.181014139	0.237597106	0.213359776	26.38571095	96.21006991	1	PRJNA827465;Yang2025_Silkie_Wenchang	CRA004775	0.749400455
ENSGALG00010007763	FZD4	2	-0.251576316	0.506549842	-0.496646713	0.619438206	0.47423819	12.63200002	92.08359722	0.5	CRA004775;Yang2025_Silkie_Wenchang	PRJNA827465	0.899230989
ENSGALG00010007763	FZD4	2	0.018843805	0.147755069	0.127534067	0.898517712	0.021827567	1.559047752	35.85828281	0.5	CRA004775;PRJNA827465	Yang2025_Silkie_Wenchang	0.975752698
ENSGALG00010007765	NUDT7	2	-0.017790051	0.061607727	-0.288763311	0.77276251	0	0.016472345	0	1	PRJNA827465;Yang2025_Silkie_Wenchang	CRA004775	0.943066211
ENSGALG00010007765	NUDT7	2	0.009175143	0.120101172	0.076395113	0.939104758	0	0.03582025	0	0.5	CRA004775;Yang2025_Silkie_Wenchang	PRJNA827465	0.985349045
ENSGALG00010007765	NUDT7	2	-0.017001641	0.067008812	-0.253722472	0.799709972	0	0.07650183	0	0.5	CRA004775;PRJNA827465	Yang2025_Silkie_Wenchang	0.949922217
ENSGALG00010007766	ENSGALG00010007766	2	0.01332161	0.100385133	0.132705007	0.894426683	0	0.200448661	0	1	PRJNA827465;Yang2025_Silkie_Wenchang	CRA004775	0.974394857
ENSGALG00010007766	ENSGALG00010007766	2	-0.111056461	0.154900421	-0.716953904	0.47340254	0	0.712383046	0	1	CRA004775;Yang2025_Silkie_Wenchang	PRJNA827465	0.854354854
ENSGALG00010007766	ENSGALG00010007766	2	-0.037181287	0.170150157	-0.218520441	0.827023633	0.020502341	1.281640078	21.97697432	0.5	CRA004775;PRJNA827465	Yang2025_Silkie_Wenchang	0.956540044
ENSGALG00010007767	VAT1L	2	0.86310823	0.260437526	3.314070151	0.000919484	0.098848707	3.609386958	72.29446409	1	PRJNA827465;Yang2025_Silkie_Wenchang	CRA004775	0.035733817
ENSGALG00010007767	VAT1L	2	0.574795655	0.196087826	2.931317388	0.003375277	0	0.00115787	0	1	CRA004775;Yang2025_Silkie_Wenchang	PRJNA827465	0.081471883
ENSGALG00010007767	VAT1L	2	0.962511158	0.235659343	4.084332691	4.42037E-05	0.049065656	1.510740629	33.8073008	1	CRA004775;PRJNA827465	Yang2025_Silkie_Wenchang	0.004466161
ENSGALG00010007772	CLEC3A	2	0.537363679	0.577186757	0.93117812	0.351761434	0	0.000190168	0	1	PRJNA827465;Yang2025_Silkie_Wenchang	CRA004775	0.810678834
ENSGALG00010007772	CLEC3A	2	-1.477921886	2.12394088	-0.695839465	0.486529371	7.91494137	7.968491279	87.45057295	0.5	CRA004775;Yang2025_Silkie_Wenchang	PRJNA827465	0.857603818
ENSGALG00010007772	CLEC3A	2	-1.479983051	2.132466219	-0.694024148	0.487667065	7.941971337	7.748947742	87.09502202	0.5	CRA004775;PRJNA827465	Yang2025_Silkie_Wenchang	0.85768885
ENSGALG00010007777	KIF13A	2	0.190603914	0.282056168	0.682855391	0.494697466	0.144862197	10.49122423	90.46822394	0.5	PRJNA827465;Yang2025_Silkie_Wenchang	CRA004775	0.866051985
ENSGALG00010007777	KIF13A	2	0.46312281	0.137148558	3.376796789	0.000733352	0	0.132848697	0	1	CRA004775;Yang2025_Silkie_Wenchang	PRJNA827465	0.030276194
ENSGALG00010007777	KIF13A	2	0.102282226	0.221859252	0.461023038	0.644782077	0.075639093	3.528875917	71.66236434	0.5	CRA004775;PRJNA827465	Yang2025_Silkie_Wenchang	0.906953008
ENSGALG00010007780	SIRT5	2	-0.227044429	0.17754628	-1.278790123	0.200970973	0.039188236	2.17326335	53.98624838	1	PRJNA827465;Yang2025_Silkie_Wenchang	CRA004775	0.713544434
ENSGALG00010007780	SIRT5	2	-0.42585592	0.218722564	-1.947014119	0.051533042	0	0.310893354	0	1	CRA004775;Yang2025_Silkie_Wenchang	PRJNA827465	0.393585899
ENSGALG00010007780	SIRT5	2	-0.107773012	0.082931814	-1.299537616	0.193759493	0	0.02305276	0	1	CRA004775;PRJNA827465	Yang2025_Silkie_Wenchang	0.70398689
ENSGALG00010007781	MBP	2	0.202991889	0.243395813	0.8339991	0.404281491	0.046516397	1.611924883	37.96236968	1	PRJNA827465;Yang2025_Silkie_Wenchang	CRA004775	0.833112102
ENSGALG00010007781	MBP	2	0.652509787	0.212654178	3.068408031	0.002152026	0	0.464439705	0	1	CRA004775;Yang2025_Silkie_Wenchang	PRJNA827465	0.061008576
ENSGALG00010007781	MBP	2	0.374280863	0.392443943	0.953718027	0.340226393	0.240006385	4.491124714	77.73386259	1	CRA004775;PRJNA827465	Yang2025_Silkie_Wenchang	0.805377063
ENSGALG00010007783	NOL7	2	0.069416475	0.217222172	0.319564406	0.74929856	0.07629491	4.837104302	79.32647432	0.5	PRJNA827465;Yang2025_Silkie_Wenchang	CRA004775	0.937362187
ENSGALG00010007783	NOL7	2	-0.061727776	0.383403455	-0.160999529	0.872093771	0.258709221	8.328000455	87.99231574	0.5	CRA004775;Yang2025_Silkie_Wenchang	PRJNA827465	0.969560463
ENSGALG00010007783	NOL7	2	-0.236956763	0.157254521	-1.50683594	0.131852715	0.031752569	2.43549825	58.94063977	1	CRA004775;PRJNA827465	Yang2025_Silkie_Wenchang	0.608454531
ENSGALG00010007784	INTS10	2	-0.099590236	0.056609814	-1.759239761	0.078536793	0	0.213643643	0	1	PRJNA827465;Yang2025_Silkie_Wenchang	CRA004775	0.482000561
ENSGALG00010007784	INTS10	2	-0.162582331	0.139543294	-1.165103153	0.243977241	0.018172593	1.785364708	43.9890351	1	CRA004775;Yang2025_Silkie_Wenchang	PRJNA827465	0.755518239
ENSGALG00010007784	INTS10	2	-0.163913776	0.093590179	-1.751399326	0.079877149	0.006986834	1.375085681	27.2772589	1	CRA004775;PRJNA827465	Yang2025_Silkie_Wenchang	0.486052267
ENSGALG00010007786	SAMSN1	2	-0.130102834	0.15522451	-0.83815909	0.40194136	0	0.116003777	0	1	PRJNA827465;Yang2025_Silkie_Wenchang	CRA004775	0.832825075
ENSGALG00010007786	SAMSN1	2	-0.123354523	0.263690714	-0.467800029	0.6399276	0	0.301672145	0	1	CRA004775;Yang2025_Silkie_Wenchang	PRJNA827465	0.906064918
ENSGALG00010007786	SAMSN1	2	-0.183921922	0.17129407	-1.07372031	0.282948056	0	0.1485494	0	1	CRA004775;PRJNA827465	Yang2025_Silkie_Wenchang	0.779541654
ENSGALG00010007787	C1orf198	2	0.05110051	0.06054177	0.844053782	0.398639353	0	0.205157218	0	1	PRJNA827465;Yang2025_Silkie_Wenchang	CRA004775	0.831980453
ENSGALG00010007787	C1orf198	2	0.001975476	0.092873131	0.02127069	0.983029724	0	0.215940783	0	0.5	CRA004775;Yang2025_Silkie_Wenchang	PRJNA827465	0.995363468
ENSGALG00010007787	C1orf198	2	0.059873434	0.07456509	0.802968713	0.421992818	0	0.433817437	0	0.5	CRA004775;PRJNA827465	Yang2025_Silkie_Wenchang	0.839649223
ENSGALG00010007788	PALM2AKAP2	2	0.127714856	0.245277006	0.520696408	0.602578277	0.080829795	2.995136418	66.61253912	0.5	PRJNA827465;Yang2025_Silkie_Wenchang	CRA004775	0.895521995
ENSGALG00010007788	PALM2AKAP2	2	0.476214388	0.208286794	2.286339804	0.022234387	0	0.766391728	0	1	CRA004775;Yang2025_Silkie_Wenchang	PRJNA827465	0.252865604
ENSGALG00010007788	PALM2AKAP2	2	0.276902703	0.47970654	0.577233536	0.563781705	0.339795558	3.261426991	69.33857471	0.5	CRA004775;PRJNA827465	Yang2025_Silkie_Wenchang	0.882651345
ENSGALG00010007789	WWOX	2	-0.178083947	0.066041381	-2.696550912	0.007006169	0.000411132	1.047867125	4.568052974	1	PRJNA827465;Yang2025_Silkie_Wenchang	CRA004775	0.12854434
ENSGALG00010007789	WWOX	2	-0.279949494	0.091730095	-3.051882755	0.002274109	0	0.30181957	0	1	CRA004775;Yang2025_Silkie_Wenchang	PRJNA827465	0.063254996
ENSGALG00010007789	WWOX	2	-0.178492741	0.10864034	-1.642969282	0.100389289	0.007897778	1.283145317	22.06650433	1	CRA004775;PRJNA827465	Yang2025_Silkie_Wenchang	0.541223839
ENSGALG00010007792	ENSGALG00010007792	2	0.188419029	0.27007188	0.697662523	0.485388271	0.093992212	2.807141603	64.37657441	0.5	PRJNA827465;Yang2025_Silkie_Wenchang	CRA004775	0.857076964
ENSGALG00010007792	ENSGALG00010007792	2	0.279279993	0.234739273	1.189745497	0.234146437	0.033869449	1.412752732	29.21620484	0.5	CRA004775;Yang2025_Silkie_Wenchang	PRJNA827465	0.747392101
ENSGALG00010007792	ENSGALG00010007792	2	-0.055767927	0.181876264	-0.306625644	0.759128332	0	0.022278887	0	1	CRA004775;PRJNA827465	Yang2025_Silkie_Wenchang	0.939081587
ENSGALG00010007795	ZNF236	2	0.04311405	0.059882783	0.719974062	0.471540966	0	0.497525083	0	1	PRJNA827465;Yang2025_Silkie_Wenchang	CRA004775	0.853998373
ENSGALG00010007795	ZNF236	2	0.255511326	0.149059884	1.714152184	0.08650078	0.016428173	1.547879214	35.39547587	1	CRA004775;Yang2025_Silkie_Wenchang	PRJNA827465	0.506036629
ENSGALG00010007795	ZNF236	2	0.189090936	0.20347038	0.929329052	0.352718584	0.064432758	3.934712874	74.58518494	1	CRA004775;PRJNA827465	Yang2025_Silkie_Wenchang	0.811035834
ENSGALG00010007796	MCUR1	2	-0.172470459	0.19566063	-0.88147758	0.378059383	0.066912181	7.89551254	87.33457777	0.5	PRJNA827465;Yang2025_Silkie_Wenchang	CRA004775	0.82325159
ENSGALG00010007796	MCUR1	2	-0.123600717	0.273254333	-0.45232848	0.651032362	0.125302712	5.959964958	83.22137786	0.5	CRA004775;Yang2025_Silkie_Wenchang	PRJNA827465	0.908769703
ENSGALG00010007796	MCUR1	2	0.044420975	0.080390788	0.552562998	0.580562685	0	0.530769772	0	1	CRA004775;PRJNA827465	Yang2025_Silkie_Wenchang	0.888441974
ENSGALG00010007798	HGSNAT	2	-0.134781442	0.053318297	-2.527864721	0.011475856	0	0.241788707	0	1	PRJNA827465;Yang2025_Silkie_Wenchang	CRA004775	0.171041171
ENSGALG00010007798	HGSNAT	2	-0.155247559	0.094875132	-1.636335629	0.101769363	0	0.301827115	0	1	CRA004775;Yang2025_Silkie_Wenchang	PRJNA827465	0.543700483
ENSGALG00010007798	HGSNAT	2	-0.113773168	0.059963373	-1.897377708	0.057778106	0	0.110808883	0	1	CRA004775;PRJNA827465	Yang2025_Silkie_Wenchang	0.416579979
ENSGALG00010007800	MAF	2	0.010021843	0.054327116	0.184472215	0.853643023	0	0.000509059	0	1	PRJNA827465;Yang2025_Silkie_Wenchang	CRA004775	0.962972345
ENSGALG00010007800	MAF	2	0.258891157	0.291688007	0.887561883	0.374776465	0.132924756	4.158875226	75.95503722	1	CRA004775;Yang2025_Silkie_Wenchang	PRJNA827465	0.822805106
ENSGALG00010007800	MAF	2	0.24997332	0.291821428	0.856596865	0.391667719	0.139235534	4.712639278	78.78046799	1	CRA004775;PRJNA827465	Yang2025_Silkie_Wenchang	0.829251057
ENSGALG00010007801	TTC13	2	0.210726657	0.168142851	1.253259687	0.210111215	0.046857708	5.803995069	82.77048846	1	PRJNA827465;Yang2025_Silkie_Wenchang	CRA004775	0.72188812
ENSGALG00010007801	TTC13	2	0.348878293	0.093933793	3.714087153	0.000203939	0	0.44664373	0	1	CRA004775;Yang2025_Silkie_Wenchang	PRJNA827465	0.012909619
ENSGALG00010007801	TTC13	2	0.082427699	0.08001988	1.030090257	0.302967638	0	0.835207915	0	1	CRA004775;PRJNA827465	Yang2025_Silkie_Wenchang	0.792084776
ENSGALG00010007802	ENSGALG00010007802	2	0.507026032	0.702198265	0.722055376	0.470260432	0	0.018087232	0	1	PRJNA827465;Yang2025_Silkie_Wenchang	CRA004775	0.853741317
ENSGALG00010007802	ENSGALG00010007802	2	2.037089246	1.761174836	1.156664974	0.247409265	4.392382349	3.39844891	70.57481143	1	CRA004775;Yang2025_Silkie_Wenchang	PRJNA827465	0.757947282
ENSGALG00010007802	ENSGALG00010007802	2	2.020575439	1.649685779	1.224824427	0.220641379	4.073967167	3.783979426	73.572795	1	CRA004775;PRJNA827465	Yang2025_Silkie_Wenchang	0.734053249
ENSGALG00010007803	ME3	2	-0.04482003	0.073362809	-0.610936667	0.541241509	0	0.159178566	0	1	PRJNA827465;Yang2025_Silkie_Wenchang	CRA004775	0.875984806
ENSGALG00010007803	ME3	2	-0.038057453	0.126822603	-0.300084151	0.764112968	0	0.824392225	0	1	CRA004775;Yang2025_Silkie_Wenchang	PRJNA827465	0.940483053
ENSGALG00010007803	ME3	2	-0.078535332	0.085852359	-0.914771971	0.360311352	0	0.610043871	0	1	CRA004775;PRJNA827465	Yang2025_Silkie_Wenchang	0.814880744
ENSGALG00010007805	ENSGALG00010007805	2	-0.165788936	0.639167751	-0.259382511	0.795340122	0	0.005311725	0	1	PRJNA827465;Yang2025_Silkie_Wenchang	CRA004775	0.948935068
ENSGALG00010007805	ENSGALG00010007805	2	-0.208598848	0.709545596	-0.293989349	0.768766042	0	0.025855579	0	1	CRA004775;Yang2025_Silkie_Wenchang	PRJNA827465	0.941623403
ENSGALG00010007805	ENSGALG00010007805	2	-0.267162838	0.713639068	-0.374366889	0.708131374	0	0.00936632	0	1	CRA004775;PRJNA827465	Yang2025_Silkie_Wenchang	0.925061251
ENSGALG00010007806	DYNLRB2	2	-0.086905649	0.229845224	-0.37810509	0.705352527	0.009978875	1.093758448	8.57213474	0.5	PRJNA827465;Yang2025_Silkie_Wenchang	CRA004775	0.924281103
ENSGALG00010007806	DYNLRB2	2	-0.335842206	0.351082779	-0.9565898	0.338774339	0	0.232274945	0	0.5	CRA004775;Yang2025_Silkie_Wenchang	PRJNA827465	0.80447392
ENSGALG00010007806	DYNLRB2	2	0.082378165	0.257038908	0.320489086	0.748597602	0	0.000366082	0	1	CRA004775;PRJNA827465	Yang2025_Silkie_Wenchang	0.937249951
ENSGALG00010007809	ARV1	2	-0.123951091	0.172738924	-0.717563173	0.473026672	0.049647106	5.918098448	83.10268055	0.5	PRJNA827465;Yang2025_Silkie_Wenchang	CRA004775	0.854341771
ENSGALG00010007809	ARV1	2	-0.278423256	0.105267419	-2.644913865	0.008171171	0	0.592381116	0	1	CRA004775;Yang2025_Silkie_Wenchang	PRJNA827465	0.140547724
ENSGALG00010007809	ARV1	2	0.040487408	0.087620615	0.462076285	0.644026616	0	0.016216618	0	0.5	CRA004775;PRJNA827465	Yang2025_Silkie_Wenchang	0.906826466
ENSGALG00010007811	PRCP	2	-0.291827835	0.141132694	-2.067755012	0.038663066	0.026256284	2.911897404	65.65813073	1	PRJNA827465;Yang2025_Silkie_Wenchang	CRA004775	0.339115038
ENSGALG00010007811	PRCP	2	-0.438543034	0.12291517	-3.567851163	0.000359921	0	0.010685268	0	1	CRA004775;Yang2025_Silkie_Wenchang	PRJNA827465	0.01893704
ENSGALG00010007811	PRCP	2	-0.170290455	0.103554042	-1.644459766	0.100081269	0	0.229484701	0	1	CRA004775;PRJNA827465	Yang2025_Silkie_Wenchang	0.54044969
ENSGALG00010007812	POMK	2	-0.091512675	0.184420282	-0.496218065	0.619740567	0.057167031	6.002877309	83.34132203	0.5	PRJNA827465;Yang2025_Silkie_Wenchang	CRA004775	0.899325257
ENSGALG00010007812	POMK	2	-0.180638717	0.186595451	-0.968076744	0.333006043	0.029891242	1.508409212	33.70499253	0.5	CRA004775;Yang2025_Silkie_Wenchang	PRJNA827465	0.802178578
ENSGALG00010007812	POMK	2	0.07926173	0.070059744	1.131344841	0.257909978	0	0.024529566	0	1	CRA004775;PRJNA827465	Yang2025_Silkie_Wenchang	0.765151037
ENSGALG00010007817	CDYL2	2	0.053191921	0.098220868	0.541554167	0.588125673	0	0.67221451	0	0.5	PRJNA827465;Yang2025_Silkie_Wenchang	CRA004775	0.891761457
ENSGALG00010007817	CDYL2	2	-0.214127208	0.530593847	-0.403561423	0.686535259	0.397956368	2.739197822	63.49296163	0.5	CRA004775;Yang2025_Silkie_Wenchang	PRJNA827465	0.919235539
ENSGALG00010007817	CDYL2	2	-0.259769493	0.421156108	-0.616800963	0.537366017	0.228487498	2.031289392	50.77018549	1	CRA004775;PRJNA827465	Yang2025_Silkie_Wenchang	0.87481821
ENSGALG00010007818	ENSGALG00010007818	2	0.372229293	0.246650713	1.509135279	0.131264216	0.075886007	2.646691394	62.21697768	1	PRJNA827465;Yang2025_Silkie_Wenchang	CRA004775	0.606878893
ENSGALG00010007818	ENSGALG00010007818	2	0.705578556	0.205839638	3.427807012	0.000608478	0	0.566058265	0	1	CRA004775;Yang2025_Silkie_Wenchang	PRJNA827465	0.026750565
ENSGALG00010007818	ENSGALG00010007818	2	0.478309675	0.433144753	1.104272121	0.269475113	0.26372577	2.975991436	66.39775277	1	CRA004775;PRJNA827465	Yang2025_Silkie_Wenchang	0.77189992
ENSGALG00010007819	NRIP1	2	-0.002295964	0.143007966	-0.016054799	0.987190674	0.027039474	2.773666253	63.94663565	0.5	PRJNA827465;Yang2025_Silkie_Wenchang	CRA004775	0.996470068
ENSGALG00010007819	NRIP1	2	0.581003264	0.798505064	0.727613751	0.466850061	1.184729079	13.53676211	92.61270907	0.5	CRA004775;Yang2025_Silkie_Wenchang	PRJNA827465	0.852842649
ENSGALG00010007819	NRIP1	2	0.710617557	0.651547133	1.090661781	0.275421734	0.769506021	9.859528151	89.8575268	1	CRA004775;PRJNA827465	Yang2025_Silkie_Wenchang	0.775135547
ENSGALG00010007821	FAM89A	2	-0.42839783	0.453475797	-0.944698333	0.344812908	0.403431134	51.99381017	98.07669414	0.5	PRJNA827465;Yang2025_Silkie_Wenchang	CRA004775	0.806961063
ENSGALG00010007821	FAM89A	2	-0.384316025	0.515720921	-0.745201541	0.456149891	0.506175707	20.40549471	95.0993592	0.5	CRA004775;Yang2025_Silkie_Wenchang	PRJNA827465	0.849188101
ENSGALG00010007821	FAM89A	2	0.028041503	0.05167033	0.5427003	0.587336167	0	0.369789585	0	1	CRA004775;PRJNA827465	Yang2025_Silkie_Wenchang	0.891660017
ENSGALG00010007826	ENSGALG00010007826	2	-2.144400992	1.364069401	-1.572061502	0.115936289	0	0.000174474	0	1	PRJNA827465;Yang2025_Silkie_Wenchang	CRA004775	0.573234866
ENSGALG00010007826	ENSGALG00010007826	2	-1.000348999	1.312216514	-0.762335322	0.445859898	0	0.702996413	0	0.5	CRA004775;Yang2025_Silkie_Wenchang	PRJNA827465	0.846013379
ENSGALG00010007826	ENSGALG00010007826	2	-0.956974432	1.346695181	-0.710609532	0.477326235	0	0.685668721	0	0.5	CRA004775;PRJNA827465	Yang2025_Silkie_Wenchang	0.854997403
ENSGALG00010007830	SMIM19	2	-0.008180863	0.056897326	-0.143782904	0.885671903	0	0.429161992	0	0.5	PRJNA827465;Yang2025_Silkie_Wenchang	CRA004775	0.972376043
ENSGALG00010007830	SMIM19	2	0.045020475	0.131770526	0.341658159	0.732608164	0	0.328261501	0	0.5	CRA004775;Yang2025_Silkie_Wenchang	PRJNA827465	0.932985777
ENSGALG00010007830	SMIM19	2	-0.027714903	0.060813839	-0.455733495	0.648581633	0	0.092443184	0	1	CRA004775;PRJNA827465	Yang2025_Silkie_Wenchang	0.907730933
ENSGALG00010007835	GUCY1A1	2	-0.106966905	0.070772943	-1.511409596	0.130684126	0.001898646	1.233789434	18.94889251	1	PRJNA827465;Yang2025_Silkie_Wenchang	CRA004775	0.606397445
ENSGALG00010007835	GUCY1A1	2	-0.140434098	0.1226689	-1.144822345	0.252282757	0.008390203	1.089820697	8.241786653	0.5	CRA004775;Yang2025_Silkie_Wenchang	PRJNA827465	0.762529875
ENSGALG00010007835	GUCY1A1	2	-0.021449812	0.088906714	-0.241261999	0.809352061	0	0.512454785	0	0.5	CRA004775;PRJNA827465	Yang2025_Silkie_Wenchang	0.95276497
ENSGALG00010007846	ENSGALG00010007846	2	0.009558966	0.132759201	0.07200229	0.942600086	0.012680212	1.502983567	33.46567307	0.5	PRJNA827465;Yang2025_Silkie_Wenchang	CRA004775	0.985827325
ENSGALG00010007846	ENSGALG00010007846	2	-0.036616791	0.313278484	-0.116882559	0.906953119	0.111397611	2.009505902	50.23652337	0.5	CRA004775;Yang2025_Silkie_Wenchang	PRJNA827465	0.978196186
ENSGALG00010007846	ENSGALG00010007846	2	-0.121353216	0.118556655	-1.023588397	0.306029728	0	0.760477796	0	1	CRA004775;PRJNA827465	Yang2025_Silkie_Wenchang	0.792366985
ENSGALG00010007847	CMC2	2	-0.283027534	0.261799034	-1.081087006	0.279658413	0.109383165	4.459150855	77.57420566	1	PRJNA827465;Yang2025_Silkie_Wenchang	CRA004775	0.777788482
ENSGALG00010007847	CMC2	2	-0.40969846	0.267318877	-1.532620758	0.125369324	0.049855278	1.435481886	30.33698234	1	CRA004775;Yang2025_Silkie_Wenchang	PRJNA827465	0.594521059
ENSGALG00010007847	CMC2	2	-0.059937443	0.087920363	-0.681724244	0.495413334	0	0.009816955	0	1	CRA004775;PRJNA827465	Yang2025_Silkie_Wenchang	0.860770265
ENSGALG00010007849	CXADR	2	0.336813848	0.428037674	0.78687898	0.431352714	0.185544127	1.999979625	49.99949062	0.5	PRJNA827465;Yang2025_Silkie_Wenchang	CRA004775	0.842503929
ENSGALG00010007849	CXADR	2	-0.195686685	0.388842755	-0.503254034	0.61478568	0	0.037670108	0	1	CRA004775;Yang2025_Silkie_Wenchang	PRJNA827465	0.898751035
ENSGALG00010007849	CXADR	2	0.35946862	0.487935273	0.736713742	0.461296448	0.223591821	1.750009759	42.85746151	0.5	CRA004775;PRJNA827465	Yang2025_Silkie_Wenchang	0.85014477
ENSGALG00010007851	AvBD7	2	0.282912011	0.352063632	0.803582039	0.421638399	0	0.057439083	0	1	PRJNA827465;Yang2025_Silkie_Wenchang	CRA004775	0.839352211
ENSGALG00010007851	AvBD7	2	0.464836731	0.367363854	1.265330615	0.205752836	0	0.801605891	0	1	CRA004775;Yang2025_Silkie_Wenchang	PRJNA827465	0.71832154
ENSGALG00010007851	AvBD7	2	0.608411351	0.401301752	1.516094431	0.129495476	0	0.397493666	0	1	CRA004775;PRJNA827465	Yang2025_Silkie_Wenchang	0.603529077
ENSGALG00010007852	SLC20A2	2	-0.23500116	0.079274007	-2.964416323	0.003032577	0.00046694	1.036640545	3.534546778	1	PRJNA827465;Yang2025_Silkie_Wenchang	CRA004775	0.075885251
ENSGALG00010007852	SLC20A2	2	0.269849572	0.653733906	0.412781974	0.67976636	0.776577305	10.29897008	90.29029124	0.5	CRA004775;Yang2025_Silkie_Wenchang	PRJNA827465	0.917701868
ENSGALG00010007852	SLC20A2	2	0.341269323	0.571193487	0.597467112	0.550195558	0.579510969	8.189973053	87.78994762	0.5	CRA004775;PRJNA827465	Yang2025_Silkie_Wenchang	0.878679315
ENSGALG00010007853	C1orf131	2	0.489356848	0.41962301	1.166182113	0.243540823	0.319134248	10.25749634	90.25103235	1	PRJNA827465;Yang2025_Silkie_Wenchang	CRA004775	0.755365463
ENSGALG00010007853	C1orf131	2	0.882864028	0.191483396	4.610655787	4.01401E-06	0	0.142104939	0	1	CRA004775;Yang2025_Silkie_Wenchang	PRJNA827465	0.000694016
ENSGALG00010007853	C1orf131	2	0.375148472	0.338844846	1.107139376	0.268233674	0.180995273	4.160064504	75.96191119	1	CRA004775;PRJNA827465	Yang2025_Silkie_Wenchang	0.771398294
ENSGALG00010007854	FAM181B	2	0.389060388	0.199102806	1.954067834	0.050693196	0.066185719	5.617582372	82.1987479	1	PRJNA827465;Yang2025_Silkie_Wenchang	CRA004775	0.389748565
ENSGALG00010007854	FAM181B	2	0.192407753	0.482251199	0.398978279	0.689909209	0.386472689	5.444854169	81.6340352	0.5	CRA004775;Yang2025_Silkie_Wenchang	PRJNA827465	0.919979266
ENSGALG00010007854	FAM181B	2	0.054385616	0.256122156	0.21234249	0.831839849	0.086480899	2.123490405	52.90772222	0.5	CRA004775;PRJNA827465	Yang2025_Silkie_Wenchang	0.957762335
ENSGALG00010007856	PDCD2	2	0.052094127	0.188076343	0.276983941	0.781792437	0.05950952	5.921668003	83.11286618	0.5	PRJNA827465;Yang2025_Silkie_Wenchang	CRA004775	0.945265125
ENSGALG00010007856	PDCD2	2	0.224634133	0.128041288	1.754388273	0.079363999	0	0.396006178	0	1	CRA004775;Yang2025_Silkie_Wenchang	PRJNA827465	0.484805397
ENSGALG00010007856	PDCD2	2	-0.10907706	0.063855258	-1.708192309	0.087600665	0	0.288514247	0	0.5	CRA004775;PRJNA827465	Yang2025_Silkie_Wenchang	0.508938499
ENSGALG00010007857	ENSGALG00010007857	2	0.670824965	0.206850642	3.243040288	0.001182615	0	0.18935078	0	1	PRJNA827465;Yang2025_Silkie_Wenchang	CRA004775	0.041723799
ENSGALG00010007857	ENSGALG00010007857	2	0.57121395	0.247052712	2.312113663	0.02077142	0	0.004818886	0	1	CRA004775;Yang2025_Silkie_Wenchang	PRJNA827465	0.243695265
ENSGALG00010007857	ENSGALG00010007857	2	0.700092714	0.249148456	2.809942013	0.004955043	0	0.155811305	0	1	CRA004775;PRJNA827465	Yang2025_Silkie_Wenchang	0.104831857
ENSGALG00010007859	CENPN	2	-0.011190773	0.082471938	-0.135691893	0.892064857	0	0.154871807	0	0.5	PRJNA827465;Yang2025_Silkie_Wenchang	CRA004775	0.974077808
ENSGALG00010007859	CENPN	2	-0.069266787	0.128534358	-0.538897051	0.589957892	0	0.366327219	0	1	CRA004775;Yang2025_Silkie_Wenchang	PRJNA827465	0.892320726
ENSGALG00010007859	CENPN	2	0.001179131	0.104029505	0.011334586	0.990956502	0	0.522764991	0	0.5	CRA004775;PRJNA827465	Yang2025_Silkie_Wenchang	0.997555603
ENSGALG00010007860	DEFB4A	2	-0.653107094	1.256437722	-0.519808569	0.603197007	2.751826627	7.781082277	87.14831736	0.5	PRJNA827465;Yang2025_Silkie_Wenchang	CRA004775	0.895538966
ENSGALG00010007860	DEFB4A	2	-0.613743448	1.292311484	-0.474919131	0.634844615	2.94479379	8.437872668	88.1486716	0.5	CRA004775;Yang2025_Silkie_Wenchang	PRJNA827465	0.903999779
ENSGALG00010007860	DEFB4A	2	0.626797078	0.425278492	1.473850876	0.140521747	0	0.007125541	0	1	CRA004775;PRJNA827465	Yang2025_Silkie_Wenchang	0.624694741
ENSGALG00010007861	INTU	2	-0.293168845	0.324153586	-0.904413395	0.365776238	0.191976521	11.55245724	91.34383292	0.5	PRJNA827465;Yang2025_Silkie_Wenchang	CRA004775	0.818968857
ENSGALG00010007861	INTU	2	-0.157272051	0.493887164	-0.31843721	0.750153316	0.438215806	9.48240046	89.45414714	0.5	CRA004775;Yang2025_Silkie_Wenchang	PRJNA827465	0.937374353
ENSGALG00010007861	INTU	2	0.100733811	0.139815008	0.720479242	0.471229976	0.008592049	1.172822763	14.73562492	1	CRA004775;PRJNA827465	Yang2025_Silkie_Wenchang	0.85393796
ENSGALG00010007862	ATMIN	2	-0.275103891	0.235187139	-1.169723365	0.242112312	0.105073221	19.80842163	94.95164219	1	PRJNA827465;Yang2025_Silkie_Wenchang	CRA004775	0.754379278
ENSGALG00010007862	ATMIN	2	-0.199740913	0.342515199	-0.583159269	0.559786088	0.208829756	8.596134821	88.36686463	0.5	CRA004775;Yang2025_Silkie_Wenchang	PRJNA827465	0.881203589
ENSGALG00010007862	ATMIN	2	-0.028472655	0.058249626	-0.48880408	0.624980409	0	0.921545246	0	0.5	CRA004775;PRJNA827465	Yang2025_Silkie_Wenchang	0.901119081
ENSGALG00010007863	AvBD1	2	0.3076935	0.343871288	0.894792647	0.370897942	0	0.041790911	0	1	PRJNA827465;Yang2025_Silkie_Wenchang	CRA004775	0.8214684

ENSGALG00010007863	AvBD1	2	0.629444873	0.373589553	1.68485673	0.092016217	0.009061297	1.029648744	2.879500844	1	CRA004775;Yang2025_Silkie_Wenchang	PRJNA827465	0.519539342
ENSGALG00010007863	AvBD1	2	0.626203733	0.463630865	1.350651521	0.176807088	0.087891678	1.250777096	20.04970323	1	CRA004775;PRJNA827465	Yang2025_Silkie_Wenchang	0.682528502
ENSGALG00010007864	TYR	2	6.758274253	0.491148481	13.76014487	4.42639E-43	0	0.096853697	0	1	PRJNA827465;Yang2025_Silkie_Wenchang	CRA004775	9.66502E-40
ENSGALG00010007864	TYR	2	6.52786476	0.630725273	10.34977515	4.1947E-25	0	0.005059038	0	1	CRA004775;Yang2025_Silkie_Wenchang	PRJNA827465	5.88801E-22
ENSGALG00010007864	TYR	2	6.768401955	0.533229358	12.69322825	6.44725E-37	0	0.114708921	0	1	CRA004775;PRJNA827465	Yang2025_Silkie_Wenchang	1.1518E-33
ENSGALG00010007866	TBP	2	0.002825333	0.045570414	0.061999287	0.9505634	0	0.019706418	0	0.5	PRJNA827465;Yang2025_Silkie_Wenchang	CRA004775	0.987277827
ENSGALG00010007866	TBP	2	-0.013833774	0.111456138	-0.124118546	0.901221414	0	0.000444499	0	1	CRA004775;Yang2025_Silkie_Wenchang	PRJNA827465	0.97660383
ENSGALG00010007866	TBP	2	0.004068743	0.046716264	0.087094795	0.930596162	0	0.006619465	0	0.5	CRA004775;PRJNA827465	Yang2025_Silkie_Wenchang	0.983903639
ENSGALG00010007867	ZNF516	2	-0.102184872	0.069438997	-1.471577605	0.141134977	0	0.022558674	0	1	PRJNA827465;Yang2025_Silkie_Wenchang	CRA004775	0.625792869
ENSGALG00010007867	ZNF516	2	-0.211864987	0.220130725	-0.962450776	0.335823218	0.031861101	1.187455606	15.78632538	1	CRA004775;Yang2025_Silkie_Wenchang	PRJNA827465	0.803504387
ENSGALG00010007867	ZNF516	2	-0.193891602	0.233887231	-0.828996102	0.407106613	0.058082012	1.361335153	26.5427035	1	CRA004775;PRJNA827465	Yang2025_Silkie_Wenchang	0.833837678
ENSGALG00010007868	INHBA	2	0.233980376	0.344473178	0.679241202	0.496985045	0.207956565	7.902669167	87.34604753	0.5	PRJNA827465;Yang2025_Silkie_Wenchang	CRA004775	0.860939846
ENSGALG00010007868	INHBA	2	0.580658669	0.194552269	2.984589554	0.002839591	0	0.055561435	0	1	CRA004775;Yang2025_Silkie_Wenchang	PRJNA827465	0.072753867
ENSGALG00010007868	INHBA	2	-0.059095979	0.130141906	-0.454088781	0.649764928	0.001314767	1.008631469	0.855760417	0.5	CRA004775;PRJNA827465	Yang2025_Silkie_Wenchang	0.90820125
ENSGALG00010007869	GCSH	2	-0.09794551	0.290541557	-0.337113601	0.736031265	0.14075725	5.891846245	83.02739144	0.5	PRJNA827465;Yang2025_Silkie_Wenchang	CRA004775	0.933592889
ENSGALG00010007869	GCSH	2	-0.307043553	0.161432956	-1.901988045	0.057172717	0	0.745540574	0	1	CRA004775;Yang2025_Silkie_Wenchang	PRJNA827465	0.414005744
ENSGALG00010007869	GCSH	2	0.119460676	0.120248319	0.993449859	0.320490774	0	0.889025987	0	0.5	CRA004775;PRJNA827465	Yang2025_Silkie_Wenchang	0.798348216
ENSGALG00010007871	HSPA4L	2	-0.002751425	0.07175991	-0.038342085	0.969414937	0	0.085189597	0	0.5	PRJNA827465;Yang2025_Silkie_Wenchang	CRA004775	0.991572914
ENSGALG00010007871	HSPA4L	2	0.015855674	0.123222947	0.128674682	0.897615069	0	0.602025581	0	0.5	CRA004775;Yang2025_Silkie_Wenchang	PRJNA827465	0.975608116
ENSGALG00010007871	HSPA4L	2	0.027030806	0.07895129	0.342373201	0.732070054	0	0.441044689	0	1	CRA004775;PRJNA827465	Yang2025_Silkie_Wenchang	0.93287651
ENSGALG00010007872	KLHL2	2	0.441284333	0.348179086	1.267406201	0.205010084	0.228959175	17.68909988	94.3468011	1	PRJNA827465;Yang2025_Silkie_Wenchang	CRA004775	0.717754438
ENSGALG00010007872	KLHL2	2	0.475199134	0.327577888	1.450644722	0.146878815	0.191847511	9.421329137	89.38578638	1	CRA004775;Yang2025_Silkie_Wenchang	PRJNA827465	0.635558525
ENSGALG00010007872	KLHL2	2	0.112042549	0.071229276	1.572984537	0.115722399	0	0.055697427	0	1	CRA004775;PRJNA827465	Yang2025_Silkie_Wenchang	0.572716099
ENSGALG00010007873	ENSGALG00010007873	2	-1.119671412	0.258002042	-4.339777324	1.42627E-05	0	0.023090908	0	1	PRJNA827465;Yang2025_Silkie_Wenchang	CRA004775	0.001887433
ENSGALG00010007873	ENSGALG00010007873	2	1.237714938	2.463519884	0.502417271	0.615374032	11.38427067	15.36476712	93.49160328	0.5	CRA004775;Yang2025_Silkie_Wenchang	PRJNA827465	0.898864379
ENSGALG00010007873	ENSGALG00010007873	2	1.207195573	2.504967762	0.481920602	0.62986235	11.75026165	15.1085343	93.38122428	0.5	CRA004775;PRJNA827465	Yang2025_Silkie_Wenchang	0.902488805
ENSGALG00010007874	ENSGALG00010007874	2	1.643976718	0.396586794	4.14531382	3.39349E-05	0	0.355256365	0	1	PRJNA827465;Yang2025_Silkie_Wenchang	CRA004775	0.003661746
ENSGALG00010007874	ENSGALG00010007874	2	0.736658299	1.148972932	0.641145042	0.521428452	2.331258937	8.534819615	88.28329074	0.5	CRA004775;Yang2025_Silkie_Wenchang	PRJNA827465	0.869518515
ENSGALG00010007874	ENSGALG00010007874	2	0.509458838	0.911951812	0.558646665	0.576402886	1.378525184	5.840036399	82.87681905	0.5	CRA004775;PRJNA827465	Yang2025_Silkie_Wenchang	0.887048016
ENSGALG00010007877	MSMO1	2	0.109504555	0.221296283	0.49483233	0.620718481	0.071774368	3.654420921	72.63588345	0.5	PRJNA827465;Yang2025_Silkie_Wenchang	CRA004775	0.899395593
ENSGALG00010007877	MSMO1	2	0.106045057	0.26971609	0.393172898	0.694191785	0.093878835	2.749727678	63.63276232	0.5	CRA004775;Yang2025_Silkie_Wenchang	PRJNA827465	0.920728119
ENSGALG00010007877	MSMO1	2	0.330672913	0.119212854	2.773802511	0.00554053	0	0.109624624	0	1	CRA004775;PRJNA827465	Yang2025_Silkie_Wenchang	0.111717753
ENSGALG00010007878	PSMB1	2	-0.013402347	0.047598867	-0.281568604	0.778274317	0	0.663773743	0	1	PRJNA827465;Yang2025_Silkie_Wenchang	CRA004775	0.944556432
ENSGALG00010007878	PSMB1	2	-0.160537434	0.130730518	-1.228002741	0.219445939	0	0.116594669	0	1	CRA004775;Yang2025_Silkie_Wenchang	PRJNA827465	0.732888275
ENSGALG00010007878	PSMB1	2	-0.00990625	0.049054538	-0.201943598	0.839960818	0	0.841260605	0	1	CRA004775;PRJNA827465	Yang2025_Silkie_Wenchang	0.95957104
ENSGALG00010007879	PLK4	2	-0.097209454	0.072779248	-1.335675448	0.181655378	0	0.000210683	0	1	PRJNA827465;Yang2025_Silkie_Wenchang	CRA004775	0.689084192
ENSGALG00010007879	PLK4	2	-0.071141449	0.120380429	-0.590971882	0.554539261	0	0.496229671	0	0.5	CRA004775;Yang2025_Silkie_Wenchang	PRJNA827465	0.880223402
ENSGALG00010007879	PLK4	2	-0.084868997	0.087541452	-0.969472118	0.332309685	0	0.52318191	0	0.5	CRA004775;PRJNA827465	Yang2025_Silkie_Wenchang	0.802178578
ENSGALG00010007880	BCO1	2	-1.079449422	0.637449962	-1.69338691	0.090381845	0.688834501	6.488347835	84.587756	1	PRJNA827465;Yang2025_Silkie_Wenchang	CRA004775	0.5160164
ENSGALG00010007880	BCO1	2	-0.778237799	0.949944196	-0.819245807	0.412646185	1.677366545	14.09739449	92.90649062	0.5	CRA004775;Yang2025_Silkie_Wenchang	PRJNA827465	0.835704442
ENSGALG00010007880	BCO1	2	-0.162231017	0.311987634	-0.519991818	0.603069278	0.103383727	2.132072842	53.09728728	0.5	CRA004775;PRJNA827465	Yang2025_Silkie_Wenchang	0.895538966
ENSGALG00010007882	CPE	2	0.122604164	0.091708077	1.336896034	0.181256575	0	0.899422015	0	1	PRJNA827465;Yang2025_Silkie_Wenchang	CRA004775	0.688706325
ENSGALG00010007882	CPE	2	0.192645805	0.119981785	1.605625431	0.108356234	0	0.083703604	0	1	CRA004775;Yang2025_Silkie_Wenchang	PRJNA827465	0.556914487
ENSGALG00010007882	CPE	2	0.02881839	0.13146555	0.219208683	0.826487492	0	0.003361327	0	1	CRA004775;PRJNA827465	Yang2025_Silkie_Wenchang	0.956393839
ENSGALG00010007883	DHTKD1	2	-0.200987942	0.078955435	-2.545587171	0.010909412	0.004825288	1.603810386	37.64848957	1	PRJNA827465;Yang2025_Silkie_Wenchang	CRA004775	0.166322288
ENSGALG00010007883	DHTKD1	2	-0.117596119	0.074826375	-1.571586466	0.116046487	0	0.984053321	0	0.5	CRA004775;Yang2025_Silkie_Wenchang	PRJNA827465	0.573367481
ENSGALG00010007883	DHTKD1	2	-0.145551391	0.231675734	-0.628254795	0.52983704	0.065677931	2.023032335	50.56925276	0.5	CRA004775;PRJNA827465	Yang2025_Silkie_Wenchang	0.871944741
ENSGALG00010007884	GLI3	2	0.094939913	0.12699451	0.747590685	0.454707085	0.012561529	1.367134762	26.85432134	1	PRJNA827465;Yang2025_Silkie_Wenchang	CRA004775	0.849072665
ENSGALG00010007884	GLI3	2	0.171856208	0.262172898	0.655507146	0.512141287	0.034450685	1.249357813	19.95887889	0.5	CRA004775;Yang2025_Silkie_Wenchang	PRJNA827465	0.865694231
ENSGALG00010007884	GLI3	2	0.01697399	0.088633981	0.191506572	0.848128732	0	0.353929462	0	0.5	CRA004775;PRJNA827465	Yang2025_Silkie_Wenchang	0.961363811
ENSGALG00010007885	USP38	2	-0.126040241	0.099409207	-1.267893041	0.20483615	0.01501672	4.054107223	75.33365683	1	PRJNA827465;Yang2025_Silkie_Wenchang	CRA004775	0.717401104
ENSGALG00010007885	USP38	2	-0.062587658	0.24402228	-0.256483376	0.797577615	0.082544433	2.603753845	61.59391173	0.5	CRA004775;Yang2025_Silkie_Wenchang	PRJNA827465	0.949828592
ENSGALG00010007885	USP38	2	-0.022752464	0.062161244	-0.366023301	0.714347688	0.000943899	1.019017642	1.866272066	0.5	CRA004775;PRJNA827465	Yang2025_Silkie_Wenchang	0.92653974
ENSGALG00010007887	CDT1	2	0.082969957	0.161880459	0.512538436	0.608274227	0.032084094	2.236512595	55.28753103	0.5	PRJNA827465;Yang2025_Silkie_Wenchang	CRA004775	0.897204334
ENSGALG00010007887	CDT1	2	0.110644751	0.238246009	0.464413868	0.642351256	0.051043373	1.762589792	43.26530176	0.5	CRA004775;Yang2025_Silkie_Wenchang	PRJNA827465	0.906634523
ENSGALG00010007887	CDT1	2	-0.046062015	0.085149401	-0.540955241	0.588538434	0	0.215310133	0	1	CRA004775;PRJNA827465	Yang2025_Silkie_Wenchang	0.892097466
ENSGALG00010007889	DCAF6	2	-0.138659181	0.046963712	-2.952474909	0.003152377	0	0.330369621	0	1	PRJNA827465;Yang2025_Silkie_Wenchang	CRA004775	0.077825294
ENSGALG00010007889	DCAF6	2	-0.154010533	0.062856623	-2.450187858	0.01427817	0	0.700763331	0	0.5	CRA004775;Yang2025_Silkie_Wenchang	PRJNA827465	0.196009401
ENSGALG00010007889	DCAF6	2	-0.10024295	0.067979132	-1.474613559	0.140316468	0	0.467799695	0	0.5	CRA004775;PRJNA827465	Yang2025_Silkie_Wenchang	0.624205787
ENSGALG00010007890	ENSGALG00010007890	2	-0.236662965	0.269997445	-0.876537794	0.38073774	0.133810805	12.03345694	91.68983605	0.5	PRJNA827465;Yang2025_Silkie_Wenchang	CRA004775	0.824392515
ENSGALG00010007890	ENSGALG00010007890	2	-0.465579047	0.117333524	-3.967996791	7.24793E-05	0	0.848473262	0	1	CRA004775;Yang2025_Silkie_Wenchang	PRJNA827465	0.006152601
ENSGALG00010007890	ENSGALG00010007890	2	0.002720919	0.083202708	0.032702289	0.973911999	0	0.751481845	0	0.5	CRA004775;PRJNA827465	Yang2025_Silkie_Wenchang	0.992472082
ENSGALG00010007891	GDI1	2	-0.080653506	0.065318472	-1.23477331	0.216914883	0.004390937	1.828805827	45.31950931	1	PRJNA827465;Yang2025_Silkie_Wenchang	CRA004775	0.730505165
ENSGALG00010007891	GDI1	2	-0.132589972	0.078089824	-1.697916126	0.089523593	0	0.683073879	0	1	CRA004775;Yang2025_Silkie_Wenchang	PRJNA827465	0.513242777
ENSGALG00010007891	GDI1	2	-0.034886698	0.042229686	-0.826117859	0.408737238	0	0.000249707	0	1	CRA004775;PRJNA827465	Yang2025_Silkie_Wenchang	0.834305842
ENSGALG00010007892	ENSGALG00010007892	2	0.670080525	0.360864344	1.856876512	0.063328725	0	0.001633966	0	1	PRJNA827465;Yang2025_Silkie_Wenchang	CRA004775	0.433398725
ENSGALG00010007892	ENSGALG00010007892	2	1.567689979	0.923198812	1.698106582	0.089487647	1.361022908	4.921830193	79.68235472	1	CRA004775;Yang2025_Silkie_Wenchang	PRJNA827465	0.513242777
ENSGALG00010007892	ENSGALG00010007892	2	1.548792949	0.937608807	1.651854096	0.098564298	1.424695058	5.218226433	80.83640078	1	CRA004775;PRJNA827465	Yang2025_Silkie_Wenchang	0.53598695
ENSGALG00010007893	MFSD8	2	0.027207247	0.073043061	0.37248229	0.709533795	0	0.045893312	0	1	PRJNA827465;Yang2025_Silkie_Wenchang	CRA004775	0.925298042
ENSGALG00010007893	MFSD8	2	-0.018858239	0.103856693	-0.181579425	0.855912797	0	0.396868508	0	0.5	CRA004775;Yang2025_Silkie_Wenchang	PRJNA827465	0.963486168
ENSGALG00010007893	MFSD8	2	0.016005503	0.090225525	0.177394405	0.859198602	0	0.57081261	0	0.5	CRA004775;PRJNA827465	Yang2025_Silkie_Wenchang	0.964605083
ENSGALG00010007894	CD88	2	-0.014184488	0.12485406	-0.113608542	0.909548116	0	0.059932102	0	0.5	PRJNA827465;Yang2025_Silkie_Wenchang	CRA004775	0.978607662
ENSGALG00010007894	CD88	2	-0.138340631	0.2017879	-0.685574462	0.49298149	0	0.374233646	0	1	CRA004775;Yang2025_Silkie_Wenchang	PRJNA827465	0.859609959
ENSGALG00010007894	CD88	2	-0.038580331	0.137550815	-0.280480569	0.779108831	0	0.694913223	0	0.5	CRA004775;PRJNA827465	Yang2025_Silkie_Wenchang	0.944723241
ENSGALG00010007895	GOLGB1	2	0.145450118	0.21151055	0.687673113	0.491658656	0.070065715	4.11039815	75.67145655	0.5	PRJNA827465;Yang2025_Silkie_Wenchang	CRA004775	0.85895707
ENSGALG00010007895	GOLGB1	2	0.108289161	0.295481209	0.366484086	0.714003885	0.132945372	4.188126463	76.12297506	0.5	CRA004775;Yang2025_Silkie_Wenchang	PRJNA827465	0.926399535
ENSGALG00010007895	GOLGB1	2	-0.048219991	0.070098842	-0.687885698	0.491524764	0	0.522466711	0	1	CRA004775;PRJNA827465	Yang2025_Silkie_Wenchang	0.85895707
ENSGALG00010007896	TIMMDC1	2	-0.245776361	0.062605254	-3.925810469	8.64382E-05	0	0.376486181	0	1	PRJNA827465;Yang2025_Silkie_Wenchang	CRA004775	0.006990289
ENSGALG00010007896	TIMMDC1	2	-0.151618254	0.13921067	-1.089128107	0.276097397	0	0.010235965	0	1	CRA004775;Yang2025_Silkie_Wenchang	PRJNA827465	0.775682505
ENSGALG00010007896	TIMMDC1	2	-0.25638041	0.066844691	-3.835464039	0.000125327	0	0.188445248	0	1	CRA004775;PRJNA827465	Yang2025_Silkie_Wenchang	0.009155659
ENSGALG00010007897	EFHC1	2	0.118517042	0.301178451	0.393511028	0.69394208	0.099999901	2.167986281	53.87424686	0.5	PRJNA827465;Yang2025_Silkie_Wenchang	CRA004775	0.920728119
ENSGALG00010007897	EFHC1	2	0.05480255	0.469920993	0.116620774	0.907160575	0.2788371	2.641270777	62.13943649	0.5	CRA004775;Yang2025_Silkie_Wenchang	PRJNA827465	0.978196186
ENSGALG00010007897	EFHC1	2	-0.208991259	0.214409922	-0.974727552	0.329695422	0	0.402478911	0	1	CRA004775;PRJNA827465	Yang2025_Silkie_Wenchang	0.801177491
ENSGALG00010007898	GAB1	2	0.00334893	0.088640536	0.037781026	0.969862273	0.005384746	1.178860257	15.17230355	0.5	PRJNA827465;Yang2025_Silkie_Wenchang	CRA004775	0.991572914
ENSGALG00010007898	GAB1	2	0.436472227	0.196558559	2.220570959	0.026380035	0.022225668	1.403553416	28.75226632	1	CRA004775;Yang2025_Silkie_Wenchang	PRJNA827465	0.276903978
ENSGALG00010007898	GAB1	2	0.261531141	0.327628768	0.798254508	0.424722812	0.188965059	7.611334038	86.86169869	0.5	CRA004775;PRJNA827465	Yang2025_Silkie_Wenchang	0.841035906
ENSGALG00010007899	APRT	2	-0.011488644	0.11468051	-0.100179568	0.920201766	0	0.802357157	0	0.5	PRJNA827465;Yang2025_Silkie_Wenchang	CRA004775	0.981364549
ENSGALG00010007899	APRT	2	-0.317662711	0.194108624	-1.63652034	0.101730732	0	0.285166589	0	1	CRA004775;Yang2025_Silkie_Wenchang	PRJNA827465	0.543700483
ENSGALG00010007899	APRT	2	-0.125434189	0.223852659	-0.560342635	0.575245752	0.060690568	2.22286296	55.01297121	0.5	CRA004775;PRJNA827465	Yang2025_Silkie_Wenchang	0.88644908
ENSGALG00010007900	FHOD3	2	-0.187930616	0.132901355	-1.41406095	0.157344008	0.006012386	1.131515494	11.6229512	0.5	PRJNA827465;Yang2025_Silkie_Wenchang	CRA004775	0.650687662
ENSGALG00010007900	FHOD3	2	-0.038744366	0.246918858	-0.156911327	0.875314733	0	0.752702812	0	0.5	CRA004775;Yang2025_Silkie_Wenchang	PRJNA827465	0.970347777
ENSGALG00010007900	FHOD3	2	-0.269216528	0.125660641	-2.14240932	0.032160559	0	0.152593269	0	1	CRA004775;PRJNA827465	Yang2025_Silkie_Wenchang	0.307618991
ENSGALG00010007901	FSTL5	2	-0.765990813	0.230954301	-3.316633672	0.00091109	0	0.106821831	0	1	PRJNA827465;Yang2025_Silkie_Wenchang	CRA004775	0.035547186
ENSGALG00010007901	FSTL5	2	0.048452895	1.020734652	0.047468649	0.962139716	1.725839011	5.318306424	81.19702175	0.5	CRA004775;Yang2025_Silkie_Wenchang	PRJNA827465	0.989885041
ENSGALG00010007901	FSTL5	2	0.115190884	0.942964463	0.122158245	0.902773695	1.420703183	4.511272992	77.83330776	0.5	CRA004775;PRJNA827465	Yang2025_Silkie_Wenchang	0.97693511
ENSGALG00010007902	C7orf25	2	0.057788871	0.093273086	0.619566414	0.535543284	0.008663306	1.822303063	45.12438572	0.5	PRJNA827465;Yang2025_Silkie_Wenchang	CRA004775	0.874171355
ENSGALG00010007902	C7orf25	2	0.189337713	0.123061168	1.538565874	0.123910315	0	0.010110606	0	1	CRA004775;Yang2025_Silkie_Wenchang	PRJNA827465	0.590384957
ENSGALG00010007902	C7orf25	2	-0.004570055	0.067456098	-0.06774858	0.945985777	0	0.292605077	0	0.5	CRA004775;PRJNA827465	Yang2025_Silkie_Wenchang	0.986321197
ENSGALG00010007903	PSMA2	2	-0.289516712	0.302456995	-0.957216123	0.338458181	0.171294719	15.06012204	93.35994757	1	PRJNA827465;Yang2025_Silkie_Wenchang	CRA004775	0.8043063
ENSGALG00010007903	PSMA2	2	-0.3952504	0.248157013	-1.592743216	0.111217808	0.085125021	3.081596446	67.5492876	1	CRA004775;Yang2025_Silkie_Wenchang	PRJNA827465	0.564247016
ENSGALG00010007903	PSMA2	2	-0.006778731	0.048428769	-0.139973216	0.888681153	0	0.172714435	0	1	CRA004775;PRJNA827465	Yang2025_Silkie_Wenchang	0.973135755
ENSGALG00010007904	ENSGALG00010007904	2	0.399829046	0.312255939	1.280452975	0.200385873	0.17726451	10.64276769	90.60394787	1	PRJNA827465;Yang2025_Silkie_Wenchang	CRA004775	0.71262386
ENSGALG00010007904	ENSGALG00010007904	2	0.288688303	0.455862166	0.633279804	0.526550939	0.373422535	9.764485254	89.75880475	0.5	CRA004775;Yang2025_Silkie_Wenchang	PRJNA827465	0.87066367
ENSGALG00010007904	ENSGALG00010007904	2	0.046141824	0.115751269	0.398629099	0.690166517	0.009664768	1.30764512	23.52665226	0.5	CRA004775;PRJNA827465	Yang2025_Silkie_Wenchang	0.919979266
ENSGALG00010007905	MRPL32	2	0.113161721	0.112818055	1.003046197	0.315838572	0.012235932	1.700952785	41.20942045	1	PRJNA827465;Yang2025_Silkie_Wenchang	CRA004775	0.796883862
ENSGALG00010007905	MRPL32	2	0.113155859	0.217723115	0.519723685	0.603256176	0.046913341	1.861604866	46.28290792	0.5	CRA004775;Yang2025_Silkie_Wenchang	PRJNA827465	0.895538966
ENSGALG00010007905	MRPL32	2	0.022431777	0.07609469	0.294787677	0.76815608	0	0.493451175	0	0.5	CRA004775;PRJNA827465	Yang2025_Silkie_Wenchang	0.941444269
ENSGALG00010007906	NAF1	2	-0.035556257	0.150002924	-0.237037091	0.812628015	0.032239306	3.138976195	68.14247902	0.5	PRJNA827465;Yang2025_Silkie_Wenchang	CRA004775	0.95348724
ENSGALG00010007906	NAF1	2	0.065899015	0.13402301	0.491699258	0.622931963	0	0.985386409	0	0.5	CRA004775;Yang2025_Silkie_Wenchang	PRJNA827465	0.900312382
ENSGALG00010007906	NAF1	2	-0.154225286	0.065509379	-2.354247409	0.018560253	0	0.004052894	0	1	CRA004775;PRJNA827465	Yang2025_Silkie_Wenchang	0.227943938
ENSGALG00010007909	GALNS	2	-0.075770355	0.079433809	-0.953880414	0.340144179	0.000622004	1.045369316	4.340027535	1	PRJNA827465;Yang2025_Silkie_Wenchang	CRA004775	0.805377063
ENSGALG00010007909	GALNS	2	-0.016829227	0.090840902	-0.185260461	0.853024749	0	0.025010183	0	0.5	CRA004775;Yang2025_Silkie_Wenchang	PRJNA827465	0.962708451
ENSGALG00010007909	GALNS	2	-0.163081381	0.128508239	-1.269034431	0.204428784	0	0.329228927	0	0.5	CRA004775;PRJNA827465	Yang2025_Silkie_Wenchang	0.71729696
ENSGALG00010007910	ANKS6	2	0.086505341	0.102752881	0.84187752	0.399856515	0.013397487	2.681977741	62.71408279	0.5	PRJNA827465;Yang2025_Silkie_Wenchang	CRA004775	0.832246851
ENSGALG00010007910	ANKS6	2	0.228729	0.093947467	2.434647867	0.014906288	0	0.537843864	0	1	CRA004775;Yang2025_Silkie_Wenchang	PRJNA827465	0.201604212
ENSGALG00010007910	ANKS6	2	0.126066787	0.189903006	0.663848296	0.506787411	0.047766613	2.3880979	58.12566981	0.5	CRA004775;PRJNA827465	Yang2025_Silkie_Wenchang	0.863929781
ENSGALG00010007911	IFI6	2	-0.604540301	0.335049832	-1.804329517	0.071179665	0	0.033479222	0	1	PRJNA827465;Yang2025_Silkie_Wenchang	CRA004775	0.460681013
ENSGALG00010007911	IFI6	2	-0.250173098	0.420742432	-0.594599163	0.552111439	0.08484308	1.315028981	23.95604856	0.5	CRA004775;Yang2025_Silkie_Wenchang	PRJNA827465	0.879061525
ENSGALG00010007911	IFI6	2	-0.246641445	0.354725985	-0.695301319	0.486866488	0.030742675	1.13591546	11.96527956	0.5	CRA004775;PRJNA827465	Yang2025_Silkie_Wenchang	0.857631005
ENSGALG00010007912	TRAPPC2L	2	-0.049211875	0.076820482	-0.640608779	0.521776894	0	0.61042071	0	1	PRJNA827465;Yang2025_Silkie_Wenchang	CRA004775	0.86956507
ENSGALG00010007912	TRAPPC2L	2	-0.342611838	0.177279325	-1.932610233	0.053284231	0	0.127759532	0	1	CRA004775;Yang2025_Silkie_Wenchang	PRJNA827465	0.400004977
ENSGALG00010007912	TRAPPC2L	2	-0.150329428	0.169234269	-0.888291885	0.374383764	0.036188161	2.252814249	55.61107621	1	CRA004775;PRJNA827465	Yang2025_Silkie_Wenchang	0.822805106
ENSGALG00010007914	PAQR8	2	-0.018184448	0.232057931	-0.078361675	0.937540359	0.086658942	4.841684332	79.34603061	0.5	PRJNA827465;Yang2025_Silkie_Wenchang	CRA004775	0.985139256
ENSGALG00010007914	PAQR8	2	-0.25871676	0.17644405	-1.466282144	0.142571438	0	0.099678083	0	1	CRA004775;Yang2025_Silkie_Wenchang	PRJNA827465	0.628893678
ENSGALG00010007914	PAQR8	2	0.17720635	0.096660876	1.83327896	0.066761097	0	0.329541706	0	0.5	CRA004775;PRJNA827465	Yang2025_Silkie_Wenchang	0.445135824
ENSGALG00010007915	HECW1	2	-0.030634857	0.081113724	-0.377677854	0.705669921	0	0.228959352	0	1	PRJNA827465;Yang2025_Silkie_Wenchang	CRA004775	0.924374044
ENSGALG00010007915	HECW1	2	-0.041609132	0.128478131	-0.323861591	0.746042824	0	0.68662734	0	0.5	CRA004775;Yang2025_Silkie_Wenchang	PRJNA827465	0.936484152
ENSGALG00010007915	HECW1	2	0.014997354	0.096137249	0.155999407	0.876033489	0	0.396894986	0	0.5	CRA004775;PRJNA827465	Yang2025_Silkie_Wenchang	0.970347777
ENSGALG00010007916	MCM3	2	-0.058295895	0.062615683	-0.93101108	0.351847833	0	0.064544607	0	1	PRJNA827465;Yang2025_Silkie_Wenchang	CRA004775	0.810678834
ENSGALG00010007916	MCM3	2	-0.249202404	0.317125221	-0.785817044	0.43197468	0.09284703	1.379884145	27.53014781	1	CRA004775;Yang2025_Silkie_Wenchang	PRJNA827465	0.842503929
ENSGALG00010007916	MCM3	2	-0.224998589	0.346783762	-0.648815238	0.516457807	0.145630383	1.628835737	38.60645508	1	CRA004775;PRJNA827465	Yang2025_Silkie_Wenchang	0.867747143
ENSGALG00010007917	POGLUT1	2	-0.265212899	0.213062032	-1.244768466	0.213216871	0.079590188	7.685773964	86.98894861	1	PRJNA827465;Yang2025_Silkie_Wenchang	CRA004775	0.72579791
ENSGALG00010007917	POGLUT1	2	-0.320165768	0.197860313	-1.618140413	0.105632341	0.05079433	2.787802301	64.12945066	1	CRA004775;Yang2025_Silkie_Wenchang	PRJNA827465	0.551567133
ENSGALG00010007917	POGLUT1	2	-0.072917132	0.059253153	-1.230603395	0.218471235	0	0.02172436	0	1	CRA004775;PRJNA827465	Yang2025_Silkie_Wenchang	0.732175117
ENSGALG00010007920	FAT3	2	-0.03600187	0.100480187	-0.358298203	0.720120163	0	0.75734776	0	0.5	PRJNA827465;Yang2025_Silkie_Wenchang	CRA004775	0.928564173
ENSGALG00010007920	FAT3	2	0.117212494	0.233029803	0.502993577	0.614968788	0	0.299579202	0	0.5	CRA004775;Yang2025_Silkie_Wenchang	PRJNA827465	0.898781283
ENSGALG00010007920	FAT3	2	-0.079548823	0.109051064	-0.729463979	0.465717894	0	0.070240095	0	1	CRA004775;PRJNA827465	Yang2025_Silkie_Wenchang	0.852424459
ENSGALG00010007921	FAM120B	2	-0.235270812	0.295648356	-0.795779198	0.426160385	0.168761118	28.77030659	96.52419415	0.5	PRJNA827465;Yang2025_Silkie_Wenchang	CRA004775	0.84125685
ENSGALG00010007921	FAM120B	2	-0.508002236	0.085074332	-5.971275061	2.35406E-09	0	0.850176061	0	1	CRA004775;Yang2025_Silkie_Wenchang	PRJNA827465	1.30312E-06
ENSGALG00010007921	FAM120B	2	-0.006106624	0.130032151	-0.046962418	0.96254318	0.016793868	1.442706237	30.68581988	0.5	CRA004775;PRJNA827465	Yang2025_Silkie_Wenchang	0.989980504
ENSGALG00010007923	YAP1	2	0.012944551	0.043516469	0.297463266	0.766112849	0	0.023863762	0	0.5	PRJNA827465;Yang2025_Silkie_Wenchang	CRA004775	0.941101213
ENSGALG00010007923	YAP1	2	0.025329768	0.123765418	0.204659492	0.837838171	0	0.231374826	0	0.5	CRA004775;Yang2025_Silkie_Wenchang	PRJNA827465	0.959032758
ENSGALG00010007923	YAP1	2	0.018783264	0.045001526	0.417391725	0.676391907	0	0.205190577	0	1	CRA004775;PRJNA827465	Yang2025_Silkie_Wenchang	0.916760849
ENSGALG00010007924	ABHD18	2	0.01499606	0.089329499	0.167873545	0.866682763	0.005888175	1.516066753	34.03984368	0.5	PRJNA827465;Yang2025_Silkie_Wenchang	CRA004775	0.96757535
ENSGALG00010007924	ABHD18	2	0.223130622	0.121868884	1.830907245	0.067114389	0.006599219	1.253056729	20.19515343	1	CRA004775;Yang2025_Silkie_Wenchang	PRJNA827465	0.446292315
ENSGALG00010007924	ABHD18	2	0.135474385	0.218310324	0.620558767	0.534889977	0.07625852	4.585856877	78.19382447	0.5	CRA004775;PRJNA827465	Yang2025_Silkie_Wenchang	0.873896294
ENSGALG00010007925	STK17A	2	-0.222697031	0.094550853	-2.355314883	0.018507016	0	0.178035472	0	1	PRJNA827465;Yang2025_Silkie_Wenchang	CRA004775	0.227601507
ENSGALG00010007925	STK17A	2	-0.268628975	0.165066857	-1.627394986	0.103653236	0	0.093512804	0	1	CRA004775;Yang2025_Silkie_Wenchang	PRJNA827465	0.547964315
ENSGALG00010007925	STK17A	2	-0.192823981	0.111224129	-1.733652422	0.082979773	0	0.028560732	0	1	CRA004775;PRJNA827465	Yang2025_Silkie_Wenchang	0.495947997
ENSGALG00010007926	EIF3E	2	-0.328800678	0.271184113	-1.212462908	0.225335233	0.134612824	11.36925255	91.20434703	1	PRJNA827465;Yang2025_Silkie_Wenchang	CRA004775	0.73852157
ENSGALG00010007926	EIF3E	2	-0.402092915	0.231866835	-1.7341545	0.082890674	0.078037456	3.588362447	72.13213507	1	CRA004775;Yang2025_Silkie_Wenchang	PRJNA827465	0.495859127
ENSGALG00010007926	EIF3E	2	-0.080958595	0.062069326	-1.304325337	0.19212268	0	0.142397322	0	1	CRA004775;PRJNA827465	Yang2025_Silkie_Wenchang	0.701849591
ENSGALG00010007927	DLL1	2	0.084789351	0.094927299	0.893203027	0.371748458	0	0.420753029	0	1	PRJNA827465;Yang2025_Silkie_Wenchang	CRA004775	0.821597118
ENSGALG00010007927	DLL1	2	0.15069087	0.115464684	1.305081915	0.191864956	0	0.236602536	0	1	CRA004775;Yang2025_Silkie_Wenchang	PRJNA827465	0.701581425
ENSGALG00010007927	DLL1	2	0.050127188	0.142757316	0.351135689	0.725486554	0	0.601749612	0	1	CRA004775;PRJNA827465	Yang2025_Silkie_Wenchang	0.930910808
ENSGALG00010007929	LRRC14B	2	-0.369455353	0.236969144	-1.559086331	0.118975948	0.059089862	2.091179826	52.18010486	1	PRJNA827465;Yang2025_Silkie_Wenchang	CRA004775	0.579372036
ENSGALG00010007929	LRRC14B	2	-0.562310537	0.233863857	-2.404435407	0.016197468	0	0.45671709	0	1	CRA004775;Yang2025_Silkie_Wenchang	PRJNA827465	0.210262467
ENSGALG00010007929	LRRC14B	2	-0.15686002	0.197804307	-0.79300609	0.427774275	0	0.002969283	0	1	CRA004775;PRJNA827465	Yang2025_Silkie_Wenchang	0.84125685
ENSGALG00010007930	CBFA2T3	2	0.110398346	0.069214226	1.59502391	0.110706889	0	0.986913832	0	1	PRJNA827465;Yang2025_Silkie_Wenchang	CRA004775	0.562970535
ENSGALG00010007930	CBFA2T3	2	0.172230509	0.120855917	1.425089584	0.154131307	0	0.907444828	0	0.5	CRA004775;Yang2025_Silkie_Wenchang	PRJNA827465	0.646788679
ENSGALG00010007930	CBFA2T3	2	0.056982076	0.078720684	0.723851382	0.46915698	0	0.26951286	0	0.5	CRA004775;PRJNA827465	Yang2025_Silkie_Wenchang	0.853472659
ENSGALG00010007931	GBE1	2	-0.129811173	0.088082025	-1.473753282	0.140548031	0.003118324	1.234477411	18.99406253	1	PRJNA827465;Yang2025_Silkie_Wenchang	CRA004775	0.624740926
ENSGALG00010007931	GBE1	2	0.194415741	0.451016078	0.431061663	0.666423535	0.376408954	13.1871661	92.41686961	0.5	CRA004775;Yang2025_Silkie_Wenchang	PRJNA827465	0.913579507
ENSGALG00010007931	GBE1	2	0.275862582	0.359846258	0.766612341	0.443312036	0.233126709	9.621307232	89.60640196	0.5	CRA004775;PRJNA827465	Yang2025_Silkie_Wenchang	0.845733906
ENSGALG00010007932	NPY5R	2	0.295478069	0.254636285	1.160392635	0.245888984	0.092660222	3.429744948	70.84331298	1	PRJNA827465;Yang2025_Silkie_Wenchang	CRA004775	0.75661406
ENSGALG00010007932	NPY5R	2	0.483063311	0.197512797	2.445731708	0.014455853	0	0.751338328	0	1	CRA004775;Yang2025_Silkie_Wenchang	PRJNA827465	0.197724547
ENSGALG00010007932	NPY5R	2	0.076389461	0.150568694	0.5073396	0.611916558	0	0.050104555	0	1	CRA004775;PRJNA827465	Yang2025_Silkie_Wenchang	0.897777844
ENSGALG00010007935	LARP1B	2	0.3425143	0.391018342	0.875954559	0.381054738	0.282078511	12.3083378	91.8754261	0.5	PRJNA827465;Yang2025_Silkie_Wenchang	CRA004775	0.824392515
ENSGALG00010007935	LARP1B	2	0.48493827	0.283327691	1.711580916	0.086973928	0.111067322	3.236825693	69.10553441	1	CRA004775;Yang2025_Silkie_Wenchang	PRJNA827465	0.507397399
ENSGALG00010007935	LARP1B	2	-0.004764267	0.07067446	-0.067411443	0.94625416	0	0.777005092	0	0.5	CRA004775;PRJNA827465	Yang2025_Silkie_Wenchang	0.98638413
ENSGALG00010007936	ENSGALG00010007936	2	-0.355463359	0.391757309	-0.907356037	0.36421855	0.264024603	6.706709279	85.08955796	1	PRJNA827465;Yang2025_Silkie_Wenchang	CRA004775	0.817449342
ENSGALG00010007936	ENSGALG00010007936	2	0.031491237	0.89519199	0.035178193	0.971937651	1.398123273	7.460921753	86.59683035	0.5	CRA004775;Yang2025_Silkie_Wenchang	PRJNA827465	0.991955348
ENSGALG00010007936	ENSGALG00010007936	2	0.333548183	0.47559723	0.701324906	0.483100274	0.325034607	2.76465774	63.82915738	0.5	CRA004775;PRJNA827465	Yang2025_Silkie_Wenchang	0.85691916
ENSGALG00010007937	ANGPTL5	2	0.298627076	0.261646686	1.141337125	0.253729658	0	0.068933335	0	1	PRJNA827465;Yang2025_Silkie_Wenchang	CRA004775	0.763418915
ENSGALG00010007937	ANGPTL5	2	-0.15516385	0.725894288	-0.213755436	0.830737779	0.663972616	2.30327089	56.58348289	0.5	CRA004775;Yang2025_Silkie_Wenchang	PRJNA827465	0.95733143
ENSGALG00010007937	ANGPTL5	2	-0.20127793	0.644075725	-0.312506624	0.75465553	0.46081264	1.900790404	47.39030681	0.5	CRA004775;PRJNA827465	Yang2025_Silkie_Wenchang	0.93838723
ENSGALG00010007938	BCL9	2	-0.005934364	0.050930382	-0.116519136	0.907241121	0	0.28380119	0	0.5	PRJNA827465;Yang2025_Silkie_Wenchang	CRA004775	0.978196186
ENSGALG00010007938	BCL9	2	0.036153611	0.139923711	0.258380872	0.796112978	0	0.213500138	0	0.5	CRA004775;Yang2025_Silkie_Wenchang	PRJNA827465	0.949321249
ENSGALG00010007938	BCL9	2	-0.016483378	0.052237793	-0.315545074	0.752347833	0	0.03102231	0	1	CRA004775;PRJNA827465	Yang2025_Silkie_Wenchang	0.93806821
ENSGALG00010007940	GLIPR2	2	-0.364373368	0.254031556	-1.434362619	0.151468809	0.114989148	9.165373324	89.08936969	1	PRJNA827465;Yang2025_Silkie_Wenchang	CRA004775	0.643006634
ENSGALG00010007940	GLIPR2	2	-0.370361981	0.34625292	-1.06962847	0.284786575	0.167318004	2.693287145	62.8706504	0.5	CRA004775;Yang2025_Silkie_Wenchang	PRJNA827465	0.780278244
ENSGALG00010007940	GLIPR2	2	-0.097466105	0.110521104	-0.881877772	0.377842909	0	0.250740119	0	0.5	CRA004775;PRJNA827465	Yang2025_Silkie_Wenchang	0.82325159
ENSGALG00010007941	MRPL19	2	0.070251664	0.186633554	0.376414969	0.706608419	0.060255483	6.971359763	85.65559613	0.5	PRJNA827465;Yang2025_Silkie_Wenchang	CRA004775	0.924548793
ENSGALG00010007941	MRPL19	2	0.126295871	0.169406842	0.745518124	0.455958559	0.034171471	2.418917561	58.65919467	0.5	CRA004775;Yang2025_Silkie_Wenchang	PRJNA827465	0.84907427
ENSGALG00010007941	MRPL19	2	-0.095713535	0.053592871	-1.785937807	0.074109329	0	0.033210111	0	1	CRA004775;PRJNA827465	Yang2025_Silkie_Wenchang	0.470243057
ENSGALG00010007942	PGRMC2	2	-0.050540099	0.084852997	-0.595619499	0.551429448	0.010788734	3.757294359	73.38510363	0.5	PRJNA827465;Yang2025_Silkie_Wenchang	CRA004775	0.878888037
ENSGALG00010007942	PGRMC2	2	-0.165577468	0.071374271	-2.319848098	0.020349096	0	0.313712061	0	1	CRA004775;Yang2025_Silkie_Wenchang	PRJNA827465	0.24026234

ENSGALG00010007942	PGRMC2	2	-0.062703889	0.129794321	-0.483101947	0.629023352	0.022370311	2.278468342	56.11086705	0.5	CRA004775;PRJNA827465	Yang2025_Silkie_Wenchang	0.902155813
ENSGALG00010007943	BLOC1S4	2	-0.18503327	0.17516441	-1.056340554	0.290812654	0.054621451	8.700136045	88.50592686	1	PRJNA827465;Yang2025_Silkie_Wenchang	CRA004775	0.784646441
ENSGALG00010007943	BLOC1S4	2	-0.348346096	0.10224491	-3.406977387	0.000656866	0	0.50854299	0	1	CRA004775;Yang2025_Silkie_Wenchang	PRJNA827465	0.028276881
ENSGALG00010007943	BLOC1S4	2	-0.025544336	0.048842429	-0.522994783	0.600977884	0	0.142025385	0	1	CRA004775;PRJNA827465	Yang2025_Silkie_Wenchang	0.895230519
ENSGALG00010007944	ACSF3	2	-0.00884088	0.119625435	-0.073904684	0.941086228	0	0.256245994	0	0.5	PRJNA827465;Yang2025_Silkie_Wenchang	CRA004775	0.985624005
ENSGALG00010007944	ACSF3	2	-0.092723114	0.246535522	-0.376104477	0.706839226	0.047091138	1.504915366	33.55108052	0.5	CRA004775;Yang2025_Silkie_Wenchang	PRJNA827465	0.924612331
ENSGALG00010007944	ACSF3	2	-0.104982864	0.137453261	-0.76377136	0.445003507	0	0.972024801	0	1	CRA004775;PRJNA827465	Yang2025_Silkie_Wenchang	0.846013379
ENSGALG00010007945	ENSGALG00010007945	2	0.005280811	0.111643228	0.047300769	0.962273515	0.017332604	3.169173486	68.44603161	0.5	PRJNA827465;Yang2025_Silkie_Wenchang	CRA004775	0.989950484
ENSGALG00010007945	ENSGALG00010007945	2	-0.095948217	0.101598853	-0.944382868	0.344974033	0	0.774438248	0	0.5	CRA004775;Yang2025_Silkie_Wenchang	PRJNA827465	0.806961063
ENSGALG00010007945	ENSGALG00010007945	2	0.107649272	0.06831523	1.575772665	0.115078204	0	0.093274995	0	1	CRA004775;PRJNA827465	Yang2025_Silkie_Wenchang	0.572141484
ENSGALG00010007946	ENSGALG00010007946	2	0.206611179	0.379841301	0.543940793	0.586482216	0.257866055	9.19020622	89.11685135	0.5	PRJNA827465;Yang2025_Silkie_Wenchang	CRA004775	0.891596903
ENSGALG00010007946	ENSGALG00010007946	2	0.078845507	0.514357739	0.153289241	0.878170185	0.496753764	16.17358603	93.81707929	0.5	CRA004775;Yang2025_Silkie_Wenchang	PRJNA827465	0.971037793
ENSGALG00010007946	ENSGALG00010007946	2	-0.283354095	0.134091177	-2.113144959	0.034588363	0.018403824	2.045630479	51.11531578	1	CRA004775;PRJNA827465	Yang2025_Silkie_Wenchang	0.320322559
ENSGALG00010007948	ENSGALG00010007948	2	-0.083696088	0.356783578	-0.234585035	0.814530826	0.171755967	2.988459298	66.53794145	0.5	PRJNA827465;Yang2025_Silkie_Wenchang	CRA004775	0.953869515
ENSGALG00010007948	ENSGALG00010007948	2	-0.954824741	0.592867743	-1.610518961	0.107284609	0.455565544	2.591887416	61.41807728	1	CRA004775;Yang2025_Silkie_Wenchang	PRJNA827465	0.554473355
ENSGALG00010007948	ENSGALG00010007948	2	-0.631602942	0.962737855	-0.656048725	0.511792774	1.619083133	7.326116436	86.35020329	0.5	CRA004775;PRJNA827465	Yang2025_Silkie_Wenchang	0.865694231
ENSGALG00010007950	ARC	2	-0.091734404	0.309172718	-0.296709246	0.766688497	0	0.316958095	0	0.5	PRJNA827465;Yang2025_Silkie_Wenchang	CRA004775	0.941271762
ENSGALG00010007950	ARC	2	-0.751577583	0.585169928	-1.284374925	0.199010802	0.247682342	1.483382675	32.5865121	1	CRA004775;Yang2025_Silkie_Wenchang	PRJNA827465	0.71096127
ENSGALG00010007950	ARC	2	-0.573877512	0.776249666	-0.73929502	0.459727873	0.827421442	2.841728947	64.81015542	0.5	CRA004775;PRJNA827465	Yang2025_Silkie_Wenchang	0.850098539
ENSGALG00010007951	TGFBR1	2	-0.077609017	0.119094124	-0.651661175	0.514619774	0.021047347	3.758256923	73.39192023	0.5	PRJNA827465;Yang2025_Silkie_Wenchang	CRA004775	0.866869697
ENSGALG00010007951	TGFBR1	2	-0.202562342	0.09879392	-2.050352321	0.040330062	0	0.061548177	0	1	CRA004775;Yang2025_Silkie_Wenchang	PRJNA827465	0.347442284
ENSGALG00010007951	TGFBR1	2	0.024127595	0.066868731	0.360820296	0.718233789	0	0.17827422	0	0.5	CRA004775;PRJNA827465	Yang2025_Silkie_Wenchang	0.927975712
ENSGALG00010007952	EMC2	2	0.120187946	0.109672208	1.09588335	0.273129827	0.016974021	3.042123017	67.12821952	1	PRJNA827465;Yang2025_Silkie_Wenchang	CRA004775	0.773904763
ENSGALG00010007952	EMC2	2	0.225792254	0.108094855	2.088834429	0.03672263	0	0.454029799	0	1	CRA004775;Yang2025_Silkie_Wenchang	PRJNA827465	0.330655103
ENSGALG00010007952	EMC2	2	0.033461237	0.051352929	0.651593534	0.51466342	0	0.008511235	0	1	CRA004775;PRJNA827465	Yang2025_Silkie_Wenchang	0.866869697
ENSGALG00010007954	PSCA	2	1.401871189	0.470142362	2.981801479	0.002865578	0	0.009112457	0	1	PRJNA827465;Yang2025_Silkie_Wenchang	CRA004775	0.073164891
ENSGALG00010007954	PSCA	2	1.982280695	0.492848683	4.022087843	5.76845E-05	0	0.646127406	0	1	CRA004775;Yang2025_Silkie_Wenchang	PRJNA827465	0.005336024
ENSGALG00010007954	PSCA	2	1.799238163	0.456964985	3.937365495	8.23811E-05	0.060075289	1.167253233	14.32878727	1	CRA004775;PRJNA827465	Yang2025_Silkie_Wenchang	0.00680215
ENSGALG00010007955	TMA16	2	0.261067052	0.291984183	0.894113677	0.371261072	0.157318984	12.54311139	92.02756001	0.5	PRJNA827465;Yang2025_Silkie_Wenchang	CRA004775	0.8214684
ENSGALG00010007955	TMA16	2	0.388214157	0.248675157	1.561129633	0.118493168	0.07196082	2.105624829	52.50815879	1	CRA004775;Yang2025_Silkie_Wenchang	PRJNA827465	0.578382634
ENSGALG00010007955	TMA16	2	-0.013794456	0.068955576	-0.200048446	0.841442693	0	0.033162362	0	0.5	CRA004775;PRJNA827465	Yang2025_Silkie_Wenchang	0.95989279
ENSGALG00010007956	HS1BP3	2	-0.190531663	0.100556683	-1.894788772	0.058123042	0	0.373850063	0	1	PRJNA827465;Yang2025_Silkie_Wenchang	CRA004775	0.417658363
ENSGALG00010007956	HS1BP3	2	-0.232711902	0.146625727	-1.587115079	0.112486583	0	0.228307465	0	1	CRA004775;Yang2025_Silkie_Wenchang	PRJNA827465	0.566223663
ENSGALG00010007956	HS1BP3	2	-0.142411473	0.112718638	-1.263424355	0.206436716	0	0.007119361	0	1	CRA004775;PRJNA827465	Yang2025_Silkie_Wenchang	0.718461193
ENSGALG00010007957	CDH15	2	0.082284997	0.171932792	0.478588149	0.632231651	0.043871403	3.798433233	73.67335586	0.5	PRJNA827465;Yang2025_Silkie_Wenchang	CRA004775	0.903058174
ENSGALG00010007957	CDH15	2	0.247315298	0.139716697	1.770119844	0.076707179	0	0.408941167	0	0.5	CRA004775;Yang2025_Silkie_Wenchang	PRJNA827465	0.477904868
ENSGALG00010007957	CDH15	2	-0.076812424	0.101544359	-0.756442059	0.449384199	0	0.004092454	0	1	CRA004775;PRJNA827465	Yang2025_Silkie_Wenchang	0.847263197
ENSGALG00010007960	TSNAX	2	-0.008597328	0.122711742	-0.070061167	0.944144975	0.025655712	6.478066575	84.56329541	0.5	PRJNA827465;Yang2025_Silkie_Wenchang	CRA004775	0.985827325
ENSGALG00010007960	TSNAX	2	-0.150076082	0.079076774	-1.897852875	0.057715466	0	0.058503524	0	1	CRA004775;Yang2025_Silkie_Wenchang	PRJNA827465	0.416294906
ENSGALG00010007960	TSNAX	2	0.023356955	0.133380956	0.17511462	0.860989571	0.022212229	1.959236367	48.9597061	0.5	CRA004775;PRJNA827465	Yang2025_Silkie_Wenchang	0.965325149
ENSGALG00010007962	ADCY8	2	0.220060765	0.173215578	1.270444423	0.203926368	0	0.359592212	0	1	PRJNA827465;Yang2025_Silkie_Wenchang	CRA004775	0.717254253
ENSGALG00010007962	ADCY8	2	0.0470295	0.493372815	0.095322439	0.92405872	0.248074898	1.838613805	45.61119921	0.5	CRA004775;Yang2025_Silkie_Wenchang	PRJNA827465	0.982291939
ENSGALG00010007962	ADCY8	2	0.027145085	0.29842927	0.090958863	0.927524484	0.066657691	1.260370745	20.65826629	0.5	CRA004775;PRJNA827465	Yang2025_Silkie_Wenchang	0.982593195
ENSGALG00010007963	LDAH	2	-0.179279243	0.161348173	-1.111132775	0.266511202	0.047218306	10.68910049	90.64467585	1	PRJNA827465;Yang2025_Silkie_Wenchang	CRA004775	0.770699087
ENSGALG00010007963	LDAH	2	-0.336374396	0.075718761	-4.442418104	8.89535E-06	0	0.215432976	0	1	CRA004775;Yang2025_Silkie_Wenchang	PRJNA827465	0.001271323
ENSGALG00010007963	LDAH	2	-0.026147525	0.060137533	-0.434795435	0.663710919	0	0.198734502	0	1	CRA004775;PRJNA827465	Yang2025_Silkie_Wenchang	0.913119244
ENSGALG00010007964	RECQL4	2	0.325998807	0.576187517	0.565785959	0.571539292	0.621593129	15.01182875	93.33588641	0.5	PRJNA827465;Yang2025_Silkie_Wenchang	CRA004775	0.883429878
ENSGALG00010007964	RECQL4	2	0.832066596	0.244507758	3.403027387	0.000666436	0	0.538525741	0	1	CRA004775;Yang2025_Silkie_Wenchang	PRJNA827465	0.028470581
ENSGALG00010007964	RECQL4	2	-0.018546614	0.327920186	-0.056558317	0.954897039	0.138496818	2.063615209	51.54135347	0.5	CRA004775;PRJNA827465	Yang2025_Silkie_Wenchang	0.988029968
ENSGALG00010007965	EGLN1	2	-0.195284	0.071200249	-2.742743217	0.006093831	0	0.504187796	0	1	PRJNA827465;Yang2025_Silkie_Wenchang	CRA004775	0.1181384
ENSGALG00010007965	EGLN1	2	0.018429514	0.340002129	0.054204114	0.956772543	0.174772804	3.421597024	70.77388153	0.5	CRA004775;Yang2025_Silkie_Wenchang	PRJNA827465	0.988540618
ENSGALG00010007965	EGLN1	2	0.054636781	0.282585101	0.193346291	0.846687775	0.10846654	2.496100645	59.93751286	0.5	CRA004775;PRJNA827465	Yang2025_Silkie_Wenchang	0.961252712
ENSGALG00010007966	TMEM74	2	0.219887953	0.175236509	1.254806741	0.209548925	0	0.00136383	0	1	PRJNA827465;Yang2025_Silkie_Wenchang	CRA004775	0.721386257
ENSGALG00010007966	TMEM74	2	-0.039997804	0.399442183	-0.10011913	0.920248748	0.167461551	1.809799591	44.74526323	0.5	CRA004775;Yang2025_Silkie_Wenchang	PRJNA827465	0.981364549
ENSGALG00010007966	TMEM74	2	-0.041983877	0.390495155	-0.107514462	0.914380853	0.158012876	1.770327215	43.51326741	0.5	CRA004775;PRJNA827465	Yang2025_Silkie_Wenchang	0.979661723
ENSGALG00010007968	SLC22A31	2	-0.086823381	0.144551619	-0.600639286	0.548080265	0	0.000743147	0	1	PRJNA827465;Yang2025_Silkie_Wenchang	CRA004775	0.878281266
ENSGALG00010007968	SLC22A31	2	-0.003155179	0.246756844	-0.012786592	0.989798054	0	0.493163669	0	0.5	CRA004775;Yang2025_Silkie_Wenchang	PRJNA827465	0.997311131
ENSGALG00010007968	SLC22A31	2	-0.054206061	0.164615461	-0.329290035	0.741936475	0	0.568550069	0	0.5	CRA004775;PRJNA827465	Yang2025_Silkie_Wenchang	0.93543545
ENSGALG00010007970	SPRTN	2	0.236433439	0.055398534	4.26786458	1.97353E-05	0	0.009911435	0	1	PRJNA827465;Yang2025_Silkie_Wenchang	CRA004775	0.002431526
ENSGALG00010007970	SPRTN	2	0.212399857	0.087918742	2.415865523	0.015697858	0	0.40656502	0	1	CRA004775;Yang2025_Silkie_Wenchang	PRJNA827465	0.207316163
ENSGALG00010007970	SPRTN	2	0.230096471	0.067778761	3.394816711	0.000686748	0	0.469864023	0	1	CRA004775;PRJNA827465	Yang2025_Silkie_Wenchang	0.029022755
ENSGALG00010007972	EXOC8	2	0.018288124	0.073576135	0.248560542	0.803700733	0.006126966	2.208250859	54.71528989	0.5	PRJNA827465;Yang2025_Silkie_Wenchang	CRA004775	0.951066707
ENSGALG00010007972	EXOC8	2	0.108432886	0.222558719	0.487210236	0.626109352	0.074317895	3.508734753	71.49969803	0.5	CRA004775;Yang2025_Silkie_Wenchang	PRJNA827465	0.901504244
ENSGALG00010007972	EXOC8	2	0.152477366	0.130489232	1.16850535	0.242602983	0.0193213	1.700417861	41.1909259	1	CRA004775;PRJNA827465	Yang2025_Silkie_Wenchang	0.7550024
ENSGALG00010007973	CARMIL1	2	-0.034679975	0.068173806	-0.508699408	0.610962937	0	0.502109198	0	0.5	PRJNA827465;Yang2025_Silkie_Wenchang	CRA004775	0.897730691
ENSGALG00010007973	CARMIL1	2	0.29035739	0.267733175	1.084502847	0.278141905	0.104063797	3.467983331	71.16479797	1	CRA004775;Yang2025_Silkie_Wenchang	PRJNA827465	0.776763074
ENSGALG00010007973	CARMIL1	2	0.226284179	0.32643341	0.693201652	0.488183012	0.182549333	6.325492055	84.19095319	0.5	CRA004775;PRJNA827465	Yang2025_Silkie_Wenchang	0.85768885
ENSGALG00010007975	ANKRD11	2	0.02945549	0.217953412	0.135145808	0.892496593	0.071789704	3.436300437	70.89893569	0.5	PRJNA827465;Yang2025_Silkie_Wenchang	CRA004775	0.974359592
ENSGALG00010007975	ANKRD11	2	0.305546845	0.176702429	1.72916041	0.08378039	0	0.008990073	0	1	CRA004775;Yang2025_Silkie_Wenchang	PRJNA827465	0.498685546
ENSGALG00010007975	ANKRD11	2	0.001586633	0.204998661	0.007739723	0.993824656	0.057661344	2.493746503	59.89969315	0.5	CRA004775;PRJNA827465	Yang2025_Silkie_Wenchang	0.998042093
ENSGALG00010007976	ENSGALG00010007976	2	0.129779975	0.704677641	0.184169282	0.853880657	0.856030945	6.939632721	85.59001549	0.5	PRJNA827465;Yang2025_Silkie_Wenchang	CRA004775	0.962975689
ENSGALG00010007976	ENSGALG00010007976	2	0.420692724	0.475886148	0.884029207	0.376680446	0.224659823	1.984159595	49.60082835	0.5	CRA004775;Yang2025_Silkie_Wenchang	PRJNA827465	0.823974013
ENSGALG00010007976	ENSGALG00010007976	2	-0.421554527	0.218558522	-1.928794736	0.05375635	0	0.740143565	0	1	CRA004775;PRJNA827465	Yang2025_Silkie_Wenchang	0.401964436
ENSGALG00010007977	APOB	2	-1.924973799	2.729006762	-0.70537524	0.480576749	13.67693006	12.22756903	91.82175952	0.5	PRJNA827465;Yang2025_Silkie_Wenchang	CRA004775	0.856719542
ENSGALG00010007977	APOB	2	-1.193529794	3.485246619	-0.342452034	0.732010735	22.89185205	17.30224058	94.22040171	0.5	CRA004775;Yang2025_Silkie_Wenchang	PRJNA827465	0.93287651
ENSGALG00010007977	APOB	2	1.471323728	0.843904419	1.743472002	0.081251201	0	0.79007917	0	1	CRA004775;PRJNA827465	Yang2025_Silkie_Wenchang	0.490992611
ENSGALG00010007978	JADE1	2	0.037627251	0.043178342	0.871438074	0.383515004	0	0.031756468	0	1	PRJNA827465;Yang2025_Silkie_Wenchang	CRA004775	0.825509017
ENSGALG00010007978	JADE1	2	0.110619164	0.126382987	0.875269423	0.381427327	0	0.639002919	0	1	CRA004775;Yang2025_Silkie_Wenchang	PRJNA827465	0.824600563
ENSGALG00010007978	JADE1	2	0.041960104	0.044822915	0.936130639	0.34920593	0	0.937455085	0	1	CRA004775;PRJNA827465	Yang2025_Silkie_Wenchang	0.809731578
ENSGALG00010007979	RAB30	2	-0.09215464	0.092147724	-1.000074548	0.895066037	0.011858476	3.201035668	68.76011067	0.5	PRJNA827465;Yang2025_Silkie_Wenchang	CRA004775	0.974641371
ENSGALG00010007979	RAB30	2	-0.081756543	0.084967156	-0.962213487	0.335942376	0.000906004	1.031781648	3.080268808	0.5	CRA004775;Yang2025_Silkie_Wenchang	PRJNA827465	0.803504387
ENSGALG00010007979	RAB30	2	0.072728363	0.055263644	1.316025469	0.188165494	0	0.061206034	0	1	CRA004775;PRJNA827465	Yang2025_Silkie_Wenchang	0.697483156
ENSGALG00010007980	MARCHF1	2	0.058121732	0.155908446	0.372793093	0.709301774	0	0.036303604	0	1	PRJNA827465;Yang2025_Silkie_Wenchang	CRA004775	0.925298042
ENSGALG00010007980	MARCHF1	2	-0.062359462	0.250312056	-0.249126883	0.803262635	0	0.795993343	0	0.5	CRA004775;Yang2025_Silkie_Wenchang	PRJNA827465	0.950951274
ENSGALG00010007980	MARCHF1	2	-0.021836035	0.164067194	-0.133092023	0.894120604	0	0.771459573	0	0.5	CRA004775;PRJNA827465	Yang2025_Silkie_Wenchang	0.974381724
ENSGALG00010007982	RECK	2	-0.121329663	0.205760636	-0.589664114	0.555415859	0.077914433	12.35737464	91.90766624	0.5	PRJNA827465;Yang2025_Silkie_Wenchang	CRA004775	0.880255849
ENSGALG00010007982	RECK	2	0.131014958	0.49214672	0.266211177	0.790075675	0.44693477	13.13042978	92.38410306	0.5	CRA004775;Yang2025_Silkie_Wenchang	PRJNA827465	0.94739019
ENSGALG00010007982	RECK	2	0.312011326	0.282194816	1.105659312	0.268874007	0.130683613	4.832341054	79.30609639	1	CRA004775;PRJNA827465	Yang2025_Silkie_Wenchang	0.771398294
ENSGALG00010007983	RCHY1	2	-0.07435421	0.073600633	-1.010238724	0.312380931	0	0.309211358	0	0.5	PRJNA827465;Yang2025_Silkie_Wenchang	CRA004775	0.796054997
ENSGALG00010007983	RCHY1	2	-0.068541307	0.168554003	-0.406643011	0.684270202	0	0.566966769	0	0.5	CRA004775;Yang2025_Silkie_Wenchang	PRJNA827465	0.918862939
ENSGALG00010007983	RCHY1	2	-0.099074032	0.074449344	-1.330755625	0.183266776	0	0.214646627	0	1	CRA004775;PRJNA827465	Yang2025_Silkie_Wenchang	0.691694865
ENSGALG00010007984	EIF3H	2	-0.131961499	0.085111959	-1.550446028	0.121034498	0.007003808	1.628008537	38.57526069	1	PRJNA827465;Yang2025_Silkie_Wenchang	CRA004775	0.583253909
ENSGALG00010007984	EIF3H	2	-0.139138761	0.145870799	-0.953849316	0.340159922	0.019107132	1.797843955	44.37782004	0.5	CRA004775;Yang2025_Silkie_Wenchang	PRJNA827465	0.805377063
ENSGALG00010007984	EIF3H	2	-0.069760225	0.052039763	-1.340517725	0.180077086	0	0.332506371	0	0.5	CRA004775;PRJNA827465	Yang2025_Silkie_Wenchang	0.68716552
ENSGALG00010007986	MYC	2	-0.042050708	0.04854647	-0.866194983	0.386383244	0	0.271770105	0	1	PRJNA827465;Yang2025_Silkie_Wenchang	CRA004775	0.826900117
ENSGALG00010007986	MYC	2	-0.092922633	0.12599139	-0.737531613	0.460799126	0	0.104949638	0	0.5	CRA004775;Yang2025_Silkie_Wenchang	PRJNA827465	0.85014477
ENSGALG00010007986	MYC	2	-0.031065569	0.051723451	-0.600608972	0.548100461	0	0.023815492	0	0.5	CRA004775;PRJNA827465	Yang2025_Silkie_Wenchang	0.878281266
ENSGALG00010007987	ENSGALG00010007987	2	-0.17340759	0.125324663	-1.383660919	0.166460469	0	0.012085637	0	1	PRJNA827465;Yang2025_Silkie_Wenchang	CRA004775	0.665350942
ENSGALG00010007987	ENSGALG00010007987	2	-0.113710138	0.177069342	-0.642178579	0.52075724	0	0.1219666	0	1	CRA004775;Yang2025_Silkie_Wenchang	PRJNA827465	0.869353339
ENSGALG00010007987	ENSGALG00010007987	2	-0.152534712	0.13980253	-1.091072619	0.27524093	0	0.209681941	0	1	CRA004775;PRJNA827465	Yang2025_Silkie_Wenchang	0.775080194
ENSGALG00010007988	TARBP1	2	0.159078917	0.08817405	1.811219607	0.069797995	0.008843504	2.318720622	56.87276896	1	PRJNA827465;Yang2025_Silkie_Wenchang	CRA004775	0.455644844
ENSGALG00010007988	TARBP1	2	-0.106391927	0.381008815	-0.279237441	0.780062612	0.264300141	10.60794099	90.5730999	0.5	CRA004775;Yang2025_Silkie_Wenchang	PRJNA827465	0.945041817
ENSGALG00010007988	TARBP1	2	-0.186304992	0.291658579	-0.638777685	0.522967567	0.145196017	6.291382301	84.10524187	0.5	CRA004775;PRJNA827465	Yang2025_Silkie_Wenchang	0.869568211
ENSGALG00010007989	SPG7	2	-0.037564039	0.056865315	-0.660579098	0.508882277	0.002768685	1.571594059	36.37033722	0.5	PRJNA827465;Yang2025_Silkie_Wenchang	CRA004775	0.865193604
ENSGALG00010007989	SPG7	2	0.033168515	0.082275221	0.403141004	0.686844497	0	0.215880357	0	0.5	CRA004775;Yang2025_Silkie_Wenchang	PRJNA827465	0.919235539
ENSGALG00010007989	SPG7	2	-0.074962371	0.042709724	-1.755159332	0.079232058	0	0.002015198	0	1	CRA004775;PRJNA827465	Yang2025_Silkie_Wenchang	0.484394238
ENSGALG00010007991	PCF11	2	0.431815389	0.376338646	1.147411762	0.251211482	0.269289359	19.63087219	94.90598283	1	PRJNA827465;Yang2025_Silkie_Wenchang	CRA004775	0.761246329
ENSGALG00010007991	PCF11	2	0.653702847	0.201414437	3.245561032	0.001172194	0.043421433	2.084946961	52.03714921	1	CRA004775;Yang2025_Silkie_Wenchang	PRJNA827465	0.041644062
ENSGALG00010007991	PCF11	2	0.16892423	0.155637857	1.085367231	0.27775904	0.031113206	2.0982319	52.34082563	1	CRA004775;PRJNA827465	Yang2025_Silkie_Wenchang	0.776763074
ENSGALG00010007992	LAPTM4A	2	-0.488140132	0.388143878	-1.257626772	0.208526767	0.290380263	27.26630215	96.33246931	1	PRJNA827465;Yang2025_Silkie_Wenchang	CRA004775	0.721011841
ENSGALG00010007992	LAPTM4A	2	-0.595598855	0.307954493	-1.934047298	0.053107313	0.160978664	6.458801802	84.51725211	1	CRA004775;Yang2025_Silkie_Wenchang	PRJNA827465	0.399631766
ENSGALG00010007992	LAPTM4A	2	-0.124016991	0.066452515	-1.875269929	0.060755598	0	0.543520941	0	1	CRA004775;PRJNA827465	Yang2025_Silkie_Wenchang	0.426940331
ENSGALG00010007993	LRRC14	2	0.086718902	0.142272621	0.609526282	0.542175657	0.028033835	3.247416212	69.20628787	0.5	PRJNA827465;Yang2025_Silkie_Wenchang	CRA004775	0.876721948
ENSGALG00010007993	LRRC14	2	0.040249911	0.25381602	0.158579082	0.87400051	0.087274593	2.593654806	61.44436809	0.5	CRA004775;Yang2025_Silkie_Wenchang	PRJNA827465	0.970074749
ENSGALG00010007993	LRRC14	2	-0.089110113	0.108836253	-0.81875396	0.412926803	0	0.55306506	0	1	CRA004775;PRJNA827465	Yang2025_Silkie_Wenchang	0.835870654
ENSGALG00010007994	FAM65B	2	-0.182574704	0.091462229	-1.996175964	0.045914773	0.002748559	1.186357222	15.70835655	1	PRJNA827465;Yang2025_Silkie_Wenchang	CRA004775	0.369489827
ENSGALG00010007994	FAM65B	2	-0.274691235	0.127653172	-2.151855934	0.031408703	0	0.208338339	0	1	CRA004775;Yang2025_Silkie_Wenchang	PRJNA827465	0.303102391
ENSGALG00010007994	FAM65B	2	-0.10893866	0.101400227	-1.07434335	0.282668822	0	0.000126195	0	1	CRA004775;PRJNA827465	Yang2025_Silkie_Wenchang	0.779532565
ENSGALG00010007996	NUDCD1	2	-0.195071755	0.119851226	-1.627615858	0.103604364	0.023342109	5.267083426	81.01416061	1	PRJNA827465;Yang2025_Silkie_Wenchang	CRA004775	0.547964315
ENSGALG00010007996	NUDCD1	2	-0.190652471	0.189705374	-1.004992377	0.314464601	0.04605855	2.266754841	55.88406907	0.5	CRA004775;Yang2025_Silkie_Wenchang	PRJNA827465	0.79653148
ENSGALG00010007996	NUDCD1	2	-0.072179559	0.060798788	-1.187187468	0.235153683	0	0.395604606	0	0.5	CRA004775;PRJNA827465	Yang2025_Silkie_Wenchang	0.748178192
ENSGALG00010007997	TMEM192	2	-0.183002022	0.110784089	-1.651880008	0.098559014	0.015654622	2.6900018	62.82530368	1	PRJNA827465;Yang2025_Silkie_Wenchang	CRA004775	0.53598695
ENSGALG00010007997	TMEM192	2	-0.297861343	0.104600846	-2.847599738	0.004405029	0	0.077910865	0	1	CRA004775;Yang2025_Silkie_Wenchang	PRJNA827465	0.097319196
ENSGALG00010007997	TMEM192	2	-0.092691135	0.07649735	-1.211690799	0.225630763	0	0.15991132	0	1	CRA004775;PRJNA827465	Yang2025_Silkie_Wenchang	0.73884362
ENSGALG00010007998	RPL13	2	-0.163387891	0.170242999	-0.959733396	0.337189411	0.033353497	1.686195235	40.69488639	1	PRJNA827465;Yang2025_Silkie_Wenchang	CRA004775	0.804086136
ENSGALG00010007998	RPL13	2	-0.497488358	0.190394532	-2.612934062	0.008976862	0	0.008639499	0	1	CRA004775;Yang2025_Silkie_Wenchang	PRJNA827465	0.148492258
ENSGALG00010007998	RPL13	2	-0.22853832	0.211529902	-1.080406687	0.279961112	0.065465633	3.055921511	67.27664646	1	CRA004775;PRJNA827465	Yang2025_Silkie_Wenchang	0.778059106
ENSGALG00010007999	SDC1	2	0.252918861	0.090155524	2.805361779	0.005026013	0	0.062204276	0	1	PRJNA827465;Yang2025_Silkie_Wenchang	CRA004775	0.105691492
ENSGALG00010007999	SDC1	2	0.20753024	0.125865164	1.648829851	0.099182498	0	0.362508957	0	0.5	CRA004775;Yang2025_Silkie_Wenchang	PRJNA827465	0.537308023
ENSGALG00010007999	SDC1	2	0.251445765	0.119966974	2.09595821	0.036085898	0	0.489184602	0	0.5	CRA004775;PRJNA827465	Yang2025_Silkie_Wenchang	0.327699644
ENSGALG00010008000	C8orf82	2	0.035853163	0.081883091	0.437857954	0.661489256	0	0.325682115	0	1	PRJNA827465;Yang2025_Silkie_Wenchang	CRA004775	0.912364181
ENSGALG00010008000	C8orf82	2	-0.107005831	0.255049149	-0.419549845	0.674814335	0.084882608	2.877158037	65.24348029	0.5	CRA004775;Yang2025_Silkie_Wenchang	PRJNA827465	0.915989029
ENSGALG00010008000	C8orf82	2	-0.123523452	0.182888643	-0.675402533	0.499420057	0.045102918	2.686418865	62.77572299	0.5	CRA004775;PRJNA827465	Yang2025_Silkie_Wenchang	0.862004589
ENSGALG00010008001	ALDH5A1	2	0.053878453	0.169423963	0.318009639	0.750477626	0.035360583	2.581901002	61.26884806	0.5	PRJNA827465;Yang2025_Silkie_Wenchang	CRA004775	0.937393444
ENSGALG00010008001	ALDH5A1	2	-0.066847898	0.143559925	-0.465644561	0.641469941	0	0.636779154	0	0.5	CRA004775;Yang2025_Silkie_Wenchang	PRJNA827465	0.906634523
ENSGALG00010008001	ALDH5A1	2	0.200731335	0.123163008	1.629802147	0.10314332	0	0.038112681	0	1	CRA004775;PRJNA827465	Yang2025_Silkie_Wenchang	0.5473214
ENSGALG00010008002	GJA8	2	0.648342505	0.242537841	2.673160204	0.007514036	0.09326749	4.799490196	79.16445374	1	PRJNA827465;Yang2025_Silkie_Wenchang	CRA004775	0.1337465
ENSGALG00010008002	GJA8	2	0.853832803	0.160735119	5.312048853	1.084E-07	0	0.685603934	0	1	CRA004775;Yang2025_Silkie_Wenchang	PRJNA827465	3.78737E-05
ENSGALG00010008002	GJA8	2	0.419135693	0.136260022	3.075999005	0.002097985	0	0.012392378	0	1	CRA004775;PRJNA827465	Yang2025_Silkie_Wenchang	0.05983826
ENSGALG00010008004	ENY2	2	-0.399428939	0.33672197	-1.186227731	0.235532378	0.206966903	11.00162883	90.91043685	1	PRJNA827465;Yang2025_Silkie_Wenchang	CRA004775	0.748604097
ENSGALG00010008004	ENY2	2	-0.552811833	0.246540546	-2.242275526	0.02494357	0.067898829	2.174488421	54.01217177	1	CRA004775;Yang2025_Silkie_Wenchang	PRJNA827465	0.269107096
ENSGALG00010008004	ENY2	2	-0.095972362	0.077026334	-1.245968181	0.212776077	0	0.345251154	0	1	CRA004775;PRJNA827465	Yang2025_Silkie_Wenchang	0.725617194
ENSGALG00010008007	CBR4	2	-0.163989848	0.064476532	-2.543403665	0.010977832	0	0.924775322	0	1	PRJNA827465;Yang2025_Silkie_Wenchang	CRA004775	0.166811819
ENSGALG00010008007	CBR4	2	-0.23295581	0.117835491	-1.976957947	0.048046384	0	0.486031305	0	1	CRA004775;Yang2025_Silkie_Wenchang	PRJNA827465	0.379037942
ENSGALG00010008007	CBR4	2	-0.125329509	0.070871875	-1.768395565	0.076994798	0	0.042481497	0	1	CRA004775;PRJNA827465	Yang2025_Silkie_Wenchang	0.478438977
ENSGALG00010008008	BTC	2	0.233837304	0.149536773	1.563744484	0.117877587	0	0.054594661	0	1	PRJNA827465;Yang2025_Silkie_Wenchang	CRA004775	0.577673664
ENSGALG00010008008	BTC	2	-0.031246772	0.278367228	-0.112250182	0.910625041	0.026008486	1.17951081	15.21908986	0.5	CRA004775;Yang2025_Silkie_Wenchang	PRJNA827465	0.97880407
ENSGALG00010008008	BTC	2	0.046129723	0.308508777	0.149524831	0.881139517	0.108726948	1.960029825	48.9803682	0.5	CRA004775;PRJNA827465	Yang2025_Silkie_Wenchang	0.971705487
ENSGALG00010008010	CERK	2	0.008007049	0.198934645	0.040249646	0.967894098	0.073946702	15.18981787	93.41664259	0.5	PRJNA827465;Yang2025_Silkie_Wenchang	CRA004775	0.991128245
ENSGALG00010008010	CERK	2	0.040657328	0.274977051	0.147857169	0.882455491	0.124171654	4.991044513	79.96411378	0.5	CRA004775;Yang2025_Silkie_Wenchang	PRJNA827465	0.972001076
ENSGALG00010008010	CERK	2	0.216567258	0.063995002	3.384127702	0.000714048	0	0.419010935	0	1	CRA004775;PRJNA827465	Yang2025_Silkie_Wenchang	0.029980946
ENSGALG00010008011	ADNP2	2	-0.055061068	0.0452927	-1.215672019	0.224109887	0	0.871496421	0	1	PRJNA827465;Yang2025_Silkie_Wenchang	CRA004775	0.737452676
ENSGALG00010008011	ADNP2	2	-0.126587755	0.087535774	-1.446125954	0.148141877	0	0.009390689	0	1	CRA004775;Yang2025_Silkie_Wenchang	PRJNA827465	0.63772401
ENSGALG00010008011	ADNP2	2	-0.032816469	0.051414265	-0.63827556	0.523294318	0	0.046871392	0	1	CRA004775;PRJNA827465	Yang2025_Silkie_Wenchang	0.869661505
ENSGALG00010008012	CCDC90B	2	-0.054770084	0.095215272	-0.575223732	0.565139994	0.010537597	2.363526533	57.6903417	0.5	PRJNA827465;Yang2025_Silkie_Wenchang	CRA004775	0.883427937
ENSGALG00010008012	CCDC90B	2	-0.131370632	0.088488026	-1.484614784	0.137645897	0	0.504127152	0	0.5	CRA004775;Yang2025_Silkie_Wenchang	PRJNA827465	0.620631442
ENSGALG00010008012	CCDC90B	2	0.030566116	0.074060451	0.412718471	0.67981289	0	0.00617279	0	1	CRA004775;PRJNA827465	Yang2025_Silkie_Wenchang	0.917701868
ENSGALG00010008013	ENSGALG00010008013	2	0.161109366	0.172705905	0.932853839	0.350895439	0.037111882	2.602247645	61.57168201	1	PRJNA827465;Yang2025_Silkie_Wenchang	CRA004775	0.810146221
ENSGALG00010008013	ENSGALG00010008013	2	0.135457037	0.286529214	0.472751226	0.636390675	0.099851128	2.289629099	56.32480385	0.5	CRA004775;Yang2025_Silkie_Wenchang	PRJNA827465	0.904566695
ENSGALG00010008013	ENSGALG00010008013	2	-0.024567012	0.122526554	-0.200503576	0.841086762	0	0.432062183	0	0.5	CRA004775;PRJNA827465	Yang2025_Silkie_Wenchang	0.959877842
ENSGALG00010008015	ARHGAP39	2	0.153664164	0.190032949	0.808618534	0.418734601	0.050196194	3.059393183	67.31377956	0.5	PRJNA827465;Yang2025_Silkie_Wenchang	CRA004775	0.838250071
ENSGALG00010008015	ARHGAP39	2	0.236948688	0.236243692	1.002984188	0.31586849	0.038963483	1.380968157	27.58703413	0.5	CRA004775;Yang2025_Silkie_Wenchang	PRJNA827465	0.796883862
ENSGALG00010008015	ARHGAP39	2	-0.014280719	0.102231947	-0.139689401	0.888905401	0	0.118537052	0	1	CRA004775;PRJNA827465	Yang2025_Silkie_Wenchang	0.973135755
ENSGALG00010008016	GSR	2	-0.147238917	0.193415692	-0.761256316	0.446503987	0.050324403	2.493293083	59.89240066	1	PRJNA827465;Yang2025_Silkie_Wenchang	CRA004775	0.846394998
ENSGALG00010008016	GSR	2	-0.489557911	0.183253528	-2.671478779	0.007551785	0	0.172973938	0	1	CRA004775;Yang2025_Silkie_Wenchang	PRJNA827465	0.134120107
ENSGALG00010008016	GSR	2	-0.231169747	0.274940935	-0.840797851	0.400461191	0.118569843	3.969889372	74.81038119	1	CRA004775;PRJNA827465	Yang2025_Silkie_Wenchang	0.832251175
ENSGALG00010008017	GMNN	2	-0.070391308	0.12270188	-0.573677506	0.566186053	0.011959569	1.488270849	32.80792937	0.5	PRJNA827465;Yang2025_Silkie_Wenchang	CRA004775	0.883487517
ENSGALG00010008017	GMNN	2	-0.265258995	0.178697864	-1.48439936	0.137703004	0	0.001623748	0	1	CRA004775;Yang2025_Silkie_Wenchang	PRJNA827465	0.62064915
ENSGALG00010008017	GMNN	2	-0.007210269	0.093749858	-0.076909655	0.938695416	0	0.481282169	0	0.5	CRA004775;PRJNA827465	Yang2025_Silkie_Wenchang	0.985349045
ENSGALG00010008018	ENSGALG00010008018	2	0.353635586	0.171963563	2.056456493	0.039738536	0.0102237	1.203182141	16.88706421	1	PRJNA827465;Yang2025_Silkie_Wenchang	CRA004775	0.344755224
ENSGALG00010008018	ENSGALG00010008018	2	0.10165151	0.59901163	0.169698726	0.865247076	0.503973505	2.854932121	64.97289751	0.5	CRA004775;Yang2025_Silkie_Wenchang	PRJNA827465	0.96700901
ENSGALG00010008018	ENSGALG00010008018	2	0.003960708	0.374925002	0.010564002	0.991571303	0.140311389	1.534536465	34.83374147	0.5	CRA004775;PRJNA827465	Yang2025_Silkie_Wenchang	0.997575757
ENSGALG00010008019	PPP2CB	2	-0.147003204	0.102970627	-1.427622705	0.153400485	0.017300178	5.109162179	80.4273193	1	PRJNA827465;Yang2025_Silkie_Wenchang	CRA004775	0.645780478
ENSGALG00010008019	PPP2CB	2	-0.26061096	0.07303505	-3.568299864	0.000359305	0	0.004137954	0	1	CRA004775;Yang2025_Silkie_Wenchang	PRJNA827465	0.018929987
ENSGALG00010008019	PPP2CB	2	-0.09359199	0.077433542	-1.208675051	0.22678771	0.00578561	1.431549317	30.14561299	1	CRA004775;PRJNA827465	Yang2025_Silkie_Wenchang	0.740380212
ENSGALG00010008020	COA6	2	-0.116702529	0.103056539	-1.132412656	0.257460987	0	0.536840359	0	0.5	PRJNA827465;Yang2025_Silkie_Wenchang	CRA004775	0.7650601
ENSGALG00010008020	COA6	2	0.009300784	0.201276895	0.046208903	0.963143747	0	0.10909781	0	0.5	CRA004775;Yang2025_Silkie_Wenchang	PRJNA827465	0.989998461
ENSGALG00010008020	COA6	2	-0.135803	0.103661595	-1.31006088	0.190175241	0	0.077192858	0	1	CRA004775;PRJNA827465	Yang2025_Silkie_Wenchang	0.699403589
ENSGALG00010008022	SLC35F3	2	-0.298970244	0.254265648	-1.175818469	0.239667424	0.038112208	1.332385171	24.9466279	1	PRJNA827465;Yang2025_Silkie_Wenchang	CRA004775	0.751887674
ENSGALG00010008022	SLC35F3	2	-0.508825546	0.352487685	-1.443527159	0.148872027	0	0.607902039	0	1	CRA004775;Yang2025_Silkie_Wenchang	PRJNA827465	0.638968865
ENSGALG00010008022	SLC35F3	2	-0.1248308	0.220462678	-0.566221915	0.571242934	0	0.006009192	0	1	CRA004775;PRJNA827465	Yang2025_Silkie_Wenchang	0.885354083
ENSGALG00010008023	ATAD2	2	0.005686645	0.080720358	0.070448705	0.943836527	0.006043799	1.841718996	45.70290027	0.5	PRJNA827465;Yang2025_Silkie_Wenchang	CRA004775	0.985827325
ENSGALG00010008023	ATAD2	2	-0.097134106	0.091862527	-1.057385512	0.290335681	0	0.405442187	0	1	CRA004775;Yang2025_Silkie_Wenchang	PRJNA827465	0.784195688
ENSGALG00010008023	ATAD2	2	0.065608252	0.073797474	0.889031137	0.373986346	0	0.968215648	0	0.5	CRA004775;PRJNA827465	Yang2025_Silkie_Wenchang	0.822579781
ENSGALG00010008024	MRS2	2	-0.139063268	0.079278349	-1.754114086	0.079410961	0	0.03956924	0	1	PRJNA827465;Yang2025_Silkie_Wenchang	CRA004775	0.484867016
ENSGALG00010008024	MRS2	2	-0.131182895	0.101942227	-1.286835686	0.198151566	0	0.219026364	0	1	CRA004775;Yang2025_Silkie_Wenchang	PRJNA827465	0.710234722
ENSGALG00010008024	MRS2	2	-0.107198541	0.098782664	-1.085195899	0.2778349	0	0.11939264	0	1	CRA004775;PRJNA827465	Yang2025_Silkie_Wenchang	0.776763074

ENSGALG00010008025	TBC1D22A	2	-0.059621768	0.046521285	-1.281601929	0.199982323	0	0.141421716	0	1	PRJNA827465;Yang2025_Silkie_Wenchang	CRA004775	0.712076938
ENSGALG00010008025	TBC1D22A	2	0.106474708	0.137374568	0.775068557	0.43829915	0.024615383	2.85998987	65.03484119	0.5	CRA004775;Yang2025_Silkie_Wenchang	PRJNA827465	0.843979791
ENSGALG00010008025	TBC1D22A	2	0.071925944	0.158532183	0.453699324	0.650045254	0.042078251	5.696794424	82.44626845	0.5	CRA004775;PRJNA827465	Yang2025_Silkie_Wenchang	0.908335059
ENSGALG00010008026	PURG	2	0.220366977	0.080900665	2.723920479	0.006451204	0	0.032576145	0	1	PRJNA827465;Yang2025_Silkie_Wenchang	CRA004775	0.122370493
ENSGALG00010008026	PURG	2	0.234120657	0.147579631	1.586402233	0.112648094	0	0.275197679	0	1	CRA004775;Yang2025_Silkie_Wenchang	PRJNA827465	0.566454459
ENSGALG00010008026	PURG	2	0.240419169	0.089027806	2.700495285	0.006923632	0	0.216041375	0	1	CRA004775;PRJNA827465	Yang2025_Silkie_Wenchang	0.127875706
ENSGALG00010008027	C6orf62	2	-0.298394684	0.143718828	-2.076239328	0.037871822	0.025500917	2.497824281	59.96515818	1	PRJNA827465;Yang2025_Silkie_Wenchang	CRA004775	0.335620339
ENSGALG00010008027	C6orf62	2	-0.122911923	0.37312217	-0.329414687	0.741842268	0.234691583	6.175974537	83.8082234	0.5	CRA004775;Yang2025_Silkie_Wenchang	PRJNA827465	0.93543545
ENSGALG00010008027	C6orf62	2	-0.010862583	0.219792901	-0.049421901	0.960583075	0.066154143	2.733992822	63.4234592	0.5	CRA004775;PRJNA827465	Yang2025_Silkie_Wenchang	0.98951084
ENSGALG00010008028	KCNK1	2	-0.032568322	0.202095825	-0.161152869	0.871973001	0.018672792	1.283391123	22.08143081	0.5	PRJNA827465;Yang2025_Silkie_Wenchang	CRA004775	0.969535896
ENSGALG00010008028	KCNK1	2	-0.126598284	0.19562546	-0.647146256	0.517537291	0	0.451376715	0	0.5	CRA004775;Yang2025_Silkie_Wenchang	PRJNA827465	0.867962259
ENSGALG00010008028	KCNK1	2	0.176218445	0.232385758	0.758301401	0.448270569	0	0.043986118	0	1	CRA004775;PRJNA827465	Yang2025_Silkie_Wenchang	0.846800937
ENSGALG00010008029	LY6E	2	-0.324366005	0.408831575	-0.793397634	0.427546189	0.319243543	22.03399815	95.46155903	0.5	PRJNA827465;Yang2025_Silkie_Wenchang	CRA004775	0.84125685
ENSGALG00010008029	LY6E	2	-0.311835676	0.458107502	-0.680708145	0.496058713	0.372572965	8.622001271	88.40176464	0.5	CRA004775;Yang2025_Silkie_Wenchang	PRJNA827465	0.860911322
ENSGALG00010008029	LY6E	2	0.086415491	0.087289708	0.989984881	0.322181509	0	0.119204736	0	1	CRA004775;PRJNA827465	Yang2025_Silkie_Wenchang	0.798513866
ENSGALG00010008030	GJA5	2	0.39454938	0.155335233	2.539986397	0.011085678	0.030870757	2.768072227	63.87377504	1	PRJNA827465;Yang2025_Silkie_Wenchang	CRA004775	0.167383943
ENSGALG00010008030	GJA5	2	0.223299928	0.129806834	1.720247861	0.085387397	0	0.038341508	0	1	CRA004775;Yang2025_Silkie_Wenchang	PRJNA827465	0.502621951
ENSGALG00010008030	GJA5	2	0.499787712	0.123039664	4.062004859	4.86531E-05	0.001506434	1.020547275	2.013358498	1	CRA004775;PRJNA827465	Yang2025_Silkie_Wenchang	0.004768607
ENSGALG00010008031	MAP3K21	2	-0.036718118	0.215041774	-0.170748769	0.864421313	0.059169702	2.728564567	63.35069318	0.5	PRJNA827465;Yang2025_Silkie_Wenchang	CRA004775	0.966676482
ENSGALG00010008031	MAP3K21	2	-0.316642928	0.198403456	-1.595954699	0.110498908	0	0.438514733	0	1	CRA004775;Yang2025_Silkie_Wenchang	PRJNA827465	0.562832796
ENSGALG00010008031	MAP3K21	2	-0.06052288	0.380587574	-0.159024844	0.873649301	0.167610874	1.759144016	43.15417094	0.5	CRA004775;PRJNA827465	Yang2025_Silkie_Wenchang	0.9699681
ENSGALG00010008032	PUM2	2	0.042798434	0.034185719	1.251938953	0.210592109	0	0.210788908	0	0.5	PRJNA827465;Yang2025_Silkie_Wenchang	CRA004775	0.722116705
ENSGALG00010008032	PUM2	2	0.003970208	0.112180375	0.035391291	0.971767729	0	0.100549807	0	0.5	CRA004775;Yang2025_Silkie_Wenchang	PRJNA827465	0.991955348
ENSGALG00010008032	PUM2	2	0.048031913	0.035376446	1.357737093	0.174547122	0	0.021202881	0	1	CRA004775;PRJNA827465	Yang2025_Silkie_Wenchang	0.678894166
ENSGALG00010008033	ACOT13	2	0.216645833	0.244212298	0.8871209	0.375013813	0.100152135	5.923404674	83.11781729	0.5	PRJNA827465;Yang2025_Silkie_Wenchang	CRA004775	0.827916144
ENSGALG00010008033	ACOT13	2	0.426263254	0.15558967	2.73966295	0.006150222	0	0.444317592	0	1	CRA004775;Yang2025_Silkie_Wenchang	PRJNA827465	0.118607543
ENSGALG00010008033	ACOT13	2	0.015930027	0.087105328	0.182882347	0.854890334	0	0.615621265	0	0.5	CRA004775;PRJNA827465	Yang2025_Silkie_Wenchang	0.963248391
ENSGALG00010008034	DPEP1	2	-0.047091563	0.181458978	-0.259516304	0.795236904	0	0.011534274	0	1	PRJNA827465;Yang2025_Silkie_Wenchang	CRA004775	0.948935068
ENSGALG00010008034	DPEP1	2	0.657615186	0.793592078	0.828656439	0.407298842	1.069112246	6.378076537	84.32129194	0.5	CRA004775;Yang2025_Silkie_Wenchang	PRJNA827465	0.833863243
ENSGALG00010008034	DPEP1	2	0.661630482	0.771437396	0.857659332	0.391080603	1.027543441	6.844241621	85.38917742	0.5	CRA004775;PRJNA827465	Yang2025_Silkie_Wenchang	0.829251057
ENSGALG00010008035	TDP2	2	-0.070687821	0.08850167	-0.798717378	0.424454308	0	0.005282988	0	1	PRJNA827465;Yang2025_Silkie_Wenchang	CRA004775	0.840671527
ENSGALG00010008035	TDP2	2	-0.005671902	0.131846224	-0.043019074	0.965686329	0	0.472808801	0	0.5	CRA004775;Yang2025_Silkie_Wenchang	PRJNA827465	0.990668014
ENSGALG00010008035	TDP2	2	-0.032659402	0.092283524	-0.353902847	0.723411693	0	0.543685046	0	0.5	CRA004775;PRJNA827465	Yang2025_Silkie_Wenchang	0.930032096
ENSGALG00010008036	SPOCK3	2	-0.064872257	0.115508076	-0.561625295	0.574371343	0	0.6161017	0	0.5	PRJNA827465;Yang2025_Silkie_Wenchang	CRA004775	0.886083039
ENSGALG00010008036	SPOCK3	2	0.102382672	0.365389617	0.280201373	0.779323013	0.17836402	2.669072923	62.53380748	0.5	CRA004775;Yang2025_Silkie_Wenchang	PRJNA827465	0.944727706
ENSGALG00010008036	SPOCK3	2	0.152771635	0.248539457	0.614677591	0.538767662	0.061491322	1.613163004	38.00998428	1	CRA004775;PRJNA827465	Yang2025_Silkie_Wenchang	0.87481821
ENSGALG00010008037	ENSGALG00010008037	2	-0.388160111	0.71007496	-0.546646668	0.584621501	0.218890661	1.266460163	21.03975876	0.5	PRJNA827465;Yang2025_Silkie_Wenchang	CRA004775	0.891016154
ENSGALG00010008037	ENSGALG00010008037	2	2.663245315	3.933703557	0.677032541	0.498385308	29.67275323	24.22323637	95.87173248	0.5	CRA004775;Yang2025_Silkie_Wenchang	PRJNA827465	0.861478809
ENSGALG00010008037	ENSGALG00010008037	2	3.348289788	3.211835385	1.042484868	0.297186937	19.54646774	18.84430719	94.69335757	1	CRA004775;PRJNA827465	Yang2025_Silkie_Wenchang	0.788466926
ENSGALG00010008038	WRN	2	-0.038893197	0.058585587	-0.66386971	0.506773704	0	0.101131634	0	1	PRJNA827465;Yang2025_Silkie_Wenchang	CRA004775	0.863929781
ENSGALG00010008038	WRN	2	-0.022175578	0.090812844	-0.244189882	0.807083763	0	0.057434855	0	1	CRA004775;Yang2025_Silkie_Wenchang	PRJNA827465	0.95234753
ENSGALG00010008038	WRN	2	-0.055352311	0.071484917	-0.774321538	0.43874067	0	0.012473243	0	1	CRA004775;PRJNA827465	Yang2025_Silkie_Wenchang	0.844002503
ENSGALG00010008039	CHMP1A	2	0.077157308	0.057923382	1.332058066	0.182841108	0	0.027246764	0	1	PRJNA827465;Yang2025_Silkie_Wenchang	CRA004775	0.691266551
ENSGALG00010008039	CHMP1A	2	-0.24205264	0.352035595	-0.687580014	0.491717297	0.217031617	8.005592156	87.50873164	0.5	CRA004775;Yang2025_Silkie_Wenchang	PRJNA827465	0.85895707
ENSGALG00010008039	CHMP1A	2	-0.240194477	0.33726845	-0.712175944	0.476355831	0.208197232	11.14983281	91.03125565	0.5	CRA004775;PRJNA827465	Yang2025_Silkie_Wenchang	0.854973661
ENSGALG00010008041	ENSGALG00010008041	2	0.524948451	0.469747269	1.117512511	0.263775253	0.396567701	9.592774222	89.57548696	1	PRJNA827465;Yang2025_Silkie_Wenchang	CRA004775	0.768643821
ENSGALG00010008041	ENSGALG00010008041	2	0.922155356	0.244853416	3.76615271	0.000165782	0	0.899999542	0	1	CRA004775;Yang2025_Silkie_Wenchang	PRJNA827465	0.011234045
ENSGALG00010008041	ENSGALG00010008041	2	0.096574172	0.142100704	0.679617828	0.496746478	0	0.221224064	0	1	CRA004775;PRJNA827465	Yang2025_Silkie_Wenchang	0.860939846
ENSGALG00010008043	IL17REL	2	0.030538551	0.314605199	0.097069442	0.922671248	0.079101702	1.637276642	38.92296671	0.5	PRJNA827465;Yang2025_Silkie_Wenchang	CRA004775	0.981804675
ENSGALG00010008043	IL17REL	2	0.424396512	0.849265861	0.49972162	0.617271107	1.148049016	4.695342117	78.7022974	0.5	CRA004775;Yang2025_Silkie_Wenchang	PRJNA827465	0.899121942
ENSGALG00010008043	IL17REL	2	0.658954851	0.506095552	1.302031294	0.192905688	0.295065858	2.061625508	51.49458541	1	CRA004775;PRJNA827465	Yang2025_Silkie_Wenchang	0.702958174
ENSGALG00010008046	RHOB	2	0.006280147	0.050357797	0.124710514	0.900752733	0	0.008092102	0	1	PRJNA827465;Yang2025_Silkie_Wenchang	CRA004775	0.976497577
ENSGALG00010008046	RHOB	2	0.100972483	0.141396533	0.714108623	0.475160014	0	0.723930798	0	1	CRA004775;Yang2025_Silkie_Wenchang	PRJNA827465	0.854462864
ENSGALG00010008046	RHOB	2	0.030765468	0.080515565	0.381360642	0.702935661	0.004369775	1.128575593	11.40701902	1	CRA004775;PRJNA827465	Yang2025_Silkie_Wenchang	0.923347492
ENSGALG00010008047	ASAP1	2	0.203350439	0.299346181	0.679315292	0.496938109	0.146440018	4.815781718	79.23493924	0.5	PRJNA827465;Yang2025_Silkie_Wenchang	CRA004775	0.860939846
ENSGALG00010008047	ASAP1	2	0.466755781	0.236391201	1.974505725	0.048324267	0	0.595922131	0	1	CRA004775;Yang2025_Silkie_Wenchang	PRJNA827465	0.38014722
ENSGALG00010008047	ASAP1	2	-0.044146642	0.07786507	-0.56693768	0.570756523	0	0.091181604	0	0.5	CRA004775;PRJNA827465	Yang2025_Silkie_Wenchang	0.885224727
ENSGALG00010008048	ENSGALG00010008048	2	-0.390634423	0.852560604	-0.458187024	0.646816213	0.990062968	3.13030821	68.60449464	0.5	PRJNA827465;Yang2025_Silkie_Wenchang	CRA004775	0.907350807
ENSGALG00010008048	ENSGALG00010008048	2	0.794194428	2.180808608	0.364174291	0.715727864	8.499301044	9.149486241	89.07042457	0.5	CRA004775;Yang2025_Silkie_Wenchang	PRJNA827465	0.927131632
ENSGALG00010008048	ENSGALG00010008048	2	1.553178386	1.313583067	1.1823983	0.237047697	2.5421874	3.534318488	71.70600206	1	CRA004775;PRJNA827465	Yang2025_Silkie_Wenchang	0.749355649
ENSGALG00010008050	CDK10	2	0.015217695	0.067257983	0.226258572	0.821000318	0	0.411756139	0	0.5	PRJNA827465;Yang2025_Silkie_Wenchang	CRA004775	0.955548641
ENSGALG00010008050	CDK10	2	-0.322956776	0.25402657	-1.27135038	0.203604027	0.088258096	3.117161719	67.91953417	1	CRA004775;Yang2025_Silkie_Wenchang	PRJNA827465	0.717254253
ENSGALG00010008050	CDK10	2	-0.245635681	0.313780285	-0.782827005	0.433728707	0.170838069	6.920032813	85.54920147	0.5	CRA004775;PRJNA827465	Yang2025_Silkie_Wenchang	0.842762754
ENSGALG00010008051	ENSGALG00010008051	2	0.066048333	0.161656753	0.408571446	0.682854189	0.045163416	7.225894243	86.16088243	0.5	PRJNA827465;Yang2025_Silkie_Wenchang	CRA004775	0.918793207
ENSGALG00010008051	ENSGALG00010008051	2	0.120858753	0.15315338	0.789135397	0.430032875	0.026789194	2.121441319	52.86223611	0.5	CRA004775;Yang2025_Silkie_Wenchang	PRJNA827465	0.843012722
ENSGALG00010008051	ENSGALG00010008051	2	-0.086562512	0.062987119	-1.374289109	0.169351946	0	0.000697142	0	1	CRA004775;PRJNA827465	Yang2025_Silkie_Wenchang	0.670729184
ENSGALG00010008052	RBFA	2	-0.201868754	0.074312235	-2.716494177	0.006597736	0	0.084033534	0	1	PRJNA827465;Yang2025_Silkie_Wenchang	CRA004775	0.124138283
ENSGALG00010008052	RBFA	2	-0.046866862	0.261587412	-0.179163294	0.857809484	0.083956073	2.255358151	55.66114413	0.5	CRA004775;Yang2025_Silkie_Wenchang	PRJNA827465	0.964081098
ENSGALG00010008052	RBFA	2	-0.044040641	0.226964166	-0.194042132	0.846142892	0.065023963	2.081091156	51.94828458	0.5	CRA004775;PRJNA827465	Yang2025_Silkie_Wenchang	0.96122283
ENSGALG00010008056	GTF2E2	2	-0.149803217	0.116685869	-1.283816276	0.199206247	0.019749913	3.337457309	70.03706992	1	PRJNA827465;Yang2025_Silkie_Wenchang	CRA004775	0.71096127
ENSGALG00010008056	GTF2E2	2	-0.295752175	0.106354807	-2.780806838	0.005422399	0	0.011473729	0	1	CRA004775;Yang2025_Silkie_Wenchang	PRJNA827465	0.110428077
ENSGALG00010008056	GTF2E2	2	-0.070586732	0.066202806	-1.066219638	0.286324367	0.001465374	1.042665779	4.09215552	1	CRA004775;PRJNA827465	Yang2025_Silkie_Wenchang	0.781394441
ENSGALG00010008057	BAG1	2	-0.222230401	0.169643562	-1.309984287	0.190201151	0.051515543	3.285138131	89.23010099	1	PRJNA827465;Yang2025_Silkie_Wenchang	CRA004775	0.699403589
ENSGALG00010008057	BAG1	2	-0.251014149	0.192422743	-1.304493145	0.192065495	0.050271538	2.773403965	63.94322599	1	CRA004775;Yang2025_Silkie_Wenchang	PRJNA827465	0.701846421
ENSGALG00010008057	BAG1	2	-0.058275038	0.053402122	-1.091249469	0.275162126	0	0.063941991	0	1	CRA004775;PRJNA827465	Yang2025_Silkie_Wenchang	0.775068889
ENSGALG00010008058	USP25	2	-0.097240971	0.051475907	-1.889057929	0.058884067	0.000605818	1.096266629	8.781315259	1	PRJNA827465;Yang2025_Silkie_Wenchang	CRA004775	0.42013793
ENSGALG00010008058	USP25	2	0.298946945	0.379727712	0.787266599	0.431125818	0.23016845	4.261381404	76.53343118	0.5	CRA004775;Yang2025_Silkie_Wenchang	PRJNA827465	0.842503929
ENSGALG00010008058	USP25	2	0.246833845	0.44066921	0.560134085	0.575387983	0.331489227	5.92905177	83.1338955	0.5	CRA004775;PRJNA827465	Yang2025_Silkie_Wenchang	0.886563976
ENSGALG00010008059	ENSGALG00010008059	2	-0.146597065	0.054513098	-2.689208122	0.007162175	0	0.87482591	0	1	PRJNA827465;Yang2025_Silkie_Wenchang	CRA004775	0.130241134
ENSGALG00010008059	ENSGALG00010008059	2	-0.195558292	0.08607718	-2.271894748	0.023092968	0	0.35056993	0	1	CRA004775;Yang2025_Silkie_Wenchang	PRJNA827465	0.225929803
ENSGALG00010008059	ENSGALG00010008059	2	-0.108544869	0.06323896	-1.716424009	0.086084465	0	0.012852593	0	1	CRA004775;PRJNA827465	Yang2025_Silkie_Wenchang	0.504831056
ENSGALG00010008062	VPS9D1	2	0.485817131	0.349653645	1.389423899	0.164703887	0.209711634	6.637872988	84.9349332	1	PRJNA827465;Yang2025_Silkie_Wenchang	CRA004775	0.663901457
ENSGALG00010008062	VPS9D1	2	0.266566992	0.618326575	0.431110375	0.666388351	0.687092738	9.818519361	89.81316496	0.5	CRA004775;Yang2025_Silkie_Wenchang	PRJNA827465	0.925357907
ENSGALG00010008062	VPS9D1	2	-0.021839923	0.256002689	-0.085311302	0.932013906	0.089087513	2.661890733	62.43271793	0.5	CRA004775;PRJNA827465	Yang2025_Silkie_Wenchang	0.98412915
ENSGALG00010008063	KCNV1	2	0.068219547	0.322268119	0.211685683	0.832352259	0.144083542	2.975045087	66.38706404	0.5	PRJNA827465;Yang2025_Silkie_Wenchang	CRA004775	0.957808251
ENSGALG00010008063	KCNV1	2	0.18611897	0.687426689	0.270747373	0.786585339	0.620457892	2.53188512	60.50373723	0.5	CRA004775;Yang2025_Silkie_Wenchang	PRJNA827465	0.946722706
ENSGALG00010008063	KCNV1	2	0.359405746	0.16396711	2.191938036	0.028383981	0	0.830393264	0	1	CRA004775;PRJNA827465	Yang2025_Silkie_Wenchang	0.286930393
ENSGALG00010008064	DDX60	2	0.317739447	0.403362611	0.787726571	0.43085666	0.270977536	5.904939952	83.06502677	0.5	PRJNA827465;Yang2025_Silkie_Wenchang	CRA004775	0.842503929
ENSGALG00010008064	DDX60	2	0.825482393	0.246805681	3.344665284	0.000823819	0	0.65378707	0	1	CRA004775;Yang2025_Silkie_Wenchang	PRJNA827465	0.03312004
ENSGALG00010008064	DDX60	2	0.483040267	0.670377068	0.720550105	0.471186361	0.711829728	4.186679079	76.11472049	0.5	CRA004775;PRJNA827465	Yang2025_Silkie_Wenchang	0.85393796
ENSGALG00010008065	CHMP5	2	0.089183713	0.112239842	0.794581598	0.426854926	0.019333475	3.723333685	73.14234811	0.5	PRJNA827465;Yang2025_Silkie_Wenchang	CRA004775	0.84125685
ENSGALG00010008065	CHMP5	2	0.178015937	0.104133138	1.709503241	0.087357772	0.000627208	1.018382235	1.805042807	0.5	CRA004775;Yang2025_Silkie_Wenchang	PRJNA827465	0.508203451
ENSGALG00010008065	CHMP5	2	-0.001726942	0.036704745	-0.047049565	0.962473724	0	0.01998671	0	1	CRA004775;PRJNA827465	Yang2025_Silkie_Wenchang	0.98997945
ENSGALG00010008066	PCNX2	2	0.22741635	0.123011649	1.84873833	0.064495606	0	0.003144498	0	1	PRJNA827465;Yang2025_Silkie_Wenchang	CRA004775	0.437650344
ENSGALG00010008066	PCNX2	2	-0.393445307	0.769908435	-0.5110287	0.609330961	0.933209545	4.125808911	75.76232876	0.5	CRA004775;Yang2025_Silkie_Wenchang	PRJNA827465	0.897238578
ENSGALG00010008066	PCNX2	2	-0.38707115	0.758819552	-0.510096438	0.609983901	0.928241351	4.330273725	76.90677164	0.5	CRA004775;PRJNA827465	Yang2025_Silkie_Wenchang	0.897271501
ENSGALG00010008067	MPC2	2	0.177248896	0.069616988	2.546058114	0.010894705	0	0.133408055	0	1	PRJNA827465;Yang2025_Silkie_Wenchang	CRA004775	0.166322288
ENSGALG00010008067	MPC2	2	0.101883171	0.144788947	0.703666772	0.481640938	0	0.144977308	0	0.5	CRA004775;Yang2025_Silkie_Wenchang	PRJNA827465	0.85691916
ENSGALG00010008067	MPC2	2	0.180493496	0.075912408	2.377654726	0.017423132	0	0.332736331	0	0.5	CRA004775;PRJNA827465	Yang2025_Silkie_Wenchang	0.219833497
ENSGALG00010008069	DLG2	2	-0.085536815	0.075322571	-1.135606681	0.256121211	0	0.720036291	0	0.5	PRJNA827465;Yang2025_Silkie_Wenchang	CRA004775	0.764570254
ENSGALG00010008069	DLG2	2	-0.158807399	0.26437661	-0.600686939	0.548048986	0.080656753	1.979551836	49.48300494	0.5	CRA004775;Yang2025_Silkie_Wenchang	PRJNA827465	0.878381266
ENSGALG00010008069	DLG2	2	-0.179152766	0.142575897	-1.25654314	0.208991115	0.015503779	1.204474809	16.97626283	1	CRA004775;PRJNA827465	Yang2025_Silkie_Wenchang	0.721011841
ENSGALG00010008071	SMIM18	2	0.469589255	0.350194914	1.34093682	0.179940966	0.16663246	3.014839865	66.83074243	1	PRJNA827465;Yang2025_Silkie_Wenchang	CRA004775	0.686823327
ENSGALG00010008071	SMIM18	2	0.193742627	0.677179496	0.286102323	0.774799752	0.798316074	7.717094442	87.04175506	0.5	CRA004775;Yang2025_Silkie_Wenchang	PRJNA827465	0.943713409
ENSGALG00010008071	SMIM18	2	-0.106767961	0.319271058	-0.334411648	0.738068951	0.123063395	2.411438526	58.5775631	0.5	CRA004775;PRJNA827465	Yang2025_Silkie_Wenchang	0.934275628
ENSGALG00010008072	NRSN1	2	-0.445801502	0.13568443	-3.285575954	0.001017741	0	0.830480563	0	1	PRJNA827465;Yang2025_Silkie_Wenchang	CRA004775	0.038351174
ENSGALG00010008072	NRSN1	2	-0.312695306	0.231130995	-1.352892136	0.176090092	0	0.335753362	0	1	CRA004775;Yang2025_Silkie_Wenchang	PRJNA827465	0.681120843
ENSGALG00010008072	NRSN1	2	-0.526025358	0.151676626	-3.468071327	0.000524208	0	0.009268488	0	1	CRA004775;PRJNA827465	Yang2025_Silkie_Wenchang	0.024184895
ENSGALG00010008073	ZNF276	2	-0.038327572	0.050779139	-0.75478972	0.450375163	0	0.344536233	0	1	PRJNA827465;Yang2025_Silkie_Wenchang	CRA004775	0.847577105
ENSGALG00010008073	ZNF276	2	-0.152632766	0.092197581	-1.655496432	0.09782384	0	0.279920654	0	1	CRA004775;Yang2025_Silkie_Wenchang	PRJNA827465	0.534130658
ENSGALG00010008073	ZNF276	2	-0.073006186	0.079127692	-0.922637619	0.356196085	0.005465846	1.518208013	34.132873	1	CRA004775;PRJNA827465	Yang2025_Silkie_Wenchang	0.812511591
ENSGALG00010008074	RHAG	2	0.17691701	0.29530497	0.599099229	0.549106649	0.123207267	3.300263516	69.6093893	0.5	PRJNA827465;Yang2025_Silkie_Wenchang	CRA004775	0.875431377
ENSGALG00010008074	RHAG	2	-0.24204102	0.247439072	-0.97818432	0.32798317	0	0.597548607	0	1	CRA004775;Yang2025_Silkie_Wenchang	PRJNA827465	0.800530298
ENSGALG00010008074	RHAG	2	0.035604736	0.530687336	0.067091738	0.946508671	0.411916168	3.122314633	67.97247819	0.5	CRA004775;PRJNA827465	Yang2025_Silkie_Wenchang	0.986445196
ENSGALG00010008075	SDHA	2	-0.062575202	0.095168673	-0.657518911	0.510847316	0.006887242	1.46883135	31.91866447	0.5	PRJNA827465;Yang2025_Silkie_Wenchang	CRA004775	0.865436876
ENSGALG00010008075	SDHA	2	0.071134318	0.135870543	0.523548816	0.600595172	0	0.028448859	0	1	CRA004775;Yang2025_Silkie_Wenchang	PRJNA827465	0.893520519
ENSGALG00010008075	SDHA	2	-0.115139195	0.075223232	-1.530633452	0.125860009	0	0.242668733	0	1	CRA004775;PRJNA827465	Yang2025_Silkie_Wenchang	0.595769716
ENSGALG00010008078	GUCY1B1	2	-0.040583566	0.104211794	-0.389433525	0.696955476	0.016295756	3.951856212	74.69543561	0.5	PRJNA827465;Yang2025_Silkie_Wenchang	CRA004775	0.921086208
ENSGALG00010008078	GUCY1B1	2	0.017276688	0.23689836	0.072929694	0.94186286	0.077792419	2.633135506	62.02246342	0.5	CRA004775;Yang2025_Silkie_Wenchang	PRJNA827465	0.985827325
ENSGALG00010008078	GUCY1B1	2	0.069800798	0.06239556	1.118682119	0.263275776	0	0.922844792	0	1	CRA004775;PRJNA827465	Yang2025_Silkie_Wenchang	0.768643821
ENSGALG00010008079	CCDC127	2	-0.014676437	0.055503534	-0.264423462	0.791453634	0	0.76546039	0	0.5	PRJNA827465;Yang2025_Silkie_Wenchang	CRA004775	0.948059392
ENSGALG00010008079	CCDC127	2	0.114234863	0.240363758	0.475258268	0.6346009	0.091972496	4.73562948	78.88348309	0.5	CRA004775;Yang2025_Silkie_Wenchang	PRJNA827465	0.903872221
ENSGALG00010008079	CCDC127	2	0.151915049	0.178306426	0.851988633	0.3942504	0.047493975	3.39666142	70.55932646	1	CRA004775;PRJNA827465	Yang2025_Silkie_Wenchang	0.830486298
ENSGALG00010008081	TOP1MT	2	0.005718454	0.072326952	0.079063938	0.936981767	0	0.010676387	0	1	PRJNA827465;Yang2025_Silkie_Wenchang	CRA004775	0.985068196
ENSGALG00010008081	TOP1MT	2	-0.041572332	0.124747747	-0.333251162	0.738944701	0	0.855258524	0	0.5	CRA004775;Yang2025_Silkie_Wenchang	PRJNA827465	0.934613258
ENSGALG00010008081	TOP1MT	2	-0.022190301	0.080563326	-0.275439237	0.782978803	0	0.873945759	0	0.5	CRA004775;PRJNA827465	Yang2025_Silkie_Wenchang	0.945580193
ENSGALG00010008083	FANCA	2	0.161875558	0.137112287	1.177171592	0.239127025	0	0.329890085	0	1	PRJNA827465;Yang2025_Silkie_Wenchang	CRA004775	0.751391866
ENSGALG00010008083	FANCA	2	0.283118817	0.244555807	1.15768593	0.246992228	0	0.017406936	0	1	CRA004775;Yang2025_Silkie_Wenchang	PRJNA827465	0.757459165
ENSGALG00010008083	FANCA	2	0.126029262	0.151300968	0.832970624	0.404861297	0	0.03886422	0	1	CRA004775;PRJNA827465	Yang2025_Silkie_Wenchang	0.833112102
ENSGALG00010008084	CYP2AC1	2	-0.709421248	1.668743726	-0.425122946	0.670747052	4.908705239	8.38858537	88.67903889	0.5	PRJNA827465;Yang2025_Silkie_Wenchang	CRA004775	0.916502771
ENSGALG00010008084	CYP2AC1	2	-0.246321915	2.097916736	-0.117412627	0.906533079	8.246203894	15.81948577	93.67868201	0.5	CRA004775;Yang2025_Silkie_Wenchang	PRJNA827465	0.978196186
ENSGALG00010008084	CYP2AC1	2	1.497433729	0.584189147	2.563268656	0.010369178	0	0.525545712	0	1	CRA004775;PRJNA827465	Yang2025_Silkie_Wenchang	0.162122532
ENSGALG00010008086	RHPN1	2	-0.106938607	0.199472913	-0.536109906	0.591883359	0.061894556	4.329044529	76.90021451	0.5	PRJNA827465;Yang2025_Silkie_Wenchang	CRA004775	0.892776493
ENSGALG00010008086	RHPN1	2	-0.305547764	0.157140063	-1.944429436	0.051843683	0	0.221878243	0	1	CRA004775;Yang2025_Silkie_Wenchang	PRJNA827465	0.394912455
ENSGALG00010008086	RHPN1	2	0.0673083	0.098428987	0.68382599	0.494085053	0	0.071866327	0	0.5	CRA004775;PRJNA827465	Yang2025_Silkie_Wenchang	0.860175061
ENSGALG00010008087	CPNE8	2	0.044966425	0.115107647	0.390646728	0.696058381	0	0.295207447	0	0.5	PRJNA827465;Yang2025_Silkie_Wenchang	CRA004775	0.921038437
ENSGALG00010008087	CPNE8	2	0.274001575	0.382488271	0.716365954	0.473765412	0.207324299	3.165984665	68.41425004	0.5	CRA004775;Yang2025_Silkie_Wenchang	PRJNA827465	0.85444425
ENSGALG00010008087	CPNE8	2	0.310308543	0.30646112	1.01255436	0.311271069	0.122749789	2.427840299	58.81112936	1	CRA004775;PRJNA827465	Yang2025_Silkie_Wenchang	0.779416404
ENSGALG00010008088	SLC35A4	2	-0.118989309	0.064642124	-1.840739461	0.065659748	0	0.014054757	0	1	PRJNA827465;Yang2025_Silkie_Wenchang	CRA004775	0.441660976
ENSGALG00010008088	SLC35A4	2	-0.25867045	0.164135943	-1.575952506	0.115036749	0	0.941799348	0	1	CRA004775;Yang2025_Silkie_Wenchang	PRJNA827465	0.572141484
ENSGALG00010008088	SLC35A4	2	-0.19546231	0.148881725	-1.31286973	0.189226848	0.02376568	1.581829442	36.78205922	1	CRA004775;PRJNA827465	Yang2025_Silkie_Wenchang	0.698184834
ENSGALG00010008091	ENSGALG00010008091	2	0.072078374	0.54210194	0.132960922	0.894224286	0.512292405	7.701104434	87.01484951	0.5	PRJNA827465;Yang2025_Silkie_Wenchang	CRA004775	0.974381724
ENSGALG00010008091	ENSGALG00010008091	2	0.60441304	1.14674693	0.527067502	0.598146713	2.418188183	12.14323692	91.76496344	0.5	CRA004775;Yang2025_Silkie_Wenchang	PRJNA827465	0.894645663
ENSGALG00010008091	ENSGALG00010008091	2	1.081564021	0.594067888	1.820606775	0.068666652	0.539956092	3.800039473	73.68448388	1	CRA004775;PRJNA827465	Yang2025_Silkie_Wenchang	0.451456245
ENSGALG00010008092	SPIRE2	2	1.219773318	0.570097738	2.139586314	0.032388214	0	0.240037681	0	1	PRJNA827465;Yang2025_Silkie_Wenchang	CRA004775	0.30926967
ENSGALG00010008092	SPIRE2	2	0.853259219	0.313868337	2.71852595	0.006557351	0	0.686386878	0	1	CRA004775;Yang2025_Silkie_Wenchang	PRJNA827465	0.123728698
ENSGALG00010008092	SPIRE2	2	0.803697673	0.302358705	2.658093382	0.007858412	0	0.094896772	0	1	CRA004775;PRJNA827465	Yang2025_Silkie_Wenchang	0.137569534
ENSGALG00010008093	EFR3A	2	-0.217219122	0.224959552	-0.965591905	0.334248428	0.096781917	22.82009622	95.61789753	0.5	PRJNA827465;Yang2025_Silkie_Wenchang	CRA004775	0.803094614
ENSGALG00010008093	EFR3A	2	-0.226464307	0.261502559	-0.866011819	0.38648368	0.110459145	4.551608047	78.02974268	0.5	CRA004775;Yang2025_Silkie_Wenchang	PRJNA827465	0.826981729
ENSGALG00010008093	EFR3A	2	0.011517984	0.059824662	0.192529024	0.847327835	0	0.110727814	0	1	CRA004775;PRJNA827465	Yang2025_Silkie_Wenchang	0.961252712
ENSGALG00010008094	PIM3	2	0.249721845	0.066557243	3.751986021	0.000175439	0	0.098017729	0	1	PRJNA827465;Yang2025_Silkie_Wenchang	CRA004775	0.01153893
ENSGALG00010008094	PIM3	2	0.018743014	0.268040889	0.06992595	0.944252598	0.106309655	3.290939723	69.61354251	0.5	CRA004775;Yang2025_Silkie_Wenchang	PRJNA827465	0.985873665
ENSGALG00010008094	PIM3	2	0.035573063	0.290403535	0.122495283	0.902506782	0.130643191	3.840232488	73.9599099	0.5	CRA004775;PRJNA827465	Yang2025_Silkie_Wenchang	0.976923607
ENSGALG00010008096	CPSF1	2	-0.146112471	0.075008479	-1.94794605	0.051421419	0	0.331382791	0	1	PRJNA827465;Yang2025_Silkie_Wenchang	CRA004775	0.393117297
ENSGALG00010008096	CPSF1	2	-0.234268222	0.113434192	-2.065234629	0.038900805	0	0.106873029	0	1	CRA004775;Yang2025_Silkie_Wenchang	PRJNA827465	0.340288973
ENSGALG00010008096	CPSF1	2	-0.144402996	0.082980439	-1.740205253	0.081822984	0	0.584544727	0	1	CRA004775;PRJNA827465	Yang2025_Silkie_Wenchang	0.493083214
ENSGALG00010008098	ENSGALG00010008098	2	0.021553318	0.0844124	0.255333549	0.79846549	0	0.514527977	0	0.5	PRJNA827465;Yang2025_Silkie_Wenchang	CRA004775	0.949922217
ENSGALG00010008098	ENSGALG00010008098	2	-0.037003648	0.179603765	-0.206029355	0.836767981	0	0.437408763	0	0.5	CRA004775;Yang2025_Silkie_Wenchang	PRJNA827465	0.958399882
ENSGALG00010008098	ENSGALG00010008098	2	0.051499883	0.08657829	0.594835986	0.55195311	0	0.048616143	0	1	CRA004775;PRJNA827465	Yang2025_Silkie_Wenchang	0.879061525
ENSGALG00010008099	PANK3	2	-0.11412228	0.15864135	-0.719372851	0.47191122	0.044170906	7.788028118	87.15977928	0.5	PRJNA827465;Yang2025_Silkie_Wenchang	CRA004775	0.853998373
ENSGALG00010008099	PANK3	2	0.02986074	0.369946086	0.080716463	0.935667444	0.226210421	5.166781416	80.64559114	0.5	CRA004775;Yang2025_Silkie_Wenchang	PRJNA827465	0.984800337
ENSGALG00010008099	PANK3	2	0.13630649	0.18452614	0.738683906	0.460098961	0.04260858	1.850230949	45.95269307	1	CRA004775;PRJNA827465	Yang2025_Silkie_Wenchang	0.850104868
ENSGALG00010008100	ENSGALG00010008100	2	0.351568397	0.131028628	2.683141865	0.007293404	0.003319727	1.091109258	8.350149841	1	PRJNA827465;Yang2025_Silkie_Wenchang	CRA004775	0.131225376
ENSGALG00010008100	ENSGALG00010008100	2	-0.104635166	0.700635111	-0.149343309	0.881282743	0.861298513	7.825522817	87.22130108	0.5	CRA004775;Yang2025_Silkie_Wenchang	PRJNA827465	0.971790895
ENSGALG00010008100	ENSGALG00010008100	2	-0.219149533	0.55596703	-0.39417721	0.69345021	0.519455894	5.658405972	82.32717827	0.5	CRA004775;PRJNA827465	Yang2025_Silkie_Wenchang	0.920721644
ENSGALG00010008101	CENPQ	2	-0.175529625	0.180536172	-0.972268456	0.330917012	0.041497136	2.647941924	62.23482129	1	PRJNA827465;Yang2025_Silkie_Wenchang	CRA004775	0.801678056
ENSGALG00010008101	CENPQ	2	-0.303281863	0.160421194	-1.890534881	0.05868646	0	0.716427748	0	1	CRA004775;Yang2025_Silkie_Wenchang	PRJNA827465	0.419602318
ENSGALG00010008101	CENPQ	2	-0.028259277	0.114582399	-0.246628427	0.805195802	0	0.030858976	0	1	CRA004775;PRJNA827465	Yang2025_Silkie_Wenchang	0.951864391
ENSGALG00010008103	RBPMS	2	0.207678256	0.227514667	0.912812606	0.361341107	0.094783918	11.78580971	91.51522021	0.5	PRJNA827465;Yang2025_Silkie_Wenchang	CRA004775	0.815424583
ENSGALG00010008103	RBPMS	2	0.351821043	0.150554819	2.336830169	0.019448016	0.01948	1.485903749	32.70088992	1	CRA004775;Yang2025_Silkie_Wenchang	PRJNA827465	0.233679423
ENSGALG00010008103	RBPMS	2	-0.005513137	0.077662991	-0.070987952	0.943407344	0	0.159481834	0	0.5	CRA004775;PRJNA827465	Yang2025_Silkie_Wenchang	0.985827325
ENSGALG00010008104	TCF25	2	-0.096874008	0.049757244	-1.946932741	0.051542799	0	0.006202	0	1	PRJNA827465;Yang2025_Silkie_Wenchang	CRA004775	0.393585899
ENSGALG00010008104	TCF25	2	-0.085598941	0.095346693	-0.897765173	0.369310755	0	0.006712381	0	1	CRA004775;Yang2025_Silkie_Wenchang	PRJNA827465	0.820606801
ENSGALG00010008104	TCF25	2	-0.097076368	0.05445386	-1.782727034	0.074630748	0	0.017193144	0	1	CRA004775;PRJNA827465	Yang2025_Silkie_Wenchang	0.471679921
ENSGALG00010008105	MMUT	2	-0.015139353	0.312334799	-0.048471553	0.961340435	0.189080595	32.14589525	96.88918292	0.5	PRJNA827465;Yang2025_Silkie_Wenchang	CRA004775	0.98973612
ENSGALG00010008105	MMUT	2	0.278348793	0.088238135	3.154518097	0.001607634	0	0.443848584	0	1	CRA004775;Yang2025_Silkie_Wenchang	PRJNA827465	0.050669962
ENSGALG00010008105	MMUT	2	-0.15332753	0.21993909	-0.697136329	0.485717481	0.073980742	3.50738632	71.48874094	0.5	CRA004775;PRJNA827465	Yang2025_Silkie_Wenchang	0.857391223
ENSGALG00010008106	ENSGALG00010008106	2	-0.368924226	0.281676582	-1.309744048	0.190282437	0.091991924	2.364078106	57.70021314	1	PRJNA827465;Yang2025_Silkie_Wenchang	CRA004775	0.699411386
ENSGALG00010008106	ENSGALG00010008106	2	-0.4770691	0.51158324	-0.932534655	0.351060286	0.351311267	2.771617336	63.91998322	1	CRA004775;Yang2025_Silkie_Wenchang	PRJNA827465	0.810161472
ENSGALG00010008106	ENSGALG00010008106	2	-0.704892765	0.219084246	-3.217450721	0.001293352	0	0.624449912	0	1	CRA004775;PRJNA827465	Yang2025_Silkie_Wenchang	0.043633159
ENSGALG00010008107	TUBB3	2	-0.010418195	0.149013812	-0.069914291	0.944261879	0.032728518	3.745443209	73.30089006	0.5	PRJNA827465;Yang2025_Silkie_Wenchang	CRA004775	0.985873665
ENSGALG00010008107	TUBB3	2	-0.174540722	0.354212653	-0.49275688	0.622184382	0.215561504	6.778198179	85.24681673	0.5	CRA004775;Yang2025_Silkie_Wenchang	PRJNA827465	0.90005936
ENSGALG00010008107	TUBB3	2	-0.293488503	0.196753044	-1.491659279	0.135788489	0.050967133	2.497397327	59.95831383	1	CRA004775;PRJNA827465	Yang2025_Silkie_Wenchang	0.617196137
ENSGALG00010008108	ENSGALG00010008108	2	0.38405747	0.599726216	0.640387997	0.521920384	0.656156352	11.27872218	91.13374739	0.5	PRJNA827465;Yang2025_Silkie_Wenchang	CRA004775	0.86956507
ENSGALG00010008108	ENSGALG00010008108	2	0.531693862	0.511796908	1.038876659	0.298862103	0.397732924	4.062357997	75.38375493	0.5	CRA004775;Yang2025_Silkie_Wenchang	PRJNA827465	0.78974322
ENSGALG00010008108	ENSGALG00010008108	2	-0.164236503	0.184336326	-0.890961144	0.372950016	0	0.137846688	0	1	CRA004775;PRJNA827465	Yang2025_Silkie_Wenchang	0.822162024
ENSGALG00010008110	DEF8	2	0.201506234	0.070160066	2.872092988	0.00407763	0	0.404044347	0	1	PRJNA827465;Yang2025_Silkie_Wenchang	CRA004775	0.0925841

ENSGALG00010008110	DEF8	2	-0.014705456	0.199291936	-0.073788513	0.941178668	0	0.366144388	0	0.5	CRA004775;Yang2025_Silkie_Wenchang	PRJNA827465	0.985668288
ENSGALG00010008110	DEF8	2	0.165657832	0.136950331	1.209619804	0.226424815	0.015833102	1.211356649	17.4479291	0.5	CRA004775;PRJNA827465	Yang2025_Silkie_Wenchang	0.739871509
ENSGALG00010008111	SYTL2	2	0.215004688	0.24299812	0.884799798	0.376264619	0	0.001337356	0	1	PRJNA827465;Yang2025_Silkie_Wenchang	CRA004775	0.822974013
ENSGALG00010008111	SYTL2	2	0.051212094	0.322225093	0.158932668	0.873721923	0	0.718917346	0	0.5	CRA004775;Yang2025_Silkie_Wenchang	PRJNA827465	0.9699681
ENSGALG00010008111	SYTL2	2	0.095276078	0.289514815	0.329088785	0.742088581	0	0.806099358	0	0.5	CRA004775;PRJNA827465	Yang2025_Silkie_Wenchang	0.935481151
ENSGALG00010008113	DBNDD1	2	0.167486413	0.137180972	1.220915781	0.222117909	0	2.43819E-06	0	1	PRJNA827465;Yang2025_Silkie_Wenchang	CRA004775	0.735459155
ENSGALG00010008113	DBNDD1	2	0.077653179	0.162876504	0.476761087	0.633532258	0	0.397628356	0	0.5	CRA004775;Yang2025_Silkie_Wenchang	PRJNA827465	0.903374754
ENSGALG00010008113	DBNDD1	2	0.097191279	0.144317755	0.673453373	0.500658903	0	0.464573856	0	0.5	CRA004775;PRJNA827465	Yang2025_Silkie_Wenchang	0.862400704
ENSGALG00010008115	ENSGALG00010008115	2	0.157779889	0.174882287	0.902206235	0.366947323	0.042471033	3.006238964	66.73584462	1	PRJNA827465;Yang2025_Silkie_Wenchang	CRA004775	0.819578942
ENSGALG00010008115	ENSGALG00010008115	2	-0.119317137	0.53796569	-0.221793209	0.824474866	0.496422654	6.685557923	85.04238522	0.5	CRA004775;Yang2025_Silkie_Wenchang	PRJNA827465	0.95619211
ENSGALG00010008115	ENSGALG00010008115	2	-0.255912652	0.350134455	-0.730898225	0.46484132	0.187125394	3.502747832	71.45098511	0.5	CRA004775;PRJNA827465	Yang2025_Silkie_Wenchang	0.85217126
ENSGALG00010008116	URAH	2	0.134820952	0.27984753	0.481765738	0.629972371	0.041508945	1.350411556	25.94850097	0.5	PRJNA827465;Yang2025_Silkie_Wenchang	CRA004775	0.902533841
ENSGALG00010008116	URAH	2	0.632974309	0.331138869	1.911507131	0.055939441	0	0.954143215	0	1	CRA004775;Yang2025_Silkie_Wenchang	PRJNA827465	0.409268031
ENSGALG00010008116	URAH	2	0.416752219	0.653762337	0.637467464	0.523820397	0.606519028	3.084959202	67.58466046	0.5	CRA004775;PRJNA827465	Yang2025_Silkie_Wenchang	0.869661505
ENSGALG00010008117	ENSGALG00010008117	2	0.003176286	0.087958886	0.036111034	0.971193824	0	0.337237933	0	0.5	PRJNA827465;Yang2025_Silkie_Wenchang	CRA004775	0.991925437
ENSGALG00010008117	ENSGALG00010008117	2	0.074182739	0.143973717	0.515251952	0.60637697	0	0.000621107	0	1	CRA004775;Yang2025_Silkie_Wenchang	PRJNA827465	0.896731537
ENSGALG00010008117	ENSGALG00010008117	2	-0.029441752	0.106771522	-0.27574536	0.782743654	0	0.047535525	0	0.5	CRA004775;PRJNA827465	Yang2025_Silkie_Wenchang	0.945490372
ENSGALG00010008118	ENSGALG00010008118	2	-0.561205888	0.436509584	-1.285666818	0.198559366	0.249249359	2.889800425	65.39553419	1	PRJNA827465;Yang2025_Silkie_Wenchang	CRA004775	0.710744879
ENSGALG00010008118	ENSGALG00010008118	2	-0.372286893	0.650473953	-0.572331746	0.567097247	0.684528238	5.212705471	80.81610393	0.5	CRA004775;Yang2025_Silkie_Wenchang	PRJNA827465	0.883487517
ENSGALG00010008118	ENSGALG00010008118	2	0.042525723	0.275473569	0.154373151	0.877315522	0	0.582029661	0	0.5	CRA004775;PRJNA827465	Yang2025_Silkie_Wenchang	0.970598664
ENSGALG00010008119	CCDC83	2	0.637767244	0.338699544	1.8829882	0.059701967	0.113594182	1.903938473	47.47729435	1	PRJNA827465;Yang2025_Silkie_Wenchang	CRA004775	0.423249156
ENSGALG00010008119	CCDC83	2	0.667475042	0.630318394	1.058949015	0.289623	0.372345773	1.592386461	37.20117419	0.5	CRA004775;Yang2025_Silkie_Wenchang	PRJNA827465	0.784014188
ENSGALG00010008119	CCDC83	2	0.31387581	0.268414286	1.169370732	0.242254297	0	0.450697057	0	0.5	CRA004775;PRJNA827465	Yang2025_Silkie_Wenchang	0.754642199
ENSGALG00010008120	CDC5L	2	-0.007483072	0.036426199	-0.205431056	0.837235359	0	0.052778518	0	0.5	PRJNA827465;Yang2025_Silkie_Wenchang	CRA004775	0.958767556
ENSGALG00010008120	CDC5L	2	0.011775299	0.076845927	0.153232575	0.878251487	0	0.001230053	0	1	CRA004775;Yang2025_Silkie_Wenchang	PRJNA827465	0.971037793
ENSGALG00010008120	CDC5L	2	-0.009900198	0.03914184	-0.252931347	0.800321266	0	0.026317877	0	0.5	CRA004775;PRJNA827465	Yang2025_Silkie_Wenchang	0.950073297
ENSGALG00010008121	MPZL1	2	-0.122356759	0.121209097	-1.009468446	0.312750027	0.023264042	4.371772626	77.12598331	1	PRJNA827465;Yang2025_Silkie_Wenchang	CRA004775	0.796054997
ENSGALG00010008121	MPZL1	2	-0.277354802	0.103317293	-2.684495428	0.00726393	0	0.14361277	0	1	CRA004775;Yang2025_Silkie_Wenchang	PRJNA827465	0.131077397
ENSGALG00010008121	MPZL1	2	-0.059918193	0.117635144	-0.509356225	0.610502554	0.01343982	1.226767532	18.48617297	1	CRA004775;PRJNA827465	Yang2025_Silkie_Wenchang	0.897438028
ENSGALG00010008123	ENSGALG00010008123	2	-0.179059346	0.067023939	-2.671572989	0.007549665	0	0.917028277	0	1	PRJNA827465;Yang2025_Silkie_Wenchang	CRA004775	0.134120107
ENSGALG00010008123	ENSGALG00010008123	2	-0.314653696	0.271651172	-1.158300529	0.246741416	0.110060646	3.498773724	71.41855751	1	CRA004775;Yang2025_Silkie_Wenchang	PRJNA827465	0.757157859
ENSGALG00010008123	ENSGALG00010008123	2	-0.357029433	0.19451951	-1.835442795	0.066440108	0.047535934	2.148238381	53.45023117	1	CRA004775;PRJNA827465	Yang2025_Silkie_Wenchang	0.243979324
ENSGALG00010008124	ENSGALG00010008124	2	0.243283716	0.255302318	0.952924037	0.34062856	0.098217242	3.970476717	74.81410744	1	PRJNA827465;Yang2025_Silkie_Wenchang	CRA004775	0.805524125
ENSGALG00010008124	ENSGALG00010008124	2	0.238898884	0.36388642	0.656520472	0.5114893	0.167905762	2.466790063	59.46148741	0.5	CRA004775;Yang2025_Silkie_Wenchang	PRJNA827465	0.865694231
ENSGALG00010008124	ENSGALG00010008124	2	-0.016920713	0.140373695	-0.120540484	0.904055012	0	0.27625925	0	0.5	CRA004775;PRJNA827465	Yang2025_Silkie_Wenchang	0.977427069
ENSGALG00010008126	CDH3	2	2.263055085	0.283345099	7.986991774	1.38271E-15	0	0.228577958	0	1	PRJNA827465;Yang2025_Silkie_Wenchang	CRA004775	1.50958E-12
ENSGALG00010008126	CDH3	2	2.026694033	0.252858045	8.015145546	1.10007E-15	0	0.263332374	0	1	CRA004775;Yang2025_Silkie_Wenchang	PRJNA827465	1.27164E-12
ENSGALG00010008126	CDH3	2	2.09935521	0.281840652	7.448731023	9.42423E-14	0	0.855742621	0	1	CRA004775;PRJNA827465	Yang2025_Silkie_Wenchang	9.26001E-11
ENSGALG00010008127	TBC1D2	2	0.444421508	0.214072792	2.076029854	0.03789119	0.068461218	3.942366535	74.63452494	1	PRJNA827465;Yang2025_Silkie_Wenchang	CRA004775	0.335640621
ENSGALG00010008127	TBC1D2	2	0.301360221	0.420709573	0.716314153	0.473779739	0.283116921	4.604468394	78.28196625	0.5	CRA004775;Yang2025_Silkie_Wenchang	PRJNA827465	0.85444425
ENSGALG00010008127	TBC1D2	2	0.156214048	0.165561997	0.943538075	0.34540575	0.013176849	1.173526854	14.78678171	0.5	CRA004775;PRJNA827465	Yang2025_Silkie_Wenchang	0.80728624
ENSGALG00010008128	RARS	2	0.055852283	0.107241269	0.520809601	0.602499414	0.018996527	5.411324234	81.52023503	0.5	PRJNA827465;Yang2025_Silkie_Wenchang	CRA004775	0.895518685
ENSGALG00010008128	RARS	2	0.14355054	0.076707227	1.871408287	0.061288511	0	0.985872947	0	0.5	CRA004775;Yang2025_Silkie_Wenchang	PRJNA827465	0.427873874
ENSGALG00010008128	RARS	2	-0.039604854	0.039837227	-0.994166948	0.320141596	0	0.000624829	0	1	CRA004775;PRJNA827465	Yang2025_Silkie_Wenchang	0.798348216
ENSGALG00010008129	TANGO6	2	0.046087787	0.055733065	0.826937963	0.408272225	0	0.607516226	0	1	PRJNA827465;Yang2025_Silkie_Wenchang	CRA004775	0.834134074
ENSGALG00010008129	TANGO6	2	0.095584972	0.166968215	0.572474057	0.567008857	0	0.82409422	0	0.5	CRA004775;Yang2025_Silkie_Wenchang	PRJNA827465	0.883487517
ENSGALG00010008129	TANGO6	2	0.027582276	0.056983105	0.484043053	0.628355315	0	0.29304382	0	0.5	CRA004775;PRJNA827465	Yang2025_Silkie_Wenchang	0.901912657
ENSGALG00010008130	RCSD1	2	-0.104583721	0.076551872	-1.366181094	0.17188213	0.003632469	1.436636239	30.39295731	1	PRJNA827465;Yang2025_Silkie_Wenchang	CRA004775	0.674565888
ENSGALG00010008130	RCSD1	2	-0.029483029	0.095925707	-0.307352743	0.758574894	0	0.215199136	0	1	CRA004775;Yang2025_Silkie_Wenchang	PRJNA827465	0.938954369
ENSGALG00010008130	RCSD1	2	-0.171731446	0.079374757	-2.163552401	0.030498718	0	0.001004756	0	1	CRA004775;PRJNA827465	Yang2025_Silkie_Wenchang	0.297737484
ENSGALG00010008132	TDO2	2	1.240602576	0.355650515	3.488263121	0.000486169	0	0.552399208	0	1	PRJNA827465;Yang2025_Silkie_Wenchang	CRA004775	0.023132362
ENSGALG00010008132	TDO2	2	0.828498054	0.349444049	2.370903315	0.017744672	0	0.670543275	0	1	CRA004775;Yang2025_Silkie_Wenchang	PRJNA827465	0.2222112
ENSGALG00010008132	TDO2	2	1.014159912	0.573741675	1.767624624	0.077123679	0.311686458	1.897940322	47.31130436	1	CRA004775;PRJNA827465	Yang2025_Silkie_Wenchang	0.478533748
ENSGALG00010008133	CREG1	2	-0.449034571	0.435191152	-1.025391722	0.305178393	0.367306375	23.44916418	95.73545568	1	PRJNA827465;Yang2025_Silkie_Wenchang	CRA004775	0.79925794
ENSGALG00010008133	CREG1	2	-0.554639323	0.399073851	-1.389816248	0.164584683	0.251747282	4.361383675	77.0714967	1	CRA004775;Yang2025_Silkie_Wenchang	PRJNA827465	0.663862048
ENSGALG00010008133	CREG1	2	-0.023725136	0.093233735	-0.254469433	0.799132916	0	0.033785152	0	1	CRA004775;PRJNA827465	Yang2025_Silkie_Wenchang	0.949922217
ENSGALG00010008134	SLC2A13	2	0.060611571	0.214093287	0.283108228	0.777093874	0.076711248	6.014746517	83.37419545	0.5	PRJNA827465;Yang2025_Silkie_Wenchang	CRA004775	0.944218792
ENSGALG00010008134	SLC2A13	2	-0.149749576	0.134371396	-1.114485141	0.265088181	0	0.104048371	0	1	CRA004775;Yang2025_Silkie_Wenchang	PRJNA827465	0.769274038
ENSGALG00010008134	SLC2A13	2	0.241720752	0.095262132	2.537427481	0.01116705	0	0.632867179	0	0.5	CRA004775;PRJNA827465	Yang2025_Silkie_Wenchang	0.167907012
ENSGALG00010008135	WWC1	2	0.432684985	0.332546662	1.301125627	0.193251457	0.21030148	20.28275507	95.06970332	1	PRJNA827465;Yang2025_Silkie_Wenchang	CRA004775	0.703208363
ENSGALG00010008135	WWC1	2	0.471077633	0.337186535	1.397093168	0.162388563	0.189033929	5.522677399	81.89284059	1	CRA004775;Yang2025_Silkie_Wenchang	PRJNA827465	0.659890202
ENSGALG00010008135	WWC1	2	0.102680875	0.085673089	1.198519583	0.230714804	0	0.002620636	0	1	CRA004775;PRJNA827465	Yang2025_Silkie_Wenchang	0.743374303
ENSGALG00010008136	ELP1	2	0.245815384	0.123620661	1.988465216	0.046760262	0	0.018824801	0	1	PRJNA827465;Yang2025_Silkie_Wenchang	CRA004775	0.373490609
ENSGALG00010008136	ELP1	2	0.110080641	0.194760924	0.565209073	0.571931566	0.03351277	1.706094215	41.38658983	0.5	CRA004775;Yang2025_Silkie_Wenchang	PRJNA827465	0.885484876
ENSGALG00010008136	ELP1	2	0.086954693	0.178649674	0.486733008	0.62644755	0.016386698	1.318744768	24.17031529	0.5	CRA004775;PRJNA827465	Yang2025_Silkie_Wenchang	0.89050244
ENSGALG00010008139	ENSGALG00010008139	2	1.043497088	0.31711409	3.290604621	0.000999723	0.076901065	1.617497213	38.17609132	1	PRJNA827465;Yang2025_Silkie_Wenchang	CRA004775	0.037890184
ENSGALG00010008139	ENSGALG00010008139	2	0.995699475	0.486207887	2.047888363	0.040570939	0.246560617	1.959279372	48.96082641	1	CRA004775;Yang2025_Silkie_Wenchang	PRJNA827465	0.348537624
ENSGALG00010008139	ENSGALG00010008139	2	0.652366357	0.298814925	2.183178625	0.029022658	0	0.273136716	0	1	CRA004775;PRJNA827465	Yang2025_Silkie_Wenchang	0.289904262
ENSGALG00010008141	APTX	2	0.52863024	0.389649553	1.356681245	0.174882514	0.276208197	11.06386147	90.96156434	1	PRJNA827465;Yang2025_Silkie_Wenchang	CRA004775	0.679636281
ENSGALG00010008141	APTX	2	0.372741141	0.56217636	0.663032399	0.507309804	0.586111438	13.58673353	92.6398792	0.5	CRA004775;Yang2025_Silkie_Wenchang	PRJNA827465	0.864086554
ENSGALG00010008141	APTX	2	0.021204215	0.163692612	0.129536787	0.896932919	0.012902317	1.275628141	21.60724837	0.5	CRA004775;PRJNA827465	Yang2025_Silkie_Wenchang	0.975244266
ENSGALG00010008142	CD247	2	0.323136124	0.201400181	1.595010479	0.110709085	0.014649976	1.210495893	17.38922822	1	PRJNA827465;Yang2025_Silkie_Wenchang	CRA004775	0.56297035
ENSGALG00010008142	CD247	2	0.008579714	0.591040905	0.014516278	0.988418093	0.57422205	5.527261051	81.90785652	0.5	CRA004775;Yang2025_Silkie_Wenchang	PRJNA827465	0.996891127
ENSGALG00010008142	CD247	2	-0.15731321	0.379672608	-0.414339108	0.678625777	0.188635848	2.705335297	63.03600515	0.5	CRA004775;PRJNA827465	Yang2025_Silkie_Wenchang	0.917701868
ENSGALG00010008143	DCTN6	2	-0.128148616	0.25265107	-0.507215804	0.612003408	0.110952728	7.037155679	85.78971326	0.5	PRJNA827465;Yang2025_Silkie_Wenchang	CRA004775	0.897777844
ENSGALG00010008143	DCTN6	2	-0.382803759	0.157870611	-2.424794309	0.015317062	0	0.09986426	0	1	CRA004775;Yang2025_Silkie_Wenchang	PRJNA827465	0.204694493
ENSGALG00010008143	DCTN6	2	0.025147428	0.152080997	0.165355492	0.868664183	0.024632445	1.469218342	31.93659708	0.5	CRA004775;PRJNA827465	Yang2025_Silkie_Wenchang	0.968294858
ENSGALG00010008144	NTPCR	2	0.007829457	0.118691844	0.065964578	0.947406026	0	0.303575946	0	0.5	PRJNA827465;Yang2025_Silkie_Wenchang	CRA004775	0.986538092
ENSGALG00010008144	NTPCR	2	-0.176478615	0.173060679	-1.019749928	0.307847076	0	0.165628476	0	1	CRA004775;Yang2025_Silkie_Wenchang	PRJNA827465	0.793203994
ENSGALG00010008144	NTPCR	2	-0.026790534	0.127234556	-0.226279201	0.820984274	0.003018287	1.07529889	7.000006331	0.5	CRA004775;PRJNA827465	Yang2025_Silkie_Wenchang	0.955548641
ENSGALG00010008145	ENSGALG00010008145	2	0.240856	0.227855884	1.05704973	0.290488892	0.076950341	3.858925061	74.08604769	1	PRJNA827465;Yang2025_Silkie_Wenchang	CRA004775	0.78440982
ENSGALG00010008145	ENSGALG00010008145	2	0.206153375	0.334663584	0.616001814	0.537893323	0.153785308	2.801363572	64.30309831	0.5	CRA004775;Yang2025_Silkie_Wenchang	PRJNA827465	0.87481821
ENSGALG00010008145	ENSGALG00010008145	2	-0.029677338	0.154762939	-0.191760107	0.847930121	0	0.319166667	0	0.5	CRA004775;PRJNA827465	Yang2025_Silkie_Wenchang	0.961328089
ENSGALG00010008146	SOX4	2	-0.138346044	0.071555864	-1.933399125	0.053187049	0.005091975	1.840172519	45.65726909	1	PRJNA827465;Yang2025_Silkie_Wenchang	CRA004775	0.399849003
ENSGALG00010008146	SOX4	2	-0.04385472	0.245945517	-0.178310712	0.858478967	0.087537592	3.109256646	67.83797178	0.5	CRA004775;Yang2025_Silkie_Wenchang	PRJNA827465	0.964381022
ENSGALG00010008146	SOX4	2	-0.004508211	0.149652892	-0.030124448	0.975967803	0.026008075	1.678845983	40.43527457	0.5	CRA004775;PRJNA827465	Yang2025_Silkie_Wenchang	0.992868007
ENSGALG00010008147	CDKAL1	2	0.039105809	0.16169178	0.241854036	0.808893267	0.043469116	5.500752562	81.82066929	0.5	PRJNA827465;Yang2025_Silkie_Wenchang	CRA004775	0.952595795
ENSGALG00010008147	CDKAL1	2	-0.177752991	0.423853141	-0.41937401	0.674942815	0.323575392	9.768732695	89.76325762	0.5	CRA004775;Yang2025_Silkie_Wenchang	PRJNA827465	0.91599715
ENSGALG00010008147	CDKAL1	2	-0.312881737	0.256324334	-1.22064781	0.222219396	0.106603127	4.526948278	77.9100635	1	CRA004775;PRJNA827465	Yang2025_Silkie_Wenchang	0.73559121
ENSGALG00010008148	ABCD2	2	-0.007836955	0.061812155	-0.126786634	0.899109275	0.002132873	1.368446846	26.92445432	0.5	PRJNA827465;Yang2025_Silkie_Wenchang	CRA004775	0.975990605
ENSGALG00010008148	ABCD2	2	-0.082158699	0.079870635	-1.028647123	0.303645523	0	0.000225244	0	1	CRA004775;Yang2025_Silkie_Wenchang	PRJNA827465	0.792205992
ENSGALG00010008148	ABCD2	2	0.033866421	0.062996015	0.537596248	0.590855821	0	0.267128179	0	0.5	CRA004775;PRJNA827465	Yang2025_Silkie_Wenchang	0.892752146
ENSGALG00010008149	DNAJA1	2	0.223916745	0.148669498	1.506137756	0.132031814	0.024353667	2.222773635	55.01116334	1	PRJNA827465;Yang2025_Silkie_Wenchang	CRA004775	0.608737092
ENSGALG00010008149	DNAJA1	2	0.346066142	0.138404584	2.50039509	0.012405487	0	0.425191184	0	1	CRA004775;Yang2025_Silkie_Wenchang	PRJNA827465	0.180211791
ENSGALG00010008149	DNAJA1	2	0.082149828	0.128215567	0.640716491	0.521706898	0	0.000955588	0	1	CRA004775;PRJNA827465	Yang2025_Silkie_Wenchang	0.86956507
ENSGALG00010008151	SIPA1L2	2	0.125884781	0.172559456	0.729515405	0.465686448	0.04422766	3.780453247	73.54814529	0.5	PRJNA827465;Yang2025_Silkie_Wenchang	CRA004775	0.852424459
ENSGALG00010008151	SIPA1L2	2	0.300177996	0.135463163	2.215938185	0.026695736	0	0.075216174	0	1	CRA004775;Yang2025_Silkie_Wenchang	PRJNA827465	0.278826072
ENSGALG00010008151	SIPA1L2	2	-0.01094399	0.095920054	-0.11409491	0.909162557	0	0.478942702	0	0.5	CRA004775;PRJNA827465	Yang2025_Silkie_Wenchang	0.978552306
ENSGALG00010008152	ENSGALG00010008152	2	-0.221192052	0.221674268	-0.997824661	0.318364389	0.068222995	3.086003996	67.59563496	0.5	PRJNA827465;Yang2025_Silkie_Wenchang	CRA004775	0.797697029
ENSGALG00010008152	ENSGALG00010008152	2	-0.269486742	0.396944729	-0.678902433	0.497199684	0.204194385	2.559400029	60.92834302	0.5	CRA004775;Yang2025_Silkie_Wenchang	PRJNA827465	0.86098656
ENSGALG00010008152	ENSGALG00010008152	2	-0.435799507	0.123234135	-3.536353846	0.000405691	0	0.595463461	0	1	CRA004775;PRJNA827465	Yang2025_Silkie_Wenchang	0.020366982
ENSGALG00010008153	SMU1	2	-0.055119001	0.089148506	-0.618282948	0.53638884	0.013347831	6.098241497	83.60183012	0.5	PRJNA827465;Yang2025_Silkie_Wenchang	CRA004775	0.874599474
ENSGALG00010008153	SMU1	2	-0.144429118	0.056384312	-2.56151248	0.01042175	0	0.071372739	0	1	CRA004775;Yang2025_Silkie_Wenchang	PRJNA827465	0.162472542
ENSGALG00010008153	SMU1	2	0.019818586	0.038154808	0.51942565	0.603463949	0	0.733747811	0	0.5	CRA004775;PRJNA827465	Yang2025_Silkie_Wenchang	0.895625088
ENSGALG00010008154	KCNQ3	2	-0.155370198	0.142597241	-1.08957366	0.275900991	0	0.014332518	0	1	PRJNA827465;Yang2025_Silkie_Wenchang	CRA004775	0.775541559
ENSGALG00010008154	KCNQ3	2	-0.100759562	0.207574937	-0.485412948	0.62738345	0	0.161672241	0	0.5	CRA004775;Yang2025_Silkie_Wenchang	PRJNA827465	0.90170598
ENSGALG00010008154	KCNQ3	2	-0.14175519	0.175277041	-0.808749332	0.418659346	0	0.222131828	0	0.5	CRA004775;PRJNA827465	Yang2025_Silkie_Wenchang	0.838193076
ENSGALG00010008155	MAP10	2	-0.278641557	0.134966627	-2.064521902	0.038968259	0	0.608991137	0	1	PRJNA827465;Yang2025_Silkie_Wenchang	CRA004775	0.340727364
ENSGALG00010008155	MAP10	2	-0.105381222	0.192627138	-0.547073602	0.584328167	0	0.320184734	0	0.5	CRA004775;Yang2025_Silkie_Wenchang	PRJNA827465	0.890874353
ENSGALG00010008155	MAP10	2	-0.306626422	0.165376699	-1.854108971	0.063723571	0	0.993932117	0	0.5	CRA004775;PRJNA827465	Yang2025_Silkie_Wenchang	0.434889309
ENSGALG00010008157	CTSO	2	-0.038136059	0.058071835	-0.656704905	0.51137068	0	0.011292849	0	1	PRJNA827465;Yang2025_Silkie_Wenchang	CRA004775	0.865676092
ENSGALG00010008157	CTSO	2	-0.038469558	0.089785712	-0.428459681	0.668316482	0	0.064541906	0	0.5	CRA004775;Yang2025_Silkie_Wenchang	PRJNA827465	0.914233499
ENSGALG00010008157	CTSO	2	-0.029359664	0.071188171	-0.412423345	0.680029155	0	0.04656593	0	0.5	CRA004775;PRJNA827465	Yang2025_Silkie_Wenchang	0.917701868
ENSGALG00010008158	CLPTM1L	2	-0.072800873	0.134231296	-0.542353948	0.587574698	0.032924475	11.55019765	91.3421395	0.5	PRJNA827465;Yang2025_Silkie_Wenchang	CRA004775	0.89167336
ENSGALG00010008158	CLPTM1L	2	-0.194802665	0.058243883	-3.344602977	0.000824004	0	0.684373155	0	1	CRA004775;Yang2025_Silkie_Wenchang	PRJNA827465	0.03312004
ENSGALG00010008158	CLPTM1L	2	0.053944192	0.048983838	1.101265133	0.270781282	0	0.195322594	0	0.5	CRA004775;PRJNA827465	Yang2025_Silkie_Wenchang	0.772743863
ENSGALG00010008159	SUPT3H	2	0.051092103	0.15877453	0.321790294	0.747611568	0.014999082	1.42043833	29.59919633	0.5	PRJNA827465;Yang2025_Silkie_Wenchang	CRA004775	0.936820578
ENSGALG00010008159	SUPT3H	2	-0.299633908	0.208922094	-1.434189664	0.151518146	0.036868304	1.704342934	41.3263622	1	CRA004775;Yang2025_Silkie_Wenchang	PRJNA827465	0.643023891
ENSGALG00010008159	SUPT3H	2	-0.148903706	0.369693656	-0.402775931	0.687113069	0.225611768	5.628140735	82.23214296	0.5	CRA004775;PRJNA827465	Yang2025_Silkie_Wenchang	0.919433643
ENSGALG00010008160	TXNL4A	2	-0.071704274	0.064177958	-1.1172726	0.263877787	0	0.217214403	0	1	PRJNA827465;Yang2025_Silkie_Wenchang	CRA004775	0.768643821
ENSGALG00010008160	TXNL4A	2	-0.15500908	0.137488943	-1.127429424	0.259560964	0	0.071250229	0	1	CRA004775;Yang2025_Silkie_Wenchang	PRJNA827465	0.76691144
ENSGALG00010008160	TXNL4A	2	-0.066456132	0.068858364	-0.96511343	0.334488001	0	0.291197883	0	1	CRA004775;PRJNA827465	Yang2025_Silkie_Wenchang	0.803094614
ENSGALG00010008161	TMEFF1	2	-0.198668413	0.142170637	-1.39739412	0.162295086	0.003525024	1.054011228	5.124350351	0.5	PRJNA827465;Yang2025_Silkie_Wenchang	CRA004775	0.659870366
ENSGALG00010008161	TMEFF1	2	0.054904283	0.264529352	0.207554597	0.835576759	0	0.065849301	0	0.5	CRA004775;Yang2025_Silkie_Wenchang	PRJNA827465	0.958339923
ENSGALG00010008161	TMEFF1	2	-0.242533896	0.137929468	-1.758390712	0.078681052	0	0.244904012	0	1	CRA004775;PRJNA827465	Yang2025_Silkie_Wenchang	0.482554155
ENSGALG00010008162	HSBP1L1	2	-0.409272059	0.182288424	-2.245189522	0.024755968	0	0.224154068	0	1	PRJNA827465;Yang2025_Silkie_Wenchang	CRA004775	0.268761753
ENSGALG00010008162	HSBP1L1	2	-0.378798578	0.249408431	-1.518788185	0.128815821	0	0.498340726	0	1	CRA004775;Yang2025_Silkie_Wenchang	PRJNA827465	0.602157487
ENSGALG00010008162	HSBP1L1	2	-0.314105001	0.183957095	-1.707490552	0.087730912	0	0.145271075	0	1	CRA004775;PRJNA827465	Yang2025_Silkie_Wenchang	0.509318767
ENSGALG00010008163	CHTF8	2	-0.123633249	0.120390233	-1.026937533	0.304449879	0.00686801	1.236675298	19.13803066	1	PRJNA827465;Yang2025_Silkie_Wenchang	CRA004775	0.79225794
ENSGALG00010008163	CHTF8	2	-0.310620899	0.183463155	-1.693096901	0.090437024	0	0.002926365	0	1	CRA004775;Yang2025_Silkie_Wenchang	PRJNA827465	0.516101897
ENSGALG00010008163	CHTF8	2	-0.070240924	0.109999866	-0.638554629	0.523112705	0	0.402447806	0	1	CRA004775;PRJNA827465	Yang2025_Silkie_Wenchang	0.869569689
ENSGALG00010008164	TG	2	0.240733418	0.765450096	0.314499168	0.753141948	0.89781989	4.252499369	76.48441744	0.5	PRJNA827465;Yang2025_Silkie_Wenchang	CRA004775	0.938087082
ENSGALG00010008164	TG	2	0.902995227	0.447220755	2.019126386	0.043474085	0	0.147523868	0	1	CRA004775;Yang2025_Silkie_Wenchang	PRJNA827465	0.360249202
ENSGALG00010008164	TG	2	-0.02825792	0.576300758	-0.049033286	0.96089277	0.321377963	1.859821912	46.23141101	0.5	CRA004775;PRJNA827465	Yang2025_Silkie_Wenchang	0.989570499
ENSGALG00010008165	MRPL1	2	-0.051833361	0.086369015	-0.60013838	0.548414016	0.007556999	1.943393983	48.54362993	0.5	PRJNA827465;Yang2025_Silkie_Wenchang	CRA004775	0.878281266
ENSGALG00010008165	MRPL1	2	-0.11377876	0.095613125	-1.189991027	0.234049919	0	0.958812245	0	0.5	CRA004775;Yang2025_Silkie_Wenchang	PRJNA827465	0.747279664
ENSGALG00010008165	MRPL1	2	0.024186354	0.066390502	0.364304426	0.715630695	0	0.078883087	0	1	CRA004775;PRJNA827465	Yang2025_Silkie_Wenchang	0.927131632
ENSGALG00010008166	RUNX2	2	0.923521861	0.532363876	1.734756813	0.082783889	0.146264183	1.346488864	25.73276864	1	PRJNA827465;Yang2025_Silkie_Wenchang	CRA004775	0.495704284
ENSGALG00010008166	RUNX2	2	0.236277257	1.154513303	0.204655292	0.837841453	2.362243271	8.711593796	88.52104422	0.5	CRA004775;Yang2025_Silkie_Wenchang	PRJNA827465	0.959032758
ENSGALG00010008166	RUNX2	2	-0.352736129	0.615003592	-0.573551332	0.566271454	0.43755674	2.301343889	56.54712863	0.5	CRA004775;PRJNA827465	Yang2025_Silkie_Wenchang	0.883487517
ENSGALG00010008167	UTP4	2	0.123019575	0.175282049	0.701837843	0.482780296	0.049617511	4.883886814	79.52450501	0.5	PRJNA827465;Yang2025_Silkie_Wenchang	CRA004775	0.85691916
ENSGALG00010008167	UTP4	2	-0.001778666	0.382866357	-0.004645658	0.996293314	0.230565817	4.242167621	76.42714552	0.5	CRA004775;Yang2025_Silkie_Wenchang	PRJNA827465	0.99878373
ENSGALG00010008167	UTP4	2	-0.10587211	0.160827481	-0.658296144	0.510347855	0.026246238	1.413746626	29.26596735	1	CRA004775;PRJNA827465	Yang2025_Silkie_Wenchang	0.865365179
ENSGALG00010008168	CAVIN4	2	-0.015618467	0.288031027	-0.054224947	0.956755945	0.147879176	9.143700565	89.06350888	0.5	PRJNA827465;Yang2025_Silkie_Wenchang	CRA004775	0.988540618
ENSGALG00010008168	CAVIN4	2	0.281626867	0.144726078	1.945930351	0.051663106	0	0.001554214	0	1	CRA004775;Yang2025_Silkie_Wenchang	PRJNA827465	0.394045231
ENSGALG00010008168	CAVIN4	2	-0.236695664	0.179110187	-1.321508662	0.186331816	0.021815309	1.138601958	12.17299487	0.5	CRA004775;PRJNA827465	Yang2025_Silkie_Wenchang	0.695347453
ENSGALG00010008169	HTR4	2	-0.003571535	0.326440304	-0.010940853	0.991270637	0	0.262316599	0	0.5	PRJNA827465;Yang2025_Silkie_Wenchang	CRA004775	0.99755571
ENSGALG00010008169	HTR4	2	0.448736458	0.700645893	0.640461126	0.521872854	0.659686146	2.997414137	66.63791007	0.5	CRA004775;Yang2025_Silkie_Wenchang	PRJNA827465	0.86956507
ENSGALG00010008169	HTR4	2	0.574024301	0.524496384	1.094429474	0.273766663	0.267290614	1.874290164	46.64646813	1	CRA004775;PRJNA827465	Yang2025_Silkie_Wenchang	0.774480038
ENSGALG00010008170	PICALM	2	0.025726228	0.044640361	0.57629973	0.564412605	0	0.012495967	0	1	PRJNA827465;Yang2025_Silkie_Wenchang	CRA004775	0.883094571
ENSGALG00010008170	PICALM	2	0.141399704	0.146033881	0.968266427	0.332911327	0.013697397	1.393224662	28.22406701	1	CRA004775;Yang2025_Silkie_Wenchang	PRJNA827465	0.802178578
ENSGALG00010008170	PICALM	2	0.11211351	0.145232922	0.771956586	0.440140141	0.026621485	1.986590276	49.66249397	1	CRA004775;PRJNA827465	Yang2025_Silkie_Wenchang	0.844421945
ENSGALG00010008171	KIF21A	2	0.640454727	0.220157532	2.909074792	0.003625001	0.057471337	2.326599174	57.01881049	1	PRJNA827465;Yang2025_Silkie_Wenchang	CRA004775	0.085672534
ENSGALG00010008171	KIF21A	2	0.765548033	0.18014487	4.249624387	2.14129E-05	0	0.671491044	0	1	CRA004775;Yang2025_Silkie_Wenchang	PRJNA827465	0.002588796
ENSGALG00010008171	KIF21A	2	0.499942729	0.132101167	3.784544393	0.00015399	0	0.260754643	0	1	CRA004775;PRJNA827465	Yang2025_Silkie_Wenchang	0.010580919
ENSGALG00010008172	SNTB2	2	0.05776222	0.082603793	0.699268373	0.484384329	0	0.842679362	0	0.5	PRJNA827465;Yang2025_Silkie_Wenchang	CRA004775	0.857004733
ENSGALG00010008172	SNTB2	2	-0.223398467	0.174874855	-1.277476212	0.201434174	0	0.252365034	0	1	CRA004775;Yang2025_Silkie_Wenchang	PRJNA827465	0.714377024
ENSGALG00010008172	SNTB2	2	-0.043034378	0.189563772	-0.227017948	0.820409786	0.044854016	2.177703436	54.08006511	0.5	CRA004775;PRJNA827465	Yang2025_Silkie_Wenchang	0.95533793
ENSGALG00010008174	SLC26A2	2	0.021032477	0.264667673	0.079467496	0.936660784	0.108887015	4.454853759	77.55257402	0.5	PRJNA827465;Yang2025_Silkie_Wenchang	CRA004775	0.985038972
ENSGALG00010008174	SLC26A2	2	-0.393710976	0.250847616	-1.569522493	0.116526243	0.043733481	1.327646801	24.67876249	1	CRA004775;Yang2025_Silkie_Wenchang	PRJNA827465	0.574705849
ENSGALG00010008174	SLC26A2	2	-0.198395512	0.555278955	-0.35728981	0.720874858	0.506169356	4.988066394	79.95215138	0.5	CRA004775;PRJNA827465	Yang2025_Silkie_Wenchang	0.928919926
ENSGALG00010008175	ENSGALG00010008175	2	0.02487048	0.142369669	0.174689456	0.861323653	0	0.166646951	0	0.5	PRJNA827465;Yang2025_Silkie_Wenchang	CRA004775	0.965343309
ENSGALG00010008175	ENSGALG00010008175	2	0.101549184	0.217532566	0.466822903	0.640626589	0	0.014660844	0	1	CRA004775;Yang2025_Silkie_Wenchang	PRJNA827465	0.906257309
ENSGALG00010008175	ENSGALG00010008175	2	-0.014341898	0.181142074	-0.079174859	0.936893541	0	0.060490504	0	0.5	CRA004775;PRJNA827465	Yang2025_Silkie_Wenchang	0.985038972
ENSGALG00010008176	SLA	2	-0.187295721	0.21067181	-0.889040264	0.373981441	0	0.154332124	0	1	PRJNA827465;Yang2025_Silkie_Wenchang	CRA004775	0.822579781
ENSGALG00010008176	SLA	2	-0.530641767	0.322246022	-1.646697653	0.099620207	0.022737374	1.10740633	9.698908838	1	CRA004775;Yang2025_Silkie_Wenchang	PRJNA827465	0.539010599
ENSGALG00010008176	SLA	2	-0.428304823	0.409830346	-1.045078353	0.295986758	0.191071924	2.068587783	51.65784077	1	CRA004775;PRJNA827465	Yang2025_Silkie_Wenchang	0.788124569
ENSGALG00010008177	NREP	2	-0.311390803	0.362713406	-0.858503705	0.390614386	0.243036837	13.08978729	92.36045646	0.5	PRJNA827465;Yang2025_Silkie_Wenchang	CRA004775	0.829251057
ENSGALG00010008177	NREP	2	-0.592816144	0.191022888	-3.103377561	0.001913254	0.024668728	1.19998244	16.66544723	1	CRA004775;Yang2025_Silkie_Wenchang	PRJNA827465	0.056369277
ENSGALG00010008177	NREP	2	0.038216215	0.142388888	0.268393243	0.78839664	0	0.131028291	0	0.5	CRA004775;PRJNA827465	Yang2025_Silkie_Wenchang	0.94704362
ENSGALG00010008178	PGR	2	-0.217634383	0.561994014	-0.387253917	0.698568238	0.494284376	4.498121258	77.76849617	0.5	PRJNA827465;Yang2025_Silkie_Wenchang	CRA004775	0.921738593
ENSGALG00010008178	PGR	2	-0.36058113	0.458575037	-0.786307803	0.431687182	0.23536449	2.270187505	55.95077509	0.5	CRA004775;Yang2025_Silkie_Wenchang	PRJNA827465	0.842503929
ENSGALG00010008178	PGR	2	0.230914937	0.246126671	0.938195507	0.348143947	0	0.163136544	0	1	CRA004775;PRJNA827465	Yang2025_Silkie_Wenchang	0.808976658
ENSGALG00010008179	SEMA6A	2	0.248031641	0.289424009	0.856983641	0.391453926	0.132498668	4.499099468	77.77332982	0.5	PRJNA827465;Yang2025_Silkie_Wenchang	CRA004775	0.829251057
ENSGALG00010008179	SEMA6A	2	0.489190658	0.201451624	2.428328187	0.01516861	0	0.40532712	0	1	CRA004775;Yang2025_Silkie_Wenchang	PRJNA827465	0.203471634
ENSGALG00010008179	SEMA6A	2	0.028413387	0.118823338	0.239122949	0.811010247	0	0.673004911	0	0.5	CRA004775;PRJNA827465	Yang2025_Silkie_Wenchang	0.953118313
ENSGALG00010008180	POU2F1	2	0.163654665	0.375706366	0.435591941	0.663132819	0.240562829	6.215231607	83.910495	0.5	PRJNA827465;Yang2025_Silkie_Wenchang	CRA004775	0.91293776
ENSGALG00010008180	POU2F1	2	0.545751718	0.242824985	2.247510559	0.024607417	0	0.082996256	0	1	CRA004775;Yang2025_Silkie_Wenchang	PRJNA827465	0.268130106
ENSGALG00010008180	POU2F1	2	-0.035900746	0.248964501	-0.144200261	0.885342335	0.068781991	1.615430215	38.09698552	0.5	CRA004775;PRJNA827465	Yang2025_Silkie_Wenchang	0.972280795
ENSGALG00010008181	DISC1	2	0.079243245	0.065877282	1.202891841	0.229018178	0	0.885675921	0	0.5	PRJNA827465;Yang2025_Silkie_Wenchang	CRA004775	0.741680452
ENSGALG00010008181	DISC1	2	-0.0355254	0.120040511	-0.295945094	0.767272013	0	0.137859535	0	1	CRA004775;Yang2025_Silkie_Wenchang	PRJNA827465	0.941444269
ENSGALG00010008181	DISC1	2	0.106830575	0.075942749	1.406725144	0.159508907	0	0.520274427	0	0.5	CRA004775;PRJNA827465	Yang2025_Silkie_Wenchang	0.655069463
ENSGALG00010008182	CLIC5	2	-0.098032879	0.417101516	-0.235033619	0.814182639	0.264704389	4.175437991	76.0504167	0.5	PRJNA827465;Yang2025_Silkie_Wenchang	CRA004775	0.953869515
ENSGALG00010008182	CLIC5	2	-0.088411056	0.527762544	-0.16752052	0.866960503	0.377237314	2.796919285	64.24637616	0.5	CRA004775;Yang2025_Silkie_Wenchang	PRJNA827465	0.967699
ENSGALG00010008182	CLIC5	2	0.378505742	0.266695279	1.419244255	0.155827818	0	0.143689509	0	1	CRA004775;PRJNA827465	Yang2025_Silkie_Wenchang	0.64910892
ENSGALG00010008183	ALG10	2	-0.359980684	0.306176665	-1.175728673	0.239703317	0.180944646	28.46857428	96.48735483	1	PRJNA827465;Yang2025_Silkie_Wenchang	CRA004775	0.751936141
ENSGALG00010008183	ALG10	2	-0.611802779	0.13231074	-4.623984247	3.76438E-06	0.011545294	1.223615217	18.27496207	1	CRA004775;Yang2025_Silkie_Wenchang	PRJNA827465	0.000669464

ENSGALG00010008222	STYXL2	2	0.043707939	0.207757407	0.210379693	0.833371341	0.032177386	1.170450605	14.56281919	0.5	CRA004775;PRJNA827465	Yang2025_Silkie_Wenchang	0.957968425
ENSGALG00010008223	ENSGALG00010008223	2	-0.130652124	0.180767263	-0.722764296	0.469824706	0.056503146	7.355281224	86.40432677	0.5	PRJNA827465;Yang2025_Silkie_Wenchang	CRA004775	0.853485658
ENSGALG00010008223	ENSGALG00010008223	2	-0.340722694	0.097768964	-3.484978034	0.000492177	0	0.641994201	0	1	CRA004775;Yang2025_Silkie_Wenchang	PRJNA827465	0.02325006
ENSGALG00010008223	ENSGALG00010008223	2	-0.194207274	0.307577285	-0.631409678	0.527772689	0.144883416	3.575477834	72.03171026	0.5	CRA004775;PRJNA827465	Yang2025_Silkie_Wenchang	0.871296643
ENSGALG00010008224	ENSGALG00010008224	2	0.032561665	0.124368554	0.261815898	0.793463382	0.00923692	1.323601449	24.44855659	0.5	PRJNA827465;Yang2025_Silkie_Wenchang	CRA004775	0.948740761
ENSGALG00010008224	ENSGALG00010008224	2	0.035021236	0.240439579	0.145655039	0.884193717	0.050622722	1.717615887	41.77976532	0.5	CRA004775;Yang2025_Silkie_Wenchang	PRJNA827465	0.972001076
ENSGALG00010008224	ENSGALG00010008224	2	-0.071245911	0.105051723	-0.678198404	0.497645905	0	0.43386267	0	1	CRA004775;PRJNA827465	Yang2025_Silkie_Wenchang	0.86098656
ENSGALG00010008226	COX20	2	-0.075242223	0.07935391	-0.948185449	0.343035053	0	0.036079444	0	1	PRJNA827465;Yang2025_Silkie_Wenchang	CRA004775	0.806218116
ENSGALG00010008226	COX20	2	-0.102811977	0.147179876	-0.698546426	0.484835535	0	0.000180557	0	1	CRA004775;Yang2025_Silkie_Wenchang	PRJNA827465	0.857004733
ENSGALG00010008226	COX20	2	-0.070136579	0.085612473	-0.819233179	0.412653388	0	0.011203827	0	1	CRA004775;PRJNA827465	Yang2025_Silkie_Wenchang	0.835704442
ENSGALG00010008227	DUSP1	2	-0.193821623	0.102520349	-1.890567331	0.058682125	0.005200419	1.32688378	24.63544923	1	PRJNA827465;Yang2025_Silkie_Wenchang	CRA004775	0.419602318
ENSGALG00010008227	DUSP1	2	0.137796362	0.523396404	0.263273422	0.792339844	0.451158362	4.994043058	79.97614381	0.5	CRA004775;Yang2025_Silkie_Wenchang	PRJNA827465	0.948368502
ENSGALG00010008227	DUSP1	2	0.21733132	0.413104807	0.526092451	0.598823973	0.255386375	3.28861708	69.59208154	0.5	CRA004775;PRJNA827465	Yang2025_Silkie_Wenchang	0.89479201
ENSGALG00010008229	PIGY	2	-0.014174702	0.162983544	-0.08697014	0.930695247	0.043336231	5.188806752	80.72774633	0.5	PRJNA827465;Yang2025_Silkie_Wenchang	CRA004775	0.983943003
ENSGALG00010008229	PIGY	2	0.032049807	0.276155249	0.116057208	0.907607203	0.111856749	3.396200602	70.55533176	0.5	CRA004775;Yang2025_Silkie_Wenchang	PRJNA827465	0.978196186
ENSGALG00010008229	PIGY	2	0.146964245	0.067816269	2.16709424	0.030227669	0	0.672268142	0	1	CRA004775;PRJNA827465	Yang2025_Silkie_Wenchang	0.295973612
ENSGALG00010008230	TRMU	2	0.245128553	0.173283449	1.414610304	0.157182786	0.049900457	5.858929981	82.93203702	1	PRJNA827465;Yang2025_Silkie_Wenchang	CRA004775	0.650687662
ENSGALG00010008230	TRMU	2	0.237274483	0.209230738	1.134032627	0.256780862	0.066556705	4.037121787	75.2298778	1	CRA004775;Yang2025_Silkie_Wenchang	PRJNA827465	0.764738822
ENSGALG00010008230	TRMU	2	0.064699717	0.078220384	0.827146496	0.408154033	0	0.141084444	0	1	CRA004775;PRJNA827465	Yang2025_Silkie_Wenchang	0.834133033
ENSGALG00010008231	ZCCHC6	2	0.378493012	0.342302581	1.105726435	0.268844945	0.215281026	12.28258658	91.85839242	1	PRJNA827465;Yang2025_Silkie_Wenchang	CRA004775	0.771398294
ENSGALG00010008231	ZCCHC6	2	0.555364638	0.202056258	2.748564403	0.005985687	0.048016761	2.321173909	56.91835084	1	CRA004775;Yang2025_Silkie_Wenchang	PRJNA827465	0.11692618
ENSGALG00010008231	ZCCHC6	2	0.113222885	0.12156887	0.931347682	0.351673745	0.003310799	1.096825615	8.827803929	1	CRA004775;PRJNA827465	Yang2025_Silkie_Wenchang	0.810678834
ENSGALG00010008232	PYURF	2	0.082457292	0.064507437	1.278260257	0.201157676	0.000762746	1.05678562	5.373428566	0.5	PRJNA827465;Yang2025_Silkie_Wenchang	CRA004775	0.713804636
ENSGALG00010008232	PYURF	2	-0.093432031	0.138232679	-0.67590407	0.499101553	0	0.406230115	0	1	CRA004775;Yang2025_Silkie_Wenchang	PRJNA827465	0.861681896
ENSGALG00010008232	PYURF	2	0.051774489	0.146315025	0.353856268	0.723446603	0.020795676	1.322090843	24.36223233	0.5	CRA004775;PRJNA827465	Yang2025_Silkie_Wenchang	0.930032096
ENSGALG00010008233	CYRIB	2	-0.054965818	0.0642494	-0.855507102	0.392270474	0.004380294	1.988429682	49.70905891	1	PRJNA827465;Yang2025_Silkie_Wenchang	CRA004775	0.82944545
ENSGALG00010008233	CYRIB	2	-0.04718465	0.115313287	-0.409186587	0.682402737	0.014902444	2.125932459	52.96181703	0.5	CRA004775;Yang2025_Silkie_Wenchang	PRJNA827465	0.918625039
ENSGALG00010008233	CYRIB	2	0.005252434	0.044666542	0.117592144	0.90639083	0	0.495097188	0	0.5	CRA004775;PRJNA827465	Yang2025_Silkie_Wenchang	0.978196186
ENSGALG00010008234	COMMD10	2	-0.139383101	0.15954413	-0.873633525	0.382317863	0	4.10347E-07	0	1	PRJNA827465;Yang2025_Silkie_Wenchang	CRA004775	0.825080207
ENSGALG00010008234	COMMD10	2	-0.29347217	0.190470105	-1.540778117	0.123370797	0	0.547200702	0	1	CRA004775;Yang2025_Silkie_Wenchang	PRJNA827465	0.588880549
ENSGALG00010008234	COMMD10	2	-0.241099043	0.154819972	-1.557286433	0.119402495	0	0.77002859	0	1	CRA004775;PRJNA827465	Yang2025_Silkie_Wenchang	0.579940221
ENSGALG00010008236	ENSGALG00010008236	2	0.608504041	0.856827384	0.710182765	0.477590807	1.325217957	10.26100966	90.25437035	0.5	PRJNA827465;Yang2025_Silkie_Wenchang	CRA004775	0.855218676
ENSGALG00010008236	ENSGALG00010008236	2	0.997778323	0.479020451	2.08295558	0.037255273	0.306544279	3.006825091	66.74232887	1	CRA004775;Yang2025_Silkie_Wenchang	PRJNA827465	0.333009775
ENSGALG00010008236	ENSGALG00010008236	2	0.114491059	0.377397728	0.303369763	0.761608086	0.128982733	1.825742168	45.22775355	0.5	CRA004775;PRJNA827465	Yang2025_Silkie_Wenchang	0.939738254
ENSGALG00010008238	NTAQ1	2	0.031461209	0.102673994	0.306418482	0.759286038	0	0.364342432	0	0.5	PRJNA827465;Yang2025_Silkie_Wenchang	CRA004775	0.939145717
ENSGALG00010008238	NTAQ1	2	-0.064910949	0.250899842	-0.258712596	0.795857	0.064965065	1.894994712	47.2294042	0.5	CRA004775;Yang2025_Silkie_Wenchang	PRJNA827465	0.949107318
ENSGALG00010008238	NTAQ1	2	-0.083714528	0.152038097	-0.550615464	0.581897306	0.012750615	1.19890237	16.5903726	1	CRA004775;PRJNA827465	Yang2025_Silkie_Wenchang	0.889192798
ENSGALG00010008240	RCAN2	2	0.177840329	0.080930603	2.197442276	0.027988875	0	0.359946295	0	1	PRJNA827465;Yang2025_Silkie_Wenchang	CRA004775	0.285133941
ENSGALG00010008240	RCAN2	2	0.219293132	0.092367737	2.374131273	0.017590295	0	0.530164495	0	1	CRA004775;Yang2025_Silkie_Wenchang	PRJNA827465	0.220991844
ENSGALG00010008240	RCAN2	2	0.140314949	0.151441941	0.926526348	0.354172511	0	0.843701398	0	1	CRA004775;PRJNA827465	Yang2025_Silkie_Wenchang	0.811853621
ENSGALG00010008241	HERC3	2	0.091126714	0.064929178	1.403478634	0.160474159	0.003164272	1.561351282	35.95291391	1	PRJNA827465;Yang2025_Silkie_Wenchang	CRA004775	0.65630165
ENSGALG00010008241	HERC3	2	-0.042094032	0.095766482	-0.439548692	0.660264009	0.004570732	1.219436846	17.99493323	0.5	CRA004775;Yang2025_Silkie_Wenchang	PRJNA827465	0.911923994
ENSGALG00010008241	HERC3	2	0.007268519	0.173930879	0.0417897	0.966666346	0.045078568	3.369327531	70.32048708	0.5	CRA004775;PRJNA827465	Yang2025_Silkie_Wenchang	0.990893423
ENSGALG00010008243	YTHDC2	2	0.271234115	0.126339636	2.146864784	0.031804048	0	0.021153457	0	1	PRJNA827465;Yang2025_Silkie_Wenchang	CRA004775	0.305444789
ENSGALG00010008243	YTHDC2	2	0.126534007	0.218132231	0.58007937	0.561861095	0.04291914	1.707069448	41.42007513	0.5	CRA004775;Yang2025_Silkie_Wenchang	PRJNA827465	0.881802301
ENSGALG00010008243	YTHDC2	2	0.11184251	0.19763131	0.565914936	0.571451608	0.026012816	1.42265462	29.70887058	0.5	CRA004775;PRJNA827465	Yang2025_Silkie_Wenchang	0.885358031
ENSGALG00010008245	FBXO32	2	-0.218018808	0.139637925	-1.561315145	0.118449412	0	0.774498289	0	1	PRJNA827465;Yang2025_Silkie_Wenchang	CRA004775	0.578338927
ENSGALG00010008245	FBXO32	2	-0.354592397	0.218313679	-1.62423353	0.104325977	0	0.112203728	0	1	CRA004775;Yang2025_Silkie_Wenchang	PRJNA827465	0.548832002
ENSGALG00010008245	FBXO32	2	-0.123889546	0.170903391	-0.724909819	0.468507357	0	2.29967E-05	0	1	CRA004775;PRJNA827465	Yang2025_Silkie_Wenchang	0.853198008
ENSGALG00010008246	TERF2	2	0.107180286	0.114922576	0.932630376	0.351010844	0.021590407	5.096664072	80.37932291	1	PRJNA827465;Yang2025_Silkie_Wenchang	CRA004775	0.810146221
ENSGALG00010008246	TERF2	2	-0.036510127	0.28258977	-0.129198334	0.897200715	0.146484194	11.96113704	91.63959082	0.5	CRA004775;Yang2025_Silkie_Wenchang	PRJNA827465	0.975346564
ENSGALG00010008246	TERF2	2	-0.136331842	0.16504589	-0.826023852	0.408790561	0.045755491	5.620929043	82.20934667	0.5	CRA004775;PRJNA827465	Yang2025_Silkie_Wenchang	0.834305842
ENSGALG00010008247	GASK1B	2	0.008580549	0.118358267	0.072496403	0.942206868	0.015023736	2.143294934	53.34286551	0.5	PRJNA827465;Yang2025_Silkie_Wenchang	CRA004775	0.985827325
ENSGALG00010008247	GASK1B	2	-0.061907575	0.156717897	-0.395025562	0.692824023	0.012605596	1.117596496	10.52226781	0.5	CRA004775;Yang2025_Silkie_Wenchang	PRJNA827465	0.92062328
ENSGALG00010008247	GASK1B	2	0.131986827	0.102561352	1.286906079	0.198127026	0	0.30230672	0	1	CRA004775;PRJNA827465	Yang2025_Silkie_Wenchang	0.710234722
ENSGALG00010008248	TPK1	2	-0.044977458	0.18497424	-0.243155252	0.807885131	0.05912672	7.320252979	86.33926993	0.5	PRJNA827465;Yang2025_Silkie_Wenchang	CRA004775	0.952496413
ENSGALG00010008248	TPK1	2	0.148968615	0.099423557	1.498323122	0.13404932	0	0.011133479	0	1	CRA004775;Yang2025_Silkie_Wenchang	PRJNA827465	0.61327389
ENSGALG00010008248	TPK1	2	-0.117509344	0.178052874	-0.659968817	0.50927384	0.035763726	1.790764095	44.15791544	0.5	CRA004775;PRJNA827465	Yang2025_Silkie_Wenchang	0.865257661
ENSGALG00010008250	KLHL38	2	0.195783702	0.186196773	1.051488161	0.293034444	0	0.010565662	0	1	PRJNA827465;Yang2025_Silkie_Wenchang	CRA004775	0.786774237
ENSGALG00010008250	KLHL38	2	0.100697999	0.288010161	0.349633494	0.726613771	0	0.675080219	0	0.5	CRA004775;Yang2025_Silkie_Wenchang	PRJNA827465	0.931201938
ENSGALG00010008250	KLHL38	2	0.163316915	0.23081247	0.707574056	0.479209809	0	0.761831368	0	0.5	CRA004775;PRJNA827465	Yang2025_Silkie_Wenchang	0.856035423
ENSGALG00010008252	ZFP92	2	0.205240777	0.08061991	2.545782748	0.010903302	0	0.79839094	0	1	PRJNA827465;Yang2025_Silkie_Wenchang	CRA004775	0.166322288
ENSGALG00010008252	ZFP92	2	-0.039208174	0.385822209	-0.101622388	0.919056408	0.255759683	6.913185012	85.53488735	0.5	CRA004775;Yang2025_Silkie_Wenchang	PRJNA827465	0.981088364
ENSGALG00010008252	ZFP92	2	-0.101182508	0.30286578	-0.334083659	0.73831643	0.151389919	5.21110946	80.81022846	0.5	CRA004775;PRJNA827465	Yang2025_Silkie_Wenchang	0.93445487
ENSGALG00010008255	MSANTD3	2	0.048774055	0.067510182	0.722469614	0.470005801	0	0.800628198	0	1	PRJNA827465;Yang2025_Silkie_Wenchang	CRA004775	0.853633918
ENSGALG00010008255	MSANTD3	2	-0.071620062	0.267477725	-0.267760846	0.788883411	0.104623324	3.489771273	71.34482688	0.5	CRA004775;Yang2025_Silkie_Wenchang	PRJNA827465	0.947144026
ENSGALG00010008255	MSANTD3	2	-0.111087005	0.185735444	-0.598092659	0.549778108	0.044432183	2.254538954	55.64503339	0.5	CRA004775;PRJNA827465	Yang2025_Silkie_Wenchang	0.878603751
ENSGALG00010008257	DESI2	2	-0.305635224	0.215410651	-1.418849169	0.155942994	0.090445225	39.16440236	97.4466609	1	PRJNA827465;Yang2025_Silkie_Wenchang	CRA004775	0.649132948
ENSGALG00010008257	DESI2	2	-0.095625668	0.442555476	-0.216076114	0.828928418	0.375497157	23.536877	95.75134798	0.5	CRA004775;Yang2025_Silkie_Wenchang	PRJNA827465	0.956840134
ENSGALG00010008257	DESI2	2	0.103337608	0.225202189	0.458865911	0.64633046	0.087783947	6.618785783	84.89148867	0.5	CRA004775;PRJNA827465	Yang2025_Silkie_Wenchang	0.907220702
ENSGALG00010008258	CCDC82	2	0.248326598	0.188128249	1.319985698	0.186839793	0.055577229	4.578267547	78.15767668	1	PRJNA827465;Yang2025_Silkie_Wenchang	CRA004775	0.695723769
ENSGALG00010008258	CCDC82	2	0.242641552	0.262225174	0.925317537	0.354800759	0.091268147	2.687137379	62.78567639	0.5	CRA004775;Yang2025_Silkie_Wenchang	PRJNA827465	0.812016118
ENSGALG00010008258	CCDC82	2	0.05487187	0.09777514	0.56120472	0.574657985	0	0.266456345	0	0.5	CRA004775;PRJNA827465	Yang2025_Silkie_Wenchang	0.88613451
ENSGALG00010008259	MCC	2	0.069659319	0.069172991	1.007030607	0.313920066	0	0.763063742	0	0.5	PRJNA827465;Yang2025_Silkie_Wenchang	CRA004775	0.796531448
ENSGALG00010008259	MCC	2	-0.041701685	0.134027902	-0.311141816	0.755692813	0	0.002160623	0	1	CRA004775;Yang2025_Silkie_Wenchang	PRJNA827465	0.938501783
ENSGALG00010008259	MCC	2	0.096724096	0.077496105	1.248115576	0.211988735	0	0.165849265	0	0.5	CRA004775;PRJNA827465	Yang2025_Silkie_Wenchang	0.724377783
ENSGALG00010008262	CYB5B	2	-0.021628754	0.045668794	-0.473600283	0.635784974	0	0.469355436	0	0.5	PRJNA827465;Yang2025_Silkie_Wenchang	CRA004775	0.904387873
ENSGALG00010008262	CYB5B	2	-0.01833318	0.12662935	-0.144778286	0.884885927	0	0.94801521	0	0.5	CRA004775;Yang2025_Silkie_Wenchang	PRJNA827465	0.972177324
ENSGALG00010008262	CYB5B	2	-0.03786207	0.046080558	-0.821649558	0.411276375	0	0.424633722	0	1	CRA004775;PRJNA827465	Yang2025_Silkie_Wenchang	0.835128673
ENSGALG00010008264	CNTNAP2	2	0.008112432	0.130204036	0.062305535	0.950319521	0	0.034810896	0	0.5	PRJNA827465;Yang2025_Silkie_Wenchang	CRA004775	0.987277827
ENSGALG00010008264	CNTNAP2	2	-0.022451881	0.226107671	-0.099297301	0.920902222	0	0.568061673	0	0.5	CRA004775;Yang2025_Silkie_Wenchang	PRJNA827465	0.981469977
ENSGALG00010008264	CNTNAP2	2	-0.033969154	0.149163765	-0.2277306	0.819855682	0	0.491763875	0	1	CRA004775;PRJNA827465	Yang2025_Silkie_Wenchang	0.955287062

ENSGALG00010008266	ECSCR	2	0.107747411	0.129614205	0.83129323	0.405807998	0.017993067	1.876456954	46.70807673	1	PRJNA827465;Yang2025_Silkie_Wenchang	CRA004775	0.833392818
ENSGALG00010008266	ECSCR	2	0.253340014	0.169587704	1.493858389	0.135212636	0	0.330714057	0	1	CRA004775;Yang2025_Silkie_Wenchang	PRJNA827465	0.616533955
ENSGALG00010008266	ECSCR	2	0.019034001	0.080693199	0.235881106	0.81352493	0	0.000853465	0	1	CRA004775;PRJNA827465	Yang2025_Silkie_Wenchang	0.953706685
ENSGALG00010008267	MAML2	2	-0.090413329	0.073593703	-1.228547085	0.219241665	0.001356965	1.063927543	6.008636893	0.5	PRJNA827465;Yang2025_Silkie_Wenchang	CRA004775	0.73285529
ENSGALG00010008267	MAML2	2	0.065253236	0.178696726	0.365161902	0.714990556	0	0.132955862	0	0.5	CRA004775;Yang2025_Silkie_Wenchang	PRJNA827465	0.926823015
ENSGALG00010008267	MAML2	2	-0.119359801	0.06946054	-1.718382861	0.085726804	0	0.003958115	0	1	CRA004775;PRJNA827465	Yang2025_Silkie_Wenchang	0.503560095
ENSGALG00010008268	ENSGALG00010008268	2	-0.457718636	0.696939253	-0.656755426	0.511338189	0.907396717	15.12479751	93.38834124	0.5	PRJNA827465;Yang2025_Silkie_Wenchang	CRA004775	0.865658376
ENSGALG00010008268	ENSGALG00010008268	2	-1.013151628	0.229683041	-4.411085913	1.02853E-05	0	0.913735762	0	1	CRA004775;Yang2025_Silkie_Wenchang	PRJNA827465	0.001443732
ENSGALG00010008268	ENSGALG00010008268	2	-0.171285032	0.457989616	-0.373993266	0.708409326	0.319754345	3.981359503	74.88295143	0.5	CRA004775;PRJNA827465	Yang2025_Silkie_Wenchang	0.9250668
ENSGALG00010008270	ENSGALG00010008270	2	0.258438699	0.341336832	0.757136865	0.44896787	0.203878757	7.843243803	87.25017321	0.5	PRJNA827465;Yang2025_Silkie_Wenchang	CRA004775	0.847095089
ENSGALG00010008270	ENSGALG00010008270	2	0.035708552	0.588754985	0.060650954	0.951637193	0.643902342	14.02393905	92.86933581	0.5	CRA004775;Yang2025_Silkie_Wenchang	PRJNA827465	0.987713746
ENSGALG00010008270	ENSGALG00010008270	2	-0.272548565	0.243751428	-1.118141405	0.263506605	0.084876121	3.286681509	69.57417391	1	CRA004775;PRJNA827465	Yang2025_Silkie_Wenchang	0.768643821
ENSGALG00010008272	DCP2	2	0.087418726	0.078318177	1.116199696	0.264336665	0	0.053095486	0	1	PRJNA827465;Yang2025_Silkie_Wenchang	CRA004775	0.768790489
ENSGALG00010008272	DCP2	2	0.039735115	0.116328991	0.341575345	0.732670494	0	0.553752616	0	0.5	CRA004775;Yang2025_Silkie_Wenchang	PRJNA827465	0.932985777
ENSGALG00010008272	DCP2	2	0.08127394	0.099213973	0.819178364	0.412684657	0	0.708917948	0	0.5	CRA004775;PRJNA827465	Yang2025_Silkie_Wenchang	0.835722417
ENSGALG00010008273	TMEM144	2	-0.341340857	0.36960156	-0.923537383	0.355727228	0.248026844	10.83905921	90.77410705	0.5	PRJNA827465;Yang2025_Silkie_Wenchang	CRA004775	0.812301302
ENSGALG00010008273	TMEM144	2	-0.363192827	0.372573122	-0.974822944	0.329648094	0.234402696	6.305398688	84.14057462	0.5	CRA004775;Yang2025_Silkie_Wenchang	PRJNA827465	0.801177491
ENSGALG00010008273	TMEM144	2	0.026727567	0.128321518	0.208285934	0.835005714	0	0.000623466	0	1	CRA004775;PRJNA827465	Yang2025_Silkie_Wenchang	0.95823248
ENSGALG00010008274	NFAT5	2	0.278636139	0.271674757	1.025623958	0.30506887	0.120365403	4.790966226	79.12738365	1	PRJNA827465;Yang2025_Silkie_Wenchang	CRA004775	0.79225794
ENSGALG00010008274	NFAT5	2	0.337945128	0.327489454	1.03192675	0.302106434	0.118984362	2.126101484	52.96555656	0.5	CRA004775;Yang2025_Silkie_Wenchang	PRJNA827465	0.791603969
ENSGALG00010008274	NFAT5	2	0.046124255	0.073392252	0.628462188	0.529701209	0	0.088650466	0	0.5	CRA004775;PRJNA827465	Yang2025_Silkie_Wenchang	0.871944741
ENSGALG00010008275	CELSR1	2	-0.21369032	0.090782666	-2.353866978	0.018579258	0	0.148119313	0	1	PRJNA827465;Yang2025_Silkie_Wenchang	CRA004775	0.228051404
ENSGALG00010008275	CELSR1	2	-0.179728173	0.143407859	-1.253265853	0.210108971	0	0.065020711	0	1	CRA004775;Yang2025_Silkie_Wenchang	PRJNA827465	0.72188812
ENSGALG00010008275	CELSR1	2	-0.242381366	0.109211938	-2.219366964	0.026461769	0	0.009098405	0	1	CRA004775;PRJNA827465	Yang2025_Silkie_Wenchang	0.277488505
ENSGALG00010008276	DNAJC18	2	0.200537337	0.25822739	0.776593045	0.437399496	0.122968055	12.39152517	91.92996838	0.5	PRJNA827465;Yang2025_Silkie_Wenchang	CRA004775	0.84377699
ENSGALG00010008276	DNAJC18	2	0.240953745	0.263363081	0.914910868	0.360238424	0.105232622	3.94950164	74.68034979	0.5	CRA004775;Yang2025_Silkie_Wenchang	PRJNA827465	0.814880744
ENSGALG00010008276	DNAJC18	2	-0.044771995	0.057154597	-0.783348969	0.433422214	0	0.003279683	0	1	CRA004775;PRJNA827465	Yang2025_Silkie_Wenchang	0.842762754
ENSGALG00010008278	CYP39A1	2	0.152311106	0.139100346	1.094972876	0.27352852	0	0.191855201	0	1	PRJNA827465;Yang2025_Silkie_Wenchang	CRA004775	0.774252172
ENSGALG00010008278	CYP39A1	2	0.032282796	0.166606535	0.193767765	0.846357728	0	0.067598847	0	0.5	CRA004775;Yang2025_Silkie_Wenchang	PRJNA827465	0.96122283
ENSGALG00010008278	CYP39A1	2	0.126348158	0.142289312	0.887966605	0.374558715	0	0.503697906	0	0.5	CRA004775;PRJNA827465	Yang2025_Silkie_Wenchang	0.822805106
ENSGALG00010008279	MAPK15	2	0.149490273	0.186386937	0.80204265	0.422528288	0.045610599	2.823981111	64.58899827	0.5	PRJNA827465;Yang2025_Silkie_Wenchang	CRA004775	0.839709737
ENSGALG00010008279	MAPK15	2	0.10198058	0.324430304	0.314337404	0.753264792	0.138502392	2.673256571	62.59244209	0.5	CRA004775;Yang2025_Silkie_Wenchang	PRJNA827465	0.938087082
ENSGALG00010008279	MAPK15	2	-0.046038776	0.119595902	-0.384952785	0.700272397	0	0.567450425	0	1	CRA004775;PRJNA827465	Yang2025_Silkie_Wenchang	0.922303014
ENSGALG00010008282	FAM83H	2	0.130641039	0.174223846	0.749845911	0.453347514	0	0.020631546	0	1	PRJNA827465;Yang2025_Silkie_Wenchang	CRA004775	0.848869458
ENSGALG00010008282	FAM83H	2	0.455359056	0.40414947	1.12670952	0.259865316	0.223352107	3.031327824	67.01115623	1	CRA004775;Yang2025_Silkie_Wenchang	PRJNA827465	0.767047318
ENSGALG00010008282	FAM83H	2	0.473827486	0.37778157	1.254236638	0.209756007	0.18586948	2.724192646	63.29187653	1	CRA004775;PRJNA827465	Yang2025_Silkie_Wenchang	0.721625118
ENSGALG00010008283	NQO1	2	0.311339078	0.371512032	0.838032286	0.402012571	0.230045986	5.999870567	83.33297379	0.5	PRJNA827465;Yang2025_Silkie_Wenchang	CRA004775	0.832832232
ENSGALG00010008283	NQO1	2	0.01804412	0.192976885	0.09350405	0.925503133	0	0.60919953	0	0.5	CRA004775;Yang2025_Silkie_Wenchang	PRJNA827465	0.982594411
ENSGALG00010008283	NQO1	2	0.60034614	0.18660925	3.216928318	0.00129571	0	0.69205861	0	1	CRA004775;PRJNA827465	Yang2025_Silkie_Wenchang	0.04366527
ENSGALG00010008284	BCKDHA	2	-0.25891376	0.066836185	-3.873856078	0.000107127	0	0.117234983	0	1	PRJNA827465;Yang2025_Silkie_Wenchang	CRA004775	0.008159691
ENSGALG00010008284	BCKDHA	2	-0.268890627	0.140320759	-1.916256926	0.055332401	0	0.973870459	0	1	CRA004775;Yang2025_Silkie_Wenchang	PRJNA827465	0.407328965
ENSGALG00010008284	BCKDHA	2	-0.28323036	0.073011329	-3.879265903	0.000104772	0	0.796356422	0	1	CRA004775;PRJNA827465	Yang2025_Silkie_Wenchang	0.008058436
ENSGALG00010008285	ADSS2	2	0.049927503	0.050501187	0.984679896	0.324783319	0	0.95221027	0	0.5	PRJNA827465;Yang2025_Silkie_Wenchang	CRA004775	0.79991637
ENSGALG00010008285	ADSS2	2	-0.032562786	0.083177476	-0.391485618	0.695438317	0	0.314495959	0	1	CRA004775;Yang2025_Silkie_Wenchang	PRJNA827465	0.921038437
ENSGALG00010008285	ADSS2	2	0.073448471	0.06083622	1.207314839	0.227310918	0	0.836094769	0	0.5	CRA004775;PRJNA827465	Yang2025_Silkie_Wenchang	0.740605003
ENSGALG00010008289	MTMR2	2	-0.107128691	0.063964199	-1.674822683	0.093969027	0.002115994	1.331799779	24.91363824	1	PRJNA827465;Yang2025_Silkie_Wenchang	CRA004775	0.525133608
ENSGALG00010008289	MTMR2	2	-0.203563936	0.085090032	-2.392335862	0.016741514	0	0.464968929	0	1	CRA004775;Yang2025_Silkie_Wenchang	PRJNA827465	0.214609179
ENSGALG00010008289	MTMR2	2	-0.109709717	0.122818993	-0.893263447	0.371716108	0.013387818	1.331015495	24.86939457	1	CRA004775;PRJNA827465	Yang2025_Silkie_Wenchang	0.821597118
ENSGALG00010008290	FAM13A	2	-0.256597248	0.144136598	-1.780236612	0.075037246	0.032721601	4.705301498	78.74737675	1	PRJNA827465;Yang2025_Silkie_Wenchang	CRA004775	0.47268425
ENSGALG00010008290	FAM13A	2	-0.37391068	0.111722302	-3.346786397	0.000817542	0.004142461	1.037829648	3.645072947	0.5	CRA004775;Yang2025_Silkie_Wenchang	PRJNA827465	0.032955739
ENSGALG00010008290	FAM13A	2	-0.106056817	0.0906368	-1.170129755	0.241948756	0	0.162610514	0	0.5	CRA004775;PRJNA827465	Yang2025_Silkie_Wenchang	0.754178236
ENSGALG00010008291	PTGFRN	2	-0.198892014	0.175254179	-1.13487744	0.256426673	0.05427499	8.552566049	88.30760272	1	PRJNA827465;Yang2025_Silkie_Wenchang	CRA004775	0.764677554
ENSGALG00010008291	PTGFRN	2	-0.262529705	0.222863544	-1.177984072	0.238802956	0.054448479	1.57926466	36.67939104	0.5	CRA004775;Yang2025_Silkie_Wenchang	PRJNA827465	0.750806421
ENSGALG00010008291	PTGFRN	2	-0.021625527	0.074686528	-0.289550575	0.772160083	0	0.203861267	0	0.5	CRA004775;PRJNA827465	Yang2025_Silkie_Wenchang	0.943066211
ENSGALG00010008292	REEP5	2	-0.063893662	0.115330607	-0.554004384	0.579575845	0.01673029	2.604242526	61.60111856	0.5	PRJNA827465;Yang2025_Silkie_Wenchang	CRA004775	0.887969027
ENSGALG00010008292	REEP5	2	-0.205687854	0.104131184	-1.97527625	0.048236807	0	0.02118044	0	1	CRA004775;Yang2025_Silkie_Wenchang	PRJNA827465	0.379964424
ENSGALG00010008292	REEP5	2	-0.027194257	0.113850807	-0.238858706	0.811215146	0.010409389	1.406399017	28.89642359	0.5	CRA004775;PRJNA827465	Yang2025_Silkie_Wenchang	0.953179464
ENSGALG00010008293	PLA2G7	2	0.211991828	0.194941254	1.087465188	0.276831276	0.064708584	6.486280934	84.58280477	1	PRJNA827465;Yang2025_Silkie_Wenchang	CRA004775	0.776800258
ENSGALG00010008293	PLA2G7	2	-0.000811164	0.103708373	-0.007821584	0.993759342	0	0.000282122	0	0.5	CRA004775;Yang2025_Silkie_Wenchang	PRJNA827465	0.998025385
ENSGALG00010008293	PLA2G7	2	0.221231453	0.195527075	1.131461987	0.257860694	0.06170042	4.808894411	79.20519948	0.5	CRA004775;PRJNA827465	Yang2025_Silkie_Wenchang	0.765151037
ENSGALG00010008294	NOB1	2	0.278631058	0.25586129	1.088992625	0.276157138	0.115933855	8.288332371	87.93484678	1	PRJNA827465;Yang2025_Silkie_Wenchang	CRA004775	0.775882505
ENSGALG00010008294	NOB1	2	0.14694442	0.435063345	0.337754079	0.735548518	0.330331187	7.661477978	86.94768812	0.5	CRA004775;Yang2025_Silkie_Wenchang	PRJNA827465	0.933592889
ENSGALG00010008294	NOB1	2	-0.041841616	0.152422228	-0.274511248	0.783691761	0.025518274	1.660561643	39.77941114	0.5	CRA004775;PRJNA827465	Yang2025_Silkie_Wenchang	0.945815798
ENSGALG00010008295	DMAC2	2	0.367715507	0.485720152	0.757052194	0.449018593	0.41763019	8.091249052	87.64096874	0.5	PRJNA827465;Yang2025_Silkie_Wenchang	CRA004775	0.847106887
ENSGALG00010008295	DMAC2	2	0.258043978	0.651252359	0.396227322	0.691937337	0.735535161	7.52204849	86.70574909	0.5	CRA004775;Yang2025_Silkie_Wenchang	PRJNA827465	0.920348122
ENSGALG00010008295	DMAC2	2	-0.10114069	0.103191773	-0.980123583	0.32702512	0	0.811865252	0	1	CRA004775;PRJNA827465	Yang2025_Silkie_Wenchang	0.800028239
ENSGALG00010008296	PALLD	2	-0.167774436	0.065018665	-2.580404184	0.009868474	0	0.870246415	0	1	PRJNA827465;Yang2025_Silkie_Wenchang	CRA004775	0.156901547
ENSGALG00010008296	PALLD	2	0.28702193	0.352294298	0.814722042	0.415231434	0.186903865	3.807719369	73.73756038	0.5	CRA004775;Yang2025_Silkie_Wenchang	PRJNA827465	0.837489728
ENSGALG00010008296	PALLD	2	0.191636803	0.439806064	0.44482516	0.656446111	0.33906064	7.280009877	86.2637549	0.5	CRA004775;PRJNA827465	Yang2025_Silkie_Wenchang	0.916050456
ENSGALG00010008297	ENSGALG00010008297	2	0.534374937	0.283419886	1.885453222	0.059368674	0.104238483	2.842495435	64.81964447	1	PRJNA827465;Yang2025_Silkie_Wenchang	CRA004775	0.422252444
ENSGALG00010008297	ENSGALG00010008297	2	0.21461977	0.62547177	0.343132624	0.731489689	0.692999843	8.576671721	88.34046548	0.5	CRA004775;Yang2025_Silkie_Wenchang	PRJNA827465	0.932851968
ENSGALG00010008297	ENSGALG00010008297	2	-0.049936858	0.338923074	-0.1473398	0.882862382	0.138391041	2.433679477	58.90995468	0.5	CRA004775;PRJNA827465	Yang2025_Silkie_Wenchang	0.972001076
ENSGALG00010008298	ENSGALG00010008298	2	0.199961904	0.05814085	3.439266946	0.000583292	0	0.270089743	0	1	PRJNA827465;Yang2025_Silkie_Wenchang	CRA004775	0.025992195
ENSGALG00010008298	ENSGALG00010008298	2	0.244552427	0.112638866	2.171119397	0.029922148	0	0.062520113	0	1	CRA004775;Yang2025_Silkie_Wenchang	PRJNA827465	0.295167651
ENSGALG00010008298	ENSGALG00010008298	2	0.1867578	0.061638594	3.029884154	0.002446476	0	0.004999085	0	1	CRA004775;PRJNA827465	Yang2025_Silkie_Wenchang	0.066177569
ENSGALG00010008300	CUL1	2	0.229391363	0.265611375	0.863631314	0.387788258	0.131986142	14.94331162	93.30804292	0.5	PRJNA827465;Yang2025_Silkie_Wenchang	CRA004775	0.823154121
ENSGALG00010008300	CUL1	2	0.266826701	0.265951216	1.003291901	0.315720043	0.112661782	4.744845258	78.9244971	0.5	CRA004775;Yang2025_Silkie_Wenchang	PRJNA827465	0.796883862
ENSGALG00010008300	CUL1	2	-0.023388734	0.047668694	-0.490651869	0.623672694	0	0.00012804	0	1	CRA004775;PRJNA827465	Yang2025_Silkie_Wenchang	0.900519204
ENSGALG00010008301	FAM91A1	2	0.003252026	0.042701717	0.076156794	0.939294356	0	0.321568625	0	0.5	PRJNA827465;Yang2025_Silkie_Wenchang	CRA004775	0.985393654
ENSGALG00010008301	FAM91A1	2	-0.030573971	0.075033413	-0.407471412	0.683661786	0	0.021111687	0	0.5	CRA004775;Yang2025_Silkie_Wenchang	PRJNA827465	0.918800744
ENSGALG00010008301	FAM91A1	2	0.019275981	0.050671543	0.380410393	0.703640801	0	0.000121489	0	1	CRA004775;PRJNA827465	Yang2025_Silkie_Wenchang	0.923639641
ENSGALG00010008302	ENSGALG00010008302	2	0.044351049	0.1232592	0.359819376	0.718982213	0.009462559	1.29789056	22.95190126	0.5	PRJNA827465;Yang2025_Silkie_Wenchang	CRA004775	0.928449671
ENSGALG00010008302	ENSGALG00010008302	2	0.17338103	0.185310133	0.935626279	0.34946564	0	0.418712856	0	1	CRA004775;Yang2025_Silkie_Wenchang	PRJNA827465	0.80990318
ENSGALG00010008302	ENSGALG00010008302	2	-0.022080129	0.097890774	-0.225558839	0.821544558	0	0.011885498	0	0.5	CRA004775;PRJNA827465	Yang2025_Silkie_Wenchang	0.955598716
ENSGALG00010008303	TEX10	2	-0.195150366	0.169465652	-1.151562947	0.249500715	0.052149387	10.46059479	90.44031415	1	PRJNA827465;Yang2025_Silkie_Wenchang	CRA004775	0.759622031
ENSGALG00010008303	TEX10	2	-0.189652173	0.213684174	-0.887534952	0.374790957	0.071105574	4.231893474	76.36991559	0.5	CRA004775;Yang2025_Silkie_Wenchang	PRJNA827465	0.822805106
ENSGALG00010008303	TEX10	2	-0.031365021	0.042604853	-0.731489935	0.46447995	0	0.229612828	0	0.5	CRA004775;PRJNA827465	Yang2025_Silkie_Wenchang	0.845315649
ENSGALG00010008306	ENSGALG00010008306	2	0.05871447	0.390831693	0.150229552	0.880583511	0.176183292	2.342101556	57.30330321	0.5	PRJNA827465;Yang2025_Silkie_Wenchang	CRA004775	0.971623434
ENSGALG00010008306	ENSGALG00010008306	2	0.17818675	0.443002351	0.402225293	0.687518229	0.132795009	1.41740841	29.44870421	0.5	CRA004775;Yang2025_Silkie_Wenchang	PRJNA827465	0.91949273
ENSGALG00010008306	ENSGALG00010008306	2	-0.332157259	0.295522509	-1.123966024	0.26102744	0	0.046648045	0	1	CRA004775;PRJNA827465	Yang2025_Silkie_Wenchang	0.767929536
ENSGALG00010008308	WWP2	2	0.346372718	0.130448518	2.655240728	0.007925183	0	0.131159904	0	1	PRJNA827465;Yang2025_Silkie_Wenchang	CRA004775	0.13825276
ENSGALG00010008308	WWP2	2	0.024003993	0.38692324	0.062038126	0.95053247	0.251389641	6.230744858	83.9505545	0.5	CRA004775;Yang2025_Silkie_Wenchang	PRJNA827465	0.987277827
ENSGALG00010008308	WWP2	2	-0.011113917	0.337730233	-0.032907676	0.973748211	0.189523665	5.832911977	82.85590449	0.5	CRA004775;PRJNA827465	Yang2025_Silkie_Wenchang	0.992472082
ENSGALG00010008309	ENSGALG00010008309	2	-0.05374896	0.2274121	-0.236348066	0.813164545	0	0.09583978	0	0.5	PRJNA827465;Yang2025_Silkie_Wenchang	CRA004775	0.955526116
ENSGALG00010008309	ENSGALG00010008309	2	0.193814388	0.307505518	0.630279383	0.528511809	0	0.325134995	0	1	CRA004775;Yang2025_Silkie_Wenchang	PRJNA827465	0.871701504
ENSGALG00010008309	ENSGALG00010008309	2	0.02350492	0.23971133	0.098055105	0.921888536	0	0.835564947	0	0.5	CRA004775;PRJNA827465	Yang2025_Silkie_Wenchang	0.98171088
ENSGALG00010008310	EXOSC5	2	0.120623498	0.18986895	0.635298708	0.525233616	0.058289742	5.034746295	80.138026	0.5	PRJNA827465;Yang2025_Silkie_Wenchang	CRA004775	0.870179017
ENSGALG00010008310	EXOSC5	2	0.07774297	0.294505146	0.263978308	0.791796634	0.128696231	3.6186424	72.36352684	0.5	CRA004775;Yang2025_Silkie_Wenchang	PRJNA827465	0.948201801
ENSGALG00010008310	EXOSC5	2	-0.071650481	0.083391404	-0.859207042	0.390226299	0	0.545751554	0	1	CRA004775;PRJNA827465	Yang2025_Silkie_Wenchang	0.82923632
ENSGALG00010008311	SRP19	2	0.393997396	0.310498064	1.268920612	0.204469376	0.144539265	3.990152688	74.93830241	1	PRJNA827465;Yang2025_Silkie_Wenchang	CRA004775	0.71729696
ENSGALG00010008311	SRP19	2	0.330042013	0.397190949	0.830940418	0.406007289	0.250401557	4.808922454	79.20532074	0.5	CRA004775;Yang2025_Silkie_Wenchang	PRJNA827465	0.833434763
ENSGALG00010008311	SRP19	2	0.026166477	0.1695485	0.154330336	0.877349279	0	0.24298762	0	0.5	CRA004775;PRJNA827465	Yang2025_Silkie_Wenchang	0.970598664
ENSGALG00010008312	ZBTB18	2	-0.073288427	0.124272088	-0.589741654	0.555363865	0.019217061	2.495441508	59.9269309	0.5	PRJNA827465;Yang2025_Silkie_Wenchang	CRA004775	0.880255849
ENSGALG00010008312	ZBTB18	2	0.36418937	0.347253212	1.048771874	0.294283168	0.183698329	3.763138373	73.42643558	1	CRA004775;Yang2025_Silkie_Wenchang	PRJNA827465	0.787343482
ENSGALG00010008312	ZBTB18	2	0.248855663	0.477049243	0.521656132	0.601909773	0.40105667	7.647784074	86.92431703	0.5	CRA004775;PRJNA827465	Yang2025_Silkie_Wenchang	0.895304733
ENSGALG00010008313	PSMD7	2	0.107225574	0.144327726	0.74293122	0.457523323	0.03373471	4.833126806	79.30946072	0.5	PRJNA827465;Yang2025_Silkie_Wenchang	CRA004775	0.849573219
ENSGALG00010008313	PSMD7	2	0.248113657	0.109586685	2.264085798	0.023568838	0	0.231299243	0	1	CRA004775;Yang2025_Silkie_Wenchang	PRJNA827465	0.262001762
ENSGALG00010008313	PSMD7	2	-0.011074059	0.050879102	-0.217654378	0.827698412	0	0.388333686	0	0.5	CRA004775;PRJNA827465	Yang2025_Silkie_Wenchang	0.956550752
ENSGALG00010008314	INVS	2	0.25963487	0.19309825	1.520805448	0.128308667	0.055162354	3.593279713	72.17027117	1	PRJNA827465;Yang2025_Silkie_Wenchang	CRA004775	0.601082638
ENSGALG00010008314	INVS	2	0.350196239	0.187549128	1.867224034	0.061870309	0.030695395	1.760151272	43.1867013	1	CRA004775;Yang2025_Silkie_Wenchang	PRJNA827465	0.429247792
ENSGALG00010008314	INVS	2	0.130959208	0.088391919	1.481574439	0.138453564	0	0.003420001	0	1	CRA004775;PRJNA827465	Yang2025_Silkie_Wenchang	0.622313717
ENSGALG00010008316	ADGRF5	2	-0.000340923	0.09383226	-0.003633321	0.997101036	0.006878379	1.554820633	35.68389956	0.5	PRJNA827465;Yang2025_Silkie_Wenchang	CRA004775	0.999096725
ENSGALG00010008316	ADGRF5	2	0.208088491	0.40439902	0.514562302	0.606858914	0.258013969	4.200462615	76.19309843	0.5	CRA004775;Yang2025_Silkie_Wenchang	PRJNA827465	0.896799223
ENSGALG00010008316	ADGRF5	2	0.273398638	0.293782919	0.93061448	0.352053021	0.121373338	2.619405064	61.82339213	1	CRA004775;PRJNA827465	Yang2025_Silkie_Wenchang	0.810678834
ENSGALG00010008317	CEP57	2	0.060580219	0.057909841	1.046112674	0.295509014	0	0.234615736	0	1	PRJNA827465;Yang2025_Silkie_Wenchang	CRA004775	0.787628307
ENSGALG00010008317	CEP57	2	-0.090280374	0.244163782	-0.369753341	0.711566284	0.085706404	3.041606366	67.12263588	0.5	CRA004775;Yang2025_Silkie_Wenchang	PRJNA827465	0.925957344
ENSGALG00010008317	CEP57	2	-0.106637412	0.209629371	-0.508694995	0.610966031	0.059909579	2.495155406	59.92233599	0.5	CRA004775;PRJNA827465	Yang2025_Silkie_Wenchang	0.897506931
ENSGALG00010008318	ADPRH	2	0.240713903	0.288751626	0.833636528	0.404485835	0.149895393	9.824329742	89.82118856	0.5	PRJNA827465;Yang2025_Silkie_Wenchang	CRA004775	0.833112102
ENSGALG00010008318	ADPRH	2	-0.128663223	0.123514092	-1.041688608	0.297556072	0	0.83086459	0	1	CRA004775;Yang2025_Silkie_Wenchang	PRJNA827465	0.788562929
ENSGALG00010008318	ADPRH	2	0.133120587	0.411795947	0.323268326	0.746492039	0.308114547	10.57171479	90.54079665	0.5	CRA004775;PRJNA827465	Yang2025_Silkie_Wenchang	0.936668296
ENSGALG00010008319	ERP44	2	0.043816326	0.03133018	1.398534161	0.161952722	0	0.427243251	0	1	PRJNA827465;Yang2025_Silkie_Wenchang	CRA004775	0.659256758
ENSGALG00010008319	ERP44	2	0.064164052	0.076694104	0.836623005	0.40280451	0	0.782444476	0	0.5	CRA004775;Yang2025_Silkie_Wenchang	PRJNA827465	0.832937979
ENSGALG00010008319	ERP44	2	0.031736863	0.033210657	0.955622865	0.339262805	0	0.411608126	0	0.5	CRA004775;PRJNA827465	Yang2025_Silkie_Wenchang	0.804824608
ENSGALG00010008320	GPRIN3	2	-0.06587492	0.125258885	-0.52591016	0.59895063	0.021224037	2.954569678	66.15412365	0.5	PRJNA827465;Yang2025_Silkie_Wenchang	CRA004775	0.894874044
ENSGALG00010008320	GPRIN3	2	-0.226128532	0.120972622	-1.869253789	0.061587514	0	0.226405787	0	1	CRA004775;Yang2025_Silkie_Wenchang	PRJNA827465	0.428647789
ENSGALG00010008320	GPRIN3	2	0.028866758	0.079331216	0.363876406	0.715950304	0.000357603	1.002971949	0.296314304	0.5	CRA004775;PRJNA827465	Yang2025_Silkie_Wenchang	0.927261187
ENSGALG00010008321	ZFHX3	2	0.092703438	0.18247221	0.508041406	0.611424305	0.041864659	2.118427908	52.79518381	0.5	PRJNA827465;Yang2025_Silkie_Wenchang	CRA004775	0.897609796
ENSGALG00010008321	ZFHX3	2	0.124516238	0.313954293	0.396606261	0.691657835	0.090407916	1.764321113	43.32097526	0.5	CRA004775;Yang2025_Silkie_Wenchang	PRJNA827465	0.920348122
ENSGALG00010008321	ZFHX3	2	-0.034864848	0.074196089	-0.469901432	0.638425441	0	0.3548033	0	1	CRA004775;PRJNA827465	Yang2025_Silkie_Wenchang	0.90561743
ENSGALG00010008323	AKT3	2	0.019935492	0.066919745	0.297901495	0.765778347	0.001927614	1.215681016	17.74157966	0.5	PRJNA827465;Yang2025_Silkie_Wenchang	CRA004775	0.94098441
ENSGALG00010008323	AKT3	2	0.129175132	0.107164686	1.205388992	0.228053169	0	0.010746553	0	1	CRA004775;Yang2025_Silkie_Wenchang	PRJNA827465	0.740938585
ENSGALG00010008323	AKT3	2	-0.013282046	0.06440669	-0.206221521	0.836617878	0	0.391074758	0	0.5	CRA004775;PRJNA827465	Yang2025_Silkie_Wenchang	0.958399882
ENSGALG00010008324	SCRIB	2	-0.038694268	0.052179338	-0.741563029	0.45835213	0	0.07927039	0	1	PRJNA827465;Yang2025_Silkie_Wenchang	CRA004775	0.850026426
ENSGALG00010008324	SCRIB	2	-0.063186359	0.090979851	-0.694509366	0.487362829	0	0.012309936	0	1	CRA004775;Yang2025_Silkie_Wenchang	PRJNA827465	0.85768885
ENSGALG00010008324	SCRIB	2	-0.030915578	0.061914471	-0.49932717	0.617548917	0	0.038768624	0	1	CRA004775;PRJNA827465	Yang2025_Silkie_Wenchang	0.899121942
ENSGALG00010008325	AADAT	2	0.186322882	0.100007904	1.863081564	0.062450792	0	0.000291503	0	1	PRJNA827465;Yang2025_Silkie_Wenchang	CRA004775	0.430841401
ENSGALG00010008325	AADAT	2	-0.054697514	0.312949793	-0.174780478	0.861252129	0.125085134	2.500583275	60.00933023	0.5	CRA004775;Yang2025_Silkie_Wenchang	PRJNA827465	0.965343309
ENSGALG00010008325	AADAT	2	-0.042330305	0.307074701	-0.137850187	0.89035882	0.131541191	2.765026387	63.83397986	0.5	CRA004775;PRJNA827465	Yang2025_Silkie_Wenchang	0.973417023
ENSGALG00010008327	TGFB1	2	0.165065715	0.11382675	1.450148708	0.147017055	0.011320094	1.713799703	41.6501241	1	PRJNA827465;Yang2025_Silkie_Wenchang	CRA004775	0.635668678
ENSGALG00010008327	TGFB1	2	0.087521372	0.243420127	0.359548624	0.71918471	0.085611254	3.503759686	71.45922981	0.5	CRA004775;Yang2025_Silkie_Wenchang	PRJNA827465	0.928449671
ENSGALG00010008327	TGFB1	2	0.016995546	0.106366188	0.159783348	0.873051742	0.004530448	1.159932301	13.78807203	0.5	CRA004775;PRJNA827465	Yang2025_Silkie_Wenchang	0.9697477
ENSGALG00010008330	EZH2	2	0.055343443	0.11874179	0.466082268	0.641156615	0.022692572	4.897691432	79.58221718	0.5	PRJNA827465;Yang2025_Silkie_Wenchang	CRA004775	0.906429868
ENSGALG00010008330	EZH2	2	0.172482668	0.091862946	1.877608707	0.060434714	0	0.574892052	0	0.5	CRA004775;Yang2025_Silkie_Wenchang	PRJNA827465	0.425903811
ENSGALG00010008330	EZH2	2	-0.05297869	0.052752206	-1.004293367	0.31523723	0	0.054014549	0	1	CRA004775;PRJNA827465	Yang2025_Silkie_Wenchang	0.79668487
ENSGALG00010008331	CCDC97	2	-0.07599066	0.102688244	-0.740013241	0.45929196	0.007823064	1.568807152	36.25730232	0.5	PRJNA827465;Yang2025_Silkie_Wenchang	CRA004775	0.850098539
ENSGALG00010008331	CCDC97	2	-0.059853046	0.174557061	-0.342885279	0.731684767	0.024152943	1.438079476	30.46281402	0.5	CRA004775;Yang2025_Silkie_Wenchang	PRJNA827465	0.932851968
ENSGALG00010008331	CCDC97	2	-0.183550448	0.097199846	-1.888382091	0.058974674	0	0.349589696	0	1	CRA004775;PRJNA827465	Yang2025_Silkie_Wenchang	0.420439254
ENSGALG00010008332	DHX38	2	-0.014300527	0.033194599	-0.430808849	0.666607365	0	0.270394169	0	0.5	PRJNA827465;Yang2025_Silkie_Wenchang	CRA004775	0.913579507
ENSGALG00010008332	DHX38	2	-0.145420526	0.181664935	-0.80048759	0.423428351	0.05415111	5.105880091	80.41473787	0.5	CRA004775;Yang2025_Silkie_Wenchang	PRJNA827465	0.840219758
ENSGALG00010008332	DHX38	2	-0.158091754	0.16146225	-0.979125174	0.327518134	0.042074535	4.471481137	77.63604566	1	CRA004775;PRJNA827465	Yang2025_Silkie_Wenchang	0.800077397
ENSGALG00010008333	APC	2	0.335469082	0.347957245	0.964110064	0.334990747	0.207822022	6.699554761	85.07363496	1	PRJNA827465;Yang2025_Silkie_Wenchang	CRA004775	0.803101658
ENSGALG00010008333	APC	2	0.560043245	0.206686873	2.709621745	0.006735998	0.008248863	1.098638042	8.978211061	1	CRA004775;Yang2025_Silkie_Wenchang	PRJNA827465	0.125769561
ENSGALG00010008333	APC	2	0.048944541	0.10996588	0.445088433	0.656255848	0	0.601070185	0	1	CRA004775;PRJNA827465	Yang2025_Silkie_Wenchang	0.910446833
ENSGALG00010008334	STX17	2	-0.000580807	0.055585074	-0.010448978	0.991663073	0	0.246317965	0	0.5	PRJNA827465;Yang2025_Silkie_Wenchang	CRA004775	0.997575757
ENSGALG00010008334	STX17	2	-0.013200897	0.11340446	-0.116405445	0.90733122	0.006067006	1.158995689	13.71840214	0.5	CRA004775;Yang2025_Silkie_Wenchang	PRJNA827465	0.978196186
ENSGALG00010008334	STX17	2	-0.034922724	0.065380762	-0.534143721	0.593242103	0	0.796992538	0	1	CRA004775;PRJNA827465	Yang2025_Silkie_Wenchang	0.892783052
ENSGALG00010008335	HNRNPUL1	2	-0.095794609	0.048226892	-1.986331789	0.046996495	0	0.007664569	0	1	PRJNA827465;Yang2025_Silkie_Wenchang	CRA004775	0.374081275
ENSGALG00010008335	HNRNPUL1	2	-0.217860446	0.134964917	-1.614200562	0.10648393	0.02266652	2.541639217	60.65531278	1	CRA004775;Yang2025_Silkie_Wenchang	PRJNA827465	0.553422082
ENSGALG00010008335	HNRNPUL1	2	-0.203882119	0.138070685	-1.4766503	0.139769401	0.028136896	3.365614895	70.28774738	1	CRA004775;PRJNA827465	Yang2025_Silkie_Wenchang	0.623597383
ENSGALG00010008336	PAIP2B	2	-0.240620907	0.130553255	-1.843086236	0.065316421	0.027398214	4.965283746	79.86016407	1	PRJNA827465;Yang2025_Silkie_Wenchang	CRA004775	0.440578993
ENSGALG00010008336	PAIP2B	2	-0.266302629	0.166851045	-1.596050108	0.110477607	0.031587664	1.991212746	49.77934919	1	CRA004775;Yang2025_Silkie_Wenchang	PRJNA827465	0.56281288
ENSGALG00010008336	PAIP2B	2	-0.113378504	0.062192001	-1.823039988	0.068297335	0	0.15266551	0	1	CRA004775;PRJNA827465	Yang2025_Silkie_Wenchang	0.450487609
ENSGALG00010008339	FAM76B	2	0.64163114	0.644723192	0.995204063	0.319637026	0.787608095	18.20712564	94.50764486	1	PRJNA827465;Yang2025_Silkie_Wenchang	CRA004775	0.798328994
ENSGALG00010008339	FAM76B	2	0.589273475	0.719091815	0.819469034	0.412518862	0.962717697	14.42773381	93.06890456	0.5	CRA004775;Yang2025_Silkie_Wenchang	PRJNA827465	0.835704442
ENSGALG00010008339	FAM76B	2	0.017199921	0.064529973	0.266541583	0.789822139	0	0.356677898	0	0.5	CRA004775;PRJNA827465	Yang2025_Silkie_Wenchang	0.947390191
ENSGALG00010008340	PDIA4	2	-0.160996309	0.27007153	-0.596124699	0.551091925	0.139630674	23.23779435	95.69666559	0.5	PRJNA827465;Yang2025_Silkie_Wenchang	CRA004775	0.878879109
ENSGALG00010008340	PDIA4	2	-0.177698061	0.301600352	-0.589183862	0.555737941	0.148776997	4.941097244	79.76158026	0.5	CRA004775;Yang2025_Silkie_Wenchang	PRJNA827465	0.880324407
ENSGALG00010008340	PDIA4	2	0.109152631	0.062194348	1.755024914	0.079255046	0	0.070009717	0	1	CRA004775;PRJNA827465	Yang2025_Silkie_Wenchang	0.484394238
ENSGALG00010008341	DHODH	2	0.177130809	0.084549256	2.095001406	0.036170867	0	0.578081827	0	1	PRJNA827465;Yang2025_Silkie_Wenchang	CRA004775	0.32816796
ENSGALG00010008341	DHODH	2	-0.037683085	0.371842378	-0.101341556	0.919279329	0.226748939	5.554601279	81.99690761	0.5	CRA004775;Yang2025_Silkie_Wenchang	PRJNA827465	0.981168728
ENSGALG00010008341	DHODH	2	-0.09238096	0.277687218	-0.332679914	0.739375913	0.127916507	5.406348467	81.50322706	0.5	CRA004775;PRJNA827465	Yang2025_Silkie_Wenchang	0.934830469
ENSGALG00010008342	PLD3	2	0.029962988	0.092041588	0.325537492	0.744774312	0.002953777	1.196769275	16.44170511	0.5	PRJNA827465;Yang2025_Silkie_Wenchang	CRA004775	0.935893304
ENSGALG00010008342	PLD3	2	-0.059804245	0.226320304	-0.264246042	0.791590335	0.074834923	3.633950055	72.48173517	0.5	CRA004775;Yang2025_Silkie_Wenchang	PRJNA827465	0.948099191
ENSGALG00010008342	PLD3	2	-0.124047587	0.122385923	-1.01357725	0.310784518	0.010948926	1.453802088	31.21484635	1	CRA004775;PRJNA827465	Yang2025_Silkie_Wenchang	0.795128493
ENSGALG00010008343	TNFRSF21	2	0.061224105	0.059867032	1.022668114	0.306464792	0	0.704722246	0	0.5	PRJNA827465;Yang2025_Silkie_Wenchang	CRA004775	0.792485409
ENSGALG00010008343	TNFRSF21	2	0.095190893	0.158301941	0.601324865	0.547623632	0.025316395	1.725251712	42.03744339	0.5	CRA004775;Yang2025_Silkie_Wenchang	PRJNA827465	0.878281266
ENSGALG00010008343	TNFRSF21	2	0.118582398	0.071043478	1.66915248	0.095087165	0	0.897444016	0	1	CRA004775;PRJNA827465	Yang2025_Silkie_Wenchang	0.527854638
ENSGALG00010008344	MFAP3L	2	-0.102486697	0.207757473	-0.493299689	0.621800848	0	0.371324653	0	0.5	PRJNA827465;Yang2025_Silkie_Wenchang	CRA004775	0.89980238
ENSGALG00010008344	MFAP3L	2	0.1331989	0.295618437	0.450577105	0.65229437	0	0.356904462	0	1	CRA004775;Yang2025_Silkie_Wenchang	PRJNA827465	0.909377929
ENSGALG00010008344	MFAP3L	2	-0.114077869	0.251433941	-0.453709108	0.650038211	0	0.920285148	0	0.5	CRA004775;PRJNA827465	Yang2025_Silkie_Wenchang	0.908335059
ENSGALG00010008346	NR4A3	2	0.136519676	0.162890867	0.838105155	0.401971648	0	0.606977386	0	1	PRJNA827465;Yang2025_Silkie_Wenchang	CRA004775	0.832825075
ENSGALG00010008346	NR4A3	2	0.315930714	0.250559671	1.260900099	0.207344843	0	0.05888793	0	1	CRA004775;Yang2025_Silkie_Wenchang	PRJNA827465	0.719377239
ENSGALG00010008346	NR4A3	2	0.063468627	0.201629844	0.314777942	0.75293026	0	0.300725843	0	1	CRA004775;PRJNA827465	Yang2025_Silkie_Wenchang	0.938087082
ENSGALG00010008347	PUF60	2	0.048020424	0.040323859	1.190868764	0.233705109	0.000216553	1.04468239	4.27712671	1	PRJNA827465;Yang2025_Silkie_Wenchang	CRA004775	0.747077014
ENSGALG00010008347	PUF60	2	-0.040073326	0.180858869	-0.221572358	0.824646803	0.053227522	5.251179015	80.9566576	0.5	CRA004775;Yang2025_Silkie_Wenchang	PRJNA827465	0.95619211
ENSGALG00010008347	PUF60	2	-0.073908809	0.127681093	-0.578854766	0.562687174	0.024828305	3.615554931	72.34172848	0.5	CRA004775;PRJNA827465	Yang2025_Silkie_Wenchang	0.882036214
ENSGALG00010008348	ENSGALG00010008348	2	0.115796868	0.163014761	0.710345907	0.477489658	0.046152718	7.468458902	86.61035679	0.5	PRJNA827465;Yang2025_Silkie_Wenchang	CRA004775	0.855134241

ENSGALG00010008348	ENSGALG00010008348	2	0.299863967	0.093230155	3.216383865	0.001298171	0	0.132182572	0	1	CRA004775;Yang2025_Silkie_Wenchang	PRJNA827465	0.043665685
ENSGALG00010008348	ENSGALG00010008348	2	0.096981431	0.200345069	0.484071962	0.628334799	0.054243596	2.402640118	58.37911834	0.5	CRA004775;PRJNA827465	Yang2025_Silkie_Wenchang	0.901912657
ENSGALG00010008351	IST1	2	-0.00063107	0.044163483	-0.014289417	0.988599083	0	0.045331947	0	0.5	PRJNA827465;Yang2025_Silkie_Wenchang	CRA004775	0.996891127
ENSGALG00010008351	IST1	2	-0.121590719	0.153157032	-0.79389576	0.427256119	0.023493926	2.003003814	50.07498272	0.5	CRA004775;Yang2025_Silkie_Wenchang	PRJNA827465	0.84125685
ENSGALG00010008351	IST1	2	-0.099980863	0.130339365	-0.767081094	0.443033302	0.024167664	2.862615849	65.06691596	1	CRA004775;PRJNA827465	Yang2025_Silkie_Wenchang	0.845720426
ENSGALG00010008352	ENSGALG00010008352	2	0.146920335	0.281926179	0.521130514	0.602275855	0.14331187	9.937567793	89.93717557	0.5	PRJNA827465;Yang2025_Silkie_Wenchang	CRA004775	0.895349419
ENSGALG00010008352	ENSGALG00010008352	2	-0.076917443	0.545883616	-0.140904472	0.887945409	0.544470953	11.2879494	91.14099502	0.5	CRA004775;Yang2025_Silkie_Wenchang	PRJNA827465	0.973135755
ENSGALG00010008352	ENSGALG00010008352	2	-0.315999259	0.25488428	-1.239775397	0.215058481	0.094552102	3.095466261	67.69468909	1	CRA004775;PRJNA827465	Yang2025_Silkie_Wenchang	0.728473987
ENSGALG00010008354	TMEM71	2	-0.313373063	0.138521894	-2.262263778	0.023681111	0	0.360326061	0	1	PRJNA827465;Yang2025_Silkie_Wenchang	CRA004775	0.262697912
ENSGALG00010008354	TMEM71	2	-0.196192781	0.221828657	-0.884433886	0.376462037	0	0.016903005	0	1	CRA004775;Yang2025_Silkie_Wenchang	PRJNA827465	0.822974013
ENSGALG00010008354	TMEM71	2	-0.365471007	0.170537901	-2.143048574	0.032109198	0	0.105395514	0	1	CRA004775;PRJNA827465	Yang2025_Silkie_Wenchang	0.30721464
ENSGALG00010008355	MAFA	2	-0.313962062	0.093783986	-3.347715061	0.000814807	0.003853855	1.252396793	20.15310116	1	PRJNA827465;Yang2025_Silkie_Wenchang	CRA004775	0.032879235
ENSGALG00010008355	MAFA	2	0.101433209	0.615547725	0.164785288	0.869112985	0.60475387	4.077074718	75.47261041	0.5	CRA004775;Yang2025_Silkie_Wenchang	PRJNA827465	0.968461055
ENSGALG00010008355	MAFA	2	0.177329679	0.507681633	0.349293075	0.726869297	0.370892201	2.836573197	64.74619443	0.5	CRA004775;PRJNA827465	Yang2025_Silkie_Wenchang	0.931407928
ENSGALG00010008356	ENSGALG00010008356	2	0.181500211	0.253619034	0.715641127	0.474212972	0.080894663	2.638252823	62.09612698	0.5	PRJNA827465;Yang2025_Silkie_Wenchang	CRA004775	0.85444425
ENSGALG00010008356	ENSGALG00010008356	2	0.410435033	0.234189694	1.752575123	0.079674964	0	0.330289146	0	1	CRA004775;Yang2025_Silkie_Wenchang	PRJNA827465	0.48542321
ENSGALG00010008356	ENSGALG00010008356	2	-0.034820532	0.178693587	-0.194861676	0.845501237	0	0.037267194	0	0.5	CRA004775;PRJNA827465	Yang2025_Silkie_Wenchang	0.961048404
ENSGALG00010008358	HPF1	2	0.112298548	0.142831703	0.786229845	0.431732844	0.032987276	5.050972064	80.20183071	0.5	PRJNA827465;Yang2025_Silkie_Wenchang	CRA004775	0.842503929
ENSGALG00010008358	HPF1	2	-0.013734282	0.329483351	-0.041684297	0.966750372	0.176408215	4.839217124	79.33550047	0.5	CRA004775;Yang2025_Silkie_Wenchang	PRJNA827465	0.990893423
ENSGALG00010008358	HPF1	2	-0.108845106	0.161913242	-0.672243388	0.501428764	0.029948997	1.708465889	41.46795636	1	CRA004775;PRJNA827465	Yang2025_Silkie_Wenchang	0.862658912
ENSGALG00010008360	ZC3H3	2	-0.281152028	0.195212824	-1.440233397	0.149801378	0.066126532	6.875558694	85.45572739	1	PRJNA827465;Yang2025_Silkie_Wenchang	CRA004775	0.639778999
ENSGALG00010008360	ZC3H3	2	-0.448486467	0.11152306	-4.021468439	5.78365E-05	0	0.344357471	0	1	CRA004775;Yang2025_Silkie_Wenchang	PRJNA827465	0.005336024
ENSGALG00010008360	ZC3H3	2	-0.182150997	0.117061513	-1.556028046	0.119701423	0.01735495	2.057949737	51.4079483	1	CRA004775;PRJNA827465	Yang2025_Silkie_Wenchang	0.580639051
ENSGALG00010008361	MATR3	2	0.094201249	0.18202582	0.517515862	0.604796099	0.058948206	8.349261901	88.02289338	0.5	PRJNA827465;Yang2025_Silkie_Wenchang	CRA004775	0.896344437
ENSGALG00010008361	MATR3	2	0.232100147	0.096890855	2.395480441	0.0165986	0	0.741897335	0	1	CRA004775;Yang2025_Silkie_Wenchang	PRJNA827465	0.013438855
ENSGALG00010008361	MATR3	2	-0.036407466	0.073224223	-0.497205221	0.61904434	0.00533081	1.4008272	28.61360771	0.5	CRA004775;PRJNA827465	Yang2025_Silkie_Wenchang	0.899168321
ENSGALG00010008362	RAMP3	2	0.74408311	0.592412107	1.256022793	0.209107706	0.678858161	30.42711613	96.71345784	1	PRJNA827465;Yang2025_Silkie_Wenchang	CRA004775	0.721011841
ENSGALG00010008362	RAMP3	2	1.031853168	0.455547127	2.265085447	0.023507437	0.279233796	2.32380106	56.96705638	1	CRA004775;Yang2025_Silkie_Wenchang	PRJNA827465	0.261669548
ENSGALG00010008362	RAMP3	2	0.160535824	0.159333698	1.007544706	0.313673086	0	0.08819813	0	1	CRA004775;PRJNA827465	Yang2025_Silkie_Wenchang	0.796531488
ENSGALG00010008366	RPRD1A	2	0.187323254	0.226042229	0.828709109	0.40726903	0.091710163	9.707407919	89.69858887	0.5	PRJNA827465;Yang2025_Silkie_Wenchang	CRA004775	0.833863243
ENSGALG00010008366	RPRD1A	2	0.102055377	0.351165159	0.290619312	0.771342489	0.210633847	6.446121592	84.48679589	0.5	CRA004775;Yang2025_Silkie_Wenchang	PRJNA827465	0.94283287
ENSGALG00010008366	RPRD1A	2	-0.062351298	0.085375126	-0.730321587	0.465193636	0	0.886668143	0	1	CRA004775;PRJNA827465	Yang2025_Silkie_Wenchang	0.852377877
ENSGALG00010008368	SNCA	2	-0.156161446	0.151277237	-1.032286474	0.301937936	0	0.96165059	0	1	PRJNA827465;Yang2025_Silkie_Wenchang	CRA004775	0.791507149
ENSGALG00010008368	SNCA	2	0.611559494	1.020154055	0.59947759	0.54885445	1.899144379	10.97947491	90.89209631	0.5	CRA004775;Yang2025_Silkie_Wenchang	PRJNA827465	0.878281266
ENSGALG00010008368	SNCA	2	0.74657186	0.865169078	0.862920183	0.388181353	1.332078801	8.474305445	88.19962289	0.5	CRA004775;PRJNA827465	Yang2025_Silkie_Wenchang	0.828154121
ENSGALG00010008372	CLCN3	2	0.000758895	0.046829461	0.016205502	0.987070446	0	0.131379022	0	0.5	PRJNA827465;Yang2025_Silkie_Wenchang	CRA004775	0.999425483
ENSGALG00010008372	CLCN3	2	-0.026136977	0.075655667	-0.345472828	0.729738934	0	0.028694424	0	1	CRA004775;Yang2025_Silkie_Wenchang	PRJNA827465	0.932444623
ENSGALG00010008372	CLCN3	2	0.009439863	0.056786517	0.166234231	0.867972621	0	0.09269859	0	0.5	CRA004775;PRJNA827465	Yang2025_Silkie_Wenchang	0.967963225
ENSGALG00010008373	MACIR	2	-0.109234336	0.117005009	-0.933586842	0.350517055	0	0.955413932	0	1	PRJNA827465;Yang2025_Silkie_Wenchang	CRA004775	0.810111037
ENSGALG00010008373	MACIR	2	-0.021347894	0.2414322	-0.088421901	0.929541355	0.075760582	2.834097191	64.71539503	0.5	CRA004775;Yang2025_Silkie_Wenchang	PRJNA827465	0.983756466
ENSGALG00010008373	MACIR	2	0.065654215	0.125121836	0.524722283	0.599776265	0	0.875915591	0	0.5	CRA004775;PRJNA827465	Yang2025_Silkie_Wenchang	0.895092498
ENSGALG00010008374	ZNF821	2	0.315566429	0.1720296	1.834372861	0.066598665	0.050692869	6.898033028	85.50311377	1	PRJNA827465;Yang2025_Silkie_Wenchang	CRA004775	0.444552877
ENSGALG00010008374	ZNF821	2	0.17277204	0.359029611	0.481219472	0.630360523	0.221575282	6.639052672	84.93761009	0.5	CRA004775;Yang2025_Silkie_Wenchang	PRJNA827465	0.902650914
ENSGALG00010008374	ZNF821	2	0.042678254	0.170639604	0.250107557	0.802504172	0.034563274	1.937546789	48.38834315	0.5	CRA004775;PRJNA827465	Yang2025_Silkie_Wenchang	0.950688375
ENSGALG00010008375	ENSGALG00010008375	2	0.028575185	0.038998416	0.732726824	0.46372506	0	0.054113112	0	1	PRJNA827465;Yang2025_Silkie_Wenchang	CRA004775	0.852159649
ENSGALG00010008375	ENSGALG00010008375	2	0.01967371	0.07398392	0.265918726	0.790301803	0	0.092294812	0	1	CRA004775;Yang2025_Silkie_Wenchang	PRJNA827465	0.947390191
ENSGALG00010008375	ENSGALG00010008375	2	0.03568576	0.044564457	0.800767297	0.423266375	0	0.054980063	0	1	CRA004775;PRJNA827465	Yang2025_Silkie_Wenchang	0.84012238
ENSGALG00010008376	MMRN1	2	-0.388935573	0.160810178	-2.418600477	0.015580342	0	0.490693905	0	1	PRJNA827465;Yang2025_Silkie_Wenchang	CRA004775	0.206527548
ENSGALG00010008376	MMRN1	2	-0.463063604	0.304564679	-1.520411381	0.128407616	0	0.639985878	0	1	CRA004775;Yang2025_Silkie_Wenchang	PRJNA827465	0.60134038
ENSGALG00010008376	MMRN1	2	-0.3089011	0.17206991	-1.795206961	0.072620732	0	0.191102528	0	1	CRA004775;PRJNA827465	Yang2025_Silkie_Wenchang	0.465466124
ENSGALG00010008377	AKT2	2	-0.090615715	0.047614439	-1.903114205	0.057025645	0	0.639895689	0	1	PRJNA827465;Yang2025_Silkie_Wenchang	CRA004775	0.413453375
ENSGALG00010008377	AKT2	2	-0.212053941	0.121202842	-1.749578946	0.080190995	0	0.156704713	0	1	CRA004775;Yang2025_Silkie_Wenchang	PRJNA827465	0.487283038
ENSGALG00010008377	AKT2	2	-0.083204517	0.050217306	-1.656889312	0.097541856	0	0.662890059	0	1	CRA004775;PRJNA827465	Yang2025_Silkie_Wenchang	0.533277943
ENSGALG00010008380	SIL1	2	0.124955902	0.062097142	2.012264952	0.044192015	0	0.204488771	0	1	PRJNA827465;Yang2025_Silkie_Wenchang	CRA004775	0.363282309
ENSGALG00010008380	SIL1	2	0.092030036	0.120767009	0.762046166	0.446032451	0.008630513	1.292953653	22.65770719	0.5	CRA004775;Yang2025_Silkie_Wenchang	PRJNA827465	0.846013379
ENSGALG00010008380	SIL1	2	0.080441766	0.072640184	1.107400356	0.268120872	0	0.875881764	0	0.5	CRA004775;PRJNA827465	Yang2025_Silkie_Wenchang	0.771398294
ENSGALG00010008381	C18orf21	2	0.094579387	0.078593604	1.203398017	0.228822335	0	0.040835317	0	1	PRJNA827465;Yang2025_Silkie_Wenchang	CRA004775	0.741553101
ENSGALG00010008381	C18orf21	2	0.092697909	0.121235915	0.764607659	0.444505207	0	0.169669056	0	1	CRA004775;Yang2025_Silkie_Wenchang	PRJNA827465	0.846013379
ENSGALG00010008381	C18orf21	2	0.115758014	0.094913515	1.219615721	0.222610584	0	0.10928165	0	1	CRA004775;PRJNA827465	Yang2025_Silkie_Wenchang	0.736208602
ENSGALG00010008382	ENSGALG00010008382	2	0.018321838	0.086223548	0.212492271	0.831723007	0	0.000530675	0	1	PRJNA827465;Yang2025_Silkie_Wenchang	CRA004775	0.957712669
ENSGALG00010008382	ENSGALG00010008382	2	-0.254845252	0.329666006	-0.773050407	0.439489945	0.157556012	3.365522074	70.28692791	0.5	CRA004775;Yang2025_Silkie_Wenchang	PRJNA827465	0.834246008
ENSGALG00010008382	ENSGALG00010008382	2	-0.24364596	0.324706506	-0.750357492	0.453039425	0.163245716	3.817990512	73.80821149	0.5	CRA004775;PRJNA827465	Yang2025_Silkie_Wenchang	0.848663482
ENSGALG00010008385	CTNNA1	2	-0.030097244	0.040932317	-0.735292953	0.462161099	0.001730715	2.0622115	51.50836856	0.5	PRJNA827465;Yang2025_Silkie_Wenchang	CRA004775	0.850747865
ENSGALG00010008385	CTNNA1	2	-0.07033335	0.041337991	-1.701421571	0.088861854	0	0.087176239	0	1	CRA004775;Yang2025_Silkie_Wenchang	PRJNA827465	0.511771357
ENSGALG00010008385	CTNNA1	2	-0.025038165	0.037296593	-0.671325005	0.502006822	0	0.022620584	0	0.5	CRA004775;PRJNA827465	Yang2025_Silkie_Wenchang	0.959493354
ENSGALG00010008386	SESN3	2	-0.046037874	0.078377871	-0.587383576	0.556946122	0	0.380188919	0	1	PRJNA827465;Yang2025_Silkie_Wenchang	CRA004775	0.880521579
ENSGALG00010008386	SESN3	2	-0.130917396	0.12078974	-1.083845332	0.278433382	0	0.659362736	0	1	CRA004775;Yang2025_Silkie_Wenchang	PRJNA827465	0.776763074
ENSGALG00010008386	SESN3	2	-0.036568806	0.118906654	-0.30754213	0.758430761	0.004857769	1.039226805	3.774614474	1	CRA004775;PRJNA827465	Yang2025_Silkie_Wenchang	0.938954369
ENSGALG00010008387	PHF20L1	2	0.28568533	0.2957547	0.965953643	0.334090778	0.159339846	10.75177155	90.69920715	1	PRJNA827465;Yang2025_Silkie_Wenchang	CRA004775	0.80304894
ENSGALG00010008387	PHF20L1	2	0.438055753	0.203303172	2.154692174	0.031185931	0.040248315	1.866113235	46.41268379	1	CRA004775;Yang2025_Silkie_Wenchang	PRJNA827465	0.302017199
ENSGALG00010008387	PHF20L1	2	0.02096496	0.067885594	0.308827817	0.757452508	0	0.442879204	0	1	CRA004775;PRJNA827465	Yang2025_Silkie_Wenchang	0.938842663
ENSGALG00010008389	GALNT1	2	-0.252188031	0.193865992	-1.300836875	0.193314297	0.070845134	17.3883082	94.24897615	1	PRJNA827465;Yang2025_Silkie_Wenchang	CRA004775	0.703242486
ENSGALG00010008389	GALNT1	2	-0.419705621	0.09091231	-4.616598361	3.90081E-06	0.004671816	1.087268948	8.026436196	1	CRA004775;Yang2025_Silkie_Wenchang	PRJNA827465	0.000684436
ENSGALG00010008389	GALNT1	2	-0.060111755	0.064701039	-0.92906939	0.352853127	0	0.020216209	0	1	CRA004775;PRJNA827465	Yang2025_Silkie_Wenchang	0.811035834
ENSGALG00010008391	ENSGALG00010008391	2	-0.000438394	0.103988816	-0.004215777	0.996636307	0.01338146	2.59305096	61.43538961	0.5	PRJNA827465;Yang2025_Silkie_Wenchang	CRA004775	0.99895145
ENSGALG00010008391	ENSGALG00010008391	2	-0.22682809	0.406009938	-0.558676201	0.576383724	0.269888708	4.830577343	79.29854075	0.5	CRA004775;Yang2025_Silkie_Wenchang	PRJNA827465	0.885704801
ENSGALG00010008391	ENSGALG00010008391	2	-0.303494642	0.291247798	-1.042049569	0.297388698	0.121301965	2.767609194	63.86773096	1	CRA004775;PRJNA827465	Yang2025_Silkie_Wenchang	0.788562929
ENSGALG00010008392	CD36	2	-0.710421678	0.205214093	-3.46185619	0.000536464	0	0.893240143	0	1	PRJNA827465;Yang2025_Silkie_Wenchang	CRA004775	0.024574165
ENSGALG00010008392	CD36	2	-0.810886284	0.241782298	-3.353786823	0.000797137	0	0.295359747	0	1	CRA004775;Yang2025_Silkie_Wenchang	PRJNA827465	0.032332193
ENSGALG00010008392	CD36	2	-0.498735225	0.273523186	-1.823374582	0.068246677	0	0.009732889	0	1	CRA004775;PRJNA827465	Yang2025_Silkie_Wenchang	0.450415709
ENSGALG00010008394	CD2AP	2	0.055400259	0.111165181	0.498359812	0.618230457	0.017609417	3.441904781	70.94632002	0.5	PRJNA827465;Yang2025_Silkie_Wenchang	CRA004775	0.899168321
ENSGALG00010008394	CD2AP	2	0.193698543	0.087750436	2.207379834	0.027287534	0	0.341776458	0	1	CRA004775;Yang2025_Silkie_Wenchang	PRJNA827465	0.281782062
ENSGALG00010008394	CD2AP	2	0.066895087	0.173666932	0.38519185	0.700095281	0.037938278	2.152431156	53.54090665	0.5	CRA004775;PRJNA827465	Yang2025_Silkie_Wenchang	0.920267298
ENSGALG00010008395	ENDOD1	2	0.052155809	0.09050775	0.576257937	0.564440849	0	0.101566181	0	0.5	PRJNA827465;Yang2025_Silkie_Wenchang	CRA004775	0.883094571
ENSGALG00010008395	ENDOD1	2	-0.115103058	0.150756199	-0.763504647	0.445162492	0	0.277957619	0	1	CRA004775;Yang2025_Silkie_Wenchang	PRJNA827465	0.846013379
ENSGALG00010008395	ENDOD1	2	3.71457E-05	0.107371562	0.000345955	0.999723968	0.006045433	1.254380509	20.27937352	0.5	CRA004775;PRJNA827465	Yang2025_Silkie_Wenchang	0.999889665
ENSGALG00010008397	ATXN1L	2	-0.079006056	0.154408892	-0.511667785	0.608885536	0.041228384	6.88987572	85.4859501	0.5	PRJNA827465;Yang2025_Silkie_Wenchang	CRA004775	0.897206691
ENSGALG00010008397	ATXN1L	2	-0.225879608	0.102016661	-2.214144292	0.026818855	0	0.361759013	0	1	CRA004775;Yang2025_Silkie_Wenchang	PRJNA827465	0.279212912
ENSGALG00010008397	ATXN1L	2	0.056440929	0.042788006	1.319082943	0.187141385	0	0.23978786	0	0.5	CRA004775;PRJNA827465	Yang2025_Silkie_Wenchang	0.695933761
ENSGALG00010008399	INO80C	2	0.009493588	0.096488565	0.09839081	0.921621972	0.010353157	2.065386892	51.58292116	0.5	PRJNA827465;Yang2025_Silkie_Wenchang	CRA004775	0.981639793
ENSGALG00010008399	INO80C	2	0.157043904	0.10984899	1.429634486	0.152821952	0	0.137107124	0	1	CRA004775;Yang2025_Silkie_Wenchang	PRJNA827465	0.644805281
ENSGALG00010008399	INO80C	2	-0.004899543	0.119557341	-0.040980695	0.967311286	0.013435821	1.418737051	29.5147752	0.5	CRA004775;PRJNA827465	Yang2025_Silkie_Wenchang	0.990935606
ENSGALG00010008400	MAP1LC3C	2	-0.330053117	0.167967448	-1.964982623	0.049416249	0	0.009788504	0	1	PRJNA827465;Yang2025_Silkie_Wenchang	CRA004775	0.384595411
ENSGALG00010008400	MAP1LC3C	2	-0.238656516	0.292892519	-0.81482626	0.415171767	0	0.502999228	0	0.5	CRA004775;Yang2025_Silkie_Wenchang	PRJNA827465	0.837489728
ENSGALG00010008400	MAP1LC3C	2	-0.277020009	0.183626175	-1.508608506	0.131398859	0	0.511900987	0	0.5	CRA004775;PRJNA827465	Yang2025_Silkie_Wenchang	0.607072924
ENSGALG00010008401	PSMD8	2	0.058469622	0.101971087	0.57339412	0.56637787	0.012434829	2.282082681	56.1803782	0.5	PRJNA827465;Yang2025_Silkie_Wenchang	CRA004775	0.883487517
ENSGALG00010008401	PSMD8	2	0.137006128	0.129256863	1.059952442	0.289164236	0.004326064	1.067224832	6.299031846	0.5	CRA004775;Yang2025_Silkie_Wenchang	PRJNA827465	0.783530627
ENSGALG00010008401	PSMD8	2	-0.026658869	0.064062488	-0.416138528	0.677306638	0	0.217868631	0	1	CRA004775;PRJNA827465	Yang2025_Silkie_Wenchang	0.917144807
ENSGALG00010008405	LRATD2	2	-0.255740931	0.352642385	-0.725213252	0.468321214	0.232650338	15.26809867	93.45039601	0.5	PRJNA827465;Yang2025_Silkie_Wenchang	CRA004775	0.853198008
ENSGALG00010008405	LRATD2	2	-0.044262761	0.602528077	-0.073461741	0.941438688	0.669940762	12.61231702	92.07124275	0.5	CRA004775;Yang2025_Silkie_Wenchang	PRJNA827465	0.985827325
ENSGALG00010008405	LRATD2	2	0.253761024	0.236319823	1.073802853	0.282911037	0.075419233	2.511391536	60.17826654	1	CRA004775;PRJNA827465	Yang2025_Silkie_Wenchang	0.775912565
ENSGALG00010008407	GNAI1	2	-0.25168141	0.181311301	-1.388117612	0.165101234	0.058802468	9.466743543	89.4367055	1	PRJNA827465;Yang2025_Silkie_Wenchang	CRA004775	0.664377375
ENSGALG00010008407	GNAI1	2	-0.041173578	0.411453614	-0.100068579	0.920289881	0.31585577	14.41462139	93.06259961	0.5	CRA004775;Yang2025_Silkie_Wenchang	PRJNA827465	0.981364549
ENSGALG00010008407	GNAI1	2	0.12431414	0.227811009	0.545689782	0.585279197	0.082827137	4.515743277	77.85525132	0.5	CRA004775;PRJNA827465	Yang2025_Silkie_Wenchang	0.891130641
ENSGALG00010008408	SMARCA4	2	0.108026234	0.20980866	0.514879768	0.60663704	0.080663624	11.89614556	91.5939159	0.5	PRJNA827465;Yang2025_Silkie_Wenchang	CRA004775	0.899399223
ENSGALG00010008408	SMARCA4	2	0.020066467	0.31246107	0.066140935	0.947265621	0.179215592	11.97704343	91.65069405	0.5	CRA004775;Yang2025_Silkie_Wenchang	PRJNA827465	0.986445196
ENSGALG00010008408	SMARCA4	2	-0.157583581	0.093243707	-1.690018404	0.091024434	0.006375218	1.431401271	30.13838813	1	CRA004775;PRJNA827465	Yang2025_Silkie_Wenchang	0.517594064
ENSGALG00010008409	CWC15	2	-0.160428734	0.133437164	-1.202279257	0.229255351	0.029452507	5.380194257	81.41331052	1	PRJNA827465;Yang2025_Silkie_Wenchang	CRA004775	0.742256776
ENSGALG00010008409	CWC15	2	-0.179255019	0.183630977	-0.976169822	0.328980313	0.040024628	2.148040874	53.44595106	0.5	CRA004775;Yang2025_Silkie_Wenchang	PRJNA827465	0.800761292
ENSGALG00010008409	CWC15	2	-0.038471965	0.046053078	-0.835383157	0.40350201	0	0.229558418	0	0.5	CRA004775;PRJNA827465	Yang2025_Silkie_Wenchang	0.83306585
ENSGALG00010008410	NAPRT	2	0.056279975	0.176363638	0.319113256	0.749640632	0.039803172	2.435042467	58.93295441	0.5	PRJNA827465;Yang2025_Silkie_Wenchang	CRA004775	0.937362187
ENSGALG00010008410	NAPRT	2	-0.37158439	0.134281926	-2.767195863	0.005654077	0.008362905	1.25669441	20.42615996	1	CRA004775;Yang2025_Silkie_Wenchang	PRJNA827465	0.127987126
ENSGALG00010008410	NAPRT	2	-0.131019782	0.32675898	-0.400967654	0.688443939	0.200049368	15.70509901	93.63300248	0.5	CRA004775;PRJNA827465	Yang2025_Silkie_Wenchang	0.919798626
ENSGALG00010008412	NEK1	2	0.482508571	0.431065243	1.119340005	0.262995115	0.350613265	16.95462976	94.101906	1	PRJNA827465;Yang2025_Silkie_Wenchang	CRA004775	0.768643821
ENSGALG00010008412	NEK1	2	0.551691208	0.387829866	1.422508312	0.15487874	0.257250653	6.900409499	85.50810644	1	CRA004775;Yang2025_Silkie_Wenchang	PRJNA827465	0.647505492
ENSGALG00010008412	NEK1	2	0.078251857	0.054052026	1.447712652	0.14769715	0	0.154883241	0	1	CRA004775;PRJNA827465	Yang2025_Silkie_Wenchang	0.634995621
ENSGALG00010008416	MSX2	2	0.938773086	0.469223406	2.00069535	0.045425231	0	0.058241381	0	1	PRJNA827465;Yang2025_Silkie_Wenchang	CRA004775	0.367431128
ENSGALG00010008416	MSX2	2	0.716362634	0.576382401	1.242860006	0.213919426	0	0.467253041	0	1	CRA004775;Yang2025_Silkie_Wenchang	PRJNA827465	0.726593954
ENSGALG00010008416	MSX2	2	0.676825401	0.475547067	1.42325639	0.154661844	0	0.294628227	0	1	CRA004775;PRJNA827465	Yang2025_Silkie_Wenchang	0.647505492
ENSGALG00010008418	TMEM245	2	-0.122727997	0.058941527	-2.082199139	0.037324284	0	0.089191179	0	1	PRJNA827465;Yang2025_Silkie_Wenchang	CRA004775	0.333247689
ENSGALG00010008418	TMEM245	2	-0.070714466	0.140950342	-0.501697727	0.615880164	0.013925342	1.349431458	25.89471705	0.5	CRA004775;Yang2025_Silkie_Wenchang	PRJNA827465	0.89901403
ENSGALG00010008418	TMEM245	2	-0.075550415	0.095596756	-0.790303118	0.429350766	0.00561688	1.155672971	13.47033073	0.5	CRA004775;PRJNA827465	Yang2025_Silkie_Wenchang	0.842008086
ENSGALG00010008422	EXO1	2	-0.146071798	0.101173645	-1.443773208	0.148803781	0	0.088303726	0	1	PRJNA827465;Yang2025_Silkie_Wenchang	CRA004775	0.63890906
ENSGALG00010008422	EXO1	2	-0.186444716	0.153501683	-1.214610234	0.224514784	0	0.018612719	0	1	CRA004775;Yang2025_Silkie_Wenchang	PRJNA827465	0.73755471
ENSGALG00010008422	EXO1	2	-0.126134942	0.129973398	-0.970467372	0.331813581	0	0.049113934	0	1	CRA004775;PRJNA827465	Yang2025_Silkie_Wenchang	0.802002658
ENSGALG00010008424	PPIP5K2	2	0.182423201	0.151247767	1.206121613	0.227770602	0.019608097	1.749544799	42.84227527	1	PRJNA827465;Yang2025_Silkie_Wenchang	CRA004775	0.740853612
ENSGALG00010008424	PPIP5K2	2	0.274708869	0.145771042	1.884522918	0.059494278	0	0.662298043	0	1	CRA004775;Yang2025_Silkie_Wenchang	PRJNA827465	0.442395132
ENSGALG00010008424	PPIP5K2	2	0.036673098	0.142004814	0.258252495	0.796212046	0	0.001386656	0	1	CRA004775;PRJNA827465	Yang2025_Silkie_Wenchang	0.949325935
ENSGALG00010008425	MAGI2	2	0.282016406	0.108240995	2.605449134	0.00917539	0.009348247	1.533428468	34.78665481	1	PRJNA827465;Yang2025_Silkie_Wenchang	CRA004775	0.150281327
ENSGALG00010008425	MAGI2	2	0.414282585	0.150798721	2.747255299	0.006009634	0	0.143466079	0	1	CRA004775;Yang2025_Silkie_Wenchang	PRJNA827465	0.117219169
ENSGALG00010008425	MAGI2	2	0.21168074	0.086525796	2.446446609	0.01442716	0	0.039863795	0	1	CRA004775;PRJNA827465	Yang2025_Silkie_Wenchang	0.197673991
ENSGALG00010008426	AP1G1	2	-0.058849728	0.117610854	-0.500376672	0.616809876	0.024403412	8.238072792	87.8612386	0.5	PRJNA827465;Yang2025_Silkie_Wenchang	CRA004775	0.899111936
ENSGALG00010008426	AP1G1	2	-0.181278281	0.064915495	-2.792527141	0.005229808	0	0.008905261	0	1	CRA004775;Yang2025_Silkie_Wenchang	PRJNA827465	0.110955433
ENSGALG00010008426	AP1G1	2	-0.007833727	0.097565544	-0.080291945	0.936005065	0.011426583	1.899988384	47.36809927	0.5	CRA004775;PRJNA827465	Yang2025_Silkie_Wenchang	0.984822128
ENSGALG00010008428	ENSGALG00010008428	2	0.608605469	0.671829288	0.905893029	0.364992474	0.755332107	5.632796334	82.24682838	1	PRJNA827465;Yang2025_Silkie_Wenchang	CRA004775	0.818001894
ENSGALG00010008428	ENSGALG00010008428	2	0.815818105	0.686886808	1.187703849	0.234950107	0.501224895	2.037553508	50.92153428	0.5	CRA004775;Yang2025_Silkie_Wenchang	PRJNA827465	0.747791642
ENSGALG00010008428	ENSGALG00010008428	2	0.016631503	0.201361006	0.082595451	0.934173219	0	0.003146998	0	1	CRA004775;PRJNA827465	Yang2025_Silkie_Wenchang	0.98432801
ENSGALG00010008429	IGFBP3	2	-0.274790788	0.159181139	-1.726314999	0.084290758	0.042116073	5.81897126	82.81483177	1	PRJNA827465;Yang2025_Silkie_Wenchang	CRA004775	0.499981841
ENSGALG00010008429	IGFBP3	2	0.021083894	0.476359611	0.044260456	0.964696792	0.431193843	19.81492296	94.95329857	0.5	CRA004775;Yang2025_Silkie_Wenchang	PRJNA827465	0.990394578
ENSGALG00010008429	IGFBP3	2	0.169903382	0.316253067	0.53723236	0.591107121	0.18112672	10.099465	90.09848542	0.5	CRA004775;PRJNA827465	Yang2025_Silkie_Wenchang	0.897276493
ENSGALG00010008430	ENSGALG00010008430	2	0.345525408	0.242283901	1.426117903	0.153834312	0	0.459041001	0	1	PRJNA827465;Yang2025_Silkie_Wenchang	CRA004775	0.646370532
ENSGALG00010008430	ENSGALG00010008430	2	0.475258123	0.3543368	1.341260977	0.179835735	0	0.225836433	0	1	CRA004775;Yang2025_Silkie_Wenchang	PRJNA827465	0.68659821
ENSGALG00010008430	ENSGALG00010008430	2	0.202594266	0.290787854	0.696708142	0.485985462	0	0.014227854	0	1	CRA004775;PRJNA827465	Yang2025_Silkie_Wenchang	0.857418308
ENSGALG00010008431	SH3RF1	2	-0.102962659	0.088715754	-1.160590465	0.245808485	0.005013194	1.344802809	25.6396556	1	PRJNA827465;Yang2025_Silkie_Wenchang	CRA004775	0.75661406
ENSGALG00010008431	SH3RF1	2	0.046267268	0.311223772	0.148662383	0.881820045	0.158812002	5.446330775	81.63901457	0.5	CRA004775;Yang2025_Silkie_Wenchang	PRJNA827465	0.971906148
ENSGALG00010008431	SH3RF1	2	0.115540654	0.207115037	0.55785739	0.57694177	0.063651608	3.350089857	70.15005439	0.5	CRA004775;PRJNA827465	Yang2025_Silkie_Wenchang	0.887113945
ENSGALG00010008433	EBAG9	2	-0.050913909	0.110080722	-0.462514309	0.643712544	0.015181501	2.104272389	52.47763525	0.5	PRJNA827465;Yang2025_Silkie_Wenchang	CRA004775	0.906643868
ENSGALG00010008433	EBAG9	2	0.13126	0.124995384	1.050118776	0.293663507	0	0.018527252	0	1	CRA004775;Yang2025_Silkie_Wenchang	PRJNA827465	0.786979185
ENSGALG00010008433	EBAG9	2	-0.05484915	0.118721554	-0.461998248	0.644082577	0.01660874	1.778188931	43.76300615	0.5	CRA004775;PRJNA827465	Yang2025_Silkie_Wenchang	0.906837811
ENSGALG00010008437	SHKBP1	2	-0.028963575	0.070558663	-0.410489277	0.681447068	0.00197937	1.203649414	16.91932982	0.5	PRJNA827465;Yang2025_Silkie_Wenchang	CRA004775	0.918242009
ENSGALG00010008437	SHKBP1	2	-0.084097624	0.194192012	-0.433064283	0.66496807	0.049438763	2.72617319	63.31854471	0.5	CRA004775;Yang2025_Silkie_Wenchang	PRJNA827465	0.913399315
ENSGALG00010008437	SHKBP1	2	-0.118917035	0.09253765	-1.285066512	0.198769043	0.00520538	1.215753126	17.74645864	1	CRA004775;PRJNA827465	Yang2025_Silkie_Wenchang	0.71096127
ENSGALG00010008438	SYBU	2	0.018240637	0.133445002	0.136690299	0.891275598	0	0.318342669	0	0.5	PRJNA827465;Yang2025_Silkie_Wenchang	CRA004775	0.973780469
ENSGALG00010008438	SYBU	2	0.028333409	0.153998437	0.183985042	0.854025189	0	0.3982063	0	0.5	CRA004775;Yang2025_Silkie_Wenchang	PRJNA827465	0.962975689
ENSGALG00010008438	SYBU	2	-0.088661879	0.168381043	-0.526554994	0.598502653	0	0.038514984	0	1	CRA004775;PRJNA827465	Yang2025_Silkie_Wenchang	0.894680883
ENSGALG00010008441	PHLPP2	2	0.055708339	0.052423872	1.06265212	0.287939742	0	0.072820675	0	1	PRJNA827465;Yang2025_Silkie_Wenchang	CRA004775	0.782840067
ENSGALG00010008441	PHLPP2	2	0.005014757	0.104293923	0.048082926	0.961650153	0	0.342802794	0	0.5	CRA004775;Yang2025_Silkie_Wenchang	PRJNA827465	0.98973612
ENSGALG00010008441	PHLPP2	2	0.054347854	0.058076292	0.935801039	0.349375637	0	0.537576258	0	0.5	CRA004775;PRJNA827465	Yang2025_Silkie_Wenchang	0.809903518
ENSGALG00010008443	RSFR	2	-0.159844328	0.595871624	-0.268252962	0.78850461	0.634510373	9.328876956	89.2805961	0.5	PRJNA827465;Yang2025_Silkie_Wenchang	CRA004775	0.947048735
ENSGALG00010008443	RSFR	2	-0.611846166	0.226618698	-2.699892684	0.006936185	0	0.576986942	0	1	CRA004775;Yang2025_Silkie_Wenchang	PRJNA827465	0.12798726
ENSGALG00010008443	RSFR	2	0.02772265	0.422380095	0.065634368	0.947668927	0.275725704	4.317395273	76.83788635	0.5	CRA004775;PRJNA827465	Yang2025_Silkie_Wenchang	0.986602877
ENSGALG00010008444	SLC7A11	2	0.609426109	0.302459328	2.014902674	0.043914848	0	0.263784887	0	1	PRJNA827465;Yang2025_Silkie_Wenchang	CRA004775	0.361888215
ENSGALG00010008444	SLC7A11	2	0.540984263	0.424103822	1.275593935	0.202099099	0	0.244959284	0	1	CRA004775;Yang2025_Silkie_Wenchang	PRJNA827465	0.71497298
ENSGALG00010008444	SLC7A11	2	0.744577013	0.331868741	2.243588867	0.024858866	0	0.020217327	0	1	CRA004775;PRJNA827465	Yang2025_Silkie_Wenchang	0.268931467
ENSGALG00010008447	FRRS1L	2	0.060924851	0.061503757	0.990587478	0.321887054	0	0.661968143	0	0.5	PRJNA827465;Yang2025_Silkie_Wenchang	CRA004775	0.798513866
ENSGALG00010008447	FRRS1L	2	-0.175482032	0.214937147	-0.816434174	0.414251858	0.057138824	2.270758156	55.96184484	1	CRA004775;Yang2025_Silkie_Wenchang	PRJNA827465	0.836914812
ENSGALG00010008447	FRRS1L	2	-0.126268175	0.274454475	-0.460069652	0.645466226	0.117784707	3.883181418	74.24791962	0.5	CRA004775;PRJNA827465	Yang2025_Silkie_Wenchang	0.906966468
ENSGALG00010008448	TNS3	2	0.005697795	0.062403174	0.09130618	0.927249308	0.000276507	1.027183877	2.646446977	0.5	PRJNA827465;Yang2025_Silkie_Wenchang	CRA004775	0.982993195
ENSGALG00010008448	TNS3	2	-0.08967663	0.11770039	-0.76190597	0.446116127	0	0.112456035	0	0.5	CRA004775;Yang2025_Silkie_Wenchang	PRJNA827465	0.846013379
ENSGALG00010008448	TNS3	2	0.040991326	0.068758902	0.596160276	0.55106816	0	0.001545768	0	1	CRA004775;PRJNA827465	Yang2025_Silkie_Wenchang	0.878879109
ENSGALG00010008449	SRA1	2	0.056492047	0.07831075	0.721383043	0.470673876	0	0.113141307	0	0.5	PRJNA827465;Yang2025_Silkie_Wenchang	CRA004775	0.853744788
ENSGALG00010008449	SRA1	2	-0.182499566	0.227406722	-0.80252494	0.422249368	0	0.476428967	0	1	CRA004775;Yang2025_Silkie_Wenchang	PRJNA827465	0.839649223
ENSGALG00010008449	SRA1	2	-0.016970508	0.165788569	-0.102362352	0.918469066	0.027394629	1.44424979	30.7598999	0.5	CRA004775;PRJNA827465	Yang2025_Silkie_Wenchang	0.981033557
ENSGALG00010008450	GIN1	2	-0.175003178	0.107886213	-1.622108819	0.10478005	0	0.245974164	0	1	PRJNA827465;Yang2025_Silkie_Wenchang	CRA004775	0.549969326
ENSGALG00010008450	GIN1	2	-0.23428548	0.146081013	-1.603805142	0.108757011	0	0.000144227	0	1	CRA004775;Yang2025_Silkie_Wenchang	PRJNA827465	0.55846313
ENSGALG00010008450	GIN1	2	-0.141967521	0.135445213	-1.048154585	0.294567391	0	0.083445646	0	1	CRA004775;PRJNA827465	Yang2025_Silkie_Wenchang	0.787408474
ENSGALG00010008451	OPN3	2	-0.30897258	0.161754188	-1.910136507	0.05611564	0	0.512228317	0	1	PRJNA827465;Yang2025_Silkie_Wenchang	CRA004775	0.409794312
ENSGALG00010008451	OPN3	2	-0.075127298	0.221671668	-0.338912497	0.73467565	0.000744611	1.00449111	0.447102981	0.5	CRA004775;Yang2025_Silkie_Wenchang	PRJNA827465	0.933520234
ENSGALG00010008451	OPN3	2	-0.146095802	0.380461958	-0.383995822	0.700981544	0.168575111	2.055818497	51.35757356	0.5	CRA004775;PRJNA827465	Yang2025_Silkie_Wenchang	0.922538094
ENSGALG00010008453	AMOTL1	2	-0.019152714	0.073183092	-0.261709549	0.793545378	0.001807478	1.192197442	16.12127618	0.5	PRJNA827465;Yang2025_Silkie_Wenchang	CRA004775	0.948743674
ENSGALG00010008453	AMOTL1	2	0.053411149	0.105642826	0.50558236	0.613149868	0	0.536632687	0	0.5	CRA004775;Yang2025_Silkie_Wenchang	PRJNA827465	0.898190375
ENSGALG00010008453	AMOTL1	2	-0.084520851	0.082539432	-1.024005728	0.305832569	0	0.178336653	0	1	CRA004775;PRJNA827465	Yang2025_Silkie_Wenchang	0.79225794
ENSGALG00010008455	FKBP1B	2	-0.09830938	0.075069265	-1.309582292	0.190337182	0	0.0445568	0	1	PRJNA827465;Yang2025_Silkie_Wenchang	CRA004775	0.699411386
ENSGALG00010008455	FKBP1B	2	-0.082331128	0.135115302	-0.609339778	0.542299246	0	0.071019295	0	1	CRA004775;Yang2025_Silkie_Wenchang	PRJNA827465	0.876721948
ENSGALG00010008455	FKBP1B	2	-0.111363986	0.088132504	-1.26359721	0.206374636	0	0.04448989	0	1	CRA004775;PRJNA827465	Yang2025_Silkie_Wenchang	0.718451597
ENSGALG00010008456	NHLRC1	2	-0.037882516	0.069884388	-0.542074095	0.587767464	0	0.068726574	0	1	PRJNA827465;Yang2025_Silkie_Wenchang	CRA004775	0.891742161
ENSGALG00010008456	NHLRC1	2	-0.10101931	0.129229035	-0.781707532	0.434386474	0	0.323434014	0	1	CRA004775;Yang2025_Silkie_Wenchang	PRJNA827465	0.842762754

ENSGALG00010008456	NHLRC1	2	-0.041202056	0.078283282	-0.526319987	0.598665898	0	0.525659756	0	1	CRA004775;PRJNA827465	Yang2025_Silkie_Wenchang	0.894762943
ENSGALG00010008457	MARVELD3	2	0.045002989	0.106637726	0.42201752	0.673012229	0	0.266568452	0	0.5	PRJNA827465;Yang2025_Silkie_Wenchang	CRA004775	0.915153756
ENSGALG00010008457	MARVELD3	2	-0.116061504	0.150635808	-0.770477519	0.441016685	0	0.079274083	0	1	CRA004775;Yang2025_Silkie_Wenchang	PRJNA827465	0.844888742
ENSGALG00010008457	MARVELD3	2	0.011728162	0.101803807	0.115203571	0.908283768	0	0.956802505	0	0.5	CRA004775;PRJNA827465	Yang2025_Silkie_Wenchang	0.978236585
ENSGALG00010008459	EPB41L4B	2	0.341557597	0.182399111	1.872583672	0.061125898	0	0.312946466	0	1	PRJNA827465;Yang2025_Silkie_Wenchang	CRA004775	0.427371325
ENSGALG00010008459	EPB41L4B	2	0.95261913	0.547663645	1.739423711	0.08196026	0.460823463	4.148461722	75.89467935	1	CRA004775;Yang2025_Silkie_Wenchang	PRJNA827465	0.493532115
ENSGALG00010008459	EPB41L4B	2	0.850086461	0.651737001	1.304339725	0.192117776	0.721555398	6.34241819	84.2331431	1	CRA004775;PRJNA827465	Yang2025_Silkie_Wenchang	0.701849591
ENSGALG00010008464	SF3B6	2	0.091316163	0.188606291	0.484162871	0.628270285	0.060808017	6.449660412	84.49530772	0.5	PRJNA827465;Yang2025_Silkie_Wenchang	CRA004775	0.901904489
ENSGALG00010008464	SF3B6	2	0.033105858	0.302280404	0.109520358	0.912789775	0.144441095	4.528255713	77.91644149	0.5	CRA004775;Yang2025_Silkie_Wenchang	PRJNA827465	0.979024575
ENSGALG00010008464	SF3B6	2	-0.090082996	0.057343389	-1.570939515	0.116196699	0	0.792153831	0	1	CRA004775;PRJNA827465	Yang2025_Silkie_Wenchang	0.573800585
ENSGALG00010008468	EPPK1	2	-0.20003559	0.240051653	-0.833302281	0.40467427	0	0.330075118	0	1	PRJNA827465;Yang2025_Silkie_Wenchang	CRA004775	0.833112102
ENSGALG00010008468	EPPK1	2	-0.003742966	0.46780085	-0.008001195	0.993616039	0.201636105	1.797102108	44.35485911	0.5	CRA004775;Yang2025_Silkie_Wenchang	PRJNA827465	0.997979742
ENSGALG00010008468	EPPK1	2	0.043070046	0.272422191	0.158100357	0.874377719	0.005954625	1.027750931	2.700161104	0.5	CRA004775;PRJNA827465	Yang2025_Silkie_Wenchang	0.970135804
ENSGALG00010008469	NOCT	2	-0.061131356	0.061743768	-0.990081393	0.322134337	0	0.115810611	0	1	PRJNA827465;Yang2025_Silkie_Wenchang	CRA004775	0.798513866
ENSGALG00010008469	NOCT	2	-0.001065553	0.168028379	-0.006341505	0.994940245	0	0.018833394	0	0.5	CRA004775;Yang2025_Silkie_Wenchang	PRJNA827465	0.998242335
ENSGALG00010008469	NOCT	2	-0.067895501	0.065876121	-1.030654202	0.302703009	0	0.049978687	0	0.5	CRA004775;PRJNA827465	Yang2025_Silkie_Wenchang	0.791852471
ENSGALG00010008474	ENSGALG00010008474	2	-0.015668578	0.194309294	-0.080637307	0.935730396	0.062596295	5.846407238	82.89547821	0.5	PRJNA827465;Yang2025_Silkie_Wenchang	CRA004775	0.984800337
ENSGALG00010008474	ENSGALG00010008474	2	0.197229357	0.108425942	1.81902369	0.068907816	0	0.269132578	0	1	CRA004775;Yang2025_Silkie_Wenchang	PRJNA827465	0.452044654
ENSGALG00010008474	ENSGALG00010008474	2	-0.014440246	0.273878259	-0.052725055	0.957950976	0.100327776	2.486178167	59.7776212	0.5	CRA004775;PRJNA827465	Yang2025_Silkie_Wenchang	0.988864712
ENSGALG00010008476	RNF166	2	-0.288177494	0.174498073	-1.651465196	0.098643622	0.055997994	12.3471326	91.90095359	1	PRJNA827465;Yang2025_Silkie_Wenchang	CRA004775	0.536014139
ENSGALG00010008476	RNF166	2	-0.382805157	0.118369676	-3.233979949	0.00122078	0.014701963	1.878197133	46.75745252	1	CRA004775;Yang2025_Silkie_Wenchang	PRJNA827465	0.042624337
ENSGALG00010008476	RNF166	2	-0.128983402	0.055670724	-2.316898235	0.020509276	0	0.315812836	0	1	CRA004775;PRJNA827465	Yang2025_Silkie_Wenchang	0.241484743
ENSGALG00010008486	CTU2	2	0.009000972	0.064137033	0.140339703	0.888391596	0	0.333253522	0	0.5	PRJNA827465;Yang2025_Silkie_Wenchang	CRA004775	0.973135755
ENSGALG00010008486	CTU2	2	-0.381981722	0.279766729	-1.365357931	0.172140579	0.092947796	2.446395006	59.12352675	1	CRA004775;Yang2025_Silkie_Wenchang	PRJNA827465	0.674809614
ENSGALG00010008486	CTU2	2	-0.280478639	0.346553046	-0.809338258	0.418320606	0.205713581	6.209092133	83.8945859	0.5	CRA004775;PRJNA827465	Yang2025_Silkie_Wenchang	0.838112595
ENSGALG00010008488	CCSER1	2	0.01669012	0.104907895	0.15909308	0.87359554	0	0.295443404	0	0.5	PRJNA827465;Yang2025_Silkie_Wenchang	CRA004775	0.9699681
ENSGALG00010008488	CCSER1	2	-0.124159313	0.167982639	-0.739119912	0.459834187	0	0.487997273	0	1	CRA004775;Yang2025_Silkie_Wenchang	PRJNA827465	0.850098539
ENSGALG00010008488	CCSER1	2	0.001346225	0.140972756	0.009549542	0.992380683	0.006713509	1.081730102	7.555498572	0.5	CRA004775;PRJNA827465	Yang2025_Silkie_Wenchang	0.997813736
ENSGALG00010008492	ANKRD49	2	-0.038243656	0.066514049	-0.5749711	0.565310842	0	0.808121398	0	1	PRJNA827465;Yang2025_Silkie_Wenchang	CRA004775	0.883427937
ENSGALG00010008492	ANKRD49	2	-0.161582835	0.149371912	-1.081748454	0.279364316	0	0.053211667	0	1	CRA004775;Yang2025_Silkie_Wenchang	PRJNA827465	0.777457036
ENSGALG00010008492	ANKRD49	2	-0.018261327	0.070033377	-0.26075177	0.79428394	0	0.084907736	0	1	CRA004775;PRJNA827465	Yang2025_Silkie_Wenchang	0.948905846
ENSGALG00010008497	PAM	2	-0.18155538	0.136964112	-1.325568991	0.184982503	0.029007945	4.199312268	76.18657685	1	PRJNA827465;Yang2025_Silkie_Wenchang	CRA004775	0.693265167
ENSGALG00010008497	PAM	2	-0.008693635	0.379947691	-0.022881135	0.981745088	0.237958996	5.162000918	80.62766714	0.5	CRA004775;Yang2025_Silkie_Wenchang	PRJNA827465	0.99503081
ENSGALG00010008497	PAM	2	0.088194815	0.225944369	0.390338627	0.696286164	0.068626219	2.305403222	56.62364004	0.5	CRA004775;PRJNA827465	Yang2025_Silkie_Wenchang	0.921038437
ENSGALG00010008498	ENSGALG00010008498	2	0.07619597	0.171426523	0.444481803	0.656694282	0.044766419	3.983208703	74.89461199	0.5	PRJNA827465;Yang2025_Silkie_Wenchang	CRA004775	0.910530553
ENSGALG00010008498	ENSGALG00010008498	2	0.235092733	0.135403155	1.736242653	0.082520938	0	0.300033768	0	1	CRA004775;Yang2025_Silkie_Wenchang	PRJNA827465	0.495285278
ENSGALG00010008498	ENSGALG00010008498	2	-0.063065958	0.082227984	-0.766964667	0.443102523	0	0.244575684	0	0.5	CRA004775;PRJNA827465	Yang2025_Silkie_Wenchang	0.845730569
ENSGALG00010008500	ENSGALG00010008500	2	-0.143441299	0.164429136	-0.872359382	0.383012352	0.030178853	2.084675618	52.03090633	0.5	PRJNA827465;Yang2025_Silkie_Wenchang	CRA004775	0.825348674
ENSGALG00010008500	ENSGALG00010008500	2	0.140282692	0.172754511	0.8120349	0.416771606	0	0.365367556	0	1	CRA004775;Yang2025_Silkie_Wenchang	PRJNA827465	0.837984974
ENSGALG00010008500	ENSGALG00010008500	2	-0.06079163	0.273618086	-0.222176945	0.824176141	0.105664148	2.908924552	65.62303415	0.5	CRA004775;PRJNA827465	Yang2025_Silkie_Wenchang	0.95619211
ENSGALG00010008501	ENSGALG00010008501	2	0.073726858	0.370755164	0.198855917	0.842375454	0.22504161	5.287972764	81.0891613	0.5	PRJNA827465;Yang2025_Silkie_Wenchang	CRA004775	0.960231061
ENSGALG00010008501	ENSGALG00010008501	2	0.027839711	0.364145783	0.076452103	0.939059419	0.184791316	3.297909897	69.67776466	0.5	CRA004775;Yang2025_Silkie_Wenchang	PRJNA827465	0.985349045
ENSGALG00010008501	ENSGALG00010008501	2	0.406148293	0.139556249	2.910283816	0.003611007	0	0.00261052	0	1	CRA004775;PRJNA827465	Yang2025_Silkie_Wenchang	0.085547559
ENSGALG00010008504	RNF139	2	-0.135991229	0.144387096	-0.941851678	0.346268583	0.034537404	5.246237007	80.93871858	1	PRJNA827465;Yang2025_Silkie_Wenchang	CRA004775	0.807744676
ENSGALG00010008504	RNF139	2	-0.081469484	0.269872522	-0.301881361	0.762742505	0.107829795	3.491352213	71.35780239	0.5	CRA004775;Yang2025_Silkie_Wenchang	PRJNA827465	0.940082621
ENSGALG00010008504	RNF139	2	-0.006133778	0.040528406	-0.151345157	0.879703449	0	0.900992502	0	0.5	CRA004775;PRJNA827465	Yang2025_Silkie_Wenchang	0.971407853
ENSGALG00010008505	PTPN3	2	0.022208824	0.118289091	0.187750401	0.851072318	0	0.00142517	0	1	PRJNA827465;Yang2025_Silkie_Wenchang	CRA004775	0.962071965
ENSGALG00010008505	PTPN3	2	0.025944507	0.156320624	0.165969826	0.868180696	0	0.032826023	0	1	CRA004775;Yang2025_Silkie_Wenchang	PRJNA827465	0.968042774
ENSGALG00010008505	PTPN3	2	0.035124555	0.165523896	0.212202321	0.831949196	0	0.027396754	0	1	CRA004775;PRJNA827465	Yang2025_Silkie_Wenchang	0.957762335
ENSGALG00010008509	MGARP	2	0.138112493	0.237011727	0.582724299	0.560078913	0	0.889027782	0	0.5	PRJNA827465;Yang2025_Silkie_Wenchang	CRA004775	0.881203589
ENSGALG00010008509	MGARP	2	0.081219107	0.264518823	0.307044717	0.758809336	0	0.708977498	0	0.5	CRA004775;Yang2025_Silkie_Wenchang	PRJNA827465	0.938996879
ENSGALG00010008509	MGARP	2	0.48348122	0.342023715	1.413589755	0.157482391	0	0.022409075	0	1	CRA004775;PRJNA827465	Yang2025_Silkie_Wenchang	0.65076873
ENSGALG00010008514	PIEZO1	2	0.025920624	0.077997262	0.332327355	0.739642086	0	0.002653801	0	1	PRJNA827465;Yang2025_Silkie_Wenchang	CRA004775	0.934914078
ENSGALG00010008514	PIEZO1	2	-0.074103555	0.160494128	-0.461721284	0.644281206	0.022323227	1.505816684	33.59085399	0.5	CRA004775;Yang2025_Silkie_Wenchang	PRJNA827465	0.906890059
ENSGALG00010008514	PIEZO1	2	-0.070765066	0.16540195	-0.427836954	0.668769831	0.025728008	1.588846264	37.06124861	0.5	CRA004775;PRJNA827465	Yang2025_Silkie_Wenchang	0.914377769
ENSGALG00010008515	TAFA5	2	0.328430532	0.199082069	1.649724323	0.098999333	0.053229762	3.044289796	67.15161608	1	PRJNA827465;Yang2025_Silkie_Wenchang	CRA004775	0.536907794
ENSGALG00010008515	TAFA5	2	0.296797949	0.339798966	0.873451595	0.382416979	0.14261796	2.139184008	53.25320326	0.5	CRA004775;Yang2025_Silkie_Wenchang	PRJNA827465	0.825106199
ENSGALG00010008515	TAFA5	2	0.0938132	0.153363455	0.611705049	0.540732921	0	0.443897675	0	0.5	CRA004775;PRJNA827465	Yang2025_Silkie_Wenchang	0.875630422
ENSGALG00010008516	MRE11	2	0.041580039	0.145772111	0.285240013	0.775460267	0.03650578	6.961163714	85.63458581	0.5	PRJNA827465;Yang2025_Silkie_Wenchang	CRA004775	0.943815265
ENSGALG00010008516	MRE11	2	0.172565049	0.086272364	2.000235546	0.045474835	0	0.512151768	0	1	CRA004775;Yang2025_Silkie_Wenchang	PRJNA827465	0.367681022
ENSGALG00010008516	MRE11	2	-0.090263507	0.059527855	-1.516323858	0.129437482	0	0.167448123	0	0.5	CRA004775;PRJNA827465	Yang2025_Silkie_Wenchang	0.603529077
ENSGALG00010008522	NUDT12	2	-0.073437678	0.125025991	-0.587379293	0.556948997	0	0.328397895	0	1	PRJNA827465;Yang2025_Silkie_Wenchang	CRA004775	0.880521579
ENSGALG00010008522	NUDT12	2	-0.122486177	0.182841875	-0.669902214	0.502920128	0.016652592	1.267700013	21.11698435	1	CRA004775;Yang2025_Silkie_Wenchang	PRJNA827465	0.863050365
ENSGALG00010008522	NUDT12	2	-0.208263871	0.152618582	-1.364603629	0.172377664	0	0.519451215	0	1	CRA004775;PRJNA827465	Yang2025_Silkie_Wenchang	0.675240867
ENSGALG00010008527	CHUK	2	-0.032895803	0.049118513	-0.66972311	0.503034317	0.0019964	1.587611249	37.01228806	0.5	PRJNA827465;Yang2025_Silkie_Wenchang	CRA004775	0.863050365
ENSGALG00010008527	CHUK	2	-0.136857995	0.062240421	-2.198860347	0.027887854	0	0.970107603	0	1	CRA004775;Yang2025_Silkie_Wenchang	PRJNA827465	0.284557362
ENSGALG00010008527	CHUK	2	-0.092306595	0.119605404	-0.771759403	0.440256941	0.021585996	3.496825834	71.40263635	0.5	CRA004775;PRJNA827465	Yang2025_Silkie_Wenchang	0.844563576
ENSGALG00010008534	RAB33B	2	-0.156637061	0.126576651	-1.237487797	0.215906042	0.029609046	13.08615112	92.3583337	1	PRJNA827465;Yang2025_Silkie_Wenchang	CRA004775	0.729267374
ENSGALG00010008534	RAB33B	2	-0.00031079	0.326452753	-0.000952022	0.999240396	0.18505351	6.801678787	85.29774735	0.5	CRA004775;Yang2025_Silkie_Wenchang	PRJNA827465	0.999889665
ENSGALG00010008534	RAB33B	2	0.09730823	0.189296741	0.51405127	0.607216146	0.051023773	2.635913685	62.06249067	0.5	CRA004775;PRJNA827465	Yang2025_Silkie_Wenchang	0.896998866
ENSGALG00010008542	MOCOS	2	0.096233779	0.116901996	0.823200476	0.410393998	0	0.037753803	0	1	PRJNA827465;Yang2025_Silkie_Wenchang	CRA004775	0.834820782
ENSGALG00010008542	MOCOS	2	0.20397018	0.168518719	1.210371054	0.226136544	0	0.692103234	0	1	CRA004775;Yang2025_Silkie_Wenchang	PRJNA827465	0.739595164
ENSGALG00010008542	MOCOS	2	0.136747418	0.135907043	1.006183452	0.314327331	0.000205337	1.002894435	0.288608108	1	CRA004775;PRJNA827465	Yang2025_Silkie_Wenchang	0.796531448
ENSGALG00010008561	EEF1D	2	-0.144881631	0.127651922	-1.134974149	0.25638615	0.015553355	1.509947915	33.77255002	1	PRJNA827465;Yang2025_Silkie_Wenchang	CRA004775	0.764677554
ENSGALG00010008561	EEF1D	2	-0.318629874	0.18765128	-1.697989342	0.089509773	0	0.178589006	0	1	CRA004775;Yang2025_Silkie_Wenchang	PRJNA827465	0.513242777
ENSGALG00010008561	EEF1D	2	-0.082780221	0.072752269	-1.137836954	0.255188571	0	0.188202761	0	1	CRA004775;PRJNA827465	Yang2025_Silkie_Wenchang	0.764467569
ENSGALG00010008580	PANX1	2	-0.228853888	0.203407798	-1.125098888	0.260547133	0.020593096	1.275166796	21.57888656	0.5	PRJNA827465;Yang2025_Silkie_Wenchang	CRA004775	0.767694434
ENSGALG00010008580	PANX1	2	-0.228732461	0.341733518	-0.669329899	0.503285058	0.095927789	1.675290714	40.30886748	0.5	CRA004775;Yang2025_Silkie_Wenchang	PRJNA827465	0.863108263
ENSGALG00010008580	PANX1	2	-0.414897698	0.180479391	-2.298864677	0.021512625	0	0.298420921	0	1	CRA004775;PRJNA827465	Yang2025_Silkie_Wenchang	0.248109211
ENSGALG00010008588	PYCR3	2	-0.090813469	0.093628873	-0.969930175	0.332081298	0	0.763608062	0	0.5	PRJNA827465;Yang2025_Silkie_Wenchang	CRA004775	0.802002658
ENSGALG00010008588	PYCR3	2	-0.350233605	0.467271309	-0.749529446	0.453538158	0.364904781	6.044984302	83.45735985	0.5	CRA004775;Yang2025_Silkie_Wenchang	PRJNA827465	0.848950336
ENSGALG00010008588	PYCR3	2	-0.425406422	0.35007008	-1.215203602	0.224288447	0.201972595	5.11333956	80.44330934	1	CRA004775;PRJNA827465	Yang2025_Silkie_Wenchang	0.737471949
ENSGALG00010008592	KIAA0513	2	-0.137439627	0.071120641	-1.932485787	0.053299575	0	0.094280843	0	1	PRJNA827465;Yang2025_Silkie_Wenchang	CRA004775	0.400004977
ENSGALG00010008592	KIAA0513	2	-0.17528209	0.099989029	-1.753013216	0.079599739	0	0.069889017	0	1	CRA004775;Yang2025_Silkie_Wenchang	PRJNA827465	0.48542321
ENSGALG00010008592	KIAA0513	2	-0.133854516	0.086939138	-1.539634726	0.123649416	0	0.196030809	0	1	CRA004775;PRJNA827465	Yang2025_Silkie_Wenchang	0.589709136
ENSGALG00010008596	ENSGALG00010008596	2	0.244567471	0.32393346	0.754992926	0.450253227	0.191080508	10.94027303	90.85946029	0.5	PRJNA827465;Yang2025_Silkie_Wenchang	CRA004775	0.847564661
ENSGALG00010008596	ENSGALG00010008596	2	0.304362317	0.381915424	0.796936436	0.425487948	0.195034012	2.545251727	60.71115523	0.5	CRA004775;Yang2025_Silkie_Wenchang	PRJNA827465	0.84125685
ENSGALG00010008596	ENSGALG00010008596	2	-0.071229176	0.096084056	-0.741321494	0.458498532	0	0.100386132	0	1	CRA004775;PRJNA827465	Yang2025_Silkie_Wenchang	0.850089963
ENSGALG00010008618	EIF4EBP3	2	0.17058133	0.230903219	0.738756827	0.460054672	0.093905205	7.790970008	87.16462778	0.5	PRJNA827465;Yang2025_Silkie_Wenchang	CRA004775	0.850104868
ENSGALG00010008618	EIF4EBP3	2	0.377792693	0.130884104	2.88646735	0.003895932	0	0.308009314	0	1	CRA004775;Yang2025_Silkie_Wenchang	PRJNA827465	0.089807509
ENSGALG00010008618	EIF4EBP3	2	0.029089692	0.1268216	0.2293749	0.818577543	0.017652673	1.600908841	37.53548143	0.5	CRA004775;PRJNA827465	Yang2025_Silkie_Wenchang	0.955256039
ENSGALG00010008623	TRPS1	2	0.209945111	0.308620053	0.680270478	0.496333214	0.161926125	6.256612635	84.01691045	0.5	PRJNA827465;Yang2025_Silkie_Wenchang	CRA004775	0.860939846
ENSGALG00010008623	TRPS1	2	0.597001207	0.201412219	2.964076418	0.003035929	0	0.169424459	0	1	CRA004775;Yang2025_Silkie_Wenchang	PRJNA827465	0.075885251
ENSGALG00010008623	TRPS1	2	0.235198041	0.396806605	0.592727131	0.553363781	0.238761995	3.385855837	70.46536982	0.5	CRA004775;PRJNA827465	Yang2025_Silkie_Wenchang	0.879487916
ENSGALG00010008636	AGTPBP1	2	0.149714468	0.162255004	0.92271094	0.356157863	0	0.700696157	0	0.5	PRJNA827465;Yang2025_Silkie_Wenchang	CRA004775	0.812471559
ENSGALG00010008636	AGTPBP1	2	0.03749239	0.217921776	0.172045176	0.863402013	0	0.177710669	0	0.5	CRA004775;Yang2025_Silkie_Wenchang	PRJNA827465	0.966256632
ENSGALG00010008636	AGTPBP1	2	0.234772307	0.180101494	1.30355558	0.192385154	0	0.039583844	0	1	CRA004775;PRJNA827465	Yang2025_Silkie_Wenchang	0.702137035
ENSGALG00010008653	ENSGALG00010008653	2	-0.128557766	0.093404383	-1.376356885	0.168711169	0	0.246435741	0	1	PRJNA827465;Yang2025_Silkie_Wenchang	CRA004775	0.669861707
ENSGALG00010008653	ENSGALG00010008653	2	0.031525831	0.279897408	0.112633522	0.910321108	0.100211609	2.694928791	62.89326815	0.5	CRA004775;Yang2025_Silkie_Wenchang	PRJNA827465	0.97880407
ENSGALG00010008653	ENSGALG00010008653	2	0.047963092	0.213945356	0.224183841	0.822614256	0.057435997	2.26301422	55.81114819	0.5	CRA004775;PRJNA827465	Yang2025_Silkie_Wenchang	0.955822987
ENSGALG00010008656	CCDC6	2	0.106702882	0.2716311	0.392822771	0.694450385	0.128399467	6.931122542	85.57232261	0.5	PRJNA827465;Yang2025_Silkie_Wenchang	CRA004775	0.920728119
ENSGALG00010008656	CCDC6	2	0.366004586	0.138861796	2.635747177	0.008395226	0	0.114608407	0	1	CRA004775;Yang2025_Silkie_Wenchang	PRJNA827465	0.142771619
ENSGALG00010008656	CCDC6	2	0.057254412	0.22368064	0.255964986	0.797977874	0.083489039	5.303233862	81.14358096	0.5	CRA004775;PRJNA827465	Yang2025_Silkie_Wenchang	0.949922217
ENSGALG00010008658	TSTA3	2	0.059485886	0.093506088	0.636171257	0.524664805	0	0.49785163	0	1	PRJNA827465;Yang2025_Silkie_Wenchang	CRA004775	0.869823294
ENSGALG00010008658	TSTA3	2	-0.007780681	0.222768977	-0.034927128	0.972137848	0.049122356	1.809127419	44.72473361	0.5	CRA004775;Yang2025_Silkie_Wenchang	PRJNA827465	0.991955348
ENSGALG00010008658	TSTA3	2	-0.026285856	0.106992571	-0.245679265	0.805930524	0	0.99905427	0	0.5	CRA004775;PRJNA827465	Yang2025_Silkie_Wenchang	0.952063038
ENSGALG00010008666	SLC16A9	2	-0.439738507	0.187509011	-2.345159327	0.01901895	0.059371003	6.418286607	84.41951784	1	PRJNA827465;Yang2025_Silkie_Wenchang	CRA004775	0.230995614
ENSGALG00010008666	SLC16A9	2	-0.345637073	0.426086123	-0.811190636	0.417256203	0.25031303	2.421024006	58.6951638	0.5	CRA004775;Yang2025_Silkie_Wenchang	PRJNA827465	0.838112595
ENSGALG00010008666	SLC16A9	2	-0.238494136	0.099279425	-2.402251395	0.016294506	0	0.857889705	0	0.5	CRA004775;PRJNA827465	Yang2025_Silkie_Wenchang	0.211002492
ENSGALG00010008668	ENSGALG00010008668	2	0.191303041	0.235160182	0.81350099	0.415930879	0	0.006190822	0	1	PRJNA827465;Yang2025_Silkie_Wenchang	CRA004775	0.837742614
ENSGALG00010008668	ENSGALG00010008668	2	0.119995354	0.310273098	0.386741082	0.698947899	0	0.037550551	0	1	CRA004775;Yang2025_Silkie_Wenchang	PRJNA827465	0.92185597
ENSGALG00010008668	ENSGALG00010008668	2	0.164204445	0.254097143	0.646227039	0.518132331	0	0.077249124	0	1	CRA004775;PRJNA827465	Yang2025_Silkie_Wenchang	0.868333404
ENSGALG00010008674	RXFP1	2	-0.277960381	0.127759501	-2.17565331	0.029581194	0	0.104949381	0	1	PRJNA827465;Yang2025_Silkie_Wenchang	CRA004775	0.293445145
ENSGALG00010008674	RXFP1	2	-0.336141221	0.190334943	-1.76605103	0.077387288	0	0.009356664	0	1	CRA004775;Yang2025_Silkie_Wenchang	PRJNA827465	0.479060101
ENSGALG00010008674	RXFP1	2	-0.256412059	0.157123472	-1.631914419	0.102697516	0	0.061884112	0	1	CRA004775;PRJNA827465	Yang2025_Silkie_Wenchang	0.546039025
ENSGALG00010008686	ENSGALG00010008686	2	-0.121237839	0.275060868	-0.44076731	0.659381462	0	0.698085462	0	0.5	PRJNA827465;Yang2025_Silkie_Wenchang	CRA004775	0.911449691
ENSGALG00010008686	ENSGALG00010008686	2	-0.122600471	0.533757958	-0.229693009	0.818330328	0.172856012	1.277412815	21.71677094	0.5	CRA004775;Yang2025_Silkie_Wenchang	PRJNA827465	0.955256039
ENSGALG00010008686	ENSGALG00010008686	2	-0.383694924	0.330727977	-1.160152605	0.245986679	0	0.531434493	0	1	CRA004775;PRJNA827465	Yang2025_Silkie_Wenchang	0.75661406
ENSGALG00010008688	HBEGF	2	0.257099817	0.321677829	0.799246307	0.424147606	0.16278742	4.59615922	78.24270326	0.5	PRJNA827465;Yang2025_Silkie_Wenchang	CRA004775	0.840447358
ENSGALG00010008688	HBEGF	2	0.521061315	0.226663644	2.298830579	0.021514562	0	0.745533464	0	1	CRA004775;Yang2025_Silkie_Wenchang	PRJNA827465	0.248109211
ENSGALG00010008688	HBEGF	2	-0.034221963	0.166441365	-0.205609724	0.837095781	0	0.024711748	0	0.5	CRA004775;PRJNA827465	Yang2025_Silkie_Wenchang	0.958691517
ENSGALG00010008690	YIF1B	2	-0.025349911	0.061733836	-0.410632362	0.68134213	0	0.779023834	0	0.5	PRJNA827465;Yang2025_Silkie_Wenchang	CRA004775	0.918242009
ENSGALG00010008690	YIF1B	2	-0.127906404	0.253539158	-0.504483823	0.613921427	0.095033261	3.831720565	73.9020635	0.5	CRA004775;Yang2025_Silkie_Wenchang	PRJNA827465	0.898256044
ENSGALG00010008690	YIF1B	2	-0.169546811	0.163582323	-1.036461695	0.299986801	0.037808109	2.898239969	65.49630083	1	CRA004775;PRJNA827465	Yang2025_Silkie_Wenchang	0.790134113
ENSGALG00010008693	C4orf46	2	-0.162897761	0.068969224	-2.361890602	0.018182007	0	0.05576025	0	1	PRJNA827465;Yang2025_Silkie_Wenchang	CRA004775	0.225144112
ENSGALG00010008693	C4orf46	2	-0.056851908	0.168741257	-0.336917653	0.736178978	0	0.066253118	0	1	CRA004775;Yang2025_Silkie_Wenchang	PRJNA827465	0.933661344
ENSGALG00010008693	C4orf46	2	-0.153889478	0.068339323	-2.251843772	0.024332146	0	0.396352175	0	1	CRA004775;PRJNA827465	Yang2025_Silkie_Wenchang	0.266745744
ENSGALG00010008696	PFN4	2	0.392535886	0.390741983	1.004591015	0.315093826	0.218068207	3.319056023	69.87095147	1	PRJNA827465;Yang2025_Silkie_Wenchang	CRA004775	0.796635323
ENSGALG00010008696	PFN4	2	0.48096132	0.429947539	1.118651176	0.263288982	0.17793239	1.881319269	46.84581099	0.5	CRA004775;Yang2025_Silkie_Wenchang	PRJNA827465	0.768643821
ENSGALG00010008696	PFN4	2	0.042986338	0.201807677	0.213006456	0.831321927	0	0.02129433	0	0.5	CRA004775;PRJNA827465	Yang2025_Silkie_Wenchang	0.957626151
ENSGALG00010008699	NAA35	2	0.159375084	0.104707378	1.522099854	0.127984063	5.52479E-05	1.002461541	0.245549718	1	PRJNA827465;Yang2025_Silkie_Wenchang	CRA004775	0.60032911
ENSGALG00010008699	NAA35	2	0.118582413	0.221478756	0.53541213	0.592364904	0.049842086	1.86718222	46.44336321	0.5	CRA004775;Yang2025_Silkie_Wenchang	PRJNA827465	0.892776493
ENSGALG00010008699	NAA35	2	0.026285957	0.124726896	0.210748106	0.833083835	0	0.585298759	0	0.5	CRA004775;PRJNA827465	Yang2025_Silkie_Wenchang	0.95792089
ENSGALG00010008700	ENSGALG00010008700	2	-0.067789662	0.20650337	-0.328273875	0.742704595	0.065984651	4.392681225	77.2348607	0.5	PRJNA827465;Yang2025_Silkie_Wenchang	CRA004775	0.935621057
ENSGALG00010008700	ENSGALG00010008700	2	0.013791663	0.173724709	0.079388032	0.936723988	0.025057871	1.633604939	38.78568947	0.5	CRA004775;Yang2025_Silkie_Wenchang	PRJNA827465	0.985038972
ENSGALG00010008700	ENSGALG00010008700	2	-0.254003968	0.111338255	-2.281371919	0.022526448	0	0.040339317	0	1	CRA004775;PRJNA827465	Yang2025_Silkie_Wenchang	0.255146108
ENSGALG00010008708	ZC3H4	2	-0.088680813	0.116136592	-0.763590631	0.445111234	0.010000876	1.417664213	29.46143448	0.5	PRJNA827465;Yang2025_Silkie_Wenchang	CRA004775	0.846013379
ENSGALG00010008708	ZC3H4	2	0.039452461	0.139636123	0.282537639	0.77753129	0	0.188775753	0	0.5	CRA004775;Yang2025_Silkie_Wenchang	PRJNA827465	0.944383704
ENSGALG00010008708	ZC3H4	2	-0.134688497	0.083728257	-1.608638492	0.107695412	0	0.410319535	0	1	CRA004775;PRJNA827465	Yang2025_Silkie_Wenchang	0.555333613
ENSGALG00010008716	TATDN1	2	0.21539461	0.249926578	0.861831547	0.388780222	0.117588882	16.94209898	94.09754363	0.5	PRJNA827465;Yang2025_Silkie_Wenchang	CRA004775	0.828400391
ENSGALG00010008716	TATDN1	2	0.359884295	0.180768335	1.990859149	0.046496374	0.035180919	1.736406305	42.40979217	1	CRA004775;Yang2025_Silkie_Wenchang	PRJNA827465	0.372340465
ENSGALG00010008716	TATDN1	2	-0.024924407	0.069951906	-0.356307763	0.721610097	0	0.094069902	0	0.5	CRA004775;PRJNA827465	Yang2025_Silkie_Wenchang	0.929182637
ENSGALG00010008729	TMEM254	2	-0.07345323	0.373852404	-0.196476549	0.844237186	0.258346789	13.18827596	92.41750777	0.5	PRJNA827465;Yang2025_Silkie_Wenchang	CRA004775	0.960723102
ENSGALG00010008729	TMEM254	2	-0.405949295	0.12502274	-3.247003665	0.001166269	0	0.259681427	0	1	CRA004775;Yang2025_Silkie_Wenchang	PRJNA827465	0.041557449
ENSGALG00010008729	TMEM254	2	0.016331541	0.304682985	0.053601749	0.957252463	0.152777986	5.488678985	81.78067978	0.5	CRA004775;PRJNA827465	Yang2025_Silkie_Wenchang	0.988541306
ENSGALG00010008731	NDUFB9	2	0.014817365	0.050260633	0.294810558	0.768138601	0	0.323740507	0	1	PRJNA827465;Yang2025_Silkie_Wenchang	CRA004775	0.941444269
ENSGALG00010008731	NDUFB9	2	0.088981335	0.171592008	0.518563396	0.60406524	0	0.120038237	0	0.5	CRA004775;Yang2025_Silkie_Wenchang	PRJNA827465	0.896058435
ENSGALG00010008731	NDUFB9	2	0.00742506	0.051107682	0.145282651	0.884487712	0	0.000600528	0	0.5	CRA004775;PRJNA827465	Yang2025_Silkie_Wenchang	0.972062774
ENSGALG00010008739	VSTM5	2	0.037379853	0.167166613	0.223608362	0.823062057	0	0.000547594	0	1	PRJNA827465;Yang2025_Silkie_Wenchang	CRA004775	0.955822987
ENSGALG00010008739	VSTM5	2	-0.078705952	0.281503998	-0.279590885	0.779791401	0	0.682933587	0	0.5	CRA004775;Yang2025_Silkie_Wenchang	PRJNA827465	0.944967824
ENSGALG00010008739	VSTM5	2	-0.01814147	0.186861719	-0.097084998	0.922658894	0	0.76445995	0	0.5	CRA004775;PRJNA827465	Yang2025_Silkie_Wenchang	0.981804675
ENSGALG00010008745	GRAMD4	2	-0.070387505	0.073463473	-0.958129284	0.337997571	0.00437932	1.479895031	32.42763986	1	PRJNA827465;Yang2025_Silkie_Wenchang	CRA004775	0.80415385
ENSGALG00010008745	GRAMD4	2	0.257228872	0.511760902	0.502634865	0.615221012	0.446181156	6.105580141	83.62154002	0.5	CRA004775;Yang2025_Silkie_Wenchang	PRJNA827465	0.8988191
ENSGALG00010008745	GRAMD4	2	0.319210987	0.424892736	0.751274285	0.452487602	0.295962579	4.638587442	78.44171286	0.5	CRA004775;PRJNA827465	Yang2025_Silkie_Wenchang	0.848321192
ENSGALG00010008750	PFDN1	2	0.35360805	0.423950064	0.834079483	0.404236196	0.34028308	18.27190487	94.52711686	0.5	PRJNA827465;Yang2025_Silkie_Wenchang	CRA004775	0.833112102
ENSGALG00010008750	PFDN1	2	0.221187145	0.579826497	0.381471261	0.702853592	0.630903313	16.16763329	93.81480281	0.5	CRA004775;Yang2025_Silkie_Wenchang	PRJNA827465	0.923345143
ENSGALG00010008750	PFDN1	2	-0.139833287	0.13880737	-1.007390946	0.313746962	0.020483054	1.732870801	42.29229326	1	CRA004775;PRJNA827465	Yang2025_Silkie_Wenchang	0.796531448
ENSGALG00010008752	GOLM1	2	-0.733061291	0.142843503	-5.13191903	2.86803E-07	0.002133687	1.054948164	5.208612715	1	PRJNA827465;Yang2025_Silkie_Wenchang	CRA004775	8.08275E-05
ENSGALG00010008752	GOLM1	2	-0.632501161	0.281533485	-2.246628537	0.024663777	0.101663107	2.730642077	63.37857647	1	CRA004775;Yang2025_Silkie_Wenchang	PRJNA827465	0.268408231
ENSGALG00010008752	GOLM1	2	-0.508313952	0.156782206	-3.2421661	0.001186249	0	0.698176432	0	1	CRA004775;PRJNA827465	Yang2025_Silkie_Wenchang	0.041814466
ENSGALG00010008756	ITSN2	2	-0.025505154	0.219890829	-0.115990074	0.907660408	0.079059452	4.935428129	79.73833326	0.5	PRJNA827465;Yang2025_Silkie_Wenchang	CRA004775	0.978196186
ENSGALG00010008756	ITSN2	2	0.094779166	0.170709099	0.555208636	0.578751963	0.012343366	1.253235675	20.20654856	0.5	CRA004775;Yang2025_Silkie_Wenchang	PRJNA827465	0.887883539
ENSGALG00010008756	ITSN2	2	-0.206670807	0.064903479	-3.18427936	0.001451149	0	0.138253335	0	1	CRA004775;PRJNA827465	Yang2025_Silkie_Wenchang	0.047213999
ENSGALG00010008762	SAE1	2	-0.00918521	0.084365246	-0.108874336	0.913302162	0.007273036	1.571245595	36.35622573	0.5	PRJNA827465;Yang2025_Silkie_Wenchang	CRA004775	0.979169142
ENSGALG00010008762	SAE1	2	-0.005283355	0.189784316	-0.027838736	0.977790771	0.034989233	1.850293952	45.95453339	0.5	CRA004775;Yang2025_Silkie_Wenchang	PRJNA827465	0.993739602
ENSGALG00010008762	SAE1	2	-0.061704147	0.043854134	-1.407031478	0.159418055	0	0.549904641	0	1	CRA004775;PRJNA827465	Yang2025_Silkie_Wenchang	0.654987226
ENSGALG00010008764	ETFDH	2	-0.070639979	0.13957925	-0.506092269	0.612791878	0.031961519	5.554453324	81.99642806	0.5	PRJNA827465;Yang2025_Silkie_Wenchang	CRA004775	0.898034557
ENSGALG00010008764	ETFDH	2	0.115098969	0.103135418	1.115998478	0.264422786	0.004757127	1.129298719	11.44947006	1	CRA004775;Yang2025_Silkie_Wenchang	PRJNA827465	0.768790489
ENSGALG00010008764	ETFDH	2	0.023711649	0.278845299	0.085035142	0.932233453	0.12497321	4.467444713	77.61583938	0.5	CRA004775;PRJNA827465	Yang2025_Silkie_Wenchang	0.984202262
ENSGALG00010008768	PRXL2C	2	0.045838402	0.117028936	0.391684346	0.695291458	0	0.018941747	0	1	PRJNA827465;Yang2025_Silkie_Wenchang	CRA004775	0.921038437
ENSGALG00010008768	PRXL2C	2	0.02892603	0.165587571	0.174687204	0.861325423	0	0.187087407	0	0.5	CRA004775;Yang2025_Silkie_Wenchang	PRJNA827465	0.965343309
ENSGALG00010008768	PRXL2C	2	0.011741006	0.138929044	0.084510811	0.932650307	0	0.133303692	0	0.5	CRA004775;PRJNA827465	Yang2025_Silkie_Wenchang	0.984224753
ENSGALG00010008775	MED17	2	0.001370706	0.044058822	0.031110815	0.975181165	0	0.92795476	0	0.5	PRJNA827465;Yang2025_Silkie_Wenchang	CRA004775	0.992684416
ENSGALG00010008775	MED17	2	-0.110042217	0.082530729	-1.333348415	0.182417492	0	0.277984592	0	1	CRA004775;Yang2025_Silkie_Wenchang	PRJNA827465	0.690508977
ENSGALG00010008775	MED17	2	-0.024912882	0.085750822	-0.290526456	0.771413514	0.007195428	1.546950863	35.35670562	0.5	CRA004775;PRJNA827465	Yang2025_Silkie_Wenchang	0.94283287
ENSGALG00010008780	KARS1	2	-0.055203543	0.123481964	-0.44705754	0.654833515	0.024330307	4.44610137	77.50838506	0.5	PRJNA827465;Yang2025_Silkie_Wenchang	CRA004775	0.910222014
ENSGALG00010008780	KARS1	2	-0.050161618	0.161507313	-0.310584193	0.756116745	0.036669168	3.333410122	70.00069108	0.5	CRA004775;Yang2025_Silkie_Wenchang	PRJNA827465	0.93854559
ENSGALG00010008780	KARS1	2	0.055101532	0.04014016	1.372728264	0.169836839	0	0.256667309	0	1	CRA004775;PRJNA827465	Yang2025_Silkie_Wenchang	0.671232541
ENSGALG00010008782	CCDC9	2	0.621988823	0.552795338	1.125170167	0.260516932	0.529703385	7.501025477	86.66848949	1	PRJNA827465;Yang2025_Silkie_Wenchang	CRA004775	0.767694434
ENSGALG00010008782	CCDC9	2	0.52813524	0.640899199	0.824035519	0.409909149	0.746883228	11.00707791	90.91493666	0.5	CRA004775;Yang2025_Silkie_Wenchang	PRJNA827465	0.834652129
ENSGALG00010008782	CCDC9	2	-0.029650963	0.19665981	-0.150772862	0.880154894	0	0.199147797	0	0.5	CRA004775;PRJNA827465	Yang2025_Silkie_Wenchang	0.971569381
ENSGALG00010008790	GPN1	2	-0.105127109	0.135988061	-0.773061314	0.439486095	0.029506799	4.896309419	79.57645413	0.5	PRJNA827465;Yang2025_Silkie_Wenchang	CRA004775	0.844246008
ENSGALG00010008790	GPN1	2	-0.219817159	0.086450709	-2.542687756	0.011000348	0	0.407788848	0	1	CRA004775;Yang2025_Silkie_Wenchang	PRJNA827465	0.166811819
ENSGALG00010008790	GPN1	2	0.005436436	0.070143272	0.077504746	0.938222016	0	0.448111203	0	0.5	CRA004775;PRJNA827465	Yang2025_Silkie_Wenchang	0.985349045
ENSGALG00010008793	C11orf54	2	0.005663013	0.152107618	0.037230304	0.970301376	0.029442847	2.60351483	61.59038587	0.5	PRJNA827465;Yang2025_Silkie_Wenchang	CRA004775	0.991681174
ENSGALG00010008793	C11orf54	2	-0.035706633	0.24052813	-0.148450963	0.881986883	0.080952186	3.28874018	69.59321973	0.5	CRA004775;Yang2025_Silkie_Wenchang	PRJNA827465	0.971928118
ENSGALG00010008793	C11orf54	2	-0.152931792	0.08951867	-1.708378737	0.08756609	0	0.55886936	0	1	CRA004775;PRJNA827465	Yang2025_Silkie_Wenchang	0.508888073

ENSGALG00010008799	ENSGALG00010008799	2	-0.007851357	0.105492832	-0.074425498	0.940671821	0	0.899381624	0	0.5	PRJNA827465;Yang2025_Silkie_Wenchang	CRA004775	0.985565295
ENSGALG00010008799	ENSGALG00010008799	2	0.456004653	0.245778207	1.855350226	0.06354623	0.003477327	1.027815034	2.706229505	1	CRA004775;Yang2025_Silkie_Wenchang	PRJNA827465	0.434131321
ENSGALG00010008799	ENSGALG00010008799	2	0.275311828	0.39839425	0.691053719	0.489531783	0.248432075	3.938990739	74.61278621	0.5	CRA004775;PRJNA827465	Yang2025_Silkie_Wenchang	0.858419004
ENSGALG00010008807	BRD1	2	-0.066039633	0.051094064	-1.292510874	0.196180282	0	0.077879971	0	1	PRJNA827465;Yang2025_Silkie_Wenchang	CRA004775	0.707577649
ENSGALG00010008807	BRD1	2	0.042085762	0.18349393	0.229357791	0.81859084	0.036319491	2.023921345	50.59096529	0.5	CRA004775;Yang2025_Silkie_Wenchang	PRJNA827465	0.955256039
ENSGALG00010008807	BRD1	2	0.035113212	0.152287808	0.230571391	0.817647796	0.029189526	2.034890192	50.85729914	0.5	CRA004775;PRJNA827465	Yang2025_Silkie_Wenchang	0.954951008
ENSGALG00010008811	TERF2IP	2	-0.161250315	0.204310405	-0.78924182	0.429970684	0.07396907	8.322223254	87.98398013	0.5	PRJNA827465;Yang2025_Silkie_Wenchang	CRA004775	0.842012722
ENSGALG00010008811	TERF2IP	2	-0.14614872	0.253640528	-0.576204132	0.564477213	0.103497146	5.019964098	80.07953881	0.5	CRA004775;Yang2025_Silkie_Wenchang	PRJNA827465	0.883116308
ENSGALG00010008811	TERF2IP	2	0.034766616	0.053210708	0.653376308	0.513513709	0	0.25829571	0	1	CRA004775;PRJNA827465	Yang2025_Silkie_Wenchang	0.866402971
ENSGALG00010008814	ENSGALG00010008814	2	0.079170758	0.163316519	0.484768829	0.627840336	0	0.945113654	0	1	PRJNA827465;Yang2025_Silkie_Wenchang	CRA004775	0.901878949
ENSGALG00010008814	ENSGALG00010008814	2	-0.75366655	1.258643558	-0.598792681	0.549311144	2.878444515	10.79204829	90.73391841	0.5	CRA004775;Yang2025_Silkie_Wenchang	PRJNA827465	0.878554604
ENSGALG00010008814	ENSGALG00010008814	2	-0.931725712	1.03159488	-0.903189547	0.366425305	1.925647718	9.785297223	89.78058635	0.5	CRA004775;PRJNA827465	Yang2025_Silkie_Wenchang	0.819255965
ENSGALG00010008821	ENSGALG00010008821	2	0.43008415	0.142949207	3.008650137	0.002624111	0.02025326	1.981963999	49.54499676	1	PRJNA827465;Yang2025_Silkie_Wenchang	CRA004775	0.069264902
ENSGALG00010008821	ENSGALG00010008821	2	0.128377978	0.473167123	0.271316353	0.78614773	0.402319559	9.574290071	89.55536136	0.5	CRA004775;Yang2025_Silkie_Wenchang	PRJNA827465	0.946639719
ENSGALG00010008821	ENSGALG00010008821	2	-0.001944932	0.328582924	-0.005919152	0.995277228	0.172793407	4.732337087	78.86879186	0.5	CRA004775;PRJNA827465	Yang2025_Silkie_Wenchang	0.998269135
ENSGALG00010008824	GREM2	2	-0.777735526	0.190072254	-4.091788839	4.28058E-05	0	0.219659295	0	1	PRJNA827465;Yang2025_Silkie_Wenchang	CRA004775	0.004381244
ENSGALG00010008824	GREM2	2	-1.831637342	1.033453294	-1.772346513	0.076337055	1.953394992	11.28203335	91.13634955	1	CRA004775;Yang2025_Silkie_Wenchang	PRJNA827465	0.476766627
ENSGALG00010008824	GREM2	2	-1.750576006	1.123726572	-1.557830927	0.119273333	2.332346275	12.74632817	92.15460338	1	CRA004775;PRJNA827465	Yang2025_Silkie_Wenchang	0.579671051
ENSGALG00010008825	PPID	2	-0.030308349	0.047601044	-0.636716051	0.524309816	0	0.483638963	0	0.5	PRJNA827465;Yang2025_Silkie_Wenchang	CRA004775	0.869790047
ENSGALG00010008825	PPID	2	0.015669973	0.130341215	0.120222702	0.904306735	0	0.506428593	0	0.5	CRA004775;Yang2025_Silkie_Wenchang	PRJNA827465	0.977586231
ENSGALG00010008825	PPID	2	-0.045079341	0.049296245	-0.914457913	0.360476283	0	0.154637646	0	1	CRA004775;PRJNA827465	Yang2025_Silkie_Wenchang	0.814880744
ENSGALG00010008830	DHX34	2	0.106135097	0.228086765	0.465327732	0.641696778	0.091112771	7.816228796	87.20610634	0.5	PRJNA827465;Yang2025_Silkie_Wenchang	CRA004775	0.906634523
ENSGALG00010008830	DHX34	2	-0.013491983	0.403450162	-0.033441512	0.973322507	0.27437492	6.003525383	83.34312031	0.5	CRA004775;Yang2025_Silkie_Wenchang	PRJNA827465	0.992392211
ENSGALG00010008830	DHX34	2	-0.167830956	0.13298617	-1.262018121	0.206942266	0.014655376	1.304603917	23.34838286	1	CRA004775;PRJNA827465	Yang2025_Silkie_Wenchang	0.718911823
ENSGALG00010008832	MTSS1	2	0.230317123	0.060232597	3.823795295	0.000131413	0	0.319191667	0	1	PRJNA827465;Yang2025_Silkie_Wenchang	CRA004775	0.009476929
ENSGALG00010008832	MTSS1	2	0.209618482	0.15956784	1.313663715	0.188959397	0.019224093	1.252950148	20.1883649	0.5	CRA004775;Yang2025_Silkie_Wenchang	PRJNA827465	0.698162702
ENSGALG00010008832	MTSS1	2	0.193094304	0.072124318	2.677242709	0.007423084	0	0.905024853	0	0.5	CRA004775;PRJNA827465	Yang2025_Silkie_Wenchang	0.132915249
ENSGALG00010008837	ENSGALG00010008837	2	0.233070203	0.259347314	0.89867984	0.368823218	0.121708142	10.3344015	90.32358091	0.5	PRJNA827465;Yang2025_Silkie_Wenchang	CRA004775	0.820458397
ENSGALG00010008837	ENSGALG00010008837	2	0.254020853	0.303629655	0.83661411	0.402809512	0.135756763	3.428086553	70.82920794	0.5	CRA004775;Yang2025_Silkie_Wenchang	PRJNA827465	0.832937979
ENSGALG00010008837	ENSGALG00010008837	2	-0.023368568	0.083500359	-0.279861883	0.779583472	0	0.099217033	0	1	CRA004775;PRJNA827465	Yang2025_Silkie_Wenchang	0.944807046
ENSGALG00010008839	ZNF512	2	0.000175087	0.054306949	0.003224032	0.997427599	0.002922014	1.828637399	45.31447292	0.5	PRJNA827465;Yang2025_Silkie_Wenchang	CRA004775	0.999290321
ENSGALG00010008839	ZNF512	2	0.055289161	0.067575004	0.818189527	0.413248974	0	0.395083177	0	0.5	CRA004775;Yang2025_Silkie_Wenchang	PRJNA827465	0.83606052
ENSGALG00010008839	ZNF512	2	-0.040214786	0.03944766	-1.019446669	0.30799096	0	0.000407462	0	1	CRA004775;PRJNA827465	Yang2025_Silkie_Wenchang	0.793278812
ENSGALG00010008846	SLC35D2	2	0.625080888	0.294080095	2.125546404	0.033541046	0.099987014	2.366974248	57.75196959	1	PRJNA827465;Yang2025_Silkie_Wenchang	CRA004775	0.314546348
ENSGALG00010008846	SLC35D2	2	0.117207412	0.906829979	0.1292496	0.89716015	1.401876008	6.32388468	84.18693492	0.5	CRA004775;Yang2025_Silkie_Wenchang	PRJNA827465	0.975346564
ENSGALG00010008846	SLC35D2	2	-0.124487254	0.598810867	-0.207890773	0.835314253	0.506494057	2.963428333	66.25530002	0.5	CRA004775;PRJNA827465	Yang2025_Silkie_Wenchang	0.9583267
ENSGALG00010008850	CYSTM1	2	-0.206923817	0.080883053	-2.558308676	0.010518268	0	0.195773864	0	1	PRJNA827465;Yang2025_Silkie_Wenchang	CRA004775	0.16333445
ENSGALG00010008850	CYSTM1	2	-0.226566796	0.161137132	-1.406049574	0.159709405	0	0.282163329	0	1	CRA004775;Yang2025_Silkie_Wenchang	PRJNA827465	0.655404348
ENSGALG00010008850	CYSTM1	2	-0.182870472	0.084696353	-2.159130432	0.030840049	0	0.087154609	0	1	CRA004775;PRJNA827465	Yang2025_Silkie_Wenchang	0.299877897
ENSGALG00010008852	KHDRBS3	2	0.07655594	0.123024201	0.622283578	0.533755417	0.02118632	3.24229762	69.15767406	0.5	PRJNA827465;Yang2025_Silkie_Wenchang	CRA004775	0.873408008
ENSGALG00010008852	KHDRBS3	2	0.223926517	0.107887497	2.075555765	0.037935056	0	0.088528027	0	1	CRA004775;Yang2025_Silkie_Wenchang	PRJNA827465	0.335802143
ENSGALG00010008852	KHDRBS3	2	-0.017475265	0.076352007	-0.228877607	0.818964046	0	0.955823904	0	0.5	CRA004775;PRJNA827465	Yang2025_Silkie_Wenchang	0.955256039
ENSGALG00010008859	ENSGALG00010008859	2	0.576889851	0.339306601	1.700202266	0.089092886	0.039702044	1.208006996	17.21902249	1	PRJNA827465;Yang2025_Silkie_Wenchang	CRA004775	0.512381871
ENSGALG00010008859	ENSGALG00010008859	2	-0.25085305	1.212102399	-0.206956978	0.836043458	2.661729179	10.54704674	90.51867291	0.5	CRA004775;Yang2025_Silkie_Wenchang	PRJNA827465	0.958399882
ENSGALG00010008859	ENSGALG00010008859	2	-0.572785304	0.871554042	-0.657199986	0.511052335	1.25397805	5.639080251	82.26661165	0.5	CRA004775;PRJNA827465	Yang2025_Silkie_Wenchang	0.865588911
ENSGALG00010008860	SQLE	2	0.198056386	0.12898955	1.535445202	0.124674508	0	0.366475684	0	1	PRJNA827465;Yang2025_Silkie_Wenchang	CRA004775	0.592226514
ENSGALG00010008860	SQLE	2	0.055345221	0.234170489	0.236345839	0.81316432	0	0.006746324	0	1	CRA004775;Yang2025_Silkie_Wenchang	PRJNA827465	0.953526116
ENSGALG00010008860	SQLE	2	0.228917179	0.144468353	1.584548966	0.113068848	0	0.15156693	0	1	CRA004775;PRJNA827465	Yang2025_Silkie_Wenchang	0.567481156
ENSGALG00010008862	ZNF367	2	0.041408975	0.108869548	0.380354068	0.703682606	0	6.15793E-05	0	1	PRJNA827465;Yang2025_Silkie_Wenchang	CRA004775	0.923645363
ENSGALG00010008862	ZNF367	2	0.020239707	0.143391261	0.141150212	0.887751277	0	0.13300359	0	0.5	CRA004775;Yang2025_Silkie_Wenchang	PRJNA827465	0.973090153
ENSGALG00010008862	ZNF367	2	0.019554504	0.140847599	0.138834484	0.889580943	0	0.130721916	0	0.5	CRA004775;PRJNA827465	Yang2025_Silkie_Wenchang	0.973153999
ENSGALG00010008864	ADAT1	2	0.370466868	0.085985283	4.308491567	1.64372E-05	0	0.58779791	0	1	PRJNA827465;Yang2025_Silkie_Wenchang	CRA004775	0.002119264
ENSGALG00010008864	ADAT1	2	0.162286448	0.336627615	0.482094873	0.629738552	0.184810471	5.279354896	81.05829178	0.5	CRA004775;Yang2025_Silkie_Wenchang	PRJNA827465	0.902457014
ENSGALG00010008864	ADAT1	2	0.111055683	0.260626073	0.426111179	0.670026835	0.104775544	3.943120285	74.6393737	0.5	CRA004775;PRJNA827465	Yang2025_Silkie_Wenchang	0.914428878
ENSGALG00010008866	WASHC5	2	0.020855433	0.056877856	0.366670524	0.713864796	0.002973232	1.804932664	44.59627109	0.5	PRJNA827465;Yang2025_Silkie_Wenchang	CRA004775	0.92636344
ENSGALG00010008866	WASHC5	2	0.092574672	0.064799107	1.42864117	0.153107395	0	0.025776281	0	1	CRA004775;Yang2025_Silkie_Wenchang	PRJNA827465	0.645187993
ENSGALG00010008866	WASHC5	2	-0.016094978	0.048567893	-0.331391311	0.740348927	0	0.623304768	0	0.5	CRA004775;PRJNA827465	Yang2025_Silkie_Wenchang	0.935128797
ENSGALG00010008871	PRR7	2	0.461455604	0.484838059	0.951772649	0.341212296	0.424459739	9.758453561	89.75247468	1	PRJNA827465;Yang2025_Silkie_Wenchang	CRA004775	0.805646109
ENSGALG00010008871	PRR7	2	0.524394845	0.469109309	1.117852141	0.263630148	0.347506641	4.746063671	78.92990762	1	CRA004775;Yang2025_Silkie_Wenchang	PRJNA827465	0.768643821
ENSGALG00010008871	PRR7	2	0.016190142	0.104166027	0.155426313	0.876485241	0	0.010340759	0	1	CRA004775;PRJNA827465	Yang2025_Silkie_Wenchang	0.970347777
ENSGALG00010008877	FNDC4	2	-0.254966488	0.162063597	-1.573284591	0.115661037	0.023987677	1.821634235	45.10423769	1	PRJNA827465;Yang2025_Silkie_Wenchang	CRA004775	0.572716099
ENSGALG00010008877	FNDC4	2	-0.217293063	0.196211817	-1.107441269	0.268103192	0.023992698	1.394608232	28.2952748	1	CRA004775;Yang2025_Silkie_Wenchang	PRJNA827465	0.771398294
ENSGALG00010008877	FNDC4	2	-0.418136368	0.135484056	-3.086240406	0.002027049	0	0.066841113	0	1	CRA004775;PRJNA827465	Yang2025_Silkie_Wenchang	0.058623319
ENSGALG00010008879	HABP4	2	0.119419093	0.206203916	0.579131062	0.562500742	0.070443906	5.569453709	82.04491765	0.5	PRJNA827465;Yang2025_Silkie_Wenchang	CRA004775	0.881955027
ENSGALG00010008879	HABP4	2	0.295358895	0.140184718	2.106926486	0.035123952	0	0.638361903	0	1	CRA004775;Yang2025_Silkie_Wenchang	PRJNA827465	0.323167978
ENSGALG00010008879	HABP4	2	-0.06051931	0.08131757	-0.744234121	0.456734849	0	0.119031022	0	0.5	CRA004775;PRJNA827465	Yang2025_Silkie_Wenchang	0.849515887
ENSGALG00010008888	FBXO46	2	0.0279041	0.064080888	0.435451207	0.663234948	0	0.54462578	0	1	PRJNA827465;Yang2025_Silkie_Wenchang	CRA004775	0.91293776
ENSGALG00010008888	FBXO46	2	0.09116111	0.119158746	0.765039187	0.44424821	0	0.14957701	0	0.5	CRA004775;Yang2025_Silkie_Wenchang	PRJNA827465	0.846013379
ENSGALG00010008888	FBXO46	2	0.000771195	0.071119291	0.010928053	0.991280849	0	0.001252723	0	0.5	CRA004775;PRJNA827465	Yang2025_Silkie_Wenchang	0.99755571
ENSGALG00010008892	ENSGALG00010008892	2	-0.050452297	0.079070124	-0.638070292	0.523427924	0	0.712725294	0	0.5	PRJNA827465;Yang2025_Silkie_Wenchang	CRA004775	0.869661505
ENSGALG00010008892	ENSGALG00010008892	2	-0.034309526	0.142563217	-0.240661841	0.809817216	0.001813649	1.045819914	4.381243223	0.5	CRA004775;Yang2025_Silkie_Wenchang	PRJNA827465	0.952818762
ENSGALG00010008892	ENSGALG00010008892	2	-0.098863938	0.080923516	-1.221669031	0.221825398	0	0.252111733	0	1	CRA004775;PRJNA827465	Yang2025_Silkie_Wenchang	0.735226622
ENSGALG00010008893	ENSGALG00010008893	2	-0.231415913	0.232239146	-0.996455235	0.319029007	0.090394718	6.164853052	83.7790132	0.5	PRJNA827465;Yang2025_Silkie_Wenchang	CRA004775	0.798079048
ENSGALG00010008893	ENSGALG00010008893	2	-0.002726336	0.13496526	-0.020200279	0.983883605	0	0.053266141	0	1	CRA004775;Yang2025_Silkie_Wenchang	PRJNA827465	0.995579629
ENSGALG00010008893	ENSGALG00010008893	2	-0.446341764	0.123037679	-3.627683537	0.000285975	0	0.279838858	0	1	CRA004775;PRJNA827465	Yang2025_Silkie_Wenchang	0.016522793
ENSGALG00010008897	NSMCE2	2	-0.212111088	0.06794039	-3.122017545	0.001796162	0	0.000213504	0	1	PRJNA827465;Yang2025_Silkie_Wenchang	CRA004775	0.054054034
ENSGALG00010008897	NSMCE2	2	0.183795917	0.433720393	0.42376591	0.671736542	0.324076744	6.82795472	85.35432584	0.5	CRA004775;Yang2025_Silkie_Wenchang	PRJNA827465	0.914674175
ENSGALG00010008897	NSMCE2	2	0.175120927	0.434107402	0.403404609	0.686650597	0.33276875	7.79831309	87.17671388	0.5	CRA004775;PRJNA827465	Yang2025_Silkie_Wenchang	0.919235539
ENSGALG00010008903	ENSGALG00010008903	2	0.07093222	0.190965082	0.371440783	0.710309254	0.002475771	1.035101885	3.391152614	0.5	PRJNA827465;Yang2025_Silkie_Wenchang	CRA004775	0.925364369
ENSGALG00010008903	ENSGALG00010008903	2	0.249954757	0.2330302	1.072630771	0.283437979	0	0.002632906	0	1	CRA004775;Yang2025_Silkie_Wenchang	PRJNA827465	0.73956117
ENSGALG00010008903	ENSGALG00010008903	2	-0.050306029	0.23907312	-0.210421101	0.833339025	0	0.366897719	0	0.5	CRA004775;PRJNA827465	Yang2025_Silkie_Wenchang	0.957968425
ENSGALG00010008905	GABARAPL2	2	-0.551530853	0.546975562	-1.008328143	0.313296957	0.579737294	31.67344192	96.84278077	1	PRJNA827465;Yang2025_Silkie_Wenchang	CRA004775	0.796531448
ENSGALG00010008905	GABARAPL2	2	-0.694263419	0.433319654	-1.60219693	0.109112069	0.329179891	8.00781292	87.51219583	1	CRA004775;Yang2025_Silkie_Wenchang	PRJNA827465	0.559179559
ENSGALG00010008905	GABARAPL2	2	-0.03640213	0.074619348	-0.487837685	0.625664816	0	0.752584737	0	1	CRA004775;PRJNA827465	Yang2025_Silkie_Wenchang	0.901501367
ENSGALG00010008906	DBN1	2	0.045780857	0.077501432	0.590709818	0.554714869	0	0.962444465	0	0.5	PRJNA827465;Yang2025_Silkie_Wenchang	CRA004775	0.880223402
ENSGALG00010008906	DBN1	2	0.142552437	0.130895955	1.089051503	0.276131174	0	0.121585617	0	0.5	CRA004775;Yang2025_Silkie_Wenchang	PRJNA827465	0.775682505
ENSGALG00010008906	DBN1	2	-0.007440634	0.092583735	-0.080366533	0.935948739	0	0.000668615	0	1	CRA004775;PRJNA827465	Yang2025_Silkie_Wenchang	0.984822128
ENSGALG00010008910	TRIB1	2	-0.048336453	0.197484472	-0.244760779	0.806641664	0.061763291	4.506897406	77.81178692	0.5	PRJNA827465;Yang2025_Silkie_Wenchang	CRA004775	0.952273023
ENSGALG00010008910	TRIB1	2	-0.219414414	0.157150044	-1.396209691	0.162651359	0	0.87870713	0	0.5	CRA004775;Yang2025_Silkie_Wenchang	PRJNA827465	0.660469716
ENSGALG00010008910	TRIB1	2	0.12651328	0.08321126	1.520386534	0.128413857	0	0.014253658	0	1	CRA004775;PRJNA827465	Yang2025_Silkie_Wenchang	0.60134038
ENSGALG00010008918	PLAC9	2	-0.137744057	0.164003153	-0.839886641	0.400971949	0.040184543	3.928731742	74.54649323	0.5	PRJNA827465;Yang2025_Silkie_Wenchang	CRA004775	0.832395176
ENSGALG00010008918	PLAC9	2	-0.282958628	0.121009419	-2.338319028	0.019370705	0	0.457118004	0	1	CRA004775;Yang2025_Silkie_Wenchang	PRJNA827465	0.23313687
ENSGALG00010008918	PLAC9	2	0.015680693	0.102508466	0.152969737	0.878422141	0	0.014164201	0	0.5	CRA004775;PRJNA827465	Yang2025_Silkie_Wenchang	0.971037793
ENSGALG00010008921	OPA3	2	0.932807352	0.852212574	1.094571274	0.273700562	1.369910748	17.1383725	94.16514024	1	PRJNA827465;Yang2025_Silkie_Wenchang	CRA004775	0.774415754
ENSGALG00010008921	OPA3	2	0.944239827	0.873430558	1.08107029	0.279665848	1.38638593	10.94670575	90.86483164	1	CRA004775;Yang2025_Silkie_Wenchang	PRJNA827465	0.777788482
ENSGALG00010008921	OPA3	2	0.106678398	0.151292027	0.705115819	0.480738163	0	0.010306226	0	1	CRA004775;PRJNA827465	Yang2025_Silkie_Wenchang	0.856722567
ENSGALG00010008931	FNIP2	2	0.01103466	0.059107422	0.186688223	0.85190509	0	0.010044374	0	1	PRJNA827465;Yang2025_Silkie_Wenchang	CRA004775	0.960255491
ENSGALG00010008931	FNIP2	2	0.042593743	0.109542213	0.388834052	0.697390909	0	0.748812877	0	1	CRA004775;Yang2025_Silkie_Wenchang	PRJNA827465	0.921328007
ENSGALG00010008931	FNIP2	2	0.028775168	0.065899013	0.436655513	0.662361196	0	0.75348541	0	1	CRA004775;PRJNA827465	Yang2025_Silkie_Wenchang	0.91272639
ENSGALG00010008932	VASP	2	0.011768007	0.149127469	0.078912403	0.937102298	0.028478876	2.507619396	60.12153991	0.5	PRJNA827465;Yang2025_Silkie_Wenchang	CRA004775	0.985108291
ENSGALG00010008932	VASP	2	0.211930101	0.161836482	1.309532304	0.190351693	0	0.015450705	0	1	CRA004775;Yang2025_Silkie_Wenchang	PRJNA827465	0.699411386
ENSGALG00010008932	VASP	2	-0.090243164	0.083156516	-1.085220602	0.277823962	0	0.704405767	0	0.5	CRA004775;PRJNA827465	Yang2025_Silkie_Wenchang	0.776763074
ENSGALG00010008936	IFT172	2	0.197600585	0.112655609	1.754023491	0.079426482	0.018316807	3.518870008	71.58178627	1	PRJNA827465;Yang2025_Silkie_Wenchang	CRA004775	0.484886459
ENSGALG00010008936	IFT172	2	0.216418252	0.153942427	1.405838896	0.159719069	0.025038868	1.838693288	45.61325452	0.5	CRA004775;Yang2025_Silkie_Wenchang	PRJNA827465	0.655404348
ENSGALG00010008936	IFT172	2	0.085149997	0.067278375	1.265656946	0.205643092	0	0.206478716	0	0.5	CRA004775;PRJNA827465	Yang2025_Silkie_Wenchang	0.71825498
ENSGALG00010008938	ZBED4	2	-0.151785589	0.199778103	-0.7597709	0.44739154	0.074063476	13.68283751	92.69159897	0.5	PRJNA827465;Yang2025_Silkie_Wenchang	CRA004775	0.84650962
ENSGALG00010008938	ZBED4	2	0.172819292	0.55176933	0.313209311	0.754121645	0.577852892	19.08175458	94.75939177	0.5	CRA004775;Yang2025_Silkie_Wenchang	PRJNA827465	0.938385954
ENSGALG00010008938	ZBED4	2	0.356983389	0.350348016	1.018939376	0.308231749	0.218814437	8.437342021	88.14792624	1	CRA004775;PRJNA827465	Yang2025_Silkie_Wenchang	0.793403631
ENSGALG00010008939	RANBP9	2	-0.058913103	0.079012441	-0.74561807	0.455898164	0.007510523	2.252071798	55.59644231	0.5	PRJNA827465;Yang2025_Silkie_Wenchang	CRA004775	0.84907427
ENSGALG00010008939	RANBP9	2	-0.144096575	0.092293217	-1.561291067	0.11845509	0	0.321290059	0	1	CRA004775;Yang2025_Silkie_Wenchang	PRJNA827465	0.578338927
ENSGALG00010008939	RANBP9	2	0.000487429	0.046988979	0.010373269	0.991723477	0	0.000587659	0	0.5	CRA004775;PRJNA827465	Yang2025_Silkie_Wenchang	0.997575757
ENSGALG00010008943	ENSGALG00010008943	2	0.018193629	0.245641692	0.074067418	0.940955558	0.064720223	2.088639161	52.12193571	0.5	PRJNA827465;Yang2025_Silkie_Wenchang	CRA004775	0.98556663
ENSGALG00010008943	ENSGALG00010008943	2	0.000172628	0.413274942	0.000417708	0.999666718	0.185988576	2.003568466	50.08905277	0.5	CRA004775;Yang2025_Silkie_Wenchang	PRJNA827465	0.999889665
ENSGALG00010008943	ENSGALG00010008943	2	-0.231864525	0.184646747	-1.255719524	0.209217678	0	0.401428938	0	1	CRA004775;PRJNA827465	Yang2025_Silkie_Wenchang	0.721178951
ENSGALG00010008946	COL22A1	2	0.096384207	0.137063395	0.703208958	0.481925537	0	0.216889672	0	1	PRJNA827465;Yang2025_Silkie_Wenchang	CRA004775	0.85691916
ENSGALG00010008946	COL22A1	2	-0.591835075	0.987742814	-0.599179328	0.549053306	1.550332853	4.321017373	76.85730203	0.5	CRA004775;Yang2025_Silkie_Wenchang	PRJNA827465	0.878431377
ENSGALG00010008946	COL22A1	2	-0.636534931	0.895414968	-0.710882611	0.477156982	1.272454461	3.978605878	74.86556772	0.5	CRA004775;PRJNA827465	Yang2025_Silkie_Wenchang	0.854986476
ENSGALG00010008955	KRTCAP3	2	-0.309437312	0.208724171	-1.482517866	0.138202553	0	0.876769288	0	1	PRJNA827465;Yang2025_Silkie_Wenchang	CRA004775	0.621626797
ENSGALG00010008955	KRTCAP3	2	0.263488696	0.849539948	0.310154569	0.756443417	1.302875263	10.25809619	90.25160243	0.5	CRA004775;Yang2025_Silkie_Wenchang	PRJNA827465	0.938640387
ENSGALG00010008955	KRTCAP3	2	0.443083911	0.688549582	0.643502776	0.519895545	0.719059345	7.44655031	86.57096295	0.5	CRA004775;PRJNA827465	Yang2025_Silkie_Wenchang	0.858931707
ENSGALG00010008956	LVRN	2	-0.290201369	0.179001124	-1.621226515	0.104969068	0	0.697641242	0	1	PRJNA827465;Yang2025_Silkie_Wenchang	CRA004775	0.550153258
ENSGALG00010008956	LVRN	2	0.465361451	0.718387567	0.64778606	0.517123233	0.858531463	5.483840891	81.76460587	0.5	CRA004775;Yang2025_Silkie_Wenchang	PRJNA827465	0.867952013
ENSGALG00010008956	LVRN	2	0.313643655	0.385194535	[illegible]	[illegible]	[illegible]	7.568884463	86.78820039	0.5	CRA004775;PRJNA827465	Yang2025_Silkie_Wenchang	0.922927298
ENSGALG00010008960	LRRC2	2	-0.164091675	0.410936474	-0.399311536	0.689663668	0.250986616	3.879352447	74.22250198	0.5	PRJNA827465;Yang2025_Silkie_Wenchang	CRA004775	0.919979266
ENSGALG00010008960	LRRC2	2	0.267580123	0.248769189	1.07561601	0.282099024	0	0.090919011	0	1	CRA004775;Yang2025_Silkie_Wenchang	PRJNA827465	0.779519046
ENSGALG00010008960	LRRC2	2	-0.202471834	0.494395028	-0.409534527	0.682147434	0.295346157	2.322868866	56.94978676	0.5	CRA004775;PRJNA827465	Yang2025_Silkie_Wenchang	0.918524893
ENSGALG00010008966	NRBP1	2	-0.006856813	0.073836993	-0.092864196	0.92601145	0.003715886	1.455172722	31.27963541	0.5	PRJNA827465;Yang2025_Silkie_Wenchang	CRA004775	0.992799417
ENSGALG00010008966	NRBP1	2	0.075064138	0.229092276	0.327658965	0.743169533	0.073704548	2.982581026	66.47199217	0.5	CRA004775;Yang2025_Silkie_Wenchang	PRJNA827465	0.93573824
ENSGALG00010008966	NRBP1	2	0.114926136	0.129222827	0.889364039	0.373807465	0.016401892	1.485674013	32.69048318	1	CRA004775;PRJNA827465	Yang2025_Silkie_Wenchang	0.822579781
ENSGALG00010008969	PPP1R37	2	0.197352911	0.149267332	1.322140024	0.186120196	0.038514036	7.262110455	86.22990823	1	PRJNA827465;Yang2025_Silkie_Wenchang	CRA004775	0.695099823
ENSGALG00010008969	PPP1R37	2	0.058515803	0.34718121	0.16854542	0.866154215	0.199530987	5.168254387	80.65110722	0.5	CRA004775;Yang2025_Silkie_Wenchang	PRJNA827465	0.967318736
ENSGALG00010008969	PPP1R37	2	-0.044058415	0.174006856	-0.253199304	0.800114205	0.036101391	1.789524894	44.1192462	0.5	CRA004775;PRJNA827465	Yang2025_Silkie_Wenchang	0.94999675
ENSGALG00010008971	ANXA11	2	-0.148309801	0.184871701	-0.802230953	0.422419375	0.061613239	10.00683049	90.00682583	0.5	PRJNA827465;Yang2025_Silkie_Wenchang	CRA004775	0.839704389
ENSGALG00010008971	ANXA11	2	0.039064688	0.08424625	0.463696456	0.642865236	0	0.009268555	0	1	CRA004775;Yang2025_Silkie_Wenchang	PRJNA827465	0.906634523
ENSGALG00010008971	ANXA11	2	-0.185315137	0.172166838	-1.076369522	0.281762027	0.04527451	3.759028469	73.39738158	0.5	CRA004775;PRJNA827465	Yang2025_Silkie_Wenchang	0.779295548
ENSGALG00010008974	SMCO4	2	-0.177075251	0.178862953	-0.990005187	0.322171584	0.043320919	2.659194362	62.39462544	1	PRJNA827465;Yang2025_Silkie_Wenchang	CRA004775	0.798513866
ENSGALG00010008974	SMCO4	2	-0.27495243	0.198816701	-1.382944331	0.166681936	0.013748464	1.182895265	15.46166179	0.5	CRA004775;Yang2025_Silkie_Wenchang	PRJNA827465	0.665626918
ENSGALG00010008974	SMCO4	2	-0.040006541	0.079150877	-0.505446588	0.613245204	0	0.021604453	0	0.5	CRA004775;PRJNA827465	Yang2025_Silkie_Wenchang	0.898236361
ENSGALG00010008980	ENSGALG00010008980	2	-0.148135919	0.522086389	-0.283738328	0.77661092	0.384800014	3.341213869	70.07075754	0.5	PRJNA827465;Yang2025_Silkie_Wenchang	CRA004775	0.944156005
ENSGALG00010008980	ENSGALG00010008980	2	0.128039982	0.3055262	0.419080203	0.675157519	0	0.725936241	0	0.5	CRA004775;Yang2025_Silkie_Wenchang	PRJNA827465	0.916130363
ENSGALG00010008980	ENSGALG00010008980	2	-0.411268318	0.262650258	-1.565840148	0.117386045	0	0.963918518	0	1	CRA004775;PRJNA827465	Yang2025_Silkie_Wenchang	0.57688585
ENSGALG00010008983	ENSGALG00010008983	2	0.366525492	0.421424589	0.869729723	0.384448125	0.169582829	1.869353757	46.50557733	1	PRJNA827465;Yang2025_Silkie_Wenchang	CRA004775	0.826099934
ENSGALG00010008983	ENSGALG00010008983	2	0.168794037	0.727813815	0.231919255	0.816600729	0.756843862	3.480981495	71.27247009	0.5	CRA004775;Yang2025_Silkie_Wenchang	PRJNA827465	0.954635885
ENSGALG00010008983	ENSGALG00010008983	2	-0.158697161	0.314035707	-0.505347504	0.613314784	0	0.738827834	0	0.5	CRA004775;PRJNA827465	Yang2025_Silkie_Wenchang	0.89823785
ENSGALG00010008988	PPM1G	2	-0.04995226	0.045477877	-1.098385912	0.272036011	0	0.025993285	0	1	PRJNA827465;Yang2025_Silkie_Wenchang	CRA004775	0.773184343
ENSGALG00010008988	PPM1G	2	-0.05526569	0.062813862	-0.879832705	0.378949945	0	0.010954836	0	1	CRA004775;Yang2025_Silkie_Wenchang	PRJNA827465	0.823912132
ENSGALG00010008988	PPM1G	2	-0.044082016	0.052724157	-0.836087637	0.403105603	0	6.80131E-08	0	1	CRA004775;PRJNA827465	Yang2025_Silkie_Wenchang	0.833046091
ENSGALG00010008991	ENSGALG00010008991	2	0.079641554	0.140576989	0.566533359	0.571031262	0.022574489	1.938743059	48.42018928	0.5	PRJNA827465;Yang2025_Silkie_Wenchang	CRA004775	0.885354083
ENSGALG00010008991	ENSGALG00010008991	2	0.047552544	0.271607614	0.175078097	0.861018268	0.09123505	2.574006989	61.15006664	0.5	CRA004775;Yang2025_Silkie_Wenchang	PRJNA827465	0.965329787
ENSGALG00010008991	ENSGALG00010008991	2	-0.03119793	0.073653301	-0.423578166	0.671873481	0	0.729561159	0	1	CRA004775;PRJNA827465	Yang2025_Silkie_Wenchang	0.914674175
ENSGALG00010008995	ECD	2	0.157195505	0.109105053	1.440771998	0.149649107	0.01810936	4.005206396	75.03249768	1	PRJNA827465;Yang2025_Silkie_Wenchang	CRA004775	0.639449755
ENSGALG00010008995	ECD	2	0.245254935	0.086014833	2.851309781	0.004353953	0	0.831466075	0	1	CRA004775;Yang2025_Silkie_Wenchang	PRJNA827465	0.096734544
ENSGALG00010008995	ECD	2	0.060620196	0.054790733	1.106395055	0.268555565	0	0.001087554	0	1	CRA004775;PRJNA827465	Yang2025_Silkie_Wenchang	0.771398294
ENSGALG00010008996	CHRM3	2	-0.132060672	0.162573423	-0.812314029	0.416611463	0.018907564	1.475367146	32.22026107	0.5	PRJNA827465;Yang2025_Silkie_Wenchang	CRA004775	0.837984974
ENSGALG00010008996	CHRM3	2	0.509400333	0.503792983	1.011130266	0.311954086	0.36878389	3.340471439	70.06410567	1	CRA004775;Yang2025_Silkie_Wenchang	PRJNA827465	0.795993742
ENSGALG00010008996	CHRM3	2	0.348831073	0.678865051	0.5138445	0.607360713	0.797446653	6.733540161	85.14897103	0.5	CRA004775;PRJNA827465	Yang2025_Silkie_Wenchang	0.896998866
ENSGALG00010008997	CHST6	2	-0.148181571	0.139463462	-1.062511778	0.288003414	0.03258131	5.972737782	83.25725929	1	PRJNA827465;Yang2025_Silkie_Wenchang	CRA004775	0.782840067
ENSGALG00010008997	CHST6	2	0.069660273	0.394447911	0.176601956	0.859821058	0.281823662	10.15149319	90.14923242	0.5	CRA004775;Yang2025_Silkie_Wenchang	PRJNA827465	0.964858345
ENSGALG00010008997	CHST6	2	0.188933798	0.251034635	0.752620441	0.45167803	0.102764605	4.699950648	78.72318084	0.5	CRA004775;PRJNA827465	Yang2025_Silkie_Wenchang	0.848006549
ENSGALG00010008999	TRAPPC9	2	-0.16843006	0.075365966	-2.234829179	0.025428565	0.004537795	1.551526487	35.54734589	1	PRJNA827465;Yang2025_Silkie_Wenchang	CRA004775	0.271950723
ENSGALG00010008999	TRAPPC9	2	-0.277117514	0.106939682	-2.59134411	0.009560184	0	0.005089067	0	1	CRA004775;Yang2025_Silkie_Wenchang	PRJNA827465	0.153870904
ENSGALG00010008999	TRAPPC9	2	-0.120291432	0.062292511	-1.931073719	0.053473936	0	0.24636555	0	1	CRA004775;PRJNA827465	Yang2025_Silkie_Wenchang	0.400855641
ENSGALG00010009000	FAM149B1	2	0.187188425	0.142584345	1.312825929	0.189241611	0.031721568	4.046378032	75.2865404	1	PRJNA827465;Yang2025_Silkie_Wenchang	CRA004775	0.698184834
ENSGALG00010009000	FAM149B1	2	0.173889523	0.217657405	0.798913884	0.424340348	0.063525467	2.887760703	65.3710919	0.5	CRA004775;Yang2025_Silkie_Wenchang	PRJNA827465	0.840617373
ENSGALG00010009000	FAM149B1	2	0.061791316	0.049072182	1.259192347	0.207960865	0	0.439829864	0	0.5	CRA004775;PRJNA827465	Yang2025_Silkie_Wenchang	0.720511802
ENSGALG00010009002	ENSGALG00010009002	2	-0.200645916	0.076108287	-2.636321533	0.008381027	0	0.643468729	0	1	PRJNA827465;Yang2025_Silkie_Wenchang	CRA004775	0.142771619
ENSGALG00010009002	ENSGALG00010009002	2	-0.106791243	0.179376052	-0.595348386	0.551610619	0	0.334194212	0	1	CRA004775;Yang2025_Silkie_Wenchang	PRJNA827465	0.879004923
ENSGALG00010009002	ENSGALG00010009002	2	-0.227167036	0.079454909	-2.859068605	0.004248869	0	0.021983946	0	1	CRA004775;PRJNA827465	Yang2025_Silkie_Wenchang	0.095261433
ENSGALG00010009003	ALG12	2	-0.148006576	0.301393103	-0.491074862	0.6233735	0.176446315	34.62401226	97.11183097	0.5	PRJNA827465;Yang2025_Silkie_Wenchang	CRA004775	0.900354575
ENSGALG00010009003	ALG12	2	-0.453409634	0.082642105	-5.486424099	4.10152E-08	0	0.003841516	0	1	CRA004775;Yang2025_Silkie_Wenchang	PRJNA827465	1.71491E-05
ENSGALG00010009003	ALG12	2	-0.010431857	0.274950148	-0.037940903	0.969734801	0.098033736	1.970645191	49.25519802	0.5	CRA004775;PRJNA827465	Yang2025_Silkie_Wenchang	0.991572914
ENSGALG00010009007	TMEM65	2	-0.313122909	0.221752832	-1.412035673	0.157939458	0.092031367	15.4169375	93.51362746	1	PRJNA827465;Yang2025_Silkie_Wenchang	CRA004775	0.651568647
ENSGALG00010009007	TMEM65	2	-0.214753524	0.341508425	-0.628838142	0.529455026	0.213936318	11.79018703	91.51837034	0.5	CRA004775;Yang2025_Silkie_Wenchang	PRJNA827465	0.871944741
ENSGALG00010009007	TMEM65	2	-0.032990283	0.106196159	-0.3106542	0.756063519	0.011552207	1.66881085	40.07709142	0.5	CRA004775;PRJNA827465	Yang2025_Silkie_Wenchang	0.938524556
ENSGALG00010009011	ACP6	2	0.059450051	0.146453885	0.405930175	0.684793906	0.017386769	1.626178569	38.50613831	0.5	PRJNA827465;Yang2025_Silkie_Wenchang	CRA004775	0.919003112
ENSGALG00010009011	ACP6	2	-0.089482878	0.130189826	-0.687326199	0.491877192	0	0.058272412	0	1	CRA004775;Yang2025_Silkie_Wenchang	PRJNA827465	0.859020186
ENSGALG00010009011	ACP6	2	0.084888116	0.115442408	0.7353287	0.462139333	0.003976291	1.164435557	14.12148192	0.5	CRA004775;PRJNA827465	Yang2025_Silkie_Wenchang	0.850747865
ENSGALG00010009013	LIPG	2	0.131848612	0.113007592	1.166723491	0.243322053	0	0.083706216	0	1	PRJNA827465;Yang2025_Silkie_Wenchang	CRA004775	0.755238542
ENSGALG00010009013	LIPG	2	0.105668908	0.191769803	0.551019537	0.581620282	0	0.126783242	0	1	CRA004775;Yang2025_Silkie_Wenchang	PRJNA827465	0.889045686
ENSGALG00010009013	LIPG	2	0.161438922	0.133304295	1.211055665	0.225874071	0	0.065057778	0	1	CRA004775;PRJNA827465	Yang2025_Silkie_Wenchang	0.739120026
ENSGALG00010009015	TMEM231	2	0.032124528	0.049063343	0.654756199	0.512624736	0	0.022316801	0	1	PRJNA827465;Yang2025_Silkie_Wenchang	CRA004775	0.865859727
ENSGALG00010009015	TMEM231	2	0.025828962	0.070560245	0.366055443	0.714323705	0	0.006984226	0	1	CRA004775;Yang2025_Silkie_Wenchang	PRJNA827465	0.92653974
ENSGALG00010009015	TMEM231	2	0.037580118	0.057018146	0.659090487	0.509837664	0	5.58469E-05	0	1	CRA004775;PRJNA827465	Yang2025_Silkie_Wenchang	0.865257661
ENSGALG00010009016	DNAJC9	2	0.042350539	0.056162823	0.754067131	0.450808913	0	0.38292696	0	1	PRJNA827465;Yang2025_Silkie_Wenchang	CRA004775	0.847631399
ENSGALG00010009016	DNAJC9	2	0.097788716	0.098336688	0.994427592	0.320014741	0	0.002894553	0	1	CRA004775;Yang2025_Silkie_Wenchang	PRJNA827465	0.798348216
ENSGALG00010009016	DNAJC9	2	0.023850746	0.065461697	0.364346585	0.715599217	0	0.083895104	0	1	CRA004775;PRJNA827465	Yang2025_Silkie_Wenchang	0.927131632
ENSGALG00010009019	ZNF513	2	0.134874619	0.161419676	0.835552534	0.403406681	0.042995222	5.328732335	81.23381065	0.5	PRJNA827465;Yang2025_Silkie_Wenchang	CRA004775	0.83306585

ENSGALG00010009019	ZNF513	2	0.053830953	0.312358212	0.172337241	0.863172408	0.150714789	4.013295938	75.0828243	0.5	CRA004775;Yang2025_Silkie_Wenchang	PRJNA827465	0.966228964
ENSGALG00010009019	ZNF513	2	-0.031707923	0.082743382	-0.383207961	0.701565576	0.00406273	1.093507222	8.551129834	1	CRA004775;PRJNA827465	Yang2025_Silkie_Wenchang	0.922874082
ENSGALG00010009020	ARHGAP35	2	-0.077006374	0.058421038	-1.31812744	0.18746099	0	0.686437364	0	1	PRJNA827465;Yang2025_Silkie_Wenchang	CRA004775	0.696584977
ENSGALG00010009020	ARHGAP35	2	-0.045135883	0.11050547	-0.408449313	0.682943836	0.00360971	1.049473614	4.714136033	1	CRA004775;Yang2025_Silkie_Wenchang	PRJNA827465	0.918800744
ENSGALG00010009020	ARHGAP35	2	-0.123220655	0.071067784	-1.733846884	0.082945254	0	0.593991922	0	1	CRA004775;PRJNA827465	Yang2025_Silkie_Wenchang	0.495859127
ENSGALG00010009021	HNF4beta	2	0.953284193	0.317823286	2.99941582	0.002704979	0	0.035271961	0	1	PRJNA827465;Yang2025_Silkie_Wenchang	CRA004775	0.070754925
ENSGALG00010009021	HNF4beta	2	1.228104024	0.348487877	3.524093968	0.000424934	0	0.987526979	0	1	CRA004775;Yang2025_Silkie_Wenchang	PRJNA827465	0.020876453
ENSGALG00010009021	HNF4beta	2	1.227589622	0.429570232	2.857715756	0.004267024	0.060035051	1.183631154	15.51422111	1	CRA004775;PRJNA827465	Yang2025_Silkie_Wenchang	0.095505045
ENSGALG00010009024	PMAIP1	2	-0.226516194	0.193509749	-1.170567347	0.241772729	0.041741136	2.206007938	54.66924743	1	PRJNA827465;Yang2025_Silkie_Wenchang	CRA004775	0.75406671
ENSGALG00010009024	PMAIP1	2	-0.408057717	0.196556243	-2.07603539	0.037890678	0	0.255532873	0	1	CRA004775;Yang2025_Silkie_Wenchang	PRJNA827465	0.335640621
ENSGALG00010009024	PMAIP1	2	-0.067099289	0.148543234	-0.451715551	0.651473914	0	0.040310676	0	1	CRA004775;PRJNA827465	Yang2025_Silkie_Wenchang	0.908927523
ENSGALG00010009025	UTP23	2	0.378287009	0.224234045	1.687018619	0.091599772	0.087222899	7.478424667	86.62819986	1	PRJNA827465;Yang2025_Silkie_Wenchang	CRA004775	0.518914432
ENSGALG00010009025	UTP23	2	0.473061406	0.1927941	2.453713087	0.014138974	0.039067502	1.885984532	46.97729577	1	CRA004775;Yang2025_Silkie_Wenchang	PRJNA827465	0.194915504
ENSGALG00010009025	UTP23	2	0.166814448	0.093311356	1.7877186	0.073821419	0	0.020642057	0	1	CRA004775;PRJNA827465	Yang2025_Silkie_Wenchang	0.469352297
ENSGALG00010009026	RAPGEF2	2	0.016024996	0.043062822	0.372130651	0.709795577	0	0.353664556	0	1	PRJNA827465;Yang2025_Silkie_Wenchang	CRA004775	0.925298042
ENSGALG00010009026	RAPGEF2	2	0.067935151	0.137699092	0.49335947	0.621758615	0	0.313332728	0	0.5	CRA004775;Yang2025_Silkie_Wenchang	PRJNA827465	0.899796991
ENSGALG00010009026	RAPGEF2	2	0.006404325	0.044567048	0.143700896	0.885736663	0	0.113243915	0	0.5	CRA004775;PRJNA827465	Yang2025_Silkie_Wenchang	0.972378092
ENSGALG00010009028	PDLIM7	2	0.23136931	0.306005956	0.756094141	0.449592754	0.178891018	22.1565331	95.48665852	0.5	PRJNA827465;Yang2025_Silkie_Wenchang	CRA004775	0.847275788
ENSGALG00010009028	PDLIM7	2	0.208600973	0.373496263	0.558508863	0.576496954	0.23947042	6.580686704	84.8040175	0.5	CRA004775;Yang2025_Silkie_Wenchang	PRJNA827465	0.887113945
ENSGALG00010009028	PDLIM7	2	-0.077573626	0.067813496	-1.143926078	0.252654295	0	0.246804663	0	1	CRA004775;PRJNA827465	Yang2025_Silkie_Wenchang	0.762687335
ENSGALG00010009029	IGSF3	2	-0.167629195	0.143609924	-1.167253559	0.243107987	0.028228768	3.145555904	68.20911691	1	PRJNA827465;Yang2025_Silkie_Wenchang	CRA004775	0.7550024
ENSGALG00010009029	IGSF3	2	-0.291662096	0.12082032	-2.414015267	0.015777801	0	0.833326167	0	0.5	CRA004775;Yang2025_Silkie_Wenchang	PRJNA827465	0.207882978
ENSGALG00010009029	IGSF3	2	-0.024633065	0.101504001	-0.242680726	0.80825274	0	0.119721366	0	0.5	CRA004775;PRJNA827465	Yang2025_Silkie_Wenchang	0.952496413
ENSGALG00010009030	ZDHHC7	2	0.08156329	0.042410972	1.923164837	0.054459352	0	0.004254226	0	1	PRJNA827465;Yang2025_Silkie_Wenchang	CRA004775	0.404097297
ENSGALG00010009030	ZDHHC7	2	0.06645746	0.065993436	1.007031368	0.3139197	0	0.474970732	0	0.5	CRA004775;Yang2025_Silkie_Wenchang	PRJNA827465	0.796531448
ENSGALG00010009030	ZDHHC7	2	0.069020914	0.05039854	1.369502242	0.170842335	0	0.464176658	0	0.5	CRA004775;PRJNA827465	Yang2025_Silkie_Wenchang	0.672739833
ENSGALG00010009031	SPIN1	2	-0.155692918	0.219094115	-0.710621178	0.477319016	0.066594598	3.264076091	69.36345931	0.5	PRJNA827465;Yang2025_Silkie_Wenchang	CRA004775	0.854997403
ENSGALG00010009031	SPIN1	2	-0.092413582	0.310912061	-0.297233829	0.766287997	0.146819078	4.027404167	75.17011061	0.5	CRA004775;Yang2025_Silkie_Wenchang	PRJNA827465	0.941123716
ENSGALG00010009031	SPIN1	2	0.121867714	0.144355327	0.844220415	0.398546249	0	0.355410338	0	1	CRA004775;PRJNA827465	Yang2025_Silkie_Wenchang	0.831980453
ENSGALG00010009034	SNX17	2	-0.103975692	0.043241652	-2.404526366	0.016193438	0.000215386	1.016541978	1.627279417	1	PRJNA827465;Yang2025_Silkie_Wenchang	CRA004775	0.210262467
ENSGALG00010009034	SNX17	2	-0.253064832	0.141921929	-1.783127061	0.074565622	0	0.000333899	0	1	CRA004775;Yang2025_Silkie_Wenchang	PRJNA827465	0.471620961
ENSGALG00010009034	SNX17	2	-0.093858088	0.042217177	-2.223220359	0.026200945	0	0.200317146	0	1	CRA004775;PRJNA827465	Yang2025_Silkie_Wenchang	0.27602655
ENSGALG00010009035	ENSGALG00010009035	2	-0.136977645	0.060533718	-2.262832162	0.023646038	0	0.001347967	0	1	PRJNA827465;Yang2025_Silkie_Wenchang	CRA004775	0.262382895
ENSGALG00010009035	ENSGALG00010009035	2	-0.150120299	0.124952022	-1.201423527	0.229586954	0	0.09687638	0	1	CRA004775;Yang2025_Silkie_Wenchang	PRJNA827465	0.742632495
ENSGALG00010009035	ENSGALG00010009035	2	-0.142706418	0.065829463	-2.167819873	0.030172394	0	0.099542847	0	1	CRA004775;PRJNA827465	Yang2025_Silkie_Wenchang	0.295973612
ENSGALG00010009037	ENSGALG00010009037	2	0.191956272	0.16400845	1.170404768	0.241838118	0	0.9567815	0	1	PRJNA827465;Yang2025_Silkie_Wenchang	CRA004775	0.75406671
ENSGALG00010009037	ENSGALG00010009037	2	-0.225416984	0.645497723	-0.349214221	0.726928491	0.71670041	6.939827721	85.59042039	0.5	CRA004775;Yang2025_Silkie_Wenchang	PRJNA827465	0.93142315
ENSGALG00010009037	ENSGALG00010009037	2	-0.364508421	0.480256933	-0.758986276	0.447860766	0.357545644	4.16568989	75.99437245	0.5	CRA004775;PRJNA827465	Yang2025_Silkie_Wenchang	0.846601815
ENSGALG00010009038	RAD21	2	-0.115420746	0.111170648	-1.038230399	0.299162803	0.022708506	12.23465975	91.8264993	1	PRJNA827465;Yang2025_Silkie_Wenchang	CRA004775	0.789767499
ENSGALG00010009038	RAD21	2	-0.224824264	0.049699593	-4.523664086	6.07782E-06	0	0.188647618	0	1	CRA004775;Yang2025_Silkie_Wenchang	PRJNA827465	0.000954316
ENSGALG00010009038	RAD21	2	-0.009023218	0.038014457	-0.237362787	0.812375356	0	0.172501059	0	1	CRA004775;PRJNA827465	Yang2025_Silkie_Wenchang	0.953371387
ENSGALG00010009039	SLC36A4	2	-0.064422225	0.22180342	-0.290447394	0.77147399	0.070196178	3.401705565	70.60298192	0.5	PRJNA827465;Yang2025_Silkie_Wenchang	CRA004775	0.94283287
ENSGALG00010009039	SLC36A4	2	-0.319023582	0.161934476	-1.970078212	0.048829408	0	0.006092744	0	1	CRA004775;Yang2025_Silkie_Wenchang	PRJNA827465	0.381918847
ENSGALG00010009039	SLC36A4	2	-0.037587483	0.22783308	-0.164978163	0.86896117	0.06287933	2.286972132	56.27406273	0.5	CRA004775;PRJNA827465	Yang2025_Silkie_Wenchang	0.9684491
ENSGALG00010009040	CRELD2	2	0.282716665	0.056541805	5.000135147	5.72901E-07	0	0.0052767	0	1	PRJNA827465;Yang2025_Silkie_Wenchang	CRA004775	0.00014241
ENSGALG00010009040	CRELD2	2	0.100632568	0.261618299	0.384654165	0.700493658	0.080924038	2.005582488	50.13917373	0.5	CRA004775;Yang2025_Silkie_Wenchang	PRJNA827465	0.922303014
ENSGALG00010009040	CRELD2	2	0.115729318	0.263131074	0.439816235	0.660070208	0.093865567	2.274663291	56.0374494	0.5	CRA004775;PRJNA827465	Yang2025_Silkie_Wenchang	0.911825461
ENSGALG00010009043	TFAM	2	-0.023435003	0.080633353	-0.29063659	0.771329273	0.005290573	1.458405546	31.43196674	0.5	PRJNA827465;Yang2025_Silkie_Wenchang	CRA004775	0.94283287
ENSGALG00010009043	TFAM	2	0.047400533	0.108035162	0.438750981	0.660841982	0	0.529333764	0	0.5	CRA004775;Yang2025_Silkie_Wenchang	PRJNA827465	0.91192683
ENSGALG00010009043	TFAM	2	-0.068901595	0.055975347	-1.230927523	0.218349973	0	0.016687269	0	1	CRA004775;PRJNA827465	Yang2025_Silkie_Wenchang	0.731986436
ENSGALG00010009047	SUPT5H	2	0.184140168	0.221582932	0.831021442	0.405961516	0.090429601	12.35576951	91.90661497	0.5	PRJNA827465;Yang2025_Silkie_Wenchang	CRA004775	0.833416425
ENSGALG00010009047	SUPT5H	2	-0.007217703	0.447972808	-0.016111923	0.987145102	0.367293792	11.38025457	91.21285034	0.5	CRA004775;Yang2025_Silkie_Wenchang	PRJNA827465	0.996462132
ENSGALG00010009047	SUPT5H	2	-0.194272466	0.218712239	-0.888256034	0.374403044	0.071967503	3.321198869	69.89039081	1	CRA004775;PRJNA827465	Yang2025_Silkie_Wenchang	0.822805106
ENSGALG00010009050	PIK3CG	2	0.087398485	0.071424446	1.223649463	0.221084492	0	0.627453496	0	0.5	PRJNA827465;Yang2025_Silkie_Wenchang	CRA004775	0.734279503
ENSGALG00010009050	PIK3CG	2	0.039370387	0.117725412	0.334425566	0.73805845	0	0.380910117	0	0.5	CRA004775;Yang2025_Silkie_Wenchang	PRJNA827465	0.934275628
ENSGALG00010009050	PIK3CG	2	0.123382567	0.076607368	1.610583549	0.107270522	0	0.012244589	0	1	CRA004775;PRJNA827465	Yang2025_Silkie_Wenchang	0.554473355
ENSGALG00010009051	TROJANZ	2	0.345061836	0.21628316	1.595417024	0.110619012	0	0.179476486	0	1	PRJNA827465;Yang2025_Silkie_Wenchang	CRA004775	0.562832796
ENSGALG00010009051	TROJANZ	2	-0.321474399	0.644989569	-0.498417981	0.618189466	0.682179606	5.351085471	81.31220282	0.5	CRA004775;Yang2025_Silkie_Wenchang	PRJNA827465	0.899168321
ENSGALG00010009051	TROJANZ	2	-0.246543867	0.739401763	-0.333436947	0.738804477	0.924386745	6.361831889	84.28125707	0.5	CRA004775;PRJNA827465	Yang2025_Silkie_Wenchang	0.934613258
ENSGALG00010009057	NXNL2	2	0.734819258	0.336235473	2.185430499	0.028857297	0.077831341	1.51591909	34.03341862	1	PRJNA827465;Yang2025_Silkie_Wenchang	CRA004775	0.289410648
ENSGALG00010009057	NXNL2	2	0.515649796	0.61414621	0.839620579	0.401121157	0.522461433	3.070826264	67.43547456	0.5	CRA004775;Yang2025_Silkie_Wenchang	PRJNA827465	0.832453107
ENSGALG00010009057	NXNL2	2	0.180730827	0.345975231	0.522380827	0.601405203	0	0.584218304	0	0.5	CRA004775;PRJNA827465	Yang2025_Silkie_Wenchang	0.895230519
ENSGALG00010009059	MED29	2	-0.389473886	0.532621091	-0.731240074	0.464632526	0.515902627	10.32580291	90.31552307	0.5	PRJNA827465;Yang2025_Silkie_Wenchang	CRA004775	0.85217126
ENSGALG00010009059	MED29	2	-0.829344154	0.265678299	-3.121610444	0.001798648	0	0.605698409	0	1	CRA004775;Yang2025_Silkie_Wenchang	PRJNA827465	0.054087414
ENSGALG00010009059	MED29	2	-0.033070551	0.255706316	-0.129330206	0.897096371	0.07496671	1.634112716	38.80471094	0.5	CRA004775;PRJNA827465	Yang2025_Silkie_Wenchang	0.975341042
ENSGALG00010009065	EIF2B4	2	-0.114316633	0.057775234	-1.978644234	0.047856076	0	0.160102218	0	1	PRJNA827465;Yang2025_Silkie_Wenchang	CRA004775	0.378353533
ENSGALG00010009065	EIF2B4	2	-0.233833426	0.134822596	-1.734378605	0.082850929	0	0.80940451	0	1	CRA004775;Yang2025_Silkie_Wenchang	PRJNA827465	0.495856563
ENSGALG00010009065	EIF2B4	2	-0.173465428	0.141822963	-1.223112426	0.221287236	0.020924665	1.470507187	31.99625211	1	CRA004775;PRJNA827465	Yang2025_Silkie_Wenchang	0.734685079
ENSGALG00010009067	ENSGALG00010009067	2	-0.28930315	1.268761685	-0.228020087	0.819630624	2.330246051	3.592827372	72.16676738	0.5	PRJNA827465;Yang2025_Silkie_Wenchang	CRA004775	0.955256039
ENSGALG00010009067	ENSGALG00010009067	2	5.564409402	4.690436887	1.186330727	0.235491717	42.91613505	40.47178244	97.52914268	1	CRA004775;Yang2025_Silkie_Wenchang	PRJNA827465	0.748604097
ENSGALG00010009067	ENSGALG00010009067	2	4.316700116	5.961200421	0.724132693	0.468984275	69.81027229	56.31503609	98.22427531	0.5	CRA004775;PRJNA827465	Yang2025_Silkie_Wenchang	0.85345437
ENSGALG00010009068	AGO2	2	0.042784919	0.077921975	0.549073852	0.582954769	0	0.80521692	0	1	PRJNA827465;Yang2025_Silkie_Wenchang	CRA004775	0.890230846
ENSGALG00010009068	AGO2	2	0.171365533	0.177254684	0.966775769	0.33365614	0	0.153076131	0	1	CRA004775;Yang2025_Silkie_Wenchang	PRJNA827465	0.802691373
ENSGALG00010009068	AGO2	2	0.012335512	0.083471133	0.147781768	0.882514998	0	0.000106216	0	1	CRA004775;PRJNA827465	Yang2025_Silkie_Wenchang	0.972001076
ENSGALG00010009070	ENSGALG00010009070	2	3.509742398	1.202409396	2.918924627	0.003512411	0.772543748	1.364567593	26.71671193	1	PRJNA827465;Yang2025_Silkie_Wenchang	CRA004775	0.083890916
ENSGALG00010009070	ENSGALG00010009070	2	2.375205704	2.085701864	1.138804038	0.254784897	7.702034635	7.907973321	87.35453498	1	CRA004775;Yang2025_Silkie_Wenchang	PRJNA827465	0.764394153
ENSGALG00010009070	ENSGALG00010009070	2	0.854753759	0.701350002	1.218726393	0.222948057	0.50522808	1.45730533	31.3802002	1	CRA004775;PRJNA827465	Yang2025_Silkie_Wenchang	0.736458649
ENSGALG00010009073	PRKAR2B	2	-0.541427377	0.244476614	-2.214638727	0.026784872	0	0.082578742	0	1	PRJNA827465;Yang2025_Silkie_Wenchang	CRA004775	0.279174307
ENSGALG00010009073	PRKAR2B	2	-0.578480027	0.299023099	-1.934566357	0.053043532	0	0.379195736	0	1	CRA004775;Yang2025_Silkie_Wenchang	PRJNA827465	0.399631766
ENSGALG00010009073	PRKAR2B	2	-0.682936693	0.298345297	-2.289081477	0.022074619	0	0.169325248	0	1	CRA004775;PRJNA827465	Yang2025_Silkie_Wenchang	0.252135642
ENSGALG00010009076	TMEM214	2	0.461252797	0.217539041	2.120321915	0.033978907	0.080812262	6.741322568	85.16611555	1	PRJNA827465;Yang2025_Silkie_Wenchang	CRA004775	0.317139152
ENSGALG00010009076	TMEM214	2	0.205445933	0.494324594	0.41560937	0.677695869	0.46034083	17.11532726	94.15728379	0.5	CRA004775;Yang2025_Silkie_Wenchang	PRJNA827465	0.917294512
ENSGALG00010009076	TMEM214	2	0.004008051	0.275362899	0.014555522	0.988386784	0.129241542	6.434888944	84.45971626	0.5	CRA004775;PRJNA827465	Yang2025_Silkie_Wenchang	0.996891127
ENSGALG00010009078	PTTG2	2	-0.220042916	0.235323094	-0.93506724	0.349753648	0.076105125	2.756043804	63.71610645	1	PRJNA827465;Yang2025_Silkie_Wenchang	CRA004775	0.809903518
ENSGALG00010009078	PTTG2	2	-0.353017268	0.221495089	-1.593792758	0.11098246	0.004947009	1.050424165	4.800362233	1	CRA004775;Yang2025_Silkie_Wenchang	PRJNA827465	0.563777127
ENSGALG00010009078	PTTG2	2	-0.04451343	0.100908807	-0.441125321	0.659122273	0	0.009173332	0	1	CRA004775;PRJNA827465	Yang2025_Silkie_Wenchang	0.911449691
ENSGALG00010009079	KCTD15	2	-0.059628446	0.121080655	-0.492468812	0.622387965	0.016169023	1.890394903	47.10099997	0.5	PRJNA827465;Yang2025_Silkie_Wenchang	CRA004775	0.900093208
ENSGALG00010009079	KCTD15	2	0.186331208	0.145476935	1.280829898	0.20025342	0	0.416891073	0	1	CRA004775;Yang2025_Silkie_Wenchang	PRJNA827465	0.712517205
ENSGALG00010009079	KCTD15	2	0.025544534	0.222031411	0.11504919	0.908406133	0.071854724	3.037601365	67.07928791	0.5	CRA004775;PRJNA827465	Yang2025_Silkie_Wenchang	0.978291088
ENSGALG00010009080	TLL1	2	0.145983965	0.139222933	1.04856263	0.294379463	0	0.240255759	0	1	PRJNA827465;Yang2025_Silkie_Wenchang	CRA004775	0.787343482
ENSGALG00010009080	TLL1	2	0.266606303	0.236465192	1.127465319	0.259545796	0	0.002015852	0	1	CRA004775;Yang2025_Silkie_Wenchang	PRJNA827465	0.76691144
ENSGALG00010009080	TLL1	2	0.123882254	0.153467996	0.807218819	0.419540425	0	0.127043465	0	1	CRA004775;PRJNA827465	Yang2025_Silkie_Wenchang	0.838527387
ENSGALG00010009081	HBP1	2	0.000840609	0.049249089	0.017068522	0.986381951	0	0.384081995	0	0.5	PRJNA827465;Yang2025_Silkie_Wenchang	CRA004775	0.99618774
ENSGALG00010009081	HBP1	2	0.073472208	0.111910935	0.656523946	0.511487066	0	0.005305949	0	1	CRA004775;Yang2025_Silkie_Wenchang	PRJNA827465	0.865694231
ENSGALG00010009081	HBP1	2	-0.008952464	0.052884443	-0.1692835	0.865573654	0	0.133523123	0	0.5	CRA004775;PRJNA827465	Yang2025_Silkie_Wenchang	0.967088846
ENSGALG00010009083	MED30	2	-0.159548453	0.138054705	-1.15569008	0.247807947	0.022952562	2.141984466	53.31432062	1	PRJNA827465;Yang2025_Silkie_Wenchang	CRA004775	0.7580874
ENSGALG00010009083	MED30	2	-0.264026957	0.147714122	-1.787418512	0.073869872	0	0.657148418	0	1	CRA004775;Yang2025_Silkie_Wenchang	PRJNA827465	0.469467289
ENSGALG00010009083	MED30	2	-0.06640253	0.071072844	-0.934288353	0.350155171	0	0.032168087	0	1	CRA004775;PRJNA827465	Yang2025_Silkie_Wenchang	0.81011
ENSGALG00010009085	PAF1	2	0.400776999	0.406327534	0.986339752	0.323966419	0.314226898	19.97754356	94.99437958	1	PRJNA827465;Yang2025_Silkie_Wenchang	CRA004775	0.799600434
ENSGALG00010009085	PAF1	2	0.325639904	0.502615814	0.647890287	0.517055913	0.471067666	14.77146222	93.2301895	0.5	CRA004775;Yang2025_Silkie_Wenchang	PRJNA827465	0.867952013
ENSGALG00010009085	PAF1	2	-0.001598438	0.050204078	-0.031838811	0.974600596	0	0.904817779	0	0.5	CRA004775;PRJNA827465	Yang2025_Silkie_Wenchang	0.992492038
ENSGALG00010009086	UBE2D1	2	-0.084530101	0.069579373	-1.214872992	0.224414536	0.001913988	1.217244863	17.84726065	0.5	PRJNA827465;Yang2025_Silkie_Wenchang	CRA004775	0.737471949
ENSGALG00010009086	UBE2D1	2	0.033615463	0.105923948	0.317354704	0.750974473	0	0.523475036	0	1	CRA004775;Yang2025_Silkie_Wenchang	PRJNA827465	0.937567095
ENSGALG00010009086	UBE2D1	2	-0.079742159	0.14466017	-0.551237832	0.581470649	0.018504857	1.244048783	19.6173001	0.5	CRA004775;PRJNA827465	Yang2025_Silkie_Wenchang	0.889045686
ENSGALG00010009088	ENSGALG00010009088	2	0.287417445	0.627945719	0.457710653	0.647160333	0.721921212	11.72254843	91.4694317	0.5	PRJNA827465;Yang2025_Silkie_Wenchang	CRA004775	0.907350807
ENSGALG00010009088	ENSGALG00010009088	2	0.788228536	0.2514613	3.134591832	0.001720934	0	0.80305214	0	1	CRA004775;Yang2025_Silkie_Wenchang	PRJNA827465	0.052473127
ENSGALG00010009088	ENSGALG00010009088	2	-0.054871726	0.363630659	-0.150899614	0.880054905	0.158971921	2.216631435	54.88650101	0.5	CRA004775;PRJNA827465	Yang2025_Silkie_Wenchang	0.971546618
ENSGALG00010009089	AGBL5	2	0.232927694	0.196489952	1.185443287	0.235842225	0.068295474	8.44124781	88.15341022	1	PRJNA827465;Yang2025_Silkie_Wenchang	CRA004775	0.748669015
ENSGALG00010009089	AGBL5	2	0.025844214	0.425844744	0.060689287	0.951606664	0.340122732	15.92040331	93.71875209	0.5	CRA004775;Yang2025_Silkie_Wenchang	PRJNA827465	0.987713746
ENSGALG00010009089	AGBL5	2	-0.152008557	0.227229605	-0.668964578	0.503518074	0.086697062	5.728466079	82.54331987	0.5	CRA004775;PRJNA827465	Yang2025_Silkie_Wenchang	0.863108263
ENSGALG00010009091	EXT1	2	-0.075196462	0.096865504	-0.776297637	0.437573268	0.014708018	4.418573937	77.36826374	0.5	PRJNA827465;Yang2025_Silkie_Wenchang	CRA004775	0.843827727
ENSGALG00010009091	EXT1	2	-0.026025673	0.184457318	-0.1410932	0.887796315	0.052442576	4.03339518	75.20699174	0.5	CRA004775;Yang2025_Silkie_Wenchang	PRJNA827465	0.973090153
ENSGALG00010009091	EXT1	2	0.027700341	0.051637827	0.536435062	0.591657905	0.000791164	1.052283263	4.968554089	1	CRA004775;PRJNA827465	Yang2025_Silkie_Wenchang	0.892776493
ENSGALG00010009092	EED	2	-0.3678641	0.256926807	-1.431785592	0.152205192	0.10882443	5.442896834	81.62743057	1	PRJNA827465;Yang2025_Silkie_Wenchang	CRA004775	0.643706371
ENSGALG00010009092	EED	2	-0.543223431	0.157912374	-3.440030808	0.000581648	0	0.923105165	0	1	CRA004775;Yang2025_Silkie_Wenchang	PRJNA827465	0.025964366
ENSGALG00010009092	EED	2	-0.161164232	0.099194577	-1.624728256	0.104220474	0	0.448351782	0	1	CRA004775;PRJNA827465	Yang2025_Silkie_Wenchang	0.548669102
ENSGALG00010009093	SLU7	2	0.191942609	0.179943071	1.066685187	0.286114019	0.055183309	6.540238457	84.71003762	1	PRJNA827465;Yang2025_Silkie_Wenchang	CRA004775	0.78194146
ENSGALG00010009093	SLU7	2	0.346416322	0.107945736	3.209170964	0.001331183	0	0.479657326	0	1	CRA004775;Yang2025_Silkie_Wenchang	PRJNA827465	0.044565154
ENSGALG00010009093	SLU7	2	0.040903451	0.067288554	0.60788127	0.543266223	0	0.568489473	0	1	CRA004775;PRJNA827465	Yang2025_Silkie_Wenchang	0.876968972
ENSGALG00010009094	DARS	2	-0.115600983	0.051986473	-2.223674288	0.026170366	0	0.05335632	0	1	PRJNA827465;Yang2025_Silkie_Wenchang	CRA004775	0.276012526
ENSGALG00010009094	DARS	2	-0.126266678	0.097658544	-1.292940408	0.19603167	0	0.623885098	0	1	CRA004775;Yang2025_Silkie_Wenchang	PRJNA827465	0.707301271
ENSGALG00010009094	DARS	2	-0.132246192	0.058141057	-2.274574937	0.022931441	0	0.526312586	0	1	CRA004775;PRJNA827465	Yang2025_Silkie_Wenchang	0.258244821
ENSGALG00010009097	C1QTNF2	2	-0.160703064	0.113991309	-1.409783483	0.158603625	0	0.082063244	0	1	PRJNA827465;Yang2025_Silkie_Wenchang	CRA004775	0.652596135
ENSGALG00010009097	C1QTNF2	2	-0.171549132	0.177932706	-0.964123664	0.33498393	0	0.343729235	0	0.5	CRA004775;Yang2025_Silkie_Wenchang	PRJNA827465	0.803101658
ENSGALG00010009097	C1QTNF2	2	-0.119292428	0.140659017	-0.848096558	0.396384198	0	0.240555509	0	0.5	CRA004775;PRJNA827465	Yang2025_Silkie_Wenchang	0.831365249
ENSGALG00010009098	CAD	2	0.070359834	0.061824744	1.138052981	0.25509836	0	0.318296006	0	1	PRJNA827465;Yang2025_Silkie_Wenchang	CRA004775	0.764467569
ENSGALG00010009098	CAD	2	-0.279068957	0.449194963	-0.621264662	0.5344255	0.349840968	6.909135148	85.52640846	0.5	CRA004775;Yang2025_Silkie_Wenchang	PRJNA827465	0.873427894
ENSGALG00010009098	CAD	2	-0.307927116	0.410488359	-0.75014823	0.453165434	0.288272128	6.136098795	83.70300034	0.5	CRA004775;PRJNA827465	Yang2025_Silkie_Wenchang	0.848737721
ENSGALG00010009099	CXCR4	2	-0.120237608	0.398955486	-0.301381011	0.763123972	0.293424245	12.58357649	92.05313369	0.5	PRJNA827465;Yang2025_Silkie_Wenchang	CRA004775	0.940184187
ENSGALG00010009099	CXCR4	2	0.224749322	0.809295715	0.277709764	0.781235165	1.19373681	10.81427826	90.75296589	0.5	CRA004775;Yang2025_Silkie_Wenchang	PRJNA827465	0.945235709
ENSGALG00010009099	CXCR4	2	0.559233872	0.394801605	1.416493413	0.156631091	0.229084108	3.082536378	67.55918252	1	CRA004775;PRJNA827465	Yang2025_Silkie_Wenchang	0.64980313
ENSGALG00010009100	ENSGALG00010009100	2	-0.074492065	0.071023171	-1.048841733	0.294250966	0.000210609	1.020877377	2.04504257	1	PRJNA827465;Yang2025_Silkie_Wenchang	CRA004775	0.787343482
ENSGALG00010009100	ENSGALG00010009100	2	-0.13016405	0.098200921	-1.325487067	0.185009656	0	0.362094392	0	0.5	CRA004775;Yang2025_Silkie_Wenchang	PRJNA827465	0.693265167
ENSGALG00010009100	ENSGALG00010009100	2	-0.010989388	0.086639102	-0.126840974	0.899066265	0	0.003561112	0	0.5	CRA004775;PRJNA827465	Yang2025_Silkie_Wenchang	0.975990605
ENSGALG00010009101	HIKESHI	2	-0.322000568	0.335574199	-0.959551028	0.337281226	0.185085002	4.802663075	79.17821874	1	PRJNA827465;Yang2025_Silkie_Wenchang	CRA004775	0.804086136
ENSGALG00010009101	HIKESHI	2	-0.253094057	0.468547584	-0.540167244	0.5890817	0.352878822	5.088616515	80.34829315	0.5	CRA004775;Yang2025_Silkie_Wenchang	PRJNA827465	0.892171138
ENSGALG00010009101	HIKESHI	2	-0.033286442	0.061703853	-0.53945484	0.58957305	0	0.77152653	0	0.5	CRA004775;PRJNA827465	Yang2025_Silkie_Wenchang	0.892320726
ENSGALG00010009102	HTRA3	2	-0.095614115	0.153365249	-0.623440551	0.532995057	0	0.145240119	0	1	PRJNA827465;Yang2025_Silkie_Wenchang	CRA004775	0.873312145
ENSGALG00010009102	HTRA3	2	0.15336882	0.205336253	0.746915449	0.4551146	0.015133472	1.213215678	17.57442491	0.5	CRA004775;Yang2025_Silkie_Wenchang	PRJNA827465	0.849072665
ENSGALG00010009102	HTRA3	2	0.082376192	0.263909557	0.312137966	0.754935675	0.0813619	2.326457965	57.01620167	0.5	CRA004775;PRJNA827465	Yang2025_Silkie_Wenchang	0.93838723
ENSGALG00010009103	AP3S1	2	-0.389033482	0.40703784	-0.955767359	0.339189783	0.306306585	12.95485377	92.28088547	0.5	PRJNA827465;Yang2025_Silkie_Wenchang	CRA004775	0.804824608
ENSGALG00010009103	AP3S1	2	-0.490413695	0.345830208	-1.418076514	0.156168427	0.184481526	4.303403277	76.76257753	1	CRA004775;Yang2025_Silkie_Wenchang	PRJNA827465	0.649132948
ENSGALG00010009103	AP3S1	2	-0.015650849	0.101014705	-0.154936341	0.876871504	0	0.175253751	0	0.5	CRA004775;PRJNA827465	Yang2025_Silkie_Wenchang	0.97046577
ENSGALG00010009104	MTR	2	-0.04703386	0.060287137	-0.780164096	0.435294293	0	0.213030117	0	1	PRJNA827465;Yang2025_Silkie_Wenchang	CRA004775	0.842762754
ENSGALG00010009104	MTR	2	-0.107192194	0.115163632	-0.930781632	0.351966533	0.007307768	1.084518767	7.793204647	1	CRA004775;Yang2025_Silkie_Wenchang	PRJNA827465	0.810678834
ENSGALG00010009104	MTR	2	-0.100694757	0.182932737	-0.550446893	0.582012893	0.032748307	1.377788566	27.41992315	1	CRA004775;PRJNA827465	Yang2025_Silkie_Wenchang	0.88927624
ENSGALG00010009106	THSD7B	2	0.298092096	0.138615458	2.15049677	0.031515941	0.023733027	2.58025765	61.2441804	1	PRJNA827465;Yang2025_Silkie_Wenchang	CRA004775	0.303670272
ENSGALG00010009106	THSD7B	2	0.459705346	0.131609485	3.492949955	0.000477716	0	0.076303661	0	1	CRA004775;Yang2025_Silkie_Wenchang	PRJNA827465	0.022813698
ENSGALG00010009106	THSD7B	2	0.189530438	0.104859302	1.807473766	0.070688441	0	0.662449397	0	1	CRA004775;PRJNA827465	Yang2025_Silkie_Wenchang	0.458542662
ENSGALG00010009109	ENSGALG00010009109	2	0.075224763	0.156901967	0.479437988	0.631627074	0	0.22009751	0	0.5	PRJNA827465;Yang2025_Silkie_Wenchang	CRA004775	0.903058174
ENSGALG00010009109	ENSGALG00010009109	2	0.093789529	0.182021866	0.515265176	0.60636773	0	0.346301504	0	0.5	CRA004775;Yang2025_Silkie_Wenchang	PRJNA827465	0.896731537
ENSGALG00010009109	ENSGALG00010009109	2	0.144773888	0.145665859	0.993876597	0.32028295	0	0.036917309	0	1	CRA004775;PRJNA827465	Yang2025_Silkie_Wenchang	0.798348216
ENSGALG00010009111	LRFN1	2	0.073225609	0.130521064	0.56102522	0.574780345	0.016387313	1.914351556	47.76299072	0.5	PRJNA827465;Yang2025_Silkie_Wenchang	CRA004775	0.886183583
ENSGALG00010009111	LRFN1	2	0.053781036	0.267709495	0.20089327	0.84078203	0.076915955	1.722656666	41.9501274	0.5	CRA004775;Yang2025_Silkie_Wenchang	PRJNA827465	0.95980036
ENSGALG00010009111	LRFN1	2	-0.07182523	0.11818818	-0.607719232	0.543373705	0	0.569942355	0	1	CRA004775;PRJNA827465	Yang2025_Silkie_Wenchang	0.876968972
ENSGALG00010009112	ENSGALG00010009112	2	0.143470538	0.135164633	1.06145028	0.288485317	0	0.405642051	0	1	PRJNA827465;Yang2025_Silkie_Wenchang	CRA004775	0.783088502
ENSGALG00010009112	ENSGALG00010009112	2	0.218468746	0.203893783	1.071483118	0.283952257	0	0.383128267	0	0.5	CRA004775;Yang2025_Silkie_Wenchang	PRJNA827465	0.77978499
ENSGALG00010009112	ENSGALG00010009112	2	0.056068257	0.175118168	0.320173842	0.748836551	0	0.205923534	0	0.5	CRA004775;PRJNA827465	Yang2025_Silkie_Wenchang	0.937249951
ENSGALG00010009113	RPLP1	2	0.081221342	0.086714141	0.936656249	0.348935408	0	0.415724847	0	1	PRJNA827465;Yang2025_Silkie_Wenchang	CRA004775	0.809415141
ENSGALG00010009113	RPLP1	2	0.123673779	0.199425437	0.620150475	0.535158723	0	0.453922564	0	0.5	CRA004775;Yang2025_Silkie_Wenchang	PRJNA827465	0.87403237
ENSGALG00010009113	RPLP1	2	0.054676365	0.087517276	0.624749385	0.532135555	0	0.077710703	0	0.5	CRA004775;PRJNA827465	Yang2025_Silkie_Wenchang	0.873104119
ENSGALG00010009114	SETD7	2	0.101919598	0.136707052	0.74553285	0.45594966	0.02728069	3.402950098	70.61373305	0.5	PRJNA827465;Yang2025_Silkie_Wenchang	CRA004775	0.84907427
ENSGALG00010009114	SETD7	2	0.293738886	0.125173536	2.346653253	0.018942874	0	0.291017398	0	1	CRA004775;Yang2025_Silkie_Wenchang	PRJNA827465	0.230641818
ENSGALG00010009114	SETD7	2	0.112403736	0.20732721	0.542156217	0.587710894	0.054075164	1.959850612	48.97570285	0.5	CRA004775;PRJNA827465	Yang2025_Silkie_Wenchang	0.891742161
ENSGALG00010009115	COG5	2	-0.094544606	0.146749898	-0.644256702	0.519408987	0.036869157	6.597568428	84.84290067	0.5	PRJNA827465;Yang2025_Silkie_Wenchang	CRA004775	0.869086345
ENSGALG00010009115	COG5	2	-0.219592119	0.096321162	-2.279790994	0.022620087	0	0.938894756	0	0.5	CRA004775;Yang2025_Silkie_Wenchang	PRJNA827465	0.255732111
ENSGALG00010009115	COG5	2	0.038372638	0.049119639	0.781207654	0.434680373	0	0.004477074	0	1	CRA004775;PRJNA827465	Yang2025_Silkie_Wenchang	0.842762754
ENSGALG00010009116	S1PR3	2	0.322359916	0.088779313	3.631025121	0.000282298	0	0.797523516	0	1	PRJNA827465;Yang2025_Silkie_Wenchang	CRA004775	0.016365461
ENSGALG00010009116	S1PR3	2	0.199134578	0.132562117	1.502198238	0.13304592	0	0.356513526	0	0.5	CRA004775;Yang2025_Silkie_Wenchang	PRJNA827465	0.611090777
ENSGALG00010009116	S1PR3	2	0.357352986	0.110715068	3.22768159	0.001247978	0	0.965612342	0	0.5	CRA004775;PRJNA827465	Yang2025_Silkie_Wenchang	0.042993261
ENSGALG00010009117	ERGIC1	2	-0.043197813	0.037786305	-1.143213472	0.25294997	0.000128292	1.046434951	4.437442623	1	PRJNA827465;Yang2025_Silkie_Wenchang	CRA004775	0.762731469
ENSGALG00010009117	ERGIC1	2	-0.074995841	0.050374581	-1.488763556	0.136549642	0	0.199605275	0	1	CRA004775;Yang2025_Silkie_Wenchang	PRJNA827465	0.618470274
ENSGALG00010009117	ERGIC1	2	-0.01187514	0.045683186	-0.259945532	0.794905788	0	0.017362018	0	1	CRA004775;PRJNA827465	Yang2025_Silkie_Wenchang	0.948905846
ENSGALG00010009118	ACTN2	2	0.116353537	0.291678859	0.398909737	0.689959714	0.157795588	13.7609468	92.73305816	0.5	PRJNA827465;Yang2025_Silkie_Wenchang	CRA004775	0.919979266
ENSGALG00010009118	ACTN2	2	0.410949309	0.11479818	3.57975458	0.000343917	0	0.002362845	0	1	CRA004775;Yang2025_Silkie_Wenchang	PRJNA827465	0.018390471
ENSGALG00010009118	ACTN2	2	-0.159946912	0.104678749	-1.527978836	0.126517793	0	0.739193378	0	0.5	CRA004775;PRJNA827465	Yang2025_Silkie_Wenchang	0.597082602
ENSGALG00010009119	CISD1	2	-0.141042693	0.117849624	-1.196802231	0.231383648	0.017840274	2.566981614	61.04374124	1	PRJNA827465;Yang2025_Silkie_Wenchang	CRA004775	0.743891332
ENSGALG00010009119	CISD1	2	-0.201659872	0.152559302	-1.321845798	0.186219504	0.012982901	1.261064409	20.7019092	0.5	CRA004775;Yang2025_Silkie_Wenchang	PRJNA827465	0.695258401
ENSGALG00010009119	CISD1	2	-0.040726733	0.06785098	-0.600237951	0.548347665	0	0.146116987	0	0.5	CRA004775;PRJNA827465	Yang2025_Silkie_Wenchang	0.878281266
ENSGALG00010009120	ATG12	2	-0.065937715	0.247793026	-0.266099964	0.790162223	0.106545061	7.192418171	86.09647025	0.5	PRJNA827465;Yang2025_Silkie_Wenchang	CRA004775	0.947390191
ENSGALG00010009120	ATG12	2	-0.303649189	0.127891442	-2.374272925	0.017583548	0	0.092147476	0	1	CRA004775;Yang2025_Silkie_Wenchang	PRJNA827465	0.220991844
ENSGALG00010009120	ATG12	2	-0.009070109	0.205659016	-0.044102658	0.964822574	0.064858084	3.842564047	73.97571029	0.5	CRA004775;PRJNA827465	Yang2025_Silkie_Wenchang	0.990451382
ENSGALG00010009123	ENSGALG00010009123	2	0.758990256	0.774891047	0.979479965	0.327342883	1.181466919	61.59266608	98.37643008	0.5	PRJNA827465;Yang2025_Silkie_Wenchang	CRA004775	0.800028239
ENSGALG00010009123	ENSGALG00010009123	2	0.847816319	0.707031816	1.199120463	0.230481109	0.950214831	19.97260812	94.99314264	1	CRA004775;Yang2025_Silkie_Wenchang	PRJNA827465	0.743335644
ENSGALG00010009123	ENSGALG00010009123	2	0.007745603	0.099674314	0.077709115	0.938059444	0	0.217743076	0	0.5	CRA004775;PRJNA827465	Yang2025_Silkie_Wenchang	0.985349045
ENSGALG00010009126	JPH3	2	0.110964005	0.115476982	0.960918815	0.33659299	0.008119623	1.349519941	25.89957588	1	PRJNA827465;Yang2025_Silkie_Wenchang	CRA004775	0.803810565
ENSGALG00010009126	JPH3	2	0.276929561	0.172215751	1.608038525	0.107826742	0	0.01018885	0	1	CRA004775;Yang2025_Silkie_Wenchang	PRJNA827465	0.555864956

ENSGALG00010009126	JPH3	2	0.045690107	0.102396307	0.446208546	0.65544661	0	0.211000956	0	1	CRA004775;PRJNA827465	Yang2025_Silkie_Wenchang	0.910411938
ENSGALG00010009127	SCOC	2	-0.157895323	0.142358964	-1.109135093	0.267371908	0.036048649	9.008032083	88.8987962	1	PRJNA827465;Yang2025_Silkie_Wenchang	CRA004775	0.771398294
ENSGALG00010009127	SCOC	2	-0.057970503	0.279435198	-0.20745598	0.835653768	0.132908868	6.127313353	83.67963343	0.5	CRA004775;Yang2025_Silkie_Wenchang	PRJNA827465	0.958339923
ENSGALG00010009127	SCOC	2	0.044085996	0.116342319	0.378933452	0.704737282	0.013807997	1.5508669	35.51993404	0.5	CRA004775;PRJNA827465	Yang2025_Silkie_Wenchang	0.924152671
ENSGALG00010009129	ENSGALG00010009129	2	0.750257341	0.552732794	1.357359919	0.174666877	0.506109007	5.728308368	82.54283925	1	PRJNA827465;Yang2025_Silkie_Wenchang	CRA004775	0.679158317
ENSGALG00010009129	ENSGALG00010009129	2	1.329924346	0.233813686	5.687966218	1.28561E-08	0	0.209996067	0	1	CRA004775;Yang2025_Silkie_Wenchang	PRJNA827465	6.31605E-06
ENSGALG00010009129	ENSGALG00010009129	2	0.810246922	0.680133899	1.191305012	0.233533867	0.73711393	4.869968229	79.46598514	1	CRA004775;PRJNA827465	Yang2025_Silkie_Wenchang	0.746809711
ENSGALG00010009130	HEATR1	2	-0.06670018	0.041245182	-1.617162947	0.105843112	0	0.043442214	0	1	PRJNA827465;Yang2025_Silkie_Wenchang	CRA004775	0.552010594
ENSGALG00010009130	HEATR1	2	-0.076596475	0.06948645	-1.102322458	0.270321509	0	0.020632135	0	1	CRA004775;Yang2025_Silkie_Wenchang	PRJNA827465	0.772430311
ENSGALG00010009130	HEATR1	2	-0.059880667	0.050024502	-1.197026744	0.231296131	0	0.008105524	0	1	CRA004775;PRJNA827465	Yang2025_Silkie_Wenchang	0.743891332
ENSGALG00010009131	CDKL2	2	0.559529522	0.441589584	1.267080434	0.205126531	0.311296341	4.923623458	79.68975474	1	PRJNA827465;Yang2025_Silkie_Wenchang	CRA004775	0.717842405
ENSGALG00010009131	CDKL2	2	0.496473101	0.607838307	0.816784818	0.41405141	0.547716684	3.631556436	72.46359742	0.5	CRA004775;Yang2025_Silkie_Wenchang	PRJNA827465	0.836767923
ENSGALG00010009131	CDKL2	2	0.078230233	0.230437785	0.339485265	0.734244195	0	0.310402293	0	0.5	CRA004775;PRJNA827465	Yang2025_Silkie_Wenchang	0.933492903
ENSGALG00010009133	BOD1L1	2	0.317126825	0.408353869	0.776598067	0.437395942	0.312553176	15.21939479	93.42943649	0.5	PRJNA827465;Yang2025_Silkie_Wenchang	CRA004775	0.84377699
ENSGALG00010009133	BOD1L1	2	0.648245356	0.163890147	3.955365023	7.64179E-05	0	0.739584417	0	1	CRA004775;Yang2025_Silkie_Wenchang	PRJNA827465	0.006417633
ENSGALG00010009133	BOD1L1	2	0.117431202	0.249367768	0.470915721	0.637700921	0.093574351	3.252545955	69.25485408	0.5	CRA004775;PRJNA827465	Yang2025_Silkie_Wenchang	0.905279419
ENSGALG00010009135	TENM4	2	-0.227706777	0.098805555	-2.304594888	0.021189273	0	0.761650135	0	1	PRJNA827465;Yang2025_Silkie_Wenchang	CRA004775	0.246205819
ENSGALG00010009135	TENM4	2	-0.346989078	0.202576899	-1.712875854	0.086735381	0	0.354831703	0	1	CRA004775;Yang2025_Silkie_Wenchang	PRJNA827465	0.50653205
ENSGALG00010009135	TENM4	2	-0.179880566	0.10763755	-1.671169272	0.094688248	0	0.0435255	0	1	CRA004775;PRJNA827465	Yang2025_Silkie_Wenchang	0.526919621
ENSGALG00010009136	KHK	2	0.1049486	0.295786422	0.354812095	0.722730367	0	0.686581863	0	0.5	PRJNA827465;Yang2025_Silkie_Wenchang	CRA004775	0.929985319
ENSGALG00010009136	KHK	2	1.006650018	0.55413753	1.816606825	0.069277338	0.40850054	2.972770282	66.36134295	1	CRA004775;Yang2025_Silkie_Wenchang	PRJNA827465	0.453272383
ENSGALG00010009136	KHK	2	0.714058401	0.808583772	0.883097615	0.377183533	1.149834708	8.274416415	87.91455554	0.5	CRA004775;PRJNA827465	Yang2025_Silkie_Wenchang	0.823122953
ENSGALG00010009137	IPMK	2	-0.118230398	0.055538274	-2.128809379	0.033270035	0	0.219191942	0	1	PRJNA827465;Yang2025_Silkie_Wenchang	CRA004775	0.313333153
ENSGALG00010009137	IPMK	2	0.100366675	0.286299327	0.350565529	0.725914321	0.137073049	5.609999668	82.174687	0.5	CRA004775;Yang2025_Silkie_Wenchang	PRJNA827465	0.931029023
ENSGALG00010009137	IPMK	2	0.12050212	0.258397092	0.466344722	0.640968772	0.109124017	4.848788395	79.37629118	0.5	CRA004775;PRJNA827465	Yang2025_Silkie_Wenchang	0.906429868
ENSGALG00010009138	ENSGALG00010009138	2	-0.027758884	0.076842575	-0.361243544	0.717917394	0	0.35754142	0	0.5	PRJNA827465;Yang2025_Silkie_Wenchang	CRA004775	0.927927766
ENSGALG00010009138	ENSGALG00010009138	2	0.160285281	0.292948796	0.54714436	0.584279558	0.133188158	4.272776422	76.5960139	0.5	CRA004775;Yang2025_Silkie_Wenchang	PRJNA827465	0.890842213
ENSGALG00010009138	ENSGALG00010009138	2	0.191288655	0.237851004	0.804237326	0.421259926	0.084542319	3.461835051	71.11358614	1	CRA004775;PRJNA827465	Yang2025_Silkie_Wenchang	0.839352211
ENSGALG00010009139	CKS2	2	-0.517457296	0.352197153	-1.469226229	0.141771438	0.13866676	2.08493507	52.03687565	1	PRJNA827465;Yang2025_Silkie_Wenchang	CRA004775	0.627129189
ENSGALG00010009139	CKS2	2	-0.211747687	0.926485049	-0.228549491	0.819219085	1.248269368	3.351659699	70.16403544	0.5	CRA004775;Yang2025_Silkie_Wenchang	PRJNA827465	0.955256039
ENSGALG00010009139	CKS2	2	-0.018730195	0.461265293	-0.040606122	0.967609904	0.207680665	1.450700186	31.06776922	0.5	CRA004775;PRJNA827465	Yang2025_Silkie_Wenchang	0.991065147
ENSGALG00010009141	MAML3	2	0.042705752	0.140626313	0.303682512	0.761369782	0.03043631	4.280971254	76.64081488	0.5	PRJNA827465;Yang2025_Silkie_Wenchang	CRA004775	0.939621206
ENSGALG00010009141	MAML3	2	0.120675794	0.166977146	0.72270845	0.469859023	0.021246382	1.251874317	20.11977672	0.5	CRA004775;Yang2025_Silkie_Wenchang	PRJNA827465	0.853485658
ENSGALG00010009141	MAML3	2	-0.096549545	0.080416662	-1.200616165	0.229900128	0	0.193047394	0	1	CRA004775;PRJNA827465	Yang2025_Silkie_Wenchang	0.742767343
ENSGALG00010009142	LGALS8	2	0.050039418	0.171967043	0.290982606	0.771064622	0.045407758	4.215530927	76.27819562	0.5	PRJNA827465;Yang2025_Silkie_Wenchang	CRA004775	0.942646827
ENSGALG00010009142	LGALS8	2	-0.11726989	0.131810408	-0.889686114	0.373634452	0	0.238042827	0	0.5	CRA004775;Yang2025_Silkie_Wenchang	PRJNA827465	0.822537408
ENSGALG00010009142	LGALS8	2	0.204168253	0.09461727	2.157832843	0.03094083	0	0.053041431	0	1	CRA004775;PRJNA827465	Yang2025_Silkie_Wenchang	0.300486152
ENSGALG00010009143	MCCC2	2	0.087086011	0.104970864	0.829620785	0.406753221	0.012900266	2.281663379	56.17232546	1	PRJNA827465;Yang2025_Silkie_Wenchang	CRA004775	0.833618122
ENSGALG00010009143	MCCC2	2	0.217201544	0.114223	1.901556985	0.057229096	0	0.001233989	0	1	CRA004775;Yang2025_Silkie_Wenchang	PRJNA827465	0.414231152
ENSGALG00010009143	MCCC2	2	0.011554615	0.072161383	0.160121861	0.872785081	0	0.420379872	0	1	CRA004775;PRJNA827465	Yang2025_Silkie_Wenchang	0.969643328
ENSGALG00010009144	CREBZF	2	0.615549275	0.444586919	1.38454203	0.166192548	0.351169039	8.749673937	88.57100268	1	PRJNA827465;Yang2025_Silkie_Wenchang	CRA004775	0.66529196
ENSGALG00010009144	CREBZF	2	0.535760758	0.568857927	0.941818217	0.346285717	0.558384046	7.230881777	86.17042802	0.5	CRA004775;Yang2025_Silkie_Wenchang	PRJNA827465	0.807744676
ENSGALG00010009144	CREBZF	2	0.151155576	0.140132488	1.078661901	0.280738476	0	0.451355786	0	0.5	CRA004775;PRJNA827465	Yang2025_Silkie_Wenchang	0.778536485
ENSGALG00010009147	HNMT	2	-0.013388524	0.247046345	-0.054194381	0.956780298	0.095119974	4.530086358	77.92536563	0.5	PRJNA827465;Yang2025_Silkie_Wenchang	CRA004775	0.988540618
ENSGALG00010009147	HNMT	2	0.306293432	0.148062461	2.068677157	0.038576392	0	0.938990724	0	1	CRA004775;Yang2025_Silkie_Wenchang	PRJNA827465	0.338965227
ENSGALG00010009147	HNMT	2	0.118144317	0.423628171	0.278886829	0.780331676	0.295597862	5.341944387	81.28022444	0.5	CRA004775;PRJNA827465	Yang2025_Silkie_Wenchang	0.945041817
ENSGALG00010009149	FUT4	2	0.284459806	0.13366694	2.128123866	0.033326815	0	0.005258648	0	1	PRJNA827465;Yang2025_Silkie_Wenchang	CRA004775	0.313584825
ENSGALG00010009149	FUT4	2	0.184673558	0.205325097	0.899420288	0.368428837	0	0.999820749	0	0.5	CRA004775;Yang2025_Silkie_Wenchang	PRJNA827465	0.820279089
ENSGALG00010009149	FUT4	2	0.206420739	0.207978654	0.992509255	0.320949167	0.022637123	1.148123135	12.90132833	0.5	CRA004775;PRJNA827465	Yang2025_Silkie_Wenchang	0.798472282
ENSGALG00010009150	ENSGALG00010009150	2	0.258428396	0.424674344	0.608533101	0.542833959	0.267801845	3.878878374	74.21935148	0.5	PRJNA827465;Yang2025_Silkie_Wenchang	CRA004775	0.876968972
ENSGALG00010009150	ENSGALG00010009150	2	-0.07979832	0.871671293	-0.091546344	0.927058484	1.254401604	5.352077719	81.31566744	0.5	CRA004775;Yang2025_Silkie_Wenchang	PRJNA827465	0.982928581
ENSGALG00010009150	ENSGALG00010009150	2	-0.396585408	0.399170322	-0.993524284	0.320454522	0.1298891	1.461535011	31.57878584	1	CRA004775;PRJNA827465	Yang2025_Silkie_Wenchang	0.798348216
ENSGALG00010009151	CCNJL	2	0.50244237	0.160318816	3.134019965	0.001724291	0	0.145511039	0	1	PRJNA827465;Yang2025_Silkie_Wenchang	CRA004775	0.052534743
ENSGALG00010009151	CCNJL	2	0.064833091	0.591622989	0.109585145	0.912738392	0.548275559	4.252700178	76.48552782	0.5	CRA004775;Yang2025_Silkie_Wenchang	PRJNA827465	0.979024575
ENSGALG00010009151	CCNJL	2	0.019838184	0.525998901	0.037715257	0.969914712	0.414834331	3.561103046	71.91881316	0.5	CRA004775;PRJNA827465	Yang2025_Silkie_Wenchang	0.991572914
ENSGALG00010009152	RPP25L	2	0.042643585	0.213945477	0.19931987	0.842012537	0.049922111	2.137791086	53.22274443	0.5	PRJNA827465;Yang2025_Silkie_Wenchang	CRA004775	0.960064308
ENSGALG00010009152	RPP25L	2	-0.228684242	0.197524342	-1.157752206	0.246965172	0	0.02317223	0	1	CRA004775;Yang2025_Silkie_Wenchang	PRJNA827465	0.757459165
ENSGALG00010009152	RPP25L	2	0.065845967	0.232118077	0.283674447	0.776659879	0.050074615	1.6820819	40.54986265	0.5	CRA004775;PRJNA827465	Yang2025_Silkie_Wenchang	0.944156005
ENSGALG00010009153	SECISBP2	2	0.173301112	0.071358904	2.428584286	0.015157901	0	0.154368006	0	1	PRJNA827465;Yang2025_Silkie_Wenchang	CRA004775	0.203466871
ENSGALG00010009153	SECISBP2	2	0.158734414	0.094558519	1.67868972	0.093212531	0	0.681279309	0	1	CRA004775;Yang2025_Silkie_Wenchang	PRJNA827465	0.523212238
ENSGALG00010009153	SECISBP2	2	0.124956145	0.082799058	1.509149364	0.131260617	0	0.337346383	0	1	CRA004775;PRJNA827465	Yang2025_Silkie_Wenchang	0.606878893
ENSGALG00010009155	PAK4	2	-0.096590542	0.070269956	-1.374563862	0.169266699	0	0.25732123	0	1	PRJNA827465;Yang2025_Silkie_Wenchang	CRA004775	0.670673091
ENSGALG00010009155	PAK4	2	-0.07518609	0.097906011	-0.76794151	0.442521935	0	0.161786866	0	1	CRA004775;Yang2025_Silkie_Wenchang	PRJNA827465	0.845535782
ENSGALG00010009155	PAK4	2	-0.123860231	0.076927404	-1.610092436	0.107377678	0	0.001767076	0	1	CRA004775;PRJNA827465	Yang2025_Silkie_Wenchang	0.554714101
ENSGALG00010009157	ABHD1	2	0.018887841	0.25226998	0.074871535	0.940316925	0.102559555	4.930228061	79.71696263	0.5	PRJNA827465;Yang2025_Silkie_Wenchang	CRA004775	0.985511899
ENSGALG00010009157	ABHD1	2	0.050316653	0.339506343	0.14820534	0.882180718	0.137581526	2.184138218	54.21535176	0.5	CRA004775;Yang2025_Silkie_Wenchang	PRJNA827465	0.971942333
ENSGALG00010009157	ABHD1	2	-0.221285924	0.10955352	-2.019888759	0.043394928	0	0.204541761	0	1	CRA004775;PRJNA827465	Yang2025_Silkie_Wenchang	0.360031851
ENSGALG00010009159	PTK2	2	-0.03900069	0.032552563	-1.198083517	0.230884505	0	0.666132204	0	0.5	PRJNA827465;Yang2025_Silkie_Wenchang	CRA004775	0.743381151
ENSGALG00010009159	PTK2	2	0.058947656	0.09279325	0.635258023	0.525260146	0	0.177943213	0	1	CRA004775;Yang2025_Silkie_Wenchang	PRJNA827465	0.870186289
ENSGALG00010009159	PTK2	2	-0.043262191	0.033903644	-1.276033665	0.201943619	0	0.744219329	0	0.5	CRA004775;PRJNA827465	Yang2025_Silkie_Wenchang	0.714969076
ENSGALG00010009160	ENSGALG00010009160	2	0.075877281	0.196724506	0.385703251	0.699716455	0	0.227256709	0	0.5	PRJNA827465;Yang2025_Silkie_Wenchang	CRA004775	0.922124474
ENSGALG00010009160	ENSGALG00010009160	2	0.280870659	0.251600665	1.116370889	0.264263409	0	0.388323987	0	1	CRA004775;Yang2025_Silkie_Wenchang	PRJNA827465	0.768790489
ENSGALG00010009160	ENSGALG00010009160	2	0.119743702	0.229339972	0.522123122	0.601584609	0	0.987345837	0	0.5	CRA004775;PRJNA827465	Yang2025_Silkie_Wenchang	0.895230519
ENSGALG00010009163	SAMD12	2	-0.411907981	0.09739248	-4.229361269	2.34356E-05	0	0.46596914	0	1	PRJNA827465;Yang2025_Silkie_Wenchang	CRA004775	0.002741334
ENSGALG00010009163	SAMD12	2	-0.185338971	0.35515668	-0.521851288	0.601773877	0.203281623	5.064391301	80.25429038	0.5	CRA004775;Yang2025_Silkie_Wenchang	PRJNA827465	0.895256914
ENSGALG00010009163	SAMD12	2	-0.135627692	0.277880092	-0.488079917	0.625493236	0.119201643	4.00663016	75.04136993	0.5	CRA004775;PRJNA827465	Yang2025_Silkie_Wenchang	0.901367193
ENSGALG00010009164	TMEM128	2	-0.1647898	0.080524096	-2.04646569	0.040710575	0.002459046	1.23330532	18.91707725	1	PRJNA827465;Yang2025_Silkie_Wenchang	CRA004775	0.349194657
ENSGALG00010009164	TMEM128	2	-0.081808158	0.184798798	-0.442687718	0.657991627	0.051042901	3.848692206	74.017148	0.5	CRA004775;Yang2025_Silkie_Wenchang	PRJNA827465	0.910881331
ENSGALG00010009164	TMEM128	2	-0.018136365	0.099029631	-0.183140797	0.854687546	0.004794126	1.277357029	21.71335206	0.5	CRA004775;PRJNA827465	Yang2025_Silkie_Wenchang	0.963242367
ENSGALG00010009166	CLGN	2	-0.161022825	0.142894621	-1.126864142	0.259799926	0	0.553380682	0	1	PRJNA827465;Yang2025_Silkie_Wenchang	CRA004775	0.579047318
ENSGALG00010009166	CLGN	2	-0.039740462	0.20794914	-0.191106642	0.848442044	0	0.00484008	0	1	CRA004775;Yang2025_Silkie_Wenchang	PRJNA827465	0.961364896
ENSGALG00010009166	CLGN	2	-0.234346292	0.177719103	-1.318633105	0.187291801	0	0.078320304	0	1	CRA004775;PRJNA827465	Yang2025_Silkie_Wenchang	0.69615374
ENSGALG00010009167	DCTN3	2	0.023390576	0.148470168	0.157543965	0.874816178	0	0.089076161	0	0.5	PRJNA827465;Yang2025_Silkie_Wenchang	CRA004775	0.970159044
ENSGALG00010009167	DCTN3	2	-0.183509788	0.316269624	-0.580232101	0.561758108	0.113546879	2.206534959	54.68007447	0.5	CRA004775;Yang2025_Silkie_Wenchang	PRJNA827465	0.881802301
ENSGALG00010009167	DCTN3	2	-0.202986421	0.262982767	-0.771862062	0.440196129	0.068106153	1.796938619	44.34979641	1	CRA004775;PRJNA827465	Yang2025_Silkie_Wenchang	0.844488137
ENSGALG00010009168	MRPS12	2	-0.228791132	0.355695596	-0.643221719	0.520080242	0.187273294	3.059885666	67.31904035	0.5	PRJNA827465;Yang2025_Silkie_Wenchang	CRA004775	0.869331539
ENSGALG00010009168	MRPS12	2	-0.390579659	0.263238107	-1.483750444	0.137875138	0	0.992971084	0	1	CRA004775;Yang2025_Silkie_Wenchang	PRJNA827465	0.620651308
ENSGALG00010009168	MRPS12	2	0.019541078	0.082696594	0.236298468	0.813201076	0	0.360173593	0	0.5	CRA004775;PRJNA827465	Yang2025_Silkie_Wenchang	0.953526116
ENSGALG00010009170	RPL26L1	2	-0.143121297	0.066951564	-2.137684137	0.032542388	0	0.949513356	0	1	PRJNA827465;Yang2025_Silkie_Wenchang	CRA004775	0.310037591
ENSGALG00010009170	RPL26L1	2	-0.379538413	0.209358896	-1.812860215	0.069853384	0	0.111053476	0	1	CRA004775;Yang2025_Silkie_Wenchang	PRJNA827465	0.455777165
ENSGALG00010009170	RPL26L1	2	-0.142239388	0.066116869	-2.151332792	0.031449098	0	0.509513377	0	1	CRA004775;PRJNA827465	Yang2025_Silkie_Wenchang	0.303358988
ENSGALG00010009172	SPOPL	2	0.014282028	0.068471227	0.208584365	0.834772717	0	0.491740165	0	0.5	PRJNA827465;Yang2025_Silkie_Wenchang	CRA004775	0.958133448
ENSGALG00010009172	SPOPL	2	0.18482362	0.113841124	1.04076943	0.297982571	0	0.46087387	0	1	CRA004775;Yang2025_Silkie_Wenchang	PRJNA827465	0.788979318
ENSGALG00010009172	SPOPL	2	0.017576019	0.100548949	0.174800626	0.861236296	0.004582583	1.097328489	8.869585553	0.5	CRA004775;PRJNA827465	Yang2025_Silkie_Wenchang	0.965343309
ENSGALG00010009173	LYAR	2	0.165335322	0.15125373	1.093099127	0.274350275	0.029964805	2.533388416	60.52717406	1	PRJNA827465;Yang2025_Silkie_Wenchang	CRA004775	0.774526342
ENSGALG00010009173	LYAR	2	0.359952877	0.150106822	2.397978136	0.016485849	0	0.0034081	0	1	CRA004775;Yang2025_Silkie_Wenchang	PRJNA827465	0.212579834
ENSGALG00010009173	LYAR	2	0.090516826	0.100316817	0.90230959	0.366892433	0.005686103	1.149285904	12.98944877	1	CRA004775;PRJNA827465	Yang2025_Silkie_Wenchang	0.819577857
ENSGALG00010009178	ENSGALG00010009178	2	-0.379223461	0.27264154	-1.391290046	0.164137491	0	0.832487544	0	1	PRJNA827465;Yang2025_Silkie_Wenchang	CRA004775	0.66287462
ENSGALG00010009178	ENSGALG00010009178	2	-0.416648423	0.305386743	-1.364330418	0.172463597	0	0.801536145	0	1	CRA004775;Yang2025_Silkie_Wenchang	PRJNA827465	0.675388277
ENSGALG00010009178	ENSGALG00010009178	2	-0.111659663	0.301445423	-0.370414192	0.711071901	0	0.010582206	0	1	CRA004775;PRJNA827465	Yang2025_Silkie_Wenchang	0.925588272
ENSGALG00010009180	NFKBIB	2	-0.06076497	0.059355	-1.143433459	0.252832737	0	0.224002142	0	1	PRJNA827465;Yang2025_Silkie_Wenchang	CRA004775	0.762731469
ENSGALG00010009180	NFKBIB	2	-0.205284402	0.177843671	-1.154296921	0.248378458	0	0.18319077	0	1	CRA004775;Yang2025_Silkie_Wenchang	PRJNA827465	0.75856854
ENSGALG00010009180	NFKBIB	2	-0.067634911	0.061063608	-1.10761407	0.268028525	0	0.593547712	0	1	CRA004775;PRJNA827465	Yang2025_Silkie_Wenchang	0.771398294
ENSGALG00010009186	PREB	2	0.116674786	0.1724364	0.676625041	0.498643887	0.037842727	2.228108703	55.11888632	0.5	PRJNA827465;Yang2025_Silkie_Wenchang	CRA004775	0.861478809
ENSGALG00010009186	PREB	2	0.077283449	0.303165059	0.25492202	0.798783328	0.121505707	2.942519962	66.01552367	0.5	CRA004775;Yang2025_Silkie_Wenchang	PRJNA827465	0.949922217
ENSGALG00010009186	PREB	2	-0.018798503	0.072555039	-0.259093007	0.79556348	0	0.749951017	0	1	CRA004775;PRJNA827465	Yang2025_Silkie_Wenchang	0.949040321
ENSGALG00010009189	ENSGALG00010009189	2	0.374599028	0.384496037	0.974259788	0.329927565	0.2328854	4.650459933	78.49675055	1	PRJNA827465;Yang2025_Silkie_Wenchang	CRA004775	0.801209935
ENSGALG00010009189	ENSGALG00010009189	2	-0.466943111	1.367892545	-0.341359497	0.732832962	3.350381565	8.923679533	88.79386024	0.5	CRA004775;Yang2025_Silkie_Wenchang	PRJNA827465	0.932985777
ENSGALG00010009189	ENSGALG00010009189	2	-0.794857386	0.971556378	-0.818142318	0.413275928	1.548274774	4.817759912	79.24346331	0.5	CRA004775;PRJNA827465	Yang2025_Silkie_Wenchang	0.83606052
ENSGALG00010009190	ZBTB49	2	0.030886322	0.061880963	0.499124784	0.617691479	0	0.002250672	0	1	PRJNA827465;Yang2025_Silkie_Wenchang	CRA004775	0.899121942
ENSGALG00010009190	ZBTB49	2	-0.019748964	0.111830561	-0.176597199	0.859824795	0	0.969529392	0	0.5	CRA004775;Yang2025_Silkie_Wenchang	PRJNA827465	0.964858345
ENSGALG00010009190	ZBTB49	2	-0.000424311	0.097002931	-0.004374213	0.996509894	0.005319183	1.117809907	10.53935073	0.5	CRA004775;PRJNA827465	Yang2025_Silkie_Wenchang	0.998999891
ENSGALG00010009192	LSM14A	2	-0.030887817	0.041285452	-0.748152567	0.454368138	0.000474515	1.110621112	9.960292524	0.5	PRJNA827465;Yang2025_Silkie_Wenchang	CRA004775	0.849072665
ENSGALG00010009192	LSM14A	2	0.026391527	0.073632067	0.630045149	0.528665045	0	0.004238863	0	1	CRA004775;Yang2025_Silkie_Wenchang	PRJNA827465	0.871792397
ENSGALG00010009192	LSM14A	2	-0.046836931	0.040492785	-1.156673497	0.247405782	0	0.4273473	0	0.5	CRA004775;PRJNA827465	Yang2025_Silkie_Wenchang	0.757947282
ENSGALG00010009193	EDARADD	2	-0.511259878	0.162069819	-3.154565624	0.001607372	0	0.612156694	0	1	PRJNA827465;Yang2025_Silkie_Wenchang	CRA004775	0.050669962
ENSGALG00010009193	EDARADD	2	-0.557810791	0.2171715	-2.56852667	0.010213185	0	0.690743641	0	1	CRA004775;Yang2025_Silkie_Wenchang	PRJNA827465	0.16043518
ENSGALG00010009193	EDARADD	2	-0.371461776	0.192232417	-1.932357618	0.053315381	0	0.113620396	0	1	CRA004775;PRJNA827465	Yang2025_Silkie_Wenchang	0.400004977
ENSGALG00010009197	FH	2	-0.13974332	0.053589716	-2.607651812	0.009116563	0	0.012336958	0	1	PRJNA827465;Yang2025_Silkie_Wenchang	CRA004775	0.149669296
ENSGALG00010009197	FH	2	-0.120413359	0.097855023	-1.230528128	0.218499401	0	0.861420576	0	0.5	CRA004775;Yang2025_Silkie_Wenchang	PRJNA827465	0.73275117
ENSGALG00010009197	FH	2	-0.125063738	0.06164154	-2.028887305	0.04246977	0	0.838104185	0	0.5	CRA004775;PRJNA827465	Yang2025_Silkie_Wenchang	0.356610291
ENSGALG00010009198	KCNN2	2	0.136996354	0.100829644	1.358691204	0.174244246	0.005678925	1.374511024	27.24685487	1	PRJNA827465;Yang2025_Silkie_Wenchang	CRA004775	0.674857591
ENSGALG00010009198	KCNN2	2	0.016557959	0.129487036	0.128041842	0.898115961	0	0.003408648	0	0.5	CRA004775;Yang2025_Silkie_Wenchang	PRJNA827465	0.975573636
ENSGALG00010009198	KCNN2	2	0.209297479	0.105791335	1.978399066	0.047883705	0	0.267810804	0	0.5	CRA004775;PRJNA827465	Yang2025_Silkie_Wenchang	0.378353533
ENSGALG00010009199	SEMA4D	2	0.230918302	0.406902654	0.567502571	0.57037278	0.313466976	18.65090513	94.63832992	0.5	PRJNA827465;Yang2025_Silkie_Wenchang	CRA004775	0.8851128
ENSGALG00010009199	SEMA4D	2	-0.160158761	0.107569862	-1.488881343	0.136518617	0	0.10417808	0	1	CRA004775;Yang2025_Silkie_Wenchang	PRJNA827465	0.618440666
ENSGALG00010009199	SEMA4D	2	0.418064604	0.318667544	1.311857487	0.189568224	0.12969332	2.322461714	56.94223961	0.5	CRA004775;PRJNA827465	Yang2025_Silkie_Wenchang	0.699754721
ENSGALG00010009200	ADGRA3	2	0.176711758	0.063414036	2.786634798	0.005325846	0.001170169	1.132336123	11.68700005	1	PRJNA827465;Yang2025_Silkie_Wenchang	CRA004775	0.109021737
ENSGALG00010009200	ADGRA3	2	0.071477975	0.10908677	0.655239634	0.51231348	0	0.006922625	0	1	CRA004775;Yang2025_Silkie_Wenchang	PRJNA827465	0.865809047
ENSGALG00010009200	ADGRA3	2	0.209731512	0.065099949	3.221684726	0.001274302	0	0.247093958	0	1	CRA004775;PRJNA827465	Yang2025_Silkie_Wenchang	0.048386199
ENSGALG00010009202	FABP6	2	0.263472241	0.452466208	0.582302582	0.560362887	0.357213232	7.832114333	87.23205564	0.5	PRJNA827465;Yang2025_Silkie_Wenchang	CRA004775	0.881203589
ENSGALG00010009202	FABP6	2	0.622221966	0.190036291	3.274227061	0.001059514	0	0.473204146	0	1	CRA004775;Yang2025_Silkie_Wenchang	PRJNA827465	0.03932209
ENSGALG00010009202	FABP6	2	0.091458853	0.31386783	0.291380141	0.770760601	0.126573422	2.715303387	63.17170285	0.5	CRA004775;PRJNA827465	Yang2025_Silkie_Wenchang	0.944291331
ENSGALG00010009205	SIRT2	2	0.024514153	0.095227557	0.257427094	0.796849089	0.012913345	3.033505848	67.03484186	0.5	PRJNA827465;Yang2025_Silkie_Wenchang	CRA004775	0.949622804
ENSGALG00010009205	SIRT2	2	-0.01106173	0.226385007	-0.048862468	0.961028899	0.06503289	2.260700243	55.76591797	0.5	CRA004775;Yang2025_Silkie_Wenchang	PRJNA827465	0.989599947
ENSGALG00010009205	SIRT2	2	-0.055053233	0.040746758	-1.351107083	0.176661133	0	0.875588792	0	1	CRA004775;PRJNA827465	Yang2025_Silkie_Wenchang	0.68212128
ENSGALG00010009208	SLC5A6	2	0.547924067	0.331667007	1.652030665	0.0985283	0.183075917	5.923921252	83.11928945	1	PRJNA827465;Yang2025_Silkie_Wenchang	CRA004775	0.53598695
ENSGALG00010009208	SLC5A6	2	0.132232303	0.779645218	0.169605738	0.865320209	1.138929388	15.66527027	93.6164523	0.5	CRA004775;Yang2025_Silkie_Wenchang	PRJNA827465	0.967024637
ENSGALG00010009208	SLC5A6	2	-0.175638468	0.445794267	-0.393989967	0.693588447	0.331317151	5.717678032	82.51038281	0.5	CRA004775;PRJNA827465	Yang2025_Silkie_Wenchang	0.920721644
ENSGALG00010009210	PCDH15	2	0.374652051	0.776830677	0.482282771	0.629605085	1.060193413	8.063129101	87.59786694	0.5	PRJNA827465;Yang2025_Silkie_Wenchang	CRA004775	0.902457014
ENSGALG00010009210	PCDH15	2	0.679123985	0.665280529	1.020808449	0.307345201	0.493349189	2.092298976	52.20568325	0.5	CRA004775;Yang2025_Silkie_Wenchang	PRJNA827465	0.792675978
ENSGALG00010009210	PCDH15	2	-0.342107185	0.276936444	-1.235327426	0.216708671	0	0.042254758	0	1	CRA004775;PRJNA827465	Yang2025_Silkie_Wenchang	0.730172415
ENSGALG00010009212	NXPH2	2	-0.011972033	0.165927997	-0.072151976	0.942480963	0.00956076	1.206991195	17.14935416	0.5	PRJNA827465;Yang2025_Silkie_Wenchang	CRA004775	0.985827325
ENSGALG00010009212	NXPH2	2	-0.218510182	0.207834651	-1.051365502	0.293090754	0	0.048342771	0	1	CRA004775;Yang2025_Silkie_Wenchang	PRJNA827465	0.786774237
ENSGALG00010009212	NXPH2	2	0.073060084	0.185526223	0.393799228	0.693729274	0	0.719769232	0	0.5	CRA004775;PRJNA827465	Yang2025_Silkie_Wenchang	0.920721644
ENSGALG00010009213	DUS4L	2	0.088692531	0.058985706	1.503627535	0.132677297	0	0.414632897	0	1	PRJNA827465;Yang2025_Silkie_Wenchang	CRA004775	0.61011066
ENSGALG00010009213	DUS4L	2	0.120846601	0.107433901	1.124846066	0.26065427	0	0.527934466	0	0.5	CRA004775;Yang2025_Silkie_Wenchang	PRJNA827465	0.76772293
ENSGALG00010009213	DUS4L	2	0.058828924	0.066483182	0.884869255	0.376227152	0	0.214340097	0	0.5	CRA004775;PRJNA827465	Yang2025_Silkie_Wenchang	0.822974013
ENSGALG00010009214	PCDH10	2	0.640988864	0.170508997	3.759267107	0.000170412	0	0.838079546	0	1	PRJNA827465;Yang2025_Silkie_Wenchang	CRA004775	0.011352027
ENSGALG00010009214	PCDH10	2	0.953662863	0.26344713	3.619940227	0.000294671	0	0.636245795	0	1	CRA004775;Yang2025_Silkie_Wenchang	PRJNA827465	0.016858017
ENSGALG00010009214	PCDH10	2	0.769481299	0.392465149	1.960635996	0.0499215	0.168085085	1.770347963	43.5139294	1	CRA004775;PRJNA827465	Yang2025_Silkie_Wenchang	0.386613739
ENSGALG00010009216	NSG1	2	-0.283542976	0.173477857	-1.634462062	0.102161866	0	0.808635203	0	1	PRJNA827465;Yang2025_Silkie_Wenchang	CRA004775	0.544664652
ENSGALG00010009216	NSG1	2	-0.033420454	0.587508454	-0.05688506	0.954636755	0.478611206	2.972327695	66.35633407	0.5	CRA004775;Yang2025_Silkie_Wenchang	PRJNA827465	0.987987175
ENSGALG00010009216	NSG1	2	0.057418384	0.38461142	0.149289337	0.881325329	0.157922127	1.72360997	41.98223394	0.5	CRA004775;PRJNA827465	Yang2025_Silkie_Wenchang	0.971790895
ENSGALG00010009219	TNFRSF11B	2	0.061565387	0.143367575	0.429423367	0.667615152	0	0.203492929	0	1	PRJNA827465;Yang2025_Silkie_Wenchang	CRA004775	0.913995707
ENSGALG00010009219	TNFRSF11B	2	0.068575634	0.17795824	0.385346778	0.699980508	0	0.28550613	0	0.5	CRA004775;Yang2025_Silkie_Wenchang	PRJNA827465	0.922239748
ENSGALG00010009219	TNFRSF11B	2	-0.014915154	0.162440118	-0.091819402	0.926841529	0	0.038944021	0	0.5	CRA004775;PRJNA827465	Yang2025_Silkie_Wenchang	0.982896295
ENSGALG00010009220	GADD45G	2	-0.090247775	0.141340647	-0.638512532	0.523140099	0	0.027277449	0	1	PRJNA827465;Yang2025_Silkie_Wenchang	CRA004775	0.869569689
ENSGALG00010009220	GADD45G	2	0.340862104	0.548234085	0.621745552	0.534109193	0.420250186	2.769660328	63.89448951	0.5	CRA004775;Yang2025_Silkie_Wenchang	PRJNA827465	0.873408008
ENSGALG00010009220	GADD45G	2	0.320536582	0.573650068	0.558766746	0.57632092	0.479327878	3.054289165	67.25915766	0.5	CRA004775;PRJNA827465	Yang2025_Silkie_Wenchang	0.887048016
ENSGALG00010009223	ENSGALG00010009223	2	0.058335287	0.081731795	0.713740435	0.475387698	0	0.968978535	0	0.5	PRJNA827465;Yang2025_Silkie_Wenchang	CRA004775	0.854462864
ENSGALG00010009223	ENSGALG00010009223	2	-0.015749426	0.113564049	-0.138683198	0.889700495	0	0.098647429	0	0.5	CRA004775;Yang2025_Silkie_Wenchang	PRJNA827465	0.9731958
ENSGALG00010009223	ENSGALG00010009223	2	0.130608468	0.107310241	1.217110945	0.223562007	0	0.010419351	0	1	CRA004775;PRJNA827465	Yang2025_Silkie_Wenchang	0.737012042
ENSGALG00010009224	ENSGALG00010009224	2	-0.237779861	0.671249126	-0.354234891	0.723162858	0.796379516	8.601094655	88.3735729	0.5	PRJNA827465;Yang2025_Silkie_Wenchang	CRA004775	0.930032096
ENSGALG00010009224	ENSGALG00010009224	2	0.241942684	0.334742289	0.722772988	0.469819366	0.043520436	1.16915568	14.46819128	0.5	CRA004775;Yang2025_Silkie_Wenchang	PRJNA827465	0.853485658
ENSGALG00010009224	ENSGALG00010009224	2	-0.79355087	0.288131291	-2.754129437	0.005884849	0	0.626002321	0	1	CRA004775;PRJNA827465	Yang2025_Silkie_Wenchang	0.115819838
ENSGALG00010009225	HNRNPL	2	0.163872803	0.202924233	0.807556597	0.419345881	0.076997773	15.28892152	93.45931628	0.5	PRJNA827465;Yang2025_Silkie_Wenchang	CRA004775	0.838527387
ENSGALG00010009225	HNRNPL	2	0.248726804	0.174915775	1.421980401	0.155031941	0.038514754	2.243263608	55.42209143	0.5	CRA004775;Yang2025_Silkie_Wenchang	PRJNA827465	0.647688616
ENSGALG00010009225	HNRNPL	2	-0.033070778	0.059755499	-0.553434882	0.579965659	0	0.018641417	0	1	CRA004775;PRJNA827465	Yang2025_Silkie_Wenchang	0.888194434
ENSGALG00010009227	ENSGALG00010009227	2	0.036650972	0.06601809	0.555165591	0.578781402	0	0.404188867	0	0.5	PRJNA827465;Yang2025_Silkie_Wenchang	CRA004775	0.887888423
ENSGALG00010009227	ENSGALG00010009227	2	-0.362766414	0.232598175	-1.559627088	0.118848031	0	0.233370147	0	1	CRA004775;Yang2025_Silkie_Wenchang	PRJNA827465	0.579179687
ENSGALG00010009227	ENSGALG00010009227	2	-0.12019023	0.230883536	-0.520566481	0.602668804	0.075715727	2.725909862	63.3150012	0.5	CRA004775;PRJNA827465	Yang2025_Silkie_Wenchang	0.895527108
ENSGALG00010009228	ATP6V0E1	2	-0.360236437	0.397861856	-0.905430947	0.365237127	0.278250384	7.628845003	86.89185585	1	PRJNA827465;Yang2025_Silkie_Wenchang	CRA004775	0.818410194
ENSGALG00010009228	ATP6V0E1	2	-0.470022935	0.316862904	-1.483363716	0.137977802	0.135186595	3.044757834	67.1566655	1	CRA004775;Yang2025_Silkie_Wenchang	PRJNA827465	0.621022596
ENSGALG00010009228	ATP6V0E1	2	-0.023073846	0.082958344	-0.278137739	0.780906627	0	0.429633564	0	1	CRA004775;PRJNA827465	Yang2025_Silkie_Wenchang	0.945235709
ENSGALG00010009232	ECH1	2	-0.087628352	0.126902965	-0.690514619	0.48987062	0	0.338399297	0	0.5	PRJNA827465;Yang2025_Silkie_Wenchang	CRA004775	0.858512413
ENSGALG00010009232	ECH1	2	-0.013176081	0.207358531	-0.063542509	0.949334511	0	0.135619428	0	0.5	CRA004775;Yang2025_Silkie_Wenchang	PRJNA827465	0.98723759
ENSGALG00010009232	ECH1	2	-0.125478055	0.133014372	-0.943342084	0.345505957	0	0.002292997	0	1	CRA004775;PRJNA827465	Yang2025_Silkie_Wenchang	0.807289734
ENSGALG00010009234	PCDH18	2	-0.104994337	0.072285955	-1.452486004	0.146366518	0	0.389409752	0	1	PRJNA827465;Yang2025_Silkie_Wenchang	CRA004775	0.6352709
ENSGALG00010009234	PCDH18	2	-0.02967583	0.139781781	-0.21230113	0.831872114	0	0.01939732	0	1	CRA004775;Yang2025_Silkie_Wenchang	PRJNA827465	0.957762335
ENSGALG00010009234	PCDH18	2	-0.126384364	0.079908148	-1.581620482	0.113736235	0	0.023555607	0	1	CRA004775;PRJNA827465	Yang2025_Silkie_Wenchang	0.568978529
ENSGALG00010009235	KLHDC4	2	-0.03319845	0.05666696	-0.585851978	0.557974991	0.000273379	1.028667248	2.786833932	0.5	PRJNA827465;Yang2025_Silkie_Wenchang	CRA004775	0.880678789
ENSGALG00010009235	KLHDC4	2	0.118423844	0.114508689	1.034190903	0.301046926	0	0.747027066	0	1	CRA004775;Yang2025_Silkie_Wenchang	PRJNA827465	0.791123786
ENSGALG00010009235	KLHDC4	2	0.045337681	0.184045518	0.246339502	0.805419433	0.041639172	1.872523034	46.59611755	0.5	CRA004775;PRJNA827465	Yang2025_Silkie_Wenchang	0.951870339
ENSGALG00010009236	DTWD2	2	0.082122021	0.124783894	0.65811395	0.510464913	0	0.072897522	0	1	PRJNA827465;Yang2025_Silkie_Wenchang	CRA004775	0.865365179
ENSGALG00010009236	DTWD2	2	0.104152245	0.166630817	0.625047914	0.531939612	0	0.632835167	0	1	CRA004775;Yang2025_Silkie_Wenchang	PRJNA827465	0.873082041
ENSGALG00010009236	DTWD2	2	0.156555379	0.158747736	0.986189679	0.324040043	0	0.42630688	0	1	CRA004775;PRJNA827465	Yang2025_Silkie_Wenchang	0.799600434

ENSGALG00010009237	DENND3	2	0.242922506	0.097063117	2.502727228	0.012324049	0	0.479462437	0	1	PRJNA827465;Yang2025_Silkie_Wenchang	CRA004775	0.179397068
ENSGALG00010009237	DENND3	2	0.324381708	0.172700069	1.878295184	0.060340795	0	0.163287912	0	1	CRA004775;Yang2025_Silkie_Wenchang	PRJNA827465	0.42554715
ENSGALG00010009237	DENND3	2	0.199884934	0.109694668	1.822193705	0.068425599	0	0.007894687	0	1	CRA004775;PRJNA827465	Yang2025_Silkie_Wenchang	0.450776288
ENSGALG00010009239	STX18	2	-0.192465806	0.194124392	-0.991456063	0.321462937	0.067942743	9.736130918	89.72897953	1	PRJNA827465;Yang2025_Silkie_Wenchang	CRA004775	0.798513866
ENSGALG00010009239	STX18	2	-0.192684346	0.227262894	-0.847847804	0.396522736	0.081816095	4.66431377	78.560619	0.5	CRA004775;Yang2025_Silkie_Wenchang	PRJNA827465	0.831365249
ENSGALG00010009239	STX18	2	-0.006016853	0.049185445	-0.122329941	0.902637721	0	0.131635602	0	0.5	CRA004775;PRJNA827465	Yang2025_Silkie_Wenchang	0.976923607
ENSGALG00010009243	BCAP29	2	-0.389083539	0.432068139	-0.900514303	0.367846617	0.361396121	30.83622855	96.75706127	0.5	PRJNA827465;Yang2025_Silkie_Wenchang	CRA004775	0.820142704
ENSGALG00010009243	BCAP29	2	-0.497346997	0.334635508	-1.48623498	0.137216978	0.203011765	10.68537306	90.64141238	1	CRA004775;Yang2025_Silkie_Wenchang	PRJNA827465	0.619615281
ENSGALG00010009243	BCAP29	2	-0.01752587	0.086308719	-0.203060253	0.839087938	0.005363806	1.392906383	28.20766617	0.5	CRA004775;PRJNA827465	Yang2025_Silkie_Wenchang	0.959403482
ENSGALG00010009246	RUSC2	2	0.008119953	0.149351638	0.054368023	0.956641955	0.019467157	1.773707247	43.62091027	0.5	PRJNA827465;Yang2025_Silkie_Wenchang	CRA004775	0.988540618
ENSGALG00010009246	RUSC2	2	0.167270772	0.148143382	1.129114032	0.258849733	0	0.067375052	0	1	CRA004775;Yang2025_Silkie_Wenchang	PRJNA827465	0.765965607
ENSGALG00010009246	RUSC2	2	-0.099823408	0.152721189	-0.653631683	0.513349127	0	0.729572864	0	0.5	CRA004775;PRJNA827465	Yang2025_Silkie_Wenchang	0.866301448
ENSGALG00010009250	ERO1B	2	-0.071220623	0.155727425	-0.457341557	0.647425564	0.03857297	4.732427301	78.86919468	0.5	PRJNA827465;Yang2025_Silkie_Wenchang	CRA004775	0.907350807
ENSGALG00010009250	ERO1B	2	-0.19588656	0.11131111	-1.759811395	0.07843979	0	0.88064906	0	0.5	CRA004775;Yang2025_Silkie_Wenchang	PRJNA827465	0.481856686
ENSGALG00010009250	ERO1B	2	0.068821417	0.073899682	0.931281641	0.351707896	0	0.010458763	0	1	CRA004775;PRJNA827465	Yang2025_Silkie_Wenchang	0.810678834
ENSGALG00010009252	SIRT1	2	-0.082432564	0.129203262	-0.638006832	0.523469232	0.026478182	4.405332673	77.30023873	0.5	PRJNA827465;Yang2025_Silkie_Wenchang	CRA004775	0.869661505
ENSGALG00010009252	SIRT1	2	-0.046174033	0.232783081	-0.198356482	0.842766163	0.07628723	3.054820628	67.26485376	0.5	CRA004775;Yang2025_Silkie_Wenchang	PRJNA827465	0.96035733
ENSGALG00010009252	SIRT1	2	0.035498427	0.047342834	0.749816282	0.453365361	0	0.726395797	0	1	CRA004775;PRJNA827465	Yang2025_Silkie_Wenchang	0.848869458
ENSGALG00010009253	SLC45A4	2	-0.127948725	0.093476201	-1.368783973	0.171066812	0	0.388353295	0	1	PRJNA827465;Yang2025_Silkie_Wenchang	CRA004775	0.673219077
ENSGALG00010009253	SLC45A4	2	-0.259073773	0.149995798	-1.727206876	0.084130516	0	0.96440497	0	1	CRA004775;Yang2025_Silkie_Wenchang	PRJNA827465	0.499714459
ENSGALG00010009253	SLC45A4	2	-0.220692876	0.250072866	-0.882514285	0.377498758	0.067434731	1.606100903	37.73741128	1	CRA004775;PRJNA827465	Yang2025_Silkie_Wenchang	0.82325159
ENSGALG00010009256	CREBRF	2	-0.039066371	0.087506708	-0.446438592	0.655280461	0	0.25248546	0	1	PRJNA827465;Yang2025_Silkie_Wenchang	CRA004775	0.910411938
ENSGALG00010009256	CREBRF	2	-0.127573545	0.152774517	-0.835044666	0.40369256	0	0.025895289	0	1	CRA004775;Yang2025_Silkie_Wenchang	PRJNA827465	0.833112102
ENSGALG00010009256	CREBRF	2	-0.027760774	0.096677017	-0.28714967	0.773997722	0	0.223823012	0	1	CRA004775;PRJNA827465	Yang2025_Silkie_Wenchang	0.943248868
ENSGALG00010009257	ENSGALG00010009257	2	0.032372637	0.258488673	0.125238124	0.900335036	0.119027077	9.121929364	89.03740689	0.5	PRJNA827465;Yang2025_Silkie_Wenchang	CRA004775	0.976475201
ENSGALG00010009257	ENSGALG00010009257	2	0.28886033	0.128872804	2.241437461	0.024997752	0	0.080078183	0	1	CRA004775;Yang2025_Silkie_Wenchang	PRJNA827465	0.269433603
ENSGALG00010009257	ENSGALG00010009257	2	-0.213088646	0.108542902	-1.963174387	0.049625914	0	0.214991204	0	0.5	CRA004775;PRJNA827465	Yang2025_Silkie_Wenchang	0.38592151
ENSGALG00010009258	DNAJC12	2	-0.025618822	0.142330282	-0.179995584	0.857156035	0.018361498	1.826692941	45.25626188	0.5	PRJNA827465;Yang2025_Silkie_Wenchang	CRA004775	0.963878155
ENSGALG00010009258	DNAJC12	2	0.111047844	0.130303447	0.852224911	0.394089273	0	5.43125E-05	0	1	CRA004775;Yang2025_Silkie_Wenchang	PRJNA827465	0.830486298
ENSGALG00010009258	DNAJC12	2	-0.116706102	0.137709886	-0.847478017	0.396728735	0	0.674290662	0	0.5	CRA004775;PRJNA827465	Yang2025_Silkie_Wenchang	0.831375233
ENSGALG00010009263	COLEC10	2	-0.149398952	0.205989577	-0.725274326	0.468283753	0.071755396	6.47355666	84.55254117	0.5	PRJNA827465;Yang2025_Silkie_Wenchang	CRA004775	0.853198008
ENSGALG00010009263	COLEC10	2	-0.259456959	0.197244139	-1.315410234	0.188372068	0.035187015	1.382674462	27.67639618	0.5	CRA004775;Yang2025_Silkie_Wenchang	PRJNA827465	0.697727584
ENSGALG00010009263	COLEC10	2	0.064171344	0.111707678	0.574457776	0.565658066	0	0.046123348	0	1	CRA004775;PRJNA827465	Yang2025_Silkie_Wenchang	0.883487517
ENSGALG00010009266	PCDH7	2	0.110618715	0.206138631	0.536622927	0.591528104	0.035191641	1.590932871	37.14379669	0.5	PRJNA827465;Yang2025_Silkie_Wenchang	CRA004775	0.892776493
ENSGALG00010009266	PCDH7	2	0.463760626	0.283718001	1.634583016	0.10213649	0	0.455330098	0	1	CRA004775;Yang2025_Silkie_Wenchang	PRJNA827465	0.54460324
ENSGALG00010009266	PCDH7	2	0.145039963	0.399784233	0.362795607	0.716757574	0.16388578	1.412606765	29.20889061	0.5	CRA004775;PRJNA827465	Yang2025_Silkie_Wenchang	0.92761444
ENSGALG00010009268	PWWP2A	2	-0.091921639	0.050945688	-1.804306552	0.071183263	0	0.526932814	0	1	PRJNA827465;Yang2025_Silkie_Wenchang	CRA004775	0.460681013
ENSGALG00010009268	PWWP2A	2	-0.15129844	0.097333764	-1.554429147	0.120082085	0	0.035038687	0	1	CRA004775;Yang2025_Silkie_Wenchang	PRJNA827465	0.581229827
ENSGALG00010009268	PWWP2A	2	-0.073246309	0.05675183	-1.290642249	0.196827759	0	0.018620757	0	1	CRA004775;PRJNA827465	Yang2025_Silkie_Wenchang	0.707994211
ENSGALG00010009273	MAL2	2	0.285108616	0.719976174	0.395997294	0.692107025	0.631384246	2.523857985	60.37811929	0.5	PRJNA827465;Yang2025_Silkie_Wenchang	CRA004775	0.920348122
ENSGALG00010009273	MAL2	2	0.265609389	0.723858094	0.366935718	0.713666968	0.60655594	2.361526697	57.65451217	0.5	CRA004775;Yang2025_Silkie_Wenchang	PRJNA827465	0.926292039
ENSGALG00010009273	MAL2	2	0.9431615	0.415797781	2.268317783	0.023309845	0	2.74502E-05	0	1	CRA004775;PRJNA827465	Yang2025_Silkie_Wenchang	0.260268986
ENSGALG00010009275	CBLL1	2	0.311468154	0.379792502	0.82010085	0.412158618	0.24294353	5.461390942	81.68964627	0.5	PRJNA827465;Yang2025_Silkie_Wenchang	CRA004775	0.835532508
ENSGALG00010009275	CBLL1	2	0.269528865	0.444904417	0.605812968	0.544638958	0.336344527	6.26014887	84.02593899	0.5	CRA004775;Yang2025_Silkie_Wenchang	PRJNA827465	0.877345822
ENSGALG00010009275	CBLL1	2	-0.022105304	0.051132263	-0.432316163	0.665511641	0	0.612437751	0	1	CRA004775;PRJNA827465	Yang2025_Silkie_Wenchang	0.91350776
ENSGALG00010009276	ENSGALG00010009276	2	-0.08419659	0.102210068	-0.823760233	0.410075807	0	0.030739754	0	1	PRJNA827465;Yang2025_Silkie_Wenchang	CRA004775	0.834652129
ENSGALG00010009276	ENSGALG00010009276	2	-0.162365239	0.171413509	-0.947213787	0.343529852	0	0.611816994	0	1	CRA004775;Yang2025_Silkie_Wenchang	PRJNA827465	0.806276913
ENSGALG00010009276	ENSGALG00010009276	2	-0.099444794	0.119166761	-0.834501105	0.403998665	0	0.791717222	0	1	CRA004775;PRJNA827465	Yang2025_Silkie_Wenchang	0.833112102
ENSGALG00010009277	ACTN4	2	0.090495728	0.147311587	0.614315073	0.539007144	0.038939376	9.648682021	89.63589019	0.5	PRJNA827465;Yang2025_Silkie_Wenchang	CRA004775	0.87481821
ENSGALG00010009277	ACTN4	2	0.223785855	0.07255998	3.08414991	0.002041347	0	0.615535507	0	1	CRA004775;Yang2025_Silkie_Wenchang	PRJNA827465	0.058691341
ENSGALG00010009277	ACTN4	2	-0.046804875	0.054811511	-0.853924188	0.393146997	0	0.178917994	0	0.5	CRA004775;PRJNA827465	Yang2025_Silkie_Wenchang	0.830298571
ENSGALG00010009279	ELMOD2	2	-0.192423967	0.202137574	-0.951945567	0.341124588	0.077665942	19.81173373	94.95248617	0.5	PRJNA827465;Yang2025_Silkie_Wenchang	CRA004775	0.805646109
ENSGALG00010009279	ELMOD2	2	-0.387086382	0.077844436	-4.972563291	6.60734E-07	0	0.340814847	0	1	CRA004775;Yang2025_Silkie_Wenchang	PRJNA827465	0.000159318
ENSGALG00010009279	ELMOD2	2	-0.001647713	0.040122264	-0.041067308	0.967242237	0	0.775378931	0	0.5	CRA004775;PRJNA827465	Yang2025_Silkie_Wenchang	0.990894822
ENSGALG00010009280	HSD17B4	2	0.059748582	0.10653546	0.560832821	0.57491151	0	0.005393849	0	1	PRJNA827465;Yang2025_Silkie_Wenchang	CRA004775	0.886211989
ENSGALG00010009280	HSD17B4	2	-0.01758861	0.112437463	-0.156430156	0.875693969	0	0.702440175	0	0.5	CRA004775;Yang2025_Silkie_Wenchang	PRJNA827465	0.970347777
ENSGALG00010009280	HSD17B4	2	-0.022749385	0.123193981	-0.184663123	0.853493272	0	0.727907534	0	0.5	CRA004775;PRJNA827465	Yang2025_Silkie_Wenchang	0.962964214
ENSGALG00010009284	CCN3	2	0.687258839	0.283140964	2.427267425	0.015213037	0	0.029753697	0	1	PRJNA827465;Yang2025_Silkie_Wenchang	CRA004775	0.20392838
ENSGALG00010009284	CCN3	2	0.115123152	0.743186817	0.154904729	0.876896426	0.784001381	3.184496011	68.59785672	0.5	CRA004775;Yang2025_Silkie_Wenchang	PRJNA827465	0.97046577
ENSGALG00010009284	CCN3	2	0.067750101	0.693008104	0.09776235	0.922121005	0.629455375	2.700359597	62.96789506	0.5	CRA004775;PRJNA827465	Yang2025_Silkie_Wenchang	0.981772229
ENSGALG00010009286	ENSGALG00010009286	2	0.637446879	0.125757851	5.068843589	4.0024E-07	0	0.010650449	0	1	PRJNA827465;Yang2025_Silkie_Wenchang	CRA004775	0.000106288
ENSGALG00010009286	ENSGALG00010009286	2	0.261917026	0.396680549	0.660271916	0.509079349	0.205678638	2.881620528	65.2973044	0.5	CRA004775;Yang2025_Silkie_Wenchang	PRJNA827465	0.865193604
ENSGALG00010009286	ENSGALG00010009286	2	0.308676223	0.373871728	0.825620659	0.409019312	0.226302282	4.828650797	79.29028124	0.5	CRA004775;PRJNA827465	Yang2025_Silkie_Wenchang	0.834316328
ENSGALG00010009289	GPR137B	2	-0.04519812	0.082087656	-0.550607998	0.581902425	0	0.006990045	0	1	PRJNA827465;Yang2025_Silkie_Wenchang	CRA004775	0.889192798
ENSGALG00010009289	GPR137B	2	-0.149519585	0.175181918	-0.853510378	0.393376335	0	0.661445188	0	1	CRA004775;Yang2025_Silkie_Wenchang	PRJNA827465	0.830346712
ENSGALG00010009289	GPR137B	2	-0.065020841	0.086398386	-0.752570088	0.451708298	0	0.958654705	0	1	CRA004775;PRJNA827465	Yang2025_Silkie_Wenchang	0.848006549
ENSGALG00010009294	ACOX3	2	-0.165217645	0.100619017	-1.642012119	0.100587493	0.008996355	1.739345252	42.50710153	1	PRJNA827465;Yang2025_Silkie_Wenchang	CRA004775	0.541393598
ENSGALG00010009294	ACOX3	2	-0.257847432	0.116322917	-2.216652037	0.026646879	0	0.329324339	0	1	CRA004775;Yang2025_Silkie_Wenchang	PRJNA827465	0.278694271
ENSGALG00010009294	ACOX3	2	-0.086917077	0.083817739	-1.036977108	0.299746526	0	0.013573033	0	1	CRA004775;PRJNA827465	Yang2025_Silkie_Wenchang	0.79004225
ENSGALG00010009297	CTNNA3	2	-0.362293508	0.272499186	-1.329521432	0.183676	0.096358751	2.826223916	64.61709936	1	PRJNA827465;Yang2025_Silkie_Wenchang	CRA004775	0.692148694
ENSGALG00010009297	CTNNA3	2	0.053278892	0.245155784	0.217326677	0.827953769	0.013424946	1.078960762	7.318223693	0.5	CRA004775;Yang2025_Silkie_Wenchang	PRJNA827465	0.95655566
ENSGALG00010009297	CTNNA3	2	-0.143756303	0.566848247	-0.253606329	0.799799707	0.502885734	4.133740899	75.80883697	0.5	CRA004775;PRJNA827465	Yang2025_Silkie_Wenchang	0.949922217
ENSGALG00010009299	MAP4K1	2	0.310446845	0.233717261	1.328300888	0.184078726	0	0.041925637	0	1	PRJNA827465;Yang2025_Silkie_Wenchang	CRA004775	0.692545576
ENSGALG00010009299	MAP4K1	2	-0.120953999	0.359293266	-0.336644212	0.736385123	0.069014731	1.364807682	26.72960352	0.5	CRA004775;Yang2025_Silkie_Wenchang	PRJNA827465	0.933661344
ENSGALG00010009299	MAP4K1	2	-0.001697633	0.406114245	-0.004180185	0.996664704	0.205369785	2.532146121	60.50780832	0.5	CRA004775;PRJNA827465	Yang2025_Silkie_Wenchang	0.99895145
ENSGALG00010009303	TTC1	2	0.037059641	0.049161582	0.753833359	0.45094929	0	0.212024805	0	1	PRJNA827465;Yang2025_Silkie_Wenchang	CRA004775	0.847738076
ENSGALG00010009303	TTC1	2	0.073359145	0.104723991	0.7004999	0.483615167	0	0.061194031	0	1	CRA004775;Yang2025_Silkie_Wenchang	PRJNA827465	0.85691916
ENSGALG00010009303	TTC1	2	0.02557687	0.053507107	0.478008839	0.632643914	0	0.003025759	0	1	CRA004775;PRJNA827465	Yang2025_Silkie_Wenchang	0.903058174
ENSGALG00010009306	TBC1D9	2	-0.057627179	0.096104594	-0.599629808	0.548752977	0.007519349	1.66845425	40.06428404	0.5	PRJNA827465;Yang2025_Silkie_Wenchang	CRA004775	0.878281266
ENSGALG00010009306	TBC1D9	2	-0.135797633	0.109403285	-1.241257367	0.214510688	0	0.582076373	0	0.5	CRA004775;Yang2025_Silkie_Wenchang	PRJNA827465	0.727205473
ENSGALG00010009306	TBC1D9	2	0.032233109	0.09034533	0.356776705	0.721258977	0	0.036423938	0	1	CRA004775;PRJNA827465	Yang2025_Silkie_Wenchang	0.928950734
ENSGALG00010009307	DIRAS2	2	0.833009355	0.355949236	2.340247631	0.019270958	0.187329291	3.826602978	73.86716088	1	PRJNA827465;Yang2025_Silkie_Wenchang	CRA004775	0.232624497
ENSGALG00010009307	DIRAS2	2	1.192091945	0.207793135	5.73691688	9.64155E-09	0	0.010733878	0	1	CRA004775;Yang2025_Silkie_Wenchang	PRJNA827465	4.85823E-06
ENSGALG00010009307	DIRAS2	2	0.790104725	0.376047658	2.10107604	0.035634292	0.172461119	2.475529453	59.604601	1	CRA004775;PRJNA827465	Yang2025_Silkie_Wenchang	0.325599715
ENSGALG00010009309	TRIM54	2	0.01676198	0.16333412	0.102623874	0.918261494	0.037436942	3.229300961	69.0335459	0.5	PRJNA827465;Yang2025_Silkie_Wenchang	CRA004775	0.981033557
ENSGALG00010009309	TRIM54	2	0.115312005	0.213373831	0.540422432	0.588905741	0.029423348	1.18770337	15.80389299	0.5	CRA004775;Yang2025_Silkie_Wenchang	PRJNA827465	0.892171138
ENSGALG00010009309	TRIM54	2	-0.136284874	0.097882655	-1.392329156	0.163822746	0	0.263200576	0	1	CRA004775;PRJNA827465	Yang2025_Silkie_Wenchang	0.662308161
ENSGALG00010009311	BNIP1	2	-0.053805572	0.066144171	-0.813459006	0.415954941	0	0.073881357	0	1	PRJNA827465;Yang2025_Silkie_Wenchang	CRA004775	0.837742614
ENSGALG00010009311	BNIP1	2	-0.086680214	0.106431474	-0.814422756	0.415402807	0	0.001038812	0	1	CRA004775;Yang2025_Silkie_Wenchang	PRJNA827465	0.837603966
ENSGALG00010009311	BNIP1	2	-0.050306409	0.070933682	-0.70920341	0.478198258	0	0.058306623	0	1	CRA004775;PRJNA827465	Yang2025_Silkie_Wenchang	0.855388572
ENSGALG00010009313	UNC13B	2	-0.2122589	0.164670794	-1.288989349	0.197401789	0	0.030594429	0	1	PRJNA827465;Yang2025_Silkie_Wenchang	CRA004775	0.708952382
ENSGALG00010009313	UNC13B	2	0.186670362	0.512287264	0.364386107	0.715569709	0.388285659	3.542664536	71.77265897	0.5	CRA004775;Yang2025_Silkie_Wenchang	PRJNA827465	0.927131632
ENSGALG00010009313	UNC13B	2	0.205518093	0.480930891	0.427333941	0.669136115	0.334508097	3.259977243	69.32493925	0.5	CRA004775;PRJNA827465	Yang2025_Silkie_Wenchang	0.914377769
ENSGALG00010009320	GPR20	2	-0.113186204	0.12765202	-0.886677733	0.37525243	0.011974932	1.523867374	34.3774913	1	PRJNA827465;Yang2025_Silkie_Wenchang	CRA004775	0.822941424
ENSGALG00010009320	GPR20	2	0.537401598	0.874303884	0.614662257	0.538777791	1.398651756	11.17630267	91.05249715	0.5	CRA004775;Yang2025_Silkie_Wenchang	PRJNA827465	0.87481821
ENSGALG00010009320	GPR20	2	0.652741116	0.740420885	0.881581177	0.378003338	0.97969093	8.618024727	88.39641296	0.5	CRA004775;PRJNA827465	Yang2025_Silkie_Wenchang	0.82325159
ENSGALG00010009322	ENSGALG00010009322	2	0.202588283	0.09878359	2.050829322	0.040283571	0	0.276630861	0	1	PRJNA827465;Yang2025_Silkie_Wenchang	CRA004775	0.347442284
ENSGALG00010009322	ENSGALG00010009322	2	0.009970887	0.224312483	0.04445088	0.964545005	0	0.110524996	0	0.5	CRA004775;Yang2025_Silkie_Wenchang	PRJNA827465	0.990364463
ENSGALG00010009322	ENSGALG00010009322	2	0.1994418	0.101694187	1.961191739	0.04985666	0	0.639562096	0	0.5	CRA004775;PRJNA827465	Yang2025_Silkie_Wenchang	0.38629001
ENSGALG00010009323	RNF150	2	-0.054062071	0.057688679	-0.937134845	0.3486892	0	0.410135041	0	0.5	PRJNA827465;Yang2025_Silkie_Wenchang	CRA004775	0.809333419
ENSGALG00010009323	RNF150	2	0.074300679	0.134840158	0.551027828	0.581614598	0	0.395555453	0	1	CRA004775;Yang2025_Silkie_Wenchang	PRJNA827465	0.889045686
ENSGALG00010009323	RNF150	2	-0.055591744	0.065637123	-0.846955832	0.39701974	0.00059034	1.011470351	1.134027453	0.5	CRA004775;PRJNA827465	Yang2025_Silkie_Wenchang	0.831438925
ENSGALG00010009326	DLD	2	0.05960681	0.133666055	0.445938278	0.655641831	0.030558333	6.890400187	85.48705485	0.5	PRJNA827465;Yang2025_Silkie_Wenchang	CRA004775	0.910411938
ENSGALG00010009326	DLD	2	0.203656094	0.074477142	2.734477816	0.006247931	0	0.190554603	0	1	CRA004775;Yang2025_Silkie_Wenchang	PRJNA827465	0.119903522
ENSGALG00010009326	DLD	2	0.015508599	0.167775758	0.09243647	0.926351265	0.031218277	1.589013194	37.06786051	0.5	CRA004775;PRJNA827465	Yang2025_Silkie_Wenchang	0.98280842
ENSGALG00010009327	NID1	2	-0.384883302	0.220349261	-1.746696588	0.080689983	0.089233055	12.31718477	91.88126168	1	PRJNA827465;Yang2025_Silkie_Wenchang	CRA004775	0.48902982
ENSGALG00010009327	NID1	2	-0.15894109	0.525425007	-0.302500048	0.762270897	0.468398775	5.795651319	82.74568388	0.5	CRA004775;Yang2025_Silkie_Wenchang	PRJNA827465	0.94006317
ENSGALG00010009327	NID1	2	0.000817832	0.276360006	0.002959299	0.997638824	0.097574817	2.009295017	50.23130044	0.5	CRA004775;PRJNA827465	Yang2025_Silkie_Wenchang	0.999290321
ENSGALG00010009328	ENSGALG00010009328	2	-0.015456536	0.116029462	-0.133212169	0.894025587	0.00339328	1.131886159	11.65189253	0.5	PRJNA827465;Yang2025_Silkie_Wenchang	CRA004775	0.974381724
ENSGALG00010009328	ENSGALG00010009328	2	-0.204040353	0.164490215	-1.240440674	0.214812445	0	0.181312997	0	1	CRA004775;Yang2025_Silkie_Wenchang	PRJNA827465	0.728013582
ENSGALG00010009328	ENSGALG00010009328	2	0.006208935	0.152116804	0.040816893	0.967441872	0.009746615	1.126128276	11.2001695	0.5	CRA004775;PRJNA827465	Yang2025_Silkie_Wenchang	0.99101772
ENSGALG00010009330	EIF3K	2	-0.080354828	0.208695261	-0.385034272	0.700212024	0.067142062	3.690708666	72.90493261	0.5	PRJNA827465;Yang2025_Silkie_Wenchang	CRA004775	0.922299095
ENSGALG00010009330	EIF3K	2	-0.353379318	0.1610329	-2.194454168	0.028202775	0	0.003530227	0	1	CRA004775;Yang2025_Silkie_Wenchang	PRJNA827465	0.286126402
ENSGALG00010009330	EIF3K	2	-0.078287485	0.215582855	-0.363143371	0.716497788	0.069387826	3.226562663	69.00726549	0.5	CRA004775;PRJNA827465	Yang2025_Silkie_Wenchang	0.927487786
ENSGALG00010009332	ENSGALG00010009332	2	0.289855747	0.112532106	2.57576045	0.010001991	0.018483762	3.466413124	71.15173627	1	PRJNA827465;Yang2025_Silkie_Wenchang	CRA004775	0.158447508
ENSGALG00010009332	ENSGALG00010009332	2	0.026515191	0.424987534	0.062390514	0.950251849	0.325310531	9.60404337	89.58771882	0.5	CRA004775;Yang2025_Silkie_Wenchang	PRJNA827465	0.987277827
ENSGALG00010009332	ENSGALG00010009332	2	-0.069639587	0.308328384	-0.225861744	0.821308953	0.161152546	5.782449425	82.7062906	0.5	CRA004775;PRJNA827465	Yang2025_Silkie_Wenchang	0.955598716
ENSGALG00010009333	ADRA1B	2	0.138042441	0.149497438	0.923376632	0.355810965	0	0.853875135	0	1	PRJNA827465;Yang2025_Silkie_Wenchang	CRA004775	0.812301302
ENSGALG00010009333	ADRA1B	2	0.389279848	0.434113827	0.896722988	0.369866751	0.288051398	3.954960734	74.7152989	1	CRA004775;Yang2025_Silkie_Wenchang	PRJNA827465	0.820829216
ENSGALG00010009333	ADRA1B	2	0.507252994	0.287000246	1.767430522	0.077156155	0.076798713	1.745367615	42.70547986	1	CRA004775;PRJNA827465	Yang2025_Silkie_Wenchang	0.478533748
ENSGALG00010009334	SS18L2	2	0.134736651	0.19832321	0.679379136	0.496897666	0.054821049	3.098151538	67.72268923	0.5	PRJNA827465;Yang2025_Silkie_Wenchang	CRA004775	0.860939846
ENSGALG00010009334	SS18L2	2	0.055245496	0.359125276	0.153833494	0.877741024	0.188137202	3.529473378	71.66716128	0.5	CRA004775;Yang2025_Silkie_Wenchang	PRJNA827465	0.970868079
ENSGALG00010009334	SS18L2	2	-0.065853483	0.106002193	-0.621246424	0.534437498	0	0.854167448	0	1	CRA004775;PRJNA827465	Yang2025_Silkie_Wenchang	0.873427894
ENSGALG00010009335	PTP4A3	2	-0.108965812	0.082571496	-1.319654078	0.186950539	0	0.096522245	0	1	PRJNA827465;Yang2025_Silkie_Wenchang	CRA004775	0.695858627
ENSGALG00010009335	PTP4A3	2	-0.069946429	0.140311234	-0.498509112	0.618125248	0	0.001023768	0	1	CRA004775;Yang2025_Silkie_Wenchang	PRJNA827465	0.899121942
ENSGALG00010009335	PTP4A3	2	-0.1235109	0.097836086	-1.262426827	0.206795241	0	0.02093133	0	1	CRA004775;PRJNA827465	Yang2025_Silkie_Wenchang	0.71868063
ENSGALG00010009336	LAMB1	2	-0.158003187	0.070795712	-2.231818611	0.025626954	0	0.008563923	0	1	PRJNA827465;Yang2025_Silkie_Wenchang	CRA004775	0.27294819
ENSGALG00010009336	LAMB1	2	0.126630936	0.371171982	0.341165126	0.732979275	0.203531043	3.118841107	67.93680839	0.5	CRA004775;Yang2025_Silkie_Wenchang	PRJNA827465	0.932985777
ENSGALG00010009336	LAMB1	2	0.120556985	0.378183113	0.318779398	0.749893802	0.214855561	3.261242616	69.33684127	0.5	CRA004775;PRJNA827465	Yang2025_Silkie_Wenchang	0.937362187
ENSGALG00010009337	ZNF330	2	0.203817361	0.331160686	0.615463641	0.538248574	0.209113294	21.15059295	95.27200016	0.5	PRJNA827465;Yang2025_Silkie_Wenchang	CRA004775	0.87481821
ENSGALG00010009337	ZNF330	2	0.195048783	0.378414907	0.515436309	0.606248165	0.2486852	7.278976296	86.26180442	0.5	CRA004775;Yang2025_Silkie_Wenchang	PRJNA827465	0.896709756
ENSGALG00010009337	ZNF330	2	-0.125116231	0.064978581	-1.925499572	0.054166889	0	0.138725144	0	1	CRA004775;PRJNA827465	Yang2025_Silkie_Wenchang	0.40335757
ENSGALG00010009338	KYNU	2	0.786308516	0.703545023	1.117637807	0.263721715	0.760153694	4.307927515	76.78698175	1	PRJNA827465;Yang2025_Silkie_Wenchang	CRA004775	0.768643821
ENSGALG00010009338	KYNU	2	0.015017185	0.433094951	0.034674116	0.972339601	0	0.107488408	0	0.5	CRA004775;Yang2025_Silkie_Wenchang	PRJNA827465	0.992078693
ENSGALG00010009338	KYNU	2	0.824901517	0.858341706	0.961040937	0.336531585	0.949127831	2.524171593	60.38304199	0.5	CRA004775;PRJNA827465	Yang2025_Silkie_Wenchang	0.803806405
ENSGALG00010009340	SYK	2	0.16882755	0.143569188	1.175931631	0.239622198	0.01501545	1.383278944	27.70800102	1	PRJNA827465;Yang2025_Silkie_Wenchang	CRA004775	0.751865818
ENSGALG00010009340	SYK	2	0.465121879	0.223694142	2.079276076	0.037591983	0	0.167309572	0	1	CRA004775;Yang2025_Silkie_Wenchang	PRJNA827465	0.334407867
ENSGALG00010009340	SYK	2	0.175313177	0.202654601	0.865083628	0.38699289	0.036604996	1.328682319	24.73746467	1	CRA004775;PRJNA827465	Yang2025_Silkie_Wenchang	0.827206927
ENSGALG00010009344	ENPP2	2	0.404399681	0.441163909	0.916665377	0.359318014	0.31918197	5.274139129	81.03955972	1	PRJNA827465;Yang2025_Silkie_Wenchang	CRA004775	0.814335519
ENSGALG00010009344	ENPP2	2	0.624826893	0.2637428	2.369076594	0.01783256	0.025906236	1.228598072	18.60641633	1	CRA004775;Yang2025_Silkie_Wenchang	PRJNA827465	0.222853134
ENSGALG00010009344	ENPP2	2	0.090229961	0.157484399	0.572945391	0.566681669	0	0.915412349	0	1	CRA004775;PRJNA827465	Yang2025_Silkie_Wenchang	0.883487517
ENSGALG00010009345	ZFAT	2	-0.055333725	0.071205277	-0.777101466	0.437098909	0	0.313220569	0	0.5	PRJNA827465;Yang2025_Silkie_Wenchang	CRA004775	0.843453774
ENSGALG00010009345	ZFAT	2	0.053854653	0.119516595	0.450603974	0.652275002	0	0.431912244	0	1	CRA004775;Yang2025_Silkie_Wenchang	PRJNA827465	0.909377929
ENSGALG00010009345	ZFAT	2	-0.039549938	0.096888551	-0.408200329	0.683126606	0.004039895	1.120250334	10.7342377	0.5	CRA004775;PRJNA827465	Yang2025_Silkie_Wenchang	0.918800744
ENSGALG00010009346	STC2	2	0.111169891	0.441431272	0.251839637	0.801165019	0.353388374	10.57294269	90.5418952	0.5	PRJNA827465;Yang2025_Silkie_Wenchang	CRA004775	0.950158029
ENSGALG00010009346	STC2	2	0.631104996	0.214898047	2.936764694	0.003316556	0	0.645849796	0	1	CRA004775;Yang2025_Silkie_Wenchang	PRJNA827465	0.080463346
ENSGALG00010009346	STC2	2	0.26559163	0.70300952	0.377792365	0.705584846	0.807767937	4.700671808	78.72644505	0.5	CRA004775;PRJNA827465	Yang2025_Silkie_Wenchang	0.924358822
ENSGALG00010009347	TESK1	2	0.305380479	0.360707494	0.846615289	0.397209589	0.240979934	13.51634613	92.60155082	0.5	PRJNA827465;Yang2025_Silkie_Wenchang	CRA004775	0.831438925
ENSGALG00010009347	TESK1	2	0.348000374	0.394742416	0.881588501	0.377999376	0.237540348	3.706929088	73.02349259	0.5	CRA004775;Yang2025_Silkie_Wenchang	PRJNA827465	0.82325159
ENSGALG00010009347	TESK1	2	-0.061372003	0.125013843	-0.490921654	0.623481861	0	0.042214707	0	1	CRA004775;PRJNA827465	Yang2025_Silkie_Wenchang	0.900411816
ENSGALG00010009348	ERGIC2	2	-0.137089389	0.080168545	-1.710014686	0.087263157	0.009492305	3.60043891	72.22560846	1	PRJNA827465;Yang2025_Silkie_Wenchang	CRA004775	0.507886922
ENSGALG00010009348	ERGIC2	2	-0.222515649	0.068283788	-3.258689286	0.001119282	0	0.062574718	0	1	CRA004775;Yang2025_Silkie_Wenchang	PRJNA827465	0.040433027
ENSGALG00010009348	ERGIC2	2	-0.074726959	0.040060856	-1.865336072	0.062134312	0	0.456991043	0	1	CRA004775;PRJNA827465	Yang2025_Silkie_Wenchang	0.429956168
ENSGALG00010009351	GTF3C2	2	0.085229565	0.052088023	1.636260326	0.101785116	0	0.119542193	0	1	PRJNA827465;Yang2025_Silkie_Wenchang	CRA004775	0.543700483
ENSGALG00010009351	GTF3C2	2	-0.087295836	0.188333675	-0.463516875	0.642993922	0.023199559	1.364576443	26.7171872	0.5	CRA004775;Yang2025_Silkie_Wenchang	PRJNA827465	0.906634523
ENSGALG00010009351	GTF3C2	2	-0.044083819	0.214787611	-0.205243769	0.837381675	0.061443442	2.18378866	54.20802304	0.5	CRA004775;PRJNA827465	Yang2025_Silkie_Wenchang	0.958828842
ENSGALG00010009352	HSF1	2	0.078262307	0.309148349	0.253154536	0.800148797	0.164267968	6.501120589	84.61803644	0.5	PRJNA827465;Yang2025_Silkie_Wenchang	CRA004775	0.94999675
ENSGALG00010009352	HSF1	2	0.087642027	0.340672606	0.257261739	0.796976727	0.178272001	4.310917997	76.80308462	0.5	CRA004775;Yang2025_Silkie_Wenchang	PRJNA827465	0.949622804
ENSGALG00010009352	HSF1	2	-0.200334079	0.072326856	-2.769843586	0.005608322	0	0.056957294	0	1	CRA004775;PRJNA827465	Yang2025_Silkie_Wenchang	0.11240381
ENSGALG00010009355	GARRE1	2	-0.030828125	0.043132222	-0.714735371	0.474772577	0	0.209616919	0	0.5	PRJNA827465;Yang2025_Silkie_Wenchang	CRA004775	0.854462864
ENSGALG00010009355	GARRE1	2	0.018417357	0.109411202	0.168331548	0.866322456	0	0.008598254	0	1	CRA004775;Yang2025_Silkie_Wenchang	PRJNA827465	0.967381009
ENSGALG00010009355	GARRE1	2	-0.035999299	0.044703781	-0.805285326	0.420655048	0	0.035241925	0	0.5	CRA004775;PRJNA827465	Yang2025_Silkie_Wenchang	0.839196251
ENSGALG00010009357	LYST	2	0.156331381	0.117725532	1.327930974	0.184200911	0.015829288	2.089114664	52.13283323	1	PRJNA827465;Yang2025_Silkie_Wenchang	CRA004775	0.692545576
ENSGALG00010009357	LYST	2	0.212761259	0.180431619	1.179179464	0.238326722	0.019699882	1.289777529	22.46724902	0.5	CRA004775;Yang2025_Silkie_Wenchang	PRJNA827465	0.750437041
ENSGALG00010009357	LYST	2	0.061345909	0.072867895	0.841878424	0.399856009	0	0.251607742	0	0.5	CRA004775;PRJNA827465	Yang2025_Silkie_Wenchang	0.832246851
ENSGALG00010009359	REEP3	2	-0.098899793	0.144509374	-0.684383237	0.493733198	0.031579362	3.933827052	74.57946202	0.5	PRJNA827465;Yang2025_Silkie_Wenchang	CRA004775	0.860083144
ENSGALG00010009359	REEP3	2	-0.229767697	0.120107884	-1.913010955	0.05574665	0	0.780085724	0	0.5	CRA004775;Yang2025_Silkie_Wenchang	PRJNA827465	0.408770632
ENSGALG00010009359	REEP3	2	0.032689312	0.075060526	0.435506033	0.663195161	0	0.050946497	0	1	CRA004775;PRJNA827465	Yang2025_Silkie_Wenchang	0.91293776
ENSGALG00010009360	TNFAIP8	2	-0.278585848	0.28537363	-0.976214402	0.328958228	0.155298524	21.44792656	95.33754465	0.5	PRJNA827465;Yang2025_Silkie_Wenchang	CRA004775	0.800761292
ENSGALG00010009360	TNFAIP8	2	-0.562292542	0.09364508	-6.004507064	1.91914E-09	0	0.042815756	0	1	CRA004775;Yang2025_Silkie_Wenchang	PRJNA827465	1.09316E-06
ENSGALG00010009360	TNFAIP8	2	-0.075655657	0.179510477	-0.421455385	0.673422583	0.032625146	1.384529272	27.77328582	0.5	CRA004775;PRJNA827465	Yang2025_Silkie_Wenchang	0.915261599
ENSGALG00010009361	LRRK2	2	0.055169446	0.125580555	0.4393152	0.660433162	0.022955758	3.514136189	71.54350468	0.5	PRJNA827465;Yang2025_Silkie_Wenchang	CRA004775	0.911923994
ENSGALG00010009361	LRRK2	2	0.030463797	0.187645744	0.16234739	0.871032298	0.035932032	1.712034835	41.58997353	0.5	CRA004775;Yang2025_Silkie_Wenchang	PRJNA827465	0.969230271
ENSGALG00010009361	LRRK2	2	0.173993988	0.068430021	2.542655754	0.011001356	0	0.284790338	0	1	CRA004775;PRJNA827465	Yang2025_Silkie_Wenchang	0.166811819
ENSGALG00010009362	IL15	2	-0.210554424	0.284709718	-0.7395407	0.459578735	0.084086161	2.07696944	51.85292663	0.5	PRJNA827465;Yang2025_Silkie_Wenchang	CRA004775	0.850098539
ENSGALG00010009362	IL15	2	0.175801224	0.223978332	0.784902817	0.43251055	0	0.447252826	0	1	CRA004775;Yang2025_Silkie_Wenchang	PRJNA827465	0.842762754
ENSGALG00010009362	IL15	2	-0.095473772	0.44173279	-0.216134673	0.828882773	0.278010053	3.411605582	70.68828809	0.5	CRA004775;PRJNA827465	Yang2025_Silkie_Wenchang	0.956840134
ENSGALG00010009363	TAF2	2	-0.027951207	0.080172816	-0.348636963	0.727361874	0.007072973	1.508797142	33.72203777	0.5	PRJNA827465;Yang2025_Silkie_Wenchang	CRA004775	0.931522817
ENSGALG00010009363	TAF2	2	0.091397304	0.117802574	0.7758515	0.437836672	0	0.167336483	0	1	CRA004775;Yang2025_Silkie_Wenchang	PRJNA827465	0.843899392
ENSGALG00010009363	TAF2	2	-0.063509824	0.028761206	-2.2081767	0.027231958	0	0.398806051	0	0.5	CRA004775;PRJNA827465	Yang2025_Silkie_Wenchang	0.281657275
ENSGALG00010009365	CA5B	2	-0.020316723	0.29748254	-0.068295515	0.945550394	0.150862802	6.689416516	85.05101308	0.5	PRJNA827465;Yang2025_Silkie_Wenchang	CRA004775	0.986321197
ENSGALG00010009365	CA5B	2	-0.309243206	0.119221416	-2.593856178	0.009490618	0	0.006324335	0	1	CRA004775;Yang2025_Silkie_Wenchang	PRJNA827465	0.153228591
ENSGALG00010009365	CA5B	2	0.006218799	0.308312005	0.020170472	0.983907383	0.141583695	3.849543464	74.02289364	0.5	CRA004775;PRJNA827465	Yang2025_Silkie_Wenchang	0.995579629
ENSGALG00010009367	DMXL1	2	0.139423982	0.161514932	0.863226575	0.388012905	0.028515319	2.160557559	53.71565103	0.5	PRJNA827465;Yang2025_Silkie_Wenchang	CRA004775	0.828154121
ENSGALG00010009367	DMXL1	2	0.31187209	0.15795245	1.974468203	0.048328529	0	0.021241703	0	1	CRA004775;Yang2025_Silkie_Wenchang	PRJNA827465	0.38014722
ENSGALG00010009367	DMXL1	2	0.03241297	0.123905913	0.261593406	0.793634928	0	0.528157575	0	0.5	CRA004775;PRJNA827465	Yang2025_Silkie_Wenchang	0.948784329
ENSGALG00010009368	DSCC1	2	0.00274522	0.083379434	0.032924421	0.973734858	0	0.246566251	0	0.5	PRJNA827465;Yang2025_Silkie_Wenchang	CRA004775	0.992472082

ENSGALG00010009368	DSCC1	2	-0.078031075	0.136214979	-0.572852376	0.566744652	0	0.576955055	0	1	CRA004775;Yang2025_Silkie_Wenchang	PRJNA827465	0.883487517
ENSGALG00010009368	DSCC1	2	0.016760394	0.102150041	0.164076235	0.86967113	0	0.837895723	0	0.5	CRA004775;PRJNA827465	Yang2025_Silkie_Wenchang	0.968586462
ENSGALG00010009370	ENSGALG00010009370	2	0.496244806	0.426836538	1.162610887	0.244987411	0.019772808	1.057199157	5.410442908	1	PRJNA827465;Yang2025_Silkie_Wenchang	CRA004775	0.756475101
ENSGALG00010009370	ENSGALG00010009370	2	0.17871476	0.732950699	0.243829169	0.807363128	0.76183324	3.41872078	70.74929296	0.5	CRA004775;Yang2025_Silkie_Wenchang	PRJNA827465	0.95234753
ENSGALG00010009370	ENSGALG00010009370	2	-0.246175375	0.382056333	-0.644343134	0.519352951	0	0.635755655	0	0.5	CRA004775;PRJNA827465	Yang2025_Silkie_Wenchang	0.869086345
ENSGALG00010009371	PHTF2	2	0.167073068	0.133235023	1.253972598	0.209851965	0.022083049	2.622459411	61.86785596	1	PRJNA827465;Yang2025_Silkie_Wenchang	CRA004775	0.72165647
ENSGALG00010009371	PHTF2	2	0.294820325	0.114049646	2.585017453	0.009737407	0	0.075855062	0	1	CRA004775;Yang2025_Silkie_Wenchang	PRJNA827465	0.155572887
ENSGALG00010009371	PHTF2	2	0.070276823	0.097071337	0.723970893	0.469083605	0	0.462819047	0	1	CRA004775;PRJNA827465	Yang2025_Silkie_Wenchang	0.85345437
ENSGALG00010009372	RAB28	2	0.181933422	0.257821171	0.705657418	0.480401208	0.124937536	16.60456251	93.97775888	0.5	PRJNA827465;Yang2025_Silkie_Wenchang	CRA004775	0.856586256
ENSGALG00010009372	RAB28	2	0.275644184	0.209443137	1.316081246	0.188146775	0.062886203	3.074972991	67.47938916	1	CRA004775;Yang2025_Silkie_Wenchang	PRJNA827465	0.697483156
ENSGALG00010009372	RAB28	2	-0.065042814	0.084538882	-0.769383414	0.441665732	0	0.11520363	0	0.5	CRA004775;PRJNA827465	Yang2025_Silkie_Wenchang	0.845083421
ENSGALG00010009373	ARHGAP15	2	-0.193795721	0.128422991	-1.509042265	0.131287982	0	0.742511202	0	1	PRJNA827465;Yang2025_Silkie_Wenchang	CRA004775	0.60691738
ENSGALG00010009373	ARHGAP15	2	-0.350488077	0.211420108	-1.657780239	0.097361831	0	0.013673479	0	1	CRA004775;Yang2025_Silkie_Wenchang	PRJNA827465	0.532942995
ENSGALG00010009373	ARHGAP15	2	-0.136631653	0.146767341	-0.93094044	0.351884374	0	0.116731096	0	1	CRA004775;PRJNA827465	Yang2025_Silkie_Wenchang	0.810678834
ENSGALG00010009376	CDO1	2	-0.269973509	0.09147475	-2.951344596	0.003163937	0	0.274118249	0	1	PRJNA827465;Yang2025_Silkie_Wenchang	CRA004775	0.078012686
ENSGALG00010009376	CDO1	2	-0.235153073	0.15329565	-1.533983989	0.125033592	0	0.708040086	0	1	CRA004775;Yang2025_Silkie_Wenchang	PRJNA827465	0.593645235
ENSGALG00010009376	CDO1	2	-0.317711747	0.11036346	-2.878776604	0.00399221	0	0.481825457	0	1	CRA004775;PRJNA827465	Yang2025_Silkie_Wenchang	0.091171317
ENSGALG00010009378	ENSGALG00010009378	2	-0.212066328	0.260309311	-0.814670546	0.415260917	0.121861986	9.2898235	89.23553284	0.5	PRJNA827465;Yang2025_Silkie_Wenchang	CRA004775	0.837489728
ENSGALG00010009378	ENSGALG00010009378	2	-0.330376596	0.197208001	-1.675269735	0.093881321	0.039762318	1.984617237	49.61245013	1	CRA004775;Yang2025_Silkie_Wenchang	PRJNA827465	0.524867361
ENSGALG00010009378	ENSGALG00010009378	2	0.020461342	0.051431444	0.397837203	0.690750192	0	0.26251589	0	0.5	CRA004775;PRJNA827465	Yang2025_Silkie_Wenchang	0.920090451
ENSGALG00010009379	RYR1	2	-0.042999492	0.188572065	-0.228026841	0.819625374	0.050254129	3.32949181	69.9653864	0.5	PRJNA827465;Yang2025_Silkie_Wenchang	CRA004775	0.955256039
ENSGALG00010009379	RYR1	2	0.153674151	0.162289485	0.94691379	0.34368271	0	0.057358913	0	1	CRA004775;Yang2025_Silkie_Wenchang	PRJNA827465	0.806337247
ENSGALG00010009379	RYR1	2	-0.203403805	0.117539866	-1.730509073	0.083539362	0	0.169333674	0	0.5	CRA004775;PRJNA827465	Yang2025_Silkie_Wenchang	0.497929561
ENSGALG00010009381	INPP4B	2	-0.010761179	0.082769684	-0.13001353	0.896555722	0	0.098331048	0	0.5	PRJNA827465;Yang2025_Silkie_Wenchang	CRA004775	0.975115773
ENSGALG00010009381	INPP4B	2	-0.05058152	0.157398601	-0.3213594	0.747998047	0	0.009905892	0	0.5	CRA004775;Yang2025_Silkie_Wenchang	PRJNA827465	0.936938957
ENSGALG00010009381	INPP4B	2	0.004239745	0.094299571	0.044960382	0.964138887	0	4.04154E-05	0	1	CRA004775;PRJNA827465	Yang2025_Silkie_Wenchang	0.990248829
ENSGALG00010009383	ASXL2	2	-0.097213174	0.05821031	-1.670033606	0.094912715	0	0.588619084	0	1	PRJNA827465;Yang2025_Silkie_Wenchang	CRA004775	0.527384122
ENSGALG00010009383	ASXL2	2	-0.179556907	0.122184583	-1.469554534	0.141682442	0	0.025144735	0	1	CRA004775;Yang2025_Silkie_Wenchang	PRJNA827465	0.627088401
ENSGALG00010009383	ASXL2	2	-0.076747987	0.063929416	-1.200531136	0.229940821	0	0.022431134	0	1	CRA004775;PRJNA827465	Yang2025_Silkie_Wenchang	0.742776697
ENSGALG00010009385	GPI	2	-0.270986425	0.171455631	-1.580504672	0.113991338	0.033866073	2.304860835	56.61343259	1	PRJNA827465;Yang2025_Silkie_Wenchang	CRA004775	0.569637833
ENSGALG00010009385	GPI	2	-0.2662446	0.32699037	-0.81422765	0.415514548	0.113269153	1.725385632	42.04194232	0.5	CRA004775;Yang2025_Silkie_Wenchang	PRJNA827465	0.837678466
ENSGALG00010009385	GPI	2	-0.096976926	0.130435599	-0.743485112	0.457188002	0	0.507944267	0	0.5	CRA004775;PRJNA827465	Yang2025_Silkie_Wenchang	0.849541335
ENSGALG00010009386	TMEM60	2	-0.019964028	0.092526776	-0.21576487	0.829171033	0.007798567	1.763201595	43.28498778	0.5	PRJNA827465;Yang2025_Silkie_Wenchang	CRA004775	0.956860127
ENSGALG00010009386	TMEM60	2	-0.127987643	0.109050875	-1.173650767	0.240534938	0	0.02759226	0	1	CRA004775;Yang2025_Silkie_Wenchang	PRJNA827465	0.753158164
ENSGALG00010009386	TMEM60	2	0.042664921	0.075589944	0.564425878	0.572464331	0	0.253027854	0	0.5	CRA004775;PRJNA827465	Yang2025_Silkie_Wenchang	0.885678394
ENSGALG00010009387	DEPTOR	2	-0.242410976	0.135089254	-1.794450477	0.072741296	0.024396799	2.939226522	65.97744364	1	PRJNA827465;Yang2025_Silkie_Wenchang	CRA004775	0.465709132
ENSGALG00010009387	DEPTOR	2	-0.252382861	0.249951252	-1.009728333	0.312625464	0.065702206	1.630061891	38.65263611	0.5	CRA004775;Yang2025_Silkie_Wenchang	PRJNA827465	0.796054997
ENSGALG00010009387	DEPTOR	2	-0.108905206	0.089558854	-1.216018306	0.223977948	0	0.483632062	0	0.5	CRA004775;PRJNA827465	Yang2025_Silkie_Wenchang	0.737452676
ENSGALG00010009388	TMED7	2	-0.045569702	0.048002957	-0.94931031	0.342462811	0	0.205482637	0	1	PRJNA827465;Yang2025_Silkie_Wenchang	CRA004775	0.806005903
ENSGALG00010009388	TMED7	2	-0.073907863	0.082226762	-0.898829784	0.368743333	0	0.025465036	0	1	CRA004775;Yang2025_Silkie_Wenchang	PRJNA827465	0.820425502
ENSGALG00010009388	TMED7	2	-0.031235056	0.056311383	-0.554684588	0.57911042	0	0.000146693	0	1	CRA004775;PRJNA827465	Yang2025_Silkie_Wenchang	0.887888423
ENSGALG00010009389	CPEB4	2	-0.116732531	0.077595573	-1.504371031	0.132485859	0	0.902187186	0	0.5	PRJNA827465;Yang2025_Silkie_Wenchang	CRA004775	0.609713444
ENSGALG00010009389	CPEB4	2	0.281820421	0.302518727	0.931580085	0.351553579	0.132813588	3.450579274	71.01935877	1	CRA004775;Yang2025_Silkie_Wenchang	PRJNA827465	0.810678834
ENSGALG00010009389	CPEB4	2	0.191212778	0.391455201	0.488466567	0.625219401	0.266235136	6.968245048	85.64918436	0.5	CRA004775;PRJNA827465	Yang2025_Silkie_Wenchang	0.901331406
ENSGALG00010009391	RSBN1L	2	0.009532601	0.123607036	0.077120215	0.938527912	0.02031612	2.7662151	63.84952132	0.5	PRJNA827465;Yang2025_Silkie_Wenchang	CRA004775	0.985349045
ENSGALG00010009391	RSBN1L	2	0.145252432	0.119365077	1.216875457	0.223651605	0	0.067148742	0	1	CRA004775;Yang2025_Silkie_Wenchang	PRJNA827465	0.737076707
ENSGALG00010009391	RSBN1L	2	-0.079574589	0.069396949	-1.146658322	0.251522862	0	0.403676595	0	0.5	CRA004775;PRJNA827465	Yang2025_Silkie_Wenchang	0.761706068
ENSGALG00010009392	MAPRE3	2	0.189528785	0.184694289	1.026175666	0.304808789	0.061490349	9.951238244	89.95099931	1	PRJNA827465;Yang2025_Silkie_Wenchang	CRA004775	0.79225794
ENSGALG00010009392	MAPRE3	2	0.179431108	0.280303762	0.640130931	0.522087481	0.109950663	2.717717432	63.20441602	0.5	CRA004775;Yang2025_Silkie_Wenchang	PRJNA827465	0.869568211
ENSGALG00010009392	MAPRE3	2	0.0059938	0.060844687	0.09850983	0.921527467	0	0.39729945	0	0.5	CRA004775;PRJNA827465	Yang2025_Silkie_Wenchang	0.981565735
ENSGALG00010009393	MARVELD2	2	-0.199253907	0.225292597	-0.884422788	0.376468026	0.0636379	2.672670047	62.58423291	0.5	PRJNA827465;Yang2025_Silkie_Wenchang	CRA004775	0.822974013
ENSGALG00010009393	MARVELD2	2	-0.2435383	0.28816251	-0.845142207	0.398031445	0.077092504	1.646981649	39.28286931	0.5	CRA004775;Yang2025_Silkie_Wenchang	PRJNA827465	0.831850795
ENSGALG00010009393	MARVELD2	2	0.042868109	0.169982783	0.25219089	0.800893519	0	0.13396966	0	1	CRA004775;PRJNA827465	Yang2025_Silkie_Wenchang	0.950158029
ENSGALG00010009394	BANP	2	0.074543607	0.054333061	1.371975107	0.170071187	0	0.22998866	0	1	PRJNA827465;Yang2025_Silkie_Wenchang	CRA004775	0.671658717
ENSGALG00010009394	BANP	2	0.023239629	0.088269137	0.263281476	0.792333636	0	0.098428466	0	0.5	CRA004775;Yang2025_Silkie_Wenchang	PRJNA827465	0.948368502
ENSGALG00010009394	BANP	2	0.080823397	0.062619982	1.290696578	0.196808912	0	0.340186081	0	0.5	CRA004775;PRJNA827465	Yang2025_Silkie_Wenchang	0.707994211
ENSGALG00010009396	AUH	2	-0.052318105	0.169100919	-0.309389831	0.757025005	0.045028293	4.673721386	78.60377379	0.5	PRJNA827465;Yang2025_Silkie_Wenchang	CRA004775	0.938795135
ENSGALG00010009396	AUH	2	0.142098524	0.110598759	1.284811192	0.198858271	0	0.14190231	0	1	CRA004775;Yang2025_Silkie_Wenchang	PRJNA827465	0.71096127
ENSGALG00010009396	AUH	2	-0.061663703	0.213776483	-0.288449423	0.77300274	0.059186339	2.382047485	58.01930875	0.5	CRA004775;PRJNA827465	Yang2025_Silkie_Wenchang	0.943100418
ENSGALG00010009398	WTIP	2	-0.09073547	0.076563835	-1.185095682	0.235979618	0	0.769794082	0	1	PRJNA827465;Yang2025_Silkie_Wenchang	CRA004775	0.748669015
ENSGALG00010009398	WTIP	2	0.163602075	0.38664135	0.423136521	0.672195658	0.265755338	8.941789243	88.81655592	0.5	CRA004775;Yang2025_Silkie_Wenchang	PRJNA827465	0.914742762
ENSGALG00010009398	WTIP	2	0.225993402	0.306173764	0.738121381	0.460440692	0.164280634	7.637312215	86.90638837	0.5	CRA004775;PRJNA827465	Yang2025_Silkie_Wenchang	0.85014477
ENSGALG00010009400	CNTFR	2	-0.140604767	0.085840541	-1.637976242	0.10142665	0	0.021403811	0	1	PRJNA827465;Yang2025_Silkie_Wenchang	CRA004775	0.54352396
ENSGALG00010009400	CNTFR	2	-0.146862141	0.130878161	-1.122128701	0.261807722	0	0.571933427	0	1	CRA004775;Yang2025_Silkie_Wenchang	PRJNA827465	0.768363052
ENSGALG00010009400	CNTFR	2	-0.165364853	0.110101472	-1.501931354	0.133114838	0	0.519843491	0	1	CRA004775;PRJNA827465	Yang2025_Silkie_Wenchang	0.611335883
ENSGALG00010009404	GNG4	2	-0.058431491	0.257895548	-0.226570374	0.820757831	0.013896467	1.112359053	10.1009699	0.5	PRJNA827465;Yang2025_Silkie_Wenchang	CRA004775	0.955454439
ENSGALG00010009404	GNG4	2	-0.390862769	0.345228727	-1.132184949	0.257556686	0	0.017040032	0	1	CRA004775;Yang2025_Silkie_Wenchang	PRJNA827465	0.7650601
ENSGALG00010009404	GNG4	2	0.06612964	0.291068466	0.227196168	0.820271207	0	0.581568278	0	0.5	CRA004775;PRJNA827465	Yang2025_Silkie_Wenchang	0.955308792
ENSGALG00010009407	PTPN12	2	0.124704195	0.104962614	1.188082026	0.234801095	0.013842463	2.139222836	53.25405175	1	PRJNA827465;Yang2025_Silkie_Wenchang	CRA004775	0.747791642
ENSGALG00010009407	PTPN12	2	0.270902059	0.132441982	2.045439476	0.04081155	0	0.031802963	0	1	CRA004775;Yang2025_Silkie_Wenchang	PRJNA827465	0.349602411
ENSGALG00010009407	PTPN12	2	0.059248927	0.044257728	1.338725002	0.180660214	0	0.344342612	0	1	CRA004775;PRJNA827465	Yang2025_Silkie_Wenchang	0.68836533
ENSGALG00010009408	ENSGALG00010009408	2	0.195729471	0.069972506	2.797233965	0.005154219	0.001402004	1.159099685	13.7261434	1	PRJNA827465;Yang2025_Silkie_Wenchang	CRA004775	0.107296762
ENSGALG00010009408	ENSGALG00010009408	2	0.046803248	0.265393352	0.176354258	0.860015638	0.114729734	5.062003893	80.24497766	0.5	CRA004775;Yang2025_Silkie_Wenchang	PRJNA827465	0.964900247
ENSGALG00010009408	ENSGALG00010009408	2	-0.007522566	0.189525294	-0.039691619	0.968338983	0.050590305	2.933219882	65.90777234	0.5	CRA004775;PRJNA827465	Yang2025_Silkie_Wenchang	0.991215679
ENSGALG00010009410	JMJD1C	2	0.07476059	0.173042935	0.432034917	0.665716036	0.043108195	2.912783477	65.66857757	0.5	PRJNA827465;Yang2025_Silkie_Wenchang	CRA004775	0.913532078
ENSGALG00010009410	JMJD1C	2	0.332998225	0.145722871	2.285147294	0.022304192	0	0.026430297	0	1	CRA004775;Yang2025_Silkie_Wenchang	PRJNA827465	0.253336926
ENSGALG00010009410	JMJD1C	2	0.103441304	0.198998185	0.519810287	0.603195809	0.060901729	3.643831995	72.5563637	0.5	CRA004775;PRJNA827465	Yang2025_Silkie_Wenchang	0.895538966
ENSGALG00010009411	SMARCA5	2	0.114207421	0.084574658	1.350374033	0.176896036	0.00581961	1.623402427	38.40097913	1	PRJNA827465;Yang2025_Silkie_Wenchang	CRA004775	0.682556882
ENSGALG00010009411	SMARCA5	2	0.195231924	0.107999663	1.807708632	0.07065186	0	0.563426683	0	0.5	CRA004775;Yang2025_Silkie_Wenchang	PRJNA827465	0.458542662
ENSGALG00010009411	SMARCA5	2	0.043214914	0.074059431	0.583516693	0.559545523	0	0.078602187	0	0.5	CRA004775;PRJNA827465	Yang2025_Silkie_Wenchang	0.881203589
ENSGALG00010009415	HMP19	2	0.047533071	0.394044177	0.120628786	0.903985068	0.214167756	2.98154006	66.4602863	0.5	PRJNA827465;Yang2025_Silkie_Wenchang	CRA004775	0.977422974
ENSGALG00010009415	HMP19	2	0.700613396	0.372060387	1.883063666	0.05969174	0	0.987388104	0	1	CRA004775;Yang2025_Silkie_Wenchang	PRJNA827465	0.423249156
ENSGALG00010009415	HMP19	2	0.400324961	0.855745282	0.467808552	0.639921505	1.14489841	3.856048992	74.06671953	0.5	CRA004775;PRJNA827465	Yang2025_Silkie_Wenchang	0.906064918
ENSGALG00010009416	COL14A1	2	-0.240053895	0.109843225	-2.185422858	0.028857856	0	0.138011702	0	1	PRJNA827465;Yang2025_Silkie_Wenchang	CRA004775	0.289410648
ENSGALG00010009416	COL14A1	2	0.221735213	0.621249279	0.356918263	0.721152997	0.619309625	4.471712563	77.63720307	0.5	CRA004775;Yang2025_Silkie_Wenchang	PRJNA827465	0.928950734
ENSGALG00010009416	COL14A1	2	0.249355494	0.574355033	0.434148706	0.664180459	0.522845075	4.073170594	75.44910097	0.5	CRA004775;PRJNA827465	Yang2025_Silkie_Wenchang	0.91312035
ENSGALG00010009431	SMN	2	0.013339445	0.051840336	0.257317876	0.796933394	0	0.446403649	0	0.5	PRJNA827465;Yang2025_Silkie_Wenchang	CRA004775	0.949622804
ENSGALG00010009431	SMN	2	0.048934338	0.094715663	0.516644623	0.60540426	0	0.357880052	0	0.5	CRA004775;Yang2025_Silkie_Wenchang	PRJNA827465	0.896474603
ENSGALG00010009431	SMN	2	-0.011453077	0.058378661	-0.196186018	0.844464572	0	0.100471677	0	1	CRA004775;PRJNA827465	Yang2025_Silkie_Wenchang	0.960752464
ENSGALG00010009432	BOP1	2	0.296066956	0.314797673	0.940499187	0.346961566	0.159247453	4.443350589	77.49446099	1	PRJNA827465;Yang2025_Silkie_Wenchang	CRA004775	0.80855929
ENSGALG00010009432	BOP1	2	-0.046383731	0.730495083	-0.063496295	0.94937131	0.971964387	11.19010559	91.06353383	0.5	CRA004775;Yang2025_Silkie_Wenchang	PRJNA827465	0.98723759
ENSGALG00010009432	BOP1	2	-0.314124068	0.4055843	-0.774497602	0.438636586	0.279527284	5.934154725	83.1484003	0.5	CRA004775;PRJNA827465	Yang2025_Silkie_Wenchang	0.844002503
ENSGALG00010009435	GTDC1	2	0.071394068	0.082863324	0.86158827	0.388914128	0	0.249152692	0	1	PRJNA827465;Yang2025_Silkie_Wenchang	CRA004775	0.828571768
ENSGALG00010009435	GTDC1	2	0.089503095	0.089212782	1.003254171	0.315738242	0	0.123317263	0	1	CRA004775;Yang2025_Silkie_Wenchang	PRJNA827465	0.796883862
ENSGALG00010009435	GTDC1	2	0.05028451	0.077555524	0.648367869	0.516747047	0	0.02096179	0	1	CRA004775;PRJNA827465	Yang2025_Silkie_Wenchang	0.867952013
ENSGALG00010009437	DPYSL5	2	0.469147438	0.203594698	2.304320508	0.021204659	0	0.224217914	0	1	PRJNA827465;Yang2025_Silkie_Wenchang	CRA004775	0.246205819
ENSGALG00010009437	DPYSL5	2	0.294088393	0.398380191	0.738210383	0.460386614	0.140285724	1.678080446	40.40810127	0.5	CRA004775;Yang2025_Silkie_Wenchang	PRJNA827465	0.85014477
ENSGALG00010009437	DPYSL5	2	0.26225727	0.25586347	1.02498911	0.305368326	0.013768672	1.073059887	6.808556371	0.5	CRA004775;PRJNA827465	Yang2025_Silkie_Wenchang	0.79225794
ENSGALG00010009439	B3GALNT2	2	-0.135393944	0.062377382	-2.170561517	0.029964334	0	0.039786145	0	1	PRJNA827465;Yang2025_Silkie_Wenchang	CRA004775	0.295175875
ENSGALG00010009439	B3GALNT2	2	0.118865949	0.263154021	0.451697258	0.651487094	0.115970344	5.857021128	82.92647443	0.5	CRA004775;Yang2025_Silkie_Wenchang	PRJNA827465	0.908927523
ENSGALG00010009439	B3GALNT2	2	0.103803529	0.275786182	0.376391334	0.706625987	0.131752962	7.00082844	85.71597621	0.5	CRA004775;PRJNA827465	Yang2025_Silkie_Wenchang	0.924548793
ENSGALG00010009440	DOK3	2	-0.241319333	0.311186844	-0.775480514	0.438055776	0.094666799	1.907938376	47.58740573	0.5	PRJNA827465;Yang2025_Silkie_Wenchang	CRA004775	0.843965988
ENSGALG00010009440	DOK3	2	0.010918673	0.600605681	0.018179437	0.985495707	0.607150029	6.281693691	84.08072649	0.5	CRA004775;Yang2025_Silkie_Wenchang	PRJNA827465	0.995939127
ENSGALG00010009440	DOK3	2	0.289564507	0.28487892	1.016447643	0.309416268	0.080400698	1.973231935	49.32172026	1	CRA004775;PRJNA827465	Yang2025_Silkie_Wenchang	0.794109153
ENSGALG00010009441	ZNF518B	2	-0.173337812	0.234404814	-0.739480596	0.459615218	0.103209645	16.23518064	93.84053666	0.5	PRJNA827465;Yang2025_Silkie_Wenchang	CRA004775	0.850098539
ENSGALG00010009441	ZNF518B	2	-0.051829049	0.379309498	-0.136640526	0.891314942	0.265548375	12.66148249	92.1020307	0.5	CRA004775;Yang2025_Silkie_Wenchang	PRJNA827465	0.973796385
ENSGALG00010009441	ZNF518B	2	0.144439329	0.134320396	1.075334301	0.282225084	0.02257859	2.153384283	53.56147029	1	CRA004775;PRJNA827465	Yang2025_Silkie_Wenchang	0.779519046
ENSGALG00010009444	NFIL3	2	-0.246172142	0.097728088	-2.518949748	0.011770545	0	0.248554436	0	1	PRJNA827465;Yang2025_Silkie_Wenchang	CRA004775	0.17378578
ENSGALG00010009444	NFIL3	2	0.008333618	0.313146123	0.026612554	0.97876876	0.120698588	2.070463282	51.7016308	0.5	CRA004775;Yang2025_Silkie_Wenchang	PRJNA827465	0.994224868
ENSGALG00010009444	NFIL3	2	-0.02371657	0.371497273	-0.063840496	0.949097233	0.192333083	2.71022808	63.10273635	0.5	CRA004775;PRJNA827465	Yang2025_Silkie_Wenchang	0.987200776
ENSGALG00010009446	ENSGALG00010009446	2	-0.26229665	0.180271213	-1.455011288	0.145660137	0.036410819	1.802638995	44.52577565	1	PRJNA827465;Yang2025_Silkie_Wenchang	CRA004775	0.634354199
ENSGALG00010009446	ENSGALG00010009446	2	-0.229907745	0.342538765	-0.671197527	0.502101077	0.141326752	2.50506848	60.08093159	0.5	CRA004775;Yang2025_Silkie_Wenchang	PRJNA827465	0.802860181
ENSGALG00010009446	ENSGALG00010009446	2	-0.131086525	0.087822301	-1.492633687	0.1355331	0	0.696761867	0	0.5	CRA004775;PRJNA827465	Yang2025_Silkie_Wenchang	0.617034338
ENSGALG00010009447	DDX41	2	0.034940559	0.044464541	0.785807252	0.431980418	0	0.000863567	0	1	PRJNA827465;Yang2025_Silkie_Wenchang	CRA004775	0.842503929
ENSGALG00010009447	DDX41	2	-0.187033949	0.236649947	-0.790340129	0.429329157	0.097446382	7.426164274	86.53409805	0.5	CRA004775;Yang2025_Silkie_Wenchang	PRJNA827465	0.842008086
ENSGALG00010009447	DDX41	2	-0.181348983	0.237752462	-0.762763851	0.445604243	0.100897263	8.708088151	88.51642309	0.5	CRA004775;PRJNA827465	Yang2025_Silkie_Wenchang	0.846013379
ENSGALG00010009451	ROR2	2	-0.062944727	0.093961958	-0.66989586	0.502924179	0	0.469059706	0	1	PRJNA827465;Yang2025_Silkie_Wenchang	CRA004775	0.863050365
ENSGALG00010009451	ROR2	2	0.075042828	0.32464607	0.231152737	0.817196146	0.131494763	2.599165644	61.52611502	0.5	CRA004775;Yang2025_Silkie_Wenchang	PRJNA827465	0.954833525
ENSGALG00010009451	ROR2	2	0.100618472	0.214964861	0.468069394	0.639734966	0.054284141	1.922422878	47.9823086	0.5	CRA004775;PRJNA827465	Yang2025_Silkie_Wenchang	0.906064918
ENSGALG00010009452	TBCE	2	0.082012976	0.163631284	0.501205968	0.616226175	0.040061591	3.741687595	73.27409158	0.5	PRJNA827465;Yang2025_Silkie_Wenchang	CRA004775	0.899111936
ENSGALG00010009452	TBCE	2	-0.110396607	0.126858699	-0.870232848	0.384173168	0	0.001790012	0	1	CRA004775;Yang2025_Silkie_Wenchang	PRJNA827465	0.825767461
ENSGALG00010009452	TBCE	2	0.125431731	0.154926714	0.809619776	0.418158739	0.026874188	1.848835602	45.79462806	0.5	CRA004775;PRJNA827465	Yang2025_Silkie_Wenchang	0.838112595
ENSGALG00010009453	GSAP	2	-0.018870187	0.06630054	-0.284615896	0.775938431	0	0.411770035	0	0.5	PRJNA827465;Yang2025_Silkie_Wenchang	CRA004775	0.949807523
ENSGALG00010009453	GSAP	2	-0.095584733	0.110495001	-0.865059337	0.387006221	0	0.030459202	0	1	CRA004775;Yang2025_Silkie_Wenchang	PRJNA827465	0.827206927
ENSGALG00010009453	GSAP	2	-0.005015327	0.074809149	-0.067041621	0.946548569	0	0.30300849	0	0.5	CRA004775;PRJNA827465	Yang2025_Silkie_Wenchang	0.986445196
ENSGALG00010009457	BDP1	2	0.228061702	0.265556534	0.858806592	0.39044723	0.12567454	9.088017383	88.99649992	0.5	PRJNA827465;Yang2025_Silkie_Wenchang	CRA004775	0.829251057
ENSGALG00010009457	BDP1	2	0.48207069	0.129275071	3.729078599	0.000192181	0	0.117146581	0	1	CRA004775;Yang2025_Silkie_Wenchang	PRJNA827465	0.001240118
ENSGALG00010009457	BDP1	2	0.083854463	0.184993417	0.453283498	0.650344614	0.036212846	1.712842462	41.61751461	0.5	CRA004775;PRJNA827465	Yang2025_Silkie_Wenchang	0.908526849
ENSGALG00010009458	SCX	2	1.175113902	0.524225921	2.241617316	0.024986116	0.426881028	4.401121683	77.27851961	1	PRJNA827465;Yang2025_Silkie_Wenchang	CRA004775	0.269418193
ENSGALG00010009458	SCX	2	0.188350224	1.690552562	0.111413409	0.911288527	5.094323351	8.659416151	88.45187733	0.5	CRA004775;Yang2025_Silkie_Wenchang	PRJNA827465	0.978848551
ENSGALG00010009458	SCX	2	-0.254346684	1.148564038	-0.221447543	0.824743977	2.118479694	4.384106065	77.19033287	0.5	CRA004775;PRJNA827465	Yang2025_Silkie_Wenchang	0.95619211
ENSGALG00010009461	ZEB2	2	0.074631427	0.1532475	0.486999313	0.626258818	0.030143441	2.145162555	53.38348613	0.5	PRJNA827465;Yang2025_Silkie_Wenchang	CRA004775	0.901504244
ENSGALG00010009461	ZEB2	2	0.069204424	0.282922575	0.244605523	0.806761887	0.089304955	2.196318945	54.46927222	0.5	CRA004775;Yang2025_Silkie_Wenchang	PRJNA827465	0.952282862
ENSGALG00010009461	ZEB2	2	-0.03176161	0.055396499	-0.573350499	0.566407399	0	0.543470918	0	1	CRA004775;PRJNA827465	Yang2025_Silkie_Wenchang	0.883487517
ENSGALG00010009462	NRBF2	2	-0.057964951	0.05765357	-1.005400892	0.314703851	0	0.106954821	0	1	PRJNA827465;Yang2025_Silkie_Wenchang	CRA004775	0.796531448
ENSGALG00010009462	NRBF2	2	0.160028278	0.240910475	0.664264508	0.506521033	0.081403588	2.936300506	65.94354025	0.5	CRA004775;Yang2025_Silkie_Wenchang	PRJNA827465	0.863929781
ENSGALG00010009462	NRBF2	2	0.137016047	0.262042513	0.522877168	0.601059734	0.10678949	3.780404791	73.54780625	0.5	CRA004775;PRJNA827465	Yang2025_Silkie_Wenchang	0.895230519
ENSGALG00010009463	MRPL13	2	0.005649235	0.051075018	0.110606619	0.911929299	0	0.686854506	0	0.5	PRJNA827465;Yang2025_Silkie_Wenchang	CRA004775	0.978928739
ENSGALG00010009463	MRPL13	2	-0.133519052	0.144787665	-0.922171462	0.356439146	0	0.008104852	0	1	CRA004775;Yang2025_Silkie_Wenchang	PRJNA827465	0.812521698
ENSGALG00010009463	MRPL13	2	0.013487201	0.052759639	0.255634814	0.798232833	0	0.351697682	0	0.5	CRA004775;PRJNA827465	Yang2025_Silkie_Wenchang	0.949922217
ENSGALG00010009464	SPTLC1	2	0.083590574	0.133700496	0.625205656	0.531834778	0	0.005965373	0	1	PRJNA827465;Yang2025_Silkie_Wenchang	CRA004775	0.883427777
ENSGALG00010009464	SPTLC1	2	0.075421231	0.141984551	0.531193221	0.59528489	0	4.4819E-05	0	1	CRA004775;Yang2025_Silkie_Wenchang	PRJNA827465	0.893711832
ENSGALG00010009464	SPTLC1	2	0.086321571	0.147175707	0.586520517	0.557525778	0	0.003322242	0	1	CRA004775;PRJNA827465	Yang2025_Silkie_Wenchang	0.880678789
ENSGALG00010009465	GGPS1	2	-0.05823944	0.119740073	-0.486382203	0.626696207	0.019016683	2.865232546	65.09881889	0.5	PRJNA827465;Yang2025_Silkie_Wenchang	CRA004775	0.901504244
ENSGALG00010009465	GGPS1	2	0.031852203	0.270513807	0.117747069	0.906268086	0.110700859	3.758766755	73.3955293	0.5	CRA004775;Yang2025_Silkie_Wenchang	PRJNA827465	0.979896186
ENSGALG00010009465	GGPS1	2	0.102663955	0.119032893	0.862483904	0.388421287	0.001117079	1.307377163	23.51097842	1	CRA004775;PRJNA827465	Yang2025_Silkie_Wenchang	0.828154121
ENSGALG00010009466	FAM193B	2	0.030388497	0.058682298	0.517847763	0.604564491	0.000223129	1.023392597	2.285789134	0.5	PRJNA827465;Yang2025_Silkie_Wenchang	CRA004775	0.896276948
ENSGALG00010009466	FAM193B	2	-0.055295406	0.201466943	-0.274463915	0.783728131	0.063178927	4.48426605	77.69980664	0.5	CRA004775;Yang2025_Silkie_Wenchang	PRJNA827465	0.945830648
ENSGALG00010009466	FAM193B	2	-0.10072372	0.127671212	-0.788930551	0.430152599	0.021891121	2.705493036	63.03816026	1	CRA004775;PRJNA827465	Yang2025_Silkie_Wenchang	0.845202082
ENSGALG00010009467	EGR2	2	0.023844246	0.129977806	0.260384809	0.79456696	0	0.145928461	0	0.5	PRJNA827465;Yang2025_Silkie_Wenchang	CRA004775	0.948905846
ENSGALG00010009467	EGR2	2	0.021049998	0.165634097	0.127087348	0.898871265	0	0.244703129	0	0.5	CRA004775;Yang2025_Silkie_Wenchang	PRJNA827465	0.975893962
ENSGALG00010009467	EGR2	2	0.106842565	0.171920311	0.621466519	0.534292309	0	0.076631361	0	1	CRA004775;PRJNA827465	Yang2025_Silkie_Wenchang	0.873408008
ENSGALG00010009469	DNMT3A	2	0.077312099	0.177748741	0.434951601	0.663597558	0.053131018	6.247367846	83.99325885	0.5	PRJNA827465;Yang2025_Silkie_Wenchang	CRA004775	0.913119244
ENSGALG00010009469	DNMT3A	2	0.332259676	0.130214622	2.551631069	0.010721999	0.009856695	1.260018929	20.63611292	1	CRA004775;Yang2025_Silkie_Wenchang	PRJNA827465	0.165128023
ENSGALG00010009469	DNMT3A	2	0.19584822	0.329743611	0.59394091	0.552551635	0.185106955	6.163658395	83.7758692	0.5	CRA004775;PRJNA827465	Yang2025_Silkie_Wenchang	0.879228216
ENSGALG00010009470	ZNF469	2	-0.022097898	0.113199293	-0.195212336	0.845226272	0	0.30687805	0	0.5	PRJNA827465;Yang2025_Silkie_Wenchang	CRA004775	0.968863849
ENSGALG00010009470	ZNF469	2	-0.147535499	0.345762168	-0.426696478	0.669600416	0.121601319	1.800885128	44.47174977	0.5	CRA004775;Yang2025_Silkie_Wenchang	PRJNA827465	0.914377769
ENSGALG00010009470	ZNF469	2	-0.15639432	0.221610071	-0.705718465	0.480363235	0.042366839	1.319226064	24.19798036	1	CRA004775;PRJNA827465	Yang2025_Silkie_Wenchang	0.856585676
ENSGALG00010009472	ADO	2	-0.0359455	0.06005741	-0.598518986	0.549493694	0	0.446165795	0	1	PRJNA827465;Yang2025_Silkie_Wenchang	CRA004775	0.878554604
ENSGALG00010009472	ADO	2	-0.07725607	0.150520152	-0.5132604	0.607720353	0	0.789925231	0	0.5	CRA004775;Yang2025_Silkie_Wenchang	PRJNA827465	0.907203929
ENSGALG00010009472	ADO	2	-0.013094645	0.063409173	-0.206510262	0.836392349	0	0.406066917	0	0.5	CRA004775;PRJNA827465	Yang2025_Silkie_Wenchang	0.958399882
ENSGALG00010009474	ACVR2A	2	-0.101395879	0.067542347	-1.501219371	0.133298831	0.002081924	1.219352639	17.98927002	0.5	PRJNA827465;Yang2025_Silkie_Wenchang	CRA004775	0.611823423
ENSGALG00010009474	ACVR2A	2	0.046859127	0.110177048	0.425307721	0.670612513	0	0.516836803	0	1	CRA004775;Yang2025_Silkie_Wenchang	PRJNA827465	0.919482883
ENSGALG00010009474	ACVR2A	2	-0.051487695	0.155872224	-0.330319885	0.74115827	0.028406908	1.796084853	44.32334318	0.5	CRA004775;PRJNA827465	Yang2025_Silkie_Wenchang	0.935384591
ENSGALG00010009477	ZNF365	2	-0.029128679	0.063591583	-0.458058718	0.646910256	0.001295203	1.149032889	12.97028921	0.5	PRJNA827465;Yang2025_Silkie_Wenchang	CRA004775	0.907350807
ENSGALG00010009477	ZNF365	2	0.07085116	0.280271106	0.252795091	0.800426561	0.106088668	2.542453414	60.66791256	0.5	CRA004775;Yang2025_Silkie_Wenchang	PRJNA827465	0.950102441
ENSGALG00010009477	ZNF365	2	0.102245294	0.181145964	0.564433947	0.572445748	0.036852772	1.572021811	36.38765105	1	CRA004775;PRJNA827465	Yang2025_Silkie_Wenchang	0.835784394
ENSGALG00010009479	MTBP	2	0.147519134	0.095263345	1.548540364	0.121492251	0	0.182083716	0	1	PRJNA827465;Yang2025_Silkie_Wenchang	CRA004775	0.584456544
ENSGALG00010009479	MTBP	2	-0.088107779	0.373243408	-0.236059839	0.813386237	0.15066664	1.522410864	34.31470941	0.5	CRA004775;Yang2025_Silkie_Wenchang	PRJNA827465	0.953587297
ENSGALG00010009479	MTBP	2	-0.065701567	0.436494597	-0.150523949	0.880344157	0.236044597	1.829853422	45.35320314	0.5	CRA004775;PRJNA827465	Yang2025_Silkie_Wenchang	0.970359092
ENSGALG00010009482	ARID4B	2	0.264620509	0.314291021	0.841960132	0.39981027	0.181246671	11.63413491	91.40460371	0.5	PRJNA827465;Yang2025_Silkie_Wenchang	CRA004775	0.832246851
ENSGALG00010009482	ARID4B	2	0.557378005	0.146012989	3.817317956	0.00013491	0	0.218830648	0	1	CRA004775;Yang2025_Silkie_Wenchang	PRJNA827465	0.009675872
ENSGALG00010009482	ARID4B	2	0.127963097	0.224322855	0.570441641	0.568378193	0.07063605	2.667083323	62.50585831	0.5	CRA004775;PRJNA827465	Yang2025_Silkie_Wenchang	0.884151198
ENSGALG00010009484	RTKN2	2	-0.055139934	0.06653902	-0.828685705	0.407282277	0	0.017437341	0	1	PRJNA827465;Yang2025_Silkie_Wenchang	CRA004775	0.833863243
ENSGALG00010009484	RTKN2	2	-0.038430704	0.099625381	-0.384848757	0.700349473	0	0.104067541	0	0.5	CRA004775;Yang2025_Silkie_Wenchang	PRJNA827465	0.922303014
ENSGALG00010009484	RTKN2	2	-0.057173557	0.086595418	-0.66023767	0.509101322	0	0.129433137	0	0.5	CRA004775;PRJNA827465	Yang2025_Silkie_Wenchang	0.865193604
ENSGALG00010009486	B4GALT1	2	-0.060444775	0.259859549	-0.232605555	0.816067713	0.128502175	20.56537449	95.13745786	0.5	PRJNA827465;Yang2025_Silkie_Wenchang	CRA004775	0.954410204
ENSGALG00010009486	B4GALT1	2	-0.32110079	0.085296551	-3.76452257	0.000166868	0	0.005503843	0	1	CRA004775;Yang2025_Silkie_Wenchang	PRJNA827465	0.011248673
ENSGALG00010009486	B4GALT1	2	0.019214132	0.238543761	0.080547621	0.935801723	0.083272324	3.040157646	67.1069689	0.5	CRA004775;PRJNA827465	Yang2025_Silkie_Wenchang	0.984801197
ENSGALG00010009489	MROH1	2	0.033812677	0.08505713	0.39752902	0.690977391	0.008640632	2.471992162	59.5467973	0.5	PRJNA827465;Yang2025_Silkie_Wenchang	CRA004775	0.920199347
ENSGALG00010009489	MROH1	2	-0.221684415	0.369561884	-0.599857356	0.548601304	0.244466224	8.848486146	88.69863179	0.5	CRA004775;Yang2025_Silkie_Wenchang	PRJNA827465	0.878281266
ENSGALG00010009489	MROH1	2	-0.297765913	0.28261244	-1.053619272	0.292057262	0.131722654	5.126435792	80.49326978	1	CRA004775;PRJNA827465	Yang2025_Silkie_Wenchang	0.785516679
ENSGALG00010009490	SNTB1	2	0.104372009	0.089130175	1.171006444	0.241596187	0	0.00556017	0	1	PRJNA827465;Yang2025_Silkie_Wenchang	CRA004775	0.754026438
ENSGALG00010009490	SNTB1	2	0.067910079	0.12404896	0.547445777	0.584072513	0	0.345658447	0	0.5	CRA004775;Yang2025_Silkie_Wenchang	PRJNA827465	0.890763231
ENSGALG00010009490	SNTB1	2	0.094828422	0.112731425	0.752482479	0.451760963	0	0.40139392	0	0.5	CRA004775;PRJNA827465	Yang2025_Silkie_Wenchang	0.848006549
ENSGALG00010009491	RBM34	2	0.249641498	0.12561436	1.987363436	0.046882037	0.015434557	1.859503582	46.22220632	1	PRJNA827465;Yang2025_Silkie_Wenchang	CRA004775	0.37376485
ENSGALG00010009491	RBM34	2	0.341945276	0.134775269	2.537151498	0.011175858	0	0.592566877	0	1	CRA004775;Yang2025_Silkie_Wenchang	PRJNA827465	0.167907012

ENSGALG00010009491	RBM34	2	0.155104226	0.093262648	1.663090521	0.096294317	0	0.013459269	0	1	CRA004775;PRJNA827465	Yang2025_Silkie_Wenchang	0.530732793
ENSGALG00010009492	CDC42SE2	2	-0.119902298	0.106979918	-1.120792583	0.262376162	0.008043474	1.534758153	34.84315439	1	PRJNA827465;Yang2025_Silkie_Wenchang	CRA004775	0.768418055
ENSGALG00010009492	CDC42SE2	2	-0.15576598	0.169722881	-0.917766528	0.35874111	0.018275787	1.239284191	19.30825815	0.5	CRA004775;Yang2025_Silkie_Wenchang	PRJNA827465	0.814232788
ENSGALG00010009492	CDC42SE2	2	-0.003195558	0.108599638	-0.029425122	0.976525537	0	0.326568356	0	0.5	CRA004775;PRJNA827465	Yang2025_Silkie_Wenchang	0.993126926
ENSGALG00010009493	ENSGALG00010009493	2	0.854311183	0.376693502	2.267921209	0.023334009	0	0.063629611	0	1	PRJNA827465;Yang2025_Silkie_Wenchang	CRA004775	0.260464803
ENSGALG00010009493	ENSGALG00010009493	2	0.840148412	0.336031688	2.500205909	0.012412114	0	0.043987602	0	1	CRA004775;Yang2025_Silkie_Wenchang	PRJNA827465	0.180211791
ENSGALG00010009493	ENSGALG00010009493	2	0.904343198	0.308525422	2.931178872	0.003376783	0	0.004573369	0	1	CRA004775;PRJNA827465	Yang2025_Silkie_Wenchang	0.081471883
ENSGALG00010009494	UBA2	2	-0.138627232	0.140033991	-0.989954156	0.322196527	0.036991496	17.41827317	94.25890276	1	PRJNA827465;Yang2025_Silkie_Wenchang	CRA004775	0.798513866
ENSGALG00010009494	UBA2	2	-0.1457521	0.183919263	-0.792478709	0.428081601	0.049212031	2.998282695	66.64757457	0.5	CRA004775;Yang2025_Silkie_Wenchang	PRJNA827465	0.84125685
ENSGALG00010009494	UBA2	2	0.000639152	0.03491408	0.018306424	0.985394403	0	0.229471873	0	0.5	CRA004775;PRJNA827465	Yang2025_Silkie_Wenchang	0.99592834
ENSGALG00010009497	HS3ST1	2	-0.056000871	0.360120691	-0.155505842	0.876422549	0.22325426	7.086611589	85.88888374	0.5	PRJNA827465;Yang2025_Silkie_Wenchang	CRA004775	0.970347777
ENSGALG00010009497	HS3ST1	2	-0.020877323	0.470225305	-0.044398554	0.964586715	0.342413034	4.185876718	76.1101421	0.5	CRA004775;Yang2025_Silkie_Wenchang	PRJNA827465	0.990364463
ENSGALG00010009497	HS3ST1	2	0.317126721	0.146422678	2.165830627	0.030324132	0	0.271341023	0	1	CRA004775;PRJNA827465	Yang2025_Silkie_Wenchang	0.296622539
ENSGALG00010009498	ORC4	2	0.001348493	0.086375687	0.015611949	0.987543973	0	0.914000791	0	0.5	PRJNA827465;Yang2025_Silkie_Wenchang	CRA004775	0.996647513
ENSGALG00010009498	ORC4	2	-0.182678187	0.142294172	-1.28380653	0.199209658	0	0.454145422	0	1	CRA004775;Yang2025_Silkie_Wenchang	PRJNA827465	0.71096127
ENSGALG00010009498	ORC4	2	-0.058254252	0.182626844	-0.318979677	0.749741923	0.035377616	1.723063783	41.96384312	0.5	CRA004775;PRJNA827465	Yang2025_Silkie_Wenchang	0.937362187
ENSGALG00010009499	MXD3	2	0.133210395	0.133340614	0.999023409	0.317783351	0	0.240949461	0	0.5	PRJNA827465;Yang2025_Silkie_Wenchang	CRA004775	0.797410398
ENSGALG00010009499	MXD3	2	-0.072361849	0.270246686	-0.267762206	0.788882364	0	0.054208788	0	1	CRA004775;Yang2025_Silkie_Wenchang	PRJNA827465	0.947144026
ENSGALG00010009499	MXD3	2	0.12969628	0.137264714	0.944862493	0.344729082	0	0.463481483	0	0.5	CRA004775;PRJNA827465	Yang2025_Silkie_Wenchang	0.806945375
ENSGALG00010009500	CCDC146	2	0.78297772	0.38206215	2.049346476	0.040428247	0.161642609	2.237946835	55.31618606	1	PRJNA827465;Yang2025_Silkie_Wenchang	CRA004775	0.347691771
ENSGALG00010009500	CCDC146	2	0.667410586	0.590696093	1.129871341	0.258530442	0.46899699	2.920363634	65.75768893	0.5	CRA004775;Yang2025_Silkie_Wenchang	PRJNA827465	0.765790021
ENSGALG00010009500	CCDC146	2	0.301300321	0.300333872	1.003217915	0.315755731	0	0.392647009	0	0.5	CRA004775;PRJNA827465	Yang2025_Silkie_Wenchang	0.796883862
ENSGALG00010009501	HGH1	2	0.164822689	0.18498649	0.890998524	0.372929962	0.039759728	1.899918545	47.36616459	1	PRJNA827465;Yang2025_Silkie_Wenchang	CRA004775	0.822162024
ENSGALG00010009501	HGH1	2	0.076577996	0.3752371	0.204078957	0.838291798	0.205824316	3.713560454	73.07166498	0.5	CRA004775;Yang2025_Silkie_Wenchang	PRJNA827465	0.959197438
ENSGALG00010009501	HGH1	2	-0.029506432	0.142856377	-0.206545563	0.836364778	0.018304731	1.460169021	31.51477769	0.5	CRA004775;PRJNA827465	Yang2025_Silkie_Wenchang	0.958399882
ENSGALG00010009502	ARID5B	2	-0.016447974	0.063100702	-0.260662297	0.794352944	0	0.575013536	0	0.5	PRJNA827465;Yang2025_Silkie_Wenchang	CRA004775	0.948905846
ENSGALG00010009502	ARID5B	2	0.191659034	0.192097162	0.997719235	0.318415523	0	0.125235458	0	1	CRA004775;Yang2025_Silkie_Wenchang	PRJNA827465	0.797723439
ENSGALG00010009502	ARID5B	2	-0.019701275	0.064627159	-0.304845133	0.760484105	0	0.810205563	0	0.5	CRA004775;PRJNA827465	Yang2025_Silkie_Wenchang	0.939327609
ENSGALG00010009503	IL11RA	2	0.002832642	0.095849792	0.029552925	0.976423261	0	0.097827075	0	0.5	PRJNA827465;Yang2025_Silkie_Wenchang	CRA004775	0.993126926
ENSGALG00010009503	IL11RA	2	-0.088036398	0.143166659	-0.614922488	0.538605911	0	0.638455524	0	1	CRA004775;Yang2025_Silkie_Wenchang	PRJNA827465	0.87481821
ENSGALG00010009503	IL11RA	2	-0.013845253	0.114174075	-0.121264424	0.903481598	0	0.994875432	0	0.5	CRA004775;PRJNA827465	Yang2025_Silkie_Wenchang	0.977284786
ENSGALG00010009506	HAS2	2	0.10728819	0.0841096	1.275576029	0.202105432	0.005704703	1.630494348	38.66890731	1	PRJNA827465;Yang2025_Silkie_Wenchang	CRA004775	0.71497298
ENSGALG00010009506	HAS2	2	0.0191032	0.105793572	0.180570515	0.856704701	0	0.270475375	0	1	CRA004775;Yang2025_Silkie_Wenchang	PRJNA827465	0.963627293
ENSGALG00010009506	HAS2	2	0.17740563	0.076434741	2.321007787	0.020286424	0	0.011691867	0	1	CRA004775;PRJNA827465	Yang2025_Silkie_Wenchang	0.239939003
ENSGALG00010009508	TOMM20	2	-0.121301732	0.063679063	-1.904891915	0.056794122	0	0.561028737	0	1	PRJNA827465;Yang2025_Silkie_Wenchang	CRA004775	0.412373803
ENSGALG00010009508	TOMM20	2	-0.174243173	0.144287585	-1.186818486	0.235299225	0	0.641090624	0	0.5	CRA004775;Yang2025_Silkie_Wenchang	PRJNA827465	0.748350272
ENSGALG00010009508	TOMM20	2	-0.094274697	0.067242166	-1.402017558	0.160910004	0	0.205468009	0	1	CRA004775;PRJNA827465	Yang2025_Silkie_Wenchang	0.656996246
ENSGALG00010009511	ZFPM1	2	0.413222745	0.201945367	2.046210573	0.040735658	0.039897812	1.906577649	47.54999878	1	PRJNA827465;Yang2025_Silkie_Wenchang	CRA004775	0.349194657
ENSGALG00010009511	ZFPM1	2	0.237697039	0.509699661	0.466347257	0.640966958	0.373639262	3.218317802	68.92786662	0.5	CRA004775;Yang2025_Silkie_Wenchang	PRJNA827465	0.906429868
ENSGALG00010009511	ZFPM1	2	0.123847262	0.246797216	0.501817905	0.615795617	0.045029792	1.291621503	22.57793806	0.5	CRA004775;PRJNA827465	Yang2025_Silkie_Wenchang	0.898956845
ENSGALG00010009514	PRELID1	2	-0.005620287	0.078133246	-0.071932085	0.942655956	0	0.038222438	0	0.5	PRJNA827465;Yang2025_Silkie_Wenchang	CRA004775	0.985827325
ENSGALG00010009514	PRELID1	2	-0.156590295	0.144921061	-1.080521316	0.2799101	0	0.879677953	0	1	CRA004775;Yang2025_Silkie_Wenchang	PRJNA827465	0.778040488
ENSGALG00010009514	PRELID1	2	-0.090120161	0.143217715	-0.629252309	0.529183509	0.021524985	1.758716118	43.18034029	0.5	CRA004775;PRJNA827465	Yang2025_Silkie_Wenchang	0.871837903
ENSGALG00010009515	MBD5	2	0.149151189	0.30040746	0.496496288	0.619544306	0.154735416	6.236541765	83.96547257	0.5	PRJNA827465;Yang2025_Silkie_Wenchang	CRA004775	0.899285366
ENSGALG00010009515	MBD5	2	0.551463584	0.147380538	3.741766667	0.000182731	0	0.088903868	0	1	CRA004775;Yang2025_Silkie_Wenchang	PRJNA827465	0.011930036
ENSGALG00010009515	MBD5	2	0.21458369	0.347867155	0.616855278	0.537330187	0.223807008	12.59948269	92.0631662	0.5	CRA004775;PRJNA827465	Yang2025_Silkie_Wenchang	0.87481821
ENSGALG00010009518	FGL2	2	-0.545030557	0.231190484	-2.357495638	0.018398671	0	0.025584411	0	1	PRJNA827465;Yang2025_Silkie_Wenchang	CRA004775	0.227043528
ENSGALG00010009518	FGL2	2	-0.191079799	0.403204127	-0.47390338	0.635568809	0.204212963	2.685587608	62.76420114	0.5	CRA004775;Yang2025_Silkie_Wenchang	PRJNA827465	0.904244739
ENSGALG00010009518	FGL2	2	-0.162671117	0.365814177	-0.444682374	0.656549308	0.152756296	2.321298781	56.92066837	0.5	CRA004775;PRJNA827465	Yang2025_Silkie_Wenchang	0.910509456
ENSGALG00010009522	TAF5L	2	-0.143367813	0.106699123	-1.3436644	0.179056934	0.018594147	5.298207467	81.12569192	1	PRJNA827465;Yang2025_Silkie_Wenchang	CRA004775	0.685713211
ENSGALG00010009522	TAF5L	2	-0.096155056	0.187548278	-0.512694953	0.608164472	0.055541098	4.426828582	77.41046482	0.5	CRA004775;Yang2025_Silkie_Wenchang	PRJNA827465	0.897204334
ENSGALG00010009522	TAF5L	2	-0.030838603	0.047486986	-0.649411675	0.51607232	0	0.945321527	0	0.5	CRA004775;PRJNA827465	Yang2025_Silkie_Wenchang	0.867498525
ENSGALG00010009524	FAM185A	2	-0.173047188	0.085031596	-2.035092789	0.041841537	0	0.124556627	0	1	PRJNA827465;Yang2025_Silkie_Wenchang	CRA004775	0.354227064
ENSGALG00010009524	FAM185A	2	-0.109856173	0.196392306	-0.55937186	0.575908508	0.019951823	1.257885742	20.45093134	0.5	CRA004775;Yang2025_Silkie_Wenchang	PRJNA827465	0.888692804
ENSGALG00010009524	FAM185A	2	-0.125556828	0.103265387	-1.215865562	0.224036139	0.002400373	1.036507031	3.522120948	0.5	CRA004775;PRJNA827465	Yang2025_Silkie_Wenchang	0.737452676
ENSGALG00010009525	ENSGALG00010009525	2	-0.383272955	0.390380331	-0.981793712	0.326201487	0.256070076	6.239098675	83.97204385	1	PRJNA827465;Yang2025_Silkie_Wenchang	CRA004775	0.800023788
ENSGALG00010009525	ENSGALG00010009525	2	-0.216432378	0.631346887	-0.342810557	0.731740983	0.66332378	5.625471125	82.22371108	0.5	CRA004775;Yang2025_Silkie_Wenchang	PRJNA827465	0.932851968
ENSGALG00010009525	ENSGALG00010009525	2	0.073759199	0.188601479	0.391082644	0.695734449	0	0.876388501	0	0.5	CRA004775;PRJNA827465	Yang2025_Silkie_Wenchang	0.921038437
ENSGALG00010009526	ENSGALG00010009526	2	0.361145922	0.129318583	2.792683872	0.005227275	0	0.282199567	0	1	PRJNA827465;Yang2025_Silkie_Wenchang	CRA004775	0.107955433
ENSGALG00010009526	ENSGALG00010009526	2	-0.203690214	0.732888602	-0.277927933	0.781067681	0.932413188	7.240612441	86.18901359	0.5	CRA004775;Yang2025_Silkie_Wenchang	PRJNA827465	0.945235709
ENSGALG00010009526	ENSGALG00010009526	2	-0.259279137	0.654154499	-0.396357645	0.691841207	0.737687109	6.603957996	84.85756571	0.5	CRA004775;PRJNA827465	Yang2025_Silkie_Wenchang	0.920848122
ENSGALG00010009530	KIF3C	2	0.229797363	0.32310496	0.711215833	0.476950499	0.191836515	12.31063303	91.87694088	0.5	PRJNA827465;Yang2025_Silkie_Wenchang	CRA004775	0.85498476
ENSGALG00010009530	KIF3C	2	0.241261685	0.377768296	0.638649902	0.52305071	0.222840801	4.06110414	75.37615472	0.5	CRA004775;Yang2025_Silkie_Wenchang	PRJNA827465	0.869568211
ENSGALG00010009530	KIF3C	2	-0.105696643	0.119551877	-0.884106925	0.376638495	0	0.104703409	0	1	CRA004775;PRJNA827465	Yang2025_Silkie_Wenchang	0.822974013
ENSGALG00010009534	XPA	2	0.332304806	0.26745147	1.24246017	0.214053166	0.083173485	2.359070415	57.61042172	1	PRJNA827465;Yang2025_Silkie_Wenchang	CRA004775	0.726832726
ENSGALG00010009534	XPA	2	0.112600245	0.543183382	0.207296925	0.835777975	0.484272766	5.499571355	81.8167647	0.5	CRA004775;Yang2025_Silkie_Wenchang	PRJNA827465	0.958339923
ENSGALG00010009534	XPA	2	-0.101787679	0.262111846	-0.388336813	0.697766798	0.057756634	1.615315559	38.0925916	0.5	CRA004775;PRJNA827465	Yang2025_Silkie_Wenchang	0.921422004
ENSGALG00010009538	CRIM1	2	0.060548952	0.117530433	0.515176793	0.606429484	0.015649469	2.162385716	53.75478145	0.5	PRJNA827465;Yang2025_Silkie_Wenchang	CRA004775	0.896775454
ENSGALG00010009538	CRIM1	2	0.241729505	0.131390546	1.839778531	0.065800759	0	0.398702623	0	1	CRA004775;Yang2025_Silkie_Wenchang	PRJNA827465	0.44200431
ENSGALG00010009538	CRIM1	2	0.085630722	0.207367414	0.412942038	0.67964908	0.050151311	1.762904801	43.27543953	0.5	CRA004775;PRJNA827465	Yang2025_Silkie_Wenchang	0.917701868
ENSGALG00010009540	RAB10	2	-0.046290525	0.044083009	-1.050076332	0.293683019	0	0.041235436	0	1	PRJNA827465;Yang2025_Silkie_Wenchang	CRA004775	0.786979185
ENSGALG00010009540	RAB10	2	0.072881871	0.157253696	0.463468236	0.643029787	0.023600933	1.640883956	39.05723825	0.5	CRA004775;Yang2025_Silkie_Wenchang	PRJNA827465	0.906634523
ENSGALG00010009540	RAB10	2	0.054057701	0.166925313	0.32384364	0.746056415	0.036422787	2.121951148	52.87356162	0.5	CRA004775;PRJNA827465	Yang2025_Silkie_Wenchang	0.936484152
ENSGALG00010009541	PMPCB	2	0.035486548	0.056502642	0.628051128	0.529970447	0	0.043072987	0	1	PRJNA827465;Yang2025_Silkie_Wenchang	CRA004775	0.87207153
ENSGALG00010009541	PMPCB	2	-0.173959966	0.230898947	-0.75340303	0.451207763	0.074009597	2.982926521	66.47587553	0.5	CRA004775;Yang2025_Silkie_Wenchang	PRJNA827465	0.847847413
ENSGALG00010009541	PMPCB	2	-0.152235666	0.243692847	-0.624703304	0.532165977	0.092911807	3.889589735	74.29034762	0.5	CRA004775;PRJNA827465	Yang2025_Silkie_Wenchang	0.873104119
ENSGALG00010009542	WDR1	2	-0.173341944	0.278234869	-0.623005823	0.533280695	0.139579254	9.487535847	89.45985537	0.5	PRJNA827465;Yang2025_Silkie_Wenchang	CRA004775	0.873379507
ENSGALG00010009542	WDR1	2	-0.069587642	0.440203358	-0.158080607	0.874393282	0.32973993	6.483641326	84.57656817	0.5	CRA004775;Yang2025_Silkie_Wenchang	PRJNA827465	0.970135804
ENSGALG00010009542	WDR1	2	0.109066719	0.090432447	1.206057263	0.227795412	0.006147317	1.132268808	11.68174965	1	CRA004775;PRJNA827465	Yang2025_Silkie_Wenchang	0.740853612
ENSGALG00010009543	MAF1	2	-0.130707497	0.067836187	-1.926810783	0.054003215	0	0.128203932	0	1	PRJNA827465;Yang2025_Silkie_Wenchang	CRA004775	0.402825653
ENSGALG00010009543	MAF1	2	-0.267940286	0.25887265	-1.035027403	0.300656114	0.08388684	2.022726806	50.56178634	1	CRA004775;Yang2025_Silkie_Wenchang	PRJNA827465	0.790625401
ENSGALG00010009543	MAF1	2	-0.280427789	0.224707205	-1.247969729	0.212042143	0.057174627	1.697841511	41.10168742	1	CRA004775;PRJNA827465	Yang2025_Silkie_Wenchang	0.724432597
ENSGALG00010009548	PIK3C3	2	0.04455988	0.094507645	0.471490971	0.637287731	0.000713741	1.041592308	3.993146639	0.5	PRJNA827465;Yang2025_Silkie_Wenchang	CRA004775	0.905149724
ENSGALG00010009548	PIK3C3	2	-0.053032787	0.108994448	-0.486564119	0.626567256	0	0.005971357	0	1	CRA004775;Yang2025_Silkie_Wenchang	PRJNA827465	0.901504244
ENSGALG00010009548	PIK3C3	2	0.079084239	0.110977718	0.712613671	0.47608485	0	0.735421811	0	0.5	CRA004775;PRJNA827465	Yang2025_Silkie_Wenchang	0.854810196
ENSGALG00010009551	HADHA	2	-0.065455024	0.092091757	-0.710758772	0.477233733	0	0.364024212	0	0.5	PRJNA827465;Yang2025_Silkie_Wenchang	CRA004775	0.854997403
ENSGALG00010009551	HADHA	2	0.011223659	0.135795613	0.082651117	0.934128955	0	0.000975407	0	1	CRA004775;Yang2025_Silkie_Wenchang	PRJNA827465	0.98432801
ENSGALG00010009551	HADHA	2	-0.086586847	0.102836499	-0.841985562	0.399796035	0	0.153081441	0	0.5	CRA004775;PRJNA827465	Yang2025_Silkie_Wenchang	0.832246851
ENSGALG00010009554	ABCB10	2	-0.194777144	0.077968967	-2.498136782	0.012484801	0.002304821	1.21210767	17.49928908	1	PRJNA827465;Yang2025_Silkie_Wenchang	CRA004775	0.180907385
ENSGALG00010009554	ABCB10	2	-0.283964131	0.114918493	-2.471004652	0.013473407	0	0.159500678	0	1	CRA004775;Yang2025_Silkie_Wenchang	PRJNA827465	0.189937338
ENSGALG00010009554	ABCB10	2	-0.136231933	0.082914202	-1.643047021	0.100373205	0	3.20888E-05	0	1	CRA004775;PRJNA827465	Yang2025_Silkie_Wenchang	0.541223839
ENSGALG00010009555	EPC2	2	-0.035268618	0.042462176	-0.830589046	0.406205825	0	0.380560172	0	0.5	PRJNA827465;Yang2025_Silkie_Wenchang	CRA004775	0.833477131
ENSGALG00010009555	EPC2	2	0.109324861	0.137254289	0.79651326	0.425733771	0	0.426786269	0	1	CRA004775;Yang2025_Silkie_Wenchang	PRJNA827465	0.84125685
ENSGALG00010009555	EPC2	2	-0.009296856	0.095985371	-0.096857011	0.922839949	0.008377679	1.211109946	17.4311132	0.5	CRA004775;PRJNA827465	Yang2025_Silkie_Wenchang	0.983819849
ENSGALG00010009557	CIDEC	2	-0.496344129	0.502291296	-0.988159923	0.323074332	0	0.502497645	0	1	PRJNA827465;Yang2025_Silkie_Wenchang	CRA004775	0.798909393
ENSGALG00010009557	CIDEC	2	-0.177751515	0.554427982	-0.320603435	0.748510935	0.258643259	1.688341555	40.77027856	0.5	CRA004775;Yang2025_Silkie_Wenchang	PRJNA827465	0.937249951
ENSGALG00010009557	CIDEC	2	0.147563691	0.418990156	0.352188922	0.724696584	0	0.217503983	0	0.5	CRA004775;PRJNA827465	Yang2025_Silkie_Wenchang	0.930474334
ENSGALG00010009558	TMEM26	2	-0.273121658	0.091335641	-2.990307567	0.002786967	0	0.570294838	0	1	PRJNA827465;Yang2025_Silkie_Wenchang	CRA004775	0.0719461
ENSGALG00010009558	TMEM26	2	-0.320169292	0.127699018	-2.507218121	0.012168559	0	0.515897035	0	0.5	CRA004775;Yang2025_Silkie_Wenchang	PRJNA827465	0.177990656
ENSGALG00010009558	TMEM26	2	-0.193330685	0.121865931	-1.586421105	0.112643816	0	0.19434805	0	0.5	CRA004775;PRJNA827465	Yang2025_Silkie_Wenchang	0.566454459
ENSGALG00010009560	RAB24	2	0.17771333	0.242773893	0.732011698	0.464161428	0.097108091	5.326640566	81.22644118	0.5	PRJNA827465;Yang2025_Silkie_Wenchang	CRA004775	0.852159649
ENSGALG00010009560	RAB24	2	0.123858421	0.362371594	0.341799476	0.732501804	0.201832737	4.177066161	76.05975195	0.5	CRA004775;Yang2025_Silkie_Wenchang	PRJNA827465	0.932985777
ENSGALG00010009560	RAB24	2	-0.057970922	0.087180306	-0.664954333	0.506079702	0	0.580901003	0	1	CRA004775;PRJNA827465	Yang2025_Silkie_Wenchang	0.863929781
ENSGALG00010009562	HADHB	2	-0.054450792	0.084696634	-0.642892044	0.520294152	0	0.00530426	0	1	PRJNA827465;Yang2025_Silkie_Wenchang	CRA004775	0.869331539
ENSGALG00010009562	HADHB	2	-0.047257367	0.141785638	-0.33330151	0.7389067	0	0.001345724	0	1	CRA004775;Yang2025_Silkie_Wenchang	PRJNA827465	0.934613258
ENSGALG00010009562	HADHB	2	-0.058771796	0.099112906	-0.59297824	0.553195715	0	3.83428E-05	0	1	CRA004775;PRJNA827465	Yang2025_Silkie_Wenchang	0.87934426
ENSGALG00010009565	TMEM8B	2	0.015121982	0.175009502	0.086406632	0.931143175	0.021685874	1.544504551	35.25431834	0.5	PRJNA827465;Yang2025_Silkie_Wenchang	CRA004775	0.983943003
ENSGALG00010009565	TMEM8B	2	0.084221245	0.216325918	0.389325726	0.697035208	0.016829009	1.144989397	12.66294671	0.5	CRA004775;Yang2025_Silkie_Wenchang	PRJNA827465	0.921116491
ENSGALG00010009565	TMEM8B	2	-0.175297334	0.173560676	-1.010006057	0.312492388	0	0.121146281	0	1	CRA004775;PRJNA827465	Yang2025_Silkie_Wenchang	0.796054997
ENSGALG00010009567	NSD1	2	-0.068758863	0.048376373	-1.421331506	0.155220408	0	0.024370689	0	1	PRJNA827465;Yang2025_Silkie_Wenchang	CRA004775	0.647874911
ENSGALG00010009567	NSD1	2	-0.069438071	0.131941786	-0.526278093	0.598695002	0	0.076157868	0	1	CRA004775;Yang2025_Silkie_Wenchang	PRJNA827465	0.894762943
ENSGALG00010009567	NSD1	2	-0.073484873	0.049681397	-1.479122516	0.139107574	0	0.047304366	0	1	CRA004775;PRJNA827465	Yang2025_Silkie_Wenchang	0.622860674
ENSGALG00010009569	ZC3H18	2	-0.053813467	0.059331902	-0.906990425	0.364411861	0	0.530663202	0	0.5	PRJNA827465;Yang2025_Silkie_Wenchang	CRA004775	0.817496251
ENSGALG00010009569	ZC3H18	2	-0.002711205	0.095351666	-0.028433742	0.977316213	0	0.091578136	0	0.5	CRA004775;Yang2025_Silkie_Wenchang	PRJNA827465	0.993494016
ENSGALG00010009569	ZC3H18	2	-0.077512849	0.065753614	-1.178837864	0.238462744	0	0.022289796	0	1	CRA004775;PRJNA827465	Yang2025_Silkie_Wenchang	0.750649855
ENSGALG00010009570	SHARPIN	2	-0.006224175	0.154142848	-0.040379263	0.967790762	0.036957207	4.489768073	77.72713459	0.5	PRJNA827465;Yang2025_Silkie_Wenchang	CRA004775	0.991065147
ENSGALG00010009570	SHARPIN	2	0.125913121	0.094580752	1.331276366	0.183098092	0	0.667466388	0	0.5	CRA004775;Yang2025_Silkie_Wenchang	PRJNA827465	0.691392093
ENSGALG00010009570	SHARPIN	2	-0.166418874	0.104702028	-1.589452249	0.111958326	0	0.006955761	0	1	CRA004775;PRJNA827465	Yang2025_Silkie_Wenchang	0.56547057
ENSGALG00010009571	DNAJC2	2	0.620241847	0.569096961	1.089870249	0.275770304	0.610094169	16.48641556	93.93440014	1	PRJNA827465;Yang2025_Silkie_Wenchang	CRA004775	0.7754953
ENSGALG00010009571	DNAJC2	2	0.897477003	0.345743075	2.595791693	0.009437326	0.150260883	2.669407863	62.53850849	1	CRA004775;Yang2025_Silkie_Wenchang	PRJNA827465	0.152954732
ENSGALG00010009571	DNAJC2	2	0.189841231	0.192344556	0.986985204	0.323649892	0.04336477	1.769906901	43.49985304	1	CRA004775;PRJNA827465	Yang2025_Silkie_Wenchang	0.799600434
ENSGALG00010009574	TAF1C	2	0.069636036	0.211431011	0.329355828	0.741886751	0.049867744	2.23887103	55.33463131	0.5	PRJNA827465;Yang2025_Silkie_Wenchang	CRA004775	0.93543545
ENSGALG00010009574	TAF1C	2	-0.139788472	0.424099621	-0.329612348	0.741692891	0.309879586	7.100327542	85.91614268	0.5	CRA004775;Yang2025_Silkie_Wenchang	PRJNA827465	0.93543545
ENSGALG00010009574	TAF1C	2	-0.34909946	0.210933217	-1.655023641	0.097919703	0.031398345	1.533806163	34.80271342	1	CRA004775;PRJNA827465	Yang2025_Silkie_Wenchang	0.534130658
ENSGALG00010009575	RHOBTB1	2	-0.454187279	0.185665329	-2.446268672	0.014434339	0	0.061039316	0	1	PRJNA827465;Yang2025_Silkie_Wenchang	CRA004775	0.197673991
ENSGALG00010009575	RHOBTB1	2	0.157252358	0.725718757	0.216684985	0.82845385	0.890032565	6.110595677	83.63498335	0.5	CRA004775;Yang2025_Silkie_Wenchang	PRJNA827465	0.956777964
ENSGALG00010009575	RHOBTB1	2	0.199907131	0.679210865	0.294322634	0.768511379	0.75799898	5.295011001	81.11429797	0.5	CRA004775;PRJNA827465	Yang2025_Silkie_Wenchang	0.941487523
ENSGALG00010009578	KIF5C	2	0.029007403	0.118857818	0.244051285	0.807191101	0	0.584788005	0	0.5	PRJNA827465;Yang2025_Silkie_Wenchang	CRA004775	0.95234753
ENSGALG00010009578	KIF5C	2	0.060314832	0.213438303	0.282586731	0.777493653	0	0.906488241	0	0.5	CRA004775;Yang2025_Silkie_Wenchang	PRJNA827465	0.944367175
ENSGALG00010009578	KIF5C	2	-0.040406489	0.124673714	-0.324097899	0.745863917	0	0.267862447	0	1	CRA004775;PRJNA827465	Yang2025_Silkie_Wenchang	0.936373614
ENSGALG00010009582	TIGD5	2	-0.183489653	0.099754193	-1.83941794	0.065853737	0.000167415	1.007291385	0.723860512	1	PRJNA827465;Yang2025_Silkie_Wenchang	CRA004775	0.4420276
ENSGALG00010009582	TIGD5	2	-0.260194938	0.154748714	-1.681402908	0.092684681	0	0.658714995	0	1	CRA004775;Yang2025_Silkie_Wenchang	PRJNA827465	0.521366255
ENSGALG00010009582	TIGD5	2	-0.106235812	0.111850849	-0.949798888	0.342214451	0	0.053892413	0	1	CRA004775;PRJNA827465	Yang2025_Silkie_Wenchang	0.806005903
ENSGALG00010009583	LYPD6B	2	-0.079418397	0.231801266	-0.342614166	0.731888744	0	0.031758421	0	1	PRJNA827465;Yang2025_Silkie_Wenchang	CRA004775	0.932851968
ENSGALG00010009583	LYPD6B	2	-0.187055286	0.395831125	-0.47256336	0.636524728	0	0.044504939	0	1	CRA004775;Yang2025_Silkie_Wenchang	PRJNA827465	0.904566695
ENSGALG00010009583	LYPD6B	2	-0.079356731	0.260877853	-0.304191139	0.760982276	0	0.104266297	0	1	CRA004775;PRJNA827465	Yang2025_Silkie_Wenchang	0.939496981
ENSGALG00010009593	NCBP1	2	-0.106115817	0.090869105	-1.167787633	0.242892438	0.012762376	4.305243156	76.77250822	1	PRJNA827465;Yang2025_Silkie_Wenchang	CRA004775	0.7550024
ENSGALG00010009593	NCBP1	2	-0.11180186	0.134807876	-0.829342195	0.406910801	0.021589848	2.077301767	51.86062921	0.5	CRA004775;Yang2025_Silkie_Wenchang	PRJNA827465	0.833618122
ENSGALG00010009593	NCBP1	2	-0.015196007	0.048421776	-0.313825902	0.753653271	0	0.301739761	0	0.5	CRA004775;PRJNA827465	Yang2025_Silkie_Wenchang	0.938110356
ENSGALG00010009594	HINT2	2	0.198546	0.348220828	0.570172671	0.56856059	0.169320885	3.222705686	68.97017298	0.5	PRJNA827465;Yang2025_Silkie_Wenchang	CRA004775	0.884190118
ENSGALG00010009594	HINT2	2	0.084761085	0.533604208	0.158846359	0.873789924	0.43426312	4.164612864	75.98816426	0.5	CRA004775;Yang2025_Silkie_Wenchang	PRJNA827465	0.969974876
ENSGALG00010009594	HINT2	2	-0.19531084	0.195362304	-0.999736573	0.317438008	0	0.61595485	0	1	CRA004775;PRJNA827465	Yang2025_Silkie_Wenchang	0.797125693
ENSGALG00010009595	ENSGALG00010009595	2	-0.17519029	0.14070196	-1.245116203	0.213089039	0	0.005170008	0	1	PRJNA827465;Yang2025_Silkie_Wenchang	CRA004775	0.72579791
ENSGALG00010009595	ENSGALG00010009595	2	-0.204583604	0.197085389	-1.038045514	0.299248866	0	0.020272709	0	1	CRA004775;Yang2025_Silkie_Wenchang	PRJNA827465	0.78978392
ENSGALG00010009595	ENSGALG00010009595	2	-0.182064898	0.16235306	-1.121413405	0.262111933	0	0.039474453	0	1	CRA004775;PRJNA827465	Yang2025_Silkie_Wenchang	0.768363052
ENSGALG00010009596	URB2	2	-0.025610069	0.142183269	-0.180120131	0.857058259	0.03174884	4.594904612	78.23676258	0.5	PRJNA827465;Yang2025_Silkie_Wenchang	CRA004775	0.963878155
ENSGALG00010009596	URB2	2	-0.174374458	0.098114739	-1.777250393	0.075527052	0	0.003163426	0	1	CRA004775;Yang2025_Silkie_Wenchang	PRJNA827465	0.474458409
ENSGALG00010009596	URB2	2	0.083357691	0.077960982	1.069223207	0.284969104	0.000317727	1.008839523	0.876207033	0.5	CRA004775;PRJNA827465	Yang2025_Silkie_Wenchang	0.780497609
ENSGALG00010009597	CDK1	2	-0.309043608	0.255675416	-1.208734155	0.226764995	0.108401595	5.511950519	81.85760201	1	PRJNA827465;Yang2025_Silkie_Wenchang	CRA004775	0.74036776
ENSGALG00010009597	CDK1	2	-0.143278333	0.540599606	-0.265035956	0.790981763	0.457696365	4.142091847	75.85760922	0.5	CRA004775;Yang2025_Silkie_Wenchang	PRJNA827465	0.947823629
ENSGALG00010009597	CDK1	2	0.002831778	0.204506093	0.013846911	0.988952117	0.039733905	1.303965347	23.31084545	0.5	CRA004775;PRJNA827465	Yang2025_Silkie_Wenchang	0.996891127
ENSGALG00010009600	LRRC17	2	-0.391416838	0.287137158	-1.363170275	0.172828853	0.136207641	5.664489939	82.34615984	1	PRJNA827465;Yang2025_Silkie_Wenchang	CRA004775	0.676043418
ENSGALG00010009600	LRRC17	2	-0.069681309	0.708908766	-0.098293762	0.921699032	0.855183754	6.157384309	83.75933757	0.5	CRA004775;Yang2025_Silkie_Wenchang	PRJNA827465	0.981649741
ENSGALG00010009600	LRRC17	2	0.147052726	0.395790179	0.371542129	0.710233784	0.207899066	2.327541037	57.03620326	0.5	CRA004775;PRJNA827465	Yang2025_Silkie_Wenchang	0.925364369
ENSGALG00010009601	ZHX2	2	-0.046030149	0.055766254	-0.825412254	0.40913758	0.000475556	1.057518297	5.438988389	1	PRJNA827465;Yang2025_Silkie_Wenchang	CRA004775	0.834316328
ENSGALG00010009601	ZHX2	2	-0.045000227	0.153715532	-0.292750034	0.769713223	0.021204572	1.620452399	38.28883833	0.5	CRA004775;Yang2025_Silkie_Wenchang	PRJNA827465	0.941878652
ENSGALG00010009601	ZHX2	2	-0.003287115	0.057915747	-0.056756836	0.954738898	0	0.682330936	0	0.5	CRA004775;PRJNA827465	Yang2025_Silkie_Wenchang	0.987987175
ENSGALG00010009602	TSTD2	2	-0.092929468	0.094013661	-0.988467714	0.322923639	0.000422024	1.023384191	2.284986525	1	PRJNA827465;Yang2025_Silkie_Wenchang	CRA004775	0.79874391
ENSGALG00010009602	TSTD2	2	-0.209451179	0.133677191	-1.566843058	0.11715138	0	6.78431E-05	0	1	CRA004775;Yang2025_Silkie_Wenchang	PRJNA827465	0.57646425
ENSGALG00010009602	TSTD2	2	-0.04153318	0.11141761	-0.37277034	0.70931938	0	0.34007889	0	1	CRA004775;PRJNA827465	Yang2025_Silkie_Wenchang	0.925298042
ENSGALG00010009603	HINT1	2	-0.041360324	0.145223935	-0.284803771	0.775794483	0	0.921360362	0	0.5	PRJNA827465;Yang2025_Silkie_Wenchang	CRA004775	0.943820051
ENSGALG00010009603	HINT1	2	-0.076270128	0.318782378	-0.23925453	0.810908221	0.092391096	1.716215574	41.73226166	0.5	CRA004775;Yang2025_Silkie_Wenchang	PRJNA827465	0.953115528
ENSGALG00010009603	HINT1	2	-0.188329585	0.163574515	-1.151338186	0.249593133	0	0.602527535	0	1	CRA004775;PRJNA827465	Yang2025_Silkie_Wenchang	0.759622031
ENSGALG00010009605	ENSGALG00010009605	2	0.1022508	0.096220287	1.062674022	0.287929806	0	0.096867343	0	1	PRJNA827465;Yang2025_Silkie_Wenchang	CRA004775	0.782840067
ENSGALG00010009605	ENSGALG00010009605	2	0.051303766	0.121650685	0.421730182	0.673221972	0	0.085102908	0	0.5	CRA004775;Yang2025_Silkie_Wenchang	PRJNA827465	0.915210237
ENSGALG00010009605	ENSGALG00010009605	2	0.099329155	0.117667007	0.844154681	0.398582975	0	0.255705116	0	0.5	CRA004775;PRJNA827465	Yang2025_Silkie_Wenchang	0.831980453
ENSGALG00010009606	CHSY3	2	0.164990155	0.129832158	1.270795753	0.20380132	0	0.464729473	0	1	PRJNA827465;Yang2025_Silkie_Wenchang	CRA004775	0.717254253
ENSGALG00010009606	CHSY3	2	0.04546761	0.181651815	0.250300884	0.802354673	0	0.022897234	0	0.5	CRA004775;Yang2025_Silkie_Wenchang	PRJNA827465	0.950688375
ENSGALG00010009606	CHSY3	2	0.202778813	0.156415729	1.296409347	0.194834494	0	0.314678771	0	0.5	CRA004775;PRJNA827465	Yang2025_Silkie_Wenchang	0.706046493
ENSGALG00010009608	AFAP1	2	0.013283834	0.068747055	0.193227674	0.846780667	0	0.24712716	0	0.5	PRJNA827465;Yang2025_Silkie_Wenchang	CRA004775	0.961252712
ENSGALG00010009608	AFAP1	2	0.066814393	0.18088322	0.36937861	0.711845539	0.028438988	1.482722966	32.55651779	0.5	CRA004775;Yang2025_Silkie_Wenchang	PRJNA827465	0.92597752
ENSGALG00010009608	AFAP1	2	0.069218572	0.101248218	0.683652245	0.494194786	0.004469005	1.081600449	7.544417083	1	CRA004775;PRJNA827465	Yang2025_Silkie_Wenchang	0.860175061
ENSGALG00010009609	MMADHC	2	-0.298868516	0.172638429	-1.731181854	0.083419335	0.056708074	20.43267354	95.10587786	1	PRJNA827465;Yang2025_Silkie_Wenchang	CRA004775	0.49751595
ENSGALG00010009609	MMADHC	2	-0.345970537	0.173203946	-1.997474913	0.045773617	0.042572348	2.858104699	65.0117786	1	CRA004775;Yang2025_Silkie_Wenchang	PRJNA827465	0.369109658
ENSGALG00010009609	MMADHC	2	-0.128509768	0.042378411	-3.032434769	0.002425895	0	0.00627388	0	1	CRA004775;PRJNA827465	Yang2025_Silkie_Wenchang	0.06593704
ENSGALG00010009612	DERL1	2	-0.325772044	0.346645532	-0.939784346	0.34732819	0.221514087	11.98912517	91.65910785	1	PRJNA827465;Yang2025_Silkie_Wenchang	CRA004775	0.808705451
ENSGALG00010009612	DERL1	2	-0.53578547	0.163316705	-3.280653196	0.00103567	0.015227478	1.39948983	28.54538999	1	CRA004775;Yang2025_Silkie_Wenchang	PRJNA827465	0.038656163
ENSGALG00010009612	DERL1	2	-0.139666929	0.177740002	-0.785793446	0.431988507	0.047845771	3.382109453	70.43265412	1	CRA004775;PRJNA827465	Yang2025_Silkie_Wenchang	0.842503929
ENSGALG00010009615	ENSGALG00010009615	2	-0.753271444	0.288571472	-2.610346197	0.009045063	0.036370276	1.268656173	21.17643685	1	PRJNA827465;Yang2025_Silkie_Wenchang	CRA004775	0.149037013
ENSGALG00010009615	ENSGALG00010009615	2	-1.040819567	0.335423367	-3.103002563	0.00191568	0	0.06049337	0	1	CRA004775;Yang2025_Silkie_Wenchang	PRJNA827465	0.056398475
ENSGALG00010009615	ENSGALG00010009615	2	-0.605473736	0.284934749	-2.124955761	0.033590304	0	0.365518889	0	1	CRA004775;PRJNA827465	Yang2025_Silkie_Wenchang	0.314632921
ENSGALG00010009616	ARMC10	2	-0.204868109	0.102153881	-2.005485328	0.044911188	0	0.304905326	0	1	PRJNA827465;Yang2025_Silkie_Wenchang	CRA004775	0.365383522
ENSGALG00010009616	ARMC10	2	-0.257401055	0.157382281	-1.635514834	0.101941168	0	0.112375492	0	1	CRA004775;Yang2025_Silkie_Wenchang	PRJNA827465	0.54407845
ENSGALG00010009616	ARMC10	2	-0.166761081	0.114696698	-1.453930967	0.145965446	0	1.65997E-06	0	1	CRA004775;PRJNA827465	Yang2025_Silkie_Wenchang	0.634891538
ENSGALG00010009617	SLC25A46	2	-0.129430654	0.065532394	-1.975063723	0.048260917	0	0.093294914	0	1	PRJNA827465;Yang2025_Silkie_Wenchang	CRA004775	0.379964424
ENSGALG00010009617	SLC25A46	2	-0.104858247	0.100330482	-1.045128514	0.295963577	0	0.720815701	0	0.5	CRA004775;Yang2025_Silkie_Wenchang	PRJNA827465	0.788124569
ENSGALG00010009617	SLC25A46	2	-0.094470126	0.07214857	-1.30938321	0.190404576	0	0.508830747	0	0.5	CRA004775;PRJNA827465	Yang2025_Silkie_Wenchang	0.699413831

ENSGALG00010009619	ENSGALG00010009619	2	-0.600777522	0.632652041	-0.949617615	0.342306584	0.744426691	14.25868097	92.98672856	0.5	PRJNA827465;Yang2025_Silkie_Wenchang	CRA004775	0.806005903
ENSGALG00010009619	ENSGALG00010009619	2	-0.253825729	1.006631356	-0.252153609	0.800922334	1.921153724	19.06500924	94.7547888	0.5	CRA004775;Yang2025_Silkie_Wenchang	PRJNA827465	0.950158029
ENSGALG00010009619	ENSGALG00010009619	2	0.332833763	0.368107142	0.904176326	0.365901911	0.181451293	2.841126185	64.80268967	1	CRA004775;PRJNA827465	Yang2025_Silkie_Wenchang	0.818968857
ENSGALG00010009620	TMOD1	2	-0.070923546	0.203673312	-0.348222088	0.727673401	0.036850999	1.788460337	44.08598393	0.5	PRJNA827465;Yang2025_Silkie_Wenchang	CRA004775	0.931639802
ENSGALG00010009620	TMOD1	2	-0.381775711	0.569931966	-0.669861903	0.502945828	0.53294381	5.140063037	80.54498568	0.5	CRA004775;Yang2025_Silkie_Wenchang	PRJNA827465	0.863050365
ENSGALG00010009620	TMOD1	2	-0.553185677	0.353873218	-1.563231261	0.11799821	0.13895965	2.022164563	50.54804053	1	CRA004775;PRJNA827465	Yang2025_Silkie_Wenchang	0.577760513
ENSGALG00010009623	XPO5	2	-0.09576583	0.065214013	-1.468485454	0.141972405	0.004757992	2.219882453	54.95256974	1	PRJNA827465;Yang2025_Silkie_Wenchang	CRA004775	0.62757683
ENSGALG00010009623	XPO5	2	-0.171010319	0.065569913	-2.60806078	0.009105678	0	0.000675882	0	1	CRA004775;Yang2025_Silkie_Wenchang	PRJNA827465	0.149588551
ENSGALG00010009623	XPO5	2	-0.050547634	0.05011934	-1.008545479	0.313192666	0	0.587155746	0	1	CRA004775;PRJNA827465	Yang2025_Silkie_Wenchang	0.796531448
ENSGALG00010009634	TBC1D31	2	0.236281227	0.227228428	1.039840082	0.298414204	0.087207592	5.997114715	83.3253148	1	PRJNA827465;Yang2025_Silkie_Wenchang	CRA004775	0.789324547
ENSGALG00010009634	TBC1D31	2	0.138428992	0.397592436	0.348168073	0.727713964	0.253164455	4.751202753	78.95269783	0.5	CRA004775;Yang2025_Silkie_Wenchang	PRJNA827465	0.931639802
ENSGALG00010009634	TBC1D31	2	0.008023223	0.090666617	0.088491481	0.929486055	0.003516844	1.063467607	5.967986828	0.5	CRA004775;PRJNA827465	Yang2025_Silkie_Wenchang	0.983724429
ENSGALG00010009635	TDRD7	2	0.346942547	0.105369073	3.292641151	0.000992511	0.004319435	1.239879524	19.34700259	1	PRJNA827465;Yang2025_Silkie_Wenchang	CRA004775	0.037653133
ENSGALG00010009635	TDRD7	2	0.140486244	0.346480128	0.405466958	0.685134302	0.202725468	6.210622448	83.89855432	0.5	CRA004775;Yang2025_Silkie_Wenchang	PRJNA827465	0.919196979
ENSGALG00010009635	TDRD7	2	0.050323012	0.238043335	0.211402736	0.832573022	0.079796887	3.141411754	68.16717838	0.5	CRA004775;PRJNA827465	Yang2025_Silkie_Wenchang	0.957861248
ENSGALG00010009637	CYBA	2	-0.076840803	0.107358472	-0.715740466	0.474151619	0.000187815	1.005300967	0.52730151	0.5	PRJNA827465;Yang2025_Silkie_Wenchang	CRA004775	0.85444425
ENSGALG00010009637	CYBA	2	0.142731809	0.19805981	0.720650034	0.471124862	0	0.00270745	0	1	CRA004775;Yang2025_Silkie_Wenchang	PRJNA827465	0.853923969
ENSGALG00010009637	CYBA	2	-0.101979631	0.113316362	-0.899955039	0.368144178	0	0.588573274	0	0.5	CRA004775;PRJNA827465	Yang2025_Silkie_Wenchang	0.820213684
ENSGALG00010009638	RND3	2	0.084651974	0.064255169	1.317434461	0.187693036	0	0.491317756	0	1	PRJNA827465;Yang2025_Silkie_Wenchang	CRA004775	0.696930043
ENSGALG00010009638	RND3	2	0.116543852	0.138786308	0.839735952	0.401056451	0	0.77767651	0	0.5	CRA004775;Yang2025_Silkie_Wenchang	PRJNA827465	0.832420876
ENSGALG00010009638	RND3	2	0.055362797	0.067948575	0.814774951	0.415201142	0	0.310633605	0	0.5	CRA004775;PRJNA827465	Yang2025_Silkie_Wenchang	0.837489728
ENSGALG00010009640	NPR2	2	0.14288211	0.351446109	0.406554822	0.684334985	0.217171757	8.25723776	87.88941255	0.5	PRJNA827465;Yang2025_Silkie_Wenchang	CRA004775	0.918862939
ENSGALG00010009640	NPR2	2	0.155097134	0.418779362	0.370355247	0.711117814	0.260738043	3.402243263	70.60762788	0.5	CRA004775;Yang2025_Silkie_Wenchang	PRJNA827465	0.925588272
ENSGALG00010009640	NPR2	2	-0.238057798	0.169948266	-1.400766263	0.16128398	0	0.108590095	0	1	CRA004775;PRJNA827465	Yang2025_Silkie_Wenchang	0.657566833
ENSGALG00010009643	PSMC2	2	-0.104291656	0.054557072	-1.911606536	0.05592668	0	0.749029413	0	1	PRJNA827465;Yang2025_Silkie_Wenchang	CRA004775	0.409250851
ENSGALG00010009643	PSMC2	2	-0.211890634	0.121560885	-1.743082351	0.081319231	0	0.002522121	0	1	CRA004775;Yang2025_Silkie_Wenchang	PRJNA827465	0.491177153
ENSGALG00010009643	PSMC2	2	-0.09050904	0.057665376	-1.569556051	0.11651843	0	0.209294352	0	1	CRA004775;PRJNA827465	Yang2025_Silkie_Wenchang	0.574705849
ENSGALG00010009644	SLC27A6	2	-0.202246703	0.182475753	-1.108348367	0.267711395	0	0.756965225	0	1	PRJNA827465;Yang2025_Silkie_Wenchang	CRA004775	0.771398294
ENSGALG00010009644	SLC27A6	2	-0.475252783	0.305250358	-1.556927846	0.119487617	0	0.033600561	0	1	CRA004775;Yang2025_Silkie_Wenchang	PRJNA827465	0.58038582
ENSGALG00010009644	SLC27A6	2	-0.141439977	0.207714177	-0.680935595	0.495912244	0	0.434110519	0	1	CRA004775;PRJNA827465	Yang2025_Silkie_Wenchang	0.860911322
ENSGALG00010009646	MVD	2	-0.111437054	0.105129735	-1.059995569	0.289146615	0	0.000529806	0	1	PRJNA827465;Yang2025_Silkie_Wenchang	CRA004775	0.783530627
ENSGALG00010009646	MVD	2	-0.23637834	0.185213803	-1.27624581	0.201868639	0	0.818129148	0	1	CRA004775;Yang2025_Silkie_Wenchang	PRJNA827465	0.714960076
ENSGALG00010009646	MVD	2	-0.1684801	0.125957838	-1.337591227	0.181029726	0.005143085	1.097664432	8.897476249	1	CRA004775;PRJNA827465	Yang2025_Silkie_Wenchang	0.688594651
ENSGALG00010009647	GALNT2	2	0.067784854	0.056312293	1.203731019	0.228693559	0	0.17372103	0	1	PRJNA827465;Yang2025_Silkie_Wenchang	CRA004775	0.74144547
ENSGALG00010009647	GALNT2	2	0.019907454	0.100325239	0.198429166	0.8427093	0	0.131351987	0	0.5	CRA004775;Yang2025_Silkie_Wenchang	PRJNA827465	0.960335284
ENSGALG00010009647	GALNT2	2	0.076165149	0.065551549	1.161915467	0.245270064	0	0.269378473	0	0.5	CRA004775;PRJNA827465	Yang2025_Silkie_Wenchang	0.75661406
ENSGALG00010009649	TENM2	2	0.327409146	0.396849887	0.825020133	0.409360161	0.271974117	7.199332206	86.10982281	0.5	PRJNA827465;Yang2025_Silkie_Wenchang	CRA004775	0.834355341
ENSGALG00010009649	TENM2	2	0.755761719	0.232069601	3.256616624	0.001127486	0	0.015717959	0	1	CRA004775;Yang2025_Silkie_Wenchang	PRJNA827465	0.040691996
ENSGALG00010009649	TENM2	2	0.130957101	0.35717416	0.366647748	0.713881787	0.132688602	1.506656837	33.68728554	0.5	CRA004775;PRJNA827465	Yang2025_Silkie_Wenchang	0.92636344
ENSGALG00010009650	KLHL29	2	-0.04857906	0.066196055	-0.733866397	0.463030169	0	0.79079872	0	1	PRJNA827465;Yang2025_Silkie_Wenchang	CRA004775	0.851430463
ENSGALG00010009650	KLHL29	2	-0.13524837	0.136676328	-0.989552264	0.322393012	0	0.298973085	0	0.5	CRA004775;Yang2025_Silkie_Wenchang	PRJNA827465	0.798652022
ENSGALG00010009650	KLHL29	2	-0.016471441	0.072489907	-0.227223922	0.820249627	0	0.031330496	0	0.5	CRA004775;PRJNA827465	Yang2025_Silkie_Wenchang	0.955308792
ENSGALG00010009652	CIAO2B	2	0.152939664	0.170456389	0.897236327	0.369592825	0.043790243	3.76827799	73.4626797	1	PRJNA827465;Yang2025_Silkie_Wenchang	CRA004775	0.820606801
ENSGALG00010009652	CIAO2B	2	0.079704622	0.300791576	0.264982892	0.791022641	0.139868999	4.27126363	76.58772469	0.5	CRA004775;Yang2025_Silkie_Wenchang	PRJNA827465	0.947823629
ENSGALG00010009652	CIAO2B	2	-0.017896052	0.077264327	-0.231621147	0.816832281	0.000254506	1.007641201	0.758325537	0.5	CRA004775;PRJNA827465	Yang2025_Silkie_Wenchang	0.954736191
ENSGALG00010009653	ABLIM2	2	-0.117462027	0.098180602	-1.196387315	0.231545449	0	0.813870307	0	1	PRJNA827465;Yang2025_Silkie_Wenchang	CRA004775	0.743924694
ENSGALG00010009653	ABLIM2	2	-0.053224085	0.127755314	-0.41660956	0.676964016	0	0.197084078	0	1	CRA004775;Yang2025_Silkie_Wenchang	PRJNA827465	0.917593503
ENSGALG00010009653	ABLIM2	2	-0.207201054	0.130746123	-1.584758689	0.113021172	0	0.000560013	0	1	CRA004775;PRJNA827465	Yang2025_Silkie_Wenchang	0.567459263
ENSGALG00010009654	NDUFA12	2	-0.107305937	0.065347907	-1.642071518	0.100575184	0	0.437266866	0	0.5	PRJNA827465;Yang2025_Silkie_Wenchang	CRA004775	0.541393598
ENSGALG00010009654	NDUFA12	2	0.007647656	0.181699884	0.042089493	0.966427356	0	0.018552723	0	0.5	CRA004775;Yang2025_Silkie_Wenchang	PRJNA827465	0.990881287
ENSGALG00010009654	NDUFA12	2	-0.119672238	0.06801588	-1.75947495	0.078496471	0	0.038673599	0	1	CRA004775;PRJNA827465	Yang2025_Silkie_Wenchang	0.481915278
ENSGALG00010009657	MSMP	2	0.364895036	0.356189684	1.024440212	0.305627397	0.109047971	1.746894967	42.75557383	0.5	PRJNA827465;Yang2025_Silkie_Wenchang	CRA004775	0.79225794
ENSGALG00010009657	MSMP	2	-0.763209945	1.584762507	-0.481592631	0.630093362	4.514287751	9.318075502	89.26817024	0.5	CRA004775;Yang2025_Silkie_Wenchang	PRJNA827465	0.90256717
ENSGALG00010009657	MSMP	2	-1.089931063	1.222470915	-0.891573799	0.372621419	2.479701455	5.420382953	81.55311902	1	CRA004775;PRJNA827465	Yang2025_Silkie_Wenchang	0.823016521
ENSGALG00010009658	NAPEPLD	2	-0.079876493	0.183065689	-0.436326948	0.662599533	0.053189082	4.80714855	79.19764722	0.5	PRJNA827465;Yang2025_Silkie_Wenchang	CRA004775	0.912778072
ENSGALG00010009658	NAPEPLD	2	-0.073454661	0.2618506	-0.280521264	0.779077614	0.090741658	2.579597117	61.23425657	0.5	CRA004775;Yang2025_Silkie_Wenchang	PRJNA827465	0.944714533
ENSGALG00010009658	NAPEPLD	2	0.113118199	0.099333447	1.138772509	0.254798051	0	0.290652545	0	1	CRA004775;PRJNA827465	Yang2025_Silkie_Wenchang	0.764394153
ENSGALG00010009659	APOLD1	2	-0.625129127	0.254627228	-2.455057224	0.014085491	0	5.08697086	80.34193575	1	PRJNA827465;Yang2025_Silkie_Wenchang	CRA004775	0.194491025
ENSGALG00010009659	APOLD1	2	0.075152286	1.012386395	0.074232809	0.94082514	1.951822434	20.55632316	95.1353168	0.5	CRA004775;Yang2025_Silkie_Wenchang	PRJNA827465	0.985565295
ENSGALG00010009659	APOLD1	2	0.312058254	0.755216518	0.413203687	0.679437387	1.059368181	13.30097292	92.48175298	0.5	CRA004775;PRJNA827465	Yang2025_Silkie_Wenchang	0.917701868
ENSGALG00010009663	FER	2	-0.039314492	0.095615961	-0.411173284	0.68094401	0	0.027585671	0	1	PRJNA827465;Yang2025_Silkie_Wenchang	CRA004775	0.918023614
ENSGALG00010009663	FER	2	0.087192856	0.174832522	0.498722174	0.617975121	0.019018696	1.327236705	24.6554894	0.5	CRA004775;Yang2025_Silkie_Wenchang	PRJNA827465	0.899121942
ENSGALG00010009663	FER	2	0.068606378	0.191810541	0.357677828	0.720584427	0.036214774	1.667938396	40.04574736	0.5	CRA004775;PRJNA827465	Yang2025_Silkie_Wenchang	0.928774793
ENSGALG00010009665	DGKQ	2	0.211299636	0.228484402	0.924788013	0.355076187	0.066285173	2.738145673	63.47893357	0.5	PRJNA827465;Yang2025_Silkie_Wenchang	CRA004775	0.812276513
ENSGALG00010009665	DGKQ	2	0.187141137	0.371753872	0.503400543	0.614682269	0.16401557	2.067742629	51.63808174	0.5	CRA004775;Yang2025_Silkie_Wenchang	PRJNA827465	0.898692812
ENSGALG00010009665	DGKQ	2	-0.056237185	0.180500499	-0.311562491	0.755373044	0	0.378936868	0	1	CRA004775;PRJNA827465	Yang2025_Silkie_Wenchang	0.938467733
ENSGALG00010009666	RRAD	2	-0.048952681	0.134672544	-0.363494144	0.716235786	0	0.495936442	0	0.5	PRJNA827465;Yang2025_Silkie_Wenchang	CRA004775	0.92742777
ENSGALG00010009666	RRAD	2	-0.134972381	0.200047749	-0.674700826	0.49986586	0	0.214015502	0	0.5	CRA004775;Yang2025_Silkie_Wenchang	PRJNA827465	0.862078151
ENSGALG00010009666	RRAD	2	0.041142633	0.172802287	0.23809079	0.811810678	0	0.050614788	0	1	CRA004775;PRJNA827465	Yang2025_Silkie_Wenchang	0.953371387
ENSGALG00010009672	DDX47	2	-0.085985693	0.058900603	-1.459844022	0.144332947	0	0.770826287	0	1	PRJNA827465;Yang2025_Silkie_Wenchang	CRA004775	0.631870428
ENSGALG00010009672	DDX47	2	-0.21392359	0.119156971	-1.795300062	0.072604472	0	0.046273115	0	1	CRA004775;Yang2025_Silkie_Wenchang	PRJNA827465	0.465466124
ENSGALG00010009672	DDX47	2	-0.076895152	0.062343346	-1.23341393	0.217421363	0	0.678129027	0	1	CRA004775;PRJNA827465	Yang2025_Silkie_Wenchang	0.730930787
ENSGALG00010009676	ACO1	2	0.004625486	0.212952347	0.021720755	0.982670707	0.072412525	4.895560849	79.57333121	0.5	PRJNA827465;Yang2025_Silkie_Wenchang	CRA004775	0.99530736
ENSGALG00010009676	ACO1	2	-0.20498394	0.107475747	-1.907257652	0.056487231	0	0.0417292	0	1	CRA004775;Yang2025_Silkie_Wenchang	PRJNA827465	0.411523553
ENSGALG00010009676	ACO1	2	0.048421384	0.237157038	0.204174349	0.838217256	0.065462112	2.18697346	54.27470803	0.5	CRA004775;PRJNA827465	Yang2025_Silkie_Wenchang	0.959165366
ENSGALG00010009677	SLC26A5	2	-0.008760734	0.417923906	-0.020962511	0.983275561	0.318844637	11.43524359	91.25510339	0.5	PRJNA827465;Yang2025_Silkie_Wenchang	CRA004775	0.995363468
ENSGALG00010009677	SLC26A5	2	-0.075203842	0.422519796	-0.177988919	0.858731678	0.270747092	3.812572205	73.77098855	0.5	CRA004775;Yang2025_Silkie_Wenchang	PRJNA827465	0.964449906
ENSGALG00010009677	SLC26A5	2	0.405841067	0.147641201	2.74883342	0.005980777	0	0.002378317	0	1	CRA004775;PRJNA827465	Yang2025_Silkie_Wenchang	0.116888359
ENSGALG00010009678	JARID2	2	0.358330757	0.354116244	1.011901496	0.311585154	0.225553493	9.370004137	89.32764612	1	PRJNA827465;Yang2025_Silkie_Wenchang	CRA004775	0.795778238
ENSGALG00010009678	JARID2	2	0.507613144	0.307421669	1.65119507	0.09869875	0.102146537	2.013991585	50.34735956	1	CRA004775;Yang2025_Silkie_Wenchang	PRJNA827465	0.536091342
ENSGALG00010009678	JARID2	2	0.03506225	0.077204076	0.454150239	0.649720696	0	0.033894081	0	1	CRA004775;PRJNA827465	Yang2025_Silkie_Wenchang	0.90820125
ENSGALG00010009679	ANK3	2	0.069449911	0.320619104	0.216611893	0.828510816	0.19315253	16.40348242	93.90373352	0.5	PRJNA827465;Yang2025_Silkie_Wenchang	CRA004775	0.956777964
ENSGALG00010009679	ANK3	2	0.400646098	0.125281728	3.197961141	0.001384029	0	0.008897498	0	1	CRA004775;Yang2025_Silkie_Wenchang	PRJNA827465	0.045672967
ENSGALG00010009679	ANK3	2	-0.040122261	0.313324627	-0.128053326	0.898106772	0.131796181	2.280925921	56.15815531	0.5	CRA004775;PRJNA827465	Yang2025_Silkie_Wenchang	0.975733636
ENSGALG00010009683	ADAMTS19	2	0.078344502	0.106160403	0.737982328	0.460525188	0	0.804955157	0	0.5	PRJNA827465;Yang2025_Silkie_Wenchang	CRA004775	0.85014477
ENSGALG00010009683	ADAMTS19	2	0.16142391	0.156132582	1.03388997	0.301187605	0	0.33063819	0	0.5	CRA004775;Yang2025_Silkie_Wenchang	PRJNA827465	0.791222412
ENSGALG00010009683	ADAMTS19	2	-0.010151937	0.13401821	-0.075750432	0.939617652	0	0.044791984	0	1	CRA004775;PRJNA827465	Yang2025_Silkie_Wenchang	0.985425793
ENSGALG00010009686	MAT2B	2	-0.06955124	0.057119112	-1.217652679	0.223355987	0.001626901	1.176030012	14.96815646	1	PRJNA827465;Yang2025_Silkie_Wenchang	CRA004775	0.736744216
ENSGALG00010009686	MAT2B	2	-0.085041252	0.143840596	-0.591218711	0.554373887	0.015566741	1.517266669	34.09200765	0.5	CRA004775;Yang2025_Silkie_Wenchang	PRJNA827465	0.880177968
ENSGALG00010009686	MAT2B	2	-0.034536215	0.047433066	-0.728104213	0.466549795	0	0.515644154	0	0.5	CRA004775;PRJNA827465	Yang2025_Silkie_Wenchang	0.852795395
ENSGALG00010009689	ENSGALG00010009689	2	-0.556296844	0.401863981	-1.384291379	0.166269253	0	0.068722832	0	1	PRJNA827465;Yang2025_Silkie_Wenchang	CRA004775	0.66529196
ENSGALG00010009689	ENSGALG00010009689	2	0.256171006	0.929415061	0.275626055	0.782835296	1.313286593	4.158011912	75.95004485	0.5	CRA004775;Yang2025_Silkie_Wenchang	PRJNA827465	0.945507994
ENSGALG00010009689	ENSGALG00010009689	2	0.303409233	0.818281469	0.370788347	0.710795181	1.036048777	4.389058136	77.21606848	0.5	CRA004775;PRJNA827465	Yang2025_Silkie_Wenchang	0.925543061
ENSGALG00010009690	RBM43	2	-0.242992021	0.355603307	-0.683323288	0.494402582	0.228176533	10.06678617	90.06634309	0.5	PRJNA827465;Yang2025_Silkie_Wenchang	CRA004775	0.860175061
ENSGALG00010009690	RBM43	2	-0.531724278	0.165096133	-3.220694936	0.001278802	0	0.798067756	0	1	CRA004775;Yang2025_Silkie_Wenchang	PRJNA827465	0.043386199
ENSGALG00010009690	RBM43	2	0.054452553	0.118393941	0.459926852	0.645568726	0.001263141	1.019395637	1.902660412	0.5	CRA004775;PRJNA827465	Yang2025_Silkie_Wenchang	0.907052464
ENSGALG00010009695	ATAD2B	2	0.195432038	0.173858093	1.124089392	0.260975105	0.044863234	3.279154542	69.50433452	1	PRJNA827465;Yang2025_Silkie_Wenchang	CRA004775	0.767929536
ENSGALG00010009695	ATAD2B	2	0.432727539	0.169735104	2.549428658	0.010789959	0	0.021571976	0	1	CRA004775;Yang2025_Silkie_Wenchang	PRJNA827465	0.165420139
ENSGALG00010009695	ATAD2B	2	0.103862536	0.127410311	0.815181557	0.414968393	0.014640274	1.196984656	16.4567403	1	CRA004775;PRJNA827465	Yang2025_Silkie_Wenchang	0.837458421
ENSGALG00010009697	ENSGALG00010009697	2	-0.250826646	0.272901762	-0.919109661	0.358038217	0.074603797	1.833031988	45.44557833	1	PRJNA827465;Yang2025_Silkie_Wenchang	CRA004775	0.813691224
ENSGALG00010009697	ENSGALG00010009697	2	-0.693865189	0.28854044	-2.404741568	0.016183906	0	0.085433673	0	1	CRA004775;Yang2025_Silkie_Wenchang	PRJNA827465	0.210262467
ENSGALG00010009697	ENSGALG00010009697	2	-0.312001409	0.355221307	-0.878329659	0.379764845	0.154436001	2.255444421	55.66284007	1	CRA004775;PRJNA827465	Yang2025_Silkie_Wenchang	0.824361963
ENSGALG00010009699	POLR1C	2	0.10062514	0.078885497	1.275584788	0.202102334	0.004355673	1.43514918	30.32083258	1	PRJNA827465;Yang2025_Silkie_Wenchang	CRA004775	0.71497298
ENSGALG00010009699	POLR1C	2	0.010062729	0.222280504	0.045270407	0.963891775	0.076704467	4.384356114	77.19163375	0.5	CRA004775;Yang2025_Silkie_Wenchang	PRJNA827465	0.990168281
ENSGALG00010009699	POLR1C	2	-0.04549448	0.130336383	-0.349054343	0.727048513	0.020757237	2.19202725	54.38012917	0.5	CRA004775;PRJNA827465	Yang2025_Silkie_Wenchang	0.931425004
ENSGALG00010009700	SH3TC1	2	-0.020890888	0.094132648	-0.221930312	0.824368133	0	0.609981664	0	0.5	PRJNA827465;Yang2025_Silkie_Wenchang	CRA004775	0.95619211
ENSGALG00010009700	SH3TC1	2	0.060765654	0.130387164	0.466040154	0.641186759	0	0.026498504	0	1	CRA004775;Yang2025_Silkie_Wenchang	PRJNA827465	0.906429868
ENSGALG00010009700	SH3TC1	2	-0.076908444	0.126693821	-0.60704179	0.543823179	0	0.204400513	0	0.5	CRA004775;PRJNA827465	Yang2025_Silkie_Wenchang	0.876968972
ENSGALG00010009702	ENSGALG00010009702	2	0.510809147	0.115772944	4.412163419	1.02343E-05	0	0.773753876	0	1	PRJNA827465;Yang2025_Silkie_Wenchang	CRA004775	0.001441713
ENSGALG00010009702	ENSGALG00010009702	2	-0.192675975	0.534373202	-0.360564441	0.718425076	0.510764395	9.451638278	89.41982363	0.5	CRA004775;Yang2025_Silkie_Wenchang	PRJNA827465	0.928100521
ENSGALG00010009702	ENSGALG00010009702	2	-0.06292012	0.651509938	-0.096575841	0.923063243	0.806926876	19.88484567	94.9710447	0.5	CRA004775;PRJNA827465	Yang2025_Silkie_Wenchang	0.981836718
ENSGALG00010009704	RGP1	2	0.057362471	0.124350029	0.461298413	0.644584523	0	0.121393239	0	1	PRJNA827465;Yang2025_Silkie_Wenchang	CRA004775	0.906953008
ENSGALG00010009704	RGP1	2	-0.14719496	0.200087481	-0.735653022	0.461941886	0.024930413	1.406424255	28.89769952	0.5	CRA004775;Yang2025_Silkie_Wenchang	PRJNA827465	0.850747865
ENSGALG00010009704	RGP1	2	-0.100670585	0.245038787	-0.410835307	0.681193302	0.071610611	2.296770204	56.46059853	0.5	CRA004775;PRJNA827465	Yang2025_Silkie_Wenchang	0.91820222
ENSGALG00010009705	DTNBP1	2	0.074315094	0.049044283	1.515265174	0.129705264	0	0.342671772	0	1	PRJNA827465;Yang2025_Silkie_Wenchang	CRA004775	0.60409121
ENSGALG00010009705	DTNBP1	2	0.088620433	0.074368214	1.191643954	0.233400882	0	0.512364649	0	0.5	CRA004775;Yang2025_Silkie_Wenchang	PRJNA827465	0.746672784
ENSGALG00010009705	DTNBP1	2	0.044173969	0.058226014	0.758663806	0.448053694	0	0.18730932	0	0.5	CRA004775;PRJNA827465	Yang2025_Silkie_Wenchang	0.846601815
ENSGALG00010009710	ENSGALG00010009710	2	0.170281512	0.177925908	0.957036074	0.338549047	0.049718205	4.287776793	76.67789047	0.5	PRJNA827465;Yang2025_Silkie_Wenchang	CRA004775	0.804408854
ENSGALG00010009710	ENSGALG00010009710	2	-0.042980201	0.137902313	-0.311671354	0.7552903	0	0.005168454	0	1	CRA004775;Yang2025_Silkie_Wenchang	PRJNA827465	0.938453928
ENSGALG00010009710	ENSGALG00010009710	2	0.227125251	0.165837299	1.369566756	0.170822183	0.031463064	1.749310443	42.83461785	0.5	CRA004775;PRJNA827465	Yang2025_Silkie_Wenchang	0.672727882
ENSGALG00010009712	UBXN2A	2	-0.113806715	0.067839958	-1.677576449	0.093429814	0	0.52366512	0	1	PRJNA827465;Yang2025_Silkie_Wenchang	CRA004775	0.52381273
ENSGALG00010009712	UBXN2A	2	0.056917871	0.172396327	0.330157099	0.741281261	0.011708698	1.110754723	9.971123336	0.5	CRA004775;Yang2025_Silkie_Wenchang	PRJNA827465	0.93538952
ENSGALG00010009712	UBXN2A	2	0.015490939	0.2641763	0.058638639	0.953239934	0.087677335	1.904007069	47.47918658	0.5	CRA004775;PRJNA827465	Yang2025_Silkie_Wenchang	0.987758913
ENSGALG00010009715	NUDCD2	2	-0.103627342	0.096615774	-1.072571669	0.283463342	0.013147344	3.195355317	68.70457584	0.5	PRJNA827465;Yang2025_Silkie_Wenchang	CRA004775	0.779568117
ENSGALG00010009715	NUDCD2	2	-0.02491764	0.087078045	-0.286152956	0.774760974	0	0.847525205	0	0.5	CRA004775;Yang2025_Silkie_Wenchang	PRJNA827465	0.943713409
ENSGALG00010009715	NUDCD2	2	-0.187651115	0.053161012	-3.529863465	0.000415774	0	0.01270222	0	1	CRA004775;PRJNA827465	Yang2025_Silkie_Wenchang	0.020580822
ENSGALG00010009717	EDN1	2	-0.335778307	0.10652412	-3.152134057	0.001620818	0	0.620214111	0	1	PRJNA827465;Yang2025_Silkie_Wenchang	CRA004775	0.050888881
ENSGALG00010009717	EDN1	2	-0.119521199	0.407935331	-0.292990555	0.769529371	0.242298908	3.265681964	69.37852458	0.5	CRA004775;Yang2025_Silkie_Wenchang	PRJNA827465	0.941843401
ENSGALG00010009717	EDN1	2	-0.066334806	0.306288475	-0.21657624	0.828538604	0.1184402	2.181470061	54.15935254	0.5	CRA004775;PRJNA827465	Yang2025_Silkie_Wenchang	0.956781335
ENSGALG00010009718	ENSGALG00010009718	2	0.640906569	0.321962607	1.99062424	0.046522213	0.13608388	2.763766497	63.81749322	1	PRJNA827465;Yang2025_Silkie_Wenchang	CRA004775	0.372346812
ENSGALG00010009718	ENSGALG00010009718	2	-0.247188743	0.506731441	-0.487810155	0.625684318	0.401311051	4.575382429	78.14390348	0.5	CRA004775;Yang2025_Silkie_Wenchang	PRJNA827465	0.901501367
ENSGALG00010009718	ENSGALG00010009718	2	0.100826669	0.832992117	0.121041564	0.903658114	1.311923337	18.10126758	94.47552501	0.5	CRA004775;PRJNA827465	Yang2025_Silkie_Wenchang	0.977333734
ENSGALG00010009719	YIPF3	2	-0.098422517	0.131434447	-0.748833501	0.453957565	0.024940361	3.055951117	67.27696349	0.5	PRJNA827465;Yang2025_Silkie_Wenchang	CRA004775	0.849072665
ENSGALG00010009719	YIPF3	2	-0.306721311	0.113722976	-2.697091841	0.006994798	0	0.151501624	0	1	CRA004775;Yang2025_Silkie_Wenchang	PRJNA827465	0.12854434
ENSGALG00010009719	YIPF3	2	-0.138508491	0.176623224	-0.784203162	0.43292091	0.048536573	3.833205458	73.91217322	0.5	CRA004775;PRJNA827465	Yang2025_Silkie_Wenchang	0.842762754
ENSGALG00010009720	RELN	2	-0.153706411	0.099556209	-1.543915866	0.122608716	0	0.033654091	0	1	PRJNA827465;Yang2025_Silkie_Wenchang	CRA004775	0.587597899
ENSGALG00010009720	RELN	2	-0.114848638	0.166941357	-0.687957977	0.491479245	0.006478039	1.05146067	4.894207771	0.5	CRA004775;Yang2025_Silkie_Wenchang	PRJNA827465	0.85895707
ENSGALG00010009720	RELN	2	-0.106884573	0.123478231	-0.865614713	0.386701484	0	0.937086511	0	0.5	CRA004775;PRJNA827465	Yang2025_Silkie_Wenchang	0.827062786
ENSGALG00010009721	ZHX1	2	0.056996108	0.080867525	0.704808359	0.480929506	0.003579745	1.250580111	20.03710985	1	PRJNA827465;Yang2025_Silkie_Wenchang	CRA004775	0.856727207
ENSGALG00010009721	ZHX1	2	0.230218145	0.133501284	1.7244639	0.084624131	0	0.155382588	0	1	CRA004775;Yang2025_Silkie_Wenchang	PRJNA827465	0.5001477
ENSGALG00010009721	ZHX1	2	0.057589709	0.110756157	0.519968467	0.603085553	0.009361595	1.238556873	19.26087356	1	CRA004775;PRJNA827465	Yang2025_Silkie_Wenchang	0.895538966
ENSGALG00010009722	GBA2	2	0.020807176	0.079595723	0.261410729	0.793775784	0	0.006416437	0	1	PRJNA827465;Yang2025_Silkie_Wenchang	CRA004775	0.948808419
ENSGALG00010009722	GBA2	2	-0.027386081	0.111955439	-0.244615902	0.80675385	0	0.324629072	0	0.5	CRA004775;Yang2025_Silkie_Wenchang	PRJNA827465	0.952282862
ENSGALG00010009722	GBA2	2	-0.002633831	0.090271515	-0.029176768	0.97672361	0	0.440757017	0	0.5	CRA004775;PRJNA827465	Yang2025_Silkie_Wenchang	0.993193912
ENSGALG00010009724	ISOC1	2	-0.048523088	0.193751696	-0.250439551	0.802247447	0.060049609	4.832598172	79.30719741	0.5	PRJNA827465;Yang2025_Silkie_Wenchang	CRA004775	0.950637102
ENSGALG00010009724	ISOC1	2	-0.206206444	0.128640066	-1.602972153	0.108940802	0	0.493771147	0	1	CRA004775;Yang2025_Silkie_Wenchang	PRJNA827465	0.558848033
ENSGALG00010009724	ISOC1	2	0.105249799	0.087386984	1.204410483	0.228430962	0	0.487243603	0	0.5	CRA004775;PRJNA827465	Yang2025_Silkie_Wenchang	0.741250174
ENSGALG00010009726	PRR16	2	-0.240635559	0.122024105	-1.972032973	0.048605843	0.005331027	1.214950863	17.69214455	1	PRJNA827465;Yang2025_Silkie_Wenchang	CRA004775	0.381308469
ENSGALG00010009726	PRR16	2	0.063016916	0.485255139	0.12986347	0.896674446	0.38460013	4.898730461	79.58654782	0.5	CRA004775;Yang2025_Silkie_Wenchang	PRJNA827465	0.975115773
ENSGALG00010009726	PRR16	2	0.164076172	0.354960179	0.462238249	0.643910477	0.170133254	2.669509089	62.539929	0.5	CRA004775;PRJNA827465	Yang2025_Silkie_Wenchang	0.906790407
ENSGALG00010009727	CDH20	2	-0.422920135	0.193832301	-2.181886778	0.02911789	0.05254631	3.317586374	69.85760468	1	PRJNA827465;Yang2025_Silkie_Wenchang	CRA004775	0.290337858
ENSGALG00010009727	CDH20	2	-0.168637636	0.523417978	-0.322185411	0.747312236	0.449067674	4.915913941	79.65790264	0.5	CRA004775;Yang2025_Silkie_Wenchang	PRJNA827465	0.93680487
ENSGALG00010009727	CDH20	2	-0.022300244	0.30809637	-0.072380742	0.94229891	0.110029524	1.939715623	48.44605115	0.5	CRA004775;PRJNA827465	Yang2025_Silkie_Wenchang	0.985827325
ENSGALG00010009728	CCDC149	2	0.01085509	0.068375877	0.158756132	0.873861012	0	0.225682244	0	0.5	PRJNA827465;Yang2025_Silkie_Wenchang	CRA004775	0.969988685
ENSGALG00010009728	CCDC149	2	-0.170327399	0.189276607	-0.899886163	0.368180835	0.033934634	1.679053311	40.44262956	1	CRA004775;Yang2025_Silkie_Wenchang	PRJNA827465	0.820213684
ENSGALG00010009728	CCDC149	2	-0.133354089	0.228779575	-0.582893333	0.559965109	0.072751221	2.646506485	62.21433782	0.5	CRA004775;PRJNA827465	Yang2025_Silkie_Wenchang	0.881203589
ENSGALG00010009730	C8orf76	2	-0.160355844	0.073598986	-2.178777887	0.029348172	0	0.552210862	0	1	PRJNA827465;Yang2025_Silkie_Wenchang	CRA004775	0.291796413
ENSGALG00010009730	C8orf76	2	-0.105074097	0.124282605	-0.845444921	0.397862473	0	0.253536574	0	1	CRA004775;Yang2025_Silkie_Wenchang	PRJNA827465	0.83172112
ENSGALG00010009730	C8orf76	2	-0.194653356	0.080674608	-2.412820607	0.015829609	0	0.004710891	0	1	CRA004775;PRJNA827465	Yang2025_Silkie_Wenchang	0.208199311
ENSGALG00010009731	NMI	2	-0.001153679	0.095088183	-0.012132723	0.990319725	0.005548427	1.436190094	30.37133423	0.5	PRJNA827465;Yang2025_Silkie_Wenchang	CRA004775	0.997468525
ENSGALG00010009731	NMI	2	-0.14661053	0.109542953	-1.338383954	0.180771307	0	0.633372741	0	1	CRA004775;Yang2025_Silkie_Wenchang	PRJNA827465	0.688484814
ENSGALG00010009731	NMI	2	-0.057011317	0.198683724	-0.286945077	0.774154374	0.048704246	2.23084476	55.1739315	0.5	CRA004775;PRJNA827465	Yang2025_Silkie_Wenchang	0.94337238
ENSGALG00010009732	HMMR	2	0.193624482	0.215080448	0.900242136	0.367991407	0.071145188	4.307928609	76.78698765	0.5	PRJNA827465;Yang2025_Silkie_Wenchang	CRA004775	0.82021134
ENSGALG00010009732	HMMR	2	0.390823321	0.153687591	2.542972527	0.010991387	0	0.489093966	0	0.5	CRA004775;Yang2025_Silkie_Wenchang	PRJNA827465	0.166811819
ENSGALG00010009732	HMMR	2	-0.014940913	0.1313529	-0.113746347	0.909438871	0	0.003046529	0	1	CRA004775;PRJNA827465	Yang2025_Silkie_Wenchang	0.978607662
ENSGALG00010009736	WISP1	2	0.173800939	0.132599763	1.310718329	0.189952944	0	0.853922528	0	1	PRJNA827465;Yang2025_Silkie_Wenchang	CRA004775	0.699277793
ENSGALG00010009736	WISP1	2	0.067968675	0.363141727	0.187168452	0.85152856	0.158852728	2.346862898	57.38992675	0.5	CRA004775;Yang2025_Silkie_Wenchang	PRJNA827465	0.96214473
ENSGALG00010009736	WISP1	2	0.013927423	0.177366761	0.078523296	0.9374118	0.012420364	1.122181196	10.88783133	0.5	CRA004775;PRJNA827465	Yang2025_Silkie_Wenchang	0.985114195
ENSGALG00010009738	ENSGALG00010009738	2	0.208261715	0.329236692	0.632559251	0.527021504	0.165922662	4.256105647	76.50434264	0.5	PRJNA827465;Yang2025_Silkie_Wenchang	CRA004775	0.87095077
ENSGALG00010009738	ENSGALG00010009738	2	-0.203781477	0.20910572	-0.974538029	0.329789466	0	0.501730627	0	1	CRA004775;Yang2025_Silkie_Wenchang	PRJNA827465	0.801177491
ENSGALG00010009738	ENSGALG00010009738	2	0.099002268	0.5051979	0.195967299	0.844635761	0.395210443	4.091136233	75.55691273	0.5	CRA004775;PRJNA827465	Yang2025_Silkie_Wenchang	0.960769014
ENSGALG00010009740	LRRC73	2	-0.117670361	0.15734226	-0.747862403	0.454543157	0	0.576052213	0	0.5	PRJNA827465;Yang2025_Silkie_Wenchang	CRA004775	0.849072665
ENSGALG00010009740	LRRC73	2	-0.76461523	0.856750946	-0.892459161	0.372146873	1.333871347	10.77927669	90.72293969	0.5	CRA004775;Yang2025_Silkie_Wenchang	PRJNA827465	0.821620523
ENSGALG00010009740	LRRC73	2	-0.872711456	0.726577226	-1.201126906	0.229701977	0.946874562	9.247936361	89.1867768	1	CRA004775;PRJNA827465	Yang2025_Silkie_Wenchang	0.742686486
ENSGALG00010009741	C2H6orf52	2	0.075400359	0.044477467	1.695248488	0.090028292	0	0.02349365	0	1	PRJNA827465;Yang2025_Silkie_Wenchang	CRA004775	0.514823507
ENSGALG00010009741	C2H6orf52	2	0.085536941	0.097894058	0.873770514	0.382243241	0	0.014119915	0	1	CRA004775;Yang2025_Silkie_Wenchang	PRJNA827465	0.825080207
ENSGALG00010009741	C2H6orf52	2	0.071663898	0.048395508	1.480796469	0.138660818	0	0.003887031	0	1	CRA004775;PRJNA827465	Yang2025_Silkie_Wenchang	0.622396264
ENSGALG00010009743	CCNG1	2	0.019267007	0.064636533	0.298082302	0.765640349	0	0.680097492	0	0.5	PRJNA827465;Yang2025_Silkie_Wenchang	CRA004775	0.940926806
ENSGALG00010009743	CCNG1	2	0.12710346	0.126728569	1.002958224	0.315881018	0	0.046979253	0	1	CRA004775;Yang2025_Silkie_Wenchang	PRJNA827465	0.796883862
ENSGALG00010009743	CCNG1	2	-0.001636292	0.072188934	-0.022666804	0.981916055	0	0.318887356	0	0.5	CRA004775;PRJNA827465	Yang2025_Silkie_Wenchang	0.995055866
ENSGALG00010009744	DAPK1	2	0.152998662	0.07043338	2.17224647	0.029837077	0	0.887829793	0	1	PRJNA827465;Yang2025_Silkie_Wenchang	CRA004775	0.29494131
ENSGALG00010009744	DAPK1	2	0.033596213	0.114185014	0.294226113	0.768585128	0	0.808041411	0	0.5	CRA004775;Yang2025_Silkie_Wenchang	PRJNA827465	0.941493634
ENSGALG00010009744	DAPK1	2	0.094608731	0.212366662	0.445497096	0.655960558	0.048955579	1.542371955	35.16479622	0.5	CRA004775;PRJNA827465	Yang2025_Silkie_Wenchang	0.910411938
ENSGALG00010009749	NDRG1	2	-0.0248432	0.101740695	-0.244181544	0.80709022	0.010624295	1.993865124	49.8461562	0.5	PRJNA827465;Yang2025_Silkie_Wenchang	CRA004775	0.95234753
ENSGALG00010009749	NDRG1	2	-0.12220417	0.109488254	-1.116139544	0.264362408	0	0.235936701	0	1	CRA004775;Yang2025_Silkie_Wenchang	PRJNA827465	0.768790489
ENSGALG00010009749	NDRG1	2	0.055489947	0.08038331	0.690316772	0.489995003	0	0.047412271	0	0.5	CRA004775;PRJNA827465	Yang2025_Silkie_Wenchang	0.858517903
ENSGALG00010009751	ENSGALG00010009751	2	0.65811406	0.588304768	1.118661781	0.263284456	0.602110159	7.593762182	86.83129685	1	PRJNA827465;Yang2025_Silkie_Wenchang	CRA004775	0.768643821

ENSGALG00010009751	ENSGALG00010009751	2	0.602924776	0.706803806	0.853029894	0.393642726	0.826345042	5.698676793	82.45206675	0.5	CRA004775;Yang2025_Silkie_Wenchang	PRJNA827465	0.83043598
ENSGALG00010009751	ENSGALG00010009751	2	0.043514622	0.221440485	0.196507073	0.844213297	0	0.193169868	0	0.5	CRA004775;PRJNA827465	Yang2025_Silkie_Wenchang	0.960723102
ENSGALG00010009761	PDP2	2	-0.098257821	0.135043509	-0.727601216	0.466857736	0.029000992	4.839372624	79.33616446	0.5	PRJNA827465;Yang2025_Silkie_Wenchang	CRA004775	0.852842649
ENSGALG00010009761	PDP2	2	0.158264761	0.424745243	0.372611026	0.709437965	0.329603354	11.06967031	90.96630729	0.5	CRA004775;Yang2025_Silkie_Wenchang	PRJNA827465	0.925298042
ENSGALG00010009761	PDP2	2	0.277668542	0.287192068	0.966839174	0.333624438	0.137705338	5.440556642	81.61952782	1	CRA004775;PRJNA827465	Yang2025_Silkie_Wenchang	0.802691373
ENSGALG00010009762	MAN2A1	2	-0.135854341	0.217750881	-0.623898009	0.532694568	0.086560358	11.3381804	91.18024264	0.5	PRJNA827465;Yang2025_Silkie_Wenchang	CRA004775	0.873183244
ENSGALG00010009762	MAN2A1	2	0.133155143	0.525151632	0.253555611	0.799858893	0.508361321	12.23003153	91.8234062	0.5	CRA004775;Yang2025_Silkie_Wenchang	PRJNA827465	0.949922217
ENSGALG00010009762	MAN2A1	2	0.323280357	0.302398917	1.069052629	0.285045955	0.147901759	4.508859832	77.82144406	1	CRA004775;PRJNA827465	Yang2025_Silkie_Wenchang	0.780501722
ENSGALG00010009765	STX10	2	0.003077827	0.097026924	0.031721375	0.974694249	0.011832032	2.446455355	59.1245351	0.5	PRJNA827465;Yang2025_Silkie_Wenchang	CRA004775	0.992522944
ENSGALG00010009765	STX10	2	-0.19096056	0.099742668	-1.914532298	0.055552176	0.001757207	1.084435735	7.786144649	1	CRA004775;Yang2025_Silkie_Wenchang	PRJNA827465	0.408228819
ENSGALG00010009765	STX10	2	-0.096856293	0.203971705	-0.474851615	0.634892741	0.06913975	5.336323924	81.2605079	0.5	CRA004775;PRJNA827465	Yang2025_Silkie_Wenchang	0.904034523
ENSGALG00010009769	TMCC3	2	-0.006113701	0.154757867	-0.039504943	0.968487812	0	0.173956929	0	0.5	PRJNA827465;Yang2025_Silkie_Wenchang	CRA004775	0.991215679
ENSGALG00010009769	TMCC3	2	0.297415074	0.457934914	0.649470186	0.516034511	0.294987564	3.176642396	68.52022118	0.5	CRA004775;Yang2025_Silkie_Wenchang	PRJNA827465	0.867498525
ENSGALG00010009769	TMCC3	2	0.333776001	0.38250862	0.872597331	0.382882596	0.193759978	2.600477128	61.54551836	1	CRA004775;PRJNA827465	Yang2025_Silkie_Wenchang	0.825204796
ENSGALG00010009774	PIAS2	2	0.072081883	0.069142901	1.04250592	0.297177182	0	0.599608149	0	0.5	PRJNA827465;Yang2025_Silkie_Wenchang	CRA004775	0.788466926
ENSGALG00010009774	PIAS2	2	-0.120148632	0.151099213	-0.795163845	0.4265182	0.019186237	1.617757204	38.18602707	1	CRA004775;Yang2025_Silkie_Wenchang	PRJNA827465	0.84125685
ENSGALG00010009774	PIAS2	2	-0.064239009	0.212055326	-0.302935137	0.761939293	0.067524165	3.544709799	71.78894588	0.5	CRA004775;PRJNA827465	Yang2025_Silkie_Wenchang	0.939881364
ENSGALG00010009775	KIAA1614	2	0.131859122	0.113190932	1.164749861	0.24412026	0	0.491695864	0	1	PRJNA827465;Yang2025_Silkie_Wenchang	CRA004775	0.755564606
ENSGALG00010009775	KIAA1614	2	0.066204113	0.168225788	0.393543192	0.69391833	0.017622476	1.424648155	29.80723019	0.5	CRA004775;Yang2025_Silkie_Wenchang	PRJNA827465	0.920728119
ENSGALG00010009775	KIAA1614	2	0.029898094	0.103376466	0.289215671	0.772416341	0	0.53883927	0	0.5	CRA004775;PRJNA827465	Yang2025_Silkie_Wenchang	0.943066211
ENSGALG00010009777	ST3GAL1	2	-0.027964686	0.058261387	-0.479986616	0.63123691	0	0.004334868	0	1	PRJNA827465;Yang2025_Silkie_Wenchang	CRA004775	0.903058174
ENSGALG00010009777	ST3GAL1	2	-0.171296448	0.232129624	-0.737934459	0.460554278	0.059890317	1.741548437	42.5798342	1	CRA004775;Yang2025_Silkie_Wenchang	PRJNA827465	0.85014477
ENSGALG00010009777	ST3GAL1	2	-0.161483303	0.23403212	-0.690004872	0.490191124	0.068594911	1.894323128	47.21069572	1	CRA004775;PRJNA827465	Yang2025_Silkie_Wenchang	0.858529056
ENSGALG00010009778	ENSGALG00010009778	2	-0.167053949	0.220869247	-0.756347709	0.449440751	0.088869254	10.78295459	90.72610394	0.5	PRJNA827465;Yang2025_Silkie_Wenchang	CRA004775	0.847263197
ENSGALG00010009778	ENSGALG00010009778	2	-0.331386087	0.103034816	-3.2162035	0.001298761	0.000400381	1.016721893	1.644687029	1	CRA004775;Yang2025_Silkie_Wenchang	PRJNA827465	0.041665685
ENSGALG00010009778	ENSGALG00010009778	2	-0.003689581	0.088514286	-0.041683447	0.96675105	0.006845788	1.384867245	27.79091254	0.5	CRA004775;PRJNA827465	Yang2025_Silkie_Wenchang	0.990893423
ENSGALG00010009779	PPP1R3E	2	-0.267797188	0.19443053	-1.377341245	0.168406769	0.007846984	1.113587608	10.20015017	1	PRJNA827465;Yang2025_Silkie_Wenchang	CRA004775	0.669138264
ENSGALG00010009779	PPP1R3E	2	-0.377932159	0.352771474	-1.071322903	0.284024265	0.155488238	2.648409392	62.24148718	1	CRA004775;Yang2025_Silkie_Wenchang	PRJNA827465	0.779864142
ENSGALG00010009779	PPP1R3E	2	-0.548335808	0.195794031	-2.800676838	0.005099556	0	0.587438588	0	1	CRA004775;PRJNA827465	Yang2025_Silkie_Wenchang	0.1064976
ENSGALG00010009780	MFSD2B	2	-0.187013389	0.202244707	-0.924688669	0.355127875	0.016349134	1.23310886	18.90415901	1	PRJNA827465;Yang2025_Silkie_Wenchang	CRA004775	0.812301302
ENSGALG00010009780	MFSD2B	2	-0.401191306	0.260081425	-1.542560398	0.122937473	0	0.078529252	0	1	CRA004775;Yang2025_Silkie_Wenchang	PRJNA827465	0.587862418
ENSGALG00010009780	MFSD2B	2	-0.06614333	0.205432461	-0.321971169	0.747474536	0	0.191694492	0	1	CRA004775;PRJNA827465	Yang2025_Silkie_Wenchang	0.93680487
ENSGALG00010009784	ENSGALG00010009784	2	0.135123771	0.159992356	0.844563914	0.398354364	0	0.403047977	0	0.5	PRJNA827465;Yang2025_Silkie_Wenchang	CRA004775	0.831980453
ENSGALG00010009784	ENSGALG00010009784	2	0.043262475	0.207333533	0.208661253	0.83471269	0	0.100724645	0	0.5	CRA004775;Yang2025_Silkie_Wenchang	PRJNA827465	0.958133448
ENSGALG00010009784	ENSGALG00010009784	2	0.17155357	0.158317886	1.083601949	0.278541326	0	0.072462599	0	1	CRA004775;PRJNA827465	Yang2025_Silkie_Wenchang	0.776763074
ENSGALG00010009786	DIPK2B	2	-0.067199193	0.122189253	-0.549960557	0.582387582	0.020935075	3.225287057	68.99500781	0.5	PRJNA827465;Yang2025_Silkie_Wenchang	CRA004775	0.889641228
ENSGALG00010009786	DIPK2B	2	0.067991203	0.109372709	0.621646876	0.53417409	0	0.009748893	0	1	CRA004775;Yang2025_Silkie_Wenchang	PRJNA827465	0.873408008
ENSGALG00010009786	DIPK2B	2	-0.165834922	0.073090325	-2.268903873	0.023274172	0	0.33804517	0	0.5	CRA004775;PRJNA827465	Yang2025_Silkie_Wenchang	0.260240338
ENSGALG00010009788	PAK1IP1	2	0.012927971	0.074789686	0.172857676	0.8627633	0.005389861	1.699755543	41.16801064	0.5	PRJNA827465;Yang2025_Silkie_Wenchang	CRA004775	0.966099097
ENSGALG00010009788	PAK1IP1	2	-0.033061922	0.128538253	-0.257214655	0.797013072	0.008152574	1.193771879	16.23190178	0.5	CRA004775;Yang2025_Silkie_Wenchang	PRJNA827465	0.949622804
ENSGALG00010009788	PAK1IP1	2	0.068635448	0.051078859	1.343715374	0.179040444	0	0.32449164	0	1	CRA004775;PRJNA827465	Yang2025_Silkie_Wenchang	0.685713211
ENSGALG00010009793	TNFAIP6	2	-0.233245918	0.141172128	-1.652209401	0.098491871	0.007551514	1.22613975	18.44322802	1	PRJNA827465;Yang2025_Silkie_Wenchang	CRA004775	0.535930498
ENSGALG00010009793	TNFAIP6	2	-0.066175321	0.428456446	-0.15445052	0.877254522	0.254703391	2.872691629	65.1894415	0.5	CRA004775;Yang2025_Silkie_Wenchang	PRJNA827465	0.970575794
ENSGALG00010009793	TNFAIP6	2	0.013797689	0.244710269	0.056383776	0.955036081	0.049351628	1.381557715	27.61793523	0.5	CRA004775;PRJNA827465	Yang2025_Silkie_Wenchang	0.988093796
ENSGALG00010009795	NDUFA6	2	-0.230755989	0.209570653	-1.101089231	0.270857824	0.061481297	2.734642499	63.43214879	1	PRJNA827465;Yang2025_Silkie_Wenchang	CRA004775	0.772743863
ENSGALG00010009795	NDUFA6	2	-0.413612269	0.218816367	-1.89022546	0.058727813	0	0.635768755	0	1	CRA004775;Yang2025_Silkie_Wenchang	PRJNA827465	0.419728393
ENSGALG00010009795	NDUFA6	2	-0.079396772	0.074773069	-1.061836464	0.28830993	0	0.010755886	0	1	CRA004775;PRJNA827465	Yang2025_Silkie_Wenchang	0.782936868
ENSGALG00010009796	ENSGALG00010009796	2	-1.389676403	0.820458593	-1.693780058	0.090307085	1.147736793	6.501668415	84.61933251	1	PRJNA827465;Yang2025_Silkie_Wenchang	CRA004775	0.515967344
ENSGALG00010009796	ENSGALG00010009796	2	-1.363155594	0.920642842	-1.480656267	0.138698193	1.370311298	5.218220044	80.83637732	1	CRA004775;Yang2025_Silkie_Wenchang	PRJNA827465	0.622396264
ENSGALG00010009796	ENSGALG00010009796	2	-0.594165024	0.267569832	-2.220597966	0.026378204	0	0.090661716	0	1	CRA004775;PRJNA827465	Yang2025_Silkie_Wenchang	0.276903978
ENSGALG00010009798	YIPF4	2	-0.249156487	0.26568664	-0.937783273	0.348355799	0.137348833	36.64083008	97.27139994	0.5	PRJNA827465;Yang2025_Silkie_Wenchang	CRA004775	0.809042603
ENSGALG00010009798	YIPF4	2	-0.131359276	0.401774607	-0.326947681	0.743707457	0.305454679	18.11091719	94.47846849	0.5	CRA004775;Yang2025_Silkie_Wenchang	PRJNA827465	0.935821071
ENSGALG00010009798	YIPF4	2	0.098189873	0.126321382	0.77730208	0.436980568	0.021130173	2.316191408	56.82567527	1	CRA004775;PRJNA827465	Yang2025_Silkie_Wenchang	0.843424215
ENSGALG00010009801	ENSGALG00010009801	2	0.663740089	0.363480507	1.826067909	0.067840033	0	0.253242353	0	1	PRJNA827465;Yang2025_Silkie_Wenchang	CRA004775	0.449101706
ENSGALG00010009801	ENSGALG00010009801	2	1.254290635	0.448909459	2.79408377	0.005204699	0.025112728	1.064170276	6.030075923	1	CRA004775;Yang2025_Silkie_Wenchang	PRJNA827465	0.107776766
ENSGALG00010009801	ENSGALG00010009801	2	1.057492995	0.634980696	1.665393928	0.09583419	0.477368052	2.378003863	57.94792366	1	CRA004775;PRJNA827465	Yang2025_Silkie_Wenchang	0.5301492
ENSGALG00010009804	XPR1	2	-0.203092519	0.177012055	-1.147337223	0.251242276	0.060315442	26.64560325	96.24703562	1	PRJNA827465;Yang2025_Silkie_Wenchang	CRA004775	0.761280947
ENSGALG00010009804	XPR1	2	-0.300936294	0.109253019	-2.754489513	0.005878377	0.0159755	2.508092904	60.12906864	1	CRA004775;Yang2025_Silkie_Wenchang	PRJNA827465	0.115750434
ENSGALG00010009804	XPR1	2	-0.040150054	0.04762332	-0.843075498	0.399186221	0	0.705225308	0	1	CRA004775;PRJNA827465	Yang2025_Silkie_Wenchang	0.832054308
ENSGALG00010009806	WDCP	2	-0.000174643	0.102501134	-0.001703812	0.998640555	0.011778816	2.227897863	55.11463893	0.5	PRJNA827465;Yang2025_Silkie_Wenchang	CRA004775	0.999657891
ENSGALG00010009806	WDCP	2	-0.116920757	0.107967232	-1.08292873	0.278840386	0	3.00423E-07	0	1	CRA004775;Yang2025_Silkie_Wenchang	PRJNA827465	0.777142287
ENSGALG00010009806	WDCP	2	0.083147257	0.081267839	1.023126225	0.306248169	0	0.201543014	0	0.5	CRA004775;PRJNA827465	Yang2025_Silkie_Wenchang	0.792485409
ENSGALG00010009809	SMDT1	2	-0.027114524	0.085826749	-0.315921601	0.752062016	0	0.054528735	0	1	PRJNA827465;Yang2025_Silkie_Wenchang	CRA004775	0.937941658
ENSGALG00010009809	SMDT1	2	0.047515622	0.188729341	0.251765951	0.801221977	0	0.531061886	0	0.5	CRA004775;Yang2025_Silkie_Wenchang	PRJNA827465	0.950158029
ENSGALG00010009809	SMDT1	2	0.00094471	0.086163844	0.010964114	0.991252078	0	0.538556839	0	0.5	CRA004775;PRJNA827465	Yang2025_Silkie_Wenchang	0.9975571
ENSGALG00010009811	ENSGALG00010009811	2	0.232982609	0.089443341	2.604806656	0.009192612	0	0.403264324	0	1	PRJNA827465;Yang2025_Silkie_Wenchang	CRA004775	0.150290025
ENSGALG00010009811	ENSGALG00010009811	2	0.241030537	0.127345345	1.892731433	0.058393594	0	0.696062035	0	1	CRA004775;Yang2025_Silkie_Wenchang	PRJNA827465	0.418650753
ENSGALG00010009811	ENSGALG00010009811	2	0.171383573	0.099373449	1.724641488	0.084592102	0	0.188053896	0	1	CRA004775;PRJNA827465	Yang2025_Silkie_Wenchang	0.500108811
ENSGALG00010009822	ENSGALG00010009822	2	0.02162821	0.155248806	0.139316741	0.889199862	0	0.410340338	0	0.5	PRJNA827465;Yang2025_Silkie_Wenchang	CRA004775	0.973155755
ENSGALG00010009822	ENSGALG00010009822	2	0.063272438	0.200014037	0.316339989	0.751744461	0	0.330606212	0	0.5	CRA004775;Yang2025_Silkie_Wenchang	PRJNA827465	0.937802658
ENSGALG00010009822	ENSGALG00010009822	2	-0.077089036	0.180077837	-0.428087308	0.668587557	0	0.015676266	0	1	CRA004775;PRJNA827465	Yang2025_Silkie_Wenchang	0.914377769
ENSGALG00010009830	RNASEL	2	-0.097383236	0.177646305	-0.548186108	0.58356412	0.037580482	2.450987726	59.20012209	0.5	PRJNA827465;Yang2025_Silkie_Wenchang	CRA004775	0.890658522
ENSGALG00010009830	RNASEL	2	-0.268480473	0.163128179	-1.645825228	0.099799747	0	0.103112032	0	1	CRA004775;Yang2025_Silkie_Wenchang	PRJNA827465	0.539684844
ENSGALG00010009830	RNASEL	2	0.047944354	0.138116051	0.347130935	0.728492954	0	0.185436895	0	0.5	CRA004775;PRJNA827465	Yang2025_Silkie_Wenchang	0.932184127
ENSGALG00010009831	N6AMT1	2	0.02469296	0.131010766	0.188480389	0.850500087	0.019045748	2.208624834	54.72295771	0.5	PRJNA827465;Yang2025_Silkie_Wenchang	CRA004775	0.961985357
ENSGALG00010009831	N6AMT1	2	-0.11043646	0.307613555	-0.359010382	0.719585323	0.154590277	5.289356122	81.09410717	0.5	CRA004775;Yang2025_Silkie_Wenchang	PRJNA827465	0.928449671
ENSGALG00010009831	N6AMT1	2	-0.211252415	0.167254714	-1.263058062	0.206568314	0.03055839	1.955878008	48.87206688	1	CRA004775;PRJNA827465	Yang2025_Silkie_Wenchang	0.71859917
ENSGALG00010009833	SOD3	2	-0.19655217	0.234515333	-0.838120763	0.401962883	0.080448261	3.663359383	72.70265088	0.5	PRJNA827465;Yang2025_Silkie_Wenchang	CRA004775	0.832825075
ENSGALG00010009833	SOD3	2	-0.368885602	0.173608896	-2.124808178	0.033602622	0	0.763160868	0	1	CRA004775;Yang2025_Silkie_Wenchang	PRJNA827465	0.314638424
ENSGALG00010009833	SOD3	2	0.005982577	0.136688623	0.043767919	0.9650894	0	0.070786048	0	0.5	CRA004775;PRJNA827465	Yang2025_Silkie_Wenchang	0.990452586
ENSGALG00010009840	ENSGALG00010009840	2	0.068724992	0.143530613	0.478817659	0.632068353	0	0.164851186	0	1	PRJNA827465;Yang2025_Silkie_Wenchang	CRA004775	0.903058174
ENSGALG00010009840	ENSGALG00010009840	2	-0.139520403	0.194616151	-0.716900433	0.473435535	0.014710087	1.217646899	17.87438534	0.5	CRA004775;Yang2025_Silkie_Wenchang	PRJNA827465	0.854373829
ENSGALG00010009840	ENSGALG00010009840	2	-0.088218682	0.256431013	-0.344025008	0.730823479	0.057906594	2.112683536	52.66683425	0.5	CRA004775;PRJNA827465	Yang2025_Silkie_Wenchang	0.932769533
ENSGALG00010009841	GABRA1	2	-0.06865668	0.296238244	-0.231761703	0.816723104	0.081752888	1.871853383	46.57701243	0.5	PRJNA827465;Yang2025_Silkie_Wenchang	CRA004775	0.954684235
ENSGALG00010009841	GABRA1	2	0.935933888	0.820703217	1.1404048	0.254117694	1.077581404	4.591091633	78.21868784	1	CRA004775;Yang2025_Silkie_Wenchang	PRJNA827465	0.763894203
ENSGALG00010009841	GABRA1	2	0.671678278	1.122469574	0.598393305	0.549577532	2.237751365	8.486555192	88.21665591	0.5	CRA004775;PRJNA827465	Yang2025_Silkie_Wenchang	0.878554604
ENSGALG00010009842	PHETA2	2	-0.287712287	0.151269035	-1.901990625	0.05717238	0.036729698	4.904018975	79.60856177	1	PRJNA827465;Yang2025_Silkie_Wenchang	CRA004775	0.414395744
ENSGALG00010009842	PHETA2	2	-0.347758728	0.164055345	-2.119764688	0.034025895	0.024486287	1.604089291	37.65933072	1	CRA004775;Yang2025_Silkie_Wenchang	PRJNA827465	0.317276331
ENSGALG00010009842	PHETA2	2	-0.145380835	0.069846044	-2.081446951	0.037393015	0	0.04440214	0	1	CRA004775;PRJNA827465	Yang2025_Silkie_Wenchang	0.333556507
ENSGALG00010009847	ENSGALG00010009847	2	-0.333439177	0.426467454	-0.781863127	0.434295017	0.33762513	13.3919207	92.53392623	0.5	PRJNA827465;Yang2025_Silkie_Wenchang	CRA004775	0.842762754
ENSGALG00010009847	ENSGALG00010009847	2	-0.388231371	0.382336188	-1.015418845	0.309906214	0.255537731	7.872424697	87.29743327	1	CRA004775;Yang2025_Silkie_Wenchang	PRJNA827465	0.794395438
ENSGALG00010009847	ENSGALG00010009847	2	0.05156192	0.074749828	0.689793168	0.490242266	0	0.233617068	0	0.5	CRA004775;PRJNA827465	Yang2025_Silkie_Wenchang	0.858529056
ENSGALG00010009850	RIF1	2	0.025548321	0.078626666	0.32493201	0.74523253	0.004001094	1.435541664	30.3398832	0.5	PRJNA827465;Yang2025_Silkie_Wenchang	CRA004775	0.93597621
ENSGALG00010009850	RIF1	2	-0.034832609	0.260058204	-0.13394158	0.893448787	0.081351325	1.938748811	48.42034233	0.5	CRA004775;Yang2025_Silkie_Wenchang	PRJNA827465	0.973481724
ENSGALG00010009850	RIF1	2	-0.058333316	0.095165132	-0.612969415	0.539896563	0.003692444	1.044292849	4.24142029	1	CRA004775;PRJNA827465	Yang2025_Silkie_Wenchang	0.87529516
ENSGALG00010009852	ENSGALG00010009852	2	-0.365948371	0.168623964	-2.170203824	0.029991409	0	0.014700228	0	1	PRJNA827465;Yang2025_Silkie_Wenchang	CRA004775	0.295278639
ENSGALG00010009852	ENSGALG00010009852	2	0.13909796	0.637345186	0.218245878	0.827237541	0.566831972	2.814717782	64.4724595	0.5	CRA004775;Yang2025_Silkie_Wenchang	PRJNA827465	0.956550752
ENSGALG00010009852	ENSGALG00010009852	2	0.115719511	0.658218091	0.175796346	0.860453941	0.634018091	3.104672771	67.79940635	0.5	CRA004775;PRJNA827465	Yang2025_Silkie_Wenchang	0.964099892
ENSGALG00010009857	ORC5	2	-0.007033801	0.1764829	-0.039855427	0.968208387	0.039395607	2.38468214	58.06569005	0.5	PRJNA827465;Yang2025_Silkie_Wenchang	CRA004775	0.991182909
ENSGALG00010009857	ORC5	2	-0.28672402	0.182418324	-1.571793964	0.115998342	0	0.125636746	0	1	CRA004775;Yang2025_Silkie_Wenchang	PRJNA827465	0.573367481
ENSGALG00010009857	ORC5	2	-0.031554912	0.234613195	-0.134497603	0.893009107	0.070342329	2.18994263	54.33670334	0.5	CRA004775;PRJNA827465	Yang2025_Silkie_Wenchang	0.974481724
ENSGALG00010009858	ST8SIA5	2	0.12876473	0.196758169	0.654431429	0.512833891	0	0.068877667	0	1	PRJNA827465;Yang2025_Silkie_Wenchang	CRA004775	0.866026915
ENSGALG00010009858	ST8SIA5	2	-0.00874051	0.249792491	-0.034991085	0.972086849	0	0.268992416	0	0.5	CRA004775;Yang2025_Silkie_Wenchang	PRJNA827465	0.991955348
ENSGALG00010009858	ST8SIA5	2	0.081841633	0.231309126	0.353819301	0.723474308	0	0.523764003	0	0.5	CRA004775;PRJNA827465	Yang2025_Silkie_Wenchang	0.930032096
ENSGALG00010009866	NAGA	2	-0.286430608	0.248761052	-1.151428673	0.249555924	0.103577286	5.490038529	81.78519159	1	PRJNA827465;Yang2025_Silkie_Wenchang	CRA004775	0.759622031
ENSGALG00010009866	NAGA	2	-0.396621629	0.24289038	-1.632924404	0.102484897	0.049011105	1.611525514	37.94699548	1	CRA004775;Yang2025_Silkie_Wenchang	PRJNA827465	0.545351191
ENSGALG00010009866	NAGA	2	-0.072585768	0.061940623	-1.171860462	0.241253083	0	0.000201935	0	1	CRA004775;PRJNA827465	Yang2025_Silkie_Wenchang	0.753793619
ENSGALG00010009868	LTN1	2	0.141277105	0.104434426	1.352782898	0.176124998	0.015909845	3.585992563	72.11371796	1	PRJNA827465;Yang2025_Silkie_Wenchang	CRA004775	0.68118882
ENSGALG00010009868	LTN1	2	0.251545403	0.083096769	3.027138187	0.002468811	0	0.022023174	0	1	CRA004775;Yang2025_Silkie_Wenchang	PRJNA827465	0.066596888
ENSGALG00010009868	LTN1	2	0.064114907	0.058750823	1.091302276	0.275139897	0	0.782898595	0	1	CRA004775;PRJNA827465	Yang2025_Silkie_Wenchang	0.775068889
ENSGALG00010009876	SELENOI	2	-0.133658287	0.146438009	-0.912729473	0.361384839	0.03631457	6.283417545	84.08509394	0.5	PRJNA827465;Yang2025_Silkie_Wenchang	CRA004775	0.815450013
ENSGALG00010009876	SELENOI	2	-0.181499501	0.151795627	-1.195683333	0.231820158	0.024969479	1.972231793	49.29602071	0.5	CRA004775;Yang2025_Silkie_Wenchang	PRJNA827465	0.744183098
ENSGALG00010009876	SELENOI	2	0.002857589	0.055069933	0.051890188	0.958616193	0	0.013463106	0	1	CRA004775;PRJNA827465	Yang2025_Silkie_Wenchang	0.989070013
ENSGALG00010009883	ENSGALG00010009883	2	0.269202217	0.139011386	1.936547965	0.052800624	0	0.471733043	0	1	PRJNA827465;Yang2025_Silkie_Wenchang	CRA004775	0.399347184
ENSGALG00010009883	ENSGALG00010009883	2	0.281405666	0.167195399	1.683094563	0.092356787	0	0.703431229	0	1	CRA004775;Yang2025_Silkie_Wenchang	PRJNA827465	0.520517459
ENSGALG00010009883	ENSGALG00010009883	2	0.132704899	0.174223507	0.761693416	0.446243006	0	0.120726245	0	1	CRA004775;PRJNA827465	Yang2025_Silkie_Wenchang	0.846054146
ENSGALG00010009884	GFOD1	2	0.059592185	0.08512263	0.70007453	0.483880761	0	0.782295627	0	0.5	PRJNA827465;Yang2025_Silkie_Wenchang	CRA004775	0.85691916
ENSGALG00010009884	GFOD1	2	0.276676189	0.378611497	0.730765417	0.46492245	0.22444396	4.167256534	76.00339716	0.5	CRA004775;Yang2025_Silkie_Wenchang	PRJNA827465	0.852233226
ENSGALG00010009884	GFOD1	2	0.330935506	0.292003724	1.133326321	0.257077243	0.120486407	2.818247259	64.51695298	1	CRA004775;PRJNA827465	Yang2025_Silkie_Wenchang	0.764870705
ENSGALG00010009910	ENSGALG00010009910	2	0.054418456	0.118983204	0.457362502	0.647410512	0	0.986681876	0	0.5	PRJNA827465;Yang2025_Silkie_Wenchang	CRA004775	0.907350807
ENSGALG00010009910	ENSGALG00010009910	2	0.125597129	0.15288082	0.821536211	0.411340907	0	0.444360331	0	0.5	CRA004775;Yang2025_Silkie_Wenchang	PRJNA827465	0.835128673
ENSGALG00010009910	ENSGALG00010009910	2	-0.067486266	0.151623658	-0.44509061	0.656254274	0	0.004767437	0	1	CRA004775;PRJNA827465	Yang2025_Silkie_Wenchang	0.910446833
ENSGALG00010009914	RWDD2B	2	0.028281033	0.157809347	0.179210124	0.857772715	0.032252583	2.719478596	63.22824525	0.5	PRJNA827465;Yang2025_Silkie_Wenchang	CRA004775	0.964081098
ENSGALG00010009914	RWDD2B	2	-0.146485764	0.142214286	-1.030035505	0.302993339	0	0.014042928	0	1	CRA004775;Yang2025_Silkie_Wenchang	PRJNA827465	0.792084776
ENSGALG00010009914	RWDD2B	2	0.131419756	0.098363147	1.336067016	0.18152737	0	0.820961459	0	0.5	CRA004775;PRJNA827465	Yang2025_Silkie_Wenchang	0.689004635
ENSGALG00010009943	DHX15	2	-0.108865669	0.115905514	-0.939262203	0.347596139	0.024726578	12.43972981	91.9612402	0.5	PRJNA827465;Yang2025_Silkie_Wenchang	CRA004775	0.808764533
ENSGALG00010009943	DHX15	2	-0.218102889	0.051506384	-4.234482657	2.29078E-05	0	0.406272906	0	1	CRA004775;Yang2025_Silkie_Wenchang	PRJNA827465	0.002711889
ENSGALG00010009943	DHX15	2	-0.000887424	0.037006126	-0.023980462	0.980868193	0	0.379770923	0	0.5	CRA004775;PRJNA827465	Yang2025_Silkie_Wenchang	0.994928779
ENSGALG00010009948	WBP2NL	2	0.010593995	0.043267847	0.244846826	0.806575035	0.000714292	1.206364933	17.10634379	0.5	PRJNA827465;Yang2025_Silkie_Wenchang	CRA004775	0.952270941
ENSGALG00010009948	WBP2NL	2	-0.056227442	0.170933152	-0.328944044	0.742197983	0.040594633	2.757728352	63.73827033	0.5	CRA004775;Yang2025_Silkie_Wenchang	PRJNA827465	0.935495071
ENSGALG00010009948	WBP2NL	2	-0.079225817	0.110258877	-0.718543658	0.472422139	0.013496896	1.620466345	38.2893694	1	CRA004775;PRJNA827465	Yang2025_Silkie_Wenchang	0.853998373
ENSGALG00010009972	PTK2B	2	0.086548957	0.230159085	0.376039717	0.706887369	0	0.651894474	0	0.5	PRJNA827465;Yang2025_Silkie_Wenchang	CRA004775	0.92461376
ENSGALG00010009972	PTK2B	2	-0.489307284	0.407832634	-1.199774719	0.230226845	0.127242013	1.575034992	36.5093471	1	CRA004775;Yang2025_Silkie_Wenchang	PRJNA827465	0.742845883
ENSGALG00010009972	PTK2B	2	-0.297787871	0.604715168	-0.492443198	0.622406068	0.557286153	3.944890637	74.65075481	0.5	CRA004775;PRJNA827465	Yang2025_Silkie_Wenchang	0.900093208
ENSGALG00010009985	USP16	2	0.274697287	0.190576551	1.441401294	0.149471345	0.062257723	6.576774473	84.79497808	1	PRJNA827465;Yang2025_Silkie_Wenchang	CRA004775	0.639246608
ENSGALG00010009985	USP16	2	0.216329365	0.283470007	0.763147281	0.445375565	0.134376235	6.027147046	83.40840214	0.5	CRA004775;Yang2025_Silkie_Wenchang	PRJNA827465	0.846013379
ENSGALG00010009985	USP16	2	0.085622194	0.056992258	1.502347813	0.133007306	0	0.888183004	0	0.5	CRA004775;PRJNA827465	Yang2025_Silkie_Wenchang	0.611056244
ENSGALG00010009989	TBC1D7	2	-0.174437233	0.246692696	-0.707103353	0.479502252	0.107147459	8.099725443	87.65390251	0.5	PRJNA827465;Yang2025_Silkie_Wenchang	CRA004775	0.856126448
ENSGALG00010009989	TBC1D7	2	-0.593099507	0.187912066	-3.156260909	0.001598058	0.036310803	1.983535476	49.58497027	1	CRA004775;Yang2025_Silkie_Wenchang	PRJNA827465	0.050611193
ENSGALG00010009989	TBC1D7	2	-0.35709968	0.437867065	-0.815543594	0.414761222	0.355783291	13.30647031	92.48485904	0.5	CRA004775;PRJNA827465	Yang2025_Silkie_Wenchang	0.837298284
ENSGALG00010009991	LHFPL3	2	-0.178166388	0.249152114	-0.715090814	0.47455293	0	0.362736468	0	0.5	PRJNA827465;Yang2025_Silkie_Wenchang	CRA004775	0.854462864
ENSGALG00010009991	LHFPL3	2	-0.016290394	0.412107505	-0.039529475	0.968468254	0	0.122945241	0	0.5	CRA004775;Yang2025_Silkie_Wenchang	PRJNA827465	0.991215679
ENSGALG00010009991	LHFPL3	2	-0.281478233	0.285569233	-0.985674224	0.324293002	0	0.00282287	0	1	CRA004775;PRJNA827465	Yang2025_Silkie_Wenchang	0.799754525
ENSGALG00010009993	ENSGALG00010009993	2	1.067050224	0.29131126	3.662921318	0.000249355	0.011807221	1.074663433	6.947610868	1	PRJNA827465;Yang2025_Silkie_Wenchang	CRA004775	0.014826635
ENSGALG00010009993	ENSGALG00010009993	2	1.30005308	0.373262745	3.482943574	0.000495933	0	0.190962488	0	1	CRA004775;Yang2025_Silkie_Wenchang	PRJNA827465	0.023287515
ENSGALG00010009993	ENSGALG00010009993	2	0.804362347	0.361092697	2.22757855	0.025908632	0	0.015460974	0	1	CRA004775;PRJNA827465	Yang2025_Silkie_Wenchang	0.274396922
ENSGALG00010009995	SEPTIN3	2	0.005881405	0.600433695	0.009795261	0.992184637	0.591761033	5.576081127	82.06625806	0.5	PRJNA827465;Yang2025_Silkie_Wenchang	CRA004775	0.997744161
ENSGALG00010009995	SEPTIN3	2	1.231571334	0.751912694	1.637917997	0.101438801	0.83738736	3.541748527	71.76535848	1	CRA004775;Yang2025_Silkie_Wenchang	PRJNA827465	0.54352396
ENSGALG00010009995	SEPTIN3	2	0.692114462	1.362294957	0.508050374	0.611418017	3.387914136	11.12704288	91.01288625	0.5	CRA004775;PRJNA827465	Yang2025_Silkie_Wenchang	0.897609796
ENSGALG00010010006	PPARGC1A	2	0.3016804	0.374780239	0.804952793	0.420846922	0.254920266	10.58098642	90.5490853	0.5	PRJNA827465;Yang2025_Silkie_Wenchang	CRA004775	0.839239169
ENSGALG00010010006	PPARGC1A	2	0.418533777	0.413107091	1.013136268	0.310995077	0.207018549	2.071696673	51.73038538	0.5	CRA004775;Yang2025_Silkie_Wenchang	PRJNA827465	0.795218958
ENSGALG00010010006	PPARGC1A	2	-0.061509483	0.114144299	-0.538874771	0.589973266	0	0.057782059	0	1	CRA004775;PRJNA827465	Yang2025_Silkie_Wenchang	0.892320726
ENSGALG00010010007	CTIF	2	0.077538118	0.066313256	1.169270258	0.242294762	0	0.05744773	0	1	PRJNA827465;Yang2025_Silkie_Wenchang	CRA004775	0.754674311
ENSGALG00010010007	CTIF	2	0.10358943	0.104437724	0.991877509	0.321257282	0	0.01891723	0	1	CRA004775;Yang2025_Silkie_Wenchang	PRJNA827465	0.798513866
ENSGALG00010010007	CTIF	2	0.070542122	0.080352237	0.877911106	0.379991962	0	0.057592679	0	1	CRA004775;PRJNA827465	Yang2025_Silkie_Wenchang	0.824392515
ENSGALG00010010009	ELF2	2	-0.109734283	0.131523617	-0.834331401	0.404094262	0.031201325	10.14204219	90.14005285	0.5	PRJNA827465;Yang2025_Silkie_Wenchang	CRA004775	0.833112102
ENSGALG00010010009	ELF2	2	-0.03420559	0.245170474	-0.139517576	0.889041168	0.10003352	5.333515689	81.25064107	0.5	CRA004775;Yang2025_Silkie_Wenchang	PRJNA827465	0.973135755
ENSGALG00010010009	ELF2	2	0.051045387	0.080398242	0.634906758	0.525489229	0.004860699	1.217883297	17.89032638	1	CRA004775;PRJNA827465	Yang2025_Silkie_Wenchang	0.870235671
ENSGALG00010010011	EPB41L4A	2	0.254660167	0.237799582	1.070902501	0.284213271	0.080405093	3.205446274	68.8030959	1	PRJNA827465;Yang2025_Silkie_Wenchang	CRA004775	0.780082638
ENSGALG00010010011	EPB41L4A	2	0.406062554	0.255692194	1.588091318	0.112265691	0.025823344	1.153683742	13.32113267	0.5	CRA004775;Yang2025_Silkie_Wenchang	PRJNA827465	0.56602666
ENSGALG00010010011	EPB41L4A	2	0.048996686	0.121102707	0.404587865	0.68578048	0	0.065891246	0	0.5	CRA004775;PRJNA827465	Yang2025_Silkie_Wenchang	0.919235539
ENSGALG00010010014	GLUL	2	-0.031491688	0.158534532	-0.198642451	0.842542444	0.02774351	2.162139351	53.74951206	0.5	PRJNA827465;Yang2025_Silkie_Wenchang	CRA004775	0.960252241
ENSGALG00010010014	GLUL	2	-0.186795133	0.173875016	-1.074306917	0.282685146	0	0.493717984	0	0.5	CRA004775;Yang2025_Silkie_Wenchang	PRJNA827465	0.779532565
ENSGALG00010010014	GLUL	2	0.108904779	0.122673491	0.887761308	0.37466916	0	0.068804169	0	1	CRA004775;PRJNA827465	Yang2025_Silkie_Wenchang	0.822805106
ENSGALG00010010017	CCDC59	2	0.001897824	0.100675838	0.018850836	0.9849601	0.008869945	1.744389641	42.67335827	0.5	PRJNA827465;Yang2025_Silkie_Wenchang	CRA004775	0.995786861
ENSGALG00010010017	CCDC59	2	0.121053575	0.106296103	1.138833606	0.254772562	0	0.000353784	0	1	CRA004775;Yang2025_Silkie_Wenchang	PRJNA827465	0.764394153
ENSGALG00010010017	CCDC59	2	-0.049866746	0.085028172	-0.586473221	0.557557552	0	0.823273506	0	0.5	CRA004775;PRJNA827465	Yang2025_Silkie_Wenchang	0.880678789
ENSGALG00010010022	KMT2E	2	0.223319609	0.263313263	0.848113788	0.396374604	0.11142203	4.264480847	76.55048678	1	PRJNA827465;Yang2025_Silkie_Wenchang	CRA004775	0.831365249
ENSGALG00010010022	KMT2E	2	0.286292854	0.299983242	0.954362826	0.339900016	0.094676797	2.075819708	51.82625946	0.5	CRA004775;Yang2025_Silkie_Wenchang	PRJNA827465	0.805201033
ENSGALG00010010022	KMT2E	2	0.010800069	0.049180585	0.219600264	0.826182485	0	0.037386198	0	0.5	CRA004775;PRJNA827465	Yang2025_Silkie_Wenchang	0.956393839
ENSGALG00010010035	ATP10B	2	0.255289228	0.291812535	0.87483983	0.381661062	0	0.013334506	0	1	PRJNA827465;Yang2025_Silkie_Wenchang	CRA004775	0.824699008
ENSGALG00010010035	ATP10B	2	0.834870034	0.716641096	1.164976497	0.244028507	0.646795763	2.623687063	61.8856984	1	CRA004775;Yang2025_Silkie_Wenchang	PRJNA827465	0.755564606
ENSGALG00010010035	ATP10B	2	0.816771107	0.670616376	1.217940892	0.223246435	0.612876067	2.852034288	64.93730794	1	CRA004775;PRJNA827465	Yang2025_Silkie_Wenchang	0.736744216
ENSGALG00010010037	NAA15	2	0.041046186	0.165595547	0.247870108	0.804234909	0.046952239	6.59869626	84.84549128	0.5	PRJNA827465;Yang2025_Silkie_Wenchang	CRA004775	0.95154709
ENSGALG00010010037	NAA15	2	0.271663013	0.103974497	2.612785064	0.008980776	0	0.839335076	0	1	CRA004775;Yang2025_Silkie_Wenchang	PRJNA827465	0.148494509
ENSGALG00010010037	NAA15	2	0.131132968	0.280736895	0.467102722	0.640426387	0.133595603	5.806935607	82.77921321	0.5	CRA004775;PRJNA827465	Yang2025_Silkie_Wenchang	0.906202415
ENSGALG00010010042	METTL25	2	-0.155572709	0.213618143	-0.72827479	0.466445391	0.078479855	7.073209547	85.86214655	0.5	PRJNA827465;Yang2025_Silkie_Wenchang	CRA004775	0.852763198
ENSGALG00010010042	METTL25	2	-0.364757173	0.116085307	-3.142147641	0.001677134	0	0.055482351	0	1	CRA004775;Yang2025_Silkie_Wenchang	PRJNA827465	0.051821081
ENSGALG00010010042	METTL25	2	-0.037209505	0.153228029	-0.242837455	0.808131319	0.021686267	1.517783442	34.11444793	0.5	CRA004775;PRJNA827465	Yang2025_Silkie_Wenchang	0.952496413
ENSGALG00010010049	ZBTB7C	2	0.1079457	0.193298027	0.558441809	0.57654273	0	0.988028417	0	0.5	PRJNA827465;Yang2025_Silkie_Wenchang	CRA004775	0.887113945
ENSGALG00010010049	ZBTB7C	2	-0.386563549	0.453085618	-0.853179915	0.39355954	0.172151262	1.434610391	30.29466355	1	CRA004775;Yang2025_Silkie_Wenchang	PRJNA827465	0.83043598
ENSGALG00010010049	ZBTB7C	2	-0.252813931	0.697310857	-0.362555563	0.71693691	0.688693325	2.821190182	64.55396711	0.5	CRA004775;PRJNA827465	Yang2025_Silkie_Wenchang	0.927632716
ENSGALG00010010052	ZNF346	2	-0.007156602	0.044494914	-0.160840897	0.872218714	0	0.724880799	0	0.5	PRJNA827465;Yang2025_Silkie_Wenchang	CRA004775	0.969617087
ENSGALG00010010052	ZNF346	2	-0.123945588	0.111017251	-1.1164534	0.264228107	0.009392251	1.360218853	26.48241876	1	CRA004775;Yang2025_Silkie_Wenchang	PRJNA827465	0.768790489
ENSGALG00010010052	ZNF346	2	-0.094947748	0.163125928	-0.582051851	0.560531756	0.036506389	2.505137974	60.08203897	0.5	CRA004775;PRJNA827465	Yang2025_Silkie_Wenchang	0.881203589
ENSGALG00010010059	ENSGALG00010010059	2	0.021267957	0.089927565	0.236500975	0.813043951	0	0.418107616	0	0.5	PRJNA827465;Yang2025_Silkie_Wenchang	CRA004775	0.953526116
ENSGALG00010010059	ENSGALG00010010059	2	-0.048056497	0.169173182	-0.284066877	0.77635913	0	0.185428905	0	0.5	CRA004775;Yang2025_Silkie_Wenchang	PRJNA827465	0.944109792

ENSGALG00010010059	ENSGALG00010010059	2	0.045973335	0.092111367	0.499105988	0.617704719	0	0.001040426	0	1	CRA004775;PRJNA827465	Yang2025_Silkie_Wenchang	0.899121942
ENSGALG00010010061	CYP2D6	2	-0.263274659	0.231303517	-1.138221595	0.255027964	0.073178405	3.109456112	67.84003491	1	PRJNA827465;Yang2025_Silkie_Wenchang	CRA004775	0.764467569
ENSGALG00010010061	CYP2D6	2	-0.391253049	0.228679766	-1.710921006	0.087095696	0.023579678	1.190243929	15.98360846	0.5	CRA004775;Yang2025_Silkie_Wenchang	PRJNA827465	0.507587624
ENSGALG00010010061	CYP2D6	2	-0.043233036	0.14927666	-0.289616851	0.772109374	0	0.027104471	0	0.5	CRA004775;PRJNA827465	Yang2025_Silkie_Wenchang	0.943066211
ENSGALG00010010062	RGS7BP	2	-0.046887903	0.096764213	-0.484558305	0.627989696	0	0.776558944	0	0.5	PRJNA827465;Yang2025_Silkie_Wenchang	CRA004775	0.901904489
ENSGALG00010010062	RGS7BP	2	0.191786218	0.201402512	0.952253357	0.340968507	0	0.891851017	0	1	CRA004775;Yang2025_Silkie_Wenchang	PRJNA827465	0.805639763
ENSGALG00010010062	RGS7BP	2	0.103784979	0.342522268	0.303002136	0.761888233	0.1431094	1.817716669	44.80373135	0.5	CRA004775;PRJNA827465	Yang2025_Silkie_Wenchang	0.939847878
ENSGALG00010010064	ENSGALG00010010064	2	0.2222532	0.220320419	1.00877259	0.31308371	0.065036913	3.026117872	66.9543606	1	PRJNA827465;Yang2025_Silkie_Wenchang	CRA004775	0.796531448
ENSGALG00010010064	ENSGALG00010010064	2	0.398569823	0.147202003	2.707638593	0.006776377	0	0.190759813	0	1	CRA004775;Yang2025_Silkie_Wenchang	PRJNA827465	0.126389007
ENSGALG00010010064	ENSGALG00010010064	2	0.114102628	0.146515251	0.778776456	0.436111409	0.003114353	1.070472368	6.58329628	1	CRA004775;PRJNA827465	Yang2025_Silkie_Wenchang	0.843095975
ENSGALG00010010071	GLT8D2	2	-0.207203067	0.222063384	-0.933080743	0.350778281	0.081378508	5.57925031	82.07644496	1	PRJNA827465;Yang2025_Silkie_Wenchang	CRA004775	0.810146221
ENSGALG00010010071	GLT8D2	2	-0.389934991	0.15776291	-2.471651861	0.013449042	0.003051242	1.025066609	2.445363892	0.5	CRA004775;Yang2025_Silkie_Wenchang	PRJNA827465	0.189751764
ENSGALG00010010071	GLT8D2	2	0.00174341	0.100336988	0.017375546	0.986137017	0	0.012848478	0	0.5	CRA004775;PRJNA827465	Yang2025_Silkie_Wenchang	0.99618277
ENSGALG00010010077	CCT8	2	0.030458876	0.061727126	0.493443936	0.621698944	0	0.319231371	0	0.5	PRJNA827465;Yang2025_Silkie_Wenchang	CRA004775	0.899796991
ENSGALG00010010077	CCT8	2	-0.052345625	0.117195649	-0.446651608	0.655126627	0	0.017415841	0	1	CRA004775;Yang2025_Silkie_Wenchang	PRJNA827465	0.91039865
ENSGALG00010010077	CCT8	2	0.035285651	0.065091499	0.542093069	0.587754393	0	0.312604272	0	0.5	CRA004775;PRJNA827465	Yang2025_Silkie_Wenchang	0.891742161
ENSGALG00010010080	CDC42EP3	2	-0.365963778	0.367469156	-0.995903389	0.319297088	0.256014251	19.0998213	94.76434892	1	PRJNA827465;Yang2025_Silkie_Wenchang	CRA004775	0.798079048
ENSGALG00010010080	CDC42EP3	2	-0.214794202	0.555765721	-0.386483357	0.699138726	0.565874417	11.5142915	91.31514084	0.5	CRA004775;Yang2025_Silkie_Wenchang	PRJNA827465	0.92185597
ENSGALG00010010080	CDC42EP3	2	0.079004747	0.157345197	0.502110956	0.615589474	0.022680038	1.464343339	31.71000454	0.5	CRA004775;PRJNA827465	Yang2025_Silkie_Wenchang	0.898886043
ENSGALG00010010084	HK3	2	0.329049062	0.518200678	0.634983851	0.525438947	0.504041411	16.25487868	93.84800084	0.5	PRJNA827465;Yang2025_Silkie_Wenchang	CRA004775	0.870215879
ENSGALG00010010084	HK3	2	0.480297086	0.4284541	1.121000093	0.262287823	0.284273236	4.13010205	75.7875124	0.5	CRA004775;Yang2025_Silkie_Wenchang	PRJNA827465	0.768366452
ENSGALG00010010084	HK3	2	-0.157080794	0.157393137	-0.998015524	0.318271831	0	0.165442165	0	1	CRA004775;PRJNA827465	Yang2025_Silkie_Wenchang	0.797617661
ENSGALG00010010085	TDG	2	0.176928453	0.043857643	4.034153259	5.47995E-05	0	0.103335155	0	1	PRJNA827465;Yang2025_Silkie_Wenchang	CRA004775	0.005164956
ENSGALG00010010085	TDG	2	0.147283534	0.089612021	1.643568936	0.100265275	0	0.017722338	0	1	CRA004775;Yang2025_Silkie_Wenchang	PRJNA827465	0.540998201
ENSGALG00010010085	TDG	2	0.182754038	0.049206259	3.714040508	0.000203976	0	0.055804138	0	1	CRA004775;PRJNA827465	Yang2025_Silkie_Wenchang	0.012909619
ENSGALG00010010094	SLC2A9	2	0.125802423	0.348236445	0.361255765	0.717908259	0.137063858	2.251110927	55.57748897	0.5	PRJNA827465;Yang2025_Silkie_Wenchang	CRA004775	0.927927766
ENSGALG00010010094	SLC2A9	2	-0.180345493	0.262420201	-0.687239367	0.4919319	0	2.94915E-06	0	1	CRA004775;Yang2025_Silkie_Wenchang	PRJNA827465	0.859039387
ENSGALG00010010094	SLC2A9	2	0.387589247	0.338034015	1.146598359	0.251547655	0	0.670141557	0	0.5	CRA004775;PRJNA827465	Yang2025_Silkie_Wenchang	0.761706068
ENSGALG00010010095	C12orf73	2	-0.110950906	0.076968357	-1.441513251	0.149439737	0	0.546641643	0	1	PRJNA827465;Yang2025_Silkie_Wenchang	CRA004775	0.639246608
ENSGALG00010010095	C12orf73	2	-0.351924195	0.185881614	-1.893270601	0.058321893	0	0.461063871	0	1	CRA004775;Yang2025_Silkie_Wenchang	PRJNA827465	0.418594841
ENSGALG00010010095	C12orf73	2	-0.199032447	0.192349463	-1.034743968	0.300788496	0.042136732	1.719912164	41.85749591	1	CRA004775;PRJNA827465	Yang2025_Silkie_Wenchang	0.790767266
ENSGALG00010010100	IRF2BP2	2	0.092015977	0.21176223	0.434524971	0.663907265	0.079339156	8.523667306	88.26796068	0.5	PRJNA827465;Yang2025_Silkie_Wenchang	CRA004775	0.913119244
ENSGALG00010010100	IRF2BP2	2	0.306143268	0.113898602	2.687858002	0.007191197	0	0.034128337	0	1	CRA004775;Yang2025_Silkie_Wenchang	PRJNA827465	0.130366981
ENSGALG00010010100	IRF2BP2	2	-0.070219983	0.112821391	-0.622399548	0.533679178	0.007986442	1.140986504	12.35654437	0.5	CRA004775;PRJNA827465	Yang2025_Silkie_Wenchang	0.873408008
ENSGALG00010010106	C7orf73	2	-0.487920728	0.421533003	-1.157491168	0.247071746	0.314768975	8.008434293	87.5131647	1	PRJNA827465;Yang2025_Silkie_Wenchang	CRA004775	0.757459165
ENSGALG00010010106	C7orf73	2	-0.843581494	0.217040953	-3.886738797	0.0001016	0	0.354793832	0	1	CRA004775;Yang2025_Silkie_Wenchang	PRJNA827465	0.007891668
ENSGALG00010010106	C7orf73	2	-0.317951734	0.280971375	-1.131616107	0.257795865	0.116518952	3.072720783	67.45555257	1	CRA004775;PRJNA827465	Yang2025_Silkie_Wenchang	0.765151037
ENSGALG00010010107	MGST2	2	0.243983201	0.320257209	0.761835156	0.446158395	0.07453575	1.545679396	35.30353044	0.5	PRJNA827465;Yang2025_Silkie_Wenchang	CRA004775	0.846013379
ENSGALG00010010107	MGST2	2	0.442983078	0.54488905	0.812978491	0.41623039	0.459586206	4.370056875	77.11700262	0.5	CRA004775;Yang2025_Silkie_Wenchang	PRJNA827465	0.837742614
ENSGALG00010010107	MGST2	2	0.735571444	0.224481998	3.276760258	0.001050092	0	0.968940669	0	1	CRA004775;PRJNA827465	Yang2025_Silkie_Wenchang	0.039083114
ENSGALG00010010111	CNOT4	2	0.262900771	0.406000185	0.647538549	0.517283454	0.286597403	6.846464682	85.39392159	0.5	PRJNA827465;Yang2025_Silkie_Wenchang	CRA004775	0.867952013
ENSGALG00010010111	CNOT4	2	0.341103025	0.389508163	0.875727539	0.38117817	0.206995329	3.145847571	68.2120644	0.5	CRA004775;Yang2025_Silkie_Wenchang	PRJNA827465	0.824395515
ENSGALG00010010111	CNOT4	2	-0.090530422	0.064552349	-1.402434211	0.160785623	0	0.015623149	0	1	CRA004775;PRJNA827465	Yang2025_Silkie_Wenchang	0.656761313
ENSGALG00010010115	NEB	2	0.213241799	0.618161894	0.344961087	0.730123625	0.736743451	27.678164	96.38704359	0.5	PRJNA827465;Yang2025_Silkie_Wenchang	CRA004775	0.932614156
ENSGALG00010010115	NEB	2	0.832925168	0.181150059	4.597984529	4.26598E-06	0	0.008895449	0	1	CRA004775;Yang2025_Silkie_Wenchang	PRJNA827465	0.00071652
ENSGALG00010010115	NEB	2	0.073809563	0.578597332	0.127566374	0.898492144	0.538518694	4.374485588	77.14016929	0.5	CRA004775;PRJNA827465	Yang2025_Silkie_Wenchang	0.97575249
ENSGALG00010010119	SRPK2	2	-0.015765762	0.059242488	-0.266122541	0.790144836	0	0.919313673	0	0.5	PRJNA827465;Yang2025_Silkie_Wenchang	CRA004775	0.947390191
ENSGALG00010010119	SRPK2	2	-0.083778432	0.219547113	-0.381596603	0.702760605	0.060808973	2.306488094	56.64404241	0.5	CRA004775;Yang2025_Silkie_Wenchang	PRJNA827465	0.923302692
ENSGALG00010010119	SRPK2	2	-0.108207544	0.126014288	-0.858692657	0.390510103	0.014149531	1.330022088	24.81327875	1	CRA004775;PRJNA827465	Yang2025_Silkie_Wenchang	0.829251057
ENSGALG00010010122	THBS4	2	0.46306396	0.13708614	3.377909012	0.000730403	0	0.181062121	0	1	PRJNA827465;Yang2025_Silkie_Wenchang	CRA004775	0.030276194
ENSGALG00010010122	THBS4	2	0.278258597	0.297319412	0.935891119	0.34932925	0.054457906	1.158575229	13.68708951	0.5	CRA004775;Yang2025_Silkie_Wenchang	PRJNA827465	0.809903518
ENSGALG00010010122	THBS4	2	0.306267554	0.406166114	0.754045067	0.450822161	0.175424517	1.522569439	34.32155051	0.5	CRA004775;PRJNA827465	Yang2025_Silkie_Wenchang	0.847631399
ENSGALG00010010124	ENSGALG00010010124	2	0.459755895	0.748075286	0.614585061	0.538828783	1.053836399	16.97366063	94.10851895	0.5	PRJNA827465;Yang2025_Silkie_Wenchang	CRA004775	0.87481821
ENSGALG00010010124	ENSGALG00010010124	2	0.615726966	0.640236565	0.961065285	0.336530345	0.690294571	6.250724405	84.0018645	0.5	CRA004775;Yang2025_Silkie_Wenchang	PRJNA827465	0.803800405
ENSGALG00010010124	ENSGALG00010010124	2	-0.230506006	0.1811281	-1.272613167	0.203155344	0	0.216984683	0	1	CRA004775;PRJNA827465	Yang2025_Silkie_Wenchang	0.716859255
ENSGALG00010010125	AHCTF1	2	0.045903481	0.045145881	1.016781159	0.309257547	0	0.332194408	0	1	PRJNA827465;Yang2025_Silkie_Wenchang	CRA004775	0.794043289
ENSGALG00010010125	AHCTF1	2	0.195571702	0.157197812	1.244112111	0.213458304	0.033667675	2.69354327	62.87418097	1	CRA004775;Yang2025_Silkie_Wenchang	PRJNA827465	0.726080554
ENSGALG00010010125	AHCTF1	2	0.173325954	0.185605806	0.933839078	0.350386908	0.053201594	3.7872112	73.59534636	1	CRA004775;PRJNA827465	Yang2025_Silkie_Wenchang	0.810011037
ENSGALG00010010127	FAM13C	2	0.436825787	0.384722312	1.135431384	0.256194616	0.230744308	4.412605199	77.33765078	1	PRJNA827465;Yang2025_Silkie_Wenchang	CRA004775	0.764613396
ENSGALG00010010127	FAM13C	2	0.775712147	0.272121117	2.850613569	0.004363496	0	0.332874	0	1	CRA004775;Yang2025_Silkie_Wenchang	PRJNA827465	0.096891808
ENSGALG00010010127	FAM13C	2	0.125855002	0.191970397	0.655595881	0.512084176	0	0.387441476	0	1	CRA004775;PRJNA827465	Yang2025_Silkie_Wenchang	0.866594231
ENSGALG00010010136	EPHX2	2	0.167605829	0.140197628	1.19549689	0.231892951	0.027194578	3.017070556	66.85526635	1	PRJNA827465;Yang2025_Silkie_Wenchang	CRA004775	0.744189487
ENSGALG00010010136	EPHX2	2	0.293420635	0.12139359	2.417101546	0.015644652	0	0.297287734	0	1	CRA004775;Yang2025_Silkie_Wenchang	PRJNA827465	0.20696121
ENSGALG00010010136	EPHX2	2	0.0662814	0.073325558	0.903933123	0.366030864	0	0.352575649	0	1	CRA004775;PRJNA827465	Yang2025_Silkie_Wenchang	0.819012587
ENSGALG00010010142	ENSGALG00010010142	2	0.074764923	0.228052196	0.327841278	0.743031675	0.053471309	2.003601263	50.08986976	0.5	PRJNA827465;Yang2025_Silkie_Wenchang	CRA004775	0.919573651
ENSGALG00010010142	ENSGALG00010010142	2	0.946808179	0.682724299	1.386808965	0.165500023	0.760599731	5.096372794	80.37820151	1	CRA004775;Yang2025_Silkie_Wenchang	PRJNA827465	0.664874006
ENSGALG00010010142	ENSGALG00010010142	2	0.727553428	0.916052924	0.794226413	0.427063635	1.520097116	10.0165835	90.01655605	0.5	CRA004775;PRJNA827465	Yang2025_Silkie_Wenchang	0.84125685
ENSGALG00010010150	NTRK2	2	-0.272172549	0.205614794	-1.323701194	0.185602302	0	0.539745987	0	1	PRJNA827465;Yang2025_Silkie_Wenchang	CRA004775	0.694265174
ENSGALG00010010150	NTRK2	2	-0.088358992	0.285709034	-0.309262156	0.757122116	0	0.000961456	0	1	CRA004775;Yang2025_Silkie_Wenchang	PRJNA827465	0.938795135
ENSGALG00010010150	NTRK2	2	-0.350138072	0.247858941	-1.412650564	0.157758494	0	0.22403088	0	1	CRA004775;PRJNA827465	Yang2025_Silkie_Wenchang	0.651232233
ENSGALG00010010153	ENSGALG00010010153	2	0.529207272	0.345054227	1.533693055	0.125105183	0	0.175147606	0	1	PRJNA827465;Yang2025_Silkie_Wenchang	CRA004775	0.593686207
ENSGALG00010010153	ENSGALG00010010153	2	0.033875525	0.366002473	0.092555434	0.926258574	0	0.564140282	0	0.5	CRA004775;Yang2025_Silkie_Wenchang	PRJNA827465	0.98280842
ENSGALG00010010153	ENSGALG00010010153	2	0.243886766	0.424234547	0.574886624	0.565367977	0.145940287	1.67790713	40.40194586	0.5	CRA004775;PRJNA827465	Yang2025_Silkie_Wenchang	0.883427937
ENSGALG00010010156	ENSGALG00010010156	2	-0.095166901	0.098656846	-0.964625416	0.334732464	0.015224712	4.518128969	77.86694433	1	PRJNA827465;Yang2025_Silkie_Wenchang	CRA004775	0.803101658
ENSGALG00010010156	ENSGALG00010010156	2	-0.190153925	0.071073921	-2.675438783	0.00746315	0	0.190624237	0	1	CRA004775;Yang2025_Silkie_Wenchang	PRJNA827465	0.133208079
ENSGALG00010010156	ENSGALG00010010156	2	-0.008312662	0.053458414	-0.155500196	0.876420693	0	0.171450209	0	1	CRA004775;PRJNA827465	Yang2025_Silkie_Wenchang	0.970347777
ENSGALG00010010161	SEPSECS	2	-0.310547774	0.314484203	-0.987482905	0.323405959	0.189747301	24.39164691	95.90023583	1	PRJNA827465;Yang2025_Silkie_Wenchang	CRA004775	0.799456433
ENSGALG00010010161	SEPSECS	2	-0.3939008	0.264497717	-1.489240832	0.136423962	0.114418547	5.199252737	80.76646683	1	CRA004775;Yang2025_Silkie_Wenchang	PRJNA827465	0.61829693
ENSGALG00010010161	SEPSECS	2	-0.010982103	0.065162989	-0.168532832	0.866164117	0	0.196350173	0	1	CRA004775;PRJNA827465	Yang2025_Silkie_Wenchang	0.967318736
ENSGALG00010010167	PUS7	2	0.094914233	0.060914934	1.55843776	0.119125911	0	0.224217908	0	1	PRJNA827465;Yang2025_Silkie_Wenchang	CRA004775	0.579471011
ENSGALG00010010167	PUS7	2	-0.122254346	0.091536204	-1.335584619	0.18168508	0	0.064354949	0	1	CRA004775;Yang2025_Silkie_Wenchang	PRJNA827465	0.689130352
ENSGALG00010010167	PUS7	2	-0.073578106	0.071391391	-1.030629956	0.302714383	0	3.66318E-05	0	1	CRA004775;PRJNA827465	Yang2025_Silkie_Wenchang	0.791852471
ENSGALG00010010168	SNCB	2	-0.133501025	0.190438707	-0.701018334	0.483291574	0	0.119927976	0	1	PRJNA827465;Yang2025_Silkie_Wenchang	CRA004775	0.85691916
ENSGALG00010010168	SNCB	2	-0.239272027	0.382603247	-0.625378977	0.531722357	0.042503809	1.1245888	11.07860933	1	CRA004775;Yang2025_Silkie_Wenchang	PRJNA827465	0.873042777
ENSGALG00010010168	SNCB	2	-0.229393686	0.208551291	-1.099938943	0.271358726	0	0.90209257	0	1	CRA004775;PRJNA827465	Yang2025_Silkie_Wenchang	0.772743863
ENSGALG00010010171	ENSGALG00010010171	2	-0.149364066	0.394419106	-0.378693791	0.704915265	0	0.001975094	0	1	PRJNA827465;Yang2025_Silkie_Wenchang	CRA004775	0.924152671
ENSGALG00010010171	ENSGALG00010010171	2	0.11798498	0.41326945	0.285491658	0.775267495	0	0.407137455	0	0.5	CRA004775;Yang2025_Silkie_Wenchang	PRJNA827465	0.943815265
ENSGALG00010010171	ENSGALG00010010171	2	0.091978779	0.379427765	0.242414465	0.808459027	0	0.420711811	0	0.5	CRA004775;PRJNA827465	Yang2025_Silkie_Wenchang	0.952496413
ENSGALG00010010178	LGI2	2	0.379132742	0.108843151	3.483294423	0.000495283	0	0.005632397	0	1	PRJNA827465;Yang2025_Silkie_Wenchang	CRA004775	0.023284834
ENSGALG00010010178	LGI2	2	-0.294903348	0.738937066	-0.399091292	0.689825939	0.952103363	7.229175966	86.16716477	0.5	CRA004775;Yang2025_Silkie_Wenchang	PRJNA827465	0.919979266
ENSGALG00010010178	LGI2	2	-0.282233835	0.747074028	-0.377786654	0.705588987	0.985224134	7.782118936	87.15002934	0.5	CRA004775;PRJNA827465	Yang2025_Silkie_Wenchang	0.924858822
ENSGALG00010010183	NKX3-2	2	1.188001944	0.99317281	1.196168413	0.231630845	1.48363343	4.027927976	75.17333959	1	PRJNA827465;Yang2025_Silkie_Wenchang	CRA004775	0.744077408
ENSGALG00010010183	NKX3-2	2	0.785964938	1.351394073	0.581595668	0.560839062	3.300294012	10.1632889	90.16066541	0.5	CRA004775;Yang2025_Silkie_Wenchang	PRJNA827465	0.881354866
ENSGALG00010010183	NKX3-2	2	-0.290458304	0.365320267	-0.79508754	0.426567817	0	0.808555545	0	0.5	CRA004775;PRJNA827465	Yang2025_Silkie_Wenchang	0.84125685
ENSGALG00010010185	PHYHIPL	2	0.408211507	0.225454636	1.810614825	0.070200496	0.022649534	1.215027592	17.69734232	1	PRJNA827465;Yang2025_Silkie_Wenchang	CRA004775	0.457181455
ENSGALG00010010185	PHYHIPL	2	1.101224103	0.427190672	2.577828061	0.009942346	0.124951988	1.466599589	31.8150634	1	CRA004775;Yang2025_Silkie_Wenchang	PRJNA827465	0.157756967
ENSGALG00010010185	PHYHIPL	2	0.849382537	0.68625375	1.237709138	0.21582393	0.759371945	4.620357948	78.35665523	1	CRA004775;PRJNA827465	Yang2025_Silkie_Wenchang	0.729213578
ENSGALG00010010186	TNPO1	2	-0.063513949	0.061901562	-1.0260476	0.30486918	0	0.006834747	0	1	PRJNA827465;Yang2025_Silkie_Wenchang	CRA004775	0.79225794
ENSGALG00010010186	TNPO1	2	0.147498391	0.216772089	0.68043073	0.496231769	0.073329994	4.467936972	77.61830558	0.5	CRA004775;Yang2025_Silkie_Wenchang	PRJNA827465	0.860939846
ENSGALG00010010186	TNPO1	2	0.131392111	0.222131001	0.591023064	0.554504968	0.083530177	6.029515402	83.41491922	0.5	CRA004775;PRJNA827465	Yang2025_Silkie_Wenchang	0.880223402
ENSGALG00010010188	DSP	2	-0.158630182	0.562077093	-0.282223197	0.777737253	0.186103048	1.40488154	28.81962133	0.5	PRJNA827465;Yang2025_Silkie_Wenchang	CRA004775	0.944388838
ENSGALG00010010188	DSP	2	0.538542956	1.323902798	0.406784363	0.684166373	3.017438473	7.167994096	86.04909565	0.5	CRA004775;Yang2025_Silkie_Wenchang	PRJNA827465	0.918806497
ENSGALG00010010188	DSP	2	1.062611664	0.755656955	1.406201405	0.159662052	0.748297411	2.896814715	65.47932476	1	CRA004775;PRJNA827465	Yang2025_Silkie_Wenchang	0.655304681
ENSGALG00010010190	RNF44	2	0.540899608	0.609313321	0.887719979	0.374691397	0.691320458	13.89631057	92.80384535	0.5	PRJNA827465;Yang2025_Silkie_Wenchang	CRA004775	0.822805106
ENSGALG00010010190	RNF44	2	0.657532638	0.519261273	1.266284763	0.20541115	0.452849147	6.230625553	83.95024719	1	CRA004775;Yang2025_Silkie_Wenchang	PRJNA827465	0.71825498
ENSGALG00010010190	RNF44	2	-0.011510798	0.099341123	-0.115871427	0.907754441	0	0.373654217	0	0.5	CRA004775;PRJNA827465	Yang2025_Silkie_Wenchang	0.978196186
ENSGALG00010010191	RINT1	2	0.056000641	0.057931466	0.966670537	0.333708761	0	0.173037295	0	1	PRJNA827465;Yang2025_Silkie_Wenchang	CRA004775	0.802691373
ENSGALG00010010191	RINT1	2	-0.12068831	0.186329872	-0.647713159	0.517170491	0.043435266	2.36903552	57.78872915	0.5	CRA004775;Yang2025_Silkie_Wenchang	PRJNA827465	0.867952013
ENSGALG00010010191	RINT1	2	-0.093901677	0.213253459	-0.440328976	0.659698858	0.068162814	3.379054712	70.40592458	0.5	CRA004775;PRJNA827465	Yang2025_Silkie_Wenchang	0.911617103
ENSGALG00010010195	ENSGALG00010010195	2	0.713822932	0.733976983	0.972541304	0.330781327	1.032755999	24.08953411	95.84881968	0.5	PRJNA827465;Yang2025_Silkie_Wenchang	CRA004775	0.801621563
ENSGALG00010010195	ENSGALG00010010195	2	1.496793238	0.217907729	6.868933387	6.46846E-12	0	0.521331395	0	1	CRA004775;Yang2025_Silkie_Wenchang	PRJNA827465	5.52674E-09
ENSGALG00010010195	ENSGALG00010010195	2	1.29266793	1.024001367	0.852965496	0.39393359	1.800138021	6.303252912	84.1151757	0.5	CRA004775;PRJNA827465	Yang2025_Silkie_Wenchang	0.830486298
ENSGALG00010010196	EFCAB10	2	0.667254611	0.413882827	1.612182404	0.106922253	0.252789769	3.71744821	73.09982699	1	PRJNA827465;Yang2025_Silkie_Wenchang	CRA004775	0.553983328
ENSGALG00010010196	EFCAB10	2	0.894654155	0.400728908	2.232567047	0.025577509	0.086431195	1.248968982	19.93396039	1	CRA004775;Yang2025_Silkie_Wenchang	PRJNA827465	0.272875365
ENSGALG00010010196	EFCAB10	2	0.282068115	0.232378416	1.213830954	0.224812284	0	0.015799951	0	1	CRA004775;PRJNA827465	Yang2025_Silkie_Wenchang	0.737885283
ENSGALG00010010197	SCCPDH	2	-0.136679312	0.155491853	-0.879012686	0.379394398	0.040918686	6.378761934	84.32297662	0.5	PRJNA827465;Yang2025_Silkie_Wenchang	CRA004775	0.824165982
ENSGALG00010010197	SCCPDH	2	-0.161160304	0.151958817	-1.060552504	0.288893317	0.032352809	3.292348751	69.62654702	0.5	CRA004775;Yang2025_Silkie_Wenchang	PRJNA827465	0.783530627
ENSGALG00010010197	SCCPDH	2	0.008441298	0.061105956	0.13814198	0.890128209	0	0.001988202	0	1	CRA004775;PRJNA827465	Yang2025_Silkie_Wenchang	0.973365672
ENSGALG00010010201	RIOK1	2	0.329894624	0.226399963	1.457131969	0.145079955	0.091451016	8.942086998	88.8169283	1	PRJNA827465;Yang2025_Silkie_Wenchang	CRA004775	0.643071776
ENSGALG00010010201	RIOK1	2	0.259184984	0.33687495	0.769380401	0.44166752	0.192892137	6.457421801	84.51394332	0.5	CRA004775;Yang2025_Silkie_Wenchang	PRJNA827465	0.845083421
ENSGALG00010010201	RIOK1	2	0.099775623	0.065631762	1.520233806	0.128452224	0	0.86259951	0	0.5	CRA004775;PRJNA827465	Yang2025_Silkie_Wenchang	0.601448386
ENSGALG00010010205	ARL5A	2	-0.113268575	0.188272958	-0.601618926	0.547427828	0.060825518	6.76840608	85.22547276	0.5	PRJNA827465;Yang2025_Silkie_Wenchang	CRA004775	0.878281266
ENSGALG00010010205	ARL5A	2	-0.191947143	0.150730204	-1.273448441	0.202854915	0.022133813	1.891592322	47.13448619	1	CRA004775;Yang2025_Silkie_Wenchang	PRJNA827465	0.716491755
ENSGALG00010010205	ARL5A	2	0.051347166	0.064243838	0.799254335	0.424142952	0	0.134023598	0	0.5	CRA004775;PRJNA827465	Yang2025_Silkie_Wenchang	0.840447358
ENSGALG00010010206	SCARA5	2	-0.005944563	0.113222268	-0.052503479	0.958127524	0.013935293	2.156130473	53.62061747	0.5	PRJNA827465;Yang2025_Silkie_Wenchang	CRA004775	0.98887765
ENSGALG00010010206	SCARA5	2	0.085289953	0.11147314	0.765116625	0.444202101	0	0.489945236	0	0.5	CRA004775;Yang2025_Silkie_Wenchang	PRJNA827465	0.846013379
ENSGALG00010010206	SCARA5	2	-0.104123351	0.08952351	-1.163083428	0.244795655	0	0.006525138	0	1	CRA004775;PRJNA827465	Yang2025_Silkie_Wenchang	0.756114592
ENSGALG00010010214	FAF2	2	-0.01397976	0.073085904	-0.191278466	0.848307431	0.00400021	1.443913331	30.74376569	0.5	PRJNA827465;Yang2025_Silkie_Wenchang	CRA004775	0.961364896
ENSGALG00010010214	FAF2	2	-0.067124976	0.184847373	-0.363137302	0.716502321	0.047851443	3.26594068	69.3809503	0.5	CRA004775;Yang2025_Silkie_Wenchang	PRJNA827465	0.927487786
ENSGALG00010010214	FAF2	2	-0.107033282	0.087823769	-1.228975466	0.219081008	0.006454117	1.413365587	29.24069765	1	CRA004775;PRJNA827465	Yang2025_Silkie_Wenchang	0.73274615
ENSGALG00010010215	ENSGALG00010010215	2	-0.470943239	0.10688501	-4.406073782	1.05261E-05	0.007781002	1.490937924	32.92812639	1	PRJNA827465;Yang2025_Silkie_Wenchang	CRA004775	0.00147227
ENSGALG00010010215	ENSGALG00010010215	2	-0.294451446	0.318644456	-0.924075219	0.355447154	0.174385987	6.997932417	85.71006491	0.5	CRA004775;Yang2025_Silkie_Wenchang	PRJNA827465	0.812301302
ENSGALG00010010215	ENSGALG00010010215	2	-0.203208564	0.207888747	-0.97748708	0.328328071	0.063636538	3.568354981	71.9758823	0.5	CRA004775;PRJNA827465	Yang2025_Silkie_Wenchang	0.800761292
ENSGALG00010010216	ATXN7L1	2	0.315382982	0.407917574	0.773153701	0.439431623	0.301903814	10.04315063	90.04296523	0.5	PRJNA827465;Yang2025_Silkie_Wenchang	CRA004775	0.842416112
ENSGALG00010010216	ATXN7L1	2	0.510588718	0.245184901	2.082463951	0.037300112	0.060086722	1.998414406	49.96032869	1	CRA004775;Yang2025_Silkie_Wenchang	PRJNA827465	0.333247689
ENSGALG00010010216	ATXN7L1	2	0.023891159	0.142518743	0.167635207	0.866870272	0.022678518	1.725519004	42.0464221	0.5	CRA004775;PRJNA827465	Yang2025_Silkie_Wenchang	0.967667423
ENSGALG00010010218	PBK	2	-0.052060609	0.156297367	-0.333086924	0.739068669	0.020724393	1.445911358	30.83946712	0.5	PRJNA827465;Yang2025_Silkie_Wenchang	CRA004775	0.934652713
ENSGALG00010010218	PBK	2	-0.267780073	0.235171214	-1.13866008	0.254844959	0	0.152722335	0	1	CRA004775;Yang2025_Silkie_Wenchang	PRJNA827465	0.764418181
ENSGALG00010010218	PBK	2	0.024923356	0.103252976	0.241381482	0.809259464	0	0.157747893	0	0.5	CRA004775;PRJNA827465	Yang2025_Silkie_Wenchang	0.95276497
ENSGALG00010010223	ENSGALG00010010223	2	-0.070029185	0.139318269	-0.502656155	0.615200041	0	0.045647688	0	1	PRJNA827465;Yang2025_Silkie_Wenchang	CRA004775	0.8988191
ENSGALG00010010223	ENSGALG00010010223	2	-0.134030592	0.224630983	-0.596669741	0.550711872	0	0.963130815	0	1	CRA004775;Yang2025_Silkie_Wenchang	PRJNA827465	0.878879109
ENSGALG00010010223	ENSGALG00010010223	2	-0.138174138	0.159261672	-0.867591909	0.385617778	0	0.843868601	0	1	CRA004775;PRJNA827465	Yang2025_Silkie_Wenchang	0.82648313
ENSGALG00010010226	BICC1	2	-0.130870194	0.112923332	-1.158929618	0.246484875	0.014054325	2.167863879	53.87164252	1	PRJNA827465;Yang2025_Silkie_Wenchang	CRA004775	0.756843363
ENSGALG00010010226	BICC1	2	-0.012498723	0.293829392	-0.042537348	0.966070339	0.132738488	3.97959522	74.87181624	0.5	CRA004775;Yang2025_Silkie_Wenchang	PRJNA827465	0.990863074
ENSGALG00010010226	BICC1	2	0.061168511	0.160722654	0.380584314	0.703511723	0.029216976	1.657174754	39.66361576	0.5	CRA004775;PRJNA827465	Yang2025_Silkie_Wenchang	0.923639641
ENSGALG00010010231	C1orf53	2	-0.207362848	0.158072485	-1.311821271	0.189580446	0.007670456	1.144920004	12.65765326	1	PRJNA827465;Yang2025_Silkie_Wenchang	CRA004775	0.698754721
ENSGALG00010010231	C1orf53	2	-0.481036659	0.220026599	-2.186265939	0.028796155	0	0.023879054	0	1	CRA004775;Yang2025_Silkie_Wenchang	PRJNA827465	0.289160772
ENSGALG00010010231	C1orf53	2	-0.196544542	0.172095429	-1.142067181	0.253426096	0.010228241	1.13251611	11.70103528	1	CRA004775;PRJNA827465	Yang2025_Silkie_Wenchang	0.763206288
ENSGALG00010010234	ENSGALG00010010234	2	-0.16991964	0.464825982	-0.365557361	0.714699889	0.406209515	16.64319859	93.99158978	0.5	PRJNA827465;Yang2025_Silkie_Wenchang	CRA004775	0.926681837
ENSGALG00010010234	ENSGALG00010010234	2	-0.497979017	0.266563601	-1.868143342	0.061742094	0.060536467	1.414318032	29.29454496	0.5	CRA004775;Yang2025_Silkie_Wenchang	PRJNA827465	0.429075295
ENSGALG00010010234	ENSGALG00010010234	2	0.268317946	0.140358759	1.911658018	0.055920072	0	0.29080792	0	1	CRA004775;PRJNA827465	Yang2025_Silkie_Wenchang	0.409250851
ENSGALG00010010243	ESCO2	2	-0.033040659	0.13226503	-0.249803463	0.802737021	0.018529899	1.876627324	46.71291485	0.5	PRJNA827465;Yang2025_Silkie_Wenchang	CRA004775	0.950504475
ENSGALG00010010243	ESCO2	2	-0.19803915	0.148240015	-1.335935852	0.181570241	0	0.045819113	0	1	CRA004775;Yang2025_Silkie_Wenchang	PRJNA827465	0.689004635
ENSGALG00010010243	ESCO2	2	0.035305578	0.081693951	0.43216881	0.665618726	0	0.692849689	0	0.5	CRA004775;PRJNA827465	Yang2025_Silkie_Wenchang	0.913532078
ENSGALG00010010246	CLTB	2	-0.063256757	0.080169994	-0.789032827	0.43009282	0.006565031	1.727493585	42.11266486	1	PRJNA827465;Yang2025_Silkie_Wenchang	CRA004775	0.842032082
ENSGALG00010010246	CLTB	2	-0.21510697	0.115887939	-1.856163155	0.063430249	0	0.332826736	0	1	CRA004775;Yang2025_Silkie_Wenchang	PRJNA827465	0.433586349
ENSGALG00010010246	CLTB	2	-0.068489477	0.133403933	-0.51339923	0.607672084	0.018498765	1.366815719	26.83724763	1	CRA004775;PRJNA827465	Yang2025_Silkie_Wenchang	0.897203929
ENSGALG00010010252	WDR7	2	0.112539276	0.091200117	1.233981713	0.217209719	0	0.998169776	0	1	PRJNA827465;Yang2025_Silkie_Wenchang	CRA004775	0.730748463
ENSGALG00010010252	WDR7	2	0.168862282	0.116534524	1.449032242	0.147328579	0	0.397678796	0	1	CRA004775;Yang2025_Silkie_Wenchang	PRJNA827465	0.636173932
ENSGALG00010010252	WDR7	2	0.029263233	0.110792632	0.264126164	0.791682703	0	0.001303718	0	1	CRA004775;PRJNA827465	Yang2025_Silkie_Wenchang	0.948152034
ENSGALG00010010253	IL10RB	2	-0.094339662	0.112296311	-0.840095828	0.400854659	0.01712584	3.007724221	66.75227094	0.5	PRJNA827465;Yang2025_Silkie_Wenchang	CRA004775	0.832353691
ENSGALG00010010253	IL10RB	2	-0.152983165	0.103859981	-1.472975095	0.140757751	0.003727844	1.172930109	14.74342825	0.5	CRA004775;Yang2025_Silkie_Wenchang	PRJNA827465	0.625201272
ENSGALG00010010253	IL10RB	2	0.003940448	0.066711677	0.059066841	0.95289887	0	7.41779E-05	0	1	CRA004775;PRJNA827465	Yang2025_Silkie_Wenchang	0.987758913
ENSGALG00010010254	WDR41	2	-0.093060797	0.12630678	-0.736783863	0.461253798	0	0.090339051	0	1	PRJNA827465;Yang2025_Silkie_Wenchang	CRA004775	0.85014477
ENSGALG00010010254	WDR41	2	-0.093944842	0.149339314	-0.629069727	0.529303408	0	0.156478766	0	1	CRA004775;Yang2025_Silkie_Wenchang	PRJNA827465	0.871886499
ENSGALG00010010254	WDR41	2	-0.146788374	0.149024161	-0.984997155	0.324625468	0	0.026258818	0	1	CRA004775;PRJNA827465	Yang2025_Silkie_Wenchang	0.799859262
ENSGALG00010010255	CACNB4	2	0.101627305	0.100906549	1.007142809	0.313866152	0	0.018365096	0	1	PRJNA827465;Yang2025_Silkie_Wenchang	CRA004775	0.796531448
ENSGALG00010010255	CACNB4	2	0.077640774	0.18023378	0.430778149	0.666629689	0	0.275853473	0	0.5	CRA004775;Yang2025_Silkie_Wenchang	PRJNA827465	0.913579507
ENSGALG00010010255	CACNB4	2	0.077822743	0.111886237	0.695552418	0.486709173	0	0.239118782	0	0.5	CRA004775;PRJNA827465	Yang2025_Silkie_Wenchang	0.857603818
ENSGALG00010010257	ENSGALG00010010257	2	0.054605011	0.118624686	0.460317432	0.64528839	0	0.804587352	0	0.5	PRJNA827465;Yang2025_Silkie_Wenchang	CRA004775	0.906966303
ENSGALG00010010257	ENSGALG00010010257	2	-0.083152547	0.158880348	-0.523365844	0.600719688	0	0.023153648	0	1	CRA004775;Yang2025_Silkie_Wenchang	PRJNA827465	0.895230519
ENSGALG00010010257	ENSGALG00010010257	2	0.094513869	0.140727667	0.6716083	0.501833093	0	0.571336557	0	0.5	CRA004775;PRJNA827465	Yang2025_Silkie_Wenchang	0.862795569
ENSGALG00010010259	IGF1	2	-0.148270351	0.170939285	-0.867386047	0.385730524	0.015492465	1.350109385	25.93192737	1	PRJNA827465;Yang2025_Silkie_Wenchang	CRA004775	0.82648313
ENSGALG00010010259	IGF1	2	-1.257529389	1.074859002	-1.169948231	0.242021803	1.932003284	5.365989048	81.36410658	1	CRA004775;Yang2025_Silkie_Wenchang	PRJNA827465	0.75421685
ENSGALG00010010259	IGF1	2	-1.105667895	1.252554389	-0.882730447	0.377381928	2.753853641	7.349799618	86.39418689	1	CRA004775;PRJNA827465	Yang2025_Silkie_Wenchang	0.82325159
ENSGALG00010010262	HSPA9	2	-0.03086978	0.118752873	-0.259949751	0.794902534	0.023957143	6.625013329	84.90569075	0.5	PRJNA827465;Yang2025_Silkie_Wenchang	CRA004775	0.948905846
ENSGALG00010010262	HSPA9	2	0.097312513	0.067548032	1.440641727	0.149685926	0	0.180379444	0	1	CRA004775;Yang2025_Silkie_Wenchang	PRJNA827465	0.639457191
ENSGALG00010010262	HSPA9	2	-0.073420998	0.145930366	-0.50312351	0.614877438	0.023058679	1.541481263	35.12733343	0.5	CRA004775;PRJNA827465	Yang2025_Silkie_Wenchang	0.898781283
ENSGALG00010010263	RAB4A	2	0.152407613	0.072335466	2.106955573	0.035121431	0.001556063	1.172956155	14.74532143	1	PRJNA827465;Yang2025_Silkie_Wenchang	CRA004775	0.323167978
ENSGALG00010010263	RAB4A	2	0.209181532	0.084474784	2.47626004	0.013276684	0	0.094980078	0	1	CRA004775;Yang2025_Silkie_Wenchang	PRJNA827465	0.188244411
ENSGALG00010010263	RAB4A	2	0.081572832	0.092833454	0.878700815	0.379563517	0	0.053054656	0	1	CRA004775;PRJNA827465	Yang2025_Silkie_Wenchang	0.82419806
ENSGALG00010010266	EFNA5	2	0.254990932	0.203703517	1.251774817	0.210651929	0.0327403	1.555791144	35.72402029	1	PRJNA827465;Yang2025_Silkie_Wenchang	CRA004775	0.722132818
ENSGALG00010010266	EFNA5	2	0.274001526	0.344554418	0.7952344	0.426477165	0.099683759	1.636457459	38.89239255	0.5	CRA004775;Yang2025_Silkie_Wenchang	PRJNA827465	0.84125685
ENSGALG00010010266	EFNA5	2	0.07227923	0.164503232	0.439378785	0.660387096	0	0.322493956	0	0.5	CRA004775;PRJNA827465	Yang2025_Silkie_Wenchang	0.911923994
ENSGALG00010010267	CCSER2	2	-0.030220641	0.089822879	-0.336447032	0.736533788	0	0.00894755	0	1	PRJNA827465;Yang2025_Silkie_Wenchang	CRA004775	0.933661344
ENSGALG00010010267	CCSER2	2	0.460285714	0.516304658	0.891500217	0.372660875	0.463484638	7.340262785	86.37650954	0.5	CRA004775;Yang2025_Silkie_Wenchang	PRJNA827465	0.822016521
ENSGALG00010010267	CCSER2	2	0.442406229	0.525206576	0.842347087	0.399593701	0.493082046	8.757658454	88.5814227	0.5	CRA004775;PRJNA827465	Yang2025_Silkie_Wenchang	0.832246851

ENSGALG00010010270	SSR1	2	-0.157281109	0.17851419	-0.881056626	0.378287172	0.057334004	9.362360034	89.31893245	0.5	PRJNA827465;Yang2025_Silkie_Wenchang	CRA004775	0.823345221
ENSGALG00010010270	SSR1	2	-0.250505433	0.15007161	-1.669239325	0.09506996	0.02046249	1.639190924	38.99429373	1	CRA004775;Yang2025_Silkie_Wenchang	PRJNA827465	0.52783368
ENSGALG00010010270	SSR1	2	0.00584257	0.037537572	0.155645928	0.876312121	0	0.022162075	0	0.5	CRA004775;PRJNA827465	Yang2025_Silkie_Wenchang	0.970347777
ENSGALG00010010271	HUNK	2	0.270959603	0.078481757	3.452517035	0.000555382	0	0.622881682	0	1	PRJNA827465;Yang2025_Silkie_Wenchang	CRA004775	0.025176695
ENSGALG00010010271	HUNK	2	0.370543402	0.124811898	2.968814735	0.002989508	0	0.035833165	0	1	CRA004775;Yang2025_Silkie_Wenchang	PRJNA827465	0.075125719
ENSGALG00010010271	HUNK	2	0.245647621	0.091181087	2.694063303	0.007058676	0	0.380190618	0	1	CRA004775;PRJNA827465	Yang2025_Silkie_Wenchang	0.128975886
ENSGALG00010010274	MIS18A	2	-0.078369513	0.147767709	-0.530356315	0.595864908	0.035516974	5.345782029	81.29366303	0.5	PRJNA827465;Yang2025_Silkie_Wenchang	CRA004775	0.893857407
ENSGALG00010010274	MIS18A	2	-0.201281868	0.090240496	-2.230504895	0.025713943	0	0.829700201	0	0.5	CRA004775;Yang2025_Silkie_Wenchang	PRJNA827465	0.273470915
ENSGALG00010010274	MIS18A	2	0.064122821	0.081508421	0.786701788	0.431456457	0	0.007978734	0	1	CRA004775;PRJNA827465	Yang2025_Silkie_Wenchang	0.842503929
ENSGALG00010010275	PDE8B	2	-0.511565497	0.389453003	-1.313548726	0.188998113	0	0.035049825	0	1	PRJNA827465;Yang2025_Silkie_Wenchang	CRA004775	0.698162702
ENSGALG00010010275	PDE8B	2	-0.104112027	0.404878176	-0.257144082	0.797067549	0	0.724236897	0	0.5	CRA004775;Yang2025_Silkie_Wenchang	PRJNA827465	0.949622804
ENSGALG00010010275	PDE8B	2	-0.193224164	0.416749203	-0.46364615	0.642901284	0.032697699	1.103348174	9.366778003	0.5	CRA004775;PRJNA827465	Yang2025_Silkie_Wenchang	0.906634523
ENSGALG00010010279	CCSAP	2	-0.138716921	0.086927766	-1.5957723	0.11053964	0	0.716222522	0	1	PRJNA827465;Yang2025_Silkie_Wenchang	CRA004775	0.562832796
ENSGALG00010010279	CCSAP	2	-0.195541437	0.115187496	-1.697592565	0.089584687	0	0.154922665	0	1	CRA004775;Yang2025_Silkie_Wenchang	PRJNA827465	0.513407255
ENSGALG00010010279	CCSAP	2	-0.058921297	0.121130493	-0.486428281	0.626663543	0	0.003371278	0	1	CRA004775;PRJNA827465	Yang2025_Silkie_Wenchang	0.901504244
ENSGALG00010010280	HSP90B1	2	0.065190539	0.126721854	0.514438009	0.606945791	0.02912029	10.71700424	90.6690342	0.5	PRJNA827465;Yang2025_Silkie_Wenchang	CRA004775	0.896864065
ENSGALG00010010280	HSP90B1	2	-0.056078596	0.053124593	-1.055605192	0.291148628	0	0.318071416	0	0.5	CRA004775;Yang2025_Silkie_Wenchang	PRJNA827465	0.78467882
ENSGALG00010010280	HSP90B1	2	0.190144581	0.054756202	3.472567033	0.000515506	0	0.049662851	0	1	CRA004775;PRJNA827465	Yang2025_Silkie_Wenchang	0.023892621
ENSGALG00010010284	CCDC25	2	0.140587795	0.155015412	0.906927855	0.36444495	0.040937889	6.278044112	84.07147223	1	PRJNA827465;Yang2025_Silkie_Wenchang	CRA004775	0.817496251
ENSGALG00010010284	CCDC25	2	0.281037858	0.10828864	2.595266293	0.009451766	0	0.702110247	0	1	CRA004775;Yang2025_Silkie_Wenchang	PRJNA827465	0.152962732
ENSGALG00010010284	CCDC25	2	0.003068365	0.046360916	0.0661843	0.947231096	0	0.010290611	0	1	CRA004775;PRJNA827465	Yang2025_Silkie_Wenchang	0.986445196
ENSGALG00010010285	ENSGALG00010010285	2	3.122090381	0.570661544	5.471001881	4.47499E-08	0.456363361	3.326229024	69.93592465	1	PRJNA827465;Yang2025_Silkie_Wenchang	CRA004775	1.82224E-05
ENSGALG00010010285	ENSGALG00010010285	2	1.767237409	1.942015829	0.910001547	0.362821694	7.21819591	23.00272896	95.65268972	0.5	CRA004775;Yang2025_Silkie_Wenchang	PRJNA827465	0.816115208
ENSGALG00010010285	ENSGALG00010010285	2	1.202865571	1.370407925	0.877742713	0.380083359	3.404204082	10.55012979	90.52144362	0.5	CRA004775;PRJNA827465	Yang2025_Silkie_Wenchang	0.824392515
ENSGALG00010010287	STAM2	2	0.033255486	0.042659391	0.779558393	0.435650853	0	0.875990168	0	0.5	PRJNA827465;Yang2025_Silkie_Wenchang	CRA004775	0.842762754
ENSGALG00010010287	STAM2	2	-0.00957683	0.067119732	-0.142682785	0.886540713	0	0.197573714	0	0.5	CRA004775;Yang2025_Silkie_Wenchang	PRJNA827465	0.972729743
ENSGALG00010010287	STAM2	2	0.064141799	0.051668956	1.24139918	0.214458322	0	0.004200636	0	1	CRA004775;PRJNA827465	Yang2025_Silkie_Wenchang	0.727205473
ENSGALG00010010288	ENSGALG00010010288	2	0.304918794	0.198242213	1.538112341	0.124021149	0.047314403	2.495394105	59.92616966	1	PRJNA827465;Yang2025_Silkie_Wenchang	CRA004775	0.590534404
ENSGALG00010010288	ENSGALG00010010288	2	0.233207573	0.324408865	0.718869297	0.472221456	0.148808047	3.27242931	69.44166229	0.5	CRA004775;Yang2025_Silkie_Wenchang	PRJNA827465	0.857399573
ENSGALG00010010288	ENSGALG00010010288	2	0.063231867	0.143464229	0.440750058	0.659393952	0	0.549840516	0	0.5	CRA004775;PRJNA827465	Yang2025_Silkie_Wenchang	0.911449691
ENSGALG00010010290	ENSGALG00010010290	2	0.252740778	0.227890249	1.109046038	0.267410322	0.087183365	6.165412703	83.78048562	1	PRJNA827465;Yang2025_Silkie_Wenchang	CRA004775	0.771398294
ENSGALG00010010290	ENSGALG00010010290	2	-0.101590853	0.62694268	-0.162041693	0.871273022	0.722480073	11.91682337	91.60850196	0.5	CRA004775;Yang2025_Silkie_Wenchang	PRJNA827465	0.969278598
ENSGALG00010010290	ENSGALG00010010290	2	-0.303724346	0.395073299	-0.768779735	0.442024081	0.258076829	5.183415463	80.70770118	0.5	CRA004775;PRJNA827465	Yang2025_Silkie_Wenchang	0.845159086
ENSGALG00010010291	TCF20	2	-0.077496606	0.050909803	-1.522233478	0.12795059	0	0.693680998	0	1	PRJNA827465;Yang2025_Silkie_Wenchang	CRA004775	0.60032911
ENSGALG00010010291	TCF20	2	0.004864004	0.094681615	0.051372217	0.959028923	0	0.086731929	0	0.5	CRA004775;Yang2025_Silkie_Wenchang	PRJNA827465	0.989102387
ENSGALG00010010291	TCF20	2	-0.094466527	0.057741237	-1.636032261	0.101832836	0	0.418332904	0	0.5	CRA004775;PRJNA827465	Yang2025_Silkie_Wenchang	0.54379819
ENSGALG00010010295	ENSGALG00010010295	2	-0.264765811	0.102253055	-2.589319329	0.009616587	0	0.023249261	0	1	PRJNA827465;Yang2025_Silkie_Wenchang	CRA004775	0.154613423
ENSGALG00010010295	ENSGALG00010010295	2	-0.250730175	0.135968042	-1.844037554	0.065177667	0	1.74758E-07	0	1	CRA004775;Yang2025_Silkie_Wenchang	PRJNA827465	0.439917301
ENSGALG00010010295	ENSGALG00010010295	2	-0.281167039	0.150524901	-1.867910469	0.061774551	0	0.001203962	0	1	CRA004775;PRJNA827465	Yang2025_Silkie_Wenchang	0.429075295
ENSGALG00010010300	DENND1B	2	0.051317118	0.266418493	0.192618452	0.847257793	0.113192426	4.812295653	79.22122922	0.5	PRJNA827465;Yang2025_Silkie_Wenchang	CRA004775	0.961252712
ENSGALG00010010300	DENND1B	2	0.333732299	0.156347501	2.134554734	0.032797398	0	0.002418388	0	1	CRA004775;Yang2025_Silkie_Wenchang	PRJNA827465	0.31117452
ENSGALG00010010300	DENND1B	2	0.024558749	0.256288452	0.095824642	0.923659846	0.095459442	3.436664327	70.90201705	0.5	CRA004775;PRJNA827465	Yang2025_Silkie_Wenchang	0.982186708
ENSGALG00010010302	WDR36	2	0.206412543	0.158696662	1.300673846	0.193370418	0.021021438	1.715157467	41.6963154	1	PRJNA827465;Yang2025_Silkie_Wenchang	CRA004775	0.703339473
ENSGALG00010010302	WDR36	2	0.180320167	0.204560552	0.881500198	0.378047147	0.046396806	2.199996981	54.54539217	0.5	CRA004775;Yang2025_Silkie_Wenchang	PRJNA827465	0.82325159
ENSGALG00010010302	WDR36	2	0.006315748	0.137939509	0.045786361	0.96348053	0	0.112080567	0	0.5	CRA004775;PRJNA827465	Yang2025_Silkie_Wenchang	0.99005635
ENSGALG00010010303	ETF1	2	-0.078026889	0.069099382	-1.129198078	0.258814285	0.006326479	2.726690924	63.32550965	1	PRJNA827465;Yang2025_Silkie_Wenchang	CRA004775	0.765919571
ENSGALG00010010303	ETF1	2	0.004022352	0.237508298	0.016935627	0.986487971	0.078728607	2.627256737	61.93748461	0.5	CRA004775;Yang2025_Silkie_Wenchang	PRJNA827465	0.99618774
ENSGALG00010010303	ETF1	2	0.035985978	0.133141901	0.270282894	0.786942624	0.019547196	1.42234728	29.69368211	0.5	CRA004775;PRJNA827465	Yang2025_Silkie_Wenchang	0.946784559
ENSGALG00010010305	URB1	2	0.084148741	0.065518229	1.284356176	0.199017359	0	0.172001143	0	1	PRJNA827465;Yang2025_Silkie_Wenchang	CRA004775	0.71096127
ENSGALG00010010305	URB1	2	0.128822502	0.095578321	1.347821363	0.177715845	0	0.156812455	0	1	CRA004775;Yang2025_Silkie_Wenchang	PRJNA827465	0.683951879
ENSGALG00010010305	URB1	2	0.074831016	0.082739721	0.90441465	0.365775573	0	0.335569056	0	1	CRA004775;PRJNA827465	Yang2025_Silkie_Wenchang	0.818968857
ENSGALG00010010308	PACRGL	2	-0.253211107	0.148669031	-1.703186645	0.088533145	0.005643622	1.1273448	11.29599388	1	PRJNA827465;Yang2025_Silkie_Wenchang	CRA004775	0.511076251
ENSGALG00010010308	PACRGL	2	-0.381963192	0.226442744	-1.686798106	0.09164218	0	0.688061282	0	0.5	CRA004775;Yang2025_Silkie_Wenchang	PRJNA827465	0.518914432
ENSGALG00010010308	PACRGL	2	-0.133443382	0.15864315	-0.840965285	0.400367382	0	0.108129834	0	0.5	CRA004775;PRJNA827465	Yang2025_Silkie_Wenchang	0.832251175
ENSGALG00010010310	NT5DC3	2	-0.425480812	0.0883841	-4.813997199	1.47941E-06	0	0.369275362	0	1	PRJNA827465;Yang2025_Silkie_Wenchang	CRA004775	0.000304425
ENSGALG00010010310	NT5DC3	2	-0.239671768	0.308155734	-0.777761833	0.436709432	0.140256585	3.5825172	72.08666577	0.5	CRA004775;Yang2025_Silkie_Wenchang	PRJNA827465	0.84331503
ENSGALG00010010310	NT5DC3	2	-0.205588465	0.244162898	-0.842013532	0.399780379	0.081526762	2.700173742	62.9653461	0.5	CRA004775;PRJNA827465	Yang2025_Silkie_Wenchang	0.832246851
ENSGALG00010010311	CTSV	2	0.182671256	0.055903728	3.267607427	0.001084619	0	0.934757071	0	1	PRJNA827465;Yang2025_Silkie_Wenchang	CRA004775	0.019839995
ENSGALG00010010311	CTSV	2	0.086876723	0.138196597	0.62864589	0.52958091	0	0.615961561	0	1	CRA004775;Yang2025_Silkie_Wenchang	PRJNA827465	0.871944741
ENSGALG00010010311	CTSV	2	0.20917626	0.060141196	3.478086139	0.000505008	0	0.2475149	0	1	CRA004775;PRJNA827465	Yang2025_Silkie_Wenchang	0.02357282
ENSGALG00010010317	ENSGALG00010010317	2	0.137922991	0.190303318	0.724753473	0.468603284	0.059167906	4.845414096	79.36192903	0.5	PRJNA827465;Yang2025_Silkie_Wenchang	CRA004775	0.853198008
ENSGALG00010010317	ENSGALG00010010317	2	0.112619554	0.318963103	0.353080193	0.724028321	0.136750231	2.738761851	63.48715024	0.5	CRA004775;Yang2025_Silkie_Wenchang	PRJNA827465	0.930355448
ENSGALG00010010317	ENSGALG00010010317	2	-0.028296018	0.052381462	-0.540191454	0.589065006	0	0.552567899	0	1	CRA004775;PRJNA827465	Yang2025_Silkie_Wenchang	0.892171138
ENSGALG00010010321	NUP133	2	-0.053901078	0.095536508	-0.564193509	0.572622445	0.010388189	2.123026974	52.89744255	0.5	PRJNA827465;Yang2025_Silkie_Wenchang	CRA004775	0.88573298
ENSGALG00010010321	NUP133	2	-0.270943091	0.161036347	-1.682496504	0.092472602	0.019868155	1.394996659	28.31524051	1	CRA004775;Yang2025_Silkie_Wenchang	PRJNA827465	0.520673653
ENSGALG00010010321	NUP133	2	-0.195848183	0.277287445	-0.706300219	0.480001457	0.118962006	3.638538398	72.5164368	0.5	CRA004775;PRJNA827465	Yang2025_Silkie_Wenchang	0.856321884
ENSGALG00010010322	ENSGALG00010010322	2	-0.021189528	0.132253397	-0.160219156	0.87270844	0.014107675	1.601977393	37.57714657	0.5	PRJNA827465;Yang2025_Silkie_Wenchang	CRA004775	0.969629321
ENSGALG00010010322	ENSGALG00010010322	2	0.135257834	0.154594731	0.87491878	0.3816181	0	0.028609143	0	1	CRA004775;Yang2025_Silkie_Wenchang	PRJNA827465	0.824699008
ENSGALG00010010322	ENSGALG00010010322	2	-0.092597504	0.108382605	-0.854357622	0.392906869	0	0.472180714	0	0.5	CRA004775;PRJNA827465	Yang2025_Silkie_Wenchang	0.830060225
ENSGALG00010010323	FBP2	2	-0.039703081	0.172754337	-0.22982393	0.818228589	0	0.248943075	0	0.5	PRJNA827465;Yang2025_Silkie_Wenchang	CRA004775	0.955256039
ENSGALG00010010323	FBP2	2	0.014777783	0.222820061	0.066321599	0.947121787	0	0.110392633	0	0.5	CRA004775;Yang2025_Silkie_Wenchang	PRJNA827465	0.986445196
ENSGALG00010010323	FBP2	2	-0.140788172	0.23812142	-0.591245306	0.55435607	0	0.008580037	0	1	CRA004775;PRJNA827465	Yang2025_Silkie_Wenchang	0.880177968
ENSGALG00010010327	FBP1	2	-0.590668033	1.306208228	-0.452200515	0.651124537	3.027463864	8.749054611	88.57019364	0.5	PRJNA827465;Yang2025_Silkie_Wenchang	CRA004775	0.908769703
ENSGALG00010010327	FBP1	2	-1.282673856	0.57952116	-2.213333947	0.026874631	0.401140569	2.479023518	59.66153637	1	CRA004775;Yang2025_Silkie_Wenchang	PRJNA827465	0.27936077
ENSGALG00010010327	FBP1	2	-0.03915047	0.722857521	-0.054160702	0.95680713	0.646602661	2.569702753	61.08499324	0.5	CRA004775;PRJNA827465	Yang2025_Silkie_Wenchang	0.988540618
ENSGALG00010010330	ALPK2	2	-0.251260713	0.100537977	-2.499162208	0.012448732	0	0.023659422	0	1	PRJNA827465;Yang2025_Silkie_Wenchang	CRA004775	0.180610003
ENSGALG00010010330	ALPK2	2	0.131284149	0.445870175	0.294444787	0.768418047	0.305427262	3.801952633	73.69772597	0.5	CRA004775;Yang2025_Silkie_Wenchang	PRJNA827465	0.941444269
ENSGALG00010010330	ALPK2	2	0.113922133	0.46168392	0.246753521	0.805098983	0.339072798	4.240400539	76.41732212	0.5	CRA004775;PRJNA827465	Yang2025_Silkie_Wenchang	0.951863117
ENSGALG00010010333	WAPL	2	0.04495414	0.096093453	0.467816886	0.639915544	0.011767436	2.189838016	54.33452189	0.5	PRJNA827465;Yang2025_Silkie_Wenchang	CRA004775	0.906064918
ENSGALG00010010333	WAPL	2	0.215756892	0.111517089	1.934742853	0.053021859	0	0.124655036	0	1	CRA004775;Yang2025_Silkie_Wenchang	PRJNA827465	0.399631766
ENSGALG00010010333	WAPL	2	0.062846087	0.131470544	0.478024087	0.632633061	0.022432816	2.098540126	52.34782564	0.5	CRA004775;PRJNA827465	Yang2025_Silkie_Wenchang	0.903058174
ENSGALG00010010334	ZBTB41	2	-0.06299529	0.064533082	-0.976170487	0.328979986	0	0.533043668	0	0.5	PRJNA827465;Yang2025_Silkie_Wenchang	CRA004775	0.800761292
ENSGALG00010010334	ZBTB41	2	0.195510078	0.178827085	1.093291197	0.274265967	0.013083679	1.225968977	18.43186748	1	CRA004775;Yang2025_Silkie_Wenchang	PRJNA827465	0.774526342
ENSGALG00010010334	ZBTB41	2	0.10936038	0.251519124	0.434799462	0.663707996	0.09446034	3.223928491	68.98194229	0.5	CRA004775;PRJNA827465	Yang2025_Silkie_Wenchang	0.913119244
ENSGALG00010010336	FMNL2	2	0.054167066	0.043053737	1.258126922	0.208345858	0	0.035332523	0	1	PRJNA827465;Yang2025_Silkie_Wenchang	CRA004775	0.720917211
ENSGALG00010010336	FMNL2	2	0.041549319	0.096393455	0.431038804	0.666440156	0	0.02116673	0	1	CRA004775;Yang2025_Silkie_Wenchang	PRJNA827465	0.913579507
ENSGALG00010010336	FMNL2	2	0.058560988	0.046958353	1.247083497	0.212366883	0	0.006893257	0	1	CRA004775;PRJNA827465	Yang2025_Silkie_Wenchang	0.725020284
ENSGALG00010010337	SCARA3	2	0.188040721	0.081967254	2.294095662	0.021785005	0	0.362731533	0	1	PRJNA827465;Yang2025_Silkie_Wenchang	CRA004775	0.249990082
ENSGALG00010010337	SCARA3	2	0.124110459	0.249584474	0.497268348	0.618999829	0.048203995	1.558532397	35.83707325	0.5	CRA004775;Yang2025_Silkie_Wenchang	PRJNA827465	0.899168321
ENSGALG00010010337	SCARA3	2	0.135194123	0.106610199	1.268116225	0.204756448	0.004710413	1.072130786	6.727797306	0.5	CRA004775;PRJNA827465	Yang2025_Silkie_Wenchang	0.717318293
ENSGALG00010010338	CNST	2	0.019190913	0.106856733	0.179594796	0.857470691	0.014315789	2.524184284	60.38324117	0.5	PRJNA827465;Yang2025_Silkie_Wenchang	CRA004775	0.963964719
ENSGALG00010010338	CNST	2	-0.108033334	0.110697335	-0.975934367	0.32909699	0	0.003791527	0	1	CRA004775;Yang2025_Silkie_Wenchang	PRJNA827465	0.800761292
ENSGALG00010010338	CNST	2	0.098214944	0.068013664	1.444047243	0.148725687	0	0.36460707	0	0.5	CRA004775;PRJNA827465	Yang2025_Silkie_Wenchang	0.638795624
ENSGALG00010010345	ENSGALG00010010345	2	0.320710471	0.157537556	2.035771533	0.041773302	0	0.187947065	0	1	PRJNA827465;Yang2025_Silkie_Wenchang	CRA004775	0.354014899
ENSGALG00010010345	ENSGALG00010010345	2	0.14970108	0.341801639	0.437976483	0.661403331	0.123720224	1.891263844	47.12530442	0.5	CRA004775;Yang2025_Silkie_Wenchang	PRJNA827465	0.912334087
ENSGALG00010010345	ENSGALG00010010345	2	0.1127321	0.251878618	0.447565186	0.654467033	0.036028531	1.263385619	20.84760306	0.5	CRA004775;PRJNA827465	Yang2025_Silkie_Wenchang	0.910177199
ENSGALG00010010346	TFB2M	2	-0.176347041	0.17988742	-0.980318919	0.326928719	0.049891435	3.807176158	73.73381324	1	PRJNA827465;Yang2025_Silkie_Wenchang	CRA004775	0.800028239
ENSGALG00010010346	TFB2M	2	-0.093772448	0.330518194	-0.283713424	0.776630007	0.170307131	4.433308305	77.44348168	0.5	CRA004775;Yang2025_Silkie_Wenchang	PRJNA827465	0.944156005
ENSGALG00010010346	TFB2M	2	0.0090875	0.100924748	0.090042335	0.928253573	0.00811999	1.228813663	18.62069654	0.5	CRA004775;PRJNA827465	Yang2025_Silkie_Wenchang	0.983161317
ENSGALG00010010347	FBXW11	2	-0.161503831	0.145443136	-1.110425936	0.266815527	0.039753173	16.4128567	93.90721543	1	PRJNA827465;Yang2025_Silkie_Wenchang	CRA004775	0.771006559
ENSGALG00010010347	FBXW11	2	-0.106722396	0.238735228	-0.447032458	0.654851625	0.094922079	5.325667779	81.22301199	0.5	CRA004775;Yang2025_Silkie_Wenchang	PRJNA827465	0.910222014
ENSGALG00010010347	FBXW11	2	-0.012415812	0.038643863	-0.321288061	0.747992104	0	0.8857278	0	0.5	CRA004775;PRJNA827465	Yang2025_Silkie_Wenchang	0.936938957
ENSGALG00010010349	RREB1	2	0.014395459	0.23740221	0.060637427	0.951647966	0.095454371	6.210201827	83.89746376	0.5	PRJNA827465;Yang2025_Silkie_Wenchang	CRA004775	0.987713746
ENSGALG00010010349	RREB1	2	0.219454154	0.149077726	1.472078762	0.14099961	0	0.451293128	0	1	CRA004775;Yang2025_Silkie_Wenchang	PRJNA827465	0.625404318
ENSGALG00010010349	RREB1	2	-0.183192245	0.082572998	-2.218549044	0.026517419	0	0.592104393	0	0.5	CRA004775;PRJNA827465	Yang2025_Silkie_Wenchang	0.277775623
ENSGALG00010010351	NAMPTP1	2	-0.141755576	0.087200582	-1.625626494	0.104029136	0	0.058106344	0	1	PRJNA827465;Yang2025_Silkie_Wenchang	CRA004775	0.548383482
ENSGALG00010010351	NAMPTP1	2	0.354835537	0.515833163	0.687888184	0.491523198	0.458826023	6.812578234	85.32126949	0.5	CRA004775;Yang2025_Silkie_Wenchang	PRJNA827465	0.85895707
ENSGALG00010010351	NAMPTP1	2	0.328441355	0.538173417	0.610289072	0.541670335	0.513234277	8.083888113	87.62971498	0.5	CRA004775;PRJNA827465	Yang2025_Silkie_Wenchang	0.876288927
ENSGALG00010010352	MALT1	2	0.266024102	0.068384586	3.890117885	0.000100196	0	0.379248482	0	1	PRJNA827465;Yang2025_Silkie_Wenchang	CRA004775	0.007844592
ENSGALG00010010352	MALT1	2	0.297602937	0.087947908	3.383854647	0.000714758	0	0.054986139	0	1	CRA004775;Yang2025_Silkie_Wenchang	PRJNA827465	0.029980946
ENSGALG00010010352	MALT1	2	0.220577188	0.096554171	2.284491565	0.022342658	0	0.001418686	0	1	CRA004775;PRJNA827465	Yang2025_Silkie_Wenchang	0.253542872
ENSGALG00010010356	STAB2	2	-0.303391736	0.276508221	-1.097225009	0.272543043	0.084301658	2.225642287	55.06914988	1	PRJNA827465;Yang2025_Silkie_Wenchang	CRA004775	0.773495131
ENSGALG00010010356	STAB2	2	-0.529177363	0.228045529	-2.320489969	0.020314387	0	0.148625065	0	1	CRA004775;Yang2025_Silkie_Wenchang	PRJNA827465	0.240053022
ENSGALG00010010356	STAB2	2	-0.084932521	0.242090948	-0.350828985	0.725716651	0	0.297609148	0	1	CRA004775;PRJNA827465	Yang2025_Silkie_Wenchang	0.931029023
ENSGALG00010010358	ASPM	2	0.004942367	0.06586556	0.075037199	0.940185115	0	0.280900219	0	0.5	PRJNA827465;Yang2025_Silkie_Wenchang	CRA004775	0.985494335
ENSGALG00010010358	ASPM	2	-0.041045331	0.120870551	-0.339580904	0.734172161	0	0.134155371	0	0.5	CRA004775;Yang2025_Silkie_Wenchang	PRJNA827465	0.933481207
ENSGALG00010010358	ASPM	2	0.02940405	0.077150384	0.381126428	0.703109438	0	0.057059704	0	1	CRA004775;PRJNA827465	Yang2025_Silkie_Wenchang	0.923459557
ENSGALG00010010361	PIGM	2	-0.337066323	0.33708221	-0.99995287	0.317333317	0.190527253	6.134469118	83.69867089	0.5	PRJNA827465;Yang2025_Silkie_Wenchang	CRA004775	0.797095376
ENSGALG00010010361	PIGM	2	-0.073243114	0.187374176	-0.390892256	0.695876879	0	0.672325264	0	0.5	CRA004775;Yang2025_Silkie_Wenchang	PRJNA827465	0.921038437
ENSGALG00010010361	PIGM	2	-0.609148675	0.152456398	-3.995559927	6.45415E-05	0	0.588049236	0	1	CRA004775;PRJNA827465	Yang2025_Silkie_Wenchang	0.005765172
ENSGALG00010010366	CLU	2	-0.017856732	0.193557374	-0.092255497	0.926495046	0.054304779	3.260525442	69.3300967	0.5	PRJNA827465;Yang2025_Silkie_Wenchang	CRA004775	0.98280842
ENSGALG00010010366	CLU	2	0.208454153	0.184915346	1.127295044	0.259617757	0	0.026710237	0	1	CRA004775;Yang2025_Silkie_Wenchang	PRJNA827465	0.76691144
ENSGALG00010010366	CLU	2	-0.164183105	0.086317012	-1.902094392	0.057158815	0	0.418533773	0	0.5	CRA004775;PRJNA827465	Yang2025_Silkie_Wenchang	0.414005744
ENSGALG00010010368	ENSGALG00010010368	2	0.327364375	0.230355724	1.421125421	0.155280301	0.080172565	3.962799676	74.76531539	1	PRJNA827465;Yang2025_Silkie_Wenchang	CRA004775	0.647874911
ENSGALG00010010368	ENSGALG00010010368	2	0.379830386	0.263842583	1.43960987	0.149977806	0.072711005	1.950975971	48.74360245	1	CRA004775;Yang2025_Silkie_Wenchang	PRJNA827465	0.640088793
ENSGALG00010010368	ENSGALG00010010368	2	0.109066243	0.119081636	0.915894735	0.359722109	0	0.052657435	0	1	CRA004775;PRJNA827465	Yang2025_Silkie_Wenchang	0.814879426
ENSGALG00010010371	SOCS1L	2	0.144835191	0.140900211	1.027927421	0.30398397	0.011679465	1.282257111	22.01252062	0.5	PRJNA827465;Yang2025_Silkie_Wenchang	CRA004775	0.792225107
ENSGALG00010010371	SOCS1L	2	-0.147434581	0.192172373	-0.76719967	0.442962809	0	0.073191267	0	1	CRA004775;Yang2025_Silkie_Wenchang	PRJNA827465	0.845720426
ENSGALG00010010371	SOCS1L	2	0.087745982	0.200880425	0.43680703	0.6622513	0.044706493	1.935340084	48.32949473	0.5	CRA004775;PRJNA827465	Yang2025_Silkie_Wenchang	0.91272639
ENSGALG00010010374	CMTM4	2	0.012655811	0.093136371	0.135884734	0.891912404	0	0.969473796	0	0.5	PRJNA827465;Yang2025_Silkie_Wenchang	CRA004775	0.974015927
ENSGALG00010010374	CMTM4	2	-0.16673902	0.157922801	-1.055826129	0.291047659	0	0.135499282	0	1	CRA004775;Yang2025_Silkie_Wenchang	PRJNA827465	0.78467882
ENSGALG00010010374	CMTM4	2	0.036931872	0.104151981	0.354595959	0.722892304	0	0.93354072	0	0.5	CRA004775;PRJNA827465	Yang2025_Silkie_Wenchang	0.930032096
ENSGALG00010010379	SLC35F6	2	-0.216651744	0.275502792	-0.786386744	0.431640947	0.117978018	3.839597728	73.95560497	0.5	PRJNA827465;Yang2025_Silkie_Wenchang	CRA004775	0.842503929
ENSGALG00010010379	SLC35F6	2	-0.492509923	0.211878555	-2.324491612	0.020099161	0	0.148645603	0	1	CRA004775;Yang2025_Silkie_Wenchang	PRJNA827465	0.23851368
ENSGALG00010010379	SLC35F6	2	-0.068165358	0.153277238	-0.444719383	0.65652256	0.022199259	1.386703686	27.88654057	0.5	CRA004775;PRJNA827465	Yang2025_Silkie_Wenchang	0.910509456
ENSGALG00010010381	MAST4	2	-0.062340497	0.271855253	-0.229315035	0.818624069	0.119039317	4.975038832	79.89965438	0.5	PRJNA827465;Yang2025_Silkie_Wenchang	CRA004775	0.955256039
ENSGALG00010010381	MAST4	2	0.482587828	0.318398364	1.515673079	0.129602038	0.119577807	2.242900551	55.41487563	1	CRA004775;Yang2025_Silkie_Wenchang	PRJNA827465	0.603882502
ENSGALG00010010381	MAST4	2	0.235284471	0.598876708	0.39287631	0.69441084	0.640401203	8.717994394	88.52947186	0.5	CRA004775;PRJNA827465	Yang2025_Silkie_Wenchang	0.920728119
ENSGALG00010010382	SEC11C	2	0.334565615	0.176578819	1.894709781	0.058130861	0.047811953	3.985330627	74.90797895	1	PRJNA827465;Yang2025_Silkie_Wenchang	CRA004775	0.417658363
ENSGALG00010010382	SEC11C	2	0.024103091	0.561943768	0.042892355	0.965787343	0.556543458	7.993205464	87.48937451	0.5	CRA004775;Yang2025_Silkie_Wenchang	PRJNA827465	0.990731137
ENSGALG00010010382	SEC11C	2	-0.119832811	0.3763946	-0.318370166	0.750204166	0.227048003	4.247926085	76.45910075	0.5	CRA004775;PRJNA827465	Yang2025_Silkie_Wenchang	0.937379569
ENSGALG00010010383	SLIT2	2	-0.065638211	0.09692351	-0.677216605	0.498268534	0	0.801579563	0	0.5	PRJNA827465;Yang2025_Silkie_Wenchang	CRA004775	0.861454634
ENSGALG00010010383	SLIT2	2	0.034281029	0.185150718	0.185152018	0.853109802	0	0.528188722	0	0.5	CRA004775;Yang2025_Silkie_Wenchang	PRJNA827465	0.962724663
ENSGALG00010010383	SLIT2	2	-0.121741081	0.107434002	-1.133170867	0.257142506	0	0.113975757	0	1	CRA004775;PRJNA827465	Yang2025_Silkie_Wenchang	0.764870705
ENSGALG00010010386	PRPF40A	2	0.068486367	0.153321942	0.446683404	0.655103666	0.039241314	5.754291561	82.62166612	0.5	PRJNA827465;Yang2025_Silkie_Wenchang	CRA004775	0.91039865
ENSGALG00010010386	PRPF40A	2	0.214697598	0.102049207	2.103863459	0.035390359	0	0.209135658	0	1	CRA004775;Yang2025_Silkie_Wenchang	PRJNA827465	0.324684241
ENSGALG00010010386	PRPF40A	2	-0.058081828	0.057103519	-1.017132199	0.309090545	0	0.663448509	0	0.5	CRA004775;PRJNA827465	Yang2025_Silkie_Wenchang	0.794043289
ENSGALG00010010390	KCNK3	2	0.073975362	0.169315158	0.436909273	0.662177147	0.006766225	1.133623833	11.78731683	0.5	PRJNA827465;Yang2025_Silkie_Wenchang	CRA004775	0.912691648
ENSGALG00010010390	KCNK3	2	0.197328998	0.221634512	0.890335154	0.373285951	0	0.772792608	0	0.5	CRA004775;Yang2025_Silkie_Wenchang	PRJNA827465	0.822187352
ENSGALG00010010390	KCNK3	2	-0.117014253	0.213718575	-0.547515596	0.584024559	0	0.250304863	0	1	CRA004775;PRJNA827465	Yang2025_Silkie_Wenchang	0.890763231
ENSGALG00010010393	FANCC	2	-0.01128564	0.126150198	-0.089461929	0.928714809	0	0.757355894	0	0.5	PRJNA827465;Yang2025_Silkie_Wenchang	CRA004775	0.98338483
ENSGALG00010010393	FANCC	2	-0.292936581	0.274334429	-1.067808304	0.285606992	0.057224659	1.359071487	26.42035321	1	CRA004775;Yang2025_Silkie_Wenchang	PRJNA827465	0.781319106
ENSGALG00010010393	FANCC	2	-0.219592864	0.417261115	-0.526272055	0.598699196	0.236386062	2.547324989	60.74313233	0.5	CRA004775;PRJNA827465	Yang2025_Silkie_Wenchang	0.894762943
ENSGALG00010010394	MRPL42	2	-0.159945294	0.195391382	-0.818589299	0.413020774	0.058525438	4.019400826	75.12066989	0.5	PRJNA827465;Yang2025_Silkie_Wenchang	CRA004775	0.835888542
ENSGALG00010010394	MRPL42	2	-0.293249767	0.148048058	-1.980774147	0.047616607	0	0.998411866	0	1	CRA004775;Yang2025_Silkie_Wenchang	PRJNA827465	0.377009572
ENSGALG00010010394	MRPL42	2	0.003321023	0.087041512	0.038154476	0.969564517	0	0.048175845	0	0.5	CRA004775;PRJNA827465	Yang2025_Silkie_Wenchang	0.991572914
ENSGALG00010010397	PICK1	2	0.039526262	0.100924659	0.39164128	0.695323283	0.0165574	5.294641012	81.11297824	0.5	PRJNA827465;Yang2025_Silkie_Wenchang	CRA004775	0.921038437
ENSGALG00010010397	PICK1	2	0.031589044	0.160452496	0.196874744	0.843925558	0.033855398	2.407382669	58.46111162	0.5	CRA004775;Yang2025_Silkie_Wenchang	PRJNA827465	0.960607206
ENSGALG00010010397	PICK1	2	-0.065742	0.052457973	-1.253231781	0.210121367	0	0.413149189	0	1	CRA004775;PRJNA827465	Yang2025_Silkie_Wenchang	0.72188812
ENSGALG00010010399	FGF18	2	0.428994628	0.583604969	0.735077066	0.462292561	0.646074715	19.3988855	94.84506468	0.5	PRJNA827465;Yang2025_Silkie_Wenchang	CRA004775	0.850823289
ENSGALG00010010399	FGF18	2	0.961942948	0.179499936	5.359015554	8.36767E-08	0	0.827063982	0	1	CRA004775;Yang2025_Silkie_Wenchang	PRJNA827465	3.04513E-05
ENSGALG00010010399	FGF18	2	-0.102635422	0.178419445	-0.575247961	0.56512361	0	0.87923578	0	0.5	CRA004775;PRJNA827465	Yang2025_Silkie_Wenchang	0.883427937
ENSGALG00010010404	COP1	2	0.028321526	0.089674998	0.31582411	0.752136017	0.009486974	2.259056266	55.73372762	0.5	PRJNA827465;Yang2025_Silkie_Wenchang	CRA004775	0.937945771
ENSGALG00010010404	COP1	2	0.163499907	0.089595985	1.824857509	0.068022535	0	0.128935989	0	1	CRA004775;Yang2025_Silkie_Wenchang	PRJNA827465	0.4497039
ENSGALG00010010404	COP1	2	0.040010709	0.11902346	0.336158176	0.736751591	0.017398866	2.161850032	53.74332238	0.5	CRA004775;PRJNA827465	Yang2025_Silkie_Wenchang	0.933777134
ENSGALG00010010405	EVA1C	2	-0.211655811	0.152033072	-1.392169536	0.163871065	0	0.016138562	0	1	PRJNA827465;Yang2025_Silkie_Wenchang	CRA004775	0.662343118
ENSGALG00010010405	EVA1C	2	-0.101666834	0.195261733	-0.520669524	0.602597008	0	0.839603075	0	0.5	CRA004775;Yang2025_Silkie_Wenchang	PRJNA827465	0.895521995
ENSGALG00010010405	EVA1C	2	-0.108707076	0.166825544	-0.651621287	0.514645512	0	0.772461498	0	0.5	CRA004775;PRJNA827465	Yang2025_Silkie_Wenchang	0.866869697
ENSGALG00010010406	PTCH1	2	0.1648543	0.078013968	2.113138261	0.034588936	0	0.696908485	0	1	PRJNA827465;Yang2025_Silkie_Wenchang	CRA004775	0.320322559
ENSGALG00010010406	PTCH1	2	0.200274155	0.128749629	1.555531902	0.119819443	0	0.915825415	0	0.5	CRA004775;Yang2025_Silkie_Wenchang	PRJNA827465	0.580639051
ENSGALG00010010406	PTCH1	2	0.108740989	0.086720853	1.253919735	0.209871181	0	0.264709382	0	0.5	CRA004775;PRJNA827465	Yang2025_Silkie_Wenchang	0.721659407
ENSGALG00010010407	SOX10	2	0.591045853	0.096862697	6.101893396	1.04819E-09	0.002370002	1.134926439	11.88856246	1	PRJNA827465;Yang2025_Silkie_Wenchang	CRA004775	6.33802E-07
ENSGALG00010010407	SOX10	2	0.886727756	0.291460425	3.042360746	0.002347304	0.092018642	1.707183367	41.42398414	1	CRA004775;Yang2025_Silkie_Wenchang	PRJNA827465	0.064424642
ENSGALG00010010407	SOX10	2	0.818568798	0.413157156	1.981252862	0.047562924	0.249876006	2.994387078	66.60418396	1	CRA004775;PRJNA827465	Yang2025_Silkie_Wenchang	0.376812254
ENSGALG00010010408	GAS8	2	0.17980285	0.186585966	0.963646165	0.335223354	0.054732331	4.528355087	77.91692611	1	PRJNA827465;Yang2025_Silkie_Wenchang	CRA004775	0.803198384
ENSGALG00010010408	GAS8	2	0.046822677	0.348480518	0.134362396	0.893116016	0.213550756	8.22531352	87.84240871	0.5	CRA004775;Yang2025_Silkie_Wenchang	PRJNA827465	0.974381724
ENSGALG00010010408	GAS8	2	-0.107744286	0.155242774	-0.69403737	0.487658773	0.029015101	2.262688869	55.8047943	0.5	CRA004775;PRJNA827465	Yang2025_Silkie_Wenchang	0.85768885
ENSGALG00010010410	CACNA2D1	2	-0.161343318	0.073989222	-2.180632717	0.029210593	0	0.972418315	0	1	PRJNA827465;Yang2025_Silkie_Wenchang	CRA004775	0.290870014
ENSGALG00010010410	CACNA2D1	2	0.3442746	0.470544579	0.7316514	0.464381367	0.362499341	4.904930734	79.61235226	0.5	CRA004775;Yang2025_Silkie_Wenchang	PRJNA827465	0.852159649
ENSGALG00010010410	CACNA2D1	2	0.272706965	0.550966626	0.49496095	0.620627686	0.529387863	7.0059994	85.7265189	0.5	CRA004775;PRJNA827465	Yang2025_Silkie_Wenchang	0.899395593
ENSGALG00010010411	CD180	2	-0.214969103	0.308463622	-0.696902609	0.485863745	0	0.408436986	0	1	PRJNA827465;Yang2025_Silkie_Wenchang	CRA004775	0.857391223
ENSGALG00010010411	CD180	2	-1.05359489	0.683581152	-1.541287214	0.123246899	0.608857234	2.795916317	64.23355042	1	CRA004775;Yang2025_Silkie_Wenchang	PRJNA827465	0.588575077
ENSGALG00010010411	CD180	2	-0.854566829	0.884467402	-0.966193697	0.333947266	1.269981418	5.127724905	80.49817378	1	CRA004775;PRJNA827465	Yang2025_Silkie_Wenchang	0.80290753
ENSGALG00010010416	SLC35B3	2	-0.304386324	0.353664554	-0.860663926	0.389423167	0.236902443	18.53401794	94.60451585	0.5	PRJNA827465;Yang2025_Silkie_Wenchang	CRA004775	0.828911149
ENSGALG00010010416	SLC35B3	2	-0.441456108	0.268008941	-1.647169331	0.099523246	0.10233474	3.269509757	69.4143748	1	CRA004775;Yang2025_Silkie_Wenchang	PRJNA827465	0.53870846
ENSGALG00010010416	SLC35B3	2	0.025925032	0.071364044	0.363278633	0.716396753	0	0.365718633	0	0.5	CRA004775;PRJNA827465	Yang2025_Silkie_Wenchang	0.927487786
ENSGALG00010010420	NPM1	2	-0.007543516	0.064536976	-0.116886734	0.906949811	0	0.350497183	0	0.5	PRJNA827465;Yang2025_Silkie_Wenchang	CRA004775	0.978196186

ENSGALG00010010420	NPM1	2	-0.044458049	0.10222512	-0.434903371	0.663632568	0	0.134081009	0	0.5	CRA004775;Yang2025_Silkie_Wenchang	PRJNA827465	0.913119244
ENSGALG00010010420	NPM1	2	0.015757091	0.070417494	0.223766712	0.822938833	0	0.000286765	0	1	CRA004775;PRJNA827465	Yang2025_Silkie_Wenchang	0.955822987
ENSGALG00010010421	POLR2F	2	-0.011945391	0.057968291	-0.206067672	0.836738051	0	0.296377948	0	1	PRJNA827465;Yang2025_Silkie_Wenchang	CRA004775	0.958399882
ENSGALG00010010421	POLR2F	2	-0.130639279	0.183779422	-0.710848245	0.47717828	0	8.12297E-06	0	1	CRA004775;Yang2025_Silkie_Wenchang	PRJNA827465	0.854986476
ENSGALG00010010421	POLR2F	2	-0.007903856	0.058959138	-0.134056507	0.893357892	0	0.155418324	0	1	CRA004775;PRJNA827465	Yang2025_Silkie_Wenchang	0.974381724
ENSGALG00010010422	CDH1	2	0.895477308	0.604783821	1.480656851	0.138698038	0.23321386	1.463299205	31.66127632	1	PRJNA827465;Yang2025_Silkie_Wenchang	CRA004775	0.622396264
ENSGALG00010010422	CDH1	2	1.441458107	1.190168674	1.211137664	0.225842648	2.299671545	5.309197986	81.16476344	1	CRA004775;Yang2025_Silkie_Wenchang	PRJNA827465	0.739120026
ENSGALG00010010422	CDH1	2	2.009439954	0.582787751	3.447979045	0.000564798	0.208035324	1.440197202	30.56506437	1	CRA004775;PRJNA827465	Yang2025_Silkie_Wenchang	0.02547712
ENSGALG00010010423	C21orf59	2	0.403545862	0.212688962	1.897352161	0.057781475	0.064390342	3.318823273	69.86883851	1	PRJNA827465;Yang2025_Silkie_Wenchang	CRA004775	0.416579979
ENSGALG00010010423	C21orf59	2	0.284541878	0.384220033	0.740570127	0.458954127	0.238941619	5.1706862	80.66020715	0.5	CRA004775;Yang2025_Silkie_Wenchang	PRJNA827465	0.850089963
ENSGALG00010010423	C21orf59	2	0.134448284	0.146441797	0.918100477	0.358566265	0.013107614	1.292406389	22.62495695	0.5	CRA004775;PRJNA827465	Yang2025_Silkie_Wenchang	0.814138066
ENSGALG00010010425	BLOC1S5	2	-0.299788447	0.276116801	-1.085730551	0.27759822	0.136641632	9.075638399	88.98149137	1	PRJNA827465;Yang2025_Silkie_Wenchang	CRA004775	0.776763074
ENSGALG00010010425	BLOC1S5	2	-0.263623366	0.348889295	-0.755607496	0.449884561	0.205156518	6.313692141	84.16140702	0.5	CRA004775;Yang2025_Silkie_Wenchang	PRJNA827465	0.84744374
ENSGALG00010010425	BLOC1S5	2	-0.034640353	0.060992525	-0.567944239	0.570072832	0	0.403284583	0	0.5	CRA004775;PRJNA827465	Yang2025_Silkie_Wenchang	0.884966131
ENSGALG00010010431	SOCS2	2	0.043123919	0.163515443	0.263729947	0.791988018	0	0.006276593	0	1	PRJNA827465;Yang2025_Silkie_Wenchang	CRA004775	0.948314332
ENSGALG00010010431	SOCS2	2	0.035953919	0.173672559	0.207021301	0.835993223	0	0.001184681	0	1	CRA004775;Yang2025_Silkie_Wenchang	PRJNA827465	0.958399882
ENSGALG00010010431	SOCS2	2	0.051252592	0.182055377	0.28152199	0.778310064	0	0.001396112	0	1	CRA004775;PRJNA827465	Yang2025_Silkie_Wenchang	0.944570649
ENSGALG00010010434	PROM1	2	0.372747374	0.129794371	2.871830048	0.004081024	0.016422859	1.794321544	44.26862882	1	PRJNA827465;Yang2025_Silkie_Wenchang	CRA004775	0.092607666
ENSGALG00010010434	PROM1	2	0.048812308	0.64533521	0.075638687	0.939706557	0.63593658	3.53191115	71.68671697	0.5	CRA004775;Yang2025_Silkie_Wenchang	PRJNA827465	0.985425793
ENSGALG00010010434	PROM1	2	-0.034460923	0.483086233	-0.07133493	0.943131196	0.321451762	2.339776676	57.26087835	0.5	CRA004775;PRJNA827465	Yang2025_Silkie_Wenchang	0.985827325
ENSGALG00010010435	GAS1	2	0.108649522	0.087794357	1.237545621	0.215884589	0	0.456271494	0	1	PRJNA827465;Yang2025_Silkie_Wenchang	CRA004775	0.729257583
ENSGALG00010010435	GAS1	2	0.151242882	0.119150024	1.26934831	0.204316863	0	0.205905242	0	0.5	CRA004775;Yang2025_Silkie_Wenchang	PRJNA827465	0.717281769
ENSGALG00010010435	GAS1	2	0.044906862	0.11656424	0.385254192	0.700049097	0	0.023541972	0	0.5	CRA004775;PRJNA827465	Yang2025_Silkie_Wenchang	0.922267298
ENSGALG00010010438	LDB3	2	0.091927277	0.433702667	0.211959216	0.832138852	0.34570977	12.05697344	91.70604459	0.5	PRJNA827465;Yang2025_Silkie_Wenchang	CRA004775	0.957762335
ENSGALG00010010438	LDB3	2	0.532415173	0.210290914	2.531803027	0.011347772	0	0.04291108	0	1	CRA004775;Yang2025_Silkie_Wenchang	PRJNA827465	0.16977597
ENSGALG00010010438	LDB3	2	-0.30328863	0.117213547	-2.587487865	0.00966786	0	0.969512079	0	0.5	CRA004775;PRJNA827465	Yang2025_Silkie_Wenchang	0.154965704
ENSGALG00010010439	HGF	2	-0.183544074	0.130519068	-1.406262521	0.159646185	0	0.033995645	0	1	PRJNA827465;Yang2025_Silkie_Wenchang	CRA004775	0.655304681
ENSGALG00010010439	HGF	2	0.088722942	0.423764679	0.209368421	0.834160643	0.202014836	1.734701945	42.35320926	0.5	CRA004775;Yang2025_Silkie_Wenchang	PRJNA827465	0.957968425
ENSGALG00010010439	HGF	2	0.072597341	0.45567082	0.159319707	0.873416095	0.253912147	1.935271652	48.32766766	0.5	CRA004775;PRJNA827465	Yang2025_Silkie_Wenchang	0.969994114
ENSGALG00010010444	TXNDC5	2	-0.194847768	0.175528087	-1.110066038	0.266970571	0.049359936	4.495761246	77.75682592	1	PRJNA827465;Yang2025_Silkie_Wenchang	CRA004775	0.771241774
ENSGALG00010010444	TXNDC5	2	-0.346777659	0.141337557	-2.453542186	0.014145695	0	0.642313398	0	1	CRA004775;Yang2025_Silkie_Wenchang	PRJNA827465	0.194938594
ENSGALG00010010444	TXNDC5	2	-0.049504739	0.056141547	-0.881784376	0.377893423	0	0.052323991	0	1	CRA004775;PRJNA827465	Yang2025_Silkie_Wenchang	0.82325159
ENSGALG00010010445	LMAN1L	2	0.092941304	0.056153791	1.655120752	0.097900006	0	0.31100363	0	1	PRJNA827465;Yang2025_Silkie_Wenchang	CRA004775	0.534130658
ENSGALG00010010445	LMAN1L	2	0.049664094	0.079866272	0.621840645	0.534046657	0	0.359500168	0	0.5	CRA004775;Yang2025_Silkie_Wenchang	PRJNA827465	0.873408008
ENSGALG00010010445	LMAN1L	2	0.110940657	0.075810173	1.463400668	0.143357776	0	0.579861158	0	0.5	CRA004775;PRJNA827465	Yang2025_Silkie_Wenchang	0.63041016
ENSGALG00010010446	C22orf23	2	0.007505138	0.319287308	0.023505907	0.981246727	0	0.104577672	0	0.5	PRJNA827465;Yang2025_Silkie_Wenchang	CRA004775	0.994962782
ENSGALG00010010446	C22orf23	2	-0.896239613	1.143441322	-0.783809012	0.433152185	2.106796026	4.979515732	79.91772586	0.5	CRA004775;Yang2025_Silkie_Wenchang	PRJNA827465	0.842762754
ENSGALG00010010446	C22orf23	2	-0.961127274	1.02935117	-0.933721457	0.350447594	1.700729347	4.686021229	78.65993449	1	CRA004775;PRJNA827465	Yang2025_Silkie_Wenchang	0.810111037
ENSGALG00010010447	BTF3	2	0.285454079	0.359128903	0.794851309	0.4267	0.218565638	6.299167308	84.12488586	0.5	PRJNA827465;Yang2025_Silkie_Wenchang	CRA004775	0.84125685
ENSGALG00010010447	BTF3	2	0.253060398	0.425318288	0.594990636	0.55184973	0.29686574	5.571851103	82.05264316	0.5	CRA004775;Yang2025_Silkie_Wenchang	PRJNA827465	0.879061525
ENSGALG00010010447	BTF3	2	-0.073530201	0.122181949	-0.601809034	0.547301261	0	0.196228655	0	1	CRA004775;PRJNA827465	Yang2025_Silkie_Wenchang	0.878281266
ENSGALG00010010448	GALNT13	2	0.111333277	0.149036542	0.747019997	0.45501149	0	0.023917268	0	1	PRJNA827465;Yang2025_Silkie_Wenchang	CRA004775	0.849072665
ENSGALG00010010448	GALNT13	2	-0.175456512	0.275078311	-0.637832444	0.523576507	0.04225596	1.380499516	27.56245197	0.5	CRA004775;Yang2025_Silkie_Wenchang	PRJNA827465	0.869061505
ENSGALG00010010448	GALNT13	2	-0.0980784	0.292775815	-0.334994885	0.737628945	0.10600127	2.345481458	57.3648303	0.5	CRA004775;PRJNA827465	Yang2025_Silkie_Wenchang	0.93407966
ENSGALG00010010449	PAPPA2	2	-0.90017672	0.349998156	-2.57194704	0.010112836	0	0.008760047	0	1	PRJNA827465;Yang2025_Silkie_Wenchang	CRA004775	0.159642386
ENSGALG00010010449	PAPPA2	2	-0.969079637	0.435006684	-2.22773505	0.025898188	0	0.035545166	0	1	CRA004775;Yang2025_Silkie_Wenchang	PRJNA827465	0.274360241
ENSGALG00010010449	PAPPA2	2	-0.920602259	0.458280786	-2.008817053	0.044556539	0	0.059209056	0	1	CRA004775;PRJNA827465	Yang2025_Silkie_Wenchang	0.364257243
ENSGALG00010010456	BMP6	2	0.19967843	0.093027827	2.146437634	0.03183808	0	0.001199096	0	1	PRJNA827465;Yang2025_Silkie_Wenchang	CRA004775	0.305575589
ENSGALG00010010456	BMP6	2	0.240145553	0.138588926	1.732790339	0.083132941	0	0.186667774	0	1	CRA004775;Yang2025_Silkie_Wenchang	PRJNA827465	0.49661996
ENSGALG00010010456	BMP6	2	0.21897768	0.104821816	2.089046814	0.036703509	0	0.23762579	0	1	CRA004775;PRJNA827465	Yang2025_Silkie_Wenchang	0.330558668
ENSGALG00010010457	AGGF1	2	0.249694457	0.242705622	1.028797166	0.303574999	0.094282011	4.899097188	79.58807589	1	PRJNA827465;Yang2025_Silkie_Wenchang	CRA004775	0.792205992
ENSGALG00010010457	AGGF1	2	0.392450214	0.130468924	3.007997627	0.002629752	0	0.883427606	0	1	CRA004775;Yang2025_Silkie_Wenchang	PRJNA827465	0.069367208
ENSGALG00010010457	AGGF1	2	0.104012226	0.113493405	0.916460533	0.359425399	0.0038237	1.151114854	13.1276956	1	CRA004775;PRJNA827465	Yang2025_Silkie_Wenchang	0.814395044
ENSGALG00010010460	SEMA3C	2	-0.284063383	0.076616292	-3.70761069	0.000209224	0.00056921	1.043401907	4.159653822	1	PRJNA827465;Yang2025_Silkie_Wenchang	CRA004775	0.003154157
ENSGALG00010010460	SEMA3C	2	0.190241702	0.624782312	0.304492779	0.760752493	0.718057523	11.96367592	91.64136502	0.5	CRA004775;Yang2025_Silkie_Wenchang	PRJNA827465	0.939390344
ENSGALG00010010460	SEMA3C	2	0.262466735	0.541059576	0.485097662	0.627607071	0.52980339	9.780795482	89.77588273	0.5	CRA004775;PRJNA827465	Yang2025_Silkie_Wenchang	0.901856848
ENSGALG00010010461	SLC7A5	2	0.306772398	0.23123378	1.326676396	0.184615752	0.08225839	4.233935064	76.38130995	1	PRJNA827465;Yang2025_Silkie_Wenchang	CRA004775	0.692938714
ENSGALG00010010461	SLC7A5	2	0.055703885	0.17568506	0.31706076	0.75119298	0	5.07061E-05	0	1	CRA004775;Yang2025_Silkie_Wenchang	PRJNA827465	0.937684688
ENSGALG00010010461	SLC7A5	2	0.495198445	0.124694241	3.971301657	7.1481E-05	0	0.677712753	0	1	CRA004775;PRJNA827465	Yang2025_Silkie_Wenchang	0.00612433
ENSGALG00010010463	ACAA2	2	0.195298424	0.232395773	0.840369947	0.400700994	0.074982187	3.243000651	69.16436018	0.5	PRJNA827465;Yang2025_Silkie_Wenchang	CRA004775	0.832353691
ENSGALG00010010463	ACAA2	2	0.152598234	0.318183915	0.479593129	0.631518041	0.145123436	3.453114342	71.0406346	0.5	CRA004775;Yang2025_Silkie_Wenchang	PRJNA827465	0.903058174
ENSGALG00010010463	ACAA2	2	-0.067785149	0.141461272	-0.479178138	0.631811905	0	0.286264637	0	1	CRA004775;PRJNA827465	Yang2025_Silkie_Wenchang	0.903058174
ENSGALG00010010465	BMPR1A	2	-0.158538911	0.165315755	-0.959006663	0.337555389	0.04797838	8.121931265	87.68765744	0.5	PRJNA827465;Yang2025_Silkie_Wenchang	CRA004775	0.80415385
ENSGALG00010010465	BMPR1A	2	0.06059422	0.41443463	0.146209355	0.883756121	0.315243452	11.66632265	91.42831867	0.5	CRA004775;Yang2025_Silkie_Wenchang	PRJNA827465	0.972001076
ENSGALG00010010465	BMPR1A	2	0.205684734	0.245598248	0.837484534	0.402320267	0.097011856	4.490030678	77.72838764	1	CRA004775;PRJNA827465	Yang2025_Silkie_Wenchang	0.832879175
ENSGALG00010010466	CRADD	2	0.162338297	0.135492201	1.198137571	0.230863464	0.018992363	1.850039061	45.94708723	0.5	PRJNA827465;Yang2025_Silkie_Wenchang	CRA004775	0.743374303
ENSGALG00010010466	CRADD	2	0.017202574	0.140201798	0.122698669	0.902345718	0	0.132052542	0	0.5	CRA004775;Yang2025_Silkie_Wenchang	PRJNA827465	0.976923607
ENSGALG00010010466	CRADD	2	0.227434351	0.084359513	2.696013082	0.007017491	0	0.665084572	0	1	CRA004775;PRJNA827465	Yang2025_Silkie_Wenchang	0.12854434
ENSGALG00010010468	ENSGALG00010010468	2	-0.790788894	0.864869819	-0.914334421	0.360535896	0.853936526	2.327487301	57.03521134	0.5	PRJNA827465;Yang2025_Silkie_Wenchang	CRA004775	0.816887387
ENSGALG00010010468	ENSGALG00010010468	2	-0.044652421	1.725924577	-0.025871594	0.979359757	4.969316944	5.94258823	83.17231547	0.5	CRA004775;Yang2025_Silkie_Wenchang	PRJNA827465	0.994390773
ENSGALG00010010468	ENSGALG00010010468	2	0.703247517	0.837302774	0.839896318	0.400966522	0.52730824	1.54765417	35.38608174	1	CRA004775;PRJNA827465	Yang2025_Silkie_Wenchang	0.832395176
ENSGALG00010010471	KCNJ3	2	-0.11012918	0.166653643	-0.660826716	0.508723447	0.03846538	3.23451191	69.08343429	0.5	PRJNA827465;Yang2025_Silkie_Wenchang	CRA004775	0.865814463
ENSGALG00010010471	KCNJ3	2	-0.306055095	0.136239513	-2.246448832	0.024675273	0	0.319312017	0	1	CRA004775;Yang2025_Silkie_Wenchang	PRJNA827465	0.268408231
ENSGALG00010010471	KCNJ3	2	-0.070248433	0.248340498	-0.282871434	0.777275393	0.061296771	1.431279686	30.13245351	0.5	CRA004775;PRJNA827465	Yang2025_Silkie_Wenchang	0.944218792
ENSGALG00010010472	MMRN2	2	-0.084882919	0.092829445	-0.914396493	0.360508544	0.005941945	1.476649451	32.2791202	0.5	PRJNA827465;Yang2025_Silkie_Wenchang	CRA004775	0.814880744
ENSGALG00010010472	MMRN2	2	0.127373637	0.198942452	0.640253678	0.52200769	0.03094633	1.294132379	22.72815238	1	CRA004775;Yang2025_Silkie_Wenchang	PRJNA827465	0.869568211
ENSGALG00010010472	MMRN2	2	0.070310633	0.332356358	0.211551941	0.832456606	0.154130025	2.532645828	60.51560037	0.5	CRA004775;PRJNA827465	Yang2025_Silkie_Wenchang	0.957861248
ENSGALG00010010473	TRIM35	2	-0.045090681	0.112237315	-0.401744114	0.687872355	0	0.170304354	0	1	PRJNA827465;Yang2025_Silkie_Wenchang	CRA004775	0.919631238
ENSGALG00010010473	TRIM35	2	-0.149878822	0.172018961	-0.871292448	0.383594493	0	0.064975815	0	1	CRA004775;Yang2025_Silkie_Wenchang	PRJNA827465	0.825509017
ENSGALG00010010473	TRIM35	2	-0.048432903	0.121079307	-0.400009747	0.689149338	0	0.36145984	0	1	CRA004775;PRJNA827465	Yang2025_Silkie_Wenchang	0.919854035
ENSGALG00010010475	CCDC71L	2	-0.039316836	0.133432449	-0.294657229	0.768255739	0.022561344	2.492825573	59.8848788	0.5	PRJNA827465;Yang2025_Silkie_Wenchang	CRA004775	0.941444269
ENSGALG00010010475	CCDC71L	2	-0.061915991	0.19421659	-0.318798671	0.749879186	0.035531793	1.765129209	43.34692356	0.5	CRA004775;Yang2025_Silkie_Wenchang	PRJNA827465	0.937362187
ENSGALG00010010475	CCDC71L	2	0.076674539	0.075900478	1.010198566	0.312400262	0	0.22363331	0	1	CRA004775;PRJNA827465	Yang2025_Silkie_Wenchang	0.796054997
ENSGALG00010010486	ENSGALG00010010486	2	-0.20865276	0.185858329	-1.122644121	0.261586669	0	0.299783263	0	1	PRJNA827465;Yang2025_Silkie_Wenchang	CRA004775	0.768310285
ENSGALG00010010486	ENSGALG00010010486	2	-0.270016415	0.270054931	-0.999857378	0.317379534	0	0.545346659	0	0.5	CRA004775;Yang2025_Silkie_Wenchang	PRJNA827465	0.797125693
ENSGALG00010010486	ENSGALG00010010486	2	-0.086264696	0.242277792	-0.356056968	0.721797903	0	0.307370607	0	0.5	CRA004775;PRJNA827465	Yang2025_Silkie_Wenchang	0.929372448
ENSGALG00010010494	ENSGALG00010010494	2	-0.525952176	0.285804631	-1.840250711	0.065731438	0.121469449	3.825694362	73.86095424	1	PRJNA827465;Yang2025_Silkie_Wenchang	CRA004775	0.44187819
ENSGALG00010010494	ENSGALG00010010494	2	-0.100752735	0.803187762	-0.125441074	0.900174373	1.129384152	7.607260153	86.85466278	0.5	CRA004775;Yang2025_Silkie_Wenchang	PRJNA827465	0.976466979
ENSGALG00010010494	ENSGALG00010010494	2	0.133919144	0.504768883	0.265307843	0.790772323	0.382350362	3.426816535	70.8183969	0.5	CRA004775;PRJNA827465	Yang2025_Silkie_Wenchang	0.947697045
ENSGALG00010010496	MRPL17	2	0.238669193	0.267034087	0.893778001	0.371440682	0.078582011	2.102090703	52.42831347	1	PRJNA827465;Yang2025_Silkie_Wenchang	CRA004775	0.821526338
ENSGALG00010010496	MRPL17	2	0.057172737	0.544690888	0.104963637	0.916404663	0.458161021	4.343954561	76.97950137	0.5	CRA004775;Yang2025_Silkie_Wenchang	PRJNA827465	0.980547001
ENSGALG00010010496	MRPL17	2	-0.125657561	0.243386558	-0.516288008	0.605653271	0.043704595	1.42023208	29.58897253	0.5	CRA004775;PRJNA827465	Yang2025_Silkie_Wenchang	0.896474603
ENSGALG00010010499	NR4A2	2	-0.077521531	0.131348102	-0.590199092	0.555057181	0	0.261610205	0	0.5	PRJNA827465;Yang2025_Silkie_Wenchang	CRA004775	0.880223402
ENSGALG00010010499	NR4A2	2	0.239791418	0.370458367	0.647283041	0.517448776	0.142872548	1.714849524	41.68584553	1	CRA004775;Yang2025_Silkie_Wenchang	PRJNA827465	0.867952013
ENSGALG00010010499	NR4A2	2	0.186595067	0.453409952	0.411537212	0.680678661	0.277741548	2.461399204	59.37270158	0.5	CRA004775;PRJNA827465	Yang2025_Silkie_Wenchang	0.917851751
ENSGALG00010010505	CD38	2	0.478255385	0.339539761	1.408540149	0.158971187	0.17552753	4.11477585	75.69733962	1	PRJNA827465;Yang2025_Silkie_Wenchang	CRA004775	0.653803724
ENSGALG00010010505	CD38	2	0.290582442	0.537991585	0.540124511	0.589111168	0.522294016	10.15386174	90.15153027	0.5	CRA004775;Yang2025_Silkie_Wenchang	PRJNA827465	0.892171138
ENSGALG00010010505	CD38	2	-0.032623412	0.197707048	-0.165008846	0.86893702	0.038528876	1.971635849	49.28069498	0.5	CRA004775;PRJNA827465	Yang2025_Silkie_Wenchang	0.9684491
ENSGALG00010010512	PLXNC1	2	-0.379777291	0.213401843	-1.779634547	0.075135789	0.077206895	6.555559521	84.74577194	1	PRJNA827465;Yang2025_Silkie_Wenchang	CRA004775	0.473095466
ENSGALG00010010512	PLXNC1	2	-0.211274474	0.49718412	-0.42494212	0.670878869	0.380332226	3.589968649	72.14460354	0.5	CRA004775;Yang2025_Silkie_Wenchang	PRJNA827465	0.914552186
ENSGALG00010010512	PLXNC1	2	-0.09138879	0.202194382	-0.451984811	0.651279925	0.034590217	1.237918097	19.21921147	0.5	CRA004775;PRJNA827465	Yang2025_Silkie_Wenchang	0.908887045
ENSGALG00010010515	RXRG	2	0.429623118	0.294328017	1.459674554	0.144379538	0.133044046	4.306780263	76.78079821	1	PRJNA827465;Yang2025_Silkie_Wenchang	CRA004775	0.631870428
ENSGALG00010010515	RXRG	2	-0.400518873	0.573382269	-0.69851981	0.484852174	0.570398834	7.288003502	86.27882108	0.5	CRA004775;Yang2025_Silkie_Wenchang	PRJNA827465	0.857004733
ENSGALG00010010515	RXRG	2	-0.119427901	0.868799402	-0.137463148	0.89066472	1.422450566	17.00772767	94.12031978	0.5	CRA004775;PRJNA827465	Yang2025_Silkie_Wenchang	0.973477891
ENSGALG00010010521	SNCG	2	-0.172062487	0.496188035	-0.346768715	0.728765083	0.404493248	5.599379439	82.14087809	0.5	PRJNA827465;Yang2025_Silkie_Wenchang	CRA004775	0.932236477
ENSGALG00010010521	SNCG	2	0.34141659	0.273342387	1.249043712	0.211649086	0	0.049674557	0	1	CRA004775;Yang2025_Silkie_Wenchang	PRJNA827465	0.724013667
ENSGALG00010010521	SNCG	2	-0.327323198	0.5391643	-0.607093604	0.543788794	0.317745521	1.820170908	45.06010421	0.5	CRA004775;PRJNA827465	Yang2025_Silkie_Wenchang	0.876968972
ENSGALG00010010526	ENSGALG00010010526	2	0.768403911	0.340617875	2.255911878	0.02407615	0.121003113	2.077052986	51.85486327	1	PRJNA827465;Yang2025_Silkie_Wenchang	CRA004775	0.265011806
ENSGALG00010010526	ENSGALG00010010526	2	0.485680225	0.741069885	0.65537709	0.512224998	0.841706915	4.052877801	75.32617441	0.5	CRA004775;Yang2025_Silkie_Wenchang	PRJNA827465	0.865723953
ENSGALG00010010526	ENSGALG00010010526	2	0.247754485	0.352491025	0.702867498	0.482138328	0.071259539	1.275948121	21.6269076	0.5	CRA004775;PRJNA827465	Yang2025_Silkie_Wenchang	0.85691916
ENSGALG00010010528	GPD2	2	0.011520736	0.199318238	0.057800713	0.95390737	0.071474868	9.953978007	89.95376523	0.5	PRJNA827465;Yang2025_Silkie_Wenchang	CRA004775	0.987838152
ENSGALG00010010528	GPD2	2	0.206904796	0.08509852	2.43135599	0.015042427	0	0.024675713	0	1	CRA004775;Yang2025_Silkie_Wenchang	PRJNA827465	0.202817325
ENSGALG00010010528	GPD2	2	-0.171318541	0.089041271	-1.92403522	0.054350169	0	0.699992998	0	0.5	CRA004775;PRJNA827465	Yang2025_Silkie_Wenchang	0.40381596
ENSGALG00010010534	SYNJ1	2	0.290348882	0.283760477	1.023218191	0.306204694	0.136444508	5.920309582	83.10899141	1	PRJNA827465;Yang2025_Silkie_Wenchang	CRA004775	0.792485409
ENSGALG00010010534	SYNJ1	2	0.241778862	0.406282691	0.595100081	0.551776575	0.255026879	4.300259752	76.74559079	0.5	CRA004775;Yang2025_Silkie_Wenchang	PRJNA827465	0.879061525
ENSGALG00010010534	SYNJ1	2	0.03224546	0.069352979	0.464947011	0.641969404	0	0.537376339	0	0.5	CRA004775;PRJNA827465	Yang2025_Silkie_Wenchang	0.906634523
ENSGALG00010010535	LMX1A	2	-0.513553901	0.522338746	-0.983181709	0.325518015	0.419400508	4.294261159	76.71310703	1	PRJNA827465;Yang2025_Silkie_Wenchang	CRA004775	0.799987949
ENSGALG00010010535	LMX1A	2	-0.646206572	0.638672288	-1.011796793	0.311635224	0.427308383	1.771741519	43.5583583	0.5	CRA004775;Yang2025_Silkie_Wenchang	PRJNA827465	0.795854399
ENSGALG00010010535	LMX1A	2	0.021179785	0.305596534	0.069306367	0.944745758	0	0.118949336	0	0.5	CRA004775;PRJNA827465	Yang2025_Silkie_Wenchang	0.986014725
ENSGALG00010010537	ENSGALG00010010537	2	0.804926815	0.212452541	3.788737061	0.000151415	0	0.081752755	0	1	PRJNA827465;Yang2025_Silkie_Wenchang	CRA004775	0.010477229
ENSGALG00010010537	ENSGALG00010010537	2	-0.048825358	1.012738995	-0.048211196	0.961547927	1.774127268	7.101492522	85.9184531	0.5	CRA004775;Yang2025_Silkie_Wenchang	PRJNA827465	0.98973612
ENSGALG00010010537	ENSGALG00010010537	2	-0.092802392	0.947641005	-0.097929903	0.921987955	1.551817619	6.859043168	85.42070701	0.5	CRA004775;PRJNA827465	Yang2025_Silkie_Wenchang	0.981720024
ENSGALG00010010544	ENSGALG00010010544	2	0.144068583	0.128161544	1.124117101	0.260963351	0	0.486417869	0	1	PRJNA827465;Yang2025_Silkie_Wenchang	CRA004775	0.767929536
ENSGALG00010010544	ENSGALG00010010544	2	-0.13374144	0.439154044	-0.304543342	0.760713977	0.298282414	4.090074435	75.55056721	0.5	CRA004775;Yang2025_Silkie_Wenchang	PRJNA827465	0.939384641
ENSGALG00010010544	ENSGALG00010010544	2	-0.201709271	0.34300475	-0.588065534	0.556488306	0.158132411	2.69789046	62.93400289	0.5	CRA004775;PRJNA827465	Yang2025_Silkie_Wenchang	0.880324407
ENSGALG00010010546	MICALL1	2	0.074733626	0.168725573	0.442930049	0.657816332	0.04053242	3.417669415	70.74029467	0.5	PRJNA827465;Yang2025_Silkie_Wenchang	CRA004775	0.910774485
ENSGALG00010010546	MICALL1	2	0.01377044	0.276935415	0.049724372	0.960342035	0.114748789	3.787483875	73.59724733	0.5	CRA004775;Yang2025_Silkie_Wenchang	PRJNA827465	0.989338243
ENSGALG00010010546	MICALL1	2	-0.117778251	0.100894313	-1.167342812	0.243071956	0	0.66025206	0	1	CRA004775;PRJNA827465	Yang2025_Silkie_Wenchang	0.7550024
ENSGALG00010010547	BST1	2	-0.0661744	0.105299141	-0.628441972	0.529714449	0.001025006	1.045586562	4.35990319	0.5	PRJNA827465;Yang2025_Silkie_Wenchang	CRA004775	0.871944741
ENSGALG00010010547	BST1	2	-0.159767376	0.155610558	-1.026712952	0.304555649	0	0.427014691	0	0.5	CRA004775;Yang2025_Silkie_Wenchang	PRJNA827465	0.79225794
ENSGALG00010010547	BST1	2	0.023271278	0.124114322	0.187498734	0.851269616	0	0.031717687	0	1	CRA004775;PRJNA827465	Yang2025_Silkie_Wenchang	0.962071965
ENSGALG00010010572	AQP3	2	-0.344783479	0.347097458	-0.993333343	0.320547534	0.13811857	2.304807464	56.61242791	1	PRJNA827465;Yang2025_Silkie_Wenchang	CRA004775	0.798348216
ENSGALG00010010572	AQP3	2	-0.397309729	0.401647018	-0.989201242	0.322564691	0.140948641	1.733262307	42.30532817	0.5	CRA004775;Yang2025_Silkie_Wenchang	PRJNA827465	0.798652022
ENSGALG00010010572	AQP3	2	-0.006293232	0.244843357	-0.025703093	0.979494157	0	0.042006024	0	0.5	CRA004775;PRJNA827465	Yang2025_Silkie_Wenchang	0.994447687
ENSGALG00010010573	CEP83	2	0.26132509	0.285586148	0.915048198	0.360166328	0.14489114	8.682179047	88.48215414	0.5	PRJNA827465;Yang2025_Silkie_Wenchang	CRA004775	0.814880744
ENSGALG00010010573	CEP83	2	0.525007145	0.163210552	3.216747561	0.001296526	0	0.673940226	0	1	CRA004775;Yang2025_Silkie_Wenchang	PRJNA827465	0.04366527
ENSGALG00010010573	CEP83	2	-0.004767225	0.091935964	-0.051853758	0.95864522	0	0.02236615	0	0.5	CRA004775;PRJNA827465	Yang2025_Silkie_Wenchang	0.989070013
ENSGALG00010010575	PAXBP1	2	0.138918513	0.054097385	2.567933992	0.010230664	0.001533998	1.314295338	23.91360061	1	PRJNA827465;Yang2025_Silkie_Wenchang	CRA004775	0.160645533
ENSGALG00010010575	PAXBP1	2	0.216861505	0.076799264	2.823744565	0.00474662	0	0.023510941	0	1	CRA004775;Yang2025_Silkie_Wenchang	PRJNA827465	0.101499671
ENSGALG00010010575	PAXBP1	2	0.108186145	0.052902432	2.045012712	0.040853605	0	0.454571523	0	1	CRA004775;PRJNA827465	Yang2025_Silkie_Wenchang	0.349666644
ENSGALG00010010577	PBX1	2	0.051781677	0.201508078	0.256970728	0.79720137	0.067158901	5.497027036	81.80834852	0.5	PRJNA827465;Yang2025_Silkie_Wenchang	CRA004775	0.949668276
ENSGALG00010010577	PBX1	2	0.279875034	0.117181135	2.388396682	0.016922066	0	0.002080092	0	1	CRA004775;Yang2025_Silkie_Wenchang	PRJNA827465	0.216007777
ENSGALG00010010577	PBX1	2	0.046092539	0.205530394	0.224261424	0.822553891	0.066762211	4.374246912	77.13892196	0.5	CRA004775;PRJNA827465	Yang2025_Silkie_Wenchang	0.955822987
ENSGALG00010010578	GLUD2	2	-0.046086621	0.096343364	-0.478358016	0.63239541	0.004516515	1.274249418	21.52242833	0.5	PRJNA827465;Yang2025_Silkie_Wenchang	CRA004775	0.903058174
ENSGALG00010010578	GLUD2	2	-0.085516513	0.089705606	-0.953301771	0.340437195	0	0.615177158	0	0.5	CRA004775;Yang2025_Silkie_Wenchang	PRJNA827465	0.805454073
ENSGALG00010010578	GLUD2	2	0.090799554	0.129542698	0.700923755	0.483350599	0	1.69442E-05	0	1	CRA004775;PRJNA827465	Yang2025_Silkie_Wenchang	0.85691916
ENSGALG00010010585	FBXL5	2	0.210904163	0.044693128	4.718939376	2.37077E-06	0	0.388044394	0	1	PRJNA827465;Yang2025_Silkie_Wenchang	CRA004775	0.000463575
ENSGALG00010010585	FBXL5	2	0.237564575	0.072652782	3.269862049	0.001075999	0	0.573610696	0	0.5	CRA004775;Yang2025_Silkie_Wenchang	PRJNA827465	0.039671676
ENSGALG00010010585	FBXL5	2	0.184971269	0.055626762	3.325220873	0.000883485	0	0.387282296	0	0.5	CRA004775;PRJNA827465	Yang2025_Silkie_Wenchang	0.034801769
ENSGALG00010010586	CYTIP	2	0.041876875	0.300951958	0.139148039	0.889333168	0	0.141811064	0	0.5	PRJNA827465;Yang2025_Silkie_Wenchang	CRA004775	0.973135755
ENSGALG00010010586	CYTIP	2	-0.258459822	0.385975377	-0.669627747	0.503095122	0.078135729	1.337947402	25.25864633	0.5	CRA004775;Yang2025_Silkie_Wenchang	PRJNA827465	0.863052662
ENSGALG00010010586	CYTIP	2	-0.269387386	0.272993194	-0.986791582	0.323744822	0	0.967710763	0	1	CRA004775;PRJNA827465	Yang2025_Silkie_Wenchang	0.799600434
ENSGALG00010010588	SLC25A3	2	-0.113978556	0.079948975	-1.425641236	0.153971927	0.004906641	1.335749614	25.1356699	1	PRJNA827465;Yang2025_Silkie_Wenchang	CRA004775	0.646534044
ENSGALG00010010588	SLC25A3	2	-0.213854485	0.141515116	-1.511177683	0.130743187	0	0.535751534	0	1	CRA004775;Yang2025_Silkie_Wenchang	PRJNA827465	0.60646754
ENSGALG00010010588	SLC25A3	2	-0.071542748	0.055095478	-1.298523056	0.194107659	0	0.025494592	0	1	CRA004775;PRJNA827465	Yang2025_Silkie_Wenchang	0.704693637
ENSGALG00010010593	EIF3L	2	0.020162396	0.137939903	0.146167973	0.883788788	0.027667012	3.401009057	70.59696157	0.5	PRJNA827465;Yang2025_Silkie_Wenchang	CRA004775	0.972001076
ENSGALG00010010593	EIF3L	2	-0.035076706	0.221342965	-0.158472196	0.874084729	0.07878079	5.099902086	80.39178041	0.5	CRA004775;Yang2025_Silkie_Wenchang	PRJNA827465	0.970074749
ENSGALG00010010593	EIF3L	2	-0.130102385	0.066238277	-1.964157134	0.049511872	0.000697886	1.055629973	5.269836455	1	CRA004775;PRJNA827465	Yang2025_Silkie_Wenchang	0.385187077
ENSGALG00010010596	SHLD2	2	-0.487106216	0.20138334	-2.418800956	0.015571758	0.065931159	5.310749066	81.17026454	1	PRJNA827465;Yang2025_Silkie_Wenchang	CRA004775	0.206527548
ENSGALG00010010596	SHLD2	2	-0.388151379	0.353165137	-1.099064822	0.271739793	0.201279985	4.808156467	79.20200794	0.5	CRA004775;Yang2025_Silkie_Wenchang	PRJNA827465	0.773086433
ENSGALG00010010596	SHLD2	2	-0.255101959	0.103594549	-2.462503682	0.013797076	0	0.964189925	0	0.5	CRA004775;PRJNA827465	Yang2025_Silkie_Wenchang	0.192703079
ENSGALG00010010599	F2RL1	2	-0.27126707	0.226235763	-1.199045928	0.230510087	0.085864554	6.143828779	83.72350474	1	PRJNA827465;Yang2025_Silkie_Wenchang	CRA004775	0.743335644
ENSGALG00010010599	F2RL1	2	0.117919888	0.650829785	0.181183914	0.856223221	0.793616325	15.45954313	93.53150354	0.5	CRA004775;Yang2025_Silkie_Wenchang	PRJNA827465	0.963499334
ENSGALG00010010599	F2RL1	2	0.325335938	0.422513895	0.770000564	0.441299558	0.310628456	7.157481504	86.02860518	0.5	CRA004775;PRJNA827465	Yang2025_Silkie_Wenchang	0.844907543
ENSGALG00010010604	HIVEP1	2	0.197666143	0.250554806	0.788913796	0.430162392	0.102882931	4.865798949	79.44839048	0.5	PRJNA827465;Yang2025_Silkie_Wenchang	CRA004775	0.842032082
ENSGALG00010010604	HIVEP1	2	0.466605922	0.167733323	2.781831984	0.005405302	0	0.042452784	0	1	CRA004775;Yang2025_Silkie_Wenchang	PRJNA827465	0.110246272
ENSGALG00010010604	HIVEP1	2	0.136897485	0.207995927	0.658173874	0.510426411	0.061529529	2.764952498	63.83301338	0.5	CRA004775;PRJNA827465	Yang2025_Silkie_Wenchang	0.865365179
ENSGALG00010010605	NUF2	2	-0.087476922	0.079239293	-1.103958898	0.269610968	0	0.224784483	0	1	PRJNA827465;Yang2025_Silkie_Wenchang	CRA004775	0.77189992
ENSGALG00010010605	NUF2	2	0.016659204	0.144241317	0.11549537	0.90805249	0	0.875190465	0	0.5	CRA004775;Yang2025_Silkie_Wenchang	PRJNA827465	0.978196186
ENSGALG00010010605	NUF2	2	-0.032296417	0.192315608	-0.167934453	0.866634845	0.034461412	1.293888177	22.71356848	0.5	CRA004775;PRJNA827465	Yang2025_Silkie_Wenchang	0.967569506
ENSGALG00010010614	IKBIP	2	-0.04605227	0.163734721	-0.28126148	0.778509853	0.038252839	3.47767724	71.24517513	0.5	PRJNA827465;Yang2025_Silkie_Wenchang	CRA004775	0.944673731
ENSGALG00010010614	IKBIP	2	-0.166943079	0.111759677	-1.493768448	0.135236151	0	0.486754625	0	1	CRA004775;Yang2025_Silkie_Wenchang	PRJNA827465	0.616533955
ENSGALG00010010614	IKBIP	2	0.075567677	0.101515362	0.744396469	0.456636655	0	0.415774311	0	0.5	CRA004775;PRJNA827465	Yang2025_Silkie_Wenchang	0.849515887
ENSGALG00010010618	APAF1	2	-0.001165194	0.091715596	-0.012704421	0.989863611	0.005423217	1.4749825	32.20258547	0.5	PRJNA827465;Yang2025_Silkie_Wenchang	CRA004775	0.997311131
ENSGALG00010010618	APAF1	2	-0.099414929	0.100213538	-0.992030927	0.321182439	0	0.106955794	0	1	CRA004775;Yang2025_Silkie_Wenchang	PRJNA827465	0.798513866
ENSGALG00010010618	APAF1	2	0.067907754	0.104689449	0.64865901	0.516558805	0	0.657727616	0	0.5	CRA004775;PRJNA827465	Yang2025_Silkie_Wenchang	0.867805544
ENSGALG00010010619	LRRC52	2	-0.079917231	0.161611784	-0.49450126	0.620952217	0	0.565245316	0	1	PRJNA827465;Yang2025_Silkie_Wenchang	CRA004775	0.899578487
ENSGALG00010010619	LRRC52	2	-0.433836906	0.263791941	-1.644617738	0.100048667	0.008068898	1.049324278	4.700575343	1	CRA004775;Yang2025_Silkie_Wenchang	PRJNA827465	0.54042825
ENSGALG00010010619	LRRC52	2	-0.30987539	0.409683898	-0.756376786	0.449423322	0.225919931	2.641588938	62.14399653	1	CRA004775;PRJNA827465	Yang2025_Silkie_Wenchang	0.847263197
ENSGALG00010010620	ANKRD54	2	-0.156323041	0.178058448	-0.877931055	0.379981135	0.052057108	5.081718636	80.32161811	1	PRJNA827465;Yang2025_Silkie_Wenchang	CRA004775	0.824392515
ENSGALG00010010620	ANKRD54	2	-0.357390043	0.13205635	-2.706345013	0.006802833	0	0.00605716	0	1	CRA004775;Yang2025_Silkie_Wenchang	PRJNA827465	0.126544334
ENSGALG00010010620	ANKRD54	2	-0.055461178	0.121981803	-0.454667635	0.649348369	0.014431081	1.345908336	25.70073508	1	CRA004775;PRJNA827465	Yang2025_Silkie_Wenchang	0.908038815
ENSGALG00010010623	SMAD2	2	0.147058359	0.124632386	1.179936957	0.23802529	0	0.188614598	0	1	PRJNA827465;Yang2025_Silkie_Wenchang	CRA004775	0.749908454
ENSGALG00010010623	SMAD2	2	-0.006778481	0.208571621	-0.032499535	0.974073687	0.055133284	2.729164952	63.35875561	0.5	CRA004775;Yang2025_Silkie_Wenchang	PRJNA827465	0.992492038
ENSGALG00010010623	SMAD2	2	-0.056094439	0.154352877	-0.363416867	0.716293504	0.017569773	1.583896428	36.86455866	0.5	CRA004775;PRJNA827465	Yang2025_Silkie_Wenchang	0.927471951
ENSGALG00010010626	ADTRP	2	-0.61593651	0.313267512	-1.966167852	0.049279225	0	0.004633578	0	1	PRJNA827465;Yang2025_Silkie_Wenchang	CRA004775	0.384061346
ENSGALG00010010626	ADTRP	2	-0.037576819	0.697330079	-0.053886703	0.957025431	0.571754681	2.363553464	57.69082378	0.5	CRA004775;Yang2025_Silkie_Wenchang	PRJNA827465	0.988540618

ENSGALG00010010626	ADTRP	2	-0.067404732	0.665149924	-0.101337653	0.919282428	0.576535699	2.635955986	62.06309949	0.5	CRA004775;PRJNA827465	Yang2025_Silkie_Wenchang	0.981168728
ENSGALG00010010635	ALDH9A1	2	-0.15629372	0.190181525	-0.821813373	0.411183122	0.058967997	5.150756466	80.58537602	0.5	PRJNA827465;Yang2025_Silkie_Wenchang	CRA004775	0.835128673
ENSGALG00010010635	ALDH9A1	2	-0.348740533	0.114995697	-3.032639846	0.002424247	0	0.042222239	0	1	CRA004775;Yang2025_Silkie_Wenchang	PRJNA827465	0.06593704
ENSGALG00010010635	ALDH9A1	2	-0.112695698	0.162319494	-0.694283199	0.487504625	0.036152006	2.832141209	64.69102611	0.5	CRA004775;PRJNA827465	Yang2025_Silkie_Wenchang	0.85768885
ENSGALG00010010638	ACVR1C	2	-0.305349792	0.269298101	-1.133872802	0.256847907	0	0.017443211	0	1	PRJNA827465;Yang2025_Silkie_Wenchang	CRA004775	0.764750282
ENSGALG00010010638	ACVR1C	2	-0.761044595	0.462521113	-1.645426714	0.099881844	0.252735718	2.413598995	58.56809677	1	CRA004775;Yang2025_Silkie_Wenchang	PRJNA827465	0.539905945
ENSGALG00010010638	ACVR1C	2	-0.734835931	0.498982308	-1.472669308	0.140840226	0.315074451	2.701893856	62.98892357	1	CRA004775;PRJNA827465	Yang2025_Silkie_Wenchang	0.625201272
ENSGALG00010010642	CC2D2A	2	-0.278063289	0.085736264	-3.243240079	0.001181786	0.006546502	1.693071926	40.93576389	1	PRJNA827465;Yang2025_Silkie_Wenchang	CRA004775	0.041723799
ENSGALG00010010642	CC2D2A	2	-0.117337414	0.108932988	-1.077152261	0.281412248	0	0.992653765	0	0.5	CRA004775;Yang2025_Silkie_Wenchang	PRJNA827465	0.779226828
ENSGALG00010010642	CC2D2A	2	-0.177542049	0.22831973	-0.777602744	0.436803243	0.074300309	2.787987406	64.13183224	0.5	CRA004775;PRJNA827465	Yang2025_Silkie_Wenchang	0.843375996
ENSGALG00010010645	FAM219A	2	-0.179897432	0.242548757	-0.741695955	0.458271571	0.096671426	5.605650052	82.16085573	0.5	PRJNA827465;Yang2025_Silkie_Wenchang	CRA004775	0.850026426
ENSGALG00010010645	FAM219A	2	-0.389811621	0.136022498	-2.865787841	0.004159729	0	0.485065148	0	1	CRA004775;Yang2025_Silkie_Wenchang	PRJNA827465	0.093756322
ENSGALG00010010645	FAM219A	2	0.045537748	0.138378941	0.329080042	0.742095189	0	0.143549447	0	0.5	CRA004775;PRJNA827465	Yang2025_Silkie_Wenchang	0.935481151
ENSGALG00010010646	MCM3AP	2	-0.077632593	0.087498673	-0.887243092	0.374948037	0.009262463	2.095549301	52.27981515	1	PRJNA827465;Yang2025_Silkie_Wenchang	CRA004775	0.822916144
ENSGALG00010010646	MCM3AP	2	-0.220209648	0.10360093	-2.125556683	0.03354019	0	0.044686719	0	1	CRA004775;Yang2025_Silkie_Wenchang	PRJNA827465	0.314546348
ENSGALG00010010646	MCM3AP	2	-0.060772266	0.09094678	-0.668217898	0.503994512	0.008157597	1.410223162	29.0892373	1	CRA004775;PRJNA827465	Yang2025_Silkie_Wenchang	0.863416281
ENSGALG00010010648	CENPM	2	-0.367752981	0.159548154	-2.304965448	0.021168509	0.00793227	1.099200247	9.024765708	1	PRJNA827465;Yang2025_Silkie_Wenchang	CRA004775	0.246205433
ENSGALG00010010648	CENPM	2	0.033957237	0.272818212	0.124468365	0.900944447	0	0.050300502	0	0.5	CRA004775;Yang2025_Silkie_Wenchang	PRJNA827465	0.976526284
ENSGALG00010010648	CENPM	2	-0.299524055	0.238295537	-1.256943616	0.208774053	0.053967008	1.580451757	36.72695191	0.5	CRA004775;PRJNA827465	Yang2025_Silkie_Wenchang	0.721011841
ENSGALG00010010651	NUAK1	2	0.014628256	0.054294945	0.26942206	0.787604912	0	0.021978232	0	1	PRJNA827465;Yang2025_Silkie_Wenchang	CRA004775	0.946919116
ENSGALG00010010651	NUAK1	2	-0.01802133	0.088996052	-0.202495837	0.839529113	0	0.887482847	0	0.5	CRA004775;Yang2025_Silkie_Wenchang	PRJNA827465	0.959493354
ENSGALG00010010651	NUAK1	2	0.007088176	0.068539147	0.10341792	0.91763129	0.000350251	1.005340148	0.531178266	0.5	CRA004775;PRJNA827465	Yang2025_Silkie_Wenchang	0.980974192
ENSGALG00010010655	C1QTNF7	2	-0.280787472	0.102647229	-2.735460805	0.0062293	0	0.685498288	0	1	PRJNA827465;Yang2025_Silkie_Wenchang	CRA004775	0.119662852
ENSGALG00010010655	C1QTNF7	2	-0.077533952	0.398476572	-0.194575937	0.845724943	0.241104055	3.99893562	74.99334585	0.5	CRA004775;Yang2025_Silkie_Wenchang	PRJNA827465	0.961182516
ENSGALG00010010655	C1QTNF7	2	-0.017917253	0.288468495	-0.062111647	0.950473922	0.115995916	2.788252719	64.13524523	0.5	CRA004775;PRJNA827465	Yang2025_Silkie_Wenchang	0.987277827
ENSGALG00010010657	TMEM170B	2	-0.318875413	0.296052259	-1.077091638	0.281439329	0.158580537	10.41084192	90.39462891	1	PRJNA827465;Yang2025_Silkie_Wenchang	CRA004775	0.779246913
ENSGALG00010010657	TMEM170B	2	-0.634269867	0.143201865	-4.429201159	9.45828E-06	0	0.09525339	0	1	CRA004775;Yang2025_Silkie_Wenchang	PRJNA827465	0.001342017
ENSGALG00010010657	TMEM170B	2	-0.202250005	0.323381486	-0.625422337	0.531693906	0.121963109	1.663833399	39.89782868	1	CRA004775;PRJNA827465	Yang2025_Silkie_Wenchang	0.873042777
ENSGALG00010010659	ADAMTS1	2	-0.083548144	0.076470685	-1.092551269	0.274590869	0	0.247591466	0	1	PRJNA827465;Yang2025_Silkie_Wenchang	CRA004775	0.774575583
ENSGALG00010010659	ADAMTS1	2	0.555428379	0.649307729	0.855416244	0.392320754	0.748389715	8.223166304	87.83923415	0.5	CRA004775;Yang2025_Silkie_Wenchang	PRJNA827465	0.829462002
ENSGALG00010010659	ADAMTS1	2	0.51626622	0.68965723	0.748583785	0.454108109	0.860266133	9.641720836	89.62840745	0.5	CRA004775;PRJNA827465	Yang2025_Silkie_Wenchang	0.849072665
ENSGALG00010010661	CPEB2	2	0.422540808	0.32372534	1.305244774	0.191809513	0.159632172	3.99184868	74.94895022	1	PRJNA827465;Yang2025_Silkie_Wenchang	CRA004775	0.701534457
ENSGALG00010010661	CPEB2	2	0.52026364	0.321912347	1.616165534	0.106058528	0.096182243	1.817832554	44.98943273	1	CRA004775;Yang2025_Silkie_Wenchang	PRJNA827465	0.552502635
ENSGALG00010010661	CPEB2	2	0.137208447	0.150381867	0.912400212	0.361558078	0	8.27775E-06	0	1	CRA004775;PRJNA827465	Yang2025_Silkie_Wenchang	0.81550034
ENSGALG00010010662	MGST3	2	-0.008427933	0.115305256	-0.073092354	0.941732626	0.0105391	1.328333085	24.71767727	0.5	PRJNA827465;Yang2025_Silkie_Wenchang	CRA004775	0.985827325
ENSGALG00010010662	MGST3	2	-0.156440066	0.175723198	-0.890264163	0.373324059	0	0.280117106	0	1	CRA004775;Yang2025_Silkie_Wenchang	PRJNA827465	0.822187352
ENSGALG00010010662	MGST3	2	0.039244372	0.073813875	0.531666596	0.594956931	0	0.155869262	0	0.5	CRA004775;PRJNA827465	Yang2025_Silkie_Wenchang	0.893390092
ENSGALG00010010663	EFR3B	2	0.143726626	0.074699491	1.924064329	0.054346521	0	0.130619013	0	1	PRJNA827465;Yang2025_Silkie_Wenchang	CRA004775	0.40381596
ENSGALG00010010663	EFR3B	2	0.118619447	0.167307137	0.708992149	0.478329349	0.014582164	1.133774677	11.79905316	0.5	CRA004775;Yang2025_Silkie_Wenchang	PRJNA827465	0.855464436
ENSGALG00010010663	EFR3B	2	0.10812954	0.088602865	1.22038423	0.222319253	0	0.924587705	0	0.5	CRA004775;PRJNA827465	Yang2025_Silkie_Wenchang	0.735815881
ENSGALG00010010666	C12orf75	2	0.049305269	0.160541857	0.307117846	0.758753675	0.023848758	1.856876787	46.14613058	0.5	PRJNA827465;Yang2025_Silkie_Wenchang	CRA004775	0.938996879
ENSGALG00010010666	C12orf75	2	0.185100296	0.166913635	1.108958508	0.267448082	0	0.422171431	0	0.5	CRA004775;Yang2025_Silkie_Wenchang	PRJNA827465	0.771398294
ENSGALG00010010666	C12orf75	2	-0.108051199	0.1529651	-0.706378114	0.479953028	0	0.012642735	0	1	CRA004775;PRJNA827465	Yang2025_Silkie_Wenchang	0.856321884
ENSGALG00010010671	SIGMAR1	2	0.063332048	0.096666025	0.655163466	0.512362513	0	0.195176453	0	1	PRJNA827465;Yang2025_Silkie_Wenchang	CRA004775	0.865809047
ENSGALG00010010671	SIGMAR1	2	-0.073135687	0.216878891	-0.337219017	0.735951803	0.058984544	2.60940044	61.67702034	0.5	CRA004775;Yang2025_Silkie_Wenchang	PRJNA827465	0.933592889
ENSGALG00010010671	SIGMAR1	2	-0.096744752	0.167935867	-0.576081536	0.564560071	0.029063241	1.908177542	47.59397498	0.5	CRA004775;PRJNA827465	Yang2025_Silkie_Wenchang	0.883173142
ENSGALG00010010678	KCNIP1	2	-0.369172685	0.219718982	-1.68020387	0.092917657	0.013248848	1.15844676	13.67751768	1	PRJNA827465;Yang2025_Silkie_Wenchang	CRA004775	0.522019616
ENSGALG00010010678	KCNIP1	2	-0.193645708	0.275817802	-0.702078351	0.482630302	0	0.249512655	0	1	CRA004775;Yang2025_Silkie_Wenchang	PRJNA827465	0.85691916
ENSGALG00010010678	KCNIP1	2	-0.570148895	0.260582793	-2.187975987	0.028671352	0	0.004252501	0	1	CRA004775;PRJNA827465	Yang2025_Silkie_Wenchang	0.288571096
ENSGALG00010010680	ENSGALG00010010680	2	0.355671421	0.290622904	1.223824468	0.221018452	0	0.522088362	0	1	PRJNA827465;Yang2025_Silkie_Wenchang	CRA004775	0.734231106
ENSGALG00010010680	ENSGALG00010010680	2	-0.0105331	0.786357313	-0.013394802	0.989312814	0.808386465	2.78737274	64.12392266	0.5	CRA004775;Yang2025_Silkie_Wenchang	PRJNA827465	0.997119019
ENSGALG00010010680	ENSGALG00010010680	2	-0.125214994	0.519619535	-0.240974378	0.809574975	0.26598741	1.711911286	41.58575809	0.5	CRA004775;PRJNA827465	Yang2025_Silkie_Wenchang	0.952770549
ENSGALG00010010682	SMIM13	2	-0.271716957	0.362215449	-0.750152868	0.453162642	0.254659427	33.79436335	97.04092665	0.5	PRJNA827465;Yang2025_Silkie_Wenchang	CRA004775	0.848737721
ENSGALG00010010682	SMIM13	2	-0.379349744	0.315840039	-1.201081867	0.229719446	0.156066371	4.019754721	75.12286024	0.5	CRA004775;Yang2025_Silkie_Wenchang	PRJNA827465	0.742686486
ENSGALG00010010682	SMIM13	2	0.082758796	0.070298746	1.177244271	0.239098023	0	0.064922489	0	1	CRA004775;PRJNA827465	Yang2025_Silkie_Wenchang	0.751360806
ENSGALG00010010685	STIM2	2	0.022638856	0.063154319	0.358468846	0.719992478	0	0.201635394	0	1	PRJNA827465;Yang2025_Silkie_Wenchang	CRA004775	0.928564173
ENSGALG00010010685	STIM2	2	0.076499806	0.105307531	0.726441923	0.467567899	0	0.03845295	0	1	CRA004775;Yang2025_Silkie_Wenchang	PRJNA827465	0.852885838
ENSGALG00010010685	STIM2	2	0.014299862	0.071819682	0.199107842	0.842178387	0	0.203171286	0	1	CRA004775;PRJNA827465	Yang2025_Silkie_Wenchang	0.960064308
ENSGALG00010010688	IQGAP2	2	0.095666625	0.093258305	1.025824182	0.304974465	0	0.00771677	0	1	PRJNA827465;Yang2025_Silkie_Wenchang	CRA004775	0.79225794
ENSGALG00010010688	IQGAP2	2	0.068302384	0.144534237	0.472568891	0.636520781	0	0.629128366	0	0.5	CRA004775;Yang2025_Silkie_Wenchang	PRJNA827465	0.904566695
ENSGALG00010010688	IQGAP2	2	0.066642607	0.114287765	0.583112348	0.559817672	0	0.598554142	0	0.5	CRA004775;PRJNA827465	Yang2025_Silkie_Wenchang	0.881203589
ENSGALG00010010689	POFUT2	2	-0.145579873	0.150421223	-0.967814713	0.333136914	0.039273045	7.440600785	86.56022506	1	PRJNA827465;Yang2025_Silkie_Wenchang	CRA004775	0.802186016
ENSGALG00010010689	POFUT2	2	-0.315278246	0.084238547	-3.742683791	0.000182065	0	0.099216741	0	1	CRA004775;Yang2025_Silkie_Wenchang	PRJNA827465	0.011906342
ENSGALG00010010689	POFUT2	2	-0.134136402	0.178701954	-0.750615194	0.452884274	0.04544025	2.84514836	64.85244797	1	CRA004775;PRJNA827465	Yang2025_Silkie_Wenchang	0.848575069
ENSGALG00010010690	MTPN	2	-0.167611679	0.141030498	-1.188478247	0.234645045	0.034972318	8.099697373	87.65385972	1	PRJNA827465;Yang2025_Silkie_Wenchang	CRA004775	0.747791642
ENSGALG00010010690	MTPN	2	-0.310953221	0.082346614	-3.776150675	0.000159271	0	0.09728017	0	1	CRA004775;Yang2025_Silkie_Wenchang	PRJNA827465	0.010924634
ENSGALG00010010690	MTPN	2	-0.036973889	0.051933381	-0.711948419	0.476496718	0	0.16764532	0	1	CRA004775;PRJNA827465	Yang2025_Silkie_Wenchang	0.854986476
ENSGALG00010010693	KCNMB1	2	0.102782451	0.216362779	0.475046823	0.6347536	0	0.377299864	0	0.5	PRJNA827465;Yang2025_Silkie_Wenchang	CRA004775	0.903935677
ENSGALG00010010693	KCNMB1	2	0.304786868	0.250318156	1.21759793	0.223376801	0	0.266367926	0	1	CRA004775;Yang2025_Silkie_Wenchang	PRJNA827465	0.736744216
ENSGALG00010010693	KCNMB1	2	0.130579766	0.265417417	0.49197889	0.622734267	0	0.937792924	0	0.5	CRA004775;PRJNA827465	Yang2025_Silkie_Wenchang	0.900255255
ENSGALG00010010697	NEDD9	2	0.010708806	0.157653256	0.067926325	0.945844283	0.032999398	2.726879303	63.3280432	0.5	PRJNA827465;Yang2025_Silkie_Wenchang	CRA004775	0.986321197
ENSGALG00010010697	NEDD9	2	0.258269513	0.151016585	1.710206284	0.087227734	0	0.354455279	0	1	CRA004775;Yang2025_Silkie_Wenchang	PRJNA827465	0.507822786
ENSGALG00010010697	NEDD9	2	0.070172387	0.25349337	0.276821391	0.781917255	0.091016724	2.816376364	64.49338189	0.5	CRA004775;PRJNA827465	Yang2025_Silkie_Wenchang	0.945328797
ENSGALG00010010698	GPRIN2	2	-0.268458422	0.306515577	-0.87583941	0.381117342	0.141530878	4.035227136	75.21824754	0.5	PRJNA827465;Yang2025_Silkie_Wenchang	CRA004775	0.824392515
ENSGALG00010010698	GPRIN2	2	-0.605410207	0.222698316	-2.718521709	0.006557435	0	0.089623209	0	1	CRA004775;Yang2025_Silkie_Wenchang	PRJNA827465	0.123728698
ENSGALG00010010698	GPRIN2	2	-0.045375946	0.239804578	-0.189220518	0.849919987	0.023055886	1.065264835	6.126629986	0.5	CRA004775;PRJNA827465	Yang2025_Silkie_Wenchang	0.961869125
ENSGALG00010010699	DNAJC27	2	0.347306346	0.401621334	0.864760699	0.387170146	0.308973236	23.28691372	95.70574267	0.5	PRJNA827465;Yang2025_Silkie_Wenchang	CRA004775	0.82737736
ENSGALG00010010699	DNAJC27	2	0.2249899	0.545250176	0.412636089	0.679873256	0.560883123	17.53181164	94.29608291	0.5	CRA004775;Yang2025_Silkie_Wenchang	PRJNA827465	0.917701868
ENSGALG00010010699	DNAJC27	2	-0.117279418	0.125432168	-0.935002717	0.349786899	0.01605514	1.637737059	38.94013728	1	CRA004775;PRJNA827465	Yang2025_Silkie_Wenchang	0.809903518
ENSGALG00010010706	SREBF2	2	0.056995734	0.06448103	0.883914752	0.376742233	0	0.335126808	0	1	PRJNA827465;Yang2025_Silkie_Wenchang	CRA004775	0.822974013
ENSGALG00010010706	SREBF2	2	0.022406602	0.136313378	0.164375665	0.86943542	0.014712247	1.429283984	30.0348978	0.5	CRA004775;Yang2025_Silkie_Wenchang	PRJNA827465	0.968577673
ENSGALG00010010706	SREBF2	2	0.00060829	0.079132729	0.007686959	0.993866754	0	0.859687653	0	0.5	CRA004775;PRJNA827465	Yang2025_Silkie_Wenchang	0.998056749
ENSGALG00010010710	UCK2	2	-0.079377701	0.067698216	-1.172522795	0.240987226	0	0.587841153	0	1	PRJNA827465;Yang2025_Silkie_Wenchang	CRA004775	0.753551568
ENSGALG00010010710	UCK2	2	-0.2262466	0.11435368	-1.978481153	0.047874453	0	0.738009798	0	1	CRA004775;Yang2025_Silkie_Wenchang	PRJNA827465	0.378353533
ENSGALG00010010710	UCK2	2	-0.156044721	0.158512967	-0.984428745	0.32490475	0.030456954	2.093376511	52.23028468	1	CRA004775;PRJNA827465	Yang2025_Silkie_Wenchang	0.799914637
ENSGALG00010010711	ENSGALG00010010711	2	-0.134724271	0.544120056	-0.247600266	0.804443705	0.463630431	4.595607943	78.24009331	0.5	PRJNA827465;Yang2025_Silkie_Wenchang	CRA004775	0.951655066
ENSGALG00010010711	ENSGALG00010010711	2	0.625550298	0.245241925	2.550747788	0.010749208	0	0.47603964	0	1	CRA004775;Yang2025_Silkie_Wenchang	PRJNA827465	0.165236469
ENSGALG00010010711	ENSGALG00010010711	2	0.055370089	0.716050351	0.077327088	0.938363342	0.917729426	9.51709371	89.49259059	0.5	CRA004775;PRJNA827465	Yang2025_Silkie_Wenchang	0.985349045
ENSGALG00010010712	ADAMTS5	2	-0.060220522	0.086495026	-0.696231039	0.486284153	0	0.050081109	0	1	PRJNA827465;Yang2025_Silkie_Wenchang	CRA004775	0.857603818
ENSGALG00010010712	ADAMTS5	2	-0.025309938	0.170824217	-0.14816364	0.882213626	0	0.000784915	0	1	CRA004775;Yang2025_Silkie_Wenchang	PRJNA827465	0.971942333
ENSGALG00010010712	ADAMTS5	2	-0.06861964	0.095114136	-0.721445227	0.470635629	0	0.00617627	0	1	CRA004775;PRJNA827465	Yang2025_Silkie_Wenchang	0.853744788
ENSGALG00010010713	NSA2	2	0.080225252	0.130476458	0.614863812	0.538644664	0	0.612288941	0	1	PRJNA827465;Yang2025_Silkie_Wenchang	CRA004775	0.87481821
ENSGALG00010010713	NSA2	2	0.173958098	0.166087424	1.047388739	0.294920323	0	0.101590834	0	1	CRA004775;Yang2025_Silkie_Wenchang	PRJNA827465	0.787569357
ENSGALG00010010713	NSA2	2	0.038000744	0.13543689	0.28057898	0.77903334	0	0.132508379	0	1	CRA004775;PRJNA827465	Yang2025_Silkie_Wenchang	0.944714533
ENSGALG00010010719	SH3BP4	2	-0.21352215	0.227744375	-0.937551808	0.348474787	0.090295521	7.666902724	86.95692334	0.5	PRJNA827465;Yang2025_Silkie_Wenchang	CRA004775	0.809147894
ENSGALG00010010719	SH3BP4	2	-0.447822918	0.120867709	-3.705066635	0.000211335	0	0.00027463	0	1	CRA004775;Yang2025_Silkie_Wenchang	PRJNA827465	0.013205256
ENSGALG00010010719	SH3BP4	2	-0.106412967	0.195387894	-0.544624156	0.586012038	0.040812752	1.676270647	40.34376241	0.5	CRA004775;PRJNA827465	Yang2025_Silkie_Wenchang	0.891402106
ENSGALG00010010722	TBC1D19	2	-0.375605009	0.270232284	-1.389933886	0.164548955	0.132517869	10.59470459	90.56132249	1	PRJNA827465;Yang2025_Silkie_Wenchang	CRA004775	0.663854194
ENSGALG00010010722	TBC1D19	2	-0.136694411	0.573721539	-0.238259159	0.811680096	0.580610869	7.929444561	87.3887762	0.5	CRA004775;Yang2025_Silkie_Wenchang	PRJNA827465	0.953348795
ENSGALG00010010722	TBC1D19	2	0.07457562	0.283684433	0.262882312	0.79264129	0.109717162	2.417430997	58.63377275	0.5	CRA004775;PRJNA827465	Yang2025_Silkie_Wenchang	0.948435155
ENSGALG00010010724	ADCY3	2	0.068035805	0.075460605	0.901606945	0.367265701	0	0.042780746	0	1	PRJNA827465;Yang2025_Silkie_Wenchang	CRA004775	0.819739734
ENSGALG00010010724	ADCY3	2	0.048911646	0.106655625	0.458594149	0.646525639	0	0.004033118	0	1	CRA004775;Yang2025_Silkie_Wenchang	PRJNA827465	0.907287363
ENSGALG00010010724	ADCY3	2	0.078212703	0.098473838	0.794248547	0.427050751	0	0.02179745	0	1	CRA004775;PRJNA827465	Yang2025_Silkie_Wenchang	0.84125685
ENSGALG00010010733	GFM2	2	0.182728876	0.163225405	1.119487964	0.262932023	0.026413347	1.980180571	49.49955502	1	PRJNA827465;Yang2025_Silkie_Wenchang	CRA004775	0.768643821
ENSGALG00010010733	GFM2	2	0.229587351	0.134242451	1.710244028	0.087220757	0.005256326	1.163287465	14.03672521	1	CRA004775;Yang2025_Silkie_Wenchang	PRJNA827465	0.507822786
ENSGALG00010010733	GFM2	2	0.035678653	0.129222244	0.276103028	0.782468935	0	0.041090013	0	1	CRA004775;PRJNA827465	Yang2025_Silkie_Wenchang	0.945424324
ENSGALG00010010734	AMN1	2	-0.067146097	0.248432893	-0.270278611	0.786945919	0.09769255	4.533946044	77.94415746	0.5	PRJNA827465;Yang2025_Silkie_Wenchang	CRA004775	0.946784559
ENSGALG00010010734	AMN1	2	-0.374216051	0.180867952	-2.069001432	0.038545952	0	0.064871473	0	1	CRA004775;Yang2025_Silkie_Wenchang	PRJNA827465	0.338965227
ENSGALG00010010734	AMN1	2	-0.050108546	0.286713767	-0.174768538	0.861261511	0.110988403	2.497309449	59.9569048	0.5	CRA004775;PRJNA827465	Yang2025_Silkie_Wenchang	0.965343309
ENSGALG00010010740	CCDC134	2	-0.312337386	0.171710239	-1.818979395	0.068914574	0.038081459	2.423052077	58.72973555	1	PRJNA827465;Yang2025_Silkie_Wenchang	CRA004775	0.452044654
ENSGALG00010010740	CCDC134	2	-0.477263968	0.166969456	-2.85839086	0.004257956	0	0.261219062	0	1	CRA004775;Yang2025_Silkie_Wenchang	PRJNA827465	0.095380758
ENSGALG00010010740	CCDC134	2	-0.203863008	0.081038603	-2.515628343	0.011882041	0	0.445883591	0	1	CRA004775;PRJNA827465	Yang2025_Silkie_Wenchang	0.174906309
ENSGALG00010010742	LCP2	2	-0.029904621	0.132854899	-0.22509235	0.821907432	0	0.051651997	0	1	PRJNA827465;Yang2025_Silkie_Wenchang	CRA004775	0.95563486
ENSGALG00010010742	LCP2	2	-0.148470037	0.204078938	-0.727512785	0.466911886	0	0.580692077	0	1	CRA004775;Yang2025_Silkie_Wenchang	PRJNA827465	0.852862288
ENSGALG00010010742	LCP2	2	-0.059054565	0.154265863	-0.382810322	0.701860407	0	0.86610305	0	1	CRA004775;PRJNA827465	Yang2025_Silkie_Wenchang	0.922907998
ENSGALG00010010743	KLHL20	2	-0.04743902	0.065189229	-0.727712549	0.466789567	0	0.014488145	0	1	PRJNA827465;Yang2025_Silkie_Wenchang	CRA004775	0.852842649
ENSGALG00010010743	KLHL20	2	-0.091714269	0.103784054	-0.883702893	0.376856618	0	0.678322929	0	1	CRA004775;Yang2025_Silkie_Wenchang	PRJNA827465	0.823064945
ENSGALG00010010743	KLHL20	2	-0.076252818	0.070755726	-1.077691124	0.281171621	0	0.687900807	0	1	CRA004775;PRJNA827465	Yang2025_Silkie_Wenchang	0.778889781
ENSGALG00010010745	ATVR1	2	-0.077153903	0.050889737	-1.516099465	0.129494204	0.003582248	3.072145877	67.44946237	1	PRJNA827465;Yang2025_Silkie_Wenchang	CRA004775	0.603529077
ENSGALG00010010745	ATVR1	2	0.009996264	0.16789713	0.059538027	0.952523578	0.047172284	5.610999291	82.17786266	0.5	CRA004775;Yang2025_Silkie_Wenchang	PRJNA827465	0.987758913
ENSGALG00010010745	ATVR1	2	0.048093223	0.112232486	0.428514283	0.668276737	0.018398098	2.958339385	66.19725224	0.5	CRA004775;PRJNA827465	Yang2025_Silkie_Wenchang	0.91421095
ENSGALG00010010748	SF3B1	2	0.134675866	0.075076256	1.793854326	0.072836423	0.005861994	2.075630375	51.82186521	1	PRJNA827465;Yang2025_Silkie_Wenchang	CRA004775	0.466160225
ENSGALG00010010748	SF3B1	2	0.020898422	0.224402318	0.093129259	0.925800873	0.08164463	4.830970947	79.30022741	0.5	CRA004775;Yang2025_Silkie_Wenchang	PRJNA827465	0.982735152
ENSGALG00010010748	SF3B1	2	-0.035513716	0.142385452	-0.249419552	0.803036263	0.025366874	2.228530447	55.12737996	0.5	CRA004775;PRJNA827465	Yang2025_Silkie_Wenchang	0.950912423
ENSGALG00010010749	NCOA4	2	-0.119168426	0.207046903	-0.575562468	0.564910956	0.08042817	15.92959598	93.72237688	0.5	PRJNA827465;Yang2025_Silkie_Wenchang	CRA004775	0.883416369
ENSGALG00010010749	NCOA4	2	-0.316375174	0.085339341	-3.707260579	0.000209513	0	0.375045557	0	1	CRA004775;Yang2025_Silkie_Wenchang	PRJNA827465	0.013154157
ENSGALG00010010749	NCOA4	2	0.074248265	0.051971533	1.428633342	0.153109646	0	0.613206174	0	0.5	CRA004775;PRJNA827465	Yang2025_Silkie_Wenchang	0.645187993
ENSGALG00010010751	SLC30A5	2	0.179290283	0.105984105	1.691671431	0.090708639	0.004370957	1.238965876	19.28752682	1	PRJNA827465;Yang2025_Silkie_Wenchang	CRA004775	0.516759185
ENSGALG00010010751	SLC30A5	2	-0.062338827	0.146956563	-0.424199	0.67142069	0.016883462	1.615724504	38.10826058	0.5	CRA004775;Yang2025_Silkie_Wenchang	PRJNA827465	0.914674175
ENSGALG00010010751	SLC30A5	2	0.03841528	0.254655782	0.150851787	0.880092633	0.10503012	5.149999764	80.58252338	0.5	CRA004775;PRJNA827465	Yang2025_Silkie_Wenchang	0.971555227
ENSGALG00010010752	ENSGALG00010010752	2	-0.339683144	0.143503676	-2.367069295	0.017929575	0	0.335734984	0	1	PRJNA827465;Yang2025_Silkie_Wenchang	CRA004775	0.22349702
ENSGALG00010010752	ENSGALG00010010752	2	-0.504890651	0.205347807	-2.458709731	0.013943731	0	0.626621035	0	1	CRA004775;Yang2025_Silkie_Wenchang	PRJNA827465	0.193650332
ENSGALG00010010752	ENSGALG00010010752	2	-0.381856568	0.241352639	-1.582152032	0.113614868	0.03557021	1.218336019	17.92083754	1	CRA004775;PRJNA827465	Yang2025_Silkie_Wenchang	0.568794964
ENSGALG00010010753	ENSGALG00010010753	2	0.140218848	0.158525872	0.88451712	0.376417125	0	0.786344232	0	0.5	PRJNA827465;Yang2025_Silkie_Wenchang	CRA004775	0.822974013
ENSGALG00010010753	ENSGALG00010010753	2	0.235759285	0.304911853	0.773204722	0.439401232	0.099725103	2.055902011	51.35954951	0.5	CRA004775;Yang2025_Silkie_Wenchang	PRJNA827465	0.844199375
ENSGALG00010010753	ENSGALG00010010753	2	0.358212654	0.186097258	1.924867986	0.054245876	0	0.642858938	0	1	CRA004775;PRJNA827465	Yang2025_Silkie_Wenchang	0.403716278
ENSGALG00010010754	DNAI1	2	0.575229033	0.176778918	3.253945888	0.00113814	0	0.001763145	0	1	PRJNA827465;Yang2025_Silkie_Wenchang	CRA004775	0.040918564
ENSGALG00010010754	DNAI1	2	0.204691105	0.583518688	0.350787573	0.725747721	0.369457934	1.763068822	43.2807167	0.5	CRA004775;Yang2025_Silkie_Wenchang	PRJNA827465	0.931029023
ENSGALG00010010754	DNAI1	2	0.229891355	0.583638615	0.393893327	0.693659797	0.417968141	1.91512618	47.78411937	0.5	CRA004775;PRJNA827465	Yang2025_Silkie_Wenchang	0.920721644
ENSGALG00010010756	CENPL	2	-0.130419836	0.09721153	-1.341608712	0.1797229	0	0.01794141	0	1	PRJNA827465;Yang2025_Silkie_Wenchang	CRA004775	0.68659821
ENSGALG00010010756	CENPL	2	0.139343485	0.39361238	0.354011946	0.723329931	0.210064649	2.602046194	61.56870687	0.5	CRA004775;Yang2025_Silkie_Wenchang	PRJNA827465	0.930032096
ENSGALG00010010756	CENPL	2	0.140380751	0.374741964	0.374606434	0.707953187	0.194450083	2.562862276	60.98112609	0.5	CRA004775;PRJNA827465	Yang2025_Silkie_Wenchang	0.925061251
ENSGALG00010010759	ENSGALG00010010759	2	0.857227281	0.661764618	1.295365842	0.195194056	0.79054206	10.11820798	90.11682699	1	PRJNA827465;Yang2025_Silkie_Wenchang	CRA004775	0.70641915
ENSGALG00010010759	ENSGALG00010010759	2	0.684720961	0.958710265	0.714210525	0.47509701	1.539041266	5.801690549	82.76364464	0.5	CRA004775;Yang2025_Silkie_Wenchang	PRJNA827465	0.854462864
ENSGALG00010010759	ENSGALG00010010759	2	0.166283752	0.217319324	0.765158612	0.444177101	0	0.638162143	0	0.5	CRA004775;PRJNA827465	Yang2025_Silkie_Wenchang	0.846013379
ENSGALG00010010761	MEI1	2	0.342893581	0.284712138	1.204351821	0.228453625	0.080331777	1.951437861	48.75573443	1	PRJNA827465;Yang2025_Silkie_Wenchang	CRA004775	0.741262514
ENSGALG00010010761	MEI1	2	-0.226205554	0.974665818	-0.232085244	0.816471805	1.641172979	6.999292214	85.71284111	0.5	CRA004775;Yang2025_Silkie_Wenchang	PRJNA827465	0.95460349
ENSGALG00010010761	MEI1	2	-0.462447072	0.677767492	-0.682309314	0.495043385	0.703449785	3.783755624	73.57123188	0.5	CRA004775;PRJNA827465	Yang2025_Silkie_Wenchang	0.860496668
ENSGALG00010010762	MRPL39	2	-0.086024353	0.062655227	-1.372979659	0.16975867	0	0.000164261	0	1	PRJNA827465;Yang2025_Silkie_Wenchang	CRA004775	0.671232541
ENSGALG00010010762	MRPL39	2	-0.063937059	0.123053683	-0.519586716	0.603351659	0	0.109445376	0	0.5	CRA004775;Yang2025_Silkie_Wenchang	PRJNA827465	0.895538966
ENSGALG00010010762	MRPL39	2	-0.080022805	0.068129641	-1.174566664	0.240168127	0	0.133806961	0	0.5	CRA004775;PRJNA827465	Yang2025_Silkie_Wenchang	0.75252297
ENSGALG00010010766	CENPO	2	0.054505895	0.0958078	0.568908744	0.569418071	0	0.292052336	0	1	PRJNA827465;Yang2025_Silkie_Wenchang	CRA004775	0.884818733
ENSGALG00010010766	CENPO	2	0.097611876	0.172195904	0.566865257	0.57080573	0	0.406026587	0	0.5	CRA004775;Yang2025_Silkie_Wenchang	PRJNA827465	0.885224727
ENSGALG00010010766	CENPO	2	0.012118574	0.109204518	0.11097136	0.911639058	0	0.183652608	0	0.5	CRA004775;PRJNA827465	Yang2025_Silkie_Wenchang	0.978895589
ENSGALG00010010768	ENSGALG00010010768	2	0.090336313	0.062882779	1.436582713	0.150836599	0	0.000434015	0	1	PRJNA827465;Yang2025_Silkie_Wenchang	CRA004775	0.642051813
ENSGALG00010010768	ENSGALG00010010768	2	0.012872625	0.188255734	0.068378395	0.94548442	0	0.252859593	0	0.5	CRA004775;Yang2025_Silkie_Wenchang	PRJNA827465	0.986321197
ENSGALG00010010768	ENSGALG00010010768	2	0.080911012	0.063367896	1.276845487	0.201656803	0	0.39967397	0	0.5	CRA004775;PRJNA827465	Yang2025_Silkie_Wenchang	0.714508754
ENSGALG00010010769	HEXB	2	0.038245942	0.105890155	0.361185062	0.717961108	0.009582105	1.6918439	40.89289207	0.5	PRJNA827465;Yang2025_Silkie_Wenchang	CRA004775	0.927927766
ENSGALG00010010769	HEXB	2	-0.098046549	0.129485226	-0.757202597	0.448928495	0	0.016248917	0	1	CRA004775;Yang2025_Silkie_Wenchang	PRJNA827465	0.847095089
ENSGALG00010010769	HEXB	2	0.109786704	0.092298796	1.189470607	0.234254531	0	0.429223631	0	0.5	CRA004775;PRJNA827465	Yang2025_Silkie_Wenchang	0.747435122
ENSGALG00010010771	ENSGALG00010010771	2	-0.219843122	0.192020689	-1.144892893	0.252253529	0.056114146	3.538256896	71.73749591	1	PRJNA827465;Yang2025_Silkie_Wenchang	CRA004775	0.762522722
ENSGALG00010010771	ENSGALG00010010771	2	-0.38064824	0.143609476	-2.65057885	0.008035397	0	0.351625925	0	1	CRA004775;Yang2025_Silkie_Wenchang	PRJNA827465	0.139297234
ENSGALG00010010771	ENSGALG00010010771	2	-0.096987485	0.073842002	-1.313446042	0.189032692	0.002821491	1.125193794	11.12642057	1	CRA004775;PRJNA827465	Yang2025_Silkie_Wenchang	0.698162702
ENSGALG00010010774	KPTN	2	0.013305446	0.347977271	0.038236538	0.969499089	0.217319871	9.562031595	89.54197139	0.5	PRJNA827465;Yang2025_Silkie_Wenchang	CRA004775	0.991572914
ENSGALG00010010774	KPTN	2	-0.013463796	0.440558382	-0.030560753	0.975619842	0.310558029	4.737115944	78.89010926	0.5	CRA004775;Yang2025_Silkie_Wenchang	PRJNA827465	0.992754467
ENSGALG00010010774	KPTN	2	-0.33754192	0.112729699	-2.994259046	0.002751123	0	0.252950608	0	1	CRA004775;PRJNA827465	Yang2025_Silkie_Wenchang	0.07160753
ENSGALG00010010776	GCAT	2	-0.21638107	0.115444137	-1.874335728	0.060884166	0.009727195	1.478140414	32.34742854	1	PRJNA827465;Yang2025_Silkie_Wenchang	CRA004775	0.427127227
ENSGALG00010010776	GCAT	2	-0.326712701	0.160281227	-2.038371598	0.041512784	0	0.600250274	0	1	CRA004775;Yang2025_Silkie_Wenchang	PRJNA827465	0.353189581
ENSGALG00010010776	GCAT	2	-0.129450819	0.098021522	-1.320636697	0.186622531	0	0.053035809	0	1	CRA004775;PRJNA827465	Yang2025_Silkie_Wenchang	0.695457578
ENSGALG00010010778	UPP2	2	-0.149518454	0.231630991	-0.645502804	0.518601402	0	0.480162639	0	0.5	PRJNA827465;Yang2025_Silkie_Wenchang	CRA004775	0.868600993
ENSGALG00010010778	UPP2	2	-0.038421223	0.28780682	-0.133496571	0.893800676	0	0.111338838	0	0.5	CRA004775;Yang2025_Silkie_Wenchang	PRJNA827465	0.974381724
ENSGALG00010010778	UPP2	2	-0.275213507	0.276353703	-0.995874141	0.319311301	0	0.027157784	0	1	CRA004775;PRJNA827465	Yang2025_Silkie_Wenchang	0.798079048
ENSGALG00010010779	DOCK2	2	-0.086257051	0.201670184	-0.427713456	0.668859752	0.048648187	2.44813378	59.15255906	0.5	PRJNA827465;Yang2025_Silkie_Wenchang	CRA004775	0.914377769
ENSGALG00010010779	DOCK2	2	-0.283619721	0.192106818	-1.476364677	0.13984602	0	0.134039808	0	1	CRA004775;Yang2025_Silkie_Wenchang	PRJNA827465	0.623665962
ENSGALG00010010779	DOCK2	2	0.080185517	0.151767154	0.528345662	0.597259445	0	0.116943248	0	0.5	CRA004775;PRJNA827465	Yang2025_Silkie_Wenchang	0.894372368
ENSGALG00010010780	PARG	2	-0.180667019	0.181664333	-0.994510131	0.319974576	0.06265173	19.58863663	94.89499949	1	PRJNA827465;Yang2025_Silkie_Wenchang	CRA004775	0.798348216
ENSGALG00010010780	PARG	2	-0.282354492	0.110653874	-2.551690979	0.010720155	0.014668109	2.23291361	55.21546397	1	CRA004775;Yang2025_Silkie_Wenchang	PRJNA827465	0.165128023
ENSGALG00010010780	PARG	2	-0.015977207	0.045081445	-0.354407611	0.723033431	0	0.817602213	0	1	CRA004775;PRJNA827465	Yang2025_Silkie_Wenchang	0.930032096
ENSGALG00010010781	NAPA	2	0.247079201	0.343564972	0.719162956	0.472040521	0.226930973	25.33165587	96.05237018	0.5	PRJNA827465;Yang2025_Silkie_Wenchang	CRA004775	0.853998373
ENSGALG00010010781	NAPA	2	0.2314592	0.39378617	0.587778895	0.556680712	0.275242215	8.600011389	88.37210842	0.5	CRA004775;Yang2025_Silkie_Wenchang	PRJNA827465	0.880358001
ENSGALG00010010781	NAPA	2	-0.091739443	0.051098075	-1.795360055	0.072596352	0	0.175078596	0	1	CRA004775;PRJNA827465	Yang2025_Silkie_Wenchang	0.465466124
ENSGALG00010010783	F2RL2	2	0.034129044	0.162674668	0.20979938	0.833824257	0	0.10228628	0	0.5	PRJNA827465;Yang2025_Silkie_Wenchang	CRA004775	0.957968425
ENSGALG00010010783	F2RL2	2	0.14500508	0.218092913	0.664877543	0.50612882	0	0.106953801	0	1	CRA004775;Yang2025_Silkie_Wenchang	PRJNA827465	0.863929781
ENSGALG00010010783	F2RL2	2	0.048228555	0.185174972	0.26044856	0.79451779	0	0.337584546	0	0.5	CRA004775;PRJNA827465	Yang2025_Silkie_Wenchang	0.948905846
ENSGALG00010010784	CCDC148	2	0.222632128	0.158427734	1.40525981	0.159944035	0.003044311	1.062462391	5.879021409	1	PRJNA827465;Yang2025_Silkie_Wenchang	CRA004775	0.655404348
ENSGALG00010010784	CCDC148	2	0.440847154	0.219229027	2.010897741	0.044336259	0	0.087392145	0	1	CRA004775;Yang2025_Silkie_Wenchang	PRJNA827465	0.363642137
ENSGALG00010010784	CCDC148	2	0.150292826	0.186648459	0.805218678	0.4206935	0	0.75376532	0	1	CRA004775;PRJNA827465	Yang2025_Silkie_Wenchang	0.839211146

ENSGALG00010010786	NABP1	2	0.351289129	0.122308694	2.872151749	0.004076871	0.015841823	2.077258793	51.85963331	1	PRJNA827465;Yang2025_Silkie_Wenchang	CRA004775	0.0925841
ENSGALG00010010786	NABP1	2	0.210989962	0.396816594	0.531706499	0.59492929	0.217682853	2.603502118	61.59019833	0.5	CRA004775;Yang2025_Silkie_Wenchang	PRJNA827465	0.893382718
ENSGALG00010010786	NABP1	2	0.152568103	0.215635595	0.707527449	0.479238761	0.044798932	1.341007269	25.4291887	0.5	CRA004775;PRJNA827465	Yang2025_Silkie_Wenchang	0.856035423
ENSGALG00010010789	ENSGALG00010010789	2	0.09748552	0.297532058	0.327647114	0.743178495	0.142794973	5.142649263	80.55476956	0.5	PRJNA827465;Yang2025_Silkie_Wenchang	CRA004775	0.93573824
ENSGALG00010010789	ENSGALG00010010789	2	-0.07087298	0.505851894	-0.140106186	0.888576093	0.442226375	7.194651955	86.100787	0.5	CRA004775;Yang2025_Silkie_Wenchang	PRJNA827465	0.973135755
ENSGALG00010010789	ENSGALG00010010789	2	-0.31063515	0.189485427	-1.639361688	0.101137956	0.022171691	1.339889108	25.36695805	1	CRA004775;PRJNA827465	Yang2025_Silkie_Wenchang	0.542813747
ENSGALG00010010791	H-10	2	-0.141939358	0.478764397	-0.296470162	0.766871051	0.413858944	10.08850717	90.08773069	0.5	PRJNA827465;Yang2025_Silkie_Wenchang	CRA004775	0.941375309
ENSGALG00010010791	H-10	2	-0.29738008	0.369472248	-0.804877989	0.420890091	0.189491711	3.23648232	69.10225667	0.5	CRA004775;Yang2025_Silkie_Wenchang	PRJNA827465	0.839239169
ENSGALG00010010791	H-10	2	0.273459113	0.140629954	1.94452964	0.051831615	0	0.373071235	0	1	CRA004775;PRJNA827465	Yang2025_Silkie_Wenchang	0.394912455
ENSGALG00010010792	XRCC6	2	-0.04998202	0.057849059	-0.864007485	0.387583779	0	0.441124904	0	0.5	PRJNA827465;Yang2025_Silkie_Wenchang	CRA004775	0.828026595
ENSGALG00010010792	XRCC6	2	-0.106471049	0.192608753	-0.552784063	0.580411283	0.038217255	1.814948042	44.90200398	0.5	CRA004775;Yang2025_Silkie_Wenchang	PRJNA827465	0.888433837
ENSGALG00010010792	XRCC6	2	-0.112697569	0.1088765	-1.035095444	0.30062434	0.009523003	1.233463367	18.92746659	1	CRA004775;PRJNA827465	Yang2025_Silkie_Wenchang	0.790594743
ENSGALG00010010796	CAVIN2	2	-0.081236969	0.069576378	-1.167594111	0.242970527	0.001307029	1.118043588	10.5580488	0.5	PRJNA827465;Yang2025_Silkie_Wenchang	CRA004775	0.7550024
ENSGALG00010010796	CAVIN2	2	0.075762187	0.118124525	0.641375591	0.521278687	0	0.597096133	0	1	CRA004775;Yang2025_Silkie_Wenchang	PRJNA827465	0.869518515
ENSGALG00010010796	CAVIN2	2	-0.01855394	0.175569961	-0.105678326	0.915837577	0.036478994	1.844116138	45.77348035	0.5	CRA004775;PRJNA827465	Yang2025_Silkie_Wenchang	0.980317634
ENSGALG00010010799	DARS2	2	-0.055610333	0.104325784	-0.533044954	0.594002462	0.01312577	2.485576181	59.76787968	0.5	PRJNA827465;Yang2025_Silkie_Wenchang	CRA004775	0.893224663
ENSGALG00010010799	DARS2	2	-0.027554115	0.128656948	-0.214167332	0.830416571	0.013149788	1.517094215	34.08451564	0.5	CRA004775;Yang2025_Silkie_Wenchang	PRJNA827465	0.957262428
ENSGALG00010010799	DARS2	2	-0.160447233	0.076106534	-2.108192609	0.035014332	0	0.099517929	0	1	CRA004775;PRJNA827465	Yang2025_Silkie_Wenchang	0.322438684
ENSGALG00010010800	BICRA	2	0.005033317	0.084300642	0.059706745	0.9523892	0.005332587	1.519962653	34.20890979	0.5	PRJNA827465;Yang2025_Silkie_Wenchang	CRA004775	0.987758913
ENSGALG00010010800	BICRA	2	-0.001400657	0.154173537	-0.009084939	0.992751367	0.023510318	1.874433342	46.65054353	0.5	CRA004775;Yang2025_Silkie_Wenchang	PRJNA827465	0.997869618
ENSGALG00010010800	BICRA	2	-0.074839235	0.07010582	-1.067518142	0.285737926	0	0.446068012	0	1	CRA004775;PRJNA827465	Yang2025_Silkie_Wenchang	0.78140535
ENSGALG00010010805	PTRHD1	2	0.135378467	0.138024355	0.98083028	0.326676443	0	8.00487E-05	0	1	PRJNA827465;Yang2025_Silkie_Wenchang	CRA004775	0.800028239
ENSGALG00010010805	PTRHD1	2	0.059488858	0.217833024	0.273093845	0.784781078	0	0.13105809	0	0.5	CRA004775;Yang2025_Silkie_Wenchang	PRJNA827465	0.946214033
ENSGALG00010010805	PTRHD1	2	0.105074279	0.140002426	0.750517562	0.452943051	0	0.205531404	0	0.5	CRA004775;PRJNA827465	Yang2025_Silkie_Wenchang	0.848618976
ENSGALG00010010809	CENPH	2	0.047277392	0.122455618	0.386077773	0.699439071	0	0.024279145	0	1	PRJNA827465;Yang2025_Silkie_Wenchang	CRA004775	0.922020923
ENSGALG00010010809	CENPH	2	0.170788773	0.205767739	0.830007529	0.406534527	0.032957441	1.51708723	34.08421216	1	CRA004775;Yang2025_Silkie_Wenchang	PRJNA827465	0.833618122
ENSGALG00010010809	CENPH	2	0.183353646	0.182374182	1.005370628	0.314718417	0.016562145	1.258471353	20.53851701	1	CRA004775;PRJNA827465	Yang2025_Silkie_Wenchang	0.796531448
ENSGALG00010010810	GALT	2	0.224299089	0.143645242	1.561479424	0.118410675	0	0.344779634	0	1	PRJNA827465;Yang2025_Silkie_Wenchang	CRA004775	0.578338927
ENSGALG00010010810	GALT	2	-0.00310303	0.375594831	-0.008261642	0.993408239	0.197335587	3.103378935	67.77705781	0.5	CRA004775;Yang2025_Silkie_Wenchang	PRJNA827465	0.997979742
ENSGALG00010010810	GALT	2	-0.066258919	0.283719947	-0.233536345	0.815344951	0.084077662	1.908661462	47.60726194	0.5	CRA004775;PRJNA827465	Yang2025_Silkie_Wenchang	0.953933933
ENSGALG00010010813	EHD2	2	0.176924713	0.379993737	0.465599025	0.641502541	0.224690694	3.744188107	73.29194016	0.5	PRJNA827465;Yang2025_Silkie_Wenchang	CRA004775	0.906634523
ENSGALG00010010813	EHD2	2	0.405689836	0.256977641	1.578697019	0.114405569	0	0.73678941	0	1	CRA004775;Yang2025_Silkie_Wenchang	PRJNA827465	0.57034745
ENSGALG00010010813	EHD2	2	-0.098217643	0.086735925	-1.132375583	0.257476566	0	0.923767054	0	0.5	CRA004775;PRJNA827465	Yang2025_Silkie_Wenchang	0.7650601
ENSGALG00010010816	AP2S1	2	-0.044259165	0.078879871	-0.561095807	0.574732226	0	0.172670191	0	1	PRJNA827465;Yang2025_Silkie_Wenchang	CRA004775	0.886144156
ENSGALG00010010816	AP2S1	2	-0.310542078	0.208698809	-1.487991613	0.136753106	0	0.716378701	0	1	CRA004775;Yang2025_Silkie_Wenchang	PRJNA827465	0.618582531
ENSGALG00010010816	AP2S1	2	-0.17533071	0.217441317	-0.806335762	0.420049276	0.060329966	2.132646099	53.10989478	1	CRA004775;PRJNA827465	Yang2025_Silkie_Wenchang	0.838603917
ENSGALG00010010821	RGS8	2	-0.249599841	0.287507237	-0.868151507	0.385311397	0	0.244308619	0	1	PRJNA827465;Yang2025_Silkie_Wenchang	CRA004775	0.826451313
ENSGALG00010010821	RGS8	2	0.03931215	0.52886227	0.074333436	0.940745073	0.226012583	1.676968135	40.36857473	0.5	CRA004775;Yang2025_Silkie_Wenchang	PRJNA827465	0.985565295
ENSGALG00010010821	RGS8	2	0.046524298	0.331591877	0.140305903	0.8884183	0.04891464	1.229848968	18.68920283	0.5	CRA004775;PRJNA827465	Yang2025_Silkie_Wenchang	0.973135755
ENSGALG00010010823	TMEFF2	2	-0.259042033	0.118736468	-2.181655196	0.02913499	0	0.582557731	0	1	PRJNA827465;Yang2025_Silkie_Wenchang	CRA004775	0.290337858
ENSGALG00010010823	TMEFF2	2	0.152332251	0.377099797	0.403957393	0.686244051	0.223104018	4.327613723	76.89257721	0.5	CRA004775;Yang2025_Silkie_Wenchang	PRJNA827465	0.919235539
ENSGALG00010010823	TMEFF2	2	0.074025458	0.469983869	0.157506381	0.87484578	0.375960121	6.435115079	84.46026236	0.5	CRA004775;PRJNA827465	Yang2025_Silkie_Wenchang	0.970159044
ENSGALG00010010824	PKP4	2	0.075560134	0.125917289	0.600077518	0.548454575	0.01884267	2.297847503	56.48101108	0.5	PRJNA827465;Yang2025_Silkie_Wenchang	CRA004775	0.878281266
ENSGALG00010010824	PKP4	2	0.16833626	0.12287733	1.369953752	0.170701339	0	0.619665323	0	1	CRA004775;Yang2025_Silkie_Wenchang	PRJNA827465	0.672386723
ENSGALG00010010824	PKP4	2	-0.01853714	0.078677796	-0.235608281	0.813736647	0	0.044117622	0	0.5	CRA004775;PRJNA827465	Yang2025_Silkie_Wenchang	0.953869515
ENSGALG00010010825	MAK	2	0.151960577	0.228783244	0.664212004	0.506554631	0	0.342513142	0	1	PRJNA827465;Yang2025_Silkie_Wenchang	CRA004775	0.863929781
ENSGALG00010010825	MAK	2	0.447072567	0.305145272	1.465113858	0.142889856	0	0.680645486	0	1	CRA004775;Yang2025_Silkie_Wenchang	PRJNA827465	0.629525841
ENSGALG00010010825	MAK	2	0.292832873	0.394280944	0.742701055	0.457662691	0.129818926	1.536880314	34.93312452	1	CRA004775;PRJNA827465	Yang2025_Silkie_Wenchang	0.849573219
ENSGALG00010010826	NCOA1	2	-0.072578383	0.222252156	-0.326558735	0.744001658	0.076621984	4.00339654	75.02121036	0.5	PRJNA827465;Yang2025_Silkie_Wenchang	CRA004775	0.935821071
ENSGALG00010010826	NCOA1	2	0.261279826	0.17097727	1.528155332	0.126473976	0	0.30429895	0	1	CRA004775;Yang2025_Silkie_Wenchang	PRJNA827465	0.597028533
ENSGALG00010010826	NCOA1	2	0.006713119	0.320014038	0.020977575	0.983263544	0.165276385	4.509168818	77.82296382	0.5	CRA004775;PRJNA827465	Yang2025_Silkie_Wenchang	0.995363468
ENSGALG00010010828	SPDL1	2	0.194006438	0.059797215	3.244405894	0.001176959	0	0.051305402	0	1	PRJNA827465;Yang2025_Silkie_Wenchang	CRA004775	0.041666301
ENSGALG00010010828	SPDL1	2	0.215239905	0.100473216	2.142261528	0.032172443	0	0.000934386	0	1	CRA004775;Yang2025_Silkie_Wenchang	PRJNA827465	0.30763011
ENSGALG00010010828	SPDL1	2	0.187113647	0.069991644	2.673371208	0.00750931	0	0.016622916	0	1	CRA004775;PRJNA827465	Yang2025_Silkie_Wenchang	0.133728332
ENSGALG00010010829	LAMB4	2	-0.34883495	0.108235163	-3.222935521	0.001268841	0	0.106696341	0	1	PRJNA827465;Yang2025_Silkie_Wenchang	CRA004775	0.043294215
ENSGALG00010010829	LAMB4	2	-0.368551583	0.122034548	-3.020059404	0.002527251	0	0.000289885	0	1	CRA004775;Yang2025_Silkie_Wenchang	PRJNA827465	0.067541822
ENSGALG00010010829	LAMB4	2	-0.291462263	0.216722454	-1.344864164	0.178669105	0	0.013630699	0	1	CRA004775;PRJNA827465	Yang2025_Silkie_Wenchang	0.685363246
ENSGALG00010010831	DESI1	2	-0.374390484	0.362462538	-1.032908081	0.301646917	0.252276565	24.74740775	95.95917273	1	PRJNA827465;Yang2025_Silkie_Wenchang	CRA004775	0.791273005
ENSGALG00010010831	DESI1	2	-0.575746726	0.183259663	-3.141699137	0.001679705	0.045628487	3.079483406	67.52702099	1	CRA004775;Yang2025_Silkie_Wenchang	PRJNA827465	0.05185975
ENSGALG00010010831	DESI1	2	-0.167339354	0.175926095	-0.951191201	0.341507324	0.048066364	4.034792803	75.21557986	1	CRA004775;PRJNA827465	Yang2025_Silkie_Wenchang	0.805758959
ENSGALG00010010834	KGD4	2	-0.087284157	0.144842561	-0.602614015	0.546765495	0	0.204142006	0	1	PRJNA827465;Yang2025_Silkie_Wenchang	CRA004775	0.878275987
ENSGALG00010010834	KGD4	2	-0.097617307	0.205734835	-0.47448118	0.635156816	0	0.345414884	0	0.5	CRA004775;Yang2025_Silkie_Wenchang	PRJNA827465	0.904051292
ENSGALG00010010834	KGD4	2	-0.016334396	0.161438874	-0.101180067	0.91940752	0	0.091648044	0	0.5	CRA004775;PRJNA827465	Yang2025_Silkie_Wenchang	0.981192162
ENSGALG00010010836	DIP2A	2	0.258630939	0.233036536	1.109830001	0.267072289	0.100701332	13.43684615	92.55777741	1	PRJNA827465;Yang2025_Silkie_Wenchang	CRA004775	0.771247817
ENSGALG00010010836	DIP2A	2	0.319857317	0.238209104	1.342758573	0.179350161	0.077145302	2.718813909	63.2192554	1	CRA004775;Yang2025_Silkie_Wenchang	PRJNA827465	0.686167565
ENSGALG00010010836	DIP2A	2	0.033819619	0.058608463	0.577043275	0.563910223	0	0.009396679	0	1	CRA004775;PRJNA827465	Yang2025_Silkie_Wenchang	0.882721184
ENSGALG00010010837	RBPJ	2	-0.099425445	0.073525658	-1.35225508	0.176293729	0	0.799935488	0	1	PRJNA827465;Yang2025_Silkie_Wenchang	CRA004775	0.681573129
ENSGALG00010010837	RBPJ	2	-0.191981258	0.126303915	-1.519994516	0.128512354	0	0.026898605	0	1	CRA004775;Yang2025_Silkie_Wenchang	PRJNA827465	0.601638797
ENSGALG00010010837	RBPJ	2	-0.059670935	0.085937123	-0.69435575	0.487459137	0	0.035420605	0	1	CRA004775;PRJNA827465	Yang2025_Silkie_Wenchang	0.85768885
ENSGALG00010010838	JAM2	2	-0.256115175	0.081269606	-3.151426305	0.001624752	0.001184558	1.080197046	7.424297862	1	PRJNA827465;Yang2025_Silkie_Wenchang	CRA004775	0.050963549
ENSGALG00010010838	JAM2	2	0.002228968	0.440600959	0.005058926	0.995963579	0.320744084	5.293666257	81.10950046	0.5	CRA004775;Yang2025_Silkie_Wenchang	PRJNA827465	0.99868243
ENSGALG00010010838	JAM2	2	0.067165069	0.345191899	0.194573131	0.845727139	0.18445451	3.709260801	73.04045054	0.5	CRA004775;PRJNA827465	Yang2025_Silkie_Wenchang	0.961182516
ENSGALG00010010840	RGS16	2	0.406576497	0.72363864	0.561850175	0.574218103	0.639979154	2.550863563	60.7975897	0.5	PRJNA827465;Yang2025_Silkie_Wenchang	CRA004775	0.886081433
ENSGALG00010010840	RGS16	2	-0.258717175	1.511616468	-0.171152657	0.864103732	3.877949975	6.523654775	84.67116924	0.5	CRA004775;Yang2025_Silkie_Wenchang	PRJNA827465	0.96655438
ENSGALG00010010840	RGS16	2	-0.871382578	0.766085986	-1.137447485	0.255351266	0.614115062	1.979237883	49.47550225	1	CRA004775;PRJNA827465	Yang2025_Silkie_Wenchang	0.764467569
ENSGALG00010010841	PMM1	2	0.173186398	0.231116457	0.749346888	0.453648154	0.079355744	3.668827924	72.74333872	0.5	PRJNA827465;Yang2025_Silkie_Wenchang	CRA004775	0.849044791
ENSGALG00010010841	PMM1	2	-0.401719995	0.324419653	-1.238272683	0.215614969	0.154513986	3.73141092	73.20048578	1	CRA004775;Yang2025_Silkie_Wenchang	PRJNA827465	0.729132243
ENSGALG00010010841	PMM1	2	-0.161276317	0.557903325	-0.28907574	0.772523418	0.582800746	15.26121224	93.44744058	0.5	CRA004775;PRJNA827465	Yang2025_Silkie_Wenchang	0.943066211
ENSGALG00010010843	ZNF395	2	-0.039847899	0.130699612	-0.304881542	0.760456374	0.022831956	2.806911844	64.37365847	0.5	PRJNA827465;Yang2025_Silkie_Wenchang	CRA004775	0.939327609
ENSGALG00010010843	ZNF395	2	0.130639337	0.115925155	1.126928292	0.2597728	0	0.023196881	0	1	CRA004775;Yang2025_Silkie_Wenchang	PRJNA827465	0.767047318
ENSGALG00010010843	ZNF395	2	-0.057138306	0.139120963	-0.410709538	0.681285533	0.021680767	1.87780231	46.74625786	0.5	CRA004775;PRJNA827465	Yang2025_Silkie_Wenchang	0.918242009
ENSGALG00010010844	CDC14B	2	0.076114729	0.064671999	1.176934846	0.239221512	0	0.070357183	0	1	PRJNA827465;Yang2025_Silkie_Wenchang	CRA004775	0.751401748
ENSGALG00010010844	CDC14B	2	0.104902377	0.113122574	0.927333716	0.353753294	0	0.712759576	0	1	CRA004775;Yang2025_Silkie_Wenchang	PRJNA827465	0.811318534
ENSGALG00010010844	CDC14B	2	0.104064388	0.069858439	1.489646632	0.136317174	0	0.580146078	0	1	CRA004775;PRJNA827465	Yang2025_Silkie_Wenchang	0.618139317
ENSGALG00010010845	FKRP	2	-0.153624157	0.155334451	-0.988989599	0.322668231	0.030010284	2.583313449	61.29002462	0.5	PRJNA827465;Yang2025_Silkie_Wenchang	CRA004775	0.798652022
ENSGALG00010010845	FKRP	2	-0.031856189	0.142837556	-0.223023902	0.823516904	0	0.645437261	0	0.5	CRA004775;Yang2025_Silkie_Wenchang	PRJNA827465	0.955934625
ENSGALG00010010845	FKRP	2	-0.28992892	0.108303056	-2.677015142	0.007428128	0	0.001386968	0	1	CRA004775;PRJNA827465	Yang2025_Silkie_Wenchang	0.132915249
ENSGALG00010010849	ACBD6	2	0.047920677	0.122948733	0.389761449	0.696712953	0.020271371	2.714819408	63.16513736	0.5	PRJNA827465;Yang2025_Silkie_Wenchang	CRA004775	0.921084006
ENSGALG00010010849	ACBD6	2	-0.077059149	0.302915102	-0.254391904	0.799192805	0.151818398	5.653602347	82.31216243	0.5	CRA004775;Yang2025_Silkie_Wenchang	PRJNA827465	0.949922217
ENSGALG00010010849	ACBD6	2	-0.166501194	0.166966474	-0.997213334	0.31866097	0.037763662	2.516733375	60.26595388	1	CRA004775;PRJNA827465	Yang2025_Silkie_Wenchang	0.797754958
ENSGALG00010010850	ENSGALG00010010850	2	-0.045341478	0.121096733	-0.374423629	0.708089166	0	0.909712561	0	0.5	PRJNA827465;Yang2025_Silkie_Wenchang	CRA004775	0.925061251
ENSGALG00010010850	ENSGALG00010010850	2	0.100564588	0.190953033	0.526645671	0.59843967	0	0.038412268	0	1	CRA004775;Yang2025_Silkie_Wenchang	PRJNA827465	0.894645663
ENSGALG00010010850	ENSGALG00010010850	2	-0.114204576	0.141583308	-0.806624576	0.41988281	0	0.086053342	0	0.5	CRA004775;PRJNA827465	Yang2025_Silkie_Wenchang	0.838584536
ENSGALG00010010851	PNOC	2	-0.959797902	0.187386679	-5.122017791	3.02283E-07	0	0.041295722	0	1	PRJNA827465;Yang2025_Silkie_Wenchang	CRA004775	8.42599E-05
ENSGALG00010010851	PNOC	2	-0.042063038	1.065009774	-0.039495448	0.968495383	1.74963058	3.79358512	73.63971103	0.5	CRA004775;Yang2025_Silkie_Wenchang	PRJNA827465	0.991215679
ENSGALG00010010851	PNOC	2	-0.091241722	1.106375951	-0.082469003	0.934273766	1.971871624	4.339295453	76.95478423	0.5	CRA004775;PRJNA827465	Yang2025_Silkie_Wenchang	0.98432801
ENSGALG00010010853	SMIM20	2	-0.293956943	0.234515402	-1.253465403	0.210036382	0.077501589	2.723677304	63.28493105	1	PRJNA827465;Yang2025_Silkie_Wenchang	CRA004775	0.721835135
ENSGALG00010010853	SMIM20	2	-0.318553606	0.346736964	-0.918718336	0.358242917	0.127278331	2.080152938	51.92661166	0.5	CRA004775;Yang2025_Silkie_Wenchang	PRJNA827465	0.813921114
ENSGALG00010010853	SMIM20	2	-0.117797131	0.075267347	-1.565049596	0.117571281	0	0.267474926	0	0.5	CRA004775;PRJNA827465	Yang2025_Silkie_Wenchang	0.577165052
ENSGALG00010010855	ATP5PF	2	-0.404997706	0.320681096	-1.26292978	0.206614417	0.167546722	4.619107642	78.35079679	1	PRJNA827465;Yang2025_Silkie_Wenchang	CRA004775	0.71859917
ENSGALG00010010855	ATP5PF	2	-0.386433652	0.417352302	-0.925917144	0.354489041	0.25375122	3.678350631	72.8139022	0.5	CRA004775;Yang2025_Silkie_Wenchang	PRJNA827465	0.81193713
ENSGALG00010010855	ATP5PF	2	-0.134234655	0.065636686	-2.045116283	0.040843395	0	0.308976758	0	0.5	CRA004775;PRJNA827465	Yang2025_Silkie_Wenchang	0.349655406
ENSGALG00010010857	SEL1L3	2	0.088516838	0.065724079	1.34679465	0.178046378	0	0.0176388	0	1	PRJNA827465;Yang2025_Silkie_Wenchang	CRA004775	0.684191679
ENSGALG00010010857	SEL1L3	2	0.132726649	0.094489297	1.404673903	0.160118269	0	0.296589452	0	1	CRA004775;Yang2025_Silkie_Wenchang	PRJNA827465	0.655509678
ENSGALG00010010857	SEL1L3	2	0.114588896	0.063028886	1.818037781	0.069058358	0	0.333748739	0	1	CRA004775;PRJNA827465	Yang2025_Silkie_Wenchang	0.452607154
ENSGALG00010010859	ENSGALG00010010859	2	-0.302936247	0.241029689	-1.256842044	0.208810838	0.085433337	3.454020412	71.04823132	1	PRJNA827465;Yang2025_Silkie_Wenchang	CRA004775	0.721011841
ENSGALG00010010859	ENSGALG00010010859	2	-0.103992655	0.534890408	-0.194418621	0.845848111	0.468505545	5.32645656	81.22579263	0.5	CRA004775;Yang2025_Silkie_Wenchang	PRJNA827465	0.96122283
ENSGALG00010010859	ENSGALG00010010859	2	0.063166132	0.263275571	0.239924014	0.810389164	0.084120721	1.975842488	49.3886782	0.5	CRA004775;PRJNA827465	Yang2025_Silkie_Wenchang	0.952997426
ENSGALG00010010861	ENSGALG00010010861	2	0.25084653	0.197365105	1.270977102	0.203736795	0.028237991	1.568263963	36.23522419	1	PRJNA827465;Yang2025_Silkie_Wenchang	CRA004775	0.717254253
ENSGALG00010010861	ENSGALG00010010861	2	0.382747837	0.20014934	1.912311263	0.055836282	0	0.432374141	0	1	CRA004775;Yang2025_Silkie_Wenchang	PRJNA827465	0.408997088
ENSGALG00010010861	ENSGALG00010010861	2	0.072275129	0.19587411	0.368987657	0.712136925	0	0.02373921	0	1	CRA004775;PRJNA827465	Yang2025_Silkie_Wenchang	0.92597752
ENSGALG00010010862	STRN4	2	0.129575888	0.19341368	0.669941692	0.502894961	0.065993839	8.367879739	88.04954145	0.5	PRJNA827465;Yang2025_Silkie_Wenchang	CRA004775	0.863050365
ENSGALG00010010862	STRN4	2	-0.057565251	0.394945472	-0.145754934	0.884114853	0.295097722	18.41940418	94.57094274	0.5	CRA004775;Yang2025_Silkie_Wenchang	PRJNA827465	0.972001076
ENSGALG00010010862	STRN4	2	-0.237438978	0.200429463	-1.184651069	0.236155436	0.067347332	5.886518193	83.01202906	1	CRA004775;PRJNA827465	Yang2025_Silkie_Wenchang	0.748669015
ENSGALG00010010865	ENSGALG00010010865	2	-0.018243916	0.040599174	-0.449366672	0.653167169	0	0.630906844	0	0.5	PRJNA827465;Yang2025_Silkie_Wenchang	CRA004775	0.909597225
ENSGALG00010010865	ENSGALG00010010865	2	0.038078332	0.074427126	0.511619007	0.608917681	0	0.037186891	0	1	CRA004775;Yang2025_Silkie_Wenchang	PRJNA827465	0.897208691
ENSGALG00010010865	ENSGALG00010010865	2	-0.034404377	0.046997026	-0.732054348	0.464135396	0	0.208514239	0	0.5	CRA004775;PRJNA827465	Yang2025_Silkie_Wenchang	0.852159649
ENSGALG00010010867	QSOX1	2	-0.057149882	0.080104028	-0.713445799	0.475569941	0	0.083980136	0	1	PRJNA827465;Yang2025_Silkie_Wenchang	CRA004775	0.854526154
ENSGALG00010010867	QSOX1	2	-0.037882963	0.159944781	-0.23685026	0.812772959	0	0.115719973	0	1	CRA004775;Yang2025_Silkie_Wenchang	PRJNA827465	0.95348724
ENSGALG00010010867	QSOX1	2	-0.07340556	0.086983666	-0.843900512	0.398725002	0	0.045148585	0	1	CRA004775;PRJNA827465	Yang2025_Silkie_Wenchang	0.832054308
ENSGALG00010010868	SLC39A10	2	0.010378867	0.078174873	0.132764743	0.894379439	0	0.104599408	0	0.5	PRJNA827465;Yang2025_Silkie_Wenchang	CRA004775	0.974381724
ENSGALG00010010868	SLC39A10	2	-0.063429731	0.14680906	-0.432055974	0.665700732	0	0.189873504	0	1	CRA004775;Yang2025_Silkie_Wenchang	PRJNA827465	0.913532078
ENSGALG00010010868	SLC39A10	2	0.01108097	0.086864822	0.127565678	0.898492694	0	0.387509176	0	0.5	CRA004775;PRJNA827465	Yang2025_Silkie_Wenchang	0.97575249
ENSGALG00010010869	ENSGALG00010010869	2	-0.241158492	0.127915954	-1.885288615	0.059390882	0	0.285786663	0	1	PRJNA827465;Yang2025_Silkie_Wenchang	CRA004775	0.42233397
ENSGALG00010010869	ENSGALG00010010869	2	0.599094194	0.990315658	0.604952764	0.545210383	1.836273635	15.45698066	93.53043119	0.5	CRA004775;Yang2025_Silkie_Wenchang	PRJNA827465	0.877422147
ENSGALG00010010869	ENSGALG00010010869	2	0.66358064	0.910097874	0.729131073	0.465921489	1.556289653	15.90871284	93.71413634	0.5	CRA004775;PRJNA827465	Yang2025_Silkie_Wenchang	0.852511753
ENSGALG00010010870	NOL6	2	-0.010315607	0.128016615	-0.080580217	0.9357758	0	0.110082652	0	0.5	PRJNA827465;Yang2025_Silkie_Wenchang	CRA004775	0.984801197
ENSGALG00010010870	NOL6	2	-0.104858037	0.135441798	-0.774192594	0.438816907	0	0.454169715	0	1	CRA004775;Yang2025_Silkie_Wenchang	PRJNA827465	0.844002503
ENSGALG00010010870	NOL6	2	-0.075791455	0.157664431	-0.480713718	0.63071998	0	0.794722061	0	0.5	CRA004775;PRJNA827465	Yang2025_Silkie_Wenchang	0.902770945
ENSGALG00010010874	ENSGALG00010010874	2	0.074000235	0.363545315	0.203551611	0.83870391	0.204086874	4.341117472	76.96445659	0.5	PRJNA827465;Yang2025_Silkie_Wenchang	CRA004775	0.959277608
ENSGALG00010010874	ENSGALG00010010874	2	-0.320571379	0.234664579	-1.366083369	0.171912798	0	0.004734168	0	1	CRA004775;Yang2025_Silkie_Wenchang	PRJNA827465	0.674589525
ENSGALG00010010874	ENSGALG00010010874	2	0.135992201	0.368727397	0.368815016	0.712265611	0.169120008	2.374710081	57.88959638	0.5	CRA004775;PRJNA827465	Yang2025_Silkie_Wenchang	0.92597752
ENSGALG00010010876	ENSGALG00010010876	2	-0.005481344	0.098568761	-0.055609343	0.955653021	0	0.03574638	0	0.5	PRJNA827465;Yang2025_Silkie_Wenchang	CRA004775	0.988317827
ENSGALG00010010876	ENSGALG00010010876	2	-0.087012298	0.165499872	-0.525754475	0.599058809	0	0.308002281	0	1	CRA004775;Yang2025_Silkie_Wenchang	PRJNA827465	0.894908299
ENSGALG00010010876	ENSGALG00010010876	2	-0.020684515	0.109361618	-0.18913871	0.849984102	0	0.515006172	0	0.5	CRA004775;PRJNA827465	Yang2025_Silkie_Wenchang	0.961869125
ENSGALG00010010880	ELP3	2	-0.221819629	0.047655483	-4.654650716	3.2453E-06	0	0.170938677	0	1	PRJNA827465;Yang2025_Silkie_Wenchang	CRA004775	0.000604503
ENSGALG00010010880	ELP3	2	-0.218334554	0.077085601	-2.832364951	0.004620508	0	0.693516785	0	1	CRA004775;Yang2025_Silkie_Wenchang	PRJNA827465	0.099889892
ENSGALG00010010880	ELP3	2	-0.245995649	0.05640672	-4.361105363	1.29407E-05	0	0.455179807	0	1	CRA004775;PRJNA827465	Yang2025_Silkie_Wenchang	0.001735865
ENSGALG00010010882	CEP350	2	-0.052623558	0.109167872	-0.482042535	0.629775731	0.008280763	1.358425957	26.38538782	0.5	PRJNA827465;Yang2025_Silkie_Wenchang	CRA004775	0.902457014
ENSGALG00010010882	CEP350	2	0.030605514	0.144302162	0.212093247	0.832034288	0	0.605873699	0	0.5	CRA004775;Yang2025_Silkie_Wenchang	PRJNA827465	0.957762335
ENSGALG00010010882	CEP350	2	-0.112803344	0.081184315	-1.389472135	0.164689229	0	0.011904178	0	1	CRA004775;PRJNA827465	Yang2025_Silkie_Wenchang	0.663901457
ENSGALG00010010885	SNRPD2	2	0.092375496	0.230455257	0.400839181	0.68853853	0.084939962	4.745554085	78.92764507	0.5	PRJNA827465;Yang2025_Silkie_Wenchang	CRA004775	0.919798626
ENSGALG00010010885	SNRPD2	2	0.063312123	0.31290577	0.202336067	0.839654006	0.145605462	3.820423845	73.82489377	0.5	CRA004775;Yang2025_Silkie_Wenchang	PRJNA827465	0.959493354
ENSGALG00010010885	SNRPD2	2	-0.134857192	0.095046302	-1.418857857	0.155940461	0	0.342211561	0	1	CRA004775;PRJNA827465	Yang2025_Silkie_Wenchang	0.649132948
ENSGALG00010010887	GABPA	2	-0.125395418	0.160095415	-0.783254272	0.433477811	0.049384632	27.28914289	96.33553899	0.5	PRJNA827465;Yang2025_Silkie_Wenchang	CRA004775	0.842762754
ENSGALG00010010887	GABPA	2	-0.024256358	0.288222985	-0.084158305	0.932930569	0.149645744	9.320652028	89.27113686	0.5	CRA004775;Yang2025_Silkie_Wenchang	PRJNA827465	0.984224753
ENSGALG00010010887	GABPA	2	0.100867016	0.11346548	0.88896655	0.374021057	0.015834542	1.89666384	47.2758441	1	CRA004775;PRJNA827465	Yang2025_Silkie_Wenchang	0.822579781
ENSGALG00010010888	GNPAT	2	-0.127174164	0.111355554	-1.142054974	0.25343117	0.018444237	3.692940424	72.921307	1	PRJNA827465;Yang2025_Silkie_Wenchang	CRA004775	0.763206288
ENSGALG00010010888	GNPAT	2	-0.243118302	0.097103954	-2.503691055	0.01229053	0	0.156790269	0	1	CRA004775;Yang2025_Silkie_Wenchang	PRJNA827465	0.179174596
ENSGALG00010010888	GNPAT	2	-0.033286018	0.056686874	-0.587190918	0.557075491	0	0.079202171	0	1	CRA004775;PRJNA827465	Yang2025_Silkie_Wenchang	0.880615293
ENSGALG00010010889	CCL19	2	-0.001059383	0.292877703	-0.003617153	0.997113936	0	0.301210828	0	0.5	PRJNA827465;Yang2025_Silkie_Wenchang	CRA004775	0.999096725
ENSGALG00010010889	CCL19	2	-0.173814694	0.429323579	-0.404857087	0.685582563	0	0.061426267	0	1	CRA004775;Yang2025_Silkie_Wenchang	PRJNA827465	0.919235539
ENSGALG00010010889	CCL19	2	0.067556106	0.305738217	0.220960621	0.825123095	0	0.036749124	0	0.5	CRA004775;PRJNA827465	Yang2025_Silkie_Wenchang	0.956321341
ENSGALG00010010890	NOL12	2	-0.094795207	0.171143867	-0.553891932	0.579652807	0.043212605	3.469569552	71.17798087	0.5	PRJNA827465;Yang2025_Silkie_Wenchang	CRA004775	0.887983094
ENSGALG00010010890	NOL12	2	-0.142538332	0.180552129	-0.78945805	0.429844339	0.029048233	1.780941224	43.84991561	0.5	CRA004775;Yang2025_Silkie_Wenchang	PRJNA827465	0.842012722
ENSGALG00010010890	NOL12	2	0.04863938	0.073480137	0.661939148	0.508010221	0	0.004672838	0	1	CRA004775;PRJNA827465	Yang2025_Silkie_Wenchang	0.864530233
ENSGALG00010010891	POLR3H	2	0.003112353	0.129898885	0.023959816	0.980884662	0.021307833	2.363865083	57.69640124	0.5	PRJNA827465;Yang2025_Silkie_Wenchang	CRA004775	0.994928779
ENSGALG00010010891	POLR3H	2	-0.088095105	0.29376919	-0.299878638	0.76426973	0.131118979	4.044806639	75.27693932	0.5	CRA004775;Yang2025_Silkie_Wenchang	PRJNA827465	0.940483208
ENSGALG00010010891	POLR3H	2	-0.165934869	0.133893408	-1.239305737	0.215232295	0.01848453	1.573694326	36.45525797	1	CRA004775;PRJNA827465	Yang2025_Silkie_Wenchang	0.728664095
ENSGALG00010010893	CCL21	2	-0.818524166	0.329163915	-2.486676485	0.01289426	0.099276742	1.834237002	45.48141822	1	PRJNA827465;Yang2025_Silkie_Wenchang	CRA004775	0.184486016
ENSGALG00010010893	CCL21	2	-0.751116015	0.584997494	-1.283964501	0.199154377	0.386023834	2.044082758	51.07830168	0.5	CRA004775;Yang2025_Silkie_Wenchang	PRJNA827465	0.71096127
ENSGALG00010010893	CCL21	2	-0.434966494	0.293361608	-1.482697399	0.138154826	0	0.460530614	0	0.5	CRA004775;PRJNA827465	Yang2025_Silkie_Wenchang	0.621483246
ENSGALG00010010894	SPAG1	2	0.30725144	0.099179918	3.097919871	0.001948841	0	0.478558017	0	1	PRJNA827465;Yang2025_Silkie_Wenchang	CRA004775	0.057160674
ENSGALG00010010894	SPAG1	2	0.157959256	0.175374826	0.900695151	0.367750427	0	0.022029761	0	1	CRA004775;Yang2025_Silkie_Wenchang	PRJNA827465	0.820067804
ENSGALG00010010894	SPAG1	2	0.316941705	0.105137276	3.014551222	0.002573598	0	0.465397052	0	1	CRA004775;PRJNA827465	Yang2025_Silkie_Wenchang	0.06852988
ENSGALG00010010896	LY86	2	-0.084890369	0.368402045	-0.230428604	0.817758736	0.241122465	8.838317431	88.68562927	0.5	PRJNA827465;Yang2025_Silkie_Wenchang	CRA004775	0.954967428
ENSGALG00010010896	LY86	2	-0.49378886	0.187320731	-2.636060921	0.008387467	0	0.095966301	0	1	CRA004775;Yang2025_Silkie_Wenchang	PRJNA827465	0.142771619
ENSGALG00010010896	LY86	2	-0.06735352	0.43378302	-0.155270069	0.876608411	0.281468646	3.349404329	70.14394496	0.5	CRA004775;PRJNA827465	Yang2025_Silkie_Wenchang	0.970434621
ENSGALG00010010897	CEP170	2	-0.047009413	0.053412915	-0.88011323	0.378797973	0	0.06935961	0	1	PRJNA827465;Yang2025_Silkie_Wenchang	CRA004775	0.823876439
ENSGALG00010010897	CEP170	2	0.225087652	0.330045523	0.681989713	0.495245456	0.178555269	5.491093057	81.78868962	0.5	CRA004775;Yang2025_Silkie_Wenchang	PRJNA827465	0.860619541
ENSGALG00010010897	CEP170	2	0.226076648	0.304117296	0.743386354	0.457247804	0.161430261	7.108412098	85.93216057	0.5	CRA004775;PRJNA827465	Yang2025_Silkie_Wenchang	0.849541335
ENSGALG00010010904	TOR1AIP1	2	-0.172492179	0.101951602	-1.691902597	0.090664547	0.013839342	2.782684749	64.06348221	1	PRJNA827465;Yang2025_Silkie_Wenchang	CRA004775	0.516732697
ENSGALG00010010904	TOR1AIP1	2	-0.298800137	0.103705161	-2.881246537	0.003961057	0	0.00402846	0	1	CRA004775;Yang2025_Silkie_Wenchang	PRJNA827465	0.090723435
ENSGALG00010010904	TOR1AIP1	2	-0.09616096	0.058769125	-1.636249653	0.101787348	0	0.477639819	0	1	CRA004775;PRJNA827465	Yang2025_Silkie_Wenchang	0.543700483
ENSGALG00010010905	ENC1	2	-0.00817012	0.154045375	-0.053037099	0.957702349	0.031360774	2.929918934	65.86936285	0.5	PRJNA827465;Yang2025_Silkie_Wenchang	CRA004775	0.988798051
ENSGALG00010010905	ENC1	2	-0.105689426	0.158101842	-0.668489532	0.503821162	0.010352078	1.115383479	10.34473626	0.5	CRA004775;Yang2025_Silkie_Wenchang	PRJNA827465	0.86324419
ENSGALG00010010905	ENC1	2	0.148262493	0.111865535	1.325363464	0.185050629	0	0.110657557	0	1	CRA004775;PRJNA827465	Yang2025_Silkie_Wenchang	0.693265167
ENSGALG00010010906	ATP6V1C1	2	-0.076835014	0.139125994	-0.552269293	0.580763866	0.031706749	5.272768755	81.03463197	0.5	PRJNA827465;Yang2025_Silkie_Wenchang	CRA004775	0.888579914
ENSGALG00010010906	ATP6V1C1	2	-0.161789922	0.150310049	-1.07637462	0.281759748	0.015947341	1.256580491	20.41894593	0.5	CRA004775;Yang2025_Silkie_Wenchang	PRJNA827465	0.779295548
ENSGALG00010010906	ATP6V1C1	2	0.051992117	0.05736411	0.906352714	0.364749193	0	0.114725897	0	1	CRA004775;PRJNA827465	Yang2025_Silkie_Wenchang	0.81764316
ENSGALG00010010908	STK17B	2	-0.091548214	0.155566387	-0.588483261	0.556207966	0.038734161	4.975051182	79.89970427	0.5	PRJNA827465;Yang2025_Silkie_Wenchang	CRA004775	0.880324407
ENSGALG00010010908	STK17B	2	-0.24969808	0.103479777	-2.413013326	0.015821241	0	0.01444029	0	1	CRA004775;Yang2025_Silkie_Wenchang	PRJNA827465	0.208176177
ENSGALG00010010908	STK17B	2	0.042809807	0.085859147	0.498605086	0.618057622	0	0.515630931	0	0.5	CRA004775;PRJNA827465	Yang2025_Silkie_Wenchang	0.899121942
ENSGALG00010010909	SLIT3	2	-0.153169181	0.140833987	-1.087586766	0.276777576	0	0.148616819	0	1	PRJNA827465;Yang2025_Silkie_Wenchang	CRA004775	0.775940673
ENSGALG00010010909	SLIT3	2	-0.305776081	0.230483294	-1.326673511	0.184616707	0	0.409763139	0	1	CRA004775;Yang2025_Silkie_Wenchang	PRJNA827465	0.692938714
ENSGALG00010010909	SLIT3	2	-0.162354721	0.160985431	-1.008505676	0.313211764	0	0.78665592	0	1	CRA004775;PRJNA827465	Yang2025_Silkie_Wenchang	0.796531448
ENSGALG00010010912	BAALC	2	0.866225221	0.528874929	1.637864027	0.101450062	0.47486155	6.57776044	84.79725723	1	PRJNA827465;Yang2025_Silkie_Wenchang	CRA004775	0.54352396

ENSGALG00010010912	BAALC	2	0.322069876	1.17951276	0.27305332	0.784812228	2.476903127	8.672298302	88.46903133	0.5	CRA004775;Yang2025_Silkie_Wenchang	PRJNA827465	0.946214033
ENSGALG00010010912	BAALC	2	-0.11819539	0.628257919	-0.188131954	0.850773213	0.550633842	2.806357909	64.36662633	0.5	CRA004775;PRJNA827465	Yang2025_Silkie_Wenchang	0.962049072
ENSGALG00010010913	IFT56	2	0.033103465	0.087772921	0.377148944	0.70606292	0.005725086	1.571484817	36.365914	0.5	PRJNA827465;Yang2025_Silkie_Wenchang	CRA004775	0.924548793
ENSGALG00010010913	IFT56	2	-0.083921385	0.107730218	-0.778995774	0.435982204	0	0.333416322	0	1	CRA004775;Yang2025_Silkie_Wenchang	PRJNA827465	0.843014708
ENSGALG00010010913	IFT56	2	0.092501563	0.086240747	1.072596957	0.283451991	0	0.943473724	0	0.5	CRA004775;PRJNA827465	Yang2025_Silkie_Wenchang	0.779568117
ENSGALG00010010915	APP	2	-0.051281269	0.043402027	-1.181540867	0.237387929	0	0.541249365	0	1	PRJNA827465;Yang2025_Silkie_Wenchang	CRA004775	0.749366344
ENSGALG00010010915	APP	2	0.011993227	0.182354103	0.065768891	0.947561824	0.039793713	2.005667387	50.14128432	0.5	CRA004775;Yang2025_Silkie_Wenchang	PRJNA827465	0.986560458
ENSGALG00010010915	APP	2	0.022812207	0.122421823	0.186341016	0.852177345	0.015599619	1.421528274	29.65317551	0.5	CRA004775;PRJNA827465	Yang2025_Silkie_Wenchang	0.962390845
ENSGALG00010010917	TANC1	2	0.197137779	0.20573956	0.958190924	0.337966493	0.076268529	9.618890077	89.60379013	1	PRJNA827465;Yang2025_Silkie_Wenchang	CRA004775	0.80415385
ENSGALG00010010917	TANC1	2	0.360972111	0.106772424	3.380761586	0.000722852	0	0.896163749	0	1	CRA004775;Yang2025_Silkie_Wenchang	PRJNA827465	0.030107556
ENSGALG00010010917	TANC1	2	0.01539185	0.049546129	0.310656952	0.756061426	0	0.643502632	0	1	CRA004775;PRJNA827465	Yang2025_Silkie_Wenchang	0.938524556
ENSGALG00010010918	TOR1AIP2	2	-0.018644321	0.159496165	-0.116895105	0.906943177	0.041592186	5.362540851	81.35212341	0.5	PRJNA827465;Yang2025_Silkie_Wenchang	CRA004775	0.978196186
ENSGALG00010010918	TOR1AIP2	2	-0.203844986	0.103242937	-1.974420635	0.048333934	0	0.082527034	0	1	CRA004775;Yang2025_Silkie_Wenchang	PRJNA827465	0.38014722
ENSGALG00010010918	TOR1AIP2	2	-0.003084819	0.187087591	-0.016488634	0.98684457	0.046768152	2.478367461	59.65085824	0.5	CRA004775;PRJNA827465	Yang2025_Silkie_Wenchang	0.996325416
ENSGALG00010010920	UBN2	2	0.199728272	0.204039013	0.978872956	0.327642756	0.064670635	4.024978091	75.15514427	1	PRJNA827465;Yang2025_Silkie_Wenchang	CRA004775	0.80013317
ENSGALG00010010920	UBN2	2	0.258204546	0.203911037	1.266260772	0.205419737	0.03941437	1.887153084	47.01012819	1	CRA004775;Yang2025_Silkie_Wenchang	PRJNA827465	0.71825498
ENSGALG00010010920	UBN2	2	0.030170346	0.072772675	0.414583444	0.67844687	0	0.000495365	0	1	CRA004775;PRJNA827465	Yang2025_Silkie_Wenchang	0.917701868
ENSGALG00010010921	ENSGALG00010010921	2	0.118611904	0.076054636	1.559561784	0.118863473	0	0.56793064	0	1	PRJNA827465;Yang2025_Silkie_Wenchang	CRA004775	0.579183126
ENSGALG00010010921	ENSGALG00010010921	2	-0.010806484	0.305659276	-0.035354675	0.971796926	0.126158539	3.035621903	67.05782104	0.5	CRA004775;Yang2025_Silkie_Wenchang	PRJNA827465	0.991955348
ENSGALG00010010921	ENSGALG00010010921	2	-0.042316163	0.204600852	-0.206823006	0.836148088	0.054663544	2.300074918	56.5231553	0.5	CRA004775;PRJNA827465	Yang2025_Silkie_Wenchang	0.958399882
ENSGALG00010010922	DYM	2	-0.118426234	0.048936043	-2.42002062	0.015519627	0	0.36768559	0	1	PRJNA827465;Yang2025_Silkie_Wenchang	CRA004775	0.206070237
ENSGALG00010010922	DYM	2	-0.087575949	0.081068417	-1.080272103	0.280021029	0	0.193031556	0	1	CRA004775;Yang2025_Silkie_Wenchang	PRJNA827465	0.778098827
ENSGALG00010010922	DYM	2	-0.142155694	0.058368026	-2.435506294	0.014870966	0	0.048087547	0	1	CRA004775;PRJNA827465	Yang2025_Silkie_Wenchang	0.201542611
ENSGALG00010010923	F13A1	2	-0.236036304	0.307987913	-0.766381713	0.443449211	0.161065507	6.551181257	84.73557728	0.5	PRJNA827465;Yang2025_Silkie_Wenchang	CRA004775	0.845733906
ENSGALG00010010923	F13A1	2	-0.770962325	0.369797799	-2.084843641	0.037083478	0.14478902	1.656435581	39.62940596	1	CRA004775;Yang2025_Silkie_Wenchang	PRJNA827465	0.332314015
ENSGALG00010010923	F13A1	2	-0.546806897	0.724084889	-0.755169601	0.450147226	0.87027856	5.100911225	80.3956596	0.5	CRA004775;PRJNA827465	Yang2025_Silkie_Wenchang	0.847564661
ENSGALG00010010924	ENSGALG00010010924	2	0.18332993	0.077929053	2.352523512	0.018646511	0	0.007445338	0	1	PRJNA827465;Yang2025_Silkie_Wenchang	CRA004775	0.228477771
ENSGALG00010010924	ENSGALG00010010924	2	0.144227643	0.103173714	1.397910737	0.162139874	0	0.96774145	0	0.5	CRA004775;Yang2025_Silkie_Wenchang	PRJNA827465	0.659645143
ENSGALG00010010924	ENSGALG00010010924	2	0.136922729	0.098543739	1.389456901	0.164691858	0	0.889190544	0	0.5	CRA004775;PRJNA827465	Yang2025_Silkie_Wenchang	0.663901457
ENSGALG00010010928	HECW2	2	0.075658709	0.09203742	0.822042914	0.411052473	0.004508381	1.256285417	20.40025407	1	PRJNA827465;Yang2025_Silkie_Wenchang	CRA004775	0.835128673
ENSGALG00010010928	HECW2	2	0.158538966	0.149546003	1.060135092	0.289083145	0	0.903173648	0	0.5	CRA004775;Yang2025_Silkie_Wenchang	PRJNA827465	0.783530627
ENSGALG00010010928	HECW2	2	0.012868168	0.080593772	0.15966703	0.873143374	0	0.170459732	0	0.5	CRA004775;PRJNA827465	Yang2025_Silkie_Wenchang	0.969794665
ENSGALG00010010929	FMC1	2	-0.096855897	0.117678173	-0.823057452	0.410475323	0	0.505994944	0	1	PRJNA827465;Yang2025_Silkie_Wenchang	CRA004775	0.834820782
ENSGALG00010010929	FMC1	2	-0.135161973	0.257735043	-0.52442218	0.599984933	0	0.574325612	0	0.5	CRA004775;Yang2025_Silkie_Wenchang	PRJNA827465	0.895092498
ENSGALG00010010929	FMC1	2	-0.060090826	0.115298473	-0.52117625	0.602234997	0	0.073125656	0	0.5	CRA004775;PRJNA827465	Yang2025_Silkie_Wenchang	0.895349419
ENSGALG00010010931	CYYR1	2	-0.133516929	0.091184123	-1.464256336	0.143123903	0	0.690505584	0	1	PRJNA827465;Yang2025_Silkie_Wenchang	CRA004775	0.630019027
ENSGALG00010010931	CYYR1	2	0.190072746	0.261173539	0.72776418	0.466757955	0.074346245	2.006721814	50.16748245	0.5	CRA004775;Yang2025_Silkie_Wenchang	PRJNA827465	0.852842649
ENSGALG00010010931	CYYR1	2	0.107569517	0.351210941	0.306281794	0.7593901	0.193866668	4.056803367	75.35005004	0.5	CRA004775;PRJNA827465	Yang2025_Silkie_Wenchang	0.939153842
ENSGALG00010010932	GHITM	2	-0.165532805	0.068330176	-2.422543226	0.015412292	0	0.067672116	0	1	PRJNA827465;Yang2025_Silkie_Wenchang	CRA004775	0.205241414
ENSGALG00010010932	GHITM	2	-0.103467489	0.135420842	-0.764044052	0.444840991	0	0.125151729	0	1	CRA004775;Yang2025_Silkie_Wenchang	PRJNA827465	0.846013379
ENSGALG00010010932	GHITM	2	-0.16358328	0.073598886	-2.222632553	0.026240587	0	0.293401229	0	1	CRA004775;PRJNA827465	Yang2025_Silkie_Wenchang	0.276215866
ENSGALG00010010933	ANAPC4	2	-0.164281945	0.190397583	-0.862836293	0.388227482	0.067130706	13.20846227	92.42909599	0.5	PRJNA827465;Yang2025_Silkie_Wenchang	CRA004775	0.828154121
ENSGALG00010010933	ANAPC4	2	-0.218262238	0.1941067	-1.12448444	0.260824443	0.050037266	2.566863978	60.95067881	0.5	CRA004775;Yang2025_Silkie_Wenchang	PRJNA827465	0.76792161
ENSGALG00010010933	ANAPC4	2	0.01933714	0.048559141	0.398218326	0.690469259	0	0.009985511	0	1	CRA004775;PRJNA827465	Yang2025_Silkie_Wenchang	0.919979266
ENSGALG00010010934	ENSGALG00010010934	2	0.420202979	0.346767414	1.211771816	0.22559974	0.229756791	22.37412124	95.53055072	1	PRJNA827465;Yang2025_Silkie_Wenchang	CRA004775	0.73884362
ENSGALG00010010934	ENSGALG00010010934	2	0.287955011	0.495101211	0.581608377	0.560839499	0.464459429	18.74088286	94.66407209	0.5	CRA004775;Yang2025_Silkie_Wenchang	PRJNA827465	0.881354866
ENSGALG00010010934	ENSGALG00010010934	2	-0.02082192	0.139141432	-0.149645724	0.881044132	0.019550072	1.79576053	44.31328769	0.5	CRA004775;PRJNA827465	Yang2025_Silkie_Wenchang	0.971685305
ENSGALG00010010935	LGALS1A	2	0.268545293	0.373240693	0.719496287	0.471835189	0.244910017	8.078290293	87.62114304	0.5	PRJNA827465;Yang2025_Silkie_Wenchang	CRA004775	0.853998373
ENSGALG00010010935	LGALS1A	2	0.637128312	0.205713941	3.097156712	0.001953866	0	0.086157722	0	1	CRA004775;Yang2025_Silkie_Wenchang	PRJNA827465	0.057181326
ENSGALG00010010935	LGALS1A	2	-0.002815479	0.202031889	-0.013935816	0.988881188	0.028632543	1.202530216	16.84200641	0.5	CRA004775;PRJNA827465	Yang2025_Silkie_Wenchang	0.996891127
ENSGALG00010010937	CDK7	2	0.040918351	0.21486989	0.190433155	0.848969719	0.063861399	3.199825369	68.74829453	0.5	PRJNA827465;Yang2025_Silkie_Wenchang	CRA004775	0.963634896
ENSGALG00010010937	CDK7	2	-0.258577256	0.151534315	-1.70639407	0.087934734	0	0.342628312	0	1	CRA004775;Yang2025_Silkie_Wenchang	PRJNA827465	0.509638136
ENSGALG00010010937	CDK7	2	-0.034532453	0.305793116	-0.112927503	0.910088031	0.146905517	4.47072555	77.63226597	0.5	CRA004775;PRJNA827465	Yang2025_Silkie_Wenchang	0.978730675
ENSGALG00010010938	FZD6	2	-0.178352952	0.099391732	-1.794444543	0.072742243	0.010591356	2.155474705	53.6065073	1	PRJNA827465;Yang2025_Silkie_Wenchang	CRA004775	0.465709132
ENSGALG00010010938	FZD6	2	-0.244615277	0.089363206	-2.737315369	0.006194288	0	0.902702853	0	1	CRA004775;Yang2025_Silkie_Wenchang	PRJNA827465	0.119165002
ENSGALG00010010938	FZD6	2	-0.072560083	0.088888677	-0.816302881	0.414326927	0	0.036961429	0	1	CRA004775;PRJNA827465	Yang2025_Silkie_Wenchang	0.836962991
ENSGALG00010010939	ZCCHC4	2	-0.009579609	0.102969264	-0.093033678	0.925876806	0	0.950190727	0	0.5	PRJNA827465;Yang2025_Silkie_Wenchang	CRA004775	0.982765528
ENSGALG00010010939	ZCCHC4	2	-0.176043585	0.144545882	-1.21790817	0.223234604	0	0.212725245	0	1	CRA004775;Yang2025_Silkie_Wenchang	PRJNA827465	0.73674216
ENSGALG00010010939	ZCCHC4	2	-0.018923459	0.15486724	-0.122191494	0.902747363	0.014614397	1.29020239	22.49278037	0.5	CRA004775;PRJNA827465	Yang2025_Silkie_Wenchang	0.97693511
ENSGALG00010010942	NRN1	2	-0.545125519	0.331530413	-1.644270021	0.100120439	0.157080171	3.496590632	71.40071272	1	PRJNA827465;Yang2025_Silkie_Wenchang	CRA004775	0.540452357
ENSGALG00010010942	NRN1	2	-0.150712967	0.220814514	-0.682519776	0.494902631	0	0.650197514	0	0.5	CRA004775;Yang2025_Silkie_Wenchang	PRJNA827465	0.860042712
ENSGALG00010010942	NRN1	2	-0.416418242	0.57570478	-0.723319062	0.469483884	0.482939251	3.324281225	69.91830918	0.5	CRA004775;PRJNA827465	Yang2025_Silkie_Wenchang	0.853485658
ENSGALG00010010943	WDSUB1	2	0.214675408	0.063970449	3.35585276	0.000791207	0	0.063200563	0	1	PRJNA827465;Yang2025_Silkie_Wenchang	CRA004775	0.032191305
ENSGALG00010010943	WDSUB1	2	0.04698389	0.195733672	0.240039895	0.810299329	0.052484617	2.781756021	64.05148429	0.5	CRA004775;Yang2025_Silkie_Wenchang	PRJNA827465	0.952997426
ENSGALG00010010943	WDSUB1	2	0.060153008	0.21303011	0.282368573	0.77766091	0.066417516	3.284202634	69.55120888	0.5	CRA004775;PRJNA827465	Yang2025_Silkie_Wenchang	0.944388893
ENSGALG00010010945	PI4K2B	2	-0.12462932	0.225695631	-0.552200853	0.58081075	0.090351066	8.777301539	88.60697681	0.5	PRJNA827465;Yang2025_Silkie_Wenchang	CRA004775	0.888613995
ENSGALG00010010945	PI4K2B	2	-0.32164975	0.114418763	-2.811162614	0.004936283	0	0.800667229	0	1	CRA004775;Yang2025_Silkie_Wenchang	PRJNA827465	0.104781471
ENSGALG00010010945	PI4K2B	2	0.086454426	0.089459636	0.966407081	0.333840522	0	0.112448573	0	0.5	CRA004775;PRJNA827465	Yang2025_Silkie_Wenchang	0.8027491
ENSGALG00010010947	LUC7L2	2	-0.038377879	0.029366102	-1.139956122	0.254304582	0	0.020485409	0	1	PRJNA827465;Yang2025_Silkie_Wenchang	CRA004775	0.764021784
ENSGALG00010010947	LUC7L2	2	-0.02360246	0.062565502	-0.377244	0.705992285	0	0.096399707	0	0.5	CRA004775;Yang2025_Silkie_Wenchang	PRJNA827465	0.924548793
ENSGALG00010010947	LUC7L2	2	-0.038307068	0.038343557	-0.999048368	0.317771261	0	0.142306602	0	0.5	CRA004775;PRJNA827465	Yang2025_Silkie_Wenchang	0.797410398
ENSGALG00010010948	PRDM15	2	0.216260543	0.215678352	1.002699349	0.316005943	0.082359885	8.270720253	87.90915459	1	PRJNA827465;Yang2025_Silkie_Wenchang	CRA004775	0.796883862
ENSGALG00010010948	PRDM15	2	0.344551364	0.160339978	2.148879949	0.031643918	0.017840209	1.416803038	29.41855902	1	CRA004775;Yang2025_Silkie_Wenchang	PRJNA827465	0.304637217
ENSGALG00010010948	PRDM15	2	0.022137028	0.057699598	0.38366	0.701230463	0	0.102323297	0	1	CRA004775;PRJNA827465	Yang2025_Silkie_Wenchang	0.922618536
ENSGALG00010010949	FZD3	2	-0.09560504	0.110797979	-0.862877114	0.388205035	0.00446514	1.209162145	17.29810559	0.5	PRJNA827465;Yang2025_Silkie_Wenchang	CRA004775	0.828154121
ENSGALG00010010949	FZD3	2	-0.055948463	0.264011473	-0.211936788	0.832141953	0.070483003	1.453759697	31.21284059	0.5	CRA004775;Yang2025_Silkie_Wenchang	PRJNA827465	0.957769021
ENSGALG00010010949	FZD3	2	0.080468295	0.157415659	0.511183549	0.609222537	0	0.614352203	0	1	CRA004775;PRJNA827465	Yang2025_Silkie_Wenchang	0.897238578
ENSGALG00010010951	ENSGALG00010010951	2	0.286406117	0.158826868	1.803259861	0.071347413	0.007931896	1.185453182	15.64407477	1	PRJNA827465;Yang2025_Silkie_Wenchang	CRA004775	0.461211741
ENSGALG00010010951	ENSGALG00010010951	2	0.312588969	0.192974272	1.619847903	0.105264953	0.012878651	1.16409345	14.09624374	1	CRA004775;Yang2025_Silkie_Wenchang	PRJNA827465	0.550749263
ENSGALG00010010951	ENSGALG00010010951	2	0.085215666	0.18234656	0.467356179	0.640265098	0	0.07243412	0	1	CRA004775;PRJNA827465	Yang2025_Silkie_Wenchang	0.906137307
ENSGALG00010010952	ENSGALG00010010952	2	0.302986768	0.346380039	0.874723521	0.381724359	0.057589055	1.29637676	22.861931	0.5	PRJNA827465;Yang2025_Silkie_Wenchang	CRA004775	0.824699008
ENSGALG00010010952	ENSGALG00010010952	2	-0.007094575	0.379699594	-0.018684705	0.985092629	0	0.141965172	0	0.5	CRA004775;Yang2025_Silkie_Wenchang	PRJNA827465	0.995835685
ENSGALG00010010952	ENSGALG00010010952	2	0.458975676	0.314770019	1.458130218	0.144804654	0	0.36635188	0	1	CRA004775;PRJNA827465	Yang2025_Silkie_Wenchang	0.64257278
ENSGALG00010010953	EXTL3	2	-0.246087332	0.149175821	-1.649646237	0.099015312	0.040544288	11.00162629	90.91043475	1	PRJNA827465;Yang2025_Silkie_Wenchang	CRA004775	0.536920367
ENSGALG00010010953	EXTL3	2	-0.352216994	0.097759852	-3.602879762	0.000314711	0.005981578	1.258658658	20.55034193	1	CRA004775;Yang2025_Silkie_Wenchang	PRJNA827465	0.017542629
ENSGALG00010010953	EXTL3	2	-0.106280368	0.044030105	-2.413811367	0.015786633	0	0.077993359	0	1	CRA004775;PRJNA827465	Yang2025_Silkie_Wenchang	0.20791309
ENSGALG00010010954	MTERF2	2	-0.426881073	0.143764951	-2.969298633	0.002984804	0	0.056776195	0	1	PRJNA827465;Yang2025_Silkie_Wenchang	CRA004775	0.075060946
ENSGALG00010010954	MTERF2	2	-0.043526074	0.414049623	-0.105122845	0.916278332	0.230295753	2.818894639	64.52510193	0.5	CRA004775;Yang2025_Silkie_Wenchang	PRJNA827465	0.98043852
ENSGALG00010010954	MTERF2	2	-0.085079741	0.449010209	-0.189482866	0.849714383	0.30426513	3.64345746	72.55354259	0.5	CRA004775;PRJNA827465	Yang2025_Silkie_Wenchang	0.961856718
ENSGALG00010010955	GTF3C3	2	0.030485837	0.055521233	0.549084299	0.5829476	0	0.907291443	0	1	PRJNA827465;Yang2025_Silkie_Wenchang	CRA004775	0.890230846
ENSGALG00010010955	GTF3C3	2	-0.138575502	0.242032674	-0.572548739	0.566950276	0.098579721	5.79182527	82.73428577	0.5	CRA004775;Yang2025_Silkie_Wenchang	PRJNA827465	0.883487517
ENSGALG00010010955	GTF3C3	2	-0.186801138	0.186491391	-1.001660918	0.316507388	0.050518237	3.315822613	69.84157125	1	CRA004775;PRJNA827465	Yang2025_Silkie_Wenchang	0.796883862
ENSGALG00010010957	ZMAT2	2	0.008844098	0.142170523	0.06220768	0.950397446	0.035279553	7.448782849	86.57498788	0.5	PRJNA827465;Yang2025_Silkie_Wenchang	CRA004775	0.987277827
ENSGALG00010010957	ZMAT2	2	0.132454001	0.088875556	1.490331045	0.136137213	0	0.838109134	0	0.5	CRA004775;Yang2025_Silkie_Wenchang	PRJNA827465	0.617782384
ENSGALG00010010957	ZMAT2	2	-0.119767199	0.042127583	-2.842963931	0.004469613	0	0.034570168	0	1	CRA004775;PRJNA827465	Yang2025_Silkie_Wenchang	0.097752851
ENSGALG00010010958	LCORL	2	0.181020339	0.156100935	1.159636479	0.246196842	0.030383238	2.437320803	58.97134268	1	PRJNA827465;Yang2025_Silkie_Wenchang	CRA004775	0.756727496
ENSGALG00010010958	LCORL	2	0.438817422	0.162653789	2.697861668	0.006978643	0	0.539453166	0	1	CRA004775;Yang2025_Silkie_Wenchang	PRJNA827465	0.128408997
ENSGALG00010010958	LCORL	2	0.268536606	0.293293678	0.915589479	0.359882252	0.121255218	2.724595613	63.29730565	1	CRA004775;PRJNA827465	Yang2025_Silkie_Wenchang	0.814880744
ENSGALG00010010959	TMEM263	2	-0.082565502	0.115622612	-0.714094765	0.475168583	0.021475965	4.787342361	79.11158374	0.5	PRJNA827465;Yang2025_Silkie_Wenchang	CRA004775	0.854462864
ENSGALG00010010959	TMEM263	2	-0.113665039	0.129617871	-0.876924134	0.380527847	0.017739166	1.973163229	49.31995562	0.5	CRA004775;Yang2025_Silkie_Wenchang	PRJNA827465	0.824392515
ENSGALG00010010959	TMEM263	2	0.022395665	0.046410857	0.48255229	0.629413662	0	0.041589021	0	1	CRA004775;PRJNA827465	Yang2025_Silkie_Wenchang	0.902390168
ENSGALG00010010960	POLDIP3	2	-0.1652017	0.172315255	-0.958717787	0.337700935	0.055790036	16.12184759	93.79723698	0.5	PRJNA827465;Yang2025_Silkie_Wenchang	CRA004775	0.80415385
ENSGALG00010010960	POLDIP3	2	-0.320512963	0.069525087	-4.610033244	4.02605E-06	0	0.470870199	0	1	CRA004775;Yang2025_Silkie_Wenchang	PRJNA827465	0.000694016
ENSGALG00010010960	POLDIP3	2	-0.062679478	0.101395293	-0.618169509	0.536463607	0.012936989	2.02499554	50.61717518	0.5	CRA004775;PRJNA827465	Yang2025_Silkie_Wenchang	0.874599474
ENSGALG00010010962	ANKDD1B	2	1.136689799	0.494108313	2.300487097	0.021420639	0.374514537	4.290729388	76.6939392	1	PRJNA827465;Yang2025_Silkie_Wenchang	CRA004775	0.247616292
ENSGALG00010010962	ANKDD1B	2	0.680792667	1.065231558	0.639102983	0.522755939	1.927420539	6.215670479	83.91163104	0.5	CRA004775;Yang2025_Silkie_Wenchang	PRJNA827465	0.869568211
ENSGALG00010010962	ANKDD1B	2	0.290743337	0.533424308	0.545050784	0.585718591	0.301850331	1.826382497	45.24695668	0.5	CRA004775;PRJNA827465	Yang2025_Silkie_Wenchang	0.891202921
ENSGALG00010010963	TSPAN14	2	-0.386196002	0.320761835	-1.203996112	0.228591081	0.196625115	22.33135082	95.52199055	1	PRJNA827465;Yang2025_Silkie_Wenchang	CRA004775	0.741280137
ENSGALG00010010963	TSPAN14	2	-0.528462903	0.271211968	-1.94852354	0.051352351	0.094610184	2.252028544	55.59558948	1	CRA004775;Yang2025_Silkie_Wenchang	PRJNA827465	0.392742061
ENSGALG00010010963	TSPAN14	2	-0.073382943	0.072848863	-1.007331346	0.313775571	0	0.023591096	0	1	CRA004775;PRJNA827465	Yang2025_Silkie_Wenchang	0.796531448
ENSGALG00010010964	INTS9	2	-0.169573579	0.097305252	-1.742697085	0.081386541	0.010896921	2.305331768	56.62229559	1	PRJNA827465;Yang2025_Silkie_Wenchang	CRA004775	0.491357177
ENSGALG00010010964	INTS9	2	-0.278989251	0.091261628	-3.057026878	0.002235442	0	0.000487751	0	1	CRA004775;Yang2025_Silkie_Wenchang	PRJNA827465	0.062480629
ENSGALG00010010964	INTS9	2	-0.110521852	0.071341572	-1.549192823	0.121335374	0	0.861925371	0	1	CRA004775;PRJNA827465	Yang2025_Silkie_Wenchang	0.583884665
ENSGALG00010010965	BAZ2B	2	0.447529851	0.449114101	0.996472499	0.319020623	0.382136327	18.17123043	94.49679534	1	PRJNA827465;Yang2025_Silkie_Wenchang	CRA004775	0.798079048
ENSGALG00010010965	BAZ2B	2	0.894693579	0.184168845	4.858007224	1.18573E-06	0	0.073522055	0	1	CRA004775;Yang2025_Silkie_Wenchang	PRJNA827465	0.000253276
ENSGALG00010010965	BAZ2B	2	0.296635477	0.370762533	0.800068646	0.423671026	0.211657474	3.417188172	70.73617402	1	CRA004775;PRJNA827465	Yang2025_Silkie_Wenchang	0.84030043
ENSGALG00010010966	ENSGALG00010010966	2	-0.460516675	0.671794789	-0.685502006	0.493027195	0.685546803	4.143909235	75.86819732	0.5	PRJNA827465;Yang2025_Silkie_Wenchang	CRA004775	0.85961529
ENSGALG00010010966	ENSGALG00010010966	2	0.170042106	0.410732173	0.413997532	0.678875914	0	0.051401319	0	1	CRA004775;Yang2025_Silkie_Wenchang	PRJNA827465	0.917701868
ENSGALG00010010966	ENSGALG00010010966	2	-0.686617549	0.552718482	-1.242255454	0.214142325	0.296945038	1.829168853	45.33036148	0.5	CRA004775;PRJNA827465	Yang2025_Silkie_Wenchang	0.726967219
ENSGALG00010010967	PRXL2A	2	0.005783225	0.213404079	0.027099882	0.978380069	0.051140729	2.169935684	53.91568481	0.5	PRJNA827465;Yang2025_Silkie_Wenchang	CRA004775	0.994035566
ENSGALG00010010967	PRXL2A	2	-0.364317956	0.208277361	-1.74919614	0.080257121	0	0.616292537	0	1	CRA004775;Yang2025_Silkie_Wenchang	PRJNA827465	0.4875341
ENSGALG00010010967	PRXL2A	2	-0.141179567	0.388043199	-0.363824356	0.715989174	0.229022898	3.759935715	73.4038006	0.5	CRA004775;PRJNA827465	Yang2025_Silkie_Wenchang	0.927261187
ENSGALG00010010968	RRP7A	2	0.073435215	0.067146855	1.093650845	0.27410814	0	0.418882845	0	1	PRJNA827465;Yang2025_Silkie_Wenchang	CRA004775	0.774526342
ENSGALG00010010968	RRP7A	2	0.013002987	0.192638615	0.067499382	0.946184154	0.035933968	1.835712306	45.52523309	0.5	CRA004775;Yang2025_Silkie_Wenchang	PRJNA827465	0.986370359
ENSGALG00010010968	RRP7A	2	0.008045598	0.103629644	0.077637993	0.938116019	0.00713705	1.210370418	17.38066423	0.5	CRA004775;PRJNA827465	Yang2025_Silkie_Wenchang	0.985349045
ENSGALG00010010970	TPM2	2	-0.11292773	0.098679181	-1.144392661	0.252460831	0	0.262969825	0	1	PRJNA827465;Yang2025_Silkie_Wenchang	CRA004775	0.762655223
ENSGALG00010010970	TPM2	2	-0.180912772	0.135661063	-1.333564452	0.18234664	0	0.248354566	0	1	CRA004775;Yang2025_Silkie_Wenchang	PRJNA827465	0.69030726
ENSGALG00010010970	TPM2	2	-0.085556799	0.134967011	-0.63390897	0.526140231	0	0.456933417	0	1	CRA004775;PRJNA827465	Yang2025_Silkie_Wenchang	0.870525481
ENSGALG00010010971	LCORL	2	0.071590636	0.08145554	0.878892167	0.379459746	0	0.093708444	0	1	PRJNA827465;Yang2025_Silkie_Wenchang	CRA004775	0.824165982
ENSGALG00010010971	LCORL	2	0.388926169	0.403470581	0.963951741	0.335070121	0.267255615	5.098415629	80.38606358	1	CRA004775;Yang2025_Silkie_Wenchang	PRJNA827465	0.803101658
ENSGALG00010010971	LCORL	2	0.406313753	0.376147934	1.080196691	0.280054602	0.230037484	4.716107728	78.79607385	1	CRA004775;PRJNA827465	Yang2025_Silkie_Wenchang	0.778098827
ENSGALG00010010972	EXOSC3	2	0.146391296	0.219143225	0.668016527	0.504123043	0.082878545	7.047605393	85.81078332	0.5	PRJNA827465;Yang2025_Silkie_Wenchang	CRA004775	0.863448292
ENSGALG00010010972	EXOSC3	2	0.312674166	0.126088845	2.479792454	0.013145888	0	0.975893758	0	1	CRA004775;Yang2025_Silkie_Wenchang	PRJNA827465	0.187132496
ENSGALG00010010972	EXOSC3	2	-0.043839199	0.077020704	-0.569187202	0.569229105	0	0.298035155	0	0.5	CRA004775;PRJNA827465	Yang2025_Silkie_Wenchang	0.884658821
ENSGALG00010010973	PGAP1	2	-0.277284704	0.265473709	-1.044490262	0.296258622	0.126129271	9.313145795	89.26248958	1	PRJNA827465;Yang2025_Silkie_Wenchang	CRA004775	0.788267543
ENSGALG00010010973	PGAP1	2	-0.535333018	0.136424548	-3.924022669	8.70825E-05	0	0.122660027	0	1	CRA004775;Yang2025_Silkie_Wenchang	PRJNA827465	0.007013535
ENSGALG00010010973	PGAP1	2	-0.092204259	0.15021484	-0.613815917	0.539336978	0.018972173	1.307366501	23.51035467	1	CRA004775;PRJNA827465	Yang2025_Silkie_Wenchang	0.874983893
ENSGALG00010010974	NCAPG	2	-0.046611794	0.213517756	-0.218304065	0.827192207	0.077372039	6.398624763	84.37164177	0.5	PRJNA827465;Yang2025_Silkie_Wenchang	CRA004775	0.956550752
ENSGALG00010010974	NCAPG	2	0.158525498	0.554286944	0.285998975	0.774878907	0.475591902	3.712251138	73.06216732	0.5	CRA004775;Yang2025_Silkie_Wenchang	PRJNA827465	0.943713409
ENSGALG00010010974	NCAPG	2	0.281785601	0.275612725	1.022396919	0.306593078	0.085582539	1.508160667	33.69406708	1	CRA004775;PRJNA827465	Yang2025_Silkie_Wenchang	0.792500392
ENSGALG00010010977	FARS2	2	-0.071091127	0.137942875	-0.515366432	0.606296985	0.029505733	4.310515753	76.80091995	0.5	PRJNA827465;Yang2025_Silkie_Wenchang	CRA004775	0.896731537
ENSGALG00010010977	FARS2	2	-0.246535957	0.100861146	-2.444310489	0.014512931	0	0.221662947	0	1	CRA004775;Yang2025_Silkie_Wenchang	PRJNA827465	0.198198336
ENSGALG00010010977	FARS2	2	-0.085359238	0.189494127	-0.45045849	0.652379879	0.050160235	2.751638781	63.65802057	0.5	CRA004775;PRJNA827465	Yang2025_Silkie_Wenchang	0.909377929
ENSGALG00010010978	SERHL	2	0.060558122	0.083355299	0.726505971	0.467528648	0	0.109668984	0	1	PRJNA827465;Yang2025_Silkie_Wenchang	CRA004775	0.852885838
ENSGALG00010010978	SERHL	2	-0.318124176	0.34429465	-0.923988149	0.355492486	0.195165986	5.592607037	82.11925148	0.5	CRA004775;Yang2025_Silkie_Wenchang	PRJNA827465	0.812301302
ENSGALG00010010978	SERHL	2	-0.272241757	0.376515772	-0.723055386	0.469645857	0.253697699	9.021195507	88.9149947	0.5	CRA004775;PRJNA827465	Yang2025_Silkie_Wenchang	0.853485658
ENSGALG00010010980	POLK	2	-0.016451831	0.114661289	-0.143481996	0.885909529	0	0.12115787	0	0.5	PRJNA827465;Yang2025_Silkie_Wenchang	CRA004775	0.97244495
ENSGALG00010010980	POLK	2	0.077279204	0.186262459	0.414894148	0.678219394	0.023845582	1.462240451	31.61179483	0.5	CRA004775;Yang2025_Silkie_Wenchang	PRJNA827465	0.917647993
ENSGALG00010010980	POLK	2	0.093014729	0.13280928	0.700363171	0.48370053	0	0.985456194	0	1	CRA004775;PRJNA827465	Yang2025_Silkie_Wenchang	0.85691916
ENSGALG00010010981	RIC8B	2	0.134964641	0.060182443	2.242591606	0.024923162	0	0.065497854	0	1	PRJNA827465;Yang2025_Silkie_Wenchang	CRA004775	0.269034616
ENSGALG00010010981	RIC8B	2	0.202534297	0.104730234	1.933866562	0.053129536	0	0.08786268	0	1	CRA004775;Yang2025_Silkie_Wenchang	PRJNA827465	0.399645964
ENSGALG00010010981	RIC8B	2	0.145877933	0.059830366	2.43819221	0.014760923	0	0.426203646	0	1	CRA004775;PRJNA827465	Yang2025_Silkie_Wenchang	0.200535277
ENSGALG00010010982	ZBTB21	2	-0.057096383	0.04346075	-1.313745925	0.188931721	0	0.036965494	0	1	PRJNA827465;Yang2025_Silkie_Wenchang	CRA004775	0.698162702
ENSGALG00010010982	ZBTB21	2	0.285705405	0.337864392	0.845621534	0.397763909	0.191707591	6.089620318	83.57861496	0.5	CRA004775;Yang2025_Silkie_Wenchang	PRJNA827465	0.831711071
ENSGALG00010010982	ZBTB21	2	0.257667333	0.351706877	0.732619546	0.463790506	0.222326162	9.02715525	88.92231304	0.5	CRA004775;PRJNA827465	Yang2025_Silkie_Wenchang	0.852159649
ENSGALG00010010983	MED28	2	-0.271979495	0.115965161	-2.345355208	0.01900896	0.01217221	1.649134236	39.36212238	1	PRJNA827465;Yang2025_Silkie_Wenchang	CRA004775	0.230995614
ENSGALG00010010983	MED28	2	-0.140433865	0.30770404	-0.45639266	0.648107643	0.155231216	5.534841981	81.93263686	0.5	CRA004775;Yang2025_Silkie_Wenchang	PRJNA827465	0.90762535
ENSGALG00010010983	MED28	2	-0.051264305	0.178520217	-0.287162459	0.77398793	0.044228936	2.917132292	65.71975831	0.5	CRA004775;PRJNA827465	Yang2025_Silkie_Wenchang	0.943248868
ENSGALG00010010984	MAT1A	2	-0.06041399	0.155433994	-0.388679389	0.697513331	0.027474136	2.170298618	53.92339138	0.5	PRJNA827465;Yang2025_Silkie_Wenchang	CRA004775	0.921342994
ENSGALG00010010984	MAT1A	2	-0.251925397	0.169966157	-1.482209175	0.138284646	0	0.003092951	0	1	CRA004775;Yang2025_Silkie_Wenchang	PRJNA827465	0.621841044
ENSGALG00010010984	MAT1A	2	0.043040856	0.105331627	0.408622339	0.682816835	0	0.492009347	0	0.5	CRA004775;PRJNA827465	Yang2025_Silkie_Wenchang	0.918793207
ENSGALG00010010985	CERT1	2	0.125535977	0.104318784	1.203388033	0.228826197	0	0.359495875	0	1	PRJNA827465;Yang2025_Silkie_Wenchang	CRA004775	0.741553101
ENSGALG00010010985	CERT1	2	0.223671466	0.12756126	1.753443526	0.079525905	0	0.151172022	0	1	CRA004775;Yang2025_Silkie_Wenchang	PRJNA827465	0.485267297
ENSGALG00010010985	CERT1	2	0.129927215	0.122563448	1.060081264	0.289107631	0	0.721734703	0	1	CRA004775;PRJNA827465	Yang2025_Silkie_Wenchang	0.783530627
ENSGALG00010010986	NARS	2	-0.036200398	0.033405113	-1.083678382	0.278507424	0	0.497687989	0	1	PRJNA827465;Yang2025_Silkie_Wenchang	CRA004775	0.776763074
ENSGALG00010010986	NARS	2	-0.100564043	0.079234405	-1.269196675	0.204370927	0	0.083789733	0	1	CRA004775;Yang2025_Silkie_Wenchang	PRJNA827465	0.717281769
ENSGALG00010010986	NARS	2	-0.029812713	0.0358076	-0.83258061	0.405081299	0	0.367147299	0	1	CRA004775;PRJNA827465	Yang2025_Silkie_Wenchang	0.833112102
ENSGALG00010010987	MARCHF7	2	0.018750003	0.03621149	0.517791542	0.60460372	0	0.048048666	0	1	PRJNA827465;Yang2025_Silkie_Wenchang	CRA004775	0.896289929
ENSGALG00010010987	MARCHF7	2	-0.086493561	0.15022706	-0.575752202	0.564782685	0.032465523	3.038235208	67.08615589	0.5	CRA004775;Yang2025_Silkie_Wenchang	PRJNA827465	0.883348079
ENSGALG00010010987	MARCHF7	2	-0.090324486	0.140481507	-0.642963536	0.520247761	0.028146043	2.837866253	64.7622576	0.5	CRA004775;PRJNA827465	Yang2025_Silkie_Wenchang	0.869331539
ENSGALG00010010988	ANKRD44	2	0.216348298	0.060817559	3.557332838	0.000374639	0	0.000692257	0	1	PRJNA827465;Yang2025_Silkie_Wenchang	CRA004775	0.019374277
ENSGALG00010010988	ANKRD44	2	0.170466258	0.133785969	1.274171425	0.20260267	0.00731478	1.075802667	7.04614972	0.5	CRA004775;Yang2025_Silkie_Wenchang	PRJNA827465	0.715444091
ENSGALG00010010988	ANKRD44	2	0.178662501	0.127598612	1.400191568	0.16145596	0.012522329	1.134804517	11.87909589	0.5	CRA004775;PRJNA827465	Yang2025_Silkie_Wenchang	0.657858553
ENSGALG00010010989	TRIQK	2	-0.071521136	0.235873238	-0.30321853	0.761723328	0.099336222	9.20995427	89.14218279	0.5	PRJNA827465;Yang2025_Silkie_Wenchang	CRA004775	0.939791938
ENSGALG00010010989	TRIQK	2	-0.307335703	0.123559388	-2.487352091	0.012869796	0	0.078178099	0	1	CRA004775;Yang2025_Silkie_Wenchang	PRJNA827465	0.184289906
ENSGALG00010010989	TRIQK	2	0.144362533	0.08701867	1.658983449	0.097119127	0	0.474463099	0	0.5	CRA004775;PRJNA827465	Yang2025_Silkie_Wenchang	0.532520426
ENSGALG00010010990	MED18	2	0.041809198	0.179206108	0.233302306	0.815526668	0.034234647	2.141321196	53.29985982	0.5	PRJNA827465;Yang2025_Silkie_Wenchang	CRA004775	0.954061336
ENSGALG00010010990	MED18	2	-0.089472528	0.336133059	-0.266181877	0.79009914	0.179247387	4.717676418	78.80312443	0.5	CRA004775;Yang2025_Silkie_Wenchang	PRJNA827465	0.947390191
ENSGALG00010010990	MED18	2	-0.238343487	0.147145493	-1.619781093	0.105279309	0.001535045	1.031706545	3.073213512	1	CRA004775;PRJNA827465	Yang2025_Silkie_Wenchang	0.550751056
ENSGALG00010010991	C2CD2	2	-0.071705322	0.185841277	-0.385841741	0.69961388	0.056552961	5.341862061	81.27993594	0.5	PRJNA827465;Yang2025_Silkie_Wenchang	CRA004775	0.922065803
ENSGALG00010010991	C2CD2	2	-0.290217189	0.131579352	-2.20564386	0.027408946	0	0.159002759	0	1	CRA004775;Yang2025_Silkie_Wenchang	PRJNA827465	0.282595432
ENSGALG00010010991	C2CD2	2	-0.008108946	0.220183721	-0.036828091	0.970622076	0.052836818	1.50838495	33.70392617	0.5	CRA004775;PRJNA827465	Yang2025_Silkie_Wenchang	0.991829886
ENSGALG00010010992	PCDHA6	2	0.186901122	0.205763261	0.908330866	0.363703439	0.035437217	1.714398112	41.67049105	0.5	PRJNA827465;Yang2025_Silkie_Wenchang	CRA004775	0.817042253
ENSGALG00010010992	PCDHA6	2	0.313716561	0.191165402	1.641073947	0.100782066	0	0.57943924	0	0.5	CRA004775;Yang2025_Silkie_Wenchang	PRJNA827465	0.541717388
ENSGALG00010010992	PCDHA6	2	-0.027663537	0.209513608	-0.132036945	0.89495507	0	0.002343533	0	1	CRA004775;PRJNA827465	Yang2025_Silkie_Wenchang	0.974641371
ENSGALG00010010993	ENSGALG00010010993	2	-9.05224E-05	0.17953802	-0.000504196	0.99959771	0	0.448819753	0	0.5	PRJNA827465;Yang2025_Silkie_Wenchang	CRA004775	0.999889665
ENSGALG00010010993	ENSGALG00010010993	2	0.075687287	0.235124164	0.32190348	0.747525817	0	0.294430587	0	0.5	CRA004775;Yang2025_Silkie_Wenchang	PRJNA827465	0.93680487

ENSGALG00010010993	ENSGALG00010010993	2	-0.15249507	0.254842201	-0.59839018	0.549579616	0	0.079584008	0	1	CRA004775;PRJNA827465	Yang2025_Silkie_Wenchang	0.878554604
ENSGALG00010010994	NAA20	2	-0.050686473	0.066845901	-0.758258511	0.44829624	0.001105923	1.073142839	6.81575989	1	PRJNA827465;Yang2025_Silkie_Wenchang	CRA004775	0.846800937
ENSGALG00010010994	NAA20	2	-0.187363357	0.138256546	-1.355186162	0.175358252	0	0.027541021	0	1	CRA004775;Yang2025_Silkie_Wenchang	PRJNA827465	0.680258136
ENSGALG00010010994	NAA20	2	-0.028098143	0.063316657	-0.443771742	0.657207621	0	0.214730274	0	1	CRA004775;PRJNA827465	Yang2025_Silkie_Wenchang	0.910711339
ENSGALG00010010995	HEBP1	2	0.101811416	0.077226804	1.318342988	0.187388857	0	0.004237591	0	1	PRJNA827465;Yang2025_Silkie_Wenchang	CRA004775	0.696382775
ENSGALG00010010995	HEBP1	2	-0.078896989	0.221020281	-0.356967191	0.721116367	0.061834522	2.485747086	59.77064579	0.5	CRA004775;Yang2025_Silkie_Wenchang	PRJNA827465	0.928950734
ENSGALG00010010995	HEBP1	2	-0.066131045	0.224549123	-0.294505914	0.768371345	0.07074603	2.883097396	65.31508088	0.5	CRA004775;PRJNA827465	Yang2025_Silkie_Wenchang	0.941444269
ENSGALG00010010997	LAP3	2	-0.056794623	0.083324057	-0.681611357	0.495484732	0	0.535832233	0	0.5	PRJNA827465;Yang2025_Silkie_Wenchang	CRA004775	0.860770265
ENSGALG00010010997	LAP3	2	-0.315423645	0.368944057	-0.854936241	0.392586446	0.232843222	6.669086567	85.0054428	0.5	CRA004775;Yang2025_Silkie_Wenchang	PRJNA827465	0.829872807
ENSGALG00010010997	LAP3	2	-0.365919939	0.303353392	-1.206249705	0.227721224	0.150997756	5.123479545	80.4820144	1	CRA004775;PRJNA827465	Yang2025_Silkie_Wenchang	0.740853612
ENSGALG00010010998	SOAT1	2	-0.374870634	0.268138675	-1.398047611	0.16209877	0.135533258	17.30352512	94.22083077	1	PRJNA827465;Yang2025_Silkie_Wenchang	CRA004775	0.659588774
ENSGALG00010010998	SOAT1	2	-0.367384919	0.323169342	-1.1368186	0.255614126	0.172219103	5.216815991	80.83121962	0.5	CRA004775;Yang2025_Silkie_Wenchang	PRJNA827465	0.764467569
ENSGALG00010010998	SOAT1	2	-0.103069496	0.072270689	-1.426159024	0.153822444	0	0.177519946	0	0.5	CRA004775;PRJNA827465	Yang2025_Silkie_Wenchang	0.646370532
ENSGALG00010011001	ANGPT1	2	0.381460525	0.273762415	1.393399911	0.163498893	0.11108358	3.76897566	73.46759199	1	PRJNA827465;Yang2025_Silkie_Wenchang	CRA004775	0.661791656
ENSGALG00010011001	ANGPT1	2	0.534348019	0.246547466	2.1673231	0.030210226	0.028400604	1.221833038	18.15575702	1	CRA004775;Yang2025_Silkie_Wenchang	PRJNA827465	0.295973612
ENSGALG00010011001	ANGPT1	2	0.131012094	0.151892444	0.862532005	0.388394829	0	0.000389667	0	1	CRA004775;PRJNA827465	Yang2025_Silkie_Wenchang	0.828154121
ENSGALG00010011003	FECH	2	-0.077849802	0.172663976	-0.450874609	0.652079925	0.045935803	4.344794577	76.98395212	0.5	PRJNA827465;Yang2025_Silkie_Wenchang	CRA004775	0.909322729
ENSGALG00010011003	FECH	2	-0.255342201	0.115268364	-2.215197574	0.026746506	0	4.363E-06	0	1	CRA004775;Yang2025_Silkie_Wenchang	PRJNA827465	0.278911632
ENSGALG00010011003	FECH	2	0.04258688	0.119232951	0.357173748	0.720961737	0.00436407	1.078561852	7.283944986	0.5	CRA004775;PRJNA827465	Yang2025_Silkie_Wenchang	0.928950734
ENSGALG00010011006	TMEM64	2	-0.276989991	0.261538218	-1.059080364	0.289563182	0.130906758	22.93362134	95.63958964	1	PRJNA827465;Yang2025_Silkie_Wenchang	CRA004775	0.784010866
ENSGALG00010011006	TMEM64	2	-0.330867407	0.261732679	-1.264142514	0.206178879	0.105081827	3.791460547	73.62493984	1	CRA004775;Yang2025_Silkie_Wenchang	PRJNA827465	0.718403416
ENSGALG00010011006	TMEM64	2	-0.020867281	0.052358711	-0.398544602	0.690228788	0	0.001772722	0	1	CRA004775;PRJNA827465	Yang2025_Silkie_Wenchang	0.919979266
ENSGALG00010011007	ENSGALG00010011007	2	0.40641731	0.396818446	1.024198853	0.305741361	0.287109094	11.29881507	91.1495144	1	PRJNA827465;Yang2025_Silkie_Wenchang	CRA004775	0.79225794
ENSGALG00010011007	ENSGALG00010011007	2	0.176321961	0.679071622	0.259651494	0.795132611	0.828939258	9.459589154	89.42871637	0.5	CRA004775;Yang2025_Silkie_Wenchang	PRJNA827465	0.948933254
ENSGALG00010011007	ENSGALG00010011007	2	-0.145289972	0.255677523	-0.568254773	0.569861984	0.067693251	1.723713001	41.98570185	0.5	CRA004775;PRJNA827465	Yang2025_Silkie_Wenchang	0.884923673
ENSGALG00010011013	ENSGALG00010011013	2	0.281997011	0.274250125	1.028247522	0.30383341	0	0.139464535	0	1	PRJNA827465;Yang2025_Silkie_Wenchang	CRA004775	0.792208658
ENSGALG00010011013	ENSGALG00010011013	2	0.196054689	0.288450165	0.679683052	0.496705169	0	0.642121949	0	0.5	CRA004775;Yang2025_Silkie_Wenchang	PRJNA827465	0.860939846
ENSGALG00010011013	ENSGALG00010011013	2	0.04256015	0.317332402	0.134118514	0.893308859	0	0.183482084	0	0.5	CRA004775;PRJNA827465	Yang2025_Silkie_Wenchang	0.974381724
ENSGALG00010011015	LYRM4	2	-0.165259164	0.116802949	-1.414854383	0.157111195	0	0.165573046	0	1	PRJNA827465;Yang2025_Silkie_Wenchang	CRA004775	0.650687662
ENSGALG00010011015	LYRM4	2	-0.21336286	0.194600332	-1.096415705	0.272896894	0	0.84186999	0	1	CRA004775;Yang2025_Silkie_Wenchang	PRJNA827465	0.773799118
ENSGALG00010011015	LYRM4	2	-0.228686482	0.122935248	-1.860218981	0.062854551	0	0.546962296	0	1	CRA004775;PRJNA827465	Yang2025_Silkie_Wenchang	0.431807797
ENSGALG00010011018	QDPR	2	-0.099012875	0.101792406	-0.972694123	0.330705347	0.004730841	1.273519522	21.47745024	1	PRJNA827465;Yang2025_Silkie_Wenchang	CRA004775	0.801575303
ENSGALG00010011018	QDPR	2	-0.43956482	0.233040158	-1.886219193	0.059265423	0.075784839	3.22872084	69.02798199	1	CRA004775;Yang2025_Silkie_Wenchang	PRJNA827465	0.421843813
ENSGALG00010011018	QDPR	2	-0.334213878	0.338345639	-0.98778834	0.323256319	0.201523223	8.03517355	87.55471809	1	CRA004775;PRJNA827465	Yang2025_Silkie_Wenchang	0.79919767
ENSGALG00010011019	NECAB1	2	0.077682567	0.121487993	0.639425886	0.522545914	0	0.854422408	0	0.5	PRJNA827465;Yang2025_Silkie_Wenchang	CRA004775	0.869568211
ENSGALG00010011019	NECAB1	2	-0.388245034	0.466007399	-0.833130623	0.404771064	0.272732009	2.163344697	53.77528132	1	CRA004775;Yang2025_Silkie_Wenchang	PRJNA827465	0.833112102
ENSGALG00010011019	NECAB1	2	-0.298795362	0.599693782	-0.498246557	0.618310271	0.547433862	3.436632851	70.90175054	0.5	CRA004775;PRJNA827465	Yang2025_Silkie_Wenchang	0.899168321
ENSGALG00010011021	PPP1R3G	2	0.139836553	0.150788836	0.927366753	0.353736146	0	0.689178777	0	1	PRJNA827465;Yang2025_Silkie_Wenchang	CRA004775	0.811318534
ENSGALG00010011021	PPP1R3G	2	0.157365099	0.200528381	0.784752253	0.43259884	0	0.956472303	0	0.5	CRA004775;Yang2025_Silkie_Wenchang	PRJNA827465	0.842762754
ENSGALG00010011021	PPP1R3G	2	0.011779048	0.162835748	0.07233699	0.942333728	0	0.144366754	0	0.5	CRA004775;PRJNA827465	Yang2025_Silkie_Wenchang	0.985827325
ENSGALG00010011022	C5orf22	2	0.174074135	0.084160244	2.068365387	0.038605677	7.97447E-05	1.005659143	0.562729683	1	PRJNA827465;Yang2025_Silkie_Wenchang	CRA004775	0.338965227
ENSGALG00010011022	C5orf22	2	0.152540983	0.16528376	0.922903636	0.356057426	0.024335124	1.568111533	36.22902587	0.5	CRA004775;Yang2025_Silkie_Wenchang	PRJNA827465	0.812424361
ENSGALG00010011022	C5orf22	2	0.055278136	0.10940436	0.505264474	0.613373091	0	0.455541539	0	0.5	CRA004775;PRJNA827465	Yang2025_Silkie_Wenchang	0.898256044
ENSGALG00010011025	POLR3B	2	-0.062995589	0.042165526	-1.494006957	0.135173801	5.22674E-05	1.012942137	1.27767779	1	PRJNA827465;Yang2025_Silkie_Wenchang	CRA004775	0.616469703
ENSGALG00010011025	POLR3B	2	-0.019792759	0.070812023	-0.279511278	0.779852484	0	0.67078616	0	1	CRA004775;Yang2025_Silkie_Wenchang	PRJNA827465	0.944978771
ENSGALG00010011025	POLR3B	2	-0.096804325	0.049450414	-1.95760392	0.050276507	0	0.21403545	0	1	CRA004775;PRJNA827465	Yang2025_Silkie_Wenchang	0.388444576
ENSGALG00010011027	FAM234B	2	0.074840669	0.130745894	0.572413152	0.567042108	0.024853866	3.609209419	72.29310124	0.5	PRJNA827465;Yang2025_Silkie_Wenchang	CRA004775	0.883487517
ENSGALG00010011027	FAM234B	2	0.070751384	0.213270095	0.331745454	0.740081475	0.054412303	2.070945453	51.71287595	0.5	CRA004775;Yang2025_Silkie_Wenchang	PRJNA827465	0.93508234
ENSGALG00010011027	FAM234B	2	-0.060702483	0.080401382	-0.754993037	0.45025316	0	0.400205858	0	1	CRA004775;PRJNA827465	Yang2025_Silkie_Wenchang	0.847564661
ENSGALG00010011028	HMBOX1	2	0.046190896	0.090906323	0.508115325	0.611372468	0	0.514976484	0	1	PRJNA827465;Yang2025_Silkie_Wenchang	CRA004775	0.897609796
ENSGALG00010011028	HMBOX1	2	0.085527422	0.170901604	0.500448327	0.616759432	0	0.822240742	0	0.5	CRA004775;Yang2025_Silkie_Wenchang	PRJNA827465	0.899111936
ENSGALG00010011028	HMBOX1	2	-0.004441782	0.098867156	-0.044926772	0.964165677	0	0.323268688	0	0.5	CRA004775;PRJNA827465	Yang2025_Silkie_Wenchang	0.990248829
ENSGALG00010011029	HMGCR	2	0.035438233	0.144929034	0.244521284	0.80682712	0.030022227	3.422394564	70.78069225	0.5	PRJNA827465;Yang2025_Silkie_Wenchang	CRA004775	0.95228484
ENSGALG00010011029	HMGCR	2	0.07197343	0.243709916	0.295324174	0.767746251	0.081430709	2.895058806	65.45838731	0.5	CRA004775;Yang2025_Silkie_Wenchang	PRJNA827465	0.941444269
ENSGALG00010011029	HMGCR	2	0.188828323	0.085815975	2.200386633	0.027779475	0	0.554852471	0	1	CRA004775;PRJNA827465	Yang2025_Silkie_Wenchang	0.284281094
ENSGALG00010011033	DROSHA	2	0.056180243	0.125474696	0.44774161	0.654339688	0.018429185	1.916907398	47.83263911	0.5	PRJNA827465;Yang2025_Silkie_Wenchang	CRA004775	0.910064502
ENSGALG00010011033	DROSHA	2	0.045590923	0.235506041	0.193587064	0.846499228	0.062456598	2.246763654	55.49153565	0.5	CRA004775;Yang2025_Silkie_Wenchang	PRJNA827465	0.96122283
ENSGALG00010011033	DROSHA	2	-0.033906475	0.058594153	-0.578666518	0.562814212	0	0.576263197	0	1	CRA004775;PRJNA827465	Yang2025_Silkie_Wenchang	0.882070607
ENSGALG00010011034	FAM180A	2	0.864331833	0.645766471	1.338458827	0.180746914	0.614748205	3.80088893	73.69036511	1	PRJNA827465;Yang2025_Silkie_Wenchang	CRA004775	0.688484814
ENSGALG00010011034	FAM180A	2	0.434040882	1.148026699	0.378075599	0.705374434	2.265317466	6.98185507	85.67715901	0.5	CRA004775;Yang2025_Silkie_Wenchang	PRJNA827465	0.924281103
ENSGALG00010011034	FAM180A	2	-0.117415023	0.478541631	-0.2453601	0.80617762	0.14222141	1.388910708	28.00113111	0.5	CRA004775;PRJNA827465	Yang2025_Silkie_Wenchang	0.952109208
ENSGALG00010011040	CDH6	2	-0.170650255	0.158829802	-1.074422136	0.282633526	0	0.842386893	0	0.5	PRJNA827465;Yang2025_Silkie_Wenchang	CRA004775	0.779532565
ENSGALG00010011040	CDH6	2	0.22474516	0.334540563	0.671802421	0.501709487	0.071446918	1.30478617	23.35908957	1	CRA004775;Yang2025_Silkie_Wenchang	PRJNA827465	0.86279037
ENSGALG00010011040	CDH6	2	0.109857928	0.521073104	0.210830165	0.8330198	0.380636423	2.768059832	63.87361326	0.5	CRA004775;PRJNA827465	Yang2025_Silkie_Wenchang	0.957916437
ENSGALG00010011045	ENSGALG00010011045	2	0.304690135	0.153597815	1.983687952	0.047290638	0	0.503514097	0	1	PRJNA827465;Yang2025_Silkie_Wenchang	CRA004775	0.375362366
ENSGALG00010011045	ENSGALG00010011045	2	0.13037286	0.299687282	0.435029673	0.663540889	0	0.07306	0	1	CRA004775;Yang2025_Silkie_Wenchang	PRJNA827465	0.913119244
ENSGALG00010011045	ENSGALG00010011045	2	0.350435647	0.163950317	2.137450249	0.032561388	0	0.023871694	0	1	CRA004775;PRJNA827465	Yang2025_Silkie_Wenchang	0.310037591
ENSGALG00010011049	ABL2	2	-0.063099925	0.058335624	-1.081670525	0.279398955	0.001149824	1.17669607	15.01628794	1	PRJNA827465;Yang2025_Silkie_Wenchang	CRA004775	0.777457036
ENSGALG00010011049	ABL2	2	-0.142071668	0.091341697	-1.555386776	0.119853982	0	0.017407508	0	1	CRA004775;Yang2025_Silkie_Wenchang	PRJNA827465	0.580639051
ENSGALG00010011049	ABL2	2	-0.024712478	0.061738002	-0.400279849	0.688950408	0	0.057199151	0	1	CRA004775;PRJNA827465	Yang2025_Silkie_Wenchang	0.919854035
ENSGALG00010011055	TOR3A	2	0.060559147	0.133772057	0.452704013	0.65076189	0.013499506	1.583582509	36.8520431	0.5	PRJNA827465;Yang2025_Silkie_Wenchang	CRA004775	0.908769703
ENSGALG00010011055	TOR3A	2	-0.08095159	0.127526409	-0.634782948	0.525569986	0	0.016554111	0	1	CRA004775;Yang2025_Silkie_Wenchang	PRJNA827465	0.870277065
ENSGALG00010011055	TOR3A	2	0.098644704	0.111089142	0.887977912	0.374552633	0.000649789	1.023571208	2.30284006	0.5	CRA004775;PRJNA827465	Yang2025_Silkie_Wenchang	0.822805106
ENSGALG00010011056	ENSGALG00010011056	2	0.060347074	0.374176404	0.161279743	0.871873077	0	0.062514599	0	0.5	PRJNA827465;Yang2025_Silkie_Wenchang	CRA004775	0.969462602
ENSGALG00010011056	ENSGALG00010011056	2	0.383611661	0.689891719	0.556046189	0.57817928	0.28993181	1.340228272	25.38584501	0.5	CRA004775;Yang2025_Silkie_Wenchang	PRJNA827465	0.88778445
ENSGALG00010011056	ENSGALG00010011056	2	0.372140112	0.509358917	0.730604883	0.465020529	0.09797218	1.127409637	11.30109525	1	CRA004775;PRJNA827465	Yang2025_Silkie_Wenchang	0.852263402
ENSGALG00010011059	ENSGALG00010011059	2	0.459472025	0.432235766	1.063012507	0.287776279	0.334816494	9.318790694	89.26899388	1	PRJNA827465;Yang2025_Silkie_Wenchang	CRA004775	0.782840067
ENSGALG00010011059	ENSGALG00010011059	2	0.709864884	0.296562825	2.393640825	0.016682076	0.068476549	1.527854857	34.54875667	1	CRA004775;Yang2025_Silkie_Wenchang	PRJNA827465	0.214133955
ENSGALG00010011059	ENSGALG00010011059	2	0.07208557	0.125948285	0.572342608	0.567089889	0	0.261996098	0	1	CRA004775;PRJNA827465	Yang2025_Silkie_Wenchang	0.883487517
ENSGALG00010011061	KIF13B	2	-0.062646753	0.063626153	-0.984606954	0.324817173	0	0.006851768	0	1	PRJNA827465;Yang2025_Silkie_Wenchang	CRA004775	0.799914637
ENSGALG00010011061	KIF13B	2	-0.175322633	0.187688249	-0.934116193	0.35024396	0.025706383	1.346846634	25.75249661	1	CRA004775;Yang2025_Silkie_Wenchang	PRJNA827465	0.810111037
ENSGALG00010011061	KIF13B	2	-0.156031706	0.186940037	-0.834661792	0.40390816	0.039092848	1.586385328	36.96361265	1	CRA004775;PRJNA827465	Yang2025_Silkie_Wenchang	0.833112102
ENSGALG00010011062	TMEM55A	2	-0.326366833	0.174953104	-1.865453234	0.062117902	0.050543058	5.546476093	81.97053439	1	PRJNA827465;Yang2025_Silkie_Wenchang	CRA004775	0.429956168
ENSGALG00010011062	TMEM55A	2	-0.37445989	0.202035562	-1.85343553	0.063819959	0.04474567	1.945865249	48.60897996	1	CRA004775;Yang2025_Silkie_Wenchang	PRJNA827465	0.43524481
ENSGALG00010011062	TMEM55A	2	-0.160209109	0.074020355	-2.164392597	0.030434232	0	0.072112152	0	1	CRA004775;PRJNA827465	Yang2025_Silkie_Wenchang	0.297477394
ENSGALG00010011064	MYLIP	2	-0.076733477	0.150123996	-0.511133991	0.609257236	0.036413679	5.053415995	80.21140549	0.5	PRJNA827465;Yang2025_Silkie_Wenchang	CRA004775	0.897238578
ENSGALG00010011064	MYLIP	2	-0.243796681	0.103945128	-2.345436144	0.01900424	0	0.006623411	0	1	CRA004775;Yang2025_Silkie_Wenchang	PRJNA827465	0.23099614
ENSGALG00010011064	MYLIP	2	-0.003029558	0.129424243	-0.023407963	0.981324853	0.015723278	1.424125034	29.78144642	0.5	CRA004775;PRJNA827465	Yang2025_Silkie_Wenchang	0.994962782
ENSGALG00010011067	TMTC2	2	-0.324556015	0.322707529	-1.005728052	0.314546405	0.194151894	14.65207602	93.17502859	1	PRJNA827465;Yang2025_Silkie_Wenchang	CRA004775	0.796531448
ENSGALG00010011067	TMTC2	2	-0.198237227	0.471569773	-0.420377299	0.674209849	0.411615369	13.23310736	92.44319589	0.5	CRA004775;Yang2025_Silkie_Wenchang	PRJNA827465	0.915800735
ENSGALG00010011067	TMTC2	2	0.075299851	0.134060923	0.561683815	0.574331463	0.015191891	1.519466196	34.18741379	0.5	CRA004775;PRJNA827465	Yang2025_Silkie_Wenchang	0.884083039
ENSGALG00010011069	ENSGALG00010011069	2	-0.153895097	0.408810165	-0.376446356	0.706585089	0	0.018594445	0	1	PRJNA827465;Yang2025_Silkie_Wenchang	CRA004775	0.924548793
ENSGALG00010011069	ENSGALG00010011069	2	-0.169291305	0.441751847	-0.383227159	0.701551343	0	0.035859275	0	1	CRA004775;Yang2025_Silkie_Wenchang	PRJNA827465	0.922874082
ENSGALG00010011069	ENSGALG00010011069	2	-0.012106735	0.432275179	-0.53227234	0.594537386	0	0.004225948	0	1	CRA004775;PRJNA827465	Yang2025_Silkie_Wenchang	0.89334442
ENSGALG00010011076	PKP2	2	0.134908665	0.126510786	1.066380739	0.286251565	0	0.040254564	0	1	PRJNA827465;Yang2025_Silkie_Wenchang	CRA004775	0.781944441
ENSGALG00010011076	PKP2	2	0.223922131	0.225253356	0.9940901	0.320179005	0	0.35240148	0	1	CRA004775;Yang2025_Silkie_Wenchang	PRJNA827465	0.798348216
ENSGALG00010011076	PKP2	2	0.141916699	0.145388824	0.976118349	0.32900882	0	0.488455968	0	1	CRA004775;PRJNA827465	Yang2025_Silkie_Wenchang	0.800761292
ENSGALG00010011077	RASGEF1A	2	0.362950486	0.225073157	1.61260486	0.106830382	0.081602643	5.091582738	80.35974172	1	PRJNA827465;Yang2025_Silkie_Wenchang	CRA004775	0.553925396
ENSGALG00010011077	RASGEF1A	2	0.227410031	0.45736707	0.497215155	0.61903704	0.322497924	3.824791127	73.85478143	0.5	CRA004775;Yang2025_Silkie_Wenchang	PRJNA827465	0.899168321
ENSGALG00010011077	RASGEF1A	2	0.101167297	0.143563829	0.704685142	0.4810062	0.007735794	1.070759207	6.608321165	0.5	CRA004775;PRJNA827465	Yang2025_Silkie_Wenchang	0.856783731
ENSGALG00010011080	EMP1	2	0.282054033	0.13634175	2.068728269	0.038571893	0.014258152	1.616472784	38.13691082	1	PRJNA827465;Yang2025_Silkie_Wenchang	CRA004775	0.338965227
ENSGALG00010011080	EMP1	2	0.392287445	0.152118098	2.578838091	0.009913324	0	0.484224896	0	1	CRA004775;Yang2025_Silkie_Wenchang	PRJNA827465	0.157550897
ENSGALG00010011080	EMP1	2	0.149631158	0.136321364	1.097635431	0.272363719	0	0.01994542	0	1	CRA004775;PRJNA827465	Yang2025_Silkie_Wenchang	0.773337076
ENSGALG00010011086	OTUD6A	2	-0.027798135	0.031208812	-0.890714313	0.373082454	0	0.983911686	0	0.5	PRJNA827465;Yang2025_Silkie_Wenchang	CRA004775	0.822162024
ENSGALG00010011086	OTUD6A	2	0.13540987	0.104775632	1.292379406	0.196225785	0	0.847838593	0	1	CRA004775;Yang2025_Silkie_Wenchang	PRJNA827465	0.707668824
ENSGALG00010011086	OTUD6A	2	0.07159196	0.163953764	0.436659448	0.662360388	0.037469887	2.425906168	58.77829023	0.5	CRA004775;PRJNA827465	Yang2025_Silkie_Wenchang	0.91272639
ENSGALG00010011091	YARS2	2	0.055000427	0.083246403	0.660694334	0.508808358	0.006451463	1.738643192	42.48388601	0.5	PRJNA827465;Yang2025_Silkie_Wenchang	CRA004775	0.865193604
ENSGALG00010011091	YARS2	2	-0.03458279	0.106317594	-0.325278149	0.744970567	0	0.283096115	0	0.5	CRA004775;Yang2025_Silkie_Wenchang	PRJNA827465	0.935928212
ENSGALG00010011091	YARS2	2	0.115686211	0.063464807	1.822840338	0.068327576	0	0.002262803	0	1	CRA004775;PRJNA827465	Yang2025_Silkie_Wenchang	0.450487609
ENSGALG00010011094	CSGALNACT2	2	-0.003420481	0.127507112	-0.026825803	0.978598673	0.026268323	4.987408356	79.94950626	0.5	PRJNA827465;Yang2025_Silkie_Wenchang	CRA004775	0.994183692
ENSGALG00010011094	CSGALNACT2	2	-0.161583046	0.093800897	-1.722612785	0.084957757	0	0.253945942	0	1	CRA004775;Yang2025_Silkie_Wenchang	PRJNA827465	0.501162525
ENSGALG00010011094	CSGALNACT2	2	0.000483587	0.180811431	0.002510184	0.997997165	0.04182591	2.039714863	50.97353957	0.5	CRA004775;PRJNA827465	Yang2025_Silkie_Wenchang	0.999913737
ENSGALG00010011095	TNN	2	0.142235201	0.49632977	0.286573986	0.774438535	0	0.119121038	0	1	PRJNA827465;Yang2025_Silkie_Wenchang	CRA004775	0.943630883
ENSGALG00010011095	TNN	2	-0.207441277	0.423494936	-0.489831776	0.624252943	0	0.703599942	0	0.5	CRA004775;Yang2025_Silkie_Wenchang	PRJNA827465	0.900886179
ENSGALG00010011095	TNN	2	-0.264241938	0.393270145	-0.671909478	0.501641326	0	0.290958548	0	0.5	CRA004775;PRJNA827465	Yang2025_Silkie_Wenchang	0.862769519
ENSGALG00010011096	LDB2	2	-0.038609676	0.054185957	-0.712540269	0.476130285	0	0.004717568	0	1	PRJNA827465;Yang2025_Silkie_Wenchang	CRA004775	0.854810196
ENSGALG00010011096	LDB2	2	0.1394857	0.241005177	0.578766407	0.562746801	0.073316611	2.283219556	56.20219715	0.5	CRA004775;Yang2025_Silkie_Wenchang	PRJNA827465	0.882059323
ENSGALG00010011096	LDB2	2	0.126546376	0.242800122	0.521195683	0.602230461	0.083148728	2.598706053	61.51931077	0.5	CRA004775;PRJNA827465	Yang2025_Silkie_Wenchang	0.895349419
ENSGALG00010011099	CCBE1	2	0.142971751	0.287599486	0.497121026	0.619103708	0.136880206	5.63138586	82.24238181	0.5	PRJNA827465;Yang2025_Silkie_Wenchang	CRA004775	0.899168321
ENSGALG00010011099	CCBE1	2	0.442993687	0.18933136	2.339779773	0.019295114	0	0.017848595	0	1	CRA004775;Yang2025_Silkie_Wenchang	PRJNA827465	0.232624497
ENSGALG00010011099	CCBE1	2	-0.078400393	0.143738161	-0.545438959	0.585451652	0.006053095	1.049348609	4.702785006	0.5	CRA004775;PRJNA827465	Yang2025_Silkie_Wenchang	0.891135366
ENSGALG00010011101	ENSGALG00010011101	2	-0.249934133	1.028547962	-0.242997062	0.808007673	1.887929554	9.268149008	89.21035906	0.5	PRJNA827465;Yang2025_Silkie_Wenchang	CRA004775	0.952496413
ENSGALG00010011101	ENSGALG00010011101	2	0.208591396	0.654905911	0.318505997	0.750101272	0.539520518	2.578602668	61.21088249	0.5	CRA004775;Yang2025_Silkie_Wenchang	PRJNA827465	0.931974353
ENSGALG00010011101	ENSGALG00010011101	2	-1.039893227	0.411086745	-2.529619939	0.011418614	0	0.714337041	0	1	CRA004775;PRJNA827465	Yang2025_Silkie_Wenchang	0.17044842
ENSGALG00010011104	ABCG1	2	-0.048716869	0.153998835	-0.316345701	0.751740127	0	0.1509171	0	0.5	PRJNA827465;Yang2025_Silkie_Wenchang	CRA004775	0.937802658
ENSGALG00010011104	ABCG1	2	-0.356354175	0.565736601	-0.629894149	0.528763841	0.405382041	2.202602776	54.59916737	0.5	CRA004775;Yang2025_Silkie_Wenchang	PRJNA827465	0.871809618
ENSGALG00010011104	ABCG1	2	-0.382027285	0.481146447	-0.793990778	0.427199003	0.274584715	1.84307089	45.74272723	1	CRA004775;PRJNA827465	Yang2025_Silkie_Wenchang	0.84125685
ENSGALG00010011106	USP40	2	-0.001412332	0.071119841	-0.019858478	0.984156268	0	0.041040565	0	0.5	PRJNA827465;Yang2025_Silkie_Wenchang	CRA004775	0.995669738
ENSGALG00010011106	USP40	2	-0.089658838	0.120764888	-0.742424722	0.457830046	0	0.905742683	0	0.5	CRA004775;Yang2025_Silkie_Wenchang	PRJNA827465	0.849740002
ENSGALG00010011106	USP40	2	-0.054135145	0.121666443	-0.444947214	0.656357002	0.010492332	1.342952069	25.53717866	0.5	CRA004775;PRJNA827465	Yang2025_Silkie_Wenchang	0.910509456
ENSGALG00010011107	ENSGALG00010011107	2	0.027869379	0.121707607	0.228986338	0.818879535	0	0.146012424	0	0.5	PRJNA827465;Yang2025_Silkie_Wenchang	CRA004775	0.955256039
ENSGALG00010011107	ENSGALG00010011107	2	-0.149061357	0.297852691	-0.500453282	0.616755944	0.105174446	2.290451225	56.34048046	0.5	CRA004775;Yang2025_Silkie_Wenchang	PRJNA827465	0.899111936
ENSGALG00010011107	ENSGALG00010011107	2	-0.16853498	0.238528965	-0.706559811	0.479840071	0.059186766	1.807565831	44.67698034	1	CRA004775;PRJNA827465	Yang2025_Silkie_Wenchang	0.856284002
ENSGALG00010011109	ENSGALG00010011109	2	0.373242308	0.458483925	0.814079377	0.415599479	0	0.17208357	0	1	PRJNA827465;Yang2025_Silkie_Wenchang	CRA004775	0.837689773
ENSGALG00010011109	ENSGALG00010011109	2	0.648173255	0.459360524	1.411033865	0.158234629	0	0.120659184	0	1	CRA004775;Yang2025_Silkie_Wenchang	PRJNA827465	0.652026484
ENSGALG00010011109	ENSGALG00010011109	2	0.489976843	0.525168581	0.932989635	0.35082532	0	0.469733324	0	1	CRA004775;PRJNA827465	Yang2025_Silkie_Wenchang	0.810146221
ENSGALG00010011111	MRPS14	2	-0.004074739	0.07089183	-0.057478266	0.954164219	0	0.006174267	0	0.5	PRJNA827465;Yang2025_Silkie_Wenchang	CRA004775	0.987925986
ENSGALG00010011111	MRPS14	2	-0.028364671	0.162974836	-0.174043255	0.861831468	0	0.128386269	0	0.5	CRA004775;Yang2025_Silkie_Wenchang	PRJNA827465	0.965635505
ENSGALG00010011111	MRPS14	2	-0.013108063	0.073676786	-0.177913064	0.858791251	0	0.131018832	0	0.5	CRA004775;PRJNA827465	Yang2025_Silkie_Wenchang	0.964472541
ENSGALG00010011116	TAPT1	2	-0.172203603	0.128906564	-1.335879239	0.181588748	0.026049909	4.615949224	78.33598353	1	PRJNA827465;Yang2025_Silkie_Wenchang	CRA004775	0.689004635
ENSGALG00010011116	TAPT1	2	0.024090132	0.370068765	0.065096347	0.948097289	0.236767655	6.770134538	85.22924479	0.5	CRA004775;Yang2025_Silkie_Wenchang	PRJNA827465	0.986705854
ENSGALG00010011116	TAPT1	2	0.127925517	0.233676575	0.547446902	0.58407174	0.078625505	2.955095097	66.16014147	0.5	CRA004775;PRJNA827465	Yang2025_Silkie_Wenchang	0.890763231
ENSGALG00010011118	GMPR2	2	-0.01299698	0.073807821	-0.176092178	0.860221526	0	0.672376303	0	0.5	PRJNA827465;Yang2025_Silkie_Wenchang	CRA004775	0.96498706
ENSGALG00010011118	GMPR2	2	-0.094239221	0.102064525	-0.923329838	0.355835343	0	0.092934115	0	1	CRA004775;Yang2025_Silkie_Wenchang	PRJNA827465	0.812301302
ENSGALG00010011118	GMPR2	2	0.014625754	0.092381321	0.158319386	0.874205133	0	0.559552847	0	0.5	CRA004775;PRJNA827465	Yang2025_Silkie_Wenchang	0.970094827
ENSGALG00010011119	ENSGALG00010011119	2	-0.448474858	0.1406912	-3.187653928	0.001434321	0	0.018914596	0	1	PRJNA827465;Yang2025_Silkie_Wenchang	CRA004775	0.04689943
ENSGALG00010011119	ENSGALG00010011119	2	-0.745262403	0.332162613	-2.243667329	0.024853814	0.059958843	1.339117523	25.32395532	1	CRA004775;Yang2025_Silkie_Wenchang	PRJNA827465	0.268931467
ENSGALG00010011119	ENSGALG00010011119	2	-0.664209446	0.340018466	-1.953451098	0.050766166	0.142130558	2.083054375	51.99357193	1	CRA004775;PRJNA827465	Yang2025_Silkie_Wenchang	0.390004426
ENSGALG00010011121	NUP35	2	-0.218438335	0.250598393	-0.871666943	0.383390099	0.115050748	11.67729887	91.43637573	0.5	PRJNA827465;Yang2025_Silkie_Wenchang	CRA004775	0.825509017
ENSGALG00010011121	NUP35	2	-0.372583854	0.145660455	-2.557892963	0.01053085	0.016618272	1.555849675	35.72643834	1	CRA004775;Yang2025_Silkie_Wenchang	PRJNA827465	0.163408236
ENSGALG00010011121	NUP35	2	-1.62603E-05	0.069506365	-0.00023394	0.999813343	0	0.787399994	0	0.5	CRA004775;PRJNA827465	Yang2025_Silkie_Wenchang	0.999889665
ENSGALG00010011124	CACYBP	2	-0.070424366	0.045887141	-1.534729872	0.124850195	0	0.533327322	0	1	PRJNA827465;Yang2025_Silkie_Wenchang	CRA004775	0.592935337
ENSGALG00010011124	CACYBP	2	-0.121462706	0.114569346	-1.060167573	0.28906837	0	0.418992575	0	0.5	CRA004775;Yang2025_Silkie_Wenchang	PRJNA827465	0.783530627
ENSGALG00010011124	CACYBP	2	-0.054410828	0.048391937	-1.124377965	0.260852716	0	0.113949504	0	0.5	CRA004775;PRJNA827465	Yang2025_Silkie_Wenchang	0.76792161
ENSGALG00010011126	ATXN1	2	0.254319789	0.286087414	0.888958329	0.374025476	0.124060613	3.638595565	72.51686861	1	PRJNA827465;Yang2025_Silkie_Wenchang	CRA004775	0.822579781
ENSGALG00010011126	ATXN1	2	0.47038594	0.223572953	2.103948321	0.035382955	0	0.57756207	0	1	CRA004775;Yang2025_Silkie_Wenchang	PRJNA827465	0.324684241
ENSGALG00010011126	ATXN1	2	0.042324391	0.10255595	0.412695618	0.679829635	0	0.407669814	0	1	CRA004775;PRJNA827465	Yang2025_Silkie_Wenchang	0.917701868
ENSGALG00010011128	GCNT4	2	0.141757664	0.200232743	0.70796448	0.478967336	0.002557008	1.032221093	3.121530159	0.5	PRJNA827465;Yang2025_Silkie_Wenchang	CRA004775	0.855875059
ENSGALG00010011128	GCNT4	2	-0.078618016	0.230272214	-0.341413384	0.732792401	0	0.003402586	0	1	CRA004775;Yang2025_Silkie_Wenchang	PRJNA827465	0.932985777
ENSGALG00010011128	GCNT4	2	0.183474715	0.20977132	0.874641562	0.381768966	0	0.73462042	0	0.5	CRA004775;PRJNA827465	Yang2025_Silkie_Wenchang	0.824727558
ENSGALG00010011129	ENSGALG00010011129	2	-0.385433571	0.106273825	-3.626796832	0.000286959	0.003636976	1.155884908	13.4861963	1	PRJNA827465;Yang2025_Silkie_Wenchang	CRA004775	0.016537164
ENSGALG00010011129	ENSGALG00010011129	2	-0.245645076	0.304393268	-0.806999043	0.419667035	0.15008699	5.260293878	80.98965527	0.5	CRA004775;Yang2025_Silkie_Wenchang	PRJNA827465	0.838527387
ENSGALG00010011129	ENSGALG00010011129	2	-0.159429738	0.185606724	-0.858965313	0.390359654	0.046535416	2.910236498	65.63853141	0.5	CRA004775;PRJNA827465	Yang2025_Silkie_Wenchang	0.829251057
ENSGALG00010011130	DNM1L	2	0.012340507	0.051453781	0.239836741	0.810456823	0.002895999	2.11667999	52.75620288	0.5	PRJNA827465;Yang2025_Silkie_Wenchang	CRA004775	0.952997426
ENSGALG00010011130	DNM1L	2	0.081098491	0.056097654	1.445666354	0.148270806	0	0.088343204	0	1	CRA004775;Yang2025_Silkie_Wenchang	PRJNA827465	0.638004061
ENSGALG00010011130	DNM1L	2	-0.020714434	0.038266526	-0.541319943	0.588287077	0	0.935219049	0	0.5	CRA004775;PRJNA827465	Yang2025_Silkie_Wenchang	0.891874519
ENSGALG00010011131	RET	2	-0.129623099	0.181969381	-0.712334668	0.476257561	0	0.018544726	0	1	PRJNA827465;Yang2025_Silkie_Wenchang	CRA004775	0.854875361
ENSGALG00010011131	RET	2	-0.177992223	0.255345257	-0.697064927	0.485762162	0	0.02751343	0	1	CRA004775;Yang2025_Silkie_Wenchang	PRJNA827465	0.857391223
ENSGALG00010011131	RET	2	-0.129770601	0.222879725	-0.582244982	0.560401679	0	0.060451766	0	1	CRA004775;PRJNA827465	Yang2025_Silkie_Wenchang	0.881203589
ENSGALG00010011133	CDC37L1	2	-0.121481637	0.065009923	-1.868662997	0.061669715	0	0.362007948	0	1	PRJNA827465;Yang2025_Silkie_Wenchang	CRA004775	0.428734062
ENSGALG00010011133	CDC37L1	2	-0.043305419	0.185290514	-0.233716329	0.81520521	0.039525443	1.834823833	45.49885489	0.5	CRA004775;Yang2025_Silkie_Wenchang	PRJNA827465	0.953933933
ENSGALG00010011133	CDC37L1	2	-0.023224034	0.124155489	-0.187056043	0.851616693	0.008808374	1.182230989	15.41416111	0.5	CRA004775;PRJNA827465	Yang2025_Silkie_Wenchang	0.96214473
ENSGALG00010011136	ENSGALG00010011136	2	-0.723442212	0.23115012	-3.129750535	0.001749548	0	0.031662126	0	1	PRJNA827465;Yang2025_Silkie_Wenchang	CRA004775	0.053057476
ENSGALG00010011136	ENSGALG00010011136	2	-0.515732478	0.329399922	-1.565672739	0.117425251	0	0.496192741	0	1	CRA004775;Yang2025_Silkie_Wenchang	PRJNA827465	0.57688585
ENSGALG00010011136	ENSGALG00010011136	2	-0.64458713	0.258283506	-2.495657347	0.012572398	0	0.790832706	0	1	CRA004775;PRJNA827465	Yang2025_Silkie_Wenchang	0.181574995
ENSGALG00010011141	MSX1	2	0.941842866	0.453991268	2.074583659	0.038025138	0	0.260950988	0	1	PRJNA827465;Yang2025_Silkie_Wenchang	CRA004775	0.336310939
ENSGALG00010011141	MSX1	2	0.669084457	0.559815892	1.195186609	0.232014129	0.105806874	1.203091151	16.88077838	1	CRA004775;Yang2025_Silkie_Wenchang	PRJNA827465	0.744396027
ENSGALG00010011141	MSX1	2	0.498656715	0.454315176	1.097600832	0.27237883	0	0.482625143	0	1	CRA004775;PRJNA827465	Yang2025_Silkie_Wenchang	0.773337076
ENSGALG00010011142	DUSP19	2	-0.648037211	0.157758289	-4.107785482	3.99471E-05	0.028980772	2.207142188	54.6925429	1	PRJNA827465;Yang2025_Silkie_Wenchang	CRA004775	0.004131685
ENSGALG00010011142	DUSP19	2	-0.426092122	0.4415463	-0.96499987	0.334544877	0.338360218	7.488285036	86.64580748	0.5	CRA004775;Yang2025_Silkie_Wenchang	PRJNA827465	0.803101658
ENSGALG00010011142	DUSP19	2	-0.296656903	0.274515082	-1.080657942	0.279849298	0.116699196	3.984558293	74.90311531	0.5	CRA004775;PRJNA827465	Yang2025_Silkie_Wenchang	0.778040488
ENSGALG00010011143	AK3	2	-0.118121791	0.251309272	-0.4700256	0.638336728	0.059813878	1.889596549	47.07865018	0.5	PRJNA827465;Yang2025_Silkie_Wenchang	CRA004775	0.905560311
ENSGALG00010011143	AK3	2	-0.074842982	0.346947332	-0.215718569	0.829207126	0.142382255	2.414121927	58.57707148	0.5	CRA004775;Yang2025_Silkie_Wenchang	PRJNA827465	0.956860127
ENSGALG00010011143	AK3	2	0.175785269	0.198376453	0.886119627	0.375553068	0	0.20497984	0	1	CRA004775;PRJNA827465	Yang2025_Silkie_Wenchang	0.822974013
ENSGALG00010011144	ATF7IP	2	0.179478214	0.261411163	0.686574406	0.492350961	0.109548681	4.283798212	76.65623004	0.5	PRJNA827465;Yang2025_Silkie_Wenchang	CRA004775	0.859228604
ENSGALG00010011144	ATF7IP	2	0.149682818	0.380347129	0.393542653	0.693918727	0.20602167	3.425893	70.81053027	0.5	CRA004775;PRJNA827465	PRJNA827465	0.920728119
ENSGALG00010011144	ATF7IP	2	-0.039246555	0.058247348	-0.673791269	0.500444027	0	0.472814601	0	1	CRA004775;PRJNA827465	Yang2025_Silkie_Wenchang	0.862400704

ENSGALG00010011146	BMS1	2	0.270612805	0.320748525	0.843691503	0.398841817	0.192647319	15.25275159	93.4438059	0.5	PRJNA827465;Yang2025_Silkie_Wenchang	CRA004775	0.832054308
ENSGALG00010011146	BMS1	2	0.32833367	0.294151472	1.116206108	0.26433392	0.140918413	5.307416606	81.15844159	1	CRA004775;Yang2025_Silkie_Wenchang	PRJNA827465	0.768790489
ENSGALG00010011146	BMS1	2	-0.031317535	0.063056516	-0.496658187	0.619430113	0	0.059183531	0	0.5	CRA004775;PRJNA827465	Yang2025_Silkie_Wenchang	0.899230989
ENSGALG00010011148	ENSGALG00010011148	2	-0.19615807	0.110753315	-1.771125953	0.076539759	0.010501748	1.656563122	39.63405397	1	PRJNA827465;Yang2025_Silkie_Wenchang	CRA004775	0.47757456
ENSGALG00010011148	ENSGALG00010011148	2	-0.38324597	0.134297163	-2.853716047	0.004321114	0	0.319368027	0	1	CRA004775;Yang2025_Silkie_Wenchang	PRJNA827465	0.096386336
ENSGALG00010011148	ENSGALG00010011148	2	-0.231254213	0.186838717	-1.237721045	0.215819513	0.040255433	1.897634274	47.30280677	1	CRA004775;PRJNA827465	Yang2025_Silkie_Wenchang	0.729213578
ENSGALG00010011150	FGD4	2	0.111183365	0.116548167	0.953969224	0.340099221	0	0.209689968	0	1	PRJNA827465;Yang2025_Silkie_Wenchang	CRA004775	0.805377063
ENSGALG00010011150	FGD4	2	0.138069276	0.20080416	0.687581751	0.491716204	0	0.434258008	0	0.5	CRA004775;Yang2025_Silkie_Wenchang	PRJNA827465	0.85895707
ENSGALG00010011150	FGD4	2	0.060712123	0.132611411	0.457819752	0.647081944	0	0.216003993	0	0.5	CRA004775;PRJNA827465	Yang2025_Silkie_Wenchang	0.907350807
ENSGALG00010011152	SLC18A3	2	-0.467331543	0.445860217	-1.048157082	0.294566241	0	0.105880055	0	1	PRJNA827465;Yang2025_Silkie_Wenchang	CRA004775	0.787408474
ENSGALG00010011152	SLC18A3	2	-0.189405855	0.500862432	-0.378159437	0.705312156	0	0.034651376	0	1	CRA004775;Yang2025_Silkie_Wenchang	PRJNA827465	0.924281103
ENSGALG00010011152	SLC18A3	2	-0.389835792	0.441873512	-0.882233899	0.377650334	0	0.287904116	0	1	CRA004775;PRJNA827465	Yang2025_Silkie_Wenchang	0.82325159
ENSGALG00010011161	ENSGALG00010011161	2	0.005358616	0.054573768	0.098190318	0.92178117	0	0.154237419	0	0.5	PRJNA827465;Yang2025_Silkie_Wenchang	CRA004775	0.981649741
ENSGALG00010011161	ENSGALG00010011161	2	0.045388568	0.098834589	0.459237691	0.646063487	0.00226857	1.126030526	11.19246089	0.5	CRA004775;Yang2025_Silkie_Wenchang	PRJNA827465	0.907220702
ENSGALG00010011161	ENSGALG00010011161	2	0.038326931	0.056719233	0.675730776	0.499211593	0	0.86365967	0	1	CRA004775;PRJNA827465	Yang2025_Silkie_Wenchang	0.861784825
ENSGALG00010011166	SOX7	2	-0.089013937	0.112143764	-0.793748436	0.427341897	0.016257582	2.792563103	64.19060329	0.5	PRJNA827465;Yang2025_Silkie_Wenchang	CRA004775	0.84125685
ENSGALG00010011166	SOX7	2	-0.183133935	0.101959616	-1.796141859	0.072471959	0	0.99204534	0	0.5	CRA004775;Yang2025_Silkie_Wenchang	PRJNA827465	0.464985342
ENSGALG00010011166	SOX7	2	0.021930578	0.081587673	0.268797694	0.788085368	0	0.193991242	0	1	CRA004775;PRJNA827465	Yang2025_Silkie_Wenchang	0.946919116
ENSGALG00010011170	RBM24	2	0.074990653	0.128707955	0.582641947	0.560134361	0.023291827	3.337792376	70.04007777	0.5	PRJNA827465;Yang2025_Silkie_Wenchang	CRA004775	0.881203589
ENSGALG00010011170	RBM24	2	0.243821006	0.104689175	2.328999212	0.019859107	0	0.995544298	0	1	CRA004775;Yang2025_Silkie_Wenchang	PRJNA827465	0.236593665
ENSGALG00010011170	RBM24	2	0.174370188	0.30071337	0.579855123	0.562012321	0.131098407	2.934997862	65.92842493	0.5	CRA004775;PRJNA827465	Yang2025_Silkie_Wenchang	0.881857644
ENSGALG00010011175	GPR52	2	-0.410615559	0.278094693	-1.476531444	0.13980128	0	0.635019294	0	1	PRJNA827465;Yang2025_Silkie_Wenchang	CRA004775	0.623607958
ENSGALG00010011175	GPR52	2	-0.28434048	0.405661612	-0.700930212	0.483346569	0	0.494831079	0	1	CRA004775;Yang2025_Silkie_Wenchang	PRJNA827465	0.85691916
ENSGALG00010011175	GPR52	2	-0.583925746	0.302771549	-1.928601772	0.053780319	0	0.026777531	0	1	CRA004775;PRJNA827465	Yang2025_Silkie_Wenchang	0.401964436
ENSGALG00010011178	RSPO2	2	-0.305192325	0.15935032	-1.915228818	0.055463329	0	0.000947348	0	1	PRJNA827465;Yang2025_Silkie_Wenchang	CRA004775	0.407910781
ENSGALG00010011178	RSPO2	2	-0.171571981	0.267089907	-0.642375382	0.52062948	0	0.943261851	0	0.5	CRA004775;Yang2025_Silkie_Wenchang	PRJNA827465	0.869331539
ENSGALG00010011178	RSPO2	2	-0.224872563	0.221448951	-1.015460047	0.309886582	0.020334155	1.099240806	9.028122488	0.5	CRA004775;PRJNA827465	Yang2025_Silkie_Wenchang	0.794395438
ENSGALG00010011181	ENSGALG00010011181	2	-0.405050586	0.153223836	-2.643522037	0.008204842	0	0.052605389	0	1	PRJNA827465;Yang2025_Silkie_Wenchang	CRA004775	0.140757277
ENSGALG00010011181	ENSGALG00010011181	2	-0.088267203	0.425677888	-0.207356796	0.835731221	0.251306048	3.073796702	67.46694408	0.5	CRA004775;Yang2025_Silkie_Wenchang	PRJNA827465	0.958339923
ENSGALG00010011181	ENSGALG00010011181	2	-0.072183836	0.384976604	-0.187501876	0.851267153	0.202404073	2.831014634	64.67697525	0.5	CRA004775;PRJNA827465	Yang2025_Silkie_Wenchang	0.962071965
ENSGALG00010011184	FAM8A1	2	-0.264754524	0.136346829	-1.941772513	0.052164646	0.031398651	6.253705647	84.00948083	1	PRJNA827465;Yang2025_Silkie_Wenchang	CRA004775	0.396562297
ENSGALG00010011184	FAM8A1	2	-0.110379005	0.353483605	-0.312260605	0.754842476	0.206840196	5.170918164	80.66107472	0.5	CRA004775;Yang2025_Silkie_Wenchang	PRJNA827465	0.93838723
ENSGALG00010011184	FAM8A1	2	-0.015047433	0.198242641	-0.075904116	0.939495382	0.051112177	2.089885254	52.150483	0.5	CRA004775;PRJNA827465	Yang2025_Silkie_Wenchang	0.985425793
ENSGALG00010011190	ENSGALG00010011190	2	-0.226638725	0.140001902	-1.618826036	0.105484698	0	0.334577467	0	1	PRJNA827465;Yang2025_Silkie_Wenchang	CRA004775	0.551018754
ENSGALG00010011190	ENSGALG00010011190	2	-0.068533822	0.305617714	-0.224246892	0.822565198	0.107588187	2.336246207	57.19629221	0.5	CRA004775;Yang2025_Silkie_Wenchang	PRJNA827465	0.955822987
ENSGALG00010011190	ENSGALG00010011190	2	-0.032741403	0.204145925	-0.160382349	0.872579893	0.033364724	1.537136932	34.94398717	0.5	CRA004775;PRJNA827465	Yang2025_Silkie_Wenchang	0.969629321
ENSGALG00010011192	RPL17	2	0.168101313	0.281331294	0.597520845	0.550159694	0.10957232	3.148968228	68.24356654	0.5	PRJNA827465;Yang2025_Silkie_Wenchang	CRA004775	0.878679315
ENSGALG00010011192	RPL17	2	0.070205128	0.435980275	0.161028221	0.872071174	0.292153349	4.28486479	76.66204072	0.5	CRA004775;Yang2025_Silkie_Wenchang	PRJNA827465	0.969560463
ENSGALG00010011192	RPL17	2	-0.15154686	0.156922692	-0.965742169	0.334173213	0	0.671686896	0	1	CRA004775;PRJNA827465	Yang2025_Silkie_Wenchang	0.803094614
ENSGALG00010011193	FAM20B	2	-0.112594449	0.175888455	-0.640146897	0.522077103	0.054301749	7.915810953	87.36705556	0.5	PRJNA827465;Yang2025_Silkie_Wenchang	CRA004775	0.869568211
ENSGALG00010011193	FAM20B	2	-0.237806945	0.117380134	-2.025955649	0.04276933	0.005711336	1.167828918	14.37101919	1	CRA004775;Yang2025_Silkie_Wenchang	PRJNA827465	0.357727807
ENSGALG00010011193	FAM20B	2	0.047505042	0.058760767	0.808448291	0.418832562	0	0.083568262	0	0.5	CRA004775;PRJNA827465	Yang2025_Silkie_Wenchang	0.838250071
ENSGALG00010011198	PINX1	2	0.051428171	0.110776309	0.464252434	0.642466899	0.014869217	2.443718796	59.07876136	0.5	PRJNA827465;Yang2025_Silkie_Wenchang	CRA004775	0.906634523
ENSGALG00010011198	PINX1	2	0.043760669	0.18361014	0.23833471	0.811621502	0.038360808	2.100673512	52.39621987	0.5	CRA004775;Yang2025_Silkie_Wenchang	PRJNA827465	0.953348795
ENSGALG00010011198	PINX1	2	-0.059288836	0.075379328	-0.786539723	0.431551357	0	0.405682806	0	1	CRA004775;PRJNA827465	Yang2025_Silkie_Wenchang	0.842503929
ENSGALG00010011200	PCDHB4	2	0.374477363	0.447701718	0.836443884	0.402905234	0	0.911918014	0	1	PRJNA827465;Yang2025_Silkie_Wenchang	CRA004775	0.833002862
ENSGALG00010011200	PCDHB4	2	0.896119182	0.531774246	1.685149643	0.091959704	0	0.022852729	0	1	CRA004775;Yang2025_Silkie_Wenchang	PRJNA827465	0.519402219
ENSGALG00010011200	PCDHB4	2	0.343218987	0.43768389	0.78417094	0.432939814	0	0.672233406	0	1	CRA004775;PRJNA827465	Yang2025_Silkie_Wenchang	0.842762754
ENSGALG00010011203	ENSGALG00010011203	2	-0.1418225	0.283428287	-0.500382305	0.616805911	0.126997457	4.718789468	78.80812427	0.5	PRJNA827465;Yang2025_Silkie_Wenchang	CRA004775	0.899111936
ENSGALG00010011203	ENSGALG00010011203	2	-0.358910535	0.156149146	-2.298511033	0.021532721	0	0.364363229	0	1	CRA004775;Yang2025_Silkie_Wenchang	PRJNA827465	0.248109211
ENSGALG00010011203	ENSGALG00010011203	2	-0.021171911	0.184207836	-0.114934908	0.908496716	0.030415973	1.745000377	42.69342211	0.5	CRA004775;PRJNA827465	Yang2025_Silkie_Wenchang	0.978291088
ENSGALG00010011205	CYTL1	2	1.984441184	0.824098905	2.408013373	0.016039593	0.575767009	1.735592041	42.38277336	1	PRJNA827465;Yang2025_Silkie_Wenchang	CRA004775	0.209265194
ENSGALG00010011205	CYTL1	2	-1.54050788	4.412952022	-0.349087838	0.727023367	37.67940552	30.56002836	96.72775173	0.5	CRA004775;Yang2025_Silkie_Wenchang	PRJNA827465	0.93142315
ENSGALG00010011205	CYTL1	2	-2.34993747	3.588461448	-0.654859333	0.512558326	24.50592035	20.48650123	95.11873702	0.5	CRA004775;PRJNA827465	Yang2025_Silkie_Wenchang	0.865859188
ENSGALG00010011210	NUP153	2	-0.047286286	0.04502698	-1.050176702	0.293636879	0	0.842290926	0	1	PRJNA827465;Yang2025_Silkie_Wenchang	CRA004775	0.786979185
ENSGALG00010011210	NUP153	2	-0.009128623	0.15109086	-0.060418099	0.951822644	0.027235309	2.300526898	56.53169712	0.5	CRA004775;Yang2025_Silkie_Wenchang	PRJNA827465	0.987719213
ENSGALG00010011210	NUP153	2	0.006883493	0.07618949	0.090347022	0.928011455	0.004360989	1.252747994	20.17548586	0.5	CRA004775;PRJNA827465	Yang2025_Silkie_Wenchang	0.983007166
ENSGALG00010011211	SLC37A1	2	-0.226129059	0.156938455	-1.440877307	0.149619348	0.03195695	2.771510419	63.91859135	1	PRJNA827465;Yang2025_Silkie_Wenchang	CRA004775	0.639445128
ENSGALG00010011211	SLC37A1	2	-0.283048807	0.171568405	-1.649772331	0.09898951	0.01981864	1.423865109	29.76862812	1	CRA004775;Yang2025_Silkie_Wenchang	PRJNA827465	0.536907794
ENSGALG00010011211	SLC37A1	2	-0.080056213	0.101038249	-0.792335711	0.428164954	0	0.029124946	0	1	CRA004775;PRJNA827465	Yang2025_Silkie_Wenchang	0.84125685
ENSGALG00010011212	NCKAP1	2	-0.025541933	0.027501687	-0.92874058	0.353023545	0	0.673159566	0	1	PRJNA827465;Yang2025_Silkie_Wenchang	CRA004775	0.811035834
ENSGALG00010011212	NCKAP1	2	0.108488048	0.254615993	0.426084971	0.670045931	0.105302899	5.263903296	81.00269052	0.5	CRA004775;Yang2025_Silkie_Wenchang	PRJNA827465	0.914428878
ENSGALG00010011212	NCKAP1	2	0.139373098	0.194896983	0.715111623	0.474540073	0.062857856	4.911445505	79.63939539	0.5	CRA004775;PRJNA827465	Yang2025_Silkie_Wenchang	0.854462864
ENSGALG00010011213	XKR6	2	0.490181733	0.531970351	0.92144559	0.356817836	0.522463436	12.991624	92.3027329	0.5	PRJNA827465;Yang2025_Silkie_Wenchang	CRA004775	0.812971699
ENSGALG00010011213	XKR6	2	0.446238129	0.590720999	0.755412674	0.450001411	0.637537247	11.52648551	91.32432866	0.5	CRA004775;Yang2025_Silkie_Wenchang	PRJNA827465	0.847526417
ENSGALG00010011213	XKR6	2	-0.077696731	0.158367096	-0.490611579	0.623701195	0	0.124924397	0	1	CRA004775;PRJNA827465	Yang2025_Silkie_Wenchang	0.900519204
ENSGALG00010011218	ENSGALG00010011218	2	0.374615448	0.326883933	1.146019766	0.251786973	0	0.111170928	0	1	PRJNA827465;Yang2025_Silkie_Wenchang	CRA004775	0.761871849
ENSGALG00010011218	ENSGALG00010011218	2	0.165896222	0.465033419	0.35674043	0.721286136	0	0.107905875	0	0.5	CRA004775;Yang2025_Silkie_Wenchang	PRJNA827465	0.928950734
ENSGALG00010011218	ENSGALG00010011218	2	0.379769207	0.391516717	0.96999487	0.332049049	0	0.286611968	0	0.5	CRA004775;PRJNA827465	Yang2025_Silkie_Wenchang	0.802002658
ENSGALG00010011222	RCL1	2	-0.103876286	0.154769713	-0.671166754	0.502114305	0.040309547	6.166029258	83.78210744	0.5	PRJNA827465;Yang2025_Silkie_Wenchang	CRA004775	0.862860181
ENSGALG00010011222	RCL1	2	-0.24950633	0.098120132	-2.542865827	0.010994744	0	0.291774724	0	1	CRA004775;Yang2025_Silkie_Wenchang	PRJNA827465	0.166811819
ENSGALG00010011222	RCL1	2	0.032996351	0.066373923	0.497128235	0.619098625	0	0.255934469	0	0.5	CRA004775;PRJNA827465	Yang2025_Silkie_Wenchang	0.899168321
ENSGALG00010011231	PLBD1	2	-0.099921055	0.148590333	-0.67246	0.501290897	0.025605754	2.37732792	57.93596703	0.5	PRJNA827465;Yang2025_Silkie_Wenchang	CRA004775	0.862658912
ENSGALG00010011231	PLBD1	2	0.025262718	0.128959989	0.195895783	0.844691738	0	0.237738487	0	0.5	CRA004775;Yang2025_Silkie_Wenchang	PRJNA827465	0.960769014
ENSGALG00010011231	PLBD1	2	-0.22561037	0.12152474	-1.856497446	0.063382687	0	0.137061184	0	1	CRA004775;PRJNA827465	Yang2025_Silkie_Wenchang	0.433558714
ENSGALG00010011235	ENSGALG00010011235	2	0.172834001	0.092811384	1.862206915	0.06257393	0.006891509	1.570273884	36.31684189	1	PRJNA827465;Yang2025_Silkie_Wenchang	CRA004775	0.431065134
ENSGALG00010011235	ENSGALG00010011235	2	-0.023072392	0.344648746	-0.066944656	0.946625762	0.205188656	7.145249004	86.00468648	0.5	CRA004775;Yang2025_Silkie_Wenchang	PRJNA827465	0.986445196
ENSGALG00010011235	ENSGALG00010011235	2	-0.100025607	0.244226897	-0.40956016	0.682128627	0.095354198	4.451088774	77.53358671	0.5	CRA004775;PRJNA827465	Yang2025_Silkie_Wenchang	0.918524893
ENSGALG00010011237	ENSGALG00010011237	2	-0.276051751	0.227395843	-1.213970083	0.224759148	0.028427666	1.376796364	27.36761762	1	PRJNA827465;Yang2025_Silkie_Wenchang	CRA004775	0.737885283
ENSGALG00010011237	ENSGALG00010011237	2	-0.44691449	0.253595296	-1.762313799	0.078016294	0	0.288781984	0	1	CRA004775;Yang2025_Silkie_Wenchang	PRJNA827465	0.480833369
ENSGALG00010011237	ENSGALG00010011237	2	-0.08711823	0.235787127	-0.369478311	0.711771237	0	0.0439252	0	1	CRA004775;PRJNA827465	Yang2025_Silkie_Wenchang	0.92597752
ENSGALG00010011239	RALGPS2	2	0.045735731	0.066021676	0.692738109	0.488473919	0.001611454	1.15857142	13.68680578	1	PRJNA827465;Yang2025_Silkie_Wenchang	CRA004775	0.857837717
ENSGALG00010011239	RALGPS2	2	0.134930091	0.118941643	1.134422633	0.25661731	0	0.351954924	0	0.5	CRA004775;Yang2025_Silkie_Wenchang	PRJNA827465	0.764677554
ENSGALG00010011239	RALGPS2	2	0.005969904	0.06337649	0.094197455	0.924952307	0	0.030792767	0	0.5	CRA004775;PRJNA827465	Yang2025_Silkie_Wenchang	0.982572225
ENSGALG00010011244	PDE9A	2	0.113072883	0.092420399	1.223462398	0.221155098	0	0.674201853	0	1	PRJNA827465;Yang2025_Silkie_Wenchang	CRA004775	0.734436741
ENSGALG00010011244	PDE9A	2	0.011330489	0.133619433	0.084796718	0.932423003	0	0.056966031	0	0.5	CRA004775;Yang2025_Silkie_Wenchang	PRJNA827465	0.984224753
ENSGALG00010011244	PDE9A	2	0.153791415	0.115263996	1.334253715	0.182120721	0	0.401045819	0	0.5	CRA004775;PRJNA827465	Yang2025_Silkie_Wenchang	0.690033956
ENSGALG00010011246	DNAJB5	2	-0.171842497	0.093493989	-1.838005826	0.066061547	0	0.483710185	0	1	PRJNA827465;Yang2025_Silkie_Wenchang	CRA004775	0.442444044
ENSGALG00010011246	DNAJB5	2	-0.28311354	0.154816403	-1.828705067	0.067443803	0	0.306198398	0	1	CRA004775;Yang2025_Silkie_Wenchang	PRJNA827465	0.447535382
ENSGALG00010011246	DNAJB5	2	-0.140614686	0.113548621	-1.238365421	0.215580596	0	0.595607736	0	1	CRA004775;PRJNA827465	Yang2025_Silkie_Wenchang	0.729108009
ENSGALG00010011247	ENSGALG00010011247	2	0.093462416	0.119611198	0.781385168	0.434575992	0.017688737	2.29736635	56.47189661	1	PRJNA827465;Yang2025_Silkie_Wenchang	CRA004775	0.842762754
ENSGALG00010011247	ENSGALG00010011247	2	0.254075523	0.133182106	1.907730181	0.056426099	0	0.000351037	0	1	CRA004775;Yang2025_Silkie_Wenchang	PRJNA827465	0.411523553
ENSGALG00010011247	ENSGALG00010011247	2	0.018023692	0.064718686	0.278492861	0.780634044	0	0.712746416	0	1	CRA004775;PRJNA827465	Yang2025_Silkie_Wenchang	0.94523121
ENSGALG00010011250	ENSGALG00010011250	2	0.217858385	0.265152148	0.821635378	0.411284448	0.120021939	6.291603343	84.10580029	0.5	PRJNA827465;Yang2025_Silkie_Wenchang	CRA004775	0.835128673
ENSGALG00010011250	ENSGALG00010011250	2	0.403231441	0.148025859	2.724060813	0.006448463	0	0.721153411	0	1	CRA004775;Yang2025_Silkie_Wenchang	PRJNA827465	0.122370493
ENSGALG00010011250	ENSGALG00010011250	2	0.047372396	0.118208779	0.400751931	0.688602773	0.012703692	1.471246381	32.03041905	0.5	CRA004775;PRJNA827465	Yang2025_Silkie_Wenchang	0.919798626
ENSGALG00010011251	FRZB	2	-0.113830861	0.216372494	-0.526087485	0.598827424	0.085410585	11.38505293	91.21655379	0.5	PRJNA827465;Yang2025_Silkie_Wenchang	CRA004775	0.89479201
ENSGALG00010011251	FRZB	2	-0.325445306	0.090781321	-3.584936875	0.00033716	0	0.110390166	0	1	CRA004775;Yang2025_Silkie_Wenchang	PRJNA827465	0.018227496
ENSGALG00010011251	FRZB	2	0.09432951	0.087810011	1.074245507	0.282712661	0	0.246766879	0	0.5	CRA004775;PRJNA827465	Yang2025_Silkie_Wenchang	0.779532565
ENSGALG00010011253	KDM4C	2	0.143270715	0.063959663	2.240016743	0.025089836	0	0.184843918	0	1	PRJNA827465;Yang2025_Silkie_Wenchang	CRA004775	0.269796394
ENSGALG00010011253	KDM4C	2	0.197444891	0.151261646	1.305320258	0.191783819	0	0.032084821	0	1	CRA004775;Yang2025_Silkie_Wenchang	PRJNA827465	0.701505763
ENSGALG00010011253	KDM4C	2	0.133248535	0.067092766	1.986034318	0.047029513	0	0.003107427	0	1	CRA004775;PRJNA827465	Yang2025_Silkie_Wenchang	0.374081275
ENSGALG00010011262	ENSGALG00010011262	2	-0.323406435	0.182538049	-1.771720676	0.076440936	0	0.070631021	0	1	PRJNA827465;Yang2025_Silkie_Wenchang	CRA004775	0.477109432
ENSGALG00010011262	ENSGALG00010011262	2	-0.276361732	0.23038601	-1.199559526	0.230310454	0	0.463379775	0	1	CRA004775;Yang2025_Silkie_Wenchang	PRJNA827465	0.742932679
ENSGALG00010011262	ENSGALG00010011262	2	-0.21878877	0.211779052	-1.033099208	0.301557474	0	0.252913117	0	1	CRA004775;PRJNA827465	Yang2025_Silkie_Wenchang	0.791273005
ENSGALG00010011267	EVC2	2	0.055096014	0.078630245	0.700697473	0.483491834	0	0.022241722	0	1	PRJNA827465;Yang2025_Silkie_Wenchang	CRA004775	0.85691916
ENSGALG00010011267	EVC2	2	0.079286599	0.147713775	0.536758327	0.591434561	0	0.000362001	0	1	CRA004775;Yang2025_Silkie_Wenchang	PRJNA827465	0.892776493
ENSGALG00010011267	EVC2	2	0.051902152	0.084878429	0.611488132	0.540876473	0	0.012980312	0	1	CRA004775;PRJNA827465	Yang2025_Silkie_Wenchang	0.875754635
ENSGALG00010011271	ANGPTL1	2	0.020313862	0.060596508	0.335231558	0.737450418	0	0.632350851	0	1	PRJNA827465;Yang2025_Silkie_Wenchang	CRA004775	0.933968189
ENSGALG00010011271	ANGPTL1	2	0.127938938	0.15749317	0.812345946	0.416593153	0	0.096135042	0	1	CRA004775;Yang2025_Silkie_Wenchang	PRJNA827465	0.837984974
ENSGALG00010011271	ANGPTL1	2	0.004311563	0.063187271	0.068234676	0.945598824	0	0.0110363	0	1	CRA004775;PRJNA827465	Yang2025_Silkie_Wenchang	0.986321197
ENSGALG00010011273	RUNX1T1	2	0.017715799	0.066564422	0.26614516	0.790127416	0	0.786374464	0	0.5	PRJNA827465;Yang2025_Silkie_Wenchang	CRA004775	0.947390191
ENSGALG00010011273	RUNX1T1	2	0.18563772	0.182247471	1.018602445	0.308391743	0	0.009541191	0	1	CRA004775;Yang2025_Silkie_Wenchang	PRJNA827465	0.793457188
ENSGALG00010011273	RUNX1T1	2	0.004055491	0.068792969	0.05895211	0.952990253	0	0.18773264	0	0.5	CRA004775;PRJNA827465	Yang2025_Silkie_Wenchang	0.987758913
ENSGALG00010011274	ENSGALG00010011274	2	-1.799534021	0.425374464	-4.230470268	2.33203E-05	0	0.043134416	0	1	PRJNA827465;Yang2025_Silkie_Wenchang	CRA004775	0.002741334
ENSGALG00010011274	ENSGALG00010011274	2	-0.904024213	0.821393507	-1.100598196	0.271071571	0.942194727	3.310173437	69.79010257	1	CRA004775;Yang2025_Silkie_Wenchang	PRJNA827465	0.772743863
ENSGALG00010011274	ENSGALG00010011274	2	-0.999095615	0.909679916	-1.098293585	0.272076312	1.263754002	4.219585735	76.30099107	1	CRA004775;PRJNA827465	Yang2025_Silkie_Wenchang	0.773184343
ENSGALG00010011275	WDR4	2	-0.002796911	0.066510916	-0.042051912	0.966457315	0	0.454408401	0	0.5	PRJNA827465;Yang2025_Silkie_Wenchang	CRA004775	0.990881287
ENSGALG00010011275	WDR4	2	-0.148855968	0.122447097	-1.215675767	0.224108459	0	0.634147379	0	1	CRA004775;Yang2025_Silkie_Wenchang	PRJNA827465	0.737452676
ENSGALG00010011275	WDR4	2	-0.068713294	0.145897299	-0.470970296	0.637661947	0.023633444	1.786490382	44.0243278	0.5	CRA004775;PRJNA827465	Yang2025_Silkie_Wenchang	0.905279419
ENSGALG00010011278	ENSGALG00010011278	2	-0.408393508	0.233396255	-1.749786036	0.080155241	0.058867354	2.131183464	53.077714	1	PRJNA827465;Yang2025_Silkie_Wenchang	CRA004775	0.487141092
ENSGALG00010011278	ENSGALG00010011278	2	0.130285158	0.886879198	0.146902936	0.883208634	1.368554762	7.243909953	86.19530051	0.5	CRA004775;Yang2025_Silkie_Wenchang	PRJNA827465	0.972001076
ENSGALG00010011278	ENSGALG00010011278	2	0.323802091	0.642988982	0.503588864	0.61455032	0.654939703	4.203615537	76.2109548	0.5	CRA004775;PRJNA827465	Yang2025_Silkie_Wenchang	0.898614239
ENSGALG00010011280	FAM199X	2	-0.228572246	0.220676474	-1.035779854	0.300304858	0.091656024	16.66654745	93.99995708	1	PRJNA827465;Yang2025_Silkie_Wenchang	CRA004775	0.790442127
ENSGALG00010011280	FAM199X	2	-0.230761264	0.283857034	-0.812948901	0.416247356	0.122538346	3.600699835	72.22762114	0.5	CRA004775;Yang2025_Silkie_Wenchang	PRJNA827465	0.837742614
ENSGALG00010011280	FAM199X	2	-0.011062195	0.049963892	-0.221403783	0.824778047	0	0.226435605	0	0.5	CRA004775;PRJNA827465	Yang2025_Silkie_Wenchang	0.956203404
ENSGALG00010011282	CRY1	2	0.001028938	0.127010578	0.008101201	0.993536248	0.022537986	3.218111285	68.92587262	0.5	PRJNA827465;Yang2025_Silkie_Wenchang	CRA004775	0.997979742
ENSGALG00010011282	CRY1	2	0.181044738	0.111888766	1.61807789	0.105645813	0	0.987919274	0	1	CRA004775;Yang2025_Silkie_Wenchang	PRJNA827465	0.551567133
ENSGALG00010011282	CRY1	2	0.106195645	0.297540065	0.356912084	0.721157623	0.129786927	3.00237391	66.69302259	0.5	CRA004775;PRJNA827465	Yang2025_Silkie_Wenchang	0.928950734
ENSGALG00010011283	LONRF3	2	-0.035634596	0.120336668	-0.296124172	0.767135255	0.005811216	1.241579836	19.45745483	0.5	PRJNA827465;Yang2025_Silkie_Wenchang	CRA004775	0.941432527
ENSGALG00010011283	LONRF3	2	-0.118940692	0.151314357	-0.786050275	0.431838034	0	0.569398231	0	0.5	CRA004775;Yang2025_Silkie_Wenchang	PRJNA827465	0.842503929
ENSGALG00010011283	LONRF3	2	0.070228697	0.126471749	0.555291582	0.578695236	0	0.008482114	0	1	CRA004775;PRJNA827465	Yang2025_Silkie_Wenchang	0.88788081
ENSGALG00010011285	ENSGALG00010011285	2	-0.103040739	0.25386646	-0.405885596	0.684826662	0	0.040543609	0	1	PRJNA827465;Yang2025_Silkie_Wenchang	CRA004775	0.919003112
ENSGALG00010011285	ENSGALG00010011285	2	0.092935603	0.307673473	0.3020592	0.762606934	0	0.122028831	0	0.5	CRA004775;Yang2025_Silkie_Wenchang	PRJNA827465	0.940082621
ENSGALG00010011285	ENSGALG00010011285	2	-0.022295531	0.24553908	-0.090802374	0.927649624	0	0.403870581	0	0.5	CRA004775;PRJNA827465	Yang2025_Silkie_Wenchang	0.982993195
ENSGALG00010011287	ENSGALG00010011287	2	0.20716368	0.147382136	1.405622726	0.159836184	0.019733013	1.827414019	45.27786316	1	PRJNA827465;Yang2025_Silkie_Wenchang	CRA004775	0.655404348
ENSGALG00010011287	ENSGALG00010011287	2	0.287312977	0.135937846	2.113561346	0.03455275	0	0.733774866	0	1	CRA004775;Yang2025_Silkie_Wenchang	PRJNA827465	0.320289326
ENSGALG00010011287	ENSGALG00010011287	2	0.078276389	0.126123932	0.62063074	0.534842609	0	0.01970853	0	1	CRA004775;PRJNA827465	Yang2025_Silkie_Wenchang	0.873896294
ENSGALG00010011288	ENSGALG00010011288	2	-0.245166141	0.207945944	-1.178989771	0.238402249	0	0.000171257	0	1	PRJNA827465;Yang2025_Silkie_Wenchang	CRA004775	0.750554597
ENSGALG00010011288	ENSGALG00010011288	2	-0.216319514	0.300975461	-0.718728076	0.472308481	0	0.062897656	0	1	CRA004775;Yang2025_Silkie_Wenchang	PRJNA827465	0.853998373
ENSGALG00010011288	ENSGALG00010011288	2	-0.220600639	0.254186847	-0.867868035	0.38546658	0	0.062710859	0	1	CRA004775;PRJNA827465	Yang2025_Silkie_Wenchang	0.82648313
ENSGALG00010011293	NDUFV3	2	-0.099825745	0.077357059	-1.290454235	0.196892992	0	0.451605796	0	1	PRJNA827465;Yang2025_Silkie_Wenchang	CRA004775	0.707994211
ENSGALG00010011293	NDUFV3	2	0.145652314	0.232696634	0.625932192	0.531359418	0.057641393	1.866443883	46.422177	0.5	CRA004775;Yang2025_Silkie_Wenchang	PRJNA827465	0.87298465
ENSGALG00010011293	NDUFV3	2	0.092755881	0.296152257	0.313203356	0.754126169	0.129782469	3.17898119	68.54338103	0.5	CRA004775;PRJNA827465	Yang2025_Silkie_Wenchang	0.938385954
ENSGALG00010011295	FAM167A	2	0.107106787	0.414416371	0.258452115	0.796058001	0.139040915	1.647479683	39.30122416	0.5	PRJNA827465;Yang2025_Silkie_Wenchang	CRA004775	0.949284493
ENSGALG00010011295	FAM167A	2	0.437894254	0.430736233	1.016618109	0.309335136	0	0.291404175	0	1	CRA004775;Yang2025_Silkie_Wenchang	PRJNA827465	0.794043289
ENSGALG00010011295	FAM167A	2	-0.168175945	0.34852081	-0.482542047	0.629420936	0	0.164323723	0	0.5	CRA004775;PRJNA827465	Yang2025_Silkie_Wenchang	0.902390168
ENSGALG00010011297	OSR2	2	-0.024318694	0.137045854	-0.177449321	0.859155469	0.026472334	3.382933256	70.43985428	0.5	PRJNA827465;Yang2025_Silkie_Wenchang	CRA004775	0.964605083
ENSGALG00010011297	OSR2	2	0.096064168	0.106724045	0.900117388	0.368057783	0	0.708254303	0	0.5	CRA004775;Yang2025_Silkie_Wenchang	PRJNA827465	0.820213684
ENSGALG00010011297	OSR2	2	-0.164531033	0.099343586	-1.656181744	0.097685019	0	0.092637008	0	1	CRA004775;PRJNA827465	Yang2025_Silkie_Wenchang	0.533757029
ENSGALG00010011300	EVC	2	-0.131919837	0.078816953	-1.673749519	0.094179837	0	0.05692748	0	1	PRJNA827465;Yang2025_Silkie_Wenchang	CRA004775	0.525712768
ENSGALG00010011300	EVC	2	0.272593384	0.410765786	0.663622418	0.506932006	0.302418988	9.26610786	89.2079823	0.5	CRA004775;Yang2025_Silkie_Wenchang	PRJNA827465	0.863929781
ENSGALG00010011300	EVC	2	0.25359022	0.429684797	0.590177317	0.555071778	0.335385196	10.46524491	90.44456189	0.5	CRA004775;PRJNA827465	Yang2025_Silkie_Wenchang	0.880223402
ENSGALG00010011304	COMMD5	2	-0.25248589	0.144908677	-1.742379374	0.081442083	0.028555725	2.698633294	62.94420578	1	PRJNA827465;Yang2025_Silkie_Wenchang	CRA004775	0.491466018
ENSGALG00010011304	COMMD5	2	-0.277041333	0.199932583	-1.385673755	0.165846546	0.038720775	1.883522608	46.90799061	1	CRA004775;Yang2025_Silkie_Wenchang	PRJNA827465	0.66529196
ENSGALG00010011304	COMMD5	2	-0.135495338	0.064285456	-2.107713721	0.03505576	0	0.175534784	0	1	CRA004775;PRJNA827465	Yang2025_Silkie_Wenchang	0.32274456
ENSGALG00010011306	ECI2	2	-0.216316561	0.09964085	-2.170962626	0.029933998	0	0.770597263	0	1	PRJNA827465;Yang2025_Silkie_Wenchang	CRA004775	0.295167651
ENSGALG00010011306	ECI2	2	-0.283516403	0.179192415	-1.582189755	0.113606259	0	0.651242722	0	1	CRA004775;Yang2025_Silkie_Wenchang	PRJNA827465	0.568794964
ENSGALG00010011306	ECI2	2	-0.163331874	0.104008784	-1.570366156	0.116329951	0	0.06339897	0	1	CRA004775;PRJNA827465	Yang2025_Silkie_Wenchang	0.57424216
ENSGALG00010011307	ENSGALG00010011307	2	-0.358262228	0.657410066	-0.544960058	0.585780989	0.684426618	4.803133743	79.18025911	0.5	PRJNA827465;Yang2025_Silkie_Wenchang	CRA004775	0.891256976
ENSGALG00010011307	ENSGALG00010011307	2	-1.093521985	0.383136772	-2.854129554	0.004315493	0	0.218885696	0	1	CRA004775;Yang2025_Silkie_Wenchang	PRJNA827465	0.096315625
ENSGALG00010011307	ENSGALG00010011307	2	-0.402911493	0.876291774	-0.459791482	0.645665898	1.081066015	3.044558231	67.15451228	0.5	CRA004775;PRJNA827465	Yang2025_Silkie_Wenchang	0.907052464
ENSGALG00010011311	DOCK8	2	-0.088785632	0.112989324	-0.785787797	0.431991817	0	0.459243776	0	1	PRJNA827465;Yang2025_Silkie_Wenchang	CRA004775	0.842503929
ENSGALG00010011311	DOCK8	2	-0.217596348	0.172948186	-1.25815918	0.208334194	0	0.572039089	0	1	CRA004775;Yang2025_Silkie_Wenchang	PRJNA827465	0.720917211
ENSGALG00010011311	DOCK8	2	-0.05701192	0.142408415	-0.400340949	0.688905411	0	0.983890002	0	1	CRA004775;PRJNA827465	Yang2025_Silkie_Wenchang	0.919854035
ENSGALG00010011312	VPS13B	2	-0.126361549	0.048949496	-2.581467815	0.009838116	0	0.387117559	0	1	PRJNA827465;Yang2025_Silkie_Wenchang	CRA004775	0.15667223
ENSGALG00010011312	VPS13B	2	-0.00829092	0.149883772	-0.055315659	0.955886987	0	0.05672878	0	0.5	CRA004775;Yang2025_Silkie_Wenchang	PRJNA827465	0.988406776
ENSGALG00010011312	VPS13B	2	-0.131041186	0.050571695	-2.591196241	0.009564293	0	0.347320902	0	0.5	CRA004775;PRJNA827465	Yang2025_Silkie_Wenchang	0.153870904
ENSGALG00010011313	RASSF9	2	-0.06891005	0.174319678	-0.395308494	0.692615231	0	0.025142816	0	1	PRJNA827465;Yang2025_Silkie_Wenchang	CRA004775	0.920559211
ENSGALG00010011313	RASSF9	2	-0.019836409	0.25223558	-0.07864239	0.937317069	0	0.580063149	0	0.5	CRA004775;Yang2025_Silkie_Wenchang	PRJNA827465	0.985114195
ENSGALG00010011313	RASSF9	2	0.006340621	0.212680148	0.02981294	0.976216238	0	0.487843268	0	0.5	CRA004775;PRJNA827465	Yang2025_Silkie_Wenchang	0.992992231
ENSGALG00010011316	PARPBP	2	-0.141870608	0.127690246	-1.111052816	0.266545616	0	0.281815283	0	1	PRJNA827465;Yang2025_Silkie_Wenchang	CRA004775	0.770699087
ENSGALG00010011316	PARPBP	2	0.077547553	0.505501142	0.153407275	0.878077108	0.305582545	1.799595268	44.43194992	0.5	CRA004775;Yang2025_Silkie_Wenchang	PRJNA827465	0.971021203
ENSGALG00010011316	PARPBP	2	0.105590429	0.394173093	0.267878328	0.788792976	0.156650343	1.410903479	29.12342943	0.5	CRA004775;PRJNA827465	Yang2025_Silkie_Wenchang	0.947144026
ENSGALG00010011318	PRP4K	2	0.359725835	0.387297728	0.928809565	0.352987786	0.275588017	11.802134	91.52695606	0.5	PRJNA827465;Yang2025_Silkie_Wenchang	CRA004775	0.811035834
ENSGALG00010011318	PRP4K	2	0.503084566	0.292031686	1.722705414	0.084941811	0.113445889	2.943651046	66.02858205	1	CRA004775;Yang2025_Silkie_Wenchang	PRJNA827465	0.501162525
ENSGALG00010011318	PRP4K	2	0.012442484	0.081475833	0.152713794	0.878623982	0	0.448627712	0	0.5	CRA004775;PRJNA827465	Yang2025_Silkie_Wenchang	0.971079508
ENSGALG00010011321	MAP2	2	0.030788539	0.099227065	0.310283677	0.756345243	0	0.272275513	0	1	PRJNA827465;Yang2025_Silkie_Wenchang	CRA004775	0.938618977
ENSGALG00010011321	MAP2	2	0.118934194	0.180183343	0.660073192	0.509206861	0	0.001368841	0	1	CRA004775;Yang2025_Silkie_Wenchang	PRJNA827465	0.865255394
ENSGALG00010011321	MAP2	2	0.009201667	0.109710787	0.083872037	0.933158173	0	0.061817808	0	1	CRA004775;PRJNA827465	Yang2025_Silkie_Wenchang	0.984259239
ENSGALG00010011326	COX6C	2	-0.15672921	0.06301143	-2.48731397	0.012871175	0	0.750157773	0	1	PRJNA827465;Yang2025_Silkie_Wenchang	CRA004775	0.184289906
ENSGALG00010011326	COX6C	2	-0.265715444	0.1986415	-1.337663299	0.18100622	0	0.426089662	0	1	CRA004775;Yang2025_Silkie_Wenchang	PRJNA827465	0.688594651
ENSGALG00010011326	COX6C	2	-0.141769021	0.063551856	-2.230761291	0.025696945	0	0.009731021	0	1	CRA004775;PRJNA827465	Yang2025_Silkie_Wenchang	0.273407428
ENSGALG00010011329	PCDHGC5	2	0.009439068	0.052194312	0.180844769	0.856489422	0	0.682170965	0	0.5	PRJNA827465;Yang2025_Silkie_Wenchang	CRA004775	0.96353738
ENSGALG00010011329	PCDHGC5	2	-0.050459597	0.093299874	-0.540832422	0.588623094	0	0.083323231	0	0.5	CRA004775;Yang2025_Silkie_Wenchang	PRJNA827465	0.89212762
ENSGALG00010011329	PCDHGC5	2	0.037419678	0.061080216	0.612631729	0.540119874	0	0.001002011	0	1	CRA004775;PRJNA827465	Yang2025_Silkie_Wenchang	0.87529516
ENSGALG00010011333	ENSGALG00010011333	2	-0.115891323	0.125656214	-0.922288831	0.356377938	0.008368452	1.297000876	22.89904974	0.5	PRJNA827465;Yang2025_Silkie_Wenchang	CRA004775	0.812521698

ENSGALG00010011333	ENSGALG00010011333	2	0.053881515	0.16128982	0.334066434	0.738329428	0	0.014841938	0	1	CRA004775;Yang2025_Silkie_Wenchang	PRJNA827465	0.93445487
ENSGALG00010011333	ENSGALG00010011333	2	-0.161017203	0.110140638	-1.461923645	0.143762135	0	0.628457492	0	0.5	CRA004775;PRJNA827465	Yang2025_Silkie_Wenchang	0.63068235
ENSGALG00010011335	VCP	2	0.151227393	0.114115516	1.32521325	0.185100432	0	0.472200138	0	1	PRJNA827465;Yang2025_Silkie_Wenchang	CRA004775	0.693279213
ENSGALG00010011335	VCP	2	0.126892991	0.163546904	0.775881341	0.437819051	0.012302433	1.206812925	17.1371155	0.5	CRA004775;Yang2025_Silkie_Wenchang	PRJNA827465	0.843899392
ENSGALG00010011335	VCP	2	0.010540232	0.150376711	0.070092185	0.944120287	0	0.389460842	0	0.5	CRA004775;PRJNA827465	Yang2025_Silkie_Wenchang	0.985827325
ENSGALG00010011339	ENSGALG00010011339	2	-1.525267208	0.28080627	-5.431741984	5.58066E-08	0	0.006334598	0	1	PRJNA827465;Yang2025_Silkie_Wenchang	CRA004775	2.15036E-05
ENSGALG00010011339	ENSGALG00010011339	2	-0.813871341	0.755062759	-1.077885688	0.281084773	0.848195167	3.903062234	74.37909159	1	CRA004775;Yang2025_Silkie_Wenchang	PRJNA827465	0.77879032
ENSGALG00010011339	ENSGALG00010011339	2	-0.847608887	0.727216079	-1.165553006	0.243795217	0.859547864	5.152261032	80.59104549	1	CRA004775;PRJNA827465	Yang2025_Silkie_Wenchang	0.755365463
ENSGALG00010011340	PKNOX1	2	0.086369947	0.064104176	1.347337283	0.17787163	0	0.063223558	0	1	PRJNA827465;Yang2025_Silkie_Wenchang	CRA004775	0.683951879
ENSGALG00010011340	PKNOX1	2	0.214132496	0.21125176	1.013636505	0.310756232	0.041786796	1.805320802	44.60818271	1	CRA004775;Yang2025_Silkie_Wenchang	PRJNA827465	0.795107883
ENSGALG00010011340	PKNOX1	2	0.197398764	0.171343912	1.152061734	0.249295706	0.035614698	1.925504749	48.06556565	1	CRA004775;PRJNA827465	Yang2025_Silkie_Wenchang	0.759540244
ENSGALG00010011342	RASAL2	2	0.109565086	0.172175357	0.636357539	0.52454341	0.043609095	3.511264999	71.52023558	0.5	PRJNA827465;Yang2025_Silkie_Wenchang	CRA004775	0.869805486
ENSGALG00010011342	RASAL2	2	0.244034428	0.147495875	1.654516966	0.098022519	0	0.832228733	0	0.5	CRA004775;Yang2025_Silkie_Wenchang	PRJNA827465	0.534130658
ENSGALG00010011342	RASAL2	2	-0.034820759	0.082204975	-0.42358457	0.67186881	0	0.003005668	0	1	CRA004775;PRJNA827465	Yang2025_Silkie_Wenchang	0.914674175
ENSGALG00010011343	PXDC1	2	-0.276138301	0.334051138	-0.826634814	0.408444079	0.214950966	26.85572977	96.27639983	0.5	PRJNA827465;Yang2025_Silkie_Wenchang	CRA004775	0.834154426
ENSGALG00010011343	PXDC1	2	-0.21038462	0.433832528	-0.484944319	0.627715844	0.340686319	10.12166894	90.1202064	0.5	CRA004775;Yang2025_Silkie_Wenchang	PRJNA827465	0.901856848
ENSGALG00010011343	PXDC1	2	0.06362334	0.061155765	1.040349013	0.298177781	0	0.613646446	0	1	CRA004775;PRJNA827465	Yang2025_Silkie_Wenchang	0.789118382
ENSGALG00010011345	CRMP1	2	0.413138513	0.308758457	1.338063797	0.180857641	0.148648685	4.430596692	77.42763878	1	PRJNA827465;Yang2025_Silkie_Wenchang	CRA004775	0.688484814
ENSGALG00010011345	CRMP1	2	0.365154596	0.4849016	0.753048857	0.451420556	0.322948095	2.85955147	65.02948065	0.5	CRA004775;Yang2025_Silkie_Wenchang	PRJNA827465	0.847847413
ENSGALG00010011345	CRMP1	2	0.09625329	0.158213679	0.608375275	0.542938606	0	0.450399658	0	0.5	CRA004775;PRJNA827465	Yang2025_Silkie_Wenchang	0.876968972
ENSGALG00010011348	SLC22A23	2	0.075569784	0.393533688	0.192028755	0.847719681	0.28648677	13.27740776	92.46840936	0.5	PRJNA827465;Yang2025_Silkie_Wenchang	CRA004775	0.961304071
ENSGALG00010011348	SLC22A23	2	-0.295246187	0.156776025	-1.883235574	0.05966845	0	0.222170644	0	1	CRA004775;Yang2025_Silkie_Wenchang	PRJNA827465	0.423235714
ENSGALG00010011348	SLC22A23	2	0.320057309	0.247233512	1.294554716	0.195473883	0.063225862	1.632897982	38.75918698	0.5	CRA004775;PRJNA827465	Yang2025_Silkie_Wenchang	0.70652106
ENSGALG00010011349	ENSGALG00010011349	2	0.218689066	0.244182946	0.895595163	0.37046902	0.108960318	11.2263788	91.09240818	0.5	PRJNA827465;Yang2025_Silkie_Wenchang	CRA004775	0.821221934
ENSGALG00010011349	ENSGALG00010011349	2	0.474804998	0.115851221	4.098403954	4.16011E-05	0	0.000981391	0	1	CRA004775;Yang2025_Silkie_Wenchang	PRJNA827465	0.004291459
ENSGALG00010011349	ENSGALG00010011349	2	0.169202675	0.232927876	0.726416599	0.467583418	0.08317111	3.581919755	72.08200997	0.5	CRA004775;PRJNA827465	Yang2025_Silkie_Wenchang	0.852885838
ENSGALG00010011351	KANK1	2	-0.06219044	0.071088426	-0.87483214	0.381665247	0	0.737585497	0	1	PRJNA827465;Yang2025_Silkie_Wenchang	CRA004775	0.824699008
ENSGALG00010011351	KANK1	2	0.229609449	0.388703599	0.590705744	0.554717599	0.269878621	8.948275764	88.82466269	0.5	CRA004775;Yang2025_Silkie_Wenchang	PRJNA827465	0.880223402
ENSGALG00010011351	KANK1	2	0.283801943	0.326240251	0.869917008	0.38434576	0.182798009	6.595957964	84.83713099	0.5	CRA004775;PRJNA827465	Yang2025_Silkie_Wenchang	0.826048809
ENSGALG00010011353	ENSGALG00010011353	2	-0.101109544	0.189897551	-0.532442592	0.594419493	0	0.142165238	0	1	PRJNA827465;Yang2025_Silkie_Wenchang	CRA004775	0.893337382
ENSGALG00010011353	ENSGALG00010011353	2	-0.068861499	0.272663275	-0.252551427	0.800614869	0	0.275101773	0	1	CRA004775;Yang2025_Silkie_Wenchang	PRJNA827465	0.950156876
ENSGALG00010011353	ENSGALG00010011353	2	-0.190371343	0.241246141	-0.789116636	0.43004384	0	0.13305764	0	1	CRA004775;PRJNA827465	Yang2025_Silkie_Wenchang	0.842012722
ENSGALG00010011357	UNC80	2	0.406046016	0.632667721	0.641799799	0.52100318	0.669286699	5.965940543	83.23818361	0.5	PRJNA827465;Yang2025_Silkie_Wenchang	CRA004775	0.869518515
ENSGALG00010011357	UNC80	2	0.476531458	0.637205589	0.747845698	0.454553235	0.584715082	3.548265082	71.81721273	0.5	CRA004775;Yang2025_Silkie_Wenchang	PRJNA827465	0.849072665
ENSGALG00010011357	UNC80	2	-0.187868212	0.250344052	-0.750440088	0.452989694	0	0.00017919	0	1	CRA004775;PRJNA827465	Yang2025_Silkie_Wenchang	0.848651221
ENSGALG00010011359	RNF19A	2	0.065365973	0.127291844	0.513512661	0.607592757	0.026535001	5.362376232	81.35155094	0.5	PRJNA827465;Yang2025_Silkie_Wenchang	CRA004775	0.897160522
ENSGALG00010011359	RNF19A	2	0.224137815	0.08723723	2.569290843	0.010190689	0	0.393766313	0	1	CRA004775;Yang2025_Silkie_Wenchang	PRJNA827465	0.160364698
ENSGALG00010011359	RNF19A	2	0.090402352	0.198971485	0.454348279	0.649578172	0.056428216	2.767732125	63.8693358	0.5	CRA004775;PRJNA827465	Yang2025_Silkie_Wenchang	0.908198602
ENSGALG00010011360	TCF4	2	0.292630157	0.247529613	1.182202619	0.237125314	0.071970185	2.407925894	58.47048273	1	PRJNA827465;Yang2025_Silkie_Wenchang	CRA004775	0.749355649
ENSGALG00010011360	TCF4	2	0.432782485	0.194788281	2.221809657	0.026296172	0	0.724625374	0	1	CRA004775;Yang2025_Silkie_Wenchang	PRJNA827465	0.276415737
ENSGALG00010011360	TCF4	2	0.104740852	0.174614264	0.599841326	0.548611989	0	0.153414465	0	1	CRA004775;PRJNA827465	Yang2025_Silkie_Wenchang	0.878281266
ENSGALG00010011361	NUP37	2	-0.144405615	0.199281044	-0.724632969	0.468677227	0.06376828	4.51174537	77.83562861	0.5	PRJNA827465;Yang2025_Silkie_Wenchang	CRA004775	0.853198008
ENSGALG00010011361	NUP37	2	-0.345317909	0.14724218	-2.345217687	0.019019953	0	0.125780847	0	1	CRA004775;Yang2025_Silkie_Wenchang	PRJNA827465	0.230995614
ENSGALG00010011361	NUP37	2	-0.019914008	0.103412974	-0.192567788	0.847297474	0.008659598	1.250725868	20.0464286	0.5	CRA004775;PRJNA827465	Yang2025_Silkie_Wenchang	0.961252712
ENSGALG00010011363	FDFT1	2	-0.063215985	0.084329802	-0.74962805	0.453478752	0	0.688113873	0	0.5	PRJNA827465;Yang2025_Silkie_Wenchang	CRA004775	0.848879567
ENSGALG00010011363	FDFT1	2	-0.179934583	0.110486443	-1.628567071	0.1034047	0	0.610225638	0	1	CRA004775;Yang2025_Silkie_Wenchang	PRJNA827465	0.547576788
ENSGALG00010011363	FDFT1	2	-0.107564956	0.165362259	-0.650480686	0.515381774	0.026074137	1.683499251	40.59991416	0.5	CRA004775;PRJNA827465	Yang2025_Silkie_Wenchang	0.867199306
ENSGALG00010011365	PSMG4	2	-0.579393923	0.127875467	-4.530923228	5.87265E-06	0.001576303	1.027824406	2.707116641	1	PRJNA827465;Yang2025_Silkie_Wenchang	CRA004775	0.000930696
ENSGALG00010011365	PSMG4	2	-0.537380247	0.324106693	-1.658035018	0.097310398	0.115864383	2.229585789	55.14861977	1	CRA004775;Yang2025_Silkie_Wenchang	PRJNA827465	0.532942995
ENSGALG00010011365	PSMG4	2	-0.471839458	0.123119341	-3.832374775	0.000126912	0	0.846157856	0	1	CRA004775;PRJNA827465	Yang2025_Silkie_Wenchang	0.009242467
ENSGALG00010011367	IER3IP1	2	0.013271926	0.085109791	0.155938889	0.876081191	0	0.138228059	0	0.5	PRJNA827465;Yang2025_Silkie_Wenchang	CRA004775	0.970347777
ENSGALG00010011367	IER3IP1	2	-0.237380119	0.198463687	-1.196088426	0.231662054	0	0.470557913	0	1	CRA004775;Yang2025_Silkie_Wenchang	PRJNA827465	0.744116843
ENSGALG00010011367	IER3IP1	2	-0.085833367	0.173802694	-0.493855216	0.621409435	0.034117341	1.840723913	45.67354762	0.5	CRA004775;PRJNA827465	Yang2025_Silkie_Wenchang	0.899693435
ENSGALG00010011371	ENSGALG00010011371	2	-0.059170913	0.170507295	-0.347029628	0.728569812	0	0.035566593	0	1	PRJNA827465;Yang2025_Silkie_Wenchang	CRA004775	0.932187391
ENSGALG00010011371	ENSGALG00010011371	2	0.022282054	0.194570655	0.114519088	0.908826316	0	0.040950607	0	0.5	CRA004775;Yang2025_Silkie_Wenchang	PRJNA827465	0.978387968
ENSGALG00010011371	ENSGALG00010011371	2	-0.028161593	0.169206736	-0.166433046	0.867816169	0	0.173865547	0	0.5	CRA004775;PRJNA827465	Yang2025_Silkie_Wenchang	0.967946362
ENSGALG00010011372	VMA21	2	-0.131491755	0.140991707	-0.932620485	0.351015953	0.033247235	5.588045263	82.10465462	1	PRJNA827465;Yang2025_Silkie_Wenchang	CRA004775	0.810146221
ENSGALG00010011372	VMA21	2	-0.238466486	0.089031168	-2.678460722	0.00739614	0	0.621423098	0	1	CRA004775;Yang2025_Silkie_Wenchang	PRJNA827465	0.132553814
ENSGALG00010011372	VMA21	2	-0.019304714	0.040573585	-0.475795125	0.634220343	0	0.853989531	0	1	CRA004775;PRJNA827465	Yang2025_Silkie_Wenchang	0.903815945
ENSGALG00010011374	ENSGALG00010011374	2	0.212006615	0.162854578	1.301815504	0.192979463	0.031448117	2.036658291	50.89996175	1	PRJNA827465;Yang2025_Silkie_Wenchang	CRA004775	0.702958174
ENSGALG00010011374	ENSGALG00010011374	2	0.040923048	0.406299167	0.100721467	0.919771569	0.282035396	6.850167792	85.40181744	0.5	CRA004775;Yang2025_Silkie_Wenchang	PRJNA827465	0.981327238
ENSGALG00010011374	ENSGALG00010011374	2	-0.089480335	0.22731107	-0.39364706	0.693841632	0.081373965	4.25876661	76.51902319	0.5	CRA004775;PRJNA827465	Yang2025_Silkie_Wenchang	0.92072583
ENSGALG00010011375	C12orf50	2	-0.287024132	0.099925807	-2.872372412	0.004074026	0	0.587859691	0	1	PRJNA827465;Yang2025_Silkie_Wenchang	CRA004775	0.0925841
ENSGALG00010011375	C12orf50	2	-0.273019581	0.141769156	-1.925803816	0.054128875	0.006765208	1.185262724	15.6305197	1	CRA004775;Yang2025_Silkie_Wenchang	PRJNA827465	0.403227287
ENSGALG00010011375	C12orf50	2	-0.189019702	0.111187478	-1.700008902	0.089129251	0	0.293548172	0	1	CRA004775;PRJNA827465	Yang2025_Silkie_Wenchang	0.512516014
ENSGALG00010011377	SEC16B	2	0.472529046	0.301828285	1.565555877	0.117452626	0.108512613	2.472363291	59.55286977	1	PRJNA827465;Yang2025_Silkie_Wenchang	CRA004775	0.57688585
ENSGALG00010011377	SEC16B	2	0.926748793	0.229183855	4.043691438	5.26162E-05	0	0.993968921	0	1	CRA004775;Yang2025_Silkie_Wenchang	PRJNA827465	0.005019351
ENSGALG00010011377	SEC16B	2	0.67760288	0.548927843	1.234411568	0.217049581	0.483599555	4.920663924	79.67753914	1	CRA004775;PRJNA827465	Yang2025_Silkie_Wenchang	0.730558054
ENSGALG00010011378	TUBB2B	2	-0.016912485	0.144750164	-0.11683914	0.906987527	0	0.255972439	0	0.5	PRJNA827465;Yang2025_Silkie_Wenchang	CRA004775	0.978196186
ENSGALG00010011378	TUBB2B	2	-0.010059019	0.154753037	-0.065000465	0.94817363	0	0.24356915	0	0.5	CRA004775;Yang2025_Silkie_Wenchang	PRJNA827465	0.98671013
ENSGALG00010011378	TUBB2B	2	-0.088057164	0.149082586	-0.590660294	0.554748057	0	0.000434564	0	1	CRA004775;PRJNA827465	Yang2025_Silkie_Wenchang	0.880223402
ENSGALG00010011381	RLIG1	2	0.125450187	0.077912347	1.61014514	0.107366174	0	0.392554015	0	1	PRJNA827465;Yang2025_Silkie_Wenchang	CRA004775	0.554714101
ENSGALG00010011381	RLIG1	2	0.107669937	0.133902006	0.804095029	0.421342095	0	0.652136718	0	1	CRA004775;Yang2025_Silkie_Wenchang	PRJNA827465	0.839352211
ENSGALG00010011381	RLIG1	2	0.167138368	0.084164568	1.985851895	0.047049771	0	0.22093527	0	1	CRA004775;PRJNA827465	Yang2025_Silkie_Wenchang	0.374104222
ENSGALG00010011382	ENSGALG00010011382	2	-0.160615177	0.141173539	-1.137714463	0.255239732	0	0.03989074	0	1	PRJNA827465;Yang2025_Silkie_Wenchang	CRA004775	0.764467569
ENSGALG00010011382	ENSGALG00010011382	2	-0.034669053	0.180774602	-0.191780555	0.847914103	0	0.754976088	0	0.5	CRA004775;Yang2025_Silkie_Wenchang	PRJNA827465	0.961328089
ENSGALG00010011382	ENSGALG00010011382	2	-0.085112542	0.177183011	-0.480365138	0.630967779	0.005498342	1.06924154	6.475762262	0.5	CRA004775;PRJNA827465	Yang2025_Silkie_Wenchang	0.902961208
ENSGALG00010011384	PUM3	2	0.167753836	0.289277157	0.579906959	0.561977363	0.149427952	9.231019269	89.16696011	0.5	PRJNA827465;Yang2025_Silkie_Wenchang	CRA004775	0.881837996
ENSGALG00010011384	PUM3	2	0.297848311	0.200688556	1.48413202	0.1377739	0.044904327	2.184493414	54.22279629	1	CRA004775;Yang2025_Silkie_Wenchang	PRJNA827465	0.62064915
ENSGALG00010011384	PUM3	2	-0.078596875	0.098960051	-0.794228319	0.427062525	0	0.464304109	0	0.5	CRA004775;PRJNA827465	Yang2025_Silkie_Wenchang	0.84125685
ENSGALG00010011386	CTSB	2	-0.023693159	0.07543054	-0.314105651	0.753440798	0	0.569009285	0	1	PRJNA827465;Yang2025_Silkie_Wenchang	CRA004775	0.938087082
ENSGALG00010011386	CTSB	2	-0.087638873	0.155701092	-0.56286614	0.573526038	0	0.359070615	0	0.5	CRA004775;Yang2025_Silkie_Wenchang	PRJNA827465	0.885917854
ENSGALG00010011386	CTSB	2	0.001264269	0.076889971	0.016442571	0.986881318	0	0.008317113	0	0.5	CRA004775;PRJNA827465	Yang2025_Silkie_Wenchang	0.996336924
ENSGALG00010011388	GPR50	2	0.092065856	0.119374062	0.77123836	0.440565661	0	0.013322993	0	1	PRJNA827465;Yang2025_Silkie_Wenchang	CRA004775	0.844784709
ENSGALG00010011388	GPR50	2	0.21636728	0.173292888	1.248564105	0.211824548	3.72555E-05	1.000489271	0.048903154	1	CRA004775;Yang2025_Silkie_Wenchang	PRJNA827465	0.724118735
ENSGALG00010011388	GPR50	2	0.185091856	0.18053144	1.025261063	0.305240023	0.021400515	1.321803585	24.34579453	1	CRA004775;PRJNA827465	Yang2025_Silkie_Wenchang	0.79225794
ENSGALG00010011389	RC3H1	2	-0.124754913	0.063211474	-1.973611826	0.048425898	0	0.27541102	0	1	PRJNA827465;Yang2025_Silkie_Wenchang	CRA004775	0.380432851
ENSGALG00010011389	RC3H1	2	0.002365814	0.139887589	0.01691225	0.98650662	0	0.493057217	0	0.5	CRA004775;Yang2025_Silkie_Wenchang	PRJNA827465	0.99618774
ENSGALG00010011389	RC3H1	2	-0.10571119	0.099707086	-1.060217419	0.289045698	0.006307412	1.131121403	11.59216003	0.5	CRA004775;PRJNA827465	Yang2025_Silkie_Wenchang	0.783530627
ENSGALG00010011394	SERPINC1	2	-0.227115308	1.21134974	-0.18748946	0.851276887	2.421213243	5.626929093	82.22831702	0.5	PRJNA827465;Yang2025_Silkie_Wenchang	CRA004775	0.962071965
ENSGALG00010011394	SERPINC1	2	-0.121330294	1.227640619	-0.098832095	0.921271585	2.714065501	10.01510545	90.01508267	0.5	CRA004775;Yang2025_Silkie_Wenchang	PRJNA827465	0.981519273
ENSGALG00010011394	SERPINC1	2	1.077414934	0.427597138	2.51969632	0.011745612	0	0.000889169	0	1	CRA004775;PRJNA827465	Yang2025_Silkie_Wenchang	0.173482822
ENSGALG00010011398	ENSGALG00010011398	2	0.278345371	0.124079219	2.243287582	0.024878276	0	0.14293574	0	1	PRJNA827465;Yang2025_Silkie_Wenchang	CRA004775	0.268993361
ENSGALG00010011398	ENSGALG00010011398	2	0.113047525	0.176663731	0.639902281	0.522236131	0	0.686819579	0	0.5	CRA004775;Yang2025_Silkie_Wenchang	PRJNA827465	0.869568211
ENSGALG00010011398	ENSGALG00010011398	2	0.195912464	0.185906641	1.053821763	0.291964528	0.024761777	1.406093957	28.88099724	0.5	CRA004775;PRJNA827465	Yang2025_Silkie_Wenchang	0.785516679
ENSGALG00010011399	JAKMIP1	2	0.583435154	0.329955531	1.768223589	0.077023532	0.145089593	2.99638058	66.62640231	1	PRJNA827465;Yang2025_Silkie_Wenchang	CRA004775	0.478453375
ENSGALG00010011399	JAKMIP1	2	0.806384394	0.28211932	2.858309719	0.004259045	0.019945697	1.05531498	5.241561121	1	CRA004775;Yang2025_Silkie_Wenchang	PRJNA827465	0.095380758
ENSGALG00010011399	JAKMIP1	2	0.228003607	0.259312412	0.879262218	0.379259116	0	0.062180212	0	1	CRA004775;PRJNA827465	Yang2025_Silkie_Wenchang	0.824128991
ENSGALG00010011400	BPHL	2	0.253432597	0.178096589	1.42300646	0.154734283	0	0.345153165	0	1	PRJNA827465;Yang2025_Silkie_Wenchang	CRA004775	0.647505492
ENSGALG00010011400	BPHL	2	0.292968428	0.219258803	1.336176352	0.181491639	0	0.284669776	0	1	CRA004775;Yang2025_Silkie_Wenchang	PRJNA827465	0.689004635
ENSGALG00010011400	BPHL	2	0.128657393	0.224539405	0.572983583	0.56665581	0	0.01897278	0	1	CRA004775;PRJNA827465	Yang2025_Silkie_Wenchang	0.883487517
ENSGALG00010011401	ANKRD46	2	-0.113836975	0.155619066	-0.731510462	0.464467417	0.045585194	16.80273794	94.04858896	0.5	PRJNA827465;Yang2025_Silkie_Wenchang	CRA004775	0.852159649
ENSGALG00010011401	ANKRD46	2	-0.212541731	0.106580612	-1.994187561	0.046131561	0.010909382	1.557618966	35.79944636	1	CRA004775;Yang2025_Silkie_Wenchang	PRJNA827465	0.370377506
ENSGALG00010011401	ANKRD46	2	0.034855053	0.038851553	0.897134098	0.369647365	0	0.113851425	0	0.5	CRA004775;PRJNA827465	Yang2025_Silkie_Wenchang	0.820606801
ENSGALG00010011406	UBE2R2	2	0.109805941	0.138593181	0.792289631	0.428191816	0.022039502	2.343505419	57.3288804	0.5	PRJNA827465;Yang2025_Silkie_Wenchang	CRA004775	0.84125685
ENSGALG00010011406	UBE2R2	2	-0.025123324	0.298784945	-0.084084974	0.932988872	0.148730657	5.827346521	82.83953088	0.5	CRA004775;Yang2025_Silkie_Wenchang	PRJNA827465	0.984224753
ENSGALG00010011406	UBE2R2	2	-0.141335236	0.154629817	-0.914023176	0.360704668	0.022049466	1.740522921	42.54600224	1	CRA004775;PRJNA827465	Yang2025_Silkie_Wenchang	0.815038264
ENSGALG00010011407	CEP290	2	0.550751566	0.402353372	1.368825526	0.17105382	0.299096697	12.79327414	92.18339271	1	PRJNA827465;Yang2025_Silkie_Wenchang	CRA004775	0.673219077
ENSGALG00010011407	CEP290	2	0.891687951	0.172892019	5.157484736	2.50289E-07	0	0.623441766	0	1	CRA004775;Yang2025_Silkie_Wenchang	PRJNA827465	7.28675E-05
ENSGALG00010011407	CEP290	2	0.305786762	0.222050974	1.377101647	0.168480823	0.058236754	1.918854763	47.88558157	1	CRA004775;PRJNA827465	Yang2025_Silkie_Wenchang	0.669314102
ENSGALG00010011411	ENSGALG00010011411	2	-0.138211217	0.406568189	-0.339945968	0.733897218	0.27405538	5.800700888	82.76070393	0.5	PRJNA827465;Yang2025_Silkie_Wenchang	CRA004775	0.933391141
ENSGALG00010011411	ENSGALG00010011411	2	0.320992342	0.241534521	1.328970871	0.18385758	0	0.104864851	0	1	CRA004775;Yang2025_Silkie_Wenchang	PRJNA827465	0.692470323
ENSGALG00010011411	ENSGALG00010011411	2	-0.162145341	0.489606549	-0.331174779	0.74051247	0.330358613	2.734477161	63.42993775	0.5	CRA004775;PRJNA827465	Yang2025_Silkie_Wenchang	0.935128797
ENSGALG00010011412	KANSL1L	2	-0.073851971	0.107375001	-0.687794839	0.491581987	0.017837493	4.31142768	76.80582688	0.5	PRJNA827465;Yang2025_Silkie_Wenchang	CRA004775	0.85895707
ENSGALG00010011412	KANSL1L	2	0.023325279	0.247136044	0.094382342	0.924805442	0.099538479	4.956491863	79.82443979	0.5	CRA004775;Yang2025_Silkie_Wenchang	PRJNA827465	0.982493782
ENSGALG00010011412	KANSL1L	2	0.097102628	0.123673671	0.785151983	0.432364465	0.017093766	1.736844766	42.42433063	1	CRA004775;PRJNA827465	Yang2025_Silkie_Wenchang	0.842762754
ENSGALG00010011413	ZBTB37	2	-0.008625092	0.08477198	-0.101744611	0.918959391	0	0.561395515	0	0.5	PRJNA827465;Yang2025_Silkie_Wenchang	CRA004775	0.981086235
ENSGALG00010011413	ZBTB37	2	-0.096266751	0.1571458	-0.612595127	0.540144081	0	0.126990533	0	0.5	CRA004775;Yang2025_Silkie_Wenchang	PRJNA827465	0.87529516
ENSGALG00010011413	ZBTB37	2	0.03003947	0.096200177	0.312260028	0.754842915	0	0.003925465	0	1	CRA004775;PRJNA827465	Yang2025_Silkie_Wenchang	0.93838723
ENSGALG00010011414	PABPC1	2	0.071374097	0.12591079	0.566862439	0.570807645	0.019414784	2.021793495	50.53896442	0.5	PRJNA827465;Yang2025_Silkie_Wenchang	CRA004775	0.885224727
ENSGALG00010011414	PABPC1	2	0.022246572	0.258793478	0.085962647	0.93149611	0.092256693	3.193635822	68.68772597	0.5	CRA004775;Yang2025_Silkie_Wenchang	PRJNA827465	0.984076328
ENSGALG00010011414	PABPC1	2	-0.034231727	0.073411158	-0.466301415	0.640999766	0.003063243	1.117802284	10.53874066	1	CRA004775;PRJNA827465	Yang2025_Silkie_Wenchang	0.906429868
ENSGALG00010011416	ENSGALG00010011416	2	0.063104531	0.67317981	0.093740974	0.925314921	0.639393779	3.303868781	69.73245409	0.5	PRJNA827465;Yang2025_Silkie_Wenchang	CRA004775	0.982594411
ENSGALG00010011416	ENSGALG00010011416	2	-0.675511852	0.481550812	-1.402784161	0.160681212	0	0.000873613	0	1	CRA004775;Yang2025_Silkie_Wenchang	PRJNA827465	0.656486694
ENSGALG00010011416	ENSGALG00010011416	2	0.165736418	0.642665626	0.257889035	0.796492548	0.485722208	2.239128171	55.33976068	0.5	CRA004775;PRJNA827465	Yang2025_Silkie_Wenchang	0.949485793
ENSGALG00010011417	WFS1	2	0.038092755	0.2206884	0.17260878	0.86295895	0.077958021	4.548156157	78.01306803	0.5	PRJNA827465;Yang2025_Silkie_Wenchang	CRA004775	0.966099097
ENSGALG00010011417	WFS1	2	0.051263753	0.251701916	0.203668505	0.838612555	0.099605701	4.572489601	78.13007601	0.5	CRA004775;Yang2025_Silkie_Wenchang	PRJNA827465	0.959226718
ENSGALG00010011417	WFS1	2	0.240800596	0.067432504	3.570986991	0.000355639	0	0.139608074	0	1	CRA004775;PRJNA827465	Yang2025_Silkie_Wenchang	0.018812466
ENSGALG00010011418	RIPK1	2	0.121886575	0.142640935	0.854499272	0.392828413	0.02747714	2.72964183	63.36515696	1	PRJNA827465;Yang2025_Silkie_Wenchang	CRA004775	0.830058498
ENSGALG00010011418	RIPK1	2	0.184815494	0.183022102	1.009798772	0.312591709	0.021456889	1.371529525	27.08870048	0.5	CRA004775;Yang2025_Silkie_Wenchang	PRJNA827465	0.796054997
ENSGALG00010011418	RIPK1	2	0.005533888	0.069612006	0.079496167	0.93663798	0	0.114036587	0	0.5	CRA004775;PRJNA827465	Yang2025_Silkie_Wenchang	0.985038972
ENSGALG00010011419	RNF180	2	0.072598515	0.1052835	0.689552629	0.490475566	0.002405651	1.121167545	10.80726476	0.5	PRJNA827465;Yang2025_Silkie_Wenchang	CRA004775	0.858670876
ENSGALG00010011419	RNF180	2	0.175703473	0.137568613	1.277206108	0.201529491	0	0.0353919	0	1	CRA004775;Yang2025_Silkie_Wenchang	PRJNA827465	0.714377024
ENSGALG00010011419	RNF180	2	-0.013718863	0.129022216	-0.106329465	0.915320955	0	0.086769247	0	0.5	CRA004775;PRJNA827465	Yang2025_Silkie_Wenchang	0.980161281
ENSGALG00010011420	HMGB3	2	0.069119094	0.069298187	0.997415615	0.318562815	0.005292328	1.846161849	45.83356813	1	PRJNA827465;Yang2025_Silkie_Wenchang	CRA004775	0.797754958
ENSGALG00010011420	HMGB3	2	0.142476844	0.098443532	1.447295127	0.147814283	0	0.878760626	0	0.5	CRA004775;Yang2025_Silkie_Wenchang	PRJNA827465	0.637081344
ENSGALG00010011420	HMGB3	2	0.021865121	0.038614176	0.566245953	0.571226595	0	0.214606906	0	0.5	CRA004775;PRJNA827465	Yang2025_Silkie_Wenchang	0.885354083
ENSGALG00010011421	ENSGALG00010011421	2	0.258901683	0.313781661	0.825101386	0.409314033	0.175866101	8.677326518	88.47571314	0.5	PRJNA827465;Yang2025_Silkie_Wenchang	CRA004775	0.834355341
ENSGALG00010011421	ENSGALG00010011421	2	0.545759115	0.159251134	3.427034407	0.000610212	0	0.206809184	0	1	CRA004775;Yang2025_Silkie_Wenchang	PRJNA827465	0.026796818
ENSGALG00010011421	ENSGALG00010011421	2	0.153148352	0.233109331	0.656980785	0.511193272	0.082694235	3.451637688	71.02824542	0.5	CRA004775;PRJNA827465	Yang2025_Silkie_Wenchang	0.865649044
ENSGALG00010011422	KATNAL2	2	0.445448401	0.190108132	2.343131759	0.019122629	0	0.646226265	0	1	PRJNA827465;Yang2025_Silkie_Wenchang	CRA004775	0.231610685
ENSGALG00010011422	KATNAL2	2	0.326809578	0.269045169	1.214701526	0.22447995	0	0.268864828	0	1	CRA004775;Yang2025_Silkie_Wenchang	PRJNA827465	0.737544559
ENSGALG00010011422	KATNAL2	2	0.58237093	0.232671287	2.502977218	0.012315347	0	0.008236146	0	1	CRA004775;PRJNA827465	Yang2025_Silkie_Wenchang	0.179397068
ENSGALG00010011423	GDF6	2	0.396890237	0.343948362	1.153923904	0.248531368	0	0.947578326	0	1	PRJNA827465;Yang2025_Silkie_Wenchang	CRA004775	0.758858636
ENSGALG00010011423	GDF6	2	0.373188355	0.422848563	0.882557936	0.377475164	0.071128212	1.241289136	19.43859245	1	CRA004775;Yang2025_Silkie_Wenchang	PRJNA827465	0.82325159
ENSGALG00010011423	GDF6	2	0.09451884	0.31995681	0.295411245	0.767679744	0	0.052143889	0	1	CRA004775;PRJNA827465	Yang2025_Silkie_Wenchang	0.941444269
ENSGALG00010011424	ACADL	2	-0.09494398	0.068747192	-1.381059756	0.167260588	0	0.198863872	0	1	PRJNA827465;Yang2025_Silkie_Wenchang	CRA004775	0.666785972
ENSGALG00010011424	ACADL	2	0.010964689	0.135994643	0.080625891	0.935739475	0	0.24711861	0	0.5	CRA004775;Yang2025_Silkie_Wenchang	PRJNA827465	0.984800337
ENSGALG00010011424	ACADL	2	-0.091281217	0.073079459	-1.24906805	0.211640185	0	0.725924401	0	0.5	CRA004775;PRJNA827465	Yang2025_Silkie_Wenchang	0.724013667
ENSGALG00010011425	WASHC3	2	-0.02413057	0.046438435	-0.519624954	0.603325002	0	0.798406751	0	0.5	PRJNA827465;Yang2025_Silkie_Wenchang	CRA004775	0.895538966
ENSGALG00010011425	WASHC3	2	0.079872892	0.108384903	0.736937434	0.461160398	0	0.006120162	0	1	CRA004775;Yang2025_Silkie_Wenchang	PRJNA827465	0.85014477
ENSGALG00010011425	WASHC3	2	-0.033071575	0.048059208	-0.688142322	0.491363161	0	0.293399067	0	0.5	CRA004775;PRJNA827465	Yang2025_Silkie_Wenchang	0.85895707
ENSGALG00010011426	NQO2	2	0.120312931	0.21033688	0.572001119	0.567321217	0.066716389	3.819680145	73.81979741	0.5	PRJNA827465;Yang2025_Silkie_Wenchang	CRA004775	0.883696329
ENSGALG00010011426	NQO2	2	0.112808329	0.277427843	0.406622231	0.684285467	0.103149817	2.97045139	66.33508283	0.5	CRA004775;Yang2025_Silkie_Wenchang	PRJNA827465	0.918862939
ENSGALG00010011426	NQO2	2	-0.079337536	0.095668305	-0.829298025	0.406935788	0	0.223624602	0	1	CRA004775;PRJNA827465	Yang2025_Silkie_Wenchang	0.833618122
ENSGALG00010011427	TMTC3	2	-0.097873802	0.036161622	-2.706565627	0.006798314	0	0.021553504	0	1	PRJNA827465;Yang2025_Silkie_Wenchang	CRA004775	0.126544334
ENSGALG00010011427	TMTC3	2	0.030578917	0.14463801	0.211416884	0.832561983	0.024001929	2.192443491	54.38879022	0.5	CRA004775;Yang2025_Silkie_Wenchang	PRJNA827465	0.957861248
ENSGALG00010011427	TMTC3	2	0.010688869	0.149578747	0.071459808	0.943031811	0.033106329	3.089796639	67.63541046	0.5	CRA004775;PRJNA827465	Yang2025_Silkie_Wenchang	0.985827325
ENSGALG00010011428	UQCRB	2	-0.077652433	0.080011091	-0.97052085	0.331786937	0	0.004221622	0	1	PRJNA827465;Yang2025_Silkie_Wenchang	CRA004775	0.802002658
ENSGALG00010011428	UQCRB	2	-0.032148315	0.202956689	-0.158399881	0.874141708	0	0.30900587	0	0.5	CRA004775;Yang2025_Silkie_Wenchang	PRJNA827465	0.970092404
ENSGALG00010011428	UQCRB	2	-0.066174109	0.083774068	-0.789911609	0.429579391	0	0.337306268	0	0.5	CRA004775;PRJNA827465	Yang2025_Silkie_Wenchang	0.842012722
ENSGALG00010011429	UBAP2	2	0.167738566	0.287511213	0.583415735	0.559613468	0.125128725	3.987236569	74.9199732	0.5	PRJNA827465;Yang2025_Silkie_Wenchang	CRA004775	0.881203589
ENSGALG00010011429	UBAP2	2	0.088237674	0.414479325	0.212887999	0.831414323	0.271151068	4.712745341	78.78094555	0.5	CRA004775;Yang2025_Silkie_Wenchang	PRJNA827465	0.957626151
ENSGALG00010011429	UBAP2	2	-0.148305725	0.139597403	-1.062381692	0.288062442	0	0.563476054	0	1	CRA004775;PRJNA827465	Yang2025_Silkie_Wenchang	0.782840067
ENSGALG00010011430	ENSGALG00010011430	2	0.030586764	0.497029641	0.061539115	0.950929864	0.41157317	5.918435978	83.10364421	0.5	PRJNA827465;Yang2025_Silkie_Wenchang	CRA004775	0.987408429
ENSGALG00010011430	ENSGALG00010011430	2	0.541183612	0.294772334	1.835937601	0.066366886	0	0.006072943	0	1	CRA004775;Yang2025_Silkie_Wenchang	PRJNA827465	0.443651804
ENSGALG00010011430	ENSGALG00010011430	2	-0.181332747	0.420314873	-0.431421199	0.666162139	0.185475311	1.731210961	42.2369646	0.5	CRA004775;PRJNA827465	Yang2025_Silkie_Wenchang	0.913579507
ENSGALG00010011431	DRAM1	2	0.180352187	0.480272781	0.375520317	0.707273539	0.350728592	4.05418011	75.33410029	0.5	PRJNA827465;Yang2025_Silkie_Wenchang	CRA004775	0.924856272
ENSGALG00010011431	DRAM1	2	-0.026434835	0.317887175	-0.083157916	0.933725975	0.045297602	1.288821198	22.40971814	0.5	CRA004775;Yang2025_Silkie_Wenchang	PRJNA827465	0.984262625
ENSGALG00010011431	DRAM1	2	0.513707545	0.219899148	2.336105206	0.019485759	0	0.481857394	0	1	CRA004775;PRJNA827465	Yang2025_Silkie_Wenchang	0.233920645
ENSGALG00010011433	RNASE6	2	-0.019258368	0.330894075	-0.058201007	0.953588519	0.143473362	2.858713653	65.01923168	0.5	PRJNA827465;Yang2025_Silkie_Wenchang	CRA004775	0.987794253
ENSGALG00010011433	RNASE6	2	-0.513184906	0.240944755	-2.129886188	0.033181011	0	0.459119903	0	1	CRA004775;Yang2025_Silkie_Wenchang	PRJNA827465	0.313112428
ENSGALG00010011433	RNASE6	2	-0.17096562	0.496974577	-0.344012809	0.730836653	0.395967125	4.886582852	79.5358018	0.5	CRA004775;PRJNA827465	Yang2025_Silkie_Wenchang	0.932769533
ENSGALG00010011434	NPL	2	0.028761713	0.077745978	0.36994471	0.711423687	0	0.04903038	0	0.5	PRJNA827465;Yang2025_Silkie_Wenchang	CRA004775	0.92587565
ENSGALG00010011434	NPL	2	-0.234411624	0.263691583	-0.888961346	0.374023854	0.097293525	3.207038185	68.81858144	1	CRA004775;Yang2025_Silkie_Wenchang	PRJNA827465	0.822579781
ENSGALG00010011434	NPL	2	-0.200612054	0.282300054	-0.710634131	0.477310987	0.128866324	4.68029662	78.63383283	0.5	CRA004775;PRJNA827465	Yang2025_Silkie_Wenchang	0.854997403
ENSGALG00010011436	UBAP2L2	2	-4.437908961	4.066649709	-1.091293639	0.275143696	29.64141148	9.595003974	89.57790947	1	PRJNA827465;Yang2025_Silkie_Wenchang	CRA004775	0.775068889
ENSGALG00010011436	UBAP2L2	2	-4.331562579	4.073761929	-1.06328319	0.287653546	30.59406133	12.48704225	91.99169843	1	CRA004775;Yang2025_Silkie_Wenchang	PRJNA827465	0.782840067
ENSGALG00010011436	UBAP2L2	2	-0.442024327	0.908061363	-0.486778036	0.626415637	0	0.000107469	0	1	CRA004775;PRJNA827465	Yang2025_Silkie_Wenchang	0.901504244
ENSGALG00010011437	DMRT1	2	-0.300202855	0.335114761	-0.895821044	0.370348349	0	0.341473498	0	1	PRJNA827465;Yang2025_Silkie_Wenchang	CRA004775	0.821109108
ENSGALG00010011437	DMRT1	2	-0.201251624	0.376284382	-0.534839164	0.592761079	0	0.825293601	0	0.5	CRA004775;Yang2025_Silkie_Wenchang	PRJNA827465	0.892776493
ENSGALG00010011437	DMRT1	2	-0.027791505	0.336372609	-0.082621189	0.934152753	0	0.170504357	0	0.5	CRA004775;PRJNA827465	Yang2025_Silkie_Wenchang	0.98432801
ENSGALG00010011438	ENSGALG00010011438	2	0.195666882	0.18193588	1.075471658	0.282163615	0.033464616	1.828864592	45.32126629	0.5	PRJNA827465;Yang2025_Silkie_Wenchang	CRA004775	0.779519046
ENSGALG00010011438	ENSGALG00010011438	2	0.57716871	0.650287714	0.887558995	0.374778019	0.762538393	10.11461922	90.11332035	0.5	CRA004775;Yang2025_Silkie_Wenchang	PRJNA827465	0.822805106
ENSGALG00010011438	ENSGALG00010011438	2	0.737108844	0.455877712	1.616900377	0.105899787	0.36019535	7.011892208	85.73851436	1	CRA004775;PRJNA827465	Yang2025_Silkie_Wenchang	0.552086395
ENSGALG00010011439	ENSGALG00010011439	2	0.437330314	1.070447883	0.408548908	0.682870731	2.181167872	20.72522805	95.17496262	0.5	PRJNA827465;Yang2025_Silkie_Wenchang	CRA004775	0.918793207
ENSGALG00010011439	ENSGALG00010011439	2	0.60659802	0.977954723	0.62027209	0.535078667	1.659238544	7.197950987	86.10715741	0.5	CRA004775;Yang2025_Silkie_Wenchang	PRJNA827465	0.873964046

ENSGALG00010011439	ENSGALG00010011439	2	-0.598638881	0.305115425	-1.962007923	0.049761562	0	0.057309304	0	1	CRA004775;PRJNA827465	Yang2025_Silkie_Wenchang	0.386162809
ENSGALG00010011440	FUNDC1	2	-0.043802772	0.064957916	-0.674325389	0.500104466	0	0.028746116	0	1	PRJNA827465;Yang2025_Silkie_Wenchang	CRA004775	0.862201423
ENSGALG00010011440	FUNDC1	2	-0.058070958	0.136007715	-0.42696812	0.669402548	0	0.016735673	0	1	CRA004775;Yang2025_Silkie_Wenchang	PRJNA827465	0.914377769
ENSGALG00010011440	FUNDC1	2	-0.037932436	0.069423648	-0.546390707	0.584797397	0	0.001981448	0	1	CRA004775;PRJNA827465	Yang2025_Silkie_Wenchang	0.891016154
ENSGALG00010011441	CD99L2	2	-0.092382347	0.079995463	-1.154844839	0.248153972	0.00547444	1.744626592	42.68114422	1	PRJNA827465;Yang2025_Silkie_Wenchang	CRA004775	0.75835437
ENSGALG00010011441	CD99L2	2	-0.155525048	0.084713534	-1.835893757	0.066373371	0	0.552753447	0	0.5	CRA004775;Yang2025_Silkie_Wenchang	PRJNA827465	0.443651804
ENSGALG00010011441	CD99L2	2	-0.011634523	0.0786656	-0.147898483	0.882422885	0	0.034109174	0	0.5	CRA004775;PRJNA827465	Yang2025_Silkie_Wenchang	0.972001076
ENSGALG00010011442	ENSGALG00010011442	2	-4.944289449	4.845565106	-1.020374165	0.307551042	42.31856006	9.978853362	89.97880855	1	PRJNA827465;Yang2025_Silkie_Wenchang	CRA004775	0.792894627
ENSGALG00010011442	ENSGALG00010011442	2	-4.70789022	5.021562678	-0.937534891	0.348483485	46.49392198	12.44631287	91.96549203	0.5	CRA004775;Yang2025_Silkie_Wenchang	PRJNA827465	0.809147894
ENSGALG00010011442	ENSGALG00010011442	2	-0.054722259	1.005294087	-0.05443408	0.956589327	0	0.028765066	0	0.5	CRA004775;PRJNA827465	Yang2025_Silkie_Wenchang	0.988540618
ENSGALG00010011443	ENSGALG00010011443	2	-0.04050521	0.252502511	-0.160415079	0.872554112	0	0.133574115	0	0.5	PRJNA827465;Yang2025_Silkie_Wenchang	CRA004775	0.969629321
ENSGALG00010011443	ENSGALG00010011443	2	-0.039282207	0.310753665	-0.126409474	0.899407803	0	0.27029561	0	0.5	CRA004775;Yang2025_Silkie_Wenchang	PRJNA827465	0.976081354
ENSGALG00010011443	ENSGALG00010011443	2	0.096008423	0.305807723	0.313950288	0.753558796	0	0.06815929	0	1	CRA004775;PRJNA827465	Yang2025_Silkie_Wenchang	0.938087082
ENSGALG00010011445	MTERF3	2	-0.24460523	0.088453395	-2.765357162	0.00568605	0.001981026	1.138802458	12.1884579	1	PRJNA827465;Yang2025_Silkie_Wenchang	CRA004775	0.113268539
ENSGALG00010011445	MTERF3	2	-0.193811952	0.209762689	-0.92395818	0.35550809	0.051662449	2.188063467	54.29748657	0.5	CRA004775;Yang2025_Silkie_Wenchang	PRJNA827465	0.812301302
ENSGALG00010011445	MTERF3	2	-0.136536533	0.097974685	-1.393589914	0.163441476	0	0.811697479	0	0.5	CRA004775;PRJNA827465	Yang2025_Silkie_Wenchang	0.661791656
ENSGALG00010011446	ENSGALG00010011446	2	0.232567751	0.556304432	0.418058418	0.675904406	0.572461316	13.29646447	92.47920376	0.5	PRJNA827465;Yang2025_Silkie_Wenchang	CRA004775	0.916522765
ENSGALG00010011446	ENSGALG00010011446	2	0.504718059	0.342232032	1.474783223	0.140270833	0.148211029	2.601845875	61.56574801	1	CRA004775;Yang2025_Silkie_Wenchang	PRJNA827465	0.624073416
ENSGALG00010011446	ENSGALG00010011446	2	-0.218354126	0.178314933	-1.224542009	0.220747829	0.00160742	1.018672727	1.833044744	0.5	CRA004775;PRJNA827465	Yang2025_Silkie_Wenchang	0.734053249
ENSGALG00010011447	LAMC1	2	-0.174267424	0.078252674	-2.226983632	0.025948368	0	0.587187102	0	1	PRJNA827465;Yang2025_Silkie_Wenchang	CRA004775	0.274669727
ENSGALG00010011447	LAMC1	2	-0.03354266	0.185104946	-0.181208883	0.856203623	0.029443337	1.316615656	24.04769036	0.5	CRA004775;Yang2025_Silkie_Wenchang	PRJNA827465	0.963499334
ENSGALG00010011447	LAMC1	2	-0.060666056	0.278000484	-0.218222843	0.827255488	0.095527595	2.021547602	50.53294818	0.5	CRA004775;PRJNA827465	Yang2025_Silkie_Wenchang	0.956550752
ENSGALG00010011449	DUSP22	2	-0.067581781	0.065691405	-1.028776609	0.303584661	0	0.29356085	0	1	PRJNA827465;Yang2025_Silkie_Wenchang	CRA004775	0.792205992
ENSGALG00010011449	DUSP22	2	-0.185879848	0.157874002	-1.177393653	0.239038422	0.01701202	1.224320838	18.32206324	1	CRA004775;Yang2025_Silkie_Wenchang	PRJNA827465	0.751233576
ENSGALG00010011449	DUSP22	2	-0.164069552	0.216504683	-0.757810638	0.448564353	0.055232622	1.758818887	43.14366264	1	CRA004775;PRJNA827465	Yang2025_Silkie_Wenchang	0.846878812
ENSGALG00010011450	PTDSS1	2	-0.20192425	0.110043185	-1.834954608	0.066512415	0.017860228	3.675922066	72.79594121	1	PRJNA827465;Yang2025_Silkie_Wenchang	CRA004775	0.44427897
ENSGALG00010011450	PTDSS1	2	-0.095850537	0.275595033	-0.347794862	0.727994249	0.119117142	4.137949658	75.8334421	0.5	CRA004775;Yang2025_Silkie_Wenchang	PRJNA827465	0.931881152
ENSGALG00010011450	PTDSS1	2	-0.026533534	0.142156641	-0.18664998	0.851935077	0.022276072	1.633250562	38.77240742	0.5	CRA004775;PRJNA827465	Yang2025_Silkie_Wenchang	0.962255491
ENSGALG00010011452	KITLG	2	0.053513132	0.280978983	0.190452438	0.84895461	0.139675857	8.639879758	88.42576485	0.5	PRJNA827465;Yang2025_Silkie_Wenchang	CRA004775	0.961364896
ENSGALG00010011452	KITLG	2	0.178277623	0.47466573	0.375585622	0.707224982	0.373092233	5.295962482	81.11769101	0.5	CRA004775;Yang2025_Silkie_Wenchang	PRJNA827465	0.924856272
ENSGALG00010011452	KITLG	2	0.365104297	0.117581036	3.105129091	0.00190196	0	0.940907275	0	1	CRA004775;PRJNA827465	Yang2025_Silkie_Wenchang	0.056193756
ENSGALG00010011453	GNPTAB	2	-0.028848841	0.049976966	-0.577242743	0.563775486	0	0.977436833	0	0.5	PRJNA827465;Yang2025_Silkie_Wenchang	CRA004775	0.882651345
ENSGALG00010011453	GNPTAB	2	0.110174008	0.227205488	0.484909098	0.627740829	0.081950615	4.265973143	76.55868974	0.5	CRA004775;Yang2025_Silkie_Wenchang	PRJNA827465	0.901856848
ENSGALG00010011453	GNPTAB	2	0.147938016	0.173347036	0.853421089	0.393425831	0.041039008	2.631128339	61.9934921	1	CRA004775;PRJNA827465	Yang2025_Silkie_Wenchang	0.830352025
ENSGALG00010011456	KIAA1328	2	0.311865396	0.197950391	1.575472495	0.115147422	0.055818306	3.452187922	71.03286314	1	PRJNA827465;Yang2025_Silkie_Wenchang	CRA004775	0.572141484
ENSGALG00010011456	KIAA1328	2	0.182963083	0.398334483	0.459320221	0.646004229	0.245677503	4.052505762	75.32390924	0.5	CRA004775;Yang2025_Silkie_Wenchang	PRJNA827465	0.907177315
ENSGALG00010011456	KIAA1328	2	0.058697405	0.150251356	0.390661401	0.696047534	0.008154485	1.106734179	9.644066376	0.5	CRA004775;PRJNA827465	Yang2025_Silkie_Wenchang	0.921038437
ENSGALG00010011457	ENSGALG00010011457	2	0.158291184	0.437222309	0.362038213	0.717323474	0.26735811	3.291547288	69.61915134	0.5	PRJNA827465;Yang2025_Silkie_Wenchang	CRA004775	0.9277944
ENSGALG00010011457	ENSGALG00010011457	2	0.301764089	0.425346198	0.70945524	0.478042019	0.143587589	1.5842014	36.87671276	0.5	CRA004775;Yang2025_Silkie_Wenchang	PRJNA827465	0.855366076
ENSGALG00010011457	ENSGALG00010011457	2	-0.251981094	0.268804376	-0.937414405	0.348545434	0	8.37932E-05	0	1	CRA004775;PRJNA827465	Yang2025_Silkie_Wenchang	0.809196124
ENSGALG00010011458	TPGS2	2	0.326221482	0.19596836	1.664664045	0.0959798	0.032819839	1.733877166	42.32578756	1	PRJNA827465;Yang2025_Silkie_Wenchang	CRA004775	0.5301492
ENSGALG00010011458	TPGS2	2	0.320574021	0.260373162	1.231209923	0.218244361	0.064682664	1.868916579	46.49306387	1	CRA004775;Yang2025_Silkie_Wenchang	PRJNA827465	0.731882092
ENSGALG00010011458	TPGS2	2	0.111326098	0.16385118	0.679434213	0.496862778	0	0.133577805	0	1	CRA004775;PRJNA827465	Yang2025_Silkie_Wenchang	0.860939846
ENSGALG00010011459	ENSGALG00010011459	2	0.612984852	0.183518768	3.340175279	0.000837255	0	0.038508567	0	1	PRJNA827465;Yang2025_Silkie_Wenchang	CRA004775	0.033578209
ENSGALG00010011459	ENSGALG00010011459	2	0.415804523	0.31910619	1.303028699	0.192564964	0	0.241769834	0	1	CRA004775;Yang2025_Silkie_Wenchang	PRJNA827465	0.702467122
ENSGALG00010011459	ENSGALG00010011459	2	0.573826333	0.190565917	3.011169784	0.002602433	0	0.55838716	0	1	CRA004775;PRJNA827465	Yang2025_Silkie_Wenchang	0.069017153
ENSGALG00010011460	LAMC2	2	-0.57694456	0.389919564	-1.479650198	0.138966623	0.084418194	1.364316784	26.70323989	1	PRJNA827465;Yang2025_Silkie_Wenchang	CRA004775	0.622711797
ENSGALG00010011460	LAMC2	2	0.421784482	1.494017315	0.282315659	0.77770148	4.111180898	12.61973495	92.0759033	0.5	CRA004775;Yang2025_Silkie_Wenchang	PRJNA827465	0.944388838
ENSGALG00010011460	LAMC2	2	0.783885384	1.095162943	0.715770551	0.474133039	2.112898341	8.234372772	87.85578419	0.5	CRA004775;PRJNA827465	Yang2025_Silkie_Wenchang	0.85444425
ENSGALG00010011461	IRF4	2	-0.06168512	0.192160235	-0.321008768	0.748203747	0	0.831839948	0	0.5	PRJNA827465;Yang2025_Silkie_Wenchang	CRA004775	0.937114463
ENSGALG00010011461	IRF4	2	-0.228125814	0.251240526	-0.907997675	0.36387945	0	0.000266228	0	1	CRA004775;Yang2025_Silkie_Wenchang	PRJNA827465	0.817044906
ENSGALG00010011461	IRF4	2	0.081747956	0.261029054	0.313175698	0.754147181	0	0.173011585	0	0.5	CRA004775;PRJNA827465	Yang2025_Silkie_Wenchang	0.938385954
ENSGALG00010011463	IMP3	2	0.197077406	0.102460136	1.923454464	0.054423	0	0.00031036	0	1	PRJNA827465;Yang2025_Silkie_Wenchang	CRA004775	0.404097297
ENSGALG00010011463	IMP3	2	-0.071378966	0.27248909	-0.261951647	0.793358721	0	0.855438458	0	0.5	CRA004775;Yang2025_Silkie_Wenchang	PRJNA827465	0.948684977
ENSGALG00010011463	IMP3	2	0.072840885	0.215406241	0.338155871	0.735245728	0.050495686	1.683839605	40.61192069	0.5	CRA004775;PRJNA827465	Yang2025_Silkie_Wenchang	0.933592889
ENSGALG00010011464	FANCG	2	0.022835603	0.136163894	0.167706739	0.866813995	0	0.2857775	0	0.5	PRJNA827465;Yang2025_Silkie_Wenchang	CRA004775	0.967632084
ENSGALG00010011464	FANCG	2	-0.320326004	0.338126107	-0.947356613	0.343457092	0.145823111	2.515719165	60.24993514	1	CRA004775;Yang2025_Silkie_Wenchang	PRJNA827465	0.806276913
ENSGALG00010011464	FANCG	2	-0.259070182	0.414898463	-0.624418273	0.532352927	0.260022118	3.763529763	73.4291991	0.5	CRA004775;PRJNA827465	Yang2025_Silkie_Wenchang	0.873104119
ENSGALG00010011465	PPP2R2C	2	0.678953553	0.620725011	1.093807309	0.274039498	0.720840707	15.48206821	93.54091465	1	PRJNA827465;Yang2025_Silkie_Wenchang	CRA004775	0.774526342
ENSGALG00010011465	PPP2R2C	2	0.620072341	0.785533948	0.789364155	0.4298992	1.029507165	5.491872287	81.79127358	0.5	CRA004775;Yang2025_Silkie_Wenchang	PRJNA827465	0.842012722
ENSGALG00010011465	PPP2R2C	2	0.025723535	0.20369479	0.126284699	0.899506567	0	0.264801724	0	0.5	CRA004775;PRJNA827465	Yang2025_Silkie_Wenchang	0.976081354
ENSGALG00010011466	MTMR1	2	-0.183094135	0.092877233	-1.971356474	0.048683116	0.014101588	5.321909724	81.20975267	1	PRJNA827465;Yang2025_Silkie_Wenchang	CRA004775	0.381461853
ENSGALG00010011466	MTMR1	2	-0.158641859	0.145838422	-1.087791937	0.27668697	0.032265047	3.860455268	74.09631946	0.5	CRA004775;Yang2025_Silkie_Wenchang	PRJNA827465	0.775851484
ENSGALG00010011466	MTMR1	2	-0.087124756	0.041127714	-2.118392276	0.034141606	0	0.598244201	0	0.5	CRA004775;PRJNA827465	Yang2025_Silkie_Wenchang	0.317827458
ENSGALG00010011467	ENSGALG00010011467	2	-5.930770781	5.932595556	-0.999692415	0.317459384	65.41010279	13.91324379	92.81260348	1	PRJNA827465;Yang2025_Silkie_Wenchang	CRA004775	0.797125693
ENSGALG00010011467	ENSGALG00010011467	2	-5.66299182	6.140768007	-0.922196021	0.356426338	71.28567035	17.6885982	94.34664076	0.5	CRA004775;Yang2025_Silkie_Wenchang	PRJNA827465	0.812521698
ENSGALG00010011467	ENSGALG00010011467	2	0.100990963	0.859506042	0.11749884	0.906464763	0	0.048492086	0	0.5	CRA004775;PRJNA827465	Yang2025_Silkie_Wenchang	0.978196186
ENSGALG00010011469	CPS1	2	0.257601865	0.18951086	1.359298696	0.174051957	0.033590034	1.878188537	46.75720885	1	PRJNA827465;Yang2025_Silkie_Wenchang	CRA004775	0.67800817
ENSGALG00010011469	CPS1	2	-0.209041671	0.719325267	-0.2892178	0.772414711	0.906293465	7.552526659	86.75893686	0.5	CRA004775;Yang2025_Silkie_Wenchang	PRJNA827465	0.943946211
ENSGALG00010011469	CPS1	2	-0.37355911	0.524859347	-0.711731842	0.476630846	0.42937945	4.084902494	75.51961151	0.5	CRA004775;PRJNA827465	Yang2025_Silkie_Wenchang	0.854986476
ENSGALG00010011471	NMNAT2	2	-0.193280171	0.328546429	-0.588288759	0.556338449	0.073788894	1.486934514	32.74754266	0.5	PRJNA827465;Yang2025_Silkie_Wenchang	CRA004775	0.880324407
ENSGALG00010011471	NMNAT2	2	0.099322966	0.35262977	0.281663589	0.778201476	0	0.170358878	0	0.5	CRA004775;Yang2025_Silkie_Wenchang	PRJNA827465	0.944526361
ENSGALG00010011471	NMNAT2	2	-0.37171934	0.283192892	-1.312601234	0.189317354	0	0.310657301	0	1	CRA004775;PRJNA827465	Yang2025_Silkie_Wenchang	0.698333172
ENSGALG00010011475	NDP	2	0.393781648	0.198022924	1.988565974	0.04674913	0	0.502072513	0	1	PRJNA827465;Yang2025_Silkie_Wenchang	CRA004775	0.373490609
ENSGALG00010011475	NDP	2	0.285522157	0.287334262	0.993693392	0.320372161	0	0.260936224	0	1	CRA004775;Yang2025_Silkie_Wenchang	PRJNA827465	0.798348216
ENSGALG00010011475	NDP	2	0.521125276	0.240909883	2.163154415	0.030529305	0	0.022119847	0	1	CRA004775;PRJNA827465	Yang2025_Silkie_Wenchang	0.297588102
ENSGALG00010011477	SDC2	2	-0.173945517	0.189221667	-0.919268495	0.357955153	0.061481755	6.871648757	85.44745176	0.5	PRJNA827465;Yang2025_Silkie_Wenchang	CRA004775	0.813641308
ENSGALG00010011477	SDC2	2	0.089696614	0.490484719	0.182873411	0.854897346	0.442677147	12.15256145	91.77128209	0.5	CRA004775;Yang2025_Silkie_Wenchang	PRJNA827465	0.963248391
ENSGALG00010011477	SDC2	2	0.255592133	0.297508764	0.859107912	0.390280983	0.146997198	5.233207451	80.89126011	1	CRA004775;PRJNA827465	Yang2025_Silkie_Wenchang	0.82923632
ENSGALG00010011478	HDAC3	2	-0.257436009	0.151625208	-1.697843397	0.089537134	0.037785876	5.204981175	80.78763464	1	PRJNA827465;Yang2025_Silkie_Wenchang	CRA004775	0.513242777
ENSGALG00010011478	HDAC3	2	-0.426502046	0.092573249	-4.607184593	4.08158E-06	0	0.00216491	0	1	CRA004775;Yang2025_Silkie_Wenchang	PRJNA827465	0.000700516
ENSGALG00010011478	HDAC3	2	-0.244432531	0.145807669	-1.676403807	0.093659124	0.032764887	3.877945495	74.21314969	1	CRA004775;PRJNA827465	Yang2025_Silkie_Wenchang	0.524147023
ENSGALG00010011480	BLTP3B	2	-0.107016154	0.122219899	-0.875603358	0.381245699	0.020353787	3.036980674	67.07255965	0.5	PRJNA827465;Yang2025_Silkie_Wenchang	CRA004775	0.824292515
ENSGALG00010011480	BLTP3B	2	-0.069748335	0.202888369	-0.343776902	0.731014072	0.058293093	3.264794711	69.37020276	0.5	CRA004775;Yang2025_Silkie_Wenchang	PRJNA827465	0.932769533
ENSGALG00010011480	BLTP3B	2	0.023968912	0.07303732	0.328173493	0.742780489	0	0.609388693	0	1	CRA004775;PRJNA827465	Yang2025_Silkie_Wenchang	0.935659163
ENSGALG00010011482	DMRT2	2	-0.175306414	0.08665366	-2.023069924	0.043065939	0	0.215361036	0	1	PRJNA827465;Yang2025_Silkie_Wenchang	CRA004775	0.358841867
ENSGALG00010011482	DMRT2	2	-0.223256447	0.136716068	-1.632993481	0.102470368	0	0.011148566	0	1	CRA004775;Yang2025_Silkie_Wenchang	PRJNA827465	0.54534714
ENSGALG00010011482	DMRT2	2	-0.14466519	0.107940139	-1.340235354	0.180168841	0	0.001296248	0	1	CRA004775;PRJNA827465	Yang2025_Silkie_Wenchang	0.687225928
ENSGALG00010011484	CHPT1	2	-0.309963742	0.131442113	-2.358176799	0.018364944	0.028090927	5.157163204	80.60949479	1	PRJNA827465;Yang2025_Silkie_Wenchang	CRA004775	0.226980313
ENSGALG00010011484	CHPT1	2	-0.245109075	0.22345919	-1.096885183	0.272691586	0.083648626	6.018471753	83.38448628	1	CRA004775;Yang2025_Silkie_Wenchang	PRJNA827465	0.773576706
ENSGALG00010011484	CHPT1	2	-0.152016416	0.074517892	-2.039998858	0.041350044	0.003446674	1.255939655	20.37834017	1	CRA004775;PRJNA827465	Yang2025_Silkie_Wenchang	0.325571164
ENSGALG00010011485	MTM1	2	0.160107633	0.080174519	1.996989005	0.045826377	0.004803588	1.458677301	31.44474111	1	PRJNA827465;Yang2025_Silkie_Wenchang	CRA004775	0.369307794
ENSGALG00010011485	MTM1	2	0.207690641	0.102947107	2.017449993	0.043648575	0	0.934528044	0	1	CRA004775;Yang2025_Silkie_Wenchang	PRJNA827465	0.361086072
ENSGALG00010011485	MTM1	2	0.103272991	0.061823845	1.670439464	0.094832447	0	0.031338774	0	1	CRA004775;PRJNA827465	Yang2025_Silkie_Wenchang	0.587185243
ENSGALG00010011487	MAN2B2	2	-0.03599172	0.231547674	-0.155439781	0.876474625	0.095720928	9.317969618	89.26804829	0.5	PRJNA827465;Yang2025_Silkie_Wenchang	CRA004775	0.970347777
ENSGALG00010011487	MAN2B2	2	-0.273044179	0.099417737	-2.74643326	0.006024714	0	0.01928487	0	1	CRA004775;Yang2025_Silkie_Wenchang	PRJNA827465	0.117234105
ENSGALG00010011487	MAN2B2	2	0.003171733	0.244450586	0.012974947	0.989647781	0.085256086	3.067873072	67.40412734	0.5	CRA004775;PRJNA827465	Yang2025_Silkie_Wenchang	0.99728544
ENSGALG00010011493	ENSGALG00010011493	2	-0.23991624	0.325378822	-0.737344361	0.460912962	0	0.064039448	0	1	PRJNA827465;Yang2025_Silkie_Wenchang	CRA004775	0.85014477
ENSGALG00010011493	ENSGALG00010011493	2	-0.226434832	0.339465286	-0.667033835	0.504750522	0	0.105619272	0	1	CRA004775;Yang2025_Silkie_Wenchang	PRJNA827465	0.863512504
ENSGALG00010011493	ENSGALG00010011493	2	-0.112205735	0.380785651	-0.29466902	0.768246732	0	0.006788074	0	1	CRA004775;PRJNA827465	Yang2025_Silkie_Wenchang	0.941444269
ENSGALG00010011496	ACTR6	2	0.0409467	0.060847212	0.67294291	0.500983612	0.001163572	1.129675859	11.47903249	0.5	PRJNA827465;Yang2025_Silkie_Wenchang	CRA004775	0.862421093
ENSGALG00010011496	ACTR6	2	-0.049489357	0.102529079	-0.48268606	0.629318663	0	0.084278464	0	0.5	CRA004775;Yang2025_Silkie_Wenchang	PRJNA827465	0.902390168
ENSGALG00010011496	ACTR6	2	0.068871315	0.05828516	1.181626939	0.23735376	0	0.126932647	0	1	CRA004775;PRJNA827465	Yang2025_Silkie_Wenchang	0.749366344
ENSGALG00010011497	CPQ	2	-0.550707751	0.499808706	-1.101837051	0.270532519	0.471715007	17.50252566	94.28653887	1	PRJNA827465;Yang2025_Silkie_Wenchang	CRA004775	0.772536273
ENSGALG00010011497	CPQ	2	-0.465395947	0.654277241	-0.71131306	0.47689026	0.745751034	7.433976898	86.54824983	0.5	CRA004775;Yang2025_Silkie_Wenchang	PRJNA827465	0.854986476
ENSGALG00010011497	CPQ	2	-0.052807695	0.097922035	-0.539283056	0.589691558	0	0.50097182	0	0.5	CRA004775;PRJNA827465	Yang2025_Silkie_Wenchang	0.892320726
ENSGALG00010011499	SMG7	2	0.003077061	0.074498965	0.041303411	0.967054014	0.004680206	1.52396822	34.38183375	0.5	PRJNA827465;Yang2025_Silkie_Wenchang	CRA004775	0.990893423
ENSGALG00010011499	SMG7	2	0.116788574	0.109891116	1.062766286	0.287887952	0	0.006617446	0	1	CRA004775;Yang2025_Silkie_Wenchang	PRJNA827465	0.782840067
ENSGALG00010011499	SMG7	2	-0.03764721	0.05428308	-0.693534891	0.487973937	0	0.291347414	0	0.5	CRA004775;PRJNA827465	Yang2025_Silkie_Wenchang	0.85768885
ENSGALG00010011503	NCF2	2	-0.093498867	0.273789316	-0.341499327	0.732727711	0	0.77151642	0	0.5	PRJNA827465;Yang2025_Silkie_Wenchang	CRA004775	0.932985777
ENSGALG00010011503	NCF2	2	0.424401793	0.961139586	0.441561037	0.658806884	1.415096737	4.012376944	75.07711728	0.5	CRA004775;Yang2025_Silkie_Wenchang	PRJNA827465	0.911311264
ENSGALG00010011503	NCF2	2	0.59678624	0.688807087	0.866405487	0.386267836	0.613711534	2.43875661	58.99549812	1	CRA004775;PRJNA827465	Yang2025_Silkie_Wenchang	0.826894808
ENSGALG00010011505	MYL1	2	-0.285282982	0.092476026	-3.084939896	0.002035933	0	0.282765839	0	1	PRJNA827465;Yang2025_Silkie_Wenchang	CRA004775	0.058631999
ENSGALG00010011505	MYL1	2	-0.226656199	0.139742134	-1.621960331	0.104811842	0	0.002999733	0	1	CRA004775;Yang2025_Silkie_Wenchang	PRJNA827465	0.550003575
ENSGALG00010011505	MYL1	2	-0.324221095	0.120492366	-2.690801971	0.007128049	0	0.031851491	0	1	CRA004775;PRJNA827465	Yang2025_Silkie_Wenchang	0.129881187
ENSGALG00010011509	EXOC2	2	-0.232451231	0.223234218	-1.041288529	0.297741659	0.095769858	25.44124571	96.06937486	1	PRJNA827465;Yang2025_Silkie_Wenchang	CRA004775	0.788858094
ENSGALG00010011509	EXOC2	2	-0.299526187	0.183213152	-1.634850905	0.102080306	0.053615693	4.616878165	78.34034245	1	CRA004775;Yang2025_Silkie_Wenchang	PRJNA827465	0.544451388
ENSGALG00010011509	EXOC2	2	-0.019504138	0.047302881	-0.412324535	0.680101568	0	0.219109836	0	1	CRA004775;PRJNA827465	Yang2025_Silkie_Wenchang	0.917706318
ENSGALG00010011510	ENSGALG00010011510	2	-0.724562586	0.297579009	-2.434857854	0.014897641	0	0.064584036	0	1	PRJNA827465;Yang2025_Silkie_Wenchang	CRA004775	0.201566194
ENSGALG00010011510	ENSGALG00010011510	2	-0.260668222	0.526342364	-0.495244616	0.62042746	0.408183562	3.797381764	73.6660662	0.5	CRA004775;Yang2025_Silkie_Wenchang	PRJNA827465	0.899395593
ENSGALG00010011510	ENSGALG00010011510	2	-0.131314208	0.445776601	-0.294574025	0.768319307	0.209468666	2.088149831	52.11071613	0.5	CRA004775;PRJNA827465	Yang2025_Silkie_Wenchang	0.941444269
ENSGALG00010011515	SERINC5	2	-0.009522114	0.101989345	-0.093363809	0.925614541	0	0.058478555	0	0.5	PRJNA827465;Yang2025_Silkie_Wenchang	CRA004775	0.982594411
ENSGALG00010011515	SERINC5	2	-0.047754326	0.136750867	-0.349206753	0.726934096	0	0.016888623	0	1	CRA004775;Yang2025_Silkie_Wenchang	PRJNA827465	0.93142315
ENSGALG00010011515	SERINC5	2	-0.005035304	0.121892651	-0.041309331	0.967049295	0	0.085283064	0	0.5	CRA004775;PRJNA827465	Yang2025_Silkie_Wenchang	0.990893423
ENSGALG00010011523	PIGO	2	-0.000805099	0.056346306	-0.01428841	0.988599886	0	0.026249234	0	0.5	PRJNA827465;Yang2025_Silkie_Wenchang	CRA004775	0.996891127
ENSGALG00010011523	PIGO	2	-0.156478345	0.22239599	-0.703602369	0.481680435	0.07056252	2.864183216	65.08603241	0.5	CRA004775;Yang2025_Silkie_Wenchang	PRJNA827465	0.85691916
ENSGALG00010011523	PIGO	2	-0.164271755	0.212318502	-0.77370438	0.439105629	0.061539515	2.59851927	61.51654477	1	CRA004775;PRJNA827465	Yang2025_Silkie_Wenchang	0.844022831
ENSGALG00010011525	ENSGALG00010011525	2	0.331394313	0.183095156	1.809956751	0.070302494	0	0.14983483	0	1	PRJNA827465;Yang2025_Silkie_Wenchang	CRA004775	0.457542463
ENSGALG00010011525	ENSGALG00010011525	2	0.268610443	0.238545146	1.126036088	0.260150244	0	0.648449288	0	1	CRA004775;Yang2025_Silkie_Wenchang	PRJNA827465	0.76744615
ENSGALG00010011525	ENSGALG00010011525	2	0.214287733	0.193805562	1.105684124	0.268863264	0	0.30714636	0	1	CRA004775;PRJNA827465	Yang2025_Silkie_Wenchang	0.771398294
ENSGALG00010011529	SCYL2	2	-0.035577995	0.094413656	-0.376831024	0.706299183	0.011234999	2.592725029	61.43054165	0.5	PRJNA827465;Yang2025_Silkie_Wenchang	CRA004775	0.924548793
ENSGALG00010011529	SCYL2	2	-0.067635492	0.145237427	-0.465689134	0.641438031	0.017338064	1.405770704	28.86464364	0.5	CRA004775;Yang2025_Silkie_Wenchang	PRJNA827465	0.906634523
ENSGALG00010011529	SCYL2	2	0.052959715	0.062720327	0.844378813	0.398457758	0	0.302994798	0	1	CRA004775;PRJNA827465	Yang2025_Silkie_Wenchang	0.831980453
ENSGALG00010011530	ARPC5	2	-0.072732621	0.142454223	-0.510568371	0.609653328	0.032987543	4.881748658	79.51553695	0.5	PRJNA827465;Yang2025_Silkie_Wenchang	CRA004775	0.897238578
ENSGALG00010011530	ARPC5	2	-0.263038407	0.113965471	-2.308053522	0.02099616	0	0.388727049	0	1	CRA004775;Yang2025_Silkie_Wenchang	PRJNA827465	0.245277621
ENSGALG00010011530	ARPC5	2	-0.095498338	0.231736013	-0.412099684	0.68026636	0.072506338	2.211988616	54.79181073	0.5	CRA004775;PRJNA827465	Yang2025_Silkie_Wenchang	0.917768536
ENSGALG00010011531	MTDH	2	-0.101676792	0.03196647	-3.180732592	0.001469032	0	0.002430546	0	1	PRJNA827465;Yang2025_Silkie_Wenchang	CRA004775	0.047481373
ENSGALG00010011531	MTDH	2	-0.090804793	0.046622469	-1.947661624	0.051455465	0.000365594	1.012823393	1.266103569	0.5	CRA004775;Yang2025_Silkie_Wenchang	PRJNA827465	0.39314816
ENSGALG00010011531	MTDH	2	-0.09029293	0.054910148	-1.644376005	0.100098558	0.000970946	1.033867129	3.275771893	0.5	CRA004775;PRJNA827465	Yang2025_Silkie_Wenchang	0.54044969
ENSGALG00010011533	ENSGALG00010011533	2	-0.657261909	0.153342119	-4.286245129	1.81718E-05	0	0.00256415	0	1	PRJNA827465;Yang2025_Silkie_Wenchang	CRA004775	0.002281815
ENSGALG00010011533	ENSGALG00010011533	2	-0.049845476	0.622852592	-0.080027725	0.936215205	0.684530294	8.385674345	88.07490061	0.5	CRA004775;Yang2025_Silkie_Wenchang	PRJNA827465	0.98485269
ENSGALG00010011533	ENSGALG00010011533	2	-0.06627911	0.6304248	-0.105134046	0.916269444	0.715624715	9.771672966	89.76633783	0.5	CRA004775;PRJNA827465	Yang2025_Silkie_Wenchang	0.98043852
ENSGALG00010011536	FCHSD1	2	0.267069759	0.140032558	1.907197602	0.056495004	0	0.093081854	0	1	PRJNA827465;Yang2025_Silkie_Wenchang	CRA004775	0.411523553
ENSGALG00010011536	FCHSD1	2	0.026140714	0.20554265	0.127179023	0.898798708	0.025889191	1.435913485	30.35792123	0.5	CRA004775;Yang2025_Silkie_Wenchang	PRJNA827465	0.975884294
ENSGALG00010011536	FCHSD1	2	0.086013226	0.247877697	0.34699865	0.728592333	0.075767427	2.525338228	60.40134391	0.5	CRA004775;PRJNA827465	Yang2025_Silkie_Wenchang	0.932187391
ENSGALG00010011538	ENSGALG00010011538	2	-0.513339896	0.319476559	-1.60681553	0.10809484	0.143669463	3.233224072	69.0711198	1	PRJNA827465;Yang2025_Silkie_Wenchang	CRA004775	0.556254984
ENSGALG00010011538	ENSGALG00010011538	2	-0.792290262	0.26425694	-2.998181468	0.002715959	0	0.334943581	0	1	CRA004775;Yang2025_Silkie_Wenchang	PRJNA827465	0.070974293
ENSGALG00010011538	ENSGALG00010011538	2	-0.267568332	0.1786092	-1.498065785	0.134116159	0	0.264161033	0	1	CRA004775;PRJNA827465	Yang2025_Silkie_Wenchang	0.61346525
ENSGALG00010011539	LANCL1	2	-0.225775116	0.159166542	-1.418483518	0.156049647	0.042518635	5.911610948	83.08413715	1	PRJNA827465;Yang2025_Silkie_Wenchang	CRA004775	0.649132948
ENSGALG00010011539	LANCL1	2	-0.384030854	0.105264398	-3.648250126	0.000264033	0	0.116378114	0	1	CRA004775;Yang2025_Silkie_Wenchang	PRJNA827465	0.015534836
ENSGALG00010011539	LANCL1	2	-0.095209394	0.057893603	-1.644558108	0.100060972	0	0.866693811	0	1	CRA004775;PRJNA827465	Yang2025_Silkie_Wenchang	0.54042825
ENSGALG00010011544	STOML2	2	0.075435637	0.067618052	1.115613877	0.264587449	0	0.014539782	0	1	PRJNA827465;Yang2025_Silkie_Wenchang	CRA004775	0.768855521
ENSGALG00010011544	STOML2	2	-0.095546858	0.236325956	-0.404301158	0.685991274	0.061720511	2.116292191	52.74754571	0.5	CRA004775;Yang2025_Silkie_Wenchang	PRJNA827465	0.919235539
ENSGALG00010011544	STOML2	2	-0.077302996	0.215712626	-0.358361019	0.72007316	0.063441262	2.482223384	59.71353722	0.5	CRA004775;PRJNA827465	Yang2025_Silkie_Wenchang	0.928564173
ENSGALG00010011545	USP12-like	2	-0.129342236	0.165271373	-0.782605197	0.433858988	0.050809839	14.10957018	92.91261189	0.5	PRJNA827465;Yang2025_Silkie_Wenchang	CRA004775	0.842762754
ENSGALG00010011545	USP12-like	2	0.020366553	0.328336422	0.062029527	0.950539318	0.204812983	19.74445236	94.93528622	0.5	CRA004775;Yang2025_Silkie_Wenchang	PRJNA827465	0.987277827
ENSGALG00010011545	USP12-like	2	0.172166463	0.161639561	1.065125775	0.286819017	0.044018472	5.79980754	82.75804855	1	CRA004775;PRJNA827465	Yang2025_Silkie_Wenchang	0.78223911
ENSGALG00010011546	MAOA	2	-0.182305535	0.201793156	-0.903427741	0.366298922	0.071959472	8.579054591	88.34370397	0.5	PRJNA827465;Yang2025_Silkie_Wenchang	CRA004775	0.819159863
ENSGALG00010011546	MAOA	2	-0.306883778	0.158486223	-1.936343561	0.052825637	0.020958442	1.368343732	26.91894758	0.5	CRA004775;Yang2025_Silkie_Wenchang	PRJNA827465	0.399347184
ENSGALG00010011546	MAOA	2	0.016268266	0.088447475	0.18393138	0.854067287	0	0.000726686	0	1	CRA004775;PRJNA827465	Yang2025_Silkie_Wenchang	0.962975689
ENSGALG00010011549	KIAA0232	2	0.133611466	0.140083133	0.953801241	0.340184261	0.030597193	4.14249526	75.85996031	1	PRJNA827465;Yang2025_Silkie_Wenchang	CRA004775	0.805377063
ENSGALG00010011549	KIAA0232	2	0.307643864	0.116477893	2.641221065	0.00826078	0	0.027156445	0	1	CRA004775;Yang2025_Silkie_Wenchang	PRJNA827465	0.141469904
ENSGALG00010011549	KIAA0232	2	0.068438339	0.122749656	0.55754404	0.577155778	0.014817505	1.358696156	26.40002729	1	CRA004775;PRJNA827465	Yang2025_Silkie_Wenchang	0.887145528
ENSGALG00010011551	GMDS	2	0.103588187	0.081574988	1.26985231	0.204137243	0.004026265	1.368017809	26.90153643	1	PRJNA827465;Yang2025_Silkie_Wenchang	CRA004775	0.717254253
ENSGALG00010011551	GMDS	2	-0.100620919	0.329258676	-0.305598384	0.759910451	0.194573474	9.668828234	89.65748511	0.5	CRA004775;Yang2025_Silkie_Wenchang	PRJNA827465	0.93917677
ENSGALG00010011551	GMDS	2	-0.173347463	0.241744055	-0.717070224	0.473330767	0.100537875	6.756806268	85.20010845	0.5	CRA004775;PRJNA827465	Yang2025_Silkie_Wenchang	0.854343014
ENSGALG00010011555	ENSGALG00010011555	2	-2.966314644	3.296329544	-0.89988413	0.368181916	16.37651099	3.847647903	74.0100959	1	PRJNA827465;Yang2025_Silkie_Wenchang	CRA004775	0.820213684
ENSGALG00010011555	ENSGALG00010011555	2	-2.924052046	3.143838916	-0.930089653	0.352324668	15.71498369	4.275002709	76.60820196	1	CRA004775;Yang2025_Silkie_Wenchang	PRJNA827465	0.810786136
ENSGALG00010011555	ENSGALG00010011555	2	-0.272590734	0.820275849	-0.332315933	0.73965071	0	0.016615198	0	1	CRA004775;PRJNA827465	Yang2025_Silkie_Wenchang	0.934914078
ENSGALG00010011556	LAPTM4B	2	-0.266784869	0.160511118	-1.662095887	0.09649355	0.037722245	3.731375163	73.20022897	1	PRJNA827465;Yang2025_Silkie_Wenchang	CRA004775	0.530885356
ENSGALG00010011556	LAPTM4B	2	0.055898702	0.538298528	0.103843313	0.917293694	0.508389566	7.488089362	86.64545852	0.5	CRA004775;Yang2025_Silkie_Wenchang	PRJNA827465	0.98082192
ENSGALG00010011556	LAPTM4B	2	0.191805893	0.371593696	0.51617101	0.605734976	0.211624132	3.68282496	72.84693107	0.5	CRA004775;PRJNA827465	Yang2025_Silkie_Wenchang	0.896474603
ENSGALG00010011558	MYBPC1	2	-0.0190295	0.265395003	-0.071702556	0.942838622	0.125498682	9.152685927	89.07424544	0.5	PRJNA827465;Yang2025_Silkie_Wenchang	CRA004775	0.985827325
ENSGALG00010011558	MYBPC1	2	0.248139096	0.13014949	1.906569867	0.05657631	0	0.010416546	0	1	CRA004775;Yang2025_Silkie_Wenchang	PRJNA827465	0.411552605
ENSGALG00010011558	MYBPC1	2	-0.275925761	0.11584887	-2.381773432	0.017229494	0	0.159067348	0	0.5	CRA004775;PRJNA827465	Yang2025_Silkie_Wenchang	0.21809044
ENSGALG00010011559	MTX3	2	0.062086837	0.210801136	0.294527999	0.768354472	0.079306866	9.278035553	89.22185635	0.5	PRJNA827465;Yang2025_Silkie_Wenchang	CRA004775	0.941444269
ENSGALG00010011559	MTX3	2	-0.002760308	0.19241369	-0.014345696	0.988554184	0.048425253	2.577539095	61.20330427	0.5	CRA004775;Yang2025_Silkie_Wenchang	PRJNA827465	0.996891127
ENSGALG00010011559	MTX3	2	0.266929975	0.085343862	3.127699728	0.001761801	0	0.009589142	0	1	CRA004775;PRJNA827465	Yang2025_Silkie_Wenchang	0.053264656
ENSGALG00010011564	ARAP3	2	-0.022068138	0.060425935	-0.365209711	0.71495487	0	0.652994339	0	0.5	PRJNA827465;Yang2025_Silkie_Wenchang	CRA004775	0.926823015
ENSGALG00010011564	ARAP3	2	-0.106670524	0.100320899	-1.063293141	0.287649035	0	0.168773402	0	1	CRA004775;Yang2025_Silkie_Wenchang	PRJNA827465	0.782840067
ENSGALG00010011564	ARAP3	2	-0.001011449	0.071523188	-0.014141548	0.988717053	0	0.564106933	0	0.5	CRA004775;PRJNA827465	Yang2025_Silkie_Wenchang	0.996891127
ENSGALG00010011570	RGL1	2	-0.034401128	0.060246333	-0.571007834	0.567994332	0	0.090128194	0	1	PRJNA827465;Yang2025_Silkie_Wenchang	CRA004775	0.88390256
ENSGALG00010011570	RGL1	2	-0.030904735	0.130227879	-0.23731274	0.81241418	0	0.313941214	0	0.5	CRA004775;Yang2025_Silkie_Wenchang	PRJNA827465	0.953371387
ENSGALG00010011570	RGL1	2	-0.019230298	0.063229594	-0.30413446	0.761025454	0	0.194559588	0	0.5	CRA004775;PRJNA827465	Yang2025_Silkie_Wenchang	0.939520776
ENSGALG00010011571	ATOSB	2	0.182215781	0.088620128	2.056144409	0.039768599	0	0.006663833	0	1	PRJNA827465;Yang2025_Silkie_Wenchang	CRA004775	0.344810333
ENSGALG00010011571	ATOSB	2	-0.145879956	0.360434145	-0.404734006	0.685673043	0.22295967	6.866859758	85.43730271	0.5	CRA004775;Yang2025_Silkie_Wenchang	PRJNA827465	0.919235539
ENSGALG00010011571	ATOSB	2	-0.147078429	0.352478975	-0.417268657	0.676481911	0.216366772	7.377028123	86.44440575	0.5	CRA004775;PRJNA827465	Yang2025_Silkie_Wenchang	0.916786558

ENSGALG00010011572	PTCD2	2	-0.083926267	0.108940381	-0.770387124	0.441070289	0	0.175604407	0	1	PRJNA827465;Yang2025_Silkie_Wenchang	CRA004775	0.844888742
ENSGALG00010011572	PTCD2	2	-0.091427216	0.153242795	-0.596616737	0.550763294	0	0.395141947	0	0.5	CRA004775;Yang2025_Silkie_Wenchang	PRJNA827465	0.878879109
ENSGALG00010011572	PTCD2	2	-0.024791047	0.130768162	-0.189580141	0.84963815	0	0.159913592	0	0.5	CRA004775;PRJNA827465	Yang2025_Silkie_Wenchang	0.961817109
ENSGALG00010011573	CMAS	2	-0.025016396	0.122444626	-0.204307834	0.838112948	0.017771335	2.454549717	59.25933	0.5	PRJNA827465;Yang2025_Silkie_Wenchang	CRA004775	0.959129781
ENSGALG00010011573	CMAS	2	-0.003422025	0.181907303	-0.018811916	0.984991148	0.041682691	2.495476911	59.9274994	0.5	CRA004775;Yang2025_Silkie_Wenchang	PRJNA827465	0.995786861
ENSGALG00010011573	CMAS	2	0.123705343	0.096538309	1.281411949	0.200049009	0	0.297802688	0	1	CRA004775;PRJNA827465	Yang2025_Silkie_Wenchang	0.712249861
ENSGALG00010011574	GAS2L3	2	0.227689979	0.078980676	2.882856792	0.003940866	0	0.93368272	0	1	PRJNA827465;Yang2025_Silkie_Wenchang	CRA004775	0.090524746
ENSGALG00010011574	GAS2L3	2	0.323705938	0.13855025	2.33637931	0.019471481	0	0.262939247	0	1	CRA004775;Yang2025_Silkie_Wenchang	PRJNA827465	0.233889863
ENSGALG00010011574	GAS2L3	2	0.174344944	0.092708065	1.880580124	0.060029058	0	0.037856041	0	1	CRA004775;PRJNA827465	Yang2025_Silkie_Wenchang	0.424082409
ENSGALG00010011575	ENSGALG00010011575	2	0.350627434	0.338245444	1.036606523	0.299919272	0.071760722	1.416912783	29.42402579	1	PRJNA827465;Yang2025_Silkie_Wenchang	CRA004775	0.790115099
ENSGALG00010011575	ENSGALG00010011575	2	0.72860308	0.419128127	1.738377916	0.082144245	0	0.058978484	0	1	CRA004775;Yang2025_Silkie_Wenchang	PRJNA827465	0.493958885
ENSGALG00010011575	ENSGALG00010011575	2	0.15041458	0.310629591	0.484224893	0.628226273	0	0.257595878	0	1	CRA004775;PRJNA827465	Yang2025_Silkie_Wenchang	0.901904489
ENSGALG00010011577	ENSGALG00010011577	2	0.118585567	0.129005136	0.919231358	0.357974572	0	0.233720406	0	1	PRJNA827465;Yang2025_Silkie_Wenchang	CRA004775	0.813641308
ENSGALG00010011577	ENSGALG00010011577	2	0.244067525	0.188651012	1.293751475	0.195751279	0	0.194886205	0	1	CRA004775;Yang2025_Silkie_Wenchang	PRJNA827465	0.706808683
ENSGALG00010011577	ENSGALG00010011577	2	0.11661619	0.150631025	0.7741844	0.438821752	0	0.575009993	0	1	CRA004775;PRJNA827465	Yang2025_Silkie_Wenchang	0.844002503
ENSGALG00010011578	MYLK4	2	0.382888681	0.163332888	2.344222802	0.019066778	0.006056639	1.115858613	10.38291157	1	PRJNA827465;Yang2025_Silkie_Wenchang	CRA004775	0.231290606
ENSGALG00010011578	MYLK4	2	0.616484341	0.24678562	2.498056169	0.012487641	0	0.019862571	0	1	CRA004775;Yang2025_Silkie_Wenchang	PRJNA827465	0.180907385
ENSGALG00010011578	MYLK4	2	0.291393114	0.18145111	1.605904281	0.108294943	0	0.373838721	0	1	CRA004775;PRJNA827465	Yang2025_Silkie_Wenchang	0.556717702
ENSGALG00010011586	ENSGALG00010011586	2	-0.094260678	0.157024346	-0.600293397	0.548310719	0	0.696759268	0	0.5	PRJNA827465;Yang2025_Silkie_Wenchang	CRA004775	0.878281266
ENSGALG00010011586	ENSGALG00010011586	2	0.03986986	0.453489666	0.087917903	0.929941927	0.23130633	1.902142506	47.4277034	0.5	CRA004775;Yang2025_Silkie_Wenchang	PRJNA827465	0.983813577
ENSGALG00010011586	ENSGALG00010011586	2	0.085183754	0.219530996	0.388026093	0.697996724	0.013434742	1.054859931	5.200683948	1	CRA004775;PRJNA827465	Yang2025_Silkie_Wenchang	0.921510422
ENSGALG00010011588	COLGALT2	2	0.096580477	0.219901436	0.439198939	0.660517394	0.081372717	6.302135589	84.13236298	0.5	PRJNA827465;Yang2025_Silkie_Wenchang	CRA004775	0.91192683
ENSGALG00010011588	COLGALT2	2	-0.115477069	0.116199559	-0.993782342	0.320328845	0	0.035353129	0	1	CRA004775;Yang2025_Silkie_Wenchang	PRJNA827465	0.798348216
ENSGALG00010011588	COLGALT2	2	0.213479452	0.168201201	1.269191004	0.204372949	0.023983333	1.494397353	33.08339323	0.5	CRA004775;PRJNA827465	Yang2025_Silkie_Wenchang	0.717281769
ENSGALG00010011589	RERG	2	-0.136291944	0.108096819	-1.260832149	0.207369328	0.004195244	1.214824649	17.68359321	1	PRJNA827465;Yang2025_Silkie_Wenchang	CRA004775	0.719377239
ENSGALG00010011589	RERG	2	-0.270434717	0.142464638	-1.898258554	0.057662031	0	0.066729341	0	1	CRA004775;Yang2025_Silkie_Wenchang	PRJNA827465	0.416138597
ENSGALG00010011589	RERG	2	-0.065483184	0.124214198	-0.527179545	0.598068912	0	0.51194905	0	1	CRA004775;PRJNA827465	Yang2025_Silkie_Wenchang	0.894645663
ENSGALG00010011593	WRNIP1	2	-0.112643139	0.203604482	-0.553244886	0.580095735	0.066573949	4.762287923	79.0016896	0.5	PRJNA827465;Yang2025_Silkie_Wenchang	CRA004775	0.888246169
ENSGALG00010011593	WRNIP1	2	-0.342416288	0.141386946	-2.421838074	0.01544223	0	0.000291408	0	1	CRA004775;Yang2025_Silkie_Wenchang	PRJNA827465	0.205526799
ENSGALG00010011593	WRNIP1	2	-0.061393794	0.188360194	-0.325938259	0.744471068	0.044857929	2.204089679	54.62979526	0.5	CRA004775;PRJNA827465	Yang2025_Silkie_Wenchang	0.935846381
ENSGALG00010011595	ENSGALG00010011595	2	0.442460848	0.278196387	1.590462236	0.111730651	0.113151532	3.658015817	72.6627754	1	PRJNA827465;Yang2025_Silkie_Wenchang	CRA004775	0.565094555
ENSGALG00010011595	ENSGALG00010011595	2	0.214355754	0.551803345	0.388464035	0.697672664	0.529439984	7.565276911	86.78171319	0.5	CRA004775;Yang2025_Silkie_Wenchang	PRJNA827465	0.921422004
ENSGALG00010011595	ENSGALG00010011595	2	-0.018561812	0.264242019	-0.070245496	0.943998263	0.080776995	2.177919272	54.08461585	0.5	CRA004775;PRJNA827465	Yang2025_Silkie_Wenchang	0.985827325
ENSGALG00010011597	TBC1D14	2	-0.033736981	0.057189687	-0.589913714	0.555248499	0.003170192	1.93840973	48.41131961	0.5	PRJNA827465;Yang2025_Silkie_Wenchang	CRA004775	0.880255849
ENSGALG00010011597	TBC1D14	2	-0.091359426	0.058898299	-1.551138615	0.120868469	0	0.017633654	0	1	CRA004775;Yang2025_Silkie_Wenchang	PRJNA827465	0.582767179
ENSGALG00010011597	TBC1D14	2	0.019382783	0.055359879	0.350123286	0.726246176	0	0.050250174	0	0.5	CRA004775;PRJNA827465	Yang2025_Silkie_Wenchang	0.931029023
ENSGALG00010011603	ENSGALG00010011603	2	-0.434888225	0.293082033	-1.483844712	0.137850122	0	0.00027909	0	1	PRJNA827465;Yang2025_Silkie_Wenchang	CRA004775	0.62064915
ENSGALG00010011603	ENSGALG00010011603	2	-0.18873965	0.266992603	-0.706909659	0.479622621	0	0.712994506	0	0.5	CRA004775;Yang2025_Silkie_Wenchang	PRJNA827465	0.856184215
ENSGALG00010011603	ENSGALG00010011603	2	-0.165827665	0.281457905	-0.589173948	0.555744592	0	0.649137349	0	0.5	CRA004775;PRJNA827465	Yang2025_Silkie_Wenchang	0.880324407
ENSGALG00010011605	APPL2	2	0.228472736	0.233751433	0.97741748	0.328362513	0.103113572	17.67776166	94.34317523	0.5	PRJNA827465;Yang2025_Silkie_Wenchang	CRA004775	0.800761292
ENSGALG00010011605	APPL2	2	0.099785321	0.410292693	0.243205211	0.80784643	0.294286827	7.268884087	86.24273013	0.5	CRA004775;Yang2025_Silkie_Wenchang	PRJNA827465	0.952496413
ENSGALG00010011605	APPL2	2	-0.068008284	0.138641408	-0.490533707	0.623756284	0.018698776	1.410162103	29.0861669	1	CRA004775;PRJNA827465	Yang2025_Silkie_Wenchang	0.900543409
ENSGALG00010011607	TSEN15	2	-0.214839902	0.097530288	-2.202801897	0.027608714	0	0.535914927	0	1	PRJNA827465;Yang2025_Silkie_Wenchang	CRA004775	0.283613514
ENSGALG00010011607	TSEN15	2	-0.252112859	0.159643784	-1.579221273	0.114285312	0	0.658463376	0	1	CRA004775;Yang2025_Silkie_Wenchang	PRJNA827465	0.57019364
ENSGALG00010011607	TSEN15	2	-0.155476344	0.106831763	-1.45533818	0.145575662	0	0.157563379	0	1	CRA004775;PRJNA827465	Yang2025_Silkie_Wenchang	0.634178701
ENSGALG00010011617	MAMLD1	2	0.037597965	0.197118791	0.190737598	0.84873118	0.059936873	3.697614422	72.95553604	0.5	PRJNA827465;Yang2025_Silkie_Wenchang	CRA004775	0.961364896
ENSGALG00010011617	MAMLD1	2	0.295243341	0.15430971	1.913316676	0.055707524	0	0.003502856	0	1	CRA004775;Yang2025_Silkie_Wenchang	PRJNA827465	0.408696004
ENSGALG00010011617	MAMLD1	2	0.03136413	0.202456581	0.154917809	0.876886114	0.060169365	3.029438883	66.99058675	0.5	CRA004775;PRJNA827465	Yang2025_Silkie_Wenchang	0.97046577
ENSGALG00010011619	PCDH1	2	0.123457702	0.141455082	0.872769647	0.382788647	0.028851014	3.418113488	70.74409602	1	PRJNA827465;Yang2025_Silkie_Wenchang	CRA004775	0.825121999
ENSGALG00010011619	PCDH1	2	0.256270354	0.124971928	2.050623355	0.04030364	0	0.460546824	0	1	CRA004775;Yang2025_Silkie_Wenchang	PRJNA827465	0.347442284
ENSGALG00010011619	PCDH1	2	0.000716372	0.078006493	0.009183486	0.992672741	0	0.001019986	0	1	CRA004775;PRJNA827465	Yang2025_Silkie_Wenchang	0.997852036
ENSGALG00010011621	WASHC4	2	0.02913296	0.065045019	0.44788918	0.654233178	0.003207212	1.556052568	35.73481896	0.5	PRJNA827465;Yang2025_Silkie_Wenchang	CRA004775	0.910008212
ENSGALG00010011621	WASHC4	2	-0.024025025	0.082031232	-0.29287656	0.769616506	0	0.751925456	0	0.5	CRA004775;Yang2025_Silkie_Wenchang	PRJNA827465	0.941843401
ENSGALG00010011621	WASHC4	2	0.084976498	0.057890635	1.467879869	0.142136857	0	0.088006787	0	1	CRA004775;PRJNA827465	Yang2025_Silkie_Wenchang	0.627968177
ENSGALG00010011622	BACH1	2	-0.188362496	0.204078946	-0.922988385	0.356013258	0.076639561	12.302502	91.87157214	0.5	PRJNA827465;Yang2025_Silkie_Wenchang	CRA004775	0.812377444
ENSGALG00010011622	BACH1	2	0.075849643	0.522603285	0.14513809	0.884601845	0.489992998	9.039589404	88.93755064	0.5	CRA004775;Yang2025_Silkie_Wenchang	PRJNA827465	0.972062774
ENSGALG00010011622	BACH1	2	0.244148222	0.309294391	0.789371645	0.429894823	0.147864151	3.56118471	71.91945711	1	CRA004775;PRJNA827465	Yang2025_Silkie_Wenchang	0.842012722
ENSGALG00010011625	ERBB4	2	-0.214225274	0.115967957	-1.847279879	0.064706588	0	0.055293368	0	1	PRJNA827465;Yang2025_Silkie_Wenchang	CRA004775	0.438551994
ENSGALG00010011625	ERBB4	2	-0.093380521	0.296864326	-0.314556224	0.753098621	0.059215883	1.334388962	25.05933211	0.5	CRA004775;Yang2025_Silkie_Wenchang	PRJNA827465	0.938087082
ENSGALG00010011625	ERBB4	2	-0.111528458	0.216651007	-0.514783936	0.606704012	0.03711953	1.236644562	19.13602088	0.5	CRA004775;PRJNA827465	Yang2025_Silkie_Wenchang	0.896799223
ENSGALG00010011628	MATN2	2	-0.150677126	0.090890203	-1.657792808	0.097359293	0	0.067283267	0	1	PRJNA827465;Yang2025_Silkie_Wenchang	CRA004775	0.532942995
ENSGALG00010011628	MATN2	2	0.444980473	0.652844056	0.68160301	0.495490011	0.794676116	14.31424398	93.01395169	0.5	CRA004775;Yang2025_Silkie_Wenchang	PRJNA827465	0.860770265
ENSGALG00010011628	MATN2	2	0.465913555	0.628910857	0.740826064	0.45879891	0.735766678	13.77115755	92.7384463	0.5	CRA004775;PRJNA827465	Yang2025_Silkie_Wenchang	0.850089963
ENSGALG00010011630	DELE1	2	-0.145638047	0.069714345	-2.089068567	0.036701551	0	0.265178528	0	1	PRJNA827465;Yang2025_Silkie_Wenchang	CRA004775	0.330558668
ENSGALG00010011630	DELE1	2	0.054227819	0.196673048	0.275725726	0.782758735	0.045617511	2.195729195	54.45704314	0.5	CRA004775;Yang2025_Silkie_Wenchang	PRJNA827465	0.945490372
ENSGALG00010011630	DELE1	2	0.016801974	0.23653254	0.071034514	0.943370287	0.083841049	3.447514318	70.993594	0.5	CRA004775;PRJNA827465	Yang2025_Silkie_Wenchang	0.985827325
ENSGALG00010011633	TADA2B	2	-0.256492916	0.173417197	-1.479051212	0.139126628	0.041891061	3.137024991	68.1226639	1	PRJNA827465;Yang2025_Silkie_Wenchang	CRA004775	0.622860674
ENSGALG00010011633	TADA2B	2	-0.481418629	0.142566568	-3.376798901	0.000733346	0	0.066661217	0	1	CRA004775;Yang2025_Silkie_Wenchang	PRJNA827465	0.030276194
ENSGALG00010011633	TADA2B	2	-0.254423162	0.204672358	-1.243075348	0.213840069	0.053094151	2.32496616	56.98862129	1	CRA004775;PRJNA827465	Yang2025_Silkie_Wenchang	0.726534945
ENSGALG00010011634	AVD	2	0.220946266	0.490604287	0.450355352	0.652454233	0.285312337	2.455043576	59.26752544	0.5	PRJNA827465;Yang2025_Silkie_Wenchang	CRA004775	0.909401809
ENSGALG00010011634	AVD	2	-0.047466771	0.803260724	-0.059092608	0.952878346	1.02659616	4.837156465	79.32669726	0.5	CRA004775;Yang2025_Silkie_Wenchang	PRJNA827465	0.987758913
ENSGALG00010011634	AVD	2	-0.496402602	0.350606048	-1.415841525	0.156821909	0	0.742844738	0	1	CRA004775;PRJNA827465	Yang2025_Silkie_Wenchang	0.650212649
ENSGALG00010011635	PCDH12	2	0.057229631	0.078698114	0.727204604	0.467100626	0	0.764938695	0	1	PRJNA827465;Yang2025_Silkie_Wenchang	CRA004775	0.852885838
ENSGALG00010011635	PCDH12	2	0.113001727	0.119927279	0.94225207	0.346063601	0	0.662722448	0	0.5	CRA004775;Yang2025_Silkie_Wenchang	PRJNA827465	0.807702909
ENSGALG00010011635	PCDH12	2	-0.010238022	0.098271421	-0.104181073	0.917025654	0	0.24647762	0	0.5	CRA004775;PRJNA827465	Yang2025_Silkie_Wenchang	0.980728718
ENSGALG00010011636	GRIK1	2	-0.419766279	0.289019998	-1.452377973	0.146396537	0	0.252934678	0	1	PRJNA827465;Yang2025_Silkie_Wenchang	CRA004775	0.6352709
ENSGALG00010011636	GRIK1	2	0.067699275	0.335436698	0.201824294	0.840054088	0	0.920339971	0	0.5	CRA004775;Yang2025_Silkie_Wenchang	PRJNA827465	0.95957104
ENSGALG00010011636	GRIK1	2	-0.117971387	0.468207434	-0.251963935	0.80106894	0.257016604	2.373906017	57.8753332	0.5	CRA004775;PRJNA827465	Yang2025_Silkie_Wenchang	0.950158029
ENSGALG00010011637	CECR2	2	0.213920743	0.067167134	3.184902069	0.00144803	0	0.03333579	0	1	PRJNA827465;Yang2025_Silkie_Wenchang	CRA004775	0.047151552
ENSGALG00010011637	CECR2	2	0.08695903	0.14402449	0.603779472	0.545990269	0	0.829362678	0	0.5	CRA004775;Yang2025_Silkie_Wenchang	PRJNA827465	0.877958239
ENSGALG00010011637	CECR2	2	0.150552595	0.128471978	1.171871078	0.24124882	0.015923259	1.496278701	33.16753095	0.5	CRA004775;PRJNA827465	Yang2025_Silkie_Wenchang	0.753793619
ENSGALG00010011638	GRPEL1	2	0.005787103	0.125270884	0.046196715	0.963153461	0.024720673	4.375733183	77.14668701	0.5	PRJNA827465;Yang2025_Silkie_Wenchang	CRA004775	0.989998461
ENSGALG00010011638	GRPEL1	2	0.116799857	0.099402323	1.175021397	0.239986155	0	0.558374226	0	0.5	CRA004775;Yang2025_Silkie_Wenchang	PRJNA827465	0.752374775
ENSGALG00010011638	GRPEL1	2	-0.100137463	0.051065667	-1.960954748	0.049884302	0	0.051306387	0	1	CRA004775;PRJNA827465	Yang2025_Silkie_Wenchang	0.386401797
ENSGALG00010011639	ENSGALG00010011639	2	0.231237933	0.17757118	1.302226707	0.192838898	0	0.577211683	0	1	PRJNA827465;Yang2025_Silkie_Wenchang	CRA004775	0.702814096
ENSGALG00010011639	ENSGALG00010011639	2	0.065945925	0.19556009	0.33721566	0.735954333	0	0.009908378	0	1	CRA004775;Yang2025_Silkie_Wenchang	PRJNA827465	0.933592889
ENSGALG00010011639	ENSGALG00010011639	2	0.22913177	0.185628019	1.234359835	0.217068849	0	0.671893441	0	1	CRA004775;PRJNA827465	Yang2025_Silkie_Wenchang	0.730558054
ENSGALG00010011641	ENSGALG00010011641	2	0.302248895	0.446758035	0.676538239	0.498698976	0.309478862	4.273104051	76.59780834	0.5	PRJNA827465;Yang2025_Silkie_Wenchang	CRA004775	0.861478809
ENSGALG00010011641	ENSGALG00010011641	2	0.853387598	0.345002712	2.4735678	0.013377142	0	0.139172631	0	1	CRA004775;Yang2025_Silkie_Wenchang	PRJNA827465	0.18898699
ENSGALG00010011641	ENSGALG00010011641	2	0.225739306	0.552648905	0.408467843	0.682930234	0.359916255	1.812550166	44.82911323	0.5	CRA004775;PRJNA827465	Yang2025_Silkie_Wenchang	0.918800744
ENSGALG00010011643	C1orf21	2	-0.279317778	0.092866704	-3.007772923	0.002632087	0	0.762848401	0	1	PRJNA827465;Yang2025_Silkie_Wenchang	CRA004775	0.069382229
ENSGALG00010011643	C1orf21	2	-0.038557118	0.204787102	-0.188279034	0.85065792	0.032900607	1.46293453	31.64424112	0.5	CRA004775;Yang2025_Silkie_Wenchang	PRJNA827465	0.961965357
ENSGALG00010011643	C1orf21	2	-0.106603599	0.30251837	-0.352387191	0.724547907	0.131781575	3.029810558	66.99463611	0.5	CRA004775;PRJNA827465	Yang2025_Silkie_Wenchang	0.930388018
ENSGALG00010011647	FBXO38	2	0.105644568	0.131744308	0.801899945	0.422616044	0.02993018	7.035328514	85.78602267	0.5	PRJNA827465;Yang2025_Silkie_Wenchang	CRA004775	0.839783527
ENSGALG00010011647	FBXO38	2	0.249314716	0.078646422	3.170070692	0.001524019	0	0.003879709	0	1	CRA004775;Yang2025_Silkie_Wenchang	PRJNA827465	0.048777281
ENSGALG00010011647	FBXO38	2	0.059846756	0.124119563	0.482170215	0.629685034	0.018838706	1.961486124	49.01824755	0.5	CRA004775;PRJNA827465	Yang2025_Silkie_Wenchang	0.902457014
ENSGALG00010011648	ENSGALG00010011648	2	-0.214324751	0.138091889	-1.552044462	0.120651585	0	0.027058747	0	1	PRJNA827465;Yang2025_Silkie_Wenchang	CRA004775	0.582479948
ENSGALG00010011648	ENSGALG00010011648	2	-0.145094762	0.231080366	-0.627897404	0.530071152	0	0.022975722	0	1	CRA004775;Yang2025_Silkie_Wenchang	PRJNA827465	0.873091192
ENSGALG00010011648	ENSGALG00010011648	2	-0.207968153	0.148858659	-1.43566256	0.151098384	0	0.095879255	0	1	CRA004775;PRJNA827465	Yang2025_Silkie_Wenchang	0.64242966
ENSGALG00010011649	MRPS27	2	0.29038599	0.113186325	2.565557181	0.010301024	0	0.19671758	0	1	PRJNA827465;Yang2025_Silkie_Wenchang	CRA004775	0.161235026
ENSGALG00010011649	MRPS27	2	0.282068944	0.143767623	1.96197821	0.049765021	0	0.293067623	0	1	CRA004775;Yang2025_Silkie_Wenchang	PRJNA827465	0.386162809
ENSGALG00010011649	MRPS27	2	0.354062886	0.134842881	2.62574401	0.008645981	0	0.056738609	0	1	CRA004775;PRJNA827465	Yang2025_Silkie_Wenchang	0.145573772
ENSGALG00010011650	RPL30	2	-0.252630276	0.251735439	-1.003554669	0.315593314	0.10561505	5.490905352	81.78806707	1	PRJNA827465;Yang2025_Silkie_Wenchang	CRA004775	0.796883862
ENSGALG00010011650	RPL30	2	-0.393463809	0.175142405	-2.246536513	0.024669664	0.011442766	1.221392221	18.12621836	1	CRA004775;Yang2025_Silkie_Wenchang	PRJNA827465	0.268408231
ENSGALG00010011650	RPL30	2	-0.046430166	0.073671482	-0.63023255	0.528542446	0	0.342530336	0	1	CRA004775;PRJNA827465	Yang2025_Silkie_Wenchang	0.871701504
ENSGALG00010011651	SERPINB6	2	-0.068094777	0.201611532	-0.337752394	0.735549788	0.072980591	9.706140556	89.69724378	0.5	PRJNA827465;Yang2025_Silkie_Wenchang	CRA004775	0.933592889
ENSGALG00010011651	SERPINB6	2	-0.277193051	0.099467296	-2.786775758	0.00532353	0	0.002754707	0	1	CRA004775;Yang2025_Silkie_Wenchang	PRJNA827465	0.109021737
ENSGALG00010011651	SERPINB6	2	0.056349463	0.158359734	0.355832011	0.721966375	0.024157605	1.370668038	27.04287453	0.5	CRA004775;PRJNA827465	Yang2025_Silkie_Wenchang	0.929385887
ENSGALG00010011653	NR2C1	2	0.153722157	0.056750109	1.331174957	0.18313145	0	0.227556065	0	1	PRJNA827465;Yang2025_Silkie_Wenchang	CRA004775	0.69141358
ENSGALG00010011653	NR2C1	2	0.037740793	0.082246199	0.45887584	0.646323329	0	0.035729261	0	0.5	CRA004775;Yang2025_Silkie_Wenchang	PRJNA827465	0.907220702
ENSGALG00010011653	NR2C1	2	0.08911656	0.070874283	1.25738922	0.208612731	0	0.172769669	0	1	CRA004775;PRJNA827465	Yang2025_Silkie_Wenchang	0.721011841
ENSGALG00010011655	SORCS2	2	-0.135679389	0.166272765	-0.816008888	0.414497734	0.038051562	3.185554579	68.60820187	0.5	PRJNA827465;Yang2025_Silkie_Wenchang	CRA004775	0.837748996
ENSGALG00010011655	SORCS2	2	-0.291149612	0.139152461	-2.092306594	0.036411098	0	0.370104934	0	0.5	CRA004775;Yang2025_Silkie_Wenchang	PRJNA827465	0.32890494
ENSGALG00010011655	SORCS2	2	0.023246181	0.117461453	0.197904763	0.843119577	0	0.004556305	0	1	CRA004775;PRJNA827465	Yang2025_Silkie_Wenchang	0.960496485
ENSGALG00010011657	ALDH1L2	2	0.113688353	0.079663612	1.427105816	0.153549583	0	0.853477519	0	1	PRJNA827465;Yang2025_Silkie_Wenchang	CRA004775	0.645911732
ENSGALG00010011657	ALDH1L2	2	0.189880798	0.125167851	1.51700936	0.129264327	0	0.316847784	0	0.5	CRA004775;Yang2025_Silkie_Wenchang	PRJNA827465	0.603386934
ENSGALG00010011657	ALDH1L2	2	0.050402214	0.099417069	0.506977467	0.612170629	0	0.079875807	0	0.5	CRA004775;PRJNA827465	Yang2025_Silkie_Wenchang	0.897833503
ENSGALG00010011658	TENM1	2	0.352083594	0.544164448	0.6470169	0.517621006	0.409392914	3.140579299	68.15874064	0.5	PRJNA827465;Yang2025_Silkie_Wenchang	CRA004775	0.867962259
ENSGALG00010011658	TENM1	2	0.769074166	0.412575037	1.86408313	0.062310031	0	0.416514655	0	1	CRA004775;Yang2025_Silkie_Wenchang	PRJNA827465	0.430323521
ENSGALG00010011658	TENM1	2	-0.024514523	0.310297527	-0.077813156	0.937976681	0	0.478936441	0	0.5	CRA004775;PRJNA827465	Yang2025_Silkie_Wenchang	0.995349045
ENSGALG00010011659	CD14	2	0.195427583	0.128243816	1.523875297	0.127539866	0.007914826	1.28459845	22.15466242	1	PRJNA827465;Yang2025_Silkie_Wenchang	CRA004775	0.599533469
ENSGALG00010011659	CD14	2	-0.057579987	0.501434544	-0.114830516	0.908579461	0.393589863	4.188306397	76.12400084	0.5	CRA004775;Yang2025_Silkie_Wenchang	PRJNA827465	0.978353385
ENSGALG00010011659	CD14	2	0.141667379	0.352411795	0.401990745	0.67492328	0.167555736	2.477716601	59.66016138	0.5	CRA004775;PRJNA827465	Yang2025_Silkie_Wenchang	0.971599715
ENSGALG00010011660	ENSGALG00010011660	2	-0.025765238	0.07845811	-0.328394838	0.742613145	0.002306977	1.18527465	15.63136862	0.5	PRJNA827465;Yang2025_Silkie_Wenchang	CRA004775	0.935598296
ENSGALG00010011660	ENSGALG00010011660	2	0.080949836	0.119157182	0.679353397	0.496913971	0	0.047089232	0	1	CRA004775;Yang2025_Silkie_Wenchang	PRJNA827465	0.860939846
ENSGALG00010011660	ENSGALG00010011660	2	-0.066213567	0.076360326	-0.867120012	0.385876257	0	0.179584046	0	0.5	CRA004775;PRJNA827465	Yang2025_Silkie_Wenchang	0.826575976
ENSGALG00010011662	RIDA	2	-0.43230569	0.548320569	-0.795945715	0.426063588	0.558322504	18.45301216	94.58080354	0.5	PRJNA827465;Yang2025_Silkie_Wenchang	CRA004775	0.84125685
ENSGALG00010011662	RIDA	2	-0.544687543	0.451132493	-1.207378213	0.227286522	0.35528166	7.831248832	87.23064454	1	CRA004775;Yang2025_Silkie_Wenchang	PRJNA827465	0.740605003
ENSGALG00010011662	RIDA	2	0.053545498	0.127536004	0.419846132	0.674597861	0	0.402593605	0	0.5	CRA004775;PRJNA827465	Yang2025_Silkie_Wenchang	0.915970608
ENSGALG00010011663	UTP20	2	-0.000336613	0.089943836	-0.003742479	0.997013964	0.007266766	1.740534962	42.5536608	0.5	PRJNA827465;Yang2025_Silkie_Wenchang	CRA004775	0.999065103
ENSGALG00010011663	UTP20	2	0.095742219	0.106793225	0.896519612	0.369975311	0	0.132093365	0	1	CRA004775;Yang2025_Silkie_Wenchang	PRJNA827465	0.820855964
ENSGALG00010011663	UTP20	2	-0.065037452	0.073638446	-0.883199683	0.377128393	0	0.089892979	0	0.5	CRA004775;PRJNA827465	Yang2025_Silkie_Wenchang	0.823064945
ENSGALG00010011664	SERPINB10B	2	-0.370041871	0.345006203	-1.07256585	0.283465954	0	0.257637919	0	1	PRJNA827465;Yang2025_Silkie_Wenchang	CRA004775	0.779568117
ENSGALG00010011664	SERPINB10B	2	-0.79412383	0.594251601	-1.336342769	0.181437263	0.476271486	3.065262401	67.37636558	1	CRA004775;Yang2025_Silkie_Wenchang	PRJNA827465	0.689004635
ENSGALG00010011664	SERPINB10B	2	-1.00252188	0.417540748	-2.401015671	0.016349636	0.085825352	1.326013513	24.58598721	1	CRA004775;PRJNA827465	Yang2025_Silkie_Wenchang	0.211447764
ENSGALG00010011667	ENSGALG00010011667	2	-0.901826428	0.243875059	-3.697903474	0.000217388	0	0.014066931	0	1	PRJNA827465;Yang2025_Silkie_Wenchang	CRA004775	0.013455089
ENSGALG00010011667	ENSGALG00010011667	2	-0.455486874	0.510199565	-0.892762175	0.371984546	0.311912337	2.35417958	57.5223569	0.5	CRA004775;Yang2025_Silkie_Wenchang	PRJNA827465	0.821597118
ENSGALG00010011667	ENSGALG00010011667	2	-0.481663165	0.540220332	-0.891605029	0.372604673	0.376837096	2.657555453	62.37143428	0.5	CRA004775;PRJNA827465	Yang2025_Silkie_Wenchang	0.822016521
ENSGALG00010011669	ENSGALG00010011669	2	0.153939005	0.744200704	0.206851464	0.836125862	0.90763147	5.320154994	81.20355514	0.5	PRJNA827465;Yang2025_Silkie_Wenchang	CRA004775	0.958399882
ENSGALG00010011669	ENSGALG00010011669	2	-0.529813287	0.524583254	-1.009969882	0.31250972	0	0.443602818	0	0.5	CRA004775;Yang2025_Silkie_Wenchang	PRJNA827465	0.796054997
ENSGALG00010011669	ENSGALG00010011669	2	0.809404523	0.314999914	2.569538873	0.010183397	0	0.092543287	0	1	CRA004775;PRJNA827465	Yang2025_Silkie_Wenchang	0.160351777
ENSGALG00010011670	TMCO6	2	-0.213981694	0.092722748	-2.307758319	0.021012582	0	0.155502407	0	1	PRJNA827465;Yang2025_Silkie_Wenchang	CRA004775	0.245277621
ENSGALG00010011670	TMCO6	2	-0.271979211	0.172891168	-1.573123798	0.115690156	0	0.892180324	0	1	CRA004775;Yang2025_Silkie_Wenchang	PRJNA827465	0.572716099
ENSGALG00010011670	TMCO6	2	-0.259870844	0.094826991	-2.740473372	0.006135075	0	0.666576932	0	1	CRA004775;PRJNA827465	Yang2025_Silkie_Wenchang	0.118495743
ENSGALG00010011671	SLC41A2	2	-0.318796855	0.395373523	-0.806318167	0.420059418	0.293045005	15.94101843	93.72687508	0.5	PRJNA827465;Yang2025_Silkie_Wenchang	CRA004775	0.838603917
ENSGALG00010011671	SLC41A2	2	-0.352033776	0.378902846	-0.929087178	0.352843909	0.254914584	8.816382791	88.65747979	0.5	CRA004775;Yang2025_Silkie_Wenchang	PRJNA827465	0.811035834
ENSGALG00010011671	SLC41A2	2	0.064337751	0.111021135	0.579509033	0.562245753	0	0.017406811	0	1	CRA004775;PRJNA827465	Yang2025_Silkie_Wenchang	0.881955027
ENSGALG00010011673	POP1	2	0.022461703	0.194689432	0.11537197	0.908150296	0.05716064	4.052217033	75.32215101	0.5	PRJNA827465;Yang2025_Silkie_Wenchang	CRA004775	0.978208122
ENSGALG00010011673	POP1	2	0.180785373	0.137813066	1.311815917	0.189582253	0	0.831579152	0	0.5	CRA004775;Yang2025_Silkie_Wenchang	PRJNA827465	0.698754721
ENSGALG00010011673	POP1	2	-0.167809335	0.122191482	-1.373330876	0.169649508	0	0.002476085	0	1	CRA004775;PRJNA827465	Yang2025_Silkie_Wenchang	0.671135844
ENSGALG00010011674	ENSGALG00010011674	2	0.371284973	0.449705821	0.82561745	0.409021133	0.036878854	1.09389144	8.583250267	1	PRJNA827465;Yang2025_Silkie_Wenchang	CRA004775	0.834316328
ENSGALG00010011674	ENSGALG00010011674	2	0.324415453	0.477970261	0.678735643	0.497305378	0.11926381	1.32205012	24.35990251	0.5	CRA004775;Yang2025_Silkie_Wenchang	PRJNA827465	0.86098656
ENSGALG00010011674	ENSGALG00010011674	2	-0.013892242	0.363592388	-0.038208288	0.969521613	0	0.007343908	0	0.5	CRA004775;PRJNA827465	Yang2025_Silkie_Wenchang	0.991572914
ENSGALG00010011677	EDEM3	2	0.044493926	0.048827983	0.911238245	0.362169857	0	0.808711123	0	1	PRJNA827465;Yang2025_Silkie_Wenchang	CRA004775	0.815579273
ENSGALG00010011677	EDEM3	2	0.118498635	0.109036067	1.086783834	0.277132354	0	0.292709765	0	0.5	CRA004775;Yang2025_Silkie_Wenchang	PRJNA827465	0.776188179
ENSGALG00010011677	EDEM3	2	0.021880132	0.053317182	0.410376747	0.681529601	0	0.057392104	0	0.5	CRA004775;PRJNA827465	Yang2025_Silkie_Wenchang	0.918277611
ENSGALG00010011681	MAP1B	2	-0.121396576	0.248715325	-0.488094473	0.625482925	0.083484387	2.649813167	62.26149026	0.5	PRJNA827465;Yang2025_Silkie_Wenchang	CRA004775	0.901367193
ENSGALG00010011681	MAP1B	2	0.227964827	0.251743566	0.905543805	0.365177364	0	0.013862088	0	1	CRA004775;Yang2025_Silkie_Wenchang	PRJNA827465	0.818322934
ENSGALG00010011681	MAP1B	2	-0.199504016	0.227383034	-0.877391829	0.380273849	0.050228109	1.41763382	29.45992216	0.5	CRA004775;PRJNA827465	Yang2025_Silkie_Wenchang	0.824392515
ENSGALG00010011682	USP15	2	-0.130778099	0.054796637	-2.38660815	0.017004605	0	0.07284827	0	1	PRJNA827465;Yang2025_Silkie_Wenchang	CRA004775	0.216579369
ENSGALG00010011682	USP15	2	0.073489576	0.28043173	0.262058706	0.793276182	0.120390526	3.692164341	72.91561513	0.5	CRA004775;Yang2025_Silkie_Wenchang	PRJNA827465	0.948644003
ENSGALG00010011682	USP15	2	0.081615823	0.262563398	0.310842348	0.755920474	0.105067799	3.465538837	71.14445842	0.5	CRA004775;PRJNA827465	Yang2025_Silkie_Wenchang	0.938501783
ENSGALG00010011685	NDUFA2	2	-0.078246687	0.092763049	-0.843511375	0.398942507	0	0.003686357	0	1	PRJNA827465;Yang2025_Silkie_Wenchang	CRA004775	0.832054308
ENSGALG00010011685	NDUFA2	2	-0.08676793	0.232097007	-0.373843383	0.708520841	0	0.002114297	0	1	CRA004775;Yang2025_Silkie_Wenchang	PRJNA827465	0.9250668
ENSGALG00010011685	NDUFA2	2	-0.076332873	0.093777753	-0.813976352	0.415658498	0	2.75814E-05	0	1	CRA004775;PRJNA827465	Yang2025_Silkie_Wenchang	0.837689773
ENSGALG00010011688	PDE3A	2	0.087590123	0.19484055	0.449547711	0.653036599	0.050893783	2.853657791	64.9572558	0.5	PRJNA827465;Yang2025_Silkie_Wenchang	CRA004775	0.909572411
ENSGALG00010011688	PDE3A	2	0.312291584	0.183833129	1.698777507	0.089361113	0	0.008682334	0	1	CRA004775;Yang2025_Silkie_Wenchang	PRJNA827465	0.513108919
ENSGALG00010011688	PDE3A	2	-0.051612737	0.112889893	-0.457195378	0.64753062	0	0.614255796	0	0.5	CRA004775;PRJNA827465	Yang2025_Silkie_Wenchang	0.907350807
ENSGALG00010011692	ARL1	2	0.234905184	0.164097265	1.431499694	0.152287054	0.048365663	9.413255893	89.37668314	1	PRJNA827465;Yang2025_Silkie_Wenchang	CRA004775	0.643791086
ENSGALG00010011692	ARL1	2	0.075800679	0.344739716	0.219878001	0.82596617	0.221558148	14.56799728	93.13563847	0.5	CRA004775;Yang2025_Silkie_Wenchang	PRJNA827465	0.956393839
ENSGALG00010011692	ARL1	2	-0.069279373	0.177991682	-0.38922815	0.697107381	0.052493505	5.169880869	80.65719452	0.5	CRA004775;PRJNA827465	Yang2025_Silkie_Wenchang	0.921116491
ENSGALG00010011696	ZFC3H1	2	0.396686569	0.396171527	1.001300048	0.31668177	0.295183656	16.42489171	93.9116798	1	PRJNA827465;Yang2025_Silkie_Wenchang	CRA004775	0.796935817
ENSGALG00010011696	ZFC3H1	2	0.518760929	0.341658207	1.518362263	0.1289231	0.169551023	3.402643032	70.61108113	1	CRA004775;Yang2025_Silkie_Wenchang	PRJNA827465	0.602361458
ENSGALG00010011696	ZFC3H1	2	0.021183911	0.084802471	0.24980299	0.802739707	0	0.084359052	0	1	CRA004775;PRJNA827465	Yang2025_Silkie_Wenchang	0.950704475
ENSGALG00010011699	TIAM1	2	0.095781603	0.072520272	1.320756245	0.186582653	0	0.543320866	0	1	PRJNA827465;Yang2025_Silkie_Wenchang	CRA004775	0.695457578
ENSGALG00010011699	TIAM1	2	-0.221837941	0.522803082	-0.424324088	0.671329474	0.425835852	3.904742449	74.3901163	0.5	CRA004775;Yang2025_Silkie_Wenchang	PRJNA827465	0.914674175
ENSGALG00010011699	TIAM1	2	-0.261794723	0.448182749	-0.584124943	0.559136255	0.303149209	3.206670343	68.81500457	0.5	CRA004775;PRJNA827465	Yang2025_Silkie_Wenchang	0.881014886
ENSGALG00010011702	NUP205	2	-0.198816372	0.072837708	-2.729580295	0.0063415	0.007276237	3.178932956	68.54290374	1	PRJNA827465;Yang2025_Silkie_Wenchang	CRA004775	0.121049048
ENSGALG00010011702	NUP205	2	-0.176834025	0.160970767	-1.098547442	0.271965513	0.031637781	1.902897432	47.44856013	0.5	CRA004775;Yang2025_Silkie_Wenchang	PRJNA827465	0.773184343
ENSGALG00010011702	NUP205	2	-0.117726596	0.054512333	-2.159632329	0.030801144	0	0.691829346	0	0.5	CRA004775;PRJNA827465	Yang2025_Silkie_Wenchang	0.299786447
ENSGALG00010011703	YWHAZ	2	-0.257608633	0.296512855	-0.868794146	0.384959736	0.170263305	30.94777292	96.76874972	0.5	PRJNA827465;Yang2025_Silkie_Wenchang	CRA004775	0.826444827
ENSGALG00010011703	YWHAZ	2	-0.346096823	0.25665182	-1.348507183	0.177495311	0.102245818	4.046650774	75.28820608	1	CRA004775;Yang2025_Silkie_Wenchang	PRJNA827465	0.683596102
ENSGALG00010011703	YWHAZ	2	0.030002387	0.035759273	0.839009974	0.401463713	0	0.08893169	0	0.5	CRA004775;PRJNA827465	Yang2025_Silkie_Wenchang	0.832623399
ENSGALG00010011708	IK	2	0.052043411	0.107448907	0.484354958	0.628133979	0.017932696	4.425639016	77.40439298	0.5	PRJNA827465;Yang2025_Silkie_Wenchang	CRA004775	0.901904489
ENSGALG00010011708	IK	2	0.004663205	0.188459319	0.024743827	0.980259297	0.054936099	4.078697005	75.48236609	0.5	CRA004775;Yang2025_Silkie_Wenchang	PRJNA827465	0.994676009
ENSGALG00010011708	IK	2	-0.069015049	0.058640182	-1.176924203	0.239225761	0	0.837305346	0	1	CRA004775;PRJNA827465	Yang2025_Silkie_Wenchang	0.751401748
ENSGALG00010011712	DCAF12L2	2	-0.26694479	0.317946582	-0.839590061	0.401138274	0.189787758	15.66827773	93.6176776	0.5	PRJNA827465;Yang2025_Silkie_Wenchang	CRA004775	0.832453107

ENSGALG00010011712	DCAF12L2	2	-0.481811922	0.126940968	-3.795558911	0.000147311	0.007059923	1.279390771	21.83779789	1	CRA004775;Yang2025_Silkie_Wenchang	PRJNA827465	0.010268856
ENSGALG00010011712	DCAF12L2	2	-0.124094846	0.188499412	-0.658330151	0.510326008	0.058397972	4.960601476	79.84115424	0.5	CRA004775;PRJNA827465	Yang2025_Silkie_Wenchang	0.865365179
ENSGALG00010011720	IKZF2	2	-0.0804112	0.168322411	-0.477721296	0.632848585	0	0.007988448	0	1	PRJNA827465;Yang2025_Silkie_Wenchang	CRA004775	0.903112631
ENSGALG00010011720	IKZF2	2	-0.101068979	0.237560901	-0.4254445	0.670512672	0	0.060939932	0	1	CRA004775;Yang2025_Silkie_Wenchang	PRJNA827465	0.914482883
ENSGALG00010011720	IKZF2	2	-0.109677464	0.183824194	-0.596643245	0.550745592	0	0.040466985	0	1	CRA004775;PRJNA827465	Yang2025_Silkie_Wenchang	0.878879109
ENSGALG00010011723	SPRY3	2	-0.245403323	0.263404114	-0.931660933	0.351511782	0.119460633	7.050965432	85.81754499	0.5	PRJNA827465;Yang2025_Silkie_Wenchang	CRA004775	0.810678834
ENSGALG00010011723	SPRY3	2	-0.022842056	0.553178003	-0.041292415	0.96706278	0.529650137	6.995713829	85.70553307	0.5	CRA004775;Yang2025_Silkie_Wenchang	PRJNA827465	0.990893423
ENSGALG00010011723	SPRY3	2	0.178205895	0.267399323	0.666441085	0.505129209	0.090072443	2.11869549	52.80114559	1	CRA004775;PRJNA827465	Yang2025_Silkie_Wenchang	0.863862536
ENSGALG00010011724	ARHGEF28	2	0.391435605	0.493062813	0.79388588	0.42726187	0.443417653	11.32466658	91.16971795	0.5	PRJNA827465;Yang2025_Silkie_Wenchang	CRA004775	0.84125685
ENSGALG00010011724	ARHGEF28	2	0.658919289	0.294121067	2.240299534	0.025071483	0.090023607	1.97684992	49.41447047	1	CRA004775;Yang2025_Silkie_Wenchang	PRJNA827465	0.269672828
ENSGALG00010011724	ARHGEF28	2	-0.014249309	0.165708896	-0.085990008	0.931474361	0	0.873496636	0	0.5	CRA004775;PRJNA827465	Yang2025_Silkie_Wenchang	0.984076328
ENSGALG00010011726	WDR55	2	0.102922706	0.195622869	0.526128188	0.598799145	0.059430453	4.221367298	76.31099287	0.5	PRJNA827465;Yang2025_Silkie_Wenchang	CRA004775	0.89479201
ENSGALG00010011726	WDR55	2	0.04089989	0.313766798	0.130351236	0.896288545	0.1511507	4.179462711	76.07347956	0.5	CRA004775;Yang2025_Silkie_Wenchang	PRJNA827465	0.975115773
ENSGALG00010011726	WDR55	2	-0.094471806	0.085539437	-1.104423988	0.26940926	0	0.744539135	0	1	CRA004775;PRJNA827465	Yang2025_Silkie_Wenchang	0.77189992
ENSGALG00010011729	FAM129A	2	0.048529887	0.078320558	0.619631528	0.535500405	0	0.124894139	0	1	PRJNA827465;Yang2025_Silkie_Wenchang	CRA004775	0.874171355
ENSGALG00010011729	FAM129A	2	-0.012211046	0.140956494	-0.086629895	0.930965702	0	0.058127128	0	0.5	CRA004775;Yang2025_Silkie_Wenchang	PRJNA827465	0.983943003
ENSGALG00010011729	FAM129A	2	0.055664765	0.088437424	0.629425449	0.529070562	0	0.177915095	0	0.5	CRA004775;PRJNA827465	Yang2025_Silkie_Wenchang	0.871809618
ENSGALG00010011734	ENSGALG00010011734	2	0.349134566	0.214374661	1.628618632	0.103393778	0.015619001	1.192173036	16.119559	1	PRJNA827465;Yang2025_Silkie_Wenchang	CRA004775	0.547576788
ENSGALG00010011734	ENSGALG00010011734	2	0.856519035	0.78259298	1.094462968	0.273751981	1.113557266	10.99597541	90.90576359	1	CRA004775;Yang2025_Silkie_Wenchang	PRJNA827465	0.774480038
ENSGALG00010011734	ENSGALG00010011734	2	1.055058839	0.561864994	1.877779983	0.06041127	0.543388549	7.07612831	85.86797814	1	CRA004775;PRJNA827465	Yang2025_Silkie_Wenchang	0.425891324
ENSGALG00010011738	FGD6	2	-0.052257994	0.124262574	-0.420544305	0.674087871	0.021051122	2.964056857	66.26245554	0.5	PRJNA827465;Yang2025_Silkie_Wenchang	CRA004775	0.915753883
ENSGALG00010011738	FGD6	2	0.103477908	0.115036724	0.899520647	0.368375403	0	0.026755235	0	1	CRA004775;Yang2025_Silkie_Wenchang	PRJNA827465	0.820251457
ENSGALG00010011738	FGD6	2	-0.123298363	0.095320654	-1.293511506	0.195834207	0.004827473	1.118681383	10.60904249	0.5	CRA004775;PRJNA827465	Yang2025_Silkie_Wenchang	0.706913285
ENSGALG00010011745	AFAP1L1	2	0.093476899	0.155375299	0.601620074	0.547427064	0.041150011	6.743524273	85.17095869	0.5	PRJNA827465;Yang2025_Silkie_Wenchang	CRA004775	0.878281266
ENSGALG00010011745	AFAP1L1	2	0.064732183	0.26556511	0.243752587	0.807432343	0.097066331	2.464403686	59.42223241	0.5	CRA004775;Yang2025_Silkie_Wenchang	PRJNA827465	0.95234753
ENSGALG00010011745	AFAP1L1	2	-0.082146266	0.088759717	-0.925490394	0.354710878	0	0.503385637	0	1	CRA004775;PRJNA827465	Yang2025_Silkie_Wenchang	0.812016118
ENSGALG00010011746	SOD1	2	0.015524041	0.057402784	0.270440554	0.786821344	0	0.761310983	0	0.5	PRJNA827465;Yang2025_Silkie_Wenchang	CRA004775	0.946784559
ENSGALG00010011746	SOD1	2	-0.042988294	0.119754233	-0.358970982	0.719616798	0	0.640676315	0	0.5	CRA004775;Yang2025_Silkie_Wenchang	PRJNA827465	0.928449671
ENSGALG00010011746	SOD1	2	0.045412349	0.062033099	0.732066419	0.464128029	0	0.170289251	0	1	CRA004775;PRJNA827465	Yang2025_Silkie_Wenchang	0.852159649
ENSGALG00010011747	UTP15	2	0.145860527	0.129527938	1.126093175	0.260126083	0	0.006488322	0	1	PRJNA827465;Yang2025_Silkie_Wenchang	CRA004775	0.76744615
ENSGALG00010011747	UTP15	2	0.036964618	0.165540638	0.223296334	0.82330488	0	0.352605852	0	0.5	CRA004775;Yang2025_Silkie_Wenchang	PRJNA827465	0.955918024
ENSGALG00010011747	UTP15	2	0.087216162	0.131463788	0.663424533	0.50705938	0	0.58401233	0	0.5	CRA004775;PRJNA827465	Yang2025_Silkie_Wenchang	0.864016158
ENSGALG00010011751	ZNF706	2	-0.359571876	0.379164029	-0.948328028	0.342962487	0.274843313	22.45041889	95.54574013	0.5	PRJNA827465;Yang2025_Silkie_Wenchang	CRA004775	0.806218116
ENSGALG00010011751	ZNF706	2	-0.21310418	0.539277207	-0.395166303	0.69272016	0.556766385	23.32627965	95.71298975	0.5	CRA004775;Yang2025_Silkie_Wenchang	PRJNA827465	0.920579378
ENSGALG00010011751	ZNF706	2	0.133936508	0.155514335	0.861248631	0.389101121	0.032310381	2.707000619	63.05874505	1	CRA004775;PRJNA827465	Yang2025_Silkie_Wenchang	0.828655722
ENSGALG00010011752	RNF2	2	0.039833749	0.106539659	0.373886583	0.708488699	0	0.334476012	0	0.5	PRJNA827465;Yang2025_Silkie_Wenchang	CRA004775	0.92350668
ENSGALG00010011752	RNF2	2	0.038466096	0.148962844	0.258226113	0.796232406	0	0.698912967	0	0.5	CRA004775;Yang2025_Silkie_Wenchang	PRJNA827465	0.949325935
ENSGALG00010011752	RNF2	2	-0.032613965	0.117728266	-0.277027477	0.781759008	0	0.21747504	0	1	CRA004775;PRJNA827465	Yang2025_Silkie_Wenchang	0.945265125
ENSGALG00010011758	SERPINB11	2	-0.184900128	0.354736597	-0.521232176	0.602205042	0	0.278071002	0	1	PRJNA827465;Yang2025_Silkie_Wenchang	CRA004775	0.895349419
ENSGALG00010011758	SERPINB11	2	-0.859833806	0.603526318	-1.42468832	0.154248797	0.290726508	1.592908069	37.22173805	1	CRA004775;Yang2025_Silkie_Wenchang	PRJNA827465	0.646936345
ENSGALG00010011758	SERPINB11	2	-0.689196839	0.800582339	-0.860869402	0.389309976	0.865136674	2.887571181	65.36881908	1	CRA004775;PRJNA827465	Yang2025_Silkie_Wenchang	0.828875947
ENSGALG00010011759	CREB3	2	-0.04511546	0.076033982	-0.593359163	0.552940813	0	0.634863257	0	1	PRJNA827465;Yang2025_Silkie_Wenchang	CRA004775	0.87928478
ENSGALG00010011759	CREB3	2	-0.267605086	0.188055424	-1.423031788	0.154732738	0.037135219	1.911430747	47.68316868	1	CRA004775;Yang2025_Silkie_Wenchang	PRJNA827465	0.647505492
ENSGALG00010011759	CREB3	2	-0.210125675	0.255938905	-0.820999352	0.411646637	0.100137181	3.741161683	73.2703346	1	CRA004775;PRJNA827465	Yang2025_Silkie_Wenchang	0.835128673
ENSGALG00010011764	PCYOX1L	2	0.094528097	0.148385603	0.637043583	0.524096455	0.023395557	1.972998853	49.31573333	0.5	PRJNA827465;Yang2025_Silkie_Wenchang	CRA004775	0.869740342
ENSGALG00010011764	PCYOX1L	2	0.083931052	0.242323456	0.346359588	0.729072492	0.064348741	2.144125124	53.36093082	0.5	CRA004775;Yang2025_Silkie_Wenchang	PRJNA827465	0.932323844
ENSGALG00010011764	PCYOX1L	2	-0.042069863	0.099303642	-0.423648743	0.671822001	0	0.388904889	0	1	CRA004775;PRJNA827465	Yang2025_Silkie_Wenchang	0.914674175
ENSGALG00010011770	CHST11	2	0.270414272	0.253216513	1.06791721	0.285557859	0	0.232482953	0	1	PRJNA827465;Yang2025_Silkie_Wenchang	CRA004775	0.781255524
ENSGALG00010011770	CHST11	2	0.170910729	0.250586065	0.682044026	0.495211112	0	0.000337874	0	1	CRA004775;Yang2025_Silkie_Wenchang	PRJNA827465	0.860619541
ENSGALG00010011770	CHST11	2	0.292098015	0.286134531	1.020841542	0.307329519	0	0.207591154	0	1	CRA004775;PRJNA827465	Yang2025_Silkie_Wenchang	0.792675978
ENSGALG00010011774	ANKRA2	2	-0.013868213	0.159999861	-0.086676409	0.930929729	0.024848286	1.922605579	47.98725172	0.5	PRJNA827465;Yang2025_Silkie_Wenchang	CRA004775	0.983943003
ENSGALG00010011774	ANKRA2	2	-0.257723543	0.146520926	-1.758953828	0.078585351	0	0.396664414	0	1	CRA004775;Yang2025_Silkie_Wenchang	PRJNA827465	0.482223273
ENSGALG00010011774	ANKRA2	2	-0.087739474	0.255234325	-0.343760482	0.731026422	0.091104046	3.143449166	68.18781068	0.5	CRA004775;PRJNA827465	Yang2025_Silkie_Wenchang	0.932769533
ENSGALG00010011775	VEZT	2	-0.003787887	0.048242459	-0.0785177	0.937416251	0	0.064671304	0	0.5	PRJNA827465;Yang2025_Silkie_Wenchang	CRA004775	0.985114195
ENSGALG00010011775	VEZT	2	-0.000877666	0.084772595	-0.010353176	0.991739509	0	0.105689109	0	0.5	CRA004775;Yang2025_Silkie_Wenchang	PRJNA827465	0.997575757
ENSGALG00010011775	VEZT	2	-0.013429532	0.050242258	-0.267295555	0.789241609	0	0.031359312	0	1	CRA004775;PRJNA827465	Yang2025_Silkie_Wenchang	0.947335786
ENSGALG00010011776	IL17RA	2	-0.120933917	0.065341989	-1.850784129	0.064200615	0	0.957370853	0	1	PRJNA827465;Yang2025_Silkie_Wenchang	CRA004775	0.43662345
ENSGALG00010011776	IL17RA	2	-0.593428243	0.500262147	-1.18623455	0.235529685	0.399651071	4.150452801	75.9062433	1	CRA004775;Yang2025_Silkie_Wenchang	PRJNA827465	0.748604097
ENSGALG00010011776	IL17RA	2	-0.540588197	0.568498756	-0.950904802	0.341652704	0.541639467	5.310278613	81.16859636	1	CRA004775;PRJNA827465	Yang2025_Silkie_Wenchang	0.805823616
ENSGALG00010011784	GRPEL2	2	-0.181352326	0.060523689	-2.99638585	0.002732005	0	0.938254823	0	1	PRJNA827465;Yang2025_Silkie_Wenchang	CRA004775	0.0712988
ENSGALG00010011784	GRPEL2	2	-0.265364328	0.108833363	-2.438262684	0.014758045	0	0.101153911	0	1	CRA004775;Yang2025_Silkie_Wenchang	PRJNA827465	0.200535277
ENSGALG00010011784	GRPEL2	2	-0.149475324	0.067932576	-2.200348244	0.027782197	0	0.022251209	0	1	CRA004775;PRJNA827465	Yang2025_Silkie_Wenchang	0.284281094
ENSGALG00010011789	TXNRD1	2	-0.046457893	0.255111052	-0.182108509	0.855497572	0.103000231	4.737241274	78.89066775	0.5	PRJNA827465;Yang2025_Silkie_Wenchang	CRA004775	0.963463555
ENSGALG00010011789	TXNRD1	2	0.065676042	0.435482154	0.150812246	0.880123826	0.300599228	4.523573818	77.89358502	0.5	CRA004775;Yang2025_Silkie_Wenchang	PRJNA827465	0.971562373
ENSGALG00010011789	TXNRD1	2	0.242840532	0.133057389	1.825081146	0.067988785	0	0.864706951	0	1	CRA004775;PRJNA827465	Yang2025_Silkie_Wenchang	0.4497039
ENSGALG00010011790	AMY2A	2	-0.362986751	0.655319801	-0.553907803	0.579641945	0.699027039	5.323371403	81.21491205	0.5	PRJNA827465;Yang2025_Silkie_Wenchang	CRA004775	0.887983094
ENSGALG00010011790	AMY2A	2	1.166913032	1.037884974	1.124318264	0.260878033	1.587686805	3.403702285	70.62022715	1	CRA004775;Yang2025_Silkie_Wenchang	PRJNA827465	0.76792161
ENSGALG00010011790	AMY2A	2	0.582128392	1.710088383	0.340408366	0.733549019	5.270155367	9.488088157	89.46046892	0.5	CRA004775;PRJNA827465	Yang2025_Silkie_Wenchang	0.933320645
ENSGALG00010011792	HDHD5	2	-0.077377428	0.069169777	-1.118659489	0.263285434	0	0.474258839	0	1	PRJNA827465;Yang2025_Silkie_Wenchang	CRA004775	0.768643821
ENSGALG00010011792	HDHD5	2	-0.116606094	0.090031647	-1.295167845	0.195262335	0	0.037034158	0	1	CRA004775;Yang2025_Silkie_Wenchang	PRJNA827465	0.70643959
ENSGALG00010011792	HDHD5	2	-0.032279689	0.093992347	-0.343428906	0.731275817	0	0.019741701	0	1	CRA004775;PRJNA827465	Yang2025_Silkie_Wenchang	0.932851968
ENSGALG00010011793	TLN1	2	0.066145857	0.142971345	0.462651149	0.643614439	0.033098775	5.219146206	80.83977799	0.5	PRJNA827465;Yang2025_Silkie_Wenchang	CRA004775	0.906643868
ENSGALG00010011793	TLN1	2	-0.065689279	0.312669889	-0.210091476	0.833596278	0.164058027	5.576328956	82.06705508	0.5	CRA004775;Yang2025_Silkie_Wenchang	PRJNA827465	0.957968425
ENSGALG00010011793	TLN1	2	-0.174676642	0.153001975	-1.141662658	0.253594268	0.022466314	1.600008182	37.50031961	1	CRA004775;PRJNA827465	Yang2025_Silkie_Wenchang	0.763346521
ENSGALG00010011796	METAP2	2	0.013550292	0.047474519	0.285422424	0.77532053	0.000763242	1.148297056	12.91452027	0.5	PRJNA827465;Yang2025_Silkie_Wenchang	CRA004775	0.943815265
ENSGALG00010011796	METAP2	2	0.101789514	0.083219117	1.223150605	0.221272818	0	0.049783702	0	1	CRA004775;Yang2025_Silkie_Wenchang	PRJNA827465	0.734685079
ENSGALG00010011796	METAP2	2	-0.007340336	0.046588452	-0.157556992	0.874805896	0	0.50148129	0	0.5	CRA004775;PRJNA827465	Yang2025_Silkie_Wenchang	0.970159044
ENSGALG00010011797	SCAF4	2	0.064057167	0.154813637	0.413769539	0.679042894	0.033347986	2.506975653	60.11129989	0.5	PRJNA827465;Yang2025_Silkie_Wenchang	CRA004775	0.917701868
ENSGALG00010011797	SCAF4	2	0.196033689	0.138308053	1.417370028	0.156374771	0	0.39277173	0	1	CRA004775;Yang2025_Silkie_Wenchang	PRJNA827465	0.649484056
ENSGALG00010011797	SCAF4	2	-0.033779883	0.039955029	-0.845447581	0.397860988	0	0.693048276	0	0.5	CRA004775;PRJNA827465	Yang2025_Silkie_Wenchang	0.83172112
ENSGALG00010011802	VPS4B	2	-0.05010322	0.103357174	-0.484758027	0.627848	0.016884528	4.579450264	78.16331781	0.5	PRJNA827465;Yang2025_Silkie_Wenchang	CRA004775	0.901878949
ENSGALG00010011802	VPS4B	2	-0.147193148	0.074366267	-1.979299935	0.047782247	0	0.216834193	0	1	CRA004775;Yang2025_Silkie_Wenchang	PRJNA827465	0.378006253
ENSGALG00010011802	VPS4B	2	0.032631837	0.047444277	0.687792907	0.491583203	0	0.514139522	0	0.5	CRA004775;PRJNA827465	Yang2025_Silkie_Wenchang	0.85895707
ENSGALG00010011808	SMARCA2	2	0.023241039	0.194738749	0.119344709	0.905002264	0.060029162	4.521757925	77.88470731	0.5	PRJNA827465;Yang2025_Silkie_Wenchang	CRA004775	0.977930936
ENSGALG00010011808	SMARCA2	2	0.215397354	0.129641698	1.661482056	0.09661667	0	0.083982271	0	1	CRA004775;Yang2025_Silkie_Wenchang	PRJNA827465	0.531114935
ENSGALG00010011808	SMARCA2	2	-0.050907781	0.145540843	-0.349783469	0.726501206	0.023348729	1.890082558	47.09225818	0.5	CRA004775;PRJNA827465	Yang2025_Silkie_Wenchang	0.931112589
ENSGALG00010011810	FGFR4	2	-0.096833386	0.078228211	-1.237832048	0.215778343	0	0.085996139	0	1	PRJNA827465;Yang2025_Silkie_Wenchang	CRA004775	0.729213578
ENSGALG00010011810	FGFR4	2	-0.103757397	0.143483791	-0.723129747	0.469600175	0	0.259868412	0	0.5	CRA004775;Yang2025_Silkie_Wenchang	PRJNA827465	0.853485658
ENSGALG00010011810	FGFR4	2	-0.074805016	0.087231941	-0.857541578	0.391145647	0	0.154829852	0	0.5	CRA004775;PRJNA827465	Yang2025_Silkie_Wenchang	0.829251057
ENSGALG00010011814	ENSGALG00010011814	2	-0.599767258	0.267163475	-2.244944818	0.024771675	0	0.152652506	0	1	PRJNA827465;Yang2025_Silkie_Wenchang	CRA004775	0.268761753
ENSGALG00010011814	ENSGALG00010011814	2	-0.50113281	0.365035017	-1.372834898	0.169803679	0	0.836762336	0	1	CRA004775;Yang2025_Silkie_Wenchang	PRJNA827465	0.671232541
ENSGALG00010011814	ENSGALG00010011814	2	-0.415646365	0.293436258	-1.416479232	0.15663524	0	0.471289644	0	1	CRA004775;PRJNA827465	Yang2025_Silkie_Wenchang	0.64980313
ENSGALG00010011817	KDSR	2	-0.290859859	0.397634774	-0.731474906	0.464489127	0.308595522	41.04877874	97.56387393	0.5	PRJNA827465;Yang2025_Silkie_Wenchang	CRA004775	0.852159649
ENSGALG00010011817	KDSR	2	-0.195852398	0.499578532	-0.392035257	0.695032163	0.485809915	37.39357253	97.32574362	0.5	CRA004775;Yang2025_Silkie_Wenchang	PRJNA827465	0.92097163
ENSGALG00010011817	KDSR	2	0.174937912	0.098063063	1.783932774	0.07443459	0.01226164	2.441754428	59.04584063	1	CRA004775;PRJNA827465	Yang2025_Silkie_Wenchang	0.47101959
ENSGALG00010011819	USP44	2	0.252008939	0.241583115	1.043156259	0.296875928	0.100381237	6.793028465	85.27902532	1	PRJNA827465;Yang2025_Silkie_Wenchang	CRA004775	0.788466926
ENSGALG00010011819	USP44	2	0.448974901	0.132163424	3.397119164	0.000680993	0	0.432883923	0	1	CRA004775;Yang2025_Silkie_Wenchang	PRJNA827465	0.028872783
ENSGALG00010011819	USP44	2	0.119778485	0.138557016	0.864470733	0.387329351	0.020410787	1.773924242	43.62780684	1	CRA004775;PRJNA827465	Yang2025_Silkie_Wenchang	0.827662996
ENSGALG00010011823	ENSGALG00010011823	2	0.328353806	0.298228939	1.101012555	0.270891193	0	0.847128187	0	1	PRJNA827465;Yang2025_Silkie_Wenchang	CRA004775	0.772743863
ENSGALG00010011823	ENSGALG00010011823	2	1.260706128	0.683561676	1.844319499	0.065136591	0.607453868	2.770307558	63.90292489	1	CRA004775;Yang2025_Silkie_Wenchang	PRJNA827465	0.439873445
ENSGALG00010011823	ENSGALG00010011823	2	0.988949206	0.965516574	1.024269528	0.305707987	1.569706213	6.094198988	83.59095261	1	CRA004775;PRJNA827465	Yang2025_Silkie_Wenchang	0.79225794
ENSGALG00010011837	VLDLR	2	0.008013775	0.088726561	0.090319911	0.928032998	0.001827638	1.126577886	11.235609	0.5	PRJNA827465;Yang2025_Silkie_Wenchang	CRA004775	0.983007166
ENSGALG00010011837	VLDLR	2	0.002930706	0.17695891	0.016561506	0.986786434	0.024214513	1.403233606	28.73602827	0.5	CRA004775;Yang2025_Silkie_Wenchang	PRJNA827465	0.996292314
ENSGALG00010011837	VLDLR	2	0.10526132	0.101800386	1.033997253	0.301137447	0	0.463543975	0	1	CRA004775;PRJNA827465	Yang2025_Silkie_Wenchang	0.791202961
ENSGALG00010011839	CBS	2	-0.499682526	0.242598106	-2.059713214	0.039425965	0.090820411	4.332223402	76.91716453	1	PRJNA827465;Yang2025_Silkie_Wenchang	CRA004775	0.3433403
ENSGALG00010011839	CBS	2	-0.751850342	0.178190456	-4.21936371	2.44993E-05	0	0.009127883	0	1	CRA004775;Yang2025_Silkie_Wenchang	PRJNA827465	0.002848802
ENSGALG00010011839	CBS	2	-0.286346705	0.140939931	-2.031693245	0.04218472	0	0.323124942	0	1	CRA004775;PRJNA827465	Yang2025_Silkie_Wenchang	0.355485861
ENSGALG00010011841	KCNMB4	2	-0.350218962	0.1830869	-1.912856473	0.055766429	0.02919727	1.576310474	36.56072099	1	PRJNA827465;Yang2025_Silkie_Wenchang	CRA004775	0.408839389
ENSGALG00010011841	KCNMB4	2	-0.547186327	0.252555289	-2.166600146	0.030265356	0	0.364264806	0	1	CRA004775;Yang2025_Silkie_Wenchang	PRJNA827465	0.296121309
ENSGALG00010011841	KCNMB4	2	-0.232737877	0.132282311	-1.75940287	0.078509105	0	0.004473162	0	1	CRA004775;PRJNA827465	Yang2025_Silkie_Wenchang	0.481915278
ENSGALG00010011843	BCL2	2	-0.270483655	0.10385789	-2.604363088	0.009204519	0.009856782	1.800837825	44.47029121	1	PRJNA827465;Yang2025_Silkie_Wenchang	CRA004775	0.150422122
ENSGALG00010011843	BCL2	2	-0.059929017	0.388917155	-0.154091986	0.877537207	0.246236331	4.870707699	79.46910261	0.5	CRA004775;Yang2025_Silkie_Wenchang	PRJNA827465	0.970706998
ENSGALG00010011843	BCL2	2	0.016946487	0.274262457	0.061789308	0.950730618	0.106278772	2.7715161	63.91866531	0.5	CRA004775;PRJNA827465	Yang2025_Silkie_Wenchang	0.98727979
ENSGALG00010011846	UBAP1	2	0.044157411	0.082867251	0.532869264	0.594124083	0	0.144642721	0	1	PRJNA827465;Yang2025_Silkie_Wenchang	CRA004775	0.893274007
ENSGALG00010011846	UBAP1	2	0.11571696	0.130961584	0.883594693	0.376915045	0	0.168140389	0	1	CRA004775;Yang2025_Silkie_Wenchang	PRJNA827465	0.823064945
ENSGALG00010011846	UBAP1	2	0.043026714	0.095550331	0.450304189	0.652491119	0	0.415691705	0	1	CRA004775;PRJNA827465	Yang2025_Silkie_Wenchang	0.909401809
ENSGALG00010011850	SPAG16	2	0.549869183	0.208943764	2.631661138	0.008496856	0	0.491942814	0	1	PRJNA827465;Yang2025_Silkie_Wenchang	CRA004775	0.14369705
ENSGALG00010011850	SPAG16	2	-0.068112393	0.902360407	-0.075482471	0.939830844	1.333045617	5.096834941	80.37998068	0.5	CRA004775;Yang2025_Silkie_Wenchang	PRJNA827465	0.985444231
ENSGALG00010011850	SPAG16	2	-0.183807188	0.746517283	-0.246219601	0.805512243	0.850456313	3.725272282	73.15632457	0.5	CRA004775;PRJNA827465	Yang2025_Silkie_Wenchang	0.951922776
ENSGALG00010011856	NFYB	2	0.036588425	0.058186407	0.62881396	0.52947086	0	0.086532468	0	1	PRJNA827465;Yang2025_Silkie_Wenchang	CRA004775	0.871944741
ENSGALG00010011856	NFYB	2	0.057913857	0.091074832	0.635893089	0.524846107	0	0.003589448	0	1	CRA004775;Yang2025_Silkie_Wenchang	PRJNA827465	0.869892816
ENSGALG00010011856	NFYB	2	0.026160785	0.068424207	0.382332314	0.702214889	0	0.007937303	0	1	CRA004775;PRJNA827465	Yang2025_Silkie_Wenchang	0.923083392
ENSGALG00010011857	ENSGALG00010011857	2	-0.20192936	0.312384029	-0.646413842	0.518011379	0	0.024994809	0	1	PRJNA827465;Yang2025_Silkie_Wenchang	CRA004775	0.868202369
ENSGALG00010011857	ENSGALG00010011857	2	-0.09828546	0.34032326	-0.288800301	0.772734202	0	0.374582131	0	0.5	CRA004775;Yang2025_Silkie_Wenchang	PRJNA827465	0.943066211
ENSGALG00010011857	ENSGALG00010011857	2	-0.012332211	0.36838545	-0.033476379	0.973294702	0	0.2055305	0	0.5	CRA004775;PRJNA827465	Yang2025_Silkie_Wenchang	0.992392211
ENSGALG00010011859	NTN4	2	0.210678862	0.204054833	1.032462005	0.301855738	0.066787678	5.037951337	80.15066178	1	PRJNA827465;Yang2025_Silkie_Wenchang	CRA004775	0.791453392
ENSGALG00010011859	NTN4	2	0.429507763	0.131364182	3.269595686	0.001077013	0	0.069553901	0	1	CRA004775;Yang2025_Silkie_Wenchang	PRJNA827465	0.039671831
ENSGALG00010011859	NTN4	2	0.075603689	0.174914908	0.432231246	0.665573352	0.020210603	1.159500698	13.7559812	1	CRA004775;PRJNA827465	Yang2025_Silkie_Wenchang	0.913532078
ENSGALG00010011860	CECR1	2	-0.075711731	0.195361536	-0.387546765	0.698351471	0.062002856	5.282091194	81.06810422	0.5	PRJNA827465;Yang2025_Silkie_Wenchang	CRA004775	0.921638221
ENSGALG00010011860	CECR1	2	-0.329382036	0.123366274	-2.669952046	0.007586208	0	0.923036446	0	1	CRA004775;Yang2025_Silkie_Wenchang	PRJNA827465	0.134479085
ENSGALG00010011860	CECR1	2	-0.187966103	0.356766587	-0.526860166	0.598290697	0.205678253	4.616740315	78.33969572	0.5	CRA004775;PRJNA827465	Yang2025_Silkie_Wenchang	0.894645663
ENSGALG00010011864	ENSGALG00010011864	2	0.55729401	0.531878366	1.047784693	0.294737817	0.494548214	7.941104728	87.40729364	1	PRJNA827465;Yang2025_Silkie_Wenchang	CRA004775	0.787482391
ENSGALG00010011864	ENSGALG00010011864	2	0.729065775	0.470519194	1.549492103	0.121263468	0.279125842	2.433619592	58.90894357	1	CRA004775;Yang2025_Silkie_Wenchang	PRJNA827465	0.583784676
ENSGALG00010011864	ENSGALG00010011864	2	0.043640176	0.238107059	0.183279641	0.854578608	0	0.021170307	0	1	CRA004775;PRJNA827465	Yang2025_Silkie_Wenchang	0.963225297
ENSGALG00010011866	KLF8	2	-0.034585727	0.170141765	-0.20327594	0.83891936	0.046469472	4.916209809	79.65912687	0.5	PRJNA827465;Yang2025_Silkie_Wenchang	CRA004775	0.959283372
ENSGALG00010011866	KLF8	2	0.166776657	0.12047515	1.384324122	0.166259231	0	0.1030012	0	1	CRA004775;Yang2025_Silkie_Wenchang	PRJNA827465	0.66529196
ENSGALG00010011866	KLF8	2	-0.069565976	0.200177072	-0.347522199	0.728199044	0.047903945	1.881725178	46.85727694	0.5	CRA004775;PRJNA827465	Yang2025_Silkie_Wenchang	0.931987594
ENSGALG00010011867	ENSGALG00010011867	2	-0.171051273	0.402576277	-0.424891586	0.670915708	0.097801653	1.429344667	30.03786819	0.5	PRJNA827465;Yang2025_Silkie_Wenchang	CRA004775	0.914552186
ENSGALG00010011867	ENSGALG00010011867	2	-0.480710421	0.284041957	-1.692392298	0.0905712	0	0.035619771	0	1	CRA004775;Yang2025_Silkie_Wenchang	PRJNA827465	0.516394163
ENSGALG00010011867	ENSGALG00010011867	2	-0.173018886	0.338323224	-0.51140115	0.60907019	0.051894555	1.271548375	21.35572507	0.5	CRA004775;PRJNA827465	Yang2025_Silkie_Wenchang	0.897212042
ENSGALG00010011868	SNRPF	2	0.006408362	0.073546873	0.087133033	0.930565768	0	0.00100096	0	1	PRJNA827465;Yang2025_Silkie_Wenchang	CRA004775	0.983903639
ENSGALG00010011868	SNRPF	2	-0.081894151	0.183669697	-0.445877311	0.655685872	0	0.657518101	0	0.5	CRA004775;Yang2025_Silkie_Wenchang	PRJNA827465	0.910411938
ENSGALG00010011868	SNRPF	2	-0.01036928	0.076178462	-0.136118259	0.891727793	0	0.83937725	0	0.5	CRA004775;PRJNA827465	Yang2025_Silkie_Wenchang	0.973985832
ENSGALG00010011871	CCT2	2	0.128011668	0.135497135	0.944755534	0.344783698	0.028523864	4.111288134	75.67672303	1	PRJNA827465;Yang2025_Silkie_Wenchang	CRA004775	0.806945375
ENSGALG00010011871	CCT2	2	0.220524835	0.111746596	1.973436706	0.048445829	0.001729302	1.056788044	5.373645623	1	CRA004775;Yang2025_Silkie_Wenchang	PRJNA827465	0.380432851
ENSGALG00010011871	CCT2	2	0.014116591	0.053982172	0.261504694	0.793703331	0	0.008920716	0	1	CRA004775;PRJNA827465	Yang2025_Silkie_Wenchang	0.948796049
ENSGALG00010011873	RFX3	2	0.312681781	0.278888869	1.121169811	0.262215588	0.130040107	5.84340077	82.88667782	1	PRJNA827465;Yang2025_Silkie_Wenchang	CRA004775	0.768363052
ENSGALG00010011873	RFX3	2	0.637263732	0.185256231	3.439904435	0.00058192	0	0.050126491	0	1	CRA004775;Yang2025_Silkie_Wenchang	PRJNA827465	0.025964366
ENSGALG00010011873	RFX3	2	0.251701805	0.30382284	0.828449255	0.407416122	0.117275773	2.0885587	52.12009123	1	CRA004775;PRJNA827465	Yang2025_Silkie_Wenchang	0.833863243
ENSGALG00010011875	PSD2	2	-0.084663031	0.123680888	-0.684527999	0.493641815	0	0.020030564	0	1	PRJNA827465;Yang2025_Silkie_Wenchang	CRA004775	0.860048738
ENSGALG00010011875	PSD2	2	-0.098956901	0.221766249	-0.44622165	0.655437146	0	0.024268162	0	1	CRA004775;Yang2025_Silkie_Wenchang	PRJNA827465	0.910411938
ENSGALG00010011875	PSD2	2	-0.070830975	0.139179033	-0.508919868	0.610808393	0	0.008957858	0	1	CRA004775;PRJNA827465	Yang2025_Silkie_Wenchang	0.897506931
ENSGALG00010011876	AMDHD1	2	0.520842406	0.321517583	1.619949993	0.105243019	0.037859053	1.218780807	17.95079192	1	PRJNA827465;Yang2025_Silkie_Wenchang	CRA004775	0.550707815
ENSGALG00010011876	AMDHD1	2	0.171605635	0.325199198	0.527693907	0.597711803	0	0.130400873	0	0.5	CRA004775;Yang2025_Silkie_Wenchang	PRJNA827465	0.894645663
ENSGALG00010011876	AMDHD1	2	0.542108208	0.444973866	1.218292241	0.223112937	0.117188842	1.387253376	27.91511507	0.5	CRA004775;PRJNA827465	Yang2025_Silkie_Wenchang	0.736705686
ENSGALG00010011880	U2AF1	2	0.270602007	0.314302485	0.860960445	0.389259829	0.178645967	10.17594301	90.17290093	0.5	PRJNA827465;Yang2025_Silkie_Wenchang	CRA004775	0.828814078
ENSGALG00010011880	U2AF1	2	0.219884183	0.391541418	0.56158601	0.574398115	0.26933323	8.188790236	87.78818395	0.5	CRA004775;Yang2025_Silkie_Wenchang	PRJNA827465	0.886083039
ENSGALG00010011880	U2AF1	2	-0.053490482	0.087012171	-0.614747128	0.538721731	0	0.436823177	0	1	CRA004775;PRJNA827465	Yang2025_Silkie_Wenchang	0.87481821
ENSGALG00010011881	RIOK2	2	0.19645737	0.105718982	1.858297976	0.063126711	0	0.023039587	0	1	PRJNA827465;Yang2025_Silkie_Wenchang	CRA004775	0.432760766
ENSGALG00010011881	RIOK2	2	0.067347892	0.150205336	0.448372166	0.653884626	0	0.796746414	0	0.5	CRA004775;Yang2025_Silkie_Wenchang	PRJNA827465	0.909861322
ENSGALG00010011881	RIOK2	2	0.121129926	0.139104507	0.870783622	0.383872309	0.01061882	1.281387915	21.95961984	0.5	CRA004775;PRJNA827465	Yang2025_Silkie_Wenchang	0.825601288
ENSGALG00010011882	ENSGALG00010011882	2	-0.22016157	0.288830713	-0.762251244	0.445910067	0	0.512609981	0	1	PRJNA827465;Yang2025_Silkie_Wenchang	CRA004775	0.846013379
ENSGALG00010011882	ENSGALG00010011882	2	-0.260286521	0.3332052	-0.78115984	0.43470849	0	0.49858914	0	0.5	CRA004775;Yang2025_Silkie_Wenchang	PRJNA827465	0.842762754
ENSGALG00010011882	ENSGALG00010011882	2	0.016102041	0.334036485	0.048204439	0.961553312	0	0.014733446	0	0.5	CRA004775;PRJNA827465	Yang2025_Silkie_Wenchang	0.98973612
ENSGALG00010011883	HCFC2	2	0.185820231	0.091128974	2.039090572	0.041440989	0	0.456348909	0	1	PRJNA827465;Yang2025_Silkie_Wenchang	CRA004775	0.352975427
ENSGALG00010011883	HCFC2	2	0.273049583	0.13086545	2.086491002	0.036934169	0	0.000404297	0	1	CRA004775;Yang2025_Silkie_Wenchang	PRJNA827465	0.331631487
ENSGALG00010011883	HCFC2	2	0.16577919	0.101738434	1.629464724	0.103214677	0	0.261055022	0	1	CRA004775;PRJNA827465	Yang2025_Silkie_Wenchang	0.54753882
ENSGALG00010011885	NCALD	2	-0.118367572	0.112361844	-1.053449888	0.29213485	0	0.292715051	0	1	PRJNA827465;Yang2025_Silkie_Wenchang	CRA004775	0.785516679
ENSGALG00010011885	NCALD	2	-0.270109987	0.210234519	-1.284803219	0.198861057	0	0.142741039	0	1	CRA004775;Yang2025_Silkie_Wenchang	PRJNA827465	0.71096127
ENSGALG00010011885	NCALD	2	-0.109120361	0.123371014	-0.884489459	0.37643205	0	0.498893524	0	1	CRA004775;PRJNA827465	Yang2025_Silkie_Wenchang	0.822974013
ENSGALG00010011886	NTNG1	2	-0.331322616	0.113308264	-2.924081662	0.003454741	0	0.096401454	0	1	PRJNA827465;Yang2025_Silkie_Wenchang	CRA004775	0.083008703
ENSGALG00010011886	NTNG1	2	-0.075800607	0.410497063	-0.184655661	0.853499126	0.216243532	2.51035831	60.16504911	0.5	CRA004775;Yang2025_Silkie_Wenchang	PRJNA827465	0.962964214
ENSGALG00010011886	NTNG1	2	-0.069882401	0.355793639	-0.196412734	0.84428713	0.168089444	2.345781468	57.37028306	0.5	CRA004775;PRJNA827465	Yang2025_Silkie_Wenchang	0.960723102
ENSGALG00010011887	PGM5	2	-0.07874563	0.071236524	-1.105410905	0.268981581	0	0.237379523	0	1	PRJNA827465;Yang2025_Silkie_Wenchang	CRA004775	0.771398294
ENSGALG00010011887	PGM5	2	-0.025971246	0.107210907	-0.242244437	0.808590764	0	0.082336938	0	0.5	CRA004775;Yang2025_Silkie_Wenchang	PRJNA827465	0.952496413
ENSGALG00010011887	PGM5	2	-0.09531527	0.088716728	-1.074377654	0.282653454	0	0.241512968	0	0.5	CRA004775;PRJNA827465	Yang2025_Silkie_Wenchang	0.779532565
ENSGALG00010011888	CAND1	2	-0.115402703	0.125741034	-0.917780769	0.358733652	0.028729772	10.77550421	90.71969181	0.5	PRJNA827465;Yang2025_Silkie_Wenchang	CRA004775	0.814232788
ENSGALG00010011888	CAND1	2	-0.237213837	0.063081249	-3.76044929	0.000169608	0	0.434943095	0	1	CRA004775;Yang2025_Silkie_Wenchang	PRJNA827465	0.011330586
ENSGALG00010011888	CAND1	2	0.004696255	0.041058574	0.114379394	0.908937048	0	0.024566034	0	0.5	CRA004775;PRJNA827465	Yang2025_Silkie_Wenchang	0.978416761
ENSGALG00010011894	VWC2L	2	1.601879704	0.198789815	8.05815782	7.74528E-16	0	0.4185583	0	1	PRJNA827465;Yang2025_Silkie_Wenchang	CRA004775	9.22463E-13
ENSGALG00010011894	VWC2L	2	0.940449132	0.906607184	1.037328127	0.299582962	1.374847443	5.647381638	82.29267891	0.5	CRA004775;Yang2025_Silkie_Wenchang	PRJNA827465	0.79004225
ENSGALG00010011894	VWC2L	2	0.83370002	0.7729157	1.078642885	0.280746956	0.941856645	4.237057986	76.39871809	0.5	CRA004775;PRJNA827465	Yang2025_Silkie_Wenchang	0.778536485
ENSGALG00010011896	SIK1	2	-0.188017284	0.071317691	-2.636334424	0.008380709	0	0.890090088	0	1	PRJNA827465;Yang2025_Silkie_Wenchang	CRA004775	0.142771619
ENSGALG00010011896	SIK1	2	0.22223836	0.422761062	0.525683133	0.599108385	0.25826077	2.914689664	65.69103008	0.5	CRA004775;Yang2025_Silkie_Wenchang	PRJNA827465	0.894908299
ENSGALG00010011896	SIK1	2	0.16391583	0.502192505	0.326400392	0.744121441	0.402431223	4.087239826	75.53361088	0.5	CRA004775;PRJNA827465	Yang2025_Silkie_Wenchang	0.935821071
ENSGALG00010011898	ENSGALG00010011898	2	-0.166233401	0.109614341	-1.516529677	0.129385472	0.016320778	2.988068952	66.53357014	1	PRJNA827465;Yang2025_Silkie_Wenchang	CRA004775	0.603517352
ENSGALG00010011898	ENSGALG00010011898	2	-0.273471334	0.099809528	-2.739932137	0.006145187	0	0.213408646	0	1	CRA004775;Yang2025_Silkie_Wenchang	PRJNA827465	0.118568625

ENSGALG00010011898	ENSGALG00010011898	2	-0.07736014	0.066321194	-1.166446742	0.24343387	0	0.118741957	0	1	CRA004775;PRJNA827465	Yang2025_Silkie_Wenchang	0.75532339
ENSGALG00010011899	PHLPP1	2	-0.01838873	0.058529205	-0.314180419	0.753384014	0.001836778	1.308832084	23.59600497	0.5	PRJNA827465;Yang2025_Silkie_Wenchang	CRA004775	0.938087082
ENSGALG00010011899	PHLPP1	2	0.117094442	0.27087077	0.432288954	0.665531415	0.112894867	3.791544803	73.62552596	0.5	CRA004775;Yang2025_Silkie_Wenchang	PRJNA827465	0.91350776
ENSGALG00010011899	PHLPP1	2	0.156498899	0.19886369	0.786965678	0.431301958	0.054276552	2.437909196	58.981245	1	CRA004775;PRJNA827465	Yang2025_Silkie_Wenchang	0.842503929
ENSGALG00010011902	TP53I3	2	-0.004742839	0.580059586	-0.008176469	0.993476194	0.619431818	12.40866558	91.94111572	0.5	PRJNA827465;Yang2025_Silkie_Wenchang	CRA004775	0.997979742
ENSGALG00010011902	TP53I3	2	-0.08100557	0.568798701	-0.14241518	0.886752072	0.520756634	4.985177151	79.94053231	0.5	CRA004775;Yang2025_Silkie_Wenchang	PRJNA827465	0.972840662
ENSGALG00010011902	TP53I3	2	0.552989855	0.163436133	3.383522628	0.000715623	0	0.001742991	0	1	CRA004775;PRJNA827465	Yang2025_Silkie_Wenchang	0.029985218
ENSGALG00010011903	LTA4H	2	-0.131263187	0.14176002	-0.925953496	0.354470148	0.027393009	2.587060886	61.34609721	1	PRJNA827465;Yang2025_Silkie_Wenchang	CRA004775	0.81193713
ENSGALG00010011903	LTA4H	2	-0.307425018	0.156667486	-1.962277081	0.049730234	0	0.061467445	0	1	CRA004775;Yang2025_Silkie_Wenchang	PRJNA827465	0.386162809
ENSGALG00010011903	LTA4H	2	-0.034629361	0.055321025	-0.625971071	0.531333916	0	0.361614964	0	1	CRA004775;PRJNA827465	Yang2025_Silkie_Wenchang	0.87298465
ENSGALG00010011904	KIF24	2	0.376029993	0.151211879	2.486775476	0.012890673	0.000338117	1.007373309	0.731934092	1	PRJNA827465;Yang2025_Silkie_Wenchang	CRA004775	0.184486016
ENSGALG00010011904	KIF24	2	-0.206667611	0.571990084	-0.361313276	0.71786527	0.449297518	2.638333252	62.09728248	0.5	CRA004775;Yang2025_Silkie_Wenchang	PRJNA827465	0.927927766
ENSGALG00010011904	KIF24	2	-0.089131626	0.738050552	-0.120766288	0.903876153	0.86577114	4.211245904	76.25405823	0.5	CRA004775;PRJNA827465	Yang2025_Silkie_Wenchang	0.977333734
ENSGALG00010011907	BIK	2	-0.079983019	0.166894477	-0.479243054	0.631765729	0	0.013609016	0	1	PRJNA827465;Yang2025_Silkie_Wenchang	CRA004775	0.903058174
ENSGALG00010011907	BIK	2	-0.172520774	0.284797257	-0.605766978	0.5446695	0	0.113767277	0	1	CRA004775;Yang2025_Silkie_Wenchang	PRJNA827465	0.877345822
ENSGALG00010011907	BIK	2	-0.097666993	0.181688023	-0.537553281	0.590885491	0	0.203060513	0	1	CRA004775;PRJNA827465	Yang2025_Silkie_Wenchang	0.892752146
ENSGALG00010011908	ENSGALG00010011908	2	0.083244148	0.110342936	0.754413024	0.450601253	0	0.49739566	0	1	PRJNA827465;Yang2025_Silkie_Wenchang	CRA004775	0.84760941
ENSGALG00010011908	ENSGALG00010011908	2	-0.191673686	0.458358234	-0.418174415	0.675819601	0.338624848	5.143112089	80.55651942	0.5	CRA004775;Yang2025_Silkie_Wenchang	PRJNA827465	0.916522765
ENSGALG00010011908	ENSGALG00010011908	2	-0.25203425	0.349893496	-0.720317049	0.47132981	0.19813604	4.779540539	79.0774868	0.5	CRA004775;PRJNA827465	Yang2025_Silkie_Wenchang	0.85394715
ENSGALG00010011909	C8orf48	2	0.141951699	0.164954853	0.860548786	0.389486603	0	0.08202297	0	1	PRJNA827465;Yang2025_Silkie_Wenchang	CRA004775	0.828911149
ENSGALG00010011909	C8orf48	2	-0.089046727	0.265846114	-0.33495591	0.737658346	0.010134159	1.059753263	5.638412751	0.5	CRA004775;Yang2025_Silkie_Wenchang	PRJNA827465	0.934083553
ENSGALG00010011909	C8orf48	2	-0.024870141	0.316075436	-0.078684195	0.937283817	0.099741718	1.682340807	40.55901182	0.5	CRA004775;PRJNA827465	Yang2025_Silkie_Wenchang	0.985114195
ENSGALG00010011911	NUDT2	2	0.045076669	0.142899991	0.315442066	0.752426032	0	0.006388696	0	1	PRJNA827465;Yang2025_Silkie_Wenchang	CRA004775	0.93806821
ENSGALG00010011911	NUDT2	2	-0.027654439	0.17881124	-0.154657161	0.877091604	0	0.40665317	0	0.5	CRA004775;Yang2025_Silkie_Wenchang	PRJNA827465	0.970509861
ENSGALG00010011911	NUDT2	2	-0.024534491	0.157005596	-0.156267761	0.875821967	0	0.374307987	0	0.5	CRA004775;PRJNA827465	Yang2025_Silkie_Wenchang	0.970347777
ENSGALG00010011912	TCP11X2	2	0.083434759	0.065769691	1.268589787	0.204587409	0	0.238232538	0	1	PRJNA827465;Yang2025_Silkie_Wenchang	CRA004775	0.71729696
ENSGALG00010011912	TCP11X2	2	0.07224431	0.148957623	0.484999081	0.627676997	0	0.948726815	0	0.5	CRA004775;Yang2025_Silkie_Wenchang	PRJNA827465	0.901856848
ENSGALG00010011912	TCP11X2	2	0.056678694	0.068771864	0.82415527	0.409851339	0	0.631563345	0	0.5	CRA004775;PRJNA827465	Yang2025_Silkie_Wenchang	0.834652129
ENSGALG00010011913	CXXC5	2	0.063049132	0.071599249	0.880583706	0.378541181	0.004031848	1.600800198	37.53358343	0.5	PRJNA827465;Yang2025_Silkie_Wenchang	CRA004775	0.823711806
ENSGALG00010011913	CXXC5	2	-0.03231395	0.09216266	-0.35061868	0.72587444	0	0.081562989	0	1	CRA004775;Yang2025_Silkie_Wenchang	PRJNA827465	0.931029023
ENSGALG00010011913	CXXC5	2	0.113263703	0.065513506	1.72886034	0.083834094	0	0.438570204	0	0.5	CRA004775;PRJNA827465	Yang2025_Silkie_Wenchang	0.498778593
ENSGALG00010011915	RRM2B	2	-0.320857629	0.160294398	-2.001677128	0.045319468	0.030586069	2.295366098	56.43396489	1	PRJNA827465;Yang2025_Silkie_Wenchang	CRA004775	0.367104503
ENSGALG00010011915	RRM2B	2	-0.280824589	0.280640484	-1.000656019	0.316993137	0.101434613	2.703633133	63.0127332	0.5	CRA004775;Yang2025_Silkie_Wenchang	PRJNA827465	0.797004943
ENSGALG00010011915	RRM2B	2	-0.165279389	0.099631197	-1.658912006	0.097133525	0	0.600894327	0	0.5	CRA004775;PRJNA827465	Yang2025_Silkie_Wenchang	0.532520426
ENSGALG00010011916	MCAT	2	0.013520659	0.166072697	0.0814141	0.935112636	0.043412869	4.510153818	77.8278072	0.5	PRJNA827465;Yang2025_Silkie_Wenchang	CRA004775	0.984512821
ENSGALG00010011916	MCAT	2	0.01257445	0.220138623	0.057120598	0.95444128	0.064644008	2.839858452	64.78697735	0.5	CRA004775;Yang2025_Silkie_Wenchang	PRJNA827465	0.987987175
ENSGALG00010011916	MCAT	2	-0.148364866	0.075936132	-1.953811213	0.050723548	0	0.217142028	0	1	CRA004775;PRJNA827465	Yang2025_Silkie_Wenchang	0.389905652
ENSGALG00010011917	HSF2BP	2	-0.463910849	0.193000996	-2.403670744	0.016231385	0.012329714	1.189282452	15.91568526	1	PRJNA827465;Yang2025_Silkie_Wenchang	CRA004775	0.210541957
ENSGALG00010011917	HSF2BP	2	-0.246767575	0.232774233	-1.060115511	0.289092052	0	0.012685403	0	1	CRA004775;Yang2025_Silkie_Wenchang	PRJNA827465	0.783530627
ENSGALG00010011917	HSF2BP	2	-0.544262358	0.196057558	-2.776033543	0.005502653	0	0.526538318	0	1	CRA004775;PRJNA827465	Yang2025_Silkie_Wenchang	0.111307656
ENSGALG00010011921	MEGF10	2	-0.030166551	0.198717308	-0.15180636	0.879339666	0.061377052	4.456158079	77.5591444	0.5	PRJNA827465;Yang2025_Silkie_Wenchang	CRA004775	0.971291607
ENSGALG00010011921	MEGF10	2	0.196448671	0.488941703	0.401783423	0.687843425	0.406498985	6.188986225	83.84226489	0.5	CRA004775;Yang2025_Silkie_Wenchang	PRJNA827465	0.919631238
ENSGALG00010011921	MEGF10	2	0.350064434	0.274689469	1.274400636	0.202521467	0.097423097	2.302529183	56.56949726	1	CRA004775;PRJNA827465	Yang2025_Silkie_Wenchang	0.715289846
ENSGALG00010011922	PIK3R1	2	0.05998143	0.191457438	0.313288585	0.754061421	0.053036146	3.343657849	70.09263372	0.5	PRJNA827465;Yang2025_Silkie_Wenchang	CRA004775	0.938385954
ENSGALG00010011922	PIK3R1	2	0.280585922	0.168602896	1.664182096	0.096076045	0	0.008927224	0	1	CRA004775;Yang2025_Silkie_Wenchang	PRJNA827465	0.530362009
ENSGALG00010011922	PIK3R1	2	-0.077779921	0.093212191	-0.834439354	0.404033448	0	0.845671272	0	0.5	CRA004775;PRJNA827465	Yang2025_Silkie_Wenchang	0.833112102
ENSGALG00010011923	BARD1	2	-0.260697036	0.238148752	-1.094681515	0.27365619	0.094410246	5.72094333	82.52036522	1	PRJNA827465;Yang2025_Silkie_Wenchang	CRA004775	0.773413073
ENSGALG00010011923	BARD1	2	-0.570823903	0.185843683	-3.071527064	0.002129669	0.006342145	1.022689272	2.21858907	1	CRA004775;Yang2025_Silkie_Wenchang	PRJNA827465	0.060478586
ENSGALG00010011923	BARD1	2	-0.450652502	0.580332173	-0.776542338	0.437428833	0.493024367	2.833214903	64.70440703	1	CRA004775;PRJNA827465	Yang2025_Silkie_Wenchang	0.843792168
ENSGALG00010011925	RRP1B	2	-0.106493319	0.173870147	-0.612487658	0.540215161	0.055396827	11.81921574	91.53920174	0.5	PRJNA827465;Yang2025_Silkie_Wenchang	CRA004775	0.875296882
ENSGALG00010011925	RRP1B	2	0.002635872	0.31437678	0.008384436	0.993310266	0.175022612	8.255213017	87.8864422	0.5	CRA004775;Yang2025_Silkie_Wenchang	PRJNA827465	0.997979742
ENSGALG00010011925	RRP1B	2	0.136694359	0.124694071	1.096237841	0.272974704	0.017809721	1.798902342	44.41054545	1	CRA004775;PRJNA827465	Yang2025_Silkie_Wenchang	0.773799118
ENSGALG00010011926	DYRK2	2	-0.131012515	0.063635308	-2.058802231	0.039513188	0.000550982	1.069873548	6.531009951	1	PRJNA827465;Yang2025_Silkie_Wenchang	CRA004775	0.343774486
ENSGALG00010011926	DYRK2	2	0.406297349	0.501952104	0.809434498	0.418262566	0.453405839	9.323036856	89.2738813	0.5	CRA004775;Yang2025_Silkie_Wenchang	PRJNA827465	0.883812956
ENSGALG00010011926	DYRK2	2	0.344953252	0.567397372	0.607957086	0.543215937	0.594444693	12.26084281	91.84395383	0.5	CRA004775;PRJNA827465	Yang2025_Silkie_Wenchang	0.876968972
ENSGALG00010011929	ELK3	2	0.019206817	0.059578034	0.322380848	0.747164192	0	0.05354928	0	1	PRJNA827465;Yang2025_Silkie_Wenchang	CRA004775	0.93678921
ENSGALG00010011929	ELK3	2	-0.053705541	0.178917521	-0.300169267	0.764048047	0.020907362	1.328663787	24.73641489	0.5	CRA004775;Yang2025_Silkie_Wenchang	PRJNA827465	0.940475427
ENSGALG00010011929	ELK3	2	-0.038529047	0.12900601	-0.29866081	0.765198816	0.015081344	1.275536454	21.60161345	0.5	CRA004775;PRJNA827465	Yang2025_Silkie_Wenchang	0.940779391
ENSGALG00010011930	TSPO	2	-0.144974724	0.167907029	-0.863422606	0.387905155	0.039090708	3.054097408	67.25710197	1	PRJNA827465;Yang2025_Silkie_Wenchang	CRA004775	0.828154121
ENSGALG00010011930	TSPO	2	-0.23839259	0.129705258	-1.837956252	0.066068852	0	0.926040388	0	1	CRA004775;Yang2025_Silkie_Wenchang	PRJNA827465	0.442444044
ENSGALG00010011930	TSPO	2	-0.020161133	0.08564687	-0.235398165	0.813909955	0	0.158012293	0	1	CRA004775;PRJNA827465	Yang2025_Silkie_Wenchang	0.953691515
ENSGALG00010011931	ENSGALG00010011931	2	-2.849620247	2.406481462	-1.18414386	0.236356121	10.17795862	8.247460657	87.87505583	1	PRJNA827465;Yang2025_Silkie_Wenchang	CRA004775	0.748669015
ENSGALG00010011931	ENSGALG00010011931	2	-2.432281944	2.80377731	-0.867501829	0.38566711	14.43024764	12.16170976	91.77747192	0.5	CRA004775;Yang2025_Silkie_Wenchang	PRJNA827465	0.82648313
ENSGALG00010011931	ENSGALG00010011931	2	-0.010947351	0.801965269	-0.013650655	0.989108692	0	0.243688324	0	0.5	CRA004775;PRJNA827465	Yang2025_Silkie_Wenchang	0.997012807
ENSGALG00010011932	VAV3	2	0.028535475	0.216522551	0.131789654	0.895150512	0.065001978	3.241798327	69.15292381	0.5	PRJNA827465;Yang2025_Silkie_Wenchang	CRA004775	0.974654419
ENSGALG00010011932	VAV3	2	-0.158491637	0.14998709	-1.056701856	0.290647678	0	0.198062424	0	0.5	CRA004775;Yang2025_Silkie_Wenchang	PRJNA827465	0.784622961
ENSGALG00010011932	VAV3	2	0.238067455	0.174048886	1.367819467	0.171368593	0	0.096345203	0	1	CRA004775;PRJNA827465	Yang2025_Silkie_Wenchang	0.673441752
ENSGALG00010011935	ST8SIA4	2	0.087327411	0.091433729	0.955089682	0.339532346	0	0.936124493	0	0.5	PRJNA827465;Yang2025_Silkie_Wenchang	CRA004775	0.80490319
ENSGALG00010011935	ST8SIA4	2	-0.108937173	0.159219372	-0.684195472	0.493851741	0	0.886878138	0	1	CRA004775;Yang2025_Silkie_Wenchang	PRJNA827465	0.860175061
ENSGALG00010011935	ST8SIA4	2	-0.033760174	0.288271189	-0.117112549	0.906770866	0.102810367	1.971743858	49.28347332	0.5	CRA004775;PRJNA827465	Yang2025_Silkie_Wenchang	0.978196186
ENSGALG00010011936	CKAP4	2	-0.123275672	0.188787903	-0.652985019	0.513765937	0.064596353	10.65800242	90.61737875	0.5	PRJNA827465;Yang2025_Silkie_Wenchang	CRA004775	0.866584405
ENSGALG00010011936	CKAP4	2	-0.29915613	0.077441416	-3.862999206	0.000112005	0	0.594340758	0	0.5	CRA004775;Yang2025_Silkie_Wenchang	PRJNA827465	0.008421586
ENSGALG00010011936	CKAP4	2	0.064166855	0.082753968	0.775393083	0.438107421	0	0.021928149	0	1	CRA004775;PRJNA827465	Yang2025_Silkie_Wenchang	0.843979791
ENSGALG00010011937	SLC1A1	2	-1.031444982	0.632576202	-1.63054661	0.102986023	0.531488769	2.969388172	66.32302879	1	PRJNA827465;Yang2025_Silkie_Wenchang	CRA004775	0.547128906
ENSGALG00010011937	SLC1A1	2	0.04414918	1.723888614	0.025606052	0.979571559	5.671185954	21.81047269	95.41504664	0.5	CRA004775;Yang2025_Silkie_Wenchang	PRJNA827465	0.994472942
ENSGALG00010011937	SLC1A1	2	0.668244526	1.090910649	0.612556607	0.540169557	2.13678416	9.779229306	89.7742453	0.5	CRA004775;PRJNA827465	Yang2025_Silkie_Wenchang	0.87529516
ENSGALG00010011941	ENSGALG00010011941	2	0.037417244	0.12707371	0.294453068	0.768411721	0	0.197138585	0	1	PRJNA827465;Yang2025_Silkie_Wenchang	CRA004775	0.941444269
ENSGALG00010011941	ENSGALG00010011941	2	0.007794091	0.270901797	0.028770908	0.977047304	0.023784873	1.108239267	9.766777813	0.5	CRA004775;Yang2025_Silkie_Wenchang	PRJNA827465	0.993374814
ENSGALG00010011941	ENSGALG00010011941	2	-0.025269529	0.142116924	-0.177808018	0.858873751	0	0.81324835	0	0.5	CRA004775;PRJNA827465	Yang2025_Silkie_Wenchang	0.964521259
ENSGALG00010011943	TTLL12	2	-0.046115863	0.046239829	-0.997319074	0.318609658	0	0.01109056	0	1	PRJNA827465;Yang2025_Silkie_Wenchang	CRA004775	0.797754958
ENSGALG00010011943	TTLL12	2	-0.19249462	0.19446549	-0.989865191	0.322240016	0.050239947	2.468576748	59.49082803	1	CRA004775;Yang2025_Silkie_Wenchang	PRJNA827465	0.798513866
ENSGALG00010011943	TTLL12	2	-0.184341003	0.199045918	-0.926123002	0.354382061	0.056542503	2.750828344	63.64731365	1	CRA004775;PRJNA827465	Yang2025_Silkie_Wenchang	0.81193713
ENSGALG00010011947	THAP1	2	0.111914674	0.154185132	0.725848083	0.467933133	0.017727788	1.534886037	34.84858318	0.5	PRJNA827465;Yang2025_Silkie_Wenchang	CRA004775	0.885315491
ENSGALG00010011947	THAP1	2	0.222907192	0.136162981	1.637061632	0.101617592	0	0.288620682	0	1	CRA004775;Yang2025_Silkie_Wenchang	PRJNA827465	0.543686036
ENSGALG00010011947	THAP1	2	0.060063782	0.11048227	0.54365087	0.586681747	0	0.563628893	0	0.5	CRA004775;PRJNA827465	Yang2025_Silkie_Wenchang	0.891596903
ENSGALG00010011950	PDXK	2	-0.171461922	0.101201237	-1.694267055	0.090214548	0	0.046748176	0	1	PRJNA827465;Yang2025_Silkie_Wenchang	CRA004775	0.515738527
ENSGALG00010011950	PDXK	2	-0.219635936	0.156312056	-1.405111939	0.159987995	0	0.092044149	0	1	CRA004775;Yang2025_Silkie_Wenchang	PRJNA827465	0.655404348
ENSGALG00010011950	PDXK	2	-0.170279987	0.121185915	-1.405113684	0.159987476	0	0.173925087	0	1	CRA004775;PRJNA827465	Yang2025_Silkie_Wenchang	0.655404348
ENSGALG00010011952	UBE2D2	2	-0.139698557	0.137738646	-1.014229197	0.3104734	0.031500229	5.810565273	82.78997046	1	PRJNA827465;Yang2025_Silkie_Wenchang	CRA004775	0.794899969
ENSGALG00010011952	UBE2D2	2	-0.060570911	0.257911945	-0.234851128	0.814324283	0.108279819	4.956032875	79.8225713	0.5	CRA004775;Yang2025_Silkie_Wenchang	PRJNA827465	0.953869515
ENSGALG00010011952	UBE2D2	2	0.028518949	0.088230329	0.323232945	0.746518832	0.004637975	1.182763959	15.45227666	0.5	CRA004775;PRJNA827465	Yang2025_Silkie_Wenchang	0.936668296
ENSGALG00010011953	CDK17	2	-0.077863027	0.045990207	-1.693034937	0.090448817	0	0.004951024	0	1	PRJNA827465;Yang2025_Silkie_Wenchang	CRA004775	0.516101897
ENSGALG00010011953	CDK17	2	0.131961328	0.229393225	0.575262535	0.565113755	0.079919513	3.960287168	74.74930586	0.5	CRA004775;Yang2025_Silkie_Wenchang	PRJNA827465	0.883427937
ENSGALG00010011953	CDK17	2	0.116253197	0.23222848	0.500598362	0.616653816	0.089647812	5.18129369	80.69980086	0.5	CRA004775;PRJNA827465	Yang2025_Silkie_Wenchang	0.899111936
ENSGALG00010011957	ENSGALG00010011957	2	-0.377428714	0.153622112	-2.456864494	0.014015554	0.030254516	2.731561955	63.39090906	1	PRJNA827465;Yang2025_Silkie_Wenchang	CRA004775	0.193842584
ENSGALG00010011957	ENSGALG00010011957	2	-0.278110208	0.339333654	-0.819577442	0.412457038	0.165576921	3.151881311	68.27291698	0.5	CRA004775;Yang2025_Silkie_Wenchang	PRJNA827465	0.835702455
ENSGALG00010011957	ENSGALG00010011957	2	-0.198922005	0.118245424	-1.682280785	0.092514405	0.003148124	1.043767634	4.19323543	1	CRA004775;PRJNA827465	Yang2025_Silkie_Wenchang	0.520781105
ENSGALG00010011960	CFAP54	2	0.377471222	0.339699416	1.111191848	0.266485779	0.167788003	3.631128393	72.46035139	1	PRJNA827465;Yang2025_Silkie_Wenchang	CRA004775	0.770699087
ENSGALG00010011960	CFAP54	2	0.725772704	0.266773553	2.720557175	0.0065172	0	0.022480032	0	1	CRA004775;Yang2025_Silkie_Wenchang	PRJNA827465	0.123206115
ENSGALG00010011960	CFAP54	2	0.084207182	0.218715067	0.3850086	0.700231044	0	0.288277815	0	1	CRA004775;PRJNA827465	Yang2025_Silkie_Wenchang	0.922299095
ENSGALG00010011961	AGPAT3	2	-0.133668791	0.15785449	-0.846784854	0.397115052	0.042122243	6.439168001	84.47004334	0.5	PRJNA827465;Yang2025_Silkie_Wenchang	CRA004775	0.831438925
ENSGALG00010011961	AGPAT3	2	-0.290436083	0.089119672	-3.25894472	0.001118275	0	0.024529299	0	1	CRA004775;Yang2025_Silkie_Wenchang	PRJNA827465	0.040433027
ENSGALG00010011961	AGPAT3	2	-0.003134361	0.07714321	-0.040630422	0.967590531	0	0.980414767	0	0.5	CRA004775;PRJNA827465	Yang2025_Silkie_Wenchang	0.991065147
ENSGALG00010011962	CPSF6	2	0.073785749	0.155251994	0.475264424	0.634598513	0.034534935	2.848113227	64.88903635	0.5	PRJNA827465;Yang2025_Silkie_Wenchang	CRA004775	0.903872221
ENSGALG00010011962	CPSF6	2	0.088574836	0.194238927	0.456009708	0.648382997	0.042073052	2.258970572	55.73204837	0.5	CRA004775;Yang2025_Silkie_Wenchang	PRJNA827465	0.90762535
ENSGALG00010011962	CPSF6	2	-0.043551105	0.046771171	-0.931152758	0.351774551	0	0.113648736	0	1	CRA004775;PRJNA827465	Yang2025_Silkie_Wenchang	0.810678834
ENSGALG00010011963	ZCCHC2	2	0.199710849	0.21571237	0.925820107	0.354539476	0.077807175	5.40494141	81.49841184	1	PRJNA827465;Yang2025_Silkie_Wenchang	CRA004775	0.81193713
ENSGALG00010011963	ZCCHC2	2	0.426770089	0.146802688	2.907099971	0.003647966	0	0.060514505	0	1	CRA004775;Yang2025_Silkie_Wenchang	PRJNA827465	0.086111715
ENSGALG00010011963	ZCCHC2	2	0.112909496	0.159994942	0.705706658	0.48037058	0.032501444	1.996085507	49.90194576	1	CRA004775;PRJNA827465	Yang2025_Silkie_Wenchang	0.856585676
ENSGALG00010011964	ENSGALG00010011964	2	-5.370436606	5.570040434	-0.964164743	0.334963337	56.1627173	10.35289099	90.34086227	1	PRJNA827465;Yang2025_Silkie_Wenchang	CRA004775	0.803101658
ENSGALG00010011964	ENSGALG00010011964	2	-5.592202382	5.237614398	-1.067700284	0.28565573	50.03912224	10.87782863	90.80698884	1	CRA004775;Yang2025_Silkie_Wenchang	PRJNA827465	0.781343668
ENSGALG00010011964	ENSGALG00010011964	2	-0.492367021	0.999587893	-0.492570012	0.622316441	0	0.089802241	0	1	CRA004775;PRJNA827465	Yang2025_Silkie_Wenchang	0.900093208
ENSGALG00010011965	ATIC	2	-0.073444997	0.254928924	-0.288099898	0.773270271	0.102517147	4.531682173	77.93313913	0.5	PRJNA827465;Yang2025_Silkie_Wenchang	CRA004775	0.943230857
ENSGALG00010011965	ATIC	2	-0.430680193	0.150386534	-2.863821523	0.004185638	0	0.255000751	0	1	CRA004775;Yang2025_Silkie_Wenchang	PRJNA827465	0.094197388
ENSGALG00010011965	ATIC	2	-0.152957994	0.331568431	-0.461316517	0.644571537	0.190120378	7.141898374	85.99812056	0.5	CRA004775;PRJNA827465	Yang2025_Silkie_Wenchang	0.906953008
ENSGALG00010011966	SLC25A24	2	0.025723276	0.222753667	0.115478573	0.908065803	0.093118252	16.10666016	93.79138822	0.5	PRJNA827465;Yang2025_Silkie_Wenchang	CRA004775	0.978196186
ENSGALG00010011966	SLC25A24	2	-0.111568175	0.147913779	-0.754278439	0.450682046	0.021761973	1.647627635	39.30667472	0.5	CRA004775;Yang2025_Silkie_Wenchang	PRJNA827465	0.847631399
ENSGALG00010011966	SLC25A24	2	0.236542793	0.061745054	3.830959367	0.000127645	0	0.202036841	0	1	CRA004775;PRJNA827465	Yang2025_Silkie_Wenchang	0.009273226
ENSGALG00010011967	MTMR9	2	-0.394004505	0.221357102	-1.77994969	0.075084195	0.091344052	14.43325871	93.07155771	1	PRJNA827465;Yang2025_Silkie_Wenchang	CRA004775	0.472846355
ENSGALG00010011967	MTMR9	2	-0.378526959	0.290661776	-1.302293559	0.192816053	0.134190584	4.385913968	77.19973517	1	CRA004775;Yang2025_Silkie_Wenchang	PRJNA827465	0.702807541
ENSGALG00010011967	MTMR9	2	-0.17486175	0.05374179	-3.253738863	0.001138969	0	0.299646794	0	1	CRA004775;PRJNA827465	Yang2025_Silkie_Wenchang	0.040918564
ENSGALG00010011968	ENSGALG00010011968	2	0.058000981	0.282858903	0.205052697	0.837530954	0.081245029	2.024010088	50.59313162	0.5	PRJNA827465;Yang2025_Silkie_Wenchang	CRA004775	0.958938418
ENSGALG00010011968	ENSGALG00010011968	2	0.385905248	0.262654054	1.469252965	0.141764189	0	0.038600387	0	1	CRA004775;Yang2025_Silkie_Wenchang	PRJNA827465	0.627129189
ENSGALG00010011968	ENSGALG00010011968	2	-0.035901915	0.299001541	-0.120072675	0.904425578	0.049400562	1.261360334	20.72051317	0.5	CRA004775;PRJNA827465	Yang2025_Silkie_Wenchang	0.977606588
ENSGALG00010011969	TMEM173	2	0.3214164	0.338054671	0.950782305	0.341714897	0.169465065	3.715703024	73.08719256	1	PRJNA827465;Yang2025_Silkie_Wenchang	CRA004775	0.805823616
ENSGALG00010011969	TMEM173	2	0.378325297	0.293071456	1.290897799	0.196739119	0.100932934	2.390094531	58.16065068	1	CRA004775;Yang2025_Silkie_Wenchang	PRJNA827465	0.707994211
ENSGALG00010011969	TMEM173	2	0.057065967	0.145957298	0.390977137	0.695814135	0	0.093877073	0	1	CRA004775;PRJNA827465	Yang2025_Silkie_Wenchang	0.921038437
ENSGALG00010011971	RNF170	2	-0.09595996	0.068846341	-1.393828038	0.163369539	0	0.01508056	0	1	PRJNA827465;Yang2025_Silkie_Wenchang	CRA004775	0.661683383
ENSGALG00010011971	RNF170	2	-0.031165503	0.113066353	-0.275639054	0.782825311	0	0.480889297	0	0.5	CRA004775;Yang2025_Silkie_Wenchang	PRJNA827465	0.945507994
ENSGALG00010011971	RNF170	2	-0.076378486	0.074795898	-1.021158766	0.307179225	0	0.733569538	0	0.5	CRA004775;PRJNA827465	Yang2025_Silkie_Wenchang	0.79266003
ENSGALG00010011972	IPO11	2	0.088817288	0.101861986	0.871937533	0.383242457	0	0.004611698	0	1	PRJNA827465;Yang2025_Silkie_Wenchang	CRA004775	0.825476201
ENSGALG00010011972	IPO11	2	0.054282386	0.115274796	0.470895529	0.637715341	0	0.108318392	0	1	CRA004775;Yang2025_Silkie_Wenchang	PRJNA827465	0.905279419
ENSGALG00010011972	IPO11	2	0.062545989	0.116600657	0.536411979	0.591673854	0	0.147387949	0	1	CRA004775;PRJNA827465	Yang2025_Silkie_Wenchang	0.892776493
ENSGALG00010011973	TRAPPC10	2	-0.066310158	0.044665318	-1.484600616	0.137649652	0	0.157163551	0	1	PRJNA827465;Yang2025_Silkie_Wenchang	CRA004775	0.620631442
ENSGALG00010011973	TRAPPC10	2	0.068993644	0.155577733	0.443467343	0.657427735	0.029348126	2.143534868	53.34808801	0.5	CRA004775;Yang2025_Silkie_Wenchang	PRJNA827465	0.910711339
ENSGALG00010011973	TRAPPC10	2	0.050637802	0.176288805	0.287243436	0.77392593	0.044756521	2.881204471	65.2922932	0.5	CRA004775;PRJNA827465	Yang2025_Silkie_Wenchang	0.943248868
ENSGALG00010011976	TNFRSF11A	2	-0.125672869	0.178357766	-0.704611142	0.481052263	0.047511026	3.731049381	73.19788891	0.5	PRJNA827465;Yang2025_Silkie_Wenchang	CRA004775	0.856783731
ENSGALG00010011976	TNFRSF11A	2	-0.032283008	0.325335663	-0.099229846	0.920955778	0.168646544	4.784191754	79.09782777	0.5	CRA004775;Yang2025_Silkie_Wenchang	PRJNA827465	0.981469977
ENSGALG00010011976	TNFRSF11A	2	0.082669873	0.113158699	0.730565777	0.465044422	0.007406035	1.209005924	17.28741936	1	CRA004775;PRJNA827465	Yang2025_Silkie_Wenchang	0.852263402
ENSGALG00010011977	GATD3A	2	0.031289497	0.05320718	0.588069067	0.556485935	0	0.556117333	0	0.5	PRJNA827465;Yang2025_Silkie_Wenchang	CRA004775	0.880324407
ENSGALG00010011977	GATD3A	2	-0.025557747	0.158333293	-0.161417393	0.871764669	0	0.688297559	0	0.5	CRA004775;Yang2025_Silkie_Wenchang	PRJNA827465	0.969462602
ENSGALG00010011977	GATD3A	2	0.048275701	0.054911495	0.879154737	0.379317383	0	0.262182319	0	1	CRA004775;PRJNA827465	Yang2025_Silkie_Wenchang	0.824165982
ENSGALG00010011978	ENSGALG00010011978	2	-3.13133469	3.166049862	-0.989035179	0.32264593	14.18381706	3.295563393	69.65617465	1	PRJNA827465;Yang2025_Silkie_Wenchang	CRA004775	0.798652022
ENSGALG00010011978	ENSGALG00010011978	2	-3.128552901	2.979017789	-1.050196112	0.293627957	13.05445994	3.453712549	71.04565056	1	CRA004775;Yang2025_Silkie_Wenchang	PRJNA827465	0.786979185
ENSGALG00010011978	ENSGALG00010011978	2	-0.546234556	1.123352401	-0.486253963	0.626787116	0	0.018305352	0	1	CRA004775;PRJNA827465	Yang2025_Silkie_Wenchang	0.901508234
ENSGALG00010011979	NEDD1	2	-0.072260307	0.170572434	-0.423634147	0.671832648	0.054505426	15.77553185	93.6610695	0.5	PRJNA827465;Yang2025_Silkie_Wenchang	CRA004775	0.914674175
ENSGALG00010011979	NEDD1	2	-0.222747823	0.060264106	-3.696193909	0.000218856	0	0.825600071	0	1	CRA004775;Yang2025_Silkie_Wenchang	PRJNA827465	0.01350344
ENSGALG00010011979	NEDD1	2	0.072518816	0.061647516	1.176346127	0.23945659	0.001209475	1.080087437	7.414903104	0.5	CRA004775;PRJNA827465	Yang2025_Silkie_Wenchang	0.751706259
ENSGALG00010011982	STK3	2	-0.29398436	0.180705638	-1.626868778	0.10376497	0.057883416	8.53657144	88.28569518	1	PRJNA827465;Yang2025_Silkie_Wenchang	CRA004775	0.548071327
ENSGALG00010011982	STK3	2	-0.190608892	0.331732269	-0.574586526	0.565570967	0.185728683	5.981525039	83.28185549	0.5	CRA004775;Yang2025_Silkie_Wenchang	PRJNA827465	0.883487517
ENSGALG00010011982	STK3	2	-0.068170137	0.118292888	-0.576282635	0.564424158	0.013539959	1.407866254	28.97052562	0.5	CRA004775;PRJNA827465	Yang2025_Silkie_Wenchang	0.883094571
ENSGALG00010011983	LYZ	2	0.287096116	0.359962032	0.797573329	0.425118133	0	0.344679276	0	1	PRJNA827465;Yang2025_Silkie_Wenchang	CRA004775	0.84125685
ENSGALG00010011983	LYZ	2	0.502734909	0.303082617	1.658738845	0.097168428	0.018256162	1.098647149	8.978965579	1	CRA004775;Yang2025_Silkie_Wenchang	PRJNA827465	0.532637481
ENSGALG00010011983	LYZ	2	0.655995124	0.290008487	2.261985952	0.023698271	0	0.117184981	0	1	CRA004775;PRJNA827465	Yang2025_Silkie_Wenchang	0.262814096
ENSGALG00010011984	FN1	2	-0.269618333	0.120892122	-2.230239071	0.025731576	0.001275662	1.037434514	3.608373692	1	PRJNA827465;Yang2025_Silkie_Wenchang	CRA004775	0.273479751
ENSGALG00010011984	FN1	2	-0.134647132	0.197467197	-0.681870881	0.495320599	0	0.29012041	0	1	CRA004775;Yang2025_Silkie_Wenchang	PRJNA827465	0.860712067
ENSGALG00010011984	FN1	2	-0.347204272	0.132697204	-2.616515348	0.008883237	0	7.32E-07	0	1	CRA004775;PRJNA827465	Yang2025_Silkie_Wenchang	0.147645798
ENSGALG00010011988	HINTW	2	-5.579931412	5.603081685	-0.995868296	0.319314141	57.91355757	12.59781772	92.06211725	1	PRJNA827465;Yang2025_Silkie_Wenchang	CRA004775	0.798079048
ENSGALG00010011988	HINTW	2	-5.605571396	5.527756903	-1.014077047	0.31054599	56.77573562	13.60304385	92.64870413	1	CRA004775;Yang2025_Silkie_Wenchang	PRJNA827465	0.794899969
ENSGALG00010011988	HINTW	2	-0.307480396	0.90729698	-0.338897189	0.734687182	0	0.005981776	0	1	CRA004775;PRJNA827465	Yang2025_Silkie_Wenchang	0.933520234
ENSGALG00010011989	H1-010	2	-0.163506133	0.145938552	-1.120376563	0.262553328	0	0.058943758	0	1	PRJNA827465;Yang2025_Silkie_Wenchang	CRA004775	0.768418419
ENSGALG00010011989	H1-010	2	-0.20128691	0.228626476	-0.8804182	0.378632801	0	0.015421581	0	1	CRA004775;Yang2025_Silkie_Wenchang	PRJNA827465	0.823771464
ENSGALG00010011989	H1-010	2	-0.144445828	0.15784333	-0.915121524	0.360127837	0	0.001777373	0	1	CRA004775;PRJNA827465	Yang2025_Silkie_Wenchang	0.814880744
ENSGALG00010011991	CSNK1A1	2	-0.261592982	0.197536834	-1.324274451	0.185411912	0.07548807	30.40477948	96.7110434	1	PRJNA827465;Yang2025_Silkie_Wenchang	CRA004775	0.693865902
ENSGALG00010011991	CSNK1A1	2	-0.247907232	0.241807067	-1.025227407	0.305255899	0.100604147	6.554930496	84.74430811	0.5	CRA004775;Yang2025_Silkie_Wenchang	PRJNA827465	0.79225794
ENSGALG00010011991	CSNK1A1	2	-0.062514061	0.038278218	-1.633149711	0.102437514	0	0.248668287	0	0.5	CRA004775;PRJNA827465	Yang2025_Silkie_Wenchang	0.545320547
ENSGALG00010011992	YEATS4	2	-0.149328879	0.064228965	-2.324946072	0.020074844	0	0.345903227	0	1	PRJNA827465;Yang2025_Silkie_Wenchang	CRA004775	0.238297069
ENSGALG00010011992	YEATS4	2	-0.127323996	0.130340842	-0.976854181	0.328641348	0.006146081	1.198205081	16.54183279	0.5	CRA004775;Yang2025_Silkie_Wenchang	PRJNA827465	0.800761292
ENSGALG00010011992	YEATS4	2	-0.110427417	0.067940715	-1.625349645	0.104088079	0	0.680762958	0	0.5	CRA004775;PRJNA827465	Yang2025_Silkie_Wenchang	0.548387904
ENSGALG00010011993	RELCH	2	-0.199273563	0.109141891	-1.825821069	0.067877219	0.018544282	4.408992915	77.31908353	1	PRJNA827465;Yang2025_Silkie_Wenchang	CRA004775	0.44912093
ENSGALG00010011993	RELCH	2	-0.301223824	0.083124793	-3.623754272	0.000290357	0	0.555906272	0	1	CRA004775;Yang2025_Silkie_Wenchang	PRJNA827465	0.016635452
ENSGALG00010011993	RELCH	2	-0.097740579	0.058029064	-1.684338365	0.092116295	0	0.008451556	0	1	CRA004775;PRJNA827465	Yang2025_Silkie_Wenchang	0.519672986
ENSGALG00010011996	LRRC70	2	0.131445268	0.14082169	0.933416349	0.350605042	0	0.222195116	0	1	PRJNA827465;Yang2025_Silkie_Wenchang	CRA004775	0.810111037
ENSGALG00010011996	LRRC70	2	0.077995633	0.17484908	0.446074028	0.655543773	0	0.003443848	0	1	CRA004775;Yang2025_Silkie_Wenchang	PRJNA827465	0.910411938
ENSGALG00010011996	LRRC70	2	0.193404497	0.199180551	0.971000912	0.331547825	0	0.033187195	0	1	CRA004775;PRJNA827465	Yang2025_Silkie_Wenchang	0.801798188
ENSGALG00010011997	DENND5B	2	-0.19933323	0.095152129	-2.094889858	0.036180785	0	0.428904651	0	1	PRJNA827465;Yang2025_Silkie_Wenchang	CRA004775	0.328182178
ENSGALG00010011997	DENND5B	2	-0.084487112	0.167243632	-0.505173867	0.613436724	0	4.56137E-05	0	1	CRA004775;Yang2025_Silkie_Wenchang	PRJNA827465	0.898256044
ENSGALG00010011997	DENND5B	2	-0.216375601	0.101818933	-2.125101825	0.033578117	0	0.207844256	0	1	CRA004775;PRJNA827465	Yang2025_Silkie_Wenchang	0.314626202
ENSGALG00010011998	MPPED1	2	-0.035237395	0.073654663	-0.478413636	0.63235583	0	0.04582996	0	1	PRJNA827465;Yang2025_Silkie_Wenchang	CRA004775	0.903058174
ENSGALG00010011998	MPPED1	2	0.106697789	0.22799191	0.467989362	0.639792197	0.06560717	2.247162359	55.4994326	0.5	CRA004775;Yang2025_Silkie_Wenchang	PRJNA827465	0.906064918
ENSGALG00010011998	MPPED1	2	0.115158328	0.207980505	0.553697705	0.579785746	0.051412129	1.989903669	49.74631106	0.5	CRA004775;PRJNA827465	Yang2025_Silkie_Wenchang	0.888013685
ENSGALG00010012001	EEIG2	2	0.12331072	0.121783968	1.012536562	0.311281574	0	0.589022376	0	1	PRJNA827465;Yang2025_Silkie_Wenchang	CRA004775	0.795416404
ENSGALG00010012001	EEIG2	2	-0.108419889	0.405269792	-0.267525217	0.789064801	0.250081952	3.983211599	74.89463025	0.5	CRA004775;Yang2025_Silkie_Wenchang	PRJNA827465	0.947210344
ENSGALG00010012001	EEIG2	2	-0.174963133	0.299949745	-0.583308158	0.559685872	0.118925694	2.590548802	61.39814084	0.5	CRA004775;PRJNA827465	Yang2025_Silkie_Wenchang	0.881203589
ENSGALG00010012004	LGR5	2	-0.346415806	0.357784392	-0.968225038	0.332931993	0.203065894	4.728138836	78.85002884	1	PRJNA827465;Yang2025_Silkie_Wenchang	CRA004775	0.802178578
ENSGALG00010012004	LGR5	2	-0.335102633	0.560905883	-0.597431126	0.550219578	0.398541627	2.293576676	56.39997518	0.5	CRA004775;Yang2025_Silkie_Wenchang	PRJNA827465	0.878679315
ENSGALG00010012004	LGR5	2	0.013264653	0.180626814	0.073436789	0.941458543	0	0.384893578	0	0.5	CRA004775;PRJNA827465	Yang2025_Silkie_Wenchang	0.985827325

ENSGALG00010012007	MREG	2	-0.160993513	0.129505331	-1.243141977	0.21381552	0	0.353708671	0	1	PRJNA827465;Yang2025_Silkie_Wenchang	CRA004775	0.72651434
ENSGALG00010012007	MREG	2	-0.239645515	0.193602064	-1.237825202	0.215780882	0	0.062587476	0	1	CRA004775;Yang2025_Silkie_Wenchang	PRJNA827465	0.729213578
ENSGALG00010012007	MREG	2	-0.107040932	0.152531289	-0.701763767	0.482826498	0	0.005809874	0	1	CRA004775;PRJNA827465	Yang2025_Silkie_Wenchang	0.85691916
ENSGALG00010012008	ZNF366	2	-0.10858853	0.155552846	-0.698081283	0.485126363	0.024943638	1.959304901	48.96149142	0.5	PRJNA827465;Yang2025_Silkie_Wenchang	CRA004775	0.857004733
ENSGALG00010012008	ZNF366	2	-0.249309648	0.182449692	-1.366456938	0.171795588	0	0.724286816	0	0.5	CRA004775;Yang2025_Silkie_Wenchang	PRJNA827465	0.674398921
ENSGALG00010012008	ZNF366	2	0.023439358	0.118013439	0.198616006	0.842563132	0	0.098756008	0	1	CRA004775;PRJNA827465	Yang2025_Silkie_Wenchang	0.960252241
ENSGALG00010012010	HENMT1	2	0.065505932	0.152267233	0.430203732	0.667047448	0	0.656093854	0	0.5	PRJNA827465;Yang2025_Silkie_Wenchang	CRA004775	0.913766816
ENSGALG00010012010	HENMT1	2	0.143424938	0.236296546	0.606970099	0.543870755	0	0.602879315	0	0.5	CRA004775;Yang2025_Silkie_Wenchang	PRJNA827465	0.876968972
ENSGALG00010012010	HENMT1	2	-0.038513295	0.171443095	-0.224641858	0.822257899	0	0.098398602	0	1	CRA004775;PRJNA827465	Yang2025_Silkie_Wenchang	0.955677851
ENSGALG00010012011	HOOK3	2	0.051312114	0.241027933	0.212888662	0.831413806	0.104221621	9.472456459	89.44307631	0.5	PRJNA827465;Yang2025_Silkie_Wenchang	CRA004775	0.957626151
ENSGALG00010012011	HOOK3	2	0.542934548	0.261363164	2.077318546	0.037772168	0.106095879	4.336245488	76.93857502	1	CRA004775;Yang2025_Silkie_Wenchang	PRJNA827465	0.335429998
ENSGALG00010012011	HOOK3	2	0.307467016	0.503582869	0.610558927	0.541491621	0.482376765	19.86993793	94.96727165	0.5	CRA004775;PRJNA827465	Yang2025_Silkie_Wenchang	0.876173124
ENSGALG00010012015	ENSGALG00010012015	2	-0.431532956	0.182667958	-2.362389988	0.01815753	0	0.179136352	0	1	PRJNA827465;Yang2025_Silkie_Wenchang	CRA004775	0.224982784
ENSGALG00010012015	ENSGALG00010012015	2	-0.427768947	0.227919032	-1.876846103	0.06053919	0	0.338509367	0	1	CRA004775;Yang2025_Silkie_Wenchang	PRJNA827465	0.426049285
ENSGALG00010012015	ENSGALG00010012015	2	-0.329792737	0.207259681	-1.591205464	0.111563344	0	0.073733624	0	1	CRA004775;PRJNA827465	Yang2025_Silkie_Wenchang	0.564762672
ENSGALG00010012016	WDR91	2	-0.067622082	0.050261968	-1.345392663	0.178498463	0.000511452	1.073341205	6.832981388	1	PRJNA827465;Yang2025_Silkie_Wenchang	CRA004775	0.684842357
ENSGALG00010012016	WDR91	2	-0.268024389	0.111459191	-2.404686303	0.016186353	0.012071695	1.937050242	48.37511293	1	CRA004775;Yang2025_Silkie_Wenchang	PRJNA827465	0.210262467
ENSGALG00010012016	WDR91	2	-0.19710798	0.172030631	-1.145772582	0.251889263	0.051157607	6.937770749	85.58614811	1	CRA004775;PRJNA827465	Yang2025_Silkie_Wenchang	0.761871849
ENSGALG00010012017	N4BP3	2	-0.026567064	0.056104424	-0.473528855	0.63583592	0	0.241716387	0	0.5	PRJNA827465;Yang2025_Silkie_Wenchang	CRA004775	0.904387873
ENSGALG00010012017	N4BP3	2	-0.006576435	0.121877947	-0.053959187	0.956967681	0	0.319764153	0	0.5	CRA004775;Yang2025_Silkie_Wenchang	PRJNA827465	0.988540618
ENSGALG00010012017	N4BP3	2	-0.042972262	0.058386508	-0.735996444	0.461732862	0	0.095750786	0	1	CRA004775;PRJNA827465	Yang2025_Silkie_Wenchang	0.850622591
ENSGALG00010012019	ENSGALG00010012019	2	-0.045953189	0.239339008	-0.192000415	0.84774188	0	0.122364319	0	0.5	PRJNA827465;Yang2025_Silkie_Wenchang	CRA004775	0.961304071
ENSGALG00010012019	ENSGALG00010012019	2	-0.11366719	0.29107658	-0.390506133	0.696162321	0	0.008694471	0	1	CRA004775;Yang2025_Silkie_Wenchang	PRJNA827465	0.921038437
ENSGALG00010012019	ENSGALG00010012019	2	0.010008065	0.290673635	0.034430591	0.97253379	0	0.025518062	0	0.5	CRA004775;PRJNA827465	Yang2025_Silkie_Wenchang	0.992191914
ENSGALG00010012021	ENSGALG00010012021	2	0.289286304	0.328182875	0.881478973	0.37805863	0.192303852	8.648047273	88.43669596	0.5	PRJNA827465;Yang2025_Silkie_Wenchang	CRA004775	0.82325159
ENSGALG00010012021	ENSGALG00010012021	2	0.42781306	0.221217296	1.933904213	0.053124906	0.052524409	2.15801863	53.66119707	1	CRA004775;Yang2025_Silkie_Wenchang	PRJNA827465	0.399645964
ENSGALG00010012021	ENSGALG00010012021	2	0.009723321	0.061150427	0.159006587	0.873663684	0	0.969232663	0	0.5	CRA004775;PRJNA827465	Yang2025_Silkie_Wenchang	0.9699681
ENSGALG00010012024	UBR5	2	-0.002224798	0.04322418	-0.05147114	0.958950099	0	0.1278611	0	0.5	PRJNA827465;Yang2025_Silkie_Wenchang	CRA004775	0.989102387
ENSGALG00010012024	UBR5	2	0.082921678	0.105815851	0.783641374	0.433250572	0	0.41949404	0	1	CRA004775;Yang2025_Silkie_Wenchang	PRJNA827465	0.842762754
ENSGALG00010012024	UBR5	2	0.002282321	0.044870344	0.050864806	0.95943325	0	0.959756002	0	0.5	CRA004775;PRJNA827465	Yang2025_Silkie_Wenchang	0.989259322
ENSGALG00010012028	TMEM140	2	-0.008336659	0.167591699	-0.049743867	0.960326499	0.036711283	2.650086191	62.26537826	0.5	PRJNA827465;Yang2025_Silkie_Wenchang	CRA004775	0.989338243
ENSGALG00010012028	TMEM140	2	0.022748485	0.229182967	0.099259055	0.920932588	0.049120865	1.782120599	43.88707472	0.5	CRA004775;Yang2025_Silkie_Wenchang	PRJNA827465	0.981469977
ENSGALG00010012028	TMEM140	2	-0.154954621	0.092428297	-1.676484651	0.0936433	0	0.168349561	0	1	CRA004775;PRJNA827465	Yang2025_Silkie_Wenchang	0.524147023
ENSGALG00010012031	GRK6	2	-0.025697218	0.061982864	-0.41458585	0.678445108	0	0.060206797	0	1	PRJNA827465;Yang2025_Silkie_Wenchang	CRA004775	0.917701868
ENSGALG00010012031	GRK6	2	-0.190282032	0.208380483	-0.913147092	0.361165186	0.03428164	1.459618758	31.48895938	1	CRA004775;Yang2025_Silkie_Wenchang	PRJNA827465	0.815240214
ENSGALG00010012031	GRK6	2	-0.157042309	0.226199673	-0.694264087	0.487516609	0.065617033	2.024680174	50.60948326	1	CRA004775;PRJNA827465	Yang2025_Silkie_Wenchang	0.85768885
ENSGALG00010012034	ARHGEF9	2	-0.069008318	0.063988973	-1.078440774	0.280837099	0	0.089295785	0	1	PRJNA827465;Yang2025_Silkie_Wenchang	CRA004775	0.778581075
ENSGALG00010012034	ARHGEF9	2	0.01160247	0.130331335	0.089022871	0.929063734	0	0.682576967	0	0.5	CRA004775;Yang2025_Silkie_Wenchang	PRJNA827465	0.983542314
ENSGALG00010012034	ARHGEF9	2	-0.062895733	0.073801467	-0.852228764	0.394087135	0.000532323	1.00835387	0.828466112	0.5	CRA004775;PRJNA827465	Yang2025_Silkie_Wenchang	0.830486298
ENSGALG00010012040	B4GALT7	2	0.212960072	0.067799176	3.141042178	0.001683478	0	0.284494271	0	1	PRJNA827465;Yang2025_Silkie_Wenchang	CRA004775	0.051935424
ENSGALG00010012040	B4GALT7	2	-0.075982969	0.387927018	-0.195869236	0.844712517	0.26151813	7.620828769	86.87806759	0.5	CRA004775;Yang2025_Silkie_Wenchang	PRJNA827465	0.960769014
ENSGALG00010012040	B4GALT7	2	-0.106408832	0.332628432	-0.319902997	0.749041866	0.198709321	9.218685064	89.15246596	0.5	CRA004775;PRJNA827465	Yang2025_Silkie_Wenchang	0.937357674
ENSGALG00010012041	ICOSLG	2	0.008131536	0.164747507	0.049357569	0.960634342	0.040302014	3.870125556	74.16104503	0.5	PRJNA827465;Yang2025_Silkie_Wenchang	CRA004775	0.989511782
ENSGALG00010012041	ICOSLG	2	-0.182580417	0.118831756	-1.536461488	0.124425236	0	0.239558638	0	1	CRA004775;Yang2025_Silkie_Wenchang	PRJNA827465	0.591542889
ENSGALG00010012041	ICOSLG	2	0.022741068	0.231830187	0.098093643	0.921857936	0.062463112	1.883457727	46.90616171	0.5	CRA004775;PRJNA827465	Yang2025_Silkie_Wenchang	0.981704892
ENSGALG00010012043	NUP50	2	0.02795066	0.052990159	0.527468892	0.597868013	0	0.850068868	0	0.5	PRJNA827465;Yang2025_Silkie_Wenchang	CRA004775	0.894645663
ENSGALG00010012043	NUP50	2	0.106652068	0.094461971	1.129047668	0.258877726	0	0.000581932	0	1	CRA004775;Yang2025_Silkie_Wenchang	PRJNA827465	0.765965607
ENSGALG00010012043	NUP50	2	0.002350707	0.061172608	0.038427452	0.969346874	0	0.149233202	0	0.5	CRA004775;PRJNA827465	Yang2025_Silkie_Wenchang	0.991572914
ENSGALG00010012045	ENSGALG00010012045	2	-0.230047117	0.312467209	-0.736228025	0.46159194	0	0.255100496	0	1	PRJNA827465;Yang2025_Silkie_Wenchang	CRA004775	0.85042452
ENSGALG00010012045	ENSGALG00010012045	2	-0.251238538	0.341766901	-0.735116647	0.462268457	0	0.382001766	0	1	CRA004775;Yang2025_Silkie_Wenchang	PRJNA827465	0.850823289
ENSGALG00010012045	ENSGALG00010012045	2	-0.416544929	0.322821471	-1.290325972	0.196937503	0	0.026332613	0	1	CRA004775;PRJNA827465	Yang2025_Silkie_Wenchang	0.707994211
ENSGALG00010012047	LMAN2	2	-0.183829999	0.240298619	-0.765006472	0.444267691	0.10074184	6.992412941	85.69878512	0.5	PRJNA827465;Yang2025_Silkie_Wenchang	CRA004775	0.846013379
ENSGALG00010012047	LMAN2	2	-0.357078383	0.094230247	-3.789424245	0.000150997	0	0.436629313	0	1	CRA004775;Yang2025_Silkie_Wenchang	PRJNA827465	0.010466718
ENSGALG00010012047	LMAN2	2	-0.130239353	0.171904683	-0.757625394	0.448675274	0.052881961	8.859364579	88.71250877	0.5	CRA004775;PRJNA827465	Yang2025_Silkie_Wenchang	0.846878812
ENSGALG00010012049	IFNAR1	2	-0.126706114	0.213664158	-0.593015297	0.553170915	0.080636022	8.341630125	88.0119355	0.5	PRJNA827465;Yang2025_Silkie_Wenchang	CRA004775	0.87934426
ENSGALG00010012049	IFNAR1	2	-0.336514908	0.121614828	-2.767054917	0.005656523	0	0.179905319	0	1	CRA004775;Yang2025_Silkie_Wenchang	PRJNA827465	0.112909246
ENSGALG00010012049	IFNAR1	2	0.065941258	0.073603882	0.895893746	0.370309514	0	0.397807357	0	0.5	CRA004775;PRJNA827465	Yang2025_Silkie_Wenchang	0.821109108
ENSGALG00010012051	ARK2C	2	0.20382923	0.259897303	0.784268353	0.432882665	0.092437643	3.123999742	67.98975408	0.5	PRJNA827465;Yang2025_Silkie_Wenchang	CRA004775	0.842762754
ENSGALG00010012051	ARK2C	2	0.455130542	0.219734846	2.071271579	0.038333424	0	0.150197714	0	1	CRA004775;Yang2025_Silkie_Wenchang	PRJNA827465	0.337684074
ENSGALG00010012051	ARK2C	2	-0.018417742	0.174288491	-0.105673882	0.915841103	0	0.135996347	0	0.5	CRA004775;PRJNA827465	Yang2025_Silkie_Wenchang	0.980317634
ENSGALG00010012052	KLF10	2	-0.020707371	0.099929664	-0.207219464	0.835838467	0.008650163	1.697110548	41.07631932	0.5	PRJNA827465;Yang2025_Silkie_Wenchang	CRA004775	0.958339923
ENSGALG00010012052	KLF10	2	0.152466159	0.121863116	1.251126373	0.210888375	0	0.953165462	0	1	CRA004775;Yang2025_Silkie_Wenchang	PRJNA827465	0.72262823
ENSGALG00010012052	KLF10	2	0.084194973	0.260308281	0.323443315	0.74635953	0.094066837	2.612991614	61.72968966	0.5	CRA004775;PRJNA827465	Yang2025_Silkie_Wenchang	0.936627825
ENSGALG00010012054	FAM60A	2	-0.163532571	0.20309773	-0.805191525	0.420709166	0.075223311	11.05011122	90.95031733	0.5	PRJNA827465;Yang2025_Silkie_Wenchang	CRA004775	0.839211146
ENSGALG00010012054	FAM60A	2	-0.28124131	0.187146412	-1.50278761	0.132893822	0.032563291	1.441650836	30.63507649	0.5	CRA004775;Yang2025_Silkie_Wenchang	PRJNA827465	0.610855703
ENSGALG00010012054	FAM60A	2	0.030852269	0.056841664	0.542775602	0.587284314	0	0.021241226	0	1	CRA004775;PRJNA827465	Yang2025_Silkie_Wenchang	0.891645821
ENSGALG00010012055	ENSGALG00010012055	2	-0.131340071	0.074899202	-1.753557666	0.07950633	0	0.087685092	0	1	PRJNA827465;Yang2025_Silkie_Wenchang	CRA004775	0.485261919
ENSGALG00010012055	ENSGALG00010012055	2	0.147550899	0.39679601	0.371855805	0.710000212	0.236772639	3.439461454	70.92568086	0.5	CRA004775;Yang2025_Silkie_Wenchang	PRJNA827465	0.925298042
ENSGALG00010012055	ENSGALG00010012055	2	0.159272494	0.368904645	0.431744345	0.665927233	0.203694579	3.20428497	68.79178945	0.5	CRA004775;PRJNA827465	Yang2025_Silkie_Wenchang	0.913546323
ENSGALG00010012059	ARHGDIB	2	0.184145726	0.233447828	0.788808907	0.430223703	0.084087498	3.846729054	74.00388783	0.5	PRJNA827465;Yang2025_Silkie_Wenchang	CRA004775	0.842040417
ENSGALG00010012059	ARHGDIB	2	0.057643112	0.471406697	0.122278942	0.902678109	0.340294959	4.038797659	75.240156	0.5	CRA004775;Yang2025_Silkie_Wenchang	PRJNA827465	0.976923607
ENSGALG00010012059	ARHGDIB	2	-0.069482536	0.161404993	-0.430485665	0.666842392	0.023467962	1.26354636	20.85767236	1	CRA004775;PRJNA827465	Yang2025_Silkie_Wenchang	0.913648
ENSGALG00010012060	ENSGALG00010012060	2	0.085992743	0.125843344	0.68333167	0.494397286	0	0.167876105	0	1	PRJNA827465;Yang2025_Silkie_Wenchang	CRA004775	0.860175061
ENSGALG00010012060	ENSGALG00010012060	2	0.08483795	0.151111715	0.561425366	0.574507596	0	0.318712469	0	0.5	CRA004775;Yang2025_Silkie_Wenchang	PRJNA827465	0.88612482
ENSGALG00010012060	ENSGALG00010012060	2	0.00959038	0.152013291	0.063089088	0.949695564	0	0.067537855	0	0.5	CRA004775;PRJNA827465	Yang2025_Silkie_Wenchang	0.98723759
ENSGALG00010012061	AZIN1	2	-0.28711176	0.22631591	-1.268632682	0.204572102	0.095743279	14.94615136	93.30931438	1	PRJNA827465;Yang2025_Silkie_Wenchang	CRA004775	0.71729696
ENSGALG00010012061	AZIN1	2	-0.164374798	0.403989602	-0.406878785	0.684097018	0.280665138	6.564905796	84.76748896	0.5	CRA004775;Yang2025_Silkie_Wenchang	PRJNA827465	0.918806497
ENSGALG00010012061	AZIN1	2	-0.010247978	0.13396998	-0.076494583	0.939025623	0.018819901	1.40692015	28.9227608	0.5	CRA004775;PRJNA827465	Yang2025_Silkie_Wenchang	0.985349045
ENSGALG00010012062	HMGA2	2	0.121522255	0.158901692	0.764763758	0.444412233	0.031443206	2.382548306	58.02813326	0.5	PRJNA827465;Yang2025_Silkie_Wenchang	CRA004775	0.846013379
ENSGALG00010012062	HMGA2	2	0.519785251	0.289816212	1.793499565	0.07289308	0.083494353	1.721924018	41.92542822	1	CRA004775;Yang2025_Silkie_Wenchang	PRJNA827465	0.466295041
ENSGALG00010012062	HMGA2	2	0.383258412	0.470305554	0.814913642	0.415121744	0.359575263	4.541863493	77.98260557	0.5	CRA004775;PRJNA827465	Yang2025_Silkie_Wenchang	0.837489728
ENSGALG00010012063	PTPRD	2	0.304618059	0.400542811	0.760513112	0.446947935	0.284124728	8.097942872	87.6511848	0.5	PRJNA827465;Yang2025_Silkie_Wenchang	CRA004775	0.84650962
ENSGALG00010012063	PTPRD	2	0.689018975	0.246706421	2.792870055	0.005224267	0	0.292237825	0	1	CRA004775;Yang2025_Silkie_Wenchang	PRJNA827465	0.107954459
ENSGALG00010012063	PTPRD	2	-0.048860323	0.085176218	-0.57363809	0.566212731	0	0.514542951	0	0.5	CRA004775;PRJNA827465	Yang2025_Silkie_Wenchang	0.883487517
ENSGALG00010012064	RGS14	2	0.281235436	0.165348346	1.700866337	0.088968089	0.031928591	2.293420054	56.39699764	1	PRJNA827465;Yang2025_Silkie_Wenchang	CRA004775	0.511998468
ENSGALG00010012064	RGS14	2	-0.078209594	0.601697164	-0.129981656	0.89658094	0.639133822	8.188073001	87.78711426	0.5	CRA004775;Yang2025_Silkie_Wenchang	PRJNA827465	0.975115773
ENSGALG00010012064	RGS14	2	-0.217202422	0.428791363	-0.506545702	0.612473615	0.300167853	4.826109731	79.27937706	0.5	CRA004775;PRJNA827465	Yang2025_Silkie_Wenchang	0.897938231
ENSGALG00010012066	STXBP3	2	0.386342761	0.119735364	3.226638705	0.001252535	0.01748032	2.552602703	60.82429911	1	PRJNA827465;Yang2025_Silkie_Wenchang	CRA004775	0.042994223
ENSGALG00010012066	STXBP3	2	0.447629196	0.150968325	2.965053739	0.003026301	0.015701785	1.154712394	13.39834878	1	CRA004775;Yang2025_Silkie_Wenchang	PRJNA827465	0.075856309
ENSGALG00010012066	STXBP3	2	0.245719445	0.10889988	2.2563794	0.024046881	0	0.290301315	0	1	CRA004775;PRJNA827465	Yang2025_Silkie_Wenchang	0.264812146
ENSGALG00010012067	ENSGALG00010012067	2	0.113478943	0.095979717	1.182322132	0.237077907	0.004655377	1.331080162	24.87304459	1	PRJNA827465;Yang2025_Silkie_Wenchang	CRA004775	0.749355649
ENSGALG00010012067	ENSGALG00010012067	2	0.040286686	0.188147116	0.214123323	0.830450888	0.048590877	2.99024996	66.55797966	0.5	CRA004775;Yang2025_Silkie_Wenchang	PRJNA827465	0.957273911
ENSGALG00010012067	ENSGALG00010012067	2	-0.053167966	0.106646713	-0.498542936	0.618101415	0	0.665030276	0	0.5	CRA004775;PRJNA827465	Yang2025_Silkie_Wenchang	0.899121942
ENSGALG00010012070	DNAJC28	2	-0.042517238	0.395320094	-0.107551421	0.914351533	0.292250469	15.32488694	93.47466638	0.5	PRJNA827465;Yang2025_Silkie_Wenchang	CRA004775	0.979657016
ENSGALG00010012070	DNAJC28	2	0.406468782	0.146600597	2.772627061	0.005560581	0	0.496055159	0	1	CRA004775;Yang2025_Silkie_Wenchang	PRJNA827465	0.111903482
ENSGALG00010012070	DNAJC28	2	0.049537258	0.530342735	0.093406122	0.925580927	0.497226869	8.023321507	87.53633393	0.5	CRA004775;PRJNA827465	Yang2025_Silkie_Wenchang	0.982594411
ENSGALG00010012071	ENSGALG00010012071	2	-0.113036781	0.207202016	-0.545538999	0.585382866	0.071575401	5.882922077	83.00164464	0.5	PRJNA827465;Yang2025_Silkie_Wenchang	CRA004775	0.891135366
ENSGALG00010012071	ENSGALG00010012071	2	-0.276522665	0.125059992	-2.211120133	0.027027519	0	0.716082341	0	1	CRA004775;Yang2025_Silkie_Wenchang	PRJNA827465	0.280280365
ENSGALG00010012071	ENSGALG00010012071	2	0.061926249	0.08926399	0.693742779	0.487843533	0	0.346480275	0	0.5	CRA004775;PRJNA827465	Yang2025_Silkie_Wenchang	0.85768885
ENSGALG00010012072	CAPRIN2	2	0.400311086	0.298995152	1.338854771	0.180617956	0.161605925	10.19087042	90.1872955	1	PRJNA827465;Yang2025_Silkie_Wenchang	CRA004775	0.688337781
ENSGALG00010012072	CAPRIN2	2	0.37032142	0.380127851	0.974202284	0.329956111	0.236398433	5.251629825	80.95829231	0.5	CRA004775;Yang2025_Silkie_Wenchang	PRJNA827465	0.801209935
ENSGALG00010012072	CAPRIN2	2	0.098890354	0.091085106	1.085691813	0.277615364	0	0.290273714	0	0.5	CRA004775;PRJNA827465	Yang2025_Silkie_Wenchang	0.776763074
ENSGALG00010012074	KIAA0930	2	-0.135456715	0.130104622	-1.04113684	0.297812044	0.028084766	5.85217813	82.91234515	1	PRJNA827465;Yang2025_Silkie_Wenchang	CRA004775	0.788858094
ENSGALG00010012074	KIAA0930	2	-0.26904858	0.068685145	-3.917129127	8.96098E-05	0	0.149906222	0	1	CRA004775;Yang2025_Silkie_Wenchang	PRJNA827465	0.007158399
ENSGALG00010012074	KIAA0930	2	-0.08714381	0.157128384	-0.554602595	0.579166514	0.025312941	1.558793402	35.84781673	1	CRA004775;PRJNA827465	Yang2025_Silkie_Wenchang	0.887888423
ENSGALG00010012075	PIGN	2	-0.169671108	0.178453651	-0.950785298	0.341713377	0.051807654	5.359492591	81.34151726	0.5	PRJNA827465;Yang2025_Silkie_Wenchang	CRA004775	0.805823616
ENSGALG00010012075	PIGN	2	-0.313536801	0.09991527	-3.138026878	0.001700893	0	0.610601416	0	1	CRA004775;Yang2025_Silkie_Wenchang	PRJNA827465	0.052226711
ENSGALG00010012075	PIGN	2	-0.015572637	0.09931474	-0.156800869	0.875401788	0	0.300585741	0	0.5	CRA004775;PRJNA827465	Yang2025_Silkie_Wenchang	0.970347777
ENSGALG00010012077	IFNGR2	2	-0.224320358	0.189733035	-1.182294678	0.237088796	0.06896229	23.48756214	95.74242744	1	PRJNA827465;Yang2025_Silkie_Wenchang	CRA004775	0.749355649
ENSGALG00010012077	IFNGR2	2	-0.225358716	0.224614191	-1.003314687	0.315709052	0.083013899	5.094522753	80.37107599	0.5	CRA004775;Yang2025_Silkie_Wenchang	PRJNA827465	0.796883862
ENSGALG00010012077	IFNGR2	2	-0.035422341	0.038607535	-0.917498134	0.35888167	0	0.149727933	0	0.5	CRA004775;PRJNA827465	Yang2025_Silkie_Wenchang	0.814241185
ENSGALG00010012078	TMBIM4	2	-0.192371565	0.308994466	-0.622572848	0.533565258	0.167107376	7.363440075	86.41939108	0.5	PRJNA827465;Yang2025_Silkie_Wenchang	CRA004775	0.873379507
ENSGALG00010012078	TMBIM4	2	-0.54058539	0.178850664	-3.022551756	0.002506532	0	0.000406594	0	1	CRA004775;Yang2025_Silkie_Wenchang	PRJNA827465	0.067383197
ENSGALG00010012078	TMBIM4	2	-0.158032404	0.306211883	-0.516088411	0.605792661	0.147605225	3.919658321	74.48757218	0.5	CRA004775;PRJNA827465	Yang2025_Silkie_Wenchang	0.896474603
ENSGALG00010012079	ENSGALG00010012079	2	-0.801233274	0.294980113	-2.716228105	0.006603041	0.080398223	1.813003179	44.84289871	1	PRJNA827465;Yang2025_Silkie_Wenchang	CRA004775	0.124138283
ENSGALG00010012079	ENSGALG00010012079	2	-1.167249736	0.291950825	-3.998103919	6.38519E-05	0	0.005286881	0	1	CRA004775;Yang2025_Silkie_Wenchang	PRJNA827465	0.005716565
ENSGALG00010012079	ENSGALG00010012079	2	-0.734428428	0.289496798	-2.536913818	0.011183448	0.048505961	1.305890117	23.42387871	1	CRA004775;PRJNA827465	Yang2025_Silkie_Wenchang	0.167908064
ENSGALG00010012081	CHIC1	2	-0.04672814	0.081310542	-0.574687355	0.565502761	0.004473165	1.507683187	33.67306816	0.5	PRJNA827465;Yang2025_Silkie_Wenchang	CRA004775	0.883487517
ENSGALG00010012081	CHIC1	2	-0.10292134	0.086509965	-1.189705024	0.23416235	0	0.426679142	0	0.5	CRA004775;Yang2025_Silkie_Wenchang	PRJNA827465	0.747392101
ENSGALG00010012081	CHIC1	2	0.023648563	0.079780275	0.296421181	0.766908452	0	0.020214283	0	1	CRA004775;PRJNA827465	Yang2025_Silkie_Wenchang	0.941375309
ENSGALG00010012082	HAUS1	2	0.134723457	0.20471633	0.658098243	0.510475005	0.037381555	1.769264802	43.47934808	0.5	PRJNA827465;Yang2025_Silkie_Wenchang	CRA004775	0.865365179
ENSGALG00010012082	HAUS1	2	-0.129176084	0.218447186	-0.591337823	0.554294092	0	0.038806976	0	1	CRA004775;Yang2025_Silkie_Wenchang	PRJNA827465	0.880177968
ENSGALG00010012082	HAUS1	2	0.215401761	0.199120849	1.081763971	0.27935742	0.013476152	1.112402132	10.10445137	0.5	CRA004775;PRJNA827465	Yang2025_Silkie_Wenchang	0.777457036
ENSGALG00010012083	MGP	2	0.068027014	0.209359941	0.324928511	0.745235178	0.009365056	1.090761457	8.320926287	0.5	PRJNA827465;Yang2025_Silkie_Wenchang	CRA004775	0.93597621
ENSGALG00010012083	MGP	2	0.044859078	0.327980622	0.136773563	0.891209781	0.057481053	1.364558766	26.71623791	0.5	CRA004775;Yang2025_Silkie_Wenchang	PRJNA827465	0.973735628
ENSGALG00010012083	MGP	2	0.237254207	0.195455685	1.213851653	0.224804378	0	0.157302484	0	1	CRA004775;PRJNA827465	Yang2025_Silkie_Wenchang	0.737885283
ENSGALG00010012084	TMEM50B	2	-0.250462311	0.220856203	-1.134051512	0.256772941	0.088455654	10.34766538	90.33598436	1	PRJNA827465;Yang2025_Silkie_Wenchang	CRA004775	0.764738822
ENSGALG00010012084	TMEM50B	2	-0.325625922	0.176974331	-1.839961311	0.065773918	0.041358281	2.883872498	65.32440319	1	CRA004775;Yang2025_Silkie_Wenchang	PRJNA827465	0.441899536
ENSGALG00010012084	TMEM50B	2	-0.052157047	0.055801776	-0.934684359	0.34995099	0	0.235047226	0	1	CRA004775;PRJNA827465	Yang2025_Silkie_Wenchang	0.809993068
ENSGALG00010012085	IPO8	2	0.173464694	0.095748085	1.811677943	0.070035974	0.011698609	2.330536244	57.09142037	1	PRJNA827465;Yang2025_Silkie_Wenchang	CRA004775	0.45646282
ENSGALG00010012085	IPO8	2	0.264927112	0.108948144	2.431680827	0.015028944	0	0.508210564	0	1	CRA004775;Yang2025_Silkie_Wenchang	PRJNA827465	0.202705079
ENSGALG00010012085	IPO8	2	0.105805164	0.045389159	2.331066836	0.019749836	0	0.006197595	0	1	CRA004775;PRJNA827465	Yang2025_Silkie_Wenchang	0.236007234
ENSGALG00010012086	CIBAR1	2	0.079315727	0.134771623	0.58851949	0.556183655	0.023127421	2.452730449	59.22911136	0.5	PRJNA827465;Yang2025_Silkie_Wenchang	CRA004775	0.880324407
ENSGALG00010012086	CIBAR1	2	0.256830625	0.142062074	1.807876082	0.070625789	0	0.000135599	0	1	CRA004775;Yang2025_Silkie_Wenchang	PRJNA827465	0.458507662
ENSGALG00010012086	CIBAR1	2	-0.006456677	0.072628408	-0.088900153	0.929161262	0	0.861929424	0	0.5	CRA004775;PRJNA827465	Yang2025_Silkie_Wenchang	0.983566083
ENSGALG00010012087	TMEM171	2	-0.209478164	0.482870181	-0.433818804	0.664420026	0.424688351	11.00428252	90.9126288	0.5	PRJNA827465;Yang2025_Silkie_Wenchang	CRA004775	0.913219726
ENSGALG00010012087	TMEM171	2	-0.647512892	0.218671114	-2.961126776	0.003065157	0	0.303214329	0	1	CRA004775;Yang2025_Silkie_Wenchang	PRJNA827465	0.076246761
ENSGALG00010012087	TMEM171	2	0.051463364	0.310658858	0.16565877	0.868425494	0.114417383	1.982654201	49.56256116	0.5	CRA004775;PRJNA827465	Yang2025_Silkie_Wenchang	0.968167735
ENSGALG00010012088	ATP5F1AZ	2	-0.075669758	0.16195846	-0.467217075	0.640344579	0	0.002939356	0	1	PRJNA827465;Yang2025_Silkie_Wenchang	CRA004775	0.906161493
ENSGALG00010012088	ATP5F1AZ	2	-0.103984331	0.198420045	-0.524061623	0.60023568	0	0.020575714	0	1	CRA004775;Yang2025_Silkie_Wenchang	PRJNA827465	0.895230519
ENSGALG00010012088	ATP5F1AZ	2	-0.089324265	0.188239588	-0.474524332	0.635126051	0	0.03545975	0	1	CRA004775;PRJNA827465	Yang2025_Silkie_Wenchang	0.904051292
ENSGALG00010012090	HELB	2	0.271730824	0.202682194	1.340674372	0.180026199	0.070276071	6.760865834	85.20899505	1	PRJNA827465;Yang2025_Silkie_Wenchang	CRA004775	0.687081928
ENSGALG00010012090	HELB	2	0.161611117	0.400828514	0.403192665	0.686806495	0.249472994	3.904847694	74.39080655	0.5	CRA004775;Yang2025_Silkie_Wenchang	PRJNA827465	0.919235539
ENSGALG00010012090	HELB	2	0.053275376	0.103685339	0.513817838	0.607379356	0.004427331	1.05480305	5.195571808	0.5	CRA004775;PRJNA827465	Yang2025_Silkie_Wenchang	0.896998866
ENSGALG00010012091	ENSGALG00010012091	2	-0.59509353	0.468004709	-1.271554579	0.203531424	0	0.796613633	0	1	PRJNA827465;Yang2025_Silkie_Wenchang	CRA004775	0.717254253
ENSGALG00010012091	ENSGALG00010012091	2	-0.098089186	0.355073422	-0.276250433	0.782355724	0	0.070037301	0	1	CRA004775;Yang2025_Silkie_Wenchang	PRJNA827465	0.945372683
ENSGALG00010012091	ENSGALG00010012091	2	-0.400112319	0.498644537	-0.802399885	0.422321679	0.196168823	1.562564934	36.0026596	1	CRA004775;PRJNA827465	Yang2025_Silkie_Wenchang	0.839704389
ENSGALG00010012092	OC3	2	0.315093965	0.390492248	0.806914777	0.419715585	0	0.147691313	0	1	PRJNA827465;Yang2025_Silkie_Wenchang	CRA004775	0.838527387
ENSGALG00010012092	OC3	2	0.648223622	0.504184448	1.285687458	0.19855216	0.173236238	1.516371845	34.05311477	1	CRA004775;Yang2025_Silkie_Wenchang	PRJNA827465	0.710744879
ENSGALG00010012092	OC3	2	0.778284001	0.397429396	1.958295003	0.050195407	0	0.786753563	0	1	CRA004775;PRJNA827465	Yang2025_Silkie_Wenchang	0.387894234
ENSGALG00010012093	ENSGALG00010012093	2	0.015332638	0.091230328	0.168065141	0.866532033	0.010229098	2.082940857	51.99095564	0.5	PRJNA827465;Yang2025_Silkie_Wenchang	CRA004775	0.967507198
ENSGALG00010012093	ENSGALG00010012093	2	0.110922193	0.103615499	1.07051739	0.284386486	0	0.28107672	0	1	CRA004775;Yang2025_Silkie_Wenchang	PRJNA827465	0.780265906
ENSGALG00010012093	ENSGALG00010012093	2	-0.040115225	0.040349579	-0.994191903	0.320129449	0	0.237001498	0	0.5	CRA004775;PRJNA827465	Yang2025_Silkie_Wenchang	0.798348216
ENSGALG00010012094	ENSGALG00010012094	2	0.4027369	0.257528544	1.563853439	0.117851991	0.015683202	1.131548736	11.62554752	1	PRJNA827465;Yang2025_Silkie_Wenchang	CRA004775	0.577620253
ENSGALG00010012094	ENSGALG00010012094	2	0.711507227	0.314711148	2.260826262	0.023770018	0	0.006327667	0	1	CRA004775;Yang2025_Silkie_Wenchang	PRJNA827465	0.263367861
ENSGALG00010012094	ENSGALG00010012094	2	0.303556129	0.281628442	1.077860341	0.281096086	0	0.717734906	0	1	CRA004775;PRJNA827465	Yang2025_Silkie_Wenchang	0.77879032
ENSGALG00010012095	RNF38	2	0.157853607	0.197991271	0.797275586	0.425290996	0.050679182	2.490788638	59.85207317	1	PRJNA827465;Yang2025_Silkie_Wenchang	CRA004775	0.84125685
ENSGALG00010012095	RNF38	2	0.026581602	0.397990583	0.066789524	0.946749263	0.255832051	5.193234304	80.74417711	0.5	CRA004775;Yang2025_Silkie_Wenchang	PRJNA827465	0.986445196
ENSGALG00010012095	RNF38	2	-0.113906157	0.178965352	-0.636470442	0.52446984	0.03596568	1.947367677	48.64862903	0.5	CRA004775;PRJNA827465	Yang2025_Silkie_Wenchang	0.869790047
ENSGALG00010012096	ENSGALG00010012096	2	-0.027756896	0.118010676	-0.235206655	0.814048341	0.015830191	2.182899446	54.18936947	0.5	PRJNA827465;Yang2025_Silkie_Wenchang	CRA004775	0.953869515
ENSGALG00010012096	ENSGALG00010012096	2	-0.169478307	0.134580552	-1.259307557	0.207919265	0	0.017250763	0	1	CRA004775;Yang2025_Silkie_Wenchang	PRJNA827465	0.720431178
ENSGALG00010012096	ENSGALG00010012096	2	0.061678992	0.082859396	0.744381389	0.456645775	0	0.133315858	0	0.5	CRA004775;PRJNA827465	Yang2025_Silkie_Wenchang	0.849515887
ENSGALG00010012097	GART	2	0.095614825	0.171696606	0.556882441	0.577607752	0.044439257	3.946600953	74.66174027	0.5	PRJNA827465;Yang2025_Silkie_Wenchang	CRA004775	0.887312588
ENSGALG00010012097	GART	2	-0.046411995	0.094692841	-0.490132041	0.624040466	0	0.563661012	0	0.5	CRA004775;Yang2025_Silkie_Wenchang	PRJNA827465	0.900755167
ENSGALG00010012097	GART	2	0.287719227	0.138747501	2.07368944	0.038108163	0	0.005003964	0	1	CRA004775;PRJNA827465	Yang2025_Silkie_Wenchang	0.33657643
ENSGALG00010012100	UBE2F	2	-0.052748515	0.065365031	-0.806983708	0.41967587	0	0.001795189	0	1	PRJNA827465;Yang2025_Silkie_Wenchang	CRA004775	0.838527387
ENSGALG00010012100	UBE2F	2	0.015536241	0.147789649	0.105124011	0.916277406	0.01349636	1.238855738	19.28035128	0.5	CRA004775;Yang2025_Silkie_Wenchang	PRJNA827465	0.98043852
ENSGALG00010012100	UBE2F	2	0.00667931	0.133710552	0.049953498	0.960159445	0.014957478	1.283128946	22.06550998	0.5	CRA004775;PRJNA827465	Yang2025_Silkie_Wenchang	0.989338243
ENSGALG00010012101	ENSGALG00010012101	2	0.012080271	0.052482395	0.230177577	0.817953785	0	0.329477126	0	0.5	PRJNA827465;Yang2025_Silkie_Wenchang	CRA004775	0.955109707
ENSGALG00010012101	ENSGALG00010012101	2	-0.007027559	0.103550682	-0.067865888	0.945892394	0	0.588844653	0	0.5	CRA004775;Yang2025_Silkie_Wenchang	PRJNA827465	0.986321197
ENSGALG00010012101	ENSGALG00010012101	2	0.034918814	0.056975218	0.612877224	0.539957524	0	0.267284306	0	1	CRA004775;PRJNA827465	Yang2025_Silkie_Wenchang	0.87529516
ENSGALG00010012102	SON	2	0.01494359	0.089221926	0.167487867	0.866986193	0.009410135	1.962200495	49.03680828	0.5	PRJNA827465;Yang2025_Silkie_Wenchang	CRA004775	0.967699
ENSGALG00010012102	SON	2	0.230602049	0.12879453	1.790464621	0.07337925	0.004859741	1.128724153	11.40439429	1	CRA004775;Yang2025_Silkie_Wenchang	PRJNA827465	0.467957919
ENSGALG00010012102	SON	2	0.143997874	0.237085554	0.607366714	0.543607573	0.089162098	3.9887086	74.92922898	0.5	CRA004775;PRJNA827465	Yang2025_Silkie_Wenchang	0.876968972
ENSGALG00010012103	ENSGALG00010012103	2	-0.068079237	0.107950847	-0.630650334	0.52826918	0.009843276	1.710927379	41.55216566	0.5	PRJNA827465;Yang2025_Silkie_Wenchang	CRA004775	0.871701504
ENSGALG00010012103	ENSGALG00010012103	2	-0.014193551	0.112020625	-0.126704799	0.899174048	0.003402969	1.060177207	5.676146056	0.5	CRA004775;Yang2025_Silkie_Wenchang	PRJNA827465	0.976033959
ENSGALG00010012103	ENSGALG00010012103	2	-0.20893289	0.12029022	-1.736906713	0.082403638	0	0.138854408	0	1	CRA004775;PRJNA827465	Yang2025_Silkie_Wenchang	0.494988567
ENSGALG00010012105	DONSON	2	-0.005093991	0.07043516	-0.072321706	0.942345891	0	0.453346303	0	0.5	PRJNA827465;Yang2025_Silkie_Wenchang	CRA004775	0.985827325
ENSGALG00010012105	DONSON	2	-0.154644294	0.338281018	-0.457147418	0.64756509	0.148535204	2.319807851	56.89298147	0.5	CRA004775;Yang2025_Silkie_Wenchang	PRJNA827465	0.907350807
ENSGALG00010012105	DONSON	2	-0.17548099	0.259316114	-0.676706847	0.498591972	0.081434897	1.772785558	43.59159825	1	CRA004775;PRJNA827465	Yang2025_Silkie_Wenchang	0.861478809
ENSGALG00010012106	TMTC1	2	-0.073233832	0.228370403	-0.32068005	0.748452867	0.091267343	7.998483778	87.49763045	0.5	PRJNA827465;Yang2025_Silkie_Wenchang	CRA004775	0.937249951
ENSGALG00010012106	TMTC1	2	-0.265331319	0.109947051	-2.413264527	0.01581034	0	0.968337676	0	0.5	CRA004775;Yang2025_Silkie_Wenchang	PRJNA827465	0.208102412
ENSGALG00010012106	TMTC1	2	0.143526914	0.106320399	1.3499471	0.177032952	0	0.08898734	0	1	CRA004775;PRJNA827465	Yang2025_Silkie_Wenchang	0.682685058
ENSGALG00010012107	CLCC1	2	-0.176366586	0.065294091	-2.701110983	0.006910827	0	0.111886489	0	1	PRJNA827465;Yang2025_Silkie_Wenchang	CRA004775	0.127699223
ENSGALG00010012107	CLCC1	2	-0.088418324	0.116861918	-0.756605105	0.449286482	0.005310576	1.143626627	12.55887403	0.5	CRA004775;Yang2025_Silkie_Wenchang	PRJNA827465	0.847263197
ENSGALG00010012107	CLCC1	2	-0.107102492	0.146531178	-0.730919477	0.464828339	0.022141082	1.643570513	39.15685442	0.5	CRA004775;PRJNA827465	Yang2025_Silkie_Wenchang	0.85217126
ENSGALG00010012108	PSTPIP2	2	0.161804531	0.230248608	0.702738366	0.482218813	0	0.214687121	0	1	PRJNA827465;Yang2025_Silkie_Wenchang	CRA004775	0.85691916

ENSGALG00010012108	PSTPIP2	2	0.155958766	0.245281721	0.635835259	0.524883804	0	0.215675063	0	1	CRA004775;Yang2025_Silkie_Wenchang	PRJNA827465	0.869892816
ENSGALG00010012108	PSTPIP2	2	0.255569606	0.223245153	1.144793527	0.252294697	0	0.000644333	0	1	CRA004775;PRJNA827465	Yang2025_Silkie_Wenchang	0.762529875
ENSGALG00010012110	ENSGALG00010012110	2	0.135965963	0.183500435	0.740957173	0.458719408	0.049659789	3.551528493	71.84310919	0.5	PRJNA827465;Yang2025_Silkie_Wenchang	CRA004775	0.850089963
ENSGALG00010012110	ENSGALG00010012110	2	0.242264019	0.160985973	1.50487657	0.132355813	0.007341805	1.139552037	12.24621894	1	CRA004775;Yang2025_Silkie_Wenchang	PRJNA827465	0.609324473
ENSGALG00010012110	ENSGALG00010012110	2	-0.017550192	0.086789923	-0.202214632	0.839748935	0	0.00785601	0	0.5	CRA004775;PRJNA827465	Yang2025_Silkie_Wenchang	0.959493354
ENSGALG00010012111	DRD1	2	-0.772619179	0.239847097	-3.221298859	0.00127611	0	0.000285797	0	1	PRJNA827465;Yang2025_Silkie_Wenchang	CRA004775	0.043386199
ENSGALG00010012111	DRD1	2	-0.553658261	0.322998691	-1.714119212	0.086506834	0	0.963625947	0	1	CRA004775;Yang2025_Silkie_Wenchang	PRJNA827465	0.506036629
ENSGALG00010012111	DRD1	2	-0.603751063	0.299205088	-2.017850257	0.043606859	0.026472086	1.124138863	11.04301851	1	CRA004775;PRJNA827465	Yang2025_Silkie_Wenchang	0.360845532
ENSGALG00010012112	MBD2	2	-0.399256134	0.174238935	-2.291428923	0.021938618	0	0.467594067	0	1	PRJNA827465;Yang2025_Silkie_Wenchang	CRA004775	0.25105736
ENSGALG00010012112	MBD2	2	-0.521994066	0.230894641	-2.260745699	0.023775009	0	0.038627847	0	1	CRA004775;Yang2025_Silkie_Wenchang	PRJNA827465	0.263367861
ENSGALG00010012112	MBD2	2	-0.345409307	0.190662379	-1.811628016	0.070043693	0	0.117207708	0	1	CRA004775;PRJNA827465	Yang2025_Silkie_Wenchang	0.45646282
ENSGALG00010012113	WDR47	2	0.078545259	0.042735073	1.837957761	0.06606863	0	0.017359145	0	1	PRJNA827465;Yang2025_Silkie_Wenchang	CRA004775	0.442444044
ENSGALG00010012113	WDR47	2	0.101871615	0.082573502	1.233708298	0.217311621	0.00268951	1.092608756	8.475930227	1	CRA004775;Yang2025_Silkie_Wenchang	PRJNA827465	0.730896703
ENSGALG00010012113	WDR47	2	0.098452034	0.059346308	1.658941169	0.097127648	0.000916775	1.032827235	3.178385855	1	CRA004775;PRJNA827465	Yang2025_Silkie_Wenchang	0.532520426
ENSGALG00010012114	TAL2	2	-0.332326952	0.259666261	-1.279823382	0.200607259	0	0.305114075	0	1	PRJNA827465;Yang2025_Silkie_Wenchang	CRA004775	0.712970384
ENSGALG00010012114	TAL2	2	-0.42771059	0.484540593	-0.88271364	0.377391011	0.160116706	1.260479218	20.6650942	1	CRA004775;Yang2025_Silkie_Wenchang	PRJNA827465	0.82325159
ENSGALG00010012114	TAL2	2	-0.592611536	0.354683713	-1.670816883	0.094757853	0	0.737711677	0	1	CRA004775;PRJNA827465	Yang2025_Silkie_Wenchang	0.526919621
ENSGALG00010012116	RBM12B	2	-0.057804538	0.056835874	-1.017043188	0.309132885	0.002247092	1.415257266	29.34146859	1	PRJNA827465;Yang2025_Silkie_Wenchang	CRA004775	0.794043289
ENSGALG00010012116	RBM12B	2	-0.146055509	0.085601894	-1.706218203	0.087967461	0	0.003238936	0	1	CRA004775;Yang2025_Silkie_Wenchang	PRJNA827465	0.509638136
ENSGALG00010012116	RBM12B	2	-0.026073666	0.047798549	-0.545490752	0.585416039	0	0.329226032	0	1	CRA004775;PRJNA827465	Yang2025_Silkie_Wenchang	0.891135366
ENSGALG00010012118	ENSGALG00010012118	2	0.123257032	0.872208768	0.141315974	0.887620331	1.042718832	3.025981653	66.952873	0.5	PRJNA827465;Yang2025_Silkie_Wenchang	CRA004775	0.973038314
ENSGALG00010012118	ENSGALG00010012118	2	-0.008939935	0.756426828	-0.011818638	0.990570311	0.602896267	2.058501624	51.4209759	0.5	CRA004775;Yang2025_Silkie_Wenchang	PRJNA827465	0.997498973
ENSGALG00010012118	ENSGALG00010012118	2	0.774192626	0.40254613	1.923239521	0.054449976	0	0.081870351	0	1	CRA004775;PRJNA827465	Yang2025_Silkie_Wenchang	0.404097297
ENSGALG00010012119	EPG5	2	-0.074748894	0.079770808	-0.93704572	0.34873504	0	0.140482524	0	1	PRJNA827465;Yang2025_Silkie_Wenchang	CRA004775	0.809333419
ENSGALG00010012119	EPG5	2	-0.132200157	0.103430272	-1.278157295	0.20119397	0	0.505658409	0	1	CRA004775;Yang2025_Silkie_Wenchang	PRJNA827465	0.713868973
ENSGALG00010012119	EPG5	2	-0.08240025	0.106508375	-0.773650426	0.439137544	0	0.813792443	0	1	CRA004775;PRJNA827465	Yang2025_Silkie_Wenchang	0.844022831
ENSGALG00010012120	ENSGALG00010012120	2	0.081599157	0.140930786	0.579001647	0.562588062	0.017207061	1.668700774	40.0731386	0.5	PRJNA827465;Yang2025_Silkie_Wenchang	CRA004775	0.881978509
ENSGALG00010012120	ENSGALG00010012120	2	0.221872554	0.184677742	1.201403871	0.229594575	0	0.636084013	0	0.5	CRA004775;Yang2025_Silkie_Wenchang	PRJNA827465	0.742632495
ENSGALG00010012120	ENSGALG00010012120	2	-0.032709307	0.116114072	-0.281699768	0.778173732	0	0.124820021	0	1	CRA004775;PRJNA827465	Yang2025_Silkie_Wenchang	0.944521855
ENSGALG00010012121	SREK1	2	0.63257977	0.486057413	1.301450719	0.193104222	0.437658256	13.18043451	92.41299671	1	PRJNA827465;Yang2025_Silkie_Wenchang	CRA004775	0.703194224
ENSGALG00010012121	SREK1	2	0.65066672	0.491969737	1.322574685	0.185976857	0.425322128	8.239974691	87.86404039	1	CRA004775;Yang2025_Silkie_Wenchang	PRJNA827465	0.695002013
ENSGALG00010012121	SREK1	2	0.167297916	0.104012998	1.608432786	0.107740426	0	0.001589561	0	1	CRA004775;PRJNA827465	Yang2025_Silkie_Wenchang	0.555492845
ENSGALG00010012122	GRIP1	2	0.33572521	0.420720636	0.79797657	0.424884087	0.312764856	8.24402344	87.87000052	0.5	PRJNA827465;Yang2025_Silkie_Wenchang	CRA004775	0.841139484
ENSGALG00010012122	GRIP1	2	0.759463551	0.238597055	3.183038247	0.001457384	0	0.068404995	0	1	CRA004775;Yang2025_Silkie_Wenchang	PRJNA827465	0.047277983
ENSGALG00010012122	GRIP1	2	0.065012423	0.259567432	0.250464486	0.802228165	0.064824596	1.387884022	27.94786996	0.5	CRA004775;PRJNA827465	Yang2025_Silkie_Wenchang	0.950637102
ENSGALG00010012123	ENSGALG00010012123	2	0.332108036	0.211320579	1.571583979	0.116047064	0.062498504	3.327700793	69.94922134	1	PRJNA827465;Yang2025_Silkie_Wenchang	CRA004775	0.573367481
ENSGALG00010012123	ENSGALG00010012123	2	0.512124061	0.155139567	3.301053822	0.000963224	0	0.277074953	0	1	CRA004775;Yang2025_Silkie_Wenchang	PRJNA827465	0.036826453
ENSGALG00010012123	ENSGALG00010012123	2	0.153398405	0.146616465	1.04625633	0.295442702	0	0.226479886	0	1	CRA004775;PRJNA827465	Yang2025_Silkie_Wenchang	0.787628307
ENSGALG00010012124	ERAP1	2	-0.016863457	0.139067397	-0.121261035	0.903484282	0	0.310724466	0	0.5	PRJNA827465;Yang2025_Silkie_Wenchang	CRA004775	0.977284786
ENSGALG00010012124	ERAP1	2	-0.133516539	0.16440554	-0.812117031	0.416724482	0	0.105479667	0	1	CRA004775;Yang2025_Silkie_Wenchang	PRJNA827465	0.837984974
ENSGALG00010012124	ERAP1	2	-0.024889442	0.161094321	-0.154502291	0.877213705	0	0.597217568	0	0.5	CRA004775;PRJNA827465	Yang2025_Silkie_Wenchang	0.970575794
ENSGALG00010012125	RNF152	2	-0.26310478	0.178182907	-1.476599435	0.139783043	0.050862072	4.989296819	79.95709543	1	PRJNA827465;Yang2025_Silkie_Wenchang	CRA004775	0.623597383
ENSGALG00010012125	RNF152	2	-0.032705996	0.464038245	-0.070481252	0.943810622	0.373177476	6.96248178	85.63730532	0.5	CRA004775;Yang2025_Silkie_Wenchang	PRJNA827465	0.985827325
ENSGALG00010012125	RNF152	2	0.109750683	0.275194637	0.398811126	0.690032378	0.106490053	2.777747055	63.99960183	0.5	CRA004775;PRJNA827465	Yang2025_Silkie_Wenchang	0.919979266
ENSGALG00010012127	ENSGALG00010012127	2	-0.095396819	0.038488847	-2.478557461	0.013191486	0	0.745244637	0	1	PRJNA827465;Yang2025_Silkie_Wenchang	CRA004775	0.187509939
ENSGALG00010012127	ENSGALG00010012127	2	-0.135136747	0.049938438	-2.706066754	0.006808536	0	0.238494393	0	1	CRA004775;Yang2025_Silkie_Wenchang	PRJNA827465	0.126544334
ENSGALG00010012127	ENSGALG00010012127	2	-0.077483125	0.052687419	-1.470619115	0.141394152	0	0.801147686	0	1	CRA004775;PRJNA827465	Yang2025_Silkie_Wenchang	0.626437222
ENSGALG00010012129	CRYZL1	2	0.004709499	0.20167703	0.023351686	0.981369647	0.060938136	3.785290834	73.58195066	0.5	PRJNA827465;Yang2025_Silkie_Wenchang	CRA004775	0.994962782
ENSGALG00010012129	CRYZL1	2	-0.256822381	0.150648677	-1.704776872	0.088236047	0	0.09368734	0	1	CRA004775;Yang2025_Silkie_Wenchang	PRJNA827465	0.510208309
ENSGALG00010012129	CRYZL1	2	-0.010776363	0.24575676	-0.043849713	0.9650242	0.086363646	3.057154363	67.28984274	0.5	CRA004775;PRJNA827465	Yang2025_Silkie_Wenchang	0.990532586
ENSGALG00010012130	TMEM67	2	-0.037819014	0.109729352	-0.344657223	0.73035208	0.011655242	1.76400748	43.31089799	0.5	PRJNA827465;Yang2025_Silkie_Wenchang	CRA004775	0.932617298
ENSGALG00010012130	TMEM67	2	-0.126419212	0.156588414	-0.807334396	0.419473852	0.004764743	1.051235834	4.873866747	0.5	CRA004775;Yang2025_Silkie_Wenchang	PRJNA827465	0.838527387
ENSGALG00010012130	TMEM67	2	0.048706884	0.079223084	0.614806717	0.538682373	0	0.262118827	0	1	CRA004775;PRJNA827465	Yang2025_Silkie_Wenchang	0.87481821
ENSGALG00010012131	SLC14A1	2	-0.490439405	0.284764977	-1.722260266	0.085022382	0	0.708118767	0	1	PRJNA827465;Yang2025_Silkie_Wenchang	CRA004775	0.50144578
ENSGALG00010012131	SLC14A1	2	-0.652619335	0.329231022	-1.982253465	0.047450879	0	0.064516658	0	1	CRA004775;Yang2025_Silkie_Wenchang	PRJNA827465	0.376379801
ENSGALG00010012131	SLC14A1	2	-0.301399947	0.363294324	-0.829630213	0.406747889	0	0.131096989	0	1	CRA004775;PRJNA827465	Yang2025_Silkie_Wenchang	0.833618122
ENSGALG00010012133	RAB3IP	2	0.239757315	0.114100685	2.101278495	0.035616526	0	0.475829087	0	1	PRJNA827465;Yang2025_Silkie_Wenchang	CRA004775	0.325599715
ENSGALG00010012133	RAB3IP	2	0.147113425	0.173632167	0.84727057	0.396844326	0	0.008352651	0	1	CRA004775;Yang2025_Silkie_Wenchang	PRJNA827465	0.831375233
ENSGALG00010012133	RAB3IP	2	0.297175679	0.142284563	2.088600986	0.036743656	0	0.029679481	0	1	CRA004775;PRJNA827465	Yang2025_Silkie_Wenchang	0.330692908
ENSGALG00010012135	CAMSAP2	2	0.191351385	0.194379878	0.984419716	0.324909188	0.059765274	4.169666006	76.01726377	1	PRJNA827465;Yang2025_Silkie_Wenchang	CRA004775	0.799914637
ENSGALG00010012135	CAMSAP2	2	0.393757328	0.162025913	2.430212059	0.015089991	0	0.150736494	0	1	CRA004775;Yang2025_Silkie_Wenchang	PRJNA827465	0.203100811
ENSGALG00010012135	CAMSAP2	2	0.039432414	0.057118914	0.690356499	0.489970026	0	0.537529835	0	1	CRA004775;PRJNA827465	Yang2025_Silkie_Wenchang	0.858512413
ENSGALG00010012136	ENSGALG00010012136	2	0.252450309	0.316697106	0.797134877	0.425372703	0.158993825	4.75588678	78.97342712	0.5	PRJNA827465;Yang2025_Silkie_Wenchang	CRA004775	0.84125685
ENSGALG00010012136	ENSGALG00010012136	2	0.567819996	0.206310978	2.752252937	0.005918679	0	0.037153718	0	1	CRA004775;Yang2025_Silkie_Wenchang	PRJNA827465	0.11619472
ENSGALG00010012136	ENSGALG00010012136	2	0.070616085	0.221552021	0.318733655	0.749928492	0.032418707	1.288227379	22.37395225	0.5	CRA004775;PRJNA827465	Yang2025_Silkie_Wenchang	0.937362187
ENSGALG00010012138	DDX11	2	0.274680633	0.108491046	2.53182768	0.011346974	0.003460639	1.151821919	13.18102361	1	PRJNA827465;Yang2025_Silkie_Wenchang	CRA004775	0.16977597
ENSGALG00010012138	DDX11	2	0.408195762	0.163377159	2.49848733	0.01247246	0	0.070503574	0	1	CRA004775;Yang2025_Silkie_Wenchang	PRJNA827465	0.180820767
ENSGALG00010012138	DDX11	2	0.204640904	0.115375467	1.773695129	0.076113593	0	0.056900122	0	1	CRA004775;PRJNA827465	Yang2025_Silkie_Wenchang	0.476155344
ENSGALG00010012141	ENSGALG00010012141	2	0.157399298	0.113484531	1.386966989	0.165451829	0.001602905	1.065797904	6.173581697	1	PRJNA827465;Yang2025_Silkie_Wenchang	CRA004775	0.664874006
ENSGALG00010012141	ENSGALG00010012141	2	0.204888389	0.126343341	1.621679362	0.10487202	0	0.547807619	0	1	CRA004775;Yang2025_Silkie_Wenchang	PRJNA827465	0.550048644
ENSGALG00010012141	ENSGALG00010012141	2	0.066866369	0.119081184	0.561519188	0.574443653	0	0.022304347	0	1	CRA004775;PRJNA827465	Yang2025_Silkie_Wenchang	0.886116367
ENSGALG00010012144	H1-01	2	-0.208632432	0.088198479	-2.36548787	0.018006333	0.001130721	1.070876928	6.61858762	1	PRJNA827465;Yang2025_Silkie_Wenchang	CRA004775	0.223885768
ENSGALG00010012144	H1-01	2	-0.308886624	0.134379506	-2.298614075	0.021526864	0	0.130509708	0	1	CRA004775;Yang2025_Silkie_Wenchang	PRJNA827465	0.248109211
ENSGALG00010012144	H1-01	2	-0.147458655	0.099959118	-1.475189644	0.140161565	0	0.015370073	0	1	CRA004775;PRJNA827465	Yang2025_Silkie_Wenchang	0.623940421
ENSGALG00010012147	ARFGAP3	2	0.048530684	0.122235931	0.397024706	0.691349242	0.02250031	3.992997011	74.95615456	0.5	PRJNA827465;Yang2025_Silkie_Wenchang	CRA004775	0.920268407
ENSGALG00010012147	ARFGAP3	2	-0.082305848	0.092069432	-0.893954122	0.371346438	0	1.32772E-05	0	1	CRA004775;Yang2025_Silkie_Wenchang	PRJNA827465	0.8214684
ENSGALG00010012147	ARFGAP3	2	0.146368333	0.071681546	2.041924896	0.041158982	0	0.735873341	0	0.5	CRA004775;PRJNA827465	Yang2025_Silkie_Wenchang	0.351699305
ENSGALG00010012148	SETBP1	2	0.378873262	0.539723762	0.70197625	0.482693975	0.538787308	13.17205327	92.40816918	0.5	PRJNA827465;Yang2025_Silkie_Wenchang	CRA004775	0.85691916
ENSGALG00010012148	SETBP1	2	0.846052705	0.212897663	3.973987741	7.06792E-05	0	0.509495607	0	1	CRA004775;Yang2025_Silkie_Wenchang	PRJNA827465	0.006103493
ENSGALG00010012148	SETBP1	2	0.113225506	0.348950349	0.324474546	0.74557879	0.16091034	2.536491406	60.57546272	0.5	CRA004775;PRJNA827465	Yang2025_Silkie_Wenchang	0.93621352
ENSGALG00010012149	SFXN1	2	-0.147118849	0.24359406	-0.603950888	0.545876295	0.095062284	4.518151082	77.86705266	0.5	PRJNA827465;Yang2025_Silkie_Wenchang	CRA004775	0.877954577
ENSGALG00010012149	SFXN1	2	-0.393892595	0.18453189	-2.134550271	0.032797763	0	0.129285008	0	1	CRA004775;Yang2025_Silkie_Wenchang	PRJNA827465	0.31117452
ENSGALG00010012149	SFXN1	2	0.040651783	0.079261732	0.512880323	0.608035038	0	0.94277834	0	0.5	CRA004775;PRJNA827465	Yang2025_Silkie_Wenchang	0.897204334
ENSGALG00010012154	CWC27	2	0.375763962	0.391288995	0.960323359	0.336892497	0.257550366	6.264466778	84.03694942	0.5	PRJNA827465;Yang2025_Silkie_Wenchang	CRA004775	0.803904536
ENSGALG00010012154	CWC27	2	0.437651399	0.378200404	1.157194427	0.247192934	0.201449212	3.296747757	69.66707575	1	CRA004775;Yang2025_Silkie_Wenchang	PRJNA827465	0.75766218
ENSGALG00010012154	CWC27	2	0.001377487	0.172795953	0.007971752	0.993639529	0	0.003193404	0	0.5	CRA004775;PRJNA827465	Yang2025_Silkie_Wenchang	0.997979742
ENSGALG00010012157	H2BC32	2	-0.147047506	0.759266437	-0.193670495	0.846433896	0.979528478	6.609282888	84.8697655	0.5	PRJNA827465;Yang2025_Silkie_Wenchang	CRA004775	0.96122283
ENSGALG00010012157	H2BC32	2	0.014398205	0.607030249	0.023719089	0.981076679	0.544451458	3.821151006	73.82987486	0.5	CRA004775;Yang2025_Silkie_Wenchang	PRJNA827465	0.994928779
ENSGALG00010012157	H2BC32	2	-0.74788218	0.277367315	-2.696360171	0.007010182	0	0.297843516	0	1	CRA004775;PRJNA827465	Yang2025_Silkie_Wenchang	0.12854434
ENSGALG00010012159	WASHC1	2	-0.130233673	0.065653283	-1.983658184	0.047293958	0	0.181780496	0	1	PRJNA827465;Yang2025_Silkie_Wenchang	CRA004775	0.375362366
ENSGALG00010012159	WASHC1	2	-0.178320554	0.126140446	-1.41366675	0.157459773	0	0.010252049	0	1	CRA004775;Yang2025_Silkie_Wenchang	PRJNA827465	0.65076873
ENSGALG00010012159	WASHC1	2	-0.119266108	0.070587338	-1.689624663	0.091099785	0	0.023424821	0	1	CRA004775;PRJNA827465	Yang2025_Silkie_Wenchang	0.517711803
ENSGALG00010012163	PDP1	2	-0.138697162	0.256721601	-0.540262922	0.589015725	0.125621669	21.25147018	95.2944432	0.5	PRJNA827465;Yang2025_Silkie_Wenchang	CRA004775	0.892171138
ENSGALG00010012163	PDP1	2	0.047553055	0.555415807	0.085617036	0.931770856	0.499467368	4.375016308	77.14294234	0.5	CRA004775;Yang2025_Silkie_Wenchang	PRJNA827465	0.98412915
ENSGALG00010012163	PDP1	2	0.200695438	0.214338073	0.936349921	0.349093053	0.048293591	1.3292204	24.76793166	1	CRA004775;PRJNA827465	Yang2025_Silkie_Wenchang	0.809654447
ENSGALG00010012171	CDH17	2	-0.13944213	0.418384596	-0.333286959	0.738917682	0.265096926	4.088342728	75.54021112	0.5	PRJNA827465;Yang2025_Silkie_Wenchang	CRA004775	0.934613258
ENSGALG00010012171	CDH17	2	0.944941985	1.613985195	0.585471284	0.558230869	4.823899316	12.96293106	92.2856953	0.5	CRA004775;Yang2025_Silkie_Wenchang	PRJNA827465	0.880678789
ENSGALG00010012171	CDH17	2	1.324178148	1.190414178	1.112367588	0.265980135	2.483250361	7.432756042	86.54604033	1	CRA004775;PRJNA827465	Yang2025_Silkie_Wenchang	0.770078617
ENSGALG00010012172	CNOT2	2	0.010869807	0.04022049	0.270255471	0.78696372	0	0.003928607	0	1	PRJNA827465;Yang2025_Silkie_Wenchang	CRA004775	0.946784559
ENSGALG00010012172	CNOT2	2	-0.003903943	0.082025825	-0.047594074	0.962039755	0	0.031445593	0	0.5	CRA004775;Yang2025_Silkie_Wenchang	PRJNA827465	0.989878931
ENSGALG00010012172	CNOT2	2	0.009029411	0.042452065	0.212696629	0.831563596	0	0.059424261	0	0.5	CRA004775;PRJNA827465	Yang2025_Silkie_Wenchang	0.957712669
ENSGALG00010012175	PACSIN2	2	0.035731715	0.058667764	0.609051931	0.542490018	0	0.061917161	0	1	PRJNA827465;Yang2025_Silkie_Wenchang	CRA004775	0.876817086
ENSGALG00010012175	PACSIN2	2	0.162259604	0.15248434	1.064106678	0.287280376	0.023983853	1.82573543	45.22755139	1	CRA004775;Yang2025_Silkie_Wenchang	PRJNA827465	0.782840067
ENSGALG00010012175	PACSIN2	2	0.144265393	0.168111747	0.85815177	0.390808664	0.037233649	2.410815817	58.52026551	1	CRA004775;PRJNA827465	Yang2025_Silkie_Wenchang	0.829251057
ENSGALG00010012179	ENSGALG00010012179	2	-0.355161239	0.352919257	-1.006352677	0.314245949	0.168340916	2.900477262	65.52291538	1	PRJNA827465;Yang2025_Silkie_Wenchang	CRA004775	0.796531448
ENSGALG00010012179	ENSGALG00010012179	2	-0.561527228	0.21476795	-2.614576468	0.008933816	0	0.527545968	0	1	CRA004775;Yang2025_Silkie_Wenchang	PRJNA827465	0.148091847
ENSGALG00010012179	ENSGALG00010012179	2	-0.219308313	0.191364705	-1.14602279	0.251785722	0.018467078	1.32320308	24.42581076	1	CRA004775;PRJNA827465	Yang2025_Silkie_Wenchang	0.761871849
ENSGALG00010012180	DDX59	2	-0.119820463	0.069417836	-1.726076036	0.084333734	0	0.097387798	0	1	PRJNA827465;Yang2025_Silkie_Wenchang	CRA004775	0.500017819
ENSGALG00010012180	DDX59	2	-0.133326579	0.108837738	-1.225003215	0.220574009	0	0.118396634	0	1	CRA004775;Yang2025_Silkie_Wenchang	PRJNA827465	0.734053249
ENSGALG00010012180	DDX59	2	-0.099213707	0.081320393	-1.22003477	0.222451694	0	0.038235336	0	1	CRA004775;PRJNA827465	Yang2025_Silkie_Wenchang	0.736006309
ENSGALG00010012186	KIF20A	2	-0.213085353	0.104104237	-2.046846114	0.040673196	0.008576324	1.625590123	38.48387819	1	PRJNA827465;Yang2025_Silkie_Wenchang	CRA004775	0.348958445
ENSGALG00010012186	KIF20A	2	-0.044478195	0.426549328	-0.104274447	0.916951556	0.249597167	2.472665574	59.55781443	0.5	CRA004775;Yang2025_Silkie_Wenchang	PRJNA827465	0.980728718
ENSGALG00010012186	KIF20A	2	0.006055958	0.274109776	0.022093184	0.982373623	0.082287941	1.496310591	33.1689553	0.5	CRA004775;PRJNA827465	Yang2025_Silkie_Wenchang	0.99513702
ENSGALG00010012187	ENSGALG00010012187	2	-0.671366815	1.123525894	-0.597553487	0.550137908	2.17174007	6.946707131	85.60469038	0.5	PRJNA827465;Yang2025_Silkie_Wenchang	CRA004775	0.878679315
ENSGALG00010012187	ENSGALG00010012187	2	-0.704679823	1.314290429	-0.536167507	0.591842788	2.55763114	3.712136799	73.06133761	0.5	CRA004775;Yang2025_Silkie_Wenchang	PRJNA827465	0.892776493
ENSGALG00010012187	ENSGALG00010012187	2	0.424788267	0.406506491	1.044972902	0.296035494	0	0.101026616	0	1	CRA004775;PRJNA827465	Yang2025_Silkie_Wenchang	0.788124569
ENSGALG00010012188	ADAMTS6	2	0.258815562	0.219512527	1.17904689	0.238379505	0.040554694	1.669363925	40.09694442	1	PRJNA827465;Yang2025_Silkie_Wenchang	CRA004775	0.750543113
ENSGALG00010012188	ADAMTS6	2	0.538308823	0.267031193	2.015902402	0.043810182	0	0.002967284	0	1	CRA004775;Yang2025_Silkie_Wenchang	PRJNA827465	0.361804689
ENSGALG00010012188	ADAMTS6	2	0.116593397	0.189652323	0.614774423	0.538703703	0	0.430974848	0	1	CRA004775;PRJNA827465	Yang2025_Silkie_Wenchang	0.87481821
ENSGALG00010012190	CAST	2	-0.043790188	0.056005671	-0.781888465	0.434280124	0	0.180846689	0	1	PRJNA827465;Yang2025_Silkie_Wenchang	CRA004775	0.842762754
ENSGALG00010012190	CAST	2	0.001445721	0.094997031	0.015218592	0.987857789	0	0.012558063	0	0.5	CRA004775;Yang2025_Silkie_Wenchang	PRJNA827465	0.996800355
ENSGALG00010012190	CAST	2	-0.050390081	0.062130253	-0.81103937	0.417343062	0	0.143931126	0	0.5	CRA004775;PRJNA827465	Yang2025_Silkie_Wenchang	0.838112595
ENSGALG00010012191	BEST3	2	0.279577637	0.141552715	1.975077887	0.04825931	0	0.085468919	0	1	PRJNA827465;Yang2025_Silkie_Wenchang	CRA004775	0.379964424
ENSGALG00010012191	BEST3	2	0.238058214	0.192660628	1.235634993	0.216594271	0	0.000226697	0	1	CRA004775;Yang2025_Silkie_Wenchang	PRJNA827465	0.730172415
ENSGALG00010012191	BEST3	2	0.313076956	0.188175843	1.663746793	0.096163041	0	0.012752292	0	1	CRA004775;PRJNA827465	Yang2025_Silkie_Wenchang	0.530381139
ENSGALG00010012193	TTLL1	2	0.022990501	0.190798067	0.120496511	0.904089843	0	0.181899189	0	0.5	PRJNA827465;Yang2025_Silkie_Wenchang	CRA004775	0.977428704
ENSGALG00010012193	TTLL1	2	-0.131270193	0.290164611	-0.452399043	0.650981536	0	0.010782967	0	1	CRA004775;Yang2025_Silkie_Wenchang	PRJNA827465	0.908769703
ENSGALG00010012193	TTLL1	2	0.029919252	0.203460871	0.147051626	0.883091272	0	0.21094184	0	0.5	CRA004775;PRJNA827465	Yang2025_Silkie_Wenchang	0.972001076
ENSGALG00010012194	LRRFIP1	2	0.338047272	0.426526249	0.792559128	0.42803473	0.346689548	20.79590342	95.19136063	0.5	PRJNA827465;Yang2025_Silkie_Wenchang	CRA004775	0.84125685
ENSGALG00010012194	LRRFIP1	2	0.409753885	0.416306691	0.984259667	0.324987855	0.280112258	4.904814825	79.61187047	0.5	CRA004775;Yang2025_Silkie_Wenchang	PRJNA827465	0.799914637
ENSGALG00010012194	LRRFIP1	2	-0.074646495	0.076500233	-0.975768209	0.329179342	0	0.00176528	0	1	CRA004775;PRJNA827465	Yang2025_Silkie_Wenchang	0.800761292
ENSGALG00010012195	ITSN1	2	0.063815408	0.252935761	0.252298874	0.800810058	0.109423245	6.378271151	84.32177033	0.5	PRJNA827465;Yang2025_Silkie_Wenchang	CRA004775	0.950158029
ENSGALG00010012195	ITSN1	2	0.291038913	0.155889192	1.86696017	0.06190715	0	0.291501265	0	1	CRA004775;Yang2025_Silkie_Wenchang	PRJNA827465	0.429324205
ENSGALG00010012195	ITSN1	2	-0.104473667	0.123888277	-0.843289365	0.399066629	0.013522938	1.333370828	25.00210902	0.5	CRA004775;PRJNA827465	Yang2025_Silkie_Wenchang	0.832054308
ENSGALG00010012196	CENPK	2	-0.012374963	0.12740232	-0.09713295	0.922620814	0	0.456711491	0	0.5	PRJNA827465;Yang2025_Silkie_Wenchang	CRA004775	0.981804675
ENSGALG00010012196	CENPK	2	-0.133718489	0.183450094	-0.728909352	0.466057114	0	0.138632261	0	1	CRA004775;Yang2025_Silkie_Wenchang	PRJNA827465	0.852630314
ENSGALG00010012196	CENPK	2	0.031196645	0.162417037	0.192077418	0.847681563	0	0.443438108	0	0.5	CRA004775;PRJNA827465	Yang2025_Silkie_Wenchang	0.961304071
ENSGALG00010012198	TMEM5	2	-0.147851595	0.08023163	-1.842809306	0.065356858	0	0.173383013	0	1	PRJNA827465;Yang2025_Silkie_Wenchang	CRA004775	0.440704235
ENSGALG00010012198	TMEM5	2	-0.042792508	0.125201622	-0.341788771	0.73250986	0	0.648679437	0	0.5	CRA004775;Yang2025_Silkie_Wenchang	PRJNA827465	0.932985777
ENSGALG00010012198	TMEM5	2	-0.108272308	0.124184594	-0.871865859	0.383281561	0.009900956	1.259301734	20.59091374	0.5	CRA004775;PRJNA827465	Yang2025_Silkie_Wenchang	0.825476201
ENSGALG00010012199	ENSGALG00010012199	2	-0.662602337	0.397436653	-1.667189808	0.095476669	0.097958766	1.445065412	30.79898032	1	PRJNA827465;Yang2025_Silkie_Wenchang	CRA004775	0.529041803
ENSGALG00010012199	ENSGALG00010012199	2	-0.334016506	0.664650406	-0.502544651	0.615284452	0.73027148	5.71242459	82.49429845	0.5	CRA004775;Yang2025_Silkie_Wenchang	PRJNA827465	0.898844961
ENSGALG00010012199	ENSGALG00010012199	2	0.131308257	0.280777455	0.467659546	0.640028075	0	0.784424383	0	0.5	CRA004775;PRJNA827465	Yang2025_Silkie_Wenchang	0.906064918
ENSGALG00010012200	GEM	2	-0.376004802	0.194322862	-1.93494887	0.05299657	0.060519101	5.016199522	80.06458883	1	PRJNA827465;Yang2025_Silkie_Wenchang	CRA004775	0.399631766
ENSGALG00010012200	GEM	2	-0.178633978	0.457278259	-0.39064612	0.69605883	0.349808859	5.56281128	82.02347789	0.5	CRA004775;Yang2025_Silkie_Wenchang	PRJNA827465	0.921038437
ENSGALG00010012200	GEM	2	-0.035123926	0.2412834	-0.145571251	0.884259865	0.068525822	1.920465457	47.92928994	0.5	CRA004775;PRJNA827465	Yang2025_Silkie_Wenchang	0.972001076
ENSGALG00010012207	GRIA3	2	-0.023782465	0.108736153	-0.218717182	0.826870364	0	0.323520077	0	0.5	PRJNA827465;Yang2025_Silkie_Wenchang	CRA004775	0.956540044
ENSGALG00010012207	GRIA3	2	0.594734498	0.848714138	0.700747722	0.483460469	1.212731759	5.78434604	82.71196099	0.5	CRA004775;Yang2025_Silkie_Wenchang	PRJNA827465	0.85691916
ENSGALG00010012207	GRIA3	2	0.646631689	0.766082524	0.84407576	0.398627072	0.981383149	5.249540041	80.95071202	1	CRA004775;PRJNA827465	Yang2025_Silkie_Wenchang	0.831980453
ENSGALG00010012209	KIF14	2	0.059297504	0.119185115	0.497524413	0.618819292	0.010985381	1.513382582	33.92285522	0.5	PRJNA827465;Yang2025_Silkie_Wenchang	CRA004775	0.899168321
ENSGALG00010012209	KIF14	2	0.267098945	0.175371616	1.523045467	0.127747331	0	0.651965794	0	1	CRA004775;Yang2025_Silkie_Wenchang	PRJNA827465	0.600026204
ENSGALG00010012209	KIF14	2	0.107353424	0.306157397	0.350647818	0.725852578	0.100768073	1.38975198	28.04471484	0.5	CRA004775;PRJNA827465	Yang2025_Silkie_Wenchang	0.931029023
ENSGALG00010012210	PPWD1	2	-0.07849508	0.110414552	-0.710912453	0.477138489	0	0.564473962	0	0.5	PRJNA827465;Yang2025_Silkie_Wenchang	CRA004775	0.854986476
ENSGALG00010012210	PPWD1	2	-0.171807731	0.133197843	-1.289868715	0.197096245	0	0.012626359	0	1	CRA004775;Yang2025_Silkie_Wenchang	PRJNA827465	0.708281403
ENSGALG00010012210	PPWD1	2	-0.051562138	0.132053589	-0.390463738	0.696193664	0	0.455057432	0	0.5	CRA004775;PRJNA827465	Yang2025_Silkie_Wenchang	0.921038437
ENSGALG00010012211	ENSGALG00010012211	2	-5.913339266	5.918334748	-0.999155931	0.31771916	65.05549687	13.78807498	92.74735595	1	PRJNA827465;Yang2025_Silkie_Wenchang	CRA004775	0.797402053
ENSGALG00010012211	ENSGALG00010012211	2	-5.497390783	6.298375908	-0.87282672	0.382757533	74.97375418	17.71075658	94.35371383	0.5	CRA004775;Yang2025_Silkie_Wenchang	PRJNA827465	0.825121999
ENSGALG00010012211	ENSGALG00010012211	2	0.293912806	0.949097991	0.309675933	0.756807406	0	0.146432168	0	0.5	CRA004775;PRJNA827465	Yang2025_Silkie_Wenchang	0.938740241
ENSGALG00010012212	RAD54B	2	0.026900838	0.08951963	0.300502108	0.763794189	0	0.672789801	0	0.5	PRJNA827465;Yang2025_Silkie_Wenchang	CRA004775	0.940361611
ENSGALG00010012212	RAD54B	2	0.121804262	0.149841146	0.812889287	0.416281537	0	0.049125939	0	0.5	CRA004775;Yang2025_Silkie_Wenchang	PRJNA827465	0.837742614
ENSGALG00010012212	RAD54B	2	-0.02535982	0.108476876	-0.233780881	0.815155093	0	0.000119287	0	1	CRA004775;PRJNA827465	Yang2025_Silkie_Wenchang	0.953933933
ENSGALG00010012215	RAB17	2	-1.157025733	0.430313522	-2.688797058	0.007171	0	0.042601186	0	1	PRJNA827465;Yang2025_Silkie_Wenchang	CRA004775	0.130241134
ENSGALG00010012215	RAB17	2	-0.208799864	0.819044452	-0.254931952	0.798776351	1.035033792	4.342712047	76.97291487	0.5	CRA004775;Yang2025_Silkie_Wenchang	PRJNA827465	0.949922217
ENSGALG00010012215	RAB17	2	-0.302973435	0.908166468	-0.333610021	0.738673855	1.344971475	5.383963729	81.42632361	0.5	CRA004775;PRJNA827465	Yang2025_Silkie_Wenchang	0.934559747
ENSGALG00010012216	BRD8	2	-0.04000107	0.04076673	-0.981218494	0.326485006	0	0.042367814	0	1	PRJNA827465;Yang2025_Silkie_Wenchang	CRA004775	0.800028239
ENSGALG00010012216	BRD8	2	-0.149627373	0.140313916	-1.066375865	0.286253767	0.027495029	3.23021125	69.04227239	1	CRA004775;Yang2025_Silkie_Wenchang	PRJNA827465	0.781944441
ENSGALG00010012216	BRD8	2	-0.147324025	0.127757361	-1.153154882	0.248864819	0.024535236	3.666611467	72.72686214	1	CRA004775;PRJNA827465	Yang2025_Silkie_Wenchang	0.725090933
ENSGALG00010012219	KDM5A	2	-0.112706899	0.081161689	-1.388671196	0.164932757	0.009122321	2.694948002	62.89353267	1	PRJNA827465;Yang2025_Silkie_Wenchang	CRA004775	0.664243485
ENSGALG00010012219	KDM5A	2	-0.208463334	0.094064134	-2.216183001	0.026678972	0	0.249176471	0	1	CRA004775;Yang2025_Silkie_Wenchang	PRJNA827465	0.278260672
ENSGALG00010012219	KDM5A	2	-0.05343897	0.031299099	-1.707354453	0.087754333	0	0.001657484	0	1	CRA004775;PRJNA827465	Yang2025_Silkie_Wenchang	0.59353637
ENSGALG00010012228	NHP2	2	0.263780008	0.101578951	2.596797928	0.009409726	0	0.22737005	0	1	PRJNA827465;Yang2025_Silkie_Wenchang	CRA004775	0.152724616
ENSGALG00010012228	NHP2	2	0.057051578	0.30799726	0.185234043	0.853045468	0.133431697	3.369533673	70.32230282	0.5	CRA004775;Yang2025_Silkie_Wenchang	PRJNA827465	0.962708451
ENSGALG00010012228	NHP2	2	0.039505233	0.240829952	0.164037873	0.86970133	0.086173924	3.573002908	72.01233736	0.5	CRA004775;PRJNA827465	Yang2025_Silkie_Wenchang	0.968586462
ENSGALG00010012229	FSBP	2	-0.152065236	0.095875891	-1.586063347	0.11272494	0.006986121	1.600877113	37.53424346	1	PRJNA827465;Yang2025_Silkie_Wenchang	CRA004775	0.566553488
ENSGALG00010012229	FSBP	2	-0.241372629	0.113172516	-2.132784854	0.032942379	0	0.361963677	0	0.5	CRA004775;Yang2025_Silkie_Wenchang	PRJNA827465	0.311683759
ENSGALG00010012229	FSBP	2	-0.062400403	0.095535743	-0.653162897	0.513651267	0	0.034347768	0	0.5	CRA004775;PRJNA827465	Yang2025_Silkie_Wenchang	0.866528862
ENSGALG00010012232	PCSK1	2	0.296942706	0.199123371	1.491249892	0.135895899	0	0.120922244	0	1	PRJNA827465;Yang2025_Silkie_Wenchang	CRA004775	0.617467165
ENSGALG00010012232	PCSK1	2	0.406145933	0.299709901	1.355130183	0.175376084	0	0.016742618	0	1	CRA004775;Yang2025_Silkie_Wenchang	PRJNA827465	0.680258136
ENSGALG00010012232	PCSK1	2	0.271032008	0.235954765	1.148660878	0.250695844	0	0.104702832	0	1	CRA004775;PRJNA827465	Yang2025_Silkie_Wenchang	0.760328632
ENSGALG00010012234	C1GALT1C1	2	-0.098170895	0.251812641	-0.389856899	0.696642367	0.122117147	26.82709525	96.27242536	0.5	PRJNA827465;Yang2025_Silkie_Wenchang	CRA004775	0.92106851
ENSGALG00010012234	C1GALT1C1	2	-0.188109398	0.181991269	-1.033617702	0.30131492	0.053966375	5.19218744	80.74003987	0.5	CRA004775;Yang2025_Silkie_Wenchang	PRJNA827465	0.791222412
ENSGALG00010012234	C1GALT1C1	2	0.131899384	0.050235097	2.625642093	0.00864857	0	0.862366569	0	1	CRA004775;PRJNA827465	Yang2025_Silkie_Wenchang	0.145573772
ENSGALG00010012240	TRIM14	2	-0.013668045	0.182972671	-0.074699925	0.940453467	0	0.170756481	0	0.5	PRJNA827465;Yang2025_Silkie_Wenchang	CRA004775	0.985539065
ENSGALG00010012240	TRIM14	2	0.118855072	0.26054953	0.456170741	0.648267204	0	0.044064931	0	1	CRA004775;Yang2025_Silkie_Wenchang	PRJNA827465	0.90762535
ENSGALG00010012240	TRIM14	2	-0.023342989	0.209174856	-0.111595578	0.911144078	0	0.254183978	0	0.5	CRA004775;PRJNA827465	Yang2025_Silkie_Wenchang	0.978845851
ENSGALG00010012242	CCDC77	2	-0.000538112	0.074535507	-0.007219739	0.994239532	0	0.418609891	0	0.5	PRJNA827465;Yang2025_Silkie_Wenchang	CRA004775	0.998227056
ENSGALG00010012242	CCDC77	2	0.061862967	0.150784656	0.410273624	0.681605238	0	0.462472242	0	0.5	CRA004775;Yang2025_Silkie_Wenchang	PRJNA827465	0.918322769

ENSGALG00010012242	CCDC77	2	-0.03279301	0.084123133	-0.389821556	0.696668503	0	0.260394214	0	1	CRA004775;PRJNA827465	Yang2025_Silkie_Wenchang	0.92106851
ENSGALG00010012243	ENSGALG00010012243	2	-2.192636697	2.260845871	-0.969830242	0.332131116	7.692610523	4.004123396	75.02574469	1	PRJNA827465;Yang2025_Silkie_Wenchang	CRA004775	0.802073687
ENSGALG00010012243	ENSGALG00010012243	2	-2.60249772	1.687741779	-1.541999939	0.123073609	3.640482791	2.654093703	62.32235513	1	CRA004775;Yang2025_Silkie_Wenchang	PRJNA827465	0.588033313
ENSGALG00010012243	ENSGALG00010012243	2	-0.728660562	0.884120312	-0.824164486	0.409846103	0	0.375426123	0	1	CRA004775;PRJNA827465	Yang2025_Silkie_Wenchang	0.834652129
ENSGALG00010012244	ENSGALG00010012244	2	-0.119745909	0.078405231	-1.527269394	0.126694037	0.009296011	3.994213608	74.96378266	1	PRJNA827465;Yang2025_Silkie_Wenchang	CRA004775	0.597476858
ENSGALG00010012244	ENSGALG00010012244	2	-0.200680552	0.064297756	-3.121112815	0.00180169	0	0.221971036	0	1	CRA004775;Yang2025_Silkie_Wenchang	PRJNA827465	0.054137478
ENSGALG00010012244	ENSGALG00010012244	2	-0.047920808	0.043972122	-1.089799755	0.275801362	0	0.005159937	0	1	CRA004775;PRJNA827465	Yang2025_Silkie_Wenchang	0.7754953
ENSGALG00010012245	ENSGALG00010012245	2	-0.033537339	0.046582017	-0.719963233	0.471547633	0	0.051477433	0	1	PRJNA827465;Yang2025_Silkie_Wenchang	CRA004775	0.853998373
ENSGALG00010012245	ENSGALG00010012245	2	-0.021909517	0.084643039	-0.258846063	0.795754016	0	0.825189229	0	0.5	CRA004775;Yang2025_Silkie_Wenchang	PRJNA827465	0.949107318
ENSGALG00010012245	ENSGALG00010012245	2	-0.016934317	0.052958042	-0.319768569	0.749143776	0	0.721533609	0	0.5	CRA004775;PRJNA827465	Yang2025_Silkie_Wenchang	0.937362187
ENSGALG00010012247	TRIM23	2	-0.116846296	0.113296759	-1.031329547	0.302386307	0.002986621	1.126321128	11.2153741	1	PRJNA827465;Yang2025_Silkie_Wenchang	CRA004775	0.791784745
ENSGALG00010012247	TRIM23	2	-0.016139337	0.284368475	-0.056755014	0.954740349	0.108805691	2.872417859	65.18612371	0.5	CRA004775;Yang2025_Silkie_Wenchang	PRJNA827465	0.987987175
ENSGALG00010012247	TRIM23	2	0.050837854	0.142255121	0.357370998	0.720814085	0.007144368	1.134645766	11.86676671	0.5	CRA004775;PRJNA827465	Yang2025_Silkie_Wenchang	0.928918486
ENSGALG00010012248	H2AC39	2	0.05696772	0.186952578	0.30471749	0.760581328	0	0.453688409	0	0.5	PRJNA827465;Yang2025_Silkie_Wenchang	CRA004775	0.939384641
ENSGALG00010012248	H2AC39	2	-0.404129205	0.648792431	-0.62289445	0.533353885	0.719963036	6.870859302	85.44577969	0.5	CRA004775;Yang2025_Silkie_Wenchang	PRJNA827465	0.873379507
ENSGALG00010012248	H2AC39	2	-0.509696762	0.515742443	-0.988277712	0.323016658	0.43508133	5.310247747	81.1684869	1	CRA004775;PRJNA827465	Yang2025_Silkie_Wenchang	0.798909393
ENSGALG00010012250	NANS	2	0.031061712	0.327153923	0.094945251	0.924358313	0.196822612	12.29011713	91.86338104	0.5	PRJNA827465;Yang2025_Silkie_Wenchang	CRA004775	0.982371283
ENSGALG00010012250	NANS	2	-0.302544629	0.134474643	-2.249826601	0.024459955	0	0.00222964	0	1	CRA004775;Yang2025_Silkie_Wenchang	PRJNA827465	0.267184364
ENSGALG00010012250	NANS	2	0.077903427	0.316081127	0.246466556	0.80532109	0.162159778	4.813056503	79.22318179	0.5	CRA004775;PRJNA827465	Yang2025_Silkie_Wenchang	0.951870339
ENSGALG00010012253	MYF6	2	-0.015111256	0.15133054	-0.099855958	0.920458682	0.030086567	2.842360817	64.81797828	0.5	PRJNA827465;Yang2025_Silkie_Wenchang	CRA004775	0.98144788
ENSGALG00010012253	MYF6	2	0.14675226	0.144176074	1.017868333	0.308740533	0	0.072453207	0	0.5	CRA004775;Yang2025_Silkie_Wenchang	PRJNA827465	0.793670558
ENSGALG00010012253	MYF6	2	-0.147612186	0.103702651	-1.423417673	0.154615112	0	0.022827818	0	1	CRA004775;PRJNA827465	Yang2025_Silkie_Wenchang	0.647505355
ENSGALG00010012259	IL1RAPL2	2	-0.031578909	0.15993074	-0.197453657	0.843472544	0	0.120401866	0	0.5	PRJNA827465;Yang2025_Silkie_Wenchang	CRA004775	0.96057435
ENSGALG00010012259	IL1RAPL2	2	0.372448162	0.421023559	0.884625465	0.376358668	0.23622335	2.763362236	63.81219998	1	CRA004775;Yang2025_Silkie_Wenchang	PRJNA827465	0.822974013
ENSGALG00010012259	IL1RAPL2	2	0.320611044	0.478694689	0.669761022	0.503010144	0.344576239	3.69219558	72.91584429	0.5	CRA004775;PRJNA827465	Yang2025_Silkie_Wenchang	0.863050365
ENSGALG00010012260	ENSGALG00010012260	2	0.427820771	0.39916443	1.071790816	0.283813999	0.06190258	1.167695433	14.36123055	0.5	PRJNA827465;Yang2025_Silkie_Wenchang	CRA004775	0.77978499
ENSGALG00010012260	ENSGALG00010012260	2	-0.227086	0.555002987	-0.409161762	0.682420954	0	0.007647673	0	1	CRA004775;Yang2025_Silkie_Wenchang	PRJNA827465	0.918625039
ENSGALG00010012260	ENSGALG00010012260	2	0.499066804	0.347977244	1.434193792	0.151516968	0	0.869825061	0	0.5	CRA004775;PRJNA827465	Yang2025_Silkie_Wenchang	0.643023891
ENSGALG00010012263	SHLD3	2	0.11402604	0.163810769	0.696083904	0.486376286	0.013059971	1.321461758	24.32622482	0.5	PRJNA827465;Yang2025_Silkie_Wenchang	CRA004775	0.857603818
ENSGALG00010012263	SHLD3	2	-0.040233343	0.170866353	-0.23546674	0.813846491	0	0.012868108	0	1	CRA004775;Yang2025_Silkie_Wenchang	PRJNA827465	0.953869515
ENSGALG00010012263	SHLD3	2	0.192484304	0.167902098	1.146407978	0.251626383	0	0.592767462	0	0.5	CRA004775;PRJNA827465	Yang2025_Silkie_Wenchang	0.761706574
ENSGALG00010012264	MYF5	2	-0.045410996	0.087835725	-0.516999153	0.60515675	0	0.001979148	0	1	PRJNA827465;Yang2025_Silkie_Wenchang	CRA004775	0.896474603
ENSGALG00010012264	MYF5	2	0.155012116	0.334460735	0.463468802	0.643028372	0.107292575	1.557804703	35.80710096	0.5	CRA004775;Yang2025_Silkie_Wenchang	PRJNA827465	0.906634523
ENSGALG00010012264	MYF5	2	0.132047309	0.329340965	0.400944077	0.688461297	0.131147422	1.743797766	42.65390061	0.5	CRA004775;PRJNA827465	Yang2025_Silkie_Wenchang	0.919798626
ENSGALG00010012268	ENSGALG00010012268	2	-0.03746075	0.153620435	-0.24385265	0.807344942	0	0.036746403	0	1	PRJNA827465;Yang2025_Silkie_Wenchang	CRA004775	0.95234753
ENSGALG00010012268	ENSGALG00010012268	2	-0.026899125	0.184328787	-0.145930136	0.883976541	0	0.145177301	0	0.5	CRA004775;Yang2025_Silkie_Wenchang	PRJNA827465	0.972001076
ENSGALG00010012268	ENSGALG00010012268	2	0.021935409	0.195439904	0.112236084	0.910636219	0	0.05891852	0	0.5	CRA004775;PRJNA827465	Yang2025_Silkie_Wenchang	0.97880407
ENSGALG00010012271	ENSGALG00010012271	2	0.211857795	0.239423957	0.884864648	0.376229637	0.074769675	2.800652841	64.2940394	0.5	PRJNA827465;Yang2025_Silkie_Wenchang	CRA004775	0.822974013
ENSGALG00010012271	ENSGALG00010012271	2	0.461279193	0.213182863	2.163772384	0.030481823	0	0.041990183	0	1	CRA004775;Yang2025_Silkie_Wenchang	PRJNA827465	0.297646478
ENSGALG00010012271	ENSGALG00010012271	2	0.035631812	0.15800745	0.225507167	0.821584751	0	0.481865546	0	0.5	CRA004775;PRJNA827465	Yang2025_Silkie_Wenchang	0.955598716
ENSGALG00010012274	B4GALNT3	2	0.044304698	0.148477249	0.298393851	0.765402581	0.034832455	4.370438947	77.1190031	0.5	PRJNA827465;Yang2025_Silkie_Wenchang	CRA004775	0.940816815
ENSGALG00010012274	B4GALNT3	2	-0.155577829	0.124262834	-1.252006119	0.210567634	0	0.373856032	0	1	CRA004775;Yang2025_Silkie_Wenchang	PRJNA827465	0.722116705
ENSGALG00010012274	B4GALNT3	2	0.026819206	0.240772849	0.111387998	0.911308677	0.075508443	2.029935734	50.7373567	0.5	CRA004775;PRJNA827465	Yang2025_Silkie_Wenchang	0.978845851
ENSGALG00010012275	VIRMA	2	-0.079999283	0.039337243	-2.033677903	0.041984082	0	0.551278348	0	1	PRJNA827465;Yang2025_Silkie_Wenchang	CRA004775	0.354529664
ENSGALG00010012275	VIRMA	2	-0.020911086	0.097628324	-0.214190774	0.83039829	0	0.117582995	0	1	CRA004775;Yang2025_Silkie_Wenchang	PRJNA827465	0.957262428
ENSGALG00010012275	VIRMA	2	-0.092238615	0.041946959	-2.198934485	0.027882581	0	0.003441479	0	1	CRA004775;PRJNA827465	Yang2025_Silkie_Wenchang	0.284557362
ENSGALG00010012277	H3C33	2	0.101750657	0.552792673	0.184066581	0.853961223	0.561447587	12.27697688	91.85467229	0.5	PRJNA827465;Yang2025_Silkie_Wenchang	CRA004775	0.962975689
ENSGALG00010012277	H3C33	2	0.179020996	0.490231637	0.365176343	0.714979776	0.415783936	7.394826664	86.47703259	0.5	CRA004775;Yang2025_Silkie_Wenchang	PRJNA827465	0.926823015
ENSGALG00010012277	H3C33	2	-0.398776749	0.164706696	-2.421132592	0.015472233	0	0.1342254	0	1	CRA004775;PRJNA827465	Yang2025_Silkie_Wenchang	0.205718931
ENSGALG00010012282	ENSGALG00010012282	2	-1.425134086	1.000029665	-1.425091811	0.154130664	1.041727358	2.08276633	51.98693268	1	PRJNA827465;Yang2025_Silkie_Wenchang	CRA004775	0.646788679
ENSGALG00010012282	ENSGALG00010012282	2	-1.004998863	1.4019899	-0.716837449	0.473474402	2.988656702	4.167084406	76.00240594	0.5	CRA004775;Yang2025_Silkie_Wenchang	PRJNA827465	0.854373829
ENSGALG00010012282	ENSGALG00010012282	2	0.027297665	0.71746623	0.038047316	0.969649956	0	0.31265898	0	0.5	CRA004775;PRJNA827465	Yang2025_Silkie_Wenchang	0.991572914
ENSGALG00010012283	CUPIN1A	2	0.862838417	0.398221935	2.1667275	0.030255638	0	0.032156719	0	1	PRJNA827465;Yang2025_Silkie_Wenchang	CRA004775	0.296099939
ENSGALG00010012283	CUPIN1A	2	0.774425936	0.43663255	1.773633085	0.076123861	0	2.77678E-07	0	1	CRA004775;Yang2025_Silkie_Wenchang	PRJNA827465	0.476155344
ENSGALG00010012283	CUPIN1A	2	0.859289487	0.390570123	2.20009017	0.027800498	0	0.034237619	0	1	CRA004775;PRJNA827465	Yang2025_Silkie_Wenchang	0.28439432
ENSGALG00010012285	FBLN1	2	-0.193565614	0.214076261	-0.904189996	0.365894664	0.076589849	5.894651551	83.03546882	0.5	PRJNA827465;Yang2025_Silkie_Wenchang	CRA004775	0.818968857
ENSGALG00010012285	FBLN1	2	-0.18289641	0.31503011	-0.580568028	0.561531626	0.135064937	2.746226908	63.58640297	0.5	CRA004775;Yang2025_Silkie_Wenchang	PRJNA827465	0.881737015
ENSGALG00010012285	FBLN1	2	0.01737756	0.088523453	0.196304586	0.844371772	0	0.352757635	0	1	CRA004775;PRJNA827465	Yang2025_Silkie_Wenchang	0.960752464
ENSGALG00010012286	ENSGALG00010012286	2	-6.876510315	6.779436501	-1.014318862	0.310430627	86.02241662	15.3867423	93.5008985	1	PRJNA827465;Yang2025_Silkie_Wenchang	CRA004775	0.794899969
ENSGALG00010012286	ENSGALG00010012286	2	-6.539147206	7.053766413	-0.927043344	0.35390403	94.6424486	19.90796633	94.97688521	0.5	CRA004775;Yang2025_Silkie_Wenchang	PRJNA827465	0.811474832
ENSGALG00010012286	ENSGALG00010012286	2	0.099365627	0.968278989	0.102620864	0.918263883	0	0.066650658	0	0.5	CRA004775;PRJNA827465	Yang2025_Silkie_Wenchang	0.981033557
ENSGALG00010012288	MCTS1	2	-0.28011976	0.245791442	-1.139664417	0.254426137	0.107548466	8.533095085	88.28092281	1	PRJNA827465;Yang2025_Silkie_Wenchang	CRA004775	0.764087626
ENSGALG00010012288	MCTS1	2	-0.178873819	0.406765016	-0.439747303	0.660120138	0.277343757	5.910151073	83.07995874	0.5	CRA004775;Yang2025_Silkie_Wenchang	PRJNA827465	0.911833064
ENSGALG00010012288	MCTS1	2	-0.024384706	0.095595778	-0.255081409	0.798660222	0.006868355	1.151101795	13.12671007	0.5	CRA004775;PRJNA827465	Yang2025_Silkie_Wenchang	0.949922217
ENSGALG00010012290	ENSGALG00010012290	2	0.062805325	0.083892425	0.748641193	0.454073497	0	0.292131649	0	0.5	PRJNA827465;Yang2025_Silkie_Wenchang	CRA004775	0.849072665
ENSGALG00010012290	ENSGALG00010012290	2	-0.179981651	0.155094137	-1.160467153	0.24585866	0	0.900354011	0	1	CRA004775;Yang2025_Silkie_Wenchang	PRJNA827465	0.75661406
ENSGALG00010012290	ENSGALG00010012290	2	-0.076068543	0.20351488	-0.373773862	0.708572567	0.058163805	2.939540639	65.98107927	0.5	CRA004775;PRJNA827465	Yang2025_Silkie_Wenchang	0.925093928
ENSGALG00010012291	NR5A2	2	0.146803158	0.090587109	1.62057449	0.105108927	0	0.878000926	0	1	PRJNA827465;Yang2025_Silkie_Wenchang	CRA004775	0.550372522
ENSGALG00010012291	NR5A2	2	0.273789259	0.185265696	1.477819506	0.139456096	0	0.344880543	0	0.5	CRA004775;Yang2025_Silkie_Wenchang	PRJNA827465	0.623080711
ENSGALG00010012291	NR5A2	2	0.097291364	0.101101826	0.96231065	0.335893581	0	0.079903966	0	0.5	CRA004775;PRJNA827465	Yang2025_Silkie_Wenchang	0.803504387
ENSGALG00010012293	SRGAP1	2	-0.041613829	0.137937422	-0.301686289	0.762891221	0.025014926	2.349264187	57.43348043	0.5	PRJNA827465;Yang2025_Silkie_Wenchang	CRA004775	0.940082621
ENSGALG00010012293	SRGAP1	2	0.096099242	0.13488571	0.712449391	0.47618654	0	0.245303001	0	1	CRA004775;Yang2025_Silkie_Wenchang	PRJNA827465	0.85482596
ENSGALG00010012293	SRGAP1	2	-0.125040144	0.053277943	-2.346940161	0.018928294	0	0.616193468	0	0.5	CRA004775;PRJNA827465	Yang2025_Silkie_Wenchang	0.230607178
ENSGALG00010012295	CUL4B	2	-0.031458179	0.040735657	-0.772251652	0.439965395	0	0.043893118	0	1	PRJNA827465;Yang2025_Silkie_Wenchang	CRA004775	0.844353254
ENSGALG00010012295	CUL4B	2	-0.014363302	0.110517321	-0.129964261	0.896594703	0	0.725210319	0	0.5	CRA004775;Yang2025_Silkie_Wenchang	PRJNA827465	0.975115773
ENSGALG00010012295	CUL4B	2	-0.02291132	0.042768089	-0.535710632	0.592158555	0	0.675342398	0	0.5	CRA004775;PRJNA827465	Yang2025_Silkie_Wenchang	0.892776493
ENSGALG00010012297	ABLIM3	2	0.244230531	0.220862237	1.105804843	0.268810998	0.079663124	5.25932731	80.98616152	1	PRJNA827465;Yang2025_Silkie_Wenchang	CRA004775	0.771398294
ENSGALG00010012297	ABLIM3	2	0.281364818	0.294308346	0.95602052	0.339061867	0.100917595	2.049407654	51.20541303	0.5	CRA004775;Yang2025_Silkie_Wenchang	PRJNA827465	0.804767595
ENSGALG00010012297	ABLIM3	2	0.03218713	0.096169033	0.334693288	0.737856465	0	0.194586709	0	0.5	CRA004775;PRJNA827465	Yang2025_Silkie_Wenchang	0.934161348
ENSGALG00010012300	NINJ2	2	0.340865866	0.328095315	1.038923296	0.298840411	0.154933369	3.441879624	70.94610768	1	PRJNA827465;Yang2025_Silkie_Wenchang	CRA004775	0.78974322
ENSGALG00010012300	NINJ2	2	0.630253805	0.264605462	2.381862415	0.017225332	0	0.332730651	0	1	CRA004775;Yang2025_Silkie_Wenchang	PRJNA827465	0.21809044
ENSGALG00010012300	NINJ2	2	0.079963916	0.183240232	0.436388425	0.662554936	0	0.234482104	0	1	CRA004775;PRJNA827465	Yang2025_Silkie_Wenchang	0.912778072
ENSGALG00010012303	CA9	2	0.176857318	0.262301357	0.674252392	0.500150866	0.118721858	7.28103307	86.26568523	0.5	PRJNA827465;Yang2025_Silkie_Wenchang	CRA004775	0.862205775
ENSGALG00010012303	CA9	2	0.452217619	0.138656314	3.261428244	0.001108525	0	0.074847423	0	1	CRA004775;Yang2025_Silkie_Wenchang	PRJNA827465	0.040192207
ENSGALG00010012303	CA9	2	0.049131019	0.268457294	0.18301242	0.854788273	0.072090501	1.453772089	31.21342693	0.5	CRA004775;PRJNA827465	Yang2025_Silkie_Wenchang	0.963248391
ENSGALG00010012307	ENSGALG00010012307	2	0.08052739	0.171814805	0.468687142	0.639293279	0.018211159	1.416126813	29.38485518	0.5	PRJNA827465;Yang2025_Silkie_Wenchang	CRA004775	0.905968982
ENSGALG00010012307	ENSGALG00010012307	2	0.122732239	0.214283699	0.572755835	0.566810026	0.018297758	1.234541384	18.99826014	0.5	CRA004775;Yang2025_Silkie_Wenchang	PRJNA827465	0.883487517
ENSGALG00010012307	ENSGALG00010012307	2	-0.077573864	0.15223408	-0.50956963	0.610353005	0	0.058413711	0	1	CRA004775;PRJNA827465	Yang2025_Silkie_Wenchang	0.897438028
ENSGALG00010012308	TRABD2A	2	-0.287372065	0.195991714	-1.466245997	0.142581282	0	0.054558759	0	1	PRJNA827465;Yang2025_Silkie_Wenchang	CRA004775	0.628893678
ENSGALG00010012308	TRABD2A	2	-0.297012185	0.28446728	-1.044099641	0.296439292	0	0.118470708	0	1	CRA004775;Yang2025_Silkie_Wenchang	PRJNA827465	0.788267543
ENSGALG00010012308	TRABD2A	2	-0.231553363	0.231902003	-0.99849661	0.318038608	0	0.048611359	0	1	CRA004775;PRJNA827465	Yang2025_Silkie_Wenchang	0.797439961
ENSGALG00010012309	TRAPPC13	2	0.082540518	0.120097427	0.687279652	0.491906519	0	0.029682979	0	1	PRJNA827465;Yang2025_Silkie_Wenchang	CRA004775	0.859033231
ENSGALG00010012309	TRAPPC13	2	0.019938262	0.136440859	0.146131171	0.88381784	0	0.641753706	0	0.5	CRA004775;Yang2025_Silkie_Wenchang	PRJNA827465	0.972001076
ENSGALG00010012309	TRAPPC13	2	-0.004070016	0.134370147	-0.030289586	0.975836102	0	0.436091972	0	0.5	CRA004775;PRJNA827465	Yang2025_Silkie_Wenchang	0.992836819
ENSGALG00010012311	ENSGALG00010012311	2	4.861861351	0.220942751	22.00507297	2.575E-107	0	0.034281497	0	1	PRJNA827465;Yang2025_Silkie_Wenchang	CRA004775	1.6868E-103
ENSGALG00010012311	ENSGALG00010012311	2	4.102529118	0.873404438	4.697169994	2.63791E-06	1.3096628	6.690558897	85.05356555	1	CRA004775;Yang2025_Silkie_Wenchang	PRJNA827465	0.000510017
ENSGALG00010012311	ENSGALG00010012311	2	4.054115596	0.832259702	4.871214581	1.10914E-06	1.148726255	5.622133748	82.21315883	1	CRA004775;PRJNA827465	Yang2025_Silkie_Wenchang	0.000240843
ENSGALG00010012312	SH3TC2	2	0.15387106	0.181080307	0.849739337	0.395470023	0.04043119	2.578051672	61.21101796	0.5	PRJNA827465;Yang2025_Silkie_Wenchang	CRA004775	0.830946177
ENSGALG00010012312	SH3TC2	2	-0.081987108	0.165921152	-0.494132946	0.621212292	0	0.408947954	0	1	CRA004775;Yang2025_Silkie_Wenchang	PRJNA827465	0.899614409
ENSGALG00010012312	SH3TC2	2	0.128207599	0.310055429	0.413498966	0.67924108	0.112973285	1.890201804	47.09559594	0.5	CRA004775;PRJNA827465	Yang2025_Silkie_Wenchang	0.917701868
ENSGALG00010012314	ADRB2	2	-0.0497355	0.199229023	-0.249639833	0.802865891	0.039890519	1.982403472	49.55618197	0.5	PRJNA827465;Yang2025_Silkie_Wenchang	CRA004775	0.950796617
ENSGALG00010012314	ADRB2	2	-0.209560465	0.168325193	-1.244973856	0.213141361	0	0.105401755	0	1	CRA004775;Yang2025_Silkie_Wenchang	PRJNA827465	0.72579791
ENSGALG00010012314	ADRB2	2	0.145458084	0.207774901	0.700075336	0.483880258	0	0.121987257	0	0.5	CRA004775;PRJNA827465	Yang2025_Silkie_Wenchang	0.85691916
ENSGALG00010012315	ATP6V1E1	2	-0.186518296	0.27276952	-0.683794494	0.494104944	0.139469113	15.68297435	93.62365851	0.5	PRJNA827465;Yang2025_Silkie_Wenchang	CRA004775	0.860175061
ENSGALG00010012315	ATP6V1E1	2	-0.374133172	0.1542277	-2.425849383	0.015272607	0.018274023	1.437395153	30.42970836	1	CRA004775;Yang2025_Silkie_Wenchang	PRJNA827465	0.204363429
ENSGALG00010012315	ATP6V1E1	2	0.064999574	0.065644002	0.990182985	0.322084688	0	0.538411691	0	0.5	CRA004775;PRJNA827465	Yang2025_Silkie_Wenchang	0.798513866
ENSGALG00010012318	POLR1D	2	0.017061735	0.122340633	0.1394609	0.889085951	0	0.282348188	0	0.5	PRJNA827465;Yang2025_Silkie_Wenchang	CRA004775	0.973135755
ENSGALG00010012318	POLR1D	2	-0.449728364	0.232527066	-1.93409039	0.053102015	0	0.829918309	0	1	CRA004775;Yang2025_Silkie_Wenchang	PRJNA827465	0.399631766
ENSGALG00010012318	POLR1D	2	-0.233627463	0.321611618	-0.726427311	0.467576854	0.161126531	4.133773219	75.80902611	0.5	CRA004775;PRJNA827465	Yang2025_Silkie_Wenchang	0.852885838
ENSGALG00010012320	DPY19L4	2	-0.195689483	0.169051718	-1.157571693	0.247038867	0.050956088	9.005982467	88.89626974	1	PRJNA827465;Yang2025_Silkie_Wenchang	CRA004775	0.757459165
ENSGALG00010012320	DPY19L4	2	-0.194146869	0.223577319	-0.868365671	0.385194181	0.072917039	3.288852041	69.59425394	0.5	CRA004775;Yang2025_Silkie_Wenchang	PRJNA827465	0.826444827
ENSGALG00010012320	DPY19L4	2	-0.028618037	0.055774564	-0.513101946	0.607880011	0	0.246647753	0	0.5	CRA004775;PRJNA827465	Yang2025_Silkie_Wenchang	0.897203929
ENSGALG00010012322	ENSGALG00010012322	2	0.3882327	0.128549009	3.020114294	0.002526793	0	0.01837051	0	1	PRJNA827465;Yang2025_Silkie_Wenchang	CRA004775	0.067541822
ENSGALG00010012322	ENSGALG00010012322	2	-0.176402001	0.718483018	-0.24552007	0.80605377	0.812314765	4.11809106	75.71690413	0.5	CRA004775;Yang2025_Silkie_Wenchang	PRJNA827465	0.952063038
ENSGALG00010012322	ENSGALG00010012322	2	-0.181537098	0.69646387	-0.260655442	0.794358231	0.772203684	4.133666466	75.80840137	0.5	CRA004775;PRJNA827465	Yang2025_Silkie_Wenchang	0.948905846
ENSGALG00010012323	SMAD7B	2	-2.874897747	2.902792421	-0.9903904	0.321983336	11.72965309	3.072700955	67.45534255	1	PRJNA827465;Yang2025_Silkie_Wenchang	CRA004775	0.798513866
ENSGALG00010012323	SMAD7B	2	-2.447217311	3.300238095	-0.741527502	0.458373663	17.34302718	4.4396316	77.47560856	0.5	CRA004775;Yang2025_Silkie_Wenchang	PRJNA827465	0.850026426
ENSGALG00010012323	SMAD7B	2	0.075945377	0.947809418	0.080127265	0.936136038	0	0.158472494	0	0.5	CRA004775;PRJNA827465	Yang2025_Silkie_Wenchang	0.984822128
ENSGALG00010012324	ENSGALG00010012324	2	0.19400198	0.296321716	0.654700515	0.512660594	0.019697481	1.124156753	11.04443423	0.5	PRJNA827465;Yang2025_Silkie_Wenchang	CRA004775	0.865883082
ENSGALG00010012324	ENSGALG00010012324	2	-0.086933893	0.572143973	-0.151944086	0.879231036	0.526726473	5.001479327	80.00591556	0.5	CRA004775;Yang2025_Silkie_Wenchang	PRJNA827465	0.971257564
ENSGALG00010012324	ENSGALG00010012324	2	-0.391864094	0.270705175	-1.447567801	0.147737962	0.041834923	1.381882007	27.63492146	1	CRA004775;PRJNA827465	Yang2025_Silkie_Wenchang	0.637081344
ENSGALG00010012325	MLPH	2	5.409756244	0.420634501	12.86094277	7.46605E-38	0	0.588699781	0	1	PRJNA827465;Yang2025_Silkie_Wenchang	CRA004775	1.39732E-34
ENSGALG00010012325	MLPH	2	5.636370766	0.497572415	11.32773964	9.56411E-30	0	0.12075229	0	1	CRA004775;Yang2025_Silkie_Wenchang	PRJNA827465	1.56624E-26
ENSGALG00010012325	MLPH	2	5.218463213	0.461876114	11.29840461	1.33623E-29	0	0.089101581	0	1	CRA004775;PRJNA827465	Yang2025_Silkie_Wenchang	2.10072E-26
ENSGALG00010012326	SGTB	2	-0.087813586	0.170116131	-0.51619788	0.605716211	0.050185388	7.473965465	86.62022183	0.5	PRJNA827465;Yang2025_Silkie_Wenchang	CRA004775	0.896474603
ENSGALG00010012326	SGTB	2	-0.124450157	0.158783978	-0.783770239	0.43317494	0.034933759	3.131046489	68.06179648	0.5	CRA004775;Yang2025_Silkie_Wenchang	PRJNA827465	0.842762754
ENSGALG00010012326	SGTB	2	0.072925339	0.068650044	1.062276656	0.288110109	0	0.013752061	0	1	CRA004775;PRJNA827465	Yang2025_Silkie_Wenchang	0.782881057
ENSGALG00010012327	UBTD2	2	-0.059502534	0.044213756	-1.345792347	0.178369494	0	0.225463487	0	1	PRJNA827465;Yang2025_Silkie_Wenchang	CRA004775	0.684689586
ENSGALG00010012327	UBTD2	2	-0.08569436	0.069336856	-1.235913549	0.2164907	0	0.007313924	0	1	CRA004775;Yang2025_Silkie_Wenchang	PRJNA827465	0.730172415
ENSGALG00010012327	UBTD2	2	-0.046183441	0.052662253	-0.876974279	0.380500609	0	0.018793459	0	1	CRA004775;PRJNA827465	Yang2025_Silkie_Wenchang	0.824392515
ENSGALG00010012329	ATXN10	2	-0.221295464	0.177922765	-1.243772621	0.21358326	0.051097122	5.064000583	80.25276689	1	PRJNA827465;Yang2025_Silkie_Wenchang	CRA004775	0.726164607
ENSGALG00010012329	ATXN10	2	-0.36232109	0.120083824	-3.01723478	0.002550922	0	0.881874383	0	1	CRA004775;Yang2025_Silkie_Wenchang	PRJNA827465	0.068018229
ENSGALG00010012329	ATXN10	2	-0.060879744	0.08456902	-0.719882335	0.471597445	0	0.033068799	0	1	CRA004775;PRJNA827465	Yang2025_Silkie_Wenchang	0.853998373
ENSGALG00010012331	ENSGALG00010012331	2	-0.289297957	0.371403178	-0.778932369	0.436019555	0	0.00209134	0	1	PRJNA827465;Yang2025_Silkie_Wenchang	CRA004775	0.843018329
ENSGALG00010012331	ENSGALG00010012331	2	-0.431507508	0.371575838	-1.161290546	0.245523763	0	0.215136833	0	1	CRA004775;Yang2025_Silkie_Wenchang	PRJNA827465	0.75661406
ENSGALG00010012331	ENSGALG00010012331	2	-0.463644748	0.388917393	-1.192141972	0.233205582	0	0.160832054	0	1	CRA004775;PRJNA827465	Yang2025_Silkie_Wenchang	0.746346811
ENSGALG00010012334	ARHGEF39	2	-0.340643613	0.177995655	-1.913774881	0.055648926	0	0.255518802	0	1	PRJNA827465;Yang2025_Silkie_Wenchang	CRA004775	0.40857092
ENSGALG00010012334	ARHGEF39	2	-0.403851954	0.234253695	-1.723993951	0.084708935	0	0.083284985	0	1	CRA004775;Yang2025_Silkie_Wenchang	PRJNA827465	0.500353159
ENSGALG00010012334	ARHGEF39	2	-0.255475391	0.224850357	-1.136201849	0.255872097	0	2.2943E-05	0	1	CRA004775;PRJNA827465	Yang2025_Silkie_Wenchang	0.764523416
ENSGALG00010012336	ENSGALG00010012336	2	-5.621198081	5.719823279	-0.9827573	0.325726902	59.88488003	11.53139595	91.32802304	1	PRJNA827465;Yang2025_Silkie_Wenchang	CRA004775	0.800023788
ENSGALG00010012336	ENSGALG00010012336	2	-5.368259934	5.908275141	-0.908600193	0.363561205	65.16987277	14.41926885	93.0648356	0.5	CRA004775;Yang2025_Silkie_Wenchang	PRJNA827465	0.817042253
ENSGALG00010012336	ENSGALG00010012336	2	0.119020208	0.857294717	0.138832313	0.889582659	0	0.039711074	0	0.5	CRA004775;PRJNA827465	Yang2025_Silkie_Wenchang	0.973153999
ENSGALG00010012337	ACSS3	2	-0.134590705	0.088916016	-1.513683492	0.130106134	0	0.128466209	0	1	PRJNA827465;Yang2025_Silkie_Wenchang	CRA004775	0.605226817
ENSGALG00010012337	ACSS3	2	-0.087031879	0.143207458	-0.607732866	0.543364661	0	0.000425488	0	1	CRA004775;Yang2025_Silkie_Wenchang	PRJNA827465	0.876968972
ENSGALG00010012337	ACSS3	2	-0.148130939	0.099776774	-1.484623461	0.137643597	0	0.039801381	0	1	CRA004775;PRJNA827465	Yang2025_Silkie_Wenchang	0.620631442
ENSGALG00010012339	WNK1	2	-0.051714985	0.118840857	-0.435161667	0.663445085	0.013924236	1.602942264	37.61472122	0.5	PRJNA827465;Yang2025_Silkie_Wenchang	CRA004775	0.913099491
ENSGALG00010012339	WNK1	2	0.174480854	0.178148367	0.979413156	0.327375879	0	0.061029856	0	1	CRA004775;Yang2025_Silkie_Wenchang	PRJNA827465	0.800028239
ENSGALG00010012339	WNK1	2	-0.10827441	0.069967915	-1.547486575	0.121745959	0	0.985366422	0	0.5	CRA004775;PRJNA827465	Yang2025_Silkie_Wenchang	0.584961056
ENSGALG00010012340	ENHO	2	0.561779099	0.134341552	4.181722557	2.89309E-05	0	0.19234668	0	1	PRJNA827465;Yang2025_Silkie_Wenchang	CRA004775	0.003203016
ENSGALG00010012340	ENHO	2	0.235765662	0.429093286	0.54945083	0.5826961	0.29401774	4.959464275	79.83653184	0.5	CRA004775;Yang2025_Silkie_Wenchang	PRJNA827465	0.890043325
ENSGALG00010012340	ENHO	2	0.202931529	0.357834354	0.567110246	0.570639282	0.211106239	5.477414653	81.74321165	0.5	CRA004775;PRJNA827465	Yang2025_Silkie_Wenchang	0.885152854
ENSGALG00010012341	GOPC	2	-0.057786565	0.125397424	-0.460827367	0.644922466	0.025889133	5.4382401	81.61169824	0.5	PRJNA827465;Yang2025_Silkie_Wenchang	CRA004775	0.906953008
ENSGALG00010012341	GOPC	2	-0.006944536	0.22755716	-0.030517766	0.975654124	0.078526664	3.730468785	73.19371753	0.5	CRA004775;Yang2025_Silkie_Wenchang	PRJNA827465	0.992754467
ENSGALG00010012341	GOPC	2	0.068289247	0.052221771	1.307677729	0.19098264	0	0.865067774	0	1	CRA004775;PRJNA827465	Yang2025_Silkie_Wenchang	0.700443419
ENSGALG00010012343	BCL2L13	2	-0.363164644	0.225711795	-1.608975039	0.1076218	0.093785476	12.4701491	91.98084969	1	PRJNA827465;Yang2025_Silkie_Wenchang	CRA004775	0.55523359
ENSGALG00010012343	BCL2L13	2	-0.55306543	0.113519006	-4.872007324	1.1047E-06	0.003781043	1.065804508	6.174163071	1	CRA004775;Yang2025_Silkie_Wenchang	PRJNA827465	0.000240843
ENSGALG00010012343	BCL2L13	2	-0.148404426	0.072267994	-2.053529078	0.040021293	0	0.09423282	0	1	CRA004775;PRJNA827465	Yang2025_Silkie_Wenchang	0.3465426
ENSGALG00010012346	NLN	2	-0.091491125	0.072344737	-1.264654887	0.205995068	0	0.09032469	0	1	PRJNA827465;Yang2025_Silkie_Wenchang	CRA004775	0.718403416
ENSGALG00010012346	NLN	2	-0.140215103	0.111011461	-1.263068709	0.206564488	0	0.202169994	0	1	CRA004775;Yang2025_Silkie_Wenchang	PRJNA827465	0.71859917
ENSGALG00010012346	NLN	2	-0.091132514	0.086865757	-1.04911897	0.294123366	0	0.370504685	0	1	CRA004775;PRJNA827465	Yang2025_Silkie_Wenchang	0.787324026
ENSGALG00010012347	ENSGALG00010012347	2	1.02248366	0.388338606	2.632969386	0.008464198	0.203138431	2.973347575	66.3678741	1	PRJNA827465;Yang2025_Silkie_Wenchang	CRA004775	0.143515254
ENSGALG00010012347	ENSGALG00010012347	2	0.779115498	0.806629313	0.965890385	0.334099034	0.975622957	3.689187644	72.89376154	0.5	CRA004775;Yang2025_Silkie_Wenchang	PRJNA827465	0.803075918
ENSGALG00010012347	ENSGALG00010012347	2	0.556070916	0.299247681	1.858229661	0.063136408	0.038576437	1.118396711	10.58628928	0.5	CRA004775;PRJNA827465	Yang2025_Silkie_Wenchang	0.432760766
ENSGALG00010012350	H2AC32	2	-0.074615533	0.164461149	-0.453697021	0.650046912	0	0.505589718	0	0.5	PRJNA827465;Yang2025_Silkie_Wenchang	CRA004775	0.908335059
ENSGALG00010012350	H2AC32	2	-0.28569234	0.393550175	-0.725936254	0.46787785	0.213810386	3.178642951	68.54003373	0.5	CRA004775;Yang2025_Silkie_Wenchang	PRJNA827465	0.853115491
ENSGALG00010012350	H2AC32	2	-0.365696236	0.264077637	-1.384805772	0.166111866	0.06658322	1.794707875	44.28062562	1	CRA004775;PRJNA827465	Yang2025_Silkie_Wenchang	0.66529196
ENSGALG00010012352	PTPRC	2	-0.071764132	0.153111531	-0.468704948	0.63928055	0.026280052	2.247212334	55.50042224	0.5	PRJNA827465;Yang2025_Silkie_Wenchang	CRA004775	0.905968982
ENSGALG00010012352	PTPRC	2	-0.234098248	0.149890165	-1.561798582	0.118335446	0	0.013946217	0	1	CRA004775;Yang2025_Silkie_Wenchang	PRJNA827465	0.578180225
ENSGALG00010012352	PTPRC	2	0.04513043	0.122402911	0.368703892	0.712348448	0	0.337936804	0	0.5	CRA004775;PRJNA827465	Yang2025_Silkie_Wenchang	0.92597752
ENSGALG00010012353	PIGG	2	0.068852487	0.172708399	0.398663223	0.69014137	0.030980272	2.078273462	51.88313674	0.5	PRJNA827465;Yang2025_Silkie_Wenchang	CRA004775	0.919979266
ENSGALG00010012353	PIGG	2	-0.239726159	0.159577593	-1.502254512	0.133031391	0.011956291	1.291211693	22.55336554	1	CRA004775;Yang2025_Silkie_Wenchang	PRJNA827465	0.611090777
ENSGALG00010012353	PIGG	2	-0.080848716	0.335094238	-0.241271579	0.809344637	0.186010177	5.740250639	82.5791579	0.5	CRA004775;PRJNA827465	Yang2025_Silkie_Wenchang	0.95276497
ENSGALG00010012354	DCBLD1	2	-0.031465029	0.089361461	-0.352109613	0.724756059	0	0.642470548	0	0.5	PRJNA827465;Yang2025_Silkie_Wenchang	CRA004775	0.930520299
ENSGALG00010012354	DCBLD1	2	0.054347426	0.120809531	0.449860418	0.65281109	0	0.248395629	0	1	CRA004775;Yang2025_Silkie_Wenchang	PRJNA827465	0.909572411
ENSGALG00010012354	DCBLD1	2	-0.078543394	0.123480891	-0.636077321	0.524726027	0	0.622624231	0	0.5	CRA004775;PRJNA827465	Yang2025_Silkie_Wenchang	0.869851408
ENSGALG00010012358	STK10	2	-0.016090698	0.084587211	-0.190226131	0.849131935	0	0.020110027	0	1	PRJNA827465;Yang2025_Silkie_Wenchang	CRA004775	0.961438996
ENSGALG00010012358	STK10	2	-0.034452322	0.118621686	-0.290438651	0.771480678	0	0.935788404	0	1	CRA004775;Yang2025_Silkie_Wenchang	PRJNA827465	0.94283287
ENSGALG00010012358	STK10	2	-0.054107777	0.112965302	-0.478976963	0.631955017	0	0.84544152	0	1	CRA004775;PRJNA827465	Yang2025_Silkie_Wenchang	0.903058174
ENSGALG00010012360	ROS1	2	0.086732776	0.185317758	0.468021935	0.639768903	0	0.123159702	0	1	PRJNA827465;Yang2025_Silkie_Wenchang	CRA004775	0.906064918
ENSGALG00010012360	ROS1	2	1.044581269	1.028092727	1.016037991	0.309611295	1.787394889	5.851276137	82.90971104	1	CRA004775;Yang2025_Silkie_Wenchang	PRJNA827465	0.794246638
ENSGALG00010012360	ROS1	2	0.988410629	1.094871916	0.902763707	0.366651317	2.065212936	6.594003726	84.83470678	1	CRA004775;PRJNA827465	Yang2025_Silkie_Wenchang	0.819420238
ENSGALG00010012361	WIF1	2	-0.027408253	0.333678257	-0.082139763	0.934535574	0	0.000367701	0	1	PRJNA827465;Yang2025_Silkie_Wenchang	CRA004775	0.984397102
ENSGALG00010012361	WIF1	2	-0.031919154	0.421291623	-0.075764987	0.939606072	0	0.002086631	0	1	CRA004775;Yang2025_Silkie_Wenchang	PRJNA827465	0.985425793
ENSGALG00010012361	WIF1	2	-0.040615009	0.39952014	-0.101659479	0.919026967	0	0.001082718	0	1	CRA004775;PRJNA827465	Yang2025_Silkie_Wenchang	0.981088364
ENSGALG00010012362	COL6A3	2	-0.04738304	0.273332286	-0.173353249	0.862373771	0.113022798	4.00392919	75.02453334	0.5	PRJNA827465;Yang2025_Silkie_Wenchang	CRA004775	0.965970249
ENSGALG00010012362	COL6A3	2	0.246030176	0.208185863	1.181781379	0.237292458	0	0.004277243	0	1	CRA004775;Yang2025_Silkie_Wenchang	PRJNA827465	0.749366344
ENSGALG00010012362	COL6A3	2	-0.256903228	0.148803767	-1.726456479	0.084265322	0	0.854372153	0	0.5	CRA004775;PRJNA827465	Yang2025_Silkie_Wenchang	0.49990641

ENSGALG00010012366	INTS8	2	-0.043875596	0.049013951	-0.895165216	0.370698648	0	0.454246365	0	1	PRJNA827465;Yang2025_Silkie_Wenchang	CRA004775	0.82145712
ENSGALG00010012366	INTS8	2	-0.096213905	0.08557444	-1.124329938	0.260873082	0	7.92061E-05	0	1	CRA004775;Yang2025_Silkie_Wenchang	PRJNA827465	0.76792161
ENSGALG00010012366	INTS8	2	-0.027394358	0.056111273	-0.48821487	0.625397653	0	0.090291624	0	1	CRA004775;PRJNA827465	Yang2025_Silkie_Wenchang	0.901367193
ENSGALG00010012367	RAD52	2	0.059540349	0.073596819	0.809007096	0.418511065	0	0.050802824	0	1	PRJNA827465;Yang2025_Silkie_Wenchang	CRA004775	0.838178696
ENSGALG00010012367	RAD52	2	0.219631879	0.236079927	0.930328474	0.352201039	0.07027198	2.49341459	59.89435515	1	CRA004775;Yang2025_Silkie_Wenchang	PRJNA827465	0.810728586
ENSGALG00010012367	RAD52	2	0.218825147	0.21073214	1.038404237	0.299081898	0.059425051	2.474702733	59.59110618	1	CRA004775;PRJNA827465	Yang2025_Silkie_Wenchang	0.789748017
ENSGALG00010012372	LEMD3	2	0.058604258	0.045672262	1.283147708	0.199440332	0	0.71965794	0	1	PRJNA827465;Yang2025_Silkie_Wenchang	CRA004775	0.711240084
ENSGALG00010012372	LEMD3	2	0.103976134	0.073908127	1.406829503	0.159477952	0	0.121502514	0	1	CRA004775;Yang2025_Silkie_Wenchang	PRJNA827465	0.655027896
ENSGALG00010012372	LEMD3	2	0.034203898	0.05239164	0.652850309	0.513852786	0	0.009385843	0	1	CRA004775;PRJNA827465	Yang2025_Silkie_Wenchang	0.866620841
ENSGALG00010012376	VGLL2	2	0.052909951	0.274876713	0.192486117	0.847361442	0.122434521	4.949379796	79.7954483	0.5	PRJNA827465;Yang2025_Silkie_Wenchang	CRA004775	0.961252712
ENSGALG00010012376	VGLL2	2	-0.403955896	0.211769718	-1.907524362	0.056452719	0	0.318340689	0	1	CRA004775;Yang2025_Silkie_Wenchang	PRJNA827465	0.411523553
ENSGALG00010012376	VGLL2	2	0.05781233	0.417406702	0.138503598	0.889842426	0.223511406	1.97787901	49.4407901	0.5	CRA004775;PRJNA827465	Yang2025_Silkie_Wenchang	0.973208458
ENSGALG00010012378	RPS19BP1	2	-0.046997343	0.058086049	-0.809098627	0.418458418	0	0.458723045	0	1	PRJNA827465;Yang2025_Silkie_Wenchang	CRA004775	0.838158675
ENSGALG00010012378	RPS19BP1	2	-0.220242755	0.148951876	-1.478616856	0.139242745	0	0.088020429	0	1	CRA004775;Yang2025_Silkie_Wenchang	PRJNA827465	0.622954765
ENSGALG00010012378	RPS19BP1	2	-0.056604273	0.067092379	-0.84367664	0.398850124	0.001282833	1.049860247	4.74922708	1	CRA004775;PRJNA827465	Yang2025_Silkie_Wenchang	0.832054308
ENSGALG00010012382	SH3PXD2B	2	0.081501272	0.107337162	0.759301542	0.447672195	0.014242202	2.515516097	60.24672625	0.5	PRJNA827465;Yang2025_Silkie_Wenchang	CRA004775	0.846601815
ENSGALG00010012382	SH3PXD2B	2	-0.408682271	0.447980385	-0.912277153	0.361622838	0.320160736	4.24465197	76.44094246	1	CRA004775;Yang2025_Silkie_Wenchang	PRJNA827465	0.815545459
ENSGALG00010012382	SH3PXD2B	2	-0.314582621	0.560829016	-0.560924296	0.574849146	0.546567138	6.758440657	85.2036875	0.5	CRA004775;PRJNA827465	Yang2025_Silkie_Wenchang	0.886211989
ENSGALG00010012384	NRK	2	0.024637081	0.071518928	0.344483359	0.730482808	0	0.071558091	0	0.5	PRJNA827465;Yang2025_Silkie_Wenchang	CRA004775	0.932653142
ENSGALG00010012384	NRK	2	-0.051497549	0.144816996	-0.355604316	0.722136911	0	0.792875181	0	1	CRA004775;Yang2025_Silkie_Wenchang	PRJNA827465	0.929404251
ENSGALG00010012384	NRK	2	0.014936952	0.097659289	0.152949626	0.878438	0.003358493	1.027769452	2.701914494	0.5	CRA004775;PRJNA827465	Yang2025_Silkie_Wenchang	0.971037793
ENSGALG00010012387	ERBIN	2	0.01400516	0.126574625	0.110647454	0.911895916	0.018309138	2.160809939	53.721057	0.5	PRJNA827465;Yang2025_Silkie_Wenchang	CRA004775	0.978928739
ENSGALG00010012387	ERBIN	2	0.192449507	0.123858585	1.553784156	0.120235911	0	0.029080565	0	1	CRA004775;Yang2025_Silkie_Wenchang	PRJNA827465	0.581544673
ENSGALG00010012387	ERBIN	2	0.010933848	0.141457029	0.077294484	0.938389279	0.021701823	1.874830637	46.66184878	0.5	CRA004775;PRJNA827465	Yang2025_Silkie_Wenchang	0.985349045
ENSGALG00010012390	MMP16	2	-0.150282034	0.096881259	-1.55119819	0.120854196	0	0.385373556	0	1	PRJNA827465;Yang2025_Silkie_Wenchang	CRA004775	0.582767179
ENSGALG00010012390	MMP16	2	-0.215329091	0.155215778	-1.387288672	0.165353756	0	0.09784523	0	1	CRA004775;Yang2025_Silkie_Wenchang	PRJNA827465	0.664874006
ENSGALG00010012390	MMP16	2	-0.107828049	0.113129429	-0.953138803	0.34051974	0	0.000155062	0	1	CRA004775;PRJNA827465	Yang2025_Silkie_Wenchang	0.805524125
ENSGALG00010012391	NEURL1B	2	0.021820334	0.066064514	0.330288267	0.741182157	0.001954189	1.232192864	18.84387343	0.5	PRJNA827465;Yang2025_Silkie_Wenchang	CRA004775	0.955384591
ENSGALG00010012391	NEURL1B	2	-0.089812219	0.107417964	-0.836100552	0.403090338	0	0.12007968	0	1	CRA004775;Yang2025_Silkie_Wenchang	PRJNA827465	0.833046091
ENSGALG00010012391	NEURL1B	2	0.057057194	0.064548752	0.883939548	0.376728847	0	0.532483863	0	0.5	CRA004775;PRJNA827465	Yang2025_Silkie_Wenchang	0.822974013
ENSGALG00010012393	ENSGALG00010012393	2	-3.684254429	3.520234372	-1.046593505	0.295287099	22.45597969	10.59588351	90.56237265	1	PRJNA827465;Yang2025_Silkie_Wenchang	CRA004775	0.787628307
ENSGALG00010012393	ENSGALG00010012393	2	-3.284905044	3.895901022	-0.843169533	0.399133635	28.27573415	14.4601778	93.08445571	0.5	CRA004775;Yang2025_Silkie_Wenchang	PRJNA827465	0.832054308
ENSGALG00010012393	ENSGALG00010012393	2	0.193293294	0.870984757	0.221925002	0.824372267	0	0.182048449	0	0.5	CRA004775;PRJNA827465	Yang2025_Silkie_Wenchang	0.95619211
ENSGALG00010012399	ERC1	2	0.081773018	0.188205611	0.434487674	0.663934344	0.048847881	2.904680078	65.57280069	0.5	PRJNA827465;Yang2025_Silkie_Wenchang	CRA004775	0.913119244
ENSGALG00010012399	ERC1	2	0.329904152	0.176919345	1.864714975	0.062221367	0	0.014204482	0	1	CRA004775;Yang2025_Silkie_Wenchang	PRJNA827465	0.430164709
ENSGALG00010012399	ERC1	2	0.019036569	0.174203188	0.109277958	0.912982029	0.028854746	1.441046886	30.6060053	0.5	CRA004775;PRJNA827465	Yang2025_Silkie_Wenchang	0.979097183
ENSGALG00010012401	RIPK2	2	-0.065036525	0.101918214	-0.638124657	0.523392537	0.012616681	2.508829307	60.14077175	0.5	PRJNA827465;Yang2025_Silkie_Wenchang	CRA004775	0.869661505
ENSGALG00010012401	RIPK2	2	-0.014211902	0.225923109	-0.062905925	0.949841416	0.067290836	2.46558998	59.44175601	0.5	CRA004775;Yang2025_Silkie_Wenchang	PRJNA827465	0.987277827
ENSGALG00010012401	RIPK2	2	0.046731509	0.076431765	0.611414752	0.540925039	0	0.837439894	0	1	CRA004775;PRJNA827465	Yang2025_Silkie_Wenchang	0.875790068
ENSGALG00010012404	VAMP7	2	-0.069201857	0.133852981	-0.516998994	0.605156861	0.033099603	12.84783582	92.21658796	0.5	PRJNA827465;Yang2025_Silkie_Wenchang	CRA004775	0.896474603
ENSGALG00010012404	VAMP7	2	-0.266412236	0.103073194	-2.584689819	0.009746664	0.009698458	1.497161819	33.20695282	1	CRA004775;Yang2025_Silkie_Wenchang	PRJNA827465	0.155657507
ENSGALG00010012404	VAMP7	2	-0.162729096	0.252688742	-0.643990289	0.519581731	0.111815427	7.198514398	86.10824477	0.5	CRA004775;PRJNA827465	Yang2025_Silkie_Wenchang	0.869094812
ENSGALG00010012405	MSRB3	2	-0.07378677	0.115686655	-0.637804713	0.523600811	0.017153545	2.779433403	64.02144413	0.5	PRJNA827465;Yang2025_Silkie_Wenchang	CRA004775	0.869661505
ENSGALG00010012405	MSRB3	2	0.036763467	0.091065901	0.403701788	0.686432025	0	0.00500672	0	1	CRA004775;Yang2025_Silkie_Wenchang	PRJNA827465	0.919235539
ENSGALG00010012405	MSRB3	2	-0.179040321	0.098730204	-1.813430074	0.069765515	0	0.274356965	0	0.5	CRA004775;PRJNA827465	Yang2025_Silkie_Wenchang	0.455632107
ENSGALG00010012406	ENSGALG00010012406	2	0.158307006	0.380733397	0.415794902	0.677560089	0.254786513	8.050328609	87.57814682	0.5	PRJNA827465;Yang2025_Silkie_Wenchang	CRA004775	0.9171739
ENSGALG00010012406	ENSGALG00010012406	2	0.503218455	0.212946492	2.363121595	0.018121723	0	0.438440187	0	1	CRA004775;Yang2025_Silkie_Wenchang	PRJNA827465	0.224751677
ENSGALG00010012406	ENSGALG00010012406	2	-0.179115472	0.133754948	-1.339131554	0.18052785	0	0.414897367	0	0.5	CRA004775;PRJNA827465	Yang2025_Silkie_Wenchang	0.688194577
ENSGALG00010012407	ATF4	2	-0.191480947	0.183529606	-1.043324568	0.296797996	0.059568757	8.30885196	87.96464295	1	PRJNA827465;Yang2025_Silkie_Wenchang	CRA004775	0.788458494
ENSGALG00010012407	ATF4	2	-0.117295376	0.305331103	-0.384157968	0.700861369	0.153888368	5.377675777	81.40460598	0.5	CRA004775;Yang2025_Silkie_Wenchang	PRJNA827465	0.922496697
ENSGALG00010012407	ATF4	2	-0.006701898	0.057193348	-0.117179682	0.906717667	0.000205754	1.006798313	0.67524084	0.5	CRA004775;PRJNA827465	Yang2025_Silkie_Wenchang	0.978196186
ENSGALG00010012412	PURA	2	0.012278668	0.070414422	0.174377181	0.861569047	0	0.105680063	0	0.5	PRJNA827465;Yang2025_Silkie_Wenchang	CRA004775	0.965479094
ENSGALG00010012412	PURA	2	0.072732563	0.158874446	0.457799007	0.64709685	0.006222565	1.124305982	11.0562413	0.5	CRA004775;Yang2025_Silkie_Wenchang	PRJNA827465	0.907350807
ENSGALG00010012412	PURA	2	0.073868288	0.038699597	0.593861211	0.552604945	6.38463E-05	1.001725774	0.172280035	1	CRA004775;PRJNA827465	Yang2025_Silkie_Wenchang	0.879277444
ENSGALG00010012413	NEK7	2	0.046287932	0.164485234	0.281410862	0.778395288	0.042308003	4.232044273	76.37075759	0.5	PRJNA827465;Yang2025_Silkie_Wenchang	CRA004775	0.944615741
ENSGALG00010012413	NEK7	2	0.138315406	0.138903129	0.995768827	0.31936248	0.007713724	1.218796429	17.95184363	0.5	CRA004775;Yang2025_Silkie_Wenchang	PRJNA827465	0.798079048
ENSGALG00010012413	NEK7	2	-0.091331392	0.065136105	-1.402162312	0.160866783	0	0.029406923	0	1	CRA004775;PRJNA827465	Yang2025_Silkie_Wenchang	0.656888018
ENSGALG00010012414	ENSGALG00010012414	2	0.241232289	0.760015284	0.317404523	0.750936676	1.092409064	18.3830961	94.56021208	0.5	PRJNA827465;Yang2025_Silkie_Wenchang	CRA004775	0.937567095
ENSGALG00010012414	ENSGALG00010012414	2	0.928292217	0.228713176	4.058761436	4.93337E-05	0	0.640223193	0	1	CRA004775;Yang2025_Silkie_Wenchang	PRJNA827465	0.004811442
ENSGALG00010012414	ENSGALG00010012414	2	-0.200760977	0.459019636	-0.437369038	0.661843735	0.247482529	2.06924306	51.6731495	0.5	CRA004775;PRJNA827465	Yang2025_Silkie_Wenchang	0.912556664
ENSGALG00010012415	BID	2	-0.271211592	0.196672896	-1.391604657	0.163969702	0.06847837	10.34428031	90.33282191	1	PRJNA827465;Yang2025_Silkie_Wenchang	CRA004775	0.661805642
ENSGALG00010012415	BID	2	-0.359369519	0.150611283	-2.386073024	0.017029369	0.026283945	2.094686568	52.26016078	1	CRA004775;Yang2025_Silkie_Wenchang	PRJNA827465	0.216673778
ENSGALG00010012415	BID	2	-0.088937527	0.074185209	-1.19885794	0.230583189	0	0.11303658	0	1	CRA004775;PRJNA827465	Yang2025_Silkie_Wenchang	0.743335644
ENSGALG00010012418	ENSGALG00010012418	2	1.148960268	0.630136208	1.823352243	0.068250058	0.615352942	4.438661699	77.47068671	1	PRJNA827465;Yang2025_Silkie_Wenchang	CRA004775	0.450415709
ENSGALG00010012418	ENSGALG00010012418	2	1.151301274	0.811845065	1.418129301	0.156153018	0.874386585	2.671703002	62.57068996	1	CRA004775;Yang2025_Silkie_Wenchang	PRJNA827465	0.649132948
ENSGALG00010012418	ENSGALG00010012418	2	0.460346867	0.374412623	1.229517486	0.218877846	0	0.165356117	0	1	CRA004775;PRJNA827465	Yang2025_Silkie_Wenchang	0.73259614
ENSGALG00010012419	HACE1	2	0.172996015	0.053935026	3.207489205	0.001338991	0	0.509339053	0	1	PRJNA827465;Yang2025_Silkie_Wenchang	CRA004775	0.044712283
ENSGALG00010012419	HACE1	2	0.253917224	0.085344594	2.975199879	0.002927977	0	0.811411416	0	1	CRA004775;Yang2025_Silkie_Wenchang	PRJNA827465	0.074041237
ENSGALG00010012419	HACE1	2	0.214881115	0.131372965	1.635657036	0.101911386	0.017703482	1.548929645	35.43928848	1	CRA004775;PRJNA827465	Yang2025_Silkie_Wenchang	0.54407845
ENSGALG00010012426	MIEF1	2	-0.257923696	0.219882986	-1.173004334	0.240794068	0.086739027	9.710351867	89.70171201	1	PRJNA827465;Yang2025_Silkie_Wenchang	CRA004775	0.753303162
ENSGALG00010012426	MIEF1	2	-0.484610535	0.092225781	-5.254610234	1.48338E-07	0	0.0274687	0	1	CRA004775;Yang2025_Silkie_Wenchang	PRJNA827465	4.9408E-05
ENSGALG00010012426	MIEF1	2	-0.232655994	0.236434497	-0.984018814	0.325106262	0.083831042	3.593863913	72.17479503	1	CRA004775;PRJNA827465	Yang2025_Silkie_Wenchang	0.799914637
ENSGALG00010012427	C7	2	0.258016287	0.376772348	0.684806857	0.493465808	0.202741973	3.452258775	71.03345765	0.5	PRJNA827465;Yang2025_Silkie_Wenchang	CRA004775	0.859868321
ENSGALG00010012427	C7	2	-0.455164687	0.479885931	-0.948485166	0.342882521	0.299966656	2.518226254	60.28950939	1	CRA004775;Yang2025_Silkie_Wenchang	PRJNA827465	0.806218116
ENSGALG00010012427	C7	2	-0.147799901	0.874084608	-0.16909107	0.865725009	1.31835213	7.039525387	85.79449686	0.5	CRA004775;PRJNA827465	Yang2025_Silkie_Wenchang	0.967088846
ENSGALG00010012428	CCNE2	2	-0.049338276	0.091024864	-0.54203076	0.587797316	0.000895803	1.046619609	4.454303078	0.5	PRJNA827465;Yang2025_Silkie_Wenchang	CRA004775	0.891742161
ENSGALG00010012428	CCNE2	2	-0.157223376	0.160925481	-0.976994907	0.328571674	0	0.676500108	0	0.5	CRA004775;Yang2025_Silkie_Wenchang	PRJNA827465	0.800761292
ENSGALG00010012428	CCNE2	2	0.01767853	0.102470683	0.172522809	0.863026531	0	0.27979669	0	1	CRA004775;PRJNA827465	Yang2025_Silkie_Wenchang	0.966099097
ENSGALG00010012429	CXCR7	2	-0.174206537	0.076793548	-2.26850487	0.023298452	0	0.001966374	0	1	PRJNA827465;Yang2025_Silkie_Wenchang	CRA004775	0.260268986
ENSGALG00010012429	CXCR7	2	0.176802612	0.377199415	0.468724514	0.639266562	0.243563592	6.82318565	85.34408924	0.5	CRA004775;Yang2025_Silkie_Wenchang	PRJNA827465	0.905968982
ENSGALG00010012429	CXCR7	2	0.168068986	0.372182001	0.451577417	0.651573442	0.246834283	8.615879102	88.39352331	0.5	CRA004775;PRJNA827465	Yang2025_Silkie_Wenchang	0.908943868
ENSGALG00010012431	LAMP2	2	-0.0537653	0.065485379	-0.821027544	0.411630579	0	0.644212361	0	1	PRJNA827465;Yang2025_Silkie_Wenchang	CRA004775	0.835128673
ENSGALG00010012431	LAMP2	2	-0.013868072	0.158620848	-0.087429059	0.930330472	0.025076129	1.940659566	48.47112717	0.5	CRA004775;Yang2025_Silkie_Wenchang	PRJNA827465	0.983813577
ENSGALG00010012431	LAMP2	2	0.002768508	0.069610881	0.039771193	0.968275543	0	0.960549581	0	0.5	CRA004775;PRJNA827465	Yang2025_Silkie_Wenchang	0.991209187
ENSGALG00010012436	ENSGALG00010012436	2	-0.369098895	0.168226723	-2.194056256	0.028231366	0	0.844926951	0	1	PRJNA827465;Yang2025_Silkie_Wenchang	CRA004775	0.286342544
ENSGALG00010012436	ENSGALG00010012436	2	-0.484912304	0.20302544	-2.38843124	0.016920474	0	0.050521887	0	1	CRA004775;Yang2025_Silkie_Wenchang	PRJNA827465	0.216007777
ENSGALG00010012436	ENSGALG00010012436	2	-0.239483821	0.222499934	-1.076332096	0.281778759	0	0.133673311	0	1	CRA004775;PRJNA827465	Yang2025_Silkie_Wenchang	0.779295548
ENSGALG00010012437	ENSGALG00010012437	2	-5.877860054	5.924224894	-0.992173687	0.321112806	64.56583084	12.22776857	91.82189298	1	PRJNA827465;Yang2025_Silkie_Wenchang	CRA004775	0.798513866
ENSGALG00010012437	ENSGALG00010012437	2	-5.656884175	6.066265343	-0.932515123	0.351070375	69.04814199	15.50301456	93.54964161	0.5	CRA004775;Yang2025_Silkie_Wenchang	PRJNA827465	0.810161472
ENSGALG00010012437	ENSGALG00010012437	2	0.027915743	0.798205479	0.034973129	0.972101168	0	0.023332379	0	0.5	CRA004775;PRJNA827465	Yang2025_Silkie_Wenchang	0.991955348
ENSGALG00010012439	TENT2	2	0.344799302	0.241839834	1.425734119	0.153945104	0.096543726	5.686567035	82.41469776	1	PRJNA827465;Yang2025_Silkie_Wenchang	CRA004775	0.646534044
ENSGALG00010012439	TENT2	2	0.267132797	0.339350854	0.787187635	0.431172034	0.198276944	7.145181811	86.00455487	0.5	CRA004775;Yang2025_Silkie_Wenchang	PRJNA827465	0.842503929
ENSGALG00010012439	TENT2	2	0.055448594	0.106995135	0.518234719	0.604294514	0	0.684090303	0	0.5	CRA004775;PRJNA827465	Yang2025_Silkie_Wenchang	0.89612415
ENSGALG00010012441	FST	2	-0.471798288	0.271497989	-1.737759787	0.082253149	0.135739682	12.58712156	92.05537187	1	PRJNA827465;Yang2025_Silkie_Wenchang	CRA004775	0.494402444
ENSGALG00010012441	FST	2	-0.524545402	0.309755071	-1.693419903	0.090375569	0.131691976	2.609650204	61.68068815	1	CRA004775;Yang2025_Silkie_Wenchang	PRJNA827465	0.5160164
ENSGALG00010012441	FST	2	-0.197662095	0.094703551	-2.087166672	0.036873071	0	0.076529868	0	1	CRA004775;PRJNA827465	Yang2025_Silkie_Wenchang	0.331459091
ENSGALG00010012442	CPLX1	2	0.38443609	0.648934834	0.592410933	0.553575447	0.51774619	2.267730646	55.90305216	0.5	PRJNA827465;Yang2025_Silkie_Wenchang	CRA004775	0.879610907
ENSGALG00010012442	CPLX1	2	1.283956766	0.626185262	2.050442329	0.040321286	0	0.000682033	0	1	CRA004775;Yang2025_Silkie_Wenchang	PRJNA827465	0.347442284
ENSGALG00010012442	CPLX1	2	0.324762642	0.637849385	0.509152551	0.610645299	0.450274213	1.863610803	46.34072745	0.5	CRA004775;PRJNA827465	Yang2025_Silkie_Wenchang	0.897438028
ENSGALG00010012445	SOBP	2	0.099264292	0.317916494	0.312233853	0.754862806	0.167232148	5.155112023	80.60177945	0.5	PRJNA827465;Yang2025_Silkie_Wenchang	CRA004775	0.93838723
ENSGALG00010012445	SOBP	2	0.360369654	0.231883842	1.55409558	0.120161619	0	0.642255557	0	1	CRA004775;Yang2025_Silkie_Wenchang	PRJNA827465	0.581455423
ENSGALG00010012445	SOBP	2	-0.163591594	0.082062841	-1.993491723	0.046207629	0	0.244348061	0	0.5	CRA004775;PRJNA827465	Yang2025_Silkie_Wenchang	0.370556711
ENSGALG00010012447	COPS8	2	-0.281726525	0.242815164	-1.160250951	0.245946648	0.103097507	7.375806207	86.44216006	1	PRJNA827465;Yang2025_Silkie_Wenchang	CRA004775	0.75661406
ENSGALG00010012447	COPS8	2	-0.327925072	0.271755794	-1.206690268	0.227551448	0.095160572	2.63692086	62.07698095	0.5	CRA004775;Yang2025_Silkie_Wenchang	PRJNA827465	0.740853612
ENSGALG00010012447	COPS8	2	-0.060995744	0.05788951	-1.053657986	0.292039531	0	0.04691694	0	0.5	CRA004775;PRJNA827465	Yang2025_Silkie_Wenchang	0.785516679
ENSGALG00010012449	SUB1	2	-0.143569193	0.157722277	-0.91026579	0.362682356	0.043469278	7.908178259	87.35486269	0.5	PRJNA827465;Yang2025_Silkie_Wenchang	CRA004775	0.815941879
ENSGALG00010012449	SUB1	2	-0.044181361	0.278032083	-0.15890742	0.873741815	0.137550522	8.723097817	88.53618266	0.5	CRA004775;Yang2025_Silkie_Wenchang	PRJNA827465	0.9699681
ENSGALG00010012449	SUB1	2	0.085407879	0.111215517	0.767949298	0.442517307	0.012132039	1.707901245	41.44860525	1	CRA004775;PRJNA827465	Yang2025_Silkie_Wenchang	0.845535782
ENSGALG00010012456	ENSGALG00010012456	2	0.032674576	0.12594945	0.259426115	0.795306483	0.024459283	4.34960703	77.00941733	0.5	PRJNA827465;Yang2025_Silkie_Wenchang	CRA004775	0.948935068
ENSGALG00010012456	ENSGALG00010012456	2	-0.095944741	0.085642978	-1.120287308	0.262591349	0	0.002435176	0	1	CRA004775;Yang2025_Silkie_Wenchang	PRJNA827465	0.768418419
ENSGALG00010012456	ENSGALG00010012456	2	0.13198356	0.075236674	1.754245012	0.079388534	0	0.862201879	0	0.5	CRA004775;PRJNA827465	Yang2025_Silkie_Wenchang	0.484805397
ENSGALG00010012458	MRPS30	2	0.060805552	0.143784504	0.422893637	0.672372865	0	0.129822156	0	1	PRJNA827465;Yang2025_Silkie_Wenchang	CRA004775	0.914742762
ENSGALG00010012458	MRPS30	2	0.047706888	0.164403514	0.290181682	0.771677249	0	0.255331333	0	0.5	CRA004775;Yang2025_Silkie_Wenchang	PRJNA827465	0.942985136
ENSGALG00010012458	MRPS30	2	-0.00826437	0.152471771	-0.054202623	0.956773731	0	0.040319324	0	0.5	CRA004775;PRJNA827465	Yang2025_Silkie_Wenchang	0.988540618
ENSGALG00010012464	ENSGALG00010012464	2	-0.335301597	0.426776774	-0.785660366	0.432066489	0	0.427428622	0	0.5	PRJNA827465;Yang2025_Silkie_Wenchang	CRA004775	0.842503929
ENSGALG00010012464	ENSGALG00010012464	2	-0.359772316	0.432253667	-0.832317558	0.405229722	0	0.355192942	0	1	CRA004775;Yang2025_Silkie_Wenchang	PRJNA827465	0.833112102
ENSGALG00010012464	ENSGALG00010012464	2	-0.018619119	0.479957142	-0.038793294	0.969055191	0	0.001752909	0	0.5	CRA004775;PRJNA827465	Yang2025_Silkie_Wenchang	0.991507463
ENSGALG00010012466	PDSS2	2	-0.025603463	0.110862666	-0.230947572	0.817355533	0.019739912	4.999937959	79.99975183	0.5	PRJNA827465;Yang2025_Silkie_Wenchang	CRA004775	0.954888783
ENSGALG00010012466	PDSS2	2	-0.131192728	0.076915373	-1.705676291	0.088068366	0	0.28119257	0	0.5	CRA004775;Yang2025_Silkie_Wenchang	PRJNA827465	0.509839669
ENSGALG00010012466	PDSS2	2	0.075160272	0.057056312	1.317299848	0.187738136	0	0.075970676	0	1	CRA004775;PRJNA827465	Yang2025_Silkie_Wenchang	0.696930043
ENSGALG00010012469	CETN2	2	-0.056779848	0.195994466	-0.289701282	0.772044775	0.053158834	3.155882562	68.31314282	0.5	PRJNA827465;Yang2025_Silkie_Wenchang	CRA004775	0.943066211
ENSGALG00010012469	CETN2	2	-0.241218668	0.139807131	-1.725367407	0.084461281	0	0.088475314	0	1	CRA004775;Yang2025_Silkie_Wenchang	PRJNA827465	0.500072565
ENSGALG00010012469	CETN2	2	0.013451057	0.146681303	0.091702599	0.926934332	0.016609565	1.527699321	34.54209307	0.5	CRA004775;PRJNA827465	Yang2025_Silkie_Wenchang	0.982896295
ENSGALG00010012470	CHD1	2	0.300552093	0.350232944	0.858149121	0.390810126	0.203660639	5.83165567	82.85221116	0.5	PRJNA827465;Yang2025_Silkie_Wenchang	CRA004775	0.829251057
ENSGALG00010012470	CHD1	2	0.669685674	0.178512066	3.751486883	0.000175789	0	0.000415871	0	1	CRA004775;Yang2025_Silkie_Wenchang	PRJNA827465	0.01153893
ENSGALG00010012470	CHD1	2	0.2856182	0.352637695	0.809948012	0.417970056	0.195568317	4.5248778	77.89995566	0.5	CRA004775;PRJNA827465	Yang2025_Silkie_Wenchang	0.838112595
ENSGALG00010012477	ENSGALG00010012477	2	-0.301494036	0.360224691	-0.836961053	0.402614461	0.220315278	6.575983011	84.79314806	0.5	PRJNA827465;Yang2025_Silkie_Wenchang	CRA004775	0.832937979
ENSGALG00010012477	ENSGALG00010012477	2	-0.193698065	0.513867781	-0.376941447	0.706217119	0.446594417	6.322695833	84.18396161	0.5	CRA004775;Yang2025_Silkie_Wenchang	PRJNA827465	0.924548793
ENSGALG00010012477	ENSGALG00010012477	2	0.109754541	0.156147776	0.702888915	0.48212498	0	0.637017367	0	1	CRA004775;PRJNA827465	Yang2025_Silkie_Wenchang	0.85691916
ENSGALG00010012483	ENSGALG00010012483	2	0.267063533	0.166133145	1.607527105	0.107938788	0	0.21489052	0	1	PRJNA827465;Yang2025_Silkie_Wenchang	CRA004775	0.556183889
ENSGALG00010012483	ENSGALG00010012483	2	0.0575296	0.241753913	0.237967604	0.81190622	0	0.879224667	0	0.5	CRA004775;Yang2025_Silkie_Wenchang	PRJNA827465	0.953371387
ENSGALG00010012483	ENSGALG00010012483	2	0.13681657	0.321145057	0.426027325	0.670087935	0.094910253	1.549337539	35.45628534	0.5	CRA004775;PRJNA827465	Yang2025_Silkie_Wenchang	0.914428878
ENSGALG00010012486	RADX	2	0.070599223	0.045188014	1.562344001	0.118206972	0	0.219041553	0	1	PRJNA827465;Yang2025_Silkie_Wenchang	CRA004775	0.578003524
ENSGALG00010012486	RADX	2	0.143682965	0.180740879	0.794966617	0.426632921	0.036559226	1.784678476	43.96749816	1	CRA004775;Yang2025_Silkie_Wenchang	PRJNA827465	0.84125685
ENSGALG00010012486	RADX	2	0.147754904	0.138160136	1.069446715	0.284868427	0.020567589	1.468454151	31.90117651	1	CRA004775;PRJNA827465	Yang2025_Silkie_Wenchang	0.780385013
ENSGALG00010012498	KCNT2	2	-0.117253243	0.148016828	-0.792161572	0.428266472	0	0.118437079	0	1	PRJNA827465;Yang2025_Silkie_Wenchang	CRA004775	0.841329286
ENSGALG00010012498	KCNT2	2	-0.089897899	0.250524774	-0.358838361	0.719716013	0	0.209945745	0	1	CRA004775;Yang2025_Silkie_Wenchang	PRJNA827465	0.928508074
ENSGALG00010012498	KCNT2	2	-0.164646204	0.168426692	-0.977554103	0.328294907	0	0.092562086	0	1	CRA004775;PRJNA827465	Yang2025_Silkie_Wenchang	0.800761292
ENSGALG00010012506	TP53INP1	2	-0.319660783	0.373274027	-0.85637028	0.391792998	0.272846558	47.67958094	97.90266613	0.5	PRJNA827465;Yang2025_Silkie_Wenchang	CRA004775	0.829251057
ENSGALG00010012506	TP53INP1	2	-0.690464772	0.089824766	-7.686797344	1.50864E-14	0	0.090414622	0	1	CRA004775;Yang2025_Silkie_Wenchang	PRJNA827465	1.52036E-11
ENSGALG00010012506	TP53INP1	2	-0.123253533	0.277939908	-0.443453889	0.657437465	0.104084687	2.190028809	54.33850022	0.5	CRA004775;PRJNA827465	Yang2025_Silkie_Wenchang	0.910711339
ENSGALG00010012508	MDM1	2	-0.113199977	0.098431082	-1.150043003	0.25012616	0.011871114	2.464305176	59.42061033	1	PRJNA827465;Yang2025_Silkie_Wenchang	CRA004775	0.75993411
ENSGALG00010012508	MDM1	2	-0.201583203	0.097306238	-2.071636997	0.038299307	0	0.396995433	0	1	CRA004775;Yang2025_Silkie_Wenchang	PRJNA827465	0.337506202
ENSGALG00010012508	MDM1	2	-0.033932351	0.065289951	-0.519717817	0.603260267	0	0.029479372	0	1	CRA004775;PRJNA827465	Yang2025_Silkie_Wenchang	0.895538966
ENSGALG00010012509	MICAL3	2	0.04232446	0.155628883	0.271957619	0.785654607	0.032095815	2.538987567	60.61422226	0.5	PRJNA827465;Yang2025_Silkie_Wenchang	CRA004775	0.946557017
ENSGALG00010012509	MICAL3	2	0.297337592	0.170138806	1.747617722	0.080530245	0	0.272774017	0	1	CRA004775;Yang2025_Silkie_Wenchang	PRJNA827465	0.488438303
ENSGALG00010012509	MICAL3	2	0.074627096	0.242591722	0.307624247	0.758368268	0.075213789	2.015230661	50.37788877	0.5	CRA004775;PRJNA827465	Yang2025_Silkie_Wenchang	0.938954369
ENSGALG00010012511	OXCT1	2	-0.36170425	0.121696488	-2.972183138	0.002956902	0.015491887	2.050831327	51.23928591	1	PRJNA827465;Yang2025_Silkie_Wenchang	CRA004775	0.074592515
ENSGALG00010012511	OXCT1	2	-0.069195877	0.447379912	-0.154669165	0.877082141	0.368573779	12.46346925	91.97655179	0.5	CRA004775;Yang2025_Silkie_Wenchang	PRJNA827465	0.970509861
ENSGALG00010012511	OXCT1	2	0.04157755	0.323558194	0.128500994	0.897752512	0.182975195	7.571839313	86.79316929	0.5	CRA004775;PRJNA827465	Yang2025_Silkie_Wenchang	0.975696709
ENSGALG00010012517	MGAT3	2	-0.406384682	0.342410147	-1.186835979	0.235292324	0.218223251	13.93169694	92.82212351	1	PRJNA827465;Yang2025_Silkie_Wenchang	CRA004775	0.748350272
ENSGALG00010012517	MGAT3	2	-0.715222295	0.15717181	-4.550576176	5.34992E-06	0	0.640290265	0	1	CRA004775;Yang2025_Silkie_Wenchang	PRJNA827465	0.0008653
ENSGALG00010012517	MGAT3	2	-0.090041371	0.069096611	-1.303122832	0.19253283	0	0.603332779	0	1	CRA004775;PRJNA827465	Yang2025_Silkie_Wenchang	0.702467122
ENSGALG00010012519	ENSGALG00010012519	2	-5.258131993	5.17606936	-1.015854237	0.309698804	50.52780132	17.34688242	94.23527539	1	PRJNA827465;Yang2025_Silkie_Wenchang	CRA004775	0.794328224
ENSGALG00010012519	ENSGALG00010012519	2	-5.231062788	5.169435732	-1.011921428	0.311575623	50.74742394	19.42901946	94.85305987	1	CRA004775;Yang2025_Silkie_Wenchang	PRJNA827465	0.795778238
ENSGALG00010012519	ENSGALG00010012519	2	-0.220451295	0.804210876	-0.274121256	0.783991439	0	4.69732E-05	0	1	CRA004775;PRJNA827465	Yang2025_Silkie_Wenchang	0.946003179
ENSGALG00010012521	HOMER1	2	0.089456145	0.348934959	0.256369109	0.797665838	0.21246902	7.643783359	86.91747329	0.5	PRJNA827465;Yang2025_Silkie_Wenchang	CRA004775	0.949842152
ENSGALG00010012521	HOMER1	2	0.539813344	0.179122345	3.013657188	0.002581193	0	0.41603866	0	1	CRA004775;Yang2025_Silkie_Wenchang	PRJNA827465	0.068626229
ENSGALG00010012521	HOMER1	2	0.19085015	0.471469954	0.404798117	0.685625913	0.38978254	7.641251021	86.91313769	0.5	CRA004775;PRJNA827465	Yang2025_Silkie_Wenchang	0.919235539
ENSGALG00010012529	BEND3	2	0.008900163	0.048704541	0.182737855	0.855003712	0.000847397	1.163565414	14.05725988	0.5	PRJNA827465;Yang2025_Silkie_Wenchang	CRA004775	0.963317625
ENSGALG00010012529	BEND3	2	-0.057854275	0.084110706	-0.687834852	0.491556786	0	0.204679234	0	0.5	CRA004775;Yang2025_Silkie_Wenchang	PRJNA827465	0.85895707
ENSGALG00010012529	BEND3	2	0.038867656	0.048076389	0.808456211	0.418828004	0	0.001863935	0	1	CRA004775;PRJNA827465	Yang2025_Silkie_Wenchang	0.838250071
ENSGALG00010012532	FAM174A	2	0.442240969	0.332328447	1.330734616	0.183276349	0.175401546	4.835848889	79.32110736	1	PRJNA827465;Yang2025_Silkie_Wenchang	CRA004775	0.691694865
ENSGALG00010012532	FAM174A	2	0.396832091	0.427057517	0.929223991	0.352773017	0.280600693	4.212434462	76.26075826	0.5	CRA004775;Yang2025_Silkie_Wenchang	PRJNA827465	0.811035834
ENSGALG00010012532	FAM174A	2	0.074523526	0.170804063	0.436310029	0.662611806	0	0.232369261	0	0.5	CRA004775;PRJNA827465	Yang2025_Silkie_Wenchang	0.912778072
ENSGALG00010012535	JMY	2	0.132579679	0.081759521	1.621580918	0.104893111	0	0.678781002	0	1	PRJNA827465;Yang2025_Silkie_Wenchang	CRA004775	0.550048644
ENSGALG00010012535	JMY	2	0.220834603	0.138972257	1.589055311	0.112047906	0	0.068616229	0	1	CRA004775;Yang2025_Silkie_Wenchang	PRJNA827465	0.56547057
ENSGALG00010012535	JMY	2	0.089593095	0.095442226	0.93871549	0.347876838	0	0.005881934	0	1	CRA004775;PRJNA827465	Yang2025_Silkie_Wenchang	0.808847403
ENSGALG00010012536	FBXO4	2	0.119527408	0.115017574	1.039209948	0.298707105	0	0.098198357	0	1	PRJNA827465;Yang2025_Silkie_Wenchang	CRA004775	0.78974322
ENSGALG00010012536	FBXO4	2	-0.037269821	0.286427012	-0.130119784	0.896471658	0.09238909	1.943148826	48.53713793	0.5	CRA004775;Yang2025_Silkie_Wenchang	PRJNA827465	0.975115773
ENSGALG00010012536	FBXO4	2	-0.056749166	0.238784693	-0.237658309	0.812146122	0.051262721	1.527453095	34.53154122	0.5	CRA004775;PRJNA827465	Yang2025_Silkie_Wenchang	0.953371387
ENSGALG00010012538	RAP1B	2	-0.227020526	0.205271805	-1.105950846	0.268747795	0.078815132	14.8970808	93.28727545	1	PRJNA827465;Yang2025_Silkie_Wenchang	CRA004775	0.771398294
ENSGALG00010012538	RAP1B	2	-0.418609395	0.095693746	-4.374469739	1.21728E-05	0	0.774609494	0	1	CRA004775;Yang2025_Silkie_Wenchang	PRJNA827465	0.001655459
ENSGALG00010012538	RAP1B	2	-0.032934766	0.037522681	-0.877729553	0.380090503	0	0.074011091	0	1	CRA004775;PRJNA827465	Yang2025_Silkie_Wenchang	0.824392515
ENSGALG00010012550	TAB1	2	-0.145308483	0.046486254	-3.125837687	0.001772994	0	0.225601382	0	1	PRJNA827465;Yang2025_Silkie_Wenchang	CRA004775	0.053520719
ENSGALG00010012550	TAB1	2	-0.364587049	0.208264238	-1.750598439	0.080015104	0.062059441	3.248047361	69.21227159	1	CRA004775;Yang2025_Silkie_Wenchang	PRJNA827465	0.486665684
ENSGALG00010012550	TAB1	2	-0.331743093	0.236078815	-1.405221779	0.15995534	0.091914771	4.958065511	79.83084335	1	CRA004775;PRJNA827465	Yang2025_Silkie_Wenchang	0.655404348
ENSGALG00010012551	PCGF3	2	0.298569809	0.271094271	1.101350491	0.270744144	0.121575976	5.663511619	82.34311029	1	PRJNA827465;Yang2025_Silkie_Wenchang	CRA004775	0.772743863
ENSGALG00010012551	PCGF3	2	0.47381926	0.171708861	2.759433941	0.005790159	0.006674573	1.106872554	9.655362206	1	CRA004775;Yang2025_Silkie_Wenchang	PRJNA827465	0.114529757
ENSGALG00010012551	PCGF3	2	0.071467357	0.117027847	0.610686763	0.541406971	0	0.279991336	0	1	CRA004775;PRJNA827465	Yang2025_Silkie_Wenchang	0.876108291
ENSGALG00010012553	ENSGALG00010012553	2	-0.38785218	0.269312986	-1.440154022	0.149823828	0.007528004	1.054499582	5.168288586	1	PRJNA827465;Yang2025_Silkie_Wenchang	CRA004775	0.639778999
ENSGALG00010012553	ENSGALG00010012553	2	-0.179921978	0.466141451	-0.385981503	0.699510368	0.300554275	3.241505299	69.15013527	0.5	CRA004775;Yang2025_Silkie_Wenchang	PRJNA827465	0.922022134
ENSGALG00010012553	ENSGALG00010012553	2	0.101158842	0.267684058	0.37790387	0.705502007	0	0.537384794	0	0.5	CRA004775;PRJNA827465	Yang2025_Silkie_Wenchang	0.924358822
ENSGALG00010012555	ENSGALG00010012555	2	0.191067946	0.112314238	1.701190786	0.088907167	0	0.045955728	0	1	PRJNA827465;Yang2025_Silkie_Wenchang	CRA004775	0.51183587
ENSGALG00010012555	ENSGALG00010012555	2	0.198628624	0.154606537	1.28473626	0.198884463	0	0.134242899	0	1	CRA004775;Yang2025_Silkie_Wenchang	PRJNA827465	0.71096127
ENSGALG00010012555	ENSGALG00010012555	2	0.155423185	0.149670759	1.038433869	0.299068108	0	0.078290878	0	1	CRA004775;PRJNA827465	Yang2025_Silkie_Wenchang	0.789748017
ENSGALG00010012561	OSGIN2	2	0.103786693	0.073138368	1.419045787	0.155885668	0	0.814404962	0	1	PRJNA827465;Yang2025_Silkie_Wenchang	CRA004775	0.649132948

ENSGALG00010012561	OSGIN2	2	-0.000823553	0.111543084	-0.007383269	0.994109057	0	0.670090032	0	0.5	CRA004775;Yang2025_Silkie_Wenchang	PRJNA827465	0.99818842
ENSGALG00010012561	OSGIN2	2	0.085980274	0.176415648	0.487373286	0.625993821	0.027350095	1.273500932	21.47630403	0.5	CRA004775;PRJNA827465	Yang2025_Silkie_Wenchang	0.901504244
ENSGALG00010012565	SYNGR1	2	-0.069498883	0.037752917	-1.840887782	0.065638005	0	0.067093426	0	1	PRJNA827465;Yang2025_Silkie_Wenchang	CRA004775	0.441590298
ENSGALG00010012565	SYNGR1	2	-0.062035389	0.05320874	-1.165887212	0.24366005	0	0.089399995	0	1	CRA004775;Yang2025_Silkie_Wenchang	PRJNA827465	0.755365463
ENSGALG00010012565	SYNGR1	2	-0.081064114	0.052326708	-1.54919195	0.121335584	0	0.059077185	0	1	CRA004775;PRJNA827465	Yang2025_Silkie_Wenchang	0.583884665
ENSGALG00010012569	C6orf203	2	-0.010119517	0.071407499	-0.141715046	0.88730509	0.003779832	1.408165885	28.98563935	0.5	PRJNA827465;Yang2025_Silkie_Wenchang	CRA004775	0.972987228
ENSGALG00010012569	C6orf203	2	0.123132649	0.118111734	1.042509872	0.297175351	0	0.15679214	0	1	CRA004775;Yang2025_Silkie_Wenchang	PRJNA827465	0.788466926
ENSGALG00010012569	C6orf203	2	-0.04659252	0.055750068	-0.835739254	0.403301607	0	0.671711022	0	0.5	CRA004775;PRJNA827465	Yang2025_Silkie_Wenchang	0.83306585
ENSGALG00010012571	AGAP1	2	-0.145022338	0.073738029	-1.966723832	0.049215058	0	0.952586876	0	0.5	PRJNA827465;Yang2025_Silkie_Wenchang	CRA004775	0.38363733
ENSGALG00010012571	AGAP1	2	0.002863652	0.149012347	0.019217548	0.984667559	0	0.021321428	0	0.5	CRA004775;Yang2025_Silkie_Wenchang	PRJNA827465	0.995703389
ENSGALG00010012571	AGAP1	2	-0.163382637	0.076883765	-2.125060296	0.033581582	0	0.306263723	0	1	CRA004775;PRJNA827465	Yang2025_Silkie_Wenchang	0.314626202
ENSGALG00010012574	PHAX	2	-0.00607322	0.117090474	-0.051867751	0.958634071	0	0.22174053	0	0.5	PRJNA827465;Yang2025_Silkie_Wenchang	CRA004775	0.989070013
ENSGALG00010012574	PHAX	2	0.131884408	0.127669247	1.033016256	0.301596291	0	0.391335786	0	1	CRA004775;Yang2025_Silkie_Wenchang	PRJNA827465	0.791273005
ENSGALG00010012574	PHAX	2	0.062357006	0.134279621	0.464381755	0.64237426	0.007275166	1.247151765	19.81729663	0.5	CRA004775;PRJNA827465	Yang2025_Silkie_Wenchang	0.906634523
ENSGALG00010012578	NDUFS4	2	0.024837396	0.159551691	0.155669901	0.876293223	0	0.52506602	0	0.5	PRJNA827465;Yang2025_Silkie_Wenchang	CRA004775	0.970347777
ENSGALG00010012578	NDUFS4	2	0.047396011	0.218879102	0.216539682	0.828567097	0	0.705978649	0	0.5	CRA004775;Yang2025_Silkie_Wenchang	PRJNA827465	0.956786127
ENSGALG00010012578	NDUFS4	2	-0.088774254	0.172376117	-0.515003212	0.606550775	0	0.111357052	0	1	CRA004775;PRJNA827465	Yang2025_Silkie_Wenchang	0.896799223
ENSGALG00010012582	CD24	2	-0.086024381	0.135084533	-0.63681887	0.524242829	0	0.242066468	0	0.5	PRJNA827465;Yang2025_Silkie_Wenchang	CRA004775	0.869790047
ENSGALG00010012582	CD24	2	-0.048040098	0.230525638	-0.208393732	0.83492155	0	0.427919928	0	0.5	CRA004775;Yang2025_Silkie_Wenchang	PRJNA827465	0.95823248
ENSGALG00010012582	CD24	2	-0.147260082	0.154034245	-0.956021709	0.339061267	0	0.193976727	0	1	CRA004775;PRJNA827465	Yang2025_Silkie_Wenchang	0.804767595
ENSGALG00010012583	NDUFAF6	2	0.068954274	0.110019442	0.626746259	0.530825576	0.011365352	1.669260392	40.093552	0.5	PRJNA827465;Yang2025_Silkie_Wenchang	CRA004775	0.872805322
ENSGALG00010012583	NDUFAF6	2	0.152335843	0.151371685	1.006369472	0.314237873	0.001687433	1.026213577	2.554397783	0.5	CRA004775;Yang2025_Silkie_Wenchang	PRJNA827465	0.796531448
ENSGALG00010012583	NDUFAF6	2	-0.011810579	0.076139666	-0.155117291	0.876728851	0	0.14679832	0	1	CRA004775;PRJNA827465	Yang2025_Silkie_Wenchang	0.97046577
ENSGALG00010012586	CDC73	2	-0.069898978	0.051582624	-1.355087672	0.175389626	0	0.000584523	0	1	PRJNA827465;Yang2025_Silkie_Wenchang	CRA004775	0.680258136
ENSGALG00010012586	CDC73	2	-0.04476578	0.09171901	-0.488075312	0.625496497	0	0.208124689	0	0.5	CRA004775;Yang2025_Silkie_Wenchang	PRJNA827465	0.90367193
ENSGALG00010012586	CDC73	2	-0.061609004	0.056624836	-1.088020881	0.276585889	0	0.261480095	0	0.5	CRA004775;PRJNA827465	Yang2025_Silkie_Wenchang	0.775851484
ENSGALG00010012587	MBLAC2	2	-0.228751971	0.326392358	-0.700849654	0.483396847	0.176996929	5.900660997	83.05274611	0.5	PRJNA827465;Yang2025_Silkie_Wenchang	CRA004775	0.85691916
ENSGALG00010012587	MBLAC2	2	-0.503615792	0.174921084	-2.879102856	0.003988082	0	0.364325992	0	1	CRA004775;Yang2025_Silkie_Wenchang	PRJNA827465	0.091148882
ENSGALG00010012587	MBLAC2	2	0.015553959	0.162810594	0.095534071	0.923890629	0	0.937186339	0	0.5	CRA004775;PRJNA827465	Yang2025_Silkie_Wenchang	0.982219519
ENSGALG00010012588	PEX26	2	-0.066250595	0.307747369	-0.215275911	0.82955221	0.18016006	20.24700164	95.06099709	0.5	PRJNA827465;Yang2025_Silkie_Wenchang	CRA004775	0.956939641
ENSGALG00010012588	PEX26	2	-0.320853253	0.097719118	-3.283423534	0.001025545	0	0.860672324	0	1	CRA004775;Yang2025_Silkie_Wenchang	PRJNA827465	0.038424195
ENSGALG00010012588	PEX26	2	0.055915207	0.207286476	0.269748457	0.787353779	0.069889291	4.885753011	79.53232597	0.5	CRA004775;PRJNA827465	Yang2025_Silkie_Wenchang	0.946919116
ENSGALG00010012599	DMGDH	2	0.143239488	0.125995694	1.136860181	0.25559674	0	0.157374656	0	1	PRJNA827465;Yang2025_Silkie_Wenchang	CRA004775	0.764467569
ENSGALG00010012599	DMGDH	2	0.030455932	0.167075733	0.182288182	0.855356573	0	0.551905724	0	0.5	CRA004775;Yang2025_Silkie_Wenchang	PRJNA827465	0.963406774
ENSGALG00010012599	DMGDH	2	0.120953769	0.157353392	0.76867595	0.442085705	0	0.976130803	0	0.5	CRA004775;PRJNA827465	Yang2025_Silkie_Wenchang	0.845184088
ENSGALG00010012600	NUP107	2	0.130936301	0.132710668	0.986629803	0.323824154	0.028209062	4.87768852	79.4984859	1	PRJNA827465;Yang2025_Silkie_Wenchang	CRA004775	0.799600434
ENSGALG00010012600	NUP107	2	0.144011157	0.186399905	0.772592437	0.439763622	0.041731827	2.141485519	53.30344329	0.5	CRA004775;Yang2025_Silkie_Wenchang	PRJNA827465	0.844353254
ENSGALG00010012600	NUP107	2	0.000951917	0.063023243	0.015104216	0.987949038	0	0.249374628	0	0.5	CRA004775;PRJNA827465	Yang2025_Silkie_Wenchang	0.996800355
ENSGALG00010012601	TUBA8A	2	-0.112292315	0.290843899	-0.386091353	0.699429013	0.127557811	3.995413415	74.97130093	0.5	PRJNA827465;Yang2025_Silkie_Wenchang	CRA004775	0.922020923
ENSGALG00010012601	TUBA8A	2	0.121462818	0.2135983	0.568650677	0.569593226	0	0.653675906	0	0.5	CRA004775;Yang2025_Silkie_Wenchang	PRJNA827465	0.88485924
ENSGALG00010012601	TUBA8A	2	-0.368626527	0.163459859	-2.255150167	0.024123905	0	0.05848439	0	1	CRA004775;PRJNA827465	Yang2025_Silkie_Wenchang	0.265075657
ENSGALG00010012604	ENSGALG00010012604	2	-4.968474073	4.955757325	-1.002566055	0.316070281	44.9764253	11.67377357	91.43378965	1	PRJNA827465;Yang2025_Silkie_Wenchang	CRA004775	0.796883862
ENSGALG00010012604	ENSGALG00010012604	2	-4.58974807	5.31584033	-0.863409694	0.387912252	52.71776213	14.57289514	93.13794555	0.5	CRA004775;Yang2025_Silkie_Wenchang	PRJNA827465	0.828154121
ENSGALG00010012604	ENSGALG00010012604	2	0.221859896	0.989946003	0.224113128	0.822669277	0	0.12907176	0	0.5	CRA004775;PRJNA827465	Yang2025_Silkie_Wenchang	0.955822987
ENSGALG00010012606	ATP5PO	2	-0.115434737	0.071558591	-1.613149926	0.106711939	0	0.536562374	0	1	PRJNA827465;Yang2025_Silkie_Wenchang	CRA004775	0.553895846
ENSGALG00010012606	ATP5PO	2	-0.179851042	0.185020463	-0.972060274	0.331020564	0	0.510038865	0	0.5	CRA004775;Yang2025_Silkie_Wenchang	PRJNA827465	0.801722786
ENSGALG00010012606	ATP5PO	2	-0.092384693	0.073464243	-1.257546387	0.208555853	0	0.103982391	0	0.5	CRA004775;PRJNA827465	Yang2025_Silkie_Wenchang	0.721011841
ENSGALG00010012607	LMNB1	2	0.038095375	0.086155941	0.442167707	0.658367852	0	0.117319682	0	0.5	PRJNA827465;Yang2025_Silkie_Wenchang	CRA004775	0.911056676
ENSGALG00010012607	LMNB1	2	0.018942112	0.135612333	0.139678386	0.888914104	0	0.215166661	0	0.5	CRA004775;Yang2025_Silkie_Wenchang	PRJNA827465	0.973135755
ENSGALG00010012607	LMNB1	2	0.06917726	0.105480738	0.655828364	0.511934564	0	0.117366947	0	1	CRA004775;PRJNA827465	Yang2025_Silkie_Wenchang	0.865694231
ENSGALG00010012610	RPL3	2	-0.272486539	0.270799698	-1.00622911	0.314305372	0.120315708	4.825573218	79.27707332	1	PRJNA827465;Yang2025_Silkie_Wenchang	CRA004775	0.796531448
ENSGALG00010012610	RPL3	2	-0.435131583	0.164539392	-2.644543515	0.008180119	0	0.828405025	0	1	CRA004775;Yang2025_Silkie_Wenchang	PRJNA827465	0.140578578
ENSGALG00010012610	RPL3	2	-0.080358542	0.084502897	-0.950956065	0.341626679	0.004592739	1.171815283	14.66231798	1	CRA004775;PRJNA827465	Yang2025_Silkie_Wenchang	0.805823616
ENSGALG00010012614	LHFPL2	2	0.010707985	0.074485806	0.143758732	0.885690991	0	0.097829803	0	0.5	PRJNA827465;Yang2025_Silkie_Wenchang	CRA004775	0.972376043
ENSGALG00010012614	LHFPL2	2	0.057101256	0.110950163	0.514656798	0.606792868	0	0.281900602	0	1	CRA004775;Yang2025_Silkie_Wenchang	PRJNA827465	0.896799223
ENSGALG00010012614	LHFPL2	2	0.007516473	0.093846472	0.080093289	0.93616306	0	0.438053077	0	0.5	CRA004775;PRJNA827465	Yang2025_Silkie_Wenchang	0.984824195
ENSGALG00010012618	B3GALT2	2	-0.766148833	0.168921806	-4.535523571	5.74608E-06	0	0.282744712	0	1	PRJNA827465;Yang2025_Silkie_Wenchang	CRA004775	0.000914324
ENSGALG00010012618	B3GALT2	2	-0.610537315	0.273731981	-2.230420107	0.025719566	0	0.11204812	0	1	CRA004775;Yang2025_Silkie_Wenchang	PRJNA827465	0.273470915
ENSGALG00010012618	B3GALT2	2	-0.803362494	0.202299337	-3.971157315	7.15243E-05	0	0.31191423	0	1	CRA004775;PRJNA827465	Yang2025_Silkie_Wenchang	0.00612433
ENSGALG00010012624	PDGFB	2	0.117983253	0.077272076	1.526854966	0.12679708	0	0.3863761	0	1	PRJNA827465;Yang2025_Silkie_Wenchang	CRA004775	0.597676535
ENSGALG00010012624	PDGFB	2	0.178787543	0.107586282	1.661806129	0.096551653	0	0.03176298	0	1	CRA004775;Yang2025_Silkie_Wenchang	PRJNA827465	0.530959789
ENSGALG00010012624	PDGFB	2	0.091533252	0.098122972	0.932842229	0.350901434	0	0.238215783	0	1	CRA004775;PRJNA827465	Yang2025_Silkie_Wenchang	0.810146221
ENSGALG00010012629	NUS1	2	0.004251191	0.051836001	0.082012322	0.934636915	0	0.064792698	0	1	PRJNA827465;Yang2025_Silkie_Wenchang	CRA004775	0.984397102
ENSGALG00010012629	NUS1	2	-0.078232798	0.14763145	-0.529919592	0.596167682	0	0.794670272	0	0.5	CRA004775;Yang2025_Silkie_Wenchang	PRJNA827465	0.894064255
ENSGALG00010012629	NUS1	2	-0.012152129	0.051764505	-0.234757938	0.814396616	0	0.94943425	0	0.5	CRA004775;PRJNA827465	Yang2025_Silkie_Wenchang	0.953869515
ENSGALG00010012631	GLRX2	2	-0.447146069	0.204720832	-2.184174736	0.028949411	0.064328562	4.125446083	75.76019708	1	PRJNA827465;Yang2025_Silkie_Wenchang	CRA004775	0.289725292
ENSGALG00010012631	GLRX2	2	-0.550264061	0.198841131	-2.767355318	0.005651312	0.025281165	1.354815385	26.18920549	1	CRA004775;Yang2025_Silkie_Wenchang	PRJNA827465	0.112909246
ENSGALG00010012631	GLRX2	2	-0.262011166	0.09994343	-2.621594692	0.008751945	0	0.006045353	0	1	CRA004775;PRJNA827465	Yang2025_Silkie_Wenchang	0.146797439
ENSGALG00010012633	USP18	2	0.229083541	0.206841227	1.107533271	0.268063436	0.058067369	3.004987601	66.72199247	0.5	PRJNA827465;Yang2025_Silkie_Wenchang	CRA004775	0.771398294
ENSGALG00010012633	USP18	2	-0.036634694	0.182748753	-0.200464809	0.841117078	0	0.132162761	0	1	CRA004775;Yang2025_Silkie_Wenchang	PRJNA827465	0.959884567
ENSGALG00010012633	USP18	2	0.264010277	0.256131672	1.03075998	0.30265339	0.069424661	1.648769829	39.34872032	0.5	CRA004775;PRJNA827465	Yang2025_Silkie_Wenchang	0.791852471
ENSGALG00010012640	PRRC1	2	0.050269315	0.078413366	0.641080946	0.521470093	0.007642502	2.572830107	61.13229563	0.5	PRJNA827465;Yang2025_Silkie_Wenchang	CRA004775	0.869518515
ENSGALG00010012640	PRRC1	2	-0.064138746	0.071840904	-0.892788677	0.371970351	0	0.491566348	0	1	CRA004775;Yang2025_Silkie_Wenchang	PRJNA827465	0.821597118
ENSGALG00010012640	PRRC1	2	0.016833051	0.136319234	0.123482585	0.901724964	0.025806014	2.78120749	64.04439426	0.5	CRA004775;PRJNA827465	Yang2025_Silkie_Wenchang	0.976674416
ENSGALG00010012648	MRPS6	2	-0.429742813	0.251881977	-1.70612768	0.08798431	0.115811353	11.45413788	91.26953063	1	PRJNA827465;Yang2025_Silkie_Wenchang	CRA004775	0.509660624
ENSGALG00010012648	MRPS6	2	-0.337852258	0.411423641	-0.821178522	0.411544588	0.276446201	4.787501383	79.11227757	0.5	CRA004775;Yang2025_Silkie_Wenchang	PRJNA827465	0.835128673
ENSGALG00010012648	MRPS6	2	-0.153453666	0.100302882	-1.529902867	0.126040774	0	0.758205934	0	0.5	CRA004775;PRJNA827465	Yang2025_Silkie_Wenchang	0.596266312
ENSGALG00010012650	DSE	2	-0.159658307	0.170082368	-0.938711689	0.34787879	0.047035652	5.301231804	81.13645966	0.5	PRJNA827465;Yang2025_Silkie_Wenchang	CRA004775	0.808847403
ENSGALG00010012650	DSE	2	-0.345698536	0.112216151	-3.080648677	0.002065502	0	0.07277697	0	1	CRA004775;Yang2025_Silkie_Wenchang	PRJNA827465	0.059057851
ENSGALG00010012650	DSE	2	-0.036437132	0.128860577	-0.282763999	0.777357753	0.009807901	1.101980437	9.254287439	0.5	CRA004775;PRJNA827465	Yang2025_Silkie_Wenchang	0.944260471
ENSGALG00010012653	CBX7	2	-0.064783975	0.125714823	-0.515324872	0.606326022	0	0.056862622	0	1	PRJNA827465;Yang2025_Silkie_Wenchang	CRA004775	0.896731537
ENSGALG00010012653	CBX7	2	-0.130605045	0.193093965	-0.676380771	0.498798922	0	0.042896451	0	1	CRA004775;Yang2025_Silkie_Wenchang	PRJNA827465	0.861571318
ENSGALG00010012653	CBX7	2	-0.064616693	0.142485074	-0.453497983	0.650190196	0	0.138928874	0	1	CRA004775;PRJNA827465	Yang2025_Silkie_Wenchang	0.908375702
ENSGALG00010012654	TENT5B	2	0.12733207	0.178145788	0.714763294	0.47475532	0.043361997	3.155921129	68.31353006	0.5	PRJNA827465;Yang2025_Silkie_Wenchang	CRA004775	0.854462864
ENSGALG00010012654	TENT5B	2	0.299202269	0.137364555	2.178162108	0.02939397	0	0.031684435	0	1	CRA004775;Yang2025_Silkie_Wenchang	PRJNA827465	0.292177846
ENSGALG00010012654	TENT5B	2	-0.042230907	0.140315533	-0.300971005	0.763436605	0	0.111207459	0	0.5	CRA004775;PRJNA827465	Yang2025_Silkie_Wenchang	0.940301633
ENSGALG00010012662	SLC35E3	2	-0.005380308	0.130928888	-0.04109336	0.967221468	0.024923238	3.558769332	71.90039852	0.5	PRJNA827465;Yang2025_Silkie_Wenchang	CRA004775	0.990894822
ENSGALG00010012662	SLC35E3	2	-0.152550635	0.102968492	-1.481527327	0.138466108	0	0.001118681	0	1	CRA004775;Yang2025_Silkie_Wenchang	PRJNA827465	0.622313717
ENSGALG00010012662	SLC35E3	2	0.059739633	0.107508962	0.555671191	0.578435655	0.008225964	1.28648553	22.26884974	0.5	CRA004775;PRJNA827465	Yang2025_Silkie_Wenchang	0.887855709
ENSGALG00010012667	SCAMP1	2	-0.296907105	0.265786148	-1.117090215	0.263955754	0.136794749	31.29213845	96.80430917	1	PRJNA827465;Yang2025_Silkie_Wenchang	CRA004775	0.768643821
ENSGALG00010012667	SCAMP1	2	-0.355007635	0.255163858	-1.391292786	0.16413666	0.103329922	4.272923356	76.59681869	1	CRA004775;Yang2025_Silkie_Wenchang	PRJNA827465	0.66287462
ENSGALG00010012667	SCAMP1	2	-0.035239348	0.049835925	-0.707107341	0.479499775	0	0.00250828	0	1	CRA004775;PRJNA827465	Yang2025_Silkie_Wenchang	0.856126448
ENSGALG00010012669	ERCC8	2	0.243557145	0.129052036	1.887278595	0.059122864	0	0.324145525	0	1	PRJNA827465;Yang2025_Silkie_Wenchang	CRA004775	0.421162606
ENSGALG00010012669	ERCC8	2	0.069934508	0.304055459	0.230005765	0.81808729	0.120364127	2.697932919	62.93458621	0.5	CRA004775;Yang2025_Silkie_Wenchang	PRJNA827465	0.95520884
ENSGALG00010012669	ERCC8	2	0.019252344	0.22177759	0.086809239	0.930823143	0.042609897	1.623333487	38.39836311	0.5	CRA004775;PRJNA827465	Yang2025_Silkie_Wenchang	0.983943003
ENSGALG00010012670	ENSGALG00010012670	2	0.943035309	0.544610403	1.731577848	0.083348753	0.471559231	4.803458692	79.18166754	1	PRJNA827465;Yang2025_Silkie_Wenchang	CRA004775	0.497321397
ENSGALG00010012670	ENSGALG00010012670	2	1.176017196	0.496734505	2.367496486	0.01790889	0.205777533	1.581137624	36.75439854	1	CRA004775;Yang2025_Silkie_Wenchang	PRJNA827465	0.223309996
ENSGALG00010012670	ENSGALG00010012670	2	0.436095906	0.273495509	1.594526753	0.110818103	0	0.001738535	0	1	CRA004775;PRJNA827465	Yang2025_Silkie_Wenchang	0.563379113
ENSGALG00010012671	NBN	2	0.353371323	0.219217502	1.611966745	0.106969177	0.066934836	3.191832595	68.6700361	1	PRJNA827465;Yang2025_Silkie_Wenchang	CRA004775	0.553983328
ENSGALG00010012671	NBN	2	0.35795571	0.262241196	1.364986566	0.172257273	0.085406716	2.611453345	61.70714664	1	CRA004775;Yang2025_Silkie_Wenchang	PRJNA827465	0.675132387
ENSGALG00010012671	NBN	2	0.139292009	0.121083166	1.150382947	0.249986181	0	0.086270257	0	1	CRA004775;PRJNA827465	Yang2025_Silkie_Wenchang	0.75993411
ENSGALG00010012672	RO60	2	-0.134270049	0.145453248	-0.923114823	0.355947371	0.032050297	3.917621959	74.47431094	1	PRJNA827465;Yang2025_Silkie_Wenchang	CRA004775	0.812321421
ENSGALG00010012672	RO60	2	-0.074661229	0.276604526	-0.269920491	0.787221423	0.110710109	3.266715164	69.38820956	0.5	CRA004775;Yang2025_Silkie_Wenchang	PRJNA827465	0.946848352
ENSGALG00010012672	RO60	2	0.010394532	0.071220012	0.145949597	0.883961178	0	0.851916568	0	0.5	CRA004775;PRJNA827465	Yang2025_Silkie_Wenchang	0.972001076
ENSGALG00010012673	ENSGALG00010012673	2	0.125093991	0.237507346	0.526695249	0.598405235	0	0.607688659	0	0.5	PRJNA827465;Yang2025_Silkie_Wenchang	CRA004775	0.894645663
ENSGALG00010012673	ENSGALG00010012673	2	0.257988333	0.264030159	0.977116909	0.328511278	0	0.060052449	0	1	CRA004775;Yang2025_Silkie_Wenchang	PRJNA827465	0.800761292
ENSGALG00010012673	ENSGALG00010012673	2	0.02779363	0.26595399	0.104505408	0.916768277	0	0.186632811	0	0.5	CRA004775;PRJNA827465	Yang2025_Silkie_Wenchang	0.980687732
ENSGALG00010012674	ENSGALG00010012674	2	0.309394803	0.456371373	0.677945247	0.49780641	0.39438958	18.64367406	94.63625036	0.5	PRJNA827465;Yang2025_Silkie_Wenchang	CRA004775	0.861072323
ENSGALG00010012674	ENSGALG00010012674	2	0.209006108	0.602867144	0.346687075	0.728826422	0.653263298	9.548228944	89.52684917	0.5	CRA004775;Yang2025_Silkie_Wenchang	PRJNA827465	0.932244113
ENSGALG00010012674	ENSGALG00010012674	2	-0.166539473	0.109807327	-1.516651724	0.129354639	0	0.645461707	0	1	CRA004775;PRJNA827465	Yang2025_Silkie_Wenchang	0.603517352
ENSGALG00010012676	ENSGALG00010012676	2	0.106982554	0.183111545	0.584248	0.559053472	0.028210134	1.662572206	39.85223641	0.5	PRJNA827465;Yang2025_Silkie_Wenchang	CRA004775	0.881013577
ENSGALG00010012676	ENSGALG00010012676	2	0.29278582	0.196766491	1.487986181	0.136754538	0	0.102971879	0	1	CRA004775;Yang2025_Silkie_Wenchang	PRJNA827465	0.618582531
ENSGALG00010012676	ENSGALG00010012676	2	0.007879198	0.14617419	0.053902804	0.957012604	0	0.423413917	0	0.5	CRA004775;PRJNA827465	Yang2025_Silkie_Wenchang	0.988540618
ENSGALG00010012682	ENSGALG00010012682	2	-0.034084788	0.139547385	-0.244252432	0.807035322	0.016225783	1.639467009	39.00456708	0.5	PRJNA827465;Yang2025_Silkie_Wenchang	CRA004775	0.95234753
ENSGALG00010012682	ENSGALG00010012682	2	-0.025707686	0.250433488	-0.102652748	0.918238577	0.062565914	1.85634197	46.13061516	0.5	CRA004775;Yang2025_Silkie_Wenchang	PRJNA827465	0.981033557
ENSGALG00010012682	ENSGALG00010012682	2	0.101340025	0.114463497	0.885295554	0.375997247	0	0.431066674	0	1	CRA004775;PRJNA827465	Yang2025_Silkie_Wenchang	0.752974013
ENSGALG00010012683	MDM2	2	-0.067142909	0.16719285	-0.401589593	0.68798609	0.048724098	7.545854796	86.74769092	0.5	PRJNA827465;Yang2025_Silkie_Wenchang	CRA004775	0.919631238
ENSGALG00010012683	MDM2	2	-0.075266742	0.212303909	-0.354523581	0.722946535	0.063001272	3.002588871	66.6954071	0.5	CRA004775;Yang2025_Silkie_Wenchang	PRJNA827465	0.930032096
ENSGALG00010012683	MDM2	2	0.095533011	0.058139219	1.643176713	0.100346377	0	0.179091587	0	1	CRA004775;PRJNA827465	Yang2025_Silkie_Wenchang	0.541223839
ENSGALG00010012685	NPTXR	2	0.011437687	0.227769642	0.050216028	0.959950239	0.067004508	2.814292637	64.46709248	0.5	PRJNA827465;Yang2025_Silkie_Wenchang	CRA004775	0.98933784
ENSGALG00010012685	NPTXR	2	0.301859532	0.181337043	1.664632484	0.095986101	0	0.336744077	0	1	CRA004775;Yang2025_Silkie_Wenchang	PRJNA827465	0.5301492
ENSGALG00010012685	NPTXR	2	0.061409286	0.347324619	0.176806601	0.859660304	0.16009782	2.641829638	62.14744564	0.5	CRA004775;PRJNA827465	Yang2025_Silkie_Wenchang	0.964839196
ENSGALG00010012688	NDUFAF2	2	0.045036297	0.113347913	0.397327679	0.691125617	0	0.823154259	0	0.5	PRJNA827465;Yang2025_Silkie_Wenchang	CRA004775	0.920268407
ENSGALG00010012688	NDUFAF2	2	0.106813386	0.145915449	0.732022463	0.464154857	0	0.395792629	0	0.5	CRA004775;Yang2025_Silkie_Wenchang	PRJNA827465	0.852159649
ENSGALG00010012688	NDUFAF2	2	-0.0236692	0.120081404	-0.197109619	0.843741756	0	0.010961846	0	1	CRA004775;PRJNA827465	Yang2025_Silkie_Wenchang	0.96057435
ENSGALG00010012690	NT5DC1	2	-0.290711266	0.191393127	-1.518922182	0.128782085	0.054058587	3.320269788	69.88196551	1	PRJNA827465;Yang2025_Silkie_Wenchang	CRA004775	0.602157487
ENSGALG00010012690	NT5DC1	2	-0.371833765	0.303574484	-1.224851837	0.22063105	0.076916228	1.399735579	28.55793517	0.5	CRA004775;Yang2025_Silkie_Wenchang	PRJNA827465	0.734053249
ENSGALG00010012690	NT5DC1	2	-0.13392287	0.073586394	-1.819940641	0.068768045	0	0.332177841	0	0.5	CRA004775;PRJNA827465	Yang2025_Silkie_Wenchang	0.451745023
ENSGALG00010012693	UCHL5	2	-0.051073573	0.110811585	-0.46090463	0.64486703	0.017425752	3.10016571	67.74365974	0.5	PRJNA827465;Yang2025_Silkie_Wenchang	CRA004775	0.906953008
ENSGALG00010012693	UCHL5	2	-0.213869214	0.101741228	-1.335439094	0.181732677	0	0.81158876	0	0.5	CRA004775;Yang2025_Silkie_Wenchang	PRJNA827465	0.689177886
ENSGALG00010012693	UCHL5	2	0.037289566	0.051426832	0.725099423	0.468391039	0	0.004663663	0	1	CRA004775;PRJNA827465	Yang2025_Silkie_Wenchang	0.853198008
ENSGALG00010012696	DNAL4	2	-0.155261431	0.060470745	-2.567546191	0.010242114	0	0.387547803	0	1	PRJNA827465;Yang2025_Silkie_Wenchang	CRA004775	0.160750741
ENSGALG00010012696	DNAL4	2	-0.125854687	0.121552633	-1.035392525	0.300485635	0	0.432426663	0	1	CRA004775;Yang2025_Silkie_Wenchang	PRJNA827465	0.790579721
ENSGALG00010012696	DNAL4	2	-0.178518278	0.063541554	-2.809472975	0.004962269	0	0.101912194	0	1	CRA004775;PRJNA827465	Yang2025_Silkie_Wenchang	0.104855593
ENSGALG00010012699	RNF128	2	-0.023853773	0.131377869	-0.181566141	0.855923223	0.017116394	1.953357299	48.80608886	0.5	PRJNA827465;Yang2025_Silkie_Wenchang	CRA004775	0.963486168
ENSGALG00010012699	RNF128	2	-0.161937057	0.140896845	-1.149330608	0.250419681	0	0.047813083	0	1	CRA004775;Yang2025_Silkie_Wenchang	PRJNA827465	0.759943035
ENSGALG00010012699	RNF128	2	0.080156239	0.112256942	0.714042598	0.475200839	0	0.145640278	0	0.5	CRA004775;PRJNA827465	Yang2025_Silkie_Wenchang	0.854462864
ENSGALG00010012704	SMIM15	2	-0.131473292	0.131529934	-0.999569361	0.317518957	0.023749592	3.027897431	66.97378221	0.5	PRJNA827465;Yang2025_Silkie_Wenchang	CRA004775	0.797325693
ENSGALG00010012704	SMIM15	2	-0.314063641	0.119905526	-2.619259112	0.008812098	0	0.287876588	0	1	CRA004775;Yang2025_Silkie_Wenchang	PRJNA827465	0.147241496
ENSGALG00010012704	SMIM15	2	-0.147078219	0.199613138	-0.736816326	0.461234053	0.05004669	2.077833411	51.87294637	1	CRA004775;PRJNA827465	Yang2025_Silkie_Wenchang	0.85014477
ENSGALG00010012705	CPM	2	0.095065648	0.095396347	0.996543176	0.318889186	0	0.011332381	0	1	PRJNA827465;Yang2025_Silkie_Wenchang	CRA004775	0.798009017
ENSGALG00010012705	CPM	2	0.141347824	0.100565717	1.40552693	0.159864647	0	0.697777567	0	0.5	CRA004775;Yang2025_Silkie_Wenchang	PRJNA827465	0.655404348
ENSGALG00010012705	CPM	2	0.170202437	0.144719963	1.176081264	0.239562404	0	0.645256417	0	1	CRA004775;PRJNA827465	Yang2025_Silkie_Wenchang	0.751810791
ENSGALG00010012706	TBC1D8B	2	-0.110266889	0.160188698	-0.688356236	0.491228476	0.040172492	4.464706046	77.60210886	0.5	PRJNA827465;Yang2025_Silkie_Wenchang	CRA004775	0.85895707
ENSGALG00010012706	TBC1D8B	2	-0.079754599	0.229831575	-0.347014236	0.728581135	0.07899523	3.803076664	73.91129665	0.5	CRA004775;Yang2025_Silkie_Wenchang	PRJNA827465	0.932187391
ENSGALG00010012706	TBC1D8B	2	0.056350079	0.076086959	0.740601013	0.458935393	0	0.428155273	0	1	CRA004775;PRJNA827465	Yang2025_Silkie_Wenchang	0.850089963
ENSGALG00010012708	SPP2	2	0.06660517	1.070135912	0.062239917	0.950371775	1.77053025	4.273974481	76.60257438	0.5	PRJNA827465;Yang2025_Silkie_Wenchang	CRA004775	0.987277827
ENSGALG00010012708	SPP2	2	0.431791773	1.366385257	0.316010269	0.751994714	3.336142289	9.350076222	89.30490002	0.5	CRA004775;Yang2025_Silkie_Wenchang	PRJNA827465	0.937903487
ENSGALG00010012708	SPP2	2	1.602114932	0.541900994	2.956471664	0.003111808	0	0.274641243	0	1	CRA004775;PRJNA827465	Yang2025_Silkie_Wenchang	0.019471028
ENSGALG00010012709	PPP1R8	2	0.008555097	0.072332972	0.118273822	0.905850699	0	0.729224337	0	0.5	PRJNA827465;Yang2025_Silkie_Wenchang	CRA004775	0.978196186
ENSGALG00010012709	PPP1R8	2	-0.117778257	0.25765256	-0.457120463	0.647584463	0.099621684	3.875570026	74.19734405	0.5	CRA004775;Yang2025_Silkie_Wenchang	PRJNA827465	0.907350807
ENSGALG00010012709	PPP1R8	2	-0.160450554	0.1799357	-0.891710503	0.372548123	0.042987563	2.53950751	60.62228617	1	CRA004775;PRJNA827465	Yang2025_Silkie_Wenchang	0.822016521
ENSGALG00010012711	ELL2	2	0.346409435	0.19912847	1.739627863	0.081924383	0.040150499	1.796881596	44.34803039	1	PRJNA827465;Yang2025_Silkie_Wenchang	CRA004775	0.493467286
ENSGALG00010012711	ELL2	2	0.443933614	0.237713886	1.867512338	0.061830076	0.010402311	1.096020453	8.760826787	1	CRA004775;Yang2025_Silkie_Wenchang	PRJNA827465	0.429075295
ENSGALG00010012711	ELL2	2	0.201108129	0.12751367	1.577149562	0.114761115	0	0.024970662	0	1	CRA004775;PRJNA827465	Yang2025_Silkie_Wenchang	0.571450161
ENSGALG00010012712	EEF1E1	2	-0.137131427	0.273732551	-0.500968653	0.616393185	0.119130028	4.717183372	78.80090891	0.5	PRJNA827465;Yang2025_Silkie_Wenchang	CRA004775	0.909911936
ENSGALG00010012712	EEF1E1	2	-0.219194305	0.271198762	-0.808242278	0.418951124	0.07514406	1.952264041	48.77742054	0.5	CRA004775;Yang2025_Silkie_Wenchang	PRJNA827465	0.838264828
ENSGALG00010012712	EEF1E1	2	0.112838459	0.124278724	0.907946708	0.363906379	0	0.000209214	0	1	CRA004775;PRJNA827465	Yang2025_Silkie_Wenchang	0.817058692
ENSGALG00010012714	RGS2	2	-0.004676254	0.080251888	-0.05827007	0.953535307	0	0.879916314	0	0.5	PRJNA827465;Yang2025_Silkie_Wenchang	CRA004775	0.987781987
ENSGALG00010012714	RGS2	2	-0.30509381	0.285393152	-1.069029299	0.285056201	0.106706797	2.429120757	58.83284119	1	CRA004775;Yang2025_Silkie_Wenchang	PRJNA827465	0.780501722
ENSGALG00010012714	RGS2	2	-0.243167045	0.3698512	-0.657472641	0.510877057	0.215083449	4.008092327	75.05047493	0.5	CRA004775;PRJNA827465	Yang2025_Silkie_Wenchang	0.865436876
ENSGALG00010012719	COL10A1	2	0.645548322	0.644208314	1.002080084	0.316304916	0	0.041834511	0	1	PRJNA827465;Yang2025_Silkie_Wenchang	CRA004775	0.796883862
ENSGALG00010012719	COL10A1	2	-2.644095981	3.738234237	-0.707313477	0.479329035	24.85328987	8.522370679	88.2663134	0.5	CRA004775;Yang2025_Silkie_Wenchang	PRJNA827465	0.85611011
ENSGALG00010012719	COL10A1	2	-2.757579884	3.604030981	-0.765137675	0.444189568	22.9559363	8.052313389	87.58120863	0.5	CRA004775;PRJNA827465	Yang2025_Silkie_Wenchang	0.846013379
ENSGALG00010012720	ACO2	2	-0.209649032	0.071194515	-2.94473572	0.003232306	0	0.545531139	0	1	PRJNA827465;Yang2025_Silkie_Wenchang	CRA004775	0.079016811
ENSGALG00010012720	ACO2	2	-0.14850118	0.210760813	-0.704598783	0.481061824	0.047180889	1.947380035	48.6489549	0.5	CRA004775;Yang2025_Silkie_Wenchang	PRJNA827465	0.856783731
ENSGALG00010012720	ACO2	2	-0.135306568	0.110777961	-1.22142117	0.22192652	0.007771172	1.182494154	15.43298574	0.5	CRA004775;PRJNA827465	Yang2025_Silkie_Wenchang	0.733599501
ENSGALG00010012722	ENSGALG00010012722	2	0.378118873	0.141867387	2.665298074	0.007692011	0.016285297	1.676838937	40.36398022	1	PRJNA827465;Yang2025_Silkie_Wenchang	CRA004775	0.135751733
ENSGALG00010012722	ENSGALG00010012722	2	0.514025159	0.137712386	3.732599334	0.000189514	0	0.00015897	0	1	CRA004775;Yang2025_Silkie_Wenchang	PRJNA827465	0.012270948
ENSGALG00010012722	ENSGALG00010012722	2	0.279829115	0.147475218	1.897468599	0.0607881	0	0.525501399	0	1	CRA004775;PRJNA827465	Yang2025_Silkie_Wenchang	0.42096726
ENSGALG00010012725	ENSGALG00010012725	2	0.614330826	0.441604681	1.391132957	0.164185113	0.317592557	5.380300619	81.41378159	1	PRJNA827465;Yang2025_Silkie_Wenchang	CRA004775	0.662998817
ENSGALG00010012725	ENSGALG00010012725	2	0.641554107	0.460672832	1.392645848	0.163726911	0.311176395	3.662800582	72.69008266	1	CRA004775;Yang2025_Silkie_Wenchang	PRJNA827465	0.66212726
ENSGALG00010012725	ENSGALG00010012725	2	0.165844079	0.216935907	0.764484226	0.444578733	0	0.0078765	0	1	CRA004775;PRJNA827465	Yang2025_Silkie_Wenchang	0.846013379
ENSGALG00010012726	IL7R	2	0.385423279	0.865079106	0.4455353	0.655932956	1.227226019	5.452311561	81.65915522	0.5	PRJNA827465;Yang2025_Silkie_Wenchang	CRA004775	0.910411938
ENSGALG00010012726	IL7R	2	-0.107829373	0.386228202	-0.279185653	0.780102353	0	0.827076703	0	0.5	CRA004775;Yang2025_Silkie_Wenchang	PRJNA827465	0.945041817
ENSGALG00010012726	IL7R	2	0.722523685	0.50740252	1.423965504	0.154456458	0.302882579	2.412876748	58.55569495	1	CRA004775;PRJNA827465	Yang2025_Silkie_Wenchang	0.647254736
ENSGALG00010012730	CAP2	2	-0.034486726	0.173923193	-0.198287101	0.842820443	0.039882863	2.496072632	59.93706324	0.5	PRJNA827465;Yang2025_Silkie_Wenchang	CRA004775	0.960378403
ENSGALG00010012730	CAP2	2	0.195121491	0.201783708	0.966983371	0.333552347	0	0.010048839	0	1	CRA004775;Yang2025_Silkie_Wenchang	PRJNA827465	0.802691373
ENSGALG00010012730	CAP2	2	-0.155715668	0.080445742	-1.935660775	0.052909262	0	0.285916889	0	0.5	CRA004775;PRJNA827465	Yang2025_Silkie_Wenchang	0.399377178
ENSGALG00010012732	MGST1	2	0.1318189	0.096906746	1.360265475	0.173745929	0	0.322076286	0	1	PRJNA827465;Yang2025_Silkie_Wenchang	CRA004775	0.677453995
ENSGALG00010012732	MGST1	2	0.090541863	0.140588389	0.644020916	0.519561871	0	0.158666291	0	1	CRA004775;Yang2025_Silkie_Wenchang	PRJNA827465	0.869094812
ENSGALG00010012732	MGST1	2	0.166084813	0.102787671	1.615804806	0.106136522	0	0.000486647	0	1	CRA004775;PRJNA827465	Yang2025_Silkie_Wenchang	0.55273403
ENSGALG00010012734	TMEM19	2	-0.162714949	0.234474604	-0.693955533	0.487710096	0.089047487	5.13386866	80.52151182	0.5	PRJNA827465;Yang2025_Silkie_Wenchang	CRA004775	0.85768885
ENSGALG00010012734	TMEM19	2	-0.391212082	0.14402373	-2.716302953	0.006601548	0	0.065142602	0	1	CRA004775;Yang2025_Silkie_Wenchang	PRJNA827465	0.124138283
ENSGALG00010012734	TMEM19	2	-0.070775548	0.182356921	-0.3881155	0.697930562	0.035805545	1.885023406	46.95026084	0.5	CRA004775;PRJNA827465	Yang2025_Silkie_Wenchang	0.921498664
ENSGALG00010012738	RAB21	2	-0.031059397	0.096930971	-0.320428001	0.748643902	0.010626457	1.894695136	47.22106048	0.5	PRJNA827465;Yang2025_Silkie_Wenchang	CRA004775	0.937249951
ENSGALG00010012738	RAB21	2	0.037335221	0.113478518	0.329006948	0.742150437	0	0.801325192	0	0.5	CRA004775;Yang2025_Silkie_Wenchang	PRJNA827465	0.935481151

ENSGALG00010012738	RAB21	2	-0.093775092	0.050287555	-1.864777316	0.062212624	0	0.000363775	0	1	CRA004775;PRJNA827465	Yang2025_Silkie_Wenchang	0.430164709
ENSGALG00010012741	ENSGALG00010012741	2	0.195268684	0.219006047	0.891613207	0.372600289	0	0.001831301	0	1	PRJNA827465;Yang2025_Silkie_Wenchang	CRA004775	0.822016521
ENSGALG00010012741	ENSGALG00010012741	2	0.007273609	0.213418611	0.034081417	0.972812227	0.000736067	1.008087019	0.802214394	0.5	CRA004775;Yang2025_Silkie_Wenchang	PRJNA827465	0.992213869
ENSGALG00010012741	ENSGALG00010012741	2	-0.034254097	0.229672565	-0.149143182	0.881440652	0	0.790755857	0	0.5	CRA004775;PRJNA827465	Yang2025_Silkie_Wenchang	0.971790895
ENSGALG00010012742	ENSGALG00010012742	2	-0.182715341	0.086549116	-2.111117367	0.034762225	0	0.01180967	0	1	PRJNA827465;Yang2025_Silkie_Wenchang	CRA004775	0.321095119
ENSGALG00010012742	ENSGALG00010012742	2	-0.109399174	0.134496134	-0.813400139	0.41598868	0.002855251	1.070085111	6.549489423	0.5	CRA004775;Yang2025_Silkie_Wenchang	PRJNA827465	0.837742614
ENSGALG00010012742	ENSGALG00010012742	2	-0.124876505	0.107148067	-1.165450648	0.243836545	0.002941573	1.084180892	7.764469211	0.5	CRA004775;PRJNA827465	Yang2025_Silkie_Wenchang	0.755365463
ENSGALG00010012743	ENSGALG00010012743	2	0.824374569	0.241930685	3.407482479	0.000655651	0.040565358	1.505837453	33.59176997	1	PRJNA827465;Yang2025_Silkie_Wenchang	CRA004775	0.028276881
ENSGALG00010012743	ENSGALG00010012743	2	0.060822706	1.051331462	0.057853025	0.953865701	2.111262487	22.25396443	95.50641863	0.5	CRA004775;Yang2025_Silkie_Wenchang	PRJNA827465	0.987838152
ENSGALG00010012743	ENSGALG00010012743	2	-0.172072543	0.805521067	-0.213616441	0.830846177	1.219810395	16.59855203	93.9753781	0.5	CRA004775;PRJNA827465	Yang2025_Silkie_Wenchang	0.957336479
ENSGALG00010012744	ARL4C	2	-0.190567156	0.189069988	-1.007918591	0.313493546	0.05498251	4.266456687	76.56134649	1	PRJNA827465;Yang2025_Silkie_Wenchang	CRA004775	0.796531448
ENSGALG00010012744	ARL4C	2	-0.324387138	0.19756067	-1.641962125	0.100597854	0.02207155	1.120964879	10.79113903	0.5	CRA004775;Yang2025_Silkie_Wenchang	PRJNA827465	0.541393598
ENSGALG00010012744	ARL4C	2	-0.004895911	0.107844361	-0.04539793	0.963790131	0	0.191119581	0	0.5	CRA004775;PRJNA827465	Yang2025_Silkie_Wenchang	0.990167386
ENSGALG00010012745	MARCHF3	2	0.302083425	0.244901197	1.233491012	0.217392628	0.004083505	1.028853411	2.80442394	0.5	PRJNA827465;Yang2025_Silkie_Wenchang	CRA004775	0.730896703
ENSGALG00010012745	MARCHF3	2	-0.098353965	0.330320703	-0.297752954	0.765891724	0	0.003219295	0	1	CRA004775;Yang2025_Silkie_Wenchang	PRJNA827465	0.941006047
ENSGALG00010012745	MARCHF3	2	0.305140585	0.255040775	1.196438433	0.231525511	0.00820478	1.051798024	4.924712079	0.5	CRA004775;PRJNA827465	Yang2025_Silkie_Wenchang	0.743924694
ENSGALG00010012747	TOB2	2	0.005137662	0.083694664	0.061385774	0.951051982	0.005371711	1.593109054	37.22965811	0.5	PRJNA827465;Yang2025_Silkie_Wenchang	CRA004775	0.987483054
ENSGALG00010012747	TOB2	2	-0.075304372	0.103146986	-0.730068564	0.465348275	0	0.410606825	0	0.5	CRA004775;Yang2025_Silkie_Wenchang	PRJNA827465	0.852411124
ENSGALG00010012747	TOB2	2	0.079178887	0.079377466	0.997498296	0.3185227	0	0.043056776	0	1	CRA004775;PRJNA827465	Yang2025_Silkie_Wenchang	0.797754958
ENSGALG00010012749	RGS4	2	-0.176737288	0.189711686	-0.931609915	0.351538157	0.053320819	3.665616531	72.71945956	1	PRJNA827465;Yang2025_Silkie_Wenchang	CRA004775	0.810678834
ENSGALG00010012749	RGS4	2	-0.30412841	0.137946154	-2.204689305	0.027475904	0	0.74322145	0	1	CRA004775;Yang2025_Silkie_Wenchang	PRJNA827465	0.282945624
ENSGALG00010012749	RGS4	2	-0.033135405	0.091924082	-0.360464905	0.718499497	0	0.29583084	0	1	CRA004775;PRJNA827465	Yang2025_Silkie_Wenchang	0.928126791
ENSGALG00010012751	AP3B1	2	0.110985271	0.100683478	1.102318608	0.270323182	0	0.09298859	0	1	PRJNA827465;Yang2025_Silkie_Wenchang	CRA004775	0.772430311
ENSGALG00010012751	AP3B1	2	0.148804871	0.11582857	1.284699202	0.198897417	0	0.02014726	0	1	CRA004775;Yang2025_Silkie_Wenchang	PRJNA827465	0.71096127
ENSGALG00010012751	AP3B1	2	0.107976503	0.12435663	0.868281038	0.3852405	0	0.135479212	0	1	CRA004775;PRJNA827465	Yang2025_Silkie_Wenchang	0.826444827
ENSGALG00010012753	GTPBP1	2	-0.093275942	0.126571126	-0.736944865	0.461155879	0.028954689	10.21834759	90.21368189	0.5	PRJNA827465;Yang2025_Silkie_Wenchang	CRA004775	0.85014477
ENSGALG00010012753	GTPBP1	2	-0.224208752	0.063015133	-3.558014413	0.000373669	0	0.001995061	0	1	CRA004775;Yang2025_Silkie_Wenchang	PRJNA827465	0.019374277
ENSGALG00010012753	GTPBP1	2	-0.025241539	0.100996771	-0.24992422	0.802645952	0.01124094	1.603272254	37.62756154	0.5	CRA004775;PRJNA827465	Yang2025_Silkie_Wenchang	0.950704475
ENSGALG00010012756	RPA2	2	-0.06701845	0.14941465	-0.448535983	0.653756423	0.027106588	1.994838636	49.87063203	0.5	PRJNA827465;Yang2025_Silkie_Wenchang	CRA004775	0.909984738
ENSGALG00010012756	RPA2	2	-0.394518709	0.180274585	-2.188432216	0.028638134	0	0.488117943	0	1	CRA004775;Yang2025_Silkie_Wenchang	PRJNA827465	0.288310597
ENSGALG00010012756	RPA2	2	-0.194439345	0.287260208	-0.67687532	0.498485064	0.127796727	3.651375809	72.61306279	0.5	CRA004775;PRJNA827465	Yang2025_Silkie_Wenchang	0.861478809
ENSGALG00010012758	NAB1	2	-0.096974637	0.119958665	-0.808400433	0.418860103	0.02554587	8.595436163	88.36591906	0.5	PRJNA827465;Yang2025_Silkie_Wenchang	CRA004775	0.838325071
ENSGALG00010012758	NAB1	2	-0.188770288	0.088867494	-2.124177023	0.033655344	0.003932378	1.140021235	12.28233573	0.5	CRA004775;Yang2025_Silkie_Wenchang	PRJNA827465	0.315016903
ENSGALG00010012758	NAB1	2	0.015565391	0.037320029	0.417078756	0.676620803	0	0.003031877	0	1	CRA004775;PRJNA827465	Yang2025_Silkie_Wenchang	0.916786558
ENSGALG00010012759	ATP1B4	2	0.075236228	0.180083058	0.417786261	0.676103397	0.048129803	3.804228646	73.71346223	0.5	PRJNA827465;Yang2025_Silkie_Wenchang	CRA004775	0.916622691
ENSGALG00010012759	ATP1B4	2	0.241784995	0.107632984	2.246383829	0.024679433	0	0.00131683	0	1	CRA004775;Yang2025_Silkie_Wenchang	PRJNA827465	0.236498231
ENSGALG00010012759	ATP1B4	2	-0.10433745	0.146850347	-0.710501892	0.477392958	0	0.273181634	0	0.5	CRA004775;PRJNA827465	Yang2025_Silkie_Wenchang	0.855077949
ENSGALG00010012760	PLEKHF2	2	-0.167025129	0.180610659	-0.924780023	0.355080344	0.060038656	12.39572853	91.9327049	0.5	PRJNA827465;Yang2025_Silkie_Wenchang	CRA004775	0.812276513
ENSGALG00010012760	PLEKHF2	2	-0.126231612	0.267749013	-0.471455006	0.637315844	0.115986484	4.708184751	78.76039168	0.5	CRA004775;Yang2025_Silkie_Wenchang	PRJNA827465	0.905150023
ENSGALG00010012760	PLEKHF2	2	0.017777372	0.054838266	0.324178231	0.745803102	0	0.563021055	0	1	CRA004775;PRJNA827465	Yang2025_Silkie_Wenchang	0.93634561
ENSGALG00010012761	SMPDL3A	2	-0.122364249	0.280395507	-0.436398752	0.662547445	0.128915912	5.500448919	81.81966573	0.5	PRJNA827465;Yang2025_Silkie_Wenchang	CRA004775	0.912778072
ENSGALG00010012761	SMPDL3A	2	-0.489405005	0.160568219	-3.047956855	0.00230403	0	0.547372482	0	1	CRA004775;Yang2025_Silkie_Wenchang	PRJNA827465	0.063678558
ENSGALG00010012761	SMPDL3A	2	-0.22450034	0.410980673	-0.54626267	0.584883393	0.284393498	5.979155338	83.27522964	0.5	CRA004775;PRJNA827465	Yang2025_Silkie_Wenchang	0.891032621
ENSGALG00010012763	SMPDL3B	2	-0.138708606	0.229440934	-0.604550388	0.545477779	0.088421093	6.105019752	83.62003661	0.5	PRJNA827465;Yang2025_Silkie_Wenchang	CRA004775	0.877672787
ENSGALG00010012763	SMPDL3B	2	-0.185746924	0.222241419	-0.83578896	0.403273638	0.064330831	2.798105879	64.26153822	0.5	CRA004775;Yang2025_Silkie_Wenchang	PRJNA827465	0.83306585
ENSGALG00010012763	SMPDL3B	2	0.073905556	0.092268491	0.800983678	0.423141095	0	0.002673669	0	1	CRA004775;PRJNA827465	Yang2025_Silkie_Wenchang	0.840074427
ENSGALG00010012765	PHF5A	2	0.009941315	0.052877017	0.188008825	0.850870184	0	0.763437026	0	0.5	PRJNA827465;Yang2025_Silkie_Wenchang	CRA004775	0.962056252
ENSGALG00010012765	PHF5A	2	-0.153764188	0.145010241	-1.060367781	0.288977314	0	0.177356397	0	1	CRA004775;Yang2025_Silkie_Wenchang	PRJNA827465	0.783530627
ENSGALG00010012765	PHF5A	2	0.017320987	0.055152744	0.314054846	0.753479384	0	0.833697301	0	0.5	CRA004775;PRJNA827465	Yang2025_Silkie_Wenchang	0.938087082
ENSGALG00010012767	JOSD1	2	-0.012320917	0.165469778	-0.074460224	0.94064419	0.042839498	3.899801416	74.35766868	0.5	PRJNA827465;Yang2025_Silkie_Wenchang	CRA004775	0.985565295
ENSGALG00010012767	JOSD1	2	-0.320213768	0.128200068	-2.497808605	0.012500615	0	0.903646258	0	1	CRA004775;Yang2025_Silkie_Wenchang	PRJNA827465	0.181028612
ENSGALG00010012767	JOSD1	2	-0.141332394	0.292041013	-0.483947074	0.628423431	0.151439953	8.108778899	87.66768693	0.5	CRA004775;PRJNA827465	Yang2025_Silkie_Wenchang	0.901912657
ENSGALG00010012768	EPS8	2	-0.194197693	0.067161208	-2.891515774	0.003833883	0	0.906431243	0	1	PRJNA827465;Yang2025_Silkie_Wenchang	CRA004775	0.088898597
ENSGALG00010012768	EPS8	2	0.034153352	0.34337072	0.099464952	0.920769114	0.20840762	8.398235336	88.09273663	0.5	CRA004775;Yang2025_Silkie_Wenchang	PRJNA827465	0.981469977
ENSGALG00010012768	EPS8	2	0.091060813	0.272779434	0.333825799	0.738511015	0.126772611	6.261801274	84.03015432	0.5	CRA004775;PRJNA827465	Yang2025_Silkie_Wenchang	0.934485814
ENSGALG00010012769	NEMP2	2	-0.058795327	0.095134708	-0.618021836	0.536560944	0.006185095	1.404925139	28.82183027	0.5	PRJNA827465;Yang2025_Silkie_Wenchang	CRA004775	0.874599474
ENSGALG00010012769	NEMP2	2	-0.125618232	0.129175417	-0.972462365	0.330820579	0	0.853870812	0	0.5	CRA004775;Yang2025_Silkie_Wenchang	PRJNA827465	0.801667256
ENSGALG00010012769	NEMP2	2	0.009627649	0.077795223	0.123756301	0.901508232	0	0.046160251	0	1	CRA004775;PRJNA827465	Yang2025_Silkie_Wenchang	0.976665597
ENSGALG00010012771	ITGA2	2	0.167092408	0.197717624	0.845106294	0.398051495	0.045826992	2.294492658	56.41738069	1	PRJNA827465;Yang2025_Silkie_Wenchang	CRA004775	0.831850795
ENSGALG00010012771	ITGA2	2	0.354796796	0.214707707	1.652464182	0.098439961	0	0.55542321	0	0.5	CRA004775;Yang2025_Silkie_Wenchang	PRJNA827465	0.535930498
ENSGALG00010012771	ITGA2	2	-0.000836309	0.137421107	-0.006085738	0.995144814	0	0.036953771	0	0.5	CRA004775;PRJNA827465	Yang2025_Silkie_Wenchang	0.998242335
ENSGALG00010012772	TOMM22	2	-0.090224641	0.063765918	-1.414935186	0.1570875	0	0.033080376	0	1	PRJNA827465;Yang2025_Silkie_Wenchang	CRA004775	0.650687662
ENSGALG00010012772	TOMM22	2	-0.172961646	0.131647581	-1.313823202	0.188905708	0	0.495643745	0	1	CRA004775;Yang2025_Silkie_Wenchang	PRJNA827465	0.698162702
ENSGALG00010012772	TOMM22	2	-0.114728028	0.061945323	-1.852085416	0.064013558	0	0.70685873	0	1	CRA004775;PRJNA827465	Yang2025_Silkie_Wenchang	0.435959952
ENSGALG00010012773	XKR8	2	-0.216617909	0.149438596	-1.449548459	0.147185556	0	0.000913121	0	1	PRJNA827465;Yang2025_Silkie_Wenchang	CRA004775	0.635975583
ENSGALG00010012773	XKR8	2	-0.447577338	0.160123768	-2.795196136	0.005186824	0	0.87783209	0	1	CRA004775;Yang2025_Silkie_Wenchang	PRJNA827465	0.107519899
ENSGALG00010012773	XKR8	2	-0.371231874	0.166907251	-2.224180622	0.026136294	0.022009803	1.649263668	39.36688113	1	CRA004775;PRJNA827465	Yang2025_Silkie_Wenchang	0.275965687
ENSGALG00010012775	KCNE2	2	0.01683042	0.163975619	0.10263977	0.918248877	0.041596114	4.25314394	76.48798126	0.5	PRJNA827465;Yang2025_Silkie_Wenchang	CRA004775	0.981033557
ENSGALG00010012775	KCNE2	2	0.180831201	0.130885177	1.377628888	0.168317897	0	0.253527513	0	0.5	CRA004775;Yang2025_Silkie_Wenchang	PRJNA827465	0.668962553
ENSGALG00010012775	KCNE2	2	-0.128234475	0.082650693	-1.551523285	0.120776332	0	0.027581558	0	1	CRA004775;PRJNA827465	Yang2025_Silkie_Wenchang	0.582724301
ENSGALG00010012778	TBC1D15	2	0.002316611	0.053309538	0.043455839	0.965338166	0	0.755916214	0	0.5	PRJNA827465;Yang2025_Silkie_Wenchang	CRA004775	0.990668014
ENSGALG00010012778	TBC1D15	2	-0.044948304	0.087655033	-0.512786349	0.608100779	0	0.405284202	0	0.5	CRA004775;Yang2025_Silkie_Wenchang	PRJNA827465	0.89170434
ENSGALG00010012778	TBC1D15	2	0.039816531	0.063701332	0.625050218	0.5319381	0	0.099681034	0	1	CRA004775;PRJNA827465	Yang2025_Silkie_Wenchang	0.873082041
ENSGALG00010012780	FABP7	2	0.73638807	0.772049791	0.953809041	0.340180312	1.10751408	14.02500737	92.86987897	0.5	PRJNA827465;Yang2025_Silkie_Wenchang	CRA004775	0.805377063
ENSGALG00010012780	FABP7	2	1.082184924	0.502623279	2.153073624	0.031312893	0.339571807	2.947012326	66.0673289	1	CRA004775;Yang2025_Silkie_Wenchang	PRJNA827465	0.302752922
ENSGALG00010012780	FABP7	2	0.087978321	0.222940399	0.394627089	0.693118119	0	0.925911884	0	0.5	CRA004775;PRJNA827465	Yang2025_Silkie_Wenchang	0.920662393
ENSGALG00010012781	SPIN1L	2	-6.373262665	6.362352522	-1.001714797	0.316481358	75.3510861	14.17915165	92.94739188	1	PRJNA827465;Yang2025_Silkie_Wenchang	CRA004775	0.796883862
ENSGALG00010012781	SPIN1L	2	-6.098458386	6.604300375	-0.92340718	0.355795051	82.19160945	16.85723658	94.06782959	0.5	CRA004775;Yang2025_Silkie_Wenchang	PRJNA827465	0.812301302
ENSGALG00010012781	SPIN1L	2	0.061319087	1.018500339	0.060205269	0.951992149	0	0.053576427	0	0.5	CRA004775;PRJNA827465	Yang2025_Silkie_Wenchang	0.987725077
ENSGALG00010012786	TEF	2	-0.032130603	0.061369628	-0.523558698	0.600585514	0	0.208079658	0	0.5	PRJNA827465;Yang2025_Silkie_Wenchang	CRA004775	0.895230519
ENSGALG00010012786	TEF	2	-0.032437388	0.101574846	-0.319344696	0.749465143	0	0.575008669	0	0.5	CRA004775;Yang2025_Silkie_Wenchang	PRJNA827465	0.937362187
ENSGALG00010012786	TEF	2	-0.062060736	0.067312838	-0.921974744	0.356541749	0	0.280309352	0	1	CRA004775;PRJNA827465	Yang2025_Silkie_Wenchang	0.812615522
ENSGALG00010012791	CFAP418	2	0.054449197	0.110362152	0.49336839	0.621752313	0	0.159773887	0	0.5	PRJNA827465;Yang2025_Silkie_Wenchang	CRA004775	0.899796991
ENSGALG00010012791	CFAP418	2	-0.167823138	0.215561876	-0.778538122	0.436251842	0.038627332	1.624703419	38.45030493	1	CRA004775;Yang2025_Silkie_Wenchang	PRJNA827465	0.843122822
ENSGALG00010012791	CFAP418	2	-0.117883944	0.262243982	-0.449520111	0.653056504	0.091938176	2.699313597	62.95354489	0.5	CRA004775;PRJNA827465	Yang2025_Silkie_Wenchang	0.909572411
ENSGALG00010012792	CBY1	2	-0.000572092	0.092708781	-0.006170851	0.995076405	0	0.00277739	0	0.5	PRJNA827465;Yang2025_Silkie_Wenchang	CRA004775	0.998242335
ENSGALG00010012792	CBY1	2	-0.072062292	0.218771281	-0.329395578	0.74185671	0	0.454775604	0	0.5	CRA004775;Yang2025_Silkie_Wenchang	PRJNA827465	0.93543545
ENSGALG00010012792	CBY1	2	-0.018396339	0.097289078	-0.189089463	0.8500227	0	0.525972285	0	1	CRA004775;PRJNA827465	Yang2025_Silkie_Wenchang	0.961869125
ENSGALG00010012795	TBCA	2	0.03861852	0.121286612	0.318407115	0.750176142	0	0.003300441	0	1	PRJNA827465;Yang2025_Silkie_Wenchang	CRA004775	0.937374353
ENSGALG00010012795	TBCA	2	-0.105802895	0.185775073	-0.569521483	0.569002296	0.00694551	1.099797209	9.074146402	0.5	CRA004775;Yang2025_Silkie_Wenchang	PRJNA827465	0.884492059
ENSGALG00010012795	TBCA	2	-0.07118347	0.182797918	-0.389410726	0.696972339	0.024836666	1.430830265	30.11050827	0.5	CRA004775;PRJNA827465	Yang2025_Silkie_Wenchang	0.921086208
ENSGALG00010012796	MOCS2	2	0.105488611	0.164967486	0.639450919	0.522529633	0.030379862	2.234326251	55.24377877	0.5	PRJNA827465;Yang2025_Silkie_Wenchang	CRA004775	0.869568211
ENSGALG00010012796	MOCS2	2	0.212661243	0.15420726	1.379061159	0.167875898	0	0.889056546	0	0.5	CRA004775;Yang2025_Silkie_Wenchang	PRJNA827465	0.668154574
ENSGALG00010012796	MOCS2	2	-0.047033031	0.127589885	-0.368626643	0.712406034	0	0.004410515	0	1	CRA004775;PRJNA827465	Yang2025_Silkie_Wenchang	0.92597752
ENSGALG00010012797	ZC3H7B	2	-0.180937693	0.163566095	-1.106205368	0.26863764	0.045068427	5.84735248	82.89824321	1	PRJNA827465;Yang2025_Silkie_Wenchang	CRA004775	0.771398294
ENSGALG00010012797	ZC3H7B	2	-0.249947579	0.128525013	-1.944738803	0.051806423	0.013980835	1.721703205	41.91797998	1	CRA004775;Yang2025_Silkie_Wenchang	PRJNA827465	0.394831847
ENSGALG00010012797	ZC3H7B	2	-0.04380919	0.0471618	-0.92891261	0.352934378	0	0.203650083	0	1	CRA004775;PRJNA827465	Yang2025_Silkie_Wenchang	0.811035834
ENSGALG00010012798	MFSD6	2	0.055283618	0.07252375	0.762282952	0.445891147	0	0.457802306	0	0.5	PRJNA827465;Yang2025_Silkie_Wenchang	CRA004775	0.846013379
ENSGALG00010012798	MFSD6	2	-0.191109539	0.189376532	-1.009151121	0.312902164	0.038398825	2.07463508	51.79875201	1	CRA004775;Yang2025_Silkie_Wenchang	PRJNA827465	0.796192786
ENSGALG00010012798	MFSD6	2	-0.125771254	0.246819473	-0.50956779	0.610354295	0.097413042	4.481747448	77.68727462	0.5	CRA004775;PRJNA827465	Yang2025_Silkie_Wenchang	0.897438028
ENSGALG00010012801	DMC1	2	-0.054980964	0.218218755	-0.251953432	0.801077059	0	0.005557205	0	1	PRJNA827465;Yang2025_Silkie_Wenchang	CRA004775	0.950158029
ENSGALG00010012801	DMC1	2	0.091872935	0.261272564	0.351636367	0.725110988	0	0.688784643	0	0.5	CRA004775;Yang2025_Silkie_Wenchang	PRJNA827465	0.930732761
ENSGALG00010012801	DMC1	2	0.090607953	0.241777782	0.374757154	0.707841082	0	0.641462112	0	0.5	CRA004775;PRJNA827465	Yang2025_Silkie_Wenchang	0.925061251
ENSGALG00010012803	NUP155	2	-0.084791518	0.103470235	-0.819477385	0.4125141	0	0.070243553	0	1	PRJNA827465;Yang2025_Silkie_Wenchang	CRA004775	0.835704442
ENSGALG00010012803	NUP155	2	-0.09530461	0.112241782	-0.849101005	0.395825091	0	0.026762109	0	1	CRA004775;Yang2025_Silkie_Wenchang	PRJNA827465	0.831296327
ENSGALG00010012803	NUP155	2	-0.057034751	0.128814353	-0.442767046	0.657934242	0	0.003475743	0	1	CRA004775;PRJNA827465	Yang2025_Silkie_Wenchang	0.910841476
ENSGALG00010012804	ENSGALG00010012804	2	0.319143069	0.347327092	0.918854521	0.35817167	0.215260653	9.147590981	89.06816011	0.5	PRJNA827465;Yang2025_Silkie_Wenchang	CRA004775	0.813853337
ENSGALG00010012804	ENSGALG00010012804	2	0.221674247	0.498596074	0.444596857	0.656611119	0.423273486	6.47813232	84.56345208	0.5	CRA004775;Yang2025_Silkie_Wenchang	PRJNA827465	0.910509456
ENSGALG00010012804	ENSGALG00010012804	2	-0.051554943	0.120271686	-0.428654033	0.668175017	0	0.692173899	0	1	CRA004775;PRJNA827465	Yang2025_Silkie_Wenchang	0.914135432
ENSGALG00010012808	EYA3	2	0.006356745	0.130347296	0.048767755	0.961104379	0.020937241	1.95883503	48.94924867	0.5	PRJNA827465;Yang2025_Silkie_Wenchang	CRA004775	0.989632838
ENSGALG00010012808	EYA3	2	-0.012746029	0.227173076	-0.056107128	0.955256465	0.064039789	2.633054858	62.0213002	0.5	CRA004775;Yang2025_Silkie_Wenchang	PRJNA827465	0.988131974
ENSGALG00010012808	EYA3	2	-0.083561499	0.047881255	-1.745181883	0.080953214	0	0.66158154	0	1	CRA004775;PRJNA827465	Yang2025_Silkie_Wenchang	0.489847627
ENSGALG00010012809	RHOBTB3	2	0.400159824	0.349281894	1.145664378	0.251934048	0.201895502	5.789045502	82.72599516	1	PRJNA827465;Yang2025_Silkie_Wenchang	CRA004775	0.761871849
ENSGALG00010012809	RHOBTB3	2	0.135860993	0.191645462	0.708918394	0.47837512	0	0.890131559	0	1	CRA004775;Yang2025_Silkie_Wenchang	PRJNA827465	0.855464436
ENSGALG00010012809	RHOBTB3	2	0.701475404	0.178231328	3.935758158	8.29345E-05	0	0.273744779	0	1	CRA004775;PRJNA827465	Yang2025_Silkie_Wenchang	0.006805537
ENSGALG00010012812	DECR1	2	-0.021808036	0.083529857	-0.26108073	0.794030252	0	0.071696663	0	0.5	PRJNA827465;Yang2025_Silkie_Wenchang	CRA004775	0.948905846
ENSGALG00010012812	DECR1	2	0.043394302	0.134050459	0.323716175	0.746152924	0	0.894430019	0	1	CRA004775;Yang2025_Silkie_Wenchang	PRJNA827465	0.936530151
ENSGALG00010012812	DECR1	2	0.007836261	0.150774914	0.051973244	0.958550013	0.013983044	1.107802064	9.731166593	0.5	CRA004775;PRJNA827465	Yang2025_Silkie_Wenchang	0.989070013
ENSGALG00010012818	RGS18	2	-0.348497306	0.188948898	-1.844399774	0.0651249	0.053491713	3.94663336	74.66194833	1	PRJNA827465;Yang2025_Silkie_Wenchang	CRA004775	0.439870073
ENSGALG00010012818	RGS18	2	-0.582879725	0.140649276	-4.144207069	3.40992E-05	0	0.459381691	0	1	CRA004775;Yang2025_Silkie_Wenchang	PRJNA827465	0.003661746
ENSGALG00010012818	RGS18	2	-0.391742723	0.318345978	-1.230556531	0.218488772	0.136423266	2.449634356	59.17758103	1	CRA004775;PRJNA827465	Yang2025_Silkie_Wenchang	0.732175117
ENSGALG00010012819	RANGAP1	2	0.111717254	0.061696236	1.81076286	0.070177568	0	0.393192174	0	1	PRJNA827465;Yang2025_Silkie_Wenchang	CRA004775	0.457107879
ENSGALG00010012819	RANGAP1	2	-0.086662418	0.176961062	-0.489725913	0.624327863	0.041622638	2.777109233	63.99133357	0.5	CRA004775;Yang2025_Silkie_Wenchang	PRJNA827465	0.900886179
ENSGALG00010012819	RANGAP1	2	-0.046769819	0.218265116	-0.214279864	0.83032882	0.076698612	4.66953572	78.5845947	0.5	CRA004775;PRJNA827465	Yang2025_Silkie_Wenchang	0.9572455
ENSGALG00010012821	ZBTB33	2	-0.015658984	0.050594237	-0.309501344	0.75694019	0.000905901	1.119194595	10.65003309	0.5	PRJNA827465;Yang2025_Silkie_Wenchang	CRA004775	0.938795135
ENSGALG00010012821	ZBTB33	2	0.043536987	0.177623836	0.245107793	0.806372969	0.045993239	3.640278014	72.52957065	0.5	CRA004775;Yang2025_Silkie_Wenchang	PRJNA827465	0.952187702
ENSGALG00010012821	ZBTB33	2	0.077746625	0.104123387	0.746677832	0.455258054	0.013416607	2.113161157	52.67753257	1	CRA004775;PRJNA827465	Yang2025_Silkie_Wenchang	0.849072665
ENSGALG00010012824	HIBCH	2	-0.081687394	0.061045151	-1.338147125	0.180848482	0	0.019144146	0	1	PRJNA827465;Yang2025_Silkie_Wenchang	CRA004775	0.688484814
ENSGALG00010012824	HIBCH	2	-0.037132673	0.111403766	-0.333316133	0.738895662	0	0.371065237	0	0.5	CRA004775;Yang2025_Silkie_Wenchang	PRJNA827465	0.934613258
ENSGALG00010012824	HIBCH	2	-0.074102137	0.068345979	-1.084220878	0.278266877	0	0.509174949	0	0.5	CRA004775;PRJNA827465	Yang2025_Silkie_Wenchang	0.776763074
ENSGALG00010012827	ENSGALG00010012827	2	-0.336466451	0.413747647	-0.813216591	0.416093889	0	0.668546754	0	0.5	PRJNA827465;Yang2025_Silkie_Wenchang	CRA004775	0.837742614
ENSGALG00010012827	ENSGALG00010012827	2	-0.440537218	0.428181464	-1.02885635	0.303547183	0	0.330132996	0	1	CRA004775;Yang2025_Silkie_Wenchang	PRJNA827465	0.792205992
ENSGALG00010012827	ENSGALG00010012827	2	-0.061919493	0.442003147	-0.140088353	0.888590183	0	0.041819532	0	0.5	CRA004775;PRJNA827465	Yang2025_Silkie_Wenchang	0.973135755
ENSGALG00010012829	RGS5	2	-0.049611639	0.130927422	-0.378924738	0.704743754	0.006880849	1.24302525	19.55111128	0.5	PRJNA827465;Yang2025_Silkie_Wenchang	CRA004775	0.924152671
ENSGALG00010012829	RGS5	2	-0.150892874	0.159125527	-0.948263155	0.342995503	0	0.321062314	0	0.5	CRA004775;Yang2025_Silkie_Wenchang	PRJNA827465	0.806218116
ENSGALG00010012829	RGS5	2	0.050880397	0.135830006	0.374588782	0.707966317	0	0.027896469	0	1	CRA004775;PRJNA827465	Yang2025_Silkie_Wenchang	0.925061251
ENSGALG00010012830	ARRDC3	2	0.281689682	0.153656573	1.833241997	0.066766591	0.028786943	2.541555882	60.65402272	1	PRJNA827465;Yang2025_Silkie_Wenchang	CRA004775	0.445135824
ENSGALG00010012830	ARRDC3	2	0.419707845	0.138919364	3.021233564	0.002517471	0	0.264677587	0	1	CRA004775;Yang2025_Silkie_Wenchang	PRJNA827465	0.067541822
ENSGALG00010012830	ARRDC3	2	0.146869425	0.11798476	1.244816919	0.213199055	0	0.049634109	0	1	CRA004775;PRJNA827465	Yang2025_Silkie_Wenchang	0.72579791
ENSGALG00010012831	ENSGALG00010012831	2	-0.081100276	0.193831639	-0.41840577	0.675650469	0	0.229668631	0	0.5	PRJNA827465;Yang2025_Silkie_Wenchang	CRA004775	0.916522765
ENSGALG00010012831	ENSGALG00010012831	2	-0.577744912	0.761470306	-0.758722838	0.448018372	0.857374999	3.355154814	70.19511601	0.5	CRA004775;Yang2025_Silkie_Wenchang	PRJNA827465	0.846601815
ENSGALG00010012831	ENSGALG00010012831	2	-0.638831926	0.654925444	-0.975426946	0.329348523	0.593254273	2.686701226	62.7796351	1	CRA004775;PRJNA827465	Yang2025_Silkie_Wenchang	0.800981213
ENSGALG00010012832	CHADL	2	1.870889247	0.362077759	5.167092428	2.37764E-07	0.001257583	1.004731902	0.470961686	1	PRJNA827465;Yang2025_Silkie_Wenchang	CRA004775	7.02619E-05
ENSGALG00010012832	CHADL	2	0.841149851	1.422761351	0.591209376	0.554380141	3.808433931	16.8042614	94.04912852	0.5	CRA004775;Yang2025_Silkie_Wenchang	PRJNA827465	0.880177968
ENSGALG00010012832	CHADL	2	0.485357965	1.057462904	0.458983443	0.646246056	2.031645201	10.90634835	90.83102825	0.5	CRA004775;PRJNA827465	Yang2025_Silkie_Wenchang	0.907220702
ENSGALG00010012833	JAK2	2	-0.103426658	0.079487037	-1.301176419	0.193198075	0	0.395224995	0	1	PRJNA827465;Yang2025_Silkie_Wenchang	CRA004775	0.703208363
ENSGALG00010012833	JAK2	2	-0.096242514	0.149747991	-0.642696529	0.520421034	0.007253341	1.141356657	12.38496801	0.5	CRA004775;Yang2025_Silkie_Wenchang	PRJNA827465	0.869331539
ENSGALG00010012833	JAK2	2	-0.049927548	0.08863424	-0.563298649	0.573231538	0	0.600383371	0	0.5	CRA004775;PRJNA827465	Yang2025_Silkie_Wenchang	0.885803753
ENSGALG00010012836	ENSGALG00010012836	2	-0.611905274	0.376953231	-1.623292292	0.104526936	0	0.492729112	0	1	PRJNA827465;Yang2025_Silkie_Wenchang	CRA004775	0.549447931
ENSGALG00010012836	ENSGALG00010012836	2	0.398681091	1.238747652	0.321842056	0.747572352	2.839441224	13.35468418	92.51199065	0.5	CRA004775;Yang2025_Silkie_Wenchang	PRJNA827465	0.93680487
ENSGALG00010012836	ENSGALG00010012836	2	0.682113272	0.972497395	0.7014037	0.483051113	1.635466111	7.344988118	86.3852741	0.5	CRA004775;PRJNA827465	Yang2025_Silkie_Wenchang	0.85691916
ENSGALG00010012837	PKIB	2	-0.207741471	0.596141855	-0.348476575	0.727482303	0.621349545	7.903067074	87.34668464	0.5	PRJNA827465;Yang2025_Silkie_Wenchang	CRA004775	0.931616336
ENSGALG00010012837	PKIB	2	-0.571624186	0.355302071	-1.608840007	0.10765133	0.078083686	1.376955351	27.37600393	1	CRA004775;Yang2025_Silkie_Wenchang	PRJNA827465	0.55523359
ENSGALG00010012837	PKIB	2	0.289995298	0.237858202	1.219194023	0.22277056	0	0.512709725	0	0.5	CRA004775;PRJNA827465	Yang2025_Silkie_Wenchang	0.736382074
ENSGALG00010012838	C2orf88	2	0.659394703	0.399559537	1.650304002	0.098880774	0.293519982	12.36594736	91.91327627	1	PRJNA827465;Yang2025_Silkie_Wenchang	CRA004775	0.536756891
ENSGALG00010012838	C2orf88	2	0.475064221	0.61305576	0.774911928	0.438391703	0.692822343	12.53327145	92.0212372	0.5	CRA004775;Yang2025_Silkie_Wenchang	PRJNA827465	0.844002503
ENSGALG00010012838	C2orf88	2	0.130814273	0.198431661	0.65924093	0.509741068	0.03543775	1.631438069	38.70438487	0.5	CRA004775;PRJNA827465	Yang2025_Silkie_Wenchang	0.865257661
ENSGALG00010012839	TGFBI	2	-0.007865269	0.099241512	-0.079253825	0.936830733	0	0.021167469	0	0.5	PRJNA827465;Yang2025_Silkie_Wenchang	CRA004775	0.985038972
ENSGALG00010012839	TGFBI	2	-0.053304373	0.170371567	-0.312871297	0.754378445	0	0.744872956	0	0.5	CRA004775;Yang2025_Silkie_Wenchang	PRJNA827465	0.93838723
ENSGALG00010012839	TGFBI	2	-0.044125043	0.112209493	-0.393238056	0.694143664	0	0.702207947	0	1	CRA004775;PRJNA827465	Yang2025_Silkie_Wenchang	0.920728119
ENSGALG00010012841	L3MBTL2	2	-0.050377932	0.068923473	-0.73092562	0.464824586	0	0.406479978	0	1	PRJNA827465;Yang2025_Silkie_Wenchang	CRA004775	0.85217126
ENSGALG00010012841	L3MBTL2	2	-0.191638739	0.116295316	-1.647862923	0.099380803	0	0.288039897	0	1	CRA004775;Yang2025_Silkie_Wenchang	PRJNA827465	0.538163666
ENSGALG00010012841	L3MBTL2	2	-0.091499494	0.104428065	-0.876196399	0.380923274	0.008949768	1.475035423	32.20501796	1	CRA004775;PRJNA827465	Yang2025_Silkie_Wenchang	0.824392515
ENSGALG00010012842	DDX17	2	0.074377189	0.064033408	1.161537259	0.24542348	0.0038272	1.709751893	41.51198169	1	PRJNA827465;Yang2025_Silkie_Wenchang	CRA004775	0.75661406
ENSGALG00010012842	DDX17	2	0.047305242	0.163409364	0.289489175	0.772207063	0.032060656	2.172006541	53.95962299	0.5	CRA004775;Yang2025_Silkie_Wenchang	PRJNA827465	0.943066211
ENSGALG00010012842	DDX17	2	0.019044631	0.047072412	0.404581575	0.685785105	0	0.898539716	0	0.5	CRA004775;PRJNA827465	Yang2025_Silkie_Wenchang	0.919235539
ENSGALG00010012843	ENSGALG00010012843	2	0.419383663	0.349928105	1.198485224	0.230728172	0.19517989	4.924751428	79.69440662	1	PRJNA827465;Yang2025_Silkie_Wenchang	CRA004775	0.743374303
ENSGALG00010012843	ENSGALG00010012843	2	0.150265036	0.657839819	0.228421922	0.819318249	0.764992155	8.34665495	88.01915251	0.5	CRA004775;Yang2025_Silkie_Wenchang	PRJNA827465	0.955256039
ENSGALG00010012843	ENSGALG00010012843	2	-0.154234461	0.296508744	-0.520168341	0.602946249	0.085530455	1.808626883	44.70943624	0.5	CRA004775;PRJNA827465	Yang2025_Silkie_Wenchang	0.895538966
ENSGALG00010012845	HDHD2	2	0.057989985	0.125840155	0.460822579	0.644925901	0	0.06688096	0	1	PRJNA827465;Yang2025_Silkie_Wenchang	CRA004775	0.906953008
ENSGALG00010012845	HDHD2	2	-0.205737838	0.29053637	-0.708131094	0.478863852	0.103515812	2.381630011	58.01195	0.5	CRA004775;Yang2025_Silkie_Wenchang	PRJNA827465	0.855840388
ENSGALG00010012845	HDHD2	2	-0.177350268	0.324822502	-0.545991325	0.585071901	0.146315232	2.999137851	66.65708447	0.5	CRA004775;PRJNA827465	Yang2025_Silkie_Wenchang	0.891086887
ENSGALG00010012848	SERINC1	2	-0.06518157	0.041883063	-1.556275156	0.119642676	0	0.191611651	0	1	PRJNA827465;Yang2025_Silkie_Wenchang	CRA004775	0.580639051
ENSGALG00010012848	SERINC1	2	-0.097084084	0.067649799	-1.435097884	0.151259207	0	0.209385565	0	1	CRA004775;Yang2025_Silkie_Wenchang	PRJNA827465	0.642835272
ENSGALG00010012848	SERINC1	2	-0.057010641	0.051564101	-1.105626585	0.268888178	0	0.353695329	0	1	CRA004775;PRJNA827465	Yang2025_Silkie_Wenchang	0.771398294
ENSGALG00010012850	CALB1	2	-0.571581414	0.464761527	-1.229838058	0.218757754	0.048016228	1.124833256	11.09793429	1	PRJNA827465;Yang2025_Silkie_Wenchang	CRA004775	0.73259614
ENSGALG00010012850	CALB1	2	0.880608788	1.200218183	0.733762277	0.463093635	1.977849815	2.908033108	65.61249605	0.5	CRA004775;Yang2025_Silkie_Wenchang	PRJNA827465	0.851447984
ENSGALG00010012850	CALB1	2	0.481606467	1.681813189	0.286361452	0.774601296	4.711365958	5.619947979	82.20624099	0.5	CRA004775;PRJNA827465	Yang2025_Silkie_Wenchang	0.943713409
ENSGALG00010012851	ENSGALG00010012851	2	0.054679408	0.28641446	0.190910083	0.84859604	0.059860541	1.565205185	36.11061288	0.5	PRJNA827465;Yang2025_Silkie_Wenchang	CRA004775	0.961364896
ENSGALG00010012851	ENSGALG00010012851	2	0.185781907	0.224265402	0.828401997	0.407442876	0	0.419592465	0	1	CRA004775;Yang2025_Silkie_Wenchang	PRJNA827465	0.833874576
ENSGALG00010012851	ENSGALG00010012851	2	-0.1166985	0.243909193	-0.478450603	0.632329554	0	0.335795606	0	0.5	CRA004775;PRJNA827465	Yang2025_Silkie_Wenchang	0.903058174
ENSGALG00010012852	MSTN	2	-0.354945309	0.10939981	-3.24447831	0.00117666	0	0.024572578	0	1	PRJNA827465;Yang2025_Silkie_Wenchang	CRA004775	0.041666301
ENSGALG00010012852	MSTN	2	-0.156907616	0.409698106	-0.382983503	0.701731996	0.179677646	1.60807599	37.81388402	0.5	CRA004775;Yang2025_Silkie_Wenchang	PRJNA827465	0.922904951
ENSGALG00010012852	MSTN	2	-0.159824563	0.372073032	-0.429551592	0.667521857	0.154656048	1.542307203	35.1620742	0.5	CRA004775;PRJNA827465	Yang2025_Silkie_Wenchang	0.913973577
ENSGALG00010012854	LECT2	2	0.213712353	0.30120641	0.709521266	0.478001059	0	0.054946345	0	1	PRJNA827465;Yang2025_Silkie_Wenchang	CRA004775	0.855366076
ENSGALG00010012854	LECT2	2	0.464012965	0.317901237	1.45961359	0.144396102	0	0.829364517	0	1	CRA004775;Yang2025_Silkie_Wenchang	PRJNA827465	0.631870428
ENSGALG00010012854	LECT2	2	0.452761163	0.381054486	1.188179589	0.234762663	0.015856925	1.056450691	5.343428857	1	CRA004775;PRJNA827465	Yang2025_Silkie_Wenchang	0.747791642

ENSGALG00010012857	NSDHL	2	0.161253334	0.136530401	1.181080051	0.237570924	0	0.003139009	0	1	PRJNA827465;Yang2025_Silkie_Wenchang	CRA004775	0.749400455
ENSGALG00010012857	NSDHL	2	0.160410362	0.237019637	0.676780893	0.498544983	0	0.015049254	0	1	CRA004775;Yang2025_Silkie_Wenchang	PRJNA827465	0.861478809
ENSGALG00010012857	NSDHL	2	0.15217243	0.154325228	0.986050255	0.324108453	0	0.01018416	0	1	CRA004775;PRJNA827465	Yang2025_Silkie_Wenchang	0.799600434
ENSGALG00010012859	FBXL21	2	-0.173845987	0.176073748	-0.987347574	0.323472276	0.051731779	5.98978631	83.30491359	1	PRJNA827465;Yang2025_Silkie_Wenchang	CRA004775	0.799456433
ENSGALG00010012859	FBXL21	2	-0.071291507	0.3129001	-0.227841112	0.819769764	0.166712245	6.425340359	84.43662212	0.5	CRA004775;Yang2025_Silkie_Wenchang	PRJNA827465	0.955256039
ENSGALG00010012859	FBXL21	2	0.059668646	0.116609645	0.511695635	0.608864041	0.009787155	1.345901039	25.70033225	0.5	CRA004775;PRJNA827465	Yang2025_Silkie_Wenchang	0.897208691
ENSGALG00010012861	WDR70	2	-0.149780089	0.135382716	-1.106345727	0.268576907	0	0.020562474	0	1	PRJNA827465;Yang2025_Silkie_Wenchang	CRA004775	0.771398294
ENSGALG00010012861	WDR70	2	-0.21394103	0.128474701	-1.665238587	0.095865165	0	0.417056094	0	1	CRA004775;Yang2025_Silkie_Wenchang	PRJNA827465	0.5301492
ENSGALG00010012861	WDR70	2	-0.242614321	0.137132852	-1.769191826	0.076861868	0	0.210971414	0	1	CRA004775;PRJNA827465	Yang2025_Silkie_Wenchang	0.477955453
ENSGALG00010012865	EP300	2	0.196094954	0.344735437	0.568827376	0.569473294	0.209005522	7.62749322	86.88953276	0.5	PRJNA827465;Yang2025_Silkie_Wenchang	CRA004775	0.884818733
ENSGALG00010012865	EP300	2	0.406833361	0.211252536	1.925815273	0.054127444	0.017103941	1.226295166	18.45356422	1	CRA004775;Yang2025_Silkie_Wenchang	PRJNA827465	0.805227287
ENSGALG00010012865	EP300	2	-0.098732075	0.07096428	-1.391292553	0.164136731	0	0.700806773	0	0.5	CRA004775;PRJNA827465	Yang2025_Silkie_Wenchang	0.66287462
ENSGALG00010012867	A4GALT	2	-0.047102923	0.055417991	-0.849957259	0.395348849	0	0.152138077	0	1	PRJNA827465;Yang2025_Silkie_Wenchang	CRA004775	0.830888664
ENSGALG00010012867	A4GALT	2	-0.066620143	0.079942434	-0.833351444	0.404646551	0	0.037606835	0	1	CRA004775;Yang2025_Silkie_Wenchang	PRJNA827465	0.833112102
ENSGALG00010012867	A4GALT	2	-0.029778668	0.067154707	-0.443433814	0.657451983	0	0.000165313	0	1	CRA004775;PRJNA827465	Yang2025_Silkie_Wenchang	0.910711339
ENSGALG00010012868	CTHRC1	2	0.678035679	0.38610046	1.756112072	0.079069274	0.219815001	3.776989503	73.52388723	1	PRJNA827465;Yang2025_Silkie_Wenchang	CRA004775	0.483757735
ENSGALG00010012868	CTHRC1	2	0.543022251	0.686964154	0.790466646	0.429255294	0.659811167	2.732686342	63.40597219	0.5	CRA004775;Yang2025_Silkie_Wenchang	PRJNA827465	0.842008086
ENSGALG00010012868	CTHRC1	2	0.191043907	0.288970822	0.661118327	0.508536433	0	0.565035699	0	0.5	CRA004775;PRJNA827465	Yang2025_Silkie_Wenchang	0.864976303
ENSGALG00010012869	ENSGALG00010012869	2	0.261941076	0.155336085	1.686286068	0.091740713	0	0.127994395	0	1	PRJNA827465;Yang2025_Silkie_Wenchang	CRA004775	0.518914432
ENSGALG00010012869	ENSGALG00010012869	2	-0.130872461	0.516344806	-0.253459431	0.799913207	0.40857713	4.240971868	76.42049909	0.5	CRA004775;Yang2025_Silkie_Wenchang	PRJNA827465	0.949922217
ENSGALG00010012869	ENSGALG00010012869	2	-0.154900488	0.447394792	-0.346227741	0.729171569	0.316577873	4.411943178	77.33425025	0.5	CRA004775;PRJNA827465	Yang2025_Silkie_Wenchang	0.932323844
ENSGALG00010012870	KCNC2	2	-0.074291047	0.383565737	-0.193685307	0.846422298	0.266907546	10.76331359	90.70918085	0.5	PRJNA827465;Yang2025_Silkie_Wenchang	CRA004775	0.96122283
ENSGALG00010012870	KCNC2	2	-0.123593168	0.473002674	-0.261294861	0.793865129	0.383766009	6.708375735	85.09326192	0.5	CRA004775;Yang2025_Silkie_Wenchang	PRJNA827465	0.948830022
ENSGALG00010012870	KCNC2	2	-0.495020647	0.147619724	-3.353350304	0.000798396	0	0.247913563	0	1	CRA004775;PRJNA827465	Yang2025_Silkie_Wenchang	0.032349849
ENSGALG00010012871	ENSGALG00010012871	2	-0.187636285	0.157228345	-1.193399862	0.232712809	0	0.005543004	0	1	PRJNA827465;Yang2025_Silkie_Wenchang	CRA004775	0.74577273
ENSGALG00010012871	ENSGALG00010012871	2	0.055607388	0.357149866	0.15569763	0.876271366	0.138946463	1.874963598	46.66563122	0.5	CRA004775;Yang2025_Silkie_Wenchang	PRJNA827465	0.970347777
ENSGALG00010012871	ENSGALG00010012871	2	0.057890637	0.363466735	0.15927355	0.873453359	0.141075019	1.868847804	46.4910948	0.5	CRA004775;PRJNA827465	Yang2025_Silkie_Wenchang	0.96994709
ENSGALG00010012873	PTAFR	2	-0.455155268	0.465758725	-0.977234015	0.328453312	0.415493296	23.49283869	95.74338369	0.5	PRJNA827465;Yang2025_Silkie_Wenchang	CRA004775	0.800761292
ENSGALG00010012873	PTAFR	2	-0.893217699	0.147656087	-6.049311746	1.45466E-09	0	0.363100396	0	1	CRA004775;Yang2025_Silkie_Wenchang	PRJNA827465	8.53321E-07
ENSGALG00010012873	PTAFR	2	-0.218554695	0.315406498	-0.692930225	0.488353341	0.136998004	2.565570084	61.02230821	0.5	CRA004775;PRJNA827465	Yang2025_Silkie_Wenchang	0.857796363
ENSGALG00010012875	HSF2	2	-0.123756041	0.078436382	-1.577788739	0.114614152	0.008471471	3.047376543	67.18488884	1	PRJNA827465;Yang2025_Silkie_Wenchang	CRA004775	0.570935361
ENSGALG00010012875	HSF2	2	-0.20389576	0.073965696	-2.756625996	0.005840111	0	0.214833875	0	1	CRA004775;Yang2025_Silkie_Wenchang	PRJNA827465	0.115170033
ENSGALG00010012875	HSF2	2	-0.058501371	0.045739968	-1.27899895	0.200897426	0	0.038045184	0	1	CRA004775;PRJNA827465	Yang2025_Silkie_Wenchang	0.71352535
ENSGALG00010012876	KDELR3	2	-0.177792531	0.21280626	-0.835466643	0.403455021	0.08021162	8.371811917	88.05515449	0.5	PRJNA827465;Yang2025_Silkie_Wenchang	CRA004775	0.83306585
ENSGALG00010012876	KDELR3	2	-0.395382379	0.126106663	-3.13530125	0.001716777	0	0.076802044	0	1	CRA004775;Yang2025_Silkie_Wenchang	PRJNA827465	0.052459682
ENSGALG00010012876	KDELR3	2	0.011492472	0.062444703	0.184042391	0.8539802	0	0.62604997	0	0.5	CRA004775;PRJNA827465	Yang2025_Silkie_Wenchang	0.965975689
ENSGALG00010012877	SLC25A32	2	0.007527348	0.044062414	0.170833759	0.864354484	0	0.238031734	0	0.5	PRJNA827465;Yang2025_Silkie_Wenchang	CRA004775	0.966643645
ENSGALG00010012877	SLC25A32	2	0.002096449	0.066864606	0.031353639	0.974987514	0	0.618699733	0	0.5	CRA004775;Yang2025_Silkie_Wenchang	PRJNA827465	0.992609821
ENSGALG00010012877	SLC25A32	2	0.033560169	0.052497367	0.639273377	0.522645105	0	0.315072192	0	1	CRA004775;PRJNA827465	Yang2025_Silkie_Wenchang	0.869568211
ENSGALG00010012878	GDNF	2	0.243332827	0.220734047	1.102380126	0.270296448	0.050887197	2.092824923	52.2176944	1	PRJNA827465;Yang2025_Silkie_Wenchang	CRA004775	0.772430311
ENSGALG00010012878	GDNF	2	0.478931363	0.198989337	2.406819228	0.016092133	0	0.043078111	0	1	CRA004775;Yang2025_Silkie_Wenchang	PRJNA827465	0.209635104
ENSGALG00010012878	GDNF	2	0.099820102	0.201570555	0.495211726	0.620450674	0	0.936580953	0	1	CRA004775;PRJNA827465	Yang2025_Silkie_Wenchang	0.899395593
ENSGALG00010012879	RCAN1	2	-0.077296389	0.212454531	-0.363825563	0.715988273	0.075940815	6.204128123	83.88169973	0.5	PRJNA827465;Yang2025_Silkie_Wenchang	CRA004775	0.927261187
ENSGALG00010012879	RCAN1	2	0.044329176	0.395389312	0.112115261	0.910732017	0.256453538	5.14522407	80.56450047	0.5	CRA004775;Yang2025_Silkie_Wenchang	PRJNA827465	0.97880407
ENSGALG00010012879	RCAN1	2	0.183311168	0.136196113	1.345935389	0.178323354	0.012311737	1.215914417	17.75736958	1	CRA004775;PRJNA827465	Yang2025_Silkie_Wenchang	0.684689586
ENSGALG00010012880	CLK4	2	0.262866378	0.172625245	1.522757453	0.127819398	0.046065896	4.278819972	76.62907048	1	PRJNA827465;Yang2025_Silkie_Wenchang	CRA004775	0.600058027
ENSGALG00010012880	CLK4	2	0.387903473	0.117054046	3.313883516	0.000920098	0	0.706662625	0	1	CRA004775;Yang2025_Silkie_Wenchang	PRJNA827465	0.035733817
ENSGALG00010012880	CLK4	2	0.126529078	0.084521387	1.497017295	0.134388758	0	0.329604967	0	1	CRA004775;PRJNA827465	Yang2025_Silkie_Wenchang	0.614100843
ENSGALG00010012883	DCAF13	2	-0.035131526	0.038855586	-0.904156387	0.365912483	0	0.573955714	0	1	PRJNA827465;Yang2025_Silkie_Wenchang	CRA004775	0.818968857
ENSGALG00010012883	DCAF13	2	-0.131995686	0.084716303	-1.558090736	0.11921174	0	0.484934714	0	1	CRA004775;Yang2025_Silkie_Wenchang	PRJNA827465	0.5795861
ENSGALG00010012883	DCAF13	2	-0.06238279	0.085892703	-0.726287423	0.467662588	0.007197237	1.406536759	28.90338675	1	CRA004775;PRJNA827465	Yang2025_Silkie_Wenchang	0.852885838
ENSGALG00010012884	ENSGALG00010012884	2	-0.279323675	0.234933097	-1.188949872	0.234459393	0	0.168329808	0	1	PRJNA827465;Yang2025_Silkie_Wenchang	CRA004775	0.747509756
ENSGALG00010012884	ENSGALG00010012884	2	-0.316128746	0.260458822	-1.21375655	0.224840703	0	0.082598137	0	1	CRA004775;Yang2025_Silkie_Wenchang	PRJNA827465	0.737885283
ENSGALG00010012884	ENSGALG00010012884	2	-0.196076416	0.262992472	-0.745559047	0.45593383	0	0.005980493	0	1	CRA004775;PRJNA827465	Yang2025_Silkie_Wenchang	0.84907427
ENSGALG00010012885	PMS1	2	0.032528009	0.115354282	0.281983546	0.777956127	0.02058343	4.407071357	77.30919427	0.5	PRJNA827465;Yang2025_Silkie_Wenchang	CRA004775	0.944432731
ENSGALG00010012885	PMS1	2	0.362623891	0.483663154	0.749744709	0.453408475	0.427932302	11.02257387	90.92770879	0.5	CRA004775;Yang2025_Silkie_Wenchang	PRJNA827465	0.848879567
ENSGALG00010012885	PMS1	2	0.465639675	0.366374371	1.270939541	0.203750158	0.231234073	6.487122615	84.58484509	1	CRA004775;PRJNA827465	Yang2025_Silkie_Wenchang	0.717254253
ENSGALG00010012887	KCNJ4	2	-0.096641068	0.345802448	-0.279469011	0.779806118	0.137488917	2.258127823	55.71552726	0.5	PRJNA827465;Yang2025_Silkie_Wenchang	CRA004775	0.944978771
ENSGALG00010012887	KCNJ4	2	0.175459709	0.347765428	0.504534651	0.613885719	0	0.626271068	0	0.5	CRA004775;Yang2025_Silkie_Wenchang	PRJNA827465	0.898256044
ENSGALG00010012887	KCNJ4	2	-0.383782611	0.243390915	-1.576815676	0.114837943	0	0.000882312	0	1	CRA004775;PRJNA827465	Yang2025_Silkie_Wenchang	0.571687865
ENSGALG00010012888	ENSGALG00010012888	2	-4.416011212	4.444849619	-0.99351195	0.32046935	34.17019257	7.116391563	85.94793455	1	PRJNA827465;Yang2025_Silkie_Wenchang	CRA004775	0.790348216
ENSGALG00010012888	ENSGALG00010012888	2	-4.130644231	4.733397898	-0.872659413	0.382848746	39.64375905	8.332229504	87.99841028	0.5	CRA004775;Yang2025_Silkie_Wenchang	PRJNA827465	0.825177092
ENSGALG00010012888	ENSGALG00010012888	2	0.00369198	1.068046774	0.003456759	0.997241911	0	0.072042922	0	0.5	CRA004775;PRJNA827465	Yang2025_Silkie_Wenchang	0.999174008
ENSGALG00010012889	RIC1	2	-0.007114147	0.047317923	-0.150347825	0.880490203	0	0.040514749	0	1	PRJNA827465;Yang2025_Silkie_Wenchang	CRA004775	0.971623434
ENSGALG00010012889	RIC1	2	0.041221253	0.125752117	0.32777988	0.743063795	0.012066697	1.326355392	24.6054258	0.5	CRA004775;Yang2025_Silkie_Wenchang	PRJNA827465	0.93573824
ENSGALG00010012889	RIC1	2	0.037140081	0.098478759	0.377137987	0.706071063	0.008007666	1.230950119	18.76193967	0.5	CRA004775;PRJNA827465	Yang2025_Silkie_Wenchang	0.924548793
ENSGALG00010012890	MFAP3	2	-0.185079089	0.182118908	-1.016254112	0.309508394	0.062804408	18.71042423	94.65538575	1	PRJNA827465;Yang2025_Silkie_Wenchang	CRA004775	0.794192328
ENSGALG00010012890	MFAP3	2	-0.367509771	0.065240737	-5.633133372	1.76964E-08	0	0.020129363	0	1	CRA004775;Yang2025_Silkie_Wenchang	PRJNA827465	8.28004E-06
ENSGALG00010012890	MFAP3	2	-0.080836815	0.137792859	-0.586654602	0.557435704	0.022106531	1.697748237	41.09845156	1	CRA004775;PRJNA827465	Yang2025_Silkie_Wenchang	0.885876789
ENSGALG00010012892	ORMDL1	2	-0.322042711	0.167076826	-1.92751274	0.053912345	0.030002559	2.119255246	52.81361214	1	PRJNA827465;Yang2025_Silkie_Wenchang	CRA004775	0.402708322
ENSGALG00010012892	ORMDL1	2	-0.611380196	0.149321145	-4.094397997	4.23267E-05	0	0.813678635	0	1	CRA004775;Yang2025_Silkie_Wenchang	PRJNA827465	0.003450883
ENSGALG00010012892	ORMDL1	2	-0.449530789	0.30771482	-1.460868177	0.144051621	0.150360626	4.614924516	78.33117321	1	CRA004775;PRJNA827465	Yang2025_Silkie_Wenchang	0.631313384
ENSGALG00010012893	ENSGALG00010012893	2	0.131139038	0.237893973	0.551249938	0.581462351	0.061950645	2.159700289	53.69727896	0.5	PRJNA827465;Yang2025_Silkie_Wenchang	CRA004775	0.889045686
ENSGALG00010012893	ENSGALG00010012893	2	-0.133312362	0.216384897	-0.616089033	0.53783576	0	0.003418914	0	1	CRA004775;Yang2025_Silkie_Wenchang	PRJNA827465	0.87481821
ENSGALG00010012893	ENSGALG00010012893	2	0.229469026	0.193613935	1.185188587	0.235942892	0.011776363	1.12944559	11.46098507	0.5	CRA004775;PRJNA827465	Yang2025_Silkie_Wenchang	0.748669015
ENSGALG00010012894	CLIC6	2	0.382085717	0.43273139	0.882962794	0.377256374	0.222926365	2.360568598	57.63732514	1	PRJNA827465;Yang2025_Silkie_Wenchang	CRA004775	0.823181514
ENSGALG00010012894	CLIC6	2	-0.040017817	1.113784075	-0.035929601	0.971338493	1.891703509	3.86073456	74.09819338	0.5	CRA004775;Yang2025_Silkie_Wenchang	PRJNA827465	0.991955348
ENSGALG00010012894	CLIC6	2	-0.309014691	0.588455337	-0.525128538	0.599493838	0.359960037	1.607630121	37.79663699	0.5	CRA004775;PRJNA827465	Yang2025_Silkie_Wenchang	0.895010988
ENSGALG00010012896	STK38L	2	-0.042717027	0.160525607	-0.266107247	0.790156614	0.046746975	10.66701014	90.62530187	0.5	PRJNA827465;Yang2025_Silkie_Wenchang	CRA004775	0.947390191
ENSGALG00010012896	STK38L	2	-0.2108562	0.077833202	-2.709077798	0.006747052	0	0.024244557	0	1	CRA004775;Yang2025_Silkie_Wenchang	PRJNA827465	0.125916131
ENSGALG00010012896	STK38L	2	0.083086833	0.102071696	0.81400463	0.415642298	0.008019806	1.127069015	11.27428873	0.5	CRA004775;PRJNA827465	Yang2025_Silkie_Wenchang	0.837689773
ENSGALG00010012897	ENSGALG00010012897	2	-0.301596666	0.382866214	-0.787733821	0.430852418	0	0.327075575	0	1	PRJNA827465;Yang2025_Silkie_Wenchang	CRA004775	0.842503929
ENSGALG00010012897	ENSGALG00010012897	2	-0.425487693	0.366307771	-1.161557923	0.245415082	0	0.053365924	0	1	CRA004775;Yang2025_Silkie_Wenchang	PRJNA827465	0.75661406
ENSGALG00010012897	ENSGALG00010012897	2	-0.22350981	0.370106572	-0.603906625	0.545905724	0	0.130538532	0	1	CRA004775;PRJNA827465	Yang2025_Silkie_Wenchang	0.877958239
ENSGALG00010012898	FAM114A2	2	-0.020077987	0.044631497	-0.449861385	0.652810392	0	0.187315259	0	0.5	PRJNA827465;Yang2025_Silkie_Wenchang	CRA004775	0.909572411
ENSGALG00010012898	FAM114A2	2	0.018125055	0.093061539	0.194764187	0.84557756	0	0.00027036	0	1	CRA004775;Yang2025_Silkie_Wenchang	PRJNA827465	0.961095892
ENSGALG00010012898	FAM114A2	2	-0.028029296	0.048902138	-0.573171181	0.566528795	0	0.029411071	0	0.5	CRA004775;PRJNA827465	Yang2025_Silkie_Wenchang	0.883487517
ENSGALG00010012899	ENSGALG00010012899	2	0.077611082	0.244556079	0.317354948	0.750974288	0.106292759	8.831969313	88.67749689	0.5	PRJNA827465;Yang2025_Silkie_Wenchang	CRA004775	0.937567095
ENSGALG00010012899	ENSGALG00010012899	2	0.327829909	0.121921981	2.688849911	0.007169865	0	0.007930456	0	1	CRA004775;Yang2025_Silkie_Wenchang	PRJNA827465	0.130241134
ENSGALG00010012899	ENSGALG00010012899	2	0.011182771	0.22253736	0.050251207	0.959922206	0.069378561	2.826803235	64.62435066	0.5	CRA004775;PRJNA827465	Yang2025_Silkie_Wenchang	0.98933784
ENSGALG00010012900	RBX1	2	-0.23357167	0.351127548	-0.665204629	0.50591962	0.214340646	6.953956873	85.61969799	0.5	PRJNA827465;Yang2025_Silkie_Wenchang	CRA004775	0.863927652
ENSGALG00010012900	RBX1	2	-0.439042106	0.195563533	-2.245010095	0.024767485	0.008308729	1.121806957	10.85810322	1	CRA004775;Yang2025_Silkie_Wenchang	PRJNA827465	0.268761753
ENSGALG00010012900	RBX1	2	0.020492101	0.121070363	0.169257782	0.865593882	0.012513705	1.333386607	25.00299653	0.5	CRA004775;PRJNA827465	Yang2025_Silkie_Wenchang	0.967088846
ENSGALG00010012903	RGMB	2	-0.30737793	0.292282904	-1.051645257	0.292962335	0.156422575	11.7799803	91.50959274	1	PRJNA827465;Yang2025_Silkie_Wenchang	CRA004775	0.786774237
ENSGALG00010012903	RGMB	2	-0.304580867	0.341230737	-0.89259505	0.372074071	0.189109693	4.991181352	79.96466308	0.5	CRA004775;Yang2025_Silkie_Wenchang	PRJNA827465	0.821620523
ENSGALG00010012903	RGMB	2	-0.008424078	0.097616247	-0.086297914	0.931229597	0	0.121336891	0	0.5	CRA004775;PRJNA827465	Yang2025_Silkie_Wenchang	0.983979913
ENSGALG00010012904	ERMP1	2	-0.192697104	0.202312871	-0.95247081	0.340858263	0.05568187	3.078971494	67.52162201	1	PRJNA827465;Yang2025_Silkie_Wenchang	CRA004775	0.805635165
ENSGALG00010012904	ERMP1	2	-0.420466631	0.164682949	-2.553188617	0.010674167	0	0.007992323	0	1	CRA004775;Yang2025_Silkie_Wenchang	PRJNA827465	0.164714096
ENSGALG00010012904	ERMP1	2	-0.096687099	0.177999498	-0.543187483	0.587000724	0.01999952	1.255967118	20.38008117	1	CRA004775;PRJNA827465	Yang2025_Silkie_Wenchang	0.891596903
ENSGALG00010012905	NKAIN1	2	-0.123908645	0.123628759	-1.002263921	0.316216143	0	0.1751424	0	1	PRJNA827465;Yang2025_Silkie_Wenchang	CRA004775	0.796883862
ENSGALG00010012905	NKAIN1	2	-0.223850226	0.200278121	-1.117696856	0.263696486	0	0.039728639	0	1	CRA004775;Yang2025_Silkie_Wenchang	PRJNA827465	0.768643821
ENSGALG00010012905	NKAIN1	2	-0.111994247	0.139806355	-0.801066922	0.423092904	0	0.211262941	0	1	CRA004775;PRJNA827465	Yang2025_Silkie_Wenchang	0.840051549
ENSGALG00010012908	STRAP	2	-0.043760204	0.038679202	-1.131362639	0.25790249	0	0.816871923	0	0.5	PRJNA827465;Yang2025_Silkie_Wenchang	CRA004775	0.765151037
ENSGALG00010012908	STRAP	2	0.005868685	0.080397322	0.072996025	0.941809281	0	0.367202183	0	0.5	CRA004775;Yang2025_Silkie_Wenchang	PRJNA827465	0.985827325
ENSGALG00010012908	STRAP	2	-0.062700612	0.042016181	-1.492296791	0.135621358	0	0.041846349	0	1	CRA004775;PRJNA827465	Yang2025_Silkie_Wenchang	0.617196137
ENSGALG00010012910	XPNPEP3	2	-0.211853514	0.13266311	-1.596928593	0.110281625	0.029501029	6.070062301	83.52570451	1	PRJNA827465;Yang2025_Silkie_Wenchang	CRA004775	0.562335482
ENSGALG00010012910	XPNPEP3	2	-0.340028965	0.084672441	-4.015816231	5.92404E-05	0	0.218267014	0	1	CRA004775;Yang2025_Silkie_Wenchang	PRJNA827465	0.005427332
ENSGALG00010012910	XPNPEP3	2	-0.092524166	0.059866479	-1.545508737	0.122223257	0	0.197422114	0	1	CRA004775;PRJNA827465	Yang2025_Silkie_Wenchang	0.586394125
ENSGALG00010012911	CSNK1E	2	-0.084932943	0.051028269	-1.664429232	0.096026682	0	0.000269041	0	1	PRJNA827465;Yang2025_Silkie_Wenchang	CRA004775	0.530281432
ENSGALG00010012911	CSNK1E	2	-0.052725347	0.11251224	-0.468618764	0.639342163	0	0.533253205	0	0.5	CRA004775;Yang2025_Silkie_Wenchang	PRJNA827465	0.906005589
ENSGALG00010012911	CSNK1E	2	-0.07643481	0.055201391	-1.38465369	0.166158386	0	0.5913227	0	0.5	CRA004775;PRJNA827465	Yang2025_Silkie_Wenchang	0.66529196
ENSGALG00010012912	NKAPL	2	0.507680553	0.539918438	0.940291195	0.347068215	0.557758236	22.55815933	95.5670142	0.5	PRJNA827465;Yang2025_Silkie_Wenchang	CRA004775	0.808602357
ENSGALG00010012912	NKAPL	2	0.564436684	0.496664917	1.136453702	0.255766731	0.452847413	12.17050597	91.78341474	1	CRA004775;Yang2025_Silkie_Wenchang	PRJNA827465	0.764478331
ENSGALG00010012912	NKAPL	2	-0.000968002	0.07687563	-0.012591795	0.989953467	0	0.174350491	0	0.5	CRA004775;PRJNA827465	Yang2025_Silkie_Wenchang	0.997312206
ENSGALG00010012913	EGFLAM	2	-0.113734329	0.063511526	-1.790766737	0.073330735	0.001546747	1.233135888	18.90593652	1	PRJNA827465;Yang2025_Silkie_Wenchang	CRA004775	0.467957919
ENSGALG00010012913	EGFLAM	2	0.354630745	0.582790613	0.60850456	0.542852883	0.609367768	8.871187654	88.72755217	0.5	CRA004775;Yang2025_Silkie_Wenchang	PRJNA827465	0.876968972
ENSGALG00010012913	EGFLAM	2	0.412012931	0.517618225	0.795978409	0.426044585	0.466666881	6.953703465	85.61917394	0.5	CRA004775;PRJNA827465	Yang2025_Silkie_Wenchang	0.84125685
ENSGALG00010012915	ENSGALG00010012915	2	-0.46016813	0.234589283	-1.961590588	0.049810169	0.042998143	1.619728038	38.26124036	1	PRJNA827465;Yang2025_Silkie_Wenchang	CRA004775	0.386284347
ENSGALG00010012915	ENSGALG00010012915	2	-0.59063576	0.245815243	-2.402762959	0.016271731	0	0.78796281	0	1	CRA004775;Yang2025_Silkie_Wenchang	PRJNA827465	0.210791192
ENSGALG00010012915	ENSGALG00010012915	2	-0.256098072	0.205692344	-1.245053984	0.213111907	0	0.000101911	0	1	CRA004775;PRJNA827465	Yang2025_Silkie_Wenchang	0.72579791
ENSGALG00010012917	RUNX1	2	0.410468534	0.268878958	1.526592255	0.126862434	0.067046301	1.82441789	45.18799636	1	PRJNA827465;Yang2025_Silkie_Wenchang	CRA004775	0.597716824
ENSGALG00010012917	RUNX1	2	-0.645360244	1.410567899	-0.457518028	0.647298747	3.766185065	18.40942174	94.56799885	0.5	CRA004775;Yang2025_Silkie_Wenchang	PRJNA827465	0.907350807
ENSGALG00010012917	RUNX1	2	-0.89789818	1.137204396	-0.789566223	0.429781141	2.400847696	13.46237673	92.57189113	0.5	CRA004775;PRJNA827465	Yang2025_Silkie_Wenchang	0.842012722
ENSGALG00010012918	GLRX	2	0.136521839	0.661373494	0.206421698	0.836461524	0.734361555	6.107313747	83.62618917	0.5	PRJNA827465;Yang2025_Silkie_Wenchang	CRA004775	0.958399882
ENSGALG00010012918	GLRX	2	-0.090221525	0.433185259	-0.208274688	0.835014494	0.217045402	2.342356973	57.30795897	0.5	CRA004775;Yang2025_Silkie_Wenchang	PRJNA827465	0.95823248
ENSGALG00010012918	GLRX	2	0.568763181	0.228839557	2.485423366	0.012939744	0	0.960705451	0	1	CRA004775;PRJNA827465	Yang2025_Silkie_Wenchang	0.184914415
ENSGALG00010012921	EBF1	2	0.405330585	0.293023994	1.383267561	0.166582841	0.145942542	6.224510397	83.9344794	1	PRJNA827465;Yang2025_Silkie_Wenchang	CRA004775	0.665626909
ENSGALG00010012921	EBF1	2	0.4437541	0.335931336	1.320966677	0.186512476	0.149227157	2.829451273	64.65745816	1	CRA004775;Yang2025_Silkie_Wenchang	PRJNA827465	0.695457578
ENSGALG00010012921	EBF1	2	0.137073893	0.089458509	1.532262206	0.125457744	0	0.064646341	0	1	CRA004775;PRJNA827465	Yang2025_Silkie_Wenchang	0.594653364
ENSGALG00010012922	SNRNP40	2	0.002433992	0.052065509	0.046748639	0.962713565	0	0.758896598	0	0.5	PRJNA827465;Yang2025_Silkie_Wenchang	CRA004775	0.989980504
ENSGALG00010012922	SNRNP40	2	-0.053021157	0.193115289	-0.274557012	0.783656597	0.046117433	2.541205758	60.64860167	0.5	CRA004775;Yang2025_Silkie_Wenchang	PRJNA827465	0.945815798
ENSGALG00010012922	SNRNP40	2	-0.07345981	0.107752924	-0.68174307	0.495401428	0.011779849	1.540528884	35.08722813	1	CRA004775;PRJNA827465	Yang2025_Silkie_Wenchang	0.860770265
ENSGALG00010012923	MLANA	2	7.659887081	0.523376996	14.63550585	1.66731E-48	0	0.098594343	0	1	PRJNA827465;Yang2025_Silkie_Wenchang	CRA004775	3.85472E-45
ENSGALG00010012923	MLANA	2	6.624275109	0.706593919	9.37493931	6.92156E-21	0.365683889	1.561178125	35.94581016	1	CRA004775;Yang2025_Silkie_Wenchang	PRJNA827465	9.38062E-18
ENSGALG00010012923	MLANA	2	6.920343922	0.885446602	7.815653599	5.46786E-15	1.103032244	3.364945575	70.28183732	1	CRA004775;PRJNA827465	Yang2025_Silkie_Wenchang	5.65535E-12
ENSGALG00010012924	GLIPR1	2	0.243342019	0.305402189	0.796791994	0.425571845	0.139120443	3.891442489	74.30258824	0.5	PRJNA827465;Yang2025_Silkie_Wenchang	CRA004775	0.84125685
ENSGALG00010012924	GLIPR1	2	0.244020681	0.379241917	0.643443328	0.519936477	0.189234254	2.791550422	64.17761284	0.5	CRA004775;Yang2025_Silkie_Wenchang	PRJNA827465	0.869253622
ENSGALG00010012924	GLIPR1	2	-0.074391161	0.171937052	-0.432665097	0.66525809	0	0.129123689	0	1	CRA004775;PRJNA827465	Yang2025_Silkie_Wenchang	0.913399315
ENSGALG00010012925	SETD4	2	-0.02982421	0.123614558	-0.241267783	0.809347579	0.006294061	1.249299044	19.95511364	0.5	PRJNA827465;Yang2025_Silkie_Wenchang	CRA004775	0.95276497
ENSGALG00010012925	SETD4	2	-0.157999212	0.154556882	-1.022272255	0.306652061	0	0.055192886	0	1	CRA004775;Yang2025_Silkie_Wenchang	PRJNA827465	0.792500392
ENSGALG00010012925	SETD4	2	0.047982553	0.128889397	0.372276962	0.709686649	0	0.213770422	0	0.5	CRA004775;PRJNA827465	Yang2025_Silkie_Wenchang	0.925298042
ENSGALG00010012926	OSGEPL1	2	0.423986663	0.195096441	2.173215769	0.029764082	0.038974315	2.014557009	50.36129554	1	PRJNA827465;Yang2025_Silkie_Wenchang	CRA004775	0.294469727
ENSGALG00010012926	OSGEPL1	2	0.365806278	0.372807202	0.98122106	0.326483741	0.169096624	2.278476603	56.11102617	0.5	CRA004775;Yang2025_Silkie_Wenchang	PRJNA827465	0.800028239
ENSGALG00010012926	OSGEPL1	2	0.207789412	0.158744017	1.308959014	0.190548235	0	0.599138992	0	0.5	CRA004775;PRJNA827465	Yang2025_Silkie_Wenchang	0.699656801
ENSGALG00010012927	ENSGALG00010012927	2	-5.170205513	5.117882943	-1.010223479	0.312388233	48.17597465	12.21765242	91.81512155	1	PRJNA827465;Yang2025_Silkie_Wenchang	CRA004775	0.796054997
ENSGALG00010012927	ENSGALG00010012927	2	-4.738234893	5.525482011	-0.857524264	0.391155211	57.3042374	15.83193399	93.68365229	0.5	CRA004775;Yang2025_Silkie_Wenchang	PRJNA827465	0.829251057
ENSGALG00010012927	ENSGALG00010012927	2	0.254284365	0.906430969	0.280533625	0.779068132	0	0.190517572	0	0.5	CRA004775;PRJNA827465	Yang2025_Silkie_Wenchang	0.944714533
ENSGALG00010012930	IL6ST	2	-0.213027229	0.136781733	-1.557424551	0.119369721	0.031177836	5.832209913	82.85384074	1	PRJNA827465;Yang2025_Silkie_Wenchang	CRA004775	0.579860319
ENSGALG00010012930	IL6ST	2	-0.20615028	0.21621714	-0.953440973	0.34036669	0.061293744	2.417288927	58.63134155	0.5	CRA004775;Yang2025_Silkie_Wenchang	PRJNA827465	0.805432415
ENSGALG00010012930	IL6ST	2	-0.077926005	0.058892155	-1.323198408	0.185769405	0	0.413831632	0	0.5	CRA004775;PRJNA827465	Yang2025_Silkie_Wenchang	0.694501564
ENSGALG00010012932	ATF6	2	0.045203187	0.11095829	0.407389005	0.683722301	0.015846124	2.416832602	58.62353068	0.5	PRJNA827465;Yang2025_Silkie_Wenchang	CRA004775	0.918800744
ENSGALG00010012932	ATF6	2	0.014533709	0.177940843	0.081677194	0.934903414	0.045396749	3.528280017	71.65757833	0.5	CRA004775;Yang2025_Silkie_Wenchang	PRJNA827465	0.984489498
ENSGALG00010012932	ATF6	2	-0.055823426	0.051251769	-1.08919999	0.276065703	0	0.770445326	0	1	CRA004775;PRJNA827465	Yang2025_Silkie_Wenchang	0.775679893
ENSGALG00010012933	ZCCHC17	2	0.185753625	0.3423409	0.542598401	0.58740634	0.220426269	16.43165608	93.91418616	0.5	PRJNA827465;Yang2025_Silkie_Wenchang	CRA004775	0.891660017
ENSGALG00010012933	ZCCHC17	2	0.132645715	0.41856026	0.316909483	0.751312287	0.318963134	11.08083925	90.97541281	0.5	CRA004775;Yang2025_Silkie_Wenchang	PRJNA827465	0.937684688
ENSGALG00010012933	ZCCHC17	2	-0.163011395	0.070543884	-2.310779971	0.020845011	0	0.503132147	0	1	CRA004775;PRJNA827465	Yang2025_Silkie_Wenchang	0.243976022
ENSGALG00010012934	NDUFA1	2	-0.36585097	0.221503367	-1.651672274	0.098601378	0.073713141	3.461473736	71.11057093	1	PRJNA827465;Yang2025_Silkie_Wenchang	CRA004775	0.536006908
ENSGALG00010012934	NDUFA1	2	-0.209087915	0.453516868	-0.46103669	0.644772282	0.342769376	5.95684049	83.21257717	0.5	CRA004775;Yang2025_Silkie_Wenchang	PRJNA827465	0.906953008
ENSGALG00010012934	NDUFA1	2	-0.051288229	0.208900421	-0.245515201	0.80605754	0.057099273	2.263170274	55.81419517	0.5	CRA004775;PRJNA827465	Yang2025_Silkie_Wenchang	0.952063038
ENSGALG00010012938	UPF3B	2	0.398992962	0.524918421	0.760104706	0.447192	0.522332507	18.45097137	94.58023114	0.5	PRJNA827465;Yang2025_Silkie_Wenchang	CRA004775	0.84650962
ENSGALG00010012938	UPF3B	2	0.738702447	0.20133289	3.669059971	0.000243444	0.029168163	1.560391298	35.91351084	1	CRA004775;Yang2025_Silkie_Wenchang	PRJNA827465	0.014585485
ENSGALG00010012938	UPF3B	2	0.187683617	0.322162583	0.582574224	0.560179961	0.183442885	7.931477692	87.39200892	0.5	CRA004775;PRJNA827465	Yang2025_Silkie_Wenchang	0.881203589
ENSGALG00010012939	GJA1	2	-0.158776789	0.128482837	-1.235782094	0.216539573	0.010376982	1.410688654	29.11263609	0.5	PRJNA827465;Yang2025_Silkie_Wenchang	CRA004775	0.730172415
ENSGALG00010012939	GJA1	2	0.026228924	0.343155627	0.076434485	0.939073435	0.181834333	3.896245012	74.33426294	0.5	CRA004775;Yang2025_Silkie_Wenchang	PRJNA827465	0.985349045
ENSGALG00010012939	GJA1	2	0.140452366	0.198061695	0.709134424	0.478241062	0.021045786	1.290437606	22.50690811	1	CRA004775;PRJNA827465	Yang2025_Silkie_Wenchang	0.855388572
ENSGALG00010012940	FBN2	2	0.046324238	0.176947026	0.261797212	0.793477789	0.039038212	2.548436197	60.76024971	0.5	PRJNA827465;Yang2025_Silkie_Wenchang	CRA004775	0.948740761
ENSGALG00010012940	FBN2	2	0.337338394	0.230072363	1.466227363	0.142586356	0.024420049	1.104283608	9.443553007	1	CRA004775;Yang2025_Silkie_Wenchang	PRJNA827465	0.628893678
ENSGALG00010012940	FBN2	2	0.240533165	0.502708656	0.478474285	0.632312673	0.356693172	2.592815261	61.43188391	0.5	CRA004775;PRJNA827465	Yang2025_Silkie_Wenchang	0.903058174
ENSGALG00010012943	ASNSD1	2	-0.227570334	0.226600831	-1.004278463	0.315244411	0.090398191	8.206302026	87.81424329	1	PRJNA827465;Yang2025_Silkie_Wenchang	CRA004775	0.79668487
ENSGALG00010012943	ASNSD1	2	-0.42211253	0.11687334	-3.611709322	0.000304185	0	0.480823212	0	1	CRA004775;Yang2025_Silkie_Wenchang	PRJNA827465	0.017177298
ENSGALG00010012943	ASNSD1	2	-0.040103255	0.081766072	-0.490463265	0.623806118	0	0.97699373	0	1	CRA004775;PRJNA827465	Yang2025_Silkie_Wenchang	0.900549196
ENSGALG00010012944	NCK1	2	-0.079571843	0.145301077	-0.547634226	0.583943084	0.036754458	7.537718663	86.73338652	0.5	PRJNA827465;Yang2025_Silkie_Wenchang	CRA004775	0.890763231
ENSGALG00010012944	NCK1	2	-0.10264082	0.1523981	-0.673504592	0.500626328	0.032281858	3.121221066	67.96125687	0.5	CRA004775;Yang2025_Silkie_Wenchang	PRJNA827465	0.862400704
ENSGALG00010012944	NCK1	2	0.059365655	0.052075153	1.139999634	0.254286454	0	0.011524784	0	1	CRA004775;PRJNA827465	Yang2025_Silkie_Wenchang	0.764021784
ENSGALG00010012945	CLINT1	2	-0.008268244	0.031457494	-0.262838624	0.792674964	0	0.196919955	0	0.5	PRJNA827465;Yang2025_Silkie_Wenchang	CRA004775	0.948435155
ENSGALG00010012945	CLINT1	2	0.049081718	0.075787561	0.647622345	0.517229242	0	0.312103762	0	1	CRA004775;Yang2025_Silkie_Wenchang	PRJNA827465	0.867952013
ENSGALG00010012945	CLINT1	2	-0.007905669	0.033133421	-0.238601061	0.811414943	0	0.741033012	0	0.5	CRA004775;PRJNA827465	Yang2025_Silkie_Wenchang	0.953213272
ENSGALG00010012948	ST13P5	2	-0.23725355	0.074578064	-3.181277962	0.001466269	0.005809812	2.092548263	52.21137702	1	PRJNA827465;Yang2025_Silkie_Wenchang	CRA004775	0.047431078
ENSGALG00010012948	ST13P5	2	-0.217438349	0.128942599	-1.686318959	0.091734381	0.02016885	2.204017695	54.62831346	1	CRA004775;Yang2025_Silkie_Wenchang	PRJNA827465	0.518914432
ENSGALG00010012948	ST13P5	2	-0.141347923	0.06798487	-2.079108513	0.037607378	0	0.442942109	0	1	CRA004775;PRJNA827465	Yang2025_Silkie_Wenchang	0.334407867
ENSGALG00010012949	ENSGALG00010012949	2	-0.376808927	0.396184406	-0.951094797	0.341556256	0.288076445	11.96998666	91.64577181	0.5	PRJNA827465;Yang2025_Silkie_Wenchang	CRA004775	0.805773441
ENSGALG00010012949	ENSGALG00010012949	2	-0.689302293	0.173390036	-3.975443497	7.02482E-05	0.002080039	1.026140718	2.547478833	1	CRA004775;Yang2025_Silkie_Wenchang	PRJNA827465	0.006081423
ENSGALG00010012949	ENSGALG00010012949	2	-0.051999537	0.137789376	-0.377384227	0.705888087	0.007395129	1.108463002	9.784990766	0.5	CRA004775;PRJNA827465	Yang2025_Silkie_Wenchang	0.924537439
ENSGALG00010012952	BRD10	2	0.394102506	0.347199832	1.135088411	0.256338277	0.219035686	10.31830884	90.30848935	1	PRJNA827465;Yang2025_Silkie_Wenchang	CRA004775	0.764677554
ENSGALG00010012952	BRD10	2	0.686167239	0.182051383	3.769085562	0.000163847	0	0.758903785	0	1	CRA004775;Yang2025_Silkie_Wenchang	PRJNA827465	0.011160602
ENSGALG00010012952	BRD10	2	0.082101617	0.06807027	1.206130318	0.227767247	0	0.607810424	0	1	CRA004775;PRJNA827465	Yang2025_Silkie_Wenchang	0.740853612
ENSGALG00010012954	RIMS2	2	0.085676044	0.375882795	0.227932869	0.819698429	0.115350725	1.647450949	39.30016546	0.5	PRJNA827465;Yang2025_Silkie_Wenchang	CRA004775	0.955256039

ENSGALG00010012954	RIMS2	2	-0.10726481	0.747824572	-0.143435792	0.885946017	0.746891855	2.896957258	65.48102333	0.5	CRA004775;Yang2025_Silkie_Wenchang	PRJNA827465	0.97244495
ENSGALG00010012954	RIMS2	2	-0.357595682	0.315603621	-1.133053165	0.257191928	0	0.821538641	0	1	CRA004775;PRJNA827465	Yang2025_Silkie_Wenchang	0.764870705
ENSGALG00010012955	RPL39L	2	-0.475660671	0.237369941	-2.003879136	0.045083009	0.087718003	3.901096923	74.36618418	1	PRJNA827465;Yang2025_Silkie_Wenchang	CRA004775	0.3659545
ENSGALG00010012955	RPL39L	2	-0.534488598	0.262849479	-2.033439823	0.042008107	0.069314358	1.982319636	49.5540486	1	CRA004775;Yang2025_Silkie_Wenchang	PRJNA827465	0.354529664
ENSGALG00010012955	RPL39L	2	-0.281601227	0.070751565	-3.98014133	6.88743E-05	0	0.020456551	0	1	CRA004775;PRJNA827465	Yang2025_Silkie_Wenchang	0.006015482
ENSGALG00010012956	FABP3	2	0.487146236	0.649659805	0.749848202	0.453346134	0.80114282	19.14024278	94.77540587	0.5	PRJNA827465;Yang2025_Silkie_Wenchang	CRA004775	0.848869458
ENSGALG00010012956	FABP3	2	0.56894662	0.615643305	0.924149773	0.355408342	0.65444092	7.251558033	86.20986007	0.5	CRA004775;Yang2025_Silkie_Wenchang	PRJNA827465	0.812301302
ENSGALG00010012956	FABP3	2	-0.132768881	0.106655503	-1.244838547	0.213191104	0	0.031748891	0	1	CRA004775;PRJNA827465	Yang2025_Silkie_Wenchang	0.72579791
ENSGALG00010012957	DERA	2	-0.030642886	0.101875984	-0.300786155	0.763577566	0	0.508898177	0	0.5	PRJNA827465;Yang2025_Silkie_Wenchang	CRA004775	0.940338057
ENSGALG00010012957	DERA	2	-0.185317758	0.176742526	-1.048518217	0.294399914	0	0.052979295	0	1	CRA004775;Yang2025_Silkie_Wenchang	PRJNA827465	0.787343482
ENSGALG00010012957	DERA	2	-0.01813473	0.110324035	-0.164376963	0.869434398	0	0.541134721	0	0.5	CRA004775;PRJNA827465	Yang2025_Silkie_Wenchang	0.968577673
ENSGALG00010012958	OLFML2B	2	-0.078205983	0.093931006	-0.832589653	0.405076197	0	0.014675076	0	1	PRJNA827465;Yang2025_Silkie_Wenchang	CRA004775	0.833112102
ENSGALG00010012958	OLFML2B	2	-0.051821921	0.123015002	-0.421265054	0.673561545	0	0.165895182	0	0.5	CRA004775;Yang2025_Silkie_Wenchang	PRJNA827465	0.915386909
ENSGALG00010012958	OLFML2B	2	-0.070479684	0.130122904	-0.541639342	0.588066984	0	0.209279779	0	0.5	CRA004775;PRJNA827465	Yang2025_Silkie_Wenchang	0.891761457
ENSGALG00010012959	TBC1D32	2	-0.150017492	0.080839904	-1.855735662	0.063491245	0	0.1247966	0	1	PRJNA827465;Yang2025_Silkie_Wenchang	CRA004775	0.433896992
ENSGALG00010012959	TBC1D32	2	-0.062739033	0.246955939	-0.2540495	0.799457317	0.054357415	1.462273646	31.61334728	0.5	CRA004775;Yang2025_Silkie_Wenchang	PRJNA827465	0.949922217
ENSGALG00010012959	TBC1D32	2	-0.065329628	0.175224993	-0.372832819	0.709272875	0.02641884	1.241111696	19.4270747	0.5	CRA004775;PRJNA827465	Yang2025_Silkie_Wenchang	0.925298042
ENSGALG00010012962	SLC25A17	2	-0.213190695	0.141850132	-1.502929121	0.132857323	0.031146658	4.073417888	75.45059143	1	PRJNA827465;Yang2025_Silkie_Wenchang	CRA004775	0.610795573
ENSGALG00010012962	SLC25A17	2	-0.310446324	0.129010957	-2.406356269	0.016112543	0.004751523	1.109947531	9.90565121	1	CRA004775;Yang2025_Silkie_Wenchang	PRJNA827465	0.209692475
ENSGALG00010012962	SLC25A17	2	-0.091207942	0.058419592	-1.561256058	0.118463347	0	0.006840949	0	1	CRA004775;PRJNA827465	Yang2025_Silkie_Wenchang	0.578338927
ENSGALG00010012963	UIMC1	2	0.126463383	0.175982534	0.718613265	0.472379238	0.052543098	6.267149252	84.04378195	0.5	PRJNA827465;Yang2025_Silkie_Wenchang	CRA004775	0.853998373
ENSGALG00010012963	UIMC1	2	-0.011282699	0.342834975	-0.032909999	0.973746359	0.211478425	9.864795539	89.86294246	0.5	CRA004775;Yang2025_Silkie_Wenchang	PRJNA827465	0.992472082
ENSGALG00010012963	UIMC1	2	-0.157796059	0.160894272	-0.980743795	0.326719101	0.037421028	3.097663351	67.71760238	1	CRA004775;PRJNA827465	Yang2025_Silkie_Wenchang	0.800028239
ENSGALG00010012964	CBR3	2	-0.239531392	0.19154815	-1.250502243	0.211116136	0.059122477	4.791993724	79.13185915	1	PRJNA827465;Yang2025_Silkie_Wenchang	CRA004775	0.723219516
ENSGALG00010012964	CBR3	2	-0.38025281	0.132008874	-2.880684031	0.003968132	0	0.754068124	0	1	CRA004775;Yang2025_Silkie_Wenchang	PRJNA827465	0.090832557
ENSGALG00010012964	CBR3	2	-0.085143897	0.071352449	-1.19328627	0.232757278	0	0.327455426	0	1	CRA004775;PRJNA827465	Yang2025_Silkie_Wenchang	0.745799878
ENSGALG00010012965	ENSGALG00010012965	2	0.067934285	0.100905005	0.673243896	0.500792139	0.012583616	2.444247207	59.08760795	0.5	PRJNA827465;Yang2025_Silkie_Wenchang	CRA004775	0.862421093
ENSGALG00010012965	ENSGALG00010012965	2	0.219887149	0.106423206	2.0661579	0.038813573	0	0.400423089	0	1	CRA004775;Yang2025_Silkie_Wenchang	PRJNA827465	0.339979907
ENSGALG00010012965	ENSGALG00010012965	2	0.090869342	0.175884442	0.516642297	0.605405884	0.037605841	1.883110381	46.89036836	0.5	CRA004775;PRJNA827465	Yang2025_Silkie_Wenchang	0.896474603
ENSGALG00010012966	GLIPR1L	2	-0.185983732	0.175121071	-1.062029432	0.288222324	0	0.426820035	0	1	PRJNA827465;Yang2025_Silkie_Wenchang	CRA004775	0.782882265
ENSGALG00010012966	GLIPR1L	2	-0.477616629	0.272718627	-1.751316494	0.079891408	0	0.460505907	0	1	CRA004775;Yang2025_Silkie_Wenchang	PRJNA827465	0.486063779
ENSGALG00010012966	GLIPR1L	2	-0.275573776	0.285866243	-0.963995511	0.335048176	0.0649777	1.420675382	29.61094333	1	CRA004775;PRJNA827465	Yang2025_Silkie_Wenchang	0.803101658
ENSGALG00010012967	ECRG4	2	0.222055519	0.130142703	1.706246394	0.087962214	0	0.409977516	0	1	PRJNA827465;Yang2025_Silkie_Wenchang	CRA004775	0.509638136
ENSGALG00010012967	ECRG4	2	-0.040753827	0.237100011	-0.171884543	0.863528299	0.013239489	1.091428382	8.376947413	0.5	CRA004775;Yang2025_Silkie_Wenchang	PRJNA827465	0.96626958
ENSGALG00010012967	ECRG4	2	0.038649234	0.347249243	0.111301133	0.911377557	0.152534935	2.204249292	54.63308058	0.5	CRA004775;PRJNA827465	Yang2025_Silkie_Wenchang	0.978845851
ENSGALG00010012968	LSM11	2	-0.159577906	0.054090975	-2.955722402	0.003119377	0	0.061190115	0	1	PRJNA827465;Yang2025_Silkie_Wenchang	CRA004775	0.077237464
ENSGALG00010012968	LSM11	2	-0.187502862	0.118552973	-1.58159561	0.113741917	0	0.575536488	0	1	CRA004775;Yang2025_Silkie_Wenchang	PRJNA827465	0.568978529
ENSGALG00010012968	LSM11	2	-0.176313147	0.056365104	-3.128055019	0.001759672	0	0.501197769	0	1	CRA004775;PRJNA827465	Yang2025_Silkie_Wenchang	0.053241265
ENSGALG00010012969	SLC12A2	2	0.094456246	0.20938697	0.451108521	0.651911336	0.069767132	4.652745298	78.50731265	0.5	PRJNA827465;Yang2025_Silkie_Wenchang	CRA004775	0.90922893
ENSGALG00010012969	SLC12A2	2	0.414153622	0.140001613	2.958206071	0.003094352	0	0.734470536	0	1	CRA004775;Yang2025_Silkie_Wenchang	PRJNA827465	0.076827108
ENSGALG00010012969	SLC12A2	2	0.209052422	0.336134148	0.621931522	0.533986897	0.194784738	6.739322941	85.16171419	0.5	CRA004775;PRJNA827465	Yang2025_Silkie_Wenchang	0.873408008
ENSGALG00010012970	UNC5A	2	0.096621673	0.270363121	0.357377414	0.720809283	0.127743397	7.779541204	87.14577153	0.5	PRJNA827465;Yang2025_Silkie_Wenchang	CRA004775	0.928918486
ENSGALG00010012970	UNC5A	2	0.358436144	0.151483032	2.366180147	0.017972697	0	0.150773171	0	1	CRA004775;Yang2025_Silkie_Wenchang	PRJNA827465	0.223750681
ENSGALG00010012970	UNC5A	2	-0.137734374	0.10278415	-1.340035145	0.180233919	0	0.730413223	0	0.5	CRA004775;PRJNA827465	Yang2025_Silkie_Wenchang	0.687301167
ENSGALG00010012971	ENSGALG00010012971	2	0.490395267	0.297976892	1.645749319	0.099815381	0.058292887	1.411977534	29.17734339	1	PRJNA827465;Yang2025_Silkie_Wenchang	CRA004775	0.53969513
ENSGALG00010012971	ENSGALG00010012971	2	0.197463649	0.820229611	0.240741917	0.809755149	0.974991578	3.459490643	71.09401056	0.5	CRA004775;Yang2025_Silkie_Wenchang	PRJNA827465	0.952818762
ENSGALG00010012971	ENSGALG00010012971	2	0.01664013	0.447420087	0.037191289	0.970332484	0.194504761	1.589747051	37.09691116	0.5	CRA004775;PRJNA827465	Yang2025_Silkie_Wenchang	0.991681174
ENSGALG00010012973	GLDC	2	0.446198102	0.17081328	2.612197969	0.008996214	0	0.870808903	0	1	PRJNA827465;Yang2025_Silkie_Wenchang	CRA004775	0.148687221
ENSGALG00010012973	GLDC	2	0.912567152	0.332458275	2.744907314	0.006052799	0.109561684	1.902844577	47.4471004	1	CRA004775;Yang2025_Silkie_Wenchang	PRJNA827465	0.117595331
ENSGALG00010012973	GLDC	2	0.759779439	0.499140039	1.522176906	0.12796476	0.396800923	4.650752765	78.49810449	1	CRA004775;PRJNA827465	Yang2025_Silkie_Wenchang	0.60032911
ENSGALG00010012974	SLC49A3	2	0.353717398	0.257229296	1.375105419	0.169098765	0.101065645	4.143237697	75.86428602	1	PRJNA827465;Yang2025_Silkie_Wenchang	CRA004775	0.67030648
ENSGALG00010012974	SLC49A3	2	0.020519398	0.187647052	0.109351029	0.912924074	0	0.374787446	0	0.5	CRA004775;Yang2025_Silkie_Wenchang	PRJNA827465	0.979071811
ENSGALG00010012974	SLC49A3	2	0.289745382	0.393078188	0.737118952	0.461050015	0.22383079	3.077933138	67.51066527	0.5	CRA004775;PRJNA827465	Yang2025_Silkie_Wenchang	0.85014477
ENSGALG00010012975	KRR1	2	-0.059701673	0.062712092	-0.951996198	0.341090891	0	0.041695244	0	1	PRJNA827465;Yang2025_Silkie_Wenchang	CRA004775	0.805646109
ENSGALG00010012975	KRR1	2	-0.050188023	0.093960842	-0.53413764	0.59324631	0	0.026476529	0	1	CRA004775;Yang2025_Silkie_Wenchang	PRJNA827465	0.892783052
ENSGALG00010012975	KRR1	2	-0.070527456	0.072940216	-0.966921399	0.333583328	0	0.002451836	0	1	CRA004775;PRJNA827465	Yang2025_Silkie_Wenchang	0.802691373
ENSGALG00010012976	SLC9A2	2	0.029821302	0.194256829	0.153514818	0.877997306	0.028445673	1.591559676	37.16855138	0.5	PRJNA827465;Yang2025_Silkie_Wenchang	CRA004775	0.970993355
ENSGALG00010012976	SLC9A2	2	0.638276576	0.40932511	1.559338922	0.118916184	0.262410045	4.570909121	78.12289696	1	CRA004775;Yang2025_Silkie_Wenchang	PRJNA827465	0.579224536
ENSGALG00010012976	SLC9A2	2	0.441682598	0.60506627	0.729973922	0.465406125	0.668613852	11.36989317	91.2048426	0.5	CRA004775;PRJNA827465	Yang2025_Silkie_Wenchang	0.852411124
ENSGALG00010012977	NKRF	2	0.058734932	0.047853872	1.22738097	0.219679435	0	0.01445632	0	1	PRJNA827465;Yang2025_Silkie_Wenchang	CRA004775	0.733094397
ENSGALG00010012977	NKRF	2	0.091011397	0.07403886	1.22923823	0.218992409	0.000896568	1.032486856	3.146466743	1	CRA004775;Yang2025_Silkie_Wenchang	PRJNA827465	0.73295614
ENSGALG00010012977	NKRF	2	0.087183693	0.085239451	1.02280918	0.306398077	0.004325725	1.160178731	13.80638403	1	CRA004775;PRJNA827465	Yang2025_Silkie_Wenchang	0.792485409
ENSGALG00010012978	HSD17B7	2	-0.065196842	0.413603835	-0.157631135	0.874747468	0.289779116	6.52806553	84.68152632	0.5	PRJNA827465;Yang2025_Silkie_Wenchang	CRA004775	0.970159044
ENSGALG00010012978	HSD17B7	2	-0.236102331	0.240152225	-0.983136138	0.325540441	0.062295121	2.171394798	53.94665212	1	CRA004775;Yang2025_Silkie_Wenchang	PRJNA827465	0.799987949
ENSGALG00010012978	HSD17B7	2	0.171694789	0.173340992	0.990503093	0.321928276	0.011547266	1.23781063	19.2121981	0.5	CRA004775;PRJNA827465	Yang2025_Silkie_Wenchang	0.798513866
ENSGALG00010012979	HTR1A	2	-0.633039967	0.187189853	-3.381807059	0.000720107	0	0.002025239	0	1	PRJNA827465;Yang2025_Silkie_Wenchang	CRA004775	0.030076904
ENSGALG00010012979	HTR1A	2	-0.532697856	0.253616553	-2.100406498	0.035693098	0	0.888158594	0	1	CRA004775;Yang2025_Silkie_Wenchang	PRJNA827465	0.325864304
ENSGALG00010012979	HTR1A	2	-0.53524797	0.233136023	-2.295861287	0.021683814	0	0.868541745	0	1	CRA004775;PRJNA827465	Yang2025_Silkie_Wenchang	0.249411453
ENSGALG00010012980	KICS2	2	-0.089441604	0.12688314	-0.704913233	0.480864235	0.026277216	5.178347592	80.68882047	0.5	PRJNA827465;Yang2025_Silkie_Wenchang	CRA004775	0.856722567
ENSGALG00010012980	KICS2	2	-0.20526979	0.086330031	-2.377733301	0.01741942	0	0.279564692	0	1	CRA004775;Yang2025_Silkie_Wenchang	PRJNA827465	0.219833497
ENSGALG00010012980	KICS2	2	0.012494474	0.050964912	0.24515836	0.806331817	0	0.632031264	0	0.5	CRA004775;PRJNA827465	Yang2025_Silkie_Wenchang	0.952177928
ENSGALG00010012982	DDR2	2	0.130333767	0.11031627	1.181455534	0.237421808	0.014623373	2.459219038	59.33668435	1	PRJNA827465;Yang2025_Silkie_Wenchang	CRA004775	0.739586344
ENSGALG00010012982	DDR2	2	-0.132571006	0.439415222	-0.301698712	0.76288175	0.329523807	6.222164486	83.9284223	0.5	CRA004775;Yang2025_Silkie_Wenchang	PRJNA827465	0.940082621
ENSGALG00010012982	DDR2	2	-0.220457475	0.322303671	-0.684005472	0.493971711	0.160177206	3.6538022	72.63124972	0.5	CRA004775;PRJNA827465	Yang2025_Silkie_Wenchang	0.860175061
ENSGALG00010012985	ARNTL2	2	0.066115013	0.075026682	0.881220004	0.378198754	0	0.063757929	0	1	PRJNA827465;Yang2025_Silkie_Wenchang	CRA004775	0.823285408
ENSGALG00010012985	ARNTL2	2	0.200568379	0.16437992	1.220153134	0.222407512	0.027445071	1.799445353	44.42732044	1	CRA004775;Yang2025_Silkie_Wenchang	PRJNA827465	0.735999809
ENSGALG00010012985	ARNTL2	2	0.184769061	0.185649584	0.995257071	0.319611251	0.042658048	2.283979771	56.2167751	1	CRA004775;PRJNA827465	Yang2025_Silkie_Wenchang	0.798328994
ENSGALG00010012986	TMEM184B	2	-0.194393849	0.113048251	-1.719565294	0.085511489	0.019057531	3.701181037	72.98159723	1	PRJNA827465;Yang2025_Silkie_Wenchang	CRA004775	0.502746158
ENSGALG00010012986	TMEM184B	2	-0.282861957	0.091329758	-3.097149982	0.00195391	0	0.715894365	0	1	CRA004775;Yang2025_Silkie_Wenchang	PRJNA827465	0.057181326
ENSGALG00010012986	TMEM184B	2	-0.100516617	0.054639236	-1.839661693	0.065820859	0	0.05237514	0	1	CRA004775;PRJNA827465	Yang2025_Silkie_Wenchang	0.643023131
ENSGALG00010012988	RPL18A	2	-0.08724416	0.060885777	-1.432915267	0.151882054	0	0.794380334	0	1	PRJNA827465;Yang2025_Silkie_Wenchang	CRA004775	0.643294424
ENSGALG00010012988	RPL18A	2	-0.329304433	0.175710626	-1.874129306	0.060912605	0	0.039541463	0	1	CRA004775;Yang2025_Silkie_Wenchang	PRJNA827465	0.427127227
ENSGALG00010012988	RPL18A	2	-0.11514843	0.102672471	-1.121532212	0.262069896	0.008348432	1.241467366	19.45015812	1	CRA004775;PRJNA827465	Yang2025_Silkie_Wenchang	0.768363052
ENSGALG00010012991	MFSD9	2	-0.012192099	0.120235042	-0.101402208	0.919231184	0.020840373	3.52578271	71.63750343	0.5	PRJNA827465;Yang2025_Silkie_Wenchang	CRA004775	0.981167324
ENSGALG00010012991	MFSD9	2	-0.140955819	0.096213802	-1.465027018	0.142913547	0	0.001305571	0	1	CRA004775;Yang2025_Silkie_Wenchang	PRJNA827465	0.629559642
ENSGALG00010012991	MFSD9	2	0.08228906	0.074433005	1.105545306	0.268923374	0	0.643787924	0	0.5	CRA004775;PRJNA827465	Yang2025_Silkie_Wenchang	0.771398294
ENSGALG00010012992	HARS1	2	-0.07663926	0.036499548	-2.099731783	0.035752442	0	0.09393946	0	1	PRJNA827465;Yang2025_Silkie_Wenchang	CRA004775	0.326178791
ENSGALG00010012992	HARS1	2	-0.126098835	0.112087139	-1.125007168	0.260585997	0.006225708	1.292739375	22.64488734	1	CRA004775;Yang2025_Silkie_Wenchang	PRJNA827465	0.767694434
ENSGALG00010012992	HARS1	2	-0.108501498	0.068977935	-1.572988491	0.115721483	0.004066644	1.263951587	20.88304564	1	CRA004775;PRJNA827465	Yang2025_Silkie_Wenchang	0.572716099
ENSGALG00010012993	DOP1B	2	-0.030000043	0.070023583	-0.428427707	0.668339756	0	0.195585579	0	1	PRJNA827465;Yang2025_Silkie_Wenchang	CRA004775	0.914233518
ENSGALG00010012993	DOP1B	2	-0.058584105	0.118351035	-0.495005555	0.620598317	0	0.175547961	0	0.5	CRA004775;Yang2025_Silkie_Wenchang	PRJNA827465	0.893095593
ENSGALG00010012993	DOP1B	2	-0.00431855	0.082114847	-0.052591587	0.958057321	0	0.062306243	0	0.5	CRA004775;PRJNA827465	Yang2025_Silkie_Wenchang	0.988864712
ENSGALG00010012995	SERINC2	2	0.071134361	0.194978024	0.36483271	0.715236286	0.060626135	4.813414558	79.22472731	0.5	PRJNA827465;Yang2025_Silkie_Wenchang	CRA004775	0.926953446
ENSGALG00010012995	SERINC2	2	0.255214174	0.14123422	1.807027891	0.070757931	0	0.355196188	0	0.5	CRA004775;Yang2025_Silkie_Wenchang	PRJNA827465	0.458683646
ENSGALG00010012995	SERINC2	2	-0.104216094	0.096611588	-1.07871216	0.280716063	0	0.048581502	0	1	CRA004775;PRJNA827465	Yang2025_Silkie_Wenchang	0.778536485
ENSGALG00010012997	UBE2A	2	-0.254711754	0.24177172	-1.053521702	0.292101953	0.109698078	15.42341684	93.51635237	1	PRJNA827465;Yang2025_Silkie_Wenchang	CRA004775	0.785516679
ENSGALG00010012997	UBE2A	2	-0.322560431	0.20199733	-1.596854925	0.110298049	0.061876865	4.074604893	75.45774311	1	CRA004775;Yang2025_Silkie_Wenchang	PRJNA827465	0.562335482
ENSGALG00010012997	UBE2A	2	-0.028954417	0.0276125	-1.048598154	0.294363106	0	0.236845018	0	1	CRA004775;PRJNA827465	Yang2025_Silkie_Wenchang	0.787343482
ENSGALG00010012998	MAFF	2	0.037004454	0.084502236	0.437910946	0.66145084	0	0.001170927	0	1	PRJNA827465;Yang2025_Silkie_Wenchang	CRA004775	0.912364181
ENSGALG00010012998	MAFF	2	0.077579544	0.212033866	0.365882797	0.714452534	0	0.109126687	0	1	CRA004775;Yang2025_Silkie_Wenchang	PRJNA827465	0.926596201
ENSGALG00010012998	MAFF	2	0.042009869	0.089116245	0.471405283	0.637351344	0	0.141724258	0	1	CRA004775;PRJNA827465	Yang2025_Silkie_Wenchang	0.905150023
ENSGALG00010013001	ADGRV1	2	0.396053932	0.247828209	1.59809867	0.11002102	0	7.50871E-05	0	1	PRJNA827465;Yang2025_Silkie_Wenchang	CRA004775	0.561870599
ENSGALG00010013001	ADGRV1	2	0.244232539	0.376043538	0.64947942	0.516028545	0	0.50390938	0	0.5	CRA004775;Yang2025_Silkie_Wenchang	PRJNA827465	0.867498525
ENSGALG00010013001	ADGRV1	2	0.309436805	0.279800537	1.105919268	0.268761464	0	0.571051885	0	0.5	CRA004775;PRJNA827465	Yang2025_Silkie_Wenchang	0.771398294
ENSGALG00010013004	UAP1	2	0.117639679	0.079650452	1.476949286	0.139689232	0	0.087871152	0	1	PRJNA827465;Yang2025_Silkie_Wenchang	CRA004775	0.623568857
ENSGALG00010013004	UAP1	2	-0.167800788	0.346846002	-0.483790464	0.628534584	0.203243625	6.130488824	83.68808706	0.5	CRA004775;Yang2025_Silkie_Wenchang	PRJNA827465	0.901942802
ENSGALG00010013004	UAP1	2	-0.18501813	0.321731767	-0.575069511	0.565244287	0.173593842	5.727929209	82.54168368	0.5	CRA004775;PRJNA827465	Yang2025_Silkie_Wenchang	0.883427937
ENSGALG00010013005	SLC40A1	2	-0.320980821	0.329404582	-0.97442731	0.329844415	0.203622433	16.19427678	93.82497895	0.5	PRJNA827465;Yang2025_Silkie_Wenchang	CRA004775	0.801177491
ENSGALG00010013005	SLC40A1	2	-0.452300205	0.290743204	-1.555669053	0.119786809	0.110930356	2.292467628	56.3788824	1	CRA004775;Yang2025_Silkie_Wenchang	PRJNA827465	0.580639051
ENSGALG00010013005	SLC40A1	2	0.007871608	0.116357754	0.067650053	0.94606421	0	0.005692871	0	0.5	CRA004775;PRJNA827465	Yang2025_Silkie_Wenchang	0.986343086
ENSGALG00010013008	STEEP1	2	0.017424979	0.065490549	0.266068597	0.790186379	0	0.111554994	0	1	PRJNA827465;Yang2025_Silkie_Wenchang	CRA004775	0.947390191
ENSGALG00010013008	STEEP1	2	-0.012366332	0.125351704	-0.098653081	0.921413723	0	0.807726561	0	0.5	CRA004775;Yang2025_Silkie_Wenchang	PRJNA827465	0.981548382
ENSGALG00010013008	STEEP1	2	-0.010795889	0.069224883	-0.155953884	0.876069371	0	0.628621264	0	0.5	CRA004775;PRJNA827465	Yang2025_Silkie_Wenchang	0.970347777
ENSGALG00010013012	TMEM182	2	-0.172800166	0.107202418	-1.611905493	0.106982507	0.013790053	2.4721428	59.54926228	1	PRJNA827465;Yang2025_Silkie_Wenchang	CRA004775	0.553983328
ENSGALG00010013012	TMEM182	2	-0.277778402	0.104804966	-2.650431677	0.008038898	0	0.370941897	0	0.5	CRA004775;Yang2025_Silkie_Wenchang	PRJNA827465	0.139297234
ENSGALG00010013012	TMEM182	2	-0.07142528	0.08488166	-0.841468941	0.400085278	0	0.057601735	0	0.5	CRA004775;PRJNA827465	Yang2025_Silkie_Wenchang	0.832246851
ENSGALG00010013015	PLA2G6	2	0.071720369	0.060983013	1.176071255	0.239566403	0	0.693139144	0	1	PRJNA827465;Yang2025_Silkie_Wenchang	CRA004775	0.751810791
ENSGALG00010013015	PLA2G6	2	-0.158298302	0.331316758	-0.477785377	0.63280297	0.180487806	4.961081584	79.84310511	0.5	CRA004775;Yang2025_Silkie_Wenchang	PRJNA827465	0.903112631
ENSGALG00010013015	PLA2G6	2	-0.199373421	0.27747117	-0.718537431	0.472425977	0.117088567	3.555851351	71.8773396	0.5	CRA004775;PRJNA827465	Yang2025_Silkie_Wenchang	0.853998373
ENSGALG00010013016	QRSL1	2	0.019138395	0.076128001	0.251397572	0.801506745	0	0.43312679	0	0.5	PRJNA827465;Yang2025_Silkie_Wenchang	CRA004775	0.950283916
ENSGALG00010013016	QRSL1	2	-0.095133622	0.254797411	-0.373369656	0.70887334	0.082073737	2.556173066	60.87901819	0.5	CRA004775;Yang2025_Silkie_Wenchang	PRJNA827465	0.925298042
ENSGALG00010013016	QRSL1	2	-0.116267964	0.177384904	-0.655455798	0.512174337	0.035982596	1.836620952	45.55218381	1	CRA004775;PRJNA827465	Yang2025_Silkie_Wenchang	0.865694231
ENSGALG00010013017	SCMH1	2	-0.103988712	0.058051202	-1.79132746	0.073240762	0	0.065784244	0	1	PRJNA827465;Yang2025_Silkie_Wenchang	CRA004775	0.467793099
ENSGALG00010013017	SCMH1	2	-0.130285432	0.09590654	-1.35846244	0.174316993	0	0.007737661	0	1	CRA004775;Yang2025_Silkie_Wenchang	PRJNA827465	0.678536276
ENSGALG00010013017	SCMH1	2	-0.098612708	0.066712908	-1.478165326	0.139363532	0	0.050715402	0	1	CRA004775;PRJNA827465	Yang2025_Silkie_Wenchang	0.623009874
ENSGALG00010013018	ID1	2	0.165160466	0.284278863	0.580980462	0.561253621	0.127728215	4.148780349	75.89653064	0.5	PRJNA827465;Yang2025_Silkie_Wenchang	CRA004775	0.881506202
ENSGALG00010013018	ID1	2	0.275944248	0.300746233	0.917531851	0.35886401	0.072573533	1.611009354	37.92711399	0.5	CRA004775;Yang2025_Silkie_Wenchang	PRJNA827465	0.814241185
ENSGALG00010013018	ID1	2	-0.068793364	0.08235843	-0.835292318	0.403553142	0	0.008675151	0	1	CRA004775;PRJNA827465	Yang2025_Silkie_Wenchang	0.83306585
ENSGALG00010013021	SLC25A5	2	-0.110983815	0.222895981	-0.497917525	0.618542175	0.080757077	4.93290251	79.72795939	0.5	PRJNA827465;Yang2025_Silkie_Wenchang	CRA004775	0.899168321
ENSGALG00010013021	SLC25A5	2	-0.147384695	0.216455876	-0.680899488	0.495935092	0.060609997	2.830194622	64.66674085	0.5	CRA004775;Yang2025_Silkie_Wenchang	PRJNA827465	0.860911322
ENSGALG00010013021	SLC25A5	2	0.080095132	0.073768132	1.085768751	0.277581315	0	0.007662709	0	1	CRA004775;PRJNA827465	Yang2025_Silkie_Wenchang	0.776763074
ENSGALG00010013027	UHMK1	2	-0.123735154	0.15943884	-0.776066571	0.43770968	0.041889588	5.155878762	80.60466419	0.5	PRJNA827465;Yang2025_Silkie_Wenchang	CRA004775	0.843889118
ENSGALG00010013027	UHMK1	2	-0.30614407	0.114531079	-2.673021798	0.007517137	0	0.001390531	0	1	CRA004775;Yang2025_Silkie_Wenchang	PRJNA827465	0.1337465
ENSGALG00010013027	UHMK1	2	-0.065925386	0.136228617	-0.483931992	0.628434135	0.022476929	1.851334257	45.98490271	0.5	CRA004775;PRJNA827465	Yang2025_Silkie_Wenchang	0.901912657
ENSGALG00010013029	MYLK2	2	-0.087584341	0.4398396	-0.199127911	0.842162689	0.363862327	16.77209759	94.03771654	0.5	PRJNA827465;Yang2025_Silkie_Wenchang	CRA004775	0.960064308
ENSGALG00010013029	MYLK2	2	0.337895265	0.141256126	2.392075114	0.016753413	0	0.183627023	0	1	CRA004775;Yang2025_Silkie_Wenchang	PRJNA827465	0.214691687
ENSGALG00010013029	MYLK2	2	-0.505926499	0.155992098	-3.243282875	0.001181608	0	0.527027954	0	0.5	CRA004775;PRJNA827465	Yang2025_Silkie_Wenchang	0.041723799
ENSGALG00010013032	BAIAP2L2	2	0.545038953	0.312299494	1.745244433	0.08094233	0	0.034479963	0	1	PRJNA827465;Yang2025_Silkie_Wenchang	CRA004775	0.489847627
ENSGALG00010013032	BAIAP2L2	2	0.229824129	0.426051821	0.539427642	0.589591812	0.126965338	1.523346591	34.3550571	0.5	CRA004775;Yang2025_Silkie_Wenchang	PRJNA827465	0.892320726
ENSGALG00010013032	BAIAP2L2	2	0.178604787	0.366341246	0.487536659	0.62587807	0.032387309	1.13263062	11.70996241	0.5	CRA004775;PRJNA827465	Yang2025_Silkie_Wenchang	0.901504244
ENSGALG00010013033	MAP3K1	2	0.085832272	0.081242003	1.056501184	0.2907393	0	0.57271988	0	1	PRJNA827465;Yang2025_Silkie_Wenchang	CRA004775	0.784646441
ENSGALG00010013033	MAP3K1	2	-0.061901069	0.369702152	-0.167434972	0.86702781	0.173447183	2.157835718	53.65726911	0.5	CRA004775;Yang2025_Silkie_Wenchang	PRJNA827465	0.967699
ENSGALG00010013033	MAP3K1	2	-0.087543846	0.267724351	-0.326992469	0.743673581	0.080383152	1.561955777	35.97770085	0.5	CRA004775;PRJNA827465	Yang2025_Silkie_Wenchang	0.935821071
ENSGALG00010013037	MORC4	2	0.061920332	0.039623463	1.562718857	0.118118737	0	0.349378186	0	1	PRJNA827465;Yang2025_Silkie_Wenchang	CRA004775	0.578003524
ENSGALG00010013037	MORC4	2	0.17882058	0.10107454	1.769195092	0.076861323	0	0.715111408	0	1	CRA004775;Yang2025_Silkie_Wenchang	PRJNA827465	0.477955453
ENSGALG00010013037	MORC4	2	0.118643495	0.114838967	1.033129247	0.301543418	0.015653709	1.775608344	43.68127391	1	CRA004775;PRJNA827465	Yang2025_Silkie_Wenchang	0.791273005
ENSGALG00010013040	SLC25A43	2	-0.156424505	0.144311069	-1.083939754	0.278391512	0.032131776	4.369273382	77.11289927	1	PRJNA827465;Yang2025_Silkie_Wenchang	CRA004775	0.776763074
ENSGALG00010013040	SLC25A43	2	-0.296316868	0.098933001	-2.99512667	0.002743309	0	0.091618715	0	1	CRA004775;Yang2025_Silkie_Wenchang	PRJNA827465	0.071498853
ENSGALG00010013040	SLC25A43	2	-0.022724497	0.090952066	-0.249851355	0.802702304	0	0.141747488	0	1	CRA004775;PRJNA827465	Yang2025_Silkie_Wenchang	0.950704475
ENSGALG00010013041	LRP12	2	-0.277973695	0.260606036	-1.066643349	0.286132918	0.131081194	28.32051961	96.46899134	1	PRJNA827465;Yang2025_Silkie_Wenchang	CRA004775	0.78194146
ENSGALG00010013041	LRP12	2	-0.068172349	0.48896949	-0.139420456	0.889117909	0.457701242	22.93907037	95.64062543	0.5	CRA004775;Yang2025_Silkie_Wenchang	PRJNA827465	0.973135755
ENSGALG00010013041	LRP12	2	0.171137254	0.22579263	0.75793995	0.448486933	0.086129422	5.704914453	82.47125337	0.5	CRA004775;PRJNA827465	Yang2025_Silkie_Wenchang	0.846878812
ENSGALG00010013042	SLC25A48	2	0.068928313	0.139551504	0.49392741	0.621357446	0	0.905016165	0	0.5	PRJNA827465;Yang2025_Silkie_Wenchang	CRA004775	0.899693435
ENSGALG00010013042	SLC25A48	2	-0.105480734	0.197163506	-0.534991168	0.592655964	0	0.175486493	0	1	CRA004775;Yang2025_Silkie_Wenchang	PRJNA827465	0.892776493
ENSGALG00010013042	SLC25A48	2	0.143416056	0.180093704	0.796341307	0.425833683	0	0.696897631	0	0.5	CRA004775;PRJNA827465	Yang2025_Silkie_Wenchang	0.84125685
ENSGALG00010013044	ENSGALG00010013044	2	0.178869659	0.231312819	0.773280354	0.43935648	0.098063904	11.89116169	91.59039271	0.5	PRJNA827465;Yang2025_Silkie_Wenchang	CRA004775	0.844199375
ENSGALG00010013044	ENSGALG00010013044	2	0.327226694	0.143515787	2.280074547	0.022603268	0.01693375	1.487362857	32.76691057	1	CRA004775;Yang2025_Silkie_Wenchang	PRJNA827465	0.255721423
ENSGALG00010013044	ENSGALG00010013044	2	-0.032871782	0.076670998	-0.428738152	0.668113793	0	0.309027006	0	0.5	CRA004775;PRJNA827465	Yang2025_Silkie_Wenchang	0.914120722
ENSGALG00010013045	FSD1L	2	0.239595187	0.245202236	0.977132962	0.328503331	0.099274705	5.670056847	82.36349252	1	PRJNA827465;Yang2025_Silkie_Wenchang	CRA004775	0.800761292
ENSGALG00010013045	FSD1L	2	0.478257114	0.152793021	3.130097903	0.001747481	0	0.093388159	0	1	CRA004775;Yang2025_Silkie_Wenchang	PRJNA827465	0.053035697
ENSGALG00010013045	FSD1L	2	0.035899305	0.118795876	0.302193188	0.762504797	0	0.69031098	0	1	CRA004775;PRJNA827465	Yang2025_Silkie_Wenchang	0.940082621
ENSGALG00010013046	SLC12A5	2	0.197587008	0.207393098	0.952717376	0.340733287	0	0.420892688	0	1	PRJNA827465;Yang2025_Silkie_Wenchang	CRA004775	0.805572689
ENSGALG00010013046	SLC12A5	2	-0.021795617	0.514454765	-0.042366441	0.966206581	0.304605168	2.141642911	53.30687506	0.5	CRA004775;Yang2025_Silkie_Wenchang	PRJNA827465	0.990863074
ENSGALG00010013046	SLC12A5	2	-0.083069977	0.328198914	-0.253108629	0.800184271	0.068127914	1.277036835	21.6937231	0.5	CRA004775;PRJNA827465	Yang2025_Silkie_Wenchang	0.94999675
ENSGALG00010013047	CTPS1	2	-0.062440672	0.046095088	-1.35460576	0.175543197	0	0.73100187	0	1	PRJNA827465;Yang2025_Silkie_Wenchang	CRA004775	0.680297576
ENSGALG00010013047	CTPS1	2	-0.20715546	0.120969717	-1.712457178	0.08681245	0	0.429937906	0	1	CRA004775;Yang2025_Silkie_Wenchang	PRJNA827465	0.506756231
ENSGALG00010013047	CTPS1	2	-0.092464816	0.112212901	-0.824012347	0.409932542	0.012160328	1.293279401	22.67718797	1	CRA004775;PRJNA827465	Yang2025_Silkie_Wenchang	0.834652129
ENSGALG00010013048	WDR75	2	0.128178077	0.115803643	1.106857039	0.268355743	0.012462063	1.670232191	40.12808487	1	PRJNA827465;Yang2025_Silkie_Wenchang	CRA004775	0.771398294
ENSGALG00010013048	WDR75	2	-0.005994457	0.327288259	-0.018315527	0.985387141	0.16995803	4.746473965	78.93172896	0.5	CRA004775;Yang2025_Silkie_Wenchang	PRJNA827465	0.99592834
ENSGALG00010013048	WDR75	2	-0.088150531	0.192584754	-0.45772331	0.647151239	0.048933451	2.443597392	59.0767283	0.5	CRA004775;PRJNA827465	Yang2025_Silkie_Wenchang	0.907350807
ENSGALG00010013051	XPOT	2	-0.273411706	0.30006919	-0.911162209	0.362209913	0.172660021	23.58736905	95.76044281	0.5	PRJNA827465;Yang2025_Silkie_Wenchang	CRA004775	0.815579273
ENSGALG00010013051	XPOT	2	-0.248757261	0.342238072	-0.726854438	0.467315131	0.215676823	12.55093895	92.03246862	0.5	CRA004775;Yang2025_Silkie_Wenchang	PRJNA827465	0.852885838
ENSGALG00010013051	XPOT	2	0.020855398	0.035480604	0.587797146	0.55666846	0	0.286213215	0	1	CRA004775;PRJNA827465	Yang2025_Silkie_Wenchang	0.880358001
ENSGALG00010013053	MRTFA	2	0.060606091	0.292884307	0.206928435	0.836065749	0.134643794	3.876558382	74.20392262	0.5	PRJNA827465;Yang2025_Silkie_Wenchang	CRA004775	0.958399882
ENSGALG00010013053	MRTFA	2	0.333912691	0.211813447	1.576447089	0.114922804	0	0.217743738	0	1	CRA004775;Yang2025_Silkie_Wenchang	PRJNA827465	0.571871165
ENSGALG00010013053	MRTFA	2	-0.073979346	0.172346264	-0.429248332	0.667742513	0.034529914	1.728874567	42.1589039	0.5	CRA004775;PRJNA827465	Yang2025_Silkie_Wenchang	0.914084637
ENSGALG00010013056	ENSGALG00010013056	2	-0.299974035	0.323997827	-0.925851997	0.354522901	0.172420879	5.29146203	81.10163138	1	PRJNA827465;Yang2025_Silkie_Wenchang	CRA004775	0.81193713
ENSGALG00010013056	ENSGALG00010013056	2	-0.35291707	0.334854037	-1.053943006	0.291909012	0.144000002	2.763649626	63.81596312	0.5	CRA004775;Yang2025_Silkie_Wenchang	PRJNA827465	0.785492255
ENSGALG00010013056	ENSGALG00010013056	2	-0.007535391	0.118786026	-0.063436677	0.949418782	0	0.003355768	0	0.5	CRA004775;PRJNA827465	Yang2025_Silkie_Wenchang	0.98723759
ENSGALG00010013058	ENSGALG00010013058	2	-0.354699426	0.412433391	-0.860016267	0.389780075	0	0.753078138	0	1	PRJNA827465;Yang2025_Silkie_Wenchang	CRA004775	0.829068422
ENSGALG00010013058	ENSGALG00010013058	2	-0.329736769	0.403316286	-0.817563735	0.413606342	0	0.823293591	0	0.5	CRA004775;Yang2025_Silkie_Wenchang	PRJNA827465	0.836267299
ENSGALG00010013058	ENSGALG00010013058	2	-0.008263272	0.392073119	-0.021075844	0.983185155	0	0.00056069	0	0.5	CRA004775;PRJNA827465	Yang2025_Silkie_Wenchang	0.995363468
ENSGALG00010013062	ENSGALG00010013062	2	0.298770835	0.213986958	1.396210487	0.16265112	0.057969036	2.428233937	58.81780643	1	PRJNA827465;Yang2025_Silkie_Wenchang	CRA004775	0.660469716
ENSGALG00010013062	ENSGALG00010013062	2	0.198753394	0.360759199	0.55093091	0.581681038	0.208548576	4.97030444	79.88050808	0.5	CRA004775;Yang2025_Silkie_Wenchang	PRJNA827465	0.889045686
ENSGALG00010013062	ENSGALG00010013062	2	0.039380733	0.132558905	0.297081008	0.766404664	0.012509998	1.47555632	32.22895079	0.5	CRA004775;PRJNA827465	Yang2025_Silkie_Wenchang	0.941152991
ENSGALG00010013063	PARP12	2	-0.047459162	0.201769258	-0.235215029	0.814041842	0.069063155	6.588302164	84.82158263	0.5	PRJNA827465;Yang2025_Silkie_Wenchang	CRA004775	0.953869515
ENSGALG00010013063	PARP12	2	-0.263874448	0.107320676	-2.458747533	0.013942263	0	0.244025302	0	1	CRA004775;Yang2025_Silkie_Wenchang	PRJNA827465	0.193650332

ENSGALG00010013063	PARP12	2	-0.027171529	0.279154752	-0.097335004	0.922460359	0.098507409	2.117637305	52.77756027	0.5	CRA004775;PRJNA827465	Yang2025_Silkie_Wenchang	0.981804675
ENSGALG00010013065	FKTN	2	0.009614581	0.090504598	0.106233064	0.915397438	0	0.007840983	0	1	PRJNA827465;Yang2025_Silkie_Wenchang	CRA004775	0.980163067
ENSGALG00010013065	FKTN	2	0.187562093	0.216322895	0.867046886	0.385916321	0.057250798	2.358938619	57.60805338	1	CRA004775;Yang2025_Silkie_Wenchang	PRJNA827465	0.826575976
ENSGALG00010013065	FKTN	2	0.177908772	0.224221876	0.793449667	0.427515883	0.066166205	2.632804526	62.01768912	1	CRA004775;PRJNA827465	Yang2025_Silkie_Wenchang	0.84125685
ENSGALG00010013069	ENSGALG00010013069	2	-2.512801562	1.959600307	-1.282303107	0.199736338	0	0.03838851	0	1	PRJNA827465;Yang2025_Silkie_Wenchang	CRA004775	0.711587863
ENSGALG00010013069	ENSGALG00010013069	2	-0.753380725	1.505967405	-0.500263633	0.616889458	0	0.263442818	0	1	CRA004775;Yang2025_Silkie_Wenchang	PRJNA827465	0.899111936
ENSGALG00010013069	ENSGALG00010013069	2	-1.091363156	1.449314648	-0.753020166	0.451437796	0	0.661283727	0	1	CRA004775;PRJNA827465	Yang2025_Silkie_Wenchang	0.847847413
ENSGALG00010013071	ENSGALG00010013071	2	-0.07778653	0.108604676	-0.716235552	0.473845914	0	0.25477391	0	1	PRJNA827465;Yang2025_Silkie_Wenchang	CRA004775	0.85444425
ENSGALG00010013071	ENSGALG00010013071	2	-0.155641294	0.16311023	-0.954209278	0.339977719	0	0.067123422	0	1	CRA004775;Yang2025_Silkie_Wenchang	PRJNA827465	0.805239502
ENSGALG00010013071	ENSGALG00010013071	2	-0.04667034	0.137528451	-0.33935044	0.734345749	0	0.197833313	0	1	CRA004775;PRJNA827465	Yang2025_Silkie_Wenchang	0.933501228
ENSGALG00010013076	RTN4IP1	2	0.082853432	0.111373722	0.743922626	0.456923286	0.007052659	1.330862859	24.86077786	1	PRJNA827465;Yang2025_Silkie_Wenchang	CRA004775	0.84954048
ENSGALG00010013076	RTN4IP1	2	-0.069934954	0.348375794	-0.200745733	0.840897398	0.188645123	4.326912938	76.88883473	0.5	CRA004775;Yang2025_Silkie_Wenchang	PRJNA827465	0.95980036
ENSGALG00010013076	RTN4IP1	2	-0.148657586	0.218108751	-0.681575522	0.495507398	0.060208417	2.290566948	56.34268621	0.5	CRA004775;PRJNA827465	Yang2025_Silkie_Wenchang	0.860770265
ENSGALG00010013078	COL5A2	2	-0.282015509	0.20367103	-1.384661869	0.166155884	0.06901622	5.811336744	82.79225514	1	PRJNA827465;Yang2025_Silkie_Wenchang	CRA004775	0.66529196
ENSGALG00010013078	COL5A2	2	-0.469617416	0.1367695	-3.433641384	0.000595532	0	0.675108787	0	1	CRA004775;Yang2025_Silkie_Wenchang	PRJNA827465	0.026382755
ENSGALG00010013078	COL5A2	2	-0.090884801	0.091254691	-0.995946626	0.319276079	0	0.00425232	0	1	CRA004775;PRJNA827465	Yang2025_Silkie_Wenchang	0.798079048
ENSGALG00010013079	ENSGALG00010013079	2	-0.199247914	0.251790789	-0.791323284	0.428755364	0	0.767657716	0	0.5	PRJNA827465;Yang2025_Silkie_Wenchang	CRA004775	0.841768924
ENSGALG00010013079	ENSGALG00010013079	2	-0.278562034	0.269947767	-1.031910867	0.302113875	0	0.355798382	0	1	CRA004775;Yang2025_Silkie_Wenchang	PRJNA827465	0.791603969
ENSGALG00010013079	ENSGALG00010013079	2	-0.014714128	0.278044567	-0.052920034	0.957795622	0	0.039978366	0	0.5	CRA004775;PRJNA827465	Yang2025_Silkie_Wenchang	0.988864712
ENSGALG00010013080	ARHGEF7	2	0.063673876	0.120319248	0.529207728	0.596661356	0.019124	2.580864379	61.25329141	0.5	PRJNA827465;Yang2025_Silkie_Wenchang	CRA004775	0.894070734
ENSGALG00010013080	ARHGEF7	2	0.264246883	0.127065817	2.079606371	0.037561652	0	0.40323459	0	1	CRA004775;Yang2025_Silkie_Wenchang	PRJNA827465	0.334303807
ENSGALG00010013080	ARHGEF7	2	0.115181237	0.208416803	0.552648519	0.580504112	0.060139941	2.512215065	60.19449075	0.5	CRA004775;PRJNA827465	Yang2025_Silkie_Wenchang	0.888433837
ENSGALG00010013081	NCOA5	2	0.093562241	0.112174007	0.8340813	0.404235172	0.019565944	4.140762251	75.84985712	0.5	PRJNA827465;Yang2025_Silkie_Wenchang	CRA004775	0.833112102
ENSGALG00010013081	NCOA5	2	0.010962886	0.227233186	0.048245092	0.961520914	0.087132962	6.27620856	84.06681374	0.5	CRA004775;Yang2025_Silkie_Wenchang	PRJNA827465	0.98973612
ENSGALG00010013081	NCOA5	2	-0.071266489	0.104796172	-0.680048594	0.496473493	0.013543993	2.084883578	52.03569109	1	CRA004775;PRJNA827465	Yang2025_Silkie_Wenchang	0.860939846
ENSGALG00010013082	CHAF1B	2	-0.112222017	0.063822431	-1.758347591	0.078688385	0.000412668	1.035548972	3.432862494	0.5	PRJNA827465;Yang2025_Silkie_Wenchang	CRA004775	0.482554155
ENSGALG00010013082	CHAF1B	2	0.003121756	0.116539683	0.026787062	0.978629573	0	0.007918643	0	0.5	CRA004775;Yang2025_Silkie_Wenchang	PRJNA827465	0.994183692
ENSGALG00010013082	CHAF1B	2	-0.13457841	0.066165917	-2.033953664	0.041956268	0	0.306709085	0	1	CRA004775;PRJNA827465	Yang2025_Silkie_Wenchang	0.354529664
ENSGALG00010013084	SGSM3	2	0.130754043	0.074343336	1.309470993	0.190374857	0.002060409	1.218406949	17.92561585	1	PRJNA827465;Yang2025_Silkie_Wenchang	CRA004775	0.699411386
ENSGALG00010013084	SGSM3	2	-0.012556076	0.09900812	-0.126818644	0.899083939	0	0.197178228	0	0.5	CRA004775;Yang2025_Silkie_Wenchang	PRJNA827465	0.975990605
ENSGALG00010013084	SGSM3	2	0.121697552	0.092696784	1.312856244	0.189231394	0.002750002	1.080796585	7.475651384	0.5	CRA004775;PRJNA827465	Yang2025_Silkie_Wenchang	0.698184834
ENSGALG00010013085	LYSMD3	2	0.17456312	0.104594764	1.668947024	0.095127879	0	0.001034498	0	1	PRJNA827465;Yang2025_Silkie_Wenchang	CRA004775	0.528006078
ENSGALG00010013085	LYSMD3	2	0.151447445	0.129608293	1.168501193	0.242604658	0	0.113655357	0	1	CRA004775;Yang2025_Silkie_Wenchang	PRJNA827465	0.7550024
ENSGALG00010013085	LYSMD3	2	0.14833206	0.124836714	1.188208626	0.234751226	0	0.101260034	0	1	CRA004775;PRJNA827465	Yang2025_Silkie_Wenchang	0.747791642
ENSGALG00010013086	CXCL14	2	0.31754471	0.337388915	0.941182997	0.346611089	0.142470772	2.640181403	62.12381472	1	PRJNA827465;Yang2025_Silkie_Wenchang	CRA004775	0.808232887
ENSGALG00010013086	CXCL14	2	0.026458189	0.736987141	0.035900475	0.971361717	0.870487012	4.791264523	79.12577027	0.5	CRA004775;Yang2025_Silkie_Wenchang	PRJNA827465	0.991955348
ENSGALG00010013086	CXCL14	2	-0.218115028	0.362036069	-0.602467673	0.546862875	0.114832301	1.546752606	35.34841992	0.5	CRA004775;PRJNA827465	Yang2025_Silkie_Wenchang	0.878275987
ENSGALG00010013087	GPN2	2	0.17993312	0.060228449	2.987510437	0.002812597	0	0.029216372	0	1	PRJNA827465;Yang2025_Silkie_Wenchang	CRA004775	0.07222107
ENSGALG00010013087	GPN2	2	-0.249939953	0.400188169	-0.624556077	0.532262455	0.269092374	6.247908598	83.99464422	0.5	CRA004775;Yang2025_Silkie_Wenchang	PRJNA827465	0.873104119
ENSGALG00010013087	GPN2	2	-0.205539266	0.418302072	-0.491365642	0.62316786	0.321946159	11.7575981	91.49486152	0.5	CRA004775;PRJNA827465	Yang2025_Silkie_Wenchang	0.900348922
ENSGALG00010013089	TBK1	2	0.056605486	0.046100516	1.227870981	0.219495405	0	0.075240654	0	1	PRJNA827465;Yang2025_Silkie_Wenchang	CRA004775	0.732888275
ENSGALG00010013089	TBK1	2	0.073506523	0.073710047	0.997238848	0.318648589	0	0.000186592	0	1	CRA004775;Yang2025_Silkie_Wenchang	PRJNA827465	0.797754958
ENSGALG00010013089	TBK1	2	0.04873156	0.055419007	0.879329369	0.379222716	0	0.00994047	0	1	CRA004775;PRJNA827465	Yang2025_Silkie_Wenchang	0.824128991
ENSGALG00010013093	GPATCH3	2	-0.032121978	0.075315078	-0.426501286	0.669742611	0	0.004382628	0	1	PRJNA827465;Yang2025_Silkie_Wenchang	CRA004775	0.914377769
ENSGALG00010013093	GPATCH3	2	0.017896383	0.104913382	0.170582458	0.864552092	0	0.447415423	0	0.5	CRA004775;Yang2025_Silkie_Wenchang	PRJNA827465	0.966723659
ENSGALG00010013093	GPATCH3	2	-0.00494584	0.085765494	-0.057667011	0.954013872	0	0.573189305	0	0.5	CRA004775;PRJNA827465	Yang2025_Silkie_Wenchang	0.987870355
ENSGALG00010013095	ADSL	2	-0.01744515	0.088460964	-0.197207129	0.843665296	0	0.011680678	0	0.5	PRJNA827465;Yang2025_Silkie_Wenchang	CRA004775	0.96057435
ENSGALG00010013095	ADSL	2	-0.071694059	0.199399216	-0.359550357	0.719183414	0	0.586016325	0	0.5	CRA004775;Yang2025_Silkie_Wenchang	PRJNA827465	0.928449671
ENSGALG00010013095	ADSL	2	-0.037446219	0.094193354	-0.397546299	0.690964652	0	0.606942138	0	1	CRA004775;PRJNA827465	Yang2025_Silkie_Wenchang	0.920199347
ENSGALG00010013099	SIM2	2	-0.060838979	0.095884776	-0.63450092	0.525753967	0	0.186326286	0	1	PRJNA827465;Yang2025_Silkie_Wenchang	CRA004775	0.87039964
ENSGALG00010013099	SIM2	2	0.299841293	0.561442481	0.534055229	0.593303324	0.465498314	3.238579685	69.12226663	0.5	CRA004775;Yang2025_Silkie_Wenchang	PRJNA827465	0.892783052
ENSGALG00010013099	SIM2	2	0.322738221	0.503502786	0.640985968	0.521531799	0.371031663	2.901361134	65.53341849	0.5	CRA004775;PRJNA827465	Yang2025_Silkie_Wenchang	0.869518515
ENSGALG00010013100	ZFPM2	2	0.120009437	0.12249598	0.979701023	0.327233721	0	0.203701035	0	1	PRJNA827465;Yang2025_Silkie_Wenchang	CRA004775	0.800028239
ENSGALG00010013100	ZFPM2	2	0.160601981	0.166799117	0.962846709	0.335624457	0	0.114734298	0	0.5	CRA004775;Yang2025_Silkie_Wenchang	PRJNA827465	0.803317552
ENSGALG00010013100	ZFPM2	2	0.05581856	0.169861081	0.328613006	0.742448216	0	0.035074345	0	0.5	CRA004775;PRJNA827465	Yang2025_Silkie_Wenchang	0.935510459
ENSGALG00010013103	AIRIM	2	0.42964661	0.400771716	1.072048234	0.283698368	0.292594278	11.193198	91.06600276	1	PRJNA827465;Yang2025_Silkie_Wenchang	CRA004775	0.779756886
ENSGALG00010013103	AIRIM	2	0.330126506	0.516487769	0.639175844	0.522708545	0.489536573	12.07316137	91.7171653	0.5	CRA004775;Yang2025_Silkie_Wenchang	PRJNA827465	0.869568211
ENSGALG00010013103	AIRIM	2	-0.036113948	0.12970835	-0.278424233	0.780684719	0	0.675335705	0	0.5	CRA004775;PRJNA827465	Yang2025_Silkie_Wenchang	0.943251709
ENSGALG00010013104	HLCS	2	-0.11380705	0.0679827	-1.674058995	0.094119006	0.00168857	1.156925773	13.56403118	1	PRJNA827465;Yang2025_Silkie_Wenchang	CRA004775	0.525712768
ENSGALG00010013104	HLCS	2	-0.208602121	0.120251827	-1.734710619	0.082792074	0	0.222587868	0	1	CRA004775;Yang2025_Silkie_Wenchang	PRJNA827465	0.495704284
ENSGALG00010013104	HLCS	2	-0.074959914	0.065141354	-1.150720696	0.249844571	0	8.58458E-07	0	1	CRA004775;PRJNA827465	Yang2025_Silkie_Wenchang	0.759798915
ENSGALG00010013106	SETD9	2	0.241860014	0.134205899	1.802156353	0.07152081	0	0.896165603	0	1	PRJNA827465;Yang2025_Silkie_Wenchang	CRA004775	0.46165999
ENSGALG00010013106	SETD9	2	-0.280147517	0.363993104	-0.769650617	0.441507171	0.181530925	3.108107407	67.8260797	0.5	CRA004775;Yang2025_Silkie_Wenchang	PRJNA827465	0.845053556
ENSGALG00010013106	SETD9	2	-0.135269343	0.502186994	-0.269360505	0.787652275	0.438785547	7.330062187	86.35755094	0.5	CRA004775;PRJNA827465	Yang2025_Silkie_Wenchang	0.946919116
ENSGALG00010013110	ANKRD10	2	-0.041435792	0.156758854	-0.264328238	0.791527003	0.036441415	3.636618466	72.50192701	0.5	PRJNA827465;Yang2025_Silkie_Wenchang	CRA004775	0.948076163
ENSGALG00010013110	ANKRD10	2	0.153663797	0.412015758	0.372956117	0.709181105	0.290764001	6.657423692	84.97917443	0.5	CRA004775;Yang2025_Silkie_Wenchang	PRJNA827465	0.925298042
ENSGALG00010013110	ANKRD10	2	0.274911219	0.244225876	1.125643292	0.260316536	0.086235208	2.964106744	66.26302335	1	CRA004775;PRJNA827465	Yang2025_Silkie_Wenchang	0.767635013
ENSGALG00010013111	RFESD	2	-0.187092325	0.400639301	-0.466984453	0.640511001	0.239291496	3.921401289	74.49891183	0.5	PRJNA827465;Yang2025_Silkie_Wenchang	CRA004775	0.90623926
ENSGALG00010013111	RFESD	2	-0.273414834	0.406874687	-0.671989782	0.50159145	0.184217763	2.051813591	51.2626291	0.5	CRA004775;Yang2025_Silkie_Wenchang	PRJNA827465	0.862759246
ENSGALG00010013111	RFESD	2	0.234411847	0.259921156	0.901857512	0.367132564	0	0.005860263	0	1	CRA004775;PRJNA827465	Yang2025_Silkie_Wenchang	0.819739734
ENSGALG00010013112	CD40	2	0.13953996	0.10336625	1.349956687	0.177029877	0	0.02912497	0	1	PRJNA827465;Yang2025_Silkie_Wenchang	CRA004775	0.682685058
ENSGALG00010013112	CD40	2	-0.087319672	0.156268523	-0.558779665	0.576312109	0.008683512	1.205511184	17.04901431	0.5	CRA004775;Yang2025_Silkie_Wenchang	PRJNA827465	0.883704016
ENSGALG00010013112	CD40	2	-0.00754944	0.179700164	-0.042011314	0.966489679	0.043217842	2.906627242	65.59586363	0.5	CRA004775;PRJNA827465	Yang2025_Silkie_Wenchang	0.990881287
ENSGALG00010013114	PIGP	2	-0.247126729	0.216692003	-1.140451543	0.25409823	0.067212843	3.23443136	69.08266435	1	PRJNA827465;Yang2025_Silkie_Wenchang	CRA004775	0.763894203
ENSGALG00010013114	PIGP	2	-0.515487799	0.171432619	-3.006941166	0.002638908	0	0.002366819	0	1	CRA004775;Yang2025_Silkie_Wenchang	PRJNA827465	0.069462139
ENSGALG00010013114	PIGP	2	-0.227712208	0.218442725	-1.042434389	0.29721033	0.061384275	2.408019668	58.47209998	1	CRA004775;PRJNA827465	Yang2025_Silkie_Wenchang	0.788466926
ENSGALG00010013115	ENSGALG00010013115	2	0.985013479	0.3268882	3.013303874	0.0025842	0.158610203	3.838545453	73.94846532	1	PRJNA827465;Yang2025_Silkie_Wenchang	CRA004775	0.068626229
ENSGALG00010013115	ENSGALG00010013115	2	0.607650879	0.742120333	0.818803705	0.412898417	1.006920951	11.57716208	91.36230457	0.5	CRA004775;Yang2025_Silkie_Wenchang	PRJNA827465	0.835870654
ENSGALG00010013115	ENSGALG00010013115	2	0.309764373	0.412137049	0.751605259	0.45228848	0.26190672	4.182382667	76.090184	0.5	CRA004775;PRJNA827465	Yang2025_Silkie_Wenchang	0.848321192
ENSGALG00010013116	TARS	2	-0.028364065	0.160206273	-0.116353057	0.907371945	0.021129774	1.694503475	40.98566248	0.5	PRJNA827465;Yang2025_Silkie_Wenchang	CRA004775	0.978196186
ENSGALG00010013116	TARS	2	-0.226636088	0.155061698	-1.461563927	0.14385455	0	0.139530888	0	1	CRA004775;Yang2025_Silkie_Wenchang	PRJNA827465	0.630832183
ENSGALG00010013116	TARS	2	-0.030462082	0.215544484	-0.141326195	0.887612257	0.045609738	1.827000894	45.2654893	0.5	CRA004775;PRJNA827465	Yang2025_Silkie_Wenchang	0.973038314
ENSGALG00010013117	ARSK	2	-0.00123546	0.133627113	-0.009245717	0.992623045	0	0.024584374	0	0.5	PRJNA827465;Yang2025_Silkie_Wenchang	CRA004775	0.997852036
ENSGALG00010013117	ARSK	2	-0.037865543	0.183761809	-0.206057739	0.83674581	0	0.016753129	0	0.5	CRA004775;Yang2025_Silkie_Wenchang	PRJNA827465	0.958399882
ENSGALG00010013117	ARSK	2	0.000682489	0.161197092	0.004233881	0.996621861	0	0.052451555	0	0.5	CRA004775;PRJNA827465	Yang2025_Silkie_Wenchang	0.99895145
ENSGALG00010013119	RNF114	2	-0.109374961	0.048811004	-2.240784933	0.025040009	0	0.24810484	0	1	PRJNA827465;Yang2025_Silkie_Wenchang	CRA004775	0.269629449
ENSGALG00010013119	RNF114	2	-0.087069742	0.095191475	-0.914680031	0.360359631	0	0.208715325	0	1	CRA004775;Yang2025_Silkie_Wenchang	PRJNA827465	0.814880744
ENSGALG00010013119	RNF114	2	-0.123506756	0.051321041	-2.406552021	0.01610391	0	0.028602145	0	1	CRA004775;PRJNA827465	Yang2025_Silkie_Wenchang	0.209649548
ENSGALG00010013121	PCBD2	2	0.002806461	0.100898608	0.027814663	0.977809971	0	0.014247185	0	0.5	PRJNA827465;Yang2025_Silkie_Wenchang	CRA004775	0.993739602
ENSGALG00010013121	PCBD2	2	-0.058809694	0.21176937	-0.277706325	0.781237805	0	0.060800753	0	1	CRA004775;Yang2025_Silkie_Wenchang	PRJNA827465	0.945235709
ENSGALG00010013121	PCBD2	2	-0.002885339	0.105886885	-0.027249926	0.978260926	0	0.132866105	0	1	CRA004775;PRJNA827465	Yang2025_Silkie_Wenchang	0.993969906
ENSGALG00010013123	PPFIBP1	2	-0.059024548	0.081957244	-0.720187077	0.47140982	0.006227206	1.44998743	31.03388491	0.5	PRJNA827465;Yang2025_Silkie_Wenchang	CRA004775	0.853998373
ENSGALG00010013123	PPFIBP1	2	0.141063633	0.126615664	1.114108859	0.265232488	0	0.191874035	0	1	CRA004775;Yang2025_Silkie_Wenchang	PRJNA827465	0.769274038
ENSGALG00010013123	PPFIBP1	2	-0.009334767	0.143460207	-0.065068688	0.948119311	0.02679174	2.168844182	53.89249223	0.5	CRA004775;PRJNA827465	Yang2025_Silkie_Wenchang	0.986705854
ENSGALG00010013129	RASSF3	2	0.004219328	0.064521058	0.06539459	0.947859832	0.000105001	1.006810237	0.676417184	0.5	PRJNA827465;Yang2025_Silkie_Wenchang	CRA004775	0.986644937
ENSGALG00010013129	RASSF3	2	0.205598781	0.120334557	1.708559748	0.08753253	0	0.246794507	0	1	CRA004775;Yang2025_Silkie_Wenchang	PRJNA827465	0.508843518
ENSGALG00010013129	RASSF3	2	0.077420315	0.140641343	0.550480488	0.581989856	0.025195982	2.342000381	57.3014587	0.5	CRA004775;PRJNA827465	Yang2025_Silkie_Wenchang	0.889275613
ENSGALG00010013131	SPATA2	2	-0.135969678	0.098224974	-1.384267897	0.16627644	0.014942065	4.374413034	77.13979013	1	PRJNA827465;Yang2025_Silkie_Wenchang	CRA004775	0.66529196
ENSGALG00010013131	SPATA2	2	-0.087387484	0.188398453	-0.463843959	0.642759546	0.053300653	3.563419253	71.93706581	0.5	CRA004775;Yang2025_Silkie_Wenchang	PRJNA827465	0.906634523
ENSGALG00010013131	SPATA2	2	-0.028118178	0.054458125	-0.516326584	0.605626332	0	0.902972023	0	0.5	CRA004775;PRJNA827465	Yang2025_Silkie_Wenchang	0.896474603
ENSGALG00010013132	SKIC3	2	-0.059799824	0.094286674	-0.634234102	0.525928055	0	0.291289877	0	1	PRJNA827465;Yang2025_Silkie_Wenchang	CRA004775	0.870414822
ENSGALG00010013132	SKIC3	2	0.019470273	0.104678664	0.186000394	0.852444452	0	0.399401684	0	0.5	CRA004775;Yang2025_Silkie_Wenchang	PRJNA827465	0.962607579
ENSGALG00010013132	SKIC3	2	-0.035319079	0.122052523	-0.289376064	0.77229361	0	0.995122128	0	0.5	CRA004775;PRJNA827465	Yang2025_Silkie_Wenchang	0.943066211
ENSGALG00010013133	TXNDC15	2	0.071926301	0.068420118	1.051244915	0.293146119	0	0.254868662	0	0.5	PRJNA827465;Yang2025_Silkie_Wenchang	CRA004775	0.786774237
ENSGALG00010013133	TXNDC15	2	-0.162620243	0.158842131	-1.023785328	0.305936682	0	0.744121738	0	1	CRA004775;Yang2025_Silkie_Wenchang	PRJNA827465	0.792366985
ENSGALG00010013133	TXNDC15	2	-0.050350909	0.185353003	-0.271648734	0.785892124	0.047030082	2.605426724	61.61857132	0.5	CRA004775;PRJNA827465	Yang2025_Silkie_Wenchang	0.946639719
ENSGALG00010013134	ENSGALG00010013134	2	-4.508032905	4.752921208	-0.948476254	0.342887056	39.37786044	7.597804373	86.83830287	1	PRJNA827465;Yang2025_Silkie_Wenchang	CRA004775	0.806218116
ENSGALG00010013134	ENSGALG00010013134	2	-4.215670712	4.940754148	-0.853244381	0.393523797	44.23665634	10.05853381	90.05819318	0.5	CRA004775;Yang2025_Silkie_Wenchang	PRJNA827465	0.830425009
ENSGALG00010013134	ENSGALG00010013134	2	0.258292819	0.892520651	0.289397023	0.772277573	0	0.035197844	0	0.5	CRA004775;PRJNA827465	Yang2025_Silkie_Wenchang	0.943066211
ENSGALG00010013135	TTC3	2	0.228291381	0.243295322	0.938330334	0.348074676	0.103771244	7.630000334	86.89384068	1	PRJNA827465;Yang2025_Silkie_Wenchang	CRA004775	0.808963336
ENSGALG00010013135	TTC3	2	0.42755429	0.130521551	3.275737115	0.001053866	0	0.472595256	0	1	CRA004775;Yang2025_Silkie_Wenchang	PRJNA827465	0.039149431
ENSGALG00010013135	TTC3	2	0.087865755	0.133944468	0.655986447	0.511832845	0.020008047	1.779649715	43.80916696	1	CRA004775;PRJNA827465	Yang2025_Silkie_Wenchang	0.865694231
ENSGALG00010013137	TMEM121	2	-0.140216417	0.111139763	-1.261622421	0.207084684	0	0.361967511	0	1	PRJNA827465;Yang2025_Silkie_Wenchang	CRA004775	0.718997292
ENSGALG00010013137	TMEM121	2	0.264975999	0.656822181	0.40342121	0.686638386	0.646443662	3.32832999	69.95490222	0.5	CRA004775;Yang2025_Silkie_Wenchang	PRJNA827465	0.919235539
ENSGALG00010013137	TMEM121	2	0.307426749	0.57195394	0.537502634	0.590920466	0.471392488	2.767210343	63.86252305	0.5	CRA004775;PRJNA827465	Yang2025_Silkie_Wenchang	0.892752146
ENSGALG00010013139	TMEM185A	2	-0.16983328	0.242604499	-0.700041756	0.483901228	0.107176594	10.70223725	90.6561593	0.5	PRJNA827465;Yang2025_Silkie_Wenchang	CRA004775	0.85691916
ENSGALG00010013139	TMEM185A	2	-0.249594741	0.192789311	-1.29465031	0.195440889	0.050519039	3.086984908	67.60593168	1	CRA004775;Yang2025_Silkie_Wenchang	PRJNA827465	0.706466776
ENSGALG00010013139	TMEM185A	2	0.047378236	0.054392907	0.871037025	0.383733937	0	0.301987798	0	0.5	CRA004775;PRJNA827465	Yang2025_Silkie_Wenchang	0.825590921
ENSGALG00010013143	ENSGALG00010013143	2	-0.35235201	0.476773778	-0.739033953	0.45988638	0	0.033477157	0	1	PRJNA827465;Yang2025_Silkie_Wenchang	CRA004775	0.850104868
ENSGALG00010013143	ENSGALG00010013143	2	-0.003214242	0.535124957	-0.006006526	0.995207514	0.051614055	1.093946754	8.587872631	0.5	CRA004775;Yang2025_Silkie_Wenchang	PRJNA827465	0.998242335
ENSGALG00010013143	ENSGALG00010013143	2	0.131671032	0.535781186	0.245755237	0.80587171	0	0.721290865	0	0.5	CRA004775;PRJNA827465	Yang2025_Silkie_Wenchang	0.952057827
ENSGALG00010013144	TEDC1	2	-0.116406242	0.092912106	-1.252864107	0.210255166	0	0.013696912	0	1	PRJNA827465;Yang2025_Silkie_Wenchang	CRA004775	0.721897568
ENSGALG00010013144	TEDC1	2	-0.382185857	0.379418921	-1.007292562	0.313794203	0.166452309	2.180226326	54.13320221	1	CRA004775;Yang2025_Silkie_Wenchang	PRJNA827465	0.796531448
ENSGALG00010013144	TEDC1	2	-0.358345754	0.349775771	-1.024501363	0.305598528	0.16881687	2.476983208	59.62830928	1	CRA004775;PRJNA827465	Yang2025_Silkie_Wenchang	0.79225794
ENSGALG00010013145	ENSGALG00010013145	2	0.415790489	0.175616345	2.367607002	0.017903542	0	0.666473488	0	1	PRJNA827465;Yang2025_Silkie_Wenchang	CRA004775	0.223309996
ENSGALG00010013145	ENSGALG00010013145	2	0.101751398	0.513747229	0.198057318	0.843000218	0.402420983	3.903826567	74.38410793	0.5	CRA004775;Yang2025_Silkie_Wenchang	PRJNA827465	0.960416186
ENSGALG00010013145	ENSGALG00010013145	2	-0.023111761	0.361574228	-0.063919823	0.949034068	0.134694136	1.915467312	47.79341867	0.5	CRA004775;PRJNA827465	Yang2025_Silkie_Wenchang	0.987161201
ENSGALG00010013146	TNRC6B	2	0.139036346	0.210777593	0.659635324	0.50948788	0.067288365	3.840744057	73.96337832	0.5	PRJNA827465;Yang2025_Silkie_Wenchang	CRA004775	0.865257661
ENSGALG00010013146	TNRC6B	2	0.208860047	0.227809948	0.916817062	0.359238511	0.045302625	1.69110135	40.86693857	0.5	CRA004775;Yang2025_Silkie_Wenchang	PRJNA827465	0.814335519
ENSGALG00010013146	TNRC6B	2	-0.045878185	0.09416769	-0.487196668	0.626118967	0	0.018995845	0	1	CRA004775;PRJNA827465	Yang2025_Silkie_Wenchang	0.901504244
ENSGALG00010013149	MCTP1	2	-0.018366216	0.107797104	-0.170377637	0.864713158	0	0.222184194	0	0.5	PRJNA827465;Yang2025_Silkie_Wenchang	CRA004775	0.966723659
ENSGALG00010013149	MCTP1	2	0.045355332	0.190660102	0.237885805	0.811969665	0	0.068603189	0	0.5	CRA004775;Yang2025_Silkie_Wenchang	PRJNA827465	0.953371387
ENSGALG00010013149	MCTP1	2	-0.053447162	0.125637815	-0.425406647	0.670540261	0	0.009798601	0	1	CRA004775;PRJNA827465	Yang2025_Silkie_Wenchang	0.914482883
ENSGALG00010013150	GNS	2	-0.232400446	0.105429745	-2.204315728	0.027502148	0.013097049	2.347323614	57.39828995	1	PRJNA827465;Yang2025_Silkie_Wenchang	CRA004775	0.283036634
ENSGALG00010013150	GNS	2	-0.096230963	0.310340665	-0.310081708	0.756498822	0.150589866	4.130790326	75.79155752	0.5	CRA004775;Yang2025_Silkie_Wenchang	PRJNA827465	0.938659623
ENSGALG00010013150	GNS	2	-0.026431632	0.186924205	-0.141402939	0.887551633	0.042402431	1.954151922	48.826906	0.5	CRA004775;PRJNA827465	Yang2025_Silkie_Wenchang	0.973038314
ENSGALG00010013152	TBXAS1	2	-0.141778231	0.146686851	-0.966536746	0.333775669	0.007655127	1.188078657	15.83048869	1	PRJNA827465;Yang2025_Silkie_Wenchang	CRA004775	0.802691373
ENSGALG00010013152	TBXAS1	2	-0.298076978	0.216077819	-1.379489016	0.167744031	0	0.256476799	0	1	CRA004775;Yang2025_Silkie_Wenchang	PRJNA827465	0.667779227
ENSGALG00010013152	TBXAS1	2	-0.039961447	0.150159971	-0.266125833	0.7901423	0	0.002067265	0	1	CRA004775;PRJNA827465	Yang2025_Silkie_Wenchang	0.947390191
ENSGALG00010013154	ENSGALG00010013154	2	-0.47726098	0.459450423	-1.038764917	0.298914082	0	0.37305052	0	1	PRJNA827465;Yang2025_Silkie_Wenchang	CRA004775	0.78974322
ENSGALG00010013154	ENSGALG00010013154	2	-0.483015027	0.542068119	-0.891059646	0.372897172	0	0.607961793	0	0.5	CRA004775;Yang2025_Silkie_Wenchang	PRJNA827465	0.822162024
ENSGALG00010013154	ENSGALG00010013154	2	-0.078754322	0.551677867	-0.142754181	0.886484324	0	0.095534702	0	0.5	CRA004775;PRJNA827465	Yang2025_Silkie_Wenchang	0.972729743
ENSGALG00010013156	ABCC9	2	-0.040921208	0.079270335	-0.516223481	0.605698332	0.000473966	1.038857637	3.74041978	0.5	PRJNA827465;Yang2025_Silkie_Wenchang	CRA004775	0.896474603
ENSGALG00010013156	ABCC9	2	0.059362218	0.110805658	0.535732728	0.592143281	0	0.170246934	0	1	CRA004775;Yang2025_Silkie_Wenchang	PRJNA827465	0.892776493
ENSGALG00010013156	ABCC9	2	-0.091307558	0.101529541	-0.899320114	0.368482177	0	0.652640216	0	0.5	CRA004775;PRJNA827465	Yang2025_Silkie_Wenchang	0.820279089
ENSGALG00010013158	CRIP1	2	0.147333476	0.108098932	1.362950339	0.172898162	0	0.027990439	0	1	PRJNA827465;Yang2025_Silkie_Wenchang	CRA004775	0.676043418
ENSGALG00010013158	CRIP1	2	0.042229919	0.160823859	0.262584914	0.792870529	0	0.455467706	0	0.5	CRA004775;Yang2025_Silkie_Wenchang	PRJNA827465	0.948451453
ENSGALG00010013158	CRIP1	2	0.111085966	0.11704282	0.949105342	0.342567038	0	0.775431162	0	0.5	CRA004775;PRJNA827465	Yang2025_Silkie_Wenchang	0.806011702
ENSGALG00010013160	CRYBG1	2	0.249596049	0.416157155	0.599763925	0.548663578	0	0.186194101	0	1	PRJNA827465;Yang2025_Silkie_Wenchang	CRA004775	0.878281266
ENSGALG00010013160	CRYBG1	2	0.94191254	0.958809865	0.982376771	0.325914266	1.320025023	3.52032075	71.59349756	1	CRA004775;Yang2025_Silkie_Wenchang	PRJNA827465	0.800023788
ENSGALG00010013160	CRYBG1	2	1.069482662	0.770075306	1.388802696	0.164892755	0.752084968	2.647843359	62.23341548	1	CRA004775;PRJNA827465	Yang2025_Silkie_Wenchang	0.664150436
ENSGALG00010013162	IDS	2	-0.040233438	0.187172156	-0.214954186	0.829803037	0.055046507	4.285725514	76.6667278	0.5	PRJNA827465;Yang2025_Silkie_Wenchang	CRA004775	0.957079054
ENSGALG00010013162	IDS	2	-0.279126439	0.140342046	-1.988901018	0.046712129	0	0.048636606	0	1	CRA004775;Yang2025_Silkie_Wenchang	PRJNA827465	0.373459483
ENSGALG00010013162	IDS	2	-0.034997011	0.212629973	-0.164591146	0.869265801	0.063575973	2.752974025	63.6756471	0.5	CRA004775;PRJNA827465	Yang2025_Silkie_Wenchang	0.968525975
ENSGALG00010013164	FAM83F	2	0.315763236	0.396655323	0.796064538	0.425994525	0	0.188858207	0	1	PRJNA827465;Yang2025_Silkie_Wenchang	CRA004775	0.84125685
ENSGALG00010013164	FAM83F	2	1.11081067	0.630374226	1.76214481	0.078044834	0.280138538	1.53453933	34.83386315	1	CRA004775;Yang2025_Silkie_Wenchang	PRJNA827465	0.480897161
ENSGALG00010013164	FAM83F	2	0.887802232	0.805873344	1.10166472	0.27060746	0.895450022	3.089109975	67.62821628	1	CRA004775;PRJNA827465	Yang2025_Silkie_Wenchang	0.772549212
ENSGALG00010013165	NUDC	2	0.074285203	0.056973339	1.303859047	0.192281646	0.0026216	1.491745578	32.96444015	1	PRJNA827465;Yang2025_Silkie_Wenchang	CRA004775	0.702082087
ENSGALG00010013165	NUDC	2	0.071794313	0.143137142	0.501577104	0.615965028	0.020318871	1.702006133	41.24580512	0.5	CRA004775;Yang2025_Silkie_Wenchang	PRJNA827465	0.899050535
ENSGALG00010013165	NUDC	2	0.031184196	0.043014929	0.724962152	0.46847525	0	0.690369474	0	0.5	CRA004775;PRJNA827465	Yang2025_Silkie_Wenchang	0.853198008
ENSGALG00010013168	VPS26C	2	0.007064377	0.050452685	0.140019834	0.88864432	0	0.220323792	0	0.5	PRJNA827465;Yang2025_Silkie_Wenchang	CRA004775	0.973135755
ENSGALG00010013168	VPS26C	2	-0.073733967	0.100134084	-0.736352336	0.461516303	0	0.335425213	0	1	CRA004775;Yang2025_Silkie_Wenchang	PRJNA827465	0.850397341
ENSGALG00010013168	VPS26C	2	0.004982006	0.054181408	0.091950473	0.92673739	0	0.849928638	0	0.5	CRA004775;PRJNA827465	Yang2025_Silkie_Wenchang	0.9828798
ENSGALG00010013170	COL3A1	2	-0.536235468	0.259751554	-2.064416783	0.038978217	0.112874798	6.061612977	83.50274088	1	PRJNA827465;Yang2025_Silkie_Wenchang	CRA004775	0.340738622
ENSGALG00010013170	COL3A1	2	-0.52232399	0.429003007	-1.217529904	0.223402666	0.237174786	2.207860106	54.70727529	0.5	CRA004775;Yang2025_Silkie_Wenchang	PRJNA827465	0.736744216
ENSGALG00010013170	COL3A1	2	-0.269072753	0.126315523	-2.130163781	0.033158094	0	0.443253141	0	0.5	CRA004775;PRJNA827465	Yang2025_Silkie_Wenchang	0.313046498
ENSGALG00010013171	SLC9A8	2	0.36168358	0.224101576	1.61392698	0.106543266	0.09134971	11.04340253	90.94481979	1	PRJNA827465;Yang2025_Silkie_Wenchang	CRA004775	0.553605232
ENSGALG00010013171	SLC9A8	2	0.166446788	0.439360675	0.378838611	0.704807713	0.360196927	14.52502213	93.1153289	0.5	CRA004775;Yang2025_Silkie_Wenchang	PRJNA827465	0.924152671
ENSGALG00010013171	SLC9A8	2	-0.036410783	0.211819245	-0.17189554	0.863519653	0.066875179	3.555911446	71.87781487	0.5	CRA004775;PRJNA827465	Yang2025_Silkie_Wenchang	0.96626958
ENSGALG00010013173	KCNJ8	2	-0.07256827	0.227461427	-0.319035499	0.749699594	0.097015164	15.90660008	93.71330143	0.5	PRJNA827465;Yang2025_Silkie_Wenchang	CRA004775	0.937362187
ENSGALG00010013173	KCNJ8	2	0.041469123	0.411700383	0.100726461	0.919767605	0.28076131	5.100836726	80.39537328	0.5	CRA004775;Yang2025_Silkie_Wenchang	PRJNA827465	0.981237238
ENSGALG00010013173	KCNJ8	2	0.177766339	0.098748405	1.800194524	0.071829928	0.006187081	1.093343353	8.537423577	1	CRA004775;PRJNA827465	Yang2025_Silkie_Wenchang	0.462429429
ENSGALG00010013174	ADAMTS12	2	0.096003051	0.13731637	0.699137697	0.484465982	0.000365616	1.009595868	0.950466244	0.5	PRJNA827465;Yang2025_Silkie_Wenchang	CRA004775	0.857004733
ENSGALG00010013174	ADAMTS12	2	0.252102107	0.191766369	1.314631487	0.188633783	0	0.000608019	0	1	CRA004775;Yang2025_Silkie_Wenchang	PRJNA827465	0.698039126
ENSGALG00010013174	ADAMTS12	2	0.008677724	0.16958591	0.051170077	0.959189995	0	0.257933118	0	0.5	CRA004775;PRJNA827465	Yang2025_Silkie_Wenchang	0.989155754
ENSGALG00010013175	HIPK2	2	0.11637922	0.334727962	0.347682993	0.72807827	0.200140202	8.734769278	88.5515007	0.5	PRJNA827465;Yang2025_Silkie_Wenchang	CRA004775	0.931924062
ENSGALG00010013175	HIPK2	2	0.338558434	0.233084352	1.452514645	0.14635856	0.0286073	1.268908412	21.19210573	0.5	CRA004775;Yang2025_Silkie_Wenchang	PRJNA827465	0.6352709
ENSGALG00010013175	HIPK2	2	-0.184265789	0.069600704	-2.647470179	0.008109651	0	0.136426578	0	1	CRA004775;PRJNA827465	Yang2025_Silkie_Wenchang	0.139734161
ENSGALG00010013177	OXR1	2	-0.106585191	0.04384784	-2.430796842	0.015065659	0	0.06355155	0	1	PRJNA827465;Yang2025_Silkie_Wenchang	CRA004775	0.20306091
ENSGALG00010013177	OXR1	2	0.032618	0.186596701	0.174804803	0.861233014	0.05227796	3.709865259	73.04484313	0.5	CRA004775;Yang2025_Silkie_Wenchang	PRJNA827465	0.965343309
ENSGALG00010013177	OXR1	2	0.03605179	0.171824418	0.209817616	0.833810024	0.045482416	3.704416297	73.00519381	0.5	CRA004775;PRJNA827465	Yang2025_Silkie_Wenchang	0.957968425
ENSGALG00010013179	TBC1D30	2	-0.119183204	0.147361899	-0.808778959	0.418642301	0	0.238870687	0	1	PRJNA827465;Yang2025_Silkie_Wenchang	CRA004775	0.838193076
ENSGALG00010013179	TBC1D30	2	0.048833184	0.21908301	0.222898088	0.823614824	0	0.101799234	0	0.5	CRA004775;Yang2025_Silkie_Wenchang	PRJNA827465	0.955934625
ENSGALG00010013179	TBC1D30	2	-0.106692862	0.156751221	-0.680650917	0.496092401	0	0.580558326	0	0.5	CRA004775;PRJNA827465	Yang2025_Silkie_Wenchang	0.860911322
ENSGALG00010013180	C5orf24	2	-0.10777132	0.087986151	-1.224866858	0.22062539	0.01199527	4.022915488	75.14240597	1	PRJNA827465;Yang2025_Silkie_Wenchang	CRA004775	0.734053249
ENSGALG00010013180	C5orf24	2	-0.137737066	0.14509656	-0.949278647	0.342478911	0.019921663	1.458300369	31.42702142	0.5	CRA004775;Yang2025_Silkie_Wenchang	PRJNA827465	0.806005903
ENSGALG00010013180	C5orf24	2	-0.031313706	0.034374341	-0.910961626	0.362315593	0	0.386542117	0	0.5	CRA004775;PRJNA827465	Yang2025_Silkie_Wenchang	0.815644888

ENSGALG00010013181	DYRK1A	2	-0.035688925	0.118605542	-0.30090436	0.763487426	0.016334015	1.809116856	44.72441088	0.5	PRJNA827465;Yang2025_Silkie_Wenchang	CRA004775	0.940301633
ENSGALG00010013181	DYRK1A	2	0.234062464	0.141343384	1.655984579	0.097724942	0	0.275575137	0	1	CRA004775;Yang2025_Silkie_Wenchang	PRJNA827465	0.533900944
ENSGALG00010013181	DYRK1A	2	0.055867038	0.204000683	0.27385711	0.784194433	0.065152078	3.882902984	74.246073	0.5	CRA004775;PRJNA827465	Yang2025_Silkie_Wenchang	0.946083234
ENSGALG00010013183	CLTA	2	0.427158917	0.132201763	3.231113608	0.001233089	0.000806478	1.023115681	2.259341877	1	PRJNA827465;Yang2025_Silkie_Wenchang	CRA004775	0.042833689
ENSGALG00010013183	CLTA	2	0.075569528	0.548064205	0.137884443	0.890331745	0.514791861	6.713460689	85.10455269	0.5	CRA004775;Yang2025_Silkie_Wenchang	PRJNA827465	0.973417023
ENSGALG00010013183	CLTA	2	-0.037243901	0.411043096	-0.090608264	0.927803866	0.261745714	4.080819674	75.49511912	0.5	CRA004775;PRJNA827465	Yang2025_Silkie_Wenchang	0.982993195
ENSGALG00010013184	LDHB	2	0.045564227	0.225299101	0.20223883	0.839730019	0.062222532	2.427444061	58.80440599	0.5	PRJNA827465;Yang2025_Silkie_Wenchang	CRA004775	0.959493354
ENSGALG00010013184	LDHB	2	-0.259198753	0.192387424	-1.347274926	0.177891705	0	0.028952667	0	1	CRA004775;Yang2025_Silkie_Wenchang	PRJNA827465	0.683951879
ENSGALG00010013184	LDHB	2	0.026848554	0.255983858	0.104883778	0.916468031	0.084216311	2.553710421	60.84129227	0.5	CRA004775;PRJNA827465	Yang2025_Silkie_Wenchang	0.980561416
ENSGALG00010013185	ENSGALG00010013185	2	-0.25484172	0.241416609	-1.055609724	0.291146557	0	0.073244999	0	1	PRJNA827465;Yang2025_Silkie_Wenchang	CRA004775	0.78467882
ENSGALG00010013185	ENSGALG00010013185	2	-0.33344355	0.277787317	-1.200355555	0.230001282	0	0.003658017	0	1	CRA004775;Yang2025_Silkie_Wenchang	PRJNA827465	0.742845187
ENSGALG00010013185	ENSGALG00010013185	2	-0.247101647	0.275057948	-0.89836214	0.368992514	0	0.081827429	0	1	CRA004775;PRJNA827465	Yang2025_Silkie_Wenchang	0.820553374
ENSGALG00010013186	DDX46	2	0.200458729	0.233046373	0.860166696	0.38969716	0.094281921	6.779476135	85.24959776	0.5	PRJNA827465;Yang2025_Silkie_Wenchang	CRA004775	0.829068422
ENSGALG00010013186	DDX46	2	0.511475834	0.142382626	3.592262954	0.000327819	0	0.182653812	0	1	CRA004775;Yang2025_Silkie_Wenchang	PRJNA827465	0.017894809
ENSGALG00010013186	DDX46	2	0.248322296	0.295247637	0.841064465	0.400311821	0.14893669	5.980310192	83.27845935	0.5	CRA004775;PRJNA827465	Yang2025_Silkie_Wenchang	0.832251175
ENSGALG00010013187	KDF1	2	-0.309639525	0.211195416	-1.466128056	0.142613404	0.024835032	1.374810759	27.26271646	1	PRJNA827465;Yang2025_Silkie_Wenchang	CRA004775	0.628905865
ENSGALG00010013187	KDF1	2	0.228959367	0.825152693	0.27747515	0.781415284	1.21418991	9.021945731	88.91591648	0.5	CRA004775;Yang2025_Silkie_Wenchang	PRJNA827465	0.945235709
ENSGALG00010013187	KDF1	2	0.416511028	0.683995886	0.608937914	0.493977763	0.613006955	5.434259104	81.59822745	0.5	CRA004775;PRJNA827465	Yang2025_Silkie_Wenchang	0.860175061
ENSGALG00010013188	ENSGALG00010013188	2	-0.328481215	0.250768561	-1.309897913	0.190230373	0.070306776	2.157147154	53.64247645	1	PRJNA827465;Yang2025_Silkie_Wenchang	CRA004775	0.699403589
ENSGALG00010013188	ENSGALG00010013188	2	-0.555507926	0.275683038	-2.015023956	0.043902139	0	0.582544626	0	1	CRA004775;Yang2025_Silkie_Wenchang	PRJNA827465	0.361888215
ENSGALG00010013188	ENSGALG00010013188	2	-0.117454589	0.180375326	-0.651167716	0.514938228	0	0.022433072	0	1	CRA004775;PRJNA827465	Yang2025_Silkie_Wenchang	0.866997453
ENSGALG00010013191	ABRA	2	-0.159704953	0.139752636	-1.142768799	0.253134596	0.014568716	1.479349215	32.4027086	0.5	PRJNA827465;Yang2025_Silkie_Wenchang	CRA004775	0.762731469
ENSGALG00010013191	ABRA	2	0.46471215	0.670395544	0.693190988	0.488189703	0.579292029	2.092020977	52.19933209	1	CRA004775;Yang2025_Silkie_Wenchang	PRJNA827465	0.85768885
ENSGALG00010013191	ABRA	2	0.360087575	0.850095342	0.423584929	0.671868548	1.087292132	3.114572329	67.89286314	0.5	CRA004775;PRJNA827465	Yang2025_Silkie_Wenchang	0.914674175
ENSGALG00010013192	B4GALT5	2	-0.076575149	0.167593198	-0.455931471	0.648439258	0.05181179	12.10780319	91.74086344	0.5	PRJNA827465;Yang2025_Silkie_Wenchang	CRA004775	0.907649027
ENSGALG00010013192	B4GALT5	2	-0.237704116	0.077993052	-3.047760144	0.002305539	0	0.366841995	0	1	CRA004775;Yang2025_Silkie_Wenchang	PRJNA827465	0.063678558
ENSGALG00010013192	B4GALT5	2	0.081845422	0.052402686	1.561742237	0.118348724	0	0.210836206	0	0.5	CRA004775;PRJNA827465	Yang2025_Silkie_Wenchang	0.578180225
ENSGALG00010013196	MRPS9	2	0.119037895	0.175855607	0.676907023	0.498464948	0.052154038	6.002859866	83.34127362	0.5	PRJNA827465;Yang2025_Silkie_Wenchang	CRA004775	0.861478809
ENSGALG00010013196	MRPS9	2	0.253378658	0.11259796	2.250293628	0.024430207	0	0.844420764	0	1	CRA004775;Yang2025_Silkie_Wenchang	PRJNA827465	0.267184364
ENSGALG00010013196	MRPS9	2	-0.030412631	0.058428722	-0.520508245	0.602709383	0	0.26933907	0	0.5	CRA004775;PRJNA827465	Yang2025_Silkie_Wenchang	0.895538966
ENSGALG00010013198	CAMLG	2	-0.063483622	0.145594729	-0.436029674	0.6628152	0.036370905	6.844509497	85.38974925	0.5	PRJNA827465;Yang2025_Silkie_Wenchang	CRA004775	0.912821987
ENSGALG00010013198	CAMLG	2	-0.161269169	0.086984454	-1.853999901	0.063739174	0.001813141	1.113898657	10.2252262	1	CRA004775;Yang2025_Silkie_Wenchang	PRJNA827465	0.434920271
ENSGALG00010013198	CAMLG	2	0.059296564	0.053794551	1.10227827	0.270340713	0	0.412023948	0	0.5	CRA004775;PRJNA827465	Yang2025_Silkie_Wenchang	0.772430311
ENSGALG00010013203	AMACR	2	-0.176913384	0.230307214	-0.768162579	0.442390602	0.072036583	2.966541414	66.29071163	0.5	PRJNA827465;Yang2025_Silkie_Wenchang	CRA004775	0.845479108
ENSGALG00010013203	AMACR	2	-0.43025415	0.181595694	-2.369297101	0.017821931	0	0.012083589	0	1	CRA004775;Yang2025_Silkie_Wenchang	PRJNA827465	0.222839658
ENSGALG00010013203	AMACR	2	-0.114255281	0.197701322	-0.577915604	0.563319043	0.037301455	1.703005609	41.26028736	0.5	CRA004775;PRJNA827465	Yang2025_Silkie_Wenchang	0.863393222
ENSGALG00010013206	AFF2	2	-0.007402278	0.126554766	-0.058490712	0.953357761	0.01074147	1.406386332	28.89578225	0.5	PRJNA827465;Yang2025_Silkie_Wenchang	CRA004775	0.987758913
ENSGALG00010013206	AFF2	2	0.094746657	0.169393822	0.559327703	0.575938092	0	0.623207659	0	0.5	CRA004775;Yang2025_Silkie_Wenchang	PRJNA827465	0.886924804
ENSGALG00010013206	AFF2	2	-0.095599902	0.105806153	-0.903538202	0.366240323	0	0.004656413	0	1	CRA004775;PRJNA827465	Yang2025_Silkie_Wenchang	0.819159863
ENSGALG00010013207	C1QTNF3	2	0.150773419	0.094946786	1.58797812	0.112291287	0	0.051989384	0	1	PRJNA827465;Yang2025_Silkie_Wenchang	CRA004775	0.56602666
ENSGALG00010013207	C1QTNF3	2	0.237390937	0.151627928	1.565614855	0.11743881	0	0.904210117	0	1	CRA004775;Yang2025_Silkie_Wenchang	PRJNA827465	0.57688585
ENSGALG00010013207	C1QTNF3	2	0.20110934	0.163240851	1.231979239	0.217958639	0.017328335	1.185708668	15.66225101	1	CRA004775;PRJNA827465	Yang2025_Silkie_Wenchang	0.73141715
ENSGALG00010013208	ENSGALG00010013208	2	0.200671618	0.339011355	0.591931849	0.553896226	0	0.005014602	0	1	PRJNA827465;Yang2025_Silkie_Wenchang	CRA004775	0.879978308
ENSGALG00010013208	ENSGALG00010013208	2	-0.29577637	0.666855562	-0.443538882	0.657376002	0.486845395	2.061967048	51.50261975	0.5	CRA004775;Yang2025_Silkie_Wenchang	PRJNA827465	0.910711339
ENSGALG00010013208	ENSGALG00010013208	2	-0.314698698	0.640730289	-0.491154889	0.623316902	0.420700522	1.915130304	47.78423181	0.5	CRA004775;PRJNA827465	Yang2025_Silkie_Wenchang	0.900348922
ENSGALG00010013209	KIAA1549	2	-0.025200452	0.149922579	-0.168089773	0.866512655	0.024568555	1.754495553	43.00356028	0.5	PRJNA827465;Yang2025_Silkie_Wenchang	CRA004775	0.967507198
ENSGALG00010013209	KIAA1549	2	0.168522269	0.209405913	0.804763659	0.420956076	0	0.18843831	0	1	CRA004775;Yang2025_Silkie_Wenchang	PRJNA827465	0.839269089
ENSGALG00010013209	KIAA1549	2	-0.111660553	0.077268266	-1.445102351	0.148429139	0	0.094844814	0	0.5	CRA004775;PRJNA827465	Yang2025_Silkie_Wenchang	0.638471099
ENSGALG00010013212	ENSGALG00010013212	2	0.007875785	0.085788784	0.091804371	0.926853471	0	0.011500809	0	1	PRJNA827465;Yang2025_Silkie_Wenchang	CRA004775	0.982896295
ENSGALG00010013212	ENSGALG00010013212	2	0.052831229	0.131981678	0.400292147	0.688941351	0	0.526951476	0	1	CRA004775;Yang2025_Silkie_Wenchang	PRJNA827465	0.919854035
ENSGALG00010013212	ENSGALG00010013212	2	0.022962839	0.104981558	0.218732123	0.826858725	0	0.619498805	0	1	CRA004775;PRJNA827465	Yang2025_Silkie_Wenchang	0.956540044
ENSGALG00010013213	SH2D1A	2	0.685349012	0.317508913	2.158518968	0.030887505	0	0.007392599	0	1	PRJNA827465;Yang2025_Silkie_Wenchang	CRA004775	0.300265056
ENSGALG00010013213	SH2D1A	2	-0.036826511	0.768577784	-0.047915138	0.961783875	0.920493269	4.521991541	77.88584983	0.5	CRA004775;Yang2025_Silkie_Wenchang	PRJNA827465	0.98973612
ENSGALG00010013213	SH2D1A	2	-0.042150194	0.740158411	-0.056947531	0.954586092	0.869304161	4.796625848	79.14331521	0.5	CRA004775;PRJNA827465	Yang2025_Silkie_Wenchang	0.987987175
ENSGALG00010013214	RAI14	2	0.079926571	0.163621042	0.488485898	0.625205712	0.036903107	2.955954894	66.16998445	0.5	PRJNA827465;Yang2025_Silkie_Wenchang	CRA004775	0.901331406
ENSGALG00010013214	RAI14	2	0.346981437	0.158294714	2.191996366	0.028379769	0	0.898255269	0	1	CRA004775;Yang2025_Silkie_Wenchang	PRJNA827465	0.286930393
ENSGALG00010013214	RAI14	2	0.217751732	0.355916942	0.611804909	0.540666842	0.192637153	3.414833496	70.71599534	0.5	CRA004775;PRJNA827465	Yang2025_Silkie_Wenchang	0.875624671
ENSGALG00010013215	SF3A3	2	-0.101069581	0.141132675	-0.716131692	0.473910037	0.033264466	5.654586994	82.31524246	0.5	PRJNA827465;Yang2025_Silkie_Wenchang	CRA004775	0.85444425
ENSGALG00010013215	SF3A3	2	-0.260142776	0.098103544	-2.651716408	0.008008377	0	1.23843E-07	0	1	CRA004775;Yang2025_Silkie_Wenchang	PRJNA827465	0.139271353
ENSGALG00010013215	SF3A3	2	-0.035477015	0.115706746	-0.306611467	0.759139124	0.014611814	1.566909511	36.18010528	0.5	CRA004775;PRJNA827465	Yang2025_Silkie_Wenchang	0.939081587
ENSGALG00010013217	TGFBRAP1	2	0.038057488	0.078926617	0.482188458	0.629672217	0	0.047544876	0	1	PRJNA827465;Yang2025_Silkie_Wenchang	CRA004775	0.902457014
ENSGALG00010013217	TGFBRAP1	2	0.131188281	0.136246212	0.962876539	0.335609484	0	0.857677886	0	1	CRA004775;Yang2025_Silkie_Wenchang	PRJNA827465	0.803317552
ENSGALG00010013217	TGFBRAP1	2	0.087089615	0.131414705	0.662708299	0.507517394	0.012310443	1.251240139	20.07929022	1	CRA004775;PRJNA827465	Yang2025_Silkie_Wenchang	0.864177979
ENSGALG00010013220	CSTF1	2	-0.018084343	0.099416638	-0.181904593	0.8556576	0.014804594	3.496279421	71.39816704	0.5	PRJNA827465;Yang2025_Silkie_Wenchang	CRA004775	0.963486168
ENSGALG00010013220	CSTF1	2	-0.107078886	0.083390709	-1.284062535	0.199120076	0	0.350879917	0	1	CRA004775;Yang2025_Silkie_Wenchang	PRJNA827465	0.71096127
ENSGALG00010013220	CSTF1	2	0.055340972	0.036833714	1.502454284	0.132979826	0	0.435474867	0	0.5	CRA004775;PRJNA827465	Yang2025_Silkie_Wenchang	0.611001415
ENSGALG00010013221	C2orf49	2	0.057093814	0.115721426	0.493372881	0.62174914	0.015085104	2.053572152	51.30436499	0.5	PRJNA827465;Yang2025_Silkie_Wenchang	CRA004775	0.899796991
ENSGALG00010013221	C2orf49	2	0.238160794	0.123191114	1.933262764	0.053203836	0	0.070454246	0	1	CRA004775;Yang2025_Silkie_Wenchang	PRJNA827465	0.399898715
ENSGALG00010013221	C2orf49	2	0.065563577	0.142742375	0.459314038	0.646008669	0.023581558	1.947080245	48.64104843	0.5	CRA004775;PRJNA827465	Yang2025_Silkie_Wenchang	0.907177315
ENSGALG00010013225	GNE	2	0.248864929	0.113503664	2.192571774	0.028338247	0.015071208	2.31648167	56.83108513	1	PRJNA827465;Yang2025_Silkie_Wenchang	CRA004775	0.286834434
ENSGALG00010013225	GNE	2	0.355807299	0.117658341	3.0495696	0.002291695	0	0.292149108	0	1	CRA004775;Yang2025_Silkie_Wenchang	PRJNA827465	0.063654063
ENSGALG00010013225	GNE	2	0.155669697	0.080925146	1.923625794	0.054401505	0	0.015116565	0	1	CRA004775;PRJNA827465	Yang2025_Silkie_Wenchang	0.404002949
ENSGALG00010013226	DUSP6	2	-0.272825712	0.231096555	-1.180570223	0.237773499	0.099548497	14.69170379	93.1934375	1	PRJNA827465;Yang2025_Silkie_Wenchang	CRA004775	0.749548355
ENSGALG00010013226	DUSP6	2	-0.201189079	0.363349369	-0.553706972	0.579779403	0.216694784	4.896967049	79.57919688	0.5	CRA004775;Yang2025_Silkie_Wenchang	PRJNA827465	0.888013685
ENSGALG00010013226	DUSP6	2	-0.027712478	0.077325241	-0.358388514	0.720052586	0	0.695318539	0	0.5	CRA004775;PRJNA827465	Yang2025_Silkie_Wenchang	0.928564173
ENSGALG00010013227	CITED4	2	0.055048344	0.204193428	0.259794571	0.795022239	0.060676649	3.603355438	72.24808884	0.5	PRJNA827465;Yang2025_Silkie_Wenchang	CRA004775	0.948905846
ENSGALG00010013227	CITED4	2	-0.312124239	0.272932066	-1.143596808	0.252790886	0.064578144	1.414548473	29.30606346	1	CRA004775;Yang2025_Silkie_Wenchang	PRJNA827465	0.762731469
ENSGALG00010013227	CITED4	2	-0.172735749	0.522481319	-0.330606555	0.740941694	0.427700787	3.875928553	74.19973082	0.5	CRA004775;PRJNA827465	Yang2025_Silkie_Wenchang	0.93535143
ENSGALG00010013228	RTFDC1	2	-0.043424972	0.116771877	-0.371878686	0.709983175	0.021795343	4.65562257	78.52059558	0.5	PRJNA827465;Yang2025_Silkie_Wenchang	CRA004775	0.925298042
ENSGALG00010013228	RTFDC1	2	-0.134374137	0.089921405	-1.49435096	0.135083912	0.000252256	1.00938589	0.92986238	0.5	CRA004775;Yang2025_Silkie_Wenchang	PRJNA827465	0.616131251
ENSGALG00010013228	RTFDC1	2	0.058659904	0.046645787	1.257560584	0.208550716	0	0.000155399	0	1	CRA004775;PRJNA827465	Yang2025_Silkie_Wenchang	0.721011841
ENSGALG00010013230	ENSGALG00010013230	2	0.152387384	0.227219704	0.670660958	0.50243654	0.084362108	5.420016769	81.54987258	0.5	PRJNA827465;Yang2025_Silkie_Wenchang	CRA004775	0.862928829
ENSGALG00010013230	ENSGALG00010013230	2	0.214552588	0.24027837	0.892933427	0.371892825	0.064840419	2.03562832	50.87511849	0.5	CRA004775;Yang2025_Silkie_Wenchang	PRJNA827465	0.821597118
ENSGALG00010013230	ENSGALG00010013230	2	-0.071568055	0.113134723	-0.632591417	0.527000493	0	0.021505099	0	1	CRA004775;PRJNA827465	Yang2025_Silkie_Wenchang	0.87095077
ENSGALG00010013232	FHL2	2	-0.19643747	0.216638282	-0.906753265	0.364537289	0.074072303	4.570927248	78.12260083	1	PRJNA827465;Yang2025_Silkie_Wenchang	CRA004775	0.817496251
ENSGALG00010013232	FHL2	2	-0.259762327	0.393263488	-0.660529988	0.508913781	0.15546856	1.388818616	27.99635685	0.5	CRA004775;Yang2025_Silkie_Wenchang	PRJNA827465	0.865193604
ENSGALG00010013232	FHL2	2	0.008396211	0.105283129	0.079749251	0.936436688	0	0.489838157	0	0.5	CRA004775;PRJNA827465	Yang2025_Silkie_Wenchang	0.985006587
ENSGALG00010013233	LRRCC1	2	0.366452996	0.572641275	0.639934654	0.522215083	0.625317109	21.25798369	95.295885	0.5	PRJNA827465;Yang2025_Silkie_Wenchang	CRA004775	0.869568211
ENSGALG00010013233	LRRCC1	2	0.537066337	0.469324848	1.14433817	0.25248342	0.346815522	4.457255611	77.56467012	1	CRA004775;Yang2025_Silkie_Wenchang	PRJNA827465	0.762655223
ENSGALG00010013233	LRRCC1	2	-0.174664616	0.119524754	-1.46132588	0.143926031	0	0.234617658	0	0.5	CRA004775;PRJNA827465	Yang2025_Silkie_Wenchang	0.630908409
ENSGALG00010013235	GCNT7	2	0.007352467	0.382469734	0.019223657	0.984662685	0.256841561	8.185452121	87.78320384	0.5	PRJNA827465;Yang2025_Silkie_Wenchang	CRA004775	0.995703389
ENSGALG00010013235	GCNT7	2	-0.167249523	0.320391375	-0.522016308	0.601658976	0.111235485	1.805558742	44.61548237	0.5	CRA004775;Yang2025_Silkie_Wenchang	PRJNA827465	0.895230519
ENSGALG00010013235	GCNT7	2	0.385011102	0.181609437	2.119995015	0.034006466	0	0.012312215	0	1	CRA004775;PRJNA827465	Yang2025_Silkie_Wenchang	0.317170413
ENSGALG00010013236	MON2	2	0.025010791	0.120102474	0.208245425	0.835037342	0.021343403	3.798077883	73.67089273	0.5	PRJNA827465;Yang2025_Silkie_Wenchang	CRA004775	0.95823248
ENSGALG00010013236	MON2	2	-0.067750741	0.090812389	-0.746051741	0.45563616	0	0.993733293	0	0.5	CRA004775;Yang2025_Silkie_Wenchang	PRJNA827465	0.84907427
ENSGALG00010013236	MON2	2	0.139767725	0.072531604	1.926990677	0.053980792	0	0.010070058	0	1	CRA004775;PRJNA827465	Yang2025_Silkie_Wenchang	0.402825653
ENSGALG00010013238	RAD1	2	0.323102287	0.131298008	2.460831607	0.013861541	0	0.356715609	0	1	PRJNA827465;Yang2025_Silkie_Wenchang	CRA004775	0.193260071
ENSGALG00010013238	RAD1	2	0.118880582	0.31654361	0.375558306	0.707245292	0.147424695	3.763356684	73.4279771	0.5	CRA004775;Yang2025_Silkie_Wenchang	PRJNA827465	0.924856272
ENSGALG00010013238	RAD1	2	0.060568782	0.233548439	0.259341413	0.795371829	0.066622414	2.47891261	59.65973161	0.5	CRA004775;PRJNA827465	Yang2025_Silkie_Wenchang	0.948935068
ENSGALG00010013241	SEC24A	2	-0.207328134	0.102035189	-2.031927774	0.042160968	0.016179798	4.306054756	76.77688612	1	PRJNA827465;Yang2025_Silkie_Wenchang	CRA004775	0.3553619
ENSGALG00010013241	SEC24A	2	-0.003509731	0.364976409	-0.009616324	0.992327402	0.226932518	6.08272463	83.55999884	0.5	CRA004775;Yang2025_Silkie_Wenchang	PRJNA827465	0.997811136
ENSGALG00010013241	SEC24A	2	0.0751888	0.25511722	0.294722561	0.768205827	0.099004149	3.33798375	70.04179544	0.5	CRA004775;PRJNA827465	Yang2025_Silkie_Wenchang	0.941444269
ENSGALG00010013242	SULT4A1	2	0.3156828	0.198666255	1.589010678	0.112057982	0.033547944	1.715257343	41.69971031	1	PRJNA827465;Yang2025_Silkie_Wenchang	CRA004775	0.56547057
ENSGALG00010013242	SULT4A1	2	0.54015086	0.194558776	2.776286278	0.005498377	0	0.000650597	0	1	CRA004775;Yang2025_Silkie_Wenchang	PRJNA827465	0.111278432
ENSGALG00010013242	SULT4A1	2	0.244165649	0.171343607	1.425005882	0.154155501	0.003801874	1.052424385	4.981297093	1	CRA004775;PRJNA827465	Yang2025_Silkie_Wenchang	0.646821143
ENSGALG00010013244	TRIM24	2	-0.046270685	0.057207996	-0.808815005	0.418621564	0	0.437055648	0	0.5	PRJNA827465;Yang2025_Silkie_Wenchang	CRA004775	0.838193076
ENSGALG00010013244	TRIM24	2	0.066771251	0.139857098	0.47742483	0.633059637	0	0.057284244	0	1	CRA004775;Yang2025_Silkie_Wenchang	PRJNA827465	0.903225139
ENSGALG00010013244	TRIM24	2	-0.053860882	0.060306521	-0.893118708	0.371793606	0	0.374569316	0	0.5	CRA004775;PRJNA827465	Yang2025_Silkie_Wenchang	0.821597118
ENSGALG00010013246	LNPEP	2	-0.204860988	0.075070794	-2.728903989	0.00635452	0	0.369299212	0	1	PRJNA827465;Yang2025_Silkie_Wenchang	CRA004775	0.1211211
ENSGALG00010013246	LNPEP	2	0.079000781	0.395390853	0.19980427	0.841633662	0.261340767	5.66615862	82.35135888	0.5	CRA004775;Yang2025_Silkie_Wenchang	PRJNA827465	0.959922457
ENSGALG00010013246	LNPEP	2	0.114499089	0.344062566	0.332785664	0.739296081	0.192930694	4.751020513	78.9518905	0.5	CRA004775;PRJNA827465	Yang2025_Silkie_Wenchang	0.93477525
ENSGALG00010013247	ATG5	2	-0.14358259	0.104577565	-1.372976988	0.169759501	0.017901739	5.507768198	81.84382559	1	PRJNA827465;Yang2025_Silkie_Wenchang	CRA004775	0.671232541
ENSGALG00010013247	ATG5	2	-0.235865438	0.059862731	-3.940104897	8.1446E-05	0	0.358884096	0	1	CRA004775;Yang2025_Silkie_Wenchang	PRJNA827465	0.006767593
ENSGALG00010013247	ATG5	2	-0.049250543	0.059194101	-0.832017758	0.405398921	0	0.239914746	0	1	CRA004775;PRJNA827465	Yang2025_Silkie_Wenchang	0.833112102
ENSGALG00010013248	ENSGALG00010013248	2	-1.836673804	1.50280164	-1.222166489	0.221644674	3.639553255	5.148577173	80.57715819	1	PRJNA827465;Yang2025_Silkie_Wenchang	CRA004775	0.734944791
ENSGALG00010013248	ENSGALG00010013248	2	-1.974836005	1.446037238	-1.365688209	0.172036847	3.07704815	3.746266702	73.30675898	1	CRA004775;Yang2025_Silkie_Wenchang	PRJNA827465	0.674672141
ENSGALG00010013248	ENSGALG00010013248	2	-0.372520893	0.734751439	-0.507002605	0.612152991	0	0.005171455	0	1	CRA004775;PRJNA827465	Yang2025_Silkie_Wenchang	0.897833503
ENSGALG00010013249	AURKA	2	-0.27904458	0.221985105	-1.257041909	0.208738461	0.083498208	6.413527722	84.407957	1	PRJNA827465;Yang2025_Silkie_Wenchang	CRA004775	0.721011841
ENSGALG00010013249	AURKA	2	-0.103255763	0.458777098	-0.225067388	0.821926851	0.358554614	6.324316504	84.18801464	0.5	CRA004775;Yang2025_Silkie_Wenchang	PRJNA827465	0.95563486
ENSGALG00010013249	AURKA	2	0.060277774	0.212981137	0.283019308	0.777162036	0.052990281	1.864244048	46.35895438	0.5	CRA004775;PRJNA827465	Yang2025_Silkie_Wenchang	0.944218792
ENSGALG00010013250	E2F5	2	0.176187094	0.165487451	1.064655312	0.28703194	0.044275106	5.134330611	80.52326436	1	PRJNA827465;Yang2025_Silkie_Wenchang	CRA004775	0.782566585
ENSGALG00010013250	E2F5	2	0.040870646	0.326568304	0.125151907	0.90040329	0.189506023	8.808505373	88.64733621	0.5	CRA004775;Yang2025_Silkie_Wenchang	PRJNA827465	0.976475201
ENSGALG00010013250	E2F5	2	-0.098374617	0.15553794	-0.632479875	0.527073355	0.031070643	2.48986982	59.83725767	0.5	CRA004775;PRJNA827465	Yang2025_Silkie_Wenchang	0.87095077
ENSGALG00010013251	MTA1	2	-0.024671216	0.039655819	-0.622133554	0.533854053	0	0.297914874	0	0.5	PRJNA827465;Yang2025_Silkie_Wenchang	CRA004775	0.873408008
ENSGALG00010013251	MTA1	2	0.106844586	0.107856089	0.99062173	0.321870323	0	0.682100545	0	1	CRA004775;Yang2025_Silkie_Wenchang	PRJNA827465	0.798513866
ENSGALG00010013251	MTA1	2	0.037929476	0.11642034	0.32579767	0.744577442	0.016644127	1.905606637	47.52327261	0.5	CRA004775;PRJNA827465	Yang2025_Silkie_Wenchang	0.935893304
ENSGALG00010013252	PPM1H	2	0.615904856	0.38872538	1.584421518	0.113097828	0.260847218	7.30558221	86.31183701	1	PRJNA827465;Yang2025_Silkie_Wenchang	CRA004775	0.567482422
ENSGALG00010013252	PPM1H	2	-0.001738764	0.331472196	-0.005245582	0.995814651	0.121089771	1.931043419	48.21452536	0.5	CRA004775;Yang2025_Silkie_Wenchang	PRJNA827465	0.998609527
ENSGALG00010013252	PPM1H	2	0.321363296	0.740689388	0.433870528	0.664382462	0.974192735	8.48391998	88.21299585	0.5	CRA004775;PRJNA827465	Yang2025_Silkie_Wenchang	0.913219726
ENSGALG00010013253	SHB	2	-0.090116951	0.118236936	-0.762172586	0.445957005	0.020982149	3.813530782	73.77758153	0.5	PRJNA827465;Yang2025_Silkie_Wenchang	CRA004775	0.846013379
ENSGALG00010013253	SHB	2	-0.244573427	0.097973602	-2.496319645	0.012548946	0	0.250476588	0	1	CRA004775;Yang2025_Silkie_Wenchang	PRJNA827465	0.181527877
ENSGALG00010013253	SHB	2	-0.090695615	0.169633512	-0.534656232	0.592887592	0.035898122	1.987525989	49.68619251	0.5	CRA004775;PRJNA827465	Yang2025_Silkie_Wenchang	0.892776493
ENSGALG00010013254	SAR1B	2	-0.256988339	0.23589517	-1.089417554	0.275969794	0.102856139	13.05997705	92.34301871	1	PRJNA827465;Yang2025_Silkie_Wenchang	CRA004775	0.775541559
ENSGALG00010013254	SAR1B	2	-0.098510429	0.457535239	-0.215306759	0.82952816	0.358609827	6.326584742	84.19368362	0.5	CRA004775;Yang2025_Silkie_Wenchang	PRJNA827465	0.956939641
ENSGALG00010013254	SAR1B	2	0.067347844	0.18398166	0.366057379	0.71432226	0.037565651	1.585801333	36.94039858	0.5	CRA004775;PRJNA827465	Yang2025_Silkie_Wenchang	0.92653974
ENSGALG00010013256	BRIX1	2	0.256711514	0.127484367	2.013670533	0.044044136	0	0.555594539	0	1	PRJNA827465;Yang2025_Silkie_Wenchang	CRA004775	0.362678958
ENSGALG00010013256	BRIX1	2	0.271094809	0.157537806	1.720823813	0.0852828	0	0.710898269	0	1	CRA004775;Yang2025_Silkie_Wenchang	PRJNA827465	0.502453887
ENSGALG00010013256	BRIX1	2	0.147119727	0.144825133	1.015843894	0.30970373	0	0.079934329	0	1	CRA004775;PRJNA827465	Yang2025_Silkie_Wenchang	0.794328224
ENSGALG00010013257	STX12	2	0.024290942	0.078434392	0.30969759	0.756790936	0.007767888	2.661393783	62.42570316	0.5	PRJNA827465;Yang2025_Silkie_Wenchang	CRA004775	0.938740241
ENSGALG00010013257	STX12	2	-0.044098113	0.070437507	-0.62606011	0.531275515	0	0.34408424	0	0.5	CRA004775;Yang2025_Silkie_Wenchang	PRJNA827465	0.872939865
ENSGALG00010013257	STX12	2	0.089776511	0.054841465	1.637018847	0.101626532	0	0.072506253	0	1	CRA004775;PRJNA827465	Yang2025_Silkie_Wenchang	0.543686036
ENSGALG00010013258	RBIS	2	-0.098087563	0.07935733	-1.236023984	0.216449649	0	0.338748118	0	1	PRJNA827465;Yang2025_Silkie_Wenchang	CRA004775	0.730172415
ENSGALG00010013258	RBIS	2	-0.198886479	0.194313776	-1.023532572	0.306056108	0	0.044683463	0	1	CRA004775;Yang2025_Silkie_Wenchang	PRJNA827465	0.792366985
ENSGALG00010013258	RBIS	2	-0.084716183	0.081908108	-1.034283226	0.301003776	0	0.025649012	0	1	CRA004775;PRJNA827465	Yang2025_Silkie_Wenchang	0.791063284
ENSGALG00010013259	PTPRB	2	-0.035682304	0.076866368	-0.464212179	0.642495736	0	0.096627051	0	1	PRJNA827465;Yang2025_Silkie_Wenchang	CRA004775	0.906634523
ENSGALG00010013259	PTPRB	2	-0.024131709	0.153858627	-0.15684339	0.875368275	0.005225198	1.03915071	3.767567993	0.5	CRA004775;Yang2025_Silkie_Wenchang	PRJNA827465	0.970347777
ENSGALG00010013259	PTPRB	2	-0.00534654	0.089268056	-0.059893092	0.952240782	0	0.88217422	0	0.5	CRA004775;PRJNA827465	Yang2025_Silkie_Wenchang	0.987758913
ENSGALG00010013260	FAM210B	2	-0.091593734	0.327286699	-0.279857795	0.779586609	0.20166707	17.03959126	94.13131463	0.5	PRJNA827465;Yang2025_Silkie_Wenchang	CRA004775	0.944807046
ENSGALG00010013260	FAM210B	2	-0.375756417	0.107739924	-3.487624684	0.000487332	0	0.925266126	0	1	CRA004775;Yang2025_Silkie_Wenchang	PRJNA827465	0.023132362
ENSGALG00010013260	FAM210B	2	0.131731687	0.165662763	0.795179828	0.426508904	0.026518243	1.627425192	38.55324321	0.5	CRA004775;PRJNA827465	Yang2025_Silkie_Wenchang	0.84125685
ENSGALG00010013261	JADE2	2	-0.122296893	0.059767682	-2.046204376	0.040736267	0.001030956	1.131376169	11.61206792	1	PRJNA827465;Yang2025_Silkie_Wenchang	CRA004775	0.349194657
ENSGALG00010013261	JADE2	2	0.023920444	0.287512155	0.083198027	0.933694081	0.129596172	4.190477488	76.13637102	0.5	CRA004775;Yang2025_Silkie_Wenchang	PRJNA827465	0.984262625
ENSGALG00010013261	JADE2	2	0.067197998	0.213011023	0.315467235	0.752406925	0.064649015	2.761741789	63.79096684	0.5	CRA004775;PRJNA827465	Yang2025_Silkie_Wenchang	0.93806821
ENSGALG00010013262	CA13	2	-0.209228555	0.476446426	-0.439143929	0.660557251	0.406469165	9.539488006	89.51725712	0.5	PRJNA827465;Yang2025_Silkie_Wenchang	CRA004775	0.91192683
ENSGALG00010013262	CA13	2	-0.608086991	0.195854908	-3.104783006	0.001904187	0	0.472725225	0	1	CRA004775;Yang2025_Silkie_Wenchang	PRJNA827465	0.056193756
ENSGALG00010013262	CA13	2	0.009381552	0.317544197	0.029544084	0.97643066	0.122268597	2.353781586	57.51517448	0.5	CRA004775;PRJNA827465	Yang2025_Silkie_Wenchang	0.993126926
ENSGALG00010013263	ATP11A	2	-0.02141002	0.113904663	-0.187964386	0.85090457	0.011843079	1.706464832	41.39931973	0.5	PRJNA827465;Yang2025_Silkie_Wenchang	CRA004775	0.962056252
ENSGALG00010013263	ATP11A	2	-0.139596522	0.152000528	-0.91839498	0.358412118	0	0.602467332	0	0.5	CRA004775;Yang2025_Silkie_Wenchang	PRJNA827465	0.814005058
ENSGALG00010013263	ATP11A	2	0.066964354	0.088260852	0.758709585	0.448026302	0	0.108211068	0	1	CRA004775;PRJNA827465	Yang2025_Silkie_Wenchang	0.846601815
ENSGALG00010013264	DNAJC21	2	0.42053305	0.324399675	1.296342389	0.194857551	0.187394729	8.897822926	88.76129579	1	PRJNA827465;Yang2025_Silkie_Wenchang	CRA004775	0.706046493
ENSGALG00010013264	DNAJC21	2	0.672182274	0.164354099	4.089841858	4.31667E-05	0	0.987054234	0	1	CRA004775;Yang2025_Silkie_Wenchang	PRJNA827465	0.004406708
ENSGALG00010013264	DNAJC21	2	0.13917767	0.104157785	1.336219562	0.181477519	0	0.566290481	0	1	CRA004775;PRJNA827465	Yang2025_Silkie_Wenchang	0.689004635
ENSGALG00010013265	PACS2	2	-0.122761705	0.061540193	-1.994821563	0.046062345	0	0.275120485	0	1	PRJNA827465;Yang2025_Silkie_Wenchang	CRA004775	0.370146868
ENSGALG00010013265	PACS2	2	-0.070098212	0.100095211	-0.700315343	0.483730392	0	0.031140462	0	1	CRA004775;Yang2025_Silkie_Wenchang	PRJNA827465	0.85691916
ENSGALG00010013265	PACS2	2	-0.13734153	0.072663564	-1.89010177	0.05874435	0	0.174356616	0	1	CRA004775;PRJNA827465	Yang2025_Silkie_Wenchang	0.419728393
ENSGALG00010013266	KIAA1755	2	0.240884238	0.258951893	0.930227755	0.352253174	0.121006366	10.18738139	90.18393479	0.5	PRJNA827465;Yang2025_Silkie_Wenchang	CRA004775	0.810764026
ENSGALG00010013266	KIAA1755	2	0.213893819	0.378491891	0.565121272	0.571991281	0.212224762	3.253805965	69.26675988	0.5	CRA004775;Yang2025_Silkie_Wenchang	PRJNA827465	0.885496034
ENSGALG00010013266	KIAA1755	2	-0.02725037	0.098197317	-0.27750626	0.7813914	0	0.384309742	0	1	CRA004775;PRJNA827465	Yang2025_Silkie_Wenchang	0.945235709
ENSGALG00010013267	PNPLA3	2	-0.03640555	0.102152904	-0.356382919	0.721553819	0.001022068	1.044380992	4.249502056	0.5	PRJNA827465;Yang2025_Silkie_Wenchang	CRA004775	0.929153713
ENSGALG00010013267	PNPLA3	2	-0.11355097	0.157630698	-0.720360762	0.471302902	0	0.756461404	0	0.5	CRA004775;Yang2025_Silkie_Wenchang	PRJNA827465	0.853939282
ENSGALG00010013267	PNPLA3	2	0.045926947	0.112657531	0.40766868	0.683516935	0	0.099041708	0	1	CRA004775;PRJNA827465	Yang2025_Silkie_Wenchang	0.918800744
ENSGALG00010013268	PTPRR	2	-0.265130509	0.213563334	-1.24146081	0.214435567	0	0.163379831	0	1	PRJNA827465;Yang2025_Silkie_Wenchang	CRA004775	0.727205473
ENSGALG00010013268	PTPRR	2	0.064819708	0.299918321	0.216124537	0.828890674	0.073539643	1.660270793	39.76886154	0.5	CRA004775;Yang2025_Silkie_Wenchang	PRJNA827465	0.956840134
ENSGALG00010013268	PTPRR	2	0.00401371	0.390372342	0.010281748	0.991796497	0.182745298	2.483636885	59.73646526	0.5	CRA004775;PRJNA827465	Yang2025_Silkie_Wenchang	0.997575757
ENSGALG00010013269	INPP5B	2	-0.034711299	0.051718293	-0.671160957	0.502117998	0	0.096009887	0	1	PRJNA827465;Yang2025_Silkie_Wenchang	CRA004775	0.862860181
ENSGALG00010013269	INPP5B	2	-0.041607058	0.073490112	-0.566158594	0.571285974	0	0.490719698	0	1	CRA004775;Yang2025_Silkie_Wenchang	PRJNA827465	0.885354083
ENSGALG00010013269	INPP5B	2	-0.061205407	0.061014354	-1.003131284	0.315797522	0	0.289537586	0	1	CRA004775;PRJNA827465	Yang2025_Silkie_Wenchang	0.796883862
ENSGALG00010013271	GULP1	2	-0.136303795	0.061602625	-2.212629653	0.026923189	0	0.002296483	0	1	PRJNA827465;Yang2025_Silkie_Wenchang	CRA004775	0.279419618
ENSGALG00010013271	GULP1	2	-0.028296961	0.187529965	-0.15089301	0.880060114	0.033864821	1.602345785	37.59149809	0.5	CRA004775;Yang2025_Silkie_Wenchang	PRJNA827465	0.971546618
ENSGALG00010013271	GULP1	2	-0.037131652	0.176645042	-0.210204895	0.833507759	0.035484819	1.685006533	40.65304908	0.5	CRA004775;PRJNA827465	Yang2025_Silkie_Wenchang	0.957968425
ENSGALG00010013273	STAG2	2	0.033769898	0.122165834	0.276426701	0.782220351	0.021885405	3.104865563	67.79248635	0.5	PRJNA827465;Yang2025_Silkie_Wenchang	CRA004775	0.945372683
ENSGALG00010013273	STAG2	2	0.329414063	0.205750235	1.601038572	0.109368376	0.040949701	1.677505834	40.3876887	1	CRA004775;Yang2025_Silkie_Wenchang	PRJNA827465	0.559628341
ENSGALG00010013273	STAG2	2	0.222768274	0.34496669	0.64576749	0.518429945	0.196520964	4.810201392	79.21084964	0.5	CRA004775;PRJNA827465	Yang2025_Silkie_Wenchang	0.868525042
ENSGALG00010013274	MTF1	2	-0.078301258	0.10095187	-0.775629587	0.437967726	0.01361843	2.787608321	64.12695455	0.5	PRJNA827465;Yang2025_Silkie_Wenchang	CRA004775	0.843947213
ENSGALG00010013274	MTF1	2	-0.176645068	0.103034671	-1.714423565	0.086450964	0	0.510280113	0	0.5	CRA004775;Yang2025_Silkie_Wenchang	PRJNA827465	0.506036629
ENSGALG00010013274	MTF1	2	0.005145902	0.057009843	0.0902634	0.928077904	0	0.010429539	0	1	CRA004775;PRJNA827465	Yang2025_Silkie_Wenchang	0.983019892
ENSGALG00010013275	MAPRE1	2	-0.029442987	0.052459714	-0.561249479	0.574627477	0	0.400541511	0	0.5	PRJNA827465;Yang2025_Silkie_Wenchang	CRA004775	0.88613451

ENSGALG00010013275	MAPRE1	2	0.005649074	0.080217209	0.070422216	0.94385761	0	0.078810897	0	0.5	CRA004775;Yang2025_Silkie_Wenchang	PRJNA827465	0.985827325
ENSGALG00010013275	MAPRE1	2	-0.050444455	0.059764933	-0.844047707	0.398642747	0	0.009397204	0	1	CRA004775;PRJNA827465	Yang2025_Silkie_Wenchang	0.831980453
ENSGALG00010013276	POC1B	2	-0.176123326	0.097002876	-1.815650547	0.069423994	0.010256265	2.171904642	53.95746293	1	PRJNA827465;Yang2025_Silkie_Wenchang	CRA004775	0.453929672
ENSGALG00010013276	POC1B	2	-0.266023119	0.099209166	-2.681436901	0.007330674	0	0.372413646	0	1	CRA004775;Yang2025_Silkie_Wenchang	PRJNA827465	0.131680746
ENSGALG00010013276	POC1B	2	-0.086648932	0.08070835	-1.073605535	0.282999516	0	0.00848247	0	1	CRA004775;PRJNA827465	Yang2025_Silkie_Wenchang	0.779568117
ENSGALG00010013277	YRDC	2	-0.112227514	0.082370184	-1.362477394	0.173047274	0	0.241296267	0	0.5	PRJNA827465;Yang2025_Silkie_Wenchang	CRA004775	0.676043418
ENSGALG00010013277	YRDC	2	-0.139170936	0.171050518	-0.813624756	0.415859952	0	0.449820421	0	0.5	CRA004775;Yang2025_Silkie_Wenchang	PRJNA827465	0.837742614
ENSGALG00010013277	YRDC	2	-0.136870203	0.078832412	-1.736217373	0.082525406	0	0.169431722	0	1	CRA004775;PRJNA827465	Yang2025_Silkie_Wenchang	0.495285278
ENSGALG00010013278	SAMM50	2	-0.264740418	0.223451206	-1.184779542	0.236104623	0.083569597	5.514770321	81.86687855	1	PRJNA827465;Yang2025_Silkie_Wenchang	CRA004775	0.748669015
ENSGALG00010013278	SAMM50	2	-0.246227972	0.303554963	-0.811147902	0.41728074	0.135725414	3.713140424	73.06861886	0.5	CRA004775;Yang2025_Silkie_Wenchang	PRJNA827465	0.838112595
ENSGALG00010013278	SAMM50	2	-0.064330485	0.05734786	-1.121759121	0.261964872	0	0.355389608	0	0.5	CRA004775;PRJNA827465	Yang2025_Silkie_Wenchang	0.768363052
ENSGALG00010013280	FRS2	2	0.144954587	0.20600943	0.703630835	0.481662703	0.064367594	3.704067502	73.00265183	0.5	PRJNA827465;Yang2025_Silkie_Wenchang	CRA004775	0.85691916
ENSGALG00010013280	FRS2	2	0.427294683	0.178829782	2.389393296	0.016876225	0	0.11415937	0	1	CRA004775;Yang2025_Silkie_Wenchang	PRJNA827465	0.215702857
ENSGALG00010013280	FRS2	2	0.13469853	0.254620718	0.529016376	0.59679409	0.083389928	2.081767965	51.96390679	0.5	CRA004775;PRJNA827465	Yang2025_Silkie_Wenchang	0.894172266
ENSGALG00010013281	BIRC8	2	-0.076713149	0.120377572	-0.637271111	0.523948265	0.01692028	2.320463849	56.90516789	0.5	PRJNA827465;Yang2025_Silkie_Wenchang	CRA004775	0.869661505
ENSGALG00010013281	BIRC8	2	0.04994887	0.123849566	0.403302748	0.686725521	0	0.097532076	0	0.5	CRA004775;Yang2025_Silkie_Wenchang	PRJNA827465	0.919235539
ENSGALG00010013281	BIRC8	2	-0.171340511	0.087068411	-1.967883745	0.049081416	0	0.124382909	0	1	CRA004775;PRJNA827465	Yang2025_Silkie_Wenchang	0.383203591
ENSGALG00010013282	SEC22B	2	-0.136745782	0.203037619	-0.673499732	0.500629419	0.075776649	12.08748689	91.72698172	0.5	PRJNA827465;Yang2025_Silkie_Wenchang	CRA004775	0.862400704
ENSGALG00010013282	SEC22B	2	-0.482885154	0.18127269	-2.663860471	0.00772496	0.040286849	2.272743308	56.00031043	1	CRA004775;Yang2025_Silkie_Wenchang	PRJNA827465	0.136027818
ENSGALG00010013282	SEC22B	2	-0.303833718	0.391624149	-0.775829884	0.437849436	0.280744851	11.0304331	90.93417284	0.5	CRA004775;PRJNA827465	Yang2025_Silkie_Wenchang	0.843899392
ENSGALG00010013283	PAWR	2	-0.196939547	0.219502213	-0.897209845	0.369606953	0.049571061	2.058954251	51.43165518	0.5	PRJNA827465;Yang2025_Silkie_Wenchang	CRA004775	0.820606801
ENSGALG00010013283	PAWR	2	0.052461608	0.491365848	0.1067669	0.914973907	0.412955282	6.791063327	85.2747655	0.5	CRA004775;Yang2025_Silkie_Wenchang	PRJNA827465	0.97997655
ENSGALG00010013283	PAWR	2	0.253345518	0.268813273	0.94245911	0.345957637	0.075865064	2.043596679	51.06666544	1	CRA004775;PRJNA827465	Yang2025_Silkie_Wenchang	0.807598873
ENSGALG00010013284	MANEAL	2	0.303620254	0.204589939	1.484042934	0.137797531	0.036682827	1.743399596	42.64080349	1	PRJNA827465;Yang2025_Silkie_Wenchang	CRA004775	0.62064915
ENSGALG00010013284	MANEAL	2	-0.216274103	0.791152149	-0.273366006	0.784571882	1.142051113	11.22931825	91.09473988	0.5	CRA004775;Yang2025_Silkie_Wenchang	PRJNA827465	0.946117405
ENSGALG00010013284	MANEAL	2	-0.400798327	0.581903057	-0.688771648	0.49096698	0.585368386	7.001471522	85.71728819	0.5	CRA004775;PRJNA827465	Yang2025_Silkie_Wenchang	0.85895707
ENSGALG00010013285	ATP2B1	2	0.085300708	0.07837356	1.088386278	0.276424616	0.005600976	1.77007716	43.50528766	1	PRJNA827465;Yang2025_Silkie_Wenchang	CRA004775	0.775851484
ENSGALG00010013285	ATP2B1	2	0.219544141	0.091740063	2.393110868	0.016706192	0	0.842297975	0	1	CRA004775;Yang2025_Silkie_Wenchang	PRJNA827465	0.214226254
ENSGALG00010013285	ATP2B1	2	0.155702991	0.184932182	0.841946429	0.39981794	0.047494747	2.669880372	62.54513833	1	CRA004775;PRJNA827465	Yang2025_Silkie_Wenchang	0.832246851
ENSGALG00010013286	RNF14	2	-0.418779515	0.305931054	-1.368868933	0.171040248	0.168953289	9.609152059	89.59325449	1	PRJNA827465;Yang2025_Silkie_Wenchang	CRA004775	0.673219077
ENSGALG00010013286	RNF14	2	-0.341951985	0.432941707	-0.789833781	0.429624848	0.319934137	6.710107104	85.09710822	0.5	CRA004775;Yang2025_Silkie_Wenchang	PRJNA827465	0.842012722
ENSGALG00010013286	RNF14	2	-0.127184625	0.057304503	-2.219452551	0.026455952	0	0.803445672	0	1	CRA004775;PRJNA827465	Yang2025_Silkie_Wenchang	0.277488505
ENSGALG00010013288	CA3A	2	0.162171432	0.567577402	0.285725667	0.775088245	0.461994354	3.500791307	71.43502961	0.5	PRJNA827465;Yang2025_Silkie_Wenchang	CRA004775	0.943803161
ENSGALG00010013288	CA3A	2	1.394858074	0.833593658	1.673306966	0.094266882	0.866094683	2.360206659	57.63082879	1	CRA004775;Yang2025_Silkie_Wenchang	PRJNA827465	0.525785636
ENSGALG00010013288	CA3A	2	0.906544961	1.426836693	0.635352991	0.52519822	3.524329101	6.90370386	85.51502179	0.5	CRA004775;PRJNA827465	Yang2025_Silkie_Wenchang	0.870179017
ENSGALG00010013289	TM9SF4	2	-0.227594722	0.25516374	-0.891955582	0.372416741	0.119464834	11.58478955	91.36799166	0.5	PRJNA827465;Yang2025_Silkie_Wenchang	CRA004775	0.821893154
ENSGALG00010013289	TM9SF4	2	-0.418342278	0.110502487	-3.785817752	0.000153204	0	0.888814488	0	1	CRA004775;Yang2025_Silkie_Wenchang	PRJNA827465	0.010545314
ENSGALG00010013289	TM9SF4	2	-0.08706806	0.139789189	-0.622852602	0.533381388	0.026376917	2.498761509	59.98017433	0.5	CRA004775;PRJNA827465	Yang2025_Silkie_Wenchang	0.873379507
ENSGALG00010013290	AKR1D1	2	0.048291449	0.110576961	0.436722518	0.662312597	0	0.754541522	0	0.5	PRJNA827465;Yang2025_Silkie_Wenchang	CRA004775	0.91272639
ENSGALG00010013290	AKR1D1	2	-0.001844224	0.140269445	-0.013147723	0.989509937	0	0.436062689	0	0.5	CRA004775;Yang2025_Silkie_Wenchang	PRJNA827465	0.997172099
ENSGALG00010013290	AKR1D1	2	0.150481486	0.138768677	1.084405281	0.278185143	0	0.011324165	0	1	CRA004775;PRJNA827465	Yang2025_Silkie_Wenchang	0.776763074
ENSGALG00010013292	PRDM1	2	0.088379753	0.167560454	0.527449949	0.597881165	0	0.525987358	0	0.5	PRJNA827465;Yang2025_Silkie_Wenchang	CRA004775	0.894645663
ENSGALG00010013292	PRDM1	2	0.009076558	0.345061377	0.026304184	0.979014718	0.104192949	1.403120017	28.63871293	0.5	CRA004775;Yang2025_Silkie_Wenchang	PRJNA827465	0.994269133
ENSGALG00010013292	PRDM1	2	-0.144838116	0.250865894	-0.57735276	0.563701179	0	0.66698957	0	1	CRA004775;PRJNA827465	Yang2025_Silkie_Wenchang	0.882640032
ENSGALG00010013293	TUBGCP3	2	-0.076757767	0.046106963	-1.664776025	0.095957449	0	0.668672244	0	1	PRJNA827465;Yang2025_Silkie_Wenchang	CRA004775	0.5301492
ENSGALG00010013293	TUBGCP3	2	-0.137813084	0.084555549	-1.629852631	0.103132647	0	0.000637308	0	1	CRA004775;Yang2025_Silkie_Wenchang	PRJNA827465	0.5473214
ENSGALG00010013293	TUBGCP3	2	-0.056333355	0.053109071	-1.060710602	0.28882144	0	0.068975614	0	1	CRA004775;PRJNA827465	Yang2025_Silkie_Wenchang	0.783550627
ENSGALG00010013295	CA3B	2	-0.380100718	0.515162542	-0.737826776	0.46061972	0.388280166	3.724375221	73.14985895	0.5	PRJNA827465;Yang2025_Silkie_Wenchang	CRA004775	0.85014477
ENSGALG00010013295	CA3B	2	0.073125966	0.33056465	0.221215325	0.824924777	0	0.115206151	0	0.5	CRA004775;Yang2025_Silkie_Wenchang	PRJNA827465	0.956260685
ENSGALG00010013295	CA3B	2	-0.728039587	0.335889263	-2.167498835	0.030196838	0	0.886627618	0	1	CRA004775;PRJNA827465	Yang2025_Silkie_Wenchang	0.295973612
ENSGALG00010013296	AGXT2	2	0.282201447	0.407427754	0.692643584	0.488534442	0.048628571	1.169243904	14.474645	0.5	PRJNA827465;Yang2025_Silkie_Wenchang	CRA004775	0.857850753
ENSGALG00010013296	AGXT2	2	-0.142776058	0.432592064	-0.330047798	0.741363846	0	0.006317064	0	1	CRA004775;Yang2025_Silkie_Wenchang	PRJNA827465	0.935433665
ENSGALG00010013296	AGXT2	2	0.376506425	0.400254636	0.940667244	0.34687341	0	0.810472249	0	0.5	CRA004775;PRJNA827465	Yang2025_Silkie_Wenchang	0.808511356
ENSGALG00010013297	FCHO2	2	0.364064033	0.388144847	0.937959206	0.348265375	0.260480861	6.973692275	85.65906342	1	PRJNA827465;Yang2025_Silkie_Wenchang	CRA004775	0.80992788
ENSGALG00010013297	FCHO2	2	0.547293854	0.323392439	1.692352041	0.090578871	0.091430568	1.678296805	40.41578359	1	CRA004775;Yang2025_Silkie_Wenchang	PRJNA827465	0.516394163
ENSGALG00010013297	FCHO2	2	0.018775548	0.116391117	0.16131427	0.871845884	0	0.054359462	0	1	CRA004775;PRJNA827465	Yang2025_Silkie_Wenchang	0.969462602
ENSGALG00010013299	PLAGL2	2	-0.070408274	0.05483193	-1.284074335	0.199115947	0	0.255720166	0	0.5	PRJNA827465;Yang2025_Silkie_Wenchang	CRA004775	0.71096127
ENSGALG00010013299	PLAGL2	2	-0.033275628	0.144656614	-0.230031644	0.818067025	0	0.312588032	0	0.5	CRA004775;Yang2025_Silkie_Wenchang	PRJNA827465	0.95520884
ENSGALG00010013299	PLAGL2	2	-0.084053911	0.057438174	-1.463380633	0.143363255	0	0.125741698	0	1	CRA004775;PRJNA827465	Yang2025_Silkie_Wenchang	0.63041016
ENSGALG00010013300	GNPDA1	2	0.211170057	0.058771439	3.593072756	0.000326801	0	9.79305E-05	0	1	PRJNA827465;Yang2025_Silkie_Wenchang	CRA004775	0.017894809
ENSGALG00010013300	GNPDA1	2	-0.122881993	0.344338914	-0.356863509	0.721191989	0.211140759	9.06961123	88.97417502	0.5	CRA004775;Yang2025_Silkie_Wenchang	PRJNA827465	0.929950734
ENSGALG00010013300	GNPDA1	2	-0.112218277	0.343076695	-0.327093849	0.743596904	0.217928196	12.89473333	92.24489584	0.5	CRA004775;PRJNA827465	Yang2025_Silkie_Wenchang	0.935821071
ENSGALG00010013301	PARVB	2	-0.209302186	0.133958917	-1.562435632	0.118185398	0.024490288	3.11250609	67.8715488	1	PRJNA827465;Yang2025_Silkie_Wenchang	CRA004775	0.578003524
ENSGALG00010013301	PARVB	2	-0.359180521	0.114128265	-3.14716535	0.001648617	0	0.02714153	0	1	CRA004775;Yang2025_Silkie_Wenchang	PRJNA827465	0.051226717
ENSGALG00010013301	PARVB	2	-0.103348334	0.091968889	-1.12373146	0.261126966	0	0.695831937	0	1	CRA004775;PRJNA827465	Yang2025_Silkie_Wenchang	0.767979536
ENSGALG00010013303	TFPI	2	0.402749049	0.123086067	3.272092926	0.001067545	0	0.641754448	0	1	PRJNA827465;Yang2025_Silkie_Wenchang	CRA004775	0.039471039
ENSGALG00010013303	TFPI	2	0.461220476	0.154302655	2.989063776	0.002798337	0	0.293357649	0	1	CRA004775;Yang2025_Silkie_Wenchang	PRJNA827465	0.072072769
ENSGALG00010013303	TFPI	2	0.273266033	0.180970496	1.510003231	0.1310426	0	0.03650728	0	1	CRA004775;PRJNA827465	Yang2025_Silkie_Wenchang	0.606767161
ENSGALG00010013304	KCNJ15	2	-0.134745887	0.21704119	-0.620830945	0.53471086	0.068400935	3.59464406	72.18083395	0.5	PRJNA827465;Yang2025_Silkie_Wenchang	CRA004775	0.87379905
ENSGALG00010013304	KCNJ15	2	0.148780015	0.614371939	0.242166033	0.808651513	0.611965407	4.703356983	78.73859025	0.5	CRA004775;Yang2025_Silkie_Wenchang	PRJNA827465	0.952539424
ENSGALG00010013304	KCNJ15	2	0.300898814	0.367836488	0.81802329	0.413343889	0.171170245	2.083151886	51.99581909	1	CRA004775;PRJNA827465	Yang2025_Silkie_Wenchang	0.836088312
ENSGALG00010013305	THOC2	2	0.32249531	0.323035481	0.998327828	0.318120418	0.19146658	11.63995938	91.40890473	1	PRJNA827465;Yang2025_Silkie_Wenchang	CRA004775	0.797501246
ENSGALG00010013305	THOC2	2	0.571140476	0.136053473	4.197911763	2.69388E-05	0	0.738148242	0	1	CRA004775;Yang2025_Silkie_Wenchang	PRJNA827465	0.003025068
ENSGALG00010013305	THOC2	2	0.171594256	0.197635986	0.868233865	0.385266318	0.057150768	3.187014362	68.62267042	1	CRA004775;PRJNA827465	Yang2025_Silkie_Wenchang	0.826444827
ENSGALG00010013306	ARSB	2	-0.056342889	0.210739381	-0.267358141	0.789193426	0.064691012	3.680338147	72.82858367	0.5	PRJNA827465;Yang2025_Silkie_Wenchang	CRA004775	0.947306881
ENSGALG00010013306	ARSB	2	-0.285180187	0.148128886	-1.925216571	0.05420227	0	0.241808039	0	1	CRA004775;Yang2025_Silkie_Wenchang	PRJNA827465	0.403544575
ENSGALG00010013306	ARSB	2	0.011476582	0.286392015	0.040072982	0.968034942	0.078117361	1.444635806	30.77840133	0.5	CRA004775;PRJNA827465	Yang2025_Silkie_Wenchang	0.991128245
ENSGALG00010013307	POFUT1	2	-0.189476611	0.299816252	-0.631975784	0.527402701	0.160107497	8.379383924	88.06594841	0.5	PRJNA827465;Yang2025_Silkie_Wenchang	CRA004775	0.871149047
ENSGALG00010013307	POFUT1	2	-0.364791489	0.142816818	-2.554261416	0.010641333	0.004479257	1.121944642	10.8690427	1	CRA004775;Yang2025_Silkie_Wenchang	PRJNA827465	0.164465713
ENSGALG00010013307	POFUT1	2	-0.030152106	0.15061217	-0.200197009	0.841326507	0.032351619	2.843729558	64.83491206	0.5	CRA004775;PRJNA827465	Yang2025_Silkie_Wenchang	0.95989279
ENSGALG00010013309	ENSGALG00010013309	2	0.097373161	0.148987331	0.653566718	0.513390993	0.004950099	1.125053561	11.1153429	0.5	PRJNA827465;Yang2025_Silkie_Wenchang	CRA004775	0.866334901
ENSGALG00010013309	ENSGALG00010013309	2	0.256168185	0.185943031	1.377670272	0.168305114	0	0.127667733	0	1	CRA004775;Yang2025_Silkie_Wenchang	PRJNA827465	0.668962553
ENSGALG00010013309	ENSGALG00010013309	2	-0.017285633	0.19714671	-0.087679031	0.930131786	0	0.573818151	0	0.5	CRA004775;PRJNA827465	Yang2025_Silkie_Wenchang	0.983813577
ENSGALG00010013310	MYO16	2	0.047820076	0.17010829	0.281115495	0.778621817	0	0.277975828	0	0.5	PRJNA827465;Yang2025_Silkie_Wenchang	CRA004775	0.944673731
ENSGALG00010013310	MYO16	2	-0.103426768	0.465980788	-0.221955006	0.82434891	0.197202463	1.611053507	37.92881518	0.5	CRA004775;Yang2025_Silkie_Wenchang	PRJNA827465	0.95619211
ENSGALG00010013310	MYO16	2	-0.109238076	0.27195988	-0.401669818	0.68792704	0.045729104	1.159666589	13.76831848	1	CRA004775;PRJNA827465	Yang2025_Silkie_Wenchang	0.919631238
ENSGALG00010013311	RSPO1	2	0.01750727	0.229220723	0.076377344	0.939118893	0	0.476282372	0	0.5	PRJNA827465;Yang2025_Silkie_Wenchang	CRA004775	0.985349045
ENSGALG00010013311	RSPO1	2	0.141850498	0.311387435	0.455543421	0.648718337	0	0.129787245	0	0.5	CRA004775;Yang2025_Silkie_Wenchang	PRJNA827465	0.907730933
ENSGALG00010013311	RSPO1	2	-0.119992486	0.283594948	-0.423112213	0.672213392	0	0.001103897	0	1	CRA004775;PRJNA827465	Yang2025_Silkie_Wenchang	0.914742762
ENSGALG00010013312	ENSGALG00010013312	2	0.306880794	0.265324026	1.156626479	0.247424999	0.045460706	1.458609926	31.44157448	1	PRJNA827465;Yang2025_Silkie_Wenchang	CRA004775	0.757947282
ENSGALG00010013312	ENSGALG00010013312	2	0.739929919	0.323491776	2.287322197	0.022177024	0	0.812670457	0	1	CRA004775;Yang2025_Silkie_Wenchang	PRJNA827465	0.252609681
ENSGALG00010013312	ENSGALG00010013312	2	0.52833832	0.63995372	0.825588325	0.40903766	0.533445509	2.346986265	57.3921665	1	CRA004775;PRJNA827465	Yang2025_Silkie_Wenchang	0.834316328
ENSGALG00010013313	GAK	2	0.055518657	0.105398296	0.526750994	0.598366519	0	0.016224936	0	1	PRJNA827465;Yang2025_Silkie_Wenchang	CRA004775	0.894645663
ENSGALG00010013313	GAK	2	0.00608241	0.162872761	0.03734455	0.970210285	0	0.234375129	0	0.5	CRA004775;Yang2025_Silkie_Wenchang	PRJNA827465	0.991681174
ENSGALG00010013313	GAK	2	0.044317344	0.126250316	0.351027585	0.725567654	0	0.314118313	0	0.5	CRA004775;PRJNA827465	Yang2025_Silkie_Wenchang	0.930954084
ENSGALG00010013314	KIT	2	0.699301701	0.089147431	7.844328146	4.35278E-15	0	0.155801364	0	1	PRJNA827465;Yang2025_Silkie_Wenchang	CRA004775	4.62371E-12
ENSGALG00010013314	KIT	2	0.626805284	0.153831641	4.07461871	4.60898E-05	0	0.126261461	0	1	CRA004775;Yang2025_Silkie_Wenchang	PRJNA827465	0.004574413
ENSGALG00010013314	KIT	2	0.71006699	0.103973385	6.829314947	8.53211E-12	0	0.275424593	0	1	CRA004775;PRJNA827465	Yang2025_Silkie_Wenchang	7.13484E-09
ENSGALG00010013316	KDM7A	2	0.015091341	0.063229476	0.238675731	0.811357036	0	0.999546804	0	0.5	PRJNA827465;Yang2025_Silkie_Wenchang	CRA004775	0.953196081
ENSGALG00010013316	KDM7A	2	0.000503069	0.186828598	0.002692676	0.997851558	0.02726543	1.463480854	31.66975863	0.5	CRA004775;Yang2025_Silkie_Wenchang	PRJNA827465	0.999290321
ENSGALG00010013316	KDM7A	2	0.05768861	0.068397698	0.843429111	0.398988497	0	0.631620532	0	1	CRA004775;PRJNA827465	Yang2025_Silkie_Wenchang	0.832054308
ENSGALG00010013317	PREP	2	-0.252813994	0.185072844	-1.366024257	0.17193135	0.065082154	19.85894963	94.96448695	1	PRJNA827465;Yang2025_Silkie_Wenchang	CRA004775	0.674594973
ENSGALG00010013317	PREP	2	-0.34159807	0.12302703	-2.776609913	0.005492906	0.020299518	2.801029901	64.29884594	1	CRA004775;Yang2025_Silkie_Wenchang	PRJNA827465	0.111278432
ENSGALG00010013317	PREP	2	-0.083808184	0.04272389	-1.96162342	0.049806344	0	0.71776742	0	1	CRA004775;PRJNA827465	Yang2025_Silkie_Wenchang	0.386284347
ENSGALG00010013319	ENSGALG00010013319	2	-4.023605971	3.634147309	-1.107166449	0.26822197	24.4422938	13.38586259	92.52943176	1	PRJNA827465;Yang2025_Silkie_Wenchang	CRA004775	0.771398294
ENSGALG00010013319	ENSGALG00010013319	2	-3.872071944	3.755413432	-1.031064093	0.302510765	26.47147473	16.19907677	93.82680869	1	CRA004775;Yang2025_Silkie_Wenchang	PRJNA827465	0.791852471
ENSGALG00010013319	ENSGALG00010013319	2	-0.278017616	0.865530491	-0.321210655	0.748050758	0	0.019290249	0	1	CRA004775;PRJNA827465	Yang2025_Silkie_Wenchang	0.936982566
ENSGALG00010013320	CALCRL	2	0.067572979	0.077805579	0.868485005	0.385128878	0	0.1025391	0	1	PRJNA827465;Yang2025_Silkie_Wenchang	CRA004775	0.826444827
ENSGALG00010013320	CALCRL	2	0.26356197	0.229104944	1.150398443	0.249979801	0.043103043	1.508100316	33.69141368	1	CRA004775;Yang2025_Silkie_Wenchang	PRJNA827465	0.75993411
ENSGALG00010013320	CALCRL	2	0.223755683	0.259605471	0.861906657	0.388738886	0.088258392	2.204318767	54.63451045	1	CRA004775;PRJNA827465	Yang2025_Silkie_Wenchang	0.828400391
ENSGALG00010013322	IRS2	2	-0.290812541	0.135346219	-2.148656558	0.031661635	0.016744046	1.836903771	45.56056686	1	PRJNA827465;Yang2025_Silkie_Wenchang	CRA004775	0.304700595
ENSGALG00010013322	IRS2	2	-0.126848793	0.145009021	-0.874764843	0.38170187	0	0.423939793	0	0.5	CRA004775;Yang2025_Silkie_Wenchang	PRJNA827465	0.824699008
ENSGALG00010013322	IRS2	2	-0.360750482	0.19596111	-1.84092896	0.06563197	0.023812376	1.10136076	9.203229627	0.5	CRA004775;PRJNA827465	Yang2025_Silkie_Wenchang	0.441590298
ENSGALG00010013323	NOTCH2	2	-0.080086594	0.071260774	-1.123852433	0.261075633	0	0.596390815	0	0.5	PRJNA827465;Yang2025_Silkie_Wenchang	CRA004775	0.767929536
ENSGALG00010013323	NOTCH2	2	-0.134273005	0.234133605	-0.57348882	0.566313767	0.056840137	1.750289033	42.86657913	0.5	CRA004775;Yang2025_Silkie_Wenchang	PRJNA827465	0.883487517
ENSGALG00010013323	NOTCH2	2	-0.144920911	0.108631826	-1.33405574	0.18218559	0.006170706	1.089640642	8.226624325	1	CRA004775;PRJNA827465	Yang2025_Silkie_Wenchang	0.690033956
ENSGALG00010013324	PARVG	2	-0.133164967	0.071820305	-1.854140922	0.063719001	0	0.326204877	0	1	PRJNA827465;Yang2025_Silkie_Wenchang	CRA004775	0.434889309
ENSGALG00010013324	PARVG	2	-0.217528246	0.114470568	-1.900298469	0.057393962	0	0.592328125	0	1	CRA004775;Yang2025_Silkie_Wenchang	PRJNA827465	0.414965948
ENSGALG00010013324	PARVG	2	-0.129330733	0.09719498	-1.330631826	0.183310185	0.00215721	1.030502934	2.960004602	1	CRA004775;PRJNA827465	Yang2025_Silkie_Wenchang	0.691756141
ENSGALG00010013325	CA2	2	0.024402627	0.250341684	0.097477282	0.922347374	0.089879139	3.508453184	71.49741075	0.5	PRJNA827465;Yang2025_Silkie_Wenchang	CRA004775	0.981804675
ENSGALG00010013325	CA2	2	-0.170922357	0.156304175	-1.093523934	0.274163826	0	0.380231041	0	0.5	CRA004775;Yang2025_Silkie_Wenchang	PRJNA827465	0.774526342
ENSGALG00010013325	CA2	2	0.237228562	0.182744241	1.29814521	0.194237441	0	0.286581487	0	1	CRA004775;PRJNA827465	Yang2025_Silkie_Wenchang	0.704982807
ENSGALG00010013327	SLF1	2	0.172486668	0.131172678	1.314958811	0.188523746	0	0.000137791	0	1	PRJNA827465;Yang2025_Silkie_Wenchang	CRA004775	0.697920084
ENSGALG00010013327	SLF1	2	0.109370706	0.162001335	0.67512225	0.499598098	0	0.380465852	0	0.5	CRA004775;Yang2025_Silkie_Wenchang	PRJNA827465	0.862078151
ENSGALG00010013327	SLF1	2	0.113889895	0.153276429	0.743035941	0.457459921	0	0.386095332	0	0.5	CRA004775;PRJNA827465	Yang2025_Silkie_Wenchang	0.849573219
ENSGALG00010013328	SLC37A3	2	-0.247687068	0.256885838	-0.964191211	0.33495007	0.116942363	8.164800023	87.7523026	1	PRJNA827465;Yang2025_Silkie_Wenchang	CRA004775	0.803101658
ENSGALG00010013328	SLC37A3	2	-0.501650274	0.133631349	-3.753986447	0.000174044	0	0.075400274	0	1	CRA004775;Yang2025_Silkie_Wenchang	PRJNA827465	0.011507636
ENSGALG00010013328	SLC37A3	2	-0.18757081	0.215301856	-0.871199229	0.383645381	0.072930842	3.99343924	74.95892788	1	CRA004775;PRJNA827465	Yang2025_Silkie_Wenchang	0.825509017
ENSGALG00010013329	LIFR	2	-0.080072959	0.077644251	-1.031279944	0.302409561	0	0.055484484	0	1	PRJNA827465;Yang2025_Silkie_Wenchang	CRA004775	0.791792883
ENSGALG00010013329	LIFR	2	0.126363175	0.308936413	0.409026486	0.682520224	0.131568249	2.742040951	63.53081453	0.5	CRA004775;Yang2025_Silkie_Wenchang	PRJNA827465	0.918667547
ENSGALG00010013329	LIFR	2	0.132944074	0.284550324	0.467207602	0.640351355	0.111718509	2.566455003	61.0357478	0.5	CRA004775;PRJNA827465	Yang2025_Silkie_Wenchang	0.906161493
ENSGALG00010013330	ERG	2	0.203216991	0.23869263	0.851375222	0.394560951	0.086962946	3.923044782	74.50959508	0.5	PRJNA827465;Yang2025_Silkie_Wenchang	CRA004775	0.830643015
ENSGALG00010013330	ERG	2	0.435835037	0.187593325	2.323297143	0.020163195	0	0.169958529	0	1	CRA004775;Yang2025_Silkie_Wenchang	PRJNA827465	0.238922186
ENSGALG00010013330	ERG	2	0.02366661	0.104081505	0.227385359	0.820124104	0	0.647764468	0	0.5	CRA004775;PRJNA827465	Yang2025_Silkie_Wenchang	0.955308792
ENSGALG00010013331	KIF3B	2	-0.030220163	0.049731529	-0.60766607	0.543408971	0.000502585	1.08745883	8.042495769	0.5	PRJNA827465;Yang2025_Silkie_Wenchang	CRA004775	0.876968972
ENSGALG00010013331	KIF3B	2	-0.085261058	0.184138354	-0.463027153	0.643344904	0.043928547	2.456562524	59.29271124	0.5	CRA004775;Yang2025_Silkie_Wenchang	PRJNA827465	0.906634523
ENSGALG00010013331	KIF3B	2	-0.107789178	0.103261903	-1.043842641	0.2965582	0.009996783	1.366103544	26.7991065	1	CRA004775;PRJNA827465	Yang2025_Silkie_Wenchang	0.788394678
ENSGALG00010013332	LUM	2	-0.465679913	0.348029637	-1.338046718	0.180881208	0.228319715	17.37043111	94.24309049	1	PRJNA827465;Yang2025_Silkie_Wenchang	CRA004775	0.688484814
ENSGALG00010013332	LUM	2	-0.300414826	0.620459746	-0.484181009	0.628257413	0.635495199	4.953073405	79.81051525	0.5	CRA004775;Yang2025_Silkie_Wenchang	PRJNA827465	0.901904489
ENSGALG00010013332	LUM	2	-0.097117246	0.112854106	-0.860555716	0.389482785	0.001401692	1.008832426	0.875509691	0.5	CRA004775;PRJNA827465	Yang2025_Silkie_Wenchang	0.828911149
ENSGALG00010013333	PPP1R12A	2	0.174941691	0.20527594	0.85222696	0.394088136	0.073009996	6.957121084	85.62623838	0.5	PRJNA827465;Yang2025_Silkie_Wenchang	CRA004775	0.830486298
ENSGALG00010013333	PPP1R12A	2	0.37970487	0.126991518	2.990001809	0.002789758	0	0.107998902	0	1	CRA004775;Yang2025_Silkie_Wenchang	PRJNA827465	0.0719461
ENSGALG00010013333	PPP1R12A	2	0.050856477	0.124125703	0.409717536	0.682013165	0.015954532	1.468318299	31.89487587	0.5	CRA004775;PRJNA827465	Yang2025_Silkie_Wenchang	0.918515969
ENSGALG00010013334	SH3BGRL3	2	-0.131486649	0.170223276	-0.772436363	0.439856024	0.04827647	5.644456106	82.28350117	0.5	PRJNA827465;Yang2025_Silkie_Wenchang	CRA004775	0.844353254
ENSGALG00010013334	SH3BGRL3	2	-0.298642008	0.111260835	-2.684161122	0.007271205	0	0.118112157	0	1	CRA004775;Yang2025_Silkie_Wenchang	PRJNA827465	0.131077397
ENSGALG00010013334	SH3BGRL3	2	-0.024352944	0.097625733	-0.249452095	0.803011092	0.007672602	1.305352364	23.39233237	0.5	CRA004775;PRJNA827465	Yang2025_Silkie_Wenchang	0.950911267
ENSGALG00010013335	POPDC3	2	-0.477362293	0.51269555	-0.931083355	0.351810448	0.510429995	34.34556939	97.0884163	0.5	PRJNA827465;Yang2025_Silkie_Wenchang	CRA004775	0.810678834
ENSGALG00010013335	POPDC3	2	-0.295750563	0.779714117	-0.379306411	0.704460339	1.075991005	7.943207744	87.41062764	0.5	CRA004775;Yang2025_Silkie_Wenchang	PRJNA827465	0.924152671
ENSGALG00010013335	POPDC3	2	0.053017939	0.106353755	0.498505565	0.618127748	0	0.975825732	0	1	CRA004775;PRJNA827465	Yang2025_Silkie_Wenchang	0.899121942
ENSGALG00010013337	RAB19	2	0.096566469	0.145101371	0.66551038	0.505724107	0	0.363459686	0	0.5	PRJNA827465;Yang2025_Silkie_Wenchang	CRA004775	0.863927652
ENSGALG00010013337	RAB19	2	-0.112525491	0.242785518	-0.463476948	0.643022534	0	0.060833437	0	1	CRA004775;Yang2025_Silkie_Wenchang	PRJNA827465	0.906634523
ENSGALG00010013337	RAB19	2	0.094634195	0.152153463	0.621965438	0.533964594	0	0.601648395	0	0.5	CRA004775;PRJNA827465	Yang2025_Silkie_Wenchang	0.873408008
ENSGALG00010013339	ATP6V0D2	2	0.453324726	0.506682786	0.89469139	0.370952083	0	0.965494242	0	0.5	PRJNA827465;Yang2025_Silkie_Wenchang	CRA004775	0.8214684
ENSGALG00010013339	ATP6V0D2	2	0.978433491	0.562836099	1.738398609	0.082140602	0	0.016224937	0	1	CRA004775;Yang2025_Silkie_Wenchang	PRJNA827465	0.493958885
ENSGALG00010013339	ATP6V0D2	2	0.358835888	0.581968951	0.616589404	0.537505585	0	0.910897799	0	0.5	CRA004775;PRJNA827465	Yang2025_Silkie_Wenchang	0.87481821
ENSGALG00010013344	OSMR	2	-0.048417903	0.12156137	-0.398300076	0.690409004	0.019752241	2.943579454	66.02775581	0.5	PRJNA827465;Yang2025_Silkie_Wenchang	CRA004775	0.919979266
ENSGALG00010013344	OSMR	2	0.360122613	0.313405782	1.149061801	0.250530498	0.159200487	4.878142424	79.50039353	1	CRA004775;Yang2025_Silkie_Wenchang	PRJNA827465	0.760096222
ENSGALG00010013344	OSMR	2	0.246236588	0.437312765	0.563067461	0.573388947	0.347197502	10.19345481	90.18978336	0.5	CRA004775;PRJNA827465	Yang2025_Silkie_Wenchang	0.885850914
ENSGALG00010013345	DCN	2	-0.282542838	0.250360749	-1.128542871	0.259090722	0.114670774	11.62628054	91.39879692	1	PRJNA827465;Yang2025_Silkie_Wenchang	CRA004775	0.766276067
ENSGALG00010013345	DCN	2	-0.05123449	0.544681455	-0.094063217	0.925058939	0.521327064	7.600051018	86.84219359	0.5	CRA004775;Yang2025_Silkie_Wenchang	PRJNA827465	0.982594411
ENSGALG00010013345	DCN	2	0.143563428	0.274416159	0.523159528	0.600863243	0.101470891	2.345259855	57.36080171	0.5	CRA004775;PRJNA827465	Yang2025_Silkie_Wenchang	0.895230519
ENSGALG00010013346	HMGCS2	2	0.225115071	1.439616786	0.156371524	0.875740182	3.343351503	5.159491358	80.6182445	0.5	PRJNA827465;Yang2025_Silkie_Wenchang	CRA004775	0.970347777
ENSGALG00010013346	HMGCS2	2	0.117626476	1.206660792	0.09748098	0.922344438	2.435917845	5.948535744	83.18914027	0.5	CRA004775;Yang2025_Silkie_Wenchang	PRJNA827465	0.981804675
ENSGALG00010013346	HMGCS2	2	1.345726765	0.458890081	2.932568866	0.003361704	0	0.180351688	0	1	CRA004775;PRJNA827465	Yang2025_Silkie_Wenchang	0.081207766
ENSGALG00010013347	ENSGALG00010013347	2	-0.559390373	0.442018099	-1.26553726	0.205678801	0	0.981525989	0	1	PRJNA827465;Yang2025_Silkie_Wenchang	CRA004775	0.71825498
ENSGALG00010013347	ENSGALG00010013347	2	-0.632673078	0.498670223	-1.268720386	0.204540809	0	0.957725656	0	0.5	CRA004775;Yang2025_Silkie_Wenchang	PRJNA827465	0.71729696
ENSGALG00010013347	ENSGALG00010013347	2	-0.037572296	0.51825014	-0.072498382	0.942205293	0	0.022675988	0	0.5	CRA004775;PRJNA827465	Yang2025_Silkie_Wenchang	0.985827325
ENSGALG00010013348	MKRN4P	2	0.064865957	0.07589234	0.854710199	0.392711603	0.008175064	3.260985191	69.33442069	0.5	PRJNA827465;Yang2025_Silkie_Wenchang	CRA004775	0.829914191
ENSGALG00010013348	MKRN4P	2	-0.049893358	0.227774682	-0.219046989	0.826613444	0.087763246	6.055867761	83.48708989	0.5	CRA004775;Yang2025_Silkie_Wenchang	PRJNA827465	0.956412853
ENSGALG00010013348	MKRN4P	2	-0.107680359	0.145668908	-0.739213059	0.459777632	0.030930952	2.986628048	66.5174242	1	CRA004775;PRJNA827465	Yang2025_Silkie_Wenchang	0.850098539
ENSGALG00010013349	ETS2	2	-0.090030611	0.079065284	-1.138686997	0.254833728	0.004425672	1.518260299	34.13514132	1	PRJNA827465;Yang2025_Silkie_Wenchang	CRA004775	0.764418181
ENSGALG00010013349	ETS2	2	-0.139457958	0.093665321	-1.488896397	0.136514653	0	0.967534306	0	0.5	CRA004775;Yang2025_Silkie_Wenchang	PRJNA827465	0.618440666
ENSGALG00010013349	ETS2	2	-0.016298686	0.073095561	-0.222977782	0.823552798	0	0.094917544	0	0.5	CRA004775;PRJNA827465	Yang2025_Silkie_Wenchang	0.955934625
ENSGALG00010013351	EDAR	2	-0.028705151	0.296669633	-0.09675797	0.922918602	0	0.01723935	0	0.5	PRJNA827465;Yang2025_Silkie_Wenchang	CRA004775	0.981827844
ENSGALG00010013351	EDAR	2	0.521003149	0.642509203	0.810888228	0.417429861	0.487273821	2.26880391	55.92391235	1	CRA004775;Yang2025_Silkie_Wenchang	PRJNA827465	0.838112595
ENSGALG00010013351	EDAR	2	0.484618655	0.683078176	0.709462946	0.478037238	0.601403546	2.599886854	61.5367877	0.5	CRA004775;PRJNA827465	Yang2025_Silkie_Wenchang	0.855366076
ENSGALG00010013352	ASXL1	2	-0.049501112	0.061526229	-0.804552995	0.421077676	0.002600265	1.393626366	28.24475598	1	PRJNA827465;Yang2025_Silkie_Wenchang	CRA004775	0.839269089
ENSGALG00010013352	ASXL1	2	-0.15521721	0.095804263	-1.620149303	0.105200209	0	0.065810754	0	1	CRA004775;Yang2025_Silkie_Wenchang	PRJNA827465	0.550557098
ENSGALG00010013352	ASXL1	2	-0.017750926	0.051269872	-0.346225286	0.729173413	0	0.578169483	0	1	CRA004775;PRJNA827465	Yang2025_Silkie_Wenchang	0.932323844
ENSGALG00010013353	NDFIP1	2	-0.227068147	0.244340983	-0.929308479	0.352729242	0.104798945	7.45827188	86.5920683	1	PRJNA827465;Yang2025_Silkie_Wenchang	CRA004775	0.811035834
ENSGALG00010013353	NDFIP1	2	-0.332028058	0.199548638	-1.663895387	0.096133337	0.0383174	1.881638712	46.85483492	1	CRA004775;Yang2025_Silkie_Wenchang	PRJNA827465	0.530364903
ENSGALG00010013353	NDFIP1	2	-0.012844154	0.046225997	-0.27785563	0.781123185	0	0.130157992	0	1	CRA004775;PRJNA827465	Yang2025_Silkie_Wenchang	0.945235709
ENSGALG00010013354	WWP1	2	-0.113939496	0.063520973	-1.793730343	0.07285622	0	0.030625363	0	1	PRJNA827465;Yang2025_Silkie_Wenchang	CRA004775	0.466211007
ENSGALG00010013354	WWP1	2	0.194473131	0.36236897	0.536671587	0.591494485	0.208727791	4.206964272	76.22989083	0.5	CRA004775;Yang2025_Silkie_Wenchang	PRJNA827465	0.892776493
ENSGALG00010013354	WWP1	2	0.183544964	0.373951811	0.490825178	0.623550101	0.22709787	4.566932444	78.10346415	0.5	CRA004775;PRJNA827465	Yang2025_Silkie_Wenchang	0.900444194
ENSGALG00010013355	FAM171B	2	-0.12352533	0.120011528	-1.029278877	0.303348646	0.009407138	1.273979647	21.5058104	1	PRJNA827465;Yang2025_Silkie_Wenchang	CRA004775	0.792084776
ENSGALG00010013355	FAM171B	2	-0.215415768	0.219893652	-0.979636135	0.327265762	0.008739962	1.081385195	7.526013413	0.5	CRA004775;Yang2025_Silkie_Wenchang	PRJNA827465	0.800028239

ENSGALG00000013355	FAM171B	2	-0.052075927	0.091125472	-0.571480623	0.567673892	0	0.211746114	0	0.5	CRA004775;PRJNA827465	Yang2025_Silkie_Wenchang	0.883720322
ENSGALG00000013356	AMER1	2	-0.149536593	0.197678758	-0.756462632	0.449371869	0.070365046	9.624359617	89.60969831	0.5	PRJNA827465;Yang2025_Silkie_Wenchang	CRA004775	0.847263197
ENSGALG00000013356	AMER1	2	-0.335624373	0.10369424	-3.236673244	0.001209318	0	0.235409174	0	1	CRA004775;Yang2025_Silkie_Wenchang	PRJNA827465	0.042380792
ENSGALG00000013356	AMER1	2	-0.010341185	0.101379347	-0.102004851	0.918752824	0.009566395	1.348831598	25.86176057	0.5	CRA004775;PRJNA827465	Yang2025_Silkie_Wenchang	0.981033557
ENSGALG00000013357	RICTOR	2	0.10239018	0.258948238	0.395407902	0.692541878	0.110667376	5.61181365	82.18044892	0.5	PRJNA827465;Yang2025_Silkie_Wenchang	CRA004775	0.920559211
ENSGALG00000013357	RICTOR	2	0.436653548	0.166038162	2.629838479	0.008542544	0	0.665219325	0	1	CRA004775;Yang2025_Silkie_Wenchang	PRJNA827465	0.144221489
ENSGALG00000013357	RICTOR	2	0.215200449	0.437015453	0.492432127	0.622413892	0.30210663	4.136772742	75.82656669	0.5	CRA004775;PRJNA827465	Yang2025_Silkie_Wenchang	0.900093208
ENSGALG00000013358	RPS16	2	-0.341992633	0.210656687	-1.62345966	0.10449118	0.063735996	2.915449656	65.69997365	1	PRJNA827465;Yang2025_Silkie_Wenchang	CRA004775	0.549406937
ENSGALG00000013358	RPS16	2	-0.449912735	0.19997212	-2.249877303	0.024456735	0.009589378	1.133868564	11.80635644	1	CRA004775;Yang2025_Silkie_Wenchang	PRJNA827465	0.267184364
ENSGALG00000013358	RPS16	2	-0.188158592	0.068449158	-2.748881037	0.005979909	0	0.016171352	0	1	CRA004775;PRJNA827465	Yang2025_Silkie_Wenchang	0.116888359
ENSGALG00000013359	BTG1	2	-0.265431296	0.185140705	-1.433673359	0.151665498	0.059418989	7.276115724	86.25640331	1	PRJNA827465;Yang2025_Silkie_Wenchang	CRA004775	0.643081249
ENSGALG00000013359	BTG1	2	-0.279382134	0.204303803	-1.367483767	0.171473722	0.061538684	3.697925508	72.95781113	1	CRA004775;Yang2025_Silkie_Wenchang	PRJNA827465	0.6736737
ENSGALG00000013359	BTG1	2	-0.087594959	0.063833027	-1.372251374	0.169985197	0	0.043387694	0	1	CRA004775;PRJNA827465	Yang2025_Silkie_Wenchang	0.671517561
ENSGALG00000013360	PRR5	2	0.094128507	0.114348984	0.823168722	0.410412053	0.012057689	1.718075899	41.79535372	0.5	PRJNA827465;Yang2025_Silkie_Wenchang	CRA004775	0.834820782
ENSGALG00000013360	PRR5	2	-0.159500217	0.164695717	-0.968453948	0.332817708	0.01088035	1.180763296	15.30902057	1	CRA004775;Yang2025_Silkie_Wenchang	PRJNA827465	0.802178578
ENSGALG00000013360	PRR5	2	-0.060795344	0.300495165	-0.202317213	0.839668744	0.139114055	3.708145892	73.03234476	0.5	CRA004775;PRJNA827465	Yang2025_Silkie_Wenchang	0.959493354
ENSGALG00000013361	ENSGALG00000013361	2	-0.565945737	0.346743367	-1.63217466	0.102642697	0.156264875	2.82329167	64.58035099	1	PRJNA827465;Yang2025_Silkie_Wenchang	CRA004775	0.545895254
ENSGALG00000013361	ENSGALG00000013361	2	-0.372248833	0.59988909	-0.620529427	0.534909287	0.578802674	5.049078379	80.1944053	0.5	CRA004775;Yang2025_Silkie_Wenchang	PRJNA827465	0.873896294
ENSGALG00000013361	ENSGALG00000013361	2	-0.105845415	0.226164863	-0.468001144	0.639783771	0.0040704	1.032866583	3.182074395	0.5	CRA004775;PRJNA827465	Yang2025_Silkie_Wenchang	0.906064918
ENSGALG00000013362	ENSGALG00000013362	2	-0.268799674	0.198143325	-1.356592123	0.174910846	0.018889608	1.312424423	23.80513632	1	PRJNA827465;Yang2025_Silkie_Wenchang	CRA004775	0.679636281
ENSGALG00000013362	ENSGALG00000013362	2	-0.793966216	0.89150402	-0.890591852	0.373148172	1.280810344	4.55038159	78.02382108	1	CRA004775;Yang2025_Silkie_Wenchang	PRJNA827465	0.822187352
ENSGALG00000013362	ENSGALG00000013362	2	-0.941969769	0.674101822	-1.397370159	0.162302288	0.646443971	2.833154668	64.70365413	1	CRA004775;PRJNA827465	Yang2025_Silkie_Wenchang	0.659870366
ENSGALG00000013363	HS3ST5	2	-0.092794638	0.121806901	-0.761817578	0.446168888	0	0.660688648	0	1	PRJNA827465;Yang2025_Silkie_Wenchang	CRA004775	0.846013379
ENSGALG00000013363	HS3ST5	2	-0.036399666	0.246758123	-0.14751152	0.882728288	0.055423164	1.652110817	39.47137263	0.5	CRA004775;Yang2025_Silkie_Wenchang	PRJNA827465	0.972001076
ENSGALG00000013363	HS3ST5	2	0.048393857	0.148753434	0.325329343	0.744991826	0	0.673596466	0	0.5	CRA004775;PRJNA827465	Yang2025_Silkie_Wenchang	0.935927651
ENSGALG00000013365	CEP85	2	-0.065099413	0.127955257	-0.508767006	0.610915549	0.019016989	2.345835097	57.37125762	0.5	PRJNA827465;Yang2025_Silkie_Wenchang	CRA004775	0.897506931
ENSGALG00000013365	CEP85	2	0.082029717	0.128916176	0.636302751	0.524579112	0	0.01525837	0	1	CRA004775;Yang2025_Silkie_Wenchang	PRJNA827465	0.869811145
ENSGALG00000013365	CEP85	2	-0.166919296	0.100322194	-1.663832185	0.09614597	0	0.391322618	0	0.5	CRA004775;PRJNA827465	Yang2025_Silkie_Wenchang	0.530364903
ENSGALG00000013367	NOL4L	2	-0.046132473	0.071403953	-0.646077292	0.518229301	0	0.294997178	0	0.5	PRJNA827465;Yang2025_Silkie_Wenchang	CRA004775	0.868375737
ENSGALG00000013367	NOL4L	2	0.124704831	0.129675654	0.961667259	0.336216774	0	0.663438081	0	1	CRA004775;Yang2025_Silkie_Wenchang	PRJNA827465	0.803727281
ENSGALG00000013367	NOL4L	2	0.038472312	0.148002645	0.259943408	0.794907427	0.026245854	2.108854906	52.5809008	0.5	CRA004775;PRJNA827465	Yang2025_Silkie_Wenchang	0.948905846
ENSGALG00000013369	MTMR8	2	-0.2190473	0.16202937	-1.35189874	0.176407711	0.045369124	7.102407367	85.92026691	1	PRJNA827465;Yang2025_Silkie_Wenchang	CRA004775	0.681691731
ENSGALG00000013369	MTMR8	2	-0.365513079	0.101907594	-3.586710907	0.000334875	0	0.78919596	0	1	CRA004775;Yang2025_Silkie_Wenchang	PRJNA827465	0.018128927
ENSGALG00000013369	MTMR8	2	-0.068917818	0.058106732	-1.186055661	0.235600319	0	0.00031259	0	1	CRA004775;PRJNA827465	Yang2025_Silkie_Wenchang	0.748626601
ENSGALG00000013370	CNKSR1	2	-0.01539404	0.279671339	-0.055043324	0.956103948	0.124316449	4.799403769	79.16407854	0.5	PRJNA827465;Yang2025_Silkie_Wenchang	CRA004775	0.988513657
ENSGALG00000013370	CNKSR1	2	0.13146174	0.317536919	0.414004583	0.678870751	0.081089964	1.318225909	24.14046838	0.5	CRA004775;Yang2025_Silkie_Wenchang	PRJNA827465	0.917701868
ENSGALG00000013370	CNKSR1	2	-0.290374568	0.147990122	-1.962121278	0.049748366	0	0.137092433	0	1	CRA004775;PRJNA827465	Yang2025_Silkie_Wenchang	0.386162809
ENSGALG00000013371	FAT1	2	-0.017060937	0.367852801	-0.046379793	0.963007542	0.239523097	8.193829535	87.79569436	0.5	PRJNA827465;Yang2025_Silkie_Wenchang	CRA004775	0.989998461
ENSGALG00000013371	FAT1	2	0.402629636	0.200583414	2.007292769	0.044718499	0	0.017078672	0	1	CRA004775;Yang2025_Silkie_Wenchang	PRJNA827465	0.364414504
ENSGALG00000013371	FAT1	2	-0.033335212	0.39016268	-0.085439264	0.931912179	0.245754227	4.469847546	77.62787232	0.5	CRA004775;PRJNA827465	Yang2025_Silkie_Wenchang	0.98412915
ENSGALG00000013372	SPRY4	2	0.259223374	0.079405338	3.264558543	0.001096348	0	0.544316442	0	1	PRJNA827465;Yang2025_Silkie_Wenchang	CRA004775	0.040009069
ENSGALG00000013372	SPRY4	2	0.146633131	0.292629565	0.501087889	0.61630927	0.105825112	2.222378721	55.00316887	0.5	CRA004775;Yang2025_Silkie_Wenchang	PRJNA827465	0.899111936
ENSGALG00000013372	SPRY4	2	0.123056156	0.197281724	0.623758521	0.532786184	0.040196499	1.505444751	33.57444708	0.5	CRA004775;PRJNA827465	Yang2025_Silkie_Wenchang	0.873183244
ENSGALG00000013373	ENSGALG00000013373	2	-0.239992059	0.396516045	-0.605251824	0.545011686	0	0.686255144	0	1	PRJNA827465;Yang2025_Silkie_Wenchang	CRA004775	0.877354247
ENSGALG00000013373	ENSGALG00000013373	2	-0.110379684	0.813747786	-0.135643606	0.892103031	0.449129234	1.504978253	33.55385716	0.5	CRA004775;Yang2025_Silkie_Wenchang	PRJNA827465	0.974088775
ENSGALG00000013373	ENSGALG00000013373	2	0.043024721	0.385845765	0.111507563	0.911213869	0	0.5590497	0	0.5	CRA004775;PRJNA827465	Yang2025_Silkie_Wenchang	0.978845851
ENSGALG00000013374	RMDN1	2	-0.099722773	0.121050468	-0.823811545	0.410046646	0	0.355855908	0	0.5	PRJNA827465;Yang2025_Silkie_Wenchang	CRA004775	0.834652129
ENSGALG00000013374	RMDN1	2	-0.181360418	0.276624858	-0.655618657	0.512069518	0.063543276	1.586051797	36.95035675	0.5	CRA004775;Yang2025_Silkie_Wenchang	PRJNA827465	0.865694231
ENSGALG00000013374	RMDN1	2	-0.192354683	0.135057643	-1.42424138	0.15437661	0	0.978243414	0	1	CRA004775;PRJNA827465	Yang2025_Silkie_Wenchang	0.646989113
ENSGALG00000013375	PSMG1	2	-0.501167747	0.462737138	-1.083050625	0.278785953	0.404997315	17.6524927	94.33507768	1	PRJNA827465;Yang2025_Silkie_Wenchang	CRA004775	0.777045905
ENSGALG00000013375	PSMG1	2	-0.551596321	0.433848047	-1.271404413	0.203584814	0.332581364	8.580044629	88.34504897	1	CRA004775;Yang2025_Silkie_Wenchang	PRJNA827465	0.717254253
ENSGALG00000013375	PSMG1	2	-0.064777427	0.052273393	-1.23920456	0.215269752	0	0.07722045	0	1	CRA004775;PRJNA827465	Yang2025_Silkie_Wenchang	0.728683754
ENSGALG00000013376	ARHGAP8	2	-0.742016326	0.369318592	-2.009149666	0.044521264	0	0.540589544	0	1	PRJNA827465;Yang2025_Silkie_Wenchang	CRA004775	0.364090561
ENSGALG00000013376	ARHGAP8	2	-0.509366521	0.483729737	-1.052998156	0.292341837	0.210879917	1.773777298	43.62313686	1	CRA004775;Yang2025_Silkie_Wenchang	PRJNA827465	0.785753005
ENSGALG00000013376	ARHGAP8	2	-0.29982624	0.317561294	-0.944152344	0.345091804	0	0.44228468	0	1	CRA004775;PRJNA827465	Yang2025_Silkie_Wenchang	0.806961063
ENSGALG00000013378	SH3RF3	2	-0.156925909	0.081864029	-1.916909183	0.05524947	0	0.007426534	0	1	PRJNA827465;Yang2025_Silkie_Wenchang	CRA004775	0.407036233
ENSGALG00000013378	SH3RF3	2	-0.060619999	0.16010209	-0.3786334	0.704960116	0.012719455	1.209184267	17.29961865	0.5	CRA004775;Yang2025_Silkie_Wenchang	PRJNA827465	0.924152671
ENSGALG00000013378	SH3RF3	2	-0.077148092	0.163040295	-0.473184203	0.636081767	0.023767433	1.427271128	29.93622723	0.5	CRA004775;PRJNA827465	Yang2025_Silkie_Wenchang	0.904450696
ENSGALG00000013379	ZNF593	2	-0.120561321	0.075212159	-1.60294988	0.10894572	0	0.412836364	0	1	PRJNA827465;Yang2025_Silkie_Wenchang	CRA004775	0.558848033
ENSGALG00000013379	ZNF593	2	-0.410525618	0.184713239	-2.222502409	0.026249371	0	0.152377471	0	1	CRA004775;Yang2025_Silkie_Wenchang	PRJNA827465	0.276215866
ENSGALG00000013379	ZNF593	2	-0.220272778	0.165124918	-1.333976609	0.182211523	0.033756872	2.144571808	53.37064507	1	CRA004775;PRJNA827465	Yang2025_Silkie_Wenchang	0.690033956
ENSGALG00000013380	PHGDH	2	0.152494845	0.196332168	0.77671859	0.437324817	0.044515935	2.321780949	56.92961472	0.5	PRJNA827465;Yang2025_Silkie_Wenchang	CRA004775	0.843715751
ENSGALG00000013380	PHGDH	2	-0.084180516	0.170496654	-0.493737053	0.621491893	0.008569472	1.06547028	6.144730755	1	CRA004775;Yang2025_Silkie_Wenchang	PRJNA827465	0.899719912
ENSGALG00000013380	PHGDH	2	0.017158604	0.449640286	0.038160735	0.969559527	0.28633584	3.042716572	67.13463195	0.5	CRA004775;PRJNA827465	Yang2025_Silkie_Wenchang	0.991572914
ENSGALG00000013381	DENND2A	2	-0.018622658	0.045123865	-0.412700861	0.679825794	0	0.217554431	0	1	PRJNA827465;Yang2025_Silkie_Wenchang	CRA004775	0.917701868
ENSGALG00000013381	DENND2A	2	-0.019941922	0.092906682	-0.214644648	0.830044381	0.000500799	1.015267111	1.503753112	0.5	CRA004775;Yang2025_Silkie_Wenchang	PRJNA827465	0.957212554
ENSGALG00000013381	DENND2A	2	0.001330175	0.04964105	0.026795877	0.978622542	0	0.728457993	0	0.5	CRA004775;PRJNA827465	Yang2025_Silkie_Wenchang	0.994183692
ENSGALG00000013382	ASB12	2	-0.206639824	0.095889339	-2.154982243	0.031163224	0	0.27229273	0	1	PRJNA827465;Yang2025_Silkie_Wenchang	CRA004775	0.301974405
ENSGALG00000013382	ASB12	2	-0.316738695	0.196825613	-1.609235147	0.107564934	0	0.106761574	0	1	CRA004775;Yang2025_Silkie_Wenchang	PRJNA827465	0.555097767
ENSGALG00000013382	ASB12	2	-0.18708078	0.10773162	-1.736544763	0.082467557	0	0.235604693	0	1	CRA004775;PRJNA827465	Yang2025_Silkie_Wenchang	0.495221142
ENSGALG00000013383	SEPTIN10	2	-0.056872828	0.101188535	-0.562048139	0.574083221	0.014862875	3.555597484	71.87533166	0.5	PRJNA827465;Yang2025_Silkie_Wenchang	CRA004775	0.886081433
ENSGALG00000013383	SEPTIN10	2	0.113675253	0.332400503	0.341982794	0.732363841	0.182298815	5.083104783	80.32698434	0.5	CRA004775;Yang2025_Silkie_Wenchang	PRJNA827465	0.932985777
ENSGALG00000013383	SEPTIN10	2	0.187415117	0.220207546	0.85108399	0.394722698	0.068003305	2.595903023	61.47775972	1	CRA004775;PRJNA827465	Yang2025_Silkie_Wenchang	0.830643015
ENSGALG00000013385	PGRMC1	2	-0.45251811	0.28640881	-1.579972734	0.114113111	0.141226404	6.988386966	85.69054626	1	PRJNA827465;Yang2025_Silkie_Wenchang	CRA004775	0.569956487
ENSGALG00000013385	PGRMC1	2	-0.448435058	0.324722395	-1.380979768	0.167285182	0.167159997	4.786002705	79.10573684	1	CRA004775;Yang2025_Silkie_Wenchang	PRJNA827465	0.666816379
ENSGALG00000013385	PGRMC1	2	-0.170482568	0.100572059	-1.695128543	0.090051039	0	0.087352131	0	1	CRA004775;PRJNA827465	Yang2025_Silkie_Wenchang	0.514878669
ENSGALG00000013386	ITGAV	2	-0.224891742	0.237795584	-0.945735567	0.344283477	0.10268812	10.71684245	90.66889333	0.5	PRJNA827465;Yang2025_Silkie_Wenchang	CRA004775	0.806639254
ENSGALG00000013386	ITGAV	2	-0.173245245	0.373018258	-0.46444173	0.642331298	0.213463269	3.70835068	73.03383401	0.5	CRA004775;Yang2025_Silkie_Wenchang	PRJNA827465	0.906634523
ENSGALG00000013386	ITGAV	2	0.016208015	0.077532307	0.209048531	0.834410354	0	0.558936222	0	1	CRA004775;PRJNA827465	Yang2025_Silkie_Wenchang	0.958051246
ENSGALG00000013387	NDST1	2	-0.050652415	0.059232525	-0.855145286	0.392470722	0	0.156896746	0	1	PRJNA827465;Yang2025_Silkie_Wenchang	CRA004775	0.829717432
ENSGALG00000013387	NDST1	2	-0.21450176	0.172263168	-1.245198046	0.213058961	0.042018848	3.105420092	67.79823758	1	CRA004775;Yang2025_Silkie_Wenchang	PRJNA827465	0.72579791
ENSGALG00000013387	NDST1	2	-0.194854729	0.196852135	-0.989853267	0.322245845	0.059822266	4.024160222	75.15009481	1	CRA004775;PRJNA827465	Yang2025_Silkie_Wenchang	0.798513866
ENSGALG00000013388	SOWAHC	2	0.420375182	0.219804928	1.912492076	0.055813108	0.041565641	1.688972901	40.79241891	1	PRJNA827465;Yang2025_Silkie_Wenchang	CRA004775	0.408997088
ENSGALG00000013388	SOWAHC	2	0.219745475	0.531243102	0.413643911	0.67913491	0.426885521	3.965378824	74.78172845	0.5	CRA004775;Yang2025_Silkie_Wenchang	PRJNA827465	0.917701868
ENSGALG00000013388	SOWAHC	2	0.072968625	0.277173929	0.26325934	0.792350697	0.069594121	1.576158681	36.55461141	0.5	CRA004775;PRJNA827465	Yang2025_Silkie_Wenchang	0.948368502
ENSGALG00000013389	NAP1L1	2	-0.137370717	0.172149184	-0.797974837	0.424885093	0.055037363	13.84655174	92.77798531	0.5	PRJNA827465;Yang2025_Silkie_Wenchang	CRA004775	0.841139484
ENSGALG00000013389	NAP1L1	2	-0.222326126	0.12340116	-1.801653451	0.071599946	0.017727617	2.153618846	53.56652817	1	CRA004775;Yang2025_Silkie_Wenchang	PRJNA827465	0.46178455
ENSGALG00000013389	NAP1L1	2	0.021663667	0.048049386	0.45086252	0.652088638	0	0.278780833	0	0.5	CRA004775;PRJNA827465	Yang2025_Silkie_Wenchang	0.909322729
ENSGALG00000013390	SLC16A2	2	-0.039238125	0.096400705	-0.407031515	0.683984841	0	0.033982848	0	1	PRJNA827465;Yang2025_Silkie_Wenchang	CRA004775	0.918806497
ENSGALG00000013390	SLC16A2	2	0.092495776	0.223457341	0.413930354	0.678925113	0.050745511	1.791039499	44.16650216	0.5	CRA004775;Yang2025_Silkie_Wenchang	PRJNA827465	0.917701868
ENSGALG00000013390	SLC16A2	2	0.095808489	0.19705165	0.486210033	0.626818259	0.037490308	1.617169951	38.1635802	0.5	CRA004775;PRJNA827465	Yang2025_Silkie_Wenchang	0.901520036
ENSGALG00000013391	HMGN1	2	0.050379248	0.050959847	0.988606743	0.322855586	0	0.478540279	0	1	PRJNA827465;Yang2025_Silkie_Wenchang	CRA004775	0.79874391
ENSGALG00000013391	HMGN1	2	0.207047268	0.155522979	1.331296958	0.183091319	0.020217848	1.455136052	31.27790365	1	CRA004775;Yang2025_Silkie_Wenchang	PRJNA827465	0.691392093
ENSGALG00000013391	HMGN1	2	0.171835648	0.206685529	0.831386935	0.405755077	0.059000399	2.47206313	59.54795864	1	CRA004775;PRJNA827465	Yang2025_Silkie_Wenchang	0.833357445
ENSGALG00000013392	HDAC2	2	0.093668486	0.043145699	2.170980826	0.029932622	0	0.376939284	0	1	PRJNA827465;Yang2025_Silkie_Wenchang	CRA004775	0.295167651
ENSGALG00000013392	HDAC2	2	0.134716543	0.107952801	1.247920773	0.212060073	0	0.229840018	0	1	CRA004775;Yang2025_Silkie_Wenchang	PRJNA827465	0.724432597
ENSGALG00000013392	HDAC2	2	0.08264723	0.044691905	1.84926619	0.064419385	0	0.022511808	0	1	CRA004775;PRJNA827465	Yang2025_Silkie_Wenchang	0.437453135
ENSGALG00000013393	ADCK2	2	0.122208267	0.079137475	1.544252799	0.122527102	0	0.725524224	0	0.5	PRJNA827465;Yang2025_Silkie_Wenchang	CRA004775	0.587278379
ENSGALG00000013393	ADCK2	2	-0.143699994	0.175768308	-0.817553492	0.413612193	0	0.559196421	0	1	CRA004775;Yang2025_Silkie_Wenchang	PRJNA827465	0.836267299
ENSGALG00000013393	ADCK2	2	-0.001124917	0.220880897	-0.005092869	0.995936496	0.064005048	2.305470344	56.62490291	0.5	CRA004775;PRJNA827465	Yang2025_Silkie_Wenchang	0.998680753
ENSGALG00000013394	DNMT3B	2	0.205734564	0.092166203	2.232212651	0.025600912	0	0.257298157	0	1	PRJNA827465;Yang2025_Silkie_Wenchang	CRA004775	0.27294819
ENSGALG00000013394	DNMT3B	2	0.889042877	0.711020444	1.25037597	0.211162239	0.828341345	4.893791825	79.56594731	1	CRA004775;Yang2025_Silkie_Wenchang	PRJNA827465	0.723314403
ENSGALG00000013394	DNMT3B	2	0.834593986	0.76697133	1.088168427	0.27652076	1.005900521	6.023258482	83.39769075	1	CRA004775;PRJNA827465	Yang2025_Silkie_Wenchang	0.775851484
ENSGALG00000013396	FYB1	2	-0.229237402	0.103745148	-2.209620469	0.027131513	0	0.549063181	0	1	PRJNA827465;Yang2025_Silkie_Wenchang	CRA004775	0.280945645
ENSGALG00000013396	FYB1	2	-0.358107142	0.16868133	-2.122980311	0.033755502	0	0.074112844	0	1	CRA004775;Yang2025_Silkie_Wenchang	PRJNA827465	0.315687663
ENSGALG00000013396	FYB1	2	-0.197106778	0.121755499	-1.618873728	0.105474435	0	0.397197227	0	1	CRA004775;PRJNA827465	Yang2025_Silkie_Wenchang	0.551018754
ENSGALG00000013397	MATN1	2	1.477306565	0.612808256	2.410715831	0.015921248	0	0.020192468	0	1	PRJNA827465;Yang2025_Silkie_Wenchang	CRA004775	0.208471156
ENSGALG00000013397	MATN1	2	-0.432294467	1.946628397	-0.222073441	0.824256712	6.996935302	12.90281605	92.24975388	0.5	CRA004775;Yang2025_Silkie_Wenchang	PRJNA827465	0.95619211
ENSGALG00000013397	MATN1	2	-0.509246652	1.859611547	-0.273845714	0.784203191	6.380400309	12.81763375	92.19824798	0.5	CRA004775;PRJNA827465	Yang2025_Silkie_Wenchang	0.946083234
ENSGALG00000013398	CHAMP1	2	-0.111222645	0.056038256	-1.984762798	0.047170868	0	0.04208148	0	1	PRJNA827465;Yang2025_Silkie_Wenchang	CRA004775	0.374763824
ENSGALG00000013398	CHAMP1	2	0.150950815	0.28321135	0.532997053	0.59403562	0.120520326	3.931366736	74.56355341	0.5	CRA004775;Yang2025_Silkie_Wenchang	PRJNA827465	0.893235212
ENSGALG00000013398	CHAMP1	2	0.128193899	0.258470394	0.495971306	0.619914656	0.119452578	8.853939189	88.70559218	0.5	CRA004775;PRJNA827465	Yang2025_Silkie_Wenchang	0.899395593
ENSGALG00000013399	FGF1	2	-0.174483829	0.146305039	-1.192603005	0.233024888	0.009267706	1.263056722	20.826992	0.5	PRJNA827465;Yang2025_Silkie_Wenchang	CRA004775	0.746055489
ENSGALG00000013399	FGF1	2	-0.252869032	0.154460771	-1.637108436	0.101607814	0	0.643472413	0	0.5	CRA004775;Yang2025_Silkie_Wenchang	PRJNA827465	0.543686036
ENSGALG00000013399	FGF1	2	0.021304381	0.192276457	0.110800779	0.911774328	0	0.044574188	0	1	CRA004775;PRJNA827465	Yang2025_Silkie_Wenchang	0.978895589
ENSGALG00000013400	BBS10	2	-0.08206529	0.122970369	-0.667358243	0.504543332	0.000237746	1.006520979	0.647873147	0.5	PRJNA827465;Yang2025_Silkie_Wenchang	CRA004775	0.863512504
ENSGALG00000013400	BBS10	2	-0.208025943	0.346985882	-0.599522785	0.548824321	0.169815043	3.332894645	69.99605129	0.5	CRA004775;Yang2025_Silkie_Wenchang	PRJNA827465	0.878281266
ENSGALG00000013400	BBS10	2	-0.288193152	0.194964698	-1.478181204	0.139359284	0.03319061	1.578200326	36.63668778	1	CRA004775;PRJNA827465	Yang2025_Silkie_Wenchang	0.623009874
ENSGALG00000013402	MARCKS	2	0.035905749	0.066129563	0.542960618	0.587156918	0	0.434825904	0	0.5	PRJNA827465;Yang2025_Silkie_Wenchang	CRA004775	0.891596903
ENSGALG00000013402	MARCKS	2	0.094326378	0.094678965	0.996275982	0.31911607	0	0.099384157	0	1	CRA004775;Yang2025_Silkie_Wenchang	PRJNA827465	0.798079048
ENSGALG00000013402	MARCKS	2	0.011290539	0.085223945	0.132480825	0.894603988	0	0.347019391	0	0.5	CRA004775;PRJNA827465	Yang2025_Silkie_Wenchang	0.974491299
ENSGALG00000013403	CPNE3	2	-0.250544301	0.171072648	-1.464549149	0.143043964	0.048684138	5.902489779	83.05799692	1	PRJNA827465;Yang2025_Silkie_Wenchang	CRA004775	0.629922346
ENSGALG00000013403	CPNE3	2	-0.218744993	0.250763128	-0.872317211	0.383035351	0.094608848	3.681702647	72.83865385	0.5	CRA004775;Yang2025_Silkie_Wenchang	PRJNA827465	0.825352982
ENSGALG00000013403	CPNE3	2	-0.066250936	0.082604165	-0.802029006	0.42253618	0	0.42309825	0	0.5	CRA004775;PRJNA827465	Yang2025_Silkie_Wenchang	0.839709737
ENSGALG00000013404	ENSGALG00000013404	2	-0.178746038	0.141970215	-1.259039008	0.208016243	0	0.163255433	0	1	PRJNA827465;Yang2025_Silkie_Wenchang	CRA004775	0.720568788
ENSGALG00000013404	ENSGALG00000013404	2	0.061449024	0.43464616	0.141377124	0.887572025	0.231654319	2.275382867	56.0513523	0.5	CRA004775;Yang2025_Silkie_Wenchang	PRJNA827465	0.973038314
ENSGALG00000013404	ENSGALG00000013404	2	0.082443951	0.353653321	0.233120815	0.815665592	0.143569952	1.862569505	46.31072842	0.5	CRA004775;PRJNA827465	Yang2025_Silkie_Wenchang	0.954197797
ENSGALG00000013405	RPS14	2	-0.109130973	0.133987918	-0.81448368	0.415367918	0.016962795	1.279878967	21.86761203	1	PRJNA827465;Yang2025_Silkie_Wenchang	CRA004775	0.837576587
ENSGALG00000013405	RPS14	2	-0.380956314	0.201118389	-1.894189368	0.058199879	0	0.062630387	0	1	CRA004775;Yang2025_Silkie_Wenchang	PRJNA827465	0.418024459
ENSGALG00000013405	RPS14	2	-0.099901416	0.102998487	-0.969931106	0.332080834	0.008732864	1.266831099	21.064125	1	CRA004775;PRJNA827465	Yang2025_Silkie_Wenchang	0.802002658
ENSGALG00000013406	ENSGALG00000013406	2	-0.315157591	0.36922096	-0.853574484	0.393340801	0	0.448042038	0	1	PRJNA827465;Yang2025_Silkie_Wenchang	CRA004775	0.830346712
ENSGALG00000013406	ENSGALG00000013406	2	-0.723644337	0.465957434	-1.553026703	0.120416755	0	0.03760302	0	1	CRA004775;Yang2025_Silkie_Wenchang	PRJNA827465	0.581775014
ENSGALG00000013406	ENSGALG00000013406	2	-0.336453658	0.387050965	-0.869274822	0.384696831	0	0.676715699	0	1	CRA004775;PRJNA827465	Yang2025_Silkie_Wenchang	0.826395909
ENSGALG00000013407	ZC3H15	2	0.154255366	0.19429593	0.779473386	0.435600082	0.071237665	10.28769769	90.27965216	0.5	PRJNA827465;Yang2025_Silkie_Wenchang	CRA004775	0.843269499
ENSGALG00000013407	ZC3H15	2	0.402324211	0.103456514	3.888824366	0.000100731	0	0.780373027	0	1	CRA004775;Yang2025_Silkie_Wenchang	PRJNA827465	0.007870834
ENSGALG00000013407	ZC3H15	2	0.242217906	0.324597084	0.746210973	0.455539981	0.177852637	5.577254836	82.07003213	0.5	CRA004775;PRJNA827465	Yang2025_Silkie_Wenchang	0.84907427
ENSGALG00000013408	RLIM	2	-0.010063739	0.032084748	-0.313661137	0.75377842	0	0.096973395	0	0.5	PRJNA827465;Yang2025_Silkie_Wenchang	CRA004775	0.938155284
ENSGALG00000013408	RLIM	2	0.058280147	0.074654766	0.780662103	0.435001256	0	0.907274331	0	1	CRA004775;Yang2025_Silkie_Wenchang	PRJNA827465	0.843762754
ENSGALG00000013408	RLIM	2	0.029239346	0.079588575	0.3673812	0.713334693	0.007014842	1.638949317	38.98530054	0.5	CRA004775;PRJNA827465	Yang2025_Silkie_Wenchang	0.926216079
ENSGALG00000013409	DAB2	2	0.252767187	0.176257195	1.434081527	0.151549999	0.039504867	2.565373473	61.01932095	1	PRJNA827465;Yang2025_Silkie_Wenchang	CRA004775	0.643023891
ENSGALG00000013409	DAB2	2	0.118301334	0.313156408	0.584431522	0.558941055	0.141508979	3.514817732	71.54902256	0.5	CRA004775;Yang2025_Silkie_Wenchang	PRJNA827465	0.880962441
ENSGALG00000013409	DAB2	2	0.070900741	0.102519386	0.691583745	0.489198773	0	0.836658395	0	0.5	CRA004775;PRJNA827465	Yang2025_Silkie_Wenchang	0.858337006
ENSGALG00000013410	KIAA0825	2	0.220880656	0.138602277	1.59362934	0.111019079	0	0.493910674	0	1	PRJNA827465;Yang2025_Silkie_Wenchang	CRA004775	0.563890263
ENSGALG00000013410	KIAA0825	2	0.31674155	0.175225798	1.807619388	0.070665759	0	7.54402E-06	0	1	CRA004775;Yang2025_Silkie_Wenchang	PRJNA827465	0.458542662
ENSGALG00000013410	KIAA0825	2	0.159742732	0.177159028	0.901691176	0.367229942	0	0.186906108	0	1	CRA004775;PRJNA827465	Yang2025_Silkie_Wenchang	0.819739734
ENSGALG00000013411	ENSGALG00000013411	2	1.181484327	0.430188616	2.746433267	0.006024714	0	0.214718932	0	1	PRJNA827465;Yang2025_Silkie_Wenchang	CRA004775	0.117234105
ENSGALG00000013411	ENSGALG00000013411	2	1.842185064	0.975318949	1.888802701	0.058918271	1.303043832	3.144488441	68.19832482	1	CRA004775;Yang2025_Silkie_Wenchang	PRJNA827465	0.42013793
ENSGALG00000013411	ENSGALG00000013411	2	1.971139048	0.759636228	2.594846027	0.00946333	0.676902519	2.310879988	56.72644164	1	CRA004775;PRJNA827465	Yang2025_Silkie_Wenchang	0.152962732
ENSGALG00000013412	COMMD7	2	-0.086832424	0.072137943	-1.203696321	0.228749509	0.001563501	1.092119589	8.434919287	1	PRJNA827465;Yang2025_Silkie_Wenchang	CRA004775	0.742152826
ENSGALG00000013412	COMMD7	2	-0.258069516	0.139105374	-1.855208819	0.063566413	0	0.012729137	0	1	CRA004775;Yang2025_Silkie_Wenchang	PRJNA827465	0.43419373
ENSGALG00000013412	COMMD7	2	-0.071439717	0.065888332	-1.084254443	0.278251998	0	0.823265525	0	1	CRA004775;PRJNA827465	Yang2025_Silkie_Wenchang	0.776763074
ENSGALG00000013413	WRB	2	-0.123598624	0.17311057	-0.714985283	0.476416281	0.05174565	7.094906371	85.9053813	0.5	PRJNA827465;Yang2025_Silkie_Wenchang	CRA004775	0.854986476
ENSGALG00000013413	WRB	2	-0.295109839	0.093145889	-3.168254047	0.001533574	0	0.047942248	0	1	CRA004775;Yang2025_Silkie_Wenchang	PRJNA827465	0.048963507
ENSGALG00000013413	WRB	2	-0.07587479	0.14689035	-0.516540327	0.605477081	0.030314519	2.93140387	65.88665211	0.5	CRA004775;PRJNA827465	Yang2025_Silkie_Wenchang	0.896474603
ENSGALG00000013414	LAPTM5	2	-0.205308667	0.316333955	-0.649025068	0.516322174	0.18841233	16.93321132	94.09444564	0.5	PRJNA827465;Yang2025_Silkie_Wenchang	CRA004775	0.867685411
ENSGALG00000013414	LAPTM5	2	-0.528784507	0.122734141	-4.30833738	1.6468E-05	0	1.10538E-05	0	1	CRA004775;Yang2025_Silkie_Wenchang	PRJNA827465	0.00211926
ENSGALG00000013414	LAPTM5	2	-0.067916648	0.280995863	-0.241699816	0.809012773	0.101207584	2.031910964	50.78524514	0.5	CRA004775;PRJNA827465	Yang2025_Silkie_Wenchang	0.952707985
ENSGALG00000013416	NDUFB2	2	0.185140107	0.162780518	1.137360351	0.255387675	0.027817652	1.503772779	33.50059171	1	PRJNA827465;Yang2025_Silkie_Wenchang	CRA004775	0.764467569
ENSGALG00000013416	NDUFB2	2	0.260945165	0.260998678	0.999794967	0.317409742	0.02571364	1.232164453	18.84200217	0.5	CRA004775;Yang2025_Silkie_Wenchang	PRJNA827465	0.797125693
ENSGALG00000013416	NDUFB2	2	0.098956124	0.071026986	1.393219676	0.163563674	0	0.108580024	0	1	CRA004775;PRJNA827465	Yang2025_Silkie_Wenchang	0.661805642
ENSGALG00000013417	AKR1B1	2	0.015720226	0.072867232	0.215737933	0.829192031	0	0.274138472	0	0.5	PRJNA827465;Yang2025_Silkie_Wenchang	CRA004775	0.956860127
ENSGALG00000013417	AKR1B1	2	-0.073446426	0.114865551	-0.639412125	0.522554863	0	0.752736898	0	1	CRA004775;Yang2025_Silkie_Wenchang	PRJNA827465	0.869568211
ENSGALG00000013417	AKR1B1	2	-0.01874182	0.141503715	-0.132449175	0.894630307	0.015742927	1.27441896	21.53287078	0.5	CRA004775;PRJNA827465	Yang2025_Silkie_Wenchang	0.974492959
ENSGALG00000013419	ENSGALG00000013419	2	-0.0810059	0.186231944	-0.434973176	0.663581898	0.024152549	1.376783629	27.36694574	0.5	PRJNA827465;Yang2025_Silkie_Wenchang	CRA004775	0.913119244
ENSGALG00000013419	ENSGALG00000013419	2	0.302292082	0.277160648	1.090674614	0.275416085	0	0.205709898	0	1	CRA004775;Yang2025_Silkie_Wenchang	PRJNA827465	0.775135547
ENSGALG00000013419	ENSGALG00000013419	2	-0.029012162	0.298132168	-0.097313087	0.922477763	0.092794456	1.598703546	37.44931621	0.5	CRA004775;PRJNA827465	Yang2025_Silkie_Wenchang	0.981804675
ENSGALG00000013420	ENSGALG00000013420	2	-6.01295569	5.98086168	-1.005366118	0.314720588	64.87972801	10.65972305	90.61889324	1	PRJNA827465;Yang2025_Silkie_Wenchang	CRA004775	0.796531448
ENSGALG00000013420	ENSGALG00000013420	2	-5.68531684	6.170560215	-0.921361536	0.356861703	71.32131218	15.2396777	93.43818144	0.5	CRA004775;Yang2025_Silkie_Wenchang	PRJNA827465	0.812971699
ENSGALG00000013420	ENSGALG00000013420	2	0.109046015	1.015789864	0.107350958	0.91451056	0	0.025026152	0	0.5	CRA004775;PRJNA827465	Yang2025_Silkie_Wenchang	0.979711523
ENSGALG00000013425	ENSGALG00000013425	2	-0.36483099	0.246417002	-1.480573834	0.136599899	0.000711086	1.005886331	0.588188441	1	PRJNA827465;Yang2025_Silkie_Wenchang	CRA004775	0.618470274
ENSGALG00000013425	ENSGALG00000013425	2	-0.35361414	0.278782315	-1.268423868	0.204646623	0.024231924	1.182759525	15.45195971	1	CRA004775;Yang2025_Silkie_Wenchang	PRJNA827465	0.71730346
ENSGALG00000013425	ENSGALG00000013425	2	-0.083755063	0.259324948	-0.322973409	0.74671538	0	0.01659082	0	1	CRA004775;PRJNA827465	Yang2025_Silkie_Wenchang	0.936683091
ENSGALG00000013427	NEXMIF	2	0.087604932	0.119730169	0.731686362	0.464360022	0	0.349708313	0	0.5	PRJNA827465;Yang2025_Silkie_Wenchang	CRA004775	0.852159649
ENSGALG00000013427	NEXMIF	2	-0.002261165	0.239599736	-0.009437262	0.992470266	0	0.283086334	0	0.5	CRA004775;Yang2025_Silkie_Wenchang	PRJNA827465	0.997852036
ENSGALG00000013427	NEXMIF	2	0.133768373	0.134090397	0.997598454	0.318474111	0	0.113748798	0	1	CRA004775;PRJNA827465	Yang2025_Silkie_Wenchang	0.797754958
ENSGALG00000013428	BPGM	2	-0.205970329	0.239477193	-0.860083277	0.389743139	0.100972274	8.196729283	87.80001186	0.5	PRJNA827465;Yang2025_Silkie_Wenchang	CRA004775	0.829068422
ENSGALG00000013428	BPGM	2	-0.079585216	0.466462085	-0.170614545	0.86452686	0.340911832	4.002610051	75.01630218	0.5	CRA004775;Yang2025_Silkie_Wenchang	PRJNA827465	0.966723659
ENSGALG00000013428	BPGM	2	0.048350913	0.109552248	0.441350254	0.65895945	0.004604552	1.042782704	4.102743909	1	CRA004775;PRJNA827465	Yang2025_Silkie_Wenchang	0.911426072
ENSGALG00000013429	PDE4DIP	2	0.242772005	0.400661116	0.605928539	0.544562208	0.311413564	33.26321584	96.99367612	0.5	PRJNA827465;Yang2025_Silkie_Wenchang	CRA004775	0.87731302
ENSGALG00000013429	PDE4DIP	2	0.52799594	0.216643509	2.437164829	0.01480293	0.049531418	1.587659687	37.01420976	1	CRA004775;Yang2025_Silkie_Wenchang	PRJNA827465	0.200897639
ENSGALG00000013429	PDE4DIP	2	-0.142044077	0.087943099	-1.615181613	0.106271371	0	0.48725769	0	1	CRA004775;PRJNA827465	Yang2025_Silkie_Wenchang	0.553122487
ENSGALG00000013431	TMEM175	2	0.037893331	0.286412192	0.132303483	0.894744252	0.138419286	6.389241274	84.34868935	0.5	PRJNA827465;Yang2025_Silkie_Wenchang	CRA004775	0.974565659
ENSGALG00000013431	TMEM175	2	-0.250026878	0.136476579	-1.832013085	0.066949471	0	0.000927514	0	1	CRA004775;Yang2025_Silkie_Wenchang	PRJNA827465	0.445834474
ENSGALG00000013431	TMEM175	2	0.065813855	0.280668511	0.23448963	0.814604883	0.120195229	4.088126175	75.53891545	0.5	CRA004775;PRJNA827465	Yang2025_Silkie_Wenchang	0.953869515
ENSGALG00000013435	SDC3	2	0.216245612	0.2940374	0.735435738	0.462074163	0.139576683	4.913653657	79.64854526	0.5	PRJNA827465;Yang2025_Silkie_Wenchang	CRA004775	0.850747865
ENSGALG00000013435	SDC3	2	0.527075163	0.187033275	2.818082302	0.004831214	0	0.017798124	0	1	CRA004775;Yang2025_Silkie_Wenchang	PRJNA827465	0.1030268
ENSGALG00000013435	SDC3	2	0.15225104	0.260366445	0.584756741	0.558711296	0.091482548	2.676946575	62.64400607	0.5	CRA004775;PRJNA827465	Yang2025_Silkie_Wenchang	0.880871906

ENSGALG00010013436	NPAS2	2	0.162398749	0.252084915	0.644222399	0.519431228	0.072232154	2.209676962	54.74451617	0.5	PRJNA827465;Yang2025_Silkie_Wenchang	CRA004775	0.869086345
ENSGALG00010013436	NPAS2	2	0.699183702	0.25161104	2.778827605	0.005455547	0.031515051	1.328083773	24.70354502	1	CRA004775;Yang2025_Silkie_Wenchang	PRJNA827465	0.110944465
ENSGALG00010013436	NPAS2	2	0.42853077	0.508159421	0.843299863	0.399060759	0.44823748	7.334628531	86.36604436	0.5	CRA004775;PRJNA827465	Yang2025_Silkie_Wenchang	0.832054308
ENSGALG00010013437	ENSGALG00010013437	2	0.330659795	0.127831842	2.586677857	0.009690614	0.007786946	1.272954706	21.44260942	1	PRJNA827465;Yang2025_Silkie_Wenchang	CRA004775	0.155237398
ENSGALG00010013437	ENSGALG00010013437	2	0.153912635	0.381552521	0.403385188	0.686664882	0.230968083	4.736106598	78.88561038	0.5	CRA004775;Yang2025_Silkie_Wenchang	PRJNA827465	0.919235539
ENSGALG00010013437	ENSGALG00010013437	2	0.057825025	0.23859329	0.242358135	0.808502671	0.072540123	2.441181859	59.03623499	0.5	CRA004775;PRJNA827465	Yang2025_Silkie_Wenchang	0.952496413
ENSGALG00010013438	RPL37	2	-0.180540876	0.121220157	-1.489363496	0.136391676	0.01368644	1.398138951	28.47635073	1	PRJNA827465;Yang2025_Silkie_Wenchang	CRA004775	0.61829693
ENSGALG00010013438	RPL37	2	-0.346572387	0.188084358	-1.842643326	0.065381104	0	0.253123618	0	1	CRA004775;Yang2025_Silkie_Wenchang	PRJNA827465	0.440704235
ENSGALG00010013438	RPL37	2	-0.128525957	0.064671981	-1.987351466	0.046883462	0	0.173003746	0	1	CRA004775;PRJNA827465	Yang2025_Silkie_Wenchang	0.37376485
ENSGALG00010013440	EEA1	2	0.36240824	0.473979636	0.764607195	0.444505484	0.42285971	16.37181056	93.89194007	0.5	PRJNA827465;Yang2025_Silkie_Wenchang	CRA004775	0.846013379
ENSGALG00010013440	EEA1	2	0.639126224	0.233864715	2.732888645	0.006278155	0.054131823	1.973309571	49.3237141	1	CRA004775;Yang2025_Silkie_Wenchang	PRJNA827465	0.12036602
ENSGALG00010013440	EEA1	2	0.092636513	0.233056697	0.397484877	0.691009936	0.081654989	3.414572978	70.71376109	0.5	CRA004775;PRJNA827465	Yang2025_Silkie_Wenchang	0.920199347
ENSGALG00010013442	ENSGALG00010013442	2	-0.068949497	0.070235789	-0.981686097	0.326254517	0	0.085308972	0	1	PRJNA827465;Yang2025_Silkie_Wenchang	CRA004775	0.800023788
ENSGALG00010013442	ENSGALG00010013442	2	-0.080296636	0.129203086	-0.621476146	0.534286385	0	0.189965484	0	0.5	CRA004775;Yang2025_Silkie_Wenchang	PRJNA827465	0.873408008
ENSGALG00010013442	ENSGALG00010013442	2	-0.050886887	0.078675146	-0.646797485	0.51776302	0	0.102190339	0	0.5	CRA004775;PRJNA827465	Yang2025_Silkie_Wenchang	0.867973554
ENSGALG00010013444	OSBPL8	2	0.076570466	0.068843929	1.112232659	0.266038129	0	0.3099898	0	1	PRJNA827465;Yang2025_Silkie_Wenchang	CRA004775	0.770081145
ENSGALG00010013444	OSBPL8	2	-0.014548941	0.132237689	-0.110021135	0.912392613	0	0.306945735	0	0.5	CRA004775;Yang2025_Silkie_Wenchang	PRJNA827465	0.978967245
ENSGALG00010013444	OSBPL8	2	0.087170468	0.077905918	1.11891971	0.263174394	0	0.605182558	0	0.5	CRA004775;PRJNA827465	Yang2025_Silkie_Wenchang	0.768643821
ENSGALG00010013446	ATP5I	2	-0.118675263	0.163391688	-0.726323744	0.467640326	0.002998116	1.051214398	4.871926978	1	PRJNA827465;Yang2025_Silkie_Wenchang	CRA004775	0.852885838
ENSGALG00010013446	ATP5I	2	-0.190767311	0.224124628	-0.851166216	0.394677027	0	0.910008879	0	0.5	CRA004775;Yang2025_Silkie_Wenchang	PRJNA827465	0.830643015
ENSGALG00010013446	ATP5I	2	0.012180573	0.168521723	0.072278951	0.942379915	0	0.047845981	0	0.5	CRA004775;PRJNA827465	Yang2025_Silkie_Wenchang	0.985827325
ENSGALG00010013449	LCA5L	2	0.62244313	0.360847823	1.724946333	0.084537145	0.178549142	3.126620383	68.01658412	1	PRJNA827465;Yang2025_Silkie_Wenchang	CRA004775	0.500084797
ENSGALG00010013449	LCA5L	2	0.874074316	0.26745564	3.268109494	0.001082685	0	0.570404175	0	1	CRA004775;Yang2025_Silkie_Wenchang	PRJNA827465	0.039806134
ENSGALG00010013449	LCA5L	2	0.357696349	0.21729865	1.64610479	0.099742186	0	0.319139813	0	1	CRA004775;PRJNA827465	Yang2025_Silkie_Wenchang	0.539447799
ENSGALG00010013450	CD74	2	-0.081426743	0.0992187	-0.820679397	0.411829909	0	0.694375288	0	0.5	PRJNA827465;Yang2025_Silkie_Wenchang	CRA004775	0.835282879
ENSGALG00010013450	CD74	2	-0.317269196	0.377797977	-0.839785323	0.401028764	0.240161627	6.221696521	83.92721348	0.5	CRA004775;Yang2025_Silkie_Wenchang	PRJNA827465	0.832407369
ENSGALG00010013450	CD74	2	-0.385700306	0.286005023	-1.348578784	0.177472299	0.129597259	4.488513706	77.72091018	1	CRA004775;PRJNA827465	Yang2025_Silkie_Wenchang	0.683574458
ENSGALG00010013451	PRKAB2	2	-0.107376278	0.122093355	-0.879460466	0.37915166	0.018987276	2.717716528	63.20440378	0.5	PRJNA827465;Yang2025_Silkie_Wenchang	CRA004775	0.824077735
ENSGALG00010013451	PRKAB2	2	0.008104975	0.113532365	0.071389111	0.943088076	0	0.404402844	0	0.5	CRA004775;Yang2025_Silkie_Wenchang	PRJNA827465	0.985827325
ENSGALG00010013451	PRKAB2	2	-0.221657179	0.090200881	-2.457372671	0.013995741	0	0.018792433	0	1	CRA004775;PRJNA827465	Yang2025_Silkie_Wenchang	0.193842584
ENSGALG00010013452	LSS	2	0.108930575	0.092706924	1.174999349	0.239994976	0	0.695335489	0	1	PRJNA827465;Yang2025_Silkie_Wenchang	CRA004775	0.752374775
ENSGALG00010013452	LSS	2	-0.018095087	0.136106591	-0.132947913	0.894234574	0	0.352600098	0	0.5	CRA004775;Yang2025_Silkie_Wenchang	PRJNA827465	0.974381724
ENSGALG00010013452	LSS	2	0.128896208	0.122711367	1.050401532	0.293533542	0.002596873	1.034953243	3.377277457	0.5	CRA004775;PRJNA827465	Yang2025_Silkie_Wenchang	0.786979185
ENSGALG00010013453	PUM1	2	0.013693729	0.107673409	0.127178378	0.898799219	0.016570695	3.030347782	67.00048734	0.5	PRJNA827465;Yang2025_Silkie_Wenchang	CRA004775	0.975884294
ENSGALG00010013453	PUM1	2	0.186527423	0.100257364	1.860486009	0.062816796	0	0.266771115	0	1	CRA004775;Yang2025_Silkie_Wenchang	PRJNA827465	0.43178313
ENSGALG00010013453	PUM1	2	0.050915023	0.160992512	0.316257089	0.751807379	0.038490284	3.163999811	68.39443554	0.5	CRA004775;PRJNA827465	Yang2025_Silkie_Wenchang	0.937802658
ENSGALG00010013457	SH3BGR	2	0.025198614	0.078422717	0.321317785	0.74796958	0	0.050271337	0	1	PRJNA827465;Yang2025_Silkie_Wenchang	CRA004775	0.936938957
ENSGALG00010013457	SH3BGR	2	0.005412383	0.131809179	0.041062259	0.967246262	0	0.032408203	0	1	CRA004775;Yang2025_Silkie_Wenchang	PRJNA827465	0.990894822
ENSGALG00010013457	SH3BGR	2	0.039571365	0.095638067	0.413761659	0.679048665	0	0.016350713	0	1	CRA004775;PRJNA827465	Yang2025_Silkie_Wenchang	0.917701868
ENSGALG00010013458	LAMA4	2	-0.094053232	0.075606474	-1.243983845	0.213505509	0	0.084961722	0	1	PRJNA827465;Yang2025_Silkie_Wenchang	CRA004775	0.726080554
ENSGALG00010013458	LAMA4	2	0.07520087	0.24008411	0.313227186	0.754108066	0.058547225	1.604357286	37.66974422	0.5	CRA004775;Yang2025_Silkie_Wenchang	PRJNA827465	0.938385954
ENSGALG00010013458	LAMA4	2	0.054797603	0.268844776	0.203826176	0.838489336	0.090757304	1.985085138	49.62432689	0.5	CRA004775;PRJNA827465	Yang2025_Silkie_Wenchang	0.959197438
ENSGALG00010013459	ATP10D	2	-0.209775148	0.246033629	-0.852627947	0.39386566	0.100409572	5.600152307	82.1433428	0.5	PRJNA827465;Yang2025_Silkie_Wenchang	CRA004775	0.830486298
ENSGALG00010013459	ATP10D	2	-0.428771755	0.170725458	-2.511469344	0.012022973	0	0.591316483	0	1	CRA004775;Yang2025_Silkie_Wenchang	PRJNA827465	0.176332839
ENSGALG00010013459	ATP10D	2	0.00820277	0.097058335	0.084513811	0.932647923	0	0.057884449	0	0.5	CRA004775;PRJNA827465	Yang2025_Silkie_Wenchang	0.984224753
ENSGALG00010013461	S100B	2	-0.008141084	0.328360534	-0.024793125	0.980219975	0	0.151469899	0	0.5	PRJNA827465;Yang2025_Silkie_Wenchang	CRA004775	0.994676009
ENSGALG00010013461	S100B	2	1.034510352	0.970567529	1.06588189	0.286477037	1.497554032	4.760009582	78.99163893	1	CRA004775;Yang2025_Silkie_Wenchang	PRJNA827465	0.782018115
ENSGALG00010013461	S100B	2	0.901423024	1.099551853	0.819809471	0.412324731	2.061906356	6.583687854	84.81094453	0.5	CRA004775;PRJNA827465	Yang2025_Silkie_Wenchang	0.835671911
ENSGALG00010013463	ENSGALG00010013463	2	-0.014121344	0.142045536	-0.099414201	0.920809408	0.019468197	1.928223532	48.13879285	0.5	PRJNA827465;Yang2025_Silkie_Wenchang	CRA004775	0.981469977
ENSGALG00010013463	ENSGALG00010013463	2	-0.020561493	0.262350957	-0.078373997	0.937530557	0.070077752	1.642610504	39.12129519	0.5	CRA004775;Yang2025_Silkie_Wenchang	PRJNA827465	0.985139256
ENSGALG00010013463	ENSGALG00010013463	2	-0.177667727	0.131863106	-1.347364945	0.177862725	0	0.460446308	0	1	CRA004775;PRJNA827465	Yang2025_Silkie_Wenchang	0.683951879
ENSGALG00010013464	ABCB7	2	0.085571277	0.06357879	1.345909178	0.178331809	0.003304977	1.582839505	36.8224007	1	PRJNA827465;Yang2025_Silkie_Wenchang	CRA004775	0.684689586
ENSGALG00010013464	ABCB7	2	-0.08043284	0.314280341	-0.255927049	0.798007168	0.154840976	3.991316117	74.94560765	0.5	CRA004775;Yang2025_Silkie_Wenchang	PRJNA827465	0.949922217
ENSGALG00010013464	ABCB7	2	-0.124143497	0.2377431	-0.522174973	0.601548509	0.080519473	2.645414014	62.19873356	0.5	CRA004775;PRJNA827465	Yang2025_Silkie_Wenchang	0.895230519
ENSGALG00010013465	CALD1	2	0.088864233	0.157473796	0.564311239	0.572542334	0.02958685	2.195779863	54.45809405	0.5	PRJNA827465;Yang2025_Silkie_Wenchang	CRA004775	0.885678394
ENSGALG00010013465	CALD1	2	0.320216695	0.159064043	2.01313062	0.04410089	0	0.020667174	0	1	CRA004775;Yang2025_Silkie_Wenchang	PRJNA827465	0.362918189
ENSGALG00010013465	CALD1	2	0.084162393	0.171961915	0.489424607	0.624541119	0.033289457	1.883398174	46.90448289	0.5	CRA004775;PRJNA827465	Yang2025_Silkie_Wenchang	0.900915348
ENSGALG00010013466	BRAF	2	-0.009599471	0.049868617	-0.192495235	0.8473543	0	0.464016137	0	0.5	PRJNA827465;Yang2025_Silkie_Wenchang	CRA004775	0.961252712
ENSGALG00010013466	BRAF	2	0.060657716	0.13901217	0.436348242	0.662584085	0	0.218517841	0	0.5	CRA004775;Yang2025_Silkie_Wenchang	PRJNA827465	0.912778072
ENSGALG00010013466	BRAF	2	-0.023187593	0.052382024	-0.442663175	0.658009382	0	0.04581903	0	1	CRA004775;PRJNA827465	Yang2025_Silkie_Wenchang	0.910881331
ENSGALG00010013468	DEPDC1B	2	-0.051413121	0.108485903	-0.473915224	0.635560362	0	0.267668836	0	1	PRJNA827465;Yang2025_Silkie_Wenchang	CRA004775	0.904244739
ENSGALG00010013468	DEPDC1B	2	-0.099517002	0.173867698	-0.572371996	0.567069984	0	0.358553129	0	0.5	CRA004775;Yang2025_Silkie_Wenchang	PRJNA827465	0.883487517
ENSGALG00010013468	DEPDC1B	2	0.002602762	0.134043203	0.019417333	0.984508183	0	0.201591225	0	0.5	CRA004775;PRJNA827465	Yang2025_Silkie_Wenchang	0.995703389
ENSGALG00010013470	RPL31	2	-0.283303556	0.309470893	-0.915444919	0.359958106	0.15694958	4.741211365	78.90834382	1	PRJNA827465;Yang2025_Silkie_Wenchang	CRA004775	0.814880744
ENSGALG00010013470	RPL31	2	-0.438846148	0.211895911	-2.071045855	0.038354511	0.013300502	1.173544181	14.78803985	1	CRA004775;Yang2025_Silkie_Wenchang	PRJNA827465	0.337765482
ENSGALG00010013470	RPL31	2	-0.038548652	0.059337226	-0.649653767	0.515915894	0	0.555899327	0	1	CRA004775;PRJNA827465	Yang2025_Silkie_Wenchang	0.867498525
ENSGALG00010013472	ENSGALG00010013472	2	-0.020417161	0.182912782	-0.111622387	0.911122821	0.027424724	1.690956921	40.86188789	0.5	PRJNA827465;Yang2025_Silkie_Wenchang	CRA004775	0.978845851
ENSGALG00010013472	ENSGALG00010013472	2	-0.156132959	0.157499267	-0.991324994	0.321526912	0	0.125039251	0	1	CRA004775;Yang2025_Silkie_Wenchang	PRJNA827465	0.798513866
ENSGALG00010013472	ENSGALG00010013472	2	0.086096486	0.167591341	0.513728725	0.607441666	0	0.49202666	0	0.5	CRA004775;PRJNA827465	Yang2025_Silkie_Wenchang	0.897023476
ENSGALG00010013476	ZDHHC17	2	0.043453401	0.053611408	0.81052527	0.417638348	0	0.153927979	0	1	PRJNA827465;Yang2025_Silkie_Wenchang	CRA004775	0.838112595
ENSGALG00010013476	ZDHHC17	2	0.037437916	0.097476822	0.38406993	0.700926618	0	0.612139225	0	1	CRA004775;Yang2025_Silkie_Wenchang	PRJNA827465	0.922496697
ENSGALG00010013476	ZDHHC17	2	0.065050544	0.060922225	1.067763754	0.285627092	0	0.417635438	0	1	CRA004775;PRJNA827465	Yang2025_Silkie_Wenchang	0.78131971
ENSGALG00010013477	RFC1	2	0.134403168	0.224520603	0.598622871	0.549424401	0.090668707	9.594442609	89.57729969	0.5	PRJNA827465;Yang2025_Silkie_Wenchang	CRA004775	0.878554604
ENSGALG00010013477	RFC1	2	0.294949266	0.117265848	2.515218804	0.011895853	0.003013572	1.103394329	9.370569223	1	CRA004775;Yang2025_Silkie_Wenchang	PRJNA827465	0.17504407
ENSGALG00010013477	RFC1	2	-0.058375667	0.061693213	-0.946225117	0.344033778	0	0.845611101	0	0.5	CRA004775;PRJNA827465	Yang2025_Silkie_Wenchang	0.806445383
ENSGALG00010013480	TBC1D8	2	-0.023673042	0.059972128	-0.394734061	0.693039163	0	0.110197227	0	0.5	PRJNA827465;Yang2025_Silkie_Wenchang	CRA004775	0.920631027
ENSGALG00010013480	TBC1D8	2	0.059309598	0.136608606	0.434157113	0.664174354	0.011664448	1.182632019	15.4428441	1	CRA004775;Yang2025_Silkie_Wenchang	PRJNA827465	0.91312035
ENSGALG00010013480	TBC1D8	2	0.046553117	0.171301395	0.271761459	0.785805442	0.030993908	1.503302035	33.47976808	0.5	CRA004775;PRJNA827465	Yang2025_Silkie_Wenchang	0.946639719
ENSGALG00010013482	ENSGALG00010013482	2	0.192027313	0.145177744	1.322704895	0.185933535	0	0.022916073	0	1	PRJNA827465;Yang2025_Silkie_Wenchang	CRA004775	0.694982951
ENSGALG00010013482	ENSGALG00010013482	2	-0.101194137	0.396372471	-0.255300619	0.798490922	0.197715483	2.531749799	60.50162617	0.5	CRA004775;Yang2025_Silkie_Wenchang	PRJNA827465	0.949922217
ENSGALG00010013482	ENSGALG00010013482	2	-0.097594323	0.365594529	-0.266946891	0.789510053	0.17775773	2.584519499	61.30808839	0.5	CRA004775;PRJNA827465	Yang2025_Silkie_Wenchang	0.947390191
ENSGALG00010013483	ENSGALG00010013483	2	-0.067063678	0.277796485	-0.241412982	0.809235052	0	0.265795137	0	0.5	PRJNA827465;Yang2025_Silkie_Wenchang	CRA004775	0.95276497
ENSGALG00010013483	ENSGALG00010013483	2	0.083350048	0.276097041	0.301886787	0.762738369	0.019057716	1.142694333	12.48753309	0.5	CRA004775;Yang2025_Silkie_Wenchang	PRJNA827465	0.940082621
ENSGALG00010013483	ENSGALG00010013483	2	0.259641365	0.283837111	0.91475482	0.360320358	0	0.204398751	0	1	CRA004775;PRJNA827465	Yang2025_Silkie_Wenchang	0.814880744
ENSGALG00010013484	TCOF1	2	0.209042324	0.151746483	1.377576065	0.168334215	0.037492619	5.008493637	80.03391693	1	PRJNA827465;Yang2025_Silkie_Wenchang	CRA004775	0.668962553
ENSGALG00010013484	TCOF1	2	0.19106957	0.197946169	0.965260257	0.334414473	0.060187903	4.292562972	76.70389447	0.5	CRA004775;Yang2025_Silkie_Wenchang	PRJNA827465	0.803094614
ENSGALG00010013484	TCOF1	2	0.063843088	0.053158735	1.200989604	0.229755234	0	0.33270625	0	0.5	CRA004775;PRJNA827465	Yang2025_Silkie_Wenchang	0.742686486
ENSGALG00010013486	PLPP6	2	0.014934066	0.080136153	0.186358664	0.852163506	0	0.000135653	0	1	PRJNA827465;Yang2025_Silkie_Wenchang	CRA004775	0.962390845
ENSGALG00010013486	PLPP6	2	-0.341724825	0.411847762	-0.829735784	0.406688185	0.208935524	2.510567989	60.16837607	0.5	CRA004775;Yang2025_Silkie_Wenchang	PRJNA827465	0.833618122
ENSGALG00010013486	PLPP6	2	-0.293251648	0.403737615	-0.726342152	0.467629045	0.251203546	3.537249609	71.72944772	0.5	CRA004775;PRJNA827465	Yang2025_Silkie_Wenchang	0.852885838
ENSGALG00010013487	ENSGALG00010013487	2	0.257309308	0.119562799	2.15208502	0.031390659	0	0.266343783	0	1	PRJNA827465;Yang2025_Silkie_Wenchang	CRA004775	0.303067885
ENSGALG00010013487	ENSGALG00010013487	2	0.339094573	0.150170341	2.25806621	0.023941532	0	0.010320097	0	1	CRA004775;Yang2025_Silkie_Wenchang	PRJNA827465	0.264351316
ENSGALG00010013487	ENSGALG00010013487	2	0.245422512	0.135683497	1.808786756	0.070484139	0	0.261274589	0	1	CRA004775;PRJNA827465	Yang2025_Silkie_Wenchang	0.458021454
ENSGALG00010013488	UPRT	2	0.070002279	0.109759368	0.637779538	0.523617201	0.015881502	2.666148957	62.4927183	0.5	PRJNA827465;Yang2025_Silkie_Wenchang	CRA004775	0.869661505
ENSGALG00010013488	UPRT	2	0.145909929	0.104910136	1.390808689	0.164283448	0	0.905080876	0	0.5	CRA004775;Yang2025_Silkie_Wenchang	PRJNA827465	0.66314125
ENSGALG00010013488	UPRT	2	-0.01672838	0.058437129	-0.286262868	0.774676795	0	0.000296329	0	1	CRA004775;PRJNA827465	Yang2025_Silkie_Wenchang	0.943713409
ENSGALG00010013491	CNOT11	2	-0.139684165	0.076580777	-1.8240108	0.068150439	0.006392492	1.933681697	48.28518046	1	PRJNA827465;Yang2025_Silkie_Wenchang	CRA004775	0.450268316
ENSGALG00010013491	CNOT11	2	-0.16060178	0.145805556	-1.101479148	0.270688175	0.017671509	1.481671466	32.50865507	0.5	CRA004775;Yang2025_Silkie_Wenchang	PRJNA827465	0.772667394
ENSGALG00010013491	CNOT11	2	-0.07964171	0.047834261	-1.664951185	0.095922495	0	0.432853056	0	0.5	CRA004775;PRJNA827465	Yang2025_Silkie_Wenchang	0.5301492
ENSGALG00010013493	ENSGALG00010013493	2	-0.322296086	0.170182957	-1.893821163	0.058248752	0.051119325	8.319685457	87.98031482	1	PRJNA827465;Yang2025_Silkie_Wenchang	CRA004775	0.418299047
ENSGALG00010013493	ENSGALG00010013493	2	0.323148349	0.857262859	0.376953634	0.706208063	1.405881876	22.28997655	95.51367855	0.5	CRA004775;Yang2025_Silkie_Wenchang	PRJNA827465	0.924548793
ENSGALG00010013493	ENSGALG00010013493	2	0.48318227	0.686091905	0.704252981	0.481275242	0.882320672	15.046786	93.35406246	0.5	CRA004775;PRJNA827465	Yang2025_Silkie_Wenchang	0.85682839
ENSGALG00010013494	ASB1	2	-0.080282799	0.227691625	-0.352594431	0.724392513	0.093363143	9.885740762	89.88442016	0.5	PRJNA827465;Yang2025_Silkie_Wenchang	CRA004775	0.930387861
ENSGALG00010013494	ASB1	2	-0.182492514	0.227066393	-0.803696715	0.421572152	0.054983123	1.701503211	41.22843886	0.5	CRA004775;Yang2025_Silkie_Wenchang	PRJNA827465	0.839352211
ENSGALG00010013494	ASB1	2	0.140555458	0.076740139	1.831576814	0.067014494	0	0.024863604	0	1	CRA004775;PRJNA827465	Yang2025_Silkie_Wenchang	0.445965231
ENSGALG00010013497	FAM172A	2	0.073453106	0.141896751	0.517651778	0.604701249	0	0.033674726	0	1	PRJNA827465;Yang2025_Silkie_Wenchang	CRA004775	0.896323417
ENSGALG00010013497	FAM172A	2	-0.020437649	0.165405993	-0.123560512	0.90166326	0	0.30304793	0	0.5	CRA004775;Yang2025_Silkie_Wenchang	PRJNA827465	0.976674416
ENSGALG00010013497	FAM172A	2	0.018098343	0.16084235	0.112522251	0.910409328	0	0.505391799	0	0.5	CRA004775;PRJNA827465	Yang2025_Silkie_Wenchang	0.97880407
ENSGALG00010013498	KDR	2	-0.007253381	0.054099923	-0.134073768	0.893344242	0	0.277336557	0	0.5	PRJNA827465;Yang2025_Silkie_Wenchang	CRA004775	0.974381724
ENSGALG00010013498	KDR	2	-0.031131414	0.079273604	-0.392708454	0.694534826	0	0.262351916	0	0.5	CRA004775;Yang2025_Silkie_Wenchang	PRJNA827465	0.920728119
ENSGALG00010013498	KDR	2	0.023179931	0.071908121	0.322354846	0.747183888	0	0.146144689	0	1	CRA004775;PRJNA827465	Yang2025_Silkie_Wenchang	0.93678921
ENSGALG00010013501	AKR1B10	2	-0.157624977	0.194134482	-0.81193704	0.416827759	0.037763302	1.870269485	46.53176947	0.5	PRJNA827465;Yang2025_Silkie_Wenchang	CRA004775	0.837984974
ENSGALG00010013501	AKR1B10	2	-0.372695911	0.550726411	-0.676735132	0.498574022	0.459400865	3.827855908	73.87571466	0.5	CRA004775;Yang2025_Silkie_Wenchang	PRJNA827465	0.861478809
ENSGALG00010013501	AKR1B10	2	-0.494334134	0.314515225	-1.571733562	0.116012355	0.112915494	1.801967588	44.50510616	1	CRA004775;PRJNA827465	Yang2025_Silkie_Wenchang	0.573367481
ENSGALG00010013502	PAFAH2	2	-0.139484578	0.171250648	-0.814505406	0.415355476	0	0.020161008	0	1	PRJNA827465;Yang2025_Silkie_Wenchang	CRA004775	0.837576587
ENSGALG00010013502	PAFAH2	2	-0.114941542	0.196385268	-0.58528597	0.558355447	0	0.071273168	0	1	CRA004775;Yang2025_Silkie_Wenchang	PRJNA827465	0.880678789
ENSGALG00010013502	PAFAH2	2	-0.10101972	0.16452335	-0.614014488	0.539205753	0	0.025718324	0	1	CRA004775;PRJNA827465	Yang2025_Silkie_Wenchang	0.874983893
ENSGALG00010013506	RNF149	2	-0.057633153	0.083560684	-0.68971614	0.490372714	0.006953574	1.950357273	48.72734274	0.5	PRJNA827465;Yang2025_Silkie_Wenchang	CRA004775	0.858529056
ENSGALG00010013506	RNF149	2	-0.006415208	0.204430032	-0.031380948	0.974965735	0.054305096	2.418353413	58.64955078	0.5	CRA004775;Yang2025_Silkie_Wenchang	PRJNA827465	0.992609821
ENSGALG00010013506	RNF149	2	0.03335299	0.069648428	0.47887642	0.632026547	0	0.943743621	0	1	CRA004775;PRJNA827465	Yang2025_Silkie_Wenchang	0.903058174
ENSGALG00010013508	TUBE1	2	0.273398115	0.117854573	2.319792153	0.020352124	0	0.530922362	0	1	PRJNA827465;Yang2025_Silkie_Wenchang	CRA004775	0.24026234
ENSGALG00010013508	TUBE1	2	-0.16689377	0.609706156	-0.273728203	0.784293503	0.631109403	6.122481206	83.66675264	0.5	CRA004775;Yang2025_Silkie_Wenchang	PRJNA827465	0.946083234
ENSGALG00010013508	TUBE1	2	-0.237525377	0.519511368	-0.457209201	0.647520686	0.437099483	4.67822317	78.62436306	0.5	CRA004775;PRJNA827465	Yang2025_Silkie_Wenchang	0.907350807
ENSGALG00010013509	ENSGALG00010013509	2	0.007991454	0.172811617	0.046243734	0.963115985	0.042129018	3.350073991	70.14991302	0.5	PRJNA827465;Yang2025_Silkie_Wenchang	CRA004775	0.989998461
ENSGALG00010013509	ENSGALG00010013509	2	-0.165828895	0.142950804	-1.160041705	0.246031826	0	0.078023495	0	1	CRA004775;Yang2025_Silkie_Wenchang	PRJNA827465	0.756693706
ENSGALG00010013509	ENSGALG00010013509	2	0.157457618	0.112967297	1.393833641	0.163367847	0	0.166865689	0	0.5	CRA004775;PRJNA827465	Yang2025_Silkie_Wenchang	0.661683383
ENSGALG00010013510	MRPS33	2	-0.21148037	0.098861279	-2.13916281	0.032422485	0.007074567	1.392100732	28.16611778	1	PRJNA827465;Yang2025_Silkie_Wenchang	CRA004775	0.309308824
ENSGALG00010013510	MRPS33	2	-0.090289252	0.13734719	-0.657379687	0.510936809	0	0.173090985	0	1	CRA004775;Yang2025_Silkie_Wenchang	PRJNA827465	0.8654765
ENSGALG00010013510	MRPS33	2	-0.259332748	0.074442964	-3.483643491	0.000494638	0	0.1945162	0	1	CRA004775;PRJNA827465	Yang2025_Silkie_Wenchang	0.023284834
ENSGALG00010013511	FAM78B	2	0.353076049	0.106856088	3.304220257	0.00095241	0.005823627	1.306116139	23.43713011	1	PRJNA827465;Yang2025_Silkie_Wenchang	CRA004775	0.036660629
ENSGALG00010013511	FAM78B	2	0.124150286	0.391083052	0.317452484	0.750900289	0.265311537	7.418070894	86.51940627	0.5	CRA004775;Yang2025_Silkie_Wenchang	PRJNA827465	0.937567095
ENSGALG00010013511	FAM78B	2	0.033053275	0.277257973	0.119214876	0.905105121	0.122648474	4.579034677	78.16133594	0.5	CRA004775;PRJNA827465	Yang2025_Silkie_Wenchang	0.977938157
ENSGALG00010013512	ENSGALG00010013512	2	-4.263483915	4.517452148	-0.943780648	0.345281752	38.06238162	14.77503109	93.23182473	0.5	PRJNA827465;Yang2025_Silkie_Wenchang	CRA004775	0.807149746
ENSGALG00010013512	ENSGALG00010013512	2	-3.939462171	4.827945397	-0.815970739	0.414516872	44.04451671	18.01448379	94.44891116	0.5	CRA004775;Yang2025_Silkie_Wenchang	PRJNA827465	0.837148996
ENSGALG00010013512	ENSGALG00010013512	2	0.519991557	0.977120942	0.532167038	0.59461031	0	0.100262247	0	1	CRA004775;PRJNA827465	Yang2025_Silkie_Wenchang	0.893379204
ENSGALG00010013514	LMBRD2	2	0.086984104	0.103733927	0.838530916	0.401732592	0	0.299949768	0	1	PRJNA827465;Yang2025_Silkie_Wenchang	CRA004775	0.832689704
ENSGALG00010013514	LMBRD2	2	0.03387461	0.146751671	0.230829467	0.817447288	0	0.046056626	0	1	CRA004775;Yang2025_Silkie_Wenchang	PRJNA827465	0.954888783
ENSGALG00010013514	LMBRD2	2	0.131479969	0.127376765	1.032213132	0.301972285	0	0.005939625	0	1	CRA004775;PRJNA827465	Yang2025_Silkie_Wenchang	0.791544398
ENSGALG00010013515	RBP4L	2	-0.143265574	0.294009259	-0.487282525	0.62605813	0.008403977	1.051092277	4.860874524	0.5	PRJNA827465;Yang2025_Silkie_Wenchang	CRA004775	0.901504244
ENSGALG00010013515	RBP4L	2	0.135135646	0.33775842	0.400095564	0.689086131	0	0.005962895	0	1	CRA004775;Yang2025_Silkie_Wenchang	PRJNA827465	0.919854035
ENSGALG00010013515	RBP4L	2	-0.271481212	0.336165911	-0.80758103	0.419331811	0	0.533402844	0	0.5	CRA004775;PRJNA827465	Yang2025_Silkie_Wenchang	0.838527387
ENSGALG00010013516	CSRP2	2	0.048484789	0.123932564	0.391219126	0.695635272	0.0109138	1.314521763	23.92670641	0.5	PRJNA827465;Yang2025_Silkie_Wenchang	CRA004775	0.921038437
ENSGALG00010013516	CSRP2	2	0.076435697	0.281455945	0.271572508	0.785950741	0.088819706	2.265747795	55.86446107	0.5	CRA004775;Yang2025_Silkie_Wenchang	PRJNA827465	0.946639719
ENSGALG00010013516	CSRP2	2	0.138836064	0.088255838	1.573109126	0.115693552	0	0.77734496	0	1	CRA004775;PRJNA827465	Yang2025_Silkie_Wenchang	0.572716099
ENSGALG00010013517	STMN1	2	0.077635992	0.068581407	1.132026829	0.257623155	0	0.472604284	0	0.5	PRJNA827465;Yang2025_Silkie_Wenchang	CRA004775	0.7650601
ENSGALG00010013517	STMN1	2	-0.061548018	0.185672309	-0.331487329	0.740276411	0	0.008100957	0	1	CRA004775;Yang2025_Silkie_Wenchang	PRJNA827465	0.935124836
ENSGALG00010013517	STMN1	2	0.089661228	0.071923794	1.24661427	0.212538967	0	0.176858704	0	0.5	CRA004775;PRJNA827465	Yang2025_Silkie_Wenchang	0.725280468
ENSGALG00010013520	CREG2	2	-0.471961052	0.101083481	-4.669022552	3.02636E-06	0	0.566271235	0	1	PRJNA827465;Yang2025_Silkie_Wenchang	CRA004775	0.000571851
ENSGALG00010013520	CREG2	2	-0.503236326	0.157522547	-3.194693946	0.001399792	0	0.942440239	0	1	CRA004775;Yang2025_Silkie_Wenchang	PRJNA827465	0.046038514
ENSGALG00010013520	CREG2	2	-0.394760985	0.116128563	-3.399344433	0.000675476	0	0.34406191	0	1	CRA004775;PRJNA827465	Yang2025_Silkie_Wenchang	0.028731851
ENSGALG00010013523	TMEM178B	2	0.078933215	0.175024752	0.450983155	0.65200169	0	0.075848696	0	1	PRJNA827465;Yang2025_Silkie_Wenchang	CRA004775	0.909322679
ENSGALG00010013523	TMEM178B	2	-0.048035989	0.256229926	-0.187472204	0.851290415	0	0.191999082	0	0.5	CRA004775;Yang2025_Silkie_Wenchang	PRJNA827465	0.962071965
ENSGALG00010013523	TMEM178B	2	0.061589501	0.20171534	0.305328793	0.760115749	0	0.408440917	0	0.5	CRA004775;PRJNA827465	Yang2025_Silkie_Wenchang	0.939294282
ENSGALG00010013524	ZDHHC15	2	-0.184552965	0.227535248	-0.811096156	0.417310453	0.085645067	5.706504392	82.47613721	0.5	PRJNA827465;Yang2025_Silkie_Wenchang	CRA004775	0.838112595
ENSGALG00010013524	ZDHHC15	2	-0.534415698	0.20212108	-2.644037422	0.00819236	0.029152389	1.335300154	25.11047071	1	CRA004775;Yang2025_Silkie_Wenchang	PRJNA827465	0.140727412
ENSGALG00010013524	ZDHHC15	2	-0.364361437	0.463024476	-0.786916147	0.431330955	0.358151408	5.400731935	81.4839912	0.5	CRA004775;PRJNA827465	Yang2025_Silkie_Wenchang	0.842503929
ENSGALG00010013526	SIRPA	2	-0.461824609	0.248371297	-1.859412157	0.06296874	0	0.17467935	0	1	PRJNA827465;Yang2025_Silkie_Wenchang	CRA004775	0.432176808
ENSGALG00010013526	SIRPA	2	-0.79932335	0.324067962	-2.466529999	0.01364293	0	0.985993721	0	1	CRA004775;Yang2025_Silkie_Wenchang	PRJNA827465	0.191338116
ENSGALG00010013526	SIRPA	2	-0.698488436	0.43569576	-1.603156377	0.108900134	0.189803438	1.843934512	45.76813909	1	CRA004775;PRJNA827465	Yang2025_Silkie_Wenchang	0.558760047
ENSGALG00010013527	ADARB1	2	0.039085691	0.146029294	0.267656505	0.788963733	0.02808193	2.698408682	62.94112131	0.5	PRJNA827465;Yang2025_Silkie_Wenchang	CRA004775	0.947175807
ENSGALG00010013527	ADARB1	2	0.094619291	0.164033133	0.576830362	0.564054057	0.016676117	1.405105428	28.83096316	0.5	CRA004775;Yang2025_Silkie_Wenchang	PRJNA827465	0.882840851
ENSGALG00010013527	ADARB1	2	-0.083577294	0.079740388	-1.048117467	0.29458449	0	0.026955369	0	1	CRA004775;PRJNA827465	Yang2025_Silkie_Wenchang	0.787408474
ENSGALG00010013528	PRDX6	2	0.000438722	0.058439081	0.007507337	0.994010068	0	0.127743609	0	0.5	PRJNA827465;Yang2025_Silkie_Wenchang	CRA004775	0.99814966
ENSGALG00010013528	PRDX6	2	0.002927357	0.114881347	0.02548157	0.979670848	0	0.799717939	0	0.5	CRA004775;Yang2025_Silkie_Wenchang	PRJNA827465	0.994472942
ENSGALG00010013528	PRDX6	2	-0.02085009	0.065468997	-0.318472723	0.750126383	0	0.625722023	0	1	CRA004775;PRJNA827465	Yang2025_Silkie_Wenchang	0.937374353
ENSGALG00010013531	ARSI	2	0.126697973	0.145474617	0.870928384	0.383793258	0.014707092	1.526080093	34.47263976	0.5	PRJNA827465;Yang2025_Silkie_Wenchang	CRA004775	0.825601288
ENSGALG00010013531	ARSI	2	-0.205061741	0.376949901	-0.544002641	0.586439656	0.157408378	1.605126673	37.69962106	1	CRA004775;Yang2025_Silkie_Wenchang	PRJNA827465	0.891596903
ENSGALG00010013531	ARSI	2	-0.124700962	0.571401072	-0.218237185	0.827244314	0.463210254	2.755126442	63.70402517	0.5	CRA004775;PRJNA827465	Yang2025_Silkie_Wenchang	0.956550752
ENSGALG00010013532	WDR19	2	0.388996775	0.349091721	1.114311087	0.265145752	0.229989699	17.63419026	94.32919808	1	PRJNA827465;Yang2025_Silkie_Wenchang	CRA004775	0.769274038
ENSGALG00010013532	WDR19	2	0.233550171	0.538873771	0.433404228	0.66472113	0.53326088	11.85407743	91.56408413	0.5	CRA004775;Yang2025_Silkie_Wenchang	PRJNA827465	0.91338442
ENSGALG00010013532	WDR19	2	-0.051398225	0.166406358	-0.308871761	0.757419078	0.028007315	1.628645353	38.59927833	0.5	CRA004775;PRJNA827465	Yang2025_Silkie_Wenchang	0.938842663
ENSGALG00010013533	SKP2	2	-0.182850904	0.213429076	-0.856729116	0.391594608	0.081138716	8.767430259	88.59414936	0.5	PRJNA827465;Yang2025_Silkie_Wenchang	CRA004775	0.829251057
ENSGALG00010013533	SKP2	2	-0.344038446	0.1055483	-3.259535642	0.001115948	0	0.785358504	0	1	CRA004775;Yang2025_Silkie_Wenchang	PRJNA827465	0.040399388
ENSGALG00010013533	SKP2	2	-0.039234255	0.098471661	-0.398431943	0.690311816	0.008897143	1.496494329	33.17716075	0.5	CRA004775;PRJNA827465	Yang2025_Silkie_Wenchang	0.919979266
ENSGALG00010013538	E2F7	2	-0.137455992	0.091013756	-1.510277098	0.130972733	0	0.157762925	0	1	PRJNA827465;Yang2025_Silkie_Wenchang	CRA004775	0.606744617
ENSGALG00010013538	E2F7	2	-0.184185451	0.156781349	-1.174791851	0.240078001	0	0.026455586	0	1	CRA004775;Yang2025_Silkie_Wenchang	PRJNA827465	0.752515007
ENSGALG00010013538	E2F7	2	-0.111842259	0.108768651	-1.028258214	0.303828381	0	0.002541545	0	1	CRA004775;PRJNA827465	Yang2025_Silkie_Wenchang	0.792208658
ENSGALG00010013539	NSFL1C	2	0.004745467	0.051530264	0.09209087	0.926625842	0	0.138302642	0	0.5	PRJNA827465;Yang2025_Silkie_Wenchang	CRA004775	0.982834319
ENSGALG00010013539	NSFL1C	2	-0.094924948	0.195750525	-0.484928192	0.627727284	0.042705798	2.018760585	50.46465601	0.5	CRA004775;Yang2025_Silkie_Wenchang	PRJNA827465	0.901856848
ENSGALG00010013539	NSFL1C	2	-0.093919484	0.155935707	-0.602296201	0.546976989	0.029245226	1.822568359	45.13237348	1	CRA004775;PRJNA827465	Yang2025_Silkie_Wenchang	0.878281266
ENSGALG00010013544	ENSGALG00010013544	2	-0.363032241	0.610335888	-0.5948073	0.551972287	0.676381164	10.78503755	90.72789505	0.5	PRJNA827465;Yang2025_Silkie_Wenchang	CRA004775	0.879061525
ENSGALG00010013544	ENSGALG00010013544	2	-0.064177799	0.365210649	-0.175728171	0.860507502	0.170953625	2.587081822	61.34641002	0.5	CRA004775;Yang2025_Silkie_Wenchang	PRJNA827465	0.965032425
ENSGALG00010013544	ENSGALG00010013544	2	-0.824131637	0.239778368	-3.437055827	0.000588074	0	0.888315779	0	1	CRA004775;PRJNA827465	Yang2025_Silkie_Wenchang	0.026163707
ENSGALG00010013546	IDUA	2	0.103668674	0.104772826	0.989461471	0.322437412	0	0.188483273	0	1	PRJNA827465;Yang2025_Silkie_Wenchang	CRA004775	0.798652022
ENSGALG00010013546	IDUA	2	0.063503212	0.154119573	0.412038595	0.680311135	0	0.062896138	0	1	CRA004775;Yang2025_Silkie_Wenchang	PRJNA827465	0.917768536
ENSGALG00010013546	IDUA	2	0.140562502	0.125615105	1.118993625	0.263142859	0	0.000534126	0	1	CRA004775;PRJNA827465	Yang2025_Silkie_Wenchang	0.768643821
ENSGALG00010013549	PCP4	2	-0.067290204	0.177563573	-0.378964014	0.704714587	0	0.12698042	0	1	PRJNA827465;Yang2025_Silkie_Wenchang	CRA004775	0.924152671

ENSGALG00010013549	PCP4	2	0.117097456	0.405108	0.289052441	0.772541247	0.129020649	1.543474778	35.21112142	0.5	CRA004775;Yang2025_Silkie_Wenchang	PRJNA827465	0.943066211
ENSGALG00010013549	PCP4	2	0.107520325	0.280704283	0.383037707	0.701691806	0.054640387	1.274173351	21.51774332	0.5	CRA004775;PRJNA827465	Yang2025_Silkie_Wenchang	0.922904951
ENSGALG00010013550	KLHL5	2	-0.016420331	0.047542729	-0.34538049	0.729808343	0	0.760256979	0	0.5	PRJNA827465;Yang2025_Silkie_Wenchang	CRA004775	0.932444623
ENSGALG00010013550	KLHL5	2	-0.059055052	0.082678648	-0.714272099	0.475058941	0	0.418055	0	0.5	CRA004775;Yang2025_Silkie_Wenchang	PRJNA827465	0.854462864
ENSGALG00010013550	KLHL5	2	0.012007376	0.053310435	0.225234997	0.821796465	0	0.046350716	0	1	CRA004775;PRJNA827465	Yang2025_Silkie_Wenchang	0.95563486
ENSGALG00010013551	UBE2N	2	-0.276145451	0.243625976	-1.133481146	0.257012254	0.112436947	18.6312734	94.63268034	1	PRJNA827465;Yang2025_Silkie_Wenchang	CRA004775	0.764848386
ENSGALG00010013551	UBE2N	2	-0.31521195	0.243118521	-1.296536144	0.194790836	0.094101263	4.571641638	78.12601951	1	CRA004775;Yang2025_Silkie_Wenchang	PRJNA827465	0.706046493
ENSGALG00010013551	UBE2N	2	-0.03951266	0.051384419	-0.768961891	0.441915934	0	0.000244884	0	1	CRA004775;PRJNA827465	Yang2025_Silkie_Wenchang	0.84513555
ENSGALG00010013552	FGF16	2	-0.199610066	0.331659789	-0.601851876	0.547272741	0.196943645	9.437451101	89.40391861	0.5	PRJNA827465;Yang2025_Silkie_Wenchang	CRA004775	0.878281266
ENSGALG00010013552	FGF16	2	-0.520262492	0.171526882	-3.033125112	0.002420352	0	0.284837801	0	1	CRA004775;Yang2025_Silkie_Wenchang	PRJNA827465	0.06593704
ENSGALG00010013552	FGF16	2	0.111668487	0.122041873	0.915001414	0.360190888	0	0.156355281	0	0.5	CRA004775;PRJNA827465	Yang2025_Silkie_Wenchang	0.814880744
ENSGALG00010013553	TADA1	2	-0.051489262	0.074479324	-0.691322892	0.489362649	0	0.760549344	0	1	PRJNA827465;Yang2025_Silkie_Wenchang	CRA004775	0.858366591
ENSGALG00010013553	TADA1	2	-0.1656322	0.142069675	-1.165851892	0.243674332	0	0.077785314	0	1	CRA004775;Yang2025_Silkie_Wenchang	PRJNA827465	0.755365463
ENSGALG00010013553	TADA1	2	-0.033856373	0.076523193	-0.442432832	0.658176025	0	0.158747354	0	1	CRA004775;PRJNA827465	Yang2025_Silkie_Wenchang	0.91095159
ENSGALG00010013554	ENSGALG00010013554	2	-5.71675338	5.725077233	-0.998546072	0.318014636	59.01282388	9.93201356	89.93154818	1	PRJNA827465;Yang2025_Silkie_Wenchang	CRA004775	0.797439961
ENSGALG00010013554	ENSGALG00010013554	2	-5.369690501	5.955426811	-0.901646628	0.367244613	65.92657614	13.72316395	92.7130507	0.5	CRA004775;Yang2025_Silkie_Wenchang	PRJNA827465	0.819739734
ENSGALG00010013554	ENSGALG00010013554	2	0.171646493	1.102752269	0.155652814	0.876306693	0	0.035668727	0	0.5	CRA004775;PRJNA827465	Yang2025_Silkie_Wenchang	0.970347777
ENSGALG00010013556	RASL11B	2	0.077614546	0.105538752	0.735412771	0.462088147	0	0.002485697	0	1	PRJNA827465;Yang2025_Silkie_Wenchang	CRA004775	0.850747865
ENSGALG00010013556	RASL11B	2	0.358921366	0.347785311	1.032019913	0.302062789	0.18099967	3.581668463	72.08005123	1	CRA004775;Yang2025_Silkie_Wenchang	PRJNA827465	0.791603969
ENSGALG00010013556	RASL11B	2	0.363496729	0.342285794	1.061968496	0.288249987	0.173362474	3.464498831	71.1357963	1	CRA004775;PRJNA827465	Yang2025_Silkie_Wenchang	0.782882265
ENSGALG00010013558	SNAPC3	2	-0.058035275	0.059543097	-0.9746768	0.329720604	0	0.118276451	0	1	PRJNA827465;Yang2025_Silkie_Wenchang	CRA004775	0.801177491
ENSGALG00010013558	SNAPC3	2	-0.173098023	0.142545119	-1.214338483	0.224618496	0.01248131	1.286387051	22.26289907	1	CRA004775;Yang2025_Silkie_Wenchang	PRJNA827465	0.73780639
ENSGALG00010013558	SNAPC3	2	-0.147962039	0.168908069	-0.875991537	0.381034634	0.034155747	1.878977446	46.77956341	1	CRA004775;PRJNA827465	Yang2025_Silkie_Wenchang	0.824392515
ENSGALG00010013561	AGK	2	-0.131110505	0.091671664	-1.430218445	0.152654333	0.002920458	1.1839251	15.53519729	1	PRJNA827465;Yang2025_Silkie_Wenchang	CRA004775	0.644378159
ENSGALG00010013561	AGK	2	-0.142858377	0.185286013	-0.77101544	0.44069778	0.032005055	1.759873106	43.17772137	1	CRA004775;Yang2025_Silkie_Wenchang	PRJNA827465	0.844848889
ENSGALG00010013561	AGK	2	-0.222412826	0.091851889	-2.421428983	0.015459621	0	0.507357725	0	1	CRA004775;PRJNA827465	Yang2025_Silkie_Wenchang	0.205620812
ENSGALG00010013562	RERGL	2	0.065644852	0.154926239	0.423716814	0.671772351	0.038385975	4.970418494	79.88096976	0.5	PRJNA827465;Yang2025_Silkie_Wenchang	CRA004775	0.914674175
ENSGALG00010013562	RERGL	2	0.220802249	0.101734761	2.170371729	0.029978697	0	0.027062322	0	1	CRA004775;Yang2025_Silkie_Wenchang	PRJNA827465	0.295227441
ENSGALG00010013562	RERGL	2	-0.070290801	0.087521176	-0.803129065	0.42190014	0	0.428741189	0	0.5	CRA004775;PRJNA827465	Yang2025_Silkie_Wenchang	0.83959196
ENSGALG00010013563	FKBP1A	2	-0.452649749	0.247281193	-1.830506167	0.067174285	0.107128292	7.564996291	86.78122287	1	PRJNA827465;Yang2025_Silkie_Wenchang	CRA004775	0.446498062
ENSGALG00010013563	FKBP1A	2	-0.44777914	0.310595705	-1.441678469	0.1493931	0.14512747	3.885007226	74.26002213	1	CRA004775;Yang2025_Silkie_Wenchang	PRJNA827465	0.639246608
ENSGALG00010013563	FKBP1A	2	-0.220836484	0.064411183	-3.42854258	0.000606831	0	0.214431358	0	1	CRA004775;PRJNA827465	Yang2025_Silkie_Wenchang	0.026737996
ENSGALG00010013569	ENSGALG00010013569	2	0.270500573	0.292002069	0.926365261	0.354256191	0	0.228176997	0	1	PRJNA827465;Yang2025_Silkie_Wenchang	CRA004775	0.811861636
ENSGALG00010013569	ENSGALG00010013569	2	0.25358131	0.360789399	0.702851333	0.482148403	0	0.877603217	0	0.5	CRA004775;Yang2025_Silkie_Wenchang	PRJNA827465	0.85691916
ENSGALG00010013569	ENSGALG00010013569	2	-0.006891199	0.397578246	-0.017332937	0.98617101	0	0.419392801	0	0.5	CRA004775;PRJNA827465	Yang2025_Silkie_Wenchang	0.99618277
ENSGALG00010013572	DENND11	2	0.040313326	0.218512968	0.184489396	0.853629545	0.085521016	9.375177373	89.33353514	0.5	PRJNA827465;Yang2025_Silkie_Wenchang	CRA004775	0.962972345
ENSGALG00010013572	DENND11	2	-0.194665996	0.114677978	-1.697501114	0.089601961	0	0.015589836	0	1	CRA004775;Yang2025_Silkie_Wenchang	PRJNA827465	0.513431384
ENSGALG00010013572	DENND11	2	0.114338335	0.215702761	0.53007358	0.596060916	0.058999309	2.037341745	50.91643302	0.5	CRA004775;PRJNA827465	Yang2025_Silkie_Wenchang	0.893989017
ENSGALG00010013573	ENSGALG00010013573	2	-0.077004918	0.498509241	-0.154470392	0.877238855	0.397633955	4.982471801	79.92964055	0.5	PRJNA827465;Yang2025_Silkie_Wenchang	CRA004775	0.970575794
ENSGALG00010013573	ENSGALG00010013573	2	-0.525651753	0.294881028	-1.782589256	0.07465319	0	0.152025311	0	1	CRA004775;Yang2025_Silkie_Wenchang	PRJNA827465	0.471681002
ENSGALG00010013573	ENSGALG00010013573	2	0.228616693	0.313328598	0.729638773	0.465611016	0.045091573	1.204010362	16.94423642	0.5	CRA004775;PRJNA827465	Yang2025_Silkie_Wenchang	0.852424459
ENSGALG00010013574	NR2F1	2	0.570536775	0.429333504	1.328889475	0.183884437	0.345079813	15.34314845	93.48243287	1	PRJNA827465;Yang2025_Silkie_Wenchang	CRA004775	0.692470323
ENSGALG00010013574	NR2F1	2	0.773291081	0.307740746	2.512800437	0.011977707	0.116044244	2.375707462	57.90727537	1	CRA004775;Yang2025_Silkie_Wenchang	PRJNA827465	0.175919213
ENSGALG00010013574	NR2F1	2	0.173509347	0.099424362	1.745139163	0.080960648	0	0.360244852	0	1	CRA004775;PRJNA827465	Yang2025_Silkie_Wenchang	0.489847627
ENSGALG00010013576	CAMK2A	2	-0.226007497	0.090520523	-2.496754223	0.012533579	0	0.009217053	0	1	PRJNA827465;Yang2025_Silkie_Wenchang	CRA004775	0.181372336
ENSGALG00010013576	CAMK2A	2	-0.235970031	0.145087751	-1.626395268	0.103865597	0	0.001558695	0	1	CRA004775;Yang2025_Silkie_Wenchang	PRJNA827465	0.548175896
ENSGALG00010013576	CAMK2A	2	-0.219294585	0.110899325	-1.977420366	0.047994134	0	5.65061E-05	0	1	CRA004775;PRJNA827465	Yang2025_Silkie_Wenchang	0.378786609
ENSGALG00010013580	RANBP3L	2	0.028286092	0.401181011	0.070507057	0.943790084	0.252352882	4.594941185	78.2369358	0.5	PRJNA827465;Yang2025_Silkie_Wenchang	CRA004775	0.985827325
ENSGALG00010013580	RANBP3L	2	0.541898369	0.261320782	2.073690291	0.038108083	0	0.535396257	0	1	CRA004775;Yang2025_Silkie_Wenchang	PRJNA827465	0.33657643
ENSGALG00010013580	RANBP3L	2	0.173069111	0.632869647	0.273467232	0.784494078	0.623930883	4.065103382	75.40037962	0.5	CRA004775;PRJNA827465	Yang2025_Silkie_Wenchang	0.946117405
ENSGALG00010013582	MAP4K4	2	0.119152297	0.194241353	0.613423946	0.539596057	0.061019439	4.700356442	78.72501772	0.5	PRJNA827465;Yang2025_Silkie_Wenchang	CRA004775	0.875051321
ENSGALG00010013582	MAP4K4	2	0.175552714	0.260159026	0.674790018	0.499809183	0.068335671	1.749172433	42.83010746	0.5	CRA004775;Yang2025_Silkie_Wenchang	PRJNA827465	0.862078151
ENSGALG00010013582	MAP4K4	2	-0.049921858	0.061548998	-0.811091313	0.417313234	0	0.186350604	0	1	CRA004775;PRJNA827465	Yang2025_Silkie_Wenchang	0.838112595
ENSGALG00010013583	FASLG	2	-0.108358745	0.400321285	-0.270679448	0.786637585	0	0.023151121	0	1	PRJNA827465;Yang2025_Silkie_Wenchang	CRA004775	0.946722706
ENSGALG00010013583	FASLG	2	-0.191452739	0.366827761	-0.521914531	0.60172984	0	0.089748896	0	1	CRA004775;Yang2025_Silkie_Wenchang	PRJNA827465	0.895248814
ENSGALG00010013583	FASLG	2	-0.218408646	0.334081422	-0.65375873	0.513267259	0	0.026879168	0	1	CRA004775;PRJNA827465	Yang2025_Silkie_Wenchang	0.866295966
ENSGALG00010013592	H3C33	2	0.755023558	0.600202718	1.257947581	0.208410714	0.524969682	3.684680222	72.86060283	1	PRJNA827465;Yang2025_Silkie_Wenchang	CRA004775	0.720989904
ENSGALG00010013592	H3C33	2	0.426997898	0.903801413	0.472446593	0.636608054	1.480598758	10.61647164	90.58067469	0.5	CRA004775;Yang2025_Silkie_Wenchang	PRJNA827465	0.904642647
ENSGALG00010013592	H3C33	2	-0.221028944	0.296017677	-0.746674813	0.455259877	0.026340418	1.166420447	14.26762083	0.5	CRA004775;PRJNA827465	Yang2025_Silkie_Wenchang	0.849072665
ENSGALG00010013595	WEE2	2	2.615793026	0.479555813	5.454616452	4.90787E-08	0	0.001906101	0	1	PRJNA827465;Yang2025_Silkie_Wenchang	CRA004775	1.94842E-05
ENSGALG00010013595	WEE2	2	0.754147958	1.924206801	0.391926667	0.695112399	6.214999054	6.137637635	83.70708635	0.5	CRA004775;Yang2025_Silkie_Wenchang	PRJNA827465	0.92097163
ENSGALG00010013595	WEE2	2	0.841765997	1.943290239	0.43316535	0.66489465	6.651444138	7.968316028	87.45029695	0.5	CRA004775;PRJNA827465	Yang2025_Silkie_Wenchang	0.913399315
ENSGALG00010013596	SDCBP2	2	-0.42845195	0.175954312	-2.435018191	0.014891041	0.043352925	3.074924272	67.4788739	1	PRJNA827465;Yang2025_Silkie_Wenchang	CRA004775	0.201566194
ENSGALG00010013596	SDCBP2	2	-0.300934011	0.352951786	-0.852620734	0.39386966	0.207637802	5.965607538	83.23724795	0.5	CRA004775;Yang2025_Silkie_Wenchang	PRJNA827465	0.830486298
ENSGALG00010013596	SDCBP2	2	-0.169535419	0.163208398	-1.038766511	0.29891334	0.0309193	2.044975164	51.09965061	0.5	CRA004775;PRJNA827465	Yang2025_Silkie_Wenchang	0.78974322
ENSGALG00010013598	PAQR7	2	0.326461196	0.156850745	2.081349348	0.037401941	0	0.105623483	0	1	PRJNA827465;Yang2025_Silkie_Wenchang	CRA004775	0.333556507
ENSGALG00010013598	PAQR7	2	0.189907511	0.205605058	0.923651943	0.355667561	0	0.497090713	0	0.5	CRA004775;Yang2025_Silkie_Wenchang	PRJNA827465	0.812301302
ENSGALG00010013598	PAQR7	2	0.284667016	0.188995097	1.506213767	0.132012306	0	0.878010987	0	0.5	CRA004775;PRJNA827465	Yang2025_Silkie_Wenchang	0.608737092
ENSGALG00010013604	FYN	2	-0.0114672	0.119910995	-0.095630929	0.9238137	0.024100359	6.07553822	83.54055289	0.5	PRJNA827465;Yang2025_Silkie_Wenchang	CRA004775	0.982219519
ENSGALG00010013604	FYN	2	-0.123332157	0.077670055	-1.58789842	0.112309311	0	0.662401071	0	0.5	CRA004775;Yang2025_Silkie_Wenchang	PRJNA827465	0.56602666
ENSGALG00010013604	FYN	2	0.102981902	0.055141644	1.867588524	0.061819447	0	0.014628489	0	1	CRA004775;PRJNA827465	Yang2025_Silkie_Wenchang	0.429075295
ENSGALG00010013609	PIGC	2	-0.176158016	0.104743934	-1.681796833	0.092608243	0	0.190738078	0	1	PRJNA827465;Yang2025_Silkie_Wenchang	CRA004775	0.52105755
ENSGALG00010013609	PIGC	2	-0.126420416	0.234501326	-0.539103202	0.589815646	0	0.202669061	0	1	CRA004775;Yang2025_Silkie_Wenchang	PRJNA827465	0.892320726
ENSGALG00010013609	PIGC	2	-0.202034084	0.11090613	-1.821667425	0.068505464	0	0.061142463	0	1	CRA004775;PRJNA827465	Yang2025_Silkie_Wenchang	0.451003838
ENSGALG00010013612	TWIST2	2	0.162892442	0.143217328	1.137379419	0.255379707	0	0.617048226	0	1	PRJNA827465;Yang2025_Silkie_Wenchang	CRA004775	0.764467569
ENSGALG00010013612	TWIST2	2	0.202088493	0.174792867	1.156159842	0.247615783	0	0.69308716	0	0.5	CRA004775;Yang2025_Silkie_Wenchang	PRJNA827465	0.757947282
ENSGALG00010013612	TWIST2	2	-0.006919746	0.20305879	-0.034077548	0.972815312	0	0.151323751	0	0.5	CRA004775;PRJNA827465	Yang2025_Silkie_Wenchang	0.992213869
ENSGALG00010013614	ATRX	2	0.205245579	0.274690077	0.747189638	0.454949098	0.130442049	6.919247378	85.54756109	0.5	PRJNA827465;Yang2025_Silkie_Wenchang	CRA004775	0.849072665
ENSGALG00010013614	ATRX	2	0.498731037	0.168135464	2.966245336	0.003014599	0	0.012265385	0	1	CRA004775;Yang2025_Silkie_Wenchang	PRJNA827465	0.075655171
ENSGALG00010013614	ATRX	2	0.10535755	0.231246184	0.455603735	0.64867208	0.068875428	2.135277455	53.16658197	0.5	CRA004775;PRJNA827465	Yang2025_Silkie_Wenchang	0.907730933
ENSGALG00010013617	PIK3C2G	2	0.087026513	0.13189233	0.659829978	0.509362943	0	0.007555547	0	1	PRJNA827465;Yang2025_Silkie_Wenchang	CRA004775	0.865257661
ENSGALG00010013617	PIK3C2G	2	0.19538572	0.196937842	0.992118719	0.321119616	0	0.431139538	0	1	CRA004775;Yang2025_Silkie_Wenchang	PRJNA827465	0.798513866
ENSGALG00010013617	PIK3C2G	2	0.131946945	0.143228655	0.921232569	0.35692861	0	0.632501758	0	1	CRA004775;PRJNA827465	Yang2025_Silkie_Wenchang	0.812971699
ENSGALG00010013619	IL1R2	2	0.557036023	0.369415138	1.507886291	0.131583632	0.236462961	7.174892642	86.06250923	1	PRJNA827465;Yang2025_Silkie_Wenchang	CRA004775	0.607498118
ENSGALG00010013619	IL1R2	2	0.796340797	0.251706716	3.163764599	0.001557427	0.028777346	1.231052005	18.76866323	1	CRA004775;Yang2025_Silkie_Wenchang	PRJNA827465	0.049644409
ENSGALG00010013619	IL1R2	2	0.231182681	0.121157047	1.908124104	0.056375178	0	0.172095421	0	1	CRA004775;PRJNA827465	Yang2025_Silkie_Wenchang	0.411307522
ENSGALG00010013621	PDGFRA	2	-0.092303017	0.078096696	-1.181906816	0.237242677	0	0.523883319	0	1	PRJNA827465;Yang2025_Silkie_Wenchang	CRA004775	0.749366344
ENSGALG00010013621	PDGFRA	2	-0.088306864	0.178120692	-0.495769823	0.620056818	0.015973418	1.256119249	20.38972409	0.5	CRA004775;Yang2025_Silkie_Wenchang	PRJNA827465	0.899395593
ENSGALG00010013621	PDGFRA	2	-0.043334897	0.084855478	-0.510690622	0.609567708	0	0.640697061	0	0.5	CRA004775;PRJNA827465	Yang2025_Silkie_Wenchang	0.897238578
ENSGALG00010013625	SSBP1	2	0.188505774	0.117422243	1.60536683	0.1084131	0.014571687	1.820698895	45.07603631	1	PRJNA827465;Yang2025_Silkie_Wenchang	CRA004775	0.557133898
ENSGALG00010013625	SSBP1	2	0.187294167	0.230975659	0.810882704	0.417433034	0.054561108	1.911652341	47.68923309	0.5	CRA004775;Yang2025_Silkie_Wenchang	PRJNA827465	0.838112595
ENSGALG00010013625	SSBP1	2	0.097448834	0.071129373	1.370022387	0.170679914	0	0.529036856	0	0.5	CRA004775;PRJNA827465	Yang2025_Silkie_Wenchang	0.672386723
ENSGALG00010013627	MYCBP	2	-0.036748697	0.106415719	-0.345331475	0.729845187	0	0.416448587	0	1	PRJNA827465;Yang2025_Silkie_Wenchang	CRA004775	0.932444623
ENSGALG00010013627	MYCBP	2	-0.117150795	0.196173857	-0.597178427	0.55038295	0	0.193454309	0	1	CRA004775;Yang2025_Silkie_Wenchang	PRJNA827465	0.878702974
ENSGALG00010013627	MYCBP	2	-0.014529963	0.104872727	-0.138548541	0.88980691	0	0.01029655	0	1	CRA004775;PRJNA827465	Yang2025_Silkie_Wenchang	0.973208458
ENSGALG00010013635	BCL6	2	0.341345737	0.357523482	0.954750538	0.339703866	0.23383797	11.33317505	91.17634736	1	PRJNA827465;Yang2025_Silkie_Wenchang	CRA004775	0.80507604
ENSGALG00010013635	BCL6	2	0.44097401	0.331618019	1.329764925	0.183595736	0.150519081	2.977247524	66.41192941	1	CRA004775;Yang2025_Silkie_Wenchang	PRJNA827465	0.692102743
ENSGALG00010013635	BCL6	2	0.006344188	0.087055432	0.072875263	0.941905379	0	0.014955547	0	1	CRA004775;PRJNA827465	Yang2025_Silkie_Wenchang	0.985827325
ENSGALG00010013638	COMMD8	2	0.004955896	0.135410406	0.036599079	0.970804678	0.026555965	3.338776132	70.04890534	0.5	PRJNA827465;Yang2025_Silkie_Wenchang	CRA004775	0.991851524
ENSGALG00010013638	COMMD8	2	0.140115597	0.115403108	1.214140588	0.224694043	0	0.153603771	0	1	CRA004775;Yang2025_Silkie_Wenchang	PRJNA827465	0.737885283
ENSGALG00010013638	COMMD8	2	-0.094313048	0.064244654	-1.454020575	0.145940602	0	0.555211621	0	0.5	CRA004775;PRJNA827465	Yang2025_Silkie_Wenchang	0.634891538
ENSGALG00010013639	ENSGALG00010013639	2	0.141640887	0.308637639	0.458922923	0.646289517	0.137066104	3.563478445	71.93753195	0.5	PRJNA827465;Yang2025_Silkie_Wenchang	CRA004775	0.907220702
ENSGALG00010013639	ENSGALG00010013639	2	0.37826769	0.209883306	1.802276215	0.071501959	0	0.636608631	0	1	CRA004775;Yang2025_Silkie_Wenchang	PRJNA827465	0.46165999
ENSGALG00010013639	ENSGALG00010013639	2	-0.148513297	0.216594372	-0.685674774	0.492918218	0	0.055200403	0	1	CRA004775;PRJNA827465	Yang2025_Silkie_Wenchang	0.859562076
ENSGALG00010013645	RRAGC	2	-0.158765743	0.187565763	-0.845682093	0.397730015	0.065890549	14.9147041	93.29520725	0.5	PRJNA827465;Yang2025_Silkie_Wenchang	CRA004775	0.831711071
ENSGALG00010013645	RRAGC	2	-0.33828117	0.081273863	-4.162238093	3.15144E-05	0	0.264070292	0	1	CRA004775;Yang2025_Silkie_Wenchang	PRJNA827465	0.003450163
ENSGALG00010013645	RRAGC	2	-0.004278277	0.076243426	-0.056113384	0.955251482	0.004582711	1.170791976	14.58773035	0.5	CRA004775;PRJNA827465	Yang2025_Silkie_Wenchang	0.988131974
ENSGALG00010013646	CLDN1	2	-0.060092978	0.321892244	-0.18668663	0.85190634	0.12287392	2.415801856	58.60587666	0.5	PRJNA827465;Yang2025_Silkie_Wenchang	CRA004775	0.962255491
ENSGALG00010013646	CLDN1	2	0.276484684	0.679203503	0.407071853	0.683994084	0.788292854	6.605461474	84.8610123	0.5	CRA004775;Yang2025_Silkie_Wenchang	PRJNA827465	0.918806497
ENSGALG00010013646	CLDN1	2	0.581040244	0.349735507	1.661379971	0.096637158	0.092741914	1.568232386	36.23394027	1	CRA004775;PRJNA827465	Yang2025_Silkie_Wenchang	0.531132738
ENSGALG00010013648	NAV3	2	0.068102687	0.141603169	0.480940416	0.630538847	0.025683286	2.428111264	58.81572583	0.5	PRJNA827465;Yang2025_Silkie_Wenchang	CRA004775	0.902756437
ENSGALG00010013648	NAV3	2	0.179893131	0.158151671	1.113066081	0.257194902	0.000775216	1.009988552	0.988976764	0.5	CRA004775;Yang2025_Silkie_Wenchang	PRJNA827465	0.764870705
ENSGALG00010013648	NAV3	2	-0.041689055	0.072630493	-0.573988324	0.565975702	0	0.077408913	0	1	CRA004775;PRJNA827465	Yang2025_Silkie_Wenchang	0.883487517
ENSGALG00010013650	BACE2	2	0.186264232	0.29643216	0.628353659	0.529772287	0.161113903	11.9869703	91.65760843	0.5	PRJNA827465;Yang2025_Silkie_Wenchang	CRA004775	0.871944741
ENSGALG00010013650	BACE2	2	-0.150511658	0.124951513	-1.204560509	0.22837301	0	0.877064224	0	1	CRA004775;Yang2025_Silkie_Wenchang	PRJNA827465	0.741167494
ENSGALG00010013650	BACE2	2	0.057561612	0.49688741	0.115844175	0.907775881	0.411953515	5.311611133	81.11581286	0.5	CRA004775;PRJNA827465	Yang2025_Silkie_Wenchang	0.978196186
ENSGALG00010013651	ENSGALG00010013651	2	0.121445734	0.073587514	1.650357878	0.098869761	0	0.779211244	0	1	PRJNA827465;Yang2025_Silkie_Wenchang	CRA004775	0.536756891
ENSGALG00010013651	ENSGALG00010013651	2	-0.025688485	0.251722105	-0.10203668	0.918727561	0.1055951	5.972406678	83.25633109	0.5	CRA004775;Yang2025_Silkie_Wenchang	PRJNA827465	0.981033557
ENSGALG00010013651	ENSGALG00010013651	2	-0.079786065	0.179908691	-0.443480884	0.657417943	0.049499584	4.029923792	75.18563497	0.5	CRA004775;PRJNA827465	Yang2025_Silkie_Wenchang	0.910711339
ENSGALG00010013657	MAGT1	2	-0.111570384	0.261287567	-0.427002942	0.669188549	0.12427954	10.48781521	90.46512567	0.5	PRJNA827465;Yang2025_Silkie_Wenchang	CRA004775	0.914377769
ENSGALG00010013657	MAGT1	2	-0.42974087	0.133164579	-3.227140968	0.001250338	0	0.160014973	0	1	CRA004775;Yang2025_Silkie_Wenchang	PRJNA827465	0.042993921
ENSGALG00010013657	MAGT1	2	-0.145658006	0.317539285	-0.458708615	0.646443427	0.174426348	6.50244101	84.62115998	0.5	CRA004775;PRJNA827465	Yang2025_Silkie_Wenchang	0.907220702
ENSGALG00010013663	OPA1	2	-0.076212687	0.039332493	-1.908538191	0.056321692	0	0.416248672	0	1	PRJNA827465;Yang2025_Silkie_Wenchang	CRA004775	0.410993587
ENSGALG00010013663	OPA1	2	-0.005924701	0.081574332	-0.072629478	0.942100968	0	0.209448162	0	0.5	CRA004775;Yang2025_Silkie_Wenchang	PRJNA827465	0.985827325
ENSGALG00010013663	OPA1	2	-0.080405311	0.043312203	-1.856412411	0.063394797	0	0.688431744	0	0.5	CRA004775;PRJNA827465	Yang2025_Silkie_Wenchang	0.433558714
ENSGALG00010013664	REV3L	2	-0.080351933	0.063916548	-1.257138186	0.208703602	0.00145174	1.099811967	9.07536653	0.5	PRJNA827465;Yang2025_Silkie_Wenchang	CRA004775	0.721011841
ENSGALG00010013664	REV3L	2	0.286888696	0.240218878	1.194280394	0.232368304	0.078306141	3.03396367	67.03981626	1	CRA004775;Yang2025_Silkie_Wenchang	PRJNA827465	0.745068586
ENSGALG00010013664	REV3L	2	0.187693723	0.329117409	0.57029412	0.568478228	0.191943061	8.028145941	87.54382385	0.5	CRA004775;PRJNA827465	Yang2025_Silkie_Wenchang	0.884151198
ENSGALG00010013666	COX7B	2	-0.41265263	0.351831397	-1.172870397	0.240847782	0.206375721	5.292827872	81.1065082	1	PRJNA827465;Yang2025_Silkie_Wenchang	CRA004775	0.753365729
ENSGALG00010013666	COX7B	2	-0.331573004	0.503536785	-0.658488147	0.510224519	0.410064183	5.221491145	80.84838273	0.5	CRA004775;Yang2025_Silkie_Wenchang	PRJNA827465	0.86365179
ENSGALG00010013666	COX7B	2	-0.098051589	0.076706131	-1.278275772	0.201152207	0	0.780498919	0	1	CRA004775;PRJNA827465	Yang2025_Silkie_Wenchang	0.713804636
ENSGALG00010013669	NPR3	2	0.060318639	0.155574086	0.38771649	0.698225852	0	0.323015564	0	1	PRJNA827465;Yang2025_Silkie_Wenchang	CRA004775	0.921624968
ENSGALG00010013669	NPR3	2	-0.019138424	0.207874064	-0.092067396	0.926644493	0	0.036035357	0	1	CRA004775;Yang2025_Silkie_Wenchang	PRJNA827465	0.982834319
ENSGALG00010013669	NPR3	2	0.123203398	0.187700622	0.656383471	0.51157866	0	0.028916863	0	1	CRA004775;PRJNA827465	Yang2025_Silkie_Wenchang	0.865694231
ENSGALG00010013672	ENSGALG00010013672	2	0.911457076	0.391176135	2.330042645	0.019803897	0.15986742	2.091295526	52.18275047	1	PRJNA827465;Yang2025_Silkie_Wenchang	CRA004775	0.236365799
ENSGALG00010013672	ENSGALG00010013672	2	0.978830541	0.526583293	1.858833262	0.063050776	0.339702668	2.475056387	59.59688008	1	CRA004775;Yang2025_Silkie_Wenchang	PRJNA827465	0.432475507
ENSGALG00010013672	ENSGALG00010013672	2	1.408324134	0.324818591	4.335725145	1.4528E-05	0	0.168977146	0	1	CRA004775;PRJNA827465	Yang2025_Silkie_Wenchang	0.001909684
ENSGALG00010013675	CHRNA6	2	0.105997132	1.370201526	0.07735879	0.938338124	3.54182291	17.62036727	94.3247494	0.5	PRJNA827465;Yang2025_Silkie_Wenchang	CRA004775	0.985349045
ENSGALG00010013675	CHRNA6	2	1.029481373	0.571504855	1.801351927	0.071647428	0.318901505	1.845468326	45.8132125	1	CRA004775;Yang2025_Silkie_Wenchang	PRJNA827465	0.461891482
ENSGALG00010013675	CHRNA6	2	-0.592255866	0.773133689	-0.766045866	0.443649012	0.831766733	3.155852769	68.31284369	0.5	CRA004775;PRJNA827465	Yang2025_Silkie_Wenchang	0.845827655
ENSGALG00010013678	ATP7A	2	-0.140595417	0.112465557	-1.250118837	0.211264079	0.019923294	4.396099931	77.25256442	1	PRJNA827465;Yang2025_Silkie_Wenchang	CRA004775	0.730201815
ENSGALG00010013678	ATP7A	2	-0.097781592	0.1894217	-0.51621114	0.60570695	0.049339626	2.889877942	65.39646241	0.5	CRA004775;Yang2025_Silkie_Wenchang	PRJNA827465	0.896474603
ENSGALG00010013678	ATP7A	2	-0.013730496	0.046733177	-0.29380618	0.768906012	0	0.577366774	0	0.5	CRA004775;PRJNA827465	Yang2025_Silkie_Wenchang	0.941677459
ENSGALG00010013679	MFSD4B	2	-0.369534008	0.209030933	-1.767843654	0.077087045	0.077885456	8.939789151	88.81405385	1	PRJNA827465;Yang2025_Silkie_Wenchang	CRA004775	0.478533748
ENSGALG00010013679	MFSD4B	2	-0.35228557	0.297016319	-1.186081529	0.235590104	0.130158775	3.396939583	70.56016943	0.5	CRA004775;Yang2025_Silkie_Wenchang	PRJNA827465	0.748626601
ENSGALG00010013679	MFSD4B	2	-0.163665526	0.066360026	-2.46632701	0.013650665	0	0.363124633	0	0.5	CRA004775;PRJNA827465	Yang2025_Silkie_Wenchang	0.191338116
ENSGALG00010013686	UQCRQ	2	-0.238566292	0.144169171	-1.654766349	0.097971902	0.016087393	1.456084022	31.3226445	1	PRJNA827465;Yang2025_Silkie_Wenchang	CRA004775	0.534130658
ENSGALG00010013686	UQCRQ	2	-0.124322206	0.388256739	-0.320206177	0.748812041	0.211699562	3.249100175	69.22224781	0.5	CRA004775;Yang2025_Silkie_Wenchang	PRJNA827465	0.937249951
ENSGALG00010013686	UQCRQ	2	-0.044996461	0.192372151	-0.233904194	0.815060134	0.034826756	1.485716061	32.69258813	0.5	CRA004775;PRJNA827465	Yang2025_Silkie_Wenchang	0.955393933
ENSGALG00010013690	CREB3L2	2	-0.159990484	0.195584807	-0.8180108	0.41335102	0.063764184	5.765978064	82.65688858	0.5	PRJNA827465;Yang2025_Silkie_Wenchang	CRA004775	0.836088312
ENSGALG00010013690	CREB3L2	2	-0.366007864	0.134052568	-2.730330855	0.006327079	0	0.027952552	0	1	CRA004775;Yang2025_Silkie_Wenchang	PRJNA827465	0.120949997
ENSGALG00010013690	CREB3L2	2	0.005525213	0.078201299	0.070653718	0.943673357	0	0.662023245	0	0.5	CRA004775;PRJNA827465	Yang2025_Silkie_Wenchang	0.985827325
ENSGALG00010013696	CETN3	2	0.251489099	0.247914909	1.014417005	0.310383813	0.067885412	2.233119493	55.21959289	1	PRJNA827465;Yang2025_Silkie_Wenchang	CRA004775	0.794899969
ENSGALG00010013696	CETN3	2	0.252670901	0.287488205	0.878891364	0.379460182	0.08849463	2.08904268	52.13118384	0.5	CRA004775;Yang2025_Silkie_Wenchang	PRJNA827465	0.824165982
ENSGALG00010013696	CETN3	2	-0.028672458	0.192654107	-0.148828687	0.881688814	0	0.045650028	0	0.5	CRA004775;PRJNA827465	Yang2025_Silkie_Wenchang	0.971870525
ENSGALG00010013697	HDAC4	2	-0.003644825	0.0972067	-0.03749562	0.970089035	0.010001517	1.632657408	38.75766522	0.5	PRJNA827465;Yang2025_Silkie_Wenchang	CRA004775	0.991660528
ENSGALG00010013697	HDAC4	2	0.180948966	0.146577474	1.234493688	0.217018997	0	0.019527223	0	1	CRA004775;Yang2025_Silkie_Wenchang	PRJNA827465	0.730558054
ENSGALG00010013697	HDAC4	2	-0.050486785	0.048802538	-1.034511457	0.300897123	0	0.890463814	0	0.5	CRA004775;PRJNA827465	Yang2025_Silkie_Wenchang	0.790941656
ENSGALG00010013709	PSMF1	2	-0.221307252	0.062856007	-3.520860796	0.000430148	0.000456848	1.029614929	2.876311193	1	PRJNA827465;Yang2025_Silkie_Wenchang	CRA004775	0.021075798
ENSGALG00010013709	PSMF1	2	-0.400524435	0.14223221	-2.815989666	0.004862723	0	0.112343351	0	1	CRA004775;Yang2025_Silkie_Wenchang	PRJNA827465	0.103587862
ENSGALG00010013709	PSMF1	2	-0.209107213	0.063002464	-3.319032303	0.0009033	0	0.954980944	0	1	CRA004775;PRJNA827465	Yang2025_Silkie_Wenchang	0.035325763
ENSGALG00010013715	IL1R1	2	0.205324889	0.119013956	1.725216895	0.084488392	0.003270636	1.127309841	11.29324309	1	PRJNA827465;Yang2025_Silkie_Wenchang	CRA004775	0.500072565
ENSGALG00010013715	IL1R1	2	0.286533467	0.134319692	2.133205213	0.032907896	0	0.135255138	0	1	CRA004775;Yang2025_Silkie_Wenchang	PRJNA827465	0.311661412
ENSGALG00010013715	IL1R1	2	0.088968956	0.154140748	0.577192967	0.563809108	0	0.076245151	0	1	CRA004775;PRJNA827465	Yang2025_Silkie_Wenchang	0.882651345
ENSGALG00010013719	NCDN	2	0.201531502	0.057269368	3.519010383	0.00043316	0	0.053914496	0	1	PRJNA827465;Yang2025_Silkie_Wenchang	CRA004775	0.021174724
ENSGALG00010013719	NCDN	2	-0.179453636	0.372508892	-0.481743227	0.629988364	0.239406701	7.101084821	85.91764462	0.5	CRA004775;Yang2025_Silkie_Wenchang	PRJNA827465	0.902533841
ENSGALG00010013719	NCDN	2	-0.150763355	0.39031085	-0.386264833	0.699300543	0.276585875	10.10096921	90.09995992	0.5	CRA004775;PRJNA827465	Yang2025_Silkie_Wenchang	0.921931076
ENSGALG00010013720	SLC16A10	2	-0.262975292	0.374215513	-0.702737547	0.482219324	0.263899733	17.19667667	94.18492294	0.5	PRJNA827465;Yang2025_Silkie_Wenchang	CRA004775	0.85691916
ENSGALG00010013720	SLC16A10	2	-0.065976189	0.643706909	-0.102494144	0.918364461	0.72753627	7.582983025	86.81257763	0.5	CRA004775;Yang2025_Silkie_Wenchang	PRJNA827465	0.981033557
ENSGALG00010013720	SLC16A10	2	0.20409024	0.211587914	0.964564736	0.334762869	0.044337624	1.423266344	29.73908191	1	CRA004775;PRJNA827465	Yang2025_Silkie_Wenchang	0.803101658
ENSGALG00010013732	FNTA	2	0.183060553	0.192094567	0.952971005	0.340604762	0.060837173	5.314819314	81.18468492	1	PRJNA827465;Yang2025_Silkie_Wenchang	CRA004775	0.805524125
ENSGALG00010013732	FNTA	2	0.017056314	0.410516108	0.041548465	0.966858657	0.292196864	7.293845176	86.28981044	0.5	CRA004775;Yang2025_Silkie_Wenchang	PRJNA827465	0.990893423
ENSGALG00010013732	FNTA	2	-0.130405746	0.204894276	-0.636453823	0.524480669	0.057768142	2.552955069	60.82970625	0.5	CRA004775;PRJNA827465	Yang2025_Silkie_Wenchang	0.869790047
ENSGALG00010013737	AFF4	2	-0.03586869	0.059030247	-0.607632393	0.543431312	0	0.589350523	0	1	PRJNA827465;Yang2025_Silkie_Wenchang	CRA004775	0.876968972
ENSGALG00010013737	AFF4	2	0.027261522	0.220038751	0.123894188	0.901399054	0.054620845	1.945123914	48.58939358	0.5	CRA004775;Yang2025_Silkie_Wenchang	PRJNA827465	0.976655087
ENSGALG00010013737	AFF4	2	0.03978415	0.127155527	0.312877865	0.754373455	0.014380981	1.276292405	21.648049	0.5	CRA004775;PRJNA827465	Yang2025_Silkie_Wenchang	0.93838723
ENSGALG00010013740	GNPDA2	2	0.128194668	0.083701721	1.531565487	0.125629695	0.00619784	1.706521184	41.40125482	1	PRJNA827465;Yang2025_Silkie_Wenchang	CRA004775	0.595034397
ENSGALG00010013740	GNPDA2	2	-0.101071849	0.378326851	-0.267154838	0.789349947	0.244593451	6.389340857	84.34893329	0.5	CRA004775;Yang2025_Silkie_Wenchang	PRJNA827465	0.947379026
ENSGALG00010013740	GNPDA2	2	-0.168479769	0.287606436	-0.585799716	0.558010114	0.131578185	4.16314758	75.97971292	0.5	CRA004775;PRJNA827465	Yang2025_Silkie_Wenchang	0.880678789
ENSGALG00010013744	ENSGALG00010013744	2	0.242685982	0.198105261	1.225035525	0.220561836	0	0.643755687	0	1	PRJNA827465;Yang2025_Silkie_Wenchang	CRA004775	0.734053249
ENSGALG00010013744	ENSGALG00010013744	2	0.31014339	0.274509268	1.129810267	0.258556181	0	0.659780483	0	0.5	CRA004775;Yang2025_Silkie_Wenchang	PRJNA827465	0.765790021
ENSGALG00010013744	ENSGALG00010013744	2	0.085694428	0.227327235	0.376965074	0.70619956	0	0.089627736	0	0.5	CRA004775;PRJNA827465	Yang2025_Silkie_Wenchang	0.924548793
ENSGALG00010013746	ENSGALG00010013746	2	0.213758447	0.270598419	0.789947142	0.429558639	0.105805057	3.58347349	72.09411475	0.5	PRJNA827465;Yang2025_Silkie_Wenchang	CRA004775	0.842012722
ENSGALG00010013746	ENSGALG00010013746	2	0.48035271	0.16830078	2.854132402	0.004315454	0	0.01453811	0	1	CRA004775;Yang2025_Silkie_Wenchang	PRJNA827465	0.096315625
ENSGALG00010013746	ENSGALG00010013746	2	0.170142477	0.247704753	0.686876108	0.492160804	0.073302159	2.411606601	58.53386703	0.5	CRA004775;PRJNA827465	Yang2025_Silkie_Wenchang	0.859171896
ENSGALG00010013749	NELL2	2	-0.047937389	0.152714115	-0.313902803	0.753594861	0	0.621402693	0	0.5	PRJNA827465;Yang2025_Silkie_Wenchang	CRA004775	0.938097071
ENSGALG00010013749	NELL2	2	0.290910411	0.597177786	0.487142051	0.626157668	0.547836975	4.012794145	75.07970846	0.5	CRA004775;Yang2025_Silkie_Wenchang	PRJNA827465	0.901504244

ENSGALG00010013749	NELL2	2	0.375180755	0.455611839	0.823465773	0.410243173	0.288821293	2.77326819	63.9414607	1	CRA004775;PRJNA827465	Yang2025_Silkie_Wenchang	0.834737228
ENSGALG00010013751	FUT10	2	0.04833575	0.172229904	0.280646674	0.778981414	0.023684906	1.663107977	39.87161303	0.5	PRJNA827465;Yang2025_Silkie_Wenchang	CRA004775	0.944714533
ENSGALG00010013751	FUT10	2	-0.105306303	0.160115427	-0.657689928	0.510737396	0	0.019687231	0	1	CRA004775;Yang2025_Silkie_Wenchang	PRJNA827465	0.865386785
ENSGALG00010013751	FUT10	2	0.151308454	0.167484973	0.903415101	0.366305628	0	0.582531947	0	0.5	CRA004775;PRJNA827465	Yang2025_Silkie_Wenchang	0.819159863
ENSGALG00010013753	PLEKHA5	2	0.004251925	0.39900834	0.01065623	0.99149772	0.29045774	11.09259165	90.98497419	0.5	PRJNA827465;Yang2025_Silkie_Wenchang	CRA004775	0.997563866
ENSGALG00010013753	PLEKHA5	2	0.503483073	0.181823935	2.769069275	0.005621668	0	0.529584841	0	1	CRA004775;Yang2025_Silkie_Wenchang	PRJNA827465	0.112499196
ENSGALG00010013753	PLEKHA5	2	0.123387816	0.547063977	0.225545497	0.821554936	0.535087739	8.790703014	88.62434553	0.5	CRA004775;PRJNA827465	Yang2025_Silkie_Wenchang	0.955598716
ENSGALG00010013755	ANGPT4	2	0.125758412	0.089613436	1.40334326	0.160514504	0.001709081	1.115277276	10.33619879	1	PRJNA827465;Yang2025_Silkie_Wenchang	CRA004775	0.65630165
ENSGALG00010013755	ANGPT4	2	0.043503472	0.241894248	0.179845006	0.85727425	0.076447363	2.59309906	61.43610495	0.5	CRA004775;Yang2025_Silkie_Wenchang	PRJNA827465	0.963936745
ENSGALG00010013755	ANGPT4	2	-0.003680819	0.119092133	-0.03090732	0.975343452	0.005061736	1.112015846	10.07322389	0.5	CRA004775;PRJNA827465	Yang2025_Silkie_Wenchang	0.992750665
ENSGALG00010013757	DGKI	2	0.268451197	0.48661986	0.551665107	0.58117782	0.404653808	6.831230992	85.36134994	0.5	PRJNA827465;Yang2025_Silkie_Wenchang	CRA004775	0.88890219
ENSGALG00010013757	DGKI	2	0.390165816	0.644464241	0.605411117	0.544905866	0.475837213	1.781835632	43.87810066	0.5	CRA004775;Yang2025_Silkie_Wenchang	PRJNA827465	0.877354247
ENSGALG00010013757	DGKI	2	-0.226407422	0.228600194	-0.990407829	0.32197482	0	0.209610039	0	1	CRA004775;PRJNA827465	Yang2025_Silkie_Wenchang	0.798513866
ENSGALG00010013760	RPF2	2	-0.151021485	0.065944666	-2.290124333	0.02201411	0.000478882	1.038864398	3.741046245	1	PRJNA827465;Yang2025_Silkie_Wenchang	CRA004775	0.251663925
ENSGALG00010013760	RPF2	2	-0.293611744	0.115555553	-2.540870911	0.011057674	0	0.048105143	0	1	CRA004775;Yang2025_Silkie_Wenchang	PRJNA827465	0.167282429
ENSGALG00010013760	RPF2	2	-0.139412429	0.067289092	-2.071842913	0.038280093	0	0.97611491	0	1	CRA004775;PRJNA827465	Yang2025_Silkie_Wenchang	0.337506202
ENSGALG00010013766	DNM3	2	-0.061483241	0.085460818	-0.71943192	0.471874835	0	0.859825435	0	1	PRJNA827465;Yang2025_Silkie_Wenchang	CRA004775	0.853998373
ENSGALG00010013766	DNM3	2	-0.146499584	0.130370978	-1.123713166	0.261134729	0	0.155322358	0	1	CRA004775;Yang2025_Silkie_Wenchang	PRJNA827465	0.767929536
ENSGALG00010013766	DNM3	2	-0.01306724	0.096823009	-0.134960074	0.892643442	0	0.030132571	0	1	CRA004775;PRJNA827465	Yang2025_Silkie_Wenchang	0.974381724
ENSGALG00010013767	PGK2	2	-0.152924074	0.095532334	-1.60075723	0.1094307	0	0.438475547	0	1	PRJNA827465;Yang2025_Silkie_Wenchang	CRA004775	0.559801483
ENSGALG00010013767	PGK2	2	-0.200088465	0.155483698	-1.28687745	0.198137006	0	0.789762544	0	0.5	CRA004775;Yang2025_Silkie_Wenchang	PRJNA827465	0.710234722
ENSGALG00010013767	PGK2	2	-0.090265537	0.115971023	-0.778345607	0.436365295	0	0.457847772	0	0.5	CRA004775;PRJNA827465	Yang2025_Silkie_Wenchang	0.843122822
ENSGALG00010013768	FAM110A	2	0.185562053	0.246329811	0.753307334	0.451265253	0.094792314	4.531404071	77.93178484	0.5	PRJNA827465;Yang2025_Silkie_Wenchang	CRA004775	0.847847413
ENSGALG00010013768	FAM110A	2	-0.224286169	0.725538999	-0.309130411	0.757222327	0.940311473	8.859523603	88.71271137	0.5	CRA004775;Yang2025_Silkie_Wenchang	PRJNA827465	0.938795135
ENSGALG00010013768	FAM110A	2	-0.434251345	0.471633925	-0.920738144	0.357187156	0.349635378	4.071765412	75.44062836	1	CRA004775;PRJNA827465	Yang2025_Silkie_Wenchang	0.813261893
ENSGALG00010013771	PELO	2	0.172735492	0.135889036	1.271151053	0.203674916	0	0.014126923	0	1	PRJNA827465;Yang2025_Silkie_Wenchang	CRA004775	0.717254253
ENSGALG00010013771	PELO	2	0.044814416	0.154932313	0.289251577	0.772388865	0	0.761433896	0	0.5	CRA004775;Yang2025_Silkie_Wenchang	PRJNA827465	0.943066211
ENSGALG00010013771	PELO	2	0.075162293	0.148430817	0.506379297	0.612590406	0.000414024	1.008787038	0.8710499	0.5	CRA004775;PRJNA827465	Yang2025_Silkie_Wenchang	0.897994639
ENSGALG00010013772	KIAA0319L	2	-0.036454978	0.104042569	-0.350385215	0.726049621	0.016973065	4.594854263	78.2365241	0.5	PRJNA827465;Yang2025_Silkie_Wenchang	CRA004775	0.931029023
ENSGALG00010013772	KIAA0319L	2	-0.148774219	0.072490508	-2.052326896	0.040137904	0	0.026033918	0	1	CRA004775;Yang2025_Silkie_Wenchang	PRJNA827465	0.347067015
ENSGALG00010013772	KIAA0319L	2	0.049527927	0.059462072	0.832933079	0.404882473	0	0.904700955	0	0.5	CRA004775;PRJNA827465	Yang2025_Silkie_Wenchang	0.833112102
ENSGALG00010013775	DBX2	2	0.143636168	0.155021668	0.926555427	0.354157406	0	0.337524556	0	1	PRJNA827465;Yang2025_Silkie_Wenchang	CRA004775	0.811853621
ENSGALG00010013775	DBX2	2	0.013470402	0.374771253	0.035942998	0.971327811	0.118501566	1.680457592	40.49239892	0.5	CRA004775;Yang2025_Silkie_Wenchang	PRJNA827465	0.991955348
ENSGALG00010013775	DBX2	2	0.012241724	0.197565498	0.061962862	0.950592407	0.0159534	1.121263359	10.81488643	0.5	CRA004775;PRJNA827465	Yang2025_Silkie_Wenchang	0.987277827
ENSGALG00010013777	CPLANE1	2	0.134238856	0.162552251	0.825819731	0.40890636	0.011768557	1.276950043	21.68840077	0.5	PRJNA827465;Yang2025_Silkie_Wenchang	CRA004775	0.834316328
ENSGALG00010013777	CPLANE1	2	0.287212118	0.170085845	1.688630343	0.091290292	0	0.065852211	0	1	CRA004775;Yang2025_Silkie_Wenchang	PRJNA827465	0.517970601
ENSGALG00010013777	CPLANE1	2	0.077437544	0.149561272	0.517764681	0.604622463	0	0.581939138	0	0.5	CRA004775;PRJNA827465	Yang2025_Silkie_Wenchang	0.896289929
ENSGALG00010013778	ENSGALG00010013778	2	-0.805198512	1.113348653	-0.723222245	0.469543354	1.932645214	4.504944014	77.80216587	0.5	PRJNA827465;Yang2025_Silkie_Wenchang	CRA004775	0.853485658
ENSGALG00010013778	ENSGALG00010013778	2	0.455127267	0.705490449	0.645121798	0.518848259	0	0.056033208	0	1	CRA004775;Yang2025_Silkie_Wenchang	PRJNA827465	0.868851946
ENSGALG00010013778	ENSGALG00010013778	2	-0.860735815	1.273119611	-0.676084013	0.498987306	2.198761099	2.800575833	64.29305758	0.5	CRA004775;PRJNA827465	Yang2025_Silkie_Wenchang	0.861571318
ENSGALG00010013779	SLC52A3	2	0.283910275	0.139682323	2.032542625	0.042098753	0	0.539292193	0	1	PRJNA827465;Yang2025_Silkie_Wenchang	CRA004775	0.355065944
ENSGALG00010013779	SLC52A3	2	-0.101196282	0.581072664	-0.174154264	0.861744228	0.569521007	6.272931684	84.05849051	0.5	CRA004775;Yang2025_Silkie_Wenchang	PRJNA827465	0.96559281
ENSGALG00010013779	SLC52A3	2	-0.1846133	0.463725295	-0.398109186	0.690549703	0.351190354	5.033273847	80.13221553	0.5	CRA004775;PRJNA827465	Yang2025_Silkie_Wenchang	0.919979266
ENSGALG00010013783	NDUFA10	2	-0.134806231	0.05199643	-2.592605489	0.009525196	0	0.684798836	0	1	PRJNA827465;Yang2025_Silkie_Wenchang	CRA004775	0.1535557
ENSGALG00010013783	NDUFA10	2	0.027035242	0.125467098	0.215476742	0.829395643	0	0.520348232	0	0.5	CRA004775;Yang2025_Silkie_Wenchang	PRJNA827465	0.956899459
ENSGALG00010013783	NDUFA10	2	-0.073715745	0.142726442	-0.516482747	0.605517286	0.023308712	1.677681546	40.39393219	0.5	CRA004775;PRJNA827465	Yang2025_Silkie_Wenchang	0.896474603
ENSGALG00010013784	ANO6	2	-0.013488624	0.17588504	-0.076690003	0.938870158	0.044563216	3.574463097	72.02377048	0.5	PRJNA827465;Yang2025_Silkie_Wenchang	CRA004775	0.985349045
ENSGALG00010013784	ANO6	2	0.218196767	0.124018148	1.759393849	0.078510636	0.000741437	1.013366049	1.318975382	1	CRA004775;Yang2025_Silkie_Wenchang	PRJNA827465	0.481915278
ENSGALG00010013784	ANO6	2	0.099576323	0.339017983	0.293719885	0.768971958	0.180901489	4.281073052	76.64137033	0.5	CRA004775;PRJNA827465	Yang2025_Silkie_Wenchang	0.941728877
ENSGALG00010013787	ZCCHC10	2	-0.184196942	0.145635304	-1.264782208	0.205949411	0.03233353	4.008382037	75.05227818	1	PRJNA827465;Yang2025_Silkie_Wenchang	CRA004775	0.718403416
ENSGALG00010013787	ZCCHC10	2	-0.235163177	0.157512597	-1.492980126	0.135442389	0.021716716	1.652642507	39.49084599	1	CRA004775;Yang2025_Silkie_Wenchang	PRJNA827465	0.616907199
ENSGALG00010013787	ZCCHC10	2	-0.051936049	0.069884531	-0.743169463	0.457379089	0	0.025986357	0	1	CRA004775;PRJNA827465	Yang2025_Silkie_Wenchang	0.849573219
ENSGALG00010013791	AEBP2	2	-0.010927698	0.045133542	-0.242119208	0.808687795	0	0.139577139	0	0.5	PRJNA827465;Yang2025_Silkie_Wenchang	CRA004775	0.952553613
ENSGALG00010013791	AEBP2	2	0.037480998	0.082391307	0.454914469	0.649170773	0	0.244437189	0	1	CRA004775;Yang2025_Silkie_Wenchang	PRJNA827465	0.907822768
ENSGALG00010013791	AEBP2	2	-0.010942406	0.050252427	-0.217748807	0.827624833	0	0.518941992	0	0.5	CRA004775;PRJNA827465	Yang2025_Silkie_Wenchang	0.956550752
ENSGALG00010013792	FGF10	2	0.133472075	0.201299472	0.663052285	0.507297069	0.034413081	1.708040365	41.45337425	0.5	PRJNA827465;Yang2025_Silkie_Wenchang	CRA004775	0.864086554
ENSGALG00010013792	FGF10	2	-0.083569275	0.227708647	-0.367000885	0.713618358	0	0.034322282	0	0.5	CRA004775;Yang2025_Silkie_Wenchang	PRJNA827465	0.926266259
ENSGALG00010013792	FGF10	2	0.277563446	0.179808576	1.543660774	0.122670534	0	0.229520578	0	1	CRA004775;PRJNA827465	Yang2025_Silkie_Wenchang	0.58762464
ENSGALG00010013793	METTL13	2	0.007210036	0.071379296	0.10101018	0.919542379	0	0.00227452	0	1	PRJNA827465;Yang2025_Silkie_Wenchang	CRA004775	0.981192162
ENSGALG00010013793	METTL13	2	-0.275349301	0.322825984	-0.852934133	0.393695832	0.16111658	4.236380932	76.39494615	0.5	CRA004775;Yang2025_Silkie_Wenchang	PRJNA827465	0.83043598
ENSGALG00010013793	METTL13	2	-0.263072654	0.316175845	-0.83204539	0.405383325	0.165853262	5.20568988	80.79025023	0.5	CRA004775;PRJNA827465	Yang2025_Silkie_Wenchang	0.833112102
ENSGALG00010013794	GTF3C6	2	-0.039926131	0.170568797	-0.234076404	0.814925664	0.04570477	4.492604646	77.7411974	0.5	PRJNA827465;Yang2025_Silkie_Wenchang	CRA004775	0.953897709
ENSGALG00010013794	GTF3C6	2	0.12407537	0.130448116	0.951147281	0.341529616	0	0.304079652	0	0.5	CRA004775;Yang2025_Silkie_Wenchang	PRJNA827465	0.805758959
ENSGALG00010013794	GTF3C6	2	-0.189328981	0.082615919	-2.291676749	0.021924303	0	0.071906095	0	1	CRA004775;PRJNA827465	Yang2025_Silkie_Wenchang	0.251002296
ENSGALG00010013797	IL1RL1	2	-0.317663089	0.351808539	-0.902943089	0.3665561	0.198911907	5.056602176	80.22387435	0.5	PRJNA827465;Yang2025_Silkie_Wenchang	CRA004775	0.819334942
ENSGALG00010013797	IL1RL1	2	-0.283470792	0.4659014	-0.608435158	0.542898899	0.318430129	3.533553179	71.69987405	0.5	CRA004775;Yang2025_Silkie_Wenchang	PRJNA827465	0.876968972
ENSGALG00010013797	IL1RL1	2	0.060321864	0.180031208	0.335063371	0.737577283	0	0.245473294	0	1	CRA004775;PRJNA827465	Yang2025_Silkie_Wenchang	0.934044334
ENSGALG00010013802	PYROXD1	2	-0.035217495	0.092789749	-0.379540798	0.704286314	0	0.537337809	0	0.5	PRJNA827465;Yang2025_Silkie_Wenchang	CRA004775	0.924071607
ENSGALG00010013802	PYROXD1	2	-0.019372144	0.137747679	-0.140634994	0.888158302	0	0.938971034	0	0.5	CRA004775;Yang2025_Silkie_Wenchang	PRJNA827465	0.973135755
ENSGALG00010013802	PYROXD1	2	-0.107436995	0.104524054	-1.027868619	0.304011633	0	0.295172754	0	1	CRA004775;PRJNA827465	Yang2025_Silkie_Wenchang	0.792240366
ENSGALG00010013805	HSPA4	2	0.004007525	0.030124255	0.133033178	0.894167142	0	0.007313458	0	0.5	PRJNA827465;Yang2025_Silkie_Wenchang	CRA004775	0.974381724
ENSGALG00010013805	HSPA4	2	0.252209966	0.329672848	0.765031054	0.444253053	0.167849753	3.594070944	72.17639787	0.5	CRA004775;Yang2025_Silkie_Wenchang	PRJNA827465	0.846013379
ENSGALG00010013805	HSPA4	2	0.251645998	0.325052065	0.774171357	0.438829464	0.165307637	3.6400811	72.52808461	1	CRA004775;PRJNA827465	Yang2025_Silkie_Wenchang	0.844002503
ENSGALG00010013808	PLAAT1	2	-0.103119238	0.154134636	-0.669020545	0.503482372	0.030791415	2.812317326	64.44213493	0.5	PRJNA827465;Yang2025_Silkie_Wenchang	CRA004775	0.863108263
ENSGALG00010013808	PLAAT1	2	-0.205377572	0.325537001	-0.630888565	0.528131389	0.157437337	3.518417998	71.57813538	0.5	CRA004775;Yang2025_Silkie_Wenchang	PRJNA827465	0.871694282
ENSGALG00010013808	PLAAT1	2	-0.294645138	0.121751528	-2.420052906	0.015518249	0.003754527	1.063694578	5.988051377	1	CRA004775;PRJNA827465	Yang2025_Silkie_Wenchang	0.206070237
ENSGALG00010013814	AMD1	2	-0.097137857	0.103094633	-0.942220307	0.346079859	0.012386058	2.233902121	55.23528133	1	PRJNA827465;Yang2025_Silkie_Wenchang	CRA004775	0.807702909
ENSGALG00010013814	AMD1	2	0.033531564	0.325773944	0.102928931	0.918019376	0.158946436	3.482731472	71.28690489	0.5	CRA004775;Yang2025_Silkie_Wenchang	PRJNA827465	0.981033557
ENSGALG00010013814	AMD1	2	0.094215518	0.194864158	0.483493316	0.628745504	0.045067224	1.76508509	43.34550748	0.5	CRA004775;PRJNA827465	Yang2025_Silkie_Wenchang	0.902047255
ENSGALG00010013815	SRXN1	2	-0.056878144	0.082882446	-0.686250789	0.492554975	0	0.002780033	0	1	PRJNA827465;Yang2025_Silkie_Wenchang	CRA004775	0.883931149
ENSGALG00010013815	SRXN1	2	-0.184397885	0.226474019	-0.814212095	0.415523458	0	0.661152833	0	1	CRA004775;Yang2025_Silkie_Wenchang	PRJNA827465	0.837678466
ENSGALG00010013815	SRXN1	2	-0.07785917	0.084113356	-0.925645747	0.354630111	0	0.911565605	0	1	CRA004775;PRJNA827465	Yang2025_Silkie_Wenchang	0.812016118
ENSGALG00010013820	MASP1	2	0.483157091	0.437815655	1.103562847	0.269782815	0	0.036662744	0	1	PRJNA827465;Yang2025_Silkie_Wenchang	CRA004775	0.77209558
ENSGALG00010013820	MASP1	2	0.549010195	0.464383878	1.182233537	0.237113049	0	0.137279762	0	1	CRA004775;Yang2025_Silkie_Wenchang	PRJNA827465	0.749355649
ENSGALG00010013820	MASP1	2	0.647578986	0.470567994	1.376164538	0.168770698	0	0.036151348	0	1	CRA004775;PRJNA827465	Yang2025_Silkie_Wenchang	0.669861707
ENSGALG00010013823	GUF1	2	0.153761661	0.201823187	0.761863207	0.446141652	0.069617173	6.859507546	85.421694	0.5	PRJNA827465;Yang2025_Silkie_Wenchang	CRA004775	0.846013379
ENSGALG00010013823	GUF1	2	0.366305415	0.103067946	3.554019769	0.000379592	0	0.015959922	0	1	CRA004775;Yang2025_Silkie_Wenchang	PRJNA827465	0.019517342
ENSGALG00010013823	GUF1	2	0.128027148	0.212533538	0.602385624	0.546917477	0.064536783	3.158765806	68.34206581	0.5	CRA004775;PRJNA827465	Yang2025_Silkie_Wenchang	0.878281266
ENSGALG00010013828	PTN	2	0.364847537	0.372995037	0.978156544	0.327996905	0.20798393	3.948531554	74.6741292	1	PRJNA827465;Yang2025_Silkie_Wenchang	CRA004775	0.800530298
ENSGALG00010013828	PTN	2	0.680961786	0.263782137	2.581531086	0.009836313	0	0.48914487	0	1	CRA004775;Yang2025_Silkie_Wenchang	PRJNA827465	0.15667223
ENSGALG00010013828	PTN	2	0.02113331	0.236221893	0.089463809	0.928713314	0	0.052512029	0	1	CRA004775;PRJNA827465	Yang2025_Silkie_Wenchang	0.98338483
ENSGALG00010013831	TCERG1	2	0.207831573	0.277880507	0.747917065	0.454510184	0.140857956	10.58906659	90.556297	0.5	PRJNA827465;Yang2025_Silkie_Wenchang	CRA004775	0.849072665
ENSGALG00010013831	TCERG1	2	0.301772656	0.219049899	1.377643437	0.168313403	0.063621039	2.95289822	66.13496554	1	CRA004775;Yang2025_Silkie_Wenchang	PRJNA827465	0.668962553
ENSGALG00010013831	TCERG1	2	-0.042754782	0.03841253	-1.113042596	0.265690136	0	0.365391879	0	0.5	CRA004775;PRJNA827465	Yang2025_Silkie_Wenchang	0.76982829
ENSGALG00010013836	IL18R1	2	-0.119563167	0.320131386	-0.373481554	0.708790072	0.155994357	3.893562464	74.31658001	0.5	PRJNA827465;Yang2025_Silkie_Wenchang	CRA004775	0.925285687
ENSGALG00010013836	IL18R1	2	-0.451399943	0.254570494	-1.773182492	0.076198472	0	0.0820876	0	1	CRA004775;Yang2025_Silkie_Wenchang	PRJNA827465	0.476428342
ENSGALG00010013836	IL18R1	2	0.118059485	0.141621669	0.833625855	0.404491851	0	0.808248848	0	0.5	CRA004775;PRJNA827465	Yang2025_Silkie_Wenchang	0.833112102
ENSGALG00010013837	IFI27L2	2	0.320561088	0.333961383	0.959874715	0.337118273	0.155522949	3.291925658	69.62264328	0.5	PRJNA827465;Yang2025_Silkie_Wenchang	CRA004775	0.830486136
ENSGALG00010013837	IFI27L2	2	0.460066783	0.149279473	3.081915916	0.002056729	0	0.795761872	0	1	CRA004775;Yang2025_Silkie_Wenchang	PRJNA827465	0.059004842
ENSGALG00010013837	IFI27L2	2	0.223957906	0.184049597	1.216834536	0.223667176	0.025071229	1.551701401	35.55461127	0.5	CRA004775;PRJNA827465	Yang2025_Silkie_Wenchang	0.737076707
ENSGALG00010013842	CDK19	2	0.200234496	0.220831898	0.906728141	0.364550578	0.081692346	5.572556786	82.05491593	1	PRJNA827465;Yang2025_Silkie_Wenchang	CRA004775	0.831749625
ENSGALG00010013842	CDK19	2	0.413976871	0.162222883	2.551901833	0.01071367	0	0.295167276	0	1	CRA004775;Yang2025_Silkie_Wenchang	PRJNA827465	0.165128023
ENSGALG00010013842	CDK19	2	0.014215404	0.059822356	0.237626943	0.812170452	0	0.311306311	0	1	CRA004775;PRJNA827465	Yang2025_Silkie_Wenchang	0.953371387
ENSGALG00010013846	YIPF7	2	-0.236625397	0.200731089	-1.178817877	0.238470704	0	0.468063169	0	1	PRJNA827465;Yang2025_Silkie_Wenchang	CRA004775	0.750649855
ENSGALG00010013846	YIPF7	2	-0.281946026	0.230721484	-1.22201895	0.221700461	0	0.402342827	0	0.5	CRA004775;Yang2025_Silkie_Wenchang	PRJNA827465	0.735005755
ENSGALG00010013846	YIPF7	2	0.001947738	0.320529241	0.006076631	0.99515158	0	0.057650526	0	0.5	CRA004775;PRJNA827465	Yang2025_Silkie_Wenchang	0.998242335
ENSGALG00010013848	ITPA	2	-0.063777796	0.137518802	-0.463775101	0.642808884	0.013648014	1.358123164	26.36897545	0.5	PRJNA827465;Yang2025_Silkie_Wenchang	CRA004775	0.906634523
ENSGALG00010013848	ITPA	2	-0.380440226	0.174876448	-2.175480058	0.029594161	0	0.152117258	0	1	CRA004775;Yang2025_Silkie_Wenchang	PRJNA827465	0.293446097
ENSGALG00010013848	ITPA	2	-0.168520091	0.223502205	-0.737442797	0.460853717	0.070540258	3.043593165	67.14609759	0.5	CRA004775;PRJNA827465	Yang2025_Silkie_Wenchang	0.85014477
ENSGALG00010013859	MYOC	2	0.188886078	0.204375398	0.924211423	0.355376249	0	0.035545927	0	1	PRJNA827465;Yang2025_Silkie_Wenchang	CRA004775	0.812301302
ENSGALG00010013859	MYOC	2	-0.016709418	0.296504723	-0.056356644	0.955059289	0.020364587	1.128992308	11.42543727	0.5	CRA004775;Yang2025_Silkie_Wenchang	PRJNA827465	0.988093796
ENSGALG00010013859	MYOC	2	0.022178776	0.229231246	0.096752848	0.92292267	0.012426543	1.104137765	9.431591661	0.5	CRA004775;PRJNA827465	Yang2025_Silkie_Wenchang	0.982327844
ENSGALG00010013861	TCF15	2	0.203746785	0.275728528	0.738939806	0.45994355	0.116728528	3.946710726	74.66224502	0.5	PRJNA827465;Yang2025_Silkie_Wenchang	CRA004775	0.850104868
ENSGALG00010013861	TCF15	2	0.471368415	0.229945188	2.049916415	0.040372588	0	0.268831065	0	1	CRA004775;Yang2025_Silkie_Wenchang	PRJNA827465	0.347442284
ENSGALG00010013861	TCF15	2	-0.017287553	0.114297011	-0.151251139	0.879771611	0	0.229433031	0	0.5	CRA004775;PRJNA827465	Yang2025_Silkie_Wenchang	0.971407853
ENSGALG00010013866	KCTD8	2	-0.020590142	0.297965853	-0.069224138	0.944914518	0.005041438	1.024651929	2.34870209	0.5	PRJNA827465;Yang2025_Silkie_Wenchang	CRA004775	0.983943003
ENSGALG00010013866	KCTD8	2	0.343637694	0.392898715	0.874621577	0.381779843	0	0.061466458	0	1	CRA004775;Yang2025_Silkie_Wenchang	PRJNA827465	0.824727558
ENSGALG00010013866	KCTD8	2	-0.091322303	0.295685909	-0.30884902	0.757436378	0	0.481793079	0	0.5	CRA004775;PRJNA827465	Yang2025_Silkie_Wenchang	0.938842663
ENSGALG00010013867	RECQL	2	0.06434799	0.0817825	0.786818566	0.431388083	0.003281959	1.221725568	18.14855756	1	PRJNA827465;Yang2025_Silkie_Wenchang	CRA004775	0.842503929
ENSGALG00010013867	RECQL	2	0.059750084	0.202159959	0.295559326	0.767566639	0.038803671	1.759821472	43.17605416	0.5	CRA004775;Yang2025_Silkie_Wenchang	PRJNA827465	0.941444269
ENSGALG00010013867	RECQL	2	0.002196031	0.072140292	0.030441122	0.975715249	0	0.653107137	0	0.5	CRA004775;PRJNA827465	Yang2025_Silkie_Wenchang	0.992765259
ENSGALG00010013871	ENSGALG00010013871	2	0.415590757	0.330761632	1.256466037	0.208947052	0.154751043	3.341323378	70.07173845	1	PRJNA827465;Yang2025_Silkie_Wenchang	CRA004775	0.721011841
ENSGALG00010013871	ENSGALG00010013871	2	0.278689644	0.490982514	0.567602779	0.570263079	0.404843178	6.229913365	83.94841242	0.5	CRA004775;Yang2025_Silkie_Wenchang	PRJNA827465	0.858008054
ENSGALG00010013871	ENSGALG00010013871	2	-0.011550508	0.164172051	-0.070356119	0.943910216	0	0.897076403	0	0.5	CRA004775;PRJNA827465	Yang2025_Silkie_Wenchang	0.985827325
ENSGALG00010013872	GLS2	2	0.119573769	0.066095014	1.809119357	0.070432462	0	0.104376028	0	1	PRJNA827465;Yang2025_Silkie_Wenchang	CRA004775	0.458021454
ENSGALG00010013872	GLS2	2	0.113549518	0.124004088	0.915691734	0.359828601	0	0.921574363	0	1	CRA004775;Yang2025_Silkie_Wenchang	PRJNA827465	0.814880744
ENSGALG00010013872	GLS2	2	0.142561405	0.076406162	1.865836483	0.062064246	0	0.784964498	0	0.5	CRA004775;PRJNA827465	Yang2025_Silkie_Wenchang	0.42991371
ENSGALG00010013878	ARID2	2	0.153010177	0.198423665	0.771128668	0.44063067	0.061057694	4.026689562	75.16570412	0.5	PRJNA827465;Yang2025_Silkie_Wenchang	CRA004775	0.844819973
ENSGALG00010013878	ARID2	2	0.398035709	0.163907361	2.428418747	0.015164823	0	0.009007293	0	1	CRA004775;Yang2025_Silkie_Wenchang	PRJNA827465	0.203471634
ENSGALG00010013878	ARID2	2	0.074483426	0.17598111	0.423245711	0.672115269	0.032708241	1.507901912	33.68268904	0.5	CRA004775;PRJNA827465	Yang2025_Silkie_Wenchang	0.912474762
ENSGALG00010013880	ZMYM4	2	0.040264695	0.042438818	0.948770404	0.342737397	0	0.144227117	0	1	PRJNA827465;Yang2025_Silkie_Wenchang	CRA004775	0.80609227
ENSGALG00010013880	ZMYM4	2	0.087084875	0.1176555	0.740168331	0.459197861	0	0.002222115	0	1	CRA004775;Yang2025_Silkie_Wenchang	PRJNA827465	0.850098539
ENSGALG00010013880	ZMYM4	2	0.036798753	0.043678772	0.842485962	0.399515994	0	0.03689451	0	1	CRA004775;PRJNA827465	Yang2025_Silkie_Wenchang	0.832246851
ENSGALG00010013889	ENSGALG00010013889	2	0.291476866	0.340522657	0.861842406	0.388774246	0.215299958	13.9116002	92.81175432	0.5	PRJNA827465;Yang2025_Silkie_Wenchang	CRA004775	0.832840391
ENSGALG00010013889	ENSGALG00010013889	2	0.148026042	0.517135506	0.286242271	0.77469257	0.488640959	11.26904073	91.12613022	0.5	CRA004775;Yang2025_Silkie_Wenchang	PRJNA827465	0.943713409
ENSGALG00010013889	ENSGALG00010013889	2	-0.131481244	0.154288822	-0.852176085	0.394116368	0.019073236	1.436007604	30.36248576	1	CRA004775;PRJNA827465	Yang2025_Silkie_Wenchang	0.830486298
ENSGALG00010013890	SFPQ	2	0.116934171	0.088239381	1.325192548	0.185107296	0.011279512	3.36705395	70.30044618	1	PRJNA827465;Yang2025_Silkie_Wenchang	CRA004775	0.693279213
ENSGALG00010013890	SFPQ	2	-0.016483169	0.260955714	-0.063164621	0.949635417	0.137891841	6.891717333	85.48982856	0.5	CRA004775;Yang2025_Silkie_Wenchang	PRJNA827465	0.98523759
ENSGALG00010013890	SFPQ	2	-0.085773966	0.166765267	-0.514339514	0.60701464	0.042312796	3.464063779	71.13217123	0.5	CRA004775;PRJNA827465	Yang2025_Silkie_Wenchang	0.89693208
ENSGALG00010013893	CSNK2A1	2	-0.109662032	0.087578302	-1.252159837	0.210511628	0.011272587	3.391053921	70.5106429	1	PRJNA827465;Yang2025_Silkie_Wenchang	CRA004775	0.72209273
ENSGALG00010013893	CSNK2A1	2	-0.187591873	0.078458969	-2.390965125	0.016804607	0	0.489065592	0	1	CRA004775;Yang2025_Silkie_Wenchang	PRJNA827465	0.215137283
ENSGALG00010013893	CSNK2A1	2	-0.040680394	0.037690527	-1.079326736	0.280442099	0	0.076737064	0	1	CRA004775;PRJNA827465	Yang2025_Silkie_Wenchang	0.778536485
ENSGALG00010013897	AMOT	2	0.039645067	0.056549821	0.701064412	0.483262818	0	0.032315122	0	1	PRJNA827465;Yang2025_Silkie_Wenchang	CRA004775	0.85691916
ENSGALG00010013897	AMOT	2	0.034874311	0.116969861	0.298147834	0.765590335	0	0.072485579	0	1	CRA004775;Yang2025_Silkie_Wenchang	PRJNA827465	0.940900467
ENSGALG00010013897	AMOT	2	0.047385072	0.060641684	0.781394404	0.434576562	0	0.037310139	0	0.5	CRA004775;PRJNA827465	Yang2025_Silkie_Wenchang	0.842762754
ENSGALG00010013898	EMB	2	-0.076593555	0.195433176	-0.391916852	0.695119651	0	0.473617574	0	0.5	PRJNA827465;Yang2025_Silkie_Wenchang	CRA004775	0.92097163
ENSGALG00010013898	EMB	2	-0.202730789	0.272737277	-0.74331896	0.457288595	0	0.072973536	0	1	CRA004775;Yang2025_Silkie_Wenchang	PRJNA827465	0.849541335
ENSGALG00010013898	EMB	2	0.015561376	0.229689034	0.067749758	0.945984839	0	0.026195579	0	0.5	CRA004775;PRJNA827465	Yang2025_Silkie_Wenchang	0.986321197
ENSGALG00010013899	WNT5B	2	-0.035322938	0.061006008	-0.579007528	0.562540093	0	0.002786436	0	1	PRJNA827465;Yang2025_Silkie_Wenchang	CRA004775	0.883979509
ENSGALG00010013899	WNT5B	2	-0.040272836	0.090021367	-0.447369746	0.654608116	0	0.164662451	0	1	CRA004775;Yang2025_Silkie_Wenchang	PRJNA827465	0.910222014
ENSGALG00010013899	WNT5B	2	-0.044600534	0.079477598	-0.561171137	0.574680877	0	0.153697424	0	1	CRA004775;PRJNA827465	Yang2025_Silkie_Wenchang	0.88613451
ENSGALG00010013901	NFXL1	2	0.189455624	0.130377027	1.453165562	0.146183541	0.024555045	3.470709232	71.18744518	1	PRJNA827465;Yang2025_Silkie_Wenchang	CRA004775	0.634916196
ENSGALG00010013901	NFXL1	2	0.299541214	0.111985928	2.674811193	0.007477135	0	0.890058323	0	0.5	CRA004775;Yang2025_Silkie_Wenchang	PRJNA827465	0.133397105
ENSGALG00010013901	NFXL1	2	0.070241803	0.074286697	0.945550221	0.344378044	0	0.047875087	0	0.5	CRA004775;PRJNA827465	Yang2025_Silkie_Wenchang	0.806764634
ENSGALG00010013908	TBC1D20	2	-0.150986324	0.122730826	-1.230223321	0.218613489	0.02226735	3.586719266	72.11936799	1	PRJNA827465;Yang2025_Silkie_Wenchang	CRA004775	0.73229743
ENSGALG00010013908	TBC1D20	2	-0.250881142	0.104540939	-2.399852168	0.016405571	0	0.627115652	0	1	CRA004775;Yang2025_Silkie_Wenchang	PRJNA827465	0.201556241
ENSGALG00010013908	TBC1D20	2	-0.050397485	0.059202751	-0.851269311	0.394619768	0	0.063345887	0	1	CRA004775;PRJNA827465	Yang2025_Silkie_Wenchang	0.830643015
ENSGALG00010013910	ADIPOR2	2	0.101273248	0.091733082	1.104011223	0.26958827	0.012640197	3.993754242	74.96090297	1	PRJNA827465;Yang2025_Silkie_Wenchang	CRA004775	0.77189992
ENSGALG00010013910	ADIPOR2	2	0.067183732	0.138071922	0.486585046	0.626552423	0.017914814	1.353619074	26.12397243	1	CRA004775;Yang2025_Silkie_Wenchang	PRJNA827465	0.901504244
ENSGALG00010013910	ADIPOR2	2	0.193547122	0.057938854	3.340600213	0.000836575	0	0.349138396	0	1	CRA004775;PRJNA827465	Yang2025_Silkie_Wenchang	0.030404019
ENSGALG00010013911	TNIK	2	-0.080973506	0.072174861	-1.121907339	0.26190184	0	0.094742172	0	1	PRJNA827465;Yang2025_Silkie_Wenchang	CRA004775	0.768363052
ENSGALG00010013911	TNIK	2	-0.09176587	0.106040718	-0.865383332	0.386828426	0	0.501825304	0	1	CRA004775;Yang2025_Silkie_Wenchang	PRJNA827465	0.827124608
ENSGALG00010013911	TNIK	2	-0.116284258	0.083607698	-1.390831952	0.164276391	0	0.307301272	0	1	CRA004775;PRJNA827465	Yang2025_Silkie_Wenchang	0.66834125
ENSGALG00010013914	NRG1	2	0.154176654	0.378827016	0.406984317	0.684019507	0.249608866	7.548845877	86.74591879	0.5	PRJNA827465;Yang2025_Silkie_Wenchang	CRA004775	0.918806497
ENSGALG00010013914	NRG1	2	0.55166685	0.216761759	2.545037709	0.010926594	0	0.000139671	0	1	CRA004775;Yang2025_Silkie_Wenchang	PRJNA827465	0.166338515
ENSGALG00010013914	NRG1	2	-0.108255758	0.252622435	-0.428535098	0.668261586	0.049711602	1.214479245	17.66018197	0.5	CRA004775;PRJNA827465	Yang2025_Silkie_Wenchang	0.91421095
ENSGALG00010013915	ENSGALG00010013915	2	0.556557159	0.427386046	1.302234272	0.192851809	0.284956033	4.407179506	77.31078074	1	PRJNA827465;Yang2025_Silkie_Wenchang	CRA004775	0.720780741
ENSGALG00010013915	ENSGALG00010013915	2	0.152284076	0.896814351	0.169805574	0.865163043	1.449874575	10.07645255	90.07587248	0.5	CRA004775;Yang2025_Silkie_Wenchang	PRJNA827465	0.966970086
ENSGALG00010013915	ENSGALG00010013915	2	-0.218714508	0.466096828	-0.474753533	0.634962657	0.312715979	3.501919356	71.44423106	0.5	CRA004775;PRJNA827465	Yang2025_Silkie_Wenchang	0.904036852
ENSGALG00010013921	PPP2R2B	2	0.006983325	0.062123622	0.112409329	0.910025717	0	0.297717258	0	0.5	PRJNA827465;Yang2025_Silkie_Wenchang	CRA004775	0.97980407
ENSGALG00010013921	PPP2R2B	2	0.087181749	0.093570284	0.931724737	0.351478799	0	0.865484334	0	1	CRA004775;Yang2025_Silkie_Wenchang	PRJNA827465	0.810678834
ENSGALG00010013921	PPP2R2B	2	0.054389107	0.133202735	0.408318243	0.683040047	0.016980242	1.523818378	34.37538134	0.5	CRA004775;PRJNA827465	Yang2025_Silkie_Wenchang	0.918800744
ENSGALG00010013928	CCNO	2	-0.969766419	0.241369912	-4.017760176	5.87539E-05	0	0.689238075	0	1	PRJNA827465;Yang2025_Silkie_Wenchang	CRA004775	0.005407978
ENSGALG00010013928	CCNO	2	-0.700125979	0.317767795	-2.203262855	0.027576227	0	0.021557468	0	1	CRA004775;Yang2025_Silkie_Wenchang	PRJNA827465	0.283513274
ENSGALG00010013928	CCNO	2	-1.024845549	0.277729356	-3.690087224	0.000224177	0	0.579334552	0	1	CRA004775;PRJNA827465	Yang2025_Silkie_Wenchang	0.013745453
ENSGALG00010013930	SLC22A16	2	0.081263496	0.075965034	1.069748696	0.284732441	0	0.041195089	0	1	PRJNA827465;Yang2025_Silkie_Wenchang	CRA004775	0.780278244
ENSGALG00010013930	SLC22A16	2	-0.085417502	0.3088641	-0.276553674	0.78212284	0.094703286	1.399500451	28.54593226	0.5	CRA004775;Yang2025_Silkie_Wenchang	PRJNA827465	0.945372683
ENSGALG00010013930	SLC22A16	2	-0.071420997	0.333795096	-0.213966585	0.830573115	0.129754072	1.562565133	36.00266775	0.5	CRA004775;PRJNA827465	Yang2025_Silkie_Wenchang	0.951795959
ENSGALG00010013934	STAT1	2	-0.040725438	0.041484701	-0.981697761	0.326248769	0	0.638855197	0	0.5	PRJNA827465;Yang2025_Silkie_Wenchang	CRA004775	0.800023788
ENSGALG00010013934	STAT1	2	-0.07995185	0.179584976	-0.445203446	0.656172737	0.03464018	1.550736941	35.51453032	0.5	CRA004775;Yang2025_Silkie_Wenchang	PRJNA827465	0.910446833
ENSGALG00010013934	STAT1	2	-0.085985043	0.091806451	-0.936590426	0.348969279	0.006638722	1.108781843	9.810932923	1	CRA004775;PRJNA827465	Yang2025_Silkie_Wenchang	0.809415141
ENSGALG00010013936	DLGAP3	2	0.221257671	0.212123198	1.043062112	0.296919527	0.041271028	1.788464926	44.08612743	1	PRJNA827465;Yang2025_Silkie_Wenchang	CRA004775	0.788466926
ENSGALG00010013936	DLGAP3	2	0.087007794	0.545314735	0.159555186	0.873231483	0.381182163	2.36373435	57.69406152	0.5	CRA004775;Yang2025_Silkie_Wenchang	PRJNA827465	0.969837713
ENSGALG00010013936	DLGAP3	2	0.000889155	0.178115006	0.00499203	0.996016953	0	0.983970123	0	0.5	CRA004775;PRJNA827465	Yang2025_Silkie_Wenchang	0.99871047
ENSGALG00010013938	ENSGALG00010013938	2	0.627708504	0.26655301	2.354910581	0.018527163	0.013475666	1.103417693	9.37248822	1	PRJNA827465;Yang2025_Silkie_Wenchang	CRA004775	0.227696405
ENSGALG00010013938	ENSGALG00010013938	2	0.281751837	0.320701143	0.878549525	0.379645574	0	0.05555775	0	1	CRA004775;Yang2025_Silkie_Wenchang	PRJNA827465	0.824239628
ENSGALG00010013938	ENSGALG00010013938	2	0.682614143	0.308873906	2.210009098	0.027104531	0.014580731	1.062889027	5.916800873	1	CRA004775;PRJNA827465	Yang2025_Silkie_Wenchang	0.280945645

ENSGALG00010013940	UPF3A	2	-0.206494136	0.196547069	-1.050609083	0.293438168	0	0.052071208	0	1	PRJNA827465;Yang2025_Silkie_Wenchang	CRA004775	0.786979185
ENSGALG00010013940	UPF3A	2	-0.672753996	0.547142611	-1.229577046	0.21885553	0.365627129	2.282556066	56.18946605	1	CRA004775;Yang2025_Silkie_Wenchang	PRJNA827465	0.73259614
ENSGALG00010013940	UPF3A	2	-0.622978516	0.594115502	-1.048581486	0.294370781	0.494544519	2.861046967	65.04776009	1	CRA004775;PRJNA827465	Yang2025_Silkie_Wenchang	0.787343482
ENSGALG00010013942	CACNA2D4	2	0.553193504	0.341156227	1.62152545	0.104904996	0	0.00165605	0	1	PRJNA827465;Yang2025_Silkie_Wenchang	CRA004775	0.550048644
ENSGALG00010013942	CACNA2D4	2	1.172958857	0.78801499	1.488498153	0.136619569	0.702182622	2.113463528	52.68430295	1	CRA004775;Yang2025_Silkie_Wenchang	PRJNA827465	0.618470274
ENSGALG00010013942	CACNA2D4	2	1.13697043	0.796483128	1.427488405	0.153439165	0.790995671	2.344615374	57.34908116	1	CRA004775;PRJNA827465	Yang2025_Silkie_Wenchang	0.645802918
ENSGALG00010013944	METTL24	2	-0.06983075	0.203855835	-0.342549675	0.731937268	0.05900689	3.343663493	70.09268421	0.5	PRJNA827465;Yang2025_Silkie_Wenchang	CRA004775	0.932853312
ENSGALG00010013944	METTL24	2	0.46796115	0.828442637	0.5648685	0.572163211	1.238532234	9.689877646	89.67995225	0.5	CRA004775;Yang2025_Silkie_Wenchang	PRJNA827465	0.885543355
ENSGALG00010013944	METTL24	2	0.644266992	0.618754442	1.041232109	0.297767837	0.650258271	5.887824385	83.01579778	1	CRA004775;PRJNA827465	Yang2025_Silkie_Wenchang	0.788858094
ENSGALG00010013945	SCAF11	2	0.311780688	0.351256632	0.887615094	0.374747832	0.230589375	14.40709598	93.05897593	0.5	PRJNA827465;Yang2025_Silkie_Wenchang	CRA004775	0.822805106
ENSGALG00010013945	SCAF11	2	0.457590408	0.210029498	2.265350401	0.023491185	0.05533202	2.682158261	62.71659229	1	CRA004775;Yang2025_Silkie_Wenchang	PRJNA827465	0.26162484
ENSGALG00010013945	SCAF11	2	0.069120455	0.130750306	0.528644695	0.597051949	0.023353921	2.377897548	57.94604353	0.5	CRA004775;PRJNA827465	Yang2025_Silkie_Wenchang	0.894212817
ENSGALG00010013947	ENSGALG00010013947	2	-0.475219096	0.477463332	-0.995299668	0.319590539	0.376452217	5.645578559	82.28702356	1	PRJNA827465;Yang2025_Silkie_Wenchang	CRA004775	0.798328994
ENSGALG00010013947	ENSGALG00010013947	2	-0.48891267	0.507806988	-0.962792324	0.335651754	0.392676934	4.178259688	76.06659053	0.5	CRA004775;Yang2025_Silkie_Wenchang	PRJNA827465	0.803317552
ENSGALG00010013947	ENSGALG00010013947	2	-0.008013182	0.197901685	-0.040490723	0.967701903	0	0.018288403	0	0.5	CRA004775;PRJNA827465	Yang2025_Silkie_Wenchang	0.991065147
ENSGALG00010013949	PRRC2C	2	0.192480637	0.346466118	0.555553999	0.578515786	0.214654238	8.756807418	88.58031298	0.5	PRJNA827465;Yang2025_Silkie_Wenchang	CRA004775	0.887867778
ENSGALG00010013949	PRRC2C	2	0.434071033	0.163072578	2.661827254	0.007771175	0	0.802711693	0	1	CRA004775;Yang2025_Silkie_Wenchang	PRJNA827465	0.136668493
ENSGALG00010013949	PRRC2C	2	0.016351065	0.191624292	0.085328768	0.932000021	0.051554975	2.72238339	63.26748086	0.5	CRA004775;PRJNA827465	Yang2025_Silkie_Wenchang	0.98412915
ENSGALG00010013953	DHX29	2	0.018632572	0.096157362	0.193771661	0.846354678	0	0.161421158	0	0.5	PRJNA827465;Yang2025_Silkie_Wenchang	CRA004775	0.96122283
ENSGALG00010013953	DHX29	2	0.109525426	0.140486448	0.779615599	0.435617172	0	0.534853093	0	1	CRA004775;Yang2025_Silkie_Wenchang	PRJNA827465	0.842762754
ENSGALG00010013953	DHX29	2	0.025713223	0.117203977	0.219388656	0.826347306	0	0.916765765	0	0.5	CRA004775;PRJNA827465	Yang2025_Silkie_Wenchang	0.956393839
ENSGALG00010013954	LPL	2	-0.301538904	0.161904852	-1.862445142	0.062540371	0.028187043	2.162140174	53.74952965	1	PRJNA827465;Yang2025_Silkie_Wenchang	CRA004775	0.431065134
ENSGALG00010013954	LPL	2	0.240598023	0.761320923	0.316027073	0.751981959	1.048090362	9.810515324	89.80685553	0.5	CRA004775;Yang2025_Silkie_Wenchang	PRJNA827465	0.937903487
ENSGALG00010013954	LPL	2	0.386433772	0.596797728	0.647512136	0.517300543	0.604861865	6.046861667	83.46249584	0.5	CRA004775;PRJNA827465	Yang2025_Silkie_Wenchang	0.867952013
ENSGALG00010013958	TUSC3	2	-0.238733965	0.116984416	-2.040733054	0.041277368	0	0.75592622	0	1	PRJNA827465;Yang2025_Silkie_Wenchang	CRA004775	0.332245589
ENSGALG00010013958	TUSC3	2	-0.359105356	0.169583919	-2.117567271	0.034211732	0	5.12584E-05	0	1	CRA004775;Yang2025_Silkie_Wenchang	PRJNA827465	0.318100902
ENSGALG00010013958	TUSC3	2	-0.171348287	0.146333891	-1.170940555	0.241622672	0	0.168431648	0	1	CRA004775;PRJNA827465	Yang2025_Silkie_Wenchang	0.75404876
ENSGALG00010013959	STK32A	2	-0.179362538	0.298182167	-0.601520004	0.547492692	0	1.547493408	0	1	PRJNA827465;Yang2025_Silkie_Wenchang	CRA004775	0.878281266
ENSGALG00010013959	STK32A	2	-0.075720548	0.466327712	-0.162376257	0.871009567	0.097967655	1.255662845	20.36205605	0.5	CRA004775;Yang2025_Silkie_Wenchang	PRJNA827465	0.969230271
ENSGALG00010013959	STK32A	2	0.092294781	0.336081992	0.274619836	0.783608325	0	0.536850519	0	0.5	CRA004775;PRJNA827465	Yang2025_Silkie_Wenchang	0.945815798
ENSGALG00010013964	SLC38A1	2	0.128653347	0.069195328	1.859277948	0.062987751	0	0.107613694	0	1	PRJNA827465;Yang2025_Silkie_Wenchang	CRA004775	0.432176808
ENSGALG00010013964	SLC38A1	2	-0.001204668	0.200665546	-0.006003365	0.995210037	0.029071053	1.253358852	20.21439044	0.5	CRA004775;Yang2025_Silkie_Wenchang	PRJNA827465	0.998242335
ENSGALG00010013964	SLC38A1	2	0.020230272	0.238263641	0.08490709	0.932335255	0.06515472	1.601884816	37.57353899	0.5	CRA004775;PRJNA827465	Yang2025_Silkie_Wenchang	0.984224753
ENSGALG00010013967	MLLT3	2	-0.028159605	0.090544805	-0.311001885	0.755799189	0	0.679079983	0	0.5	PRJNA827465;Yang2025_Silkie_Wenchang	CRA004775	0.938501783
ENSGALG00010013967	MLLT3	2	0.227413282	0.451040063	0.504197522	0.614122581	0.324161173	4.648439659	78.48740495	0.5	CRA004775;Yang2025_Silkie_Wenchang	PRJNA827465	0.898282836
ENSGALG00010013967	MLLT3	2	0.285322641	0.352318993	0.809843783	0.418031115	0.189159653	3.52853635	71.66138236	1	CRA004775;PRJNA827465	Yang2025_Silkie_Wenchang	0.883812595
ENSGALG00010013971	ENSGALG00010013971	2	-0.222903193	0.181971745	-1.224932988	0.22060047	0	0.743426672	0	1	PRJNA827465;Yang2025_Silkie_Wenchang	CRA004775	0.734053249
ENSGALG00010013971	ENSGALG00010013971	2	-0.034201377	0.229312253	-0.149147622	0.881437149	0	0.00221282	0	1	CRA004775;Yang2025_Silkie_Wenchang	PRJNA827465	0.971790895
ENSGALG00010013971	ENSGALG00010013971	2	-0.26274958	0.196847286	-1.334788939	0.181945435	0	0.471252518	0	1	CRA004775;PRJNA827465	Yang2025_Silkie_Wenchang	0.689745682
ENSGALG00010013973	ENSGALG00010013973	2	0.099825361	0.234991779	0.424803634	0.670979828	0	0.129357429	0	0.5	PRJNA827465;Yang2025_Silkie_Wenchang	CRA004775	0.914566332
ENSGALG00010013973	ENSGALG00010013973	2	0.196455279	0.299534334	0.655868983	0.511908427	0	0.618228082	0	0.5	CRA004775;Yang2025_Silkie_Wenchang	PRJNA827465	0.865694231
ENSGALG00010013973	ENSGALG00010013973	2	0.246934347	0.241550617	1.022288206	0.306644514	0	0.308583323	0	1	CRA004775;PRJNA827465	Yang2025_Silkie_Wenchang	0.792500392
ENSGALG00010013976	CDC40	2	-0.152091059	0.078863884	-1.928526093	0.053789722	0.009474856	4.153906459	75.92658837	1	PRJNA827465;Yang2025_Silkie_Wenchang	CRA004775	0.401964436
ENSGALG00010013976	CDC40	2	-0.234509644	0.058631573	-3.999716048	6.34185E-05	0	0.003976419	0	1	CRA004775;Yang2025_Silkie_Wenchang	PRJNA827465	0.005697237
ENSGALG00010013976	CDC40	2	-0.08429978	0.046803157	-1.801155854	0.071678318	0	0.421147266	0	1	CRA004775;PRJNA827465	Yang2025_Silkie_Wenchang	0.461921937
ENSGALG00010013977	SLC38A2	2	-0.050847103	0.066925441	-0.759757463	0.447399574	0.002629198	1.346134715	25.71322995	1	PRJNA827465;Yang2025_Silkie_Wenchang	CRA004775	0.84650962
ENSGALG00010013977	SLC38A2	2	-0.120304668	0.101678293	-1.183189299	0.236734133	0	0.864400837	0	0.5	CRA004775;Yang2025_Silkie_Wenchang	PRJNA827465	0.749020867
ENSGALG00010013977	SLC38A2	2	0.001593642	0.062302842	0.02557896	0.979593168	0	0.324941182	0	0.5	CRA004775;PRJNA827465	Yang2025_Silkie_Wenchang	0.994472942
ENSGALG00010013981	RLF	2	-0.275231323	0.129857877	-2.11948116	0.034049824	0.028112988	5.712625801	82.49491504	1	PRJNA827465;Yang2025_Silkie_Wenchang	CRA004775	0.31730678
ENSGALG00010013981	RLF	2	-0.112091769	0.332705968	-0.336909403	0.736185197	0.195478845	8.147697648	87.72659415	0.5	CRA004775;Yang2025_Silkie_Wenchang	PRJNA827465	0.933661344
ENSGALG00010013981	RLF	2	-0.007319025	0.196327752	-0.037279627	0.970262049	0.058739654	3.459424546	71.09345827	0.5	CRA004775;PRJNA827465	Yang2025_Silkie_Wenchang	0.991681174
ENSGALG00010013989	ENSGALG00010013989	2	0.26476923	0.180479454	1.467032531	0.142367206	0.020393628	1.433439592	30.23772992	1	PRJNA827465;Yang2025_Silkie_Wenchang	CRA004775	0.628759193
ENSGALG00010013989	ENSGALG00010013989	2	-0.071874198	0.501378917	-0.143353052	0.886011359	0.453344543	10.08849353	90.08771729	0.5	CRA004775;Yang2025_Silkie_Wenchang	PRJNA827465	0.972489512
ENSGALG00010013989	ENSGALG00010013989	2	-0.266291869	0.337945179	-0.787543477	0.402280026	0.139883077	3.244151829	69.17530212	0.5	CRA004775;PRJNA827465	Yang2025_Silkie_Wenchang	0.832879175
ENSGALG00010013995	REM1	2	-0.152760941	0.105252686	-1.451373319	0.146675935	0	0.488321746	0	1	PRJNA827465;Yang2025_Silkie_Wenchang	CRA004775	0.635558525
ENSGALG00010013995	REM1	2	-0.131396437	0.263144203	-0.499332441	0.617545205	0.015307402	1.123641365	11.00363234	0.5	CRA004775;Yang2025_Silkie_Wenchang	PRJNA827465	0.899121942
ENSGALG00010013995	REM1	2	-0.105178445	0.105971933	-0.992512279	0.320947692	0	0.534181109	0	0.5	CRA004775;PRJNA827465	Yang2025_Silkie_Wenchang	0.798472282
ENSGALG00010014001	CDC16	2	-0.105772861	0.096789769	-1.092816037	0.274477082	0.013735199	3.643738685	72.55566092	1	PRJNA827465;Yang2025_Silkie_Wenchang	CRA004775	0.774326342
ENSGALG00010014001	CDC16	2	-0.208583941	0.078672235	-2.651303117	0.008018185	0	0.02030819	0	1	CRA004775;Yang2025_Silkie_Wenchang	PRJNA827465	0.139297234
ENSGALG00010014001	CDC16	2	-0.028700414	0.055388567	-0.518164949	0.604343188	0	0.509745087	0	1	CRA004775;PRJNA827465	Yang2025_Silkie_Wenchang	0.89612415
ENSGALG00010014002	ZDHHC18	2	0.084337657	0.131932161	0.639255173	0.522660198	0	4.46873E-06	0	1	PRJNA827465;Yang2025_Silkie_Wenchang	CRA004775	0.869568211
ENSGALG00010014002	ZDHHC18	2	0.039581769	0.173948099	0.227549306	0.819996634	0	0.074252035	0	0.5	CRA004775;Yang2025_Silkie_Wenchang	PRJNA827465	0.955308792
ENSGALG00010014002	ZDHHC18	2	0.057831723	0.134538523	0.42985252	0.667302926	0	0.10163636	0	0.5	CRA004775;PRJNA827465	Yang2025_Silkie_Wenchang	0.913832992
ENSGALG00010014010	EIF5A2	2	0.150878191	0.185036078	0.815398771	0.414844088	0.055576643	4.654561948	78.51570113	0.5	PRJNA827465;Yang2025_Silkie_Wenchang	CRA004775	0.837379548
ENSGALG00010014010	EIF5A2	2	0.298102939	0.148651033	2.005387608	0.044921625	0	0.951628444	0	1	CRA004775;Yang2025_Silkie_Wenchang	PRJNA827465	0.365383522
ENSGALG00010014010	EIF5A2	2	-0.002819611	0.048709573	-0.057886178	0.953839293	0	0.003919823	0	0.5	CRA004775;PRJNA827465	Yang2025_Silkie_Wenchang	0.987838152
ENSGALG00010014014	PARP8	2	0.398987402	0.137423346	2.903345126	0.003691995	0.006858691	1.218332675	17.9206123	1	PRJNA827465;Yang2025_Silkie_Wenchang	CRA004775	0.086890114
ENSGALG00010014014	PARP8	2	0.215538349	0.430563077	0.500596454	0.616655159	0.263580551	3.034695943	67.04776957	0.5	CRA004775;Yang2025_Silkie_Wenchang	PRJNA827465	0.899111936
ENSGALG00010014014	PARP8	2	0.132339353	0.259155108	0.510656934	0.609591301	0.060914433	1.486698413	32.73686236	0.5	CRA004775;PRJNA827465	Yang2025_Silkie_Wenchang	0.897725578
ENSGALG00010014018	DPYSL3	2	-0.080401352	0.094500587	-0.850802667	0.39487898	0.002812095	1.185060156	15.61609803	0.5	PRJNA827465;Yang2025_Silkie_Wenchang	CRA004775	0.830643015
ENSGALG00010014018	DPYSL3	2	-0.188173924	0.123177283	-1.527667432	0.12659513	0	0.011273017	0	1	CRA004775;Yang2025_Silkie_Wenchang	PRJNA827465	0.597235434
ENSGALG00010014018	DPYSL3	2	-0.012931162	0.111690889	-0.115776339	0.907829003	0	0.324673941	0	0.5	CRA004775;PRJNA827465	Yang2025_Silkie_Wenchang	0.978196186
ENSGALG00010014019	IRAG2	2	-0.246374707	0.184941333	-1.332177628	0.182801826	0	0.03052039	0	1	PRJNA827465;Yang2025_Silkie_Wenchang	CRA004775	0.691266551
ENSGALG00010014019	IRAG2	2	-0.003975755	0.270979887	-0.014671771	0.98829404	0.050816354	1.481572414	32.50414288	0.5	CRA004775;Yang2025_Silkie_Wenchang	PRJNA827465	0.996891127
ENSGALG00010014019	IRAG2	2	-0.026903405	0.306185132	-0.087866465	0.929982811	0.087638504	1.819594503	45.04270055	0.5	CRA004775;PRJNA827465	Yang2025_Silkie_Wenchang	0.983813577
ENSGALG00010014020	PDXP	2	-0.267329254	0.294931303	-0.906411902	0.364717856	0.142558774	4.841042148	79.34329078	1	PRJNA827465;Yang2025_Silkie_Wenchang	CRA004775	0.837619546
ENSGALG00010014020	PDXP	2	-0.518249084	0.146223597	-3.54422332	0.000393772	0	0.218809867	0	1	CRA004775;Yang2025_Silkie_Wenchang	PRJNA827465	0.019995353
ENSGALG00010014020	PDXP	2	-0.218107704	0.223911543	-0.974079771	0.330016933	0.083884785	5.653049427	82.3104324	1	CRA004775;PRJNA827465	Yang2025_Silkie_Wenchang	0.801212404
ENSGALG00010014026	SH3BP1	2	0.099948097	0.29264516	0.341533403	0.732702063	0	0.500739744	0	0.5	PRJNA827465;Yang2025_Silkie_Wenchang	CRA004775	0.932985777
ENSGALG00010014026	SH3BP1	2	-0.217260185	0.496233478	-0.437816441	0.661517876	0.352562116	3.464526167	71.13602404	0.5	CRA004775;Yang2025_Silkie_Wenchang	PRJNA827465	0.919744181
ENSGALG00010014026	SH3BP1	2	-0.434933083	0.286047028	-1.520495026	0.128386608	0.036001092	1.271528323	21.35448483	1	CRA004775;PRJNA827465	Yang2025_Silkie_Wenchang	0.60134038
ENSGALG00010014030	WASF1	2	0.010266764	0.104548642	0.098200836	0.921772819	0	0.308025646	0	0.5	PRJNA827465;Yang2025_Silkie_Wenchang	CRA004775	0.981649741
ENSGALG00010014030	WASF1	2	0.128335672	0.246905873	0.519775698	0.603219919	0	0.062938296	0	1	CRA004775;Yang2025_Silkie_Wenchang	PRJNA827465	0.895538966
ENSGALG00010014030	WASF1	2	-0.004139681	0.105923123	-0.039081937	0.968825062	0	0.028254185	0	0.5	CRA004775;PRJNA827465	Yang2025_Silkie_Wenchang	0.991349425
ENSGALG00010014031	RPL22L1	2	-0.777480891	0.679252817	-1.14461195	0.25236994	0.897617842	36.38669348	97.25174259	1	PRJNA827465;Yang2025_Silkie_Wenchang	CRA004775	0.762639993
ENSGALG00010014031	RPL22L1	2	-0.711224309	0.780342995	-0.911425249	0.362071355	1.140953044	15.60040455	93.58990982	0.5	CRA004775;Yang2025_Silkie_Wenchang	PRJNA827465	0.815579273
ENSGALG00010014031	RPL22L1	2	-0.090664794	0.101415482	-0.893993621	0.371325304	0	0.321051739	0	0.5	CRA004775;PRJNA827465	Yang2025_Silkie_Wenchang	0.8214684
ENSGALG00010014032	PSD3	2	0.068838612	0.097781813	0.70400221	0.481431397	0	0.211559418	0	0.5	PRJNA827465;Yang2025_Silkie_Wenchang	CRA004775	0.85686777
ENSGALG00010014032	PSD3	2	0.013541207	0.160629283	0.084300987	0.932817127	0	0.033247347	0	0.5	CRA004775;Yang2025_Silkie_Wenchang	PRJNA827465	0.984224753
ENSGALG00010014032	PSD3	2	0.093727524	0.109008806	0.85981607	0.389890439	0	0.007799764	0	1	CRA004775;PRJNA827465	Yang2025_Silkie_Wenchang	0.829168548
ENSGALG00010014033	SLC38A4	2	0.547312623	0.241064518	2.270398932	0.023183389	0	0.647415438	0	1	PRJNA827465;Yang2025_Silkie_Wenchang	CRA004775	0.259609281
ENSGALG00010014033	SLC38A4	2	0.233971661	0.369183051	0.633755153	0.526240625	0.101150981	1.361730311	26.56401993	0.5	CRA004775;Yang2025_Silkie_Wenchang	PRJNA827465	0.870537576
ENSGALG00010014033	SLC38A4	2	0.319283681	0.656451107	0.486378464	0.626698857	0.517195068	2.431147467	58.86715991	0.5	CRA004775;PRJNA827465	Yang2025_Silkie_Wenchang	0.901504244
ENSGALG00010014035	PSIP1	2	0.359146292	0.339568307	1.057655514	0.290212524	0.210422571	10.94505839	90.86345669	1	PRJNA827465;Yang2025_Silkie_Wenchang	CRA004775	0.784195688
ENSGALG00010014035	PSIP1	2	0.389525853	0.335403408	1.161365219	0.245493407	0.186522614	5.845610427	82.8931467	1	CRA004775;Yang2025_Silkie_Wenchang	PRJNA827465	0.75661406
ENSGALG00010014035	PSIP1	2	0.042141981	0.073306359	0.574874834	0.565375951	0	0.002006764	0	1	CRA004775;PRJNA827465	Yang2025_Silkie_Wenchang	0.883427937
ENSGALG00010014041	SKIV2L2	2	0.009092382	0.102925029	0.088339851	0.929606566	0	0.203287708	0	0.5	PRJNA827465;Yang2025_Silkie_Wenchang	CRA004775	0.983767889
ENSGALG00010014041	SKIV2L2	2	0.069685091	0.117778367	0.591662908	0.554076339	0	0.11631134	0	1	CRA004775;Yang2025_Silkie_Wenchang	PRJNA827465	0.880092687
ENSGALG00010014041	SKIV2L2	2	0.010140246	0.134557133	0.075360155	0.939928161	0	0.417227097	0	0.5	CRA004775;PRJNA827465	Yang2025_Silkie_Wenchang	0.985444231
ENSGALG00010014045	GOLT1B	2	-0.266413366	0.327682855	-0.813021988	0.416205452	0.209239744	38.81321805	97.42355813	0.5	PRJNA827465;Yang2025_Silkie_Wenchang	CRA004775	0.837742614
ENSGALG00010014045	GOLT1B	2	-0.582490784	0.083175607	-7.003144355	2.50281E-12	0	0.296366405	0	1	CRA004775;Yang2025_Silkie_Wenchang	PRJNA827465	2.28762E-09
ENSGALG00010014045	GOLT1B	2	-0.126023376	0.242163686	-0.520405756	0.602780799	0.089117957	3.394274971	70.53862729	0.5	CRA004775;PRJNA827465	Yang2025_Silkie_Wenchang	0.895538966
ENSGALG00010014047	HM13	2	0.091678476	0.132901913	0.689820591	0.490307018	0.028676585	4.809198432	79.20651406	0.5	PRJNA827465;Yang2025_Silkie_Wenchang	CRA004775	0.858529056
ENSGALG00010014047	HM13	2	-0.076622286	0.326853208	-0.234424152	0.814655711	0.198516535	14.08768039	92.90159933	0.5	CRA004775;Yang2025_Silkie_Wenchang	PRJNA827465	0.953869515
ENSGALG00010014047	HM13	2	-0.194362476	0.191293881	-1.016041259	0.309609739	0.064448789	7.764901682	87.12153687	1	CRA004775;PRJNA827465	Yang2025_Silkie_Wenchang	0.794246638
ENSGALG00010014056	FMO3	2	0.173045112	0.17297104	1.000428235	0.317103312	0	0.02867221	0	1	PRJNA827465;Yang2025_Silkie_Wenchang	CRA004775	0.797007794
ENSGALG00010014056	FMO3	2	0.110787474	0.160093388	0.6920178	0.488926151	0	0.143774747	0	1	CRA004775;Yang2025_Silkie_Wenchang	PRJNA827465	0.858232954
ENSGALG00010014056	FMO3	2	0.102282137	0.211578598	0.483423833	0.628794829	0	0.197453884	0	1	CRA004775;PRJNA827465	Yang2025_Silkie_Wenchang	0.902084638
ENSGALG00010014062	GGA1	2	0.02643112	0.044636225	0.592145051	0.553753462	0	0.870492488	0	0.5	PRJNA827465;Yang2025_Silkie_Wenchang	CRA004775	0.879822626
ENSGALG00010014062	GGA1	2	-0.098913578	0.230711523	-0.428732717	0.668117748	0.082317836	3.897327114	74.34138909	0.5	CRA004775;Yang2025_Silkie_Wenchang	PRJNA827465	0.914120722
ENSGALG00010014062	GGA1	2	-0.128287975	0.179303728	-0.715478569	0.474313379	0.045563508	2.719081942	63.22288106	1	CRA004775;PRJNA827465	Yang2025_Silkie_Wenchang	0.854462864
ENSGALG00010014066	BCL2L1	2	0.134743685	0.070069402	1.923003216	0.054479646	0	2.88381E-05	0	1	PRJNA827465;Yang2025_Silkie_Wenchang	CRA004775	0.404097297
ENSGALG00010014066	BCL2L1	2	0.071010392	0.100722355	0.705011236	0.480803244	0	0.730417682	0	0.5	CRA004775;Yang2025_Silkie_Wenchang	PRJNA827465	0.856722567
ENSGALG00010014066	BCL2L1	2	0.098584661	0.075441966	1.306761566	0.191293702	0	0.901070141	0	0.5	CRA004775;PRJNA827465	Yang2025_Silkie_Wenchang	0.700691181
ENSGALG00010014076	PRRX1	2	0.326554319	0.379936191	0.859497797	0.390065935	0.24693415	6.705232902	85.08627493	0.5	PRJNA827465;Yang2025_Silkie_Wenchang	CRA004775	0.829204867
ENSGALG00010014076	PRRX1	2	0.102566843	0.669375956	0.153227558	0.878218826	0.783786618	7.801667504	87.1822274	0.5	CRA004775;Yang2025_Silkie_Wenchang	PRJNA827465	0.971037793
ENSGALG00010014076	PRRX1	2	-0.192135523	0.261503831	-0.734733111	0.462502051	0.073370733	1.778233043	43.76440119	1	CRA004775;PRJNA827465	Yang2025_Silkie_Wenchang	0.850823289
ENSGALG00010014077	SLITRK2	2	-0.228499569	0.072919748	-3.133575976	0.001726902	0	0.028438974	0	1	PRJNA827465;Yang2025_Silkie_Wenchang	CRA004775	0.052573533
ENSGALG00010014077	SLITRK2	2	-0.122986328	0.124270342	-0.989667576	0.322336628	0	0.816045453	0	0.5	CRA004775;Yang2025_Silkie_Wenchang	PRJNA827465	0.798652022
ENSGALG00010014077	SLITRK2	2	-0.172878966	0.112241097	-1.540246582	0.123500259	0.009621982	1.378634536	27.46446041	0.5	CRA004775;PRJNA827465	Yang2025_Silkie_Wenchang	0.589140755
ENSGALG00010014080	MYO1B	2	0.176943854	0.129811399	1.363084094	0.172856009	0.023004273	3.012119656	66.80078768	1	PRJNA827465;Yang2025_Silkie_Wenchang	CRA004775	0.676043418
ENSGALG00010014080	MYO1B	2	0.288019835	0.119814053	2.403890258	0.016221642	0	0.732937932	0	1	CRA004775;Yang2025_Silkie_Wenchang	PRJNA827465	0.210485045
ENSGALG00010014080	MYO1B	2	0.062492068	0.077817092	0.803063528	0.421938017	0	0.016712057	0	1	CRA004775;PRJNA827465	Yang2025_Silkie_Wenchang	0.839624822
ENSGALG00010014082	HNRNPR	2	0.127905007	0.209268602	0.61120018	0.541067064	0.074821904	6.166457574	83.78323392	0.5	PRJNA827465;Yang2025_Silkie_Wenchang	CRA004775	0.875829389
ENSGALG00010014082	HNRNPR	2	0.159656904	0.217676528	0.733459439	0.463278258	0.064139882	3.083257799	67.56677303	0.5	CRA004775;Yang2025_Silkie_Wenchang	PRJNA827465	0.851647585
ENSGALG00010014082	HNRNPR	2	-0.054848176	0.044476912	-1.233183095	0.217507461	0	0.004665393	0	1	CRA004775;PRJNA827465	Yang2025_Silkie_Wenchang	0.731013651
ENSGALG00010014083	ENSGALG00010014083	2	0.068351288	0.147666245	0.462876861	0.643452634	0	0.087133266	0	1	PRJNA827465;Yang2025_Silkie_Wenchang	CRA004775	0.906634523
ENSGALG00010014083	ENSGALG00010014083	2	-0.131086329	0.196984014	-0.665466836	0.505751949	0.018951477	1.300396189	23.10035904	0.5	CRA004775;Yang2025_Silkie_Wenchang	PRJNA827465	0.863927652
ENSGALG00010014083	ENSGALG00010014083	2	-0.095021486	0.242923826	-0.391157537	0.695680793	0.058466483	1.932663166	48.2579263	0.5	CRA004775;PRJNA827465	Yang2025_Silkie_Wenchang	0.921038437
ENSGALG00010014089	FIG4	2	0.061800855	0.176447172	0.350251321	0.726150094	0.05226706	6.084702268	83.56534213	0.5	PRJNA827465;Yang2025_Silkie_Wenchang	CRA004775	0.931029023
ENSGALG00010014089	FIG4	2	-0.143762084	0.113379406	-1.26797351	0.204807411	0	0.178352373	0	1	CRA004775;Yang2025_Silkie_Wenchang	PRJNA827465	0.717364376
ENSGALG00010014089	FIG4	2	0.08224469	0.228656343	0.359686895	0.719081293	0.067087498	2.099242993	52.3637805	0.5	CRA004775;PRJNA827465	Yang2025_Silkie_Wenchang	0.928449671
ENSGALG00010014091	CHIC2	2	-0.190353254	0.208004792	-0.915138792	0.360118773	0.080510163	14.13479663	92.92526079	0.5	PRJNA827465;Yang2025_Silkie_Wenchang	CRA004775	0.814880744
ENSGALG00010014091	CHIC2	2	-0.25090288	0.184427166	-1.360444266	0.173689378	0.048830218	3.294129016	69.64296191	1	CRA004775;Yang2025_Silkie_Wenchang	PRJNA827465	0.677435114
ENSGALG00010014091	CHIC2	2	0.006917764	0.053060662	0.13037462	0.896270045	0	0.047911036	0	0.5	CRA004775;PRJNA827465	Yang2025_Silkie_Wenchang	0.975115773
ENSGALG00010014092	TYRP1	2	8.774892254	0.322950867	27.17098218	1.431E-162	0	0.013539579	0	1	PRJNA827465;Yang2025_Silkie_Wenchang	CRA004775	1.8747E-158
ENSGALG00010014092	TYRP1	2	8.852864863	0.414889629	21.3378794	5.0535E-101	0	0.256919442	0	1	CRA004775;Yang2025_Silkie_Wenchang	PRJNA827465	2.48273E-97
ENSGALG00010014092	TYRP1	2	8.898178667	0.389632223	22.83737876	1.9508E-115	0	0.191021266	0	1	CRA004775;PRJNA827465	Yang2025_Silkie_Wenchang	1.5335E-111
ENSGALG00010014093	PLD1	2	-0.057235677	0.107228572	-0.533772629	0.593498853	0.013672444	2.306781535	56.64955762	0.5	PRJNA827465;Yang2025_Silkie_Wenchang	CRA004775	0.892915939
ENSGALG00010014093	PLD1	2	-0.20890697	0.118049327	-1.769658294	0.076784082	0	0.182028283	0	1	CRA004775;Yang2025_Silkie_Wenchang	PRJNA827465	0.477904868
ENSGALG00010014093	PLD1	2	-0.004509885	0.107228724	-0.04205855	0.966452023	0.007385103	1.114641817	10.28508128	0.5	CRA004775;PRJNA827465	Yang2025_Silkie_Wenchang	0.990881287
ENSGALG00010014096	JAKMIP2	2	0.361269391	0.193983839	1.862368501	0.062551166	0.054934787	3.350580798	70.15442814	1	PRJNA827465;Yang2025_Silkie_Wenchang	CRA004775	0.431065134
ENSGALG00010014096	JAKMIP2	2	0.564284255	0.180139894	3.132478009	0.001733374	0	0.180369538	0	1	CRA004775;Yang2025_Silkie_Wenchang	PRJNA827465	0.052667801
ENSGALG00010014096	JAKMIP2	2	0.209707483	0.087299975	2.402148261	0.016299101	0	0.173747902	0	1	CRA004775;PRJNA827465	Yang2025_Silkie_Wenchang	0.211002492
ENSGALG00010014099	TPX2	2	-0.132359487	0.061624909	-2.147824481	0.031727701	0	0.037098159	0	1	PRJNA827465;Yang2025_Silkie_Wenchang	CRA004775	0.305037635
ENSGALG00010014099	TPX2	2	-0.107388314	0.098341423	-1.091994714	0.274835425	0	0.037513523	0	1	CRA004775;Yang2025_Silkie_Wenchang	PRJNA827465	0.774719194
ENSGALG00010014099	TPX2	2	-0.134245918	0.072198826	-1.859391964	0.0629716	0	0.088848162	0	1	CRA004775;PRJNA827465	Yang2025_Silkie_Wenchang	0.432176808
ENSGALG00010014102	TRIM2	2	-0.087249163	0.116089486	-0.751568171	0.45231079	0.011559813	1.627212147	38.54519821	0.5	PRJNA827465;Yang2025_Silkie_Wenchang	CRA004775	0.848321192
ENSGALG00010014102	TRIM2	2	0.190673831	0.203234399	0.938196642	0.348143364	0.026903284	1.320142583	24.25060649	1	CRA004775;Yang2025_Silkie_Wenchang	PRJNA827465	0.808976658
ENSGALG00010014102	TRIM2	2	0.095602753	0.346047066	0.276270953	0.782339964	0.181798453	3.440505964	70.93450758	0.5	CRA004775;PRJNA827465	Yang2025_Silkie_Wenchang	0.945372683
ENSGALG00010014104	ALDH1A1	2	-0.240431915	0.468471871	-0.513225937	0.607793285	0	0.038397084	0	1	PRJNA827465;Yang2025_Silkie_Wenchang	CRA004775	0.897203929
ENSGALG00010014104	ALDH1A1	2	0.968875391	1.269569397	0.763152761	0.445372297	2.832770337	8.177002784	87.77058017	0.5	CRA004775;Yang2025_Silkie_Wenchang	PRJNA827465	0.846013379
ENSGALG00010014104	ALDH1A1	2	1.039710387	1.17749956	0.88298155	0.37724624	2.438741764	8.27442519	87.91456836	0.5	CRA004775;PRJNA827465	Yang2025_Silkie_Wenchang	0.823181514
ENSGALG00010014109	AMIGO2	2	0.259316461	0.439365215	0.590207081	0.555051826	0.350920909	10.92357583	90.84548855	0.5	PRJNA827465;Yang2025_Silkie_Wenchang	CRA004775	0.880223402
ENSGALG00010014109	AMIGO2	2	0.577219157	0.196738526	2.933940643	0.003346882	0.011871487	1.150722794	13.09809754	1	CRA004775;Yang2025_Silkie_Wenchang	PRJNA827465	0.08099908
ENSGALG00010014109	AMIGO2	2	-0.039337792	0.205368151	-0.191547673	0.848096534	0.032713277	1.467456768	31.85489196	0.5	CRA004775;PRJNA827465	Yang2025_Silkie_Wenchang	0.961363811
ENSGALG00010014111	GORAB	2	0.17005344	0.088593007	1.919490556	0.05492228	0.010513981	2.98193521	66.4647308	1	PRJNA827465;Yang2025_Silkie_Wenchang	CRA004775	0.405677577
ENSGALG00010014111	GORAB	2	-0.058943166	0.343296668	-0.17169746	0.863675383	0.217257854	12.37552681	91.91953591	0.5	CRA004775;Yang2025_Silkie_Wenchang	PRJNA827465	0.966335868
ENSGALG00010014111	GORAB	2	-0.138860661	0.253394419	-0.54800205	0.583690495	0.112169735	7.332743494	86.36253947	0.5	CRA004775;PRJNA827465	Yang2025_Silkie_Wenchang	0.890558522
ENSGALG00010014114	ANXA1	2	-0.264425255	0.170226493	-1.553373097	0.120334026	0.013585018	1.304946398	23.36849992	1	PRJNA827465;Yang2025_Silkie_Wenchang	CRA004775	0.58173287
ENSGALG00010014114	ANXA1	2	-0.445119082	0.19082662	-2.332583795	0.01967	0	0.079055175	0	1	CRA004775;Yang2025_Silkie_Wenchang	PRJNA827465	0.235251604
ENSGALG00010014114	ANXA1	2	-0.152731682	0.203336641	-0.7511272	0.452576107	0	0.72435172	0	1	CRA004775;PRJNA827465	Yang2025_Silkie_Wenchang	0.848321192
ENSGALG00010014120	SOX5	2	0.493494032	0.266492327	1.851813285	0.064052639	0.111348721	4.378328769	77.16023504	1	PRJNA827465;Yang2025_Silkie_Wenchang	CRA004775	0.436074984
ENSGALG00010014120	SOX5	2	0.642116832	0.236717477	2.712587345	0.006676018	0.028590951	1.270453225	21.28793252	1	CRA004775;Yang2025_Silkie_Wenchang	PRJNA827465	0.124953874
ENSGALG00010014120	SOX5	2	0.260190604	0.116589852	2.231674534	0.025636482	0	0.002050145	0	1	CRA004775;PRJNA827465	Yang2025_Silkie_Wenchang	0.27294819
ENSGALG00010014122	ABCA1	2	-0.158422426	0.076917573	-2.059638903	0.039433074	0	0.089126321	0	1	PRJNA827465;Yang2025_Silkie_Wenchang	CRA004775	0.3433403
ENSGALG00010014122	ABCA1	2	-0.180486885	0.141462186	-1.275866644	0.202002664	0	0.095343744	0	1	CRA004775;Yang2025_Silkie_Wenchang	PRJNA827465	0.71497298
ENSGALG00010014122	ABCA1	2	-0.141114389	0.086969387	-1.622575421	0.104680198	0	0.036700597	0	1	CRA004775;PRJNA827465	Yang2025_Silkie_Wenchang	0.549804368
ENSGALG00010014125	PLPP1	2	-0.317571422	0.170316508	-1.864595664	0.062238101	0.045443902	4.614249335	78.32800251	1	PRJNA827465;Yang2025_Silkie_Wenchang	CRA004775	0.430204729
ENSGALG00010014125	PLPP1	2	-0.292787446	0.259236549	-1.12942194	0.258719882	0.092646667	2.737149344	63.46563982	0.5	CRA004775;Yang2025_Silkie_Wenchang	PRJNA827465	0.765833156
ENSGALG00010014125	PLPP1	2	-0.122222934	0.106590339	-1.146660525	0.251521951	0	0.371473577	0	0.5	CRA004775;PRJNA827465	Yang2025_Silkie_Wenchang	0.761706068
ENSGALG00010014128	COL4A1	2	-0.245404484	0.19588461	-1.252801254	0.210278045	0.065420794	6.767484689	85.22346121	1	PRJNA827465;Yang2025_Silkie_Wenchang	CRA004775	0.721897568
ENSGALG00010014128	COL4A1	2	0.106338463	0.610171993	0.17427621	0.861648395	0.663252514	8.440234707	88.15198825	0.5	CRA004775;Yang2025_Silkie_Wenchang	PRJNA827465	0.965512954
ENSGALG00010014128	COL4A1	2	0.27141972	0.406902305	0.667039032	0.504747202	0.260670782	3.967680665	74.79635877	0.5	CRA004775;PRJNA827465	Yang2025_Silkie_Wenchang	0.863512504
ENSGALG00010014131	CDC23	2	0.24808545	0.190742879	1.300627583	0.193385961	0.068126268	15.56307542	93.57453477	1	PRJNA827465;Yang2025_Silkie_Wenchang	CRA004775	0.703339473
ENSGALG00010014131	CDC23	2	0.065462255	0.405757192	0.161333566	0.871830688	0.30102456	11.05657535	90.95560815	0.5	CRA004775;Yang2025_Silkie_Wenchang	PRJNA827465	0.969462602
ENSGALG00010014131	CDC23	2	-0.095851271	0.20793543	-0.460966519	0.644822626	0.065052851	3.302189976	69.71706633	0.5	CRA004775;PRJNA827465	Yang2025_Silkie_Wenchang	0.906953008
ENSGALG00010014134	RPAP3	2	0.013435416	0.046449941	0.289245055	0.772393855	0	0.023157549	0	1	PRJNA827465;Yang2025_Silkie_Wenchang	CRA004775	0.943066211
ENSGALG00010014134	RPAP3	2	0.014889361	0.073153041	0.203537147	0.838715214	0	0.277162089	0	1	CRA004775;Yang2025_Silkie_Wenchang	PRJNA827465	0.959277608
ENSGALG00010014134	RPAP3	2	0.024806263	0.057463234	0.431689306	0.665967241	0	0.232605741	0	1	CRA004775;PRJNA827465	Yang2025_Silkie_Wenchang	0.913546323
ENSGALG00010014135	ZBTB24	2	0.060219485	0.076672518	0.785411598	0.432212283	0	0.000494175	0	1	PRJNA827465;Yang2025_Silkie_Wenchang	CRA004775	0.842704601
ENSGALG00010014135	ZBTB24	2	0.259453189	0.23813447	1.089523871	0.275922934	0.074080828	2.74719221	63.59919789	1	CRA004775;Yang2025_Silkie_Wenchang	PRJNA827465	0.775541559
ENSGALG00010014135	ZBTB24	2	0.244736156	0.232760453	1.051450763	0.293051612	0.080022364	3.315025551	69.83431999	1	CRA004775;PRJNA827465	Yang2025_Silkie_Wenchang	0.786774237
ENSGALG00010014143	NFATC2	2	0.216826839	0.237630702	0.912452967	0.361530318	0.074670718	2.524803396	60.39295567	1	PRJNA827465;Yang2025_Silkie_Wenchang	CRA004775	0.81550034
ENSGALG00010014143	NFATC2	2	0.186242264	0.423834615	0.439422024	0.660355771	0.21845765	2.425961607	58.77923224	0.5	CRA004775;Yang2025_Silkie_Wenchang	PRJNA827465	0.911923994
ENSGALG00010014143	NFATC2	2	0.021686601	0.109140575	0.198703382	0.842494778	0	0.569848617	0	0.5	CRA004775;PRJNA827465	Yang2025_Silkie_Wenchang	0.960252241
ENSGALG00010014145	NTMT2	2	-0.428503622	0.499700626	-0.857520682	0.39115719	0.439819374	8.313785657	87.97178516	0.5	PRJNA827465;Yang2025_Silkie_Wenchang	CRA004775	0.829251057

ENSGALG00010014145	NTMT2	2	-0.132729585	0.914361987	-0.145160874	0.884583857	1.419261367	6.135855635	83.7023545	0.5	CRA004775;Yang2025_Silkie_Wenchang	PRJNA827465	0.972062774
ENSGALG00010014145	NTMT2	2	0.218710166	0.333251276	0.656292058	0.511636227	0.094412035	1.363532872	26.66110068	1	CRA004775;PRJNA827465	Yang2025_Silkie_Wenchang	0.865694231
ENSGALG00010014147	ENSGALG00010014147	2	-0.264271439	0.179277787	-1.474089141	0.140457592	0	0.189319573	0	1	PRJNA827465;Yang2025_Silkie_Wenchang	CRA004775	0.624550824
ENSGALG00010014147	ENSGALG00010014147	2	-0.084649023	0.272372644	-0.310783864	0.755964937	0	0.690389661	0	0.5	CRA004775;Yang2025_Silkie_Wenchang	PRJNA827465	0.938501783
ENSGALG00010014147	ENSGALG00010014147	2	-0.233342099	0.269026801	-0.86735633	0.385746801	0.028747357	1.081268366	7.516021776	0.5	CRA004775;PRJNA827465	Yang2025_Silkie_Wenchang	0.82648313
ENSGALG00010014148	KCNG1	2	0.121394975	0.311271322	0.3899973	0.696538543	0.146317261	4.03220204	75.19965542	0.5	PRJNA827465;Yang2025_Silkie_Wenchang	CRA004775	0.921041503
ENSGALG00010014148	KCNG1	2	0.395739284	0.239072728	1.655309191	0.097861795	0	0.939990154	0	0.5	CRA004775;Yang2025_Silkie_Wenchang	PRJNA827465	0.534130658
ENSGALG00010014148	KCNG1	2	-0.183526249	0.185969553	-0.986861808	0.323710389	0	0.132827433	0	1	CRA004775;PRJNA827465	Yang2025_Silkie_Wenchang	0.799600434
ENSGALG00010014151	ATP23	2	-0.177723973	0.131917401	-1.347236767	0.17790399	0.023704139	2.787187389	64.12153686	1	PRJNA827465;Yang2025_Silkie_Wenchang	CRA004775	0.683951879
ENSGALG00010014151	ATP23	2	-0.280963658	0.124506102	-2.256625605	0.024031479	0	0.641962751	0	1	CRA004775;Yang2025_Silkie_Wenchang	PRJNA827465	0.26471671
ENSGALG00010014151	ATP23	2	-0.078895127	0.063132062	-1.249683983	0.211415011	0	0.053576608	0	1	CRA004775;PRJNA827465	Yang2025_Silkie_Wenchang	0.723738713
ENSGALG00010014153	FOCAD	2	0.066674901	0.104476365	0.638181672	0.523355426	0	0.087559014	0	1	PRJNA827465;Yang2025_Silkie_Wenchang	CRA004775	0.869661505
ENSGALG00010014153	FOCAD	2	0.121734646	0.117429282	1.036663457	0.299892728	0	0.134183744	0	1	CRA004775;Yang2025_Silkie_Wenchang	PRJNA827465	0.790115099
ENSGALG00010014153	FOCAD	2	0.085873271	0.127495549	0.673539539	0.500604218	0	0.341865921	0	1	CRA004775;PRJNA827465	Yang2025_Silkie_Wenchang	0.862400704
ENSGALG00010014156	CD164	2	-0.342781475	0.318417502	-1.076515813	0.281696633	0.197819836	40.67312883	97.5413743	1	PRJNA827465;Yang2025_Silkie_Wenchang	CRA004775	0.779295548
ENSGALG00010014156	CD164	2	-0.054948772	0.628057275	-0.087490065	0.930281982	0.759149799	25.9396209	96.14489354	0.5	CRA004775;Yang2025_Silkie_Wenchang	PRJNA827465	0.983813577
ENSGALG00010014156	CD164	2	0.24082541	0.307155047	0.784051613	0.433009826	0.163947756	6.831550509	85.3620346	0.5	CRA004775;PRJNA827465	Yang2025_Silkie_Wenchang	0.842762754
ENSGALG00010014159	CDC42EP1	2	0.089473867	0.084134776	1.063458794	0.287573942	0.009880568	3.152113565	68.27525471	0.5	PRJNA827465;Yang2025_Silkie_Wenchang	CRA004775	0.782840067
ENSGALG00010014159	CDC42EP1	2	-0.059962023	0.111840651	-0.536138004	0.591863176	0.00847126	1.245575238	19.71580927	1	CRA004775;Yang2025_Silkie_Wenchang	PRJNA827465	0.892776493
ENSGALG00010014159	CDC42EP1	2	-0.005084654	0.222459945	-0.022856493	0.981764745	0.074947706	3.315866296	69.84196856	0.5	CRA004775;PRJNA827465	Yang2025_Silkie_Wenchang	0.99503081
ENSGALG00010014161	GFRA3	2	0.824080271	0.191863693	4.295133996	1.74588E-05	0.004775605	1.059819094	5.644273997	1	PRJNA827465;Yang2025_Silkie_Wenchang	CRA004775	0.002206375
ENSGALG00010014161	GFRA3	2	0.94644387	0.19835932	4.771360728	1.82985E-06	0	0.008468103	0	1	CRA004775;Yang2025_Silkie_Wenchang	PRJNA827465	0.000363226
ENSGALG00010014161	GFRA3	2	0.676871344	0.26523865	2.551933304	0.010712702	0	0.439810228	0	1	CRA004775;PRJNA827465	Yang2025_Silkie_Wenchang	0.165128023
ENSGALG00010014162	COL18A1	2	-0.120242593	0.130679184	-0.920135778	0.35750181	0.017450572	1.915698645	47.79972297	1	PRJNA827465;Yang2025_Silkie_Wenchang	CRA004775	0.813459945
ENSGALG00010014162	COL18A1	2	-0.074166294	0.371919837	-0.199414729	0.84193834	0.156432595	1.740909937	42.55877466	0.5	CRA004775;Yang2025_Silkie_Wenchang	PRJNA827465	0.960041272
ENSGALG00010014162	COL18A1	2	-0.005205172	0.097725719	-0.053263069	0.957522305	0	0.854004219	0	0.5	CRA004775;PRJNA827465	Yang2025_Silkie_Wenchang	0.98874203
ENSGALG00010014165	LUZP1	2	0.081158752	0.129071039	0.628791341	0.529485669	0.017999938	1.797815805	44.37694913	0.5	PRJNA827465;Yang2025_Silkie_Wenchang	CRA004775	0.871944741
ENSGALG00010014165	LUZP1	2	0.287106768	0.171620302	1.67291844	0.094343353	0	0.001101352	0	1	CRA004775;Yang2025_Silkie_Wenchang	PRJNA827465	0.526028771
ENSGALG00010014165	LUZP1	2	0.012435732	0.071941975	0.172857804	0.8627632	0	0.742143665	0	0.5	CRA004775;PRJNA827465	Yang2025_Silkie_Wenchang	0.966099097
ENSGALG00010014166	FGF12	2	0.45374545	0.478278053	0.9487064	0.342769958	0.413398414	10.28242075	90.27466368	0.5	PRJNA827465;Yang2025_Silkie_Wenchang	CRA004775	0.806120611
ENSGALG00010014166	FGF12	2	0.692355788	0.349939876	1.978499269	0.047872411	0.128656958	1.90690519	47.5590079	1	CRA004775;Yang2025_Silkie_Wenchang	PRJNA827465	0.378353533
ENSGALG00010014166	FGF12	2	0.016351752	0.163735056	0.099867144	0.920449801	0	0.191927956	0	0.5	CRA004775;PRJNA827465	Yang2025_Silkie_Wenchang	0.98144788
ENSGALG00010014167	SLC38A9	2	0.194259878	0.187446759	1.036346958	0.300040306	0.037922635	2.075561853	51.82027466	1	PRJNA827465;Yang2025_Silkie_Wenchang	CRA004775	0.790169134
ENSGALG00010014167	SLC38A9	2	0.046290284	0.38525293	0.120155566	0.904359916	0.240995886	5.305806986	81.15272564	0.5	CRA004775;Yang2025_Silkie_Wenchang	PRJNA827465	0.977586231
ENSGALG00010014167	SLC38A9	2	-0.099573361	0.186565561	-0.533717798	0.593536793	0.033928797	1.81991876	45.05249235	0.5	CRA004775;PRJNA827465	Yang2025_Silkie_Wenchang	0.892915939
ENSGALG00010014170	CEMIP2	2	-0.008292548	0.11822774	-0.070140459	0.944081864	0.019440452	3.240595322	69.14147246	0.5	PRJNA827465;Yang2025_Silkie_Wenchang	CRA004775	0.985827325
ENSGALG00010014170	CEMIP2	2	-0.147042483	0.100299027	-1.466040976	0.142637124	0	0.16808597	0	1	CRA004775;Yang2025_Silkie_Wenchang	PRJNA827465	0.628905865
ENSGALG00010014170	CEMIP2	2	0.075830908	0.097567746	0.777212877	0.437033186	0.003621165	1.047583	4.54216994	0.5	CRA004775;PRJNA827465	Yang2025_Silkie_Wenchang	0.843442932
ENSGALG00010014171	DLC1	2	-0.029607048	0.061787527	-0.479175159	0.631814024	0	0.194753001	0	1	PRJNA827465;Yang2025_Silkie_Wenchang	CRA004775	0.903058174
ENSGALG00010014171	DLC1	2	0.04337708	0.178521993	0.242978913	0.808021733	0.031112919	1.719450323	41.84187895	0.5	CRA004775;Yang2025_Silkie_Wenchang	PRJNA827465	0.952496413
ENSGALG00010014171	DLC1	2	0.045050239	0.124624357	0.361488237	0.717734497	0.013887789	1.363324171	26.64987382	0.5	CRA004775;PRJNA827465	Yang2025_Silkie_Wenchang	0.927927766
ENSGALG00010014173	MND1	2	0.018540441	0.111219083	0.166701976	0.86760455	0.009307956	1.512281588	33.87474874	0.5	PRJNA827465;Yang2025_Silkie_Wenchang	CRA004775	0.96788231
ENSGALG00010014173	MND1	2	-0.124051048	0.149841268	-0.827883062	0.407736728	0	0.030761231	0	1	CRA004775;Yang2025_Silkie_Wenchang	PRJNA827465	0.833982683
ENSGALG00010014173	MND1	2	0.088179985	0.094239789	0.935698029	0.349428686	0	0.181892666	0	0.5	CRA004775;PRJNA827465	Yang2025_Silkie_Wenchang	0.809903518
ENSGALG00010014176	ABHD17B	2	-0.050334702	0.122064682	-0.412360896	0.680074921	0.012525175	1.720564135	41.8795278	0.5	PRJNA827465;Yang2025_Silkie_Wenchang	CRA004775	0.917701868
ENSGALG00010014176	ABHD17B	2	0.026403011	0.230499725	0.11454682	0.908804334	0.076540161	3.467106848	71.15750844	0.5	CRA004775;Yang2025_Silkie_Wenchang	PRJNA827465	0.978387968
ENSGALG00010014176	ABHD17B	2	0.123056575	0.10690907	1.151039614	0.249715939	0	0.822365078	0	1	CRA004775;PRJNA827465	Yang2025_Silkie_Wenchang	0.759757983
ENSGALG00010014178	LRTM2	2	-0.0525551	0.169401393	-0.310240072	0.7563784	0	0.501358182	0	0.5	PRJNA827465;Yang2025_Silkie_Wenchang	CRA004775	0.938618977
ENSGALG00010014178	LRTM2	2	0.046052843	0.253995254	0.181313795	0.856121279	0	0.452704929	0	0.5	CRA004775;Yang2025_Silkie_Wenchang	PRJNA827465	0.963499334
ENSGALG00010014178	LRTM2	2	-0.175153382	0.217331407	-0.805927616	0.420284587	0	0.203616535	0	1	CRA004775;PRJNA827465	Yang2025_Silkie_Wenchang	0.838712623
ENSGALG00010014179	CTBPL	2	-0.161950579	0.19228194	-0.842255797	0.399644787	0.0707125	22.55899142	95.56717771	0.5	PRJNA827465;Yang2025_Silkie_Wenchang	CRA004775	0.832246851
ENSGALG00010014179	CTBPL	2	-0.341402465	0.06821089	-5.005102038	5.58324E-07	0	0.623235869	0	1	CRA004775;Yang2025_Silkie_Wenchang	PRJNA827465	0.00013977
ENSGALG00010014179	CTBPL	2	0.019651675	0.036875174	0.532924257	0.594086013	0	0.852339887	0	0.5	CRA004775;PRJNA827465	Yang2025_Silkie_Wenchang	0.893274007
ENSGALG00010014180	SLC19A1	2	-0.01947979	0.070042901	-0.278112272	0.780926175	0	0.159823803	0	0.5	PRJNA827465;Yang2025_Silkie_Wenchang	CRA004775	0.945235709
ENSGALG00010014180	SLC19A1	2	-0.167904973	0.222320056	-0.755239881	0.450105063	0.064230974	2.842982124	64.82566697	0.5	CRA004775;Yang2025_Silkie_Wenchang	PRJNA827465	0.847564661
ENSGALG00010014180	SLC19A1	2	-0.170820773	0.178939844	-0.954626814	0.339766452	0.04547218	2.999220233	66.65800033	1	CRA004775;PRJNA827465	Yang2025_Silkie_Wenchang	0.805078728
ENSGALG00010014184	SLC44A1	2	-0.19945698	0.137660767	-1.448902143	0.147364913	0.021454052	2.285499125	56.24588131	1	PRJNA827465;Yang2025_Silkie_Wenchang	CRA004775	0.636260921
ENSGALG00010014184	SLC44A1	2	0.199688451	0.597329475	0.334302022	0.738151665	0.637654923	8.85214278	88.70330015	0.5	CRA004775;Yang2025_Silkie_Wenchang	PRJNA827465	0.934320147
ENSGALG00010014184	SLC44A1	2	0.319148766	0.456098679	0.699736221	0.484092051	0.347660091	5.447639789	81.64342653	0.5	CRA004775;PRJNA827465	Yang2025_Silkie_Wenchang	0.857004733
ENSGALG00010014186	MOCS3	2	-0.200080654	0.173359672	-1.154136089	0.24844438	0.046496801	3.994560639	74.9659577	1	PRJNA827465;Yang2025_Silkie_Wenchang	CRA004775	0.758710913
ENSGALG00010014186	MOCS3	2	-0.305125976	0.145104249	-2.102805245	0.035482797	0.005031705	1.118319389	10.5801071	1	CRA004775;Yang2025_Silkie_Wenchang	PRJNA827465	0.325304499
ENSGALG00010014186	MOCS3	2	-0.058137778	0.065465235	-0.888071013	0.374502554	0	0.030325718	0	1	CRA004775;PRJNA827465	Yang2025_Silkie_Wenchang	0.822805106
ENSGALG00010014190	HACD4	2	-0.590674384	0.457715734	-1.290483022	0.196883003	0.369986993	8.543037568	88.2945616	1	PRJNA827465;Yang2025_Silkie_Wenchang	CRA004775	0.707994211
ENSGALG00010014190	HACD4	2	-0.436285722	0.677597388	-0.643871611	0.519658692	0.785985303	6.58767697	84.82014215	0.5	CRA004775;Yang2025_Silkie_Wenchang	PRJNA827465	0.869100572
ENSGALG00010014190	HACD4	2	-0.062793605	0.194846371	-0.322272388	0.747246349	0	0.724920822	0	0.5	CRA004775;PRJNA827465	Yang2025_Silkie_Wenchang	0.93680487
ENSGALG00010014192	KDM1A	2	-0.114009202	0.130823319	-0.871474616	0.38349506	0.030740857	9.668451866	89.6570825	0.5	PRJNA827465;Yang2025_Silkie_Wenchang	CRA004775	0.825509017
ENSGALG00010014192	KDM1A	2	-0.251772116	0.068028436	-3.70098345	0.000214765	0	0.006565102	0	1	CRA004775;Yang2025_Silkie_Wenchang	PRJNA827465	0.013365444
ENSGALG00010014192	KDM1A	2	-0.010022516	0.075268512	-0.133156826	0.894069354	0.003990775	1.109705778	9.886023851	0.5	CRA004775;PRJNA827465	Yang2025_Silkie_Wenchang	0.974381724
ENSGALG00010014193	CARD10	2	0.312498532	0.241124562	1.296004561	0.194973913	0	0.130145217	0	1	PRJNA827465;Yang2025_Silkie_Wenchang	CRA004775	0.706113528
ENSGALG00010014193	CARD10	2	-0.309360704	0.609404221	-0.507644505	0.611702674	0.426003205	2.212926277	54.81096634	0.5	CRA004775;Yang2025_Silkie_Wenchang	PRJNA827465	0.897777844
ENSGALG00010014193	CARD10	2	-0.19654929	0.701554757	-0.280162436	0.779352885	0.72980816	3.436026601	70.89661647	0.5	CRA004775;PRJNA827465	Yang2025_Silkie_Wenchang	0.944727706
ENSGALG00010014194	LRIG3	2	0.087424666	0.062226378	1.404945433	0.160037505	0	0.005796824	0	1	PRJNA827465;Yang2025_Silkie_Wenchang	CRA004775	0.655476664
ENSGALG00010014194	LRIG3	2	0.167262439	0.168682514	0.991581377	0.321401777	0.026408596	1.432161546	30.17547478	1	CRA004775;Yang2025_Silkie_Wenchang	PRJNA827465	0.798513866
ENSGALG00010014194	LRIG3	2	0.165806297	0.157719433	1.051273733	0.293132888	0.023801836	1.398443161	28.4919096	1	CRA004775;PRJNA827465	Yang2025_Silkie_Wenchang	0.786774237
ENSGALG00010014197	SH3GL2	2	-0.308913605	0.145090175	-2.129114566	0.033244783	0	0.00119237	0	1	PRJNA827465;Yang2025_Silkie_Wenchang	CRA004775	0.313333153
ENSGALG00010014197	SH3GL2	2	-0.338612255	0.320051179	-1.057994087	0.290058138	0	0.056735002	0	1	CRA004775;Yang2025_Silkie_Wenchang	PRJNA827465	0.784195688
ENSGALG00010014197	SH3GL2	2	-0.319919844	0.154014112	-2.077211233	0.037782068	0	0.059425581	0	1	CRA004775;PRJNA827465	Yang2025_Silkie_Wenchang	0.335429998
ENSGALG00010014198	KDM3B	2	-0.065998856	0.063607888	-1.037589168	0.299461363	0.004703738	1.992649352	49.81555591	1	PRJNA827465;Yang2025_Silkie_Wenchang	CRA004775	0.78996778
ENSGALG00010014198	KDM3B	2	-0.138946798	0.082413609	-1.685969102	0.091801751	0	0.311680542	0	1	CRA004775;Yang2025_Silkie_Wenchang	PRJNA827465	0.51907412
ENSGALG00010014198	KDM3B	2	-0.023830634	0.033648313	-0.70822671	0.478804482	0	0.008759379	0	1	CRA004775;PRJNA827465	Yang2025_Silkie_Wenchang	0.855773195
ENSGALG00010014199	LNX1	2	0.385775066	0.212471855	1.815652551	0.069423687	0.035913912	1.660249167	39.76807697	1	PRJNA827465;Yang2025_Silkie_Wenchang	CRA004775	0.453929672
ENSGALG00010014199	LNX1	2	0.535169748	0.197162934	2.714352737	0.006640542	0	0.253416464	0	1	CRA004775;Yang2025_Silkie_Wenchang	PRJNA827465	0.124602584
ENSGALG00010014199	LNX1	2	0.225747124	0.200724519	1.124661426	0.260732534	0	0.200300552	0	1	CRA004775;PRJNA827465	Yang2025_Silkie_Wenchang	0.767895899
ENSGALG00010014200	DCP1B	2	-0.208960145	0.177607954	-1.176524702	0.239385267	0.052971282	6.002889092	83.34135473	1	PRJNA827465;Yang2025_Silkie_Wenchang	CRA004775	0.751706259
ENSGALG00010014200	DCP1B	2	-0.36634961	0.118731158	-3.085538939	0.002031836	0	0.669715177	0	1	CRA004775;Yang2025_Silkie_Wenchang	PRJNA827465	0.058631999
ENSGALG00010014200	DCP1B	2	-0.048554494	0.070027221	-0.693366006	0.48807989	0	0.029443217	0	1	CRA004775;PRJNA827465	Yang2025_Silkie_Wenchang	0.85768885
ENSGALG00010014203	TMEM38B	2	-0.295640536	0.199240532	-1.483837316	0.137852085	0.06508563	5.456101197	81.6718942	1	PRJNA827465;Yang2025_Silkie_Wenchang	CRA004775	0.62064915
ENSGALG00010014203	TMEM38B	2	-0.53175849	0.131036451	-4.058095948	4.94744E-05	0	0.255707533	0	1	CRA004775;Yang2025_Silkie_Wenchang	PRJNA827465	0.004813104
ENSGALG00010014203	TMEM38B	2	-0.29851976	0.280656737	-1.063647225	0.287488541	0.10524451	2.361446151	57.65306783	1	CRA004775;PRJNA827465	Yang2025_Silkie_Wenchang	0.782840067
ENSGALG00010014204	KIFAP3	2	-0.072595785	0.059741645	-1.215162123	0.224304264	0	0.264759591	0	1	PRJNA827465;Yang2025_Silkie_Wenchang	CRA004775	0.737471949
ENSGALG00010014204	KIFAP3	2	-0.104299851	0.097359985	-1.071280478	0.284043335	0	0.171416729	0	0.5	CRA004775;Yang2025_Silkie_Wenchang	PRJNA827465	0.779864142
ENSGALG00010014204	KIFAP3	2	-0.046235478	0.073312348	-0.630664258	0.528260074	0	0.072047147	0	0.5	CRA004775;PRJNA827465	Yang2025_Silkie_Wenchang	0.871701504
ENSGALG00010014205	DPM1	2	0.031748319	0.055077174	0.576433333	0.564322319	0	0.946580836	0	0.5	PRJNA827465;Yang2025_Silkie_Wenchang	CRA004775	0.883084891
ENSGALG00010014205	DPM1	2	-0.112624253	0.12924314	-0.871413781	0.383528264	0	0.001131485	0	1	CRA004775;Yang2025_Silkie_Wenchang	PRJNA827465	0.825509017
ENSGALG00010014205	DPM1	2	0.040342665	0.056544684	0.713465224	0.475557925	0	0.499675913	0	0.5	CRA004775;PRJNA827465	Yang2025_Silkie_Wenchang	0.854526154
ENSGALG00010014209	CCDC50	2	-0.267297531	0.220246576	-1.213628543	0.224889602	0.092762698	22.59095037	95.57344873	1	PRJNA827465;Yang2025_Silkie_Wenchang	CRA004775	0.737888351
ENSGALG00010014209	CCDC50	2	-0.087033276	0.421145476	-0.206658461	0.8362766	0.335186542	17.69808257	94.34967039	0.5	CRA004775;Yang2025_Silkie_Wenchang	PRJNA827465	0.958399882
ENSGALG00010014209	CCDC50	2	0.110370246	0.197308522	0.559379013	0.575903081	0.062881404	4.482710021	77.69206584	0.5	CRA004775;PRJNA827465	Yang2025_Silkie_Wenchang	0.886924804
ENSGALG00010014210	ENSGALG00010014210	2	0.038755925	0.221815768	0.174721237	0.86129868	0.056460949	2.329618615	57.07451882	0.5	PRJNA827465;Yang2025_Silkie_Wenchang	CRA004775	0.965343309
ENSGALG00010014210	ENSGALG00010014210	2	0.317572925	0.2128052	1.492317509	0.135615929	0	0.372730027	0	1	CRA004775;Yang2025_Silkie_Wenchang	PRJNA827465	0.617196137
ENSGALG00010014210	ENSGALG00010014210	2	0.018499606	0.36547899	0.050617426	0.959630377	0.132887407	1.481794704	32.51426819	0.5	CRA004775;PRJNA827465	Yang2025_Silkie_Wenchang	0.989259322
ENSGALG00010014213	ETFRF1	2	-0.050566715	0.213658429	-0.236670815	0.812912178	0.075775474	5.665183834	82.34832215	0.5	PRJNA827465;Yang2025_Silkie_Wenchang	CRA004775	0.953526116
ENSGALG00010014213	ETFRF1	2	0.190776543	0.136910042	1.393444487	0.163485421	0	0.011069171	0	1	CRA004775;Yang2025_Silkie_Wenchang	PRJNA827465	0.661791656
ENSGALG00010014213	ETFRF1	2	-0.093384345	0.217746759	-0.42886675	0.668020199	0.062627007	2.402999074	58.38533561	0.5	CRA004775;PRJNA827465	Yang2025_Silkie_Wenchang	0.914120722
ENSGALG00010014214	REEP2	2	0.231974772	0.172926052	1.341468041	0.179768539	0.042716786	3.377469909	70.39203822	1	PRJNA827465;Yang2025_Silkie_Wenchang	CRA004775	0.68659821
ENSGALG00010014214	REEP2	2	0.339299854	0.131127551	2.587555779	0.009665954	0	0.955516799	0	1	CRA004775;Yang2025_Silkie_Wenchang	PRJNA827465	0.154965704
ENSGALG00010014214	REEP2	2	0.090881919	0.094369863	0.963039642	0.33552763	0	0.083667982	0	1	CRA004775;PRJNA827465	Yang2025_Silkie_Wenchang	0.803316425
ENSGALG00010014217	RASA3	2	-0.071156873	0.055379448	-1.284896762	0.198828363	0	0.27303421	0	1	PRJNA827465;Yang2025_Silkie_Wenchang	CRA004775	0.71096127
ENSGALG00010014217	RASA3	2	-0.110982748	0.082371914	-1.347337253	0.177871639	0	0.032830504	0	1	CRA004775;Yang2025_Silkie_Wenchang	PRJNA827465	0.683951879
ENSGALG00010014217	RASA3	2	-0.054773571	0.069386461	-0.789398548	0.429879104	0	0.16032603	0	1	CRA004775;PRJNA827465	Yang2025_Silkie_Wenchang	0.842012722
ENSGALG00010014218	IL31RA	2	0.18224055	0.104608346	1.742122467	0.081487017	0	0.216621562	0	1	PRJNA827465;Yang2025_Silkie_Wenchang	CRA004775	0.491594971
ENSGALG00010014218	IL31RA	2	0.08571143	0.152208297	0.563119302	0.573353649	0.003535169	1.025554196	2.491745022	0.5	CRA004775;Yang2025_Silkie_Wenchang	PRJNA827465	0.885850914
ENSGALG00010014218	IL31RA	2	0.100666008	0.255108833	0.394600243	0.693137934	0.060175064	1.435070524	30.31701345	0.5	CRA004775;PRJNA827465	Yang2025_Silkie_Wenchang	0.920662393
ENSGALG00010014220	C9orf85	2	-0.074418374	0.195597986	-0.38046595	0.703599568	0.033005949	1.750375999	42.86941773	0.5	PRJNA827465;Yang2025_Silkie_Wenchang	CRA004775	0.923639641
ENSGALG00010014220	C9orf85	2	-0.226595016	0.188670047	-1.20101214	0.229746492	0	0.244704705	0	1	CRA004775;Yang2025_Silkie_Wenchang	PRJNA827465	0.742686486
ENSGALG00010014220	C9orf85	2	0.062536717	0.170344345	0.367119416	0.713529945	0	0.215412412	0	0.5	CRA004775;PRJNA827465	Yang2025_Silkie_Wenchang	0.926253108
ENSGALG00010014221	SCYL3	2	-0.073148559	0.07708625	-0.948918379	0.342662126	0.00615057	1.872717234	46.6016555	1	PRJNA827465;Yang2025_Silkie_Wenchang	CRA004775	0.806011702
ENSGALG00010014221	SCYL3	2	-0.156371066	0.098370935	-1.589606377	0.111923559	0	0.364188738	0	1	CRA004775;Yang2025_Silkie_Wenchang	PRJNA827465	0.56547057
ENSGALG00010014221	SCYL3	2	-0.01714031	0.052638405	-0.325623657	0.744709112	0	0.001426758	0	1	CRA004775;PRJNA827465	Yang2025_Silkie_Wenchang	0.935893304
ENSGALG00010014222	TBX22	2	-0.604787323	0.216265208	-2.796507715	0.005165818	0	0.816700124	0	1	PRJNA827465;Yang2025_Silkie_Wenchang	CRA004775	0.107367602
ENSGALG00010014222	TBX22	2	-0.698943205	0.302982188	-2.306878862	0.021061574	0	0.877216582	0	1	CRA004775;Yang2025_Silkie_Wenchang	PRJNA827465	0.245414483
ENSGALG00010014222	TBX22	2	-0.396460571	0.259956586	-1.525103006	0.127233408	0	0.182425914	0	1	CRA004775;PRJNA827465	Yang2025_Silkie_Wenchang	0.598737382
ENSGALG00010014225	ZMPSTE24	2	0.067815901	0.0481592	1.408160883	0.159083436	0.001535424	1.30243877	23.2209588	0.5	PRJNA827465;Yang2025_Silkie_Wenchang	CRA004775	0.654091044
ENSGALG00010014225	ZMPSTE24	2	-0.053602909	0.085594005	-0.626246065	0.531153557	0	0.775278903	0	1	CRA004775;Yang2025_Silkie_Wenchang	PRJNA827465	0.872848946
ENSGALG00010014225	ZMPSTE24	2	0.002984802	0.14502018	0.020581976	0.983579118	0.027832322	2.155536533	53.60783801	0.5	CRA004775;PRJNA827465	Yang2025_Silkie_Wenchang	0.995457849
ENSGALG00010014226	SLC16A7	2	-0.002215746	0.129338692	-0.017131349	0.98633183	0.006778372	1.21026436	17.37342407	0.5	PRJNA827465;Yang2025_Silkie_Wenchang	CRA004775	0.99618774
ENSGALG00010014226	SLC16A7	2	-0.276519831	0.200150237	-1.381561345	0.16710643	0	0.971866615	0	1	CRA004775;Yang2025_Silkie_Wenchang	PRJNA827465	0.666416556
ENSGALG00010014226	SLC16A7	2	-0.181131486	0.389519437	-0.465012702	0.641922361	0.204899835	2.413030521	58.55833603	0.5	CRA004775;PRJNA827465	Yang2025_Silkie_Wenchang	0.906634523
ENSGALG00010014236	WDR44	2	0.035946612	0.045898879	0.783169704	0.433527463	0	0.552281845	0	1	PRJNA827465;Yang2025_Silkie_Wenchang	CRA004775	0.842762754
ENSGALG00010014236	WDR44	2	0.189750873	0.104906127	1.808768267	0.070487012	0	0.448581518	0	1	CRA004775;Yang2025_Silkie_Wenchang	PRJNA827465	0.458021454
ENSGALG00010014236	WDR44	2	0.100001798	0.115291878	0.867379384	0.385734174	0.016729891	2.140801167	53.28851575	1	CRA004775;PRJNA827465	Yang2025_Silkie_Wenchang	0.82648313
ENSGALG00010014238	PHLDA1	2	0.290903641	0.078605563	3.700802186	0.000214919	0	0.102769958	0	1	PRJNA827465;Yang2025_Silkie_Wenchang	CRA004775	0.013365444
ENSGALG00010014238	PHLDA1	2	0.301053054	0.122485324	2.457870415	0.01397636	0	0.526785516	0	1	CRA004775;Yang2025_Silkie_Wenchang	PRJNA827465	0.193829519
ENSGALG00010014238	PHLDA1	2	0.327131916	0.089819149	3.642117735	0.000270404	0	0.334577807	0	1	CRA004775;PRJNA827465	Yang2025_Silkie_Wenchang	0.015815033
ENSGALG00010014240	PIGV	2	0.235882719	0.103173465	2.286273104	0.022238286	0	0.66500824	0	1	PRJNA827465;Yang2025_Silkie_Wenchang	CRA004775	0.252865604
ENSGALG00010014240	PIGV	2	0.027145393	0.371562818	0.073057345	0.941760485	0.226963805	5.611969782	82.18094468	0.5	CRA004775;Yang2025_Silkie_Wenchang	PRJNA827465	0.985827325
ENSGALG00010014240	PIGV	2	-0.042696542	0.264755393	-0.161267886	0.871882416	0.112722679	4.852342085	79.39139528	0.5	CRA004775;PRJNA827465	Yang2025_Silkie_Wenchang	0.969462602
ENSGALG00010014241	ENSGALG00010014241	2	-1.013667019	0.686803473	-1.475920054	0.139965355	0	0.697049997	0	1	PRJNA827465;Yang2025_Silkie_Wenchang	CRA004775	0.623792706
ENSGALG00010014241	ENSGALG00010014241	2	0.190212551	0.774067494	0.245731222	0.805890302	0	0.731566496	0	0.5	CRA004775;Yang2025_Silkie_Wenchang	PRJNA827465	0.952057827
ENSGALG00010014241	ENSGALG00010014241	2	-0.397337883	1.240628029	-0.320271567	0.748762475	1.970690002	2.741689757	63.52614305	0.5	CRA004775;PRJNA827465	Yang2025_Silkie_Wenchang	0.937249951
ENSGALG00010014243	ENSGALG00010014243	2	0.112217609	0.091112852	1.23163315	0.218086152	0	0.546455092	0	0.5	PRJNA827465;Yang2025_Silkie_Wenchang	CRA004775	0.73160572
ENSGALG00010014243	ENSGALG00010014243	2	0.00547262	0.295364877	0.018528337	0.985217372	0	0.402161542	0	0.5	CRA004775;Yang2025_Silkie_Wenchang	PRJNA827465	0.995859331
ENSGALG00010014243	ENSGALG00010014243	2	0.123099198	0.089222843	1.379682524	0.167684416	0	4.5337E-05	0	1	CRA004775;PRJNA827465	Yang2025_Silkie_Wenchang	0.667779227
ENSGALG00010014244	SESN1	2	-0.343946486	0.080470609	-4.274187706	1.91835E-05	0	0.034359478	0	1	PRJNA827465;Yang2025_Silkie_Wenchang	CRA004775	0.002370977
ENSGALG00010014244	SESN1	2	-0.345120826	0.134953097	-2.557339061	0.010547635	0	0.482896502	0	0.5	CRA004775;Yang2025_Silkie_Wenchang	PRJNA827465	0.163532035
ENSGALG00010014244	SESN1	2	-0.323592099	0.09800869	-3.301667408	0.00096112	0	0.428895377	0	0.5	CRA004775;PRJNA827465	Yang2025_Silkie_Wenchang	0.036786652
ENSGALG00010014246	KRAS	2	0.153613137	0.171059607	0.898009409	0.369180533	0.045668843	4.199871567	76.18974809	1	PRJNA827465;Yang2025_Silkie_Wenchang	CRA004775	0.820603014
ENSGALG00010014246	KRAS	2	0.395292495	0.125893596	3.139893588	0.001690092	0	0.230051276	0	1	CRA004775;Yang2025_Silkie_Wenchang	PRJNA827465	0.052036292
ENSGALG00010014246	KRAS	2	0.198734369	0.233076643	0.852656734	0.393849691	0.08598784	4.198419149	76.18151108	1	CRA004775;PRJNA827465	Yang2025_Silkie_Wenchang	0.830486298
ENSGALG00010014249	ENSGALG00010014249	2	0.244607538	0.364044228	0.671917088	0.501636481	0.238565543	9.243946273	89.18210935	0.5	PRJNA827465;Yang2025_Silkie_Wenchang	CRA004775	0.862769519
ENSGALG00010014249	ENSGALG00010014249	2	0.387191493	0.2367241	1.635623469	0.101918415	0.068138306	2.521642697	60.34331109	1	CRA004775;Yang2025_Silkie_Wenchang	PRJNA827465	0.54407845
ENSGALG00010014249	ENSGALG00010014249	2	-0.019847987	0.112204123	-0.176891778	0.859593397	0.013632022	1.710279136	41.53001232	0.5	CRA004775;PRJNA827465	Yang2025_Silkie_Wenchang	0.964833198
ENSGALG00010014250	ENSGALG00010014250	2	-0.25580122	0.116942854	-2.187403593	0.028713074	0	0.073476249	0	1	PRJNA827465;Yang2025_Silkie_Wenchang	CRA004775	0.288695306
ENSGALG00010014250	ENSGALG00010014250	2	-0.19992985	0.194818335	-1.026237338	0.304779726	0	0.020342249	0	1	CRA004775;Yang2025_Silkie_Wenchang	PRJNA827465	0.79225794
ENSGALG00010014250	ENSGALG00010014250	2	-0.268967492	0.13794541	-1.949811106	0.051198637	0	0.067148534	0	1	CRA004775;PRJNA827465	Yang2025_Silkie_Wenchang	0.391986846
ENSGALG00010014257	NOP16	2	0.106578644	0.09632176	1.106485639	0.268516377	0	0.008917095	0	1	PRJNA827465;Yang2025_Silkie_Wenchang	CRA004775	0.771398294
ENSGALG00010014257	NOP16	2	-0.224352271	0.295185833	-0.760037392	0.447232234	0.102740136	2.43169358	58.87639758	0.5	CRA004775;Yang2025_Silkie_Wenchang	PRJNA827465	0.84650962
ENSGALG00010014257	NOP16	2	-0.155192961	0.306248673	-0.506754722	0.612326929	0.153543711	5.044556535	80.17665194	0.5	CRA004775;PRJNA827465	Yang2025_Silkie_Wenchang	0.897895209
ENSGALG00010014258	ENSGALG00010014258	2	0.196407301	0.401187283	0.489565121	0.624441663	0.219585015	3.116705433	67.91483759	0.5	PRJNA827465;Yang2025_Silkie_Wenchang	CRA004775	0.900892482
ENSGALG00010014258	ENSGALG00010014258	2	0.694949063	0.256787169	2.706323165	0.00680328	0	0.926700339	0	1	CRA004775;Yang2025_Silkie_Wenchang	PRJNA827465	0.126544334
ENSGALG00010014258	ENSGALG00010014258	2	0.406907975	0.708067827	0.574673724	0.565511981	0.784801509	4.435440181	77.45432338	0.5	CRA004775;PRJNA827465	Yang2025_Silkie_Wenchang	0.883487517
ENSGALG00010014260	ADNP	2	0.021502927	0.03625475	0.593106489	0.553109888	0	0.655213248	0	1	PRJNA827465;Yang2025_Silkie_Wenchang	CRA004775	0.87934426
ENSGALG00010014260	ADNP	2	0.218759485	0.109965879	1.989339676	0.046663723	0	0.964895813	0	1	CRA004775;Yang2025_Silkie_Wenchang	PRJNA827465	0.373148384
ENSGALG00010014260	ADNP	2	0.140100466	0.164074573	0.853882863	0.393169896	0.041489905	3.577151103	72.04479287	1	CRA004775;PRJNA827465	Yang2025_Silkie_Wenchang	0.830302317
ENSGALG00010014263	ZNF131	2	0.252861182	0.213749112	1.182981206	0.236816596	0.063013122	3.17307718	68.48485104	1	PRJNA827465;Yang2025_Silkie_Wenchang	CRA004775	0.749042546
ENSGALG00010014263	ZNF131	2	0.489365725	0.151238564	3.235720519	0.001213361	0	0.001298821	0	1	CRA004775;Yang2025_Silkie_Wenchang	PRJNA827465	0.042465482
ENSGALG00010014263	ZNF131	2	0.231368394	0.215485589	1.073707041	0.282954004	0.05576522	2.355607976	57.54811454	1	CRA004775;PRJNA827465	Yang2025_Silkie_Wenchang	0.779541654
ENSGALG00010014265	ZNF462	2	0.521581546	0.530646639	0.982916894	0.325648342	0.534283491	19.11162626	94.7675829	1	PRJNA827465;Yang2025_Silkie_Wenchang	CRA004775	0.800023788
ENSGALG00010014265	ZNF462	2	0.515286548	0.563434035	0.914546363	0.360429828	0.577099741	10.95503463	90.87177692	0.5	CRA004775;Yang2025_Silkie_Wenchang	PRJNA827465	0.814880744
ENSGALG00010014265	ZNF462	2	-0.000908601	0.097047821	-0.00936241	0.992529987	0	0.0538323	0	0.5	CRA004775;PRJNA827465	Yang2025_Silkie_Wenchang	0.997852036
ENSGALG00010014267	SCFD2	2	-0.005718861	0.114634534	-0.049887768	0.960211825	0.018605356	3.327086274	69.9436709	0.5	PRJNA827465;Yang2025_Silkie_Wenchang	CRA004775	0.989338243
ENSGALG00010014267	SCFD2	2	-0.146426933	0.093598538	-1.564414739	0.117720202	0	0.075990023	0	1	CRA004775;Yang2025_Silkie_Wenchang	PRJNA827465	0.577330692
ENSGALG00010014267	SCFD2	2	0.015969961	0.131844837	0.121126939	0.903590492	0.018862403	1.760018031	43.18240028	0.5	CRA004775;PRJNA827465	Yang2025_Silkie_Wenchang	0.97732491
ENSGALG00010014272	PARD6B	2	-0.21711399	0.243183444	-0.892799226	0.371964701	0.094754371	4.917194379	79.66319972	0.5	PRJNA827465;Yang2025_Silkie_Wenchang	CRA004775	0.821597118
ENSGALG00010014272	PARD6B	2	0.046340644	0.548724411	0.084451582	0.932697398	0.544244469	10.21346658	90.20900502	0.5	CRA004775;Yang2025_Silkie_Wenchang	PRJNA827465	0.984224753
ENSGALG00010014272	PARD6B	2	0.256738975	0.299976338	0.855864086	0.392072962	0.136334396	3.717924602	73.10327381	1	CRA004775;PRJNA827465	Yang2025_Silkie_Wenchang	0.829251057
ENSGALG00010014274	FIRRM	2	-0.174248717	0.086220276	-2.020971455	0.04328272	0	0.506475961	0	1	PRJNA827465;Yang2025_Silkie_Wenchang	CRA004775	0.359703576
ENSGALG00010014274	FIRRM	2	-0.305362654	0.129771896	-2.353072293	0.018619014	0	0.344378922	0	1	CRA004775;Yang2025_Silkie_Wenchang	PRJNA827465	0.22832546
ENSGALG00010014274	FIRRM	2	-0.192261374	0.122479795	-1.569739521	0.116475723	0.007630398	1.194212074	16.26277931	1	CRA004775;PRJNA827465	Yang2025_Silkie_Wenchang	0.574631355
ENSGALG00010014278	G3BP1	2	-0.115015768	0.153987664	-0.746915463	0.455114592	0.03949582	5.506950995	81.84113131	0.5	PRJNA827465;Yang2025_Silkie_Wenchang	CRA004775	0.849072665
ENSGALG00010014278	G3BP1	2	-0.273134151	0.113795173	-2.400226169	0.016384945	0	0.144132653	0	1	CRA004775;Yang2025_Silkie_Wenchang	PRJNA827465	0.211695425
ENSGALG00010014278	G3BP1	2	0.01502414	0.048513779	0.309688106	0.756798149	0	0.377227302	0	0.5	CRA004775;PRJNA827465	Yang2025_Silkie_Wenchang	0.938740241
ENSGALG00010014284	TMEM131L	2	0.095795571	0.049487985	1.935733892	0.052900301	0	0.022525979	0	1	PRJNA827465;Yang2025_Silkie_Wenchang	CRA004775	0.399377178
ENSGALG00010014284	TMEM131L	2	0.00281688	0.212811818	0.013236485	0.989439121	0.041868695	1.445433081	30.81658269	0.5	CRA004775;Yang2025_Silkie_Wenchang	PRJNA827465	0.997141349
ENSGALG00010014284	TMEM131L	2	0.011373565	0.184613253	0.061607524	0.950875385	0.037770649	1.430822684	30.11013793	0.5	CRA004775;PRJNA827465	Yang2025_Silkie_Wenchang	0.987377946
ENSGALG00010014288	ARMC2	2	0.055020549	0.159688262	0.344549737	0.730432898	0	0.007894591	0	1	PRJNA827465;Yang2025_Silkie_Wenchang	CRA004775	0.932653142
ENSGALG00010014288	ARMC2	2	-0.073937646	0.251403338	-0.2940997	0.76868172	0	0.396679904	0	0.5	CRA004775;Yang2025_Silkie_Wenchang	PRJNA827465	0.941578808

ENSGALG00010014288	ARMC2	2	0.008588634	0.173550449	0.049487822	0.960530542	0	0.584724098	0	0.5	CRA004775;PRJNA827465	Yang2025_Silkie_Wenchang	0.989482659
ENSGALG00010014291	OSTN	2	-0.27626058	0.334432835	-0.826056985	0.408771767	0	0.02872502	0	1	PRJNA827465;Yang2025_Silkie_Wenchang	CRA004775	0.834305842
ENSGALG00010014291	OSTN	2	-1.099795404	1.063957534	-1.033683553	0.301284124	1.339457911	2.087643417	52.0990993	1	CRA004775;Yang2025_Silkie_Wenchang	PRJNA827465	0.791222412
ENSGALG00010014291	OSTN	2	-1.022068939	1.122026051	-0.910913733	0.362340829	1.709589864	2.4958169	59.93295822	1	CRA004775;PRJNA827465	Yang2025_Silkie_Wenchang	0.815644888
ENSGALG00010014292	NIM1K	2	-0.151370327	0.38125536	-0.397031341	0.691344349	0.202055867	3.278587341	69.49905871	0.5	PRJNA827465;Yang2025_Silkie_Wenchang	CRA004775	0.920268407
ENSGALG00010014292	NIM1K	2	0.323197568	0.274917573	1.175616257	0.239748256	0	0.522433352	0	1	CRA004775;Yang2025_Silkie_Wenchang	PRJNA827465	0.751961193
ENSGALG00010014292	NIM1K	2	-0.022263298	0.630459499	-0.035312812	0.971830307	0.568470204	3.147396474	68.22770794	0.5	CRA004775;PRJNA827465	Yang2025_Silkie_Wenchang	0.991955348
ENSGALG00010014294	TMPO	2	-0.141946822	0.104699105	-1.355759645	0.175175655	0.017133752	4.473449042	77.64588373	1	PRJNA827465;Yang2025_Silkie_Wenchang	CRA004775	0.680194504
ENSGALG00010014294	TMPO	2	-0.007761711	0.267579294	-0.029007145	0.976858892	0.126232689	8.08287021	87.62815715	0.5	CRA004775;Yang2025_Silkie_Wenchang	PRJNA827465	0.993272855
ENSGALG00010014294	TMPO	2	0.078528954	0.158502933	0.495441644	0.620288405	0.037312316	3.325104058	69.92575323	0.5	CRA004775;PRJNA827465	Yang2025_Silkie_Wenchang	0.899395593
ENSGALG00010014295	CACNA1C	2	-0.131306321	0.257798414	-0.509337195	0.610515891	0.096818209	3.664174291	72.70872179	0.5	PRJNA827465;Yang2025_Silkie_Wenchang	CRA004775	0.897438028
ENSGALG00010014295	CACNA1C	2	0.053819098	0.182556857	0.294807322	0.768141073	0	0.812088135	0	0.5	CRA004775;Yang2025_Silkie_Wenchang	PRJNA827465	0.941444269
ENSGALG00010014295	CACNA1C	2	-0.360946638	0.160624869	-2.247140436	0.024631053	0	0.062213411	0	1	CRA004775;PRJNA827465	Yang2025_Silkie_Wenchang	0.268313274
ENSGALG00010014298	METTL18	2	-0.043061808	0.080975058	-0.53179101	0.59487075	0	0.920571657	0	0.5	PRJNA827465;Yang2025_Silkie_Wenchang	CRA004775	0.893382718
ENSGALG00010014298	METTL18	2	0.047338549	0.142536015	0.332116406	0.739801362	0	0.364657811	0	0.5	CRA004775;Yang2025_Silkie_Wenchang	PRJNA827465	0.93499302
ENSGALG00010014298	METTL18	2	-0.095306517	0.09222422	-1.033421781	0.301406558	0	0.033398011	0	1	CRA004775;PRJNA827465	Yang2025_Silkie_Wenchang	0.791222412
ENSGALG00010014300	ELFN2	2	0.463170446	0.338908588	1.366653023	0.171734089	0	0.836387952	0	1	PRJNA827465;Yang2025_Silkie_Wenchang	CRA004775	0.674292198
ENSGALG00010014300	ELFN2	2	0.131026837	0.960082383	0.136474577	0.891446121	1.245218941	3.075549296	67.48548296	0.5	CRA004775;Yang2025_Silkie_Wenchang	PRJNA827465	0.973846899
ENSGALG00010014300	ELFN2	2	-0.058197608	0.537524376	-0.108269709	0.91378175	0.273826782	1.684005453	40.61776948	0.5	CRA004775;PRJNA827465	Yang2025_Silkie_Wenchang	0.979364626
ENSGALG00010014301	ARID1A	2	0.082921982	0.187926095	0.441247829	0.659033591	0.05747972	4.817822666	79.24373583	0.5	PRJNA827465;Yang2025_Silkie_Wenchang	CRA004775	0.911449691
ENSGALG00010014301	ARID1A	2	0.182424924	0.16945395	1.076545716	0.281683267	0.016741677	1.354804016	26.18858611	0.5	CRA004775;Yang2025_Silkie_Wenchang	PRJNA827465	0.779295548
ENSGALG00010014301	ARID1A	2	-0.075585219	0.056149466	-1.34614314	0.178256358	0	0.007668866	0	1	CRA004775;PRJNA827465	Yang2025_Silkie_Wenchang	0.684689586
ENSGALG00010014302	FOXO3	2	-0.183212048	0.163622125	-1.11972661	0.262830282	0.039660814	3.684979494	72.86280693	0.5	PRJNA827465;Yang2025_Silkie_Wenchang	CRA004775	0.768643821
ENSGALG00010014302	FOXO3	2	0.025631821	0.142325626	0.180092803	0.857079713	0	0.354589853	0	1	CRA004775;Yang2025_Silkie_Wenchang	PRJNA827465	0.963878155
ENSGALG00010014302	FOXO3	2	-0.212089593	0.252147217	-0.841133983	0.400272879	0.068989539	1.448109221	30.90443532	0.5	CRA004775;PRJNA827465	Yang2025_Silkie_Wenchang	0.832251175
ENSGALG00010014303	GABRA2	2	-0.342942461	0.732481125	-0.468192899	0.63964665	0.922482383	6.98126788	85.67595432	0.5	PRJNA827465;Yang2025_Silkie_Wenchang	CRA004775	0.906064918
ENSGALG00010014303	GABRA2	2	0.665212163	0.397482814	1.673562075	0.094216697	0	0.910449647	0	1	CRA004775;Yang2025_Silkie_Wenchang	PRJNA827465	0.525712768
ENSGALG00010014303	GABRA2	2	0.020497513	1.155185597	0.017743913	0.985841149	2.354759396	7.999042697	87.49850403	0.5	CRA004775;PRJNA827465	Yang2025_Silkie_Wenchang	0.996013375
ENSGALG00010014305	ARL10	2	0.113402573	0.221988629	0.510848567	0.609457098	0	0.042262371	0	1	PRJNA827465;Yang2025_Silkie_Wenchang	CRA004775	0.897238578
ENSGALG00010014305	ARL10	2	-0.129014931	0.321238041	-0.401617848	0.687965292	0.071522064	1.52538566	34.44280838	0.5	CRA004775;Yang2025_Silkie_Wenchang	PRJNA827465	0.919631238
ENSGALG00010014305	ARL10	2	-0.138303716	0.268727206	-0.514662129	0.606789142	0.034175117	1.289606675	22.45697702	0.5	CRA004775;PRJNA827465	Yang2025_Silkie_Wenchang	0.896799223
ENSGALG00010014306	ENSGALG00010014306	2	0.486793142	0.236465457	2.058622634	0.039530403	0	0.551065207	0	1	PRJNA827465;Yang2025_Silkie_Wenchang	CRA004775	0.343800909
ENSGALG00010014306	ENSGALG00010014306	2	0.39727402	0.365463286	1.087042215	0.277018153	0.088126522	1.459868075	31.50065975	0.5	CRA004775;Yang2025_Silkie_Wenchang	PRJNA827465	0.776173801
ENSGALG00010014306	ENSGALG00010014306	2	0.227128907	0.264429989	0.858937778	0.390374846	0	0.443147334	0	0.5	CRA004775;PRJNA827465	Yang2025_Silkie_Wenchang	0.829251057
ENSGALG00010014308	ENSGALG00010014308	2	0.101433298	0.193290446	0.524771399	0.599742116	0.046048467	2.491769246	59.86787294	0.5	PRJNA827465;Yang2025_Silkie_Wenchang	CRA004775	0.895092498
ENSGALG00010014308	ENSGALG00010014308	2	0.316016274	0.192998811	1.637400109	0.101546695	0	0.032458594	0	1	CRA004775;Yang2025_Silkie_Wenchang	PRJNA827465	0.543672202
ENSGALG00010014308	ENSGALG00010014308	2	-0.041645985	0.12911408	-0.322551844	0.747034669	0	0.328508512	0	0.5	CRA004775;PRJNA827465	Yang2025_Silkie_Wenchang	0.936729365
ENSGALG00010014310	COL6A1	2	-0.155836139	0.068939919	-2.260463042	0.023792528	0	0.000236104	0	1	PRJNA827465;Yang2025_Silkie_Wenchang	CRA004775	0.263413441
ENSGALG00010014310	COL6A1	2	-0.14122092	0.138731092	-1.017947149	0.308703073	0	0.174799164	0	0.5	CRA004775;Yang2025_Silkie_Wenchang	PRJNA827465	0.793670558
ENSGALG00010014310	COL6A1	2	-0.150144084	0.077549048	-1.93611769	0.052853288	0	0.180381869	0	1	CRA004775;PRJNA827465	Yang2025_Silkie_Wenchang	0.399377178
ENSGALG00010014312	KIAA1191	2	-0.008105415	0.18064274	-0.044869865	0.964211037	0.053530651	5.1384355	80.53882354	0.5	PRJNA827465;Yang2025_Silkie_Wenchang	CRA004775	0.990263304
ENSGALG00010014312	KIAA1191	2	-0.274798739	0.127143269	-2.161331401	0.030669749	0	0.628150331	0	1	CRA004775;Yang2025_Silkie_Wenchang	PRJNA827465	0.298814255
ENSGALG00010014312	KIAA1191	2	-0.098576025	0.29299658	-0.336440873	0.736538432	0.143480213	5.351604156	81.31401406	0.5	CRA004775;PRJNA827465	Yang2025_Silkie_Wenchang	0.933661344
ENSGALG00010014314	ENSGALG00010014314	2	-0.252027216	0.174016709	-1.448293196	0.14753507	0.015189097	1.322377947	24.37865419	1	PRJNA827465;Yang2025_Silkie_Wenchang	CRA004775	0.636640065
ENSGALG00010014314	ENSGALG00010014314	2	0.062856507	0.208351056	0.301685571	0.762891769	0	0.839438149	0	0.5	CRA004775;Yang2025_Silkie_Wenchang	PRJNA827465	0.940082621
ENSGALG00010014314	ENSGALG00010014314	2	-0.0887105	0.388447476	-0.228371931	0.819357109	0.210484235	2.971240121	66.34401935	0.5	CRA004775;PRJNA827465	Yang2025_Silkie_Wenchang	0.955256039
ENSGALG00010014317	RAD23B	2	-0.015632247	0.051553408	-0.303226325	0.761717445	0	0.17166302	0	1	PRJNA827465;Yang2025_Silkie_Wenchang	CRA004775	0.939791938
ENSGALG00010014317	RAD23B	2	-0.028973507	0.096437006	-0.300439718	0.763841771	0	0.433972081	0	0.5	CRA004775;Yang2025_Silkie_Wenchang	PRJNA827465	0.940361611
ENSGALG00010014317	RAD23B	2	0.003160694	0.058255246	0.054255963	0.956731235	0	0.263660124	0	0.5	CRA004775;PRJNA827465	Yang2025_Silkie_Wenchang	0.988540618
ENSGALG00010014318	ENSGALG00010014318	2	-0.032100484	0.221260555	-0.145080013	0.884647698	0	0.147336753	0	0.5	PRJNA827465;Yang2025_Silkie_Wenchang	CRA004775	0.972062774
ENSGALG00010014318	ENSGALG00010014318	2	-0.319671483	0.286767929	-1.11473931	0.26496171	0	0.930135344	0	1	CRA004775;Yang2025_Silkie_Wenchang	PRJNA827465	0.769274038
ENSGALG00010014318	ENSGALG00010014318	2	-0.228517073	0.366629596	-0.623291396	0.533093051	0.122828144	1.706784739	41.41030342	0.5	CRA004775;PRJNA827465	Yang2025_Silkie_Wenchang	0.873335913
ENSGALG00010014319	ENSGALG00010014319	2	0.106688681	0.233044627	0.457803651	0.647093513	0.074402572	2.561626132	60.96229706	0.5	PRJNA827465;Yang2025_Silkie_Wenchang	CRA004775	0.907350807
ENSGALG00010014319	ENSGALG00010014319	2	0.109092251	0.315454324	0.345825823	0.729437467	0.118028596	2.452960631	59.23307019	0.5	CRA004775;Yang2025_Silkie_Wenchang	PRJNA827465	0.9313457
ENSGALG00010014319	ENSGALG00010014319	2	-0.070463897	0.080828534	-0.871770078	0.383333821	0	0.231055228	0	1	CRA004775;PRJNA827465	Yang2025_Silkie_Wenchang	0.825498284
ENSGALG00010014323	KLF4	2	0.047960174	0.116224319	0.412651798	0.679861745	0	0.722873241	0	0.5	PRJNA827465;Yang2025_Silkie_Wenchang	CRA004775	0.917701868
ENSGALG00010014323	KLF4	2	0.002867016	0.162853411	0.017604886	0.985954059	0	0.969312026	0	0.5	CRA004775;Yang2025_Silkie_Wenchang	PRJNA827465	0.996066018
ENSGALG00010014323	KLF4	2	0.16354023	0.14438231	1.132688876	0.257344011	0	0.304929428	0	1	CRA004775;PRJNA827465	Yang2025_Silkie_Wenchang	0.764987374
ENSGALG00010014324	COL4A2	2	-0.193458133	0.113340906	-1.706869478	0.087846315	0.014286855	2.251418261	55.58355293	1	PRJNA827465;Yang2025_Silkie_Wenchang	CRA004775	0.509491082
ENSGALG00010014324	COL4A2	2	0.14898864	0.521544299	0.285668237	0.775132235	0.469973031	6.646827681	84.95522905	0.5	CRA004775;Yang2025_Silkie_Wenchang	PRJNA827465	0.943803161
ENSGALG00010014324	COL4A2	2	0.243087237	0.403278751	0.602777203	0.546669015	0.257721678	4.11655122	75.70782078	0.5	CRA004775;PRJNA827465	Yang2025_Silkie_Wenchang	0.878275987
ENSGALG00010014328	RASSF8	2	0.078783739	0.132673457	0.593816888	0.552634593	0.027984304	4.727306397	78.84630451	0.5	PRJNA827465;Yang2025_Silkie_Wenchang	CRA004775	0.87928478
ENSGALG00010014328	RASSF8	2	0.14493072	0.16469764	0.879980559	0.378869841	0.02267254	1.341282027	25.44446433	0.5	CRA004775;Yang2025_Silkie_Wenchang	PRJNA827465	0.823876439
ENSGALG00010014328	RASSF8	2	-0.047745943	0.064538751	-0.739802715	0.459419711	0	0.191732679	0	1	CRA004775;PRJNA827465	Yang2025_Silkie_Wenchang	0.736936675
ENSGALG00010014329	IER5	2	0.189264194	0.097501499	1.941141366	0.05224131	0.000340562	1.011756265	1.160012321	1	PRJNA827465;Yang2025_Silkie_Wenchang	CRA004775	0.396636711
ENSGALG00010014329	IER5	2	0.20634619	0.223753526	0.922203076	0.356422659	0.034595087	1.484395515	32.63251002	0.5	CRA004775;Yang2025_Silkie_Wenchang	PRJNA827465	0.812521698
ENSGALG00010014329	IER5	2	0.116612969	0.101716179	1.146454481	0.251607151	0	0.440711052	0	0.5	CRA004775;PRJNA827465	Yang2025_Silkie_Wenchang	0.761706574
ENSGALG00010014334	HMGCS1	2	0.144123846	0.07649677	1.884051387	0.059558025	0	0.233189893	0	1	PRJNA827465;Yang2025_Silkie_Wenchang	CRA004775	0.422736519
ENSGALG00010014334	HMGCS1	2	0.087168765	0.122656824	0.710671956	0.477287542	0	0.000887273	0	1	CRA004775;Yang2025_Silkie_Wenchang	PRJNA827465	0.854997403
ENSGALG00010014334	HMGCS1	2	0.157417391	0.084020173	1.873566605	0.060990185	0	0.088872905	0	1	CRA004775;PRJNA827465	Yang2025_Silkie_Wenchang	0.427213905
ENSGALG00010014338	BHLHE41	2	0.078491129	0.298020551	0.263378488	0.792261644	0.152708539	7.099690983	85.91487992	0.5	PRJNA827465;Yang2025_Silkie_Wenchang	CRA004775	0.948368502
ENSGALG00010014338	BHLHE41	2	0.366885735	0.146999137	2.495846175	0.012565708	0	0.037582674	0	1	CRA004775;Yang2025_Silkie_Wenchang	PRJNA827465	0.183574995
ENSGALG00010014338	BHLHE41	2	-0.002876956	0.259846296	-0.011071762	0.991166193	0.090734274	2.788564584	64.13925624	0.5	CRA004775;PRJNA827465	Yang2025_Silkie_Wenchang	0.99755571
ENSGALG00010014340	EFNB1	2	0.043927863	0.096258333	0.456353869	0.648135532	0.012479911	3.054169677	67.25787675	0.5	PRJNA827465;Yang2025_Silkie_Wenchang	CRA004775	0.90762535
ENSGALG00010014340	EFNB1	2	0.151741684	0.070885547	2.140657577	0.032301662	0	0.583152857	0	1	CRA004775;Yang2025_Silkie_Wenchang	PRJNA827465	0.30859315
ENSGALG00010014340	EFNB1	2	0.18664471	0.358397942	0.520783488	0.720045531	0.042618253	2.098535174	52.3477132	0.5	CRA004775;PRJNA827465	Yang2025_Silkie_Wenchang	0.928564173
ENSGALG00010014344	LSG1	2	0.156030979	0.195062325	0.799903204	0.423766881	0.068781959	10.09173477	90.0909009	0.5	PRJNA827465;Yang2025_Silkie_Wenchang	CRA004775	0.84030043
ENSGALG00010014344	LSG1	2	0.009595334	0.367512075	0.026108895	0.979170483	0.247728236	11.84493632	91.55757386	0.5	CRA004775;Yang2025_Silkie_Wenchang	PRJNA827465	0.994324552
ENSGALG00010014344	LSG1	2	-0.156330213	0.166643181	-0.938113469	0.348186101	0.040979314	3.216825154	68.91344876	1	CRA004775;PRJNA827465	Yang2025_Silkie_Wenchang	0.808983112
ENSGALG00010014346	AFG1L	2	0.000941019	0.089944174	0.010521026	0.991605591	0	0.512853448	0	0.5	PRJNA827465;Yang2025_Silkie_Wenchang	CRA004775	0.997575757
ENSGALG00010014346	AFG1L	2	-0.0949917	0.105957671	-0.89650611	0.369982518	0	0.1734587	0	1	CRA004775;Yang2025_Silkie_Wenchang	PRJNA827465	0.820855964
ENSGALG00010014346	AFG1L	2	-0.004946679	0.103958963	-0.047582999	0.962048582	0	0.975803471	0	0.5	CRA004775;PRJNA827465	Yang2025_Silkie_Wenchang	0.989878931
ENSGALG00010014347	KIAA0040	2	0.293372479	0.246606809	1.189636573	0.234189265	0.018684487	1.180848644	15.25744342	1	PRJNA827465;Yang2025_Silkie_Wenchang	CRA004775	0.747408905
ENSGALG00010014347	KIAA0040	2	0.06941352	0.476869181	0.145560927	0.884268015	0.355401654	4.55974294	78.06893912	0.5	CRA004775;Yang2025_Silkie_Wenchang	PRJNA827465	0.972001076
ENSGALG00010014347	KIAA0040	2	-0.171905577	0.229487259	-0.749085496	0.453805677	0.015728317	1.175250751	14.9117753	0.5	CRA004775;PRJNA827465	Yang2025_Silkie_Wenchang	0.849044791
ENSGALG00010014350	PTGER4	2	0.184358712	0.13804234	1.335522945	0.181705251	0.005786162	1.166211744	14.25227839	1	PRJNA827465;Yang2025_Silkie_Wenchang	CRA004775	0.689140352
ENSGALG00010014350	PTGER4	2	0.007233189	0.388401878	0.018622951	0.985141894	0.231923317	4.209734995	76.24553562	0.5	CRA004775;Yang2025_Silkie_Wenchang	PRJNA827465	0.995309331
ENSGALG00010014350	PTGER4	2	-0.094725201	0.235670574	-0.401939026	0.687728901	0.060316032	1.969580257	49.22776076	0.5	CRA004775;PRJNA827465	Yang2025_Silkie_Wenchang	0.919631238
ENSGALG00010014355	RAB20	2	0.087456459	0.138409142	0.631869095	0.527472419	0.025331896	2.623204387	61.87868529	0.5	PRJNA827465;Yang2025_Silkie_Wenchang	CRA004775	0.871149047
ENSGALG00010014355	RAB20	2	0.251916237	0.143102605	1.760388892	0.078341891	0	0.040048951	0	1	CRA004775;Yang2025_Silkie_Wenchang	PRJNA827465	0.481707034
ENSGALG00010014355	RAB20	2	-0.00830085	0.070518448	-0.117711756	0.906296051	0	0.457080649	0	0.5	CRA004775;PRJNA827465	Yang2025_Silkie_Wenchang	0.978196186
ENSGALG00010014361	SSPN	2	-0.023290305	0.055689214	-0.41821932	0.675786772	0	0.306126405	0	0.5	PRJNA827465;Yang2025_Silkie_Wenchang	CRA004775	0.916522765
ENSGALG00010014361	SSPN	2	0.017561305	0.078098533	0.224860876	0.822087506	0	0.456245551	0	1	CRA004775;Yang2025_Silkie_Wenchang	PRJNA827465	0.955636543
ENSGALG00010014361	SSPN	2	-0.042449369	0.076940504	-0.551716808	0.581142393	0	0.663128548	0	0.5	CRA004775;PRJNA827465	Yang2025_Silkie_Wenchang	0.88890219
ENSGALG00010014362	NAXD	2	0.055167307	0.047537544	1.160499737	0.245845401	0	0.105955007	0	1	PRJNA827465;Yang2025_Silkie_Wenchang	CRA004775	0.75661406
ENSGALG00010014362	NAXD	2	0.059466319	0.076137946	0.781033926	0.434782541	0	0.756992519	0	0.5	CRA004775;Yang2025_Silkie_Wenchang	PRJNA827465	0.842762754
ENSGALG00010014362	NAXD	2	0.034870579	0.058829836	0.59273629	0.553357651	0	0.61327154	0	0.5	CRA004775;PRJNA827465	Yang2025_Silkie_Wenchang	0.879487916
ENSGALG00010014367	OPHN1	2	0.058666418	0.051771312	1.13318392	0.257137025	0.000576472	1.081267992	7.51598983	1	PRJNA827465;Yang2025_Silkie_Wenchang	CRA004775	0.764870705
ENSGALG00010014367	OPHN1	2	-0.071673604	0.264425526	-0.271054029	0.786349478	0.109806893	4.281490635	76.64364855	0.5	CRA004775;Yang2025_Silkie_Wenchang	PRJNA827465	0.946639719
ENSGALG00010014367	OPHN1	2	-0.110909484	0.195481441	-0.567365802	0.570465679	0.055387166	2.893330741	65.43775706	0.5	CRA004775;PRJNA827465	Yang2025_Silkie_Wenchang	0.885121495
ENSGALG00010014371	TTC33	2	0.026846475	0.098852573	0.271580945	0.785944252	0	0.00387966	0	1	PRJNA827465;Yang2025_Silkie_Wenchang	CRA004775	0.946639719
ENSGALG00010014371	TTC33	2	-0.038660621	0.127073435	-0.304238418	0.760946258	0	0.938169756	0	0.5	CRA004775;Yang2025_Silkie_Wenchang	PRJNA827465	0.939493404
ENSGALG00010014371	TTC33	2	-0.038032849	0.117695439	-0.323146327	0.746584427	0	0.899653085	0	0.5	CRA004775;PRJNA827465	Yang2025_Silkie_Wenchang	0.936668296
ENSGALG00010014372	CCDC171	2	0.158207048	0.205708364	0.769084178	0.44184334	0.049182761	2.348236626	57.4148538	0.5	PRJNA827465;Yang2025_Silkie_Wenchang	CRA004775	0.845135451
ENSGALG00010014372	CCDC171	2	0.348336225	0.155197512	2.24447043	0.024802149	0	0.071698222	0	1	CRA004775;Yang2025_Silkie_Wenchang	PRJNA827465	0.268761753
ENSGALG00010014372	CCDC171	2	0.103568105	0.161442075	0.64151867	0.521185755	0.015780184	1.405664109	28.85924927	0.5	CRA004775;PRJNA827465	Yang2025_Silkie_Wenchang	0.869518515
ENSGALG00010014375	H2BC35	2	0.537284815	0.485956265	1.105623806	0.268889381	0.310281799	2.911851784	65.65759269	1	PRJNA827465;Yang2025_Silkie_Wenchang	CRA004775	0.771398294
ENSGALG00010014375	H2BC35	2	0.274795338	0.760450746	0.361358496	0.71783147	0.982588421	6.647630961	84.95704702	0.5	CRA004775;Yang2025_Silkie_Wenchang	PRJNA827465	0.927927766
ENSGALG00010014375	H2BC35	2	-0.191867314	0.284494952	-0.674413774	0.500048288	0	0.92519651	0	0.5	CRA004775;PRJNA827465	Yang2025_Silkie_Wenchang	0.862142388
ENSGALG00010014379	CARS2	2	-0.002615598	0.047715077	-0.054817013	0.956284246	0	0.14539984	0	0.5	PRJNA827465;Yang2025_Silkie_Wenchang	CRA004775	0.988528437
ENSGALG00010014379	CARS2	2	-0.082517384	0.115886204	-0.712055283	0.476430543	0.00864712	1.221032616	18.10210583	1	CRA004775;Yang2025_Silkie_Wenchang	PRJNA827465	0.854986476
ENSGALG00010014379	CARS2	2	-0.06823356	0.145045982	-0.470427096	0.638049909	0.023795861	1.643207573	39.1434158	0.5	CRA004775;PRJNA827465	Yang2025_Silkie_Wenchang	0.905365679
ENSGALG00010014380	SNX3	2	-0.07083567	0.052309351	-1.354168393	0.17568266	0	0.298512504	0	1	PRJNA827465;Yang2025_Silkie_Wenchang	CRA004775	0.6803934
ENSGALG00010014380	SNX3	2	-0.123257908	0.122538846	-1.005868032	0.314479056	0	0.083883749	0	1	CRA004775;Yang2025_Silkie_Wenchang	PRJNA827465	0.796531448
ENSGALG00010014380	SNX3	2	-0.062051068	0.053402002	-1.161961447	0.245251126	0	0.007812355	0	1	CRA004775;PRJNA827465	Yang2025_Silkie_Wenchang	0.75661406
ENSGALG00010014388	MFNG	2	-0.283880715	0.234432079	-1.210929481	0.225922433	0.064710673	2.417884614	58.64153341	1	PRJNA827465;Yang2025_Silkie_Wenchang	CRA004775	0.739213234
ENSGALG00010014388	MFNG	2	-0.681187295	0.237612351	-2.866800876	0.004146438	0.025726616	1.2242977	18.32051961	1	CRA004775;Yang2025_Silkie_Wenchang	PRJNA827465	0.093615215
ENSGALG00010014388	MFNG	2	-0.494489465	0.495073699	-0.998819904	0.317881942	0.391951335	4.636315522	78.43114871	1	CRA004775;PRJNA827465	Yang2025_Silkie_Wenchang	0.797439961
ENSGALG00010014389	COL6A2	2	-0.171240082	0.068439816	-2.502053525	0.012347526	0	0.003224651	0	1	PRJNA827465;Yang2025_Silkie_Wenchang	CRA004775	0.179672269
ENSGALG00010014389	COL6A2	2	-0.147286599	0.149716971	-0.98376689	0.325230142	0	0.122281047	0	0.5	CRA004775;Yang2025_Silkie_Wenchang	PRJNA827465	0.799957462
ENSGALG00010014389	COL6A2	2	-0.168882346	0.075278111	-2.243445591	0.024868095	0	0.144905889	0	0.5	CRA004775;PRJNA827465	Yang2025_Silkie_Wenchang	0.268957272
ENSGALG00010014390	SPARC	2	-0.001239403	0.103412421	-0.011985054	0.99043754	0	2.67258E-07	0	1	PRJNA827465;Yang2025_Silkie_Wenchang	CRA004775	0.997468525
ENSGALG00010014390	SPARC	2	0.004858947	0.177182743	0.027423363	0.978122064	0	0.008287275	0	0.5	CRA004775;Yang2025_Silkie_Wenchang	PRJNA827465	0.993901897
ENSGALG00010014390	SPARC	2	0.00149223	0.12000649	0.012434577	0.990078899	0	0.008953669	0	0.5	CRA004775;PRJNA827465	Yang2025_Silkie_Wenchang	0.997368295
ENSGALG00010014393	ATP8A1	2	-0.018040821	0.082167026	-0.219562789	0.826211674	0	0.10321732	0	0.5	PRJNA827465;Yang2025_Silkie_Wenchang	CRA004775	0.956393839
ENSGALG00010014393	ATP8A1	2	0.043604596	0.157621547	0.276641084	0.782055715	0	0.047460769	0	1	CRA004775;Yang2025_Silkie_Wenchang	PRJNA827465	0.945372683
ENSGALG00010014393	ATP8A1	2	-0.02499328	0.091620773	-0.27279054	0.785014232	0	0.13969324	0	0.5	CRA004775;PRJNA827465	Yang2025_Silkie_Wenchang	0.946325593
ENSGALG00010014396	ING1	2	-0.035251108	0.043698599	-0.80668739	0.419846611	0	0.068681357	0	1	PRJNA827465;Yang2025_Silkie_Wenchang	CRA004775	0.838582908
ENSGALG00010014396	ING1	2	-0.039560431	0.093725099	-0.422090042	0.672959296	0	0.332262603	0	0.5	CRA004775;Yang2025_Silkie_Wenchang	PRJNA827465	0.915153756
ENSGALG00010014396	ING1	2	-0.025493905	0.047940217	-0.531785342	0.594874676	0	0.250817274	0	0.5	CRA004775;PRJNA827465	Yang2025_Silkie_Wenchang	0.893382718
ENSGALG00010014397	PRKAA1	2	-0.035131145	0.143948006	-0.244054404	0.807188685	0.030532446	3.76053659	73.40805026	0.5	PRJNA827465;Yang2025_Silkie_Wenchang	CRA004775	0.95234753
ENSGALG00010014397	PRKAA1	2	-0.131104327	0.101014909	-1.297871064	0.194331645	0	0.964138027	0	0.5	CRA004775;Yang2025_Silkie_Wenchang	PRJNA827465	0.705116012
ENSGALG00010014397	PRKAA1	2	0.09030923	0.084171642	1.07291753	0.28330812	0	0.069151939	0	1	CRA004775;PRJNA827465	Yang2025_Silkie_Wenchang	0.779568117
ENSGALG00010014399	GAS6	2	-0.022552932	0.07326763	-0.307815771	0.75822252	0	0.467609735	0	0.5	PRJNA827465;Yang2025_Silkie_Wenchang	CRA004775	0.938954369
ENSGALG00010014399	GAS6	2	0.377064588	0.39380653	0.957486886	0.338321562	0.269627428	7.089585797	85.89480361	1	CRA004775;Yang2025_Silkie_Wenchang	PRJNA827465	0.804154375
ENSGALG00010014399	GAS6	2	0.33255603	0.444821205	0.747617304	0.454691024	0.353504739	8.848569718	88.69873853	0.5	CRA004775;PRJNA827465	Yang2025_Silkie_Wenchang	0.849072665
ENSGALG00010014400	RIPOR3	2	0.178252721	0.077452757	2.301438038	0.021366884	0	0.814390248	0	1	PRJNA827465;Yang2025_Silkie_Wenchang	CRA004775	0.247158031
ENSGALG00010014400	RIPOR3	2	0.212113691	0.147212096	1.440871348	0.149621032	0.002307423	1.051589323	4.905843213	1	CRA004775;Yang2025_Silkie_Wenchang	PRJNA827465	0.639445128
ENSGALG00010014400	RIPOR3	2	0.129801326	0.081038481	1.601724574	0.109216529	0	0.253164206	0	1	CRA004775;PRJNA827465	Yang2025_Silkie_Wenchang	0.559361118
ENSGALG00010014407	YIPF6	2	-0.066928706	0.039978467	-1.674118894	0.094107235	0	0.434138487	0	1	PRJNA827465;Yang2025_Silkie_Wenchang	CRA004775	0.525712768
ENSGALG00010014407	YIPF6	2	-0.153974752	0.090689254	-1.697827969	0.089540235	0	0.464698099	0	1	CRA004775;Yang2025_Silkie_Wenchang	PRJNA827465	0.513242777
ENSGALG00010014407	YIPF6	2	-0.073015631	0.062546211	-1.167386949	0.243054139	0.002316453	1.08846254	8.127293	1	CRA004775;PRJNA827465	Yang2025_Silkie_Wenchang	0.7550024
ENSGALG00010014408	ENSGALG00010014408	2	-0.222767278	0.338034279	-0.659007952	0.509890662	0.150474064	2.862768215	65.06877523	0.5	PRJNA827465;Yang2025_Silkie_Wenchang	CRA004775	0.865257661
ENSGALG00010014408	ENSGALG00010014408	2	1.177944778	1.093544679	1.077180293	0.281399727	2.138143514	9.10087356	89.01204381	1	CRA004775;Yang2025_Silkie_Wenchang	PRJNA827465	0.779226828
ENSGALG00010014408	ENSGALG00010014408	2	0.846252005	1.434543124	0.589910468	0.555250676	3.885100782	17.26504557	94.20795042	0.5	CRA004775;PRJNA827465	Yang2025_Silkie_Wenchang	0.880255849
ENSGALG00010014411	ATP13A3	2	0.019663758	0.097607647	0.201457144	0.840341136	0.010708466	2.233618957	55.22960634	0.5	PRJNA827465;Yang2025_Silkie_Wenchang	CRA004775	0.959662809
ENSGALG00010014411	ATP13A3	2	-0.097219657	0.10242659	-0.949164244	0.342537084	0	0.040959259	0	1	CRA004775;Yang2025_Silkie_Wenchang	PRJNA827465	0.806011702
ENSGALG00010014411	ATP13A3	2	0.09483171	0.076860035	1.233823398	0.217268719	0	0.47407468	0	0.5	CRA004775;PRJNA827465	Yang2025_Silkie_Wenchang	0.730855225
ENSGALG00010014414	TMEM255B	2	0.433205435	0.655508387	0.6608694	0.508696071	0.705110089	5.569563168	82.04527052	0.5	PRJNA827465;Yang2025_Silkie_Wenchang	CRA004775	0.865144635
ENSGALG00010014414	TMEM255B	2	-0.050745348	1.231256858	-0.041214266	0.967125081	2.664055614	7.928340461	87.38701996	0.5	CRA004775;Yang2025_Silkie_Wenchang	PRJNA827465	0.990893423
ENSGALG00010014414	TMEM255B	2	-0.59856226	0.543918441	-1.100463259	0.27113033	0.291551674	1.768944039	43.46909918	1	CRA004775;PRJNA827465	Yang2025_Silkie_Wenchang	0.772743863
ENSGALG00010014416	H2BC32	2	0.21282034	1.10573615	0.19246937	0.847374559	2.265714567	13.6087985	92.65181272	0.5	PRJNA827465;Yang2025_Silkie_Wenchang	CRA004775	0.961252712
ENSGALG00010014416	H2BC32	2	0.400845001	0.938517412	0.427104491	0.669303222	1.540713725	7.962350357	87.44089427	0.5	CRA004775;Yang2025_Silkie_Wenchang	PRJNA827465	0.914377769
ENSGALG00010014416	H2BC32	2	-0.752471652	0.313855449	-2.397510237	0.01650692	0	0.271974322	0	1	CRA004775;PRJNA827465	Yang2025_Silkie_Wenchang	0.212735188
ENSGALG00010014418	RAC2	2	-0.108176622	0.732527611	-0.147675828	0.882598609	1.038617631	30.96385922	96.77042841	0.5	PRJNA827465;Yang2025_Silkie_Wenchang	CRA004775	0.972001076
ENSGALG00010014418	RAC2	2	-0.861373689	0.193439552	-4.452934686	8.47045E-06	0	0.028723311	0	1	CRA004775;Yang2025_Silkie_Wenchang	PRJNA827465	0.001233016
ENSGALG00010014418	RAC2	2	-0.090637367	0.774707988	-0.116995524	0.906863601	1.077034801	9.102012891	89.01341921	0.5	CRA004775;PRJNA827465	Yang2025_Silkie_Wenchang	0.978196186
ENSGALG00010014427	TMEM267	2	0.00658216	0.111666698	0.058944702	0.952996153	0.006790179	1.306830622	23.47898932	0.5	PRJNA827465;Yang2025_Silkie_Wenchang	CRA004775	0.987758913
ENSGALG00010014427	TMEM267	2	-0.216013156	0.14212836	-1.519845555	0.128549797	0	0.26323496	0	1	CRA004775;Yang2025_Silkie_Wenchang	PRJNA827465	0.601638797
ENSGALG00010014427	TMEM267	2	-0.060394392	0.187959792	-0.321315487	0.747971321	0.044266791	2.386969721	58.1058783	0.5	CRA004775;PRJNA827465	Yang2025_Silkie_Wenchang	0.936938957
ENSGALG00010014431	ENSGALG00010014431	2	0.005789865	0.347415164	0.016665551	0.98670343	0	0.56824311	0	0.5	PRJNA827465;Yang2025_Silkie_Wenchang	CRA004775	0.996259695
ENSGALG00010014431	ENSGALG00010014431	2	-0.463825051	0.327306113	-1.417098651	0.156454087	0	0.226067284	0	1	CRA004775;Yang2025_Silkie_Wenchang	PRJNA827465	0.649484056
ENSGALG00010014431	ENSGALG00010014431	2	-0.197955396	0.420121294	-0.471186296	0.637507704	0.151451631	1.746661423	42.74791974	0.5	CRA004775;PRJNA827465	Yang2025_Silkie_Wenchang	0.905201059
ENSGALG00010014439	ENSGALG00010014439	2	0.337574551	0.559441161	0.603413861	0.546233403	0.561238323	9.636008947	89.62225953	0.5	PRJNA827465;Yang2025_Silkie_Wenchang	CRA004775	0.878012469
ENSGALG00010014439	ENSGALG00010014439	2	0.925624296	0.259345037	3.569084285	0.000358231	0	0.025558159	0	1	CRA004775;Yang2025_Silkie_Wenchang	PRJNA827465	0.018920289
ENSGALG00010014439	ENSGALG00010014439	2	0.235633058	0.594570775	0.396307838	0.691877946	0.50048682	2.876082331	65.23048074	0.5	CRA004775;PRJNA827465	Yang2025_Silkie_Wenchang	0.920348122
ENSGALG00010014449	ENSGALG00010014449	2	-0.171498599	0.390770632	-0.438872795	0.66075371	0	0.007084774	0	1	PRJNA827465;Yang2025_Silkie_Wenchang	CRA004775	0.91192683
ENSGALG00010014449	ENSGALG00010014449	2	-0.825311957	0.628498879	-1.313147858	0.18913313	0.458897466	2.350733643	57.46008898	1	CRA004775;Yang2025_Silkie_Wenchang	PRJNA827465	0.698162702
ENSGALG00010014449	ENSGALG00010014449	2	-0.786502421	0.59763459	-1.316025601	0.18816545	0.466514359	2.881639238	65.29752973	1	CRA004775;PRJNA827465	Yang2025_Silkie_Wenchang	0.697483156
ENSGALG00010014453	NR2E1	2	-0.370659315	0.23788102	-1.558171035	0.119192709	0.070146132	2.627441418	61.94015999	1	PRJNA827465;Yang2025_Silkie_Wenchang	CRA004775	0.579565885
ENSGALG00010014453	NR2E1	2	-0.149489244	0.51785554	-0.288669778	0.772834092	0.438588707	5.2190909	80.83957495	0.5	CRA004775;Yang2025_Silkie_Wenchang	PRJNA827465	0.943066211
ENSGALG00010014453	NR2E1	2	0.036559566	0.261632903	0.139736117	0.888868489	0.058971712	1.58348004	36.84795674	0.5	CRA004775;PRJNA827465	Yang2025_Silkie_Wenchang	0.973135755
ENSGALG00010014465	SIMC1	2	0.201647515	0.233736356	0.862713522	0.388294996	0.091703556	5.829589469	82.84613342	0.5	PRJNA827465;Yang2025_Silkie_Wenchang	CRA004775	0.828154121
ENSGALG00010014465	SIMC1	2	0.376700961	0.156288031	2.410299486	0.01593943	0.000426819	1.006344537	0.630453793	1	CRA004775;Yang2025_Silkie_Wenchang	PRJNA827465	0.208475018
ENSGALG00010014465	SIMC1	2	-0.000886288	0.07776137	-0.01139753	0.990906284	0	0.096102654	0	0.5	CRA004775;PRJNA827465	Yang2025_Silkie_Wenchang	0.997555603
ENSGALG00010014469	TMCO3	2	0.12437168	0.106153027	1.171626316	0.241347118	0.014123265	2.527779434	60.43958636	1	PRJNA827465;Yang2025_Silkie_Wenchang	CRA004775	0.753847713
ENSGALG00010014469	TMCO3	2	0.207623777	0.104451008	1.987762301	0.046837985	0	0.74626237	0	1	CRA004775;Yang2025_Silkie_Wenchang	PRJNA827465	0.37376485
ENSGALG00010014469	TMCO3	2	0.035916703	0.066656367	0.538833793	0.590001544	0	0.001395136	0	1	CRA004775;PRJNA827465	Yang2025_Silkie_Wenchang	0.892320726
ENSGALG00010014470	RPS6KA1	2	-0.134929262	0.126641277	-1.065444583	0.286674791	0.021862848	2.696462967	62.91438035	1	PRJNA827465;Yang2025_Silkie_Wenchang	CRA004775	0.782018115
ENSGALG00010014470	RPS6KA1	2	-0.359776091	0.140423293	-2.562082709	0.010404654	0.000379993	1.004639305	0.461788083	1	CRA004775;Yang2025_Silkie_Wenchang	PRJNA827465	0.162401701
ENSGALG00010014470	RPS6KA1	2	-0.271212597	0.318369186	-0.851880803	0.394280253	0.151718525	3.099402794	67.73571986	1	CRA004775;PRJNA827465	Yang2025_Silkie_Wenchang	0.830486298
ENSGALG00010014474	PTPN1	2	-0.143635838	0.071902392	-1.997650333	0.045754582	0.006554356	2.616137845	61.77571446	1	PRJNA827465;Yang2025_Silkie_Wenchang	CRA004775	0.369031879
ENSGALG00010014474	PTPN1	2	-0.22343987	0.07431023	-3.00685206	0.002639682	0	0.095632085	0	1	CRA004775;Yang2025_Silkie_Wenchang	PRJNA827465	0.069462139
ENSGALG00010014474	PTPN1	2	-0.082874654	0.048206441	-1.719161428	0.085584982	0	0.005284881	0	1	CRA004775;PRJNA827465	Yang2025_Silkie_Wenchang	0.502952534
ENSGALG00010014476	C5orf34	2	0.125605962	0.230902264	0.54397891	0.586455986	0.075319801	3.351425436	70.16194992	0.5	PRJNA827465;Yang2025_Silkie_Wenchang	CRA004775	0.891596903
ENSGALG00010014476	C5orf34	2	-0.125362518	0.570625128	-0.2196933	0.826110023	0.536412171	5.267627351	81.01612105	0.5	CRA004775;Yang2025_Silkie_Wenchang	PRJNA827465	0.956393839
ENSGALG00010014476	C5orf34	2	-0.294538568	0.315527567	-0.933479666	0.350572365	0.119344122	2.020874088	50.51646185	1	CRA004775;PRJNA827465	Yang2025_Silkie_Wenchang	0.810111037

ENSGALG00010014477	ITPR2	2	-0.11461459	0.144503813	-0.793159621	0.42768483	0.02910867	3.04210038	67.12797492	0.5	PRJNA827465;Yang2025_Silkie_Wenchang	CRA004775	0.84125685
ENSGALG00010014477	ITPR2	2	-0.301768029	0.144218107	-2.09244203	0.036398992	0	0.075629065	0	1	CRA004775;Yang2025_Silkie_Wenchang	PRJNA827465	0.32890494
ENSGALG00010014477	ITPR2	2	-0.005377411	0.076116122	-0.070647466	0.943678332	0	0.724549201	0	0.5	CRA004775;PRJNA827465	Yang2025_Silkie_Wenchang	0.985827325
ENSGALG00010014482	THOC3	2	-0.034748301	0.064861377	-0.535731791	0.592143929	0	0.000641758	0	1	PRJNA827465;Yang2025_Silkie_Wenchang	CRA004775	0.892776493
ENSGALG00010014482	THOC3	2	-0.24421924	0.221649197	-1.101827769	0.270536555	0.060769223	2.596624332	61.48846071	1	CRA004775;Yang2025_Silkie_Wenchang	PRJNA827465	0.772536273
ENSGALG00010014482	THOC3	2	-0.213471427	0.220064899	-0.97003851	0.332027297	0.075726424	4.000890557	75.00556474	1	CRA004775;PRJNA827465	Yang2025_Silkie_Wenchang	0.802002658
ENSGALG00010014487	OSTM1	2	-0.235600953	0.333901093	-0.705601023	0.480436288	0.213572228	23.69010314	95.778828	0.5	PRJNA827465;Yang2025_Silkie_Wenchang	CRA004775	0.856586256
ENSGALG00010014487	OSTM1	2	-0.571904277	0.098259273	-5.820359344	5.87212E-09	0	0.000855797	0	1	CRA004775;Yang2025_Silkie_Wenchang	PRJNA827465	3.07723E-06
ENSGALG00010014487	OSTM1	2	-0.156303198	0.328149134	-0.47631757	0.63384815	0.163345231	3.441652326	70.94418886	0.5	CRA004775;PRJNA827465	Yang2025_Silkie_Wenchang	0.903596555
ENSGALG00010014488	ENSGALG00010014488	2	0.028245916	0.156169929	0.180866549	0.856472326	0.033922151	3.202072657	68.77022768	0.5	PRJNA827465;Yang2025_Silkie_Wenchang	CRA004775	0.96353738
ENSGALG00010014488	ENSGALG00010014488	2	0.066083251	0.312964411	0.211152606	0.832768191	0.095773654	1.35262374	26.06961046	0.5	CRA004775;Yang2025_Silkie_Wenchang	PRJNA827465	0.957861248
ENSGALG00010014488	ENSGALG00010014488	2	-0.12263545	0.100034046	-1.225937123	0.220222337	0	0.553569138	0	1	CRA004775;PRJNA827465	Yang2025_Silkie_Wenchang	0.733812488
ENSGALG00010014492	CPLX2	2	0.373553396	0.394291024	0.947405277	0.343432303	0.249536814	4.753336294	78.96214494	1	PRJNA827465;Yang2025_Silkie_Wenchang	CRA004775	0.806276913
ENSGALG00010014492	CPLX2	2	0.543086758	0.324456295	1.673836401	0.094162756	0.076198832	1.546959444	35.35706422	1	CRA004775;Yang2025_Silkie_Wenchang	PRJNA827465	0.525712768
ENSGALG00010014492	CPLX2	2	0.044906586	0.151230658	0.296941018	0.76651154	0	0.096658208	0	1	CRA004775;PRJNA827465	Yang2025_Silkie_Wenchang	0.941179139
ENSGALG00010014494	KLHL9	2	-0.137937944	0.116139815	-1.187688682	0.234956084	0.022907237	6.554249331	84.74272263	1	PRJNA827465;Yang2025_Silkie_Wenchang	CRA004775	0.747791642
ENSGALG00010014494	KLHL9	2	-0.24745404	0.069619792	-3.554363367	0.000378895	0	0.290911295	0	1	CRA004775;Yang2025_Silkie_Wenchang	PRJNA827465	0.01951733
ENSGALG00010014494	KLHL9	2	-0.032291113	0.052506029	-0.614998188	0.538555917	0	0.190131568	0	1	CRA004775;PRJNA827465	Yang2025_Silkie_Wenchang	0.87481821
ENSGALG00010014504	SEC63	2	0.072596347	0.114890383	0.631874875	0.527468642	0.019756789	3.318043015	69.86175298	0.5	PRJNA827465;Yang2025_Silkie_Wenchang	CRA004775	0.871149047
ENSGALG00010014504	SEC63	2	0.230985521	0.09028742	2.5583356	0.010517454	0	0.010143247	0	1	CRA004775;Yang2025_Silkie_Wenchang	PRJNA827465	0.16333445
ENSGALG00010014504	SEC63	2	0.081903553	0.124334136	0.658737455	0.510064377	0.023745313	3.630404283	72.45485841	0.5	CRA004775;PRJNA827465	Yang2025_Silkie_Wenchang	0.865290927
ENSGALG00010014505	CYTH4	2	-0.102856467	0.288253035	-0.356827004	0.721221319	0.137744811	5.716066286	82.5054513	0.5	PRJNA827465;Yang2025_Silkie_Wenchang	CRA004775	0.928950734
ENSGALG00010014505	CYTH4	2	-0.430518022	0.179719877	-2.395494746	0.016597952	0	0.044049943	0	1	CRA004775;Yang2025_Silkie_Wenchang	PRJNA827465	0.213438855
ENSGALG00010014505	CYTH4	2	-0.064604805	0.31899115	-0.202528518	0.839503566	0.137273797	2.556866026	60.88962073	0.5	CRA004775;PRJNA827465	Yang2025_Silkie_Wenchang	0.959493354
ENSGALG00010014512	HMGN4	2	-0.321711737	0.228418931	-1.408453181	0.158994021	0.096009946	12.01587756	91.67767818	1	PRJNA827465;Yang2025_Silkie_Wenchang	CRA004775	0.653803724
ENSGALG00010014512	HMGN4	2	-0.417237965	0.192768667	-2.16444909	0.0304299	0.043773967	2.211822831	54.78842221	1	CRA004775;Yang2025_Silkie_Wenchang	PRJNA827465	0.297477394
ENSGALG00010014512	HMGN4	2	-0.108991722	0.048159843	-2.263124547	0.023628013	0	0.056236709	0	1	CRA004775;PRJNA827465	Yang2025_Silkie_Wenchang	0.262343574
ENSGALG00010014515	ETNK1	2	-0.029349514	0.151836184	-0.193297234	0.846726192	0.032433689	3.128796213	68.03882605	0.5	PRJNA827465;Yang2025_Silkie_Wenchang	CRA004775	0.961252712
ENSGALG00010014515	ETNK1	2	-0.212823088	0.145503705	-1.462664191	0.143559234	0	0.000493172	0	1	CRA004775;Yang2025_Silkie_Wenchang	PRJNA827465	0.630564924
ENSGALG00010014515	ETNK1	2	0.086251952	0.079905585	1.07942332	0.28039906	0	0.523464172	0	0.5	CRA004775;PRJNA827465	Yang2025_Silkie_Wenchang	0.778536485
ENSGALG00010014517	DCUN1D2	2	0.193665075	0.074957247	2.583673815	0.00977542	0	0.571134051	0	1	PRJNA827465;Yang2025_Silkie_Wenchang	CRA004775	0.155979876
ENSGALG00010014517	DCUN1D2	2	0.127772663	0.198155838	0.644808974	0.519050985	0.043858799	1.941945625	48.50525231	0.5	CRA004775;Yang2025_Silkie_Wenchang	PRJNA827465	0.869093943
ENSGALG00010014517	DCUN1D2	2	0.106532479	0.105512582	1.009666117	0.312655281	0.003686607	1.084497904	7.791430807	0.5	CRA004775;PRJNA827465	Yang2025_Silkie_Wenchang	0.796054997
ENSGALG00010014537	GKAP1	2	0.40050447	0.384814332	1.040773268	0.297980789	0.271279812	11.76372365	91.49929028	1	PRJNA827465;Yang2025_Silkie_Wenchang	CRA004775	0.788979318
ENSGALG00010014537	GKAP1	2	0.581100697	0.281668911	2.063062958	0.039106647	0.0915229	2.170759667	53.93317762	1	CRA004775;Yang2025_Silkie_Wenchang	PRJNA827465	0.341557454
ENSGALG00010014537	GKAP1	2	0.049307399	0.11717568	0.420798909	0.673901928	0	0.256708713	0	1	CRA004775;PRJNA827465	Yang2025_Silkie_Wenchang	0.915627873
ENSGALG00010014539	PAIP1	2	-0.142607217	0.123374771	-1.155886371	0.247727638	0.025308181	5.644914001	82.28493827	1	PRJNA827465;Yang2025_Silkie_Wenchang	CRA004775	0.7580874
ENSGALG00010014539	PAIP1	2	-0.117066748	0.175088979	-0.668612886	0.50374245	0.048369975	4.634545881	78.42291293	0.5	CRA004775;Yang2025_Silkie_Wenchang	PRJNA827465	0.86322274
ENSGALG00010014539	PAIP1	2	-0.019289485	0.045518625	-0.423771267	0.671732635	0	0.522082949	0	0.5	CRA004775;PRJNA827465	Yang2025_Silkie_Wenchang	0.914674175
ENSGALG00010014547	DHDDS	2	0.151113326	0.055422218	2.726583683	0.006399368	0	0.180180572	0	1	PRJNA827465;Yang2025_Silkie_Wenchang	CRA004775	0.12162204
ENSGALG00010014547	DHDDS	2	-0.008888995	0.124522095	-0.071384884	0.94309144	0.001251065	1.039744789	3.822552383	0.5	CRA004775;Yang2025_Silkie_Wenchang	PRJNA827465	0.985827325
ENSGALG00010014547	DHDDS	2	0.051341219	0.153806741	0.33380344	0.738527888	0.031968683	2.504526189	60.07228815	0.5	CRA004775;PRJNA827465	Yang2025_Silkie_Wenchang	0.934485814
ENSGALG00010014567	UTP11	2	-0.097834692	0.150354101	-0.650695198	0.515242263	0.023383645	1.93123302	48.21960947	0.5	PRJNA827465;Yang2025_Silkie_Wenchang	CRA004775	0.8671413
ENSGALG00010014567	UTP11	2	-0.022305906	0.152109417	-0.146643817	0.883413166	0	0.998538175	0	0.5	CRA004775;Yang2025_Silkie_Wenchang	PRJNA827465	0.972001076
ENSGALG00010014567	UTP11	2	-0.213162955	0.101671576	-2.096583556	0.036030456	0	6.55009E-06	0	1	CRA004775;PRJNA827465	Yang2025_Silkie_Wenchang	0.327574606
ENSGALG00010014570	KIF27	2	0.10576717	0.218312971	0.484474968	0.628048826	0.068831339	3.414287425	70.71131175	0.5	PRJNA827465;Yang2025_Silkie_Wenchang	CRA004775	0.901904489
ENSGALG00010014570	KIF27	2	0.305523643	0.192671158	1.585725886	0.112801504	0	0.512092879	0	0.5	CRA004775;Yang2025_Silkie_Wenchang	PRJNA827465	0.566790786
ENSGALG00010014570	KIF27	2	-0.081766097	0.117479875	-0.696000891	0.486428272	0	0.002461481	0	1	CRA004775;PRJNA827465	Yang2025_Silkie_Wenchang	0.857603818
ENSGALG00010014572	IL1RAP	2	0.070057587	0.160823135	0.435618837	0.663113302	0.035936355	3.238080159	69.11750325	0.5	PRJNA827465;Yang2025_Silkie_Wenchang	CRA004775	0.91293776
ENSGALG00010014572	IL1RAP	2	-0.105761808	0.103711651	-1.01976786	0.307838569	0	0.428550238	0	1	CRA004775;Yang2025_Silkie_Wenchang	PRJNA827465	0.793203994
ENSGALG00010014572	IL1RAP	2	0.040934532	0.255483624	0.160223701	0.872730486	0.086254018	2.669496964	62.53849588	0.5	CRA004775;PRJNA827465	Yang2025_Silkie_Wenchang	0.969629321
ENSGALG00010014573	CEBPB	2	0.014857815	0.14620784	0.101621191	0.919057359	0.007167202	1.185485651	15.64638516	0.5	PRJNA827465;Yang2025_Silkie_Wenchang	CRA004775	0.981088364
ENSGALG00010014573	CEBPB	2	-0.161070962	0.205126831	-0.785226202	0.432320955	0	0.025013403	0	1	CRA004775;Yang2025_Silkie_Wenchang	PRJNA827465	0.842762754
ENSGALG00010014573	CEBPB	2	0.08300085	0.155704927	0.683811631	0.494094121	0	0.165988706	0	0.5	CRA004775;PRJNA827465	Yang2025_Silkie_Wenchang	0.860175061
ENSGALG00010014576	HRH2	2	0.084945482	0.218746621	0.388328202	0.69777317	0	0.453254077	0	0.5	PRJNA827465;Yang2025_Silkie_Wenchang	CRA004775	0.921422004
ENSGALG00010014576	HRH2	2	-0.455916039	0.452666157	-1.007179424	0.313848559	0.279174043	3.053861298	67.25457045	1	CRA004775;Yang2025_Silkie_Wenchang	PRJNA827465	0.796531448
ENSGALG00010014576	HRH2	2	-0.326753708	0.60235684	-0.542458699	0.587502552	0.584752772	5.086904789	80.34168042	0.5	CRA004775;PRJNA827465	Yang2025_Silkie_Wenchang	0.89167336
ENSGALG00010014578	ENSGALG00010014578	2	0.186313223	0.366227925	0.508735708	0.61093749	0.24188632	10.16092467	90.15837601	0.5	PRJNA827465;Yang2025_Silkie_Wenchang	CRA004775	0.897506931
ENSGALG00010014578	ENSGALG00010014578	2	0.506932113	0.16171152	3.134792834	0.001719755	0	0.682739987	0	1	CRA004775;Yang2025_Silkie_Wenchang	PRJNA827465	0.052473127
ENSGALG00010014578	ENSGALG00010014578	2	-0.142976719	0.143821603	-0.994125477	0.320161784	0	0.460487867	0	0.5	CRA004775;PRJNA827465	Yang2025_Silkie_Wenchang	0.798348216
ENSGALG00010014579	NNT	2	-0.136203475	0.072590361	-1.877018846	0.060515311	0	0.058935025	0	1	PRJNA827465;Yang2025_Silkie_Wenchang	CRA004775	0.426049285
ENSGALG00010014579	NNT	2	0.161592727	0.313831844	0.514902263	0.606621319	0.158606438	4.773492664	79.05097859	0.5	CRA004775;Yang2025_Silkie_Wenchang	PRJNA827465	0.896799223
ENSGALG00010014579	NNT	2	0.14042941	0.332072972	0.422887201	0.672377561	0.186052557	5.816266436	82.8068399	0.5	CRA004775;PRJNA827465	Yang2025_Silkie_Wenchang	0.914742762
ENSGALG00010014592	QNG1	2	0.136653864	0.10655404	1.282484115	0.199672874	0	0.228015758	0	1	PRJNA827465;Yang2025_Silkie_Wenchang	CRA004775	0.71155526
ENSGALG00010014592	QNG1	2	-0.068311061	0.232927813	-0.293274198	0.769304019	0.072010189	2.936596158	65.946969	0.5	CRA004775;Yang2025_Silkie_Wenchang	PRJNA827465	0.986321197
ENSGALG00010014592	QNG1	2	-0.049263373	0.177849927	-0.276994057	0.78178467	0.033678799	2.041494287	51.01627243	0.5	CRA004775;PRJNA827465	Yang2025_Silkie_Wenchang	0.945265125
ENSGALG00010014599	SSTR3	2	0.316853117	0.320169823	0.989640791	0.322349725	0	0.94291703	0	1	PRJNA827465;Yang2025_Silkie_Wenchang	CRA004775	0.798652022
ENSGALG00010014599	SSTR3	2	0.079376244	0.674945036	0.117604011	0.906381426	0.573941535	2.641669072	62.14514487	0.5	CRA004775;Yang2025_Silkie_Wenchang	PRJNA827465	0.978196186
ENSGALG00010014599	SSTR3	2	-0.153608949	0.357631325	-0.429517602	0.667546587	0	0.839374268	0	0.5	CRA004775;PRJNA827465	Yang2025_Silkie_Wenchang	0.919375598
ENSGALG00010014603	ENSGALG00010014603	2	-0.227064638	0.216715797	-1.047753055	0.294752397	0.058115954	2.209809272	54.7472258	1	PRJNA827465;Yang2025_Silkie_Wenchang	CRA004775	0.787482391
ENSGALG00010014603	ENSGALG00010014603	2	-0.190448052	0.377439482	-0.504579041	0.613854534	0.177548073	2.619157608	61.81978523	0.5	CRA004775;Yang2025_Silkie_Wenchang	PRJNA827465	0.898256044
ENSGALG00010014603	ENSGALG00010014603	2	-0.050658835	0.105058923	-0.481690019	0.630025167	0	0.599474201	0	0.5	CRA004775;PRJNA827465	Yang2025_Silkie_Wenchang	0.902533841
ENSGALG00010014611	TMEM189	2	-0.106862359	0.046318942	-2.307098422	0.021049334	0	0.845070269	0	1	PRJNA827465;Yang2025_Silkie_Wenchang	CRA004775	0.245414483
ENSGALG00010014611	TMEM189	2	-0.170024123	0.099763154	-1.704277738	0.088329212	0	0.471552505	0	0.5	CRA004775;Yang2025_Silkie_Wenchang	PRJNA827465	0.510441217
ENSGALG00010014611	TMEM189	2	-0.082989324	0.05075393	-1.635130993	0.10202159	0	0.129475797	0	0.5	CRA004775;PRJNA827465	Yang2025_Silkie_Wenchang	0.544433749
ENSGALG00010014614	GHR	2	-0.076573718	0.145320482	-0.526929776	0.598242216	0.026304912	2.646414449	62.21302373	0.5	PRJNA827465;Yang2025_Silkie_Wenchang	CRA004775	0.894645663
ENSGALG00010014614	GHR	2	-0.044149667	0.258927017	-0.170510081	0.864609007	0.083020986	2.219317813	54.94110874	0.5	CRA004775;Yang2025_Silkie_Wenchang	PRJNA827465	0.966723659
ENSGALG00010014614	GHR	2	0.092345189	0.113970439	0.810255622	0.417792376	0	0.502681525	0	1	CRA004775;PRJNA827465	Yang2025_Silkie_Wenchang	0.838112595
ENSGALG00010014617	GRTP1	2	-0.168575584	0.220606922	-0.764144582	0.444781088	0.087012465	9.282790277	89.227377	0.5	PRJNA827465;Yang2025_Silkie_Wenchang	CRA004775	0.846013379
ENSGALG00010014617	GRTP1	2	-0.368065823	0.114374335	-3.218080552	0.001291956	0	0.579394201	0	1	CRA004775;Yang2025_Silkie_Wenchang	PRJNA827465	0.043612329
ENSGALG00010014617	GRTP1	2	0.034252488	0.077216851	0.443588257	0.657340297	0	0.221708037	0	0.5	CRA004775;PRJNA827465	Yang2025_Silkie_Wenchang	0.910711339
ENSGALG00010014626	C2CD5	2	0.200452851	0.202583054	0.989484793	0.322426007	0.073959104	9.908418417	89.90757195	1	PRJNA827465;Yang2025_Silkie_Wenchang	CRA004775	0.798652022
ENSGALG00010014626	C2CD5	2	0.290914855	0.151502339	1.920200417	0.054832589	0.025639494	2.146977891	53.28800787	1	CRA004775;Yang2025_Silkie_Wenchang	PRJNA827465	0.408539602
ENSGALG00010014626	C2CD5	2	0.018547379	0.062523636	0.296645875	0.766736883	0	0.244523002	0	1	CRA004775;PRJNA827465	Yang2025_Silkie_Wenchang	0.941279392
ENSGALG00010014632	NWD2	2	0.291381468	0.215415013	1.352651627	0.176166951	0.054471084	2.215720339	54.86795051	1	PRJNA827465;Yang2025_Silkie_Wenchang	CRA004775	0.681284038
ENSGALG00010014632	NWD2	2	0.45203768	0.233718819	1.934109035	0.053099723	0	0.912427901	0	1	CRA004775;Yang2025_Silkie_Wenchang	PRJNA827465	0.399631766
ENSGALG00010014632	NWD2	2	0.11872076	0.134313846	0.883905594	0.376747177	0	0.031922753	0	1	CRA004775;PRJNA827465	Yang2025_Silkie_Wenchang	0.822974013
ENSGALG00010014634	CCDC152	2	-1.191548338	0.270943046	-4.397781586	1.09363E-05	0	0.027716032	0	1	PRJNA827465;Yang2025_Silkie_Wenchang	CRA004775	0.001508173
ENSGALG00010014634	CCDC152	2	-0.401908374	0.825450877	-0.486895569	0.62633234	1.228612954	10.14401464	90.14197006	0.5	CRA004775;Yang2025_Silkie_Wenchang	PRJNA827465	0.901504244
ENSGALG00010014634	CCDC152	2	-0.359556211	0.780343993	-0.460756296	0.644966285	1.088971342	9.439443289	89.4061549	0.5	CRA004775;PRJNA827465	Yang2025_Silkie_Wenchang	0.906966303
ENSGALG00010014641	DOCK11	2	-0.050761251	0.059068377	-0.85936424	0.390139592	0	0.146227195	0	1	PRJNA827465;Yang2025_Silkie_Wenchang	CRA004775	0.829204867
ENSGALG00010014641	DOCK11	2	-0.250864919	0.360524775	-0.695832676	0.486533623	0.173388136	2.255544689	55.66481105	1	CRA004775;Yang2025_Silkie_Wenchang	PRJNA827465	0.857603818
ENSGALG00010014641	DOCK11	2	-0.260436816	0.325906364	-0.799115466	0.424223462	0.139088782	2.030987784	50.76287469	1	CRA004775;PRJNA827465	Yang2025_Silkie_Wenchang	0.840555291
ENSGALG00010014645	SLC36A1	2	-0.010832099	0.085668386	-0.126442193	0.899381905	0.000698336	1.043992914	4.213909276	0.5	PRJNA827465;Yang2025_Silkie_Wenchang	CRA004775	0.976081354
ENSGALG00010014645	SLC36A1	2	-0.139920325	0.301969596	-0.463358983	0.643107074	0.132654941	3.366676662	70.29711789	0.5	CRA004775;Yang2025_Silkie_Wenchang	PRJNA827465	0.906634523
ENSGALG00010014645	SLC36A1	2	-0.194285796	0.195138137	-0.995632111	0.319428927	0.042985635	1.852067211	46.00627915	1	CRA004775;PRJNA827465	Yang2025_Silkie_Wenchang	0.798176306
ENSGALG00010014648	C1QTNF6	2	0.119439092	0.273597246	0.436550784	0.662437162	0.13189486	8.361200089	88.03999439	0.5	PRJNA827465;Yang2025_Silkie_Wenchang	CRA004775	0.912767065
ENSGALG00010014648	C1QTNF6	2	-0.653038636	0.660656862	-0.98846871	0.322923151	0.656382145	3.225096409	68.99317499	1	CRA004775;Yang2025_Silkie_Wenchang	PRJNA827465	0.79874391
ENSGALG00010014648	C1QTNF6	2	-0.43358504	0.952597427	-0.455160835	0.648993535	1.565150773	6.376855114	84.31828884	0.5	CRA004775;PRJNA827465	Yang2025_Silkie_Wenchang	0.907783669
ENSGALG00010014653	FAM162B	2	0.326467489	0.200965187	1.62449772	0.104269627	0.043034926	2.072635232	51.75224348	1	PRJNA827465;Yang2025_Silkie_Wenchang	CRA004775	0.548786638
ENSGALG00010014653	FAM162B	2	0.612922657	0.207739096	2.950444423	0.003173171	0	0.209081014	0	1	CRA004775;Yang2025_Silkie_Wenchang	PRJNA827465	0.078092601
ENSGALG00010014653	FAM162B	2	0.334554914	0.287648738	1.163067556	0.244802094	0.08819511	1.741363509	42.57373634	1	CRA004775;PRJNA827465	Yang2025_Silkie_Wenchang	0.756114592
ENSGALG00010014654	LAMP1	2	-0.125009497	0.151341859	-0.826007415	0.408799886	0.041483375	10.41796891	90.4012	0.5	PRJNA827465;Yang2025_Silkie_Wenchang	CRA004775	0.834305842
ENSGALG00010014654	LAMP1	2	-0.035828179	0.266327112	-0.134526968	0.892985887	0.125603129	8.359050236	88.03691841	0.5	CRA004775;Yang2025_Silkie_Wenchang	PRJNA827465	0.974381724
ENSGALG00010014654	LAMP1	2	0.081013526	0.101820725	0.795648681	0.426236263	0.011573774	1.763467562	43.29354156	1	CRA004775;PRJNA827465	Yang2025_Silkie_Wenchang	0.84125685
ENSGALG00010014655	FHL3	2	-0.226610699	0.13923021	-1.627597191	0.103610325	0.022733757	2.179446607	54.11679292	1	PRJNA827465;Yang2025_Silkie_Wenchang	CRA004775	0.547964315
ENSGALG00010014655	FHL3	2	-0.295168539	0.172241744	-1.713687593	0.086586117	0.012326497	1.218159189	17.9089228	1	CRA004775;Yang2025_Silkie_Wenchang	PRJNA827465	0.506111562
ENSGALG00010014655	FHL3	2	-0.115033865	0.0834565	-1.378369149	0.168089343	0	0.073460997	0	1	CRA004775;PRJNA827465	Yang2025_Silkie_Wenchang	0.668665533
ENSGALG00010014658	SELENOP1	2	-0.208297502	0.0860593	-2.420395018	0.015503655	0	0.008584843	0	1	PRJNA827465;Yang2025_Silkie_Wenchang	CRA004775	0.20599735
ENSGALG00010014658	SELENOP1	2	-0.224176198	0.11284024	-1.986668931	0.046959097	0	0.047890804	0	1	CRA004775;Yang2025_Silkie_Wenchang	PRJNA827465	0.374003609
ENSGALG00010014658	SELENOP1	2	-0.210670289	0.117600316	-1.791409202	0.073227653	0	0.067681423	0	1	CRA004775;PRJNA827465	Yang2025_Silkie_Wenchang	0.467793099
ENSGALG00010014668	GP5	2	0.165706625	0.14099223	1.175289051	0.239879093	0.016273102	1.649764131	39.38527446	1	PRJNA827465;Yang2025_Silkie_Wenchang	CRA004775	0.75219148
ENSGALG00010014668	GP5	2	-0.016174919	0.403878974	-0.040048927	0.96805412	0.255037504	4.311593312	76.8067179	0.5	CRA004775;Yang2025_Silkie_Wenchang	PRJNA827465	0.991128245
ENSGALG00010014668	GP5	2	-0.115299584	0.244634869	-0.47131296	0.637417262	0.070122601	2.016493315	50.40896031	0.5	CRA004775;PRJNA827465	Yang2025_Silkie_Wenchang	0.905170743
ENSGALG00010014676	UBE2V1	2	-0.081802498	0.040459239	-2.021849641	0.043191888	0.000577342	1.109233198	9.847631537	1	PRJNA827465;Yang2025_Silkie_Wenchang	CRA004775	0.359495531
ENSGALG00010014676	UBE2V1	2	-0.128226601	0.087786216	-1.460668956	0.144106313	0.000434504	1.01827283	1.79449254	0.5	CRA004775;Yang2025_Silkie_Wenchang	PRJNA827465	0.63137387
ENSGALG00010014676	UBE2V1	2	-0.059732632	0.036619478	-1.631171009	0.102854241	0	0.31418547	0	0.5	CRA004775;PRJNA827465	Yang2025_Silkie_Wenchang	0.546724401
ENSGALG00010014677	ENSGALG00010014677	2	0.239920094	0.198080629	1.211224414	0.225809408	0.054893063	3.324216492	69.9177234	1	PRJNA827465;Yang2025_Silkie_Wenchang	CRA004775	0.739089539
ENSGALG00010014677	ENSGALG00010014677	2	0.147126989	0.320749861	0.458696968	0.646451792	0.164738605	4.882888212	79.52031755	0.5	CRA004775;Yang2025_Silkie_Wenchang	PRJNA827465	0.907220702
ENSGALG00010014677	ENSGALG00010014677	2	-0.020296061	0.126447362	-0.160509961	0.872479376	0	0.752535346	0	0.5	CRA004775;PRJNA827465	Yang2025_Silkie_Wenchang	0.969629321
ENSGALG00010014682	IL13RA1	2	-0.353168546	0.273077098	-1.293292439	0.195909935	0.135122546	10.48703028	90.464412	1	PRJNA827465;Yang2025_Silkie_Wenchang	CRA004775	0.707121697
ENSGALG00010014682	IL13RA1	2	-0.593884214	0.133625551	-4.444391131	8.81411E-06	0	0.860726833	0	1	CRA004775;Yang2025_Silkie_Wenchang	PRJNA827465	0.001268942
ENSGALG00010014682	IL13RA1	2	-0.097617033	0.089756366	-1.08757782	0.276781527	0	0.124456163	0	1	CRA004775;PRJNA827465	Yang2025_Silkie_Wenchang	0.775940673
ENSGALG00010014687	CUL4A	2	0.081162089	0.166031938	0.488834196	0.624959086	0.044598586	4.821080361	79.25776123	0.5	PRJNA827465;Yang2025_Silkie_Wenchang	CRA004775	0.901119081
ENSGALG00010014687	CUL4A	2	0.280698398	0.129515779	2.167291123	0.030212663	0	0.021857388	0	1	CRA004775;Yang2025_Silkie_Wenchang	PRJNA827465	0.295973612
ENSGALG00010014687	CUL4A	2	-0.009497624	0.133792514	-0.07098771	0.943407537	0.017061351	1.290175556	22.49116831	0.5	CRA004775;PRJNA827465	Yang2025_Silkie_Wenchang	0.985827325
ENSGALG00010014690	CSF3R	2	-0.120748263	0.169221109	-0.713553196	0.475503508	0	0.992514299	0	0.5	PRJNA827465;Yang2025_Silkie_Wenchang	CRA004775	0.854497479
ENSGALG00010014690	CSF3R	2	-0.311172003	0.498714924	-0.623947646	0.532661968	0.323391894	2.455252443	59.27099054	0.5	CRA004775;Yang2025_Silkie_Wenchang	PRJNA827465	0.873183244
ENSGALG00010014690	CSF3R	2	-0.402047691	0.265799505	-1.512597592	0.130381906	0.03044217	1.138665413	12.17788929	1	CRA004775;PRJNA827465	Yang2025_Silkie_Wenchang	0.605608015
ENSGALG00010014691	PABIR2	2	-0.043116987	0.035171131	-1.225919831	0.220228845	0	0.019215596	0	1	PRJNA827465;Yang2025_Silkie_Wenchang	CRA004775	0.733812488
ENSGALG00010014691	PABIR2	2	-0.041298012	0.060407209	-0.683660335	0.494189676	0	0.038862574	0	1	CRA004775;Yang2025_Silkie_Wenchang	PRJNA827465	0.860175061
ENSGALG00010014691	PABIR2	2	-0.047670011	0.039091863	-1.219435652	0.222678885	0	0.017163668	0	1	CRA004775;PRJNA827465	Yang2025_Silkie_Wenchang	0.736291702
ENSGALG00010014696	SPINK5	2	0.268430318	0.538230194	0.498727721	0.617971213	0.404448516	3.078299837	67.51453552	0.5	PRJNA827465;Yang2025_Silkie_Wenchang	CRA004775	0.899121942
ENSGALG00010014696	SPINK5	2	0.57052518	0.800019634	0.713138974	0.475759765	1.116797639	7.625459842	86.88603677	0.5	CRA004775;Yang2025_Silkie_Wenchang	PRJNA827465	0.854613502
ENSGALG00010014696	SPINK5	2	1.193183684	0.370585056	3.219729625	0.001283116	0	0.467030514	0	1	CRA004775;PRJNA827465	Yang2025_Silkie_Wenchang	0.043419476
ENSGALG00010014702	ENSGALG00010014702	2	0.015797673	0.329380028	0.047961843	0.961746652	0.095209307	1.777515513	43.74170054	0.5	PRJNA827465;Yang2025_Silkie_Wenchang	CRA004775	0.98973612
ENSGALG00010014702	ENSGALG00010014702	2	-0.021561111	0.413559577	-0.05213544	0.958420774	0.185172208	2.167852941	53.87140976	0.5	CRA004775;Yang2025_Silkie_Wenchang	PRJNA827465	0.989046151
ENSGALG00010014702	ENSGALG00010014702	2	-0.35944477	0.262188769	-1.370938851	0.170394019	0	0.097944105	0	1	CRA004775;PRJNA827465	Yang2025_Silkie_Wenchang	0.672186703
ENSGALG00010014703	PCID2	2	-0.26430496	0.252015273	-1.048765641	0.294285994	0.11584258	10.91498561	90.83828384	1	PRJNA827465;Yang2025_Silkie_Wenchang	CRA004775	0.787343482
ENSGALG00010014703	PCID2	2	-0.461722745	0.11744126	-3.931520697	8.44102E-05	0	0.853455839	0	1	CRA004775;Yang2025_Silkie_Wenchang	PRJNA827465	0.006879732
ENSGALG00010014703	PCID2	2	-0.082737124	0.108344922	-0.763645608	0.445078462	0.011097274	1.446389313	30.86232102	1	CRA004775;PRJNA827465	Yang2025_Silkie_Wenchang	0.846013379
ENSGALG00010014705	ARAP2	2	0.107873293	0.172151115	0.626619774	0.530908503	0	0.251771672	0	0.5	PRJNA827465;Yang2025_Silkie_Wenchang	CRA004775	0.872840748
ENSGALG00010014705	ARAP2	2	0.067350945	0.256082162	0.263005219	0.792546556	0	0.338788006	0	0.5	CRA004775;Yang2025_Silkie_Wenchang	PRJNA827465	0.948435155
ENSGALG00010014705	ARAP2	2	0.196930497	0.201914958	0.975314057	0.3294045	0	0.100055578	0	1	CRA004775;PRJNA827465	Yang2025_Silkie_Wenchang	0.801001364
ENSGALG00010014707	KPNA5	2	-0.233421292	0.231676126	-1.007532785	0.313678811	0.102984056	24.39343531	95.90053641	1	PRJNA827465;Yang2025_Silkie_Wenchang	CRA004775	0.796531448
ENSGALG00010014707	KPNA5	2	-0.01551713	0.492104101	-0.031532209	0.974845106	0.441859663	10.68509518	90.641169	0.5	CRA004775;Yang2025_Silkie_Wenchang	PRJNA827465	0.992599409
ENSGALG00010014707	KPNA5	2	0.177213544	0.249892966	0.709157792	0.478226563	0.09368588	3.149499525	68.24892362	0.5	CRA004775;PRJNA827465	Yang2025_Silkie_Wenchang	0.855388572
ENSGALG00010014710	NPEPL1	2	-0.220059911	0.143688468	-1.531507112	0.12564411	0.033912377	5.469581194	81.71706453	1	PRJNA827465;Yang2025_Silkie_Wenchang	CRA004775	0.595034397
ENSGALG00010014710	NPEPL1	2	-0.355874273	0.098707395	-3.605345592	0.000311738	0	0.437110134	0	1	CRA004775;Yang2025_Silkie_Wenchang	PRJNA827465	0.017418181
ENSGALG00010014710	NPEPL1	2	-0.087488976	0.067067287	-1.304495521	0.192064686	0	0.010730122	0	1	CRA004775;PRJNA827465	Yang2025_Silkie_Wenchang	0.701846421
ENSGALG00010014713	ST8SIA1	2	-0.171784855	0.182589682	-0.940824552	0.346794777	0.044510145	2.834610515	64.72178472	1	PRJNA827465;Yang2025_Silkie_Wenchang	CRA004775	0.80837881
ENSGALG00010014713	ST8SIA1	2	-0.409530552	0.182671621	-2.241894774	0.024968174	0	0.137762805	0	1	CRA004775;Yang2025_Silkie_Wenchang	PRJNA827465	0.269298611
ENSGALG00010014713	ST8SIA1	2	-0.036388025	0.109148188	-0.333381849	0.738846063	0	0.895763654	0	1	CRA004775;PRJNA827465	Yang2025_Silkie_Wenchang	0.934613258
ENSGALG00010014716	TMEM35A	2	-0.19028248	0.402664746	-0.472558082	0.636528494	0.27364286	5.85785367	82.92890099	0.5	PRJNA827465;Yang2025_Silkie_Wenchang	CRA004775	0.904566695
ENSGALG00010014716	TMEM35A	2	-0.484779969	0.218836291	-2.215263142	0.026742008	0	0.525993195	0	1	CRA004775;Yang2025_Silkie_Wenchang	PRJNA827465	0.278911632
ENSGALG00010014716	TMEM35A	2	-0.004772092	0.234043983	-0.020389725	0.98373248	0.069789999	2.327419921	57.03396749	0.5	CRA004775;PRJNA827465	Yang2025_Silkie_Wenchang	0.995510523
ENSGALG00010014717	ELOVL7	2	-0.247364575	0.240112002	-1.030204956	0.302913804	0.090909633	4.601340061	78.26720071	1	PRJNA827465;Yang2025_Silkie_Wenchang	CRA004775	0.792084776
ENSGALG00010014717	ELOVL7	2	0.257449296	0.782220099	0.329126414	0.74206014	1.164029139	20.32937387	95.08100935	0.5	CRA004775;Yang2025_Silkie_Wenchang	PRJNA827465	0.935481151
ENSGALG00010014717	ELOVL7	2	0.482157146	0.540428993	0.892174833	0.37229923	0.535902415	11.65167378	91.41754207	0.5	CRA004775;PRJNA827465	Yang2025_Silkie_Wenchang	0.82179465
ENSGALG00010014722	KCTD17	2	-0.036486032	0.185414009	-0.196781419	0.843998592	0	0.302312235	0	0.5	PRJNA827465;Yang2025_Silkie_Wenchang	CRA004775	0.960662516
ENSGALG00010014722	KCTD17	2	0.108242132	0.304327474	0.355676505	0.722082843	0	0.002849121	0	1	CRA004775;Yang2025_Silkie_Wenchang	PRJNA827465	0.929404251
ENSGALG00010014722	KCTD17	2	-0.090635645	0.214076764	-0.423379182	0.672018631	0	0.050470712	0	0.5	CRA004775;PRJNA827465	Yang2025_Silkie_Wenchang	0.914714609
ENSGALG00010014726	PWP2	2	-0.107794346	0.059447121	-1.813281194	0.069788462	0	0.006583772	0	1	PRJNA827465;Yang2025_Silkie_Wenchang	CRA004775	0.455644844
ENSGALG00010014726	PWP2	2	-0.163025294	0.106574484	-1.529684096	0.126094942	0.002223246	1.058101001	5.491063786	1	CRA004775;Yang2025_Silkie_Wenchang	PRJNA827465	0.596422219
ENSGALG00010014726	PWP2	2	-0.150417984	0.106880326	-1.407349599	0.159323749	0.008411948	1.239644692	19.33172413	1	CRA004775;PRJNA827465	Yang2025_Silkie_Wenchang	0.654827591
ENSGALG00010014728	INTS13	2	-0.049672881	0.039330906	-1.262947792	0.206607943	0	0.073853262	0	1	PRJNA827465;Yang2025_Silkie_Wenchang	CRA004775	0.71859917
ENSGALG00010014728	INTS13	2	0.14228099	0.239185727	0.594855687	0.55193994	0.094644177	5.496016814	81.80500472	0.5	CRA004775;Yang2025_Silkie_Wenchang	PRJNA827465	0.879061525
ENSGALG00010014728	INTS13	2	0.147048683	0.223751641	0.657195998	0.511054899	0.084987712	5.846840424	82.89674546	0.5	CRA004775;PRJNA827465	Yang2025_Silkie_Wenchang	0.865588911
ENSGALG00010014729	MRPS15	2	0.232950766	0.195292869	1.192827817	0.232936814	0.06339297	5.270839634	81.02769066	1	PRJNA827465;Yang2025_Silkie_Wenchang	CRA004775	0.746005924
ENSGALG00010014729	MRPS15	2	0.085906033	0.417929792	0.20555135	0.837141383	0.288907757	5.491742312	81.79084263	0.5	CRA004775;Yang2025_Silkie_Wenchang	PRJNA827465	0.958715806
ENSGALG00010014729	MRPS15	2	-0.044820575	0.192687995	-0.232606992	0.816066598	0.047042839	1.905640767	47.52421248	0.5	CRA004775;PRJNA827465	Yang2025_Silkie_Wenchang	0.954410204
ENSGALG00010014734	ZUP1	2	-0.060177602	0.065070646	-0.924804128	0.355067803	0	0.226577254	0	1	PRJNA827465;Yang2025_Silkie_Wenchang	CRA004775	0.812276513
ENSGALG00010014734	ZUP1	2	-0.169590216	0.130977409	-1.294805096	0.195387474	0.011452925	1.306163743	23.43992052	1	CRA004775;Yang2025_Silkie_Wenchang	PRJNA827465	0.70643959
ENSGALG00010014734	ZUP1	2	-0.14728204	0.171987482	-0.856353254	0.391802413	0.035154397	1.989432073	49.73439841	1	CRA004775;PRJNA827465	Yang2025_Silkie_Wenchang	0.829251057
ENSGALG00010014735	STX16	2	-0.001355877	0.041333045	-0.032803712	0.973831118	0	0.473938041	0	0.5	PRJNA827465;Yang2025_Silkie_Wenchang	CRA004775	0.992472082
ENSGALG00010014735	STX16	2	0.019653386	0.118146681	0.166347335	0.867883617	0.011279887	1.536930942	34.93526788	0.5	CRA004775;Yang2025_Silkie_Wenchang	PRJNA827465	0.967946362
ENSGALG00010014735	STX16	2	0.023179915	0.044446748	0.521521055	0.602003842	0	0.937995889	0	1	CRA004775;PRJNA827465	Yang2025_Silkie_Wenchang	0.89532296
ENSGALG00010014736	KIF2A	2	-0.005283321	0.049888023	-0.105903586	0.915658849	0	0.11393261	0	0.5	PRJNA827465;Yang2025_Silkie_Wenchang	CRA004775	0.980229333
ENSGALG00010014736	KIF2A	2	0.044860835	0.09154697	0.4900308	0.624112104	0	0.270428577	0	1	CRA004775;Yang2025_Silkie_Wenchang	PRJNA827465	0.900792406
ENSGALG00010014736	KIF2A	2	-0.004873166	0.055811509	-0.087314714	0.930421359	0	0.513198443	0	0.5	CRA004775;PRJNA827465	Yang2025_Silkie_Wenchang	0.983813577
ENSGALG00010014737	RMI1	2	-0.009531311	0.121280259	-0.078589135	0.93735943	0	0.722788851	0	0.5	PRJNA827465;Yang2025_Silkie_Wenchang	CRA004775	0.985114195
ENSGALG00010014737	RMI1	2	0.062658782	0.225849888	0.277435525	0.781445707	0.053048457	2.008851368	50.22030918	0.5	CRA004775;Yang2025_Silkie_Wenchang	PRJNA827465	0.945235709
ENSGALG00010014737	RMI1	2	0.150601574	0.139230474	1.081671053	0.27939872	0	0.644668768	0	1	CRA004775;PRJNA827465	Yang2025_Silkie_Wenchang	0.777457036
ENSGALG00010014738	BRWD1	2	0.172113564	0.144048209	1.194833066	0.232152258	0.030786016	3.362780289	70.26270187	1	PRJNA827465;Yang2025_Silkie_Wenchang	CRA004775	0.744717614
ENSGALG00010014738	BRWD1	2	0.356364901	0.127820029	2.788020819	0.005303114	0	0.000722302	0	1	CRA004775;Yang2025_Silkie_Wenchang	PRJNA827465	0.108783023
ENSGALG00010014738	BRWD1	2	0.122886264	0.124359173	0.988156005	0.323076251	0.016702335	1.568561503	36.24731971	1	CRA004775;PRJNA827465	Yang2025_Silkie_Wenchang	0.798909393
ENSGALG00010014739	TRMT2B	2	0.105807884	0.229390026	0.461257562	0.644613828	0.092808151	8.391279383	88.0828661	0.5	PRJNA827465;Yang2025_Silkie_Wenchang	CRA004775	0.906953008

ENSGALG00010014739	TRMT2B	2	-0.065934936	0.446200125	-0.147769873	0.882524386	0.35033322	7.889376162	87.32472658	0.5	CRA004775;Yang2025_Silkie_Wenchang	PRJNA827465	0.972001076
ENSGALG00010014739	TRMT2B	2	-0.246469244	0.199966118	-1.232555028	0.217741824	0.048130794	2.019250132	50.47666535	1	CRA004775;PRJNA827465	Yang2025_Silkie_Wenchang	0.731070128
ENSGALG00010014744	IL2RB	2	1.240360474	0.317032254	3.912410988	9.13792E-05	0	0.366183458	0	1	PRJNA827465;Yang2025_Silkie_Wenchang	CRA004775	0.007245791
ENSGALG00010014744	IL2RB	2	0.601838694	0.804458155	0.748129272	0.454382188	1.129709811	7.841409157	87.24719014	0.5	CRA004775;Yang2025_Silkie_Wenchang	PRJNA827465	0.849072665
ENSGALG00010014744	IL2RB	2	0.396347081	0.611812516	0.647824409	0.517098526	0.575115642	4.281137611	76.64172258	0.5	CRA004775;PRJNA827465	Yang2025_Silkie_Wenchang	0.867952013
ENSGALG00010014745	LSM10	2	-0.157922054	0.179700825	-0.878805388	0.379506805	0	0.098893651	0	1	PRJNA827465;Yang2025_Silkie_Wenchang	CRA004775	0.824165982
ENSGALG00010014745	LSM10	2	-0.17716893	0.222320294	-0.79690849	0.42550418	0	0.077768234	0	1	CRA004775;Yang2025_Silkie_Wenchang	PRJNA827465	0.84125685
ENSGALG00010014745	LSM10	2	-0.128140678	0.165081891	-0.77622492	0.437616195	0	0.000144805	0	1	CRA004775;PRJNA827465	Yang2025_Silkie_Wenchang	0.843827727
ENSGALG00010014748	FGFR1OP2	2	0.219817152	0.109860332	2.000878278	0.045405509	0.011265724	1.782329015	43.89363627	1	PRJNA827465;Yang2025_Silkie_Wenchang	CRA004775	0.367363248
ENSGALG00010014748	FGFR1OP2	2	0.22811804	0.175734652	1.298082291	0.194259059	0.027808521	1.71336232	41.63522868	0.5	CRA004775;Yang2025_Silkie_Wenchang	PRJNA827465	0.704982807
ENSGALG00010014748	FGFR1OP2	2	0.116891384	0.084420688	1.38462961	0.166165753	0	0.294908784	0	0.5	CRA004775;PRJNA827465	Yang2025_Silkie_Wenchang	0.66529196
ENSGALG00010014750	LONRF1	2	0.04879885	0.075322122	0.647868756	0.51706984	0	0.189964571	0	1	PRJNA827465;Yang2025_Silkie_Wenchang	CRA004775	0.867952013
ENSGALG00010014750	LONRF1	2	0.109208887	0.180283059	0.605763444	0.544671848	0.027518932	1.467838726	31.87262454	1	CRA004775;Yang2025_Silkie_Wenchang	PRJNA827465	0.877345822
ENSGALG00010014750	LONRF1	2	0.112875238	0.110365519	1.022740067	0.306430762	0.004930021	1.089180445	8.187848562	1	CRA004775;PRJNA827465	Yang2025_Silkie_Wenchang	0.792485409
ENSGALG00010014754	DIMT1	2	-0.00446574	0.116137919	-0.038452043	0.969327268	0	0.227165281	0	0.5	PRJNA827465;Yang2025_Silkie_Wenchang	CRA004775	0.991572914
ENSGALG00010014754	DIMT1	2	-0.035225602	0.143165143	-0.246048731	0.80564451	0	0.099459718	0	0.5	CRA004775;Yang2025_Silkie_Wenchang	PRJNA827465	0.951984168
ENSGALG00010014754	DIMT1	2	0.03943926	0.13075826	0.301619648	0.762942028	0	0.003165159	0	1	CRA004775;PRJNA827465	Yang2025_Silkie_Wenchang	0.940115078
ENSGALG00010014758	FXN	2	0.149820531	0.101501393	1.476044085	0.139932058	0	0.020427218	0	1	PRJNA827465;Yang2025_Silkie_Wenchang	CRA004775	0.623792706
ENSGALG00010014758	FXN	2	-0.08148096	0.250758972	-0.324937365	0.745228477	0.086993625	3.088268632	67.61939717	0.5	CRA004775;Yang2025_Silkie_Wenchang	PRJNA827465	0.93597621
ENSGALG00010014758	FXN	2	-0.065941715	0.265325265	-0.24853161	0.803723116	0.103816577	3.593973144	72.17564072	0.5	CRA004775;PRJNA827465	Yang2025_Silkie_Wenchang	0.951066707
ENSGALG00010014762	STK40	2	0.005122421	0.041255249	0.124164113	0.901185336	0	0.603488338	0	0.5	PRJNA827465;Yang2025_Silkie_Wenchang	CRA004775	0.97660383
ENSGALG00010014762	STK40	2	-0.004914006	0.093011643	-0.052832157	0.957865639	0.006459957	1.439496461	30.53126373	0.5	CRA004775;Yang2025_Silkie_Wenchang	PRJNA827465	0.988864712
ENSGALG00010014762	STK40	2	-0.0285056	0.046974456	-0.606831936	0.543962452	0	0.70485501	0	1	CRA004775;PRJNA827465	Yang2025_Silkie_Wenchang	0.876968972
ENSGALG00010014766	F10	2	-0.38168667	0.820515889	-0.46517889	0.641803355	1.269538247	17.28499709	94.21463599	0.5	PRJNA827465;Yang2025_Silkie_Wenchang	CRA004775	0.906634523
ENSGALG00010014766	F10	2	-0.465538338	0.739189941	-0.629799383	0.528828532	1.015826128	13.98489105	92.88942588	0.5	CRA004775;Yang2025_Silkie_Wenchang	PRJNA827465	0.871809618
ENSGALG00010014766	F10	2	0.380516343	0.239787905	1.586887144	0.112538207	0	0.114692699	0	1	CRA004775;PRJNA827465	Yang2025_Silkie_Wenchang	0.566223663
ENSGALG00010014767	LRRC15	2	0.080401982	0.18046008	0.445538882	0.655930368	0.034612407	2.127912566	53.00558792	0.5	PRJNA827465;Yang2025_Silkie_Wenchang	CRA004775	0.910411938
ENSGALG00010014767	LRRC15	2	-0.019629261	0.426411184	-0.046033646	0.963283433	0.231861954	2.228772417	55.13225163	0.5	CRA004775;Yang2025_Silkie_Wenchang	PRJNA827465	0.989998461
ENSGALG00010014767	LRRC15	2	-0.132163181	0.159232381	-0.830002485	0.406479334	0	0.832985789	0	1	CRA004775;PRJNA827465	Yang2025_Silkie_Wenchang	0.833613812
ENSGALG00010014769	CCDC69	2	0.059882679	0.117066042	0.511529028	0.608980668	0	0.625056866	0	0.5	PRJNA827465;Yang2025_Silkie_Wenchang	CRA004775	0.897208691
ENSGALG00010014769	CCDC69	2	-0.043732207	0.333566188	-0.131105034	0.895692219	0.125162015	1.993948913	49.84826374	0.5	CRA004775;Yang2025_Silkie_Wenchang	PRJNA827465	0.974927462
ENSGALG00010014769	CCDC69	2	-0.075899349	0.176455343	-0.430133469	0.667098555	0.015584907	1.134865411	11.88382426	1	CRA004775;PRJNA827465	Yang2025_Silkie_Wenchang	0.913766816
ENSGALG00010014777	APCDD1L	2	0.313368824	0.230583574	1.359024923	0.174138691	0	0.734355342	0	1	PRJNA827465;Yang2025_Silkie_Wenchang	CRA004775	0.678178061
ENSGALG00010014777	APCDD1L	2	0.653907768	0.274285223	2.384043003	0.017123601	0	0.071273841	0	1	CRA004775;Yang2025_Silkie_Wenchang	PRJNA827465	0.217380136
ENSGALG00010014777	APCDD1L	2	0.386272668	0.273198918	1.413887985	0.157394794	0.040363579	1.34694451	25.75789184	1	CRA004775;PRJNA827465	Yang2025_Silkie_Wenchang	0.650687662
ENSGALG00010014780	EVA1B	2	0.039531503	0.072794646	0.543055094	0.58709187	0	0.034752297	0	1	PRJNA827465;Yang2025_Silkie_Wenchang	CRA004775	0.891596903
ENSGALG00010014780	EVA1B	2	0.053198374	0.170991972	0.311116205	0.755712282	0	0.036773947	0	1	CRA004775;Yang2025_Silkie_Wenchang	PRJNA827465	0.938501783
ENSGALG00010014780	EVA1B	2	0.032651192	0.075268672	0.433795239	0.664437139	0	0.008599608	0	1	CRA004775;PRJNA827465	Yang2025_Silkie_Wenchang	0.913219726
ENSGALG00010014783	ENSGALG00010014783	2	0.058349056	0.069683393	0.837345216	0.40239855	0.004821977	1.90946789	47.62938904	1	PRJNA827465;Yang2025_Silkie_Wenchang	CRA004775	0.832879175
ENSGALG00010014783	ENSGALG00010014783	2	-0.213008773	0.381914383	-0.557739595	0.577022216	0.265866002	10.77601108	90.72012832	0.5	CRA004775;Yang2025_Silkie_Wenchang	PRJNA827465	0.887134413
ENSGALG00010014783	ENSGALG00010014783	2	-0.274958717	0.309660073	-0.887937263	0.374574499	0.16909396	7.722765853	87.05127128	0.5	CRA004775;PRJNA827465	Yang2025_Silkie_Wenchang	0.822805106
ENSGALG00010014792	ENSGALG00010014792	2	0.023981501	0.141008902	0.170070827	0.864954436	0	0.003238904	0	1	PRJNA827465;Yang2025_Silkie_Wenchang	CRA004775	0.966819413
ENSGALG00010014792	ENSGALG00010014792	2	-0.006540931	0.205448458	-0.031837335	0.974601772	0	0.23396598	0	0.5	CRA004775;Yang2025_Silkie_Wenchang	PRJNA827465	0.992492038
ENSGALG00010014792	ENSGALG00010014792	2	-0.009651197	0.173341805	-0.055677261	0.955598915	0	0.217774142	0	0.5	CRA004775;PRJNA827465	Yang2025_Silkie_Wenchang	0.988287876
ENSGALG00010014793	TLR2A	2	-0.26561838	0.111590624	-2.380292981	0.017298878	0	0.796141179	0	1	PRJNA827465;Yang2025_Silkie_Wenchang	CRA004775	0.218613945
ENSGALG00010014793	TLR2A	2	-0.569216362	0.346612245	-1.642228082	0.100542745	0.125052117	1.566972642	36.18267651	1	CRA004775;Yang2025_Silkie_Wenchang	PRJNA827465	0.541393598
ENSGALG00010014793	TLR2A	2	-0.509085096	0.480925618	-1.058552667	0.289803553	0.317409727	2.46686855	59.4627772	1	CRA004775;PRJNA827465	Yang2025_Silkie_Wenchang	0.784067533
ENSGALG00010014795	THRAP3	2	0.13059379	0.152777907	0.854794999	0.392664647	0.042801744	11.48302127	91.29149048	0.5	PRJNA827465;Yang2025_Silkie_Wenchang	CRA004775	0.829914191
ENSGALG00010014795	THRAP3	2	0.078901527	0.23167531	0.340569422	0.733427753	0.092675643	7.064534806	85.84478628	0.5	CRA004775;Yang2025_Silkie_Wenchang	PRJNA827465	0.933209588
ENSGALG00010014795	THRAP3	2	-0.020999345	0.035794142	-0.586669866	0.55742545	0.000352766	1.028914918	2.810234136	1	CRA004775;PRJNA827465	Yang2025_Silkie_Wenchang	0.880678789
ENSGALG00010014796	PRLR	2	0.176733676	0.309839133	0.570404631	0.568403289	0.109303959	2.26185631	55.78852663	0.5	PRJNA827465;Yang2025_Silkie_Wenchang	CRA004775	0.884451198
ENSGALG00010014796	PRLR	2	0.606832612	0.316753907	1.91578572	0.055392376	0	0.378133907	0	1	CRA004775;Yang2025_Silkie_Wenchang	PRJNA827465	0.407541475
ENSGALG00010014796	PRLR	2	0.206671872	0.524507816	0.394030109	0.69335881	0.305396618	1.696230791	41.04575831	0.5	CRA004775;PRJNA827465	Yang2025_Silkie_Wenchang	0.920721644
ENSGALG00010014797	ZDHHC9	2	-0.194241024	0.050392427	-3.854567765	0.000115934	0	0.355710136	0	1	PRJNA827465;Yang2025_Silkie_Wenchang	CRA004775	0.000581101
ENSGALG00010014797	ZDHHC9	2	-0.282156546	0.104553808	-2.698673057	0.006961653	0	0.314983064	0	1	CRA004775;Yang2025_Silkie_Wenchang	PRJNA827465	0.128156372
ENSGALG00010014797	ZDHHC9	2	-0.190340003	0.054779814	-3.474637612	0.000511544	0	0.798582369	0	1	CRA004775;PRJNA827465	Yang2025_Silkie_Wenchang	0.023736966
ENSGALG00010014801	MPST	2	0.014185332	0.136354723	0.104032571	0.917143501	0.020561213	2.017150137	50.4251081	0.5	PRJNA827465;Yang2025_Silkie_Wenchang	CRA004775	0.98075015
ENSGALG00010014801	MPST	2	-0.210645105	0.138836082	-1.517216018	0.12921074	0	0.120716415	0	1	CRA004775;Yang2025_Silkie_Wenchang	PRJNA827465	0.603386934
ENSGALG00010014801	MPST	2	-0.02569798	0.184868576	-0.139006749	0.889444816	0.045745483	2.637126452	62.07993745	0.5	CRA004775;PRJNA827465	Yang2025_Silkie_Wenchang	0.973135755
ENSGALG00010014802	ENSGALG00010014802	2	0.344750666	0.173618326	1.985680965	0.04706876	0	0.099379957	0	1	PRJNA827465;Yang2025_Silkie_Wenchang	CRA004775	0.374179505
ENSGALG00010014802	ENSGALG00010014802	2	0.125226353	0.349581889	0.358217507	0.720180547	0.007288066	1.03065777	2.974582886	0.5	CRA004775;Yang2025_Silkie_Wenchang	PRJNA827465	0.920564173
ENSGALG00010014802	ENSGALG00010014802	2	0.23833105	0.195978342	1.216104531	0.223945105	0.011548917	1.088866824	8.161404344	0.5	CRA004775;PRJNA827465	Yang2025_Silkie_Wenchang	0.737452676
ENSGALG00010014806	MAP7D1	2	0.233639587	0.237001829	0.985813437	0.324224671	0.093329179	5.55403187	81.99506191	1	PRJNA827465;Yang2025_Silkie_Wenchang	CRA004775	0.799686366
ENSGALG00010014806	MAP7D1	2	0.168788646	0.388694868	0.434244597	0.664110831	0.226922574	3.726787837	73.16724096	0.5	CRA004775;Yang2025_Silkie_Wenchang	PRJNA827465	0.91312035
ENSGALG00010014806	MAP7D1	2	0.005731177	0.084261891	0.068016242	0.945772705	0	0.680161343	0	0.5	CRA004775;PRJNA827465	Yang2025_Silkie_Wenchang	0.986321197
ENSGALG00010014807	HES1	2	0.223177279	0.271740956	0.821286869	0.411482886	0.133140284	9.713045097	89.70456752	0.5	PRJNA827465;Yang2025_Silkie_Wenchang	CRA004775	0.835128673
ENSGALG00010014807	HES1	2	0.506900036	0.155732788	3.254934573	0.001134185	0	0.042021774	0	1	CRA004775;Yang2025_Silkie_Wenchang	PRJNA827465	0.040858726
ENSGALG00010014807	HES1	2	-0.025484437	0.069170733	-0.368428037	0.712554095	0	0.386278659	0	0.5	CRA004775;PRJNA827465	Yang2025_Silkie_Wenchang	0.92597752
ENSGALG00010014810	ENTREP1	2	0.062466058	0.089850054	0.6952256	0.48691393	0	0.5013903	0	0.5	PRJNA827465;Yang2025_Silkie_Wenchang	CRA004775	0.857631005
ENSGALG00010014810	ENTREP1	2	0.102314098	0.121689191	0.840782132	0.400469999	0	0.451081262	0	0.5	CRA004775;Yang2025_Silkie_Wenchang	PRJNA827465	0.832251175
ENSGALG00010014810	ENTREP1	2	-0.014008985	0.121390159	-0.115404619	0.908124418	0	0.124069181	0	1	CRA004775;PRJNA827465	Yang2025_Silkie_Wenchang	0.978208122
ENSGALG00010014812	RWDD1	2	0.12358402	0.107417202	1.150508493	0.249935965	0.014318066	2.376382058	57.91922445	1	PRJNA827465;Yang2025_Silkie_Wenchang	CRA004775	0.67599411
ENSGALG00010014812	RWDD1	2	0.241227162	0.109665537	2.199662444	0.027830853	0	0.10376697	0	1	CRA004775;Yang2025_Silkie_Wenchang	PRJNA827465	0.284423997
ENSGALG00010014812	RWDD1	2	0.053545178	0.060655181	0.882779965	0.377355167	0	0.445605388	0	1	CRA004775;PRJNA827465	Yang2025_Silkie_Wenchang	0.82325159
ENSGALG00010014813	SASH3	2	0.650153352	0.211925179	3.067843822	0.002156093	0	0.219441502	0	1	PRJNA827465;Yang2025_Silkie_Wenchang	CRA004775	0.061008576
ENSGALG00010014813	SASH3	2	-0.092284918	0.60809821	-0.151759891	0.879376318	0.568341478	4.306926636	76.78158733	0.5	CRA004775;Yang2025_Silkie_Wenchang	PRJNA827465	0.971291607
ENSGALG00010014813	SASH3	2	0.043426322	0.715339967	0.06070725	0.951592358	0.89865952	7.974752622	87.46042608	0.5	CRA004775;PRJNA827465	Yang2025_Silkie_Wenchang	0.987713746
ENSGALG00010014816	TST	2	0.00367436	0.172303705	0.021324903	0.982986479	0.043110385	3.523563358	71.61963903	0.5	PRJNA827465;Yang2025_Silkie_Wenchang	CRA004775	0.995363468
ENSGALG00010014816	TST	2	-0.140786421	0.142714058	-0.986493016	0.323891241	0	0.650393244	0	0.5	CRA004775;Yang2025_Silkie_Wenchang	PRJNA827465	0.799600434
ENSGALG00010014816	TST	2	0.154919332	0.096733447	1.601507404	0.109264582	0	0.005788457	0	1	CRA004775;PRJNA827465	Yang2025_Silkie_Wenchang	0.559358543
ENSGALG00010014818	ENSGALG00010014818	2	-0.133452093	0.263970738	-0.505556389	0.613168103	0	0.47100706	0	1	PRJNA827465;Yang2025_Silkie_Wenchang	CRA004775	0.898190375
ENSGALG00010014818	ENSGALG00010014818	2	0.06188706	0.366863194	0.168692475	0.866038538	0	0.005415556	0	1	CRA004775;Yang2025_Silkie_Wenchang	PRJNA827465	0.967288433
ENSGALG00010014818	ENSGALG00010014818	2	-0.277565416	0.354497793	-0.782985077	0.43363764	0	0.106553001	0	0.5	CRA004775;PRJNA827465	Yang2025_Silkie_Wenchang	0.843762754
ENSGALG00010014821	C3AR1L	2	-0.165271163	0.204502369	-0.808162581	0.418996996	0.062390088	3.925625793	74.52635445	0.5	PRJNA827465;Yang2025_Silkie_Wenchang	CRA004775	0.838313934
ENSGALG00010014821	C3AR1L	2	-0.350435487	0.134068401	-2.613855955	0.008952677	0	0.203939743	0	1	CRA004775;Yang2025_Silkie_Wenchang	PRJNA827465	0.148216965
ENSGALG00010014821	C3AR1L	2	0.038278952	0.149717145	0.255675143	0.79820169	0	0.036294858	0	0.5	CRA004775;PRJNA827465	Yang2025_Silkie_Wenchang	0.949922217
ENSGALG00010014824	ANXA6	2	0.142344528	0.121699675	1.1696377	0.242146799	0.023835411	5.117982387	80.46105038	1	PRJNA827465;Yang2025_Silkie_Wenchang	CRA004775	0.754426923
ENSGALG00010014824	ANXA6	2	0.099035759	0.211882824	0.467408151	0.640207892	0.066251421	3.242037517	69.15519963	0.5	CRA004775;Yang2025_Silkie_Wenchang	PRJNA827465	0.906137307
ENSGALG00010014824	ANXA6	2	5.18966E-06	0.073599493	7.05122E-05	0.999943739	0	0.633793857	0	0.5	CRA004775;PRJNA827465	Yang2025_Silkie_Wenchang	0.999969182
ENSGALG00010014829	ENSGALG00010014829	2	0.345215395	0.275241611	1.254226766	0.209759594	0	0.695591978	0	1	PRJNA827465;Yang2025_Silkie_Wenchang	CRA004775	0.721625118
ENSGALG00010014829	ENSGALG00010014829	2	0.074941008	0.626462935	0.119625605	0.904779735	0.516514561	2.898327456	65.49734234	0.5	CRA004775;Yang2025_Silkie_Wenchang	PRJNA827465	0.977793401
ENSGALG00010014829	ENSGALG00010014829	2	-0.073404678	0.362282196	-0.202617403	0.839434086	0.078263021	1.354035619	26.14669908	0.5	CRA004775;PRJNA827465	Yang2025_Silkie_Wenchang	0.959493354
ENSGALG00010014831	VAPB	2	-0.192874328	0.131340791	-1.468502863	0.141967679	0.028494207	5.547254169	81.97306326	1	PRJNA827465;Yang2025_Silkie_Wenchang	CRA004775	0.62757683
ENSGALG00010014831	VAPB	2	0.00664025	0.350493828	0.018945413	0.984884652	0.231047958	16.64646446	93.99271838	0.5	CRA004775;Yang2025_Silkie_Wenchang	PRJNA827465	0.995759046
ENSGALG00010014831	VAPB	2	0.126883154	0.217882755	0.582346013	0.560333638	0.083938283	8.136055883	87.70903231	0.5	CRA004775;PRJNA827465	Yang2025_Silkie_Wenchang	0.881203589
ENSGALG00010014837	APBA1	2	0.116966534	0.097212631	1.203203051	0.228897755	0	0.872734146	0	1	PRJNA827465;Yang2025_Silkie_Wenchang	CRA004775	0.741601554
ENSGALG00010014837	APBA1	2	-0.122820582	0.482568922	-0.254514074	0.799098433	0.337749118	3.024076498	66.93205345	0.5	CRA004775;Yang2025_Silkie_Wenchang	PRJNA827465	0.949922217
ENSGALG00010014837	APBA1	2	-0.177831262	0.36558386	-0.486430833	0.626661734	0.170952226	2.063435879	51.537142	0.5	CRA004775;PRJNA827465	Yang2025_Silkie_Wenchang	0.901504244
ENSGALG00010014839	TM7SF3	2	-0.121260645	0.183626647	-0.660365185	0.509019508	0.053046764	4.47522766	77.65476807	0.5	PRJNA827465;Yang2025_Silkie_Wenchang	CRA004775	0.865193604
ENSGALG00010014839	TM7SF3	2	-0.378446207	0.138102361	-2.740331178	0.00613773	0	0.676488783	0	1	CRA004775;Yang2025_Silkie_Wenchang	PRJNA827465	0.118495743
ENSGALG00010014839	TM7SF3	2	-0.215512356	0.326761694	-0.65953984	0.509549171	0.165882804	3.77668281	73.52173719	0.5	CRA004775;PRJNA827465	Yang2025_Silkie_Wenchang	0.865257661
ENSGALG00010014840	NCF4	2	-0.333593705	0.14909825	-2.237408593	0.025259647	0.011878189	1.303564494	23.28726316	1	PRJNA827465;Yang2025_Silkie_Wenchang	CRA004775	0.270438551
ENSGALG00010014840	NCF4	2	-0.543852832	0.212465923	-2.559717931	0.010475715	0	0.003609399	0	1	CRA004775;Yang2025_Silkie_Wenchang	PRJNA827465	0.162995658
ENSGALG00010014840	NCF4	2	-0.256998211	0.13777592	-1.865334753	0.062134497	0	0.357838082	0	1	CRA004775;PRJNA827465	Yang2025_Silkie_Wenchang	0.429956168
ENSGALG00010014847	PAICS	2	-0.195014168	0.244465598	-0.797716202	0.4250352	0.111606283	15.0882986	93.37234749	0.5	PRJNA827465;Yang2025_Silkie_Wenchang	CRA004775	0.841201577
ENSGALG00010014847	PAICS	2	-0.333893662	0.159662071	-2.091252224	0.03650546	0.028492136	1.908591632	47.60534504	1	CRA004775;Yang2025_Silkie_Wenchang	PRJNA827465	0.329454443
ENSGALG00010014847	PAICS	2	0.031267974	0.081289734	0.384648499	0.700497857	0	0.330057565	0	0.5	CRA004775;PRJNA827465	Yang2025_Silkie_Wenchang	0.922303014
ENSGALG00010014856	MMGT1	2	-0.084126552	0.072579707	-1.159091926	0.246418716	0.006527658	2.606389245	61.6327453	1	PRJNA827465;Yang2025_Silkie_Wenchang	CRA004775	0.756843363
ENSGALG00010014856	MMGT1	2	-0.141906173	0.065259699	-2.174484019	0.029668804	0	0.717789129	0	0.5	CRA004775;Yang2025_Silkie_Wenchang	PRJNA827465	0.29397955
ENSGALG00010014856	MMGT1	2	-0.014479999	0.054950067	-0.26351195	0.792156014	0	0.0166681	0	0.5	CRA004775;PRJNA827465	Yang2025_Silkie_Wenchang	0.948314332
ENSGALG00010014859	TRAPPC3	2	-0.093124383	0.165066938	-0.564161329	0.572644343	0.045128977	5.333022753	81.24890805	0.5	PRJNA827465;Yang2025_Silkie_Wenchang	CRA004775	0.88573298
ENSGALG00010014859	TRAPPC3	2	-0.155065546	0.187402387	-0.827447014	0.407983743	0.033354934	1.698202278	41.11419982	0.5	CRA004775;Yang2025_Silkie_Wenchang	PRJNA827465	0.833982683
ENSGALG00010014859	TRAPPC3	2	0.053019226	0.051792058	1.023694135	0.305979766	0	0.060190728	0	1	CRA004775;PRJNA827465	Yang2025_Silkie_Wenchang	0.792366985
ENSGALG00010014861	MED21	2	-0.169793171	0.183647716	-0.924559122	0.355195285	0.049059078	3.246499648	69.19759407	1	PRJNA827465;Yang2025_Silkie_Wenchang	CRA004775	0.812301302
ENSGALG00010014861	MED21	2	-0.257951665	0.17749394	-1.453298436	0.146140911	0.014095281	1.270124476	21.26755927	1	CRA004775;Yang2025_Silkie_Wenchang	PRJNA827465	0.634916196
ENSGALG00010014861	MED21	2	-0.024073327	0.075392409	-0.319307036	0.749493698	0	0.002129989	0	1	CRA004775;PRJNA827465	Yang2025_Silkie_Wenchang	0.937362187
ENSGALG00010014862	ENSGALG00010014862	2	0.503995954	0.60553323	0.832317582	0.405229709	0.59711038	5.381978237	81.4194715	0.5	PRJNA827465;Yang2025_Silkie_Wenchang	CRA004775	0.833112102
ENSGALG00010014862	ENSGALG00010014862	2	0.403183593	0.842301861	0.478668767	0.632174289	1.07300524	3.769608556	73.47204663	0.5	CRA004775;Yang2025_Silkie_Wenchang	PRJNA827465	0.903058174
ENSGALG00010014862	ENSGALG00010014862	2	-0.182853373	0.329726281	-0.554561112	0.579194894	0	0.324217607	0	1	CRA004775;PRJNA827465	Yang2025_Silkie_Wenchang	0.887888423
ENSGALG00010014865	TLR2	2	-0.681870506	0.252099921	-2.704762871	0.006835316	0.041848315	1.46766446	31.86453528	1	PRJNA827465;Yang2025_Silkie_Wenchang	CRA004775	0.126900539
ENSGALG00010014865	TLR2	2	-0.868028878	0.285952972	-3.035565153	0.002400853	0	0.430733203	0	1	CRA004775;Yang2025_Silkie_Wenchang	PRJNA827465	0.065665096
ENSGALG00010014865	TLR2	2	-0.488520694	0.232183856	-2.104025245	0.035376245	0	0.023900039	0	1	CRA004775;PRJNA827465	Yang2025_Silkie_Wenchang	0.324684241
ENSGALG00010014866	FAM26E	2	-0.245267736	0.314254645	-0.780474498	0.435111633	0.185998719	17.15516395	94.17085139	0.5	PRJNA827465;Yang2025_Silkie_Wenchang	CRA004775	0.842762754
ENSGALG00010014866	FAM26E	2	0.080569433	0.682682374	0.118018914	0.906052671	0.864327778	13.06549854	92.34625455	0.5	CRA004775;Yang2025_Silkie_Wenchang	PRJNA827465	0.978196186
ENSGALG00010014866	FAM26E	2	0.357436593	0.360794702	0.990692466	0.321835771	0.201989029	3.822239508	73.83732762	1	CRA004775;PRJNA827465	Yang2025_Silkie_Wenchang	0.798513866
ENSGALG00010014867	TP63	2	0.260458529	0.337215912	0.772379119	0.439889917	0.18797043	5.762479572	82.6463593	0.5	PRJNA827465;Yang2025_Silkie_Wenchang	CRA004775	0.844353254
ENSGALG00010014867	TP63	2	0.575444342	0.190289803	3.024041922	0.002494219	0	0.125390503	0	1	CRA004775;Yang2025_Silkie_Wenchang	PRJNA827465	0.067144027
ENSGALG00010014867	TP63	2	-0.023815648	0.183847566	-0.129540189	0.896930227	0	0.620741093	0	0.5	CRA004775;PRJNA827465	Yang2025_Silkie_Wenchang	0.975244266
ENSGALG00010014876	PCNT	2	0.342621165	0.418413715	0.818857396	0.41286778	0.328150283	15.33427825	93.47866275	0.5	PRJNA827465;Yang2025_Silkie_Wenchang	CRA004775	0.835870654
ENSGALG00010014876	PCNT	2	0.622128052	0.171911898	3.618877217	0.000295884	0.014680031	1.328434653	24.72343314	1	CRA004775;Yang2025_Silkie_Wenchang	PRJNA827465	0.016889382
ENSGALG00010014876	PCNT	2	0.147364157	0.242656506	0.607295305	0.543654954	0.094778182	4.524188039	77.89658627	0.5	CRA004775;PRJNA827465	Yang2025_Silkie_Wenchang	0.876968972
ENSGALG00010014877	ENSGALG00010014877	2	-0.152996634	0.241590102	-0.63329016	0.526544178	0.09979338	6.884338049	85.47427519	0.5	PRJNA827465;Yang2025_Silkie_Wenchang	CRA004775	0.87066367
ENSGALG00010014877	ENSGALG00010014877	2	-0.146214503	0.265374717	-0.550973748	0.58165167	0.115130698	5.327692433	81.23014771	0.5	CRA004775;Yang2025_Silkie_Wenchang	PRJNA827465	0.889045686
ENSGALG00010014877	ENSGALG00010014877	2	0.108029139	0.111504036	0.968836127	0.332626958	0	0.042956108	0	1	CRA004775;PRJNA827465	Yang2025_Silkie_Wenchang	0.802178578
ENSGALG00010014878	TMCO1	2	-0.159050935	0.104224865	-1.526036376	0.127000806	0.013004086	2.01809031	50.44820367	1	PRJNA827465;Yang2025_Silkie_Wenchang	CRA004775	0.598144118
ENSGALG00010014878	TMCO1	2	-0.273006164	0.120937031	-2.257424062	0.02398159	0	0.237543964	0	1	CRA004775;Yang2025_Silkie_Wenchang	PRJNA827465	0.264463641
ENSGALG00010014878	TMCO1	2	-0.09677661	0.049001257	-1.97498221	0.048270167	0	0.255671892	0	1	CRA004775;PRJNA827465	Yang2025_Silkie_Wenchang	0.379964424
ENSGALG00010014880	SFRP2	2	-0.358777174	0.260562395	-1.37693382	0.16853271	0.124239709	11.61968471	91.39391451	1	PRJNA827465;Yang2025_Silkie_Wenchang	CRA004775	0.669363714
ENSGALG00010014880	SFRP2	2	-0.610135773	0.12416016	-4.914102662	8.919E-07	0	0.387081976	0	1	CRA004775;Yang2025_Silkie_Wenchang	PRJNA827465	0.000202626
ENSGALG00010014880	SFRP2	2	-0.110906393	0.083473135	-1.328647755	0.183964209	0	0.099473634	0	1	CRA004775;PRJNA827465	Yang2025_Silkie_Wenchang	0.692470323
ENSGALG00010014882	XPNPEP2	2	0.358677713	0.8564582	0.418791849	0.675368262	1.251813654	6.67694655	85.02309413	0.5	PRJNA827465;Yang2025_Silkie_Wenchang	CRA004775	0.91635305
ENSGALG00010014882	XPNPEP2	2	0.485251906	1.026363105	0.472787753	0.636364613	1.79192835	6.364187255	84.28707453	0.5	CRA004775;Yang2025_Silkie_Wenchang	PRJNA827465	0.904566695
ENSGALG00010014882	XPNPEP2	2	1.394548759	0.442546059	3.151194621	0.001626041	0	0.141793652	0	1	CRA004775;PRJNA827465	Yang2025_Silkie_Wenchang	0.050963549
ENSGALG00010014883	PPAT	2	-0.198464738	0.263230322	-0.753958495	0.450874144	0.128029027	13.12178055	92.37908303	0.5	PRJNA827465;Yang2025_Silkie_Wenchang	CRA004775	0.847637353
ENSGALG00010014883	PPAT	2	-0.459751236	0.093177725	-4.934132461	8.05078E-07	0	0.000171772	0	1	CRA004775;Yang2025_Silkie_Wenchang	PRJNA827465	0.000187231
ENSGALG00010014883	PPAT	2	-0.095901266	0.244973523	-0.391476044	0.695445393	0.07480195	2.128411309	53.01659996	0.5	CRA004775;PRJNA827465	Yang2025_Silkie_Wenchang	0.921038437
ENSGALG00010014886	RABGAP1L	2	-0.174126425	0.123410185	-1.410956685	0.158257386	0.023466353	4.284060834	76.65766107	1	PRJNA827465;Yang2025_Silkie_Wenchang	CRA004775	0.652026484
ENSGALG00010014886	RABGAP1L	2	-0.28281287	0.087665331	-3.226051479	0.001255108	0	0.348885708	0	1	CRA004775;Yang2025_Silkie_Wenchang	PRJNA827465	0.043044471
ENSGALG00010014886	RABGAP1L	2	-0.070345289	0.066966574	-1.050453745	0.293509547	0	0.249146133	0	1	CRA004775;PRJNA827465	Yang2025_Silkie_Wenchang	0.786979185
ENSGALG00010014887	MTMR12	2	0.332064032	0.115792358	2.867754291	0.004133964	0.015298927	2.317625361	56.85238792	1	PRJNA827465;Yang2025_Silkie_Wenchang	CRA004775	0.093485158
ENSGALG00010014887	MTMR12	2	0.436393516	0.107786351	4.048689947	5.15051E-05	0	0.216584716	0	1	CRA004775;Yang2025_Silkie_Wenchang	PRJNA827465	0.004949403
ENSGALG00010014887	MTMR12	2	0.23021556	0.095284536	2.416085229	0.015688389	0	0.056464175	0	1	CRA004775;PRJNA827465	Yang2025_Silkie_Wenchang	0.207264008
ENSGALG00010014889	PVALB	2	0.180304545	0.604436364	0.298301949	0.765472717	0.617424939	6.334828685	84.21425346	0.5	PRJNA827465;Yang2025_Silkie_Wenchang	CRA004775	0.940873598
ENSGALG00010014889	PVALB	2	-0.841267078	1.757125373	-0.478774646	0.632098956	5.692063287	12.33913659	91.89570524	0.5	CRA004775;Yang2025_Silkie_Wenchang	PRJNA827465	0.903058174
ENSGALG00010014889	PVALB	2	-1.377070046	1.142190788	-1.205639251	0.227956618	2.205773496	5.801390971	82.76275457	1	CRA004775;PRJNA827465	Yang2025_Silkie_Wenchang	0.740853612
ENSGALG00010014893	SLC9A6	2	-0.023254182	0.071174836	-0.326719154	0.743880312	0.003136251	1.375314542	27.28936041	0.5	PRJNA827465;Yang2025_Silkie_Wenchang	CRA004775	0.935821071
ENSGALG00010014893	SLC9A6	2	-0.189965089	0.124790476	-1.522272327	0.127940859	0.007463448	1.191039714	16.03974342	1	CRA004775;Yang2025_Silkie_Wenchang	PRJNA827465	0.60032911
ENSGALG00010014893	SLC9A6	2	-0.134619106	0.218206431	-0.616934639	0.537277838	0.069160536	2.969667497	66.32619642	0.5	CRA004775;PRJNA827465	Yang2025_Silkie_Wenchang	0.87481821
ENSGALG00010014894	RAB22A	2	-0.077484159	0.09352628	-0.828474727	0.407401702	0	0.578121821	0	1	PRJNA827465;Yang2025_Silkie_Wenchang	CRA004775	0.833863243
ENSGALG00010014894	RAB22A	2	-0.098797844	0.127574758	-0.774430968	0.438675977	0	0.790735318	0	0.5	CRA004775;Yang2025_Silkie_Wenchang	PRJNA827465	0.844002503
ENSGALG00010014894	RAB22A	2	0.002735411	0.109651158	0.024946485	0.980097649	0	0.173544927	0	0.5	CRA004775;PRJNA827465	Yang2025_Silkie_Wenchang	0.994658414
ENSGALG00010014897	COL8A2	2	0.182732869	0.185532144	0.984912185	0.324667208	0	0.543478434	0	1	PRJNA827465;Yang2025_Silkie_Wenchang	CRA004775	0.799874335
ENSGALG00010014897	COL8A2	2	0.1543067	0.250550488	0.615870682	0.537979873	0.006320454	1.052627983	4.999675445	0.5	CRA004775;Yang2025_Silkie_Wenchang	PRJNA827465	0.87481821
ENSGALG00010014897	COL8A2	2	0.032423127	0.190385008	0.17030294	0.864771899	0	0.239647883	0	0.5	CRA004775;PRJNA827465	Yang2025_Silkie_Wenchang	0.96675285
ENSGALG00010014898	ENSGALG00010014898	2	0.066324115	0.221698765	0.299163212	0.764815517	0	0.010196321	0	1	PRJNA827465;Yang2025_Silkie_Wenchang	CRA004775	0.940711384
ENSGALG00010014898	ENSGALG00010014898	2	0.119877119	0.324595152	0.369312722	0.711894644	0	0.139840175	0	1	CRA004775;Yang2025_Silkie_Wenchang	PRJNA827465	0.92597752
ENSGALG00010014898	ENSGALG00010014898	2	0.119985976	0.24036714	0.499177951	0.617654027	0	0.113301995	0	1	CRA004775;PRJNA827465	Yang2025_Silkie_Wenchang	0.899121942
ENSGALG00010014900	UBLCP1	2	-0.093462295	0.085406911	-1.094317709	0.273815661	0.009976667	2.992634017	66.58462096	1	PRJNA827465;Yang2025_Silkie_Wenchang	CRA004775	0.774526342
ENSGALG00010014900	UBLCP1	2	-0.185078321	0.079795843	-2.319398039	0.020373464	0	0.07539548	0	1	CRA004775;Yang2025_Silkie_Wenchang	PRJNA827465	0.240387846
ENSGALG00010014900	UBLCP1	2	-0.027049753	0.049325686	-0.548390807	0.583423587	0	0.261987288	0	1	CRA004775;PRJNA827465	Yang2025_Silkie_Wenchang	0.890496981
ENSGALG00010014902	AASDH	2	0.298149789	0.141557364	2.106211786	0.03518596	0.026675002	2.880266108	65.28098576	1	PRJNA827465;Yang2025_Silkie_Wenchang	CRA004775	0.323488604
ENSGALG00010014902	AASDH	2	0.07474591	0.412227111	0.181322159	0.856114714	0.299193379	8.102953307	87.65882065	0.5	CRA004775;Yang2025_Silkie_Wenchang	PRJNA827465	0.963499334
ENSGALG00010014902	AASDH	2	-0.043448271	0.265178088	-0.163845631	0.869852669	0.109525261	4.004040219	75.02522589	0.5	CRA004775;PRJNA827465	Yang2025_Silkie_Wenchang	0.968610705
ENSGALG00010014903	MCF2L	2	-0.070547564	0.090297457	-0.781279631	0.434638047	0	0.522171835	0	0.5	PRJNA827465;Yang2025_Silkie_Wenchang	CRA004775	0.842762754
ENSGALG00010014903	MCF2L	2	-0.098450793	0.113484582	-0.867525716	0.385654028	0	0.84266548	0	0.5	CRA004775;Yang2025_Silkie_Wenchang	PRJNA827465	0.82648313
ENSGALG00010014903	MCF2L	2	0.034856588	0.136164435	0.255988926	0.797959388	0	0.404854783	0	1	CRA004775;PRJNA827465	Yang2025_Silkie_Wenchang	0.949922217
ENSGALG00010014906	IFT27	2	-0.366184163	0.201513887	-1.817165901	0.069191715	0.069010597	6.554120303	84.74242227	1	PRJNA827465;Yang2025_Silkie_Wenchang	CRA004775	0.453013819
ENSGALG00010014906	IFT27	2	-0.295383064	0.294055326	-1.004515264	0.315130318	0.149942725	7.450575153	86.57821739	0.5	CRA004775;Yang2025_Silkie_Wenchang	PRJNA827465	0.796635323
ENSGALG00010014906	IFT27	2	-0.135036017	0.080169821	-1.684374674	0.092109282	0	0.899733466	0	0.5	CRA004775;PRJNA827465	Yang2025_Silkie_Wenchang	0.519672986
ENSGALG00010014907	ENSGALG00010014907	2	0.217583878	0.262105166	0.830139601	0.40645986	0.112912122	5.296329226	81.11899851	0.5	PRJNA827465;Yang2025_Silkie_Wenchang	CRA004775	0.833618122
ENSGALG00010014907	ENSGALG00010014907	2	0.285263501	0.226236598	1.260907842	0.207342052	0.060212104	2.428386299	58.82039029	1	CRA004775;Yang2025_Silkie_Wenchang	PRJNA827465	0.719377239
ENSGALG00010014907	ENSGALG00010014907	2	-0.00133171	0.091610367	-0.014536675	0.98840182	0	0.107174148	0	0.5	CRA004775;PRJNA827465	Yang2025_Silkie_Wenchang	0.996891127
ENSGALG00010014908	ENSGALG00010014908	2	-0.111174649	0.206116494	-0.539377741	0.589626237	0.078628803	13.28378967	92.47202775	0.5	PRJNA827465;Yang2025_Silkie_Wenchang	CRA004775	0.892320726
ENSGALG00010014908	ENSGALG00010014908	2	-0.307221535	0.08980256	-3.421077691	0.000623735	0	0.392072479	0	1	CRA004775;Yang2025_Silkie_Wenchang	PRJNA827465	0.027274131
ENSGALG00010014908	ENSGALG00010014908	2	0.082513815	0.062948499	1.310814645	0.189920394	0	0.330133891	0	0.5	CRA004775;PRJNA827465	Yang2025_Silkie_Wenchang	0.699271855
ENSGALG00010014909	FBXL14	2	0.243965803	0.307035461	0.794585102	0.426854487	0.175825313	14.47679437	93.09239342	0.5	PRJNA827465;Yang2025_Silkie_Wenchang	CRA004775	0.84125685
ENSGALG00010014909	FBXL14	2	0.217584947	0.357451938	0.608711057	0.542715977	0.226914309	8.858408735	88.71129082	0.5	CRA004775;Yang2025_Silkie_Wenchang	PRJNA827465	0.87692674

ENSGALG00010014909	FBXL14	2	-0.062837274	0.067205148	-0.935006848	0.34978477	0	0.241411745	0	1	CRA004775;PRJNA827465	Yang2025_Silkie_Wenchang	0.809903518
ENSGALG00010014912	ENSGALG00010014912	2	-0.650230237	0.442123132	-1.470699426	0.141372421	0.292623853	3.904605964	74.38922111	1	PRJNA827465;Yang2025_Silkie_Wenchang	CRA004775	0.626421677
ENSGALG00010014912	ENSGALG00010014912	2	-1.056479561	0.296740117	-3.560285588	0.000370452	0	0.119159462	0	1	CRA004775;Yang2025_Silkie_Wenchang	PRJNA827465	0.01934739
ENSGALG00010014912	ENSGALG00010014912	2	-0.42964567	0.300855568	-1.428079503	0.153268976	0.056023315	1.333554473	25.01243707	1	CRA004775;PRJNA827465	Yang2025_Silkie_Wenchang	0.645602272
ENSGALG00010014914	PTAR1	2	0.184165471	0.087830412	2.096830325	0.036008598	0	0.621164904	0	1	PRJNA827465;Yang2025_Silkie_Wenchang	CRA004775	0.327557399
ENSGALG00010014914	PTAR1	2	0.262723016	0.126531325	2.076347617	0.037861812	0	0.017316869	0	1	CRA004775;Yang2025_Silkie_Wenchang	PRJNA827465	0.335620339
ENSGALG00010014914	PTAR1	2	0.141965871	0.104765159	1.355086673	0.175389944	0	0.103244527	0	1	CRA004775;PRJNA827465	Yang2025_Silkie_Wenchang	0.680258136
ENSGALG00010014916	DYNLT2B	2	-0.223602414	0.365406756	-0.611927422	0.540585778	0.225899508	6.22863893	83.94512812	0.5	PRJNA827465;Yang2025_Silkie_Wenchang	CRA004775	0.875579799
ENSGALG00010014916	DYNLT2B	2	-0.366084504	0.25516253	-1.434711064	0.15136945	0.064113268	1.969859622	49.23496128	1	CRA004775;Yang2025_Silkie_Wenchang	PRJNA827465	0.64289984
ENSGALG00010014916	DYNLT2B	2	0.059545383	0.122965772	0.484243553	0.628213031	0	0.585722463	0	0.5	CRA004775;PRJNA827465	Yang2025_Silkie_Wenchang	0.901904489
ENSGALG00010014917	EIF3D	2	-0.019378693	0.076720299	-0.252588859	0.80058594	0.002575842	1.214561175	17.66573632	0.5	PRJNA827465;Yang2025_Silkie_Wenchang	CRA004775	0.950156876
ENSGALG00010014917	EIF3D	2	-0.259147444	0.162787846	-1.591933619	0.111399621	0.021762285	1.584038125	36.87020631	1	CRA004775;Yang2025_Silkie_Wenchang	PRJNA827465	0.564582759
ENSGALG00010014917	EIF3D	2	-0.17954206	0.252443701	-0.711216242	0.476950245	0.101416115	4.286125366	76.66890455	0.5	CRA004775;PRJNA827465	Yang2025_Silkie_Wenchang	0.854986476
ENSGALG00010014918	PLRG1	2	-0.071892488	0.041604941	-1.727979586	0.083991884	0	0.166772065	0	1	PRJNA827465;Yang2025_Silkie_Wenchang	CRA004775	0.499490548
ENSGALG00010014918	PLRG1	2	-0.100682413	0.088048639	-1.143486309	0.252836736	0	0.029919263	0	1	CRA004775;Yang2025_Silkie_Wenchang	PRJNA827465	0.762731469
ENSGALG00010014918	PLRG1	2	-0.064195118	0.044799936	-1.432928789	0.151878189	0	0.000716114	0	1	CRA004775;PRJNA827465	Yang2025_Silkie_Wenchang	0.643294424
ENSGALG00010014922	MAN1A1	2	-0.026919568	0.24437438	-0.110157078	0.912284802	0.100534224	6.318260852	84.17285985	0.5	PRJNA827465;Yang2025_Silkie_Wenchang	CRA004775	0.978967245
ENSGALG00010014922	MAN1A1	2	-0.295697047	0.132503139	-2.231622957	0.025639893	0	0.499681266	0	1	CRA004775;Yang2025_Silkie_Wenchang	PRJNA827465	0.27294819
ENSGALG00010014922	MAN1A1	2	-0.072024951	0.409704808	-0.175797183	0.860453283	0.232776907	2.606796181	61.63873465	0.5	CRA004775;PRJNA827465	Yang2025_Silkie_Wenchang	0.964999892
ENSGALG00010014928	SUCO	2	0.135208473	0.04562098	2.96373448	0.003039304	0	0.709230481	0	1	PRJNA827465;Yang2025_Silkie_Wenchang	CRA004775	0.075885251
ENSGALG00010014928	SUCO	2	0.086100903	0.178592549	0.482108036	0.629729201	0.036868259	1.905426381	47.51830823	0.5	CRA004775;Yang2025_Silkie_Wenchang	PRJNA827465	0.902457014
ENSGALG00010014928	SUCO	2	0.074524377	0.10087075	0.738810576	0.460022029	0.00879861	1.230782677	18.75088766	0.5	CRA004775;PRJNA827465	Yang2025_Silkie_Wenchang	0.850104868
ENSGALG00010014929	FHL1	2	-0.244433399	0.138729629	-1.761940839	0.078079294	0	0.026770624	0	1	PRJNA827465;Yang2025_Silkie_Wenchang	CRA004775	0.480995373
ENSGALG00010014929	FHL1	2	-0.260049267	0.223574907	-1.163141567	0.24477207	0	0.182229823	0	1	CRA004775;Yang2025_Silkie_Wenchang	PRJNA827465	0.756114592
ENSGALG00010014929	FHL1	2	-0.278787825	0.156922932	-1.776590722	0.075635603	0	0.128564393	0	1	CRA004775;PRJNA827465	Yang2025_Silkie_Wenchang	0.474784631
ENSGALG00010014931	RNF145	2	-0.04033162	0.099471077	-0.405460774	0.685138846	0.012613765	2.649830652	62.26173929	0.5	PRJNA827465;Yang2025_Silkie_Wenchang	CRA004775	0.919196979
ENSGALG00010014931	RNF145	2	-0.044380832	0.127396621	-0.34836742	0.727564267	0.018026309	2.203338278	54.61432273	0.5	CRA004775;Yang2025_Silkie_Wenchang	PRJNA827465	0.931630234
ENSGALG00010014931	RNF145	2	0.055643539	0.060705482	0.916614725	0.359344565	0	0.130136703	0	1	CRA004775;PRJNA827465	Yang2025_Silkie_Wenchang	0.814335519
ENSGALG00010014934	MAMDC2	2	-0.158239515	0.223975314	-0.706504266	0.479874601	0.077714719	4.421896206	77.38526746	0.5	PRJNA827465;Yang2025_Silkie_Wenchang	CRA004775	0.856284002
ENSGALG00010014934	MAMDC2	2	-0.365576108	0.152131185	-2.403032018	0.016259764	0	0.209840536	0	1	CRA004775;Yang2025_Silkie_Wenchang	PRJNA827465	0.210770941
ENSGALG00010014934	MAMDC2	2	0.041252468	0.134271464	0.307231836	0.758666915	0	0.197598014	0	0.5	CRA004775;PRJNA827465	Yang2025_Silkie_Wenchang	0.938968566
ENSGALG00010014935	PCYT1A	2	-0.104364332	0.078085964	-1.336531262	0.181375689	0	0.016832537	0	1	PRJNA827465;Yang2025_Silkie_Wenchang	CRA004775	0.689004635
ENSGALG00010014935	PCYT1A	2	-0.159342862	0.116752475	-1.364792157	0.172318385	0	0.940583267	0	1	CRA004775;Yang2025_Silkie_Wenchang	PRJNA827465	0.67516992
ENSGALG00010014935	PCYT1A	2	-0.147074121	0.087414635	-1.682488527	0.092474148	0	0.917707482	0	1	CRA004775;PRJNA827465	Yang2025_Silkie_Wenchang	0.520673653
ENSGALG00010014936	FOXRED2	2	0.030234853	0.092039907	0.328497208	0.742535755	0.0025847	1.144487667	12.62465916	0.5	PRJNA827465;Yang2025_Silkie_Wenchang	CRA004775	0.935535551
ENSGALG00010014936	FOXRED2	2	-0.378039745	0.298398454	-1.266895788	0.205192555	0.132919101	3.788202202	73.60225388	1	CRA004775;Yang2025_Silkie_Wenchang	PRJNA827465	0.717945605
ENSGALG00010014936	FOXRED2	2	-0.275199173	0.399954129	-0.688076838	0.491404395	0.283505786	8.237857957	87.86092204	0.5	CRA004775;PRJNA827465	Yang2025_Silkie_Wenchang	0.85895707
ENSGALG00010014940	VAMP4	2	0.086197479	0.082354542	1.046663325	0.295254884	0	0.340767527	0	1	PRJNA827465;Yang2025_Silkie_Wenchang	CRA004775	0.787628307
ENSGALG00010014940	VAMP4	2	-0.014955383	0.194000189	-0.07708953	0.938552322	0.045908009	2.490069977	59.84048604	0.5	CRA004775;Yang2025_Silkie_Wenchang	PRJNA827465	0.985349045
ENSGALG00010014940	VAMP4	2	-0.039045611	0.134571152	-0.29014845	0.771702671	0.01623022	1.629945784	38.64826611	0.5	CRA004775;PRJNA827465	Yang2025_Silkie_Wenchang	0.942986883
ENSGALG00010014942	PMEPA1	2	0.26116426	0.180659066	1.445619453	0.148283967	0.057241432	8.119895656	87.68457081	1	PRJNA827465;Yang2025_Silkie_Wenchang	CRA004775	0.638004061
ENSGALG00010014942	PMEPA1	2	0.23936581	0.306044094	0.782128505	0.434139056	0.126691888	2.361465705	57.65341847	0.5	CRA004775;Yang2025_Silkie_Wenchang	PRJNA827465	0.842762754
ENSGALG00010014942	PMEPA1	2	0.070061264	0.084451406	0.829604478	0.406762444	0	0.492007089	0	0.5	CRA004775;PRJNA827465	Yang2025_Silkie_Wenchang	0.833618122
ENSGALG00010014943	APLN	2	0.19823139	0.129285348	1.533285811	0.125205449	0.003414508	1.092721368	8.485362354	1	PRJNA827465;Yang2025_Silkie_Wenchang	CRA004775	0.593887252
ENSGALG00010014943	APLN	2	0.408289959	0.21139617	1.931397147	0.053433958	0	0.011182353	0	1	CRA004775;Yang2025_Silkie_Wenchang	PRJNA827465	0.400632362
ENSGALG00010014943	APLN	2	0.138417763	0.137392036	1.007465692	0.313711036	0	0.4020716	0	1	CRA004775;PRJNA827465	Yang2025_Silkie_Wenchang	0.796531448
ENSGALG00010014944	FGB	2	-0.538685499	1.462668945	-0.368289421	0.71265744	3.695262237	7.3107154	86.3214481	0.5	PRJNA827465;Yang2025_Silkie_Wenchang	CRA004775	0.926028213
ENSGALG00010014944	FGB	2	-0.202507336	1.733489628	-0.116820622	0.907002202	5.636188893	15.98801962	93.74531666	0.5	CRA004775;Yang2025_Silkie_Wenchang	PRJNA827465	0.978196186
ENSGALG00010014944	FGB	2	1.347862123	0.433195139	3.111443319	0.001861752	0	0.312777827	0	1	CRA004775;PRJNA827465	Yang2025_Silkie_Wenchang	0.055391706
ENSGALG00010014945	TXN2	2	0.102056495	0.093993999	1.085776705	0.277577795	0	0.069883742	0	1	PRJNA827465;Yang2025_Silkie_Wenchang	CRA004775	0.776763074
ENSGALG00010014945	TXN2	2	-0.003708166	0.205821227	-0.018016443	0.985625736	0.017962539	1.247884119	19.86435403	0.5	CRA004775;Yang2025_Silkie_Wenchang	PRJNA827465	0.995939127
ENSGALG00010014945	TXN2	2	0.027569105	0.136794782	0.201536235	0.8402793	0.011932293	1.225326391	18.38909149	0.5	CRA004775;PRJNA827465	Yang2025_Silkie_Wenchang	0.959662809
ENSGALG00010014946	PCK1	2	-0.566862761	1.049584438	-0.540083047	0.589139762	1.838583733	6.039831341	83.44324628	0.5	PRJNA827465;Yang2025_Silkie_Wenchang	CRA004775	0.892171138
ENSGALG00010014946	PCK1	2	0.555916412	2.234429103	0.248795727	0.803518797	9.365982208	15.97905729	93.74180853	0.5	CRA004775;Yang2025_Silkie_Wenchang	PRJNA827465	0.951064358
ENSGALG00010014946	PCK1	2	1.550025505	1.180371288	1.313167748	0.189126429	2.202938023	4.624736734	78.37714755	1	CRA004775;PRJNA827465	Yang2025_Silkie_Wenchang	0.698162702
ENSGALG00010014948	RBM38	2	0.084241769	0.402788516	0.209146401	0.834333953	0.293761046	10.07379213	90.07325159	0.5	PRJNA827465;Yang2025_Silkie_Wenchang	CRA004775	0.958013011
ENSGALG00010014948	RBM38	2	0.557383293	0.202248583	2.755931764	0.00585252	0	0.111525371	0	1	CRA004775;Yang2025_Silkie_Wenchang	PRJNA827465	0.115356877
ENSGALG00010014948	RBM38	2	0.113913985	0.47436402	0.240140442	0.810221383	0.379679444	5.640718195	82.27176105	0.5	CRA004775;PRJNA827465	Yang2025_Silkie_Wenchang	0.952960588
ENSGALG00010014949	AGO3	2	-0.040993437	0.070640766	-0.580308496	0.561706599	0	0.580584753	0	1	PRJNA827465;Yang2025_Silkie_Wenchang	CRA004775	0.881802301
ENSGALG00010014949	AGO3	2	-0.122246406	0.111400677	-1.097357829	0.272485	0	0.053062904	0	1	CRA004775;Yang2025_Silkie_Wenchang	PRJNA827465	0.773470892
ENSGALG00010014949	AGO3	2	-0.013469813	0.085206702	-0.158083963	0.874390638	0	0.315111233	0	1	CRA004775;PRJNA827465	Yang2025_Silkie_Wenchang	0.970135804
ENSGALG00010014956	FGG	2	-1.408857511	2.864511049	-0.49183176	0.622838282	13.81116266	6.247095397	83.99256076	0.5	PRJNA827465;Yang2025_Silkie_Wenchang	CRA004775	0.900255255
ENSGALG00010014956	FGG	2	-1.224120965	2.942447821	-0.416021299	0.677394418	15.40434875	8.64176511	88.42828997	0.5	CRA004775;Yang2025_Silkie_Wenchang	PRJNA827465	0.917144807
ENSGALG00010014956	FGG	2	1.455269321	0.703393124	2.068927419	0.038552898	0	0.010965631	0	1	CRA004775;PRJNA827465	Yang2025_Silkie_Wenchang	0.338965227
ENSGALG00010014965	ABHD13	2	-0.396027112	0.370953874	-1.067591256	0.285704929	0.267294572	34.40509812	97.09345401	1	PRJNA827465;Yang2025_Silkie_Wenchang	CRA004775	0.781404649
ENSGALG00010014965	ABHD13	2	-0.36227877	0.42905121	-0.844371864	0.39846164	0.340387447	12.96489916	92.28686635	0.5	CRA004775;Yang2025_Silkie_Wenchang	PRJNA827465	0.831980453
ENSGALG00010014965	ABHD13	2	-0.023469708	0.054877856	-0.427671746	0.668890123	0	0.288355239	0	0.5	CRA004775;PRJNA827465	Yang2025_Silkie_Wenchang	0.914377769
ENSGALG00010014967	HNRNPH1	2	-0.041172294	0.049170228	-0.837341936	0.402400393	0.001172092	1.224640677	18.343395	0.5	PRJNA827465;Yang2025_Silkie_Wenchang	CRA004775	0.832879175
ENSGALG00010014967	HNRNPH1	2	0.027241798	0.084740518	0.321473105	0.74785189	0	0.215102653	0	0.5	CRA004775;Yang2025_Silkie_Wenchang	PRJNA827465	0.936938957
ENSGALG00010014967	HNRNPH1	2	-0.069800817	0.044554551	-1.566637208	0.117199516	0	3.63692E-06	0	1	CRA004775;PRJNA827465	Yang2025_Silkie_Wenchang	0.57646425
ENSGALG00010014971	SMC5	2	0.510799532	0.452280477	1.129386647	0.258734763	0.363700967	8.955596564	88.83379803	1	PRJNA827465;Yang2025_Silkie_Wenchang	CRA004775	0.765833156
ENSGALG00010014971	SMC5	2	0.588706073	0.405816174	1.450671786	0.146871275	0.257487409	4.54456667	77.99570185	1	CRA004775;Yang2025_Silkie_Wenchang	PRJNA827465	0.635558525
ENSGALG00010014971	SMC5	2	0.096601848	0.157697452	0.612577104	0.540156001	0	0.069283838	0	1	CRA004775;PRJNA827465	Yang2025_Silkie_Wenchang	0.87529516
ENSGALG00010014972	FGA	2	-0.86764537	2.505747404	-0.346262105	0.729145745	9.998124338	4.880142555	79.50879531	0.5	PRJNA827465;Yang2025_Silkie_Wenchang	CRA004775	0.932323844
ENSGALG00010014972	FGA	2	-0.647122972	2.582525039	-0.250577618	0.802140688	11.584379	7.231908037	86.17239054	0.5	CRA004775;Yang2025_Silkie_Wenchang	PRJNA827465	0.950630607
ENSGALG00010014972	FGA	2	1.675791726	0.714823554	2.344343182	0.019060624	0	0.010412943	0	1	CRA004775;PRJNA827465	Yang2025_Silkie_Wenchang	0.231290606
ENSGALG00010014974	LIG4	2	0.15427364	0.113774756	1.355956678	0.175112952	0	0.115436503	0	1	PRJNA827465;Yang2025_Silkie_Wenchang	CRA004775	0.680094025
ENSGALG00010014974	LIG4	2	0.152784931	0.169718843	0.900223735	0.368001198	0	0.283125318	0	1	CRA004775;Yang2025_Silkie_Wenchang	PRJNA827465	0.82021134
ENSGALG00010014974	LIG4	2	0.200069256	0.128695614	1.554592651	0.120043115	0	0.118233039	0	1	CRA004775;PRJNA827465	Yang2025_Silkie_Wenchang	0.581146887
ENSGALG00010014976	ITGB2	2	-0.233681254	0.168165965	-1.389587096	0.164654297	0.031918251	2.233763553	55.23250441	1	PRJNA827465;Yang2025_Silkie_Wenchang	CRA004775	0.663901457
ENSGALG00010014976	ITGB2	2	-0.415384422	0.174425151	-2.381447979	0.017244726	0	0.044101487	0	1	CRA004775;Yang2025_Silkie_Wenchang	PRJNA827465	0.218142734
ENSGALG00010014976	ITGB2	2	-0.102131324	0.127988677	-0.797971559	0.424886995	0	0.175392268	0	1	CRA004775;PRJNA827465	Yang2025_Silkie_Wenchang	0.841139484
ENSGALG00010014980	ENSGALG00010014980	2	-0.425282333	0.22944851	-1.853497905	0.063811026	0	0.198023987	0	1	PRJNA827465;Yang2025_Silkie_Wenchang	CRA004775	0.43524481
ENSGALG00010014980	ENSGALG00010014980	2	-0.55872212	0.295928765	-1.888029101	0.059022044	0	0.009310923	0	1	CRA004775;Yang2025_Silkie_Wenchang	PRJNA827465	0.42070065
ENSGALG00010014980	ENSGALG00010014980	2	-0.396322511	0.269018028	-1.473219154	0.140691952	0	0.17604166	0	1	CRA004775;PRJNA827465	Yang2025_Silkie_Wenchang	0.625168546
ENSGALG00010014984	LPP	2	0.065812412	0.125902209	0.522726425	0.601164647	0	3.21127E-05	0	1	PRJNA827465;Yang2025_Silkie_Wenchang	CRA004775	0.895230519
ENSGALG00010014984	LPP	2	0.171529773	0.217909838	0.78715938	0.431188572	0	0.450182898	0	1	CRA004775;Yang2025_Silkie_Wenchang	PRJNA827465	0.842503929
ENSGALG00010014984	LPP	2	0.10571166	0.132768486	0.796210486	0.425909704	0	0.595217407	0	1	CRA004775;PRJNA827465	Yang2025_Silkie_Wenchang	0.84125685
ENSGALG00010014985	ENSGALG00010014985	2	0.090780929	0.088496437	1.025814513	0.304979024	0.00963142	2.452483069	59.22499883	1	PRJNA827465;Yang2025_Silkie_Wenchang	CRA004775	0.79225794
ENSGALG00010014985	ENSGALG00010014985	2	0.1977519	0.089123397	2.218855058	0.026496587	0	0.000144289	0	1	CRA004775;Yang2025_Silkie_Wenchang	PRJNA827465	0.277705427
ENSGALG00010014985	ENSGALG00010014985	2	0.031372552	0.05695197	0.550859827	0.581729768	0	0.680938014	0	1	CRA004775;PRJNA827465	Yang2025_Silkie_Wenchang	0.889085592
ENSGALG00010014987	KLF9	2	-0.183124873	0.119395862	-1.533762313	0.125088138	0	0.02431019	0	1	PRJNA827465;Yang2025_Silkie_Wenchang	CRA004775	0.593686207
ENSGALG00010014987	KLF9	2	0.050421196	0.367915579	0.137045558	0.890994785	0.173696747	2.232361586	55.20438954	0.5	CRA004775;Yang2025_Silkie_Wenchang	PRJNA827465	0.973636056
ENSGALG00010014987	KLF9	2	0.070687846	0.34776783	0.203261601	0.838930567	0.137604497	1.932924392	48.26491898	0.5	CRA004775;PRJNA827465	Yang2025_Silkie_Wenchang	0.959283372
ENSGALG00010014990	AGO1	2	-0.15042415	0.123837818	-1.214686691	0.22448561	0.025677483	5.951137025	83.1964884	1	PRJNA827465;Yang2025_Silkie_Wenchang	CRA004775	0.737544559
ENSGALG00010014990	AGO1	2	-0.14181292	0.181901198	-0.779615096	0.435617467	0.045812103	2.881022703	65.29010344	0.5	CRA004775;Yang2025_Silkie_Wenchang	PRJNA827465	0.842762754
ENSGALG00010014990	AGO1	2	-0.028080226	0.050782452	-0.552951349	0.580296724	0	0.363062121	0	0.5	CRA004775;PRJNA827465	Yang2025_Silkie_Wenchang	0.888433837
ENSGALG00010014992	ARL15	2	-0.308582576	0.231470465	-1.333140173	0.182485808	0.101093216	17.29402775	94.21765702	1	PRJNA827465;Yang2025_Silkie_Wenchang	CRA004775	0.690634542
ENSGALG00010014992	ARL15	2	-0.229721073	0.335204444	-0.68531631	0.493144342	0.203949288	10.55862467	90.52906954	0.5	CRA004775;Yang2025_Silkie_Wenchang	PRJNA827465	0.859667262
ENSGALG00010014992	ARL15	2	-0.054087976	0.07432071	-0.727764528	0.466757742	0.004176802	1.239279908	19.30797928	0.5	CRA004775;PRJNA827465	Yang2025_Silkie_Wenchang	0.852842649
ENSGALG00010014994	ENSGALG00010014994	2	-0.285544199	0.175705909	-1.625125763	0.104135765	0.050432225	5.22548757	80.86302978	1	PRJNA827465;Yang2025_Silkie_Wenchang	CRA004775	0.548509371
ENSGALG00010014994	ENSGALG00010014994	2	-0.367527559	0.134119676	-2.740295604	0.006138395	0.012421906	1.502773265	33.45636209	1	CRA004775;Yang2025_Silkie_Wenchang	PRJNA827465	0.118495743
ENSGALG00010014994	ENSGALG00010014994	2	-0.139050491	0.06945774	-2.001943794	0.045290777	0	0.17613146	0	1	CRA004775;PRJNA827465	Yang2025_Silkie_Wenchang	0.366947722
ENSGALG00010014995	LRAT	2	1.051923037	0.249987926	4.20789537	2.5776E-05	0.008055478	1.059208673	5.589896914	1	PRJNA827465;Yang2025_Silkie_Wenchang	CRA004775	0.002953571
ENSGALG00010014995	LRAT	2	1.162978999	0.33808866	3.43986397	0.000582007	0	0.876894661	0	1	CRA004775;Yang2025_Silkie_Wenchang	PRJNA827465	0.025964366
ENSGALG00010014995	LRAT	2	0.856430079	0.254723767	3.362191482	0.000773265	0	0.033034943	0	1	CRA004775;PRJNA827465	Yang2025_Silkie_Wenchang	0.031625002
ENSGALG00010014997	RAE1	2	0.008804613	0.050485192	0.174399902	0.861551191	0	0.596268332	0	0.5	PRJNA827465;Yang2025_Silkie_Wenchang	CRA004775	0.965479094
ENSGALG00010014997	RAE1	2	-0.065842893	0.096246447	-0.684107258	0.493907439	0	0.000105007	0	1	CRA004775;Yang2025_Silkie_Wenchang	PRJNA827465	0.860175061
ENSGALG00010014997	RAE1	2	0.022102729	0.054714212	0.40396687	0.686237082	0	0.19897181	0	0.5	CRA004775;PRJNA827465	Yang2025_Silkie_Wenchang	0.919235539
ENSGALG00010015002	ADAM19	2	-0.071074997	0.206137055	-0.344794858	0.730248599	0.069978699	5.467572379	81.71034729	0.5	PRJNA827465;Yang2025_Silkie_Wenchang	CRA004775	0.932615145
ENSGALG00010015002	ADAM19	2	-0.316966407	0.147109344	-2.154631358	0.031190693	0	0.28426182	0	1	CRA004775;Yang2025_Silkie_Wenchang	PRJNA827465	0.302017199
ENSGALG00010015002	ADAM19	2	-0.027883841	0.291720909	-0.095583964	0.923851002	0.09814359	1.582994035	36.82856804	0.5	CRA004775;PRJNA827465	Yang2025_Silkie_Wenchang	0.982219519
ENSGALG00010015006	HSPB3	2	-0.102960277	0.400335452	-0.25718501	0.797035955	0.26551165	5.825160933	82.83309231	0.5	PRJNA827465;Yang2025_Silkie_Wenchang	CRA004775	0.949622804
ENSGALG00010015006	HSPB3	2	0.266718173	0.221394784	1.204717507	0.228312375	0	0.165603884	0	0.5	CRA004775;Yang2025_Silkie_Wenchang	PRJNA827465	0.741167494
ENSGALG00010015006	HSPB3	2	-0.453571309	0.223639995	-2.028131454	0.042546833	0	0.453009436	0	1	CRA004775;PRJNA827465	Yang2025_Silkie_Wenchang	0.356625761
ENSGALG00010015009	AGO4	2	-0.154961993	0.0380461	-4.073006035	4.64102E-05	0	0.006442044	0	1	PRJNA827465;Yang2025_Silkie_Wenchang	CRA004775	0.004594612
ENSGALG00010015009	AGO4	2	-0.164469942	0.082195047	-2.000971455	0.045395466	0	0.017845117	0	1	CRA004775;Yang2025_Silkie_Wenchang	PRJNA827465	0.367363248
ENSGALG00010015009	AGO4	2	-0.154489392	0.041969012	-3.68103475	0.000232289	0	0.027185326	0	1	CRA004775;PRJNA827465	Yang2025_Silkie_Wenchang	0.01411077
ENSGALG00010015013	SNX18	2	-0.134921384	0.172962282	-0.780062463	0.43535411	0.051205894	6.935221764	85.58085042	0.5	PRJNA827465;Yang2025_Silkie_Wenchang	CRA004775	0.842762754
ENSGALG00010015013	SNX18	2	-0.222332074	0.165917814	-1.340013278	0.180241028	0.026072401	1.466066838	31.79028583	0.5	CRA004775;Yang2025_Silkie_Wenchang	PRJNA827465	0.687301167
ENSGALG00010015013	SNX18	2	0.041613523	0.087799314	0.473961821	0.635527133	0	0.031356632	0	1	CRA004775;PRJNA827465	Yang2025_Silkie_Wenchang	0.904244739
ENSGALG00010015016	ARGLU1	2	0.419905309	0.351189361	1.195666371	0.231826779	0.236467741	23.81976594	95.80180593	1	PRJNA827465;Yang2025_Silkie_Wenchang	CRA004775	0.744183098
ENSGALG00010015016	ARGLU1	2	0.454437337	0.334596916	1.358163553	0.174411793	0.201396807	9.882614966	89.88122067	1	CRA004775;Yang2025_Silkie_Wenchang	PRJNA827465	0.678690684
ENSGALG00010015016	ARGLU1	2	0.081701193	0.059470679	1.37380628	0.169501831	0	0.033670526	0	1	CRA004775;PRJNA827465	Yang2025_Silkie_Wenchang	0.670890582
ENSGALG00010015017	FAM184A	2	0.185334643	0.233055593	0.795237912	0.426475122	0.073756318	2.862026161	65.0597184	0.5	PRJNA827465;Yang2025_Silkie_Wenchang	CRA004775	0.84125685
ENSGALG00010015017	FAM184A	2	0.363042064	0.207815086	1.746947592	0.080646429	0	0.606726908	0	1	CRA004775;Yang2025_Silkie_Wenchang	PRJNA827465	0.488841242
ENSGALG00010015017	FAM184A	2	0.009387202	0.121206954	0.077447714	0.938267385	0	0.103914042	0	0.5	CRA004775;PRJNA827465	Yang2025_Silkie_Wenchang	0.985349045
ENSGALG00010015018	MAP7D3	2	0.194552969	0.207656279	0.936899041	0.348810492	0.072773154	5.673767906	82.37502808	1	PRJNA827465;Yang2025_Silkie_Wenchang	CRA004775	0.809333419
ENSGALG00010015018	MAP7D3	2	0.186366566	0.251736647	0.740323541	0.4591037	0.097062192	4.270030041	76.58096102	0.5	CRA004775;Yang2025_Silkie_Wenchang	PRJNA827465	0.850098539
ENSGALG00010015018	MAP7D3	2	0.011761139	0.04274807	0.275126782	0.783218836	0	0.215214993	0	0.5	CRA004775;PRJNA827465	Yang2025_Silkie_Wenchang	0.945729563
ENSGALG00010015024	CRACD	2	0.159016185	0.241365569	0.658818843	0.510012106	0.083768822	3.270733255	69.42581611	0.5	PRJNA827465;Yang2025_Silkie_Wenchang	CRA004775	0.865276949
ENSGALG00010015024	CRACD	2	0.639091285	0.227909647	2.804143192	0.005045049	0.028383736	1.358380844	26.38294299	1	CRA004775;Yang2025_Silkie_Wenchang	PRJNA827465	0.105958126
ENSGALG00010015024	CRACD	2	0.392172334	0.475492778	0.824770328	0.409501995	0.399274594	8.024929335	87.53883108	0.5	CRA004775;PRJNA827465	Yang2025_Silkie_Wenchang	0.83448182
ENSGALG00010015025	TRAF3IP1	2	0.513406438	0.531215044	0.966475712	0.333806195	0.51061273	9.806066828	89.80223144	1	PRJNA827465;Yang2025_Silkie_Wenchang	CRA004775	0.802715667
ENSGALG00010015025	TRAF3IP1	2	0.597507211	0.470680742	1.269453278	0.204279444	0.359627601	5.251903292	80.95928382	1	CRA004775;Yang2025_Silkie_Wenchang	PRJNA827465	0.717281769
ENSGALG00010015025	TRAF3IP1	2	0.041803319	0.088716904	0.471199029	0.637498612	0	0.21533721	0	1	CRA004775;PRJNA827465	Yang2025_Silkie_Wenchang	0.905201059
ENSGALG00010015027	CLSPN	2	0.217218281	0.318275724	0.682484604	0.494932575	0.182580104	9.587477261	89.56972754	0.5	PRJNA827465;Yang2025_Silkie_Wenchang	CRA004775	0.860456274
ENSGALG00010015027	CLSPN	2	0.630382907	0.175224785	3.597567021	0.000321208	0	0.735466355	0	1	CRA004775;Yang2025_Silkie_Wenchang	PRJNA827465	0.01770607
ENSGALG00010015027	CLSPN	2	0.357658257	0.510761065	0.700245735	0.483773854	0.444551165	5.892688077	83.02981616	0.5	CRA004775;PRJNA827465	Yang2025_Silkie_Wenchang	0.85691916
ENSGALG00010015028	MCM9	2	-0.604944613	0.185597293	-3.259447392	0.001116295	0	0.014616165	0	1	PRJNA827465;Yang2025_Silkie_Wenchang	CRA004775	0.040399388
ENSGALG00010015028	MCM9	2	-0.61894639	0.27113492	-2.28279851	0.02244224	0	0.013042467	0	1	CRA004775;Yang2025_Silkie_Wenchang	PRJNA827465	0.254447309
ENSGALG00010015028	MCM9	2	-0.582959218	0.222540666	-2.61956266	0.00880426	0	0.002631778	0	1	CRA004775;PRJNA827465	Yang2025_Silkie_Wenchang	0.147185801
ENSGALG00010015029	TFRC	2	0.013610306	0.110135833	0.123577455	0.901649844	0	0.990487004	0	0.5	PRJNA827465;Yang2025_Silkie_Wenchang	CRA004775	0.976674416
ENSGALG00010015029	TFRC	2	-0.093300815	0.137506461	-0.678519499	0.497442365	0	0.000738254	0	1	CRA004775;Yang2025_Silkie_Wenchang	PRJNA827465	0.86098656
ENSGALG00010015029	TFRC	2	0.078240653	0.138240789	0.565973717	0.571411647	0	0.393400426	0	0.5	CRA004775;PRJNA827465	Yang2025_Silkie_Wenchang	0.885354083
ENSGALG00010015031	MYH9	2	0.253078075	0.321381238	0.787469974	0.431006799	0.193412199	15.13898558	93.3945376	0.5	PRJNA827465;Yang2025_Silkie_Wenchang	CRA004775	0.842503929
ENSGALG00010015031	MYH9	2	0.308864285	0.369124227	0.836748883	0.402733736	0.191790106	2.901099746	65.53031307	0.5	CRA004775;Yang2025_Silkie_Wenchang	PRJNA827465	0.832937979
ENSGALG00010015031	MYH9	2	-0.054804728	0.05695808	-0.962194082	0.335952121	0	0.081564245	0	1	CRA004775;PRJNA827465	Yang2025_Silkie_Wenchang	0.803504387
ENSGALG00010015032	C1orf216	2	1.423805743	0.409288575	3.478733172	0.00050379	0.270957232	5.170237237	80.65852776	1	PRJNA827465;Yang2025_Silkie_Wenchang	CRA004775	0.023568345
ENSGALG00010015032	C1orf216	2	0.757177769	1.097398048	0.689975502	0.490209593	2.326767548	29.44545321	96.60388994	0.5	CRA004775;Yang2025_Silkie_Wenchang	PRJNA827465	0.858529056
ENSGALG00010015032	C1orf216	2	0.3598198	0.687453102	0.523409959	0.600688995	0.881044373	14.67263694	93.18459249	0.5	CRA004775;PRJNA827465	Yang2025_Silkie_Wenchang	0.895230519
ENSGALG00010015033	BMP7	2	0.00123231	0.14808924	0.008321404	0.993360557	0	0.010690874	0	0.5	PRJNA827465;Yang2025_Silkie_Wenchang	CRA004775	0.997979742
ENSGALG00010015033	BMP7	2	0.023880355	0.198677764	0.120196417	0.904327556	0	0.004311453	0	1	CRA004775;Yang2025_Silkie_Wenchang	PRJNA827465	0.977586231
ENSGALG00010015033	BMP7	2	-0.002957429	0.185296896	-0.015960489	0.987265913	0	0.015905981	0	0.5	CRA004775;PRJNA827465	Yang2025_Silkie_Wenchang	0.996492495
ENSGALG00010015035	SLC35F1	2	-0.088614	0.220497267	-0.401882531	0.68777048	0.086219253	8.781522364	88.61245285	0.5	PRJNA827465;Yang2025_Silkie_Wenchang	CRA004775	0.919631238
ENSGALG00010015035	SLC35F1	2	0.261512144	0.641163403	0.40787129	0.683368171	0.729603687	8.153729258	87.73567323	0.5	CRA004775;Yang2025_Silkie_Wenchang	PRJNA827465	0.918800744
ENSGALG00010015035	SLC35F1	2	0.44353925	0.410505123	1.0804719	0.279932093	0.260436879	3.616146212	72.34625091	1	CRA004775;PRJNA827465	Yang2025_Silkie_Wenchang	0.778040488
ENSGALG00010015037	CYFIP2	2	0.068603949	0.132894089	0.51623025	0.605693605	0	0.584596134	0	0.5	PRJNA827465;Yang2025_Silkie_Wenchang	CRA004775	0.896474603
ENSGALG00010015037	CYFIP2	2	0.132346499	0.209060982	0.633052128	0.526699602	0	0.863500136	0	0.5	CRA004775;Yang2025_Silkie_Wenchang	PRJNA827465	0.87066367
ENSGALG00010015037	CYFIP2	2	-0.03367933	0.159091399	-0.211697997	0.832342651	0	0.371419268	0	1	CRA004775;PRJNA827465	Yang2025_Silkie_Wenchang	0.957808251
ENSGALG00010015038	PCSK5	2	0.028918034	0.08395028	0.344466204	0.730495708	0	0.004314287	0	1	PRJNA827465;Yang2025_Silkie_Wenchang	CRA004775	0.932653142
ENSGALG00010015038	PCSK5	2	0.073116723	0.144989705	0.504289065	0.614058261	0	0.637190545	0	1	CRA004775;Yang2025_Silkie_Wenchang	PRJNA827465	0.898282836
ENSGALG00010015038	PCSK5	2	0.054197333	0.094971255	0.570670915	0.568222738	0	0.657342421	0	1	CRA004775;PRJNA827465	Yang2025_Silkie_Wenchang	0.884049492
ENSGALG00010015039	FAM114A1	2	-0.079642671	0.19590549	-0.406536186	0.684348674	0.071281779	13.94932427	92.83119396	0.5	PRJNA827465;Yang2025_Silkie_Wenchang	CRA004775	0.918862939
ENSGALG00010015039	FAM114A1	2	-0.256839881	0.077324744	-3.321574301	0.000895111	0	0.504377803	0	1	CRA004775;Yang2025_Silkie_Wenchang	PRJNA827465	0.035180566
ENSGALG00010015039	FAM114A1	2	0.074434519	0.086702924	0.858500677	0.390616057	0.004943488	1.217041719	17.83354799	0.5	CRA004775;PRJNA827465	Yang2025_Silkie_Wenchang	0.829251057
ENSGALG00010015040	ZC4H2	2	0.046512958	0.05072528	0.91695813	0.359164581	0	0.164716961	0	0.5	PRJNA827465;Yang2025_Silkie_Wenchang	CRA004775	0.814273507
ENSGALG00010015040	ZC4H2	2	-0.043444334	0.133403302	-0.325661612	0.744680392	0	0.245394196	0	1	CRA004775;Yang2025_Silkie_Wenchang	PRJNA827465	0.935893304
ENSGALG00010015040	ZC4H2	2	0.046691078	0.053080374	0.879629781	0.3790599	0	0.573488706	0	0.5	CRA004775;PRJNA827465	Yang2025_Silkie_Wenchang	0.823923861
ENSGALG00010015042	EFNB2	2	-0.009253457	0.120000184	-0.077112027	0.938534426	0.020413803	3.180224075	68.55567481	0.5	PRJNA827465;Yang2025_Silkie_Wenchang	CRA004775	0.985349045
ENSGALG00010015042	EFNB2	2	0.484907639	0.415959754	1.165756143	0.243713054	0.28079537	4.71159734	78.77577544	1	CRA004775;Yang2025_Silkie_Wenchang	PRJNA827465	0.755365463
ENSGALG00010015042	EFNB2	2	0.374105678	0.540765442	0.691807665	0.489058122	0.522013954	8.42973272	88.13722768	0.5	CRA004775;PRJNA827465	Yang2025_Silkie_Wenchang	0.858232954
ENSGALG00010015043	COQ10B	2	0.1128049	0.097776837	1.153697578	0.248624177	0.004672316	1.247616109	19.84713944	0.5	PRJNA827465;Yang2025_Silkie_Wenchang	CRA004775	0.758965129
ENSGALG00010015043	COQ10B	2	-0.026021858	0.158225672	-0.164460405	0.869368714	0	0.068957556	0	0.5	CRA004775;Yang2025_Silkie_Wenchang	PRJNA827465	0.968558268
ENSGALG00010015043	COQ10B	2	0.169843112	0.090939829	1.867642745	0.061811884	0	0.049589204	0	1	CRA004775;PRJNA827465	Yang2025_Silkie_Wenchang	0.429075295
ENSGALG00010015044	ZC3H12B	2	0.081670556	0.083691849	0.975848395	0.329139598	0	0.882148639	0	1	PRJNA827465;Yang2025_Silkie_Wenchang	CRA004775	0.800761292
ENSGALG00010015044	ZC3H12B	2	0.213942534	0.160000868	1.33713358	0.181179038	0	0.055929706	0	1	CRA004775;Yang2025_Silkie_Wenchang	PRJNA827465	0.688657314
ENSGALG00010015044	ZC3H12B	2	0.049578335	0.090316298	0.548941182	0.583045815	0	0.096965313	0	1	CRA004775;PRJNA827465	Yang2025_Silkie_Wenchang	0.890269423
ENSGALG00010015046	PSMB2	2	-0.117876702	0.081965483	-1.438126127	0.150398271	0.00578279	1.357426393	26.33118045	1	PRJNA827465;Yang2025_Silkie_Wenchang	CRA004775	0.641187031
ENSGALG00010015046	PSMB2	2	-0.347671482	0.140789866	-2.469435425	0.013532644	0	0.355183083	0	1	CRA004775;Yang2025_Silkie_Wenchang	PRJNA827465	0.190294637
ENSGALG00010015046	PSMB2	2	-0.199063045	0.179342702	-1.109958995	0.267016696	0.044233453	2.439642017	59.01037967	1	CRA004775;PRJNA827465	Yang2025_Silkie_Wenchang	0.771247817
ENSGALG00010015047	NPY2R	2	0.128792829	0.178237869	0.722589596	0.469932062	0	0.027908526	0	1	PRJNA827465;Yang2025_Silkie_Wenchang	CRA004775	0.853539435
ENSGALG00010015047	NPY2R	2	0.030461625	0.247813208	0.122921716	0.902169089	0	0.185223906	0	0.5	CRA004775;Yang2025_Silkie_Wenchang	PRJNA827465	0.976923607
ENSGALG00010015047	NPY2R	2	0.099221714	0.207463897	0.47826015	0.632465056	0	0.316192228	0	0.5	CRA004775;PRJNA827465	Yang2025_Silkie_Wenchang	0.903058174
ENSGALG00010015049	DCK2	2	-0.262181125	0.264554097	-0.991030298	0.321670786	0.109934513	4.099159367	75.60475428	1	PRJNA827465;Yang2025_Silkie_Wenchang	CRA004775	0.798513866
ENSGALG00010015049	DCK2	2	0.029139351	0.666274438	0.043734757	0.965115833	0.756192206	6.486913298	84.58434769	0.5	CRA004775;Yang2025_Silkie_Wenchang	PRJNA827465	0.990532586
ENSGALG00010015049	DCK2	2	0.226033009	0.377765849	0.598341565	0.549612048	0.208599155	2.901185924	65.53133697	0.5	CRA004775;PRJNA827465	Yang2025_Silkie_Wenchang	0.878554604

ENSGALG00010015050	HIVEP3	2	0.241015949	0.304781286	0.790783295	0.42907046	0.152055863	4.990155481	79.96054424	0.5	PRJNA827465;Yang2025_Silkie_Wenchang	CRA004775	0.841924927
ENSGALG00010015050	HIVEP3	2	0.270676712	0.366818282	0.737904093	0.460572732	0.173531444	2.745033562	63.57057292	0.5	CRA004775;Yang2025_Silkie_Wenchang	PRJNA827465	0.85014477
ENSGALG00010015050	HIVEP3	2	-0.028540791	0.092955137	-0.307038343	0.758814187	0	0.105744143	0	1	CRA004775;PRJNA827465	Yang2025_Silkie_Wenchang	0.938996879
ENSGALG00010015052	ENSGALG00010015052	2	-0.155439003	0.105761148	-1.469717441	0.141638297	0.007745589	1.338191916	25.27230301	1	PRJNA827465;Yang2025_Silkie_Wenchang	CRA004775	0.627088401
ENSGALG00010015052	ENSGALG00010015052	2	-0.301119625	0.169640374	-1.775046931	0.075890138	0	0.179203025	0	1	CRA004775;Yang2025_Silkie_Wenchang	PRJNA827465	0.47539798
ENSGALG00010015052	ENSGALG00010015052	2	-0.100919839	0.081013443	-1.245717202	0.212868236	0	0.059213092	0	1	CRA004775;PRJNA827465	Yang2025_Silkie_Wenchang	0.725659838
ENSGALG00010015054	ENSGALG00010015054	2	-0.284309956	0.082140471	-3.461265249	0.000537643	0	0.675749037	0	1	PRJNA827465;Yang2025_Silkie_Wenchang	CRA004775	0.024599505
ENSGALG00010015054	ENSGALG00010015054	2	-0.242925695	0.105262198	-2.307815157	0.02100942	0	0.3602448	0	1	CRA004775;Yang2025_Silkie_Wenchang	PRJNA827465	0.245277621
ENSGALG00010015054	ENSGALG00010015054	2	-0.370623823	0.118041152	-3.139784875	0.001690719	0	0.0644056	0	1	CRA004775;PRJNA827465	Yang2025_Silkie_Wenchang	0.052036292
ENSGALG00010015055	ZNF335	2	-0.073836979	0.031700502	-2.32920534	0.01984819	0	0.75894846	0	1	PRJNA827465;Yang2025_Silkie_Wenchang	CRA004775	0.2365353
ENSGALG00010015055	ZNF335	2	-0.119896694	0.052878743	-2.267389252	0.023366457	0	0.465252406	0	1	CRA004775;Yang2025_Silkie_Wenchang	PRJNA827465	0.260752947
ENSGALG00010015055	ZNF335	2	-0.059472489	0.038638621	-1.539198024	0.12375596	0	0.847662573	0	1	CRA004775;PRJNA827465	Yang2025_Silkie_Wenchang	0.590002486
ENSGALG00010015056	ENSGALG00010015056	2	0.451187488	0.366980114	1.229460319	0.218899267	0.107087381	1.639140806	38.99242846	1	PRJNA827465;Yang2025_Silkie_Wenchang	CRA004775	0.73259614
ENSGALG00010015056	ENSGALG00010015056	2	0.271786852	0.606359587	0.448227186	0.653989244	0.523677289	3.473482967	71.21045333	0.5	CRA004775;Yang2025_Silkie_Wenchang	PRJNA827465	0.909866877
ENSGALG00010015056	ENSGALG00010015056	2	-0.043781806	0.284932349	-0.153656844	0.877880314	0	0.640621243	0	0.5	CRA004775;PRJNA827465	Yang2025_Silkie_Wenchang	0.970941655
ENSGALG00010015057	LAS1L	2	0.140865764	0.268279797	0.525070339	0.599534295	0.13529867	16.61629118	93.98180984	0.5	PRJNA827465;Yang2025_Silkie_Wenchang	CRA004775	0.895010988
ENSGALG00010015057	LAS1L	2	-0.017899413	0.450186858	-0.039759963	0.968284496	0.377245888	14.02382059	92.86927558	0.5	CRA004775;Yang2025_Silkie_Wenchang	PRJNA827465	0.991209187
ENSGALG00010015057	LAS1L	2	-0.251454557	0.173678926	-1.447812712	0.147669437	0.039004149	2.417285975	58.63129103	1	CRA004775;PRJNA827465	Yang2025_Silkie_Wenchang	0.636995621
ENSGALG00010015059	ENSGALG00010015059	2	0.184934952	0.257188224	0.719064617	0.472101107	0.12076871	11.41786798	91.24179749	0.5	PRJNA827465;Yang2025_Silkie_Wenchang	CRA004775	0.853998373
ENSGALG00010015059	ENSGALG00010015059	2	0.026435265	0.452890945	0.058370045	0.953453875	0.368275314	9.361584674	89.3180478	0.5	CRA004775;Yang2025_Silkie_Wenchang	PRJNA827465	0.987758913
ENSGALG00010015059	ENSGALG00010015059	2	-0.180763019	0.178686884	-1.011618843	0.311720333	0.036445764	1.888737732	47.05458661	1	CRA004775;PRJNA827465	Yang2025_Silkie_Wenchang	0.795968311
ENSGALG00010015061	MMP9	2	0.309076996	0.490929392	0.629575253	0.528972519	0	0.135288325	0	1	PRJNA827465;Yang2025_Silkie_Wenchang	CRA004775	0.871809618
ENSGALG00010015061	MMP9	2	0.472836271	0.379199613	1.246932368	0.212422297	0	0.000556132	0	1	CRA004775;Yang2025_Silkie_Wenchang	PRJNA827465	0.725098108
ENSGALG00010015061	MMP9	2	0.37338224	0.400466483	0.932368264	0.35114624	0	0.184714913	0	1	CRA004775;PRJNA827465	Yang2025_Silkie_Wenchang	0.810161472
ENSGALG00010015062	ENSGALG00010015062	2	-0.304514569	0.136088318	-2.237624617	0.025245545	0	0.581687088	0	1	PRJNA827465;Yang2025_Silkie_Wenchang	CRA004775	0.270438551
ENSGALG00010015062	ENSGALG00010015062	2	0.022409319	0.570827383	0.039257611	0.968685002	0.505827595	4.214381869	76.27172783	0.5	CRA004775;Yang2025_Silkie_Wenchang	PRJNA827465	0.991279382
ENSGALG00010015062	ENSGALG00010015062	2	0.096128623	0.442248513	0.21736336	0.827925183	0.284918544	3.111375672	67.85987597	0.5	CRA004775;PRJNA827465	Yang2025_Silkie_Wenchang	0.956550752
ENSGALG00010015063	EEF1AKMT4	2	-0.022313835	0.138087623	-0.161591852	0.871627275	0	0.563627524	0	0.5	PRJNA827465;Yang2025_Silkie_Wenchang	CRA004775	0.969425739
ENSGALG00010015063	EEF1AKMT4	2	-0.033851246	0.201044187	-0.168377144	0.866286588	0.00430517	1.055226754	5.233638528	0.5	CRA004775;Yang2025_Silkie_Wenchang	PRJNA827465	0.967373047
ENSGALG00010015063	EEF1AKMT4	2	-0.128157346	0.143129203	-0.895396208	0.370575327	0	0.267547934	0	1	CRA004775;PRJNA827465	Yang2025_Silkie_Wenchang	0.821334353
ENSGALG00010015064	RFK	2	-0.181613248	0.18050242	-1.00615409	0.314341453	0.045392403	3.295923702	69.65949183	1	PRJNA827465;Yang2025_Silkie_Wenchang	CRA004775	0.796531448
ENSGALG00010015064	RFK	2	-0.319777245	0.123882895	-2.581286503	0.009843285	0	0.415925722	0	1	CRA004775;Yang2025_Silkie_Wenchang	PRJNA827465	0.15667223
ENSGALG00010015064	RFK	2	-0.036572871	0.125158693	-0.292211995	0.770124541	0	0.299903834	0	1	CRA004775;PRJNA827465	Yang2025_Silkie_Wenchang	0.942170227
ENSGALG00010015066	RUFY1	2	0.107739291	0.202677761	0.531579244	0.595017444	0.076584763	14.6018916	93.15157222	0.5	PRJNA827465;Yang2025_Silkie_Wenchang	CRA004775	0.893412691
ENSGALG00010015066	RUFY1	2	0.297086358	0.083012284	3.578824041	0.000345144	0	0.378549631	0	1	CRA004775;Yang2025_Silkie_Wenchang	PRJNA827465	0.018430957
ENSGALG00010015066	RUFY1	2	-0.07483695	0.058678356	-1.275375699	0.202176296	0.000226637	1.007420598	0.73659381	0.5	CRA004775;PRJNA827465	Yang2025_Silkie_Wenchang	0.715159298
ENSGALG00010015068	ESM1	2	0.137870292	0.117947037	1.168916958	0.24243709	0	0.117297461	0	1	PRJNA827465;Yang2025_Silkie_Wenchang	CRA004775	0.754698112
ENSGALG00010015068	ESM1	2	0.504690844	0.451898084	1.116824484	0.264069378	0.279614356	2.591124229	61.4067134	1	CRA004775;Yang2025_Silkie_Wenchang	PRJNA827465	0.768790489
ENSGALG00010015068	ESM1	2	0.474615867	0.497534436	0.953935712	0.340116185	0.361761571	3.07796989	67.51105321	1	CRA004775;PRJNA827465	Yang2025_Silkie_Wenchang	0.805377063
ENSGALG00010015069	HSPD1	2	0.230427597	0.144618982	1.593342684	0.111083336	0.030676043	3.520168441	71.59226847	1	PRJNA827465;Yang2025_Silkie_Wenchang	CRA004775	0.56392513
ENSGALG00010015069	HSPD1	2	0.350306394	0.121276201	2.888500732	0.003870831	0	0.591157159	0	1	CRA004775;Yang2025_Silkie_Wenchang	PRJNA827465	0.089438731
ENSGALG00010015069	HSPD1	2	0.111668142	0.071712721	1.557159457	0.119432631	0	0.060180137	0	1	CRA004775;PRJNA827465	Yang2025_Silkie_Wenchang	0.580011679
ENSGALG00010015070	EDN2	2	0.102612046	0.255098294	0.402245129	0.687503633	0	0.091990231	0	1	PRJNA827465;Yang2025_Silkie_Wenchang	CRA004775	0.91949273
ENSGALG00010015070	EDN2	2	0.04166788	0.35514517	0.117326331	0.90660146	0	0.031899699	0	1	CRA004775;Yang2025_Silkie_Wenchang	PRJNA827465	0.978196186
ENSGALG00010015070	EDN2	2	0.159847696	0.292396055	0.546682124	0.584597138	0	0.000473809	0	1	CRA004775;PRJNA827465	Yang2025_Silkie_Wenchang	0.891016154
ENSGALG00010015071	SNAI1	2	0.125255555	0.06821524	1.836181398	0.066330833	0	0.176540313	0	1	PRJNA827465;Yang2025_Silkie_Wenchang	CRA004775	0.443593793
ENSGALG00010015071	SNAI1	2	-0.005510423	0.15483409	-0.035589208	0.971609914	0	0.713190145	0	0.5	CRA004775;Yang2025_Silkie_Wenchang	PRJNA827465	0.991955348
ENSGALG00010015071	SNAI1	2	0.076873367	0.145514508	0.528286613	0.597300422	0.019345763	1.300075018	23.08136177	0.5	CRA004775;PRJNA827465	Yang2025_Silkie_Wenchang	0.894380466
ENSGALG00010015072	MAP9	2	0.411591294	0.290515105	1.416763833	0.156551987	0.141758196	5.798479452	82.75409944	1	PRJNA827465;Yang2025_Silkie_Wenchang	CRA004775	0.649800692
ENSGALG00010015072	MAP9	2	0.547580435	0.272477011	2.009639029	0.044469407	0.060877984	1.571437298	36.36398975	1	CRA004775;Yang2025_Silkie_Wenchang	PRJNA827465	0.363948415
ENSGALG00010015072	MAP9	2	0.15464729	0.091558125	1.689061342	0.091207675	0	0.000259952	0	1	CRA004775;PRJNA827465	Yang2025_Silkie_Wenchang	0.517862098
ENSGALG00010015075	ENSGALG00010015075	2	-0.195644577	0.098506494	-1.986108423	0.047021286	0.002091825	1.070230443	6.562179528	1	PRJNA827465;Yang2025_Silkie_Wenchang	CRA004775	0.374081275
ENSGALG00010015075	ENSGALG00010015075	2	-0.326027359	0.195812396	-1.664998568	0.095913042	0	0.49200033	0	1	CRA004775;Yang2025_Silkie_Wenchang	PRJNA827465	0.5301492
ENSGALG00010015075	ENSGALG00010015075	2	-0.144225759	0.09644136	-1.495476204	0.134790206	0	0.03124114	0	1	CRA004775;PRJNA827465	Yang2025_Silkie_Wenchang	0.615292791
ENSGALG00010015080	ASF1A	2	-0.319323936	0.300542251	-1.06249266	0.288012088	0.167951681	13.75103357	92.72781937	1	PRJNA827465;Yang2025_Silkie_Wenchang	CRA004775	0.782840067
ENSGALG00010015080	ASF1A	2	-0.395965998	0.286010072	-1.384447741	0.166221399	0.116818497	3.24849499	69.21651401	1	CRA004775;Yang2025_Silkie_Wenchang	PRJNA827465	0.66529196
ENSGALG00010015080	ASF1A	2	-0.034711392	0.061197042	-0.567207012	0.570573544	0	0.005105541	0	1	CRA004775;PRJNA827465	Yang2025_Silkie_Wenchang	0.885152854
ENSGALG00010015083	CEP135	2	0.133857432	0.201213662	0.665250215	0.505890468	0.065902063	5.355193674	81.32653904	0.5	PRJNA827465;Yang2025_Silkie_Wenchang	CRA004775	0.863927652
ENSGALG00010015083	CEP135	2	0.318511413	0.129619372	2.45728249	0.013999256	0	0.912186762	0	0.5	CRA004775;Yang2025_Silkie_Wenchang	PRJNA827465	0.193842584
ENSGALG00010015083	CEP135	2	-0.069216357	0.112365976	-0.615990351	0.537900888	0	0.175671288	0	1	CRA004775;PRJNA827465	Yang2025_Silkie_Wenchang	0.87481821
ENSGALG00010015084	FOXO6	2	-0.142747081	0.091909408	-1.553128061	0.120392544	0	0.474905982	0	1	PRJNA827465;Yang2025_Silkie_Wenchang	CRA004775	0.581775014
ENSGALG00010015084	FOXO6	2	0.1649549	0.457264619	0.360742759	0.718291757	0.331191944	4.178852226	76.06998415	0.5	CRA004775;Yang2025_Silkie_Wenchang	PRJNA827465	0.928010944
ENSGALG00010015084	FOXO6	2	0.212862242	0.388379569	0.548077858	0.583638444	0.221645933	3.142484837	68.17804852	0.5	CRA004775;PRJNA827465	Yang2025_Silkie_Wenchang	0.890558522

ENSGALG00010015149	ENSGALG00010015149	2	-0.093406887	0.104385168	-0.894829116	0.370878443	0	0.049334123	0	1	CRA004775;Yang2025_Silkie_Wenchang	PRJNA827465	0.8214684
ENSGALG00010015149	ENSGALG00010015149	2	0.098975774	0.081219991	1.21861346	0.222990938	0	0.527371234	0	0.5	CRA004775;PRJNA827465	Yang2025_Silkie_Wenchang	0.736488473
ENSGALG00010015152	BOLL	2	0.084705932	0.157170119	0.538944248	0.589925324	0.011729304	1.26890481	21.19188202	0.5	PRJNA827465;Yang2025_Silkie_Wenchang	CRA004775	0.892320726
ENSGALG00010015152	BOLL	2	0.265346098	0.218057543	1.216862733	0.223656447	0	0.113908529	0	1	CRA004775;Yang2025_Silkie_Wenchang	PRJNA827465	0.737076707
ENSGALG00010015152	BOLL	2	-0.009813291	0.150616226	-0.065154277	0.948051165	0	0.107306938	0	0.5	CRA004775;PRJNA827465	Yang2025_Silkie_Wenchang	0.986697717
ENSGALG00010015153	ERCC6L	2	-0.064141894	0.120589927	-0.531900928	0.594794615	0.012016688	1.597082939	37.38584418	0.5	PRJNA827465;Yang2025_Silkie_Wenchang	CRA004775	0.893382718
ENSGALG00010015153	ERCC6L	2	-0.147716761	0.227228539	-0.650080144	0.515640455	0.028365916	1.126951569	11.26504212	0.5	CRA004775;Yang2025_Silkie_Wenchang	PRJNA827465	0.867300757
ENSGALG00010015153	ERCC6L	2	0.034430255	0.099832548	0.344880055	0.730184546	0	0.487150624	0	1	CRA004775;PRJNA827465	Yang2025_Silkie_Wenchang	0.932614156
ENSGALG00010015155	HMOX1	2	0.08094286	0.221440353	0.365528951	0.7147166	0.069924113	3.445282792	70.97480642	0.5	PRJNA827465;Yang2025_Silkie_Wenchang	CRA004775	0.926681837
ENSGALG00010015155	HMOX1	2	-0.291899708	0.156914048	-1.860252235	0.062849848	0.007860445	1.185364473	15.63776181	1	CRA004775;Yang2025_Silkie_Wenchang	PRJNA827465	0.431807797
ENSGALG00010015155	HMOX1	2	-0.074408819	0.380046041	-0.195788959	0.844775352	0.253766635	8.095975992	87.64818472	0.5	CRA004775;PRJNA827465	Yang2025_Silkie_Wenchang	0.960769014
ENSGALG00010015156	CNOT6	2	-0.181736862	0.208574569	-0.871328001	0.383575086	0.081029948	14.32739363	93.02036347	0.5	PRJNA827465;Yang2025_Silkie_Wenchang	CRA004775	0.825509017
ENSGALG00010015156	CNOT6	2	-0.260341374	0.195171933	-1.333907858	0.182234056	0.04785882	2.254351355	55.64134234	0.5	CRA004775;Yang2025_Silkie_Wenchang	PRJNA827465	0.690033956
ENSGALG00010015156	CNOT6	2	0.019662497	0.054282531	0.362225141	0.717183794	0	9.88351E-05	0	1	CRA004775;PRJNA827465	Yang2025_Silkie_Wenchang	0.9277944
ENSGALG00010015157	CDC42BPB	2	0.008619352	0.105167863	0.081958045	0.934680077	0.014939981	2.586685147	61.34048239	0.5	PRJNA827465;Yang2025_Silkie_Wenchang	CRA004775	0.984397102
ENSGALG00010015157	CDC42BPB	2	0.213946909	0.113632029	1.882804627	0.05972685	0	0.975647074	0	1	CRA004775;Yang2025_Silkie_Wenchang	PRJNA827465	0.423249156
ENSGALG00010015157	CDC42BPB	2	0.119906422	0.237119525	0.505679244	0.613081842	0.089611154	4.095523905	75.58309942	0.5	CRA004775;PRJNA827465	Yang2025_Silkie_Wenchang	0.898130964
ENSGALG00010015158	PIN4	2	0.085458418	0.067928924	1.258056402	0.208371359	0	0.196640685	0	1	PRJNA827465;Yang2025_Silkie_Wenchang	CRA004775	0.720917211
ENSGALG00010015158	PIN4	2	0.094890765	0.215612876	0.44009786	0.659866232	0	0.342096225	0	0.5	CRA004775;Yang2025_Silkie_Wenchang	PRJNA827465	0.911773501
ENSGALG00010015158	PIN4	2	0.072823289	0.068154047	1.068510117	0.285290469	0	0.133814424	0	0.5	CRA004775;PRJNA827465	Yang2025_Silkie_Wenchang	0.780888036
ENSGALG00010015161	GFPT2	2	-0.046472328	0.081169981	-0.572530961	0.566962317	0	0.067861	0	1	PRJNA827465;Yang2025_Silkie_Wenchang	CRA004775	0.883487517
ENSGALG00010015161	GFPT2	2	-0.069830544	0.127987109	-0.545606075	0.585336747	0	0.012211752	0	1	CRA004775;Yang2025_Silkie_Wenchang	PRJNA827465	0.891135366
ENSGALG00010015161	GFPT2	2	-0.031220545	0.096657075	-0.323003203	0.746692816	0	5.89845E-05	0	1	CRA004775;PRJNA827465	Yang2025_Silkie_Wenchang	0.936683091
ENSGALG00010015163	PLK2	2	-0.021308546	0.099177643	-0.214852317	0.829882462	0	0.452769948	0	0.5	PRJNA827465;Yang2025_Silkie_Wenchang	CRA004775	0.957079054
ENSGALG00010015163	PLK2	2	-0.098391823	0.136223883	-0.722280272	0.47012218	0	0.030632944	0	1	CRA004775;Yang2025_Silkie_Wenchang	PRJNA827465	0.853648049
ENSGALG00010015163	PLK2	2	0.024047638	0.13143569	0.18296125	0.854828423	0	0.213940971	0	0.5	CRA004775;PRJNA827465	Yang2025_Silkie_Wenchang	0.963248391
ENSGALG00010015164	ENSGALG00010015164	2	0.095235893	0.784332168	0.121422908	0.903356073	0.794452057	2.558656402	60.91698755	0.5	PRJNA827465;Yang2025_Silkie_Wenchang	CRA004775	0.977253688
ENSGALG00010015164	ENSGALG00010015164	2	-0.023181609	0.833578591	-0.027809746	0.977813893	0.651408766	1.877211865	46.72950781	0.5	CRA004775;Yang2025_Silkie_Wenchang	PRJNA827465	0.993739602
ENSGALG00010015164	ENSGALG00010015164	2	0.723272325	0.39173061	1.846351313	0.064841212	0	0.003460681	0	1	CRA004775;PRJNA827465	Yang2025_Silkie_Wenchang	0.439161492
ENSGALG00010015165	KLF3	2	-0.030922364	0.06217572	-0.497338246	0.618950545	0	0.031411092	0	0.5	PRJNA827465;Yang2025_Silkie_Wenchang	CRA004775	0.899168321
ENSGALG00010015165	KLF3	2	-0.159666889	0.190779693	-0.836917634	0.402638868	0.012380556	1.200877899	16.72758732	0.5	CRA004775;Yang2025_Silkie_Wenchang	PRJNA827465	0.832937979
ENSGALG00010015165	KLF3	2	-0.107690855	0.140763597	-0.76504762	0.444243189	0.021063265	1.559402787	35.87288621	1	CRA004775;PRJNA827465	Yang2025_Silkie_Wenchang	0.846013379
ENSGALG00010015166	YTHDF2	2	-0.078503045	0.103336702	-0.75968212	0.447444619	0.016336742	3.934690103	74.58503786	0.5	PRJNA827465;Yang2025_Silkie_Wenchang	CRA004775	0.84650962
ENSGALG00010015166	YTHDF2	2	-0.053786037	0.181126794	-0.296952402	0.766502848	0.045066619	2.904381973	65.56926708	0.5	CRA004775;Yang2025_Silkie_Wenchang	PRJNA827465	0.941179139
ENSGALG00010015166	YTHDF2	2	0.01803408	0.044686985	0.403564477	0.686533012	0	0.645116804	0	1	CRA004775;PRJNA827465	Yang2025_Silkie_Wenchang	0.919235539
ENSGALG00010015171	NHSL2	2	0.003627563	0.081692946	0.044404855	0.964581692	0	0.094333873	0	0.5	PRJNA827465;Yang2025_Silkie_Wenchang	CRA004775	0.990364463
ENSGALG00010015171	NHSL2	2	-0.149435297	0.282241603	-0.529458789	0.596487225	0.092783608	2.032124638	50.79041996	0.5	CRA004775;Yang2025_Silkie_Wenchang	PRJNA827465	0.894064255
ENSGALG00010015171	NHSL2	2	-0.152457601	0.239435557	-0.636737514	0.524295834	0.06798173	1.827122304	45.26912631	1	CRA004775;PRJNA827465	Yang2025_Silkie_Wenchang	0.869790047
ENSGALG00010015172	PPP4R3A	2	-0.04790457	0.048697242	-0.983722432	0.325252007	0	0.279856907	0	0.5	PRJNA827465;Yang2025_Silkie_Wenchang	CRA004775	0.799961179
ENSGALG00010015172	PPP4R3A	2	0.122523219	0.129290582	0.947657728	0.343303727	0	0.601645684	0	1	CRA004775;Yang2025_Silkie_Wenchang	PRJNA827465	0.806218116
ENSGALG00010015172	PPP4R3A	2	0.029549448	0.134304938	0.220017581	0.825857462	0.022710426	2.062718254	51.52028164	0.5	CRA004775;PRJNA827465	Yang2025_Silkie_Wenchang	0.956393839
ENSGALG00010015173	ENS-1	2	-0.326070917	0.410836288	-0.793676038	0.427384054	0	0.156289286	0	1	PRJNA827465;Yang2025_Silkie_Wenchang	CRA004775	0.84125685
ENSGALG00010015173	ENS-1	2	0.991618506	1.195698571	0.829321478	0.40692252	2.309001215	5.12512116	80.48826615	0.5	CRA004775;Yang2025_Silkie_Wenchang	PRJNA827465	0.833618122
ENSGALG00010015173	ENS-1	2	0.816975274	1.359581827	0.600901878	0.547905342	3.200478223	7.289374171	86.28140117	0.5	CRA004775;PRJNA827465	Yang2025_Silkie_Wenchang	0.878281266
ENSGALG00010015174	ST6GAL2	2	0.103041262	0.148811118	0.692429862	0.488667418	0	0.169868313	0	1	PRJNA827465;Yang2025_Silkie_Wenchang	CRA004775	0.857953638
ENSGALG00010015174	ST6GAL2	2	0.017240079	0.207058275	0.083261966	0.933643242	0	0.033240362	0	0.5	CRA004775;Yang2025_Silkie_Wenchang	PRJNA827465	0.984262625
ENSGALG00010015174	ST6GAL2	2	0.128728959	0.185557913	0.693740069	0.487845233	0	0.164277792	0	0.5	CRA004775;PRJNA827465	Yang2025_Silkie_Wenchang	0.85768885
ENSGALG00010015175	MAPK9	2	-0.102016465	0.05772394	-1.767316396	0.077175255	0	0.196775846	0	1	PRJNA827465;Yang2025_Silkie_Wenchang	CRA004775	0.478576691
ENSGALG00010015175	MAPK9	2	0.128893022	0.335191551	0.384535415	0.700581652	0.175033049	4.036525707	75.22621996	0.5	CRA004775;Yang2025_Silkie_Wenchang	PRJNA827465	0.922366238
ENSGALG00010015175	MAPK9	2	0.144927574	0.300744584	0.481895873	0.629879918	0.140421577	3.674153775	72.78284848	0.5	CRA004775;PRJNA827465	Yang2025_Silkie_Wenchang	0.902488805
ENSGALG00010015177	TC2N	2	-0.097954026	0.216429033	-0.452591893	0.650842638	0.074420414	4.733441979	78.87372435	0.5	PRJNA827465;Yang2025_Silkie_Wenchang	CRA004775	0.908769703
ENSGALG00010015177	TC2N	2	0.177412332	0.602239788	0.294587532	0.768308988	0.595805051	4.988228453	79.9528027	0.5	CRA004775;Yang2025_Silkie_Wenchang	PRJNA827465	0.941444269
ENSGALG00010015177	TC2N	2	0.329521765	0.357087496	0.922803988	0.356109362	0.16778604	2.186311026	54.26085363	1	CRA004775;PRJNA827465	Yang2025_Silkie_Wenchang	0.812455231
ENSGALG00010015180	PITHD1	2	0.059941441	0.188398221	0.318163517	0.750360905	0.053965733	3.840993324	73.965068	0.5	PRJNA827465;Yang2025_Silkie_Wenchang	CRA004775	0.937393444
ENSGALG00010015180	PITHD1	2	0.12650187	0.221393937	0.57138814	0.567736567	0.042309993	1.619417399	38.24939756	0.5	CRA004775;Yang2025_Silkie_Wenchang	PRJNA827465	0.883747882
ENSGALG00010015180	PITHD1	2	-0.105135434	0.081004338	-1.297898814	0.194322108	0	0.069322648	0	1	CRA004775;PRJNA827465	Yang2025_Silkie_Wenchang	0.705116012
ENSGALG00010015181	VPS13A	2	-0.003695399	0.188248857	-0.019630391	0.98433822	0.057447858	5.13577202	80.52873071	0.5	PRJNA827465;Yang2025_Silkie_Wenchang	CRA004775	0.995703389
ENSGALG00010015181	VPS13A	2	0.380376269	0.259313256	1.466860103	0.142414116	0.080002584	2.09257167	52.21191157	1	CRA004775;Yang2025_Silkie_Wenchang	PRJNA827465	0.628788228
ENSGALG00010015181	VPS13A	2	0.224602101	0.461275053	0.486915778	0.626318017	0.36619077	6.426937412	84.44048952	0.5	CRA004775;PRJNA827465	Yang2025_Silkie_Wenchang	0.901504244
ENSGALG00010015182	PHYKPL	2	0.196869846	0.072992497	2.69712441	0.006994113	0	0.585060099	0	1	PRJNA827465;Yang2025_Silkie_Wenchang	CRA004775	0.12854434
ENSGALG00010015182	PHYKPL	2	-0.129139981	0.471671636	-0.273792127	0.784244374	0.381950989	6.662342108	84.99026343	0.5	CRA004775;Yang2025_Silkie_Wenchang	PRJNA827465	0.946083234
ENSGALG00010015182	PHYKPL	2	-0.177749005	0.403039912	-0.441020851	0.659197902	0.273607227	5.59175736	82.11653447	0.5	CRA004775;PRJNA827465	Yang2025_Silkie_Wenchang	0.911449691
ENSGALG00010015186	ENSGALG00010015186	2	0.332079277	0.194102566	1.710844338	0.087109852	0.003983979	1.055475268	5.255951521	1	PRJNA827465;Yang2025_Silkie_Wenchang	CRA004775	0.507587624
ENSGALG00010015186	ENSGALG00010015186	2	0.229547631	0.234535746	0.978731964	0.327712434	0	0.54244152	0	1	CRA004775;Yang2025_Silkie_Wenchang	PRJNA827465	0.800253607
ENSGALG00010015186	ENSGALG00010015186	2	0.555875577	0.250743128	2.216912507	0.026629071	0	0.013566075	0	1	CRA004775;PRJNA827465	Yang2025_Silkie_Wenchang	0.278694271
ENSGALG00010015190	FBLN5	2	0.357602397	0.170204234	2.101019397	0.035639264	0.004489092	1.076237431	7.083700004	1	PRJNA827465;Yang2025_Silkie_Wenchang	CRA004775	0.325599715
ENSGALG00010015190	FBLN5	2	0.517965172	0.186151988	2.782485315	0.005394431	0	0.209472434	0	1	CRA004775;Yang2025_Silkie_Wenchang	PRJNA827465	0.110081676
ENSGALG00010015190	FBLN5	2	0.28754809	0.265676638	1.082323583	0.279108771	0.01876856	1.115965562	10.39150009	1	CRA004775;PRJNA827465	Yang2025_Silkie_Wenchang	0.777370468
ENSGALG00010015191	TOM1	2	-0.091861415	0.09409483	-0.976264209	0.328933551	0	0.34748969	0	1	PRJNA827465;Yang2025_Silkie_Wenchang	CRA004775	0.800761292
ENSGALG00010015191	TOM1	2	-0.005685284	0.160787	-0.0353591	0.971793397	0	0.000711404	0	0.5	CRA004775;Yang2025_Silkie_Wenchang	PRJNA827465	0.991955348
ENSGALG00010015191	TOM1	2	-0.120686334	0.109309549	-1.1040786	0.269559043	0	0.07995035	0	0.5	CRA004775;PRJNA827465	Yang2025_Silkie_Wenchang	0.77189992
ENSGALG00010015192	ENSGALG00010015192	2	0.100023769	0.192701184	0.519061516	0.603717844	0.02009248	1.359207558	26.42771931	0.5	PRJNA827465;Yang2025_Silkie_Wenchang	CRA004775	0.895856405
ENSGALG00010015192	ENSGALG00010015192	2	0.370197204	0.191472866	1.933418616	0.05318465	0.00329367	1.032945374	3.189459434	1	CRA004775;Yang2025_Silkie_Wenchang	PRJNA827465	0.399849003
ENSGALG00010015192	ENSGALG00010015192	2	0.245431852	0.418968798	0.585799834	0.558010035	0.24496644	3.196159436	68.71244942	0.5	CRA004775;PRJNA827465	Yang2025_Silkie_Wenchang	0.880678789
ENSGALG00010015193	LYPLA2	2	-0.032722615	0.087504085	-0.373955286	0.708437583	0.008017178	1.939279566	48.43445898	0.5	PRJNA827465;Yang2025_Silkie_Wenchang	CRA004775	0.9250668
ENSGALG00010015193	LYPLA2	2	-0.246593619	0.126905754	-1.943124019	0.052001176	0.012647459	1.581192017	36.75657419	1	CRA004775;Yang2025_Silkie_Wenchang	PRJNA827465	0.395759125
ENSGALG00010015193	LYPLA2	2	-0.159005654	0.22013589	-0.722306819	0.470105861	0.081458888	5.726877323	82.53847702	0.5	CRA004775;PRJNA827465	Yang2025_Silkie_Wenchang	0.853648049
ENSGALG00010015194	TMLHE	2	-0.213700243	0.221941063	-0.96286933	0.335613103	0.093256496	18.44753955	94.5792229	0.5	PRJNA827465;Yang2025_Silkie_Wenchang	CRA004775	0.803317552
ENSGALG00010015194	TMLHE	2	-0.407450675	0.083602468	-4.873668027	1.09545E-06	0	0.960099317	0	1	CRA004775;Yang2025_Silkie_Wenchang	PRJNA827465	0.000240843
ENSGALG00010015194	TMLHE	2	-0.009190656	0.048094042	-0.191097593	0.848449133	0	0.976021697	0	0.5	CRA004775;PRJNA827465	Yang2025_Silkie_Wenchang	0.961364896
ENSGALG00010015196	PDE4D	2	-0.008628175	0.39293663	-0.021958186	0.982481311	0.26756717	7.305819601	86.31228179	0.5	PRJNA827465;Yang2025_Silkie_Wenchang	CRA004775	0.995218117
ENSGALG00010015196	PDE4D	2	0.626479295	0.275741747	2.271978407	0.023087815	0.061358626	1.61582421	38.11207964	1	CRA004775;Yang2025_Silkie_Wenchang	PRJNA827465	0.259429803
ENSGALG00010015196	PDE4D	2	0.257370961	0.676103625	0.38066792	0.703449676	0.837452903	11.42323709	91.24591399	0.5	CRA004775;PRJNA827465	Yang2025_Silkie_Wenchang	0.923639641
ENSGALG00010015198	NME5	2	-0.137108584	0.185494413	-0.739152096	0.459814646	0.040962305	2.432078558	58.8829071	0.5	PRJNA827465;Yang2025_Silkie_Wenchang	CRA004775	0.850098539
ENSGALG00010015198	NME5	2	-0.150017323	0.261534274	-0.573604829	0.566235243	0.069806205	1.872106996	46.58424962	0.5	CRA004775;Yang2025_Silkie_Wenchang	PRJNA827465	0.883487517
ENSGALG00010015198	NME5	2	0.055647994	0.135070096	0.411993445	0.680344228	0	0.208936317	0	1	CRA004775;PRJNA827465	Yang2025_Silkie_Wenchang	0.91777228
ENSGALG00010015199	ENSGALG00010015199	2	-0.06397519	0.098583439	-0.648944602	0.516374184	0	0.015765249	0	1	PRJNA827465;Yang2025_Silkie_Wenchang	CRA004775	0.867685411
ENSGALG00010015199	ENSGALG00010015199	2	-0.338682811	0.247137047	-1.370425094	0.170554243	0.074417612	2.558487509	60.91440757	1	CRA004775;Yang2025_Silkie_Wenchang	PRJNA827465	0.672201941
ENSGALG00010015199	ENSGALG00010015199	2	-0.287839671	0.26064099	-1.104353045	0.269440021	0.108721884	4.713266993	78.78329402	1	CRA004775;PRJNA827465	Yang2025_Silkie_Wenchang	0.77189992
ENSGALG00010015200	TBC1D1	2	-0.05201728	0.053525744	-0.971817972	0.331141114	0	0.03350408	0	1	PRJNA827465;Yang2025_Silkie_Wenchang	CRA004775	0.801725552
ENSGALG00010015200	TBC1D1	2	-0.03376036	0.096415827	-0.350153714	0.726223341	0	0.429557026	0	0.5	CRA004775;Yang2025_Silkie_Wenchang	PRJNA827465	0.931029023
ENSGALG00010015200	TBC1D1	2	-0.035527726	0.058290312	-0.609496243	0.542195563	0	0.367462946	0	0.5	CRA004775;PRJNA827465	Yang2025_Silkie_Wenchang	0.876721948
ENSGALG00010015201	GCC2	2	0.289263551	0.263295103	1.098628678	0.271930063	0.119904039	7.085128339	85.88592962	1	PRJNA827465;Yang2025_Silkie_Wenchang	CRA004775	0.773184343
ENSGALG00010015201	GCC2	2	0.558753124	0.15415718	3.624567638	0.000289445	0	0.036762961	0	1	CRA004775;Yang2025_Silkie_Wenchang	PRJNA827465	0.016607397
ENSGALG00010015201	GCC2	2	0.177745997	0.207254939	0.857620077	0.391102285	0.051929617	1.9703547	49.24771668	1	CRA004775;PRJNA827465	Yang2025_Silkie_Wenchang	0.829251057
ENSGALG00010015203	HMGXB4	2	0.057981944	0.070127894	0.82680288	0.408348798	0.006176381	2.616032522	61.77417553	0.5	PRJNA827465;Yang2025_Silkie_Wenchang	CRA004775	0.834134074
ENSGALG00010015203	HMGXB4	2	0.132656365	0.064695052	2.050487025	0.040316929	0	0.027921491	0	1	CRA004775;Yang2025_Silkie_Wenchang	PRJNA827465	0.347442284
ENSGALG00010015203	HMGXB4	2	0.007397539	0.047931411	0.154335937	0.877344863	0	0.493552744	0	0.5	CRA004775;PRJNA827465	Yang2025_Silkie_Wenchang	0.970598664
ENSGALG00010015204	ENSGALG00010015204	2	0.294967387	0.130272256	2.264237961	0.023559483	0.017301055	1.907662552	47.57982753	1	PRJNA827465;Yang2025_Silkie_Wenchang	CRA004775	0.262001762
ENSGALG00010015204	ENSGALG00010015204	2	0.187706696	0.329117199	0.570333901	0.568451252	0.156541476	3.349459565	70.14443731	0.5	CRA004775;Yang2025_Silkie_Wenchang	PRJNA827465	0.884151198
ENSGALG00010015204	ENSGALG00010015204	2	0.115104899	0.159463186	0.721827413	0.470400593	0.022119093	1.3863785	27.86962578	0.5	CRA004775;PRJNA827465	Yang2025_Silkie_Wenchang	0.853744788
ENSGALG00010015207	AKR1E2	2	-0.171077782	0.091634974	-1.866948545	0.061908774	0	0.714268424	0	1	PRJNA827465;Yang2025_Silkie_Wenchang	CRA004775	0.429324205
ENSGALG00010015207	AKR1E2	2	-0.064380281	0.130844844	-0.492035289	0.622694397	0	0.00911109	0	1	CRA004775;Yang2025_Silkie_Wenchang	PRJNA827465	0.900255255
ENSGALG00010015207	AKR1E2	2	-0.20178782	0.106611907	-1.892732491	0.058393453	0	0.413737671	0	1	CRA004775;PRJNA827465	Yang2025_Silkie_Wenchang	0.418650753
ENSGALG00010015212	GNAQ	2	-0.030403337	0.071443225	-0.425559419	0.670428916	0	0.179976768	0	1	PRJNA827465;Yang2025_Silkie_Wenchang	CRA004775	0.914482883
ENSGALG00010015212	GNAQ	2	-0.021538689	0.143775976	-0.149807289	0.880916658	0	0.805007241	0	0.5	CRA004775;Yang2025_Silkie_Wenchang	PRJNA827465	0.971685305
ENSGALG00010015212	GNAQ	2	-0.000621172	0.076607794	-0.008108469	0.993530448	0	0.545584101	0	0.5	CRA004775;PRJNA827465	Yang2025_Silkie_Wenchang	0.997979742
ENSGALG00010015213	RUBCN	2	0.110596859	0.07037355	1.57156857	0.11605064	0	0.758583022	0	1	PRJNA827465;Yang2025_Silkie_Wenchang	CRA004775	0.573367481
ENSGALG00010015213	RUBCN	2	-0.169137405	0.379354117	-0.445856253	0.655701084	0.257183461	9.043154815	88.94191219	0.5	CRA004775;Yang2025_Silkie_Wenchang	PRJNA827465	0.910411938
ENSGALG00010015213	RUBCN	2	-0.223384876	0.315916083	-0.707101941	0.47950313	0.171957859	6.736485258	85.15546369	0.5	CRA004775;PRJNA827465	Yang2025_Silkie_Wenchang	0.856126448
ENSGALG00010015218	PLCL1	2	0.079159414	0.132528493	0.597301098	0.550306372	0.017372918	1.917327097	47.84405844	0.5	PRJNA827465;Yang2025_Silkie_Wenchang	CRA004775	0.878679315
ENSGALG00010015218	PLCL1	2	0.213079995	0.150014143	1.420399375	0.155491445	0	0.211521093	0	1	CRA004775;Yang2025_Silkie_Wenchang	PRJNA827465	0.648167852
ENSGALG00010015218	PLCL1	2	-0.027913116	0.107847585	-0.258820041	0.795774094	0	0.014861689	0	0.5	CRA004775;PRJNA827465	Yang2025_Silkie_Wenchang	0.949107318
ENSGALG00010015227	FYTTD1	2	-0.159616264	0.118426442	-1.347809335	0.177719715	0.026078678	14.02813397	92.87146813	1	PRJNA827465;Yang2025_Silkie_Wenchang	CRA004775	0.683951879
ENSGALG00010015227	FYTTD1	2	-0.27787166	0.052317809	-5.311225101	1.08891E-07	0	0.089153997	0	1	CRA004775;Yang2025_Silkie_Wenchang	PRJNA827465	3.78737E-05
ENSGALG00010015227	FYTTD1	2	-0.049444569	0.032331754	-1.529288149	0.126193026	0	0.714137197	0	1	CRA004775;PRJNA827465	Yang2025_Silkie_Wenchang	0.596623553
ENSGALG00010015234	TRIP11	2	0.265393652	0.216037994	1.22845823	0.219275	0.071938688	4.066489069	75.40876213	1	PRJNA827465;Yang2025_Silkie_Wenchang	CRA004775	0.73285529
ENSGALG00010015234	TRIP11	2	0.614607846	0.160895147	3.819927808	0.000133491	0	0.843262698	0	1	CRA004775;Yang2025_Silkie_Wenchang	PRJNA827465	0.009591566
ENSGALG00010015234	TRIP11	2	0.408193481	0.375089701	1.088255637	0.276482269	0.239574285	6.136782877	83.704817	1	CRA004775;PRJNA827465	Yang2025_Silkie_Wenchang	0.775851484
ENSGALG00010015241	ENSGALG00010015241	2	0.082252259	0.170016498	0.483789866	0.628535008	0.016042772	1.382427382	27.66346985	0.5	PRJNA827465;Yang2025_Silkie_Wenchang	CRA004775	0.901942802
ENSGALG00010015241	ENSGALG00010015241	2	-0.212013942	0.555490691	-0.381669659	0.702706408	0.488121828	4.37449602	77.1402238	0.5	CRA004775;Yang2025_Silkie_Wenchang	PRJNA827465	0.923272306
ENSGALG00010015241	ENSGALG00010015241	2	-0.339776992	0.36969943	-0.919062797	0.358062727	0.166233334	2.175568168	54.03499578	1	CRA004775;PRJNA827465	Yang2025_Silkie_Wenchang	0.813699876
ENSGALG00010015248	ENSGALG00010015248	2	0.077022042	0.080940463	0.951588855	0.341305536	0.003633702	1.34292092	25.53545148	1	PRJNA827465;Yang2025_Silkie_Wenchang	CRA004775	0.805665554
ENSGALG00010015248	ENSGALG00010015248	2	0.033416109	0.187619061	0.178106153	0.85863961	0.043611634	2.452844005	59.23099887	0.5	CRA004775;Yang2025_Silkie_Wenchang	PRJNA827465	0.964449906
ENSGALG00010015248	ENSGALG00010015248	2	-0.010830754	0.075868944	-0.142756088	0.886482819	0	0.888856325	0	0.5	CRA004775;PRJNA827465	Yang2025_Silkie_Wenchang	0.972729743
ENSGALG00010015253	ENSGALG00010015253	2	0.014691062	0.111281033	0.132017666	0.894970319	0.017268472	2.999795954	66.66439934	0.5	PRJNA827465;Yang2025_Silkie_Wenchang	CRA004775	0.974641371
ENSGALG00010015253	ENSGALG00010015253	2	-0.150160191	0.111261486	-1.349615186	0.177139451	0	0.43999737	0	1	CRA004775;Yang2025_Silkie_Wenchang	PRJNA827465	0.682961725
ENSGALG00010015253	ENSGALG00010015253	2	-0.01267609	0.199054019	-0.06368166	0.949223708	0.050180713	1.940716851	48.47264817	0.5	CRA004775;PRJNA827465	Yang2025_Silkie_Wenchang	0.98723759
ENSGALG00010015260	RASGEF1C	2	-0.176841919	0.096699728	-1.828773702	0.067433516	0	0.110997797	0	1	PRJNA827465;Yang2025_Silkie_Wenchang	CRA004775	0.447535382
ENSGALG00010015260	RASGEF1C	2	-0.056765362	0.175357234	-0.323712692	0.746155561	0	0.460751225	0	0.5	CRA004775;Yang2025_Silkie_Wenchang	PRJNA827465	0.936530151
ENSGALG00010015260	RASGEF1C	2	-0.165891774	0.106313446	-1.560402564	0.118664779	0	0.860953023	0	0.5	CRA004775;PRJNA827465	Yang2025_Silkie_Wenchang	0.578789008
ENSGALG00010015261	IQCG	2	-0.117316037	0.20776479	-0.564657934	0.572306452	0.02397477	1.30254911	23.22746279	0.5	PRJNA827465;Yang2025_Silkie_Wenchang	CRA004775	0.885651577
ENSGALG00010015261	IQCG	2	-0.290111571	0.273377653	-1.061211724	0.288593693	0	0.462504003	0	1	CRA004775;Yang2025_Silkie_Wenchang	PRJNA827465	0.783274491
ENSGALG00010015261	IQCG	2	0.006427094	0.177372894	0.036234927	0.971095036	0	0.022104867	0	0.5	CRA004775;PRJNA827465	Yang2025_Silkie_Wenchang	0.991916113
ENSGALG00010015285	ALG13	2	0.463517715	0.532001358	0.871271677	0.383605831	0.459809585	5.323539535	81.21550534	0.5	PRJNA827465;Yang2025_Silkie_Wenchang	CRA004775	0.825509017
ENSGALG00010015285	ALG13	2	0.558915516	0.618880848	0.903106822	0.366469204	0.465976803	2.202095956	54.5887182	0.5	CRA004775;Yang2025_Silkie_Wenchang	PRJNA827465	0.819255965
ENSGALG00010015285	ALG13	2	-0.092600171	0.292785862	-0.316272685	0.75179542	0	0.077423516	0	1	CRA004775;PRJNA827465	Yang2025_Silkie_Wenchang	0.937802658
ENSGALG00010015291	ZFP64	2	0.136348854	0.110047388	1.234218032	0.21712167	0.019406351	4.531869347	77.93405053	1	PRJNA827465;Yang2025_Silkie_Wenchang	CRA004775	0.730610701
ENSGALG00010015291	ZFP64	2	0.174780705	0.110842729	1.576835102	0.114833473	0.011182798	1.765265851	43.35130885	1	CRA004775;Yang2025_Silkie_Wenchang	PRJNA827465	0.571687865
ENSGALG00010015291	ZFP64	2	0.040095405	0.042607226	0.941047061	0.346680743	0	0.0007106	0	1	CRA004775;PRJNA827465	Yang2025_Silkie_Wenchang	0.808232887
ENSGALG00010015296	RPL35A	2	-0.097840019	0.074579203	-1.311894125	0.189558456	0	0.795032472	0	1	PRJNA827465;Yang2025_Silkie_Wenchang	CRA004775	0.698754721
ENSGALG00010015296	RPL35A	2	-0.278679396	0.194800152	-1.43059127	0.152547391	0	0.158288251	0	1	CRA004775;Yang2025_Silkie_Wenchang	PRJNA827465	0.644200076
ENSGALG00010015296	RPL35A	2	-0.099520961	0.072966403	-1.304997331	0.191893757	0	0.304766184	0	1	CRA004775;PRJNA827465	Yang2025_Silkie_Wenchang	0.701581425
ENSGALG00010015298	ENSGALG00010015298	2	0.131754261	0.176212784	0.747699786	0.45464126	0	0.046178481	0	1	PRJNA827465;Yang2025_Silkie_Wenchang	CRA004775	0.849072665
ENSGALG00010015298	ENSGALG00010015298	2	0.35393426	0.299606749	1.181329398	0.237471893	0.068445907	1.610654252	37.9134287	1	CRA004775;Yang2025_Silkie_Wenchang	PRJNA827465	0.749366344
ENSGALG00010015298	ENSGALG00010015298	2	0.339863116	0.247925433	1.370827963	0.170428592	0.049432315	1.578608258	36.65306167	1	CRA004775;PRJNA827465	Yang2025_Silkie_Wenchang	0.672201941
ENSGALG00010015299	GALE	2	0.048457653	0.065319668	0.74185393	0.458175841	0	0.090292681	0	0.5	PRJNA827465;Yang2025_Silkie_Wenchang	CRA004775	0.850026426
ENSGALG00010015299	GALE	2	-0.120195596	0.151632499	-0.79267701	0.427966028	0.001295025	1.026924591	2.621866404	1	CRA004775;Yang2025_Silkie_Wenchang	PRJNA827465	0.84125685
ENSGALG00010015299	GALE	2	-0.193565788	0.171476889	-1.128436663	0.259143434	0.037117279	2.125961954	52.96246962	0.5	CRA004775;PRJNA827465	Yang2025_Silkie_Wenchang	0.693510459
ENSGALG00010015300	ATXN3	2	-0.246200559	0.085369558	-2.883938527	0.003927354	0.003839312	1.251531126	20.09787219	1	PRJNA827465;Yang2025_Silkie_Wenchang	CRA004775	0.090319952
ENSGALG00010015300	ATXN3	2	-0.333847975	0.145062311	-2.301410845	0.021368419	0	0.944484399	0	0.5	CRA004775;Yang2025_Silkie_Wenchang	PRJNA827465	0.247158031
ENSGALG00010015300	ATXN3	2	-0.187970425	0.075705747	-2.482969747	0.013031465	0	0.280304793	0	0.5	CRA004775;PRJNA827465	Yang2025_Silkie_Wenchang	0.103372819
ENSGALG00010015306	BCAT1	2	-0.349035941	0.244967186	-1.424827325	0.154207122	0.107995018	9.900750294	89.89975537	1	PRJNA827465;Yang2025_Silkie_Wenchang	CRA004775	0.646830576
ENSGALG00010015306	BCAT1	2	-0.334448262	0.325085374	-1.028801322	0.303573045	0.161246473	3.762068317	73.41887718	0.5	CRA004775;Yang2025_Silkie_Wenchang	PRJNA827465	0.792205992
ENSGALG00010015306	BCAT1	2	-0.097999002	0.088501748	-1.107311481	0.268159282	0	0.272128038	0	0.5	CRA004775;PRJNA827465	Yang2025_Silkie_Wenchang	0.771398294
ENSGALG00010015310	PGM2	2	-0.057483385	0.232728205	-0.246999793	0.804909826	0.101712214	16.35451499	93.88548055	1	PRJNA827465;Yang2025_Silkie_Wenchang	CRA004775	0.95184424
ENSGALG00010015310	PGM2	2	-0.276426462	0.084711821	-3.263139195	0.001101854	0	0.460313737	0	1	CRA004775;Yang2025_Silkie_Wenchang	PRJNA827465	0.040098299
ENSGALG00010015310	PGM2	2	0.159720034	0.07216276	2.213330455	0.026874871	0	0.585668141	0	1	CRA004775;PRJNA827465	Yang2025_Silkie_Wenchang	0.27936077
ENSGALG00010015314	GMEB1	2	0.136126286	0.074181189	1.835051286	0.066498091	0.005784613	1.945468669	48.598504	1	PRJNA827465;Yang2025_Silkie_Wenchang	CRA004775	0.44425879
ENSGALG00010015314	GMEB1	2	0.14474942	0.109197109	1.325579237	0.184970107	0.010584387	1.745489307	42.70947429	1	CRA004775;Yang2025_Silkie_Wenchang	PRJNA827465	0.649251167
ENSGALG00010015314	GMEB1	2	0.071249486	0.049786843	1.431090677	0.152402229	0	0.213647427	0	1	CRA004775;PRJNA827465	Yang2025_Silkie_Wenchang	0.643965957
ENSGALG00010015318	SALL4	2	0.071599799	0.207171648	0.345606166	0.729638712	0.058722897	3.1627425	68.38187111	0.5	PRJNA827465;Yang2025_Silkie_Wenchang	CRA004775	0.932372802
ENSGALG00010015318	SALL4	2	0.36721941	0.512340114	0.590063077	0.548766465	0.434321705	5.274706095	81.04159054	0.5	CRA004775;Yang2025_Silkie_Wenchang	PRJNA827465	0.838781266
ENSGALG00010015318	SALL4	2	0.464764827	0.285817366	1.626090231	0.103930462	0.092413269	1.927240671	48.1123445	1	CRA004775;PRJNA827465	Yang2025_Silkie_Wenchang	0.548175896
ENSGALG00010015327	LARGE1	2	-0.15954537	0.078575584	-2.03047006	0.042308781	0	0.004273441	0	1	PRJNA827465;Yang2025_Silkie_Wenchang	CRA004775	0.356149505
ENSGALG00010015327	LARGE1	2	-0.004730759	0.24632205	-0.019205586	0.984677101	0.069051586	1.807147566	44.6641758	0.5	CRA004775;Yang2025_Silkie_Wenchang	PRJNA827465	0.995703389
ENSGALG00010015327	LARGE1	2	-0.009937237	0.252119601	-0.039414774	0.968559701	0.07620623	1.905112598	47.50966422	0.5	CRA004775;PRJNA827465	Yang2025_Silkie_Wenchang	0.993215679
ENSGALG00010015333	MED7	2	0.050831513	0.123294893	0.4122759	0.680137211	0.021383249	3.251951134	69.24923042	0.5	PRJNA827465;Yang2025_Silkie_Wenchang	CRA004775	0.917722906
ENSGALG00010015333	MED7	2	0.030235015	0.201668578	0.149934189	0.880816538	0.053028958	2.622557605	61.8692837	0.5	CRA004775;Yang2025_Silkie_Wenchang	PRJNA827465	0.971676583
ENSGALG00010015333	MED7	2	-0.071253234	0.071470072	-1.038849491	0.298831877	0	0.474612407	0	0.5	CRA004775;PRJNA827465	Yang2025_Silkie_Wenchang	0.70474322
ENSGALG00010015339	ENSGALG00010015339	2	-1.621431753	1.675751417	-0.967584892	0.333251726	4.93554481	8.192343242	87.7934802	0.5	PRJNA827465;Yang2025_Silkie_Wenchang	CRA004775	0.802360424
ENSGALG00010015339	ENSGALG00010015339	2	-0.990118693	2.37761515	-0.41643354	0.67709279	10.36199161	11.96826563	91.64457048	0.5	CRA004775;Yang2025_Silkie_Wenchang	PRJNA827465	0.917109899
ENSGALG00010015339	ENSGALG00010015339	2	0.51954394	0.676439006	0.768057335	0.442453123	0.213926824	1.278121705	21.76018948	1	CRA004775;PRJNA827465	Yang2025_Silkie_Wenchang	0.845535782
ENSGALG00010015348	CPSF2	2	0.026940525	0.041032644	0.656563226	0.511461801	0	0.18006131	0	0.5	PRJNA827465;Yang2025_Silkie_Wenchang	CRA004775	0.865694231
ENSGALG00010015348	CPSF2	2	0.017474913	0.092521267	0.188874551	0.85019114	0	0.437604399	0	0.5	CRA004775;Yang2025_Silkie_Wenchang	PRJNA827465	0.961982453
ENSGALG00010015348	CPSF2	2	0.039162611	0.043658183	0.897027958	0.369703999	0	0.246059949	0	1	CRA004775;PRJNA827465	Yang2025_Silkie_Wenchang	0.820606801
ENSGALG00010015353	RELL1	2	-0.280003662	0.330621652	-0.846900559	0.397050551	0.21311336	39.59895474	97.47468082	0.5	PRJNA827465;Yang2025_Silkie_Wenchang	CRA004775	0.831438925
ENSGALG00010015353	RELL1	2	-0.155793768	0.474019273	-0.328665471	0.742408555	0.42669939	19.34408785	94.83046186	0.5	CRA004775;Yang2025_Silkie_Wenchang	PRJNA827465	0.935510459
ENSGALG00010015353	RELL1	2	0.130015924	0.129753756	1.002020507	0.316333689	0.019732872	1.915244744	47.78735181	1	CRA004775;PRJNA827465	Yang2025_Silkie_Wenchang	0.796883862
ENSGALG00010015356	ENSGALG00010015356	2	-0.348637805	0.197983135	-1.760946991	0.078247375	0.055768317	3.17272473	68.4813501	1	PRJNA827465;Yang2025_Silkie_Wenchang	CRA004775	0.481684356
ENSGALG00010015356	ENSGALG00010015356	2	-0.47737405	0.151425789	-3.152528075	0.001618633	0	0.679523556	0	1	CRA004775;Yang2025_Silkie_Wenchang	PRJNA827465	0.050888881
ENSGALG00010015356	ENSGALG00010015356	2	-0.210536549	0.091225926	-2.307858713	0.021006996	0	0.379093907	0	1	CRA004775;PRJNA827465	Yang2025_Silkie_Wenchang	0.245277621
ENSGALG00010015362	ATP9A	2	0.094300943	0.182658923	0.516267923	0.605667297	0.021709505	1.470544136	31.99796081	0.5	PRJNA827465;Yang2025_Silkie_Wenchang	CRA004775	0.896474603
ENSGALG00010015362	ATP9A	2	-0.222894906	0.615913096	-0.361893434	0.717431667	0.600150126	4.370596324	77.119827	0.5	CRA004775;Yang2025_Silkie_Wenchang	PRJNA827465	0.9277944
ENSGALG00010015362	ATP9A	2	-0.355793563	0.413198962	-0.861070806	0.389199047	0.21714741	2.282275214	56.18407482	1	CRA004775;PRJNA827465	Yang2025_Silkie_Wenchang	0.828774456
ENSGALG00010015364	CLOCK	2	0.167056807	0.27498646	0.607487117	0.54352769	0.133514603	7.989900302	87.48419927	0.5	PRJNA827465;Yang2025_Silkie_Wenchang	CRA004775	0.876969872
ENSGALG00010015364	CLOCK	2	0.630119341	0.211345056	2.981471881	0.002868664	0.036918365	1.622534872	38.36804267	1	CRA004775;Yang2025_Silkie_Wenchang	PRJNA827465	0.073164891

ENSGALG00010015364	CLOCK	2	0.372006282	0.493917904	0.75317432	0.451345169	0.446354301	10.95308221	90.87014978	0.5	CRA004775;PRJNA827465	Yang2025_Silkie_Wenchang	0.847847413
ENSGALG00010015370	ENSGALG00010015370	2	-0.105849602	0.052354481	-2.021786865	0.043198375	0	0.020359356	0	1	PRJNA827465;Yang2025_Silkie_Wenchang	CRA004775	0.359495531
ENSGALG00010015370	ENSGALG00010015370	2	0.294386267	0.470806203	0.625281199	0.531786517	0.380472196	6.438797708	84.46915021	0.5	CRA004775;Yang2025_Silkie_Wenchang	PRJNA827465	0.873042777
ENSGALG00010015370	ENSGALG00010015370	2	0.297124501	0.460592564	0.645091832	0.518867677	0.368041235	6.662335899	84.99024944	0.5	CRA004775;PRJNA827465	Yang2025_Silkie_Wenchang	0.868851946
ENSGALG00010015374	LIMS1	2	-0.020639357	0.078599102	-0.262590233	0.792866429	0	0.237504807	0	0.5	PRJNA827465;Yang2025_Silkie_Wenchang	CRA004775	0.948451453
ENSGALG00010015374	LIMS1	2	-0.174423719	0.21031876	-0.829330292	0.406917535	0.045297695	1.574905814	36.50413943	1	CRA004775;Yang2025_Silkie_Wenchang	PRJNA827465	0.833618122
ENSGALG00010015374	LIMS1	2	-0.153991555	0.263125493	-0.585239969	0.558386373	0.086348615	2.09521677	52.27258108	0.5	CRA004775;PRJNA827465	Yang2025_Silkie_Wenchang	0.880678789
ENSGALG00010015384	TAF12	2	-0.003073548	0.060086336	-0.051152196	0.959204244	0	0.136660411	0	0.5	PRJNA827465;Yang2025_Silkie_Wenchang	CRA004775	0.989155754
ENSGALG00010015384	TAF12	2	-0.15341047	0.159851801	-0.959704356	0.33720403	0.02066535	1.535054628	34.85573858	1	CRA004775;Yang2025_Silkie_Wenchang	PRJNA827465	0.804086136
ENSGALG00010015384	TAF12	2	-0.120093339	0.186246213	-0.644809561	0.519050605	0.046645423	2.429454024	58.83848839	0.5	CRA004775;PRJNA827465	Yang2025_Silkie_Wenchang	0.868939943
ENSGALG00010015386	SLC24A4	2	1.149395604	0.206776387	5.558640521	2.71884E-08	0	0.448216584	0	1	PRJNA827465;Yang2025_Silkie_Wenchang	CRA004775	1.20066E-05
ENSGALG00010015386	SLC24A4	2	0.952848749	0.321249946	2.96606664	0.003016351	0	0.010576455	0	1	CRA004775;Yang2025_Silkie_Wenchang	PRJNA827465	0.075655171
ENSGALG00010015386	SLC24A4	2	1.22405743	0.248735377	4.921123172	8.6049E-07	0	0.170743712	0	1	CRA004775;PRJNA827465	Yang2025_Silkie_Wenchang	0.000196627
ENSGALG00010015388	PSMD1	2	-0.033713561	0.063540325	-0.530585279	0.595706199	0	0.549882069	0	0.5	PRJNA827465;Yang2025_Silkie_Wenchang	CRA004775	0.893802528
ENSGALG00010015388	PSMD1	2	-0.112272313	0.101564171	-1.105432283	0.268972322	0	0.221216549	0	1	CRA004775;Yang2025_Silkie_Wenchang	PRJNA827465	0.771398294
ENSGALG00010015388	PSMD1	2	-0.012591477	0.077050584	-0.163418323	0.870189077	0	0.585622559	0	0.5	CRA004775;PRJNA827465	Yang2025_Silkie_Wenchang	0.968813136
ENSGALG00010015390	CCNB3	2	-0.059798237	0.088948222	-0.672281415	0.501404559	0	0.408180534	0	1	PRJNA827465;Yang2025_Silkie_Wenchang	CRA004775	0.862658912
ENSGALG00010015390	CCNB3	2	-0.102484054	0.181606179	-0.564320303	0.572536167	0	0.790732353	0	0.5	CRA004775;Yang2025_Silkie_Wenchang	PRJNA827465	0.885678394
ENSGALG00010015390	CCNB3	2	-0.017545417	0.097238178	-0.18043753	0.856809093	0	0.41353299	0	0.5	CRA004775;PRJNA827465	Yang2025_Silkie_Wenchang	0.963689555
ENSGALG00010015391	SYN3	2	0.290323022	0.341884914	0.849183484	0.395779202	0.065919923	1.367358095	26.86626832	1	PRJNA827465;Yang2025_Silkie_Wenchang	CRA004775	0.831256879
ENSGALG00010015391	SYN3	2	0.896935658	0.392514932	2.285099458	0.022306997	0	0.412654742	0	1	CRA004775;Yang2025_Silkie_Wenchang	PRJNA827465	0.253336926
ENSGALG00010015391	SYN3	2	0.499375433	0.602117205	0.829365825	0.406897433	0.469896062	2.615427982	61.76533986	1	CRA004775;PRJNA827465	Yang2025_Silkie_Wenchang	0.833618122
ENSGALG00010015392	TMEM165	2	-0.07942608	0.078911199	-1.00652481	0.314163183	0.005268526	1.553962314	35.64837507	1	PRJNA827465;Yang2025_Silkie_Wenchang	CRA004775	0.796531448
ENSGALG00010015392	TMEM165	2	-0.178323747	0.104620259	-1.704485816	0.088290364	0	0.101149574	0	1	CRA004775;Yang2025_Silkie_Wenchang	PRJNA827465	0.510380375
ENSGALG00010015392	TMEM165	2	-0.03618344	0.057696866	-0.627130077	0.530573973	0	0.232674809	0	1	CRA004775;PRJNA827465	Yang2025_Silkie_Wenchang	0.872626224
ENSGALG00010015393	DCX	2	-0.129215192	0.136123908	-0.949246855	0.342495076	0	0.73268787	0	1	PRJNA827465;Yang2025_Silkie_Wenchang	CRA004775	0.806005903
ENSGALG00010015393	DCX	2	-0.41900846	0.273689272	-1.530964138	0.125778256	0	0.445630967	0	1	CRA004775;Yang2025_Silkie_Wenchang	PRJNA827465	0.595474757
ENSGALG00010015393	DCX	2	-0.138549951	0.233005386	-0.594621237	0.552096681	0.035621645	1.112617124	10.12182193	1	CRA004775;PRJNA827465	Yang2025_Silkie_Wenchang	0.879061525
ENSGALG00010015397	SRD5A3	2	-0.026985686	0.193665672	-0.139341609	0.889180211	0.051873807	2.827437352	64.63228445	0.5	PRJNA827465;Yang2025_Silkie_Wenchang	CRA004775	0.973135755
ENSGALG00010015397	SRD5A3	2	-0.248811229	0.161501237	-1.540615003	0.123410514	0	0.038697239	0	1	CRA004775;Yang2025_Silkie_Wenchang	PRJNA827465	0.588993243
ENSGALG00010015397	SRD5A3	2	0.024892298	0.15267334	0.163042859	0.870484689	0.024897465	1.781066291	43.85385849	0.5	CRA004775;PRJNA827465	Yang2025_Silkie_Wenchang	0.968950119
ENSGALG00010015407	TIMP3	2	-0.069137333	0.109188402	-0.633193006	0.526607612	0.016353212	3.136004173	68.11228734	0.5	PRJNA827465;Yang2025_Silkie_Wenchang	CRA004775	0.87066367
ENSGALG00010015407	TIMP3	2	0.068455733	0.345631454	0.198059904	0.842998195	0.175474326	3.052558492	67.24059498	0.5	CRA004775;Yang2025_Silkie_Wenchang	PRJNA827465	0.960416186
ENSGALG00010015407	TIMP3	2	0.12836025	0.198537528	0.646528901	0.517936886	0.04316323	1.516923104	34.07708026	1	CRA004775;PRJNA827465	Yang2025_Silkie_Wenchang	0.86819096
ENSGALG00010015409	FAM13B	2	0.141778995	0.063066808	2.248076278	0.024571326	0	0.450926224	0	1	PRJNA827465;Yang2025_Silkie_Wenchang	CRA004775	0.267885393
ENSGALG00010015409	FAM13B	2	0.017500137	0.235541122	0.074297588	0.940773596	0.077625336	2.954778478	66.15651537	0.5	CRA004775;Yang2025_Silkie_Wenchang	PRJNA827465	0.985565295
ENSGALG00010015409	FAM13B	2	-0.00658255	0.180908146	-0.036386145	0.970974461	0.041067419	2.12622576	52.96830567	0.5	CRA004775;PRJNA827465	Yang2025_Silkie_Wenchang	0.991885126
ENSGALG00010015410	HTR2B	2	-0.225370378	0.137733516	-1.636278408	0.101781333	0	0.750218253	0	1	PRJNA827465;Yang2025_Silkie_Wenchang	CRA004775	0.543700483
ENSGALG00010015410	HTR2B	2	0.006016471	0.475226663	0.012660214	0.989898881	0.330347561	3.476895796	71.23871239	0.5	CRA004775;Yang2025_Silkie_Wenchang	PRJNA827465	0.997311131
ENSGALG00010015410	HTR2B	2	0.083963004	0.330379767	0.254140879	0.799386723	0.132573585	2.122904565	52.89472656	0.5	CRA004775;PRJNA827465	Yang2025_Silkie_Wenchang	0.949922217
ENSGALG00010015413	CARNMT1	2	0.304858861	0.212318221	1.435858211	0.151042692	0.084349077	15.52002456	93.55671123	1	PRJNA827465;Yang2025_Silkie_Wenchang	CRA004775	0.642401354
ENSGALG00010015413	CARNMT1	2	0.48769848	0.078908257	6.180576014	6.38681E-10	0.00117746	1.043043673	4.126737363	1	CRA004775;Yang2025_Silkie_Wenchang	PRJNA827465	3.98446E-07
ENSGALG00010015413	CARNMT1	2	0.112221989	0.07134154	1.57302447	0.115713152	0	0.633613482	0	1	CRA004775;PRJNA827465	Yang2025_Silkie_Wenchang	0.572716099
ENSGALG00010015416	CAPN6	2	-0.575693472	0.279484824	-2.059838038	0.039414027	0.131002028	6.192907784	83.85249652	1	PRJNA827465;Yang2025_Silkie_Wenchang	CRA004775	0.343326573
ENSGALG00010015416	CAPN6	2	-0.315849071	0.636888287	-0.495925388	0.619947053	0.672153322	5.177738734	80.68654964	0.5	CRA004775;Yang2025_Silkie_Wenchang	PRJNA827465	0.899395593
ENSGALG00010015416	CAPN6	2	-0.117366139	0.315454777	-0.372053771	0.709852815	0.10780397	1.673968558	40.26172147	0.5	CRA004775;PRJNA827465	Yang2025_Silkie_Wenchang	0.925298042
ENSGALG00010015422	ENSGALG00010015422	2	-0.025187322	0.290143146	-0.086809986	0.93082255	0	0.654957194	0	0.5	PRJNA827465;Yang2025_Silkie_Wenchang	CRA004775	0.983943003
ENSGALG00010015422	ENSGALG00010015422	2	-0.208850213	0.503411427	-0.41486983	0.678237197	0.262788601	1.984308295	49.60460516	0.5	CRA004775;Yang2025_Silkie_Wenchang	PRJNA827465	0.917647993
ENSGALG00010015422	ENSGALG00010015422	2	-0.34127016	0.253138471	-1.348156043	0.177608199	0	0.816913099	0	1	CRA004775;PRJNA827465	Yang2025_Silkie_Wenchang	0.683896841
ENSGALG00010015430	HMGCL	2	0.074562631	0.072755297	1.024841271	0.305438089	0	0.304699348	0	1	PRJNA827465;Yang2025_Silkie_Wenchang	CRA004775	0.79225794
ENSGALG00010015430	HMGCL	2	-0.038547166	0.24869726	-0.154996344	0.876824199	0.070183821	2.222294922	55.00147212	0.5	CRA004775;Yang2025_Silkie_Wenchang	PRJNA827465	0.97046577
ENSGALG00010015430	HMGCL	2	-0.045309968	0.172787604	-0.262229278	0.793144683	0.034220857	1.796117504	44.32435531	0.5	CRA004775;PRJNA827465	Yang2025_Silkie_Wenchang	0.948573334
ENSGALG00010015431	RIN3	2	-0.162345449	0.08065596	-2.012814037	0.044134197	0	0.71832198	0	1	PRJNA827465;Yang2025_Silkie_Wenchang	CRA004775	0.363116251
ENSGALG00010015431	RIN3	2	-0.289219168	0.143052513	-2.021769228	0.043200198	0	0.103185119	0	1	CRA004775;Yang2025_Silkie_Wenchang	PRJNA827465	0.359495531
ENSGALG00010015431	RIN3	2	-0.134673868	0.092565652	-1.454901083	0.145696648	0	0.483395757	0	1	CRA004775;PRJNA827465	Yang2025_Silkie_Wenchang	0.634354199
ENSGALG00010015436	SLC30A9	2	-0.016307002	0.072346523	-0.225401321	0.821667084	0.005277957	1.857353158	46.15994294	0.5	PRJNA827465;Yang2025_Silkie_Wenchang	CRA004775	0.955598716
ENSGALG00010015436	SLC30A9	2	0.081624977	0.085451979	0.955214589	0.33946919	0	5.41498E-05	0	1	CRA004775;Yang2025_Silkie_Wenchang	PRJNA827465	0.80490319
ENSGALG00010015436	SLC30A9	2	-0.057445447	0.050998589	-1.126412488	0.259990963	0	0.673656141	0	0.5	CRA004775;PRJNA827465	Yang2025_Silkie_Wenchang	0.767264217
ENSGALG00010015440	ENSGALG00010015440	2	0.080716161	0.300560087	0.268552494	0.788274074	0	0.039902857	0	1	PRJNA827465;Yang2025_Silkie_Wenchang	CRA004775	0.947013172
ENSGALG00010015440	ENSGALG00010015440	2	0.02964508	0.351830012	0.084259668	0.932849979	0	0.169260477	0	0.5	CRA004775;Yang2025_Silkie_Wenchang	PRJNA827465	0.984224753
ENSGALG00010015440	ENSGALG00010015440	2	-0.031397922	0.323506477	-0.097055002	0.922682715	0	0.064905954	0	0.5	CRA004775;PRJNA827465	Yang2025_Silkie_Wenchang	0.981804675
ENSGALG00010015443	PAK3	2	-0.057633373	0.052673602	-1.094160469	0.273884606	0	0.120484291	0	1	PRJNA827465;Yang2025_Silkie_Wenchang	CRA004775	0.774526342
ENSGALG00010015443	PAK3	2	0.181397273	0.240229524	0.755099828	0.450189086	0.080800866	3.079695518	67.52925755	0.5	CRA004775;Yang2025_Silkie_Wenchang	PRJNA827465	0.847564661
ENSGALG00010015443	PAK3	2	0.149120101	0.263485504	0.565951821	0.571426533	0.112316454	4.472000621	77.63864354	0.5	CRA004775;PRJNA827465	Yang2025_Silkie_Wenchang	0.885354083
ENSGALG00010015449	HDX	2	-0.055507214	0.078301512	-0.7088907	0.478392307	0.006355857	1.969572449	49.22755949	0.5	PRJNA827465;Yang2025_Silkie_Wenchang	CRA004775	0.855464436
ENSGALG00010015449	HDX	2	-0.000441014	0.221534868	-0.001990719	0.998411637	0.063167283	2.293311566	56.39493496	0.5	CRA004775;Yang2025_Silkie_Wenchang	PRJNA827465	0.999547764
ENSGALG00010015449	HDX	2	0.02786495	0.07832119	0.355777922	0.722006885	0.00291604	1.063465357	5.967787893	1	CRA004775;PRJNA827465	Yang2025_Silkie_Wenchang	0.929389074
ENSGALG00010015466	NMRK1	2	0.274189568	0.148325019	1.848572609	0.064519551	0.015316845	1.53298751	34.76789643	1	PRJNA827465;Yang2025_Silkie_Wenchang	CRA004775	0.437737253
ENSGALG00010015466	NMRK1	2	0.408233768	0.156627462	2.606399684	0.009149962	0	0.066605584	0	1	CRA004775;Yang2025_Silkie_Wenchang	PRJNA827465	0.150154891
ENSGALG00010015466	NMRK1	2	0.164647914	0.149894996	1.098421678	0.272020401	0	0.239950343	0	1	CRA004775;PRJNA827465	Yang2025_Silkie_Wenchang	0.773184343
ENSGALG00010015469	TMEM33	2	-0.182394831	0.068680591	-2.655696859	0.007914472	0.003908104	1.693043267	40.93476407	1	PRJNA827465;Yang2025_Silkie_Wenchang	CRA004775	0.138127225
ENSGALG00010015469	TMEM33	2	-0.255323264	0.077020113	-3.315020658	0.000916364	0	0.018178303	0	1	CRA004775;Yang2025_Silkie_Wenchang	PRJNA827465	0.035659243
ENSGALG00010015469	TMEM33	2	-0.129212424	0.065062837	-1.985963583	0.047037368	0	0.191879594	0	1	CRA004775;PRJNA827465	Yang2025_Silkie_Wenchang	0.374081275
ENSGALG00010015472	LGMN	2	-0.120299139	0.082794956	-1.452976657	0.146230235	0.006637665	1.871139052	46.55661754	1	PRJNA827465;Yang2025_Silkie_Wenchang	CRA004775	0.634919017
ENSGALG00010015472	LGMN	2	-0.194314723	0.097608472	-1.990756736	0.046507638	0	0.820876645	0	0.5	CRA004775;Yang2025_Silkie_Wenchang	PRJNA827465	0.372346812
ENSGALG00010015472	LGMN	2	-0.046288795	0.068023401	-0.680483398	0.49619843	0	0.187950817	0	0.5	CRA004775;PRJNA827465	Yang2025_Silkie_Wenchang	0.860939846
ENSGALG00010015477	ARMC9	2	0.013532695	0.069438373	0.194887852	0.845480745	0	0.6241758	0	0.5	PRJNA827465;Yang2025_Silkie_Wenchang	CRA004775	0.961048404
ENSGALG00010015477	ARMC9	2	-0.056357706	0.101577684	-0.554823693	0.579015259	0	0.141659308	0	1	CRA004775;Yang2025_Silkie_Wenchang	PRJNA827465	0.887888423
ENSGALG00010015477	ARMC9	2	0.05137908	0.091358007	0.562392738	0.573848466	0	0.374547896	0	0.5	CRA004775;PRJNA827465	Yang2025_Silkie_Wenchang	0.886032852
ENSGALG00010015478	FBXO7	2	-0.062849057	0.043337158	-1.450234856	0.146993038	0	0.150531876	0	1	PRJNA827465;Yang2025_Silkie_Wenchang	CRA004775	0.635668678
ENSGALG00010015478	FBXO7	2	-0.15105341	0.078435543	-1.92582857	0.054125783	0	0.475927708	0	1	CRA004775;Yang2025_Silkie_Wenchang	PRJNA827465	0.403227287
ENSGALG00010015478	FBXO7	2	-0.096499731	0.068097529	-1.417081239	0.156459177	0.004079786	1.525193524	34.43454983	1	CRA004775;PRJNA827465	Yang2025_Silkie_Wenchang	0.649484056
ENSGALG00010015481	GOLGA5	2	0.160039483	0.063022039	2.539420883	0.011103616	0.002009353	1.245535221	19.71322986	1	PRJNA827465;Yang2025_Silkie_Wenchang	CRA004775	0.167461782
ENSGALG00010015481	GOLGA5	2	0.098238255	0.200925615	0.488928476	0.624892334	0.055197149	2.944345839	66.03659846	0.5	CRA004775;Yang2025_Silkie_Wenchang	PRJNA827465	0.90106521
ENSGALG00010015481	GOLGA5	2	0.065076489	0.109245789	0.595688767	0.551383163	0.011800877	1.500031384	33.33472814	0.5	CRA004775;PRJNA827465	Yang2025_Silkie_Wenchang	0.878888037
ENSGALG00010015483	SGCD	2	-0.374694901	0.081366709	-4.605014802	4.12437E-06	0.000625463	1.049427225	4.709923992	1	PRJNA827465;Yang2025_Silkie_Wenchang	CRA004775	0.000704782
ENSGALG00010015483	SGCD	2	-0.212356625	0.350397885	-0.606044253	0.544485368	0.171020047	2.63031122	61.98168519	0.5	CRA004775;Yang2025_Silkie_Wenchang	PRJNA827465	0.877225185
ENSGALG00010015483	SGCD	2	-0.166733532	0.242410734	-0.687814145	0.491569827	0.063951516	1.613830801	38.03563549	0.5	CRA004775;PRJNA827465	Yang2025_Silkie_Wenchang	0.85895707
ENSGALG00010015485	BCAS1	2	0.37682475	0.345166136	1.09171993	0.274956223	0.19661756	5.711205485	82.49056171	1	PRJNA827465;Yang2025_Silkie_Wenchang	CRA004775	0.774932927
ENSGALG00010015485	BCAS1	2	0.786184951	0.172450394	4.558904931	5.1421E-06	0	0.627345352	0	1	CRA004775;Yang2025_Silkie_Wenchang	PRJNA827465	0.000835124
ENSGALG00010015485	BCAS1	2	0.491813717	0.511106213	0.962253451	0.335982306	0.432704914	5.594777711	82.12491091	1	CRA004775;PRJNA827465	Yang2025_Silkie_Wenchang	0.883504387
ENSGALG00010015486	MTFR1L	2	-0.261531877	0.262452644	-0.996491684	0.319011305	0.12239201	8.439428372	88.15085624	1	PRJNA827465;Yang2025_Silkie_Wenchang	CRA004775	0.798079048
ENSGALG00010015486	MTFR1L	2	-0.381978439	0.231498546	-1.650025223	0.098937777	0.052538436	1.786337424	44.0195348	1	CRA004775;Yang2025_Silkie_Wenchang	PRJNA827465	0.536907794
ENSGALG00010015486	MTFR1L	2	-0.022778079	0.063551595	-0.358418682	0.720030013	0	0.013502787	0	1	CRA004775;PRJNA827465	Yang2025_Silkie_Wenchang	0.929856173
ENSGALG00010015487	RPS6KA6	2	0.144697408	0.101417003	1.426756896	0.15364998	0.009037675	1.721158474	41.89959757	1	PRJNA827465;Yang2025_Silkie_Wenchang	CRA004775	0.646148636
ENSGALG00010015487	RPS6KA6	2	0.287918738	0.117857258	2.442944481	0.014567978	0	0.088224386	0	1	CRA004775;Yang2025_Silkie_Wenchang	PRJNA827465	0.198669413
ENSGALG00010015487	RPS6KA6	2	0.102439027	0.100518562	1.019105573	0.308152848	0.003753841	1.093223331	8.52738213	1	CRA004775;PRJNA827465	Yang2025_Silkie_Wenchang	0.793353295
ENSGALG00010015490	RANBP2	2	-0.048047489	0.050756398	-0.946629208	0.343827755	0	0.385821789	0	0.5	PRJNA827465;Yang2025_Silkie_Wenchang	CRA004775	0.806388726
ENSGALG00010015490	RANBP2	2	0.103065527	0.141938899	0.726126016	0.467761522	0.019224752	1.703758206	41.30622548	1	CRA004775;Yang2025_Silkie_Wenchang	PRJNA827465	0.853026683
ENSGALG00010015490	RANBP2	2	0.068436198	0.180970854	0.378161433	0.705310674	0.047464319	2.985518241	66.50497772	0.5	CRA004775;PRJNA827465	Yang2025_Silkie_Wenchang	0.924281103
ENSGALG00010015493	OSTF1	2	0.220107832	0.099663961	2.208499746	0.027209455	0.011743184	2.372727898	57.93347414	1	PRJNA827465;Yang2025_Silkie_Wenchang	CRA004775	0.283149612
ENSGALG00010015493	OSTF1	2	-0.026349345	0.426121788	-0.061835245	0.950694036	0.300759574	5.156152987	80.60569571	0.5	CRA004775;Yang2025_Silkie_Wenchang	PRJNA827465	0.98727979
ENSGALG00010015493	OSTF1	2	-0.101250267	0.316931969	-0.319470035	0.749370111	0.14995814	3.157569476	68.33007135	0.5	CRA004775;PRJNA827465	Yang2025_Silkie_Wenchang	0.937362187
ENSGALG00010015494	CHRDL1	2	-0.212749429	0.172353631	-1.234377416	0.2170623	0.012095113	1.231784761	18.81698556	1	PRJNA827465;Yang2025_Silkie_Wenchang	CRA004775	0.730558054
ENSGALG00010015494	CHRDL1	2	-0.023709924	0.496694261	-0.048198511	0.961558037	0.351066857	3.230163689	69.04181657	0.5	CRA004775;Yang2025_Silkie_Wenchang	PRJNA827465	0.989731612
ENSGALG00010015494	CHRDL1	2	0.08022476	0.281733729	0.284753835	0.775832743	0.071454799	1.511370609	33.8348917	0.5	CRA004775;PRJNA827465	Yang2025_Silkie_Wenchang	0.943837382
ENSGALG00010015498	MID2	2	-0.119873767	0.045553636	-2.63148627	0.00850123	0	0.420416346	0	1	PRJNA827465;Yang2025_Silkie_Wenchang	CRA004775	0.143709183
ENSGALG00010015498	MID2	2	-0.164244489	0.085539467	-1.920101856	0.054845035	0	0.044842555	0	1	CRA004775;Yang2025_Silkie_Wenchang	PRJNA827465	0.405411775
ENSGALG00010015498	MID2	2	-0.102251302	0.052260938	-1.956553142	0.050400029	0	9.52735E-06	0	1	CRA004775;PRJNA827465	Yang2025_Silkie_Wenchang	0.38897029
ENSGALG00010015505	CHGA	2	0.234282497	0.710383762	0.329797089	0.741553287	0.737227316	3.580714225	72.07261074	0.5	PRJNA827465;Yang2025_Silkie_Wenchang	CRA004775	0.93543545
ENSGALG00010015505	CHGA	2	1.682398561	0.613867213	2.740655512	0.006131676	0.396324502	2.101286808	52.41011383	1	CRA004775;Yang2025_Silkie_Wenchang	PRJNA827465	0.118495743
ENSGALG00010015505	CHGA	2	0.903761863	1.329768469	0.679642401	0.496730915	3.296178741	14.62589395	93.16281109	0.5	CRA004775;PRJNA827465	Yang2025_Silkie_Wenchang	0.860939846
ENSGALG00010015507	AMMECR1	2	0.022373766	0.065312059	0.342567143	0.731924124	0.00262381	1.299902218	23.07113673	0.5	PRJNA827465;Yang2025_Silkie_Wenchang	CRA004775	0.932853312
ENSGALG00010015507	AMMECR1	2	-0.07248453	0.113338215	-0.639548139	0.522470505	0	0.209559112	0	0.5	CRA004775;Yang2025_Silkie_Wenchang	PRJNA827465	0.869568211
ENSGALG00010015507	AMMECR1	2	0.060473194	0.05464415	1.106672789	0.268435424	0	0.00187668	0	1	CRA004775;PRJNA827465	Yang2025_Silkie_Wenchang	0.771398294
ENSGALG00010015508	FUCA1	2	-0.122024467	0.060309105	-2.023317494	0.043040424	0	0.306974242	0	1	PRJNA827465;Yang2025_Silkie_Wenchang	CRA004775	0.358773658
ENSGALG00010015508	FUCA1	2	-0.10997367	0.105622892	-1.041191619	0.297786625	0	0.582018636	0	0.5	CRA004775;Yang2025_Silkie_Wenchang	PRJNA827465	0.788858094
ENSGALG00010015508	FUCA1	2	-0.151561947	0.065863854	-2.301139979	0.02138372	0	0.236618801	0	1	CRA004775;PRJNA827465	Yang2025_Silkie_Wenchang	0.247262241
ENSGALG00010015516	SELENON	2	-0.221962715	0.345155025	-0.643081222	0.520171398	0.230836322	31.76585712	96.85196595	0.5	PRJNA827465;Yang2025_Silkie_Wenchang	CRA004775	0.869331539
ENSGALG00010015516	SELENON	2	-0.438815291	0.180085887	-2.436700054	0.014821968	0.038779163	2.220603515	54.96881959	1	CRA004775;Yang2025_Silkie_Wenchang	PRJNA827465	0.201086578
ENSGALG00010015516	SELENON	2	0.036796429	0.137228793	0.268139275	0.788592115	0.022016466	1.79970283	44.435271	0.5	CRA004775;PRJNA827465	Yang2025_Silkie_Wenchang	0.947048735
ENSGALG00010015520	UBE3A	2	-0.242378298	0.152281692	-1.591644367	0.111464635	0.044123025	20.47779625	95.11666203	1	PRJNA827465;Yang2025_Silkie_Wenchang	CRA004775	0.564762672
ENSGALG00010015520	UBE3A	2	-0.02886511	0.394298053	-0.073206321	0.941641937	0.287927272	12.74830695	92.15582113	0.5	CRA004775;Yang2025_Silkie_Wenchang	PRJNA827465	0.985422731
ENSGALG00010015520	UBE3A	2	0.104979746	0.238595806	0.439989903	0.65994442	0.094181336	4.946321635	79.78295643	0.5	CRA004775;PRJNA827465	Yang2025_Silkie_Wenchang	0.911773501
ENSGALG00010015521	DKC1	2	0.092477654	0.093160897	0.992665987	0.320872756	0.010895751	2.632395245	62.01178368	1	PRJNA827465;Yang2025_Silkie_Wenchang	CRA004775	0.798436461
ENSGALG00010015521	DKC1	2	0.072426425	0.167400514	0.432653538	0.665256488	0.034813979	2.318349767	56.86587009	0.5	CRA004775;Yang2025_Silkie_Wenchang	PRJNA827465	0.913399315
ENSGALG00010015521	DKC1	2	-0.006681915	0.065442972	-0.102102065	0.918675027	0	0.53918489	0	0.5	CRA004775;PRJNA827465	Yang2025_Silkie_Wenchang	0.981033557
ENSGALG00010015523	MRPL22	2	-0.111845807	0.080277301	-1.393243247	0.163546246	0.003423573	1.278762295	21.79938336	1	PRJNA827465;Yang2025_Silkie_Wenchang	CRA004775	0.661805642
ENSGALG00010015523	MRPL22	2	-0.207899656	0.118298601	-1.757414322	0.078847214	0	0.183315772	0	1	CRA004775;Yang2025_Silkie_Wenchang	PRJNA827465	0.482993365
ENSGALG00010015523	MRPL22	2	-0.066630671	0.071388713	-0.933350222	0.350639173	0	0.064855003	0	1	CRA004775;PRJNA827465	Yang2025_Silkie_Wenchang	0.810111037
ENSGALG00010015532	NXT2	2	-0.112878296	0.208841763	-0.540496758	0.588854496	0.079182188	10.6262288	90.58932365	0.5	PRJNA827465;Yang2025_Silkie_Wenchang	CRA004775	0.892171138
ENSGALG00010015532	NXT2	2	-0.308016405	0.103987235	-2.962059778	0.003055884	0	0.401654316	0	1	CRA004775;Yang2025_Silkie_Wenchang	PRJNA827465	0.076064236
ENSGALG00010015532	NXT2	2	0.077910724	0.064024726	1.216884925	0.223648002	0	0.430673438	0	0.5	CRA004775;PRJNA827465	Yang2025_Silkie_Wenchang	0.737076707
ENSGALG00010015533	PSAT1	2	0.003233582	0.261070967	0.012385836	0.990117786	0.118246705	7.543586357	86.74370581	0.5	PRJNA827465;Yang2025_Silkie_Wenchang	CRA004775	0.997375486
ENSGALG00010015533	PSAT1	2	-0.228279207	0.122405856	-1.864936982	0.062190238	0	0.309213556	0	1	CRA004775;Yang2025_Silkie_Wenchang	PRJNA827465	0.430100813
ENSGALG00010015533	PSAT1	2	0.212729184	0.124775371	1.704897232	0.088213593	0	0.941689655	0	0.5	CRA004775;PRJNA827465	Yang2025_Silkie_Wenchang	0.510208309
ENSGALG00010015540	ACSL4	2	0.030509154	0.053148004	0.574041382	0.565939799	0	0.00296891	0	1	PRJNA827465;Yang2025_Silkie_Wenchang	CRA004775	0.883487517
ENSGALG00010015540	ACSL4	2	0.032407335	0.091564709	0.353928224	0.723392674	0	0.022061686	0	0.5	CRA004775;Yang2025_Silkie_Wenchang	PRJNA827465	0.930032096
ENSGALG00010015540	ACSL4	2	0.027455445	0.064404266	0.426298543	0.66989032	0	0.019221733	0	0.5	CRA004775;PRJNA827465	Yang2025_Silkie_Wenchang	0.914381442
ENSGALG00010015542	CNGA3	2	0.206155159	0.205555649	1.002916534	0.315901134	0	0.065321544	0	1	PRJNA827465;Yang2025_Silkie_Wenchang	CRA004775	0.796883862
ENSGALG00010015542	CNGA3	2	0.458136153	0.335915811	1.363842184	0.17261724	0.018078328	1.057969974	5.479359123	1	CRA004775;Yang2025_Silkie_Wenchang	PRJNA827465	0.675574638
ENSGALG00010015542	CNGA3	2	0.39592269	0.397677234	0.995588019	0.319450358	0.146202712	1.521411871	34.27157897	1	CRA004775;PRJNA827465	Yang2025_Silkie_Wenchang	0.798179112
ENSGALG00010015544	RTCB	2	-0.01313858	0.046742347	-0.28108515	0.778645091	0	0.703403289	0	0.5	PRJNA827465;Yang2025_Silkie_Wenchang	CRA004775	0.944673731
ENSGALG00010015544	RTCB	2	-0.082568448	0.206920911	-0.399033849	0.689868264	0.055188297	2.639566483	62.11499099	0.5	CRA004775;Yang2025_Silkie_Wenchang	PRJNA827465	0.919979266
ENSGALG00010015544	RTCB	2	-0.102136795	0.130668886	-0.781645867	0.434422723	0.020011049	1.754042985	42.98885439	1	CRA004775;PRJNA827465	Yang2025_Silkie_Wenchang	0.842762754
ENSGALG00010015555	PNRC2	2	-0.29363879	0.392396594	-0.748321456	0.454266286	0.294641285	22.52854759	95.56118744	0.5	PRJNA827465;Yang2025_Silkie_Wenchang	CRA004775	0.849072665
ENSGALG00010015555	PNRC2	2	-0.274482237	0.450208758	-0.609677692	0.542075335	0.359290385	8.583867506	88.35023957	0.5	CRA004775;Yang2025_Silkie_Wenchang	PRJNA827465	0.876685104
ENSGALG00010015555	PNRC2	2	0.089887784	0.052791157	1.702705307	0.088623231	0	0.185696836	0	1	CRA004775;PRJNA827465	Yang2025_Silkie_Wenchang	0.511326903
ENSGALG00010015557	IRS4	2	-0.082796189	0.120274712	-0.688392334	0.49120575	0.019017661	2.706488542	63.05175564	0.5	PRJNA827465;Yang2025_Silkie_Wenchang	CRA004775	0.85895707
ENSGALG00010015557	IRS4	2	-0.055789014	0.270709546	-0.206084398	0.836724986	0.086537413	1.960847915	49.00165421	0.5	CRA004775;Yang2025_Silkie_Wenchang	PRJNA827465	0.958399882
ENSGALG00010015557	IRS4	2	0.025701934	0.070010143	0.367117294	0.713531528	0	0.723989251	0	1	CRA004775;PRJNA827465	Yang2025_Silkie_Wenchang	0.926253108
ENSGALG00010015558	MPP1	2	0.016279035	0.087682315	0.185659275	0.852711967	0	0.188584611	0	0.5	PRJNA827465;Yang2025_Silkie_Wenchang	CRA004775	0.962662677
ENSGALG00010015558	MPP1	2	-0.087540379	0.150317705	-0.582369052	0.560318123	0	0.456355898	0	1	CRA004775;Yang2025_Silkie_Wenchang	PRJNA827465	0.881203589
ENSGALG00010015558	MPP1	2	0.015132004	0.100154581	0.15108649	0.879907489	0	0.852871604	0	0.5	CRA004775;PRJNA827465	Yang2025_Silkie_Wenchang	0.971487276
ENSGALG00010015562	IRF9	2	-0.042952984	0.382945661	-0.112164697	0.91069282	0.208525085	3.158915135	68.34356235	0.5	PRJNA827465;Yang2025_Silkie_Wenchang	CRA004775	0.97880407
ENSGALG00010015562	IRF9	2	0.132784422	0.261911949	0.506981154	0.612168042	0	0.966609736	0	0.5	CRA004775;Yang2025_Silkie_Wenchang	PRJNA827465	0.897833503
ENSGALG00010015562	IRF9	2	-0.296266385	0.167690618	-1.766743947	0.07727112	0	0.433351772	0	1	CRA004775;PRJNA827465	Yang2025_Silkie_Wenchang	0.478793445
ENSGALG00010015563	B3GNT7	2	0.64339546	0.393755748	1.633996359	0.102259615	0.217455193	3.321578863	69.89383539	1	PRJNA827465;Yang2025_Silkie_Wenchang	CRA004775	0.54496402
ENSGALG00010015563	B3GNT7	2	1.064519489	0.298505611	3.566162407	0.000362247	0	0.000698045	0	1	CRA004775;Yang2025_Silkie_Wenchang	PRJNA827465	0.019033945
ENSGALG00010015563	B3GNT7	2	0.449513247	0.334853561	1.342417399	0.179460696	0.068010683	1.262782198	20.80978009	1	CRA004775;PRJNA827465	Yang2025_Silkie_Wenchang	0.686323221
ENSGALG00010015564	PKD2L2	2	-0.067603497	0.203984477	-0.331414912	0.740331103	0.03950624	1.845890346	45.82560105	0.5	PRJNA827465;Yang2025_Silkie_Wenchang	CRA004775	0.935128797
ENSGALG00010015564	PKD2L2	2	0.09752525	0.243512613	0.400493627	0.688792977	0.003124411	1.0095725	0.948173615	0.5	CRA004775;Yang2025_Silkie_Wenchang	PRJNA827465	0.919802608
ENSGALG00010015564	PKD2L2	2	-0.256133146	0.170685743	-1.500612428	0.133455835	0	0.25339014	0	1	CRA004775;PRJNA827465	Yang2025_Silkie_Wenchang	0.612342914
ENSGALG00010015565	ENSGALG00010015565	2	3.154597543	1.287238588	2.45067043	0.014259044	0	0.625997728	0	1	PRJNA827465;Yang2025_Silkie_Wenchang	CRA004775	0.195883682
ENSGALG00010015565	ENSGALG00010015565	2	1.554281574	2.634483717	0.589975776	0.55520689	10.1584252	3.68281501	72.84685771	0.5	CRA004775;Yang2025_Silkie_Wenchang	PRJNA827465	0.880255849
ENSGALG00010015565	ENSGALG00010015565	2	0.460572803	1.603453245	0.287238062	0.773930044	0.818238193	1.188029894	15.82703387	0.5	CRA004775;PRJNA827465	Yang2025_Silkie_Wenchang	0.943248868
ENSGALG00010015567	ENSGALG00010015567	2	-0.314157379	0.24373189	-1.288946555	0.197416668	0	0.056305077	0	1	PRJNA827465;Yang2025_Silkie_Wenchang	CRA004775	0.708952382
ENSGALG00010015567	ENSGALG00010015567	2	0.248941502	0.520630196	0.478154176	0.632540475	0.426378198	4.67890465	78.62747641	0.5	CRA004775;Yang2025_Silkie_Wenchang	PRJNA827465	0.903058174
ENSGALG00010015567	ENSGALG00010015567	2	0.203707117	0.578924533	0.351871627	0.724934539	0.540451152	5.160940189	80.62368554	0.5	CRA004775;PRJNA827465	Yang2025_Silkie_Wenchang	0.930597452
ENSGALG00010015570	LIMCH1	2	0.033239091	0.141469332	0.234956157	0.814242762	0.023658186	2.020870161	50.51636571	0.5	PRJNA827465;Yang2025_Silkie_Wenchang	CRA004775	0.953869515
ENSGALG00010015570	LIMCH1	2	0.312900298	0.170777004	1.832215645	0.066919299	0	0.393287626	0	1	CRA004775;Yang2025_Silkie_Wenchang	PRJNA827465	0.445709067
ENSGALG00010015570	LIMCH1	2	0.121591863	0.256429247	0.474173147	0.63537644	0.093939146	2.76927024	63.88940358	0.5	CRA004775;PRJNA827465	Yang2025_Silkie_Wenchang	0.90419692
ENSGALG00010015571	DUSP15	2	0.245444306	0.152201829	1.612623896	0.106826244	0	0.538948347	0	1	PRJNA827465;Yang2025_Silkie_Wenchang	CRA004775	0.553925396
ENSGALG00010015571	DUSP15	2	0.037706505	0.413219651	0.091250512	0.92729354	0.233455159	3.015462214	66.8375881	0.5	CRA004775;Yang2025_Silkie_Wenchang	PRJNA827465	0.982993195
ENSGALG00010015571	DUSP15	2	-0.031819482	0.281401593	-0.113074989	0.909971103	0.079938899	1.782209524	43.88987454	0.5	CRA004775;PRJNA827465	Yang2025_Silkie_Wenchang	0.978730675
ENSGALG00010015573	MAN1C1	2	-0.057525317	0.069078872	-0.832748349	0.40498667	0.004373286	1.817814751	44.98889399	0.5	PRJNA827465;Yang2025_Silkie_Wenchang	CRA004775	0.833112102
ENSGALG00010015573	MAN1C1	2	0.008012654	0.078239794	0.102411494	0.918430061	0	0.343203597	0	0.5	CRA004775;Yang2025_Silkie_Wenchang	PRJNA827465	0.981033557
ENSGALG00010015573	MAN1C1	2	-0.119288791	0.061900451	-1.927106986	0.053966298	0	0.010377421	0	1	CRA004775;PRJNA827465	Yang2025_Silkie_Wenchang	0.402825653
ENSGALG00010015574	C14orf132	2	-0.207566135	0.332177608	-0.624864922	0.532059716	0.150558607	3.050086443	67.21404397	0.5	PRJNA827465;Yang2025_Silkie_Wenchang	CRA004775	0.873104119
ENSGALG00010015574	C14orf132	2	-0.362414853	0.313373547	-1.15649472	0.247478858	0.046145298	1.26289806	20.81704521	0.5	CRA004775;Yang2025_Silkie_Wenchang	PRJNA827465	0.757947282
ENSGALG00010015574	C14orf132	2	0.085842552	0.197474889	0.434701102	0.663779398	0	4.10063E-05	0	1	CRA004775;PRJNA827465	Yang2025_Silkie_Wenchang	0.913119244
ENSGALG00010015580	GEMIN5	2	-0.113580811	0.099620087	-1.14013965	0.254228125	0.016861256	6.453568227	84.50469624	1	PRJNA827465;Yang2025_Silkie_Wenchang	CRA004775	0.764008204
ENSGALG00010015580	GEMIN5	2	-0.206516328	0.064914431	-3.181362373	0.001465842	0	0.622031082	0	1	CRA004775;Yang2025_Silkie_Wenchang	PRJNA827465	0.047431078
ENSGALG00010015580	GEMIN5	2	-0.020955084	0.040021244	-0.523599017	0.600557466	0	9.77869E-06	0	1	CRA004775;PRJNA827465	Yang2025_Silkie_Wenchang	0.895230519
ENSGALG00010015583	PRDM4	2	0.045996779	0.181913542	0.252849668	0.800384385	0.050333956	3.652703943	72.62302076	0.5	PRJNA827465;Yang2025_Silkie_Wenchang	CRA004775	0.950102441
ENSGALG00010015583	PRDM4	2	0.217589758	0.167888858	1.296034528	0.194963589	0	0.531677204	0	0.5	CRA004775;Yang2025_Silkie_Wenchang	PRJNA827465	0.706113528
ENSGALG00010015583	PRDM4	2	-0.099650056	0.064486899	-1.545275996	0.122279519	0	0.009565193	0	1	CRA004775;PRJNA827465	Yang2025_Silkie_Wenchang	0.586575409

ENSGALG00010015584	TTLL9	2	0.171607641	0.188880998	0.908548996	0.36358824	0	0.104774455	0	1	PRJNA827465;Yang2025_Silkie_Wenchang	CRA004775	0.817042253
ENSGALG00010015584	TTLL9	2	0.082021165	0.268014868	0.306032146	0.75958017	0	0.682883912	0	0.5	CRA004775;Yang2025_Silkie_Wenchang	PRJNA827465	0.939163539
ENSGALG00010015584	TTLL9	2	0.06158338	0.197966879	0.311079208	0.755740407	0	0.442391286	0	0.5	CRA004775;PRJNA827465	Yang2025_Silkie_Wenchang	0.938501783
ENSGALG00010015585	BDKRB1	2	-0.952251218	0.558557839	-1.704839056	0.088224445	0.575785625	12.890277	92.24221481	1	PRJNA827465;Yang2025_Silkie_Wenchang	CRA004775	0.510208309
ENSGALG00010015585	BDKRB1	2	-0.703154538	0.873916615	-0.804601407	0.42104973	1.373321937	9.547541598	89.52609957	0.5	CRA004775;Yang2025_Silkie_Wenchang	PRJNA827465	0.839269089
ENSGALG00010015585	BDKRB1	2	-0.264324413	0.263143466	-1.004487848	0.315143526	0.05261843	1.354308285	26.16156818	0.5	CRA004775;PRJNA827465	Yang2025_Silkie_Wenchang	0.796635323
ENSGALG00010015588	BDKRB2	2	0.816139253	0.364255733	2.240566662	0.025054158	0.002761366	1.00975804	0.966374126	1	PRJNA827465;Yang2025_Silkie_Wenchang	CRA004775	0.269672828
ENSGALG00010015588	BDKRB2	2	1.196258827	0.458058205	2.611586941	0.009012307	0	0.065319189	0	1	CRA004775;Yang2025_Silkie_Wenchang	PRJNA827465	0.148703065
ENSGALG00010015588	BDKRB2	2	0.689805323	0.382879178	1.801626631	0.071604168	0	0.378059178	0	1	CRA004775;PRJNA827465	Yang2025_Silkie_Wenchang	0.46178455
ENSGALG00010015589	FRMD3	2	-0.333795128	0.214667928	-1.554937113	0.119961047	0.057053205	2.623793467	61.88724408	1	PRJNA827465;Yang2025_Silkie_Wenchang	CRA004775	0.580930143
ENSGALG00010015589	FRMD3	2	0.099198018	0.702678219	0.141171329	0.887734596	0.881154408	8.861016076	88.71461251	0.5	CRA004775;Yang2025_Silkie_Wenchang	PRJNA827465	0.973090153
ENSGALG00010015589	FRMD3	2	0.288909186	0.483686168	0.597307107	0.55030236	0.369642147	4.342324302	76.97085868	0.5	CRA004775;PRJNA827465	Yang2025_Silkie_Wenchang	0.878679315
ENSGALG00010015596	UCHL1	2	-0.456486336	0.168296925	-2.712386665	0.006680062	0.002431358	1.039753594	3.823366813	1	PRJNA827465;Yang2025_Silkie_Wenchang	CRA004775	0.124962626
ENSGALG00010015596	UCHL1	2	-0.764108105	0.265684951	-2.875993175	0.004027584	0	0.427532592	0	1	CRA004775;Yang2025_Silkie_Wenchang	PRJNA827465	0.091819108
ENSGALG00010015596	UCHL1	2	-0.49595687	0.332739012	-1.49052817	0.136085415	0.095805391	1.354324825	26.16246993	1	CRA004775;PRJNA827465	Yang2025_Silkie_Wenchang	0.617688541
ENSGALG00010015605	LDLRAP1	2	-0.056832261	0.045991758	-1.235705341	0.216568111	0	0.90759701	0	1	PRJNA827465;Yang2025_Silkie_Wenchang	CRA004775	0.730172415
ENSGALG00010015605	LDLRAP1	2	-0.139311201	0.082541488	-1.687771866	0.09145503	0	0.04709923	0	1	CRA004775;Yang2025_Silkie_Wenchang	PRJNA827465	0.51833799
ENSGALG00010015605	LDLRAP1	2	-0.040837146	0.051577443	-0.791763693	0.428498476	0	0.509528363	0	1	CRA004775;PRJNA827465	Yang2025_Silkie_Wenchang	0.841538794
ENSGALG00010015606	TMEM131	2	0.084173741	0.285101034	0.295241794	0.767809177	0.134118378	4.967265757	79.86820016	0.5	PRJNA827465;Yang2025_Silkie_Wenchang	CRA004775	0.941444269
ENSGALG00010015606	TMEM131	2	0.481292776	0.155599719	3.093146819	0.001980461	0	0.078048721	0	1	CRA004775;Yang2025_Silkie_Wenchang	PRJNA827465	0.057700571
ENSGALG00010015606	TMEM131	2	0.154420528	0.333517822	0.463005325	0.64336055	0.202441514	10.45490701	90.4351134	0.5	CRA004775;PRJNA827465	Yang2025_Silkie_Wenchang	0.906634523
ENSGALG00010015610	ENSGALG00010015610	2	0.256990866	0.33865047	0.75886759	0.447931768	0.212964452	13.67850323	92.68925859	0.5	PRJNA827465;Yang2025_Silkie_Wenchang	CRA004775	0.846601815
ENSGALG00010015610	ENSGALG00010015610	2	0.599976565	0.158949469	3.774637107	0.000160241	0	0.095760969	0	1	CRA004775;Yang2025_Silkie_Wenchang	PRJNA827465	0.010945128
ENSGALG00010015610	ENSGALG00010015610	2	-0.063123601	0.084894513	-0.743553367	0.457146724	0	0.280212404	0	0.5	CRA004775;PRJNA827465	Yang2025_Silkie_Wenchang	0.849541335
ENSGALG00010015613	PWP1	2	-0.083487614	0.063511229	-1.314533125	0.188666858	0	0.68793494	0	0.5	PRJNA827465;Yang2025_Silkie_Wenchang	CRA004775	0.698095794
ENSGALG00010015613	PWP1	2	-0.015403721	0.113322123	-0.135928626	0.891877705	0	0.166161656	0	0.5	CRA004775;Yang2025_Silkie_Wenchang	PRJNA827465	0.974015927
ENSGALG00010015613	PWP1	2	-0.111737727	0.069508934	-1.607530446	0.107938056	0	0.003685994	0	1	CRA004775;PRJNA827465	Yang2025_Silkie_Wenchang	0.556183889
ENSGALG00010015614	ATG2B	2	0.098208252	0.116722356	0.841383397	0.400133184	0.019116711	2.899443827	65.51062688	1	PRJNA827465;Yang2025_Silkie_Wenchang	CRA004775	0.832246851
ENSGALG00010015614	ATG2B	2	0.246614446	0.121943102	2.022373074	0.043137825	0	0.004459026	0	1	CRA004775;Yang2025_Silkie_Wenchang	PRJNA827465	0.359280763
ENSGALG00010015614	ATG2B	2	0.014144418	0.049883812	0.283547249	0.776757368	0	0.483618334	0	1	CRA004775;PRJNA827465	Yang2025_Silkie_Wenchang	0.944156005
ENSGALG00010015616	NADK2	2	0.325717703	0.114718843	2.839269411	0.004521696	0.007670262	1.405894451	28.870905	1	PRJNA827465;Yang2025_Silkie_Wenchang	CRA004775	0.098457744
ENSGALG00010015616	NADK2	2	0.32108033	0.137842537	2.329326913	0.019841754	0.014497889	1.564722159	36.09089037	1	CRA004775;Yang2025_Silkie_Wenchang	PRJNA827465	0.236530314
ENSGALG00010015616	NADK2	2	0.179242655	0.115339235	1.554047545	0.120173076	0	0.049625643	0	1	CRA004775;PRJNA827465	Yang2025_Silkie_Wenchang	0.581455423
ENSGALG00010015617	SRSF10	2	0.004701969	0.037880488	0.124126405	0.901215191	0	0.857188882	0	0.5	PRJNA827465;Yang2025_Silkie_Wenchang	CRA004775	0.97660383
ENSGALG00010015617	SRSF10	2	-0.053607526	0.067311152	-0.79641374	0.425791595	0	0.004141322	0	1	CRA004775;Yang2025_Silkie_Wenchang	PRJNA827465	0.84125685
ENSGALG00010015617	SRSF10	2	0.019400745	0.041989168	0.462041659	0.644051446	0	0.205853283	0	0.5	CRA004775;PRJNA827465	Yang2025_Silkie_Wenchang	0.906826466
ENSGALG00010015618	NCL	2	0.095325848	0.18415492	0.517639434	0.604709863	0.05581761	5.107213731	80.41985214	0.5	PRJNA827465;Yang2025_Silkie_Wenchang	CRA004775	0.896323417
ENSGALG00010015618	NCL	2	0.126146883	0.209966994	0.600793872	0.547977286	0.053723873	2.490387454	59.84560561	0.5	CRA004775;Yang2025_Silkie_Wenchang	PRJNA827465	0.878281266
ENSGALG00010015618	NCL	2	-0.065954849	0.05406533	-1.219910222	0.22249891	0	0.051149342	0	1	CRA004775;PRJNA827465	Yang2025_Silkie_Wenchang	0.73602412
ENSGALG00010015620	PDRG1	2	-0.199225338	0.08641004	-2.305580904	0.021134061	0	0.660647571	0	1	PRJNA827465;Yang2025_Silkie_Wenchang	CRA004775	0.246040288
ENSGALG00010015620	PDRG1	2	-0.303355538	0.248958606	-1.218497896	0.223034824	0	0.479231498	0	1	CRA004775;Yang2025_Silkie_Wenchang	PRJNA827465	0.736571521
ENSGALG00010015620	PDRG1	2	-0.179071531	0.086204914	-2.077277528	0.037775952	0	0.01530996	0	1	CRA004775;PRJNA827465	Yang2025_Silkie_Wenchang	0.335429998
ENSGALG00010015621	IDNK	2	-0.295460706	0.267062911	-1.106333728	0.268582099	0.108392063	4.01839474	75.11444085	1	PRJNA827465;Yang2025_Silkie_Wenchang	CRA004775	0.771398294
ENSGALG00010015621	IDNK	2	-0.4053616	0.260331112	-1.557100095	0.119446722	0.05155162	1.520068115	34.21347436	1	CRA004775;Yang2025_Silkie_Wenchang	PRJNA827465	0.580011679
ENSGALG00010015621	IDNK	2	-0.054203546	0.133967453	-0.404602344	0.685769835	0	0.000823208	0	1	CRA004775;PRJNA827465	Yang2025_Silkie_Wenchang	0.919235539
ENSGALG00010015624	ITGA1	2	-0.0815287	0.080402513	-1.01400687	0.310579474	0	0.149185449	0	1	PRJNA827465;Yang2025_Silkie_Wenchang	CRA004775	0.794899969
ENSGALG00010015624	ITGA1	2	0.352566578	0.431421952	0.817219838	0.413802807	0.30846877	5.461287658	81.68929999	0.5	CRA004775;Yang2025_Silkie_Wenchang	PRJNA827465	0.836538492
ENSGALG00010015624	ITGA1	2	0.313211416	0.466227042	0.671800193	0.501710906	0.379537071	7.172802728	86.05844831	0.5	CRA004775;PRJNA827465	Yang2025_Silkie_Wenchang	0.86279037
ENSGALG00010015626	XKR7	2	-0.191653901	0.35347779	-0.542195031	0.587684158	0.108608552	1.750240532	42.86499589	0.5	PRJNA827465;Yang2025_Silkie_Wenchang	CRA004775	0.891742161
ENSGALG00010015626	XKR7	2	0.184282472	0.342021172	0.538804284	0.590021908	0	0.008199721	0	1	CRA004775;Yang2025_Silkie_Wenchang	PRJNA827465	0.892320726
ENSGALG00010015626	XKR7	2	-0.347751849	0.294793257	-1.179646551	0.23814082	0	0.887631116	0	0.5	CRA004775;PRJNA827465	Yang2025_Silkie_Wenchang	0.750031946
ENSGALG00010015628	UBQLN1	2	0.022175356	0.071912827	0.30836441	0.75780506	0	0.050250404	0	0.5	PRJNA827465;Yang2025_Silkie_Wenchang	CRA004775	0.938954369
ENSGALG00010015628	UBQLN1	2	0.02558277	0.106806343	0.239524817	0.810698655	0	0.178942431	0	0.5	CRA004775;Yang2025_Silkie_Wenchang	PRJNA827465	0.953020781
ENSGALG00010015628	UBQLN1	2	0.044070547	0.081998735	0.537453985	0.590954062	0	0.092792523	0	1	CRA004775;PRJNA827465	Yang2025_Silkie_Wenchang	0.892766566
ENSGALG00010015631	GSKIP	2	-0.410739075	0.361280414	-1.136898262	0.255580819	0.252138984	29.1241603	96.56642461	1	PRJNA827465;Yang2025_Silkie_Wenchang	CRA004775	0.764467569
ENSGALG00010015631	GSKIP	2	-0.343269557	0.449197906	-0.764183341	0.444757992	0.37742363	15.18691464	93.41538407	0.5	CRA004775;Yang2025_Silkie_Wenchang	PRJNA827465	0.846013379
ENSGALG00010015631	GSKIP	2	-0.034984337	0.067310896	-0.519742553	0.603243024	0	0.683194994	0	0.5	CRA004775;PRJNA827465	Yang2025_Silkie_Wenchang	0.895538966
ENSGALG00010015632	COL4A5	2	-0.181788239	0.206292345	-0.881216599	0.378200597	0.072085509	6.350430157	84.25303522	0.5	PRJNA827465;Yang2025_Silkie_Wenchang	CRA004775	0.823285408
ENSGALG00010015632	COL4A5	2	0.199214344	0.688889209	0.289181978	0.772442123	0.818236874	6.531090275	84.68862077	0.5	CRA004775;Yang2025_Silkie_Wenchang	PRJNA827465	0.943066211
ENSGALG00010015632	COL4A5	2	0.360626212	0.468388467	0.769929743	0.441341569	0.334940653	3.362701501	70.26200513	1	CRA004775;PRJNA827465	Yang2025_Silkie_Wenchang	0.844912212
ENSGALG00010015633	CCM2L	2	0.145544177	0.142303059	1.022776164	0.30641369	0	0.99311441	0	1	PRJNA827465;Yang2025_Silkie_Wenchang	CRA004775	0.792485409
ENSGALG00010015633	CCM2L	2	0.116058633	0.290185881	0.399945831	0.689196415	0.076033735	1.731546372	42.24815367	0.5	CRA004775;Yang2025_Silkie_Wenchang	PRJNA827465	0.919854035
ENSGALG00010015633	CCM2L	2	-0.009752557	0.160276777	-0.060848225	0.951480084	0	0.505339029	0	0.5	CRA004775;PRJNA827465	Yang2025_Silkie_Wenchang	0.987713746
ENSGALG00010015635	ABTB3	2	0.114300966	0.100563807	1.13660142	0.255704946	0	0.047981868	0	1	PRJNA827465;Yang2025_Silkie_Wenchang	CRA004775	0.764478331
ENSGALG00010015635	ABTB3	2	-0.111102025	0.295305113	-0.37622791	0.706747468	0.088638148	1.731525257	42.2474494	0.5	CRA004775;Yang2025_Silkie_Wenchang	PRJNA827465	0.924553846
ENSGALG00010015635	ABTB3	2	-0.081176831	0.319383357	-0.254167379	0.799366251	0.131681343	2.188619604	54.30909975	0.5	CRA004775;PRJNA827465	Yang2025_Silkie_Wenchang	0.949922217
ENSGALG00010015636	APBB2	2	-0.064360592	0.058581615	-1.098648319	0.271921493	0	0.588397345	0	1	PRJNA827465;Yang2025_Silkie_Wenchang	CRA004775	0.773184343
ENSGALG00010015636	APBB2	2	-0.099323024	0.091814091	-1.081784094	0.279348476	0	0.727640408	0	0.5	CRA004775;Yang2025_Silkie_Wenchang	PRJNA827465	0.777457036
ENSGALG00010015636	APBB2	2	-0.019895114	0.072211343	-0.275512308	0.782922671	0	0.345771038	0	0.5	CRA004775;PRJNA827465	Yang2025_Silkie_Wenchang	0.945555411
ENSGALG00010015643	ENSGALG00010015643	2	0.198595752	0.151145969	1.3139335	0.188868584	0	0.012942333	0	1	PRJNA827465;Yang2025_Silkie_Wenchang	CRA004775	0.698162702
ENSGALG00010015643	ENSGALG00010015643	2	0.227083344	0.187991794	1.207942852	0.227069245	0	0.005485847	0	1	CRA004775;Yang2025_Silkie_Wenchang	PRJNA827465	0.740605003
ENSGALG00010015643	ENSGALG00010015643	2	0.200346086	0.174049708	1.15108545	0.249697083	0	0.026953966	0	1	CRA004775;PRJNA827465	Yang2025_Silkie_Wenchang	0.759757983
ENSGALG00010015645	MACO1	2	0.090495858	0.165070436	0.54822572	0.583536924	0.045616046	5.817558375	82.81065809	0.5	PRJNA827465;Yang2025_Silkie_Wenchang	CRA004775	0.890558522
ENSGALG00010015645	MACO1	2	0.259859785	0.108445635	2.3962217	0.016565068	0	0.055556308	0	1	CRA004775;Yang2025_Silkie_Wenchang	PRJNA827465	0.213278058
ENSGALG00010015645	MACO1	2	-0.012882823	0.101149609	-0.127364037	0.898652278	0.008518873	1.303122787	23.26126054	0.5	CRA004775;PRJNA827465	Yang2025_Silkie_Wenchang	0.975817944
ENSGALG00010015647	PDZD2	2	-0.188281253	0.095433961	-1.972895715	0.048507445	0	0.540843595	0	1	PRJNA827465;Yang2025_Silkie_Wenchang	CRA004775	0.380764551
ENSGALG00010015647	PDZD2	2	-0.335283951	0.34956632	-0.959142606	0.337486909	0.156828949	2.412010585	58.54081213	1	CRA004775;Yang2025_Silkie_Wenchang	PRJNA827465	0.80415385
ENSGALG00010015647	PDZD2	2	-0.367277446	0.243888451	-1.505923897	0.132086711	0.064782375	1.637637848	38.93643815	1	CRA004775;PRJNA827465	Yang2025_Silkie_Wenchang	0.608786928
ENSGALG00010015653	HCK	2	0.841280899	0.313019924	2.687627319	0.007196166	0	0.508525601	0	1	PRJNA827465;Yang2025_Silkie_Wenchang	CRA004775	0.130396922
ENSGALG00010015653	HCK	2	0.589029197	0.448313393	1.313878205	0.188887194	0.232451788	2.321142461	56.91776714	1	CRA004775;Yang2025_Silkie_Wenchang	PRJNA827465	0.698162702
ENSGALG00010015653	HCK	2	0.384081493	0.268963454	1.428006248	0.15329006	0	0.69113992	0	1	CRA004775;PRJNA827465	Yang2025_Silkie_Wenchang	0.645602272
ENSGALG00010015655	ENSGALG00010015655	2	-0.221929178	0.20444979	-1.085494773	0.277702578	0	0.029278783	0	1	PRJNA827465;Yang2025_Silkie_Wenchang	CRA004775	0.776763074
ENSGALG00010015655	ENSGALG00010015655	2	-0.030359015	0.242066181	-0.125416175	0.900194083	0	0.976401815	0	0.5	CRA004775;Yang2025_Silkie_Wenchang	PRJNA827465	0.976466979
ENSGALG00010015655	ENSGALG00010015655	2	-0.055383926	0.280500558	-0.197446758	0.843477942	0.035774371	1.256541558	20.4164802	0.5	CRA004775;PRJNA827465	Yang2025_Silkie_Wenchang	0.96057435
ENSGALG00010015660	KLHL3	2	0.210608921	0.182585223	1.153482837	0.248712258	0.052731004	4.547849141	78.01158374	1	PRJNA827465;Yang2025_Silkie_Wenchang	CRA004775	0.759154375
ENSGALG00010015660	KLHL3	2	0.164231269	0.281144301	0.584152936	0.559117423	0.119627109	3.960606166	74.75133961	0.5	CRA004775;Yang2025_Silkie_Wenchang	PRJNA827465	0.881014886
ENSGALG00010015660	KLHL3	2	0.026950496	0.078941281	0.341399276	0.73280302	0	0.603086733	0	0.5	CRA004775;PRJNA827465	Yang2025_Silkie_Wenchang	0.932985777
ENSGALG00010015668	ZNF217	2	0.019911231	0.054379251	0.366154926	0.714249474	0	0.032383446	0	0.5	PRJNA827465;Yang2025_Silkie_Wenchang	CRA004775	0.92653974
ENSGALG00010015668	ZNF217	2	-0.027383201	0.142106413	-0.19269504	0.847197808	0	0.152828825	0	1	CRA004775;Yang2025_Silkie_Wenchang	PRJNA827465	0.961252712
ENSGALG00010015668	ZNF217	2	0.019456186	0.057571189	0.33795005	0.735400829	0	0.239378737	0	0.5	CRA004775;PRJNA827465	Yang2025_Silkie_Wenchang	0.933592889
ENSGALG00010015669	INPP4A	2	-0.102920367	0.050545946	-2.036174518	0.041732834	0.000225388	1.029199208	2.837080335	1	PRJNA827465;Yang2025_Silkie_Wenchang	CRA004775	0.354014899
ENSGALG00010015669	INPP4A	2	-0.16173479	0.112186202	-1.441663833	0.149397231	0.001873419	1.032987271	3.193386008	0.5	CRA004775;Yang2025_Silkie_Wenchang	PRJNA827465	0.639246608
ENSGALG00010015669	INPP4A	2	-0.071357844	0.053745887	-1.327689375	0.184280745	0	0.450231005	0	0.5	CRA004775;PRJNA827465	Yang2025_Silkie_Wenchang	0.692559392
ENSGALG00010015670	ENSGALG00010015670	2	0.483640898	0.545095787	0.88725855	0.374939716	0.561229411	17.18668609	94.18154265	0.5	PRJNA827465;Yang2025_Silkie_Wenchang	CRA004775	0.822916144
ENSGALG00010015670	ENSGALG00010015670	2	0.634175714	0.411227803	1.542151841	0.1230367	0.282901708	6.085400938	83.56722901	1	CRA004775;Yang2025_Silkie_Wenchang	PRJNA827465	0.58801364
ENSGALG00010015670	ENSGALG00010015670	2	0.022930803	0.109520945	0.209373673	0.834156543	0.011801051	1.484813254	32.65146325	0.5	CRA004775;PRJNA827465	Yang2025_Silkie_Wenchang	0.957968425
ENSGALG00010015674	RFKL	2	0.136401427	0.07980115	1.709266443	0.087401606	0	0.751718376	0	1	PRJNA827465;Yang2025_Silkie_Wenchang	CRA004775	0.508252579
ENSGALG00010015674	RFKL	2	0.074317665	0.114105079	0.651309003	0.514847038	0	0.172342476	0	1	CRA004775;Yang2025_Silkie_Wenchang	PRJNA827465	0.866930857
ENSGALG00010015674	RFKL	2	0.19522837	0.099326751	1.965516527	0.049354485	0	6.33686E-05	0	1	CRA004775;PRJNA827465	Yang2025_Silkie_Wenchang	0.384419206
ENSGALG00010015676	TGM2	2	-0.027637921	0.180841412	-0.152829603	0.878532653	0.048899455	3.958286105	74.73654068	0.5	PRJNA827465;Yang2025_Silkie_Wenchang	CRA004775	0.971079508
ENSGALG00010015676	TGM2	2	0.117753153	0.11655892	1.010245752	0.312377564	0	0.592439363	0	0.5	CRA004775;Yang2025_Silkie_Wenchang	PRJNA827465	0.796054997
ENSGALG00010015676	TGM2	2	-0.201540938	0.124224994	-1.622386374	0.104720645	0	0.046027315	0	1	CRA004775;PRJNA827465	Yang2025_Silkie_Wenchang	0.549804368
ENSGALG00010015677	GRHL3	2	0.003912346	0.13367279	0.029268082	0.976650783	0	0.034669042	0	0.5	PRJNA827465;Yang2025_Silkie_Wenchang	CRA004775	0.993151507
ENSGALG00010015677	GRHL3	2	-0.017110303	0.203463411	-0.084095232	0.932980716	0	0.017255414	0	0.5	CRA004775;Yang2025_Silkie_Wenchang	PRJNA827465	0.984224753
ENSGALG00010015677	GRHL3	2	0.024000688	0.156386289	0.15347054	0.878027221	0	0.001065528	0	1	CRA004775;PRJNA827465	Yang2025_Silkie_Wenchang	0.970993355
ENSGALG00010015680	ENSGALG00010015680	2	0.25318346	0.304171792	0.832369954	0.405200156	0.176523029	21.39632587	95.32630038	0.5	PRJNA827465;Yang2025_Silkie_Wenchang	CRA004775	0.833112102
ENSGALG00010015680	ENSGALG00010015680	2	0.31019429	0.297642978	1.042169018	0.297333324	0.140206413	4.408901359	77.31861254	0.5	CRA004775;Yang2025_Silkie_Wenchang	PRJNA827465	0.788535199
ENSGALG00010015680	ENSGALG00010015680	2	-0.042514221	0.058661653	-0.724736163	0.468613905	0	0.000557791	0	1	CRA004775;PRJNA827465	Yang2025_Silkie_Wenchang	0.853198008
ENSGALG00010015684	PDE6D	2	-0.255377804	0.297640954	-0.858006269	0.390889002	0.167984458	18.90004297	94.70900674	0.5	PRJNA827465;Yang2025_Silkie_Wenchang	CRA004775	0.829251057
ENSGALG00010015684	PDE6D	2	-0.384708535	0.218217571	-1.762958564	0.077907479	0.064197493	2.83388148	64.71270916	1	CRA004775;Yang2025_Silkie_Wenchang	PRJNA827465	0.480724545
ENSGALG00010015684	PDE6D	2	0.023460334	0.058109493	0.40372636	0.686413953	0	0.385878732	0	0.5	CRA004775;PRJNA827465	Yang2025_Silkie_Wenchang	0.919235539
ENSGALG00010015686	CNOT8	2	-0.123937387	0.222613263	-0.556738556	0.57770607	0.094788043	22.57671069	95.5706568	0.5	PRJNA827465;Yang2025_Silkie_Wenchang	CRA004775	0.887389782
ENSGALG00010015686	CNOT8	2	-0.329153239	0.077466079	-4.248998328	2.14729E-05	0	0.550955794	0	1	CRA004775;Yang2025_Silkie_Wenchang	PRJNA827465	0.002588796
ENSGALG00010015686	CNOT8	2	0.025603501	0.119703697	0.213890642	0.830632338	0.017061675	1.769185069	43.47680086	0.5	CRA004775;PRJNA827465	Yang2025_Silkie_Wenchang	0.957295959
ENSGALG00010015693	COA5	2	-0.00245802	0.114817745	-0.021408014	0.982920181	0.013817723	1.819329677	45.03470081	0.5	PRJNA827465;Yang2025_Silkie_Wenchang	CRA004775	0.995363468
ENSGALG00010015693	COA5	2	0.030549286	0.168470987	0.181332623	0.856106501	0.018863303	1.467957906	31.87815563	0.5	CRA004775;Yang2025_Silkie_Wenchang	PRJNA827465	0.963499334
ENSGALG00010015693	COA5	2	-0.088817401	0.069704256	-1.274203413	0.202591336	0	0.160976221	0	1	CRA004775;PRJNA827465	Yang2025_Silkie_Wenchang	0.715444091
ENSGALG00010015695	ASAH1	2	-0.120506251	0.063188152	-1.907102003	0.05650738	0	0.545300699	0	1	PRJNA827465;Yang2025_Silkie_Wenchang	CRA004775	0.411523553
ENSGALG00010015695	ASAH1	2	0.048528405	0.283387676	0.17124388	0.864032005	0.118701791	3.181134863	68.5646776	0.5	CRA004775;Yang2025_Silkie_Wenchang	PRJNA827465	0.966530523
ENSGALG00010015695	ASAH1	2	0.080480778	0.22909449	0.351299494	0.725363674	0.068043319	2.24808014	55.51760002	0.5	CRA004775;PRJNA827465	Yang2025_Silkie_Wenchang	0.930910808
ENSGALG00010015697	FAXDC2	2	-0.103658827	0.106520787	-0.973132379	0.330487513	0.000906612	1.041463188	3.981243697	1	PRJNA827465;Yang2025_Silkie_Wenchang	CRA004775	0.80156474
ENSGALG00010015697	FAXDC2	2	0.104295921	0.350821744	0.297290355	0.766244844	0.202869999	5.484439868	81.76659743	0.5	CRA004775;Yang2025_Silkie_Wenchang	PRJNA827465	0.941123716
ENSGALG00010015697	FAXDC2	2	0.194679446	0.240849138	0.808304517	0.418915303	0.076648641	2.7459902	63.58326406	0.5	CRA004775;PRJNA827465	Yang2025_Silkie_Wenchang	0.838264828
ENSGALG00010015698	TNFAIP2	2	0.012527671	0.079644909	0.157294062	0.875013099	0	0.061003571	0	0.5	PRJNA827465;Yang2025_Silkie_Wenchang	CRA004775	0.970198884
ENSGALG00010015698	TNFAIP2	2	-0.065697862	0.131277063	-0.500451945	0.616756885	0	0.416474945	0	1	CRA004775;Yang2025_Silkie_Wenchang	PRJNA827465	0.899111936
ENSGALG00010015698	TNFAIP2	2	-0.001263504	0.088811821	-0.014226758	0.988649072	0	0.720829541	0	0.5	CRA004775;PRJNA827465	Yang2025_Silkie_Wenchang	0.996891127
ENSGALG00010015701	UNC50	2	-0.001067529	0.07004651	-0.015240284	0.987840483	0	0.327378682	0	0.5	PRJNA827465;Yang2025_Silkie_Wenchang	CRA004775	0.996800355
ENSGALG00010015701	UNC50	2	-0.074911944	0.140162522	-0.534464871	0.593019948	0	0.076477248	0	1	CRA004775;Yang2025_Silkie_Wenchang	PRJNA827465	0.892776493
ENSGALG00010015701	UNC50	2	0.003855331	0.068127036	0.056590322	0.954871544	0	0.084502311	0	0.5	CRA004775;PRJNA827465	Yang2025_Silkie_Wenchang	0.988029599
ENSGALG00010015703	GK5	2	-0.055721638	0.327346877	-0.170221994	0.864835556	0.195048506	11.08637835	90.97992177	0.5	PRJNA827465;Yang2025_Silkie_Wenchang	CRA004775	0.966769016
ENSGALG00010015703	GK5	2	-0.349846529	0.143732039	-2.434019115	0.014932207	0	0.580333765	0	1	CRA004775;Yang2025_Silkie_Wenchang	PRJNA827465	0.201815857
ENSGALG00010015703	GK5	2	0.241207478	0.118920323	2.028311656	0.04252845	0	0.493984433	0	0.5	CRA004775;PRJNA827465	Yang2025_Silkie_Wenchang	0.356625761
ENSGALG00010015706	ENSGALG00010015706	2	0.121527379	0.359720395	0.337838444	0.735484937	0.21848364	6.319912684	84.17699658	0.5	PRJNA827465;Yang2025_Silkie_Wenchang	CRA004775	0.933592889
ENSGALG00010015706	ENSGALG00010015706	2	-0.141436907	0.197526055	-0.716041775	0.473965554	0	0.848569794	0	0.5	CRA004775;Yang2025_Silkie_Wenchang	PRJNA827465	0.85444425
ENSGALG00010015706	ENSGALG00010015706	2	0.408494477	0.150051972	2.722353275	0.006481881	0	0.636756781	0	1	CRA004775;PRJNA827465	Yang2025_Silkie_Wenchang	0.122833832
ENSGALG00010015709	HNRNPA0	2	0.024292248	0.041367021	0.587237057	0.557044508	0	0.340362465	0	1	PRJNA827465;Yang2025_Silkie_Wenchang	CRA004775	0.880601733
ENSGALG00010015709	HNRNPA0	2	-0.028882086	0.148760254	-0.194151901	0.846056944	0.024006447	1.864522821	46.3669745	0.5	CRA004775;Yang2025_Silkie_Wenchang	PRJNA827465	0.96122283
ENSGALG00010015709	HNRNPA0	2	-0.033447921	0.099173691	-0.337266071	0.735916334	0.009704073	1.390754545	28.09658586	0.5	CRA004775;PRJNA827465	Yang2025_Silkie_Wenchang	0.933592889
ENSGALG00010015711	LBFABP	2	0.416913326	0.199177573	2.09317404	0.036333621	0	0.297626761	0	1	PRJNA827465;Yang2025_Silkie_Wenchang	CRA004775	0.3286583
ENSGALG00010015711	LBFABP	2	0.616483288	0.388113995	1.588407777	0.11219416	0.163643567	2.112696458	52.66712376	1	CRA004775;Yang2025_Silkie_Wenchang	PRJNA827465	0.5658803
ENSGALG00010015711	LBFABP	2	0.678978745	0.259545121	2.616033554	0.008895782	0.02642413	1.195374794	16.34422905	1	CRA004775;PRJNA827465	Yang2025_Silkie_Wenchang	0.147648184
ENSGALG00010015716	MGAT4A	2	-0.089256791	0.163314081	-0.546534569	0.584698532	0.036041915	2.938469938	65.96868367	0.5	PRJNA827465;Yang2025_Silkie_Wenchang	CRA004775	0.891016154
ENSGALG00010015716	MGAT4A	2	-0.14775168	0.226206102	-0.653172831	0.513644864	0.041651853	1.447088286	30.89571592	0.5	CRA004775;Yang2025_Silkie_Wenchang	PRJNA827465	0.866528862
ENSGALG00010015716	MGAT4A	2	0.061951909	0.097871729	0.632990856	0.526739615	0	0.210042943	0	1	CRA004775;PRJNA827465	Yang2025_Silkie_Wenchang	0.870692142
ENSGALG00010015719	XRN1	2	-0.075658245	0.05214324	-1.45096939	0.146788384	0	0.122331693	0	1	PRJNA827465;Yang2025_Silkie_Wenchang	CRA004775	0.635558525
ENSGALG00010015719	XRN1	2	-0.08608575	0.115279719	-0.746755373	0.455211238	0	0.463410621	0	0.5	CRA004775;Yang2025_Silkie_Wenchang	PRJNA827465	0.849072665
ENSGALG00010015719	XRN1	2	-0.06152741	0.056801099	-1.083208087	0.278716071	0	0.331639287	0	0.5	CRA004775;PRJNA827465	Yang2025_Silkie_Wenchang	0.776992195
ENSGALG00010015729	RHCE	2	0.15975518	0.102988521	1.551194046	0.120855189	0	0.986838349	0	1	PRJNA827465;Yang2025_Silkie_Wenchang	CRA004775	0.582767179
ENSGALG00010015729	RHCE	2	0.083982453	0.356792535	0.235381756	0.813912445	0.139623086	1.608534654	37.83161605	0.5	CRA004775;Yang2025_Silkie_Wenchang	PRJNA827465	0.953869515
ENSGALG00010015729	RHCE	2	0.040967008	0.135144966	0.303133806	0.761787892	0	0.939763894	0	0.5	CRA004775;PRJNA827465	Yang2025_Silkie_Wenchang	0.939811773
ENSGALG00010015731	PHF6	2	0.087968844	0.041319687	2.128981374	0.033255801	0	0.051254575	0	1	PRJNA827465;Yang2025_Silkie_Wenchang	CRA004775	0.313333153
ENSGALG00010015731	PHF6	2	0.033512607	0.115528096	0.290081876	0.771753601	0	0.105475	0	0.5	CRA004775;Yang2025_Silkie_Wenchang	PRJNA827465	0.943019797
ENSGALG00010015731	PHF6	2	0.086254671	0.042414656	2.033605353	0.041991402	0	0.283943478	0	0.5	CRA004775;PRJNA827465	Yang2025_Silkie_Wenchang	0.354529664
ENSGALG00010015733	COL4A6	2	-0.170788753	0.082104702	-2.080133651	0.037513276	0.00114963	1.086483684	7.959961627	1	PRJNA827465;Yang2025_Silkie_Wenchang	CRA004775	0.334024531
ENSGALG00010015733	COL4A6	2	-0.182703886	0.228020731	-0.801259977	0.422981156	0.043996641	1.27099411	21.32142926	0.5	CRA004775;Yang2025_Silkie_Wenchang	PRJNA827465	0.840012882
ENSGALG00010015733	COL4A6	2	-0.093472566	0.096334239	-0.97029433	0.331899803	0	0.703586946	0	0.5	CRA004775;PRJNA827465	Yang2025_Silkie_Wenchang	0.802002658
ENSGALG00010015734	TSHZ2	2	0.16025809	0.172795594	0.927443153	0.353696494	0.038507775	2.519082791	60.30301173	1	PRJNA827465;Yang2025_Silkie_Wenchang	CRA004775	0.811318534
ENSGALG00010015734	TSHZ2	2	0.346542387	0.17784639	1.948548898	0.05134932	0	0.143933255	0	1	CRA004775;Yang2025_Silkie_Wenchang	PRJNA827465	0.392742061
ENSGALG00010015734	TSHZ2	2	0.03992701	0.092211168	0.43299538	0.665018126	0	0.281949484	0	1	CRA004775;PRJNA827465	Yang2025_Silkie_Wenchang	0.913399315
ENSGALG00010015742	HPRT1	2	-0.007405342	0.076746689	-0.096490698	0.923130861	0	0.103368822	0	0.5	PRJNA827465;Yang2025_Silkie_Wenchang	CRA004775	0.981836718
ENSGALG00010015742	HPRT1	2	-0.035751939	0.11671769	-0.306311232	0.759367688	0	0.010312804	0	1	CRA004775;Yang2025_Silkie_Wenchang	PRJNA827465	0.939153842
ENSGALG00010015742	HPRT1	2	0.007120325	0.087990863	0.08092119	0.935504628	0	0.008098476	0	0.5	CRA004775;PRJNA827465	Yang2025_Silkie_Wenchang	0.984736493
ENSGALG00010015746	PAPOLB	2	-0.06385226	0.036324309	-1.757838282	0.07877503	0	0.147626262	0	1	PRJNA827465;Yang2025_Silkie_Wenchang	CRA004775	0.482840119
ENSGALG00010015746	PAPOLB	2	0.019997725	0.153437952	0.130331025	0.896304535	0.025328561	2.087424758	52.09408166	0.5	CRA004775;Yang2025_Silkie_Wenchang	PRJNA827465	0.975115773
ENSGALG00010015746	PAPOLB	2	0.014146733	0.118095208	0.119790917	0.904648777	0.017914743	2.07339536	51.76993162	0.5	CRA004775;PRJNA827465	Yang2025_Silkie_Wenchang	0.977683364
ENSGALG00010015749	ATR	2	-0.01643258	0.064082622	-0.256428023	0.797620352	0	0.197362673	0	0.5	PRJNA827465;Yang2025_Silkie_Wenchang	CRA004775	0.949842152
ENSGALG00010015749	ATR	2	0.030827953	0.099207826	0.310741144	0.755997416	0	0.140205491	0	1	CRA004775;Yang2025_Silkie_Wenchang	PRJNA827465	0.938501783
ENSGALG00010015749	ATR	2	-0.028650218	0.079399625	-0.360835686	0.718222284	0	0.29725222	0	0.5	CRA004775;PRJNA827465	Yang2025_Silkie_Wenchang	0.927975712
ENSGALG00010015751	MYOM3	2	0.453503081	0.284279556	1.595271527	0.11065153	0.130584435	5.127001966	80.4954239	1	PRJNA827465;Yang2025_Silkie_Wenchang	CRA004775	0.562832796
ENSGALG00010015751	MYOM3	2	0.607154575	0.360982504	1.681950147	0.092578507	0.11086012	1.266559325	21.04594073	0.5	CRA004775;Yang2025_Silkie_Wenchang	PRJNA827465	0.520992708
ENSGALG00010015751	MYOM3	2	0.173699024	0.145675966	1.192365692	0.233117887	0	0.256938511	0	0.5	CRA004775;PRJNA827465	Yang2025_Silkie_Wenchang	0.746170885
ENSGALG00010015753	RPRD1B	2	-0.185068444	0.162691673	-1.137540972	0.255312207	0.050742433	23.921152	95.81959932	1	PRJNA827465;Yang2025_Silkie_Wenchang	CRA004775	0.764467569
ENSGALG00010015753	RPRD1B	2	-0.220480382	0.170166006	-1.295678191	0.195086378	0.042741193	3.206167883	68.81011736	0.5	CRA004775;Yang2025_Silkie_Wenchang	PRJNA827465	0.706113528
ENSGALG00010015753	RPRD1B	2	-0.024544709	0.034197455	-0.71773497	0.472920717	0	0.019872627	0	0.5	CRA004775;PRJNA827465	Yang2025_Silkie_Wenchang	0.854269829
ENSGALG00010015756	PCM1	2	0.120941401	0.205239831	0.589268665	0.555681061	0.07546896	9.017572187	88.91054067	0.5	PRJNA827465;Yang2025_Silkie_Wenchang	CRA004775	0.880324407
ENSGALG00010015756	PCM1	2	0.319164104	0.114263627	2.793225739	0.005218526	0	0.17729757	0	1	CRA004775;Yang2025_Silkie_Wenchang	PRJNA827465	0.107949331
ENSGALG00010015756	PCM1	2	-0.015626726	0.112324964	-0.139120689	0.88935478	0.013211346	1.431684314	30.15219972	0.5	CRA004775;PRJNA827465	Yang2025_Silkie_Wenchang	0.973135755
ENSGALG00010015758	PCDH19	2	-0.106436086	0.067752734	-1.570948947	0.116194508	0	0.96792	0	1	PRJNA827465;Yang2025_Silkie_Wenchang	CRA004775	0.573800585
ENSGALG00010015758	PCDH19	2	-0.179519581	0.106670679	-1.682932767	0.092388108	0	0.201582	0	0.5	CRA004775;Yang2025_Silkie_Wenchang	PRJNA827465	0.520517459
ENSGALG00010015758	PCDH19	2	-0.052134164	0.084201812	-0.619157266	0.535812761	0	0.018886624	0	0.5	CRA004775;PRJNA827465	Yang2025_Silkie_Wenchang	0.874321378
ENSGALG00010015759	EIF5	2	-0.145908767	0.214866304	-0.679067702	0.497094966	0.083384128	9.884255626	89.88290026	0.5	PRJNA827465;Yang2025_Silkie_Wenchang	CRA004775	0.86098656
ENSGALG00010015759	EIF5	2	0.06932088	0.482879561	0.143557288	0.88585007	0.415767725	8.75135946	88.57320392	0.5	CRA004775;Yang2025_Silkie_Wenchang	PRJNA827465	0.97244495
ENSGALG00010015759	EIF5	2	0.241584172	0.256219209	0.942880795	0.345741879	0.097530974	3.062495298	67.34688864	1	CRA004775;PRJNA827465	Yang2025_Silkie_Wenchang	0.807408977
ENSGALG00010015764	TTI1	2	0.008596942	0.054804312	0.156866159	0.875350331	0	0.033677249	0	1	PRJNA827465;Yang2025_Silkie_Wenchang	CRA004775	0.970347777
ENSGALG00010015764	TTI1	2	-0.063017824	0.126791263	-0.497020244	0.619174775	0.014596071	1.640720021	39.05114905	0.5	CRA004775;Yang2025_Silkie_Wenchang	PRJNA827465	0.899168321
ENSGALG00010015764	TTI1	2	-0.060903404	0.108747474	-0.56004431	0.575449214	0.011144778	1.539525202	35.04490872	0.5	CRA004775;PRJNA827465	Yang2025_Silkie_Wenchang	0.886623563
ENSGALG00010015766	TLE1	2	0.086423474	0.135773561	0.636526531	0.524433294	0.025000337	3.035539657	67.05692848	0.5	PRJNA827465;Yang2025_Silkie_Wenchang	CRA004775	0.869790047

ENSGALG00010015766	TLE1	2	0.117368357	0.178052984	0.659176578	0.509782386	0.029886636	1.680303152	40.48692947	0.5	CRA004775;Yang2025_Silkie_Wenchang	PRJNA827465	0.865257661
ENSGALG00010015766	TLE1	2	-0.047343473	0.08650786	-0.547273657	0.584190739	0	0.154460183	0	1	CRA004775;PRJNA827465	Yang2025_Silkie_Wenchang	0.890837612
ENSGALG00010015769	TMEM50A	2	-0.175439038	0.256109341	-0.685016165	0.493333721	0.112890979	6.407630168	84.39360616	0.5	PRJNA827465;Yang2025_Silkie_Wenchang	CRA004775	0.859868321
ENSGALG00010015769	TMEM50A	2	-0.219576094	0.249683047	-0.879419314	0.379173964	0.08558687	3.189064548	68.64284228	0.5	CRA004775;Yang2025_Silkie_Wenchang	PRJNA827465	0.824080641
ENSGALG00010015769	TMEM50A	2	0.046244144	0.046472386	0.995088642	0.319693154	0	0.007784665	0	1	CRA004775;PRJNA827465	Yang2025_Silkie_Wenchang	0.798328994
ENSGALG00010015774	PLS1	2	0.598224676	0.336228058	1.779222947	0.075203218	0.144090266	2.741600875	63.52496057	1	PRJNA827465;Yang2025_Silkie_Wenchang	CRA004775	0.473260364
ENSGALG00010015774	PLS1	2	0.967555706	0.7544335	1.282493031	0.199669748	0.971299915	6.486490088	84.58334189	1	CRA004775;Yang2025_Silkie_Wenchang	PRJNA827465	0.71155526
ENSGALG00010015774	PLS1	2	1.263012427	0.405797584	3.112419784	0.001855604	0.164565264	1.86673656	46.43057723	1	CRA004775;PRJNA827465	Yang2025_Silkie_Wenchang	0.055292508
ENSGALG00010015776	SPOCK1	2	0.085221429	0.188405878	0.452328931	0.651032037	0	0.123081561	0	1	PRJNA827465;Yang2025_Silkie_Wenchang	CRA004775	0.908769703
ENSGALG00010015776	SPOCK1	2	0.663218404	0.480065342	1.381516945	0.167120072	0.270288244	2.405468178	58.42805118	1	CRA004775;Yang2025_Silkie_Wenchang	PRJNA827465	0.666416556
ENSGALG00010015776	SPOCK1	2	0.533924721	0.555841478	0.96057013	0.336768353	0.497348937	4.747762238	78.93744569	1	CRA004775;PRJNA827465	Yang2025_Silkie_Wenchang	0.803887433
ENSGALG00010015778	PABIR3	2	-0.249789917	0.212117154	-1.17760357	0.238954686	0.082242476	11.44410066	91.26187344	1	PRJNA827465;Yang2025_Silkie_Wenchang	CRA004775	0.751090532
ENSGALG00010015778	PABIR3	2	-0.397769957	0.117660701	-3.380652623	0.000723139	0.007784694	1.288454698	22.38764763	1	CRA004775;Yang2025_Silkie_Wenchang	PRJNA827465	0.030107556
ENSGALG00010015778	PABIR3	2	-0.060775657	0.063523195	-0.956747486	0.338694724	0	0.548700501	0	1	CRA004775;PRJNA827465	Yang2025_Silkie_Wenchang	0.804450436
ENSGALG00010015782	ATG4A	2	-0.051931878	0.052669663	-0.985992228	0.324136927	0	0.129455529	0	1	PRJNA827465;Yang2025_Silkie_Wenchang	CRA004775	0.799620489
ENSGALG00010015782	ATG4A	2	0.047830892	0.174114244	0.27470982	0.783539186	0.035064746	2.219455266	54.94389929	0.5	CRA004775;Yang2025_Silkie_Wenchang	PRJNA827465	0.945815798
ENSGALG00010015782	ATG4A	2	0.047801746	0.140658095	0.339843548	0.733974351	0.024546417	2.056935525	51.38398905	0.5	CRA004775;PRJNA827465	Yang2025_Silkie_Wenchang	0.933391141
ENSGALG00010015785	FGL1	2	-0.016499998	0.194005996	-0.085048908	0.932222508	0.052541706	3.310403001	69.79219752	0.5	PRJNA827465;Yang2025_Silkie_Wenchang	CRA004775	0.984202262
ENSGALG00010015785	FGL1	2	0.421337095	0.311149219	1.354131938	0.175694288	0.13195727	2.779648787	64.02423196	1	CRA004775;Yang2025_Silkie_Wenchang	PRJNA827465	0.6803934
ENSGALG00010015785	FGL1	2	0.256402517	0.512608693	0.500191512	0.616940235	0.457063472	7.222519327	86.15441573	0.5	CRA004775;PRJNA827465	Yang2025_Silkie_Wenchang	0.899111936
ENSGALG00010015788	TRPC1	2	0.017136927	0.065811589	0.260393754	0.79456006	0	0.099880219	0	1	PRJNA827465;Yang2025_Silkie_Wenchang	CRA004775	0.948905846
ENSGALG00010015788	TRPC1	2	0.056244637	0.103558522	0.543119348	0.587047632	0	0.015318679	0	1	CRA004775;Yang2025_Silkie_Wenchang	PRJNA827465	0.891596903
ENSGALG00010015788	TRPC1	2	0.013017017	0.073553661	0.176973064	0.859529547	0	0.115169627	0	1	CRA004775;PRJNA827465	Yang2025_Silkie_Wenchang	0.964789085
ENSGALG00010015792	MARK3	2	-0.13987015	0.041168219	-3.397527332	0.000679978	0	0.09242553	0	1	PRJNA827465;Yang2025_Silkie_Wenchang	CRA004775	0.028867656
ENSGALG00010015792	MARK3	2	0.06504266	0.228309698	0.284887853	0.775730063	0.096692778	13.78044657	92.74334112	0.5	CRA004775;Yang2025_Silkie_Wenchang	PRJNA827465	0.943815265
ENSGALG00010015792	MARK3	2	0.074149699	0.213826682	0.346774771	0.728760532	0.086177619	17.04525878	94.13326595	0.5	CRA004775;PRJNA827465	Yang2025_Silkie_Wenchang	0.932236477
ENSGALG00010015793	RSRP1	2	0.379217614	0.199628294	1.899618571	0.057483193	0.055905972	3.133180599	68.08355062	1	PRJNA827465;Yang2025_Silkie_Wenchang	CRA004775	0.415229176
ENSGALG00010015793	RSRP1	2	0.516763326	0.172976396	2.987478854	0.002812888	0	0.961648063	0	1	CRA004775;Yang2025_Silkie_Wenchang	PRJNA827465	0.07222107
ENSGALG00010015793	RSRP1	2	0.212930733	0.104839303	2.031020111	0.042252955	0	0.002285604	0	1	CRA004775;PRJNA827465	Yang2025_Silkie_Wenchang	0.355831985
ENSGALG00010015795	PSMD10	2	0.079468671	0.078670521	1.010145472	0.312425599	0.001399319	1.067790516	6.348671841	0.5	PRJNA827465;Yang2025_Silkie_Wenchang	CRA004775	0.796054997
ENSGALG00010015795	PSMD10	2	-0.317724282	0.211591747	-1.501591094	0.133202745	0.059132784	2.929657571	65.86631796	1	CRA004775;Yang2025_Silkie_Wenchang	PRJNA827465	0.611633901
ENSGALG00010015795	PSMD10	2	-0.186125269	0.316454511	-0.588158054	0.556426209	0.184734986	12.50981705	92.00627798	0.5	CRA004775;PRJNA827465	Yang2025_Silkie_Wenchang	0.880324407
ENSGALG00010015796	LARP1	2	-0.006238866	0.06844017	-0.09115796	0.927367079	0	0.552150441	0	0.5	PRJNA827465;Yang2025_Silkie_Wenchang	CRA004775	0.982993195
ENSGALG00010015796	LARP1	2	0.111846853	0.158682328	0.704847567	0.480905103	0	0.010570378	0	1	CRA004775;Yang2025_Silkie_Wenchang	PRJNA827465	0.856722567
ENSGALG00010015796	LARP1	2	-0.020424792	0.07162549	-0.285160941	0.775520843	0	0.123704456	0	0.5	CRA004775;PRJNA827465	Yang2025_Silkie_Wenchang	0.943815265
ENSGALG00010015797	PIP5K1B	2	-0.02376386	0.081697456	-0.29087638	0.771145867	0	0.341809595	0	0.5	PRJNA827465;Yang2025_Silkie_Wenchang	CRA004775	0.942716827
ENSGALG00010015797	PIP5K1B	2	0.054396925	0.111132207	0.489479386	0.624502345	0	0.197785733	0	1	CRA004775;Yang2025_Silkie_Wenchang	PRJNA827465	0.900892482
ENSGALG00010015797	PIP5K1B	2	-0.031497262	0.100134501	-0.314549548	0.753103691	0	0.643666886	0	0.5	CRA004775;PRJNA827465	Yang2025_Silkie_Wenchang	0.938087082
ENSGALG00010015798	IL22RA1	2	0.127435567	0.226808158	0.561865007	0.574207997	0.065003254	2.712938502	63.13959939	0.5	PRJNA827465;Yang2025_Silkie_Wenchang	CRA004775	0.886081433
ENSGALG00010015798	IL22RA1	2	0.450435227	0.220761453	2.04037082	0.041313406	0.023819731	1.164958916	14.16006298	1	CRA004775;Yang2025_Silkie_Wenchang	PRJNA827465	0.352374305
ENSGALG00010015798	IL22RA1	2	0.308660513	0.506538149	0.609352945	0.54229052	0.387328067	3.634182328	72.48349395	0.5	CRA004775;PRJNA827465	Yang2025_Silkie_Wenchang	0.876721948
ENSGALG00010015800	BVES	2	-0.36724456	0.242657554	-1.513427272	0.130171162	0.092456205	4.611824267	78.31660657	1	PRJNA827465;Yang2025_Silkie_Wenchang	CRA004775	0.605390875
ENSGALG00010015800	BVES	2	-0.468432748	0.297382743	-1.575184707	0.115213816	0.080647825	1.436035189	30.3638234	0.5	CRA004775;Yang2025_Silkie_Wenchang	PRJNA827465	0.572203827
ENSGALG00010015800	BVES	2	-0.12291293	0.136628143	-0.899616485	0.368324381	0	0.164741392	0	0.5	CRA004775;PRJNA827465	Yang2025_Silkie_Wenchang	0.820213684
ENSGALG00010015801	VSTM2L	2	-0.082780206	0.202028757	-0.409744668	0.68199326	0.035285108	1.747032053	42.76006566	0.5	PRJNA827465;Yang2025_Silkie_Wenchang	CRA004775	0.918515969
ENSGALG00010015801	VSTM2L	2	0.190563124	0.1884302	1.011319438	0.311863566	0	0.119095874	0	1	CRA004775;Yang2025_Silkie_Wenchang	PRJNA827465	0.795993742
ENSGALG00010015801	VSTM2L	2	-0.048584656	0.265180617	-0.183213451	0.854630541	0.078410705	2.150339126	53.49570736	0.5	CRA004775;PRJNA827465	Yang2025_Silkie_Wenchang	0.963225297
ENSGALG00010015803	SYF2	2	0.160571825	0.125832707	1.276073839	0.201929418	0.022713626	3.344210257	70.09757392	1	PRJNA827465;Yang2025_Silkie_Wenchang	CRA004775	0.714969076
ENSGALG00010015803	SYF2	2	0.125587427	0.221178895	0.567809272	0.570164483	0.067271178	2.952337751	66.12853663	0.5	CRA004775;Yang2025_Silkie_Wenchang	PRJNA827465	0.885038495
ENSGALG00010015803	SYF2	2	0.036485712	0.066465995	0.548938021	0.583047985	0	0.652701086	0	0.5	CRA004775;PRJNA827465	Yang2025_Silkie_Wenchang	0.890269423
ENSGALG00010015804	CKB	2	-0.047108407	0.211760827	-0.222460441	0.823955465	0.055873128	2.525944659	60.41085079	0.5	PRJNA827465;Yang2025_Silkie_Wenchang	CRA004775	0.956170356
ENSGALG00010015804	CKB	2	-0.259707514	0.202935796	-1.279752116	0.200632329	0	0.137913401	0	1	CRA004775;Yang2025_Silkie_Wenchang	PRJNA827465	0.712970384
ENSGALG00010015804	CKB	2	0.106145607	0.136005933	0.780448351	0.435127017	0	0.294173924	0	0.5	CRA004775;PRJNA827465	Yang2025_Silkie_Wenchang	0.842762754
ENSGALG00010015807	ENSGALG00010015807	2	0.102861479	0.511774396	0.20098989	0.84070648	0	0.039439517	0	0.5	PRJNA827465;Yang2025_Silkie_Wenchang	CRA004775	0.959778278
ENSGALG00010015807	ENSGALG00010015807	2	-0.757746558	1.102026248	-0.687593929	0.491708532	1.692547135	3.101154629	67.75394588	0.5	CRA004775;Yang2025_Silkie_Wenchang	PRJNA827465	0.85895707
ENSGALG00010015807	ENSGALG00010015807	2	-0.94708931	1.003925526	-0.943386024	0.345483489	1.028164996	2.018442927	50.45686024	1	CRA004775;PRJNA827465	Yang2025_Silkie_Wenchang	0.807289734
ENSGALG00010015809	PCOLCE2	2	0.344854187	0.291635622	1.182483074	0.237014078	0.113046743	2.652128365	62.29443441	0.5	PRJNA827465;Yang2025_Silkie_Wenchang	CRA004775	0.749355649
ENSGALG00010015809	PCOLCE2	2	0.027451869	0.235388134	0.116623843	0.907158143	0	0.060005404	0	0.5	CRA004775;Yang2025_Silkie_Wenchang	PRJNA827465	0.978196186
ENSGALG00010015809	PCOLCE2	2	0.414329507	0.227400565	1.82202497	0.068451197	0.053154256	1.811863802	44.80821358	1	CRA004775;PRJNA827465	Yang2025_Silkie_Wenchang	0.450797217
ENSGALG00010015810	LIPT1	2	-0.007012798	0.138533755	-0.050621582	0.959627065	0.027260853	3.134323618	68.09518984	0.5	PRJNA827465;Yang2025_Silkie_Wenchang	CRA004775	0.989259322
ENSGALG00010015810	LIPT1	2	-0.177087934	0.12448541	-1.422559747	0.15486382	0	3.1417E-06	0	1	CRA004775;Yang2025_Silkie_Wenchang	PRJNA827465	0.647505492
ENSGALG00010015810	LIPT1	2	0.062388049	0.101814548	0.612761639	0.540033959	0.007601275	1.235408642	19.05512349	0.5	CRA004775;PRJNA827465	Yang2025_Silkie_Wenchang	0.87529516
ENSGALG00010015811	CTNNBL1	2	-0.033447584	0.050924274	-0.656810223	0.51130295	0	0.236386041	0	1	PRJNA827465;Yang2025_Silkie_Wenchang	CRA004775	0.865658376
ENSGALG00010015811	CTNNBL1	2	-0.140347829	0.104049624	-1.34885474	0.177383628	0	0.271992296	0	1	CRA004775;Yang2025_Silkie_Wenchang	PRJNA827465	0.683500856
ENSGALG00010015811	CTNNBL1	2	-0.050440826	0.064573632	-0.78113657	0.434722175	0.001881955	1.129077878	11.43215014	1	CRA004775;PRJNA827465	Yang2025_Silkie_Wenchang	0.842762754
ENSGALG00010015813	ENSGALG00010015813	2	-0.030934063	0.06249356	-0.494996013	0.620602935	0	0.258629833	0	0.5	PRJNA827465;Yang2025_Silkie_Wenchang	CRA004775	0.899395593
ENSGALG00010015813	ENSGALG00010015813	2	0.084920327	0.153133067	0.554552513	0.579200778	0	0.135509867	0	1	CRA004775;Yang2025_Silkie_Wenchang	PRJNA827465	0.887888423
ENSGALG00010015813	ENSGALG00010015813	2	-0.032884538	0.065334309	-0.503327243	0.614734216	0	0.513447395	0	0.5	CRA004775;PRJNA827465	Yang2025_Silkie_Wenchang	0.898709228
ENSGALG00010015814	PAQR9	2	0.399054485	0.29841416	1.337250501	0.181140882	0	0.21062586	0	1	PRJNA827465;Yang2025_Silkie_Wenchang	CRA004775	0.688594651
ENSGALG00010015814	PAQR9	2	0.384410436	0.299548349	1.283300129	0.199386947	0	0.165061035	0	1	CRA004775;Yang2025_Silkie_Wenchang	PRJNA827465	0.711181159
ENSGALG00010015814	PAQR9	2	0.536285606	0.316949933	1.692019939	0.090642172	0	0.002307563	0	1	CRA004775;PRJNA827465	Yang2025_Silkie_Wenchang	0.516680099
ENSGALG00010015816	MITD1	2	-0.069598258	0.075205534	-0.925440647	0.354736744	0	0.163122791	0	1	PRJNA827465;Yang2025_Silkie_Wenchang	CRA004775	0.812701118
ENSGALG00010015816	MITD1	2	-0.166997765	0.144380935	-1.156646928	0.247416641	0.002787479	1.024860696	2.42576341	1	CRA004775;Yang2025_Silkie_Wenchang	PRJNA827465	0.757947282
ENSGALG00010015816	MITD1	2	-0.145309193	0.206866885	-0.702428488	0.482411985	0.043903048	1.414812698	29.31926597	1	CRA004775;PRJNA827465	Yang2025_Silkie_Wenchang	0.85691916
ENSGALG00010015820	ENSGALG00010015820	2	0.075887462	0.166103134	0.456869537	0.647764821	0	0.029427467	0	1	PRJNA827465;Yang2025_Silkie_Wenchang	CRA004775	0.907471066
ENSGALG00010015820	ENSGALG00010015820	2	-0.218901214	0.314836842	-0.695284695	0.486850904	0.067761334	1.480542771	32.45720289	0.5	CRA004775;Yang2025_Silkie_Wenchang	PRJNA827465	0.857631005
ENSGALG00010015820	ENSGALG00010015820	2	-0.15513956	0.33847103	-0.458354027	0.646698115	0.137720269	2.213822229	54.82925471	0.5	CRA004775;PRJNA827465	Yang2025_Silkie_Wenchang	0.907350807
ENSGALG00010015825	RUNX3	2	-0.086556684	0.551969702	-0.156814193	0.875391287	0	0.224929079	0	0.5	PRJNA827465;Yang2025_Silkie_Wenchang	CRA004775	0.970347777
ENSGALG00010015825	RUNX3	2	-0.302418615	0.420515738	-0.719161229	0.472041585	0	0.026832146	0	1	CRA004775;Yang2025_Silkie_Wenchang	PRJNA827465	0.853998373
ENSGALG00010015825	RUNX3	2	-0.146797446	0.389701421	-0.37669321	0.706402435	0	0.202731724	0	0.5	CRA004775;PRJNA827465	Yang2025_Silkie_Wenchang	0.924548793
ENSGALG00010015827	ENSGALG00010015827	2	0.1303259	0.099955997	1.303832726	0.192290622	0.001609249	1.086736176	7.981346175	1	PRJNA827465;Yang2025_Silkie_Wenchang	CRA004775	0.702082087
ENSGALG00010015827	ENSGALG00010015827	2	0.20145599	0.135149227	1.490618882	0.136061584	0	0.684943253	0	0.5	CRA004775;Yang2025_Silkie_Wenchang	PRJNA827465	0.617651702
ENSGALG00010015827	ENSGALG00010015827	2	0.025106894	0.123145268	0.203880558	0.838447044	0	0.138066783	0	0.5	CRA004775;PRJNA827465	Yang2025_Silkie_Wenchang	0.959597438
ENSGALG00010015829	SMAD5	2	-0.170341161	0.182069349	-0.935583952	0.34948744	0.058646247	8.078883102	87.62205137	1	PRJNA827465;Yang2025_Silkie_Wenchang	CRA004775	0.809903518
ENSGALG00010015829	SMAD5	2	-0.009678652	0.364365674	-0.026563018	0.97880827	0.248567516	15.64303095	93.60737697	0.5	CRA004775;Yang2025_Silkie_Wenchang	PRJNA827465	0.994239305
ENSGALG00010015829	SMAD5	2	0.152306177	0.179854936	0.846827895	0.397091058	0.055373558	6.289248377	84.09984882	0.5	CRA004775;PRJNA827465	Yang2025_Silkie_Wenchang	0.831438925
ENSGALG00010015831	LYG2	2	0.196244696	0.275431651	0.712498711	0.476156009	0	0.23181158	0	1	PRJNA827465;Yang2025_Silkie_Wenchang	CRA004775	0.854810196
ENSGALG00010015831	LYG2	2	0.134607416	0.294524011	0.45703376	0.64764678	0	0.054399447	0	1	CRA004775;Yang2025_Silkie_Wenchang	PRJNA827465	0.907376718
ENSGALG00010015831	LYG2	2	0.309379592	0.336193912	0.920241507	0.357446568	0	0.032094075	0	1	CRA004775;PRJNA827465	Yang2025_Silkie_Wenchang	0.813381338
ENSGALG00010015832	RAB39B	2	0.079215753	0.07812162	1.01400552	0.310580018	0.008493236	3.083165332	67.56580032	1	PRJNA827465;Yang2025_Silkie_Wenchang	CRA004775	0.794899969
ENSGALG00010015832	RAB39B	2	0.11490934	0.118717544	0.967922148	0.333083252	0.011135113	1.317524241	24.1000682	0.5	CRA004775;Yang2025_Silkie_Wenchang	PRJNA827465	0.802178578
ENSGALG00010015832	RAB39B	2	0.009156488	0.043359387	0.211176597	0.832749472	0	0.32113884	0	0.5	CRA004775;PRJNA827465	Yang2025_Silkie_Wenchang	0.957861248
ENSGALG00010015834	STARD8	2	-0.09232123	0.066215195	-1.39426048	0.16323896	0	0.396463283	0	1	PRJNA827465;Yang2025_Silkie_Wenchang	CRA004775	0.661668506
ENSGALG00010015834	STARD8	2	-0.067188744	0.099308948	-0.676562846	0.498683359	0	0.468643403	0	1	CRA004775;Yang2025_Silkie_Wenchang	PRJNA827465	0.861478809
ENSGALG00010015834	STARD8	2	-0.135014052	0.079947028	-1.688793892	0.091266299	0	0.151808534	0	1	CRA004775;PRJNA827465	Yang2025_Silkie_Wenchang	0.51792097
ENSGALG00010015837	GDA	2	-0.16778958	0.12990563	-1.291626756	0.196486434	0	0.00782942	0	1	PRJNA827465;Yang2025_Silkie_Wenchang	CRA004775	0.707837424
ENSGALG00010015837	GDA	2	-0.18063669	0.215770916	-0.837168852	0.402497663	0	0.002383907	0	1	CRA004775;Yang2025_Silkie_Wenchang	PRJNA827465	0.832905052
ENSGALG00010015837	GDA	2	-0.15961595	0.15150168	-1.053558942	0.292084895	0	9.98076E-05	0	1	CRA004775;PRJNA827465	Yang2025_Silkie_Wenchang	0.785516679
ENSGALG00010015838	IFNLR1	2	0.356134424	1.075033828	0.331277412	0.740434952	0	0.002119581	0	1	PRJNA827465;Yang2025_Silkie_Wenchang	CRA004775	0.935128797
ENSGALG00010015838	IFNLR1	2	0.212027798	1.14872137	0.184577221	0.853560654	0	0.006586593	0	1	CRA004775;Yang2025_Silkie_Wenchang	PRJNA827465	0.962964214
ENSGALG00010015838	IFNLR1	2	0.276632209	1.092879542	0.253122324	0.800173689	0	0.016837712	0	1	CRA004775;PRJNA827465	Yang2025_Silkie_Wenchang	0.934999675
ENSGALG00010015839	U2SURP	2	0.369661679	0.351660406	1.051189366	0.293171626	0.227476077	11.75880153	91.49573196	1	PRJNA827465;Yang2025_Silkie_Wenchang	CRA004775	0.786788967
ENSGALG00010015839	U2SURP	2	0.555488083	0.187778004	2.958216992	0.003094242	0.033131232	1.885220359	46.95580305	1	CRA004775;Yang2025_Silkie_Wenchang	PRJNA827465	0.076827108
ENSGALG00010015839	U2SURP	2	0.157803261	0.157161117	1.004085891	0.315337215	0.034760299	2.780417041	64.03417239	1	CRA004775;PRJNA827465	Yang2025_Silkie_Wenchang	0.796766221
ENSGALG00010015840	MTUS1	2	-0.061731817	0.170241926	-0.362612306	0.716894516	0.048239566	5.93154416	83.14098365	0.5	PRJNA827465;Yang2025_Silkie_Wenchang	CRA004775	0.907632716
ENSGALG00010015840	MTUS1	2	0.136981513	0.101097266	1.35494775	0.175434205	0	0.386400382	0	1	CRA004775;Yang2025_Silkie_Wenchang	PRJNA827465	0.680258136
ENSGALG00010015840	MTUS1	2	-0.032294192	0.257317187	-0.125503438	0.900125003	0.096050213	3.041584708	67.12240177	0.5	CRA004775;PRJNA827465	Yang2025_Silkie_Wenchang	0.976466979
ENSGALG00010015843	ENSGALG00010015843	2	0.171405036	0.205550009	0.833884839	0.404345881	0	0.071221689	0	1	PRJNA827465;Yang2025_Silkie_Wenchang	CRA004775	0.833112102
ENSGALG00010015843	ENSGALG00010015843	2	-0.003676209	0.25752759	-0.014275011	0.988610576	0	0.310285197	0	0.5	CRA004775;Yang2025_Silkie_Wenchang	PRJNA827465	0.996891127
ENSGALG00010015843	ENSGALG00010015843	2	0.101664625	0.226014657	0.4498143	0.652844346	0	0.663699461	0	0.5	CRA004775;PRJNA827465	Yang2025_Silkie_Wenchang	0.909572411
ENSGALG00010015845	SAP30L	2	-0.140826892	0.106372713	-1.323900539	0.185536079	0.015461641	3.043634425	67.14454299	1	PRJNA827465;Yang2025_Silkie_Wenchang	CRA004775	0.694091426
ENSGALG00010015845	SAP30L	2	-0.203050357	0.147132653	-1.380049384	0.167571378	0.014559342	1.192155217	16.11830525	0.5	CRA004775;Yang2025_Silkie_Wenchang	PRJNA827465	0.667448579
ENSGALG00010015845	SAP30L	2	-0.041046323	0.066333077	-0.618791188	0.536053928	0	0.304364868	0	0.5	CRA004775;PRJNA827465	Yang2025_Silkie_Wenchang	0.874575656
ENSGALG00010015848	UBE2B	2	-0.261917499	0.210920755	-1.241781535	0.214317177	0.074468021	5.924093902	83.11978141	1	PRJNA827465;Yang2025_Silkie_Wenchang	CRA004775	0.727123478
ENSGALG00010015848	UBE2B	2	-0.165745069	0.410652799	-0.403613635	0.686496858	0.251950921	3.424288888	70.79685644	0.5	CRA004775;Yang2025_Silkie_Wenchang	PRJNA827465	0.919235539
ENSGALG00010015848	UBE2B	2	-0.051061362	0.085948209	-0.594094541	0.552448882	0	0.915562449	0	0.5	CRA004775;PRJNA827465	Yang2025_Silkie_Wenchang	0.879228216
ENSGALG00010015852	ZFAND5	2	-0.259183666	0.241843106	-1.071701691	0.283854041	0.088311659	4.057304132	75.35309241	1	PRJNA827465;Yang2025_Silkie_Wenchang	CRA004775	0.77978499
ENSGALG00010015852	ZFAND5	2	-0.400564884	0.224689911	-1.782745309	0.074627772	0.028877038	1.220686856	18.07890817	0.5	CRA004775;Yang2025_Silkie_Wenchang	PRJNA827465	0.471679921
ENSGALG00010015852	ZFAND5	2	-0.019953768	0.146783356	-0.135940266	0.891868503	0	0.026124399	0	0.5	CRA004775;PRJNA827465	Yang2025_Silkie_Wenchang	0.974015927
ENSGALG00010015855	NIPAL3	2	0.117770939	0.119038029	0.989355587	0.322489196	0.022166083	4.338643941	76.95132365	1	PRJNA827465;Yang2025_Silkie_Wenchang	CRA004775	0.798652022
ENSGALG00010015855	NIPAL3	2	-0.00940255	0.304638289	-0.030864636	0.975377492	0.151377288	4.943190582	79.77015081	0.5	CRA004775;Yang2025_Silkie_Wenchang	PRJNA827465	0.992754467
ENSGALG00010015855	NIPAL3	2	-0.090578815	0.167996207	-0.539153931	0.589780645	0.035974543	2.035870352	50.88095865	0.5	CRA004775;PRJNA827465	Yang2025_Silkie_Wenchang	0.892320726
ENSGALG00010015856	TXNDC9	2	-0.144198212	0.131024819	-1.100541203	0.271096388	0.029492639	6.972585954	85.65793302	1	PRJNA827465;Yang2025_Silkie_Wenchang	CRA004775	0.772741863
ENSGALG00010015856	TXNDC9	2	0.028554441	0.334871687	0.085269799	0.932046901	0.202030684	9.600933867	89.58434655	0.5	CRA004775;Yang2025_Silkie_Wenchang	PRJNA827465	0.98412915
ENSGALG00010015856	TXNDC9	2	0.139343732	0.199503584	0.698452273	0.484894396	0.062040362	3.862430895	74.10956915	0.5	CRA004775;PRJNA827465	Yang2025_Silkie_Wenchang	0.857004733
ENSGALG00010015858	CHST2	2	0.061702792	0.064286607	0.959807891	0.33715191	0	0.082051949	0	1	PRJNA827465;Yang2025_Silkie_Wenchang	CRA004775	0.804086136
ENSGALG00010015858	CHST2	2	-0.247464532	0.299391616	-0.82655799	0.408487637	0.14429052	4.95381582	79.81354099	0.5	CRA004775;Yang2025_Silkie_Wenchang	PRJNA827465	0.834164004
ENSGALG00010015858	CHST2	2	-0.215283807	0.321251876	-0.670140233	0.5027684	0.180014732	7.18079299	86.07396145	0.5	CRA004775;PRJNA827465	Yang2025_Silkie_Wenchang	0.863050365
ENSGALG00010015861	PPP2CA	2	-0.279175028	0.235794173	-1.183979637	0.236421915	0.105939701	20.51677405	95.1259394	1	PRJNA827465;Yang2025_Silkie_Wenchang	CRA004775	0.748697972
ENSGALG00010015861	PPP2CA	2	-0.290087359	0.306206609	-0.780819947	0.434909755	0.165365263	8.169903916	87.74645541	0.5	CRA004775;Yang2025_Silkie_Wenchang	PRJNA827465	0.834706254
ENSGALG00010015861	PPP2CA	2	-0.048344372	0.032332778	-1.495212446	0.134859006	0	0.537860667	0	1	CRA004775;PRJNA827465	Yang2025_Silkie_Wenchang	0.615462555
ENSGALG00010015862	PDGFRL	2	0.054296557	0.077613794	0.699573545	0.484193668	0	0.453950915	0	1	PRJNA827465;Yang2025_Silkie_Wenchang	CRA004775	0.857004733
ENSGALG00010015862	PDGFRL	2	-0.03358274	0.129799599	-0.258727609	0.795845416	0	0.135137025	0	1	CRA004775;Yang2025_Silkie_Wenchang	PRJNA827465	0.949107318
ENSGALG00010015862	PDGFRL	2	0.079873381	0.093055793	0.858338612	0.390705515	0	0.364489677	0	0.5	CRA004775;PRJNA827465	Yang2025_Silkie_Wenchang	0.829251057
ENSGALG00010015863	ENSGALG00010015863	2	0.256803257	0.189798235	1.353032906	0.176045118	0	0.203773902	0	1	PRJNA827465;Yang2025_Silkie_Wenchang	CRA004775	0.681107371
ENSGALG00010015863	ENSGALG00010015863	2	0.187925802	0.291754345	0.644123404	0.519495415	0	0.145833407	0	1	CRA004775;Yang2025_Silkie_Wenchang	PRJNA827465	0.869086345
ENSGALG00010015863	ENSGALG00010015863	2	0.334282555	0.228552499	1.462607308	0.143574862	0	0.032356847	0	1	CRA004775;PRJNA827465	Yang2025_Silkie_Wenchang	0.630564924
ENSGALG00010015865	VRK1	2	-0.013458355	0.065808092	-0.20451533	0.837958299	0	0.435575899	0	0.5	PRJNA827465;Yang2025_Silkie_Wenchang	CRA004775	0.959932758
ENSGALG00010015865	VRK1	2	-0.056094357	0.091166704	-0.615294342	0.538360354	0	0.002868995	0	1	CRA004775;Yang2025_Silkie_Wenchang	PRJNA827465	0.87481821
ENSGALG00010015865	VRK1	2	0.026819391	0.090639744	0.295889966	0.767314114	0	0.021470325	0	0.5	CRA004775;PRJNA827465	Yang2025_Silkie_Wenchang	0.941444269
ENSGALG00010015866	ARHGEF6	2	-0.056036051	0.121548998	-0.461016149	0.644787019	0.024936753	6.294698956	84.11361676	0.5	PRJNA827465;Yang2025_Silkie_Wenchang	CRA004775	0.906953008
ENSGALG00010015866	ARHGEF6	2	-0.179390472	0.076365422	-2.349106012	0.018818548	0	0.049968706	0	1	CRA004775;Yang2025_Silkie_Wenchang	PRJNA827465	0.229831303
ENSGALG00010015866	ARHGEF6	2	0.050456772	0.053563012	0.942007748	0.346188673	0	0.505925666	0	0.5	CRA004775;PRJNA827465	Yang2025_Silkie_Wenchang	0.807744676
ENSGALG00010015867	PICALML	2	-0.084500999	0.030246166	-2.793775526	0.005209663	0.000100432	1.045517345	4.353571498	1	PRJNA827465;Yang2025_Silkie_Wenchang	CRA004775	0.107822736
ENSGALG00010015867	PICALML	2	-0.0851607	0.077680981	-1.096287643	0.272952915	0.00515518	1.529907768	34.63658259	0.5	CRA004775;Yang2025_Silkie_Wenchang	PRJNA827465	0.773799118
ENSGALG00010015867	PICALML	2	-0.059011884	0.032864429	-1.795615671	0.072555662	0	0.630395458	0	0.5	CRA004775;PRJNA827465	Yang2025_Silkie_Wenchang	0.465348429
ENSGALG00010015869	SLC7A2	2	0.026692692	0.096705454	0.276020539	0.782532291	0.002984983	1.188301669	15.84628502	0.5	PRJNA827465;Yang2025_Silkie_Wenchang	CRA004775	0.945432561
ENSGALG00010015869	SLC7A2	2	0.15149029	0.127616865	1.187071085	0.235199583	0	0.333522716	0	1	CRA004775;Yang2025_Silkie_Wenchang	PRJNA827465	0.748263656
ENSGALG00010015869	SLC7A2	2	-0.035368013	0.116139584	-0.304530219	0.760723974	0	0.923196684	0	0.5	CRA004775;PRJNA827465	Yang2025_Silkie_Wenchang	0.939384641
ENSGALG00010015870	EIF5B	2	0.175784595	0.346811605	0.506859033	0.612253732	0.220625394	11.26395164	91.12212098	0.5	PRJNA827465;Yang2025_Silkie_Wenchang	CRA004775	0.897895209
ENSGALG00010015870	EIF5B	2	0.508012735	0.155518409	3.266576212	0.001088565	0	0.113744299	0	1	CRA004775;Yang2025_Silkie_Wenchang	PRJNA827465	0.039877802
ENSGALG00010015870	EIF5B	2	0.102017604	0.290602298	0.351055739	0.725546533	0.143031471	5.681443898	82.39884054	0.5	CRA004775;PRJNA827465	Yang2025_Silkie_Wenchang	0.930954084
ENSGALG00010015874	CLIC4	2	-0.082264764	0.146516191	-0.561472173	0.574475695	0.037130285	7.252439801	86.2115367	0.5	PRJNA827465;Yang2025_Silkie_Wenchang	CRA004775	0.88612482
ENSGALG00010015874	CLIC4	2	-0.201405375	0.099397766	-2.026256561	0.0427385	0.002814457	1.061512599	5.794806323	0.5	CRA004775;Yang2025_Silkie_Wenchang	PRJNA827465	0.357546035
ENSGALG00010015874	CLIC4	2	0.05701071	0.058182202	0.979865106	0.327152709	0	0.00381109	0	1	CRA004775;PRJNA827465	Yang2025_Silkie_Wenchang	0.800028239
ENSGALG00010015875	DNTTIP1	2	-0.025978488	0.107939658	-0.240676026	0.809806221	0.011198843	1.51932532	34.18131147	0.5	PRJNA827465;Yang2025_Silkie_Wenchang	CRA004775	0.952818762
ENSGALG00010015875	DNTTIP1	2	0.091589047	0.169944046	0.538936485	0.589930681	0	0.609653488	0	0.5	CRA004775;Yang2025_Silkie_Wenchang	PRJNA827465	0.892320726
ENSGALG00010015875	DNTTIP1	2	-0.086771963	0.0618107	-1.403834015	0.160368282	0	0.018200441	0	1	CRA004775;PRJNA827465	Yang2025_Silkie_Wenchang	0.65607938
ENSGALG00010015876	SKP1	2	-0.413761574	0.401080598	-1.031617025	0.302251561	0.313649142	39.29398028	97.45508092	1	PRJNA827465;Yang2025_Silkie_Wenchang	CRA004775	0.791647454
ENSGALG00010015876	SKP1	2	-0.469729265	0.381603303	-1.230936055	0.218346781	0.255810175	7.864972401	87.28539721	1	CRA004775;Yang2025_Silkie_Wenchang	PRJNA827465	0.731986436
ENSGALG00010015876	SKP1	2	-0.021312051	0.046910379	-0.454314181	0.64960271	0	0.021470452	0	1	CRA004775;PRJNA827465	Yang2025_Silkie_Wenchang	0.908200019
ENSGALG00010015877	REV1	2	0.07750367	0.061174777	1.266921978	0.205183189	0	0.949854641	0	1	PRJNA827465;Yang2025_Silkie_Wenchang	CRA004775	0.717945605
ENSGALG00010015877	REV1	2	0.018102885	0.084868171	0.213305941	0.83108834	0	0.047318465	0	1	CRA004775;Yang2025_Silkie_Wenchang	PRJNA827465	0.957470197
ENSGALG00010015877	REV1	2	0.121813017	0.0765082	1.592156353	0.111349578	0	0.088047787	0	1	CRA004775;PRJNA827465	Yang2025_Silkie_Wenchang	0.564466729
ENSGALG00010015879	TRMT61A	2	-0.067816642	0.088407099	-0.767094985	0.443025043	0.005050055	1.254509223	20.28755297	1	PRJNA827465;Yang2025_Silkie_Wenchang	CRA004775	0.845720426
ENSGALG00010015879	TRMT61A	2	-0.527452599	0.292402433	-1.803858449	0.0712535	0.128888809	4.03771217	75.23349962	1	CRA004775;Yang2025_Silkie_Wenchang	PRJNA827465	0.461027938
ENSGALG00010015879	TRMT61A	2	-0.400601384	0.403271808	-0.993378104	0.320525728	0.299570755	11.9947888	91.66304621	1	CRA004775;PRJNA827465	Yang2025_Silkie_Wenchang	0.798348216
ENSGALG00010015881	GAB3	2	-0.195798676	0.156440438	-1.251586094	0.210720724	0.023960347	1.930734623	48.20624296	1	PRJNA827465;Yang2025_Silkie_Wenchang	CRA004775	0.722305653
ENSGALG00010015881	GAB3	2	-0.354639214	0.163558854	-2.168266688	0.030138401	0	0.065026872	0	1	CRA004775;Yang2025_Silkie_Wenchang	PRJNA827465	0.295973612
ENSGALG00010015881	GAB3	2	-0.078872383	0.132452151	-0.595478308	0.551523795	0	0.218790299	0	1	CRA004775;PRJNA827465	Yang2025_Silkie_Wenchang	0.878944924
ENSGALG00010015886	AFF3	2	0.137218863	0.155079991	0.884826354	0.376250293	0.014368183	1.332535433	24.95509121	1	PRJNA827465;Yang2025_Silkie_Wenchang	CRA004775	0.822974013
ENSGALG00010015886	AFF3	2	-0.171070066	0.635188717	-0.269321638	0.787682182	0.635882441	4.359083285	77.05939679	0.5	CRA004775;Yang2025_Silkie_Wenchang	PRJNA827465	0.946919116
ENSGALG00010015886	AFF3	2	-0.281735656	0.446683949	-0.630727066	0.528218999	0.280876951	2.692602946	62.86121571	0.5	CRA004775;PRJNA827465	Yang2025_Silkie_Wenchang	0.871701504
ENSGALG00010015887	SLC9A9	2	-0.176114544	0.136602323	-1.289249994	0.19731119	0.013325845	1.478490823	32.36346247	1	PRJNA827465;Yang2025_Silkie_Wenchang	CRA004775	0.708859387
ENSGALG00010015887	SLC9A9	2	-0.374302399	0.181511783	-2.062138296	0.039194571	0	0.015544041	0	1	CRA004775;Yang2025_Silkie_Wenchang	PRJNA827465	0.342173308
ENSGALG00010015887	SLC9A9	2	-0.103827844	0.11851041	-0.876107376	0.380971663	0	0.617776507	0	1	CRA004775;PRJNA827465	Yang2025_Silkie_Wenchang	0.824392515
ENSGALG00010015889	SH3BP5	2	-0.201142846	0.107465425	-1.871698243	0.061248363	0.017690417	4.240190912	76.41615624	1	PRJNA827465;Yang2025_Silkie_Wenchang	CRA004775	0.427802453
ENSGALG00010015889	SH3BP5	2	-0.291277238	0.084109059	-3.463089936	0.00053401	0.001022186	1.018448312	1.811413719	0.5	CRA004775;Yang2025_Silkie_Wenchang	PRJNA827465	0.024517568
ENSGALG00010015889	SH3BP5	2	-0.093831779	0.064674308	-1.450835462	0.146825683	0	0.13429444	0	0.5	CRA004775;PRJNA827465	Yang2025_Silkie_Wenchang	0.635558525
ENSGALG00010015892	UBE2C	2	-0.004141527	0.147704543	-0.028039263	0.977630836	0.016999267	1.524153606	34.38981503	0.5	PRJNA827465;Yang2025_Silkie_Wenchang	CRA004775	0.993711039
ENSGALG00010015892	UBE2C	2	0.067066851	0.273647211	0.24508509	0.806390547	0.045078516	1.246402739	19.76911082	0.5	CRA004775;Yang2025_Silkie_Wenchang	PRJNA827465	0.952187702
ENSGALG00010015892	UBE2C	2	-0.124361765	0.121568631	-1.022975779	0.306319298	0	0.392255263	0	1	CRA004775;PRJNA827465	Yang2025_Silkie_Wenchang	0.792485409
ENSGALG00010015893	TCF7	2	-0.333704371	0.210287248	-1.586897802	0.112535793	0	0.695849869	0	1	PRJNA827465;Yang2025_Silkie_Wenchang	CRA004775	0.566223663
ENSGALG00010015893	TCF7	2	-0.485759888	0.298634406	-1.626603893	0.103821252	0	0.184912995	0	1	CRA004775;Yang2025_Silkie_Wenchang	PRJNA827465	0.548157801
ENSGALG00010015893	TCF7	2	-0.195941612	0.251452499	-0.779239072	0.435838898	0	0.00237681	0	1	CRA004775;PRJNA827465	Yang2025_Silkie_Wenchang	0.842834885
ENSGALG00010015894	CEP78	2	0.422188835	0.20292671	2.080499084	0.03747978	0.050583026	2.580865067	61.25330174	1	PRJNA827465;Yang2025_Silkie_Wenchang	CRA004775	0.333894819
ENSGALG00010015894	CEP78	2	0.570496744	0.176520213	3.231906042	0.001229675	0	0.836591532	0	1	CRA004775;Yang2025_Silkie_Wenchang	PRJNA827465	0.042769831

ENSGALG00010015894	CEP78	2	0.223105689	0.156938436	1.421612799	0.155138687	0	0.01329556	0	1	CRA004775;PRJNA827465	Yang2025_Silkie_Wenchang	0.647765412
ENSGALG00010015895	RBMXL1	2	0.26681674	0.146518353	1.821046541	0.068599783	0.031708988	3.440371131	70.93336846	1	PRJNA827465;Yang2025_Silkie_Wenchang	CRA004775	0.45121266
ENSGALG00010015895	RBMXL1	2	0.166749127	0.320076449	0.520966561	0.602390066	0.158573881	4.161783345	75.97183906	0.5	CRA004775;Yang2025_Silkie_Wenchang	PRJNA827465	0.895451466
ENSGALG00010015895	RBMXL1	2	0.080546734	0.136595415	0.589673772	0.555409382	0.018974246	1.459813408	31.49809459	0.5	CRA004775;PRJNA827465	Yang2025_Silkie_Wenchang	0.880255849
ENSGALG00010015896	LONRF2	2	-0.219128709	0.057590821	-3.804924236	0.000141848	0	0.006983895	0	1	PRJNA827465;Yang2025_Silkie_Wenchang	CRA004775	0.010027044
ENSGALG00010015896	LONRF2	2	0.170742262	0.4147828	0.411642581	0.680601416	0.308874056	9.259666088	89.2004745	0.5	CRA004775;Yang2025_Silkie_Wenchang	PRJNA827465	0.917851751
ENSGALG00010015896	LONRF2	2	0.163109941	0.419693065	0.388641021	0.697541718	0.319885305	10.15886369	90.15637939	0.5	CRA004775;PRJNA827465	Yang2025_Silkie_Wenchang	0.921342994
ENSGALG00010015898	ENSGALG00010015898	2	0.644620444	0.436511273	1.476755548	0.139741176	0.336186431	8.42510854	88.13071671	1	PRJNA827465;Yang2025_Silkie_Wenchang	CRA004775	0.623597383
ENSGALG00010015898	ENSGALG00010015898	2	0.517733622	0.700352185	0.739247529	0.459756705	0.766562384	4.048575535	75.29995448	0.5	CRA004775;Yang2025_Silkie_Wenchang	PRJNA827465	0.850098539
ENSGALG00010015898	ENSGALG00010015898	2	0.183232894	0.175899997	1.041687873	0.297556413	0	0.639648013	0	0.5	CRA004775;PRJNA827465	Yang2025_Silkie_Wenchang	0.788562929
ENSGALG00010015899	SMARCA1	2	-0.157521864	0.105075434	-1.49913122	0.133839594	0.01686725	4.185508027	76.1080377	1	PRJNA827465;Yang2025_Silkie_Wenchang	CRA004775	0.613104446
ENSGALG00010015899	SMARCA1	2	-0.175336786	0.138037196	-1.270214051	0.204008394	0.02122128	1.928106152	48.13563563	0.5	CRA004775;Yang2025_Silkie_Wenchang	PRJNA827465	0.717254253
ENSGALG00010015899	SMARCA1	2	-0.050656947	0.059957371	-0.844882716	0.398176326	0	0.173390958	0	0.5	CRA004775;PRJNA827465	Yang2025_Silkie_Wenchang	0.831980453
ENSGALG00010015900	ENSGALG00010015900	2	0.07843773	0.109514831	0.716229292	0.473849779	0	0.374275153	0	1	PRJNA827465;Yang2025_Silkie_Wenchang	CRA004775	0.85444425
ENSGALG00010015900	ENSGALG00010015900	2	0.178337295	0.19543301	0.912523912	0.361492988	0	0.05345664	0	1	CRA004775;Yang2025_Silkie_Wenchang	PRJNA827465	0.81550034
ENSGALG00010015900	ENSGALG00010015900	2	0.054133226	0.114538058	0.472622172	0.636482761	0	0.045086308	0	1	CRA004775;PRJNA827465	Yang2025_Silkie_Wenchang	0.904566695
ENSGALG00010015904	MTMR7	2	0.288599428	0.464405857	0.621437959	0.534311503	0.387924015	9.887691221	89.88641557	0.5	PRJNA827465;Yang2025_Silkie_Wenchang	CRA004775	0.873408008
ENSGALG00010015904	MTMR7	2	0.825507471	0.211453949	3.903958631	9.4632E-05	0	0.433146476	0	1	CRA004775;Yang2025_Silkie_Wenchang	PRJNA827465	0.007453547
ENSGALG00010015904	MTMR7	2	0.395679292	0.645796988	0.61269919	0.540075258	0.697978841	5.56873761	82.04260876	0.5	CRA004775;PRJNA827465	Yang2025_Silkie_Wenchang	0.87529516
ENSGALG00010015905	ANPEP	2	0.146972279	0.098045577	1.49901998	0.133868449	0	0.823638955	0	1	PRJNA827465;Yang2025_Silkie_Wenchang	CRA004775	0.613104446
ENSGALG00010015905	ANPEP	2	0.288159937	0.163081102	1.766973204	0.077232716	0	0.003286337	0	1	CRA004775;Yang2025_Silkie_Wenchang	PRJNA827465	0.47878193
ENSGALG00010015905	ANPEP	2	0.107474352	0.111944464	0.960068487	0.337020747	0	0.294211888	0	1	CRA004775;PRJNA827465	Yang2025_Silkie_Wenchang	0.804050409
ENSGALG00010015906	BAG5	2	0.067240493	0.159407991	0.421813814	0.673160922	0.036891435	3.315366541	69.83742257	0.5	PRJNA827465;Yang2025_Silkie_Wenchang	CRA004775	0.915210237
ENSGALG00010015906	BAG5	2	0.157766006	0.173050939	0.911673793	0.361940464	0.014468306	1.231141003	18.77453538	0.5	CRA004775;Yang2025_Silkie_Wenchang	PRJNA827465	0.815579273
ENSGALG00010015906	BAG5	2	-0.067278888	0.073091531	-0.920474463	0.357324872	0	0.043618847	0	1	CRA004775;PRJNA827465	Yang2025_Silkie_Wenchang	0.813292764
ENSGALG00010015908	CHRD	2	0.061509459	0.0933593	0.658846619	0.509994268	0	0.108749709	0	0.5	PRJNA827465;Yang2025_Silkie_Wenchang	CRA004775	0.865276949
ENSGALG00010015908	CHRD	2	-0.072376868	0.187753863	-0.385488035	0.699875869	0	0.421335029	0	1	CRA004775;Yang2025_Silkie_Wenchang	PRJNA827465	0.922198312
ENSGALG00010015908	CHRD	2	0.051077148	0.099844564	0.511566636	0.608954341	0	0.850729462	0	0.5	CRA004775;PRJNA827465	Yang2025_Silkie_Wenchang	0.897208691
ENSGALG00010015909	TNNC2	2	0.061053084	0.217230207	0.281052459	0.778670164	0.073810248	4.441952782	77.48737889	0.5	PRJNA827465;Yang2025_Silkie_Wenchang	CRA004775	0.944673731
ENSGALG00010015909	TNNC2	2	0.277706147	0.17065413	1.627303995	0.10367255	0	0.347492116	0	0.5	CRA004775;Yang2025_Silkie_Wenchang	PRJNA827465	0.547964315
ENSGALG00010015909	TNNC2	2	-0.136948203	0.109832194	-1.2468858	0.212439374	0	0.002016522	0	1	CRA004775;PRJNA827465	Yang2025_Silkie_Wenchang	0.725098108
ENSGALG00010015910	C5orf15	2	-0.362221616	0.399248095	-0.907259472	0.364269601	0.315356412	92.38125812	98.91752936	0.5	PRJNA827465;Yang2025_Silkie_Wenchang	CRA004775	0.817496251
ENSGALG00010015910	C5orf15	2	-0.416536267	0.361634092	-1.151816922	0.249396313	0.246081418	16.45984993	93.92461047	1	CRA004775;Yang2025_Silkie_Wenchang	PRJNA827465	0.759618659
ENSGALG00010015910	C5orf15	2	0.030333226	0.040269876	0.753248548	0.451300571	0	0.196213814	0	0.5	CRA004775;PRJNA827465	Yang2025_Silkie_Wenchang	0.847847413
ENSGALG00010015911	TM9SF2L	2	-0.284253144	0.297614115	-0.955106392	0.339523897	0.172742864	40.02004712	97.50125232	0.5	PRJNA827465;Yang2025_Silkie_Wenchang	CRA004775	0.80490319
ENSGALG00010015911	TM9SF2L	2	-0.351252409	0.248766182	-1.411978134	0.1579564	0.110219882	8.862301613	88.71624953	1	CRA004775;Yang2025_Silkie_Wenchang	PRJNA827465	0.651570149
ENSGALG00010015911	TM9SF2L	2	0.0001779	0.047104862	0.003776672	0.996986659	0	0.388273448	0	0.5	CRA004775;PRJNA827465	Yang2025_Silkie_Wenchang	0.999065103
ENSGALG00010015913	CHST10	2	-0.131414398	0.076642785	-1.714634953	0.086412176	0.005570975	1.688900609	40.78988458	1	PRJNA827465;Yang2025_Silkie_Wenchang	CRA004775	0.506036629
ENSGALG00010015913	CHST10	2	-0.244341947	0.104615817	-2.335611898	0.019511478	0	0.000164613	0	1	CRA004775;Yang2025_Silkie_Wenchang	PRJNA827465	0.234012699
ENSGALG00010015913	CHST10	2	-0.08684913	0.053364533	-1.62746912	0.103637502	0	0.389217332	0	1	CRA004775;PRJNA827465	Yang2025_Silkie_Wenchang	0.547964315
ENSGALG00010015914	SRRM1	2	0.274365448	0.32645541	0.840437743	0.400662995	0.189158446	8.000638207	87.50099712	0.5	PRJNA827465;Yang2025_Silkie_Wenchang	CRA004775	0.832353691
ENSGALG00010015914	SRRM1	2	0.173119852	0.458927047	0.377227389	0.706004628	0.377907538	9.684076508	89.67377014	0.5	CRA004775;Yang2025_Silkie_Wenchang	PRJNA827465	0.924548793
ENSGALG00010015914	SRRM1	2	-0.082409216	0.113838999	-0.723910235	0.469120846	0.016023852	1.894487503	47.21527599	1	CRA004775;PRJNA827465	Yang2025_Silkie_Wenchang	0.85345437
ENSGALG00010015916	COA8	2	-0.218926125	0.258035029	-0.848435678	0.39619538	0.106180798	4.84066911	79.3416989	0.5	PRJNA827465;Yang2025_Silkie_Wenchang	CRA004775	0.831365249
ENSGALG00010015916	COA8	2	-0.142962294	0.393609158	-0.363208758	0.716448946	0.242363867	4.403497662	77.29077936	0.5	CRA004775;Yang2025_Silkie_Wenchang	PRJNA827465	0.927487786
ENSGALG00010015916	COA8	2	0.062038057	0.124652412	0.497688383	0.618703698	0	0.610053843	0	1	CRA004775;PRJNA827465	Yang2025_Silkie_Wenchang	0.899168321
ENSGALG00010015917	TMEM63A	2	-0.072586284	0.108930421	-0.66635457	0.505184493	0.0136328	2.1337934	53.13510672	0.5	PRJNA827465;Yang2025_Silkie_Wenchang	CRA004775	0.863862536
ENSGALG00010015917	TMEM63A	2	-0.258970547	0.12448565	-2.080324492	0.03749578	0	0.498403254	0	1	CRA004775;Yang2025_Silkie_Wenchang	PRJNA827465	0.333944399
ENSGALG00010015917	TMEM63A	2	-0.130085399	0.208921366	-0.62265244	0.533512943	0.058178355	2.321705385	56.92821292	0.5	CRA004775;PRJNA827465	Yang2025_Silkie_Wenchang	0.873379507
ENSGALG00010015918	BICD1	2	0.47705327	0.473392016	1.007734086	0.313582137	0.417107406	14.40843969	93.05962324	1	PRJNA827465;Yang2025_Silkie_Wenchang	CRA004775	0.796531448
ENSGALG00010015918	BICD1	2	0.895468622	0.17205596	5.204519643	1.94499E-07	0	0.809837411	0	1	CRA004775;Yang2025_Silkie_Wenchang	PRJNA827465	6.01921E-05
ENSGALG00010015918	BICD1	2	0.056251531	0.159681491	0.352273332	0.724633286	0	0.828838084	0	1	CRA004775;PRJNA827465	Yang2025_Silkie_Wenchang	0.930453854
ENSGALG00010015919	ENSGALG00010015919	2	0.104871785	0.178079847	0.588903164	0.555926235	0	0.878677811	0	0.5	PRJNA827465;Yang2025_Silkie_Wenchang	CRA004775	0.880324407
ENSGALG00010015919	ENSGALG00010015919	2	-0.098751843	0.240500344	-0.410609987	0.68135854	0	0.03140327	0	1	CRA004775;Yang2025_Silkie_Wenchang	PRJNA827465	0.918242009
ENSGALG00010015919	ENSGALG00010015919	2	0.189391088	0.221754399	0.854057865	0.393072929	0	0.518087591	0	0.5	CRA004775;PRJNA827465	Yang2025_Silkie_Wenchang	0.830282579
ENSGALG00010015921	SNX21	2	0.505654599	0.433897539	1.16537789	0.243866063	0.361480585	24.74111944	95.9581457	1	PRJNA827465;Yang2025_Silkie_Wenchang	CRA004775	0.755365463
ENSGALG00010015921	SNX21	2	0.532147572	0.505231821	1.053274062	0.292215403	0.405686956	4.272398767	76.59394512	0.5	CRA004775;Yang2025_Silkie_Wenchang	PRJNA827465	0.785617484
ENSGALG00010015921	SNX21	2	0.075955831	0.083344686	0.911345821	0.36211319	0	0.112142301	0	0.5	CRA004775;PRJNA827465	Yang2025_Silkie_Wenchang	0.815579273
ENSGALG00010015923	RCAN3	2	-0.094509957	0.235648986	-0.401062439	0.688374154	0.103009631	13.66781938	92.68354394	0.5	PRJNA827465;Yang2025_Silkie_Wenchang	CRA004775	0.919798626
ENSGALG00010015923	RCAN3	2	-0.295364576	0.094151484	-3.137120783	0.001706158	0	0.753298612	0	1	CRA004775;Yang2025_Silkie_Wenchang	PRJNA827465	0.052265891
ENSGALG00010015923	RCAN3	2	0.072647553	0.11323149	0.641584365	0.521143088	0.011551595	1.472936297	32.10840129	0.5	CRA004775;PRJNA827465	Yang2025_Silkie_Wenchang	0.869518515
ENSGALG00010015927	GLA	2	-0.117134409	0.074051069	-1.581805772	0.113693917	0	0.004636568	0	1	PRJNA827465;Yang2025_Silkie_Wenchang	CRA004775	0.56894729
ENSGALG00010015927	GLA	2	-0.14818576	0.110754055	-1.337971425	0.180905752	0	0.789626834	0	1	CRA004775;Yang2025_Silkie_Wenchang	PRJNA827465	0.688484814
ENSGALG00010015927	GLA	2	-0.145385945	0.090772621	-1.601649737	0.109233086	0	0.770350684	0	1	CRA004775;PRJNA827465	Yang2025_Silkie_Wenchang	0.559373025
ENSGALG00010015928	CAPN7	2	0.019327999	0.076350523	0.253148219	0.800153679	0.005748031	1.870703127	46.54416375	0.5	PRJNA827465;Yang2025_Silkie_Wenchang	CRA004775	0.94999675
ENSGALG00010015928	CAPN7	2	-0.06664415	0.086828138	-0.76754093	0.442759967	0	0.063169164	0	1	CRA004775;Yang2025_Silkie_Wenchang	PRJNA827465	0.845624071
ENSGALG00010015928	CAPN7	2	0.069777129	0.05780144	1.207186689	0.227360256	0	0.268206525	0	0.5	CRA004775;PRJNA827465	Yang2025_Silkie_Wenchang	0.740649824
ENSGALG00010015931	RBM47	2	0.054334428	0.149423905	0.363626073	0.716137254	0	0.545202732	0	0.5	PRJNA827465;Yang2025_Silkie_Wenchang	CRA004775	0.927361289
ENSGALG00010015931	RBM47	2	-0.070457788	0.198979245	-0.354096167	0.723266815	0	0.089179228	0	1	CRA004775;Yang2025_Silkie_Wenchang	PRJNA827465	0.930032096
ENSGALG00010015931	RBM47	2	0.129456845	0.205035704	0.631386835	0.527787622	0	0.366167271	0	0.5	CRA004775;PRJNA827465	Yang2025_Silkie_Wenchang	0.871296643
ENSGALG00010015932	ACOT8	2	0.132917517	0.105789481	1.25643416	0.208958603	0	0.920737528	0	0.5	PRJNA827465;Yang2025_Silkie_Wenchang	CRA004775	0.721011841
ENSGALG00010015932	ACOT8	2	-0.22299949	0.228326473	-0.976669445	0.328732827	0.044505696	1.694294461	40.97838226	1	CRA004775;Yang2025_Silkie_Wenchang	PRJNA827465	0.800761292
ENSGALG00010015932	ACOT8	2	-0.103751774	0.344459775	-0.301201422	0.763260906	0.191934497	4.799793699	79.16577123	0.5	CRA004775;PRJNA827465	Yang2025_Silkie_Wenchang	0.940229325
ENSGALG00010015935	EPHX1	2	-0.048067324	0.168213778	-0.285751406	0.775068529	0.04115638	3.590305583	72.14721764	0.5	PRJNA827465;Yang2025_Silkie_Wenchang	CRA004775	0.943803161
ENSGALG00010015935	EPHX1	2	0.078048529	0.340465644	0.229240544	0.818681962	0.191078093	5.465384869	81.70302689	0.5	CRA004775;Yang2025_Silkie_Wenchang	PRJNA827465	0.955256039
ENSGALG00010015935	EPHX1	2	0.201114943	0.154638381	1.300549977	0.193412539	0.022200919	1.588788201	37.05894849	1	CRA004775;PRJNA827465	Yang2025_Silkie_Wenchang	0.703339473
ENSGALG00010015936	RPL36AL	2	0.002499153	0.074889321	0.033371291	0.973378503	0.00093135	1.022725616	2.222063852	0.5	PRJNA827465;Yang2025_Silkie_Wenchang	CRA004775	0.992392211
ENSGALG00010015936	RPL36AL	2	0.029330613	0.198075834	0.148077697	0.88228145	0.02086016	1.343239735	25.55312549	0.5	CRA004775;Yang2025_Silkie_Wenchang	PRJNA827465	0.971967286
ENSGALG00010015936	RPL36AL	2	-0.030133726	0.067735079	-0.444876224	0.656409206	0	0.26337197	0	1	CRA004775;PRJNA827465	Yang2025_Silkie_Wenchang	0.910509456
ENSGALG00010015937	ENSGALG00010015937	2	-0.090470529	0.449087937	-0.201453929	0.84034365	0.336616321	5.917727958	83.10162266	0.5	PRJNA827465;Yang2025_Silkie_Wenchang	CRA004775	0.959662809
ENSGALG00010015937	ENSGALG00010015937	2	0.228882939	0.23120973	0.989936447	0.322205184	0	0.619310914	0	1	CRA004775;Yang2025_Silkie_Wenchang	PRJNA827465	0.798513866
ENSGALG00010015937	ENSGALG00010015937	2	-0.320264175	0.25563801	-1.252803426	0.210277255	0.059683669	1.724092931	41.99848615	0.5	CRA004775;PRJNA827465	Yang2025_Silkie_Wenchang	0.721897568
ENSGALG00010015939	SUN2	2	-0.021942822	0.05514819	-0.397888351	0.690712487	0	0.370936589	0	1	PRJNA827465;Yang2025_Silkie_Wenchang	CRA004775	0.920083812
ENSGALG00010015939	SUN2	2	-0.123181829	0.122204891	-1.007994259	0.313457218	0	0.044282177	0	1	CRA004775;Yang2025_Silkie_Wenchang	PRJNA827465	0.796531448
ENSGALG00010015939	SUN2	2	-0.019126425	0.057308138	-0.333747102	0.738570403	0	0.460213326	0	1	CRA004775;PRJNA827465	Yang2025_Silkie_Wenchang	0.934489024
ENSGALG00010015942	AP3S2	2	-0.003812013	0.078661777	-0.048460809	0.961348998	0	0.283980449	0	0.5	PRJNA827465;Yang2025_Silkie_Wenchang	CRA004775	0.98973612
ENSGALG00010015942	AP3S2	2	-0.108182339	0.226754393	-0.477090377	0.633297767	0.046562707	1.826055135	45.23714095	0.5	CRA004775;Yang2025_Silkie_Wenchang	PRJNA827465	0.9032522
ENSGALG00010015942	AP3S2	2	-0.09919501	0.139511843	-0.711014977	0.477074955	0.018891177	1.568168403	36.23133854	1	CRA004775;PRJNA827465	Yang2025_Silkie_Wenchang	0.854986476
ENSGALG00010015946	TLE4	2	0.290208441	0.17079072	1.699204973	0.08928057	0.036141749	2.500443645	60.00709706	1	PRJNA827465;Yang2025_Silkie_Wenchang	CRA004775	0.512891552
ENSGALG00010015946	TLE4	2	0.368244667	0.158547247	2.322617862	0.02019969	0.00631702	1.135520349	11.93464734	1	CRA004775;Yang2025_Silkie_Wenchang	PRJNA827465	0.239129045
ENSGALG00010015946	TLE4	2	0.151467352	0.10446631	1.449915784	0.147082005	0	0.006556098	0	1	CRA004775;PRJNA827465	Yang2025_Silkie_Wenchang	0.635807749
ENSGALG00010015954	CTSA	2	-0.015056801	0.049821177	-0.302216889	0.76248673	0	0.017484261	0	0.5	PRJNA827465;Yang2025_Silkie_Wenchang	CRA004775	0.940082621
ENSGALG00010015954	CTSA	2	-0.055858234	0.111658828	-0.500258108	0.616893347	0	0.988430506	0	0.5	CRA004775;Yang2025_Silkie_Wenchang	PRJNA827465	0.899111936
ENSGALG00010015954	CTSA	2	-0.033535998	0.061542388	-0.544925188	0.585804972	0.001080863	1.033530842	3.244300074	1	CRA004775;PRJNA827465	Yang2025_Silkie_Wenchang	0.891258964
ENSGALG00010015957	ENSGALG00010015957	2	-0.211819429	0.14695492	-1.441390524	0.149474386	0.012426845	1.397736101	28.45573645	1	PRJNA827465;Yang2025_Silkie_Wenchang	CRA004775	0.639246608
ENSGALG00010015957	ENSGALG00010015957	2	-0.64483653	0.411887412	-1.565565036	0.117450481	0.20660729	2.047838888	51.16803348	1	CRA004775;Yang2025_Silkie_Wenchang	PRJNA827465	0.57688585
ENSGALG00010015957	ENSGALG00010015957	2	-0.537673456	0.580698348	-0.925908362	0.354493605	0.52043535	3.702161937	72.98875584	1	CRA004775;PRJNA827465	Yang2025_Silkie_Wenchang	0.81193713
ENSGALG00010015959	TRNAU1AP	2	0.006193718	0.068052949	0.091013222	0.927482085	0	0.969915509	0	0.5	PRJNA827465;Yang2025_Silkie_Wenchang	CRA004775	0.982993195
ENSGALG00010015959	TRNAU1AP	2	-0.089780284	0.120798405	-0.743224084	0.457346025	0	0.06473378	0	1	CRA004775;Yang2025_Silkie_Wenchang	PRJNA827465	0.849573219
ENSGALG00010015959	TRNAU1AP	2	0.046001829	0.078501609	0.585998553	0.557876487	0	0.017731451	0	0.5	CRA004775;PRJNA827465	Yang2025_Silkie_Wenchang	0.880678789
ENSGALG00010015963	DIPK2A	2	-0.325117574	0.331112603	-0.981894289	0.326151929	0.211662977	28.47695192	96.48838821	1	PRJNA827465;Yang2025_Silkie_Wenchang	CRA004775	0.800023788
ENSGALG00010015963	DIPK2A	2	-0.589524839	0.179664703	-3.281250173	0.00103348	0.025435279	1.241004864	19.42013853	1	CRA004775;Yang2025_Silkie_Wenchang	PRJNA827465	0.038647838
ENSGALG00010015963	DIPK2A	2	-0.003167815	0.055550131	-0.057026241	0.954524292	0	0.112254408	0	1	CRA004775;PRJNA827465	Yang2025_Silkie_Wenchang	0.987987175
ENSGALG00010015964	KLC1	2	0.143716233	0.272572203	0.527259315	0.598013523	0.130431746	7.843214506	87.25012558	0.5	PRJNA827465;Yang2025_Silkie_Wenchang	CRA004775	0.894645663
ENSGALG00010015964	KLC1	2	0.215905422	0.258805287	0.834238839	0.404146409	0.085791336	2.673445335	62.59508331	0.5	CRA004775;Yang2025_Silkie_Wenchang	PRJNA827465	0.833112102
ENSGALG00010015964	KLC1	2	-0.106276953	0.08219624	-1.292966117	0.196022778	0	0.006804787	0	1	CRA004775;PRJNA827465	Yang2025_Silkie_Wenchang	0.707301271
ENSGALG00010015967	ZSWIM1	2	0.070078291	0.160650512	0.436215799	0.662680166	0	0.071516156	0	0.5	PRJNA827465;Yang2025_Silkie_Wenchang	CRA004775	0.912778072
ENSGALG00010015967	ZSWIM1	2	-0.244401757	0.235339656	-1.038506478	0.299034321	0.010824291	1.108246331	9.767352997	1	CRA004775;Yang2025_Silkie_Wenchang	PRJNA827465	0.789748017
ENSGALG00010015967	ZSWIM1	2	-0.134472973	0.279433229	-0.481234725	0.630349684	0.096243038	2.456998774	59.299939	0.5	CRA004775;PRJNA827465	Yang2025_Silkie_Wenchang	0.902650914
ENSGALG00010015970	THPO	2	-0.124616417	0.217673804	-0.572491567	0.566988997	0	0.040910848	0	1	PRJNA827465;Yang2025_Silkie_Wenchang	CRA004775	0.883487517
ENSGALG00010015970	THPO	2	-0.050402668	0.309564618	-0.162817921	0.870661798	0	0.003975138	0	1	CRA004775;Yang2025_Silkie_Wenchang	PRJNA827465	0.969038318
ENSGALG00010015970	THPO	2	-0.130852107	0.24162329	-0.5415542	0.58812565	0	0.047807501	0	1	CRA004775;PRJNA827465	Yang2025_Silkie_Wenchang	0.891761457
ENSGALG00010015971	ENSGALG00010015971	2	-0.156920728	0.159005384	-0.986889394	0.323696864	0.040332	4.904024236	79.60858365	1	PRJNA827465;Yang2025_Silkie_Wenchang	CRA004775	0.799600434
ENSGALG00010015971	ENSGALG00010015971	2	-0.335060478	0.108215148	-3.096243774	0.001959892	0	0.170966956	0	1	CRA004775;Yang2025_Silkie_Wenchang	PRJNA827465	0.057238421
ENSGALG00010015971	ENSGALG00010015971	2	-0.085964257	0.183799868	-0.467705759	0.639995022	0.032270466	1.353421295	26.11317674	1	CRA004775;PRJNA827465	Yang2025_Silkie_Wenchang	0.906064918
ENSGALG00010015972	FSTL4	2	0.004865927	0.13221666	0.036802679	0.970642338	0	0.442212883	0	0.5	PRJNA827465;Yang2025_Silkie_Wenchang	CRA004775	0.991829886
ENSGALG00010015972	FSTL4	2	-0.107003383	0.396170574	-0.270094223	0.787087766	0.157631758	1.727302777	42.10627034	0.5	CRA004775;Yang2025_Silkie_Wenchang	PRJNA827465	0.946848017
ENSGALG00010015972	FSTL4	2	-0.122709815	0.210436349	-0.583120815	0.559811973	0.026158234	1.133825721	11.80302394	1	CRA004775;PRJNA827465	Yang2025_Silkie_Wenchang	0.881203589
ENSGALG00010015974	VPS37A	2	0.022192374	0.115908141	0.191465192	0.848161148	0.01759088	2.403548498	58.39484825	0.5	PRJNA827465;Yang2025_Silkie_Wenchang	CRA004775	0.961363811
ENSGALG00010015974	VPS37A	2	0.274121336	0.139210117	1.969119358	0.048939386	0.004841038	1.114533746	10.27638205	1	CRA004775;Yang2025_Silkie_Wenchang	PRJNA827465	0.382398549
ENSGALG00010015974	VPS37A	2	0.162179059	0.270905368	0.598655759	0.549402465	0.120208447	4.714069327	78.7869051	0.5	CRA004775;PRJNA827465	Yang2025_Silkie_Wenchang	0.878554604
ENSGALG00010015975	BTK	2	-0.198875822	0.210657961	-0.944069814	0.345133974	0.063552061	3.426332587	70.81427519	1	PRJNA827465;Yang2025_Silkie_Wenchang	CRA004775	0.806961063
ENSGALG00010015975	BTK	2	-0.440505007	0.172041475	-2.560458206	0.010453424	0	0.009426598	0	1	CRA004775;Yang2025_Silkie_Wenchang	PRJNA827465	0.162777697
ENSGALG00010015975	BTK	2	-0.074453007	0.16204565	-0.459456992	0.64590603	0.013741589	1.143331385	12.53629413	1	CRA004775;PRJNA827465	Yang2025_Silkie_Wenchang	0.907098003
ENSGALG00010015979	ENSGALG00010015979	2	-0.112666736	0.465800682	-0.241877568	0.808875032	0.418295337	27.72254425	96.39282747	0.5	PRJNA827465;Yang2025_Silkie_Wenchang	CRA004775	0.952595795
ENSGALG00010015979	ENSGALG00010015979	2	-0.4861032	0.225299665	-2.157585099	0.030960104	0.045142159	1.26953714	21.23113467	0.5	CRA004775;Yang2025_Silkie_Wenchang	PRJNA827465	0.300524815
ENSGALG00010015979	ENSGALG00010015979	2	0.340685159	0.128907151	2.642872461	0.008220599	0	0.230649622	0	1	CRA004775;PRJNA827465	Yang2025_Silkie_Wenchang	0.140904587
ENSGALG00010015980	EXOSC7	2	0.2352225	0.161957556	1.452371255	0.146398405	0.036427087	2.98210866	66.46668133	1	PRJNA827465;Yang2025_Silkie_Wenchang	CRA004775	0.6352709
ENSGALG00010015980	EXOSC7	2	0.063033453	0.396673488	0.158905133	0.873743617	0.265994043	6.318902211	84.17446628	0.5	CRA004775;Yang2025_Silkie_Wenchang	PRJNA827465	0.9699681
ENSGALG00010015980	EXOSC7	2	-0.059849894	0.221709562	-0.269947283	0.78720081	0.068934455	2.782460674	64.06058819	0.5	CRA004775;PRJNA827465	Yang2025_Silkie_Wenchang	0.946848352
ENSGALG00010015983	CNOT7	2	-0.265811353	0.258691486	-1.027522617	0.304174441	0.130397791	38.43859026	97.39844778	1	PRJNA827465;Yang2025_Silkie_Wenchang	CRA004775	0.79225794
ENSGALG00010015983	CNOT7	2	-0.098129833	0.435324185	-0.22541783	0.821654242	0.369334325	38.99071748	97.435287	0.5	CRA004775;Yang2025_Silkie_Wenchang	PRJNA827465	0.955598716
ENSGALG00010015983	CNOT7	2	0.147505047	0.175734486	0.839363121	0.401265572	0.054928816	8.362859126	88.04236703	0.5	CRA004775;PRJNA827465	Yang2025_Silkie_Wenchang	0.83248071
ENSGALG00010015984	PDCL3	2	0.037900457	0.062395387	0.607424019	0.543569552	0	0.022232752	0	1	PRJNA827465;Yang2025_Silkie_Wenchang	CRA004775	0.876968972
ENSGALG00010015984	PDCL3	2	0.024540215	0.103381101	0.237376223	0.812364934	0	0.37508783	0	0.5	CRA004775;Yang2025_Silkie_Wenchang	PRJNA827465	0.953371387
ENSGALG00010015984	PDCL3	2	0.019972687	0.07203815	0.277251534	0.781586972	0	0.321274815	0	0.5	CRA004775;PRJNA827465	Yang2025_Silkie_Wenchang	0.945249331
ENSGALG00010015985	TIMM8A	2	-0.050232014	0.081752231	-0.614442114	0.538923214	0	0.069471761	0	0.5	PRJNA827465;Yang2025_Silkie_Wenchang	CRA004775	0.87481821
ENSGALG00010015985	TIMM8A	2	-0.167735648	0.188626216	-0.889248866	0.373869346	0.010102379	1.164592404	14.13304807	0.5	CRA004775;Yang2025_Silkie_Wenchang	PRJNA827465	0.822579781
ENSGALG00010015985	TIMM8A	2	-0.119692308	0.124011812	-0.965168613	0.334460365	0.01159191	1.314375741	23.918255	1	CRA004775;PRJNA827465	Yang2025_Silkie_Wenchang	0.803094614
ENSGALG00010015986	NEURL2	2	-0.171195849	0.128967274	-1.327436365	0.184364377	0	0.022836755	0	1	PRJNA827465;Yang2025_Silkie_Wenchang	CRA004775	0.692608786
ENSGALG00010015986	NEURL2	2	-0.180918919	0.229944338	-0.786794408	0.431402227	0	0.159654845	0	1	CRA004775;Yang2025_Silkie_Wenchang	PRJNA827465	0.842503929
ENSGALG00010015986	NEURL2	2	-0.196441245	0.144437446	-1.360043745	0.173816081	0	0.11987016	0	1	CRA004775;PRJNA827465	Yang2025_Silkie_Wenchang	0.677480397
ENSGALG00010015987	POLR2H	2	-0.213219972	0.308481822	-0.691191364	0.48944529	0.151861683	4.266885102	76.56369984	0.5	PRJNA827465;Yang2025_Silkie_Wenchang	CRA004775	0.858419004
ENSGALG00010015987	POLR2H	2	-0.530000156	0.195483764	-2.711223402	0.006703544	0	0.078475672	0	1	CRA004775;Yang2025_Silkie_Wenchang	PRJNA827465	0.125282647
ENSGALG00010015987	POLR2H	2	-0.161826298	0.252858141	-0.639988484	0.522180086	0.097420501	3.593715446	72.17364549	0.5	CRA004775;PRJNA827465	Yang2025_Silkie_Wenchang	0.869568211
ENSGALG00010015989	ENAH	2	0.045607026	0.17573024	0.259528619	0.795227403	0.042970355	2.900376571	65.52171845	0.5	PRJNA827465;Yang2025_Silkie_Wenchang	CRA004775	0.948935068
ENSGALG00010015989	ENAH	2	0.727123075	0.482914958	1.505695906	0.132145255	0.403372417	7.254600771	86.21564395	1	CRA004775;Yang2025_Silkie_Wenchang	PRJNA827465	0.608786928
ENSGALG00010015989	ENAH	2	0.541472705	0.664001286	0.815469361	0.414803696	0.835115057	18.16048604	94.49353945	0.5	CRA004775;PRJNA827465	Yang2025_Silkie_Wenchang	0.83734102
ENSGALG00010015992	ATP10A	2	0.153044336	0.115636512	1.323494917	0.185670846	0	0.934487535	0	1	PRJNA827465;Yang2025_Silkie_Wenchang	CRA004775	0.694265174
ENSGALG00010015992	ATP10A	2	0.02496984	0.159570382	0.156481674	0.875653363	0	0.003934311	0	1	CRA004775;Yang2025_Silkie_Wenchang	PRJNA827465	0.970347777
ENSGALG00010015992	ATP10A	2	0.223001006	0.145919636	1.52824535	0.126451633	0	0.322178022	0	1	CRA004775;PRJNA827465	Yang2025_Silkie_Wenchang	0.597028533
ENSGALG00010015994	ENSGALG00010015994	2	-0.14964909	0.161023662	-0.929360871	0.352702099	0.028547222	1.950853098	48.7403741	1	PRJNA827465;Yang2025_Silkie_Wenchang	CRA004775	0.811035834
ENSGALG00010015994	ENSGALG00010015994	2	-0.398981377	0.159184898	-2.506402183	0.01219668	0	0.016761905	0	1	CRA004775;Yang2025_Silkie_Wenchang	PRJNA827465	0.178269287
ENSGALG00010015994	ENSGALG00010015994	2	-0.174292085	0.184007984	-0.947198492	0.343537644	0.042663716	2.382255326	58.02297138	1	CRA004775;PRJNA827465	Yang2025_Silkie_Wenchang	0.806276913
ENSGALG00010016004	XRCC3	2	0.022473039	0.066583962	0.337514299	0.735729235	0	0.012453953	0	1	PRJNA827465;Yang2025_Silkie_Wenchang	CRA004775	0.933592889
ENSGALG00010016004	XRCC3	2	0.041054407	0.104241757	0.393838405	0.693700348	0	0.128627319	0	1	CRA004775;Yang2025_Silkie_Wenchang	PRJNA827465	0.920721644
ENSGALG00010016004	XRCC3	2	0.023022102	0.083330489	0.276274652	0.782337123	0	0.157487759	0	1	CRA004775;PRJNA827465	Yang2025_Silkie_Wenchang	0.945372683
ENSGALG00010016006	PLTP	2	0.581403166	0.095619164	6.08040418	1.1988E-09	0	0.380042325	0	1	PRJNA827465;Yang2025_Silkie_Wenchang	CRA004775	7.13885E-07
ENSGALG00010016006	PLTP	2	0.647926487	0.181178895	3.576169774	0.000348665	0	0.408200711	0	1	CRA004775;Yang2025_Silkie_Wenchang	PRJNA827465	0.018543425
ENSGALG00010016006	PLTP	2	0.53832314	0.109307939	4.924831114	8.44332E-07	0	0.202724172	0	1	CRA004775;PRJNA827465	Yang2025_Silkie_Wenchang	0.000194063
ENSGALG00010016008	TAF7	2	0.146753807	0.195491721	0.750690649	0.452838852	0.066699863	7.408243204	86.50152307	0.5	PRJNA827465;Yang2025_Silkie_Wenchang	CRA004775	0.848530412
ENSGALG00010016008	TAF7	2	0.12372085	0.269881249	0.458426994	0.646645702	0.111740231	4.079480683	75.48707599	0.5	CRA004775;Yang2025_Silkie_Wenchang	PRJNA827465	0.907350468
ENSGALG00010016008	TAF7	2	-0.039562961	0.054972517	-0.719686193	0.471718229	0	0.39419835	0	1	CRA004775;PRJNA827465	Yang2025_Silkie_Wenchang	0.853998373
ENSGALG00010016011	ADD1	2	-0.120662126	0.100770476	-1.197395623	0.231152389	0.015499882	4.014629315	75.09110003	1	PRJNA827465;Yang2025_Silkie_Wenchang	CRA004775	0.743891332
ENSGALG00010016011	ADD1	2	-0.098745954	0.161408213	-0.611777752	0.540684812	0.036895884	3.209201683	68.83960254	0.5	CRA004775;Yang2025_Silkie_Wenchang	PRJNA827465	0.875624671
ENSGALG00010016011	ADD1	2	-0.020826806	0.047931325	-0.434513473	0.663915613	0	0.54787116	0	0.5	CRA004775;PRJNA827465	Yang2025_Silkie_Wenchang	0.913119244
ENSGALG00010016015	ZDHHC3	2	-0.150268996	0.140586937	-1.068868834	0.285128777	0.035771668	10.45513994	90.43532649	1	PRJNA827465;Yang2025_Silkie_Wenchang	CRA004775	0.78060855
ENSGALG00010016015	ZDHHC3	2	-0.271763905	0.064676494	-4.201896025	2.64689E-05	0	0.850730905	0	1	CRA004775;Yang2025_Silkie_Wenchang	PRJNA827465	0.002998
ENSGALG00010016015	ZDHHC3	2	-0.021115302	0.050613138	-0.417190141	0.676539336	0	0.289881032	0	1	CRA004775;PRJNA827465	Yang2025_Silkie_Wenchang	0.916786558
ENSGALG00010016016	ZDHHC2	2	-0.318257803	0.21839414	-1.457263476	0.145043665	0.075541732	4.702550214	78.73494265	1	PRJNA827465;Yang2025_Silkie_Wenchang	CRA004775	0.632983695
ENSGALG00010016016	ZDHHC2	2	-0.60670471	0.155225832	-3.908529293	9.28597E-05	0	0.777215688	0	1	CRA004775;Yang2025_Silkie_Wenchang	PRJNA827465	0.007343389
ENSGALG00010016016	ZDHHC2	2	-0.436414851	0.410219538	-1.063856813	0.28739357	0.257918505	3.591404796	72.15574248	1	CRA004775;PRJNA827465	Yang2025_Silkie_Wenchang	0.782840067
ENSGALG00010016017	RAPGEF6	2	-0.058523432	0.046591455	-1.25609796	0.209080456	0	0.242183626	0	1	PRJNA827465;Yang2025_Silkie_Wenchang	CRA004775	0.721011841
ENSGALG00010016017	RAPGEF6	2	-0.000799561	0.121166957	-0.006598836	0.994734929	0	0.000181104	0	1	CRA004775;Yang2025_Silkie_Wenchang	PRJNA827465	0.998242335
ENSGALG00010016017	RAPGEF6	2	-0.066590657	0.049680632	-1.340374577	0.180123597	0	0.023576738	0	1	CRA004775;PRJNA827465	Yang2025_Silkie_Wenchang	0.687225928
ENSGALG00010016021	ENSGALG00010016021	2	0.880983633	0.57310404	1.537214138	0.124240879	0.493263846	4.01099963	75.06855916	1	PRJNA827465;Yang2025_Silkie_Wenchang	CRA004775	0.590809351
ENSGALG00010016021	ENSGALG00010016021	2	0.837359838	0.701028575	1.19447319	0.232292922	0.707322928	3.389338109	70.49571428	1	CRA004775;Yang2025_Silkie_Wenchang	PRJNA827465	0.744986431
ENSGALG00010016021	ENSGALG00010016021	2	0.221153082	0.353720253	0.625220298	0.531826482	0	0.118859602	0	1	CRA004775;PRJNA827465	Yang2025_Silkie_Wenchang	0.873042777
ENSGALG00010016022	EPHB3	2	0.028693172	0.073053934	0.392766962	0.694491608	0.004188856	1.627862	38.56973135	0.5	PRJNA827465;Yang2025_Silkie_Wenchang	CRA004775	0.920728119
ENSGALG00010016022	EPHB3	2	0.026608941	0.146184785	0.182022645	0.855564954	0.020573766	1.575850585	36.54220716	0.5	CRA004775;Yang2025_Silkie_Wenchang	PRJNA827465	0.963475815
ENSGALG00010016022	EPHB3	2	-0.049728306	0.069614733	-0.71433594	0.475019473	0	0.5161686	0	1	CRA004775;PRJNA827465	Yang2025_Silkie_Wenchang	0.854462864
ENSGALG00010016025	PCIF1	2	-0.051294263	0.105284823	-0.487195229	0.626119986	0.016028639	3.044880518	67.15798882	0.5	PRJNA827465;Yang2025_Silkie_Wenchang	CRA004775	0.901504244
ENSGALG00010016025	PCIF1	2	-0.152240481	0.084712429	-1.797144551	0.072312675	0	0.199333519	0	1	CRA004775;Yang2025_Silkie_Wenchang	PRJNA827465	0.46442847
ENSGALG00010016025	PCIF1	2	0.003013945	0.05495081	0.054848054	0.956259517	0.00221153	1.276810967	21.67987074	0.5	CRA004775;PRJNA827465	Yang2025_Silkie_Wenchang	0.988528437
ENSGALG00010016027	COPS2	2	-0.02165517	0.058729063	-0.36873005	0.712328948	0	0.007424759	0	1	PRJNA827465;Yang2025_Silkie_Wenchang	CRA004775	0.92597752
ENSGALG00010016027	COPS2	2	0.01248171	0.113905806	0.10957923	0.912743084	0	0.51326584	0	0.5	CRA004775;Yang2025_Silkie_Wenchang	PRJNA827465	0.979024575
ENSGALG00010016027	COPS2	2	-0.015587063	0.066478717	-0.234466964	0.814622477	0	0.590929631	0	0.5	CRA004775;PRJNA827465	Yang2025_Silkie_Wenchang	0.953869515

ENSGALG00010016040	RPSA2	2	0.015169914	0.101832531	0.148969234	0.88157791	0.011777742	1.833620202	45.46307907	0.5	PRJNA827465;Yang2025_Silkie_Wenchang	CRA004775	0.971802788
ENSGALG00010016040	RPSA2	2	-0.105927371	0.313465453	-0.337923589	0.73542077	0.154207212	4.504319573	77.79908855	0.5	CRA004775;Yang2025_Silkie_Wenchang	PRJNA827465	0.933592889
ENSGALG00010016040	RPSA2	2	-0.174496084	0.18675487	-0.934358947	0.350118767	0.048365759	2.491120298	59.85741834	1	CRA004775;PRJNA827465	Yang2025_Silkie_Wenchang	0.810073462
ENSGALG00010016041	ENSGALG00010016041	2	-0.006538295	0.141357352	-0.046253662	0.963108072	0.028188074	3.17851748	68.53879187	0.5	PRJNA827465;Yang2025_Silkie_Wenchang	CRA004775	0.989998461
ENSGALG00010016041	ENSGALG00010016041	2	-0.078023235	0.158641864	-0.491819961	0.622846625	0.014533431	1.293103044	22.66664247	0.5	CRA004775;Yang2025_Silkie_Wenchang	PRJNA827465	0.900255255
ENSGALG00010016041	ENSGALG00010016041	2	0.117775157	0.074813267	1.57425497	0.115428516	0	0.059574419	0	1	CRA004775;PRJNA827465	Yang2025_Silkie_Wenchang	0.572524855
ENSGALG00010016043	ADAMTSL1	2	0.101629201	0.123145413	0.825278007	0.409213775	0.005590579	1.217878015	17.88997028	0.5	PRJNA827465;Yang2025_Silkie_Wenchang	CRA004775	0.834316328
ENSGALG00010016043	ADAMTSL1	2	-0.000209866	0.163390728	-0.001284444	0.998975162	0	0.460120147	0	0.5	CRA004775;Yang2025_Silkie_Wenchang	PRJNA827465	0.999814637
ENSGALG00010016043	ADAMTSL1	2	0.211884137	0.135467517	1.564095524	0.117795137	0	0.023113716	0	1	CRA004775;PRJNA827465	Yang2025_Silkie_Wenchang	0.577557669
ENSGALG00010016047	SRP9	2	-0.20801903	0.187293093	-1.110660443	0.266714535	0.062454173	9.017681807	88.91067548	1	PRJNA827465;Yang2025_Silkie_Wenchang	CRA004775	0.770949983
ENSGALG00010016047	SRP9	2	-0.316132721	0.151189462	-2.090970607	0.036530699	0.019978936	1.475683204	32.23477792	1	CRA004775;Yang2025_Silkie_Wenchang	PRJNA827465	0.329606533
ENSGALG00010016047	SRP9	2	-0.028712308	0.070485743	-0.407349165	0.683751558	0	0.006538564	0	1	CRA004775;PRJNA827465	Yang2025_Silkie_Wenchang	0.918800744
ENSGALG00010016049	ZFYVE21	2	-0.317367167	0.324382441	-0.978373448	0.327889655	0.195418448	13.40102144	92.53788225	1	PRJNA827465;Yang2025_Silkie_Wenchang	CRA004775	0.80043771
ENSGALG00010016049	ZFYVE21	2	-0.277551979	0.405285357	-0.684831007	0.493450567	0.283431382	7.154559277	86.02289867	0.5	CRA004775;Yang2025_Silkie_Wenchang	PRJNA827465	0.859868321
ENSGALG00010016049	ZFYVE21	2	-0.003447189	0.056441457	-0.061075474	0.951299102	0	0.389420055	0	0.5	CRA004775;PRJNA827465	Yang2025_Silkie_Wenchang	0.987687454
ENSGALG00010016053	DRP2	2	0.169188033	0.160957671	1.051133708	0.293197185	0	0.545234057	0	1	PRJNA827465;Yang2025_Silkie_Wenchang	CRA004775	0.786803834
ENSGALG00010016053	DRP2	2	0.771898686	0.601264677	1.28379184	0.199214799	0.576977511	4.457305371	77.56492058	1	CRA004775;Yang2025_Silkie_Wenchang	PRJNA827465	0.71096127
ENSGALG00010016053	DRP2	2	0.675261865	0.727696354	0.92794455	0.353436334	0.891931489	5.984245309	83.28945509	1	CRA004775;PRJNA827465	Yang2025_Silkie_Wenchang	0.811254349
ENSGALG00010016057	SOHO1	2	0.082783142	0.241840989	0.342304018	0.732122112	0	0.859840198	0	0.5	PRJNA827465;Yang2025_Silkie_Wenchang	CRA004775	0.93287651
ENSGALG00010016057	SOHO1	2	0.381490154	0.373368883	1.021751331	0.306898609	0	0.014861023	0	1	CRA004775;Yang2025_Silkie_Wenchang	PRJNA827465	0.79266003
ENSGALG00010016057	SOHO1	2	-0.027276694	0.275637644	-0.098958523	0.921171202	0	0.19431159	0	0.5	CRA004775;PRJNA827465	Yang2025_Silkie_Wenchang	0.981519273
ENSGALG00010016059	EPDR1	2	-0.116389664	0.074564626	-1.560923324	0.118541843	0	0.13976517	0	1	PRJNA827465;Yang2025_Silkie_Wenchang	CRA004775	0.578415575
ENSGALG00010016059	EPDR1	2	0.0033574	0.246287903	0.013632015	0.989123562	0.068942107	1.908097445	47.59177512	0.5	CRA004775;Yang2025_Silkie_Wenchang	PRJNA827465	0.997012807
ENSGALG00010016059	EPDR1	2	0.009922941	0.199541473	0.049728715	0.960338574	0.043185555	1.606251596	37.74325252	0.5	CRA004775;PRJNA827465	Yang2025_Silkie_Wenchang	0.989338243
ENSGALG00010016060	ENSGALG00010016060	2	0.830168886	0.644405192	1.288271566	0.197651447	0.432376156	1.966575535	49.15018608	1	PRJNA827465;Yang2025_Silkie_Wenchang	CRA004775	0.709468779
ENSGALG00010016060	ENSGALG00010016060	2	1.441391282	0.469833224	3.067878576	0.002155842	0	0.5918747	0	1	CRA004775;Yang2025_Silkie_Wenchang	PRJNA827465	0.061008576
ENSGALG00010016060	ENSGALG00010016060	2	0.940012983	1.274801609	0.737379822	0.460891403	1.834455637	2.048161972	51.17573641	1	CRA004775;PRJNA827465	Yang2025_Silkie_Wenchang	0.85014477
ENSGALG00010016066	C14orf2	2	-0.200976477	0.123623887	-1.625709083	0.104011557	0.016517081	1.839595661	45.64022838	1	PRJNA827465;Yang2025_Silkie_Wenchang	CRA004775	0.548383482
ENSGALG00010016066	C14orf2	2	-0.137715476	0.300424478	-0.458402981	0.646662951	0.119219672	2.773146294	63.93987573	0.5	CRA004775;Yang2025_Silkie_Wenchang	PRJNA827465	0.907350468
ENSGALG00010016066	C14orf2	2	-0.087897404	0.09896592	-0.888158304	0.374455604	0.005385983	1.101680299	9.229564963	0.5	CRA004775;PRJNA827465	Yang2025_Silkie_Wenchang	0.822805106
ENSGALG00010016067	AHSG	2	0.154839887	0.330251296	0.468854744	0.639173467	0.142157884	2.665922946	62.48953851	0.5	PRJNA827465;Yang2025_Silkie_Wenchang	CRA004775	0.905968982
ENSGALG00010016067	AHSG	2	0.671705624	0.923716066	0.727177592	0.467117171	1.431417607	5.471042182	81.72194681	0.5	CRA004775;Yang2025_Silkie_Wenchang	PRJNA827465	0.852885838
ENSGALG00010016067	AHSG	2	0.962930064	0.565521102	1.702730562	0.088618503	0.350283356	1.956288447	48.88279377	1	CRA004775;PRJNA827465	Yang2025_Silkie_Wenchang	0.511326903
ENSGALG00010016069	GALK2	2	-0.229481355	0.085277021	-2.691010441	0.007123597	0	0.517110524	0	1	PRJNA827465;Yang2025_Silkie_Wenchang	CRA004775	0.129860257
ENSGALG00010016069	GALK2	2	-0.096125205	0.118615327	-0.810394468	0.417713496	0	0.692838064	0	0.5	CRA004775;Yang2025_Silkie_Wenchang	PRJNA827465	0.838112595
ENSGALG00010016069	GALK2	2	-0.16459944	0.165594231	-0.993992596	0.320226472	0.028473503	1.826751894	45.25802857	0.5	CRA004775;PRJNA827465	Yang2025_Silkie_Wenchang	0.798348216
ENSGALG00010016076	RAB3GAP2	2	-0.077796598	0.040249455	-1.932860904	0.053253335	0	0.354856358	0	1	PRJNA827465;Yang2025_Silkie_Wenchang	CRA004775	0.400004977
ENSGALG00010016076	RAB3GAP2	2	-0.081433528	0.072390149	-1.12492555	0.260620584	8.73273E-05	1.005773639	0.574049527	0.5	CRA004775;Yang2025_Silkie_Wenchang	PRJNA827465	0.76772293
ENSGALG00010016076	RAB3GAP2	2	-0.054309191	0.044322018	-1.225332099	0.220450119	0	0.546188202	0	0.5	CRA004775;PRJNA827465	Yang2025_Silkie_Wenchang	0.734053249
ENSGALG00010016083	CDH22	2	0.362478531	0.293843995	1.233574744	0.217361409	0	0.472289958	0	1	PRJNA827465;Yang2025_Silkie_Wenchang	CRA004775	0.730896703
ENSGALG00010016083	CDH22	2	-0.163212222	0.842329337	-0.193762955	0.846361495	1.085690781	4.123658232	75.74968769	0.5	CRA004775;Yang2025_Silkie_Wenchang	PRJNA827465	0.96122283
ENSGALG00010016083	CDH22	2	-0.319213328	0.628190108	-0.508147652	0.611349799	0.50469712	2.578071508	61.21131641	0.5	CRA004775;PRJNA827465	Yang2025_Silkie_Wenchang	0.897609796
ENSGALG00010016087	IARS2	2	0.097027242	0.16870178	0.575140595	0.565196215	0.053196275	15.11989136	93.38619587	0.5	PRJNA827465;Yang2025_Silkie_Wenchang	CRA004775	0.883427937
ENSGALG00010016087	IARS2	2	0.075687655	0.238664571	0.317129829	0.751145092	0.089553196	4.072799922	75.44686655	0.5	CRA004775;Yang2025_Silkie_Wenchang	PRJNA827465	0.937684688
ENSGALG00010016087	IARS2	2	-0.073062746	0.045953228	-1.58993719	0.111848964	0	0.406334061	0	1	CRA004775;PRJNA827465	Yang2025_Silkie_Wenchang	0.565401905
ENSGALG00010016094	FGF7	2	-0.057745757	0.197527203	-0.292343315	0.770024144	0.049726574	2.606332768	61.63191391	0.5	PRJNA827465;Yang2025_Silkie_Wenchang	CRA004775	0.942078099
ENSGALG00010016094	FGF7	2	0.226893837	0.196940069	1.152095853	0.249281687	0	0.330734207	0	1	CRA004775;Yang2025_Silkie_Wenchang	PRJNA827465	0.759540244
ENSGALG00010016094	FGF7	2	-0.031792087	0.317481856	-0.10013828	0.920234545	0.120714442	1.872743629	46.60240813	0.5	CRA004775;PRJNA827465	Yang2025_Silkie_Wenchang	0.981364549
ENSGALG00010016097	FNIP1	2	-0.085751569	0.074156484	-1.1563597	0.247534059	0	0.022933348	0	1	PRJNA827465;Yang2025_Silkie_Wenchang	CRA004775	0.757947282
ENSGALG00010016097	FNIP1	2	-0.057503602	0.115476374	-0.497968547	0.618506212	0	0.050871343	0	1	CRA004775;Yang2025_Silkie_Wenchang	PRJNA827465	0.899168321
ENSGALG00010016097	FNIP1	2	-0.084344173	0.086146093	-0.979082976	0.327538982	0	0.102907565	0	1	CRA004775;PRJNA827465	Yang2025_Silkie_Wenchang	0.800078597
ENSGALG00010016100	SFRP4	2	-0.442879958	0.24802204	-1.785647592	0.074156336	0	0.000191586	0	1	PRJNA827465;Yang2025_Silkie_Wenchang	CRA004775	0.470243057
ENSGALG00010016100	SFRP4	2	-0.241144808	0.350235822	-0.688521254	0.491124591	0	0.581024526	0	0.5	CRA004775;Yang2025_Silkie_Wenchang	PRJNA827465	0.85895707
ENSGALG00010016100	SFRP4	2	-0.327861411	0.268762071	-1.219894645	0.222504816	0	0.729584201	0	0.5	CRA004775;PRJNA827465	Yang2025_Silkie_Wenchang	0.73602412
ENSGALG00010016103	BPNT1	2	-0.029079946	0.088807445	-0.32744942	0.743327993	0	0.032740869	0	1	PRJNA827465;Yang2025_Silkie_Wenchang	CRA004775	0.935767527
ENSGALG00010016103	BPNT1	2	0.062205329	0.150779934	0.412557078	0.679931154	0.018685416	1.57818899	36.63623263	0.5	CRA004775;Yang2025_Silkie_Wenchang	PRJNA827465	0.917701868
ENSGALG00010016103	BPNT1	2	0.073295791	0.131571891	0.557077888	0.577474214	0.008676188	1.26632772	21.03150042	0.5	CRA004775;PRJNA827465	Yang2025_Silkie_Wenchang	0.88731056
ENSGALG00010016107	UVSSA	2	0.00873673	0.058523684	0.14928537	0.881328459	0	0.708902528	0	0.5	PRJNA827465;Yang2025_Silkie_Wenchang	CRA004775	0.971790895
ENSGALG00010016107	UVSSA	2	-0.011954521	0.120844041	-0.098925198	0.921197662	0.012399056	1.673188665	40.23387674	0.5	CRA004775;Yang2025_Silkie_Wenchang	PRJNA827465	0.981519273
ENSGALG00010016107	UVSSA	2	-0.046345275	0.064109278	-0.722910574	0.469734827	0	0.653392677	0	1	CRA004775;PRJNA827465	Yang2025_Silkie_Wenchang	0.853485658
ENSGALG00010016109	MLH1	2	-0.092581768	0.093250251	-0.992831296	0.320792176	0.005127664	1.406545569	28.90383206	1	PRJNA827465;Yang2025_Silkie_Wenchang	CRA004775	0.798348216
ENSGALG00010016109	MLH1	2	-0.181844905	0.113553504	-1.601402849	0.109287722	0	0.14894989	0	1	CRA004775;Yang2025_Silkie_Wenchang	PRJNA827465	0.559426991
ENSGALG00010016109	MLH1	2	-0.018234167	0.095297256	-0.191339892	0.848259309	0	0.042055114	0	1	CRA004775;PRJNA827465	Yang2025_Silkie_Wenchang	0.961363811
ENSGALG00010016111	EPB41	2	0.032283472	0.088721603	0.363873863	0.715952203	0.004564273	1.381937543	27.63782958	0.5	PRJNA827465;Yang2025_Silkie_Wenchang	CRA004775	0.927261187
ENSGALG00010016111	EPB41	2	-0.093660548	0.121715825	-0.769501815	0.441595467	0	0.337802761	0	1	CRA004775;Yang2025_Silkie_Wenchang	PRJNA827465	0.845068977
ENSGALG00010016111	EPB41	2	0.0904462	0.090392293	1.000596363	0.317021989	0	0.81293516	0	0.5	CRA004775;PRJNA827465	Yang2025_Silkie_Wenchang	0.797007794
ENSGALG00010016113	DTWD1	2	-0.036697082	0.061107451	-0.600533671	0.548150628	0	0.001038219	0	1	PRJNA827465;Yang2025_Silkie_Wenchang	CRA004775	0.878281266
ENSGALG00010016113	DTWD1	2	-0.043097685	0.104169907	-0.413724908	0.679075583	0	0.050464107	0	1	CRA004775;Yang2025_Silkie_Wenchang	PRJNA827465	0.917701868
ENSGALG00010016113	DTWD1	2	-0.042015276	0.070459383	-0.596304915	0.550971549	0	0.048118757	0	1	CRA004775;PRJNA827465	Yang2025_Silkie_Wenchang	0.878879109
ENSGALG00010016114	HAUS6	2	-0.021222309	0.120905486	-0.175528092	0.860664699	0	0.658828389	0	0.5	PRJNA827465;Yang2025_Silkie_Wenchang	CRA004775	0.965114242
ENSGALG00010016114	HAUS6	2	-0.146962772	0.146105681	-1.00586624	0.314479918	0	0.30812133	0	1	CRA004775;Yang2025_Silkie_Wenchang	PRJNA827465	0.796531448
ENSGALG00010016114	HAUS6	2	-0.012470967	0.176163068	-0.070792178	0.943563157	0.008244591	1.110608688	9.959285287	0.5	CRA004775;PRJNA827465	Yang2025_Silkie_Wenchang	0.985827325
ENSGALG00010016117	ENSGALG00010016117	2	-0.260041122	0.153362737	-1.695595205	0.089962567	0	0.118157248	0	1	PRJNA827465;Yang2025_Silkie_Wenchang	CRA004775	0.514545335
ENSGALG00010016117	ENSGALG00010016117	2	-0.308241664	0.197192131	-1.563153974	0.118016383	0	0.985505517	0	1	CRA004775;Yang2025_Silkie_Wenchang	PRJNA827465	0.577777516
ENSGALG00010016117	ENSGALG00010016117	2	-0.383707975	0.188582975	-2.034690431	0.041882032	0	0.608546447	0	1	CRA004775;PRJNA827465	Yang2025_Silkie_Wenchang	0.354378792
ENSGALG00010016118	EPRS	2	0.054094467	0.180875533	0.299070116	0.764886547	0.062067621	19.17691565	94.7853971	0.5	PRJNA827465;Yang2025_Silkie_Wenchang	CRA004775	0.940711384
ENSGALG00010016118	EPRS	2	0.226814164	0.067186615	3.375883174	0.000735792	0	0.244833932	0	1	CRA004775;Yang2025_Silkie_Wenchang	PRJNA827465	0.030345032
ENSGALG00010016118	EPRS	2	-0.048372161	0.118678814	-0.407588843	0.683575557	0.01788225	2.034810855	50.85538307	0.5	CRA004775;PRJNA827465	Yang2025_Silkie_Wenchang	0.918800744
ENSGALG00010016121	SLC35C2	2	0.177682158	0.059030413	3.010010396	0.002612387	0	0.446341933	0	1	PRJNA827465;Yang2025_Silkie_Wenchang	CRA004775	0.069138993
ENSGALG00010016121	SLC35C2	2	0.035515395	0.214833128	0.165316192	0.868695115	0.074808002	5.120017582	80.46881707	0.5	CRA004775;Yang2025_Silkie_Wenchang	PRJNA827465	0.968301875
ENSGALG00010016121	SLC35C2	2	0.005454755	0.17131792	0.031839955	0.974599683	0.04485511	3.87162978	74.1710841	0.5	CRA004775;PRJNA827465	Yang2025_Silkie_Wenchang	0.992492038
ENSGALG00010016122	AFG2B	2	-0.255390233	0.077681115	-3.287674646	0.001010185	0	0.788081771	0	1	PRJNA827465;Yang2025_Silkie_Wenchang	CRA004775	0.038212998
ENSGALG00010016122	AFG2B	2	-0.258719756	0.146437084	-1.766763912	0.077267775	0.011975391	1.313897731	23.89057563	1	CRA004775;Yang2025_Silkie_Wenchang	PRJNA827465	0.478793445
ENSGALG00010016122	AFG2B	2	-0.186045471	0.087419955	-2.128180814	0.033322095	0	0.428948158	0	1	CRA004775;PRJNA827465	Yang2025_Silkie_Wenchang	0.313584825
ENSGALG00010016127	ENSGALG00010016127	2	0.108485892	0.16938712	0.640461281	0.521872754	0	0.074525434	0	1	PRJNA827465;Yang2025_Silkie_Wenchang	CRA004775	0.86956507
ENSGALG00010016127	ENSGALG00010016127	2	-0.96643891	1.22503075	-0.788909919	0.430164658	2.715207913	10.00726147	90.0072562	0.5	CRA004775;Yang2025_Silkie_Wenchang	PRJNA827465	0.842032082
ENSGALG00010016127	ENSGALG00010016127	2	-1.002612964	1.174693837	-0.853510023	0.393376532	2.500549848	9.94995209	89.94970035	0.5	CRA004775;PRJNA827465	Yang2025_Silkie_Wenchang	0.830346712
ENSGALG00010016129	CENPI	2	-0.312987454	0.272154508	-1.150035897	0.250129087	0.131694708	8.847328813	88.69715344	1	PRJNA827465;Yang2025_Silkie_Wenchang	CRA004775	0.75993411
ENSGALG00010016129	CENPI	2	-0.339717806	0.396922309	-0.85587985	0.392064242	0.200624451	2.141724184	53.30864695	0.5	CRA004775;Yang2025_Silkie_Wenchang	PRJNA827465	0.829251057
ENSGALG00010016129	CENPI	2	-0.044319101	0.096916555	-0.457291337	0.647461655	0	0.308697939	0	0.5	CRA004775;PRJNA827465	Yang2025_Silkie_Wenchang	0.907350807
ENSGALG00010016131	SMYD1	2	0.066255089	0.306151225	0.216412948	0.828665874	0.177309958	18.47311951	94.58672911	0.5	PRJNA827465;Yang2025_Silkie_Wenchang	CRA004775	0.956837266
ENSGALG00010016131	SMYD1	2	0.372651363	0.10111974	3.685248432	0.00022848	0	0.000206606	0	1	CRA004775;Yang2025_Silkie_Wenchang	PRJNA827465	0.013965686
ENSGALG00010016131	SMYD1	2	-0.22876193	0.098485134	-2.322806706	0.020189539	0	0.651184865	0	0.5	CRA004775;PRJNA827465	Yang2025_Silkie_Wenchang	0.239129045
ENSGALG00010016137	STARD3NL	2	0.115720816	0.070831692	1.633743487	0.102312723	0.002084452	1.249301647	19.95528039	1	PRJNA827465;Yang2025_Silkie_Wenchang	CRA004775	0.545029796
ENSGALG00010016137	STARD3NL	2	0.092783882	0.18097943	0.512676397	0.608177703	0.034320139	1.698541335	41.1259544	0.5	CRA004775;Yang2025_Silkie_Wenchang	PRJNA827465	0.897204334
ENSGALG00010016137	STARD3NL	2	0.040154582	0.076603705	0.524185896	0.60014925	0	0.740524043	0	0.5	CRA004775;PRJNA827465	Yang2025_Silkie_Wenchang	0.895230519
ENSGALG00010016139	LURAP1L	2	0.062987665	0.156830503	0.401628916	0.687957146	0	0.023603768	0	1	PRJNA827465;Yang2025_Silkie_Wenchang	CRA004775	0.919631238
ENSGALG00010016139	LURAP1L	2	-0.0043368	0.230573342	-0.018808768	0.984993659	0	0.086433197	0	0.5	CRA004775;Yang2025_Silkie_Wenchang	PRJNA827465	0.995786861
ENSGALG00010016139	LURAP1L	2	0.052158333	0.182818232	0.285301592	0.775413093	0	0.161730774	0	0.5	CRA004775;PRJNA827465	Yang2025_Silkie_Wenchang	0.943815265
ENSGALG00010016141	RRP15	2	0.298682891	0.331534792	0.900909644	0.367636363	0.198751471	9.824117466	89.82096862	0.5	PRJNA827465;Yang2025_Silkie_Wenchang	CRA004775	0.819975483
ENSGALG00010016141	RRP15	2	0.328936847	0.342531247	0.960311941	0.336898242	0.185326699	4.729195371	78.85475389	0.5	CRA004775;Yang2025_Silkie_Wenchang	PRJNA827465	0.803904536
ENSGALG00010016141	RRP15	2	-0.008550519	0.065622372	-0.130298833	0.896330003	0	0.006510436	0	1	CRA004775;PRJNA827465	Yang2025_Silkie_Wenchang	0.975115773
ENSGALG00010016147	FABP1	2	0.001238168	0.225370891	0.005493912	0.995616515	0	0.305026483	0	0.5	PRJNA827465;Yang2025_Silkie_Wenchang	CRA004775	0.998484999
ENSGALG00010016147	FABP1	2	0.859500241	0.766743895	1.120974353	0.26229878	0.954277385	5.146208042	80.5682166	1	CRA004775;Yang2025_Silkie_Wenchang	PRJNA827465	0.768366452
ENSGALG00010016147	FABP1	2	0.72885827	0.894276781	0.815025377	0.415057784	1.401427162	7.752047611	87.10018243	0.5	CRA004775;PRJNA827465	Yang2025_Silkie_Wenchang	0.837489728
ENSGALG00010016150	POLG	2	0.280685509	0.313415719	0.895569342	0.370482816	0.18851922	24.72560642	95.95560981	0.5	PRJNA827465;Yang2025_Silkie_Wenchang	CRA004775	0.821221934
ENSGALG00010016150	POLG	2	-0.044159991	0.648455399	-0.068100274	0.945705812	0.821079835	41.94568235	97.6159644	0.5	CRA004775;Yang2025_Silkie_Wenchang	PRJNA827465	0.986321197
ENSGALG00010016150	POLG	2	-0.347998446	0.33460289	-1.040034192	0.298324016	0.20562219	11.85151003	91.56225665	1	CRA004775;PRJNA827465	Yang2025_Silkie_Wenchang	0.789245341
ENSGALG00010016159	ASPG	2	0.118215871	0.117497848	1.00611095	0.314362202	0	0.988071103	0	0.5	PRJNA827465;Yang2025_Silkie_Wenchang	CRA004775	0.796531448
ENSGALG00010016159	ASPG	2	-0.093112114	0.180958062	-0.514550791	0.60686696	0	0.017431809	0	1	CRA004775;Yang2025_Silkie_Wenchang	PRJNA827465	0.896799223
ENSGALG00010016159	ASPG	2	0.134371371	0.124804248	1.076657026	0.281633518	0	0.905304359	0	0.5	CRA004775;PRJNA827465	Yang2025_Silkie_Wenchang	0.779295548
ENSGALG00010016168	PLIN2	2	-0.012460317	0.132095523	-0.094328077	0.924848547	0	0.000645766	0	1	PRJNA827465;Yang2025_Silkie_Wenchang	CRA004775	0.982493782
ENSGALG00010016168	PLIN2	2	-0.038882633	0.178410261	-0.217939446	0.827476292	0	0.096874903	0	1	CRA004775;Yang2025_Silkie_Wenchang	PRJNA827465	0.956550752
ENSGALG00010016168	PLIN2	2	-0.030696684	0.169053656	-0.181579533	0.855912712	0	0.107131201	0	1	CRA004775;PRJNA827465	Yang2025_Silkie_Wenchang	0.963486168
ENSGALG00010016169	RHOH	2	-0.031519235	0.084056584	-0.374976402	0.707678017	0	0.545794433	0	0.5	PRJNA827465;Yang2025_Silkie_Wenchang	CRA004775	0.925061251
ENSGALG00010016169	RHOH	2	-0.070224043	0.111619307	-0.629138852	0.529258157	0	0.274410719	0	0.5	CRA004775;Yang2025_Silkie_Wenchang	PRJNA827465	0.871885042
ENSGALG00010016169	RHOH	2	0.02704456	0.102014692	0.265104561	0.790928914	0	0.004065929	0	1	CRA004775;PRJNA827465	Yang2025_Silkie_Wenchang	0.947798009
ENSGALG00010016181	FANCI	2	-0.08075707	0.077439669	-1.042838521	0.297023087	0	0.375215285	0	1	PRJNA827465;Yang2025_Silkie_Wenchang	CRA004775	0.788466926
ENSGALG00010016181	FANCI	2	-0.134569977	0.117943087	-1.140973844	0.253880808	0	0.015164452	0	1	CRA004775;Yang2025_Silkie_Wenchang	PRJNA827465	0.76368264
ENSGALG00010016181	FANCI	2	-0.043142252	0.098244512	-0.439131422	0.660566313	0	0.005380854	0	1	CRA004775;PRJNA827465	Yang2025_Silkie_Wenchang	0.91192683
ENSGALG00010016182	ELMO2	2	0.078544648	0.03819705	2.056301424	0.039753471	0	0.246123586	0	1	PRJNA827465;Yang2025_Silkie_Wenchang	CRA004775	0.344755224
ENSGALG00010016182	ELMO2	2	0.015417454	0.071150946	0.216686561	0.828452622	0	0.390607276	0	0.5	CRA004775;Yang2025_Silkie_Wenchang	PRJNA827465	0.956777964
ENSGALG00010016182	ELMO2	2	0.073635206	0.044202629	1.665855794	0.09574214	0.000319265	1.030337428	2.94441674	0.5	CRA004775;PRJNA827465	Yang2025_Silkie_Wenchang	0.529918791
ENSGALG00010016190	THNSL2	2	0.205660664	0.190623277	1.078885364	0.280638834	0.052458281	3.53493411	71.71092957	0.5	PRJNA827465;Yang2025_Silkie_Wenchang	CRA004775	0.778536485
ENSGALG00010016190	THNSL2	2	-0.107864861	0.187444161	-0.575450631	0.56498657	0.019938185	1.265846016	21.00144982	1	CRA004775;Yang2025_Silkie_Wenchang	PRJNA827465	0.883420877
ENSGALG00010016190	THNSL2	2	0.038696492	0.403703014	0.09585386	0.92363664	0.265675542	4.845576382	79.36262023	0.5	CRA004775;PRJNA827465	Yang2025_Silkie_Wenchang	0.982186708
ENSGALG00010016193	TCAIM	2	-0.104242304	0.070186283	-1.485223327	0.137484675	0	0.852934189	0	1	PRJNA827465;Yang2025_Silkie_Wenchang	CRA004775	0.620332372
ENSGALG00010016193	TCAIM	2	-0.030149849	0.224328226	-0.134400601	0.893085806	0.064788495	2.599784405	61.535272	0.5	CRA004775;Yang2025_Silkie_Wenchang	PRJNA827465	0.974381724
ENSGALG00010016193	TCAIM	2	0.001811409	0.128148817	0.0141352	0.988722117	0.014163441	1.408580771	29.00655607	0.5	CRA004775;PRJNA827465	Yang2025_Silkie_Wenchang	0.996891127
ENSGALG00010016197	SNRPN	2	0.186866493	0.068186998	2.740500361	0.006134571	0	0.447150658	0	1	PRJNA827465;Yang2025_Silkie_Wenchang	CRA004775	0.118495743
ENSGALG00010016197	SNRPN	2	-0.061501682	0.394019585	-0.15608788	0.875963752	0.257854482	5.89477324	83.03581903	0.5	CRA004775;Yang2025_Silkie_Wenchang	PRJNA827465	0.970347777
ENSGALG00010016197	SNRPN	2	-0.103546873	0.313287124	-0.330517488	0.741008981	0.169052772	6.549857037	84.73249119	0.5	CRA004775;PRJNA827465	Yang2025_Silkie_Wenchang	0.935376284
ENSGALG00010016200	MICU3	2	-0.109423982	0.11731037	-0.932773307	0.350937026	0.020959992	4.067846879	75.41697046	1	PRJNA827465;Yang2025_Silkie_Wenchang	CRA004775	0.810146221
ENSGALG00010016200	MICU3	2	-0.00942803	0.279745635	-0.030137477	0.975965387	0.121808461	3.995990632	74.97491631	0.5	CRA004775;Yang2025_Silkie_Wenchang	PRJNA827465	0.992580007
ENSGALG00010016200	MICU3	2	0.062806424	0.133015909	0.472172276	0.636803827	0.017941821	1.473093692	32.11565526	0.5	CRA004775;PRJNA827465	Yang2025_Silkie_Wenchang	0.904812056
ENSGALG00010016206	KNG1	2	-0.635885172	1.591570314	-0.399533195	0.68950037	4.053888014	4.985063575	79.94007529	0.5	PRJNA827465;Yang2025_Silkie_Wenchang	CRA004775	0.919965816
ENSGALG00010016206	KNG1	2	-0.228252694	1.87942298	-0.121448283	0.903335976	6.476747877	11.8423072	91.55569955	0.5	CRA004775;Yang2025_Silkie_Wenchang	PRJNA827465	0.977253688
ENSGALG00010016206	KNG1	2	1.4548037	0.511293587	2.845339529	0.004436415	0	0.219953064	0	1	CRA004775;PRJNA827465	Yang2025_Silkie_Wenchang	0.097602941
ENSGALG00010016209	ADAMTS7	2	-0.061856061	0.165727133	-0.373240399	0.708969531	0.040806084	3.888567859	74.28359138	0.5	PRJNA827465;Yang2025_Silkie_Wenchang	CRA004775	0.925298042
ENSGALG00010016209	ADAMTS7	2	-0.39659529	0.320841163	-1.236110998	0.216417307	0.11672981	1.621051986	38.31166374	1	CRA004775;Yang2025_Silkie_Wenchang	PRJNA827465	0.730172415
ENSGALG00010016209	ADAMTS7	2	-0.296825025	0.533618456	-0.556249547	0.578040272	0.430727911	3.295570472	69.65623984	0.5	CRA004775;PRJNA827465	Yang2025_Silkie_Wenchang	0.886907604
ENSGALG00010016211	TACC3	2	-0.112688265	0.079519791	-1.417109666	0.156450867	0.006645429	2.09517471	52.27128339	1	PRJNA827465;Yang2025_Silkie_Wenchang	CRA004775	0.649484056
ENSGALG00010016211	TACC3	2	-0.155249479	0.138172455	-1.123592101	0.261186109	0.013947381	1.132928511	11.73317732	0.5	CRA004775;Yang2025_Silkie_Wenchang	PRJNA827465	0.768023165
ENSGALG00010016211	TACC3	2	-0.030606993	0.070856684	-0.431953633	0.665731153	0	0.517623873	0	0.5	CRA004775;PRJNA827465	Yang2025_Silkie_Wenchang	0.913532078
ENSGALG00010016218	N4BP2	2	0.14265194	0.189469253	0.752731102	0.451611516	0.053328331	3.471448537	71.19354813	0.5	PRJNA827465;Yang2025_Silkie_Wenchang	CRA004775	0.848006549
ENSGALG00010016218	N4BP2	2	0.33985374	0.175775655	1.933451711	0.053180576	0	0.21476657	0	1	CRA004775;Yang2025_Silkie_Wenchang	PRJNA827465	0.399849003
ENSGALG00010016218	N4BP2	2	-0.006196409	0.078203464	-0.079234462	0.936846134	0	0.138520589	0	0.5	CRA004775;PRJNA827465	Yang2025_Silkie_Wenchang	0.985038972
ENSGALG00010016221	TMEM200B	2	-0.061106751	0.10071893	-0.606705724	0.544046223	0	0.001821688	0	1	PRJNA827465;Yang2025_Silkie_Wenchang	CRA004775	0.87969872
ENSGALG00010016221	TMEM200B	2	-0.067460344	0.183708567	-0.367213939	0.713459742	0	0.020904288	0	1	CRA004775;Yang2025_Silkie_Wenchang	PRJNA827465	0.926253108
ENSGALG00010016221	TMEM200B	2	-0.068149651	0.112608811	-0.605189324	0.545053208	0	0.024439048	0	1	CRA004775;PRJNA827465	Yang2025_Silkie_Wenchang	0.877354247
ENSGALG00010016224	MOSPD1	2	-0.071691705	0.060236488	-1.190170733	0.233979294	0	0.823780015	0	1	PRJNA827465;Yang2025_Silkie_Wenchang	CRA004775	0.747279664
ENSGALG00010016224	MOSPD1	2	-0.10933158	0.172145198	-0.635112577	0.525354995	0.013545618	1.198126783	16.5363788	0.5	CRA004775;Yang2025_Silkie_Wenchang	PRJNA827465	0.870196703
ENSGALG00010016224	MOSPD1	2	-0.040209956	0.063881635	-0.629444684	0.529057972	0	0.535034857	0	0.5	CRA004775;PRJNA827465	Yang2025_Silkie_Wenchang	0.871809618
ENSGALG00010016225	P4HA2	2	-0.249863976	0.132419226	-1.886916148	0.059171605	0.024579557	3.294850657	69.64961074	1	PRJNA827465;Yang2025_Silkie_Wenchang	CRA004775	0.421308258
ENSGALG00010016225	P4HA2	2	-0.097824406	0.333376312	-0.293435384	0.769189382	0.184886407	5.546629645	81.97103351	0.5	CRA004775;Yang2025_Silkie_Wenchang	PRJNA827465	0.941790377
ENSGALG00010016225	P4HA2	2	0.001671283	0.189101008	0.008838044	0.992948353	0.044531896	2.175780004	54.03947099	0.5	CRA004775;PRJNA827465	Yang2025_Silkie_Wenchang	0.99794341
ENSGALG00010016229	ENSGALG00010016229	2	0.874310474	0.236302275	3.699966387	0.000215628	0	0.078527578	0	1	PRJNA827465;Yang2025_Silkie_Wenchang	CRA004775	0.013388354
ENSGALG00010016229	ENSGALG00010016229	2	0.823516815	0.261468373	3.149584803	0.001635026	0	2.67867E-05	0	1	CRA004775;Yang2025_Silkie_Wenchang	PRJNA827465	0.051122869
ENSGALG00010016229	ENSGALG00010016229	2	0.915144035	0.32028219	2.85730541	0.004272545	0	0.042903548	0	1	CRA004775;PRJNA827465	Yang2025_Silkie_Wenchang	0.095574185
ENSGALG00010016235	DDX27	2	0.183104043	0.170519145	1.073803429	0.282910793	0.050602971	7.341598994	86.37898909	1	PRJNA827465;Yang2025_Silkie_Wenchang	CRA004775	0.779532565
ENSGALG00010016235	DDX27	2	0.248053642	0.146433032	1.693973267	0.090270363	0.023053084	2.07553626	51.81968057	1	CRA004775;Yang2025_Silkie_Wenchang	PRJNA827465	0.515967344
ENSGALG00010016235	DDX27	2	0.030577318	0.051949139	0.588601043	0.556128934	0	0.058970204	0	1	CRA004775;PRJNA827465	Yang2025_Silkie_Wenchang	0.880324407
ENSGALG00010016236	GPATCH2	2	-0.134866378	0.169552935	-0.795423438	0.42636723	0.051926952	9.897844417	89.89679007	0.5	PRJNA827465;Yang2025_Silkie_Wenchang	CRA004775	0.84125685
ENSGALG00010016236	GPATCH2	2	-0.183058154	0.203755628	-0.898420112	0.368961618	0.049525112	2.002964553	50.07400413	0.5	CRA004775;Yang2025_Silkie_Wenchang	PRJNA827465	0.820553374
ENSGALG00010016236	GPATCH2	2	0.02574277	0.043272026	0.594905584	0.551906584	0	0.120960758	0	1	CRA004775;PRJNA827465	Yang2025_Silkie_Wenchang	0.879061525
ENSGALG00010016240	EIF4A2	2	-0.180701249	0.13452811	-1.343222979	0.179199783	0.027475743	3.807833905	73.73835033	1	PRJNA827465;Yang2025_Silkie_Wenchang	CRA004775	0.685873949
ENSGALG00010016240	EIF4A2	2	-0.113198973	0.234375855	-0.482980522	0.629109566	0.090409915	5.621203483	82.21021525	0.5	CRA004775;Yang2025_Silkie_Wenchang	PRJNA827465	0.902174382
ENSGALG00010016240	EIF4A2	2	-0.019181537	0.084348409	-0.227408398	0.82010619	0.005944527	1.448196972	30.94861959	0.5	CRA004775;PRJNA827465	Yang2025_Silkie_Wenchang	0.955308792
ENSGALG00010016242	PDS5A	2	-0.092875882	0.041612414	-2.231927252	0.025619771	0	0.058081221	0	1	PRJNA827465;Yang2025_Silkie_Wenchang	CRA004775	0.27294819
ENSGALG00010016242	PDS5A	2	0.082638174	0.246820274	0.334811126	0.737767567	0.088273185	3.280531017	69.51713016	0.5	CRA004775;Yang2025_Silkie_Wenchang	PRJNA827465	0.934134811
ENSGALG00010016242	PDS5A	2	0.082529362	0.22787421	0.362170699	0.717224474	0.079345473	3.395377873	70.54819707	0.5	CRA004775;PRJNA827465	Yang2025_Silkie_Wenchang	0.9277944
ENSGALG00010016245	FAM96A	2	0.030507931	0.0801819	0.380484011	0.703586163	0	0.4386571	0	0.5	PRJNA827465;Yang2025_Silkie_Wenchang	CRA004775	0.923639641
ENSGALG00010016245	FAM96A	2	-0.154861448	0.175051378	-0.884662831	0.376338508	0	0.130458247	0	1	CRA004775;Yang2025_Silkie_Wenchang	PRJNA827465	0.822974013
ENSGALG00010016245	FAM96A	2	0.027330472	0.082395448	0.331698808	0.740116701	0	0.926959713	0	0.5	CRA004775;PRJNA827465	Yang2025_Silkie_Wenchang	0.93508234
ENSGALG00010016247	KIF26A	2	-0.045135753	0.130026129	-0.347128331	0.728494911	0.022398766	2.898463627	65.49896328	0.5	PRJNA827465;Yang2025_Silkie_Wenchang	CRA004775	0.932184127
ENSGALG00010016247	KIF26A	2	-0.163822254	0.119914639	-1.366157255	0.171889611	0	0.645882469	0	0.5	CRA004775;Yang2025_Silkie_Wenchang	PRJNA827465	0.674565888
ENSGALG00010016247	KIF26A	2	0.076310349	0.088739067	0.859940855	0.389821646	0	0.068001857	0	1	CRA004775;PRJNA827465	Yang2025_Silkie_Wenchang	0.829111974
ENSGALG00010016251	MPDZ	2	0.12991819	0.250392721	0.518857694	0.603859982	0.112252437	9.437637204	89.40412756	0.5	PRJNA827465;Yang2025_Silkie_Wenchang	CRA004775	0.895892991
ENSGALG00010016251	MPDZ	2	0.435176918	0.118054614	3.686233885	0.000227597	0	0.53572877	0	1	CRA004775;Yang2025_Silkie_Wenchang	PRJNA827465	0.013933415
ENSGALG00010016251	MPDZ	2	0.204776093	0.353012411	0.580081852	0.561859422	0.215937165	6.948377077	85.60815009	0.5	CRA004775;PRJNA827465	Yang2025_Silkie_Wenchang	0.881802301
ENSGALG00010016253	EDN3	2	1.881749937	0.207400428	9.073028233	1.15754E-19	0.068507635	4.908903943	79.62885378	1	PRJNA827465;Yang2025_Silkie_Wenchang	CRA004775	1.46758E-16
ENSGALG00010016253	EDN3	2	2.106155241	0.120664777	17.45459855	3.17603E-68	0	0.141130337	0	1	CRA004775;Yang2025_Silkie_Wenchang	PRJNA827465	1.24827E-64
ENSGALG00010016253	EDN3	2	1.867454003	0.261090454	7.152517338	8.52008E-13	0.08789575	2.450177289	59.18662684	1	CRA004775;PRJNA827465	Yang2025_Silkie_Wenchang	7.97297E-10
ENSGALG00010016257	ENSGALG00010016257	2	0.098128494	0.062298782	1.575126993	0.115227134	0	0.028122999	0	1	PRJNA827465;Yang2025_Silkie_Wenchang	CRA004775	0.572203827
ENSGALG00010016257	ENSGALG00010016257	2	-0.125197624	0.296852437	-0.421750366	0.673207238	0.11428345	2.309741159	56.70510541	0.5	CRA004775;Yang2025_Silkie_Wenchang	PRJNA827465	0.915210237
ENSGALG00010016257	ENSGALG00010016257	2	-0.109067503	0.308335423	-0.353730044	0.723541205	0.135361961	2.652782033	62.30372539	0.5	CRA004775;PRJNA827465	Yang2025_Silkie_Wenchang	0.930032096
ENSGALG00010016258	CTNNB1	2	-0.192585346	0.131185389	-1.468039595	0.142093468	0.028558781	5.838924845	82.87355932	1	PRJNA827465;Yang2025_Silkie_Wenchang	CRA004775	0.627847057

ENSGALG00010016258	CTNNB1	2	-0.302558433	0.079639174	-3.79911567	0.000145213	0	0.885008956	0	1	CRA004775;Yang2025_Silkie_Wenchang	PRJNA827465	0.010211774
ENSGALG00010016258	CTNNB1	2	-0.067299426	0.066048674	-1.018936823	0.308232961	0	0.005905102	0	1	CRA004775;PRJNA827465	Yang2025_Silkie_Wenchang	0.793403631
ENSGALG00010016260	INTS6L	2	0.036770602	0.050985509	0.721197107	0.470788251	0	0.62747332	0	0.5	PRJNA827465;Yang2025_Silkie_Wenchang	CRA004775	0.853762555
ENSGALG00010016260	INTS6L	2	0.120086851	0.226453828	0.530292873	0.595908888	0.062426314	1.865477092	46.39441007	0.5	CRA004775;Yang2025_Silkie_Wenchang	PRJNA827465	0.893857407
ENSGALG00010016260	INTS6L	2	0.13490156	0.155580689	0.86708422	0.385895866	0.024137086	1.339804206	25.36222862	1	CRA004775;PRJNA827465	Yang2025_Silkie_Wenchang	0.826575976
ENSGALG00010016262	SPG11	2	-0.048709839	0.069103163	-0.704885819	0.480881296	0.006025057	2.47280353	59.56007066	0.5	PRJNA827465;Yang2025_Silkie_Wenchang	CRA004775	0.856722567
ENSGALG00010016262	SPG11	2	-0.125934665	0.07449712	-1.690463541	0.090939308	0	0.122954266	0	1	CRA004775;Yang2025_Silkie_Wenchang	PRJNA827465	0.517431489
ENSGALG00010016262	SPG11	2	0.00273414	0.040342754	0.067772774	0.945966517	0	0.114175626	0	0.5	CRA004775;PRJNA827465	Yang2025_Silkie_Wenchang	0.986321197
ENSGALG00010016266	PDLIM4	2	-0.082657831	0.113121557	-0.730699198	0.464962905	0.019211091	3.880928846	74.23297257	0.5	PRJNA827465;Yang2025_Silkie_Wenchang	CRA004775	0.852233226
ENSGALG00010016266	PDLIM4	2	-0.18645046	0.093161058	-2.00137765	0.045351707	0	0.675126077	0	0.5	CRA004775;Yang2025_Silkie_Wenchang	PRJNA827465	0.367138652
ENSGALG00010016266	PDLIM4	2	0.021180654	0.061757487	0.342964963	0.731624819	0	0.032081128	0	1	CRA004775;PRJNA827465	Yang2025_Silkie_Wenchang	0.932851968
ENSGALG00010016270	RFC4	2	-0.081197113	0.0533711	-1.521368545	0.128167378	0	0.000945385	0	1	PRJNA827465;Yang2025_Silkie_Wenchang	CRA004775	0.600696128
ENSGALG00010016270	RFC4	2	-0.166174568	0.133076136	-1.248718015	0.211768229	0	0.8553548	0	1	CRA004775;Yang2025_Silkie_Wenchang	PRJNA827465	0.724064961
ENSGALG00010016270	RFC4	2	-0.115401926	0.091230574	-1.264947925	0.205889996	0.006322866	1.20250979	16.84059385	1	CRA004775;PRJNA827465	Yang2025_Silkie_Wenchang	0.718403277
ENSGALG00010016274	ZNF831	2	1.80097298	0.570743598	3.155485207	0.001602314	0.450837961	3.190788615	68.65978538	1	PRJNA827465;Yang2025_Silkie_Wenchang	CRA004775	0.050669962
ENSGALG00010016274	ZNF831	2	1.21521135	1.250314987	0.971924166	0.331088277	2.762089879	8.506716094	88.24458241	0.5	CRA004775;Yang2025_Silkie_Wenchang	PRJNA827465	0.801722786
ENSGALG00010016274	ZNF831	2	0.720192719	0.667392421	1.079114322	0.280536768	0.609512337	2.966716514	66.2927012	0.5	CRA004775;PRJNA827465	Yang2025_Silkie_Wenchang	0.778536485
ENSGALG00010016276	ACTR2	2	-0.254331253	0.329582214	-0.771677725	0.440305327	0.209240395	26.78223868	96.26618218	0.5	PRJNA827465;Yang2025_Silkie_Wenchang	CRA004775	0.844615172
ENSGALG00010016276	ACTR2	2	-0.547731012	0.110230296	-4.968969782	6.73096E-07	0.001107554	1.021779695	2.131545132	1	CRA004775;Yang2025_Silkie_Wenchang	PRJNA827465	0.000161309
ENSGALG00010016276	ACTR2	2	0.026361392	0.110903404	0.237696869	0.812116212	0.010745715	1.233473403	18.92812626	0.5	CRA004775;PRJNA827465	Yang2025_Silkie_Wenchang	0.953371387
ENSGALG00010016279	PRELID3B	2	1.284702683	0.065171805	19.71255325	1.68259E-86	0.003092288	1.545285752	35.28704974	1	PRJNA827465;Yang2025_Silkie_Wenchang	CRA004775	7.34788E-83
ENSGALG00010016279	PRELID3B	2	1.208280706	0.078679132	15.35706699	3.17622E-53	0	0.001819515	0	1	CRA004775;Yang2025_Silkie_Wenchang	PRJNA827465	8.9168E-50
ENSGALG00010016279	PRELID3B	2	1.328753351	0.061491382	21.60877349	1.4854E-103	0	0.313190089	0	1	CRA004775;PRJNA827465	Yang2025_Silkie_Wenchang	8.34021E-100
ENSGALG00010016281	SLC22A4	2	-0.287475561	0.279892405	-1.027093111	0.304376622	0.125236661	4.982961152	79.93161156	1	PRJNA827465;Yang2025_Silkie_Wenchang	CRA004775	0.79225794
ENSGALG00010016281	SLC22A4	2	-0.603177916	0.172283266	-3.501082432	0.000463372	0	0.641993622	0	1	CRA004775;Yang2025_Silkie_Wenchang	PRJNA827465	0.022279651
ENSGALG00010016281	SLC22A4	2	-0.376364006	0.53062272	-0.709287393	0.47814615	0.386054463	2.511622472	60.18509903	1	CRA004775;PRJNA827465	Yang2025_Silkie_Wenchang	0.855366076
ENSGALG00010016286	ULK4	2	-0.092457154	0.095502108	-0.968116374	0.332986253	0.003615226	1.237277318	19.17737553	1	PRJNA827465;Yang2025_Silkie_Wenchang	CRA004775	0.802178578
ENSGALG00010016286	ULK4	2	-0.227054755	0.132665944	-1.711477322	0.086993034	0	0.390934722	0	1	CRA004775;Yang2025_Silkie_Wenchang	PRJNA827465	0.507433544
ENSGALG00010016286	ULK4	2	-0.032795571	0.106867216	-0.306881495	0.758933575	0	0.939336186	0	1	CRA004775;PRJNA827465	Yang2025_Silkie_Wenchang	0.939031852
ENSGALG00010016287	BCL11B	2	0.547965387	0.62964034	0.870283164	0.384145677	0.547606243	3.163275787	68.38720152	0.5	PRJNA827465;Yang2025_Silkie_Wenchang	CRA004775	0.825767461
ENSGALG00010016287	BCL11B	2	0.414904173	0.831166028	0.499183267	0.617650282	1.04476271	4.100867737	75.61491704	0.5	CRA004775;Yang2025_Silkie_Wenchang	PRJNA827465	0.899121942
ENSGALG00010016287	BCL11B	2	-0.156027264	0.33940726	-0.459705146	0.645727876	0	0.305623043	0	1	CRA004775;PRJNA827465	Yang2025_Silkie_Wenchang	0.907074688
ENSGALG00010016289	IRF1	2	-0.261749314	0.12434894	-2.104958149	0.035294951	0	0.954261796	0	1	PRJNA827465;Yang2025_Silkie_Wenchang	CRA004775	0.324168275
ENSGALG00010016289	IRF1	2	-0.486313131	0.180034993	-2.701214492	0.006908677	0	0.830773636	0	1	CRA004775;Yang2025_Silkie_Wenchang	PRJNA827465	0.127699223
ENSGALG00010016289	IRF1	2	-0.385732903	0.320567907	-1.203279851	0.228868044	0.126581475	2.174623421	54.01502668	1	CRA004775;PRJNA827465	Yang2025_Silkie_Wenchang	0.741566423
ENSGALG00010016292	PHKA1	2	0.069358883	0.075625743	0.917133251	0.359072819	0	0.095679464	0	1	PRJNA827465;Yang2025_Silkie_Wenchang	CRA004775	0.814241185
ENSGALG00010016292	PHKA1	2	0.097545521	0.111418848	0.87548491	0.381310117	0	0.006489419	0	1	CRA004775;Yang2025_Silkie_Wenchang	PRJNA827465	0.824392515
ENSGALG00010016292	PHKA1	2	0.051625707	0.099491277	0.518896816	0.603832699	0	0.027516233	0	1	CRA004775;PRJNA827465	Yang2025_Silkie_Wenchang	0.895892991
ENSGALG00010016295	UBE2K	2	-0.115243088	0.10299594	-1.118909043	0.263178945	0.016878527	4.550715698	78.02543454	1	PRJNA827465;Yang2025_Silkie_Wenchang	CRA004775	0.768643821
ENSGALG00010016295	UBE2K	2	-0.092719235	0.217305285	-0.426677313	0.669614377	0.059333742	2.099853507	52.37763032	0.5	CRA004775;Yang2025_Silkie_Wenchang	PRJNA827465	0.914377769
ENSGALG00010016295	UBE2K	2	-0.02104628	0.04152586	-0.506823467	0.612278689	0	0.704792628	0	0.5	CRA004775;PRJNA827465	Yang2025_Silkie_Wenchang	0.897895209
ENSGALG00010016299	SRSF4	2	0.287718167	0.375665145	0.765889971	0.443741774	0.267082326	1.629478604	94.52488975	0.5	PRJNA827465;Yang2025_Silkie_Wenchang	CRA004775	0.845840387
ENSGALG00010016299	SRSF4	2	0.445129436	0.273723699	1.626199644	0.103907149	0.10344954	3.036156001	67.06361598	1	CRA004775;Yang2025_Silkie_Wenchang	PRJNA827465	0.54817589
ENSGALG00010016299	SRSF4	2	-0.061163534	0.075775711	-0.807165427	0.419571182	0	0.463777748	0	0.5	CRA004775;PRJNA827465	Yang2025_Silkie_Wenchang	0.838527387
ENSGALG00010016303	ENSGALG00010016303	2	0.565634199	0.493915162	1.145205175	0.252124175	0.450388304	12.40640142	91.93964498	1	PRJNA827465;Yang2025_Silkie_Wenchang	CRA004775	0.762248958
ENSGALG00010016303	ENSGALG00010016303	2	0.691229077	0.655184116	1.055015011	0.29141846	0.783675525	11.46592227	91.27850358	1	CRA004775;Yang2025_Silkie_Wenchang	PRJNA827465	0.784817029
ENSGALG00010016303	ENSGALG00010016303	2	1.079036311	0.116188876	9.286918141	1.5882E-20	0.007562847	1.172999114	14.71956249	1	CRA004775;PRJNA827465	Yang2025_Silkie_Wenchang	2.08075E-17
ENSGALG00010016304	EIF3J	2	0.200292888	0.284485711	0.704052545	0.481400052	0.15448348	21.63161973	95.37713767	0.5	PRJNA827465;Yang2025_Silkie_Wenchang	CRA004775	0.85686777
ENSGALG00010016304	EIF3J	2	0.480503033	0.1025026	4.687715551	2.76272E-06	0	0.119406545	0	1	CRA004775;Yang2025_Silkie_Wenchang	PRJNA827465	0.000524556
ENSGALG00010016304	EIF3J	2	0.032788036	0.193272966	0.169646259	0.86528834	0.046076481	1.834551268	45.49075747	0.5	CRA004775;PRJNA827465	Yang2025_Silkie_Wenchang	0.967024637
ENSGALG00010016305	DENND4C	2	0.228393588	0.280759166	0.813485774	0.415939599	0.13123058	5.710128548	82.48725941	0.5	PRJNA827465;Yang2025_Silkie_Wenchang	CRA004775	0.83774261
ENSGALG00010016305	DENND4C	2	0.445676391	0.181488866	2.455667949	0.014062303	0	0.833799649	0	1	CRA004775;Yang2025_Silkie_Wenchang	PRJNA827465	0.194199114
ENSGALG00010016305	DENND4C	2	-0.012497393	0.108019534	-0.115695675	0.907893734	0	0.19418098	0	0.5	CRA004775;PRJNA827465	Yang2025_Silkie_Wenchang	0.978196186
ENSGALG00010016307	RAD50	2	0.289030767	0.305484418	0.946139149	0.344077618	0.170391383	11.04941751	90.94974917	1	PRJNA827465;Yang2025_Silkie_Wenchang	CRA004775	0.806845383
ENSGALG00010016307	RAD50	2	0.279823534	0.380545376	0.735322386	0.462143178	0.227951959	4.421433816	77.38290243	0.5	CRA004775;Yang2025_Silkie_Wenchang	PRJNA827465	0.850747865
ENSGALG00010016307	RAD50	2	-0.00594977	0.072042403	-0.082587052	0.934179897	0	0.219136548	0	0.5	CRA004775;PRJNA827465	Yang2025_Silkie_Wenchang	0.98432801
ENSGALG00010016308	RPS6	2	0.050645936	0.142928295	0.354345063	0.7230803	0	0.154423679	0	1	PRJNA827465;Yang2025_Silkie_Wenchang	CRA004775	0.930032096
ENSGALG00010016308	RPS6	2	-0.178813942	0.325598894	-0.552578964	0.580055175	0.140348986	3.010280017	66.78049901	0.5	CRA004775;Yang2025_Silkie_Wenchang	PRJNA827465	0.880581974
ENSGALG00010016308	RPS6	2	-0.209046979	0.261212451	-0.800294851	0.423539986	0.084020904	2.474920007	59.59465367	0.5	CRA004775;PRJNA827465	Yang2025_Silkie_Wenchang	0.84030043
ENSGALG00010016310	HDAC8	2	-0.002074605	0.058911979	-0.035215336	0.971908034	0	0.965307394	0	0.5	PRJNA827465;Yang2025_Silkie_Wenchang	CRA004775	0.991955348
ENSGALG00010016310	HDAC8	2	-0.116565449	0.123264778	-0.945650905	0.344326671	0	0.019690106	0	1	CRA004775;Yang2025_Silkie_Wenchang	PRJNA827465	0.806692367
ENSGALG00010016310	HDAC8	2	0.019065881	0.063236771	0.30149991	0.763033318	0	0.153342697	0	0.5	CRA004775;PRJNA827465	Yang2025_Silkie_Wenchang	0.940139746
ENSGALG00010016312	MECR	2	0.09198354	0.080448862	1.143379016	0.252881261	0	0.637968123	0	0.5	PRJNA827465;Yang2025_Silkie_Wenchang	CRA004775	0.762731469
ENSGALG00010016312	MECR	2	-0.227896146	0.247094472	-0.922303702	0.356310184	0.053431883	1.636412081	39.14024942	1	CRA004775;Yang2025_Silkie_Wenchang	PRJNA827465	0.812521698
ENSGALG00010016312	MECR	2	-0.140519021	0.336547451	-0.417531082	0.676289994	0.173761206	3.570557904	71.9931723	0.5	CRA004775;PRJNA827465	Yang2025_Silkie_Wenchang	0.91671756
ENSGALG00010016314	ACER2	2	0.176562286	0.069135958	2.553841596	0.010654171	0	0.013496807	0	1	PRJNA827465;Yang2025_Silkie_Wenchang	CRA004775	0.16459941
ENSGALG00010016314	ACER2	2	0.184360408	0.123895869	1.488027078	0.136743753	0	0.359636641	0	1	CRA004775;Yang2025_Silkie_Wenchang	PRJNA827465	0.618582531
ENSGALG00010016314	ACER2	2	0.189829928	0.080796665	2.349477262	0.018799792	0	0.330953694	0	1	CRA004775;PRJNA827465	Yang2025_Silkie_Wenchang	0.229831303
ENSGALG00010016315	RPS4Y1	2	-0.294602859	0.311584919	-0.944855934	0.344723431	0.165048991	5.909354514	83.07767798	1	PRJNA827465;Yang2025_Silkie_Wenchang	CRA004775	0.806945375
ENSGALG00010016315	RPS4Y1	2	-0.539642968	0.175671856	-3.071880618	0.002127148	0	0.42179976	0	1	CRA004775;Yang2025_Silkie_Wenchang	PRJNA827465	0.06045068
ENSGALG00010016315	RPS4Y1	2	-0.157673757	0.188449636	-0.836689103	0.402767346	0.048953559	2.535692824	60.56304649	1	CRA004775;PRJNA827465	Yang2025_Silkie_Wenchang	0.832937979
ENSGALG00010016317	CTDSPL2	2	-0.174473075	0.075712766	-2.304403613	0.021199773	0.007020637	2.181128161	54.15216686	1	PRJNA827465;Yang2025_Silkie_Wenchang	CRA004775	0.246265819
ENSGALG00010016317	CTDSPL2	2	0.149200017	0.452183545	0.329954547	0.741434307	0.380384894	13.91896605	92.81555831	0.5	CRA004775;Yang2025_Silkie_Wenchang	PRJNA827465	0.93543545
ENSGALG00010016317	CTDSPL2	2	0.219228982	0.370814524	0.591209266	0.554380215	0.251969036	11.06615665	90.96343896	0.5	CRA004775;PRJNA827465	Yang2025_Silkie_Wenchang	0.880177968
ENSGALG00010016319	RBM41	2	-0.022436359	0.089042172	-0.251974522	0.801060757	0	0.401548806	0	0.5	PRJNA827465;Yang2025_Silkie_Wenchang	CRA004775	0.950158029
ENSGALG00010016319	RBM41	2	-0.16235043	0.124185551	-1.307321406	0.191103577	0	0.920242893	0	1	CRA004775;Yang2025_Silkie_Wenchang	PRJNA827465	0.70443419
ENSGALG00010016319	RBM41	2	-0.110838611	0.185339072	-0.598031544	0.549818886	0.03916297	2.048766605	51.19014545	0.5	CRA004775;PRJNA827465	Yang2025_Silkie_Wenchang	0.878603751
ENSGALG00010016322	EBF4	2	0.157479007	0.17451262	0.902393232	0.366848015	0.050940311	5.973997682	83.26079029	0.5	PRJNA827465;Yang2025_Silkie_Wenchang	CRA004775	0.819577857
ENSGALG00010016322	EBF4	2	0.274228588	0.146192523	1.875804462	0.060682135	0.011924167	1.203021052	16.87593511	0.5	CRA004775;Yang2025_Silkie_Wenchang	PRJNA827465	0.426652944
ENSGALG00010016322	EBF4	2	-0.007226091	0.076239112	-0.094781942	0.924488031	0	0.006239482	0	1	CRA004775;PRJNA827465	Yang2025_Silkie_Wenchang	0.982361364
ENSGALG00010016324	TUBB1	2	0.17338	0.350048406	0.495302926	0.620386306	0.212264381	7.466619868	86.60705891	0.5	PRJNA827465;Yang2025_Silkie_Wenchang	CRA004775	0.899395593
ENSGALG00010016324	TUBB1	2	-0.09402073	0.153109014	-0.614077037	0.539164422	0	0.887912466	0	0.5	CRA004775;Yang2025_Silkie_Wenchang	PRJNA827465	0.874983893
ENSGALG00010016324	TUBB1	2	0.434097106	0.16136853	2.690097672	0.007143111	0	0.9459059	0	1	CRA004775;PRJNA827465	Yang2025_Silkie_Wenchang	0.130035057
ENSGALG00010016328	USH2A	2	-0.07703769	0.18090644	-0.425842716	0.670222459	0	0.650442223	0	0.5	PRJNA827465;Yang2025_Silkie_Wenchang	CRA004775	0.914482883
ENSGALG00010016328	USH2A	2	-0.152105016	0.250221722	-0.607880943	0.54326644	0	0.829625321	0	0.5	CRA004775;Yang2025_Silkie_Wenchang	PRJNA827465	0.876968972
ENSGALG00010016328	USH2A	2	0.100743729	0.234006389	0.430517	0.666819603	0	0.29368784	0	1	CRA004775;PRJNA827465	Yang2025_Silkie_Wenchang	0.913648
ENSGALG00010016332	ENSGALG00010016332	2	0.093827264	0.101713127	0.92246956	0.356283701	0	0.19143883	0	1	PRJNA827465;Yang2025_Silkie_Wenchang	CRA004775	0.812521698
ENSGALG00010016332	ENSGALG00010016332	2	0.111889108	0.146485584	0.763823341	0.444972525	0	0.416914036	0	0.5	CRA004775;Yang2025_Silkie_Wenchang	PRJNA827465	0.846013379
ENSGALG00010016332	ENSGALG00010016332	2	0.037193973	0.128751596	0.288881644	0.772671951	0	0.211459141	0	0.5	CRA004775;PRJNA827465	Yang2025_Silkie_Wenchang	0.943066211
ENSGALG00010016335	ABHD4	2	-0.354941069	0.285957707	-1.241236239	0.214518491	0.156294845	22.37374297	95.53047516	1	PRJNA827465;Yang2025_Silkie_Wenchang	CRA004775	0.727205473
ENSGALG00010016335	ABHD4	2	-0.194477952	0.507943239	-0.382873393	0.70181364	0.451878067	7.350134293	86.39480641	0.5	CRA004775;Yang2025_Silkie_Wenchang	PRJNA827465	0.922904951
ENSGALG00010016335	ABHD4	2	0.009157637	0.175300841	0.052239552	0.958337818	0.033656524	1.495442348	33.13015367	0.5	CRA004775;PRJNA827465	Yang2025_Silkie_Wenchang	0.989012479
ENSGALG00010016338	PTPRU	2	0.157046635	0.078085708	2.01120843	0.044303446	0	0.488558683	0	1	PRJNA827465;Yang2025_Silkie_Wenchang	CRA004775	0.363642137
ENSGALG00010016338	PTPRU	2	0.041307753	0.131981891	0.312980464	0.754295505	0	0.275101251	0	0.5	CRA004775;Yang2025_Silkie_Wenchang	PRJNA827465	0.93838723
ENSGALG00010016338	PTPRU	2	0.168972068	0.089241356	1.893427836	0.058300997	0	0.823215834	0	0.5	CRA004775;PRJNA827465	Yang2025_Silkie_Wenchang	0.418521291
ENSGALG00010016339	SETD3	2	-0.050740845	0.057388608	-0.884162332	0.376608589	0	0.018063564	0	1	PRJNA827465;Yang2025_Silkie_Wenchang	CRA004775	0.822974013
ENSGALG00010016339	SETD3	2	-0.058912322	0.101831387	-0.578528132	0.56290761	0	0.014409552	0	1	CRA004775;Yang2025_Silkie_Wenchang	PRJNA827465	0.882100307
ENSGALG00010016339	SETD3	2	-0.044843055	0.066563842	-0.673684895	0.500511667	0	0.005308373	0	1	CRA004775;PRJNA827465	Yang2025_Silkie_Wenchang	0.862400704
ENSGALG00010016340	RGS12	2	0.207406157	0.080227649	2.585220409	0.009731676	0	0.073512554	0	1	PRJNA827465;Yang2025_Silkie_Wenchang	CRA004775	0.155544563
ENSGALG00010016340	RGS12	2	0.504943941	0.28153373	1.793546872	0.072885523	0.103619184	2.810474997	64.41882596	1	CRA004775;Yang2025_Silkie_Wenchang	PRJNA827465	0.466295041
ENSGALG00010016340	RGS12	2	0.455056108	0.307665039	1.47906343	0.139123363	0.153205845	4.632787401	78.41472286	1	CRA004775;PRJNA827465	Yang2025_Silkie_Wenchang	0.622860674
ENSGALG00010016341	SLX4	2	0.086957547	0.043028944	2.020908255	0.043289263	0	0.202725852	0	1	PRJNA827465;Yang2025_Silkie_Wenchang	CRA004775	0.359703576
ENSGALG00010016341	SLX4	2	0.115630064	0.083083075	1.391740315	0.164001049	0	0.048498312	0	1	CRA004775;Yang2025_Silkie_Wenchang	PRJNA827465	0.662664053
ENSGALG00010016341	SLX4	2	0.075070018	0.049154788	1.527216794	0.126707112	0	0.00814113	0	1	CRA004775;PRJNA827465	Yang2025_Silkie_Wenchang	0.597476858
ENSGALG00010016344	SMIM14	2	-0.141224273	0.326376245	-0.432703897	0.665229898	0.207481952	38.23116651	97.38433302	0.5	PRJNA827465;Yang2025_Silkie_Wenchang	CRA004775	0.913399315
ENSGALG00010016344	SMIM14	2	-0.102590305	0.376306562	-0.272624277	0.785142048	0.269466895	20.37645206	95.09237429	0.5	CRA004775;Yang2025_Silkie_Wenchang	PRJNA827465	0.946325593
ENSGALG00010016344	SMIM14	2	0.198907487	0.057790534	3.441869657	0.000577709	0	0.406358596	0	1	CRA004775;PRJNA827465	Yang2025_Silkie_Wenchang	0.025895023
ENSGALG00010016345	CTSZ	2	0.091102145	0.205090457	0.4442047	0.656894594	0.070384657	6.050337169	83.47199549	0.5	PRJNA827465;Yang2025_Silkie_Wenchang	CRA004775	0.910653178
ENSGALG00010016345	CTSZ	2	-0.005559633	0.174544563	-0.031852226	0.974589897	0.026517507	1.602993556	37.61671739	0.5	CRA004775;Yang2025_Silkie_Wenchang	PRJNA827465	0.992492038
ENSGALG00010016345	CTSZ	2	0.28215919	0.092960736	3.035251239	0.002403354	0	0.015507132	0	1	CRA004775;PRJNA827465	Yang2025_Silkie_Wenchang	0.065687774
ENSGALG00010016346	TRAP1	2	0.066197374	0.102313821	0.647003245	0.517629843	0.016534855	4.603250489	78.2762202	0.5	PRJNA827465;Yang2025_Silkie_Wenchang	CRA004775	0.867962259
ENSGALG00010016346	TRAP1	2	0.09140255	0.15652545	0.583946888	0.559256046	0.025533452	1.605052971	37.69676029	0.5	CRA004775;Yang2025_Silkie_Wenchang	PRJNA827465	0.881014886
ENSGALG00010016346	TRAP1	2	-0.032143035	0.050147685	-0.640967476	0.521543814	0	0.328422529	0	1	CRA004775;PRJNA827465	Yang2025_Silkie_Wenchang	0.869518515
ENSGALG00010016347	ANO10	2	-0.137015666	0.220862078	-0.620367552	0.53501583	0.092342184	18.68785869	94.64893214	0.5	PRJNA827465;Yang2025_Silkie_Wenchang	CRA004775	0.87393405
ENSGALG00010016347	ANO10	2	-0.184165707	0.212403204	-0.867057103	0.385910723	0.070182808	3.924232182	74.51730801	0.5	CRA004775;Yang2025_Silkie_Wenchang	PRJNA827465	0.826575976
ENSGALG00010016347	ANO10	2	0.084113424	0.071169127	1.181880784	0.237253008	0	0.001214962	0	1	CRA004775;PRJNA827465	Yang2025_Silkie_Wenchang	0.749366344
ENSGALG00010016350	CASC4	2	0.153184611	0.178113207	0.86004072	0.389766597	0.053470493	5.642387318	82.2770054	1	PRJNA827465;Yang2025_Silkie_Wenchang	CRA004775	0.829068422
ENSGALG00010016350	CASC4	2	0.264205665	0.105997212	2.492571843	0.012682167	0	0.914513934	0	1	CRA004775;Yang2025_Silkie_Wenchang	PRJNA827465	0.182648301
ENSGALG00010016350	CASC4	2	0.010208778	0.037721562	0.27063509	0.786671705	0	0.992539037	0	1	CRA004775;PRJNA827465	Yang2025_Silkie_Wenchang	0.946722706
ENSGALG00010016353	C1orf232	2	0.113730216	0.149626954	0.760091768	0.447199733	0	0.757547467	0	0.5	PRJNA827465;Yang2025_Silkie_Wenchang	CRA004775	0.84650962
ENSGALG00010016353	C1orf232	2	-0.223680213	0.208462357	-1.073000498	0.283270893	0	0.12955005	0	1	CRA004775;Yang2025_Silkie_Wenchang	PRJNA827465	0.779568117
ENSGALG00010016353	C1orf232	2	-0.003535136	0.229352477	-0.015413553	0.987702251	0.058466652	2.133890379	53.13723658	0.5	CRA004775;PRJNA827465	Yang2025_Silkie_Wenchang	0.996756062
ENSGALG00010016355	MCF2L2	2	-0.073738678	0.133006692	-0.554398257	0.579306319	0.001609504	1.036903586	3.559018027	0.5	PRJNA827465;Yang2025_Silkie_Wenchang	CRA004775	0.887888423
ENSGALG00010016355	MCF2L2	2	0.133801584	0.239309444	0.559115354	0.576082997	0	0.02264106	0	1	CRA004775;Yang2025_Silkie_Wenchang	PRJNA827465	0.887008933
ENSGALG00010016355	MCF2L2	2	-0.145834605	0.14677512	-0.993592136	0.320421474	0	0.067806886	0	0.5	CRA004775;PRJNA827465	Yang2025_Silkie_Wenchang	0.798348216
ENSGALG00010016361	NELFCD	2	-0.199801304	0.209867844	-0.952033908	0.341079786	0.081080211	11.95359027	91.63431256	1	PRJNA827465;Yang2025_Silkie_Wenchang	CRA004775	0.805646109
ENSGALG00010016361	NELFCD	2	-0.237989238	0.208794916	-1.139822952	0.25436007	0.064953652	3.777133548	73.52489693	1	CRA004775;Yang2025_Silkie_Wenchang	PRJNA827465	0.764087626
ENSGALG00010016361	NELFCD	2	-0.003503735	0.037922257	-0.092392576	0.926386138	0	3.16794E-06	0	1	CRA004775;PRJNA827465	Yang2025_Silkie_Wenchang	0.98280842
ENSGALG00010016362	NUP62	2	0.149241673	0.275271704	0.542161331	0.587707371	0.138665528	11.29222644	91.14435045	0.5	PRJNA827465;Yang2025_Silkie_Wenchang	CRA004775	0.891742161
ENSGALG00010016362	NUP62	2	0.168162049	0.277314043	0.60639572	0.54425201	0.129065778	6.212269794	83.90282404	0.5	CRA004775;Yang2025_Silkie_Wenchang	PRJNA827465	0.877028977
ENSGALG00010016362	NUP62	2	-0.109798489	0.058989028	-1.861337503	0.06269653	0	0.000304955	0	1	CRA004775;PRJNA827465	Yang2025_Silkie_Wenchang	0.431400862
ENSGALG00010016366	KIF3A	2	0.051975493	0.04915406	1.057399788	0.290329168	0	0.952327134	0	1	PRJNA827465;Yang2025_Silkie_Wenchang	CRA004775	0.784195688
ENSGALG00010016366	KIF3A	2	0.112045369	0.107443365	1.042831912	0.297026149	0	0.613536268	0	0.5	CRA004775;Yang2025_Silkie_Wenchang	PRJNA827465	0.788466926
ENSGALG00010016366	KIF3A	2	0.029274942	0.051134644	0.572507011	0.566978538	0	0.048365618	0	0.5	CRA004775;PRJNA827465	Yang2025_Silkie_Wenchang	0.883487517
ENSGALG00010016368	CCNK	2	0.24994245	0.327645914	0.762843178	0.445556927	0.168187203	3.74264139	73.28090256	0.5	PRJNA827465;Yang2025_Silkie_Wenchang	CRA004775	0.846013379
ENSGALG00010016368	CCNK	2	0.254667472	0.341564231	0.745591748	0.45591407	0.173509451	3.480345838	71.26722324	0.5	CRA004775;Yang2025_Silkie_Wenchang	PRJNA827465	0.84907427
ENSGALG00010016368	CCNK	2	-0.005405658	0.051333466	-0.105304751	0.916133993	0	0.01765599	0	1	CRA004775;PRJNA827465	Yang2025_Silkie_Wenchang	0.980406784
ENSGALG00010016370	TMEM255A	2	0.244748965	0.205513144	1.19091636	0.233686422	0.073222847	7.509321923	86.6832184	1	PRJNA827465;Yang2025_Silkie_Wenchang	CRA004775	0.747077014
ENSGALG00010016370	TMEM255A	2	0.448384893	0.102654851	4.367887991	1.25454E-05	0	0.003801382	0	1	CRA004775;Yang2025_Silkie_Wenchang	PRJNA827465	0.00169752
ENSGALG00010016370	TMEM255A	2	0.057045945	0.104388892	0.546475237	0.584739305	0	0.608757032	0	1	CRA004775;PRJNA827465	Yang2025_Silkie_Wenchang	0.891016154
ENSGALG00010016372	DNAAF6	2	0.149575897	0.217036511	0.689173892	0.490713844	0.072925843	4.408228532	77.31515068	0.5	PRJNA827465;Yang2025_Silkie_Wenchang	CRA004775	0.858798485
ENSGALG00010016372	DNAAF6	2	-0.106427611	0.142482525	-0.746952026	0.45509252	0	0.224790594	0	1	CRA004775;Yang2025_Silkie_Wenchang	PRJNA827465	0.849072665
ENSGALG00010016372	DNAAF6	2	0.132571094	0.295293901	0.448946264	0.653470421	0.120438606	2.812400883	64.44319136	0.5	CRA004775;PRJNA827465	Yang2025_Silkie_Wenchang	0.909648135
ENSGALG00010016373	SRL	2	-0.269779162	0.076629124	-3.5205826	0.0004306	0	0.670094104	0	1	PRJNA827465;Yang2025_Silkie_Wenchang	CRA004775	0.021075798
ENSGALG00010016373	SRL	2	-0.342208357	0.116015378	-2.949680994	0.003181022	0	0.000742001	0	1	CRA004775;Yang2025_Silkie_Wenchang	PRJNA827465	0.078139815
ENSGALG00010016373	SRL	2	-0.217861144	0.100105054	-2.176325134	0.029530957	0	0.021205876	0	1	CRA004775;PRJNA827465	Yang2025_Silkie_Wenchang	0.293094753
ENSGALG00010016374	FRMD5	2	0.117924299	0.156170228	0.755101022	0.45018837	0	0.05677812	0	1	PRJNA827465;Yang2025_Silkie_Wenchang	CRA004775	0.847564661
ENSGALG00010016374	FRMD5	2	0.116240138	0.233073952	0.498726422	0.617972128	0	0.253494445	0	1	CRA004775;Yang2025_Silkie_Wenchang	PRJNA827465	0.899121942
ENSGALG00010016374	FRMD5	2	0.171466172	0.19157296	0.895043703	0.370763726	0	0.163193738	0	1	CRA004775;PRJNA827465	Yang2025_Silkie_Wenchang	0.8214684
ENSGALG00010016375	CCNI2	2	0.109905247	0.201864286	0.544451171	0.586131041	0.069895248	7.021555635	85.75814176	0.5	PRJNA827465;Yang2025_Silkie_Wenchang	CRA004775	0.891409988
ENSGALG00010016375	CCNI2	2	-0.110342613	0.102293305	-1.078688516	0.280726607	0	0.721555581	0	1	CRA004775;Yang2025_Silkie_Wenchang	PRJNA827465	0.778536485
ENSGALG00010016375	CCNI2	2	0.034890203	0.382452755	0.091227484	0.927311837	0.211551845	2.897025815	65.48184021	0.5	CRA004775;PRJNA827465	Yang2025_Silkie_Wenchang	0.982993195
ENSGALG00010016377	FRMPD3	2	-0.369588682	0.412885446	-0.895136133	0.37071432	0.307708313	10.251584	90.24540988	0.5	PRJNA827465;Yang2025_Silkie_Wenchang	CRA004775	0.82145712
ENSGALG00010016377	FRMPD3	2	-0.310140052	0.594318733	-0.521841286	0.601780841	0.54344736	3.734593534	73.22332428	0.5	CRA004775;Yang2025_Silkie_Wenchang	PRJNA827465	0.895256914
ENSGALG00010016377	FRMPD3	2	0.070873401	0.168156041	0.421474009	0.673408987	0	0.392802623	0	1	CRA004775;PRJNA827465	Yang2025_Silkie_Wenchang	0.915261599
ENSGALG00010016378	WDR76	2	0.048944764	0.104132082	0.470025787	0.638336594	0.00679259	1.415566649	29.35691156	0.5	PRJNA827465;Yang2025_Silkie_Wenchang	CRA004775	0.905560311
ENSGALG00010016378	WDR76	2	-0.045217274	0.265125638	-0.170550366	0.864577329	0.100435664	3.322277459	69.90016601	0.5	CRA004775;Yang2025_Silkie_Wenchang	PRJNA827465	0.966723659
ENSGALG00010016378	WDR76	2	-0.108329936	0.136841632	-0.791644579	0.428567946	0.014049737	1.375113893	27.27875088	1	CRA004775;PRJNA827465	Yang2025_Silkie_Wenchang	0.841586523
ENSGALG00010016379	TFAP4	2	0.256413025	0.2517161	1.01865961	0.308364594	0.117561518	13.83114833	92.76994234	1	PRJNA827465;Yang2025_Silkie_Wenchang	CRA004775	0.793457188
ENSGALG00010016379	TFAP4	2	0.328629504	0.210368932	1.562157969	0.118250779	0.067180337	3.866996761	74.14013867	1	CRA004775;Yang2025_Silkie_Wenchang	PRJNA827465	0.578003524
ENSGALG00010016379	TFAP4	2	0.020338569	0.083956515	0.242251226	0.808585504	0	0.131548558	0	1	CRA004775;PRJNA827465	Yang2025_Silkie_Wenchang	0.952496413
ENSGALG00010016382	UGDH	2	-0.204742322	0.430736633	-0.475330646	0.634551319	0.365039787	61.40994188	98.37159918	0.5	PRJNA827465;Yang2025_Silkie_Wenchang	CRA004775	0.903872221
ENSGALG00010016382	UGDH	2	-0.326957435	0.340611258	-0.959913766	0.337098617	0.204515274	7.953373123	87.42671839	0.5	CRA004775;Yang2025_Silkie_Wenchang	PRJNA827465	0.804086136
ENSGALG00010016382	UGDH	2	0.211101746	0.058941296	3.581559316	0.00034155	0	0.58122177	0	1	CRA004775;PRJNA827465	Yang2025_Silkie_Wenchang	0.018373991
ENSGALG00010016383	FAM110D	2	0.06556034	0.093915454	0.698078293	0.485128232	0	0.582179039	0	0.5	PRJNA827465;Yang2025_Silkie_Wenchang	CRA004775	0.857004733
ENSGALG00010016383	FAM110D	2	-0.280210768	0.233381299	-1.200656475	0.229884484	0.01331319	1.119812466	10.69933311	1	CRA004775;Yang2025_Silkie_Wenchang	PRJNA827465	0.742767343
ENSGALG00010016383	FAM110D	2	-0.160255218	0.340631238	-0.470465418	0.638022536	0.170224143	3.061062465	67.33160425	0.5	CRA004775;PRJNA827465	Yang2025_Silkie_Wenchang	0.905365679
ENSGALG00010016384	GNAS	2	0.015638053	0.049642047	0.315016273	0.752749299	0	0.017199496	0	1	PRJNA827465;Yang2025_Silkie_Wenchang	CRA004775	0.938087082
ENSGALG00010016384	GNAS	2	0.152176282	0.163966337	0.928094656	0.353358471	0.035353271	2.880986057	65.28966194	1	CRA004775;Yang2025_Silkie_Wenchang	PRJNA827465	0.811170375
ENSGALG00010016384	GNAS	2	0.141927985	0.152481484	0.930788324	0.351963071	0.035478439	3.697919105	72.95776431	1	CRA004775;PRJNA827465	Yang2025_Silkie_Wenchang	0.810678834
ENSGALG00010016385	HTT	2	-0.017888651	0.051302024	-0.348692892	0.727319881	0	0.069801877	0	1	PRJNA827465;Yang2025_Silkie_Wenchang	CRA004775	0.931522817
ENSGALG00010016385	HTT	2	0.043605074	0.161066069	0.27072787	0.78660034	0.022790344	1.424500239	29.7999416	0.5	CRA004775;Yang2025_Silkie_Wenchang	PRJNA827465	0.946722706
ENSGALG00010016385	HTT	2	0.040149687	0.124611147	0.322199804	0.747301333	0.014505665	1.288414187	22.38520734	0.5	CRA004775;PRJNA827465	Yang2025_Silkie_Wenchang	0.93680487
ENSGALG00010016387	CCDC85C	2	0.000735787	0.176524397	0.00416819	0.996674276	0.028232399	1.635965792	38.87402749	0.5	PRJNA827465;Yang2025_Silkie_Wenchang	CRA004775	0.99895145
ENSGALG00010016387	CCDC85C	2	0.252043792	0.201824265	1.248827996	0.211727992	0	0.003920488	0	1	CRA004775;Yang2025_Silkie_Wenchang	PRJNA827465	0.72405336
ENSGALG00010016387	CCDC85C	2	-0.048776462	0.140836009	-0.346335167	0.729090843	0.007595381	1.136525611	12.01254152	0.5	CRA004775;PRJNA827465	Yang2025_Silkie_Wenchang	0.932323844
ENSGALG00010016390	ENSGALG00010016390	2	-0.024931275	0.306977762	-0.081215246	0.935270775	0	0.008931748	0	0.5	PRJNA827465;Yang2025_Silkie_Wenchang	CRA004775	0.984597077
ENSGALG00010016390	ENSGALG00010016390	2	0.091656194	0.341119046	0.26869269	0.788166178	0	0.071331416	0	1	CRA004775;Yang2025_Silkie_Wenchang	PRJNA827465	0.946941439
ENSGALG00010016390	ENSGALG00010016390	2	0.045462028	0.31793687	0.142990739	0.886297495	0	0.138030446	0	0.5	CRA004775;PRJNA827465	Yang2025_Silkie_Wenchang	0.972694919
ENSGALG00010016392	STMN3	2	-0.191750338	0.472126592	-0.406141787	0.684638423	0.293904316	2.928724299	65.85544088	0.5	PRJNA827465;Yang2025_Silkie_Wenchang	CRA004775	0.919003112
ENSGALG00010016392	STMN3	2	0.324762545	0.324503473	1.000798362	0.316924302	0	0.109577654	0	1	CRA004775;Yang2025_Silkie_Wenchang	PRJNA827465	0.796964476
ENSGALG00010016392	STMN3	2	-0.197565495	0.587437032	-0.33631774	0.736631274	0.410442763	2.336722262	57.20501249	0.5	CRA004775;PRJNA827465	Yang2025_Silkie_Wenchang	0.933718804
ENSGALG00010016393	BLM	2	-0.126983539	0.123944942	-1.024515701	0.305591759	0.018314784	2.333339409	57.14296874	1	PRJNA827465;Yang2025_Silkie_Wenchang	CRA004775	0.79225794
ENSGALG00010016393	BLM	2	-0.20251439	0.14694701	-1.378145703	0.168158307	0.007237823	1.127378444	11.29864107	0.5	CRA004775;Yang2025_Silkie_Wenchang	PRJNA827465	0.668761111
ENSGALG00010016393	BLM	2	-0.018627679	0.081846988	-0.227591504	0.819963825	0	0.109074039	0	0.5	CRA004775;PRJNA827465	Yang2025_Silkie_Wenchang	0.955308792
ENSGALG00010016394	PDIK1L	2	0.064374312	0.051144382	1.258678073	0.208146634	0.000596763	1.072313271	6.743670277	0.5	PRJNA827465;Yang2025_Silkie_Wenchang	CRA004775	0.72083771
ENSGALG00010016394	PDIK1L	2	-0.078169753	0.104979376	-0.744620092	0.456501418	0	0.388229706	0	1	CRA004775;Yang2025_Silkie_Wenchang	PRJNA827465	0.849515887

ENSGALG00010016394	PDIK1L	2	0.026956131	0.119577895	0.225427372	0.821646819	0.015475108	1.556234541	35.74233358	0.5	CRA004775;PRJNA827465	Yang2025_Silkie_Wenchang	0.955598716
ENSGALG00010016395	ENSGALG00010016395	2	0.007574268	0.086132141	0.087937762	0.929926143	0	0.003369607	0	1	PRJNA827465;Yang2025_Silkie_Wenchang	CRA004775	0.983813577
ENSGALG00010016395	ENSGALG00010016395	2	0.145995367	0.152865651	0.955056721	0.339549014	0.014194006	1.420876329	29.62089805	1	CRA004775;Yang2025_Silkie_Wenchang	PRJNA827465	0.80490319
ENSGALG00010016395	ENSGALG00010016395	2	0.116581811	0.152924724	0.762347699	0.445852513	0.025613747	1.99270041	49.81684176	1	CRA004775;PRJNA827465	Yang2025_Silkie_Wenchang	0.846013379
ENSGALG00010016397	LIAS	2	0.008124544	0.05461658	0.148756	0.881746171	0.001219106	1.217049838	17.83409614	0.5	PRJNA827465;Yang2025_Silkie_Wenchang	CRA004775	0.971900875
ENSGALG00010016397	LIAS	2	0.010384882	0.135927411	0.076400203	0.939100708	0.016358934	1.483540867	32.59370051	0.5	CRA004775;Yang2025_Silkie_Wenchang	PRJNA827465	0.985349045
ENSGALG00010016397	LIAS	2	-0.039602247	0.054930206	-0.720955744	0.470936744	0	0.636471082	0	1	CRA004775;PRJNA827465	Yang2025_Silkie_Wenchang	0.853786007
ENSGALG00010016398	SEPTIN6	2	0.009419415	0.087981291	0.107061572	0.914740133	0.010451886	2.753548376	63.68322385	0.5	PRJNA827465;Yang2025_Silkie_Wenchang	CRA004775	0.97982748
ENSGALG00010016398	SEPTIN6	2	-0.007488629	0.153331607	-0.048839432	0.961047258	0.030300523	2.671331236	62.56548096	0.5	CRA004775;Yang2025_Silkie_Wenchang	PRJNA827465	0.989599947
ENSGALG00010016398	SEPTIN6	2	-0.068975499	0.04363139	-1.580868695	0.113908064	0	0.594637162	0	1	CRA004775;PRJNA827465	Yang2025_Silkie_Wenchang	0.569481237
ENSGALG00010016399	ACSM5	2	-0.349367271	0.219562111	-1.591200179	0.111564533	0	0.055371636	0	1	PRJNA827465;Yang2025_Silkie_Wenchang	CRA004775	0.564762672
ENSGALG00010016399	ACSM5	2	-0.411081738	0.301369596	-1.364045161	0.172553353	0	0.00018045	0	1	CRA004775;Yang2025_Silkie_Wenchang	PRJNA827465	0.675484504
ENSGALG00010016399	ACSM5	2	-0.322145476	0.267277736	-1.205283615	0.228093833	0	0.023766467	0	1	CRA004775;PRJNA827465	Yang2025_Silkie_Wenchang	0.740938585
ENSGALG00010016400	ENSGALG00010016400	2	-0.19046199	0.148246795	-1.284762951	0.198875133	0.034306354	4.117553288	75.71373264	1	PRJNA827465;Yang2025_Silkie_Wenchang	CRA004775	0.71096127
ENSGALG00010016400	ENSGALG00010016400	2	-0.322924053	0.127125727	-2.540194356	0.011079088	0	0.724797991	0	1	CRA004775;Yang2025_Silkie_Wenchang	PRJNA827465	0.167383943
ENSGALG00010016400	ENSGALG00010016400	2	-0.065751551	0.055487474	-1.184980074	0.236025325	0	0.000647634	0	1	CRA004775;PRJNA827465	Yang2025_Silkie_Wenchang	0.748669015
ENSGALG00010016402	SEPTIN8	2	0.044309716	0.054813911	0.808366267	0.418879765	0	0.00729415	0	1	PRJNA827465;Yang2025_Silkie_Wenchang	CRA004775	0.838250071
ENSGALG00010016402	SEPTIN8	2	-0.062486302	0.151195908	-0.413280377	0.679401205	0.023479063	1.769307876	43.48072408	0.5	CRA004775;Yang2025_Silkie_Wenchang	PRJNA827465	0.917701868
ENSGALG00010016402	SEPTIN8	2	-0.05205964	0.154299504	-0.33739344	0.735820329	0.029304055	2.045008351	51.10044418	0.5	CRA004775;PRJNA827465	Yang2025_Silkie_Wenchang	0.933592889
ENSGALG00010016403	GRK4	2	0.018450443	0.227727708	0.081019756	0.935426241	0.089479155	7.048419374	85.81242195	0.5	PRJNA827465;Yang2025_Silkie_Wenchang	CRA004775	0.984701764
ENSGALG00010016403	GRK4	2	0.083371414	0.317297385	0.26275481	0.792739569	0.110704928	1.681159395	40.51724049	0.5	CRA004775;Yang2025_Silkie_Wenchang	PRJNA827465	0.948435155
ENSGALG00010016403	GRK4	2	-0.199818166	0.083856639	-2.382854466	0.017178984	0	0.257665719	0	1	CRA004775;PRJNA827465	Yang2025_Silkie_Wenchang	0.217801813
ENSGALG00010016404	ESRRG	2	-0.061547132	0.062711784	-0.981428502	0.326381477	0	0.706060086	0	0.5	PRJNA827465;Yang2025_Silkie_Wenchang	CRA004775	0.800028239
ENSGALG00010016404	ESRRG	2	0.279913604	0.338276384	0.827470132	0.407970644	0.178968049	3.968569323	74.80200247	1	CRA004775;Yang2025_Silkie_Wenchang	PRJNA827465	0.833982683
ENSGALG00010016404	ESRRG	2	0.232787477	0.394935238	0.589432025	0.555571498	0.262125745	5.501080293	81.82175233	0.5	CRA004775;PRJNA827465	Yang2025_Silkie_Wenchang	0.880255849
ENSGALG00010016407	RPL9	2	-0.301158998	0.211657008	-1.422863339	0.154775776	0.068825283	3.676692482	72.80164156	1	PRJNA827465;Yang2025_Silkie_Wenchang	CRA004775	0.647505492
ENSGALG00010016407	RPL9	2	-0.450128118	0.157276517	-2.862017338	0.004209539	0	0.617951712	0	1	CRA004775;Yang2025_Silkie_Wenchang	PRJNA827465	0.094595485
ENSGALG00010016407	RPL9	2	-0.147181537	0.061643818	-2.387612299	0.016958221	0	0.627339153	0	1	CRA004775;PRJNA827465	Yang2025_Silkie_Wenchang	0.216258584
ENSGALG00010016409	GMEB2	2	0.181768145	0.221441556	0.820840264	0.411737261	0.088314008	9.873515721	89.8718954	0.5	PRJNA827465;Yang2025_Silkie_Wenchang	CRA004775	0.835183193
ENSGALG00010016409	GMEB2	2	0.19940251	0.299601192	0.665559802	0.505692508	0.121184618	2.523958436	60.3796962	0.5	CRA004775;Yang2025_Silkie_Wenchang	PRJNA827465	0.863927652
ENSGALG00010016409	GMEB2	2	-0.037120927	0.073352432	-0.506062669	0.612812656	0	0.240795121	0	1	CRA004775;PRJNA827465	Yang2025_Silkie_Wenchang	0.898034557
ENSGALG00010016410	TRAK1	2	-0.103251988	0.058165878	-1.775129888	0.075876443	0	0.050730826	0	1	PRJNA827465;Yang2025_Silkie_Wenchang	CRA004775	0.47539798
ENSGALG00010016410	TRAK1	2	-0.086507336	0.10120537	-0.854770217	0.392678369	0	0.870960705	0	0.5	CRA004775;Yang2025_Silkie_Wenchang	PRJNA827465	0.829914191
ENSGALG00010016410	TRAK1	2	-0.080391126	0.066740922	-1.20452526	0.228386625	0	0.759232343	0	0.5	CRA004775;PRJNA827465	Yang2025_Silkie_Wenchang	0.741167494
ENSGALG00010016411	CTSH	2	0.07792479	0.101562899	0.767256457	0.442929052	0	0.225379118	0	1	PRJNA827465;Yang2025_Silkie_Wenchang	CRA004775	0.845720426
ENSGALG00010016411	CTSH	2	0.11547599	0.138264088	0.835184259	0.403613971	0	0.065182064	0	1	CRA004775;Yang2025_Silkie_Wenchang	PRJNA827465	0.83306585
ENSGALG00010016411	CTSH	2	0.033679738	0.128203641	0.262705007	0.792777957	0	4.03932E-06	0	1	CRA004775;PRJNA827465	Yang2025_Silkie_Wenchang	0.948451453
ENSGALG00010016412	TMSB15B	2	-0.152548638	0.114969511	-1.326861671	0.184554445	0	0.039676156	0	1	PRJNA827465;Yang2025_Silkie_Wenchang	CRA004775	0.692925427
ENSGALG00010016412	TMSB15B	2	-0.063762724	0.20910934	-0.30492528	0.760423061	0	0.645802566	0	0.5	CRA004775;Yang2025_Silkie_Wenchang	PRJNA827465	0.939327609
ENSGALG00010016412	TMSB15B	2	-0.105240479	0.118573575	-0.887554241	0.374780577	0	0.64158979	0	0.5	CRA004775;PRJNA827465	Yang2025_Silkie_Wenchang	0.822805106
ENSGALG00010016414	KLB	2	1.54295214	0.380450022	4.05559745	5.00063E-05	0	0.511749908	0	1	PRJNA827465;Yang2025_Silkie_Wenchang	CRA004775	0.004840883
ENSGALG00010016414	KLB	2	1.248211079	0.415352915	3.005181943	0.00265422	0	0.092884222	0	1	CRA004775;Yang2025_Silkie_Wenchang	PRJNA827465	0.069655625
ENSGALG00010016414	KLB	2	1.572298925	0.484058133	3.24816136	0.001161534	0	0.705996036	0	1	CRA004775;PRJNA827465	Yang2025_Silkie_Wenchang	0.041501602
ENSGALG00010016415	RASGRF1	2	0.591473171	0.548611109	1.078128316	0.280976497	0.473769149	4.687760702	78.66785308	1	PRJNA827465;Yang2025_Silkie_Wenchang	CRA004775	0.778623652
ENSGALG00010016415	RASGRF1	2	0.730634248	0.437279526	1.670863152	0.094748711	0.227286284	2.460268536	59.35403045	1	CRA004775;Yang2025_Silkie_Wenchang	PRJNA827465	0.526919621
ENSGALG00010016415	RASGRF1	2	0.144760022	0.261925455	0.552676417	0.580485005	0	0.173598113	0	1	CRA004775;PRJNA827465	Yang2025_Silkie_Wenchang	0.888433837
ENSGALG00010016416	CYP46A1	2	0.243397763	0.245805364	0.990205255	0.322073805	0.11310608	15.56741293	93.57632508	1	PRJNA827465;Yang2025_Silkie_Wenchang	CRA004775	0.798513866
ENSGALG00010016416	CYP46A1	2	0.475011833	0.091612659	5.185002153	2.16012E-07	0	0.257359686	0	1	CRA004775;Yang2025_Silkie_Wenchang	PRJNA827465	6.58134E-05
ENSGALG00010016416	CYP46A1	2	0.096674511	0.157035319	0.615622726	0.538143549	0.02786759	1.79979503	44.43811748	1	CRA004775;PRJNA827465	Yang2025_Silkie_Wenchang	0.87481821
ENSGALG00010016417	NOP14	2	0.236587962	0.156640127	1.51039179	0.130943482	0.032254542	2.620280727	61.83615023	1	PRJNA827465;Yang2025_Silkie_Wenchang	CRA004775	0.606744617
ENSGALG00010016417	NOP14	2	0.355917521	0.144630084	2.460881656	0.013859607	0	0.56808632	0	1	CRA004775;Yang2025_Silkie_Wenchang	PRJNA827465	0.193260071
ENSGALG00010016417	NOP14	2	0.124913881	0.080123713	1.559012638	0.118993389	0	0.139519698	0	1	CRA004775;PRJNA827465	Yang2025_Silkie_Wenchang	0.579385179
ENSGALG00010016418	NFIB	2	0.064324871	0.247838258	0.259543751	0.79521573	0.091567521	3.563684318	71.93915312	0.5	PRJNA827465;Yang2025_Silkie_Wenchang	CRA004775	0.948935068
ENSGALG00010016418	NFIB	2	0.336415792	0.189765836	1.772794297	0.076262799	0	0.031718226	0	1	CRA004775;Yang2025_Silkie_Wenchang	PRJNA827465	0.47652731
ENSGALG00010016418	NFIB	2	-0.014224074	0.197733764	-0.071935481	0.942653253	0.043180056	1.836706756	45.55472742	0.5	CRA004775;PRJNA827465	Yang2025_Silkie_Wenchang	0.985827325
ENSGALG00010016421	ENSGALG00010016421	2	0.126069057	0.246456543	0.511526518	0.608982425	0	0.033081064	0	1	PRJNA827465;Yang2025_Silkie_Wenchang	CRA004775	0.897208691
ENSGALG00010016421	ENSGALG00010016421	2	0.336072122	0.353585095	0.950470274	0.341873351	0.023152203	1.094627179	8.644694841	1	CRA004775;Yang2025_Silkie_Wenchang	PRJNA827465	0.805844328
ENSGALG00010016421	ENSGALG00010016421	2	0.304310493	0.267689368	1.136804556	0.255619998	3.02459E-05	1.000148809	0.014878709	1	CRA004775;PRJNA827465	Yang2025_Silkie_Wenchang	0.764467569
ENSGALG00010016422	KCTD3	2	-0.095002191	0.065876362	-1.442128688	0.149266073	0.001978424	1.293715657	22.70326214	1	PRJNA827465;Yang2025_Silkie_Wenchang	CRA004775	0.639246608
ENSGALG00010016422	KCTD3	2	-0.175826598	0.082701562	-2.126037214	0.033500161	0	0.252871266	0	1	CRA004775;Yang2025_Silkie_Wenchang	PRJNA827465	0.314508586
ENSGALG00010016422	KCTD3	2	-0.049231318	0.076544439	-0.643173016	0.52011184	0	0.792654129	0	1	CRA004775;PRJNA827465	Yang2025_Silkie_Wenchang	0.869331539
ENSGALG00010016423	TRMT44	2	-0.032617051	0.062825763	-0.519166815	0.603644419	0	0.235957197	0	0.5	PRJNA827465;Yang2025_Silkie_Wenchang	CRA004775	0.895825275
ENSGALG00010016423	TRMT44	2	-0.000856695	0.100026981	-0.008564634	0.993166494	0	0.070644358	0	0.5	CRA004775;Yang2025_Silkie_Wenchang	PRJNA827465	0.99794341
ENSGALG00010016423	TRMT44	2	-0.051463515	0.069853988	-0.736729798	0.461286682	0	0.000889881	0	1	CRA004775;PRJNA827465	Yang2025_Silkie_Wenchang	0.85014477
ENSGALG00010016425	TRIM63	2	-0.033129132	0.148685359	-0.222813681	0.82368052	0.027791232	2.589273545	61.37912883	0.5	PRJNA827465;Yang2025_Silkie_Wenchang	CRA004775	0.955934625
ENSGALG00010016425	TRIM63	2	0.128759843	0.153105499	0.840987706	0.400354822	0	0.137474096	0	0.5	CRA004775;Yang2025_Silkie_Wenchang	PRJNA827465	0.832251175
ENSGALG00010016425	TRIM63	2	-0.160316235	0.101785532	-1.575039523	0.115247322	0	0.000252627	0	1	CRA004775;PRJNA827465	Yang2025_Silkie_Wenchang	0.572203827
ENSGALG00010016426	HELZ2	2	0.079316618	0.135518446	0.58528282	0.558357564	0.019109766	1.968854863	49.20905453	0.5	PRJNA827465;Yang2025_Silkie_Wenchang	CRA004775	0.880678789
ENSGALG00010016426	HELZ2	2	-0.12872462	0.16151186	-0.796997946	0.425452224	0	0.4915672	0	1	CRA004775;Yang2025_Silkie_Wenchang	PRJNA827465	0.84125685
ENSGALG00010016426	HELZ2	2	0.053690781	0.266533931	0.201440698	0.840353995	0.077921116	1.535377626	34.86944301	0.5	CRA004775;PRJNA827465	Yang2025_Silkie_Wenchang	0.959662809
ENSGALG00010016428	ADRA1D	2	0.010586168	0.129639144	0.081658729	0.934918098	0	0.154474967	0	0.5	PRJNA827465;Yang2025_Silkie_Wenchang	CRA004775	0.984489498
ENSGALG00010016428	ADRA1D	2	0.340965287	0.501348682	0.680096108	0.496443609	0.363190863	3.191445751	68.6662385	0.5	CRA004775;Yang2025_Silkie_Wenchang	PRJNA827465	0.860939846
ENSGALG00010016428	ADRA1D	2	0.369112205	0.439157947	0.840499887	0.400628165	0.270097064	2.746117901	63.58495753	1	CRA004775;PRJNA827465	Yang2025_Silkie_Wenchang	0.832353691
ENSGALG00010016429	ENSGALG00010016429	2	0.942936924	0.292583794	3.222792735	0.001269474	0	0.153616357	0	1	PRJNA827465;Yang2025_Silkie_Wenchang	CRA004775	0.043294215
ENSGALG00010016429	ENSGALG00010016429	2	0.219750666	0.555838123	0.395350116	0.692584518	0.373511981	2.527635381	60.43733176	0.5	CRA004775;Yang2025_Silkie_Wenchang	PRJNA827465	0.920559211
ENSGALG00010016429	ENSGALG00010016429	2	0.387571288	0.675853498	0.573454585	0.56633694	0.733663264	5.008763245	80.03499165	0.5	CRA004775;PRJNA827465	Yang2025_Silkie_Wenchang	0.883487517
ENSGALG00010016431	B3GNT5	2	-0.165134031	0.373164328	-0.442523625	0.658110338	0.261632356	16.50642689	93.94175368	0.5	PRJNA827465;Yang2025_Silkie_Wenchang	CRA004775	0.910938006
ENSGALG00010016431	B3GNT5	2	-0.510931314	0.122128739	-4.18354694	2.86996E-05	0	0.546172427	0	1	CRA004775;Yang2025_Silkie_Wenchang	PRJNA827465	0.003186383
ENSGALG00010016431	B3GNT5	2	0.173830845	0.12669777	1.372011874	0.170059741	0	0.824310958	0	0.5	CRA004775;PRJNA827465	Yang2025_Silkie_Wenchang	0.671658717
ENSGALG00010016433	TMED3	2	0.074959386	0.059730042	1.254969586	0.209489801	0	0.776717073	0	1	PRJNA827465;Yang2025_Silkie_Wenchang	CRA004775	0.721386257
ENSGALG00010016433	TMED3	2	0.149744462	0.105908216	1.413907894	0.157388948	0	0.097536988	0	1	CRA004775;Yang2025_Silkie_Wenchang	PRJNA827465	0.650687662
ENSGALG00010016433	TMED3	2	0.0496548	0.065266087	0.760805531	0.44677323	0	0.042349188	0	1	CRA004775;PRJNA827465	Yang2025_Silkie_Wenchang	0.84650962
ENSGALG00010016441	ENSGALG00010016441	2	-0.276321409	0.343234942	-0.805050345	0.420790628	0.088075933	1.581597641	36.77279389	0.5	PRJNA827465;Yang2025_Silkie_Wenchang	CRA004775	0.839211146
ENSGALG00010016441	ENSGALG00010016441	2	0.107123679	0.372116149	0.287876995	0.773440898	0	0.001042789	0	1	CRA004775;Yang2025_Silkie_Wenchang	PRJNA827465	0.943248868
ENSGALG00010016441	ENSGALG00010016441	2	-0.463603178	0.316263283	-1.465877332	0.142681708	0	0.629650825	0	0.5	CRA004775;PRJNA827465	Yang2025_Silkie_Wenchang	0.628961327
ENSGALG00010016445	ZDHHC21	2	-0.112053204	0.279714092	-0.400599067	0.688715333	0.127948366	5.460996686	81.68832436	0.5	PRJNA827465;Yang2025_Silkie_Wenchang	CRA004775	0.919799842
ENSGALG00010016445	ZDHHC21	2	-0.281255304	0.19096215	-1.472832727	0.140796145	0.017171988	1.234832906	19.01738322	0.5	CRA004775;Yang2025_Silkie_Wenchang	PRJNA827465	0.625201272
ENSGALG00010016445	ZDHHC21	2	0.135618128	0.140265864	0.96686481	0.333611621	0	0.132938267	0	1	CRA004775;PRJNA827465	Yang2025_Silkie_Wenchang	0.802691373
ENSGALG00010016447	ID3	2	0.234615754	0.150337536	1.560593318	0.118619736	0.033513905	3.719817159	73.11695825	1	PRJNA827465;Yang2025_Silkie_Wenchang	CRA004775	0.578712946
ENSGALG00010016447	ID3	2	0.37455809	0.121129794	3.092204479	0.001986759	0	0.28146844	0	1	CRA004775;Yang2025_Silkie_Wenchang	PRJNA827465	0.057841189
ENSGALG00010016447	ID3	2	0.110942163	0.080090776	1.385205248	0.165989716	0	0.189229143	0	1	CRA004775;PRJNA827465	Yang2025_Silkie_Wenchang	0.66529196
ENSGALG00010016449	FMR1	2	0.205695093	0.322139576	0.638527856	0.523130127	0.188242276	10.11197575	90.11073578	0.5	PRJNA827465;Yang2025_Silkie_Wenchang	CRA004775	0.869569689
ENSGALG00010016449	FMR1	2	0.434364172	0.142585142	3.046349475	0.002316384	0	0.847504354	0	1	CRA004775;Yang2025_Silkie_Wenchang	PRJNA827465	0.063799725
ENSGALG00010016449	FMR1	2	0.046950759	0.183959652	0.255223137	0.798550762	0.04915923	3.036071812	67.06270266	0.5	CRA004775;PRJNA827465	Yang2025_Silkie_Wenchang	0.949922217
ENSGALG00010016450	ENSGALG00010016450	2	0.542458195	0.197161088	2.751345108	0.005935108	0.0285388	1.566142751	36.14886004	1	PRJNA827465;Yang2025_Silkie_Wenchang	CRA004775	0.116400973
ENSGALG00010016450	ENSGALG00010016450	2	0.425693288	0.421627033	1.009644199	0.312665786	0.221740112	2.198315675	54.51062777	0.5	CRA004775;Yang2025_Silkie_Wenchang	PRJNA827465	0.796054997
ENSGALG00010016450	ENSGALG00010016450	2	0.241333882	0.225535357	1.070048994	0.284597256	0	0.653629645	0	0.5	CRA004775;PRJNA827465	Yang2025_Silkie_Wenchang	0.780265906
ENSGALG00010016451	USP46	2	0.028837948	0.107136896	0.269169158	0.787799513	0.005365521	1.280604276	21.91186468	0.5	PRJNA827465;Yang2025_Silkie_Wenchang	CRA004775	0.946919116
ENSGALG00010016451	USP46	2	0.179179437	0.143737201	1.246576636	0.212552773	0	0.031589215	0	1	CRA004775;Yang2025_Silkie_Wenchang	PRJNA827465	0.725280468
ENSGALG00010016451	USP46	2	-0.024671487	0.108015725	-0.228406437	0.819330286	0	0.652136702	0	0.5	CRA004775;PRJNA827465	Yang2025_Silkie_Wenchang	0.955256039
ENSGALG00010016452	GLCE	2	-0.210864307	0.178142231	-1.183685115	0.236537733	0.053377481	5.814746587	82.80234598	1	PRJNA827465;Yang2025_Silkie_Wenchang	CRA004775	0.7487803
ENSGALG00010016452	GLCE	2	-0.342586959	0.130105282	-2.633151821	0.008459652	0.002749752	1.060059687	5.665689183	1	CRA004775;Yang2025_Silkie_Wenchang	PRJNA827465	0.143515254
ENSGALG00010016452	GLCE	2	-0.055566748	0.054352636	-1.022337686	0.306621102	0	0.029874254	0	1	CRA004775;PRJNA827465	Yang2025_Silkie_Wenchang	0.792500392
ENSGALG00010016453	MYCL	2	-0.262068484	0.298543912	-0.877822237	0.380040195	0.159714093	9.378865575	89.33772969	0.5	PRJNA827465;Yang2025_Silkie_Wenchang	CRA004775	0.824392515
ENSGALG00010016453	MYCL	2	-0.516479969	0.154723075	-3.338092717	0.000843556	0	0.77461091	0	1	CRA004775;Yang2025_Silkie_Wenchang	PRJNA827465	0.033796412
ENSGALG00010016453	MYCL	2	0.006615264	0.094096148	0.070303239	0.943952304	0	0.348497116	0	0.5	CRA004775;PRJNA827465	Yang2025_Silkie_Wenchang	0.985827325
ENSGALG00010016454	ANKS3	2	0.017176389	0.06182806	0.277808958	0.781159014	0.002105893	1.201061884	16.74034341	0.5	PRJNA827465;Yang2025_Silkie_Wenchang	CRA004775	0.945235709
ENSGALG00010016454	ANKS3	2	-0.160980474	0.105589069	-1.52459412	0.127360365	0	0.335733558	0	1	CRA004775;Yang2025_Silkie_Wenchang	PRJNA827465	0.599191336
ENSGALG00010016454	ANKS3	2	-0.049685963	0.134480619	-0.3694656	0.711780709	0.024802149	2.600130769	61.54039589	0.5	CRA004775;PRJNA827465	Yang2025_Silkie_Wenchang	0.92597752
ENSGALG00010016455	RBM27	2	0.1032762	0.130300005	0.792603188	0.428009051	0.024202206	2.941258237	66.00094519	1	PRJNA827465;Yang2025_Silkie_Wenchang	CRA004775	0.84125685
ENSGALG00010016455	RBM27	2	0.165984382	0.149847347	1.107689829	0.267995793	0.012009122	1.320610976	24.27747323	0.5	CRA004775;Yang2025_Silkie_Wenchang	PRJNA827465	0.771398294
ENSGALG00010016455	RBM27	2	0.002720321	0.04878023	0.055766871	0.955527527	0	0.034781034	0	0.5	CRA004775;PRJNA827465	Yang2025_Silkie_Wenchang	0.988263618
ENSGALG00010016456	LAMP3	2	0.623371765	0.47652336	1.308166224	0.190816935	0.275460881	2.439129915	59.00177379	1	PRJNA827465;Yang2025_Silkie_Wenchang	CRA004775	0.700380838
ENSGALG00010016456	LAMP3	2	1.174540567	0.425561366	2.759979315	0.005780502	0	0.001575057	0	1	CRA004775;Yang2025_Silkie_Wenchang	PRJNA827465	0.114502839
ENSGALG00010016456	LAMP3	2	0.447440366	0.399827899	1.119082402	0.263104987	0.097258572	1.28995394	22.47785218	1	CRA004775;PRJNA827465	Yang2025_Silkie_Wenchang	0.768643821
ENSGALG00010016457	SMOX	2	-0.0192233	0.102332534	-0.187851304	0.850993217	0.009722227	1.760555081	43.19973225	0.5	PRJNA827465;Yang2025_Silkie_Wenchang	CRA004775	0.962056252
ENSGALG00010016457	SMOX	2	-0.102816498	0.1138708	-0.902922418	0.366567072	0	0.442660208	0	0.5	CRA004775;Yang2025_Silkie_Wenchang	PRJNA827465	0.819334942
ENSGALG00010016457	SMOX	2	0.05222448	0.076797815	0.680025603	0.49648825	0	0.046749173	0	1	CRA004775;PRJNA827465	Yang2025_Silkie_Wenchang	0.860939846
ENSGALG00010016458	ZC3H12A	2	-0.45514329	0.357759756	-1.272203714	0.203300748	0.237812655	13.5067248	92.59628063	1	PRJNA827465;Yang2025_Silkie_Wenchang	CRA004775	0.717188086
ENSGALG00010016458	ZC3H12A	2	-0.668513026	0.206510025	-3.237194059	0.001207113	0.036309166	1.682129012	40.55152769	1	CRA004775;Yang2025_Silkie_Wenchang	PRJNA827465	0.042380792
ENSGALG00010016458	ZC3H12A	2	-0.147526025	0.089171455	-1.654408647	0.098044511	0.004565797	1.118555239	10.59896148	1	CRA004775;PRJNA827465	Yang2025_Silkie_Wenchang	0.534161825
ENSGALG00010016459	PAQR5	2	-0.042234205	0.146273895	-0.288733716	0.77278516	0	0.382561643	0	0.5	PRJNA827465;Yang2025_Silkie_Wenchang	CRA004775	0.943066211
ENSGALG00010016459	PAQR5	2	0.11564757	0.198312682	0.583157711	0.559787136	0	0.000564937	0	1	CRA004775;Yang2025_Silkie_Wenchang	PRJNA827465	0.881203589
ENSGALG00010016459	PAQR5	2	-0.035796432	0.144437131	-0.247834	0.804262847	0	0.448170436	0	0.5	CRA004775;PRJNA827465	Yang2025_Silkie_Wenchang	0.95154709
ENSGALG00010016460	LBR	2	-0.099135443	0.20480052	-0.484058551	0.628344316	0.082101865	47.0045499	97.87254638	0.5	PRJNA827465;Yang2025_Silkie_Wenchang	CRA004775	0.901912657
ENSGALG00010016460	LBR	2	-0.304494253	0.04179159	-7.286017368	3.19252E-13	0	0.022357153	0	1	CRA004775;Yang2025_Silkie_Wenchang	PRJNA827465	3.06038E-10
ENSGALG00010016460	LBR	2	0.061455459	0.137942502	0.44551504	0.655947594	0.019181175	1.20573428	17.06298669	0.5	CRA004775;PRJNA827465	Yang2025_Silkie_Wenchang	0.910411938
ENSGALG00010016462	RNF24	2	-0.002084853	0.075128712	-0.027750421	0.977861209	0	0.023409623	0	0.5	PRJNA827465;Yang2025_Silkie_Wenchang	CRA004775	0.993739602
ENSGALG00010016462	RNF24	2	0.006735372	0.151356856	0.044499944	0.964505896	0	0.154845421	0	0.5	CRA004775;Yang2025_Silkie_Wenchang	PRJNA827465	0.990364463
ENSGALG00010016462	RNF24	2	0.010814465	0.080679995	0.134041463	0.893369788	0	0.11814761	0	1	CRA004775;PRJNA827465	Yang2025_Silkie_Wenchang	0.974381724
ENSGALG00010016463	FREM1	2	0.067274679	0.124227287	0.541545106	0.588131917	0.009312825	1.372982702	27.16587043	0.5	PRJNA827465;Yang2025_Silkie_Wenchang	CRA004775	0.891761457
ENSGALG00010016463	FREM1	2	-0.061013874	0.179944481	-0.33907055	0.734556584	0	0.566700058	0	0.5	CRA004775;Yang2025_Silkie_Wenchang	PRJNA827465	0.933520234
ENSGALG00010016463	FREM1	2	0.164839796	0.116536616	1.414489296	0.157218288	0	0.112447804	0	1	CRA004775;PRJNA827465	Yang2025_Silkie_Wenchang	0.650687662
ENSGALG00010016464	PANK2	2	0.00515192	0.06142259	0.083876629	0.933154522	0	0.04990017	0	0.5	PRJNA827465;Yang2025_Silkie_Wenchang	CRA004775	0.984259239
ENSGALG00010016464	PANK2	2	-0.233946848	0.225382805	-1.037997763	0.299271097	0.077092354	4.097336723	75.59390239	1	CRA004775;Yang2025_Silkie_Wenchang	PRJNA827465	0.78978392
ENSGALG00010016464	PANK2	2	-0.203583492	0.241344487	-0.843539018	0.398927053	0.100022062	6.571017026	84.78165562	0.5	CRA004775;PRJNA827465	Yang2025_Silkie_Wenchang	0.832054308
ENSGALG00010016465	STK35	2	-0.26819789	0.164593468	-1.629456463	0.103216424	0.023940576	1.713853275	41.65194799	1	PRJNA827465;Yang2025_Silkie_Wenchang	CRA004775	0.54753882
ENSGALG00010016465	STK35	2	-0.517374678	0.203432899	-2.543220298	0.010983596	0	0.257888962	0	1	CRA004775;Yang2025_Silkie_Wenchang	PRJNA827465	0.166811819
ENSGALG00010016465	STK35	2	-0.226105625	0.221411163	-1.021202459	0.307158528	0.036790162	1.209814424	17.34269483	1	CRA004775;PRJNA827465	Yang2025_Silkie_Wenchang	0.79266003
ENSGALG00010016466	TMEM164	2	0.055741318	0.182853221	0.304841869	0.760486592	0.046600409	3.249018062	69.22146997	0.5	PRJNA827465;Yang2025_Silkie_Wenchang	CRA004775	0.939327609
ENSGALG00010016466	TMEM164	2	0.228694953	0.467540274	0.489144928	0.624739096	0.330324204	3.532555992	71.69188536	0.5	CRA004775;Yang2025_Silkie_Wenchang	PRJNA827465	0.901035584
ENSGALG00010016466	TMEM164	2	0.335711543	0.231713463	1.448821915	0.147387322	0.050909065	1.407512961	28.95269687	1	CRA004775;PRJNA827465	Yang2025_Silkie_Wenchang	0.636287777
ENSGALG00010016468	TTC39B	2	0.094669975	0.155905341	0.607227275	0.543700094	0.028245943	2.308105268	56.67441976	0.5	PRJNA827465;Yang2025_Silkie_Wenchang	CRA004775	0.876968972
ENSGALG00010016468	TTC39B	2	0.235367384	0.150667724	1.562161938	0.118249845	0	0.261933041	0	1	CRA004775;Yang2025_Silkie_Wenchang	PRJNA827465	0.578003524
ENSGALG00010016468	TTC39B	2	-0.022383652	0.110004184	-0.203480003	0.838759874	0	0.153702275	0	0.5	CRA004775;PRJNA827465	Yang2025_Silkie_Wenchang	0.959283372
ENSGALG00010016469	MAVS	2	0.019910119	0.070974796	0.280523786	0.779075679	0	0.000875287	0	1	PRJNA827465;Yang2025_Silkie_Wenchang	CRA004775	0.944714533
ENSGALG00010016469	MAVS	2	-0.28706922	0.334041608	-0.859381626	0.390130004	0.180446068	5.023462135	80.09341022	0.5	CRA004775;Yang2025_Silkie_Wenchang	PRJNA827465	0.829204867
ENSGALG00010016469	MAVS	2	-0.272575344	0.33521684	-0.813131417	0.416142716	0.192117138	6.249661419	83.99913319	0.5	CRA004775;PRJNA827465	Yang2025_Silkie_Wenchang	0.837742614
ENSGALG00010016470	TNIP1	2	0.196997435	0.211796703	0.930125127	0.352306302	0.085072928	19.20159533	94.79209939	0.5	PRJNA827465;Yang2025_Silkie_Wenchang	CRA004775	0.810786136
ENSGALG00010016470	TNIP1	2	-0.05566306	0.514481733	-0.10819249	0.913843002	0.477782606	9.491409876	89.46415745	0.5	CRA004775;Yang2025_Silkie_Wenchang	PRJNA827465	0.979364626
ENSGALG00010016470	TNIP1	2	-0.231196252	0.292938404	-0.789231623	0.429976643	0.131347022	3.405883498	70.63904268	1	CRA004775;PRJNA827465	Yang2025_Silkie_Wenchang	0.842012722
ENSGALG00010016471	POMGNT2	2	-0.166058642	0.121669326	-1.364835717	0.17230469	0.019612167	2.943345537	66.02505593	1	PRJNA827465;Yang2025_Silkie_Wenchang	CRA004775	0.67516992
ENSGALG00010016471	POMGNT2	2	-0.279942807	0.102870503	-2.721312716	0.006502322	0	0.185014446	0	1	CRA004775;Yang2025_Silkie_Wenchang	PRJNA827465	0.123006701
ENSGALG00010016471	POMGNT2	2	-0.058527704	0.088095523	-0.664366381	0.506455844	0	0.085902329	0	1	CRA004775;PRJNA827465	Yang2025_Silkie_Wenchang	0.863929781
ENSGALG00010016472	TFDP1	2	-0.176376886	0.217056299	-0.812585889	0.416455525	0.090428066	24.64147567	95.94180148	0.5	PRJNA827465;Yang2025_Silkie_Wenchang	CRA004775	0.837921137
ENSGALG00010016472	TFDP1	2	-0.03076725	0.37807412	-0.081378884	0.935140641	0.27172998	19.82348543	94.95547842	0.5	CRA004775;Yang2025_Silkie_Wenchang	PRJNA827465	0.984512821
ENSGALG00010016472	TFDP1	2	0.165352412	0.157779484	1.047996914	0.294640029	0.039090506	4.038466256	75.23812416	1	CRA004775;PRJNA827465	Yang2025_Silkie_Wenchang	0.78744982
ENSGALG00010016473	RAB9B	2	-0.141870778	0.238535517	-0.594757459	0.552005607	0.091210944	4.419990238	77.37551564	0.5	PRJNA827465;Yang2025_Silkie_Wenchang	CRA004775	0.879061525
ENSGALG00010016473	RAB9B	2	-0.261407045	0.135869534	-1.923956302	0.054360061	0	0.940910967	0	1	CRA004775;Yang2025_Silkie_Wenchang	PRJNA827465	0.40381596
ENSGALG00010016473	RAB9B	2	0.007212727	0.089600381	0.08049884	0.935840518	0.005649165	1.327511766	24.67110083	0.5	CRA004775;PRJNA827465	Yang2025_Silkie_Wenchang	0.984801197
ENSGALG00010016474	MAPK8IP3	2	0.415443846	0.167845712	2.475153175	0.013317904	0.037345821	2.954037416	66.14802525	1	PRJNA827465;Yang2025_Silkie_Wenchang	CRA004775	0.188498106
ENSGALG00010016474	MAPK8IP3	2	0.168394297	0.44200833	0.380975394	0.703221507	0.352318058	9.984728717	89.98470536	0.5	CRA004775;Yang2025_Silkie_Wenchang	PRJNA827465	0.923537772
ENSGALG00010016474	MAPK8IP3	2	0.017147726	0.271984757	0.063046643	0.949729363	0.113953099	4.106630067	75.64913363	0.5	CRA004775;PRJNA827465	Yang2025_Silkie_Wenchang	0.98723759
ENSGALG00010016475	MCCC1	2	0.041745919	0.162738472	0.25652151	0.797548173	0.048264422	11.26095174	91.11975592	0.5	PRJNA827465;Yang2025_Silkie_Wenchang	CRA004775	0.949822309
ENSGALG00010016475	MCCC1	2	0.199936652	0.067106074	2.979412152	0.00288802	0	0.07016497	0	1	CRA004775;Yang2025_Silkie_Wenchang	PRJNA827465	0.073467871
ENSGALG00010016475	MCCC1	2	-0.099744257	0.070139079	-1.422092482	0.154999405	0.000809114	1.024947148	2.433993627	0.5	CRA004775;PRJNA827465	Yang2025_Silkie_Wenchang	0.647665491
ENSGALG00010016477	CNIH3	2	0.113702959	0.213310361	0.533040019	0.594005878	0.024924941	1.329275404	24.77104465	0.5	PRJNA827465;Yang2025_Silkie_Wenchang	CRA004775	0.893224663
ENSGALG00010016477	CNIH3	2	-0.169321915	0.291679127	-0.580507481	0.561572444	0	0.005194982	0	1	CRA004775;Yang2025_Silkie_Wenchang	PRJNA827465	0.88176588
ENSGALG00010016477	CNIH3	2	0.230463332	0.20072455	1.148157172	0.250903686	0	0.339520301	0	0.5	CRA004775;PRJNA827465	Yang2025_Silkie_Wenchang	0.760665503
ENSGALG00010016479	ENSGALG00010016479	2	0.436983138	0.383667932	1.138961851	0.254719066	0.231144973	4.497923856	77.76752048	1	PRJNA827465;Yang2025_Silkie_Wenchang	CRA004775	0.764394153
ENSGALG00010016479	ENSGALG00010016479	2	0.188782538	0.828811319	0.227775048	0.819821126	1.04249111	3.650415832	72.60586065	0.5	CRA004775;Yang2025_Silkie_Wenchang	PRJNA827465	0.955275118
ENSGALG00010016479	ENSGALG00010016479	2	-0.018414493	0.296151909	-0.062179213	0.950420116	0.062143379	1.170553817	14.57035245	0.5	CRA004775;PRJNA827465	Yang2025_Silkie_Wenchang	0.987277827
ENSGALG00010016480	GPX3	2	-0.314855478	0.12506237	-2.51758764	0.011816157	0.011028915	1.502771482	33.45628313	1	PRJNA827465;Yang2025_Silkie_Wenchang	CRA004775	0.174328234
ENSGALG00010016480	GPX3	2	-0.426226477	0.152185837	-2.800697398	0.005099231	0	0.340295198	0	1	CRA004775;Yang2025_Silkie_Wenchang	PRJNA827465	0.10649726
ENSGALG00010016480	GPX3	2	-0.220111709	0.113095709	-1.946242801	0.051625581	0	0.012613743	0	1	CRA004775;PRJNA827465	Yang2025_Silkie_Wenchang	0.393961017
ENSGALG00010016482	EML1	2	-0.134160043	0.066717908	-2.010855068	0.044340768	0	0.113497693	0	1	PRJNA827465;Yang2025_Silkie_Wenchang	CRA004775	0.363642137
ENSGALG00010016482	EML1	2	-0.165902113	0.094956529	-1.747137497	0.080613491	0	0.003560888	0	1	CRA004775;Yang2025_Silkie_Wenchang	PRJNA827465	0.488792351
ENSGALG00010016482	EML1	2	-0.125745005	0.077983261	-1.612461494	0.106861553	0	0.076615272	0	1	CRA004775;PRJNA827465	Yang2025_Silkie_Wenchang	0.553927143
ENSGALG00010016483	WDR26	2	-0.168575285	0.086253676	-1.954412772	0.050652421	0.009950253	2.903152112	65.5546812	1	PRJNA827465;Yang2025_Silkie_Wenchang	CRA004775	0.389740039
ENSGALG00010016483	WDR26	2	-0.269671887	0.083039458	-3.247515038	0.001164175	0	0.006603614	0	1	CRA004775;Yang2025_Silkie_Wenchang	PRJNA827465	0.041520485
ENSGALG00010016483	WDR26	2	-0.099248794	0.054882889	-1.808374075	0.0705483	0	0.362590805	0	1	CRA004775;PRJNA827465	Yang2025_Silkie_Wenchang	0.458155954
ENSGALG00010016491	DNAJC8	2	0.160755662	0.24522651	0.655539495	0.512120466	0.09972827	5.018565871	80.07398875	0.5	PRJNA827465;Yang2025_Silkie_Wenchang	CRA004775	0.865694231
ENSGALG00010016491	DNAJC8	2	0.153837268	0.293958585	0.523329735	0.600744812	0.130144213	4.038831792	75.24036525	0.5	CRA004775;Yang2025_Silkie_Wenchang	PRJNA827465	0.895230519
ENSGALG00010016491	DNAJC8	2	-0.046506087	0.040869884	-1.137906035	0.255159721	0	0.200809836	0	1	CRA004775;PRJNA827465	Yang2025_Silkie_Wenchang	0.764467569
ENSGALG00010016496	PLP1	2	0.303785579	0.393916576	0.771192676	0.440592735	0.192921282	2.250100918	55.55754892	1	PRJNA827465;Yang2025_Silkie_Wenchang	CRA004775	0.844795407
ENSGALG00010016496	PLP1	2	1.296339902	0.455633907	2.84513484	0.004439263	0.133702359	1.474111169	32.16251114	1	CRA004775;Yang2025_Silkie_Wenchang	PRJNA827465	0.097602941
ENSGALG00010016496	PLP1	2	0.822374178	0.869518748	0.945780847	0.344260376	1.354029147	9.021402911	88.91524955	1	CRA004775;PRJNA827465	Yang2025_Silkie_Wenchang	0.806633216

ENSGALG00010016498	KIF23	2	0.052744398	0.070867207	0.744270872	0.456712619	0	0.465407749	0	1	PRJNA827465;Yang2025_Silkie_Wenchang	CRA004775	0.849515887
ENSGALG00010016498	KIF23	2	0.129640265	0.111117103	1.166699462	0.24333176	0	0.291472371	0	1	CRA004775;Yang2025_Silkie_Wenchang	PRJNA827465	0.755238542
ENSGALG00010016498	KIF23	2	0.028346003	0.088354381	0.320821698	0.748345515	0	0.58401091	0	1	CRA004775;PRJNA827465	Yang2025_Silkie_Wenchang	0.937232292
ENSGALG00010016500	ENSGALG00010016500	2	-0.095851893	0.061359135	-1.562145443	0.11825373	0	0.542055471	0	1	PRJNA827465;Yang2025_Silkie_Wenchang	CRA004775	0.578003524
ENSGALG00010016500	ENSGALG00010016500	2	-0.120871288	0.231298046	-0.522578077	0.6012679	0.007676154	1.070452375	6.581551528	0.5	CRA004775;Yang2025_Silkie_Wenchang	PRJNA827465	0.895230519
ENSGALG00010016500	ENSGALG00010016500	2	-0.078042131	0.062092575	-1.256867371	0.208801665	0	0.518372712	0	0.5	CRA004775;PRJNA827465	Yang2025_Silkie_Wenchang	0.721011841
ENSGALG00010016502	YIPF5	2	-0.040674537	0.168085046	-0.241987841	0.808789585	0.044432465	4.213208707	76.26512073	0.5	PRJNA827465;Yang2025_Silkie_Wenchang	CRA004775	0.952595795
ENSGALG00010016502	YIPF5	2	-0.262416144	0.140293885	-1.870474571	0.061417945	0	0.10794277	0	1	CRA004775;Yang2025_Silkie_Wenchang	PRJNA827465	0.427938152
ENSGALG00010016502	YIPF5	2	-0.014805359	0.199834406	-0.074088139	0.940940253	0.049485182	1.865367752	46.39126795	0.5	CRA004775;PRJNA827465	Yang2025_Silkie_Wenchang	0.98556663
ENSGALG00010016504	SNRK	2	-0.023805164	0.093491026	-0.254625121	0.799012655	0.008385596	1.699484573	41.15863032	0.5	PRJNA827465;Yang2025_Silkie_Wenchang	CRA004775	0.949922217
ENSGALG00010016504	SNRK	2	0.108969734	0.126942125	0.858420591	0.390660262	0	0.010777724	0	1	CRA004775;Yang2025_Silkie_Wenchang	PRJNA827465	0.829251057
ENSGALG00010016504	SNRK	2	-0.078980902	0.06412975	-1.231579763	0.218106104	0	0.300252533	0	0.5	CRA004775;PRJNA827465	Yang2025_Silkie_Wenchang	0.73160572
ENSGALG00010016507	SPATA18	2	-0.292884805	0.100817009	-2.90511301	0.003671205	0.00466402	1.295501836	22.80983535	1	PRJNA827465;Yang2025_Silkie_Wenchang	CRA004775	0.086556316
ENSGALG00010016507	SPATA18	2	-0.363842949	0.124204509	-2.929385993	0.003396324	0	0.747876224	0	1	CRA004775;Yang2025_Silkie_Wenchang	PRJNA827465	0.081842865
ENSGALG00010016507	SPATA18	2	-0.186518008	0.113604164	-1.641823694	0.100626547	0	0.127977319	0	1	CRA004775;PRJNA827465	Yang2025_Silkie_Wenchang	0.541412814
ENSGALG00010016508	CREBBP	2	0.240293064	0.421488599	0.570105726	0.568605991	0.331935319	14.5510192	93.12762916	0.5	PRJNA827465;Yang2025_Silkie_Wenchang	CRA004775	0.884199349
ENSGALG00010016508	CREBBP	2	0.592364096	0.159366074	3.717002492	0.0002016	0	0.460100181	0	1	CRA004775;Yang2025_Silkie_Wenchang	PRJNA827465	0.012862829
ENSGALG00010016508	CREBBP	2	0.11116421	0.309906843	0.358702016	0.71981802	0.164100129	6.160892232	83.76858477	0.5	CRA004775;PRJNA827465	Yang2025_Silkie_Wenchang	0.928564173
ENSGALG00010016510	THG1L	2	-0.036296454	0.095434945	-0.380326667	0.703702943	0.00168372	1.100728887	9.151107791	0.5	PRJNA827465;Yang2025_Silkie_Wenchang	CRA004775	0.923645363
ENSGALG00010016510	THG1L	2	0.012572637	0.120784195	0.104091743	0.917096544	0	0.808865418	0	0.5	CRA004775;Yang2025_Silkie_Wenchang	PRJNA827465	0.980728718
ENSGALG00010016510	THG1L	2	-0.135729897	0.110528399	-1.228009255	0.219443494	0	0.06443098	0	1	CRA004775;PRJNA827465	Yang2025_Silkie_Wenchang	0.732888275
ENSGALG00010016514	GASK1A	2	0.054823976	0.122605518	0.447157495	0.654761349	0	0.649004581	0	0.5	PRJNA827465;Yang2025_Silkie_Wenchang	CRA004775	0.910222014
ENSGALG00010016514	GASK1A	2	-0.097818754	0.196446661	-0.497940528	0.61852596	0	0.094515478	0	1	CRA004775;Yang2025_Silkie_Wenchang	PRJNA827465	0.899168321
ENSGALG00010016514	GASK1A	2	0.106160233	0.148484168	0.714959948	0.474633792	0	0.38904719	0	0.5	CRA004775;PRJNA827465	Yang2025_Silkie_Wenchang	0.854462864
ENSGALG00010016518	TMEM42	2	-0.211694103	0.073182464	-2.892688925	0.003819594	0.00074024	1.044363297	4.247879703	1	PRJNA827465;Yang2025_Silkie_Wenchang	CRA004775	0.088674731
ENSGALG00010016518	TMEM42	2	-0.411017949	0.128501098	-3.198555934	0.001381177	0	0.209960015	0	1	CRA004775;Yang2025_Silkie_Wenchang	PRJNA827465	0.045617155
ENSGALG00010016518	TMEM42	2	-0.27198184	0.142162796	-1.91317171	0.055726074	0.023497911	1.995882613	49.89685298	1	CRA004775;PRJNA827465	Yang2025_Silkie_Wenchang	0.408696004
ENSGALG00010016519	MEX3B	2	0.180591218	0.139898124	1.290876626	0.196746462	0.028456225	3.620587028	72.38016951	1	PRJNA827465;Yang2025_Silkie_Wenchang	CRA004775	0.707994211
ENSGALG00010016519	MEX3B	2	0.339682122	0.109529206	3.101292647	0.001926778	0	0.063191484	0	1	CRA004775;Yang2025_Silkie_Wenchang	PRJNA827465	0.056682741
ENSGALG00010016519	MEX3B	2	0.095634387	0.121843648	0.784894321	0.432515532	0.008701122	1.146639042	12.78859666	1	CRA004775;PRJNA827465	Yang2025_Silkie_Wenchang	0.842762754
ENSGALG00010016520	CHMP1B	2	0.087958733	0.099320279	0.885606987	0.375829345	0.01260379	2.296961901	56.46423219	1	PRJNA827465;Yang2025_Silkie_Wenchang	CRA004775	0.822974013
ENSGALG00010016520	CHMP1B	2	0.024156118	0.222977584	0.108334291	0.913730522	0.074604298	3.925487881	74.5254595	0.5	CRA004775;Yang2025_Silkie_Wenchang	PRJNA827465	0.979364626
ENSGALG00010016520	CHMP1B	2	-0.032927405	0.097345262	-0.338253801	0.735171935	0.009991759	1.558384711	35.83099262	0.5	CRA004775;PRJNA827465	Yang2025_Silkie_Wenchang	0.933592889
ENSGALG00010016526	EIF1B	2	-0.013404116	0.063970794	-0.209534932	0.834030669	0	0.283833329	0	0.5	PRJNA827465;Yang2025_Silkie_Wenchang	CRA004775	0.957968425
ENSGALG00010016526	EIF1B	2	0.03587782	0.120769317	0.297077279	0.76640751	0	0.052374027	0	0.5	CRA004775;Yang2025_Silkie_Wenchang	PRJNA827465	0.941152991
ENSGALG00010016526	EIF1B	2	-0.032662673	0.07164656	-0.455886138	0.648471858	0	1.2717E-06	0	1	CRA004775;PRJNA827465	Yang2025_Silkie_Wenchang	0.907652758
ENSGALG00010016527	MRPS34	2	0.29625983	0.265861283	1.114339879	0.265133405	0.13231697	15.2741594	93.45299487	1	PRJNA827465;Yang2025_Silkie_Wenchang	CRA004775	0.769274038
ENSGALG00010016527	MRPS34	2	0.218141301	0.369458427	0.590435312	0.554898843	0.247453646	10.51836126	90.49281561	0.5	CRA004775;Yang2025_Silkie_Wenchang	PRJNA827465	0.880223402
ENSGALG00010016527	MRPS34	2	0.017695786	0.065165214	0.271552645	0.785966015	0.001116534	1.054763248	5.191994326	0.5	CRA004775;PRJNA827465	Yang2025_Silkie_Wenchang	0.946639719
ENSGALG00010016529	DCUN1D1	2	-0.100784738	0.058461003	-1.723965248	0.084714117	0.002514265	1.515563642	34.01794735	1	PRJNA827465;Yang2025_Silkie_Wenchang	CRA004775	0.500353159
ENSGALG00010016529	DCUN1D1	2	-0.014555262	0.187088301	-0.077798887	0.937988032	0.054892493	4.353460389	77.02976688	0.5	CRA004775;Yang2025_Silkie_Wenchang	PRJNA827465	0.985349045
ENSGALG00010016529	DCUN1D1	2	0.026406898	0.11983425	0.22036186	0.825589346	0.018410382	2.283629391	56.21005737	0.5	CRA004775;PRJNA827465	Yang2025_Silkie_Wenchang	0.956386623
ENSGALG00010016533	CNIH4	2	-0.038370882	0.073767936	-0.52015665	0.602954397	0	0.015710551	0	1	PRJNA827465;Yang2025_Silkie_Wenchang	CRA004775	0.895538966
ENSGALG00010016533	CNIH4	2	-0.181663305	0.203101242	-0.894447038	0.371082755	0	0.487093533	0	1	CRA004775;Yang2025_Silkie_Wenchang	PRJNA827465	0.8214684
ENSGALG00010016533	CNIH4	2	-0.052658412	0.074912323	-0.702933909	0.482096938	0	0.935317934	0	1	CRA004775;PRJNA827465	Yang2025_Silkie_Wenchang	0.85691916
ENSGALG00010016534	TRIT1	2	0.142342897	0.081100602	1.755139848	0.07923539	0	0.367492234	0	1	PRJNA827465;Yang2025_Silkie_Wenchang	CRA004775	0.484394238
ENSGALG00010016534	TRIT1	2	-0.164359785	0.222014501	-0.740311034	0.459111287	0.060083053	2.545406781	60.71354853	0.5	CRA004775;Yang2025_Silkie_Wenchang	PRJNA827465	0.850098539
ENSGALG00010016534	TRIT1	2	-0.084397846	0.281939667	-0.299347187	0.764675155	0.134456678	6.050319867	83.47194823	0.5	CRA004775;PRJNA827465	Yang2025_Silkie_Wenchang	0.940711384
ENSGALG00010016535	BNC2	2	0.060849809	0.195456474	0.311321531	0.755556201	0.038375404	1.797273592	44.36016841	0.5	PRJNA827465;Yang2025_Silkie_Wenchang	CRA004775	0.938501652
ENSGALG00010016535	BNC2	2	0.277675074	0.239578711	1.159013971	0.24645049	0	0.190744624	0	1	CRA004775;Yang2025_Silkie_Wenchang	PRJNA827465	0.756843363
ENSGALG00010016535	BNC2	2	-0.062200276	0.130857142	-0.47532962	0.634552051	0	0.132636395	0	0.5	CRA004775;PRJNA827465	Yang2025_Silkie_Wenchang	0.903872221
ENSGALG00010016541	UBE2J2	2	-0.119604907	0.095742298	-1.249237897	0.211578075	0.015690513	6.723753662	85.12735519	1	PRJNA827465;Yang2025_Silkie_Wenchang	CRA004775	0.724013667
ENSGALG00010016541	UBE2J2	2	-0.105655831	0.13678887	-0.772400786	0.439877089	0.028303825	3.81833224	73.81055558	0.5	CRA004775;Yang2025_Silkie_Wenchang	PRJNA827465	0.844353254
ENSGALG00010016541	UBE2J2	2	-0.02382412	0.035796238	-0.665548141	0.505699963	0	0.414874051	0	0.5	CRA004775;PRJNA827465	Yang2025_Silkie_Wenchang	0.863927652
ENSGALG00010016542	ENOX2	2	0.091038883	0.041125111	2.21370546	0.026849047	0	0.186051685	0	1	PRJNA827465;Yang2025_Silkie_Wenchang	CRA004775	0.279313949
ENSGALG00010016542	ENOX2	2	0.083253184	0.078907569	1.055072219	0.291392297	0	0.441948775	0	1	CRA004775;Yang2025_Silkie_Wenchang	PRJNA827465	0.784817029
ENSGALG00010016542	ENOX2	2	0.106103477	0.045043002	2.355604042	0.018492618	0	0.235271809	0	1	CRA004775;PRJNA827465	Yang2025_Silkie_Wenchang	0.227601507
ENSGALG00010016543	TCEA3	2	-0.174367137	0.223905007	-0.778754969	0.436124069	0.073177168	3.683027489	72.84842421	0.5	PRJNA827465;Yang2025_Silkie_Wenchang	CRA004775	0.843095975
ENSGALG00010016543	TCEA3	2	0.026901086	0.145750821	0.184569017	0.853567089	0	0.101505143	0	0.5	CRA004775;Yang2025_Silkie_Wenchang	PRJNA827465	0.962964214
ENSGALG00010016543	TCEA3	2	-0.380718714	0.168531172	-2.259040329	0.023880876	0	0.235790335	0	1	CRA004775;PRJNA827465	Yang2025_Silkie_Wenchang	0.264093469
ENSGALG00010016544	ADCY9	2	-0.099457355	0.039769592	-2.500839229	0.012389941	0	0.710516129	0	1	PRJNA827465;Yang2025_Silkie_Wenchang	CRA004775	0.180135188
ENSGALG00010016544	ADCY9	2	-0.136486752	0.054891536	-2.48648081	0.012901353	0	0.14571051	0	1	CRA004775;Yang2025_Silkie_Wenchang	PRJNA827465	0.18452033
ENSGALG00010016544	ADCY9	2	-0.071915239	0.055618046	-1.293019883	0.196004182	0	0.36524092	0	1	CRA004775;PRJNA827465	Yang2025_Silkie_Wenchang	0.707301271
ENSGALG00010016546	C1QTNF12	2	0.366632984	0.250138347	1.46572082	0.14272436	0	0.449761881	0	1	PRJNA827465;Yang2025_Silkie_Wenchang	CRA004775	0.629032597
ENSGALG00010016546	C1QTNF12	2	0.599558319	0.305999906	1.959341515	0.050072805	0	0.126256804	0	1	CRA004775;Yang2025_Silkie_Wenchang	PRJNA827465	0.387273603
ENSGALG00010016546	C1QTNF12	2	0.348710876	0.304371404	1.145675553	0.251929423	0	0.698981597	0	1	CRA004775;PRJNA827465	Yang2025_Silkie_Wenchang	0.761871849
ENSGALG00010016547	SGCB	2	-0.309613133	0.083657338	-3.700968049	0.000214779	0	0.389304476	0	1	PRJNA827465;Yang2025_Silkie_Wenchang	CRA004775	0.013365444
ENSGALG00010016547	SGCB	2	-0.334207844	0.111018561	-3.010378083	0.002609227	0	0.514145387	0	1	CRA004775;Yang2025_Silkie_Wenchang	PRJNA827465	0.069130135
ENSGALG00010016547	SGCB	2	-0.239464531	0.113339063	-2.112815525	0.03461656	0	0.189112619	0	1	CRA004775;PRJNA827465	Yang2025_Silkie_Wenchang	0.320497684
ENSGALG00010016548	EVL	2	-0.143068247	0.048022643	-2.979183128	0.00289018	0	0.747807822	0	1	PRJNA827465;Yang2025_Silkie_Wenchang	CRA004775	0.073475253
ENSGALG00010016548	EVL	2	0.031859951	0.125463762	0.253937478	0.799543861	0	0.489430953	0	0.5	CRA004775;Yang2025_Silkie_Wenchang	PRJNA827465	0.949922217
ENSGALG00010016548	EVL	2	-0.073797746	0.143828054	-0.513097022	0.607883455	0.02509045	1.844033439	45.77104847	0.5	CRA004775;PRJNA827465	Yang2025_Silkie_Wenchang	0.897203929
ENSGALG00010016549	PTPRA	2	-0.000377202	0.043390157	-0.008693252	0.993063875	0	0.622639578	0	0.5	PRJNA827465;Yang2025_Silkie_Wenchang	CRA004775	0.99794341
ENSGALG00010016549	PTPRA	2	0.07313422	0.095481312	0.765953236	0.443704128	0	0.000218009	0	1	CRA004775;Yang2025_Silkie_Wenchang	PRJNA827465	0.845840387
ENSGALG00010016549	PTPRA	2	-0.013561881	0.047183036	-0.287431285	0.773782109	0	0.11690808	0	0.5	CRA004775;PRJNA827465	Yang2025_Silkie_Wenchang	0.943248868
ENSGALG00010016550	FBLIM1	2	-0.257369684	0.115228705	-2.233555298	0.025512348	0	0.203790561	0	1	PRJNA827465;Yang2025_Silkie_Wenchang	CRA004775	0.272402014
ENSGALG00010016550	FBLIM1	2	-0.279345593	0.243039495	-1.14938353	0.250397868	0	0.577242868	0	1	CRA004775;Yang2025_Silkie_Wenchang	PRJNA827465	0.759943035
ENSGALG00010016550	FBLIM1	2	-0.299028717	0.117476168	-2.545441533	0.010913964	0	0.309407186	0	1	CRA004775;PRJNA827465	Yang2025_Silkie_Wenchang	0.166322288
ENSGALG00010016552	ENSGALG00010016552	2	0.646092256	0.748751497	0.862892774	0.388196424	1.031591711	12.13844126	91.76170994	0.5	PRJNA827465;Yang2025_Silkie_Wenchang	CRA004775	0.828154121
ENSGALG00010016552	ENSGALG00010016552	2	1.293629345	0.325561333	3.973534976	7.08138E-05	0	0.447164152	0	1	CRA004775;Yang2025_Silkie_Wenchang	PRJNA827465	0.006103493
ENSGALG00010016552	ENSGALG00010016552	2	0.286581773	0.485496498	0.590285973	0.554998942	0.324454287	2.642265175	62.15368504	0.5	CRA004775;PRJNA827465	Yang2025_Silkie_Wenchang	0.880223402
ENSGALG00010016553	SLC25A14	2	0.100860144	0.062232346	1.620702919	0.105081367	0	0.179119365	0	1	PRJNA827465;Yang2025_Silkie_Wenchang	CRA004775	0.55030153
ENSGALG00010016553	SLC25A14	2	-0.170618631	0.244504923	-0.697812659	0.485294361	0.070668646	2.330000236	57.08154942	0.5	CRA004775;Yang2025_Silkie_Wenchang	PRJNA827465	0.857076964
ENSGALG00010016553	SLC25A14	2	-0.115139876	0.281431447	-0.409122282	0.682449925	0.125881224	4.128217535	75.77647031	0.5	CRA004775;PRJNA827465	Yang2025_Silkie_Wenchang	0.918625039
ENSGALG00010016556	ASAP3	2	-0.087683503	0.207186605	-0.423210289	0.672141841	0.068595621	4.934211186	79.73333604	0.5	PRJNA827465;Yang2025_Silkie_Wenchang	CRA004775	0.914742762
ENSGALG00010016556	ASAP3	2	-0.301026239	0.137279191	-2.192803118	0.028321567	0	0.004912515	0	1	CRA004775;Yang2025_Silkie_Wenchang	PRJNA827465	0.286739453
ENSGALG00010016556	ASAP3	2	0.080331612	0.11164056	0.719555797	0.471798536	0	0.85260916	0	0.5	CRA004775;PRJNA827465	Yang2025_Silkie_Wenchang	0.853998373
ENSGALG00010016558	NR2F2	2	0.04430298	0.113586022	0.390039011	0.6965077	0.017514549	2.615301008	61.76348355	0.5	PRJNA827465;Yang2025_Silkie_Wenchang	CRA004775	0.921041503
ENSGALG00010016558	NR2F2	2	0.216946641	0.121910948	1.77955011	0.075149617	0	0.07083149	0	1	CRA004775;Yang2025_Silkie_Wenchang	PRJNA827465	0.473106744
ENSGALG00010016558	NR2F2	2	0.020506896	0.123334707	0.166270281	0.867944253	0.01642413	1.494831434	33.10282501	0.5	CRA004775;PRJNA827465	Yang2025_Silkie_Wenchang	0.967959054
ENSGALG00010016559	ROGDI	2	-0.034087232	0.067589238	-0.504329294	0.614029995	0	0.250640777	0	0.5	PRJNA827465;Yang2025_Silkie_Wenchang	CRA004775	0.898282836
ENSGALG00010016559	ROGDI	2	-0.359780249	0.431763498	-0.833280838	0.404686361	0.318199229	6.489190026	84.58975626	0.5	CRA004775;Yang2025_Silkie_Wenchang	PRJNA827465	0.833112102
ENSGALG00010016559	ROGDI	2	-0.386747938	0.387549658	-0.997931309	0.318312669	0.257110288	6.190077113	83.8451124	1	CRA004775;PRJNA827465	Yang2025_Silkie_Wenchang	0.797669141
ENSGALG00010016560	DCUN1D4	2	-0.004141889	0.109305211	-0.037892879	0.969773091	0.013524752	2.291671443	56.36372732	0.5	PRJNA827465;Yang2025_Silkie_Wenchang	CRA004775	0.991572914
ENSGALG00010016560	DCUN1D4	2	-0.098028631	0.10299649	-0.951766717	0.341215305	0	0.365460392	0	0.5	CRA004775;Yang2025_Silkie_Wenchang	PRJNA827465	0.805646109
ENSGALG00010016560	DCUN1D4	2	0.09599634	0.090097031	1.06547729	0.286659997	0	0.006688983	0	1	CRA004775;PRJNA827465	Yang2025_Silkie_Wenchang	0.782018115
ENSGALG00010016562	GLIS2	2	0.224518012	0.256394552	0.875673881	0.381207349	0.105127316	4.535694713	77.95266077	1	PRJNA827465;Yang2025_Silkie_Wenchang	CRA004775	0.824392515
ENSGALG00010016562	GLIS2	2	0.332269022	0.255872805	1.298571066	0.194091173	0.051645589	1.574066588	36.47028609	0.5	CRA004775;Yang2025_Silkie_Wenchang	PRJNA827465	0.704693637
ENSGALG00010016562	GLIS2	2	0.005196241	0.088653094	0.058613191	0.953260204	0	0.00120118	0	0.5	CRA004775;PRJNA827465	Yang2025_Silkie_Wenchang	0.987758913
ENSGALG00010016564	SPEN	2	-0.110732891	0.163263415	-0.678246816	0.497615214	0.031456106	2.029462076	50.72585925	0.5	PRJNA827465;Yang2025_Silkie_Wenchang	CRA004775	0.86098656
ENSGALG00010016564	SPEN	2	0.336957193	0.251771966	1.338342781	0.180784722	0.051892069	1.647434953	39.29957609	1	CRA004775;Yang2025_Silkie_Wenchang	PRJNA827465	0.688484814
ENSGALG00010016564	SPEN	2	0.154911139	0.429227966	0.360906444	0.718169387	0.31811145	6.588724042	84.82255451	0.5	CRA004775;PRJNA827465	Yang2025_Silkie_Wenchang	0.927975712
ENSGALG00010016565	NVL	2	-0.114580302	0.049728741	-2.304106219	0.021216682	0	0.927430634	0	1	PRJNA827465;Yang2025_Silkie_Wenchang	CRA004775	0.246272665
ENSGALG00010016565	NVL	2	-0.186140871	0.073592384	-2.529349647	0.011427412	0	0.145837159	0	1	CRA004775;Yang2025_Silkie_Wenchang	PRJNA827465	0.17044842
ENSGALG00010016565	NVL	2	-0.093848966	0.060435304	-1.552858156	0.120451052	0	0.769251715	0	1	CRA004775;PRJNA827465	Yang2025_Silkie_Wenchang	0.581869184
ENSGALG00010016566	CNTLN	2	0.374754085	0.383766549	0.976515764	0.32880894	0.23650905	5.005533779	80.02211064	1	PRJNA827465;Yang2025_Silkie_Wenchang	CRA004775	0.800761292
ENSGALG00010016566	CNTLN	2	0.573161063	0.415483587	1.3795035	0.167739568	0.139803837	1.351563792	26.01163145	0.5	CRA004775;Yang2025_Silkie_Wenchang	PRJNA827465	0.667779227
ENSGALG00010016566	CNTLN	2	-0.002026317	0.198855069	-0.010189917	0.991869763	0	0.094357896	0	0.5	CRA004775;PRJNA827465	Yang2025_Silkie_Wenchang	0.997575757
ENSGALG00010016567	E2F2	2	-0.021566909	0.10814254	-0.199430396	0.841926086	0	0.139199972	0	0.5	PRJNA827465;Yang2025_Silkie_Wenchang	CRA004775	0.960041272
ENSGALG00010016567	E2F2	2	-0.052858393	0.188829667	-0.279926318	0.779534035	0	0.186549288	0	0.5	CRA004775;Yang2025_Silkie_Wenchang	PRJNA827465	0.944807046
ENSGALG00010016567	E2F2	2	0.012440939	0.123632389	0.100628475	0.919845392	0	0.077513624	0	1	CRA004775;PRJNA827465	Yang2025_Silkie_Wenchang	0.981237238
ENSGALG00010016568	PAM16	2	-0.095911584	0.08817309	-1.087768806	0.276698095	0	0.742185201	0	1	PRJNA827465;Yang2025_Silkie_Wenchang	CRA004775	0.775851484
ENSGALG00010016568	PAM16	2	-0.135794542	0.235747293	-0.576017395	0.564603424	0	0.893632618	0	0.5	CRA004775;Yang2025_Silkie_Wenchang	PRJNA827465	0.883173142
ENSGALG00010016568	PAM16	2	-0.06713002	0.087024017	-0.771396479	0.440471962	0	0.154600889	0	0.5	CRA004775;PRJNA827465	Yang2025_Silkie_Wenchang	0.844733855
ENSGALG00010016569	YY1	2	-0.09742232	0.102967294	-0.946148204	0.344073	0.01531033	2.995156771	66.61276599	1	PRJNA827465;Yang2025_Silkie_Wenchang	CRA004775	0.806445383
ENSGALG00010016569	YY1	2	0.113325185	0.365606641	0.309964788	0.756587733	0.240548266	9.766910022	89.75507564	0.5	CRA004775;Yang2025_Silkie_Wenchang	PRJNA827465	0.938700918
ENSGALG00010016569	YY1	2	0.200600508	0.256026902	0.783513399	0.433325689	0.112958306	6.363256419	84.284776	0.5	CRA004775;PRJNA827465	Yang2025_Silkie_Wenchang	0.842762754
ENSGALG00010016572	RAB33A	2	0.296256883	0.295810035	1.001510591	0.316580022	0.166189258	19.37978927	94.83998517	1	PRJNA827465;Yang2025_Silkie_Wenchang	CRA004775	0.796883862
ENSGALG00010016572	RAB33A	2	0.297941255	0.321216784	0.927539501	0.353646493	0.180835938	7.932325165	87.39335593	0.5	CRA004775;Yang2025_Silkie_Wenchang	PRJNA827465	0.811318534
ENSGALG00010016572	RAB33A	2	0.007248878	0.050809231	0.142668531	0.886551971	0	0.066411125	0	0.5	CRA004775;PRJNA827465	Yang2025_Silkie_Wenchang	0.972729743
ENSGALG00010016573	DEGS1	2	-0.079296426	0.136884759	-0.579293313	0.562391277	0.032750315	7.884734206	87.3172643	0.5	PRJNA827465;Yang2025_Silkie_Wenchang	CRA004775	0.881950027
ENSGALG00010016573	DEGS1	2	-0.232464032	0.073692943	-3.154495151	0.001607761	0	0.347353788	0	1	CRA004775;Yang2025_Silkie_Wenchang	PRJNA827465	0.050669962
ENSGALG00010016573	DEGS1	2	-0.086626832	0.205280014	-0.421993503	0.673029759	0.058155209	2.492746463	59.88360571	0.5	CRA004775;PRJNA827465	Yang2025_Silkie_Wenchang	0.919153756
ENSGALG00010016576	MRPS26	2	-0.044576927	0.067575871	-0.659657446	0.509473681	0.00068924	1.055236104	5.234478177	1	PRJNA827465;Yang2025_Silkie_Wenchang	CRA004775	0.865257661
ENSGALG00010016576	MRPS26	2	-0.169598976	0.128422579	-1.320632071	0.186624074	0	0.000324328	0	1	CRA004775;Yang2025_Silkie_Wenchang	PRJNA827465	0.695457578
ENSGALG00010016576	MRPS26	2	-0.016081119	0.070232145	-0.228970926	0.818891514	0	0.232483574	0	1	CRA004775;PRJNA827465	Yang2025_Silkie_Wenchang	0.955256039
ENSGALG00010016577	ZBTB17	2	0.108160937	0.167808313	0.644550529	0.519218503	0.050557193	9.635175032	89.62136135	0.5	PRJNA827465;Yang2025_Silkie_Wenchang	CRA004775	0.869024024
ENSGALG00010016577	ZBTB17	2	0.14211495	0.208990938	0.680005319	0.496501093	0.055360344	2.194586148	54.43333461	0.5	CRA004775;Yang2025_Silkie_Wenchang	PRJNA827465	0.860939846
ENSGALG00010016577	ZBTB17	2	-0.057637238	0.058508009	-0.985117066	0.324566571	0	0.150389221	0	1	CRA004775;PRJNA827465	Yang2025_Silkie_Wenchang	0.799859262
ENSGALG00010016580	MARS2	2	-0.090330175	0.067489412	-1.338434761	0.180754745	0	0.515293863	0	1	PRJNA827465;Yang2025_Silkie_Wenchang	CRA004775	0.688484814
ENSGALG00010016580	MARS2	2	-0.22727084	0.14425008	-1.575533546	0.115133342	0	0.000418234	0	1	CRA004775;Yang2025_Silkie_Wenchang	PRJNA827465	0.572141484
ENSGALG00010016580	MARS2	2	-0.090354895	0.06732481	-1.342074274	0.179571914	0	0.496621573	0	1	CRA004775;PRJNA827465	Yang2025_Silkie_Wenchang	0.686481369
ENSGALG00010016581	H2AFYMACROH2A1	2	0.084526344	0.078642733	1.074814416	0.282457825	0.00565072	1.654151457	39.54604363	1	PRJNA827465;Yang2025_Silkie_Wenchang	CRA004775	0.779532565
ENSGALG00010016581	H2AFYMACROH2A1	2	0.167070392	0.105128652	1.589199416	0.112015378	0	0.384796417	0	1	CRA004775;Yang2025_Silkie_Wenchang	PRJNA827465	0.56547057
ENSGALG00010016581	H2AFYMACROH2A1	2	0.031398398	0.056573367	0.555003165	0.578892496	0	0.000865193	0	1	CRA004775;PRJNA827465	Yang2025_Silkie_Wenchang	0.887888423
ENSGALG00010016583	VPS41	2	-0.005137854	0.068268597	-0.0752594	0.940008324	0	0.369267337	0	0.5	PRJNA827465;Yang2025_Silkie_Wenchang	CRA004775	0.985444231
ENSGALG00010016583	VPS41	2	-0.002726094	0.095641878	-0.028503145	0.97726086	0	0.769844398	0	0.5	CRA004775;Yang2025_Silkie_Wenchang	PRJNA827465	0.993487352
ENSGALG00010016583	VPS41	2	-0.055374292	0.077682968	-0.712824098	0.475954612	0	0.255232725	0	1	CRA004775;PRJNA827465	Yang2025_Silkie_Wenchang	0.835800041
ENSGALG00010016584	EHBP1	2	0.06840112	0.127458947	0.536652164	0.591507005	0.01887154	1.970530627	49.25224778	0.5	PRJNA827465;Yang2025_Silkie_Wenchang	CRA004775	0.892776493
ENSGALG00010016584	EHBP1	2	0.2389699	0.152834297	1.56358818	0.117914313	0	0.043141358	0	1	CRA004775;Yang2025_Silkie_Wenchang	PRJNA827465	0.577693705
ENSGALG00010016584	EHBP1	2	-0.003883327	0.065139311	-0.059614848	0.952462393	0	0.563683994	0	0.5	CRA004775;PRJNA827465	Yang2025_Silkie_Wenchang	0.987758913
ENSGALG00010016588	ATP11B	2	-0.120202815	0.042250646	-2.845419558	0.004435296	0	0.368620332	0	1	PRJNA827465;Yang2025_Silkie_Wenchang	CRA004775	0.097602941
ENSGALG00010016588	ATP11B	2	-0.012613745	0.122395578	-0.103057191	0.917917581	0	0.11319595	0	0.5	CRA004775;Yang2025_Silkie_Wenchang	PRJNA827465	0.981033557
ENSGALG00010016588	ATP11B	2	-0.121994307	0.043482202	-2.805614711	0.00502207	0	0.58037046	0	1	CRA004775;PRJNA827465	Yang2025_Silkie_Wenchang	0.10566511
ENSGALG00010016589	FBXO28	2	-0.327003887	0.326886141	-1.000360206	0.317136221	0.202818905	19.04507923	94.74929987	1	PRJNA827465;Yang2025_Silkie_Wenchang	CRA004775	0.797007794
ENSGALG00010016589	FBXO28	2	-0.499465453	0.192447342	-2.595335679	0.009449858	0.045341778	2.505525624	60.08821501	1	CRA004775;Yang2025_Silkie_Wenchang	PRJNA827465	0.152962732
ENSGALG00010016589	FBXO28	2	-0.074966406	0.112351345	-0.667249743	0.504612623	0.013862654	1.6457064	39.23581996	1	CRA004775;PRJNA827465	Yang2025_Silkie_Wenchang	0.863512504
ENSGALG00010016591	HOMER2	2	0.618652748	0.587265406	1.053446606	0.292136353	0.635623883	12.69342553	92.12190596	1	PRJNA827465;Yang2025_Silkie_Wenchang	CRA004775	0.785516679
ENSGALG00010016591	HOMER2	2	0.872899131	0.399662468	2.184090828	0.028954573	0.211114308	2.82047575	64.54772655	1	CRA004775;Yang2025_Silkie_Wenchang	PRJNA827465	0.209752292
ENSGALG00010016591	HOMER2	2	0.116245062	0.18300612	0.635197675	0.5252995	0	0.648747699	0	1	CRA004775;PRJNA827465	Yang2025_Silkie_Wenchang	0.870188344
ENSGALG00010016592	TMEM129	2	-0.22002365	0.261392286	-0.841737351	0.399934987	0.126888801	13.64177883	92.66957768	0.5	PRJNA827465;Yang2025_Silkie_Wenchang	CRA004775	0.832246851
ENSGALG00010016592	TMEM129	2	-0.38021828	0.145109839	-2.620210196	0.008787558	0.017699335	1.653802309	39.5332807	1	CRA004775;Yang2025_Silkie_Wenchang	PRJNA827465	0.147090871
ENSGALG00010016592	TMEM129	2	-0.006095263	0.082299715	-0.07406178	0.940961227	0.004160667	1.198657455	16.57332993	0.5	CRA004775;PRJNA827465	Yang2025_Silkie_Wenchang	0.98556663
ENSGALG00010016597	FRYL	2	-0.036733153	0.045567915	-0.806118793	0.420174356	0	0.144453255	0	1	PRJNA827465;Yang2025_Silkie_Wenchang	CRA004775	0.838662794
ENSGALG00010016597	FRYL	2	-0.07307587	0.12189356	-0.599505582	0.548835789	0	0.051158404	0	0.5	CRA004775;Yang2025_Silkie_Wenchang	PRJNA827465	0.878281266
ENSGALG00010016597	FRYL	2	-0.02956974	0.048305769	-0.612136822	0.540447297	0	0.009706524	0	1	CRA004775;PRJNA827465	Yang2025_Silkie_Wenchang	0.875506901
ENSGALG00010016598	AIFM1	2	-0.111474807	0.052110111	-2.139216462	0.032418142	0	0.04822815	0	1	PRJNA827465;Yang2025_Silkie_Wenchang	CRA004775	0.309308824
ENSGALG00010016598	AIFM1	2	-0.070243942	0.149054109	-0.471264711	0.637451713	0	6.98315E-05	0	1	CRA004775;Yang2025_Silkie_Wenchang	PRJNA827465	0.905186959
ENSGALG00010016598	AIFM1	2	-0.112485173	0.052839877	-2.128793244	0.03327137	0	0.035210472	0	1	CRA004775;PRJNA827465	Yang2025_Silkie_Wenchang	0.313333153
ENSGALG00010016599	ENSGALG00010016599	2	-0.200318941	0.213339146	-0.961818603	0.336140732	0	0.810053761	0	1	PRJNA827465;Yang2025_Silkie_Wenchang	CRA004775	0.803727281
ENSGALG00010016599	ENSGALG00010016599	2	-0.500736646	0.309942799	-1.615577609	0.106185668	0	0.219216235	0	0.5	CRA004775;Yang2025_Silkie_Wenchang	PRJNA827465	0.552843465
ENSGALG00010016599	ENSGALG00010016599	2	-0.13303745	0.260672694	-0.510362062	0.60979783	0	0.893042602	0	1	CRA004775;PRJNA827465	Yang2025_Silkie_Wenchang	0.897238578
ENSGALG00010016600	GNL2	2	0.094133182	0.096195038	0.978557171	0.342799167	0.000710958	1.977108804	49.42109417	1	PRJNA827465;Yang2025_Silkie_Wenchang	CRA004775	0.806005903
ENSGALG00010016600	GNL2	2	0.195345279	0.10930574	1.78714565	0.073913951	0	0.132379446	0	1	CRA004775;Yang2025_Silkie_Wenchang	PRJNA827465	0.469539359
ENSGALG00010016600	GNL2	2	0.023300264	0.069362329	0.33592101	0.736930433	0	0.132718702	0	1	CRA004775;PRJNA827465	Yang2025_Silkie_Wenchang	0.93386802
ENSGALG00010016602	HSPB7	2	0.07970762	0.253857495	0.313985688	0.753531909	0	0.549571018	0	0.5	PRJNA827465;Yang2025_Silkie_Wenchang	CRA004775	0.938087082
ENSGALG00010016602	HSPB7	2	0.187062891	0.404799478	0.463594696	0.642938155	0	0.55386531	0	0.5	CRA004775;Yang2025_Silkie_Wenchang	PRJNA827465	0.906634523
ENSGALG00010016602	HSPB7	2	-0.069190376	0.276413945	-0.250314347	0.802344263	0	0.087412349	0	1	CRA004775;PRJNA827465	Yang2025_Silkie_Wenchang	0.950688375
ENSGALG00010016603	ENSGALG00010016603	2	0.120822911	0.065054459	1.857257952	0.063274464	0	0.442217329	0	1	PRJNA827465;Yang2025_Silkie_Wenchang	CRA004775	0.433102794
ENSGALG00010016603	ENSGALG00010016603	2	0.086020615	0.12012099	0.716113113	0.473948473	0	0.589809617	0	0.5	CRA004775;Yang2025_Silkie_Wenchang	PRJNA827465	0.863512504
ENSGALG00010016603	ENSGALG00010016603	2	0.140757322	0.073038938	1.927154565	0.05396037	0	0.006822502	0	1	CRA004775;PRJNA827465	Yang2025_Silkie_Wenchang	0.402825653
ENSGALG00010016604	TP53BP2	2	0.040885021	0.03970574	1.029700501	0.30315062	0	0.108365049	0	1	PRJNA827465;Yang2025_Silkie_Wenchang	CRA004775	0.792084776
ENSGALG00010016604	TP53BP2	2	0.018076163	0.078907658	0.229079963	0.818806767	0	0.006483867	0	1	CRA004775;Yang2025_Silkie_Wenchang	PRJNA827465	0.955256039
ENSGALG00010016604	TP53BP2	2	0.046418728	0.043052178	1.078196989	0.280945856	0	0.008781861	0	1	CRA004775;PRJNA827465	Yang2025_Silkie_Wenchang	0.778593637
ENSGALG00010016605	WDPCP	2	-0.102382255	0.091360898	-1.120635391	0.262443094	0	0.452816924	0	1	PRJNA827465;Yang2025_Silkie_Wenchang	CRA004775	0.768418419
ENSGALG00010016605	WDPCP	2	-0.106492289	0.163382449	-0.651797605	0.514531747	0.009779461	1.154368635	13.37255969	1	CRA004775;Yang2025_Silkie_Wenchang	PRJNA827465	0.866869697
ENSGALG00010016605	WDPCP	2	-0.172966635	0.105274323	-1.643008766	0.10038112	0	0.556373173	0	1	CRA004775;PRJNA827465	Yang2025_Silkie_Wenchang	0.541223839
ENSGALG00010016606	VASN	2	0.106466115	0.132305417	0.804699592	0.420993055	0.024848484	3.271137073	69.42959045	0.5	PRJNA827465;Yang2025_Silkie_Wenchang	CRA004775	0.839269089
ENSGALG00010016606	VASN	2	0.220919518	0.117587189	1.878772002	0.060275632	0	0.636480375	0	0.5	CRA004775;Yang2025_Silkie_Wenchang	PRJNA827465	0.425316544
ENSGALG00010016606	VASN	2	-0.007373451	0.073039486	-0.100951573	0.919588903	0	0.000103702	0	1	CRA004775;PRJNA827465	Yang2025_Silkie_Wenchang	0.981192162
ENSGALG00010016607	FAM103A1	2	-0.330103287	0.414028027	-0.79729696	0.425278596	0.328830309	24.1338293	95.85643875	0.5	PRJNA827465;Yang2025_Silkie_Wenchang	CRA004775	0.84125685
ENSGALG00010016607	FAM103A1	2	-0.634562333	0.178929791	-3.54643198	0.000390486	0.023755154	1.46465286	31.72443607	1	CRA004775;Yang2025_Silkie_Wenchang	PRJNA827465	0.019924928
ENSGALG00010016607	FAM103A1	2	-0.063125733	0.204194038	-0.309145818	0.757210607	0.054665587	2.279776185	56.13604499	0.5	CRA004775;PRJNA827465	Yang2025_Silkie_Wenchang	0.938795135
ENSGALG00010016611	DNALI1	2	0.184416269	0.124525436	1.480952613	0.138619202	0	0.870957845	0	1	PRJNA827465;Yang2025_Silkie_Wenchang	CRA004775	0.622371164
ENSGALG00010016611	DNALI1	2	0.03246416	0.171893878	0.188861641	0.850201259	0	0.001899421	0	1	CRA004775;Yang2025_Silkie_Wenchang	PRJNA827465	0.961982453
ENSGALG00010016611	DNALI1	2	0.22828693	0.143014701	1.596247991	0.110433437	0	0.485998737	0	1	CRA004775;PRJNA827465	Yang2025_Silkie_Wenchang	0.562733744
ENSGALG00010016612	GRIA1	2	0.278749294	0.684678593	0.407124302	0.683916695	0.868275639	13.46028568	92.57073718	0.5	PRJNA827465;Yang2025_Silkie_Wenchang	CRA004775	0.918806497

ENSGALG00010016612	GRIA1	2	0.184244427	0.894170264	0.20605072	0.836751292	1.340038092	5.750708777	82.61083914	0.5	CRA004775;Yang2025_Silkie_Wenchang	PRJNA827465	0.958399882
ENSGALG00010016612	GRIA1	2	-0.433609129	0.213790696	-2.028194572	0.042540394	0	0.354084983	0	1	CRA004775;PRJNA827465	Yang2025_Silkie_Wenchang	0.356625761
ENSGALG00010016613	SNIP1	2	0.110816583	0.167185276	0.662836978	0.507434968	0.048700376	7.283721548	86.27075468	0.5	PRJNA827465;Yang2025_Silkie_Wenchang	CRA004775	0.864149944
ENSGALG00010016613	SNIP1	2	0.12185893	0.191488092	0.636378632	0.524529665	0.053462939	3.597027625	72.19926828	0.5	CRA004775;Yang2025_Silkie_Wenchang	PRJNA827465	0.869805486
ENSGALG00010016613	SNIP1	2	-0.04417287	0.045005586	-0.981497499	0.326347467	0	0.079363792	0	1	CRA004775;PRJNA827465	Yang2025_Silkie_Wenchang	0.800028239
ENSGALG00010016614	DNAJC19	2	0.307702463	0.544415225	0.565198122	0.571939014	0.571417505	27.67010247	96.38599098	0.5	PRJNA827465;Yang2025_Silkie_Wenchang	CRA004775	0.885484876
ENSGALG00010016614	DNAJC19	2	0.519449041	0.410683004	1.252666988	0.210326925	0.258915818	3.899770894	74.35746799	1	CRA004775;Yang2025_Silkie_Wenchang	PRJNA827465	0.721900195
ENSGALG00010016614	DNAJC19	2	-0.207777409	0.117234737	-1.772319485	0.076341539	0	0.389889626	0	0.5	CRA004775;PRJNA827465	Yang2025_Silkie_Wenchang	0.476766627
ENSGALG00010016615	BCORL1	2	0.100896362	0.192659354	0.523703416	0.600484839	0.058554284	4.600801382	78.26465616	0.5	PRJNA827465;Yang2025_Silkie_Wenchang	CRA004775	0.895230519
ENSGALG00010016615	BCORL1	2	0.15937274	0.171677154	0.928328179	0.353237361	0.029716748	1.986941147	49.67138298	0.5	CRA004775;Yang2025_Silkie_Wenchang	PRJNA827465	0.811157428
ENSGALG00010016615	BCORL1	2	-0.067066556	0.087652203	-0.765143981	0.444185813	0	0.035583928	0	1	CRA004775;PRJNA827465	Yang2025_Silkie_Wenchang	0.846013379
ENSGALG00010016616	SLBP	2	-0.178836191	0.171269427	-1.044180474	0.296401899	0.041867088	3.033353703	67.03318841	1	PRJNA827465;Yang2025_Silkie_Wenchang	CRA004775	0.788267543
ENSGALG00010016616	SLBP	2	-0.353587091	0.180009851	-1.964265224	0.049499342	0	0.57933317	0	0.5	CRA004775;Yang2025_Silkie_Wenchang	PRJNA827465	0.385165839
ENSGALG00010016616	SLBP	2	-0.044013532	0.071563509	-0.615027587	0.538536502	0	0.033036287	0	0.5	CRA004775;PRJNA827465	Yang2025_Silkie_Wenchang	0.87481821
ENSGALG00010016619	SLC25A47	2	-0.042916402	0.282929516	-0.151685844	0.879434723	0.125924755	4.680347437	78.63406481	0.5	PRJNA827465;Yang2025_Silkie_Wenchang	CRA004775	0.971291607
ENSGALG00010016619	SLC25A47	2	0.224921762	0.179064535	1.256093293	0.209082148	0	0.081885729	0	1	CRA004775;Yang2025_Silkie_Wenchang	PRJNA827465	0.721011841
ENSGALG00010016619	SLC25A47	2	-0.274720942	0.167499296	-1.640131918	0.100977741	0	0.608374202	0	0.5	CRA004775;PRJNA827465	Yang2025_Silkie_Wenchang	0.542398273
ENSGALG00010016620	ENSGALG00010016620	2	0.043288272	0.099006022	0.437228671	0.661941519	0	0.132503888	0	0.5	PRJNA827465;Yang2025_Silkie_Wenchang	CRA004775	0.912556664
ENSGALG00010016620	ENSGALG00010016620	2	-0.036162362	0.145698442	-0.248200059	0.80397962	0	0.153849563	0	1	CRA004775;Yang2025_Silkie_Wenchang	PRJNA827465	0.951341593
ENSGALG00010016620	ENSGALG00010016620	2	0.040399994	0.114428259	0.353059586	0.72404377	0	0.412564695	0	0.5	CRA004775;PRJNA827465	Yang2025_Silkie_Wenchang	0.930355448
ENSGALG00010016622	KIAA1143	2	-0.319121796	0.367438897	-0.868503033	0.385119013	0.255962703	19.17569332	94.7850647	0.5	PRJNA827465;Yang2025_Silkie_Wenchang	CRA004775	0.826444827
ENSGALG00010016622	KIAA1143	2	-0.440035851	0.308240343	-1.427573843	0.153414557	0.140907996	3.446232411	70.98280439	1	CRA004775;Yang2025_Silkie_Wenchang	PRJNA827465	0.645780478
ENSGALG00010016622	KIAA1143	2	0.033929025	0.101904166	0.332950322	0.739171783	0	0.099643761	0	0.5	CRA004775;PRJNA827465	Yang2025_Silkie_Wenchang	0.934708297
ENSGALG00010016623	DNAJA3	2	0.095326324	0.224442532	0.424724864	0.671037256	0.093556204	13.67816268	92.68907657	0.5	PRJNA827465;Yang2025_Silkie_Wenchang	CRA004775	0.914582784
ENSGALG00010016623	DNAJA3	2	0.296345396	0.100094975	2.960642081	0.003069985	0	0.651761103	0	1	CRA004775;Yang2025_Silkie_Wenchang	PRJNA827465	0.076318541
ENSGALG00010016623	DNAJA3	2	-0.110395263	0.053954153	-2.046093893	0.040747134	0	0.670535871	0	0.5	CRA004775;PRJNA827465	Yang2025_Silkie_Wenchang	0.349211644
ENSGALG00010016624	MDH1	2	-0.146061238	0.18024025	-0.810369703	0.417727725	0.04606148	2.969181876	66.32068893	1	PRJNA827465;Yang2025_Silkie_Wenchang	CRA004775	0.838112595
ENSGALG00010016624	MDH1	2	-0.122243483	0.291186592	-0.419811511	0.674623155	0.110063139	2.772544991	63.93205509	0.5	CRA004775;Yang2025_Silkie_Wenchang	PRJNA827465	0.915970608
ENSGALG00010016624	MDH1	2	0.006814059	0.073351529	0.092895931	0.925986238	0	0.515478961	0	0.5	CRA004775;PRJNA827465	Yang2025_Silkie_Wenchang	0.982799198
ENSGALG00010016626	CAPN2	2	-0.260882958	0.231873182	-1.125110527	0.260542201	0.060238747	2.119788922	52.82549174	1	PRJNA827465;Yang2025_Silkie_Wenchang	CRA004775	0.767694434
ENSGALG00010016626	CAPN2	2	-0.372844815	0.223517741	-1.668077057	0.095300431	0.002731613	1.027031466	2.631999821	1	CRA004775;Yang2025_Silkie_Wenchang	PRJNA827465	0.528530665
ENSGALG00010016626	CAPN2	2	-0.083075623	0.147593496	-0.562867777	0.573524924	0	0.003267979	0	1	CRA004775;PRJNA827465	Yang2025_Silkie_Wenchang	0.838917854
ENSGALG00010016629	LARS1	2	0.013721586	0.090089299	0.15231094	0.878941697	0.012117572	3.874235724	74.18845751	0.5	PRJNA827465;Yang2025_Silkie_Wenchang	CRA004775	0.971127951
ENSGALG00010016629	LARS1	2	0.068477141	0.077940847	0.87857835	0.379629939	0.002398392	1.163224551	14.03207582	0.5	CRA004775;Yang2025_Silkie_Wenchang	PRJNA827465	0.824239628
ENSGALG00010016629	LARS1	2	-0.069983721	0.05108196	-1.370028094	0.170678132	0	0.000642332	0	1	CRA004775;PRJNA827465	Yang2025_Silkie_Wenchang	0.672386723
ENSGALG00010016630	ENSGALG00010016630	2	-0.028902092	0.312310603	-0.092542781	0.926266803	0	0.052345342	0	0.5	PRJNA827465;Yang2025_Silkie_Wenchang	CRA004775	0.98280842
ENSGALG00010016630	ENSGALG00010016630	2	-0.442333309	0.3898757	-1.134549573	0.256564092	0.066116868	1.246122206	19.75104885	0.5	CRA004775;Yang2025_Silkie_Wenchang	PRJNA827465	0.764677554
ENSGALG00010016630	ENSGALG00010016630	2	-0.373106323	0.326794887	-1.141714079	0.253572887	0.065178409	1.435059845	30.31649492	1	CRA004775;PRJNA827465	Yang2025_Silkie_Wenchang	0.763346521
ENSGALG00010016631	FAM53A	2	-0.135855417	0.054796021	-2.47929349	0.013164294	0	0.848921276	0	1	PRJNA827465;Yang2025_Silkie_Wenchang	CRA004775	0.187266073
ENSGALG00010016631	FAM53A	2	0.230469587	0.451726387	0.510197309	0.609913237	0.383917706	16.44285549	93.91833105	0.5	CRA004775;Yang2025_Silkie_Wenchang	PRJNA827465	0.897252272
ENSGALG00010016631	FAM53A	2	0.278393981	0.398794962	0.698088009	0.485122156	0.296114986	13.86730752	92.78879481	0.5	CRA004775;PRJNA827465	Yang2025_Silkie_Wenchang	0.857004733
ENSGALG00010016640	OCIAD1	2	-0.226057218	0.195019701	-1.15915067	0.246394775	0.068484308	9.583410946	89.5653019	1	PRJNA827465;Yang2025_Silkie_Wenchang	CRA004775	0.756843363
ENSGALG00010016640	OCIAD1	2	-0.146693614	0.314973912	-0.465732563	0.641406926	0.169232563	6.519877732	84.66228906	0.5	CRA004775;Yang2025_Silkie_Wenchang	PRJNA827465	0.906634523
ENSGALG00010016640	OCIAD1	2	-0.018035366	0.075549739	-0.238721751	0.811321349	0.003950198	1.157097847	13.57688525	0.5	CRA004775;PRJNA827465	Yang2025_Silkie_Wenchang	0.953196081
ENSGALG00010016643	BTBD1	2	-0.095778369	0.113683375	-0.84250111	0.399507519	0.013350818	2.033373809	50.8206511	0.5	PRJNA827465;Yang2025_Silkie_Wenchang	CRA004775	0.832246851
ENSGALG00010016643	BTBD1	2	0.073087131	0.346177393	0.21112624	0.832788765	0.192397819	4.650818338	78.49840765	0.5	CRA004775;Yang2025_Silkie_Wenchang	PRJNA827465	0.957861248
ENSGALG00010016643	BTBD1	2	0.155281336	0.219786364	0.706510327	0.479870833	0.061515744	2.24606999	55.47778989	1	CRA004775;PRJNA827465	Yang2025_Silkie_Wenchang	0.856284002
ENSGALG00010016644	NMRAL1	2	0.046487083	0.132288825	0.351405968	0.725283806	0	0.082045527	0	0.5	PRJNA827465;Yang2025_Silkie_Wenchang	CRA004775	0.930868485
ENSGALG00010016644	NMRAL1	2	0.008434414	0.192662952	0.043778078	0.965081302	0	0.011010827	0	0.5	CRA004775;Yang2025_Silkie_Wenchang	PRJNA827465	0.990532586
ENSGALG00010016644	NMRAL1	2	0.074335047	0.159830979	0.465085333	0.641870334	0	0.002139276	0	1	CRA004775;PRJNA827465	Yang2025_Silkie_Wenchang	0.906634523
ENSGALG00010016647	EGR1	2	-0.090306834	0.111407603	-0.810598482	0.417596289	0	0.643526818	0	1	PRJNA827465;Yang2025_Silkie_Wenchang	CRA004775	0.838112595
ENSGALG00010016647	EGR1	2	-0.171324196	0.221442938	-0.773671979	0.439124794	0	0.942653715	0	0.5	CRA004775;Yang2025_Silkie_Wenchang	PRJNA827465	0.844022831
ENSGALG00010016647	EGR1	2	-0.025746308	0.122923652	-0.209449583	0.834097289	0	0.434948253	0	0.5	CRA004775;PRJNA827465	Yang2025_Silkie_Wenchang	0.957968425
ENSGALG00010016648	ADRA2C	2	0.033248695	0.159287707	0.20873359	0.834656517	0.019983031	1.60928697	37.86067877	0.5	PRJNA827465;Yang2025_Silkie_Wenchang	CRA004775	0.958133448
ENSGALG00010016648	ADRA2C	2	0.406857079	0.277293321	1.467244425	0.142309576	0.0658628	1.553038929	35.61011372	1	CRA004775;Yang2025_Silkie_Wenchang	PRJNA827465	0.628589937
ENSGALG00010016648	ADRA2C	2	0.276768855	0.457294159	0.605231555	0.545025152	0.326613652	4.003951681	75.02467363	0.5	CRA004775;PRJNA827465	Yang2025_Silkie_Wenchang	0.877354247
ENSGALG00010016651	ENSGALG00010016651	2	-0.077439088	0.105316453	-0.735299055	0.462157383	0.008294548	1.569373356	36.28029964	0.5	PRJNA827465;Yang2025_Silkie_Wenchang	CRA004775	0.850747865
ENSGALG00010016651	ENSGALG00010016651	2	-0.236491001	0.356992599	-0.662453513	0.507680618	0.189817344	3.418916646	70.75096869	0.5	CRA004775;Yang2025_Silkie_Wenchang	PRJNA827465	0.906434571
ENSGALG00010016651	ENSGALG00010016651	2	-0.302062303	0.228206546	-1.323635577	0.185624104	0.059984194	1.804819817	44.59280695	1	CRA004775;PRJNA827465	Yang2025_Silkie_Wenchang	0.694265174
ENSGALG00010016652	FXR1	2	-0.036832261	0.106485995	-0.345883816	0.72942665	0.017822883	4.542129638	77.98389567	0.5	PRJNA827465;Yang2025_Silkie_Wenchang	CRA004775	0.9323457
ENSGALG00010016652	FXR1	2	0.080185921	0.081380034	0.984993698	0.324627166	0	0.001218289	0	0.5	CRA004775;Yang2025_Silkie_Wenchang	PRJNA827465	0.799659262
ENSGALG00010016652	FXR1	2	-0.128426541	0.054282132	-2.365908179	0.017985905	0	0.442061505	0	0.5	CRA004775;PRJNA827465	Yang2025_Silkie_Wenchang	0.223844207
ENSGALG00010016653	EPHA2	2	0.174920652	0.083945172	2.083748816	0.037183021	0	0.02206797	0	1	PRJNA827465;Yang2025_Silkie_Wenchang	CRA004775	0.332590871
ENSGALG00010016653	EPHA2	2	0.147382628	0.151312223	0.974029893	0.330041697	0	0.78566239	0	0.5	CRA004775;Yang2025_Silkie_Wenchang	PRJNA827465	0.801212404
ENSGALG00010016653	EPHA2	2	0.149210637	0.096176376	1.551427113	0.120799362	0	0.737303891	0	0.5	CRA004775;PRJNA827465	Yang2025_Silkie_Wenchang	0.582763879
ENSGALG00010016654	SMIM3	2	-0.242643187	0.117982925	-2.05659579	0.039725124	0	0.781106612	0	0.5	PRJNA827465;Yang2025_Silkie_Wenchang	CRA004775	0.344755224
ENSGALG00010016654	SMIM3	2	0.053130121	0.219074058	0.242521279	0.808376271	0	0.005620151	0	1	CRA004775;Yang2025_Silkie_Wenchang	PRJNA827465	0.952496413
ENSGALG00010016654	SMIM3	2	-0.204742374	0.144915156	-1.412843064	0.157701873	0.011062028	1.221532962	18.13565161	0.5	CRA004775;PRJNA827465	Yang2025_Silkie_Wenchang	0.651135279
ENSGALG00010016655	TRANK1	2	-0.135901084	0.066667197	-2.038500059	0.041499949	0	0.007621617	0	1	PRJNA827465;Yang2025_Silkie_Wenchang	CRA004775	0.353189581
ENSGALG00010016655	TRANK1	2	-0.110141085	0.15490591	-0.711019254	0.477072305	0	0.049221349	0	0.5	CRA004775;Yang2025_Silkie_Wenchang	PRJNA827465	0.854986476
ENSGALG00010016655	TRANK1	2	-0.135355556	0.071948104	-1.881294286	0.059931898	0	0.071685552	0	1	CRA004775;PRJNA827465	Yang2025_Silkie_Wenchang	0.423882851
ENSGALG00010016657	SLC25A29	2	0.141172925	0.116525333	1.211513318	0.225695669	0.014754027	2.161071323	53.72665448	1	PRJNA827465;Yang2025_Silkie_Wenchang	CRA004775	0.713901865
ENSGALG00010016657	SLC25A29	2	0.244544626	0.119617358	2.044390794	0.040914956	0	0.529097837	0	0.5	CRA004775;Yang2025_Silkie_Wenchang	PRJNA827465	0.350050212
ENSGALG00010016657	SLC25A29	2	0.031974	0.096361507	0.331812999	0.740030468	0	0.036179027	0	0.5	CRA004775;PRJNA827465	Yang2025_Silkie_Wenchang	0.935072094
ENSGALG00010016658	ZAR1	2	-0.128703332	0.422170163	-0.304861269	0.760471815	0	0.349946665	0	0.5	PRJNA827465;Yang2025_Silkie_Wenchang	CRA004775	0.939327609
ENSGALG00010016658	ZAR1	2	0.584795563	0.463565297	1.261517131	0.207122592	0	0.804478232	0	1	CRA004775;Yang2025_Silkie_Wenchang	PRJNA827465	0.719065386
ENSGALG00010016658	ZAR1	2	0.283977626	0.667667975	0.425327613	0.670597867	0.529262545	2.443485853	59.07486025	0.5	CRA004775;PRJNA827465	Yang2025_Silkie_Wenchang	0.914482883
ENSGALG00010016661	UBE4B	2	0.034156664	0.043124692	0.79204425	0.428334875	0	0.408214612	0	1	PRJNA827465;Yang2025_Silkie_Wenchang	CRA004775	0.841329286
ENSGALG00010016661	UBE4B	2	-0.045135448	0.123420109	-0.365705789	0.714584626	0.009859501	1.099151994	9.020771916	1	CRA004775;Yang2025_Silkie_Wenchang	PRJNA827465	0.926596201
ENSGALG00010016661	UBE4B	2	-0.033692513	0.194305457	-0.173397725	0.862337241	0.04240624	1.435772684	30.32682843	0.5	CRA004775;PRJNA827465	Yang2025_Silkie_Wenchang	0.959709249
ENSGALG00010016664	TM6SF1	2	-0.210148916	0.192590995	-1.091166883	0.275199457	0.039563941	2.010973727	50.27284612	0.5	PRJNA827465;Yang2025_Silkie_Wenchang	CRA004775	0.775068889
ENSGALG00010016664	TM6SF1	2	-0.121452616	0.190402618	-0.637872617	0.523556604	0.002974841	1.041186354	3.955713936	0.5	CRA004775;Yang2025_Silkie_Wenchang	PRJNA827465	0.869661505
ENSGALG00010016664	TM6SF1	2	-0.359305017	0.12817602	-2.803215578	0.005059584	0	0.000743476	0	1	CRA004775;PRJNA827465	Yang2025_Silkie_Wenchang	0.106040882
ENSGALG00010016667	WARS1	2	-0.043498772	0.039461328	-1.102313957	0.270325204	0	0.479585786	0	1	PRJNA827465;Yang2025_Silkie_Wenchang	CRA004775	0.772430311
ENSGALG00010016667	WARS1	2	-0.105059705	0.073964822	-1.420400974	0.155490979	0	0.146961378	0	1	CRA004775;Yang2025_Silkie_Wenchang	PRJNA827465	0.648167852
ENSGALG00010016667	WARS1	2	-0.036406481	0.043862521	-0.830013417	0.406531198	0	0.561877969	0	1	CRA004775;PRJNA827465	Yang2025_Silkie_Wenchang	0.833618122
ENSGALG00010016671	CASS4	2	0.081620833	0.16345111	0.499359307	0.617526281	0.000192875	1.003410183	0.339859345	0.5	PRJNA827465;Yang2025_Silkie_Wenchang	CRA004775	0.899121942
ENSGALG00010016671	CASS4	2	-0.201096144	0.236905434	-0.848845638	0.395967192	0	0.381845277	0	1	CRA004775;Yang2025_Silkie_Wenchang	PRJNA827465	0.831296327
ENSGALG00010016671	CASS4	2	0.026335393	0.30815089	0.085462653	0.931893586	0.085611519	1.532693942	34.75540209	0.5	CRA004775;PRJNA827465	Yang2025_Silkie_Wenchang	0.98412915
ENSGALG00010016672	DCTN4	2	-0.139588364	0.085789568	-1.627101838	0.103715471	0.01244395	6.260180867	84.02602063	1	PRJNA827465;Yang2025_Silkie_Wenchang	CRA004775	0.547964315
ENSGALG00010016672	DCTN4	2	-0.294653872	0.11232521	-2.623221208	0.00871027	0.012979482	1.587833695	37.02111227	1	CRA004775;Yang2025_Silkie_Wenchang	PRJNA827465	0.146236539
ENSGALG00010016672	DCTN4	2	-0.233571302	0.213740431	-1.092780162	0.274490334	0.074516079	4.56091799	78.07458932	1	CRA004775;PRJNA827465	Yang2025_Silkie_Wenchang	0.774526342
ENSGALG00010016673	LRPAP1	2	0.083519565	0.10092866	0.827510887	0.407947554	0.010082767	1.750914266	42.88698086	1	PRJNA827465;Yang2025_Silkie_Wenchang	CRA004775	0.833982683
ENSGALG00010016673	LRPAP1	2	0.088710674	0.181409501	0.489007872	0.624836123	0.031295706	1.815896057	44.93076868	0.5	CRA004775;Yang2025_Silkie_Wenchang	PRJNA827465	0.901043264
ENSGALG00010016673	LRPAP1	2	0.001990018	0.066680043	0.029844275	0.976191248	0	0.410816268	0	0.5	CRA004775;PRJNA827465	Yang2025_Silkie_Wenchang	0.992992231
ENSGALG00010016674	SLC10A4	2	-0.089708464	0.258342289	-0.347246531	0.728406117	0	5.04173E-05	0	1	PRJNA827465;Yang2025_Silkie_Wenchang	CRA004775	0.932161553
ENSGALG00010016674	SLC10A4	2	-0.0321105	0.406390675	-0.079013871	0.93702159	0	0.066101231	0	0.5	CRA004775;Yang2025_Silkie_Wenchang	PRJNA827465	0.985068196
ENSGALG00010016674	SLC10A4	2	-0.058436698	0.289155775	-0.202094177	0.8398431	0	0.074472803	0	0.5	CRA004775;PRJNA827465	Yang2025_Silkie_Wenchang	0.959517263
ENSGALG00010016675	SKIL	2	0.10716967	0.070582259	1.518365555	0.12892227	0	0.395350613	0	1	PRJNA827465;Yang2025_Silkie_Wenchang	CRA004775	0.602361458
ENSGALG00010016675	SKIL	2	0.210821813	0.11298614	1.865908623	0.062054151	0	0.737739033	0	1	CRA004775;Yang2025_Silkie_Wenchang	PRJNA827465	0.42991371
ENSGALG00010016675	SKIL	2	0.152740094	0.149172121	1.023918493	0.305873775	0.020701589	1.445926797	30.84020559	1	CRA004775;PRJNA827465	Yang2025_Silkie_Wenchang	0.792312461
ENSGALG00010016677	MEAF6	2	0.019600312	0.048309898	0.405720422	0.684948035	0	0.48367356	0	0.5	PRJNA827465;Yang2025_Silkie_Wenchang	CRA004775	0.919102514
ENSGALG00010016677	MEAF6	2	-0.120191228	0.113085146	-1.062838334	0.287855272	0	0.757119042	0	1	CRA004775;Yang2025_Silkie_Wenchang	PRJNA827465	0.782840067
ENSGALG00010016677	MEAF6	2	-0.054050836	0.144441095	-0.374206774	0.708250485	0.025741464	1.932507826	48.25376712	0.5	CRA004775;PRJNA827465	Yang2025_Silkie_Wenchang	0.925061251
ENSGALG00010016678	NOX1	2	0.169053645	0.143422667	1.178709395	0.238513913	0	0.421286258	0	1	PRJNA827465;Yang2025_Silkie_Wenchang	CRA004775	0.750652917
ENSGALG00010016678	NOX1	2	0.029329105	0.33229909	0.088261165	0.929669104	0.123147193	1.917150084	47.83924282	0.5	CRA004775;Yang2025_Silkie_Wenchang	PRJNA827465	0.983767889
ENSGALG00010016678	NOX1	2	-0.024746939	0.199418424	-0.124095548	0.901239623	0.001990467	1.014358639	1.415538697	0.5	CRA004775;PRJNA827465	Yang2025_Silkie_Wenchang	0.97660383
ENSGALG00010016681	SUSD4	2	-0.141814298	0.124102828	-1.142716087	0.253156488	0	0.016241939	0	1	PRJNA827465;Yang2025_Silkie_Wenchang	CRA004775	0.762731469
ENSGALG00010016681	SUSD4	2	-0.271095595	0.22511041	-1.20427836	0.228482008	0.030179595	1.341010144	25.42934856	1	CRA004775;Yang2025_Silkie_Wenchang	PRJNA827465	0.741270737
ENSGALG00010016681	SUSD4	2	-0.259701125	0.192178352	-1.351354733	0.176581827	0.024239975	1.307217763	23.5016515	1	CRA004775;PRJNA827465	Yang2025_Silkie_Wenchang	0.682016071
ENSGALG00010016682	GPR141	2	-0.055125478	0.812134913	-0.067877242	0.945883355	0.976952963	3.642798653	72.54857885	0.5	PRJNA827465;Yang2025_Silkie_Wenchang	CRA004775	0.986321197
ENSGALG00010016682	GPR141	2	-0.790599873	0.606464786	-1.303620411	0.192363038	0	0.210704958	0	1	CRA004775;Yang2025_Silkie_Wenchang	PRJNA827465	0.702137035
ENSGALG00010016682	GPR141	2	0.497432438	0.389799898	1.276122544	0.201912203	0	0.897896465	0	0.5	CRA004775;PRJNA827465	Yang2025_Silkie_Wenchang	0.714969076
ENSGALG00010016683	RBM22	2	-0.194450707	0.144478538	-1.345879527	0.178341373	0.035214752	5.773922972	82.68075267	1	PRJNA827465;Yang2025_Silkie_Wenchang	CRA004775	0.684689586
ENSGALG00010016683	RBM22	2	-0.368759679	0.093364059	-3.949696288	7.82504E-05	0	0.013234779	0	1	CRA004775;Yang2025_Silkie_Wenchang	PRJNA827465	0.006543566
ENSGALG00010016683	RBM22	2	-0.188622012	0.152633259	-1.235785788	0.216538199	0.036209775	3.739658138	73.25958783	1	CRA004775;PRJNA827465	Yang2025_Silkie_Wenchang	0.730172415
ENSGALG00010016691	ENSGALG00010016691	2	-0.034609754	0.219148813	-0.157928091	0.874513463	0	6.89827E-05	0	1	PRJNA827465;Yang2025_Silkie_Wenchang	CRA004775	0.970143165
ENSGALG00010016691	ENSGALG00010016691	2	-0.820550478	0.828768702	-0.990083814	0.322133154	1.160022032	6.32001083	84.1772423	1	CRA004775;Yang2025_Silkie_Wenchang	PRJNA827465	0.798513866
ENSGALG00010016691	ENSGALG00010016691	2	-0.798419411	0.829308125	-0.962753634	0.335671174	1.200213007	7.514516269	86.69242352	1	CRA004775;PRJNA827465	Yang2025_Silkie_Wenchang	0.803317552
ENSGALG00010016692	PRKCI	2	-0.261163918	0.204507519	-1.277038225	0.201588752	0.07842136	15.96744269	93.73725637	1	PRJNA827465;Yang2025_Silkie_Wenchang	CRA004775	0.714495693
ENSGALG00010016692	PRKCI	2	-0.152932371	0.328856597	-0.465042733	0.641900855	0.20130708	14.10340217	92.90951227	0.5	CRA004775;Yang2025_Silkie_Wenchang	PRJNA827465	0.906634523
ENSGALG00010016692	PRKCI	2	0.024698772	0.117812638	0.209644504	0.833945143	0.016785245	2.175926929	54.04257439	0.5	CRA004775;PRJNA827465	Yang2025_Silkie_Wenchang	0.957968425
ENSGALG00010016696	DOK7	2	-0.21543184	0.072693457	-2.963565773	0.003040971	0.000719607	1.071113565	6.639218047	1	PRJNA827465;Yang2025_Silkie_Wenchang	CRA004775	0.075885251
ENSGALG00010016696	DOK7	2	0.111637049	0.516292492	0.216228303	0.828809793	0.424723657	4.127170968	75.77032772	0.5	CRA004775;Yang2025_Silkie_Wenchang	PRJNA827465	0.956840134
ENSGALG00010016696	DOK7	2	0.164463236	0.434430861	0.378571714	0.70500593	0.281381593	3.098197314	67.72316614	0.5	CRA004775;PRJNA827465	Yang2025_Silkie_Wenchang	0.924152671
ENSGALG00010016699	SYNPO	2	0.353657694	0.360187202	0.981871903	0.326162959	0.238911677	12.04679657	91.69903804	1	PRJNA827465;Yang2025_Silkie_Wenchang	CRA004775	0.800023788
ENSGALG00010016699	SYNPO	2	0.282743094	0.531366878	0.532105229	0.594653116	0.451026112	4.540443722	77.97572085	0.5	CRA004775;Yang2025_Silkie_Wenchang	PRJNA827465	0.893379204
ENSGALG00010016699	SYNPO	2	0.00796519	0.069471265	0.11465445	0.90871902	0	0.565069854	0	0.5	CRA004775;PRJNA827465	Yang2025_Silkie_Wenchang	0.978387968
ENSGALG00010016700	CLUAP1	2	0.144150754	0.242255093	0.595037042	0.551818711	0.101801154	7.533401813	86.72578438	0.5	PRJNA827465;Yang2025_Silkie_Wenchang	CRA004775	0.879061525
ENSGALG00010016700	CLUAP1	2	0.128611511	0.28354309	0.453587181	0.650125981	0.131342083	5.27999132	81.06057493	0.5	CRA004775;Yang2025_Silkie_Wenchang	PRJNA827465	0.908335059
ENSGALG00010016700	CLUAP1	2	-0.116988988	0.105024158	-1.113924554	0.265311555	0	0.120738372	0	1	CRA004775;PRJNA827465	Yang2025_Silkie_Wenchang	0.76929776
ENSGALG00010016702	WDR25	2	-0.302490555	0.227198997	-1.331390363	0.183060599	0.086009902	5.898255576	83.0458347	1	PRJNA827465;Yang2025_Silkie_Wenchang	CRA004775	0.691392093
ENSGALG00010016702	WDR25	2	-0.431831941	0.163834005	-2.635789446	0.00839418	0.015769863	1.33958368	25.34994155	1	CRA004775;Yang2025_Silkie_Wenchang	PRJNA827465	0.142771619
ENSGALG00010016702	WDR25	2	-0.102551741	0.100101668	-1.024475845	0.305610574	0	0.129160721	0	1	CRA004775;PRJNA827465	Yang2025_Silkie_Wenchang	0.79225794
ENSGALG00010016704	CSTF2	2	0.208609856	0.220221475	0.947272998	0.343499687	0.088912162	11.85316735	91.56343642	0.5	PRJNA827465;Yang2025_Silkie_Wenchang	CRA004775	0.806276913
ENSGALG00010016704	CSTF2	2	0.035453971	0.413645681	0.085710966	0.931696185	0.320370499	15.45039051	93.52767168	0.5	CRA004775;Yang2025_Silkie_Wenchang	PRJNA827465	0.98412915
ENSGALG00010016704	CSTF2	2	-0.161722419	0.190101861	-0.850714549	0.394927939	0.055976161	3.960075652	74.74795716	1	CRA004775;PRJNA827465	Yang2025_Silkie_Wenchang	0.830643015
ENSGALG00010016706	RTN4	2	-0.09226469	0.108268598	-0.852183295	0.394112367	0.015722156	2.835145284	64.72843894	0.5	PRJNA827465;Yang2025_Silkie_Wenchang	CRA004775	0.830486298
ENSGALG00010016706	RTN4	2	-0.010651837	0.103471768	-0.102944376	0.918007118	2.36332E-05	1.000663521	0.066308086	0.5	CRA004775;Yang2025_Silkie_Wenchang	PRJNA827465	0.981033557
ENSGALG00010016706	RTN4	2	-0.184934174	0.061434546	-3.01026355	0.002610211	0	0.034901542	0	1	CRA004775;PRJNA827465	Yang2025_Silkie_Wenchang	0.069130135
ENSGALG00010016707	KIF1B	2	-0.076384553	0.060851984	-1.255251656	0.209387419	0	0.631773	0	0.5	PRJNA827465;Yang2025_Silkie_Wenchang	CRA004775	0.721294679
ENSGALG00010016707	KIF1B	2	0.179302736	0.248397359	0.721838336	0.470393877	0.070713158	1.927819402	48.12792118	1	CRA004775;Yang2025_Silkie_Wenchang	PRJNA827465	0.853744788
ENSGALG00010016707	KIF1B	2	0.12997537	0.315067782	0.412531454	0.679949931	0.14815199	3.120872479	67.95767828	0.5	CRA004775;PRJNA827465	Yang2025_Silkie_Wenchang	0.917701868
ENSGALG00010016712	ENSGALG00010016712	2	0.189328912	0.071706743	2.640322284	0.008282722	0	0.456742808	0	1	PRJNA827465;Yang2025_Silkie_Wenchang	CRA004775	0.141783895
ENSGALG00010016712	ENSGALG00010016712	2	0.144966447	0.099315829	1.459650983	0.14438602	0	0.04889001	0	1	CRA004775;Yang2025_Silkie_Wenchang	PRJNA827465	0.631870428
ENSGALG00010016712	ENSGALG00010016712	2	0.231856009	0.093131156	2.489564388	0.012789975	0	0.007223381	0	1	CRA004775;PRJNA827465	Yang2025_Silkie_Wenchang	0.183473076
ENSGALG00010016720	INTS15	2	0.051429787	0.049215159	1.044998884	0.296023485	0	0.549607969	0	1	PRJNA827465;Yang2025_Silkie_Wenchang	CRA004775	0.788124569
ENSGALG00010016720	INTS15	2	0.071532473	0.081703532	0.875512621	0.381295046	0	0.927638619	0	0.5	CRA004775;Yang2025_Silkie_Wenchang	PRJNA827465	0.824392515
ENSGALG00010016720	INTS15	2	0.016919424	0.056481008	0.299559531	0.764513157	0	0.39696788	0	0.5	CRA004775;PRJNA827465	Yang2025_Silkie_Wenchang	0.940627228
ENSGALG00010016724	PGD	2	0.027265773	0.074252908	0.367201416	0.713468783	0.003063626	1.321874239	24.34983827	0.5	PRJNA827465;Yang2025_Silkie_Wenchang	CRA004775	0.926253108
ENSGALG00010016724	PGD	2	-0.127965117	0.102170984	-1.25246045	0.210402131	0	0.658870082	0	1	CRA004775;Yang2025_Silkie_Wenchang	PRJNA827465	0.721968882
ENSGALG00010016724	PGD	2	-0.044116597	0.166983336	-0.264197603	0.791627658	0.03787193	2.623618616	61.88470405	0.5	CRA004775;PRJNA827465	Yang2025_Silkie_Wenchang	0.948115
ENSGALG00010016729	ELMO1	2	-0.029161502	0.06279472	-0.464394165	0.64236537	0	0.596579398	0	0.5	PRJNA827465;Yang2025_Silkie_Wenchang	CRA004775	0.906634523
ENSGALG00010016729	ELMO1	2	0.06900246	0.143951287	0.479345905	0.63169257	0	0.04696373	0	1	CRA004775;Yang2025_Silkie_Wenchang	PRJNA827465	0.903058174
ENSGALG00010016729	ELMO1	2	-0.047991372	0.067015991	-0.716118224	0.473918352	0	0.022770223	0	0.5	CRA004775;PRJNA827465	Yang2025_Silkie_Wenchang	0.85444425
ENSGALG00010016731	C4orf48	2	-0.00579798	0.272490071	-0.021277767	0.983024079	0.095565515	2.685640093	62.76492884	0.5	PRJNA827465;Yang2025_Silkie_Wenchang	CRA004775	0.995363468
ENSGALG00010016731	C4orf48	2	0.348837091	0.728628505	0.478758501	0.632110442	0.904423256	6.519378746	84.66111513	0.5	CRA004775;Yang2025_Silkie_Wenchang	PRJNA827465	0.903058174
ENSGALG00010016731	C4orf48	2	0.563921781	0.439422791	1.283323925	0.199378614	0.273988004	2.957846606	66.19162069	1	CRA004775;PRJNA827465	Yang2025_Silkie_Wenchang	0.711181159
ENSGALG00010016732	ARHGAP26	2	0.171940746	0.088106374	1.951513133	0.050996036	0.007027491	1.450289295	31.04823957	1	PRJNA827465;Yang2025_Silkie_Wenchang	CRA004775	0.390929819
ENSGALG00010016732	ARHGAP26	2	0.134784956	0.241255939	0.558680366	0.576379881	0.071185442	2.461811998	59.37951392	0.5	CRA004775;Yang2025_Silkie_Wenchang	PRJNA827465	0.887048016
ENSGALG00010016732	ARHGAP26	2	0.106234734	0.069901679	1.519773721	0.128567856	0.002322993	1.064505782	6.059693005	0.5	CRA004775;PRJNA827465	Yang2025_Silkie_Wenchang	0.601638797
ENSGALG00010016733	PHC3	2	0.494326391	0.44807793	1.103215219	0.269933714	0.345778176	6.556830558	84.74872896	1	PRJNA827465;Yang2025_Silkie_Wenchang	CRA004775	0.772196285
ENSGALG00010016733	PHC3	2	0.339049395	0.680732047	0.498065863	0.61843762	0.790422719	6.771173938	85.23151216	0.5	CRA004775;Yang2025_Silkie_Wenchang	PRJNA827465	0.899168321
ENSGALG00010016733	PHC3	2	0.033168761	0.161276462	0.20566399	0.837053389	0.019505585	1.227773095	18.55172557	0.5	CRA004775;PRJNA827465	Yang2025_Silkie_Wenchang	0.958670902
ENSGALG00010016735	ENSGALG00010016735	2	0.170915795	0.469172044	0.364292368	0.715639699	0.333362147	4.041105781	75.25429785	0.5	PRJNA827465;Yang2025_Silkie_Wenchang	CRA004775	0.927131632
ENSGALG00010016735	ENSGALG00010016735	2	-0.227048024	0.342248522	-0.663401035	0.507073743	0	0.440433213	0	0.5	CRA004775;Yang2025_Silkie_Wenchang	PRJNA827465	0.864016158
ENSGALG00010016735	ENSGALG00010016735	2	0.565663266	0.258356079	2.189471474	0.02856259	0	0.187754575	0	1	CRA004775;PRJNA827465	Yang2025_Silkie_Wenchang	0.287842636
ENSGALG00010016736	CENPS	2	-0.269008239	0.439057007	-0.61269547	0.540077718	0.362334064	16.30702283	93.86767278	0.5	PRJNA827465;Yang2025_Silkie_Wenchang	CRA004775	0.87529516
ENSGALG00010016736	CENPS	2	-0.515768108	0.291778241	-1.76767159	0.077115822	0.091265212	1.918174303	47.86709432	1	CRA004775;Yang2025_Silkie_Wenchang	PRJNA827465	0.478533748
ENSGALG00010016736	CENPS	2	0.139462005	0.098411953	1.417124659	0.156446484	0	0.396294044	0	0.5	CRA004775;PRJNA827465	Yang2025_Silkie_Wenchang	0.649484056
ENSGALG00010016738	NAA60	2	-0.129961207	0.056800227	-2.288040294	0.022135175	0	0.294503329	0	1	PRJNA827465;Yang2025_Silkie_Wenchang	CRA004775	0.252387227
ENSGALG00010016738	NAA60	2	-0.156339274	0.084996541	-1.839360424	0.065862191	0	0.123702076	0	1	CRA004775;Yang2025_Silkie_Wenchang	PRJNA827465	0.4420276
ENSGALG00010016738	NAA60	2	-0.105821327	0.066264159	-1.596961741	0.110274236	0	0.002432854	0	1	CRA004775;PRJNA827465	Yang2025_Silkie_Wenchang	0.562335482
ENSGALG00010016741	NELFA	2	-0.055241221	0.10789752	-0.511978594	0.608665991	0.018580268	4.534863667	77.94862044	0.5	PRJNA827465;Yang2025_Silkie_Wenchang	CRA004775	0.897208691
ENSGALG00010016741	NELFA	2	-0.174177046	0.090481497	-1.925001807	0.054229132	0	0.0672937	0	1	CRA004775;Yang2025_Silkie_Wenchang	PRJNA827465	0.403668103
ENSGALG00010016741	NELFA	2	0.035284947	0.040015448	0.88178313	0.377894096	0	0.162878619	0	0.5	CRA004775;PRJNA827465	Yang2025_Silkie_Wenchang	0.82325159
ENSGALG00010016742	TRMT12	2	-0.00135815	0.097452125	-0.013936583	0.988880576	0	0.023729275	0	0.5	PRJNA827465;Yang2025_Silkie_Wenchang	CRA004775	0.996891127
ENSGALG00010016742	TRMT12	2	-0.108779417	0.175024937	-0.621508105	0.534265363	0	0.64418663	0	0.5	CRA004775;Yang2025_Silkie_Wenchang	PRJNA827465	0.873408008
ENSGALG00010016742	TRMT12	2	-0.045227756	0.097203505	-0.465289351	0.64172426	0	0.800692145	0	1	CRA004775;PRJNA827465	Yang2025_Silkie_Wenchang	0.906634523
ENSGALG00010016745	BEGAIN	2	0.086188502	0.26954831	0.319751595	0.749156645	0	0.071293619	0	0.5	PRJNA827465;Yang2025_Silkie_Wenchang	CRA004775	0.937362187
ENSGALG00010016745	BEGAIN	2	0.471092356	0.608017748	0.774800338	0.43845765	0.401683495	2.056282582	51.36855173	0.5	CRA004775;Yang2025_Silkie_Wenchang	PRJNA827465	0.844002503
ENSGALG00010016745	BEGAIN	2	0.488301366	0.511707063	0.954259578	0.339952264	0.267594382	1.789800714	44.12785781	1	CRA004775;PRJNA827465	Yang2025_Silkie_Wenchang	0.805227735
ENSGALG00010016746	HDGFL3	2	0.04992389	0.099175862	0.503387505	0.614691855	0.012564979	2.678330196	62.66330412	0.5	PRJNA827465;Yang2025_Silkie_Wenchang	CRA004775	0.898692812
ENSGALG00010016746	HDGFL3	2	0.135434295	0.095468048	1.418634794	0.156005517	0	0.712303122	0	0.5	CRA004775;Yang2025_Silkie_Wenchang	PRJNA827465	0.649132948
ENSGALG00010016746	HDGFL3	2	-0.039498541	0.066861736	-0.590749552	0.554688241	0	0.03691223	0	1	CRA004775;PRJNA827465	Yang2025_Silkie_Wenchang	0.880223402
ENSGALG00010016750	SYTL4	2	0.108033734	0.094660321	1.141277923	0.253754286	0.004183426	1.273155233	21.45498257	1	PRJNA827465;Yang2025_Silkie_Wenchang	CRA004775	0.763418915
ENSGALG00010016750	SYTL4	2	0.093478847	0.18092991	0.51665779	0.605395066	0.031862117	1.845723784	45.82071225	0.5	CRA004775;Yang2025_Silkie_Wenchang	PRJNA827465	0.896474603

ENSGALG00010016750	SYTL4	2	0.010705656	0.091016058	0.117623819	0.906365731	0	0.479045837	0	0.5	CRA004775;PRJNA827465	Yang2025_Silkie_Wenchang	0.978196186
ENSGALG00010016751	DFFA	2	-0.109290937	0.070872774	-1.542072233	0.123056041	0	0.046035284	0	1	PRJNA827465;Yang2025_Silkie_Wenchang	CRA004775	0.588020863
ENSGALG00010016751	DFFA	2	-0.165061355	0.116143894	-1.421179796	0.155264497	0	0.753139515	0	1	CRA004775;Yang2025_Silkie_Wenchang	PRJNA827465	0.647874911
ENSGALG00010016751	DFFA	2	-0.144942166	0.073291415	-1.977614519	0.04797221	0	0.721195492	0	1	CRA004775;PRJNA827465	Yang2025_Silkie_Wenchang	0.378772122
ENSGALG00010016752	ENSGALG00010016752	2	-0.351593469	0.361629286	-0.972248329	0.330927023	0.243382448	14.30796843	93.01088757	0.5	PRJNA827465;Yang2025_Silkie_Wenchang	CRA004775	0.801678056
ENSGALG00010016752	ENSGALG00010016752	2	-0.703619898	0.144394704	-4.872892676	1.09976E-06	0	0.100099966	0	1	CRA004775;Yang2025_Silkie_Wenchang	PRJNA827465	0.000240843
ENSGALG00010016752	ENSGALG00010016752	2	-0.161320127	0.262980575	-0.613429823	0.539592172	0.083262036	1.940522722	48.46749338	0.5	CRA004775;PRJNA827465	Yang2025_Silkie_Wenchang	0.875051321
ENSGALG00010016754	ENSGALG00010016754	2	-0.017811509	0.124645162	-0.142897714	0.886370964	0	0.091934915	0	0.5	PRJNA827465;Yang2025_Silkie_Wenchang	CRA004775	0.972729743
ENSGALG00010016754	ENSGALG00010016754	2	-0.026169359	0.211237451	-0.123885982	0.901405552	0	0.517223206	0	0.5	CRA004775;Yang2025_Silkie_Wenchang	PRJNA827465	0.976655087
ENSGALG00010016754	ENSGALG00010016754	2	-0.066418737	0.142397848	-0.466430764	0.640907195	0	0.363839641	0	1	CRA004775;PRJNA827465	Yang2025_Silkie_Wenchang	0.906429868
ENSGALG00010016756	NUDT16L1	2	0.025170597	0.051263175	0.491007373	0.623421233	0	0.001074587	0	1	PRJNA827465;Yang2025_Silkie_Wenchang	CRA004775	0.803357342
ENSGALG00010016756	NUDT16L1	2	-0.058548501	0.157543783	-0.371633204	0.710165964	0	0.838164469	0	0.5	CRA004775;Yang2025_Silkie_Wenchang	PRJNA827465	0.925364369
ENSGALG00010016756	NUDT16L1	2	0.00376299	0.078511777	0.047928993	0.961772833	0.003756583	1.081632117	7.547123995	0.5	CRA004775;PRJNA827465	Yang2025_Silkie_Wenchang	0.98973612
ENSGALG00010016757	SRPX2	2	-0.088360297	0.230379269	-0.383542743	0.701317384	0.086572274	5.26713737	81.01435505	0.5	PRJNA827465;Yang2025_Silkie_Wenchang	CRA004775	0.922671124
ENSGALG00010016757	SRPX2	2	-0.373363225	0.159906356	-2.334886703	0.01954934	0	0.356452593	0	1	CRA004775;Yang2025_Silkie_Wenchang	PRJNA827465	0.234180956
ENSGALG00010016757	SRPX2	2	-0.120131936	0.354076279	-0.339282644	0.734396816	0.173366871	2.524524843	60.38858549	0.5	CRA004775;PRJNA827465	Yang2025_Silkie_Wenchang	0.933520234
ENSGALG00010016758	NR3C1	2	-0.081020147	0.048732804	-1.662538161	0.096404918	0	0.120602235	0	1	PRJNA827465;Yang2025_Silkie_Wenchang	CRA004775	0.530885356
ENSGALG00010016758	NR3C1	2	0.040770957	0.221491349	0.184074715	0.853954842	0.05992566	2.232918518	55.21556241	0.5	CRA004775;Yang2025_Silkie_Wenchang	PRJNA827465	0.962975689
ENSGALG00010016758	NR3C1	2	0.041475531	0.187222916	0.221530205	0.824679621	0.045490218	2.069790971	51.68594249	0.5	CRA004775;PRJNA827465	Yang2025_Silkie_Wenchang	0.95619211
ENSGALG00010016760	DLK1	2	-0.451364091	0.091323066	-4.942498196	7.71278E-07	0	0.104144075	0	1	PRJNA827465;Yang2025_Silkie_Wenchang	CRA004775	0.000181518
ENSGALG00010016760	DLK1	2	-0.109236988	0.5215087	-0.209463404	0.834086501	0.393447449	2.830256513	64.66751352	0.5	CRA004775;Yang2025_Silkie_Wenchang	PRJNA827465	0.957968425
ENSGALG00010016760	DLK1	2	-0.090118215	0.486314294	-0.185308587	0.852987003	0.330387922	2.538549203	60.60740101	0.5	CRA004775;PRJNA827465	Yang2025_Silkie_Wenchang	0.962708451
ENSGALG00010016761	GPR160	2	-0.080252494	0.13284288	-0.604115889	0.545766597	0.02025254	2.153828142	53.5710403	0.5	PRJNA827465;Yang2025_Silkie_Wenchang	CRA004775	0.877885919
ENSGALG00010016761	GPR160	2	-0.296249866	0.142238178	-2.082773199	0.037271902	0	0.262774085	0	1	CRA004775;Yang2025_Silkie_Wenchang	PRJNA827465	0.333082662
ENSGALG00010016761	GPR160	2	-0.119218145	0.205835004	-0.579192763	0.562459114	0.054445076	2.266243658	55.87411811	0.5	CRA004775;PRJNA827465	Yang2025_Silkie_Wenchang	0.881955027
ENSGALG00010016762	EML6	2	0.246290458	0.426020241	0.578119145	0.563183682	0.332951046	11.77449097	91.50706385	0.5	PRJNA827465;Yang2025_Silkie_Wenchang	CRA004775	0.882321851
ENSGALG00010016762	EML6	2	0.660631397	0.204519482	3.230163655	0.001237194	0	0.159490878	0	1	CRA004775;Yang2025_Silkie_Wenchang	PRJNA827465	0.042841786
ENSGALG00010016762	EML6	2	-0.081029335	0.205656987	-0.394002343	0.693579309	0.035293378	1.226811833	18.48790717	0.5	CRA004775;PRJNA827465	Yang2025_Silkie_Wenchang	0.920721644
ENSGALG00010016763	UBALD1	2	0.08010817	0.065156125	1.229480272	0.21889179	0	0.65223117	0	1	PRJNA827465;Yang2025_Silkie_Wenchang	CRA004775	0.73259614
ENSGALG00010016763	UBALD1	2	0.201002132	0.135446381	1.4839978	0.137809504	0	0.001099162	0	1	CRA004775;Yang2025_Silkie_Wenchang	PRJNA827465	0.62064915
ENSGALG00010016763	UBALD1	2	0.067709322	0.068682465	0.985831274	0.324215916	0	0.329093946	0	1	CRA004775;PRJNA827465	Yang2025_Silkie_Wenchang	0.799686366
ENSGALG00010016766	AOAH	2	-0.002434766	0.205267337	-0.011861441	0.990536161	0	0.92855506	0	0.5	PRJNA827465;Yang2025_Silkie_Wenchang	CRA004775	0.997490142
ENSGALG00010016766	AOAH	2	0.116459394	0.278691445	0.417879328	0.676035347	0	0.54563181	0	0.5	CRA004775;Yang2025_Silkie_Wenchang	PRJNA827465	0.91657106
ENSGALG00010016766	AOAH	2	-0.172876761	0.236164233	-0.732019236	0.464156827	0	0.009014524	0	1	CRA004775;PRJNA827465	Yang2025_Silkie_Wenchang	0.852159649
ENSGALG00010016767	SLAIN2	2	-0.011030823	0.044822679	-0.24609915	0.805605481	0	0.455648517	0	0.5	PRJNA827465;Yang2025_Silkie_Wenchang	CRA004775	0.951975713
ENSGALG00010016767	SLAIN2	2	0.225314275	0.339215572	0.664221498	0.506548556	0.194577842	6.014079715	83.37235209	0.5	CRA004775;Yang2025_Silkie_Wenchang	PRJNA827465	0.865929781
ENSGALG00010016767	SLAIN2	2	0.252261175	0.297415887	0.84817653	0.396339666	0.148110305	5.306091495	81.15373621	1	CRA004775;PRJNA827465	Yang2025_Silkie_Wenchang	0.831365249
ENSGALG00010016768	GALNT10	2	-0.071124339	0.163249711	-0.435678195	0.663070228	0.036478871	2.870141362	65.15851055	0.5	PRJNA827465;Yang2025_Silkie_Wenchang	CRA004775	0.91293776
ENSGALG00010016768	GALNT10	2	-0.187833624	0.161784064	-1.161014378	0.245636052	0.00201974	1.027677536	2.693212081	0.5	CRA004775;Yang2025_Silkie_Wenchang	PRJNA827465	0.75661406
ENSGALG00010016768	GALNT10	2	0.062695866	0.082555505	0.759438949	0.447590022	0	0.022839462	0	1	CRA004775;PRJNA827465	Yang2025_Silkie_Wenchang	0.846601815
ENSGALG00010016772	TSPAN6	2	-0.231415502	0.287000021	-0.80632573	0.420055058	0.155651292	17.65076501	94.33452318	0.5	PRJNA827465;Yang2025_Silkie_Wenchang	CRA004775	0.838603917
ENSGALG00010016772	TSPAN6	2	-0.473998385	0.111106756	-4.266152704	1.98873E-05	0	0.881007394	0	1	CRA004775;Yang2025_Silkie_Wenchang	PRJNA827465	0.002442592
ENSGALG00010016772	TSPAN6	2	-0.037286885	0.140804421	-0.264813314	0.79115328	0.023713488	1.820457414	45.05999874	0.5	CRA004775;PRJNA827465	Yang2025_Silkie_Wenchang	0.947893469
ENSGALG00010016773	RPS27A	2	-0.332519056	0.338905912	-0.981154486	0.326516565	0.191762423	5.272146658	81.03239411	1	PRJNA827465;Yang2025_Silkie_Wenchang	CRA004775	0.800028239
ENSGALG00010016773	RPS27A	2	-0.498603125	0.206655267	-2.412728852	0.015833594	0.013375821	1.18166577	15.37370167	1	CRA004775;Yang2025_Silkie_Wenchang	PRJNA827465	0.208199311
ENSGALG00010016773	RPS27A	2	-0.07670236	0.084100717	-0.912029806	0.361753027	0.003553978	1.107310999	9.691134544	1	CRA004775;PRJNA827465	Yang2025_Silkie_Wenchang	0.815545459
ENSGALG00010016774	PEX14	2	0.044417797	0.125834811	0.352984971	0.724099708	0.021987178	2.765119804	63.8352017	0.5	PRJNA827465;Yang2025_Silkie_Wenchang	CRA004775	0.930355448
ENSGALG00010016774	PEX14	2	-0.002479215	0.241584761	-0.014336496	0.988561523	0.082942682	3.355736859	70.2002856	0.5	CRA004775;Yang2025_Silkie_Wenchang	PRJNA827465	0.996891127
ENSGALG00010016774	PEX14	2	-0.061350287	0.05038796	-1.217558474	0.223391803	0	0.983340071	0	1	CRA004775;PRJNA827465	Yang2025_Silkie_Wenchang	0.736744216
ENSGALG00010016777	TNMD	2	0.780931335	0.618857557	1.261891894	0.206987689	0.693129144	10.47270289	90.45136666	1	PRJNA827465;Yang2025_Silkie_Wenchang	CRA004775	0.718911823
ENSGALG00010016777	TNMD	2	0.14174054	1.375668263	0.10303438	0.917935685	3.418909908	9.77289918	89.76762185	0.5	CRA004775;Yang2025_Silkie_Wenchang	PRJNA827465	0.981033557
ENSGALG00010016777	TNMD	2	-0.378188083	0.732002845	-0.516648378	0.605401638	0.78438443	3.098483416	67.72614644	0.5	CRA004775;PRJNA827465	Yang2025_Silkie_Wenchang	0.896474603
ENSGALG00010016778	MTIF2	2	0.106195189	0.126018345	0.842696268	0.399398335	0.027361317	7.012924028	85.74061268	0.5	PRJNA827465;Yang2025_Silkie_Wenchang	CRA004775	0.832237344
ENSGALG00010016778	MTIF2	2	0.237230947	0.078435755	3.024525574	0.002490234	0	0.0265281	0	1	CRA004775;Yang2025_Silkie_Wenchang	PRJNA827465	0.06708271
ENSGALG00010016778	MTIF2	2	-0.004716541	0.047774919	-0.098724198	0.921357255	0	0.591774048	0	0.5	CRA004775;PRJNA827465	Yang2025_Silkie_Wenchang	0.981519273
ENSGALG00010016779	CTNNA2	2	-0.088406	0.09548168	-0.925894897	0.354500604	0	0.115254147	0	1	PRJNA827465;Yang2025_Silkie_Wenchang	CRA004775	0.81193713
ENSGALG00010016779	CTNNA2	2	0.09324323	0.28500927	0.327158586	0.743547943	0.096516423	2.390088797	58.1605503	0.5	CRA004775;Yang2025_Silkie_Wenchang	PRJNA827465	0.935816088
ENSGALG00010016779	CTNNA2	2	0.092576646	0.23332191	0.396776479	0.691532297	0.070165392	2.337736695	57.2242599	0.5	CRA004775;PRJNA827465	Yang2025_Silkie_Wenchang	0.920271344
ENSGALG00010016780	PRORSD1P	2	-0.020705191	0.069904786	-0.296191328	0.767083971	0	0.458444238	0	0.5	PRJNA827465;Yang2025_Silkie_Wenchang	CRA004775	0.941432527
ENSGALG00010016780	PRORSD1P	2	-0.112053858	0.110559837	-1.013513239	0.310815076	0	0.178799773	0	1	CRA004775;Yang2025_Silkie_Wenchang	PRJNA827465	0.795154913
ENSGALG00010016780	PRORSD1P	2	-0.010424131	0.080506587	-0.129480931	0.896975821	0	0.682816423	0	0.5	CRA004775;PRJNA827465	Yang2025_Silkie_Wenchang	0.975244266
ENSGALG00010016781	CDIP1	2	0.093700775	0.161260482	0.581052306	0.561205201	0.039895996	4.217609878	76.28988861	0.5	PRJNA827465;Yang2025_Silkie_Wenchang	CRA004775	0.881506202
ENSGALG00010016781	CDIP1	2	0.043309359	0.316125201	0.137000654	0.891030278	0.133866173	2.476717684	59.62398111	0.5	CRA004775;Yang2025_Silkie_Wenchang	PRJNA827465	0.973638442
ENSGALG00010016781	CDIP1	2	-0.07310619	0.090090791	-0.811472401	0.417094436	0	0.692480544	0	1	CRA004775;PRJNA827465	Yang2025_Silkie_Wenchang	0.838112595
ENSGALG00010016783	CLBA1	2	0.264528175	0.241902918	1.093530321	0.274161024	0.038335598	1.364865095	26.73268562	0.5	PRJNA827465;Yang2025_Silkie_Wenchang	CRA004775	0.774526342
ENSGALG00010016783	CLBA1	2	-0.045866246	0.310506455	-0.147714309	0.882568239	0	0.074317097	0	0.5	CRA004775;Yang2025_Silkie_Wenchang	PRJNA827465	0.972001076
ENSGALG00010016783	CLBA1	2	0.35925441	0.19132857	1.877683037	0.060424539	0	0.486489032	0	1	CRA004775;PRJNA827465	Yang2025_Silkie_Wenchang	0.425903811
ENSGALG00010016785	PDGFRB	2	-0.03560256	0.075614639	-0.470842162	0.637753454	0	0.186315459	0	1	PRJNA827465;Yang2025_Silkie_Wenchang	CRA004775	0.905279419
ENSGALG00010016785	PDGFRB	2	-0.075140027	0.122304689	-0.614367506	0.538972503	0	0.017601759	0	1	CRA004775;Yang2025_Silkie_Wenchang	PRJNA827465	0.87481821
ENSGALG00010016785	PDGFRB	2	-0.01198983	0.09171844	-0.130724317	0.895993395	0	0.000408293	0	1	CRA004775;PRJNA827465	Yang2025_Silkie_Wenchang	0.97500494
ENSGALG00010016786	DISP1	2	-0.142733657	0.17863803	-0.799010478	0.424284336	0.053823354	6.374840892	84.31333398	0.5	PRJNA827465;Yang2025_Silkie_Wenchang	CRA004775	0.840617373
ENSGALG00010016786	DISP1	2	-0.04178061	0.297566764	-0.140407516	0.88833802	0.157259535	8.73697863	88.55439572	0.5	CRA004775;Yang2025_Silkie_Wenchang	PRJNA827465	0.973135755
ENSGALG00010016786	DISP1	2	0.109372656	0.111224643	0.983349123	0.325435641	0.009102551	1.469847785	31.96574432	1	CRA004775;PRJNA827465	Yang2025_Silkie_Wenchang	0.799987949
ENSGALG00010016787	PPP2R5C	2	-0.040638705	0.046495309	-0.874038828	0.382097109	0	0.071425531	0	1	PRJNA827465;Yang2025_Silkie_Wenchang	CRA004775	0.825001735
ENSGALG00010016787	PPP2R5C	2	0.037864273	0.092989187	0.407190066	0.683868397	0.001347602	1.062993484	5.926046081	0.5	CRA004775;Yang2025_Silkie_Wenchang	PRJNA827465	0.918806497
ENSGALG00010016787	PPP2R5C	2	0.013714346	0.105447133	0.130058972	0.89651977	0.012073669	1.684832458	40.64691744	0.5	CRA004775;PRJNA827465	Yang2025_Silkie_Wenchang	0.975115773
ENSGALG00010016788	ANLN	2	-0.206307572	0.156455478	-1.318634377	0.187291375	0.036423328	3.894396982	74.32208363	1	PRJNA827465;Yang2025_Silkie_Wenchang	CRA004775	0.69615374
ENSGALG00010016788	ANLN	2	0.102157616	0.597498666	0.170975471	0.864243053	0.563781136	3.934417847	74.58327918	0.5	CRA004775;Yang2025_Silkie_Wenchang	PRJNA827465	0.966629047
ENSGALG00010016788	ANLN	2	0.207529821	0.414256597	0.50096926	0.616392757	0.22942393	2.204011581	54.62818759	0.5	CRA004775;PRJNA827465	Yang2025_Silkie_Wenchang	0.899111936
ENSGALG00010016789	DISP3	2	-0.226584313	0.180103925	-1.258075372	0.208364499	0.051101366	4.375465244	77.14528755	1	PRJNA827465;Yang2025_Silkie_Wenchang	CRA004775	0.720917211
ENSGALG00010016789	DISP3	2	-0.330685752	0.174304102	-1.897177101	0.057804568	0.016551827	1.267413434	21.09914785	1	CRA004775;Yang2025_Silkie_Wenchang	PRJNA827465	0.416631751
ENSGALG00010016789	DISP3	2	-0.0689994	0.072978467	-0.945476153	0.34441584	0	0.009848069	0	1	CRA004775;PRJNA827465	Yang2025_Silkie_Wenchang	0.806768255
ENSGALG00010016792	HMOX2	2	0.074329223	0.068692846	1.082051874	0.279229478	0	0.038969646	0	1	PRJNA827465;Yang2025_Silkie_Wenchang	CRA004775	0.777442633
ENSGALG00010016792	HMOX2	2	0.071342008	0.125414016	0.568851954	0.569456613	0	0.059300548	0	1	CRA004775;Yang2025_Silkie_Wenchang	PRJNA827465	0.884818733
ENSGALG00010016792	HMOX2	2	0.083847242	0.069849614	1.200396636	0.229985335	0	0.015301445	0	1	CRA004775;PRJNA827465	Yang2025_Silkie_Wenchang	0.742845187
ENSGALG00010016793	SLC9A1	2	0.054790631	0.065768686	0.833080819	0.404799151	0	0.711094324	0	1	PRJNA827465;Yang2025_Silkie_Wenchang	CRA004775	0.833112102
ENSGALG00010016793	SLC9A1	2	0.090332486	0.089452864	1.009833354	0.312575137	0	0.420330151	0	0.5	CRA004775;Yang2025_Silkie_Wenchang	PRJNA827465	0.796054997
ENSGALG00010016793	SLC9A1	2	-0.002312822	0.08280766	-0.027930048	0.977717943	0	0.038491211	0	0.5	CRA004775;PRJNA827465	Yang2025_Silkie_Wenchang	0.993739602
ENSGALG00010016796	BROX	2	-0.056167498	0.067317638	-0.834365247	0.404075195	0.001989357	1.262203009	20.77344191	1	PRJNA827465;Yang2025_Silkie_Wenchang	CRA004775	0.833112102
ENSGALG00010016796	BROX	2	-0.150180936	0.091616947	-1.639226596	0.101166077	0	0.05490473	0	1	CRA004775;Yang2025_Silkie_Wenchang	PRJNA827465	0.542825462
ENSGALG00010016796	BROX	2	-0.019806039	0.070127019	-0.282430922	0.777613107	0	0.624976935	0	1	CRA004775;PRJNA827465	Yang2025_Silkie_Wenchang	0.944388838
ENSGALG00010016797	SEC62	2	0.171241894	0.23238068	0.736902454	0.461181672	0.098029061	10.33267819	90.32196705	0.5	PRJNA827465;Yang2025_Silkie_Wenchang	CRA004775	0.85014477
ENSGALG00010016797	SEC62	2	0.288284456	0.170882371	1.687034504	0.091596718	0.028582035	1.846878433	45.85458457	1	CRA004775;Yang2025_Silkie_Wenchang	PRJNA827465	0.518914432
ENSGALG00010016797	SEC62	2	-0.039134251	0.052421152	-0.746535498	0.455343996	0	0.243341684	0	0.5	CRA004775;PRJNA827465	Yang2025_Silkie_Wenchang	0.84907427
ENSGALG00010016799	WDTC1	2	-0.361516471	0.197101958	-1.834159715	0.066630289	0.070243629	10.12764034	90.12603167	1	PRJNA827465;Yang2025_Silkie_Wenchang	CRA004775	0.444612948
ENSGALG00010016799	WDTC1	2	-0.561466466	0.104267527	-5.384864126	7.24994E-08	0	0.061441914	0	1	CRA004775;Yang2025_Silkie_Wenchang	PRJNA827465	2.71376E-05
ENSGALG00010016799	WDTC1	2	-0.183963504	0.057138655	-3.219598092	0.001283704	0	0.811151552	0	1	CRA004775;PRJNA827465	Yang2025_Silkie_Wenchang	0.043419476
ENSGALG00010016800	RPL14	2	-0.002433592	0.06223223	-0.039105004	0.968806672	0	0.244983746	0	0.5	PRJNA827465;Yang2025_Silkie_Wenchang	CRA004775	0.991349425
ENSGALG00010016800	RPL14	2	-0.030782571	0.152896384	-0.201329621	0.840440842	0.00493996	1.104103862	9.428810624	0.5	CRA004775;Yang2025_Silkie_Wenchang	PRJNA827465	0.959662809
ENSGALG00010016800	RPL14	2	-0.029115622	0.064464082	-0.451656503	0.651516459	0	0.775765186	0	1	CRA004775;PRJNA827465	Yang2025_Silkie_Wenchang	0.908936226
ENSGALG00010016802	TMEM222	2	0.077945633	0.054691419	1.425189428	0.154102452	0.00082483	1.074802682	6.959666448	0.5	PRJNA827465;Yang2025_Silkie_Wenchang	CRA004775	0.646788679
ENSGALG00010016802	TMEM222	2	-0.295376435	0.250710826	-1.178155886	0.238734466	0.10213552	5.304543392	81.14823603	1	CRA004775;Yang2025_Silkie_Wenchang	PRJNA827465	0.75079325
ENSGALG00010016802	TMEM222	2	-0.207717933	0.327267819	-0.634703203	0.525622004	0.199571751	13.97006589	92.84183763	0.5	CRA004775;PRJNA827465	Yang2025_Silkie_Wenchang	0.870277065
ENSGALG00010016804	CSF1R	2	-0.297585154	0.228915691	-1.299977095	0.19360882	0.095439894	11.05664689	90.95566667	1	PRJNA827465;Yang2025_Silkie_Wenchang	CRA004775	0.703672794
ENSGALG00010016804	CSF1R	2	-0.55732214	0.111183087	-5.012652163	5.36849E-07	0	0.686538386	0	1	CRA004775;Yang2025_Silkie_Wenchang	PRJNA827465	0.000136128
ENSGALG00010016804	CSF1R	2	-0.380911876	0.408898951	-0.931555033	0.351566531	0.2540323	3.303054449	69.72499196	1	CRA004775;PRJNA827465	Yang2025_Silkie_Wenchang	0.810678834
ENSGALG00010016807	VSIG4	2	0.096350798	0.173776128	0.554453589	0.57926846	0	0.694254649	0	0.5	PRJNA827465;Yang2025_Silkie_Wenchang	CRA004775	0.887888423
ENSGALG00010016807	VSIG4	2	-0.13456824	0.228391341	-0.58920027	0.555726936	0	0.155662289	0	1	CRA004775;Yang2025_Silkie_Wenchang	PRJNA827465	0.880324407
ENSGALG00010016807	VSIG4	2	0.101323945	0.209074458	0.484630912	0.627938182	0.005892347	1.050987344	4.851375607	0.5	CRA004775;PRJNA827465	Yang2025_Silkie_Wenchang	0.901904489
ENSGALG00010016810	SH3GL3	2	-0.052346488	0.192342377	-0.27215265	0.785504648	0.057585329	4.509500239	77.8245937	0.5	PRJNA827465;Yang2025_Silkie_Wenchang	CRA004775	0.946492402
ENSGALG00010016810	SH3GL3	2	-0.218967718	0.123587118	-1.771768133	0.076433054	0	0.459388954	0	0.5	CRA004775;Yang2025_Silkie_Wenchang	PRJNA827465	0.477109432
ENSGALG00010016810	SH3GL3	2	0.130289891	0.120641064	1.079979625	0.280151253	0	0.051858294	0	1	CRA004775;PRJNA827465	Yang2025_Silkie_Wenchang	0.77825733
ENSGALG00010016812	LRRC31	2	0.070550418	0.108932145	0.647654723	0.517208295	0	0.000285978	0	1	PRJNA827465;Yang2025_Silkie_Wenchang	CRA004775	0.867952013
ENSGALG00010016812	LRRC31	2	0.040259617	0.192036824	0.209645297	0.833944523	0	0.056294267	0	0.5	CRA004775;Yang2025_Silkie_Wenchang	PRJNA827465	0.957968425
ENSGALG00010016812	LRRC31	2	0.060878271	0.118471366	0.513864854	0.607346481	0	0.074359339	0	0.5	CRA004775;PRJNA827465	Yang2025_Silkie_Wenchang	0.896998866
ENSGALG00010016813	ENSGALG00010016813	2	-0.349267615	0.453429218	-0.770280347	0.441133612	0.18129863	1.686680097	40.71193452	1	PRJNA827465;Yang2025_Silkie_Wenchang	CRA004775	0.844898121
ENSGALG00010016813	ENSGALG00010016813	2	-0.732470871	0.559248035	-1.309742413	0.19028299	0	0.64514674	0	0.5	CRA004775;Yang2025_Silkie_Wenchang	PRJNA827465	0.699411386
ENSGALG00010016813	ENSGALG00010016813	2	0.007576214	0.359902815	0.021050722	0.983205194	0	0.02168551	0	0.5	CRA004775;PRJNA827465	Yang2025_Silkie_Wenchang	0.995363468
ENSGALG00010016814	HMGXB3	2	0.060299317	0.067147422	0.89801388	0.369178149	0.003406507	1.556563126	35.75589814	1	PRJNA827465;Yang2025_Silkie_Wenchang	CRA004775	0.820603014
ENSGALG00010016814	HMGXB3	2	0.013001598	0.169058269	0.076906018	0.93869831	0.037754269	2.647417373	62.22733861	0.5	CRA004775;Yang2025_Silkie_Wenchang	PRJNA827465	0.985349045
ENSGALG00010016814	HMGXB3	2	-0.018042853	0.068398464	-0.263790316	0.791941497	0.001400437	1.06760852	6.332707037	0.5	CRA004775;PRJNA827465	Yang2025_Silkie_Wenchang	0.948314332
ENSGALG00010016816	MAP3K6	2	0.28125476	0.607580152	0.462909723	0.643429078	0.660753791	9.517855786	89.4934319	0.5	PRJNA827465;Yang2025_Silkie_Wenchang	CRA004775	0.906634523
ENSGALG00010016816	MAP3K6	2	0.239308506	0.637339092	0.375480664	0.707303024	0.750272287	13.04427261	92.33380021	0.5	CRA004775;Yang2025_Silkie_Wenchang	PRJNA827465	0.924856272
ENSGALG00010016816	MAP3K6	2	-0.366149655	0.176823516	-2.070706792	0.038386205	0	0.026909895	0	1	CRA004775;PRJNA827465	Yang2025_Silkie_Wenchang	0.337817514
ENSGALG00010016817	GGCT	2	-0.000363034	0.09757876	-0.003720424	0.997031538	0	0.48217084	0	0.5	PRJNA827465;Yang2025_Silkie_Wenchang	CRA004775	0.999065103
ENSGALG00010016817	GGCT	2	-0.36077587	0.350481363	-1.029372479	0.303304676	0.137240041	1.923557167	48.01298255	1	CRA004775;Yang2025_Silkie_Wenchang	PRJNA827465	0.792084776
ENSGALG00010016817	GGCT	2	-0.289266008	0.438973026	-0.658960779	0.509920955	0.28654978	3.137563851	68.12813866	0.5	CRA004775;PRJNA827465	Yang2025_Silkie_Wenchang	0.865268882
ENSGALG00010016820	EIF2AK3	2	0.061585462	0.100500999	0.612784572	0.540018793	0.010740563	1.961175298	49.01016747	0.5	PRJNA827465;Yang2025_Silkie_Wenchang	CRA004775	0.87529516
ENSGALG00010016820	EIF2AK3	2	-0.043893137	0.117364602	-0.373989572	0.708412075	0	0.229761018	0	0.5	CRA004775;Yang2025_Silkie_Wenchang	PRJNA827465	0.9250668
ENSGALG00010016820	EIF2AK3	2	0.13214102	0.068518387	1.928548322	0.05378696	0	0.066996464	0	1	CRA004775;PRJNA827465	Yang2025_Silkie_Wenchang	0.401964436
ENSGALG00010016823	HSF3	2	-0.015770804	0.040479587	-0.389598936	0.696833139	0	0.733878185	0	0.5	PRJNA827465;Yang2025_Silkie_Wenchang	CRA004775	0.921086208
ENSGALG00010016823	HSF3	2	0.120397713	0.208386662	0.577761131	0.563425401	0.071636648	5.201862541	80.7761164	0.5	CRA004775;Yang2025_Silkie_Wenchang	PRJNA827465	0.882407972
ENSGALG00010016823	HSF3	2	0.147559768	0.170527627	0.865312974	0.386867032	0.044943998	3.769341137	73.47016458	1	CRA004775;PRJNA827465	Yang2025_Silkie_Wenchang	0.827124787
ENSGALG00010016825	ENSGALG00010016825	2	0.138808311	0.265921565	0.521989675	0.601677519	0.098271383	3.164359105	68.39802415	0.5	PRJNA827465;Yang2025_Silkie_Wenchang	CRA004775	0.895230519
ENSGALG00010016825	ENSGALG00010016825	2	0.339457218	0.227089347	1.494817885	0.134961977	0	0.862486424	0	0.5	CRA004775;Yang2025_Silkie_Wenchang	PRJNA827465	0.61578043
ENSGALG00010016825	ENSGALG00010016825	2	-0.096208674	0.15799471	-0.608936044	0.542566832	0	0.000105679	0	1	CRA004775;PRJNA827465	Yang2025_Silkie_Wenchang	0.876817086
ENSGALG00010016828	LACTBL1	2	-0.178865881	0.203474179	-0.879059359	0.379369092	0	0.568494709	0	1	PRJNA827465;Yang2025_Silkie_Wenchang	CRA004775	0.824165982
ENSGALG00010016828	LACTBL1	2	-0.068730878	0.270639914	-0.253956916	0.799528843	0	0.194457414	0	1	CRA004775;Yang2025_Silkie_Wenchang	PRJNA827465	0.949922217
ENSGALG00010016828	LACTBL1	2	-0.338623615	0.26936097	-1.257136899	0.208704068	0	0.005234792	0	1	CRA004775;PRJNA827465	Yang2025_Silkie_Wenchang	0.721011841
ENSGALG00010016831	AIDA	2	-0.021530487	0.047397823	-0.454250543	0.649648509	0	0.189082807	0	1	PRJNA827465;Yang2025_Silkie_Wenchang	CRA004775	0.908200019
ENSGALG00010016831	AIDA	2	-0.042584948	0.097978066	-0.434637565	0.663825524	0	0.162764347	0	0.5	CRA004775;Yang2025_Silkie_Wenchang	PRJNA827465	0.913119244
ENSGALG00010016831	AIDA	2	-0.009877882	0.049998586	-0.197563232	0.843386804	0	0.028951804	0	0.5	CRA004775;PRJNA827465	Yang2025_Silkie_Wenchang	0.96057435
ENSGALG00010016832	DYNC1H1	2	-0.133693461	0.046470805	-2.876934447	0.00401559	0	0.135121471	0	1	PRJNA827465;Yang2025_Silkie_Wenchang	CRA004775	0.091651995
ENSGALG00010016832	DYNC1H1	2	-0.039173402	0.18600819	-0.210600414	0.833199089	0.039338928	2.031021901	50.76370177	0.5	CRA004775;Yang2025_Silkie_Wenchang	PRJNA827465	0.957968425
ENSGALG00010016832	DYNC1H1	2	-0.039461789	0.150440468	-0.262308335	0.793083736	0.027522981	1.832659605	45.43449329	0.5	CRA004775;PRJNA827465	Yang2025_Silkie_Wenchang	0.948571413
ENSGALG00010016834	ENSGALG00010016834	2	-0.197447137	0.125156042	-1.577607715	0.114655759	0.022926978	3.61032425	72.30165684	1	PRJNA827465;Yang2025_Silkie_Wenchang	CRA004775	0.57103367
ENSGALG00010016834	ENSGALG00010016834	2	0.126474657	0.476551415	0.265395617	0.790704712	0.429818584	18.383577	94.56036222	0.5	CRA004775;Yang2025_Silkie_Wenchang	PRJNA827465	0.947644914
ENSGALG00010016834	ENSGALG00010016834	2	0.242739391	0.35007701	0.693388552	0.488065745	0.224895721	11.58597707	91.36887641	0.5	CRA004775;PRJNA827465	Yang2025_Silkie_Wenchang	0.85768885
ENSGALG00010016835	CCDC88A	2	0.200623959	0.208223326	0.963503769	0.335294774	0.068904929	4.716917424	78.79971367	1	PRJNA827465;Yang2025_Silkie_Wenchang	CRA004775	0.803198384
ENSGALG00010016835	CCDC88A	2	0.320472386	0.228772236	1.400836008	0.161263118	0.037551525	1.273440539	21.47258007	0.5	CRA004775;Yang2025_Silkie_Wenchang	PRJNA827465	0.657549988
ENSGALG00010016835	CCDC88A	2	0.003555606	0.100610211	0.035340409	0.971808302	0	0.11598034	0	0.5	CRA004775;PRJNA827465	Yang2025_Silkie_Wenchang	0.991955348
ENSGALG00010016836	HEPH	2	-0.060787277	0.091855178	-0.661772997	0.508116714	0	0.35197178	0	1	PRJNA827465;Yang2025_Silkie_Wenchang	CRA004775	0.864674022
ENSGALG00010016836	HEPH	2	-0.037592523	0.12401219	-0.30313571	0.761786441	0	0.720980884	0	1	CRA004775;Yang2025_Silkie_Wenchang	PRJNA827465	0.939811773
ENSGALG00010016836	HEPH	2	-0.138528391	0.125318775	-1.105408116	0.268982789	0	0.375138698	0	1	CRA004775;PRJNA827465	Yang2025_Silkie_Wenchang	0.771398294
ENSGALG00010016840	LRRC34	2	0.112540999	0.056887661	1.978302444	0.047894598	0	0.095998009	0	1	PRJNA827465;Yang2025_Silkie_Wenchang	CRA004775	0.378353533
ENSGALG00010016840	LRRC34	2	0.157960948	0.139085317	1.135712606	0.256076863	0	0.000193091	0	1	CRA004775;Yang2025_Silkie_Wenchang	PRJNA827465	0.764570254
ENSGALG00010016840	LRRC34	2	0.108090491	0.059426721	1.818886994	0.068928672	0	0.029316658	0	1	CRA004775;PRJNA827465	Yang2025_Silkie_Wenchang	0.452044654
ENSGALG00010016842	NPY7R	2	0.071781297	0.15507619	0.462877618	0.643452091	0	0.199239981	0	0.5	PRJNA827465;Yang2025_Silkie_Wenchang	CRA004775	0.906634523
ENSGALG00010016842	NPY7R	2	-0.255515556	0.305087428	-0.837515845	0.402302674	0.078992759	1.653240055	39.51271646	1	CRA004775;Yang2025_Silkie_Wenchang	PRJNA827465	0.832879175
ENSGALG00010016842	NPY7R	2	-0.17828163	0.378649417	-0.470835614	0.63775813	0.196904552	2.8713517	65.17319701	0.5	CRA004775;PRJNA827465	Yang2025_Silkie_Wenchang	0.905279419
ENSGALG00010016845	MIA3	2	0.077871881	0.171141408	0.455014843	0.649098561	0.045557273	3.8604259	74.0961224	0.5	PRJNA827465;Yang2025_Silkie_Wenchang	CRA004775	0.907786383
ENSGALG00010016845	MIA3	2	0.264538042	0.145270033	1.821009028	0.068605485	0	0.087590184	0	1	CRA004775;Yang2025_Silkie_Wenchang	PRJNA827465	0.45121266
ENSGALG00010016845	MIA3	2	-0.05098429	0.049774929	-1.024296574	0.305695216	0	0.832218768	0	0.5	CRA004775;PRJNA827465	Yang2025_Silkie_Wenchang	0.79225794
ENSGALG00010016847	EPHB2	2	-0.142748671	0.129137879	-1.105397361	0.268987447	0.022584543	3.047352178	67.18462647	1	PRJNA827465;Yang2025_Silkie_Wenchang	CRA004775	0.771398294
ENSGALG00010016847	EPHB2	2	-0.125913196	0.214816271	-0.586143662	0.557778977	0.057088403	2.291165314	56.35408787	0.5	CRA004775;Yang2025_Silkie_Wenchang	PRJNA827465	0.880678789
ENSGALG00010016847	EPHB2	2	-0.005911514	0.085282433	-0.069316899	0.944737375	0	0.441458032	0	0.5	CRA004775;PRJNA827465	Yang2025_Silkie_Wenchang	0.986014725
ENSGALG00010016848	ENSGALG00010016848	2	0.093064848	0.083222527	1.118265101	0.263453787	0	0.17661414	0	1	PRJNA827465;Yang2025_Silkie_Wenchang	CRA004775	0.768643821
ENSGALG00010016848	ENSGALG00010016848	2	-0.234330135	0.271599367	-0.862778651	0.38825918	0.098828309	2.996917048	66.63237641	0.5	CRA004775;Yang2025_Silkie_Wenchang	PRJNA827465	0.828154121
ENSGALG00010016848	ENSGALG00010016848	2	-0.167768869	0.31531305	-0.532070806	0.594676956	0.167806622	5.883358598	83.00290585	0.5	CRA004775;PRJNA827465	Yang2025_Silkie_Wenchang	0.893379268
ENSGALG00010016849	WASF2	2	-0.051554541	0.049738766	-1.036506235	0.299966032	0	0.012238084	0	1	PRJNA827465;Yang2025_Silkie_Wenchang	CRA004775	0.790132361
ENSGALG00010016849	WASF2	2	0.043524053	0.127305799	0.34188586	0.732436791	0.012710295	1.61579027	38.1107797	0.5	CRA004775;Yang2025_Silkie_Wenchang	PRJNA827465	0.932985777
ENSGALG00010016849	WASF2	2	0.024795918	0.111930961	0.221528677	0.82468081	0.015030574	2.029753356	50.73293034	0.5	CRA004775;PRJNA827465	Yang2025_Silkie_Wenchang	0.95619211
ENSGALG00010016851	TEC	2	-0.031582935	0.082546846	-0.382606194	0.702011777	0	0.727288355	0	0.5	PRJNA827465;Yang2025_Silkie_Wenchang	CRA004775	0.923051857
ENSGALG00010016851	TEC	2	0.126090387	0.329216679	0.38300121	0.701718867	0.153408236	2.963759421	66.25906972	0.5	CRA004775;Yang2025_Silkie_Wenchang	PRJNA827465	0.922904951
ENSGALG00010016851	TEC	2	0.167524118	0.238485176	0.702450863	0.482398035	0.068302589	1.929240207	48.1661228	1	CRA004775;PRJNA827465	Yang2025_Silkie_Wenchang	0.85691916
ENSGALG00010016852	AHDC1	2	0.117340915	0.155157393	0.756270219	0.4494872	0.038981191	5.052152073	80.20645488	0.5	PRJNA827465;Yang2025_Silkie_Wenchang	CRA004775	0.847263197
ENSGALG00010016852	AHDC1	2	0.274464364	0.115221971	2.382048863	0.017216613	0	0.183970458	0	1	CRA004775;Yang2025_Silkie_Wenchang	PRJNA827465	0.218077656
ENSGALG00010016852	AHDC1	2	-0.020311994	0.06925626	-0.293287474	0.769302428	0	0.078171787	0	0.5	CRA004775;PRJNA827465	Yang2025_Silkie_Wenchang	0.941790377
ENSGALG00010016854	TAF1A	2	0.218458343	0.134830813	1.620240495	0.105180626	0.022570317	2.493223706	59.89128462	1	PRJNA827465;Yang2025_Silkie_Wenchang	CRA004775	0.550557098
ENSGALG00010016854	TAF1A	2	0.063031297	0.345684507	0.18233764	0.855317762	0.198723375	5.744509372	82.59207296	0.5	CRA004775;Yang2025_Silkie_Wenchang	PRJNA827465	0.963406774
ENSGALG00010016854	TAF1A	2	-0.038768925	0.199612169	-0.194221251	0.846002643	0.053033753	2.523353516	60.37019809	0.5	CRA004775;PRJNA827465	Yang2025_Silkie_Wenchang	0.96122283
ENSGALG00010016856	NOD1	2	0.313344582	0.090826452	3.449926492	0.000560739	0	0.887024279	0	1	PRJNA827465;Yang2025_Silkie_Wenchang	CRA004775	0.025361028
ENSGALG00010016856	NOD1	2	0.200541162	0.136847654	1.465433688	0.142802632	0	0.000648843	0	1	CRA004775;Yang2025_Silkie_Wenchang	PRJNA827465	0.62921209
ENSGALG00010016856	NOD1	2	0.360595431	0.108729203	3.31645428	0.000911675	0	0.263034276	0	1	CRA004775;PRJNA827465	Yang2025_Silkie_Wenchang	0.035547186
ENSGALG00010016858	ENSGALG00010016858	2	0.351864718	0.330017364	1.06620062	0.286332962	0.185948149	6.577627959	84.79695103	1	PRJNA827465;Yang2025_Silkie_Wenchang	CRA004775	0.781944441
ENSGALG00010016858	ENSGALG00010016858	2	0.354512626	0.420616646	0.842840219	0.399317811	0.252065237	3.253252893	69.26153506	0.5	CRA004775;Yang2025_Silkie_Wenchang	PRJNA827465	0.832152064
ENSGALG00010016858	ENSGALG00010016858	2	0.032120877	0.118987803	0.269951002	0.787197949	0	0.193944908	0	0.5	CRA004775;PRJNA827465	Yang2025_Silkie_Wenchang	0.946848352

ENSGALG00010016859	RPIA	2	-0.349674416	0.240223308	-1.45562235	0.145497046	0.104151601	10.0503158	90.0500639	1	PRJNA827465;Yang2025_Silkie_Wenchang	CRA004775	0.634047057
ENSGALG00010016859	RPIA	2	-0.185835727	0.457889194	-0.405853053	0.684850575	0.365377423	7.306226348	86.3130438	0.5	CRA004775;Yang2025_Silkie_Wenchang	PRJNA827465	0.919003112
ENSGALG00010016859	RPIA	2	-0.006407927	0.192457473	-0.033295287	0.973439112	0.043821263	1.832883857	45.44116932	0.5	CRA004775;PRJNA827465	Yang2025_Silkie_Wenchang	0.992392211
ENSGALG00010016861	FGR	2	-0.012817256	0.057958334	-0.221146041	0.824978722	0.001368368	1.198689693	16.57557364	0.5	PRJNA827465;Yang2025_Silkie_Wenchang	CRA004775	0.956283606
ENSGALG00010016861	FGR	2	-0.168159605	0.116606598	-1.442110547	0.14927119	0.007164294	1.227743841	18.54978486	1	CRA004775;Yang2025_Silkie_Wenchang	PRJNA827465	0.639246608
ENSGALG00010016861	FGR	2	-0.119046763	0.194009554	-0.613612893	0.539471162	0.054693712	2.960656505	66.22370754	0.5	CRA004775;PRJNA827465	Yang2025_Silkie_Wenchang	0.874993194
ENSGALG00010016863	MYNN	2	0.01380574	0.111869226	0.123409628	0.901782733	0.017542106	3.202277279	68.77222324	0.5	PRJNA827465;Yang2025_Silkie_Wenchang	CRA004775	0.976680723
ENSGALG00010016863	MYNN	2	-0.112357128	0.102490212	-1.096271794	0.272959849	0	0.005220651	0	1	CRA004775;Yang2025_Silkie_Wenchang	PRJNA827465	0.773799118
ENSGALG00010016863	MYNN	2	0.10446529	0.065728306	1.589350109	0.111981372	0	0.303114582	0	0.5	CRA004775;PRJNA827465	Yang2025_Silkie_Wenchang	0.56547057
ENSGALG00010016864	ENSGALG00010016864	2	0.559087918	0.413172857	1.353157422	0.176005344	0.265430914	4.393763095	77.24046613	1	PRJNA827465;Yang2025_Silkie_Wenchang	CRA004775	0.68110013
ENSGALG00010016864	ENSGALG00010016864	2	0.470681835	0.567545178	0.829329283	0.406918105	0.503822275	4.53631902	77.95569501	0.5	CRA004775;Yang2025_Silkie_Wenchang	PRJNA827465	0.833618122
ENSGALG00010016864	ENSGALG00010016864	2	0.11006269	0.198629955	0.554109223	0.579504098	0	0.412673722	0	0.5	CRA004775;PRJNA827465	Yang2025_Silkie_Wenchang	0.887969027
ENSGALG00010016865	CFAP36	2	0.166426189	0.171739698	0.969060685	0.332514912	0.045316734	3.734815267	73.22491399	1	PRJNA827465;Yang2025_Silkie_Wenchang	CRA004775	0.802178578
ENSGALG00010016865	CFAP36	2	0.282744522	0.131669608	2.147378773	0.03176139	0	0.755336087	0	1	CRA004775;Yang2025_Silkie_Wenchang	PRJNA827465	0.305303658
ENSGALG00010016865	CFAP36	2	0.03719376	0.054316703	0.684757324	0.493497069	0	0.325246568	0	1	CRA004775;PRJNA827465	Yang2025_Silkie_Wenchang	0.859868321
ENSGALG00010016866	ZNRF2	2	0.06004642	0.230519836	0.260482659	0.79449149	0.08990148	6.301638892	84.13111228	0.5	PRJNA827465;Yang2025_Silkie_Wenchang	CRA004775	0.948905846
ENSGALG00010016866	ZNRF2	2	0.23926975	0.137829675	1.735981387	0.082567126	0	0.815150861	0	1	CRA004775;Yang2025_Silkie_Wenchang	PRJNA827465	0.495344636
ENSGALG00010016866	ZNRF2	2	-0.13702802	0.090372784	-1.516253166	0.129455349	0	0.316628251	0	0.5	CRA004775;PRJNA827465	Yang2025_Silkie_Wenchang	0.603529077
ENSGALG00010016867	FLT4	2	-0.063798471	0.112907073	-0.565052918	0.572037771	0.020138041	4.704596043	78.74418992	0.5	PRJNA827465;Yang2025_Silkie_Wenchang	CRA004775	0.885533125
ENSGALG00010016867	FLT4	2	-0.179225708	0.080552448	-2.224956693	0.026084146	0	0.042239183	0	1	CRA004775;Yang2025_Silkie_Wenchang	PRJNA827465	0.275587412
ENSGALG00010016867	FLT4	2	0.03824645	0.062424674	0.612681606	0.540086887	0	0.180871504	0	0.5	CRA004775;PRJNA827465	Yang2025_Silkie_Wenchang	0.87529516
ENSGALG00010016869	HSP90AA1	2	0.123503998	0.095997914	1.286527932	0.198258877	0.004206329	1.217018131	17.83195544	0.5	PRJNA827465;Yang2025_Silkie_Wenchang	CRA004775	0.710234722
ENSGALG00010016869	HSP90AA1	2	0.324508525	0.381006166	0.851714629	0.394372499	0.244632784	6.249835426	83.99957868	0.5	CRA004775;Yang2025_Silkie_Wenchang	PRJNA827465	0.830486298
ENSGALG00010016869	HSP90AA1	2	0.404471636	0.268404711	1.50694686	0.131824279	0.113483337	4.190579048	76.13694937	1	CRA004775;PRJNA827465	Yang2025_Silkie_Wenchang	0.608454531
ENSGALG00010016872	EDA2R	2	-0.111877592	0.139924186	-0.799558646	0.423966555	0	0.959795003	0	0.5	PRJNA827465;Yang2025_Silkie_Wenchang	CRA004775	0.85030043
ENSGALG00010016872	EDA2R	2	0.116731147	0.2165157	0.539134791	0.589793851	0.004846205	1.017005187	1.672084619	1	CRA004775;Yang2025_Silkie_Wenchang	PRJNA827465	0.892320726
ENSGALG00010016872	EDA2R	2	0.055379754	0.463053763	0.119596811	0.904802546	0.261279186	1.937938462	48.39877429	0.5	CRA004775;PRJNA827465	Yang2025_Silkie_Wenchang	0.977793401
ENSGALG00010016873	ENTPD6	2	0.254013033	0.087229688	2.912002096	0.003591202	0	0.001245065	0	1	PRJNA827465;Yang2025_Silkie_Wenchang	CRA004775	0.085232501
ENSGALG00010016873	ENTPD6	2	0.128221055	0.171941891	0.745723183	0.455834652	0.018765804	1.391455698	28.1328179	0.5	CRA004775;Yang2025_Silkie_Wenchang	PRJNA827465	0.84907427
ENSGALG00010016873	ENTPD6	2	0.150102524	0.16787077	0.89415521	0.371238853	0.02854694	1.702506216	41.26306321	0.5	CRA004775;PRJNA827465	Yang2025_Silkie_Wenchang	0.8214684
ENSGALG00010016874	ADAMTSL3	2	0.036170827	0.118057019	0.30638438	0.759312	0.015666189	2.263506276	55.82075427	0.5	PRJNA827465;Yang2025_Silkie_Wenchang	CRA004775	0.939145717
ENSGALG00010016874	ADAMTSL3	2	0.18578094	0.117064854	1.586991606	0.112514546	0	0.455355444	0	1	CRA004775;Yang2025_Silkie_Wenchang	PRJNA827465	0.566623663
ENSGALG00010016874	ADAMTSL3	2	0.029339318	0.213652372	0.137322686	0.890775739	0.045937482	1.436318601	30.3775639	0.5	CRA004775;PRJNA827465	Yang2025_Silkie_Wenchang	0.973477891
ENSGALG00010016875	SESN2	2	0.01458117	0.080070352	0.182104482	0.855500731	0	0.997282339	0	0.5	PRJNA827465;Yang2025_Silkie_Wenchang	CRA004775	0.963463555
ENSGALG00010016875	SESN2	2	0.098680648	0.160054416	0.616544363	0.537535302	0	0.84808966	0	0.5	CRA004775;Yang2025_Silkie_Wenchang	PRJNA827465	0.87481821
ENSGALG00010016875	SESN2	2	-0.034674907	0.086355498	-0.401536761	0.688024979	0	0.191031566	0	1	CRA004775;PRJNA827465	Yang2025_Silkie_Wenchang	0.919651943
ENSGALG00010016876	MTURN	2	0.248450225	0.138249644	1.797112941	0.072317692	0.021826887	2.158300218	53.66724279	1	PRJNA827465;Yang2025_Silkie_Wenchang	CRA004775	0.46442847
ENSGALG00010016876	MTURN	2	-0.141283905	0.624448823	-0.226253777	0.821004047	0.682949567	7.558638152	86.77010356	0.5	CRA004775;Yang2025_Silkie_Wenchang	PRJNA827465	0.955548641
ENSGALG00010016876	MTURN	2	-0.255940518	0.476675138	-0.536928607	0.59131693	0.37575891	4.990722406	79.96282064	0.5	CRA004775;PRJNA827465	Yang2025_Silkie_Wenchang	0.892776493
ENSGALG00010016877	UBE2D3	2	-0.103580014	0.042234595	-2.452492173	0.014187047	0	0.016115978	0	1	PRJNA827465;Yang2025_Silkie_Wenchang	CRA004775	0.19521731
ENSGALG00010016877	UBE2D3	2	-0.081511243	0.098538568	-0.827201425	0.408122904	0	0.028138355	0	1	CRA004775;Yang2025_Silkie_Wenchang	PRJNA827465	0.834133033
ENSGALG00010016877	UBE2D3	2	-0.103238177	0.04454406	-2.317664269	0.020467574	0	0.066710307	0	1	CRA004775;PRJNA827465	Yang2025_Silkie_Wenchang	0.241210517
ENSGALG00010016878	SUCLG1	2	-0.336225113	0.21484032	-1.565000056	0.117582896	0.075624677	4.645578429	78.47415526	1	PRJNA827465;Yang2025_Silkie_Wenchang	CRA004775	0.577165052
ENSGALG00010016878	SUCLG1	2	-0.254716074	0.374963443	-0.679309088	0.496942039	0.214492968	3.964471849	74.77595912	0.5	CRA004775;Yang2025_Silkie_Wenchang	PRJNA827465	0.860939846
ENSGALG00010016878	SUCLG1	2	-0.124829108	0.087935699	-1.419549844	0.155738776	0	0.869468627	0	0.5	CRA004775;PRJNA827465	Yang2025_Silkie_Wenchang	0.648892306
ENSGALG00010016879	RNF130	2	-0.152629616	0.22980977	-0.664156342	0.506590252	0.091783084	7.214078949	86.13821658	0.5	PRJNA827465;Yang2025_Silkie_Wenchang	CRA004775	0.863929781
ENSGALG00010016879	RNF130	2	-0.389553118	0.13115048	-2.970275958	0.002975323	0	0.031710553	0	1	CRA004775;Yang2025_Silkie_Wenchang	PRJNA827465	0.074960985
ENSGALG00010016879	RNF130	2	-0.085816444	0.195542925	-0.438862432	0.660761219	0.053707036	2.749542167	63.63030863	0.5	CRA004775;PRJNA827465	Yang2025_Silkie_Wenchang	0.91192683
ENSGALG00010016880	AR	2	-0.086635116	0.0766944	-1.129614627	0.258638645	0	0.16208972	0	1	PRJNA827465;Yang2025_Silkie_Wenchang	CRA004775	0.765798911
ENSGALG00010016880	AR	2	-0.133893323	0.101846484	-1.314658275	0.188624775	0	0.569856053	0	1	CRA004775;Yang2025_Silkie_Wenchang	PRJNA827465	0.698039126
ENSGALG00010016880	AR	2	-0.078559307	0.108989665	-0.720795934	0.471035077	0	0.791328304	0	1	CRA004775;PRJNA827465	Yang2025_Silkie_Wenchang	0.853855613
ENSGALG00010016881	C1QB	2	0.248255599	0.446413864	0.556110861	0.578135071	0.286439306	3.39568498	70.5508607	0.5	PRJNA827465;Yang2025_Silkie_Wenchang	CRA004775	0.88776881
ENSGALG00010016881	C1QB	2	-0.500865978	0.285302957	-1.755558311	0.079163856	0	0.606358291	0	1	CRA004775;Yang2025_Silkie_Wenchang	PRJNA827465	0.484261016
ENSGALG00010016881	C1QB	2	-0.002718918	0.672429115	-0.004043426	0.996773821	0.797858946	8.347075852	88.00975663	0.5	CRA004775;PRJNA827465	Yang2025_Silkie_Wenchang	0.998959673
ENSGALG00010016882	ENSGALG00010016882	2	-0.074964561	0.085405979	-0.877743714	0.380082816	0.012377296	6.55576724	84.74625527	0.5	PRJNA827465;Yang2025_Silkie_Wenchang	CRA004775	0.824392515
ENSGALG00010016882	ENSGALG00010016882	2	-0.160408932	0.050973533	-3.146906319	0.001650078	0	0.067915534	0	1	CRA004775;Yang2025_Silkie_Wenchang	PRJNA827465	0.051226717
ENSGALG00010016882	ENSGALG00010016882	2	0.004424973	0.041009992	0.107899881	0.914075111	0	0.196249119	0	0.5	CRA004775;PRJNA827465	Yang2025_Silkie_Wenchang	0.979467654
ENSGALG00010016883	TMEM251	2	-0.154728148	0.207917128	-0.744186425	0.456764682	0.077207879	8.75130167	88.57312846	0.5	PRJNA827465;Yang2025_Silkie_Wenchang	CRA004775	0.849515887
ENSGALG00010016883	TMEM251	2	-0.233954112	0.178141433	-1.313305429	0.18908005	0.035925406	2.210078246	54.75273322	1	CRA004775;Yang2025_Silkie_Wenchang	PRJNA827465	0.698162702
ENSGALG00010016883	TMEM251	2	0.032550619	0.044676326	0.72858765	0.466253936	0	0.070017869	0	0.5	CRA004775;PRJNA827465	Yang2025_Silkie_Wenchang	0.852690821
ENSGALG00010016884	SBK1	2	0.078315639	0.103753613	0.754823244	0.450355049	0	0.917155039	0	1	PRJNA827465;Yang2025_Silkie_Wenchang	CRA004775	0.843757105
ENSGALG00010016884	SBK1	2	0.430094354	0.277883332	1.547751534	0.121682129	0.089315775	2.157547427	53.65107679	1	CRA004775;Yang2025_Silkie_Wenchang	PRJNA827465	0.5849268
ENSGALG00010016884	SBK1	2	0.335064562	0.388219548	0.86308009	0.388093434	0.241420289	4.49767448	77.76628779	1	CRA004775;PRJNA827465	Yang2025_Silkie_Wenchang	0.828154121
ENSGALG00010016885	CDCA4	2	-0.082013542	0.051808737	-1.583006005	0.113420096	0.00149098	1.335624226	25.12864169	1	PRJNA827465;Yang2025_Silkie_Wenchang	CRA004775	0.568518049
ENSGALG00010016885	CDCA4	2	-0.1523601	0.075992193	-2.00496411	0.044969022	0	0.001734285	0	1	CRA004775;Yang2025_Silkie_Wenchang	PRJNA827465	0.365622153
ENSGALG00010016885	CDCA4	2	-0.047729286	0.050206533	-0.950658884	0.341777566	0	0.125632944	0	1	CRA004775;PRJNA827465	Yang2025_Silkie_Wenchang	0.805823616
ENSGALG00010016887	TJP2	2	0.089481201	0.161821098	0.552963752	0.580288231	0.043887652	6.092552038	83.58651689	0.5	PRJNA827465;Yang2025_Silkie_Wenchang	CRA004775	0.888433837
ENSGALG00010016887	TJP2	2	0.267739973	0.099644401	2.686954523	0.007210677	0	0.048605191	0	1	CRA004775;Yang2025_Silkie_Wenchang	PRJNA827465	0.130599648
ENSGALG00010016887	TJP2	2	0.032498918	0.169081977	0.192208057	0.847579235	0.032003098	1.71981396	41.65630954	0.5	CRA004775;PRJNA827465	Yang2025_Silkie_Wenchang	0.96156308
ENSGALG00010016888	ENSGALG00010016888	2	-0.046219998	0.07454288	-0.620045771	0.535227653	0	0.047724812	0	1	PRJNA827465;Yang2025_Silkie_Wenchang	CRA004775	0.874062095
ENSGALG00010016888	ENSGALG00010016888	2	-0.020253168	0.125111521	-0.161880915	0.871399632	0	0.83061296	0	0.5	CRA004775;Yang2025_Silkie_Wenchang	PRJNA827465	0.969337138
ENSGALG00010016888	ENSGALG00010016888	2	-0.014699292	0.08529526	-0.172332157	0.863239295	0	0.717554365	0	0.5	CRA004775;PRJNA827465	Yang2025_Silkie_Wenchang	0.966235427
ENSGALG00010016889	FUNDC2	2	-0.3569593	0.183599557	-1.944227458	0.051868028	0.05543793	5.254550917	80.96887792	1	PRJNA827465;Yang2025_Silkie_Wenchang	CRA004775	0.394994982
ENSGALG00010016889	FUNDC2	2	-0.515096808	0.135711275	-3.795534373	0.000147326	0	0.801765009	0	1	CRA004775;Yang2025_Silkie_Wenchang	PRJNA827465	0.010268856
ENSGALG00010016889	FUNDC2	2	-0.195669416	0.06627888	-2.952213678	0.003155045	0	0.00425908	0	1	CRA004775;PRJNA827465	Yang2025_Silkie_Wenchang	0.077842273
ENSGALG00010016890	UBR7	2	-0.179064211	0.228983334	-0.781996695	0.434216516	0.098956253	17.47860492	94.27871958	0.5	PRJNA827465;Yang2025_Silkie_Wenchang	CRA004775	0.842762754
ENSGALG00010016890	UBR7	2	-0.31227698	0.128311912	-2.433733345	0.014944	0.017865082	2.06883899	51.66371067	1	CRA004775;Yang2025_Silkie_Wenchang	PRJNA827465	0.201836433
ENSGALG00010016890	UBR7	2	0.000412593	0.080714006	0.005111792	0.995921398	0.005487477	1.404606616	28.80568917	0.5	CRA004775;PRJNA827465	Yang2025_Silkie_Wenchang	0.998680753
ENSGALG00010016891	ENSGALG00010016891	2	-0.257188192	0.259360494	-0.991624391	0.321380787	0.066515785	1.856033391	46.12165896	1	PRJNA827465;Yang2025_Silkie_Wenchang	CRA004775	0.798513866
ENSGALG00010016891	ENSGALG00010016891	2	-0.021413949	0.583886143	-0.036674871	0.970744245	0.555195677	5.376652317	81.4010663	0.5	CRA004775;Yang2025_Silkie_Wenchang	PRJNA827465	0.991843322
ENSGALG00010016891	ENSGALG00010016891	2	0.179297308	0.305669473	0.586572505	0.557490853	0.109521457	2.223896717	55.03388299	0.5	CRA004775;PRJNA827465	Yang2025_Silkie_Wenchang	0.880678789
ENSGALG00010016892	MAL	2	0.068723353	0.183067528	0.375398924	0.707363805	0	0.052543481	0	1	PRJNA827465;Yang2025_Silkie_Wenchang	CRA004775	0.924867585
ENSGALG00010016892	MAL	2	0.688896111	0.618580139	1.11367318	0.265419419	0.58817743	4.176564057	76.05687387	1	CRA004775;Yang2025_Silkie_Wenchang	PRJNA827465	0.769476127
ENSGALG00010016892	MAL	2	0.626575655	0.662085415	0.9463668	0.343961533	0.733192737	5.684153071	82.40722958	1	CRA004775;PRJNA827465	Yang2025_Silkie_Wenchang	0.806445383
ENSGALG00010016893	TBC1D9B	2	-0.124986546	0.140941338	-0.886798348	0.375187477	0.035376471	8.624278015	88.40482649	0.5	PRJNA827465;Yang2025_Silkie_Wenchang	CRA004775	0.822941424
ENSGALG00010016893	TBC1D9B	2	-0.240447877	0.078890825	-3.047856052	0.002304803	0	0.797697979	0	1	CRA004775;Yang2025_Silkie_Wenchang	PRJNA827465	0.063678558
ENSGALG00010016893	TBC1D9B	2	-0.000458789	0.034555166	-0.013276993	0.989406804	0	0.451376477	0	0.5	CRA004775;PRJNA827465	Yang2025_Silkie_Wenchang	0.997141349
ENSGALG00010016894	BTBD7	2	-0.046427133	0.038583303	-1.203295948	0.228861816	0	0.362175941	0	1	PRJNA827465;Yang2025_Silkie_Wenchang	CRA004775	0.741566423
ENSGALG00010016894	BTBD7	2	0.043158343	0.245356928	0.175900241	0.860372316	0.068371159	2.077793165	51.87201417	0.5	CRA004775;Yang2025_Silkie_Wenchang	PRJNA827465	0.964999892
ENSGALG00010016894	BTBD7	2	0.046463815	0.169171808	0.274654601	0.783581613	0.034604744	1.666780364	40.00409282	0.5	CRA004775;PRJNA827465	Yang2025_Silkie_Wenchang	0.945815798
ENSGALG00010016895	ENSGALG00010016895	2	0.485109229	0.382101464	1.269582231	0.204233482	0.26177808	9.275156395	89.21851064	1	PRJNA827465;Yang2025_Silkie_Wenchang	CRA004775	0.717271785
ENSGALG00010016895	ENSGALG00010016895	2	0.422545119	0.467367892	0.904095308	0.365944866	0.389371961	9.190673672	89.11940478	0.5	CRA004775;Yang2025_Silkie_Wenchang	PRJNA827465	0.818968857
ENSGALG00010016895	ENSGALG00010016895	2	0.093586703	0.094620312	0.989076247	0.322625838	0	0.523320302	0	0.5	CRA004775;PRJNA827465	Yang2025_Silkie_Wenchang	0.798652022
ENSGALG00010016896	SPTBN1	2	0.153698432	0.188359684	0.815983697	0.41450946	0.056564288	4.334612046	76.92988462	0.5	PRJNA827465;Yang2025_Silkie_Wenchang	CRA004775	0.837148996
ENSGALG00010016896	SPTBN1	2	0.390381538	0.14586981	2.676232578	0.007445496	0	0.008803323	0	1	CRA004775;Yang2025_Silkie_Wenchang	PRJNA827465	0.132977243
ENSGALG00010016896	SPTBN1	2	0.124391395	0.190463606	0.653097974	0.513693118	0.049082097	2.353476261	57.50966277	0.5	CRA004775;PRJNA827465	Yang2025_Silkie_Wenchang	0.866547089
ENSGALG00010016899	SAXO2	2	-0.303036979	0.230753212	-1.313251403	0.189098248	0.075938233	3.395253056	70.54711435	1	PRJNA827465;Yang2025_Silkie_Wenchang	CRA004775	0.698162702
ENSGALG00010016899	SAXO2	2	-0.090314525	0.552107328	-0.163581464	0.870060638	0.481656904	4.333265582	76.92271611	0.5	CRA004775;Yang2025_Silkie_Wenchang	PRJNA827465	0.968752464
ENSGALG00010016899	SAXO2	2	0.060354376	0.27862996	0.21661122	0.828511341	0.081181146	1.608623958	37.83506737	0.5	CRA004775;PRJNA827465	Yang2025_Silkie_Wenchang	0.956777964
ENSGALG00010016900	CISD2	2	0.269808639	0.36493886	0.739325591	0.459709313	0.254232331	21.77297952	95.40715133	0.5	PRJNA827465;Yang2025_Silkie_Wenchang	CRA004775	0.850098539
ENSGALG00010016900	CISD2	2	0.257262205	0.417710166	0.615886866	0.53796919	0.305036669	7.594685316	86.8328975	0.5	CRA004775;Yang2025_Silkie_Wenchang	PRJNA827465	0.87481821
ENSGALG00010016900	CISD2	2	-0.096815661	0.079058971	-1.224600567	0.220725754	0	0.143274487	0	1	CRA004775;PRJNA827465	Yang2025_Silkie_Wenchang	0.734053249
ENSGALG00010016901	MECOM	2	0.142531144	0.13490038	1.056565918	0.290709742	0.013734233	1.60317564	37.62380271	1	PRJNA827465;Yang2025_Silkie_Wenchang	CRA004775	0.784646441
ENSGALG00010016901	MECOM	2	0.31266537	0.135423098	2.30880385	0.020954468	0	0.734241586	0	1	CRA004775;Yang2025_Silkie_Wenchang	PRJNA827465	0.245184122
ENSGALG00010016901	MECOM	2	0.211262171	0.297512801	0.710094392	0.477645604	0.108354674	2.199950095	54.54442343	1	CRA004775;PRJNA827465	Yang2025_Silkie_Wenchang	0.855218676
ENSGALG00010016902	MRNIP	2	0.025765967	0.100908173	0.255340738	0.798459938	0	0.164242975	0	1	PRJNA827465;Yang2025_Silkie_Wenchang	CRA004775	0.949922217
ENSGALG00010016902	MRNIP	2	0.071985066	0.178107772	0.404165777	0.686090818	0	0.068350891	0	0.5	CRA004775;Yang2025_Silkie_Wenchang	PRJNA827465	0.919235539
ENSGALG00010016902	MRNIP	2	8.4196E-05	0.112072186	0.000751266	0.999400577	0	0.002749036	0	0.5	CRA004775;PRJNA827465	Yang2025_Silkie_Wenchang	0.999889665
ENSGALG00010016903	PPM1A	2	-0.385406162	0.112777523	-3.417402255	0.000632218	0	0.037291058	0	1	PRJNA827465;Yang2025_Silkie_Wenchang	CRA004775	0.027578314
ENSGALG00010016903	PPM1A	2	-0.033887597	0.362619804	-0.09345214	0.92554437	0.220007347	5.896601192	83.04107795	0.5	CRA004775;Yang2025_Silkie_Wenchang	PRJNA827465	0.982594411
ENSGALG00010016903	PPM1A	2	-0.049194861	0.38519778	-0.127713252	0.898375903	0.249224781	6.114044012	83.64421326	0.5	CRA004775;PRJNA827465	Yang2025_Silkie_Wenchang	0.97575249
ENSGALG00010016904	EAF1	2	-0.042193709	0.039078233	-1.079724083	0.280265066	0	0.202900112	0	1	PRJNA827465;Yang2025_Silkie_Wenchang	CRA004775	0.778461864
ENSGALG00010016904	EAF1	2	-0.060559722	0.067400883	-0.898500426	0.368918818	0	0.115275897	0	1	CRA004775;Yang2025_Silkie_Wenchang	PRJNA827465	0.820553374
ENSGALG00010016904	EAF1	2	-0.029287877	0.044880352	-0.652576796	0.514029149	0	0.021211272	0	1	CRA004775;PRJNA827465	Yang2025_Silkie_Wenchang	0.866816306
ENSGALG00010016905	C1QC	2	0.558624683	0.307337861	1.817624033	0.069121616	0.046398702	1.301055124	23.13930581	1	PRJNA827465;Yang2025_Silkie_Wenchang	CRA004775	0.452781144
ENSGALG00010016905	C1QC	2	-0.358267741	0.494745822	-0.724145056	0.468976686	0.317069502	2.821159337	64.55357955	0.5	CRA004775;Yang2025_Silkie_Wenchang	PRJNA827465	0.85345437
ENSGALG00010016905	C1QC	2	-0.000817351	0.812111281	-0.001006452	0.999196967	1.193490038	10.48252228	90.46031124	0.5	CRA004775;PRJNA827465	Yang2025_Silkie_Wenchang	0.999889665
ENSGALG00010016907	SQSTM1	2	-0.008692731	0.109713351	-0.0792313	0.936848649	0	0.430302828	0	0.5	PRJNA827465;Yang2025_Silkie_Wenchang	CRA004775	0.985038972
ENSGALG00010016907	SQSTM1	2	-0.163418113	0.160619861	-1.017421581	0.30895292	0	0.406422057	0	1	CRA004775;Yang2025_Silkie_Wenchang	PRJNA827465	0.794008802
ENSGALG00010016907	SQSTM1	2	-0.036731546	0.158493304	-0.231754562	0.81672865	0.013094762	1.20787596	17.21004203	0.5	CRA004775;PRJNA827465	Yang2025_Silkie_Wenchang	0.954684235
ENSGALG00010016908	CMC4	2	-0.240088311	0.140823081	-1.704893188	0.088214347	0.033753352	6.695833894	85.06534039	1	PRJNA827465;Yang2025_Silkie_Wenchang	CRA004775	0.510208309
ENSGALG00010016908	CMC4	2	-0.293958378	0.15317426	-1.919110807	0.054970312	0.024796473	1.674528053	40.28168128	1	CRA004775;Yang2025_Silkie_Wenchang	PRJNA827465	0.405768983
ENSGALG00010016908	CMC4	2	-0.097242783	0.068856553	-1.412251696	0.157875864	0	0.066566897	0	1	CRA004775;PRJNA827465	Yang2025_Silkie_Wenchang	0.651374667
ENSGALG00010016909	MRPS5	2	0.030812727	0.120776956	0.255120906	0.798629718	0.023428924	4.86877272	79.46094309	0.5	PRJNA827465;Yang2025_Silkie_Wenchang	CRA004775	0.949922217
ENSGALG00010016909	MRPS5	2	0.090346552	0.130346693	0.693125001	0.488231109	0.013435376	1.414287405	29.2930138	0.5	CRA004775;Yang2025_Silkie_Wenchang	PRJNA827465	0.85768885
ENSGALG00010016909	MRPS5	2	-0.08123451	0.05360257	-1.515496546	0.129646704	0	0.061287577	0	1	CRA004775;PRJNA827465	Yang2025_Silkie_Wenchang	0.604019016
ENSGALG00010016910	NSD2	2	0.110870367	0.064764248	1.711906964	0.086913815	0	0.039615593	0	1	PRJNA827465;Yang2025_Silkie_Wenchang	CRA004775	0.507197279
ENSGALG00010016910	NSD2	2	-0.047981348	0.181923542	-0.263744579	0.791976742	0.038470261	2.180371358	54.13625315	0.5	CRA004775;Yang2025_Silkie_Wenchang	PRJNA827465	0.948314332
ENSGALG00010016910	NSD2	2	-0.027468854	0.194729496	-0.141061598	0.887821281	0.053471812	2.870155327	65.15868007	0.5	CRA004775;PRJNA827465	Yang2025_Silkie_Wenchang	0.973090153
ENSGALG00010016911	AXIN1	2	-0.02803676	0.093467047	-0.299964109	0.764204532	0.010604672	2.273308028	56.01124056	0.5	PRJNA827465;Yang2025_Silkie_Wenchang	CRA004775	0.940483053
ENSGALG00010016911	AXIN1	2	0.157398841	0.094535314	1.664974011	0.095917941	0	0.803023103	0	1	CRA004775;Yang2025_Silkie_Wenchang	PRJNA827465	0.5301492
ENSGALG00010016911	AXIN1	2	0.057388239	0.183996193	0.311899055	0.755117241	0.055026081	4.771599108	79.04266521	0.5	CRA004775;PRJNA827465	Yang2025_Silkie_Wenchang	0.93838723
ENSGALG00010016912	CCDC88C	2	0.109832247	0.337972274	0.324974133	0.745200649	0.221063106	30.69459311	96.74209723	0.5	PRJNA827465;Yang2025_Silkie_Wenchang	CRA004775	0.93597621
ENSGALG00010016912	CCDC88C	2	0.443336217	0.095597126	4.637547526	3.52568E-06	0	0.050609472	0	1	CRA004775;Yang2025_Silkie_Wenchang	PRJNA827465	0.000647522
ENSGALG00010016912	CCDC88C	2	0.047367644	0.307103713	0.154239894	0.877420588	0.16349228	6.789228794	85.27078656	0.5	CRA004775;PRJNA827465	Yang2025_Silkie_Wenchang	0.97065023
ENSGALG00010016913	BRCC3	2	0.017219143	0.072769717	0.236625125	0.812947626	0	0.930107662	0	0.5	PRJNA827465;Yang2025_Silkie_Wenchang	CRA004775	0.953526116
ENSGALG00010016913	BRCC3	2	-0.314564399	0.200536676	-1.568612812	0.116738185	0.049840985	2.618458156	61.80958639	1	CRA004775;Yang2025_Silkie_Wenchang	PRJNA827465	0.575245849
ENSGALG00010016913	BRCC3	2	-0.212230735	0.287776965	-0.737483401	0.460828434	0.146088981	8.043876182	87.56818259	0.5	CRA004775;PRJNA827465	Yang2025_Silkie_Wenchang	0.85014477
ENSGALG00010016915	PLOD2	2	-0.150396248	0.191186882	-0.786645224	0.431489578	0.049787034	3.076836514	67.49908565	0.5	PRJNA827465;Yang2025_Silkie_Wenchang	CRA004775	0.842503929
ENSGALG00010016915	PLOD2	2	-0.20493849	0.227812781	-0.89959171	0.36833757	0.04518575	1.606950136	37.77031546	0.5	CRA004775;Yang2025_Silkie_Wenchang	PRJNA827465	0.820213684
ENSGALG00010016915	PLOD2	2	0.03680082	0.121969762	0.301720849	0.762864874	0	0.08177177	0	1	CRA004775;PRJNA827465	Yang2025_Silkie_Wenchang	0.940082621
ENSGALG00010016916	ACYP2	2	-0.486600572	0.171718422	-2.833712101	0.004601076	0.023952672	1.594295046	37.27635281	1	PRJNA827465;Yang2025_Silkie_Wenchang	CRA004775	0.099689141
ENSGALG00010016916	ACYP2	2	-0.399675196	0.411400607	-0.971498801	0.33129995	0.211368483	2.406014026	58.43748253	0.5	CRA004775;Yang2025_Silkie_Wenchang	PRJNA827465	0.801798188
ENSGALG00010016916	ACYP2	2	-0.317940831	0.141653269	-2.244500493	0.024800217	0	0.917703176	0	0.5	CRA004775;PRJNA827465	Yang2025_Silkie_Wenchang	0.268761753
ENSGALG00010016917	METTL6	2	0.067460328	0.069474549	0.97100778	0.331544404	0.001610329	1.193760415	16.23109736	1	PRJNA827465;Yang2025_Silkie_Wenchang	CRA004775	0.801798188
ENSGALG00010016917	METTL6	2	0.297946343	0.221583172	1.344625318	0.178746263	0.061205009	2.142096327	53.31675858	1	CRA004775;Yang2025_Silkie_Wenchang	PRJNA827465	0.685472119
ENSGALG00010016917	METTL6	2	0.245581735	0.301285157	0.815113953	0.415007086	0.140251886	3.699230079	72.96734784	1	CRA004775;PRJNA827465	Yang2025_Silkie_Wenchang	0.837489728
ENSGALG00010016918	RCOR1	2	-0.012695215	0.05970808	-0.212621383	0.831622292	0.002152732	1.304264653	23.32844432	0.5	PRJNA827465;Yang2025_Silkie_Wenchang	CRA004775	0.957712669
ENSGALG00010016918	RCOR1	2	-0.10491083	0.101573053	-1.032860845	0.301669025	0	0.056624612	0	1	CRA004775;Yang2025_Silkie_Wenchang	PRJNA827465	0.791277208
ENSGALG00010016918	RCOR1	2	0.021426087	0.051060199	0.419624045	0.67476012	0	0.029473592	0	0.5	CRA004775;PRJNA827465	Yang2025_Silkie_Wenchang	0.915970608
ENSGALG00010016919	ARHGDIG	2	0.105707816	0.160948008	0.65678238	0.511320855	0	0.664061574	0	0.5	PRJNA827465;Yang2025_Silkie_Wenchang	CRA004775	0.865658376
ENSGALG00010016919	ARHGDIG	2	0.214588965	0.197234644	1.087988196	0.276600318	0	0.100159276	0	1	CRA004775;Yang2025_Silkie_Wenchang	PRJNA827465	0.775851484
ENSGALG00010016919	ARHGDIG	2	0.039981145	0.173960437	0.229828953	0.818224686	0	0.135916164	0	0.5	CRA004775;PRJNA827465	Yang2025_Silkie_Wenchang	0.955256039
ENSGALG00010016920	C1QA	2	0.261356703	0.300191908	0.870632071	0.383955079	0.059467777	1.447958852	30.93726396	0.5	PRJNA827465;Yang2025_Silkie_Wenchang	CRA004775	0.825614755
ENSGALG00010016920	C1QA	2	-0.458594941	0.27917206	-1.642696412	0.100445762	0.007737342	1.050587391	4.81515306	1	CRA004775;Yang2025_Silkie_Wenchang	PRJNA827465	0.541278811
ENSGALG00010016920	C1QA	2	-0.082504528	0.593222168	-0.139078633	0.889388013	0.599462085	6.712312265	85.10200419	0.5	CRA004775;PRJNA827465	Yang2025_Silkie_Wenchang	0.973135755
ENSGALG00010016921	EFL1	2	-0.026352854	0.059198328	-0.445162125	0.656202595	0	0.316472683	0	1	PRJNA827465;Yang2025_Silkie_Wenchang	CRA004775	0.910446833
ENSGALG00010016921	EFL1	2	0.03215001	0.137564747	0.233708204	0.815211518	0.018810611	1.916321011	47.8166761	0.5	CRA004775;Yang2025_Silkie_Wenchang	PRJNA827465	0.953933933
ENSGALG00010016921	EFL1	2	0.040046352	0.085723769	0.467155761	0.640388442	0.004651389	1.281880895	21.98963226	0.5	CRA004775;PRJNA827465	Yang2025_Silkie_Wenchang	0.906181348
ENSGALG00010016922	ENSGALG00010016922	2	-0.049596333	0.078772602	-0.629613999	0.528947162	0	0.071656	0	1	PRJNA827465;Yang2025_Silkie_Wenchang	CRA004775	0.871809618
ENSGALG00010016922	ENSGALG00010016922	2	-0.066695101	0.123086117	-0.541857221	0.587916868	0	0.081689226	0	0.5	CRA004775;Yang2025_Silkie_Wenchang	PRJNA827465	0.891761457
ENSGALG00010016922	ENSGALG00010016922	2	-0.028878543	0.097710997	-0.295550595	0.767573308	0	0.038807291	0	0.5	CRA004775;PRJNA827465	Yang2025_Silkie_Wenchang	0.941444269
ENSGALG00010016923	ENSGALG00010016923	2	-0.175509605	0.29113365	-0.602848913	0.546609204	0.151342265	9.159898259	89.08284818	0.5	PRJNA827465;Yang2025_Silkie_Wenchang	CRA004775	0.878275987
ENSGALG00010016923	ENSGALG00010016923	2	-0.433389217	0.152593566	-2.84015394	0.004509177	0	0.694384049	0	1	CRA004775;Yang2025_Silkie_Wenchang	PRJNA827465	0.098239565
ENSGALG00010016923	ENSGALG00010016923	2	0.092213289	0.100230416	0.920013033	0.357565949	0	0.184010988	0	0.5	CRA004775;PRJNA827465	Yang2025_Silkie_Wenchang	0.813486201
ENSGALG00010016924	LETM1	2	0.179393097	0.055346576	3.241268212	0.001189992	0	8.36057E-05	0	1	PRJNA827465;Yang2025_Silkie_Wenchang	CRA004775	0.041908818
ENSGALG00010016924	LETM1	2	0.209244944	0.103085651	2.029816394	0.042375206	0	0.254149247	0	1	CRA004775;Yang2025_Silkie_Wenchang	PRJNA827465	0.356238117
ENSGALG00010016924	LETM1	2	0.189047063	0.060105654	3.145245926	0.001659473	0	0.312163054	0	1	CRA004775;PRJNA827465	Yang2025_Silkie_Wenchang	0.051437109
ENSGALG00010016925	PHACTR4	2	0.058892449	0.068053119	0.865389417	0.386825088	0.003863832	1.260790133	20.68465848	1	PRJNA827465;Yang2025_Silkie_Wenchang	CRA004775	0.827124608
ENSGALG00010016925	PHACTR4	2	0.122638915	0.121815497	1.006759549	0.314050338	0	0.778357922	0	1	CRA004775;Yang2025_Silkie_Wenchang	PRJNA827465	0.796531448
ENSGALG00010016925	PHACTR4	2	0.029834246	0.038466923	0.775581811	0.437995943	0	0.014664565	0	1	CRA004775;PRJNA827465	Yang2025_Silkie_Wenchang	0.843947213
ENSGALG00010016926	ENSGALG00010016926	2	0.118783667	0.282535343	0.420420559	0.674178252	0.12236757	4.271264691	76.58773051	0.5	PRJNA827465;Yang2025_Silkie_Wenchang	CRA004775	0.915800735
ENSGALG00010016926	ENSGALG00010016926	2	0.357075089	0.190836046	1.871109237	0.061329942	0	0.503611251	0	1	CRA004775;Yang2025_Silkie_Wenchang	PRJNA827465	0.427938152
ENSGALG00010016926	ENSGALG00010016926	2	-0.139131078	0.171861335	-0.809554272	0.418196398	0	0.082825654	0	0.5	CRA004775;PRJNA827465	Yang2025_Silkie_Wenchang	0.838112595
ENSGALG00010016927	MGAT4B	2	-0.089275561	0.078377796	-1.139041476	0.254685855	0.000549025	1.042256776	4.054353684	1	PRJNA827465;Yang2025_Silkie_Wenchang	CRA004775	0.764394153
ENSGALG00010016927	MGAT4B	2	0.118935475	0.368623713	0.322647381	0.746962306	0.218339751	4.636085817	78.43008004	0.5	CRA004775;Yang2025_Silkie_Wenchang	PRJNA827465	0.936729365
ENSGALG00010016927	MGAT4B	2	0.177850572	0.279320602	0.636725578	0.52430361	0.113206856	3.024269462	66.93416335	0.5	CRA004775;PRJNA827465	Yang2025_Silkie_Wenchang	0.869790047
ENSGALG00010016928	RGS11	2	0.636044549	0.665503213	0.955734753	0.33920626	0.788050333	9.05767496	88.95963915	0.5	PRJNA827465;Yang2025_Silkie_Wenchang	CRA004775	0.804824608
ENSGALG00010016928	RGS11	2	0.701727839	0.616458467	1.138321357	0.254986319	0.642797466	6.435096498	84.46021749	1	CRA004775;Yang2025_Silkie_Wenchang	PRJNA827465	0.764467569
ENSGALG00010016928	RGS11	2	0.005660303	0.247361834	0.022882686	0.981743851	0	0.037661109	0	0.5	CRA004775;PRJNA827465	Yang2025_Silkie_Wenchang	0.99503081
ENSGALG00010016930	C10H15ORF48	2	-0.057156325	0.414821641	-0.137785302	0.8904101	0	0.594291193	0	0.5	PRJNA827465;Yang2025_Silkie_Wenchang	CRA004775	0.973417023
ENSGALG00010016930	C10H15ORF48	2	-0.478863119	0.356585589	-1.342912148	0.179300422	0	0.012949558	0	1	CRA004775;Yang2025_Silkie_Wenchang	PRJNA827465	0.686044052
ENSGALG00010016930	C10H15ORF48	2	-0.221704348	0.356761036	-0.621436553	0.534312428	0.020806977	1.084330976	7.777235762	0.5	CRA004775;PRJNA827465	Yang2025_Silkie_Wenchang	0.873408008
ENSGALG00010016931	SLC30A4	2	-0.017269591	0.03420122	-0.504940801	0.613600416	0	0.756181887	0	0.5	PRJNA827465;Yang2025_Silkie_Wenchang	CRA004775	0.898256044
ENSGALG00010016931	SLC30A4	2	0.124235269	0.105609313	1.176366605	0.23944841	0	0.118225535	0	1	CRA004775;Yang2025_Silkie_Wenchang	PRJNA827465	0.751706259
ENSGALG00010016931	SLC30A4	2	0.00186833	0.066696932	0.017749403	0.985838769	0.003888508	1.255353712	20.34117535	0.5	CRA004775;PRJNA827465	Yang2025_Silkie_Wenchang	0.996013375
ENSGALG00010016932	OGT	2	0.543920959	0.391709125	1.388583834	0.164959336	0.294408182	24.57936726	95.93154702	1	PRJNA827465;Yang2025_Silkie_Wenchang	CRA004775	0.664282458

ENSGALG00010016932	OGT	2	0.286049052	0.6607127	0.432940144	0.665058255	0.847748452	34.30067215	97.08460524	0.5	CRA004775;Yang2025_Silkie_Wenchang	PRJNA827465	0.913399315
ENSGALG00010016932	OGT	2	-0.089418567	0.267953953	-0.333708706	0.738599379	0.121898648	6.331855297	84.2068406	0.5	CRA004775;PRJNA827465	Yang2025_Silkie_Wenchang	0.934495603
ENSGALG00010016934	SLC25A38	2	-0.144434932	0.141594234	-1.020062243	0.307698942	0.031520781	4.627256197	78.38892083	1	PRJNA827465;Yang2025_Silkie_Wenchang	CRA004775	0.793040073
ENSGALG00010016934	SLC25A38	2	-0.298135374	0.097665243	-3.05262513	0.002268491	0	0.015053974	0	1	CRA004775;Yang2025_Silkie_Wenchang	PRJNA827465	0.063175788
ENSGALG00010016934	SLC25A38	2	-0.055199859	0.111423507	-0.495405862	0.620313658	0.007884996	1.181149537	15.33671488	1	CRA004775;PRJNA827465	Yang2025_Silkie_Wenchang	0.899395593
ENSGALG00010016935	PRIMA1	2	0.287766593	0.149571444	1.923940721	0.054362014	0	0.008166534	0	1	PRJNA827465;Yang2025_Silkie_Wenchang	CRA004775	0.40381596
ENSGALG00010016935	PRIMA1	2	0.424129123	0.349089279	1.214958889	0.224381772	0	0.417323402	0	1	CRA004775;Yang2025_Silkie_Wenchang	PRJNA827465	0.737471949
ENSGALG00010016935	PRIMA1	2	0.323079664	0.153194077	2.108956626	0.034948326	0	0.510488808	0	1	CRA004775;PRJNA827465	Yang2025_Silkie_Wenchang	0.322057221
ENSGALG00010016936	RCC1	2	0.023962983	0.096415717	0.248538136	0.803718068	0	0.424425856	0	0.5	PRJNA827465;Yang2025_Silkie_Wenchang	CRA004775	0.951066707
ENSGALG00010016936	RCC1	2	0.008334371	0.143085655	0.058247425	0.953551545	0	0.935720442	0	0.5	CRA004775;Yang2025_Silkie_Wenchang	PRJNA827465	0.987781987
ENSGALG00010016936	RCC1	2	-0.040987366	0.098953013	-0.414210391	0.678720033	0	0.306592991	0	1	CRA004775;PRJNA827465	Yang2025_Silkie_Wenchang	0.917701868
ENSGALG00010016937	DHRS7	2	0.049808546	0.219757411	0.226652407	0.820694038	0.064153628	2.808271023	64.39090131	0.5	PRJNA827465;Yang2025_Silkie_Wenchang	CRA004775	0.955442469
ENSGALG00010016937	DHRS7	2	-0.131825226	0.123102014	-1.07086165	0.284231642	0	0.002223742	0	1	CRA004775;Yang2025_Silkie_Wenchang	PRJNA827465	0.780082638
ENSGALG00010016937	DHRS7	2	0.144651991	0.209893458	0.689168646	0.490717145	0.019172514	1.260737535	20.68134945	0.5	CRA004775;PRJNA827465	Yang2025_Silkie_Wenchang	0.858798485
ENSGALG00010016938	PSME4	2	-0.046793395	0.051253257	-0.912983824	0.361251049	0.000867645	1.186613874	15.72658793	1	PRJNA827465;Yang2025_Silkie_Wenchang	CRA004775	0.815335362
ENSGALG00010016938	PSME4	2	-0.105014152	0.075697031	-1.387295514	0.16535167	0	0.267679811	0	0.5	CRA004775;Yang2025_Silkie_Wenchang	PRJNA827465	0.664874006
ENSGALG00010016938	PSME4	2	-0.003644246	0.058315925	-0.062491431	0.950171486	0	0.101465703	0	0.5	CRA004775;PRJNA827465	Yang2025_Silkie_Wenchang	0.987277827
ENSGALG00010016939	FAM234A	2	-0.152701913	0.254818956	-0.599256492	0.549001856	0.110349378	6.026285666	83.40603059	0.5	PRJNA827465;Yang2025_Silkie_Wenchang	CRA004775	0.878431377
ENSGALG00010016939	FAM234A	2	-0.493122096	0.13466277	-3.661903702	0.000250348	0	0.107114506	0	1	CRA004775;Yang2025_Silkie_Wenchang	PRJNA827465	0.014863182
ENSGALG00010016939	FAM234A	2	-0.207629994	0.300481528	-0.690990874	0.489571276	0.163142061	9.765375353	89.75973822	0.5	CRA004775;PRJNA827465	Yang2025_Silkie_Wenchang	0.858419004
ENSGALG00010016940	FGFR3	2	0.363949431	0.148478024	2.451200657	0.014238056	0	0.129247429	0	1	PRJNA827465;Yang2025_Silkie_Wenchang	CRA004775	0.195732181
ENSGALG00010016940	FGFR3	2	0.310165334	0.229440426	1.351833848	0.176428473	0	0.039351236	0	1	CRA004775;Yang2025_Silkie_Wenchang	PRJNA827465	0.681691731
ENSGALG00010016940	FGFR3	2	0.392825216	0.159200225	2.467491581	0.013606342	0	0.003104531	0	1	CRA004775;PRJNA827465	Yang2025_Silkie_Wenchang	0.190989308
ENSGALG00010016941	SLC8A1	2	-0.269562012	0.12491926	-2.157889919	0.030936392	0	0.523372331	0	1	PRJNA827465;Yang2025_Silkie_Wenchang	CRA004775	0.300486152
ENSGALG00010016941	SLC8A1	2	-0.199990886	0.163797673	-1.220942922	0.222100059	0	0.093914959	0	1	CRA004775;Yang2025_Silkie_Wenchang	PRJNA827465	0.735459155
ENSGALG00010016941	SLC8A1	2	-0.361123803	0.169307024	-2.132952281	0.032928641	0	0.001338118	0	1	CRA004775;PRJNA827465	Yang2025_Silkie_Wenchang	0.311661412
ENSGALG00010016942	ZBTB40	2	0.406089058	0.249330125	1.62872039	0.103372224	0.096953924	4.387649916	77.20875596	1	PRJNA827465;Yang2025_Silkie_Wenchang	CRA004775	0.547576788
ENSGALG00010016942	ZBTB40	2	0.207347242	0.495934333	0.418094148	0.675878284	0.429744393	7.806000416	87.18934221	0.5	CRA004775;Yang2025_Silkie_Wenchang	PRJNA827465	0.916522765
ENSGALG00010016942	ZBTB40	2	0.004793416	0.235619862	0.020343854	0.983769072	0.070241814	2.386811251	58.10109677	0.5	CRA004775;PRJNA827465	Yang2025_Silkie_Wenchang	0.99552192
ENSGALG00010016943	PCNX4	2	0.132211342	0.055096795	2.39961947	0.016412123	0	0.138547082	0	1	PRJNA827465;Yang2025_Silkie_Wenchang	CRA004775	0.211917183
ENSGALG00010016943	PCNX4	2	0.049518486	0.12646454	0.391531868	0.695400137	0.010741437	1.222889828	18.22648476	0.5	CRA004775;Yang2025_Silkie_Wenchang	PRJNA827465	0.921038437
ENSGALG00010016943	PCNX4	2	0.062631288	0.159999947	0.391445681	0.695467832	0.02806821	1.604824014	37.6878716	0.5	CRA004775;PRJNA827465	Yang2025_Silkie_Wenchang	0.921038437
ENSGALG00010016945	BDH2	2	0.012146756	0.064127208	0.189416566	0.849766342	0	0.990918257	0	0.5	PRJNA827465;Yang2025_Silkie_Wenchang	CRA004775	0.961856718
ENSGALG00010016945	BDH2	2	-0.119955289	0.113988664	-1.052344018	0.292641743	0	0.280426211	0	1	CRA004775;Yang2025_Silkie_Wenchang	PRJNA827465	0.786263139
ENSGALG00010016945	BDH2	2	0.019196193	0.093520647	0.205261551	0.837367782	0.004127581	1.09411515	8.60194193	0.5	CRA004775;PRJNA827465	Yang2025_Silkie_Wenchang	0.958828842
ENSGALG00010016946	TAF1	2	0.022914027	0.200585131	0.114235922	0.909050776	0.072839879	9.831310513	89.82841607	0.5	PRJNA827465;Yang2025_Silkie_Wenchang	CRA004775	0.978512384
ENSGALG00010016946	TAF1	2	0.217466597	0.102310132	2.12556267	0.033539691	0	0.122320987	0	1	CRA004775;Yang2025_Silkie_Wenchang	PRJNA827465	0.314546348
ENSGALG00010016946	TAF1	2	-0.046768681	0.155423461	-0.300911333	0.763482108	0.036007478	3.108757802	67.83281093	0.5	CRA004775;PRJNA827465	Yang2025_Silkie_Wenchang	0.940301633
ENSGALG00010016947	CANX	2	-0.068069638	0.136953717	-0.497026584	0.619170305	0.034984237	14.78434269	93.23608752	0.5	PRJNA827465;Yang2025_Silkie_Wenchang	CRA004775	0.899168321
ENSGALG00010016947	CANX	2	0.093172853	0.322203337	0.28917408	0.772448166	0.190918425	11.76656127	91.5013403	0.5	CRA004775;Yang2025_Silkie_Wenchang	PRJNA827465	0.943066211
ENSGALG00010016947	CANX	2	0.211897575	0.181484853	1.167577194	0.242977354	0.052075932	4.039426688	75.24402392	1	CRA004775;PRJNA827465	Yang2025_Silkie_Wenchang	0.7550024
ENSGALG00010016949	LUC7L	2	0.334937058	0.25918236	1.292283387	0.196239023	0.127580105	19.36852834	94.83698512	1	PRJNA827465;Yang2025_Silkie_Wenchang	CRA004775	0.707666824
ENSGALG00010016949	LUC7L	2	0.409928787	0.219924238	1.86395456	0.062328086	0.074094966	4.061759064	75.37588141	1	CRA004775;Yang2025_Silkie_Wenchang	PRJNA827465	0.823572585
ENSGALG00010016949	LUC7L	2	0.08901257	0.044543662	1.99832179	0.045681785	0	0.146470645	0	1	CRA004775;PRJNA827465	Yang2025_Silkie_Wenchang	0.368613377
ENSGALG00010016950	ENSGALG00010016950	2	0.155352172	0.590043554	0.263289331	0.792327583	0	0.030092028	0	1	PRJNA827465;Yang2025_Silkie_Wenchang	CRA004775	0.948368502
ENSGALG00010016950	ENSGALG00010016950	2	-0.1618471	0.380259565	-0.425622694	0.670382801	0	0.31261447	0	0.5	CRA004775;Yang2025_Silkie_Wenchang	PRJNA827465	0.914482883
ENSGALG00010016950	ENSGALG00010016950	2	-0.200486062	0.378214991	-0.530088917	0.596053056	0	0.12248833	0	0.5	CRA004775;PRJNA827465	Yang2025_Silkie_Wenchang	0.893989017
ENSGALG00010016952	GINS1	2	0.272360141	0.294103564	0.926068823	0.354410215	0.13850117	4.615183542	78.33238937	1	PRJNA827465;Yang2025_Silkie_Wenchang	CRA004775	0.81193713
ENSGALG00010016952	GINS1	2	0.503652466	0.226609625	2.222555492	0.026245788	0	0.930394562	0	1	CRA004775;Yang2025_Silkie_Wenchang	PRJNA827465	0.276215866
ENSGALG00010016952	GINS1	2	0.021005803	0.108441285	0.193706696	0.846405549	0	0.009174176	0	1	CRA004775;PRJNA827465	Yang2025_Silkie_Wenchang	0.96122283
ENSGALG00010016953	GJA4	2	-0.08296753	0.07145699	-1.161115975	0.245594739	0	0.978972223	0	0.5	PRJNA827465;Yang2025_Silkie_Wenchang	CRA004775	0.75661406
ENSGALG00010016953	GJA4	2	0.019059548	0.112286894	0.169739735	0.865214823	0	0.027568725	0	1	CRA004775;Yang2025_Silkie_Wenchang	PRJNA827465	0.96700046
ENSGALG00010016953	GJA4	2	-0.121033852	0.085828102	-1.410189083	0.158483859	0	0.374364499	0	1	CRA004775;PRJNA827465	Yang2025_Silkie_Wenchang	0.652239905
ENSGALG00010016955	ENSGALG00010016955	2	0.016717995	0.259798549	0.064342147	0.948697776	0.085563569	2.643681935	62.17596704	0.5	PRJNA827465;Yang2025_Silkie_Wenchang	CRA004775	0.989692018
ENSGALG00010016955	ENSGALG00010016955	2	0.288050916	0.25775247	1.117548614	0.263759826	0	0.159857502	0	0.5	CRA004775;Yang2025_Silkie_Wenchang	PRJNA827465	0.768643821
ENSGALG00010016955	ENSGALG00010016955	2	-0.205964164	0.178874288	-1.15144645	0.249548613	0	0.010996253	0	1	CRA004775;PRJNA827465	Yang2025_Silkie_Wenchang	0.759622031
ENSGALG00010016956	CENPE	2	0.066065737	0.127396797	0.518582401	0.604051984	0.019715091	2.517805741	60.28287713	0.5	PRJNA827465;Yang2025_Silkie_Wenchang	CRA004775	0.896058435
ENSGALG00010016956	CENPE	2	0.177278953	0.09984706	1.775504995	0.075814541	0	0.163138059	0	1	CRA004775;Yang2025_Silkie_Wenchang	PRJNA827465	0.475313276
ENSGALG00010016956	CENPE	2	-0.07403768	0.123914703	-0.597489069	0.550180903	0	0.002982519	0	1	CRA004775;PRJNA827465	Yang2025_Silkie_Wenchang	0.878679315
ENSGALG00010016957	WNT4	2	-0.138165855	0.1319251	-1.047305284	0.2949588	0	0.146381469	0	1	PRJNA827465;Yang2025_Silkie_Wenchang	CRA004775	0.787569357
ENSGALG00010016957	WNT4	2	-0.189076248	0.186330011	-0.587539935	0.556841007	0	0.128094091	0	1	CRA004775;Yang2025_Silkie_Wenchang	PRJNA827465	0.880511839
ENSGALG00010016957	WNT4	2	-0.189105521	0.158748519	-1.191226994	0.233564485	0	0.021122194	0	1	CRA004775;PRJNA827465	Yang2025_Silkie_Wenchang	0.746809711
ENSGALG00010016958	TLR15	2	0.938489881	0.331621497	2.830003148	0.004654755	0	0.70994495	0	1	PRJNA827465;Yang2025_Silkie_Wenchang	CRA004775	0.100408405
ENSGALG00010016958	TLR15	2	1.016947949	0.36421553	2.792159766	0.00523575	0	0.524617243	0	1	CRA004775;Yang2025_Silkie_Wenchang	PRJNA827465	0.10802135
ENSGALG00010016958	TLR15	2	0.791210241	0.294801033	2.683878794	0.007277348	0	0.011631266	0	1	CRA004775;PRJNA827465	Yang2025_Silkie_Wenchang	0.131092321
ENSGALG00010016961	EIF4A3	2	-0.047988823	0.041838262	-1.147008043	0.251378297	0	0.004583453	0	1	PRJNA827465;Yang2025_Silkie_Wenchang	CRA004775	0.761634382
ENSGALG00010016961	EIF4A3	2	-0.080147431	0.085668036	-0.935558173	0.349500718	0	0.421465481	0	1	CRA004775;Yang2025_Silkie_Wenchang	PRJNA827465	0.809903518
ENSGALG00010016961	EIF4A3	2	-0.058626521	0.043697492	-1.341645007	0.179711126	0	0.500009031	0	1	CRA004775;PRJNA827465	Yang2025_Silkie_Wenchang	0.68659821
ENSGALG00010016962	SQOR	2	-0.154950564	0.099615649	-1.555484102	0.119830806	0.009361155	1.834711683	45.49552341	1	PRJNA827465;Yang2025_Silkie_Wenchang	CRA004775	0.588639051
ENSGALG00010016962	SQOR	2	-0.155303437	0.150995519	-1.028530104	0.303700538	0.022152587	1.856467112	46.1342464	0.5	CRA004775;Yang2025_Silkie_Wenchang	PRJNA827465	0.792208658
ENSGALG00010016962	SQOR	2	-0.054753766	0.078201793	-0.700159983	0.48382474	0	0.260487622	0	0.5	CRA004775;PRJNA827465	Yang2025_Silkie_Wenchang	0.85691916
ENSGALG00010016964	MAML1	2	0.032226596	0.065722079	0.490346571	0.623888677	0.003615229	1.499199558	33.29773916	0.5	PRJNA827465;Yang2025_Silkie_Wenchang	CRA004775	0.900614985
ENSGALG00010016964	MAML1	2	-0.086871536	0.105808315	-0.821027494	0.411630608	0	0.153732907	0	1	CRA004775;Yang2025_Silkie_Wenchang	PRJNA827465	0.835128673
ENSGALG00010016964	MAML1	2	0.067740844	0.047922856	1.413539374	0.157497193	0	0.627814421	0	0.5	CRA004775;PRJNA827465	Yang2025_Silkie_Wenchang	0.65076873
ENSGALG00010016965	SLC25A33	2	-0.098438613	0.096370629	-1.021458661	0.307037185	0	0.105561662	0	1	PRJNA827465;Yang2025_Silkie_Wenchang	CRA004775	0.79266003
ENSGALG00010016965	SLC25A33	2	-0.235493786	0.239357231	-0.983859084	0.325184804	0	0.314058867	0	1	CRA004775;Yang2025_Silkie_Wenchang	PRJNA827465	0.799914637
ENSGALG00010016965	SLC25A33	2	-0.099225089	0.102189929	-0.97128054	0.331408596	0	0.565092768	0	1	CRA004775;PRJNA827465	Yang2025_Silkie_Wenchang	0.801798188
ENSGALG00010016966	KDM3A	2	-0.02231721	0.03882296	-0.574845668	0.565395678	0	0.007854767	0	1	PRJNA827465;Yang2025_Silkie_Wenchang	CRA004775	0.883427937
ENSGALG00010016966	KDM3A	2	-0.010123591	0.089115187	-0.11360119	0.909553944	0	0.010870176	0	0.5	CRA004775;Yang2025_Silkie_Wenchang	PRJNA827465	0.978607662
ENSGALG00010016966	KDM3A	2	-0.022535188	0.041495888	-0.543070185	0.587081345	0	0.024180312	0	0.5	CRA004775;PRJNA827465	Yang2025_Silkie_Wenchang	0.891596903
ENSGALG00010016967	ERLEC1	2	0.0124516	0.066175044	0.18816157	0.850749997	0.004282416	1.759958207	43.18047475	0.5	PRJNA827465;Yang2025_Silkie_Wenchang	CRA004775	0.962049072
ENSGALG00010016967	ERLEC1	2	-0.05018994	0.084096825	-0.596811355	0.550633336	0	0.519360903	0	0.5	CRA004775;Yang2025_Silkie_Wenchang	PRJNA827465	0.878857433
ENSGALG00010016967	ERLEC1	2	0.059201946	0.045827675	1.291833118	0.196431142	0	0.001148317	0	1	CRA004775;PRJNA827465	Yang2025_Silkie_Wenchang	0.70782911
ENSGALG00010016968	C15orf61	2	-0.247238578	0.083098666	-2.975241253	0.002927582	0	0.559234002	0	1	PRJNA827465;Yang2025_Silkie_Wenchang	CRA004775	0.074041237
ENSGALG00010016968	C15orf61	2	-0.2944596	0.198910167	-1.480364748	0.138775932	0	0.717127343	0	1	CRA004775;Yang2025_Silkie_Wenchang	PRJNA827465	0.622497285
ENSGALG00010016968	C15orf61	2	-0.215309216	0.084986369	-2.533455869	0.011294396	0	0.1952725	0	1	CRA004775;PRJNA827465	Yang2025_Silkie_Wenchang	0.169299638
ENSGALG00010016969	CHMP3	2	0.12653517	0.180433329	0.701284902	0.483125234	0.05313672	5.193699221	80.7459008	0.5	PRJNA827465;Yang2025_Silkie_Wenchang	CRA004775	0.85691916
ENSGALG00010016969	CHMP3	2	0.296553558	0.122252198	2.425752367	0.01527669	0	0.200765385	0	1	CRA004775;Yang2025_Silkie_Wenchang	PRJNA827465	0.204363429
ENSGALG00010016969	CHMP3	2	-0.016743765	0.073578389	-0.227563622	0.819985504	0	0.767379792	0	0.5	CRA004775;PRJNA827465	Yang2025_Silkie_Wenchang	0.955308792
ENSGALG00010016970	SYNE3	2	0.100699029	0.091298896	1.102959989	0.270044541	0	0.324976487	0	1	PRJNA827465;Yang2025_Silkie_Wenchang	CRA004775	0.772336005
ENSGALG00010016970	SYNE3	2	-0.169588695	0.390756697	-0.434000737	0.664287906	0.247882294	5.084348607	80.3317971	0.5	CRA004775;Yang2025_Silkie_Wenchang	PRJNA827465	0.913214881
ENSGALG00010016970	SYNE3	2	-0.207221697	0.329693438	-0.628528422	0.529657834	0.172734896	4.33077334	76.90943576	0.5	CRA004775;PRJNA827465	Yang2025_Silkie_Wenchang	0.871944741
ENSGALG00010016972	ASB3	2	0.060327325	0.150374216	0.401181307	0.688286642	0.032159736	3.344245504	70.09788908	0.5	PRJNA827465;Yang2025_Silkie_Wenchang	CRA004775	0.919768134
ENSGALG00010016972	ASB3	2	0.055167279	0.229868947	0.239994484	0.810334533	0.064163288	2.291536244	56.3611528	0.5	CRA004775;Yang2025_Silkie_Wenchang	PRJNA827465	0.952997426
ENSGALG00010016972	ASB3	2	-0.089299831	0.086454838	-1.032907284	0.30164729	0	0.34344532	0	1	CRA004775;PRJNA827465	Yang2025_Silkie_Wenchang	0.791273005
ENSGALG00010016973	PLSCR1	2	0.127730093	0.079485569	1.606959537	0.108063245	0	0.537276511	0	1	PRJNA827465;Yang2025_Silkie_Wenchang	CRA004775	0.556208709
ENSGALG00010016973	PLSCR1	2	0.053479062	0.165211387	0.323700826	0.746164546	0.032622055	2.409848864	58.50362174	0.5	CRA004775;Yang2025_Silkie_Wenchang	PRJNA827465	0.936530151
ENSGALG00010016973	PLSCR1	2	0.017345103	0.097127889	0.178580052	0.858267459	0.002251088	1.10692739	9.6598378	0.5	CRA004775;PRJNA827465	Yang2025_Silkie_Wenchang	0.964291891
ENSGALG00010016975	MALL	2	-0.155745344	0.236274143	-0.659172188	0.509785204	0.05266457	1.856868797	46.14589887	0.5	PRJNA827465;Yang2025_Silkie_Wenchang	CRA004775	0.865257661
ENSGALG00010016975	MALL	2	0.309096092	0.27809088	1.11149309	0.266356163	0.035463279	1.249343905	19.95798789	1	CRA004775;Yang2025_Silkie_Wenchang	PRJNA827465	0.770542931
ENSGALG00010016975	MALL	2	0.102295943	0.531654325	0.192410629	0.847420567	0.451206109	4.597595185	78.24949871	0.5	CRA004775;PRJNA827465	Yang2025_Silkie_Wenchang	0.961252712
ENSGALG00010016977	CLCN7	2	0.044350324	0.180288776	0.245996036	0.805685301	0.047494989	3.033048282	67.02986873	0.5	PRJNA827465;Yang2025_Silkie_Wenchang	CRA004775	0.951984168
ENSGALG00010016977	CLCN7	2	-0.347179605	0.167619468	-2.07123677	0.038336675	0.006647822	1.132145132	11.6721018	1	CRA004775;Yang2025_Silkie_Wenchang	PRJNA827465	0.337684074
ENSGALG00010016977	CLCN7	2	-0.140736576	0.354975653	-0.396468251	0.691759625	0.22491395	8.509574512	88.24853113	0.5	CRA004775;PRJNA827465	Yang2025_Silkie_Wenchang	0.920348122
ENSGALG00010016978	FAM181A	2	-0.573849818	0.118460585	-4.844225768	1.27106E-06	0	0.947934313	0	1	PRJNA827465;Yang2025_Silkie_Wenchang	CRA004775	0.000268584
ENSGALG00010016978	FAM181A	2	-0.064385327	0.895551255	-0.071894631	0.942685762	1.233344101	3.582039466	72.08294299	0.5	CRA004775;Yang2025_Silkie_Wenchang	PRJNA827465	0.985827325
ENSGALG00010016978	FAM181A	2	0.017099561	0.746654796	0.022901562	0.981728795	0.807036906	2.744870263	63.56840564	0.5	CRA004775;PRJNA827465	Yang2025_Silkie_Wenchang	0.99503081
ENSGALG00010016980	NPHP1	2	0.216937057	0.091422712	2.372901146	0.017648987	0.008527232	2.032608716	50.80213954	1	PRJNA827465;Yang2025_Silkie_Wenchang	CRA004775	0.221474498
ENSGALG00010016980	NPHP1	2	0.311715744	0.086863878	3.58855432	0.000332517	0	0.004089131	0	1	CRA004775;Yang2025_Silkie_Wenchang	PRJNA827465	0.018046299
ENSGALG00010016980	NPHP1	2	0.157283652	0.077937266	2.018080173	0.043582912	0	0.61421316	0	1	CRA004775;PRJNA827465	Yang2025_Silkie_Wenchang	0.360845532
ENSGALG00010016981	ENSGALG00010016981	2	0.533005727	0.46646385	1.142651733	0.253183217	0.126494435	1.391429004	28.13143916	1	PRJNA827465;Yang2025_Silkie_Wenchang	CRA004775	0.762751801
ENSGALG00010016981	ENSGALG00010016981	2	1.387445647	0.54659117	2.538360886	0.011137307	0	0.82925871	0	1	CRA004775;Yang2025_Silkie_Wenchang	PRJNA827465	0.167712484
ENSGALG00010016981	ENSGALG00010016981	2	0.967899779	1.018444476	0.95037069	0.341923932	1.473682886	3.11942947	67.94285591	1	CRA004775;PRJNA827465	Yang2025_Silkie_Wenchang	0.805904173
ENSGALG00010016982	GLRX5	2	-0.104204527	0.203306994	-0.512547671	0.608267766	0.063289346	3.812993105	73.77388386	0.5	PRJNA827465;Yang2025_Silkie_Wenchang	CRA004775	0.897204334
ENSGALG00010016982	GLRX5	2	-0.192190994	0.193688045	-0.992270813	0.321065437	0.023815432	1.439017113	30.50812313	0.5	CRA004775;Yang2025_Silkie_Wenchang	PRJNA827465	0.798513866
ENSGALG00010016982	GLRX5	2	0.061856324	0.075096703	0.823688941	0.410116325	0	0.004810309	0	1	CRA004775;PRJNA827465	Yang2025_Silkie_Wenchang	0.834652129
ENSGALG00010016983	ZFAND2A	2	0.417981985	0.1076112	3.88418663	0.000102673	0	0.003310318	0	1	PRJNA827465;Yang2025_Silkie_Wenchang	CRA004775	0.007959286
ENSGALG00010016983	ZFAND2A	2	0.283403452	0.202656436	1.398442893	0.16198011	0	0.89133162	0	0.5	CRA004775;Yang2025_Silkie_Wenchang	PRJNA827465	0.659256758
ENSGALG00010016983	ZFAND2A	2	0.347997182	0.163133957	2.133198931	0.032908411	0.01747384	1.22370964	18.28126811	0.5	CRA004775;PRJNA827465	Yang2025_Silkie_Wenchang	0.311661412
ENSGALG00010016984	RNF103	2	-0.178812789	0.143535057	-1.245777814	0.212845977	0.034570507	5.962348363	83.22808499	1	PRJNA827465;Yang2025_Silkie_Wenchang	CRA004775	0.725659838
ENSGALG00010016984	RNF103	2	-0.057783678	0.324376885	-0.178137473	0.858615014	0.171841105	4.937647092	79.74743878	0.5	CRA004775;Yang2025_Silkie_Wenchang	PRJNA827465	0.964449906
ENSGALG00010016984	RNF103	2	0.036944133	0.154868866	0.238551065	0.811453714	0.027880267	1.698323971	41.11841926	0.5	CRA004775;PRJNA827465	Yang2025_Silkie_Wenchang	0.953213272
ENSGALG00010016985	ENSGALG00010016985	2	-0.134562569	0.132454424	-1.015916005	0.309669387	0	0.625972525	0	1	PRJNA827465;Yang2025_Silkie_Wenchang	CRA004775	0.794328224
ENSGALG00010016985	ENSGALG00010016985	2	-0.098444425	0.198929539	-0.494870825	0.620691306	0.022780687	1.384103848	27.75108593	0.5	CRA004775;Yang2025_Silkie_Wenchang	PRJNA827465	0.899395593
ENSGALG00010016985	ENSGALG00010016985	2	0.004968294	0.145081681	0.034244809	0.972681935	0	0.351620011	0	0.5	CRA004775;PRJNA827465	Yang2025_Silkie_Wenchang	0.992213869
ENSGALG00010016986	GFER	2	-0.166870266	0.119526847	-1.396090245	0.162687321	0.018499258	2.801480788	64.3045919	1	PRJNA827465;Yang2025_Silkie_Wenchang	CRA004775	0.660479266
ENSGALG00010016986	GFER	2	-0.156805659	0.159911158	-0.980579847	0.326799976	0.032498315	2.66083013	62.41774367	0.5	CRA004775;Yang2025_Silkie_Wenchang	PRJNA827465	0.800028239
ENSGALG00010016986	GFER	2	-0.03778209	0.078016036	-0.484286205	0.628182765	0	0.199427951	0	0.5	CRA004775;PRJNA827465	Yang2025_Silkie_Wenchang	0.901904489
ENSGALG00010016987	ASB2	2	-0.432436868	0.106544203	-4.058755478	4.93349E-05	0	0.119715204	0	1	PRJNA827465;Yang2025_Silkie_Wenchang	CRA004775	0.004811442
ENSGALG00010016987	ASB2	2	-0.03777531	0.534369447	-0.070691372	0.943643387	0.338822395	1.877659573	46.74220958	0.5	CRA004775;Yang2025_Silkie_Wenchang	PRJNA827465	0.985827325
ENSGALG00010016987	ASB2	2	-0.077862451	0.586400309	-0.132780372	0.894367078	0.469242196	2.277848966	56.09893302	0.5	CRA004775;PRJNA827465	Yang2025_Silkie_Wenchang	0.974381724
ENSGALG00010016988	ECI1	2	-0.047849244	0.069413808	-0.689333224	0.490613595	0	0.192122266	0	1	PRJNA827465;Yang2025_Silkie_Wenchang	CRA004775	0.858798485
ENSGALG00010016988	ECI1	2	-0.161619464	0.156333546	-1.033811801	0.301224154	0	0.303639274	0	1	CRA004775;Yang2025_Silkie_Wenchang	PRJNA827465	0.791222412
ENSGALG00010016988	ECI1	2	-0.048159854	0.07388931	-0.651783779	0.514540667	0	0.701550338	0	1	CRA004775;PRJNA827465	Yang2025_Silkie_Wenchang	0.866869697
ENSGALG00010016989	RHOG2	2	0.069283719	0.048818808	1.419201387	0.155840312	0	0.140725891	0	1	PRJNA827465;Yang2025_Silkie_Wenchang	CRA004775	0.64910892
ENSGALG00010016989	RHOG2	2	0.104970603	0.077685117	1.351231827	0.176621182	0	0.117503171	0	1	CRA004775;Yang2025_Silkie_Wenchang	PRJNA827465	0.682034027
ENSGALG00010016989	RHOG2	2	0.064633499	0.057990728	1.114548843	0.265043803	0	0.277944517	0	1	CRA004775;PRJNA827465	Yang2025_Silkie_Wenchang	0.769274038
ENSGALG00010016991	PDLIM3	2	0.075945515	0.463522746	0.163844203	0.869853793	0.400483527	14.57247986	93.13775	0.5	PRJNA827465;Yang2025_Silkie_Wenchang	CRA004775	0.968610705
ENSGALG00010016991	PDLIM3	2	0.543453612	0.194201182	2.798405276	0.005135563	0	0.022986948	0	1	CRA004775;Yang2025_Silkie_Wenchang	PRJNA827465	0.107021757
ENSGALG00010016991	PDLIM3	2	-0.289927001	0.249729954	-1.160962058	0.24565733	0.05128338	1.184216153	15.55595676	0.5	CRA004775;PRJNA827465	Yang2025_Silkie_Wenchang	0.75661406
ENSGALG00010016993	ENSGALG00010016993	2	-0.37148065	0.080550157	-4.611792996	3.9921E-06	0	0.547604822	0	1	PRJNA827465;Yang2025_Silkie_Wenchang	CRA004775	0.000694016
ENSGALG00010016993	ENSGALG00010016993	2	-0.169197621	0.327301429	-0.516947393	0.605192883	0.166590605	4.071451179	75.43873287	0.5	CRA004775;Yang2025_Silkie_Wenchang	PRJNA827465	0.896474603
ENSGALG00010016993	ENSGALG00010016993	2	-0.126111514	0.2609319	-0.483311983	0.628874233	0.095830943	2.857148066	65.00006381	0.5	CRA004775;PRJNA827465	Yang2025_Silkie_Wenchang	0.902084638
ENSGALG00010016994	ATL2	2	0.075618261	0.159069529	0.475378669	0.634517095	0.046448318	12.07422266	91.71789333	0.5	PRJNA827465;Yang2025_Silkie_Wenchang	CRA004775	0.903872221
ENSGALG00010016994	ATL2	2	0.237616284	0.07178635	3.310048288	0.000932799	0	0.004872385	0	1	CRA004775;Yang2025_Silkie_Wenchang	PRJNA827465	0.036084439
ENSGALG00010016994	ATL2	2	-0.045348956	0.094763371	-0.478549419	0.632259209	0.007291732	1.195185627	16.33098848	0.5	CRA004775;PRJNA827465	Yang2025_Silkie_Wenchang	0.903058174
ENSGALG00010016996	ZIC4	2	0.26236815	0.424609832	0.617904084	0.536638567	0	0.295204195	0	1	PRJNA827465;Yang2025_Silkie_Wenchang	CRA004775	0.874599474
ENSGALG00010016996	ZIC4	2	0.432293773	0.63312344	0.682795401	0.494736136	0	0.176260416	0	0.5	CRA004775;Yang2025_Silkie_Wenchang	PRJNA827465	0.860356282
ENSGALG00010016996	ZIC4	2	0.079363545	0.482439918	0.164504516	0.869333992	0	0.008506929	0	0.5	CRA004775;PRJNA827465	Yang2025_Silkie_Wenchang	0.968558268
ENSGALG00010016999	ITGB1BP2	2	-0.039613633	0.139293489	-0.284389697	0.776111754	0.0249952	2.71779613	63.20548149	0.5	PRJNA827465;Yang2025_Silkie_Wenchang	CRA004775	0.944001494
ENSGALG00010016999	ITGB1BP2	2	0.109391823	0.13794517	0.793009449	0.427772318	0	0.126182688	0	0.5	CRA004775;Yang2025_Silkie_Wenchang	PRJNA827465	0.84125685
ENSGALG00010016999	ITGB1BP2	2	-0.159907106	0.094542099	-1.691385185	0.09076326	0	0.005808843	0	1	CRA004775;PRJNA827465	Yang2025_Silkie_Wenchang	0.516920505
ENSGALG00010017001	TPCN3	2	-0.172928839	0.149355117	-1.157836724	0.246930674	0.029671416	2.702634079	62.99906051	1	PRJNA827465;Yang2025_Silkie_Wenchang	CRA004775	0.757459165
ENSGALG00010017001	TPCN3	2	-0.325186707	0.136394381	-2.384164971	0.017117927	0	0.137767807	0	1	CRA004775;Yang2025_Silkie_Wenchang	PRJNA827465	0.217378311
ENSGALG00010017001	TPCN3	2	-0.076362736	0.076778681	-0.994582541	0.319939342	0	0.655460908	0	1	CRA004775;PRJNA827465	Yang2025_Silkie_Wenchang	0.798348216
ENSGALG00010017003	CPZ	2	-0.26011098	0.310637632	-0.837345359	0.40239847	0.153371226	4.809225862	79.20663265	0.5	PRJNA827465;Yang2025_Silkie_Wenchang	CRA004775	0.832879175
ENSGALG00010017003	CPZ	2	-0.598962689	0.220719511	-2.713682569	0.006653989	0	0.035306892	0	1	CRA004775;Yang2025_Silkie_Wenchang	PRJNA827465	0.124771821
ENSGALG00010017003	CPZ	2	0.008507868	0.16798787	0.050645729	0.959607823	0	0.6832422	0	0.5	CRA004775;PRJNA827465	Yang2025_Silkie_Wenchang	0.989259322
ENSGALG00010017004	CX3CR1	2	-0.119317136	0.705496646	-0.169125023	0.865698303	0.645592821	2.712318989	63.13118022	0.5	PRJNA827465;Yang2025_Silkie_Wenchang	CRA004775	0.967088846
ENSGALG00010017004	CX3CR1	2	-0.403821225	0.329808638	-1.2244107	0.220797335	0	0.688206782	0	1	CRA004775;Yang2025_Silkie_Wenchang	PRJNA827465	0.734054953
ENSGALG00010017004	CX3CR1	2	0.0562665	0.369749043	0.152174835	0.879049041	0.105235964	1.607742783	37.80099586	0.5	CRA004775;PRJNA827465	Yang2025_Silkie_Wenchang	0.971139658
ENSGALG00010017006	DNAH3	2	0.427905445	0.403769494	1.059776561	0.289246263	0.291252069	9.348102236	89.3026416	1	PRJNA827465;Yang2025_Silkie_Wenchang	CRA004775	0.783530627
ENSGALG00010017006	DNAH3	2	0.523820153	0.383551944	1.365708507	0.172030473	0.205849651	3.054186751	67.25805978	1	CRA004775;Yang2025_Silkie_Wenchang	PRJNA827465	0.674672141
ENSGALG00010017006	DNAH3	2	0.034397272	0.158420773	0.217126018	0.828110138	0	0.003355334	0	1	CRA004775;PRJNA827465	Yang2025_Silkie_Wenchang	0.95664866
ENSGALG00010017008	RTN1	2	0.061798988	0.310817657	0.198827147	0.842397959	0.095135512	1.83420969	45.48060641	0.5	PRJNA827465;Yang2025_Silkie_Wenchang	CRA004775	0.960231061
ENSGALG00010017008	RTN1	2	0.348443364	0.324058984	1.075246732	0.282264278	0	0.237983392	0	1	CRA004775;Yang2025_Silkie_Wenchang	PRJNA827465	0.779519046
ENSGALG00010017008	RTN1	2	-0.11666412	0.215775582	-0.54067341	0.588732709	0	0.318744319	0	0.5	CRA004775;PRJNA827465	Yang2025_Silkie_Wenchang	0.892171138
ENSGALG00010017010	NONO	2	0.000745267	0.042047143	0.017724567	0.985858582	0	0.03991712	0	0.5	PRJNA827465;Yang2025_Silkie_Wenchang	CRA004775	0.996013375
ENSGALG00010017010	NONO	2	-0.0314345	0.05646545	-0.556703255	0.577730192	0	0.867074352	0	1	CRA004775;Yang2025_Silkie_Wenchang	PRJNA827465	0.887389782
ENSGALG00010017010	NONO	2	-0.019914042	0.061058801	-0.326145315	0.744314413	0.001238415	1.108042753	9.750774791	0.5	CRA004775;PRJNA827465	Yang2025_Silkie_Wenchang	0.935821071
ENSGALG00010017012	SYNGR3	2	0.152679668	0.134473758	1.13538634	0.256213481	0	0.00429881	0	1	PRJNA827465;Yang2025_Silkie_Wenchang	CRA004775	0.764613396
ENSGALG00010017012	SYNGR3	2	-0.172652567	0.419585714	-0.411483426	0.680718092	0.256128694	3.221557009	68.95910899	0.5	CRA004775;Yang2025_Silkie_Wenchang	PRJNA827465	0.917851751
ENSGALG00010017012	SYNGR3	2	-0.187613495	0.411759819	-0.455638181	0.648650183	0.234011299	2.916343954	65.71049178	0.5	CRA004775;PRJNA827465	Yang2025_Silkie_Wenchang	0.907730933
ENSGALG00010017017	ENSGALG00010017017	2	0.691060844	0.344107585	2.008269719	0.044614638	0.160546933	3.103000236	67.77312524	1	PRJNA827465;Yang2025_Silkie_Wenchang	CRA004775	0.364257243
ENSGALG00010017017	ENSGALG00010017017	2	0.472706155	0.595632226	0.793620852	0.427416189	0.597450193	6.256309552	84.01613616	0.5	CRA004775;Yang2025_Silkie_Wenchang	PRJNA827465	0.84125685
ENSGALG00010017017	ENSGALG00010017017	2	0.174649987	0.241020258	0.724627831	0.46868038	0.017508816	1.159710323	13.77157037	0.5	CRA004775;PRJNA827465	Yang2025_Silkie_Wenchang	0.853198008
ENSGALG00010017018	HP1BP3	2	-0.118532687	0.065471081	-1.810458667	0.070224688	0.004542486	1.909022371	47.61716702	1	PRJNA827465;Yang2025_Silkie_Wenchang	CRA004775	0.457187499
ENSGALG00010017018	HP1BP3	2	-0.19998582	0.080682632	-2.478672474	0.013187234	0	0.060410077	0	1	CRA004775;Yang2025_Silkie_Wenchang	PRJNA827465	0.187509939
ENSGALG00010017018	HP1BP3	2	-0.076185464	0.043063241	-1.769153024	0.076868341	0	0.211308481	0	1	CRA004775;PRJNA827465	Yang2025_Silkie_Wenchang	0.477955453
ENSGALG00010017022	CCR4	2	0.308004869	0.471843856	0.652768634	0.513905448	0.371217674	5.914777883	83.09319437	0.5	PRJNA827465;Yang2025_Silkie_Wenchang	CRA004775	0.866644891
ENSGALG00010017022	CCR4	2	0.33632182	0.522304324	0.643919272	0.519627784	0.393879932	3.501386033	71.43988151	0.5	CRA004775;Yang2025_Silkie_Wenchang	PRJNA827465	0.869100572
ENSGALG00010017022	CCR4	2	-0.157290901	0.201064724	-0.78228989	0.434044227	0	0.041700262	0	1	CRA004775;PRJNA827465	Yang2025_Silkie_Wenchang	0.842762754
ENSGALG00010017024	BUB1	2	-0.121952867	0.084485518	-1.443476582	0.148886265	0.002235299	1.181191856	15.33974816	1	PRJNA827465;Yang2025_Silkie_Wenchang	CRA004775	0.638968865
ENSGALG00010017024	BUB1	2	0.07864163	0.433186046	0.18154239	0.855941863	0.262113892	2.559555881	60.93072211	0.5	CRA004775;Yang2025_Silkie_Wenchang	PRJNA827465	0.963486168
ENSGALG00010017024	BUB1	2	0.12388828	0.317589998	0.390088732	0.696470934	0.120414772	1.72542713	42.04333626	0.5	CRA004775;PRJNA827465	Yang2025_Silkie_Wenchang	0.921041503
ENSGALG00010017033	LDAF1	2	-0.137018572	0.058875908	-2.327243469	0.019952311	0	0.900955023	0	1	PRJNA827465;Yang2025_Silkie_Wenchang	CRA004775	0.237416187
ENSGALG00010017033	LDAF1	2	-0.296082051	0.137889751	-2.147237555	0.031774374	0	0.000451592	0	1	CRA004775;Yang2025_Silkie_Wenchang	PRJNA827465	0.305336976

ENSGALG00010017033	LDAF1	2	-0.131301758	0.059825712	-2.194737908	0.028182404	0	0.613689327	0	1	CRA004775;PRJNA827465	Yang2025_Silkie_Wenchang	0.285993549
ENSGALG00010017034	ZMYM3	2	0.210365132	0.265465589	0.792438423	0.428105083	0.131823528	15.10574553	93.38000235	0.5	PRJNA827465;Yang2025_Silkie_Wenchang	CRA004775	0.84125685
ENSGALG00010017034	ZMYM3	2	0.055821822	0.443350341	0.125909054	0.899803915	0.367190006	14.98715335	93.32761882	0.5	CRA004775;Yang2025_Silkie_Wenchang	PRJNA827465	0.976319826
ENSGALG00010017034	ZMYM3	2	-0.175032797	0.171386702	-1.021274079	0.307124604	0.042648104	3.068616825	67.41202773	1	CRA004775;PRJNA827465	Yang2025_Silkie_Wenchang	0.79266003
ENSGALG00010017035	PIK3CD	2	0.10664416	0.091060787	1.171131543	0.241545907	0	0.015618901	0	1	PRJNA827465;Yang2025_Silkie_Wenchang	CRA004775	0.75398926
ENSGALG00010017035	PIK3CD	2	-0.222222732	0.474007532	-0.468816879	0.639200534	0.30169109	2.324081041	56.97224054	0.5	CRA004775;Yang2025_Silkie_Wenchang	PRJNA827465	0.905968982
ENSGALG00010017035	PIK3CD	2	-0.210960797	0.486894691	-0.433278081	0.66481276	0.33030074	2.476936345	59.62754545	0.5	CRA004775;PRJNA827465	Yang2025_Silkie_Wenchang	0.913393112
ENSGALG00010017038	ZNF598	2	0.024419041	0.057863395	0.422011902	0.67301633	0.002355895	1.234405717	18.98935768	0.5	PRJNA827465;Yang2025_Silkie_Wenchang	CRA004775	0.915153756
ENSGALG00010017038	ZNF598	2	0.227298628	0.115344002	1.970615063	0.048767922	0	0.967777096	0	1	CRA004775;Yang2025_Silkie_Wenchang	PRJNA827465	0.381741815
ENSGALG00010017038	ZNF598	2	0.148277348	0.197390598	0.751187489	0.452539829	0.059585803	3.421150713	70.77006879	0.5	CRA004775;PRJNA827465	Yang2025_Silkie_Wenchang	0.848321192
ENSGALG00010017039	MAP2K5	2	-0.195678706	0.29884336	-0.654786863	0.51260499	0.169999151	20.08004103	95.0199305	0.5	PRJNA827465;Yang2025_Silkie_Wenchang	CRA004775	0.865859727
ENSGALG00010017039	MAP2K5	2	-0.333891325	0.192435399	-1.735082667	0.082726164	0.050797244	3.107096175	67.81560841	1	CRA004775;Yang2025_Silkie_Wenchang	PRJNA827465	0.495704284
ENSGALG00010017039	MAP2K5	2	0.057703585	0.077212041	0.747339203	0.454858834	0.005434793	1.322495704	24.38538763	0.5	CRA004775;PRJNA827465	Yang2025_Silkie_Wenchang	0.849072665
ENSGALG00010017041	JKAMP	2	-0.070795054	0.110640459	-0.639865877	0.5222598	0.015615882	2.468996128	59.49770886	0.5	PRJNA827465;Yang2025_Silkie_Wenchang	CRA004775	0.869568211
ENSGALG00010017041	JKAMP	2	-0.406433558	0.217505134	-1.868615925	0.061676269	0.066503923	3.241466903	69.14976984	1	CRA004775;Yang2025_Silkie_Wenchang	PRJNA827465	0.428734062
ENSGALG00010017041	JKAMP	2	-0.291261456	0.332623193	-0.87564987	0.381220405	0.200036292	9.748056577	89.74154497	0.5	CRA004775;PRJNA827465	Yang2025_Silkie_Wenchang	0.824392515
ENSGALG00010017042	HNRNPLL	2	-0.289591279	0.248820889	-1.163854372	0.244483033	0.1133182	11.11576668	91.00376943	1	PRJNA827465;Yang2025_Silkie_Wenchang	CRA004775	0.756012326
ENSGALG00010017042	HNRNPLL	2	-0.37028561	0.215136466	-1.721166186	0.085220672	0.060919851	2.823400459	64.58171575	1	CRA004775;Yang2025_Silkie_Wenchang	PRJNA827465	0.502163128
ENSGALG00010017042	HNRNPLL	2	-0.061269706	0.042593763	-1.438466611	0.150301704	0	0.086835427	0	1	CRA004775;PRJNA827465	Yang2025_Silkie_Wenchang	0.640914385
ENSGALG00010017045	GLB1	2	0.2136619	0.191610408	1.115085042	0.264813981	0.065980075	9.503362748	89.47740893	1	PRJNA827465;Yang2025_Silkie_Wenchang	CRA004775	0.769252319
ENSGALG00010017045	GLB1	2	0.226682792	0.216744056	1.045854713	0.295628116	0.071088289	3.922798342	74.50799372	0.5	CRA004775;Yang2025_Silkie_Wenchang	PRJNA827465	0.787628307
ENSGALG00010017045	GLB1	2	0.030081223	0.052434979	0.573686177	0.566180184	0	0.071342638	0	0.5	CRA004775;PRJNA827465	Yang2025_Silkie_Wenchang	0.883487517
ENSGALG00010017049	TRAF3	2	0.281928932	0.213604092	1.319866715	0.186879522	0.073370969	4.920389538	79.67640585	1	PRJNA827465;Yang2025_Silkie_Wenchang	CRA004775	0.695805785
ENSGALG00010017049	TRAF3	2	0.631286151	0.205945217	3.065311062	0.002174437	0.028022742	1.312446713	23.80643036	1	CRA004775;Yang2025_Silkie_Wenchang	PRJNA827465	0.061310839
ENSGALG00010017049	TRAF3	2	0.467315253	0.450540405	1.037232727	0.299627409	0.337092285	5.165184158	80.63960607	1	CRA004775;PRJNA827465	Yang2025_Silkie_Wenchang	0.79004225
ENSGALG00010017050	L3HYPDH	2	-0.246245127	0.154282354	-1.596067991	0.110473614	0	0.097638006	0	1	PRJNA827465;Yang2025_Silkie_Wenchang	CRA004775	0.56281288
ENSGALG00010017050	L3HYPDH	2	-0.730565575	0.395999811	-1.844863444	0.065057406	0.199144662	2.738938289	63.48950234	1	CRA004775;Yang2025_Silkie_Wenchang	PRJNA827465	0.439716459
ENSGALG00010017050	L3HYPDH	2	-0.627800914	0.45225077	-1.388169918	0.16508531	0.339658974	5.588194114	82.10513129	1	CRA004775;PRJNA827465	Yang2025_Silkie_Wenchang	0.664377375
ENSGALG00010017054	GALM	2	0.060655984	0.157375957	0.385420908	0.699925594	0.038480818	4.334355782	76.92852063	0.5	PRJNA827465;Yang2025_Silkie_Wenchang	CRA004775	0.922198312
ENSGALG00010017054	GALM	2	-0.227829054	0.141873189	-1.605864058	0.108303782	0.01681402	1.664989172	39.93954936	1	CRA004775;Yang2025_Silkie_Wenchang	PRJNA827465	0.556717702
ENSGALG00010017054	GALM	2	-0.077372191	0.302383763	-0.255874157	0.79804801	0.163550743	9.03755893	88.93506523	0.5	CRA004775;PRJNA827465	Yang2025_Silkie_Wenchang	0.949922217
ENSGALG00010017055	GPR135	2	0.072003671	0.188045636	0.382905305	0.701789978	0	0.582155999	0	0.5	PRJNA827465;Yang2025_Silkie_Wenchang	CRA004775	0.922904951
ENSGALG00010017055	GPR135	2	-0.205426361	0.261382991	-0.785920922	0.431913817	0	0.35934306	0	1	CRA004775;Yang2025_Silkie_Wenchang	PRJNA827465	0.842503929
ENSGALG00010017055	GPR135	2	0.013119315	0.286438666	0.045801481	0.963468478	0.054324442	1.345647958	25.68635843	0.5	CRA004775;PRJNA827465	Yang2025_Silkie_Wenchang	0.99005635
ENSGALG00010017056	AGTR1	2	-0.176266902	0.097878803	-1.800869002	0.07172353	0	0.411647022	0	1	PRJNA827465;Yang2025_Silkie_Wenchang	CRA004775	0.462047188
ENSGALG00010017056	AGTR1	2	0.091990505	0.242324276	0.379617375	0.704229461	0.039423574	1.373823332	27.210437	0.5	CRA004775;Yang2025_Silkie_Wenchang	PRJNA827465	0.92402786
ENSGALG00010017056	AGTR1	2	0.02064681	0.325022142	0.06352432	0.949348994	0.14655012	2.620310249	61.83658022	0.5	CRA004775;PRJNA827465	Yang2025_Silkie_Wenchang	0.98723759
ENSGALG00010017057	NDUFAB1	2	-0.048992959	0.085739329	-0.571417571	0.567716621	0	3.15843E-07	0	1	PRJNA827465;Yang2025_Silkie_Wenchang	CRA004775	0.883747882
ENSGALG00010017057	NDUFAB1	2	-0.057750172	0.226857793	-0.254565522	0.799058692	0	0.003652594	0	1	CRA004775;Yang2025_Silkie_Wenchang	PRJNA827465	0.949922217
ENSGALG00010017057	NDUFAB1	2	-0.050295353	0.088526695	-0.568137703	0.569941469	0	0.004926035	0	1	CRA004775;PRJNA827465	Yang2025_Silkie_Wenchang	0.884923673
ENSGALG00010017060	CLSTN1	2	-0.168992982	0.113885063	-1.483890656	0.137837931	0.012098695	1.87274383	46.60241384	1	PRJNA827465;Yang2025_Silkie_Wenchang	CRA004775	0.62064915
ENSGALG00010017060	CLSTN1	2	-0.024095727	0.278953409	-0.086379036	0.931165112	0.129997229	5.84450387	82.88990781	0.5	CRA004775;Yang2025_Silkie_Wenchang	PRJNA827465	0.983943003
ENSGALG00010017060	CLSTN1	2	0.075296961	0.161439622	0.466409426	0.640922466	0.027586817	1.999954031	49.99885074	0.5	CRA004775;PRJNA827465	Yang2025_Silkie_Wenchang	0.906429868
ENSGALG00010017063	TMPPE	2	-0.389827612	0.3264171	-1.194262224	0.23237541	0.185984237	7.513813624	86.69117907	1	PRJNA827465;Yang2025_Silkie_Wenchang	CRA004775	0.745068586
ENSGALG00010017063	TMPPE	2	-0.594777873	0.244733986	-2.430303543	0.015086182	0.036085689	1.312898447	23.83264659	1	CRA004775;Yang2025_Silkie_Wenchang	PRJNA827465	0.203100811
ENSGALG00010017063	TMPPE	2	-0.095024167	0.10177485	-0.933670419	0.350473928	0	0.056116189	0	1	CRA004775;PRJNA827465	Yang2025_Silkie_Wenchang	0.810111037
ENSGALG00010017065	VRK2	2	0.258396844	0.316412064	0.816646626	0.414130401	0.191962684	24.2076398	95.86907271	0.5	PRJNA827465;Yang2025_Silkie_Wenchang	CRA004775	0.836841499
ENSGALG00010017065	VRK2	2	0.257890769	0.363443976	0.709575026	0.477967711	0.224258852	6.011571946	83.36541575	0.5	CRA004775;Yang2025_Silkie_Wenchang	PRJNA827465	0.855366076
ENSGALG00010017065	VRK2	2	-0.06618426	0.084429637	-0.783898439	0.433099706	0	0.114394182	0	1	CRA004775;PRJNA827465	Yang2025_Silkie_Wenchang	0.842762754
ENSGALG00010017070	BLOC1S6	2	0.546186517	0.375364515	1.455082977	0.145646291	0.260178546	12.63097437	92.08295441	1	PRJNA827465;Yang2025_Silkie_Wenchang	CRA004775	0.634345765
ENSGALG00010017070	BLOC1S6	2	0.4963829	0.457749805	1.08439784	0.278188441	0.37066424	8.605300531	88.37925536	1	CRA004775;Yang2025_Silkie_Wenchang	PRJNA827465	0.776763074
ENSGALG00010017070	BLOC1S6	2	0.170959587	0.083138803	2.056315235	0.039752141	0	0.39419011	0	1	CRA004775;PRJNA827465	Yang2025_Silkie_Wenchang	0.344755224
ENSGALG00010017077	CTNNBIP1	2	-0.177936476	0.144457005	-1.231760797	0.218038452	0.035328404	6.390167765	84.35095859	1	PRJNA827465;Yang2025_Silkie_Wenchang	CRA004775	0.731566098
ENSGALG00010017077	CTNNBIP1	2	-0.140734086	0.231724681	-0.607333172	0.543629828	0.080945973	3.643212584	72.5516978	0.5	CRA004775;Yang2025_Silkie_Wenchang	PRJNA827465	0.876968972
ENSGALG00010017077	CTNNBIP1	2	-0.028558566	0.061468341	-0.464606105	0.64221356	0	0.594686099	0	0.5	CRA004775;PRJNA827465	Yang2025_Silkie_Wenchang	0.906634523
ENSGALG00010017080	PALB2	2	-0.044245883	0.075134955	-0.588885467	0.555938107	0	0.977316245	0	0.5	PRJNA827465;Yang2025_Silkie_Wenchang	CRA004775	0.880324407
ENSGALG00010017080	PALB2	2	-0.175523801	0.116251371	-1.509864351	0.131078041	0	0.319363206	0	1	CRA004775;Yang2025_Silkie_Wenchang	PRJNA827465	0.606767161
ENSGALG00010017080	PALB2	2	-0.047637353	0.125664415	-0.379083868	0.704625585	0.010457303	1.234645791	19.00511004	0.5	CRA004775;PRJNA827465	Yang2025_Silkie_Wenchang	0.924152671
ENSGALG00010017083	ENSGALG00010017083	2	-0.140404736	0.330952772	-0.424244026	0.671387856	0	0.177622609	0	0.5	PRJNA827465;Yang2025_Silkie_Wenchang	CRA004775	0.914674175
ENSGALG00010017083	ENSGALG00010017083	2	0.77697727	0.447671606	1.735596495	0.082635206	0.13865308	1.428150761	29.97938122	1	CRA004775;Yang2025_Silkie_Wenchang	PRJNA827465	0.495622083
ENSGALG00010017083	ENSGALG00010017083	2	0.428198943	0.648591232	0.660198476	0.50912647	0.693507222	5.689245438	82.42297663	0.5	CRA004775;PRJNA827465	Yang2025_Silkie_Wenchang	0.865193604
ENSGALG00010017085	ENSGALG00010017085	2	-0.107145634	0.131437644	-0.815182248	0.414967998	0.024131637	3.092988018	67.66880459	0.5	PRJNA827465;Yang2025_Silkie_Wenchang	CRA004775	0.837458421
ENSGALG00010017085	ENSGALG00010017085	2	-0.294781609	0.116536009	-2.529532383	0.011421463	0	0.220118364	0	1	CRA004775;Yang2025_Silkie_Wenchang	PRJNA827465	0.17044842
ENSGALG00010017085	ENSGALG00010017085	2	-0.129356347	0.186553148	-0.693402116	0.488057234	0.047036491	2.509508988	60.1515673	0.5	CRA004775;PRJNA827465	Yang2025_Silkie_Wenchang	0.85768885
ENSGALG00010017089	CRTAP	2	0.091894664	0.103626664	0.886785894	0.375194184	0.011899445	2.242300805	55.40295049	0.5	PRJNA827465;Yang2025_Silkie_Wenchang	CRA004775	0.822941424
ENSGALG00010017089	CRTAP	2	-0.027941994	0.089990083	-0.31050081	0.756180144	0	0.147320412	0	1	CRA004775;Yang2025_Silkie_Wenchang	PRJNA827465	0.93854559
ENSGALG00010017089	CRTAP	2	0.109858924	0.135086101	0.813251128	0.416074091	0.016034436	1.554922227	35.68810179	0.5	CRA004775;PRJNA827465	Yang2025_Silkie_Wenchang	0.837742614
ENSGALG00010017092	ANKS4B	2	0.227457043	0.318109464	0.715027588	0.474591996	0	0.429663568	0	1	PRJNA827465;Yang2025_Silkie_Wenchang	CRA004775	0.854462864
ENSGALG00010017092	ANKS4B	2	1.366055488	0.934730824	1.461442646	0.143894005	1.363915054	4.476597343	77.66160494	1	CRA004775;Yang2025_Silkie_Wenchang	PRJNA827465	0.630838378
ENSGALG00010017092	ANKS4B	2	1.140445169	1.149592212	0.992043229	0.321176438	2.315389084	7.819308114	87.2111447	1	CRA004775;PRJNA827465	Yang2025_Silkie_Wenchang	0.798513866
ENSGALG00010017094	SORBS2	2	0.603354625	0.503513557	1.198288738	0.23080463	0.47034913	13.48425701	92.58394438	1	PRJNA827465;Yang2025_Silkie_Wenchang	CRA004775	0.743374303
ENSGALG00010017094	SORBS2	2	0.942417739	0.187895287	5.015653961	5.28534E-07	0.010363563	1.172016843	14.67699414	1	CRA004775;Yang2025_Silkie_Wenchang	PRJNA827465	0.000134889
ENSGALG00010017094	SORBS2	2	0.401806528	0.314075896	1.2793294	0.200781084	0.161819195	5.256417813	80.97563711	1	CRA004775;PRJNA827465	Yang2025_Silkie_Wenchang	0.713340819
ENSGALG00010017096	DCTN5	2	-0.174475779	0.166859535	-1.045644647	0.295725128	0.047458909	6.445807668	84.48604036	1	PRJNA827465;Yang2025_Silkie_Wenchang	CRA004775	0.787778548
ENSGALG00010017096	DCTN5	2	-0.383346351	0.090328337	-4.24392127	2.19647E-05	0	0.11332386	0	1	CRA004775;Yang2025_Silkie_Wenchang	PRJNA827465	0.002631952
ENSGALG00010017096	DCTN5	2	-0.200603296	0.197395187	-1.016252216	0.309509297	0.067204329	6.815974229	85.32858303	1	CRA004775;PRJNA827465	Yang2025_Silkie_Wenchang	0.794192328
ENSGALG00010017098	DAAM1	2	0.112475913	0.056195739	2.001502522	0.045338262	0	4.17924E-05	0	1	PRJNA827465;Yang2025_Silkie_Wenchang	CRA004775	0.367105422
ENSGALG00010017098	DAAM1	2	0.165183009	0.114916511	1.437417543	0.150599387	0	0.908111545	0	1	CRA004775;Yang2025_Silkie_Wenchang	PRJNA827465	0.641696412
ENSGALG00010017098	DAAM1	2	0.134223617	0.074211752	1.808657162	0.070504282	0.002058822	1.056752823	5.370491762	1	CRA004775;PRJNA827465	Yang2025_Silkie_Wenchang	0.458021454
ENSGALG00010017101	CSMD2	2	-0.294657099	0.12702323	-2.319710328	0.020356553	0	0.01199177	0	1	PRJNA827465;Yang2025_Silkie_Wenchang	CRA004775	0.24026234
ENSGALG00010017101	CSMD2	2	-0.601148491	0.385745639	-1.558406448	0.119136929	0.207622206	3.107084117	67.81548351	1	CRA004775;Yang2025_Silkie_Wenchang	PRJNA827465	0.579471011
ENSGALG00010017101	CSMD2	2	-0.598088941	0.367284771	-1.628406587	0.103438702	0.196524366	3.256332757	69.29060773	1	CRA004775;PRJNA827465	Yang2025_Silkie_Wenchang	0.547683053
ENSGALG00010017104	FANCL	2	-0.012811628	0.08659637	-0.147946479	0.882385007	0	0.079159737	0	0.5	PRJNA827465;Yang2025_Silkie_Wenchang	CRA004775	0.972001076
ENSGALG00010017104	FANCL	2	0.015066408	0.135579399	0.111126087	0.911516363	0	0.008040925	0	0.5	CRA004775;Yang2025_Silkie_Wenchang	PRJNA827465	0.978851978
ENSGALG00010017104	FANCL	2	-0.031144963	0.106252862	-0.293121172	0.769429535	0	0.000262913	0	1	CRA004775;PRJNA827465	Yang2025_Silkie_Wenchang	0.941790377
ENSGALG00010017105	PLK1	2	-0.1362272	0.091325379	-1.491668598	0.135786045	0	0.55008338	0	1	PRJNA827465;Yang2025_Silkie_Wenchang	CRA004775	0.617196137
ENSGALG00010017105	PLK1	2	-0.099883351	0.290770707	-0.343512427	0.731212994	0.073308372	1.470554758	31.998452	0.5	CRA004775;Yang2025_Silkie_Wenchang	PRJNA827465	0.932837714
ENSGALG00010017105	PLK1	2	-0.081408635	0.101213077	-0.804329214	0.42120687	0	0.925571195	0	0.5	CRA004775;PRJNA827465	Yang2025_Silkie_Wenchang	0.839352211
ENSGALG00010017106	CRYM	2	-0.182345617	0.194213626	-0.93889199	0.347786202	0.050889369	2.525475941	60.4035032	1	PRJNA827465;Yang2025_Silkie_Wenchang	CRA004775	0.808847403
ENSGALG00010017106	CRYM	2	-0.355023988	0.202688999	-1.751570088	0.07984776	0	0.501412521	0	1	CRA004775;Yang2025_Silkie_Wenchang	PRJNA827465	0.486023928
ENSGALG00010017106	CRYM	2	-0.048205063	0.076381945	-0.631105468	0.527971566	0	0.079907554	0	1	CRA004775;PRJNA827465	Yang2025_Silkie_Wenchang	0.871518961
ENSGALG00010017109	ITGB1BP3	2	-0.08815791	0.063849534	-1.380713448	0.167367085	0	0.037513503	0	1	PRJNA827465;Yang2025_Silkie_Wenchang	CRA004775	0.666945317
ENSGALG00010017109	ITGB1BP3	2	-0.11971254	0.134126343	-0.892535627	0.372105906	0	0.000932051	0	1	CRA004775;Yang2025_Silkie_Wenchang	PRJNA827465	0.821620523
ENSGALG00010017109	ITGB1BP3	2	-0.086171375	0.066744947	-1.291054674	0.196684719	0	0.029565399	0	1	CRA004775;PRJNA827465	Yang2025_Silkie_Wenchang	0.707994211
ENSGALG00010017110	BRF1	2	0.014728114	0.064532962	0.228226219	0.81947038	0	0.380746815	0	0.5	PRJNA827465;Yang2025_Silkie_Wenchang	CRA004775	0.955256039
ENSGALG00010017110	BRF1	2	0.065151766	0.107291123	0.607242833	0.54368977	0	0.05825354	0	1	CRA004775;Yang2025_Silkie_Wenchang	PRJNA827465	0.876968972
ENSGALG00010017110	BRF1	2	-0.006017308	0.071117984	-0.084610212	0.93257128	0	0.018275857	0	0.5	CRA004775;PRJNA827465	Yang2025_Silkie_Wenchang	0.984224753
ENSGALG00010017111	GJB1	2	0.237938859	0.226389889	1.051013629	0.29325233	0.019482578	1.206645269	17.12560221	1	PRJNA827465;Yang2025_Silkie_Wenchang	CRA004775	0.78684437
ENSGALG00010017111	GJB1	2	0.452595495	0.449975583	1.005822343	0.314501037	0.286966071	3.201182777	68.76154629	1	CRA004775;Yang2025_Silkie_Wenchang	PRJNA827465	0.796531448
ENSGALG00010017111	GJB1	2	0.722352561	0.281352375	2.56743012	0.010245544	0	0.573630358	0	1	CRA004775;PRJNA827465	Yang2025_Silkie_Wenchang	0.160750741
ENSGALG00010017112	LZIC	2	-0.170535308	0.169815829	-1.004236821	0.315264478	0.053522923	13.81556368	92.76178647	1	PRJNA827465;Yang2025_Silkie_Wenchang	CRA004775	0.79668487
ENSGALG00010017112	LZIC	2	-0.23856316	0.137680103	-1.732735196	0.083142746	0.024683673	2.541329571	60.65051888	1	CRA004775;Yang2025_Silkie_Wenchang	PRJNA827465	0.49661996
ENSGALG00010017112	LZIC	2	-0.009664416	0.051832463	-0.186454879	0.85208806	0	0.099816376	0	1	CRA004775;PRJNA827465	Yang2025_Silkie_Wenchang	0.962372971
ENSGALG00010017115	LGALSL	2	-0.290837024	0.086685968	-3.355064615	0.000793464	0	0.447909147	0	1	PRJNA827465;Yang2025_Silkie_Wenchang	CRA004775	0.032216461
ENSGALG00010017115	LGALSL	2	-0.31852326	0.126245616	-2.523044122	0.01163438	0	0.832271235	0	0.5	CRA004775;Yang2025_Silkie_Wenchang	PRJNA827465	0.172635381
ENSGALG00010017115	LGALSL	2	-0.220960497	0.108989332	-2.027358948	0.042625717	0	0.39714916	0	0.5	CRA004775;PRJNA827465	Yang2025_Silkie_Wenchang	0.357019514
ENSGALG00010017116	ENSGALG00010017116	2	0.054929907	0.177410103	0.309621076	0.756849127	0	0.605595326	0	0.5	PRJNA827465;Yang2025_Silkie_Wenchang	CRA004775	0.938758521
ENSGALG00010017116	ENSGALG00010017116	2	-0.25786446	0.731685452	-0.352425293	0.724519336	0.701866331	2.389106647	58.14335031	0.5	CRA004775;Yang2025_Silkie_Wenchang	PRJNA827465	0.930388018
ENSGALG00010017116	ENSGALG00010017116	2	-0.323356555	0.525891997	-0.614872554	0.53863889	0.311386257	1.650697617	39.4195527	1	CRA004775;PRJNA827465	Yang2025_Silkie_Wenchang	0.87481821
ENSGALG00010017119	BTBD6	2	0.187893543	0.138223395	1.359346898	0.17403669	0.028483542	3.836505265	73.93461156	1	PRJNA827465;Yang2025_Silkie_Wenchang	CRA004775	0.67800817
ENSGALG00010017119	BTBD6	2	0.298675375	0.106283665	2.810171963	0.004951504	0	0.739146372	0	1	CRA004775;Yang2025_Silkie_Wenchang	PRJNA827465	0.104831857
ENSGALG00010017119	BTBD6	2	0.064341127	0.07740617	0.831214451	0.405852493	0	0.012823167	0	1	CRA004775;PRJNA827465	Yang2025_Silkie_Wenchang	0.833416425
ENSGALG00010017120	ERN2	2	0.010312876	0.065925363	0.156432594	0.875692047	0	0.290079736	0	0.5	PRJNA827465;Yang2025_Silkie_Wenchang	CRA004775	0.970347777
ENSGALG00010017120	ERN2	2	-0.068029493	0.109091747	-0.623598893	0.532891038	0	0.190162428	0	1	CRA004775;Yang2025_Silkie_Wenchang	PRJNA827465	0.873237026
ENSGALG00010017120	ERN2	2	0.015143503	0.074806173	0.202436543	0.839575463	0	0.582349131	0	0.5	CRA004775;PRJNA827465	Yang2025_Silkie_Wenchang	0.959493354
ENSGALG00010017122	NCBP2	2	0.604187339	0.608741113	0.992519357	0.320944241	0.724761647	45.22555565	97.78886078	0.5	PRJNA827465;Yang2025_Silkie_Wenchang	CRA004775	0.798472282
ENSGALG00010017122	NCBP2	2	0.456444774	0.767528355	0.594694347	0.552047801	1.145886315	36.31913519	97.24663047	0.5	CRA004775;Yang2025_Silkie_Wenchang	PRJNA827465	0.879061525
ENSGALG00010017122	NCBP2	2	-0.109836086	0.150564146	-0.729496955	0.46569773	0.021658552	1.752280252	42.93150319	1	CRA004775;PRJNA827465	Yang2025_Silkie_Wenchang	0.852424459
ENSGALG00010017127	SOX8	2	0.163202331	0.145191941	1.12404538	0.260993775	0.023786852	2.092652577	52.21375919	1	PRJNA827465;Yang2025_Silkie_Wenchang	CRA004775	0.767929536
ENSGALG00010017127	SOX8	2	0.406027033	0.166188873	2.443166174	0.014559032	0	0.390135934	0	1	CRA004775;Yang2025_Silkie_Wenchang	PRJNA827465	0.198616324
ENSGALG00010017127	SOX8	2	0.217392637	0.256431546	0.847760896	0.396571145	0.083291676	2.090656356	52.16813145	1	CRA004775;PRJNA827465	Yang2025_Silkie_Wenchang	0.831365249
ENSGALG00010017131	TLR3	2	0.207078026	0.264976338	0.781496292	0.434510657	0	0.0492902	0	1	PRJNA827465;Yang2025_Silkie_Wenchang	CRA004775	0.842762754
ENSGALG00010017131	TLR3	2	0.347332609	0.319529637	1.087012185	0.277031424	0	0.046834003	0	1	CRA004775;Yang2025_Silkie_Wenchang	PRJNA827465	0.776173801
ENSGALG00010017131	TLR3	2	0.249004718	0.272670692	0.913206756	0.361133812	0	0.20320856	0	1	CRA004775;PRJNA827465	Yang2025_Silkie_Wenchang	0.815240214
ENSGALG00010017132	TMEM108	2	0.376025978	0.511660558	0.734912966	0.462392501	0.462282582	8.253315153	87.88365667	0.5	PRJNA827465;Yang2025_Silkie_Wenchang	CRA004775	0.850823289
ENSGALG00010017132	TMEM108	2	-0.167188759	1.183062297	-0.141318644	0.887618222	2.49019164	8.602531564	88.3755149	0.5	CRA004775;Yang2025_Silkie_Wenchang	PRJNA827465	0.973038314
ENSGALG00010017132	TMEM108	2	-0.580447806	0.645091021	-0.899792104	0.368230897	0.617018282	3.114210688	67.88913468	1	CRA004775;PRJNA827465	Yang2025_Silkie_Wenchang	0.820213684
ENSGALG00010017133	WDR20	2	0.112978257	0.237297471	0.476103922	0.634000343	0.09962327	8.636342235	88.42102394	0.5	PRJNA827465;Yang2025_Silkie_Wenchang	CRA004775	0.903714339
ENSGALG00010017133	WDR20	2	0.309021324	0.107661497	2.870304911	0.004100762	0	0.562300482	0	1	CRA004775;Yang2025_Silkie_Wenchang	PRJNA827465	0.09294823
ENSGALG00010017133	WDR20	2	-0.038598313	0.129257139	-0.298616489	0.765232683	0.012543936	1.435610545	30.34322552	0.5	CRA004775;PRJNA827465	Yang2025_Silkie_Wenchang	0.940779391
ENSGALG00010017135	ENSGALG00010017135	2	-0.176776376	0.130642964	-1.35312588	0.176015419	0.01117353	1.457233388	31.37681253	1	PRJNA827465;Yang2025_Silkie_Wenchang	CRA004775	0.68110013
ENSGALG00010017135	ENSGALG00010017135	2	0.056374643	0.424216976	0.132891059	0.894279538	0.303313256	6.175141035	83.80603788	0.5	CRA004775;Yang2025_Silkie_Wenchang	PRJNA827465	0.974381724
ENSGALG00010017135	ENSGALG00010017135	2	0.162933986	0.286033284	0.569632961	0.568926668	0.119119214	3.319269263	69.87288705	0.5	CRA004775;PRJNA827465	Yang2025_Silkie_Wenchang	0.884492059
ENSGALG00010017136	ENSGALG00010017136	2	0.098795907	0.219698953	0.449687658	0.652935672	0	0.036615325	0	1	PRJNA827465;Yang2025_Silkie_Wenchang	CRA004775	0.909572411
ENSGALG00010017136	ENSGALG00010017136	2	0.522946112	0.55054293	0.94987345	0.342176559	0.325271353	2.140915138	53.29100242	1	CRA004775;Yang2025_Silkie_Wenchang	PRJNA827465	0.806005903
ENSGALG00010017136	ENSGALG00010017136	2	0.483346101	0.481237153	1.004382347	0.315194356	0.30270184	2.527502404	60.4352503	1	CRA004775;PRJNA827465	Yang2025_Silkie_Wenchang	0.796662552
ENSGALG00010017139	COL9A2	2	1.518544105	1.097184501	1.384037145	0.166347081	1.674687603	3.280578787	69.51757403	1	PRJNA827465;Yang2025_Silkie_Wenchang	CRA004775	0.665303687
ENSGALG00010017139	COL9A2	2	-0.229188678	2.859256142	-0.080156749	0.936112588	15.62906925	22.64920951	95.58483487	0.5	CRA004775;Yang2025_Silkie_Wenchang	PRJNA827465	0.984822128
ENSGALG00010017139	COL9A2	2	-1.317936733	1.761778779	-0.748071635	0.45441695	5.543606042	9.346947957	89.30132055	0.5	CRA004775;PRJNA827465	Yang2025_Silkie_Wenchang	0.849072665
ENSGALG00010017140	UQCRC2	2	-0.134992312	0.066821818	-2.020183162	0.043364392	0	0.001031178	0	1	PRJNA827465;Yang2025_Silkie_Wenchang	CRA004775	0.360023385
ENSGALG00010017140	UQCRC2	2	-0.121886124	0.149770555	-0.813819003	0.415748647	0	0.06013427	0	1	CRA004775;Yang2025_Silkie_Wenchang	PRJNA827465	0.837701508
ENSGALG00010017140	UQCRC2	2	-0.130227684	0.071307421	-1.826285147	0.067807321	0	0.062627077	0	1	CRA004775;PRJNA827465	Yang2025_Silkie_Wenchang	0.449101706
ENSGALG00010017141	AFTPH	2	-0.103240796	0.045554371	-2.2663203	0.023431779	0	5.3764E-06	0	1	PRJNA827465;Yang2025_Silkie_Wenchang	CRA004775	0.26118526
ENSGALG00010017141	AFTPH	2	0.024927276	0.160382378	0.155424034	0.876487038	0.033664981	2.646646879	62.2163422	0.5	CRA004775;Yang2025_Silkie_Wenchang	PRJNA827465	0.970347777
ENSGALG00010017141	AFTPH	2	0.016197422	0.158304928	0.102317865	0.918504375	0.036588249	3.078639878	67.5181236	0.5	CRA004775;PRJNA827465	Yang2025_Silkie_Wenchang	0.981033557
ENSGALG00010017142	RBP7	2	-0.933264109	0.467253656	-1.997339342	0.045788332	0.176908491	1.675255805	40.30762364	1	PRJNA827465;Yang2025_Silkie_Wenchang	CRA004775	0.36915258
ENSGALG00010017142	RBP7	2	-0.546563375	0.841968241	-0.649149634	0.516241663	1.153426999	5.348623291	81.30360009	0.5	CRA004775;Yang2025_Silkie_Wenchang	PRJNA827465	0.867680725
ENSGALG00010017142	RBP7	2	-0.061647355	0.38508834	-0.160086267	0.872813119	0	0.941950225	0	0.5	CRA004775;PRJNA827465	Yang2025_Silkie_Wenchang	0.969647069
ENSGALG00010017143	FAM149A	2	-0.026179641	0.288464457	-0.090755171	0.927687131	0.124979027	3.968782013	74.80335285	0.5	PRJNA827465;Yang2025_Silkie_Wenchang	CRA004775	0.982993195
ENSGALG00010017143	FAM149A	2	-0.213587992	0.203151232	-1.051374339	0.293086697	0.003052137	1.029116752	2.829295313	0.5	CRA004775;Yang2025_Silkie_Wenchang	PRJNA827465	0.786774237
ENSGALG00010017143	FAM149A	2	0.225733898	0.162305764	1.390794093	0.164287875	0	0.068331648	0	1	CRA004775;PRJNA827465	Yang2025_Silkie_Wenchang	0.66314125
ENSGALG00010017144	CPB1	2	-0.00855927	0.320455309	-0.026709715	0.978691264	0	0.449075546	0	0.5	PRJNA827465;Yang2025_Silkie_Wenchang	CRA004775	0.994183692
ENSGALG00010017144	CPB1	2	0.983346955	1.404619175	0.700080828	0.483876828	3.213650702	4.867127873	79.45400191	0.5	CRA004775;Yang2025_Silkie_Wenchang	PRJNA827465	0.85691916
ENSGALG00010017144	CPB1	2	1.161386355	1.179113061	0.984966068	0.324640739	2.077200271	3.479912834	71.26364803	1	CRA004775;PRJNA827465	Yang2025_Silkie_Wenchang	0.799859262
ENSGALG00010017147	SRSF7L	2	0.047633693	0.049885264	0.954865016	0.339645963	0	0.971379546	0	0.5	PRJNA827465;Yang2025_Silkie_Wenchang	CRA004775	0.8050359
ENSGALG00010017147	SRSF7L	2	-0.278123913	0.168327962	-1.65227399	0.098478709	0.018701251	1.48615985	32.71248714	1	CRA004775;Yang2025_Silkie_Wenchang	PRJNA827465	0.535930498
ENSGALG00010017147	SRSF7L	2	-0.16148741	0.259381157	-0.622587285	0.533555769	0.114376931	5.919007334	83.1052752	0.5	CRA004775;PRJNA827465	Yang2025_Silkie_Wenchang	0.873379507
ENSGALG00010017152	CYP4V2	2	-0.145787608	0.547257878	-0.266396544	0.789933826	0.518566712	7.338685188	86.3735809	0.5	PRJNA827465;Yang2025_Silkie_Wenchang	CRA004775	0.947390191
ENSGALG00010017152	CYP4V2	2	-0.255493941	0.432777601	-0.590358513	0.554950319	0.303985489	5.249510836	80.95060604	0.5	CRA004775;Yang2025_Silkie_Wenchang	PRJNA827465	0.880223402
ENSGALG00010017152	CYP4V2	2	0.302207971	0.22295134	1.355488474	0.175261978	0	0.26136262	0	1	CRA004775;PRJNA827465	Yang2025_Silkie_Wenchang	0.680258136
ENSGALG00010017154	PIGZ	2	-0.22635558	0.16133277	-1.403035355	0.160606296	0	0.149083116	0	1	PRJNA827465;Yang2025_Silkie_Wenchang	CRA004775	0.65630165
ENSGALG00010017154	PIGZ	2	-0.181210849	0.204286488	-0.887042752	0.375055884	0	0.612496268	0	0.5	CRA004775;Yang2025_Silkie_Wenchang	PRJNA827465	0.822916144
ENSGALG00010017154	PIGZ	2	-0.120181098	0.176214571	-0.682015664	0.495229046	0	0.266305414	0	0.5	CRA004775;PRJNA827465	Yang2025_Silkie_Wenchang	0.860619541
ENSGALG00010017162	BCL11A	2	-0.09786568	0.076766456	-1.27484952	0.202362509	0	0.498816751	0	1	PRJNA827465;Yang2025_Silkie_Wenchang	CRA004775	0.715289846
ENSGALG00010017162	BCL11A	2	0.096046193	0.19551428	0.491248993	0.623250351	0.031821677	1.452905703	31.17240866	0.5	CRA004775;Yang2025_Silkie_Wenchang	PRJNA827465	0.900348922
ENSGALG00010017162	BCL11A	2	0.051393439	0.266791101	0.192635505	0.847244436	0.097053327	2.493890076	59.90200171	0.5	CRA004775;PRJNA827465	Yang2025_Silkie_Wenchang	0.961252712
ENSGALG00010017163	CDV3	2	-0.039583334	0.055427174	-0.714150311	0.475134239	0.00228104	1.526184376	34.4771172	0.5	PRJNA827465;Yang2025_Silkie_Wenchang	CRA004775	0.854462864
ENSGALG00010017163	CDV3	2	-0.105222634	0.071929242	-1.462863099	0.143504843	0	0.056405174	0	1	CRA004775;Yang2025_Silkie_Wenchang	PRJNA827465	0.630564924
ENSGALG00010017163	CDV3	2	-0.002161164	0.048868092	-0.044224437	0.964725503	0	0.124370168	0	0.5	CRA004775;PRJNA827465	Yang2025_Silkie_Wenchang	0.990394578
ENSGALG00010017167	SMAP2	2	-0.193555253	0.23195213	-0.834462063	0.404020657	0.09726927	9.899324406	89.89830054	0.5	PRJNA827465;Yang2025_Silkie_Wenchang	CRA004775	0.833112102
ENSGALG00010017167	SMAP2	2	-0.472026394	0.122456218	-3.854654351	0.000115893	0	0.206720971	0	1	CRA004775;Yang2025_Silkie_Wenchang	PRJNA827465	0.008581101
ENSGALG00010017167	SMAP2	2	-0.222111237	0.295581354	-0.751438593	0.452388744	0.143949503	4.856820783	79.41039942	0.5	CRA004775;PRJNA827465	Yang2025_Silkie_Wenchang	0.848321192
ENSGALG00010017174	DHX57	2	-0.105756188	0.109507614	-0.965742788	0.334172903	0.017858987	3.704331682	73.00457719	1	PRJNA827465;Yang2025_Silkie_Wenchang	CRA004775	0.803094614
ENSGALG00010017174	DHX57	2	-0.247235902	0.093371695	-2.647867777	0.00810012	0	0.181416492	0	1	CRA004775;Yang2025_Silkie_Wenchang	PRJNA827465	0.139692415
ENSGALG00010017174	DHX57	2	-0.086497177	0.141353588	-0.611920632	0.540590271	0.022554981	1.644616104	39.19553644	1	CRA004775;PRJNA827465	Yang2025_Silkie_Wenchang	0.875579799
ENSGALG00010017183	NFYC	2	0.072609853	0.143884729	0.504639051	0.613812376	0.035283041	6.284385733	84.08754582	0.5	PRJNA827465;Yang2025_Silkie_Wenchang	CRA004775	0.898256044
ENSGALG00010017183	NFYC	2	0.114673066	0.139964099	0.819303425	0.412613319	0.022353765	2.254362986	55.64157119	0.5	CRA004775;Yang2025_Silkie_Wenchang	PRJNA827465	0.835704442
ENSGALG00010017183	NFYC	2	-0.055143124	0.041497778	-1.328821106	0.183906997	0	0.001492928	0	1	CRA004775;PRJNA827465	Yang2025_Silkie_Wenchang	0.692470323
ENSGALG00010017184	CARD11	2	0.099299415	0.124738809	0.796058704	0.425997915	0	0.410528145	0	1	PRJNA827465;Yang2025_Silkie_Wenchang	CRA004775	0.84125685
ENSGALG00010017184	CARD11	2	-0.125345143	0.419042284	-0.2991229	0.764846274	0.241848467	2.861087876	65.04825985	0.5	CRA004775;Yang2025_Silkie_Wenchang	PRJNA827465	0.940711384
ENSGALG00010017184	CARD11	2	-0.171282438	0.319519418	-0.536062686	0.591915228	0.121516714	2.004060037	50.10129529	0.5	CRA004775;PRJNA827465	Yang2025_Silkie_Wenchang	0.892776493
ENSGALG00010017185	NAA30	2	-0.259799554	0.198428162	-1.309287711	0.190436911	0.070967338	9.756199378	89.75010697	1	PRJNA827465;Yang2025_Silkie_Wenchang	CRA004775	0.699443221
ENSGALG00010017185	NAA30	2	-0.255618242	0.250959486	-1.018563775	0.308410109	0.096361097	3.965037734	74.77955906	0.5	CRA004775;Yang2025_Silkie_Wenchang	PRJNA827465	0.793457188
ENSGALG00010017185	NAA30	2	-0.067613693	0.053570017	-1.26215553	0.206892827	0	0.220495531	0	0.5	CRA004775;PRJNA827465	Yang2025_Silkie_Wenchang	0.71890273
ENSGALG00010017187	ZNF839	2	0.290074469	0.246427768	1.177117626	0.239148561	0.110308008	10.65645859	90.61601946	1	PRJNA827465;Yang2025_Silkie_Wenchang	CRA004775	0.751399463
ENSGALG00010017187	ZNF839	2	0.511630555	0.121009695	4.228012909	2.35764E-05	0	0.525557593	0	1	CRA004775;Yang2025_Silkie_Wenchang	PRJNA827465	0.002749627
ENSGALG00010017187	ZNF839	2	0.068683973	0.07280592	0.943384457	0.345484291	0	0.598886445	0	1	CRA004775;PRJNA827465	Yang2025_Silkie_Wenchang	0.807289734

ENSGALG00010017188	ENSGALG00010017188	2	0.146460995	0.109984707	1.331648726	0.182975645	0.011967465	1.941503898	48.49353633	1	PRJNA827465;Yang2025_Silkie_Wenchang	CRA004775	0.691392093
ENSGALG00010017188	ENSGALG00010017188	2	0.243885228	0.122699328	1.987665556	0.046848691	0	0.765604443	0	0.5	CRA004775;Yang2025_Silkie_Wenchang	PRJNA827465	0.37376485
ENSGALG00010017188	ENSGALG00010017188	2	0.042762083	0.094203318	0.453933935	0.649876378	0	0.178318848	0	0.5	CRA004775;PRJNA827465	Yang2025_Silkie_Wenchang	0.908292425
ENSGALG00010017190	STK26	2	-0.130785542	0.181737474	-0.719639927	0.471746722	0.062486398	18.49816287	94.59405776	0.5	PRJNA827465;Yang2025_Silkie_Wenchang	CRA004775	0.853998373
ENSGALG00010017190	STK26	2	0.083121764	0.407540554	0.203959491	0.838385155	0.319246366	25.18196746	96.02890441	0.5	CRA004775;Yang2025_Silkie_Wenchang	PRJNA827465	0.959197438
ENSGALG00010017190	STK26	2	0.255159908	0.224960788	1.134241709	0.256693173	0.0890848	7.832622719	87.23288436	1	CRA004775;PRJNA827465	Yang2025_Silkie_Wenchang	0.764677554
ENSGALG00010017198	TOPBP1	2	-0.151193361	0.122407485	-1.235164344	0.216769346	0.024932873	5.823293852	82.82758821	1	PRJNA827465;Yang2025_Silkie_Wenchang	CRA004775	0.730300498
ENSGALG00010017198	TOPBP1	2	-0.288464341	0.081787795	-3.526985194	0.00042032	0	0.081982495	0	1	CRA004775;Yang2025_Silkie_Wenchang	PRJNA827465	0.020727538
ENSGALG00010017198	TOPBP1	2	-0.066717629	0.096079867	-0.694397601	0.487432898	0.006977161	1.139347751	12.23048459	1	CRA004775;PRJNA827465	Yang2025_Silkie_Wenchang	0.85768885
ENSGALG00010017200	KCNQ4	2	-0.112393499	0.141593318	-0.793776856	0.427325349	0	0.069464036	0	1	PRJNA827465;Yang2025_Silkie_Wenchang	CRA004775	0.84125685
ENSGALG00010017200	KCNQ4	2	-0.23600702	0.226407813	-1.042397865	0.297227256	0	0.591651305	0	1	CRA004775;Yang2025_Silkie_Wenchang	PRJNA827465	0.788466926
ENSGALG00010017200	KCNQ4	2	-0.13059029	0.16680503	-0.782891796	0.433690655	0	0.854957474	0	1	CRA004775;PRJNA827465	Yang2025_Silkie_Wenchang	0.842762754
ENSGALG00010017201	AP5M1	2	-0.202479256	0.427836595	-0.47326306	0.636025513	0.338998032	13.16904351	92.40643408	0.5	PRJNA827465;Yang2025_Silkie_Wenchang	CRA004775	0.90443615
ENSGALG00010017201	AP5M1	2	-0.544548888	0.180978604	-3.008913081	0.002621841	0	0.942224278	0	1	CRA004775;Yang2025_Silkie_Wenchang	PRJNA827465	0.069251493
ENSGALG00010017201	AP5M1	2	0.098635279	0.194461879	0.507221671	0.611999291	0.038308424	1.571188564	36.35391555	0.5	CRA004775;PRJNA827465	Yang2025_Silkie_Wenchang	0.897777844
ENSGALG00010017207	ENSGALG00010017207	2	0.049509424	0.30915848	0.160142538	0.872768793	0.147657389	4.380517783	77.17164843	0.5	PRJNA827465;Yang2025_Silkie_Wenchang	CRA004775	0.969643328
ENSGALG00010017207	ENSGALG00010017207	2	0.33424028	0.211641481	1.579275847	0.114272799	0	0.244677803	0	1	CRA004775;Yang2025_Silkie_Wenchang	PRJNA827465	0.57019364
ENSGALG00010017207	ENSGALG00010017207	2	-0.230762258	0.187115934	-1.23325819	0.217479451	0	0.135835407	0	0.5	CRA004775;PRJNA827465	Yang2025_Silkie_Wenchang	0.731013651
ENSGALG00010017208	CINP	2	-0.055744253	0.082322372	-0.677145853	0.498313418	0	0.067007204	0	1	PRJNA827465;Yang2025_Silkie_Wenchang	CRA004775	0.861478809
ENSGALG00010017208	CINP	2	-0.185134809	0.149759524	-1.236213928	0.216379055	0	0.971936602	0	1	CRA004775;Yang2025_Silkie_Wenchang	PRJNA827465	0.730172415
ENSGALG00010017208	CINP	2	-0.139302946	0.16581323	-0.840119609	0.400841327	0.027801627	1.60237781	37.59274537	1	CRA004775;PRJNA827465	Yang2025_Silkie_Wenchang	0.832353691
ENSGALG00010017211	UBFD1	2	0.154677477	0.137128209	1.127977081	0.259329598	0.030748842	5.068178701	80.26904616	1	PRJNA827465;Yang2025_Silkie_Wenchang	CRA004775	0.766578761
ENSGALG00010017211	UBFD1	2	0.316100697	0.10701655	2.95375526	0.003139329	0	0.014663166	0	1	CRA004775;Yang2025_Silkie_Wenchang	PRJNA827465	0.077551875
ENSGALG00010017211	UBFD1	2	0.041532767	0.048323256	0.859477818	0.390076953	0	0.879930541	0	1	CRA004775;PRJNA827465	Yang2025_Silkie_Wenchang	0.829204867
ENSGALG00010017212	TINAGL1	2	0.096676258	0.180318682	0.536141108	0.591861031	0.050948836	4.566277305	78.10032258	0.5	PRJNA827465;Yang2025_Silkie_Wenchang	CRA004775	0.892776493
ENSGALG00010017212	TINAGL1	2	0.297802518	0.126972129	2.345416421	0.019005839	0	0.059108218	0	1	CRA004775;Yang2025_Silkie_Wenchang	PRJNA827465	0.230995614
ENSGALG00010017212	TINAGL1	2	-0.001711741	0.167933085	-0.010192993	0.991867309	0.022771776	1.269273062	21.21474643	0.5	CRA004775;PRJNA827465	Yang2025_Silkie_Wenchang	0.997575757
ENSGALG00010017219	EARS2	2	-0.013757734	0.127002945	-0.108326101	0.913737019	0.019689246	2.540331731	60.63506243	0.5	PRJNA827465;Yang2025_Silkie_Wenchang	CRA004775	0.979364626
ENSGALG00010017219	EARS2	2	-0.253785499	0.433515153	-0.585413214	0.558269906	0.31522194	5.647870637	82.29421203	0.5	CRA004775;Yang2025_Silkie_Wenchang	PRJNA827465	0.880678789
ENSGALG00010017219	EARS2	2	-0.352377396	0.297256717	-1.185431232	0.235846989	0.127266724	2.952074835	66.12551999	1	CRA004775;PRJNA827465	Yang2025_Silkie_Wenchang	0.748669015
ENSGALG00010017221	SPPL2A	2	-0.207333241	0.181213746	-1.14413639	0.252567078	0.060676204	12.98363452	92.29799639	1	PRJNA827465;Yang2025_Silkie_Wenchang	CRA004775	0.762655223
ENSGALG00010017221	SPPL2A	2	-0.12634836	0.274623767	-0.460078022	0.645460219	0.14008596	13.91862772	92.81538367	0.5	CRA004775;Yang2025_Silkie_Wenchang	PRJNA827465	0.906966468
ENSGALG00010017221	SPPL2A	2	0.030838219	0.088134881	0.349898004	0.726415245	0.008635465	1.979650478	49.48603246	0.5	CRA004775;PRJNA827465	Yang2025_Silkie_Wenchang	0.93109166
ENSGALG00010017223	NOC2L	2	0.092762583	0.11239811	0.825303769	0.409199153	0.013753562	1.848980553	45.91614289	1	PRJNA827465;Yang2025_Silkie_Wenchang	CRA004775	0.834316328
ENSGALG00010017223	NOC2L	2	0.013903132	0.253109181	0.054929386	0.95619472	0.098340024	4.298587227	76.73654279	0.5	CRA004775;Yang2025_Silkie_Wenchang	PRJNA827465	0.98851389
ENSGALG00010017223	NOC2L	2	-0.059906142	0.119944679	-0.499448099	0.617463742	0.016228813	1.903405146	47.46257767	0.5	CRA004775;PRJNA827465	Yang2025_Silkie_Wenchang	0.899121942
ENSGALG00010017226	PEF1	2	0.21082004	0.075202641	2.803359538	0.005057326	0	0.126086273	0	1	PRJNA827465;Yang2025_Silkie_Wenchang	CRA004775	0.106040882
ENSGALG00010017226	PEF1	2	-0.076526957	0.217776923	-0.35140067	0.72528778	0.059997848	2.71770717	63.20427709	0.5	CRA004775;Yang2025_Silkie_Wenchang	PRJNA827465	0.930868485
ENSGALG00010017226	PEF1	2	-0.008024832	0.25395929	-0.031598891	0.974791928	0.110777378	6.688352543	85.04863502	0.5	CRA004775;PRJNA827465	Yang2025_Silkie_Wenchang	0.992572967
ENSGALG00010017228	RAP2C	2	-0.171294542	0.191211492	-0.89583811	0.370339232	0.067386092	12.36142145	91.91031546	0.5	PRJNA827465;Yang2025_Silkie_Wenchang	CRA004775	0.821109108
ENSGALG00010017228	RAP2C	2	-0.19506203	0.213448709	-0.913859027	0.360790925	0.06738062	3.507839056	71.49242072	0.5	CRA004775;Yang2025_Silkie_Wenchang	PRJNA827465	0.815095283
ENSGALG00010017228	RAP2C	2	0.012732073	0.045416915	0.280337692	0.779218435	0	0.056814037	0	1	CRA004775;PRJNA827465	Yang2025_Silkie_Wenchang	0.944727706
ENSGALG00010017240	MELTF	2	0.68716272	0.159926294	4.296746343	1.73323E-05	0	0.711079593	0	1	PRJNA827465;Yang2025_Silkie_Wenchang	CRA004775	0.00219746
ENSGALG00010017240	MELTF	2	0.651232846	0.203121818	3.206119623	0.00134538	0.02014987	1.238088765	19.23034697	1	CRA004775;Yang2025_Silkie_Wenchang	PRJNA827465	0.044788793
ENSGALG00010017240	MELTF	2	0.41523693	0.2288341	1.814576282	0.06958905	0	0.12019075	0	1	CRA004775;PRJNA827465	Yang2025_Silkie_Wenchang	0.454857548
ENSGALG00010017246	GGA2	2	0.206033742	0.165200183	1.247176231	0.212332886	0.044637186	5.341707594	81.27939461	1	PRJNA827465;Yang2025_Silkie_Wenchang	CRA004775	0.724986485
ENSGALG00010017246	GGA2	2	-0.006774975	0.416477615	-0.016267321	0.987021128	0.31213208	9.644841579	89.63176334	0.5	CRA004775;Yang2025_Silkie_Wenchang	PRJNA827465	0.99640129
ENSGALG00010017246	GGA2	2	-0.146952142	0.246275927	-0.596697141	0.550709602	0.094762375	3.978966012	74.86784262	0.5	CRA004775;PRJNA827465	Yang2025_Silkie_Wenchang	0.878879109
ENSGALG00010017248	TECPR2	2	-0.212430468	0.132936186	-1.597988283	0.110045585	0.03080244	7.402124955	86.49036586	1	PRJNA827465;Yang2025_Silkie_Wenchang	CRA004775	0.561923037
ENSGALG00010017248	TECPR2	2	-0.214836539	0.196225376	-1.094845855	0.273584173	0.050942577	2.491029145	59.85594942	0.5	CRA004775;Yang2025_Silkie_Wenchang	PRJNA827465	0.774328808
ENSGALG00010017248	TECPR2	2	-0.086584955	0.040805654	-2.121886205	0.033847295	0	0.341605461	0	0.5	CRA004775;PRJNA827465	Yang2025_Silkie_Wenchang	0.316060879
ENSGALG00010017250	COL16A1	2	0.234162363	0.245971153	0.95199116	0.341101465	0.094184036	4.255841765	76.50288579	1	PRJNA827465;Yang2025_Silkie_Wenchang	CRA004775	0.805646109
ENSGALG00010017250	COL16A1	2	0.31773426	0.273317297	1.162510617	0.245028114	0.068473151	1.702069527	41.24799346	0.5	CRA004775;Yang2025_Silkie_Wenchang	PRJNA827465	0.756475101
ENSGALG00010017250	COL16A1	2	0.012227805	0.108667868	0.112524569	0.91040749	0	0.039293894	0	0.5	CRA004775;PRJNA827465	Yang2025_Silkie_Wenchang	0.97880407
ENSGALG00010017252	EXOC5	2	-0.127692306	0.167964585	-0.760233512	0.447115017	0.051465549	11.35107838	91.19026434	0.5	PRJNA827465;Yang2025_Silkie_Wenchang	CRA004775	0.84650962
ENSGALG00010017252	EXOC5	2	-0.28751396	0.071222704	-4.036830182	5.41783E-05	0	0.150523574	0	1	CRA004775;Yang2025_Silkie_Wenchang	PRJNA827465	0.005118673
ENSGALG00010017252	EXOC5	2	-0.021483105	0.107428953	-0.199975003	0.841500131	0.010940514	1.512199472	33.87115795	0.5	CRA004775;PRJNA827465	Yang2025_Silkie_Wenchang	0.959903632
ENSGALG00010017256	CARHSP1	2	-0.109434842	0.224417866	-0.487638726	0.62580576	0.088486727	7.698560252	87.01055824	0.5	PRJNA827465;Yang2025_Silkie_Wenchang	CRA004775	0.901504244
ENSGALG00010017256	CARHSP1	2	-0.03338427	0.356066441	-0.093758542	0.925300965	0.208337793	5.380631988	81.4148226	0.5	CRA004775;Yang2025_Silkie_Wenchang	PRJNA827465	0.982594411
ENSGALG00010017256	CARHSP1	2	0.105560621	0.056000138	1.885006436	0.059428969	0	0.928972326	0	1	CRA004775;PRJNA827465	Yang2025_Silkie_Wenchang	0.422451938
ENSGALG00010017263	COG7	2	-0.125568998	0.130384949	-0.963063597	0.335515609	0.025945405	3.79136071	73.62424531	1	PRJNA827465;Yang2025_Silkie_Wenchang	CRA004775	0.803316425
ENSGALG00010017263	COG7	2	-0.333899663	0.103413288	-3.228788781	0.001243157	0	0.493158904	0	1	CRA004775;Yang2025_Silkie_Wenchang	PRJNA827465	0.042969436
ENSGALG00010017263	COG7	2	-0.192186384	0.20706106	-0.928162852	0.353323101	0.070625742	4.998236805	79.99294473	1	CRA004775;PRJNA827465	Yang2025_Silkie_Wenchang	0.811157428
ENSGALG00010017264	PIAS1	2	0.161931833	0.215285016	0.752174195	0.45194631	0.073030233	4.046476207	75.28714	0.5	PRJNA827465;Yang2025_Silkie_Wenchang	CRA004775	0.848151928
ENSGALG00010017264	PIAS1	2	0.264202048	0.195000576	1.354878298	0.175456335	0.020629277	1.357747368	26.34859594	1	CRA004775;Yang2025_Silkie_Wenchang	PRJNA827465	0.680258136
ENSGALG00010017264	PIAS1	2	-0.009769077	0.058005112	-0.168417512	0.866254833	0	0.026896439	0	0.5	CRA004775;PRJNA827465	Yang2025_Silkie_Wenchang	0.967365071
ENSGALG00010017266	PMM2	2	-0.317608816	0.278229238	-1.141536445	0.253646755	0.143898725	13.71173086	92.70697471	1	PRJNA827465;Yang2025_Silkie_Wenchang	CRA004775	0.763418915
ENSGALG00010017266	PMM2	2	-0.44530011	0.226049172	-1.969925863	0.048846868	0.059151915	2.105078257	52.49582781	1	CRA004775;Yang2025_Silkie_Wenchang	PRJNA827465	0.381979396
ENSGALG00010017266	PMM2	2	-0.056234198	0.05786911	-0.971748113	0.331175875	0	0.074394788	0	1	CRA004775;PRJNA827465	Yang2025_Silkie_Wenchang	0.801725552
ENSGALG00010017272	NRXN1	2	-0.092918621	0.155148502	-0.598901179	0.549238785	0	0.068020605	0	1	PRJNA827465;Yang2025_Silkie_Wenchang	CRA004775	0.878545114
ENSGALG00010017272	NRXN1	2	0.429394848	0.504598857	0.850962785	0.394790026	0.403384696	4.703053861	78.73721991	0.5	CRA004775;Yang2025_Silkie_Wenchang	PRJNA827465	0.830643015
ENSGALG00010017272	NRXN1	2	0.375221468	0.546235193	0.686922909	0.49213131	0.509159164	6.488275193	84.58758345	0.5	CRA004775;PRJNA827465	Yang2025_Silkie_Wenchang	0.859158569
ENSGALG00010017278	CCR5	2	-0.230450788	0.442098266	-0.521265984	0.602181494	0.36517986	15.15636166	93.40211047	0.5	PRJNA827465;Yang2025_Silkie_Wenchang	CRA004775	0.895349419
ENSGALG00010017278	CCR5	2	-0.71658485	0.169425493	-4.229498399	2.34213E-05	0	0.579209959	0	1	CRA004775;Yang2025_Silkie_Wenchang	PRJNA827465	0.002741334
ENSGALG00010017278	CCR5	2	-0.349714291	0.668830872	-0.522874027	0.60106192	0.735296324	4.856069743	79.40721504	0.5	CRA004775;PRJNA827465	Yang2025_Silkie_Wenchang	0.895230519
ENSGALG00010017280	SOS1	2	0.03889526	0.176442939	0.220441011	0.825527708	0.041801689	2.273458656	56.01415503	0.5	PRJNA827465;Yang2025_Silkie_Wenchang	CRA004775	0.956386623
ENSGALG00010017280	SOS1	2	0.239973418	0.156451369	1.533853106	0.125065795	0	0.129123774	0	1	CRA004775;Yang2025_Silkie_Wenchang	PRJNA827465	0.593686207
ENSGALG00010017280	SOS1	2	-0.009390617	0.116452091	-0.080639316	0.935728796	0.015500363	1.731144211	42.23473737	0.5	CRA004775;PRJNA827465	Yang2025_Silkie_Wenchang	0.984800337
ENSGALG00010017283	INF2	2	0.091295963	0.076449652	1.194197231	0.232400826	0	0.135007816	0	1	PRJNA827465;Yang2025_Silkie_Wenchang	CRA004775	0.745089295
ENSGALG00010017283	INF2	2	0.237563769	0.187304544	1.268328914	0.204603516	0	0.93037862	0	1	CRA004775;Yang2025_Silkie_Wenchang	PRJNA827465	0.71730346
ENSGALG00010017283	INF2	2	0.180415786	0.219993568	0.820095732	0.412161535	0.051693659	1.439191748	30.51655545	1	CRA004775;PRJNA827465	Yang2025_Silkie_Wenchang	0.835532508
ENSGALG00010017284	ENSGALG00010017284	2	-0.159391151	0.225598319	-0.673494164	0.500632961	0.063307771	2.565247829	61.0174117	0.5	PRJNA827465;Yang2025_Silkie_Wenchang	CRA004775	0.862400704
ENSGALG00010017284	ENSGALG00010017284	2	0.131956952	0.57449891	0.229696517	0.818332264	0.570170956	7.196091664	86.10356779	0.5	CRA004775;Yang2025_Silkie_Wenchang	PRJNA827465	0.955256039
ENSGALG00010017284	ENSGALG00010017284	2	0.317954303	0.339641559	0.936146639	0.349197693	0.163972118	3.109083996	67.8361858	1	CRA004775;PRJNA827465	Yang2025_Silkie_Wenchang	0.809731578
ENSGALG00010017289	ADGRB2	2	0.046407868	0.199663002	0.232430982	0.816203287	0.022244474	1.355641358	26.23417737	0.5	PRJNA827465;Yang2025_Silkie_Wenchang	CRA004775	0.95448249
ENSGALG00010017289	ADGRB2	2	0.165142641	0.212899338	0.775684145	0.437935503	0	0.498162057	0	1	CRA004775;Yang2025_Silkie_Wenchang	PRJNA827465	0.843941117
ENSGALG00010017289	ADGRB2	2	-0.076605028	0.171605028	-0.446384507	0.655319522	0	0.085757767	0	0.5	CRA004775;PRJNA827465	Yang2025_Silkie_Wenchang	0.910411938
ENSGALG00010017290	DACT1	2	0.031921288	0.128219971	0.248957226	0.803393868	0.026745371	5.1760225	80.68014581	0.5	PRJNA827465;Yang2025_Silkie_Wenchang	CRA004775	0.951020698
ENSGALG00010017290	DACT1	2	-0.101225441	0.093138772	-1.08682387	0.277114656	0	0.072282943	0	1	CRA004775;Yang2025_Silkie_Wenchang	PRJNA827465	0.776188179
ENSGALG00010017290	DACT1	2	0.144301555	0.057599498	2.505257151	0.012236239	0	0.227402018	0	0.5	CRA004775;PRJNA827465	Yang2025_Silkie_Wenchang	0.178627415
ENSGALG00010017296	SCNN1G	2	-0.076544814	0.080637504	-0.949293824	0.342495601	0	0.363688106	0	1	PRJNA827465;Yang2025_Silkie_Wenchang	CRA004775	0.806005903
ENSGALG00010017296	SCNN1G	2	-0.308156834	0.438196008	-0.703239711	0.481906375	0.260777085	2.37283324	57.85628828	1	CRA004775;Yang2025_Silkie_Wenchang	PRJNA827465	0.85691916
ENSGALG00010017296	SCNN1G	2	-0.333102887	0.370845101	-0.898226472	0.369064823	0.174113225	1.930961437	48.21232672	1	CRA004775;PRJNA827465	Yang2025_Silkie_Wenchang	0.820577053
ENSGALG00010017299	USP31	2	-0.075926569	0.091070341	-0.833713458	0.404442473	0.009466081	2.280712797	56.15405844	0.5	PRJNA827465;Yang2025_Silkie_Wenchang	CRA004775	0.833112102
ENSGALG00010017299	USP31	2	-0.184053809	0.095359285	-1.930108946	0.05359334	0	0.051486591	0	1	CRA004775;Yang2025_Silkie_Wenchang	PRJNA827465	0.401444454
ENSGALG00010017299	USP31	2	-0.003059101	0.071589312	-0.042731254	0.965915765	0	0.400993656	0	0.5	CRA004775;PRJNA827465	Yang2025_Silkie_Wenchang	0.990745532
ENSGALG00010017300	TRPM7	2	0.158944671	0.078091818	2.035356259	0.041815039	0.005079303	1.564156746	36.06778842	1	PRJNA827465;Yang2025_Silkie_Wenchang	CRA004775	0.354116889
ENSGALG00010017300	TRPM7	2	0.267976457	0.10832265	2.473872592	0.013365728	0	0.012891254	0	1	CRA004775;Yang2025_Silkie_Wenchang	PRJNA827465	0.188936172
ENSGALG00010017300	TRPM7	2	0.112927577	0.060374166	1.870461894	0.061419704	0	0.252360297	0	1	CRA004775;PRJNA827465	Yang2025_Silkie_Wenchang	0.427938152
ENSGALG00010017304	ANKRD9	2	0.013082527	0.186333959	0.070210104	0.944026432	0	0.015209445	0	0.5	PRJNA827465;Yang2025_Silkie_Wenchang	CRA004775	0.985827325
ENSGALG00010017304	ANKRD9	2	-0.055311962	0.264391009	-0.20920286	0.834283988	0	0.420415231	0	0.5	CRA004775;Yang2025_Silkie_Wenchang	PRJNA827465	0.957399093
ENSGALG00010017304	ANKRD9	2	-0.062022637	0.215386551	-0.287959656	0.773377621	0	0.361912605	0	1	CRA004775;PRJNA827465	Yang2025_Silkie_Wenchang	0.943245453
ENSGALG00010017305	HS3ST2	2	0.099397341	0.472939389	0.210169302	0.833535538	0.260593682	2.336526471	57.20142645	0.5	PRJNA827465;Yang2025_Silkie_Wenchang	CRA004775	0.957968425
ENSGALG00010017305	HS3ST2	2	0.556524845	0.288530354	1.92882599	0.053752468	0	0.038337684	0	1	CRA004775;Yang2025_Silkie_Wenchang	PRJNA827465	0.401964436
ENSGALG00010017305	HS3ST2	2	0.110197678	0.535277438	0.205870209	0.836892297	0.335254471	2.406600107	58.44760427	0.5	CRA004775;PRJNA827465	Yang2025_Silkie_Wenchang	0.958514336
ENSGALG00010017307	TMEM186	2	0.243067302	0.094563707	2.570407926	0.010157882	0.002243878	1.141576656	12.40200621	1	PRJNA827465;Yang2025_Silkie_Wenchang	CRA004775	0.160142501
ENSGALG00010017307	TMEM186	2	0.159689162	0.274895995	0.58090756	0.561302757	0.09338365	2.164716229	53.80456864	0.5	CRA004775;Yang2025_Silkie_Wenchang	PRJNA827465	0.881518511
ENSGALG00010017307	TMEM186	2	0.119932677	0.117426721	1.021340596	0.307093099	0.00134252	1.017159886	1.687039165	0.5	CRA004775;PRJNA827465	Yang2025_Silkie_Wenchang	0.79266003
ENSGALG00010017310	SAMD11	2	0.363096839	0.16843052	2.155766104	0.031101918	0.044010812	4.249321063	76.46682582	1	PRJNA827465;Yang2025_Silkie_Wenchang	CRA004775	0.301529028
ENSGALG00010017310	SAMD11	2	0.15729058	0.461068293	0.341162166	0.732981503	0.348724459	5.032733236	80.13008135	0.5	CRA004775;Yang2025_Silkie_Wenchang	PRJNA827465	0.932985777
ENSGALG00010017310	SAMD11	2	0.039686523	0.27022288	0.146865888	0.883237877	0.096609128	2.213484165	54.82235583	0.5	CRA004775;PRJNA827465	Yang2025_Silkie_Wenchang	0.972001076
ENSGALG00010017312	PTP4A2	2	0.086722653	0.258451427	0.335547201	0.737212345	0.119156957	8.744189003	88.56383365	0.5	PRJNA827465;Yang2025_Silkie_Wenchang	CRA004775	0.933895493
ENSGALG00010017312	PTP4A2	2	0.315297313	0.149974576	2.102338423	0.035523641	0	0.658923071	0	1	CRA004775;Yang2025_Silkie_Wenchang	PRJNA827465	0.325417923
ENSGALG00010017312	PTP4A2	2	-0.145742936	0.063021963	-2.312573732	0.020746087	0	0.230784876	0	0.5	CRA004775;PRJNA827465	Yang2025_Silkie_Wenchang	0.243543447
ENSGALG00010017313	MAP4K3	2	0.037986779	0.042771705	0.888128695	0.374471529	0	0.025899817	0	1	PRJNA827465;Yang2025_Silkie_Wenchang	CRA004775	0.822805106
ENSGALG00010017313	MAP4K3	2	0.233035603	0.246875612	0.943939343	0.345200646	0.093403052	3.97163298	74.82143982	1	CRA004775;Yang2025_Silkie_Wenchang	PRJNA827465	0.803702174
ENSGALG00010017313	MAP4K3	2	0.230617161	0.234817842	0.982110898	0.326045217	0.088994604	4.375019302	77.14295799	1	CRA004775;PRJNA827465	Yang2025_Silkie_Wenchang	0.800023788
ENSGALG00010017314	CALML4	2	0.099784406	0.073628974	1.355232872	0.175343374	0	0.498854162	0	1	PRJNA827465;Yang2025_Silkie_Wenchang	CRA004775	0.680258136
ENSGALG00010017314	CALML4	2	0.085973406	0.181847468	0.472777584	0.636371658	0.022872959	1.323588179	24.4477991	0.5	CRA004775;Yang2025_Silkie_Wenchang	PRJNA827465	0.904566695
ENSGALG00010017314	CALML4	2	0.040098919	0.083686198	0.596262227	0.551000061	0	0.773086526	0	0.5	CRA004775;PRJNA827465	Yang2025_Silkie_Wenchang	0.878879109
ENSGALG00010017318	ADSS1	2	-0.252361018	0.126581883	-1.993658275	0.046189412	0.017765279	2.238746243	55.33214168	1	PRJNA827465;Yang2025_Silkie_Wenchang	CRA004775	0.370491759
ENSGALG00010017318	ADSS1	2	0.17822036	0.397773813	0.448044478	0.654121097	0.231512808	3.070249497	67.4293571	0.5	CRA004775;Yang2025_Silkie_Wenchang	PRJNA827465	0.909981418
ENSGALG00010017318	ADSS1	2	0.071384826	0.533479247	0.145058667	0.884665973	0.475001077	5.301269205	81.13659274	0.5	CRA004775;PRJNA827465	Yang2025_Silkie_Wenchang	0.972962774
ENSGALG00010017322	KHDRBS1	2	0.074923506	0.07022509	1.066905082	0.2860147	0.007055358	3.338018155	70.04210422	1	PRJNA827465;Yang2025_Silkie_Wenchang	CRA004775	0.781835842
ENSGALG00010017322	KHDRBS1	2	0.052280573	0.145345832	0.359697782	0.719073151	0.027439702	2.388174975	58.12702124	0.5	CRA004775;Yang2025_Silkie_Wenchang	PRJNA827465	0.928449671
ENSGALG00010017322	KHDRBS1	2	0.007141338	0.038689128	0.184582556	0.853556468	0	0.746314022	0	0.5	CRA004775;PRJNA827465	Yang2025_Silkie_Wenchang	0.962964214
ENSGALG00010017323	ENSGALG00010017323	2	0.155992477	0.132356773	1.178575699	0.238561173	0.025941571	3.438602427	70.91841755	1	PRJNA827465;Yang2025_Silkie_Wenchang	CRA004775	0.738652917
ENSGALG00010017323	ENSGALG00010017323	2	0.139331795	0.246543565	0.565140666	0.57197809	0.074916407	2.264797638	55.84594477	0.5	CRA004775;Yang2025_Silkie_Wenchang	PRJNA827465	0.885496034
ENSGALG00010017323	ENSGALG00010017323	2	0.041048981	0.050051187	0.745596978	0.455919009	0	0.592170235	0	0.5	CRA004775;PRJNA827465	Yang2025_Silkie_Wenchang	0.84907427
ENSGALG00010017326	STON1	2	0.041164533	0.094021956	0.437818302	0.661518803	0.003798024	1.26393562	20.81940001	0.5	PRJNA827465;Yang2025_Silkie_Wenchang	CRA004775	0.912364181
ENSGALG00010017326	STON1	2	-0.047019797	0.125658859	-0.374186085	0.708265876	0	0.312411408	0	0.5	CRA004775;Yang2025_Silkie_Wenchang	PRJNA827465	0.925061251
ENSGALG00010017326	STON1	2	0.122538812	0.102262912	1.198272274	0.230811037	0	0.008881744	0	1	CRA004775;PRJNA827465	Yang2025_Silkie_Wenchang	0.743374303
ENSGALG00010017327	CLN6	2	0.012984318	0.0791061	0.164138011	0.869623499	0	0.135823247	0	0.5	PRJNA827465;Yang2025_Silkie_Wenchang	CRA004775	0.968586462
ENSGALG00010017327	CLN6	2	0.023671385	0.174186002	0.135897172	0.891902571	0	0.578745825	0	0.5	CRA004775;Yang2025_Silkie_Wenchang	PRJNA827465	0.97401927
ENSGALG00010017327	CLN6	2	-0.010762295	0.085885637	-0.125309606	0.900278448	0	0.418849712	0	1	CRA004775;PRJNA827465	Yang2025_Silkie_Wenchang	0.976475201
ENSGALG00010017332	FGFR1	2	-0.092664575	0.083734828	-1.106643164	0.268448238	0.008933376	2.580873721	61.25343167	1	PRJNA827465;Yang2025_Silkie_Wenchang	CRA004775	0.771398294
ENSGALG00010017332	FGFR1	2	-0.08796175	0.158672363	-0.554360875	0.579331897	0.028081638	1.918759732	47.8830005	0.5	CRA004775;Yang2025_Silkie_Wenchang	PRJNA827465	0.887888423
ENSGALG00010017332	FGFR1	2	-0.015377164	0.051472591	-0.298744711	0.765134839	0	0.532025972	0	0.5	CRA004775;PRJNA827465	Yang2025_Silkie_Wenchang	0.940779391
ENSGALG00010017334	KDM8	2	-0.064765875	0.115831254	-0.559139898	0.576066248	0	0.170964141	0	0.5	PRJNA827465;Yang2025_Silkie_Wenchang	CRA004775	0.887008933
ENSGALG00010017334	KDM8	2	-0.085902754	0.172887745	-0.496870115	0.619280647	0	0.515394775	0	0.5	CRA004775;Yang2025_Silkie_Wenchang	PRJNA827465	0.899168321
ENSGALG00010017334	KDM8	2	-0.12276097	0.122067701	-1.005679382	0.314569824	0	0.227415924	0	1	CRA004775;PRJNA827465	Yang2025_Silkie_Wenchang	0.796531448
ENSGALG00010017335	THUMPD2	2	-0.035540534	0.071526283	-0.496887748	0.619268212	0	0.332517088	0	0.5	PRJNA827465;Yang2025_Silkie_Wenchang	CRA004775	0.899168321
ENSGALG00010017335	THUMPD2	2	-0.011639356	0.11035652	-0.105470492	0.916002483	0	0.667658503	0	0.5	CRA004775;Yang2025_Silkie_Wenchang	PRJNA827465	0.980383574
ENSGALG00010017335	THUMPD2	2	-0.081276725	0.08826557	-0.920820257	0.357144277	0	0.367400445	0	1	CRA004775;PRJNA827465	Yang2025_Silkie_Wenchang	0.813260191
ENSGALG00010017337	ABAT	2	0.103759641	0.070740639	1.466761433	0.142440965	0	0.274855448	0	1	PRJNA827465;Yang2025_Silkie_Wenchang	CRA004775	0.628816941
ENSGALG00010017337	ABAT	2	0.235850244	0.179122324	1.316699328	0.187939429	0	0.311466531	0	1	CRA004775;Yang2025_Silkie_Wenchang	PRJNA827465	0.697242154
ENSGALG00010017337	ABAT	2	0.098323339	0.075222836	1.307094295	0.191180689	0	0.649842695	0	1	CRA004775;PRJNA827465	Yang2025_Silkie_Wenchang	0.700473068
ENSGALG00010017338	MBNL3	2	0.089647844	0.243941856	0.367496771	0.7132485	0.098451672	5.193896814	80.74663329	0.5	PRJNA827465;Yang2025_Silkie_Wenchang	CRA004775	0.926216079
ENSGALG00010017338	MBNL3	2	0.296348439	0.174106637	1.702108801	0.088734974	0	0.511582864	0	1	CRA004775;Yang2025_Silkie_Wenchang	PRJNA827465	0.511667361
ENSGALG00010017338	MBNL3	2	-0.110064449	0.066644921	-1.651505434	0.098635412	0	0.424463935	0	0.5	CRA004775;PRJNA827465	Yang2025_Silkie_Wenchang	0.536014139
ENSGALG00010017340	SIVA1	2	0.028004305	0.102456053	0.27332992	0.784599618	0	0.446284291	0	0.5	PRJNA827465;Yang2025_Silkie_Wenchang	CRA004775	0.946117405
ENSGALG00010017340	SIVA1	2	-0.238464493	0.207560855	-1.148889529	0.250601536	0	0.009413835	0	1	CRA004775;Yang2025_Silkie_Wenchang	PRJNA827465	0.760152524
ENSGALG00010017340	SIVA1	2	-0.016110668	0.122809402	-0.131184318	0.895629501	0.007790738	1.209469607	17.31912946	0.5	CRA004775;PRJNA827465	Yang2025_Silkie_Wenchang	0.974927462
ENSGALG00010017341	TA3	2	0.009591555	0.053314284	0.179905911	0.857226434	0	0.001017462	0	1	PRJNA827465;Yang2025_Silkie_Wenchang	CRA004775	0.963910696
ENSGALG00010017341	TA3	2	-0.027364651	0.112722364	-0.242761509	0.808190155	0	0.798967382	0	0.5	CRA004775;Yang2025_Silkie_Wenchang	PRJNA827465	0.952496413
ENSGALG00010017341	TA3	2	-0.001869479	0.058227326	-0.032106555	0.974387076	0	0.867085591	0	0.5	CRA004775;PRJNA827465	Yang2025_Silkie_Wenchang	0.992492038
ENSGALG00010017343	DLG1	2	0.036240424	0.130834285	0.276994859	0.781784054	0.02866724	5.688480263	82.42061229	0.5	PRJNA827465;Yang2025_Silkie_Wenchang	CRA004775	0.945265125
ENSGALG00010017343	DLG1	2	0.208921931	0.092573354	2.256825782	0.024018963	0	0.309560844	0	1	CRA004775;Yang2025_Silkie_Wenchang	PRJNA827465	0.264653015
ENSGALG00010017343	DLG1	2	0.062425653	0.190259654	0.328107674	0.742830252	0.054474713	3.228214934	69.02312825	0.5	CRA004775;PRJNA827465	Yang2025_Silkie_Wenchang	0.935661872
ENSGALG00010017344	RERE	2	0.085097294	0.38078338	0.223479538	0.823162307	0.244702167	5.657079581	82.3230346	0.5	PRJNA827465;Yang2025_Silkie_Wenchang	CRA004775	0.955880995
ENSGALG00010017344	RERE	2	0.413868146	0.220798744	1.874413498	0.060873455	0	0.272404554	0	1	CRA004775;Yang2025_Silkie_Wenchang	PRJNA827465	0.427127227
ENSGALG00010017344	RERE	2	-0.053147949	0.257132712	-0.206694624	0.836248356	0.094995355	2.859385422	65.02744988	0.5	CRA004775;PRJNA827465	Yang2025_Silkie_Wenchang	0.958399882
ENSGALG00010017346	GM2A	2	0.178117644	0.135943359	1.310234247	0.190116604	0	0.769592657	0	1	PRJNA827465;Yang2025_Silkie_Wenchang	CRA004775	0.699403589
ENSGALG00010017346	GM2A	2	0.146184902	0.242637063	0.602483811	0.546852136	0.043856376	1.551420381	35.54293782	0.5	CRA004775;Yang2025_Silkie_Wenchang	PRJNA827465	0.878275987
ENSGALG00010017346	GM2A	2	0.039045774	0.149513089	0.261152884	0.793974611	0	0.452072235	0	0.5	CRA004775;PRJNA827465	Yang2025_Silkie_Wenchang	0.948872932
ENSGALG00010017347	TMEM39B	2	-0.251245199	0.198071005	-1.268460263	0.204633633	0.070182761	9.145377884	89.0655147	1	PRJNA827465;Yang2025_Silkie_Wenchang	CRA004775	0.71730346
ENSGALG00010017347	TMEM39B	2	-0.486357597	0.104922343	-4.635405411	3.56239E-06	0	0.204391285	0	1	CRA004775;Yang2025_Silkie_Wenchang	PRJNA827465	0.000651221
ENSGALG00010017347	TMEM39B	2	-0.27340541	0.252921998	-1.080987068	0.279702866	0.103635131	4.533516567	77.94206804	1	CRA004775;PRJNA827465	Yang2025_Silkie_Wenchang	0.777836393
ENSGALG00010017349	ENSGALG00010017349	2	-0.067444706	0.094847628	-0.711084788	0.477031696	0.008972108	1.95940676	48.96414463	0.5	PRJNA827465;Yang2025_Silkie_Wenchang	CRA004775	0.854986476
ENSGALG00010017349	ENSGALG00010017349	2	-0.19588973	0.103694376	-1.889106592	0.058877547	0	0.370108348	0	1	CRA004775;Yang2025_Silkie_Wenchang	PRJNA827465	0.42013793
ENSGALG00010017349	ENSGALG00010017349	2	-0.053920242	0.154071106	-0.349969851	0.726361324	0.021656122	1.343071242	25.54378584	0.5	CRA004775;PRJNA827465	Yang2025_Silkie_Wenchang	0.931090933
ENSGALG00010017351	CCR2	2	-0.392068691	0.336505361	-1.165118707	0.243970946	0.204854651	10.28438881	90.27652476	1	PRJNA827465;Yang2025_Silkie_Wenchang	CRA004775	0.755518239
ENSGALG00010017351	CCR2	2	-0.765017727	0.166286404	-4.600602993	4.2127E-06	0	0.099709876	0	1	CRA004775;Yang2025_Silkie_Wenchang	PRJNA827465	0.000712352
ENSGALG00010017351	CCR2	2	-0.370058046	0.396884711	-0.932406907	0.351126276	0.235451088	3.242618548	69.16072658	1	CRA004775;PRJNA827465	Yang2025_Silkie_Wenchang	0.810161472
ENSGALG00010017352	GPCPD1	2	0.139344984	0.061363781	2.270801776	0.02315898	0	0.274743429	0	1	PRJNA827465;Yang2025_Silkie_Wenchang	CRA004775	0.259577892
ENSGALG00010017352	GPCPD1	2	0.189857721	0.103170603	1.840230787	0.065734362	0	0.055042821	0	1	CRA004775;Yang2025_Silkie_Wenchang	PRJNA827465	0.441784819
ENSGALG00010017352	GPCPD1	2	0.120900594	0.074427623	1.624404871	0.104289428	0	0.14377617	0	1	CRA004775;PRJNA827465	Yang2025_Silkie_Wenchang	0.548786638
ENSGALG00010017354	FEM1B	2	-0.223849165	0.16644374	-1.344893863	0.178659512	0.051272639	13.12292531	92.37974783	1	PRJNA827465;Yang2025_Silkie_Wenchang	CRA004775	0.685363246
ENSGALG00010017354	FEM1B	2	-0.374940136	0.068334963	-5.486797992	4.09285E-08	0	0.270049882	0	1	CRA004775;Yang2025_Silkie_Wenchang	PRJNA827465	1.71491E-05
ENSGALG00010017354	FEM1B	2	-0.162919016	0.124003518	-1.313825754	0.188904849	0.02319806	3.528096162	71.65610136	1	CRA004775;PRJNA827465	Yang2025_Silkie_Wenchang	0.698162702
ENSGALG00010017356	NSMCE1	2	-0.16317778	0.097246164	-1.677986801	0.093349676	0.006280516	1.314501823	23.92555245	0.5	PRJNA827465;Yang2025_Silkie_Wenchang	CRA004775	0.523579592
ENSGALG00010017356	NSMCE1	2	-0.169721899	0.206208248	-0.823060673	0.410473491	0.041680529	1.931723188	48.23274856	0.5	CRA004775;Yang2025_Silkie_Wenchang	PRJNA827465	0.834820782
ENSGALG00010017356	NSMCE1	2	-0.235596239	0.074099553	-3.179455595	0.00147552	0	0.564199776	0	1	CRA004775;PRJNA827465	Yang2025_Silkie_Wenchang	0.047564941
ENSGALG00010017359	EHD3	2	-0.032555513	0.059752725	-0.54483729	0.585865429	0.002194874	1.308695248	23.58801627	0.5	PRJNA827465;Yang2025_Silkie_Wenchang	CRA004775	0.891316442
ENSGALG00010017359	EHD3	2	-0.024125428	0.124440031	-0.193871927	0.846276166	0.015885523	2.018829095	50.46633701	0.5	CRA004775;Yang2025_Silkie_Wenchang	PRJNA827465	0.96122283
ENSGALG00010017359	EHD3	2	0.016277866	0.048092346	0.338471025	0.735008259	0	0.590212102	0	1	CRA004775;PRJNA827465	Yang2025_Silkie_Wenchang	0.93359216
ENSGALG00010017361	METTL22	2	-0.183289913	0.061270721	-2.991476344	0.00277632	0.00118864	1.13991647	12.2742739	1	PRJNA827465;Yang2025_Silkie_Wenchang	CRA004775	0.071882558

ENSGALG00010017361	METTL22	2	-0.252982076	0.106958989	-2.365225006	0.01801912	0	0.656873764	0	1	CRA004775;Yang2025_Silkie_Wenchang	PRJNA827465	0.2239074
ENSGALG00010017361	METTL22	2	-0.141655951	0.061191471	-2.314962356	0.020614092	0	0.157602149	0	1	CRA004775;PRJNA827465	Yang2025_Silkie_Wenchang	0.242254693
ENSGALG00010017362	ENO1	2	-0.302374205	0.258266718	-1.170782695	0.241686135	0.109293696	5.036911423	80.14656372	1	PRJNA827465;Yang2025_Silkie_Wenchang	CRA004775	0.75406671
ENSGALG00010017362	ENO1	2	-0.335825803	0.28403456	-1.182341341	0.237070288	0.104341368	2.808587802	64.39491764	1	CRA004775;Yang2025_Silkie_Wenchang	PRJNA827465	0.749355649
ENSGALG00010017362	ENO1	2	-0.075390325	0.079519134	-0.948077783	0.343089858	0	0.029420588	0	1	CRA004775;PRJNA827465	Yang2025_Silkie_Wenchang	0.806218116
ENSGALG00010017363	TIMM9	2	-0.067042519	0.064092535	-1.046026953	0.295548589	0	0.503598	0	0.5	PRJNA827465;Yang2025_Silkie_Wenchang	CRA004775	0.787628307
ENSGALG00010017363	TIMM9	2	-0.016547638	0.114388156	-0.144662159	0.884977617	0	0.270572474	0	0.5	CRA004775;Yang2025_Silkie_Wenchang	PRJNA827465	0.972194923
ENSGALG00010017363	TIMM9	2	-0.09841196	0.072637403	-1.354838644	0.175468972	0	0.044949405	0	1	CRA004775;PRJNA827465	Yang2025_Silkie_Wenchang	0.680258136
ENSGALG00010017364	AKT1	2	-0.00182085	0.047980727	-0.037949616	0.969727853	0	0.007302941	0	1	PRJNA827465;Yang2025_Silkie_Wenchang	CRA004775	0.991572914
ENSGALG00010017364	AKT1	2	0.080586515	0.102992943	0.782446962	0.433951944	0.00310075	1.168850624	14.44586847	0.5	CRA004775;Yang2025_Silkie_Wenchang	PRJNA827465	0.842762754
ENSGALG00010017364	AKT1	2	0.051592644	0.086241095	0.598237349	0.549681573	0.007426427	1.635665806	38.86281682	0.5	CRA004775;PRJNA827465	Yang2025_Silkie_Wenchang	0.878554604
ENSGALG00010017365	CD8BP	2	-0.12513414	0.527003624	-0.237444553	0.812311929	0.149661629	1.331264866	24.88346794	0.5	PRJNA827465;Yang2025_Silkie_Wenchang	CRA004775	0.953371387
ENSGALG00010017365	CD8BP	2	0.339298498	0.468088794	0.724859263	0.468538375	0	0.139101125	0	1	CRA004775;Yang2025_Silkie_Wenchang	PRJNA827465	0.853198008
ENSGALG00010017365	CD8BP	2	-0.187568657	0.393782331	-0.476325731	0.633842337	0	0.856994262	0	0.5	CRA004775;PRJNA827465	Yang2025_Silkie_Wenchang	0.903596555
ENSGALG00010017367	TIMD4	2	-0.138993699	0.256195492	-0.54252984	0.587453557	0.070093616	2.144100898	53.36040385	0.5	PRJNA827465;Yang2025_Silkie_Wenchang	CRA004775	0.89167336
ENSGALG00010017367	TIMD4	2	-0.363003457	0.235264536	-1.542958677	0.122840803	0	0.162495763	0	1	CRA004775;Yang2025_Silkie_Wenchang	PRJNA827465	0.587706885
ENSGALG00010017367	TIMD4	2	0.079058621	0.224339119	0.352406755	0.724533237	0	0.132403678	0	0.5	CRA004775;PRJNA827465	Yang2025_Silkie_Wenchang	0.930388018
ENSGALG00010017368	ERLIN2	2	-0.121941142	0.146521727	-0.832239314	0.405273877	0.040129067	14.85364511	93.2676458	0.5	PRJNA827465;Yang2025_Silkie_Wenchang	CRA004775	0.833112102
ENSGALG00010017368	ERLIN2	2	-0.020403176	0.269163961	-0.075802035	0.939576597	0.132556029	11.37634425	91.20983	0.5	CRA004775;Yang2025_Silkie_Wenchang	PRJNA827465	0.985425793
ENSGALG00010017368	ERLIN2	2	0.100782583	0.116135016	0.867805298	0.385500929	0.019381482	2.790648391	64.16603384	1	CRA004775;PRJNA827465	Yang2025_Silkie_Wenchang	0.82648313
ENSGALG00010017372	ENSGALG00010017372	2	-0.256595259	0.300326081	-0.854388864	0.392889564	0.174600868	30.95024005	96.76900729	0.5	PRJNA827465;Yang2025_Silkie_Wenchang	CRA004775	0.830060225
ENSGALG00010017372	ENSGALG00010017372	2	-0.445608308	0.177718509	-2.507382653	0.012162896	0.036500997	1.935893261	48.34425946	1	CRA004775;Yang2025_Silkie_Wenchang	PRJNA827465	0.177974052
ENSGALG00010017372	ENSGALG00010017372	2	0.030079136	0.055103503	0.545866142	0.585157954	0	0.618994229	0	0.5	CRA004775;PRJNA827465	Yang2025_Silkie_Wenchang	0.891086887
ENSGALG00010017373	RP1-27O5.3	2	0.258439142	0.145029019	1.781982285	0.074752121	0.032150333	4.232825972	76.37512133	1	PRJNA827465;Yang2025_Silkie_Wenchang	CRA004775	0.47211676
ENSGALG00010017373	RP1-27O5.3	2	0.082530138	0.376927208	0.218955108	0.826685018	0.236503312	5.37991033	81.4123296	0.5	CRA004775;Yang2025_Silkie_Wenchang	PRJNA827465	0.956467508
ENSGALG00010017373	RP1-27O5.3	2	-0.025589827	0.216810123	-0.118028747	0.906044881	0.058526224	2.106299508	52.52337115	0.5	CRA004775;PRJNA827465	Yang2025_Silkie_Wenchang	0.978196186
ENSGALG00010017375	BDH1A	2	0.020480097	0.083077083	0.246519217	0.805280331	0.001429408	1.071930785	6.710394538	0.5	PRJNA827465;Yang2025_Silkie_Wenchang	CRA004775	0.951870339
ENSGALG00010017375	BDH1A	2	-0.096421383	0.156695849	-0.61534102	0.538329533	0	0.28393067	0	0.5	CRA004775;Yang2025_Silkie_Wenchang	PRJNA827465	0.87481821
ENSGALG00010017375	BDH1A	2	0.061633824	0.082144187	0.750312659	0.45306642	0	0.001382878	0	1	CRA004775;PRJNA827465	Yang2025_Silkie_Wenchang	0.848673602
ENSGALG00010017376	GALNT14	2	0.118132148	0.180467728	0.654588773	0.512732555	0	0.135130319	0	1	PRJNA827465;Yang2025_Silkie_Wenchang	CRA004775	0.865930199
ENSGALG00010017376	GALNT14	2	0.01800843	0.278248703	0.064720625	0.948396441	0	0.00332178	0	1	CRA004775;Yang2025_Silkie_Wenchang	PRJNA827465	0.986778445
ENSGALG00010017376	GALNT14	2	0.135561759	0.189419055	0.715671185	0.474194407	0	0.058519958	0	1	CRA004775;PRJNA827465	Yang2025_Silkie_Wenchang	0.85444425
ENSGALG00010017377	USP8	2	0.173232042	0.277706586	0.623795226	0.532762075	0.139376645	9.741169246	89.7342919	0.5	PRJNA827465;Yang2025_Silkie_Wenchang	CRA004775	0.873183244
ENSGALG00010017377	USP8	2	0.43649633	0.123233235	3.542034166	0.000397054	0	0.108954467	0	1	CRA004775;Yang2025_Silkie_Wenchang	PRJNA827465	0.020084189
ENSGALG00010017377	USP8	2	0.125249771	0.235985321	0.530752379	0.595590383	0.09573358	6.469465386	84.54277223	0.5	CRA004775;PRJNA827465	Yang2025_Silkie_Wenchang	0.893802528
ENSGALG00010017378	ENSGALG00010017378	2	0.147796651	0.308818169	0.478587938	0.632231801	0	0.707393513	0	0.5	PRJNA827465;Yang2025_Silkie_Wenchang	CRA004775	0.903058174
ENSGALG00010017378	ENSGALG00010017378	2	0.290871712	0.432020055	0.673282892	0.500767334	0	0.532334486	0	0.5	CRA004775;Yang2025_Silkie_Wenchang	PRJNA827465	0.862421093
ENSGALG00010017378	ENSGALG00010017378	2	-0.07819	0.349081422	-0.223987857	0.822766752	0	0.030754977	0	1	CRA004775;PRJNA827465	Yang2025_Silkie_Wenchang	0.955822987
ENSGALG00010017380	MARCKSL1	2	0.069293359	0.097052493	0.713978145	0.475240694	0.008726974	1.510424114	33.79342988	0.5	PRJNA827465;Yang2025_Silkie_Wenchang	CRA004775	0.854462864
ENSGALG00010017380	MARCKSL1	2	-0.072874969	0.155751539	-0.467892447	0.639861505	0	0.148988144	0	0.5	CRA004775;Yang2025_Silkie_Wenchang	PRJNA827465	0.906064918
ENSGALG00010017380	MARCKSL1	2	0.1214616	0.059627007	2.037023268	0.04164771	0	0.050545993	0	1	CRA004775;PRJNA827465	Yang2025_Silkie_Wenchang	0.35369057
ENSGALG00010017381	CA6	2	0.252072066	0.35562404	0.708816158	0.478438569	0.179313361	3.374882428	70.36933815	0.5	PRJNA827465;Yang2025_Silkie_Wenchang	CRA004775	0.855469318
ENSGALG00010017381	CA6	2	0.31409749	0.368748377	0.851793553	0.394328685	0.144584387	2.098636555	52.35001517	0.5	CRA004775;Yang2025_Silkie_Wenchang	PRJNA827465	0.830486298
ENSGALG00010017381	CA6	2	-0.08137467	0.201280536	-0.404284843	0.68600327	0	0.00392116	0	1	CRA004775;PRJNA827465	Yang2025_Silkie_Wenchang	0.919235539
ENSGALG00010017383	ENSGALG00010017383	2	0.153586222	0.111891794	1.37263169	0.169866875	0.012076176	1.903940138	47.47734029	1	PRJNA827465;Yang2025_Silkie_Wenchang	CRA004775	0.671232541
ENSGALG00010017383	ENSGALG00010017383	2	0.016027186	0.120415847	0.133098649	0.894115364	0	0.056427105	0	0.5	CRA004775;Yang2025_Silkie_Wenchang	PRJNA827465	0.974381724
ENSGALG00010017383	ENSGALG00010017383	2	0.227422184	0.096825946	2.348773274	0.018835372	0	0.763201946	0	0.5	CRA004775;PRJNA827465	Yang2025_Silkie_Wenchang	0.229831303
ENSGALG00010017384	ARID4A	2	0.239496638	0.263844187	0.907719973	0.364026189	0.123530936	8.24645895	87.873583	1	PRJNA827465;Yang2025_Silkie_Wenchang	CRA004775	0.817187797
ENSGALG00010017384	ARID4A	2	0.295340886	0.249916937	1.181756186	0.237302457	0.086981008	3.265564351	69.37742171	1	CRA004775;Yang2025_Silkie_Wenchang	PRJNA827465	0.749366344
ENSGALG00010017384	ARID4A	2	0.002319761	0.055239773	0.041994403	0.96650316	0	0.017671626	0	1	CRA004775;PRJNA827465	Yang2025_Silkie_Wenchang	0.990881287
ENSGALG00010017387	HS6ST2	2	-0.132760079	0.056434874	-2.352476386	0.018648874	0.001309609	1.216012153	17.76397975	1	PRJNA827465;Yang2025_Silkie_Wenchang	CRA004775	0.228477771
ENSGALG00010017387	HS6ST2	2	-0.228280646	0.088861249	-2.568956095	0.010200538	0	0.174190551	0	1	CRA004775;Yang2025_Silkie_Wenchang	PRJNA827465	0.160364698
ENSGALG00010017387	HS6ST2	2	-0.104450419	0.056630422	-1.844422409	0.065121604	0	0.732542064	0	1	CRA004775;PRJNA827465	Yang2025_Silkie_Wenchang	0.439870073
ENSGALG00010017388	PLPBP	2	-0.012670967	0.051383142	-0.246597743	0.805219551	0	0.200407061	0	1	PRJNA827465;Yang2025_Silkie_Wenchang	CRA004775	0.951584391
ENSGALG00010017388	PLPBP	2	-0.199041707	0.147111312	-1.353000691	0.17605541	0.00578857	1.143328899	12.53610391	1	CRA004775;Yang2025_Silkie_Wenchang	PRJNA827465	0.681107371
ENSGALG00010017388	PLPBP	2	-0.133282345	0.180863494	-0.736922315	0.461169593	0.046167398	2.683457316	62.73464109	1	CRA004775;PRJNA827465	Yang2025_Silkie_Wenchang	0.85014477
ENSGALG00010017390	DDX24	2	0.098544422	0.079262165	1.243271895	0.213767657	0.005195263	1.632721316	38.75256053	1	PRJNA827465;Yang2025_Silkie_Wenchang	CRA004775	0.726416385
ENSGALG00010017390	DDX24	2	0.180944704	0.103241553	1.752634454	0.079664773	0	0.438529468	0	0.5	CRA004775;Yang2025_Silkie_Wenchang	PRJNA827465	0.48542321
ENSGALG00010017390	DDX24	2	0.034062507	0.068445821	0.497656486	0.618726183	0	0.057943282	0	0.5	CRA004775;PRJNA827465	Yang2025_Silkie_Wenchang	0.899168321
ENSGALG00010017391	CRAMP1	2	-0.056556371	0.055190391	-1.024750325	0.305481011	0	0.010003251	0	1	PRJNA827465;Yang2025_Silkie_Wenchang	CRA004775	0.79225794
ENSGALG00010017391	CRAMP1	2	-0.046191059	0.11437615	-0.403851219	0.686321415	0	9.21094E-05	0	1	CRA004775;Yang2025_Silkie_Wenchang	PRJNA827465	0.919152539
ENSGALG00010017391	CRAMP1	2	-0.059104991	0.061148471	-0.96658167	0.333753201	0	0.000748089	0	1	CRA004775;PRJNA827465	Yang2025_Silkie_Wenchang	0.802691373
ENSGALG00010017393	VDAC1	2	-0.380302855	0.328137628	-1.158973622	0.246466937	0.204520453	19.82034621	94.95467945	1	PRJNA827465;Yang2025_Silkie_Wenchang	CRA004775	0.756843363
ENSGALG00010017393	VDAC1	2	-0.427948148	0.365916452	-1.169524208	0.242192494	0.201319932	3.408664502	70.66299721	0.5	CRA004775;Yang2025_Silkie_Wenchang	PRJNA827465	0.754509479
ENSGALG00010017393	VDAC1	2	-0.051673622	0.087067628	-0.59348834	0.552854384	0	0.06014356	0	0.5	CRA004775;PRJNA827465	Yang2025_Silkie_Wenchang	0.86792478
ENSGALG00010017395	BRF2	2	-0.252114247	0.292039679	-0.863287644	0.387979336	0.149127293	7.582186387	86.81119206	0.5	PRJNA827465;Yang2025_Silkie_Wenchang	CRA004775	0.828154121
ENSGALG00010017395	BRF2	2	-0.434813218	0.151614528	-2.867886233	0.004132241	0.00413335	1.097333745	8.870022063	1	CRA004775;Yang2025_Silkie_Wenchang	PRJNA827465	0.093485158
ENSGALG00010017395	BRF2	2	-0.053529135	0.12143404	-0.440808317	0.659351772	0.011388168	1.405943349	28.87337882	0.5	CRA004775;PRJNA827465	Yang2025_Silkie_Wenchang	0.911449691
ENSGALG00010017396	CCR8L	2	0.14799039	0.164900523	0.897452518	0.369477499	0	0.973799237	0	1	PRJNA827465;Yang2025_Silkie_Wenchang	CRA004775	0.829060801
ENSGALG00010017396	CCR8L	2	0.321997354	0.208257565	1.546149613	0.122068439	0	0.002051208	0	1	CRA004775;Yang2025_Silkie_Wenchang	PRJNA827465	0.585937452
ENSGALG00010017396	CCR8L	2	0.075355521	0.193387262	0.389661244	0.696787059	0	0.461600766	0	1	CRA004775;PRJNA827465	Yang2025_Silkie_Wenchang	0.921086208
ENSGALG00010017398	SLC22A5	2	-0.14960103	0.294448109	-0.508072649	0.611402396	0.160388566	13.32503884	92.49533144	0.5	PRJNA827465;Yang2025_Silkie_Wenchang	CRA004775	0.897609796
ENSGALG00010017398	SLC22A5	2	-0.462191719	0.108086622	-4.276123248	1.90176E-05	0	0.542128916	0	1	CRA004775;Yang2025_Silkie_Wenchang	PRJNA827465	0.002357882
ENSGALG00010017398	SLC22A5	2	-0.217144778	0.456616252	-0.475552013	0.63439357	0.32667454	3.900911149	74.36496342	0.5	CRA004775;PRJNA827465	Yang2025_Silkie_Wenchang	0.903815945
ENSGALG00010017399	CPXM1	2	-0.11697375	0.103187382	-1.133605176	0.256960201	0	0.002061893	0	1	PRJNA827465;Yang2025_Silkie_Wenchang	CRA004775	0.764848386
ENSGALG00010017399	CPXM1	2	-0.160255073	0.172713987	-0.927863898	0.353478173	0	0.848372584	0	1	CRA004775;Yang2025_Silkie_Wenchang	PRJNA827465	0.811303004
ENSGALG00010017399	CPXM1	2	-0.145019455	0.122692178	-1.181977995	0.237214432	0	0.85819664	0	1	CRA004775;PRJNA827465	Yang2025_Silkie_Wenchang	0.749366344
ENSGALG00010017400	GPC4	2	0.015255582	0.048769808	0.312807915	0.754426601	0	0.6459567	0	0.5	PRJNA827465;Yang2025_Silkie_Wenchang	CRA004775	0.93838723
ENSGALG00010017400	GPC4	2	0.052884292	0.080299422	0.658588708	0.510159916	0	0.456903707	0	0.5	CRA004775;Yang2025_Silkie_Wenchang	PRJNA827465	0.865365179
ENSGALG00010017400	GPC4	2	-0.017533646	0.058154855	-0.301499268	0.763033808	0	0.142580233	0	1	CRA004775;PRJNA827465	Yang2025_Silkie_Wenchang	0.940339746
ENSGALG00010017402	SLC2A5	2	-0.077545811	0.157539387	-0.492231259	0.622555869	0	0.055005178	0	1	PRJNA827465;Yang2025_Silkie_Wenchang	CRA004775	0.90016604
ENSGALG00010017402	SLC2A5	2	-0.067818241	0.210132773	-0.322739952	0.746892193	0	0.155774827	0	0.5	CRA004775;Yang2025_Silkie_Wenchang	PRJNA827465	0.936729365
ENSGALG00010017402	SLC2A5	2	-0.025454322	0.176711984	-0.144044005	0.885465721	0	0.058221236	0	0.5	CRA004775;PRJNA827465	Yang2025_Silkie_Wenchang	0.972325079
ENSGALG00010017403	ITGA11	2	0.381956279	0.152414796	2.50603149	0.012209475	0.002195692	1.046887078	4.478714004	1	PRJNA827465;Yang2025_Silkie_Wenchang	CRA004775	0.178358966
ENSGALG00010017403	ITGA11	2	0.069984006	0.606161912	0.115454311	0.908085033	0.563754684	3.872785226	74.17879016	0.5	CRA004775;Yang2025_Silkie_Wenchang	PRJNA827465	0.978196186
ENSGALG00010017403	ITGA11	2	-0.036227312	0.430227185	-0.084205075	0.932893383	0.237941864	2.28598431	56.25516783	0.5	CRA004775;PRJNA827465	Yang2025_Silkie_Wenchang	0.984224753
ENSGALG00010017406	IFT140	2	0.116843041	0.065300155	1.789323953	0.073562881	0	0.206948604	0	1	PRJNA827465;Yang2025_Silkie_Wenchang	CRA004775	0.468672702
ENSGALG00010017406	IFT140	2	0.162894172	0.125997866	1.292832785	0.196068899	0	0.028446137	0	1	CRA004775;Yang2025_Silkie_Wenchang	PRJNA827465	0.707370656
ENSGALG00010017406	IFT140	2	0.102292012	0.071507803	1.430501399	0.152573165	0	0.003633127	0	1	CRA004775;PRJNA827465	Yang2025_Silkie_Wenchang	0.6442129
ENSGALG00010017407	EPN2	2	0.036212355	0.034966371	1.035633775	0.300373029	0	0.022427157	0	1	PRJNA827465;Yang2025_Silkie_Wenchang	CRA004775	0.790532865
ENSGALG00010017407	EPN2	2	0.100694303	0.097559429	1.03213297	0.302009701	0	0.630009707	0	1	CRA004775;Yang2025_Silkie_Wenchang	PRJNA827465	0.791590021
ENSGALG00010017407	EPN2	2	0.04390637	0.041229738	1.064919937	0.286912162	0.000440086	1.024768649	2.416999102	1	CRA004775;PRJNA827465	Yang2025_Silkie_Wenchang	0.782438851
ENSGALG00010017409	GPC3	2	0.117352862	0.276997161	0.423660837	0.671813181	0	0.150098302	0	1	PRJNA827465;Yang2025_Silkie_Wenchang	CRA004775	0.914674175
ENSGALG00010017409	GPC3	2	0.118379505	0.381425163	0.311671901	0.755298985	0	0.278257916	0	0.5	CRA004775;Yang2025_Silkie_Wenchang	PRJNA827465	0.938453928
ENSGALG00010017409	GPC3	2	-0.003464554	0.302638434	-0.011447831	0.990866152	0	0.072321296	0	0.5	CRA004775;PRJNA827465	Yang2025_Silkie_Wenchang	0.997555603
ENSGALG00010017411	RAB11FIP1	2	-0.133195679	0.197393933	-0.674770853	0.499821342	0.066931726	6.874332903	85.45313394	0.5	PRJNA827465;Yang2025_Silkie_Wenchang	CRA004775	0.862078151
ENSGALG00010017411	RAB11FIP1	2	-0.239292338	0.183671119	-1.302830514	0.192632631	0.027977428	1.450412594	31.05410115	0.5	CRA004775;Yang2025_Silkie_Wenchang	PRJNA827465	0.702583546
ENSGALG00010017411	RAB11FIP1	2	0.052088842	0.074288573	0.701168967	0.483197574	0	0.00327921	0	1	CRA004775;PRJNA827465	Yang2025_Silkie_Wenchang	0.85691916
ENSGALG00010017414	ENSGALG00010017414	2	-0.006251021	0.096268214	-0.064933583	0.948227041	0	0.027608845	0	0.5	PRJNA827465;Yang2025_Silkie_Wenchang	CRA004775	0.98671346
ENSGALG00010017414	ENSGALG00010017414	2	-0.037571312	0.20061504	-0.187280636	0.851440604	0	0.244042337	0	0.5	CRA004775;Yang2025_Silkie_Wenchang	PRJNA827465	0.962139388
ENSGALG00010017414	ENSGALG00010017414	2	-0.026725783	0.100431677	-0.266109095	0.79015519	0	0.207844532	0	1	CRA004775;PRJNA827465	Yang2025_Silkie_Wenchang	0.947390191
ENSGALG00010017415	HDAC1	2	-0.072741585	0.047496392	-1.531518119	0.125641392	0	0.82989218	0	1	PRJNA827465;Yang2025_Silkie_Wenchang	CRA004775	0.595034397
ENSGALG00010017415	HDAC1	2	-0.129064479	0.190863501	-0.676213516	0.498905093	0.044123622	2.037593781	50.92250432	1	CRA004775;Yang2025_Silkie_Wenchang	PRJNA827465	0.861571318
ENSGALG00010017415	HDAC1	2	-0.144669573	0.111000223	-1.303326862	0.192463194	0.011171424	1.278444504	21.77994453	1	CRA004775;PRJNA827465	Yang2025_Silkie_Wenchang	0.702356631
ENSGALG00010017418	CHGB	2	0.909082902	0.500411453	1.816670856	0.069267527	0.430651874	6.850498273	85.40252168	1	PRJNA827465;Yang2025_Silkie_Wenchang	CRA004775	0.453272383
ENSGALG00010017418	CHGB	2	0.996357143	0.497203262	2.003923184	0.04507829	0.340185781	3.154618764	68.30044849	1	CRA004775;Yang2025_Silkie_Wenchang	PRJNA827465	0.3659545
ENSGALG00010017418	CHGB	2	0.449268046	0.166058151	2.705486261	0.006820447	0	0.000164354	0	1	CRA004775;PRJNA827465	Yang2025_Silkie_Wenchang	0.126684323
ENSGALG00010017419	B9D1	2	-0.010058469	0.122273469	-0.082262077	0.93443831	0.021245628	3.368619849	70.31425198	0.5	PRJNA827465;Yang2025_Silkie_Wenchang	CRA004775	0.984353495
ENSGALG00010017419	B9D1	2	-0.072854443	0.125022101	-0.582732516	0.56007338	0.008859011	1.272078162	21.38847831	0.5	CRA004775;Yang2025_Silkie_Wenchang	PRJNA827465	0.881203589
ENSGALG00010017419	B9D1	2	0.104527075	0.072818701	1.435442722	0.151160979	0	0.029904601	0	1	CRA004775;PRJNA827465	Yang2025_Silkie_Wenchang	0.642626282
ENSGALG00010017421	GPR157	2	-0.37435087	0.106439283	-3.51703676	0.000436393	0	0.471276678	0	1	PRJNA827465;Yang2025_Silkie_Wenchang	CRA004775	0.02125169
ENSGALG00010017421	GPR157	2	-0.566346078	0.267405735	-2.117927944	0.034181171	0	0.000335918	0	1	CRA004775;Yang2025_Silkie_Wenchang	PRJNA827465	0.317969834
ENSGALG00010017421	GPR157	2	-0.354956546	0.111499263	-3.183487816	0.001455122	0	0.130788724	0	1	CRA004775;PRJNA827465	Yang2025_Silkie_Wenchang	0.04726502
ENSGALG00010017422	ENSGALG00010017422	2	0.233872763	0.171343911	1.36493186	0.172274467	0	0.228928349	0	1	PRJNA827465;Yang2025_Silkie_Wenchang	CRA004775	0.675132455
ENSGALG00010017422	ENSGALG00010017422	2	-0.045960843	0.225831959	-0.203517885	0.838730268	0	0.650500404	0	0.5	CRA004775;Yang2025_Silkie_Wenchang	PRJNA827465	0.959277608
ENSGALG00010017422	ENSGALG00010017422	2	0.086440584	0.26273583	0.329001886	0.742154262	0.067305116	1.843780836	45.76361895	0.5	CRA004775;PRJNA827465	Yang2025_Silkie_Wenchang	0.935481151
ENSGALG00010017423	TMEM204	2	-0.072466289	0.114268552	-0.634175261	0.525966451	0.022621733	7.471209673	86.61528663	0.5	PRJNA827465;Yang2025_Silkie_Wenchang	CRA004775	0.870439151
ENSGALG00010017423	TMEM204	2	-0.019725125	0.239366815	-0.082405431	0.934324318	0.08182307	2.71788083	63.20662816	0.5	CRA004775;Yang2025_Silkie_Wenchang	PRJNA827465	0.98432801
ENSGALG00010017423	TMEM204	2	0.050142138	0.055784893	0.898847965	0.368733647	0	0.829422568	0	1	CRA004775;PRJNA827465	Yang2025_Silkie_Wenchang	0.820425502
ENSGALG00010017424	APOD	2	0.208882283	0.312975295	0.667408215	0.50451142	0	0.045075261	0	1	PRJNA827465;Yang2025_Silkie_Wenchang	CRA004775	0.863512504
ENSGALG00010017424	APOD	2	-0.281176769	0.405365489	-0.693637659	0.487909471	0.085711079	1.35279177	26.07879336	0.5	CRA004775;Yang2025_Silkie_Wenchang	PRJNA827465	0.85768885
ENSGALG00010017424	APOD	2	-0.169296161	0.471452467	-0.359094867	0.719524121	0.246181492	2.220536657	54.96584139	0.5	CRA004775;PRJNA827465	Yang2025_Silkie_Wenchang	0.928449671
ENSGALG00010017425	H6PD	2	-0.056868056	0.087791335	-0.647763886	0.517137677	0.004743841	1.43356621	30.24389156	0.5	PRJNA827465;Yang2025_Silkie_Wenchang	CRA004775	0.867952013
ENSGALG00010017425	H6PD	2	-0.166906243	0.11057368	-1.509457257	0.131181971	0	0.125055931	0	1	CRA004775;Yang2025_Silkie_Wenchang	PRJNA827465	0.606878893
ENSGALG00010017425	H6PD	2	0.005849259	0.092262427	0.063398062	0.949449531	0	0.547547533	0	0.5	CRA004775;PRJNA827465	Yang2025_Silkie_Wenchang	0.98723759
ENSGALG00010017428	PSMA3	2	-0.024032194	0.059156446	-0.406248112	0.684560306	0	0.067420546	0	1	PRJNA827465;Yang2025_Silkie_Wenchang	CRA004775	0.919003112
ENSGALG00010017428	PSMA3	2	-0.059903032	0.150795305	-0.397247326	0.691185086	0	0.00977949	0	1	CRA004775;Yang2025_Silkie_Wenchang	PRJNA827465	0.920268407
ENSGALG00010017428	PSMA3	2	-0.020504117	0.060145616	-0.340907922	0.7331729	0	0.008079041	0	1	CRA004775;PRJNA827465	Yang2025_Silkie_Wenchang	0.933089653
ENSGALG00010017429	TELO2	2	-0.071224086	0.140290845	-0.507688767	0.611671627	0.02573964	2.659045737	62.39252353	0.5	PRJNA827465;Yang2025_Silkie_Wenchang	CRA004775	0.897777844
ENSGALG00010017429	TELO2	2	-0.208799099	0.113577603	-1.83838269	0.066006034	0	0.118283247	0	1	CRA004775;Yang2025_Silkie_Wenchang	PRJNA827465	0.442444044
ENSGALG00010017429	TELO2	2	-0.013188277	0.091937324	-0.143448568	0.885935928	0.004777736	1.28007776	21.87974583	0.5	CRA004775;PRJNA827465	Yang2025_Silkie_Wenchang	0.97244495
ENSGALG00010017430	CACNA1H	2	0.015475921	0.137035303	0.112933829	0.910083016	0.013037767	1.458839083	31.45234375	0.5	PRJNA827465;Yang2025_Silkie_Wenchang	CRA004775	0.978730675
ENSGALG00010017430	CACNA1H	2	0.182880624	0.17799741	1.027434181	0.304216063	0	0.045365561	0	1	CRA004775;Yang2025_Silkie_Wenchang	PRJNA827465	0.79225794
ENSGALG00010017430	CACNA1H	2	-0.064152435	0.118756405	-0.540201895	0.589057806	0	0.254421589	0	0.5	CRA004775;PRJNA827465	Yang2025_Silkie_Wenchang	0.892171138
ENSGALG00010017431	FYCO1	2	0.118273855	0.196911408	0.600645014	0.54807645	0.067612007	7.668585768	86.95978593	0.5	PRJNA827465;Yang2025_Silkie_Wenchang	CRA004775	0.878281266
ENSGALG00010017431	FYCO1	2	0.172587	0.281929319	0.612164072	0.54042921	0.088760745	1.645553939	39.23019012	0.5	CRA004775;Yang2025_Silkie_Wenchang	PRJNA827465	0.875506901
ENSGALG00010017431	FYCO1	2	-0.075258059	0.077631348	-0.969428734	0.332331321	0	0.290133464	0	1	CRA004775;PRJNA827465	Yang2025_Silkie_Wenchang	0.802178578
ENSGALG00010017433	RNPS1	2	-0.224639898	0.237615397	-0.945392851	0.344458351	0.104991595	13.58915496	92.64119069	1	PRJNA827465;Yang2025_Silkie_Wenchang	CRA004775	0.806768255
ENSGALG00010017433	RNPS1	2	-0.40116499	0.101016612	-3.971277415	7.14883E-05	0.000803427	1.037664625	3.629749334	1	CRA004775;Yang2025_Silkie_Wenchang	PRJNA827465	0.00612433
ENSGALG00010017433	RNPS1	2	-0.084847841	0.123518553	-0.686923858	0.492130711	0.020774941	2.430738103	58.86023267	1	CRA004775;PRJNA827465	Yang2025_Silkie_Wenchang	0.859158569
ENSGALG00010017434	FGF13	2	-0.430083618	0.087286184	-4.927281728	8.33815E-07	0	0.032486242	0	1	PRJNA827465;Yang2025_Silkie_Wenchang	CRA004775	0.000192773
ENSGALG00010017434	FGF13	2	-0.301522782	0.243352967	-1.239034748	0.21533263	0.062655075	1.488853201	32.83421098	0.5	CRA004775;Yang2025_Silkie_Wenchang	PRJNA827465	0.728757551
ENSGALG00010017434	FGF13	2	-0.296408924	0.279951168	-1.058787954	0.289696361	0.076728303	1.563134222	36.0259672	0.5	CRA004775;PRJNA827465	Yang2025_Silkie_Wenchang	0.784049259
ENSGALG00010017435	ACTR10	2	-0.123655854	0.176991434	-0.698654459	0.484768001	0.044326722	2.968809973	66.31646993	0.5	PRJNA827465;Yang2025_Silkie_Wenchang	CRA004775	0.857004733
ENSGALG00010017435	ACTR10	2	-0.471313777	0.142651122	-3.303961229	0.00095329	0	0.750286979	0	1	CRA004775;Yang2025_Silkie_Wenchang	PRJNA827465	0.036660629
ENSGALG00010017435	ACTR10	2	-0.26331167	0.305012324	-0.863282069	0.3879824	0.163815527	7.819766013	87.21189357	0.5	CRA004775;PRJNA827465	Yang2025_Silkie_Wenchang	0.828154121
ENSGALG00010017437	DRG2	2	-0.075136294	0.051595113	-1.456267653	0.145318644	0	0.014597751	0	1	PRJNA827465;Yang2025_Silkie_Wenchang	CRA004775	0.633761502
ENSGALG00010017437	DRG2	2	-0.102581028	0.091468076	-1.121495409	0.262077045	0	0.12920333	0	1	CRA004775;Yang2025_Silkie_Wenchang	PRJNA827465	0.768363052
ENSGALG00010017437	DRG2	2	-0.079214412	0.057234123	-1.384041685	0.166345691	0	0.206936391	0	1	CRA004775;PRJNA827465	Yang2025_Silkie_Wenchang	0.665303687
ENSGALG00010017439	PPP4R4	2	0.261903258	0.236012702	1.109699841	0.267128391	0	0.498055672	0	1	PRJNA827465;Yang2025_Silkie_Wenchang	CRA004775	0.771247817
ENSGALG00010017439	PPP4R4	2	0.20250164	0.421098024	0.480889552	0.630594999	0.106395139	1.32518854	24.53903953	0.5	CRA004775;Yang2025_Silkie_Wenchang	PRJNA827465	0.902756437
ENSGALG00010017439	PPP4R4	2	0.038743869	0.278722169	0.139005338	0.88944593	0	0.576551962	0	0.5	CRA004775;PRJNA827465	Yang2025_Silkie_Wenchang	0.973135755
ENSGALG00010017441	SPSB1	2	-0.101651732	0.081757044	-1.243339131	0.21374289	0	0.911022573	0	1	PRJNA827465;Yang2025_Silkie_Wenchang	CRA004775	0.726416385
ENSGALG00010017441	SPSB1	2	-0.187186069	0.139121749	-1.345483868	0.178469027	0	0.482271821	0	0.5	CRA004775;Yang2025_Silkie_Wenchang	PRJNA827465	0.684796269
ENSGALG00010017441	SPSB1	2	-0.041192182	0.096880879	-0.425183818	0.67070268	0	0.136652721	0	0.5	CRA004775;PRJNA827465	Yang2025_Silkie_Wenchang	0.914482883
ENSGALG00010017442	LCK	2	0.37040711	0.418922684	0.88418967	0.376593834	0.291635344	5.91269309	83.0872331	0.5	PRJNA827465;Yang2025_Silkie_Wenchang	CRA004775	0.822974013
ENSGALG00010017442	LCK	2	0.032064375	0.793217393	0.040423187	0.967755745	1.144515243	10.84028048	90.77514644	0.5	CRA004775;Yang2025_Silkie_Wenchang	PRJNA827465	0.991065147
ENSGALG00010017442	LCK	2	-0.345852124	0.367295046	-0.941619353	0.346387558	0.166545129	2.450835968	59.19759571	1	CRA004775;PRJNA827465	Yang2025_Silkie_Wenchang	0.807812863
ENSGALG00010017447	CAPN13	2	-0.373768966	0.326846989	-1.143559457	0.252806384	0	0.197578431	0	1	PRJNA827465;Yang2025_Silkie_Wenchang	CRA004775	0.762731469
ENSGALG00010017447	CAPN13	2	0.661609214	1.289544138	0.513056664	0.607911684	2.886181434	7.522014208	86.7056885	0.5	CRA004775;Yang2025_Silkie_Wenchang	PRJNA827465	0.897204334
ENSGALG00010017447	CAPN13	2	0.771508544	1.129799967	0.682871806	0.494687851	2.219905878	7.389099246	86.4665507	0.5	CRA004775;PRJNA827465	Yang2025_Silkie_Wenchang	0.860351985
ENSGALG00010017449	TRMT6	2	0.15880028	0.2403014	0.660837929	0.508716256	0.102797553	8.675600943	88.47342096	0.5	PRJNA827465;Yang2025_Silkie_Wenchang	CRA004775	0.865144635
ENSGALG00010017449	TRMT6	2	0.121654501	0.325108897	0.37419616	0.708258381	0.171425499	5.109627888	80.42910322	0.5	CRA004775;Yang2025_Silkie_Wenchang	PRJNA827465	0.925061251
ENSGALG00010017449	TRMT6	2	-0.074927089	0.063852698	-1.173436543	0.24062079	0	0.446418822	0	1	CRA004775;PRJNA827465	Yang2025_Silkie_Wenchang	0.753158164
ENSGALG00010017450	ENSGALG00010017450	2	-0.236929919	0.11275144	-2.101347172	0.035610502	0	0.013943311	0	1	PRJNA827465;Yang2025_Silkie_Wenchang	CRA004775	0.325599715
ENSGALG00010017450	ENSGALG00010017450	2	-0.208775976	0.145930796	-1.430650565	0.152530388	0	0.204257764	0	1	CRA004775;Yang2025_Silkie_Wenchang	PRJNA827465	0.644197489
ENSGALG00010017450	ENSGALG00010017450	2	-0.198130872	0.128401493	-1.543057385	0.122816853	0	0.147744084	0	1	CRA004775;PRJNA827465	Yang2025_Silkie_Wenchang	0.587706885
ENSGALG00010017451	GABPB1	2	0.029043652	0.047817601	0.607384128	0.543596019	0	0.677959534	0	1	PRJNA827465;Yang2025_Silkie_Wenchang	CRA004775	0.876968972
ENSGALG00010017451	GABPB1	2	0.09126791	0.089764847	1.016744441	0.309275019	0	0.038160459	0	1	CRA004775;Yang2025_Silkie_Wenchang	PRJNA827465	0.794043289
ENSGALG00010017451	GABPB1	2	0.008732388	0.053603788	0.16290618	0.870592305	0	0.027985837	0	1	CRA004775;PRJNA827465	Yang2025_Silkie_Wenchang	0.969018906
ENSGALG00010017452	ARMH4	2	-1.259852268	0.373000727	-3.37761344	0.000731178	0	0.045322953	0	1	PRJNA827465;Yang2025_Silkie_Wenchang	CRA004775	0.030276194
ENSGALG00010017452	ARMH4	2	-1.302102166	0.553428385	-2.352792522	0.018633028	0	0.243397267	0	1	CRA004775;Yang2025_Silkie_Wenchang	PRJNA827465	0.228426039
ENSGALG00010017452	ARMH4	2	-1.384889609	0.428292419	-3.233514179	0.001222773	0	0.150206083	0	1	CRA004775;PRJNA827465	Yang2025_Silkie_Wenchang	0.042624337
ENSGALG00010017453	PPP1R2	2	0.620792904	0.627568804	0.989202938	0.322563861	0.766378366	36.37415136	97.25079497	1	PRJNA827465;Yang2025_Silkie_Wenchang	CRA004775	0.798652022
ENSGALG00010017453	PPP1R2	2	0.807175731	0.473652641	1.704151228	0.088352838	0.392644031	7.913047407	87.36264364	1	CRA004775;Yang2025_Silkie_Wenchang	PRJNA827465	0.510441217
ENSGALG00010017453	PPP1R2	2	0.045810157	0.102686411	0.446117037	0.655512707	0.007226228	1.18065956	15.30157942	1	CRA004775;PRJNA827465	Yang2025_Silkie_Wenchang	0.910411938
ENSGALG00010017454	ENSGALG00010017454	2	0.121466515	0.077198924	1.573422377	0.115621049	0	0.7395706	0	1	PRJNA827465;Yang2025_Silkie_Wenchang	CRA004775	0.572716099
ENSGALG00010017454	ENSGALG00010017454	2	-0.025926586	0.309315282	-0.083819285	0.933200114	0.146626616	4.230207206	76.36049604	0.5	CRA004775;Yang2025_Silkie_Wenchang	PRJNA827465	0.984260547
ENSGALG00010017454	ENSGALG00010017454	2	-0.075961181	0.21540931	-0.352636483	0.724360983	0.067020934	3.096604002	67.70655857	0.5	CRA004775;PRJNA827465	Yang2025_Silkie_Wenchang	0.930387861
ENSGALG00010017455	CCR9	2	0.059454128	0.113359406	0.52447459	0.599948489	0.009257162	1.523763521	34.37301877	0.5	PRJNA827465;Yang2025_Silkie_Wenchang	CRA004775	0.895092498
ENSGALG00010017455	CCR9	2	0.052273254	0.232109901	0.225209068	0.821816635	0.052356214	1.665229881	39.94823107	0.5	CRA004775;Yang2025_Silkie_Wenchang	PRJNA827465	0.95563486
ENSGALG00010017455	CCR9	2	-0.054643448	0.104611687	-0.522345537	0.601429769	0	0.536968107	0	1	CRA004775;PRJNA827465	Yang2025_Silkie_Wenchang	0.895230519
ENSGALG00010017457	ZMAT4	2	-0.149391069	0.141255353	-1.057595804	0.290239757	0	0.392220973	0	1	PRJNA827465;Yang2025_Silkie_Wenchang	CRA004775	0.784195688
ENSGALG00010017457	ZMAT4	2	-0.100137767	0.223802328	-0.447438451	0.654558518	0	0.811424859	0	1	CRA004775;Yang2025_Silkie_Wenchang	PRJNA827465	0.910222014
ENSGALG00010017457	ZMAT4	2	-0.244132602	0.170431115	-1.432441494	0.152017511	0	0.43765922	0	1	CRA004775;PRJNA827465	Yang2025_Silkie_Wenchang	0.643414195
ENSGALG00010017458	ENSGALG00010017458	2	0.20503057	0.068767047	2.981523524	0.00286818	0	0.042328444	0	1	PRJNA827465;Yang2025_Silkie_Wenchang	CRA004775	0.073164891
ENSGALG00010017458	ENSGALG00010017458	2	0.16948009	0.093041958	1.821544743	0.068524092	0	0.543362368	0	0.5	CRA004775;Yang2025_Silkie_Wenchang	PRJNA827465	0.451003838

ENSGALG00010017458	ENSGALG00010017458	2	0.195448957	0.092706606	2.108252747	0.035009133	0	0.678506105	0	0.5	CRA004775;PRJNA827465	Yang2025_Silkie_Wenchang	0.322438684
ENSGALG00010017459	FAM167B	2	0.637722909	0.367639919	1.734639999	0.08280459	0.208731783	4.386763628	77.20415129	1	PRJNA827465;Yang2025_Silkie_Wenchang	CRA004775	0.495704284
ENSGALG00010017459	FAM167B	2	0.308641357	0.71328998	0.432701097	0.665231933	0.935766015	12.40043838	91.93576897	0.5	CRA004775;Yang2025_Silkie_Wenchang	PRJNA827465	0.913399315
ENSGALG00010017459	FAM167B	2	-0.041336153	0.344450869	-0.120005947	0.904478437	0.160456418	3.041797379	67.12470045	0.5	CRA004775;PRJNA827465	Yang2025_Silkie_Wenchang	0.977633684
ENSGALG00010017462	MCM8	2	0.323336536	0.097921079	3.302011584	0.000959941	0	0.004049571	0	1	PRJNA827465;Yang2025_Silkie_Wenchang	CRA004775	0.036786652
ENSGALG00010017462	MCM8	2	-0.095620117	0.438705809	-0.217959542	0.827460635	0.351441692	11.39658477	91.22544148	0.5	CRA004775;Yang2025_Silkie_Wenchang	PRJNA827465	0.956550752
ENSGALG00010017462	MCM8	2	-0.098213584	0.432270595	-0.227203944	0.820265161	0.343501438	12.13098506	91.78373817	0.5	CRA004775;PRJNA827465	Yang2025_Silkie_Wenchang	0.955308792
ENSGALG00010017463	PPP1R36	2	-0.354137581	0.171012052	-2.070834054	0.038374307	0	0.787838439	0	1	PRJNA827465;Yang2025_Silkie_Wenchang	CRA004775	0.337788438
ENSGALG00010017463	PPP1R36	2	-0.489663134	0.227740937	-2.150088341	0.031548228	0	0.039117168	0	1	CRA004775;Yang2025_Silkie_Wenchang	PRJNA827465	0.303832392
ENSGALG00010017463	PPP1R36	2	-0.219967444	0.228163821	-0.964076793	0.335007427	0	0.049095687	0	1	CRA004775;PRJNA827465	Yang2025_Silkie_Wenchang	0.803101658
ENSGALG00010017464	SH3BP2	2	0.037804895	0.061223467	0.617490263	0.536911402	0	0.105188509	0	0.5	PRJNA827465;Yang2025_Silkie_Wenchang	CRA004775	0.874713452
ENSGALG00010017464	SH3BP2	2	-0.021958446	0.148927767	-0.147443599	0.882781895	0	0.00396007	0	1	CRA004775;Yang2025_Silkie_Wenchang	PRJNA827465	0.972001076
ENSGALG00010017464	SH3BP2	2	0.040824004	0.063519348	0.642701882	0.520417559	0	0.08279004	0	0.5	CRA004775;PRJNA827465	Yang2025_Silkie_Wenchang	0.869331539
ENSGALG00010017465	TMEM234	2	0.139053541	0.096993828	1.433632892	0.151657052	0	0.056759359	0	1	PRJNA827465;Yang2025_Silkie_Wenchang	CRA004775	0.643081249
ENSGALG00010017465	TMEM234	2	-0.211275661	0.360473581	-0.586105811	0.557804411	0.203624135	4.375737835	77.1467113	0.5	CRA004775;Yang2025_Silkie_Wenchang	PRJNA827465	0.880678789
ENSGALG00010017465	TMEM234	2	-0.18116121	0.384306353	-0.471397906	0.637356611	0.246875298	5.599843388	82.14235773	0.5	CRA004775;PRJNA827465	Yang2025_Silkie_Wenchang	0.905150023
ENSGALG00010017467	SFRP1	2	-0.028657372	0.155569444	-0.184209518	0.853849093	0.036800694	4.101821879	75.62058935	0.5	PRJNA827465;Yang2025_Silkie_Wenchang	CRA004775	0.962975689
ENSGALG00010017467	SFRP1	2	0.419169527	0.65097937	0.643906008	0.519636386	0.786869487	13.41444917	92.54535175	0.5	CRA004775;Yang2025_Silkie_Wenchang	PRJNA827465	0.869100572
ENSGALG00010017467	SFRP1	2	0.559999949	0.493302575	1.135205808	0.256289096	0.432270644	8.226430253	87.8440591	1	CRA004775;PRJNA827465	Yang2025_Silkie_Wenchang	0.764677554
ENSGALG00010017468	AMDHD2	2	-0.025159563	0.288141641	-0.087316652	0.930419819	0.146063523	7.611276705	86.86159972	0.5	PRJNA827465;Yang2025_Silkie_Wenchang	CRA004775	0.983813577
ENSGALG00010017468	AMDHD2	2	-0.47397004	0.155467474	-3.048676539	0.002298518	0	0.954523154	0	1	CRA004775;Yang2025_Silkie_Wenchang	PRJNA827465	0.063663608
ENSGALG00010017468	AMDHD2	2	-0.180680707	0.443314111	-0.407568139	0.68359076	0.363858098	12.67162085	92.10834974	0.5	CRA004775;PRJNA827465	Yang2025_Silkie_Wenchang	0.918800744
ENSGALG00010017470	HSPA2	2	-0.052469582	0.081014584	-0.647655998	0.51720747	0	0.916931843	0	0.5	PRJNA827465;Yang2025_Silkie_Wenchang	CRA004775	0.867952013
ENSGALG00010017470	HSPA2	2	0.405839158	0.461144783	0.880068848	0.378822014	0.344852292	4.588769302	78.20766454	1	CRA004775;Yang2025_Silkie_Wenchang	PRJNA827465	0.823876439
ENSGALG00010017470	HSPA2	2	0.340859563	0.541843507	0.629073816	0.529300732	0.50252684	6.22599358	83.9383066	0.5	CRA004775;PRJNA827465	Yang2025_Silkie_Wenchang	0.871886499
ENSGALG00010017471	CRLS1	2	-0.008501083	0.048351751	-0.175817475	0.86043734	0	0.011241685	0	1	PRJNA827465;Yang2025_Silkie_Wenchang	CRA004775	0.964999892
ENSGALG00010017471	CRLS1	2	-0.031655609	0.13376626	-0.236648677	0.812929354	0	0.016024867	0	1	CRA004775;Yang2025_Silkie_Wenchang	PRJNA827465	0.953526116
ENSGALG00010017471	CRLS1	2	-0.008363596	0.050427737	-0.165853091	0.868272564	0	0.03711362	0	1	CRA004775;PRJNA827465	Yang2025_Silkie_Wenchang	0.968105435
ENSGALG00010017472	LZTFL1	2	-0.13963061	0.261370071	-0.534225702	0.593185389	0.125228658	11.90692489	91.66152592	0.5	PRJNA827465;Yang2025_Silkie_Wenchang	CRA004775	0.892776493
ENSGALG00010017472	LZTFL1	2	-0.286316587	0.181163665	-1.580432019	0.114007964	0.032675833	1.739633638	42.51663234	1	CRA004775;Yang2025_Silkie_Wenchang	PRJNA827465	0.569648487
ENSGALG00010017472	LZTFL1	2	0.102387831	0.085615201	1.195907142	0.231732798	0	0.209594449	0	0.5	CRA004775;PRJNA827465	Yang2025_Silkie_Wenchang	0.744150932
ENSGALG00010017473	EIF3I	2	0.004049801	0.045354366	0.089292425	0.928849514	0	0.031558932	0	1	PRJNA827465;Yang2025_Silkie_Wenchang	CRA004775	0.983474473
ENSGALG00010017473	EIF3I	2	-0.090594937	0.137425559	-0.65922917	0.509748618	0.007017914	1.224613685	18.34159522	0.5	CRA004775;Yang2025_Silkie_Wenchang	PRJNA827465	0.865257661
ENSGALG00010017473	EIF3I	2	-0.053720415	0.102875099	-0.522190651	0.601537595	0.011430978	1.604737159	37.68449901	0.5	CRA004775;PRJNA827465	Yang2025_Silkie_Wenchang	0.895230519
ENSGALG00010017475	STUM	2	-0.052642106	0.082219499	-0.640263041	0.522001604	0	0.299120044	0	1	PRJNA827465;Yang2025_Silkie_Wenchang	CRA004775	0.869568211
ENSGALG00010017475	STUM	2	-0.116637341	0.224247059	-0.520128742	0.602973847	0.048863978	1.408672576	29.01118282	1	CRA004775;Yang2025_Silkie_Wenchang	PRJNA827465	0.895538966
ENSGALG00010017475	STUM	2	-0.130292404	0.116762835	-1.115872221	0.264476834	0	0.997375478	0	1	CRA004775;PRJNA827465	Yang2025_Silkie_Wenchang	0.768790489
ENSGALG00010017476	ATP6V0C	2	-0.365829557	0.331686441	-1.102937931	0.27005412	0.202064723	11.6020622	91.38084263	1	PRJNA827465;Yang2025_Silkie_Wenchang	CRA004775	0.772336005
ENSGALG00010017476	ATP6V0C	2	-0.420256919	0.30144758	-1.394129352	0.163278547	0.147143201	5.252929004	80.9630018	1	CRA004775;Yang2025_Silkie_Wenchang	PRJNA827465	0.661668506
ENSGALG00010017476	ATP6V0C	2	-0.062423994	0.056525717	-1.104346801	0.269442728	0	0.101239321	0	1	CRA004775;PRJNA827465	Yang2025_Silkie_Wenchang	0.77189992
ENSGALG00010017477	MCF2	2	0.166623611	0.236391463	0.704863068	0.480895456	0.092319453	5.733088619	82.55739504	0.5	PRJNA827465;Yang2025_Silkie_Wenchang	CRA004775	0.856722567
ENSGALG00010017477	MCF2	2	0.373630061	0.14274764	2.617416728	0.00885981	0	0.691532576	0	0.5	CRA004775;Yang2025_Silkie_Wenchang	PRJNA827465	0.147549629
ENSGALG00010017477	MCF2	2	-0.062003743	0.125729701	-0.493150802	0.621906037	0	0.004742973	0	1	CRA004775;PRJNA827465	Yang2025_Silkie_Wenchang	0.899853801
ENSGALG00010017480	DCDC2B	2	0.377273774	0.22770087	1.656883326	0.097543066	0.069378823	2.963015096	66.25059382	1	PRJNA827465;Yang2025_Silkie_Wenchang	CRA004775	0.533277943
ENSGALG00010017480	DCDC2B	2	0.202393573	0.443070273	0.456797906	0.647816311	0.332383035	6.465020065	84.53214391	0.5	CRA004775;Yang2025_Silkie_Wenchang	PRJNA827465	0.907478507
ENSGALG00010017480	DCDC2B	2	0.015897466	0.206196488	0.077098628	0.938545084	0.041630563	1.825146693	45.20988347	0.5	CRA004775;PRJNA827465	Yang2025_Silkie_Wenchang	0.985349045
ENSGALG00010017482	CORO2B	2	-0.163921856	0.238834539	-0.686339531	0.492499026	0.10037372	8.152269187	87.73347669	0.5	PRJNA827465;Yang2025_Silkie_Wenchang	CRA004775	0.859304325
ENSGALG00010017482	CORO2B	2	-0.385732202	0.136638347	-2.823015724	0.004757424	0	0.624566702	0	0.5	CRA004775;Yang2025_Silkie_Wenchang	PRJNA827465	0.101675383
ENSGALG00010017482	CORO2B	2	0.060725333	0.087818324	0.691488179	0.489258807	0	0.013772069	0	1	CRA004775;PRJNA827465	Yang2025_Silkie_Wenchang	0.858337006
ENSGALG00010017484	IQCC	2	0.25715041	0.104140436	2.469265727	0.013539064	0	0.213332471	0	1	PRJNA827465;Yang2025_Silkie_Wenchang	CRA004775	0.190316819
ENSGALG00010017484	IQCC	2	-0.129509857	0.335132369	-0.386443892	0.699167949	0.181996646	5.216240668	80.82910541	0.5	CRA004775;Yang2025_Silkie_Wenchang	PRJNA827465	0.92185597
ENSGALG00010017484	IQCC	2	-0.070533863	0.385640844	-0.182900396	0.854876172	0.263385427	8.472466157	88.19706115	0.5	CRA004775;PRJNA827465	Yang2025_Silkie_Wenchang	0.963248391
ENSGALG00010017485	LBH	2	-0.217064597	0.189688247	-1.14432286	0.252489766	0.065187797	10.3095685	90.300273	1	PRJNA827465;Yang2025_Silkie_Wenchang	CRA004775	0.762655223
ENSGALG00010017485	LBH	2	-0.383188704	0.095433573	-4.015240066	5.93853E-05	0	0.61369343	0	1	CRA004775;Yang2025_Silkie_Wenchang	PRJNA827465	0.005427956
ENSGALG00010017485	LBH	2	-0.047624458	0.052884028	-0.900545209	0.367830178	0	0.614456776	0	1	CRA004775;PRJNA827465	Yang2025_Silkie_Wenchang	0.820142704
ENSGALG00010017487	C1QTNF8	2	0.452497439	0.333475285	1.356914466	0.17480839	0	0.923574295	0	1	PRJNA827465;Yang2025_Silkie_Wenchang	CRA004775	0.679506889
ENSGALG00010017487	C1QTNF8	2	0.10499718	0.782468595	0.134187085	0.893254638	0.814033993	2.748270822	63.61348409	0.5	CRA004775;Yang2025_Silkie_Wenchang	PRJNA827465	0.974381724
ENSGALG00010017487	C1QTNF8	2	-0.149794685	0.431955253	-0.346782876	0.728754443	0	0.928498209	0	0.5	CRA004775;PRJNA827465	Yang2025_Silkie_Wenchang	0.932236477
ENSGALG00010017489	TNFRSF1B	2	-0.088270172	0.111310982	-0.793005061	0.427774874	0.008036604	1.378918047	27.47937399	1	PRJNA827465;Yang2025_Silkie_Wenchang	CRA004775	0.84125685
ENSGALG00010017489	TNFRSF1B	2	-0.299700705	0.170024782	-1.762683869	0.077953064	0	0.501662338	0	1	CRA004775;Yang2025_Silkie_Wenchang	PRJNA827465	0.480745215
ENSGALG00010017489	TNFRSF1B	2	-0.128770843	0.240411052	-0.535627802	0.59222151	0.061051823	1.464640321	31.72285154	1	CRA004775;PRJNA827465	Yang2025_Silkie_Wenchang	0.892776493
ENSGALG00010017491	CCDC28B	2	0.613735552	0.331540485	1.851163223	0.064146075	0.114919628	2.015599875	50.38697847	1	PRJNA827465;Yang2025_Silkie_Wenchang	CRA004775	0.436408721
ENSGALG00010017491	CCDC28B	2	0.446159147	0.559188248	0.797869319	0.42494633	0.480460019	4.310561738	76.80116744	0.5	CRA004775;Yang2025_Silkie_Wenchang	PRJNA827465	0.841139484
ENSGALG00010017491	CCDC28B	2	0.188233289	0.214178363	0.878862302	0.379475941	0	0.964754656	0	0.5	CRA004775;PRJNA827465	Yang2025_Silkie_Wenchang	0.824165982
ENSGALG00010017493	YPEL5	2	-0.361599701	0.359643378	-1.00543962	0.31468521	0.252390495	40.83371432	97.55104326	1	PRJNA827465;Yang2025_Silkie_Wenchang	CRA004775	0.79651448
ENSGALG00010017493	YPEL5	2	-0.3898066	0.403393888	-0.966317567	0.333885298	0.266027074	4.776167083	79.06270902	0.5	CRA004775;Yang2025_Silkie_Wenchang	PRJNA827465	0.802807652
ENSGALG00010017493	YPEL5	2	-0.004047708	0.055239699	-0.073275348	0.941587009	0	0.07585023	0	0.5	CRA004775;PRJNA827465	Yang2025_Silkie_Wenchang	0.985827325
ENSGALG00010017494	TBC1D24	2	-0.122542578	0.108485847	-1.129572024	0.258656605	0.016515434	3.183818329	68.59117272	1	PRJNA827465;Yang2025_Silkie_Wenchang	CRA004775	0.765798911
ENSGALG00010017494	TBC1D24	2	-0.261963633	0.101665134	-2.576730328	0.009973973	0	0.21175016	0	1	CRA004775;Yang2025_Silkie_Wenchang	PRJNA827465	0.158194941
ENSGALG00010017494	TBC1D24	2	-0.058538009	0.103869709	-0.563571514	0.573045778	0.007689122	1.107741887	9.726262802	1	CRA004775;PRJNA827465	Yang2025_Silkie_Wenchang	0.88573298
ENSGALG00010017495	NT5M	2	-0.193157243	0.11283855	-1.711801893	0.086933183	0.018154959	3.249259312	69.2237552	1	PRJNA827465;Yang2025_Silkie_Wenchang	CRA004775	0.50723499
ENSGALG00010017495	NT5M	2	-0.34246109	0.103697902	-3.302488125	0.000958311	0	0.130642212	0	1	CRA004775;Yang2025_Silkie_Wenchang	PRJNA827465	0.036781753
ENSGALG00010017495	NT5M	2	-0.159425924	0.12979993	-1.228243532	0.219355562	0.016999198	1.430003364	30.07009456	1	CRA004775;PRJNA827465	Yang2025_Silkie_Wenchang	0.732888275
ENSGALG00010017496	PARP1	2	-0.003155733	0.112812583	-0.027973236	0.977683498	0.019811896	4.413097504	77.34017888	0.5	PRJNA827465;Yang2025_Silkie_Wenchang	CRA004775	0.993738867
ENSGALG00010017496	PARP1	2	0.023775003	0.142103888	0.167307196	0.867128342	0.021421741	1.831225771	45.39176895	0.5	CRA004775;Yang2025_Silkie_Wenchang	PRJNA827465	0.967699
ENSGALG00010017496	PARP1	2	-0.113704191	0.058839169	-1.932457436	0.053303071	0	0.137838733	0	1	CRA004775;PRJNA827465	Yang2025_Silkie_Wenchang	0.400004977
ENSGALG00010017499	SNRPD1	2	-0.019078316	0.108438759	-0.175936319	0.860343972	0.010086144	1.373458598	27.19110707	0.5	PRJNA827465;Yang2025_Silkie_Wenchang	CRA004775	0.964999892
ENSGALG00010017499	SNRPD1	2	-0.298586345	0.169631461	-1.760206174	0.078372856	0	0.17960132	0	1	CRA004775;Yang2025_Silkie_Wenchang	PRJNA827465	0.481746691
ENSGALG00010017499	SNRPD1	2	-0.097496432	0.194425322	-0.501459538	0.616047747	0.050715323	2.382002352	58.01851333	0.5	CRA004775;PRJNA827465	Yang2025_Silkie_Wenchang	0.899057762
ENSGALG00010017500	ACAP2	2	0.042571895	0.056088489	0.759013047	0.447844752	0	0.026147109	0	1	PRJNA827465;Yang2025_Silkie_Wenchang	CRA004775	0.846601815
ENSGALG00010017500	ACAP2	2	0.023899521	0.08567877	0.278931537	0.780297366	0	0.025104342	0	0.5	CRA004775;Yang2025_Silkie_Wenchang	PRJNA827465	0.945041817
ENSGALG00010017500	ACAP2	2	0.043478764	0.065849379	0.660276	0.509076729	0	0.064650643	0	0.5	CRA004775;PRJNA827465	Yang2025_Silkie_Wenchang	0.865193604
ENSGALG00010017502	NAT8B	2	-0.013451814	0.108896437	-0.123528508	0.901689601	0	0.162754821	0	0.5	PRJNA827465;Yang2025_Silkie_Wenchang	CRA004775	0.976674416
ENSGALG00010017502	NAT8B	2	-0.019093295	0.249542929	-0.076513068	0.939010918	0	0.323967048	0	0.5	CRA004775;Yang2025_Silkie_Wenchang	PRJNA827465	0.985349045
ENSGALG00010017502	NAT8B	2	-0.040551321	0.109033814	-0.371915094	0.709956067	0	0.131192036	0	1	CRA004775;PRJNA827465	Yang2025_Silkie_Wenchang	0.925298042
ENSGALG00010017503	MED9	2	-0.128892123	0.072960425	-1.766603239	0.077294698	0.002535592	1.122635901	10.92392472	1	PRJNA827465;Yang2025_Silkie_Wenchang	CRA004775	0.478832063
ENSGALG00010017503	MED9	2	-0.317195291	0.176341701	-1.798755725	0.072057646	0	0.000258398	0	1	CRA004775;Yang2025_Silkie_Wenchang	PRJNA827465	0.463464923
ENSGALG00010017503	MED9	2	-0.104881152	0.059380984	-1.766241387	0.07735536	0	0.276093525	0	1	CRA004775;PRJNA827465	Yang2025_Silkie_Wenchang	0.479060101
ENSGALG00010017505	TNFRSF8	2	-0.311141399	0.21940254	-1.418130345	0.156152713	0	0.806445717	0	1	PRJNA827465;Yang2025_Silkie_Wenchang	CRA004775	0.649132948
ENSGALG00010017505	TNFRSF8	2	-0.166791972	0.329390731	-0.506365103	0.612600368	0	0.542711497	0	1	CRA004775;Yang2025_Silkie_Wenchang	PRJNA827465	0.897994639
ENSGALG00010017505	TNFRSF8	2	-0.48096238	0.256204877	-1.877256927	0.060482889	0	0.061804186	0	1	CRA004775;PRJNA827465	Yang2025_Silkie_Wenchang	0.426049285
ENSGALG00010017509	RASD1	2	0.480459149	0.219477318	2.189106156	0.028589125	0	0.572187818	0	1	PRJNA827465;Yang2025_Silkie_Wenchang	CRA004775	0.287890952
ENSGALG00010017509	RASD1	2	0.815883438	0.2740962	2.9766317	0.002914339	0	0.312798532	0	1	CRA004775;Yang2025_Silkie_Wenchang	PRJNA827465	0.073993706
ENSGALG00010017509	RASD1	2	0.592788721	0.316215193	1.874637063	0.060842671	0.081044217	1.609788576	37.88004121	1	CRA004775;PRJNA827465	Yang2025_Silkie_Wenchang	0.427127227
ENSGALG00010017510	HDC	2	-0.752526291	1.035583764	-0.726668684	0.467428942	1.98563123	13.44143775	92.56031967	0.5	PRJNA827465;Yang2025_Silkie_Wenchang	CRA004775	0.852885838
ENSGALG00010017510	HDC	2	-1.024527272	0.829564468	-1.235018267	0.216823706	1.104633513	4.979249625	79.9166526	1	CRA004775;Yang2025_Silkie_Wenchang	PRJNA827465	0.730358425
ENSGALG00010017510	HDC	2	0.161905149	0.311429144	0.519877961	0.603148637	0	0.334388054	0	0.5	CRA004775;PRJNA827465	Yang2025_Silkie_Wenchang	0.895538966
ENSGALG00010017513	TNIP2	2	-0.015813124	0.105470748	-0.149929	0.880820632	0.003823629	1.166382007	14.2047954	0.5	PRJNA827465;Yang2025_Silkie_Wenchang	CRA004775	0.971676583
ENSGALG00010017513	TNIP2	2	0.104704845	0.160696147	0.65157035	0.51467838	0	0.225039235	0	0.5	CRA004775;Yang2025_Silkie_Wenchang	PRJNA827465	0.866869697
ENSGALG00010017513	TNIP2	2	-0.078501353	0.103602366	-0.757717615	0.448620051	0	0.023922897	0	1	CRA004775;PRJNA827465	Yang2025_Silkie_Wenchang	0.846878812
ENSGALG00010017514	TXLNA	2	0.054398761	0.076976735	0.706690937	0.479758563	0.007448028	2.664364191	62.46759346	0.5	PRJNA827465;Yang2025_Silkie_Wenchang	CRA004775	0.856274132
ENSGALG00010017514	TXLNA	2	0.126162259	0.067818299	1.860298196	0.062843349	0	0.163328313	0	1	CRA004775;Yang2025_Silkie_Wenchang	PRJNA827465	0.431807797
ENSGALG00010017514	TXLNA	2	-0.008841432	0.05641155	-0.156730886	0.875456944	0	0.173721129	0	0.5	CRA004775;PRJNA827465	Yang2025_Silkie_Wenchang	0.970347777
ENSGALG00010017516	AAK1	2	0.068011601	0.153192136	0.443962742	0.657069522	0.035725775	3.580670492	72.07226964	0.5	PRJNA827465;Yang2025_Silkie_Wenchang	CRA004775	0.910695405
ENSGALG00010017516	AAK1	2	0.172789917	0.144184802	1.198392031	0.230764433	0.003430371	1.070538153	6.589036805	0.5	CRA004775;Yang2025_Silkie_Wenchang	PRJNA827465	0.743374303
ENSGALG00010017516	AAK1	2	-0.053385439	0.048364546	-1.1038135	0.269674047	0	0.000821569	0	1	CRA004775;PRJNA827465	Yang2025_Silkie_Wenchang	0.77189992
ENSGALG00010017517	LIN9	2	-0.023033204	0.061502	-0.374511464	0.708023829	0.001110123	1.117341609	10.5018562	0.5	PRJNA827465;Yang2025_Silkie_Wenchang	CRA004775	0.925061251
ENSGALG00010017517	LIN9	2	-0.126735009	0.116339341	-1.089356428	0.275996738	0	0.038594547	0	1	CRA004775;Yang2025_Silkie_Wenchang	PRJNA827465	0.775541559
ENSGALG00010017517	LIN9	2	0.008901367	0.060952716	0.146037244	0.883891987	0	0.036787788	0	0.5	CRA004775;PRJNA827465	Yang2025_Silkie_Wenchang	0.972001076
ENSGALG00010017518	PEMT	2	0.05580956	0.052596316	1.061092566	0.288647836	0	0.45596469	0	1	PRJNA827465;Yang2025_Silkie_Wenchang	CRA004775	0.783313256
ENSGALG00010017518	PEMT	2	0.09291867	0.090604683	1.025539374	0.305108757	0	0.550471025	0	0.5	CRA004775;Yang2025_Silkie_Wenchang	PRJNA827465	0.79225794
ENSGALG00010017518	PEMT	2	0.041525189	0.063199521	0.410416869	0.681500174	0	0.332670863	0	0.5	CRA004775;PRJNA827465	Yang2025_Silkie_Wenchang	0.918260442
ENSGALG00010017519	NTN3	2	0.046383293	0.096792686	0.479202454	0.631794609	0	0.213592612	0	1	PRJNA827465;Yang2025_Silkie_Wenchang	CRA004775	0.903058174
ENSGALG00010017519	NTN3	2	0.407799901	0.401342522	1.016089447	0.309586794	0.240117391	3.361859227	70.25455462	1	CRA004775;Yang2025_Silkie_Wenchang	PRJNA827465	0.794246638
ENSGALG00010017519	NTN3	2	0.371637082	0.44916795	0.827390026	0.408016032	0.319165244	4.169540025	76.01653914	1	CRA004775;PRJNA827465	Yang2025_Silkie_Wenchang	0.830400531
ENSGALG00010017520	ZBTB1	2	-0.055018636	0.034136636	-1.61171813	0.107022291	0	0.455047408	0	1	PRJNA827465;Yang2025_Silkie_Wenchang	CRA004775	0.554048524
ENSGALG00010017520	ZBTB1	2	-0.075730018	0.05814982	-1.302325917	0.192804995	0	0.464795857	0	0.5	CRA004775;Yang2025_Silkie_Wenchang	PRJNA827465	0.702807541
ENSGALG00010017520	ZBTB1	2	-0.035712821	0.039923172	-0.894538677	0.371033746	0	0.181694902	0	0.5	CRA004775;PRJNA827465	Yang2025_Silkie_Wenchang	0.8214684
ENSGALG00010017521	MIIP	2	0.00043714	0.067348037	0.006490758	0.994821161	0	0.760927541	0	0.5	PRJNA827465;Yang2025_Silkie_Wenchang	CRA004775	0.998242335
ENSGALG00010017521	MIIP	2	-0.073136497	0.210003073	-0.348263935	0.727641976	0.05606543	2.530980136	60.48961485	0.5	CRA004775;Yang2025_Silkie_Wenchang	PRJNA827465	0.931639802
ENSGALG00010017521	MIIP	2	-0.101322169	0.123614467	-0.819662709	0.412408414	0.013234797	1.41816139	29.48616375	1	CRA004775;PRJNA827465	Yang2025_Silkie_Wenchang	0.835702455
ENSGALG00010017522	XXYLT1	2	-0.052388751	0.096592129	-0.542370812	0.587563083	0.008899497	1.470567407	31.99903688	1	PRJNA827465;Yang2025_Silkie_Wenchang	CRA004775	0.89167336
ENSGALG00010017522	XXYLT1	2	-0.263292827	0.140005068	-1.880594974	0.060025706	0	0.042051817	0	1	CRA004775;Yang2025_Silkie_Wenchang	PRJNA827465	0.424082489
ENSGALG00010017522	XXYLT1	2	-0.077788992	0.12845529	-0.605572507	0.544798663	0.01926955	1.807437927	44.67306539	1	CRA004775;PRJNA827465	Yang2025_Silkie_Wenchang	0.877345822
ENSGALG00010017524	ACBD3	2	-0.002226308	0.066725274	-0.033365286	0.973383292	0.004314554	1.57586849	36.54292816	0.5	PRJNA827465;Yang2025_Silkie_Wenchang	CRA004775	0.992392211
ENSGALG00010017524	ACBD3	2	-0.094206848	0.214569408	-0.439050695	0.660624804	0.075504925	5.522087782	81.89090722	0.5	CRA004775;Yang2025_Silkie_Wenchang	PRJNA827465	0.91192683
ENSGALG00010017524	ACBD3	2	-0.145463874	0.133776043	-1.087368636	0.276873926	0.027177346	3.50640948	71.48079807	1	CRA004775;PRJNA827465	Yang2025_Silkie_Wenchang	0.776859465
ENSGALG00010017526	PLEKHA2	2	0.097513843	0.094743654	1.029238783	0.303367482	0	0.419301673	0	1	PRJNA827465;Yang2025_Silkie_Wenchang	CRA004775	0.792084776
ENSGALG00010017526	PLEKHA2	2	-0.018048597	0.13835033	-0.130455758	0.896203855	0	0.579251175	0	0.5	CRA004775;Yang2025_Silkie_Wenchang	PRJNA827465	0.975115773
ENSGALG00010017526	PLEKHA2	2	0.07846216	0.15947731	0.491991558	0.622722544	0.015187212	1.19509561	16.11157423	0.5	CRA004775;PRJNA827465	Yang2025_Silkie_Wenchang	0.900255255
ENSGALG00010017528	RAI1	2	-0.042570181	0.077831215	-0.546955111	0.584409572	0.004838693	1.608485353	37.82971054	0.5	PRJNA827465;Yang2025_Silkie_Wenchang	CRA004775	0.890963902
ENSGALG00010017528	RAI1	2	0.043707986	0.100109823	0.436600377	0.662401189	0	0.167587288	0	0.5	CRA004775;Yang2025_Silkie_Wenchang	PRJNA827465	0.912749498
ENSGALG00010017528	RAI1	2	-0.103831499	0.069430444	-1.495470084	0.134790498	0	0.000580078	0	1	CRA004775;PRJNA827465	Yang2025_Silkie_Wenchang	0.615292791
ENSGALG00010017529	MFN2	2	0.015066116	0.069307859	0.217379621	0.827912512	0.004359309	1.825605954	45.22166682	0.5	PRJNA827465;Yang2025_Silkie_Wenchang	CRA004775	0.956550752
ENSGALG00010017529	MFN2	2	-0.051847739	0.07478253	-0.693313523	0.488112819	0	0.173545859	0	0.5	CRA004775;Yang2025_Silkie_Wenchang	PRJNA827465	0.85768885
ENSGALG00010017529	MFN2	2	0.081033494	0.067530075	1.199961563	0.230154269	0	0.001942142	0	1	CRA004775;PRJNA827465	Yang2025_Silkie_Wenchang	0.742845187
ENSGALG00010017530	TLR1B	2	-0.051173972	0.092562443	-0.552858918	0.58036002	0	0.175611815	0	1	PRJNA827465;Yang2025_Silkie_Wenchang	CRA004775	0.888433837
ENSGALG00010017530	TLR1B	2	-0.14528926	0.144214018	-1.007622761	0.313635598	0	0.936794388	0	1	CRA004775;Yang2025_Silkie_Wenchang	PRJNA827465	0.796531448
ENSGALG00010017530	TLR1B	2	-0.113751616	0.209508229	-0.542945814	0.587167111	0.038763566	1.318530081	24.15796846	1	CRA004775;PRJNA827465	Yang2025_Silkie_Wenchang	0.891596903
ENSGALG00010017531	SACM1L	2	0.04049087	0.069253024	0.584680181	0.558762783	0.005776147	2.406465205	58.44527491	0.5	PRJNA827465;Yang2025_Silkie_Wenchang	CRA004775	0.880871906
ENSGALG00010017531	SACM1L	2	-0.026607977	0.070698989	-0.376355833	0.706652376	0	0.289466515	0	0.5	CRA004775;Yang2025_Silkie_Wenchang	PRJNA827465	0.924552541
ENSGALG00010017531	SACM1L	2	0.096810732	0.047537063	2.036531603	0.041697003	0	0.024213805	0	1	CRA004775;PRJNA827465	Yang2025_Silkie_Wenchang	0.353826658
ENSGALG00010017532	MIXL1	2	-0.675638481	0.327660159	-2.062009868	0.039206797	0.056207334	1.352525058	26.06421643	1	PRJNA827465;Yang2025_Silkie_Wenchang	CRA004775	0.342204025
ENSGALG00010017532	MIXL1	2	-0.171970052	0.362606141	-0.474261279	0.63531136	0	0.561680181	0	0.5	CRA004775;Yang2025_Silkie_Wenchang	PRJNA827465	0.904143478
ENSGALG00010017532	MIXL1	2	-0.490809114	0.626812118	-0.783026249	0.433612848	0.489326404	2.423618963	58.75938868	0.5	CRA004775;PRJNA827465	Yang2025_Silkie_Wenchang	0.843762754
ENSGALG00010017533	FAM193A	2	0.085753928	0.142489474	0.601826403	0.547289698	0.028954044	2.779047788	64.01645181	0.5	PRJNA827465;Yang2025_Silkie_Wenchang	CRA004775	0.878281266
ENSGALG00010017533	FAM193A	2	0.226977673	0.142932077	1.588010734	0.112283912	0	0.317875321	0	1	CRA004775;Yang2025_Silkie_Wenchang	PRJNA827465	0.56602666
ENSGALG00010017533	FAM193A	2	-0.012788133	0.041989079	-0.304558541	0.7607024	0	0.269595169	0	0.5	CRA004775;PRJNA827465	Yang2025_Silkie_Wenchang	0.939384641
ENSGALG00010017534	ATP11C	2	-0.097016002	0.108230063	-0.896386819	0.370046204	0.016281685	3.18889521	68.64117714	1	PRJNA827465;Yang2025_Silkie_Wenchang	CRA004775	0.820855964
ENSGALG00010017534	ATP11C	2	-0.219183293	0.097521707	-2.247533406	0.024605958	0	0.002437124	0	1	CRA004775;Yang2025_Silkie_Wenchang	PRJNA827465	0.268130106
ENSGALG00010017534	ATP11C	2	-0.006750262	0.068356378	-0.098751019	0.921335959	0	0.325274126	0	1	CRA004775;PRJNA827465	Yang2025_Silkie_Wenchang	0.981519273
ENSGALG00010017535	LIMD1	2	-0.251605438	0.211420798	-1.190069473	0.234019087	0.071422103	4.501351507	77.78444988	1	PRJNA827465;Yang2025_Silkie_Wenchang	CRA004775	0.747279664
ENSGALG00010017535	LIMD1	2	-0.414818325	0.156909294	-2.643682311	0.008200959	0	0.689377168	0	1	CRA004775;Yang2025_Silkie_Wenchang	PRJNA827465	0.140757277
ENSGALG00010017535	LIMD1	2	-0.081305067	0.071667207	-1.134480763	0.256592939	0	0.242240369	0	1	CRA004775;PRJNA827465	Yang2025_Silkie_Wenchang	0.764677554
ENSGALG00010017536	GSC	2	0.206544615	0.330241448	0.625435166	0.531685488	0	0.03072545	0	1	PRJNA827465;Yang2025_Silkie_Wenchang	CRA004775	0.873042777
ENSGALG00010017536	GSC	2	0.392467831	0.415632211	0.944267121	0.345033163	0	0.037719108	0	1	CRA004775;Yang2025_Silkie_Wenchang	PRJNA827465	0.806961063
ENSGALG00010017536	GSC	2	0.267281179	0.317554957	0.841684796	0.399964411	0	0.187406216	0	1	CRA004775;PRJNA827465	Yang2025_Silkie_Wenchang	0.832246851
ENSGALG00010017537	MANBA	2	0.021490682	0.161359491	0.133185113	0.894046984	0.035481413	2.918535308	65.73623771	0.5	PRJNA827465;Yang2025_Silkie_Wenchang	CRA004775	0.974381724
ENSGALG00010017537	MANBA	2	-0.145014911	0.158808568	-0.913142867	0.361167407	0	0.240370557	0	0.5	CRA004775;Yang2025_Silkie_Wenchang	PRJNA827465	0.815240214
ENSGALG00010017537	MANBA	2	0.15159317	0.089696156	1.690074324	0.091013736	0	0.035182554	0	1	CRA004775;PRJNA827465	Yang2025_Silkie_Wenchang	0.517594064
ENSGALG00010017538	H3-3A	2	-0.063944719	0.044632457	-1.432695458	0.151944888	0	0.123694846	0	1	PRJNA827465;Yang2025_Silkie_Wenchang	CRA004775	0.643294424
ENSGALG00010017538	H3-3A	2	-0.034278149	0.082848058	-0.413747163	0.679059283	0	0.000308245	0	1	CRA004775;Yang2025_Silkie_Wenchang	PRJNA827465	0.917701868
ENSGALG00010017538	H3-3A	2	-0.069403367	0.04885191	-1.420688932	0.155407211	0	0.048696409	0	1	CRA004775;PRJNA827465	Yang2025_Silkie_Wenchang	0.648167852
ENSGALG00010017539	KPNA6	2	-0.069249411	0.059618681	-1.161538795	0.245422856	0.003687019	1.634768424	38.82925648	1	PRJNA827465;Yang2025_Silkie_Wenchang	CRA004775	0.75661406
ENSGALG00010017539	KPNA6	2	-0.157995811	0.093492001	-1.68993935	0.091039558	0	0.118231976	0	1	CRA004775;Yang2025_Silkie_Wenchang	PRJNA827465	0.517594064
ENSGALG00010017539	KPNA6	2	-0.035963477	0.032040847	-1.122425891	0.261681401	0	0.05408006	0	1	CRA004775;PRJNA827465	Yang2025_Silkie_Wenchang	0.768310285
ENSGALG00010017540	EIF4G1	2	0.038775734	0.164897086	0.235151114	0.814091448	0.044535297	5.506941442	81.84109981	0.5	PRJNA827465;Yang2025_Silkie_Wenchang	CRA004775	0.953869515
ENSGALG00010017540	EIF4G1	2	0.153397869	0.108743935	1.410633786	0.158352624	0.003539238	1.106229314	9.602829444	0.5	CRA004775;Yang2025_Silkie_Wenchang	PRJNA827465	0.652042151
ENSGALG00010017540	EIF4G1	2	-0.114232412	0.08587573	-1.330206009	0.183450405	0	0.053383309	0	1	CRA004775;PRJNA827465	Yang2025_Silkie_Wenchang	0.691847354
ENSGALG00010017541	SREBF1	2	0.119542845	0.26837125	0.445438344	0.656003007	0.134784997	15.45719538	93.53052106	0.5	PRJNA827465;Yang2025_Silkie_Wenchang	CRA004775	0.910411938
ENSGALG00010017541	SREBF1	2	0.114911445	0.34781983	0.330376349	0.74111561	0.186299836	3.749334789	73.3286021	0.5	CRA004775;Yang2025_Silkie_Wenchang	PRJNA827465	0.935384591
ENSGALG00010017541	SREBF1	2	-0.151938378	0.077647047	-1.956782448	0.050373052	0	0.239012178	0	1	CRA004775;PRJNA827465	Yang2025_Silkie_Wenchang	0.388961109
ENSGALG00010017542	PLOD1	2	-0.167535539	0.148478762	-1.128346821	0.259173477	0.038285691	7.502634957	86.67134939	1	PRJNA827465;Yang2025_Silkie_Wenchang	CRA004775	0.766314541
ENSGALG00010017542	PLOD1	2	-0.104148538	0.257106714	-0.405079027	0.685419422	0.106011561	4.5634532	78.0867699	0.5	CRA004775;Yang2025_Silkie_Wenchang	PRJNA827465	0.919235539
ENSGALG00010017542	PLOD1	2	-0.009270238	0.060862311	-0.152314926	0.878938553	0	0.899525826	0	0.5	CRA004775;PRJNA827465	Yang2025_Silkie_Wenchang	0.971127951
ENSGALG00010017543	ZBTB25	2	0.076915334	0.105859581	0.726578862	0.467483981	0	0.312996745	0	1	PRJNA827465;Yang2025_Silkie_Wenchang	CRA004775	0.852885838
ENSGALG00010017543	ZBTB25	2	-0.217815163	0.444217881	-0.490334074	0.623897519	0.311906951	4.667824819	78.5767453	0.5	CRA004775;Yang2025_Silkie_Wenchang	PRJNA827465	0.900614985
ENSGALG00010017543	ZBTB25	2	-0.257173047	0.365774196	-0.70309237	0.481998187	0.212187553	4.283483348	76.65451413	0.5	CRA004775;PRJNA827465	Yang2025_Silkie_Wenchang	0.85691916
ENSGALG00010017544	SDE2	2	-0.077052713	0.040391825	-1.907631387	0.056438875	0.000573064	1.194211847	16.2627634	1	PRJNA827465;Yang2025_Silkie_Wenchang	CRA004775	0.411523553
ENSGALG00010017544	SDE2	2	-0.017992293	0.05936159	-0.303096546	0.761816286	0	0.083295682	0	0.5	CRA004775;Yang2025_Silkie_Wenchang	PRJNA827465	0.939811773
ENSGALG00010017544	SDE2	2	-0.100141262	0.043438604	-2.30535179	0.021146879	0	0.53009189	0	0.5	CRA004775;PRJNA827465	Yang2025_Silkie_Wenchang	0.246043752
ENSGALG00010017545	DICER1	2	0.021352744	0.088453245	0.241401477	0.809243968	0.004796999	1.275640051	21.60798028	0.5	PRJNA827465;Yang2025_Silkie_Wenchang	CRA004775	0.95276497
ENSGALG00010017545	DICER1	2	0.227135712	0.156117261	1.454904545	0.145695689	0	0.366377206	0	1	CRA004775;Yang2025_Silkie_Wenchang	PRJNA827465	0.634354199
ENSGALG00010017545	DICER1	2	0.056265013	0.17707359	0.317749322	0.750675095	0.032906883	1.449571516	31.01409697	0.5	CRA004775;PRJNA827465	Yang2025_Silkie_Wenchang	0.937473087
ENSGALG00010017546	NFU1	2	-0.035312966	0.080780799	-0.437145539	0.662005803	0	0.10879013	0	0.5	PRJNA827465;Yang2025_Silkie_Wenchang	CRA004775	0.912556664
ENSGALG00010017546	NFU1	2	-0.12120959	0.215090007	-0.563529622	0.573074295	0.021968597	1.268506839	21.16715739	0.5	CRA004775;Yang2025_Silkie_Wenchang	PRJNA827465	0.885742225
ENSGALG00010017546	NFU1	2	-0.091587401	0.123204624	-0.743376324	0.457253874	0.009702714	1.157049479	13.57327251	1	CRA004775;PRJNA827465	Yang2025_Silkie_Wenchang	0.849541335

ENSGALG00010017547	RNF4	2	-0.059746064	0.153957001	-0.388069811	0.697964372	0.044840861	18.45994143	94.58286472	0.5	PRJNA827465;Yang2025_Silkie_Wenchang	CRA004775	0.921498664
ENSGALG00010017547	RNF4	2	-0.139207792	0.114647767	-1.214221575	0.224663124	0.015916092	2.052352271	51.27542117	0.5	CRA004775;Yang2025_Silkie_Wenchang	PRJNA827465	0.737857004
ENSGALG00010017547	RNF4	2	0.088574378	0.044713436	1.980934297	0.047598642	0	0.117353413	0	1	CRA004775;PRJNA827465	Yang2025_Silkie_Wenchang	0.376943268
ENSGALG00010017550	TM2D2	2	-0.115533527	0.06239791	-1.851560826	0.064088913	0	0.027120122	0	1	PRJNA827465;Yang2025_Silkie_Wenchang	CRA004775	0.436198289
ENSGALG00010017550	TM2D2	2	-0.221957157	0.144534066	-1.535673654	0.12461844	0	0.746441432	0	1	CRA004775;Yang2025_Silkie_Wenchang	PRJNA827465	0.592140711
ENSGALG00010017550	TM2D2	2	-0.153992625	0.105619606	-1.457992798	0.144842528	0.008473047	1.222474439	18.19869861	1	CRA004775;PRJNA827465	Yang2025_Silkie_Wenchang	0.632667913
ENSGALG00010017551	NALF2	2	0.103998769	0.248478266	0.418542719	0.675555036	0.089647593	3.613233697	72.32396009	0.5	PRJNA827465;Yang2025_Silkie_Wenchang	CRA004775	0.91647357
ENSGALG00010017551	NALF2	2	-0.08569523	0.506844248	-0.169076063	0.865736813	0.421045397	5.253088919	80.96358132	0.5	CRA004775;Yang2025_Silkie_Wenchang	PRJNA827465	0.967088846
ENSGALG00010017551	NALF2	2	-0.266650925	0.229661234	-1.161061973	0.245616698	0.044847725	1.490860894	32.9246609	1	CRA004775;PRJNA827465	Yang2025_Silkie_Wenchang	0.75661406
ENSGALG00010017552	LARS2	2	-0.042815777	0.07610971	-0.562553412	0.573739024	0.004766742	1.650691527	39.41932918	0.5	PRJNA827465;Yang2025_Silkie_Wenchang	CRA004775	0.886016235
ENSGALG00010017552	LARS2	2	-0.094472391	0.08932527	-1.057622225	0.290227706	0	0.968810259	0	0.5	CRA004775;Yang2025_Silkie_Wenchang	PRJNA827465	0.784195688
ENSGALG00010017552	LARS2	2	0.026174544	0.066008208	0.396534683	0.691710627	0	0.103956353	0	1	CRA004775;PRJNA827465	Yang2025_Silkie_Wenchang	0.920348122
ENSGALG00010017553	EDA	2	-0.22226541	0.186659012	-1.190756384	0.233749236	0.05554815	4.517930604	77.86597255	1	PRJNA827465;Yang2025_Silkie_Wenchang	CRA004775	0.747082975
ENSGALG00010017553	EDA	2	-0.095020551	0.396291431	-0.239774428	0.810505133	0.249328996	4.518112454	77.86686343	0.5	CRA004775;Yang2025_Silkie_Wenchang	PRJNA827465	0.952997426
ENSGALG00010017553	EDA	2	0.018733053	0.166246452	0.112682421	0.910282338	0.029258717	1.487609515	32.77805837	0.5	CRA004775;PRJNA827465	Yang2025_Silkie_Wenchang	0.97880407
ENSGALG00010017554	FAM131A	2	0.307658162	0.215448091	1.427992054	0.153294145	0	0.384767699	0	1	PRJNA827465;Yang2025_Silkie_Wenchang	CRA004775	0.645602272
ENSGALG00010017554	FAM131A	2	0.290245959	0.299797033	0.968141531	0.332973691	0.005252268	1.023766788	2.321504123	0.5	CRA004775;Yang2025_Silkie_Wenchang	PRJNA827465	0.802178578
ENSGALG00010017554	FAM131A	2	0.124216558	0.248522282	0.499820609	0.617201398	0	0.397954308	0	0.5	CRA004775;PRJNA827465	Yang2025_Silkie_Wenchang	0.899121942
ENSGALG00010017556	TOM1L2	2	-0.049626529	0.049976654	-0.992994233	0.320712765	0	0.006398707	0	1	PRJNA827465;Yang2025_Silkie_Wenchang	CRA004775	0.798348216
ENSGALG00010017556	TOM1L2	2	-0.162737703	0.164267258	-0.990688618	0.321837651	0.024475199	1.594276666	37.27562968	1	CRA004775;Yang2025_Silkie_Wenchang	PRJNA827465	0.798513866
ENSGALG00010017556	TOM1L2	2	-0.145736325	0.164074816	-0.888230924	0.374416548	0.033461926	1.93003044	48.18734568	1	CRA004775;PRJNA827465	Yang2025_Silkie_Wenchang	0.822805106
ENSGALG00010017558	ENSGALG00010017558	2	0.067115333	0.129053691	0.520057443	0.603023538	0.028475073	6.644320478	84.94955198	0.5	PRJNA827465;Yang2025_Silkie_Wenchang	CRA004775	0.895538966
ENSGALG00010017558	ENSGALG00010017558	2	0.172987342	0.078520399	2.203087926	0.027588552	0	0.746444047	0	1	CRA004775;Yang2025_Silkie_Wenchang	PRJNA827465	0.283554618
ENSGALG00010017558	ENSGALG00010017558	2	-0.045703209	0.047102759	-0.970287305	0.331903303	0	0.238122551	0	0.5	CRA004775;PRJNA827465	Yang2025_Silkie_Wenchang	0.802002658
ENSGALG00010017559	DUSP10	2	-0.00179479	0.087652985	-0.020476085	0.983665589	0	0.188594611	0	0.5	PRJNA827465;Yang2025_Silkie_Wenchang	CRA004775	0.995492071
ENSGALG00010017559	DUSP10	2	0.274775534	0.408100759	0.673303168	0.500754438	0.251467536	3.531530961	71.68366889	0.5	CRA004775;Yang2025_Silkie_Wenchang	PRJNA827465	0.862421093
ENSGALG00010017559	DUSP10	2	0.298324726	0.364364365	0.81875385	0.412926866	0.194080129	3.03826785	67.08650951	1	CRA004775;PRJNA827465	Yang2025_Silkie_Wenchang	0.835870654
ENSGALG00010017562	SLC30A10	2	0.347076658	0.941483521	0.368648681	0.712389605	1.549395273	7.935044568	87.39767633	0.5	PRJNA827465;Yang2025_Silkie_Wenchang	CRA004775	0.92597752
ENSGALG00010017562	SLC30A10	2	0.205405131	0.901177659	0.227929723	0.819700723	1.22736823	3.934094746	74.58119175	0.5	CRA004775;Yang2025_Silkie_Wenchang	PRJNA827465	0.955256039
ENSGALG00010017562	SLC30A10	2	1.266725571	0.399300253	3.172363558	0.001512036	0	0.005750152	0	1	CRA004775;PRJNA827465	Yang2025_Silkie_Wenchang	0.048472714
ENSGALG00010017563	ALK	2	0.084397399	0.257566411	0.32767238	0.743159389	0.113292249	6.808679679	85.31286465	0.5	PRJNA827465;Yang2025_Silkie_Wenchang	CRA004775	0.93573824
ENSGALG00010017563	ALK	2	0.341476089	0.121168658	2.818188256	0.004829548	0	0.053785197	0	1	CRA004775;Yang2025_Silkie_Wenchang	PRJNA827465	0.1030268
ENSGALG00010017563	ALK	2	-0.042960323	0.26190134	-0.164032466	0.869705586	0.064195497	1.466572184	31.81378927	0.5	CRA004775;PRJNA827465	Yang2025_Silkie_Wenchang	0.968586462
ENSGALG00010017565	ENSGALG00010017565	2	-0.094370171	0.063949135	-1.475706772	0.140022627	0	0.243551367	0	1	PRJNA827465;Yang2025_Silkie_Wenchang	CRA004775	0.623792706
ENSGALG00010017565	ENSGALG00010017565	2	-0.120983009	0.091685381	-1.319545246	0.186986894	0	0.116675461	0	0.5	CRA004775;Yang2025_Silkie_Wenchang	PRJNA827465	0.695858627
ENSGALG00010017565	ENSGALG00010017565	2	-0.061798412	0.084841242	-0.728381874	0.466394336	0	0.033617873	0	0.5	CRA004775;PRJNA827465	Yang2025_Silkie_Wenchang	0.855763198
ENSGALG00010017568	CDCP1	2	0.372050326	0.26522518	1.402771509	0.160684986	0.070424929	1.975413241	49.37768063	1	PRJNA827465;Yang2025_Silkie_Wenchang	CRA004775	0.656486694
ENSGALG00010017568	CDCP1	2	0.716651421	0.198947505	3.602213672	0.000315519	0	0.737999556	0	1	CRA004775;Yang2025_Silkie_Wenchang	PRJNA827465	0.017542629
ENSGALG00010017568	CDCP1	2	0.502976481	0.454993799	1.105457881	0.268961655	0.302315061	3.671843234	72.76572184	1	CRA004775;PRJNA827465	Yang2025_Silkie_Wenchang	0.771398294
ENSGALG00010017569	CLMN	2	0.31817622	0.23957503	1.328085905	0.184149729	0.088614181	4.257156551	76.51014267	1	PRJNA827465;Yang2025_Silkie_Wenchang	CRA004775	0.692545576
ENSGALG00010017569	CLMN	2	0.378885586	0.259705668	1.458903802	0.144591587	0.070379353	1.964061036	49.08508536	1	CRA004775;Yang2025_Silkie_Wenchang	PRJNA827465	0.63213383
ENSGALG00010017569	CLMN	2	0.093086337	0.118984184	0.782342109	0.434013546	0	0.023667451	0	1	CRA004775;PRJNA827465	Yang2025_Silkie_Wenchang	0.842762754
ENSGALG00010017571	ADAM9	2	-0.063820801	0.039902583	-1.599415276	0.109752836	0	0.051306833	0	1	PRJNA827465;Yang2025_Silkie_Wenchang	CRA004775	0.56081231
ENSGALG00010017571	ADAM9	2	-0.060400119	0.07331082	-0.823890923	0.410001539	0	0.170966091	0	1	CRA004775;Yang2025_Silkie_Wenchang	PRJNA827465	0.834652129
ENSGALG00010017571	ADAM9	2	-0.072596606	0.045072197	-1.610673809	0.107250837	0	0.110220539	0	1	CRA004775;PRJNA827465	Yang2025_Silkie_Wenchang	0.554473355
ENSGALG00010017574	DYNC1LI1	2	-0.020936131	0.038558523	-0.542970245	0.587150289	0	0.041475224	0	1	PRJNA827465;Yang2025_Silkie_Wenchang	CRA004775	0.891596903
ENSGALG00010017574	DYNC1LI1	2	-0.002060916	0.071544788	-0.028805958	0.977019349	0	0.9690618	0	0.5	CRA004775;Yang2025_Silkie_Wenchang	PRJNA827465	0.993372089
ENSGALG00010017574	DYNC1LI1	2	-0.005973271	0.042746506	-0.139737062	0.888867742	0	0.894439239	0	0.5	CRA004775;PRJNA827465	Yang2025_Silkie_Wenchang	0.973135755
ENSGALG00010017576	ENSGALG00010017576	2	-0.171526708	0.127271072	-1.347727379	0.177746083	0	0.009592743	0	1	PRJNA827465;Yang2025_Silkie_Wenchang	CRA004775	0.683951879
ENSGALG00010017576	ENSGALG00010017576	2	-0.491506618	0.361075156	-1.361230785	0.173440768	0.161549414	2.491305595	59.86040403	1	CRA004775;Yang2025_Silkie_Wenchang	PRJNA827465	0.676684485
ENSGALG00010017576	ENSGALG00010017576	2	-0.45621415	0.370774359	-1.230436148	0.218533824	0.200590076	3.224723202	68.98967302	1	CRA004775;PRJNA827465	Yang2025_Silkie_Wenchang	0.732214932
ENSGALG00010017577	CLPX	2	-0.154068743	0.053556619	-2.876745158	0.004017999	0	0.015688486	0	1	PRJNA827465;Yang2025_Silkie_Wenchang	CRA004775	0.091653762
ENSGALG00010017577	CLPX	2	-0.020620319	0.180608531	-0.114171344	0.909101968	0.037386491	1.980921413	49.51844161	0.5	CRA004775;Yang2025_Silkie_Wenchang	PRJNA827465	0.978513889
ENSGALG00010017577	CLPX	2	-0.033251256	0.186591891	-0.178203111	0.858563466	0.046460816	2.328850053	57.06050016	0.5	CRA004775;PRJNA827465	Yang2025_Silkie_Wenchang	0.964820815
ENSGALG00010017578	BSDC1	2	-0.136145511	0.144135679	-0.944564955	0.344881026	0.030331303	3.105642447	67.80054314	1	PRJNA827465;Yang2025_Silkie_Wenchang	CRA004775	0.806961063
ENSGALG00010017578	BSDC1	2	-0.317696762	0.129005815	-2.462654584	0.013791271	0	0.006466117	0	1	CRA004775;Yang2025_Silkie_Wenchang	PRJNA827465	0.192701185
ENSGALG00010017578	BSDC1	2	-0.088346752	0.11551925	-0.764779484	0.444402866	0.014703572	1.617006468	38.15732838	1	CRA004775;PRJNA827465	Yang2025_Silkie_Wenchang	0.846013379
ENSGALG00010017579	P3H2	2	-0.00736618	0.059568131	-0.12369975	0.901584682	0	0.036735563	0	0.5	PRJNA827465;Yang2025_Silkie_Wenchang	CRA004775	0.976674416
ENSGALG00010017579	P3H2	2	-0.009103446	0.078526698	-0.11592804	0.907709573	0	0.524118892	0	0.5	CRA004775;Yang2025_Silkie_Wenchang	PRJNA827465	0.978196186
ENSGALG00010017579	P3H2	2	-0.033435063	0.08651695	-0.386456792	0.699158396	0	0.437541639	0	1	CRA004775;PRJNA827465	Yang2025_Silkie_Wenchang	0.92185597
ENSGALG00010017580	LYPLAL1	2	-0.219895258	0.158655304	-1.385993739	0.165748815	0.03370072	3.00360688	66.70669501	1	PRJNA827465;Yang2025_Silkie_Wenchang	CRA004775	0.66629196
ENSGALG00010017580	LYPLAL1	2	-0.338664295	0.111756865	-3.030366813	0.002442569	0	0.708381235	0	1	CRA004775;Yang2025_Silkie_Wenchang	PRJNA827465	0.066177569
ENSGALG00010017580	LYPLAL1	2	-0.051441432	0.131093334	-0.392403106	0.694760392	0	3.1985E-06	0	1	CRA004775;PRJNA827465	Yang2025_Silkie_Wenchang	0.92082578
ENSGALG00010017582	CLCN6	2	0.013746907	0.060212574	0.228306253	0.819408164	0.003653966	1.857391493	46.16105414	0.5	PRJNA827465;Yang2025_Silkie_Wenchang	CRA004775	0.955256039
ENSGALG00010017582	CLCN6	2	-0.080170234	0.075956868	-1.055470503	0.291210193	0	0.207726323	0	1	CRA004775;Yang2025_Silkie_Wenchang	PRJNA827465	0.78467882
ENSGALG00010017582	CLCN6	2	0.041534893	0.060339528	0.688352964	0.491230536	0.001958166	1.078181121	7.251204793	0.5	CRA004775;PRJNA827465	Yang2025_Silkie_Wenchang	0.85895707
ENSGALG00010017583	MTHFD1	2	0.019574893	0.086814374	0.225479866	0.821605987	0	0.439221837	0	0.5	PRJNA827465;Yang2025_Silkie_Wenchang	CRA004775	0.955598716
ENSGALG00010017583	MTHFD1	2	-0.170733497	0.197561158	-0.864205791	0.387474852	0.048665409	2.332144232	57.12100537	1	CRA004775;Yang2025_Silkie_Wenchang	PRJNA827465	0.827883887
ENSGALG00010017583	MTHFD1	2	-0.132625357	0.26339759	-0.503517731	0.614600318	0.100748787	3.462656156	71.12043602	0.5	CRA004775;PRJNA827465	Yang2025_Silkie_Wenchang	0.889647183
ENSGALG00010017585	ENSGALG00010017585	2	0.006208143	0.12059685	0.051478486	0.958944244	0.01304787	1.527392134	34.52892827	0.5	PRJNA827465;Yang2025_Silkie_Wenchang	CRA004775	0.989102387
ENSGALG00010017585	ENSGALG00010017585	2	0.003094136	0.202376715	0.015288994	0.987801623	0.040737169	1.988543512	49.71193771	0.5	CRA004775;Yang2025_Silkie_Wenchang	PRJNA827465	0.996800355
ENSGALG00010017585	ENSGALG00010017585	2	-0.083890334	0.075243731	-1.114914407	0.264887017	0	0.365751284	0	1	CRA004775;PRJNA827465	Yang2025_Silkie_Wenchang	0.769738038
ENSGALG00010017586	ZFYVE28	2	0.187737713	0.187447438	1.001548567	0.316561672	0.043478737	2.48246795	59.71750612	1	PRJNA827465;Yang2025_Silkie_Wenchang	CRA004775	0.796883862
ENSGALG00010017586	ZFYVE28	2	0.382973533	0.173905444	2.202194048	0.027651604	0	0.076631593	0	1	CRA004775;Yang2025_Silkie_Wenchang	PRJNA827465	0.28376061
ENSGALG00010017586	ZFYVE28	2	0.069314889	0.116822062	0.593337318	0.552955429	0	0.644821578	0	1	CRA004775;PRJNA827465	Yang2025_Silkie_Wenchang	0.87928478
ENSGALG00010017587	MB21D2	2	-0.040198815	0.087047661	-0.46180236	0.644232058	0	0.001857318	0	1	PRJNA827465;Yang2025_Silkie_Wenchang	CRA004775	0.906873169
ENSGALG00010017587	MB21D2	2	0.041262246	0.158454095	0.260405043	0.794551353	0.012449392	1.212021064	17.49318313	0.5	CRA004775;Yang2025_Silkie_Wenchang	PRJNA827465	0.948905846
ENSGALG00010017587	MB21D2	2	0.032242205	0.144421293	0.223251046	0.823340125	0.013530004	1.247138915	19.81647049	0.5	CRA004775;PRJNA827465	Yang2025_Silkie_Wenchang	0.955918024
ENSGALG00010017589	ATPAF2	2	0.095770853	0.160802922	0.595579057	0.551456471	0.041275256	4.605057775	78.28474584	0.5	PRJNA827465;Yang2025_Silkie_Wenchang	CRA004775	0.878888037
ENSGALG00010017589	ATPAF2	2	0.061697002	0.281274471	0.219348032	0.826278949	0.108289748	2.801949833	64.31056729	0.5	CRA004775;Yang2025_Silkie_Wenchang	PRJNA827465	0.956393839
ENSGALG00010017589	ATPAF2	2	-0.054183185	0.063172601	-0.85770072	0.391057743	0	0.618614061	0	1	CRA004775;PRJNA827465	Yang2025_Silkie_Wenchang	0.829251057
ENSGALG00010017590	ENSGALG00010017590	2	0.196127572	0.11652867	1.683084275	0.092358778	0	0.748720596	0	1	PRJNA827465;Yang2025_Silkie_Wenchang	CRA004775	0.520517459
ENSGALG00010017590	ENSGALG00010017590	2	0.031859217	0.418062497	0.076206028	0.939254455	0.231238824	2.608050598	61.65718561	0.5	CRA004775;Yang2025_Silkie_Wenchang	PRJNA827465	0.985393654
ENSGALG00010017590	ENSGALG00010017590	2	-0.01923855	0.272571076	-0.070581774	0.943730616	0.077577683	1.594853432	37.29831343	0.5	CRA004775;PRJNA827465	Yang2025_Silkie_Wenchang	0.985827325
ENSGALG00010017591	ESR2	2	0.158502399	0.508943336	0.311434275	0.755470501	0.398448983	4.30099607	76.74957187	0.5	PRJNA827465;Yang2025_Silkie_Wenchang	CRA004775	0.938501652
ENSGALG00010017591	ESR2	2	-0.051481371	0.504981872	-0.101946968	0.918798769	0.214672422	1.531257506	34.69419768	0.5	CRA004775;Yang2025_Silkie_Wenchang	PRJNA827465	0.981033557
ENSGALG00010017591	ESR2	2	0.6563434	0.290543472	2.259019608	0.023882165	0	0.011490236	0	1	CRA004775;PRJNA827465	Yang2025_Silkie_Wenchang	0.264093469
ENSGALG00010017592	GID4	2	-0.053020999	0.070567678	-0.751349639	0.452442263	0	0.204532766	0	1	PRJNA827465;Yang2025_Silkie_Wenchang	CRA004775	0.848321192
ENSGALG00010017592	GID4	2	-0.034756307	0.15061321	-0.230765326	0.81749712	0	0.355869095	0	0.5	CRA004775;Yang2025_Silkie_Wenchang	PRJNA827465	0.954888783
ENSGALG00010017592	GID4	2	-0.074559009	0.074391139	-1.00225659	0.316219683	0	0.147602944	0	1	CRA004775;PRJNA827465	Yang2025_Silkie_Wenchang	0.796883862
ENSGALG00010017593	MTHFR	2	-0.167460094	0.21588108	-0.775705284	0.43792302	0.08541277	11.49395791	91.29977674	0.5	PRJNA827465;Yang2025_Silkie_Wenchang	CRA004775	0.843941117
ENSGALG00010017593	MTHFR	2	-0.348150841	0.088904504	-3.916009037	9.00269E-05	0	0.401772024	0	1	CRA004775;Yang2025_Silkie_Wenchang	PRJNA827465	0.007177132
ENSGALG00010017593	MTHFR	2	-0.098454558	0.157324218	-0.625806754	0.531441701	0.040278733	4.840838694	79.3424226	0.5	CRA004775;PRJNA827465	Yang2025_Silkie_Wenchang	0.873030436
ENSGALG00010017594	CEP68	2	-0.129453718	0.083192233	-1.556079377	0.119689218	0	0.003395978	0	1	PRJNA827465;Yang2025_Silkie_Wenchang	CRA004775	0.580639051
ENSGALG00010017594	CEP68	2	-0.164513298	0.148782347	-1.105731304	0.268842836	0	0.162430191	0	1	CRA004775;Yang2025_Silkie_Wenchang	PRJNA827465	0.771398294
ENSGALG00010017594	CEP68	2	-0.137921633	0.093478861	-1.475431248	0.140096639	0	0.208124176	0	1	CRA004775;PRJNA827465	Yang2025_Silkie_Wenchang	0.623792706
ENSGALG00010017595	CCNF	2	-0.098139118	0.148825846	-0.659422545	0.509624469	0.030325792	2.945254762	66.04707977	0.5	PRJNA827465;Yang2025_Silkie_Wenchang	CRA004775	0.865257661
ENSGALG00010017595	CCNF	2	-0.270406034	0.143528086	-1.883993865	0.059565805	0	0.016383436	0	1	CRA004775;Yang2025_Silkie_Wenchang	PRJNA827465	0.422736519
ENSGALG00010017595	CCNF	2	0.01287302	0.0814735	0.158002547	0.874454792	0	0.435271473	0	0.5	CRA004775;PRJNA827465	Yang2025_Silkie_Wenchang	0.970135804
ENSGALG00010017596	TDP1	2	-0.02281317	0.044737675	-0.509931957	0.610099133	0	0.971578997	0	1	PRJNA827465;Yang2025_Silkie_Wenchang	CRA004775	0.897407418
ENSGALG00010017596	TDP1	2	-0.193477457	0.122958056	-1.573524044	0.115597525	0	0.901828931	0	1	CRA004775;Yang2025_Silkie_Wenchang	PRJNA827465	0.572716099
ENSGALG00010017596	TDP1	2	-0.133721443	0.209642467	-0.637854746	0.523568239	0.057743537	2.061444775	51.4903328	1	CRA004775;PRJNA827465	Yang2025_Silkie_Wenchang	0.869661505
ENSGALG00010017597	CNOT10	2	0.080652794	0.056033235	1.439374234	0.15004452	0.000705777	1.123067948	10.9581925	1	PRJNA827465;Yang2025_Silkie_Wenchang	CRA004775	0.640234479
ENSGALG00010017597	CNOT10	2	-0.025674231	0.194599594	-0.131933629	0.895036789	0.060717225	4.75373308	78.96390094	0.5	CRA004775;Yang2025_Silkie_Wenchang	PRJNA827465	0.974641371
ENSGALG00010017597	CNOT10	2	-0.068980514	0.134197215	-0.514023438	0.607235604	0.023668971	2.561662583	60.96285254	0.5	CRA004775;PRJNA827465	Yang2025_Silkie_Wenchang	0.896998866
ENSGALG00010017598	ENSGALG00010017598	2	0.340657076	0.419023654	0.812978151	0.416230585	0.336150261	22.98712326	95.64973838	0.5	PRJNA827465;Yang2025_Silkie_Wenchang	CRA004775	0.837742614
ENSGALG00010017598	ENSGALG00010017598	2	0.360636692	0.434725098	0.829574123	0.406779612	0.332733698	8.16606999	87.75420733	0.5	CRA004775;Yang2025_Silkie_Wenchang	PRJNA827465	0.833618122
ENSGALG00010017598	ENSGALG00010017598	2	-0.069165334	0.070838691	-0.976377925	0.328877217	0	0.014394578	0	1	CRA004775;PRJNA827465	Yang2025_Silkie_Wenchang	0.800761292
ENSGALG00010017599	KCNK13	2	-0.088793522	0.143354872	-0.61939661	0.535655114	0	0.464028421	0	1	PRJNA827465;Yang2025_Silkie_Wenchang	CRA004775	0.874215303
ENSGALG00010017599	KCNK13	2	-0.382373222	0.346582537	-1.103267423	0.269911049	0.099312445	1.363260538	26.64645004	1	CRA004775;Yang2025_Silkie_Wenchang	PRJNA827465	0.772187652
ENSGALG00010017599	KCNK13	2	-0.323078251	0.484938685	-0.66622495	0.505267328	0.299587894	2.130696551	53.06699119	1	CRA004775;PRJNA827465	Yang2025_Silkie_Wenchang	0.863862536
ENSGALG00010017600	MXD4	2	-0.068344564	0.074992471	-0.911352334	0.36210976	0.006768767	2.470030828	59.51467533	1	PRJNA827465;Yang2025_Silkie_Wenchang	CRA004775	0.815579273
ENSGALG00010017600	MXD4	2	-0.137603591	0.073544753	-1.871018454	0.061342523	0	0.507128675	0	0.5	CRA004775;Yang2025_Silkie_Wenchang	PRJNA827465	0.427938152
ENSGALG00010017600	MXD4	2	0.001511174	0.057190787	0.026423377	0.978919648	0	0.04040015	0	0.5	CRA004775;PRJNA827465	Yang2025_Silkie_Wenchang	0.994259589
ENSGALG00010017601	ENSGALG00010017601	2	0.255658359	0.303772037	0.841612552	0.400004861	0.161248776	7.654111091	86.93512561	0.5	PRJNA827465;Yang2025_Silkie_Wenchang	CRA004775	0.832246851
ENSGALG00010017601	ENSGALG00010017601	2	0.224539119	0.369576332	0.607558167	0.543480553	0.226819935	5.859925619	82.93493698	0.5	CRA004775;Yang2025_Silkie_Wenchang	PRJNA827465	0.876968972
ENSGALG00010017601	ENSGALG00010017601	2	-0.048974204	0.096326102	-0.508420904	0.611158196	0	0.253787201	0	1	CRA004775;PRJNA827465	Yang2025_Silkie_Wenchang	0.897609796
ENSGALG00010017604	GATM	2	-0.125529132	0.11680763	-1.074665515	0.282524509	0	0.254000251	0	1	PRJNA827465;Yang2025_Silkie_Wenchang	CRA004775	0.779532565
ENSGALG00010017604	GATM	2	-0.032366979	0.19796198	-0.163500984	0.870123998	0	0.022756072	0	0.5	CRA004775;Yang2025_Silkie_Wenchang	PRJNA827465	0.968789456
ENSGALG00010017604	GATM	2	-0.157912812	0.139609586	-1.13110293	0.258011771	0	0.101048294	0	0.5	CRA004775;PRJNA827465	Yang2025_Silkie_Wenchang	0.765157824
ENSGALG00010017605	GFPT1	2	0.056203595	0.047230991	1.189972807	0.23405708	0	0.828728905	0	1	PRJNA827465;Yang2025_Silkie_Wenchang	CRA004775	0.747279664
ENSGALG00010017605	GFPT1	2	-0.050667853	0.181405589	-0.279307008	0.780009228	0.055065179	6.026529647	83.40670239	0.5	CRA004775;Yang2025_Silkie_Wenchang	PRJNA827465	0.945041817
ENSGALG00010017605	GFPT1	2	-0.086287594	0.133384182	-0.646910252	0.517690031	0.02755435	4.088816218	75.54304359	0.5	CRA004775;PRJNA827465	Yang2025_Silkie_Wenchang	0.867962259
ENSGALG00010017606	ENSGALG00010017606	2	-0.059320354	0.187121863	-0.317014552	0.75123256	0	0.047028005	0	1	PRJNA827465;Yang2025_Silkie_Wenchang	CRA004775	0.937684688
ENSGALG00010017606	ENSGALG00010017606	2	-0.521577436	0.605687973	-0.861132232	0.389165219	0.53958226	3.518182813	71.57623543	1	CRA004775;Yang2025_Silkie_Wenchang	PRJNA827465	0.828747323
ENSGALG00010017606	ENSGALG00010017606	2	-0.541045351	0.558953406	-0.967961452	0.333063622	0.457720523	3.321170568	69.89013423	1	CRA004775;PRJNA827465	Yang2025_Silkie_Wenchang	0.802178578
ENSGALG00010017608	RBBP4	2	-0.240018474	0.134903934	-1.779180693	0.075210143	0.031372965	6.956086654	85.62410088	1	PRJNA827465;Yang2025_Silkie_Wenchang	CRA004775	0.473260364
ENSGALG00010017608	RBBP4	2	-0.409189716	0.076783759	-5.329118069	9.86908E-08	0	0.221435018	0	1	CRA004775;Yang2025_Silkie_Wenchang	PRJNA827465	3.49446E-05
ENSGALG00010017608	RBBP4	2	-0.264819057	0.173399289	-1.527221129	0.126706034	0.050024055	5.355277708	81.32683206	1	CRA004775;PRJNA827465	Yang2025_Silkie_Wenchang	0.597476858
ENSGALG00010017609	TRIM71	2	-0.095712921	0.118828431	-0.805471554	0.420547615	0	0.280925598	0	0.5	PRJNA827465;Yang2025_Silkie_Wenchang	CRA004775	0.839067105
ENSGALG00010017609	TRIM71	2	0.012341388	0.215505097	0.057267269	0.954332293	0	0.002489545	0	1	CRA004775;Yang2025_Silkie_Wenchang	PRJNA827465	0.987987175
ENSGALG00010017609	TRIM71	2	-0.125568955	0.135578415	-0.926172176	0.35435651	0	0.075010165	0	0.5	CRA004775;PRJNA827465	Yang2025_Silkie_Wenchang	0.81193713
ENSGALG00010017610	PSMC1	2	0.056659907	0.042719257	1.32633173	0.184729841	0	0.160715344	0	1	PRJNA827465;Yang2025_Silkie_Wenchang	CRA004775	0.69308359
ENSGALG00010017610	PSMC1	2	-0.147845666	0.217028766	-0.68122613	0.495728416	0.057120223	2.221914867	54.99377519	0.5	CRA004775;Yang2025_Silkie_Wenchang	PRJNA827465	0.860911322
ENSGALG00010017610	PSMC1	2	-0.116003643	0.242778902	-0.477815998	0.632781173	0.088913917	3.208908981	68.83676022	0.5	CRA004775;PRJNA827465	Yang2025_Silkie_Wenchang	0.903112631
ENSGALG00010017612	CLDN11	2	-0.298289504	0.420347405	-0.709626133	0.477936009	0.187053848	2.071702362	51.73051794	0.5	PRJNA827465;Yang2025_Silkie_Wenchang	CRA004775	0.855366076
ENSGALG00010017612	CLDN11	2	-0.025879213	0.379626915	-0.068170122	0.945650211	0	0.55388335	0	0.5	CRA004775;Yang2025_Silkie_Wenchang	PRJNA827465	0.986321197
ENSGALG00010017612	CLDN11	2	-0.596788084	0.302866244	-1.97046748	0.048784818	0	0.138493571	0	1	CRA004775;PRJNA827465	Yang2025_Silkie_Wenchang	0.381798033
ENSGALG00010017613	HAUS3	2	-0.377769482	0.288181999	-1.310871196	0.189901284	0.153952135	13.50200527	92.59369272	1	PRJNA827465;Yang2025_Silkie_Wenchang	CRA004775	0.699271855
ENSGALG00010017613	HAUS3	2	-0.676182403	0.128926899	-5.244696094	1.5654E-07	0	0.000826743	0	1	CRA004775;Yang2025_Silkie_Wenchang	PRJNA827465	5.17017E-05
ENSGALG00010017613	HAUS3	2	-0.150702962	0.167807911	-0.898068279	0.369149149	0.023852236	1.157088934	13.57621953	1	CRA004775;PRJNA827465	Yang2025_Silkie_Wenchang	0.820603014
ENSGALG00010017615	AGTRAP	2	-0.073491216	0.129351214	-0.568152502	0.56993142	0.018935533	2.287405849	56.28235363	0.5	PRJNA827465;Yang2025_Silkie_Wenchang	CRA004775	0.884923673
ENSGALG00010017615	AGTRAP	2	-0.232118446	0.09824788	-2.362579693	0.018148239	0	0.569506748	0	1	CRA004775;Yang2025_Silkie_Wenchang	PRJNA827465	0.22493858
ENSGALG00010017615	AGTRAP	2	-0.12250895	0.218076339	-0.56177094	0.574272094	0.065809895	3.097021377	67.71091064	0.5	CRA004775;PRJNA827465	Yang2025_Silkie_Wenchang	0.886083039
ENSGALG00010017616	PDCD7	2	0.005362933	0.122668612	0.043718869	0.965128498	0.020537071	2.972780787	66.36146182	0.5	PRJNA827465;Yang2025_Silkie_Wenchang	CRA004775	0.990532586
ENSGALG00010017616	PDCD7	2	0.131347576	0.115567791	1.136541371	0.255730061	0	0.165737451	0	1	CRA004775;Yang2025_Silkie_Wenchang	PRJNA827465	0.764478331
ENSGALG00010017616	PDCD7	2	-0.092406135	0.070689631	-1.307209187	0.191141676	0	0.128773119	0	0.5	CRA004775;PRJNA827465	Yang2025_Silkie_Wenchang	0.700443419
ENSGALG00010017619	POLN	2	0.269148466	0.257348138	1.045853559	0.295628649	0	0.075287746	0	1	PRJNA827465;Yang2025_Silkie_Wenchang	CRA004775	0.787628307
ENSGALG00010017619	POLN	2	0.222401977	0.395882539	0.561787792	0.57426061	0	0.062218601	0	1	CRA004775;Yang2025_Silkie_Wenchang	PRJNA827465	0.886083039
ENSGALG00010017619	POLN	2	0.327585723	0.290063222	1.129359731	0.258746113	0	0.008126687	0	1	CRA004775;PRJNA827465	Yang2025_Silkie_Wenchang	0.765833156
ENSGALG00010017620	UBAP1L	2	-0.249506742	0.193593664	-1.288816671	0.197461829	0	0.030018479	0	1	PRJNA827465;Yang2025_Silkie_Wenchang	CRA004775	0.708952382
ENSGALG00010017620	UBAP1L	2	-0.028690297	0.299868999	-0.095676102	0.923777822	0.016440467	1.100057576	9.095667191	0.5	CRA004775;Yang2025_Silkie_Wenchang	PRJNA827465	0.982219519
ENSGALG00010017620	UBAP1L	2	-0.090121754	0.231796868	-0.388796255	0.697426871	0.023575742	1.207792327	17.20430924	0.5	CRA004775;PRJNA827465	Yang2025_Silkie_Wenchang	0.921328007
ENSGALG00010017621	SYNE2	2	0.003338274	0.420321335	0.007942196	0.993663111	0.333805287	17.85328861	94.39879105	0.5	PRJNA827465;Yang2025_Silkie_Wenchang	CRA004775	0.997979742
ENSGALG00010017621	SYNE2	2	0.428510645	0.158954893	2.695800286	0.007021975	0	0.011178291	0	1	CRA004775;Yang2025_Silkie_Wenchang	PRJNA827465	0.12854434
ENSGALG00010017621	SYNE2	2	-0.111002152	0.38332953	-0.289573704	0.772142387	0.223659874	3.45425095	71.05016356	0.5	CRA004775;PRJNA827465	Yang2025_Silkie_Wenchang	0.943066211
ENSGALG00010017622	RAB1B	2	-0.13011509	0.122322616	-1.063704284	0.287462683	0.027340831	11.26222221	91.12075769	1	PRJNA827465;Yang2025_Silkie_Wenchang	CRA004775	0.782840067
ENSGALG00010017622	RAB1B	2	-0.249309475	0.063419219	-3.931134439	8.4546E-05	0	0.4951253	0	1	CRA004775;Yang2025_Silkie_Wenchang	PRJNA827465	0.006879732
ENSGALG00010017622	RAB1B	2	-0.014268782	0.033271889	-0.428853976	0.668029496	0	0.008356744	0	1	CRA004775;PRJNA827465	Yang2025_Silkie_Wenchang	0.914120722
ENSGALG00010017625	YBX1	2	0.032685663	0.102583154	0.318626024	0.750010117	0.013709695	2.478935113	59.66009781	0.5	PRJNA827465;Yang2025_Silkie_Wenchang	CRA004775	0.937374353
ENSGALG00010017625	YBX1	2	0.238007449	0.104266209	2.282690167	0.022448625	0	0.88766083	0	1	CRA004775;Yang2025_Silkie_Wenchang	PRJNA827465	0.254447309
ENSGALG00010017625	YBX1	2	0.132112986	0.209412239	0.630875191	0.528122134	0.072058816	4.914335253	79.65136792	0.5	CRA004775;PRJNA827465	Yang2025_Silkie_Wenchang	0.871694282
ENSGALG00010017626	COPS7B	2	-0.030230182	0.048301586	-0.625863143	0.531404711	0	0.133144475	0	1	PRJNA827465;Yang2025_Silkie_Wenchang	CRA004775	0.87300616
ENSGALG00010017626	COPS7B	2	-0.051970071	0.083370858	-0.623360162	0.533047871	0	0.030917828	0	1	CRA004775;Yang2025_Silkie_Wenchang	PRJNA827465	0.873332797
ENSGALG00010017626	COPS7B	2	-0.018920411	0.055282184	-0.342251503	0.732161629	0	0.000108405	0	1	CRA004775;PRJNA827465	Yang2025_Silkie_Wenchang	0.93287651
ENSGALG00010017628	NRDE2	2	0.013664065	0.055977682	0.244098437	0.807154583	0	0.881459953	0	0.5	PRJNA827465;Yang2025_Silkie_Wenchang	CRA004775	0.95234753
ENSGALG00010017628	NRDE2	2	0.07785098	0.094934072	0.820053107	0.412185833	0	0.181760337	0	0.5	CRA004775;Yang2025_Silkie_Wenchang	PRJNA827465	0.835532508
ENSGALG00010017628	NRDE2	2	-0.021623145	0.06513949	-0.331951409	0.73992595	0	0.000967935	0	1	CRA004775;PRJNA827465	Yang2025_Silkie_Wenchang	0.935072094
ENSGALG00010017629	ENSGALG00010017629	2	0.158577229	0.103512098	1.531968077	0.125530313	0.013251907	2.476469619	59.6199367	1	PRJNA827465;Yang2025_Silkie_Wenchang	CRA004775	0.594853855
ENSGALG00010017629	ENSGALG00010017629	2	0.287479383	0.093496755	3.074752505	0.002106773	0	0.002769198	0	1	CRA004775;Yang2025_Silkie_Wenchang	PRJNA827465	0.060045322
ENSGALG00010017629	ENSGALG00010017629	2	0.13591323	0.099746009	1.362593161	0.173010766	0.009126851	1.594616849	37.28901079	1	CRA004775;PRJNA827465	Yang2025_Silkie_Wenchang	0.676043418
ENSGALG00010017631	NAT8L	2	0.213828424	0.13486728	1.58547295	0.112858918	0	0.233472406	0	1	PRJNA827465;Yang2025_Silkie_Wenchang	CRA004775	0.566980581
ENSGALG00010017631	NAT8L	2	0.244463454	0.236728881	1.032672707	0.30175709	0	0.715607286	0	0.5	CRA004775;Yang2025_Silkie_Wenchang	PRJNA827465	0.791367646
ENSGALG00010017631	NAT8L	2	0.148410748	0.155765226	0.952784848	0.340699093	0	0.456724694	0	0.5	CRA004775;PRJNA827465	Yang2025_Silkie_Wenchang	0.805540303
ENSGALG00010017632	AWAT1	2	0.550048959	0.556771349	0.987926121	0.323188832	0.446556342	3.423347213	70.7888234	1	PRJNA827465;Yang2025_Silkie_Wenchang	CRA004775	0.799115433
ENSGALG00010017632	AWAT1	2	-0.224341224	0.328215425	-0.683518222	0.49427944	0.088168382	1.671452632	40.17180141	0.5	CRA004775;Yang2025_Silkie_Wenchang	PRJNA827465	0.860175061
ENSGALG00010017632	AWAT1	2	0.262743238	0.892393993	0.294425153	0.768433049	1.380489255	7.450763018	86.57855581	0.5	CRA004775;PRJNA827465	Yang2025_Silkie_Wenchang	0.941444269
ENSGALG00010017633	HLX	2	0.008145674	0.087966629	0.092599592	0.926221668	0	0.169225281	0	0.5	PRJNA827465;Yang2025_Silkie_Wenchang	CRA004775	0.98280842
ENSGALG00010017633	HLX	2	0.010198173	0.132525326	0.076952633	0.938661227	0	0.623018836	0	0.5	CRA004775;Yang2025_Silkie_Wenchang	PRJNA827465	0.985349045
ENSGALG00010017633	HLX	2	-0.040745814	0.10537222	-0.386684594	0.698989723	0	0.364596355	0	1	CRA004775;PRJNA827465	Yang2025_Silkie_Wenchang	0.92185597
ENSGALG00010017634	PPIH	2	0.036206759	0.07562944	0.478738949	0.632124353	0	0.000544063	0	1	PRJNA827465;Yang2025_Silkie_Wenchang	CRA004775	0.903058174
ENSGALG00010017634	PPIH	2	-0.154951233	0.198695278	-0.779843563	0.435482961	0.019465525	1.325299448	24.54535452	0.5	CRA004775;Yang2025_Silkie_Wenchang	PRJNA827465	0.842762754
ENSGALG00010017634	PPIH	2	-0.092580562	0.188505173	-0.491130088	0.623334442	0.045887171	2.284346069	56.22379579	0.5	CRA004775;PRJNA827465	Yang2025_Silkie_Wenchang	0.900348922
ENSGALG00010017636	ZBTB8OS	2	0.306533854	0.331243303	0.92540393	0.354755835	0.205219622	15.2301064	93.43405769	0.5	PRJNA827465;Yang2025_Silkie_Wenchang	CRA004775	0.812016118
ENSGALG00010017636	ZBTB8OS	2	0.061303519	0.608043895	0.100820877	0.919692654	0.692753669	15.54149433	93.5656123	0.5	CRA004775;Yang2025_Silkie_Wenchang	PRJNA827465	0.981233519
ENSGALG00010017636	ZBTB8OS	2	-0.232031574	0.270481848	-0.857845273	0.390977907	0.112445215	3.73830214	73.24988825	1	CRA004775;PRJNA827465	Yang2025_Silkie_Wenchang	0.829251057
ENSGALG00010017638	GFRA2	2	0.306839477	0.127031137	2.415466658	0.015715061	0	0.13748395	0	1	PRJNA827465;Yang2025_Silkie_Wenchang	CRA004775	0.207334357

ENSGALG00010017638	GFRA2	2	0.263070826	0.183558316	1.433173018	0.151808398	0	0.028691733	0	1	CRA004775;Yang2025_Silkie_Wenchang	PRJNA827465	0.643294424
ENSGALG00010017638	GFRA2	2	0.348737005	0.162163042	2.150533197	0.031513063	0	0.00027363	0	1	CRA004775;PRJNA827465	Yang2025_Silkie_Wenchang	0.303670272
ENSGALG00010017639	ENSGALG00010017639	2	0.404268275	0.447640891	0.903108458	0.366468335	0.383180781	22.68832171	95.59244614	0.5	PRJNA827465;Yang2025_Silkie_Wenchang	CRA004775	0.819255965
ENSGALG00010017639	ENSGALG00010017639	2	0.634850566	0.279742874	2.269407467	0.023243559	0.104782454	2.75442148	63.69473564	1	CRA004775;Yang2025_Silkie_Wenchang	PRJNA827465	0.259971992
ENSGALG00010017639	ENSGALG00010017639	2	-0.001522559	0.10280621	-0.014809993	0.988183767	0	0.959883237	0	0.5	CRA004775;PRJNA827465	Yang2025_Silkie_Wenchang	0.996891127
ENSGALG00010017640	P2RY4	2	0.275603333	0.372283115	0.740305757	0.459114489	0	0.19682306	0	1	PRJNA827465;Yang2025_Silkie_Wenchang	CRA004775	0.850098539
ENSGALG00010017640	P2RY4	2	0.333589171	0.434842468	0.767149475	0.442992649	0	0.603941519	0	1	CRA004775;Yang2025_Silkie_Wenchang	PRJNA827465	0.845720426
ENSGALG00010017640	P2RY4	2	0.574225855	0.446558643	1.285891257	0.198481015	0	0.182008274	0	1	CRA004775;PRJNA827465	Yang2025_Silkie_Wenchang	0.710609673
ENSGALG00010017641	GPRC5B	2	-0.227273775	0.165430852	-1.373829447	0.169494637	0.043795178	4.91802093	79.66661765	1	PRJNA827465;Yang2025_Silkie_Wenchang	CRA004775	0.670890582
ENSGALG00010017641	GPRC5B	2	-0.111708891	0.391173568	-0.285573719	0.775204634	0.223642025	3.049230121	67.20483662	0.5	CRA004775;Yang2025_Silkie_Wenchang	PRJNA827465	0.943815265
ENSGALG00010017641	GPRC5B	2	-0.03257524	0.133034609	-0.244862902	0.806562587	0.011725898	1.111130101	10.00153818	0.5	CRA004775;PRJNA827465	Yang2025_Silkie_Wenchang	0.952270941
ENSGALG00010017642	MTARC1	2	-0.053810192	0.074851823	-0.718889525	0.472208991	0	0.755924982	0	1	PRJNA827465;Yang2025_Silkie_Wenchang	CRA004775	0.853998373
ENSGALG00010017642	MTARC1	2	-0.129284682	0.122725827	-1.053443151	0.292137936	0	0.163595436	0	0.5	CRA004775;Yang2025_Silkie_Wenchang	PRJNA827465	0.785516679
ENSGALG00010017642	MTARC1	2	-0.005182896	0.090044353	-0.057559372	0.954099612	0	0.008998184	0	0.5	CRA004775;PRJNA827465	Yang2025_Silkie_Wenchang	0.987907083
ENSGALG00010017643	CALM1	2	-0.182685865	0.11225223	-1.627458679	0.103639718	0.021401025	6.544008708	84.71884674	1	PRJNA827465;Yang2025_Silkie_Wenchang	CRA004775	0.547964315
ENSGALG00010017643	CALM1	2	-0.069129145	0.301223298	-0.229494684	0.818484452	0.139200489	3.522916834	71.61443068	0.5	CRA004775;Yang2025_Silkie_Wenchang	PRJNA827465	0.955256039
ENSGALG00010017643	CALM1	2	-0.005114328	0.152259969	-0.033589447	0.973220538	0.025549012	1.473636718	32.14067023	0.5	CRA004775;PRJNA827465	Yang2025_Silkie_Wenchang	0.992392211
ENSGALG00010017644	RBBP6	2	0.22799261	0.340612148	0.66936136	0.503264994	0.213443699	11.91009273	91.60375974	0.5	PRJNA827465;Yang2025_Silkie_Wenchang	CRA004775	0.863108263
ENSGALG00010017644	RBBP6	2	0.446757276	0.135113139	3.306542061	0.000944552	0.0045433	1.14137132	12.38609362	1	CRA004775;Yang2025_Silkie_Wenchang	PRJNA827465	0.036431516
ENSGALG00010017644	RBBP6	2	0.08410471	0.203623592	0.413040109	0.679577228	0.067719502	4.967128455	79.86764367	0.5	CRA004775;PRJNA827465	Yang2025_Silkie_Wenchang	0.917701868
ENSGALG00010017645	SYNC	2	0.247027234	0.376665554	0.655826453	0.511935794	0.265857522	15.74745316	93.64976679	0.5	PRJNA827465;Yang2025_Silkie_Wenchang	CRA004775	0.865694231
ENSGALG00010017645	SYNC	2	0.087781938	0.601202167	0.146010681	0.883892957	0.633353367	7.545554263	86.7471631	0.5	CRA004775;Yang2025_Silkie_Wenchang	PRJNA827465	0.972001076
ENSGALG00010017645	SYNC	2	-0.175998383	0.147564112	-1.192690963	0.232990426	0.012801631	1.141200616	12.37298809	1	CRA004775;PRJNA827465	Yang2025_Silkie_Wenchang	0.746005924
ENSGALG00010017646	MAEA	2	-0.109185729	0.155345309	-0.702858228	0.482144106	0.044069202	11.0435366	90.94492973	0.5	PRJNA827465;Yang2025_Silkie_Wenchang	CRA004775	0.85691916
ENSGALG00010017646	MAEA	2	-0.258581592	0.079509533	-3.252208671	0.001145519	0	0.230358217	0	1	CRA004775;Yang2025_Silkie_Wenchang	PRJNA827465	0.041064434
ENSGALG00010017646	MAEA	2	0.03035532	0.035932532	0.844786568	0.398230016	0	0.967567729	0	0.5	CRA004775;PRJNA827465	Yang2025_Silkie_Wenchang	0.831980453
ENSGALG00010017647	ITGA9	2	-0.185995862	0.093025615	-1.999404811	0.045564572	0.00538044	1.395724009	28.35259737	1	PRJNA827465;Yang2025_Silkie_Wenchang	CRA004775	0.367927862
ENSGALG00010017647	ITGA9	2	-0.082272972	0.305648195	-0.269175389	0.787794718	0.127106217	2.689258479	62.81502846	0.5	CRA004775;Yang2025_Silkie_Wenchang	PRJNA827465	0.946919116
ENSGALG00010017647	ITGA9	2	-0.036487705	0.170836427	-0.213582099	0.830872492	0.027776496	1.39899949	28.52034564	0.5	CRA004775;PRJNA827465	Yang2025_Silkie_Wenchang	0.957338734
ENSGALG00010017648	ENSGALG00010017648	2	0.58708912	0.46443868	1.264083171	0.206200176	0.406006062	16.83340942	94.05943279	1	PRJNA827465;Yang2025_Silkie_Wenchang	CRA004775	0.718403416
ENSGALG00010017648	ENSGALG00010017648	2	0.555213806	0.543330773	1.021870718	0.306842093	0.516844734	7.770873646	87.13143405	0.5	CRA004775;Yang2025_Silkie_Wenchang	PRJNA827465	0.79266003
ENSGALG00010017648	ENSGALG00010017648	2	0.114845497	0.116729022	0.983864125	0.325182325	0	0.193740318	0	0.5	CRA004775;PRJNA827465	Yang2025_Silkie_Wenchang	0.927994637
ENSGALG00010017649	ENSGALG00010017649	2	-0.134426815	0.081938988	-1.640572066	0.100886277	0	0.000244434	0	1	PRJNA827465;Yang2025_Silkie_Wenchang	CRA004775	0.542203386
ENSGALG00010017649	ENSGALG00010017649	2	-0.167056517	0.126493073	-1.320677194	0.186609021	0	0.3583455	0	1	CRA004775;Yang2025_Silkie_Wenchang	PRJNA827465	0.695457578
ENSGALG00010017649	ENSGALG00010017649	2	-0.155028027	0.096309002	-1.609694048	0.107464065	0	0.379056262	0	1	CRA004775;PRJNA827465	Yang2025_Silkie_Wenchang	0.554966053
ENSGALG00010017651	PPCS	2	0.115397386	0.067856937	1.700598215	0.08901846	0	0.412830118	0	0.5	PRJNA827465;Yang2025_Silkie_Wenchang	CRA004775	0.512103706
ENSGALG00010017651	PPCS	2	-0.174956885	0.209552168	-0.834908493	0.403769233	0.01663107	1.207044641	17.15302265	1	CRA004775;Yang2025_Silkie_Wenchang	PRJNA827465	0.833112102
ENSGALG00010017651	PPCS	2	-0.078045862	0.282345546	-0.276419668	0.782225752	0.117602742	3.042616355	67.13354944	0.5	CRA004775;PRJNA827465	Yang2025_Silkie_Wenchang	0.945372683
ENSGALG00010017652	C1orf115	2	-0.21536157	0.246294415	-0.874407037	0.381896626	0.093735493	4.342992563	76.9744022	0.5	PRJNA827465;Yang2025_Silkie_Wenchang	CRA004775	0.824934493
ENSGALG00010017652	C1orf115	2	-0.178878172	0.353738195	-0.504266078	0.614074412	0.178937833	3.278750398	69.50057557	0.5	CRA004775;Yang2025_Silkie_Wenchang	PRJNA827465	0.898828836
ENSGALG00010017652	C1orf115	2	0.048823349	0.132756159	0.367767107	0.713046897	0	0.353105179	0	1	CRA004775;PRJNA827465	Yang2025_Silkie_Wenchang	0.926138858
ENSGALG00010017653	ENSGALG00010017653	2	-0.244224731	0.24865021	-0.982201989	0.326000348	0.085333054	2.803141635	64.32574125	1	PRJNA827465;Yang2025_Silkie_Wenchang	CRA004775	0.800023788
ENSGALG00010017653	ENSGALG00010017653	2	-0.144937503	0.478831854	-0.302689769	0.762126295	0.327109294	3.365460302	70.28638254	0.5	CRA004775;Yang2025_Silkie_Wenchang	PRJNA827465	0.939973436
ENSGALG00010017653	ENSGALG00010017653	2	-0.026108664	0.109447523	-0.238549611	0.811454842	0	0.974779072	0	0.5	CRA004775;PRJNA827465	Yang2025_Silkie_Wenchang	0.953213272
ENSGALG00010017654	KBTBD13	2	0.235412351	0.136472221	1.72498366	0.084530417	0	0.521810745	0	1	PRJNA827465;Yang2025_Silkie_Wenchang	CRA004775	0.500084797
ENSGALG00010017654	KBTBD13	2	-0.120823152	0.542246218	-0.222819723	0.823675817	0.468471486	4.49688933	77.76240582	0.5	CRA004775;Yang2025_Silkie_Wenchang	PRJNA827465	0.955934625
ENSGALG00010017654	KBTBD13	2	-0.197420941	0.43690582	-0.451861549	0.651368726	0.274126386	3.098712034	67.72852757	0.5	CRA004775;PRJNA827465	Yang2025_Silkie_Wenchang	0.908927523
ENSGALG00010017655	ENSGALG00010017655	2	0.223630027	0.229074652	0.976232096	0.328949461	0.090101841	6.870732255	85.44551057	1	PRJNA827465;Yang2025_Silkie_Wenchang	CRA004775	0.800761292
ENSGALG00010017655	ENSGALG00010017655	2	0.407080609	0.136502201	2.982227441	0.002861593	0	0.967340043	0	1	CRA004775;Yang2025_Silkie_Wenchang	PRJNA827465	0.073126917
ENSGALG00010017655	ENSGALG00010017655	2	0.017348554	0.086715302	0.200063348	0.841431038	0	0.099126455	0	1	CRA004775;PRJNA827465	Yang2025_Silkie_Wenchang	0.95989279
ENSGALG00010017656	FSD2	2	0.053342016	0.236309662	0.225729306	0.821411963	0.091836651	5.591635356	82.11620824	0.5	PRJNA827465;Yang2025_Silkie_Wenchang	CRA004775	0.955599716
ENSGALG00010017656	FSD2	2	0.273097594	0.143588932	1.901940417	0.057178945	0	0.18423331	0	1	CRA004775;Yang2025_Silkie_Wenchang	PRJNA827465	0.414005744
ENSGALG00010017656	FSD2	2	-0.144301066	0.119437372	-1.208173487	0.226980538	0	0.565619394	0	0.5	CRA004775;PRJNA827465	Yang2025_Silkie_Wenchang	0.740517645
ENSGALG00010017657	DIS3L2	2	-0.051784508	0.059312164	0.24252206	0.808375666	0	0.306451991	0	0.5	PRJNA827465;Yang2025_Silkie_Wenchang	CRA004775	0.952496413
ENSGALG00010017657	DIS3L2	2	-0.101922014	0.210082636	-0.485152014	0.627568518	0.059889835	2.669185978	62.53539438	0.5	CRA004775;Yang2025_Silkie_Wenchang	PRJNA827465	0.901840054
ENSGALG00010017657	DIS3L2	2	-0.119969077	0.168405024	-0.712384194	0.4762269	0.034332426	2.003523276	50.08792701	1	CRA004775;PRJNA827465	Yang2025_Silkie_Wenchang	0.854859368
ENSGALG00010017658	WHAMM	2	0.30947048	0.337203968	0.917754562	0.358747375	0.209056364	12.17682377	91.78767781	0.5	PRJNA827465;Yang2025_Silkie_Wenchang	CRA004775	0.814232788
ENSGALG00010017658	WHAMM	2	0.633801026	0.150501203	4.211268845	2.53946E-05	0	0.139815624	0	1	CRA004775;Yang2025_Silkie_Wenchang	PRJNA827465	0.002926867
ENSGALG00010017658	WHAMM	2	0.139151488	0.240058969	0.579655442	0.562146997	0.071526362	2.051707574	51.26011071	0.5	CRA004775;PRJNA827465	Yang2025_Silkie_Wenchang	0.881955027
ENSGALG00010017659	TNRC6A	2	0.17807305	0.264188527	0.67403779	0.500287289	0.121641771	7.229989795	86.16872183	0.5	PRJNA827465;Yang2025_Silkie_Wenchang	CRA004775	0.862289669
ENSGALG00010017659	TNRC6A	2	0.307306289	0.198810129	1.545726272	0.122176069	0.032149671	1.645961953	39.24525421	0.5	CRA004775;Yang2025_Silkie_Wenchang	PRJNA827465	0.558285056
ENSGALG00010017659	TNRC6A	2	-0.051161402	0.065615873	-0.779710761	0.435561144	0	0.22984166	0	0.5	CRA004775;PRJNA827465	Yang2025_Silkie_Wenchang	0.842762754
ENSGALG00010017660	CLIP4	2	0.105011977	0.234252156	0.448286063	0.653946758	0.093392691	6.585640957	84.81544915	0.5	PRJNA827465;Yang2025_Silkie_Wenchang	CRA004775	0.909861322
ENSGALG00010017660	CLIP4	2	0.336879357	0.144049254	2.338640062	0.01935407	0	0.114849963	0	1	CRA004775;Yang2025_Silkie_Wenchang	PRJNA827465	0.233120747
ENSGALG00010017660	CLIP4	2	-0.101508093	0.099439342	-1.02080415	0.307347238	0	0.431622816	0	0.5	CRA004775;PRJNA827465	Yang2025_Silkie_Wenchang	0.792675978
ENSGALG00010017661	NHSL3	2	0.112980926	0.194713348	0.580242324	0.561751216	0.051607368	2.759707433	63.76427486	0.5	PRJNA827465;Yang2025_Silkie_Wenchang	CRA004775	0.881802301
ENSGALG00010017661	NHSL3	2	0.215873456	0.222861313	0.968644818	0.332722434	0.022151573	1.234938592	19.02431373	0.5	CRA004775;Yang2025_Silkie_Wenchang	PRJNA827465	0.800178578
ENSGALG00010017661	NHSL3	2	-0.042526393	0.091910868	-0.462691671	0.643585389	0	0.043533398	0	1	CRA004775;PRJNA827465	Yang2025_Silkie_Wenchang	0.906643868
ENSGALG00010017662	GOLGA4	2	0.538253655	0.688167733	0.782154742	0.434123639	0.907857502	23.46435447	95.73821644	0.5	PRJNA827465;Yang2025_Silkie_Wenchang	CRA004775	0.842762754
ENSGALG00010017662	GOLGA4	2	0.806494773	0.446275671	1.807167241	0.070736207	0.323288081	5.307854673	81.15999662	1	CRA004775;Yang2025_Silkie_Wenchang	PRJNA827465	0.456618467
ENSGALG00010017662	GOLGA4	2	0.044179037	0.231513042	0.190827421	0.848660804	0.074971209	2.766480442	63.85298863	0.5	CRA004775;PRJNA827465	Yang2025_Silkie_Wenchang	0.961364896
ENSGALG00010017663	ZMYND12	2	-0.182347115	0.148788375	-1.225546786	0.220369274	0	0.338320396	0	1	PRJNA827465;Yang2025_Silkie_Wenchang	CRA004775	0.734655249
ENSGALG00010017663	ZMYND12	2	-0.386960743	0.193999202	-1.994651208	0.046080934	0	0.766374733	0	1	CRA004775;Yang2025_Silkie_Wenchang	PRJNA827465	0.370220557
ENSGALG00010017663	ZMYND12	2	-0.296708081	0.263285152	-1.126945739	0.259765423	0.066431184	1.755139388	43.02446822	1	CRA004775;PRJNA827465	Yang2025_Silkie_Wenchang	0.767047318
ENSGALG00010017664	ENSGALG00010017664	2	0.100769352	0.127159081	0.792466815	0.428088534	0.023140158	2.919873261	65.75193816	1	PRJNA827465;Yang2025_Silkie_Wenchang	CRA004775	0.84125685
ENSGALG00010017664	ENSGALG00010017664	2	0.345234585	0.132887209	2.597951951	0.009378162	0.001064876	1.022286784	2.180091226	1	CRA004775;Yang2025_Silkie_Wenchang	PRJNA827465	0.152372834
ENSGALG00010017664	ENSGALG00010017664	2	0.232996911	0.282237098	0.825536092	0.409067301	0.129202728	4.421852378	77.38504332	1	CRA004775;PRJNA827465	Yang2025_Silkie_Wenchang	0.834316328
ENSGALG00010017665	CTBP1	2	-0.03431362	0.041572398	-0.825394283	0.40914778	0	0.213283984	0	1	PRJNA827465;Yang2025_Silkie_Wenchang	CRA004775	0.834316328
ENSGALG00010017665	CTBP1	2	0.025616263	0.11760773	0.217811054	0.827576332	0.015763998	2.156888717	53.63692192	0.5	CRA004775;Yang2025_Silkie_Wenchang	PRJNA827465	0.956550752
ENSGALG00010017665	CTBP1	2	0.030506125	0.088100567	0.346256667	0.729149832	0.00824549	1.71375101	41.6484662	0.5	CRA004775;PRJNA827465	Yang2025_Silkie_Wenchang	0.932323844
ENSGALG00010017667	SLC2A1	2	-0.060208162	0.09213763	-0.653459202	0.513460283	0.010094463	2.416674471	58.62082328	0.5	PRJNA827465;Yang2025_Silkie_Wenchang	CRA004775	0.8664
ENSGALG00010017667	SLC2A1	2	-0.149226175	0.086068484	-1.733807401	0.082952262	0	0.123985495	0	1	CRA004775;Yang2025_Silkie_Wenchang	PRJNA827465	0.495859127
ENSGALG00010017667	SLC2A1	2	0.008972478	0.067049637	0.133816459	0.893546146	0	0.269842448	0	0.5	CRA004775;PRJNA827465	Yang2025_Silkie_Wenchang	0.974381724
ENSGALG00010017669	ENSGALG00010017669	2	0.065556858	0.275962935	0.237556751	0.812224898	0.133162676	7.128263655	85.97133821	0.5	PRJNA827465;Yang2025_Silkie_Wenchang	CRA004775	0.953371387
ENSGALG00010017669	ENSGALG00010017669	2	0.443422323	0.163467312	2.712605453	0.006675654	0	0.270074771	0	1	CRA004775;Yang2025_Silkie_Wenchang	PRJNA827465	0.124953874
ENSGALG00010017669	ENSGALG00010017669	2	0.140525016	0.363455663	0.386635924	0.699025759	0.231067234	7.07633595	85.86839281	0.5	CRA004775;PRJNA827465	Yang2025_Silkie_Wenchang	0.92185597
ENSGALG00010017670	FAM179A	2	-0.053789184	0.166877819	-0.322326745	0.747205174	0.022879982	1.682762505	40.57390767	0.5	PRJNA827465;Yang2025_Silkie_Wenchang	CRA004775	0.93678921
ENSGALG00010017670	FAM179A	2	0.083423544	0.159773151	0.522137436	0.601574643	0	0.084402499	0	0.5	CRA004775;Yang2025_Silkie_Wenchang	PRJNA827465	0.895230519
ENSGALG00010017670	FAM179A	2	-0.227299629	0.19025848	-1.194688557	0.232208735	0	0.047614254	0	1	CRA004775;PRJNA827465	Yang2025_Silkie_Wenchang	0.744836098
ENSGALG00010017671	ANTXR1	2	-0.04419072	0.098443513	-0.448894179	0.653507996	0	0.370519279	0	0.5	PRJNA827465;Yang2025_Silkie_Wenchang	CRA004775	0.909648135
ENSGALG00010017671	ANTXR1	2	0.028674502	0.188298381	0.152282257	0.878964318	0	0.222150847	0	0.5	CRA004775;Yang2025_Silkie_Wenchang	PRJNA827465	0.971127951
ENSGALG00010017671	ANTXR1	2	-0.083102336	0.110160019	-0.754378371	0.450622055	0	0.052502044	0	1	CRA004775;PRJNA827465	Yang2025_Silkie_Wenchang	0.84760941
ENSGALG00010017672	SPRED2	2	-0.204044505	0.201902666	-1.010608274	0.312203954	0.076484652	15.81734298	93.67782565	1	PRJNA827465;Yang2025_Silkie_Wenchang	CRA004775	0.796054997
ENSGALG00010017672	SPRED2	2	-0.008828943	0.436824322	-0.020211656	0.98387453	0.346517907	10.28677108	90.27877658	0.5	CRA004775;Yang2025_Silkie_Wenchang	PRJNA827465	0.995579629
ENSGALG00010017672	SPRED2	2	0.158277683	0.225994344	0.700361256	0.483701726	0.077203222	3.256028695	69.28773996	0.5	CRA004775;PRJNA827465	Yang2025_Silkie_Wenchang	0.85691916
ENSGALG00010017673	WDR89	2	-0.066215188	0.054280423	-1.219872378	0.222513258	0	0.318680978	0	1	PRJNA827465;Yang2025_Silkie_Wenchang	CRA004775	0.73602412
ENSGALG00010017673	WDR89	2	-0.060899399	0.094046754	-0.647543871	0.517280011	0	0.925102553	0	0.5	CRA004775;Yang2025_Silkie_Wenchang	PRJNA827465	0.867952013
ENSGALG00010017673	WDR89	2	-0.03500539	0.057888694	-0.604701681	0.545377231	0	0.46359822	0	0.5	CRA004775;PRJNA827465	Yang2025_Silkie_Wenchang	0.877636727
ENSGALG00010017676	SPON2	2	-0.651634387	0.377001437	-1.728466585	0.083904607	0.256258774	10.06266743	90.06227715	1	PRJNA827465;Yang2025_Silkie_Wenchang	CRA004775	0.499047028
ENSGALG00010017676	SPON2	2	-0.133908337	0.931266983	-0.143791566	0.885665063	1.652122652	20.75266281	95.18134126	0.5	CRA004775;Yang2025_Silkie_Wenchang	PRJNA827465	0.972376043
ENSGALG00010017676	SPON2	2	0.218313004	0.552165036	0.395376364	0.69256515	0.539962326	8.219120047	87.83324742	0.5	CRA004775;PRJNA827465	Yang2025_Silkie_Wenchang	0.920559211
ENSGALG00010017677	WDR43	2	-0.104746409	0.14084918	-0.743677804	0.457071421	0.03369764	6.138194376	83.70856414	0.5	PRJNA827465;Yang2025_Silkie_Wenchang	CRA004775	0.849541335
ENSGALG00010017677	WDR43	2	-0.076817686	0.227241154	-0.33804478	0.735329443	0.075152701	3.338405751	70.0455824	0.5	CRA004775;Yang2025_Silkie_Wenchang	PRJNA827465	0.933592889
ENSGALG00010017677	WDR43	2	0.025800168	0.040937739	0.630229426	0.528544489	0	0.58977512	0	1	CRA004775;PRJNA827465	Yang2025_Silkie_Wenchang	0.871701504
ENSGALG00010017678	YARS	2	0.044063825	0.063394976	0.695068097	0.487012627	0	0.006188092	0	1	PRJNA827465;Yang2025_Silkie_Wenchang	CRA004775	0.85768885
ENSGALG00010017678	YARS	2	-0.238907348	0.312663489	-0.764103761	0.444805411	0.156646948	4.964243831	79.85594515	0.5	CRA004775;Yang2025_Silkie_Wenchang	PRJNA827465	0.846013379
ENSGALG00010017678	YARS	2	-0.226364608	0.303715362	-0.745318271	0.456079339	0.159595563	6.756556406	85.19956114	0.5	CRA004775;PRJNA827465	Yang2025_Silkie_Wenchang	0.849162679
ENSGALG00010017679	RASL12	2	0.251032362	0.136985029	1.832553262	0.066869034	0	0.115490671	0	1	PRJNA827465;Yang2025_Silkie_Wenchang	CRA004775	0.445476026
ENSGALG00010017679	RASL12	2	0.209569284	0.186869631	1.121473204	0.262086491	0	0.012660704	0	1	CRA004775;Yang2025_Silkie_Wenchang	PRJNA827465	0.768363052
ENSGALG00010017679	RASL12	2	0.291577549	0.177346341	1.644113705	0.100152717	0	0.002780586	0	1	CRA004775;PRJNA827465	Yang2025_Silkie_Wenchang	0.540552356
ENSGALG00010017684	TRMT61B	2	0.127470985	0.276291192	0.461364636	0.644537019	0.135772995	8.674841943	88.47241245	0.5	PRJNA827465;Yang2025_Silkie_Wenchang	CRA004775	0.906953008
ENSGALG00010017684	TRMT61B	2	0.150012092	0.294773088	0.508907012	0.610817405	0.13345145	4.236803833	76.39730232	0.5	CRA004775;Yang2025_Silkie_Wenchang	PRJNA827465	0.897506931
ENSGALG00010017684	TRMT61B	2	-0.13375529	0.077081603	-1.735242717	0.082697823	0	0.022722742	0	1	CRA004775;PRJNA827465	Yang2025_Silkie_Wenchang	0.495704284
ENSGALG00010017685	PHC2	2	0.227774285	0.290368951	0.784430582	0.432787499	0.160750277	21.21301843	95.28591368	0.5	PRJNA827465;Yang2025_Silkie_Wenchang	CRA004775	0.842762754
ENSGALG00010017685	PHC2	2	0.254180808	0.338328148	0.751284839	0.452481252	0.175937672	3.715614106	73.08654851	0.5	CRA004775;Yang2025_Silkie_Wenchang	PRJNA827465	0.848321192
ENSGALG00010017685	PHC2	2	-0.060601881	0.065128402	-0.930498522	0.352113029	0	0.104262174	0	1	CRA004775;PRJNA827465	Yang2025_Silkie_Wenchang	0.810678834
ENSGALG00010017687	TTC7B	2	-0.1399019	0.063492887	-2.203426328	0.027564714	0	0.320378126	0	1	PRJNA827465;Yang2025_Silkie_Wenchang	CRA004775	0.283513274
ENSGALG00010017687	TTC7B	2	-0.154544901	0.099291707	-1.556473407	0.119595561	0	0.560988266	0	1	CRA004775;Yang2025_Silkie_Wenchang	PRJNA827465	0.580519248
ENSGALG00010017687	TTC7B	2	-0.102925743	0.073253582	-1.405060888	0.160003173	0	0.218261298	0	1	CRA004775;PRJNA827465	Yang2025_Silkie_Wenchang	0.655404348
ENSGALG00010017688	SPDYA	2	0.397718536	0.294045865	1.35257313	0.176192041	0.152862365	8.617457681	88.39564943	1	PRJNA827465;Yang2025_Silkie_Wenchang	CRA004775	0.681314031
ENSGALG00010017688	SPDYA	2	0.396152581	0.403290933	0.982299745	0.3259522	0.229970674	2.814691903	64.47213285	0.5	CRA004775;Yang2025_Silkie_Wenchang	PRJNA827465	0.800023788
ENSGALG00010017688	SPDYA	2	0.085431131	0.133358673	0.64061173	0.521774977	0	0.261312507	0	0.5	CRA004775;PRJNA827465	Yang2025_Silkie_Wenchang	0.86956507
ENSGALG00010017691	LZTS1	2	0.154515095	0.154518552	0.999977632	0.317321333	0.016308224	1.391567561	28.13859506	1	PRJNA827465;Yang2025_Silkie_Wenchang	CRA004775	0.797095376
ENSGALG00010017691	LZTS1	2	0.058421548	0.434293546	0.134520875	0.892990705	0.236721325	2.435997161	58.94904904	0.5	CRA004775;Yang2025_Silkie_Wenchang	PRJNA827465	0.974381724
ENSGALG00010017691	LZTS1	2	-0.000450059	0.167504807	-0.002686845	0.99785621	0.013883414	1.098794266	8.991152339	0.5	CRA004775;PRJNA827465	Yang2025_Silkie_Wenchang	0.999290321
ENSGALG00010017692	EPCAM	2	-0.181901938	0.880322088	-0.206631119	0.836297955	0.815432549	2.073729279	51.77769779	0.5	PRJNA827465;Yang2025_Silkie_Wenchang	CRA004775	0.958399882
ENSGALG00010017692	EPCAM	2	0.682354829	1.892751253	0.360509511	0.718466145	6.096768687	6.697869574	85.06987947	0.5	CRA004775;Yang2025_Silkie_Wenchang	PRJNA827465	0.928114212
ENSGALG00010017692	EPCAM	2	1.444998528	0.993681324	1.454187065	0.14589445	1.150664704	2.319826089	56.89332038	1	CRA004775;PRJNA827465	Yang2025_Silkie_Wenchang	0.634793487
ENSGALG00010017693	MTFMT	2	0.488689243	0.280910724	1.739660334	0.081918678	0.12771941	5.189322546	80.72966189	1	PRJNA827465;Yang2025_Silkie_Wenchang	CRA004775	0.493467286
ENSGALG00010017693	MTFMT	2	0.398204119	0.39156181	1.01696363	0.309170731	0.263724828	7.134772768	85.98413667	1	CRA004775;Yang2025_Silkie_Wenchang	PRJNA827465	0.794043289
ENSGALG00010017693	MTFMT	2	0.150156432	0.123988337	1.211052876	0.22587514	0	0.692183769	0	1	CRA004775;PRJNA827465	Yang2025_Silkie_Wenchang	0.739120026
ENSGALG00010017694	ATP6V1B2	2	-0.195644846	0.214570332	-0.911798214	0.361874951	0.086654979	16.53070556	93.95065143	0.5	PRJNA827465;Yang2025_Silkie_Wenchang	CRA004775	0.815579273
ENSGALG00010017694	ATP6V1B2	2	-0.425188695	0.091755424	-4.633935292	3.58779E-06	0	0.039241473	0	1	CRA004775;Yang2025_Silkie_Wenchang	PRJNA827465	0.000652829
ENSGALG00010017694	ATP6V1B2	2	-0.150031653	0.230036067	-0.65220926	0.514266187	0.076820666	2.770297643	63.9027957	0.5	CRA004775;PRJNA827465	Yang2025_Silkie_Wenchang	0.866866841
ENSGALG00010017695	FNDC5	2	0.041071375	0.098218244	0.418164424	0.675826905	0	0.540117411	0	0.5	PRJNA827465;Yang2025_Silkie_Wenchang	CRA004775	0.916522765
ENSGALG00010017695	FNDC5	2	-0.083389047	0.311485155	-0.267714354	0.7889192	0.117928296	2.093148349	52.22507757	0.5	CRA004775;Yang2025_Silkie_Wenchang	PRJNA827465	0.947151276
ENSGALG00010017695	FNDC5	2	-0.115149715	0.202499842	-0.568641011	0.569599787	0.033571382	1.321126601	24.30702708	1	CRA004775;PRJNA827465	Yang2025_Silkie_Wenchang	0.88485924
ENSGALG00010017696	SLC18A1	2	0.344273031	0.153221991	2.24689047	0.024647028	0.005513687	1.131295472	11.60576303	1	PRJNA827465;Yang2025_Silkie_Wenchang	CRA004775	0.268408231
ENSGALG00010017696	SLC18A1	2	0.236212893	0.329357875	0.71719218	0.473255524	0.139097243	2.657316263	62.36804727	0.5	CRA004775;Yang2025_Silkie_Wenchang	PRJNA827465	0.854343014
ENSGALG00010017696	SLC18A1	2	0.122676401	0.168049204	0.730002872	0.465388429	0	0.820643019	0	0.5	CRA004775;PRJNA827465	Yang2025_Silkie_Wenchang	0.852411124
ENSGALG00010017699	RNF19B	2	0.325577745	0.335976429	0.969049363	0.332520561	0.199223645	8.426087309	88.13209544	1	PRJNA827465;Yang2025_Silkie_Wenchang	CRA004775	0.802178578
ENSGALG00010017699	RNF19B	2	0.142552252	0.551017622	0.258707248	0.795861127	0.550940314	10.6471587	90.60782291	0.5	CRA004775;Yang2025_Silkie_Wenchang	PRJNA827465	0.949107318
ENSGALG00010017699	RNF19B	2	-0.143387645	0.203364075	-0.705078538	0.480761362	0.043614344	1.890080171	47.09219137	0.5	CRA004775;PRJNA827465	Yang2025_Silkie_Wenchang	0.856722567
ENSGALG00010017700	VPS35L	2	-0.038320174	0.060882484	-0.629412125	0.529079282	0.002453872	1.463967463	31.69247094	0.5	PRJNA827465;Yang2025_Silkie_Wenchang	CRA004775	0.871809618
ENSGALG00010017700	VPS35L	2	-0.143434754	0.074730006	-1.919372989	0.054937147	0	0.694386309	0	1	CRA004775;Yang2025_Silkie_Wenchang	PRJNA827465	0.405711139
ENSGALG00010017700	VPS35L	2	-0.085079788	0.134214911	-0.633907121	0.526141438	0.023565911	2.41783847	58.64074409	0.5	CRA004775;PRJNA827465	Yang2025_Silkie_Wenchang	0.870525481
ENSGALG00010017701	PPP2R5E	2	-0.282493026	0.231587336	-1.219812062	0.222536128	0.104561097	39.49754764	97.46819724	1	PRJNA827465;Yang2025_Silkie_Wenchang	CRA004775	0.736037821
ENSGALG00010017701	PPP2R5E	2	-0.272191942	0.267723242	-1.016691488	0.309300216	0.127531656	8.492120033	88.22437747	0.5	CRA004775;Yang2025_Silkie_Wenchang	PRJNA827465	0.794043289
ENSGALG00010017701	PPP2R5E	2	-0.049585286	0.038901572	-1.274634511	0.202438636	0	0.174484611	0	0.5	CRA004775;PRJNA827465	Yang2025_Silkie_Wenchang	0.715289846
ENSGALG00010017703	RPS6KA5	2	-0.069414815	0.064104126	-1.082844735	0.278877345	0	0.000763143	0	1	PRJNA827465;Yang2025_Silkie_Wenchang	CRA004775	0.777190407
ENSGALG00010017703	RPS6KA5	2	-0.037874364	0.130841405	-0.289467727	0.772223474	0	0.2061106	0	0.5	CRA004775;Yang2025_Silkie_Wenchang	PRJNA827465	0.943066211
ENSGALG00010017703	RPS6KA5	2	-0.062138103	0.069652424	-0.892116879	0.372330288	0	0.252842295	0	0.5	CRA004775;PRJNA827465	Yang2025_Silkie_Wenchang	0.82179465
ENSGALG00010017704	ZNF362	2	0.353629452	0.486355937	0.727100103	0.467164635	0.429303317	10.01103685	90.01102468	0.5	PRJNA827465;Yang2025_Silkie_Wenchang	CRA004775	0.852885838
ENSGALG00010017704	ZNF362	2	0.421726904	0.440256725	0.957911329	0.338107473	0.318731401	5.552869161	81.99129187	1	CRA004775;Yang2025_Silkie_Wenchang	PRJNA827465	0.80415385
ENSGALG00010017704	ZNF362	2	-0.082484877	0.065698721	-1.255502018	0.209296576	0	0.166295402	0	0.5	CRA004775;PRJNA827465	Yang2025_Silkie_Wenchang	0.721187439
ENSGALG00010017705	MAK16	2	-0.138708084	0.049686206	-2.791681968	0.005243487	0	0.01704883	0	1	PRJNA827465;Yang2025_Silkie_Wenchang	CRA004775	0.108124213
ENSGALG00010017705	MAK16	2	-0.048596937	0.135132469	-0.359624431	0.719128011	0.013818831	1.398572707	28.4985332	0.5	CRA004775;Yang2025_Silkie_Wenchang	PRJNA827465	0.928449671
ENSGALG00010017705	MAK16	2	-0.063898616	0.139531291	-0.457951869	0.646987021	0.022393708	1.72260183	41.94827947	0.5	CRA004775;PRJNA827465	Yang2025_Silkie_Wenchang	0.907350807
ENSGALG00010017706	FGFRL1	2	0.126976836	0.202947447	0.62566363	0.531535594	0.066509656	5.112342483	80.43949514	0.5	PRJNA827465;Yang2025_Silkie_Wenchang	CRA004775	0.873042777
ENSGALG00010017706	FGFRL1	2	0.338035426	0.138071044	2.448271669	0.014354338	0	0.010121535	0	1	CRA004775;Yang2025_Silkie_Wenchang	PRJNA827465	0.196848758
ENSGALG00010017706	FGFRL1	2	-0.043785168	0.102510873	-0.427127064	0.669286781	0	0.653323362	0	0.5	CRA004775;PRJNA827465	Yang2025_Silkie_Wenchang	0.914377769
ENSGALG00010017707	AK2	2	0.020774208	0.055942209	0.371351229	0.710375946	0.000612115	1.064476912	6.057145182	0.5	PRJNA827465;Yang2025_Silkie_Wenchang	CRA004775	0.925364369
ENSGALG00010017707	AK2	2	-0.05831424	0.208117661	-0.280198421	0.779325278	0.065231177	3.989350259	74.93326143	0.5	CRA004775;Yang2025_Silkie_Wenchang	PRJNA827465	0.944727706
ENSGALG00010017707	AK2	2	-0.099186852	0.130041238	-0.762733835	0.445622148	0.022251407	2.427615785	58.80732008	1	CRA004775;PRJNA827465	Yang2025_Silkie_Wenchang	0.846013379
ENSGALG00010017708	MEIS1	2	0.052476635	0.315272199	0.16644866	0.867803883	0.182340721	11.74293538	91.48424165	0.5	PRJNA827465;Yang2025_Silkie_Wenchang	CRA004775	0.967946362
ENSGALG00010017708	MEIS1	2	0.282389629	0.129255216	2.184744557	0.028907581	0	0.993459232	0	1	CRA004775;Yang2025_Silkie_Wenchang	PRJNA827465	0.289667055
ENSGALG00010017708	MEIS1	2	-0.113726886	0.176358903	-0.644860476	0.519017607	0.042097809	2.677396927	62.65028954	0.5	CRA004775;PRJNA827465	Yang2025_Silkie_Wenchang	0.868939943
ENSGALG00010017710	RAB41	2	0.262325088	0.132761173	1.975917224	0.048164153	0.022110094	2.67801073	62.65885013	1	PRJNA827465;Yang2025_Silkie_Wenchang	CRA004775	0.379586066
ENSGALG00010017710	RAB41	2	0.142818909	0.269167086	0.53059574	0.595698948	0.121959029	6.101840453	83.61150201	0.5	CRA004775;Yang2025_Silkie_Wenchang	PRJNA827465	0.893802528
ENSGALG00010017710	RAB41	2	0.027147487	0.131546489	0.206371813	0.836500487	0.012642203	1.505229568	33.56495108	0.5	CRA004775;PRJNA827465	Yang2025_Silkie_Wenchang	0.958399882
ENSGALG00010017711	LRRFIP2	2	0.493040918	0.467744854	1.054080902	0.291845879	0.423490346	30.46585729	96.71763709	1	PRJNA827465;Yang2025_Silkie_Wenchang	CRA004775	0.785409532
ENSGALG00010017711	LRRFIP2	2	0.898363576	0.139985387	6.417552524	1.38483E-10	0	0.91606299	0	1	CRA004775;Yang2025_Silkie_Wenchang	PRJNA827465	9.54874E-08
ENSGALG00010017711	LRRFIP2	2	0.283432376	0.303653743	0.933406495	0.350610128	0.149760549	4.492503146	77.7406945	1	CRA004775;PRJNA827465	Yang2025_Silkie_Wenchang	0.810111037
ENSGALG00010017712	MSH2	2	-0.24275799	0.131282413	-1.849128026	0.064439328	0.028460574	5.533430167	81.9280271	1	PRJNA827465;Yang2025_Silkie_Wenchang	CRA004775	0.437495064
ENSGALG00010017712	MSH2	2	-0.362439111	0.089079132	-4.068731952	4.72697E-05	0	0.402151206	0	1	CRA004775;Yang2025_Silkie_Wenchang	PRJNA827465	0.004644601
ENSGALG00010017712	MSH2	2	-0.128579864	0.055134567	-2.332109815	0.019694915	0	0.196771809	0	1	CRA004775;PRJNA827465	Yang2025_Silkie_Wenchang	0.235422518
ENSGALG00010017713	ALKBH5	2	-0.067548383	0.10249951	-0.659011765	0.509888214	0.013699178	2.856447658	64.99148173	0.5	PRJNA827465;Yang2025_Silkie_Wenchang	CRA004775	0.865257661
ENSGALG00010017713	ALKBH5	2	-0.118240559	0.144328616	-0.819245428	0.412646401	0.015602342	1.216639906	17.80641129	0.5	CRA004775;Yang2025_Silkie_Wenchang	PRJNA827465	0.835704442
ENSGALG00010017713	ALKBH5	2	0.038694645	0.076990038	0.502592885	0.615250533	0	0.321129273	0	1	CRA004775;PRJNA827465	Yang2025_Silkie_Wenchang	0.898828818
ENSGALG00010017714	ATAD3A	2	0.072031955	0.085988928	0.837688727	0.402205547	0.008311312	2.281824564	56.17542137	0.5	PRJNA827465;Yang2025_Silkie_Wenchang	CRA004775	0.832879175
ENSGALG00010017714	ATAD3A	2	0.010837587	0.172986511	0.062649895	0.950045297	0.045343336	3.862909061	74.11277397	0.5	CRA004775;Yang2025_Silkie_Wenchang	PRJNA827465	0.987277827
ENSGALG00010017714	ATAD3A	2	-0.048372038	0.071648624	-0.675128636	0.499594042	0.00028948	1.018658873	1.831709638	1	CRA004775;PRJNA827465	Yang2025_Silkie_Wenchang	0.862078151
ENSGALG00010017715	CILP	2	-0.134064491	0.117464755	-1.141316735	0.25373814	0	0.147553988	0	1	PRJNA827465;Yang2025_Silkie_Wenchang	CRA004775	0.763418915
ENSGALG00010017715	CILP	2	-0.118402799	0.156569907	-0.756229603	0.449511547	0	0.44125585	0	1	CRA004775;Yang2025_Silkie_Wenchang	PRJNA827465	0.847263197
ENSGALG00010017715	CILP	2	-0.205320597	0.164712546	-1.246538904	0.212566616	0	0.272119882	0	1	CRA004775;PRJNA827465	Yang2025_Silkie_Wenchang	0.725280468
ENSGALG00010017717	LLGL1	2	-0.084970451	0.053913956	-1.576038155	0.11501701	0	0.12982837	0	1	PRJNA827465;Yang2025_Silkie_Wenchang	CRA004775	0.572141484
ENSGALG00010017717	LLGL1	2	-0.115404092	0.081811868	-1.410603318	0.158361613	0	0.041330706	0	1	CRA004775;PRJNA827465	Yang2025_Silkie_Wenchang	0.652042151

ENSGALG00010017717	LLGL1	2	-0.076384025	0.066206902	-1.153716949	0.248616233	0	0.133180932	0	1	CRA004775;PRJNA827465	Yang2025_Silkie_Wenchang	0.758965129
ENSGALG00010017718	ATOH8	2	0.298613387	0.439047807	0.68013866	0.496416667	0.358710998	14.30019479	93.00708826	0.5	PRJNA827465;Yang2025_Silkie_Wenchang	CRA004775	0.860939846
ENSGALG00010017718	ATOH8	2	0.12162194	0.742462686	0.163808825	0.869881644	0.907091859	4.970283777	79.88042444	0.5	CRA004775;Yang2025_Silkie_Wenchang	PRJNA827465	0.968610705
ENSGALG00010017718	ATOH8	2	-0.159094673	0.135613469	-1.173148027	0.24073645	0	0.895511065	0	1	CRA004775;PRJNA827465	Yang2025_Silkie_Wenchang	0.753303162
ENSGALG00010017720	ENSGALG00010017720	2	0.017386828	0.228355121	0.076139428	0.939308172	0	0.525938987	0	0.5	PRJNA827465;Yang2025_Silkie_Wenchang	CRA004775	0.985393654
ENSGALG00010017720	ENSGALG00010017720	2	-0.29793215	0.647506797	-0.460122042	0.645428624	0.55567742	2.683672525	62.73762948	0.5	CRA004775;Yang2025_Silkie_Wenchang	PRJNA827465	0.906966468
ENSGALG00010017720	ENSGALG00010017720	2	-0.395771642	0.444241461	-0.890893077	0.372986535	0.183059379	1.587070297	36.99081874	1	CRA004775;PRJNA827465	Yang2025_Silkie_Wenchang	0.822162024
ENSGALG00010017721	VWA1	2	-0.038799343	0.076673491	-0.506033346	0.612833241	0.000760515	1.069152268	6.467953136	0.5	PRJNA827465;Yang2025_Silkie_Wenchang	CRA004775	0.898034557
ENSGALG00010017721	VWA1	2	0.077930683	0.262561437	0.296809325	0.766612086	0.095224282	2.694730312	62.89053507	0.5	CRA004775;Yang2025_Silkie_Wenchang	PRJNA827465	0.941271762
ENSGALG00010017721	VWA1	2	0.123026616	0.162574247	0.756741105	0.449204983	0.022513885	1.400112559	28.57717093	1	CRA004775;PRJNA827465	Yang2025_Silkie_Wenchang	0.847263197
ENSGALG00010017723	KCNK12	2	-0.443975215	0.34516097	-1.286284527	0.198343781	0.166068436	3.189282852	68.64498866	1	PRJNA827465;Yang2025_Silkie_Wenchang	CRA004775	0.710296638
ENSGALG00010017723	KCNK12	2	-0.765998186	0.29980987	-2.554946527	0.010620411	0	0.299055767	0	1	CRA004775;Yang2025_Silkie_Wenchang	PRJNA827465	0.164336229
ENSGALG00010017723	KCNK12	2	-0.155539777	0.20703651	-0.751267382	0.452491755	0	0.084603291	0	1	CRA004775;PRJNA827465	Yang2025_Silkie_Wenchang	0.848321192
ENSGALG00010017724	FLII	2	0.049236053	0.18863243	0.261015842	0.794080291	0.066289931	14.55436026	93.12920676	0.5	PRJNA827465;Yang2025_Silkie_Wenchang	CRA004775	0.948905846
ENSGALG00010017724	FLII	2	0.229569678	0.060360372	3.803317835	0.000142771	0	0.173174727	0	1	CRA004775;Yang2025_Silkie_Wenchang	PRJNA827465	0.010074198
ENSGALG00010017724	FLII	2	-0.124543641	0.074518222	-1.671317925	0.094658898	0	0.762194126	0	0.5	CRA004775;PRJNA827465	Yang2025_Silkie_Wenchang	0.526916621
ENSGALG00010017725	ENSGALG00010017725	2	0.248115784	0.385388064	0.64380765	0.519700172	0.062806623	1.257702064	20.48991344	0.5	PRJNA827465;Yang2025_Silkie_Wenchang	CRA004775	0.869101946
ENSGALG00010017725	ENSGALG00010017725	2	-0.304724003	0.275147319	-1.107493995	0.268080407	0	0.464324106	0	1	CRA004775;Yang2025_Silkie_Wenchang	PRJNA827465	0.771398294
ENSGALG00010017725	ENSGALG00010017725	2	0.051873204	0.578267978	0.089704437	0.92852209	0.467857552	3.209245302	68.84002605	0.5	CRA004775;PRJNA827465	Yang2025_Silkie_Wenchang	0.983311758
ENSGALG00010017726	TTI2	2	0.110417274	0.105191099	1.049682673	0.293864033	0	0.556751686	0	1	PRJNA827465;Yang2025_Silkie_Wenchang	CRA004775	0.787195888
ENSGALG00010017726	TTI2	2	-0.015705163	0.269061289	-0.058370205	0.953453747	0.097152489	2.993717195	66.59671122	0.5	CRA004775;Yang2025_Silkie_Wenchang	PRJNA827465	0.987758913
ENSGALG00010017726	TTI2	2	-0.060768234	0.164598567	-0.369190541	0.711985706	0.03168473	2.276246857	56.0680338	0.5	CRA004775;PRJNA827465	Yang2025_Silkie_Wenchang	0.92597752
ENSGALG00010017727	AZIN2	2	0.043899366	0.106331235	0.412854852	0.679712961	0	0.75273382	0	0.5	PRJNA827465;Yang2025_Silkie_Wenchang	CRA004775	0.917701868
ENSGALG00010017727	AZIN2	2	-0.031127456	0.196415197	-0.15847784	0.874080281	0.046785139	2.534882364	60.55043761	0.5	CRA004775;Yang2025_Silkie_Wenchang	PRJNA827465	0.970074749
ENSGALG00010017727	AZIN2	2	-0.097965188	0.102740457	-0.95352105	0.340326136	0	0.879004626	0	1	CRA004775;PRJNA827465	Yang2025_Silkie_Wenchang	0.805432415
ENSGALG00010017729	MIEF2	2	-0.242392733	0.086269355	-2.809720001	0.004958462	0	0.059530567	0	1	PRJNA827465;Yang2025_Silkie_Wenchang	CRA004775	0.104831857
ENSGALG00010017729	MIEF2	2	-0.241533519	0.159010161	-1.518981665	0.128767111	0	0.34638115	0	1	CRA004775;Yang2025_Silkie_Wenchang	PRJNA827465	0.602157487
ENSGALG00010017729	MIEF2	2	-0.218692051	0.097388687	-2.245559099	0.024732263	0	0.257995378	0	1	CRA004775;PRJNA827465	Yang2025_Silkie_Wenchang	0.268758652
ENSGALG00010017730	TARP	2	-0.380189401	0.519724713	-0.731520728	0.464461148	0.155898277	1.391741934	28.14759867	0.5	PRJNA827465;Yang2025_Silkie_Wenchang	CRA004775	0.853159649
ENSGALG00010017730	TARP	2	-0.565045814	0.359510935	-1.571706888	0.116018544	0	0.505416499	0	1	CRA004775;Yang2025_Silkie_Wenchang	PRJNA827465	0.573367481
ENSGALG00010017730	TARP	2	-0.239379208	0.335402228	-0.713707864	0.475407842	0	0.461948156	0	0.5	CRA004775;PRJNA827465	Yang2025_Silkie_Wenchang	0.854462864
ENSGALG00010017732	RHOJ	2	0.118813983	0.101458857	1.171055794	0.241576351	0	0.626623522	0	1	PRJNA827465;Yang2025_Silkie_Wenchang	CRA004775	0.754024407
ENSGALG00010017732	RHOJ	2	0.26808327	0.165237655	1.622410276	0.10471553	0	0.409218051	0	1	CRA004775;Yang2025_Silkie_Wenchang	PRJNA827465	0.549804368
ENSGALG00010017732	RHOJ	2	0.090903842	0.120962246	0.751505901	0.452348251	0	0.967443391	0	1	CRA004775;PRJNA827465	Yang2025_Silkie_Wenchang	0.848321192
ENSGALG00010017733	TRIM62	2	0.090852595	0.080174738	1.13318232	0.257137697	0	0.611770349	0	1	PRJNA827465;Yang2025_Silkie_Wenchang	CRA004775	0.764870705
ENSGALG00010017733	TRIM62	2	0.065138541	0.211209948	0.308406597	0.757772963	0.029176048	1.481984225	32.52289849	0.5	CRA004775;Yang2025_Silkie_Wenchang	PRJNA827465	0.938954369
ENSGALG00010017733	TRIM62	2	0.045226659	0.08113551	0.557421265	0.57723964	0	0.71093291	0	0.5	CRA004775;PRJNA827465	Yang2025_Silkie_Wenchang	0.887145528
ENSGALG00010017734	TOP3A	2	-0.218872942	0.095637271	-2.28857369	0.022104134	0	0.00274105	0	1	PRJNA827465;Yang2025_Silkie_Wenchang	CRA004775	0.252326105
ENSGALG00010017734	TOP3A	2	-0.225085606	0.136466511	-1.649383462	0.099069101	0	6.14925E-05	0	1	CRA004775;Yang2025_Silkie_Wenchang	PRJNA827465	0.536989775
ENSGALG00010017734	TOP3A	2	-0.216747398	0.106738212	-2.03064482	0.042291038	0	0.000883161	0	1	CRA004775;PRJNA827465	Yang2025_Silkie_Wenchang	0.356076404
ENSGALG00010017737	ENSGALG00010017737	2	0.540692519	0.559412019	0.966537187	0.333775448	0.558069681	9.201378217	89.13206287	0.5	PRJNA827465;Yang2025_Silkie_Wenchang	CRA004775	0.802691373
ENSGALG00010017737	ENSGALG00010017737	2	0.545526628	0.592461936	0.9207792	0.357165716	0.59064876	6.231452783	83.9523778	0.5	CRA004775;Yang2025_Silkie_Wenchang	PRJNA827465	0.813260191
ENSGALG00010017737	ENSGALG00010017737	2	-0.026705577	0.20112394	-0.132781662	0.894366034	0	0.022376796	0	1	CRA004775;PRJNA827465	Yang2025_Silkie_Wenchang	0.974381724
ENSGALG00010017738	NDUFS5	2	0.161695881	0.213872261	0.756039519	0.449625502	0.073131396	4.413140357	77.34039892	0.5	PRJNA827465;Yang2025_Silkie_Wenchang	CRA004775	0.847275788
ENSGALG00010017738	NDUFS5	2	0.265524164	0.205245335	1.293691593	0.19577197	0.027001171	1.416722346	29.41453895	0.5	CRA004775;Yang2025_Silkie_Wenchang	PRJNA827465	0.706818459
ENSGALG00010017738	NDUFS5	2	-0.016236278	0.064605169	-0.251315461	0.801570222	0	0.000428905	0	1	CRA004775;PRJNA827465	Yang2025_Silkie_Wenchang	0.950313523
ENSGALG00010017739	MRPL20	2	0.283497861	0.330989295	0.856516706	0.391712036	0.204559959	14.5333932	93.1192944	0.5	PRJNA827465;Yang2025_Silkie_Wenchang	CRA004775	0.829251057
ENSGALG00010017739	MRPL20	2	0.118935989	0.538175082	0.220998691	0.825093452	0.526339689	10.69763279	90.65213754	0.5	CRA004775;Yang2025_Silkie_Wenchang	PRJNA827465	0.956315185
ENSGALG00010017739	MRPL20	2	-0.144047746	0.184646342	-0.780127809	0.435315649	0.042905137	1.995217167	49.88014254	1	CRA004775;PRJNA827465	Yang2025_Silkie_Wenchang	0.842762754
ENSGALG00010017741	SMCR8	2	-0.176991137	0.097044302	-1.823817917	0.068179604	0.013089925	3.146154083	68.21516132	1	PRJNA827465;Yang2025_Silkie_Wenchang	CRA004775	0.450274056
ENSGALG00010017741	SMCR8	2	-0.276107917	0.089619571	-3.080888612	0.002063838	0	0.215676656	0	1	CRA004775;Yang2025_Silkie_Wenchang	PRJNA827465	0.03905785
ENSGALG00010017741	SMCR8	2	-0.093615536	0.058131203	-1.610418014	0.107306631	0	0.01423712	0	1	CRA004775;PRJNA827465	Yang2025_Silkie_Wenchang	0.554492834
ENSGALG00010017742	DGLUCY	2	-0.087851899	0.182735329	-0.480760341	0.630686884	0.036672716	2.136987192	53.20514771	0.5	PRJNA827465;Yang2025_Silkie_Wenchang	CRA004775	0.902770945
ENSGALG00010017742	DGLUCY	2	0.061398276	0.18918521	0.324540571	0.745528812	0	0.554581461	0	0.5	CRA004775;Yang2025_Silkie_Wenchang	PRJNA827465	0.936204195
ENSGALG00010017742	DGLUCY	2	-0.239289523	0.134634864	-1.777322126	0.075515255	0	0.00110159	0	1	CRA004775;PRJNA827465	Yang2025_Silkie_Wenchang	0.474458409
ENSGALG00010017744	ETAA1	2	-0.124841359	0.095751249	-1.303809199	0.192298646	0.014266237	4.488660158	77.72163708	1	PRJNA827465;Yang2025_Silkie_Wenchang	CRA004775	0.702082087
ENSGALG00010017744	ETAA1	2	-0.127221166	0.140000379	-0.908720156	0.363497862	0.02423308	2.173500487	53.99126865	0.5	CRA004775;Yang2025_Silkie_Wenchang	PRJNA827465	0.817042253
ENSGALG00010017744	ETAA1	2	-0.022697825	0.056517949	-0.401603829	0.687976612	0	0.290711907	0	0.5	CRA004775;PRJNA827465	Yang2025_Silkie_Wenchang	0.979631238
ENSGALG00010017745	SHMT1	2	0.199625856	0.195703485	1.020042414	0.307708346	0.046224172	2.378559977	57.95775554	1	PRJNA827465;Yang2025_Silkie_Wenchang	CRA004775	0.793040073
ENSGALG00010017745	SHMT1	2	0.015957971	0.126274327	0.126375418	0.899434759	0	0.325965718	0	0.5	CRA004775;Yang2025_Silkie_Wenchang	PRJNA827465	0.976081354
ENSGALG00010017745	SHMT1	2	0.206677601	0.308687478	0.669536719	0.503151166	0.09998545	1.938036144	48.40137511	0.5	CRA004775;PRJNA827465	Yang2025_Silkie_Wenchang	0.863065896
ENSGALG00010017746	MSH6	2	0.027620477	0.146697428	0.188281946	0.850655638	0.036285205	6.027441052	83.40921145	0.5	PRJNA827465;Yang2025_Silkie_Wenchang	CRA004775	0.961985357
ENSGALG00010017746	MSH6	2	0.153320756	0.101024415	1.517660417	0.129100028	0	0.914273008	0	0.5	CRA004775;Yang2025_Silkie_Wenchang	PRJNA827465	0.60297307
ENSGALG00010017746	MSH6	2	-0.104469268	0.051401419	-2.032419931	0.042111162	0	0.000394335	0	1	CRA004775;PRJNA827465	Yang2025_Silkie_Wenchang	0.355094401
ENSGALG00010017747	DUSP26	2	-0.096240728	0.099976027	-0.962638048	0.335729197	0	3.33765E-05	0	1	PRJNA827465;Yang2025_Silkie_Wenchang	CRA004775	0.80340749
ENSGALG00010017747	DUSP26	2	-0.130096123	0.182720197	-0.711996401	0.476467005	0	0.365422097	0	1	CRA004775;Yang2025_Silkie_Wenchang	PRJNA827465	0.854986476
ENSGALG00010017747	DUSP26	2	-0.110352201	0.115083177	-0.958890805	0.337613757	0	0.384701991	0	1	CRA004775;PRJNA827465	Yang2025_Silkie_Wenchang	0.80415385
ENSGALG00010017749	TMEM88B	2	-0.049314514	0.45271375	-0.108930895	0.913257301	0.282763205	3.223979775	68.98243569	0.5	PRJNA827465;Yang2025_Silkie_Wenchang	CRA004775	0.979169142
ENSGALG00010017749	TMEM88B	2	-0.155885889	0.72182493	-0.214856069	0.829885726	0.705415319	2.726482614	63.32720762	0.5	CRA004775;Yang2025_Silkie_Wenchang	PRJNA827465	0.957079054
ENSGALG00010017749	TMEM88B	2	-0.596025678	0.329503939	-1.80885752	0.070473141	0	0.420275698	0	1	CRA004775;PRJNA827465	Yang2025_Silkie_Wenchang	0.458021454
ENSGALG00010017751	ST3GAL5	2	0.150311711	0.154533302	0.972681676	0.330711535	0.018850368	1.646946304	39.28156628	0.5	PRJNA827465;Yang2025_Silkie_Wenchang	CRA004775	0.801575303
ENSGALG00010017751	ST3GAL5	2	0.172504298	0.248850601	0.693204263	0.488181373	0.064736799	1.935305419	48.3285692	0.5	CRA004775;Yang2025_Silkie_Wenchang	PRJNA827465	0.85768885
ENSGALG00010017751	ST3GAL5	2	0.335291176	0.14413359	2.32625286	0.020005065	0	0.327525212	0	1	CRA004775;PRJNA827465	Yang2025_Silkie_Wenchang	0.237827913
ENSGALG00010017753	GNRHR	2	0.334092486	0.251999753	1.325765136	0.184917505	0	0.505607299	0	1	PRJNA827465;Yang2025_Silkie_Wenchang	CRA004775	0.693265167
ENSGALG00010017753	GNRHR	2	0.487763773	0.316798358	1.539666357	0.123641701	0	0.024647563	0	1	CRA004775;Yang2025_Silkie_Wenchang	PRJNA827465	0.589709136
ENSGALG00010017753	GNRHR	2	0.204770702	0.316561977	0.646858173	0.517723738	0	0.092580054	0	1	CRA004775;PRJNA827465	Yang2025_Silkie_Wenchang	0.867973554
ENSGALG00010017754	PDZD11	2	0.041832679	0.075955589	0.550751827	0.581803811	0	0.0644548	0	1	PRJNA827465;Yang2025_Silkie_Wenchang	CRA004775	0.889164179
ENSGALG00010017754	PDZD11	2	-0.15370793	0.193774075	-0.793232688	0.427642266	0.045460421	2.44380872	59.08026714	0.5	CRA004775;Yang2025_Silkie_Wenchang	PRJNA827465	0.84125685
ENSGALG00010017754	PDZD11	2	-0.122442181	0.213473146	-0.573571822	0.566257584	0.068627562	3.659904414	72.67688205	0.5	CRA004775;PRJNA827465	Yang2025_Silkie_Wenchang	0.883487517
ENSGALG00010017755	C1D	2	-0.363829239	0.322345657	-1.128698149	0.259022585	0.195869144	17.06097257	94.11801817	1	PRJNA827465;Yang2025_Silkie_Wenchang	CRA004775	0.759069756
ENSGALG00010017755	C1D	2	-0.465578686	0.301053234	-1.546499532	0.121983973	0.124826032	2.819545278	64.53328812	1	CRA004775;Yang2025_Silkie_Wenchang	PRJNA827465	0.585707157
ENSGALG00010017755	C1D	2	-0.054269462	0.064999946	-0.834915488	0.403765294	0	0.004419798	0	1	CRA004775;PRJNA827465	Yang2025_Silkie_Wenchang	0.833112102
ENSGALG00010017756	EIF2B5	2	-0.079557383	0.076265455	-1.043164084	0.296852305	0.005652241	1.812393572	44.82534637	1	PRJNA827465;Yang2025_Silkie_Wenchang	CRA004775	0.758466926
ENSGALG00010017756	EIF2B5	2	-0.197237936	0.087688888	-2.249292239	0.024493909	0	0.094194195	0	1	CRA004775;Yang2025_Silkie_Wenchang	PRJNA827465	0.267263775
ENSGALG00010017756	EIF2B5	2	-0.074057806	0.093276567	-0.793959387	0.427219075	0.007936309	1.489579776	32.86697252	1	CRA004775;PRJNA827465	Yang2025_Silkie_Wenchang	0.84125685
ENSGALG00010017757	ENSGALG00010017757	2	-0.469562895	0.380673875	-1.233504386	0.217387641	0.269784047	13.79558983	92.75130667	1	PRJNA827465;Yang2025_Silkie_Wenchang	CRA004775	0.730896703
ENSGALG00010017757	ENSGALG00010017757	2	-0.508945695	0.400393841	-1.271112697	0.203688559	0.254901058	4.720991657	78.81531719	1	CRA004775;Yang2025_Silkie_Wenchang	PRJNA827465	0.717254253
ENSGALG00010017757	ENSGALG00010017757	2	-0.109550501	0.059455792	-1.842553894	0.065394171	0	0.016886695	0	1	CRA004775;PRJNA827465	Yang2025_Silkie_Wenchang	0.440704235
ENSGALG00010017759	ENSGALG00010017759	2	-0.330903545	0.472240137	-0.700710336	0.483483804	0.269682087	2.448393949	59.15689956	0.5	PRJNA827465;Yang2025_Silkie_Wenchang	CRA004775	0.85691916
ENSGALG00010017759	ENSGALG00010017759	2	1.326511163	1.216373797	1.090545658	0.275472852	2.457983954	5.672083549	82.36979425	1	CRA004775;Yang2025_Silkie_Wenchang	PRJNA827465	0.775135547
ENSGALG00010017759	ENSGALG00010017759	2	0.860746488	1.669752527	0.507523839	0.611787314	5.313585339	12.57455555	92.04743264	0.5	CRA004775;PRJNA827465	Yang2025_Silkie_Wenchang	0.897777844
ENSGALG00010017761	PARP16	2	-0.154534618	0.128755799	-1.20021482	0.230055921	0.018888982	2.205399154	54.65673421	1	PRJNA827465;Yang2025_Silkie_Wenchang	CRA004775	0.742845187
ENSGALG00010017761	PARP16	2	-0.245186134	0.141744334	-1.729777316	0.083670069	0.001985141	1.03056524	2.965871392	0.5	CRA004775;Yang2025_Silkie_Wenchang	PRJNA827465	0.498330764
ENSGALG00010017761	PARP16	2	-0.041839192	0.089738594	-0.466236178	0.641066456	0	0.091433074	0	0.5	CRA004775;PRJNA827465	Yang2025_Silkie_Wenchang	0.906429868
ENSGALG00010017762	FBXO11	2	-0.019495947	0.031909651	-0.610973373	0.541217208	0	0.354616839	0	0.5	PRJNA827465;Yang2025_Silkie_Wenchang	CRA004775	0.875981549
ENSGALG00010017762	FBXO11	2	0.110264276	0.104562895	1.054525849	0.291642233	0	0.292462736	0	1	CRA004775;Yang2025_Silkie_Wenchang	PRJNA827465	0.785137868
ENSGALG00010017762	FBXO11	2	0.014707656	0.078772653	0.186710177	0.851838776	0.00662724	1.50262736	33.44990073	0.5	CRA004775;PRJNA827465	Yang2025_Silkie_Wenchang	0.962255491
ENSGALG00010017763	POLR1A	2	-0.068368033	0.059256732	-1.153759765	0.248598674	0	0.001290085	0	1	PRJNA827465;Yang2025_Silkie_Wenchang	CRA004775	0.758965129
ENSGALG00010017763	POLR1A	2	-0.08590873	0.088166271	-0.974394504	0.329860697	0	0.205122162	0	1	CRA004775;Yang2025_Silkie_Wenchang	PRJNA827465	0.801177491
ENSGALG00010017763	POLR1A	2	-0.077233477	0.073001684	-1.057968424	0.290069838	0	0.229688014	0	1	CRA004775;PRJNA827465	Yang2025_Silkie_Wenchang	0.784195688
ENSGALG00010017764	IGDCC3	2	-0.348364474	0.144051727	-2.418329028	0.015591971	0	0.148335901	0	1	PRJNA827465;Yang2025_Silkie_Wenchang	CRA004775	0.206610535
ENSGALG00010017764	IGDCC3	2	-0.472929458	0.19519939	-2.422801923	0.015401322	0	0.396885034	0	1	CRA004775;Yang2025_Silkie_Wenchang	PRJNA827465	0.205241414
ENSGALG00010017764	IGDCC3	2	-0.367409557	0.17559796	-2.092333859	0.036408661	0	0.773305405	0	1	CRA004775;PRJNA827465	Yang2025_Silkie_Wenchang	0.32890494
ENSGALG00010017765	SYT16	2	-0.273635123	0.272946547	-1.002522749	0.316091185	0.112177608	3.924914058	74.52173512	1	PRJNA827465;Yang2025_Silkie_Wenchang	CRA004775	0.796883862
ENSGALG00010017765	SYT16	2	-0.544316802	0.213408707	-2.550583847	0.010754265	0	0.122856428	0	1	CRA004775;Yang2025_Silkie_Wenchang	PRJNA827465	0.165236469
ENSGALG00010017765	SYT16	2	-0.050856908	0.146761836	-0.346526792	0.728946853	0	0.349412748	0	1	CRA004775;PRJNA827465	Yang2025_Silkie_Wenchang	0.932323844
ENSGALG00010017766	DNAAF10	2	-0.170168479	0.067626448	-2.516300706	0.011859395	0	0.090654904	0	1	PRJNA827465;Yang2025_Silkie_Wenchang	CRA004775	0.174703824
ENSGALG00010017766	DNAAF10	2	-0.161549387	0.11681054	-1.383003516	0.166663788	0	0.978632684	0	0.5	CRA004775;Yang2025_Silkie_Wenchang	PRJNA827465	0.665626918
ENSGALG00010017766	DNAAF10	2	-0.140883841	0.078841842	-1.786917166	0.073950878	0	0.809800428	0	0.5	CRA004775;PRJNA827465	Yang2025_Silkie_Wenchang	0.469669846
ENSGALG00010017767	KIF4B	2	0.035384203	0.097732254	0.362052458	0.71731283	0.005642707	1.399920209	28.56735739	0.5	PRJNA827465;Yang2025_Silkie_Wenchang	CRA004775	0.9277944
ENSGALG00010017767	KIF4B	2	0.165266301	0.130139593	1.269915615	0.204114691	0	0.206845057	0	1	CRA004775;Yang2025_Silkie_Wenchang	PRJNA827465	0.717254253
ENSGALG00010017767	KIF4B	2	-0.033285944	0.101277523	-0.328660719	0.742412148	0	0.592864098	0	0.5	CRA004775;PRJNA827465	Yang2025_Silkie_Wenchang	0.935510459
ENSGALG00010017768	FOXN2	2	-0.105439204	0.074460255	-1.416046765	0.156761813	0	0.275965165	0	1	PRJNA827465;Yang2025_Silkie_Wenchang	CRA004775	0.650122352
ENSGALG00010017768	FOXN2	2	-0.146042225	0.122557033	-1.191626641	0.233407674	0	0.112746135	0	1	CRA004775;Yang2025_Silkie_Wenchang	PRJNA827465	0.746672784
ENSGALG00010017768	FOXN2	2	-0.076600691	0.08656085	-0.884934602	0.376191904	0	0.009173641	0	1	CRA004775;PRJNA827465	Yang2025_Silkie_Wenchang	0.822974013
ENSGALG00010017770	PTCD3	2	-0.08463781	0.114500154	-0.739193853	0.459789293	0.01884004	3.220846908	68.9522654	0.5	PRJNA827465;Yang2025_Silkie_Wenchang	CRA004775	0.850098539
ENSGALG00010017770	PTCD3	2	-0.213891438	0.104368749	-2.049382021	0.040424774	0	0.040086172	0	1	CRA004775;Yang2025_Silkie_Wenchang	PRJNA827465	0.347691771
ENSGALG00010017770	PTCD3	2	-0.001709624	0.05313301	-0.032176311	0.974331448	0	0.641384106	0	0.5	CRA004775;PRJNA827465	Yang2025_Silkie_Wenchang	0.992492038
ENSGALG00010017771	PNO1	2	-0.08137829	0.065242409	-1.247321969	0.212279465	0	0.419351161	0	1	PRJNA827465;Yang2025_Silkie_Wenchang	CRA004775	0.72493004
ENSGALG00010017771	PNO1	2	-0.114355907	0.142036292	-0.805117519	0.420751867	0	0.712900483	0	0.5	CRA004775;Yang2025_Silkie_Wenchang	PRJNA827465	0.839211146
ENSGALG00010017771	PNO1	2	-0.053329259	0.06969254	-0.765207571	0.444147952	0	0.325798295	0	0.5	CRA004775;PRJNA827465	Yang2025_Silkie_Wenchang	0.846013379
ENSGALG00010017772	ENSGALG00010017772	2	0.028929961	0.186033955	0.155509035	0.876420032	0	0.622617666	0	0.5	PRJNA827465;Yang2025_Silkie_Wenchang	CRA004775	0.970347777
ENSGALG00010017772	ENSGALG00010017772	2	0.242412009	0.253175864	0.957484669	0.338322681	0	0.048017427	0	1	CRA004775;Yang2025_Silkie_Wenchang	PRJNA827465	0.804154375
ENSGALG00010017772	ENSGALG00010017772	2	-0.009361375	0.215786525	-0.043382574	0.965396568	0	0.600953125	0	0.5	CRA004775;PRJNA827465	Yang2025_Silkie_Wenchang	0.990668014
ENSGALG00010017773	DVL3	2	-0.024415096	0.058653799	-0.416257706	0.677221438	0.000613034	1.06919129	6.471366769	0.5	PRJNA827465;Yang2025_Silkie_Wenchang	CRA004775	0.917144807
ENSGALG00010017773	DVL3	2	0.032317298	0.096749177	0.334031762	0.738355591	0	0.519154325	0	0.5	CRA004775;Yang2025_Silkie_Wenchang	PRJNA827465	0.934457891
ENSGALG00010017773	DVL3	2	-0.058596534	0.058663591	-0.998856933	0.317864002	0	0.004010276	0	1	CRA004775;PRJNA827465	Yang2025_Silkie_Wenchang	0.797439961
ENSGALG00010017774	ASH2L	2	-0.134334288	0.06530674	-2.056974329	0.039688695	0.004163652	1.708055544	41.45389455	1	PRJNA827465;Yang2025_Silkie_Wenchang	CRA004775	0.344755224
ENSGALG00010017774	ASH2L	2	-0.219312742	0.090971299	-2.410790495	0.015917989	0	0.11940482	0	1	CRA004775;Yang2025_Silkie_Wenchang	PRJNA827465	0.208471156
ENSGALG00010017774	ASH2L	2	-0.092921358	0.043707562	-2.125978965	0.033505011	0	0.053441559	0	1	CRA004775;PRJNA827465	Yang2025_Silkie_Wenchang	0.314508586
ENSGALG00010017775	PRPSAP2	2	0.057806297	0.137639616	0.419982986	0.674497883	0.032174953	6.535748654	84.69953401	0.5	PRJNA827465;Yang2025_Silkie_Wenchang	CRA004775	0.915962625
ENSGALG00010017775	PRPSAP2	2	-0.102294348	0.332045899	-0.308072916	0.75802685	0.195485794	8.340920604	88.01091573	0.5	CRA004775;Yang2025_Silkie_Wenchang	PRJNA827465	0.938954369
ENSGALG00010017775	PRPSAP2	2	-0.215193838	0.188039156	-1.144409722	0.252453758	0.051688705	3.092511721	67.66382506	1	CRA004775;PRJNA827465	Yang2025_Silkie_Wenchang	0.762655223
ENSGALG00010017776	GPR68	2	0.362983028	0.306226768	1.185340621	0.235882799	0.14552563	4.451116121	77.53372474	1	PRJNA827465;Yang2025_Silkie_Wenchang	CRA004775	0.748669015
ENSGALG00010017776	GPR68	2	0.611816107	0.182636791	3.349906143	0.000808389	0	0.310919327	0	1	CRA004775;Yang2025_Silkie_Wenchang	PRJNA827465	0.032687378
ENSGALG00010017776	GPR68	2	0.162543794	0.168698956	0.963513927	0.335289679	0.000976883	1.013044155	1.28761955	1	CRA004775;PRJNA827465	Yang2025_Silkie_Wenchang	0.803198384
ENSGALG00010017778	PPP3R1	2	-0.205760138	0.048074258	-4.280048107	1.86853E-05	0.000610701	1.143401752	12.54167676	1	PRJNA827465;Yang2025_Silkie_Wenchang	CRA004775	0.002338815
ENSGALG00010017778	PPP3R1	2	-0.084517756	0.233777687	-0.361530464	0.717702935	0.082311443	3.415054435	70.71788989	0.5	CRA004775;Yang2025_Silkie_Wenchang	PRJNA827465	0.927927766
ENSGALG00010017778	PPP3R1	2	-0.053342824	0.175460431	-0.304016258	0.761115505	0.04066403	2.232777071	55.21272531	0.5	CRA004775;PRJNA827465	Yang2025_Silkie_Wenchang	0.939602434
ENSGALG00010017779	SNAPC1	2	-0.01441485	0.061060977	-0.236073029	0.813376002	0	0.057339645	0	0.5	PRJNA827465;Yang2025_Silkie_Wenchang	CRA004775	0.953587297
ENSGALG00010017779	SNAPC1	2	-0.001321241	0.115729984	-0.011416582	0.990891084	0	0.052492563	0	0.5	CRA004775;Yang2025_Silkie_Wenchang	PRJNA827465	0.997555603
ENSGALG00010017779	SNAPC1	2	-0.023940307	0.065734811	-0.364195274	0.715712196	0	0.010781379	0	1	CRA004775;PRJNA827465	Yang2025_Silkie_Wenchang	0.927131632
ENSGALG00010017780	TAF9	2	0.057883639	0.056688818	1.02107685	0.30721803	0	0.085219314	0	1	PRJNA827465;Yang2025_Silkie_Wenchang	CRA004775	0.79266003
ENSGALG00010017780	TAF9	2	-0.017023178	0.116941299	-0.145570286	0.884260626	0	0.142932166	0	0.5	CRA004775;Yang2025_Silkie_Wenchang	PRJNA827465	0.972001076
ENSGALG00010017780	TAF9	2	0.051593342	0.058329432	0.88451644	0.376417492	0	0.481140836	0	0.5	CRA004775;PRJNA827465	Yang2025_Silkie_Wenchang	0.822974013
ENSGALG00010017781	CYP26C1	2	-0.232652845	0.309684927	-0.751256598	0.452498244	0.080248872	1.658594651	39.70799317	0.5	PRJNA827465;Yang2025_Silkie_Wenchang	CRA004775	0.848321192
ENSGALG00010017781	CYP26C1	2	-0.421150385	0.284722412	-1.479161343	0.139097199	0	0.468206107	0	1	CRA004775;Yang2025_Silkie_Wenchang	PRJNA827465	0.622860674
ENSGALG00010017781	CYP26C1	2	-0.066834753	0.22658267	-0.294968512	0.768017934	0	0.259116068	0	0.5	CRA004775;PRJNA827465	Yang2025_Silkie_Wenchang	0.941444269
ENSGALG00010017782	IGDCC4	2	-0.181063519	0.133775676	-1.353486108	0.175900385	0.022521599	2.667530226	62.51213987	1	PRJNA827465;Yang2025_Silkie_Wenchang	CRA004775	0.680990232
ENSGALG00010017782	IGDCC4	2	-0.469067531	0.231082213	-2.02987294	0.042369456	0.057408758	1.755459882	43.03487021	1	CRA004775;Yang2025_Silkie_Wenchang	PRJNA827465	0.356238117
ENSGALG00010017782	IGDCC4	2	-0.370579353	0.384321852	-0.964242211	0.334924506	0.235002397	4.2157854	76.27962752	1	CRA004775;PRJNA827465	Yang2025_Silkie_Wenchang	0.803101658
ENSGALG00010017783	STAR	2	0.298645564	0.270202626	1.105265215	0.269044686	0	0.447415872	0	1	PRJNA827465;Yang2025_Silkie_Wenchang	CRA004775	0.771461882
ENSGALG00010017783	STAR	2	0.169528617	0.343295028	0.493827766	0.621427822	0.061616361	1.353816285	26.134734	0.5	CRA004775;Yang2025_Silkie_Wenchang	PRJNA827465	0.899693435
ENSGALG00010017783	STAR	2	0.007765085	0.272711231	0.028473654	0.977284381	0	0.338016018	0	0.5	CRA004775;PRJNA827465	Yang2025_Silkie_Wenchang	0.993487352
ENSGALG00010017786	KAZALD1	2	-0.084280265	0.080386997	-1.048431562	0.294439818	0	0.009411834	0	1	PRJNA827465;Yang2025_Silkie_Wenchang	CRA004775	0.787396624
ENSGALG00010017786	KAZALD1	2	-0.116518243	0.144948095	-0.803861847	0.421476767	0	0.859999126	0	1	CRA004775;Yang2025_Silkie_Wenchang	PRJNA827465	0.839352211
ENSGALG00010017786	KAZALD1	2	-0.106684713	0.092574077	-1.152425353	0.249146328	0	0.846691543	0	1	CRA004775;PRJNA827465	Yang2025_Silkie_Wenchang	0.759411487
ENSGALG00010017787	CNRIP1	2	0.145572656	0.120034549	1.212756304	0.225223006	0	0.529804288	0	1	PRJNA827465;Yang2025_Silkie_Wenchang	CRA004775	0.738276881
ENSGALG00010017787	CNRIP1	2	-0.076248602	0.353965144	-0.21541274	0.829445538	0.192072434	4.042852168	75.26498723	0.5	CRA004775;Yang2025_Silkie_Wenchang	PRJNA827465	0.95692599
ENSGALG00010017787	CNRIP1	2	-0.148073545	0.262243101	-0.56464229	0.572317094	0.082500105	2.306942862	56.65258918	0.5	CRA004775;PRJNA827465	Yang2025_Silkie_Wenchang	0.885651577
ENSGALG00010017789	IMMT	2	0.053448409	0.179389883	0.297945506	0.765744755	0.058951839	11.8954078	91.59339455	0.5	PRJNA827465;Yang2025_Silkie_Wenchang	CRA004775	0.940972552
ENSGALG00010017789	IMMT	2	0.231165901	0.073394341	3.149642042	0.001634706	0	0.020159768	0	1	CRA004775;Yang2025_Silkie_Wenchang	PRJNA827465	0.051122869
ENSGALG00010017789	IMMT	2	-0.110802296	0.070059944	-1.581535612	0.113755623	0	0.871329712	0	0.5	CRA004775;PRJNA827465	Yang2025_Silkie_Wenchang	0.568978529
ENSGALG00010017790	R3HCC1L	2	0.479821975	0.332028375	1.445123399	0.148423227	0.21056226	22.20579345	95.49667071	1	PRJNA827465;Yang2025_Silkie_Wenchang	CRA004775	0.638471099
ENSGALG00010017790	R3HCC1L	2	0.407628806	0.430586053	0.946683718	0.343799969	0.33912764	11.28652346	91.13987577	0.5	CRA004775;Yang2025_Silkie_Wenchang	PRJNA827465	0.806388726
ENSGALG00010017790	R3HCC1L	2	0.122137996	0.086976206	1.404269075	0.16023874	0	0.619885989	0	0.5	CRA004775;PRJNA827465	Yang2025_Silkie_Wenchang	0.655685911
ENSGALG00010017791	CCNL2	2	0.385854276	0.225927717	1.707866043	0.087661201	0.093721613	12.18744799	91.79483678	1	PRJNA827465;Yang2025_Silkie_Wenchang	CRA004775	0.509064449
ENSGALG00010017791	CCNL2	2	0.235667292	0.397291324	0.593185094	0.553057287	0.291687718	12.79691302	92.1856154	0.5	CRA004775;Yang2025_Silkie_Wenchang	PRJNA827465	0.87934426
ENSGALG00010017791	CCNL2	2	0.038162456	0.165092063	0.231158634	0.817191565	0.035434389	2.501236092	60.0197677	0.5	CRA004775;PRJNA827465	Yang2025_Silkie_Wenchang	0.954833525
ENSGALG00010017792	LSM1	2	0.432925986	0.267593843	1.617847333	0.105695503	0.106966539	3.668238983	72.73896263	1	PRJNA827465;Yang2025_Silkie_Wenchang	CRA004775	0.551679993
ENSGALG00010017792	LSM1	2	0.257132961	0.499386352	0.514897854	0.6066244	0.426396609	6.879107789	85.46323113	0.5	CRA004775;Yang2025_Silkie_Wenchang	PRJNA827465	0.896799223
ENSGALG00010017792	LSM1	2	0.048339153	0.216966801	0.222795161	0.823694935	0.055486802	2.138034971	53.22808029	0.5	CRA004775;PRJNA827465	Yang2025_Silkie_Wenchang	0.955934625
ENSGALG00010017793	FAM83G	2	-0.083000476	0.637643415	-0.130167542	0.896433874	0.478524142	2.292463895	56.37881137	0.5	PRJNA827465;Yang2025_Silkie_Wenchang	CRA004775	0.975115773
ENSGALG00010017793	FAM83G	2	0.414195824	1.27457642	0.32496743	0.745205722	2.724123691	6.188045364	83.83980819	0.5	CRA004775;Yang2025_Silkie_Wenchang	PRJNA827465	0.93597621
ENSGALG00010017793	FAM83G	2	0.933968389	0.610641494	1.529487266	0.126143693	0.425355625	2.21087529	54.76904535	1	CRA004775;PRJNA827465	Yang2025_Silkie_Wenchang	0.596525734
ENSGALG00010017795	MACF1	2	0.099076651	0.077991448	1.270352754	0.203959004	0.00374783	1.234318175	18.98361218	1	PRJNA827465;Yang2025_Silkie_Wenchang	CRA004775	0.717254253
ENSGALG00010017795	MACF1	2	0.014377785	0.305840847	0.047010676	0.962504719	0.125634678	2.779819383	64.02643977	0.5	CRA004775;Yang2025_Silkie_Wenchang	PRJNA827465	0.989980504
ENSGALG00010017795	MACF1	2	-0.024968695	0.171616552	-0.145491181	0.884323078	0.033126137	1.567639428	36.20982082	0.5	CRA004775;PRJNA827465	Yang2025_Silkie_Wenchang	0.972001076
ENSGALG00010017796	MXI1	2	-0.122395872	0.045821202	-2.671162399	0.007558907	0	0.506229125	0	1	PRJNA827465;Yang2025_Silkie_Wenchang	CRA004775	0.134185957
ENSGALG00010017796	MXI1	2	-0.16664873	0.076745142	-2.171456395	0.02989669	0	0.004880564	0	1	CRA004775;Yang2025_Silkie_Wenchang	PRJNA827465	0.295167651
ENSGALG00010017796	MXI1	2	-0.100072345	0.055697368	-1.796715872	0.072380738	0	0.014571782	0	1	CRA004775;PRJNA827465	Yang2025_Silkie_Wenchang	0.4646815
ENSGALG00010017797	PPP1R21	2	0.169836807	0.108858191	1.56016562	0.118720748	0.017691775	3.779042431	73.53827013	1	PRJNA827465;Yang2025_Silkie_Wenchang	CRA004775	0.578883823
ENSGALG00010017797	PPP1R21	2	0.099088042	0.231859012	0.427363342	0.669114703	0.082016951	3.82872703	73.88165852	0.5	CRA004775;Yang2025_Silkie_Wenchang	PRJNA827465	0.914377769
ENSGALG00010017797	PPP1R21	2	0.037465567	0.088218348	0.424691326	0.671061708	0.005650782	1.216830996	17.81931896	0.5	CRA004775;PRJNA827465	Yang2025_Silkie_Wenchang	0.914582784
ENSGALG00010017798	BAG4	2	0.232626736	0.139168441	1.671548053	0.094613476	0.02912723	3.662426997	72.69570149	1	PRJNA827465;Yang2025_Silkie_Wenchang	CRA004775	0.526919621
ENSGALG00010017798	BAG4	2	0.222184876	0.198799681	1.117631956	0.263724215	0.05288448	2.947948273	66.07810221	0.5	CRA004775;Yang2025_Silkie_Wenchang	PRJNA827465	0.768643821
ENSGALG00010017798	BAG4	2	0.106171767	0.05746852	1.847476962	0.064678044	0	0.344923677	0	0.5	CRA004775;PRJNA827465	Yang2025_Silkie_Wenchang	0.438519901

ENSGALG00010017799	GRAPL	2	0.847330528	0.229164946	3.697470072	0.000217759	0	0.283228871	0	1	PRJNA827465;Yang2025_Silkie_Wenchang	CRA004775	0.013456883
ENSGALG00010017799	GRAPL	2	0.229930723	0.482517972	0.476522609	0.633702104	0.332491057	3.494271489	71.38173141	0.5	CRA004775;Yang2025_Silkie_Wenchang	PRJNA827465	0.90355138
ENSGALG00010017799	GRAPL	2	0.365986583	0.605783035	0.60415456	0.545740889	0.618793944	6.332712007	84.20897715	0.5	CRA004775;PRJNA827465	Yang2025_Silkie_Wenchang	0.877885919
ENSGALG00010017801	SMNDC1	2	-0.26593595	0.321761789	-0.826499477	0.408520815	0.20213586	41.68719766	97.601182	0.5	PRJNA827465;Yang2025_Silkie_Wenchang	CRA004775	0.834164004
ENSGALG00010017801	SMNDC1	2	-0.243600953	0.365866258	-0.665819674	0.505526368	0.247542938	12.90758692	92.25261851	0.5	CRA004775;Yang2025_Silkie_Wenchang	PRJNA827465	0.863927652
ENSGALG00010017801	SMNDC1	2	0.056231009	0.043422547	1.294972591	0.195329685	0	0.222190124	0	1	CRA004775;PRJNA827465	Yang2025_Silkie_Wenchang	0.70643959
ENSGALG00010017803	ENSGALG00010017803	2	0.055021	0.051999547	1.058105365	0.290007409	0	0.631277628	0	1	PRJNA827465;Yang2025_Silkie_Wenchang	CRA004775	0.784195688
ENSGALG00010017803	ENSGALG00010017803	2	0.178349514	0.12109122	1.472852558	0.140790797	0	0.366447153	0	1	CRA004775;Yang2025_Silkie_Wenchang	PRJNA827465	0.625201272
ENSGALG00010017803	ENSGALG00010017803	2	0.046289959	0.055927293	0.827681007	0.407851178	0	0.950057576	0	1	CRA004775;PRJNA827465	Yang2025_Silkie_Wenchang	0.833982683
ENSGALG00010017804	DUSP5	2	-0.079390543	0.109068732	-0.727894621	0.466678096	0.010547813	1.714120384	41.6610403	0.5	PRJNA827465;Yang2025_Silkie_Wenchang	CRA004775	0.852842649
ENSGALG00010017804	DUSP5	2	-0.257160652	0.13850957	-1.856627318	0.063364195	0	0.786115965	0	1	CRA004775;Yang2025_Silkie_Wenchang	PRJNA827465	0.433558714
ENSGALG00010017804	DUSP5	2	-0.164219503	0.289461577	-0.567327468	0.570491719	0.10403777	1.896739385	47.27794403	0.5	CRA004775;PRJNA827465	Yang2025_Silkie_Wenchang	0.885126955
ENSGALG00010017805	DPP8	2	-0.104536513	0.131853707	-0.792821948	0.427881567	0.027985942	5.023568947	80.09383348	0.5	PRJNA827465;Yang2025_Silkie_Wenchang	CRA004775	0.84125685
ENSGALG00010017805	DPP8	2	-0.182279117	0.13328831	-1.367555174	0.171451356	0.012422467	1.268318984	21.15548118	0.5	CRA004775;Yang2025_Silkie_Wenchang	PRJNA827465	0.673653167
ENSGALG00010017805	DPP8	2	0.022434841	0.065432362	0.342870715	0.731695724	0	0.069971974	0	1	CRA004775;PRJNA827465	Yang2025_Silkie_Wenchang	0.932851968
ENSGALG00010017806	ENSGALG00010017806	2	-0.312915829	0.22347458	-1.400230079	0.161444431	0	4.6599E-05	0	1	PRJNA827465;Yang2025_Silkie_Wenchang	CRA004775	0.657858553
ENSGALG00010017806	ENSGALG00010017806	2	0.932666304	1.33583047	0.698192117	0.485057055	3.232034653	10.13809745	90.13621634	0.5	CRA004775;Yang2025_Silkie_Wenchang	PRJNA827465	0.857004733
ENSGALG00010017806	ENSGALG00010017806	2	0.931273958	1.334092881	0.698057812	0.48514104	3.231381503	10.33514821	90.32428003	0.5	CRA004775;PRJNA827465	Yang2025_Silkie_Wenchang	0.857004733
ENSGALG00010017807	MRPL35	2	0.006990485	0.115591653	0.060475689	0.951776778	0.017348163	2.615387837	61.76475298	0.5	PRJNA827465;Yang2025_Silkie_Wenchang	CRA004775	0.987713746
ENSGALG00010017807	MRPL35	2	-0.127427536	0.118421089	-1.076054422	0.281902918	0	0.035857673	0	1	CRA004775;Yang2025_Silkie_Wenchang	PRJNA827465	0.779432317
ENSGALG00010017807	MRPL35	2	0.090010396	0.065638279	1.371309518	0.17027849	0	0.365587938	0	0.5	CRA004775;PRJNA827465	Yang2025_Silkie_Wenchang	0.672068235
ENSGALG00010017808	SFRP5	2	0.327367525	0.137959703	2.372921356	0.017648021	0	0.874710011	0	1	PRJNA827465;Yang2025_Silkie_Wenchang	CRA004775	0.221474498
ENSGALG00010017808	SFRP5	2	0.49122269	0.176205306	2.787880525	0.005306958	0	0.725022754	0	1	CRA004775;Yang2025_Silkie_Wenchang	PRJNA827465	0.108805103
ENSGALG00010017808	SFRP5	2	0.38438398	0.339268788	1.132977728	0.257223606	0.11278456	1.605674307	37.72086931	1	CRA004775;PRJNA827465	Yang2025_Silkie_Wenchang	0.764898192
ENSGALG00010017809	MOSMO	2	-0.066868664	0.246390964	-0.271392517	0.786089157	0.110668684	11.18156629	91.05670911	0.5	PRJNA827465;Yang2025_Silkie_Wenchang	CRA004775	0.946639719
ENSGALG00010017809	MOSMO	2	-0.027312784	0.331486664	-0.082394821	0.934332755	0.181526217	5.379676254	81.41152083	0.5	CRA004775;Yang2025_Silkie_Wenchang	PRJNA827465	0.98432801
ENSGALG00010017809	MOSMO	2	0.188198679	0.079839702	2.357206673	0.018412996	0	0.406050614	0	1	CRA004775;PRJNA827465	Yang2025_Silkie_Wenchang	0.227145629
ENSGALG00010017810	HIF1A	2	0.005680089	0.047006076	0.120837329	0.903819883	0	0.855871491	0	0.5	PRJNA827465;Yang2025_Silkie_Wenchang	CRA004775	0.977333734
ENSGALG00010017810	HIF1A	2	-0.092907147	0.105351375	-0.881878828	0.377842337	0	0.020738397	0	1	CRA004775;Yang2025_Silkie_Wenchang	PRJNA827465	0.63232159
ENSGALG00010017810	HIF1A	2	0.021884231	0.051205316	0.427382009	0.66910111	0	0.245442229	0	0.5	CRA004775;PRJNA827465	Yang2025_Silkie_Wenchang	0.914377769
ENSGALG00010017811	HACD3	2	-0.045460001	0.159131169	-0.285676281	0.775126073	0.04613334	11.18640377	91.06057656	0.5	PRJNA827465;Yang2025_Silkie_Wenchang	CRA004775	0.943803161
ENSGALG00010017811	HACD3	2	-0.192400685	0.070795034	-2.717714456	0.006573454	0	0.519904041	0	1	CRA004775;Yang2025_Silkie_Wenchang	PRJNA827465	0.123859732
ENSGALG00010017811	HACD3	2	0.103628552	0.05838452	1.774932001	0.075909116	0	0.260091168	0	0.5	CRA004775;PRJNA827465	Yang2025_Silkie_Wenchang	0.47539798
ENSGALG00010017813	DDHD2	2	-0.028046356	0.060904173	-0.460499745	0.645157554	0	0.775630661	0	0.5	PRJNA827465;Yang2025_Silkie_Wenchang	CRA004775	0.906966303
ENSGALG00010017813	DDHD2	2	-0.011066248	0.12651081	-0.08747275	0.930295744	0.004732733	1.162046421	13.94491803	0.5	CRA004775;Yang2025_Silkie_Wenchang	PRJNA827465	0.983813577
ENSGALG00010017813	DDHD2	2	-0.065681541	0.063366816	-1.036528974	0.29995543	0	0.342117713	0	1	CRA004775;PRJNA827465	Yang2025_Silkie_Wenchang	0.799012361
ENSGALG00010017814	ENSGALG00010017814	2	-0.21695652	0.463944532	-0.467634611	0.64004591	0.367288031	6.794666531	85.28257427	0.5	PRJNA827465;Yang2025_Silkie_Wenchang	CRA004775	0.906064918
ENSGALG00010017814	ENSGALG00010017814	2	-0.205531493	0.536806761	-0.382877988	0.701810233	0.450720498	4.284087788	76.65780793	0.5	CRA004775;Yang2025_Silkie_Wenchang	PRJNA827465	0.922904951
ENSGALG00010017814	ENSGALG00010017814	2	0.295296504	0.233217498	1.266185024	0.205446848	0	0.08361431	0	1	CRA004775;PRJNA827465	Yang2025_Silkie_Wenchang	0.71825498
ENSGALG00010017815	VWA9	2	-0.100717257	0.081531576	-1.23531646	0.216712175	0.006350321	1.794896211	44.28647218	1	PRJNA827465;Yang2025_Silkie_Wenchang	CRA004775	0.730172415
ENSGALG00010017815	VWA9	2	-0.229138407	0.092625026	-2.473828252	0.013367394	0	0.133150564	0	1	CRA004775;Yang2025_Silkie_Wenchang	PRJNA827465	0.188936172
ENSGALG00010017815	VWA9	2	-0.1064213	0.109444513	-0.972376748	0.330863155	0.012418824	1.706741493	41.40881884	1	CRA004775;PRJNA827465	Yang2025_Silkie_Wenchang	0.801677893
ENSGALG00010017816	PRKCH	2	0.315830177	0.261557292	1.20749903	0.227240018	0	0.112372132	0	1	PRJNA827465;Yang2025_Silkie_Wenchang	CRA004775	0.740605003
ENSGALG00010017816	PRKCH	2	0.202889241	0.303370494	0.668783698	0.503633467	0	0.505095243	0	1	CRA004775;Yang2025_Silkie_Wenchang	PRJNA827465	0.86313636
ENSGALG00010017816	PRKCH	2	0.159016362	0.253471463	0.627354102	0.530427146	0	0.224821805	0	1	CRA004775;PRJNA827465	Yang2025_Silkie_Wenchang	0.872494272
ENSGALG00010017817	REEP1	2	-0.150049527	0.068790974	-2.181238583	0.029165774	0.000230194	1.024550152	2.396188416	1	PRJNA827465;Yang2025_Silkie_Wenchang	CRA004775	0.290570959
ENSGALG00010017817	REEP1	2	-0.08782589	0.08751588	-1.003519139	0.315610254	0	0.003344611	0	1	CRA004775;Yang2025_Silkie_Wenchang	PRJNA827465	0.79688386
ENSGALG00010017817	REEP1	2	-0.214406592	0.098271632	-2.181775017	0.029126141	0	0.201643089	0	1	CRA004775;PRJNA827465	Yang2025_Silkie_Wenchang	0.290337858
ENSGALG00010017818	SLC38A6	2	0.343238182	0.227805026	1.506719091	0.131882676	0.086762377	6.07389847	83.53610939	1	PRJNA827465;Yang2025_Silkie_Wenchang	CRA004775	0.608521345
ENSGALG00010017818	SLC38A6	2	0.107487658	0.497080595	0.216237888	0.828802322	0.447808495	10.33589017	90.32497459	0.5	CRA004775;Yang2025_Silkie_Wenchang	PRJNA827465	0.956840134
ENSGALG00010017818	SLC38A6	2	-0.091489864	0.263879067	-0.346711337	0.728808192	0.100564664	3.223193944	68.97487346	0.5	CRA004775;PRJNA827465	Yang2025_Silkie_Wenchang	0.932244113
ENSGALG00010017820	FLRT2	2	-0.192352254	0.217448444	-0.884587862	0.376378956	0.078971835	5.662888738	82.34116815	0.5	PRJNA827465;Yang2025_Silkie_Wenchang	CRA004775	0.822974013
ENSGALG00010017820	FLRT2	2	0.079404081	0.538707988	0.14739726	0.882818468	0.528837185	11.05537531	90.9546264	0.5	CRA004775;Yang2025_Silkie_Wenchang	PRJNA827465	0.972001076
ENSGALG00010017820	FLRT2	2	0.265260185	0.315317514	0.841247863	0.400209092	0.163796393	4.970423186	79.88098875	1	CRA004775;PRJNA827465	Yang2025_Silkie_Wenchang	0.832246851
ENSGALG00010017821	ENSGALG00010017821	2	-0.177642558	0.368267961	-0.482373099	0.629540927	0	0.336390582	0	0.5	PRJNA827465;Yang2025_Silkie_Wenchang	CRA004775	0.902457014
ENSGALG00010017821	ENSGALG00010017821	2	-0.261708206	0.343454726	-0.761987494	0.446067468	0	0.116790807	0	1	CRA004775;Yang2025_Silkie_Wenchang	PRJNA827465	0.846013379
ENSGALG00010017821	ENSGALG00010017821	2	-0.079850358	0.337067232	-0.236897421	0.812736371	0	0.077730197	0	0.5	CRA004775;PRJNA827465	Yang2025_Silkie_Wenchang	0.95348724
ENSGALG00010017823	PPIE	2	0.182642697	0.253004984	0.721893673	0.470330985	0.11596711	10.11492542	90.11361965	0.5	PRJNA827465;Yang2025_Silkie_Wenchang	CRA004775	0.853744788
ENSGALG00010017823	PPIE	2	0.1667194	0.309998272	0.537807514	0.590709944	0.156967883	5.325566829	81.22265606	0.5	CRA004775;Yang2025_Silkie_Wenchang	PRJNA827465	0.892708037
ENSGALG00010017823	PPIE	2	-0.060080528	0.056901181	-1.05587489	0.291025377	0	0.242031798	0	1	CRA004775;PRJNA827465	Yang2025_Silkie_Wenchang	0.78467882
ENSGALG00010017824	YAE1	2	0.060330121	0.094155061	0.640752823	0.521668288	0	0.371661472	0	1	PRJNA827465;Yang2025_Silkie_Wenchang	CRA004775	0.86956507
ENSGALG00010017824	YAE1	2	0.059650041	0.140784746	0.423696764	0.671786975	0	0.655695398	0	0.5	CRA004775;Yang2025_Silkie_Wenchang	PRJNA827465	0.914674175
ENSGALG00010017824	YAE1	2	0.00643028	0.097361602	0.066045341	0.947341727	0	0.167892282	0	0.5	CRA004775;PRJNA827465	Yang2025_Silkie_Wenchang	0.986497414
ENSGALG00010017825	PLPP5	2	0.019235794	0.092066408	0.208933908	0.834499833	0	0.008640519	0	1	PRJNA827465;Yang2025_Silkie_Wenchang	CRA004775	0.958093639
ENSGALG00010017825	PLPP5	2	-0.011736683	0.154314683	-0.07605681	0.939371901	0	0.078164882	0	0.5	CRA004775;Yang2025_Silkie_Wenchang	PRJNA827465	0.985406156
ENSGALG00010017825	PLPP5	2	0.003179282	0.089282139	0.035609389	0.971593822	0	0.079994604	0	0.5	CRA004775;PRJNA827465	Yang2025_Silkie_Wenchang	0.991955348
ENSGALG00010017826	PABPC4	2	-0.255303843	0.154777586	-1.649488464	0.099047605	0.028128267	2.421766483	58.70782724	1	PRJNA827465;Yang2025_Silkie_Wenchang	CRA004775	0.536947311
ENSGALG00010017826	PABPC4	2	-0.089708645	0.134244608	-0.668247658	0.503975519	0	0.061477175	0	0.5	CRA004775;Yang2025_Silkie_Wenchang	PRJNA827465	0.863416281
ENSGALG00010017826	PABPC4	2	-0.370983247	0.133836405	-2.771915808	0.005572745	0	0.837422118	0	0.5	CRA004775;PRJNA827465	Yang2025_Silkie_Wenchang	0.112033549
ENSGALG00010017828	ENSGALG00010017828	2	-0.005654909	0.063460385	-0.089109277	0.928995065	0	0.064748874	0	0.5	PRJNA827465;Yang2025_Silkie_Wenchang	CRA004775	0.983522601
ENSGALG00010017828	ENSGALG00010017828	2	-0.31548765	0.368818117	-0.855401715	0.392328795	0.229257625	6.270833725	84.05315714	0.5	CRA004775;Yang2025_Silkie_Wenchang	PRJNA827465	0.829462002
ENSGALG00010017828	ENSGALG00010017828	2	-0.319604497	0.34361164	-0.930394843	0.352166688	0.208053014	7.714860047	87.03800301	1	CRA004775;PRJNA827465	Yang2025_Silkie_Wenchang	0.810717746
ENSGALG00010017830	DENND4A	2	0.062889054	0.064404323	0.976472563	0.328830338	0	0.02597473	0	1	PRJNA827465;Yang2025_Silkie_Wenchang	CRA004775	0.800761292
ENSGALG00010017830	DENND4A	2	0.216999486	0.241548664	0.898366825	0.368990017	0.064396382	1.700218069	41.18401526	1	CRA004775;Yang2025_Silkie_Wenchang	PRJNA827465	0.820553374
ENSGALG00010017830	DENND4A	2	0.205078104	0.253997945	0.807400643	0.419435697	0.080732233	1.913401508	47.73705383	1	CRA004775;PRJNA827465	Yang2025_Silkie_Wenchang	0.838527387
ENSGALG00010017831	PLEK	2	-0.097679827	0.093226676	-1.047766924	0.294746005	0	8.07637E-05	0	1	PRJNA827465;Yang2025_Silkie_Wenchang	CRA004775	0.787462391
ENSGALG00010017831	PLEK	2	-0.102055222	0.122245359	-0.834839236	0.403808231	0	0.017044321	0	1	CRA004775;Yang2025_Silkie_Wenchang	PRJNA827465	0.833112102
ENSGALG00010017831	PLEK	2	-0.101280161	0.135211236	-0.749051366	0.453826246	0	0.017624936	0	1	CRA004775;PRJNA827465	Yang2025_Silkie_Wenchang	0.849044791
ENSGALG00010017833	ENSGALG00010017833	2	-0.13225871	0.13441936	-0.983926053	0.325151873	0	0.00461428	0	1	PRJNA827465;Yang2025_Silkie_Wenchang	CRA004775	0.799914637
ENSGALG00010017833	ENSGALG00010017833	2	0.181212466	0.408852324	0.443259333	0.657346751	0.237317178	3.048853765	67.20078832	0.5	CRA004775;Yang2025_Silkie_Wenchang	PRJNA827465	0.910711339
ENSGALG00010017833	ENSGALG00010017833	2	0.192201692	0.399530801	0.481068522	0.6304678	0.21877347	2.836422444	64.74432072	0.5	CRA004775;PRJNA827465	Yang2025_Silkie_Wenchang	0.902672978
ENSGALG00010017838	B3GNT2	2	0.051023327	0.076083716	0.670620857	0.502462092	0	8.02809E-05	0	1	PRJNA827465;Yang2025_Silkie_Wenchang	CRA004775	0.862935006
ENSGALG00010017838	B3GNT2	2	0.095914628	0.123236446	0.778295766	0.436393598	0	0.559085872	0	1	CRA004775;Yang2025_Silkie_Wenchang	PRJNA827465	0.843122822
ENSGALG00010017838	B3GNT2	2	0.072714827	0.087289437	0.833031228	0.404827118	0	0.630005207	0	1	CRA004775;PRJNA827465	Yang2025_Silkie_Wenchang	0.833112102
ENSGALG00010017841	GALC	2	-0.191879419	0.175704778	-1.092055785	0.274808582	0.037543304	2.514499321	60.23065142	0.5	PRJNA827465;Yang2025_Silkie_Wenchang	CRA004775	0.774719194
ENSGALG00010017841	GALC	2	0.223533777	0.645426432	0.346335022	0.729090952	0.72824007	7.269878906	86.2446127	0.5	CRA004775;Yang2025_Silkie_Wenchang	PRJNA827465	0.932323844
ENSGALG00010017841	GALC	2	0.378506354	0.463275644	0.817021916	0.413915904	0.325680108	3.665049362	72.71523788	1	CRA004775;PRJNA827465	Yang2025_Silkie_Wenchang	0.836666158
ENSGALG00010017843	NSD3	2	-0.004686067	0.034535691	-0.135687659	0.892068204	0	0.0025795	0	0.5	PRJNA827465;Yang2025_Silkie_Wenchang	CRA004775	0.974077808
ENSGALG00010017843	NSD3	2	-0.000224947	0.114229416	-0.001969255	0.998428763	0	0.001134037	0	0.5	CRA004775;Yang2025_Silkie_Wenchang	PRJNA827465	0.999547764
ENSGALG00010017843	NSD3	2	-0.005262154	0.035742059	-0.147225815	0.882953785	0	0.000226941	0	1	CRA004775;PRJNA827465	Yang2025_Silkie_Wenchang	0.972001076
ENSGALG00010017848	CYP3A4	2	0.409468415	0.128007926	3.198773907	0.001380134	0	0.414827112	0	1	PRJNA827465;Yang2025_Silkie_Wenchang	CRA004775	0.045617155
ENSGALG00010017848	CYP3A4	2	0.544842657	0.169836094	3.208049862	0.001336383	0	0.094452152	0	1	CRA004775;Yang2025_Silkie_Wenchang	PRJNA827465	0.044663156
ENSGALG00010017848	CYP3A4	2	0.404512645	0.145758483	2.775225401	0.005516346	0	0.644358657	0	1	CRA004775;PRJNA827465	Yang2025_Silkie_Wenchang	0.111469898
ENSGALG00010017849	FBXO48	2	-0.047926236	0.13292341	-0.36055527	0.718431933	0	0.023190339	0	1	PRJNA827465;Yang2025_Silkie_Wenchang	CRA004775	0.928100521
ENSGALG00010017849	FBXO48	2	-0.002193007	0.210725772	-0.010406926	0.991696624	0	0.198599277	0	0.5	CRA004775;Yang2025_Silkie_Wenchang	PRJNA827465	0.997575757
ENSGALG00010017849	FBXO48	2	-0.011223886	0.136081299	-0.082479267	0.934265604	0	0.16005793	0	0.5	CRA004775;PRJNA827465	Yang2025_Silkie_Wenchang	0.98432801
ENSGALG00010017850	TRMT5	2	0.047684831	0.098330695	0.484943501	0.627716424	0.008200799	1.657794515	39.67889319	0.5	PRJNA827465;Yang2025_Silkie_Wenchang	CRA004775	0.901856848
ENSGALG00010017850	TRMT5	2	-0.198003559	0.156620216	-1.264227339	0.206148441	0.020801403	1.645258129	39.219264	1	CRA004775;Yang2025_Silkie_Wenchang	PRJNA827465	0.718403416
ENSGALG00010017850	TRMT5	2	-0.103578753	0.261850239	-0.39556486	0.692426065	0.112930188	5.183485264	80.70796098	0.5	CRA004775;PRJNA827465	Yang2025_Silkie_Wenchang	0.920559211
ENSGALG00010017852	LETM2	2	0.129973707	0.090108512	1.442413197	0.149185843	0.007128228	1.700598462	41.19717133	1	PRJNA827465;Yang2025_Silkie_Wenchang	CRA004775	0.639246608
ENSGALG00010017852	LETM2	2	0.122973432	0.151846574	0.809853188	0.418024559	0.023740166	1.971364361	49.27371013	0.5	CRA004775;Yang2025_Silkie_Wenchang	PRJNA827465	0.838112595
ENSGALG00010017852	LETM2	2	0.041858303	0.070889314	0.590474089	0.554872852	0	0.383967502	0	0.5	CRA004775;PRJNA827465	Yang2025_Silkie_Wenchang	0.880223402
ENSGALG00010017856	FASTKD5	2	-0.061942492	0.069453114	-0.891860545	0.372467685	0	0.39339835	0	0.5	PRJNA827465;Yang2025_Silkie_Wenchang	CRA004775	0.821959428
ENSGALG00010017856	FASTKD5	2	-0.171619629	0.231009631	-0.742911146	0.457535477	0.068271104	2.429972578	58.84727223	0.5	CRA004775;Yang2025_Silkie_Wenchang	PRJNA827465	0.849573219
ENSGALG00010017856	FASTKD5	2	-0.19182368	0.169744904	-1.130070334	0.258446589	0.031642074	1.721702014	41.91793982	1	CRA004775;PRJNA827465	Yang2025_Silkie_Wenchang	0.765696237
ENSGALG00010017858	GPR65	2	-0.104003572	0.143151079	-0.726530126	0.467513846	0	0.240927418	0	1	PRJNA827465;Yang2025_Silkie_Wenchang	CRA004775	0.852885838
ENSGALG00010017858	GPR65	2	-0.108923621	0.218165124	-0.499271466	0.617588154	0	0.645979372	0	0.5	CRA004775;Yang2025_Silkie_Wenchang	PRJNA827465	0.899121942
ENSGALG00010017858	GPR65	2	-0.0198824	0.163916645	-0.121295796	0.90345675	0	0.303098301	0	0.5	CRA004775;PRJNA827465	Yang2025_Silkie_Wenchang	0.977284786
ENSGALG00010017859	EXOC6	2	0.044620259	0.178768569	0.249597897	0.802898325	0.052270827	5.37276106	81.3875959	0.5	PRJNA827465;Yang2025_Silkie_Wenchang	CRA004775	0.950806377
ENSGALG00010017859	EXOC6	2	0.223168943	0.122870977	1.816286882	0.069326377	0	0.130743193	0	1	CRA004775;Yang2025_Silkie_Wenchang	PRJNA827465	0.453517739
ENSGALG00010017859	EXOC6	2	-0.114774985	0.084967115	-1.350816552	0.176754204	0	0.191280268	0	0.5	CRA004775;PRJNA827465	Yang2025_Silkie_Wenchang	0.682413605
ENSGALG00010017860	CYSLTR1	2	-0.371844775	0.338642266	-1.098045968	0.272184417	0.167183749	3.388767925	70.49074997	1	PRJNA827465;Yang2025_Silkie_Wenchang	CRA004775	0.773286758
ENSGALG00010017860	CYSLTR1	2	-0.489535041	0.268903515	-1.82048584	0.068685051	0.0428963	1.4185519	29.50557536	1	CRA004775;Yang2025_Silkie_Wenchang	PRJNA827465	0.45150168
ENSGALG00010017860	CYSLTR1	2	-0.123865046	0.144338637	-0.858155847	0.390806413	0	0.188004999	0	1	CRA004775;PRJNA827465	Yang2025_Silkie_Wenchang	0.829251057
ENSGALG00010017861	PRSS56	2	0.173452112	0.323034882	0.536945455	0.591305292	0	0.141512797	0	1	PRJNA827465;Yang2025_Silkie_Wenchang	CRA004775	0.892776493
ENSGALG00010017861	PRSS56	2	0.397966685	0.493019459	0.807202795	0.419549656	0.144622508	1.365177255	26.74943882	1	CRA004775;Yang2025_Silkie_Wenchang	PRJNA827465	0.838527387
ENSGALG00010017861	PRSS56	2	0.490418095	0.381051772	1.287011715	0.198090205	0	0.831101263	0	1	CRA004775;PRJNA827465	Yang2025_Silkie_Wenchang	0.710234722
ENSGALG00010017865	UBOX5	2	-0.069255699	0.073148217	-0.946785882	0.343747897	0.001293332	1.100178477	9.105656891	1	PRJNA827465;Yang2025_Silkie_Wenchang	CRA004775	0.806388726
ENSGALG00010017865	UBOX5	2	-0.226157419	0.121558805	-1.860477477	0.062818002	0	0.18269384	0	1	CRA004775;Yang2025_Silkie_Wenchang	PRJNA827465	0.43178313
ENSGALG00010017865	UBOX5	2	-0.081471744	0.112684473	-0.723007723	0.46967514	0.009826237	1.31328289	23.85494337	1	CRA004775;PRJNA827465	Yang2025_Silkie_Wenchang	0.853485658
ENSGALG00010017866	RALA	2	-0.264645754	0.237032746	-1.116494485	0.26421053	0.106422132	18.75630421	94.66845926	1	PRJNA827465;Yang2025_Silkie_Wenchang	CRA004775	0.768790489
ENSGALG00010017866	RALA	2	-0.095966757	0.425583701	-0.225494437	0.821594653	0.341421073	17.03795044	94.13074945	0.5	CRA004775;Yang2025_Silkie_Wenchang	PRJNA827465	0.955598716
ENSGALG00010017866	RALA	2	0.115875768	0.184377798	0.628469205	0.529696614	0.052272306	3.751136739	73.34141436	0.5	CRA004775;PRJNA827465	Yang2025_Silkie_Wenchang	0.871944741
ENSGALG00010017867	APLF	2	-0.027503837	0.06740689	-0.408027089	0.683253787	0.000573599	1.064868918	6.091727993	0.5	PRJNA827465;Yang2025_Silkie_Wenchang	CRA004775	0.918800744
ENSGALG00010017867	APLF	2	-0.020687759	0.130189506	-0.158904964	0.87374375	0.0141358	1.562036799	35.98102166	0.5	CRA004775;Yang2025_Silkie_Wenchang	PRJNA827465	0.9699681
ENSGALG00010017867	APLF	2	0.049955005	0.077529543	0.644335085	0.519358169	0	0.437585193	0	1	CRA004775;PRJNA827465	Yang2025_Silkie_Wenchang	0.869086345
ENSGALG00010017868	CHRND	2	0.091235412	0.423272916	0.215547483	0.829340496	0.329246868	12.14005509	91.76280509	0.5	PRJNA827465;Yang2025_Silkie_Wenchang	CRA004775	0.95688454
ENSGALG00010017868	CHRND	2	0.253414251	0.435021339	0.58253292	0.560207774	0.223211998	1.938247847	48.40701091	0.5	CRA004775;Yang2025_Silkie_Wenchang	PRJNA827465	0.881203589
ENSGALG00010017868	CHRND	2	-0.320926344	0.125712369	-2.55286211	0.010684179	0	0.02851426	0	1	CRA004775;PRJNA827465	Yang2025_Silkie_Wenchang	0.164803873
ENSGALG00010017870	RAB11A	2	-0.105893317	0.136773291	-0.774225117	0.438797677	0.033305506	8.665889459	88.46050362	0.5	PRJNA827465;Yang2025_Silkie_Wenchang	CRA004775	0.844002503
ENSGALG00010017870	RAB11A	2	-0.177388207	0.111828935	-1.586246061	0.112683503	0.011042786	1.622852439	38.38010309	1	CRA004775;Yang2025_Silkie_Wenchang	PRJNA827465	0.566553488
ENSGALG00010017870	RAB11A	2	0.01884762	0.03604364	0.522911124	0.601036103	0	0.040414734	0	0.5	CRA004775;PRJNA827465	Yang2025_Silkie_Wenchang	0.895230519
ENSGALG00010017873	HSPG2	2	-0.151532869	0.100407497	-1.509178834	0.131253088	0	0.75422732	0	1	PRJNA827465;Yang2025_Silkie_Wenchang	CRA004775	0.606878893
ENSGALG00010017873	HSPG2	2	-0.029719424	0.138255118	-0.214960754	0.829797917	0	0.19458974	0	0.5	CRA004775;Yang2025_Silkie_Wenchang	PRJNA827465	0.957079054
ENSGALG00010017873	HSPG2	2	-0.187374978	0.126701807	-1.47886587	0.139176167	0	0.815007006	0	0.5	CRA004775;PRJNA827465	Yang2025_Silkie_Wenchang	0.622913323
ENSGALG00010017876	COMMD1	2	-0.035636863	0.105609184	-0.337440949	0.73578452	0.006513051	1.149103246	12.97561788	0.5	PRJNA827465;Yang2025_Silkie_Wenchang	CRA004775	0.933592889
ENSGALG00010017876	COMMD1	2	-0.234729731	0.197221923	-1.190180719	0.23397537	0	0.142011099	0	1	CRA004775;Yang2025_Silkie_Wenchang	PRJNA827465	0.747279664
ENSGALG00010017876	COMMD1	2	-0.00830234	0.076042494	-0.109180276	0.913059504	0	0.348687658	0	0.5	CRA004775;PRJNA827465	Yang2025_Silkie_Wenchang	0.979129568
ENSGALG00010017878	CHRNG	2	0.074382925	0.129325341	0.575161249	0.565182247	0	0.341465162	0	0.5	PRJNA827465;Yang2025_Silkie_Wenchang	CRA004775	0.883427937
ENSGALG00010017878	CHRNG	2	-0.406751761	0.679498634	-0.598605708	0.549435849	0.731696971	4.104895645	75.63884477	0.5	CRA004775;Yang2025_Silkie_Wenchang	PRJNA827465	0.878554604
ENSGALG00010017878	CHRNG	2	-0.468388948	0.597444916	-0.783986833	0.433047836	0.526716792	3.19312352	68.68270226	1	CRA004775;PRJNA827465	Yang2025_Silkie_Wenchang	0.842762754
ENSGALG00010017880	CDK13	2	0.124882416	0.185771857	0.672235383	0.501433858	0.054593946	4.072903349	75.44749005	0.5	PRJNA827465;Yang2025_Silkie_Wenchang	CRA004775	0.862658912
ENSGALG00010017880	CDK13	2	0.344357643	0.158458066	2.173178388	0.029766894	0	0.016378334	0	1	CRA004775;Yang2025_Silkie_Wenchang	PRJNA827465	0.294469727
ENSGALG00010017880	CDK13	2	-0.007475663	0.073334371	-0.101939421	0.918804759	0.003441786	1.066039153	6.194814956	0.5	CRA004775;PRJNA827465	Yang2025_Silkie_Wenchang	0.981033557
ENSGALG00010017882	LZTS3	2	0.155092065	0.112773076	1.375257917	0.169051498	0	0.371461335	0	1	PRJNA827465;Yang2025_Silkie_Wenchang	CRA004775	0.67030648
ENSGALG00010017882	LZTS3	2	0.261546245	0.189338277	1.381370158	0.167165177	0	0.004808509	0	1	CRA004775;Yang2025_Silkie_Wenchang	PRJNA827465	0.666473213
ENSGALG00010017882	LZTS3	2	0.119346057	0.132205311	0.902732696	0.366667779	0	0.109310822	0	1	CRA004775;PRJNA827465	Yang2025_Silkie_Wenchang	0.819420238
ENSGALG00010017886	DDRGK1	2	0.212562288	0.274748527	0.773661247	0.439131143	0.137203953	10.30644974	90.29733783	0.5	PRJNA827465;Yang2025_Silkie_Wenchang	CRA004775	0.844022831
ENSGALG00010017886	DDRGK1	2	0.108331298	0.401835802	0.269590956	0.787474959	0.296111048	12.0353351	91.69113289	0.5	CRA004775;Yang2025_Silkie_Wenchang	PRJNA827465	0.946919116
ENSGALG00010017886	DDRGK1	2	-0.119313571	0.116759821	-1.021871819	0.306841572	0.017182645	2.171136243	53.94116775	1	CRA004775;PRJNA827465	Yang2025_Silkie_Wenchang	0.79266003
ENSGALG00010017887	PROKR1	2	-0.470864862	0.211796911	-2.223190412	0.026202963	0	0.674957296	0	1	PRJNA827465;Yang2025_Silkie_Wenchang	CRA004775	0.27602655
ENSGALG00010017887	PROKR1	2	-0.549483395	0.278507298	-1.972958692	0.048500269	0	0.706407957	0	0.5	CRA004775;Yang2025_Silkie_Wenchang	PRJNA827465	0.380764551
ENSGALG00010017887	PROKR1	2	-0.254967419	0.27451841	-0.928780767	0.353002714	0	0.156149336	0	0.5	CRA004775;PRJNA827465	Yang2025_Silkie_Wenchang	0.811035834
ENSGALG00010017888	MPLKIP	2	0.030277141	0.065276428	0.463829623	0.642769818	0.002219769	1.33531386	25.11123938	0.5	PRJNA827465;Yang2025_Silkie_Wenchang	CRA004775	0.906634523
ENSGALG00010017888	MPLKIP	2	-0.058262706	0.085951922	-0.677852273	0.497865364	0	0.098417135	0	1	CRA004775;Yang2025_Silkie_Wenchang	PRJNA827465	0.861098503
ENSGALG00010017888	MPLKIP	2	0.069184555	0.067872137	1.019336623	0.308043183	0	0.66412252	0	0.5	CRA004775;PRJNA827465	Yang2025_Silkie_Wenchang	0.793278812
ENSGALG00010017890	ANAPC1	2	-0.03247818	0.086808393	-0.374136402	0.708302837	0.011463291	4.121555709	75.73731691	0.5	PRJNA827465;Yang2025_Silkie_Wenchang	CRA004775	0.925061251
ENSGALG00010017890	ANAPC1	2	-0.133173314	0.065307862	-2.03916205	0.041433857	0	0.164372055	0	1	CRA004775;Yang2025_Silkie_Wenchang	PRJNA827465	0.352975427
ENSGALG00010017890	ANAPC1	2	0.003124224	0.101099325	0.030902519	0.975347281	0.009625291	1.361517151	26.55252271	0.5	CRA004775;PRJNA827465	Yang2025_Silkie_Wenchang	0.992750665
ENSGALG00010017891	GOLGA7B	2	0.078019283	0.414923365	0.188032995	0.850850786	0.292959629	6.696733098	85.06734574	0.5	PRJNA827465;Yang2025_Silkie_Wenchang	CRA004775	0.962056252
ENSGALG00010017891	GOLGA7B	2	-0.015824854	0.689831669	-0.022940167	0.981698	0.682304877	2.857521022	65.00463191	0.5	CRA004775;Yang2025_Silkie_Wenchang	PRJNA827465	0.99503081
ENSGALG00010017891	GOLGA7B	2	-0.389195993	0.227700124	-1.709248049	0.087405012	0	0.514973191	0	1	CRA004775;PRJNA827465	Yang2025_Silkie_Wenchang	0.508252579
ENSGALG00010017894	KAT6A	2	0.04233599	0.105619141	0.400836345	0.688540618	0.013028674	1.81913349	45.028773	0.5	PRJNA827465;Yang2025_Silkie_Wenchang	CRA004775	0.919798626
ENSGALG00010017894	KAT6A	2	0.151789073	0.143897903	1.054838674	0.291499114	0	0.517003662	0	0.5	CRA004775;Yang2025_Silkie_Wenchang	PRJNA827465	0.784926669
ENSGALG00010017894	KAT6A	2	-0.019937466	0.044737745	-0.445652012	0.655848634	0	0.00347252	0	1	CRA004775;PRJNA827465	Yang2025_Silkie_Wenchang	0.910411938
ENSGALG00010017895	MNAT1	2	0.156932263	0.161954621	0.968989102	0.332550627	0.045916876	7.74493152	87.08833	1	PRJNA827465;Yang2025_Silkie_Wenchang	CRA004775	0.802178578
ENSGALG00010017895	MNAT1	2	0.25660812	0.132203688	1.941005758	0.052257579	0.012434461	1.35001605	25.92680656	1	CRA004775;Yang2025_Silkie_Wenchang	PRJNA827465	0.396884952
ENSGALG00010017895	MNAT1	2	0.006821784	0.053998038	0.126333932	0.899467597	0	0.003549263	0	1	CRA004775;PRJNA827465	Yang2025_Silkie_Wenchang	0.976081354
ENSGALG00010017898	NHERF2	2	-0.115783548	0.071033342	-1.629988733	0.103103878	0.005924312	2.082959928	51.99139519	1	PRJNA827465;Yang2025_Silkie_Wenchang	CRA004775	0.5473214
ENSGALG00010017898	NHERF2	2	-0.040385499	0.225205203	-0.179327559	0.857680509	0.07251255	3.066276862	67.38715892	0.5	CRA004775;Yang2025_Silkie_Wenchang	PRJNA827465	0.964067753
ENSGALG00010017898	NHERF2	2	-0.004014311	0.124594843	-0.032218918	0.974297469	0.017728295	1.5674938	36.20389439	0.5	CRA004775;PRJNA827465	Yang2025_Silkie_Wenchang	0.992492038
ENSGALG00010017905	EIF4E2	2	0.316077424	0.274720589	1.150541445	0.249920934	0.141931463	16.56885194	93.96457881	1	PRJNA827465;Yang2025_Silkie_Wenchang	CRA004775	0.75993411
ENSGALG00010017905	EIF4E2	2	0.184572638	0.435633503	0.423687887	0.67179345	0.347715366	11.58037567	91.36470155	0.5	CRA004775;Yang2025_Silkie_Wenchang	PRJNA827465	0.914674175
ENSGALG00010017905	EIF4E2	2	-0.03933253	0.14366637	-0.273776879	0.784256093	0.023222677	1.804078128	44.57002806	0.5	CRA004775;PRJNA827465	Yang2025_Silkie_Wenchang	0.946083234
ENSGALG00010017909	NTHL1	2	0.011315061	0.118989641	0.09509282	0.924241101	0	0.003418034	0	1	PRJNA827465;Yang2025_Silkie_Wenchang	CRA004775	0.98232425
ENSGALG00010017909	NTHL1	2	-0.09366343	0.184337373	-0.508108739	0.611377087	0	0.737750333	0	0.5	CRA004775;Yang2025_Silkie_Wenchang	PRJNA827465	0.897609796
ENSGALG00010017909	NTHL1	2	-0.04569163	0.12715329	-0.35934288	0.7193386	0	0.859501215	0	0.5	CRA004775;PRJNA827465	Yang2025_Silkie_Wenchang	0.928449671
ENSGALG00010017915	BFSP1	2	-0.697740116	0.503115074	-1.386840012	0.165490553	0.237694642	1.8404369	45.66507551	1	PRJNA827465;Yang2025_Silkie_Wenchang	CRA004775	0.664874006
ENSGALG00010017915	BFSP1	2	0.194889509	1.477793972	0.131878674	0.895080257	4.026779721	12.80361754	92.18970735	0.5	CRA004775;Yang2025_Silkie_Wenchang	PRJNA827465	0.974641371
ENSGALG00010017915	BFSP1	2	0.665484511	0.967199071	0.688053299	0.491419218	1.617794557	7.345038728	86.38536791	0.5	CRA004775;PRJNA827465	Yang2025_Silkie_Wenchang	0.85895707
ENSGALG00010017918	SIX4	2	-0.126991385	0.066458011	-1.910851407	0.056023679	0	0.055975296	0	1	PRJNA827465;Yang2025_Silkie_Wenchang	CRA004775	0.409427048
ENSGALG00010017918	SIX4	2	0.014785064	0.205837725	0.071828739	0.942738202	0.022494474	1.206339432	17.10459153	0.5	CRA004775;Yang2025_Silkie_Wenchang	PRJNA827465	0.985827325
ENSGALG00010017918	SIX4	2	-0.018112257	0.225851043	-0.080195589	0.936081698	0.059149605	1.614195725	38.0496439	0.5	CRA004775;PRJNA827465	Yang2025_Silkie_Wenchang	0.984822128
ENSGALG00010017920	AP3M2	2	0.111068914	0.083600231	1.328571845	0.183989266	0.006466567	1.640104181	39.02826348	0.5	PRJNA827465;Yang2025_Silkie_Wenchang	CRA004775	0.692470323
ENSGALG00010017920	AP3M2	2	-0.209497206	0.232487508	-0.901111668	0.36752895	0.074859377	3.027965942	66.97452946	1	CRA004775;Yang2025_Silkie_Wenchang	PRJNA827465	0.819975483
ENSGALG00010017920	AP3M2	2	-0.12038406	0.324852253	-0.370580959	0.710949666	0.184049097	7.061923555	85.83955218	0.5	CRA004775;PRJNA827465	Yang2025_Silkie_Wenchang	0.925563881
ENSGALG00010017921	SPATA7	2	0.038228805	0.204524033	0.186915955	0.851726529	0.066635401	4.908423269	79.62685887	0.5	PRJNA827465;Yang2025_Silkie_Wenchang	CRA004775	0.962215236

ENSGALG00010017921	SPATA7	2	0.295304309	0.134421692	2.196850116	0.028031152	0.005587256	1.084555974	7.796367916	1	CRA004775;Yang2025_Silkie_Wenchang	PRJNA827465	0.285342757
ENSGALG00010017921	SPATA7	2	0.161786471	0.388898996	0.416011543	0.677401556	0.2427261	4.595565125	78.23989057	0.5	CRA004775;PRJNA827465	Yang2025_Silkie_Wenchang	0.917144807
ENSGALG00010017922	MERTK	2	0.033812633	0.055785565	0.606117967	0.544436421	0	0.442819134	0	0.5	PRJNA827465;Yang2025_Silkie_Wenchang	CRA004775	0.877218245
ENSGALG00010017922	MERTK	2	-0.054887382	0.118432003	-0.463450598	0.643041418	0	0.053301725	0	1	CRA004775;Yang2025_Silkie_Wenchang	PRJNA827465	0.906634523
ENSGALG00010017922	MERTK	2	0.044852212	0.060664163	0.739352681	0.459692868	0	0.304905953	0	0.5	CRA004775;PRJNA827465	Yang2025_Silkie_Wenchang	0.850098539
ENSGALG00010017927	PCSK2	2	-0.078727788	0.211228411	-0.372714009	0.709361309	0.068543512	4.310179731	76.79911135	0.5	PRJNA827465;Yang2025_Silkie_Wenchang	CRA004775	0.925298042
ENSGALG00010017927	PCSK2	2	0.168559316	0.119985032	1.40466951	0.160119576	0	0.293526922	0	1	CRA004775;Yang2025_Silkie_Wenchang	PRJNA827465	0.655509678
ENSGALG00010017927	PCSK2	2	-0.036539401	0.2835974	-0.128842509	0.897482269	0.124192743	4.240457518	76.417639	0.5	CRA004775;PRJNA827465	Yang2025_Silkie_Wenchang	0.97554471
ENSGALG00010017930	TSC2	2	0.035168302	0.106716359	0.329549307	0.741740531	0.017534144	4.089290275	75.5458788	0.5	PRJNA827465;Yang2025_Silkie_Wenchang	CRA004775	0.93543545
ENSGALG00010017930	TSC2	2	0.091902116	0.114282374	0.804167017	0.421300525	0.008531623	1.322669803	24.39534059	0.5	CRA004775;Yang2025_Silkie_Wenchang	PRJNA827465	0.839352211
ENSGALG00010017930	TSC2	2	-0.060479693	0.048684585	-1.242276038	0.214134733	0	0.051627854	0	1	CRA004775;PRJNA827465	Yang2025_Silkie_Wenchang	0.726967219
ENSGALG00010017933	SIX1	2	-0.051655378	0.073206174	-0.705615053	0.48042756	0	0.202574595	0	1	PRJNA827465;Yang2025_Silkie_Wenchang	CRA004775	0.856586256
ENSGALG00010017933	SIX1	2	-0.000752161	0.125265691	-0.006004524	0.995209112	0	0.003898111	0	0.5	CRA004775;Yang2025_Silkie_Wenchang	PRJNA827465	0.998242335
ENSGALG00010017933	SIX1	2	-0.070217295	0.086751444	-0.809407797	0.418280619	0	0.048178421	0	0.5	CRA004775;PRJNA827465	Yang2025_Silkie_Wenchang	0.838112595
ENSGALG00010017937	PTPN21	2	0.258256534	0.488710708	0.528444599	0.597190789	0.4413237	12.54826481	92.03077067	0.5	PRJNA827465;Yang2025_Silkie_Wenchang	CRA004775	0.894350498
ENSGALG00010017937	PTPN21	2	0.680064317	0.226237829	3.005970841	0.002647344	0	0.571923661	0	1	CRA004775;Yang2025_Silkie_Wenchang	PRJNA827465	0.069551173
ENSGALG00010017937	PTPN21	2	-0.078616324	0.237445555	-0.331092002	0.740574992	0.063003948	1.587915594	37.02436052	0.5	CRA004775;PRJNA827465	Yang2025_Silkie_Wenchang	0.935128797
ENSGALG00010017941	DIS3L	2	0.177212406	0.083316476	2.126979118	0.033421818	0.007934026	2.249832773	55.55225207	1	PRJNA827465;Yang2025_Silkie_Wenchang	CRA004775	0.314027665
ENSGALG00010017941	DIS3L	2	0.217339851	0.110381386	1.96899006	0.048954233	0.006871427	1.239307666	19.30978665	1	CRA004775;Yang2025_Silkie_Wenchang	PRJNA827465	0.382438521
ENSGALG00010017941	DIS3L	2	0.099473483	0.060525935	1.643485285	0.100282567	0	0.181536204	0	1	CRA004775;PRJNA827465	Yang2025_Silkie_Wenchang	0.540998201
ENSGALG00010017946	PTGDR	2	0.157420117	0.141741506	1.110614112	0.266734486	0	0.352331568	0	1	PRJNA827465;Yang2025_Silkie_Wenchang	CRA004775	0.770949983
ENSGALG00010017946	PTGDR	2	0.288849484	0.179857228	1.60599319	0.108275406	0	0.02243151	0	1	CRA004775;Yang2025_Silkie_Wenchang	PRJNA827465	0.556717702
ENSGALG00010017946	PTGDR	2	0.157769131	0.151308856	1.042695947	0.297089136	0	0.466048643	0	1	CRA004775;PRJNA827465	Yang2025_Silkie_Wenchang	0.788466926
ENSGALG00010017948	PLAT	2	0.007218646	0.086471054	0.083480488	0.933469491	0	0.482878711	0	0.5	PRJNA827465;Yang2025_Silkie_Wenchang	CRA004775	0.984260547
ENSGALG00010017948	PLAT	2	0.260731757	0.214799407	1.213838349	0.224809459	0.059302156	2.645053798	62.1935856	1	CRA004775;Yang2025_Silkie_Wenchang	PRJNA827465	0.737885283
ENSGALG00010017948	PLAT	2	0.202011844	0.277420271	0.728179824	0.466503515	0.123819745	4.779239958	79.07617092	0.5	CRA004775;PRJNA827465	Yang2025_Silkie_Wenchang	0.852792382
ENSGALG00010017949	SFXN3	2	-0.058983525	0.058993784	-0.999826109	0.317394668	0	0.266730867	0	1	PRJNA827465;Yang2025_Silkie_Wenchang	CRA004775	0.797125693
ENSGALG00010017949	SFXN3	2	-0.028347948	0.095844403	-0.29577051	0.767405346	0	0.14548053	0	1	CRA004775;Yang2025_Silkie_Wenchang	PRJNA827465	0.941444269
ENSGALG00010017949	SFXN3	2	-0.084301388	0.07119655	-1.184065631	0.236387084	0	0.03911453	0	1	CRA004775;PRJNA827465	Yang2025_Silkie_Wenchang	0.748669015
ENSGALG00010017950	FBLN7	2	0.480101977	0.237705806	2.019731807	0.043411214	0	0.66289342	0	1	PRJNA827465;Yang2025_Silkie_Wenchang	CRA004775	0.360031851
ENSGALG00010017950	FBLN7	2	-0.330905102	1.135263419	-0.291478697	0.770685234	2.269516918	8.123431246	87.6899309	0.5	CRA004775;Yang2025_Silkie_Wenchang	PRJNA827465	0.942491331
ENSGALG00010017950	FBLN7	2	-0.498899348	0.930872324	-0.535948202	0.59199435	1.468920414	6.131913016	83.69187565	0.5	CRA004775;PRJNA827465	Yang2025_Silkie_Wenchang	0.892776493
ENSGALG00010017954	PTGER2	2	0.058079567	0.138075549	0.420636145	0.674020796	0	0.47678406	0	0.5	PRJNA827465;Yang2025_Silkie_Wenchang	CRA004775	0.915694412
ENSGALG00010017954	PTGER2	2	0.032718206	0.215372943	0.151914191	0.879254615	0	0.58511903	0	0.5	CRA004775;Yang2025_Silkie_Wenchang	PRJNA827465	0.971257564
ENSGALG00010017954	PTGER2	2	0.127896933	0.139710102	0.91544513	0.359957995	0	0.079524626	0	1	CRA004775;PRJNA827465	Yang2025_Silkie_Wenchang	0.814880744
ENSGALG00010017955	IKBKB	2	0.132336462	0.157304494	0.84127579	0.40019345	0.044599077	10.08625612	90.08551847	0.5	PRJNA827465;Yang2025_Silkie_Wenchang	CRA004775	0.832246851
ENSGALG00010017955	IKBKB	2	0.155317304	0.155197311	1.000773163	0.316936487	0.037099207	4.145348442	75.87657554	0.5	CRA004775;Yang2025_Silkie_Wenchang	PRJNA827465	0.796964476
ENSGALG00010017955	IKBKB	2	-0.021633066	0.056909958	-0.380127963	0.703850429	0	0.000176192	0	1	CRA004775;PRJNA827465	Yang2025_Silkie_Wenchang	0.923724717
ENSGALG00010017956	TIPIN	2	0.020829855	0.073432339	0.283660518	0.776670554	0	0.089533471	0	1	PRJNA827465;Yang2025_Silkie_Wenchang	CRA004775	0.944156005
ENSGALG00010017956	TIPIN	2	0.042870549	0.114621069	0.3740198	0.708389585	0	0.028644518	0	1	CRA004775;Yang2025_Silkie_Wenchang	PRJNA827465	0.9250668
ENSGALG00010017956	TIPIN	2	0.008455262	0.079305493	0.106616343	0.915093353	0	0.001257603	0	1	CRA004775;PRJNA827465	Yang2025_Silkie_Wenchang	0.980051066
ENSGALG00010017957	THUMPD1	2	-0.116888151	0.04895245	-2.387789598	0.016950043	0	0.34080558	0	1	PRJNA827465;Yang2025_Silkie_Wenchang	CRA004775	0.216224448
ENSGALG00010017957	THUMPD1	2	-0.141344162	0.075897646	-1.862299694	0.062560859	0	0.242545828	0	1	CRA004775;Yang2025_Silkie_Wenchang	PRJNA827465	0.431065134
ENSGALG00010017957	THUMPD1	2	-0.090706938	0.059904111	-1.514202206	0.129974562	0	0.069549865	0	1	CRA004775;PRJNA827465	Yang2025_Silkie_Wenchang	0.604798801
ENSGALG00010017958	CAPN10	2	-0.126682196	0.183069932	-0.691988003	0.488944863	0.049298661	3.627641907	72.43388334	0.5	PRJNA827465;Yang2025_Silkie_Wenchang	CRA004775	0.858232954
ENSGALG00010017958	CAPN10	2	-0.358521956	0.139970484	-2.561411133	0.010424791	0	0.080596248	0	1	CRA004775;Yang2025_Silkie_Wenchang	PRJNA827465	0.162472542
ENSGALG00010017958	CAPN10	2	-0.128101196	0.219202409	-0.584396845	0.558953349	0.064294867	2.590706605	61.40049213	0.5	CRA004775;PRJNA827465	Yang2025_Silkie_Wenchang	0.880926437
ENSGALG00010017959	PDZD7	2	-0.170509193	0.220981891	-0.771598034	0.440352539	0.063991143	2.898838557	65.50342559	0.5	PRJNA827465;Yang2025_Silkie_Wenchang	CRA004775	0.84462329
ENSGALG00010017959	PDZD7	2	-0.125472482	0.33437613	-0.375243538	0.707479353	0.108766309	1.510443535	33.79428117	0.5	CRA004775;Yang2025_Silkie_Wenchang	PRJNA827465	0.92489559
ENSGALG00010017959	PDZD7	2	-0.415326392	0.172301771	-2.410459212	0.015932453	0	0.306855728	0	1	CRA004775;PRJNA827465	Yang2025_Silkie_Wenchang	0.208471156
ENSGALG00010017962	TXNDC16	2	-0.051647856	0.128085695	-0.403228919	0.686779827	0.025813397	4.40175342	77.28178059	0.5	PRJNA827465;Yang2025_Silkie_Wenchang	CRA004775	0.919235539
ENSGALG00010017962	TXNDC16	2	-0.193103652	0.101733773	-1.89812731	0.057679313	0	0.014600667	0	1	CRA004775;Yang2025_Silkie_Wenchang	PRJNA827465	0.416173603
ENSGALG00010017962	TXNDC16	2	0.051334867	0.054969565	0.933877997	0.35036683	0	0.642532063	0	0.5	CRA004775;PRJNA827465	Yang2025_Silkie_Wenchang	0.810111037
ENSGALG00010017963	LPAR4	2	-0.152112986	0.165907168	-0.916856018	0.359218094	0.04737467	6.985709147	85.68506104	0.5	PRJNA827465;Yang2025_Silkie_Wenchang	CRA004775	0.814335519
ENSGALG00010017963	LPAR4	2	-0.330861185	0.103868386	-3.185388717	0.001445597	0	0.003165458	0	1	CRA004775;Yang2025_Silkie_Wenchang	PRJNA827465	0.047151552
ENSGALG00010017963	LPAR4	2	-0.005841959	0.064584278	-0.090454821	0.927925794	0	0.754269763	0	0.5	CRA004775;PRJNA827465	Yang2025_Silkie_Wenchang	0.982993195
ENSGALG00010017972	RNPEPL1	2	-0.19590773	0.103109821	-1.89999099	0.057434302	0.016617084	4.435805711	77.45618124	1	PRJNA827465;Yang2025_Silkie_Wenchang	CRA004775	0.415104887
ENSGALG00010017972	RNPEPL1	2	-0.283214571	0.076968096	-3.679635949	0.000233567	0	0.725721383	0	1	CRA004775;Yang2025_Silkie_Wenchang	PRJNA827465	0.014132351
ENSGALG00010017972	RNPEPL1	2	-0.10305516	0.051430392	-2.003779379	0.045093699	0	0.005434564	0	1	CRA004775;PRJNA827465	Yang2025_Silkie_Wenchang	0.3659545
ENSGALG00010017973	PKD1	2	-0.105176074	0.086898634	-1.210330583	0.226152067	0	8.13986E-06	0	1	PRJNA827465;Yang2025_Silkie_Wenchang	CRA004775	0.739595164
ENSGALG00010017973	PKD1	2	-0.101344851	0.159720469	-0.634513859	0.525745525	0	0.002007561	0	1	CRA004775;Yang2025_Silkie_Wenchang	PRJNA827465	0.87039964
ENSGALG00010017973	PKD1	2	-0.103621556	0.0936445	-1.106541835	0.268492067	0	0.002302773	0	1	CRA004775;PRJNA827465	Yang2025_Silkie_Wenchang	0.771398294
ENSGALG00010017974	MXRA8	2	0.008953866	0.101364349	0.088333483	0.929611628	0.010446923	1.94416342	48.5639947	0.5	PRJNA827465;Yang2025_Silkie_Wenchang	CRA004775	0.983767889
ENSGALG00010017974	MXRA8	2	-0.111245662	0.11962742	-0.929934474	0.352405012	0	0.022741708	0	1	CRA004775;Yang2025_Silkie_Wenchang	PRJNA827465	0.810828603
ENSGALG00010017974	MXRA8	2	0.085523185	0.079225075	1.079496411	0.280366493	0	0.113650514	0	0.5	CRA004775;PRJNA827465	Yang2025_Silkie_Wenchang	0.7785251
ENSGALG00010017976	GPR137C	2	-0.117891662	0.162041184	-0.727541347	0.466894396	0.0389719	3.591223182	72.15433435	0.5	PRJNA827465;Yang2025_Silkie_Wenchang	CRA004775	0.852862288
ENSGALG00010017976	GPR137C	2	-0.232445718	0.132986895	-1.747884388	0.080484049	0	0.937575368	0	1	CRA004775;Yang2025_Silkie_Wenchang	PRJNA827465	0.488261768
ENSGALG00010017976	GPR137C	2	0.01605318	0.074475278	0.21555046	0.829338175	0	0.018325003	0	0.5	CRA004775;PRJNA827465	Yang2025_Silkie_Wenchang	0.95688454
ENSGALG00010017979	ENSGALG00010017979	2	0.187140089	0.098290456	1.903949754	0.056916728	0	0.199933843	0	1	PRJNA827465;Yang2025_Silkie_Wenchang	CRA004775	0.412958865
ENSGALG00010017979	ENSGALG00010017979	2	0.007668238	0.305263424	0.02512007	0.979959192	0.121407689	2.665314274	62.48097233	0.5	CRA004775;Yang2025_Silkie_Wenchang	PRJNA827465	0.994637196
ENSGALG00010017979	ENSGALG00010017979	2	-0.010198296	0.245886953	-0.041475548	0.966916786	0.075612148	2.202690164	54.60096857	0.5	CRA004775;PRJNA827465	Yang2025_Silkie_Wenchang	0.990893423
ENSGALG00010017983	SLC4A11	2	-0.161961793	0.225397594	-0.718560436	0.472411798	0.08237623	5.261828339	80.99519909	0.5	PRJNA827465;Yang2025_Silkie_Wenchang	CRA004775	0.853998373
ENSGALG00010017983	SLC4A11	2	-0.240294582	0.267362316	-0.898760101	0.368780456	0.072771268	1.616021122	38.1196207	0.5	CRA004775;Yang2025_Silkie_Wenchang	PRJNA827465	0.820456146
ENSGALG00010017983	SLC4A11	2	0.068329796	0.122676469	0.556991872	0.577532983	0	0.121110826	0	1	CRA004775;PRJNA827465	Yang2025_Silkie_Wenchang	0.88731056
ENSGALG00010017985	ABCC5	2	0.733690046	0.444042828	1.65229568	0.09847429	0.367835862	14.84486989	93.26366612	1	PRJNA827465;Yang2025_Silkie_Wenchang	CRA004775	0.535930498
ENSGALG00010017985	ABCC5	2	0.663502808	0.598818052	1.108020719	0.267852868	0.59725186	5.441628345	81.62314777	0.5	CRA004775;Yang2025_Silkie_Wenchang	PRJNA827465	0.771398294
ENSGALG00010017985	ABCC5	2	0.26782706	0.141490527	1.892897462	0.058371507	0	0.398475238	0	0.5	CRA004775;PRJNA827465	Yang2025_Silkie_Wenchang	0.418650753
ENSGALG00010017987	ERI2	2	0.359809877	0.244371392	1.472389518	0.140915722	0.084213892	3.303875924	69.73251953	1	PRJNA827465;Yang2025_Silkie_Wenchang	CRA004775	0.625271605
ENSGALG00010017987	ERI2	2	0.318441921	0.425185545	0.748948134	0.453888467	0.229456696	2.372517334	57.85067677	0.5	CRA004775;Yang2025_Silkie_Wenchang	PRJNA827465	0.849063008
ENSGALG00010017987	ERI2	2	0.109051947	0.148407917	0.734812191	0.462453882	0	0.515739618	0	0.5	CRA004775;PRJNA827465	Yang2025_Silkie_Wenchang	0.850823289
ENSGALG00010017991	RAB26	2	-0.21841543	0.15001953	-1.455913314	0.145416585	0.030537852	3.06372523	67.35999723	1	PRJNA827465;Yang2025_Silkie_Wenchang	CRA004775	0.633907279
ENSGALG00010017991	RAB26	2	-0.39841028	0.13023232	-3.059227372	0.002219087	0	0.167646911	0	1	CRA004775;Yang2025_Silkie_Wenchang	PRJNA827465	0.062298448
ENSGALG00010017991	RAB26	2	-0.141565245	0.161361469	-0.877317527	0.380314195	0.018789683	1.187444063	15.78550677	1	CRA004775;PRJNA827465	Yang2025_Silkie_Wenchang	0.824392515
ENSGALG00010017993	REXO5	2	0.158562594	0.106748246	1.485388291	0.137440996	0.014116295	2.55968775	60.93273486	1	PRJNA827465;Yang2025_Silkie_Wenchang	CRA004775	0.620259898
ENSGALG00010017993	REXO5	2	0.057732142	0.277972989	0.207689754	0.835471218	0.114628346	3.398241545	70.57301588	0.5	CRA004775;Yang2025_Silkie_Wenchang	PRJNA827465	0.958339923
ENSGALG00010017993	REXO5	2	-0.003457346	0.139718291	-0.024745124	0.980258262	0.018064203	1.402832848	28.71566979	0.5	CRA004775;PRJNA827465	Yang2025_Silkie_Wenchang	0.994676009
ENSGALG00010017996	ZC3H14	2	0.170451771	0.198699541	0.857836761	0.390982608	0.068925204	7.127870948	85.9705653	0.5	PRJNA827465;Yang2025_Silkie_Wenchang	CRA004775	0.829251057
ENSGALG00010017996	ZC3H14	2	0.26939297	0.157842498	1.706720138	0.087874082	0.01966077	1.600068996	37.50269504	1	CRA004775;Yang2025_Silkie_Wenchang	PRJNA827465	0.509491082
ENSGALG00010017996	ZC3H14	2	-0.002829338	0.036820779	-0.076840802	0.938750191	0	0.128120142	0	0.5	CRA004775;PRJNA827465	Yang2025_Silkie_Wenchang	0.985349045
ENSGALG00010017997	ENSGALG00010017997	2	2.780553778	0.874588376	3.179271361	0.001476458	1.237059663	5.225623387	80.86352716	1	PRJNA827465;Yang2025_Silkie_Wenchang	CRA004775	0.047564941
ENSGALG00010017997	ENSGALG00010017997	2	2.520774355	1.239766451	2.033265502	0.042025707	2.530542144	5.452704067	81.66047547	1	CRA004775;Yang2025_Silkie_Wenchang	PRJNA827465	0.354602049
ENSGALG00010017997	ENSGALG00010017997	2	1.713731239	0.467364493	3.666798105	0.000245607	0	0.480742576	0	1	CRA004775;PRJNA827465	Yang2025_Silkie_Wenchang	0.014692655
ENSGALG00010018000	MAP2K1	2	-0.140373878	0.214161285	-0.655458703	0.512172466	0.080792767	7.760686134	87.11454139	0.5	PRJNA827465;Yang2025_Silkie_Wenchang	CRA004775	0.865694231
ENSGALG00010018000	MAP2K1	2	-0.251617009	0.157676694	-1.595778064	0.110538352	0.019247098	1.584823926	36.90150788	1	CRA004775;Yang2025_Silkie_Wenchang	PRJNA827465	0.562832796
ENSGALG00010018000	MAP2K1	2	0.047224874	0.046343783	1.019012074	0.308197235	0	0.249796223	0	0.5	CRA004775;PRJNA827465	Yang2025_Silkie_Wenchang	0.793403631
ENSGALG00010018001	TRAF7	2	-0.177123987	0.155997568	-1.135427868	0.256196089	0.040927801	5.910775882	83.0817473	1	PRJNA827465;Yang2025_Silkie_Wenchang	CRA004775	0.764613396
ENSGALG00010018001	TRAF7	2	-0.287883591	0.087866388	-3.27637902	0.001051473	0	0.668182412	0	1	CRA004775;Yang2025_Silkie_Wenchang	PRJNA827465	0.039097505
ENSGALG00010018001	TRAF7	2	-0.07847801	0.072533812	-1.081950709	0.279274431	0.00401439	1.390307265	28.0734536	1	CRA004775;PRJNA827465	Yang2025_Silkie_Wenchang	0.777457036
ENSGALG00010018002	ZC3H8	2	0.044245941	0.047868011	0.924332129	0.35531342	0	0.135191102	0	1	PRJNA827465;Yang2025_Silkie_Wenchang	CRA004775	0.812301302
ENSGALG00010018002	ZC3H8	2	0.221058883	0.158752789	1.392472439	0.163779382	0.012654299	1.301411727	23.16036658	1	CRA004775;Yang2025_Silkie_Wenchang	PRJNA827465	0.662244964
ENSGALG00010018002	ZC3H8	2	0.166116422	0.184062925	0.902498002	0.366792382	0.047567131	2.593622641	61.44388994	1	CRA004775;PRJNA827465	Yang2025_Silkie_Wenchang	0.819577857
ENSGALG00010018004	CDC42BPA	2	-0.176271449	0.122245842	-1.44194228	0.149318657	0.01242885	1.546498446	35.33779471	0.5	PRJNA827465;Yang2025_Silkie_Wenchang	CRA004775	0.639246608
ENSGALG00010018004	CDC42BPA	2	0.118503338	0.170869983	0.693529293	0.487977449	0.005091029	1.078348263	7.265580711	1	CRA004775;Yang2025_Silkie_Wenchang	PRJNA827465	0.85768885
ENSGALG00010018004	CDC42BPA	2	-0.003735255	0.312895417	-0.011937709	0.990475312	0.15318166	3.983227819	74.89473248	0.5	CRA004775;PRJNA827465	Yang2025_Silkie_Wenchang	0.997468525
ENSGALG00010018006	DCUN1D3	2	-0.200306314	0.078692729	-2.54542341	0.01091453	0.004332497	1.520177388	34.21820323	1	PRJNA827465;Yang2025_Silkie_Wenchang	CRA004775	0.166322288
ENSGALG00010018006	DCUN1D3	2	-0.17486237	0.171411091	-1.020134512	0.307664671	0.032816144	1.936461412	48.35941508	0.5	CRA004775;Yang2025_Silkie_Wenchang	PRJNA827465	0.793031517
ENSGALG00010018006	DCUN1D3	2	-0.112122341	0.076353198	-1.468469466	0.141976744	0	0.660081377	0	0.5	CRA004775;PRJNA827465	Yang2025_Silkie_Wenchang	0.62757683
ENSGALG00010018007	DNAAF9	2	0.516529599	0.209659576	2.463658512	0.013752707	0.075354601	6.88106686	85.46736981	1	PRJNA827465;Yang2025_Silkie_Wenchang	CRA004775	0.192493818
ENSGALG00010018007	DNAAF9	2	0.502416909	0.285863356	1.757542191	0.078825437	0.119558446	3.384495242	70.45349665	1	CRA004775;Yang2025_Silkie_Wenchang	PRJNA827465	0.482974778
ENSGALG00010018007	DNAAF9	2	0.303343168	0.085827595	3.534331446	0.000408808	0	0.291287089	0	1	CRA004775;PRJNA827465	Yang2025_Silkie_Wenchang	0.020366982
ENSGALG00010018008	ABCF3	2	-0.03752886	0.052907251	-0.709333024	0.478117839	0	0.97535242	0	0.5	PRJNA827465;Yang2025_Silkie_Wenchang	CRA004775	0.855366076
ENSGALG00010018008	ABCF3	2	0.055512695	0.129422872	0.428924919	0.667977865	0	0.549073751	0	0.5	CRA004775;Yang2025_Silkie_Wenchang	PRJNA827465	0.914120722
ENSGALG00010018008	ABCF3	2	-0.06294475	0.056933942	-1.105575116	0.268910465	0	0.187329867	0	1	CRA004775;PRJNA827465	Yang2025_Silkie_Wenchang	0.771398294
ENSGALG00010018010	ENSGALG00010018010	2	-0.069710438	0.057081926	-1.221234867	0.221997106	0	0.910646036	0	1	PRJNA827465;Yang2025_Silkie_Wenchang	CRA004775	0.735359501
ENSGALG00010018010	ENSGALG00010018010	2	-0.180098629	0.135008312	-1.333981784	0.182209826	0	0.175754686	0	1	CRA004775;Yang2025_Silkie_Wenchang	PRJNA827465	0.690033956
ENSGALG00010018010	ENSGALG00010018010	2	-0.054866151	0.058251874	-0.941877867	0.346255173	0	0.067707098	0	1	CRA004775;PRJNA827465	Yang2025_Silkie_Wenchang	0.807744676
ENSGALG00010018011	LIPA	2	-0.286223842	0.38625874	-0.741015833	0.45868384	0.288355662	29.66227692	96.62871464	0.5	PRJNA827465;Yang2025_Silkie_Wenchang	CRA004775	0.850089963
ENSGALG00010018011	LIPA	2	0.219176821	0.965210944	0.227076601	0.820364178	1.720206029	12.19554314	91.8002832	0.5	CRA004775;Yang2025_Silkie_Wenchang	PRJNA827465	0.955325326
ENSGALG00010018011	LIPA	2	0.554799073	0.56842988	0.976020249	0.32905443	0.525746698	4.48297904	77.69340452	1	CRA004775;PRJNA827465	Yang2025_Silkie_Wenchang	0.800761292
ENSGALG00010018012	DVL1	2	-0.152669739	0.126475731	-1.207106984	0.227390946	0.026315655	5.584679224	82.0938686	1	PRJNA827465;Yang2025_Silkie_Wenchang	CRA004775	0.740688408
ENSGALG00010018012	DVL1	2	-0.085123416	0.25059353	-0.339687206	0.734092097	0.095542294	3.609418055	72.29470278	0.5	CRA004775;Yang2025_Silkie_Wenchang	PRJNA827465	0.933420308
ENSGALG00010018012	DVL1	2	-0.015700135	0.063642629	-0.24669212	0.805146505	0	0.967269138	0	0.5	CRA004775;PRJNA827465	Yang2025_Silkie_Wenchang	0.951863117
ENSGALG00010018014	LYRM1	2	0.088061786	0.041933437	2.100037423	0.035725549	0	0.032307432	0	1	PRJNA827465;Yang2025_Silkie_Wenchang	CRA004775	0.326084826
ENSGALG00010018014	LYRM1	2	0.073950525	0.067842596	1.090030892	0.275699536	0	0.015867893	0	1	CRA004775;Yang2025_Silkie_Wenchang	PRJNA827465	0.775427142
ENSGALG00010018014	LYRM1	2	0.090417179	0.049373583	1.831286583	0.067057779	0	0.045894824	0	1	CRA004775;PRJNA827465	Yang2025_Silkie_Wenchang	0.446111942
ENSGALG00010018016	IFIT5	2	-0.592111896	0.252079048	-2.348913569	0.018828277	0	0.036793844	0	1	PRJNA827465;Yang2025_Silkie_Wenchang	CRA004775	0.229831303
ENSGALG00010018016	IFIT5	2	-0.304357973	0.380603514	-0.799671998	0.423900861	0.114720173	1.644324285	39.1847454	0.5	CRA004775;Yang2025_Silkie_Wenchang	PRJNA827465	0.84030043
ENSGALG00010018016	IFIT5	2	-0.288870843	0.325801549	-0.886646621	0.375269186	0.063187391	1.395929958	28.36316791	0.5	CRA004775;PRJNA827465	Yang2025_Silkie_Wenchang	0.822941424
ENSGALG00010018019	NAPB	2	0.914555394	0.448216276	2.040433252	0.041307193	0.31558229	4.659034649	78.53632618	1	PRJNA827465;Yang2025_Silkie_Wenchang	CRA004775	0.352374305
ENSGALG00010018019	NAPB	2	0.542895064	0.881669136	0.615758272	0.538054072	1.360271939	7.674206526	86.96933687	0.5	CRA004775;Yang2025_Silkie_Wenchang	PRJNA827465	0.87481821
ENSGALG00010018019	NAPB	2	0.160888778	0.414742062	0.387924911	0.698071602	0.172849675	1.83297395	45.44385096	0.5	CRA004775;PRJNA827465	Yang2025_Silkie_Wenchang	0.921516414
ENSGALG00010018020	SNAPC5	2	-0.19371766	0.305086865	-0.634959029	0.525455137	0.169693257	10.91317936	90.83676748	0.5	PRJNA827465;Yang2025_Silkie_Wenchang	CRA004775	0.870215879
ENSGALG00010018020	SNAPC5	2	-0.449365994	0.14061801	-3.195650351	0.001395161	0	0.551163202	0	1	CRA004775;Yang2025_Silkie_Wenchang	PRJNA827465	0.045924624
ENSGALG00010018020	SNAPC5	2	-0.019131627	0.174668058	-0.109531346	0.912781061	0.037209326	2.02887967	50.7117147	0.5	CRA004775;PRJNA827465	Yang2025_Silkie_Wenchang	0.979024575
ENSGALG00010018021	ATRN	2	-0.081928582	0.058001804	-1.412517813	0.15779755	0	0.147590755	0	1	PRJNA827465;Yang2025_Silkie_Wenchang	CRA004775	0.651321256
ENSGALG00010018021	ATRN	2	-0.146379483	0.175359016	-0.834741699	0.403863158	0.031494535	1.481977974	32.52261386	1	CRA004775;Yang2025_Silkie_Wenchang	PRJNA827465	0.833112102
ENSGALG00010018021	ATRN	2	-0.153393043	0.130943287	-1.171446401	0.24141939	0.012987965	1.199114902	16.60515618	1	CRA004775;PRJNA827465	Yang2025_Silkie_Wenchang	0.75393977
ENSGALG00010018022	XPO6	2	0.068415057	0.051809894	1.320501759	0.186667549	0	0.00028254	0	1	PRJNA827465;Yang2025_Silkie_Wenchang	CRA004775	0.695457578
ENSGALG00010018022	XPO6	2	-0.193325296	0.318184198	-0.607589244	0.543459937	0.159737186	4.132356797	75.80073432	0.5	CRA004775;Yang2025_Silkie_Wenchang	PRJNA827465	0.876968972
ENSGALG00010018022	XPO6	2	-0.188311611	0.318322	-0.591575862	0.554134642	0.163748985	4.394919486	77.24645461	0.5	CRA004775;PRJNA827465	Yang2025_Silkie_Wenchang	0.880108051
ENSGALG00010018025	CPTP	2	-0.10256956	0.180186249	-0.569241887	0.569191999	0.054849499	6.311186391	84.15511858	0.5	PRJNA827465;Yang2025_Silkie_Wenchang	CRA004775	0.884646991
ENSGALG00010018025	CPTP	2	-0.312473865	0.10313376	-3.029792224	0.00244722	0	0.09205339	0	1	CRA004775;Yang2025_Silkie_Wenchang	PRJNA827465	0.066177569
ENSGALG00010018025	CPTP	2	-0.108805792	0.212511725	-0.511999009	0.608651703	0.06969676	3.913256685	74.44583679	0.5	CRA004775;PRJNA827465	Yang2025_Silkie_Wenchang	0.897208691
ENSGALG00010018030	POLB	2	-0.023179224	0.046935348	-0.493854309	0.621409075	0	0.462156439	0	0.5	PRJNA827465;Yang2025_Silkie_Wenchang	CRA004775	0.899693435
ENSGALG00010018030	POLB	2	-0.02419379	0.13051355	-0.185373779	0.852935874	0.011672294	1.20632397	17.103529	0.5	CRA004775;Yang2025_Silkie_Wenchang	PRJNA827465	0.962708451
ENSGALG00010018030	POLB	2	-0.050666083	0.054021217	-0.937892297	0.348299762	0	0.819091082	0	1	CRA004775;PRJNA827465	Yang2025_Silkie_Wenchang	0.809042603
ENSGALG00010018032	ERO1A	2	0.035067261	0.088794603	0.394925586	0.692897807	0.006605188	1.592541042	37.20726979	0.5	PRJNA827465;Yang2025_Silkie_Wenchang	CRA004775	0.92062328
ENSGALG00010018032	ERO1A	2	-0.027004133	0.23134766	-0.116725336	0.907077712	0.071700708	2.789308166	64.1488161	0.5	CRA004775;Yang2025_Silkie_Wenchang	PRJNA827465	0.978196186
ENSGALG00010018032	ERO1A	2	-0.068132267	0.099360461	-0.685708035	0.492897239	0.006297597	1.184475426	15.57444097	1	CRA004775;PRJNA827465	Yang2025_Silkie_Wenchang	0.859562076
ENSGALG00010018033	COQ8A	2	-0.07681875	0.116116888	-0.661563975	0.508250702	0.010193271	1.539943462	35.06255103	0.5	PRJNA827465;Yang2025_Silkie_Wenchang	CRA004775	0.864714831
ENSGALG00010018033	COQ8A	2	0.110040227	0.150285981	0.732205533	0.464043128	0	0.278359692	0	1	CRA004775;Yang2025_Silkie_Wenchang	PRJNA827465	0.852159649
ENSGALG00010018033	COQ8A	2	-0.081596482	0.177344031	-0.460102779	0.64544245	0.027341851	1.347032985	25.76276818	0.5	CRA004775;PRJNA827465	Yang2025_Silkie_Wenchang	0.906966468
ENSGALG00010018035	ITM2A	2	-0.302613897	0.319263712	-0.947849335	0.343206161	0.196293749	26.34034359	96.20354231	0.5	PRJNA827465;Yang2025_Silkie_Wenchang	CRA004775	0.806218116
ENSGALG00010018035	ITM2A	2	-0.407143924	0.238191267	-1.709315077	0.087392602	0.093260001	5.524039485	81.89730536	1	CRA004775;Yang2025_Silkie_Wenchang	PRJNA827465	0.508252579
ENSGALG00010018035	ITM2A	2	-0.001974869	0.039588588	-0.049884804	0.960214187	0	0.898411589	0	0.5	CRA004775;PRJNA827465	Yang2025_Silkie_Wenchang	0.989338243
ENSGALG00010018036	RPL4	2	-0.130106563	0.165268175	-0.787245107	0.431138397	0.044978606	5.21193404	80.81326447	0.5	PRJNA827465;Yang2025_Silkie_Wenchang	CRA004775	0.842503929
ENSGALG00010018036	RPL4	2	-0.306655016	0.107502361	-2.852542158	0.004337106	0	0.025119921	0	1	CRA004775;Yang2025_Silkie_Wenchang	PRJNA827465	0.096523938
ENSGALG00010018036	RPL4	2	-0.087520364	0.141340339	-0.619217163	0.535773307	0.027180464	2.570542702	61.09770909	0.5	CRA004775;PRJNA827465	Yang2025_Silkie_Wenchang	0.874321378
ENSGALG00010018037	GZF1	2	0.311609232	0.127479153	2.444393644	0.014509585	0.02283564	3.001054784	66.67838237	1	PRJNA827465;Yang2025_Silkie_Wenchang	CRA004775	0.198198336
ENSGALG00010018037	GZF1	2	0.231242554	0.281922535	0.820234374	0.41208251	0.118305549	3.649835221	72.60150282	0.5	CRA004775;Yang2025_Silkie_Wenchang	PRJNA827465	0.835532508
ENSGALG00010018037	GZF1	2	0.163210234	0.111084978	1.469237669	0.141768336	0.010876846	1.294649391	22.75901054	0.5	CRA004775;PRJNA827465	Yang2025_Silkie_Wenchang	0.627129189
ENSGALG00010018039	TTC14	2	0.454096203	0.396356769	1.145675408	0.251929483	0.295362408	16.29027406	93.86136785	1	PRJNA827465;Yang2025_Silkie_Wenchang	CRA004775	0.761871849
ENSGALG00010018039	TTC14	2	0.572353668	0.332191434	1.722963359	0.084895151	0.165111652	3.802663849	73.70264531	1	CRA004775;Yang2025_Silkie_Wenchang	PRJNA827465	0.501146611
ENSGALG00010018039	TTC14	2	0.08227231	0.080466629	1.022440121	0.306572639	0	0.164878838	0	1	CRA004775;PRJNA827465	Yang2025_Silkie_Wenchang	0.792500392
ENSGALG00010018042	ZWILCH	2	-0.209602563	0.082205906	-2.54972634	0.010780751	0	0.120611385	0	1	PRJNA827465;Yang2025_Silkie_Wenchang	CRA004775	0.165420139
ENSGALG00010018042	ZWILCH	2	-0.185548106	0.124783442	-1.486960952	0.137025124	0	0.0937214	0	1	CRA004775;Yang2025_Silkie_Wenchang	PRJNA827465	0.619307551
ENSGALG00010018042	ZWILCH	2	-0.237481136	0.103588104	-2.292552201	0.021873799	0	0.034838504	0	1	CRA004775;PRJNA827465	Yang2025_Silkie_Wenchang	0.25071622
ENSGALG00010018043	PSMC6	2	-0.042537617	0.067550123	-0.629719316	0.528878242	0.004334071	1.6961346	41.04241489	0.5	PRJNA827465;Yang2025_Silkie_Wenchang	CRA004775	0.871809618
ENSGALG00010018043	PSMC6	2	-0.082559473	0.202846123	-0.407005426	0.684004003	0.050478886	2.14943518	53.47614995	0.5	CRA004775;Yang2025_Silkie_Wenchang	PRJNA827465	0.918806497
ENSGALG00010018043	PSMC6	2	-0.102672902	0.062587111	-1.64047997	0.10090541	0.001785568	1.044611405	4.270622051	1	CRA004775;PRJNA827465	Yang2025_Silkie_Wenchang	0.542232064
ENSGALG00010018044	INTS11	2	0.070507368	0.05746442	1.226974331	0.219832239	0	0.93991508	0	0.5	PRJNA827465;Yang2025_Silkie_Wenchang	CRA004775	0.733380873
ENSGALG00010018044	INTS11	2	-0.07562717	0.097257272	-0.777599124	0.436805378	0	0.764054878	0	1	CRA004775;Yang2025_Silkie_Wenchang	PRJNA827465	0.843375996
ENSGALG00010018044	INTS11	2	-0.008197875	0.152431903	-0.053780575	0.957109986	0.03120597	2.561332491	60.95782163	0.5	CRA004775;PRJNA827465	Yang2025_Silkie_Wenchang	0.988541306
ENSGALG00010018045	PSEN2	2	0.264639199	0.148906918	1.777212249	0.075533325	0.038218848	7.082947893	85.88158469	1	PRJNA827465;Yang2025_Silkie_Wenchang	CRA004775	0.474458409
ENSGALG00010018045	PSEN2	2	0.142849689	0.31142184	0.458701577	0.646448482	0.166341952	6.570295473	84.77998434	0.5	CRA004775;Yang2025_Silkie_Wenchang	PRJNA827465	0.907220702
ENSGALG00010018045	PSEN2	2	0.032105747	0.147464454	0.21771855	0.827648409	0.026587896	1.976283096	49.39996187	0.5	CRA004775;PRJNA827465	Yang2025_Silkie_Wenchang	0.956550752
ENSGALG00010018046	CAPN15	2	0.099219912	0.069049539	1.436938074	0.15073559	0	0.19180962	0	1	PRJNA827465;Yang2025_Silkie_Wenchang	CRA004775	0.642051813
ENSGALG00010018046	CAPN15	2	-0.144770157	0.210804611	-0.68675043	0.492240012	0.053361614	2.428221136	58.81758934	0.5	CRA004775;Yang2025_Silkie_Wenchang	PRJNA827465	0.859228604
ENSGALG00010018046	CAPN15	2	-0.092830272	0.24721112	-0.375510097	0.707281139	0.098015386	4.486181063	77.7093259	0.5	CRA004775;PRJNA827465	Yang2025_Silkie_Wenchang	0.924856272
ENSGALG00010018048	GFRA4	2	0.232522243	0.108882949	2.13552484	0.032718163	0	0.002432977	0	1	PRJNA827465;Yang2025_Silkie_Wenchang	CRA004775	0.310759298
ENSGALG00010018048	GFRA4	2	0.483384745	0.349506537	1.383049224	0.166649773	0.155918842	2.293016471	56.38932329	1	CRA004775;Yang2025_Silkie_Wenchang	PRJNA827465	0.665626918
ENSGALG00010018048	GFRA4	2	0.476751564	0.354796968	1.343730663	0.179035498	0.166304814	2.405995669	58.43716542	1	CRA004775;PRJNA827465	Yang2025_Silkie_Wenchang	0.685713211
ENSGALG00010018049	SUGCT	2	-0.395027814	0.149351774	-2.644948917	0.008170325	0	0.245194707	0	1	PRJNA827465;Yang2025_Silkie_Wenchang	CRA004775	0.140547724
ENSGALG00010018049	SUGCT	2	-0.480213374	0.21454757	-2.238260604	0.025204066	0	0.000172984	0	1	CRA004775;Yang2025_Silkie_Wenchang	PRJNA827465	0.270285761
ENSGALG00010018049	SUGCT	2	-0.3451133	0.187092434	-1.844613872	0.065093728	0	0.04921127	0	1	CRA004775;PRJNA827465	Yang2025_Silkie_Wenchang	0.439870073
ENSGALG00010018051	GNPNAT1	2	-0.009699443	0.050921475	-0.190478446	0.848934231	0	0.156087012	0	0.5	PRJNA827465;Yang2025_Silkie_Wenchang	CRA004775	0.961364896
ENSGALG00010018051	GNPNAT1	2	-0.035122445	0.093558208	-0.375407412	0.707357493	0	0.051904849	0	0.5	CRA004775;Yang2025_Silkie_Wenchang	PRJNA827465	0.924867585

ENSGALG00010018051	GNPNAT1	2	0.001863512	0.05605584	0.033243858	0.973480124	0	0.000630216	0	1	CRA004775;PRJNA827465	Yang2025_Silkie_Wenchang	0.992392211
ENSGALG00010018052	PPP1RL	2	-0.053653432	0.132064488	-0.406266915	0.684546492	0.025555308	3.107054674	67.81517852	0.5	PRJNA827465;Yang2025_Silkie_Wenchang	CRA004775	0.919003112
ENSGALG00010018052	PPP1RL	2	-0.252432669	0.134813463	-1.872458897	0.061143143	0	0.13717792	0	1	CRA004775;Yang2025_Silkie_Wenchang	PRJNA827465	0.427371325
ENSGALG00010018052	PPP1RL	2	-0.037467248	0.163221343	-0.229548704	0.818442471	0.031785493	1.647775745	39.31213014	0.5	CRA004775;PRJNA827465	Yang2025_Silkie_Wenchang	0.955256039
ENSGALG00010018053	DIAPH2	2	-0.124514965	0.107869057	-1.15431588	0.248370688	0.014388533	2.376577887	57.9226919	1	PRJNA827465;Yang2025_Silkie_Wenchang	CRA004775	0.75856854
ENSGALG00010018053	DIAPH2	2	-0.039496409	0.265611744	-0.148699783	0.881790533	0.104243957	3.549528351	71.82724291	0.5	CRA004775;Yang2025_Silkie_Wenchang	PRJNA827465	0.971900875
ENSGALG00010018053	DIAPH2	2	0.02049779	0.125134704	0.163805794	0.869884030	0.015242175	1.439970856	30.55415007	0.5	CRA004775;PRJNA827465	Yang2025_Silkie_Wenchang	0.968610705
ENSGALG00010018054	SMAD6	2	0.034950077	0.115062343	0.303749044	0.761319091	0	0.027719849	0	1	PRJNA827465;Yang2025_Silkie_Wenchang	CRA004775	0.939618592
ENSGALG00010018054	SMAD6	2	0.013287383	0.136215914	0.097546484	0.922292422	0	0.179667686	0	0.5	CRA004775;Yang2025_Silkie_Wenchang	PRJNA827465	0.981804675
ENSGALG00010018054	SMAD6	2	-0.00916661	0.12998821	-0.070518777	0.943780756	0	0.087832763	0	0.5	CRA004775;PRJNA827465	Yang2025_Silkie_Wenchang	0.985827325
ENSGALG00010018057	SSTR4	2	0.190895025	0.081193348	2.351116573	0.018717169	0	0.478563671	0	1	PRJNA827465;Yang2025_Silkie_Wenchang	CRA004775	0.229171617
ENSGALG00010018057	SSTR4	2	0.117030988	0.236393349	0.495068869	0.620551508	0.059692265	1.649673036	39.38192732	0.5	CRA004775;Yang2025_Silkie_Wenchang	PRJNA827465	0.899395593
ENSGALG00010018057	SSTR4	2	0.09916621	0.125382969	0.790906537	0.428998534	0.004973533	1.054695462	5.185900963	0.5	CRA004775;PRJNA827465	Yang2025_Silkie_Wenchang	0.84190989
ENSGALG00010018058	ITPKB	2	0.024473161	0.052020471	0.470452512	0.638031754	0.000873892	1.1837361	15.52171133	0.5	PRJNA827465;Yang2025_Silkie_Wenchang	CRA004775	0.905365679
ENSGALG00010018058	ITPKB	2	0.079543192	0.073470983	1.082647718	0.278964818	0	0.15236488	0	0.5	CRA004775;Yang2025_Silkie_Wenchang	PRJNA827465	0.777307224
ENSGALG00010018058	ITPKB	2	-0.01814378	0.05884537	-0.308329786	0.757831403	0	5.06916E-08	0	1	CRA004775;PRJNA827465	Yang2025_Silkie_Wenchang	0.938954369
ENSGALG00010018060	CASKIN1	2	0.025818385	0.353859457	0.072962259	0.94183615	0.203458694	5.015731905	80.06273025	0.5	PRJNA827465;Yang2025_Silkie_Wenchang	CRA004775	0.985827325
ENSGALG00010018060	CASKIN1	2	-0.186829215	0.685319951	-0.272616046	0.785148376	0.762994893	5.050134645	80.19854775	0.5	CRA004775;Yang2025_Silkie_Wenchang	PRJNA827465	0.946325593
ENSGALG00010018060	CASKIN1	2	-0.414288347	0.261445933	-1.584604288	0.11305627	0.064344975	1.407863858	28.97040473	1	CRA004775;PRJNA827465	Yang2025_Silkie_Wenchang	0.567481156
ENSGALG00010018061	NDUFAF5	2	0.00878331	0.102893189	0.08536338	0.931972505	0.00743817	1.522635955	34.32441965	0.5	PRJNA827465;Yang2025_Silkie_Wenchang	CRA004775	0.98412915
ENSGALG00010018061	NDUFAF5	2	0.094795246	0.125947149	0.752658923	0.4516549	0	0.694310845	0	0.5	CRA004775;Yang2025_Silkie_Wenchang	PRJNA827465	0.848006549
ENSGALG00010018061	NDUFAF5	2	-0.088785546	0.101412336	-0.87549059	0.381307028	0	0.127386112	0	1	CRA004775;PRJNA827465	Yang2025_Silkie_Wenchang	0.824392515
ENSGALG00010018062	GOLGA7	2	-0.066806953	0.144345467	-0.462826818	0.643488507	0.035029068	5.93430972	83.14884043	0.5	PRJNA827465;Yang2025_Silkie_Wenchang	CRA004775	0.906643868
ENSGALG00010018062	GOLGA7	2	-0.238232135	0.098178352	-2.426524082	0.015244239	0	0.067601515	0	1	CRA004775;Yang2025_Silkie_Wenchang	PRJNA827465	0.204219788
ENSGALG00010018062	GOLGA7	2	-0.037139928	0.161342438	-0.230191172	0.817941667	0.033390371	2.066095907	51.59953627	0.5	CRA004775;PRJNA827465	Yang2025_Silkie_Wenchang	0.955109707
ENSGALG00010018063	DECR2	2	-0.13338129	0.347133204	-0.384236625	0.700803075	0.210407228	7.171776924	86.0564542	0.5	PRJNA827465;Yang2025_Silkie_Wenchang	CRA004775	0.922496697
ENSGALG00010018063	DECR2	2	-0.470788672	0.171795462	-2.740402252	0.006136403	0	0.086375881	0	1	CRA004775;Yang2025_Silkie_Wenchang	PRJNA827465	0.118495743
ENSGALG00010018063	DECR2	2	-0.08243328	0.296049224	-0.278444507	0.780671158	0.148779749	5.948781398	83.18983447	0.5	CRA004775;PRJNA827465	Yang2025_Silkie_Wenchang	0.945235709
ENSGALG00010018064	TTC8	2	0.115814296	0.136195202	0.850355184	0.395127645	0.021738943	2.278774234	56.11675852	0.5	PRJNA827465;Yang2025_Silkie_Wenchang	CRA004775	0.830643015
ENSGALG00010018064	TTC8	2	-0.143548682	0.121438482	-1.182069147	0.237178265	0	0.819724855	0	1	CRA004775;Yang2025_Silkie_Wenchang	PRJNA827465	0.749366344
ENSGALG00010018064	TTC8	2	0.002262241	0.249527486	0.009066097	0.9927664	0.103450545	5.593090342	82.12079657	0.5	CRA004775;PRJNA827465	Yang2025_Silkie_Wenchang	0.997869618
ENSGALG00010018067	ENSGALG00010018067	2	-0.293175501	0.169854026	-1.726043869	0.084339521	0.029260748	1.936150169	48.35111367	1	PRJNA827465;Yang2025_Silkie_Wenchang	CRA004775	0.500017819
ENSGALG00010018067	ENSGALG00010018067	2	-0.496769634	0.197401718	-2.516541598	0.011851291	0	0.011624794	0	1	CRA004775;Yang2025_Silkie_Wenchang	PRJNA827465	0.174649902
ENSGALG00010018067	ENSGALG00010018067	2	-0.171820036	0.131264711	-1.308958328	0.190548467	0	0.233915454	0	1	CRA004775;PRJNA827465	Yang2025_Silkie_Wenchang	0.699656801
ENSGALG00010018068	ADAM33	2	-0.125461898	0.10617203	-1.181685027	0.237330702	0.014836382	2.924542547	65.80661817	1	PRJNA827465;Yang2025_Silkie_Wenchang	CRA004775	0.749366344
ENSGALG00010018068	ADAM33	2	-0.250425045	0.086317438	-2.901210358	0.003717242	0	0.942897111	0	1	CRA004775;Yang2025_Silkie_Wenchang	PRJNA827465	0.087190443
ENSGALG00010018068	ADAM33	2	-0.199976165	0.279590488	-0.715246668	0.474456637	0.103041168	2.206082106	54.67077144	1	CRA004775;PRJNA827465	Yang2025_Silkie_Wenchang	0.854462864
ENSGALG00010018069	ENSGALG00010018069	2	0.058460413	0.264499378	0.221022874	0.825074623	0	0.000112052	0	1	PRJNA827465;Yang2025_Silkie_Wenchang	CRA004775	0.956315185
ENSGALG00010018069	ENSGALG00010018069	2	-0.01542372	0.270417362	-0.057036722	0.954515943	0	0.039865518	0	0.5	CRA004775;Yang2025_Silkie_Wenchang	PRJNA827465	0.987987175
ENSGALG00010018069	ENSGALG00010018069	2	0.003816431	0.237097918	0.016096435	0.987157458	0	0.061367387	0	0.5	CRA004775;PRJNA827465	Yang2025_Silkie_Wenchang	0.996462132
ENSGALG00010018071	GPAT4	2	-0.103808172	0.119472218	-0.868889635	0.384907499	0.019108441	2.481516772	59.70206563	1	PRJNA827465;Yang2025_Silkie_Wenchang	CRA004775	0.826444827
ENSGALG00010018071	GPAT4	2	-0.523095584	0.285195684	-1.834163745	0.066629691	0.121016016	3.732192922	73.20610106	1	CRA004775;Yang2025_Silkie_Wenchang	PRJNA827465	0.444612948
ENSGALG00010018071	GPAT4	2	-0.394976405	0.412260323	-0.958075232	0.338024824	0.309021855	10.15062076	90.14838577	1	CRA004775;PRJNA827465	Yang2025_Silkie_Wenchang	0.80415385
ENSGALG00010018075	MLST8	2	0.096793137	0.148128928	0.653438448	0.513473659	0.031283736	3.023519181	66.92595813	0.5	PRJNA827465;Yang2025_Silkie_Wenchang	CRA004775	0.8664
ENSGALG00010018075	MLST8	2	0.169831782	0.197355906	0.860535593	0.389493872	0.025770023	1.344061519	25.59864369	0.5	CRA004775;Yang2025_Silkie_Wenchang	PRJNA827465	0.828911149
ENSGALG00010018075	MLST8	2	-0.022673683	0.061472525	-0.368842561	0.712245079	0	0.161315589	0	1	CRA004775;PRJNA827465	Yang2025_Silkie_Wenchang	0.92597752
ENSGALG00010018078	NKX6-3	2	0.261423227	0.354401109	0.737647882	0.460728451	0	0.005955754	0	1	PRJNA827465;Yang2025_Silkie_Wenchang	CRA004775	0.85014477
ENSGALG00010018078	NKX6-3	2	0.479772701	0.422637857	1.135186289	0.256297273	0	0.309826924	0	1	CRA004775;Yang2025_Silkie_Wenchang	PRJNA827465	0.764677554
ENSGALG00010018078	NKX6-3	2	0.411754843	0.386428051	1.065540769	0.286631287	0	0.424573599	0	1	CRA004775;PRJNA827465	Yang2025_Silkie_Wenchang	0.782018115
ENSGALG00010018080	BRICD5	2	-0.267704699	0.335785735	-0.797248575	0.42530668	0.156668903	2.676435552	62.63687353	1	PRJNA827465;Yang2025_Silkie_Wenchang	CRA004775	0.84125685
ENSGALG00010018080	BRICD5	2	-0.393644444	0.29855508	-1.318498563	0.187336806	0.031892927	1.213726985	17.60914833	1	CRA004775;Yang2025_Silkie_Wenchang	PRJNA827465	0.696255175
ENSGALG00010018080	BRICD5	2	-0.036174175	0.11574402	-0.312536015	0.754633197	0	0.078542233	0	1	CRA004775;PRJNA827465	Yang2025_Silkie_Wenchang	0.93838723
ENSGALG00010018081	DIO3	2	-0.036198256	0.135192465	-0.267753503	0.788889063	0	0.874177815	0	0.5	PRJNA827465;Yang2025_Silkie_Wenchang	CRA004775	0.947144026
ENSGALG00010018081	DIO3	2	-0.177150213	0.167703896	-1.056327357	0.290818682	0	0.24409146	0	1	CRA004775;Yang2025_Silkie_Wenchang	PRJNA827465	0.784646441
ENSGALG00010018081	DIO3	2	0.029555398	0.189206547	0.156207056	0.875869815	0	0.951513628	0	0.5	CRA004775;PRJNA827465	Yang2025_Silkie_Wenchang	0.970347777
ENSGALG00010018082	RAB11FIP3	2	0.612342654	0.35660424	1.717149115	0.08595193	0.240317746	17.69876374	94.34988785	1	PRJNA827465;Yang2025_Silkie_Wenchang	CRA004775	0.504285479
ENSGALG00010018082	RAB11FIP3	2	0.504077096	0.50683375	0.99456103	0.319949809	0.461994643	9.647354062	89.63446357	0.5	CRA004775;Yang2025_Silkie_Wenchang	PRJNA827465	0.798348216
ENSGALG00010018082	RAB11FIP3	2	0.247840081	0.077339091	3.204590057	0.00135255	0.001830898	1.041953793	4.02645427	0.5	CRA004775;PRJNA827465	Yang2025_Silkie_Wenchang	0.044973994
ENSGALG00010018085	PGP	2	-0.009962288	0.104487844	-0.095343987	0.924041605	0	0.797881934	0	0.5	PRJNA827465;Yang2025_Silkie_Wenchang	CRA004775	0.982291939
ENSGALG00010018085	PGP	2	-0.00090168	0.191393291	-0.004711135	0.996241072	0.024906993	1.356679808	26.29064029	0.5	CRA004775;Yang2025_Silkie_Wenchang	PRJNA827465	0.99878373
ENSGALG00010018085	PGP	2	0.108984712	0.129260018	0.84314325	0.399148333	0	0.454869977	0	1	CRA004775;PRJNA827465	Yang2025_Silkie_Wenchang	0.832054308
ENSGALG00010018087	ENSGALG00010018087	2	0.025715661	0.052179359	0.492832065	0.622131252	0	0.010325533	0	1	PRJNA827465;Yang2025_Silkie_Wenchang	CRA004775	0.900048758
ENSGALG00010018087	ENSGALG00010018087	2	0.004177611	0.102670772	0.040689386	0.967543523	0	0.159646748	0	0.5	CRA004775;Yang2025_Silkie_Wenchang	PRJNA827465	0.991065147
ENSGALG00010018087	ENSGALG00010018087	2	0.024376175	0.058979014	0.413302514	0.679384988	0	0.197722711	0	0.5	CRA004775;PRJNA827465	Yang2025_Silkie_Wenchang	0.917701868
ENSGALG00010018089	TASP1	2	-0.098101064	0.189571523	-0.517488395	0.604815268	0.056675915	4.666590899	78.57108066	0.5	PRJNA827465;Yang2025_Silkie_Wenchang	CRA004775	0.896344437
ENSGALG00010018089	TASP1	2	-0.233270209	0.124099363	-1.879705126	0.060148277	0	0.998965166	0	1	CRA004775;Yang2025_Silkie_Wenchang	PRJNA827465	0.424646617
ENSGALG00010018089	TASP1	2	0.069265581	0.097990046	0.706863441	0.479651345	0	0.081274862	0	0.5	CRA004775;PRJNA827465	Yang2025_Silkie_Wenchang	0.856196603
ENSGALG00010018092	ENSGALG00010018092	2	-0.116293655	0.104819763	-1.109463063	0.26723047	0	0.507381401	0	0.5	PRJNA827465;Yang2025_Silkie_Wenchang	CRA004775	0.771362152
ENSGALG00010018092	ENSGALG00010018092	2	-0.00911533	0.290951538	-0.031329376	0.975006863	0	0.745866491	0	0.5	CRA004775;Yang2025_Silkie_Wenchang	PRJNA827465	0.992609821
ENSGALG00010018092	ENSGALG00010018092	2	-0.152545648	0.109972915	-1.387120172	0.165405122	0	0.377806856	0	1	CRA004775;PRJNA827465	Yang2025_Silkie_Wenchang	0.664874006
ENSGALG00010018095	E4F1	2	0.144773776	0.12647282	1.144702684	0.252332339	0.027466986	6.987098875	85.68790827	1	PRJNA827465;Yang2025_Silkie_Wenchang	CRA004775	0.762585001
ENSGALG00010018095	E4F1	2	0.161709499	0.14668512	1.102426064	0.270276486	0.028795354	2.716331515	63.1856423	0.5	CRA004775;Yang2025_Silkie_Wenchang	PRJNA827465	0.772430311
ENSGALG00010018095	E4F1	2	0.018820016	0.053212615	0.353675835	0.723581834	0	0.077289403	0	0.5	CRA004775;PRJNA827465	Yang2025_Silkie_Wenchang	0.930032096
ENSGALG00010018099	SMAD3	2	0.006917449	0.056550523	0.122323339	0.902642949	0	0.463794831	0	0.5	PRJNA827465;Yang2025_Silkie_Wenchang	CRA004775	0.976923607
ENSGALG00010018099	SMAD3	2	0.001148022	0.163257511	0.007031968	0.994389347	0.016181908	1.278953248	21.81105902	0.5	CRA004775;Yang2025_Silkie_Wenchang	PRJNA827465	0.99823774
ENSGALG00010018099	SMAD3	2	-0.021397586	0.060932909	-0.351166335	0.725463565	0	0.791347184	0	1	CRA004775;PRJNA827465	Yang2025_Silkie_Wenchang	0.930910808
ENSGALG00010018102	ZDHHC4	2	-0.217838926	0.304422659	-0.715580524	0.474250403	0.156839642	6.361419329	84.28023766	0.5	PRJNA827465;Yang2025_Silkie_Wenchang	CRA004775	0.854462864
ENSGALG00010018102	ZDHHC4	2	-0.590589214	0.161118561	-3.665556653	0.000246801	0	0.155271779	0	1	CRA004775;Yang2025_Silkie_Wenchang	PRJNA827465	0.014719311
ENSGALG00010018102	ZDHHC4	2	-0.267160278	0.369141585	-0.723733897	0.469229119	0.231048079	6.244840207	83.98678002	0.5	CRA004775;PRJNA827465	Yang2025_Silkie_Wenchang	0.853485658
ENSGALG00010018103	BTRC	2	0.084718179	0.102120179	0.829592933	0.406768974	0.010947669	1.997678059	49.94188401	1	PRJNA827465;Yang2025_Silkie_Wenchang	CRA004775	0.833618122
ENSGALG00010018103	BTRC	2	0.059866597	0.187713054	0.318926123	0.749782535	0.042722731	2.386455928	58.09685868	0.5	CRA004775;Yang2025_Silkie_Wenchang	PRJNA827465	0.937362187
ENSGALG00010018103	BTRC	2	-0.017496412	0.073605949	-0.237703769	0.81211086	0	0.583351169	0	0.5	CRA004775;PRJNA827465	Yang2025_Silkie_Wenchang	0.953371387
ENSGALG00010018115	KLHL4	2	-0.079606443	0.10031334	-0.793577835	0.42744124	0	0.995298589	0	0.5	PRJNA827465;Yang2025_Silkie_Wenchang	CRA004775	0.84125685
ENSGALG00010018115	KLHL4	2	-0.231233093	0.157445427	-1.468655507	0.141926251	0	0.715989613	0	1	CRA004775;Yang2025_Silkie_Wenchang	PRJNA827465	0.62757683
ENSGALG00010018115	KLHL4	2	-0.098434954	0.281437296	-0.349758029	0.7265203	0.072484306	1.218190638	17.91104207	0.5	CRA004775;PRJNA827465	Yang2025_Silkie_Wenchang	0.931112589
ENSGALG00010018121	FERMT2	2	0.009503364	0.124219896	0.07650436	0.939017846	0.022139586	3.459838934	71.09692043	0.5	PRJNA827465;Yang2025_Silkie_Wenchang	CRA004775	0.985349045
ENSGALG00010018121	FERMT2	2	0.175247727	0.099342572	1.764074796	0.077719387	0	0.40906959	0	1	CRA004775;Yang2025_Silkie_Wenchang	PRJNA827465	0.480343171
ENSGALG00010018121	FERMT2	2	0.044377003	0.199548329	0.222387244	0.824012441	0.055624211	2.77179601	63.92230899	0.5	CRA004775;PRJNA827465	Yang2025_Silkie_Wenchang	0.956170356
ENSGALG00010018123	USP13	2	-0.056245712	0.165961677	-0.338907832	0.734679164	0.043817732	4.761788588	78.99948766	0.5	PRJNA827465;Yang2025_Silkie_Wenchang	CRA004775	0.933520234
ENSGALG00010018123	USP13	2	0.132244642	0.124220434	1.064596517	0.287058557	0	0.083365068	0	1	CRA004775;Yang2025_Silkie_Wenchang	PRJNA827465	0.782566585
ENSGALG00010018123	USP13	2	-0.19796156	0.083096037	-2.382322524	0.017203822	0	0.779523714	0	0.5	CRA004775;PRJNA827465	Yang2025_Silkie_Wenchang	0.218046383
ENSGALG00010018128	SEPTIN12	2	0.190784221	0.294847648	0.647060344	0.51759289	0.159889945	12.32362467	91.88550425	0.5	PRJNA827465;Yang2025_Silkie_Wenchang	CRA004775	0.867962259
ENSGALG00010018128	SEPTIN12	2	-0.28084274	0.836393478	-0.335778252	0.737038092	1.28105231	11.14962746	91.03109047	0.5	CRA004775;Yang2025_Silkie_Wenchang	PRJNA827465	0.93387966
ENSGALG00010018128	SEPTIN12	2	-0.536631265	0.534009937	-1.004908764	0.314940784	0.470288141	4.877509518	79.4977335	1	CRA004775;PRJNA827465	Yang2025_Silkie_Wenchang	0.796572019
ENSGALG00010018137	NDUFB5	2	-0.085956745	0.074546615	-1.153060343	0.248885617	0	0.000456778	0	1	PRJNA827465;Yang2025_Silkie_Wenchang	CRA004775	0.759350366
ENSGALG00010018137	NDUFB5	2	0.01026792	0.191493551	0.053620189	0.957237771	0	0.599971007	0	0.5	CRA004775;Yang2025_Silkie_Wenchang	PRJNA827465	0.988541306
ENSGALG00010018137	NDUFB5	2	-0.071382307	0.077208135	-0.924543854	0.35520323	0	0.816482802	0	0.5	CRA004775;PRJNA827465	Yang2025_Silkie_Wenchang	0.812301302
ENSGALG00010018140	CHPF	2	0.118506827	0.170036967	0.696947433	0.485835692	0.048156001	5.979552979	83.27634183	0.5	PRJNA827465;Yang2025_Silkie_Wenchang	CRA004775	0.857391223
ENSGALG00010018140	CHPF	2	0.021173218	0.291266039	0.072693741	0.94204983	0.146828862	7.119603731	85.95427445	0.5	CRA004775;Yang2025_Silkie_Wenchang	PRJNA827465	0.985827325
ENSGALG00010018140	CHPF	2	-0.114251277	0.107248738	-1.065292503	0.286743584	0.006183544	1.261039462	20.70034049	1	CRA004775;PRJNA827465	Yang2025_Silkie_Wenchang	0.782141931
ENSGALG00010018142	DDHD1	2	0.205888338	0.065127865	3.16129416	0.001570698	0.00161079	1.217540882	17.86723431	1	PRJNA827465;Yang2025_Silkie_Wenchang	CRA004775	0.049945905
ENSGALG00010018142	DDHD1	2	0.183025226	0.198857456	0.920384027	0.357372113	0.041251988	1.564156238	36.06776769	0.5	CRA004775;Yang2025_Silkie_Wenchang	PRJNA827465	0.813353185
ENSGALG00010018142	DDHD1	2	0.14241527	0.070871406	2.009488415	0.044485362	0	0.834142258	0	0.5	CRA004775;PRJNA827465	Yang2025_Silkie_Wenchang	0.363948415
ENSGALG00010018143	MRPL47	2	-0.118432511	0.108684547	-1.089690436	0.275849531	0.011906178	1.711054796	41.55651811	1	PRJNA827465;Yang2025_Silkie_Wenchang	CRA004775	0.77551603
ENSGALG00010018143	MRPL47	2	-0.206638907	0.14824512	-1.393900228	0.163347736	0	0.984163956	0	0.5	CRA004775;Yang2025_Silkie_Wenchang	PRJNA827465	0.661683383
ENSGALG00010018143	MRPL47	2	-0.043685546	0.067194445	-0.650136279	0.515604197	0	0.117278956	0	0.5	CRA004775;PRJNA827465	Yang2025_Silkie_Wenchang	0.867300757
ENSGALG00010018145	ENSGALG00010018145	2	0.391570624	0.182980258	2.139961048	0.032357915	0	0.576922643	0	1	PRJNA827465;Yang2025_Silkie_Wenchang	CRA004775	0.309055437
ENSGALG00010018145	ENSGALG00010018145	2	-0.165310786	0.468726284	-0.352680855	0.724327714	0.286689893	2.698279698	62.9393498	0.5	CRA004775;Yang2025_Silkie_Wenchang	PRJNA827465	0.930387861
ENSGALG00010018145	ENSGALG00010018145	2	-0.033171018	0.613729113	-0.054048304	0.956896679	0.610939316	4.935673857	79.73934201	0.5	CRA004775;PRJNA827465	Yang2025_Silkie_Wenchang	0.988540618
ENSGALG00010018147	ISM1	2	0.011103505	0.109410978	0.101484374	0.919165961	0	0.101669447	0	0.5	PRJNA827465;Yang2025_Silkie_Wenchang	CRA004775	0.981150999
ENSGALG00010018147	ISM1	2	0.441121301	0.424180966	1.039936575	0.298369369	0.235347543	2.722919206	63.27470908	1	CRA004775;Yang2025_Silkie_Wenchang	PRJNA827465	0.789312196
ENSGALG00010018147	ISM1	2	0.37359064	0.467065305	0.799868104	0.423787219	0.346872577	4.180758357	76.08089455	0.5	CRA004775;PRJNA827465	Yang2025_Silkie_Wenchang	0.84030043
ENSGALG00010018148	XRN2	2	-0.070280878	0.036588873	-1.920826541	0.054753579	0	0.014995688	0	1	PRJNA827465;Yang2025_Silkie_Wenchang	CRA004775	0.405183476
ENSGALG00010018148	XRN2	2	-0.072087269	0.065276049	-1.104344858	0.269443571	0	0.080126265	0	1	CRA004775;Yang2025_Silkie_Wenchang	PRJNA827465	0.77189992
ENSGALG00010018148	XRN2	2	-0.075686807	0.040406223	-1.87314728	0.06104805	0	0.055407788	0	1	CRA004775;PRJNA827465	Yang2025_Silkie_Wenchang	0.42722323
ENSGALG00010018149	ENSGALG00010018149	2	0.033665866	0.183212114	0.183753493	0.854206842	0.042415022	2.67113899	62.56278674	0.5	PRJNA827465;Yang2025_Silkie_Wenchang	CRA004775	0.963077783
ENSGALG00010018149	ENSGALG00010018149	2	0.235346467	0.165309578	1.42367109	0.154541706	0	8.46892E-05	0	1	CRA004775;Yang2025_Silkie_Wenchang	PRJNA827465	0.647391159
ENSGALG00010018149	ENSGALG00010018149	2	-0.098763727	0.130231543	-0.758370243	0.448229367	0	0.652405479	0	0.5	CRA004775;PRJNA827465	Yang2025_Silkie_Wenchang	0.846796712
ENSGALG00010018150	AAGAB	2	0.006812451	0.053950127	0.126273127	0.899515727	0.000746799	1.147074149	12.82167755	0.5	PRJNA827465;Yang2025_Silkie_Wenchang	CRA004775	0.976081354
ENSGALG00010018150	AAGAB	2	-0.05684084	0.146467293	-0.388078722	0.697957777	0.030373539	3.105629097	67.80040472	0.5	CRA004775;Yang2025_Silkie_Wenchang	PRJNA827465	0.921498664
ENSGALG00010018150	AAGAB	2	-0.09273555	0.082299182	-1.126810107	0.259822777	0.004029492	1.282041162	21.99938429	1	CRA004775;PRJNA827465	Yang2025_Silkie_Wenchang	0.767047318
ENSGALG00010018157	FBXL2	2	0.01999669	0.097339158	0.205433153	0.83723372	0	0.165439199	0	0.5	PRJNA827465;Yang2025_Silkie_Wenchang	CRA004775	0.958767556
ENSGALG00010018157	FBXL2	2	-0.204792407	0.298351132	-0.686414046	0.49245205	0.139587853	4.328073819	76.89503364	0.5	CRA004775;Yang2025_Silkie_Wenchang	PRJNA827465	0.859260507
ENSGALG00010018157	FBXL2	2	-0.243256164	0.257706379	-0.943927603	0.345206646	0.092070579	3.068491009	67.41069154	1	CRA004775;PRJNA827465	Yang2025_Silkie_Wenchang	0.807022174
ENSGALG00010018160	ENSGALG00010018160	2	0.450296832	0.308422099	1.46000184	0.144289568	0.165101218	7.537441788	86.7328992	1	PRJNA827465;Yang2025_Silkie_Wenchang	CRA004775	0.631870428
ENSGALG00010018160	ENSGALG00010018160	2	0.098434895	0.691955444	0.142256117	0.886877707	0.894852016	14.98818259	93.32807701	0.5	CRA004775;Yang2025_Silkie_Wenchang	PRJNA827465	0.972850601
ENSGALG00010018160	ENSGALG00010018160	2	-0.184337862	0.380560011	-0.48438579	0.628112101	0.23529049	4.957965905	79.83043815	0.5	CRA004775;PRJNA827465	Yang2025_Silkie_Wenchang	0.901904489
ENSGALG00010018162	ENSGALG00010018162	2	0.52523844	0.222067678	2.365217869	0.018019467	0	0.003966778	0	1	PRJNA827465;Yang2025_Silkie_Wenchang	CRA004775	0.2239074
ENSGALG00010018162	ENSGALG00010018162	2	0.461458846	0.3499158	1.318771103	0.187245648	0	0.116555382	0	1	CRA004775;Yang2025_Silkie_Wenchang	PRJNA827465	0.696113856
ENSGALG00010018162	ENSGALG00010018162	2	0.481923674	0.237091855	2.032645418	0.042088359	0	0.114519538	0	1	CRA004775;PRJNA827465	Yang2025_Silkie_Wenchang	0.355054473
ENSGALG00010018163	CCT7	2	0.022942817	0.047111987	0.486984698	0.626269175	0	0.569316302	0	0.5	PRJNA827465;Yang2025_Silkie_Wenchang	CRA004775	0.901504244
ENSGALG00010018163	CCT7	2	-0.161389515	0.12554494	-1.28551191	0.198613457	0.011936275	1.568556427	36.24711342	1	CRA004775;Yang2025_Silkie_Wenchang	PRJNA827465	0.710809025
ENSGALG00010018163	CCT7	2	-0.103487377	0.173649612	-0.595955131	0.551205202	0.04761297	4.129082663	75.78154564	0.5	CRA004775;PRJNA827465	Yang2025_Silkie_Wenchang	0.878879109
ENSGALG00010018165	DACH2	2	0.113231766	0.139287722	0.812934293	0.416255732	0.020268959	1.857454341	46.16287583	1	PRJNA827465;Yang2025_Silkie_Wenchang	CRA004775	0.837742614
ENSGALG00010018165	DACH2	2	0.281728361	0.173359526	1.625110359	0.104139046	0	0.106550361	0	1	CRA004775;Yang2025_Silkie_Wenchang	PRJNA827465	0.548509371
ENSGALG00010018165	DACH2	2	0.025204511	0.090254399	0.279260752	0.780044724	0	0.177644193	0	1	CRA004775;PRJNA827465	Yang2025_Silkie_Wenchang	0.945041817
ENSGALG00010018166	MSS51	2	-0.396338297	0.14028867	-2.825162556	0.004725663	0	0.364535063	0	1	PRJNA827465;Yang2025_Silkie_Wenchang	CRA004775	0.101336428
ENSGALG00010018166	MSS51	2	-0.489311927	0.2486976	-1.967497584	0.049125874	0	0.213054121	0	1	CRA004775;Yang2025_Silkie_Wenchang	PRJNA827465	0.383342347
ENSGALG00010018166	MSS51	2	-0.335460076	0.160782761	-2.086418187	0.036940759	0	0.047914791	0	1	CRA004775;PRJNA827465	Yang2025_Silkie_Wenchang	0.331631487
ENSGALG00010018172	ACTL6A	2	-0.216324638	0.254641767	-0.849525358	0.395589026	0.121220868	15.2551355	93.44483043	0.5	PRJNA827465;Yang2025_Silkie_Wenchang	CRA004775	0.831031125
ENSGALG00010018172	ACTL6A	2	-0.449871556	0.097738322	-4.60281643	4.16816E-06	0	0.536893314	0	1	CRA004775;Yang2025_Silkie_Wenchang	PRJNA827465	0.000709182
ENSGALG00010018172	ACTL6A	2	0.014407775	0.076197618	0.189084328	0.850026724	0	0.885861967	0	0.5	CRA004775;PRJNA827465	Yang2025_Silkie_Wenchang	0.961869125
ENSGALG00010018174	BMP4	2	0.080522171	0.157806549	0.510258742	0.609870203	0.023939814	1.87784786	46.74754962	0.5	PRJNA827465;Yang2025_Silkie_Wenchang	CRA004775	0.897238578
ENSGALG00010018174	BMP4	2	0.255325111	0.166468683	1.533772637	0.125085597	0	0.017077824	0	1	CRA004775;Yang2025_Silkie_Wenchang	PRJNA827465	0.593686207
ENSGALG00010018174	BMP4	2	-0.01753778	0.127681165	-0.137356044	0.890749374	0	0.505557936	0	0.5	CRA004775;PRJNA827465	Yang2025_Silkie_Wenchang	0.973477891
ENSGALG00010018177	KIF20B	2	0.057155113	0.137440883	0.415852343	0.677518054	0.02483706	2.914322342	65.68670577	0.5	PRJNA827465;Yang2025_Silkie_Wenchang	CRA004775	0.917148587
ENSGALG00010018177	KIF20B	2	0.33786042	0.254437405	1.327872446	0.184220249	0.070063536	1.623080758	38.38877117	1	CRA004775;Yang2025_Silkie_Wenchang	PRJNA827465	0.692545576
ENSGALG00010018177	KIF20B	2	0.250058046	0.424772193	0.588687419	0.556070979	0.275108817	3.470505428	71.18575318	0.5	CRA004775;PRJNA827465	Yang2025_Silkie_Wenchang	0.880324407
ENSGALG00010018179	PRADC1	2	-0.035042492	0.079012577	-0.44350524	0.65740033	0	0.027547786	0	1	PRJNA827465;Yang2025_Silkie_Wenchang	CRA004775	0.910711339
ENSGALG00010018179	PRADC1	2	-0.237284533	0.236698287	-1.002476764	0.316113383	0.062944905	2.281783566	56.17463395	1	CRA004775;Yang2025_Silkie_Wenchang	PRJNA827465	0.796883862
ENSGALG00010018179	PRADC1	2	-0.208682783	0.211559851	-0.986400692	0.323936526	0.066041013	3.34540703	70.1082711	1	CRA004775;PRJNA827465	Yang2025_Silkie_Wenchang	0.799600434
ENSGALG00010018185	GNB4	2	-0.366046867	0.345367222	-1.059877266	0.289200439	0.233315517	44.9681352	97.77620309	1	PRJNA827465;Yang2025_Silkie_Wenchang	CRA004775	0.783530627
ENSGALG00010018185	GNB4	2	-0.387482652	0.370860053	-1.044821756	0.296105358	0.23698091	6.652835318	84.96881477	0.5	CRA004775;Yang2025_Silkie_Wenchang	PRJNA827465	0.788203784
ENSGALG00010018185	GNB4	2	-0.025321018	0.036674016	-0.690434827	0.489920782	0	0.041332044	0	0.5	CRA004775;PRJNA827465	Yang2025_Silkie_Wenchang	0.858512413
ENSGALG00010018188	UBP1	2	-0.054112698	0.026852149	-2.015209234	0.04388273	0	0.115096786	0	1	PRJNA827465;Yang2025_Silkie_Wenchang	CRA004775	0.361804689
ENSGALG00010018188	UBP1	2	-0.065065892	0.045395813	-1.4333016	0.151771665	0	0.037055801	0	1	CRA004775;Yang2025_Silkie_Wenchang	PRJNA827465	0.643294424
ENSGALG00010018188	UBP1	2	-0.047135341	0.03251095	-1.449829714	0.147106011	0	0.01096701	0	1	CRA004775;PRJNA827465	Yang2025_Silkie_Wenchang	0.635841588
ENSGALG00010018189	MIS18BP1	2	0.345890018	0.300122564	1.152495879	0.249117362	0.165017243	11.74268731	91.48406175	1	PRJNA827465;Yang2025_Silkie_Wenchang	CRA004775	0.759411487
ENSGALG00010018189	MIS18BP1	2	0.349842363	0.375807227	0.930909089	0.351900593	0.213731529	3.65840208	72.66566172	0.5	CRA004775;Yang2025_Silkie_Wenchang	PRJNA827465	0.810678834
ENSGALG00010018189	MIS18BP1	2	0.046305378	0.091483379	0.506161653	0.612743173	0	0.187121837	0	0.5	CRA004775;PRJNA827465	Yang2025_Silkie_Wenchang	0.898034557
ENSGALG00010018192	IQCH	2	0.340700515	0.404616982	0.84203217	0.399769947	0.28740335	8.180016621	87.77508596	0.5	PRJNA827465;Yang2025_Silkie_Wenchang	CRA004775	0.832246851
ENSGALG00010018192	IQCH	2	0.255175428	0.565583223	0.451172201	0.651865443	0.52025854	4.930698753	79.71889888	0.5	CRA004775;Yang2025_Silkie_Wenchang	PRJNA827465	0.909197187
ENSGALG00010018192	IQCH	2	-0.112690522	0.182777491	-0.616544854	0.537534978	0	0.421912302	0	1	CRA004775;PRJNA827465	Yang2025_Silkie_Wenchang	0.87481821
ENSGALG00010018193	ANK1	2	0.111725808	0.22334365	0.500241703	0.616904897	0.080277754	4.976330708	79.90487251	0.5	PRJNA827465;Yang2025_Silkie_Wenchang	CRA004775	0.899111936
ENSGALG00010018193	ANK1	2	-0.066669111	0.495122609	-0.13465172	0.892887247	0.394016985	4.608689026	78.30185559	0.5	CRA004775;Yang2025_Silkie_Wenchang	PRJNA827465	0.974381724
ENSGALG00010018193	ANK1	2	-0.210768167	0.226431216	-0.93082646	0.35194334	0.053191864	1.527758579	34.544632	1	CRA004775;PRJNA827465	Yang2025_Silkie_Wenchang	0.810678834
ENSGALG00010018198	BTBD3	2	-0.119752611	0.087093095	-1.374995467	0.16913285	0.010425701	3.167959208	68.43393698	1	PRJNA827465;Yang2025_Silkie_Wenchang	CRA004775	0.670373981
ENSGALG00010018198	BTBD3	2	0.056217977	0.31350321	0.179321854	0.857684988	0.164802143	5.52396238	81.89705267	0.5	CRA004775;Yang2025_Silkie_Wenchang	PRJNA827465	0.964067753
ENSGALG00010018198	BTBD3	2	0.123015682	0.21939896	0.560694006	0.575006154	0.070289065	2.983374369	66.48090798	0.5	CRA004775;PRJNA827465	Yang2025_Silkie_Wenchang	0.886281107
ENSGALG00010018199	BORCS7	2	-0.037863595	0.048434455	-0.781749176	0.434361995	0	0.303000516	0	1	PRJNA827465;Yang2025_Silkie_Wenchang	CRA004775	0.842762754
ENSGALG00010018199	BORCS7	2	-0.287844499	0.23451954	-1.227379598	0.219679951	0.090202802	5.221220791	80.84739106	1	CRA004775;Yang2025_Silkie_Wenchang	PRJNA827465	0.733094397
ENSGALG00010018199	BORCS7	2	-0.2569977	0.263962018	-0.973616212	0.330247134	0.122132384	7.442476065	86.56361147	1	CRA004775;PRJNA827465	Yang2025_Silkie_Wenchang	0.801364642
ENSGALG00010018206	KIZ	2	0.166461301	0.159289383	1.045024458	0.296011666	0.031203106	2.45724132	59.30395636	1	PRJNA827465;Yang2025_Silkie_Wenchang	CRA004775	0.788124569
ENSGALG00010018206	KIZ	2	0.297040753	0.155404712	1.911401192	0.055953044	0	0.538708648	0	1	CRA004775;Yang2025_Silkie_Wenchang	PRJNA827465	0.409290467
ENSGALG00010018206	KIZ	2	0.040049383	0.101806293	0.393388091	0.694032864	0	0.024034199	0	1	CRA004775;PRJNA827465	Yang2025_Silkie_Wenchang	0.920728119
ENSGALG00010018209	GMPPA	2	0.002756345	0.124776301	0.022090291	0.982375931	0.022825872	3.329250159	69.96320636	0.5	PRJNA827465;Yang2025_Silkie_Wenchang	CRA004775	0.99513702
ENSGALG00010018209	GMPPA	2	-0.442392026	0.311637634	-1.419571891	0.155732353	0.1645436	6.362374312	84.28259717	1	CRA004775;Yang2025_Silkie_Wenchang	PRJNA827465	0.648892306
ENSGALG00010018209	GMPPA	2	-0.313221394	0.439514332	-0.712653424	0.476060244	0.363752477	16.33594013	93.87852801	0.5	CRA004775;PRJNA827465	Yang2025_Silkie_Wenchang	0.854810196
ENSGALG00010018215	CFAP70	2	-0.282731003	0.358553258	-0.788532794	0.430385124	0.222769925	7.475193546	86.62241996	0.5	PRJNA827465;Yang2025_Silkie_Wenchang	CRA004775	0.842150081
ENSGALG00010018215	CFAP70	2	-0.043733608	0.218647942	-0.200018384	0.841466204	0.030184284	1.27096476	21.31961233	0.5	CRA004775;Yang2025_Silkie_Wenchang	PRJNA827465	0.95989279
ENSGALG00010018215	CFAP70	2	-0.615830624	0.17827324	-3.454419882	0.000551478	0	0.148791541	0	1	CRA004775;PRJNA827465	Yang2025_Silkie_Wenchang	0.025057505
ENSGALG00010018216	FANCM	2	0.117393675	0.110155359	1.065710065	0.286554726	0.01983027	5.457720953	81.67733366	1	PRJNA827465;Yang2025_Silkie_Wenchang	CRA004775	0.782018115
ENSGALG00010018216	FANCM	2	0.163521474	0.125910187	1.298715211	0.194041682	0.015302358	1.488687133	32.82671838	0.5	CRA004775;Yang2025_Silkie_Wenchang	PRJNA827465	0.704584278
ENSGALG00010018216	FANCM	2	0.004051356	0.06077526	0.066661276	0.946851363	0	0.117191351	0	0.5	CRA004775;PRJNA827465	Yang2025_Silkie_Wenchang	0.986445196
ENSGALG00010018226	JAG1	2	0.003784696	0.056517586	0.066964932	0.946609621	0	0.012126195	0	1	PRJNA827465;Yang2025_Silkie_Wenchang	CRA004775	0.986445196
ENSGALG00010018226	JAG1	2	0.210798543	0.254905115	0.826968668	0.408254821	0.081295248	2.373205659	57.86290175	1	CRA004775;Yang2025_Silkie_Wenchang	PRJNA827465	0.834134074
ENSGALG00010018226	JAG1	2	0.188995221	0.259864581	0.727283497	0.467052305	0.099047359	2.952347299	66.12864617	1	CRA004775;PRJNA827465	Yang2025_Silkie_Wenchang	0.852885838

ENSGALG00010018229	AS3MT	2	-0.500511564	0.111004181	-4.508943332	6.51513E-06	0	0.972871825	0	1	PRJNA827465;Yang2025_Silkie_Wenchang	CRA004775	0.001012111
ENSGALG00010018229	AS3MT	2	-0.401375169	0.188384319	-2.130618791	0.03312056	0	0.561448733	0	1	CRA004775;Yang2025_Silkie_Wenchang	PRJNA827465	0.312842437
ENSGALG00010018229	AS3MT	2	-0.563919381	0.117128181	-4.814549133	1.47533E-06	0	0.009308335	0	1	CRA004775;PRJNA827465	Yang2025_Silkie_Wenchang	0.000304425
ENSGALG00010018231	SMYD5	2	-0.021764427	0.062931899	-0.345840937	0.729462258	0	0.002523223	0	1	PRJNA827465;Yang2025_Silkie_Wenchang	CRA004775	0.9323457
ENSGALG00010018231	SMYD5	2	-0.288370742	0.250781	-1.149890706	0.250188889	0.104433441	5.860475329	82.93653767	1	CRA004775;Yang2025_Silkie_Wenchang	PRJNA827465	0.75993411
ENSGALG00010018231	SMYD5	2	-0.263325845	0.254991775	-1.03268368	0.301751953	0.11934352	11.84486626	91.55752392	1	CRA004775;PRJNA827465	Yang2025_Silkie_Wenchang	0.791367646
ENSGALG00010018233	RASSF2	2	0.375944818	0.076103724	4.939900408	7.81625E-07	0	0.261885947	0	1	PRJNA827465;Yang2025_Silkie_Wenchang	CRA004775	0.000182858
ENSGALG00010018233	RASSF2	2	0.022576607	0.468707671	0.048167778	0.961582529	0.371204472	5.948855165	83.19004292	0.5	CRA004775;Yang2025_Silkie_Wenchang	PRJNA827465	0.98973612
ENSGALG00010018233	RASSF2	2	-0.007647141	0.423642246	-0.01805094	0.985598216	0.299817248	5.331323893	81.2429329	0.5	CRA004775;PRJNA827465	Yang2025_Silkie_Wenchang	0.995939127
ENSGALG00010018240	SOUL	2	0.181831655	0.28717317	0.633177727	0.526617589	0	0.282587721	0	1	PRJNA827465;Yang2025_Silkie_Wenchang	CRA004775	0.87066367
ENSGALG00010018240	SOUL	2	0.082470786	0.366891455	0.224782521	0.822148464	0	0.096607662	0	1	CRA004775;Yang2025_Silkie_Wenchang	PRJNA827465	0.955636543
ENSGALG00010018240	SOUL	2	0.317043478	0.349143723	0.908060084	0.363846478	0	0.001926222	0	1	CRA004775;PRJNA827465	Yang2025_Silkie_Wenchang	0.817042253
ENSGALG00010018241	RALGAPA2	2	-0.050104804	0.058341469	-0.858819724	0.390439984	0	0.406832206	0	1	PRJNA827465;Yang2025_Silkie_Wenchang	CRA004775	0.829251057
ENSGALG00010018241	RALGAPA2	2	-0.115493481	0.108609884	-1.063379107	0.287610064	0	0.007388525	0	1	CRA004775;Yang2025_Silkie_Wenchang	PRJNA827465	0.782840067
ENSGALG00010018241	RALGAPA2	2	-0.031961772	0.066737577	-0.478917176	0.631997551	0	0.102431945	0	1	CRA004775;PRJNA827465	Yang2025_Silkie_Wenchang	0.903058174
ENSGALG00010018244	CHM	2	-0.168397354	0.061167747	-2.753041638	0.005904439	0.001820798	1.294695082	22.76173645	1	PRJNA827465;Yang2025_Silkie_Wenchang	CRA004775	0.11608912
ENSGALG00010018244	CHM	2	0.009753873	0.27876445	0.03498966	0.972087986	0.135785717	7.585890135	86.81763139	0.5	CRA004775;Yang2025_Silkie_Wenchang	PRJNA827465	0.991955348
ENSGALG00010018244	CHM	2	0.06181813	0.21381549	0.289119046	0.772490279	0.075074902	5.039494307	80.15673917	0.5	CRA004775;PRJNA827465	Yang2025_Silkie_Wenchang	0.943066211
ENSGALG00010018251	USP48	2	0.014957479	0.066995465	0.223261072	0.823332323	0.006301028	3.066735739	67.3920388	0.5	PRJNA827465;Yang2025_Silkie_Wenchang	CRA004775	0.955918024
ENSGALG00010018251	USP48	2	0.08580153	0.06330587	1.355348723	0.175306479	0	0.137704498	0	1	CRA004775;Yang2025_Silkie_Wenchang	PRJNA827465	0.680258136
ENSGALG00010018251	USP48	2	-0.035820685	0.033469522	-1.07024788	0.284507748	0	0.254128804	0	0.5	CRA004775;PRJNA827465	Yang2025_Silkie_Wenchang	0.780265906
ENSGALG00010018255	MFN1	2	-0.116904865	0.044138308	-2.648603201	0.008082517	0	0.393731582	0	1	PRJNA827465;Yang2025_Silkie_Wenchang	CRA004775	0.139633915
ENSGALG00010018255	MFN1	2	0.020442151	0.22609327	0.090414681	0.92795769	0.074608351	2.975218217	66.38901999	0.5	CRA004775;Yang2025_Silkie_Wenchang	PRJNA827465	0.982993195
ENSGALG00010018255	MFN1	2	0.038463122	0.193072386	0.199216068	0.842093731	0.049981465	2.327053231	57.02719703	0.5	CRA004775;PRJNA827465	Yang2025_Silkie_Wenchang	0.960064308
ENSGALG00010018258	OSCP1	2	0.129995448	0.10900835	1.192537437	0.233054499	0	0.216023361	0	1	PRJNA827465;Yang2025_Silkie_Wenchang	CRA004775	0.746089515
ENSGALG00010018258	OSCP1	2	-0.157875784	0.313349417	-0.503833024	0.614378719	0.105554483	1.867067197	46.44006379	0.5	CRA004775;Yang2025_Silkie_Wenchang	PRJNA827465	0.898523733
ENSGALG00010018258	OSCP1	2	-0.107398522	0.372929595	-0.287986053	0.773357415	0.195575983	2.755048762	63.70300178	0.5	CRA004775;PRJNA827465	Yang2025_Silkie_Wenchang	0.943245453
ENSGALG00010018259	WDR90	2	-0.210653022	0.070177587	-3.001713675	0.002684646	0	0.076504173	0	1	PRJNA827465;Yang2025_Silkie_Wenchang	CRA004775	0.070390009
ENSGALG00010018259	WDR90	2	-0.308621346	0.171661311	-1.797858285	0.072195189	0.02646477	1.725236875	42.03694493	1	CRA004775;Yang2025_Silkie_Wenchang	PRJNA827465	0.463869134
ENSGALG00010018259	WDR90	2	-0.301979436	0.135694881	-2.225429825	0.026052397	0.018409557	1.625373495	38.47567942	1	CRA004775;PRJNA827465	Yang2025_Silkie_Wenchang	0.275325993
ENSGALG00010018261	PRNP	2	-0.137779696	0.138327596	-0.996039116	0.31923114	0.029746	4.377444397	77.15562074	1	PRJNA827465;Yang2025_Silkie_Wenchang	CRA004775	0.798079048
ENSGALG00010018261	PRNP	2	-0.182551512	0.17589121	-1.037866035	0.299332428	0.030054028	1.612073997	37.96810807	0.5	CRA004775;Yang2025_Silkie_Wenchang	PRJNA827465	0.793858093
ENSGALG00010018261	PRNP	2	-0.003645061	0.071883014	-0.050708241	0.95955801	0	0.15776779	0	0.5	CRA004775;PRJNA827465	Yang2025_Silkie_Wenchang	0.989259322
ENSGALG00010018263	SLX4IP	2	0.176745035	0.155454268	1.136958397	0.255555678	0.029851163	2.452258584	59.2212662	1	PRJNA827465;Yang2025_Silkie_Wenchang	CRA004775	0.764467569
ENSGALG00010018263	SLX4IP	2	0.366760539	0.16723441	2.193092551	0.028300712	0	0.00031584	0	1	CRA004775;Yang2025_Silkie_Wenchang	PRJNA827465	0.286649306
ENSGALG00010018263	SLX4IP	2	0.060222929	0.099964474	0.602443315	0.546879084	0	0.298252117	0	1	CRA004775;PRJNA827465	Yang2025_Silkie_Wenchang	0.878275987
ENSGALG00010018264	CFAP61	2	0.331131119	0.297769783	1.112037346	0.266122093	0	0.069251855	0	1	PRJNA827465;Yang2025_Silkie_Wenchang	CRA004775	0.770092601
ENSGALG00010018264	CFAP61	2	0.26830845	0.461747742	0.581071493	0.561192271	0	0.037600038	0	1	CRA004775;Yang2025_Silkie_Wenchang	PRJNA827465	0.881506202
ENSGALG00010018264	CFAP61	2	0.377674382	0.312819495	1.207323675	0.227307516	0	2.40161E-05	0	1	CRA004775;PRJNA827465	Yang2025_Silkie_Wenchang	0.740605003
ENSGALG00010018265	PDCD4	2	0.039911067	0.080655779	0.494832078	0.620718659	0.006594609	1.979517189	49.48263113	0.5	PRJNA827465;Yang2025_Silkie_Wenchang	CRA004775	0.899393593
ENSGALG00010018265	PDCD4	2	0.026432455	0.145363503	0.181836946	0.855710689	0.023941077	2.07652672	51.84266158	0.5	CRA004775;Yang2025_Silkie_Wenchang	PRJNA827465	0.963486168
ENSGALG00010018265	PDCD4	2	-0.045442562	0.064317357	-0.70653653	0.479854544	0	0.489993474	0	1	CRA004775;PRJNA827465	Yang2025_Silkie_Wenchang	0.856284002
ENSGALG00010018267	ADPRHL2	2	-0.109845743	0.099232497	-1.106953335	0.268314105	0.009430143	1.383472762	27.70261304	1	PRJNA827465;Yang2025_Silkie_Wenchang	CRA004775	0.771398294
ENSGALG00010018267	ADPRHL2	2	-0.223092853	0.146885831	-1.518818064	0.128808298	0	0.332059756	0	1	CRA004775;Yang2025_Silkie_Wenchang	PRJNA827465	0.602157487
ENSGALG00010018267	ADPRHL2	2	-0.057541685	0.071815052	-0.801248258	0.422987939	0	0.039423542	0	1	CRA004775;PRJNA827465	Yang2025_Silkie_Wenchang	0.840012882
ENSGALG00010018274	ZNF711	2	0.283007074	0.257250365	1.10012312	0.271278482	0.124219737	15.9146906	93.71649739	1	PRJNA827465;Yang2025_Silkie_Wenchang	CRA004775	0.772743863
ENSGALG00010018274	ZNF711	2	0.301206336	0.265207079	1.13574018	0.256065319	0.118980155	6.34856428	84.24840711	1	CRA004775;Yang2025_Silkie_Wenchang	PRJNA827465	0.764570254
ENSGALG00010018274	ZNF711	2	0.033812172	0.05441017	0.621431107	0.53431601	0	0.008001813	0	1	CRA004775;PRJNA827465	Yang2025_Silkie_Wenchang	0.873408008
ENSGALG00010018275	ZNF639	2	-0.102191677	0.091737796	-1.113953915	0.265298958	0.010055826	2.149154194	53.47006731	1	PRJNA827465;Yang2025_Silkie_Wenchang	CRA004775	0.76929776
ENSGALG00010018275	ZNF639	2	-0.110196367	0.170481855	-0.646381911	0.518032052	0.028712639	1.781516345	43.86804237	0.5	CRA004775;Yang2025_Silkie_Wenchang	PRJNA827465	0.868202369
ENSGALG00010018275	ZNF639	2	-0.029368845	0.050411347	-0.582584018	0.560173367	0	0.459762954	0	0.5	CRA004775;PRJNA827465	Yang2025_Silkie_Wenchang	0.881203589
ENSGALG00010018276	ARHGAP17	2	0.363425592	0.435074565	0.835317945	0.403538716	0.344961905	10.42508223	90.40774952	0.5	PRJNA827465;Yang2025_Silkie_Wenchang	CRA004775	0.83306585
ENSGALG00010018276	ARHGAP17	2	0.580877682	0.239861797	2.42171821	0.015447324	0.053736741	1.867562446	46.45426705	1	CRA004775;Yang2025_Silkie_Wenchang	PRJNA827465	0.205526799
ENSGALG00010018276	ARHGAP17	2	0.097000317	0.185372989	0.523271042	0.60078565	0.048853612	2.732081104	63.39786552	0.5	CRA004775;PRJNA827465	Yang2025_Silkie_Wenchang	0.893520519
ENSGALG00010018278	PUSL1	2	0.45850372	0.514694764	0.89082647	0.373022272	0.504225157	20.64108126	95.15529256	0.5	PRJNA827465;Yang2025_Silkie_Wenchang	CRA004775	0.822162024
ENSGALG00010018278	PUSL1	2	0.426701595	0.572910462	0.744796305	0.456394869	0.60168271	11.8180744	91.53838463	0.5	CRA004775;Yang2025_Silkie_Wenchang	PRJNA827465	0.849515887
ENSGALG00010018278	PUSL1	2	-0.073047534	0.124674712	-0.585904977	0.557939373	0	0.139111604	0	1	CRA004775;PRJNA827465	Yang2025_Silkie_Wenchang	0.880678789
ENSGALG00010018283	POF1B	2	0.001576156	0.11662693	0.013514508	0.989217311	0	0.057632613	0	0.5	PRJNA827465;Yang2025_Silkie_Wenchang	CRA004775	0.997081732
ENSGALG00010018283	POF1B	2	0.490501605	0.610981808	0.802808854	0.422085224	0.611791871	5.063362867	80.25027978	0.5	CRA004775;Yang2025_Silkie_Wenchang	PRJNA827465	0.839649223
ENSGALG00010018283	POF1B	2	0.506975025	0.578748667	0.875984782	0.381038307	0.550448842	4.924359373	79.69278998	1	CRA004775;PRJNA827465	Yang2025_Silkie_Wenchang	0.824392515
ENSGALG00010018284	ENSGALG00010018284	2	0.182443429	0.267394582	0.682315353	0.495039567	0.130649475	11.54035178	91.33475288	0.5	PRJNA827465;Yang2025_Silkie_Wenchang	CRA004775	0.869696668
ENSGALG00010018284	ENSGALG00010018284	2	0.256372556	0.330756146	0.775110483	0.438274377	0.133772895	1.90672833	47.5541437	0.5	CRA004775;Yang2025_Silkie_Wenchang	PRJNA827465	0.843979791
ENSGALG00010018284	ENSGALG00010018284	2	-0.087567002	0.097596096	-0.897238773	0.36959152	0	0.159697903	0	1	CRA004775;PRJNA827465	Yang2025_Silkie_Wenchang	0.820606801
ENSGALG00010018285	BBIP1	2	0.141804908	0.19022084	0.745475141	0.455984534	0.056498987	4.262570317	76.53997645	0.5	PRJNA827465;Yang2025_Silkie_Wenchang	CRA004775	0.84907427
ENSGALG00010018285	BBIP1	2	0.297397147	0.141184764	2.368507327	0.017860027	0	0.090460698	0	1	CRA004775;Yang2025_Silkie_Wenchang	PRJNA827465	0.223125437
ENSGALG00010018285	BBIP1	2	0.010202971	0.092744141	0.110012023	0.91239984	0.002753172	1.070277989	6.566330435	0.5	CRA004775;PRJNA827465	Yang2025_Silkie_Wenchang	0.978967245
ENSGALG00010018287	MKKS	2	-0.150626569	0.186670557	-0.80691123	0.419717628	0.064993712	14.62667751	93.16317736	0.5	PRJNA827465;Yang2025_Silkie_Wenchang	CRA004775	0.838527387
ENSGALG00010018287	MKKS	2	-0.275766683	0.092697023	-2.974924922	0.002930603	0.006160114	1.481440125	32.49811562	1	CRA004775;Yang2025_Silkie_Wenchang	PRJNA827465	0.074041237
ENSGALG00010018287	MKKS	2	-0.014373689	0.077334037	-0.185869377	0.85255065	0.005427795	1.51391746	33.94620076	0.5	CRA004775;PRJNA827465	Yang2025_Silkie_Wenchang	0.965057579
ENSGALG00010018288	CRNKL1	2	0.030974672	0.034705555	0.892498969	0.372125545	0	0.499014845	0	0.5	PRJNA827465;Yang2025_Silkie_Wenchang	CRA004775	0.821620523
ENSGALG00010018288	CRNKL1	2	0.094455126	0.12975863	0.727929433	0.466656785	0.02302364	2.802495309	64.31751387	0.5	CRA004775;Yang2025_Silkie_Wenchang	PRJNA827465	0.852842649
ENSGALG00010018288	CRNKL1	2	0.107634446	0.096449715	1.115964375	0.264437384	0.011277561	1.972107681	49.29282971	1	CRA004775;PRJNA827465	Yang2025_Silkie_Wenchang	0.768790489
ENSGALG00010018289	HEYL	2	0.194636118	0.209611578	0.928556141	0.35311916	0.075792352	7.19538003	86.10219341	0.5	PRJNA827465;Yang2025_Silkie_Wenchang	CRA004775	0.81107437
ENSGALG00010018289	HEYL	2	0.334500787	0.159395692	2.098556017	0.035856057	0.015700517	1.241617223	19.45988008	1	CRA004775;Yang2025_Silkie_Wenchang	PRJNA827465	0.326896453
ENSGALG00010018289	HEYL	2	-0.005187909	0.089308661	-0.058089657	0.953677213	0	0.002608262	0	0.5	CRA004775;PRJNA827465	Yang2025_Silkie_Wenchang	0.987834059
ENSGALG00010018290	RHOT2	2	0.057624768	0.057059538	1.009905973	0.312540341	0	0.879721854	0	1	PRJNA827465;Yang2025_Silkie_Wenchang	CRA004775	0.796054997
ENSGALG00010018290	RHOT2	2	-0.104200561	0.304905285	-0.341747309	0.732541066	0.152195584	5.335022447	81.25593641	0.5	CRA004775;Yang2025_Silkie_Wenchang	PRJNA827465	0.932985777
ENSGALG00010018290	RHOT2	2	-0.151279457	0.22944085	-0.659339681	0.509677666	0.082809247	4.00853938	75.05325743	0.5	CRA004775;PRJNA827465	Yang2025_Silkie_Wenchang	0.865257661
ENSGALG00010018294	FKBP3	2	0.176612778	0.293280013	0.602198479	0.547042028	0.157866143	11.71841807	91.46642495	0.5	PRJNA827465;Yang2025_Silkie_Wenchang	CRA004775	0.878281266
ENSGALG00010018294	FKBP3	2	0.392419787	0.170940016	2.295657838	0.021693453	0.013577814	1.200773791	16.7203675	1	CRA004775;Yang2025_Silkie_Wenchang	PRJNA827465	0.249472519
ENSGALG00010018294	FKBP3	2	-0.09258294	0.06799278	-1.361658396	0.173305717	0	0.294939677	0	0.5	CRA004775;PRJNA827465	Yang2025_Silkie_Wenchang	0.676811862
ENSGALG00010018296	PIK3CA	2	-0.106531371	0.050389592	-2.114154273	0.034502093	0	0.402972843	0	1	PRJNA827465;Yang2025_Silkie_Wenchang	CRA004775	0.319895202
ENSGALG00010018296	PIK3CA	2	0.101375281	0.325677286	0.311275259	0.755591374	0.16981284	4.665987405	78.56179416	0.5	CRA004775;Yang2025_Silkie_Wenchang	PRJNA827465	0.938501652
ENSGALG00010018296	PIK3CA	2	0.126836424	0.276651314	0.458470346	0.646641452	0.121913017	4.097128643	75.59266288	0.5	CRA004775;PRJNA827465	Yang2025_Silkie_Wenchang	0.907347358
ENSGALG00010018298	ENSGALG00010018298	2	0.460721121	0.308516691	1.493342609	0.135347527	0.166267922	7.870658182	87.29458227	1	PRJNA827465;Yang2025_Silkie_Wenchang	CRA004775	0.616776509
ENSGALG00010018298	ENSGALG00010018298	2	1.158573163	0.464454101	2.494483652	0.012614054	0.333093195	3.896689151	74.33718828	1	CRA004775;Yang2025_Silkie_Wenchang	PRJNA827465	0.181934001
ENSGALG00010018298	ENSGALG00010018298	2	0.883156175	0.779772412	1.12750974	0.25738984	1.112259169	11.0595536	90.95771813	1	CRA004775;PRJNA827465	Yang2025_Silkie_Wenchang	0.764987374
ENSGALG00010018299	SHOC2	2	0.025294235	0.045273613	0.558697071	0.576368479	0	0.248288278	0	0.5	PRJNA827465;Yang2025_Silkie_Wenchang	CRA004775	0.887048016
ENSGALG00010018299	SHOC2	2	0.011215236	0.080362808	0.139557546	0.889009586	0	0.511388814	0	0.5	CRA004775;Yang2025_Silkie_Wenchang	PRJNA827465	0.973135755
ENSGALG00010018299	SHOC2	2	0.045728223	0.051685289	0.884743493	0.376294992	0	0.274070657	0	1	CRA004775;PRJNA827465	Yang2025_Silkie_Wenchang	0.822974013
ENSGALG00010018301	ENSGALG00010018301	2	-0.228420928	0.433388676	-0.527057907	0.598153376	0.24383569	2.733761229	63.42036059	0.5	PRJNA827465;Yang2025_Silkie_Wenchang	CRA004775	0.894645663
ENSGALG00010018301	ENSGALG00010018301	2	-0.542213211	0.322695837	-1.680260946	0.092906556	0	0.38533226	0	1	CRA004775;Yang2025_Silkie_Wenchang	PRJNA827465	0.522019616
ENSGALG00010018301	ENSGALG00010018301	2	0.013342298	0.24461688	0.054543653	0.95650203	0	0.752270836	0	0.5	CRA004775;PRJNA827465	Yang2025_Silkie_Wenchang	0.988540618
ENSGALG00010018302	RAB11FIP5	2	-0.073786636	0.138582252	-0.532439288	0.59442178	0.02909017	4.093807346	75.57286126	0.5	PRJNA827465;Yang2025_Silkie_Wenchang	CRA004775	0.909337382
ENSGALG00010018302	RAB11FIP5	2	-0.274718322	0.118565611	-2.317015184	0.020502905	0	0.566588312	0	1	CRA004775;Yang2025_Silkie_Wenchang	PRJNA827465	0.241482068
ENSGALG00010018302	RAB11FIP5	2	-0.136164332	0.253043472	-0.53810648	0.590503539	0.093169456	2.833390234	64.70659113	0.5	CRA004775;PRJNA827465	Yang2025_Silkie_Wenchang	0.892602616
ENSGALG00010018306	DES	2	0.156616966	0.325975463	0.480456305	0.630902966	0.186304372	8.063225732	87.59801557	0.5	PRJNA827465;Yang2025_Silkie_Wenchang	CRA004775	0.902901332
ENSGALG00010018306	DES	2	0.207898586	0.436383295	0.476412796	0.63378032	0.23873847	2.09968399	52.37378553	0.5	CRA004775;Yang2025_Silkie_Wenchang	PRJNA827465	0.903596555
ENSGALG00010018306	DES	2	-0.175209763	0.139740116	-1.253825801	0.209905329	0	0.216755621	0	1	CRA004775;PRJNA827465	Yang2025_Silkie_Wenchang	0.721713686
ENSGALG00010018308	ACAP3	2	0.211853551	0.066476344	3.18690137	0.001438058	0	0.291746443	0	1	PRJNA827465;Yang2025_Silkie_Wenchang	CRA004775	0.04698254
ENSGALG00010018308	ACAP3	2	0.232466414	0.093349953	2.490308247	0.012756454	0	0.619511034	0	0.5	CRA004775;Yang2025_Silkie_Wenchang	PRJNA827465	0.183357994
ENSGALG00010018308	ACAP3	2	0.164920527	0.090310125	1.826157659	0.067826517	0	0.388104865	0	0.5	CRA004775;PRJNA827465	Yang2025_Silkie_Wenchang	0.449101706
ENSGALG00010018309	DPYSL2	2	-0.012820178	0.066646681	-0.192360338	0.847459958	0.001236684	1.161473838	13.90249465	0.5	PRJNA827465;Yang2025_Silkie_Wenchang	CRA004775	0.961252712
ENSGALG00010018309	DPYSL2	2	-0.061818306	0.084977124	-0.727469972	0.466938104	0	0.531439554	0	0.5	CRA004775;Yang2025_Silkie_Wenchang	PRJNA827465	0.85287054
ENSGALG00010018309	DPYSL2	2	0.058280528	0.081766371	0.712769833	0.475988759	0	0.072325982	0	1	CRA004775;PRJNA827465	Yang2025_Silkie_Wenchang	0.854810196
ENSGALG00010018310	APOOL	2	-0.004997732	0.074084854	-0.067459563	0.946215853	0.006068789	2.095560294	52.28006549	0.5	PRJNA827465;Yang2025_Silkie_Wenchang	CRA004775	0.986370359
ENSGALG00010018310	APOOL	2	-0.055540578	0.078507433	-0.7074563	0.47928296	0	0.904631129	0	0.5	CRA004775;Yang2025_Silkie_Wenchang	PRJNA827465	0.856035423
ENSGALG00010018310	APOOL	2	0.055450454	0.050380156	1.100640767	0.271053035	0	0.013947008	0	1	CRA004775;PRJNA827465	Yang2025_Silkie_Wenchang	0.772743863
ENSGALG00010018312	PRPF39	2	0.260961891	0.211967848	1.231139028	0.218270871	0.085582439	20.97962951	95.23347159	1	PRJNA827465;Yang2025_Silkie_Wenchang	CRA004775	0.731988544
ENSGALG00010018312	PRPF39	2	0.29143825	0.230628049	1.263672185	0.206347713	0.081751634	3.689958583	72.89942482	1	CRA004775;Yang2025_Silkie_Wenchang	PRJNA827465	0.718451597
ENSGALG00010018312	PRPF39	2	0.047589917	0.058160013	0.818258369	0.413209672	0	0.041472465	0	1	CRA004775;PRJNA827465	Yang2025_Silkie_Wenchang	0.836055586
ENSGALG00010018313	MYEF2	2	0.201558081	0.201796218	0.998819911	0.317881939	0.07341797	9.562282901	89.54236624	1	PRJNA827465;Yang2025_Silkie_Wenchang	CRA004775	0.797439961
ENSGALG00010018313	MYEF2	2	0.167573732	0.287643356	0.582574666	0.560179664	0.131478111	4.579142927	78.16185221	0.5	CRA004775;Yang2025_Silkie_Wenchang	PRJNA827465	0.881203589
ENSGALG00010018313	MYEF2	2	0.010216405	0.042505309	0.240355969	0.810054309	0	0.510413618	0	0.5	CRA004775;PRJNA827465	Yang2025_Silkie_Wenchang	0.952908565
ENSGALG00010018314	SLC24A3	2	-0.125591432	0.05890545	-2.132085089	0.032999852	0	0.573696326	0	1	PRJNA827465;Yang2025_Silkie_Wenchang	CRA004775	0.312002211
ENSGALG00010018314	SLC24A3	2	-0.410085409	0.233247052	-1.758159023	0.078720455	0.053915217	1.792432352	44.20988894	1	CRA004775;Yang2025_Silkie_Wenchang	PRJNA827465	0.482600227
ENSGALG00010018314	SLC24A3	2	-0.340724517	0.303568959	-1.122395773	0.261694201	0.141977288	3.534497513	71.70743518	1	CRA004775;PRJNA827465	Yang2025_Silkie_Wenchang	0.768310285
ENSGALG00010018315	CLASP2	2	0.013907546	0.116872042	0.118998056	0.905276896	0.019820893	3.579831912	72.06572753	0.5	PRJNA827465;Yang2025_Silkie_Wenchang	CRA004775	0.977980705
ENSGALG00010018315	CLASP2	2	0.136234119	0.092334977	1.475433506	0.140096033	0	0.014794533	0	1	CRA004775;Yang2025_Silkie_Wenchang	PRJNA827465	0.623792706
ENSGALG00010018315	CLASP2	2	-0.076894935	0.070511201	-1.090535035	0.275477529	0	0.628178162	0	0.5	CRA004775;PRJNA827465	Yang2025_Silkie_Wenchang	0.775135547
ENSGALG00010018317	ZMAT3	2	-0.123936053	0.103367674	-1.198982697	0.230534674	0	0.028217463	0	1	PRJNA827465;Yang2025_Silkie_Wenchang	CRA004775	0.743335644
ENSGALG00010018317	ZMAT3	2	-0.140475759	0.134301009	-1.045976948	0.295571676	0	0.000897499	0	1	CRA004775;Yang2025_Silkie_Wenchang	PRJNA827465	0.787628307
ENSGALG00010018317	ZMAT3	2	-0.112607753	0.126859328	-0.887658441	0.374724507	0	0.006071597	0	1	CRA004775;PRJNA827465	Yang2025_Silkie_Wenchang	0.822805106
ENSGALG00010018319	STUB1	2	0.121277971	0.126916501	0.955572913	0.339288051	0.027870979	7.297237379	86.29618377	1	PRJNA827465;Yang2025_Silkie_Wenchang	CRA004775	0.804824608
ENSGALG00010018319	STUB1	2	0.232802128	0.076101662	3.059093893	0.002220076	0.000226089	1.005557097	0.55263865	0.5	CRA004775;Yang2025_Silkie_Wenchang	PRJNA827465	0.062298448
ENSGALG00010018319	STUB1	2	-0.000568547	0.052008343	-0.010931834	0.991277832	0	0.013018178	0	0.5	CRA004775;PRJNA827465	Yang2025_Silkie_Wenchang	0.99755571
ENSGALG00010018322	NT5C1A	2	-0.0621593	0.110670735	-0.561659774	0.574347846	0	0.966043751	0	0.5	PRJNA827465;Yang2025_Silkie_Wenchang	CRA004775	0.886083039
ENSGALG00010018322	NT5C1A	2	-0.159484192	0.142516106	-1.119060833	0.263114188	0	0.023636078	0	1	CRA004775;Yang2025_Silkie_Wenchang	PRJNA827465	0.768643821
ENSGALG00010018322	NT5C1A	2	0.047161138	0.165757783	0.284518395	0.776013139	0	0.206940657	0	0.5	CRA004775;PRJNA827465	Yang2025_Silkie_Wenchang	0.943969187
ENSGALG00010018323	GOT1	2	-0.269736356	0.090664109	-2.975117272	0.002928766	0	0.720240454	0	1	PRJNA827465;Yang2025_Silkie_Wenchang	CRA004775	0.074041237
ENSGALG00010018323	GOT1	2	-0.395852079	0.216209022	-1.83087679	0.067118935	0	0.353126986	0	1	CRA004775;Yang2025_Silkie_Wenchang	PRJNA827465	0.446292315
ENSGALG00010018323	GOT1	2	-0.234581501	0.095917188	-2.445667	0.014458447	0	0.042178307	0	1	CRA004775;PRJNA827465	Yang2025_Silkie_Wenchang	0.197724547
ENSGALG00010018325	ENSGALG00010018325	2	0.054448343	0.161449237	0.337247449	0.735930371	0	0.328797296	0	0.5	PRJNA827465;Yang2025_Silkie_Wenchang	CRA004775	0.933592889
ENSGALG00010018325	ENSGALG00010018325	2	0.200339975	0.241998847	0.827855082	0.407752576	0	0.131969216	0	1	CRA004775;Yang2025_Silkie_Wenchang	PRJNA827465	0.833982683
ENSGALG00010018325	ENSGALG00010018325	2	0.015408288	0.199466671	0.077247433	0.938426708	0	0.388039947	0	0.5	CRA004775;PRJNA827465	Yang2025_Silkie_Wenchang	0.985349045
ENSGALG00010018326	LCMT1	2	-0.073726161	0.061019649	-1.208236407	0.226956342	0	0.530664551	0	1	PRJNA827465;Yang2025_Silkie_Wenchang	CRA004775	0.740500532
ENSGALG00010018326	LCMT1	2	-0.206660392	0.126133051	-1.63843173	0.101331665	0	0.184530043	0	1	CRA004775;Yang2025_Silkie_Wenchang	PRJNA827465	0.543185817
ENSGALG00010018326	LCMT1	2	-0.071364467	0.064386288	-1.108379891	0.267697786	0	0.941507249	0	1	CRA004775;PRJNA827465	Yang2025_Silkie_Wenchang	0.771398294
ENSGALG00010018329	ENSGALG00010018329	2	-0.274139878	0.408249296	-0.671501164	0.501901318	0.283190674	6.591561674	84.82908832	0.5	PRJNA827465;Yang2025_Silkie_Wenchang	CRA004775	0.862795569
ENSGALG00010018329	ENSGALG00010018329	2	-0.750811758	0.187325266	-4.008064547	6.12184E-05	0	0.095893752	0	1	CRA004775;Yang2025_Silkie_Wenchang	PRJNA827465	0.005543932
ENSGALG00010018329	ENSGALG00010018329	2	-0.326564152	0.466372912	-0.700221096	0.483789239	0.37795099	7.516230477	86.69545854	0.5	CRA004775;PRJNA827465	Yang2025_Silkie_Wenchang	0.85691916
ENSGALG00010018333	ENSGALG00010018333	2	0.466972264	0.226067387	2.065633038	0.038863142	0.00196832	1.019363028	1.899522266	1	PRJNA827465;Yang2025_Silkie_Wenchang	CRA004775	0.340110908
ENSGALG00010018333	ENSGALG00010018333	2	0.6086848	0.199642402	3.048875365	0.002296998	0	0.171155308	0	1	CRA004775;Yang2025_Silkie_Wenchang	PRJNA827465	0.063663608
ENSGALG00010018333	ENSGALG00010018333	2	0.433323467	0.190825311	2.270786121	0.023159929	0	0.527362676	0	1	CRA004775;PRJNA827465	Yang2025_Silkie_Wenchang	0.259577892
ENSGALG00010018336	JMJD8	2	0.082591873	0.076292345	1.082570903	0.278998928	0	0.557861779	0	0.5	PRJNA827465;Yang2025_Silkie_Wenchang	CRA004775	0.777308774
ENSGALG00010018336	JMJD8	2	-0.114322528	0.139066741	-0.822069516	0.411037334	0	0.4271318	0	1	CRA004775;Yang2025_Silkie_Wenchang	PRJNA827465	0.835128673
ENSGALG00010018336	JMJD8	2	0.003294397	0.155347116	0.02120668	0.983080786	0.028260615	2.030001285	50.73894744	0.5	CRA004775;PRJNA827465	Yang2025_Silkie_Wenchang	0.995363468
ENSGALG00010018337	TMEM151B	2	-0.054175714	0.118224833	-0.458243099	0.646777799	0	0.031176405	0	1	PRJNA827465;Yang2025_Silkie_Wenchang	CRA004775	0.907350807
ENSGALG00010018337	TMEM151B	2	-0.038630451	0.186320512	-0.207333326	0.835749549	0	0.062809957	0	1	CRA004775;Yang2025_Silkie_Wenchang	PRJNA827465	0.958339923
ENSGALG00010018337	TMEM151B	2	-0.076078582	0.147774934	-0.51482738	0.60667365	0	0.040400223	0	1	CRA004775;PRJNA827465	Yang2025_Silkie_Wenchang	0.896799223
ENSGALG00010018338	PDCD6IP	2	0.085498736	0.108918558	0.784978588	0.432466123	0.012994261	1.804725237	44.58990323	1	PRJNA827465;Yang2025_Silkie_Wenchang	CRA004775	0.842762754
ENSGALG00010018338	PDCD6IP	2	0.188172395	0.118220656	1.591704881	0.111451031	0	0.333248131	0	1	CRA004775;Yang2025_Silkie_Wenchang	PRJNA827465	0.564762672
ENSGALG00010018338	PDCD6IP	2	0.029024571	0.056286626	0.515656604	0.606094269	0	0.356916423	0	1	CRA004775;PRJNA827465	Yang2025_Silkie_Wenchang	0.896709756
ENSGALG00010018339	FAM76A	2	-0.122415743	0.156719608	-0.781113127	0.434735962	0.0435493	8.59522046	88.3656271	0.5	PRJNA827465;Yang2025_Silkie_Wenchang	CRA004775	0.842762754
ENSGALG00010018339	FAM76A	2	-0.277896273	0.087480837	-3.176653124	0.001489851	0	0.122058101	0	1	CRA004775;Yang2025_Silkie_Wenchang	PRJNA827465	0.047917856
ENSGALG00010018339	FAM76A	2	0.017344095	0.052958951	0.327500736	0.743289187	0	0.605542039	0	0.5	CRA004775;PRJNA827465	Yang2025_Silkie_Wenchang	0.935760111
ENSGALG00010018340	DNPEP	2	0.061837255	0.148860217	0.415404847	0.677845559	0.03599893	5.057416798	80.22705978	0.5	PRJNA827465;Yang2025_Silkie_Wenchang	CRA004775	0.917408612
ENSGALG00010018340	DNPEP	2	0.109718921	0.204825303	0.535670736	0.592186132	0.042149906	1.606720491	37.76142114	0.5	CRA004775;Yang2025_Silkie_Wenchang	PRJNA827465	0.892776493
ENSGALG00010018340	DNPEP	2	-0.076544973	0.061444549	-1.245756922	0.212853649	0	0.231808993	0	1	CRA004775;PRJNA827465	Yang2025_Silkie_Wenchang	0.725659838
ENSGALG00010018342	BNIP3L	2	-0.131810353	0.148099075	-0.890014691	0.373457998	0.037913169	7.130321486	85.97538692	0.5	PRJNA827465;Yang2025_Silkie_Wenchang	CRA004775	0.822254022
ENSGALG00010018342	BNIP3L	2	-0.112134821	0.221874688	-0.505397088	0.613279964	0.071861455	3.301289044	69.70880203	0.5	CRA004775;Yang2025_Silkie_Wenchang	PRJNA827465	0.89823785
ENSGALG00010018342	BNIP3L	2	0.014315009	0.053225535	0.26895003	0.787968136	0	0.440113995	0	1	CRA004775;PRJNA827465	Yang2025_Silkie_Wenchang	0.946919116
ENSGALG00010018344	SFXN5	2	0.044421962	0.076715743	0.579046237	0.562557975	0	0.422248525	0	1	PRJNA827465;Yang2025_Silkie_Wenchang	CRA004775	0.881978509
ENSGALG00010018344	SFXN5	2	0.090022309	0.160286044	0.561635357	0.574364486	0	0.445497289	0	0.5	CRA004775;Yang2025_Silkie_Wenchang	PRJNA827465	0.886083039
ENSGALG00010018344	SFXN5	2	0.01481289	0.080943128	0.18300368	0.854795131	0	0.110055056	0	0.5	CRA004775;PRJNA827465	Yang2025_Silkie_Wenchang	0.963248391
ENSGALG00010018345	WDR24	2	-0.186072296	0.107265522	-1.734688758	0.082795949	0.014335454	2.615343488	61.76410461	1	PRJNA827465;Yang2025_Silkie_Wenchang	CRA004775	0.495704284
ENSGALG00010018345	WDR24	2	-0.287940882	0.099488381	-2.89421618	0.003801064	0	0.222961917	0	1	CRA004775;Yang2025_Silkie_Wenchang	PRJNA827465	0.088450684
ENSGALG00010018345	WDR24	2	-0.09218992	0.079335149	-1.162031223	0.245222782	0	0.034718726	0	1	CRA004775;PRJNA827465	Yang2025_Silkie_Wenchang	0.75661406
ENSGALG00010018348	TOGARAM1	2	0.082519779	0.047582325	1.734252754	0.082873246	0	0.92819236	0	1	PRJNA827465;Yang2025_Silkie_Wenchang	CRA004775	0.495859127
ENSGALG00010018348	TOGARAM1	2	0.144775982	0.083031668	1.74362368	0.081224731	0	0.097006239	0	1	CRA004775;Yang2025_Silkie_Wenchang	PRJNA827465	0.490908137
ENSGALG00010018348	TOGARAM1	2	0.054003665	0.055138507	0.979418343	0.327373317	0	0.00531604	0	1	CRA004775;PRJNA827465	Yang2025_Silkie_Wenchang	0.800028239
ENSGALG00010018349	KCNMB2	2	-0.08152847	0.201888541	-0.40382911	0.686338388	0.054471157	2.884288308	65.32940216	0.5	PRJNA827465;Yang2025_Silkie_Wenchang	CRA004775	0.919235539
ENSGALG00010018349	KCNMB2	2	0.144346126	0.180951844	0.797704644	0.425041908	0	0.009006249	0	1	CRA004775;Yang2025_Silkie_Wenchang	PRJNA827465	0.841201577
ENSGALG00010018349	KCNMB2	2	-0.220500546	0.122029158	-1.806949664	0.070770128	0	0.77224523	0	0.5	CRA004775;PRJNA827465	Yang2025_Silkie_Wenchang	0.458687061
ENSGALG00010018350	CNNM1	2	0.100023947	0.274982291	0.363746868	0.716047042	0	0.786706146	0	0.5	PRJNA827465;Yang2025_Silkie_Wenchang	CRA004775	0.927275021
ENSGALG00010018350	CNNM1	2	-0.278084684	0.723198866	-0.384520355	0.700592812	0.810952319	4.443713358	77.49629827	0.5	CRA004775;Yang2025_Silkie_Wenchang	PRJNA827465	0.922366238
ENSGALG00010018350	CNNM1	2	-0.473209275	0.462020908	-1.024216147	0.305733193	0.251623824	2.349909354	57.44516705	1	CRA004775;PRJNA827465	Yang2025_Silkie_Wenchang	0.79225794
ENSGALG00010018351	RIN2	2	-0.151558833	0.10820748	-1.400631755	0.16132422	0.018721603	4.916439375	79.66007666	1	PRJNA827465;Yang2025_Silkie_Wenchang	CRA004775	0.657655531
ENSGALG00010018351	RIN2	2	-0.113785533	0.177258741	-0.641917755	0.520926586	0.048539633	4.08883012	75.54312675	0.5	CRA004775;Yang2025_Silkie_Wenchang	PRJNA827465	0.869518515
ENSGALG00010018351	RIN2	2	-0.032939301	0.054554421	-0.603787926	0.545984648	0	0.701426784	0	0.5	CRA004775;PRJNA827465	Yang2025_Silkie_Wenchang	0.877958239
ENSGALG00010018354	AARS2	2	-0.061187209	0.152422414	-0.401431831	0.688102218	0.029288589	2.176020559	54.04455183	0.5	PRJNA827465;Yang2025_Silkie_Wenchang	CRA004775	0.919679555
ENSGALG00010018354	AARS2	2	-0.434212144	0.117922723	-3.682175348	0.000231252	0	0.679811918	0	1	CRA004775;Yang2025_Silkie_Wenchang	PRJNA827465	0.014069516
ENSGALG00010018354	AARS2	2	-0.214341451	0.26966838	-0.79483346	0.426710384	0.13361418	11.91117077	91.60451966	0.5	CRA004775;PRJNA827465	Yang2025_Silkie_Wenchang	0.84125685
ENSGALG00010018355	PRKCB	2	-0.091880116	0.104932701	-0.875609939	0.381242121	0	0.044696021	0	1	PRJNA827465;Yang2025_Silkie_Wenchang	CRA004775	0.824392515
ENSGALG00010018355	PRKCB	2	-0.161181188	0.160518205	-1.004130269	0.315315827	0	0.428062744	0	1	CRA004775;Yang2025_Silkie_Wenchang	PRJNA827465	0.796763402
ENSGALG00010018355	PRKCB	2	-0.103837284	0.12694958	-0.817941136	0.4133908	0	0.594148307	0	1	CRA004775;PRJNA827465	Yang2025_Silkie_Wenchang	0.83612076
ENSGALG00010018359	CACNG3	2	0.219065552	0.338286067	0.647574857	0.517259964	0	0.749021935	0	1	PRJNA827465;Yang2025_Silkie_Wenchang	CRA004775	0.867952013

ENSGALG00010018359	CACNG3	2	0.770686153	0.48852539	1.577576455	0.114662945	0	0.12254111	0	1	CRA004775;Yang2025_Silkie_Wenchang	PRJNA827465	0.57103367
ENSGALG00010018359	CACNG3	2	0.243036769	0.414097757	0.58690675	0.557266336	0.053831828	1.12326584	10.97387955	1	CRA004775;PRJNA827465	Yang2025_Silkie_Wenchang	0.880678789
ENSGALG00010018361	ASIC4	2	0.220663385	0.299099559	0.737758977	0.460660926	0.140966225	4.622676492	78.36751065	0.5	PRJNA827465;Yang2025_Silkie_Wenchang	CRA004775	0.85014477
ENSGALG00010018361	ASIC4	2	0.518710403	0.213011078	2.435133455	0.014886298	0	0.084538354	0	1	CRA004775;Yang2025_Silkie_Wenchang	PRJNA827465	0.201566194
ENSGALG00010018361	ASIC4	2	-0.029249452	0.155050171	-0.188645079	0.850370999	0	0.451669364	0	0.5	CRA004775;PRJNA827465	Yang2025_Silkie_Wenchang	0.961985357
ENSGALG00010018363	TCTE1	2	-0.071149364	0.120484927	-0.590525022	0.554838715	0	0.153856032	0	1	PRJNA827465;Yang2025_Silkie_Wenchang	CRA004775	0.880223402
ENSGALG00010018363	TCTE1	2	-0.185156568	0.193542691	-0.95667042	0.338733633	0	0.695270885	0	1	CRA004775;Yang2025_Silkie_Wenchang	PRJNA827465	0.804450436
ENSGALG00010018363	TCTE1	2	-0.06967562	0.146047391	-0.477075415	0.633308421	0	0.997068043	0	1	CRA004775;PRJNA827465	Yang2025_Silkie_Wenchang	0.9032522
ENSGALG00010018365	HPSE2	2	-0.08506061	0.135452923	-0.62797176	0.53002244	0	0.012252792	0	1	PRJNA827465;Yang2025_Silkie_Wenchang	CRA004775	0.872072262
ENSGALG00010018365	HPSE2	2	-0.135309965	0.192699874	-0.702179829	0.482567024	0	0.399934632	0	1	CRA004775;Yang2025_Silkie_Wenchang	PRJNA827465	0.85691916
ENSGALG00010018365	HPSE2	2	-0.137521627	0.156450551	-0.879010178	0.379395757	0	0.352362909	0	1	CRA004775;PRJNA827465	Yang2025_Silkie_Wenchang	0.824165982
ENSGALG00010018367	PAK5	2	-0.097609624	0.112411721	-0.868322473	0.385217823	0	0.000221745	0	1	PRJNA827465;Yang2025_Silkie_Wenchang	CRA004775	0.826444827
ENSGALG00010018367	PAK5	2	-0.110383426	0.203417368	-0.542645042	0.587374221	0	0.008880067	0	1	CRA004775;Yang2025_Silkie_Wenchang	PRJNA827465	0.891660017
ENSGALG00010018367	PAK5	2	-0.100613102	0.124031884	-0.8111874	0.417258061	0	0.012122599	0	1	CRA004775;PRJNA827465	Yang2025_Silkie_Wenchang	0.838112595
ENSGALG00010018370	ZBTB8A	2	-0.256655847	0.346490148	-0.740730576	0.458856816	0.229261386	21.87542678	95.42866061	0.5	PRJNA827465;Yang2025_Silkie_Wenchang	CRA004775	0.850089963
ENSGALG00010018370	ZBTB8A	2	-0.187674688	0.42897645	-0.437494151	0.661753017	0.346925582	17.38606998	94.24826887	0.5	CRA004775;Yang2025_Silkie_Wenchang	PRJNA827465	0.912545506
ENSGALG00010018370	ZBTB8A	2	0.110092258	0.066453759	1.656674642	0.097585272	0	0.875372564	0	1	CRA004775;PRJNA827465	Yang2025_Silkie_Wenchang	0.533292282
ENSGALG00010018371	ENSGALG00010018371	2	-0.045817336	0.103945073	-0.440784108	0.6593693	0	0.65393105	0	0.5	PRJNA827465;Yang2025_Silkie_Wenchang	CRA004775	0.911449691
ENSGALG00010018371	ENSGALG00010018371	2	0.061242672	0.24374046	0.251261823	0.801611689	0.075857828	2.622608744	61.87002723	0.5	CRA004775;Yang2025_Silkie_Wenchang	PRJNA827465	0.950313523
ENSGALG00010018371	ENSGALG00010018371	2	0.113791408	0.143883673	0.790856984	0.429027453	0.009315715	1.213608107	17.60107776	1	CRA004775;PRJNA827465	Yang2025_Silkie_Wenchang	0.841924604
ENSGALG00010018372	NLGN3	2	0.114838282	0.163017749	0.704452632	0.481150939	0.016952393	1.44265007	30.68312124	0.5	PRJNA827465;Yang2025_Silkie_Wenchang	CRA004775	0.85681099
ENSGALG00010018372	NLGN3	2	-0.02934244	0.429915775	-0.068251601	0.945585351	0.253265806	2.86673996	65.11717093	0.5	CRA004775;Yang2025_Silkie_Wenchang	PRJNA827465	0.986321197
ENSGALG00010018372	NLGN3	2	-0.116554009	0.210018874	-0.554969214	0.578915718	0.026106725	1.209020224	17.28839767	1	CRA004775;PRJNA827465	Yang2025_Silkie_Wenchang	0.887888423
ENSGALG00010018374	HPS1	2	0.074510568	0.093180537	0.799636598	0.423921377	0	0.00885522	0	1	PRJNA827465;Yang2025_Silkie_Wenchang	CRA004775	0.84030043
ENSGALG00010018374	HPS1	2	-0.179532848	0.274602646	-0.653791399	0.513246208	0.081918079	2.188751961	54.31186275	0.5	CRA004775;Yang2025_Silkie_Wenchang	PRJNA827465	0.866295966
ENSGALG00010018374	HPS1	2	-0.135074494	0.255428692	-0.528814885	0.596933871	0.097899579	3.534029117	71.70368531	0.5	CRA004775;PRJNA827465	Yang2025_Silkie_Wenchang	0.894172266
ENSGALG00010018376	PYGB	2	0.010506363	0.174111135	0.060342857	0.95188257	0.048757832	4.894634072	79.56946351	0.5	PRJNA827465;Yang2025_Silkie_Wenchang	CRA004775	0.987719213
ENSGALG00010018376	PYGB	2	0.217502495	0.120541954	1.804371731	0.071173052	0	0.045451522	0	1	CRA004775;Yang2025_Silkie_Wenchang	PRJNA827465	0.460681013
ENSGALG00010018376	PYGB	2	-0.010807651	0.193413083	-0.055878594	0.955438523	0.049611622	2.417906116	58.64190121	0.5	CRA004775;PRJNA827465	Yang2025_Silkie_Wenchang	0.988226014
ENSGALG00010018377	RAP1GAP	2	0.173030475	0.252968988	0.683998763	0.493975947	0.082130459	2.769930981	63.89801743	0.5	PRJNA827465;Yang2025_Silkie_Wenchang	CRA004775	0.860175061
ENSGALG00010018377	RAP1GAP	2	0.630941713	0.285187361	2.212376143	0.026940686	0.092128688	2.13228481	53.10194983	1	CRA004775;Yang2025_Silkie_Wenchang	PRJNA827465	0.279527396
ENSGALG00010018377	RAP1GAP	2	0.419122916	0.547108396	0.766069244	0.443635102	0.510939518	6.692882484	85.05875455	0.5	CRA004775;PRJNA827465	Yang2025_Silkie_Wenchang	0.845827655
ENSGALG00010018378	ARPP21	2	0.365675388	0.6172162	0.592459155	0.553543164	0.72320073	19.06627308	94.75513649	0.5	PRJNA827465;Yang2025_Silkie_Wenchang	CRA004775	0.879610907
ENSGALG00010018378	ARPP21	2	0.636625053	0.432750694	1.471112727	0.141260634	0.254254296	2.974355879	66.37927535	1	CRA004775;Yang2025_Silkie_Wenchang	PRJNA827465	0.62620874
ENSGALG00010018378	ARPP21	2	-0.210991217	0.089114572	-2.367639897	0.017901951	0	0.668663905	0	0.5	CRA004775;PRJNA827465	Yang2025_Silkie_Wenchang	0.223309996
ENSGALG00010018379	SRBD1	2	-0.252717887	0.305886724	-0.826181285	0.408701263	0.177114952	18.67044777	94.64394206	0.5	PRJNA827465;Yang2025_Silkie_Wenchang	CRA004775	0.834305842
ENSGALG00010018379	SRBD1	2	-0.376214439	0.286507004	-1.313107301	0.189146794	0.104950159	2.120949616	52.85130808	0.5	CRA004775;Yang2025_Silkie_Wenchang	PRJNA827465	0.698162702
ENSGALG00010018379	SRBD1	2	0.052524364	0.092900793	0.565381225	0.571814492	0	0.001830339	0	1	CRA004775;PRJNA827465	Yang2025_Silkie_Wenchang	0.885470687
ENSGALG00010018380	SLC20A1	2	0.157352599	0.105826894	1.486886671	0.137044745	0.005070679	1.280906265	21.93027489	1	PRJNA827465;Yang2025_Silkie_Wenchang	CRA004775	0.619325011
ENSGALG00010018380	SLC20A1	2	-0.30401939	0.340602463	-0.892593046	0.372075144	0.205984305	8.89107657	88.7527681	0.5	CRA004775;Yang2025_Silkie_Wenchang	PRJNA827465	0.821620523
ENSGALG00010018380	SLC20A1	2	-0.193331328	0.448083769	-0.431462465	0.666132139	0.379705189	18.24808132	94.51997181	0.5	CRA004775;PRJNA827465	Yang2025_Silkie_Wenchang	0.913579507
ENSGALG00010018381	HAPSTR1	2	-0.095319485	0.043361215	-2.198266019	0.027930154	0	0.132566904	0	1	PRJNA827465;Yang2025_Silkie_Wenchang	CRA004775	0.284609505
ENSGALG00010018381	HAPSTR1	2	0.006551769	0.163554993	0.04005851	0.96804648	0.035981172	2.744993095	63.57003587	0.5	CRA004775;Yang2025_Silkie_Wenchang	PRJNA827465	0.991128245
ENSGALG00010018381	HAPSTR1	2	0.011837125	0.140749813	0.084100467	0.932976553	0.026874906	2.477028485	59.62904722	0.5	CRA004775;PRJNA827465	Yang2025_Silkie_Wenchang	0.984224753
ENSGALG00010018383	METRN	2	-0.028298831	0.090785471	-0.311711017	0.755260154	0	0.069711703	0	0.5	PRJNA827465;Yang2025_Silkie_Wenchang	CRA004775	0.938453928
ENSGALG00010018383	METRN	2	0.173946701	0.20427368	0.851537511	0.394470836	0.023389586	1.349169055	25.88030418	1	CRA004775;Yang2025_Silkie_Wenchang	PRJNA827465	0.830595052
ENSGALG00010018383	METRN	2	0.118416943	0.227681788	0.520098441	0.602994965	0.066725815	2.305136831	56.6186273	0.5	CRA004775;PRJNA827465	Yang2025_Silkie_Wenchang	0.895538966
ENSGALG00010018384	LAMP5	2	-0.052940204	0.130718591	-0.404993688	0.685482151	0	0.365968851	0	0.5	PRJNA827465;Yang2025_Silkie_Wenchang	CRA004775	0.919235539
ENSGALG00010018384	LAMP5	2	0.03494431	0.187447687	0.186421666	0.852114103	0	0.034469582	0	0.5	CRA004775;Yang2025_Silkie_Wenchang	PRJNA827465	0.96237473
ENSGALG00010018384	LAMP5	2	-0.092199838	0.144864466	-0.636455855	0.524479345	0	0.060831521	0	1	CRA004775;PRJNA827465	Yang2025_Silkie_Wenchang	0.869790047
ENSGALG00010018385	SPR	2	0.329620452	0.112126416	2.939721637	0.003285072	0	0.285627693	0	1	PRJNA827465;Yang2025_Silkie_Wenchang	CRA004775	0.079946251
ENSGALG00010018385	SPR	2	0.02882006	0.180481624	0.159684181	0.873129862	0.001107876	1.017299692	1.700550182	0.5	CRA004775;Yang2025_Silkie_Wenchang	PRJNA827465	0.969794665
ENSGALG00010018385	SPR	2	0.151009428	0.251358699	0.600772636	0.547991432	0.090911037	3.274684682	69.46270872	0.5	CRA004775;PRJNA827465	Yang2025_Silkie_Wenchang	0.878281266
ENSGALG00010018387	NT5DC4	2	0.17422832	0.554490523	0.314213342	0.75335901	0.563949196	12.04948873	91.70089269	0.5	PRJNA827465;Yang2025_Silkie_Wenchang	CRA004775	0.938087082
ENSGALG00010018387	NT5DC4	2	-0.021775476	0.387800339	-0.056151255	0.955221313	0.230208712	4.141129846	75.85200085	0.5	CRA004775;Yang2025_Silkie_Wenchang	PRJNA827465	0.988131974
ENSGALG00010018387	NT5DC4	2	0.615635338	0.191779142	3.210126664	0.001326765	0	0.68239884	0	1	CRA004775;PRJNA827465	Yang2025_Silkie_Wenchang	0.044531035
ENSGALG00010018388	SMC3	2	0.252325898	0.352863787	0.715080174	0.474559504	0.233130974	14.99038438	93.32905698	0.5	PRJNA827465;Yang2025_Silkie_Wenchang	CRA004775	0.854462864
ENSGALG00010018388	SMC3	2	0.395005289	0.22175707	1.781252289	0.074871245	0.07170837	3.677103157	72.80467919	1	CRA004775;Yang2025_Silkie_Wenchang	PRJNA827465	0.472257613
ENSGALG00010018388	SMC3	2	0.011526981	0.125731836	0.091679094	0.926953008	0.021653185	2.675069967	62.61780019	0.5	CRA004775;PRJNA827465	Yang2025_Silkie_Wenchang	0.982896295
ENSGALG00010018391	STAC	2	0.207634378	0.240835017	0.862143639	0.38860848	0.08100867	3.313772725	69.82291536	0.5	PRJNA827465;Yang2025_Silkie_Wenchang	CRA004775	0.828338548
ENSGALG00010018391	STAC	2	0.435144911	0.185854967	2.341314406	0.019215977	0	0.140787661	0	1	CRA004775;Yang2025_Silkie_Wenchang	PRJNA827465	0.232311767
ENSGALG00010018391	STAC	2	-0.022322503	0.178866769	-0.124799608	0.900682198	0	0.039492135	0	0.5	CRA004775;PRJNA827465	Yang2025_Silkie_Wenchang	0.976497577
ENSGALG00010018393	PRKCE	2	0.067630282	0.120693714	0.560346353	0.575243216	0.014392773	1.760731789	43.20543273	0.5	PRJNA827465;Yang2025_Silkie_Wenchang	CRA004775	0.88644908
ENSGALG00010018393	PRKCE	2	0.240614418	0.15154204	1.587773389	0.112337591	0	0.000410033	0	1	CRA004775;Yang2025_Silkie_Wenchang	PRJNA827465	0.56602666
ENSGALG00010018393	PRKCE	2	0.002502291	0.081268891	0.030790272	0.975436798	0	0.642401493	0	0.5	CRA004775;PRJNA827465	Yang2025_Silkie_Wenchang	0.992754467
ENSGALG00010018394	KLHL28	2	-0.021254546	0.120526875	-0.176346941	0.860021386	0.022043319	3.747126806	73.31288607	0.5	PRJNA827465;Yang2025_Silkie_Wenchang	CRA004775	0.964900247
ENSGALG00010018394	KLHL28	2	-0.128160911	0.096464625	-1.328579371	0.183986782	0	0.344536259	0	1	CRA004775;Yang2025_Silkie_Wenchang	PRJNA827465	0.692470323
ENSGALG00010018394	KLHL28	2	0.0692806	0.048043625	1.44203522	0.149292438	0	0.470364813	0	0.5	CRA004775;PRJNA827465	Yang2025_Silkie_Wenchang	0.639246608
ENSGALG00010018395	MED12	2	0.048652114	0.107392314	0.45303162	0.650525973	0.017548788	3.873706643	74.18493211	0.5	PRJNA827465;Yang2025_Silkie_Wenchang	CRA004775	0.908618725
ENSGALG00010018395	MED12	2	0.152170383	0.090753025	1.676752735	0.093590844	0	0.312857266	0	1	CRA004775;Yang2025_Silkie_Wenchang	PRJNA827465	0.52406339
ENSGALG00010018395	MED12	2	-0.039345848	0.047610214	-0.826416123	0.408568081	0	0.121243419	0	0.5	CRA004775;PRJNA827465	Yang2025_Silkie_Wenchang	0.834179287
ENSGALG00010018396	TBL1XR1	2	0.023123837	0.152334592	0.151796362	0.879347551	0.035582924	3.634155731	72.48329257	0.5	PRJNA827465;Yang2025_Silkie_Wenchang	CRA004775	0.971291607
ENSGALG00010018396	TBL1XR1	2	0.198671483	0.132045712	1.504565957	0.132435704	0	0.041663231	0	1	CRA004775;Yang2025_Silkie_Wenchang	PRJNA827465	0.609570264
ENSGALG00010018396	TBL1XR1	2	-0.079020132	0.065135571	-1.213164032	0.225067113	0.002524804	1.084458401	7.788072019	0.5	CRA004775;PRJNA827465	Yang2025_Silkie_Wenchang	0.738087485
ENSGALG00010018397	FAM173A	2	-0.277829836	0.103816122	-2.676172353	0.007446834	0	0.640296134	0	1	PRJNA827465;Yang2025_Silkie_Wenchang	CRA004775	0.132977243
ENSGALG00010018397	FAM173A	2	-0.518742033	0.255255577	-2.032245636	0.042128795	0	0.016400642	0	1	CRA004775;Yang2025_Silkie_Wenchang	PRJNA827465	0.355166888
ENSGALG00010018397	FAM173A	2	-0.261803751	0.108279422	-2.417853235	0.015612372	0	0.402585574	0	1	CRA004775;PRJNA827465	Yang2025_Silkie_Wenchang	0.206742949
ENSGALG00010018398	LRFN5	2	-0.273607885	0.152240883	-1.797203751	0.072303279	0.006428623	1.149437539	13.00092735	1	PRJNA827465;Yang2025_Silkie_Wenchang	CRA004775	0.46442847
ENSGALG00010018398	LRFN5	2	-0.418574422	0.203703955	-2.054817353	0.039896649	0	0.191656136	0	1	CRA004775;Yang2025_Silkie_Wenchang	PRJNA827465	0.345768029
ENSGALG00010018398	LRFN5	2	-0.172691211	0.160814272	-1.073855009	0.282887671	0	0.060297246	0	1	CRA004775;PRJNA827465	Yang2025_Silkie_Wenchang	0.779532565
ENSGALG00010018399	ABHD12	2	-0.339765403	0.283770612	-1.197324134	0.231180241	0.151695505	16.78364275	94.04181789	1	PRJNA827465;Yang2025_Silkie_Wenchang	CRA004775	0.743891332
ENSGALG00010018399	ABHD12	2	-0.22449407	0.437421616	-0.513221253	0.607796561	0.34387064	9.523419739	89.49957024	0.5	CRA004775;Yang2025_Silkie_Wenchang	PRJNA827465	0.897203929
ENSGALG00010018399	ABHD12	2	-0.012340309	0.117867959	-0.104696045	0.916617001	0.013688611	1.403415762	28.745278	0.5	CRA004775;PRJNA827465	Yang2025_Silkie_Wenchang	0.980643874
ENSGALG00010018401	ENSGALG00010018401	2	-0.086248966	0.140042232	-0.615878257	0.537974873	0.027962265	2.938636072	65.9706076	0.5	PRJNA827465;Yang2025_Silkie_Wenchang	CRA004775	0.87481821
ENSGALG00010018401	ENSGALG00010018401	2	-0.262234484	0.110978802	-2.362924092	0.018131383	0	0.006396718	0	1	CRA004775;Yang2025_Silkie_Wenchang	PRJNA827465	0.22480055
ENSGALG00010018401	ENSGALG00010018401	2	-0.079530569	0.131418394	-0.605170755	0.545065545	0.024244563	2.847402675	64.88027462	0.5	CRA004775;PRJNA827465	Yang2025_Silkie_Wenchang	0.877354247
ENSGALG00010018404	IL2RG	2	0.012688012	0.105010376	0.120826268	0.903828643	0	0.098012464	0	0.5	PRJNA827465;Yang2025_Silkie_Wenchang	CRA004775	0.977333734
ENSGALG00010018404	IL2RG	2	0.017054374	0.15250239	0.111830206	0.910958037	0	0.385814768	0	0.5	CRA004775;Yang2025_Silkie_Wenchang	PRJNA827465	0.978845851
ENSGALG00010018404	IL2RG	2	-0.03506541	0.131673942	-0.266304856	0.790004434	0	0.239244313	0	1	CRA004775;PRJNA827465	Yang2025_Silkie_Wenchang	0.947390191
ENSGALG00010018405	DCLK3	2	0.429948845	0.233174782	1.84389084	0.06519905	0.07363697	2.809667651	64.40860186	1	PRJNA827465;Yang2025_Silkie_Wenchang	CRA004775	0.439917301
ENSGALG00010018405	DCLK3	2	0.20400924	0.578583735	0.352601063	0.724387541	0.536997238	4.814458937	79.229234	0.5	CRA004775;Yang2025_Silkie_Wenchang	PRJNA827465	0.930387861
ENSGALG00010018405	DCLK3	2	0.046540542	0.310488852	0.149894406	0.880847925	0.119862333	2.037713908	50.92539753	0.5	CRA004775;PRJNA827465	Yang2025_Silkie_Wenchang	0.971679419
ENSGALG00010018406	TMEM260	2	0.172178796	0.099657974	1.727697133	0.084042538	0	0.052542688	0	1	PRJNA827465;Yang2025_Silkie_Wenchang	CRA004775	0.499567879
ENSGALG00010018406	TMEM260	2	0.06270982	0.208434365	0.300861231	0.763520314	0.022614449	1.308287854	23.56422198	0.5	CRA004775;Yang2025_Silkie_Wenchang	PRJNA827465	0.940301633
ENSGALG00010018406	TMEM260	2	0.085417899	0.15165469	0.563239412	0.573271869	0.015693024	1.271235806	21.33638814	0.5	CRA004775;PRJNA827465	Yang2025_Silkie_Wenchang	0.885803753
ENSGALG00010018410	EXOC6B	2	0.345521333	0.444608277	0.777136528	0.437078225	0.373251611	17.38563888	94.24812624	0.5	PRJNA827465;Yang2025_Silkie_Wenchang	CRA004775	0.843453774
ENSGALG00010018410	EXOC6B	2	0.283591987	0.546588644	0.51883988	0.603872405	0.535565346	9.515365423	89.49068212	0.5	CRA004775;Yang2025_Silkie_Wenchang	PRJNA827465	0.895892991
ENSGALG00010018410	EXOC6B	2	-0.095089524	0.077175461	-1.232121227	0.217903803	0	0.447642207	0	1	CRA004775;PRJNA827465	Yang2025_Silkie_Wenchang	0.73141715
ENSGALG00010018413	SLC15A2	2	0.076244249	0.214505931	0.355441216	0.722259076	0	0.724211405	0	0.5	PRJNA827465;Yang2025_Silkie_Wenchang	CRA004775	0.929500605
ENSGALG00010018413	SLC15A2	2	0.190988848	0.265321	0.719840677	0.471623097	0	0.186994994	0	0.5	CRA004775;Yang2025_Silkie_Wenchang	PRJNA827465	0.853998373
ENSGALG00010018413	SLC15A2	2	-0.152694259	0.318479054	-0.479448356	0.631619699	0	0.002119243	0	1	CRA004775;PRJNA827465	Yang2025_Silkie_Wenchang	0.903058174
ENSGALG00010018414	EPAS1	2	-0.071653657	0.139170244	-0.514863339	0.606648521	0.025735916	2.864764626	65.09311827	0.5	PRJNA827465;Yang2025_Silkie_Wenchang	CRA004775	0.896799223
ENSGALG00010018414	EPAS1	2	0.056838441	0.322653292	0.176159496	0.86016864	0.168255917	4.961258537	79.84382405	0.5	CRA004775;Yang2025_Silkie_Wenchang	PRJNA827465	0.964955275
ENSGALG00010018414	EPAS1	2	0.155023549	0.166687342	0.930025923	0.352357663	0.030862014	1.837502255	45.57829807	1	CRA004775;PRJNA827465	Yang2025_Silkie_Wenchang	0.810814591
ENSGALG00010018415	PELI2	2	0.217958064	0.058809149	3.706193094	0.000210398	0	0.285787101	0	1	PRJNA827465;Yang2025_Silkie_Wenchang	CRA004775	0.013167626
ENSGALG00010018415	PELI2	2	0.276433331	0.103041084	2.682748662	0.007301984	0	0.023033327	0	1	CRA004775;Yang2025_Silkie_Wenchang	PRJNA827465	0.131225376
ENSGALG00010018415	PELI2	2	0.206562664	0.066477026	3.107278952	0.001888181	0	0.185130556	0	1	CRA004775;PRJNA827465	Yang2025_Silkie_Wenchang	0.055924033
ENSGALG00010018416	DUT	2	-0.195602884	0.063077875	-3.100974513	0.001928849	0	0.061320312	0	1	PRJNA827465;Yang2025_Silkie_Wenchang	CRA004775	0.056701233
ENSGALG00010018416	DUT	2	-0.154035975	0.171809095	-0.896553091	0.369957439	0	0.264956942	0	1	CRA004775;Yang2025_Silkie_Wenchang	PRJNA827465	0.820855964
ENSGALG00010018416	DUT	2	-0.183168677	0.062322663	-2.939038032	0.003292327	0	0.229134652	0	1	CRA004775;PRJNA827465	Yang2025_Silkie_Wenchang	0.080073216
ENSGALG00010018418	PLCB4	2	0.251869578	0.222438438	1.132311393	0.257503542	0.078257739	4.506583991	77.81024381	1	PRJNA827465;Yang2025_Silkie_Wenchang	CRA004775	0.7650601
ENSGALG00010018418	PLCB4	2	0.556797884	0.161248633	3.453039396	0.000554308	0	0.300890356	0	1	CRA004775;Yang2025_Silkie_Wenchang	PRJNA827465	0.025157008
ENSGALG00010018418	PLCB4	2	0.311380353	0.317641793	0.980287733	0.326944108	0.156522483	3.843317842	73.98081446	1	CRA004775;PRJNA827465	Yang2025_Silkie_Wenchang	0.800028239
ENSGALG00010018419	USP7	2	-0.164589625	0.051838962	-3.175017783	0.001498273	0.001971495	1.439479636	30.53045179	1	PRJNA827465;Yang2025_Silkie_Wenchang	CRA004775	0.048147232
ENSGALG00010018419	USP7	2	0.026779563	0.30120923	0.088906847	0.929155942	0.157287363	7.018056302	85.7510405	0.5	CRA004775;Yang2025_Silkie_Wenchang	PRJNA827465	0.983566083
ENSGALG00010018419	USP7	2	0.070979593	0.242050514	0.293242894	0.769336499	0.097557549	5.152887309	80.59340443	0.5	CRA004775;PRJNA827465	Yang2025_Silkie_Wenchang	0.941790377
ENSGALG00010018420	RHOQ	2	-0.08225377	0.063555049	-1.294212994	0.19559186	0	0.376866824	0	1	PRJNA827465;Yang2025_Silkie_Wenchang	CRA004775	0.706675375
ENSGALG00010018420	RHOQ	2	0.247236793	0.440968392	0.560667834	0.575023999	0.339891386	7.586046687	86.81790343	0.5	CRA004775;Yang2025_Silkie_Wenchang	PRJNA827465	0.886281107
ENSGALG00010018420	RHOQ	2	0.281874243	0.39092333	0.721047382	0.470880363	0.266315009	7.015228026	85.74529586	0.5	CRA004775;PRJNA827465	Yang2025_Silkie_Wenchang	0.853762555
ENSGALG00010018422	ENSGALG00010018422	2	-0.074103816	0.090394396	-0.8197833	0.412339653	0	0.849107571	0	0.5	PRJNA827465;Yang2025_Silkie_Wenchang	CRA004775	0.835671911
ENSGALG00010018422	ENSGALG00010018422	2	-0.024562328	0.235848504	-0.104144516	0.917054664	0.028972799	1.190024554	15.96812041	0.5	CRA004775;Yang2025_Silkie_Wenchang	PRJNA827465	0.980728718
ENSGALG00010018422	ENSGALG00010018422	2	-0.131375989	0.099548942	-1.319712561	0.186931005	0	0.570506248	0	1	CRA004775;PRJNA827465	Yang2025_Silkie_Wenchang	0.695858627
ENSGALG00010018423	IGFALS	2	-0.829288189	0.814284669	-1.018425398	0.308475837	0.701771059	2.12340854	52.90590665	1	PRJNA827465;Yang2025_Silkie_Wenchang	CRA004775	0.793457188
ENSGALG00010018423	IGFALS	2	-0.898487616	0.719388369	-1.248960443	0.211679542	0.450093233	1.769030546	43.47186359	1	CRA004775;Yang2025_Silkie_Wenchang	PRJNA827465	0.724013667
ENSGALG00010018423	IGFALS	2	-0.103065358	0.549410528	-0.187592616	0.851196015	0	0.029700081	0	1	CRA004775;PRJNA827465	Yang2025_Silkie_Wenchang	0.962071965
ENSGALG00010018425	CYP26B1	2	0.203147975	0.275916241	0.736266824	0.461568332	0.129149852	6.577657861	84.79702014	0.5	PRJNA827465;Yang2025_Silkie_Wenchang	CRA004775	0.85042452
ENSGALG00010018425	CYP26B1	2	0.446804128	0.145612369	3.068448986	0.002151731	0	0.300121838	0	1	CRA004775;Yang2025_Silkie_Wenchang	PRJNA827465	0.061008576
ENSGALG00010018425	CYP26B1	2	-0.01773948	0.132367734	-0.134016648	0.89338941	0	0.840879128	0	0.5	CRA004775;PRJNA827465	Yang2025_Silkie_Wenchang	0.974381724
ENSGALG00010018426	ENSGALG00010018426	2	0.330346778	0.172628336	1.913630093	0.055667437	0	0.013787714	0	1	PRJNA827465;Yang2025_Silkie_Wenchang	CRA004775	0.40857092
ENSGALG00010018426	ENSGALG00010018426	2	1.035487148	0.804893582	1.286489507	0.198272279	1.114673621	6.712117673	85.10157228	1	CRA004775;Yang2025_Silkie_Wenchang	PRJNA827465	0.710234722
ENSGALG00010018426	ENSGALG00010018426	2	1.053853335	0.784303778	1.343680045	0.179051873	1.049478393	6.367031265	84.29409314	1	CRA004775;PRJNA827465	Yang2025_Silkie_Wenchang	0.685713211
ENSGALG00010018427	PIGF	2	0.236505937	0.115989273	2.039032847	0.041446749	0.007963677	1.407637253	28.95897024	1	PRJNA827465;Yang2025_Silkie_Wenchang	CRA004775	0.352975427
ENSGALG00010018427	PIGF	2	0.296284277	0.126044155	2.350638763	0.018741218	0	0.725908743	0	1	CRA004775;Yang2025_Silkie_Wenchang	PRJNA827465	0.229376786
ENSGALG00010018427	PIGF	2	0.137773826	0.108435498	1.270560179	0.203885161	0	0.000459632	0	1	CRA004775;PRJNA827465	Yang2025_Silkie_Wenchang	0.717254253
ENSGALG00010018428	PPP2R5D	2	-0.003648173	0.03954307	-0.092258207	0.926492893	0	0.108921293	0	0.5	PRJNA827465;Yang2025_Silkie_Wenchang	CRA004775	0.98280842
ENSGALG00010018428	PPP2R5D	2	-0.079545339	0.078045291	-1.019220222	0.308098428	0	0.960615317	0	1	CRA004775;Yang2025_Silkie_Wenchang	PRJNA827465	0.79331712
ENSGALG00010018428	PPP2R5D	2	-0.051196444	0.089580037	-0.571516219	0.567649769	0.008996984	1.774090632	43.63309394	0.5	CRA004775;PRJNA827465	Yang2025_Silkie_Wenchang	0.883717772
ENSGALG00010018430	LOXL4	2	0.093529641	0.201267852	0.464702335	0.642144636	0	0.007507303	0	1	PRJNA827465;Yang2025_Silkie_Wenchang	CRA004775	0.906634523
ENSGALG00010018430	LOXL4	2	-0.701977892	0.836799437	-0.838884278	0.401534251	1.193233948	6.526118539	84.67695623	0.5	CRA004775;Yang2025_Silkie_Wenchang	PRJNA827465	0.832623399
ENSGALG00010018430	LOXL4	2	-0.673950702	0.853934078	-0.789230363	0.429977379	1.273736896	7.490931914	86.65052611	0.5	CRA004775;PRJNA827465	Yang2025_Silkie_Wenchang	0.842012722
ENSGALG00010018434	HSPBAP1	2	0.026717071	0.092850947	0.287741505	0.773544616	0	0.232800285	0	0.5	PRJNA827465;Yang2025_Silkie_Wenchang	CRA004775	0.943248868
ENSGALG00010018434	HSPBAP1	2	0.04752742	0.140330349	0.338682402	0.734848999	0	0.315560881	0	0.5	CRA004775;Yang2025_Silkie_Wenchang	PRJNA827465	0.933537081
ENSGALG00010018434	HSPBAP1	2	-0.018018196	0.107337284	-0.167865215	0.866689316	0	0.091568539	0	1	CRA004775;PRJNA827465	Yang2025_Silkie_Wenchang	0.96757535
ENSGALG00010018436	FOXO4	2	-0.32885709	0.280266375	-1.17337333	0.240646128	0.149340566	20.00412785	95.00103175	1	PRJNA827465;Yang2025_Silkie_Wenchang	CRA004775	0.753158164
ENSGALG00010018436	FOXO4	2	-0.595755288	0.104570437	-5.697167434	1.21814E-08	0	0.470883607	0	1	CRA004775;Yang2025_Silkie_Wenchang	PRJNA827465	6.06034E-06
ENSGALG00010018436	FOXO4	2	-0.063175142	0.06151094	-1.027055387	0.304394384	0	0.565455394	0	1	CRA004775;PRJNA827465	Yang2025_Silkie_Wenchang	0.79225794
ENSGALG00010018438	PPP2R2A	2	-0.069774208	0.036486535	-1.912327589	0.055834189	0	0.668087948	0	1	PRJNA827465;Yang2025_Silkie_Wenchang	CRA004775	0.408997088
ENSGALG00010018438	PPP2R2A	2	0.161656844	0.197706509	0.817660707	0.413550953	0.06203375	4.509993375	77.82701843	0.5	CRA004775;Yang2025_Silkie_Wenchang	PRJNA827465	0.836246395
ENSGALG00010018438	PPP2R2A	2	0.123557805	0.23511815	0.525513684	0.599226143	0.09699823	7.383964127	86.45713897	0.5	CRA004775;PRJNA827465	Yang2025_Silkie_Wenchang	0.894908299
ENSGALG00010018440	MEA1	2	0.219777358	0.188532006	1.165729692	0.243723751	0.048994171	2.867834343	65.13048243	1	PRJNA827465;Yang2025_Silkie_Wenchang	CRA004775	0.755365463
ENSGALG00010018440	MEA1	2	0.201859969	0.341967767	0.590289462	0.554996603	0.139292197	2.221671876	54.98885272	0.5	CRA004775;Yang2025_Silkie_Wenchang	PRJNA827465	0.880223402
ENSGALG00010018440	MEA1	2	0.05687706	0.090601649	0.627770696	0.530154165	0	0.544245871	0	0.5	CRA004775;PRJNA827465	Yang2025_Silkie_Wenchang	0.872191258
ENSGALG00010018441	CRIPT	2	-0.280574762	0.422855713	-0.663523642	0.506995243	0.345122082	28.37822819	96.47617183	0.5	PRJNA827465;Yang2025_Silkie_Wenchang	CRA004775	0.864000088
ENSGALG00010018441	CRIPT	2	-0.240416011	0.488820676	-0.491828645	0.622840485	0.441447713	12.88880319	92.24132772	0.5	CRA004775;Yang2025_Silkie_Wenchang	PRJNA827465	0.900255255
ENSGALG00010018441	CRIPT	2	0.148209665	0.074787579	1.981741714	0.047508156	0	0.285474712	0	1	CRA004775;PRJNA827465	Yang2025_Silkie_Wenchang	0.376702342
ENSGALG00010018443	PLCB1	2	0.043013184	0.251139279	0.17127223	0.864009715	0.102334872	5.205590683	80.78988417	0.5	PRJNA827465;Yang2025_Silkie_Wenchang	CRA004775	0.966530523
ENSGALG00010018443	PLCB1	2	0.411110174	0.172055543	2.389403829	0.016875741	0.007150442	1.100565372	9.137610076	1	CRA004775;Yang2025_Silkie_Wenchang	PRJNA827465	0.215702857
ENSGALG00010018443	PLCB1	2	0.205007636	0.446445062	0.459200142	0.646090449	0.341100676	6.419504001	84.42247252	0.5	CRA004775;PRJNA827465	Yang2025_Silkie_Wenchang	0.907220702
ENSGALG00010018444	NUBP2	2	-0.087688786	0.085448591	-1.026216873	0.30478937	0	0.081723403	0	1	PRJNA827465;Yang2025_Silkie_Wenchang	CRA004775	0.79225794
ENSGALG00010018444	NUBP2	2	-0.24182539	0.192514703	-1.256139852	0.209065269	0.0270824	1.561695611	35.96703527	1	CRA004775;Yang2025_Silkie_Wenchang	PRJNA827465	0.721011841
ENSGALG00010018444	NUBP2	2	-0.205919442	0.149690174	-1.375637666	0.168933839	0.025910897	2.098518539	52.34733544	1	CRA004775;PRJNA827465	Yang2025_Silkie_Wenchang	0.670135297
ENSGALG00010018446	FBN1	2	-0.218837455	0.223500159	-0.979137804	0.327511895	0.06487588	2.749249929	63.62644264	1	PRJNA827465;Yang2025_Silkie_Wenchang	CRA004775	0.800077397
ENSGALG00010018446	FBN1	2	-0.036051318	0.519992115	-0.06933051	0.944726541	0.41299916	3.893407689	74.31555902	0.5	CRA004775;Yang2025_Silkie_Wenchang	PRJNA827465	0.986014725
ENSGALG00010018446	FBN1	2	0.100908493	0.245104341	0.411696068	0.680562207	0.053364212	1.416100656	29.38355084	0.5	CRA004775;PRJNA827465	Yang2025_Silkie_Wenchang	0.917851751
ENSGALG00010018448	KLHDC3	2	-0.083552435	0.220681463	-0.378611024	0.704976735	0.087259714	9.503580525	89.47765006	0.5	PRJNA827465;Yang2025_Silkie_Wenchang	CRA004775	0.924152671
ENSGALG00010018448	KLHDC3	2	0.109308504	0.120837173	0.904593359	0.365680855	0.002186466	1.022636847	2.213576301	0.5	CRA004775;Yang2025_Silkie_Wenchang	PRJNA827465	0.818968857
ENSGALG00010018448	KLHDC3	2	-0.296708219	0.081124893	-3.657425084	0.000254762	0	4.29989E-05	0	1	CRA004775;PRJNA827465	Yang2025_Silkie_Wenchang	0.01505699
ENSGALG00010018451	PIK3R4	2	0.05329027	0.050117806	1.063300131	0.287645866	0	0.648238441	0	0.5	PRJNA827465;Yang2025_Silkie_Wenchang	CRA004775	0.782840067
ENSGALG00010018451	PIK3R4	2	-0.002187548	0.084781458	-0.025802201	0.979415106	0	0.019799454	0	0.5	CRA004775;Yang2025_Silkie_Wenchang	PRJNA827465	0.994401035
ENSGALG00010018451	PIK3R4	2	0.0785772	0.059178173	1.327807141	0.184241827	0	0.026989934	0	1	CRA004775;PRJNA827465	Yang2025_Silkie_Wenchang	0.692545576
ENSGALG00010018452	SPSB3	2	-0.139903309	0.092915706	-1.505701404	0.132143843	0.008161934	1.802708439	44.52791264	1	PRJNA827465;Yang2025_Silkie_Wenchang	CRA004775	0.608786928
ENSGALG00010018452	SPSB3	2	-0.239017252	0.110991746	-2.153468703	0.031281861	0	0.171054415	0	1	CRA004775;Yang2025_Silkie_Wenchang	PRJNA827465	0.30260177
ENSGALG00010018452	SPSB3	2	-0.071599799	0.07324214	-0.977576556	0.328283797	0	0.049178007	0	1	CRA004775;PRJNA827465	Yang2025_Silkie_Wenchang	0.800761292
ENSGALG00010018453	TMX4	2	-0.075317719	0.074149358	-1.015756863	0.309745183	0.006233465	2.190817765	54.3549438	1	PRJNA827465;Yang2025_Silkie_Wenchang	CRA004775	0.794371602
ENSGALG00010018453	TMX4	2	-0.180684182	0.08513847	-2.122239016	0.033817672	0	0.653555538	0	1	CRA004775;Yang2025_Silkie_Wenchang	PRJNA827465	0.315934384
ENSGALG00010018453	TMX4	2	-0.090095394	0.178125106	-0.5057984	0.612998182	0.034466352	1.386252936	27.86309235	1	CRA004775;PRJNA827465	Yang2025_Silkie_Wenchang	0.898130964
ENSGALG00010018455	RBM20	2	-0.119568248	0.171546411	-0.697002331	0.485801335	0.040579797	3.18574422	68.61016043	0.5	PRJNA827465;Yang2025_Silkie_Wenchang	CRA004775	0.857391223
ENSGALG00010018455	RBM20	2	0.0970332	0.145440269	0.66716873	0.504664362	0	0.686983346	0	1	CRA004775;Yang2025_Silkie_Wenchang	PRJNA827465	0.863512504
ENSGALG00010018455	RBM20	2	-0.04830681	0.369635407	-0.130687724	0.896022344	0.17565582	2.082209456	51.9740919	0.5	CRA004775;PRJNA827465	Yang2025_Silkie_Wenchang	0.975009446
ENSGALG00010018458	SEMA5B	2	-0.180886428	0.087112479	-2.07646975	0.037850526	0	0.265154516	0	1	PRJNA827465;Yang2025_Silkie_Wenchang	CRA004775	0.335620339
ENSGALG00010018458	SEMA5B	2	-0.098159687	0.231241921	-0.424489152	0.671209115	0.05220542	1.657199086	39.65721993	0.5	CRA004775;Yang2025_Silkie_Wenchang	PRJNA827465	0.914656815
ENSGALG00010018458	SEMA5B	2	-0.089027811	0.146391676	-0.608148042	0.543089291	0.013531341	1.18294782	15.46541759	0.5	CRA004775;PRJNA827465	Yang2025_Silkie_Wenchang	0.876968972
ENSGALG00010018459	SNX12	2	-0.194814738	0.272964947	-0.71369874	0.475413486	0.134578861	9.462908882	89.43242493	0.5	PRJNA827465;Yang2025_Silkie_Wenchang	CRA004775	0.854462864
ENSGALG00010018459	SNX12	2	-0.247537872	0.257381676	-0.961754063	0.336173158	0.097319832	3.754574004	73.36581996	0.5	CRA004775;Yang2025_Silkie_Wenchang	PRJNA827465	0.803727281
ENSGALG00010018459	SNX12	2	0.051577035	0.032326128	1.595521598	0.110595644	0	0.027414821	0	1	CRA004775;PRJNA827465	Yang2025_Silkie_Wenchang	0.562832796
ENSGALG00010018462	KTN1	2	0.193329102	0.286800586	0.674088937	0.500254773	0.152370449	12.92390336	92.26239959	0.5	PRJNA827465;Yang2025_Silkie_Wenchang	CRA004775	0.862271438
ENSGALG00010018462	KTN1	2	0.430489099	0.123758689	3.478455544	0.000504312	0	0.598737771	0	1	CRA004775;Yang2025_Silkie_Wenchang	PRJNA827465	0.023568345

ENSGALG00010018462	KTN1	2	0.051864945	0.176706235	0.293509425	0.769132797	0.045112066	2.875175253	65.21951143	0.5	CRA004775;PRJNA827465	Yang2025_Silkie_Wenchang	0.941790377
ENSGALG00010018465	NAALADL2	2	0.223220812	0.098184841	2.273475307	0.022997552	0	0.015882273	0	1	PRJNA827465;Yang2025_Silkie_Wenchang	CRA004775	0.258544853
ENSGALG00010018465	NAALADL2	2	0.65305864	0.551345343	1.18448201	0.236222314	0.411510019	2.594186904	61.45227629	1	CRA004775;Yang2025_Silkie_Wenchang	PRJNA827465	0.748669015
ENSGALG00010018465	NAALADL2	2	0.623439893	0.564087456	1.105218501	0.269064922	0.471280006	2.992496174	66.58308175	1	CRA004775;PRJNA827465	Yang2025_Silkie_Wenchang	0.771461882
ENSGALG00010018466	DYSF	2	-0.014856274	0.221268473	-0.067141395	0.94646914	0.081129053	5.725755508	82.53505588	0.5	PRJNA827465;Yang2025_Silkie_Wenchang	CRA004775	0.986445196
ENSGALG00010018466	DYSF	2	0.203205081	0.14516631	1.399808821	0.161570576	0	0.138170839	0	1	CRA004775;Yang2025_Silkie_Wenchang	PRJNA827465	0.658189089
ENSGALG00010018466	DYSF	2	-0.210677436	0.102757197	-2.050245072	0.040340522	0	0.283114563	0	0.5	CRA004775;PRJNA827465	Yang2025_Silkie_Wenchang	0.347442284
ENSGALG00010018467	TLX1	2	0.591390098	0.682416608	0.866611526	0.386154896	0.744030015	4.966980904	79.86704561	0.5	PRJNA827465;Yang2025_Silkie_Wenchang	CRA004775	0.826894808
ENSGALG00010018467	TLX1	2	1.496479152	0.455921654	3.282316466	0.00102958	0.088101728	1.161341465	13.89268097	1	CRA004775;Yang2025_Silkie_Wenchang	PRJNA827465	0.038538644
ENSGALG00010018467	TLX1	2	1.000557401	1.237247316	0.80869636	0.418689822	2.543243364	5.543632901	81.96128752	0.5	CRA004775;PRJNA827465	Yang2025_Silkie_Wenchang	0.838211394
ENSGALG00010018469	KLHDC7A	2	0.210998209	0.17181259	1.228071874	0.219419988	0.037018736	2.642293435	62.15408982	1	PRJNA827465;Yang2025_Silkie_Wenchang	CRA004775	0.732888275
ENSGALG00010018469	KLHDC7A	2	0.166922369	0.251218926	0.664449814	0.50640246	0.089187043	3.361313565	70.24972587	0.5	CRA004775;Yang2025_Silkie_Wenchang	PRJNA827465	0.863929781
ENSGALG00010018469	KLHDC7A	2	0.012721839	0.111803669	0.113787313	0.909406396	0	0.391361758	0	0.5	CRA004775;PRJNA827465	Yang2025_Silkie_Wenchang	0.978607662
ENSGALG00010018470	IQCB1	2	-0.049928218	0.201841027	-0.2225921	0.823852985	0.061308069	4.035261099	75.21845612	0.5	PRJNA827465;Yang2025_Silkie_Wenchang	CRA004775	0.956089818
ENSGALG00010018470	IQCB1	2	0.124346791	0.133922515	0.928498029	0.35314929	0	0.351067653	0	0.5	CRA004775;Yang2025_Silkie_Wenchang	PRJNA827465	0.81107437
ENSGALG00010018470	IQCB1	2	-0.213488115	0.125909041	-1.695574155	0.089966556	0	0.307094563	0	1	CRA004775;PRJNA827465	Yang2025_Silkie_Wenchang	0.514545335
ENSGALG00010018471	HHEX	2	-0.086327661	0.166875074	-0.517319085	0.604933434	0.043284215	4.472275322	77.64001704	0.5	PRJNA827465;Yang2025_Silkie_Wenchang	CRA004775	0.896474603
ENSGALG00010018471	HHEX	2	-0.271198438	0.114386072	-2.370904363	0.017744622	0	0.140807901	0	1	CRA004775;Yang2025_Silkie_Wenchang	PRJNA827465	0.2222112
ENSGALG00010018471	HHEX	2	-0.015367347	0.190851868	-0.080519764	0.935823878	0.033948208	1.389486911	28.03098812	0.5	CRA004775;PRJNA827465	Yang2025_Silkie_Wenchang	0.984801197
ENSGALG00010018472	LMF1	2	-0.258646163	0.131229771	-1.970941208	0.048730601	0.019850931	2.28945142	56.32141432	1	PRJNA827465;Yang2025_Silkie_Wenchang	CRA004775	0.381601676
ENSGALG00010018472	LMF1	2	-0.224530336	0.215010456	-1.044276359	0.296357547	0.060448341	2.786481633	64.11244961	0.5	CRA004775;Yang2025_Silkie_Wenchang	PRJNA827465	0.778267543
ENSGALG00010018472	LMF1	2	-0.116129352	0.091073935	-1.275110733	0.202270051	0	0.49927848	0	0.5	CRA004775;PRJNA827465	Yang2025_Silkie_Wenchang	0.715289846
ENSGALG00010018476	ENSGALG00010018476	2	0.336741373	0.567108407	0.593786599	0.552654853	0.617019754	24.49077663	95.91683018	0.5	PRJNA827465;Yang2025_Silkie_Wenchang	CRA004775	0.87928478
ENSGALG00010018476	ENSGALG00010018476	2	0.451979725	0.470653546	0.960323636	0.336892358	0.398204167	9.813239763	89.80968544	0.5	CRA004775;Yang2025_Silkie_Wenchang	PRJNA827465	0.803904536
ENSGALG00010018476	ENSGALG00010018476	2	-0.171403692	0.121737663	-1.440833402	0.149631754	0	0.477334283	0	1	CRA004775;PRJNA827465	Yang2025_Silkie_Wenchang	0.649445128
ENSGALG00010018480	EME2	2	0.247926926	0.113143273	2.191265285	0.028432601	0	0.002142857	0	1	PRJNA827465;Yang2025_Silkie_Wenchang	CRA004775	0.287193179
ENSGALG00010018480	EME2	2	-0.605238835	0.907345827	-0.667043168	0.50474456	1.440127842	7.663266691	86.95073472	0.5	CRA004775;Yang2025_Silkie_Wenchang	PRJNA827465	0.863512504
ENSGALG00010018480	EME2	2	-0.578056677	0.913127906	-0.633051156	0.526700237	1.504652103	9.409389496	89.37231793	0.5	CRA004775;PRJNA827465	Yang2025_Silkie_Wenchang	0.87066367
ENSGALG00010018482	TWNK	2	0.009692124	0.078050099	0.124178237	0.901174154	0	0.162332254	0	0.5	PRJNA827465;Yang2025_Silkie_Wenchang	CRA004775	0.979660383
ENSGALG00010018482	TWNK	2	-0.168290025	0.308407454	-0.545674309	0.585289835	0.120183152	2.519435923	60.30857578	0.5	CRA004775;Yang2025_Silkie_Wenchang	PRJNA827465	0.891130641
ENSGALG00010018482	TWNK	2	-0.173318465	0.252688912	-0.685896595	0.492778318	0.085093919	2.320597264	56.90764547	1	CRA004775;PRJNA827465	Yang2025_Silkie_Wenchang	0.859474364
ENSGALG00010018485	ATP2C1	2	-0.034709946	0.050679381	-0.684892851	0.493411538	0.002738855	1.991082924	49.77607471	0.5	PRJNA827465;Yang2025_Silkie_Wenchang	CRA004775	0.893868321
ENSGALG00010018485	ATP2C1	2	-0.092821903	0.062071437	-1.495404437	0.134808923	0	0.187813238	0	1	CRA004775;Yang2025_Silkie_Wenchang	PRJNA827465	0.615305436
ENSGALG00010018485	ATP2C1	2	0.004089348	0.036184305	0.113014406	0.910019134	0	0.000490992	0	0.5	CRA004775;PRJNA827465	Yang2025_Silkie_Wenchang	0.978730675
ENSGALG00010018486	ENSGALG00010018486	2	0.039794286	0.092748976	0.429053642	0.667884188	0.008569547	1.482805153	32.56025596	0.5	PRJNA827465;Yang2025_Silkie_Wenchang	CRA004775	0.914120722
ENSGALG00010018486	ENSGALG00010018486	2	0.243813629	0.124237312	1.962483132	0.049706263	0	0.026689403	0	1	CRA004775;Yang2025_Silkie_Wenchang	PRJNA827465	0.346162809
ENSGALG00010018486	ENSGALG00010018486	2	0.078495192	0.125801219	0.623962093	0.53265248	0.02094489	2.347872086	57.40824187	0.5	CRA004775;PRJNA827465	Yang2025_Silkie_Wenchang	0.873183244
ENSGALG00010018487	ENSGALG00010018487	2	-0.052524872	0.122911817	-0.427337851	0.669133267	0.017255117	2.167501769	53.86393616	0.5	PRJNA827465;Yang2025_Silkie_Wenchang	CRA004775	0.914377769
ENSGALG00010018487	ENSGALG00010018487	2	-0.241354775	0.139623839	-1.728607216	0.083879417	0	0.352925617	0	1	CRA004775;Yang2025_Silkie_Wenchang	PRJNA827465	0.498972717
ENSGALG00010018487	ENSGALG00010018487	2	-0.06327687	0.205042656	-0.308603445	0.7576232	0.046378739	1.591657019	37.17239405	0.5	CRA004775;PRJNA827465	Yang2025_Silkie_Wenchang	0.938948211
ENSGALG00010018488	ENSGALG00010018488	2	-0.053927027	0.236991489	-0.227548371	0.819997361	0.08218883	3.666784386	72.7281483	0.5	PRJNA827465;Yang2025_Silkie_Wenchang	CRA004775	0.955308792
ENSGALG00010018488	ENSGALG00010018488	2	0.093347588	0.183219315	0.509485523	0.610411944	0.00584225	1.071941887	6.711360771	0.5	CRA004775;Yang2025_Silkie_Wenchang	PRJNA827465	0.897438028
ENSGALG00010018488	ENSGALG00010018488	2	-0.264360786	0.136763901	-1.932971962	0.053239652	0	0.023342453	0	1	CRA004775;PRJNA827465	Yang2025_Silkie_Wenchang	0.400004977
ENSGALG00010018489	SLC7A3	2	0.051689048	0.089330307	0.578628344	0.562839975	0.00450039	1.341226665	25.44138692	0.5	PRJNA827465;Yang2025_Silkie_Wenchang	CRA004775	0.882070607
ENSGALG00010018489	SLC7A3	2	-0.364735514	0.337545521	-1.080552079	0.279896409	0.181049985	4.520661284	77.87934249	1	CRA004775;Yang2025_Silkie_Wenchang	PRJNA827465	0.778040488
ENSGALG00010018489	SLC7A3	2	-0.274701897	0.4327559	-0.634773314	0.52557627	0.332524846	8.245506315	87.87218199	0.5	CRA004775;PRJNA827465	Yang2025_Silkie_Wenchang	0.870277065
ENSGALG00010018490	NME3	2	-0.177086546	0.249165809	-0.710717683	0.4772592	0.103744205	5.585097539	82.09520974	0.5	PRJNA827465;Yang2025_Silkie_Wenchang	CRA004775	0.854997403
ENSGALG00010018490	NME3	2	-0.553574178	0.166342201	-3.327923852	0.000874958	0	0.725818669	0	1	CRA004775;Yang2025_Silkie_Wenchang	PRJNA827465	0.034721966
ENSGALG00010018490	NME3	2	-0.311679239	0.402988741	-0.773419222	0.439274317	0.280905253	6.653999301	84.97144417	0.5	CRA004775;PRJNA827465	Yang2025_Silkie_Wenchang	0.844161867
ENSGALG00010018491	TIA1	2	0.219515944	0.303505807	0.723267692	0.469515438	0.177276093	26.25050927	96.19056185	0.5	PRJNA827465;Yang2025_Silkie_Wenchang	CRA004775	0.853485658
ENSGALG00010018491	TIA1	2	0.30633616	0.250814881	1.221363572	0.221948393	0.101904428	4.960115383	79.83917867	1	CRA004775;Yang2025_Silkie_Wenchang	PRJNA827465	0.735359501
ENSGALG00010018491	TIA1	2	-0.070203388	0.057185884	-1.227634927	0.219584044	0	0.233046738	0	0.5	CRA004775;PRJNA827465	Yang2025_Silkie_Wenchang	0.733059687
ENSGALG00010018492	ENSGALG00010018492	2	-0.062475901	0.115092001	-0.542834833	0.587243803	0.014364015	2.035702918	50.87691866	0.5	PRJNA827465;Yang2025_Silkie_Wenchang	CRA004775	0.891645821
ENSGALG00010018492	ENSGALG00010018492	2	-0.245338428	0.139795243	-1.754993097	0.079259582	0	0.8934689	0	1	CRA004775;Yang2025_Silkie_Wenchang	PRJNA827465	0.484394238
ENSGALG00010018492	ENSGALG00010018492	2	-0.169964524	0.338065343	-0.502756428	0.615135531	0.146159943	1.936219959	48.35297533	0.5	CRA004775;PRJNA827465	Yang2025_Silkie_Wenchang	0.8988191
ENSGALG00010018494	HAO1	2	0.366485111	0.849044524	0.431644161	0.666000057	1.06775981	3.848343761	74.0147954	0.5	PRJNA827465;Yang2025_Silkie_Wenchang	CRA004775	0.913546323
ENSGALG00010018494	HAO1	2	-0.109099149	0.432875206	-0.25203372	0.801015	0	0.819197599	0	0.5	CRA004775;Yang2025_Silkie_Wenchang	PRJNA827465	0.950158029
ENSGALG00010018494	HAO1	2	0.785801849	0.455330421	1.725783767	0.084386321	0.000220347	1.000511635	0.055135277	1	CRA004775;PRJNA827465	Yang2025_Silkie_Wenchang	0.500837819
ENSGALG00010018495	ENSGALG00010018495	2	0.240155468	0.26956331	0.890905622	0.372979804	0	0.006053327	0	1	PRJNA827465;Yang2025_Silkie_Wenchang	CRA004775	0.822162024
ENSGALG00010018495	ENSGALG00010018495	2	0.245883746	0.337924715	0.727628773	0.466840862	0	0.034718263	0	1	CRA004775;Yang2025_Silkie_Wenchang	PRJNA827465	0.852842649
ENSGALG00010018495	ENSGALG00010018495	2	0.286172243	0.369398325	0.774698268	0.438517975	0	0.02011579	0	1	CRA004775;PRJNA827465	Yang2025_Silkie_Wenchang	0.844802503
ENSGALG00010018497	ASTE1	2	-0.190198384	0.092616275	-2.053617279	0.040012748	0	0.257057685	0	1	PRJNA827465;Yang2025_Silkie_Wenchang	CRA004775	0.3465426
ENSGALG00010018497	ASTE1	2	-0.292694005	0.171567481	-1.705999309	0.088008208	0	7.35974E-05	0	1	CRA004775;Yang2025_Silkie_Wenchang	PRJNA827465	0.509723933
ENSGALG00010018497	ASTE1	2	-0.183839272	0.095641823	-1.922164036	0.054583121	0	0.186405896	0	1	CRA004775;PRJNA827465	Yang2025_Silkie_Wenchang	0.404326987
ENSGALG00010018498	ENSGALG00010018498	2	-0.339250447	0.316459453	-1.072018686	0.283711639	0.172610588	7.235112494	86.17889689	1	PRJNA827465;Yang2025_Silkie_Wenchang	CRA004775	0.779756886
ENSGALG00010018498	ENSGALG00010018498	2	-0.440104634	0.359429834	-1.22445215	0.220781705	0.147934748	1.805748403	44.62129951	0.5	CRA004775;Yang2025_Silkie_Wenchang	PRJNA827465	0.734054953
ENSGALG00010018498	ENSGALG00010018498	2	-0.00948195	0.159495684	-0.05944957	0.952594032	0	0.089673565	0	0.5	CRA004775;PRJNA827465	Yang2025_Silkie_Wenchang	0.987758913
ENSGALG00010018501	ATG14	2	0.108272785	0.067814114	1.596611359	0.110352365	0.001894206	1.204741247	16.99462417	1	PRJNA827465;Yang2025_Silkie_Wenchang	CRA004775	0.562436811
ENSGALG00010018501	ATG14	2	0.195299135	0.097269477	2.007815215	0.044662931	0	0.079778295	0	1	CRA004775;Yang2025_Silkie_Wenchang	PRJNA827465	0.364322144
ENSGALG00010018501	ATG14	2	0.076983733	0.063043934	1.22111246	0.222043443	0	0.239278798	0	1	CRA004775;PRJNA827465	Yang2025_Silkie_Wenchang	0.735359501
ENSGALG00010018502	ARHGEF10L	2	0.160798358	0.091238877	1.762388614	0.078003661	0.009659808	2.08739338	52.09336153	1	PRJNA827465;Yang2025_Silkie_Wenchang	CRA004775	0.480830912
ENSGALG00010018502	ARHGEF10L	2	-0.108083013	0.426452273	-0.253446914	0.799922878	0.323824436	8.727376812	88.54180332	0.5	CRA004775;Yang2025_Silkie_Wenchang	PRJNA827465	0.949923217
ENSGALG00010018502	ARHGEF10L	2	-0.186221909	0.327569449	-0.568495961	0.569698247	0.183590472	6.102636989	83.61364109	0.5	CRA004775;PRJNA827465	Yang2025_Silkie_Wenchang	0.884908912
ENSGALG00010018503	BMP2	2	0.21171571	0.128929575	1.642103521	0.100568552	0	0.664642606	0	1	PRJNA827465;Yang2025_Silkie_Wenchang	CRA004775	0.541393598
ENSGALG00010018503	BMP2	2	0.283470373	0.223439771	1.268665696	0.204560322	0	0.796449896	0	0.5	CRA004775;Yang2025_Silkie_Wenchang	PRJNA827465	0.71729696
ENSGALG00010018503	BMP2	2	0.127636466	0.143015277	0.892467252	0.372142485	0	0.23507541	0	0.5	CRA004775;PRJNA827465	Yang2025_Silkie_Wenchang	0.821620523
ENSGALG00010018504	SLCO3A1	2	-0.010601377	0.060611978	-0.174905638	0.86115378	0	9.87047E-06	0	1	PRJNA827465;Yang2025_Silkie_Wenchang	CRA004775	0.965343309
ENSGALG00010018504	SLCO3A1	2	-0.077189727	0.153692506	-0.502234814	0.615502357	0.01974567	1.284508028	22.14918253	1	CRA004775;Yang2025_Silkie_Wenchang	PRJNA827465	0.898886043
ENSGALG00010018504	SLCO3A1	2	-0.075143884	0.15219105	-0.493747061	0.621484825	0.020746723	1.302590371	23.22989466	1	CRA004775;PRJNA827465	Yang2025_Silkie_Wenchang	0.899719912
ENSGALG00010018505	CHTF18	2	-0.015634401	0.045541111	-0.343303027	0.731370504	0	0.003620988	0	1	PRJNA827465;Yang2025_Silkie_Wenchang	CRA004775	0.932851968
ENSGALG00010018505	CHTF18	2	-0.051377271	0.096068903	-0.534796061	0.592790887	0	0.945787798	0	1	CRA004775;Yang2025_Silkie_Wenchang	PRJNA827465	0.892776493
ENSGALG00010018505	CHTF18	2	-0.031755292	0.073371905	-0.432799074	0.665160746	0.003086607	1.062057129	5.843106527	1	CRA004775;PRJNA827465	Yang2025_Silkie_Wenchang	0.913399315
ENSGALG00010018506	ENSGALG00010018506	2	0.419892064	0.416466158	1.008226134	0.313345915	0.307585808	8.681492099	88.48124276	1	PRJNA827465;Yang2025_Silkie_Wenchang	CRA004775	0.796531448
ENSGALG00010018506	ENSGALG00010018506	2	0.345235717	0.530172234	0.651176533	0.514932537	0.485920265	7.30869294	86.31766298	0.5	CRA004775;Yang2025_Silkie_Wenchang	PRJNA827465	0.866997453
ENSGALG00010018506	ENSGALG00010018506	2	-0.022503425	0.13884763	-0.162072809	0.871248519	0	0.423445447	0	0.5	CRA004775;PRJNA827465	Yang2025_Silkie_Wenchang	0.969278598
ENSGALG00010018508	ENSGALG00010018508	2	0.176596205	0.175576566	1.005807372	0.31450824	0.051857866	5.86666315	82.95453524	1	PRJNA827465;Yang2025_Silkie_Wenchang	CRA004775	0.796531448
ENSGALG00010018508	ENSGALG00010018508	2	-0.062812878	0.455528167	-0.137890217	0.890327182	0.38043162	11.77665097	91.50862157	0.5	CRA004775;Yang2025_Silkie_Wenchang	PRJNA827465	0.973417023
ENSGALG00010018508	ENSGALG00010018508	2	-0.214451425	0.275614526	-0.778084625	0.436519126	0.127852725	5.57073052	82.04903295	0.5	CRA004775;PRJNA827465	Yang2025_Silkie_Wenchang	0.843196108
ENSGALG00010018509	ENSGALG00010018509	2	0.069625399	0.089320785	0.779498285	0.435686247	0.008767589	2.16710319	53.85545069	0.5	PRJNA827465;Yang2025_Silkie_Wenchang	CRA004775	0.842762754
ENSGALG00010018509	ENSGALG00010018509	2	-0.058491731	0.244966546	-0.238774364	0.81128055	0.103588397	7.151516	86.01695081	0.5	CRA004775;Yang2025_Silkie_Wenchang	PRJNA827465	0.953179464
ENSGALG00010018509	ENSGALG00010018509	2	-0.13298674	0.152224909	-0.873620097	0.382325178	0.033606801	3.310574634	69.7937636	1	CRA004775;PRJNA827465	Yang2025_Silkie_Wenchang	0.825080207
ENSGALG00010018510	ENSGALG00010018510	2	-0.161933939	0.066775811	-2.425038894	0.015306747	0	0.155688807	0	1	PRJNA827465;Yang2025_Silkie_Wenchang	CRA004775	0.204675996
ENSGALG00010018510	ENSGALG00010018510	2	-0.192974961	0.146788412	-1.314647101	0.188628533	0	0.142006549	0	1	CRA004775;Yang2025_Silkie_Wenchang	PRJNA827465	0.698039126
ENSGALG00010018510	ENSGALG00010018510	2	-0.147105859	0.071168362	-2.067012019	0.038733021	0	0.038470084	0	1	CRA004775;PRJNA827465	Yang2025_Silkie_Wenchang	0.339652821
ENSGALG00010018511	SOCS5	2	-0.143739236	0.280935339	-0.511645266	0.608899299	0.151888229	26.28269269	96.19521481	0.5	PRJNA827465;Yang2025_Silkie_Wenchang	CRA004775	0.897208691
ENSGALG00010018511	SOCS5	2	-0.127636165	0.321591363	-0.39688928	0.69144911	0.185848341	9.517163227	89.49266734	0.5	CRA004775;Yang2025_Silkie_Wenchang	PRJNA827465	0.920268407
ENSGALG00010018511	SOCS5	2	0.139535692	0.054895136	2.54185892	0.011026467	0	0.178130537	0	1	CRA004775;PRJNA827465	Yang2025_Silkie_Wenchang	0.166938842
ENSGALG00010018512	FERMT1	2	0.046297253	0.081504589	0.568032476	0.570012916	0	0.049402749	0	1	PRJNA827465;Yang2025_Silkie_Wenchang	CRA004775	0.884943026
ENSGALG00010018512	FERMT1	2	-0.07018484	0.190198269	-0.369008827	0.712121145	0.040434521	1.99952723	49.98817795	0.5	CRA004775;Yang2025_Silkie_Wenchang	PRJNA827465	0.92597752
ENSGALG00010018512	FERMT1	2	-0.079568446	0.167276628	-0.475669836	0.634309614	0.027328279	1.689807823	40.82167297	0.5	CRA004775;PRJNA827465	Yang2025_Silkie_Wenchang	0.903815945
ENSGALG00010018513	RPUSD1	2	0.055855939	0.113617217	0.491615098	0.622991468	0.015239175	2.248522059	55.52634248	0.5	PRJNA827465;Yang2025_Silkie_Wenchang	CRA004775	0.900334887
ENSGALG00010018513	RPUSD1	2	-0.325950931	0.279096434	-1.16787924	0.242855479	0.114768546	3.527193904	71.64885098	1	CRA004775;Yang2025_Silkie_Wenchang	PRJNA827465	0.7550024
ENSGALG00010018513	RPUSD1	2	-0.214176893	0.397989218	-0.538147475	0.590475239	0.281503672	8.26822529	87.90550614	0.5	CRA004775;PRJNA827465	Yang2025_Silkie_Wenchang	0.892594166
ENSGALG00010018514	EAF2	2	0.275449579	0.123629154	2.228030921	0.025878453	0	0.14392583	0	1	PRJNA827465;Yang2025_Silkie_Wenchang	CRA004775	0.274225085
ENSGALG00010018514	EAF2	2	0.292045472	0.189330516	1.54251664	0.122948097	0	0.420879325	0	0.5	CRA004775;Yang2025_Silkie_Wenchang	PRJNA827465	0.587862418
ENSGALG00010018514	EAF2	2	0.217955833	0.151213543	1.441377726	0.149477999	0	0.249161234	0	0.5	CRA004775;PRJNA827465	Yang2025_Silkie_Wenchang	0.639246608
ENSGALG00010018515	SEMA4G	2	0.297652793	0.106471263	2.795616241	0.005180087	0	5.15721E-05	0	1	PRJNA827465;Yang2025_Silkie_Wenchang	CRA004775	0.107436917
ENSGALG00010018515	SEMA4G	2	0.028577702	0.268555375	0.106412696	0.91525492	0.088598723	2.592150734	61.42199654	0.5	CRA004775;Yang2025_Silkie_Wenchang	PRJNA827465	0.980117272
ENSGALG00010018515	SEMA4G	2	0.067411127	0.26668489	0.252774451	0.800442512	0.11004007	4.124034743	75.75190166	0.5	CRA004775;PRJNA827465	Yang2025_Silkie_Wenchang	0.950102441
ENSGALG00010018516	LRRN4	2	-0.171058024	0.108775539	-1.572578035	0.115816557	0	0.409759451	0	1	PRJNA827465;Yang2025_Silkie_Wenchang	CRA004775	0.572808488
ENSGALG00010018516	LRRN4	2	0.097923545	0.231259837	0.423435155	0.6719778	0.020667476	1.178845797	15.17126306	0.5	CRA004775;Yang2025_Silkie_Wenchang	PRJNA827465	0.914714609
ENSGALG00010018516	LRRN4	2	0.01693824	0.323163731	0.052413801	0.958198978	0.140044847	2.443889747	59.08162382	0.5	CRA004775;PRJNA827465	Yang2025_Silkie_Wenchang	0.988921128
ENSGALG00010018517	ECT2	2	-0.335664643	0.244031333	-1.37549813	0.168977065	0.103948558	7.832940495	87.23340231	1	PRJNA827465;Yang2025_Silkie_Wenchang	CRA004775	0.670229648
ENSGALG00010018517	ECT2	2	-0.001764183	0.675379208	-0.002612137	0.997915819	0.778650419	6.078942023	83.54976908	0.5	CRA004775;Yang2025_Silkie_Wenchang	PRJNA827465	0.999290321
ENSGALG00010018517	ECT2	2	0.184086824	0.409294979	0.449765654	0.652879426	0.233965277	2.552917051	60.82912292	0.5	CRA004775;PRJNA827465	Yang2025_Silkie_Wenchang	0.909572411
ENSGALG00010018519	OGDH	2	-0.116852579	0.092596759	-1.261951066	0.206966395	0.009320326	2.19052228	54.34878662	1	PRJNA827465;Yang2025_Silkie_Wenchang	CRA004775	0.718911823
ENSGALG00010018519	OGDH	2	-0.029303999	0.087086787	-0.336491911	0.73649995	0	0.089391099	0	1	CRA004775;Yang2025_Silkie_Wenchang	PRJNA827465	0.933661344
ENSGALG00010018519	OGDH	2	-0.206182561	0.085868575	-2.40114106	0.016344035	0	0.020589954	0	1	CRA004775;PRJNA827465	Yang2025_Silkie_Wenchang	0.211447764
ENSGALG00010018520	ENSGALG00010018520	2	0.391647991	0.09243296	4.23710323	2.26422E-05	0	0.055889121	0	1	PRJNA827465;Yang2025_Silkie_Wenchang	CRA004775	0.002696687
ENSGALG00010018520	ENSGALG00010018520	2	-0.238411374	0.690436222	-0.345305426	0.729864768	0.887823525	14.42838219	93.06921603	0.5	CRA004775;Yang2025_Silkie_Wenchang	PRJNA827465	0.932444623
ENSGALG00010018520	ENSGALG00010018520	2	-0.252838334	0.662466761	-0.381661917	0.702712152	0.829792568	17.70800895	94.35283773	0.5	CRA004775;PRJNA827465	Yang2025_Silkie_Wenchang	0.923272306
ENSGALG00010018521	ENSGALG00010018521	2	0.301871331	0.43211185	0.698595353	0.484804948	0.364870674	43.50337509	97.70132778	0.5	PRJNA827465;Yang2025_Silkie_Wenchang	CRA004775	0.857004733
ENSGALG00010018521	ENSGALG00010018521	2	0.334267887	0.432919535	0.772124747	0.440040546	0.338449787	9.81346019	89.80991434	0.5	CRA004775;Yang2025_Silkie_Wenchang	PRJNA827465	0.844353254
ENSGALG00010018521	ENSGALG00010018521	2	-0.127956226	0.075874264	-1.686424608	0.091714045	0	0.000212757	0	1	CRA004775;PRJNA827465	Yang2025_Silkie_Wenchang	0.518914432
ENSGALG00010018522	NEK11	2	-0.075031068	0.168216199	-0.446039491	0.65556872	0	0.161808327	0	0.5	PRJNA827465;Yang2025_Silkie_Wenchang	CRA004775	0.910411938
ENSGALG00010018522	NEK11	2	0.093836915	0.263725517	0.3558128	0.721980762	0	0.36547922	0	1	CRA004775;Yang2025_Silkie_Wenchang	PRJNA827465	0.929385887
ENSGALG00010018522	NEK11	2	-0.068495109	0.196061668	-0.349354924	0.726822869	0	0.721094495	0	0.5	CRA004775;PRJNA827465	Yang2025_Silkie_Wenchang	0.931378802
ENSGALG00010018523	TEX11	2	-0.005744893	0.169756374	-0.033841987	0.973003154	0.034251834	2.444621226	59.09386741	0.5	PRJNA827465;Yang2025_Silkie_Wenchang	CRA004775	0.992343539
ENSGALG00010018523	TEX11	2	0.172833746	0.148138867	1.166700882	0.243331187	0	0.003237593	0	1	CRA004775;Yang2025_Silkie_Wenchang	PRJNA827465	0.755238542
ENSGALG00010018523	TEX11	2	-0.111562749	0.127406739	-0.875642455	0.381224438	0	0.853760162	0	0.5	CRA004775;PRJNA827465	Yang2025_Silkie_Wenchang	0.824392515
ENSGALG00010018524	SEC22A	2	0.027673435	0.041084458	0.673574303	0.500581995	0	0.12939651	0	1	PRJNA827465;Yang2025_Silkie_Wenchang	CRA004775	0.862400704
ENSGALG00010018524	SEC22A	2	-0.063263356	0.130189401	-0.485933232	0.627014506	0.021980917	2.8349812	64.72639748	0.5	CRA004775;Yang2025_Silkie_Wenchang	PRJNA827465	0.901573209
ENSGALG00010018524	SEC22A	2	-0.063046063	0.107073251	-0.588812448	0.555987095	0.016555398	3.145854688	68.21213632	0.5	CRA004775;PRJNA827465	Yang2025_Silkie_Wenchang	0.880324407
ENSGALG00010018525	ST8SIA2	2	-0.022562994	0.08484626	-0.265927975	0.79029468	0	0.097596328	0	1	PRJNA827465;Yang2025_Silkie_Wenchang	CRA004775	0.947390191
ENSGALG00010018525	ST8SIA2	2	-0.065528098	0.150240184	-0.4361556	0.662723839	0	0.007308404	0	1	CRA004775;Yang2025_Silkie_Wenchang	PRJNA827465	0.912778072
ENSGALG00010018525	ST8SIA2	2	-0.007542053	0.100619377	-0.074956267	0.940249508	0	0.028534844	0	1	CRA004775;PRJNA827465	Yang2025_Silkie_Wenchang	0.985508732
ENSGALG00010018526	ENSGALG00010018526	2	0.004555016	0.06415476	0.071000429	0.943397414	0	0.931812847	0	0.5	PRJNA827465;Yang2025_Silkie_Wenchang	CRA004775	0.985827325
ENSGALG00010018526	ENSGALG00010018526	2	0.111140064	0.11382367	0.976423124	0.328854827	0	0.004818814	0	1	CRA004775;Yang2025_Silkie_Wenchang	PRJNA827465	0.800761292
ENSGALG00010018526	ENSGALG00010018526	2	-0.021891649	0.072308415	-0.302753818	0.76207748	0	0.310406912	0	0.5	CRA004775;PRJNA827465	Yang2025_Silkie_Wenchang	0.939963321
ENSGALG00010018527	CCT4	2	-0.144132055	0.169128495	-0.852204443	0.394100631	0.046114425	4.506646851	77.81055332	1	PRJNA827465;Yang2025_Silkie_Wenchang	CRA004775	0.830486298
ENSGALG00010018527	CCT4	2	-0.2657892	0.121613807	-2.185518296	0.028850866	0	0.724937102	0	1	CRA004775;Yang2025_Silkie_Wenchang	PRJNA827465	0.289410648
ENSGALG00010018527	CCT4	2	-0.010985179	0.044729947	-0.245588919	0.806000468	0	0.432722194	0	1	CRA004775;PRJNA827465	Yang2025_Silkie_Wenchang	0.952063038
ENSGALG00010018529	UBR2	2	0.055214042	0.037205238	1.484039456	0.137798453	0	0.073866661	0	1	PRJNA827465;Yang2025_Silkie_Wenchang	CRA004775	0.62064915
ENSGALG00010018529	UBR2	2	0.082195327	0.102509211	0.801833572	0.422649236	0.005783783	1.189346375	15.92020448	1	CRA004775;Yang2025_Silkie_Wenchang	PRJNA827465	0.839807023
ENSGALG00010018529	UBR2	2	0.075074964	0.058311646	1.287478041	0.197927719	0.001892025	1.06774069	6.344301665	1	CRA004775;PRJNA827465	Yang2025_Silkie_Wenchang	0.710035882
ENSGALG00010018530	FAM161A	2	0.249992355	0.160510867	1.557479317	0.119356727	0.014515885	1.364062383	26.6895699	1	PRJNA827465;Yang2025_Silkie_Wenchang	CRA004775	0.579860319
ENSGALG00010018530	FAM161A	2	0.048745112	0.490265429	0.099425962	0.92080007	0.351207952	3.376924526	70.38725645	0.5	CRA004775;Yang2025_Silkie_Wenchang	PRJNA827465	0.981469977
ENSGALG00010018530	FAM161A	2	-0.050445468	0.293167904	-0.172070228	0.863382319	0.088598392	1.654904082	39.57353718	0.5	CRA004775;PRJNA827465	Yang2025_Silkie_Wenchang	0.966256632
ENSGALG00010018531	ADCY5	2	0.021873305	0.082487005	0.265172734	0.790876398	0	0.94432414	0	0.5	PRJNA827465;Yang2025_Silkie_Wenchang	CRA004775	0.947777321
ENSGALG00010018531	ADCY5	2	0.06919832	0.237302169	0.291604244	0.77058923	0.060377763	1.732478898	42.27923925	0.5	CRA004775;Yang2025_Silkie_Wenchang	PRJNA827465	0.942491331
ENSGALG00010018531	ADCY5	2	0.11471637	0.103078233	1.112905869	0.265748859	0	0.863614268	0	1	CRA004775;PRJNA827465	Yang2025_Silkie_Wenchang	0.769919462
ENSGALG00010018532	MFSD13A	2	0.034408837	0.0678521	0.507115287	0.61207393	0	0.516745055	0	0.5	PRJNA827465;Yang2025_Silkie_Wenchang	CRA004775	0.897825694
ENSGALG00010018532	MFSD13A	2	-0.313830313	0.3216109	-0.975807453	0.329159891	0.166177567	4.675075428	78.60997078	1	CRA004775;Yang2025_Silkie_Wenchang	PRJNA827465	0.800761292
ENSGALG00010018532	MFSD13A	2	-0.263273857	0.374478877	-0.703040606	0.482030445	0.243218397	6.864197476	85.43165456	0.5	CRA004775;PRJNA827465	Yang2025_Silkie_Wenchang	0.85691916
ENSGALG00010018533	PCYOX1	2	-0.064884938	0.079280395	-0.818423499	0.413115408	0	0.046234338	0	1	PRJNA827465;Yang2025_Silkie_Wenchang	CRA004775	0.836037016
ENSGALG00010018533	PCYOX1	2	0.008357491	0.113072864	0.073912442	0.941080056	0	0.546949851	0	0.5	CRA004775;Yang2025_Silkie_Wenchang	PRJNA827465	0.985624005
ENSGALG00010018533	PCYOX1	2	-0.039421882	0.09027241	-0.436699125	0.662329564	0	0.874029571	0	0.5	CRA004775;PRJNA827465	Yang2025_Silkie_Wenchang	0.91272639
ENSGALG00010018534	NCEH1	2	0.01133181	0.08014245	0.141395856	0.887557228	0	0.799362414	0	0.5	PRJNA827465;Yang2025_Silkie_Wenchang	CRA004775	0.973038314
ENSGALG00010018534	NCEH1	2	-0.07090492	0.12288436	-0.577005242	0.563935914	0	0.050287993	0	0.5	CRA004775;Yang2025_Silkie_Wenchang	PRJNA827465	0.882726243
ENSGALG00010018534	NCEH1	2	0.062697661	0.097823504	0.640926352	0.521570534	0	0.025909363	0	1	CRA004775;PRJNA827465	Yang2025_Silkie_Wenchang	0.869518515
ENSGALG00010018535	FAM174B	2	0.021760672	0.166944071	0.130347077	0.896291836	0.051554246	13.30694003	92.48512432	0.5	PRJNA827465;Yang2025_Silkie_Wenchang	CRA004775	0.975115773
ENSGALG00010018535	FAM174B	2	-0.147896459	0.065680568	-2.251753672	0.024337843	0	0.009412475	0	1	CRA004775;Yang2025_Silkie_Wenchang	PRJNA827465	0.266745744
ENSGALG00010018535	FAM174B	2	0.107039695	0.150409072	0.711657174	0.476677094	0.025300925	1.632006365	38.72572917	0.5	CRA004775;PRJNA827465	Yang2025_Silkie_Wenchang	0.854986476
ENSGALG00010018536	ENSGALG00010018536	2	0.682798857	0.744193617	0.917501631	0.358879839	0.984137735	8.548216491	88.30165332	0.5	PRJNA827465;Yang2025_Silkie_Wenchang	CRA004775	0.814241185
ENSGALG00010018536	ENSGALG00010018536	2	0.460840137	1.002399213	0.45973713	0.645704915	1.841783629	11.89733006	91.59475282	0.5	CRA004775;Yang2025_Silkie_Wenchang	PRJNA827465	0.907074688
ENSGALG00010018536	ENSGALG00010018536	2	-0.185893903	0.24485249	-0.75920773	0.447728302	0.049762417	1.610492066	37.90717626	1	CRA004775;PRJNA827465	Yang2025_Silkie_Wenchang	0.846601815
ENSGALG00010018539	CHD2	2	-0.102110425	0.048582076	-2.101812694	0.035569687	0.000189099	1.021789298	2.132464888	0.5	PRJNA827465;Yang2025_Silkie_Wenchang	CRA004775	0.325417923
ENSGALG00010018539	CHD2	2	0.032761053	0.100511334	0.325943871	0.744466822	0	0.111517512	0	1	CRA004775;Yang2025_Silkie_Wenchang	PRJNA827465	0.935846381
ENSGALG00010018539	CHD2	2	-0.092190034	0.073509466	-1.254124649	0.209796702	0.003748588	1.215985928	17.76220619	0.5	CRA004775;PRJNA827465	Yang2025_Silkie_Wenchang	0.721625118
ENSGALG00010018540	TNFSF10	2	-0.10905691	0.0573068	-1.90303612	0.057035832	0	0.032673709	0	1	PRJNA827465;Yang2025_Silkie_Wenchang	CRA004775	0.413453375
ENSGALG00010018540	TNFSF10	2	-0.078061891	0.127533425	-0.612089665	0.540478435	0	0.041960795	0	1	CRA004775;Yang2025_Silkie_Wenchang	PRJNA827465	0.875506901
ENSGALG00010018540	TNFSF10	2	-0.11109616	0.06251087	-1.777229477	0.075530491	0	0.079099634	0	1	CRA004775;PRJNA827465	Yang2025_Silkie_Wenchang	0.474458409
ENSGALG00010018542	ZMIZ2	2	-0.119331751	0.293798425	-0.406168792	0.684618583	0.156517734	10.70538026	90.65890258	0.5	PRJNA827465;Yang2025_Silkie_Wenchang	CRA004775	0.919003112
ENSGALG00010018542	ZMIZ2	2	0.01437	0.265728736	0.054077705	0.956873255	0.08070937	1.788600326	44.09036018	0.5	CRA004775;Yang2025_Silkie_Wenchang	PRJNA827465	0.988540618
ENSGALG00010018542	ZMIZ2	2	-0.41679704	0.126300054	-3.30005433	0.000966661	0	0.001470172	0	1	CRA004775;PRJNA827465	Yang2025_Silkie_Wenchang	0.036921946
ENSGALG00010018543	MRPL3	2	-0.07176888	0.126322901	-0.568138318	0.56994105	0.024528828	4.0226475	75.14074997	0.5	PRJNA827465;Yang2025_Silkie_Wenchang	CRA004775	0.884923673
ENSGALG00010018543	MRPL3	2	-0.191262309	0.103865439	-1.84144323	0.065556633	0	0.339365003	0	1	CRA004775;Yang2025_Silkie_Wenchang	PRJNA827465	0.441431509
ENSGALG00010018543	MRPL3	2	0.032327733	0.055500182	0.582479756	0.560243573	0	0.133163079	0	0.5	CRA004775;PRJNA827465	Yang2025_Silkie_Wenchang	0.881203589
ENSGALG00010018544	HACD2	2	-0.05379255	0.170967113	-0.314636826	0.753037415	0.047902426	5.533515143	81.92830463	0.5	PRJNA827465;Yang2025_Silkie_Wenchang	CRA004775	0.938087082
ENSGALG00010018544	HACD2	2	-0.203940723	0.102016664	-1.999092263	0.045598372	0	0.591540793	0	0.5	CRA004775;Yang2025_Silkie_Wenchang	PRJNA827465	0.368074106
ENSGALG00010018544	HACD2	2	0.111497089	0.095654666	1.165621013	0.243767708	0	0.018409831	0	1	CRA004775;PRJNA827465	Yang2025_Silkie_Wenchang	0.755365463
ENSGALG00010018546	SNRPGP15	2	-0.223124873	0.312672296	-0.713606149	0.475470754	0.158861748	4.631992418	78.4110182	0.5	PRJNA827465;Yang2025_Silkie_Wenchang	CRA004775	0.854497479
ENSGALG00010018546	SNRPGP15	2	-0.492689948	0.218999816	-2.24972768	0.024466237	0	0.347959316	0	1	CRA004775;Yang2025_Silkie_Wenchang	PRJNA827465	0.267184364
ENSGALG00010018546	SNRPGP15	2	-0.003733735	0.114858427	-0.032507278	0.974067512	0.008862539	1.145009593	12.66448716	0.5	CRA004775;PRJNA827465	Yang2025_Silkie_Wenchang	0.992492038
ENSGALG00010018548	RGMA	2	-0.065348027	0.050982037	-1.281785335	0.19991796	0	0.353284416	0	1	PRJNA827465;Yang2025_Silkie_Wenchang	CRA004775	0.711976765
ENSGALG00010018548	RGMA	2	-0.08361359	0.072143393	-1.158991658	0.246459585	0	0.693921529	0	0.5	CRA004775;Yang2025_Silkie_Wenchang	PRJNA827465	0.756843363
ENSGALG00010018548	RGMA	2	-0.026846925	0.068796593	-0.390236254	0.696361856	0	0.427247314	0	0.5	CRA004775;PRJNA827465	Yang2025_Silkie_Wenchang	0.921041503

ENSGALG00010018551	FBXO34	2	-0.020160262	0.057790831	-0.348848794	0.727202829	0	0.448431988	0	0.5	PRJNA827465;Yang2025_Silkie_Wenchang	CRA004775	0.931522817
ENSGALG00010018551	FBXO34	2	0.064108886	0.094480556	0.67854053	0.497429035	0	0.407136717	0	1	CRA004775;Yang2025_Silkie_Wenchang	PRJNA827465	0.86098656
ENSGALG00010018551	FBXO34	2	-0.023275476	0.071526778	-0.325409266	0.744871344	0.000787141	1.026760024	2.606258873	0.5	CRA004775;PRJNA827465	Yang2025_Silkie_Wenchang	0.935893304
ENSGALG00010018552	DTD1	2	-0.059377485	0.048610038	-1.221506653	0.221894248	0	0.042247122	0	1	PRJNA827465;Yang2025_Silkie_Wenchang	CRA004775	0.735359501
ENSGALG00010018552	DTD1	2	-0.010802114	0.113667657	-0.095032435	0.924289064	0	0.119396282	0	0.5	CRA004775;Yang2025_Silkie_Wenchang	PRJNA827465	0.98232425
ENSGALG00010018552	DTD1	2	-0.05713074	0.050890234	-1.1226268	0.261596028	0	0.269251697	0	0.5	CRA004775;PRJNA827465	Yang2025_Silkie_Wenchang	0.768310285
ENSGALG00010018554	PPIA	2	-0.117407424	0.053434974	-2.19720187	0.028006032	0	0.157375481	0	1	PRJNA827465;Yang2025_Silkie_Wenchang	CRA004775	0.285254797
ENSGALG00010018554	PPIA	2	-0.13095912	0.11421759	-1.146575759	0.251557	0	0.18107331	0	1	CRA004775;Yang2025_Silkie_Wenchang	PRJNA827465	0.761706068
ENSGALG00010018554	PPIA	2	-0.106220382	0.054116502	-1.962809445	0.04966832	0	0.033425051	0	1	CRA004775;PRJNA827465	Yang2025_Silkie_Wenchang	0.386022143
ENSGALG00010018555	UBN1	2	0.043675296	0.04679919	0.93324899	0.350691426	0	0.679645089	0	1	PRJNA827465;Yang2025_Silkie_Wenchang	CRA004775	0.810111037
ENSGALG00010018555	UBN1	2	0.031583798	0.148866649	0.212161679	0.831980902	0.018117998	1.58442611	36.88566518	0.5	CRA004775;Yang2025_Silkie_Wenchang	PRJNA827465	0.957762335
ENSGALG00010018555	UBN1	2	0.017581894	0.049131303	0.357855245	0.720451645	0	0.836279873	0	0.5	CRA004775;PRJNA827465	Yang2025_Silkie_Wenchang	0.928755937
ENSGALG00010018556	MRPL2	2	-0.024004436	0.091326519	-0.262841896	0.792672442	0	0.438730205	0	0.5	PRJNA827465;Yang2025_Silkie_Wenchang	CRA004775	0.948435155
ENSGALG00010018556	MRPL2	2	0.071482005	0.223365107	0.320023151	0.748950781	0	0.260267769	0	0.5	CRA004775;Yang2025_Silkie_Wenchang	PRJNA827465	0.937333223
ENSGALG00010018556	MRPL2	2	-0.051219787	0.09586534	-0.534288899	0.593141672	0	0.037595183	0	1	CRA004775;PRJNA827465	Yang2025_Silkie_Wenchang	0.892776493
ENSGALG00010018557	TBCC	2	0.00616638	0.079763199	0.077308589	0.938378058	0	0.337170955	0	0.5	PRJNA827465;Yang2025_Silkie_Wenchang	CRA004775	0.985349045
ENSGALG00010018557	TBCC	2	-0.023971638	0.125732016	-0.190656594	0.848794647	0	0.91884012	0	0.5	CRA004775;Yang2025_Silkie_Wenchang	PRJNA827465	0.961364896
ENSGALG00010018557	TBCC	2	-0.042098207	0.079287959	-0.530953343	0.595451111	0	0.40021984	0	1	CRA004775;PRJNA827465	Yang2025_Silkie_Wenchang	0.893794893
ENSGALG00010018558	ENSGALG00010018558	2	0.229302856	0.161987479	1.415559134	0.156904623	0	0.230722574	0	1	PRJNA827465;Yang2025_Silkie_Wenchang	CRA004775	0.650302901
ENSGALG00010018558	ENSGALG00010018558	2	0.153152783	0.206941756	0.740076755	0.459253423	0	0.018102208	0	1	CRA004775;Yang2025_Silkie_Wenchang	PRJNA827465	0.850098539
ENSGALG00010018558	ENSGALG00010018558	2	0.264535968	0.17770877	1.488592646	0.136594669	0	0.063865943	0	1	CRA004775;PRJNA827465	Yang2025_Silkie_Wenchang	0.618470274
ENSGALG00010018559	DLG3	2	0.124595264	0.066718377	1.867480421	0.061834528	0	0.170597467	0	1	PRJNA827465;Yang2025_Silkie_Wenchang	CRA004775	0.429075295
ENSGALG00010018559	DLG3	2	-0.008376938	0.239784547	-0.034935271	0.972131356	0.073408943	2.367810815	57.76689617	0.5	CRA004775;Yang2025_Silkie_Wenchang	PRJNA827465	0.991955348
ENSGALG00010018559	DLG3	2	-0.018730849	0.1998016	-0.093747241	0.925309942	0.049110724	1.989300999	49.73108642	0.5	CRA004775;PRJNA827465	Yang2025_Silkie_Wenchang	0.982594411
ENSGALG00010018560	ENSGALG00010018560	2	0.011682191	0.061318769	0.190515749	0.848905003	0.003196332	1.612869127	37.99868922	0.5	PRJNA827465;Yang2025_Silkie_Wenchang	CRA004775	0.961364896
ENSGALG00010018560	ENSGALG00010018560	2	0.077178922	0.085254453	0.905277309	0.365318494	0	0.611066898	0	0.5	CRA004775;Yang2025_Silkie_Wenchang	PRJNA827465	0.818452533
ENSGALG00010018560	ENSGALG00010018560	2	-0.035129563	0.04905024	-0.716195537	0.473870618	0	0.133735784	0	1	CRA004775;PRJNA827465	Yang2025_Silkie_Wenchang	0.85444425
ENSGALG00010018561	ACTR1A	2	-0.157469265	0.113101656	-1.392280808	0.163837381	0.018839543	3.317164219	69.85376864	1	PRJNA827465;Yang2025_Silkie_Wenchang	CRA004775	0.662308161
ENSGALG00010018561	ACTR1A	2	-0.291778936	0.091009669	-3.206021275	0.00134584	0	0.011114736	0	1	CRA004775;Yang2025_Silkie_Wenchang	PRJNA827465	0.044788793
ENSGALG00010018561	ACTR1A	2	-0.132307452	0.097861713	-1.351983809	0.176380495	0.011738532	2.076591928	51.84417379	1	CRA004775;PRJNA827465	Yang2025_Silkie_Wenchang	0.681691731
ENSGALG00010018562	ENSGALG00010018562	2	0.045178872	0.270006245	0.167325284	0.867114111	0.063424023	1.694967003	41.00180132	0.5	PRJNA827465;Yang2025_Silkie_Wenchang	CRA004775	0.967699
ENSGALG00010018562	ENSGALG00010018562	2	0.457773591	0.343868588	1.331245735	0.183108167	0	0.456961433	0	1	CRA004775;Yang2025_Silkie_Wenchang	PRJNA827465	0.691392093
ENSGALG00010018562	ENSGALG00010018562	2	0.032878964	0.493180273	0.066667233	0.94684662	0.228696456	1.293204606	22.67271587	0.5	CRA004775;PRJNA827465	Yang2025_Silkie_Wenchang	0.986445196
ENSGALG00010018564	DLGAP5	2	-0.237626093	0.159156406	-1.493035049	0.135428013	0.043347419	6.845238756	85.39130576	1	PRJNA827465;Yang2025_Silkie_Wenchang	CRA004775	0.616907199
ENSGALG00010018564	DLGAP5	2	-0.109243483	0.382256678	-0.285785674	0.775042281	0.223357154	3.508547788	71.49817929	0.5	CRA004775;Yang2025_Silkie_Wenchang	PRJNA827465	0.943803161
ENSGALG00010018564	DLGAP5	2	-0.022540622	0.160376966	-0.140547755	0.888227224	0.024534222	1.282763837	22.04332775	0.5	CRA004775;PRJNA827465	Yang2025_Silkie_Wenchang	0.973135755
ENSGALG00010018566	ENSGALG00010018566	2	0.206788953	0.082624635	2.502751794	0.012323193	0	0.745468286	0	1	PRJNA827465;Yang2025_Silkie_Wenchang	CRA004775	0.179397068
ENSGALG00010018566	ENSGALG00010018566	2	0.195005478	0.226945216	0.859262344	0.390195794	0.043109739	1.426247337	29.88593393	0.5	CRA004775;Yang2025_Silkie_Wenchang	PRJNA827465	0.829234633
ENSGALG00010018566	ENSGALG00010018566	2	0.142574472	0.095155482	1.498331667	0.1340471	0	0.764730699	0	0.5	CRA004775;PRJNA827465	Yang2025_Silkie_Wenchang	0.61327389
ENSGALG00010018568	FAM136A	2	0.701233824	0.867759044	0.808097396	0.419034517	1.477314494	52.30631759	98.08818505	0.5	PRJNA827465;Yang2025_Silkie_Wenchang	CRA004775	0.838346328
ENSGALG00010018568	FAM136A	2	0.982158894	0.618982267	1.586731876	0.112573384	0.694887917	10.55696722	90.5275826	1	CRA004775;Yang2025_Silkie_Wenchang	PRJNA827465	0.566223663
ENSGALG00010018568	FAM136A	2	0.000238796	0.231984165	0.001029361	0.999178689	0.062653648	2.033476294	50.82312969	0.5	CRA004775;PRJNA827465	Yang2025_Silkie_Wenchang	0.999889665
ENSGALG00010018573	SEC23B	2	0.204044781	0.057357782	3.55740359	0.000374538	0.00093525	1.15279107	13.25401229	1	PRJNA827465;Yang2025_Silkie_Wenchang	CRA004775	0.019374277
ENSGALG00010018573	SEC23B	2	0.189277002	0.138351771	1.368085133	0.17128543	0.019214905	1.718089712	41.79582167	0.5	CRA004775;Yang2025_Silkie_Wenchang	PRJNA827465	0.673243267
ENSGALG00010018573	SEC23B	2	0.144219796	0.062599051	2.303865536	0.021230193	0	0.682344849	0	0.5	CRA004775;PRJNA827465	Yang2025_Silkie_Wenchang	0.246356738
ENSGALG00010018574	ENSGALG00010018574	2	0.321622561	0.182489335	1.762418392	0.077998633	0	0.040822582	0	1	PRJNA827465;Yang2025_Silkie_Wenchang	CRA004775	0.480830912
ENSGALG00010018574	ENSGALG00010018574	2	0.27755321	0.233918731	1.186536917	0.235410332	0	0.222805334	0	1	CRA004775;Yang2025_Silkie_Wenchang	PRJNA827465	0.748510012
ENSGALG00010018574	ENSGALG00010018574	2	0.252887691	0.192052504	1.316763309	0.187917975	0	0.118195715	0	1	CRA004775;PRJNA827465	Yang2025_Silkie_Wenchang	0.697228375
ENSGALG00010018576	PTK7	2	0.031418898	0.129512348	0.242593839	0.808320055	0.028286113	6.312569915	84.15859129	0.5	PRJNA827465;Yang2025_Silkie_Wenchang	CRA004775	0.952496413
ENSGALG00010018576	PTK7	2	0.081216769	0.124755114	0.651009538	0.51504033	0.016083636	1.815987399	44.93353862	0.5	CRA004775;Yang2025_Silkie_Wenchang	PRJNA827465	0.867107735
ENSGALG00010018576	PTK7	2	-0.093454714	0.058767874	-1.590234721	0.111781907	0	0.001883123	0	1	CRA004775;PRJNA827465	Yang2025_Silkie_Wenchang	0.565281045
ENSGALG00010018577	NARFL	2	-0.09366788	0.068019724	-1.377069391	0.168490795	8.85647E-05	1.00596116	0.592583508	1	PRJNA827465;Yang2025_Silkie_Wenchang	CRA004775	0.669314102
ENSGALG00010018577	NARFL	2	-0.132815153	0.1068233	-1.243316333	0.213751288	0	0.799554582	0	1	CRA004775;Yang2025_Silkie_Wenchang	PRJNA827465	0.726416385
ENSGALG00010018577	NARFL	2	-0.056775781	0.066872762	-0.849012049	0.395874588	0	0.012403281	0	1	CRA004775;PRJNA827465	Yang2025_Silkie_Wenchang	0.831296327
ENSGALG00010018579	FNDC3B	2	-0.026035381	0.068115978	-0.382221347	0.702297189	0	0.067378814	0	0.5	PRJNA827465;Yang2025_Silkie_Wenchang	CRA004775	0.923141374
ENSGALG00010018579	FNDC3B	2	0.074044466	0.154056085	0.480633181	0.630777228	0	0.646877956	0	1	CRA004775;Yang2025_Silkie_Wenchang	PRJNA827465	0.902787131
ENSGALG00010018579	FNDC3B	2	-0.016462869	0.077602947	-0.212142322	0.831996003	0.000803406	1.01212811	1.198278175	0.5	CRA004775;PRJNA827465	Yang2025_Silkie_Wenchang	0.957762335
ENSGALG00010018581	SUFU	2	-0.059598283	0.094958334	-0.627625621	0.530249221	0.014467626	4.95717755	79.82723052	0.5	PRJNA827465;Yang2025_Silkie_Wenchang	CRA004775	0.872246963
ENSGALG00010018581	SUFU	2	-0.151507596	0.064751467	-2.33983263	0.019292383	0	0.134995887	0	1	CRA004775;Yang2025_Silkie_Wenchang	PRJNA827465	0.232624497
ENSGALG00010018581	SUFU	2	0.019223814	0.046860436	0.410235486	0.681633212	0	0.502564304	0	0.5	CRA004775;PRJNA827465	Yang2025_Silkie_Wenchang	0.918322769
ENSGALG00010018582	RCC2	2	-0.011263651	0.037320023	-0.301812533	0.762794976	0	0.631362771	0	0.5	PRJNA827465;Yang2025_Silkie_Wenchang	CRA004775	0.940082621
ENSGALG00010018582	RCC2	2	-0.183864642	0.165278188	-1.112455577	0.26594232	0.031428902	1.94671396	48.63138497	1	CRA004775;Yang2025_Silkie_Wenchang	PRJNA827465	0.770078617
ENSGALG00010018582	RCC2	2	-0.150729496	0.208506858	-0.722899463	0.469741653	0.065282153	3.164991386	68.40433739	0.5	CRA004775;PRJNA827465	Yang2025_Silkie_Wenchang	0.853485658
ENSGALG00010018583	PPL	2	0.116621963	0.559919158	0.208283573	0.835007557	0.572162868	11.34775434	91.18768375	0.5	PRJNA827465;Yang2025_Silkie_Wenchang	CRA004775	0.95823248
ENSGALG00010018583	PPL	2	0.554276673	0.229347809	2.416751549	0.015659702	0	0.978082735	0	1	CRA004775;Yang2025_Silkie_Wenchang	PRJNA827465	0.207039858
ENSGALG00010018583	PPL	2	-0.254164306	0.275097624	-0.923905854	0.355535335	0.067415544	1.573773106	36.45843887	0.5	CRA004775;PRJNA827465	Yang2025_Silkie_Wenchang	0.812301302
ENSGALG00010018585	GDPD2	2	0.175151504	0.296011481	0.591705104	0.554048078	0	0.082057533	0	1	PRJNA827465;Yang2025_Silkie_Wenchang	CRA004775	0.880092687
ENSGALG00010018585	GDPD2	2	1.068031161	1.050571362	1.016619336	0.309334552	1.856392534	6.265139925	84.03866455	1	CRA004775;Yang2025_Silkie_Wenchang	PRJNA827465	0.794043289
ENSGALG00010018585	GDPD2	2	1.113960004	0.955365472	1.166004045	0.24361281	1.568410634	6.847466577	85.39605869	1	CRA004775;PRJNA827465	Yang2025_Silkie_Wenchang	0.755365463
ENSGALG00010018587	SRF	2	0.036135209	0.087771324	0.411697212	0.680561369	0.004977096	1.446953567	30.88928197	0.5	PRJNA827465;Yang2025_Silkie_Wenchang	CRA004775	0.917851751
ENSGALG00010018587	SRF	2	0.152044607	0.114280774	1.330447818	0.183370768	0	0.037623757	0	1	CRA004775;Yang2025_Silkie_Wenchang	PRJNA827465	0.691847354
ENSGALG00010018587	SRF	2	-0.020661148	0.085618545	-0.241316275	0.809309998	0	0.463091482	0	0.5	CRA004775;PRJNA827465	Yang2025_Silkie_Wenchang	0.95276497
ENSGALG00010018588	POLR3F	2	0.073186278	0.130967635	0.55881194	0.576290073	0.023088996	2.919643591	65.74924408	0.5	PRJNA827465;Yang2025_Silkie_Wenchang	CRA004775	0.887048016
ENSGALG00010018588	POLR3F	2	0.208609691	0.113186298	1.843064884	0.065319538	0	0.065169956	0	1	CRA004775;Yang2025_Silkie_Wenchang	PRJNA827465	0.440578993
ENSGALG00010018588	POLR3F	2	-0.015164691	0.076701148	-0.197711394	0.843270875	0	0.715551248	0	0.5	CRA004775;PRJNA827465	Yang2025_Silkie_Wenchang	0.96057435
ENSGALG00010018590	TGFA	2	0.732691381	0.24038127	3.048038562	0.002303404	0.006054349	1.05522121	5.233140637	1	PRJNA827465;Yang2025_Silkie_Wenchang	CRA004775	0.063678558
ENSGALG00010018590	TGFA	2	0.378850225	0.616691743	0.614326735	0.538999439	0.629610227	5.790400205	82.73003653	0.5	CRA004775;Yang2025_Silkie_Wenchang	PRJNA827465	0.87481821
ENSGALG00010018590	TGFA	2	0.152723247	0.375235967	0.407005887	0.684003665	0.155669313	2.219870104	54.95231913	0.5	CRA004775;PRJNA827465	Yang2025_Silkie_Wenchang	0.918806497
ENSGALG00010018593	LGALS3	2	0.553938605	0.274395631	2.018758838	0.043512291	0	0.012222693	0	1	PRJNA827465;Yang2025_Silkie_Wenchang	CRA004775	0.360489794
ENSGALG00010018593	LGALS3	2	0.972738102	0.548101048	1.774742278	0.075940451	0.300838108	1.943439992	48.5448481	1	CRA004775;Yang2025_Silkie_Wenchang	PRJNA827465	0.47539798
ENSGALG00010018593	LGALS3	2	0.953988264	0.503829366	1.89347491	0.058294742	0.279136684	2.025848072	50.63795683	1	CRA004775;PRJNA827465	Yang2025_Silkie_Wenchang	0.418521291
ENSGALG00010018594	BICRAL	2	0.168962697	0.16140156	1.046846737	0.295170271	0.032560219	2.353770737	57.51497865	1	PRJNA827465;Yang2025_Silkie_Wenchang	CRA004775	0.787628307
ENSGALG00010018594	BICRAL	2	0.316719243	0.163419584	1.938073976	0.052614198	0	0.346774789	0	1	CRA004775;Yang2025_Silkie_Wenchang	PRJNA827465	0.398438501
ENSGALG00010018594	BICRAL	2	0.060301445	0.085636385	0.704156826	0.481335115	0	0.198150721	0	1	CRA004775;PRJNA827465	Yang2025_Silkie_Wenchang	0.85682839
ENSGALG00010018598	ENSGALG00010018598	2	0.014744222	0.092138917	0.160021659	0.872864012	0.010851117	2.287702599	56.28802449	0.5	PRJNA827465;Yang2025_Silkie_Wenchang	CRA004775	0.969664257
ENSGALG00010018598	ENSGALG00010018598	2	-0.087624368	0.261155788	-0.335525277	0.73722888	0.108084889	4.613249039	78.32330335	0.5	CRA004775;Yang2025_Silkie_Wenchang	PRJNA827465	0.933895493
ENSGALG00010018598	ENSGALG00010018598	2	-0.148721075	0.15097863	-0.985047192	0.324600891	0.030863175	2.327069427	57.02749611	1	CRA004775;PRJNA827465	Yang2025_Silkie_Wenchang	0.799859262
ENSGALG00010018602	MCTP2	2	-0.054700453	0.100776373	-0.542790445	0.587274093	0	0.28566649	0	0.5	PRJNA827465;Yang2025_Silkie_Wenchang	CRA004775	0.891645821
ENSGALG00010018602	MCTP2	2	-0.033165776	0.166981931	-0.198618948	0.84256083	0	0.978616045	0	0.5	CRA004775;Yang2025_Silkie_Wenchang	PRJNA827465	0.960252241
ENSGALG00010018602	MCTP2	2	-0.11481141	0.119580512	-0.960118069	0.336995796	0	0.643719878	0	1	CRA004775;PRJNA827465	Yang2025_Silkie_Wenchang	0.804050409
ENSGALG00010018604	FAM43A	2	0.13533305	0.18760865	0.721358264	0.470689118	0.051090751	3.427889902	70.82753447	0.5	PRJNA827465;Yang2025_Silkie_Wenchang	CRA004775	0.853744788
ENSGALG00010018604	FAM43A	2	0.134734899	0.291060656	0.462910036	0.643428853	0.100872888	2.227445014	55.10551354	0.5	CRA004775;Yang2025_Silkie_Wenchang	PRJNA827465	0.906634523
ENSGALG00010018604	FAM43A	2	-0.040697394	0.095696838	-0.425274179	0.670636814	0	0.357393955	0	1	CRA004775;PRJNA827465	Yang2025_Silkie_Wenchang	0.914482883
ENSGALG00010018606	DZANK1	2	0.322056291	0.259919208	1.239063068	0.215322143	0.075836843	2.269069268	55.92906686	1	PRJNA827465;Yang2025_Silkie_Wenchang	CRA004775	0.728757551
ENSGALG00010018606	DZANK1	2	0.250169218	0.460138932	0.54368192	0.586660376	0.264834555	2.374214117	57.88079967	0.5	CRA004775;Yang2025_Silkie_Wenchang	PRJNA827465	0.891596903
ENSGALG00010018606	DZANK1	2	0.016133075	0.210863895	0.076509423	0.939013818	0	0.514367861	0	0.5	CRA004775;PRJNA827465	Yang2025_Silkie_Wenchang	0.985349045
ENSGALG00010018607	HAGHL	2	-0.092126708	0.098199468	-0.938158932	0.34816274	0	0.496073822	0	1	PRJNA827465;Yang2025_Silkie_Wenchang	CRA004775	0.808976658
ENSGALG00010018607	HAGHL	2	-0.258949549	0.128375016	-2.017133537	0.04368158	0	0.067563525	0	1	CRA004775;Yang2025_Silkie_Wenchang	PRJNA827465	0.361207053
ENSGALG00010018607	HAGHL	2	-0.138207043	0.111521805	-1.23928269	0.215240827	0.006953425	1.348559215	25.84678605	1	CRA004775;PRJNA827465	Yang2025_Silkie_Wenchang	0.728664095
ENSGALG00010018608	TRIM8	2	0.278724372	0.331672773	0.840359517	0.40070684	0.198860009	10.04359165	90.04340245	0.5	PRJNA827465;Yang2025_Silkie_Wenchang	CRA004775	0.832353691
ENSGALG00010018608	TRIM8	2	0.266489801	0.369567767	0.721085075	0.470857173	0.234214435	7.007197542	85.72895949	0.5	CRA004775;Yang2025_Silkie_Wenchang	PRJNA827465	0.853762555
ENSGALG00010018608	TRIM8	2	-0.045479425	0.083757935	-0.54298647	0.587139118	0	0.109494221	0	1	CRA004775;PRJNA827465	Yang2025_Silkie_Wenchang	0.891596903
ENSGALG00010018609	ENSGALG00010018609	2	0.006943974	0.121463497	0.057169227	0.954410391	0.01748172	2.214415495	54.84135646	0.5	PRJNA827465;Yang2025_Silkie_Wenchang	CRA004775	0.987987175
ENSGALG00010018609	ENSGALG00010018609	2	-0.190754099	0.126392025	-1.509225757	0.1312411	0	0.192751004	0	1	CRA004775;Yang2025_Silkie_Wenchang	PRJNA827465	0.606878893
ENSGALG00010018609	ENSGALG00010018609	2	-0.025655493	0.17575357	-0.145974233	0.88394173	0.040667129	2.413051727	58.55870022	0.5	CRA004775;PRJNA827465	Yang2025_Silkie_Wenchang	0.972001076
ENSGALG00010018611	NUDCD3	2	0.02811568	0.05135283	0.547500113	0.584035193	0.001366134	1.201457607	16.76776654	1	PRJNA827465;Yang2025_Silkie_Wenchang	CRA004775	0.890763231
ENSGALG00010018611	NUDCD3	2	-0.0456597	0.236623353	-0.192963627	0.846987454	0.075172438	2.595724355	61.47510818	0.5	CRA004775;Yang2025_Silkie_Wenchang	PRJNA827465	0.961252712
ENSGALG00010018611	NUDCD3	2	-0.071328756	0.14686111	-0.485688523	0.627188022	0.025030555	1.592153174	37.19197273	0.5	CRA004775;PRJNA827465	Yang2025_Silkie_Wenchang	0.901583369
ENSGALG00010018613	PADI3	2	0.056695789	0.245320485	0.231109071	0.817230069	0.050085797	1.710413899	41.53461916	0.5	PRJNA827465;Yang2025_Silkie_Wenchang	CRA004775	0.954833525
ENSGALG00010018613	PADI3	2	0.575856689	0.32195347	1.788633276	0.073673895	0.097143939	1.818058679	44.9962748	1	CRA004775;Yang2025_Silkie_Wenchang	PRJNA827465	0.469151831
ENSGALG00010018613	PADI3	2	0.353806569	0.572560969	0.617936932	0.536616913	0.545628965	5.80499764	82.77346414	0.5	CRA004775;PRJNA827465	Yang2025_Silkie_Wenchang	0.874599474
ENSGALG00010018616	ENSGALG00010018616	2	-0.092337118	0.803301999	-0.114946954	0.908487168	1.171983275	10.81112309	90.75026718	0.5	PRJNA827465;Yang2025_Silkie_Wenchang	CRA004775	0.978291088
ENSGALG00010018616	ENSGALG00010018616	2	2.387957312	1.844301107	1.294776272	0.195397419	6.196076045	10.47652992	90.45485473	1	CRA004775;Yang2025_Silkie_Wenchang	PRJNA827465	0.70643959
ENSGALG00010018616	ENSGALG00010018616	2	1.63704975	2.652320117	0.617214242	0.537093423	13.40291283	20.51051849	95.12445285	0.5	CRA004775;PRJNA827465	Yang2025_Silkie_Wenchang	0.87481821
ENSGALG00010018618	FAHD1	2	0.018567641	0.083592313	0.222121387	0.82421939	0	0.127482197	0	1	PRJNA827465;Yang2025_Silkie_Wenchang	CRA004775	0.95619211
ENSGALG00010018618	FAHD1	2	-0.004349642	0.17246646	-0.025220221	0.979879308	0	0.394230975	0	0.5	CRA004775;Yang2025_Silkie_Wenchang	PRJNA827465	0.994607486
ENSGALG00010018618	FAHD1	2	-0.006862482	0.08417317	-0.081528141	0.935021946	0	0.224832983	0	0.5	CRA004775;PRJNA827465	Yang2025_Silkie_Wenchang	0.984512821
ENSGALG00010018619	MAPK1IP1L	2	-0.131351538	0.07936548	-1.655021013	0.097920235	0.00848452	2.706295265	63.04911689	1	PRJNA827465;Yang2025_Silkie_Wenchang	CRA004775	0.534130658
ENSGALG00010018619	MAPK1IP1L	2	-0.237612594	0.070298574	-3.380048555	0.00072473	0	0.003583297	0	1	CRA004775;Yang2025_Silkie_Wenchang	PRJNA827465	0.030141876
ENSGALG00010018619	MAPK1IP1L	2	-0.125977027	0.08078202	-1.55946863	0.118885504	0.008081344	2.18563741	54.24675678	1	CRA004775;PRJNA827465	Yang2025_Silkie_Wenchang	0.579218663
ENSGALG00010018623	ARRDC4	2	0.024738429	0.127807839	0.193559555	0.846520769	0.018644831	2.113700974	52.68961824	0.5	PRJNA827465;Yang2025_Silkie_Wenchang	CRA004775	0.96122283
ENSGALG00010018623	ARRDC4	2	0.205318229	0.142931957	1.436475324	0.150867133	0	0.018755138	0	1	CRA004775;Yang2025_Silkie_Wenchang	PRJNA827465	0.642051813
ENSGALG00010018623	ARRDC4	2	-0.045930397	0.085538789	-0.536954029	0.591299369	0.001029771	1.021541833	2.108756835	0.5	CRA004775;PRJNA827465	Yang2025_Silkie_Wenchang	0.892776493
ENSGALG00010018624	ENSGALG00010018624	2	0.286942039	0.440937813	0.650753987	0.515205307	0.206257054	2.127931446	53.00600488	0.5	PRJNA827465;Yang2025_Silkie_Wenchang	CRA004775	0.8671413
ENSGALG00010018624	ENSGALG00010018624	2	0.385168662	0.47312071	0.814102308	0.415586344	0.156395583	1.474376745	32.17473053	0.5	CRA004775;Yang2025_Silkie_Wenchang	PRJNA827465	0.837689773
ENSGALG00010018624	ENSGALG00010018624	2	-0.170624239	0.354965557	-0.480678296	0.630745159	0	0.016864071	0	1	CRA004775;PRJNA827465	Yang2025_Silkie_Wenchang	0.902774108
ENSGALG00010018627	MYLK	2	0.129320666	0.238715752	0.54173495	0.588001109	0.085147717	3.916981872	74.47013969	0.5	PRJNA827465;Yang2025_Silkie_Wenchang	CRA004775	0.891761457
ENSGALG00010018627	MYLK	2	0.497850043	0.154179671	3.229025192	0.00124213	0.001523558	1.030468158	2.956729657	1	CRA004775;Yang2025_Silkie_Wenchang	PRJNA827465	0.042969436
ENSGALG00010018627	MYLK	2	0.277347801	0.398763375	0.695519746	0.486729641	0.275859544	7.338475255	86.37319109	0.5	CRA004775;PRJNA827465	Yang2025_Silkie_Wenchang	0.857603818
ENSGALG00010018628	XDH	2	1.489251101	0.466245189	3.194137194	0.001402494	0	0.182437403	0	1	PRJNA827465;Yang2025_Silkie_Wenchang	CRA004775	0.04608883
ENSGALG00010018628	XDH	2	0.418450436	0.860974202	0.486019715	0.626953188	0.929468625	2.677256007	62.6483236	0.5	CRA004775;Yang2025_Silkie_Wenchang	PRJNA827465	0.901573209
ENSGALG00010018628	XDH	2	0.647988748	1.061620148	0.610377214	0.541611959	1.773960611	4.655015663	78.51779516	0.5	CRA004775;PRJNA827465	Yang2025_Silkie_Wenchang	0.876288927
ENSGALG00010018631	KAT14	2	-0.090220396	0.082145665	-1.098297716	0.272074509	0.007321418	2.023228164	50.57403719	1	PRJNA827465;Yang2025_Silkie_Wenchang	CRA004775	0.773184343
ENSGALG00010018631	KAT14	2	-0.197597559	0.087276225	-2.264047964	0.023571165	0	7.14942E-06	0	1	CRA004775;Yang2025_Silkie_Wenchang	PRJNA827465	0.262001762
ENSGALG00010018631	KAT14	2	-0.048812805	0.05665954	-0.861510788	0.388956782	0.000286577	1.020073194	1.967819025	1	CRA004775;PRJNA827465	Yang2025_Silkie_Wenchang	0.828615121
ENSGALG00010018633	ENSGALG00010018633	2	0.228880251	0.235061293	0.973704554	0.330203256	0.074382161	2.728914951	63.35539884	1	PRJNA827465;Yang2025_Silkie_Wenchang	CRA004775	0.801307641
ENSGALG00010018633	ENSGALG00010018633	2	0.251147251	0.375944095	0.668044142	0.504105416	0.147552976	1.91548226	47.79382607	0.5	CRA004775;Yang2025_Silkie_Wenchang	PRJNA827465	0.863448292
ENSGALG00010018633	ENSGALG00010018633	2	0.028788817	0.1173336	0.245358676	0.806178723	0	0.341808265	0	0.5	CRA004775;PRJNA827465	Yang2025_Silkie_Wenchang	0.952109208
ENSGALG00010018638	SEC14L5	2	-0.205397703	0.197016422	-1.042541025	0.297160915	0.0538181	3.14250265	68.17822891	1	PRJNA827465;Yang2025_Silkie_Wenchang	CRA004775	0.788466926
ENSGALG00010018638	SEC14L5	2	-0.010497097	0.462045864	-0.022718734	0.981874632	0.353878558	5.585975521	82.09802395	0.5	CRA004775;Yang2025_Silkie_Wenchang	PRJNA827465	0.995055866
ENSGALG00010018638	SEC14L5	2	0.135300516	0.24683844	0.548133897	0.583599967	0.074051115	2.105070855	52.49566078	0.5	CRA004775;PRJNA827465	Yang2025_Silkie_Wenchang	0.890558522
ENSGALG00010018644	ESF1	2	0.289669949	0.305387941	0.948531068	0.342859164	0.175190495	16.09966672	93.7886913	0.5	PRJNA827465;Yang2025_Silkie_Wenchang	CRA004775	0.806218116
ENSGALG00010018644	ESF1	2	0.428537723	0.237117027	1.807283634	0.070718066	0.069337076	2.321069963	56.91642147	1	CRA004775;Yang2025_Silkie_Wenchang	PRJNA827465	0.4585765
ENSGALG00010018644	ESF1	2	0.001507247	0.065476581	0.023019642	0.981634605	0	0.152359543	0	0.5	CRA004775;PRJNA827465	Yang2025_Silkie_Wenchang	0.99503081
ENSGALG00010018649	HAGH	2	0.056856832	0.07183508	0.791491174	0.428657425	0	0.247180034	0	0.5	PRJNA827465;Yang2025_Silkie_Wenchang	CRA004775	0.841695436
ENSGALG00010018649	HAGH	2	0.147316876	0.185549037	0.793951177	0.427223855	0.030249857	1.780536456	43.83715105	0.5	CRA004775;Yang2025_Silkie_Wenchang	PRJNA827465	0.84125685
ENSGALG00010018649	HAGH	2	0.140135132	0.117550349	1.19212859	0.233210828	0.012482705	1.520229961	34.22047811	1	CRA004775;PRJNA827465	Yang2025_Silkie_Wenchang	0.746346811
ENSGALG00010018654	ENSGALG00010018654	2	0.049022998	0.382559363	0.128144813	0.898034372	0.22141352	4.015593323	75.09707982	0.5	PRJNA827465;Yang2025_Silkie_Wenchang	CRA004775	0.975733636
ENSGALG00010018654	ENSGALG00010018654	2	0.183759882	0.590986213	0.31093768	0.755847999	0.554745656	4.769765075	79.03460685	0.5	CRA004775;Yang2025_Silkie_Wenchang	PRJNA827465	0.938501783
ENSGALG00010018654	ENSGALG00010018654	2	0.48286943	0.196906768	2.452274416	0.014195636	0	0.71193104	0	1	CRA004775;PRJNA827465	Yang2025_Silkie_Wenchang	0.19521731
ENSGALG00010018655	BIRC5	2	-0.665098779	0.569303987	-1.168266505	0.242699282	0.606276658	15.02803966	93.34577215	1	PRJNA827465;Yang2025_Silkie_Wenchang	CRA004775	0.7550024
ENSGALG00010018655	BIRC5	2	-0.759079923	0.546977287	-1.387772292	0.165206395	0.478314909	4.837683306	79.32894866	1	CRA004775;Yang2025_Silkie_Wenchang	PRJNA827465	0.664732487
ENSGALG00010018655	BIRC5	2	-0.12366543	0.115286363	-1.072680472	0.283414505	0	0.00903215	0	1	CRA004775;PRJNA827465	Yang2025_Silkie_Wenchang	0.779568117
ENSGALG00010018665	IL13RA2	2	-0.067632055	0.150142417	-0.450452686	0.652384063	0	0.02921356	0	1	PRJNA827465;Yang2025_Silkie_Wenchang	CRA004775	0.909377929
ENSGALG00010018665	IL13RA2	2	0.122752928	0.237551418	0.516742559	0.605335883	0.004246654	1.031870758	3.088638557	0.5	CRA004775;Yang2025_Silkie_Wenchang	PRJNA827465	0.896474603
ENSGALG00010018665	IL13RA2	2	0.068567801	0.255717996	0.268138349	0.788592828	0.056584383	1.505981981	33.59814309	0.5	CRA004775;PRJNA827465	Yang2025_Silkie_Wenchang	0.947048735
ENSGALG00010018670	MRAS	2	-0.015533663	0.087574939	-0.177375661	0.859213324	0	0.110386371	0	0.5	PRJNA827465;Yang2025_Silkie_Wenchang	CRA004775	0.964605083
ENSGALG00010018670	MRAS	2	-0.299231066	0.411173433	-0.727749027	0.466767232	0.251300018	3.399141632	70.58080809	0.5	CRA004775;Yang2025_Silkie_Wenchang	PRJNA827465	0.852842649
ENSGALG00010018670	MRAS	2	-0.314270162	0.374279473	-0.839667106	0.401095062	0.207009893	3.115493045	67.90235172	1	CRA004775;PRJNA827465	Yang2025_Silkie_Wenchang	0.832453107
ENSGALG00010018672	PKDCC	2	-0.081517241	0.130879518	-0.622841845	0.533388457	0.025592303	3.744579199	73.29472961	0.5	PRJNA827465;Yang2025_Silkie_Wenchang	CRA004775	0.873379507
ENSGALG00010018672	PKDCC	2	0.33943567	0.616485367	0.550598097	0.581909213	0.692231626	10.57373761	90.54260625	0.5	CRA004775;Yang2025_Silkie_Wenchang	PRJNA827465	0.889192798
ENSGALG00010018672	PKDCC	2	0.453846805	0.481813349	0.941955646	0.346215349	0.405168127	7.001449512	85.71724329	1	CRA004775;PRJNA827465	Yang2025_Silkie_Wenchang	0.807744676
ENSGALG00010018673	NAGPA	2	0.025991044	0.104931248	0.247695939	0.804369674	0.014271389	2.696090092	62.90925133	0.5	PRJNA827465;Yang2025_Silkie_Wenchang	CRA004775	0.951602591
ENSGALG00010018673	NAGPA	2	-0.304055582	0.219509637	-1.385158239	0.166004087	0.078041945	5.160395913	80.62164189	1	CRA004775;Yang2025_Silkie_Wenchang	PRJNA827465	0.66529196
ENSGALG00010018673	NAGPA	2	-0.196396931	0.326118549	-0.602225576	0.547023993	0.198332896	14.3327943	93.02299343	0.5	CRA004775;PRJNA827465	Yang2025_Silkie_Wenchang	0.878281266
ENSGALG00010018685	ARL3	2	-0.077407222	0.148261552	-0.522099095	0.601601336	0.035041009	4.842623272	79.35003522	0.5	PRJNA827465;Yang2025_Silkie_Wenchang	CRA004775	0.895230519
ENSGALG00010018685	ARL3	2	-0.246235965	0.096231754	-2.558780804	0.010503995	0	0.037840127	0	1	CRA004775;Yang2025_Silkie_Wenchang	PRJNA827465	0.1632418
ENSGALG00010018685	ARL3	2	-0.060248279	0.163491084	-0.3685111	0.71249217	0.035324523	2.537019905	60.58367543	0.5	CRA004775;PRJNA827465	Yang2025_Silkie_Wenchang	0.92597752
ENSGALG00010018693	ENSGALG00010018693	2	0.561238126	0.486924358	1.152618711	0.24906692	0.447543533	17.79383895	94.38007727	1	PRJNA827465;Yang2025_Silkie_Wenchang	CRA004775	0.759411487
ENSGALG00010018693	ENSGALG00010018693	2	0.762697625	0.324628	2.349451145	0.018801111	0.160642777	3.98435831	74.90185565	1	CRA004775;Yang2025_Silkie_Wenchang	PRJNA827465	0.229831303
ENSGALG00010018693	ENSGALG00010018693	2	0.153701345	0.14381345	1.06875501	0.285180077	0	0.927372826	0	1	CRA004775;PRJNA827465	Yang2025_Silkie_Wenchang	0.780667164
ENSGALG00010018694	MGME1	2	-0.176642281	0.083697144	-2.110493536	0.034815867	0	0.343334665	0	1	PRJNA827465;Yang2025_Silkie_Wenchang	CRA004775	0.321263903
ENSGALG00010018694	MGME1	2	-0.084603497	0.129242523	-0.654610378	0.512718641	0	0.106840868	0	1	CRA004775;Yang2025_Silkie_Wenchang	PRJNA827465	0.865930199
ENSGALG00010018694	MGME1	2	-0.186506277	0.09597656	-1.943248205	0.051986176	0	0.486309248	0	1	CRA004775;PRJNA827465	Yang2025_Silkie_Wenchang	0.395741369
ENSGALG00010018698	MRPS16	2	0.177306534	0.195331781	0.907719845	0.364026256	0.045920976	1.843013992	45.74105219	1	PRJNA827465;Yang2025_Silkie_Wenchang	CRA004775	0.817187797
ENSGALG00010018698	MRPS16	2	0.117689961	0.376394398	0.312677239	0.754525889	0.192791344	3.116080331	67.90840114	0.5	CRA004775;Yang2025_Silkie_Wenchang	PRJNA827465	0.93838723
ENSGALG00010018698	MRPS16	2	0.030227729	0.09452511	0.319785176	0.749131186	0.004708687	1.112283941	10.09489903	0.5	CRA004775;PRJNA827465	Yang2025_Silkie_Wenchang	0.937362187
ENSGALG00010018701	SFXN2	2	-0.011257734	0.070052179	-0.160704985	0.872325763	0	0.307688915	0	0.5	PRJNA827465;Yang2025_Silkie_Wenchang	CRA004775	0.969626388
ENSGALG00010018701	SFXN2	2	-0.169563346	0.191459209	-0.88563693	0.375813205	0.02927424	1.353852085	26.13668723	1	CRA004775;Yang2025_Silkie_Wenchang	PRJNA827465	0.822974013
ENSGALG00010018701	SFXN2	2	-0.139132595	0.249066962	-0.558615217	0.576424352	0.078445863	2.009109786	50.226712	0.5	CRA004775;PRJNA827465	Yang2025_Silkie_Wenchang	0.887048016
ENSGALG00010018704	PGPEP1L	2	0.146277604	0.170471948	0.858074339	0.390851416	0.005431272	1.102720398	9.315180722	0.5	PRJNA827465;Yang2025_Silkie_Wenchang	CRA004775	0.829251057
ENSGALG00010018704	PGPEP1L	2	0.484595471	0.369279548	1.312272706	0.189428138	0.118160906	1.357946758	26.35941032	1	CRA004775;Yang2025_Silkie_Wenchang	PRJNA827465	0.698610689
ENSGALG00010018704	PGPEP1L	2	0.394921886	0.607165449	0.650435375	0.515411033	0.496525184	2.489345191	59.82879339	0.5	CRA004775;PRJNA827465	Yang2025_Silkie_Wenchang	0.867199306
ENSGALG00010018707	SLC45A1	2	0.144733268	0.078328282	1.847777889	0.064634481	0	0.332018223	0	1	PRJNA827465;Yang2025_Silkie_Wenchang	CRA004775	0.43836566
ENSGALG00010018707	SLC45A1	2	0.00214395	0.230960785	0.009282747	0.992593546	0.077602324	3.63634883	72.49988803	0.5	CRA004775;Yang2025_Silkie_Wenchang	PRJNA827465	0.997852036
ENSGALG00010018707	SLC45A1	2	-0.024540471	0.176090965	-0.139362468	0.889163729	0.04292499	2.955437408	66.16406095	0.5	CRA004775;PRJNA827465	Yang2025_Silkie_Wenchang	0.973135755
ENSGALG00010018709	SNX5	2	-0.11788719	0.183582058	-0.642149843	0.520775896	0.058440502	7.392687406	86.47311938	0.5	PRJNA827465;Yang2025_Silkie_Wenchang	CRA004775	0.869353339

ENSGALG00010018822	TRAF5	2	-0.192461742	0.122633831	-1.569401688	0.116554371	0	0.417024823	0	1	PRJNA827465;Yang2025_Silkie_Wenchang	CRA004775	0.57477217
ENSGALG00010018822	TRAF5	2	-0.237709668	0.182477591	-1.302678686	0.192684482	0	0.569221356	0	0.5	CRA004775;Yang2025_Silkie_Wenchang	PRJNA827465	0.702642251
ENSGALG00010018822	TRAF5	2	-0.106935819	0.15031789	-0.711397821	0.476837749	0	0.217770478	0	0.5	CRA004775;PRJNA827465	Yang2025_Silkie_Wenchang	0.854986476
ENSGALG00010018823	RAB40C	2	0.043616636	0.04435712	0.983306312	0.325456705	0	0.025664771	0	1	PRJNA827465;Yang2025_Silkie_Wenchang	CRA004775	0.799987949
ENSGALG00010018823	RAB40C	2	0.049345635	0.067157705	0.734772508	0.462478053	0	0.143803811	0	1	CRA004775;Yang2025_Silkie_Wenchang	PRJNA827465	0.850823289
ENSGALG00010018823	RAB40C	2	0.054690399	0.049025128	1.115558521	0.264611156	0	0.090245778	0	1	CRA004775;PRJNA827465	Yang2025_Silkie_Wenchang	0.768855521
ENSGALG00010018829	PTPN4	2	-0.063662094	0.146934833	-0.433267543	0.664820415	0.031757019	3.726349397	73.16408384	0.5	PRJNA827465;Yang2025_Silkie_Wenchang	CRA004775	0.913393112
ENSGALG00010018829	PTPN4	2	0.160736285	0.153041144	1.050281521	0.293588698	0.013061918	1.174943197	14.8895025	1	CRA004775;Yang2025_Silkie_Wenchang	PRJNA827465	0.786979185
ENSGALG00010018829	PTPN4	2	0.068072836	0.345647646	0.196942861	0.843872253	0.183009721	3.558319848	71.896849	0.5	CRA004775;PRJNA827465	Yang2025_Silkie_Wenchang	0.96057435
ENSGALG00010018831	WBP1L	2	-0.300092975	0.246956813	-1.215163786	0.224303629	0.115683562	19.22919934	94.79957547	1	PRJNA827465;Yang2025_Silkie_Wenchang	CRA004775	0.737471949
ENSGALG00010018831	WBP1L	2	-0.418956659	0.261806358	-1.600253951	0.109542258	0.077407046	1.585917851	36.94503157	0.5	CRA004775;Yang2025_Silkie_Wenchang	PRJNA827465	0.560210558
ENSGALG00010018831	WBP1L	2	-0.056058903	0.060969796	-0.919453661	0.357858333	0	0.090720255	0	0.5	CRA004775;PRJNA827465	Yang2025_Silkie_Wenchang	0.813641308
ENSGALG00010018832	EEPD1	2	-0.285471431	0.079709138	-3.581414108	0.000341739	0	0.04818552	0	1	PRJNA827465;Yang2025_Silkie_Wenchang	CRA004775	0.018373991
ENSGALG00010018832	EEPD1	2	-0.286248942	0.117276994	-2.440793651	0.014655026	0	0.275173019	0	1	CRA004775;Yang2025_Silkie_Wenchang	PRJNA827465	0.199510385
ENSGALG00010018832	EEPD1	2	-0.258434582	0.100809475	-2.563594167	0.01035946	0	0.19542094	0	1	CRA004775;PRJNA827465	Yang2025_Silkie_Wenchang	0.162085126
ENSGALG00010018834	SAMD4A	2	-0.014237775	0.123527526	-0.115259939	0.908239091	0.019820443	2.621360923	61.85187659	0.5	PRJNA827465;Yang2025_Silkie_Wenchang	CRA004775	0.978236585
ENSGALG00010018834	SAMD4A	2	0.144487987	0.13139995	1.099604575	0.271504448	0	0.01043445	0	1	CRA004775;Yang2025_Silkie_Wenchang	PRJNA827465	0.772805786
ENSGALG00010018834	SAMD4A	2	-0.104299058	0.071227627	-1.464306243	0.143110292	0	0.505754428	0	0.5	CRA004775;PRJNA827465	Yang2025_Silkie_Wenchang	0.630019027
ENSGALG00010018835	CAMK2B	2	0.426691057	0.492521398	0.866340141	0.38630366	0.459954845	18.9428455	94.72096207	0.5	PRJNA827465;Yang2025_Silkie_Wenchang	CRA004775	0.826894808
ENSGALG00010018835	CAMK2B	2	0.489894921	0.453217769	1.080926111	0.279729983	0.362628742	8.500549284	88.23605432	1	CRA004775;Yang2025_Silkie_Wenchang	PRJNA827465	0.777856764
ENSGALG00010018835	CAMK2B	2	-0.038358425	0.096348144	-0.398123131	0.690539425	0	0.091963051	0	0.5	CRA004775;PRJNA827465	Yang2025_Silkie_Wenchang	0.919979266
ENSGALG00010018837	RCOR3	2	0.038486441	0.053902715	0.713998189	0.475228299	0	0.3202713	0	1	PRJNA827465;Yang2025_Silkie_Wenchang	CRA004775	0.854462864
ENSGALG00010018837	RCOR3	2	0.14123545	0.122220774	1.155576461	0.247854441	0	0.05601805	0	1	CRA004775;Yang2025_Silkie_Wenchang	PRJNA827465	0.7580874
ENSGALG00010018837	RCOR3	2	0.038339861	0.055418645	0.691822411	0.489048861	0	0.522065081	0	1	CRA004775;PRJNA827465	Yang2025_Silkie_Wenchang	0.858232954
ENSGALG00010018838	WFIKKN1	2	0.170605601	0.261734645	0.651826587	0.514513048	0.066894874	1.897419285	47.29683588	0.5	PRJNA827465;Yang2025_Silkie_Wenchang	CRA004775	0.866869697
ENSGALG00010018838	WFIKKN1	2	0.423906519	0.299942766	1.413291357	0.157570074	0	0.371050739	0	0.5	CRA004775;Yang2025_Silkie_Wenchang	PRJNA827465	0.650796198
ENSGALG00010018838	WFIKKN1	2	-0.052674837	0.216854663	-0.242903868	0.808079869	0	0.007743099	0	1	CRA004775;PRJNA827465	Yang2025_Silkie_Wenchang	0.952496413
ENSGALG00010018839	RPL3L	2	0.201116947	0.289569514	0.69453771	0.48734506	0.032158647	1.232430859	18.85954551	0.5	PRJNA827465;Yang2025_Silkie_Wenchang	CRA004775	0.85768885
ENSGALG00010018839	RPL3L	2	-0.187300735	0.349660592	-0.535664411	0.592190504	0	0.091301653	0	1	CRA004775;Yang2025_Silkie_Wenchang	PRJNA827465	0.892776493
ENSGALG00010018839	RPL3L	2	0.279139154	0.34020385	0.820505571	0.411927955	0.030425662	1.096281637	8.782564037	0.5	CRA004775;PRJNA827465	Yang2025_Silkie_Wenchang	0.835413801
ENSGALG00010018843	MCRIP2	2	0.037576742	0.148728185	0.252653806	0.800535747	0.018228302	1.392566614	28.19014978	0.5	PRJNA827465;Yang2025_Silkie_Wenchang	CRA004775	0.950155719
ENSGALG00010018843	MCRIP2	2	0.321330745	0.231435693	1.388423456	0.165008138	0	0.000287284	0	1	CRA004775;Yang2025_Silkie_Wenchang	PRJNA827465	0.664342845
ENSGALG00010018843	MCRIP2	2	-0.018244241	0.09728951	-0.187525264	0.851248817	0	0.835801924	0	0.5	CRA004775;PRJNA827465	Yang2025_Silkie_Wenchang	0.962071965
ENSGALG00010018847	CGRRF1	2	-0.216939428	0.390687789	-0.555275681	0.57870611	0.27683933	10.51302798	90.4879926	0.5	PRJNA827465;Yang2025_Silkie_Wenchang	CRA004775	0.88788081
ENSGALG00010018847	CGRRF1	2	-0.473153402	0.176123151	-2.686491808	0.007220672	0.011095084	1.21270092	17.53943749	1	CRA004775;Yang2025_Silkie_Wenchang	PRJNA827465	0.130635564
ENSGALG00010018847	CGRRF1	2	-0.003281943	0.206301395	-0.015908486	0.9873074	0.053084012	2.401259022	58.3551799	0.5	CRA004775;PRJNA827465	Yang2025_Silkie_Wenchang	0.996492495
ENSGALG00010018848	MEF2A	2	-0.002436508	0.209592082	-0.011625002	0.990724799	0.070339459	4.410430346	77.32647561	0.5	PRJNA827465;Yang2025_Silkie_Wenchang	CRA004775	0.997555603
ENSGALG00010018848	MEF2A	2	0.307132305	0.143617155	2.138548877	0.032472222	0	0.19255171	0	1	CRA004775;Yang2025_Silkie_Wenchang	PRJNA827465	0.309572925
ENSGALG00010018848	MEF2A	2	0.062257926	0.273983788	0.227232153	0.820243227	0.128157823	6.152133188	83.74547544	0.5	CRA004775;PRJNA827465	Yang2025_Silkie_Wenchang	0.955308792
ENSGALG00010018852	NDUFB10	2	0.1705062	0.248468921	0.686227475	0.492569675	0.101873316	5.263703418	81.00196913	0.5	PRJNA827465;Yang2025_Silkie_Wenchang	CRA004775	0.859313149
ENSGALG00010018852	NDUFB10	2	0.41812698	0.17728485	2.358503729	0.018348775	0	0.144978741	0	1	CRA004775;Yang2025_Silkie_Wenchang	PRJNA827465	0.22688766
ENSGALG00010018852	NDUFB10	2	-0.02989196	0.08110249	-0.368570191	0.712448118	0	0.815361044	0	0.5	CRA004775;PRJNA827465	Yang2025_Silkie_Wenchang	0.92597752
ENSGALG00010018854	LYSMD4	2	0.273403374	0.285878091	0.956363508	0.338888614	0.157077993	25.58168524	96.09095339	0.5	PRJNA827465;Yang2025_Silkie_Wenchang	CRA004775	0.804646102
ENSGALG00010018854	LYSMD4	2	0.408547429	0.279703191	1.460646291	0.144112536	0.093824697	1.744484468	42.67647444	0.5	CRA004775;Yang2025_Silkie_Wenchang	PRJNA827465	0.63137387
ENSGALG00010018854	LYSMD4	2	-0.01146832	0.068609626	-0.167153217	0.867249494	0	0.03350645	0	1	CRA004775;PRJNA827465	Yang2025_Silkie_Wenchang	0.967705955
ENSGALG00010018857	ENSGALG00010018857	2	0.01270374	0.099545445	0.127617495	0.898451685	0.00959097	1.584518065	36.8893279	0.5	PRJNA827465;Yang2025_Silkie_Wenchang	CRA004775	0.97575249
ENSGALG00010018857	ENSGALG00010018857	2	0.203086129	0.146368371	1.387500095	0.165289322	0	0.05633811	0	1	CRA004775;Yang2025_Silkie_Wenchang	PRJNA827465	0.664874006
ENSGALG00010018857	ENSGALG00010018857	2	-0.018695365	0.086971002	-0.214960902	0.829797802	0.004562546	1.107272288	9.687977323	0.5	CRA004775;PRJNA827465	Yang2025_Silkie_Wenchang	0.957079054
ENSGALG00010018858	EPB41L5	2	0.046042177	0.074709225	0.616285032	0.537706416	0.002791923	1.219102025	17.97241091	1	PRJNA827465;Yang2025_Silkie_Wenchang	CRA004775	0.87481821
ENSGALG00010018858	EPB41L5	2	0.120184698	0.130596721	0.920273477	0.357429866	0	0.939342515	0	0.5	CRA004775;Yang2025_Silkie_Wenchang	PRJNA827465	0.813381338
ENSGALG00010018858	EPB41L5	2	-0.002316626	0.065497081	-0.035369916	0.971784773	0	0.229804042	0	0.5	CRA004775;PRJNA827465	Yang2025_Silkie_Wenchang	0.991955348
ENSGALG00010018860	AGTR2	2	-0.209583424	0.073284195	-2.859872106	0.004238119	0	0.578948242	0	1	PRJNA827465;Yang2025_Silkie_Wenchang	CRA004775	0.095151436
ENSGALG00010018860	AGTR2	2	-0.124762191	0.249896475	-0.499255507	0.617599395	0.070338228	1.597340744	37.39594988	0.5	CRA004775;Yang2025_Silkie_Wenchang	PRJNA827465	0.899121942
ENSGALG00010018860	AGTR2	2	-0.103061219	0.129071246	-0.798483182	0.424590149	0.00399538	1.033193002	3.212662313	0.5	CRA004775;PRJNA827465	Yang2025_Silkie_Wenchang	0.84081557
ENSGALG00010018864	ABCA3	2	0.012461122	0.053346411	0.233588761	0.815304253	0	0.02050389	0	1	PRJNA827465;Yang2025_Silkie_Wenchang	CRA004775	0.953933933
ENSGALG00010018864	ABCA3	2	-0.005954848	0.082839602	-0.071884078	0.942694161	0	0.14163807	0	0.5	CRA004775;Yang2025_Silkie_Wenchang	PRJNA827465	0.985827325
ENSGALG00010018864	ABCA3	2	0.012143366	0.066379985	0.182937166	0.85484732	0	0.186296744	0	0.5	CRA004775;PRJNA827465	Yang2025_Silkie_Wenchang	0.963248391
ENSGALG00010018865	ENSGALG00010018865	2	-0.062722142	0.111920194	-0.560418457	0.575194045	0.011196783	1.793622819	44.24691807	0.5	PRJNA827465;Yang2025_Silkie_Wenchang	CRA004775	0.88644908
ENSGALG00010018865	ENSGALG00010018865	2	-0.194486964	0.127256848	-1.528302543	0.126437439	0	0.145313655	0	1	CRA004775;Yang2025_Silkie_Wenchang	PRJNA827465	0.597028533
ENSGALG00010018865	ENSGALG00010018865	2	0.028664485	0.106590199	0.26892233	0.787989453	0	0.447425958	0	0.5	CRA004775;PRJNA827465	Yang2025_Silkie_Wenchang	0.946919116
ENSGALG00010018867	RPS2	2	-0.40079196	0.353181099	-1.134805801	0.256456695	0.227752698	10.93812998	90.85766944	1	PRJNA827465;Yang2025_Silkie_Wenchang	CRA004775	0.764677554
ENSGALG00010018867	RPS2	2	-0.628249488	0.209463461	-2.99932735	0.002705764	0.02645121	1.372404553	27.13518781	1	CRA004775;Yang2025_Silkie_Wenchang	PRJNA827465	0.070754925
ENSGALG00010018867	RPS2	2	-0.085118033	0.072871655	-1.168054062	0.242784959	0	0.66117991	0	1	CRA004775;PRJNA827465	Yang2025_Silkie_Wenchang	0.7550024
ENSGALG00010018872	KCTD9	2	-0.119064455	0.118902035	-1.001365998	0.316649896	0.02003999	3.243758296	69.17156247	1	PRJNA827465;Yang2025_Silkie_Wenchang	CRA004775	0.796935817
ENSGALG00010018872	KCTD9	2	-0.184432357	0.097887489	-1.88412593	0.059547944	0.001583385	1.082576129	7.627743421	1	CRA004775;Yang2025_Silkie_Wenchang	PRJNA827465	0.422736519
ENSGALG00010018872	KCTD9	2	-0.02295152	0.061395045	-0.373833422	0.708528252	0	0.046507083	0	1	CRA004775;PRJNA827465	Yang2025_Silkie_Wenchang	0.9250668
ENSGALG00010018874	ENSGALG00010018874	2	-0.107044656	0.141051167	-0.758906565	0.447908452	0.014344652	1.552479168	35.58689734	0.5	PRJNA827465;Yang2025_Silkie_Wenchang	CRA004775	0.846601815
ENSGALG00010018874	ENSGALG00010018874	2	-0.010980329	0.148729691	-0.073827415	0.941147713	0	0.456839203	0	0.5	CRA004775;Yang2025_Silkie_Wenchang	PRJNA827465	0.985662134
ENSGALG00010018874	ENSGALG00010018874	2	-0.220518273	0.12997346	-1.696640777	0.089764596	0	0.034155139	0	1	CRA004775;PRJNA827465	Yang2025_Silkie_Wenchang	0.514063515
ENSGALG00010018876	GMFB	2	-0.389539865	0.376002224	-1.036004151	0.300200206	0.278035561	59.90511863	98.33069357	1	PRJNA827465;Yang2025_Silkie_Wenchang	CRA004775	0.790325453
ENSGALG00010018876	GMFB	2	-0.700979831	0.167077105	-4.195546907	2.72214E-05	0.027593772	1.28466585	22.15874656	1	CRA004775;Yang2025_Silkie_Wenchang	PRJNA827465	0.003048098
ENSGALG00010018876	GMFB	2	-0.01967034	0.069785277	-0.281869484	0.778043589	0	0.32909343	0	1	CRA004775;PRJNA827465	Yang2025_Silkie_Wenchang	0.944480563
ENSGALG00010018880	TMEM17	2	-0.211281665	0.210905699	-1.001782628	0.316448589	0.060812598	3.159846293	68.35289102	1	PRJNA827465;Yang2025_Silkie_Wenchang	CRA004775	0.796883862
ENSGALG00010018880	TMEM17	2	-0.410492152	0.161777614	-2.537385379	0.011168393	0	0.066477912	0	1	CRA004775;Yang2025_Silkie_Wenchang	PRJNA827465	0.167907012
ENSGALG00010018880	TMEM17	2	-0.027204981	0.157961909	-0.172224947	0.863260686	0	0.255318795	0	1	CRA004775;PRJNA827465	Yang2025_Silkie_Wenchang	0.966235427
ENSGALG00010018881	ENSGALG00010018881	2	-0.910218905	0.580797639	-1.567187681	0.117070829	0	0.33490352	0	1	PRJNA827465;Yang2025_Silkie_Wenchang	CRA004775	0.576306963
ENSGALG00010018881	ENSGALG00010018881	2	1.063295971	1.66418175	0.638930195	0.522868344	4.497852076	5.26714409	81.01437927	0.5	CRA004775;Yang2025_Silkie_Wenchang	PRJNA827465	0.869568211
ENSGALG00010018881	ENSGALG00010018881	2	0.712981372	2.003601513	0.355849887	0.721952987	7.089128166	8.412942389	88.11355227	0.5	CRA004775;PRJNA827465	Yang2025_Silkie_Wenchang	0.929385887
ENSGALG00010018882	HHAT	2	-0.107822314	0.082581397	-1.305648951	0.191671966	0	0.020209636	0	1	PRJNA827465;Yang2025_Silkie_Wenchang	CRA004775	0.70135772
ENSGALG00010018882	HHAT	2	-0.33416619	0.268846613	-1.242962249	0.213881745	0.093470959	2.542926849	60.67523529	1	CRA004775;Yang2025_Silkie_Wenchang	PRJNA827465	0.726593954
ENSGALG00010018882	HHAT	2	-0.313663678	0.279976119	-1.120322973	0.262576156	0.114204985	3.102423922	67.76713869	1	CRA004775;PRJNA827465	Yang2025_Silkie_Wenchang	0.768418419
ENSGALG00010018883	TBL3	2	-0.058990508	0.086667547	-0.680652788	0.496091216	0.0044419	1.208808904	17.27393829	1	PRJNA827465;Yang2025_Silkie_Wenchang	CRA004775	0.860911322
ENSGALG00010018883	TBL3	2	-0.278924599	0.14967939	-1.863480333	0.062394717	0	0.072327247	0	1	CRA004775;Yang2025_Silkie_Wenchang	PRJNA827465	0.430605718
ENSGALG00010018883	TBL3	2	-0.09174356	0.131183574	-0.6993525	0.484331765	0.017850696	1.599185076	37.4681508	1	CRA004775;PRJNA827465	Yang2025_Silkie_Wenchang	0.857004733
ENSGALG00010018885	EFEMP1	2	-0.306750028	0.150538412	-2.037686095	0.041581336	0.026218582	2.369432139	57.79579488	1	PRJNA827465;Yang2025_Silkie_Wenchang	CRA004775	0.353355943
ENSGALG00010018885	EFEMP1	2	-0.034358606	0.459834097	-0.074719571	0.940437836	0.373701483	8.264025106	87.89935912	0.5	CRA004775;Yang2025_Silkie_Wenchang	PRJNA827465	0.985539065
ENSGALG00010018885	EFEMP1	2	0.097783601	0.306061718	0.319489812	0.749355115	0.14267056	3.848321535	74.01464532	0.5	CRA004775;PRJNA827465	Yang2025_Silkie_Wenchang	0.937362187
ENSGALG00010018887	ADAMTS17	2	-0.17512372	0.098809683	-1.772333592	0.076339198	0	0.007376802	0	1	PRJNA827465;Yang2025_Silkie_Wenchang	CRA004775	0.476766627
ENSGALG00010018887	ADAMTS17	2	-0.182403903	0.133875986	-1.362484102	0.173045158	0	0.25567367	0	1	CRA004775;Yang2025_Silkie_Wenchang	PRJNA827465	0.676043418
ENSGALG00010018887	ADAMTS17	2	-0.199561468	0.138450846	-1.441388581	0.149474934	0	0.227736795	0	1	CRA004775;PRJNA827465	Yang2025_Silkie_Wenchang	0.639246608
ENSGALG00010018892	APMAP	2	-0.242975937	0.206158985	-1.178585242	0.238563371	0.075797301	8.927634071	88.79882405	1	PRJNA827465;Yang2025_Silkie_Wenchang	CRA004775	0.750652917
ENSGALG00010018892	APMAP	2	-0.429370317	0.112120426	-3.829545887	0.00012838	0	0.44994632	0	1	CRA004775;Yang2025_Silkie_Wenchang	PRJNA827465	0.009309441
ENSGALG00010018892	APMAP	2	-0.06041937	0.06202959	-0.974041088	0.330036139	0	0.55697005	0	1	CRA004775;PRJNA827465	Yang2025_Silkie_Wenchang	0.801212404
ENSGALG00010018894	SERTAD4	2	-0.050966394	0.080358561	-0.634237271	0.525925987	0.007178621	1.884111656	46.92458926	0.5	PRJNA827465;Yang2025_Silkie_Wenchang	CRA004775	0.870414822
ENSGALG00010018894	SERTAD4	2	0.093782698	0.299271357	0.31337011	0.75399949	0.14654941	5.191706585	80.73851086	0.5	CRA004775;Yang2025_Silkie_Wenchang	PRJNA827465	0.938363002

ENSGALG00010018894	SERTAD4	2	0.152995892	0.204373943	0.748607625	0.454093735	0.062495118	3.160693795	68.36137681	1	CRA004775;PRJNA827465	Yang2025_Silkie_Wenchang	0.849072665
ENSGALG00010018895	SEPTIN7	2	0.054269857	0.104434737	0.519653309	0.603305235	0.013153158	1.87340371	46.62122238	0.5	PRJNA827465;Yang2025_Silkie_Wenchang	CRA004775	0.895538966
ENSGALG00010018895	SEPTIN7	2	0.171590739	0.102687122	1.671005434	0.094720604	0	0.187494312	0	1	CRA004775;Yang2025_Silkie_Wenchang	PRJNA827465	0.526919621
ENSGALG00010018895	SEPTIN7	2	0.012826839	0.050877979	0.252109828	0.800956174	0.001396818	1.152149367	13.20569813	0.5	CRA004775;PRJNA827465	Yang2025_Silkie_Wenchang	0.950158029
ENSGALG00010018897	CCDC85A	2	-0.119258655	0.159702092	-0.746756995	0.455210259	0.043488107	6.730917833	85.14318515	0.5	PRJNA827465;Yang2025_Silkie_Wenchang	CRA004775	0.849072665
ENSGALG00010018897	CCDC85A	2	0.059886091	0.411139681	0.145658749	0.884190788	0.278441688	4.96474845	79.8579926	0.5	CRA004775;Yang2025_Silkie_Wenchang	PRJNA827465	0.972001076
ENSGALG00010018897	CCDC85A	2	0.170966482	0.227278321	0.752234006	0.451910347	0.064467167	1.942771044	48.52713072	1	CRA004775;PRJNA827465	Yang2025_Silkie_Wenchang	0.848124933
ENSGALG00010018899	ACSS1	2	-0.164277063	0.28752458	-0.571349632	0.567762664	0.154106474	14.61486814	93.15765294	0.5	PRJNA827465;Yang2025_Silkie_Wenchang	CRA004775	0.883753504
ENSGALG00010018899	ACSS1	2	-0.422856685	0.11727943	-3.605548613	0.000311494	0	0.965278157	0	1	CRA004775;Yang2025_Silkie_Wenchang	PRJNA827465	0.017418181
ENSGALG00010018899	ACSS1	2	0.109241416	0.085031225	1.284721184	0.198889733	0	0.165867889	0	0.5	CRA004775;PRJNA827465	Yang2025_Silkie_Wenchang	0.71096127
ENSGALG00010018901	TCF7L2	2	0.382756789	0.416226574	0.919585783	0.357788318	0.296308366	6.21144981	83.90069902	1	PRJNA827465;Yang2025_Silkie_Wenchang	CRA004775	0.813618706
ENSGALG00010018901	TCF7L2	2	0.556395185	0.265314188	2.0971181	0.035983122	0.057609209	1.671091114	40.1588584	1	CRA004775;Yang2025_Silkie_Wenchang	PRJNA827465	0.327447244
ENSGALG00010018901	TCF7L2	2	0.077613859	0.119084634	0.651753769	0.514560029	0.009165004	1.245802576	19.73045974	1	CRA004775;PRJNA827465	Yang2025_Silkie_Wenchang	0.866869697
ENSGALG00010018903	CAMTA1	2	-0.005314396	0.127634373	-0.041637542	0.966787645	0.018420031	2.086324463	52.06881682	0.5	PRJNA827465;Yang2025_Silkie_Wenchang	CRA004775	0.990893423
ENSGALG00010018903	CAMTA1	2	0.175329983	0.147252496	1.190675797	0.233780883	0	0.020193972	0	1	CRA004775;Yang2025_Silkie_Wenchang	PRJNA827465	0.747082975
ENSGALG00010018903	CAMTA1	2	-0.082105924	0.081231913	-1.010759458	0.312131572	0	0.855987007	0	0.5	CRA004775;PRJNA827465	Yang2025_Silkie_Wenchang	0.796054997
ENSGALG00010018904	UGP2	2	0.061337997	0.082669752	0.741964212	0.458109019	0	0.009687104	0	1	PRJNA827465;Yang2025_Silkie_Wenchang	CRA004775	0.850026426
ENSGALG00010018904	UGP2	2	0.050349671	0.113249454	0.444590852	0.65661546	0	0.010726952	0	1	CRA004775;Yang2025_Silkie_Wenchang	PRJNA827465	0.910509456
ENSGALG00010018904	UGP2	2	0.065657317	0.113756181	0.577175816	0.563820692	0	0.018745479	0	1	CRA004775;PRJNA827465	Yang2025_Silkie_Wenchang	0.882651345
ENSGALG00010018908	ENSGALG00010018908	2	0.069178246	0.115983731	0.596447839	0.55087609	0	0.546589698	0	0.5	PRJNA827465;Yang2025_Silkie_Wenchang	CRA004775	0.878879109
ENSGALG00010018908	ENSGALG00010018908	2	-0.163899244	0.213063517	-0.769250625	0.441744542	0	0.464596791	0	1	CRA004775;Yang2025_Silkie_Wenchang	PRJNA827465	0.845135451
ENSGALG00010018908	ENSGALG00010018908	2	0.010259094	0.218048605	0.047049573	0.962473718	0.041272968	1.366825261	26.8377584	0.5	CRA004775;PRJNA827465	Yang2025_Silkie_Wenchang	0.98997945
ENSGALG00010018909	TTBK1	2	0.042109511	0.078446004	0.536796123	0.59140845	0	0.000561393	0	1	PRJNA827465;Yang2025_Silkie_Wenchang	CRA004775	0.892776493
ENSGALG00010018909	TTBK1	2	0.073325187	0.122219305	0.599947565	0.548541115	0	0.567419596	0	1	CRA004775;Yang2025_Silkie_Wenchang	PRJNA827465	0.878281266
ENSGALG00010018909	TTBK1	2	0.06348476	0.094763523	0.669928241	0.502903536	0	0.58161978	0	1	CRA004775;PRJNA827465	Yang2025_Silkie_Wenchang	0.863050365
ENSGALG00010018911	CNIH1	2	-0.078480051	0.03706056	-2.117616416	0.034207567	0	0.06803253	0	1	PRJNA827465;Yang2025_Silkie_Wenchang	CRA004775	0.318100902
ENSGALG00010018911	CNIH1	2	-0.077242714	0.102099773	-0.756541482	0.449324611	0	0.307998568	0	1	CRA004775;Yang2025_Silkie_Wenchang	PRJNA827465	0.847263197
ENSGALG00010018911	CNIH1	2	-0.084857085	0.038730263	-2.190976224	0.028453513	0	0.227705709	0	1	CRA004775;PRJNA827465	Yang2025_Silkie_Wenchang	0.287193179
ENSGALG00010018913	ENSGALG00010018913	2	0.117476219	0.282014866	0.416476693	0.677061219	0.110794874	3.280747026	69.51913719	0.5	PRJNA827465;Yang2025_Silkie_Wenchang	CRA004775	0.971709899
ENSGALG00010018913	ENSGALG00010018913	2	0.161512566	0.26798818	0.602685407	0.546717991	0.079051926	2.211947258	54.79096546	0.5	CRA004775;Yang2025_Silkie_Wenchang	PRJNA827465	0.878275987
ENSGALG00010018913	ENSGALG00010018913	2	-0.142635095	0.163485114	-0.872465333	0.382954573	0	0.006448104	0	1	CRA004775;PRJNA827465	Yang2025_Silkie_Wenchang	0.825269413
ENSGALG00010018915	DOCK5	2	0.060865024	0.090437289	0.673008049	0.50094217	0	0.05073233	0	1	PRJNA827465;Yang2025_Silkie_Wenchang	CRA004775	0.862421093
ENSGALG00010018915	DOCK5	2	-0.014353172	0.198261952	-0.07239499	0.942287572	0	0.259440358	0	0.5	CRA004775;Yang2025_Silkie_Wenchang	PRJNA827465	0.985827325
ENSGALG00010018915	DOCK5	2	0.061189847	0.099157003	0.61710061	0.537168367	0	0.37171888	0	0.5	CRA004775;PRJNA827465	Yang2025_Silkie_Wenchang	0.87481821
ENSGALG00010018916	CERS3	2	0.084096253	0.218569673	0.384757187	0.700417321	0	0.472461268	0	0.5	PRJNA827465;Yang2025_Silkie_Wenchang	CRA004775	0.922303014
ENSGALG00010018916	CERS3	2	-0.024730306	0.263074742	-0.094004868	0.92510529	0	0.072808188	0	0.5	CRA004775;Yang2025_Silkie_Wenchang	PRJNA827465	0.982594411
ENSGALG00010018916	CERS3	2	0.187897801	0.257073908	0.730909652	0.46483434	0	0.065490159	0	1	CRA004775;PRJNA827465	Yang2025_Silkie_Wenchang	0.85217126
ENSGALG00010018917	DNAJC11	2	-0.040138399	0.061686825	-0.650680262	0.515252907	0	0.763320817	0	0.5	PRJNA827465;Yang2025_Silkie_Wenchang	CRA004775	0.8671413
ENSGALG00010018917	DNAJC11	2	0.017522236	0.102128187	0.17157101	0.8637748	0	0.262309322	0	0.5	CRA004775;Yang2025_Silkie_Wenchang	PRJNA827465	0.96638033
ENSGALG00010018917	DNAJC11	2	-0.074620953	0.069038259	-1.080863776	0.279757714	0	0.000672099	0	1	CRA004775;PRJNA827465	Yang2025_Silkie_Wenchang	0.777878842
ENSGALG00010018919	RALB	2	0.046367792	0.081855121	0.566461709	0.571079955	0.008539291	2.294737285	56.42202677	0.5	PRJNA827465;Yang2025_Silkie_Wenchang	CRA004775	0.885354083
ENSGALG00010018919	RALB	2	0.140636494	0.099188656	1.417868727	0.156229094	0	0.261382498	0	1	CRA004775;Yang2025_Silkie_Wenchang	PRJNA827465	0.649146008
ENSGALG00010018919	RALB	2	-0.010336909	0.037918242	-0.272610449	0.785152679	0	0.020419349	0	0.5	CRA004775;PRJNA827465	Yang2025_Silkie_Wenchang	0.946325593
ENSGALG00010018920	ACAD11	2	0.123962423	0.15481564	0.800709947	0.423299582	0.029288447	2.561058108	60.95363878	0.5	PRJNA827465;Yang2025_Silkie_Wenchang	CRA004775	0.84012238
ENSGALG00010018920	ACAD11	2	0.139969398	0.175652093	0.796855853	0.425534753	0.031710601	1.897393191	47.29611106	0.5	CRA004775;Yang2025_Silkie_Wenchang	PRJNA827465	0.84125685
ENSGALG00010018920	ACAD11	2	-0.054344831	0.123273879	-0.440846281	0.659324286	0	0.041888908	0	1	CRA004775;PRJNA827465	Yang2025_Silkie_Wenchang	0.911449691
ENSGALG00010018922	EEF2K	2	-0.111699483	0.109241177	-1.022504373	0.306542067	0.014005356	2.107309651	52.54612916	0.5	PRJNA827465;Yang2025_Silkie_Wenchang	CRA004775	0.792500392
ENSGALG00010018922	EEF2K	2	0.098127563	0.131675567	0.745222255	0.456137371	0	0.82910565	0	1	CRA004775;Yang2025_Silkie_Wenchang	PRJNA827465	0.849188101
ENSGALG00010018922	EEF2K	2	-0.003101615	0.256787628	-0.01207852	0.990362969	0.096771852	2.97657103	66.40429576	0.5	CRA004775;PRJNA827465	Yang2025_Silkie_Wenchang	0.997468525
ENSGALG00010018923	VPS54	2	0.106555465	0.09957675	1.070083778	0.2845816	0.012591723	2.602806139	61.56929684	1	PRJNA827465;Yang2025_Silkie_Wenchang	CRA004775	0.780265906
ENSGALG00010018923	VPS54	2	0.135210743	0.144849114	0.933459234	0.350582909	0.017673974	1.512415659	33.88061051	0.5	CRA004775;Yang2025_Silkie_Wenchang	PRJNA827465	0.810111037
ENSGALG00010018923	VPS54	2	0.014653473	0.063240669	0.231709645	0.81676354	0	0.235738543	0	0.5	CRA004775;PRJNA827465	Yang2025_Silkie_Wenchang	0.954684235
ENSGALG00010018925	LINS1	2	-0.141672722	0.200455543	-0.706753825	0.479719474	0.049249565	2.398190382	58.30189265	0.5	PRJNA827465;Yang2025_Silkie_Wenchang	CRA004775	0.856274132
ENSGALG00010018925	LINS1	2	-0.371066437	0.20138322	-1.842588655	0.065389092	0	0.034585549	0	1	CRA004775;Yang2025_Silkie_Wenchang	PRJNA827465	0.440704235
ENSGALG00010018925	LINS1	2	-0.01123018	0.122765869	-0.091476405	0.927114054	0	0.581175285	0	0.5	CRA004775;PRJNA827465	Yang2025_Silkie_Wenchang	0.982960983
ENSGALG00010018932	CDKN3	2	-0.264813183	0.128690635	-2.05775022	0.039614117	0.006776957	1.216947984	17.82721917	1	PRJNA827465;Yang2025_Silkie_Wenchang	CRA004775	0.344306421
ENSGALG00010018932	CDKN3	2	0.477207267	0.971409664	0.491252336	0.623247987	1.762444213	14.71230692	93.20296942	0.5	CRA004775;Yang2025_Silkie_Wenchang	PRJNA827465	0.900348922
ENSGALG00010018932	CDKN3	2	0.599796673	0.832455979	0.720514584	0.471208224	1.277869382	12.12226016	91.75071326	0.5	CRA004775;PRJNA827465	Yang2025_Silkie_Wenchang	0.853917796
ENSGALG00010018933	PHF13	2	-1.01053E-05	0.052449583	-0.000192667	0.999846274	0	0.028902805	0	0.5	PRJNA827465;Yang2025_Silkie_Wenchang	CRA004775	0.999897156
ENSGALG00010018933	PHF13	2	-0.01447813	0.082053777	-0.17644684	0.859942909	0	0.000121814	0	1	CRA004775;Yang2025_Silkie_Wenchang	PRJNA827465	0.964879591
ENSGALG00010018933	PHF13	2	0.002049614	0.057738581	0.03549893	0.96317909	0	0.017509069	0	0.5	CRA004775;PRJNA827465	Yang2025_Silkie_Wenchang	0.99069926
ENSGALG00010018936	SYT14	2	-0.021069747	0.328123454	-0.064212863	0.948800735	0.173467308	5.123305705	80.48135213	0.5	PRJNA827465;Yang2025_Silkie_Wenchang	CRA004775	0.986996858
ENSGALG00010018936	SYT14	2	0.348327521	0.209215645	1.664920998	0.095928518	0	0.192074583	0	1	CRA004775;Yang2025_Silkie_Wenchang	PRJNA827465	0.5301492
ENSGALG00010018936	SYT14	2	-0.064334452	0.433711766	-0.148334579	0.882078727	0.228254918	1.984538639	49.61045453	0.5	CRA004775;PRJNA827465	Yang2025_Silkie_Wenchang	0.971928118
ENSGALG00010018937	INHBB	2	0.434574918	0.402485616	1.079727823	0.2802634	0.25844665	4.795622221	79.14777899	1	PRJNA827465;Yang2025_Silkie_Wenchang	CRA004775	0.778461864
ENSGALG00010018937	INHBB	2	0.858712306	0.28716475	2.990312379	0.002786923	0	0.016856887	0	1	CRA004775;Yang2025_Silkie_Wenchang	PRJNA827465	0.0719461
ENSGALG00010018937	INHBB	2	0.112862506	0.191429007	0.589578913	0.555472992	0	0.836402537	0	1	CRA004775;PRJNA827465	Yang2025_Silkie_Wenchang	0.880255849
ENSGALG00010018941	KLHL21	2	-0.34678152	0.067558021	-5.13309171	2.85021E-07	0	0.106330657	0	1	PRJNA827465;Yang2025_Silkie_Wenchang	CRA004775	8.08275E-05
ENSGALG00010018941	KLHL21	2	-0.356450643	0.104385332	-3.414757955	0.000638387	0	0.41976149	0	1	CRA004775;Yang2025_Silkie_Wenchang	PRJNA827465	0.027785747
ENSGALG00010018941	KLHL21	2	-0.319301786	0.084760408	-3.767110082	0.000165148	0	0.300221008	0	1	CRA004775;PRJNA827465	Yang2025_Silkie_Wenchang	0.011210396
ENSGALG00010018943	ACKR4	2	0.048180396	0.21417736	0.224955598	0.822013817	0	0.435554388	0	0.5	PRJNA827465;Yang2025_Silkie_Wenchang	CRA004775	0.955636543
ENSGALG00010018943	ACKR4	2	0.069612524	0.267580257	0.26015568	0.79474369	0	0.549763898	0	0.5	CRA004775;Yang2025_Silkie_Wenchang	PRJNA827465	0.948905846
ENSGALG00010018943	ACKR4	2	-0.106464107	0.237716405	-0.447861842	0.654252908	0	0.058457402	0	1	CRA004775;PRJNA827465	Yang2025_Silkie_Wenchang	0.910008212
ENSGALG00010018944	NEFL	2	0.158940529	0.63028073	0.252174184	0.800906431	0.613190633	3.998218991	74.98886373	0.5	PRJNA827465;Yang2025_Silkie_Wenchang	CRA004775	0.950158029
ENSGALG00010018944	NEFL	2	0.654758563	0.399831183	1.637587538	0.101507764	0	0.346257147	0	1	CRA004775;Yang2025_Silkie_Wenchang	PRJNA827465	0.543637124
ENSGALG00010018944	NEFL	2	-0.113581875	0.376900884	-0.301357413	0.763141965	0.142453307	1.776809965	43.71936112	0.5	CRA004775;PRJNA827465	Yang2025_Silkie_Wenchang	0.940184187
ENSGALG00010018945	HABP2	2	0.663151296	0.27530317	2.408803704	0.016004904	0	0.018593705	0	1	PRJNA827465;Yang2025_Silkie_Wenchang	CRA004775	0.209024249
ENSGALG00010018945	HABP2	2	1.104394512	0.506594298	2.180037391	0.02925469	0.362488082	3.351976848	70.16685838	1	CRA004775;Yang2025_Silkie_Wenchang	PRJNA827465	0.291235329
ENSGALG00010018945	HABP2	2	1.204521173	0.467828804	2.574705024	0.010032561	0.22478835	2.051967799	51.26629177	1	CRA004775;PRJNA827465	Yang2025_Silkie_Wenchang	0.158867741
ENSGALG00010018947	ENSGALG00010018947	2	-0.319311521	0.327912224	-0.973771324	0.330170094	0.159570739	3.700830687	72.97903945	1	PRJNA827465;Yang2025_Silkie_Wenchang	CRA004775	0.801276642
ENSGALG00010018947	ENSGALG00010018947	2	-0.037980184	0.680586839	-0.055805051	0.955497111	0.804303633	7.511533666	86.68713948	0.5	CRA004775;Yang2025_Silkie_Wenchang	PRJNA827465	0.988260604
ENSGALG00010018947	ENSGALG00010018947	2	0.227441107	0.338192873	0.672518924	0.501253397	0.15018801	2.573247048	61.13859334	0.5	CRA004775;PRJNA827465	Yang2025_Silkie_Wenchang	0.862658912
ENSGALG00010018948	POLR3E	2	-0.052396976	0.040098709	-1.306699821	0.19131468	0	0.776916455	0	1	PRJNA827465;Yang2025_Silkie_Wenchang	CRA004775	0.700702716
ENSGALG00010018948	POLR3E	2	0.033964812	0.212519706	0.159819588	0.873023194	0.064006547	3.142089416	68.17404384	0.5	CRA004775;Yang2025_Silkie_Wenchang	PRJNA827465	0.9697477
ENSGALG00010018948	POLR3E	2	0.057373083	0.150020498	0.382434956	0.702138766	0.029777978	2.190564553	54.3496676	0.5	CRA004775;PRJNA827465	Yang2025_Silkie_Wenchang	0.923083392
ENSGALG00010018950	TTL	2	-0.08396587	0.086390432	-0.971934824	0.331082974	0.012464035	6.017813855	83.38266979	1	PRJNA827465;Yang2025_Silkie_Wenchang	CRA004775	0.801722786
ENSGALG00010018950	TTL	2	-0.030866255	0.203528359	-0.151655797	0.879458423	0.058772469	2.683461998	62.73470611	0.5	CRA004775;Yang2025_Silkie_Wenchang	PRJNA827465	0.971291607
ENSGALG00010018950	TTL	2	0.006477771	0.043186383	0.149995685	0.88076802	0	0.983759585	0	0.5	CRA004775;PRJNA827465	Yang2025_Silkie_Wenchang	0.971676583
ENSGALG00010018951	YKT6	2	-0.011602096	0.05234197	-0.221659515	0.824578948	0	0.336939037	0	0.5	PRJNA827465;Yang2025_Silkie_Wenchang	CRA004775	0.95619211
ENSGALG00010018951	YKT6	2	-0.143287808	0.234589242	-0.610802982	0.541330019	0.07574651	3.207072503	68.8189151	0.5	CRA004775;Yang2025_Silkie_Wenchang	PRJNA827465	0.876088431
ENSGALG00010018951	YKT6	2	-0.15536425	0.172835925	-0.898911785	0.36869965	0.045091685	3.450430824	71.01811191	1	CRA004775;PRJNA827465	Yang2025_Silkie_Wenchang	0.820425502
ENSGALG00010018955	ZBTB48	2	-0.008797538	0.058149554	-0.151291585	0.879745707	0	0.443569468	0	0.5	PRJNA827465;Yang2025_Silkie_Wenchang	CRA004775	0.971407853
ENSGALG00010018955	ZBTB48	2	0.018807605	0.09226934	0.203833738	0.838483427	0	0.628110161	0	0.5	CRA004775;Yang2025_Silkie_Wenchang	PRJNA827465	0.959197438
ENSGALG00010018955	ZBTB48	2	-0.047172375	0.070425231	-0.669822093	0.502971208	0	0.29446274	0	1	CRA004775;PRJNA827465	Yang2025_Silkie_Wenchang	0.863050365
ENSGALG00010018958	PELI3	2	-0.157224054	0.158638662	-0.991082828	0.321645137	0.046960307	14.71735975	93.20530301	1	PRJNA827465;Yang2025_Silkie_Wenchang	CRA004775	0.798513866
ENSGALG00010018958	PELI3	2	-0.136207017	0.232605016	-0.585572141	0.558163075	0.083225356	3.700868847	72.97931807	0.5	CRA004775;Yang2025_Silkie_Wenchang	PRJNA827465	0.880678789
ENSGALG00010018958	PELI3	2	0.001410504	0.041947647	0.033625348	0.973175909	0	0.439006071	0	0.5	CRA004775;PRJNA827465	Yang2025_Silkie_Wenchang	0.992392211
ENSGALG00010018959	UBA5	2	-0.015509289	0.110516139	-0.140335059	0.888395265	0.020457945	6.120019552	83.66018292	0.5	PRJNA827465;Yang2025_Silkie_Wenchang	CRA004775	0.973135755
ENSGALG00010018959	UBA5	2	-0.126172141	0.067319429	-1.874230726	0.060898631	0	0.047394044	0	1	CRA004775;Yang2025_Silkie_Wenchang	PRJNA827465	0.427127227
ENSGALG00010018959	UBA5	2	0.085667422	0.055498934	1.543586816	0.122688461	0	0.283576916	0	0.5	CRA004775;PRJNA827465	Yang2025_Silkie_Wenchang	0.58762464
ENSGALG00010018960	RBFOX1	2	0.068853854	0.454546314	0.151478192	0.879598512	0.366812989	8.760565117	88.58521127	0.5	PRJNA827465;Yang2025_Silkie_Wenchang	CRA004775	0.971364438
ENSGALG00010018960	RBFOX1	2	0.510852286	0.223403182	2.286683128	0.022214325	0	0.070193234	0	1	CRA004775;Yang2025_Silkie_Wenchang	PRJNA827465	0.252849587
ENSGALG00010018960	RBFOX1	2	-0.092938082	0.36908285	-0.251808183	0.801189332	0.182052274	2.566546354	61.03713465	0.5	CRA004775;PRJNA827465	Yang2025_Silkie_Wenchang	0.950158029
ENSGALG00010018961	ASB7	2	-0.053244449	0.072159721	-0.737869385	0.460593824	0.006090575	2.157989266	53.66056654	0.5	PRJNA827465;Yang2025_Silkie_Wenchang	CRA004775	0.85014477
ENSGALG00010018961	ASB7	2	-0.129762295	0.088088024	-1.473098025	0.140724606	0	0.550451285	0	0.5	CRA004775;Yang2025_Silkie_Wenchang	PRJNA827465	0.625201272
ENSGALG00010018961	ASB7	2	0.002220957	0.044215638	0.050230117	0.959939012	0	0.06948555	0	1	CRA004775;PRJNA827465	Yang2025_Silkie_Wenchang	0.98933784
ENSGALG00010018968	GLI2	2	0.076154206	0.188312415	0.404403533	0.685916003	0.048981764	2.754315883	63.69334374	0.5	PRJNA827465;Yang2025_Silkie_Wenchang	CRA004775	0.919235539
ENSGALG00010018968	GLI2	2	0.213238136	0.196956838	1.082664298	0.278957456	0.002351722	1.023256702	2.272812121	0.5	CRA004775;Yang2025_Silkie_Wenchang	PRJNA827465	0.777307224
ENSGALG00010018968	GLI2	2	-0.067807533	0.078828092	-0.860195036	0.38968154	0	0.024694681	0	1	CRA004775;PRJNA827465	Yang2025_Silkie_Wenchang	0.829068422
ENSGALG00010018969	DIEXF	2	0.061929754	0.062104188	0.997191276	0.318671675	0	0.286017829	0	1	PRJNA827465;Yang2025_Silkie_Wenchang	CRA004775	0.797754958
ENSGALG00010018969	DIEXF	2	0.05828073	0.096010063	0.607027303	0.543832793	0	0.933032763	0	0.5	CRA004775;Yang2025_Silkie_Wenchang	PRJNA827465	0.876968972
ENSGALG00010018969	DIEXF	2	0.020546778	0.070141863	0.292931734	0.769574332	0	0.481840794	0	0.5	CRA004775;PRJNA827465	Yang2025_Silkie_Wenchang	0.941843401
ENSGALG00010018971	POLR1B	2	-0.076009268	0.113294071	-0.670902434	0.502282685	0.022412979	7.740485845	87.0809143	0.5	PRJNA827465;Yang2025_Silkie_Wenchang	CRA004775	0.862860181
ENSGALG00010018971	POLR1B	2	-0.052820657	0.148524937	-0.355634937	0.722113976	0.037704376	6.807164207	85.30959487	0.5	CRA004775;Yang2025_Silkie_Wenchang	PRJNA827465	0.929404251
ENSGALG00010018971	POLR1B	2	0.045321983	0.040262743	1.12565564	0.260311307	0	0.478828243	0	1	CRA004775;PRJNA827465	Yang2025_Silkie_Wenchang	0.767635013
ENSGALG00010018974	TAS1R1	2	0.678320498	0.384531562	1.764017743	0.077728992	0.252917374	6.847385544	85.39588587	1	PRJNA827465;Yang2025_Silkie_Wenchang	CRA004775	0.480343171
ENSGALG00010018974	TAS1R1	2	0.386939053	0.697654315	0.554628624	0.579148706	0.909592943	15.22615861	93.43235529	0.5	CRA004775;Yang2025_Silkie_Wenchang	PRJNA827465	0.887888423
ENSGALG00010018974	TAS1R1	2	0.028547758	0.311116106	0.091759178	0.926889378	0.143267647	3.728680838	73.1808636	0.5	CRA004775;PRJNA827465	Yang2025_Silkie_Wenchang	0.982896295
ENSGALG00010018977	NPHP3	2	0.299640579	0.172262832	1.739438364	0.081957685	0.048351987	5.352366535	81.31667565	1	PRJNA827465;Yang2025_Silkie_Wenchang	CRA004775	0.493532115
ENSGALG00010018977	NPHP3	2	0.157604659	0.339523765	0.464193305	0.642509257	0.205238779	8.904358948	88.76954528	0.5	CRA004775;Yang2025_Silkie_Wenchang	PRJNA827465	0.906634523
ENSGALG00010018977	NPHP3	2	0.011459043	0.161608593	0.070906147	0.943472451	0.03278388	2.402200448	58.37150056	0.5	CRA004775;PRJNA827465	Yang2025_Silkie_Wenchang	0.985827325
ENSGALG00010018983	MDGA2	2	0.428387173	0.256339678	1.671169972	0.09468811	0.092953309	3.223048862	68.97347689	1	PRJNA827465;Yang2025_Silkie_Wenchang	CRA004775	0.526919621
ENSGALG00010018983	MDGA2	2	0.82147875	0.230865096	3.558263087	0.000373315	0	0.87931966	0	1	CRA004775;Yang2025_Silkie_Wenchang	PRJNA827465	0.019374277
ENSGALG00010018983	MDGA2	2	0.623721155	0.539821872	1.155420309	0.247918349	0.442236231	3.422058253	70.77782065	1	CRA004775;PRJNA827465	Yang2025_Silkie_Wenchang	0.75810588
ENSGALG00010018985	NOL9	2	0.067268504	0.05524966	1.217536989	0.223399972	0	0.006360187	0	1	PRJNA827465;Yang2025_Silkie_Wenchang	CRA004775	0.736744216
ENSGALG00010018985	NOL9	2	-0.028712162	0.127205678	-0.22571447	0.821423503	0.00091303	1.024645741	2.405293847	0.5	CRA004775;Yang2025_Silkie_Wenchang	PRJNA827465	0.955598716
ENSGALG00010018985	NOL9	2	0.008193326	0.117649276	0.069641956	0.944478642	0.014008309	1.507971508	33.6857497	0.5	CRA004775;PRJNA827465	Yang2025_Silkie_Wenchang	0.985935636
ENSGALG00010018986	CTDSP1	2	-0.216374955	0.132733584	-1.630144743	0.103070909	0.023481733	2.726952784	63.32903137	1	PRJNA827465;Yang2025_Silkie_Wenchang	CRA004775	0.54728397
ENSGALG00010018986	CTDSP1	2	-0.341584278	0.130976593	-2.607979565	0.009107839	0	0.362354041	0	1	CRA004775;Yang2025_Silkie_Wenchang	PRJNA827465	0.149588551
ENSGALG00010018986	CTDSP1	2	-0.115618134	0.070691051	-1.635541304	0.101935623	0	0.076269646	0	1	CRA004775;PRJNA827465	Yang2025_Silkie_Wenchang	0.54407845
ENSGALG00010018987	MRPS10	2	-0.15767341	0.202916082	-0.777037525	0.437136632	0.057762146	3.038329417	67.08717644	0.5	PRJNA827465;Yang2025_Silkie_Wenchang	CRA004775	0.843453774
ENSGALG00010018987	MRPS10	2	-0.337372188	0.182566717	-1.847939169	0.064611143	0	0.389439897	0	1	CRA004775;Yang2025_Silkie_Wenchang	PRJNA827465	0.438283012
ENSGALG00010018987	MRPS10	2	-0.006424092	0.097658706	-0.065781051	0.947552142	0	0.201179989	0	0.5	CRA004775;PRJNA827465	Yang2025_Silkie_Wenchang	0.986560458
ENSGALG00010018990	GCK	2	0.452777635	0.51967347	0.871273329	0.38360493	0.448231467	5.76682528	82.65943649	0.5	PRJNA827465;Yang2025_Silkie_Wenchang	CRA004775	0.825509017
ENSGALG00010018990	GCK	2	0.32910849	0.687858218	0.478453963	0.632327134	0.803497886	6.617481086	84.88850989	0.5	CRA004775;Yang2025_Silkie_Wenchang	PRJNA827465	0.903058174
ENSGALG00010018990	GCK	2	-0.12722231	0.208464611	-0.610282529	0.541674668	0	0.513413851	0	1	CRA004775;PRJNA827465	Yang2025_Silkie_Wenchang	0.876288927
ENSGALG00010018991	RYBP	2	-0.224130425	0.212603962	-1.054215654	0.291784195	0.081603557	10.18334278	90.18004184	1	PRJNA827465;Yang2025_Silkie_Wenchang	CRA004775	0.78534312
ENSGALG00010018991	RYBP	2	-0.278452652	0.225904032	-1.232614795	0.217719515	0.066553518	2.449894056	59.1819084	0.5	CRA004775;Yang2025_Silkie_Wenchang	PRJNA827465	0.731070128
ENSGALG00010018991	RYBP	2	-0.014650693	0.075016627	-0.195299272	0.845158667	0	0.027683074	0	0.5	CRA004775;PRJNA827465	Yang2025_Silkie_Wenchang	0.960863849
ENSGALG00010018998	RPS29	2	-0.019656554	0.076130557	-0.25819532	0.796256169	0	0.868820376	0	0.5	PRJNA827465;Yang2025_Silkie_Wenchang	CRA004775	0.949325935
ENSGALG00010018998	RPS29	2	0.125047973	0.175842854	0.711134802	0.477000706	0	0.156894449	0	1	CRA004775;Yang2025_Silkie_Wenchang	PRJNA827465	0.854986476
ENSGALG00010018998	RPS29	2	-0.036809786	0.077144026	-0.477156668	0.633250565	0	0.101361788	0	0.5	CRA004775;PRJNA827465	Yang2025_Silkie_Wenchang	0.9032522
ENSGALG00010018999	THUMPD3	2	0.188455423	0.186164561	1.012305574	0.311391971	0.064756462	14.94811227	93.31019207	1	PRJNA827465;Yang2025_Silkie_Wenchang	CRA004775	0.795543332
ENSGALG00010018999	THUMPD3	2	0.028450601	0.373326789	0.076208302	0.939253378	0.256766292	12.22575594	91.82054668	0.5	CRA004775;Yang2025_Silkie_Wenchang	PRJNA827465	0.985393654
ENSGALG00010018999	THUMPD3	2	-0.130387532	0.181249935	-0.719379743	0.471906974	0.049973069	3.443754605	70.96192631	0.5	CRA004775;PRJNA827465	Yang2025_Silkie_Wenchang	0.853998373
ENSGALG00010019000	TRERF1	2	0.059992354	0.175664622	0.341516428	0.73271484	0.04090857	2.4984967	59.97593272	0.5	PRJNA827465;Yang2025_Silkie_Wenchang	CRA004775	0.932985777
ENSGALG00010019000	TRERF1	2	-0.022353063	0.336204017	-0.066486604	0.946990422	0.169661567	3.99151423	74.94685119	0.5	CRA004775;Yang2025_Silkie_Wenchang	PRJNA827465	0.986445196
ENSGALG00010019000	TRERF1	2	-0.122556549	0.116789224	-1.049382339	0.294002183	0.010391861	1.291325533	22.56019304	1	CRA004775;PRJNA827465	Yang2025_Silkie_Wenchang	0.787324026
ENSGALG00010019001	ALDH1A3	2	-0.335430254	0.242177172	-1.385061409	0.166033691	0.101208401	7.274768676	86.25385845	1	PRJNA827465;Yang2025_Silkie_Wenchang	CRA004775	0.66529196
ENSGALG00010019001	ALDH1A3	2	-0.535557218	0.111870346	-4.787302795	1.69038E-06	0	0.951078709	0	1	CRA004775;Yang2025_Silkie_Wenchang	PRJNA827465	0.000340702
ENSGALG00010019001	ALDH1A3	2	-0.10650168	0.12565964	-0.84754087	0.396693717	0	0.126915173	0	1	CRA004775;PRJNA827465	Yang2025_Silkie_Wenchang	0.831375233
ENSGALG00010019004	ECE2	2	0.144017715	0.133400134	1.079591983	0.280323913	0	0.573875089	0	1	PRJNA827465;Yang2025_Silkie_Wenchang	CRA004775	0.778461864
ENSGALG00010019004	ECE2	2	0.020212908	0.310843947	0.065025902	0.948153377	0.113664872	2.285848496	56.25256873	0.5	CRA004775;Yang2025_Silkie_Wenchang	PRJNA827465	0.98671013
ENSGALG00010019004	ECE2	2	-0.03126248	0.178065985	-0.175566828	0.860634265	0.011840552	1.148429902	12.92459399	0.5	CRA004775;PRJNA827465	Yang2025_Silkie_Wenchang	0.965114242
ENSGALG00010019005	ENSGALG00010019005	2	-0.063184772	0.241815444	-0.261293367	0.793866281	0.107230732	11.97028806	91.64598216	0.5	PRJNA827465;Yang2025_Silkie_Wenchang	CRA004775	0.948830022
ENSGALG00010019005	ENSGALG00010019005	2	-0.289737558	0.107460646	-2.696220151	0.00701313	0	0.540576357	0	0.5	CRA004775;Yang2025_Silkie_Wenchang	PRJNA827465	0.12854434
ENSGALG00010019005	ENSGALG00010019005	2	0.167262973	0.083303306	2.007879167	0.044656133	0	0.136270205	0	1	CRA004775;PRJNA827465	Yang2025_Silkie_Wenchang	0.364322144
ENSGALG00010019006	ENSGALG00010019006	2	0.006760181	0.132165514	0.051149359	0.959206505	0.028760999	5.604269471	82.15646116	0.5	PRJNA827465;Yang2025_Silkie_Wenchang	CRA004775	0.989155754
ENSGALG00010019006	ENSGALG00010019006	2	-0.115781762	0.069255934	-1.67179554	0.094564647	0	0.081780909	0	1	CRA004775;Yang2025_Silkie_Wenchang	PRJNA827465	0.526888901
ENSGALG00010019006	ENSGALG00010019006	2	0.03828539	0.110648934	0.346007768	0.729336877	0.016567295	2.946496682	66.06139059	0.5	CRA004775;PRJNA827465	Yang2025_Silkie_Wenchang	0.9323457
ENSGALG00010019008	HERPUD2	2	-0.133857166	0.100535044	-1.331447827	0.183041701	0.015633211	4.295408092	76.71932495	1	PRJNA827465;Yang2025_Silkie_Wenchang	CRA004775	0.691392093
ENSGALG00010019008	HERPUD2	2	-0.213825275	0.066574359	-3.211826249	0.001318941	0	0.421777909	0	1	CRA004775;Yang2025_Silkie_Wenchang	PRJNA827465	0.044306278
ENSGALG00010019008	HERPUD2	2	-0.061264201	0.049071281	-1.248473648	0.211857653	0	0.724227822	0	1	CRA004775;PRJNA827465	Yang2025_Silkie_Wenchang	0.724118735
ENSGALG00010019009	NRAP	2	0.183159666	0.335829451	0.545394888	0.585481956	0.201120751	9.226608451	89.16178133	0.5	PRJNA827465;Yang2025_Silkie_Wenchang	CRA004775	0.891139408
ENSGALG00010019009	NRAP	2	0.799082302	0.443326567	1.802468794	0.07147168	0.24850066	2.053271029	51.2972235	1	CRA004775;Yang2025_Silkie_Wenchang	PRJNA827465	0.461559554
ENSGALG00010019009	NRAP	2	0.549263026	0.81808013	0.671404923	0.50196261	1.136311417	5.839106468	82.87409203	0.5	CRA004775;PRJNA827465	Yang2025_Silkie_Wenchang	0.862795569
ENSGALG00010019011	VIL1	2	0.235471982	0.28840215	0.816470967	0.414230822	0	0.067115857	0	1	PRJNA827465;Yang2025_Silkie_Wenchang	CRA004775	0.836914812
ENSGALG00010019011	VIL1	2	1.205372974	0.92298891	1.305945241	0.19157118	1.387147249	5.306063756	81.15363769	1	CRA004775;Yang2025_Silkie_Wenchang	PRJNA827465	0.701184773
ENSGALG00010019011	VIL1	2	1.108619374	0.999329359	1.109363359	0.267273462	1.732268852	7.292110321	86.28654867	1	CRA004775;PRJNA827465	Yang2025_Silkie_Wenchang	0.77137971
ENSGALG00010019012	ADAM28	2	-0.21975959	0.270640312	-0.811998733	0.416792359	0.093927786	2.725590684	63.31070524	0.5	PRJNA827465;Yang2025_Silkie_Wenchang	CRA004775	0.837984974
ENSGALG00010019012	ADAM28	2	-0.117573506	0.647179295	-0.181670686	0.855841172	0.511872832	1.977779261	49.43824016	0.5	CRA004775;Yang2025_Silkie_Wenchang	PRJNA827465	0.963486168
ENSGALG00010019012	ADAM28	2	0.055376337	0.190729596	0.290339511	0.771556515	0	0.788935148	0	1	CRA004775;PRJNA827465	Yang2025_Silkie_Wenchang	0.942896231
ENSGALG00010019013	LRRK1	2	0.106465215	0.061884104	1.720396798	0.085360339	0.000147216	1.016204062	1.594567746	1	PRJNA827465;Yang2025_Silkie_Wenchang	CRA004775	0.502621951
ENSGALG00010019013	LRRK1	2	-0.114756439	0.189670786	-0.605029598	0.54515933	0.038762583	1.836953143	45.56203005	0.5	CRA004775;Yang2025_Silkie_Wenchang	PRJNA827465	0.877408247
ENSGALG00010019013	LRRK1	2	-0.059939322	0.265653032	-0.225630107	0.821489122	0.107857764	3.53764929	71.7326417	0.5	CRA004775;PRJNA827465	Yang2025_Silkie_Wenchang	0.955598716
ENSGALG00010019016	PLEKHG5	2	0.182790056	0.133675403	1.367417286	0.171494547	0.025990409	3.50893652	71.50133682	1	PRJNA827465;Yang2025_Silkie_Wenchang	CRA004775	0.673688175
ENSGALG00010019016	PLEKHG5	2	0.306585604	0.115751909	2.648644037	0.00808154	0	0.485101992	0	1	CRA004775;Yang2025_Silkie_Wenchang	PRJNA827465	0.139633915
ENSGALG00010019016	PLEKHG5	2	0.066044155	0.073435589	0.899348065	0.368467293	0	0.001355091	0	1	CRA004775;PRJNA827465	Yang2025_Silkie_Wenchang	0.820279089
ENSGALG00010019017	IRAK2	2	-0.044014822	0.144873729	-0.303815068	0.761268787	0.036228778	7.248588578	86.2042108	0.5	PRJNA827465;Yang2025_Silkie_Wenchang	CRA004775	0.939618592
ENSGALG00010019017	IRAK2	2	-0.02338036	0.187739973	-0.124535867	0.900891003	0.057100926	5.066841684	80.26383964	0.5	CRA004775;Yang2025_Silkie_Wenchang	PRJNA827465	0.976522218
ENSGALG00010019017	IRAK2	2	0.111025445	0.059987795	1.85080056	0.06419825	0	0.302505868	0	1	CRA004775;PRJNA827465	Yang2025_Silkie_Wenchang	0.43662345
ENSGALG00010019018	SH3BGRL	2	-0.280271621	0.288582059	-0.971202515	0.331447442	0.158508283	20.55391875	95.13474772	0.5	PRJNA827465;Yang2025_Silkie_Wenchang	CRA004775	0.801798188
ENSGALG00010019018	SH3BGRL	2	-0.563702687	0.102369787	-5.50653374	3.65968E-08	0	0.165231015	0	1	CRA004775;Yang2025_Silkie_Wenchang	PRJNA827465	1.56344E-05
ENSGALG00010019018	SH3BGRL	2	-0.008108884	0.070209855	-0.115494955	0.908052819	0	0.886855578	0	0.5	CRA004775;PRJNA827465	Yang2025_Silkie_Wenchang	0.978196186
ENSGALG00010019019	TBX20	2	-0.41321997	0.273316689	-1.511872439	0.130566317	0.099903477	2.971707765	66.34931565	1	PRJNA827465;Yang2025_Silkie_Wenchang	CRA004775	0.606115935
ENSGALG00010019019	TBX20	2	-0.667974828	0.189001688	-3.534226788	0.00040897	0	0.012214719	0	1	CRA004775;Yang2025_Silkie_Wenchang	PRJNA827465	0.020366982
ENSGALG00010019019	TBX20	2	-0.163138253	0.243226109	-0.670726733	0.502394629	0	0.492375114	0	1	CRA004775;PRJNA827465	Yang2025_Silkie_Wenchang	0.8629188

ENSGALG00010019020	KCNK2	2	0.236477573	0.244434922	0.96744594	0.333321154	0.08926062	3.931415466	74.5638687	0.5	PRJNA827465;Yang2025_Silkie_Wenchang	CRA004775	0.802433009
ENSGALG00010019020	KCNK2	2	-1.20701606	1.286171266	-0.938456714	0.348009752	3.03128919	11.17693395	91.05300251	1	CRA004775;Yang2025_Silkie_Wenchang	PRJNA827465	0.808860276
ENSGALG00010019020	KCNK2	2	-0.976923062	1.532362661	-0.637527321	0.523781419	4.418643886	16.09078126	93.78526136	0.5	CRA004775;PRJNA827465	Yang2025_Silkie_Wenchang	0.869661505
ENSGALG00010019021	LRR1	2	-0.044211716	0.075994266	-0.581777	0.560716898	0	0.712521671	0	1	PRJNA827465;Yang2025_Silkie_Wenchang	CRA004775	0.88130295
ENSGALG00010019021	LRR1	2	-0.243814468	0.152785455	-1.595796321	0.110534275	0.01595307	1.423417638	29.74654992	1	CRA004775;Yang2025_Silkie_Wenchang	PRJNA827465	0.562832796
ENSGALG00010019021	LRR1	2	-0.181510643	0.226937595	-0.799826236	0.42381148	0.075738883	3.316361999	69.84647634	1	CRA004775;PRJNA827465	Yang2025_Silkie_Wenchang	0.84030043
ENSGALG00010019023	AP2M1	2	-0.210140667	0.204782059	-1.026167371	0.304812699	0.074028336	7.76666639	87.12446306	1	PRJNA827465;Yang2025_Silkie_Wenchang	CRA004775	0.79225794
ENSGALG00010019023	AP2M1	2	-0.340509893	0.098985845	-3.439985796	0.000581745	0	0.91085802	0	1	CRA004775;Yang2025_Silkie_Wenchang	PRJNA827465	0.025964366
ENSGALG00010019023	AP2M1	2	-0.099967338	0.10448951	-0.956721281	0.338707954	0.014892431	2.491093463	59.85698591	1	CRA004775;PRJNA827465	Yang2025_Silkie_Wenchang	0.804450436
ENSGALG00010019024	CNPY3	2	0.380041127	0.296076928	1.283589129	0.199285756	0.161907544	12.57138677	92.0454281	1	PRJNA827465;Yang2025_Silkie_Wenchang	CRA004775	0.711142914
ENSGALG00010019024	CNPY3	2	0.32714362	0.378047103	0.86535148	0.386845903	0.25313487	8.674832809	88.47240031	0.5	CRA004775;Yang2025_Silkie_Wenchang	PRJNA827465	0.827124608
ENSGALG00010019024	CNPY3	2	0.086476764	0.059885382	1.444037948	0.148728301	0	0.582185091	0	0.5	CRA004775;PRJNA827465	Yang2025_Silkie_Wenchang	0.638795624
ENSGALG00010019025	DNAAF2	2	0.25934367	0.139287337	1.861932866	0.062612554	0	0.055594904	0	1	PRJNA827465;Yang2025_Silkie_Wenchang	CRA004775	0.431124947
ENSGALG00010019025	DNAAF2	2	0.056129133	0.200275208	0.280260017	0.779278024	0	0.94223198	0	0.5	CRA004775;Yang2025_Silkie_Wenchang	PRJNA827465	0.944727706
ENSGALG00010019025	DNAAF2	2	0.126766481	0.221239361	0.572983398	0.566655935	0.044998925	1.678641079	40.42800377	0.5	CRA004775;PRJNA827465	Yang2025_Silkie_Wenchang	0.883487517
ENSGALG00010019026	CHSY1	2	-0.036045545	0.135752646	-0.2655237	0.790606055	0.033217453	10.1153314	90.11401643	0.5	PRJNA827465;Yang2025_Silkie_Wenchang	CRA004775	0.947584465
ENSGALG00010019026	CHSY1	2	-0.176900858	0.062946841	-2.810321447	0.004949204	0	0.202264393	0	1	CRA004775;Yang2025_Silkie_Wenchang	PRJNA827465	0.104831857
ENSGALG00010019026	CHSY1	2	0.090545921	0.06133558	1.476238117	0.13987998	0.000563022	1.004651845	0.463030512	0.5	CRA004775;PRJNA827465	Yang2025_Silkie_Wenchang	0.623746637
ENSGALG00010019027	GNLY	2	0.284271026	0.192856941	1.473999453	0.140481738	0	0.166705045	0	1	PRJNA827465;Yang2025_Silkie_Wenchang	CRA004775	0.62458753
ENSGALG00010019027	GNLY	2	-0.050080647	0.473362941	-0.105797566	0.915742968	0.270554451	2.510545138	60.16801352	0.5	CRA004775;Yang2025_Silkie_Wenchang	PRJNA827465	0.980265984
ENSGALG00010019027	GNLY	2	-0.052443253	0.364861742	-0.14373459	0.885710056	0.177501383	2.789397964	64.14997025	0.5	CRA004775;PRJNA827465	Yang2025_Silkie_Wenchang	0.972376043
ENSGALG00010019028	TNFRSF25	2	0.910028994	0.267683669	3.399643311	0.000674738	0.011963798	1.091085997	8.348195916	1	PRJNA827465;Yang2025_Silkie_Wenchang	CRA004775	0.028531562
ENSGALG00010019028	TNFRSF25	2	0.504971795	0.671808633	0.751660175	0.452255446	0.773100649	6.967211049	85.64705457	0.5	CRA004775;Yang2025_Silkie_Wenchang	PRJNA827465	0.848321192
ENSGALG00010019028	TNFRSF25	2	0.236670518	0.404119979	0.585644189	0.55811646	0.19743323	2.528091728	60.44447325	0.5	CRA004775;PRJNA827465	Yang2025_Silkie_Wenchang	0.880678789
ENSGALG00010019029	CENPF	2	0.055321661	0.193738655	0.285545874	0.775224432	0.047607413	2.723896775	63.28788928	0.5	PRJNA827465;Yang2025_Silkie_Wenchang	CRA004775	0.949815265
ENSGALG00010019029	CENPF	2	0.246620716	0.17198764	1.433944413	0.151588126	0	0.09040485	0	1	CRA004775;Yang2025_Silkie_Wenchang	PRJNA827465	0.643051067
ENSGALG00010019029	CENPF	2	-0.124616433	0.152716071	-0.816000784	0.414499687	0	0.030667739	0	0.5	CRA004775;PRJNA827465	Yang2025_Silkie_Wenchang	0.837148996
ENSGALG00010019030	CDR2	2	0.098546861	0.10549589	0.934129856	0.350236913	0.011131597	1.826604811	45.25362059	0.5	PRJNA827465;Yang2025_Silkie_Wenchang	CRA004775	0.810111037
ENSGALG00010019030	CDR2	2	-0.018281313	0.13626641	-0.134158614	0.893277151	0	0.321830837	0	0.5	CRA004775;Yang2025_Silkie_Wenchang	PRJNA827465	0.974381724
ENSGALG00010019030	CDR2	2	0.175888886	0.075291625	2.336101591	0.019485947	0	0.001946145	0	1	CRA004775;PRJNA827465	Yang2025_Silkie_Wenchang	0.233920645
ENSGALG00010019032	GNMT	2	0.059978037	0.097823547	0.613124741	0.539793862	0	0.005740112	0	1	PRJNA827465;Yang2025_Silkie_Wenchang	CRA004775	0.875269925
ENSGALG00010019032	GNMT	2	0.023745976	0.199405965	0.11908358	0.905209139	0	0.04215083	0	0.5	CRA004775;Yang2025_Silkie_Wenchang	PRJNA827465	0.977980705
ENSGALG00010019032	GNMT	2	0.056606951	0.105527601	0.536418442	0.591669388	0	0.070594073	0	0.5	CRA004775;PRJNA827465	Yang2025_Silkie_Wenchang	0.892776493
ENSGALG00010019034	CASP7	2	-0.301034352	0.420188543	-0.716426844	0.473727824	0.321265477	11.01802558	90.92396371	0.5	PRJNA827465;Yang2025_Silkie_Wenchang	CRA004775	0.85444425
ENSGALG00010019034	CASP7	2	-0.228214751	0.530293187	-0.430355805	0.666936839	0.49395379	8.05912776	87.5917093	0.5	CRA004775;Yang2025_Silkie_Wenchang	PRJNA827465	0.913745549
ENSGALG00010019034	CASP7	2	0.150998973	0.137361022	1.099285451	0.271643576	0	0.40030136	0	1	CRA004775;PRJNA827465	Yang2025_Silkie_Wenchang	0.773036527
ENSGALG00010019035	ENSGALG00010019035	2	0.046951339	0.218851498	0.214535151	0.830129758	0	0.177736974	0	0.5	PRJNA827465;Yang2025_Silkie_Wenchang	CRA004775	0.957212554
ENSGALG00010019035	ENSGALG00010019035	2	-0.134824433	0.299686383	-0.449885082	0.652793305	0	0.036281235	0	1	CRA004775;Yang2025_Silkie_Wenchang	PRJNA827465	0.909572411
ENSGALG00010019035	ENSGALG00010019035	2	0.034721511	0.231200461	0.150179248	0.880623198	0	0.336918435	0	0.5	CRA004775;PRJNA827465	Yang2025_Silkie_Wenchang	0.971623434
ENSGALG00010019036	REL	2	0.125197735	0.175536073	0.713230808	0.475702945	0.04715624	3.836251448	73.932887	0.5	PRJNA827465;Yang2025_Silkie_Wenchang	CRA004775	0.85458236
ENSGALG00010019036	REL	2	0.25535361	0.146240007	1.746126896	0.080788904	0	0.947638544	0	1	CRA004775;Yang2025_Silkie_Wenchang	PRJNA827465	0.489478387
ENSGALG00010019036	REL	2	-0.019132974	0.068093882	-0.280979342	0.778726245	0	0.007644869	0	0.5	CRA004775;PRJNA827465	Yang2025_Silkie_Wenchang	0.944673731
ENSGALG00010019040	HMGN5	2	-0.214385264	0.237914968	-0.901100361	0.367534961	0.104181501	12.25062581	91.83715171	0.5	PRJNA827465;Yang2025_Silkie_Wenchang	CRA004775	0.819975483
ENSGALG00010019040	HMGN5	2	-0.162644661	0.314684757	-0.516849505	0.605261219	0.175047282	8.476352158	88.20247223	0.5	CRA004775;Yang2025_Silkie_Wenchang	PRJNA827465	0.896474603
ENSGALG00010019040	HMGN5	2	0.030216591	0.0603442	0.500737282	0.61655603	0	0.646974545	0	1	CRA004775;PRJNA827465	Yang2025_Silkie_Wenchang	0.899111936
ENSGALG00010019041	KATNIP	2	0.161405409	0.068617837	2.352236904	0.018660886	0.003015576	1.453074792	31.18041785	1	PRJNA827465;Yang2025_Silkie_Wenchang	CRA004775	0.22855369
ENSGALG00010019041	KATNIP	2	0.246997949	0.087311846	2.828916829	0.004670584	0	0.037851409	0	1	CRA004775;Yang2025_Silkie_Wenchang	PRJNA827465	0.100537004
ENSGALG00010019041	KATNIP	2	0.11265487	0.069642405	1.617618903	0.105744753	0	0.381864541	0	1	CRA004775;PRJNA827465	Yang2025_Silkie_Wenchang	0.551748176
ENSGALG00010019042	PEX6	2	0.105926774	0.064898482	1.632191878	0.102639071	0	0.044389193	0	1	PRJNA827465;Yang2025_Silkie_Wenchang	CRA004775	0.545895254
ENSGALG00010019042	PEX6	2	-0.000491487	0.178738441	-0.002749758	0.997806013	0.024855606	1.570974093	36.34522654	0.5	CRA004775;Yang2025_Silkie_Wenchang	PRJNA827465	0.999290321
ENSGALG00010019042	PEX6	2	0.017763524	0.14347307	0.123810856	0.901465036	0.022307628	1.675107589	40.30234197	0.5	CRA004775;PRJNA827465	Yang2025_Silkie_Wenchang	0.9766596
ENSGALG00010019043	METTL9	2	-0.308971854	0.180473081	-1.712010749	0.086894688	0.056555334	7.563752561	86.77904926	1	PRJNA827465;Yang2025_Silkie_Wenchang	CRA004775	0.50716096
ENSGALG00010019043	METTL9	2	-0.23048343	0.290434139	-0.793582432	0.427438563	0.143447782	6.325978552	84.19216898	0.5	CRA004775;Yang2025_Silkie_Wenchang	PRJNA827465	0.84125685
ENSGALG00010019043	METTL9	2	-0.100930994	0.078661955	-1.283097965	0.199457757	0	0.971779813	0	0.5	CRA004775;PRJNA827465	Yang2025_Silkie_Wenchang	0.711240084
ENSGALG00010019045	LBX1	2	-0.211553337	0.147431217	-1.434929056	0.151307315	0.031370448	3.589599872	72.14174182	1	PRJNA827465;Yang2025_Silkie_Wenchang	CRA004775	0.64289984
ENSGALG00010019045	LBX1	2	-0.332328707	0.119943151	-2.770718484	0.005593276	0.003594967	1.019104946	1.874678929	0.5	CRA004775;Yang2025_Silkie_Wenchang	PRJNA827465	0.112216709
ENSGALG00010019045	LBX1	2	-0.050562568	0.111422911	-0.453789684	0.649980209	0	0.278522917	0	0.5	CRA004775;PRJNA827465	Yang2025_Silkie_Wenchang	0.908335059
ENSGALG00010019046	SELENOS	2	-0.031237381	0.050733242	-0.615718203	0.538080521	0	1.12635E-05	0	1	PRJNA827465;Yang2025_Silkie_Wenchang	CRA004775	0.87481821
ENSGALG00010019046	SELENOS	2	-0.071528945	0.101812958	-0.702552468	0.482334693	0	0.34146094	0	1	CRA004775;Yang2025_Silkie_Wenchang	PRJNA827465	0.85691916
ENSGALG00010019046	SELENOS	2	-0.042139979	0.053304434	-0.790552982	0.429204893	0	0.456230106	0	1	CRA004775;PRJNA827465	Yang2025_Silkie_Wenchang	0.842008086
ENSGALG00010019049	POLD2	2	0.162492532	0.218634525	0.743215335	0.45735132	0.08047998	5.885140044	83.00805091	0.5	PRJNA827465;Yang2025_Silkie_Wenchang	CRA004775	0.849573219
ENSGALG00010019049	POLD2	2	0.145600974	0.278288938	0.523200726	0.600834576	0.118725656	4.239071288	76.40992727	0.5	CRA004775;Yang2025_Silkie_Wenchang	PRJNA827465	0.895230519
ENSGALG00010019049	POLD2	2	-0.043351655	0.067255203	-0.644584396	0.519196549	0	0.272065675	0	1	CRA004775;PRJNA827465	Yang2025_Silkie_Wenchang	0.869042024
ENSGALG00010019050	TNS1	2	0.0528011	0.221606663	0.238264948	0.811675606	0.083274869	6.323578312	84.1861688	0.5	PRJNA827465;Yang2025_Silkie_Wenchang	CRA004775	0.953348795
ENSGALG00010019050	TNS1	2	0.242483687	0.145850895	1.662545077	0.096403533	0	0.984881052	0	0.5	CRA004775;Yang2025_Silkie_Wenchang	PRJNA827465	0.530885356
ENSGALG00010019050	TNS1	2	-0.151432583	0.084012357	-1.802503688	0.071466195	0	0.00102577	0	1	CRA004775;PRJNA827465	Yang2025_Silkie_Wenchang	0.461559554
ENSGALG00010019051	IGSF6	2	-0.303792998	0.160798794	-1.889274114	0.058855108	0	0.441479562	0	1	PRJNA827465;Yang2025_Silkie_Wenchang	CRA004775	0.42013793
ENSGALG00010019051	IGSF6	2	-0.539393774	0.232825469	-2.31673011	0.020518438	0	0.561706321	0	1	CRA004775;Yang2025_Silkie_Wenchang	PRJNA827465	0.24152027
ENSGALG00010019051	IGSF6	2	-0.38743737	0.278880677	-1.389258566	0.164754138	0.064149262	1.463926786	31.69057295	1	CRA004775;PRJNA827465	Yang2025_Silkie_Wenchang	0.663999614
ENSGALG00010019059	BRWD3	2	0.06331159	0.08964068	0.706281898	0.480012849	0.004924347	1.262702119	20.8047579	1	PRJNA827465;Yang2025_Silkie_Wenchang	CRA004775	0.856321884
ENSGALG00010019059	BRWD3	2	0.217404937	0.157986393	1.376099118	0.168790949	0	0.037065011	0	1	CRA004775;Yang2025_Silkie_Wenchang	PRJNA827465	0.669861707
ENSGALG00010019059	BRWD3	2	0.022761943	0.071349211	0.319021643	0.749710101	0	0.178905233	0	1	CRA004775;PRJNA827465	Yang2025_Silkie_Wenchang	0.937362187
ENSGALG00010019062	PUS10	2	0.152634813	0.173276534	0.880874108	0.378385963	0.048024589	4.936805744	79.74398727	0.5	PRJNA827465;Yang2025_Silkie_Wenchang	CRA004775	0.823415288
ENSGALG00010019062	PUS10	2	0.066160191	0.306876946	0.215591924	0.829305852	0.151275483	4.753906829	78.96466978	0.5	CRA004775;Yang2025_Silkie_Wenchang	PRJNA827465	0.95688454
ENSGALG00010019062	PUS10	2	-0.044446307	0.088761658	-0.500737681	0.61655575	0	0.997641303	0	1	CRA004775;PRJNA827465	Yang2025_Silkie_Wenchang	0.899111936
ENSGALG00010019064	ENSGALG00010019064	2	-0.271204985	0.22606717	-1.199665503	0.230269276	0.093156415	11.13849469	91.02212617	1	PRJNA827465;Yang2025_Silkie_Wenchang	CRA004775	0.742860818
ENSGALG00010019064	ENSGALG00010019064	2	-0.300107057	0.275846454	-1.087949667	0.276617328	0.105430856	2.746821375	63.59428359	0.5	CRA004775;Yang2025_Silkie_Wenchang	PRJNA827465	0.775851484
ENSGALG00010019064	ENSGALG00010019064	2	-0.047118034	0.07241452	-0.650671084	0.515258833	0	0.13432591	0	0.5	CRA004775;PRJNA827465	Yang2025_Silkie_Wenchang	0.8671413
ENSGALG00010019065	CAND2	2	-0.272727654	0.084545612	-3.225804959	0.001256189	0.006812022	1.908668212	47.60744725	1	PRJNA827465;Yang2025_Silkie_Wenchang	CRA004775	0.043044471
ENSGALG00010019065	CAND2	2	-0.285602445	0.175873713	-1.623906381	0.104395791	0.028518225	1.31009667	23.66975486	0.5	CRA004775;Yang2025_Silkie_Wenchang	PRJNA827465	0.549118786
ENSGALG00010019065	CAND2	2	-0.178253514	0.082435683	-2.162334421	0.030592408	0	0.565354424	0	0.5	CRA004775;PRJNA827465	Yang2025_Silkie_Wenchang	0.298355689
ENSGALG00010019067	GTF3C1	2	0.095429231	0.104579321	0.912505743	0.361502548	0.018904784	7.177593011	86.06775282	0.5	PRJNA827465;Yang2025_Silkie_Wenchang	CRA004775	0.81550034
ENSGALG00010019067	GTF3C1	2	0.026923377	0.218038802	0.123479749	0.90172721	0.07548628	4.264880877	76.55268625	0.5	CRA004775;Yang2025_Silkie_Wenchang	PRJNA827465	0.976674416
ENSGALG00010019067	GTF3C1	2	-0.034308649	0.080176176	-0.427915754	0.668712457	0.005789154	1.266728389	21.05647834	1	CRA004775;PRJNA827465	Yang2025_Silkie_Wenchang	0.914377769
ENSGALG00010019068	SNRPA1	2	-0.138842275	0.073275207	-1.894805639	0.058118156	0	0.269440055	0	1	PRJNA827465;Yang2025_Silkie_Wenchang	CRA004775	0.417658363
ENSGALG00010019068	SNRPA1	2	-0.087886057	0.155720016	-0.564385101	0.572492076	0	0.219330399	0	1	CRA004775;Yang2025_Silkie_Wenchang	PRJNA827465	0.885678394
ENSGALG00010019068	SNRPA1	2	-0.161715575	0.080511056	-2.008613247	0.044578165	0	0.082171583	0	1	CRA004775;PRJNA827465	Yang2025_Silkie_Wenchang	0.364257243
ENSGALG00010019072	AEBP1	2	-0.146906627	0.123125149	-1.193148828	0.232811091	0.006837421	1.262194217	20.77289004	0.5	PRJNA827465;Yang2025_Silkie_Wenchang	CRA004775	0.745799878
ENSGALG00010019072	AEBP1	2	0.014202697	0.172069709	0.082540365	0.934217021	0	0.001794856	0	0.5	CRA004775;Yang2025_Silkie_Wenchang	PRJNA827465	0.98432801
ENSGALG00010019072	AEBP1	2	-0.219228724	0.124165216	-1.765621088	0.07745944	0	0.280457384	0	1	CRA004775;PRJNA827465	Yang2025_Silkie_Wenchang	0.479280283
ENSGALG00010019076	ESPN	2	0.235009825	0.242069072	0.970837881	0.331629016	0	0.491280664	0	1	PRJNA827465;Yang2025_Silkie_Wenchang	CRA004775	0.801846521
ENSGALG00010019076	ESPN	2	0.659549399	0.267138377	2.468942898	0.013551284	0	0.790029454	0	1	CRA004775;Yang2025_Silkie_Wenchang	PRJNA827465	0.190420492
ENSGALG00010019076	ESPN	2	0.469262484	0.408151284	1.149726837	0.250256397	0.20927734	2.667439917	62.51087068	1	CRA004775;PRJNA827465	Yang2025_Silkie_Wenchang	0.75993411
ENSGALG00010019079	CAPG	2	0.030074448	0.364916793	0.082414536	0.934317078	0.198291883	3.63456178	72.4863667	0.5	PRJNA827465;Yang2025_Silkie_Wenchang	CRA004775	0.98432801
ENSGALG00010019079	CAPG	2	0.004210566	0.466249597	0.009030713	0.992794632	0.30542594	3.357155035	70.21287401	0.5	CRA004775;Yang2025_Silkie_Wenchang	PRJNA827465	0.997872476
ENSGALG00010019079	CAPG	2	-0.31457273	0.158821099	-1.980673421	0.047627909	0	0.218991543	0	1	CRA004775;PRJNA827465	Yang2025_Silkie_Wenchang	0.377023106
ENSGALG00010019081	PCSK6	2	-0.150888831	0.083701621	-1.802699031	0.071435494	0	0.321967857	0	1	PRJNA827465;Yang2025_Silkie_Wenchang	CRA004775	0.461553385
ENSGALG00010019081	PCSK6	2	-0.092201646	0.213227062	-0.432410621	0.665443	0.033169397	1.497627103	33.22770415	0.5	CRA004775;Yang2025_Silkie_Wenchang	PRJNA827465	0.91350776
ENSGALG00010019081	PCSK6	2	-0.099751945	0.091501295	-1.090169765	0.275638369	0.000513681	1.009775081	0.968045331	0.5	CRA004775;PRJNA827465	Yang2025_Silkie_Wenchang	0.775366077
ENSGALG00010019082	POLR3K	2	0.14207773	0.405291327	0.350557045	0.725920687	0.297309968	10.25727671	90.25082361	0.5	PRJNA827465;Yang2025_Silkie_Wenchang	CRA004775	0.931029023
ENSGALG00010019082	POLR3K	2	0.474232933	0.192409617	2.464704933	0.013712612	0	0.729873947	0	1	CRA004775;Yang2025_Silkie_Wenchang	PRJNA827465	0.192069423
ENSGALG00010019082	POLR3K	2	-0.180417193	0.15158867	-1.190175976	0.233977233	0.011976921	1.147114715	12.82476051	0.5	CRA004775;PRJNA827465	Yang2025_Silkie_Wenchang	0.747279664
ENSGALG00010019083	OTOA	2	0.634137258	0.586275952	1.081636141	0.279414239	0.648262476	17.48006541	94.27919761	1	PRJNA827465;Yang2025_Silkie_Wenchang	CRA004775	0.777457036
ENSGALG00010019083	OTOA	2	0.854334066	0.461100939	1.852813547	0.063909088	0.30348681	3.165777456	68.41218266	1	CRA004775;Yang2025_Silkie_Wenchang	PRJNA827465	0.435607433
ENSGALG00010019083	OTOA	2	0.082409861	0.160146552	0.514590291	0.606839351	0	0.20535809	0	1	CRA004775;PRJNA827465	Yang2025_Silkie_Wenchang	0.896799223
ENSGALG00010019087	PEX13	2	-0.096045715	0.064890291	-1.480124578	0.138840003	0.003578633	1.65954764	39.74261565	1	PRJNA827465;Yang2025_Silkie_Wenchang	CRA004775	0.622497285
ENSGALG00010019087	PEX13	2	-0.132983378	0.092586221	-1.43631932	0.150911499	0.003086203	1.133780786	11.7995284	0.5	CRA004775;Yang2025_Silkie_Wenchang	PRJNA827465	0.642051813
ENSGALG00010019087	PEX13	2	-0.038890893	0.053834774	-0.72241212	0.470041138	0	0.214977263	0	0.5	CRA004775;PRJNA827465	Yang2025_Silkie_Wenchang	0.853648049
ENSGALG00010019093	RPL32	2	-0.13190108	0.083950678	-1.571173481	0.116142358	0	0.207647016	0	1	PRJNA827465;Yang2025_Silkie_Wenchang	CRA004775	0.573676398
ENSGALG00010019093	RPL32	2	-0.202388052	0.184301972	-1.098132864	0.272146477	0	0.046462277	0	1	CRA004775;Yang2025_Silkie_Wenchang	PRJNA827465	0.773286758
ENSGALG00010019093	RPL32	2	-0.121885916	0.08491695	-1.43535438	0.151186139	0	0.018958657	0	1	CRA004775;PRJNA827465	Yang2025_Silkie_Wenchang	0.642663727
ENSGALG00010019094	SNRNP25	2	0.307585884	0.4515576	0.681166443	0.495766179	0.382009833	15.39945861	93.50626522	0.5	PRJNA827465;Yang2025_Silkie_Wenchang	CRA004775	0.860911322
ENSGALG00010019094	SNRNP25	2	0.224187655	0.567807259	0.394830554	0.692967944	0.586039872	10.91030619	90.83435439	0.5	CRA004775;Yang2025_Silkie_Wenchang	PRJNA827465	0.92062328
ENSGALG00010019094	SNRNP25	2	-0.150900868	0.090712228	-1.663511868	0.096210017	0	0.643028257	0	1	CRA004775;PRJNA827465	Yang2025_Silkie_Wenchang	0.530565779
ENSGALG00010019101	POLE2	2	-0.180360819	0.23847078	-0.756322512	0.449455854	0.096608757	6.511872359	84.64343364	0.5	PRJNA827465;Yang2025_Silkie_Wenchang	CRA004775	0.847263197
ENSGALG00010019101	POLE2	2	-0.471800414	0.134310101	-3.512769402	0.000443462	0	0.236474489	0	1	CRA004775;Yang2025_Silkie_Wenchang	PRJNA827465	0.021438369
ENSGALG00010019101	POLE2	2	-0.221868033	0.310022119	-0.71565227	0.47420609	0.156234957	4.859796582	79.42300705	0.5	CRA004775;PRJNA827465	Yang2025_Silkie_Wenchang	0.85444425
ENSGALG00010019104	POLL	2	-0.261798215	0.102141253	-2.563099709	0.010374225	0	0.242097622	0	1	PRJNA827465;Yang2025_Silkie_Wenchang	CRA004775	0.162122532
ENSGALG00010019104	POLL	2	-0.295518263	0.121936176	-2.423548725	0.015369691	0	0.047686449	0	1	CRA004775;Yang2025_Silkie_Wenchang	PRJNA827465	0.205049211
ENSGALG00010019104	POLL	2	-0.224834521	0.120431817	-1.866903004	0.061915134	0	0.025685803	0	1	CRA004775;PRJNA827465	Yang2025_Silkie_Wenchang	0.429324205
ENSGALG00010019108	KDELR2	2	-0.131229765	0.240576188	-0.545481106	0.585422672	0.108322603	15.34192909	93.48191486	0.5	PRJNA827465;Yang2025_Silkie_Wenchang	CRA004775	0.891135366
ENSGALG00010019108	KDELR2	2	-0.340165104	0.096276205	-3.533220944	0.000410529	0	0.768011449	0	1	CRA004775;Yang2025_Silkie_Wenchang	PRJNA827465	0.020398268
ENSGALG00010019108	KDELR2	2	0.06161905	0.094269188	0.653649944	0.513337359	0.006692822	1.242422279	19.51206789	0.5	CRA004775;PRJNA827465	Yang2025_Silkie_Wenchang	0.866301448
ENSGALG00010019110	MBD4	2	0.384637465	0.343929671	1.11836081	0.263412925	0.222069117	16.1626785	93.81290669	1	PRJNA827465;Yang2025_Silkie_Wenchang	CRA004775	0.768643821
ENSGALG00010019110	MBD4	2	0.220965597	0.525750236	0.420286254	0.674276351	0.522335081	18.02364565	94.45173291	0.5	CRA004775;Yang2025_Silkie_Wenchang	PRJNA827465	0.915851652
ENSGALG00010019110	MBD4	2	-0.088743466	0.176379493	-0.503139364	0.614866293	0.041176455	2.651461538	62.2849517	0.5	CRA004775;PRJNA827465	Yang2025_Silkie_Wenchang	0.898781283
ENSGALG00010019113	KLHDC1	2	0.117473944	0.237832088	0.493936479	0.621351041	0.097408841	7.161933782	86.03729062	0.5	PRJNA827465;Yang2025_Silkie_Wenchang	CRA004775	0.899693435
ENSGALG00010019113	KLHDC1	2	0.237549544	0.162078852	1.465641826	0.14274589	0.022794187	1.680768055	40.50339088	1	CRA004775;Yang2025_Silkie_Wenchang	PRJNA827465	0.629032597
ENSGALG00010019113	KLHDC1	2	-0.084529749	0.100015645	-0.845165265	0.398018573	0	0.324962901	0	0.5	CRA004775;PRJNA827465	Yang2025_Silkie_Wenchang	0.831850795
ENSGALG00010019114	TENT5D	2	0.837242559	0.252550557	3.31514833	0.000915945	0	5.89845E-06	0	1	PRJNA827465;Yang2025_Silkie_Wenchang	CRA004775	0.035659243
ENSGALG00010019114	TENT5D	2	0.315031322	0.558701287	0.563863605	0.572846961	0.409852779	2.885020482	65.33820102	0.5	CRA004775;Yang2025_Silkie_Wenchang	PRJNA827465	0.88573298
ENSGALG00010019114	TENT5D	2	0.348405185	0.556111016	0.626502937	0.530985111	0.448038529	3.479910366	71.26362765	0.5	CRA004775;PRJNA827465	Yang2025_Silkie_Wenchang	0.872840748
ENSGALG00010019115	DBNL	2	0.367268065	0.375851626	0.977162369	0.328488775	0.277411627	55.021831	98.18253958	0.5	PRJNA827465;Yang2025_Silkie_Wenchang	CRA004775	0.800761292
ENSGALG00010019115	DBNL	2	0.244398007	0.517111667	0.472621336	0.636483358	0.511748909	22.73208034	95.60093056	0.5	CRA004775;Yang2025_Silkie_Wenchang	PRJNA827465	0.904566695
ENSGALG00010019115	DBNL	2	-0.082016069	0.125632559	-0.65282495	0.513869137	0.018737129	1.879746588	46.80133981	1	CRA004775;PRJNA827465	Yang2025_Silkie_Wenchang	0.866620841
ENSGALG00010019120	DAGLB	2	-0.144657029	0.069789954	-2.072748583	0.038195684	0.0013389	1.132102264	11.66875715	1	PRJNA827465;Yang2025_Silkie_Wenchang	CRA004775	0.33719788
ENSGALG00010019120	DAGLB	2	-0.034052525	0.109073779	-0.312197169	0.754890684	0	0.010909868	0	1	CRA004775;Yang2025_Silkie_Wenchang	PRJNA827465	0.93838723
ENSGALG00010019120	DAGLB	2	-0.176310601	0.072223147	-2.441192446	0.014638851	0	0.466873947	0	1	CRA004775;PRJNA827465	Yang2025_Silkie_Wenchang	0.199389056
ENSGALG00010019123	PLEKHO2	2	-0.036246541	0.079485858	-0.456012454	0.648381023	0.001787682	1.151117687	13.12790943	0.5	PRJNA827465;Yang2025_Silkie_Wenchang	CRA004775	0.90762535
ENSGALG00010019123	PLEKHO2	2	-0.132748841	0.108284645	-1.225924883	0.220226944	0	0.01772899	0	1	CRA004775;Yang2025_Silkie_Wenchang	PRJNA827465	0.733812488
ENSGALG00010019123	PLEKHO2	2	0.005094753	0.083610754	0.060934189	0.951411621	0	0.305788577	0	0.5	CRA004775;PRJNA827465	Yang2025_Silkie_Wenchang	0.987713746
ENSGALG00010019124	BMPER	2	-0.14852162	0.093238193	-1.59292684	0.111176604	0	0.183496802	0	1	PRJNA827465;Yang2025_Silkie_Wenchang	CRA004775	0.564179998
ENSGALG00010019124	BMPER	2	-0.163536563	0.139366765	-1.173425833	0.240625083	0	0.494809652	0	0.5	CRA004775;Yang2025_Silkie_Wenchang	PRJNA827465	0.753158164
ENSGALG00010019124	BMPER	2	-0.098524676	0.115808833	-0.850752689	0.394906748	0	0.283222964	0	0.5	CRA004775;PRJNA827465	Yang2025_Silkie_Wenchang	0.830643015
ENSGALG00010019125	ENSGALG00010019125	2	0.058907754	0.087911204	0.670082442	0.502805237	0	0.019079086	0	1	PRJNA827465;Yang2025_Silkie_Wenchang	CRA004775	0.863050365
ENSGALG00010019125	ENSGALG00010019125	2	0.010881407	0.125808994	0.086491485	0.931075725	0	0.980614608	0	0.5	CRA004775;Yang2025_Silkie_Wenchang	PRJNA827465	0.983943003
ENSGALG00010019125	ENSGALG00010019125	2	0.019907295	0.151159282	0.13169747	0.895223587	0.01122206	1.095857342	8.747246425	0.5	CRA004775;PRJNA827465	Yang2025_Silkie_Wenchang	0.974706982
ENSGALG00010019127	TMBIM1	2	-0.050340451	0.053958061	-0.932955163	0.350843119	0	0.137858392	0	1	PRJNA827465;Yang2025_Silkie_Wenchang	CRA004775	0.810146221
ENSGALG00010019127	TMBIM1	2	-0.155722719	0.119279839	-1.305524217	0.191714407	0	0.387583096	0	1	CRA004775;Yang2025_Silkie_Wenchang	PRJNA827465	0.701447712
ENSGALG00010019127	TMBIM1	2	-0.062641952	0.062638544	-1.000054412	0.317284176	0.000970608	1.043926866	4.207848976	1	CRA004775;PRJNA827465	Yang2025_Silkie_Wenchang	0.797095376
ENSGALG00010019130	RPS6KC1	2	-0.04319819	0.04049898	-1.066648848	0.286130434	0	0.113133126	0	1	PRJNA827465;Yang2025_Silkie_Wenchang	CRA004775	0.78194146
ENSGALG00010019130	RPS6KC1	2	-0.077064312	0.065006793	-1.185480906	0.23582736	0	0.13568447	0	1	CRA004775;Yang2025_Silkie_Wenchang	PRJNA827465	0.748669015
ENSGALG00010019130	RPS6KC1	2	-0.044042224	0.0455871	-0.966111556	0.333988363	0	0.361365428	0	1	CRA004775;PRJNA827465	Yang2025_Silkie_Wenchang	0.80295722
ENSGALG00010019132	ENSGALG00010019132	2	-0.246993232	0.387501592	-0.637399271	0.523864803	0.266506457	8.12501883	87.69233622	0.5	PRJNA827465;Yang2025_Silkie_Wenchang	CRA004775	0.869661505
ENSGALG00010019132	ENSGALG00010019132	2	-0.380250195	0.287687208	-1.321748709	0.186251843	0.101678674	2.586403906	61.33627862	1	CRA004775;Yang2025_Silkie_Wenchang	PRJNA827465	0.695313088
ENSGALG00010019132	ENSGALG00010019132	2	0.086146116	0.063579418	1.354937157	0.17543758	0	0.669323347	0	0.5	CRA004775;PRJNA827465	Yang2025_Silkie_Wenchang	0.680258136
ENSGALG00010019135	KIAA1841	2	0.120455971	0.152162076	0.791629386	0.428576807	0	0.925419952	0	1	PRJNA827465;Yang2025_Silkie_Wenchang	CRA004775	0.841586523
ENSGALG00010019135	KIAA1841	2	0.226598125	0.201648394	1.123728887	0.261128058	0	0.352795177	0	1	CRA004775;Yang2025_Silkie_Wenchang	PRJNA827465	0.767929536
ENSGALG00010019135	KIAA1841	2	0.034312147	0.160276341	0.214081174	0.830483756	0	0.033839755	0	1	CRA004775;PRJNA827465	Yang2025_Silkie_Wenchang	0.957283722
ENSGALG00010019139	DPCD	2	0.05123681	0.078777816	0.650396432	0.515436181	0	0.080106542	0	1	PRJNA827465;Yang2025_Silkie_Wenchang	CRA004775	0.867199306
ENSGALG00010019139	DPCD	2	0.036629098	0.14090721	0.259951912	0.794900868	0	0.673299459	0	0.5	CRA004775;Yang2025_Silkie_Wenchang	PRJNA827465	0.948905846
ENSGALG00010019139	DPCD	2	0.020847508	0.088435469	0.235736956	0.813636791	0	0.527436281	0	0.5	CRA004775;PRJNA827465	Yang2025_Silkie_Wenchang	0.953782677
ENSGALG00010019141	ACOT7	2	0.314568257	0.101552507	3.097592252	0.001950997	0	0.398401556	0	1	PRJNA827465;Yang2025_Silkie_Wenchang	CRA004775	0.057181223
ENSGALG00010019141	ACOT7	2	0.190892716	0.245672383	0.777021467	0.437146105	0.075447919	2.558230087	60.91047458	0.5	CRA004775;Yang2025_Silkie_Wenchang	PRJNA827465	0.843453774
ENSGALG00010019141	ACOT7	2	0.155893382	0.167471823	0.93086335	0.351924255	0.024163494	1.573212483	36.43579549	0.5	CRA004775;PRJNA827465	Yang2025_Silkie_Wenchang	0.810678834
ENSGALG00010019144	IFT122	2	0.002102818	0.049087056	0.042838537	0.965830244	0	0.382545083	0	0.5	PRJNA827465;Yang2025_Silkie_Wenchang	CRA004775	0.990731137
ENSGALG00010019144	IFT122	2	0.014386785	0.070217567	0.20488869	0.837659092	0	0.737215268	0	0.5	CRA004775;Yang2025_Silkie_Wenchang	PRJNA827465	0.959001319
ENSGALG00010019144	IFT122	2	-0.035790785	0.061636382	-0.580676276	0.561458654	0	0.334170671	0	1	CRA004775;PRJNA827465	Yang2025_Silkie_Wenchang	0.881692882
ENSGALG00010019145	RAC1	2	0.026633921	0.088048728	0.302490696	0.762278025	0.007214113	1.227910869	18.56086424	0.5	PRJNA827465;Yang2025_Silkie_Wenchang	CRA004775	0.94006317
ENSGALG00010019145	RAC1	2	0.053647021	0.099046658	0.541633831	0.588070781	0	0.935179765	0	1	CRA004775;Yang2025_Silkie_Wenchang	PRJNA827465	0.891761457
ENSGALG00010019145	RAC1	2	-0.002763257	0.036667976	-0.075358877	0.939929177	0	0.021223901	0	0.5	CRA004775;PRJNA827465	Yang2025_Silkie_Wenchang	0.985444231
ENSGALG00010019146	KLHDC2	2	-0.158576207	0.09891385	-1.603174959	0.108896032	0.011108551	1.979075775	49.47136371	1	PRJNA827465;Yang2025_Silkie_Wenchang	CRA004775	0.558760047
ENSGALG00010019146	KLHDC2	2	-0.235479534	0.144053595	-1.634666143	0.102119054	0.005849758	1.089930865	8.251061414	0.5	CRA004775;Yang2025_Silkie_Wenchang	PRJNA827465	0.544584147
ENSGALG00010019146	KLHDC2	2	-0.085849347	0.056566664	-1.517666764	0.129098427	0	0.23778442	0	0.5	CRA004775;PRJNA827465	Yang2025_Silkie_Wenchang	0.60297307
ENSGALG00010019149	COX5B	2	0.239783204	0.384359878	0.623850764	0.532725597	0.278673337	17.36582297	94.24156286	0.5	PRJNA827465;Yang2025_Silkie_Wenchang	CRA004775	0.873183244
ENSGALG00010019149	COX5B	2	0.578566508	0.147106384	3.932980293	8.38991E-05	0	0.855841793	0	1	CRA004775;Yang2025_Silkie_Wenchang	PRJNA827465	0.006869765
ENSGALG00010019149	COX5B	2	-0.112375544	0.090492679	-1.241819183	0.214303283	0	0.954436091	0	0.5	CRA004775;PRJNA827465	Yang2025_Silkie_Wenchang	0.727123478
ENSGALG00010019157	ENSGALG00010019157	2	0.263557003	0.382976732	0.688180196	0.491339313	0.127098701	1.74803107	42.79277886	0.5	PRJNA827465;Yang2025_Silkie_Wenchang	CRA004775	0.85895707
ENSGALG00010019157	ENSGALG00010019157	2	0.527758788	0.39396171	1.339619498	0.180369082	0	0.563636393	0	0.5	CRA004775;Yang2025_Silkie_Wenchang	PRJNA827465	0.687722741
ENSGALG00010019157	ENSGALG00010019157	2	-0.066398217	0.336735084	-0.197182357	0.843684837	0	0.006736622	0	1	CRA004775;PRJNA827465	Yang2025_Silkie_Wenchang	0.96057435
ENSGALG00010019158	ENSGALG00010019158	2	0.47453139	0.283055562	1.676460219	0.093648082	0.100211746	2.542033988	60.66142291	1	PRJNA827465;Yang2025_Silkie_Wenchang	CRA004775	0.524147023
ENSGALG00010019158	ENSGALG00010019158	2	0.370992591	0.436444574	0.850033688	0.395306357	0.291119576	4.238819146	76.40852404	0.5	CRA004775;Yang2025_Silkie_Wenchang	PRJNA827465	0.830885382
ENSGALG00010019158	ENSGALG00010019158	2	0.148430842	0.159919588	0.928159234	0.353324977	0	0.725362357	0	0.5	CRA004775;PRJNA827465	Yang2025_Silkie_Wenchang	0.811157428
ENSGALG00010019166	PNPT1	2	-0.02069526	0.043427092	-0.476551829	0.633681292	0	0.937216169	0	0.5	PRJNA827465;Yang2025_Silkie_Wenchang	CRA004775	0.90355138
ENSGALG00010019166	PNPT1	2	0.035641848	0.076453975	0.466186984	0.641081665	0	0.136489107	0	0.5	CRA004775;Yang2025_Silkie_Wenchang	PRJNA827465	0.906429868
ENSGALG00010019166	PNPT1	2	-0.04683194	0.049979542	-0.937022188	0.348747145	0	0.000805057	0	1	CRA004775;PRJNA827465	Yang2025_Silkie_Wenchang	0.809333419
ENSGALG00010019169	GPR153	2	0.56174898	0.180124882	3.118663974	0.00181673	0	0.977610567	0	1	PRJNA827465;Yang2025_Silkie_Wenchang	CRA004775	0.054422978

ENSGALG00010019169	GPR153	2	0.461613031	0.468368114	0.985577407	0.324340529	0.262092663	2.467868449	59.47920156	0.5	CRA004775;Yang2025_Silkie_Wenchang	PRJNA827465	0.79980407
ENSGALG00010019169	GPR153	2	0.378603482	0.193145004	1.96020334	0.049972028	0.003864045	1.031038154	3.01037877	0.5	CRA004775;PRJNA827465	Yang2025_Silkie_Wenchang	0.386928808
ENSGALG00010019172	NEMF	2	0.246334413	0.312113688	0.789245787	0.429968366	0.186307638	22.33680174	95.52308333	0.5	PRJNA827465;Yang2025_Silkie_Wenchang	CRA004775	0.842012722
ENSGALG00010019172	NEMF	2	0.274458575	0.787458044	0.348546247	0.727429988	1.004840253	5.18939019	80.72991308	0.5	CRA004775;Yang2025_Silkie_Wenchang	PRJNA827465	0.931579695
ENSGALG00010019172	NEMF	2	0.224499639	0.35527011	0.631906334	0.527444026	0.229670408	10.98159391	90.89385377	0.5	CRA004775;PRJNA827465	Yang2025_Silkie_Wenchang	0.871149047
ENSGALG00010019172	NEMF	2	-0.061790079	0.046637651	-1.324896891	0.185205352	0	0.224215292	0	1	CRA004775;PRJNA827465	Yang2025_Silkie_Wenchang	0.693580367
ENSGALG00010019174	ENSGALG00010019174	2	0.669856411	0.315623721	2.122325942	0.033810376	0.054717023	1.374337702	27.23767975	1	PRJNA827465;Yang2025_Silkie_Wenchang	CRA004775	0.315934384
ENSGALG00010019174	ENSGALG00010019174	2	0.274458575	0.787458044	0.348546247	0.727429988	1.004840253	5.18939019	80.72991308	0.5	CRA004775;Yang2025_Silkie_Wenchang	PRJNA827465	0.931579695
ENSGALG00010019174	ENSGALG00010019174	2	0.008943543	0.461407701	0.019383167	0.984535439	0.223850593	2.009954348	50.24762623	0.5	CRA004775;PRJNA827465	Yang2025_Silkie_Wenchang	0.995703389
ENSGALG00010019183	ANGEL2	2	0.0518349	0.084484546	0.613542978	0.539517374	0.009863397	3.148579507	68.23964592	0.5	PRJNA827465;Yang2025_Silkie_Wenchang	CRA004775	0.874998368
ENSGALG00010019183	ANGEL2	2	0.001347627	0.096257747	0.014000188	0.988829831	0.005001561	1.182378176	15.42469062	0.5	CRA004775;Yang2025_Silkie_Wenchang	PRJNA827465	0.996891127
ENSGALG00010019183	ANGEL2	2	0.132282094	0.053016938	2.495091157	0.012592478	0	0.138731577	0	1	CRA004775;PRJNA827465	Yang2025_Silkie_Wenchang	0.181734907
ENSGALG00010019184	PNKD	2	0.020183149	0.106940322	0.188732826	0.850302222	0	0.296947883	0	0.5	PRJNA827465;Yang2025_Silkie_Wenchang	CRA004775	0.961985357
ENSGALG00010019184	PNKD	2	-0.14145505	0.271280727	-0.521434206	0.602064328	0.098993274	2.973063199	66.36465715	0.5	CRA004775;Yang2025_Silkie_Wenchang	PRJNA827465	0.89532296
ENSGALG00010019184	PNKD	2	-0.175317193	0.203544418	-0.861321543	0.389060974	0.047351154	2.1160003	52.74102749	1	CRA004775;PRJNA827465	Yang2025_Silkie_Wenchang	0.828615121
ENSGALG00010019185	ANKDD1A	2	0.189187867	0.133024268	1.422205654	0.154966558	0	0.354130268	0	1	PRJNA827465;Yang2025_Silkie_Wenchang	CRA004775	0.647628882
ENSGALG00010019185	ANKDD1A	2	-0.203965343	0.540877961	-0.377100488	0.706098929	0.482333561	5.299544836	81.13045495	0.5	CRA004775;Yang2025_Silkie_Wenchang	PRJNA827465	0.924548793
ENSGALG00010019185	ANKDD1A	2	-0.269559424	0.458617095	-0.587765757	0.556689531	0.323888769	3.935986731	74.59341028	0.5	CRA004775;PRJNA827465	Yang2025_Silkie_Wenchang	0.880358001
ENSGALG00010019189	ICMT	2	-0.246440499	0.230298644	-1.070090968	0.284578364	0.085109908	4.331808402	76.91495313	1	PRJNA827465;Yang2025_Silkie_Wenchang	CRA004775	0.780265906
ENSGALG00010019189	ICMT	2	-0.396377633	0.149128503	-2.657960259	0.007861516	0	0.686543783	0	1	CRA004775;Yang2025_Silkie_Wenchang	PRJNA827465	0.137569534
ENSGALG00010019189	ICMT	2	-0.084219211	0.070464997	-1.195192141	0.232011968	0.002564166	1.115424009	10.34799393	1	CRA004775;PRJNA827465	Yang2025_Silkie_Wenchang	0.744396027
ENSGALG00010019194	ENSGALG00010019194	2	0.067197125	0.280401141	0.239646403	0.810604388	0.09288724	2.09373145	52.23838281	0.5	PRJNA827465;Yang2025_Silkie_Wenchang	CRA004775	0.953011794
ENSGALG00010019194	ENSGALG00010019194	2	0.107150592	0.322319844	0.332435604	0.739560358	0.087820031	1.71712219	41.76302719	0.5	CRA004775;Yang2025_Silkie_Wenchang	PRJNA827465	0.934898869
ENSGALG00010019194	ENSGALG00010019194	2	-0.139863729	0.140115252	-0.998204888	0.318180017	0	0.021946356	0	1	CRA004775;PRJNA827465	Yang2025_Silkie_Wenchang	0.797512752
ENSGALG00010019204	ARF6	2	0.181417141	0.182353783	0.994863602	0.319802608	0.054276506	4.961379011	79.84431349	1	PRJNA827465;Yang2025_Silkie_Wenchang	CRA004775	0.798348216
ENSGALG00010019204	ARF6	2	0.155993264	0.235233907	0.663149067	0.507240211	0.087758829	4.829459457	79.29374894	0.5	CRA004775;Yang2025_Silkie_Wenchang	PRJNA827465	0.860048203
ENSGALG00010019204	ARF6	2	0.009616837	0.056288369	0.170849448	0.864342147	0	0.400933385	0	0.5	CRA004775;PRJNA827465	Yang2025_Silkie_Wenchang	0.966643645
ENSGALG00010019206	RNF207	2	0.231312616	0.401033867	0.576790728	0.564080834	0.295875445	12.47460737	91.98371564	0.5	PRJNA827465;Yang2025_Silkie_Wenchang	CRA004775	0.882847603
ENSGALG00010019206	RNF207	2	0.34507584	0.387334584	0.890898603	0.37298357	0.207106733	2.703949956	63.01706702	0.5	CRA004775;Yang2025_Silkie_Wenchang	PRJNA827465	0.822162024
ENSGALG00010019206	RNF207	2	-0.17215205	0.15493755	-1.111106057	0.266522701	0	0.000306203	0	1	CRA004775;PRJNA827465	Yang2025_Silkie_Wenchang	0.770699087
ENSGALG00010019208	PLXND1	2	-0.122066122	0.06408729	-1.904685353	0.056820984	0	0.036918363	0	1	PRJNA827465;Yang2025_Silkie_Wenchang	CRA004775	0.412416462
ENSGALG00010019208	PLXND1	2	-0.117999514	0.12319712	-0.957810653	0.338158247	0	0.257091066	0	1	CRA004775;Yang2025_Silkie_Wenchang	PRJNA827465	0.80415385
ENSGALG00010019208	PLXND1	2	-0.134557759	0.073157259	-1.839295696	0.065871706	0	0.210683702	0	1	CRA004775;PRJNA827465	Yang2025_Silkie_Wenchang	0.4420276
ENSGALG00010019209	VCPKMT	2	0.439215491	0.290979491	1.509437969	0.131186896	0.161190056	20.75188672	95.18116105	1	PRJNA827465;Yang2025_Silkie_Wenchang	CRA004775	0.606878893
ENSGALG00010019209	VCPKMT	2	0.153465919	0.586791633	0.261533925	0.793680791	0.670237426	37.17734406	97.31018978	0.5	CRA004775;Yang2025_Silkie_Wenchang	PRJNA827465	0.948796049
ENSGALG00010019209	VCPKMT	2	-0.127589691	0.295331135	-0.432022487	0.66572507	0.157993139	10.26015139	90.25355512	0.5	CRA004775;PRJNA827465	Yang2025_Silkie_Wenchang	0.913532078
ENSGALG00010019212	RPL22	2	0.151452765	0.153967426	0.983665758	0.325278986	0.033510513	2.881008409	65.28993123	1	PRJNA827465;Yang2025_Silkie_Wenchang	CRA004775	0.799977473
ENSGALG00010019212	RPL22	2	0.088604408	0.296453611	0.298881189	0.765030701	0.127335753	3.530186828	71.67288734	0.5	CRA004775;Yang2025_Silkie_Wenchang	PRJNA827465	0.940771616
ENSGALG00010019212	RPL22	2	0.015567611	0.071389163	0.218066863	0.827377016	0.001851347	1.051207935	4.871342149	0.5	CRA004775;PRJNA827465	Yang2025_Silkie_Wenchang	0.956550752
ENSGALG00010019213	RHBDF1	2	0.156992293	0.049226341	3.189192803	0.001426707	0	0.343052256	0	1	PRJNA827465;Yang2025_Silkie_Wenchang	CRA004775	0.046689309
ENSGALG00010019213	RHBDF1	2	0.16896292	0.077973655	2.166923176	0.030240712	0.000631429	1.015454865	1.52196473	0.5	CRA004775;Yang2025_Silkie_Wenchang	PRJNA827465	0.29602757
ENSGALG00010019213	RHBDF1	2	0.121461023	0.06219786	1.952816759	0.050841312	0	0.658951282	0	0.5	CRA004775;PRJNA827465	Yang2025_Silkie_Wenchang	0.390049394
ENSGALG00010019215	ENSGALG00010019215	2	-0.054201113	0.146941752	-0.368861216	0.712231173	0.030213097	2.773853839	63.94907382	0.5	PRJNA827465;Yang2025_Silkie_Wenchang	CRA004775	0.92597752
ENSGALG00010019215	ENSGALG00010019215	2	-0.286896501	0.124331551	-2.307515645	0.021026314	0	0.074657777	0	1	CRA004775;Yang2025_Silkie_Wenchang	PRJNA827465	0.245277621
ENSGALG00010019215	ENSGALG00010019215	2	-0.096241707	0.183982273	-0.523103153	0.600902472	0.053371974	4.127784451	75.7739288	0.5	CRA004775;PRJNA827465	Yang2025_Silkie_Wenchang	0.895230519
ENSGALG00010019218	RMND5A	2	0.00780503	0.04693543	0.166292931	0.867926429	0	0.000601854	0	1	PRJNA827465;Yang2025_Silkie_Wenchang	CRA004775	0.967959054
ENSGALG00010019218	RMND5A	2	-0.001964579	0.083113844	-0.0236372	0.981141999	0	0.133226494	0	0.5	CRA004775;Yang2025_Silkie_Wenchang	PRJNA827465	0.994938438
ENSGALG00010019218	RMND5A	2	0.004951135	0.055214406	0.089674697	0.928545724	0	0.144129107	0	0.5	CRA004775;PRJNA827465	Yang2025_Silkie_Wenchang	0.983311758
ENSGALG00010019221	VASH2	2	-0.044548581	0.105221784	-0.423377924	0.672019549	0.008207591	1.469854823	31.9660701	0.5	PRJNA827465;Yang2025_Silkie_Wenchang	CRA004775	0.914714609
ENSGALG00010019221	VASH2	2	-0.186540777	0.156019253	-1.195626651	0.231842287	0	0.067151273	0	1	CRA004775;Yang2025_Silkie_Wenchang	PRJNA827465	0.744183098
ENSGALG00010019221	VASH2	2	0.023910764	0.087736797	0.272528339	0.785215804	0	0.032181012	0	0.5	CRA004775;PRJNA827465	Yang2025_Silkie_Wenchang	0.946325593
ENSGALG00010019223	AAMP	2	0.115574444	0.078188132	1.47815839	0.139365388	0.005137868	1.381242623	27.6014232	1	PRJNA827465;Yang2025_Silkie_Wenchang	CRA004775	0.623009874
ENSGALG00010019223	AAMP	2	-0.058504266	0.343250295	-0.170441998	0.864662546	0.200412695	6.601426993	84.85176006	0.5	CRA004775;Yang2025_Silkie_Wenchang	PRJNA827465	0.966723659
ENSGALG00010019223	AAMP	2	-0.126447948	0.243024662	-0.520309121	0.60284814	0.097317982	4.912354577	79.64316329	0.5	CRA004775;PRJNA827465	Yang2025_Silkie_Wenchang	0.895538966
ENSGALG00010019227	CLCN5	2	0.088440857	0.060512993	1.46151847	0.14387321	0	0.991850254	0	1	PRJNA827465;Yang2025_Silkie_Wenchang	CRA004775	0.640832183
ENSGALG00010019227	CLCN5	2	0.100981639	0.160487918	0.629216452	0.52920736	0.019044245	1.475831943	32.24160753	0.5	CRA004775;Yang2025_Silkie_Wenchang	PRJNA827465	0.871837903
ENSGALG00010019227	CLCN5	2	0.045675078	0.065021605	0.702460029	0.482392321	0	0.556688443	0	0.5	CRA004775;PRJNA827465	Yang2025_Silkie_Wenchang	0.85691916
ENSGALG00010019230	SPG21	2	-0.26191184	0.420244022	-0.623237517	0.533128451	0.337240404	21.41523506	95.33042716	0.5	PRJNA827465;Yang2025_Silkie_Wenchang	CRA004775	0.873355599
ENSGALG00010019230	SPG21	2	-0.504730798	0.193382127	-2.610017825	0.009053751	0.044699093	2.48521844	59.76208837	1	CRA004775;Yang2025_Silkie_Wenchang	PRJNA827465	0.149037013
ENSGALG00010019230	SPG21	2	-0.05522067	0.225212714	-0.245193394	0.806306691	0.087072191	6.38978476	84.35002058	0.5	CRA004775;PRJNA827465	Yang2025_Silkie_Wenchang	0.952174506
ENSGALG00010019231	ENSGALG00010019231	2	-0.072409512	0.144621004	-0.50068462	0.616593098	0	0.085658293	0	1	PRJNA827465;Yang2025_Silkie_Wenchang	CRA004775	0.899111936
ENSGALG00010019231	ENSGALG00010019231	2	-0.073964784	0.222466439	-0.332475978	0.739529876	0	0.318583722	0	1	CRA004775;Yang2025_Silkie_Wenchang	PRJNA827465	0.934890406
ENSGALG00010019231	ENSGALG00010019231	2	-0.128056318	0.168746572	-0.758867671	0.44793172	0	0.183170698	0	1	CRA004775;PRJNA827465	Yang2025_Silkie_Wenchang	0.846601815
ENSGALG00010019232	SOS2	2	-0.10415251	0.04048593	-2.572560934	0.010094918	0	0.796018985	0	1	PRJNA827465;Yang2025_Silkie_Wenchang	CRA004775	0.15959798
ENSGALG00010019232	SOS2	2	-0.040498258	0.198086283	-0.204447562	0.838003766	0.051120029	2.585445949	61.32195297	0.5	CRA004775;Yang2025_Silkie_Wenchang	PRJNA827465	0.959032758
ENSGALG00010019232	SOS2	2	-0.021771325	0.12642693	-0.172204807	0.863276519	0.018718297	1.689131515	40.79797865	0.5	CRA004775;PRJNA827465	Yang2025_Silkie_Wenchang	0.966235427
ENSGALG00010019233	ARPC2	2	0.052277583	0.040316374	1.296683658	0.194740054	0	0.219761815	0	1	PRJNA827465;Yang2025_Silkie_Wenchang	CRA004775	0.705946169
ENSGALG00010019233	ARPC2	2	-0.051686773	0.101505685	-0.509200772	0.610611502	0.003655272	1.158576481	13.68718279	0.5	CRA004775;Yang2025_Silkie_Wenchang	PRJNA827465	0.897438028
ENSGALG00010019233	ARPC2	2	-0.021121386	0.128166542	-0.164796409	0.869104231	0.021152503	2.120861268	52.84934404	0.5	CRA004775;PRJNA827465	Yang2025_Silkie_Wenchang	0.968461055
ENSGALG00010019236	MPG	2	0.117535023	0.088887248	1.322293416	0.186070464	0	0.109864188	0	1	PRJNA827465;Yang2025_Silkie_Wenchang	CRA004775	0.6950992
ENSGALG00010019236	MPG	2	-0.057184583	0.302030025	-0.189334101	0.84983097	0.1148678	2.281091536	56.16133838	0.5	CRA004775;Yang2025_Silkie_Wenchang	PRJNA827465	0.961856718
ENSGALG00010019236	MPG	2	-0.067935191	0.260417996	-0.260869801	0.794192914	0.082443341	1.975265817	49.37390241	0.5	CRA004775;PRJNA827465	Yang2025_Silkie_Wenchang	0.948905846
ENSGALG00010019237	SORCS3	2	-0.237505756	0.183235289	-1.296179119	0.194913782	0	0.387169172	0	1	PRJNA827465;Yang2025_Silkie_Wenchang	CRA004775	0.706054964
ENSGALG00010019237	SORCS3	2	-0.52762124	0.297286724	-1.774789112	0.075932715	0	0.49923883	0	1	CRA004775;Yang2025_Silkie_Wenchang	PRJNA827465	0.47539798
ENSGALG00010019237	SORCS3	2	-0.311847899	0.299069391	-1.042727567	0.297074487	0.063763889	1.290624151	22.51810883	1	CRA004775;PRJNA827465	Yang2025_Silkie_Wenchang	0.788466926
ENSGALG00010019239	CYTH3	2	0.05816935	0.092402097	0.629524127	0.529005978	0.008924326	1.831252507	45.39256624	0.5	PRJNA827465;Yang2025_Silkie_Wenchang	CRA004775	0.871809618
ENSGALG00010019239	CYTH3	2	0.355846717	0.216995527	1.639880428	0.101030031	0.055657517	2.194759349	54.43691808	0.5	CRA004775;Yang2025_Silkie_Wenchang	PRJNA827465	0.542487465
ENSGALG00010019239	CYTH3	2	0.265103762	0.320544031	0.827043202	0.408212575	0.172114077	5.362056406	81.35043863	0.5	CRA004775;PRJNA827465	Yang2025_Silkie_Wenchang	0.834134074
ENSGALG00010019240	ENSGALG00010019240	2	-0.032465751	0.194705864	-0.166742545	0.867572628	0	0.159638378	0	0.5	PRJNA827465;Yang2025_Silkie_Wenchang	CRA004775	0.96788231
ENSGALG00010019240	ENSGALG00010019240	2	-0.245076723	0.253928257	-0.965141593	0.334473897	0	0.10420343	0	1	CRA004775;Yang2025_Silkie_Wenchang	PRJNA827465	0.803094614
ENSGALG00010019240	ENSGALG00010019240	2	-0.086018919	0.191458507	-0.449282203	0.653222022	0	0.655774134	0	0.5	CRA004775;PRJNA827465	Yang2025_Silkie_Wenchang	0.904597225
ENSGALG00010019243	SERTAD2	2	-0.161166312	0.066747309	-2.414573911	0.015753626	0	0.132631433	0	1	PRJNA827465;Yang2025_Silkie_Wenchang	CRA004775	0.207703716
ENSGALG00010019243	SERTAD2	2	0.006735648	0.189598657	0.03552582	0.971660458	0.02346987	1.344885868	25.64424803	0.5	CRA004775;Yang2025_Silkie_Wenchang	PRJNA827465	0.991955348
ENSGALG00010019243	SERTAD2	2	-0.033042192	0.22164374	-0.149077939	0.881492133	0.063129528	2.090102563	52.15545794	0.5	CRA004775;PRJNA827465	Yang2025_Silkie_Wenchang	0.971790895
ENSGALG00010019246	IL17RC	2	0.15243939	0.075900389	2.008413807	0.044590337	0	0.074956977	0	1	PRJNA827465;Yang2025_Silkie_Wenchang	CRA004775	0.364257243
ENSGALG00010019246	IL17RC	2	-0.000607132	0.22587489	-0.002687913	0.997855359	0	0.25072352	0	0.5	CRA004775;Yang2025_Silkie_Wenchang	PRJNA827465	0.999290321
ENSGALG00010019246	IL17RC	2	0.145869968	0.077361306	1.885567543	0.059353255	0	0.638927369	0	0.5	CRA004775;PRJNA827465	Yang2025_Silkie_Wenchang	0.422252444
ENSGALG00010019248	RMI2	2	0.331963464	0.242122454	1.371057474	0.170357474	0.068951887	2.329957753	57.08076687	1	PRJNA827465;Yang2025_Silkie_Wenchang	CRA004775	0.672114378
ENSGALG00010019248	RMI2	2	0.220322205	0.431556998	0.510528635	0.609681158	0.27331569	3.695805909	72.94230204	0.5	CRA004775;Yang2025_Silkie_Wenchang	PRJNA827465	0.897238578
ENSGALG00010019248	RMI2	2	0.056135923	0.158516279	0.354133492	0.723238844	0	0.86763391	0	0.5	CRA004775;PRJNA827465	Yang2025_Silkie_Wenchang	0.930032096
ENSGALG00010019250	CXCR1	2	-0.372024433	0.24530169	-1.516599554	0.129367818	0	0.021837363	0	1	PRJNA827465;Yang2025_Silkie_Wenchang	CRA004775	0.603517352
ENSGALG00010019250	CXCR1	2	-0.495902758	0.238482136	-2.079412599	0.037579443	0	0.321783705	0	1	CRA004775;Yang2025_Silkie_Wenchang	PRJNA827465	0.334386431
ENSGALG00010019250	CXCR1	2	-0.509259281	0.222462888	-2.289187586	0.022068456	0	0.207607794	0	1	CRA004775;PRJNA827465	Yang2025_Silkie_Wenchang	0.252135642
ENSGALG00010019252	TATDN3	2	-0.037862497	0.184952694	-0.204714492	0.837759198	0.050752285	3.696252319	72.9455699	0.5	PRJNA827465;Yang2025_Silkie_Wenchang	CRA004775	0.959032758
ENSGALG00010019252	TATDN3	2	-0.259854861	0.138988077	-1.869619797	0.061536634	0	0.015274079	0	1	CRA004775;Yang2025_Silkie_Wenchang	PRJNA827465	0.42852132
ENSGALG00010019252	TATDN3	2	-0.015496892	0.194559952	-0.079650986	0.936514843	0.047633077	2.303198745	56.58212291	0.5	CRA004775;PRJNA827465	Yang2025_Silkie_Wenchang	0.985038972
ENSGALG00010019253	HBAD	2	-0.18396637	0.46058019	-0.399423107	0.689581471	0.372059661	7.959690275	87.43669709	0.5	PRJNA827465;Yang2025_Silkie_Wenchang	CRA004775	0.919979266
ENSGALG00010019253	HBAD	2	-0.631851738	0.211253817	-2.990960103	0.002781018	0	0.04617574	0	1	CRA004775;Yang2025_Silkie_Wenchang	PRJNA827465	0.071912707
ENSGALG00010019253	HBAD	2	-0.116772792	0.412647809	-0.282984457	0.777188982	0.276092092	5.01725765	80.06879316	0.5	CRA004775;PRJNA827465	Yang2025_Silkie_Wenchang	0.944183792
ENSGALG00010019256	BBS9	2	-0.040954941	0.104808601	-0.390759349	0.695975126	0.005155384	1.242435668	19.51293529	0.5	PRJNA827465;Yang2025_Silkie_Wenchang	CRA004775	0.921038437
ENSGALG00010019256	BBS9	2	-0.080580474	0.199867587	-0.403169294	0.686823687	0.022840474	1.29360884	22.69687953	0.5	CRA004775;Yang2025_Silkie_Wenchang	PRJNA827465	0.919235539
ENSGALG00010019256	BBS9	2	0.043832843	0.098031045	0.447132267	0.654779562	0	0.357392882	0	0.5	CRA004775;PRJNA827465	Yang2025_Silkie_Wenchang	0.910222014
ENSGALG00010019257	SLC1A4	2	0.055803484	0.140376833	0.397526306	0.690979392	0.027988547	3.424738276	70.80068842	0.5	PRJNA827465;Yang2025_Silkie_Wenchang	CRA004775	0.920199347
ENSGALG00010019257	SLC1A4	2	-0.282435265	0.27982145	-1.00934101	0.312811119	0.100638716	2.247084731	55.49789528	1	CRA004775;Yang2025_Silkie_Wenchang	PRJNA827465	0.796054997
ENSGALG00010019257	SLC1A4	2	-0.174776609	0.434711009	-0.40205241	0.687645456	0.311684168	4.969498081	79.87724346	0.5	CRA004775;PRJNA827465	Yang2025_Silkie_Wenchang	0.919564865
ENSGALG00010019259	L2HGDH	2	-0.208532755	0.302571115	-0.689205715	0.490695834	0.169928845	13.49697134	92.59043632	0.5	PRJNA827465;Yang2025_Silkie_Wenchang	CRA004775	0.858799485
ENSGALG00010019259	L2HGDH	2	-0.458506202	0.129813127	-3.532048045	0.000412354	0	0.813161814	0	1	CRA004775;Yang2025_Silkie_Wenchang	PRJNA827465	0.020443294
ENSGALG00010019259	L2HGDH	2	0.005358342	0.139379606	0.038444234	0.969333494	0.020233986	1.561148843	35.94460869	0.5	CRA004775;PRJNA827465	Yang2025_Silkie_Wenchang	0.991572914
ENSGALG00010019260	TRAIL-LIKE	2	-0.104548775	0.062879358	-1.662688348	0.096374836	0	0.519092694	0	1	PRJNA827465;Yang2025_Silkie_Wenchang	CRA004775	0.530885356
ENSGALG00010019260	TRAIL-LIKE	2	-0.157903956	0.117472574	-1.34417721	0.178891089	0	0.424426459	0	0.5	CRA004775;Yang2025_Silkie_Wenchang	PRJNA827465	0.665601971
ENSGALG00010019260	TRAIL-LIKE	2	-0.072021323	0.072215039	-0.997317499	0.318610423	0	0.183005922	0	0.5	CRA004775;PRJNA827465	Yang2025_Silkie_Wenchang	0.797754958
ENSGALG00010019263	SNRPB2	2	-0.035489112	0.064698511	-0.548530581	0.583327637	0.003404405	1.118783408	30.34926742	0.5	PRJNA827465;Yang2025_Silkie_Wenchang	CRA004775	0.890479822
ENSGALG00010019263	SNRPB2	2	-0.145711532	0.099652222	-1.462200529	0.143606267	0	0.001615895	0	1	CRA004775;Yang2025_Silkie_Wenchang	PRJNA827465	0.630564924
ENSGALG00010019263	SNRPB2	2	-0.0059234	0.045235349	-0.130946273	0.89581781	0	0.494597775	0	0.5	CRA004775;PRJNA827465	Yang2025_Silkie_Wenchang	0.974975835
ENSGALG00010019264	HK2	2	0.348952317	0.203222746	1.717092815	0.085962215	0.075323091	11.35201403	91.19099045	1	PRJNA827465;Yang2025_Silkie_Wenchang	CRA004775	0.504285479
ENSGALG00010019264	HK2	2	0.203473022	0.40169014	0.506542238	0.612476046	0.275559687	6.126258559	83.67682346	0.5	CRA004775;Yang2025_Silkie_Wenchang	PRJNA827465	0.897938231
ENSGALG00010019264	HK2	2	0.057514971	0.166932071	0.34454117	0.73043934	0.02814642	1.522082052	34.30051956	0.5	CRA004775;PRJNA827465	Yang2025_Silkie_Wenchang	0.932653142
ENSGALG00010019266	ATP5S	2	0.030194977	0.118267158	0.255311594	0.798482446	0.004620181	1.166478329	14.27187502	0.5	PRJNA827465;Yang2025_Silkie_Wenchang	CRA004775	0.949922217
ENSGALG00010019266	ATP5S	2	-0.157677419	0.167017403	-0.944077779	0.345129904	0	0.016038735	0	1	CRA004775;Yang2025_Silkie_Wenchang	PRJNA827465	0.806961063
ENSGALG00010019266	ATP5S	2	0.067160181	0.116355896	0.577196199	0.563806925	0	0.824368153	0	0.5	CRA004775;PRJNA827465	Yang2025_Silkie_Wenchang	0.882651345
ENSGALG00010019267	STARD5	2	0.143598866	0.169242664	0.848479116	0.396171198	0	0.237652142	0	1	PRJNA827465;Yang2025_Silkie_Wenchang	CRA004775	0.831365249
ENSGALG00010019267	STARD5	2	0.227060831	0.18764286	1.210069123	0.22625237	0	0.002645891	0	1	CRA004775;Yang2025_Silkie_Wenchang	PRJNA827465	0.739677001
ENSGALG00010019267	STARD5	2	0.128355525	0.175991545	0.72932779	0.465801177	0	0.166537839	0	1	CRA004775;PRJNA827465	Yang2025_Silkie_Wenchang	0.852511753
ENSGALG00010019268	NSL1	2	-0.064022986	0.053796189	-1.190102643	0.234006052	0	0.607032672	0	1	PRJNA827465;Yang2025_Silkie_Wenchang	CRA004775	0.747279664
ENSGALG00010019268	NSL1	2	-0.152927154	0.116443027	-1.313321696	0.189074571	0	0.000229492	0	1	CRA004775;Yang2025_Silkie_Wenchang	PRJNA827465	0.698162702
ENSGALG00010019268	NSL1	2	-0.048604423	0.058184424	-0.835351098	0.403520055	0	0.123582156	0	1	CRA004775;PRJNA827465	Yang2025_Silkie_Wenchang	0.83306585
ENSGALG00010019269	SOCS1	2	0.001197698	0.140685747	0.008513285	0.993207463	0	0.020687525	0	0.5	PRJNA827465;Yang2025_Silkie_Wenchang	CRA004775	0.99794341
ENSGALG00010019269	SOCS1	2	-0.004368975	0.211943648	-0.020613852	0.983553691	0	0.045463192	0	0.5	CRA004775;Yang2025_Silkie_Wenchang	PRJNA827465	0.995457747
ENSGALG00010019269	SOCS1	2	-0.019153747	0.143116369	-0.133833376	0.893534334	0	0.015056045	0	1	CRA004775;PRJNA827465	Yang2025_Silkie_Wenchang	0.974381724
ENSGALG00010019270	ENSGALG00010019270	2	1.09436954	0.396246661	2.761839142	0.005747679	0.209321789	2.958694487	66.20130925	1	PRJNA827465;Yang2025_Silkie_Wenchang	CRA004775	0.114206785
ENSGALG00010019270	ENSGALG00010019270	2	0.657318269	0.884127665	0.743465333	0.457200002	1.401331382	9.618789411	89.60368132	0.5	CRA004775;Yang2025_Silkie_Wenchang	PRJNA827465	0.849541335
ENSGALG00010019270	ENSGALG00010019270	2	0.300248421	0.483295189	0.621252658	0.534433397	0.337288701	3.472529018	71.20254446	0.5	CRA004775;PRJNA827465	Yang2025_Silkie_Wenchang	0.873427894
ENSGALG00010019271	CDKL1	2	0.406064417	0.13569219	2.992540826	0.002766657	0.003184546	1.075106314	6.985942962	1	PRJNA827465;Yang2025_Silkie_Wenchang	CRA004775	0.071732358
ENSGALG00010019271	CDKL1	2	0.034359394	0.631370846	0.054420304	0.956600302	0.683000963	6.781414242	85.25381337	0.5	CRA004775;Yang2025_Silkie_Wenchang	PRJNA827465	0.988540618
ENSGALG00010019271	CDKL1	2	-0.082082085	0.475484577	-0.17262828	0.862943622	0.366365693	4.732565247	78.86981061	0.5	CRA004775;PRJNA827465	Yang2025_Silkie_Wenchang	0.966099097
ENSGALG00010019275	CTAGE1	2	0.187896219	0.30855066	0.60896392	0.542548354	0	0.216402446	0	1	PRJNA827465;Yang2025_Silkie_Wenchang	CRA004775	0.876817086
ENSGALG00010019275	CTAGE1	2	1.428031929	1.530116187	0.933283329	0.3506737	4.120317219	8.039519092	87.56144505	1	CRA004775;Yang2025_Silkie_Wenchang	PRJNA827465	0.810111037
ENSGALG00010019275	CTAGE1	2	1.546226511	1.378592879	1.121597634	0.262033558	3.305540171	7.196570738	86.10449287	1	CRA004775;PRJNA827465	Yang2025_Silkie_Wenchang	0.768363052
ENSGALG00010019277	IGFBP5	2	-0.022017102	0.079153987	-0.278155314	0.780893136	0	0.453623484	0	0.5	PRJNA827465;Yang2025_Silkie_Wenchang	CRA004775	0.945235709
ENSGALG00010019277	IGFBP5	2	0.406013906	0.374535004	1.084047957	0.278343536	0.230564869	5.356395682	81.33072948	1	CRA004775;Yang2025_Silkie_Wenchang	PRJNA827465	0.776763074
ENSGALG00010019277	IGFBP5	2	0.341674393	0.435222338	0.785057116	0.432420082	0.33722447	8.472821864	88.19755666	0.5	CRA004775;PRJNA827465	Yang2025_Silkie_Wenchang	0.842762754
ENSGALG00010019278	JPT2	2	-0.2385527	0.216274653	-1.103008132	0.270023634	0.089394561	22.48052365	95.55170504	1	PRJNA827465;Yang2025_Silkie_Wenchang	CRA004775	0.772336005
ENSGALG00010019278	JPT2	2	-0.448553089	0.067697949	-6.625800231	3.45372E-11	0	0.176215544	0	1	CRA004775;Yang2025_Silkie_Wenchang	PRJNA827465	2.56116E-08
ENSGALG00010019278	JPT2	2	-0.098410354	0.136482318	-0.721048378	0.47087975	0.020782026	1.648795137	39.34965129	1	CRA004775;PRJNA827465	Yang2025_Silkie_Wenchang	0.853762555
ENSGALG00010019280	TLNRD1	2	0.047853012	0.045235242	1.057870136	0.290114652	0	0.766723173	0	1	PRJNA827465;Yang2025_Silkie_Wenchang	CRA004775	0.784195688
ENSGALG00010019280	TLNRD1	2	0.153213838	0.100254388	1.528250704	0.126450304	0	0.018707704	0	1	CRA004775;Yang2025_Silkie_Wenchang	PRJNA827465	0.597028533
ENSGALG00010019280	TLNRD1	2	0.04089691	0.047277885	0.865032548	0.387020925	0	0.555715818	0	1	CRA004775;PRJNA827465	Yang2025_Silkie_Wenchang	0.827206927
ENSGALG00010019282	IGFBP2	2	-0.072496068	0.068396641	-1.059936077	0.289173681	0	0.687384316	0	1	PRJNA827465;Yang2025_Silkie_Wenchang	CRA004775	0.783530627
ENSGALG00010019282	IGFBP2	2	-0.284946418	0.176529585	-1.614156729	0.106493435	0	0.029384955	0	1	CRA004775;Yang2025_Silkie_Wenchang	PRJNA827465	0.553422082
ENSGALG00010019282	IGFBP2	2	-0.07275545	0.069090879	-1.053039864	0.292322722	0	0.907291526	0	1	CRA004775;PRJNA827465	Yang2025_Silkie_Wenchang	0.785753005
ENSGALG00010019283	NT5C3A	2	-0.174604639	0.144315497	-1.209881421	0.226324397	0.022616106	2.136040063	53.18439868	1	PRJNA827465;Yang2025_Silkie_Wenchang	CRA004775	0.739727883
ENSGALG00010019283	NT5C3A	2	-0.046920332	0.344084999	-0.13636262	0.891534623	0.179049654	3.817190195	73.80272009	0.5	CRA004775;Yang2025_Silkie_Wenchang	PRJNA827465	0.973846899
ENSGALG00010019283	NT5C3A	2	0.045035012	0.171394082	0.262757101	0.792737802	0.024515213	1.426547278	29.90067591	0.5	CRA004775;PRJNA827465	Yang2025_Silkie_Wenchang	0.948435155
ENSGALG00010019284	CHD5	2	0.251500497	0.188902417	1.331377865	0.183064709	0.054098011	4.087731973	75.53655654	1	PRJNA827465;Yang2025_Silkie_Wenchang	CRA004775	0.691392093
ENSGALG00010019284	CHD5	2	0.35516521	0.124490086	2.852959803	0.00433141	0.003106114	1.103878821	9.410346387	1	CRA004775;Yang2025_Silkie_Wenchang	PRJNA827465	0.096509898
ENSGALG00010019284	CHD5	2	0.104906405	0.099241236	1.05708483	0.290472874	0	0.287700456	0	1	CRA004775;PRJNA827465	Yang2025_Silkie_Wenchang	0.78440982
ENSGALG00010019285	BATF3	2	-0.560459746	0.361335993	-1.551076444	0.120883366	0.197497318	3.891204642	74.3010175	1	PRJNA827465;Yang2025_Silkie_Wenchang	CRA004775	0.582767179
ENSGALG00010019285	BATF3	2	-0.663266665	0.329875424	-2.010658018	0.044361591	0.101416368	1.866436706	46.42197097	1	CRA004775;Yang2025_Silkie_Wenchang	PRJNA827465	0.363642137
ENSGALG00010019285	BATF3	2	-0.258030771	0.162377636	-1.589078257	0.112042726	0	0.028361945	0	1	CRA004775;PRJNA827465	Yang2025_Silkie_Wenchang	0.56547057
ENSGALG00010019287	AHSA2	2	0.26378766	0.219070396	1.204122807	0.228542116	0.088304298	12.41940351	91.94808351	1	PRJNA827465;Yang2025_Silkie_Wenchang	CRA004775	0.741270737
ENSGALG00010019287	AHSA2	2	0.465957113	0.082245067	5.665471825	1.46621E-08	0	0.151443887	0	1	CRA004775;Yang2025_Silkie_Wenchang	PRJNA827465	6.94292E-06
ENSGALG00010019287	AHSA2	2	0.213868825	0.183739609	1.163977795	0.244433011	0.055203156	5.1854763	80.71536842	1	CRA004775;PRJNA827465	Yang2025_Silkie_Wenchang	0.755976598
ENSGALG00010019288	RPL37A	2	-0.543036448	0.609472071	-0.890994803	0.372931958	0.691317726	13.65400763	92.67614295	0.5	PRJNA827465;Yang2025_Silkie_Wenchang	CRA004775	0.822162024
ENSGALG00010019288	RPL37A	2	-0.659071585	0.519081243	-1.269688694	0.204195542	0.451997556	6.17714653	83.81129547	1	CRA004775;Yang2025_Silkie_Wenchang	PRJNA827465	0.717271785
ENSGALG00010019288	RPL37A	2	0.012517393	0.080051019	0.156367697	0.875743198	0	0.421142438	0	0.5	CRA004775;PRJNA827465	Yang2025_Silkie_Wenchang	0.970347777
ENSGALG00010019289	FKBP9	2	-0.082394939	0.134195384	-0.61399235	0.539220382	0.026719426	3.848764312	74.01763478	0.5	PRJNA827465;Yang2025_Silkie_Wenchang	CRA004775	0.874983893
ENSGALG00010019289	FKBP9	2	-0.234602906	0.096122928	-2.440655012	0.014660653	0	0.070626782	0	1	CRA004775;Yang2025_Silkie_Wenchang	PRJNA827465	0.199517877
ENSGALG00010019289	FKBP9	2	-0.052554609	0.153123807	-0.343216444	0.731435635	0.025412373	1.809163168	44.72582586	0.5	CRA004775;PRJNA827465	Yang2025_Silkie_Wenchang	0.932851968
ENSGALG00010019291	ADD2	2	0.258847996	0.190522721	1.358620083	0.174267008	0	0.212533031	0	1	PRJNA827465;Yang2025_Silkie_Wenchang	CRA004775	0.678487591
ENSGALG00010019291	ADD2	2	0.030366623	0.230317415	0.131846837	0.895105439	0	0.451843175	0	0.5	CRA004775;Yang2025_Silkie_Wenchang	PRJNA827465	0.974641371
ENSGALG00010019291	ADD2	2	0.159093996	0.236399015	0.67298925	0.50095413	0.023972262	1.242919124	19.5442422	0.5	CRA004775;PRJNA827465	Yang2025_Silkie_Wenchang	0.862421093
ENSGALG00010019292	ENSGALG00010019292	2	-0.028841213	0.117859554	-0.244708318	0.806682287	0.013513214	1.931868494	48.23664225	0.5	PRJNA827465;Yang2025_Silkie_Wenchang	CRA004775	0.952273023
ENSGALG00010019292	ENSGALG00010019292	2	-0.029337564	0.205885235	-0.142494747	0.886689229	0.04188312	1.664089083	39.90706324	0.5	CRA004775;Yang2025_Silkie_Wenchang	PRJNA827465	0.972826027
ENSGALG00010019292	ENSGALG00010019292	2	0.100586348	0.103772053	0.969300932	0.332395065	0	0.405178863	0	1	CRA004775;PRJNA827465	Yang2025_Silkie_Wenchang	0.802178578
ENSGALG00010019294	SMARCAL1	2	0.086387527	0.066293251	1.303111951	0.192536544	0	0.384077729	0	1	PRJNA827465;Yang2025_Silkie_Wenchang	CRA004775	0.702467122
ENSGALG00010019294	SMARCAL1	2	0.114270201	0.083987417	1.360563348	0.173651721	0	0.096561783	0	1	CRA004775;Yang2025_Silkie_Wenchang	PRJNA827465	0.677420535
ENSGALG00010019294	SMARCAL1	2	0.046076302	0.086482313	0.532782955	0.594183834	0	0.003115263	0	1	CRA004775;PRJNA827465	Yang2025_Silkie_Wenchang	0.893287198
ENSGALG00010019295	MAP4K5	2	-0.068059077	0.085120725	-0.799559413	0.423966111	0	0.00419714	0	1	PRJNA827465;Yang2025_Silkie_Wenchang	CRA004775	0.84030043
ENSGALG00010019295	MAP4K5	2	-0.03078614	0.126174059	-0.243997379	0.80723285	0	0.347556308	0	0.5	CRA004775;Yang2025_Silkie_Wenchang	PRJNA827465	0.95234753
ENSGALG00010019295	MAP4K5	2	-0.050896881	0.103606938	-0.491249735	0.623249826	0	0.406460951	0	0.5	CRA004775;PRJNA827465	Yang2025_Silkie_Wenchang	0.900348922
ENSGALG00010019296	ENSGALG00010019296	2	0.06163551	0.097607725	0.631461395	0.527738884	0.005549633	1.340955305	25.42629899	0.5	PRJNA827465;Yang2025_Silkie_Wenchang	CRA004775	0.871296643
ENSGALG00010019296	ENSGALG00010019296	2	0.179634501	0.132295919	1.357823454	0.174519711	0	0.085683522	0	1	CRA004775;Yang2025_Silkie_Wenchang	PRJNA827465	0.67885473
ENSGALG00010019296	ENSGALG00010019296	2	0.008643004	0.086636169	0.099762078	0.920533215	0	0.204414771	0	0.5	CRA004775;PRJNA827465	Yang2025_Silkie_Wenchang	0.98144788
ENSGALG00010019297	SLC25A26	2	-0.215990984	0.106218692	-2.0334555	0.042006525	0	0.725093704	0	1	PRJNA827465;Yang2025_Silkie_Wenchang	CRA004775	0.354529664
ENSGALG00010019297	SLC25A26	2	-0.397841567	0.154082148	-2.582009479	0.009822689	0	0.359867656	0	1	CRA004775;Yang2025_Silkie_Wenchang	PRJNA827465	0.156553582
ENSGALG00010019297	SLC25A26	2	-0.262307287	0.17866147	-1.468180503	0.142055199	0.027224987	1.526881424	34.50702952	1	CRA004775;PRJNA827465	Yang2025_Silkie_Wenchang	0.627783206
ENSGALG00010019298	PGAP6	2	-0.213911589	0.129885226	-1.646927802	0.099572887	0.022674721	2.770968788	63.91153865	1	PRJNA827465;Yang2025_Silkie_Wenchang	CRA004775	0.538828743
ENSGALG00010019298	PGAP6	2	-0.388630727	0.124633019	-3.118200372	0.001819591	0	0.028357201	0	1	CRA004775;Yang2025_Silkie_Wenchang	PRJNA827465	0.054467104

ENSGALG00010019298	PGAP6	2	-0.176119284	0.128130359	-1.374532039	0.169276571	0.015811314	1.46787635	31.87437074	1	CRA004775;PRJNA827465	Yang2025_Silkie_Wenchang	0.670673091
ENSGALG00010019300	MESDC2	2	-0.042792197	0.094724514	-0.451754199	0.651446069	0.013648342	3.979489738	74.87115018	0.5	PRJNA827465;Yang2025_Silkie_Wenchang	CRA004775	0.908927523
ENSGALG00010019300	MESDC2	2	-0.048003042	0.125720054	-0.381824861	0.702591277	0.019389222	2.425499685	58.77138199	0.5	CRA004775;Yang2025_Silkie_Wenchang	PRJNA827465	0.923265404
ENSGALG00010019300	MESDC2	2	0.047482324	0.045519176	1.043127936	0.296889044	0	0.191856298	0	1	CRA004775;PRJNA827465	Yang2025_Silkie_Wenchang	0.788466926
ENSGALG00010019302	USP34	2	0.109044253	0.139901254	0.779437279	0.435722171	0.029035474	3.195397085	68.70498491	1	PRJNA827465;Yang2025_Silkie_Wenchang	CRA004775	0.842762754
ENSGALG00010019302	USP34	2	0.271970756	0.131616649	2.06638566	0.038792079	0	0.06506275	0	1	CRA004775;Yang2025_Silkie_Wenchang	PRJNA827465	0.339943161
ENSGALG00010019302	USP34	2	0.00952044	0.041210123	0.23102188	0.817297804	0	0.790958463	0	1	CRA004775;PRJNA827465	Yang2025_Silkie_Wenchang	0.954854362
ENSGALG00010019305	SNRNP27	2	-0.004884064	0.057442536	-0.085025208	0.93224135	0	0.081489799	0	0.5	PRJNA827465;Yang2025_Silkie_Wenchang	CRA004775	0.984202262
ENSGALG00010019305	SNRNP27	2	-0.037993883	0.127561174	-0.297848336	0.765818921	0	0.006323015	0	1	CRA004775;Yang2025_Silkie_Wenchang	PRJNA827465	0.941000908
ENSGALG00010019305	SNRNP27	2	0.000592787	0.060376139	0.009818232	0.99216631	0	0.008227369	0	0.5	CRA004775;PRJNA827465	Yang2025_Silkie_Wenchang	0.997744161
ENSGALG00010019307	ATF3	2	0.129788767	0.152640575	0.850290076	0.395163833	0	0.001507591	0	1	PRJNA827465;Yang2025_Silkie_Wenchang	CRA004775	0.83067466
ENSGALG00010019307	ATF3	2	0.737128349	0.743894074	0.990904988	0.321731976	0.876027265	4.222553852	76.31764958	1	CRA004775;Yang2025_Silkie_Wenchang	PRJNA827465	0.798513866
ENSGALG00010019307	ATF3	2	0.737331802	0.73664309	1.000934933	0.316858267	0.864925349	4.265592178	76.55659617	1	CRA004775;PRJNA827465	Yang2025_Silkie_Wenchang	0.796959041
ENSGALG00010019310	CRELD1	2	0.271202795	0.346507406	0.782675322	0.433817797	0.227991009	19.54187629	94.8827841	0.5	PRJNA827465;Yang2025_Silkie_Wenchang	CRA004775	0.842762754
ENSGALG00010019310	CRELD1	2	0.109761801	0.583767332	0.188023198	0.850858466	0.58950472	6.765977669	85.22016996	0.5	CRA004775;Yang2025_Silkie_Wenchang	PRJNA827465	0.962056252
ENSGALG00010019310	CRELD1	2	-0.121957694	0.153749834	-0.793221628	0.427648708	0.020446038	1.212558846	17.5297757	1	CRA004775;PRJNA827465	Yang2025_Silkie_Wenchang	0.84125685
ENSGALG00010019312	KBTBD2	2	-0.105225324	0.160167746	-0.656969502	0.511200527	0.048664772	19.14771932	94.7774459	0.5	PRJNA827465;Yang2025_Silkie_Wenchang	CRA004775	0.865649044
ENSGALG00010019312	KBTBD2	2	-0.060587164	0.241825846	-0.250540481	0.802169404	0.098181963	5.622876575	82.21550862	0.5	CRA004775;Yang2025_Silkie_Wenchang	PRJNA827465	0.950630607
ENSGALG00010019312	KBTBD2	2	0.055968686	0.035026986	1.597873303	0.110071177	0	0.714032437	0	1	CRA004775;PRJNA827465	Yang2025_Silkie_Wenchang	0.561937076
ENSGALG00010019313	MRPL28	2	0.169914504	0.107888944	1.574901918	0.115279086	0.012534667	1.940392175	48.46402636	1	PRJNA827465;Yang2025_Silkie_Wenchang	CRA004775	0.572216963
ENSGALG00010019313	MRPL28	2	0.029343427	0.322758455	0.090914509	0.927560521	0.166904106	4.810462665	79.21197877	0.5	CRA004775;Yang2025_Silkie_Wenchang	PRJNA827465	0.982993195
ENSGALG00010019313	MRPL28	2	-0.046466936	0.198095768	-0.23456804	0.814544018	0.053299125	2.496042779	59.93658408	0.5	CRA004775;PRJNA827465	Yang2025_Silkie_Wenchang	0.953869515
ENSGALG00010019314	GKN2	2	-1.099411172	0.558826411	-1.967357215	0.049142043	0	0.056600966	0	1	PRJNA827465;Yang2025_Silkie_Wenchang	CRA004775	0.383342347
ENSGALG00010019314	GKN2	2	0.478071471	1.389917536	0.343956716	0.730878838	3.093400107	5.012308223	80.049112	0.5	CRA004775;Yang2025_Silkie_Wenchang	PRJNA827465	0.932769533
ENSGALG00010019314	GKN2	2	0.28775537	1.526530528	0.18850286	0.850482474	4.050346824	7.609853427	86.85914243	0.5	CRA004775;PRJNA827465	Yang2025_Silkie_Wenchang	0.961985357
ENSGALG00010019316	CLEC16A	2	0.135425817	0.069045511	1.961399299	0.049832462	0	0.011514633	0	1	PRJNA827465;Yang2025_Silkie_Wenchang	CRA004775	0.386284807
ENSGALG00010019316	CLEC16A	2	0.116645873	0.139934093	0.833577226	0.404519263	0	0.218233422	0	0.5	CRA004775;Yang2025_Silkie_Wenchang	PRJNA827465	0.833112102
ENSGALG00010019316	CLEC16A	2	0.123330697	0.07444498	1.656669092	0.097586395	0	0.202746307	0	0.5	CRA004775;PRJNA827465	Yang2025_Silkie_Wenchang	0.533292282
ENSGALG00010019318	NOG2	2	-0.053685824	0.182257315	-0.2945606	0.768329564	0.017151661	1.317828131	24.11757068	0.5	PRJNA827465;Yang2025_Silkie_Wenchang	CRA004775	0.941444269
ENSGALG00010019318	NOG2	2	0.081742314	0.245319619	0.333207407	0.738977727	0	0.856768528	0	0.5	CRA004775;Yang2025_Silkie_Wenchang	PRJNA827465	0.934613258
ENSGALG00010019318	NOG2	2	-0.214773689	0.180291396	-1.191258674	0.233552052	0	0.135902315	0	1	CRA004775;PRJNA827465	Yang2025_Silkie_Wenchang	0.746809711
ENSGALG00010019319	ACTR3	2	0.112016627	0.066661646	1.680375953	0.092884192	0.004570173	2.003471405	50.08663476	1	PRJNA827465;Yang2025_Silkie_Wenchang	CRA004775	0.522019616
ENSGALG00010019319	ACTR3	2	0.182260587	0.076259288	2.390011646	0.016847839	0	0.320035006	0	0.5	CRA004775;Yang2025_Silkie_Wenchang	PRJNA827465	0.215550325
ENSGALG00010019319	ACTR3	2	0.053539681	0.055027759	0.972957693	0.330574329	0	0.059214065	0	0.5	CRA004775;PRJNA827465	Yang2025_Silkie_Wenchang	0.80156474
ENSGALG00010019322	EGR3	2	0.890745465	0.684710302	1.300908518	0.19328977	0.876205917	14.8794501	93.27932153	1	PRJNA827465;Yang2025_Silkie_Wenchang	CRA004775	0.703242486
ENSGALG00010019322	EGR3	2	1.123585779	0.51717641	2.172538727	0.029815052	0.396428363	3.80639851	73.72844705	1	CRA004775;Yang2025_Silkie_Wenchang	PRJNA827465	0.294871913
ENSGALG00010019322	EGR3	2	0.272864095	0.143924222	1.895887229	0.057974958	0	0.54813568	0	1	CRA004775;PRJNA827465	Yang2025_Silkie_Wenchang	0.417400582
ENSGALG00010019323	LRIG1	2	-0.083959779	0.057528794	-1.45943923	0.144444255	0	0.001862956	0	1	PRJNA827465;Yang2025_Silkie_Wenchang	CRA004775	0.631870428
ENSGALG00010019323	LRIG1	2	0.080677035	0.237032311	0.340363026	0.733583159	0.065967906	2.018339364	50.45431814	0.5	CRA004775;Yang2025_Silkie_Wenchang	PRJNA827465	0.933320645
ENSGALG00010019323	LRIG1	2	0.067578971	0.236060294	0.286278435	0.774664874	0.074888741	2.269408221	55.93564919	0.5	CRA004775;PRJNA827465	Yang2025_Silkie_Wenchang	0.943713409
ENSGALG00010019325	SLITRK4	2	-0.462957858	0.251529645	-1.84056976	0.065684633	0.076247003	2.455638184	59.2773884	1	PRJNA827465;Yang2025_Silkie_Wenchang	CRA004775	0.441718274
ENSGALG00010019325	SLITRK4	2	0.914276956	1.229052344	0.743887728	0.4569444	2.632678395	7.103124884	85.92168917	0.5	CRA004775;Yang2025_Silkie_Wenchang	PRJNA827465	0.84954048
ENSGALG00010019325	SLITRK4	2	0.680080571	1.485927718	0.45768079	0.647181791	4.033294157	10.71978243	90.67145246	0.5	CRA004775;PRJNA827465	Yang2025_Silkie_Wenchang	0.907350807
ENSGALG00010019331	CEMIP	2	0.248022297	0.195927963	1.265885144	0.205554206	0	0.543832288	0	1	PRJNA827465;Yang2025_Silkie_Wenchang	CRA004775	0.71825498
ENSGALG00010019331	CEMIP	2	0.092139075	0.515082196	0.178882274	0.858030141	0.279379539	1.700313722	41.18732403	0.5	CRA004775;Yang2025_Silkie_Wenchang	PRJNA827465	0.96417997
ENSGALG00010019331	CEMIP	2	0.033239961	0.255536164	0.130079283	0.896503701	0	0.972658428	0	0.5	CRA004775;PRJNA827465	Yang2025_Silkie_Wenchang	0.975115773
ENSGALG00010019334	ATL1	2	-0.074647401	0.173251995	-0.430860265	0.666569977	0.053560367	9.117396587	89.03195676	0.5	PRJNA827465;Yang2025_Silkie_Wenchang	CRA004775	0.913579507
ENSGALG00010019334	ATL1	2	-0.225825352	0.090611461	-2.492238271	0.012694085	0	0.798529776	0	1	CRA004775;Yang2025_Silkie_Wenchang	PRJNA827465	0.182752973
ENSGALG00010019334	ATL1	2	0.085464056	0.060301921	1.417269217	0.156404231	0	0.139383585	0	0.5	CRA004775;PRJNA827465	Yang2025_Silkie_Wenchang	0.649484056
ENSGALG00010019335	HIPK3	2	0.101567107	0.334414002	0.30371667	0.761343757	0.194930886	7.216569767	86.14300101	0.5	PRJNA827465;Yang2025_Silkie_Wenchang	CRA004775	0.939618592
ENSGALG00010019335	HIPK3	2	0.515375844	0.204076826	2.525401118	0.01155663	0	0.120845425	0	1	CRA004775;Yang2025_Silkie_Wenchang	PRJNA827465	0.171919086
ENSGALG00010019335	HIPK3	2	0.124963228	0.407203612	0.306881433	0.758933622	0.263505485	4.065107193	75.40040268	0.5	CRA004775;PRJNA827465	Yang2025_Silkie_Wenchang	0.939031852
ENSGALG00010019336	DEXI	2	0.134459632	0.198780496	0.676422662	0.498772333	0.031945204	1.672445522	40.20731995	0.5	PRJNA827465;Yang2025_Silkie_Wenchang	CRA004775	0.861567662
ENSGALG00010019336	DEXI	2	0.237439094	0.187690692	1.26505524	0.205851527	0	0.680086529	0	1	CRA004775;Yang2025_Silkie_Wenchang	PRJNA827465	0.71833282
ENSGALG00010019336	DEXI	2	-0.029758663	0.1724979	-0.172516091	0.863031812	0	0.035301943	0	0.5	CRA004775;PRJNA827465	Yang2025_Silkie_Wenchang	0.966099097
ENSGALG00010019337	XPO1	2	-0.056436553	0.080805181	-0.698427408	0.484909941	0.008328475	2.466469121	59.45621247	0.5	PRJNA827465;Yang2025_Silkie_Wenchang	CRA004775	0.857004733
ENSGALG00010019337	XPO1	2	0.04977557	0.258694912	0.192410318	0.847420811	0.102407404	3.775612825	73.51423342	0.5	CRA004775;Yang2025_Silkie_Wenchang	PRJNA827465	0.961252712
ENSGALG00010019337	XPO1	2	0.099095767	0.158055508	0.626968135	0.530680123	0.031713291	1.953788372	48.81738398	1	CRA004775;PRJNA827465	Yang2025_Silkie_Wenchang	0.872665695
ENSGALG00010019344	DNAJC24	2	0.204057871	0.059526479	3.428018483	0.000608004	0	0.179768146	0	1	PRJNA827465;Yang2025_Silkie_Wenchang	CRA004775	0.026750565
ENSGALG00010019344	DNAJC24	2	0.086975579	0.162117616	0.536496779	0.591615262	0.022465043	1.381642466	27.62237519	0.5	CRA004775;Yang2025_Silkie_Wenchang	PRJNA827465	0.892776493
ENSGALG00010019344	DNAJC24	2	0.105281337	0.199240313	0.528413828	0.597212142	0.04798831	1.85251254	46.01925879	0.5	CRA004775;PRJNA827465	Yang2025_Silkie_Wenchang	0.894350498
ENSGALG00010019345	ENSGALG00010019345	2	0.088016792	0.095441341	0.922208248	0.356419962	0	0.954609979	0	1	PRJNA827465;Yang2025_Silkie_Wenchang	CRA004775	0.812521698
ENSGALG00010019345	ENSGALG00010019345	2	0.175715283	0.2439596	0.720263862	0.471362551	0	0.920652732	0	0.5	CRA004775;Yang2025_Silkie_Wenchang	PRJNA827465	0.853967103
ENSGALG00010019345	ENSGALG00010019345	2	0.052909935	0.095172401	0.555937802	0.578253375	0	0.09990012	0	0.5	CRA004775;PRJNA827465	Yang2025_Silkie_Wenchang	0.88778445
ENSGALG00010019346	POU3F4	2	0.540404957	0.380335179	1.420865036	0.155355999	0.199613616	3.13061095	68.05735315	1	PRJNA827465;Yang2025_Silkie_Wenchang	CRA004775	0.648121944
ENSGALG00010019346	POU3F4	2	0.93433448	0.302663098	3.087044589	0.002021573	0	0.031282868	0	1	CRA004775;Yang2025_Silkie_Wenchang	PRJNA827465	0.058563901
ENSGALG00010019346	POU3F4	2	0.322300133	0.2516502	1.280746581	0.200282693	0.018298885	1.095749162	8.738237286	1	CRA004775;PRJNA827465	Yang2025_Silkie_Wenchang	0.712517205
ENSGALG00010019350	SAV1	2	0.006109858	0.036152455	0.169002577	0.865794615	0	0.003289684	0	1	PRJNA827465;Yang2025_Silkie_Wenchang	CRA004775	0.967098441
ENSGALG00010019350	SAV1	2	0.055802618	0.093215719	0.598639574	0.54941326	0.004691949	1.254900489	20.31240656	1	CRA004775;Yang2025_Silkie_Wenchang	PRJNA827465	0.878554604
ENSGALG00010019350	SAV1	2	0.044458751	0.080216984	0.554231149	0.579420663	0.006217822	1.394607373	28.29523066	1	CRA004775;PRJNA827465	Yang2025_Silkie_Wenchang	0.887951482
ENSGALG00010019355	ENTPD4	2	-0.009771111	0.060955145	-0.160300025	0.872644739	0	0.723713907	0	0.5	PRJNA827465;Yang2025_Silkie_Wenchang	CRA004775	0.969629321
ENSGALG00010019355	ENTPD4	2	-0.109033325	0.086349169	-1.262702652	0.206696061	0	0.343503501	0	1	CRA004775;Yang2025_Silkie_Wenchang	PRJNA827465	0.71868063
ENSGALG00010019355	ENTPD4	2	-0.032241875	0.099406764	-0.324342867	0.745678469	0.007846737	1.468154676	31.88728569	0.5	CRA004775;PRJNA827465	Yang2025_Silkie_Wenchang	0.93624895
ENSGALG00010019356	AVL9	2	-0.140693938	0.118083629	-1.191477084	0.233466348	0.01924082	2.947120977	66.06857989	1	PRJNA827465;Yang2025_Silkie_Wenchang	CRA004775	0.746738921
ENSGALG00010019356	AVL9	2	-0.265529856	0.11691721	-2.27109299	0.023141349	0	0.193647322	0	1	CRA004775;Yang2025_Silkie_Wenchang	PRJNA827465	0.259577892
ENSGALG00010019356	AVL9	2	-0.048398365	0.061128768	-0.791744484	0.428509678	0	0.084095533	0	1	CRA004775;PRJNA827465	Yang2025_Silkie_Wenchang	0.841538794
ENSGALG00010019362	ENSGALG00010019362	2	0.001251081	0.181801373	0.006881584	0.994509333	0	0.030711821	0	0.5	PRJNA827465;Yang2025_Silkie_Wenchang	CRA004775	0.99823774
ENSGALG00010019362	ENSGALG00010019362	2	0.190078007	0.283724639	0.669938316	0.502897113	0.005426713	1.022610395	2.211046812	1	CRA004775;Yang2025_Silkie_Wenchang	PRJNA827465	0.863050365
ENSGALG00010019362	ENSGALG00010019362	2	0.144757151	0.316439039	0.457456677	0.647342834	0.077491814	1.356809591	26.29769081	0.5	CRA004775;PRJNA827465	Yang2025_Silkie_Wenchang	0.907350807
ENSGALG00010019365	ENSGALG00010019365	2	0.628085976	0.510964783	1.229215782	0.218990913	0.47568801	10.99398893	90.90412037	1	PRJNA827465;Yang2025_Silkie_Wenchang	CRA004775	0.73259614
ENSGALG00010019365	ENSGALG00010019365	2	0.953586252	0.358957887	2.656540741	0.007894691	0.101036916	1.423288104	29.74015609	1	CRA004775;Yang2025_Silkie_Wenchang	PRJNA827465	0.137977634
ENSGALG00010019365	ENSGALG00010019365	2	0.151046758	0.14885481	1.014725413	0.310236737	0	0.092038711	0	1	CRA004775;PRJNA827465	Yang2025_Silkie_Wenchang	0.794813537
ENSGALG00010019371	TMEM87A	2	-0.141620258	0.142989103	-0.990426927	0.32196549	0.035981982	7.852990042	87.26599684	1	PRJNA827465;Yang2025_Silkie_Wenchang	CRA004775	0.798513866
ENSGALG00010019371	TMEM87A	2	-0.150625752	0.172326694	-0.874070922	0.382079632	0.042492504	3.317844542	69.85995012	0.5	CRA004775;Yang2025_Silkie_Wenchang	PRJNA827465	0.825001735
ENSGALG00010019371	TMEM87A	2	-0.007916567	0.0377461	-0.209732046	0.833876813	0	0.130162287	0	0.5	CRA004775;PRJNA827465	Yang2025_Silkie_Wenchang	0.957968425
ENSGALG00010019373	DENND5A	2	0.024045349	0.117893181	0.203958776	0.838385714	0.016155282	1.839695416	45.64317597	0.5	PRJNA827465;Yang2025_Silkie_Wenchang	CRA004775	0.959197438
ENSGALG00010019373	DENND5A	2	0.043731013	0.227971021	0.191827071	0.847877665	0.046950613	1.72862631	42.15059702	0.5	CRA004775;Yang2025_Silkie_Wenchang	PRJNA827465	0.961328089
ENSGALG00010019373	DENND5A	2	-0.053443538	0.052510025	-1.017777809	0.30878356	0	0.441374345	0	1	CRA004775;PRJNA827465	Yang2025_Silkie_Wenchang	0.793729253
ENSGALG00010019376	ENSGALG00010019376	2	0.478418644	0.306577182	1.56051615	0.118637956	0	0.349450269	0	1	PRJNA827465;Yang2025_Silkie_Wenchang	CRA004775	0.578729999
ENSGALG00010019376	ENSGALG00010019376	2	-0.16097465	0.364095149	-0.442122478	0.65840058	0	0.813630282	0	0.5	CRA004775;Yang2025_Silkie_Wenchang	PRJNA827465	0.911069886
ENSGALG00010019376	ENSGALG00010019376	2	0.121721876	0.52420018	0.232204949	0.816378834	0.367374132	2.967454989	66.30108953	0.5	CRA004775;PRJNA827465	Yang2025_Silkie_Wenchang	0.954546835
ENSGALG00010019388	GPR75	2	-0.234670073	0.176165601	-1.332099299	0.182827561	0.050055187	5.098074762	80.38475215	1	PRJNA827465;Yang2025_Silkie_Wenchang	CRA004775	0.691266551
ENSGALG00010019388	GPR75	2	-0.301506159	0.190745499	-1.580672466	0.113952947	0.034945651	1.637235552	38.92143385	0.5	CRA004775;Yang2025_Silkie_Wenchang	PRJNA827465	0.569567735
ENSGALG00010019388	GPR75	2	-0.062248738	0.089127867	-0.698420595	0.4849142	0	0.04893134	0	0.5	CRA004775;PRJNA827465	Yang2025_Silkie_Wenchang	0.857004733
ENSGALG00010019390	TVP23A	2	0.569251784	0.3765294	1.511838874	0.130574857	0.142339753	1.973070238	49.31756708	1	PRJNA827465;Yang2025_Silkie_Wenchang	CRA004775	0.606115935
ENSGALG00010019390	TVP23A	2	0.561702311	0.44399529	1.265108715	0.205832359	0.204250188	2.074481645	51.7951869	1	CRA004775;Yang2025_Silkie_Wenchang	PRJNA827465	0.718329712
ENSGALG00010019390	TVP23A	2	0.194073951	0.263814996	0.735644121	0.461947304	0	0.05421981	0	1	CRA004775;PRJNA827465	Yang2025_Silkie_Wenchang	0.850747865
ENSGALG00010019391	GANC	2	0.441989305	0.34820369	1.269341245	0.204319382	0.229195561	18.21701148	94.5106254	1	PRJNA827465;Yang2025_Silkie_Wenchang	CRA004775	0.717281769
ENSGALG00010019391	GANC	2	0.767074356	0.113751077	6.743446971	1.54672E-11	0	0.287094768	0	1	CRA004775;Yang2025_Silkie_Wenchang	PRJNA827465	1.24063E-08
ENSGALG00010019391	GANC	2	0.291214581	0.254488794	1.144311998	0.252494269	0.091298731	2.910819169	65.6454097	1	CRA004775;PRJNA827465	Yang2025_Silkie_Wenchang	0.762655223
ENSGALG00010019392	NIN	2	0.535011978	0.28280689	1.891792589	0.058518621	0.140805222	7.878899555	87.30787221	1	PRJNA827465;Yang2025_Silkie_Wenchang	CRA004775	0.419012091
ENSGALG00010019392	NIN	2	0.589239016	0.346881082	1.698677289	0.089380005	0.150514331	2.327050928	57.02715451	1	CRA004775;Yang2025_Silkie_Wenchang	PRJNA827465	0.513108919
ENSGALG00010019392	NIN	2	0.271947227	0.075699532	3.592455866	0.000327576	0	0.127242347	0	1	CRA004775;PRJNA827465	Yang2025_Silkie_Wenchang	0.017894809
ENSGALG00010019398	SLC35F5	2	-0.321378937	0.250307199	-1.283938051	0.199163632	0.115225456	12.28220243	91.85813778	1	PRJNA827465;Yang2025_Silkie_Wenchang	CRA004775	0.71096127
ENSGALG00010019398	SLC35F5	2	-0.187903164	0.423320783	-0.443878901	0.65713014	0.318604156	8.600784534	88.37315368	0.5	CRA004775;Yang2025_Silkie_Wenchang	PRJNA827465	0.910711339
ENSGALG00010019398	SLC35F5	2	0.004061585	0.148442107	0.027361408	0.978171479	0.022782594	1.601725795	37.56734124	0.5	CRA004775;PRJNA827465	Yang2025_Silkie_Wenchang	0.993926412
ENSGALG00010019399	CD93	2	0.124932655	0.070173801	1.78033187	0.075021664	0	0.418210388	0	1	PRJNA827465;Yang2025_Silkie_Wenchang	CRA004775	0.47268425
ENSGALG00010019399	CD93	2	0.121220608	0.146698032	0.826327429	0.408618378	0.013160641	1.158294289	13.66615465	0.5	CRA004775;Yang2025_Silkie_Wenchang	PRJNA827465	0.834238643
ENSGALG00010019399	CD93	2	0.067456888	0.090258099	0.747377673	0.454835618	0	0.737640925	0	0.5	CRA004775;PRJNA827465	Yang2025_Silkie_Wenchang	0.849072665
ENSGALG00010019403	PACC1	2	0.067961459	0.070984594	0.957411387	0.338359653	0	0.091464492	0	1	PRJNA827465;Yang2025_Silkie_Wenchang	CRA004775	0.804169405
ENSGALG00010019403	PACC1	2	-0.081585998	0.249372488	-0.327165192	0.743542946	0.079209173	2.352854302	57.49843077	0.5	CRA004775;Yang2025_Silkie_Wenchang	PRJNA827465	0.935816088
ENSGALG00010019403	PACC1	2	-0.088175841	0.218443201	-0.403655689	0.686465928	0.060246177	2.106121575	52.51936014	0.5	CRA004775;PRJNA827465	Yang2025_Silkie_Wenchang	0.919235539
ENSGALG00010019411	LSM5	2	-0.046208255	0.108999129	-0.423932329	0.671615166	0	0.55293165	0	1	PRJNA827465;Yang2025_Silkie_Wenchang	CRA004775	0.914674175
ENSGALG00010019411	LSM5	2	-0.209730696	0.212133979	-0.988670917	0.322824177	0	0.040417547	0	1	CRA004775;Yang2025_Silkie_Wenchang	PRJNA827465	0.79874391
ENSGALG00010019411	LSM5	2	-0.033930383	0.109317049	-0.310385095	0.756268127	0	0.21826625	0	1	CRA004775;PRJNA827465	Yang2025_Silkie_Wenchang	0.938571038
ENSGALG00010019418	ZNF703	2	0.19381455	0.259347855	0.747315027	0.454873424	0.108968173	4.834135826	79.31377942	0.5	PRJNA827465;Yang2025_Silkie_Wenchang	CRA004775	0.849072665
ENSGALG00010019418	ZNF703	2	0.167496677	0.372514805	0.449637637	0.652971745	0.19695495	3.303981403	69.73348581	0.5	CRA004775;Yang2025_Silkie_Wenchang	PRJNA827465	0.909572411
ENSGALG00010019418	ZNF703	2	-0.04528204	0.089535487	-0.505744048	0.613036342	0	0.393863685	0	1	CRA004775;PRJNA827465	Yang2025_Silkie_Wenchang	0.898130964
ENSGALG00010019422	IMMP1L	2	-0.005218695	0.109985024	-0.047449141	0.962155264	0	0.444930598	0	0.5	PRJNA827465;Yang2025_Silkie_Wenchang	CRA004775	0.989885041
ENSGALG00010019422	IMMP1L	2	-0.125807661	0.194124085	-0.648078577	0.516934129	0	0.007226871	0	1	CRA004775;Yang2025_Silkie_Wenchang	PRJNA827465	0.867952013
ENSGALG00010019422	IMMP1L	2	0.023500804	0.120125175	0.195635957	0.844895114	0	0.105790147	0	0.5	CRA004775;PRJNA827465	Yang2025_Silkie_Wenchang	0.960769014
ENSGALG00010019423	LYPD1	2	0.06990252	0.102679022	0.680786775	0.49600642	0	0.008455594	0	1	PRJNA827465;Yang2025_Silkie_Wenchang	CRA004775	0.860911322
ENSGALG00010019423	LYPD1	2	-0.000247382	0.150913068	-0.001639237	0.998692079	0	0.682177361	0	0.5	CRA004775;Yang2025_Silkie_Wenchang	PRJNA827465	0.999658596
ENSGALG00010019423	LYPD1	2	0.036716936	0.122522184	0.29967582	0.764424444	0	0.822864396	0	0.5	CRA004775;PRJNA827465	Yang2025_Silkie_Wenchang	0.940585246
ENSGALG00010019426	GRM7	2	-0.324819685	0.239151114	-1.358219413	0.174394072	0.078376801	3.15218732	68.275997	1	PRJNA827465;Yang2025_Silkie_Wenchang	CRA004775	0.678690684
ENSGALG00010019426	GRM7	2	0.434191435	0.587691689	0.738808194	0.460023476	0.557309869	4.730492221	78.8605508	0.5	CRA004775;Yang2025_Silkie_Wenchang	PRJNA827465	0.850104868
ENSGALG00010019426	GRM7	2	0.216158883	0.831255786	0.260038952	0.794833727	1.248594123	9.758802015	89.75284058	0.5	CRA004775;PRJNA827465	Yang2025_Silkie_Wenchang	0.948905846
ENSGALG00010019430	ENSGALG00010019430	2	-0.144632554	0.174044281	-0.831010093	0.405967928	0	0.34286198	0	1	PRJNA827465;Yang2025_Silkie_Wenchang	CRA004775	0.833416425
ENSGALG00010019430	ENSGALG00010019430	2	-0.455258757	0.217409909	-2.094011076	0.036258994	0.022963914	1.31846073	24.15397915	1	CRA004775;Yang2025_Silkie_Wenchang	PRJNA827465	0.328436789
ENSGALG00010019430	ENSGALG00010019430	2	-0.351961039	0.319688119	-1.100951265	0.270917868	0.137002635	3.014277262	66.82455152	1	CRA004775;PRJNA827465	Yang2025_Silkie_Wenchang	0.772743863
ENSGALG00010019441	LMCD1	2	-0.0360694	0.112732558	-0.31995548	0.74900208	0	0.672071609	0	0.5	PRJNA827465;Yang2025_Silkie_Wenchang	CRA004775	0.937357674
ENSGALG00010019441	LMCD1	2	0.243521753	0.254890295	0.955398293	0.339376317	0.070374826	1.931565875	48.22853247	1	CRA004775;Yang2025_Silkie_Wenchang	PRJNA827465	0.804824608
ENSGALG00010019441	LMCD1	2	0.171347868	0.356128726	0.48114026	0.630416816	0.189335701	3.564693048	71.94709372	0.5	CRA004775;PRJNA827465	Yang2025_Silkie_Wenchang	0.902665749
ENSGALG00010019443	NCKAP5	2	-1.167026178	0.69699994	-1.721920425	0.085083934	0.004002391	1.157200459	13.5845486	1	PRJNA827465;Yang2025_Silkie_Wenchang	CRA004775	0.501658247
ENSGALG00010019443	NCKAP5	2	0.474302859	0.511282104	0.927673499	0.353576959	0.4180494	4.65860733	78.53435739	1	CRA004775;Yang2025_Silkie_Wenchang	PRJNA827465	0.811318534
ENSGALG00010019443	NCKAP5	2	0.35215812	0.633456813	0.555930749	0.578258197	0.715689095	8.412234709	88.11255232	0.5	CRA004775;PRJNA827465	Yang2025_Silkie_Wenchang	0.88778445
ENSGALG00010019451	CAV3	2	-0.021348488	0.187568141	-0.11381724	0.909382672	0.049900361	2.750639419	63.64481679	0.5	PRJNA827465;Yang2025_Silkie_Wenchang	CRA004775	0.978607662
ENSGALG00010019451	CAV3	2	0.145797793	0.166798962	0.874092925	0.38206765	0	0.638399524	0	0.5	CRA004775;Yang2025_Silkie_Wenchang	PRJNA827465	0.825801735
ENSGALG00010019451	CAV3	2	-0.208580913	0.145647684	-1.432092207	0.152117434	0	0.098839936	0	1	CRA004775;PRJNA827465	Yang2025_Silkie_Wenchang	0.643667715
ENSGALG00010019458	ELP4	2	-0.229702528	0.057614218	-3.986907013	6.69402E-05	0	0.637634879	0	1	PRJNA827465;Yang2025_Silkie_Wenchang	CRA004775	0.005898997
ENSGALG00010019458	ELP4	2	-0.220783684	0.134199972	-1.645184428	0.099931814	0.01300365	1.403554476	28.75232013	1	CRA004775;Yang2025_Silkie_Wenchang	PRJNA827465	0.540027512
ENSGALG00010019458	ELP4	2	-0.184950364	0.065144911	-2.839060805	0.004524653	0	0.687554629	0	1	CRA004775;PRJNA827465	Yang2025_Silkie_Wenchang	0.098467582
ENSGALG00010019461	MGAT5	2	-0.201993923	0.217312989	-0.929506903	0.352626449	0.08433627	9.172524799	89.0978763	0.5	PRJNA827465;Yang2025_Silkie_Wenchang	CRA004775	0.811035834
ENSGALG00010019461	MGAT5	2	-0.434176075	0.118067844	-3.677343976	0.000235675	0	0.025347719	0	1	CRA004775;Yang2025_Silkie_Wenchang	PRJNA827465	0.014206656
ENSGALG00010019461	MGAT5	2	-0.009946803	0.087887214	-0.113176903	0.909890307	0.002055064	1.015354931	1.529728829	0.5	CRA004775;PRJNA827465	Yang2025_Silkie_Wenchang	0.979730675
ENSGALG00010019470	TGFB2	2	0.001240232	0.191156391	0.006488047	0.994823324	0.062214401	6.475419296	84.55698459	0.5	PRJNA827465;Yang2025_Silkie_Wenchang	CRA004775	0.998242335
ENSGALG00010019470	TGFB2	2	0.177314865	0.123987621	1.430101353	0.152687932	0	0.488543357	0	0.5	CRA004775;Yang2025_Silkie_Wenchang	PRJNA827465	0.644378159
ENSGALG00010019470	TGFB2	2	-0.170424461	0.071545823	-2.382032301	0.017217387	0	0.093758679	0	1	CRA004775;PRJNA827465	Yang2025_Silkie_Wenchang	0.218077656
ENSGALG00010019471	NUBP1	2	-0.231756199	0.124063532	-1.868044504	0.061755868	0.015261086	1.703145274	41.2851026	1	PRJNA827465;Yang2025_Silkie_Wenchang	CRA004775	0.429075295
ENSGALG00010019471	NUBP1	2	-0.437330609	0.145129194	-3.01338826	0.002583482	0	0.012489623	0	1	CRA004775;Yang2025_Silkie_Wenchang	PRJNA827465	0.068626229
ENSGALG00010019471	NUBP1	2	-0.23378701	0.135329595	-1.727537938	0.084071098	0.018513924	1.663546099	39.88744884	1	CRA004775;PRJNA827465	Yang2025_Silkie_Wenchang	0.49965921
ENSGALG00010019472	CAPN3	2	0.080019131	0.350569031	0.228256939	0.819448053	0.276745828	12.88977806	92.24792864	0.5	PRJNA827465;Yang2025_Silkie_Wenchang	CRA004775	0.955256039
ENSGALG00010019472	CAPN3	2	0.449170517	0.134461984	3.340501933	0.000836271	0	0.161642749	0	1	CRA004775;Yang2025_Silkie_Wenchang	PRJNA827465	0.033572991
ENSGALG00010019472	CAPN3	2	0.075104595	0.432356436	0.173709904	0.862093454	0.287370006	3.706667766	73.02159073	0.5	CRA004775;PRJNA827465	Yang2025_Silkie_Wenchang	0.965763853
ENSGALG00010019473	OXTR	2	0.654940343	0.300198604	2.181690171	0.029132407	0	0.041533504	0	1	PRJNA827465;Yang2025_Silkie_Wenchang	CRA004775	0.290337858
ENSGALG00010019473	OXTR	2	0.024884231	0.724848185	0.034330267	0.97261379	0.793272012	4.071464999	75.43381624	0.5	CRA004775;Yang2025_Silkie_Wenchang	PRJNA827465	0.99221869
ENSGALG00010019473	OXTR	2	-0.004367652	0.66030422	-0.006614606	0.994722346	0.661185421	4.058282924	75.35903685	0.5	CRA004775;PRJNA827465	Yang2025_Silkie_Wenchang	0.998242335
ENSGALG00010019474	TMEM163	2	1.670981024	0.581344123	2.874340613	0.004048721	0.356940345	2.118638711	52.79988067	1	PRJNA827465;Yang2025_Silkie_Wenchang	CRA004775	0.09224746
ENSGALG00010019474	TMEM163	2	0.72812357	1.693786054	0.429879304	0.667283441	4.88673134	6.372661496	84.30796927	0.5	CRA004775;Yang2025_Silkie_Wenchang	PRJNA827465	0.913832992
ENSGALG00010019474	TMEM163	2	0.239128089	1.089628482	0.219458369	0.826293006	1.651290924	2.78880037	64.14228818	0.5	CRA004775;PRJNA827465	Yang2025_Silkie_Wenchang	0.951242839
ENSGALG00010019477	NCAPH	2	-0.034758839	0.068292103	-0.508973036	0.610771124	0	0.931389375	0	1	PRJNA827465;Yang2025_Silkie_Wenchang	CRA004775	0.897506931
ENSGALG00010019477	NCAPH	2	-0.129327156	0.145386672	-0.889539281	0.373713322	0	0.579223934	0	0.5	CRA004775;Yang2025_Silkie_Wenchang	PRJNA827465	0.822579781
ENSGALG00010019477	NCAPH	2	0.000349505	0.075081867	0.004631169	0.996441081	0	0.179601711	0	1	CRA004775;PRJNA827465	Yang2025_Silkie_Wenchang	0.999345523
ENSGALG00010019481	PYGL	2	-0.028219716	0.070759574	-0.398811281	0.690032264	0	0.222891526	0	1	PRJNA827465;Yang2025_Silkie_Wenchang	CRA004775	0.919979266
ENSGALG00010019481	PYGL	2	-0.240293346	0.225712944	-1.064597099	0.287058293	0.066011656	2.475530337	59.60461542	1	CRA004775;Yang2025_Silkie_Wenchang	PRJNA827465	0.782566585
ENSGALG00010019481	PYGL	2	-0.209240872	0.262383537	-0.797461892	0.425182827	0.103621914	3.465916607	71.14760356	1	CRA004775;PRJNA827465	Yang2025_Silkie_Wenchang	0.84125685
ENSGALG00010019483	SCUBE2	2	0.352410221	0.147255942	2.393181661	0.016702969	0	0.020567117	0	1	PRJNA827465;Yang2025_Silkie_Wenchang	CRA004775	0.214226254
ENSGALG00010019483	SCUBE2	2	0.2827238	0.245110968	1.15345226	0.248724802	0	0.121953661	0	1	CRA004775;Yang2025_Silkie_Wenchang	PRJNA827465	0.759154375
ENSGALG00010019483	SCUBE2	2	0.344802687	0.169368682	2.035811357	0.041769302	0	0.192641816	0	1	CRA004775;PRJNA827465	Yang2025_Silkie_Wenchang	0.354014899
ENSGALG00010019495	EMP2	2	-0.125196927	0.184545959	-0.678401441	0.497535185	0.058528803	7.034026352	85.78339133	0.5	PRJNA827465;Yang2025_Silkie_Wenchang	CRA004775	0.85909656
ENSGALG00010019495	EMP2	2	0.193830001	0.523018258	0.370598919	0.710936287	0.522467647	21.9739303	95.44915276	0.5	CRA004775;Yang2025_Silkie_Wenchang	PRJNA827465	0.925563881
ENSGALG00010019495	EMP2	2	0.367929692	0.337705602	1.089498336	0.275934188	0.206980721	10.35747397	90.34513624	1	CRA004775;PRJNA827465	Yang2025_Silkie_Wenchang	0.775541559
ENSGALG00010019498	ENSGALG00010019498	2	0.63178718	0.515614969	1.225308065	0.220459171	0.477497997	9.831619235	89.82873547	1	PRJNA827465;Yang2025_Silkie_Wenchang	CRA004775	0.734053249
ENSGALG00010019498	ENSGALG00010019498	2	0.07547424	1.154695142	0.065362914	0.947885051	2.434946969	10.96484191	90.87994147	0.5	CRA004775;Yang2025_Silkie_Wenchang	PRJNA827465	0.986644937
ENSGALG00010019498	ENSGALG00010019498	2	-0.371502045	0.624430997	-0.594944913	0.551880294	0.585516767	3.457509801	71.07745003	0.5	CRA004775;PRJNA827465	Yang2025_Silkie_Wenchang	0.879061525
ENSGALG00010019509	ZNF106	2	-0.069125015	0.122216526	-0.565594664	0.571669356	0.012252949	1.63729398	38.92361346	0.5	PRJNA827465;Yang2025_Silkie_Wenchang	CRA004775	0.885445282
ENSGALG00010019509	ZNF106	2	0.239294289	0.285271486	0.838830033	0.401564695	0.078952978	1.516447358	34.05639869	1	CRA004775;Yang2025_Silkie_Wenchang	PRJNA827465	0.832623399
ENSGALG00010019509	ZNF106	2	0.156246529	0.443296056	0.352465417	0.72448925	0.288397476	2.978476415	66.42578753	0.5	CRA004775;PRJNA827465	Yang2025_Silkie_Wenchang	0.930388018
ENSGALG00010019515	ENSGALG00010019515	2	-0.399100753	0.371843641	-1.073302617	0.283135361	0.264291043	22.44311128	95.54428979	1	PRJNA827465;Yang2025_Silkie_Wenchang	CRA004775	0.779568117
ENSGALG00010019515	ENSGALG00010019515	2	-0.390909546	0.390921594	-0.999969181	0.317325422	0.285106902	14.85832552	93.26976651	0.5	CRA004775;Yang2025_Silkie_Wenchang	PRJNA827465	0.797095376
ENSGALG00010019515	ENSGALG00010019515	2	-0.024429263	0.075991589	-0.321473255	0.747851777	0	0.045809442	0	0.5	CRA004775;PRJNA827465	Yang2025_Silkie_Wenchang	0.936938957
ENSGALG00010019524	HHIPL2	2	-0.534663514	0.281281128	-1.900815452	0.057326189	0.004671373	1.030412845	2.951520405	1	PRJNA827465;Yang2025_Silkie_Wenchang	CRA004775	0.414689094
ENSGALG00010019524	HHIPL2	2	-0.033795495	0.322187112	-0.104894001	0.916459919	0	0.892896783	0	0.5	CRA004775;Yang2025_Silkie_Wenchang	PRJNA827465	0.980561416
ENSGALG00010019524	HHIPL2	2	-0.273912592	0.600460454	-0.45617091	0.648267082	0.498716306	3.157969094	68.33407896	0.5	CRA004775;PRJNA827465	Yang2025_Silkie_Wenchang	0.90762535
ENSGALG00010019529	SNAP23	2	-0.006385847	0.105534575	-0.060509521	0.951749833	0.018620408	5.921289642	83.11178712	0.5	PRJNA827465;Yang2025_Silkie_Wenchang	CRA004775	0.987713746
ENSGALG00010019529	SNAP23	2	-0.028837059	0.131946408	-0.218551297	0.826999595	0.020283755	2.043469384	51.06361721	0.5	CRA004775;Yang2025_Silkie_Wenchang	PRJNA827465	0.956540044
ENSGALG00010019529	SNAP23	2	0.095546093	0.04428858	2.157352807	0.030978185	0	0.138827801	0	1	CRA004775;PRJNA827465	Yang2025_Silkie_Wenchang	0.300626079

ENSGALG00010019534	PPP2R5A	2	-0.180132214	0.039248232	-4.589562531	4.44176E-06	0	0.081296427	0	1	PRJNA827465;Yang2025_Silkie_Wenchang	CRA004775	0.00074287
ENSGALG00010019534	PPP2R5A	2	-0.120327253	0.146524729	-0.821207819	0.411527903	0.022523481	1.537375881	34.95409858	0.5	CRA004775;Yang2025_Silkie_Wenchang	PRJNA827465	0.835128673
ENSGALG00010019534	PPP2R5A	2	-0.119365856	0.122236958	-0.976511995	0.328810807	0.015452298	1.379541345	27.51213989	0.5	CRA004775;PRJNA827465	Yang2025_Silkie_Wenchang	0.800761292
ENSGALG00010019540	UNC5D	2	-0.088190354	0.18063256	-0.488230657	0.625386472	0	0.113775741	0	1	PRJNA827465;Yang2025_Silkie_Wenchang	CRA004775	0.901367193
ENSGALG00010019540	UNC5D	2	-0.148799455	0.292503458	-0.508710071	0.610955462	0	0.047398315	0	1	CRA004775;Yang2025_Silkie_Wenchang	PRJNA827465	0.897506931
ENSGALG00010019540	UNC5D	2	-0.04365289	0.208332939	-0.209534267	0.834031188	0	0.002489099	0	1	CRA004775;PRJNA827465	Yang2025_Silkie_Wenchang	0.957968425
ENSGALG00010019546	HSD17B12	2	-0.208796428	0.166156604	-1.256624313	0.208889707	0.049984031	10.41346255	90.39704618	1	PRJNA827465;Yang2025_Silkie_Wenchang	CRA004775	0.721011841
ENSGALG00010019546	HSD17B12	2	-0.315726428	0.108212666	-2.917647625	0.003526827	0.008626523	1.407397518	28.94686917	1	CRA004775;Yang2025_Silkie_Wenchang	PRJNA827465	0.084110962
ENSGALG00010019546	HSD17B12	2	-0.056249024	0.05441687	-1.033668867	0.301290992	0	0.204621029	0	1	CRA004775;PRJNA827465	Yang2025_Silkie_Wenchang	0.791222412
ENSGALG00010019550	ACMSD	2	0.871912132	0.274196495	3.179880655	0.001473357	0	0.45596322	0	1	PRJNA827465;Yang2025_Silkie_Wenchang	CRA004775	0.047542986
ENSGALG00010019550	ACMSD	2	0.147828009	0.965325235	0.153138034	0.878289423	1.596679967	6.768350403	85.22535122	0.5	CRA004775;Yang2025_Silkie_Wenchang	PRJNA827465	0.971037793
ENSGALG00010019550	ACMSD	2	-0.027174943	0.778190636	-0.034920676	0.972142994	0.93781103	4.27578105	76.61246008	0.5	CRA004775;PRJNA827465	Yang2025_Silkie_Wenchang	0.991955348
ENSGALG00010019557	TRIM9	2	0.133224374	0.112886513	1.180162002	0.237935788	0.002056467	1.083649042	7.719200508	1	PRJNA827465;Yang2025_Silkie_Wenchang	CRA004775	0.74978532
ENSGALG00010019557	TRIM9	2	0.262765323	0.157387828	1.669540303	0.09501035	0	0.018276283	0	1	CRA004775;Yang2025_Silkie_Wenchang	PRJNA827465	0.527726369
ENSGALG00010019557	TRIM9	2	0.062901264	0.129420148	0.486023735	0.626950338	0	0.193855309	0	1	CRA004775;PRJNA827465	Yang2025_Silkie_Wenchang	0.901573209
ENSGALG00010019572	NFKBIE	2	-0.005171601	0.144769832	-0.03572292	0.971503295	0.008014443	1.200710836	16.71600102	0.5	PRJNA827465;Yang2025_Silkie_Wenchang	CRA004775	0.991955348
ENSGALG00010019572	NFKBIE	2	-0.262199948	0.184276907	-1.422858416	0.154777203	0	0.09737921	0	1	CRA004775;Yang2025_Silkie_Wenchang	PRJNA827465	0.647505492
ENSGALG00010019572	NFKBIE	2	-0.04696573	0.201028262	-0.233627496	0.815274179	0.037568194	1.701659667	41.23384252	0.5	CRA004775;PRJNA827465	Yang2025_Silkie_Wenchang	0.953933933
ENSGALG00010019577	MXD1	2	-0.077441825	0.068773247	-1.126045784	0.260146141	0	0.057099292	0	1	PRJNA827465;Yang2025_Silkie_Wenchang	CRA004775	0.76744615
ENSGALG00010019577	MXD1	2	-0.150247398	0.130823101	-1.148477573	0.250771466	0	0.074477739	0	1	CRA004775;Yang2025_Silkie_Wenchang	PRJNA827465	0.760383127
ENSGALG00010019577	MXD1	2	-0.086065226	0.069436265	-1.239485246	0.215165849	0	0.328533563	0	1	CRA004775;PRJNA827465	Yang2025_Silkie_Wenchang	0.72864582
ENSGALG00010019581	SLC35B2	2	-0.065545469	0.058069615	-1.128739512	0.259007736	0	0.870834851	0	1	PRJNA827465;Yang2025_Silkie_Wenchang	CRA004775	0.766195756
ENSGALG00010019581	SLC35B2	2	-0.378231355	0.262623071	-1.440206123	0.149809091	0.119861063	7.372259152	86.4356369	1	CRA004775;Yang2025_Silkie_Wenchang	PRJNA827465	0.639778999
ENSGALG00010019581	SLC35B2	2	-0.323323739	0.318467152	-1.015249883	0.309986728	0.18633926	11.84950921	91.56083191	1	CRA004775;PRJNA827465	Yang2025_Silkie_Wenchang	0.794395438
ENSGALG00010019584	RAD18	2	0.211641724	0.16235682	1.303559179	0.192383926	0.039862345	3.915374039	74.46030825	1	PRJNA827465;Yang2025_Silkie_Wenchang	CRA004775	0.702137035
ENSGALG00010019584	RAD18	2	0.0890733	0.336437228	0.264754587	0.791198523	0.186772096	5.489946981	81.78488784	0.5	CRA004775;Yang2025_Silkie_Wenchang	PRJNA827465	0.947918779
ENSGALG00010019584	RAD18	2	-0.025973121	0.154807868	-0.167776492	0.866759117	0.025774144	1.736609814	42.41654101	0.5	CRA004775;PRJNA827465	Yang2025_Silkie_Wenchang	0.967598306
ENSGALG00010019585	PDE1C	2	-0.375317281	0.127464042	-2.944495365	0.003234818	0	0.019641599	0	1	PRJNA827465;Yang2025_Silkie_Wenchang	CRA004775	0.079016811
ENSGALG00010019585	PDE1C	2	-0.383427872	0.190826326	-2.009302806	0.04450503	0	0.02647694	0	1	CRA004775;Yang2025_Silkie_Wenchang	PRJNA827465	0.364033551
ENSGALG00010019585	PDE1C	2	-0.357279499	0.147714562	-2.418715489	0.015575417	0	0.007510094	0	1	CRA004775;PRJNA827465	Yang2025_Silkie_Wenchang	0.206527548
ENSGALG00010019586	GLYR1	2	-0.073176136	0.136966614	-0.53426258	0.593159878	0.032533197	6.871442031	85.44701395	0.5	PRJNA827465;Yang2025_Silkie_Wenchang	CRA004775	0.892776493
ENSGALG00010019586	GLYR1	2	-0.225802559	0.089506725	-2.522744048	0.011644312	0	0.001879437	0	1	CRA004775;Yang2025_Silkie_Wenchang	PRJNA827465	0.172635381
ENSGALG00010019586	GLYR1	2	-0.01508386	0.11334834	-0.133075259	0.894133862	0.015899771	1.811675561	44.80247889	0.5	CRA004775;PRJNA827465	Yang2025_Silkie_Wenchang	0.974381724
ENSGALG00010019587	CCNT2	2	0.26345385	0.151226512	1.742114177	0.081488467	0.029244042	2.623890522	61.88865383	1	PRJNA827465;Yang2025_Silkie_Wenchang	CRA004775	0.491594971
ENSGALG00010019587	CCNT2	2	0.16555971	0.326232947	0.507489241	0.611811584	0.157866538	3.621891648	72.39011828	0.5	CRA004775;Yang2025_Silkie_Wenchang	PRJNA827465	0.897777844
ENSGALG00010019587	CCNT2	2	0.080307352	0.129846276	0.618480208	0.53625884	0.010552131	1.203892316	16.93609248	0.5	CRA004775;PRJNA827465	Yang2025_Silkie_Wenchang	0.874599474
ENSGALG00010019588	CHMP7	2	-0.17091718	0.050061126	-3.414169698	0.000639767	0	0.201782049	0	1	PRJNA827465;Yang2025_Silkie_Wenchang	CRA004775	0.027815009
ENSGALG00010019588	CHMP7	2	-0.238844839	0.133874468	-1.784095521	0.074408145	0	0.004918814	0	1	CRA004775;Yang2025_Silkie_Wenchang	PRJNA827465	0.470928071
ENSGALG00010019588	CHMP7	2	-0.165789124	0.052320773	-3.168705544	0.001531194	0	0.09625176	0	1	CRA004775;PRJNA827465	Yang2025_Silkie_Wenchang	0.048927262
ENSGALG00010019591	ENSGALG00010019591	2	0.053767502	0.051843129	1.037119168	0.299680323	0	0.186531798	0	1	PRJNA827465;Yang2025_Silkie_Wenchang	CRA004775	0.79004225
ENSGALG00010019591	ENSGALG00010019591	2	0.075272566	0.116979581	0.643467564	0.519920755	0	0.492576081	0	0.5	CRA004775;Yang2025_Silkie_Wenchang	PRJNA827465	0.869253622
ENSGALG00010019591	ENSGALG00010019591	2	0.038438248	0.056529282	0.67997058	0.49652309	0	0.332585699	0	0.5	CRA004775;PRJNA827465	Yang2025_Silkie_Wenchang	0.860939846
ENSGALG00010019592	ALKBH3	2	-0.125221659	0.085721861	-1.460813595	0.144066604	0	0.008483844	0	1	PRJNA827465;Yang2025_Silkie_Wenchang	CRA004775	0.631313384
ENSGALG00010019592	ALKBH3	2	-0.134723016	0.12367034	-1.089372087	0.275989835	0	8.37211E-05	0	1	CRA004775;Yang2025_Silkie_Wenchang	PRJNA827465	0.775541559
ENSGALG00010019592	ALKBH3	2	-0.120915473	0.101025653	-1.196878912	0.231353755	0	0.002133151	0	1	CRA004775;PRJNA827465	Yang2025_Silkie_Wenchang	0.743891332
ENSGALG00010019594	TMX1	2	-0.551858425	0.236873722	-2.329757903	0.01981895	0	0.187612334	0	1	PRJNA827465;Yang2025_Silkie_Wenchang	CRA004775	0.236401882
ENSGALG00010019594	TMX1	2	-0.318149251	0.280476188	-1.134318122	0.256661089	0	0.358192971	0	1	CRA004775;Yang2025_Silkie_Wenchang	PRJNA827465	0.764677554
ENSGALG00010019594	TMX1	2	-0.470025732	0.267079569	-1.759871539	0.07842959	0	0.944872043	0	1	CRA004775;PRJNA827465	Yang2025_Silkie_Wenchang	0.481856686
ENSGALG00010019595	GRIN2A	2	-0.040174609	0.325142801	-0.123559891	0.901663752	0.177990359	6.158017988	83.76100879	0.5	PRJNA827465;Yang2025_Silkie_Wenchang	CRA004775	0.976674416
ENSGALG00010019595	GRIN2A	2	0.31428875	0.218152415	1.440684258	0.149673904	0	0.015357455	0	1	CRA004775;Yang2025_Silkie_Wenchang	PRJNA827465	0.639457191
ENSGALG00010019595	GRIN2A	2	-0.330595162	0.138478038	-2.38734724	0.016970454	0	0.507327654	0	0.5	CRA004775;PRJNA827465	Yang2025_Silkie_Wenchang	0.216344387
ENSGALG00010019598	KCNAB2	2	0.065464701	0.166249681	0.393773393	0.69374835	0.041138458	3.816390189	73.79722852	0.5	PRJNA827465;Yang2025_Silkie_Wenchang	CRA004775	0.920721644
ENSGALG00010019598	KCNAB2	2	-0.131211083	0.452036895	-0.290266313	0.771612508	0.328201589	4.516547501	77.85919444	0.5	CRA004775;Yang2025_Silkie_Wenchang	PRJNA827465	0.94293534
ENSGALG00010019598	KCNAB2	2	-0.243605902	0.259216281	-0.939773555	0.347331161	0.082467104	1.934720734	48.31295377	1	CRA004775;PRJNA827465	Yang2025_Silkie_Wenchang	0.808705451
ENSGALG00010019602	SLC29A1	2	-0.067442037	0.068089779	-0.990486937	0.321936171	0	0.021090776	0	1	PRJNA827465;Yang2025_Silkie_Wenchang	CRA004775	0.798513866
ENSGALG00010019602	SLC29A1	2	-0.125429697	0.112214155	-1.11777072	0.26366493	0	0.658212439	0	1	CRA004775;Yang2025_Silkie_Wenchang	PRJNA827465	0.768643821
ENSGALG00010019602	SLC29A1	2	-0.08347457	0.07781053	-1.072792719	0.283364128	0	0.874412382	0	1	CRA004775;PRJNA827465	Yang2025_Silkie_Wenchang	0.779568117
ENSGALG00010019610	CORO7	2	0.163223238	0.077691967	2.100902383	0.035649536	0	0.058274827	0	1	PRJNA827465;Yang2025_Silkie_Wenchang	CRA004775	0.325617876
ENSGALG00010019610	CORO7	2	-0.017722577	0.205947129	-0.086054015	0.931423478	0.050522112	2.235596693	55.26921277	0.5	CRA004775;Yang2025_Silkie_Wenchang	PRJNA827465	0.984076328
ENSGALG00010019610	CORO7	2	0.004213293	0.225406705	0.018691958	0.985086844	0.071293423	2.901560475	65.53578639	0.5	CRA004775;PRJNA827465	Yang2025_Silkie_Wenchang	0.995835685
ENSGALG00010019613	FRMD6	2	0.090028989	0.079202786	1.237696221	0.215827221	0.00760677	2.35458898	57.5282996	1	PRJNA827465;Yang2025_Silkie_Wenchang	CRA004775	0.729213578
ENSGALG00010019613	FRMD6	2	0.118882881	0.167203168	0.711008548	0.477078939	0.025567141	1.374020285	27.22087067	0.5	CRA004775;Yang2025_Silkie_Wenchang	PRJNA827465	0.854986476
ENSGALG00010019613	FRMD6	2	0.029672972	0.050718338	0.585054107	0.558511336	0	0.56091517	0	0.5	CRA004775;PRJNA827465	Yang2025_Silkie_Wenchang	0.880794921
ENSGALG00010019614	MICALL2	2	-0.131600726	0.132514406	-0.993105049	0.320658764	0.021751068	2.471472579	59.53829274	1	PRJNA827465;Yang2025_Silkie_Wenchang	CRA004775	0.798348216
ENSGALG00010019614	MICALL2	2	-0.273059467	0.136768939	-1.996502045	0.045879303	0	0.126055997	0	1	CRA004775;Yang2025_Silkie_Wenchang	PRJNA827465	0.369489827
ENSGALG00010019614	MICALL2	2	-0.030628192	0.084650262	-0.361820406	0.717486242	0	0.133815408	0	1	CRA004775;PRJNA827465	Yang2025_Silkie_Wenchang	0.9277944
ENSGALG00010019617	ANO3	2	0.324019876	0.248901974	1.301797133	0.192985744	0.104075713	6.173760823	83.80241754	1	PRJNA827465;Yang2025_Silkie_Wenchang	CRA004775	0.702958174
ENSGALG00010019617	ANO3	2	0.31484066	0.341392313	0.922225394	0.35641102	0.163551305	2.983306655	66.48014717	0.5	CRA004775;Yang2025_Silkie_Wenchang	PRJNA827465	0.812521698
ENSGALG00010019617	ANO3	2	0.006044662	0.113289413	0.600626839	0.548088557	0	0.269556525	0	0.5	CRA004775;PRJNA827465	Yang2025_Silkie_Wenchang	0.878261266
ENSGALG00010019618	NPHP4	2	-0.020251241	0.068007532	-0.29777939	0.765871546	0	0.70707812	0	0.5	PRJNA827465;Yang2025_Silkie_Wenchang	CRA004775	0.941006047
ENSGALG00010019618	NPHP4	2	-0.190641445	0.270023615	-0.706017677	0.480177145	0.117750768	4.8203753	79.25472732	0.5	CRA004775;Yang2025_Silkie_Wenchang	PRJNA827465	0.85643503
ENSGALG00010019618	NPHP4	2	-0.235340946	0.209030359	-1.126826492	0.259815848	0.062744452	3.1191375	67.93961105	1	CRA004775;PRJNA827465	Yang2025_Silkie_Wenchang	0.767047318
ENSGALG00010019621	ENSGALG00010019621	2	0.151813155	0.111745663	1.358559708	0.17428615	0.013299507	2.12743652	52.9950722	1	PRJNA827465;Yang2025_Silkie_Wenchang	CRA004775	0.678487591
ENSGALG00010019621	ENSGALG00010019621	2	0.19073882	0.161454243	1.181380039	0.237451784	0.020281331	1.231502925	18.79840643	0.5	CRA004775;Yang2025_Silkie_Wenchang	PRJNA827465	0.749366344
ENSGALG00010019621	ENSGALG00010019621	2	0.013775522	0.111877231	0.123130705	0.902003597	0	0.323614415	0	0.5	CRA004775;PRJNA827465	Yang2025_Silkie_Wenchang	0.976866093
ENSGALG00010019622	C2orf42	2	0.15070738	0.089866262	1.677018456	0.093538873	0.009937836	2.469564802	59.51703544	1	PRJNA827465;Yang2025_Silkie_Wenchang	CRA004775	0.523999859
ENSGALG00010019622	C2orf42	2	0.183727352	0.12145836	1.512677702	0.130361546	0.010576138	1.401077846	28.62637841	0.5	CRA004775;Yang2025_Silkie_Wenchang	PRJNA827465	0.605608015
ENSGALG00010019622	C2orf42	2	0.068758852	0.058627621	1.17280646	0.240873427	0	0.169934235	0	0.5	CRA004775;PRJNA827465	Yang2025_Silkie_Wenchang	0.753385986
ENSGALG00010019624	SRGAP3	2	0.059989812	0.073978128	0.810912817	0.41741574	0	0.449129759	0	0.5	PRJNA827465;Yang2025_Silkie_Wenchang	CRA004775	0.838112595
ENSGALG00010019624	SRGAP3	2	-0.193423263	0.234885491	-0.823478974	0.410235669	0.059335277	1.906147415	47.53816036	1	CRA004775;Yang2025_Silkie_Wenchang	PRJNA827465	0.834737228
ENSGALG00010019624	SRGAP3	2	-0.138482886	0.296945164	-0.466358449	0.640958948	0.132156457	3.295016575	69.65113901	0.5	CRA004775;PRJNA827465	Yang2025_Silkie_Wenchang	0.906429868
ENSGALG00010019625	INTS1	2	-0.077244761	0.041377451	-1.866832257	0.061925016	0	0.077764542	0	1	PRJNA827465;Yang2025_Silkie_Wenchang	CRA004775	0.429324205
ENSGALG00010019625	INTS1	2	-0.110085008	0.068010314	-1.618651663	0.105522233	0	0.622227974	0	1	CRA004775;Yang2025_Silkie_Wenchang	PRJNA827465	0.551141569
ENSGALG00010019625	INTS1	2	-0.079033444	0.049630477	-1.592437725	0.111286385	0	0.828826543	0	1	CRA004775;PRJNA827465	Yang2025_Silkie_Wenchang	0.56444558
ENSGALG00010019630	AJAP1	2	-0.07243854	0.273691533	-0.264672199	0.791261996	0	0.333184275	0	0.5	PRJNA827465;Yang2025_Silkie_Wenchang	CRA004775	0.947937032
ENSGALG00010019630	AJAP1	2	0.193325585	0.35597974	0.543080303	0.587074514	0	0.026423773	0	1	CRA004775;Yang2025_Silkie_Wenchang	PRJNA827465	0.891596903
ENSGALG00010019630	AJAP1	2	-0.064899788	0.288706238	-0.22479524	0.822138569	0	0.480630675	0	0.5	CRA004775;PRJNA827465	Yang2025_Silkie_Wenchang	0.955636543
ENSGALG00010019634	GNG2	2	0.05743459	0.194007357	0.296043361	0.767196967	0.057209074	4.014019182	75.08731387	0.5	PRJNA827465;Yang2025_Silkie_Wenchang	CRA004775	0.941432527
ENSGALG00010019634	GNG2	2	0.086726088	0.293037503	0.295955594	0.767263994	0.093613755	1.814426858	44.88617737	0.5	CRA004775;Yang2025_Silkie_Wenchang	PRJNA827465	0.941444269
ENSGALG00010019634	GNG2	2	-0.129140369	0.100559682	-1.284216161	0.199066332	0	0.306139946	0	1	CRA004775;PRJNA827465	Yang2025_Silkie_Wenchang	0.71096127
ENSGALG00010019635	LRRC57	2	-0.185953282	0.170918961	-1.087961691	0.27661202	0.049401163	6.168950603	83.78978753	1	PRJNA827465;Yang2025_Silkie_Wenchang	CRA004775	0.775851484
ENSGALG00010019635	LRRC57	2	-0.242894939	0.170161077	-1.427441241	0.153452751	0.030974646	2.000059798	50.0014949	1	CRA004775;Yang2025_Silkie_Wenchang	PRJNA827465	0.645802918
ENSGALG00010019635	LRRC57	2	-0.029895244	0.060405437	-0.494909826	0.620663774	0	0.001456351	0	1	CRA004775;PRJNA827465	Yang2025_Silkie_Wenchang	0.899395593
ENSGALG00010019640	MGRN1	2	-0.079962671	0.063699712	-1.255306639	0.209367466	0.00181244	1.165380263	14.19109869	1	PRJNA827465;Yang2025_Silkie_Wenchang	CRA004775	0.721294679
ENSGALG00010019640	MGRN1	2	-0.179650751	0.112615107	-1.595263339	0.11065336	0	0.135388006	0	1	CRA004775;Yang2025_Silkie_Wenchang	PRJNA827465	0.562832796
ENSGALG00010019640	MGRN1	2	-0.053695189	0.054782147	-0.980158533	0.32700787	0	0.117978013	0	1	CRA004775;PRJNA827465	Yang2025_Silkie_Wenchang	0.800028239
ENSGALG00010019641	CAPN11	2	0.193196008	0.127363776	1.516883486	0.129296104	0.027914976	7.164694212	86.0426702	1	PRJNA827465;Yang2025_Silkie_Wenchang	CRA004775	0.603386934
ENSGALG00010019641	CAPN11	2	-0.001187961	0.359561402	-0.003303916	0.997363861	0.228185165	7.78466662	87.15423474	0.5	CRA004775;Yang2025_Silkie_Wenchang	PRJNA827465	0.999270721
ENSGALG00010019641	CAPN11	2	-0.106786595	0.226083676	-0.472332176	0.636689707	0.076239579	3.262750825	69.35101534	0.5	CRA004775;PRJNA827465	Yang2025_Silkie_Wenchang	0.904725968
ENSGALG00010019643	RAB3GAP1	2	-0.021721616	0.034792507	-0.624318802	0.532418239	0	0.89594675	0	0.5	PRJNA827465;Yang2025_Silkie_Wenchang	CRA004775	0.873138364
ENSGALG00010019643	RAB3GAP1	2	-0.026991165	0.094218925	-0.286472855	0.774515981	0.007916303	1.612901982	37.99995218	0.5	CRA004775;Yang2025_Silkie_Wenchang	PRJNA827465	0.943695992
ENSGALG00010019643	RAB3GAP1	2	-0.049672045	0.038628876	-1.2858786	0.198485433	0	0.723982295	0	1	CRA004775;PRJNA827465	Yang2025_Silkie_Wenchang	0.710609673
ENSGALG00010019645	ENSGALG00010019645	2	0.075777295	0.09311339	0.81381738	0.415749577	0.003326284	1.236017556	19.09500027	0.5	PRJNA827465;Yang2025_Silkie_Wenchang	CRA004775	0.837701508
ENSGALG00010019645	ENSGALG00010019645	2	0.090136161	0.156767884	0.574965728	0.565314476	0.016577397	1.311792771	23.76844711	0.5	CRA004775;Yang2025_Silkie_Wenchang	PRJNA827465	0.883427937
ENSGALG00010019645	ENSGALG00010019645	2	-0.034454748	0.106359585	-0.323945864	0.74597902	0	0.335690103	0	1	CRA004775;PRJNA827465	Yang2025_Silkie_Wenchang	0.936476729
ENSGALG00010019646	VPS37C	2	0.133165313	0.104449963	1.274919685	0.202337671	0.010145336	1.560703738	35.92634043	1	PRJNA827465;Yang2025_Silkie_Wenchang	CRA004775	0.715289846
ENSGALG00010019646	VPS37C	2	0.245236098	0.160099564	1.531772428	0.125578602	0	0.678481656	0	0.5	CRA004775;Yang2025_Silkie_Wenchang	PRJNA827465	0.595010947
ENSGALG00010019646	VPS37C	2	0.067713609	0.06691609	1.011918197	0.311577168	0	0.070881355	0	0.5	CRA004775;PRJNA827465	Yang2025_Silkie_Wenchang	0.795778238
ENSGALG00010019648	PRDM16	2	0.299008684	0.229190894	1.304627244	0.192019807	0.060986318	2.365701844	57.72924628	1	PRJNA827465;Yang2025_Silkie_Wenchang	CRA004775	0.701846421
ENSGALG00010019648	PRDM16	2	0.550833989	0.197371625	2.790846914	0.005257033	0	0.001765941	0	1	CRA004775;Yang2025_Silkie_Wenchang	PRJNA827465	0.108331963
ENSGALG00010019648	PRDM16	2	0.206429633	0.207527468	0.994709929	0.319877364	0.020708655	1.220718451	18.08102853	1	CRA004775;PRJNA827465	Yang2025_Silkie_Wenchang	0.798348216
ENSGALG00010019649	TMEM63B	2	0.124913208	0.047645195	2.62173779	0.008748271	0	0.256467791	0	1	PRJNA827465;Yang2025_Silkie_Wenchang	CRA004775	0.146797439
ENSGALG00010019649	TMEM63B	2	0.088070778	0.095998963	0.917413852	0.358925817	0	0.064148032	0	1	CRA004775;Yang2025_Silkie_Wenchang	PRJNA827465	0.814241185
ENSGALG00010019649	TMEM63B	2	0.138120414	0.052767385	2.617533806	0.008856771	0	0.002869825	0	1	CRA004775;PRJNA827465	Yang2025_Silkie_Wenchang	0.147549629
ENSGALG00010019650	KBTBD8	2	-0.178425481	0.280003225	-0.637226522	0.523977305	0.149375568	20.88041548	95.21082327	0.5	PRJNA827465;Yang2025_Silkie_Wenchang	CRA004775	0.869661505
ENSGALG00010019650	KBTBD8	2	-0.436756125	0.100675435	-4.33825913	1.43616E-05	0	0.766697743	0	1	CRA004775;Yang2025_Silkie_Wenchang	PRJNA827465	0.001894138
ENSGALG00010019650	KBTBD8	2	0.086302419	0.060958953	1.415746426	0.15684976	0	0.588748495	0	0.5	CRA004775;PRJNA827465	Yang2025_Silkie_Wenchang	0.650212649
ENSGALG00010019652	TNFRSF10B	2	-0.328244186	0.346743921	-0.946647268	0.343818549	0.102953752	1.746121646	42.73022144	0.5	PRJNA827465;Yang2025_Silkie_Wenchang	CRA004775	0.806388726
ENSGALG00010019652	TNFRSF10B	2	-0.573651928	0.308781441	-1.857792772	0.063198448	0	0.257068649	0	1	CRA004775;Yang2025_Silkie_Wenchang	PRJNA827465	0.432959491
ENSGALG00010019652	TNFRSF10B	2	-0.115686718	0.293355041	-0.394357354	0.693317225	0	0.34414828	0	0.5	CRA004775;PRJNA827465	Yang2025_Silkie_Wenchang	0.920721644
ENSGALG00010019655	RTRAF	2	-0.095053733	0.117520839	-0.80882449	0.418616107	0.01826323	2.424430041	58.75319217	1	PRJNA827465;Yang2025_Silkie_Wenchang	CRA004775	0.838193076
ENSGALG00010019655	RTRAF	2	-0.205280199	0.138867234	-1.478247907	0.139341435	0	0.803139486	0	0.5	CRA004775;Yang2025_Silkie_Wenchang	PRJNA827465	0.623009874
ENSGALG00010019655	RTRAF	2	-0.010252665	0.048947351	-0.209463126	0.834086718	0	0.059029626	0	0.5	CRA004775;PRJNA827465	Yang2025_Silkie_Wenchang	0.957968425
ENSGALG00010019657	PPP1R17	2	0.497682133	0.295298421	1.685353179	0.091920451	0.078578692	1.818235145	45.00161308	1	PRJNA827465;Yang2025_Silkie_Wenchang	CRA004775	0.519297038
ENSGALG00010019657	PPP1R17	2	0.551317334	0.292855615	1.882556815	0.059760453	0.047221632	1.36838191	26.92098658	1	CRA004775;Yang2025_Silkie_Wenchang	PRJNA827465	0.423353479
ENSGALG00010019657	PPP1R17	2	0.211975291	0.238385517	0.889212119	0.373889091	0	7.39666E-06	0	1	CRA004775;PRJNA827465	Yang2025_Silkie_Wenchang	0.822579781
ENSGALG00010019659	MRPL14	2	-0.210706404	0.32053049	-0.657367742	0.510944488	0.187007385	10.52784144	90.5013767	0.5	PRJNA827465;Yang2025_Silkie_Wenchang	CRA004775	0.8654765
ENSGALG00010019659	MRPL14	2	-0.318456383	0.286268639	-1.112438947	0.265949467	0.103379855	2.560523103	60.94548029	0.5	CRA004775;Yang2025_Silkie_Wenchang	PRJNA827465	0.770078617
ENSGALG00010019659	MRPL14	2	0.084206347	0.063287064	1.330545958	0.183338455	0	0.035916487	0	1	CRA004775;PRJNA827465	Yang2025_Silkie_Wenchang	0.691796398
ENSGALG00010019670	ENSGALG00010019670	2	0.020084407	0.075536347	0.265890631	0.790323441	0	0.437388334	0	0.5	PRJNA827465;Yang2025_Silkie_Wenchang	CRA004775	0.947390191
ENSGALG00010019670	ENSGALG00010019670	2	0.047103556	0.114015753	0.413131999	0.679509906	0	0.408829849	0	0.5	CRA004775;Yang2025_Silkie_Wenchang	PRJNA827465	0.917701868
ENSGALG00010019670	ENSGALG00010019670	2	-0.020946479	0.082861699	-0.252788433	0.800431707	0	0.048296352	0	1	CRA004775;PRJNA827465	Yang2025_Silkie_Wenchang	0.950102441
ENSGALG00010019673	NID2	2	0.041391404	0.064376901	0.642954282	0.520253766	0	0.206169964	0	1	PRJNA827465;Yang2025_Silkie_Wenchang	CRA004775	0.869331539
ENSGALG00010019673	NID2	2	0.075961655	0.084110887	0.903113233	0.366465802	0	0.232734526	0	1	CRA004775;Yang2025_Silkie_Wenchang	PRJNA827465	0.819255965
ENSGALG00010019673	NID2	2	0.022553194	0.094370166	0.238986484	0.811116064	0	0.386646343	0	1	CRA004775;PRJNA827465	Yang2025_Silkie_Wenchang	0.953179464
ENSGALG00010019675	MAFK	2	0.016340935	0.081174356	0.20130661	0.840458834	0.008415062	2.704625616	63.02630597	0.5	PRJNA827465;Yang2025_Silkie_Wenchang	CRA004775	0.959662809
ENSGALG00010019675	MAFK	2	0.094333943	0.069859621	1.350335744	0.176908312	0	0.107465837	0	1	CRA004775;Yang2025_Silkie_Wenchang	PRJNA827465	0.682556882
ENSGALG00010019675	MAFK	2	-0.039989851	0.054014993	-0.740347249	0.459089318	0	0.517956541	0	0.5	CRA004775;PRJNA827465	Yang2025_Silkie_Wenchang	0.850098539
ENSGALG00010019679	RHOBTB2	2	0.019530814	0.074871175	0.260858921	0.794201305	0	0.18000264	0	0.5	PRJNA827465;Yang2025_Silkie_Wenchang	CRA004775	0.948905846
ENSGALG00010019679	RHOBTB2	2	-0.020398792	0.104607068	-0.195003958	0.845389849	0	0.007523143	0	1	CRA004775;Yang2025_Silkie_Wenchang	PRJNA827465	0.960993702
ENSGALG00010019679	RHOBTB2	2	0.036783379	0.093560014	0.393152776	0.694206646	0	0.096160938	0	0.5	CRA004775;PRJNA827465	Yang2025_Silkie_Wenchang	0.920728119
ENSGALG00010019680	CCDC34	2	-0.100122663	0.083386508	-1.200705786	0.229865349	0	0.289988073	0	1	PRJNA827465;Yang2025_Silkie_Wenchang	CRA004775	0.742767343
ENSGALG00010019680	CCDC34	2	-0.112842786	0.146298393	-0.771319381	0.440517647	0	0.789507595	0	0.5	CRA004775;Yang2025_Silkie_Wenchang	PRJNA827465	0.844733855
ENSGALG00010019680	CCDC34	2	-0.055565546	0.093455672	-0.594565794	0.552133751	0	0.433097861	0	0.5	CRA004775;PRJNA827465	Yang2025_Silkie_Wenchang	0.879061525
ENSGALG00010019682	TMEM158	2	-0.093823224	0.170954772	-0.54881898	0.583129684	0	0.109272187	0	1	PRJNA827465;Yang2025_Silkie_Wenchang	CRA004775	0.890299155
ENSGALG00010019682	TMEM158	2	0.533936219	0.613945187	0.869680601	0.384474976	0.586456062	4.302277283	76.75649582	0.5	CRA004775;Yang2025_Silkie_Wenchang	PRJNA827465	0.826099934
ENSGALG00010019682	TMEM158	2	0.463119935	0.673338109	0.687797006	0.491580622	0.76557655	5.951045593	83.19623024	0.5	CRA004775;PRJNA827465	Yang2025_Silkie_Wenchang	0.85895707
ENSGALG00010019683	TMEM184A	2	-0.152078951	0.306471546	-0.496225354	0.619735425	0.146295591	4.518291034	77.86773821	0.5	PRJNA827465;Yang2025_Silkie_Wenchang	CRA004775	0.899325257
ENSGALG00010019683	TMEM184A	2	-0.351567736	0.177005002	-1.986202265	0.047010869	0	0.978923604	0	1	CRA004775;Yang2025_Silkie_Wenchang	PRJNA827465	0.374081275
ENSGALG00010019683	TMEM184A	2	0.09130461	0.170127386	0.536683788	0.591486056	0	0.340380863	0	0.5	CRA004775;PRJNA827465	Yang2025_Silkie_Wenchang	0.892776493
ENSGALG00010019684	PIGQ	2	-0.074484803	0.131925114	-0.564599122	0.572346462	0.017473166	1.946670398	48.63023546	0.5	PRJNA827465;Yang2025_Silkie_Wenchang	CRA004775	0.885662153
ENSGALG00010019684	PIGQ	2	-0.230706055	0.129403853	-1.782837598	0.074612743	0	0.000279463	0	1	CRA004775;Yang2025_Silkie_Wenchang	PRJNA827465	0.471679921
ENSGALG00010019684	PIGQ	2	-0.021050183	0.108418878	-0.194156072	0.846053678	0.002886103	1.090709219	8.31653549	0.5	CRA004775;PRJNA827465	Yang2025_Silkie_Wenchang	0.96122283
ENSGALG00010019690	ENSGALG00010019690	2	-0.253427252	0.141794158	-1.787289805	0.073890661	0.023745982	1.873580083	46.6262473	1	PRJNA827465;Yang2025_Silkie_Wenchang	CRA004775	0.469467289
ENSGALG00010019690	ENSGALG00010019690	2	-0.398796099	0.187478079	-2.127161221	0.03340669	0	0.49622062	0	1	CRA004775;Yang2025_Silkie_Wenchang	PRJNA827465	0.314027665
ENSGALG00010019690	ENSGALG00010019690	2	-0.168086686	0.060806388	-2.764293243	0.005704625	0	0.008700855	0	1	CRA004775;PRJNA827465	Yang2025_Silkie_Wenchang	0.113466024
ENSGALG00010019692	MMEL1	2	0.734473926	0.492914684	1.490062986	0.136207675	0.396731628	5.120734387	80.47155106	1	PRJNA827465;Yang2025_Silkie_Wenchang	CRA004775	0.617958013
ENSGALG00010019692	MMEL1	2	1.223217984	0.314252904	3.892463587	9.92314E-05	0	0.079885456	0	1	CRA004775;Yang2025_Silkie_Wenchang	PRJNA827465	0.007784614
ENSGALG00010019692	MMEL1	2	0.55703814	0.377441891	1.475824898	0.139990905	0.172633988	2.120016238	52.83054996	1	CRA004775;PRJNA827465	Yang2025_Silkie_Wenchang	0.623792706
ENSGALG00010019693	HAUS2	2	0.004393123	0.056151965	0.078236317	0.937640074	0	0.402669819	0	0.5	PRJNA827465;Yang2025_Silkie_Wenchang	CRA004775	0.985191355
ENSGALG00010019693	HAUS2	2	-0.116721661	0.1380469	-0.845521779	0.397819578	0	0.019572317	0	1	CRA004775;Yang2025_Silkie_Wenchang	PRJNA827465	0.831719925
ENSGALG00010019693	HAUS2	2	0.005962926	0.057254409	0.104147894	0.917051983	0	0.467866222	0	0.5	CRA004775;PRJNA827465	Yang2025_Silkie_Wenchang	0.980728718
ENSGALG00010019697	EIF4EBP1	2	-0.004653781	0.18043211	-0.025792423	0.979422905	0.04646925	2.998050488	66.64499134	0.5	PRJNA827465;Yang2025_Silkie_Wenchang	CRA004775	0.994401035
ENSGALG00010019697	EIF4EBP1	2	0.081094675	0.221956614	0.365362734	0.714840655	0.030271486	1.357182943	26.31796584	0.5	CRA004775;Yang2025_Silkie_Wenchang	PRJNA827465	0.926755613
ENSGALG00010019697	EIF4EBP1	2	-0.147561119	0.071463536	-2.064844916	0.038937676	0	0.084396423	0	1	CRA004775;PRJNA827465	Yang2025_Silkie_Wenchang	0.340535713
ENSGALG00010019700	HES5B	2	-0.221850759	0.220461414	-1.006301988	0.314270324	0.064647749	2.927869482	65.84547207	1	PRJNA827465;Yang2025_Silkie_Wenchang	CRA004775	0.796531448
ENSGALG00010019700	HES5B	2	-0.445960065	0.201519928	-2.21298246	0.026898855	0	0.141004174	0	1	CRA004775;Yang2025_Silkie_Wenchang	PRJNA827465	0.279388398
ENSGALG00010019700	HES5B	2	-0.026954807	0.15137613	-0.178065111	0.858671842	0	0.025215424	0	1	CRA004775;PRJNA827465	Yang2025_Silkie_Wenchang	0.964449906
ENSGALG00010019706	NPRL3	2	-0.124118471	0.080675018	-1.538499445	0.123926544	0.005938014	1.828180685	45.30081143	1	PRJNA827465;Yang2025_Silkie_Wenchang	CRA004775	0.590386056
ENSGALG00010019706	NPRL3	2	-0.061711399	0.083925807	-0.735308978	0.462151342	0	0.52501978	0	1	CRA004775;Yang2025_Silkie_Wenchang	PRJNA827465	0.850747865
ENSGALG00010019706	NPRL3	2	-0.19940843	0.073425273	-2.715801007	0.006611564	0	0.001700969	0	1	CRA004775;PRJNA827465	Yang2025_Silkie_Wenchang	0.124213344
ENSGALG00010019713	NUDT14	2	-0.23321063	0.077164223	-3.022263685	0.002508919	0	0.25610863	0	1	PRJNA827465;Yang2025_Silkie_Wenchang	CRA004775	0.067401258
ENSGALG00010019713	NUDT14	2	-0.276741412	0.109737736	-2.521843657	0.011674158	0	0.014705377	0	1	CRA004775;Yang2025_Silkie_Wenchang	PRJNA827465	0.172876725
ENSGALG00010019713	NUDT14	2	-0.212118091	0.088341381	-2.401118124	0.016345059	0	0.04628411	0	1	CRA004775;PRJNA827465	Yang2025_Silkie_Wenchang	0.211447764
ENSGALG00010019716	VEGFA	2	0.333907296	0.250325631	1.333891762	0.182239332	0.119895239	23.06365378	95.66417355	1	PRJNA827465;Yang2025_Silkie_Wenchang	CRA004775	0.690033956
ENSGALG00010019716	VEGFA	2	0.527001594	0.116866674	4.509425802	6.50033E-06	0.010993588	1.348056649	25.81914115	1	CRA004775;Yang2025_Silkie_Wenchang	PRJNA827465	0.001012111
ENSGALG00010019716	VEGFA	2	0.10101749	0.066386017	1.521668182	0.128092244	0	0.705097973	0	1	CRA004775;PRJNA827465	Yang2025_Silkie_Wenchang	0.600574823
ENSGALG00010019717	ZRANB3	2	0.171678774	0.173283122	0.990741464	0.321811839	0.042278687	3.163917341	68.39361171	1	PRJNA827465;Yang2025_Silkie_Wenchang	CRA004775	0.798513866
ENSGALG00010019717	ZRANB3	2	0.309012609	0.150744102	2.04991509	0.040372717	0	0.640579891	0	1	CRA004775;Yang2025_Silkie_Wenchang	PRJNA827465	0.347442284
ENSGALG00010019717	ZRANB3	2	0.031731772	0.091081634	0.348388265	0.727548614	0	0.047421006	0	1	CRA004775;PRJNA827465	Yang2025_Silkie_Wenchang	0.931630234
ENSGALG00010019726	PEBP4	2	-0.129871396	0.182448976	-0.711823105	0.476574323	0	0.210209384	0	0.5	PRJNA827465;Yang2025_Silkie_Wenchang	CRA004775	0.854986476
ENSGALG00010019726	PEBP4	2	-0.088205694	0.346144639	-0.254823227	0.798859635	0	0.353191958	0	0.5	CRA004775;Yang2025_Silkie_Wenchang	PRJNA827465	0.949922217
ENSGALG00010019726	PEBP4	2	-0.194150823	0.19568882	-0.992140598	0.321128945	0	0.135457282	0	1	CRA004775;PRJNA827465	Yang2025_Silkie_Wenchang	0.798513866
ENSGALG00010019731	PANK4	2	0.211542994	0.193520742	1.093128271	0.274337485	0.067812806	10.39280312	90.37795686	1	PRJNA827465;Yang2025_Silkie_Wenchang	CRA004775	0.774526342
ENSGALG00010019731	PANK4	2	0.173788568	0.287419486	0.604651307	0.545410708	0.129636498	4.152355843	75.91728557	0.5	CRA004775;Yang2025_Silkie_Wenchang	PRJNA827465	0.877636727
ENSGALG00010019731	PANK4	2	0.015406734	0.062090068	0.248135244	0.804029767	0	0.511024116	0	0.5	CRA004775;PRJNA827465	Yang2025_Silkie_Wenchang	0.951372288
ENSGALG00010019732	HBA1	2	0.052495804	0.17192303	0.305344804	0.760103556	0.021116315	1.511178978	33.82650139	0.5	PRJNA827465;Yang2025_Silkie_Wenchang	CRA004775	0.939294282

ENSGALG00010019732	HBA1	2	-0.212353956	0.181177705	-1.172075535	0.241166731	0	0.098682994	0	1	CRA004775;Yang2025_Silkie_Wenchang	PRJNA827465	0.753758453
ENSGALG00010019732	HBA1	2	0.010492208	0.227605277	0.046098265	0.963231929	0.056551183	2.058056102	51.41045965	0.5	CRA004775;PRJNA827465	Yang2025_Silkie_Wenchang	0.989998461
ENSGALG00010019735	ENSGALG00010019735	2	0.08936172	0.266612457	0.335174587	0.737493391	0.107989142	3.774688169	73.5077454	0.5	PRJNA827465;Yang2025_Silkie_Wenchang	CRA004775	0.933968189
ENSGALG00010019735	ENSGALG00010019735	2	0.333584466	0.214888951	1.552357461	0.120576716	0	0.297831746	0	1	CRA004775;Yang2025_Silkie_Wenchang	PRJNA827465	0.582190009
ENSGALG00010019735	ENSGALG00010019735	2	-0.112026161	0.107694273	-1.040223941	0.298235871	0	0.475514579	0	0.5	CRA004775;PRJNA827465	Yang2025_Silkie_Wenchang	0.789118382
ENSGALG00010019738	ADRA2A	2	-0.394136557	0.180426532	-2.184471164	0.028927644	0.020244545	1.449990589	31.03403513	1	PRJNA827465;Yang2025_Silkie_Wenchang	CRA004775	0.289667055
ENSGALG00010019738	ADRA2A	2	-0.521503683	0.206331701	-2.527501496	0.011487733	0	0.708373409	0	1	CRA004775;Yang2025_Silkie_Wenchang	PRJNA827465	0.171088438
ENSGALG00010019738	ADRA2A	2	-0.202468502	0.194873975	-1.038971477	0.298818002	0	0.087057002	0	1	CRA004775;PRJNA827465	Yang2025_Silkie_Wenchang	0.78974322
ENSGALG00010019752	GPAM	2	0.18978361	0.090425067	2.098794254	0.035835042	0	0.020703057	0	1	PRJNA827465;Yang2025_Silkie_Wenchang	CRA004775	0.326780659
ENSGALG00010019752	GPAM	2	0.071127169	0.162596893	0.437444823	0.661788783	0.009782255	1.171318718	14.62614024	0.5	CRA004775;Yang2025_Silkie_Wenchang	PRJNA827465	0.912545506
ENSGALG00010019752	GPAM	2	0.097842426	0.171793435	0.569535302	0.56899292	0.027813325	1.558873155	35.85109881	0.5	CRA004775;PRJNA827465	Yang2025_Silkie_Wenchang	0.884492059
ENSGALG00010019761	MRPS18A	2	-0.151398473	0.173648257	-0.87186866	0.383280032	0.045890454	3.788981224	73.6076813	1	PRJNA827465;Yang2025_Silkie_Wenchang	CRA004775	0.825476201
ENSGALG00010019761	MRPS18A	2	-0.362523351	0.141277493	-2.566037542	0.010286769	0	0.000906009	0	1	CRA004775;Yang2025_Silkie_Wenchang	PRJNA827465	0.161140254
ENSGALG00010019761	MRPS18A	2	-0.096924591	0.15672612	-0.618432916	0.536290006	0.027508883	1.719662903	41.84906832	1	CRA004775;PRJNA827465	Yang2025_Silkie_Wenchang	0.874599474
ENSGALG00010019767	SUCLG2	2	-0.102293174	0.063259092	-1.617050929	0.105867288	0	0.011033574	0	1	PRJNA827465;Yang2025_Silkie_Wenchang	CRA004775	0.552063422
ENSGALG00010019767	SUCLG2	2	-0.097987007	0.094387978	-1.03813016	0.299209462	0	0.122160096	0	1	CRA004775;Yang2025_Silkie_Wenchang	PRJNA827465	0.78978392
ENSGALG00010019767	SUCLG2	2	-0.08962486	0.077581698	-1.155231994	0.247995436	0	0.095198083	0	1	CRA004775;PRJNA827465	Yang2025_Silkie_Wenchang	0.758223619
ENSGALG00010019771	VWCE	2	0.353729909	0.201940812	1.751651416	0.079833766	0	0.014171779	0	1	PRJNA827465;Yang2025_Silkie_Wenchang	CRA004775	0.486023928
ENSGALG00010019771	VWCE	2	0.411165495	0.268893038	1.529104275	0.126238596	0	0.066136088	0	1	CRA004775;Yang2025_Silkie_Wenchang	PRJNA827465	0.596627648
ENSGALG00010019771	VWCE	2	0.367038407	0.254036415	1.444825963	0.148506776	0	0.108054338	0	1	CRA004775;PRJNA827465	Yang2025_Silkie_Wenchang	0.63857057
ENSGALG00010019772	ENSGALG00010019772	2	-0.016449449	0.069161704	-0.237840422	0.812004866	0	0.07417489	0	1	PRJNA827465;Yang2025_Silkie_Wenchang	CRA004775	0.953371387
ENSGALG00010019772	ENSGALG00010019772	2	-0.162726291	0.22750823	-0.715254525	0.474451783	0.050707768	1.923432589	48.00961544	0.5	CRA004775;Yang2025_Silkie_Wenchang	PRJNA827465	0.854462864
ENSGALG00010019772	ENSGALG00010019772	2	-0.144005781	0.184077595	-0.782310205	0.434032291	0.043000137	2.143041609	53.33735024	1	CRA004775;PRJNA827465	Yang2025_Silkie_Wenchang	0.842762754
ENSGALG00010019778	ABHD17C	2	-0.089570121	0.061345571	-1.460091082	0.144265043	0	0.92132542	0	1	PRJNA827465;Yang2025_Silkie_Wenchang	CRA004775	0.631870428
ENSGALG00010019778	ABHD17C	2	0.087209193	0.32004631	0.272489294	0.785245822	0.167686079	5.224453952	80.85961004	0.5	CRA004775;Yang2025_Silkie_Wenchang	PRJNA827465	0.946325593
ENSGALG00010019778	ABHD17C	2	0.135972407	0.244702297	0.555664613	0.578440152	0.092647294	3.749159544	73.32735542	0.5	CRA004775;PRJNA827465	Yang2025_Silkie_Wenchang	0.887855709
ENSGALG00010019785	ACSL5	2	-0.125568958	0.345863725	-0.363059056	0.716560769	0.119873841	1.973969372	49.34065269	0.5	PRJNA827465;Yang2025_Silkie_Wenchang	CRA004775	0.927487786
ENSGALG00010019785	ACSL5	2	-0.19056142	0.34955061	-0.545161172	0.585642674	0.086954581	1.450024289	31.035638	0.5	CRA004775;Yang2025_Silkie_Wenchang	PRJNA827465	0.891161809
ENSGALG00010019785	ACSL5	2	0.283682007	0.317171953	0.894410759	0.37110218	0	0.005940445	0	1	CRA004775;PRJNA827465	Yang2025_Silkie_Wenchang	0.8214684
ENSGALG00010019786	R3HDM1	2	0.144655868	0.274587038	0.526812444	0.598323841	0.130516206	6.662301558	84.99017207	0.5	PRJNA827465;Yang2025_Silkie_Wenchang	CRA004775	0.894645663
ENSGALG00010019786	R3HDM1	2	0.471393499	0.151593998	3.109578903	0.001873542	0	0.007457994	0	1	CRA004775;Yang2025_Silkie_Wenchang	PRJNA827465	0.056658228
ENSGALG00010019786	R3HDM1	2	0.155290189	0.287480091	0.540177193	0.589074839	0.142978	6.615239654	84.88338968	0.5	CRA004775;PRJNA827465	Yang2025_Silkie_Wenchang	0.892171138
ENSGALG00010019789	ARNT2	2	0.064982469	0.056603174	1.148035777	0.250953795	0	0.936100012	0	1	PRJNA827465;Yang2025_Silkie_Wenchang	CRA004775	0.760758735
ENSGALG00010019789	ARNT2	2	0.191646037	0.14020964	1.366853505	0.171671228	0	0.023644549	0	1	CRA004775;Yang2025_Silkie_Wenchang	PRJNA827465	0.674247454
ENSGALG00010019789	ARNT2	2	0.045147336	0.060305196	0.748647525	0.45406968	0	0.059434948	0	1	CRA004775;PRJNA827465	Yang2025_Silkie_Wenchang	0.849072665
ENSGALG00010019794	RSPH9	2	0.034255714	0.12850939	0.266561953	0.789806453	0	0.088775295	0	0.5	PRJNA827465;Yang2025_Silkie_Wenchang	CRA004775	0.947390191
ENSGALG00010019794	RSPH9	2	-0.066389062	0.2062808	-0.321838301	0.747575197	0	0.209784198	0	1	CRA004775;Yang2025_Silkie_Wenchang	PRJNA827465	0.93680487
ENSGALG00010019794	RSPH9	2	0.029726353	0.148102069	0.200715311	0.840921187	0	0.414510953	0	0.5	CRA004775;PRJNA827465	Yang2025_Silkie_Wenchang	0.95980036
ENSGALG00010019799	DOK2	2	-0.049407659	0.337478854	-0.146402236	0.883603862	0.161369194	3.386188084	70.4682677	0.5	PRJNA827465;Yang2025_Silkie_Wenchang	CRA004775	0.972001076
ENSGALG00010019799	DOK2	2	-0.608919019	0.337992561	-1.80157521	0.071612264	0.068577208	1.293156045	22.66981205	1	CRA004775;Yang2025_Silkie_Wenchang	PRJNA827465	0.46178455
ENSGALG00010019799	DOK2	2	-0.348602516	0.71824429	-0.485353689	0.627425477	0.840621761	4.869009752	79.46194296	0.5	CRA004775;PRJNA827465	Yang2025_Silkie_Wenchang	0.901733409
ENSGALG00010019800	EXT2	2	-0.285759614	0.319321771	-0.894895495	0.370842955	0.200263202	15.44775083	98.19650034	0.5	PRJNA827465;Yang2025_Silkie_Wenchang	CRA004775	0.8214684
ENSGALG00010019800	EXT2	2	-0.282126175	0.358470232	-0.787029801	0.431264421	0.229146039	8.555225149	88.3112369	0.5	CRA004775;Yang2025_Silkie_Wenchang	PRJNA827465	0.842503929
ENSGALG00010019800	EXT2	2	0.034834055	0.04759853	0.731830475	0.464272046	0	0.115104602	0	1	CRA004775;PRJNA827465	Yang2025_Silkie_Wenchang	0.852159649
ENSGALG00010019801	FIBIN	2	0.068331953	0.141294639	0.483613206	0.6286604	0	0.04809874	0	1	PRJNA827465;Yang2025_Silkie_Wenchang	CRA004775	0.901991009
ENSGALG00010019801	FIBIN	2	0.003247092	0.183033265	0.017740445	0.985845915	0	0.078877877	0	0.5	CRA004775;Yang2025_Silkie_Wenchang	PRJNA827465	0.996013375
ENSGALG00010019801	FIBIN	2	0.057077868	0.167537957	0.34068619	0.733319836	0	0.195262897	0	0.5	CRA004775;PRJNA827465	Yang2025_Silkie_Wenchang	0.933182392
ENSGALG00010019803	FAH	2	-0.013186189	1.046096484	-0.012605136	0.989942823	1.829130183	6.036799626	83.43493139	0.5	PRJNA827465;Yang2025_Silkie_Wenchang	CRA004775	0.997312206
ENSGALG00010019803	FAH	2	-0.805377669	0.412248726	-1.953620759	0.050746084	0	0.43335847	0	1	CRA004775;Yang2025_Silkie_Wenchang	PRJNA827465	0.38992636
ENSGALG00010019803	FAH	2	0.307790821	0.767288695	0.401140826	0.688316444	0.72704951	2.614164997	61.7468675	0.5	CRA004775;PRJNA827465	Yang2025_Silkie_Wenchang	0.919768134
ENSGALG00010019804	ELFN1	2	-0.270774903	0.396133681	-0.683544256	0.494262995	0.259007861	5.715979517	82.56018573	0.5	PRJNA827465;Yang2025_Silkie_Wenchang	CRA004775	0.86037061
ENSGALG00010019804	ELFN1	2	0.176943921	0.220015297	0.804234628	0.421261484	0	0.173162188	0	1	CRA004775;Yang2025_Silkie_Wenchang	PRJNA827465	0.839352211
ENSGALG00010019804	ELFN1	2	-0.245832261	0.502507981	-0.489210661	0.624692563	0.374660321	3.509837121	71.50864939	0.5	CRA004775;PRJNA827465	Yang2025_Silkie_Wenchang	0.901001534
ENSGALG00010019807	ZDHHC6	2	-0.17309996	0.172821768	-1.001035246	0.3168001	0.048311767	5.198493576	80.76365806	1	PRJNA827465;Yang2025_Silkie_Wenchang	CRA004775	0.796959041
ENSGALG00010019807	ZDHHC6	2	-0.296319697	0.123599782	-2.397412776	0.016511312	0.004714176	1.095433909	8.711973197	1	CRA004775;Yang2025_Silkie_Wenchang	PRJNA827465	0.212735188
ENSGALG00010019807	ZDHHC6	2	-0.00805881	0.091652038	-0.087928324	0.929933645	0	0.003029828	0	1	CRA004775;PRJNA827465	Yang2025_Silkie_Wenchang	0.983813577
ENSGALG00010019810	ZFAND6	2	-0.330803713	0.261589317	-1.264591831	0.206017683	0.12657803	13.01849749	92.31862201	1	PRJNA827465;Yang2025_Silkie_Wenchang	CRA004775	0.718403416
ENSGALG00010019810	ZFAND6	2	-0.346952119	0.304628831	-1.138959965	0.254730708	0.142389731	3.926951989	74.53490732	0.5	CRA004775;Yang2025_Silkie_Wenchang	PRJNA827465	0.754394153
ENSGALG00010019810	ZFAND6	2	-0.075060103	0.066335665	-1.13151957	0.257836471	0	0.104316066	0	0.5	CRA004775;PRJNA827465	Yang2025_Silkie_Wenchang	0.765151037
ENSGALG00010019811	UBXN4	2	0.062370922	0.108230043	0.576281039	0.564425237	0.016752265	3.273135666	69.4482569	0.5	PRJNA827465;Yang2025_Silkie_Wenchang	CRA004775	0.883094571
ENSGALG00010019811	UBXN4	2	0.159214541	0.101802846	1.563949909	0.117829356	0	0.909578151	0	0.5	CRA004775;Yang2025_Silkie_Wenchang	PRJNA827465	0.617762053
ENSGALG00010019811	UBXN4	2	-0.032057876	0.056395193	-0.568450504	0.569729105	0	0.10893544	0	1	CRA004775;PRJNA827465	Yang2025_Silkie_Wenchang	0.884921871
ENSGALG00010019813	ENSGALG00010019813	2	-0.377184018	0.552720922	-0.682413136	0.494977752	0.522029353	6.850389419	85.40228973	0.5	PRJNA827465;Yang2025_Silkie_Wenchang	CRA004775	0.860496668
ENSGALG00010019813	ENSGALG00010019813	2	-0.683370686	0.268518911	-2.544962971	0.010928933	0.050798653	1.529493822	34.61889252	1	CRA004775;Yang2025_Silkie_Wenchang	PRJNA827465	0.166338515
ENSGALG00010019813	ENSGALG00010019813	2	-0.083056824	0.281720737	-0.294819368	0.768131614	0.050511058	1.465717886	31.76473592	0.5	CRA004775;PRJNA827465	Yang2025_Silkie_Wenchang	0.941444269
ENSGALG00010019820	BBOX1	2	-0.016638907	0.106878841	-0.155680085	0.876285195	0	0.380463205	0	0.5	PRJNA827465;Yang2025_Silkie_Wenchang	CRA004775	0.970347777
ENSGALG00010019820	BBOX1	2	0.064529697	0.151180435	0.426838945	0.669496639	0	0.010080028	0	1	CRA004775;Yang2025_Silkie_Wenchang	PRJNA827465	0.914377769
ENSGALG00010019820	BBOX1	2	-0.055880539	0.134491054	-0.415496327	0.677778603	0	0.16308394	0	0.5	CRA004775;PRJNA827465	Yang2025_Silkie_Wenchang	0.917374904
ENSGALG00010019823	LCT	2	0.141592232	0.183223817	0.772782897	0.439650877	0	0.01732004	0	1	PRJNA827465;Yang2025_Silkie_Wenchang	CRA004775	0.844278492
ENSGALG00010019823	LCT	2	0.395927145	0.314631737	1.258382732	0.208253374	0.091190459	1.839028161	45.62345366	1	CRA004775;Yang2025_Silkie_Wenchang	PRJNA827465	0.720917211
ENSGALG00010019823	LCT	2	0.387769863	0.284169868	1.364570656	0.172388033	0.078675431	1.864876861	46.37715654	1	CRA004775;PRJNA827465	Yang2025_Silkie_Wenchang	0.675240867
ENSGALG00010019825	BCL2A1	2	0.070320296	0.204832498	0.343306344	0.731368009	0	0.483870199	0	0.5	PRJNA827465;Yang2025_Silkie_Wenchang	CRA004775	0.932851968
ENSGALG00010019825	BCL2A1	2	-0.387189067	0.334845705	-1.156320841	0.247549947	0.094885604	1.70664655	41.40555933	1	CRA004775;Yang2025_Silkie_Wenchang	PRJNA827465	0.757947282
ENSGALG00010019825	BCL2A1	2	-0.242621138	0.480445049	-0.504992482	0.613564116	0.34670121	3.891967972	74.30605783	0.5	CRA004775;PRJNA827465	Yang2025_Silkie_Wenchang	0.898256044
ENSGALG00010019830	JAG2	2	-0.110298493	0.055139875	-2.00033992	0.045463571	0	0.077243732	0	1	PRJNA827465;Yang2025_Silkie_Wenchang	CRA004775	0.367665584
ENSGALG00010019830	JAG2	2	-0.011008159	0.153954363	-0.071502142	0.942997643	0.023471793	1.516622858	34.06402951	0.5	CRA004775;Yang2025_Silkie_Wenchang	PRJNA827465	0.985827325
ENSGALG00010019830	JAG2	2	-0.020394244	0.17576524	-0.116031154	0.907627851	0.036182124	1.810049416	44.75288954	0.5	CRA004775;PRJNA827465	Yang2025_Silkie_Wenchang	0.978196186
ENSGALG00010019832	MAD2L1BP	2	-0.257172635	0.177243311	-1.450958195	0.146791501	0.051575455	5.459694294	81.68395617	1	PRJNA827465;Yang2025_Silkie_Wenchang	CRA004775	0.635558525
ENSGALG00010019832	MAD2L1BP	2	-0.45860263	0.12191811	-3.761562837	0.000168855	0	0.113659865	0	1	CRA004775;Yang2025_Silkie_Wenchang	PRJNA827465	0.011325098
ENSGALG00010019832	MAD2L1BP	2	-0.152106983	0.165632251	-0.918345181	0.358440064	0.023912556	1.218811687	17.95258705	1	CRA004775;PRJNA827465	Yang2025_Silkie_Wenchang	0.814400058
ENSGALG00010019833	MTHFS	2	-0.064715087	0.127255058	-0.508546282	0.61107029	0.014718177	1.798230203	44.38976732	0.5	PRJNA827465;Yang2025_Silkie_Wenchang	CRA004775	0.897559444
ENSGALG00010019833	MTHFS	2	-0.199535697	0.140166576	-1.423561182	0.154573539	0	0.04776051	0	1	CRA004775;Yang2025_Silkie_Wenchang	PRJNA827465	0.647400237
ENSGALG00010019833	MTHFS	2	0.026915528	0.109756189	0.245230165	0.806278221	0	0.225682168	0	0.5	CRA004775;PRJNA827465	Yang2025_Silkie_Wenchang	0.952174506
ENSGALG00010019834	PLCH2	2	0.358518888	0.248107924	1.445011838	0.148454561	0.079542385	2.697598879	62.92999646	1	PRJNA827465;Yang2025_Silkie_Wenchang	CRA004775	0.638510571
ENSGALG00010019834	PLCH2	2	0.861402319	0.294705901	2.922921857	0.003467635	0.064134265	1.494447897	33.08565644	1	CRA004775;Yang2025_Silkie_Wenchang	PRJNA827465	0.083153429
ENSGALG00010019834	PLCH2	2	0.636738536	0.557880119	1.141353697	0.253722765	0.523962131	5.792141911	82.73522964	1	CRA004775;PRJNA827465	Yang2025_Silkie_Wenchang	0.763418915
ENSGALG00010019838	ALX4	2	-0.308127065	0.170827803	-1.803725912	0.071272799	0.01861459	1.466957383	31.83169384	1	PRJNA827465;Yang2025_Silkie_Wenchang	CRA004775	0.461027938
ENSGALG00010019838	ALX4	2	0.436995423	0.964776755	0.452949785	0.650584901	1.741503205	15.08777897	93.37211924	0.5	CRA004775;Yang2025_Silkie_Wenchang	PRJNA827465	0.908636451
ENSGALG00010019838	ALX4	2	0.597322824	0.792948351	0.753293481	0.451273575	1.142050536	10.43552286	90.41734647	0.5	CRA004775;PRJNA827465	Yang2025_Silkie_Wenchang	0.847847413
ENSGALG00010019843	MCM6	2	-0.120211398	0.095406764	-1.259988209	0.207673616	0.010549004	2.075588797	51.8209001	1	PRJNA827465;Yang2025_Silkie_Wenchang	CRA004775	0.720024358
ENSGALG00010019843	MCM6	2	-0.223609086	0.116093222	-1.92611664	0.054089811	0	0.31119583	0	1	CRA004775;Yang2025_Silkie_Wenchang	PRJNA827465	0.403227287
ENSGALG00010019843	MCM6	2	-0.053469664	0.055311438	-0.966701749	0.333693152	0	0.013929185	0	1	CRA004775;PRJNA827465	Yang2025_Silkie_Wenchang	0.802691373
ENSGALG00010019846	ENSGALG00010019846	2	-0.018526541	0.063933209	-0.289779621	0.77198484	0	0.56767328	0	0.5	PRJNA827465;Yang2025_Silkie_Wenchang	CRA004775	0.943066211
ENSGALG00010019846	ENSGALG00010019846	2	-0.077572874	0.154576271	-0.501842059	0.615778625	0.027762406	2.246482059	55.48595654	0.5	CRA004775;Yang2025_Silkie_Wenchang	PRJNA827465	0.898956845
ENSGALG00010019846	ENSGALG00010019846	2	-0.099858815	0.088239388	-1.131680734	0.257768684	0.003607423	1.184202634	15.55499279	1	CRA004775;PRJNA827465	Yang2025_Silkie_Wenchang	0.765151037
ENSGALG00010019848	MAD1L1	2	-0.040753395	0.207726066	-0.19618816	0.844462895	0.075481001	7.789865907	87.16280855	0.5	PRJNA827465;Yang2025_Silkie_Wenchang	CRA004775	0.960752464
ENSGALG00010019848	MAD1L1	2	-0.07190701	0.263810214	-0.272570985	0.785183018	0.116622273	6.094671145	83.59222383	0.5	CRA004775;Yang2025_Silkie_Wenchang	PRJNA827465	0.946325593
ENSGALG00010019848	MAD1L1	2	-0.255641298	0.070830193	-3.609213647	0.000307127	0	0.352443585	0	1	CRA004775;PRJNA827465	Yang2025_Silkie_Wenchang	0.017244281
ENSGALG00010019854	CCDC14	2	0.236169952	0.149558766	1.5791114	0.114310507	0.032989905	3.545627588	71.79624833	1	PRJNA827465;Yang2025_Silkie_Wenchang	CRA004775	0.57019364
ENSGALG00010019854	CCDC14	2	0.098403939	0.334252291	0.294400194	0.768452118	0.189877167	6.511931762	84.64357373	0.5	CRA004775;Yang2025_Silkie_Wenchang	PRJNA827465	0.941444269
ENSGALG00010019854	CCDC14	2	-0.0154966	0.17371339	-0.08920786	0.928916719	0.04024152	2.491500695	59.86354722	0.5	CRA004775;PRJNA827465	Yang2025_Silkie_Wenchang	0.983492641
ENSGALG00010019855	TTC7A	2	-0.029346484	0.063637723	-0.461149176	0.644691582	0	0.009760555	0	1	PRJNA827465;Yang2025_Silkie_Wenchang	CRA004775	0.906953008
ENSGALG00010019855	TTC7A	2	-0.045951152	0.129600018	-0.354561308	0.722918267	0	0.01128641	0	1	CRA004775;Yang2025_Silkie_Wenchang	PRJNA827465	0.930032096
ENSGALG00010019855	TTC7A	2	-0.027892412	0.070789811	-0.394017321	0.693568251	0	0.021745318	0	1	CRA004775;PRJNA827465	Yang2025_Silkie_Wenchang	0.920721644
ENSGALG00010019859	BIN3	2	0.072924061	0.074514501	0.978655955	0.327750002	0.001267065	1.106627595	9.635363816	1	PRJNA827465;Yang2025_Silkie_Wenchang	CRA004775	0.800295621
ENSGALG00010019859	BIN3	2	-0.124338069	0.316855339	-0.39241273	0.694753281	0.179631584	9.453235864	89.42161166	0.5	CRA004775;Yang2025_Silkie_Wenchang	PRJNA827465	0.92082578
ENSGALG00010019859	BIN3	2	-0.19216184	0.234999527	-0.817711603	0.413521884	0.095174448	6.924755093	85.55905607	0.5	CRA004775;PRJNA827465	Yang2025_Silkie_Wenchang	0.836246395
ENSGALG00010019865	ENSGALG00010019865	2	-0.186256637	0.067275976	-2.768546064	0.005630703	0	0.765223413	0	1	PRJNA827465;Yang2025_Silkie_Wenchang	CRA004775	0.112565362
ENSGALG00010019865	ENSGALG00010019865	2	-0.194300886	0.181542382	-1.070278377	0.284494024	0.021534997	1.377475891	27.40344814	0.5	CRA004775;Yang2025_Silkie_Wenchang	PRJNA827465	0.780265906
ENSGALG00010019865	ENSGALG00010019865	2	-0.14473943	0.071980207	-2.010822639	0.044344194	0	0.624526904	0	0.5	CRA004775;PRJNA827465	Yang2025_Silkie_Wenchang	0.363642137
ENSGALG00010019867	PEX11A	2	-0.203382735	0.54658152	-0.372099545	0.709818735	0.453955904	4.116530083	75.70769605	0.5	PRJNA827465;Yang2025_Silkie_Wenchang	CRA004775	0.925298042
ENSGALG00010019867	PEX11A	2	-0.736822648	0.337509798	-2.183114838	0.029027354	0	0.032268061	0	1	CRA004775;Yang2025_Silkie_Wenchang	PRJNA827465	0.289904262
ENSGALG00010019867	PEX11A	2	-0.07182159	0.474104994	-0.151488786	0.879590157	0.261770713	2.256583413	55.6852189	0.5	CRA004775;PRJNA827465	Yang2025_Silkie_Wenchang	0.971364438
ENSGALG00010019872	VTI1A	2	0.272229674	0.20817275	1.307710421	0.190971547	0.078025788	9.870358843	89.86865609	1	PRJNA827465;Yang2025_Silkie_Wenchang	CRA004775	0.700443419
ENSGALG00010019872	VTI1A	2	0.185025819	0.328667148	0.562958057	0.573463445	0.188166504	7.450565256	86.57819956	0.5	CRA004775;Yang2025_Silkie_Wenchang	PRJNA827465	0.885890802
ENSGALG00010019872	VTI1A	2	0.035233625	0.087516211	0.402595409	0.687245888	0.003866544	1.151279801	13.14014202	0.5	CRA004775;PRJNA827465	Yang2025_Silkie_Wenchang	0.919451777
ENSGALG00010019873	LGR4	2	-0.126184701	0.113687589	-1.109925033	0.267031333	0.01758871	2.909291108	65.62736546	1	PRJNA827465;Yang2025_Silkie_Wenchang	CRA004775	0.771247817
ENSGALG00010019873	LGR4	2	-0.106164965	0.223284312	-0.475469878	0.634452098	0.06042866	2.164577058	53.8015985	0.5	CRA004775;Yang2025_Silkie_Wenchang	PRJNA827465	0.903866565
ENSGALG00010019873	LGR4	2	-0.021457837	0.063073309	-0.340204721	0.733702363	0	0.627405858	0	0.5	CRA004775;PRJNA827465	Yang2025_Silkie_Wenchang	0.933377698
ENSGALG00010019875	PLEKHH1	2	0.12481732	0.444265964	0.280951796	0.778747373	0.322420962	5.3711046	81.38185579	0.5	PRJNA827465;Yang2025_Silkie_Wenchang	CRA004775	0.944673731
ENSGALG00010019875	PLEKHH1	2	-0.476409036	1.197555023	-0.397818077	0.690764291	2.488087934	6.997528765	85.7092406	0.5	CRA004775;Yang2025_Silkie_Wenchang	PRJNA827465	0.920090451
ENSGALG00010019875	PLEKHH1	2	-0.828643251	0.725768659	-1.141745707	0.253559736	0.763292677	2.952252282	66.12755603	1	CRA004775;PRJNA827465	Yang2025_Silkie_Wenchang	0.763346521
ENSGALG00010019876	MRM2	2	-0.238971297	0.17631149	-1.355392645	0.175292492	0.039348643	2.573973612	61.14956286	1	PRJNA827465;Yang2025_Silkie_Wenchang	CRA004775	0.680258136
ENSGALG00010019876	MRM2	2	-0.127345395	0.367744598	-0.346287601	0.729126586	0.20336946	3.845652664	73.99661157	0.5	CRA004775;Yang2025_Silkie_Wenchang	PRJNA827465	0.932323844
ENSGALG00010019876	MRM2	2	-0.022695325	0.14785263	-0.153499637	0.878004277	0.013581155	1.227750615	18.55023424	0.5	CRA004775;PRJNA827465	Yang2025_Silkie_Wenchang	0.970993355
ENSGALG00010019878	MCFD2	2	-0.282988406	0.328335811	-0.86188712	0.388749637	0.205708649	21.26989957	95.29852035	0.5	PRJNA827465;Yang2025_Silkie_Wenchang	CRA004775	0.828400391
ENSGALG00010019878	MCFD2	2	-0.267177337	0.371100321	-0.719959864	0.471549708	0.246610696	9.408149262	89.37091693	0.5	CRA004775;Yang2025_Silkie_Wenchang	PRJNA827465	0.853998373
ENSGALG00010019878	MCFD2	2	0.039906982	0.051435351	0.775866809	0.437827632	0	0.167390309	0	1	CRA004775;PRJNA827465	Yang2025_Silkie_Wenchang	0.843899392
ENSGALG00010019891	PIGH	2	-0.022017512	0.109085876	-0.201836506	0.840044541	0.00689895	1.345290299	25.66660141	0.5	PRJNA827465;Yang2025_Silkie_Wenchang	CRA004775	0.95957104
ENSGALG00010019891	PIGH	2	-0.016808051	0.203915964	-0.082426364	0.934307672	0.034991381	1.621760614	38.33861844	0.5	CRA004775;Yang2025_Silkie_Wenchang	PRJNA827465	0.98432801
ENSGALG00010019891	PIGH	2	-0.121238917	0.098117091	-1.235655435	0.216586669	0	0.404582741	0	1	CRA004775;PRJNA827465	Yang2025_Silkie_Wenchang	0.730172415
ENSGALG00010019896	DDB1	2	-0.169194882	0.04867234	-3.476201917	0.000508569	0.00022129	1.045035564	4.309476693	1	PRJNA827465;Yang2025_Silkie_Wenchang	CRA004775	0.023657862
ENSGALG00010019896	DDB1	2	-0.119005561	0.07473956	-1.592270025	0.111324045	0	0.281000288	0	1	CRA004775;Yang2025_Silkie_Wenchang	PRJNA827465	0.564466729
ENSGALG00010019896	DDB1	2	-0.207227425	0.056962521	-3.637960888	0.000274805	0	0.008724948	0	1	CRA004775;PRJNA827465	Yang2025_Silkie_Wenchang	0.016024728
ENSGALG00010019898	NUDT1	2	-0.04833324	0.253889917	-0.190370852	0.849018537	0.110243116	6.686038315	85.04345993	0.5	PRJNA827465;Yang2025_Silkie_Wenchang	CRA004775	0.961364896
ENSGALG00010019898	NUDT1	2	0.161498127	0.1547862	1.043362569	0.296780403	0	0.804241688	0	0.5	CRA004775;Yang2025_Silkie_Wenchang	PRJNA827465	0.788458494
ENSGALG00010019898	NUDT1	2	-0.274617927	0.095592527	-2.872797033	0.004068554	0	0.13771814	0	1	CRA004775;PRJNA827465	Yang2025_Silkie_Wenchang	0.092538416
ENSGALG00010019899	ENSGALG00010019899	2	0.173183506	0.302328484	0.572832252	0.566758279	0.121708709	2.984072137	66.48874578	0.5	PRJNA827465;Yang2025_Silkie_Wenchang	CRA004775	0.883487517
ENSGALG00010019899	ENSGALG00010019899	2	0.268198061	0.339223869	0.790622613	0.429164247	0.099026814	1.478492625	32.36354491	0.5	CRA004775;Yang2025_Silkie_Wenchang	PRJNA827465	0.841982849
ENSGALG00010019899	ENSGALG00010019899	2	-0.16867652	0.239210067	-0.705139722	0.480723289	0	0.073490481	0	1	CRA004775;PRJNA827465	Yang2025_Silkie_Wenchang	0.856722567
ENSGALG00010019905	ENSGALG00010019905	2	-0.168842348	0.153800259	-1.097802752	0.272290629	0.043700379	13.10259594	92.36792461	1	PRJNA827465;Yang2025_Silkie_Wenchang	CRA004775	0.773331629
ENSGALG00010019905	ENSGALG00010019905	2	-0.008170044	0.346637414	-0.023569424	0.981196062	0.215777394	9.090535995	88.99954853	0.5	CRA004775;Yang2025_Silkie_Wenchang	PRJNA827465	0.994962782
ENSGALG00010019905	ENSGALG00010019905	2	0.118970002	0.185095436	0.642749515	0.520386646	0.049207944	2.86745931	65.12592187	0.5	CRA004775;PRJNA827465	Yang2025_Silkie_Wenchang	0.869331539
ENSGALG00010019906	ENSGALG00010019906	2	-0.10965383	0.13875484	-0.790270309	0.429369923	0.029729642	4.276466139	76.61620676	0.5	PRJNA827465;Yang2025_Silkie_Wenchang	CRA004775	0.842008086
ENSGALG00010019906	ENSGALG00010019906	2	0.05401067	0.380360765	0.141998531	0.887081166	0.231523085	4.395718104	77.2505885	0.5	CRA004775;Yang2025_Silkie_Wenchang	PRJNA827465	0.972957277
ENSGALG00010019906	ENSGALG00010019906	2	0.14600103	0.21723614	0.672084442	0.50152994	0.058043748	1.895970267	47.25655686	1	CRA004775;PRJNA827465	Yang2025_Silkie_Wenchang	0.862728958
ENSGALG00010019910	ENSGALG00010019910	2	0.854560798	0.719291666	1.188058806	0.234810242	0.891303904	6.437464607	84.46593401	1	PRJNA827465;Yang2025_Silkie_Wenchang	CRA004775	0.747791642
ENSGALG00010019910	ENSGALG00010019910	2	0.620214163	0.990140674	0.626389946	0.531059203	1.797071911	11.370294	91.20515266	0.5	CRA004775;Yang2025_Silkie_Wenchang	PRJNA827465	0.872848946
ENSGALG00010019910	ENSGALG00010019910	2	-0.019251459	0.265611598	-0.072479737	0.94222013	0.115370195	5.378618441	81.40786503	0.5	CRA004775;PRJNA827465	Yang2025_Silkie_Wenchang	0.985827325
ENSGALG00010019912	ARG2	2	-0.049280461	0.150921583	-0.326530244	0.744023211	0	0.654350715	0	0.5	PRJNA827465;Yang2025_Silkie_Wenchang	CRA004775	0.935821071
ENSGALG00010019912	ARG2	2	0.282242487	0.317098348	0.890078706	0.373423626	0	0.131869366	0	1	CRA004775;Yang2025_Silkie_Wenchang	PRJNA827465	0.822254022
ENSGALG00010019912	ARG2	2	-0.068788503	0.160776595	-0.427851472	0.66875926	0	0.781830485	0	0.5	CRA004775;PRJNA827465	Yang2025_Silkie_Wenchang	0.914377769
ENSGALG00010019916	NOC3L	2	-0.009965395	0.044291329	-0.224996525	0.821981978	0	0.307929864	0	0.5	PRJNA827465;Yang2025_Silkie_Wenchang	CRA004775	0.955636543
ENSGALG00010019916	NOC3L	2	0.029517373	0.073666452	0.400689486	0.688648753	0	0.066252788	0	1	CRA004775;Yang2025_Silkie_Wenchang	PRJNA827465	0.919799842
ENSGALG00010019916	NOC3L	2	-0.023035822	0.053459762	-0.430900191	0.666540945	0	0.193685274	0	0.5	CRA004775;PRJNA827465	Yang2025_Silkie_Wenchang	0.913579507
ENSGALG00010019919	LIN7C	2	-0.248434831	0.289441127	-0.858325952	0.390712503	0.158765797	18.5458824	94.60796753	0.5	PRJNA827465;Yang2025_Silkie_Wenchang	CRA004775	0.829251057
ENSGALG00010019919	LIN7C	2	-0.120098657	0.439165177	-0.273470354	0.784491678	0.36161362	15.87416507	93.70045608	0.5	CRA004775;Yang2025_Silkie_Wenchang	PRJNA827465	0.946117405
ENSGALG00010019919	LIN7C	2	0.128383323	0.140642984	0.912831333	0.361331256	0.027085071	2.531975884	60.50515306	1	CRA004775;PRJNA827465	Yang2025_Silkie_Wenchang	0.815424583
ENSGALG00010019921	CALM2	2	-0.009370281	0.079371094	-0.118056598	0.906022813	0.006510688	1.949701594	48.71009989	0.5	PRJNA827465;Yang2025_Silkie_Wenchang	CRA004775	0.978196186
ENSGALG00010019921	CALM2	2	0.106978361	0.096356438	1.110235731	0.26689746	0	0.292184321	0	1	CRA004775;Yang2025_Silkie_Wenchang	PRJNA827465	0.771143928
ENSGALG00010019921	CALM2	2	-0.055254176	0.074727378	-0.739410079	0.459658024	0.002195093	1.0352373	3.403789613	0.5	CRA004775;PRJNA827465	Yang2025_Silkie_Wenchang	0.850098539
ENSGALG00010019925	RDH11	2	-0.213384467	0.182195816	-1.171182037	0.241525614	0.048552166	3.665802943	72.72084682	1	PRJNA827465;Yang2025_Silkie_Wenchang	CRA004775	0.7539858
ENSGALG00010019925	RDH11	2	-0.359967107	0.142814301	-2.520525645	0.01171797	0	0.880629138	0	0.5	CRA004775;Yang2025_Silkie_Wenchang	PRJNA827465	0.173335104
ENSGALG00010019925	RDH11	2	-0.041080222	0.110677601	-0.371170155	0.710510801	0	0.011164786	0	0.5	CRA004775;PRJNA827465	Yang2025_Silkie_Wenchang	0.925404854
ENSGALG00010019926	KIF7	2	0.061528766	0.053206249	1.156419926	0.247509435	0.001159212	1.233808859	18.95016863	1	PRJNA827465;Yang2025_Silkie_Wenchang	CRA004775	0.757947282
ENSGALG00010019926	KIF7	2	-0.121658253	0.267094693	-0.455487348	0.648758667	0.129015667	10.18360162	90.18029144	0.5	CRA004775;Yang2025_Silkie_Wenchang	PRJNA827465	0.907730933
ENSGALG00010019926	KIF7	2	-0.169412375	0.211065775	-0.802652043	0.422175879	0.077636048	7.271332744	86.24736296	0.5	CRA004775;PRJNA827465	Yang2025_Silkie_Wenchang	0.839649223
ENSGALG00010019931	ZFYVE26	2	0.002579984	0.097508673	0.026459019	0.978891221	0.008848736	1.7306624	42.21865571	0.5	PRJNA827465;Yang2025_Silkie_Wenchang	CRA004775	0.994259589
ENSGALG00010019931	ZFYVE26	2	-0.113819344	0.12764315	-0.891699585	0.372553976	0	0.133583788	0	0.5	CRA004775;Yang2025_Silkie_Wenchang	PRJNA827465	0.822016521
ENSGALG00010019931	ZFYVE26	2	0.071343342	0.074150966	0.962136376	0.335981104	0	0.025771743	0	1	CRA004775;PRJNA827465	Yang2025_Silkie_Wenchang	0.803504387
ENSGALG00010019932	KALRN	2	0.032745445	0.069962021	0.468046008	0.639751688	0	0.977200406	0	0.5	PRJNA827465;Yang2025_Silkie_Wenchang	CRA004775	0.906064918
ENSGALG00010019932	KALRN	2	-0.056636625	0.094410669	-0.599896451	0.548575248	0	0.055833339	0	1	CRA004775;Yang2025_Silkie_Wenchang	PRJNA827465	0.878281266
ENSGALG00010019932	KALRN	2	0.061876482	0.085384325	0.724681979	0.468647153	0	0.71628989	0	0.5	CRA004775;PRJNA827465	Yang2025_Silkie_Wenchang	0.853198008
ENSGALG00010019934	TKFC	2	0.397327031	0.135812364	2.925558618	0.003438384	0.006679497	1.19744779	16.48905209	1	PRJNA827465;Yang2025_Silkie_Wenchang	CRA004775	0.082704286
ENSGALG00010019934	TKFC	2	0.354899944	0.245673569	1.444599619	0.14857038	0.066075702	2.193397576	54.4086302	1	CRA004775;Yang2025_Silkie_Wenchang	PRJNA827465	0.638659263
ENSGALG00010019934	TKFC	2	0.245275114	0.127547483	1.923010225	0.054478766	0	0.501004259	0	1	CRA004775;PRJNA827465	Yang2025_Silkie_Wenchang	0.404097297
ENSGALG00010019935	ENSGALG00010019935	2	1.50775919	0.442462537	3.407653902	0.000655239	0	0.111926273	0	1	PRJNA827465;Yang2025_Silkie_Wenchang	CRA004775	0.028276881
ENSGALG00010019935	ENSGALG00010019935	2	1.446527801	0.597129478	2.422469253	0.01541543	0	0.107562721	0	1	CRA004775;Yang2025_Silkie_Wenchang	PRJNA827465	0.205241414
ENSGALG00010019935	ENSGALG00010019935	2	1.655071436	0.509190315	3.250398501	0.001152434	0	0.011319768	0	1	CRA004775;PRJNA827465	Yang2025_Silkie_Wenchang	0.041251469
ENSGALG00010019947	ENSGALG00010019947	2	-0.363949216	0.570817559	-0.637592887	0.523738727	0.525888804	5.065552442	80.25881656	0.5	PRJNA827465;Yang2025_Silkie_Wenchang	CRA004775	0.869661505
ENSGALG00010019947	ENSGALG00010019947	2	-0.816293407	0.323467994	-2.523567776	0.011617066	0	0.360313135	0	1	CRA004775;Yang2025_Silkie_Wenchang	PRJNA827465	0.172556902
ENSGALG00010019947	ENSGALG00010019947	2	-0.093375224	0.356845057	-0.261668819	0.793576782	0.113743345	1.678474627	40.42209612	0.5	CRA004775;PRJNA827465	Yang2025_Silkie_Wenchang	0.948743674
ENSGALG00010019948	TICRR	2	-0.094798135	0.088556486	-1.070482121	0.284402352	0.007155294	1.773905647	43.62721593	1	PRJNA827465;Yang2025_Silkie_Wenchang	CRA004775	0.780265906
ENSGALG00010019948	TICRR	2	-0.180347017	0.105481677	-1.709747344	0.087312604	0	0.370249809	0	1	CRA004775;Yang2025_Silkie_Wenchang	PRJNA827465	0.508091098
ENSGALG00010019948	TICRR	2	-0.02227261	0.073950105	-0.301184288	0.76327397	0	0.002300878	0	1	CRA004775;PRJNA827465	Yang2025_Silkie_Wenchang	0.940229325
ENSGALG00010019954	CYB561A3	2	0.080333625	0.149065185	0.538916079	0.589944762	0.020800586	1.691267424	40.87274514	0.5	PRJNA827465;Yang2025_Silkie_Wenchang	CRA004775	0.892320726
ENSGALG00010019954	CYB561A3	2	0.254548101	0.177012894	1.438020108	0.150428349	0	0.104993738	0	1	CRA004775;Yang2025_Silkie_Wenchang	PRJNA827465	0.641245704

ENSGALG00010019954	CYB561A3	2	0.001057934	0.102762598	0.010294937	0.991785974	0	0.389358931	0	0.5	CRA004775;PRJNA827465	Yang2025_Silkie_Wenchang	0.997575757
ENSGALG00010019956	SLC29A3	2	0.074701001	0.068432684	1.091598286	0.27500971	0.000368971	1.027783352	2.703230361	0.5	PRJNA827465;Yang2025_Silkie_Wenchang	CRA004775	0.774984342
ENSGALG00010019956	SLC29A3	2	-0.066571702	0.136451308	-0.487878814	0.625635682	0	0.066467851	0	1	CRA004775;Yang2025_Silkie_Wenchang	PRJNA827465	0.901501367
ENSGALG00010019956	SLC29A3	2	0.102073646	0.073839672	1.382368639	0.16685854	0	0.441428583	0	0.5	CRA004775;PRJNA827465	Yang2025_Silkie_Wenchang	0.665858586
ENSGALG00010019961	HTATSF1	2	-0.074400075	0.073785165	-1.008333787	0.313294248	0.007372326	2.928405995	65.85172952	1	PRJNA827465;Yang2025_Silkie_Wenchang	CRA004775	0.796531448
ENSGALG00010019961	HTATSF1	2	-0.10337443	0.110022083	-0.93957892	0.347433593	0.009819096	1.383730215	27.73157733	0.5	CRA004775;Yang2025_Silkie_Wenchang	PRJNA827465	0.808705451
ENSGALG00010019961	HTATSF1	2	-0.007136042	0.043449652	-0.164237021	0.869544558	0	0.285794071	0	0.5	CRA004775;PRJNA827465	Yang2025_Silkie_Wenchang	0.968578601
ENSGALG00010019962	SNX8	2	-0.122445533	0.243248775	-0.503375745	0.614700121	0.105303141	9.07158481	88.97656781	0.5	PRJNA827465;Yang2025_Silkie_Wenchang	CRA004775	0.898692812
ENSGALG00010019962	SNX8	2	-0.37668339	0.107497913	-3.504099562	0.000458154	0	0.030562428	0	1	CRA004775;Yang2025_Silkie_Wenchang	PRJNA827465	0.022067191
ENSGALG00010019962	SNX8	2	-0.100415946	0.263242436	-0.381458048	0.702863395	0.105292619	3.76862761	73.4651416	0.5	CRA004775;PRJNA827465	Yang2025_Silkie_Wenchang	0.923345143
ENSGALG00010019967	CEP170B	2	0.184401306	0.110552608	1.667995983	0.095316524	0	0.861641278	0	1	PRJNA827465;Yang2025_Silkie_Wenchang	CRA004775	0.528530665
ENSGALG00010019967	CEP170B	2	0.346620545	0.177153624	1.95660996	0.050393343	0	0.044811233	0	1	CRA004775;Yang2025_Silkie_Wenchang	PRJNA827465	0.38897029
ENSGALG00010019967	CEP170B	2	0.162322039	0.112891053	1.437864515	0.1504725	0	0.354303527	0	1	CRA004775;PRJNA827465	Yang2025_Silkie_Wenchang	0.641364348
ENSGALG00010019972	PPP1CB	2	-0.118034892	0.047677802	-2.475678146	0.01329834	0	0.350874908	0	1	PRJNA827465;Yang2025_Silkie_Wenchang	CRA004775	0.188414806
ENSGALG00010019972	PPP1CB	2	-0.167494549	0.094508752	-1.77226496	0.076350585	0	0.018955658	0	1	CRA004775;Yang2025_Silkie_Wenchang	PRJNA827465	0.476766627
ENSGALG00010019972	PPP1CB	2	-0.106138439	0.051724293	-2.052003677	0.040169305	0	0.031110162	0	1	CRA004775;PRJNA827465	Yang2025_Silkie_Wenchang	0.347135927
ENSGALG00010019975	ENSGALG00010019975	2	0.368787372	0.350414171	1.052432814	0.29260102	0.233903115	20.88347719	95.2115254	1	PRJNA827465;Yang2025_Silkie_Wenchang	CRA004775	0.786223963
ENSGALG00010019975	ENSGALG00010019975	2	0.38239679	0.394076163	0.970362649	0.331865759	0.256875581	5.30807848	81.16079098	0.5	CRA004775;Yang2025_Silkie_Wenchang	PRJNA827465	0.802002658
ENSGALG00010019975	ENSGALG00010019975	2	0.01874049	0.081767122	0.229193462	0.818718554	0	0.081445694	0	0.5	CRA004775;PRJNA827465	Yang2025_Silkie_Wenchang	0.955256039
ENSGALG00010019977	ZMIZ1	2	-0.119700592	0.070380445	-1.700764919	0.088987139	0	0.95666085	0	0.5	PRJNA827465;Yang2025_Silkie_Wenchang	CRA004775	0.511998468
ENSGALG00010019977	ZMIZ1	2	0.050858444	0.174493545	0.291463181	0.770691	0	0.040887835	0	0.5	CRA004775;Yang2025_Silkie_Wenchang	PRJNA827465	0.942491331
ENSGALG00010019977	ZMIZ1	2	-0.136239471	0.072586875	-1.876916039	0.060529602	0	0.200632762	0	1	CRA004775;PRJNA827465	Yang2025_Silkie_Wenchang	0.426049285
ENSGALG00010019982	PLCE1	2	0.168969829	0.301730739	0.56000204	0.575478046	0.159859427	7.507181703	86.67942192	0.5	PRJNA827465;Yang2025_Silkie_Wenchang	CRA004775	0.886633228
ENSGALG00010019982	PLCE1	2	0.440445298	0.190057943	2.317426421	0.020480514	0	0.477815057	0	1	CRA004775;Yang2025_Silkie_Wenchang	PRJNA827465	0.241290666
ENSGALG00010019982	PLCE1	2	-0.094607721	0.060826282	-1.555375704	0.119856617	0	0.435867076	0	0.5	CRA004775;PRJNA827465	Yang2025_Silkie_Wenchang	0.580639051
ENSGALG00010019985	UMPS	2	-0.131585729	0.265024085	-0.496504795	0.619538305	0.129959317	12.99457279	92.3044796	0.5	PRJNA827465;Yang2025_Silkie_Wenchang	CRA004775	0.899285366
ENSGALG00010019985	UMPS	2	-0.38649005	0.119711443	-3.228513842	0.001244352	0	0.165822705	0	1	CRA004775;Yang2025_Silkie_Wenchang	PRJNA827465	0.042969436
ENSGALG00010019985	UMPS	2	0.010770699	0.180155425	0.059785593	0.952326401	0.040495231	1.980400074	49.50515236	0.5	CRA004775;PRJNA827465	Yang2025_Silkie_Wenchang	0.987758913
ENSGALG00010019989	ENSGALG00010019989	2	-0.005598059	0.271182601	-0.020643134	0.983530152	0.082011267	2.199679546	54.53883263	0.5	PRJNA827465;Yang2025_Silkie_Wenchang	CRA004775	0.995457747
ENSGALG00010019989	ENSGALG00010019989	2	0.23650463	0.197773335	1.195836789	0.231760256	0	0.010263689	0	1	CRA004775;Yang2025_Silkie_Wenchang	PRJNA827465	0.744150932
ENSGALG00010019989	ENSGALG00010019989	2	-0.156572462	0.268598943	-0.582922851	0.559945237	0.008364513	1.048018236	4.58181299	0.5	CRA004775;PRJNA827465	Yang2025_Silkie_Wenchang	0.881203589
ENSGALG00010019990	MARCHF8	2	-0.037269894	0.061471738	-0.606293149	0.544320107	0	0.27388668	0	1	PRJNA827465;Yang2025_Silkie_Wenchang	CRA004775	0.877102749
ENSGALG00010019990	MARCHF8	2	-0.059648608	0.110924156	-0.537742275	0.590754989	0	0.491414481	0	0.5	CRA004775;Yang2025_Silkie_Wenchang	PRJNA827465	0.892708037
ENSGALG00010019990	MARCHF8	2	-0.009653773	0.069669786	-0.138564706	0.889794134	0	0.245886714	0	0.5	CRA004775;PRJNA827465	Yang2025_Silkie_Wenchang	0.973208458
ENSGALG00010019991	EIF3B	2	-0.180261558	0.147258468	-1.224116753	0.220908187	0.037631911	7.311608095	86.32311816	1	PRJNA827465;Yang2025_Silkie_Wenchang	CRA004775	0.73419654
ENSGALG00010019991	EIF3B	2	-0.228647924	0.123847166	-1.852139486	0.064005796	0.017482096	1.975139657	49.37066873	1	CRA004775;Yang2025_Silkie_Wenchang	PRJNA827465	0.43595992
ENSGALG00010019991	EIF3B	2	-0.046002868	0.050643085	-0.908374123	0.363680593	0	0.028717106	0	1	CRA004775;PRJNA827465	Yang2025_Silkie_Wenchang	0.817042253
ENSGALG00010019992	CHAC2	2	-0.183855346	0.143499663	-1.281224931	0.200114672	0.023660959	2.211387476	54.77952141	1	PRJNA827465;Yang2025_Silkie_Wenchang	CRA004775	0.712419107
ENSGALG00010019992	CHAC2	2	-0.299625412	0.147715848	-2.028390429	0.042520416	0	0.562671178	0	1	CRA004775;Yang2025_Silkie_Wenchang	PRJNA827465	0.356625761
ENSGALG00010019992	CHAC2	2	-0.072253297	0.095766604	-0.754472799	0.450565373	0	0.012829996	0	1	CRA004775;PRJNA827465	Yang2025_Silkie_Wenchang	0.84760941
ENSGALG00010019996	ITGB5	2	-0.187539935	0.066714342	-2.811088741	0.004937417	0	0.314116131	0	1	PRJNA827465;Yang2025_Silkie_Wenchang	CRA004775	0.104781471
ENSGALG00010019996	ITGB5	2	-0.13972207	0.16277054	-0.858390011	0.390672173	0.023972295	1.522683714	34.32647956	0.5	CRA004775;Yang2025_Silkie_Wenchang	PRJNA827465	0.829251057
ENSGALG00010019996	ITGB5	2	-0.132342612	0.083361793	-1.587569163	0.112383796	0.000369189	1.008848132	0.837735911	0.5	CRA004775;PRJNA827465	Yang2025_Silkie_Wenchang	0.56602666
ENSGALG00010019997	FSHR	2	-0.189677262	0.306549922	-0.618748361	0.536082146	0.092980824	1.976252453	49.39917729	0.5	PRJNA827465;Yang2025_Silkie_Wenchang	CRA004775	0.874585387
ENSGALG00010019997	FSHR	2	0.580852981	0.686441331	0.846180082	0.397452292	0.573006092	2.053726884	51.30803381	1	CRA004775;Yang2025_Silkie_Wenchang	PRJNA827465	0.831571329
ENSGALG00010019997	FSHR	2	0.35659092	1.032337002	0.34542104	0.729777862	1.685426632	4.141171052	75.85224113	0.5	CRA004775;PRJNA827465	Yang2025_Silkie_Wenchang	0.932444623
ENSGALG00010019999	ERLIN1	2	-0.165391584	0.040242248	-4.109899221	3.95823E-05	0	0.062312232	0	1	PRJNA827465;Yang2025_Silkie_Wenchang	CRA004775	0.004104849
ENSGALG00010019999	ERLIN1	2	-0.22580543	0.068243367	-3.308826041	0.00093688	0.000261758	1.023582697	2.303936614	1	CRA004775;Yang2025_Silkie_Wenchang	PRJNA827465	0.036206692
ENSGALG00010019999	ERLIN1	2	-0.206768238	0.077322145	-2.674114078	0.007492696	0.006033494	1.66651366	39.99449124	1	CRA004775;PRJNA827465	Yang2025_Silkie_Wenchang	0.133545368
ENSGALG00010020003	ENSGALG00010020003	2	-0.170830026	0.10593055	-1.612660613	0.106818262	0	0.964834659	0	0.5	PRJNA827465;Yang2025_Silkie_Wenchang	CRA004775	0.553925396
ENSGALG00010020003	ENSGALG00010020003	2	0.02254638	0.214077713	0.105317672	0.916122947	0	0.101436736	0	0.5	CRA004775;Yang2025_Silkie_Wenchang	PRJNA827465	0.980406784
ENSGALG00010020003	ENSGALG00010020003	2	-0.214906224	0.116013148	-1.852429898	0.063964115	0	0.117698966	0	1	CRA004775;PRJNA827465	Yang2025_Silkie_Wenchang	0.435774246
ENSGALG00010020005	EOGT	2	-0.048432149	0.049066347	-0.987074689	0.323606025	0	0.314680253	0	1	PRJNA827465;Yang2025_Silkie_Wenchang	CRA004775	0.799600434
ENSGALG00010020005	EOGT	2	0.103846095	0.190072681	0.546349401	0.584825785	0.042166882	1.834976114	45.50337783	0.5	CRA004775;Yang2025_Silkie_Wenchang	PRJNA827465	0.891016154
ENSGALG00010020005	EOGT	2	0.083432924	0.226056174	0.369080491	0.71206773	0.070469843	2.443166626	59.06951293	0.5	CRA004775;PRJNA827465	Yang2025_Silkie_Wenchang	0.92597752
ENSGALG00010020006	PLB1	2	-0.014738922	0.261438521	-0.056376246	0.95504208	0	0.054292663	0	0.5	PRJNA827465;Yang2025_Silkie_Wenchang	CRA004775	0.988093796
ENSGALG00010020006	PLB1	2	0.464800959	0.503566239	0.923018508	0.35599756	0.234819172	1.786093816	44.01189953	1	CRA004775;Yang2025_Silkie_Wenchang	PRJNA827465	0.812377444
ENSGALG00010020006	PLB1	2	0.384300873	0.563885925	0.681522371	0.495541016	0.407333423	2.544565378	60.69746866	0.5	CRA004775;PRJNA827465	Yang2025_Silkie_Wenchang	0.86097062
ENSGALG00010020008	EIF2S1	2	-0.009834208	0.043108947	-0.228124533	0.819549429	0	0.436618813	0	0.5	PRJNA827465;Yang2025_Silkie_Wenchang	CRA004775	0.955256039
ENSGALG00010020008	EIF2S1	2	0.034663032	0.092011907	0.376723333	0.706379221	0	0.138868539	0	0.5	CRA004775;Yang2025_Silkie_Wenchang	PRJNA827465	0.924548793
ENSGALG00010020008	EIF2S1	2	-0.023304351	0.046114269	-0.505360966	0.61330833	0	0.001693181	0	1	CRA004775;PRJNA827465	Yang2025_Silkie_Wenchang	0.89823785
ENSGALG00010020009	MACROD2	2	0.09226364	0.280799773	0.328574481	0.742477339	0.130443584	5.123821851	80.48331833	0.5	PRJNA827465;Yang2025_Silkie_Wenchang	CRA004775	0.935517163
ENSGALG00010020009	MACROD2	2	0.380469906	0.169822838	2.240393055	0.025065417	0	0.063567894	0	1	CRA004775;Yang2025_Silkie_Wenchang	PRJNA827465	0.269672828
ENSGALG00010020009	MACROD2	2	0.042369721	0.235777825	0.179701891	0.857386609	0.085937839	3.767992525	73.46066922	0.5	CRA004775;PRJNA827465	Yang2025_Silkie_Wenchang	0.963952912
ENSGALG00010020010	SORBS3	2	0.684970584	0.611130859	1.120824736	0.262362473	0.689971679	12.45146186	91.9688145	1	PRJNA827465;Yang2025_Silkie_Wenchang	CRA004775	0.768481855
ENSGALG00010020010	SORBS3	2	0.795924693	0.545438886	1.459237164	0.144499843	0.48143297	5.237985101	80.90868949	1	CRA004775;Yang2025_Silkie_Wenchang	PRJNA827465	0.631943623
ENSGALG00010020010	SORBS3	2	0.124159799	0.099388406	1.249238256	0.211577943	0	0.140046505	0	1	CRA004775;PRJNA827465	Yang2025_Silkie_Wenchang	0.724013667
ENSGALG00010020011	TLE3	2	0.254051515	0.411291665	0.617691862	0.536778477	0.322212629	20.90248992	95.21588096	0.5	PRJNA827465;Yang2025_Silkie_Wenchang	CRA004775	0.874651173
ENSGALG00010020011	TLE3	2	0.225300678	0.465038532	0.484477441	0.628047071	0.394156879	11.08435305	90.97827365	0.5	CRA004775;Yang2025_Silkie_Wenchang	PRJNA827465	0.901904489
ENSGALG00010020011	TLE3	2	-0.168462552	0.095879529	-1.757023146	0.078913864	0	0.172754429	0	1	CRA004775;PRJNA827465	Yang2025_Silkie_Wenchang	0.483032487
ENSGALG00010020013	ADAM8	2	0.616966977	0.483539456	1.275939263	0.20197699	0	0.300110717	0	1	PRJNA827465;Yang2025_Silkie_Wenchang	CRA004775	0.714969076
ENSGALG00010020013	ADAM8	2	0.374322157	0.486215293	0.769663484	0.44149953	0	0.827342605	0	1	CRA004775;Yang2025_Silkie_Wenchang	PRJNA827465	0.845053556
ENSGALG00010020013	ADAM8	2	0.242854772	0.422168013	0.575256211	0.565118032	0	0.194409582	0	1	CRA004775;PRJNA827465	Yang2025_Silkie_Wenchang	0.883427937
ENSGALG00010020015	ATP6V1D	2	0.094163931	0.149954783	0.627948836	0.530037458	0.037188648	5.426282363	81.57117649	0.5	PRJNA827465;Yang2025_Silkie_Wenchang	CRA004775	0.872072262
ENSGALG00010020015	ATP6V1D	2	0.11532247	0.196142426	0.587952704	0.556564039	0.046489081	2.259551649	55.74343254	0.5	CRA004775;Yang2025_Silkie_Wenchang	PRJNA827465	0.880358001
ENSGALG00010020015	ATP6V1D	2	-0.044458324	0.054259334	-0.8193673	0.412576886	0	0.179265321	0	1	CRA004775;PRJNA827465	Yang2025_Silkie_Wenchang	0.835704442
ENSGALG00010020017	ENSGALG00010020017	2	0.413760525	0.694218087	0.596009428	0.551168928	0.877971943	10.63918394	90.60078287	0.5	PRJNA827465;Yang2025_Silkie_Wenchang	CRA004775	0.878879109
ENSGALG00010020017	ENSGALG00010020017	2	0.821256023	0.30063467	2.731740892	0.006300067	0.043698935	1.312706391	23.82150292	1	CRA004775;Yang2025_Silkie_Wenchang	PRJNA827465	0.120550885
ENSGALG00010020017	ENSGALG00010020017	2	0.099913469	0.388326097	0.257292698	0.796952829	0.240343524	4.487912936	77.71792782	0.5	CRA004775;PRJNA827465	Yang2025_Silkie_Wenchang	0.949622804
ENSGALG00010020018	ENSGALG00010020018	2	0.063073935	0.434489506	0.145167914	0.884575299	0.316183167	6.099327703	83.60098629	0.5	PRJNA827465;Yang2025_Silkie_Wenchang	CRA004775	0.972962774
ENSGALG00010020018	ENSGALG00010020018	2	-0.553027103	1.211442701	-0.456502897	0.648028388	2.532500952	6.641182684	84.94244102	0.5	CRA004775;Yang2025_Silkie_Wenchang	PRJNA827465	0.907581504
ENSGALG00010020018	ENSGALG00010020018	2	-0.89384986	0.746574137	-1.197268719	0.231201833	0.802534816	2.851980163	64.93664252	1	CRA004775;PRJNA827465	Yang2025_Silkie_Wenchang	0.743891332
ENSGALG00010020019	PEX10	2	0.127714883	0.152228145	0.838947904	0.401498544	0.033993732	3.448913169	71.00335876	1	PRJNA827465;Yang2025_Silkie_Wenchang	CRA004775	0.838253399
ENSGALG00010020019	PEX10	2	0.241014988	0.134450121	1.792597777	0.073037262	0.000411142	1.007569867	0.751299477	0.5	CRA004775;Yang2025_Silkie_Wenchang	PRJNA827465	0.466989347
ENSGALG00010020019	PEX10	2	-0.000610225	0.070836719	-0.008614525	0.993126688	0	0.005001635	0	0.5	CRA004775;PRJNA827465	Yang2025_Silkie_Wenchang	0.99794341
ENSGALG00010020023	FOSL2	2	-0.036158076	0.181531317	-0.199183682	0.842119064	0.024834319	1.57640647	36.56458414	0.5	PRJNA827465;Yang2025_Silkie_Wenchang	CRA004775	0.960064308
ENSGALG00010020023	FOSL2	2	-0.24270341	0.208619564	-1.163377993	0.244676174	0	0.007939657	0	1	CRA004775;Yang2025_Silkie_Wenchang	PRJNA827465	0.756093682
ENSGALG00010020023	FOSL2	2	0.074778126	0.165358613	0.452217908	0.651112009	0	0.427688139	0	0.5	CRA004775;PRJNA827465	Yang2025_Silkie_Wenchang	0.908769703
ENSGALG00010020025	LOXL2	2	-0.093078209	0.098709778	-0.942948214	0.345707392	0	0.055538011	0	1	PRJNA827465;Yang2025_Silkie_Wenchang	CRA004775	0.807376412
ENSGALG00010020025	LOXL2	2	0.109898038	0.288890194	0.380414566	0.703637704	0.090936195	1.751671422	42.91166782	0.5	CRA004775;Yang2025_Silkie_Wenchang	PRJNA827465	0.923639641
ENSGALG00010020025	LOXL2	2	0.091726905	0.317745113	0.288680775	0.772825676	0.12605193	2.071117285	51.71688213	0.5	CRA004775;PRJNA827465	Yang2025_Silkie_Wenchang	0.943066211
ENSGALG00010020027	RER1	2	-0.18521606	0.208007142	-0.890431255	0.373234366	0.077765829	9.398835863	89.36038447	0.5	PRJNA827465;Yang2025_Silkie_Wenchang	CRA004775	0.822187352
ENSGALG00010020027	RER1	2	-0.24620774	0.188217733	-1.308100661	0.190839169	0.046288161	2.779937212	64.02796452	1	CRA004775;Yang2025_Silkie_Wenchang	PRJNA827465	0.700389407
ENSGALG00010020027	RER1	2	0.005128712	0.050318804	0.101924362	0.918816712	0	0.035057361	0	0.5	CRA004775;PRJNA827465	Yang2025_Silkie_Wenchang	0.981033557
ENSGALG00010020030	MRPL33	2	0.080723565	0.088197823	0.915255751	0.360057383	0.006385707	1.506970462	33.64169868	1	PRJNA827465;Yang2025_Silkie_Wenchang	CRA004775	0.814880744
ENSGALG00010020030	MRPL33	2	0.072399465	0.221559595	0.326771968	0.743840362	0.050075494	1.764496587	43.32661183	0.5	CRA004775;Yang2025_Silkie_Wenchang	PRJNA827465	0.935821071
ENSGALG00010020030	MRPL33	2	0.013392167	0.065994454	0.202928678	0.839190779	0	0.711733688	0	0.5	CRA004775;PRJNA827465	Yang2025_Silkie_Wenchang	0.959413439
ENSGALG00010020032	CHST12	2	0.01658384	0.072645336	0.228284995	0.819424689	0.001265518	1.105076536	9.508530182	0.5	PRJNA827465;Yang2025_Silkie_Wenchang	CRA004775	0.955256039
ENSGALG00010020032	CHST12	2	-0.137345867	0.120917908	-1.135860426	0.256014983	0	0.35714123	0	1	CRA004775;Yang2025_Silkie_Wenchang	PRJNA827465	0.764570254
ENSGALG00010020032	CHST12	2	-0.011090731	0.140700932	-0.078824857	0.937171933	0.019011851	1.463230156	31.65805146	0.5	CRA004775;PRJNA827465	Yang2025_Silkie_Wenchang	0.985114195
ENSGALG00010020036	TMF1	2	0.165286343	0.183825819	0.899146504	0.368574631	0.056445432	5.519954506	81.88390866	1	PRJNA827465;Yang2025_Silkie_Wenchang	CRA004775	0.82033738
ENSGALG00010020036	TMF1	2	0.3707047	0.127996695	2.896205254	0.003777053	0	0.006708528	0	1	CRA004775;Yang2025_Silkie_Wenchang	PRJNA827465	0.088100594
ENSGALG00010020036	TMF1	2	0.092640763	0.150531488	0.615424481	0.538274428	0.027391231	1.831261443	45.3928327	1	CRA004775;PRJNA827465	Yang2025_Silkie_Wenchang	0.87481821
ENSGALG00010020037	MPP5	2	-0.034527552	0.062475653	-0.552656121	0.580498905	0	0.363213469	0	0.5	PRJNA827465;Yang2025_Silkie_Wenchang	CRA004775	0.888433837
ENSGALG00010020037	MPP5	2	0.295217254	0.28775342	1.025938301	0.304920668	0.129889056	4.425433311	77.40334269	1	CRA004775;Yang2025_Silkie_Wenchang	PRJNA827465	0.79225794
ENSGALG00010020037	MPP5	2	0.243246092	0.333720548	0.728891564	0.466067996	0.194716082	7.272644619	86.24984373	0.5	CRA004775;PRJNA827465	Yang2025_Silkie_Wenchang	0.852630314
ENSGALG00010020038	SKI	2	-0.019153621	0.159744369	-0.119901695	0.904561022	0.032835935	2.327177347	57.02948892	0.5	PRJNA827465;Yang2025_Silkie_Wenchang	CRA004775	0.977669174
ENSGALG00010020038	SKI	2	0.194871018	0.188460358	1.03401596	0.301128702	0	0.019085773	0	1	CRA004775;Yang2025_Silkie_Wenchang	PRJNA827465	0.791202961
ENSGALG00010020038	SKI	2	-0.123831649	0.071993758	-1.720033121	0.085426421	0	0.400504056	0	0.5	CRA004775;PRJNA827465	Yang2025_Silkie_Wenchang	0.502621951
ENSGALG00010020041	CDH23	2	-0.096489587	0.083088867	-1.161281775	0.245527329	0	0.114965058	0	1	PRJNA827465;Yang2025_Silkie_Wenchang	CRA004775	0.75661406
ENSGALG00010020041	CDH23	2	-0.015328205	0.150666805	-0.101735779	0.918966402	0	0.318235401	0	0.5	CRA004775;Yang2025_Silkie_Wenchang	PRJNA827465	0.981086235
ENSGALG00010020041	CDH23	2	-0.096622331	0.09411328	-1.026659904	0.304580636	0	0.553765912	0	0.5	CRA004775;PRJNA827465	Yang2025_Silkie_Wenchang	0.79225794
ENSGALG00010020046	BDNF	2	-0.096049746	0.169272324	-0.567427352	0.570423871	0	0.335480199	0	0.5	PRJNA827465;Yang2025_Silkie_Wenchang	CRA004775	0.8851128
ENSGALG00010020046	BDNF	2	0.042681825	0.263195822	0.162167564	0.871173902	0	0.002850441	0	1	CRA004775;Yang2025_Silkie_Wenchang	PRJNA827465	0.969278071
ENSGALG00010020046	BDNF	2	-0.147270637	0.201083338	-0.732386077	0.463932955	0	0.116838242	0	0.5	CRA004775;PRJNA827465	Yang2025_Silkie_Wenchang	0.852159649
ENSGALG00010020048	ENSGALG00010020048	2	-0.510328986	0.141202424	-3.614165887	0.000301316	0	0.62547935	0	1	PRJNA827465;Yang2025_Silkie_Wenchang	CRA004775	0.017064304
ENSGALG00010020048	ENSGALG00010020048	2	-0.20643893	0.236863593	-0.871551967	0.383452844	0.015470648	1.1212973	10.81758603	0.5	CRA004775;Yang2025_Silkie_Wenchang	PRJNA827465	0.825509017
ENSGALG00010020048	ENSGALG00010020048	2	-0.307745273	0.371629615	-0.82809674	0.407615715	0.187063719	2.673968227	62.60239781	0.5	CRA004775;PRJNA827465	Yang2025_Silkie_Wenchang	0.833982683
ENSGALG00010020053	LFNG	2	0.035217089	0.088334558	0.398678499	0.690130112	0.006030789	1.602506744	37.59776651	0.5	PRJNA827465;Yang2025_Silkie_Wenchang	CRA004775	0.919979266
ENSGALG00010020053	LFNG	2	-0.057439733	0.102132075	-0.562406405	0.573839156	0	0.061030306	0	1	CRA004775;Yang2025_Silkie_Wenchang	PRJNA827465	0.886032852
ENSGALG00010020053	LFNG	2	0.097606472	0.080979544	1.205322568	0.228078801	0	0.190170105	0	0.5	CRA004775;PRJNA827465	Yang2025_Silkie_Wenchang	0.740938585
ENSGALG00010020055	R3HCC1	2	-0.070873178	0.042697219	-1.659901501	0.096934269	0	0.062635873	0	1	PRJNA827465;Yang2025_Silkie_Wenchang	CRA004775	0.532111212
ENSGALG00010020055	R3HCC1	2	-0.110816792	0.06398982	-1.731787844	0.083311343	0	0.793251645	0	1	CRA004775;Yang2025_Silkie_Wenchang	PRJNA827465	0.497316548
ENSGALG00010020055	R3HCC1	2	-0.088876176	0.062035223	-1.432672785	0.151951371	0.001645378	1.123175804	10.96674298	1	CRA004775;PRJNA827465	Yang2025_Silkie_Wenchang	0.643294424
ENSGALG00010020056	UACA	2	0.52571649	0.473997875	1.109111491	0.267382088	0.417965556	14.1136376	92.91465441	1	PRJNA827465;Yang2025_Silkie_Wenchang	CRA004775	0.771398294
ENSGALG00010020056	UACA	2	0.677068344	0.405200153	1.670947899	0.094731969	0.232088067	3.168997632	68.44428061	1	CRA004775;Yang2025_Silkie_Wenchang	PRJNA827465	0.526919621
ENSGALG00010020056	UACA	2	0.078660523	0.124302188	0.63281688	0.526853232	0	0.084770728	0	1	CRA004775;PRJNA827465	Yang2025_Silkie_Wenchang	0.870806702
ENSGALG00010020057	RBKS	2	0.041890061	0.151524947	0.276456528	0.782197445	0.027356547	2.433440099	58.90591264	0.5	PRJNA827465;Yang2025_Silkie_Wenchang	CRA004775	0.945372683
ENSGALG00010020057	RBKS	2	-0.333883521	0.243605866	-1.370589004	0.170503113	0.078287973	2.798546814	64.26716912	1	CRA004775;Yang2025_Silkie_Wenchang	PRJNA827465	0.672201941
ENSGALG00010020057	RBKS	2	-0.19043536	0.398002836	-0.478477394	0.63231046	0.280080086	8.254584526	87.8855199	0.5	CRA004775;PRJNA827465	Yang2025_Silkie_Wenchang	0.903058174
ENSGALG00010020059	PRKCZ	2	0.095302852	0.123659286	0.770689002	0.440891292	0.010332478	1.476278225	32.26209105	0.5	PRJNA827465;Yang2025_Silkie_Wenchang	CRA004775	0.844888742
ENSGALG00010020059	PRKCZ	2	0.255152569	0.130038801	1.962126431	0.049747766	0	0.003581569	0	1	CRA004775;Yang2025_Silkie_Wenchang	PRJNA827465	0.386162809
ENSGALG00010020059	PRKCZ	2	0.080445939	0.124966204	0.643741564	0.519743033	0.007916296	1.280264317	21.89112927	0.5	CRA004775;PRJNA827465	Yang2025_Silkie_Wenchang	0.869105702
ENSGALG00010020061	UBA3	2	-0.030155565	0.037930936	-0.795012414	0.426606281	0	0.24166168	0	1	PRJNA827465;Yang2025_Silkie_Wenchang	CRA004775	0.84125685
ENSGALG00010020061	UBA3	2	-0.053746587	0.087089	-0.617145527	0.537138742	0	0.223764668	0	0.5	CRA004775;Yang2025_Silkie_Wenchang	PRJNA827465	0.87481821
ENSGALG00010020061	UBA3	2	-0.020067247	0.040321549	-0.497680472	0.618709274	0	0.066531429	0	0.5	CRA004775;PRJNA827465	Yang2025_Silkie_Wenchang	0.899168321
ENSGALG00010020063	ENSGALG00010020063	2	-0.041314614	0.057637423	-0.716801883	0.473496349	0	0.105567569	0	1	PRJNA827465;Yang2025_Silkie_Wenchang	CRA004775	0.854373829
ENSGALG00010020063	ENSGALG00010020063	2	-0.233064204	0.307783356	-0.757234592	0.448909329	0.132500881	2.617475188	61.79524434	1	CRA004775;Yang2025_Silkie_Wenchang	PRJNA827465	0.847095089
ENSGALG00010020063	ENSGALG00010020063	2	-0.24259692	0.283172023	-0.856712174	0.391603974	0.109675193	2.367333308	57.75837746	1	CRA004775;PRJNA827465	Yang2025_Silkie_Wenchang	0.829251057
ENSGALG00010020064	TMEM138	2	-0.132421349	0.166835535	-0.793723884	0.427356193	0.033111802	2.038439374	50.94286283	1	PRJNA827465;Yang2025_Silkie_Wenchang	CRA004775	0.84125685
ENSGALG00010020064	TMEM138	2	-0.3477369	0.147563302	-2.356526962	0.018446728	0	0.031844115	0	1	CRA004775;Yang2025_Silkie_Wenchang	PRJNA827465	0.227285201
ENSGALG00010020064	TMEM138	2	-0.127714052	0.146167487	-0.873751436	0.382253633	0.025610602	2.199970999	54.54485534	1	CRA004775;PRJNA827465	Yang2025_Silkie_Wenchang	0.825080207
ENSGALG00010020065	TTYH3	2	0.059757891	0.077329248	0.772772173	0.439657225	0	0.021318995	0	1	PRJNA827465;Yang2025_Silkie_Wenchang	CRA004775	0.844278492
ENSGALG00010020065	TTYH3	2	0.043718652	0.164575568	0.265644848	0.790512742	0	0.54747779	0	0.5	CRA004775;Yang2025_Silkie_Wenchang	PRJNA827465	0.947530415
ENSGALG00010020065	TTYH3	2	0.04440119	0.085422348	0.519784244	0.603213962	0	0.511893306	0	0.5	CRA004775;PRJNA827465	Yang2025_Silkie_Wenchang	0.895538966
ENSGALG00010020066	ENSGALG00010020066	2	0.31029118	0.367505497	0.844317112	0.398492226	0.078956742	1.395776212	28.35527707	0.5	PRJNA827465;Yang2025_Silkie_Wenchang	CRA004775	0.831980453
ENSGALG00010020066	ENSGALG00010020066	2	-0.109508143	0.344646582	-0.317740402	0.750681862	0	0.00248241	0	1	CRA004775;Yang2025_Silkie_Wenchang	PRJNA827465	0.937473087
ENSGALG00010020066	ENSGALG00010020066	2	0.31694834	0.351822271	0.900876282	0.367654103	0.063611181	1.33517631	25.10352434	0.5	CRA004775;PRJNA827465	Yang2025_Silkie_Wenchang	0.819975483
ENSGALG00010020069	TMEM216	2	0.159491839	0.177759972	0.89723146	0.369595421	0	0.250108911	0	1	PRJNA827465;Yang2025_Silkie_Wenchang	CRA004775	0.820606801
ENSGALG00010020069	TMEM216	2	0.138256076	0.207557154	0.666110869	0.505340238	0	0.405865302	0	1	CRA004775;Yang2025_Silkie_Wenchang	PRJNA827465	0.863862536
ENSGALG00010020069	TMEM216	2	0.070383577	0.169320209	0.415683262	0.677641791	0	0.047160402	0	1	CRA004775;PRJNA827465	Yang2025_Silkie_Wenchang	0.917252904
ENSGALG00010020070	ARL6IP5	2	-0.220412886	0.103995327	-2.119449912	0.034052463	0.014088945	2.602640549	61.57748329	1	PRJNA827465;Yang2025_Silkie_Wenchang	CRA004775	0.31730678
ENSGALG00010020070	ARL6IP5	2	-0.328417741	0.103180942	-3.182930259	0.001457927	0	0.164272172	0	1	CRA004775;Yang2025_Silkie_Wenchang	PRJNA827465	0.047277983
ENSGALG00010020070	ARL6IP5	2	-0.147106668	0.056466879	-2.605185049	0.009182465	0	0.293866139	0	1	CRA004775;PRJNA827465	Yang2025_Silkie_Wenchang	0.150281327
ENSGALG00010020071	ENSGALG00010020071	2	0.361278315	0.061512296	5.873269832	4.27282E-09	0	0.259505969	0	1	PRJNA827465;Yang2025_Silkie_Wenchang	CRA004775	2.30048E-06
ENSGALG00010020071	ENSGALG00010020071	2	0.201576817	0.299605107	0.67280835	0.501069225	0.110506392	2.570027461	61.08990993	0.5	CRA004775;Yang2025_Silkie_Wenchang	PRJNA827465	0.86247641
ENSGALG00010020071	ENSGALG00010020071	2	0.199526854	0.223078659	0.894423763	0.371095203	0.069171335	2.534354109	60.54221483	0.5	CRA004775;PRJNA827465	Yang2025_Silkie_Wenchang	0.8214684
ENSGALG00010020074	ENSGALG00010020074	2	-0.149441481	0.23775187	-0.628560695	0.529636699	0.099060007	8.074685892	87.61561733	0.5	PRJNA827465;Yang2025_Silkie_Wenchang	CRA004775	0.871944741
ENSGALG00010020074	ENSGALG00010020074	2	-0.440720637	0.109334512	-4.030937987	5.55547E-05	0	0.84268507	0	1	CRA004775;Yang2025_Silkie_Wenchang	PRJNA827465	0.005223604
ENSGALG00010020074	ENSGALG00010020074	2	-0.244575807	0.360313221	-0.678786659	0.497273048	0.22622055	7.398985627	86.48463384	0.5	CRA004775;PRJNA827465	Yang2025_Silkie_Wenchang	0.86098656
ENSGALG00010020075	CPSF7	2	-0.01504909	0.047542634	-0.316538829	0.751593558	0	0.040271502	0	1	PRJNA827465;Yang2025_Silkie_Wenchang	CRA004775	0.937716403
ENSGALG00010020075	CPSF7	2	-0.182657062	0.190058806	-0.961055507	0.33652426	0.062654687	7.364724842	86.42176019	1	CRA004775;Yang2025_Silkie_Wenchang	PRJNA827465	0.803806405
ENSGALG00010020075	CPSF7	2	-0.189353297	0.180117285	-1.051277764	0.293131036	0.056318648	7.275835108	86.25587324	1	CRA004775;PRJNA827465	Yang2025_Silkie_Wenchang	0.786774237
ENSGALG00010020080	SLC4A1AP	2	0.127276088	0.04151842	3.065533025	0.002172824	0	0.352112674	0	1	PRJNA827465;Yang2025_Silkie_Wenchang	CRA004775	0.061310839
ENSGALG00010020080	SLC4A1AP	2	0.06568777	0.144164092	0.455645853	0.648644665	0.02473867	2.167269156	53.85898437	0.5	CRA004775;Yang2025_Silkie_Wenchang	PRJNA827465	0.907730933
ENSGALG00010020080	SLC4A1AP	2	0.057596704	0.102866907	0.5599148	0.575537553	0.011394068	1.605457372	37.71245395	0.5	CRA004775;PRJNA827465	Yang2025_Silkie_Wenchang	0.886655396
ENSGALG00010020084	ENSGALG00010020084	2	0.085183411	0.066519736	1.280573491	0.200343516	0	0.539036719	0	1	PRJNA827465;Yang2025_Silkie_Wenchang	CRA004775	0.712588345
ENSGALG00010020084	ENSGALG00010020084	2	0.02372117	0.107472761	0.220717976	0.825312034	0	0.02902159	0	1	CRA004775;Yang2025_Silkie_Wenchang	PRJNA827465	0.956381808
ENSGALG00010020084	ENSGALG00010020084	2	0.11763928	0.079441147	1.480835615	0.138650383	0	0.017717317	0	1	CRA004775;PRJNA827465	Yang2025_Silkie_Wenchang	0.622396264
ENSGALG00010020087	ENSGALG00010020087	2	0.174010923	0.35531586	0.489735873	0.624320814	0	0.04300888	0	1	PRJNA827465;Yang2025_Silkie_Wenchang	CRA004775	0.900886179
ENSGALG00010020087	ENSGALG00010020087	2	1.130745785	0.867829092	1.302959069	0.192588736	1.228041077	5.413056343	81.52614832	1	CRA004775;Yang2025_Silkie_Wenchang	PRJNA827465	0.702488639
ENSGALG00010020087	ENSGALG00010020087	2	1.039349673	0.942024531	1.10331487	0.269890451	1.528303998	7.192810138	86.09722791	1	CRA004775;PRJNA827465	Yang2025_Silkie_Wenchang	0.772187652
ENSGALG00010020088	LMOD3	2	-0.004228777	0.223289543	-0.018938534	0.98489014	0.088890798	9.209391047	89.14151875	0.5	PRJNA827465;Yang2025_Silkie_Wenchang	CRA004775	0.995759046
ENSGALG00010020088	LMOD3	2	0.210128046	0.100356566	2.093814623	0.036276497	0	0.222608424	0	0.5	CRA004775;Yang2025_Silkie_Wenchang	PRJNA827465	0.328451985
ENSGALG00010020088	LMOD3	2	-0.224100254	0.10476628	-2.139049454	0.032431663	0	0.061032099	0	1	CRA004775;PRJNA827465	Yang2025_Silkie_Wenchang	0.309308824
ENSGALG00010020090	MORF4L2	2	-0.056868722	0.039790603	-1.429199819	0.15294681	0	0.061292347	0	1	PRJNA827465;Yang2025_Silkie_Wenchang	CRA004775	0.645068129
ENSGALG00010020090	MORF4L2	2	-0.021740408	0.072677295	-0.299136171	0.764836149	0	0.418628927	0	0.5	CRA004775;Yang2025_Silkie_Wenchang	PRJNA827465	0.940711384
ENSGALG00010020090	MORF4L2	2	-0.054444721	0.045038363	-1.208852143	0.226719655	0	0.619887427	0	0.5	CRA004775;PRJNA827465	Yang2025_Silkie_Wenchang	0.740342522
ENSGALG00010020091	SUPT7L	2	-0.109962402	0.104897	-1.048289291	0.294505342	0.016831794	3.953772829	74.70770216	1	PRJNA827465;Yang2025_Silkie_Wenchang	CRA004775	0.787408474
ENSGALG00010020091	SUPT7L	2	-0.223267494	0.090895991	-2.456296383	0.014037733	0	0.093259297	0	1	CRA004775;Yang2025_Silkie_Wenchang	PRJNA827465	0.193996137
ENSGALG00010020091	SUPT7L	2	-0.023000276	0.046974987	-0.489628161	0.624397046	0	0.155863178	0	1	CRA004775;PRJNA827465	Yang2025_Silkie_Wenchang	0.900886179
ENSGALG00010020092	SDHAF2	2	0.029247819	0.077215388	0.378782263	0.70484956	0	0.02474118	0	0.5	PRJNA827465;Yang2025_Silkie_Wenchang	CRA004775	0.924152671
ENSGALG00010020092	SDHAF2	2	-0.02723812	0.186790507	-0.145821755	0.884062101	0	0.011234525	0	1	CRA004775;Yang2025_Silkie_Wenchang	PRJNA827465	0.972001076
ENSGALG00010020092	SDHAF2	2	0.026581268	0.077727367	0.341980809	0.732365335	0	0.081316456	0	0.5	CRA004775;PRJNA827465	Yang2025_Silkie_Wenchang	0.932985777
ENSGALG00010020094	CLEC3B	2	-0.168385606	0.082048933	-2.052258313	0.040144565	0	0.044201571	0	1	PRJNA827465;Yang2025_Silkie_Wenchang	CRA004775	0.347067015
ENSGALG00010020094	CLEC3B	2	-0.146929649	0.119802912	-1.226428024	0.220037646	0	0.0003001	0	1	CRA004775;Yang2025_Silkie_Wenchang	PRJNA827465	0.733691086
ENSGALG00010020094	CLEC3B	2	-0.177410633	0.09602914	-1.847466648	0.064679538	0	0.012005992	0	1	CRA004775;PRJNA827465	Yang2025_Silkie_Wenchang	0.438519901

ENSGALG00010020106	ENSGALG00010020106	2	-0.008768209	0.070432629	-0.124490731	0.900926739	0	0.944504923	0	0.5	PRJNA827465;Yang2025_Silkie_Wenchang	CRA004775	0.976526284
ENSGALG00010020106	ENSGALG00010020106	2	0.051935512	0.100456387	0.516995618	0.605159218	0	0.272021758	0	0.5	CRA004775;Yang2025_Silkie_Wenchang	PRJNA827465	0.896474603
ENSGALG00010020106	ENSGALG00010020106	2	-0.076709986	0.093659132	-0.819033714	0.412767179	0	0.041109997	0	1	CRA004775;PRJNA827465	Yang2025_Silkie_Wenchang	0.835805689
ENSGALG00010020108	RPAP1	2	-0.002307307	0.090351482	-0.025537008	0.97962663	0.010388463	2.632531098	62.01374408	0.5	PRJNA827465;Yang2025_Silkie_Wenchang	CRA004775	0.994472942
ENSGALG00010020108	RPAP1	2	-0.012060327	0.173221025	-0.069623922	0.944492996	0.034395291	1.959122759	48.9567463	0.5	CRA004775;Yang2025_Silkie_Wenchang	PRJNA827465	0.985935636
ENSGALG00010020108	RPAP1	2	-0.089924267	0.059069652	-1.522342922	0.127923179	0	0.554206856	0	1	CRA004775;PRJNA827465	Yang2025_Silkie_Wenchang	0.60032911
ENSGALG00010020109	WDFY4	2	0.027199821	0.131799914	0.206372066	0.836500289	0	0.203995654	0	0.5	PRJNA827465;Yang2025_Silkie_Wenchang	CRA004775	0.958399882
ENSGALG00010020109	WDFY4	2	0.126598647	0.204726393	0.618379706	0.536325072	0	0.060126265	0	1	CRA004775;Yang2025_Silkie_Wenchang	PRJNA827465	0.874599474
ENSGALG00010020109	WDFY4	2	0.004385404	0.157856652	0.027780927	0.977836879	0	0.217308028	0	0.5	CRA004775;PRJNA827465	Yang2025_Silkie_Wenchang	0.993739602
ENSGALG00010020111	ADRA2B	2	0.256201345	0.280264365	0.914141705	0.360642391	0.145844145	13.79200299	92.74942153	0.5	PRJNA827465;Yang2025_Silkie_Wenchang	CRA004775	0.815038117
ENSGALG00010020111	ADRA2B	2	0.306042402	0.285796944	1.070838609	0.284242003	0.122648739	3.669584805	72.74896062	0.5	CRA004775;Yang2025_Silkie_Wenchang	PRJNA827465	0.780082638
ENSGALG00010020111	ADRA2B	2	-0.017147678	0.077689206	-0.220721495	0.825309294	0	0.004808033	0	1	CRA004775;PRJNA827465	Yang2025_Silkie_Wenchang	0.956381808
ENSGALG00010020113	CIAO1	2	0.080115081	0.205288261	0.390256512	0.696346877	0.069059916	5.128863925	80.50250475	0.5	PRJNA827465;Yang2025_Silkie_Wenchang	CRA004775	0.921041503
ENSGALG00010020113	CIAO1	2	-0.014167509	0.35722485	-0.03965992	0.968364256	0.207631704	5.207332759	80.79631077	0.5	CRA004775;Yang2025_Silkie_Wenchang	PRJNA827465	0.991215679
ENSGALG00010020113	CIAO1	2	-0.143183272	0.106259458	-1.347487323	0.177823333	0.007805418	1.205361706	17.0373511	1	CRA004775;PRJNA827465	Yang2025_Silkie_Wenchang	0.683951879
ENSGALG00010020116	GNA12	2	-0.213726366	0.125476604	-1.703316478	0.088508859	0.024567918	4.30832045	76.78909887	1	PRJNA827465;Yang2025_Silkie_Wenchang	CRA004775	0.511042113
ENSGALG00010020116	GNA12	2	-0.205660668	0.241847549	-0.850373176	0.395117645	0.069675518	1.92027119	47.92402213	0.5	CRA004775;Yang2025_Silkie_Wenchang	PRJNA827465	0.830643015
ENSGALG00010020116	GNA12	2	-0.096342075	0.057659355	-1.670883696	0.094744652	0	0.571578774	0	0.5	CRA004775;PRJNA827465	Yang2025_Silkie_Wenchang	0.526919621
ENSGALG00010020117	AKAP13	2	-0.024258006	0.060110598	-0.403556221	0.686539084	0	0.39396959	0	1	PRJNA827465;Yang2025_Silkie_Wenchang	CRA004775	0.919235539
ENSGALG00010020117	AKAP13	2	-0.148339382	0.139968887	-1.059802547	0.289234438	0	0.803003825	0	1	CRA004775;Yang2025_Silkie_Wenchang	PRJNA827465	0.783530627
ENSGALG00010020117	AKAP13	2	-0.079925146	0.183165266	-0.436355362	0.66257892	0.034478479	1.317724419	24.11159833	1	CRA004775;PRJNA827465	Yang2025_Silkie_Wenchang	0.912778072
ENSGALG00010020118	GNB1	2	-0.222089155	0.226657029	-0.979846757	0.327161768	0.100028461	37.57769939	97.3388472	0.5	PRJNA827465;Yang2025_Silkie_Wenchang	CRA004775	0.800028239
ENSGALG00010020118	GNB1	2	-0.237135913	0.257937688	-0.919353488	0.357910709	0.108505876	4.723890226	78.83100681	0.5	CRA004775;Yang2025_Silkie_Wenchang	PRJNA827465	0.813641308
ENSGALG00010020118	GNB1	2	0.003572437	0.03744562	0.095403345	0.923994459	0	0.091366056	0	1	CRA004775;PRJNA827465	Yang2025_Silkie_Wenchang	0.982276764
ENSGALG00010020120	DAAM2	2	0.12239279	0.096803469	1.264343022	0.206106934	0	0.662443461	0	1	PRJNA827465;Yang2025_Silkie_Wenchang	CRA004775	0.718403416
ENSGALG00010020120	DAAM2	2	0.205213004	0.17608878	1.165395113	0.243859095	0	0.405522909	0	0.5	CRA004775;Yang2025_Silkie_Wenchang	PRJNA827465	0.755365463
ENSGALG00010020120	DAAM2	2	0.071352999	0.106559311	0.669608304	0.503107519	0	0.050816302	0	0.5	CRA004775;PRJNA827465	Yang2025_Silkie_Wenchang	0.863052662
ENSGALG00010020121	ENSGALG00010020121	2	0.054752837	0.179445761	0.305121929	0.76027329	0	0.755355274	0	0.5	PRJNA827465;Yang2025_Silkie_Wenchang	CRA004775	0.939327609
ENSGALG00010020121	ENSGALG00010020121	2	0.190129609	0.278629835	0.682373474	0.495002824	0	0.386468354	0	0.5	CRA004775;Yang2025_Silkie_Wenchang	PRJNA827465	0.860496668
ENSGALG00010020121	ENSGALG00010020121	2	-0.068016704	0.208219739	-0.326658292	0.74392635	0	0.027175213	0	1	CRA004775;PRJNA827465	Yang2025_Silkie_Wenchang	0.935821071
ENSGALG00010020123	ENSGALG00010020123	2	-0.259017542	0.095960433	-2.699211906	0.00695039	0	0.057177884	0	1	PRJNA827465;Yang2025_Silkie_Wenchang	CRA004775	0.128069009
ENSGALG00010020123	ENSGALG00010020123	2	-0.186761231	0.166740925	-1.120068342	0.26268464	0	0.227216803	0	1	CRA004775;Yang2025_Silkie_Wenchang	PRJNA827465	0.768519756
ENSGALG00010020123	ENSGALG00010020123	2	-0.254649124	0.108841541	-2.339631734	0.019302763	0	0.387886362	0	1	CRA004775;PRJNA827465	Yang2025_Silkie_Wenchang	0.23264535
ENSGALG00010020125	NAV2	2	0.11182725	0.24448489	0.457399432	0.647383973	0.099641562	5.56021679	82.01508974	0.5	PRJNA827465;Yang2025_Silkie_Wenchang	CRA004775	0.907350807
ENSGALG00010020125	NAV2	2	0.311411102	0.172824	1.80189732	0.071561562	0	0.828448159	0	0.5	CRA004775;Yang2025_Silkie_Wenchang	PRJNA827465	0.461684844
ENSGALG00010020125	NAV2	2	-0.099422971	0.078791334	-1.261851596	0.207002192	0	0.060862759	0	1	CRA004775;PRJNA827465	Yang2025_Silkie_Wenchang	0.718911823
ENSGALG00010020126	NADK	2	0.163623167	0.124388744	1.315417797	0.188369527	0.022056413	3.296150669	69.66158103	1	PRJNA827465;Yang2025_Silkie_Wenchang	CRA004775	0.697727584
ENSGALG00010020126	NADK	2	0.013506615	0.115153557	0.117292213	0.906628496	0	0.025862732	0	0.5	CRA004775;Yang2025_Silkie_Wenchang	PRJNA827465	0.978196186
ENSGALG00010020126	NADK	2	0.261702531	0.068614755	3.814085315	0.000136688	0	0.583982469	0	0.5	CRA004775;PRJNA827465	Yang2025_Silkie_Wenchang	0.009757782
ENSGALG00010020127	FRMD4B	2	-0.036303404	0.086478594	-0.419796412	0.674634186	0	0.011666032	0	1	PRJNA827465;Yang2025_Silkie_Wenchang	CRA004775	0.915970608
ENSGALG00010020127	FRMD4B	2	0.005620093	0.148951106	0.037731129	0.969902056	0	0.157236016	0	0.5	CRA004775;Yang2025_Silkie_Wenchang	PRJNA827465	0.991572914
ENSGALG00010020127	FRMD4B	2	-0.028918271	0.097991345	-0.29511046	0.7679095	0	0.225621298	0	0.5	CRA004775;PRJNA827465	Yang2025_Silkie_Wenchang	0.941444269
ENSGALG00010020129	MGAT2	2	-0.158324905	0.220021631	-0.719587908	0.471778759	0.087999583	10.47059015	90.44943995	0.5	PRJNA827465;Yang2025_Silkie_Wenchang	CRA004775	0.853998373
ENSGALG00010020129	MGAT2	2	-0.310806835	0.105134658	-2.956273794	0.003113805	0.001944893	1.091040133	8.344343215	1	CRA004775;Yang2025_Silkie_Wenchang	PRJNA827465	0.077163868
ENSGALG00010020129	MGAT2	2	-0.012140749	0.099029158	-0.12259772	0.90242566	0.010776016	1.738922217	42.49311498	0.5	CRA004775;PRJNA827465	Yang2025_Silkie_Wenchang	0.976923607
ENSGALG00010020130	PDXDC1	2	0.229771023	0.091121098	2.521600684	0.011682224	0.00885304	2.056556415	51.37502707	1	PRJNA827465;Yang2025_Silkie_Wenchang	CRA004775	0.172876725
ENSGALG00010020130	PDXDC1	2	0.148683956	0.215747653	0.689156773	0.490724616	0.071594955	4.13783963	75.83279949	0.5	CRA004775;Yang2025_Silkie_Wenchang	PRJNA827465	0.858798485
ENSGALG00010020130	PDXDC1	2	0.088202017	0.110099951	0.80110859	0.423068784	0.011441607	1.585786991	36.93982824	0.5	CRA004775;PRJNA827465	Yang2025_Silkie_Wenchang	0.840046095
ENSGALG00010020131	SNRNP200	2	0.000930371	0.106936409	0.008700224	0.993058313	0.016185636	3.336449806	70.02802206	0.5	PRJNA827465;Yang2025_Silkie_Wenchang	CRA004775	0.99794341
ENSGALG00010020131	SNRNP200	2	-0.073624866	0.213456747	-0.344917025	0.730156751	0.073543296	4.973972683	79.89534596	0.5	CRA004775;Yang2025_Silkie_Wenchang	PRJNA827465	0.932614156
ENSGALG00010020131	SNRNP200	2	-0.14850671	0.092359158	-1.607926188	0.107851346	0.006772127	1.419592364	29.55724298	1	CRA004775;PRJNA827465	Yang2025_Silkie_Wenchang	0.555918877
ENSGALG00010020132	SHC4	2	0.106583924	0.097263818	1.09582295	0.273156263	0	0.003776797	0	1	PRJNA827465;Yang2025_Silkie_Wenchang	CRA004775	0.773904763
ENSGALG00010020132	SHC4	2	0.16224314	0.155754566	1.041658964	0.297569821	0	0.82801816	0	1	CRA004775;Yang2025_Silkie_Wenchang	PRJNA827465	0.788562929
ENSGALG00010020132	SHC4	2	0.143313718	0.112824379	1.270237147	0.204000169	0	0.848402858	0	1	CRA004775;PRJNA827465	Yang2025_Silkie_Wenchang	0.717254253
ENSGALG00010020136	KCNK17	2	1.438779455	0.280398685	5.131191883	2.87913E-07	0.070653501	1.779630783	43.8085692	1	PRJNA827465;Yang2025_Silkie_Wenchang	CRA004775	8.08275E-05
ENSGALG00010020136	KCNK17	2	0.782544581	0.370798711	2.110429613	0.034821368	0.128541892	1.852793392	46.02744136	1	CRA004775;Yang2025_Silkie_Wenchang	PRJNA827465	0.321263903
ENSGALG00010020136	KCNK17	2	1.065903823	0.656059499	1.624706029	0.104225212	0.739771446	6.907149065	85.52224672	1	CRA004775;PRJNA827465	Yang2025_Silkie_Wenchang	0.548669102
ENSGALG00010020137	NTAN1	2	0.129798846	0.151648648	0.855918253	0.392042998	0.037783962	5.296056706	81.11802695	0.5	PRJNA827465;Yang2025_Silkie_Wenchang	CRA004775	0.829251057
ENSGALG00010020137	NTAN1	2	0.134597027	0.207598544	0.64835246	0.516757011	0.056278302	2.628113263	61.94988952	0.5	CRA004775;Yang2025_Silkie_Wenchang	PRJNA827465	0.867952013
ENSGALG00010020137	NTAN1	2	-0.013093918	0.057963253	-0.225900335	0.821278937	0	0.2495618	0	1	CRA004775;PRJNA827465	Yang2025_Silkie_Wenchang	0.955598716
ENSGALG00010020138	ENSGALG00010020138	2	-0.08860076	0.21899965	-0.404570325	0.685793375	0.088378296	12.36499557	91.91265379	0.5	PRJNA827465;Yang2025_Silkie_Wenchang	CRA004775	0.919235539
ENSGALG00010020138	ENSGALG00010020138	2	-0.280228296	0.096319959	-2.909348148	0.003621833	0	0.457001399	0	1	CRA004775;Yang2025_Silkie_Wenchang	PRJNA827465	0.085650848
ENSGALG00010020138	ENSGALG00010020138	2	0.031696676	0.133327242	0.23773593	0.812085914	0.022507593	2.155581096	53.6087971	0.5	CRA004775;PRJNA827465	Yang2025_Silkie_Wenchang	0.953371387
ENSGALG00010020140	SECISBP2L	2	0.032318919	0.05559995	0.581276052	0.561054418	0	0.106782863	0	0.5	PRJNA827465;Yang2025_Silkie_Wenchang	CRA004775	0.881417858
ENSGALG00010020140	SECISBP2L	2	0.229510952	0.317327195	0.723262791	0.469518448	0.137338626	2.734677318	63.43261439	0.5	CRA004775;Yang2025_Silkie_Wenchang	PRJNA827465	0.853485658
ENSGALG00010020140	SECISBP2L	2	0.232978595	0.282379145	0.825055954	0.409339825	0.113507252	2.621738454	61.85736993	1	CRA004775;PRJNA827465	Yang2025_Silkie_Wenchang	0.834355341
ENSGALG00010020141	THOC7	2	-0.122391979	0.067214146	-1.820925893	0.068618123	0	0.245868519	0	1	PRJNA827465;Yang2025_Silkie_Wenchang	CRA004775	0.45121266
ENSGALG00010020141	THOC7	2	-0.034510184	0.129745229	-0.265984226	0.790251357	0	0.040663307	0	0.5	CRA004775;Yang2025_Silkie_Wenchang	PRJNA827465	0.947390191
ENSGALG00010020141	THOC7	2	-0.124886944	0.07083946	-1.762957312	0.07790769	0	0.342851373	0	0.5	CRA004775;PRJNA827465	Yang2025_Silkie_Wenchang	0.480724545
ENSGALG00010020142	SLC35E2B	2	-0.184304552	0.279731948	-0.658861289	0.509984846	0.149291727	21.46103317	95.34039209	0.5	PRJNA827465;Yang2025_Silkie_Wenchang	CRA004775	0.865276949
ENSGALG00010020142	SLC35E2B	2	-0.429877103	0.095923483	-4.481458453	7.41347E-06	0	0.922596506	0	1	CRA004775;Yang2025_Silkie_Wenchang	PRJNA827465	0.001106098
ENSGALG00010020142	SLC35E2B	2	0.017492631	0.130799187	0.133736545	0.893610906	0.018622337	1.62570347	38.48816722	0.5	CRA004775;PRJNA827465	Yang2025_Silkie_Wenchang	0.974381724
ENSGALG00010020143	KCNK5	2	-0.041582133	0.416995084	-0.099718521	0.920567797	0.307177346	8.522982714	88.26701832	0.5	PRJNA827465;Yang2025_Silkie_Wenchang	CRA004775	0.98144788
ENSGALG00010020143	KCNK5	2	-0.305033521	0.286010719	-1.066510801	0.286192799	0.058945002	1.368732237	26.93969115	0.5	CRA004775;Yang2025_Silkie_Wenchang	PRJNA827465	0.781941993
ENSGALG00010020143	KCNK5	2	0.346446974	0.168433764	2.056873664	0.03969838	0	0.099002458	0	1	CRA004775;PRJNA827465	Yang2025_Silkie_Wenchang	0.344755224
ENSGALG00010020147	ABHD2	2	-0.174490172	0.260048831	-0.670990027	0.502226883	0.117971675	7.691992703	86.99946765	0.5	PRJNA827465;Yang2025_Silkie_Wenchang	CRA004775	0.862860181
ENSGALG00010020147	ABHD2	2	-0.234914596	0.321970302	-0.729615727	0.465625106	0.124597147	2.03812353	50.93526054	0.5	CRA004775;Yang2025_Silkie_Wenchang	PRJNA827465	0.852424459
ENSGALG00010020147	ABHD2	2	0.081155762	0.101157634	0.802270266	0.422396638	0	0.141107145	0	1	CRA004775;PRJNA827465	Yang2025_Silkie_Wenchang	0.839704389
ENSGALG00010020149	FAHD2A	2	0.110353463	0.090547373	1.218737327	0.222943906	0	0.008374056	0	1	PRJNA827465;Yang2025_Silkie_Wenchang	CRA004775	0.736458649
ENSGALG00010020149	FAHD2A	2	-0.094952683	0.244509641	-0.388339221	0.697765016	0.003458318	1.027027027	2.631578924	0.5	CRA004775;Yang2025_Silkie_Wenchang	PRJNA827465	0.921422004
ENSGALG00010020149	FAHD2A	2	-0.011317285	0.227667275	-0.049709757	0.960353681	0.058251526	1.660262293	39.76855319	0.5	CRA004775;PRJNA827465	Yang2025_Silkie_Wenchang	0.989338243
ENSGALG00010020152	ATXN7	2	-0.043398859	0.062469693	-0.694718627	0.487231652	0	0.004968737	0	1	PRJNA827465;Yang2025_Silkie_Wenchang	CRA004775	0.85768885
ENSGALG00010020152	ATXN7	2	0.122105255	0.219715005	0.555743814	0.578386	0.047316064	1.771198574	43.54105662	0.5	CRA004775;Yang2025_Silkie_Wenchang	PRJNA827465	0.887855709
ENSGALG00010020152	ATXN7	2	0.096644846	0.218512879	0.442284437	0.658283391	0.063271152	2.237351395	55.30429408	0.5	CRA004775;PRJNA827465	Yang2025_Silkie_Wenchang	0.911003948
ENSGALG00010020153	AXL	2	-0.173477264	0.227940965	-0.761062248	0.446619888	0.094429088	10.53678137	90.50943581	0.5	PRJNA827465;Yang2025_Silkie_Wenchang	CRA004775	0.84648396
ENSGALG00010020153	AXL	2	-0.37935292	0.120535522	-3.147229236	0.001648257	0	0.695036369	0	1	CRA004775;Yang2025_Silkie_Wenchang	PRJNA827465	0.051226717
ENSGALG00010020153	AXL	2	0.036617305	0.056765134	0.645066837	0.518883874	0	0.171473197	0	0.5	CRA004775;PRJNA827465	Yang2025_Silkie_Wenchang	0.868851946
ENSGALG00010020155	MFGE8	2	0.003819432	0.086363563	0.044225045	0.964725019	0	0.003402945	0	0.5	PRJNA827465;Yang2025_Silkie_Wenchang	CRA004775	0.990394578
ENSGALG00010020155	MFGE8	2	-0.002655358	0.1392606	-0.019067548	0.98478722	0	0.000315083	0	0.5	CRA004775;Yang2025_Silkie_Wenchang	PRJNA827465	0.995706218
ENSGALG00010020155	MFGE8	2	0.006402138	0.0962339	0.066526848	0.946958383	0	0.000324583	0	1	CRA004775;PRJNA827465	Yang2025_Silkie_Wenchang	0.986445196
ENSGALG00010020160	SYT7	2	0.199922232	0.445092489	0.449170086	0.653308965	0.274240318	3.024743714	66.93934777	0.5	PRJNA827465;Yang2025_Silkie_Wenchang	CRA004775	0.909597225
ENSGALG00010020160	SYT7	2	0.275118856	0.554642275	0.496029366	0.619873693	0.309005932	1.941188992	48.48518079	0.5	CRA004775;Yang2025_Silkie_Wenchang	PRJNA827465	0.899395593
ENSGALG00010020160	SYT7	2	-0.197546842	0.231939343	-0.851717692	0.394370799	0	0.098592978	0	1	CRA004775;PRJNA827465	Yang2025_Silkie_Wenchang	0.830486298
ENSGALG00010020163	CDC2L1	2	0.463565402	0.423297369	1.095129419	0.273459942	0.308411306	6.400245524	84.3755994	1	PRJNA827465;Yang2025_Silkie_Wenchang	CRA004775	0.774252172
ENSGALG00010020163	CDC2L1	2	0.445982101	0.466818404	0.955365293	0.339392999	0.364597367	5.931618434	83.14119475	1	CRA004775;Yang2025_Silkie_Wenchang	PRJNA827465	0.804824608
ENSGALG00010020163	CDC2L1	2	0.085246185	0.071923741	1.185230126	0.235926472	0	0.145857794	0	1	CRA004775;PRJNA827465	Yang2025_Silkie_Wenchang	0.748669015

ENSGALG00010020215	PRMT3	2	-0.114942509	0.101960597	-1.127322832	0.259606012	0	0.308760931	0	1	CRA004775;Yang2025_Silkie_Wenchang	PRJNA827465	0.76691144
ENSGALG00010020215	PRMT3	2	-0.010372225	0.091411622	-0.113467242	0.909660132	0.007034646	1.300684592	23.11741014	0.5	CRA004775;PRJNA827465	Yang2025_Silkie_Wenchang	0.978659044
ENSGALG00010020224	MRPL46	2	-0.056898448	0.072587344	-0.783861823	0.433121193	0	0.00050071	0	1	PRJNA827465;Yang2025_Silkie_Wenchang	CRA004775	0.842762754
ENSGALG00010020224	MRPL46	2	-0.094541486	0.189963745	-0.497681734	0.618708385	0	0.030641651	0	1	CRA004775;Yang2025_Silkie_Wenchang	PRJNA827465	0.899168321
ENSGALG00010020224	MRPL46	2	-0.06156121	0.072795907	-0.845668559	0.397737668	0	0.065381204	0	1	CRA004775;PRJNA827465	Yang2025_Silkie_Wenchang	0.831711071
ENSGALG00010020226	TCIM	2	0.060764795	0.094004987	0.646399697	0.518020537	0	2.072E-06	0	1	PRJNA827465;Yang2025_Silkie_Wenchang	CRA004775	0.868202369
ENSGALG00010020226	TCIM	2	0.20346641	0.192792177	1.055366526	0.291257726	0.023901981	1.416081658	29.38260347	1	CRA004775;Yang2025_Silkie_Wenchang	PRJNA827465	0.78467882
ENSGALG00010020226	TCIM	2	0.176638407	0.182649773	0.967088019	0.333500034	0.034811572	1.746072118	42.72859696	1	CRA004775;PRJNA827465	Yang2025_Silkie_Wenchang	0.802691373
ENSGALG00010020228	TM9SF3	2	-0.108258667	0.087634272	-1.235348502	0.21670083	0.011939019	4.292920203	76.70583303	1	PRJNA827465;Yang2025_Silkie_Wenchang	CRA004775	0.730172415
ENSGALG00010020228	TM9SF3	2	-0.158814106	0.097886	-1.62243943	0.104709292	0.00645299	1.264797191	20.9359408	0.5	CRA004775;Yang2025_Silkie_Wenchang	PRJNA827465	0.549804368
ENSGALG00010020228	TM9SF3	2	-0.027244181	0.041998539	-0.648693553	0.516536472	0	0.114298016	0	0.5	CRA004775;PRJNA827465	Yang2025_Silkie_Wenchang	0.86780512
ENSGALG00010020230	ENSGALG00010020230	2	0.100069405	0.119531018	0.837183575	0.402489388	0	0.373262002	0	1	PRJNA827465;Yang2025_Silkie_Wenchang	CRA004775	0.832905052
ENSGALG00010020230	ENSGALG00010020230	2	0.002133977	0.289806116	0.007363464	0.994124859	0.076776371	1.75491299	43.01171792	0.5	CRA004775;Yang2025_Silkie_Wenchang	PRJNA827465	0.99818842
ENSGALG00010020230	ENSGALG00010020230	2	-0.007835343	0.154809831	-0.050612694	0.959634148	0.009453067	1.115341591	10.34136916	0.5	CRA004775;PRJNA827465	Yang2025_Silkie_Wenchang	0.989259322
ENSGALG00010020236	SLC35G1	2	0.37196861	0.161273766	2.306442145	0.021085939	0	0.865234872	0	1	PRJNA827465;Yang2025_Silkie_Wenchang	CRA004775	0.245552792
ENSGALG00010020236	SLC35G1	2	0.212325094	0.222635396	0.953689744	0.340240713	0	0.038674005	0	1	CRA004775;Yang2025_Silkie_Wenchang	PRJNA827465	0.805377063
ENSGALG00010020236	SLC35G1	2	0.458726458	0.189068859	2.426240149	0.015256171	0	0.16677266	0	1	CRA004775;PRJNA827465	Yang2025_Silkie_Wenchang	0.204297547
ENSGALG00010020237	MIB2	2	0.053477384	0.068394871	0.781891722	0.43427821	0.004452623	1.747996518	42.79164805	1	PRJNA827465;Yang2025_Silkie_Wenchang	CRA004775	0.842762754
ENSGALG00010020237	MIB2	2	0.138807982	0.088487566	1.568672164	0.116724348	0	0.066019811	0	1	CRA004775;Yang2025_Silkie_Wenchang	PRJNA827465	0.575245849
ENSGALG00010020237	MIB2	2	0.008286175	0.05016647	0.165173572	0.868807366	0	0.123721342	0	1	CRA004775;PRJNA827465	Yang2025_Silkie_Wenchang	0.968372069
ENSGALG00010020244	SLC25A37	2	-0.14594616	0.391807732	-0.37249433	0.70952483	0.277147176	10.01673835	90.01671038	0.5	PRJNA827465;Yang2025_Silkie_Wenchang	CRA004775	0.925298042
ENSGALG00010020244	SLC25A37	2	-0.39221587	0.218082532	-1.79847449	0.072101848	0.028393649	1.385101856	27.80314344	1	CRA004775;Yang2025_Silkie_Wenchang	PRJNA827465	0.463486013
ENSGALG00010020244	SLC25A37	2	0.183764184	0.112526803	1.633070343	0.102454203	0	0.96323936	0	0.5	CRA004775;PRJNA827465	Yang2025_Silkie_Wenchang	0.545335529
ENSGALG00010020246	ADAMTS9	2	0.14046428	0.160328438	0.876103338	0.380973858	0.039905859	4.293074244	76.70666886	0.5	PRJNA827465;Yang2025_Silkie_Wenchang	CRA004775	0.824392515
ENSGALG00010020246	ADAMTS9	2	0.324045949	0.124550555	2.601722249	0.009275694	0	0.01015	0	1	CRA004775;Yang2025_Silkie_Wenchang	PRJNA827465	0.151020136
ENSGALG00010020246	ADAMTS9	2	0.016165364	0.081453254	0.198461862	0.842683721	0.000545293	1.008474063	0.840285677	0.5	CRA004775;PRJNA827465	Yang2025_Silkie_Wenchang	0.960333979
ENSGALG00010020248	NOLC1	2	-0.066741922	0.049758689	-1.341311916	0.179819203	0.000333425	1.060039924	5.663930434	1	PRJNA827465;Yang2025_Silkie_Wenchang	CRA004775	0.68659821
ENSGALG00010020248	NOLC1	2	-0.088419785	0.102248657	-0.864752535	0.387174628	0.005492624	1.219380614	17.99115151	0.5	CRA004775;Yang2025_Silkie_Wenchang	PRJNA827465	0.82737736
ENSGALG00010020248	NOLC1	2	-0.024314857	0.054595472	-0.445363989	0.656056734	0	0.422306976	0	0.5	CRA004775;PRJNA827465	Yang2025_Silkie_Wenchang	0.910420668
ENSGALG00010020250	ENSGALG00010020250	2	0.087386016	0.178596301	0.489293539	0.624633895	0.023691989	1.590336189	37.12021353	0.5	PRJNA827465;Yang2025_Silkie_Wenchang	CRA004775	0.90097623
ENSGALG00010020250	ENSGALG00010020250	2	0.493537526	0.330809439	1.491908839	0.135723045	0.117841989	1.863808599	46.34642202	1	CRA004775;Yang2025_Silkie_Wenchang	PRJNA827465	0.617196137
ENSGALG00010020250	ENSGALG00010020250	2	0.357969996	0.527581964	0.678510678	0.497447956	0.437871967	4.243551015	76.43483025	0.5	CRA004775;PRJNA827465	Yang2025_Silkie_Wenchang	0.860986656
ENSGALG00010020252	DMTN	2	0.345580653	0.172264049	2.006110122	0.0448445	0.027783745	1.741798952	42.58809269	1	PRJNA827465;Yang2025_Silkie_Wenchang	CRA004775	0.36506284
ENSGALG00010020252	DMTN	2	0.333982063	0.279836554	1.193489763	0.232677619	0.082000157	2.062789588	51.52195815	1	CRA004775;Yang2025_Silkie_Wenchang	PRJNA827465	0.745733382
ENSGALG00010020252	DMTN	2	0.187750745	0.122822138	1.52863928	0.126353893	0	0.357906092	0	1	CRA004775;PRJNA827465	Yang2025_Silkie_Wenchang	0.596957212
ENSGALG00010020254	ENSGALG00010020254	2	0.264627706	0.13526766	1.956327359	0.050426603	0.024983842	2.902446175	65.54630336	1	PRJNA827465;Yang2025_Silkie_Wenchang	CRA004775	0.38897029
ENSGALG00010020254	ENSGALG00010020254	2	0.288575296	0.220748817	1.307256462	0.191125659	0.048056946	1.687965627	40.75708747	0.5	CRA004775;Yang2025_Silkie_Wenchang	PRJNA827465	0.700443419
ENSGALG00010020254	ENSGALG00010020254	2	0.144874334	0.072609676	1.995248314	0.046015804	0	0.352640317	0	0.5	CRA004775;PRJNA827465	Yang2025_Silkie_Wenchang	0.369999825
ENSGALG00010020257	ZNF710	2	0.135281394	0.103929582	1.301663987	0.193031276	0.014737339	3.029133292	66.98725663	1	PRJNA827465;Yang2025_Silkie_Wenchang	CRA004775	0.703058866
ENSGALG00010020257	ZNF710	2	0.135347201	0.156796865	0.863200939	0.388026998	0.028505583	2.162425765	53.75563793	0.5	CRA004775;Yang2025_Silkie_Wenchang	PRJNA827465	0.828154121
ENSGALG00010020257	ZNF710	2	0.032927679	0.062256286	0.528905291	0.596871152	0	0.311444286	0	0.5	CRA004775;PRJNA827465	Yang2025_Silkie_Wenchang	0.894172266
ENSGALG00010020259	ENSGALG00010020259	2	-0.054210733	0.141109766	-0.384174215	0.700849328	0	0.185173843	0	1	PRJNA827465;Yang2025_Silkie_Wenchang	CRA004775	0.922496697
ENSGALG00010020259	ENSGALG00010020259	2	-0.176423239	0.203257014	-0.867981065	0.385404699	0	0.178937369	0	1	CRA004775;Yang2025_Silkie_Wenchang	PRJNA827465	0.82048313
ENSGALG00010020259	ENSGALG00010020259	2	-0.054159946	0.166014541	-0.326236159	0.744245686	0	0.495929524	0	1	CRA004775;PRJNA827465	Yang2025_Silkie_Wenchang	0.935821071
ENSGALG00010020261	SSU72	2	0.087984159	0.049117681	1.791293025	0.073246285	0	0.976816376	0	1	PRJNA827465;Yang2025_Silkie_Wenchang	CRA004775	0.467793099
ENSGALG00010020261	SSU72	2	0.054622018	0.193228771	0.282680566	0.777421715	0.044109039	2.326568495	57.01824372	0.5	CRA004775;Yang2025_Silkie_Wenchang	PRJNA827465	0.94430898
ENSGALG00010020261	SSU72	2	0.035835334	0.084829887	0.422437602	0.672705636	0.005554469	1.220288719	18.05218024	0.5	CRA004775;PRJNA827465	Yang2025_Silkie_Wenchang	0.914956406
ENSGALG00010020265	ENSGALG00010020265	2	0.135577685	0.37566084	0.360904494	0.718170844	0.245800858	7.648631404	86.92576558	0.5	PRJNA827465;Yang2025_Silkie_Wenchang	CRA004775	0.927975712
ENSGALG00010020265	ENSGALG00010020265	2	0.760560977	0.324572671	2.343268689	0.019115611	0.11030184	1.885311968	46.95838052	1	CRA004775;Yang2025_Silkie_Wenchang	PRJNA827465	0.231597063
ENSGALG00010020265	ENSGALG00010020265	2	0.433587265	0.716127544	0.605460953	0.544872762	0.920746764	9.194473836	89.12390184	0.5	CRA004775;PRJNA827465	Yang2025_Silkie_Wenchang	0.857345822
ENSGALG00010020268	CPLANE2	2	0.02906214	0.195288614	0.152714482	0.87862344	0.047974385	1.975182317	49.37176223	0.5	PRJNA827465;Yang2025_Silkie_Wenchang	CRA004775	0.971079508
ENSGALG00010020268	CPLANE2	2	0.090026476	0.260395357	0.345729958	0.729545668	0.042420109	1.455153455	31.27872553	0.5	CRA004775;Yang2025_Silkie_Wenchang	PRJNA827465	0.9323457
ENSGALG00010020268	CPLANE2	2	-0.091820289	0.062918343	-1.459356436	0.14446703	0	0.06641711	0	1	CRA004775;PRJNA827465	Yang2025_Silkie_Wenchang	0.631870428
ENSGALG00010020269	ENSGALG00010020269	2	-0.145166192	0.483267749	-0.300384606	0.763830804	0	0.265570837	0	0.5	PRJNA827465;Yang2025_Silkie_Wenchang	CRA004775	0.940361611
ENSGALG00010020269	ENSGALG00010020269	2	-0.504626784	0.551361181	-0.915238144	0.360066624	0	0.006523894	0	1	CRA004775;Yang2025_Silkie_Wenchang	PRJNA827465	0.814880744
ENSGALG00010020269	ENSGALG00010020269	2	-0.179030051	0.48352714	-0.370258536	0.711189865	0	0.365398507	0	0.5	CRA004775;PRJNA827465	Yang2025_Silkie_Wenchang	0.925651399
ENSGALG00010020272	ENSGALG00010020272	2	-0.032269389	0.04985416	-0.64727575	0.517453493	0	0.023636947	0	1	PRJNA827465;Yang2025_Silkie_Wenchang	CRA004775	0.86795013
ENSGALG00010020272	ENSGALG00010020272	2	-0.067108653	0.085117629	-0.788422489	0.430449662	0	0.449736235	0	1	CRA004775;Yang2025_Silkie_Wenchang	PRJNA827465	0.842234352
ENSGALG00010020272	ENSGALG00010020272	2	-0.03885839	0.057461741	-0.676248049	0.49888317	0	0.594436298	0	1	CRA004775;PRJNA827465	Yang2025_Silkie_Wenchang	0.861571318
ENSGALG00010020277	IDH2	2	-0.207018227	0.071277447	-2.904400162	0.003679575	0	0.006530958	0	1	PRJNA827465;Yang2025_Silkie_Wenchang	CRA004775	0.086700498
ENSGALG00010020277	IDH2	2	-0.205896764	0.160382437	-1.283786228	0.199216764	0	0.02510092	0	1	CRA004775;Yang2025_Silkie_Wenchang	PRJNA827465	0.71096127
ENSGALG00010020277	IDH2	2	-0.21161198	0.076217237	-2.776432053	0.005495912	0	0.017095231	0	1	CRA004775;PRJNA827465	Yang2025_Silkie_Wenchang	0.111278432
ENSGALG00010020282	MAGI1	2	0.331159266	0.327935089	1.009831752	0.312575905	0.196038294	10.88255839	90.81098429	1	PRJNA827465;Yang2025_Silkie_Wenchang	CRA004775	0.796054997
ENSGALG00010020282	MAGI1	2	0.622216335	0.162363964	3.832231739	0.000126986	0	0.502554779	0	1	CRA004775;Yang2025_Silkie_Wenchang	PRJNA827465	0.009242467
ENSGALG00010020282	MAGI1	2	0.040434654	0.080192696	0.504218666	0.614107725	0	0.977345306	0	1	CRA004775;PRJNA827465	Yang2025_Silkie_Wenchang	0.898282836
ENSGALG00010020283	FBXO42	2	-0.06094211	0.05220136	-1.167442959	0.243031531	0	0.974593954	0	1	PRJNA827465;Yang2025_Silkie_Wenchang	CRA004775	0.7550024
ENSGALG00010020283	FBXO42	2	-0.12216215	0.079004256	-1.546273032	0.122038642	0	0.033543261	0	1	CRA004775;Yang2025_Silkie_Wenchang	PRJNA827465	0.585865977
ENSGALG00010020283	FBXO42	2	-0.02804198	0.062434007	-0.449145922	0.653326395	0	0.100497814	0	1	CRA004775;PRJNA827465	Yang2025_Silkie_Wenchang	0.909597225
ENSGALG00010020284	HEG1	2	-0.124861386	0.091251662	-1.368319032	0.171212236	0	0.54547895	0	0.5	PRJNA827465;Yang2025_Silkie_Wenchang	CRA004775	0.673243267
ENSGALG00010020284	HEG1	2	0.001740035	0.215508631	0.008074083	0.993557884	0	0.125642953	0	0.5	CRA004775;Yang2025_Silkie_Wenchang	PRJNA827465	0.997979742
ENSGALG00010020284	HEG1	2	-0.151454334	0.09613859	-1.575375023	0.115169906	0	0.000597175	0	1	CRA004775;PRJNA827465	Yang2025_Silkie_Wenchang	0.572180865
ENSGALG00010020285	ADAMTS14	2	0.008475265	0.103399623	0.081966115	0.934673659	0.004067088	1.178045176	15.11361194	0.5	PRJNA827465;Yang2025_Silkie_Wenchang	CRA004775	0.984397102
ENSGALG00010020285	ADAMTS14	2	-0.124829102	0.177470072	-0.703381146	0.481818253	0	0.350920798	0	0.5	CRA004775;Yang2025_Silkie_Wenchang	PRJNA827465	0.85691916
ENSGALG00010020285	ADAMTS14	2	0.075759464	0.101465996	0.746648796	0.455275585	0	0.042290594	0	1	CRA004775;PRJNA827465	Yang2025_Silkie_Wenchang	0.849072665
ENSGALG00010020287	GALNT16	2	-0.07744251	0.173112645	-0.447353283	0.654620001	0.042424841	3.421363048	70.77188284	0.5	PRJNA827465;Yang2025_Silkie_Wenchang	CRA004775	0.910222014
ENSGALG00010020287	GALNT16	2	-0.242093087	0.127930666	-1.892377285	0.05844073	0	0.059485197	0	1	CRA004775;Yang2025_Silkie_Wenchang	PRJNA827465	0.418835891
ENSGALG00010020287	GALNT16	2	0.069662866	0.122964967	0.566526121	0.571036181	0	0.347465498	0	0.5	CRA004775;PRJNA827465	Yang2025_Silkie_Wenchang	0.885354083
ENSGALG00010020291	SNX29	2	-0.044836491	0.084796533	-0.528753821	0.596976236	0	0.348339248	0	0.5	PRJNA827465;Yang2025_Silkie_Wenchang	CRA004775	0.894201647
ENSGALG00010020291	SNX29	2	0.00371872	0.158449916	0.02346937	0.981275871	0	0.415689208	0	0.5	CRA004775;Yang2025_Silkie_Wenchang	PRJNA827465	0.994962782
ENSGALG00010020291	SNX29	2	-0.082638319	0.095889145	-0.861810996	0.388791533	0	0.181537457	0	1	CRA004775;PRJNA827465	Yang2025_Silkie_Wenchang	0.828400391
ENSGALG00010020292	NPM2	2	-0.048694862	0.239023096	-0.203724505	0.838568792	0	0.491768739	0	0.5	PRJNA827465;Yang2025_Silkie_Wenchang	CRA004775	0.959226718
ENSGALG00010020292	NPM2	2	0.195287585	0.266131229	0.733801839	0.46306952	0	0.002159215	0	1	CRA004775;Yang2025_Silkie_Wenchang	PRJNA827465	0.851447984
ENSGALG00010020292	NPM2	2	-0.01246125	0.216348408	-0.057598066	0.95406879	0	0.611023076	0	0.5	CRA004775;PRJNA827465	Yang2025_Silkie_Wenchang	0.987901195
ENSGALG00010020293	SEMA4B	2	0.100049985	0.077138777	1.297012845	0.194626767	0	0.004585198	0	1	PRJNA827465;Yang2025_Silkie_Wenchang	CRA004775	0.705730772
ENSGALG00010020293	SEMA4B	2	0.020539974	0.134603399	0.152596247	0.878716685	0	0.421635221	0	0.5	CRA004775;Yang2025_Silkie_Wenchang	PRJNA827465	0.971100041
ENSGALG00010020293	SEMA4B	2	0.076337858	0.080233216	0.951449563	0.341376211	0	0.680483452	0	0.5	CRA004775;PRJNA827465	Yang2025_Silkie_Wenchang	0.805758959
ENSGALG00010020295	LGI1	2	0.19805297	0.087098085	2.27390729	0.022971561	0	0.693105593	0	1	PRJNA827465;Yang2025_Silkie_Wenchang	CRA004775	0.258400479
ENSGALG00010020295	LGI1	2	0.314620359	0.150264258	2.093780404	0.036279547	0	0.002669309	0	1	CRA004775;Yang2025_Silkie_Wenchang	PRJNA827465	0.328451985
ENSGALG00010020295	LGI1	2	0.162820409	0.100166693	1.625494498	0.104057235	0	0.189554613	0	1	CRA004775;PRJNA827465	Yang2025_Silkie_Wenchang	0.548383482
ENSGALG00010020298	ENSGALG00010020298	2	0.078837213	0.256112675	0.30782238	0.758217491	0.0934566	2.805164558	64.35146747	0.5	PRJNA827465;Yang2025_Silkie_Wenchang	CRA004775	0.938954369
ENSGALG00010020298	ENSGALG00010020298	2	0.07104554	0.357580399	0.198684099	0.842509863	0.161292301	2.707537095	63.06606466	0.5	CRA004775;Yang2025_Silkie_Wenchang	PRJNA827465	0.960252241
ENSGALG00010020298	ENSGALG00010020298	2	-0.11866857	0.078556279	-1.510618524	0.130885672	0	0.315051316	0	1	CRA004775;PRJNA827465	Yang2025_Silkie_Wenchang	0.606627305
ENSGALG00010020300	EHD4	2	-0.093676018	0.10577205	-0.88564056	0.375811248	0.015902066	3.400001243	70.58824604	0.5	PRJNA827465;Yang2025_Silkie_Wenchang	CRA004775	0.822974013
ENSGALG00010020300	EHD4	2	-0.197934329	0.089900565	-2.201702843	0.027686306	0	0.283223045	0	0.5	CRA004775;Yang2025_Silkie_Wenchang	PRJNA827465	0.283891176
ENSGALG00010020300	EHD4	2	0.0041064	0.068147659	0.060257386	0.951950641	0	0.001135507	0	1	CRA004775;PRJNA827465	Yang2025_Silkie_Wenchang	0.987725077
ENSGALG00010020303	EXD2	2	-0.057932703	0.069657342	-0.831681218	0.405588905	0.005380904	2.017360728	50.43028319	1	PRJNA827465;Yang2025_Silkie_Wenchang	CRA004775	0.833257341
ENSGALG00010020303	EXD2	2	-0.185838142	0.083636612	-2.221971197	0.026285252	0	0.719286869	0	1	CRA004775;Yang2025_Silkie_Wenchang	PRJNA827465	0.276374871
ENSGALG00010020303	EXD2	2	-0.116429721	0.156094137	-0.745894259	0.455731294	0.034290602	2.64824204	62.23910107	1	CRA004775;PRJNA827465	Yang2025_Silkie_Wenchang	0.84907427
ENSGALG00010020304	ZFP36L2	2	-0.069997797	0.073853949	-0.947786778	0.343238013	0.001414178	1.059003936	5.57164467	0.5	PRJNA827465;Yang2025_Silkie_Wenchang	CRA004775	0.806218116
ENSGALG00010020304	ZFP36L2	2	0.26733685	0.18942201	1.411329393	0.158147511	0.012238909	1.184286991	15.56100779	1	CRA004775;Yang2025_Silkie_Wenchang	PRJNA827465	0.65200722
ENSGALG00010020304	ZFP36L2	2	0.145128432	0.303378657	0.478373902	0.632384106	0.146146787	4.093318747	75.56994552	0.5	CRA004775;PRJNA827465	Yang2025_Silkie_Wenchang	0.903058174
ENSGALG00010020305	XPO7	2	-0.059034845	0.039342375	-1.500540951	0.133474334	0.000345493	1.108307868	9.772362992	1	PRJNA827465;Yang2025_Silkie_Wenchang	CRA004775	0.612342914
ENSGALG00010020305	XPO7	2	-0.09602781	0.062031629	-1.548045909	0.121611244	0	0.671095129	0	0.5	CRA004775;Yang2025_Silkie_Wenchang	PRJNA827465	0.584814231
ENSGALG00010020305	XPO7	2	-0.027693068	0.042672969	-0.64896042	0.51636396	0	0.139495753	0	0.5	CRA004775;PRJNA827465	Yang2025_Silkie_Wenchang	0.867685411
ENSGALG00010020307	SHQ1	2	0.399251616	0.395197156	1.010259336	0.312371058	0.258902882	5.701105943	82.45954364	1	PRJNA827465;Yang2025_Silkie_Wenchang	CRA004775	0.796054997
ENSGALG00010020307	SHQ1	2	0.182075406	0.668534962	0.272349864	0.785353019	0.781263555	7.846944752	87.25618656	0.5	CRA004775;Yang2025_Silkie_Wenchang	PRJNA827465	0.946396741
ENSGALG00010020307	SHQ1	2	-0.133266518	0.250521347	-0.531956736	0.594755961	0.057329076	1.625915894	38.49620367	0.5	CRA004775;PRJNA827465	Yang2025_Silkie_Wenchang	0.893382718
ENSGALG00010020309	GXYLT2	2	-0.295239475	0.271193636	-1.088666678	0.276300901	0.141194204	24.90548702	95.98482054	1	PRJNA827465;Yang2025_Silkie_Wenchang	CRA004775	0.77582325
ENSGALG00010020309	GXYLT2	2	-0.192717544	0.406521139	-0.474065247	0.63545338	0.298601952	9.63458916	89.62073023	0.5	CRA004775;Yang2025_Silkie_Wenchang	PRJNA827465	0.90421144
ENSGALG00010020309	GXYLT2	2	0.010298372	0.088299184	0.116630434	0.90715292	0.001808091	1.050639109	4.819838611	0.5	CRA004775;PRJNA827465	Yang2025_Silkie_Wenchang	0.978196186
ENSGALG00010020311	SGPL1	2	-0.132191268	0.114000704	-1.159565372	0.246225806	0.01799767	3.07861529	67.51786418	1	PRJNA827465;Yang2025_Silkie_Wenchang	CRA004775	0.756728888
ENSGALG00010020311	SGPL1	2	-0.04640989	0.289269198	-0.160438408	0.872535736	0.118968727	2.934961228	65.92799965	0.5	CRA004775;Yang2025_Silkie_Wenchang	PRJNA827465	0.969629321
ENSGALG00010020311	SGPL1	2	0.007141472	0.117472678	0.060792625	0.951524364	0.011120688	1.194155625	16.25882092	0.5	CRA004775;PRJNA827465	Yang2025_Silkie_Wenchang	0.987713746
ENSGALG00010020313	FRA10AC1	2	0.180791095	0.184116571	0.981938207	0.326130291	0.053558832	4.568113299	78.10912439	1	PRJNA827465;Yang2025_Silkie_Wenchang	CRA004775	0.800023788
ENSGALG00010020313	FRA10AC1	2	0.310020051	0.126066786	2.459173113	0.013925745	0	0.893680547	0	1	CRA004775;Yang2025_Silkie_Wenchang	PRJNA827465	0.193621431
ENSGALG00010020313	FRA10AC1	2	0.028355138	0.082398551	0.344121804	0.730754686	0	0.180633929	0	1	CRA004775;PRJNA827465	Yang2025_Silkie_Wenchang	0.932769533
ENSGALG00010020314	THADA	2	-0.046523667	0.073804676	-0.630362048	0.528457735	0	0.042937805	0	1	PRJNA827465;Yang2025_Silkie_Wenchang	CRA004775	0.871701504
ENSGALG00010020314	THADA	2	-0.099297669	0.117113925	-0.847872432	0.396509019	0	0.230843053	0	1	CRA004775;Yang2025_Silkie_Wenchang	PRJNA827465	0.831365249
ENSGALG00010020314	THADA	2	-0.055287247	0.083671816	-0.660763078	0.508764264	0	0.406577142	0	1	CRA004775;PRJNA827465	Yang2025_Silkie_Wenchang	0.865176613
ENSGALG00010020315	ANO5	2	-0.033971255	0.06351466	-0.534856918	0.592748801	0	0.854744307	0	0.5	PRJNA827465;Yang2025_Silkie_Wenchang	CRA004775	0.892776493
ENSGALG00010020315	ANO5	2	-0.027866347	0.144876188	-0.192345944	0.847471232	0.016858775	1.459667221	31.49123408	0.5	CRA004775;Yang2025_Silkie_Wenchang	PRJNA827465	0.961252712
ENSGALG00010020315	ANO5	2	0.026764719	0.074658641	0.358494593	0.719973213	0	0.611373212	0	1	CRA004775;PRJNA827465	Yang2025_Silkie_Wenchang	0.928564173
ENSGALG00010020316	PDPK1	2	-0.047696924	0.043554692	-1.095104146	0.273471013	0	0.211085392	0	1	PRJNA827465;Yang2025_Silkie_Wenchang	CRA004775	0.774252172
ENSGALG00010020316	PDPK1	2	0.053708984	0.185964802	0.288812634	0.772724763	0.044565476	2.552753748	60.82661711	0.5	CRA004775;Yang2025_Silkie_Wenchang	PRJNA827465	0.943066211
ENSGALG00010020316	PDPK1	2	0.059348926	0.150540869	0.394237968	0.693405356	0.030007227	2.242252747	55.40199466	0.5	CRA004775;PRJNA827465	Yang2025_Silkie_Wenchang	0.920721644
ENSGALG00010020319	NTRK3	2	0.002091524	0.089676168	0.02332308	0.981392562	0	0.738948674	0	0.5	PRJNA827465;Yang2025_Silkie_Wenchang	CRA004775	0.994962782
ENSGALG00010020319	NTRK3	2	0.380553443	0.355688435	1.06990671	0.284661302	0.159752833	2.277484805	56.09191342	1	CRA004775;Yang2025_Silkie_Wenchang	PRJNA827465	0.780265906
ENSGALG00010020319	NTRK3	2	0.307025646	0.450436396	0.681618201	0.495480403	0.315567454	3.730020774	73.19049784	0.5	CRA004775;PRJNA827465	Yang2025_Silkie_Wenchang	0.860770265
ENSGALG00010020321	SZRD1	2	-0.120646346	0.143555107	-0.840418351	0.400673863	0.031697984	3.897043663	74.33952282	1	PRJNA827465;Yang2025_Silkie_Wenchang	CRA004775	0.832353691
ENSGALG00010020321	SZRD1	2	-0.219681255	0.131163979	-1.674859635	0.093961775	0.003897153	1.090007568	8.257517677	0.5	CRA004775;Yang2025_Silkie_Wenchang	PRJNA827465	0.525133608
ENSGALG00010020321	SZRD1	2	-0.000636021	0.05487501	-0.011590353	0.990752444	0	0.003255371	0	0.5	CRA004775;PRJNA827465	Yang2025_Silkie_Wenchang	0.997555603
ENSGALG00010020323	ARHGAP25	2	-0.057879246	0.079087163	-0.731841213	0.464265491	0	0.011328448	0	1	PRJNA827465;Yang2025_Silkie_Wenchang	CRA004775	0.852159649
ENSGALG00010020323	ARHGAP25	2	-0.056031428	0.118052972	-0.474629539	0.635051048	0	0.05184892	0	1	CRA004775;Yang2025_Silkie_Wenchang	PRJNA827465	0.904051292
ENSGALG00010020323	ARHGAP25	2	-0.069737345	0.099768111	-0.698994341	0.484555567	0	0.035932624	0	1	CRA004775;PRJNA827465	Yang2025_Silkie_Wenchang	0.857004733
ENSGALG00010020324	PLEKHH2	2	0.218756166	0.192263046	1.137796218	0.255205585	0.051969524	3.19314211	68.68288459	1	PRJNA827465;Yang2025_Silkie_Wenchang	CRA004775	0.764467569
ENSGALG00010020324	PLEKHH2	2	0.45584759	0.164308585	2.774338234	0.005531414	0	0.021112231	0	1	CRA004775;Yang2025_Silkie_Wenchang	PRJNA827465	0.111704511
ENSGALG00010020324	PLEKHH2	2	0.159685962	0.189706684	0.841751902	0.39992684	0.034983607	1.544230517	35.24282877	1	CRA004775;PRJNA827465	Yang2025_Silkie_Wenchang	0.832246851
ENSGALG00010020329	PPP4R2	2	-0.046047352	0.055887311	-0.823932156	0.409978108	0.002581573	1.47103146	32.02048852	1	PRJNA827465;Yang2025_Silkie_Wenchang	CRA004775	0.834652129
ENSGALG00010020329	PPP4R2	2	0.029157636	0.200214625	0.1456319	0.884211984	0.059281223	3.550317255	71.83350309	0.5	CRA004775;Yang2025_Silkie_Wenchang	PRJNA827465	0.972001076
ENSGALG00010020329	PPP4R2	2	0.063351248	0.12431461	0.5096042	0.610328781	0.019580736	2.008337051	50.20756105	0.5	CRA004775;PRJNA827465	Yang2025_Silkie_Wenchang	0.897438028
ENSGALG00010020336	OSBP	2	0.114123664	0.125992662	0.905796117	0.365043777	0.02779491	7.694343587	87.00343975	0.5	PRJNA827465;Yang2025_Silkie_Wenchang	CRA004775	0.818070222
ENSGALG00010020336	OSBP	2	0.131802855	0.189625594	0.69506891	0.487012118	0.043370019	1.952465951	48.78271758	0.5	CRA004775;Yang2025_Silkie_Wenchang	PRJNA827465	0.85768885
ENSGALG00010020336	OSBP	2	-0.005503518	0.040026104	-0.137498221	0.890636999	0	0.331598608	0	1	CRA004775;PRJNA827465	Yang2025_Silkie_Wenchang	0.973477891
ENSGALG00010020338	NECAP2	2	0.133167886	0.096557658	1.379154057	0.16784726	0.011611929	2.493793808	59.9004538	1	PRJNA827465;Yang2025_Silkie_Wenchang	CRA004775	0.66810825
ENSGALG00010020338	NECAP2	2	0.03156297	0.281016139	0.112317286	0.910571837	0.115493642	3.205395514	68.80260187	0.5	CRA004775;Yang2025_Silkie_Wenchang	PRJNA827465	0.97880407
ENSGALG00010020338	NECAP2	2	-0.020739098	0.151616074	-0.136786932	0.891199213	0.024573987	1.50579665	33.58997046	0.5	CRA004775;PRJNA827465	Yang2025_Silkie_Wenchang	0.973735628
ENSGALG00010020340	DCAF5	2	-0.007461401	0.040622626	-0.183675988	0.854267647	0	0.149936549	0	0.5	PRJNA827465;Yang2025_Silkie_Wenchang	CRA004775	0.963091083
ENSGALG00010020340	DCAF5	2	-0.006617152	0.087501152	-0.075623595	0.939718564	0	0.515163682	0	0.5	CRA004775;Yang2025_Silkie_Wenchang	PRJNA827465	0.985425793
ENSGALG00010020340	DCAF5	2	-0.020483483	0.043200967	-0.474144084	0.635397164	0	0.324138956	0	1	CRA004775;PRJNA827465	Yang2025_Silkie_Wenchang	0.90419692
ENSGALG00010020341	DYNC2LI1	2	-0.255670318	0.31191354	-0.819683292	0.412396677	0.179058988	12.50860538	92.00550365	0.5	PRJNA827465;Yang2025_Silkie_Wenchang	CRA004775	0.835702455
ENSGALG00010020341	DYNC2LI1	2	-0.532099368	0.128369375	-4.145064709	3.39718E-05	0	0.889942164	0	1	CRA004775;Yang2025_Silkie_Wenchang	PRJNA827465	0.003661746
ENSGALG00010020341	DYNC2LI1	2	0.036640596	0.109288904	0.335263643	0.737426217	0	0.257092021	0	0.5	CRA004775;PRJNA827465	Yang2025_Silkie_Wenchang	0.933968189
ENSGALG00010020342	CPPED1	2	-0.127094296	0.116303616	-1.092780267	0.274490288	0	0.236065819	0	1	PRJNA827465;Yang2025_Silkie_Wenchang	CRA004775	0.774526342
ENSGALG00010020342	CPPED1	2	-0.070613259	0.198422224	-0.35587374	0.721935123	0.017954773	1.245760983	19.72777975	0.5	CRA004775;Yang2025_Silkie_Wenchang	PRJNA827465	0.929385887
ENSGALG00010020342	CPPED1	2	-0.036719063	0.131200182	-0.279870523	0.779576843	0	0.744295044	0	0.5	CRA004775;PRJNA827465	Yang2025_Silkie_Wenchang	0.944807046
ENSGALG00010020343	PATL1	2	0.217941135	0.363491579	0.599576849	0.54878828	0.244715998	12.9502897	92.27816502	0.5	PRJNA827465;Yang2025_Silkie_Wenchang	CRA004775	0.878281266
ENSGALG00010020343	PATL1	2	0.154716174	0.475859669	0.325129832	0.745082811	0.391818648	7.260132414	86.22614654	0.5	CRA004775;Yang2025_Silkie_Wenchang	PRJNA827465	0.93597621
ENSGALG00010020343	PATL1	2	-0.135745164	0.066650571	-2.036669196	0.041683203	0	0.541207377	0	1	CRA004775;PRJNA827465	Yang2025_Silkie_Wenchang	0.353826658
ENSGALG00010020345	AGBL1	2	0.023840481	0.118817881	0.20064725	0.840974409	0	0.010135196	0	1	PRJNA827465;Yang2025_Silkie_Wenchang	CRA004775	0.959805361
ENSGALG00010020345	AGBL1	2	-0.018882229	0.185290657	-0.101905994	0.918831292	0	0.376720386	0	0.5	CRA004775;Yang2025_Silkie_Wenchang	PRJNA827465	0.981033557
ENSGALG00010020345	AGBL1	2	0.014533168	0.149121343	0.097458669	0.922362156	0	0.423721069	0	0.5	CRA004775;PRJNA827465	Yang2025_Silkie_Wenchang	0.981804675
ENSGALG00010020346	PCBD1	2	0.509001634	0.182782635	2.784737373	0.005357109	0	0.027327294	0	1	PRJNA827465;Yang2025_Silkie_Wenchang	CRA004775	0.109433716
ENSGALG00010020346	PCBD1	2	0.348745408	0.201392567	1.731669707	0.083332387	0	0.985666188	0	1	CRA004775;Yang2025_Silkie_Wenchang	PRJNA827465	0.497316548
ENSGALG00010020346	PCBD1	2	0.351193597	0.240682286	1.459158476	0.144521495	0.017540881	1.168348354	14.409089	1	CRA004775;PRJNA827465	Yang2025_Silkie_Wenchang	0.631965661
ENSGALG00010020350	PDZRN3	2	0.013186133	0.220565916	0.059783185	0.952328318	0.088887508	11.46132866	91.27500808	0.5	PRJNA827465;Yang2025_Silkie_Wenchang	CRA004775	0.987758913
ENSGALG00010020350	PDZRN3	2	0.225531158	0.10268643	2.196309279	0.028069814	0	0.262917052	0	1	CRA004775;Yang2025_Silkie_Wenchang	PRJNA827465	0.285514467
ENSGALG00010020350	PDZRN3	2	-0.191872308	0.073465419	-2.611736384	0.009008369	0	0.387577847	0	0.5	CRA004775;PRJNA827465	Yang2025_Silkie_Wenchang	0.14870051
ENSGALG00010020351	CROCC	2	1.014844763	0.898554385	1.129419409	0.258720949	1.588774767	61.6000205	98.37662392	1	PRJNA827465;Yang2025_Silkie_Wenchang	CRA004775	0.765833156
ENSGALG00010020351	CROCC	2	0.84703086	1.074845791	0.788048729	0.430668203	2.269717698	56.53091221	98.23105632	0.5	CRA004775;Yang2025_Silkie_Wenchang	PRJNA827465	0.842473342
ENSGALG00010020351	CROCC	2	-0.008619982	0.171263863	-0.050331585	0.959858155	0.038865249	2.671264706	62.56454862	0.5	CRA004775;PRJNA827465	Yang2025_Silkie_Wenchang	0.98933455
ENSGALG00010020356	STX3	2	0.501561748	0.287799328	1.742748156	0.081377616	0.071425331	1.750388395	42.86982232	1	PRJNA827465;Yang2025_Silkie_Wenchang	CRA004775	0.491357177
ENSGALG00010020356	STX3	2	0.426219535	0.378786134	1.125224757	0.260493805	0.184532804	2.799941363	64.28496635	1	CRA004775;Yang2025_Silkie_Wenchang	PRJNA827465	0.767694434
ENSGALG00010020356	STX3	2	0.151901524	0.211332376	0.718780186	0.472276368	0	0.181054902	0	1	CRA004775;PRJNA827465	Yang2025_Silkie_Wenchang	0.853998373
ENSGALG00010020357	RBP4	2	-0.074653455	1.062895037	-0.070235962	0.944005852	1.829083878	5.109709147	80.42941445	0.5	PRJNA827465;Yang2025_Silkie_Wenchang	CRA004775	0.985827325
ENSGALG00010020357	RBP4	2	-0.041548097	1.008554113	-0.041195705	0.967139878	1.78255037	8.077320561	87.61965688	0.5	CRA004775;Yang2025_Silkie_Wenchang	PRJNA827465	0.990893423
ENSGALG00010020357	RBP4	2	0.993828605	0.417443693	2.380748882	0.017277485	0	0.015363603	0	1	CRA004775;PRJNA827465	Yang2025_Silkie_Wenchang	0.218416536
ENSGALG00010020362	CDS2	2	-0.184720711	0.317961834	-0.580952465	0.561272491	0.196600161	36.09897538	97.22983827	0.5	PRJNA827465;Yang2025_Silkie_Wenchang	CRA004775	0.881506202
ENSGALG00010020362	CDS2	2	-0.5143756	0.077120922	-6.669728347	2.56277E-11	0	0.391577894	0	1	CRA004775;Yang2025_Silkie_Wenchang	PRJNA827465	2.01449E-08
ENSGALG00010020362	CDS2	2	-0.229320287	0.426437689	-0.537758019	0.590744119	0.307611226	5.654876777	82.31614871	0.5	CRA004775;PRJNA827465	Yang2025_Silkie_Wenchang	0.892708037
ENSGALG00010020364	MRPL16	2	0.122352064	0.118799758	1.029901622	0.303056189	0.010672278	1.273715924	21.48955812	0.5	PRJNA827465;Yang2025_Silkie_Wenchang	CRA004775	0.792084776
ENSGALG00010020364	MRPL16	2	-0.139262946	0.208646866	-0.667457647	0.504479854	0	9.19044E-06	0	1	CRA004775;Yang2025_Silkie_Wenchang	PRJNA827465	0.863512504
ENSGALG00010020364	MRPL16	2	0.159249943	0.077574654	2.052860503	0.040086109	0	0.853176466	0	0.5	CRA004775;PRJNA827465	Yang2025_Silkie_Wenchang	0.346798223
ENSGALG00010020365	UNC5B	2	-0.073508705	0.099245726	-0.740673763	0.458891271	0.004595424	1.265770934	20.99676385	0.5	PRJNA827465;Yang2025_Silkie_Wenchang	CRA004775	0.850089963
ENSGALG00010020365	UNC5B	2	-0.006010547	0.180435345	-0.033311361	0.973426294	0.013272725	1.102607497	9.305895096	0.5	CRA004775;Yang2025_Silkie_Wenchang	PRJNA827465	0.992392211
ENSGALG00010020365	UNC5B	2	-0.158812179	0.099665418	-1.593453195	0.11105856	0	0.431009852	0	1	CRA004775;PRJNA827465	Yang2025_Silkie_Wenchang	0.56392513
ENSGALG00010020370	CEP55	2	-0.080581883	0.108494585	-0.742727236	0.457646837	0	0.538874245	0	1	PRJNA827465;Yang2025_Silkie_Wenchang	CRA004775	0.849573219
ENSGALG00010020370	CEP55	2	-0.196813766	0.175111924	-1.123931264	0.261042187	0	0.057154322	0	1	CRA004775;Yang2025_Silkie_Wenchang	PRJNA827465	0.767929536

ENSGALG00010020370	CEP55	2	-0.032284936	0.133471974	-0.241885502	0.808868884	0	0.217590269	0	1	CRA004775;PRJNA827465	Yang2025_Silkie_Wenchang	0.952595795
ENSGALG00010020373	MS4A4A	2	0.045113428	0.066351974	0.67991086	0.496560905	0	0.389226529	0	1	PRJNA827465;Yang2025_Silkie_Wenchang	CRA004775	0.860939846
ENSGALG00010020373	MS4A4A	2	-0.041381684	0.283536847	-0.145948168	0.883962306	0.085363177	1.797475204	44.3664092	0.5	CRA004775;Yang2025_Silkie_Wenchang	PRJNA827465	0.972001076
ENSGALG00010020373	MS4A4A	2	-0.046727818	0.178910823	-0.261179379	0.793954179	0.032346932	1.340085308	25.37788496	0.5	CRA004775;PRJNA827465	Yang2025_Silkie_Wenchang	0.948872932
ENSGALG00010020374	PCNA	2	0.020751561	0.045585605	0.455221808	0.648949673	0	0.133615032	0	1	PRJNA827465;Yang2025_Silkie_Wenchang	CRA004775	0.907783669
ENSGALG00010020374	PCNA	2	-0.079719035	0.214130414	-0.372291976	0.709675472	0.046058132	1.82264917	45.13480618	0.5	CRA004775;Yang2025_Silkie_Wenchang	PRJNA827465	0.925298042
ENSGALG00010020374	PCNA	2	-0.071333142	0.163111467	-0.437327576	0.661873799	0.031876478	1.702484771	41.26232335	0.5	CRA004775;PRJNA827465	Yang2025_Silkie_Wenchang	0.912556664
ENSGALG00010020375	LRPPRC	2	-0.15827147	0.040298101	-3.92751686	8.58274E-05	0	0.782457664	0	1	PRJNA827465;Yang2025_Silkie_Wenchang	CRA004775	0.006955205
ENSGALG00010020375	LRPPRC	2	-0.101839951	0.059954189	-1.698629455	0.089389023	0	0.553803961	0	0.5	CRA004775;Yang2025_Silkie_Wenchang	PRJNA827465	0.513108919
ENSGALG00010020375	LRPPRC	2	-0.156006215	0.079706898	-1.957248611	0.050318246	0.004621987	1.211790552	17.4774883	0.5	CRA004775;PRJNA827465	Yang2025_Silkie_Wenchang	0.388614271
ENSGALG00010020377	TMEM230	2	-0.142417153	0.089796577	-1.585997561	0.112739863	0	0.001440851	0	1	PRJNA827465;Yang2025_Silkie_Wenchang	CRA004775	0.566553488
ENSGALG00010020377	TMEM230	2	-0.335451293	0.220209975	-1.523324697	0.127677491	0.028359555	1.395954676	28.36443638	1	CRA004775;Yang2025_Silkie_Wenchang	PRJNA827465	0.599965141
ENSGALG00010020377	TMEM230	2	-0.282385043	0.211164778	-1.337273409	0.181133407	0.055112689	2.120112963	52.83270197	1	CRA004775;PRJNA827465	Yang2025_Silkie_Wenchang	0.688594651
ENSGALG00010020379	COL13A1	2	0.298769437	0.398228571	0.750246111	0.453106492	0.289274303	11.14888	91.03048916	0.5	PRJNA827465;Yang2025_Silkie_Wenchang	CRA004775	0.848708214
ENSGALG00010020379	COL13A1	2	-0.20103614	1.015544113	-0.197959042	0.843077109	1.827668568	8.108525583	87.66730166	0.5	CRA004775;Yang2025_Silkie_Wenchang	PRJNA827465	0.960475945
ENSGALG00010020379	COL13A1	2	-0.521147933	0.594650231	-0.876394065	0.380815844	0.531439775	3.184843399	68.60128192	1	CRA004775;PRJNA827465	Yang2025_Silkie_Wenchang	0.824392515
ENSGALG00010020382	MFAP2	2	-0.003227632	0.062376756	-0.051744152	0.958732556	0	0.070372386	0	0.5	PRJNA827465;Yang2025_Silkie_Wenchang	CRA004775	0.989102387
ENSGALG00010020382	MFAP2	2	-0.06540902	0.158390748	-0.412959854	0.679636027	0	0.088988153	0	1	CRA004775;Yang2025_Silkie_Wenchang	PRJNA827465	0.917701868
ENSGALG00010020382	MFAP2	2	-0.001477968	0.066080129	-0.022366305	0.982155758	0	0.190616399	0	0.5	CRA004775;PRJNA827465	Yang2025_Silkie_Wenchang	0.995105937
ENSGALG00010020383	BAAT	2	0.390519686	0.302336117	1.291673948	0.196470083	0.166535084	10.73183708	90.68193085	1	PRJNA827465;Yang2025_Silkie_Wenchang	CRA004775	0.707837424
ENSGALG00010020383	BAAT	2	0.334703567	0.395075098	0.847189735	0.396889374	0.270433551	7.399734602	86.48600181	0.5	CRA004775;Yang2025_Silkie_Wenchang	PRJNA827465	0.831375233
ENSGALG00010020383	BAAT	2	0.093248049	0.066075929	1.411225698	0.158178075	0	0.571102219	0	0.5	CRA004775;PRJNA827465	Yang2025_Silkie_Wenchang	0.65200722
ENSGALG00010020385	SLC12A8	2	-0.021571315	0.218395695	-0.098771704	0.921319536	0	0.040117941	0	0.5	PRJNA827465;Yang2025_Silkie_Wenchang	CRA004775	0.981519273
ENSGALG00010020385	SLC12A8	2	0.49845763	0.594271775	0.838770494	0.401598111	0.485060894	2.892361046	65.42616969	1	CRA004775;Yang2025_Silkie_Wenchang	PRJNA827465	0.832623399
ENSGALG00010020385	SLC12A8	2	0.46382925	0.640835234	0.723788621	0.469195516	0.595108372	3.318862553	69.86919512	0.5	CRA004775;PRJNA827465	Yang2025_Silkie_Wenchang	0.893485658
ENSGALG00010020386	PPM1B	2	-0.128872309	0.069091456	-1.865242335	0.062147444	0.006486398	2.917926645	65.72909049	1	PRJNA827465;Yang2025_Silkie_Wenchang	CRA004775	0.429956168
ENSGALG00010020386	PPM1B	2	0.041891373	0.265881316	0.157556664	0.874806154	0.129883648	11.99367111	91.66226928	0.5	CRA004775;Yang2025_Silkie_Wenchang	PRJNA827465	0.970159044
ENSGALG00010020386	PPM1B	2	0.103112359	0.194745727	0.529471739	0.596478244	0.066743675	7.65159047	86.93082172	0.5	CRA004775;PRJNA827465	Yang2025_Silkie_Wenchang	0.894064255
ENSGALG00010020388	MYOF	2	-0.039048663	0.119486642	-0.326803498	0.74381654	0	0.799727599	0	0.5	PRJNA827465;Yang2025_Silkie_Wenchang	CRA004775	0.935821071
ENSGALG00010020388	MYOF	2	0.217756514	0.185722481	1.172483337	0.241003058	0.003667261	1.041477724	3.982583923	1	CRA004775;Yang2025_Silkie_Wenchang	PRJNA827465	0.753551568
ENSGALG00010020388	MYOF	2	0.126005225	0.316002861	0.398747102	0.690079558	0.1365449	2.749633664	63.63151888	0.5	CRA004775;PRJNA827465	Yang2025_Silkie_Wenchang	0.919979266
ENSGALG00010020389	ACTN1	2	0.192962777	0.188928826	1.021351694	0.307087843	0.055636622	4.278523747	76.62745238	1	PRJNA827465;Yang2025_Silkie_Wenchang	CRA004775	0.79266003
ENSGALG00010020389	ACTN1	2	0.303224751	0.238645133	1.270609406	0.203867639	0.041591284	1.247432917	19.8353686	0.5	CRA004775;Yang2025_Silkie_Wenchang	PRJNA827465	0.717254253
ENSGALG00010020389	ACTN1	2	0.020758655	0.085930443	0.241575101	0.809109418	0	0.223808725	0	0.5	CRA004775;PRJNA827465	Yang2025_Silkie_Wenchang	0.952716213
ENSGALG00010020390	KLHL25	2	-0.0534462	0.214891257	-0.248712771	0.803582969	0.067281533	3.580840213	72.07359333	0.5	PRJNA827465;Yang2025_Silkie_Wenchang	CRA004775	0.951064358
ENSGALG00010020390	KLHL25	2	-0.163923425	0.228308093	-0.717992179	0.472762109	0.032769448	1.296245362	22.85411162	0.5	CRA004775;Yang2025_Silkie_Wenchang	PRJNA827465	0.854086678
ENSGALG00010020390	KLHL25	2	0.148713621	0.12286755	1.210357177	0.226141867	0	0.052958619	0	1	CRA004775;PRJNA827465	Yang2025_Silkie_Wenchang	0.739595164
ENSGALG00010020392	SLC23A2	2	0.202718114	0.08702268	2.329485995	0.019833334	0	0.026770879	0	1	PRJNA827465;Yang2025_Silkie_Wenchang	CRA004775	0.236501678
ENSGALG00010020392	SLC23A2	2	0.22733088	0.123679139	1.838069707	0.066052134	0	0.043148584	0	1	CRA004775;Yang2025_Silkie_Wenchang	PRJNA827465	0.442444044
ENSGALG00010020392	SLC23A2	2	0.199479132	0.111738625	1.78522988	0.074224037	0	0.078927373	0	1	CRA004775;PRJNA827465	Yang2025_Silkie_Wenchang	0.47044466
ENSGALG00010020393	SHISA9	2	-0.182537736	0.26363318	-0.692392879	0.488690637	0.086361278	2.481621044	59.70375886	0.5	PRJNA827465;Yang2025_Silkie_Wenchang	CRA004775	0.857953638
ENSGALG00010020393	SHISA9	2	0.56173393	0.589638797	0.952674642	0.340754945	0.439074647	2.303776486	56.59301766	1	CRA004775;Yang2025_Silkie_Wenchang	PRJNA827465	0.805575436
ENSGALG00010020393	SHISA9	2	0.33728187	0.877991489	0.38415164	0.700866059	1.282301468	5.160609542	80.62263182	0.5	CRA004775;PRJNA827465	Yang2025_Silkie_Wenchang	0.922096697
ENSGALG00010020396	CHL1	2	0.51799444	0.179410389	2.887204259	0.003886818	0	0.058760939	0	1	PRJNA827465;Yang2025_Silkie_Wenchang	CRA004775	0.08970265
ENSGALG00010020396	CHL1	2	0.787112592	0.293256151	2.68404461	0.00727374	0.030545132	1.187059454	15.75822118	1	CRA004775;Yang2025_Silkie_Wenchang	PRJNA827465	0.131077397
ENSGALG00010020396	CHL1	2	0.719121921	0.334656288	2.148837318	0.031647298	0.117705966	1.852236276	46.01120749	1	CRA004775;PRJNA827465	Yang2025_Silkie_Wenchang	0.304637217
ENSGALG00010020399	ATP13A2	2	0.461179044	0.434456684	1.061507537	0.288459309	0.367750818	38.70087481	97.41607908	1	PRJNA827465;Yang2025_Silkie_Wenchang	CRA004775	0.783088502
ENSGALG00010020399	ATP13A2	2	0.226761663	0.680884049	0.333040057	0.739104046	0.901303661	35.41448487	97.17629664	0.5	CRA004775;Yang2025_Silkie_Wenchang	PRJNA827465	0.934652713
ENSGALG00010020399	ATP13A2	2	-0.18634449	0.244268979	-0.762865962	0.445543338	0.095482892	4.662007729	78.55001411	0.5	CRA004775;PRJNA827465	Yang2025_Silkie_Wenchang	0.846013379
ENSGALG00010020402	SLC3A1	2	0.472460796	0.179132177	2.637498201	0.008352007	0.020655789	1.474603654	32.18516738	1	PRJNA827465;Yang2025_Silkie_Wenchang	CRA004775	0.142597274
ENSGALG00010020402	SLC3A1	2	0.729179381	0.184762419	3.946568457	7.92738E-05	0	0.609322171	0	1	CRA004775;Yang2025_Silkie_Wenchang	PRJNA827465	0.006601058
ENSGALG00010020402	SLC3A1	2	0.568244628	0.344057667	1.65159705	0.098616721	0.150279333	2.519301594	60.30645945	1	CRA004775;PRJNA827465	Yang2025_Silkie_Wenchang	0.536014139
ENSGALG00010020405	UBE2I	2	-0.161335499	0.186460547	-0.865252739	0.386900085	0.063253145	10.63168666	90.5941547	0.5	PRJNA827465;Yang2025_Silkie_Wenchang	CRA004775	0.827149575
ENSGALG00010020405	UBE2I	2	-0.33069113	0.09535396	-3.468032274	0.000524274	0	0.468012242	0	1	CRA004775;Yang2025_Silkie_Wenchang	PRJNA827465	0.024184895
ENSGALG00010020405	UBE2I	2	0.005509731	0.044967588	0.122526711	0.902481893	0	0.789106441	0	0.5	CRA004775;PRJNA827465	Yang2025_Silkie_Wenchang	0.976923607
ENSGALG00010020413	CYP26A1	2	0.316928452	0.22159598	1.43020849	0.152657189	0	0.007052195	0	1	PRJNA827465;Yang2025_Silkie_Wenchang	CRA004775	0.644378159
ENSGALG00010020413	CYP26A1	2	0.526791158	0.275696892	1.910762046	0.056035167	0	0.862237839	0	1	CRA004775;Yang2025_Silkie_Wenchang	PRJNA827465	0.409434872
ENSGALG00010020413	CYP26A1	2	0.47304047	0.263032529	1.798410529	0.072111975	0.014125401	1.0936696	8.564707277	1	CRA004775;PRJNA827465	Yang2025_Silkie_Wenchang	0.463486013
ENSGALG00010020415	TSR3	2	0.204467029	0.156313972	1.308053441	0.190855184	0.040448464	5.647643964	82.29350139	1	PRJNA827465;Yang2025_Silkie_Wenchang	CRA004775	0.700389407
ENSGALG00010020415	TSR3	2	0.068425531	0.349977396	0.195514145	0.844990465	0.202897918	5.335427151	81.25735819	0.5	CRA004775;Yang2025_Silkie_Wenchang	PRJNA827465	0.960789801
ENSGALG00010020415	TSR3	2	-0.040067119	0.171028889	-0.234271058	0.814773479	0.034213991	1.794416689	44.27158384	0.5	CRA004775;PRJNA827465	Yang2025_Silkie_Wenchang	0.953485608
ENSGALG00010020422	ERCC4	2	-0.038739676	0.202838418	-0.190987861	0.848535103	0.076803616	14.98132811	93.32502437	0.5	PRJNA827465;Yang2025_Silkie_Wenchang	CRA004775	0.961364896
ENSGALG00010020422	ERCC4	2	-0.036771665	0.234734599	-0.156652088	0.875519049	0.091571653	5.494971733	81.80154424	0.5	CRA004775;Yang2025_Silkie_Wenchang	PRJNA827465	0.970347777
ENSGALG00010020422	ERCC4	2	0.169811339	0.063588025	2.670492417	0.007574008	0	0.116937352	0	1	CRA004775;PRJNA827465	Yang2025_Silkie_Wenchang	0.134392245
ENSGALG00010020428	SEMA4C	2	0.113809899	0.058210022	1.955159847	0.050566206	0	0.107011571	0	1	PRJNA827465;Yang2025_Silkie_Wenchang	CRA004775	0.389297528
ENSGALG00010020428	SEMA4C	2	0.127647281	0.093464813	1.365725533	0.172025127	0	0.111317814	0	1	CRA004775;Yang2025_Silkie_Wenchang	PRJNA827465	0.674672141
ENSGALG00010020428	SEMA4C	2	0.096802822	0.067915706	1.425337791	0.154059581	0	0.033425312	0	1	CRA004775;PRJNA827465	Yang2025_Silkie_Wenchang	0.646788679
ENSGALG00010020429	ZNF148	2	0.138683174	0.080187972	1.729476021	0.083723935	0.005040457	1.382340442	27.65892034	1	PRJNA827465;Yang2025_Silkie_Wenchang	CRA004775	0.4985005
ENSGALG00010020429	ZNF148	2	0.242075022	0.13346905	1.813716532	0.069721379	0	0.365849223	0	1	CRA004775;Yang2025_Silkie_Wenchang	PRJNA827465	0.455495236
ENSGALG00010020429	ZNF148	2	0.094949143	0.056244194	1.688159011	0.091380709	0	3.00997E-05	0	1	CRA004775;PRJNA827465	Yang2025_Silkie_Wenchang	0.518259165
ENSGALG00010020433	GNPTG	2	0.055250223	0.063563626	0.869211322	0.384731556	0	0.948759893	0	1	PRJNA827465;Yang2025_Silkie_Wenchang	CRA004775	0.826425334
ENSGALG00010020433	GNPTG	2	-0.073734685	0.28088652	-0.262507028	0.792930568	0.126204627	4.948452469	79.79166202	0.5	CRA004775;Yang2025_Silkie_Wenchang	PRJNA827465	0.948490432
ENSGALG00010020433	GNPTG	2	-0.126596834	0.193171428	-0.655360037	0.512235975	0.056241131	3.528085034	71.65601196	0.5	CRA004775;PRJNA827465	Yang2025_Silkie_Wenchang	0.865723953
ENSGALG00010020436	CNTN4	2	-0.168015187	0.112597979	-1.492168766	0.135654908	0	0.334364051	0	1	PRJNA827465;Yang2025_Silkie_Wenchang	CRA004775	0.617196137
ENSGALG00010020436	CNTN4	2	-0.007455987	0.177450655	-0.042017242	0.966484953	0	0.405416477	0	0.5	CRA004775;Yang2025_Silkie_Wenchang	PRJNA827465	0.990881287
ENSGALG00010020436	CNTN4	2	-0.149059666	0.146596233	-1.016804202	0.309246583	0.007069102	1.093281393	8.53224002	0.5	CRA004775;PRJNA827465	Yang2025_Silkie_Wenchang	0.794043289
ENSGALG00010020437	SNX4	2	-0.09755699	0.034195146	-2.852948504	0.004331564	0	0.624799767	0	1	PRJNA827465;Yang2025_Silkie_Wenchang	CRA004775	0.096509898
ENSGALG00010020437	SNX4	2	-0.093988049	0.089845641	-1.046105825	0.295512176	0.006252168	1.310270604	23.67988744	0.5	CRA004775;Yang2025_Silkie_Wenchang	PRJNA827465	0.787628307
ENSGALG00010020437	SNX4	2	-0.071492839	0.040430814	-1.76827601	0.077014772	0	0.769231211	0	0.5	CRA004775;PRJNA827465	Yang2025_Silkie_Wenchang	0.478453375
ENSGALG00010020438	HTR7	2	-0.140110505	0.225263285	-0.621985538	0.533951377	0.059785847	2.432932249	58.89733467	0.5	PRJNA827465;Yang2025_Silkie_Wenchang	CRA004775	0.873408008
ENSGALG00010020438	HTR7	2	-0.263815147	0.175412761	-1.503967821	0.132589653	0	0.884614041	0	1	CRA004775;Yang2025_Silkie_Wenchang	PRJNA827465	0.609921713
ENSGALG00010020438	HTR7	2	0.058345947	0.171000541	0.341203291	0.732950546	0	0.050410111	0	0.5	CRA004775;PRJNA827465	Yang2025_Silkie_Wenchang	0.932985777
ENSGALG00010020440	PAX7	2	0.104958183	0.15943971	0.658293861	0.510349322	0.029340099	2.14266153	53.32907294	0.5	PRJNA827465;Yang2025_Silkie_Wenchang	CRA004775	0.865365179
ENSGALG00010020440	PAX7	2	0.296830896	0.189637718	1.565252416	0.117523736	0	0.079471654	0	1	CRA004775;Yang2025_Silkie_Wenchang	PRJNA827465	0.577090877
ENSGALG00010020440	PAX7	2	-0.009586202	0.10082866	-0.095074179	0.924255908	0	0.089133157	0	0.5	CRA004775;PRJNA827465	Yang2025_Silkie_Wenchang	0.98232425
ENSGALG00010020443	PREPL	2	-0.033461253	0.309158379	-0.108233369	0.913810576	0.152705523	4.688841306	78.67276935	0.5	PRJNA827465;Yang2025_Silkie_Wenchang	CRA004775	0.979364626
ENSGALG00010020443	PREPL	2	0.2680286	0.206262214	1.299455654	0.193787603	0	0.10296969	0	1	CRA004775;Yang2025_Silkie_Wenchang	PRJNA827465	0.70398689
ENSGALG00010020443	PREPL	2	-0.172550577	0.216648577	-0.796453774	0.425768334	0.04742139	1.679559253	40.46057034	0.5	CRA004775;PRJNA827465	Yang2025_Silkie_Wenchang	0.84125685
ENSGALG00010020449	PRPF19	2	-0.115543291	0.041975373	-2.752644764	0.0059116	0	0.670233362	0	1	PRJNA827465;Yang2025_Silkie_Wenchang	CRA004775	0.116113758
ENSGALG00010020449	PRPF19	2	-0.243616684	0.093275821	-2.611788148	0.009007005	0.001178812	1.052105344	4.952483516	1	CRA004775;Yang2025_Silkie_Wenchang	PRJNA827465	0.14870051
ENSGALG00010020449	PRPF19	2	-0.201522207	0.145294412	-1.386992139	0.16544416	0.029308318	2.580215371	61.24354535	1	CRA004775;PRJNA827465	Yang2025_Silkie_Wenchang	0.664874006
ENSGALG00010020450	ANKRD39	2	0.119690001	0.21237708	0.563573063	0.573044723	0.040684773	1.687608777	40.74456038	0.5	PRJNA827465;Yang2025_Silkie_Wenchang	CRA004775	0.88573298
ENSGALG00010020450	ANKRD39	2	0.26309191	0.249082133	1.056245614	0.290856016	0	0.7935217	0	0.5	CRA004775;Yang2025_Silkie_Wenchang	PRJNA827465	0.784646441
ENSGALG00010020450	ANKRD39	2	-0.038268731	0.156988643	-0.243767512	0.807410883	0	0.004496838	0	1	CRA004775;PRJNA827465	Yang2025_Silkie_Wenchang	0.95234753
ENSGALG00010020453	ENSGALG00010020453	2	0.052224255	0.08383251	0.622959466	0.533311158	0	0.326715889	0	1	PRJNA827465;Yang2025_Silkie_Wenchang	CRA004775	0.873379507
ENSGALG00010020453	ENSGALG00010020453	2	0.157119763	0.109660807	1.432779557	0.151920845	0	0.624122728	0	1	CRA004775;Yang2025_Silkie_Wenchang	PRJNA827465	0.643294424
ENSGALG00010020453	ENSGALG00010020453	2	0.098788108	0.135565263	0.728712549	0.466177515	0.013780861	1.459633089	31.48963205	1	CRA004775;PRJNA827465	Yang2025_Silkie_Wenchang	0.852631007
ENSGALG00010020460	MAPK8	2	0.109542326	0.170176898	0.643696809	0.519772059	0.051698994	9.168053505	89.09255929	0.5	PRJNA827465;Yang2025_Silkie_Wenchang	CRA004775	0.869117262
ENSGALG00010020460	MAPK8	2	0.255959776	0.086634069	2.954493296	0.00313183	0	0.736230259	0	1	CRA004775;Yang2025_Silkie_Wenchang	PRJNA827465	0.077464001
ENSGALG00010020460	MAPK8	2	-0.043999775	0.059607968	-0.738152576	0.460421738	0	0.32753543	0	0.5	CRA004775;PRJNA827465	Yang2025_Silkie_Wenchang	0.85014477
ENSGALG00010020462	CNNM4	2	-0.036856472	0.057703084	-0.638726211	0.523001058	0	0.852755633	0	1	PRJNA827465;Yang2025_Silkie_Wenchang	CRA004775	0.869568211
ENSGALG00010020462	CNNM4	2	-0.216652832	0.11625552	-1.863591781	0.062379053	0	0.498549129	0	1	CRA004775;Yang2025_Silkie_Wenchang	PRJNA827465	0.430573219
ENSGALG00010020462	CNNM4	2	-0.115881876	0.148521804	-0.780234778	0.435252695	0.028590575	2.307001299	56.65368717	1	CRA004775;PRJNA827465	Yang2025_Silkie_Wenchang	0.842762754
ENSGALG00010020467	ALDH4A1	2	0.039748386	0.063029691	0.630629559	0.528282767	0	0.842675106	0	0.5	PRJNA827465;Yang2025_Silkie_Wenchang	CRA004775	0.871701504
ENSGALG00010020467	ALDH4A1	2	-0.087175086	0.107815736	-0.808556241	0.418770444	0	0.782177278	0	1	CRA004775;Yang2025_Silkie_Wenchang	PRJNA827465	0.838250071
ENSGALG00010020467	ALDH4A1	2	-0.026498539	0.177086762	-0.149635913	0.881051873	0.036480596	1.793893885	44.25534261	0.5	CRA004775;PRJNA827465	Yang2025_Silkie_Wenchang	0.971685305
ENSGALG00010020473	TMEM109	2	0.218938896	0.159685539	1.371062767	0.17035539	0	0.332218483	0	1	PRJNA827465;Yang2025_Silkie_Wenchang	CRA004775	0.672114378
ENSGALG00010020473	TMEM109	2	0.205929992	0.272503901	0.755695574	0.449831738	0.013410416	1.070234288	6.562515247	0.5	CRA004775;Yang2025_Silkie_Wenchang	PRJNA827465	0.84742064
ENSGALG00010020473	TMEM109	2	0.110188809	0.180516436	0.610408732	0.541591085	0	0.576509586	0	0.5	CRA004775;PRJNA827465	Yang2025_Silkie_Wenchang	0.876288927
ENSGALG00010020476	TMEM132A	2	0.079033824	0.070154262	1.12657196	0.2599235	0	0.213128059	0	1	PRJNA827465;Yang2025_Silkie_Wenchang	CRA004775	0.767122724
ENSGALG00010020476	TMEM132A	2	0.087322992	0.126017352	0.692944192	0.488344575	0	0.369069628	0	0.5	CRA004775;Yang2025_Silkie_Wenchang	PRJNA827465	0.857796363
ENSGALG00010020476	TMEM132A	2	0.053232355	0.073655411	0.722721589	0.469850949	0	0.123390913	0	0.5	CRA004775;PRJNA827465	Yang2025_Silkie_Wenchang	0.853485658
ENSGALG00010020481	RPP30	2	-0.413819112	0.314344503	-1.316450927	0.188022739	0.16640075	5.887749289	83.01558115	1	PRJNA827465;Yang2025_Silkie_Wenchang	CRA004775	0.697353751
ENSGALG00010020481	RPP30	2	-0.465204482	0.288816007	-1.610729565	0.107238679	0.11724909	3.347295502	70.12513537	1	CRA004775;Yang2025_Silkie_Wenchang	PRJNA827465	0.554473355
ENSGALG00010020481	RPP30	2	-0.145557217	0.090852129	-1.602133258	0.109126145	0	0.057546238	0	1	CRA004775;PRJNA827465	Yang2025_Silkie_Wenchang	0.559179559
ENSGALG00010020484	TRNT1	2	-0.087370581	0.145198029	-0.601733937	0.547351256	0.032552152	4.1437666	75.86736666	0.5	PRJNA827465;Yang2025_Silkie_Wenchang	CRA004775	0.878281266
ENSGALG00010020484	TRNT1	2	0.083493504	0.115832831	0.72081035	0.471026207	0	0.010232779	0	1	CRA004775;Yang2025_Silkie_Wenchang	PRJNA827465	0.853855613
ENSGALG00010020484	TRNT1	2	-0.173049855	0.108466454	-1.595422807	0.110617719	0.008776767	1.199598919	16.63880452	0.5	CRA004775;PRJNA827465	Yang2025_Silkie_Wenchang	0.562832796
ENSGALG00010020485	LMAN2L	2	-0.031541111	0.187209026	-0.168480718	0.866205111	0.065872842	16.45265987	93.92195543	0.5	PRJNA827465;Yang2025_Silkie_Wenchang	CRA004775	0.967337032
ENSGALG00010020485	LMAN2L	2	-0.224582182	0.412043615	-0.545044684	0.585722786	0.31192386	11.67445158	91.43428714	0.5	CRA004775;Yang2025_Silkie_Wenchang	PRJNA827465	0.891202921
ENSGALG00010020485	LMAN2L	2	-0.38472048	0.218866672	-1.757784662	0.078784156	0.074397461	3.703402948	72.99780731	1	CRA004775;PRJNA827465	Yang2025_Silkie_Wenchang	0.482840119
ENSGALG00010020486	MRTFB	2	0.354249404	0.42272011	0.838023541	0.402017482	0.318241109	8.349483495	88.02321125	0.5	PRJNA827465;Yang2025_Silkie_Wenchang	CRA004775	0.832832232
ENSGALG00010020486	MRTFB	2	0.568190745	0.292976378	1.939373914	0.052455825	0.069722179	1.664382104	39.91764285	1	CRA004775;Yang2025_Silkie_Wenchang	PRJNA827465	0.39754556
ENSGALG00010020486	MRTFB	2	-0.0164519	0.066145682	-0.248722206	0.803575671	0	0.508051432	0	0.5	CRA004775;PRJNA827465	Yang2025_Silkie_Wenchang	0.951064358
ENSGALG00010020489	IFFO2	2	0.283640101	0.149581193	1.896228363	0.057929854	0.028791288	2.772788461	63.9352221	1	PRJNA827465;Yang2025_Silkie_Wenchang	CRA004775	0.417375481
ENSGALG00010020489	IFFO2	2	0.274420846	0.236633497	1.159687235	0.246176169	0.065483214	2.116093523	52.74310946	0.5	CRA004775;Yang2025_Silkie_Wenchang	PRJNA827465	0.756727496
ENSGALG00010020489	IFFO2	2	0.122690914	0.105898361	1.158572358	0.246630541	0	0.356457982	0	0.5	CRA004775;PRJNA827465	Yang2025_Silkie_Wenchang	0.757054059
ENSGALG00010020493	DEPDC7	2	-0.110519093	0.216542986	-0.510379466	0.60978564	0.074527703	4.652681995	78.50702023	0.5	PRJNA827465;Yang2025_Silkie_Wenchang	CRA004775	0.897238578
ENSGALG00010020493	DEPDC7	2	0.15441772	0.550085138	0.280716037	0.778928207	0.528566831	7.549850794	86.75470513	0.5	CRA004775;Yang2025_Silkie_Wenchang	PRJNA827465	0.944714533
ENSGALG00010020493	DEPDC7	2	0.327198342	0.321649652	1.017250727	0.30903417	0.152952226	3.17717194	68.52546797	1	CRA004775;PRJNA827465	Yang2025_Silkie_Wenchang	0.794014496
ENSGALG00010020497	OSBPL11	2	-0.187435778	0.066975083	-2.798589686	0.005132631	0.004382903	1.926140952	48.08271955	1	PRJNA827465;Yang2025_Silkie_Wenchang	CRA004775	0.107017406
ENSGALG00010020497	OSBPL11	2	-0.257029869	0.073659908	-3.489413392	0.000484082	0	0.090599094	0	1	CRA004775;Yang2025_Silkie_Wenchang	PRJNA827465	0.023061661
ENSGALG00010020497	OSBPL11	2	-0.130920657	0.058421189	-2.240978992	0.025027436	0	0.036270933	0	1	CRA004775;PRJNA827465	Yang2025_Silkie_Wenchang	0.269567912
ENSGALG00010020507	ANKRD1	2	0.124705708	0.271405779	0.459480665	0.645889033	0	0.065632587	0	1	PRJNA827465;Yang2025_Silkie_Wenchang	CRA004775	0.907098003
ENSGALG00010020507	ANKRD1	2	0.256804868	0.444693501	0.577487341	0.563610288	0.048685738	1.085682502	7.89204042	1	CRA004775;Yang2025_Silkie_Wenchang	PRJNA827465	0.882532874
ENSGALG00010020507	ANKRD1	2	0.285386394	0.322613572	0.884607527	0.376368346	0	0.886125807	0	1	CRA004775;PRJNA827465	Yang2025_Silkie_Wenchang	0.822974013
ENSGALG00010020509	CRBN	2	-0.114769394	0.158765454	-0.722886445	0.469749652	0.036300557	3.48849219	71.33432023	0.5	PRJNA827465;Yang2025_Silkie_Wenchang	CRA004775	0.853485658
ENSGALG00010020509	CRBN	2	-0.013820748	0.300093929	-0.046054742	0.963266619	0.146657463	5.222295893	80.85133396	0.5	CRA004775;Yang2025_Silkie_Wenchang	PRJNA827465	0.989998461
ENSGALG00010020509	CRBN	2	0.099860651	0.122498817	0.815196864	0.414959633	0.010309389	1.345748066	25.69188654	1	CRA004775;PRJNA827465	Yang2025_Silkie_Wenchang	0.837458421
ENSGALG00010020514	CAMKMT	2	-0.229422317	0.266149549	-0.862005283	0.38868461	0.126553587	9.184407104	89.1119809	0.5	PRJNA827465;Yang2025_Silkie_Wenchang	CRA004775	0.828400391
ENSGALG00010020514	CAMKMT	2	-0.286581569	0.277145581	-1.034047044	0.301114171	0.10383013	2.810988324	64.4253236	0.5	CRA004775;Yang2025_Silkie_Wenchang	PRJNA827465	0.791202961
ENSGALG00010020514	CAMKMT	2	0.026838072	0.088033835	0.304860874	0.760472116	0	0.012024602	0	1	CRA004775;PRJNA827465	Yang2025_Silkie_Wenchang	0.939327609
ENSGALG00010020517	UBR4	2	-0.015829091	0.145011991	-0.10915712	0.913077871	0.031671787	3.763561945	73.42942631	0.5	PRJNA827465;Yang2025_Silkie_Wenchang	CRA004775	0.979129568
ENSGALG00010020517	UBR4	2	0.140504053	0.119031632	1.180392565	0.237844118	0	0.045809145	0	1	CRA004775;Yang2025_Silkie_Wenchang	PRJNA827465	0.74963812
ENSGALG00010020517	UBR4	2	-0.123527969	0.065810044	-1.877038238	0.060512853	0	0.798892942	0	0.5	CRA004775;PRJNA827465	Yang2025_Silkie_Wenchang	0.426049285
ENSGALG00010020520	ARHGAP22	2	-0.03455274	0.140029216	-0.246753789	0.805098775	0.027377222	3.173836923	68.49239503	0.5	PRJNA827465;Yang2025_Silkie_Wenchang	CRA004775	0.951863117
ENSGALG00010020520	ARHGAP22	2	-0.187030455	0.126934275	-1.47344328	0.140631547	0	0.035056571	0	1	CRA004775;Yang2025_Silkie_Wenchang	PRJNA827465	0.624970795
ENSGALG00010020520	ARHGAP22	2	0.077910025	0.082311395	0.946527816	0.343879442	0	0.275732942	0	0.5	CRA004775;PRJNA827465	Yang2025_Silkie_Wenchang	0.806413705
ENSGALG00010020524	ENSGALG00010020524	2	-0.296138179	0.335952321	-0.881488715	0.378053359	0.142060535	2.686846947	62.78165375	0.5	PRJNA827465;Yang2025_Silkie_Wenchang	CRA004775	0.82325159
ENSGALG00010020524	ENSGALG00010020524	2	0.223297185	0.888257299	0.251387954	0.80151418	1.454661628	12.7449105	92.1537307	0.5	CRA004775;Yang2025_Silkie_Wenchang	PRJNA827465	0.950283916
ENSGALG00010020524	ENSGALG00010020524	2	0.539791087	0.550872596	0.979883718	0.327143521	0.496792356	5.407833897	81.50830778	1	CRA004775;PRJNA827465	Yang2025_Silkie_Wenchang	0.800028239
ENSGALG00010020525	ENSGALG00010020525	2	-0.545966082	0.849976877	-0.642330511	0.520658607	1.045849425	3.612569463	72.31887137	0.5	PRJNA827465;Yang2025_Silkie_Wenchang	CRA004775	0.869342166
ENSGALG00010020525	ENSGALG00010020525	2	-0.604317018	0.785501384	-0.769339215	0.441691963	0.84747828	3.188493746	68.63722874	0.5	CRA004775;Yang2025_Silkie_Wenchang	PRJNA827465	0.845089048
ENSGALG00010020525	ENSGALG00010020525	2	0.262716018	0.463268284	0.567092604	0.570651267	0	0.019438275	0	1	CRA004775;PRJNA827465	Yang2025_Silkie_Wenchang	0.885152854
ENSGALG00010020530	SRSF5A	2	0.065089958	0.078661006	0.827474271	0.4079683	0.010329007	5.810451798	82.78963436	0.5	PRJNA827465;Yang2025_Silkie_Wenchang	CRA004775	0.833982683
ENSGALG00010020530	SRSF5A	2	0.037424775	0.146122373	0.256119404	0.79785864	0.031022041	3.152066224	68.27477823	0.5	CRA004775;Yang2025_Silkie_Wenchang	PRJNA827465	0.949922217
ENSGALG00010020530	SRSF5A	2	-0.01230956	0.031379107	-0.392285203	0.694847495	0	0.774507897	0	1	CRA004775;PRJNA827465	Yang2025_Silkie_Wenchang	0.92087205
ENSGALG00010020533	ENSGALG00010020533	2	0.353794659	0.319442088	1.107539279	0.26806084	0.176458129	6.764205678	85.21629815	1	PRJNA827465;Yang2025_Silkie_Wenchang	CRA004775	0.771398294
ENSGALG00010020533	ENSGALG00010020533	2	0.317206916	0.406849042	0.779667354	0.4355867	0.267651991	5.208382828	80.80018245	0.5	CRA004775;Yang2025_Silkie_Wenchang	PRJNA827465	0.842762754
ENSGALG00010020533	ENSGALG00010020533	2	0.058000356	0.072981829	0.794723246	0.426774507	0	0.374771816	0	0.5	CRA004775;PRJNA827465	Yang2025_Silkie_Wenchang	0.84125685
ENSGALG00010020535	LMLN	2	0.278768712	0.110954706	2.512455049	0.011989438	0	0.049041869	0	1	PRJNA827465;Yang2025_Silkie_Wenchang	CRA004775	0.176025728
ENSGALG00010020535	LMLN	2	-0.209622736	0.527056909	-0.397723153	0.690834269	0.461823494	5.444148248	81.63165376	0.5	CRA004775;Yang2025_Silkie_Wenchang	PRJNA827465	0.920151714
ENSGALG00010020535	LMLN	2	-0.185258405	0.552084261	-0.335561831	0.737201311	0.51881533	6.145513478	83.72796669	0.5	CRA004775;PRJNA827465	Yang2025_Silkie_Wenchang	0.933895493
ENSGALG00010020537	CCDC167	2	-0.212027044	0.272575422	-0.777865599	0.43664825	0.13013597	7.719703473	87.04613456	0.5	PRJNA827465;Yang2025_Silkie_Wenchang	CRA004775	0.843275468
ENSGALG00010020537	CCDC167	2	-0.500438947	0.152705143	-3.277158426	0.001048575	0	0.005152335	0	1	CRA004775;Yang2025_Silkie_Wenchang	PRJNA827465	0.039063649
ENSGALG00010020537	CCDC167	2	-0.137928341	0.247249948	-0.557849826	0.576946936	0.085100564	2.673652649	62.59798369	0.5	CRA004775;PRJNA827465	Yang2025_Silkie_Wenchang	0.887113945
ENSGALG00010020540	PCGF5	2	0.127740893	0.16316978	0.782871026	0.433702854	0.031282352	2.352173036	57.48612094	0.5	PRJNA827465;Yang2025_Silkie_Wenchang	CRA004775	0.842762754
ENSGALG00010020540	PCGF5	2	0.352853237	0.152627623	2.311856996	0.020785565	0	0.165779912	0	1	CRA004775;Yang2025_Silkie_Wenchang	PRJNA827465	0.243715714
ENSGALG00010020540	PCGF5	2	0.141577663	0.22193936	0.637911465	0.523531314	0.058002374	2.075047762	51.80833817	0.5	CRA004775;PRJNA827465	Yang2025_Silkie_Wenchang	0.869661505
ENSGALG00010020544	BMERB1	2	-0.045870943	0.057821469	-0.793320256	0.427591258	0	0.018435139	0	1	PRJNA827465;Yang2025_Silkie_Wenchang	CRA004775	0.84125685
ENSGALG00010020544	BMERB1	2	0.328164522	0.43904959	0.747442953	0.454796226	0.29440798	3.700282215	72.97503429	0.5	CRA004775;Yang2025_Silkie_Wenchang	PRJNA827465	0.849072665
ENSGALG00010020544	BMERB1	2	0.308214192	0.448752372	0.686824653	0.492193232	0.325116321	4.285504946	76.66552687	0.5	CRA004775;PRJNA827465	Yang2025_Silkie_Wenchang	0.859190345
ENSGALG00010020545	STYX	2	0.101229262	0.189695941	0.533639579	0.59359092	0.062533412	7.613450588	86.86535115	0.5	PRJNA827465;Yang2025_Silkie_Wenchang	CRA004775	0.892935613
ENSGALG00010020545	STYX	2	0.300031083	0.087396819	3.432974882	0.000596997	0	0.239808519	0	1	CRA004775;Yang2025_Silkie_Wenchang	PRJNA827465	0.026393463
ENSGALG00010020545	STYX	2	0.095460211	0.257498931	0.370720806	0.710845492	0.090662506	2.620666637	61.8417701	0.5	CRA004775;PRJNA827465	Yang2025_Silkie_Wenchang	0.92554556
ENSGALG00010020546	LRRC20	2	-0.396888599	0.08508091	-4.664837233	3.08861E-06	0	0.211564503	0	1	PRJNA827465;Yang2025_Silkie_Wenchang	CRA004775	0.000578056
ENSGALG00010020546	LRRC20	2	-0.302766762	0.171145779	-1.769057723	0.076884242	0	0.123189703	0	1	CRA004775;Yang2025_Silkie_Wenchang	PRJNA827465	0.477978704
ENSGALG00010020546	LRRC20	2	-0.408334057	0.094820936	-4.30637024	1.65955E-05	0	0.293017942	0	1	CRA004775;PRJNA827465	Yang2025_Silkie_Wenchang	0.002131548
ENSGALG00010020548	CMTR1	2	-0.093519534	0.15216502	-0.614592851	0.538823637	0.033662752	3.522731537	71.61293759	0.5	PRJNA827465;Yang2025_Silkie_Wenchang	CRA004775	0.87481821
ENSGALG00010020548	CMTR1	2	-0.266659599	0.125834507	-2.119129363	0.034079536	0	0.000407695	0	1	CRA004775;Yang2025_Silkie_Wenchang	PRJNA827465	0.317399999
ENSGALG00010020548	CMTR1	2	0.017448503	0.083906605	0.20795148	0.835266852	0	0.973517366	0	0.5	CRA004775;PRJNA827465	Yang2025_Silkie_Wenchang	0.958309459
ENSGALG00010020550	HECTD2	2	0.216858924	0.131863707	1.644568691	0.100058788	0.010872486	1.434159548	30.27275094	1	PRJNA827465;Yang2025_Silkie_Wenchang	CRA004775	0.54042825
ENSGALG00010020550	HECTD2	2	0.1094556	0.16445673	0.665558654	0.505693241	0	0.579827262	0	1	CRA004775;Yang2025_Silkie_Wenchang	PRJNA827465	0.863927652
ENSGALG00010020550	HECTD2	2	0.335188736	0.13041594	2.570151589	0.010165402	0	0.040924972	0	1	CRA004775;PRJNA827465	Yang2025_Silkie_Wenchang	0.160196794
ENSGALG00010020554	ZFP36L1	2	0.128498876	0.221383614	0.580435352	0.561621071	0.073644635	3.50912376	71.50285745	0.5	PRJNA827465;Yang2025_Silkie_Wenchang	CRA004775	0.881802301
ENSGALG00010020554	ZFP36L1	2	0.312393065	0.176410646	1.770828872	0.076589163	0	0.413709203	0	1	CRA004775;Yang2025_Silkie_Wenchang	PRJNA827465	0.47762877
ENSGALG00010020554	ZFP36L1	2	-0.030835162	0.078621561	-0.392197281	0.694912453	0	0.586906878	0	0.5	CRA004775;PRJNA827465	Yang2025_Silkie_Wenchang	0.92087205

ENSGALG00010020556	EMC1	2	0.011099791	0.055402441	0.200348417	0.841208099	0	0.386230781	0	0.5	PRJNA827465;Yang2025_Silkie_Wenchang	CRA004775	0.95989279
ENSGALG00010020556	EMC1	2	-0.092082518	0.093624921	-0.983525719	0.325348764	0.000213426	1.008141357	0.807561064	1	CRA004775;Yang2025_Silkie_Wenchang	PRJNA827465	0.799987949
ENSGALG00010020556	EMC1	2	-0.059703232	0.140618256	-0.424576676	0.671145299	0.024033183	2.048913768	51.19365121	0.5	CRA004775;PRJNA827465	Yang2025_Silkie_Wenchang	0.914621672
ENSGALG00010020557	ENSGALG00010020557	2	-0.012778544	0.146216431	-0.087394718	0.930357768	0.01396651	1.453125058	31.18279846	0.5	PRJNA827465;Yang2025_Silkie_Wenchang	CRA004775	0.983813577
ENSGALG00010020557	ENSGALG00010020557	2	-0.185496172	0.182628869	-1.015700167	0.309772189	0	0.011039983	0	1	CRA004775;Yang2025_Silkie_Wenchang	PRJNA827465	0.794371602
ENSGALG00010020557	ENSGALG00010020557	2	0.080207421	0.138429794	0.579408658	0.562313463	0	0.289658325	0	0.5	CRA004775;PRJNA827465	Yang2025_Silkie_Wenchang	0.881955027
ENSGALG00010020558	EIF4EBP2	2	-0.15555982	0.056031045	-2.776314806	0.005497895	0	0.230724281	0	1	PRJNA827465;Yang2025_Silkie_Wenchang	CRA004775	0.111278432
ENSGALG00010020558	EIF4EBP2	2	-0.216475548	0.113901803	-1.900545404	0.057361582	0	0.07113257	0	1	CRA004775;Yang2025_Silkie_Wenchang	PRJNA827465	0.414808146
ENSGALG00010020558	EIF4EBP2	2	-0.14625024	0.062438894	-2.342293886	0.019165617	0	0.204957994	0	1	CRA004775;PRJNA827465	Yang2025_Silkie_Wenchang	0.231912888
ENSGALG00010020562	LRRN1	2	-0.051378827	0.132346551	-0.388214327	0.697857431	0	0.049276196	0	1	PRJNA827465;Yang2025_Silkie_Wenchang	CRA004775	0.921450333
ENSGALG00010020562	LRRN1	2	-0.033370756	0.25112032	-0.132885719	0.894282338	0	0.294203995	0	0.5	CRA004775;Yang2025_Silkie_Wenchang	PRJNA827465	0.974381724
ENSGALG00010020562	LRRN1	2	-0.017126518	0.143580465	-0.119281672	0.905052204	0	0.214256109	0	0.5	CRA004775;PRJNA827465	Yang2025_Silkie_Wenchang	0.977931125
ENSGALG00010020568	PLA2G4F	2	0.216613326	0.505947429	0.428134058	0.668553522	0.246105168	1.895731105	47.24990283	0.5	PRJNA827465;Yang2025_Silkie_Wenchang	CRA004775	0.914377769
ENSGALG00010020568	PLA2G4F	2	0.428289785	0.437217097	0.979581513	0.327292734	0	0.880064957	0	0.5	CRA004775;Yang2025_Silkie_Wenchang	PRJNA827465	0.800028239
ENSGALG00010020568	PLA2G4F	2	-0.163990525	0.373919841	-0.438571338	0.660972169	0	0.059415799	0	1	CRA004775;PRJNA827465	Yang2025_Silkie_Wenchang	0.912027424
ENSGALG00010020569	DTX3L	2	-0.200131073	0.170613378	-1.173009263	0.240792091	0.040031022	3.197265063	68.72326879	1	PRJNA827465;Yang2025_Silkie_Wenchang	CRA004775	0.753303162
ENSGALG00010020569	DTX3L	2	-0.359331306	0.129651591	-2.771514823	0.005579613	0	0.086138947	0	1	CRA004775;Yang2025_Silkie_Wenchang	PRJNA827465	0.112114283
ENSGALG00010020569	DTX3L	2	-0.062287741	0.121510056	-0.512613877	0.608221444	0	0.396978567	0	1	CRA004775;PRJNA827465	Yang2025_Silkie_Wenchang	0.897204334
ENSGALG00010020571	MRTO4	2	0.278741347	0.185173816	1.505295693	0.132248072	0.053376616	4.134221593	75.81164973	1	PRJNA827465;Yang2025_Silkie_Wenchang	CRA004775	0.608991913
ENSGALG00010020571	MRTO4	2	0.073694606	0.426501376	0.172788671	0.862817543	0.332377764	11.56672048	91.35450708	0.5	CRA004775;Yang2025_Silkie_Wenchang	PRJNA827465	0.966099097
ENSGALG00010020571	MRTO4	2	-0.087349892	0.236975364	-0.36860326	0.712423465	0.094027908	5.67573113	82.38112453	0.5	CRA004775;PRJNA827465	Yang2025_Silkie_Wenchang	0.92597752
ENSGALG00010020572	PPP1R3C	2	-0.030219726	0.232372698	-0.130048523	0.896528037	0.083523491	4.322148561	76.86335891	0.5	PRJNA827465;Yang2025_Silkie_Wenchang	CRA004775	0.975115773
ENSGALG00010020572	PPP1R3C	2	-0.305810368	0.172100289	-1.776931167	0.075579566	0	0.144045175	0	1	CRA004775;Yang2025_Silkie_Wenchang	PRJNA827465	0.474597167
ENSGALG00010020572	PPP1R3C	2	0.02294043	0.285735089	0.080285659	0.936010064	0.091164498	1.69515134	41.00821699	0.5	CRA004775;PRJNA827465	Yang2025_Silkie_Wenchang	0.984822128
ENSGALG00010020574	DTL	2	-0.040466262	0.07565255	-0.534896211	0.592721629	0	0.04058801	0	1	PRJNA827465;Yang2025_Silkie_Wenchang	CRA004775	0.892776493
ENSGALG00010020574	DTL	2	-0.047884579	0.157060202	-0.304880413	0.760457234	0	0.210748454	0	0.5	CRA004775;Yang2025_Silkie_Wenchang	PRJNA827465	0.939327609
ENSGALG00010020574	DTL	2	-0.027155579	0.084199266	-0.322515621	0.747062106	0	0.164638953	0	0.5	CRA004775;PRJNA827465	Yang2025_Silkie_Wenchang	0.936729365
ENSGALG00010020576	AKR7L	2	-0.041915172	0.078221134	-0.535854823	0.592058889	0	0.015364976	0	1	PRJNA827465;Yang2025_Silkie_Wenchang	CRA004775	0.892776493
ENSGALG00010020576	AKR7L	2	-0.066870571	0.139469477	-0.479463836	0.631608689	0	0.001577033	0	1	CRA004775;Yang2025_Silkie_Wenchang	PRJNA827465	0.903058174
ENSGALG00010020576	AKR7L	2	-0.042248919	0.080510781	-0.524761012	0.599749338	0	0.023905697	0	1	CRA004775;PRJNA827465	Yang2025_Silkie_Wenchang	0.895092498
ENSGALG00010020578	TNKS2	2	-0.205973139	0.200315816	-1.02824202	0.303835997	0.073088001	11.08111382	90.97563641	1	PRJNA827465;Yang2025_Silkie_Wenchang	CRA004775	0.792208658
ENSGALG00010020578	TNKS2	2	-0.125747125	0.320382128	-0.392491071	0.694669407	0.175247509	6.390557123	84.35191204	0.5	CRA004775;Yang2025_Silkie_Wenchang	PRJNA827465	0.92082578
ENSGALG00010020578	TNKS2	2	0.006819608	0.06538637	0.104297089	0.916933588	0	0.993807562	0	0.5	CRA004775;PRJNA827465	Yang2025_Silkie_Wenchang	0.980728718
ENSGALG00010020580	ENSGALG00010020580	2	0.000365849	0.077839366	0.004700053	0.996249914	0.002876419	1.204189598	16.95659872	0.5	PRJNA827465;Yang2025_Silkie_Wenchang	CRA004775	0.99878373
ENSGALG00010020580	ENSGALG00010020580	2	-0.235352006	0.113471426	-2.074108126	0.03806927	0	0.86708441	0	1	CRA004775;Yang2025_Silkie_Wenchang	PRJNA827465	0.33638411
ENSGALG00010020580	ENSGALG00010020580	2	-0.124960096	0.196114861	-0.637224001	0.523979946	0.061734768	4.628516714	78.39480633	0.5	CRA004775;PRJNA827465	Yang2025_Silkie_Wenchang	0.869961505
ENSGALG00010020582	KANSL3	2	0.015480869	0.05064452	0.305677087	0.75985052	0	0.946055969	0	0.5	PRJNA827465;Yang2025_Silkie_Wenchang	CRA004775	0.939165042
ENSGALG00010020582	KANSL3	2	-0.114644111	0.124992416	-0.917208534	0.359033376	0	0.002427359	0	1	CRA004775;Yang2025_Silkie_Wenchang	PRJNA827465	0.814241185
ENSGALG00010020582	KANSL3	2	0.026886229	0.052635312	0.510802121	0.609489624	0	0.318756705	0	0.5	CRA004775;PRJNA827465	Yang2025_Silkie_Wenchang	0.897238578
ENSGALG00010020584	PARP9	2	-0.246234742	0.210159283	-1.1716577	0.241334512	0.077424423	7.951858668	87.42432377	1	PRJNA827465;Yang2025_Silkie_Wenchang	CRA004775	0.753847713
ENSGALG00010020584	PARP9	2	-0.470188986	0.119444988	-3.936448017	8.26965E-05	0	0.005972157	0	1	CRA004775;Yang2025_Silkie_Wenchang	PRJNA827465	0.006805537
ENSGALG00010020584	PARP9	2	-0.100095965	0.146444636	-0.68350721	0.494236396	0.017675387	1.207465549	17.18190211	1	CRA004775;PRJNA827465	Yang2025_Silkie_Wenchang	0.860175061
ENSGALG00010020586	C11orf16	2	0.020992143	0.122299928	0.171644769	0.863716809	0.023239038	4.435789992	77.45410135	0.5	PRJNA827465;Yang2025_Silkie_Wenchang	CRA004775	0.966342957
ENSGALG00010020586	C11orf16	2	-0.089037556	0.084389355	-1.055080418	0.291388547	0	0.287123261	0	0.5	CRA004775;Yang2025_Silkie_Wenchang	PRJNA827465	0.784817029
ENSGALG00010020586	C11orf16	2	0.125138806	0.068049172	1.838946799	0.065923012	0	0.270330269	0	1	CRA004775;PRJNA827465	Yang2025_Silkie_Wenchang	0.442269089
ENSGALG00010020588	PALD1	2	0.058081049	0.068217435	0.851410623	0.394541293	0.003613303	1.470727457	32.006437	0.5	PRJNA827465;Yang2025_Silkie_Wenchang	CRA004775	0.830643015
ENSGALG00010020588	PALD1	2	-0.075469836	0.104398624	-0.722900681	0.469740905	0	0.481485456	0	1	CRA004775;Yang2025_Silkie_Wenchang	PRJNA827465	0.853485658
ENSGALG00010020588	PALD1	2	0.032350772	0.144899543	0.22326345	0.823330472	0.022863874	1.493521223	33.04413861	0.5	CRA004775;PRJNA827465	Yang2025_Silkie_Wenchang	0.955918024
ENSGALG00010020590	INTS7	2	-0.09569051	0.080929131	-1.182398829	0.237047488	0.006385306	1.863912535	46.34941387	1	PRJNA827465;Yang2025_Silkie_Wenchang	CRA004775	0.749355649
ENSGALG00010020590	INTS7	2	-0.269432702	0.136743517	-1.970350826	0.048798177	0.013486271	1.327068937	24.64596432	1	CRA004775;Yang2025_Silkie_Wenchang	PRJNA827465	0.381826549
ENSGALG00010020590	INTS7	2	-0.211382923	0.238881078	-0.88488768	0.376217213	0.085230169	3.232392042	69.06315858	1	CRA004775;PRJNA827465	Yang2025_Silkie_Wenchang	0.822974013
ENSGALG00010020594	PQLC2	2	-0.015749844	0.089146408	-0.176674507	0.859764066	0	0.044777026	0	1	PRJNA827465;Yang2025_Silkie_Wenchang	CRA004775	0.964858345
ENSGALG00010020594	PQLC2	2	-0.01456202	0.170567063	-0.085374161	0.931963935	0	0.112579383	0	0.5	CRA004775;Yang2025_Silkie_Wenchang	PRJNA827465	0.98412915
ENSGALG00010020594	PQLC2	2	0.000380554	0.093146017	0.004085565	0.996740199	0	0.053856189	0	0.5	CRA004775;PRJNA827465	Yang2025_Silkie_Wenchang	0.99989145
ENSGALG00010020595	ZFYVE27	2	0.051508741	0.074050525	0.695589145	0.486686166	0	0.136677096	0	1	PRJNA827465;Yang2025_Silkie_Wenchang	CRA004775	0.857603818
ENSGALG00010020595	ZFYVE27	2	-0.130092163	0.236470925	-0.550140203	0.582223214	0.088075284	4.615096493	78.33198067	0.5	CRA004775;Yang2025_Silkie_Wenchang	PRJNA827465	0.889493857
ENSGALG00010020595	ZFYVE27	2	-0.147178876	0.206153596	-0.713928251	0.475271547	0.066252694	4.240365884	76.41712939	0.5	CRA004775;PRJNA827465	Yang2025_Silkie_Wenchang	0.854462864
ENSGALG00010020596	SUMF1	2	-0.110467058	0.077703294	-1.42165219	0.155127246	0.001337321	1.08234075	7.607085141	1	PRJNA827465;Yang2025_Silkie_Wenchang	CRA004775	0.647765412
ENSGALG00010020596	SUMF1	2	-0.353129344	0.124567357	-2.834846576	0.00458477	0	0.880649042	0	1	CRA004775;Yang2025_Silkie_Wenchang	PRJNA827465	0.099390627
ENSGALG00010020596	SUMF1	2	-0.244513242	0.209494318	-1.167159302	0.243146043	0.068226303	4.051393402	75.31713411	1	CRA004775;PRJNA827465	Yang2025_Silkie_Wenchang	0.7550024
ENSGALG00010020597	ARID5A	2	0.240317851	0.169930525	1.414213376	0.157299955	0.043107513	3.823265681	73.84434974	1	PRJNA827465;Yang2025_Silkie_Wenchang	CRA004775	0.650657662
ENSGALG00010020597	ARID5A	2	0.344597145	0.197728707	1.742777519	0.081372485	0.024888545	1.19831208	16.54928492	0.5	CRA004775;Yang2025_Silkie_Wenchang	PRJNA827465	0.491357177
ENSGALG00010020597	ARID5A	2	0.079136301	0.094228552	0.839833564	0.401001711	0	0.178888227	0	0.5	CRA004775;PRJNA827465	Yang2025_Silkie_Wenchang	0.832395176
ENSGALG00010020599	PARN	2	-0.069987527	0.068458306	-1.022337993	0.306620957	0.004619208	1.882676805	46.88413872	1	PRJNA827465;Yang2025_Silkie_Wenchang	CRA004775	0.792500392
ENSGALG00010020599	PARN	2	-0.151918855	0.083514228	-1.819077528	0.068899603	0	0.032341225	0	1	CRA004775;Yang2025_Silkie_Wenchang	PRJNA827465	0.452044654
ENSGALG00010020599	PARN	2	-0.017183052	0.054624032	-0.314569451	0.753088577	0	0.047392651	0	1	CRA004775;PRJNA827465	Yang2025_Silkie_Wenchang	0.938087082
ENSGALG00010020601	AKIP1	2	-0.273962527	0.177538181	-1.543118923	0.122801924	0.036225093	2.175912148	54.04226218	1	PRJNA827465;Yang2025_Silkie_Wenchang	CRA004775	0.587706885
ENSGALG00010020601	AKIP1	2	-0.400482323	0.176110852	-2.274035463	0.022963854	0	0.63455557	0	1	CRA004775;Yang2025_Silkie_Wenchang	PRJNA827465	0.258400479
ENSGALG00010020601	AKIP1	2	-0.145261143	0.111630311	-1.301269714	0.19316615	0	0.047313597	0	1	CRA004775;PRJNA827465	Yang2025_Silkie_Wenchang	0.703208363
ENSGALG00010020602	DENND2B	2	0.081354683	0.074215772	1.096191298	0.272995067	0.007435178	3.036873337	67.07139584	1	PRJNA827465;Yang2025_Silkie_Wenchang	CRA004775	0.773801033
ENSGALG00010020602	DENND2B	2	0.15989027	0.06507456	2.457031914	0.014009024	0	0.011156228	0	1	CRA004775;Yang2025_Silkie_Wenchang	PRJNA827465	0.193842584
ENSGALG00010020602	DENND2B	2	0.017723289	0.051572065	0.343660649	0.731101508	0	0.214810007	0	1	CRA004775;PRJNA827465	Yang2025_Silkie_Wenchang	0.932769533
ENSGALG00010020603	VPS39	2	0.075587383	0.073897212	1.022871921	0.306368407	0	0.112164836	0	1	PRJNA827465;Yang2025_Silkie_Wenchang	CRA004775	0.792485409
ENSGALG00010020603	VPS39	2	0.135740445	0.117043937	1.159739224	0.246154995	0	0.155225231	0	1	CRA004775;Yang2025_Silkie_Wenchang	PRJNA827465	0.756727496
ENSGALG00010020603	VPS39	2	0.076861109	0.084331655	0.911414691	0.362076916	0	0.377712641	0	1	CRA004775;PRJNA827465	Yang2025_Silkie_Wenchang	0.815579273
ENSGALG00010020605	STAMBP	2	0.021445548	0.072438139	0.296053273	0.767189398	0.006281505	2.328067931	57.04592694	0.5	PRJNA827465;Yang2025_Silkie_Wenchang	CRA004775	0.941432527
ENSGALG00010020605	STAMBP	2	-0.054147892	0.177051036	-0.305832112	0.759732477	0.051743913	5.612777196	82.183508	0.5	CRA004775;Yang2025_Silkie_Wenchang	PRJNA827465	0.939163539
ENSGALG00010020605	STAMBP	2	-0.108484354	0.09832868	-1.10328293	0.269904317	0.012479879	2.41300746	58.55793997	1	CRA004775;PRJNA827465	Yang2025_Silkie_Wenchang	0.772187652
ENSGALG00010020607	ENSGALG00010020607	2	-0.227585329	0.096622438	-2.35540868	0.018502344	0	0.183268274	0	1	PRJNA827465;Yang2025_Silkie_Wenchang	CRA004775	0.227601507
ENSGALG00010020607	ENSGALG00010020607	2	-0.318550369	0.19245443	-1.65519894	0.09788415	0	0.026572175	0	1	CRA004775;Yang2025_Silkie_Wenchang	PRJNA827465	0.534130658
ENSGALG00010020607	ENSGALG00010020607	2	-0.213877543	0.106892814	-2.000859882	0.045407492	0	0.129983494	0	1	CRA004775;PRJNA827465	Yang2025_Silkie_Wenchang	0.367363248
ENSGALG00010020608	CAPZB	2	0.081018619	0.166467707	0.48669271	0.626476112	0.04521017	5.020476474	80.08157184	0.5	PRJNA827465;Yang2025_Silkie_Wenchang	CRA004775	0.901504244
ENSGALG00010020608	CAPZB	2	0.195763041	0.146664575	1.334767037	0.181952605	0.008405515	1.149930087	13.03819148	0.5	CRA004775;Yang2025_Silkie_Wenchang	PRJNA827465	0.689745682
ENSGALG00010020608	CAPZB	2	-0.065134381	0.058964551	-1.104636269	0.269317229	0	0.005488406	0	1	CRA004775;PRJNA827465	Yang2025_Silkie_Wenchang	0.77189992
ENSGALG00010020609	AVPI1	2	0.317925252	0.271658496	1.1703122	0.241875354	0.105455259	3.225811414	69.00004769	1	PRJNA827465;Yang2025_Silkie_Wenchang	CRA004775	0.754119232
ENSGALG00010020609	AVPI1	2	0.182756338	0.527536463	0.346433566	0.729016903	0.416786791	3.789636339	73.61224374	0.5	CRA004775;Yang2025_Silkie_Wenchang	PRJNA827465	0.932323844
ENSGALG00010020609	AVPI1	2	0.03658701	0.159762388	0.229008908	0.818861993	0.009580849	1.079105736	7.33067512	0.5	CRA004775;PRJNA827465	Yang2025_Silkie_Wenchang	0.955256039
ENSGALG00010020611	LPGAT1	2	-0.134235194	0.062623296	-2.143534469	0.032070206	0	0.161761693	0	1	PRJNA827465;Yang2025_Silkie_Wenchang	CRA004775	0.306978889
ENSGALG00010020611	LPGAT1	2	-0.156544188	0.094601602	-1.654773117	0.097970529	0	0.07093041	0	1	CRA004775;Yang2025_Silkie_Wenchang	PRJNA827465	0.534130658
ENSGALG00010020611	LPGAT1	2	-0.112604621	0.076032534	-1.481005769	0.138605036	0	0.006755075	0	1	CRA004775;PRJNA827465	Yang2025_Silkie_Wenchang	0.622371164
ENSGALG00010020612	RPL27A	2	-0.02686848	0.069058446	-0.389068703	0.697225324	0	0.350816639	0	1	PRJNA827465;Yang2025_Silkie_Wenchang	CRA004775	0.921172748
ENSGALG00010020612	RPL27A	2	-0.283758456	0.18785524	-1.510516588	0.13091166	0	0.206758991	0	1	CRA004775;Yang2025_Silkie_Wenchang	PRJNA827465	0.606676214
ENSGALG00010020612	RPL27A	2	-0.103242612	0.149319706	-0.691419868	0.489301722	0.024743397	1.720114613	41.86433902	1	CRA004775;PRJNA827465	Yang2025_Silkie_Wenchang	0.858337006
ENSGALG00010020613	ACTG2	2	-0.280963641	0.188679074	-1.489108646	0.136458761	0	0.926843239	0	1	PRJNA827465;Yang2025_Silkie_Wenchang	CRA004775	0.618333765
ENSGALG00010020613	ACTG2	2	0.487459918	0.629882474	0.77389027	0.438995684	0.649318787	5.296127309	81.11827867	0.5	CRA004775;Yang2025_Silkie_Wenchang	PRJNA827465	0.844022511
ENSGALG00010020613	ACTG2	2	0.315496734	0.813259215	0.387941173	0.698059568	1.181391362	9.082759489	88.99013013	0.5	CRA004775;PRJNA827465	Yang2025_Silkie_Wenchang	0.921516414
ENSGALG00010020614	FAIM	2	-0.127284331	0.089743405	-1.418314038	0.1560991	0.005787272	1.550550269	35.50676685	1	PRJNA827465;Yang2025_Silkie_Wenchang	CRA004775	0.649132948
ENSGALG00010020614	FAIM	2	-0.035691472	0.105349627	-0.33879068	0.734767423	0	0.060733031	0	1	CRA004775;Yang2025_Silkie_Wenchang	PRJNA827465	0.933533809
ENSGALG00010020614	FAIM	2	-0.201275692	0.090170106	-2.232177618	0.025603226	0	0.068729209	0	1	CRA004775;PRJNA827465	Yang2025_Silkie_Wenchang	0.27294819
ENSGALG00010020616	PI4K2A	2	-0.082973394	0.109181551	-0.75995801	0.447279684	0.010329992	1.363083955	26.63694733	1	PRJNA827465;Yang2025_Silkie_Wenchang	CRA004775	0.84650962
ENSGALG00010020616	PI4K2A	2	-0.359177811	0.175474223	-2.046897863	0.040668114	0	0.117491902	0	1	CRA004775;Yang2025_Silkie_Wenchang	PRJNA827465	0.348958445
ENSGALG00010020616	PI4K2A	2	-0.14304781	0.180231635	-0.793688691	0.427376685	0.040848212	2.036705667	50.90110386	1	CRA004775;PRJNA827465	Yang2025_Silkie_Wenchang	0.84125685
ENSGALG00010020617	DGUOK	2	0.099606432	0.194619478	0.511800941	0.608790331	0.048303523	2.316745238	56.83599631	0.5	PRJNA827465;Yang2025_Silkie_Wenchang	CRA004775	0.897208691
ENSGALG00010020617	DGUOK	2	0.235034916	0.205594015	1.143199214	0.252955889	0	0.857223306	0	0.5	CRA004775;Yang2025_Silkie_Wenchang	PRJNA827465	0.762731469
ENSGALG00010020617	DGUOK	2	-0.037768002	0.089969419	-0.419787105	0.674640986	0	0.001159611	0	1	CRA004775;PRJNA827465	Yang2025_Silkie_Wenchang	0.915970608
ENSGALG00010020619	QARS	2	0.105910426	0.13961702	0.758578187	0.448104926	0.028857103	3.520461865	71.59463621	0.5	PRJNA827465;Yang2025_Silkie_Wenchang	CRA004775	0.846602312
ENSGALG00010020619	QARS	2	-0.089983628	0.391364529	-0.229922799	0.81815176	0.266590547	7.437867675	86.55528649	0.5	CRA004775;Yang2025_Silkie_Wenchang	PRJNA827465	0.955255737
ENSGALG00010020619	QARS	2	-0.201014081	0.243221014	-0.826466751	0.408539371	0.09135279	3.677828728	72.81004435	1	CRA004775;PRJNA827465	Yang2025_Silkie_Wenchang	0.834164004
ENSGALG00010020620	MARF1	2	-0.322150273	0.263553113	-1.222335298	0.221580856	0.133339873	24.28077596	95.88151548	1	PRJNA827465;Yang2025_Silkie_Wenchang	CRA004775	0.734944791
ENSGALG00010020620	MARF1	2	-0.256414313	0.355500635	-0.721276668	0.470739309	0.230376264	11.00330432	90.91182094	0.5	CRA004775;Yang2025_Silkie_Wenchang	PRJNA827465	0.853762555
ENSGALG00010020620	MARF1	2	-0.06100422	0.034189281	-1.784308367	0.074373571	0	0.919693859	0	0.5	CRA004775;PRJNA827465	Yang2025_Silkie_Wenchang	0.470928071
ENSGALG00010020622	ABCG2	2	-0.040910127	0.187570769	-0.218105024	0.827347283	0.060796818	7.342530773	86.38071762	0.5	PRJNA827465;Yang2025_Silkie_Wenchang	CRA004775	0.956550752
ENSGALG00010020622	ABCG2	2	-0.031631127	0.267922722	-0.118060636	0.906019613	0.101017862	2.827521612	64.63333841	0.5	CRA004775;Yang2025_Silkie_Wenchang	PRJNA827465	0.978196186
ENSGALG00010020622	ABCG2	2	0.158204575	0.088496269	1.78769769	0.073824795	0	0.312083326	0	1	CRA004775;PRJNA827465	Yang2025_Silkie_Wenchang	0.469352297
ENSGALG00010020623	NEK2	2	-0.060739855	0.104388613	-0.581862839	0.560659073	0	0.012443994	0	1	PRJNA827465;Yang2025_Silkie_Wenchang	CRA004775	0.88130295
ENSGALG00010020623	NEK2	2	0.381968126	0.542534671	0.704043717	0.481405549	0.444622837	3.487531594	71.32642463	0.5	CRA004775;Yang2025_Silkie_Wenchang	PRJNA827465	0.85686777
ENSGALG00010020623	NEK2	2	0.365912715	0.554229203	0.660219116	0.509113227	0.479861868	3.803235656	73.70659905	0.5	CRA004775;PRJNA827465	Yang2025_Silkie_Wenchang	0.865193604
ENSGALG00010020624	MINOS1	2	-0.454650148	0.30449886	-1.493109522	0.13540852	0.150629225	4.885589754	79.53164202	1	PRJNA827465;Yang2025_Silkie_Wenchang	CRA004775	0.616905631
ENSGALG00010020624	MINOS1	2	-0.276934677	0.527574809	-0.524920205	0.599638663	0.493012499	8.743389577	88.56278802	0.5	CRA004775;Yang2025_Silkie_Wenchang	PRJNA827465	0.895092498
ENSGALG00010020624	MINOS1	2	-0.028181783	0.212429959	-0.132663881	0.894459209	0.06037253	2.663355077	62.45337286	0.5	CRA004775;PRJNA827465	Yang2025_Silkie_Wenchang	0.974394857
ENSGALG00010020625	SLC30A1	2	-0.108893037	0.169904455	-0.640907485	0.521582793	0.053918341	15.12661969	93.38913769	0.5	PRJNA827465;Yang2025_Silkie_Wenchang	CRA004775	0.869518515
ENSGALG00010020625	SLC30A1	2	-0.27643782	0.06093829	-4.536356689	5.72343E-06	0	0.038060534	0	1	CRA004775;Yang2025_Silkie_Wenchang	PRJNA827465	0.000914324
ENSGALG00010020625	SLC30A1	2	0.048695703	0.059887307	0.813122279	0.416147954	0	0.911827677	0	0.5	CRA004775;PRJNA827465	Yang2025_Silkie_Wenchang	0.837742614
ENSGALG00010020626	MORN4	2	0.04787128	0.068883087	0.694964209	0.487077732	0	0.575586761	0	0.5	PRJNA827465;Yang2025_Silkie_Wenchang	CRA004775	0.85768885
ENSGALG00010020626	MORN4	2	-0.133310969	0.203852643	-0.653957519	0.513139175	0	0.018125177	0	1	CRA004775;Yang2025_Silkie_Wenchang	PRJNA827465	0.866263853
ENSGALG00010020626	MORN4	2	0.051969429	0.069063695	0.752485509	0.451759141	0	0.297692863	0	0.5	CRA004775;PRJNA827465	Yang2025_Silkie_Wenchang	0.848006549
ENSGALG00010020629	TRIM66	2	0.071687726	0.329683866	0.217443839	0.82786247	0.084192262	1.61591942	38.11572608	0.5	PRJNA827465;Yang2025_Silkie_Wenchang	CRA004775	0.956550752
ENSGALG00010020629	TRIM66	2	0.169455891	0.33872743	0.500272125	0.616883479	0.026629827	1.125341236	11.13806478	0.5	CRA004775;Yang2025_Silkie_Wenchang	PRJNA827465	0.899111936
ENSGALG00010020629	TRIM66	2	-0.226705545	0.277085803	-0.818178133	0.413255479	0	0.001951375	0	1	CRA004775;PRJNA827465	Yang2025_Silkie_Wenchang	0.83606052
ENSGALG00010020630	QRICH1	2	-0.007394282	0.038064592	-0.194256187	0.845975289	0	0.190618617	0	0.5	PRJNA827465;Yang2025_Silkie_Wenchang	CRA004775	0.96122283
ENSGALG00010020630	QRICH1	2	0.035506639	0.065278389	0.543926397	0.586492123	0	0.709835618	0	1	CRA004775;Yang2025_Silkie_Wenchang	PRJNA827465	0.891596903
ENSGALG00010020630	QRICH1	2	-0.002003728	0.058233823	-0.034408322	0.972551548	0.00157043	1.067746555	6.344816034	0.5	CRA004775;PRJNA827465	Yang2025_Silkie_Wenchang	0.992191914
ENSGALG00010020632	BLOC1S2	2	-0.134316063	0.067344833	-1.994452398	0.046102637	0	0.361949005	0	1	PRJNA827465;Yang2025_Silkie_Wenchang	CRA004775	0.370319221
ENSGALG00010020632	BLOC1S2	2	-0.180442645	0.132868745	-1.358051856	0.17444723	0	0.225993955	0	1	CRA004775;Yang2025_Silkie_Wenchang	PRJNA827465	0.678707136
ENSGALG00010020632	BLOC1S2	2	-0.111634383	0.071879519	-1.553076372	0.12040489	0	0.022204335	0	1	CRA004775;PRJNA827465	Yang2025_Silkie_Wenchang	0.581775014
ENSGALG00010020633	HOGA1	2	-0.080489912	0.409845542	-0.196390845	0.844304261	0.263696244	3.908482684	74.41462376	0.5	PRJNA827465;Yang2025_Silkie_Wenchang	CRA004775	0.960723102
ENSGALG00010020633	HOGA1	2	-0.24360504	0.256758675	-0.948770438	0.34273738	0.019632135	1.155524671	13.45922547	1	CRA004775;Yang2025_Silkie_Wenchang	PRJNA827465	0.80609227
ENSGALG00010020633	HOGA1	2	0.211490069	0.094165409	2.245942239	0.024707708	7.00518E-05	1.001541199	0.153882702	0.5	CRA004775;PRJNA827465	Yang2025_Silkie_Wenchang	0.268627122
ENSGALG00010020635	NDE1	2	0.035142179	0.062389872	0.563267363	0.573252839	0	0.265166526	0	0.5	PRJNA827465;Yang2025_Silkie_Wenchang	CRA004775	0.885803753
ENSGALG00010020635	NDE1	2	0.161755871	0.263197628	0.614579519	0.538832443	0.085537188	2.314656525	56.79704573	0.5	CRA004775;Yang2025_Silkie_Wenchang	PRJNA827465	0.87481821
ENSGALG00010020635	NDE1	2	0.170863497	0.203713071	0.838745874	0.40161193	0.051433512	1.915328723	47.78964111	1	CRA004775;PRJNA827465	Yang2025_Silkie_Wenchang	0.832623399
ENSGALG00010020636	NBL1	2	-0.128436893	0.096284149	-1.333936001	0.182224832	0	0.516364809	0	1	PRJNA827465;Yang2025_Silkie_Wenchang	CRA004775	0.690033956
ENSGALG00010020636	NBL1	2	-0.064715493	0.146306161	-0.442329241	0.658250974	0	0.182312548	0	1	CRA004775;Yang2025_Silkie_Wenchang	PRJNA827465	0.910991163
ENSGALG00010020636	NBL1	2	-0.179092859	0.111146454	-1.611323186	0.1071093	0	0.000419638	0	1	CRA004775;PRJNA827465	Yang2025_Silkie_Wenchang	0.554420757
ENSGALG00010020638	IMPDH2	2	-0.077486384	0.072484958	-1.068999492	0.285069898	0	0.332357008	0	1	PRJNA827465;Yang2025_Silkie_Wenchang	CRA004775	0.780501722
ENSGALG00010020638	IMPDH2	2	-0.058101758	0.122501184	-0.474295477	0.635289217	0	0.740389435	0	1	CRA004775;Yang2025_Silkie_Wenchang	PRJNA827465	0.904141515
ENSGALG00010020638	IMPDH2	2	-0.118862994	0.083252457	-1.42774158	0.153366253	0	0.382837701	0	1	CRA004775;PRJNA827465	Yang2025_Silkie_Wenchang	0.645715464
ENSGALG00010020640	PTPN14	2	-0.019203138	0.071762946	-0.267591273	0.789013949	0	0.005908545	0	1	PRJNA827465;Yang2025_Silkie_Wenchang	CRA004775	0.94717823
ENSGALG00010020640	PTPN14	2	0.121046873	0.224343221	0.53956109	0.589499757	0.029217263	1.269894794	21.25331922	0.5	CRA004775;Yang2025_Silkie_Wenchang	PRJNA827465	0.892320726
ENSGALG00010020640	PTPN14	2	0.088260867	0.217326024	0.40612194	0.684653005	0.053280295	1.574848705	36.50183687	0.5	CRA004775;PRJNA827465	Yang2025_Silkie_Wenchang	0.919003112
ENSGALG00010020642	ANKRD2	2	0.174908511	0.394462619	0.443409599	0.657469494	0.082009903	1.341552616	25.45950208	0.5	PRJNA827465;Yang2025_Silkie_Wenchang	CRA004775	0.910711339
ENSGALG00010020642	ANKRD2	2	-0.468183382	1.158870762	-0.40399965	0.686212977	2.294159564	6.80482125	85.30453684	0.5	CRA004775;Yang2025_Silkie_Wenchang	PRJNA827465	0.919235539
ENSGALG00010020642	ANKRD2	2	-0.808539854	0.751355654	-1.076108031	0.281878944	0.814076879	3.423467385	70.78984879	1	CRA004775;PRJNA827465	Yang2025_Silkie_Wenchang	0.779420863
ENSGALG00010020646	CEP70	2	-0.098817631	0.101819847	-0.970514429	0.331790136	0.006794051	1.45303157	31.17837076	0.5	PRJNA827465;Yang2025_Silkie_Wenchang	CRA004775	0.802002658
ENSGALG00010020646	CEP70	2	0.025516295	0.129440641	0.197127387	0.843727852	0	0.003019084	0	1	CRA004775;Yang2025_Silkie_Wenchang	PRJNA827465	0.96057435
ENSGALG00010020646	CEP70	2	-0.164056754	0.096577318	-1.69870894	0.089374038	0	0.299521244	0	0.5	CRA004775;PRJNA827465	Yang2025_Silkie_Wenchang	0.513108919
ENSGALG00010020648	TET3	2	-0.129699908	0.069827503	-1.85743299	0.063249577	0	0.330413471	0	1	PRJNA827465;Yang2025_Silkie_Wenchang	CRA004775	0.433102794
ENSGALG00010020648	TET3	2	-0.178425922	0.125962089	-1.416504938	0.156627719	0	0.153756539	0	1	CRA004775;Yang2025_Silkie_Wenchang	PRJNA827465	0.64980313
ENSGALG00010020648	TET3	2	-0.101690788	0.080618688	-1.261379837	0.207172029	0	0.036348785	0	1	CRA004775;PRJNA827465	Yang2025_Silkie_Wenchang	0.719173491
ENSGALG00010020649	MYH11	2	0.153452595	0.540960251	0.283667044	0.776665553	0.544900954	14.39344207	93.05239153	0.5	PRJNA827465;Yang2025_Silkie_Wenchang	CRA004775	0.944156005
ENSGALG00010020649	MYH11	2	0.790127854	0.203812786	3.876733502	0.000105868	0	0.548634491	0	1	CRA004775;Yang2025_Silkie_Wenchang	PRJNA827465	0.00809521
ENSGALG00010020649	MYH11	2	0.2919702	0.717811353	0.406750602	0.684191171	0.933163619	10.0717914	90.07127967	0.5	CRA004775;PRJNA827465	Yang2025_Silkie_Wenchang	0.918808405
ENSGALG00010020651	LMO1	2	0.167379209	0.142429212	1.175174716	0.239924824	0	0.670652774	0	0.5	PRJNA827465;Yang2025_Silkie_Wenchang	CRA004775	0.752274858
ENSGALG00010020651	LMO1	2	-0.079069722	0.219895534	-0.359578572	0.719162311	0	0.049490452	0	1	CRA004775;Yang2025_Silkie_Wenchang	PRJNA827465	0.928449671
ENSGALG00010020651	LMO1	2	0.164009086	0.147644762	1.110835794	0.266639036	0	0.962312707	0	0.5	CRA004775;PRJNA827465	Yang2025_Silkie_Wenchang	0.770816805
ENSGALG00010020653	NDUFAF3	2	-0.122956494	0.092832784	-1.32449431	0.185338932	0	0.76619952	0	0.5	PRJNA827465;Yang2025_Silkie_Wenchang	CRA004775	0.693795574
ENSGALG00010020653	NDUFAF3	2	-0.064530406	0.225616012	-0.286018736	0.774863772	0	0.978532041	0	0.5	CRA004775;Yang2025_Silkie_Wenchang	PRJNA827465	0.943713409
ENSGALG00010020653	NDUFAF3	2	-0.16319911	0.094338373	-1.729933486	0.08364216	0	0.251406293	0	1	CRA004775;PRJNA827465	Yang2025_Silkie_Wenchang	0.498257654
ENSGALG00010020654	SAYSD1	2	-0.105095539	0.084602421	-1.242228507	0.214152265	0	0.421234911	0	1	PRJNA827465;Yang2025_Silkie_Wenchang	CRA004775	0.726967219
ENSGALG00010020654	SAYSD1	2	-0.356020035	0.214815178	-1.657331843	0.097452403	0	0.355845431	0	1	CRA004775;Yang2025_Silkie_Wenchang	PRJNA827465	0.533219726
ENSGALG00010020654	SAYSD1	2	-0.166628593	0.172838396	-0.964071624	0.335010018	0.028323493	1.382412524	27.66269238	1	CRA004775;PRJNA827465	Yang2025_Silkie_Wenchang	0.803101658
ENSGALG00010020655	RIC3	2	-0.211223967	0.129087687	-1.636282839	0.101780406	0.01197914	1.522755805	34.3295887	1	PRJNA827465;Yang2025_Silkie_Wenchang	CRA004775	0.543700483
ENSGALG00010020655	RIC3	2	-0.34588768	0.162135863	-2.133320008	0.032898484	0	0.160610992	0	1	CRA004775;Yang2025_Silkie_Wenchang	PRJNA827465	0.311661412
ENSGALG00010020655	RIC3	2	-0.112407527	0.119968992	-0.936971507	0.348773215	0	0.024707017	0	1	CRA004775;PRJNA827465	Yang2025_Silkie_Wenchang	0.809333419
ENSGALG00010020657	DALRD3	2	0.105320842	0.098808715	1.065906406	0.286465953	0.007979166	1.499038057	33.29055286	1	PRJNA827465;Yang2025_Silkie_Wenchang	CRA004775	0.782018115
ENSGALG00010020657	DALRD3	2	-0.115183307	0.377039229	-0.305494223	0.759989769	0.258058203	10.82743197	90.76419965	0.5	CRA004775;Yang2025_Silkie_Wenchang	PRJNA827465	0.939215731
ENSGALG00010020657	DALRD3	2	-0.205400051	0.266878057	-0.769640085	0.44151342	0.127243642	8.982283131	88.86697307	0.5	CRA004775;PRJNA827465	Yang2025_Silkie_Wenchang	0.845053556
ENSGALG00010020659	RAD51B	2	-0.220521079	0.213597153	-1.032415816	0.301877366	0.066327544	3.541678269	71.76479838	1	PRJNA827465;Yang2025_Silkie_Wenchang	CRA004775	0.791453946
ENSGALG00010020659	RAD51B	2	-0.255795728	0.252963498	-1.011196201	0.311922534	0.069061963	2.063316215	51.53433136	0.5	CRA004775;Yang2025_Silkie_Wenchang	PRJNA827465	0.795993742
ENSGALG00010020659	RAD51B	2	-0.017187927	0.115838481	-0.148378384	0.882044158	0	0.070645586	0	0.5	CRA004775;PRJNA827465	Yang2025_Silkie_Wenchang	0.971928118
ENSGALG00010020662	BFAR	2	-0.241833965	0.199823205	-1.210239647	0.226186949	0.072179346	10.15375561	90.15142733	1	PRJNA827465;Yang2025_Silkie_Wenchang	CRA004775	0.739647696
ENSGALG00010020662	BFAR	2	-0.388009732	0.136043031	-2.852110309	0.004343003	0.010664612	1.206519907	17.11699125	1	CRA004775;Yang2025_Silkie_Wenchang	PRJNA827465	0.096545833
ENSGALG00010020662	BFAR	2	-0.05379617	0.060588166	-0.887898981	0.374595093	0	0.023374402	0	1	CRA004775;PRJNA827465	Yang2025_Silkie_Wenchang	0.822805106
ENSGALG00010020664	ESYT3	2	0.176158992	0.329603305	0.534457602	0.593024975	0	0.173901343	0	0.5	PRJNA827465;Yang2025_Silkie_Wenchang	CRA004775	0.892776493
ENSGALG00010020664	ESYT3	2	0.238507755	0.371216375	0.642503326	0.52054643	0	0.367146441	0	0.5	CRA004775;Yang2025_Silkie_Wenchang	PRJNA827465	0.869331539
ENSGALG00010020664	ESYT3	2	0.345240515	0.318552599	1.08377868	0.27846294	0	0.064550538	0	1	CRA004775;PRJNA827465	Yang2025_Silkie_Wenchang	0.776763074
ENSGALG00010020666	TMCO4	2	0.009534093	0.124736588	0.076433812	0.939073971	0	0.11211315	0	0.5	PRJNA827465;Yang2025_Silkie_Wenchang	CRA004775	0.985349045

ENSGALG00010020666	TMCO4	2	-0.283973289	0.264441855	-1.073859085	0.282885844	0.072296509	2.029916849	50.73689837	1	CRA004775;Yang2025_Silkie_Wenchang	PRJNA827465	0.779532565
ENSGALG00010020666	TMCO4	2	-0.221190815	0.307566915	-0.719163227	0.472040354	0.138978692	3.481896094	71.28001603	0.5	CRA004775;PRJNA827465	Yang2025_Silkie_Wenchang	0.853998373
ENSGALG00010020667	UBTD1	2	0.130671011	0.119247939	1.095792614	0.273169542	0	0.276782626	0	1	PRJNA827465;Yang2025_Silkie_Wenchang	CRA004775	0.773904763
ENSGALG00010020667	UBTD1	2	0.079564737	0.209043365	0.380613548	0.703490027	0.00487132	1.057045794	5.396719273	0.5	CRA004775;Yang2025_Silkie_Wenchang	PRJNA827465	0.923639641
ENSGALG00010020667	UBTD1	2	0.058399776	0.123325987	0.473539902	0.63582804	0	0.608597486	0	0.5	CRA004775;PRJNA827465	Yang2025_Silkie_Wenchang	0.904387873
ENSGALG00010020670	ENSGALG00010020670	2	0.141902713	0.211061134	0.672329908	0.501373694	0.06942672	4.202503965	76.20466254	0.5	PRJNA827465;Yang2025_Silkie_Wenchang	CRA004775	0.862658912
ENSGALG00010020670	ENSGALG00010020670	2	0.244257514	0.159640544	1.530046867	0.126005129	0.010657265	1.260253743	20.65090024	1	CRA004775;Yang2025_Silkie_Wenchang	PRJNA827465	0.596169444
ENSGALG00010020670	ENSGALG00010020670	2	-0.02413218	0.083254203	-0.289861404	0.771922269	0	0.203388469	0	0.5	CRA004775;PRJNA827465	Yang2025_Silkie_Wenchang	0.943066211
ENSGALG00010020673	MMS19	2	-0.113271278	0.059749748	-1.895761601	0.057991576	0	0.296520177	0	1	PRJNA827465;Yang2025_Silkie_Wenchang	CRA004775	0.417443754
ENSGALG00010020673	MMS19	2	-0.199675618	0.161928443	-1.233110219	0.217534646	0.032251008	2.538577195	60.60785538	1	CRA004775;Yang2025_Silkie_Wenchang	PRJNA827465	0.731013651
ENSGALG00010020673	MMS19	2	-0.211957157	0.116880338	-1.81345435	0.069761773	0.015353188	1.956683865	48.89312382	1	CRA004775;PRJNA827465	Yang2025_Silkie_Wenchang	0.455632107
ENSGALG00010020675	SMYD2	2	-0.166139384	0.181927176	-0.913219164	0.361127287	0.048110193	3.650021834	72.60290361	0.5	PRJNA827465;Yang2025_Silkie_Wenchang	CRA004775	0.815240214
ENSGALG00010020675	SMYD2	2	-0.371815785	0.112482986	-3.305529102	0.000947973	0	0.260801456	0	1	CRA004775;Yang2025_Silkie_Wenchang	PRJNA827465	0.036527614
ENSGALG00010020675	SMYD2	2	-0.191117966	0.249474809	-0.76608122	0.443627977	0.087262872	3.161833027	68.37277644	0.5	CRA004775;PRJNA827465	Yang2025_Silkie_Wenchang	0.845827655
ENSGALG00010020677	CYR61	2	0.235623491	0.15517701	1.518417514	0.128909179	0	0.20964559	0	1	PRJNA827465;Yang2025_Silkie_Wenchang	CRA004775	0.602361458
ENSGALG00010020677	CYR61	2	0.190064465	0.214905727	0.884408563	0.376475702	0	0.124580405	0	1	CRA004775;Yang2025_Silkie_Wenchang	PRJNA827465	0.822974013
ENSGALG00010020677	CYR61	2	0.300772068	0.184547427	1.629781965	0.103147587	0	0.005980784	0	1	CRA004775;PRJNA827465	Yang2025_Silkie_Wenchang	0.5473214
ENSGALG00010020679	OTUD3	2	0.016252628	0.072223246	0.225033195	0.82195345	0.001729571	1.138979201	12.20208422	0.5	PRJNA827465;Yang2025_Silkie_Wenchang	CRA004775	0.95563486
ENSGALG00010020679	OTUD3	2	0.129242506	0.124179136	1.040774718	0.297980116	0	0.048481273	0	1	CRA004775;Yang2025_Silkie_Wenchang	PRJNA827465	0.788979318
ENSGALG00010020679	OTUD3	2	-0.017718084	0.069356717	-0.255463133	0.798365415	0	0.137774671	0	0.5	CRA004775;PRJNA827465	Yang2025_Silkie_Wenchang	0.949922217
ENSGALG00010020681	BCL10	2	-0.337007429	0.334159607	-1.008522343	0.313203767	0.215916138	29.92220511	96.65800032	1	PRJNA827465;Yang2025_Silkie_Wenchang	CRA004775	0.796531448
ENSGALG00010020681	BCL10	2	-0.242847452	0.442778353	-0.548462793	0.583374171	0.373107953	20.46618486	95.11389149	0.5	CRA004775;Yang2025_Silkie_Wenchang	PRJNA827465	0.890479822
ENSGALG00010020681	BCL10	2	0.046723997	0.094398525	0.494965331	0.620624593	0.007997573	1.511949293	33.86021579	0.5	CRA004775;PRJNA827465	Yang2025_Silkie_Wenchang	0.899395593
ENSGALG00010020682	ZDHHC16	2	0.245419668	0.165112656	1.486377083	0.137179408	0.038768401	3.060908632	67.32996243	1	PRJNA827465;Yang2025_Silkie_Wenchang	CRA004775	0.619615281
ENSGALG00010020682	ZDHHC16	2	0.015737098	0.454680633	0.034611323	0.972389672	0.366892367	8.785750607	88.61793323	0.5	CRA004775;Yang2025_Silkie_Wenchang	PRJNA827465	0.992104026
ENSGALG00010020682	ZDHHC16	2	-0.12085878	0.280957936	-0.430166814	0.667074301	0.128938623	4.808005895	79.20135662	0.5	CRA004775;PRJNA827465	Yang2025_Silkie_Wenchang	0.913766816
ENSGALG00010020684	P4HTM	2	0.028112909	0.120726857	0.23286375	0.815867208	0	0.051899861	0	0.5	PRJNA827465;Yang2025_Silkie_Wenchang	CRA004775	0.954260895
ENSGALG00010020684	P4HTM	2	-0.092018628	0.181687633	-0.506466105	0.612529478	0	0.638509045	0	1	CRA004775;Yang2025_Silkie_Wenchang	PRJNA827465	0.897957706
ENSGALG00010020684	P4HTM	2	-0.007038877	0.137591312	-0.051157857	0.959199733	0	0.9933805	0	0.5	CRA004775;PRJNA827465	Yang2025_Silkie_Wenchang	0.989155754
ENSGALG00010020685	RPL6	2	-0.399654384	0.425330222	-0.939633169	0.347405757	0.345354902	21.40842564	95.3289419	0.5	PRJNA827465;Yang2025_Silkie_Wenchang	CRA004775	0.808705451
ENSGALG00010020685	RPL6	2	-0.511508021	0.339725752	-1.505649831	0.132157089	0.193750442	6.192417542	83.85121815	1	CRA004775;Yang2025_Silkie_Wenchang	PRJNA827465	0.608786928
ENSGALG00010020685	RPL6	2	-0.003173279	0.060148728	-0.052757201	0.957925363	0	0.603829778	0	0.5	CRA004775;PRJNA827465	Yang2025_Silkie_Wenchang	0.988864712
ENSGALG00010020686	C1orf52	2	-0.139492442	0.065348485	-2.134593363	0.03279424	0	0.520409353	0	1	PRJNA827465;Yang2025_Silkie_Wenchang	CRA004775	0.31117452
ENSGALG00010020686	C1orf52	2	-0.127810663	0.140973759	-0.906627333	0.364603902	0.011671534	1.326319846	24.60340521	0.5	CRA004775;Yang2025_Silkie_Wenchang	PRJNA827465	0.817496251
ENSGALG00010020686	C1orf52	2	-0.092872164	0.072095646	-1.288179937	0.197683334	0	0.660189255	0	0.5	CRA004775;PRJNA827465	Yang2025_Silkie_Wenchang	0.709468779
ENSGALG00010020691	ENSGALG00010020691	2	0.325798167	0.100777114	3.232858664	0.001225582	0	0.064238976	0	1	PRJNA827465;Yang2025_Silkie_Wenchang	CRA004775	0.042665228
ENSGALG00010020691	ENSGALG00010020691	2	0.340565923	0.248095408	1.372721592	0.169838914	0	0.156384435	0	1	CRA004775;Yang2025_Silkie_Wenchang	PRJNA827465	0.671232541
ENSGALG00010020691	ENSGALG00010020691	2	0.34196835	0.10048611	3.403140489	0.00066616	0	0.080020397	0	1	CRA004775;PRJNA827465	Yang2025_Silkie_Wenchang	0.028470581
ENSGALG00010020692	TUB	2	0.063471361	0.081301685	0.780689358	0.434985221	0	0.084586881	0	1	PRJNA827465;Yang2025_Silkie_Wenchang	CRA004775	0.842762754
ENSGALG00010020692	TUB	2	0.068660623	0.141296626	0.485932502	0.627015024	0	0.191645015	0	0.5	CRA004775;Yang2025_Silkie_Wenchang	PRJNA827465	0.901573209
ENSGALG00010020692	TUB	2	0.041162535	0.088857831	0.463240373	0.64319208	0	0.087154249	0	0.5	CRA004775;PRJNA827465	Yang2025_Silkie_Wenchang	0.906634523
ENSGALG00010020693	PTGFR	2	-0.356539601	0.414464373	-0.860241855	0.389655737	0.316615922	12.72504664	92.14148263	0.5	PRJNA827465;Yang2025_Silkie_Wenchang	CRA004775	0.829068422
ENSGALG00010020693	PTGFR	2	-0.492145516	0.353223517	-1.393297707	0.163529784	0.174115013	2.99277113	66.58615188	1	CRA004775;Yang2025_Silkie_Wenchang	PRJNA827465	0.661805642
ENSGALG00010020693	PTGFR	2	0.038193431	0.139567948	0.273654747	0.784349957	0	0.067536392	0	0.5	CRA004775;PRJNA827465	Yang2025_Silkie_Wenchang	0.946087232
ENSGALG00010020696	MOB1A	2	-0.004675582	0.057426079	-0.081419136	0.935108631	0	0.656833931	0	0.5	PRJNA827465;Yang2025_Silkie_Wenchang	CRA004775	0.984512821
ENSGALG00010020696	MOB1A	2	-0.364537762	0.299358217	-1.217730938	0.223326236	0.129983529	3.351572472	70.16325893	1	CRA004775;Yang2025_Silkie_Wenchang	PRJNA827465	0.736744216
ENSGALG00010020696	MOB1A	2	-0.297020973	0.364877925	-0.814028342	0.415628714	0.226573497	5.894837813	83.03600486	0.5	CRA004775;PRJNA827465	Yang2025_Silkie_Wenchang	0.837689773
ENSGALG00010020699	PLA2G10	2	0.201400699	0.135426642	1.48715715	0.13697331	0	0.000435505	0	1	PRJNA827465;Yang2025_Silkie_Wenchang	CRA004775	0.619215783
ENSGALG00010020699	PLA2G10	2	0.236733487	0.20179593	1.173133114	0.240742429	0	0.12380448	0	1	CRA004775;Yang2025_Silkie_Wenchang	PRJNA827465	0.753303162
ENSGALG00010020699	PLA2G10	2	0.225866905	0.155319511	1.454208191	0.145888595	0	0.129513518	0	1	CRA004775;PRJNA827465	Yang2025_Silkie_Wenchang	0.634793487
ENSGALG00010020700	MTHFD2	2	-0.105312886	0.075140799	-1.401540677	0.161052453	0	0.630976319	0	1	PRJNA827465;Yang2025_Silkie_Wenchang	CRA004775	0.65730473
ENSGALG00010020700	MTHFD2	2	-0.025264051	0.118882853	-0.212512152	0.831707499	0	0.009256934	0	0.5	CRA004775;Yang2025_Silkie_Wenchang	PRJNA827465	0.957712669
ENSGALG00010020700	MTHFD2	2	-0.145003969	0.093193124	-1.555951375	0.119719656	0	0.123126304	0	0.5	CRA004775;PRJNA827465	Yang2025_Silkie_Wenchang	0.580639051
ENSGALG00010020701	PTPN11	2	-0.133886271	0.090267671	-1.483213969	0.138017571	0.012562608	3.882627263	74.24424411	1	PRJNA827465;Yang2025_Silkie_Wenchang	CRA004775	0.621022596
ENSGALG00010020701	PTPN11	2	-0.044879985	0.232383566	-0.193128911	0.846858011	0.085856889	4.458370048	77.57027817	0.5	CRA004775;Yang2025_Silkie_Wenchang	PRJNA827465	0.961252712
ENSGALG00010020701	PTPN11	2	0.012263506	0.124189403	0.098748407	0.921338033	0.019475038	1.902622225	47.44095876	0.5	CRA004775;PRJNA827465	Yang2025_Silkie_Wenchang	0.981519273
ENSGALG00010020704	PROX1	2	0.447766784	0.214849386	2.08409618	0.037151419	0	0.033650764	0	1	PRJNA827465;Yang2025_Silkie_Wenchang	CRA004775	0.332383847
ENSGALG00010020704	PROX1	2	0.319031713	0.283412083	1.125681411	0.260300395	0	0.260144241	0	1	CRA004775;Yang2025_Silkie_Wenchang	PRJNA827465	0.767635013
ENSGALG00010020704	PROX1	2	0.398727719	0.254507639	1.566663065	0.117193468	0	0.424703292	0	1	CRA004775;PRJNA827465	Yang2025_Silkie_Wenchang	0.57646425
ENSGALG00010020705	EXOSC1	2	0.290349902	0.258069984	1.125082033	0.260554274	0.09325779	3.000884997	66.67649706	1	PRJNA827465;Yang2025_Silkie_Wenchang	CRA004775	0.767694434
ENSGALG00010020705	EXOSC1	2	0.343701844	0.299795169	1.146455582	0.251606696	0.085003396	1.877263168	46.7309636	1	CRA004775;Yang2025_Silkie_Wenchang	PRJNA827465	0.761706574
ENSGALG00010020705	EXOSC1	2	0.074639033	0.118859323	0.627961119	0.530029411	0	0.041549414	0	1	CRA004775;PRJNA827465	Yang2025_Silkie_Wenchang	0.872072262
ENSGALG00010020708	FAM214A	2	0.022702972	0.053662022	0.423073368	0.672241733	0	0.382027477	0	0.5	PRJNA827465;Yang2025_Silkie_Wenchang	CRA004775	0.914742762
ENSGALG00010020708	FAM214A	2	-0.016690477	0.125049451	-0.133471014	0.893820886	0	0.560494478	0	0.5	CRA004775;Yang2025_Silkie_Wenchang	PRJNA827465	0.974381724
ENSGALG00010020708	FAM214A	2	0.04240373	0.057699565	0.734905536	0.462397026	0	0.283071857	0	1	CRA004775;PRJNA827465	Yang2025_Silkie_Wenchang	0.850823289
ENSGALG00010020711	PLA2G2E	2	0.141701489	0.145836021	0.971649448	0.331224974	0	0.342025583	0	1	PRJNA827465;Yang2025_Silkie_Wenchang	CRA004775	0.801757415
ENSGALG00010020711	PLA2G2E	2	0.793771072	0.797356632	0.995503191	0.319491593	1.148661848	9.992495083	89.99248945	1	CRA004775;Yang2025_Silkie_Wenchang	PRJNA827465	0.798231395
ENSGALG00010020711	PLA2G2E	2	0.869823982	0.710343874	1.224511134	0.220759469	0.894259052	8.334126676	88.0011423	1	CRA004775;PRJNA827465	Yang2025_Silkie_Wenchang	0.734053249
ENSGALG00010020712	ZC3H7A	2	0.028435546	0.034306968	0.828856276	0.407185739	0	0.002549112	0	1	PRJNA827465;Yang2025_Silkie_Wenchang	CRA004775	0.833863243
ENSGALG00010020712	ZC3H7A	2	0.031034669	0.072279619	0.429369571	0.667654295	0	0.001235659	0	1	CRA004775;Yang2025_Silkie_Wenchang	PRJNA827465	0.913995707
ENSGALG00010020712	ZC3H7A	2	0.027444797	0.037941308	0.723348732	0.46946566	0	0.000336985	0	1	CRA004775;PRJNA827465	Yang2025_Silkie_Wenchang	0.853485658
ENSGALG00010020714	PGAM1	2	-0.00117956	0.084624207	-0.013938805	0.988878803	0.001917421	1.146729356	12.79546521	0.5	PRJNA827465;Yang2025_Silkie_Wenchang	CRA004775	0.996891127
ENSGALG00010020714	PGAM1	2	0.096441901	0.124783887	0.772871428	0.439598476	0	0.078696953	0	0.5	CRA004775;Yang2025_Silkie_Wenchang	PRJNA827465	0.844278492
ENSGALG00010020714	PGAM1	2	-0.06967663	0.098338899	-0.708535801	0.478612588	0	7.93291E-05	0	1	CRA004775;PRJNA827465	Yang2025_Silkie_Wenchang	0.85563306
ENSGALG00010020716	RRAS	2	-0.138828686	0.138111909	-1.005189827	0.314805453	0.030872978	4.914716656	79.65294706	1	PRJNA827465;Yang2025_Silkie_Wenchang	CRA004775	0.796531448
ENSGALG00010020716	RRAS	2	-0.21760026	0.171870435	-1.266071503	0.205487484	0.022860359	1.280106737	21.88151419	0.5	CRA004775;Yang2025_Silkie_Wenchang	PRJNA827465	0.71825498
ENSGALG00010020716	RRAS	2	-0.013553817	0.05446139	-0.248870196	0.80346119	0	0.207996078	0	0.5	CRA004775;PRJNA827465	Yang2025_Silkie_Wenchang	0.951064358
ENSGALG00010020719	RPH3A	2	-0.172522964	0.29668876	-0.581494778	0.560907037	0	0.542214529	0	0.5	PRJNA827465;Yang2025_Silkie_Wenchang	CRA004775	0.881354866
ENSGALG00010020719	RPH3A	2	-0.00710259	0.316934861	-0.02241025	0.982120704	0	0.019866155	0	0.5	CRA004775;Yang2025_Silkie_Wenchang	PRJNA827465	0.995105937
ENSGALG00010020719	RPH3A	2	-0.264508706	0.333736592	-0.792567288	0.428029974	0	0.262955218	0	1	CRA004775;PRJNA827465	Yang2025_Silkie_Wenchang	0.84125685
ENSGALG00010020720	ENSGALG00010020720	2	0.246537105	0.242052745	1.018526377	0.308427872	0	0.167647995	0	1	PRJNA827465;Yang2025_Silkie_Wenchang	CRA004775	0.793457188
ENSGALG00010020720	ENSGALG00010020720	2	0.449567553	0.358045483	1.255615764	0.209255313	0	0.220748317	0	1	CRA004775;Yang2025_Silkie_Wenchang	PRJNA827465	0.721187439
ENSGALG00010020720	ENSGALG00010020720	2	0.241698035	0.28958766	0.834628229	0.403927063	0	0.501509815	0	1	CRA004775;PRJNA827465	Yang2025_Silkie_Wenchang	0.833112102
ENSGALG00010020721	PTDSS2	2	-0.103365357	0.168473512	-0.613540703	0.539518878	0.044876784	4.401991869	77.2830112	0.5	PRJNA827465;Yang2025_Silkie_Wenchang	CRA004775	0.874998368

ENSGALG00010020782	PPIF	2	0.068077977	0.078207889	0.870474547	0.384041123	0	0.0493047	0	1	CRA004775;PRJNA827465	Yang2025_Silkie_Wenchang	0.825664256
ENSGALG00010020786	DDX19	2	0.004926767	0.116121361	0.042427743	0.966157713	0.021429004	4.604562236	78.28240886	0.5	PRJNA827465;Yang2025_Silkie_Wenchang	CRA004775	0.990863074
ENSGALG00010020786	DDX19	2	0.159569407	0.081744413	1.95205277	0.05093194	0	0.211181334	0	1	CRA004775;Yang2025_Silkie_Wenchang	PRJNA827465	0.390514638
ENSGALG00010020786	DDX19	2	0.027526067	0.155266164	0.177283104	0.859286022	0.037207303	3.807970778	73.73929427	0.5	CRA004775;PRJNA827465	Yang2025_Silkie_Wenchang	0.964605083
ENSGALG00010020790	PRPH2L	2	0.181442376	0.249565443	0.727033252	0.467205586	0	0.00559805	0	1	PRJNA827465;Yang2025_Silkie_Wenchang	CRA004775	0.852885838
ENSGALG00010020790	PRPH2L	2	0.211681634	0.294547798	0.718666497	0.472346431	0	0.001637752	0	1	CRA004775;Yang2025_Silkie_Wenchang	PRJNA827465	0.853998373
ENSGALG00010020790	PRPH2L	2	0.185243228	0.274037919	0.675976626	0.499055486	0	0.011361072	0	1	CRA004775;PRJNA827465	Yang2025_Silkie_Wenchang	0.86164021
ENSGALG00010020794	STEAP3	2	-0.119926842	0.134868035	-0.889216207	0.373886894	0.022249973	2.393753051	58.22459633	1	PRJNA827465;Yang2025_Silkie_Wenchang	CRA004775	0.822579781
ENSGALG00010020794	STEAP3	2	-0.282013742	0.144741071	-1.948401653	0.051366922	0	0.012215262	0	1	CRA004775;Yang2025_Silkie_Wenchang	PRJNA827465	0.392777071
ENSGALG00010020794	STEAP3	2	-0.023046818	0.085000567	-0.271137224	0.786285493	0	0.335399335	0	1	CRA004775;PRJNA827465	Yang2025_Silkie_Wenchang	0.946639719
ENSGALG00010020797	DGCR8	2	0.230627635	0.219019357	1.053001153	0.292340464	0.082775406	6.693702759	85.06058551	1	PRJNA827465;Yang2025_Silkie_Wenchang	CRA004775	0.785753005
ENSGALG00010020797	DGCR8	2	0.41193346	0.142256895	2.895701197	0.003783124	0	0.731152539	0	1	CRA004775;Yang2025_Silkie_Wenchang	PRJNA827465	0.088142287
ENSGALG00010020797	DGCR8	2	0.041046036	0.053643863	0.765158099	0.444177407	0	0.241073208	0	1	CRA004775;PRJNA827465	Yang2025_Silkie_Wenchang	0.846013379
ENSGALG00010020798	C1QL2	2	0.132230482	0.354642705	0.372855495	0.709255997	0	0.585653769	0	0.5	PRJNA827465;Yang2025_Silkie_Wenchang	CRA004775	0.925298042
ENSGALG00010020798	C1QL2	2	-0.195813027	0.383681168	-0.510353501	0.609803827	0	0.072353961	0	1	CRA004775;Yang2025_Silkie_Wenchang	PRJNA827465	0.897238578
ENSGALG00010020798	C1QL2	2	0.119503675	0.403901116	0.295873594	0.767326617	0	0.851212391	0	0.5	CRA004775;PRJNA827465	Yang2025_Silkie_Wenchang	0.941444269
ENSGALG00010020803	ABCC6	2	0.481095112	0.252386666	1.906182757	0.056626499	0.088795132	3.258207493	69.30827757	1	PRJNA827465;Yang2025_Silkie_Wenchang	CRA004775	0.411670741
ENSGALG00010020803	ABCC6	2	0.367991149	0.416159727	0.884254591	0.376558795	0.272616528	4.594510848	78.2348974	0.5	CRA004775;Yang2025_Silkie_Wenchang	PRJNA827465	0.822974013
ENSGALG00010020803	ABCC6	2	0.173214649	0.151851325	1.140685796	0.2540007	0	0.815031396	0	0.5	CRA004775;PRJNA827465	Yang2025_Silkie_Wenchang	0.763867893
ENSGALG00010020806	HTD2	2	-0.068584096	0.086693681	-0.791108363	0.42888076	0	0.337389319	0	1	PRJNA827465;Yang2025_Silkie_Wenchang	CRA004775	0.841876188
ENSGALG00010020806	HTD2	2	-0.287258937	0.210564364	-1.364233391	0.172494122	0	0.175728926	0	1	CRA004775;Yang2025_Silkie_Wenchang	PRJNA827465	0.675388277
ENSGALG00010020806	HTD2	2	-0.075221618	0.088366998	-0.851241074	0.394635451	0	0.981065635	0	1	CRA004775;PRJNA827465	Yang2025_Silkie_Wenchang	0.83064301
ENSGALG00010020809	ENSGALG00010020809	2	0.059548634	0.105743713	0.563141136	0.573338782	0.005842763	1.344742358	25.63631286	0.5	PRJNA827465;Yang2025_Silkie_Wenchang	CRA004775	0.885850914
ENSGALG00010020809	ENSGALG00010020809	2	0.152893056	0.138211292	1.106226952	0.2686283	0	0.812030119	0	0.5	CRA004775;Yang2025_Silkie_Wenchang	PRJNA827465	0.771398294
ENSGALG00010020809	ENSGALG00010020809	2	-0.04489859	0.115588639	-0.389189634	0.697133458	0	0.330172906	0	1	CRA004775;PRJNA827465	Yang2025_Silkie_Wenchang	0.921116491
ENSGALG00010020810	IFITM5	2	0.393018439	0.347325458	1.131556671	0.257820865	0.212737847	8.119125605	87.68340276	1	PRJNA827465;Yang2025_Silkie_Wenchang	CRA004775	0.765151037
ENSGALG00010020810	IFITM5	2	0.32797136	0.43186907	0.759423128	0.447599482	0.331536733	8.970693856	88.85259027	0.5	CRA004775;Yang2025_Silkie_Wenchang	PRJNA827465	0.846601815
ENSGALG00010020810	IFITM5	2	0.028765887	0.093953751	0.306170714	0.759474669	0	0.595492252	0	0.5	CRA004775;PRJNA827465	Yang2025_Silkie_Wenchang	0.939163539
ENSGALG00010020811	MCOLN2	2	0.206889062	1.156299949	0.178927834	0.862990196	2.02649494	4.076394988	75.46852052	0.5	PRJNA827465;Yang2025_Silkie_Wenchang	CRA004775	0.966099097
ENSGALG00010020811	MCOLN2	2	-0.631663007	0.634356501	-0.995753973	0.319369699	0	0.378740571	0	1	CRA004775;Yang2025_Silkie_Wenchang	PRJNA827465	0.798079048
ENSGALG00010020811	MCOLN2	2	0.625101594	0.761485674	0.820897379	0.411704724	0.513976985	1.773941561	43.62835721	0.5	CRA004775;PRJNA827465	Yang2025_Silkie_Wenchang	0.835183193
ENSGALG00010020813	NPY4R	2	0.19897562	0.256719673	0.775069622	0.438298521	0	0.004727146	0	1	PRJNA827465;Yang2025_Silkie_Wenchang	CRA004775	0.843979791
ENSGALG00010020813	NPY4R	2	-0.026497328	0.344065238	-0.077012511	0.938613592	0.032208301	1.119475145	10.67242496	0.5	CRA004775;Yang2025_Silkie_Wenchang	PRJNA827465	0.985349045
ENSGALG00010020813	NPY4R	2	-0.01958702	0.369007346	-0.053080298	0.957667929	0.057902318	1.21292247	17.55449957	0.5	CRA004775;PRJNA827465	Yang2025_Silkie_Wenchang	0.988788489
ENSGALG00010020816	TRMT2A	2	-0.109062008	0.050297867	-2.168322734	0.03013414	0	0.002121205	0	1	PRJNA827465;Yang2025_Silkie_Wenchang	CRA004775	0.295973612
ENSGALG00010020816	TRMT2A	2	-0.181365177	0.12894478	-1.406533687	0.159565709	0	0.663469634	0	1	CRA004775;Yang2025_Silkie_Wenchang	PRJNA827465	0.651752935
ENSGALG00010020816	TRMT2A	2	-0.121969683	0.051375974	-2.37406073	0.017593656	0	0.913055785	0	1	CRA004775;PRJNA827465	Yang2025_Silkie_Wenchang	0.220991844
ENSGALG00010020818	TMEM62	2	0.285651948	0.093446379	3.056854102	0.002236731	0	0.00983171	0	1	PRJNA827465;Yang2025_Silkie_Wenchang	CRA004775	0.062480629
ENSGALG00010020818	TMEM62	2	0.017871706	0.298706165	0.059830389	0.952290722	0.147291413	5.589904483	82.11060667	0.5	CRA004775;Yang2025_Silkie_Wenchang	PRJNA827465	0.987758913
ENSGALG00010020818	TMEM62	2	0.011955888	0.289106242	0.041354651	0.967013165	0.137884799	5.516930649	81.87397915	0.5	CRA004775;PRJNA827465	Yang2025_Silkie_Wenchang	0.990893423
ENSGALG00010020820	VWA5B1	2	0.402884292	0.548261163	0.734840107	0.462436878	0.434576938	3.555133655	71.8716623	0.5	PRJNA827465;Yang2025_Silkie_Wenchang	CRA004775	0.850823289
ENSGALG00010020820	VWA5B1	2	0.582905132	0.502409581	1.160218982	0.245959661	0.205832314	1.644487085	39.19076599	0.5	CRA004775;Yang2025_Silkie_Wenchang	PRJNA827465	0.75661406
ENSGALG00010020820	VWA5B1	2	-0.088284033	0.309871476	-0.284905323	0.775716678	0	0.010225337	0	1	CRA004775;PRJNA827465	Yang2025_Silkie_Wenchang	0.943815265
ENSGALG00010020827	ENSGALG00010020827	2	-0.046614628	0.052214526	-0.8927521	0.371989943	0.000756345	1.110503619	9.950766256	0.5	PRJNA827465;Yang2025_Silkie_Wenchang	CRA004775	0.821597118
ENSGALG00010020827	ENSGALG00010020827	2	-0.006683981	0.134028676	-0.04986978	0.960226159	0.010047559	1.15763042	13.61664463	0.5	CRA004775;Yang2025_Silkie_Wenchang	PRJNA827465	0.989338243
ENSGALG00010020827	ENSGALG00010020827	2	-0.081614674	0.05232506	-1.559762658	0.118815979	0	0.569924109	0	1	CRA004775;PRJNA827465	Yang2025_Silkie_Wenchang	0.579158002
ENSGALG00010020828	CAMK2N1	2	-0.130643793	0.121157641	-1.078295951	0.280901703	0	0.663718754	0	1	PRJNA827465;Yang2025_Silkie_Wenchang	CRA004775	0.778581075
ENSGALG00010020828	CAMK2N1	2	-0.067398225	0.321049347	-0.209931047	0.83372149	0.096471542	1.841178541	45.68696203	0.5	CRA004775;Yang2025_Silkie_Wenchang	PRJNA827465	0.957968425
ENSGALG00010020828	CAMK2N1	2	-0.042145966	0.12624498	-0.333842706	0.738498256	0	0.931716173	0	0.5	CRA004775;PRJNA827465	Yang2025_Silkie_Wenchang	0.934485814
ENSGALG00010020831	MUL1	2	-0.02695006	0.072363585	-0.372425718	0.709575908	0	0.527237192	0	1	PRJNA827465;Yang2025_Silkie_Wenchang	CRA004775	0.925298042
ENSGALG00010020831	MUL1	2	-0.148537855	0.154326221	-0.962492662	0.335802187	0	0.005985391	0	1	CRA004775;Yang2025_Silkie_Wenchang	PRJNA827465	0.803504387
ENSGALG00010020831	MUL1	2	-0.016710931	0.074576865	-0.22407661	0.822697692	0	0.227377134	0	1	CRA004775;PRJNA827465	Yang2025_Silkie_Wenchang	0.955822987
ENSGALG00010020833	RANBP1	2	-0.138652807	0.1995976	-0.694661691	0.487267341	0.072814054	11.32733679	91.17179953	0.5	PRJNA827465;Yang2025_Silkie_Wenchang	CRA004775	0.85768885
ENSGALG00010020833	RANBP1	2	-0.11552128	0.245932186	-0.469728385	0.638549228	0.1036838	6.872139636	85.44849126	0.5	CRA004775;Yang2025_Silkie_Wenchang	PRJNA827465	0.905665632
ENSGALG00010020833	RANBP1	2	0.062284895	0.053142667	1.172031788	0.241184294	0	0.313137532	0	1	CRA004775;PRJNA827465	Yang2025_Silkie_Wenchang	0.753758453
ENSGALG00010020835	PTGR2	2	-0.051544359	0.21142048	-0.243800219	0.80738555	0.077289202	7.382852948	86.45510066	0.5	PRJNA827465;Yang2025_Silkie_Wenchang	CRA004775	0.95234753
ENSGALG00010020835	PTGR2	2	0.162752514	0.102278425	1.591269271	0.111548989	0	0.002577336	0	1	CRA004775;Yang2025_Silkie_Wenchang	PRJNA827465	0.564762672
ENSGALG00010020835	PTGR2	2	-0.107251922	0.209155997	-0.512784435	0.608101278	0.055956946	2.412819127	58.5547052	0.5	CRA004775;PRJNA827465	Yang2025_Silkie_Wenchang	0.897204334
ENSGALG00010020839	PGGHG	2	-0.019349368	0.09061568	-0.213532225	0.830911857	0	0.242258288	0	0.5	PRJNA827465;Yang2025_Silkie_Wenchang	CRA004775	0.957356025
ENSGALG00010020839	PGGHG	2	-0.163455599	0.255337543	-0.640154974	0.522071852	0.084285294	2.520367416	60.32324518	0.5	CRA004775;Yang2025_Silkie_Wenchang	PRJNA827465	0.869568211
ENSGALG00010020839	PGGHG	2	-0.188740284	0.200702229	-0.940399539	0.347012659	0.04318899	1.816335583	44.94989462	0.5	CRA004775;PRJNA827465	Yang2025_Silkie_Wenchang	0.808598952
ENSGALG00010020843	HMG20A	2	-0.010742645	0.091636141	-0.11723153	0.906676582	0.010107522	2.349644555	57.44037123	0.5	PRJNA827465;Yang2025_Silkie_Wenchang	CRA004775	0.978196186
ENSGALG00010020843	HMG20A	2	-0.193465563	0.371487985	-0.520785519	0.602516192	0.214698701	3.841541423	73.96878258	0.5	CRA004775;Yang2025_Silkie_Wenchang	PRJNA827465	0.895518685
ENSGALG00010020843	HMG20A	2	-0.253151449	0.261557445	-0.967861758	0.333113415	0.093118772	2.296917976	56.46339963	1	CRA004775;PRJNA827465	Yang2025_Silkie_Wenchang	0.802178578
ENSGALG00010020845	RAB35	2	-0.357267436	0.320094935	-1.116129614	0.264366658	0.198885986	33.46169431	97.0061399	0.5	PRJNA827465;Yang2025_Silkie_Wenchang	CRA004775	0.765390489
ENSGALG00010020845	RAB35	2	-0.336379532	0.353259465	-0.952216613	0.340987137	0.235328539	17.44804052	94.2686974	0.5	CRA004775;Yang2025_Silkie_Wenchang	PRJNA827465	0.805639763
ENSGALG00010020845	RAB35	2	-0.038966563	0.037790366	-1.031124259	0.302482553	0	0.225688602	0	0.5	CRA004775;PRJNA827465	Yang2025_Silkie_Wenchang	0.791852471
ENSGALG00010020847	MIDEAS	2	-0.032102323	0.105449023	-0.304434519	0.760796872	0.008298251	1.280573666	21.90999813	0.5	PRJNA827465;Yang2025_Silkie_Wenchang	CRA004775	0.939415629
ENSGALG00010020847	MIDEAS	2	0.187206357	0.207093434	0.903970511	0.366011038	0	0.004097945	0	1	CRA004775;Yang2025_Silkie_Wenchang	PRJNA827465	0.81592587
ENSGALG00010020847	MIDEAS	2	-0.075388811	0.071509436	-1.054249832	0.291768551	0	0.273194466	0	0.5	CRA004775;PRJNA827465	Yang2025_Silkie_Wenchang	0.78534312
ENSGALG00010020854	ENSGALG00010020854	2	-0.438455379	0.303605947	-1.444159392	0.148694145	0.132051666	3.119473099	67.94330426	1	PRJNA827465;Yang2025_Silkie_Wenchang	CRA004775	0.638795624
ENSGALG00010020854	ENSGALG00010020854	2	-0.355370417	0.610294885	-0.582293963	0.560369365	0.482790276	2.517813115	60.28299344	0.5	CRA004775;Yang2025_Silkie_Wenchang	PRJNA827465	0.881203589
ENSGALG00010020854	ENSGALG00010020854	2	-0.176099585	0.132692609	-1.327124291	0.184467571	0	0.752461381	0	0.5	CRA004775;PRJNA827465	Yang2025_Silkie_Wenchang	0.692797797
ENSGALG00010020855	DNAL1	2	0.098129053	0.101633924	0.965514753	0.33428705	0.00829511	1.623896824	38.41973302	0.5	PRJNA827465;Yang2025_Silkie_Wenchang	CRA004775	0.803094614
ENSGALG00010020855	DNAL1	2	-0.019175818	0.11624488	-0.164960542	0.868975039	0	0.01568135	0	0.5	CRA004775;Yang2025_Silkie_Wenchang	PRJNA827465	0.9684491
ENSGALG00010020855	DNAL1	2	0.156935991	0.0872809	1.798056507	0.072168054	0	0.463543187	0	1	CRA004775;PRJNA827465	Yang2025_Silkie_Wenchang	0.463770604
ENSGALG00010020861	CDA	2	0.2702178	0.159959145	1.689292602	0.09116337	0.013420931	1.26822968	21.14992922	1	PRJNA827465;Yang2025_Silkie_Wenchang	CRA004775	0.517862098
ENSGALG00010020861	CDA	2	0.122791636	0.203181083	0.604345808	0.545613757	0	0.324371344	0	1	CRA004775;Yang2025_Silkie_Wenchang	PRJNA827465	0.877802934
ENSGALG00010020861	CDA	2	0.344933905	0.132911465	2.595215588	0.00945316	0	0.14085243	0	1	CRA004775;PRJNA827465	Yang2025_Silkie_Wenchang	0.152962732
ENSGALG00010020866	LCOR	2	0.068673829	0.132905002	0.516713652	0.605356065	0.025838975	3.489606609	71.34347472	0.5	PRJNA827465;Yang2025_Silkie_Wenchang	CRA004775	0.896474603
ENSGALG00010020866	LCOR	2	0.169512319	0.113027011	1.499750527	0.133679037	0	0.915993609	0	0.5	CRA004775;Yang2025_Silkie_Wenchang	PRJNA827465	0.612847937
ENSGALG00010020866	LCOR	2	-0.044735839	0.066119175	-0.676594031	0.498663568	0	1.15771E-05	0	1	CRA004775;PRJNA827465	Yang2025_Silkie_Wenchang	0.861478809
ENSGALG00010020870	RIOX1	2	-0.036868408	0.050373242	-0.7319046	0.464262598	0	0.97283437	0	1	PRJNA827465;Yang2025_Silkie_Wenchang	CRA004775	0.853159649
ENSGALG00010020870	RIOX1	2	-0.140912901	0.111461499	-1.264229373	0.206147711	0	0.002520217	0	1	CRA004775;Yang2025_Silkie_Wenchang	PRJNA827465	0.718403416
ENSGALG00010020870	RIOX1	2	-0.0168449	0.05481231	-0.307319646	0.758600083	0	0.11746427	0	1	CRA004775;PRJNA827465	Yang2025_Silkie_Wenchang	0.938954369
ENSGALG00010020873	PINK1	2	-0.257582868	0.081703793	-3.15264271	0.001617997	0	0.279230132	0	1	PRJNA827465;Yang2025_Silkie_Wenchang	CRA004775	0.050888881
ENSGALG00010020873	PINK1	2	-0.341253045	0.201467295	-1.693838428	0.09029599	0	0.07522637	0	1	CRA004775;Yang2025_Silkie_Wenchang	PRJNA827465	0.515967344
ENSGALG00010020873	PINK1	2	-0.243117215	0.084339333	-2.88260774	0.003943982	0	0.002158055	0	1	CRA004775;PRJNA827465	Yang2025_Silkie_Wenchang	0.090543422
ENSGALG00010020875	ENSGALG00010020875	2	-0.086289273	0.063273318	-1.363758497	0.172644846	0	0.217710145	0	1	PRJNA827465;Yang2025_Silkie_Wenchang	CRA004775	0.675574638
ENSGALG00010020875	ENSGALG00010020875	2	-0.17643636	0.150214287	-1.174546308	0.240176275	0	0.168448516	0	1	CRA004775;Yang2025_Silkie_Wenchang	PRJNA827465	0.75252297
ENSGALG00010020875	ENSGALG00010020875	2	-0.079888822	0.068139307	-1.172433735	0.241022962	0	0.370653038	0	1	CRA004775;PRJNA827465	Yang2025_Silkie_Wenchang	0.753553852
ENSGALG00010020878	XYLT1	2	0.266540655	0.240526689	1.108154178	0.267795236	0.088257624	3.997788235	74.98616882	1	PRJNA827465;Yang2025_Silkie_Wenchang	CRA004775	0.771398294
ENSGALG00010020878	XYLT1	2	-0.028617448	0.638739794	-0.044802982	0.964264348	0.68734715	5.92782755	83.13041343	0.5	CRA004775;Yang2025_Silkie_Wenchang	PRJNA827465	0.990266302
ENSGALG00010020878	XYLT1	2	-0.207450423	0.37606348	-0.551636716	0.581197276	0.196446601	2.582084617	61.27160228	0.5	CRA004775;PRJNA827465	Yang2025_Silkie_Wenchang	0.88890219
ENSGALG00010020880	PXK	2	-0.053182785	0.085058265	-0.625251229	0.531806184	0.00403746	1.176958424	15.03523155	0.5	PRJNA827465;Yang2025_Silkie_Wenchang	CRA004775	0.873042777
ENSGALG00010020880	PXK	2	0.107949717	0.146142797	0.73865917	0.460113985	0	0.056742643	0	1	CRA004775;Yang2025_Silkie_Wenchang	PRJNA827465	0.850104868
ENSGALG00010020880	PXK	2	-0.07525057	0.064443546	-1.167697535	0.242928791	0	0.53492938	0	0.5	CRA004775;PRJNA827465	Yang2025_Silkie_Wenchang	0.7550024
ENSGALG00010020886	GBF1	2	0.089735408	0.104620641	0.857721831	0.391046082	0.015889285	3.357211632	70.21337617	1	PRJNA827465;Yang2025_Silkie_Wenchang	CRA004775	0.829251057
ENSGALG00010020886	GBF1	2	0.154718008	0.09856423	1.569717619	0.11648082	0.002558489	1.117157066	10.48707201	0.5	CRA004775;Yang2025_Silkie_Wenchang	PRJNA827465	0.574631355
ENSGALG00010020886	GBF1	2	0.001977787	0.049529299	0.039931649	0.968147619	0	0.001870223	0	0.5	CRA004775;PRJNA827465	Yang2025_Silkie_Wenchang	0.991182909
ENSGALG00010020887	ARPP19	2	-0.239197701	0.192629088	-1.241752754	0.214327799	0.06988462	16.76205164	94.03414318	1	PRJNA827465;Yang2025_Silkie_Wenchang	CRA004775	0.727123478
ENSGALG00010020887	ARPP19	2	-0.164488702	0.291622636	-0.564046414	0.572722545	0.153296636	9.813065096	89.80950406	0.5	CRA004775;Yang2025_Silkie_Wenchang	PRJNA827465	0.88573298
ENSGALG00010020887	ARPP19	2	-0.019564572	0.074981661	-0.260924763	0.794150528	0.005167667	1.354385919	26.16580063	0.5	CRA004775;PRJNA827465	Yang2025_Silkie_Wenchang	0.948905846
ENSGALG00010020889	PPRC1	2	0.05570082	0.17103049	0.32567772	0.744668203	0.051587775	8.455189943	88.17294459	0.5	PRJNA827465;Yang2025_Silkie_Wenchang	CRA004775	0.935893304
ENSGALG00010020889	PPRC1	2	0.219539699	0.078602549	2.793035379	0.005221598	0	0.145912044	0	1	CRA004775;Yang2025_Silkie_Wenchang	PRJNA827465	0.107954459
ENSGALG00010020889	PPRC1	2	-0.109771314	0.084518117	-1.298790345	0.194015889	0	0.169156033	0	0.5	CRA004775;PRJNA827465	Yang2025_Silkie_Wenchang	0.704555714
ENSGALG00010020890	RPS15A	2	-0.033940886	0.063646099	-0.533275191	0.593843099	0	0.00911474	0	1	PRJNA827465;Yang2025_Silkie_Wenchang	CRA004775	0.893190313
ENSGALG00010020890	RPS15A	2	-0.198580106	0.199101976	-0.997378884	0.318580637	0	0.4982832	0	1	CRA004775;Yang2025_Silkie_Wenchang	PRJNA827465	0.797754958
ENSGALG00010020890	RPS15A	2	-0.063450517	0.092662053	-0.684751906	0.493500489	0.005270825	1.124528915	11.07387401	1	CRA004775;PRJNA827465	Yang2025_Silkie_Wenchang	0.859868321
ENSGALG00010020894	PSMD13	2	0.065425961	0.110808156	0.59044355	0.554893321	0.017001492	2.819013701	64.52660022	0.5	PRJNA827465;Yang2025_Silkie_Wenchang	CRA004775	0.880223402
ENSGALG00010020894	PSMD13	2	-0.167604106	0.428861252	-0.39081196	0.695936234	0.310388893	5.894601128	83.03532371	0.5	CRA004775;Yang2025_Silkie_Wenchang	PRJNA827465	0.921038437
ENSGALG00010020894	PSMD13	2	-0.251834394	0.305917848	-0.823209225	0.410389024	0.14512253	3.56650425	71.96134002	1	CRA004775;PRJNA827465	Yang2025_Silkie_Wenchang	0.834820782
ENSGALG00010020896	MARCO	2	0.665262368	0.314329855	2.116446646	0.034306838	0	0.200084661	0	1	PRJNA827465;Yang2025_Silkie_Wenchang	CRA004775	0.318385282
ENSGALG00010020896	MARCO	2	0.393842909	0.37654046	1.045951102	0.295583609	0	0.161899686	0	1	CRA004775;Yang2025_Silkie_Wenchang	PRJNA827465	0.787628307
ENSGALG00010020896	MARCO	2	0.604115635	0.349665785	1.727694445	0.08404302	0	0.628650359	0	1	CRA004775;PRJNA827465	Yang2025_Silkie_Wenchang	0.499567879
ENSGALG00010020898	ENSGALG00010020898	2	0.017614299	0.170205602	0.103488364	0.917575384	0.023762973	1.61122224	37.93531547	0.5	PRJNA827465;Yang2025_Silkie_Wenchang	CRA004775	0.980974192
ENSGALG00010020898	ENSGALG00010020898	2	-0.336544881	0.183014442	-1.838897942	0.065930199	0	0.734767825	0	1	CRA004775;Yang2025_Silkie_Wenchang	PRJNA827465	0.442269089
ENSGALG00010020898	ENSGALG00010020898	2	-0.145166364	0.333828675	-0.43485289	0.663669212	0.174564947	4.325789513	76.88283267	0.5	CRA004775;PRJNA827465	Yang2025_Silkie_Wenchang	0.913119244
ENSGALG00010020900	PDHB	2	-0.24610139	0.270033818	-0.91137248	0.362099149	0.126418429	6.922930417	85.55524988	1	PRJNA827465;Yang2025_Silkie_Wenchang	CRA004775	0.815579273
ENSGALG00010020900	PDHB	2	-0.206278135	0.351860564	-0.586249657	0.557707756	0.203012187	5.534232574	81.93064735	0.5	CRA004775;Yang2025_Silkie_Wenchang	PRJNA827465	0.880678789
ENSGALG00010020900	PDHB	2	0.007536068	0.064157149	0.117462641	0.906493447	0	0.460697692	0	0.5	CRA004775;PRJNA827465	Yang2025_Silkie_Wenchang	0.978196186
ENSGALG00010020903	AGMAT	2	0.272276363	0.364366934	0.747258704	0.454907415	0	0.018852624	0	1	PRJNA827465;Yang2025_Silkie_Wenchang	CRA004775	0.849072665
ENSGALG00010020903	AGMAT	2	0.332130082	0.360426991	0.921490595	0.356794349	0	0.002722157	0	1	CRA004775;Yang2025_Silkie_Wenchang	PRJNA827465	0.812971699
ENSGALG00010020903	AGMAT	2	0.285597869	0.389233592	0.733744145	0.463104688	0	0.031874171	0	1	CRA004775;PRJNA827465	Yang2025_Silkie_Wenchang	0.851447984
ENSGALG00010020905	KCTD6	2	-0.109826206	0.103452482	-1.06161016	0.2884127	0.013207573	2.355105639	57.53905967	1	PRJNA827465;Yang2025_Silkie_Wenchang	CRA004775	0.783088502
ENSGALG00010020905	KCTD6	2	-0.196772063	0.105295778	-1.868755491	0.06165684	0	0.498769106	0	1	CRA004775;Yang2025_Silkie_Wenchang	PRJNA827465	0.428734062
ENSGALG00010020905	KCTD6	2	-0.035188071	0.05863402	-0.600130624	0.548419184	0	0.062926452	0	1	CRA004775;PRJNA827465	Yang2025_Silkie_Wenchang	0.878281266
ENSGALG00010020906	SIRT3	2	0.100704262	0.064864111	1.552542078	0.120532573	0	0.157672803	0	1	PRJNA827465;Yang2025_Silkie_Wenchang	CRA004775	0.582048372
ENSGALG00010020906	SIRT3	2	-0.137047462	0.286473592	-0.47839475	0.63236927	0.117338112	2.86358038	65.07868237	0.5	CRA004775;Yang2025_Silkie_Wenchang	PRJNA827465	0.903058174
ENSGALG00010020906	SIRT3	2	-0.117552055	0.31515828	-0.372993707	0.709153128	0.150188526	3.405376446	70.63467091	0.5	CRA004775;PRJNA827465	Yang2025_Silkie_Wenchang	0.925298042
ENSGALG00010020912	PDE8A	2	0.349848923	0.231283311	1.512642316	0.130370539	0.083762002	4.59308767	78.22815344	1	PRJNA827465;Yang2025_Silkie_Wenchang	CRA004775	0.605608015
ENSGALG00010020912	PDE8A	2	0.554614995	0.152422603	3.63866634	0.000274054	0	0.348734736	0	1	CRA004775;Yang2025_Silkie_Wenchang	PRJNA827465	0.016004648
ENSGALG00010020912	PDE8A	2	0.142858345	0.135238101	1.056346869	0.29080977	0	0.163013929	0	1	CRA004775;PRJNA827465	Yang2025_Silkie_Wenchang	0.784646441
ENSGALG00010020915	EN1	2	0.180758464	0.324838798	0.556455894	0.577899238	0.124845625	2.446495001	59.1251975	0.5	PRJNA827465;Yang2025_Silkie_Wenchang	CRA004775	0.887510696
ENSGALG00010020915	EN1	2	0.320542614	0.361806948	0.885949306	0.375644845	0.076817543	1.253196321	20.20404278	0.5	CRA004775;Yang2025_Silkie_Wenchang	PRJNA827465	0.822974013
ENSGALG00010020915	EN1	2	-0.16690524	0.265140456	-0.629497445	0.529023441	0	0.082681526	0	1	CRA004775;PRJNA827465	Yang2025_Silkie_Wenchang	0.871809618
ENSGALG00010020916	ARL6IP1	2	-0.381869844	0.547850812	-0.697032542	0.485782429	0.569705827	19.11773116	94.76925378	0.5	PRJNA827465;Yang2025_Silkie_Wenchang	CRA004775	0.857391223
ENSGALG00010020916	ARL6IP1	2	-0.627332075	0.335278542	-1.871077319	0.061334365	0.162844794	3.615578255	72.3419069	1	CRA004775;Yang2025_Silkie_Wenchang	PRJNA827465	0.427938152
ENSGALG00010020916	ARL6IP1	2	0.005185254	0.200055791	0.025919038	0.979321915	0.051135683	2.297942941	56.48281852	0.5	CRA004775;PRJNA827465	Yang2025_Silkie_Wenchang	0.994390773
ENSGALG00010020918	ENSGALG00010020918	2	-0.171568922	0.275209012	-0.62341317	0.533013046	0.040158052	1.340321005	25.39100737	0.5	PRJNA827465;Yang2025_Silkie_Wenchang	CRA004775	0.873312145
ENSGALG00010020918	ENSGALG00010020918	2	-0.105731929	0.483776165	-0.218555474	0.82699634	0.24742901	2.049929835	51.21784254	0.5	CRA004775;Yang2025_Silkie_Wenchang	PRJNA827465	0.956540044
ENSGALG00010020918	ENSGALG00010020918	2	0.140343378	0.259824804	0.540146191	0.589096218	0	0.43522263	0	1	CRA004775;PRJNA827465	Yang2025_Silkie_Wenchang	0.892171138
ENSGALG00010020920	ACOX2	2	-0.065166269	0.130320989	-0.500044309	0.617043878	0	0.803192956	0	0.5	PRJNA827465;Yang2025_Silkie_Wenchang	CRA004775	0.899121942
ENSGALG00010020920	ACOX2	2	0.009618287	0.169168138	0.056856377	0.954659604	0	0.368067314	0	0.5	CRA004775;Yang2025_Silkie_Wenchang	PRJNA827465	0.987987175
ENSGALG00010020920	ACOX2	2	-0.202098923	0.179529482	-1.125714397	0.260286428	0	0.035169542	0	1	CRA004775;PRJNA827465	Yang2025_Silkie_Wenchang	0.767635013
ENSGALG00010020921	LDB1	2	-0.006444132	0.055299002	-0.116532515	0.907230518	0	0.648391762	0	0.5	PRJNA827465;Yang2025_Silkie_Wenchang	CRA004775	0.978196186
ENSGALG00010020921	LDB1	2	-0.157775809	0.161465136	-0.97715094	0.328494432	0.030682626	1.965248107	49.11583954	1	CRA004775;Yang2025_Silkie_Wenchang	PRJNA827465	0.800761292
ENSGALG00010020921	LDB1	2	-0.127092545	0.213573487	-0.595076415	0.551792393	0.06642214	3.106801367	67.8125544	0.5	CRA004775;PRJNA827465	Yang2025_Silkie_Wenchang	0.879061525
ENSGALG00010020922	INSIG2	2	-0.288002226	0.378803578	-0.760294365	0.447078649	0.27455389	22.51242975	95.55800946	0.5	PRJNA827465;Yang2025_Silkie_Wenchang	CRA004775	0.84650962
ENSGALG00010020922	INSIG2	2	-0.354153501	0.331501948	-1.068330074	0.285371647	0.193480593	8.33613806	88.00403745	1	CRA004775;Yang2025_Silkie_Wenchang	PRJNA827465	0.781055839
ENSGALG00010020922	INSIG2	2	0.069575309	0.052188795	1.333146497	0.182483733	0	0.27142113	0	0.5	CRA004775;PRJNA827465	Yang2025_Silkie_Wenchang	0.690634542
ENSGALG00010020924	DNAJC16	2	-0.095886791	0.06286128	-1.525371276	0.127166519	0.001854599	1.255343088	20.34050217	1	PRJNA827465;Yang2025_Silkie_Wenchang	CRA004775	0.598494276
ENSGALG00010020924	DNAJC16	2	-0.190118363	0.094844392	-2.004529301	0.045013391	0	0.002679567	0	1	CRA004775;Yang2025_Silkie_Wenchang	PRJNA827465	0.365831539
ENSGALG00010020924	DNAJC16	2	-0.062210155	0.060760968	-1.023850618	0.305905838	0	0.34773405	0	1	CRA004775;PRJNA827465	Yang2025_Silkie_Wenchang	0.792343294
ENSGALG00010020925	RIC8A	2	-0.229987025	0.22528738	-1.020860667	0.307320457	0.082358513	4.521609678	77.88398223	1	PRJNA827465;Yang2025_Silkie_Wenchang	CRA004775	0.792675978
ENSGALG00010020925	RIC8A	2	-0.563372107	0.171440524	-3.286108172	0.00101582	0	0.217492983	0	1	CRA004775;Yang2025_Silkie_Wenchang	PRJNA827465	0.038315518
ENSGALG00010020925	RIC8A	2	-0.293449408	0.307104537	-0.955535892	0.339306764	0.153288388	4.460343311	77.58020111	1	CRA004775;PRJNA827465	Yang2025_Silkie_Wenchang	0.804824608
ENSGALG00010020927	ENSGALG00010020927	2	0.147656754	0.080912813	1.824887162	0.068018059	0	0.017136793	0	1	PRJNA827465;Yang2025_Silkie_Wenchang	CRA004775	0.4497039
ENSGALG00010020927	ENSGALG00010020927	2	0.128278568	0.140151913	0.915282318	0.360043439	0	0.535824325	0	0.5	CRA004775;Yang2025_Silkie_Wenchang	PRJNA827465	0.814880744
ENSGALG00010020927	ENSGALG00010020927	2	0.12580043	0.094151177	1.336153559	0.181499087	0	0.494107609	0	0.5	CRA004775;PRJNA827465	Yang2025_Silkie_Wenchang	0.689004635
ENSGALG00010020928	AACS	2	0.336502562	0.131303056	2.56279307	0.010383391	0.006107669	1.17904001	15.18523616	1	PRJNA827465;Yang2025_Silkie_Wenchang	CRA004775	0.162201284
ENSGALG00010020928	AACS	2	0.264604835	0.309537187	0.854840214	0.392639612	0.117945147	2.515241285	60.24238287	0.5	CRA004775;Yang2025_Silkie_Wenchang	PRJNA827465	0.829914191
ENSGALG00010020928	AACS	2	0.196601426	0.126736395	1.551262575	0.120838772	0	0.934267361	0	0.5	CRA004775;PRJNA827465	Yang2025_Silkie_Wenchang	0.582767179
ENSGALG00010020932	CASP9	2	-0.13721847	0.237290839	-0.578271249	0.563081002	0.089131665	4.50765772	77.81552943	0.5	PRJNA827465;Yang2025_Silkie_Wenchang	CRA004775	0.882244362
ENSGALG00010020932	CASP9	2	-0.42222368	0.15370302	-2.747009667	0.006014136	0	0.012874123	0	1	CRA004775;Yang2025_Silkie_Wenchang	PRJNA827465	0.117225517
ENSGALG00010020932	CASP9	2	-0.140649483	0.252992841	-0.55594254	0.578250136	0.098270976	3.903215527	74.38009782	0.5	CRA004775;PRJNA827465	Yang2025_Silkie_Wenchang	0.88778445
ENSGALG00010020934	ABCD3	2	-0.102514957	0.117147372	-0.875093959	0.381522784	0.022142746	5.142130817	80.55280903	0.5	PRJNA827465;Yang2025_Silkie_Wenchang	CRA004775	0.824670845
ENSGALG00010020934	ABCD3	2	-0.208314094	0.078308531	-2.660171154	0.007810095	0	0.909271692	0	0.5	CRA004775;Yang2025_Silkie_Wenchang	PRJNA827465	0.13708394
ENSGALG00010020934	ABCD3	2	0.014032312	0.064428816	0.217795585	0.827588385	0	0.117649224	0	1	CRA004775;PRJNA827465	Yang2025_Silkie_Wenchang	0.956550752
ENSGALG00010020935	BRI3BP	2	0.046370347	0.04037292	1.148550762	0.25074127	0	0.235035365	0	1	PRJNA827465;Yang2025_Silkie_Wenchang	CRA004775	0.760383127
ENSGALG00010020935	BRI3BP	2	0.068404598	0.073832543	0.926483027	0.354195014	0	0.24915364	0	0.5	CRA004775;Yang2025_Silkie_Wenchang	PRJNA827465	0.811857855
ENSGALG00010020935	BRI3BP	2	0.031417009	0.047045404	0.667801876	0.504260069	0	0.134374247	0	0.5	CRA004775;PRJNA827465	Yang2025_Silkie_Wenchang	0.863512504
ENSGALG00010020936	BET1L	2	-0.03270501	0.11486921	-0.284715198	0.775862346	0.02079446	4.224478335	76.3284382	0.5	PRJNA827465;Yang2025_Silkie_Wenchang	CRA004775	0.943844181
ENSGALG00010020936	BET1L	2	-0.204316312	0.097999905	-2.084862346	0.037081797	0	0.703289168	0	1	CRA004775;Yang2025_Silkie_Wenchang	PRJNA827465	0.332143015
ENSGALG00010020936	BET1L	2	-0.101032827	0.222269633	-0.454550745	0.649432477	0.074798295	3.247592773	69.20796202	0.5	CRA004775;PRJNA827465	Yang2025_Silkie_Wenchang	0.908043243
ENSGALG00010020938	SMG1	2	-0.112370445	0.047126479	-2.384443912	0.017104955	0	0.006877756	0	1	PRJNA827465;Yang2025_Silkie_Wenchang	CRA004775	0.217354043
ENSGALG00010020938	SMG1	2	0.002933407	0.172078479	0.017046912	0.986399191	0.025626497	1.526109315	34.47389449	0.5	CRA004775;Yang2025_Silkie_Wenchang	PRJNA827465	0.99618774
ENSGALG00010020938	SMG1	2	-0.016043218	0.171043203	-0.093796293	0.925270976	0.036247699	1.854601722	46.0800673	0.5	CRA004775;PRJNA827465	Yang2025_Silkie_Wenchang	0.982594411
ENSGALG00010020940	ENSGALG00010020940	2	0.519989796	0.377926962	1.375900235	0.168852522	0.269386296	17.38573296	94.24815737	1	PRJNA827465;Yang2025_Silkie_Wenchang	CRA004775	0.669938491
ENSGALG00010020940	ENSGALG00010020940	2	0.716636745	0.291521262	2.458265786	0.013960981	0.097916378	1.935656784	48.33794873	1	CRA004775;Yang2025_Silkie_Wenchang	PRJNA827465	0.193808158
ENSGALG00010020940	ENSGALG00010020940	2	0.156951279	0.095020797	1.651757141	0.098584069	0	0.073177113	0	1	CRA004775;PRJNA827465	Yang2025_Silkie_Wenchang	0.53598695
ENSGALG00010020941	ENSGALG00010020941	2	0.417780128	0.149317516	2.797931148	0.005143107	0.028673058	2.794741448	64.21851471	1	PRJNA827465;Yang2025_Silkie_Wenchang	CRA004775	0.10712218
ENSGALG00010020941	ENSGALG00010020941	2	0.556347416	0.113411685	4.905556393	9.31629E-07	0	0.019861083	0	1	CRA004775;Yang2025_Silkie_Wenchang	PRJNA827465	0.000210436
ENSGALG00010020941	ENSGALG00010020941	2	0.293936772	0.124001997	2.370419663	0.017767905	0	0.434158711	0	1	CRA004775;PRJNA827465	Yang2025_Silkie_Wenchang	0.222257149
ENSGALG00010020943	FAM107A	2	0.162607741	0.341700136	0.47587848	0.634160954	0.075508781	1.47139113	32.0371056	0.5	PRJNA827465;Yang2025_Silkie_Wenchang	CRA004775	0.903815945
ENSGALG00010020943	FAM107A	2	-0.187709954	0.336968528	-0.557054854	0.577489952	0	0.066658695	0	1	CRA004775;Yang2025_Silkie_Wenchang	PRJNA827465	0.88731056
ENSGALG00010020943	FAM107A	2	0.336433871	0.380009562	0.885330014	0.375978667	0.002567025	1.00615916	0.612145736	0.5	CRA004775;PRJNA827465	Yang2025_Silkie_Wenchang	0.822974013

ENSGALG00010020944	MYO5A	2	0.285576647	0.094977975	3.006767047	0.00264042	0.005879456	1.393529277	28.23975668	1	PRJNA827465;Yang2025_Silkie_Wenchang	CRA004775	0.069462139
ENSGALG00010020944	MYO5A	2	0.365690232	0.162908914	2.244752744	0.02478401	0.007496627	1.061787511	5.819197358	0.5	CRA004775;Yang2025_Silkie_Wenchang	PRJNA827465	0.268761753
ENSGALG00010020944	MYO5A	2	0.211029246	0.084252492	2.504724078	0.012254695	0	0.405078569	0	0.5	CRA004775;PRJNA827465	Yang2025_Silkie_Wenchang	0.178784814
ENSGALG00010020945	NUMB	2	0.314465132	0.287704259	1.093015215	0.27438712	0.142994614	6.678380768	85.0263105	1	PRJNA827465;Yang2025_Silkie_Wenchang	CRA004775	0.774526342
ENSGALG00010020945	NUMB	2	0.601086044	0.131195658	4.581600134	4.61432E-06	0	0.054126325	0	1	CRA004775;Yang2025_Silkie_Wenchang	PRJNA827465	0.000762002
ENSGALG00010020945	NUMB	2	0.296639879	0.257430376	1.152311098	0.249193258	0.117763724	8.457582773	88.17629071	1	CRA004775;PRJNA827465	Yang2025_Silkie_Wenchang	0.759411487
ENSGALG00010020946	CTBS	2	0.022950783	0.112211946	0.204530656	0.837938838	0.011135724	1.701301992	41.22148773	0.5	PRJNA827465;Yang2025_Silkie_Wenchang	CRA004775	0.959032758
ENSGALG00010020946	CTBS	2	0.138783663	0.141740627	0.979138199	0.3275117	0	0.332851507	0	0.5	CRA004775;Yang2025_Silkie_Wenchang	PRJNA827465	0.800077397
ENSGALG00010020946	CTBS	2	-0.063853313	0.091597347	-0.697108762	0.485734731	0	0.001564331	0	1	CRA004775;PRJNA827465	Yang2025_Silkie_Wenchang	0.857391223
ENSGALG00010020949	HPS6	2	0.032823563	0.093264219	0.351941652	0.724882021	0.007260809	1.422608819	29.70660757	0.5	PRJNA827465;Yang2025_Silkie_Wenchang	CRA004775	0.930564843
ENSGALG00010020949	HPS6	2	-0.327816576	0.226560075	-1.446930027	0.147916522	0.057407896	2.188035675	54.29690606	1	CRA004775;Yang2025_Silkie_Wenchang	PRJNA827465	0.637382203
ENSGALG00010020949	HPS6	2	-0.210719972	0.337386165	-0.624566133	0.532255853	0.196642431	6.586556541	84.81755992	0.5	CRA004775;PRJNA827465	Yang2025_Silkie_Wenchang	0.873104119
ENSGALG00010020950	EFHD2	2	0.013587584	0.315480262	0.043069521	0.965646116	0.139088958	3.013487948	66.81586198	0.5	PRJNA827465;Yang2025_Silkie_Wenchang	CRA004775	0.990668014
ENSGALG00010020950	EFHD2	2	-0.202024226	0.636449604	-0.317423759	0.750922082	0.659345923	5.31580899	81.18818788	0.5	CRA004775;Yang2025_Silkie_Wenchang	PRJNA827465	0.937567095
ENSGALG00010020950	EFHD2	2	-0.423126797	0.285105504	-1.484106028	0.137780794	0.088734771	1.812425067	44.82530515	1	CRA004775;PRJNA827465	Yang2025_Silkie_Wenchang	0.62064915
ENSGALG00010020952	GNG5	2	-0.034626169	0.067574027	-0.512418315	0.608358276	0.002574331	1.189701652	15.94531297	1	PRJNA827465;Yang2025_Silkie_Wenchang	CRA004775	0.897204334
ENSGALG00010020952	GNG5	2	-0.231067055	0.135387967	-1.706703037	0.087877262	0	0.381773505	0	1	CRA004775;Yang2025_Silkie_Wenchang	PRJNA827465	0.509491082
ENSGALG00010020952	GNG5	2	-0.094602094	0.160459571	-0.589569658	0.555479199	0.031027054	1.791557314	44.18263975	1	CRA004775;PRJNA827465	Yang2025_Silkie_Wenchang	0.880255849
ENSGALG00010020954	PLIN1	2	-0.939407749	0.608740835	-1.54319818	0.122782699	0	0.510524125	0	1	PRJNA827465;Yang2025_Silkie_Wenchang	CRA004775	0.587706885
ENSGALG00010020954	PLIN1	2	-1.951596618	0.527691914	-3.698363696	0.000216994	0.007297607	1.012893416	1.272929189	1	CRA004775;Yang2025_Silkie_Wenchang	PRJNA827465	0.013451906
ENSGALG00010020954	PLIN1	2	-1.518438082	0.968726704	-1.567457648	0.117007758	1.240328783	2.894460941	65.45125257	1	CRA004775;PRJNA827465	Yang2025_Silkie_Wenchang	0.57615266
ENSGALG00010020957	RPF1	2	-0.058420352	0.136208185	-0.428904857	0.667992465	0.029870337	4.942408714	79.76695053	0.5	PRJNA827465;Yang2025_Silkie_Wenchang	CRA004775	0.914120722
ENSGALG00010020957	RPF1	2	-0.173878114	0.097977044	-1.774682181	0.075950379	0	0.763497558	0	0.5	CRA004775;Yang2025_Silkie_Wenchang	PRJNA827465	0.47539798
ENSGALG00010020957	RPF1	2	0.063891767	0.061483857	1.039163295	0.298728798	0	0.009598018	0	1	CRA004775;PRJNA827465	Yang2025_Silkie_Wenchang	0.78974322
ENSGALG00010020959	ENSGALG00010020959	2	0.103858922	0.137356543	0.7561265	0.449573355	0	0.042954696	0	1	PRJNA827465;Yang2025_Silkie_Wenchang	CRA004775	0.847275788
ENSGALG00010020959	ENSGALG00010020959	2	0.137659415	0.221865913	0.620462212	0.534953525	0	0.981061764	0	1	CRA004775;Yang2025_Silkie_Wenchang	PRJNA827465	0.873904916
ENSGALG00010020959	ENSGALG00010020959	2	0.16162005	0.165009011	0.979461963	0.327351774	0	0.868436927	0	1	CRA004775;PRJNA827465	Yang2025_Silkie_Wenchang	0.800028239
ENSGALG00010020960	CLEC19A	2	0.128084795	0.1180165	1.085312604	0.277783226	0	0.549744194	0	1	PRJNA827465;Yang2025_Silkie_Wenchang	CRA004775	0.776763074
ENSGALG00010020960	CLEC19A	2	0.109624	0.287183954	0.381720492	0.702668699	0.064623933	1.235125863	19.03659134	0.5	CRA004775;Yang2025_Silkie_Wenchang	PRJNA827465	0.923272306
ENSGALG00010020960	CLEC19A	2	0.020241817	0.154440858	0.131065171	0.895723753	0	0.772520869	0	0.5	CRA004775;PRJNA827465	Yang2025_Silkie_Wenchang	0.974927462
ENSGALG00010020963	CCDC93	2	0.278266851	0.33283513	0.836110212	0.403092904	0.202151373	10.98455927	90.89631203	0.5	PRJNA827465;Yang2025_Silkie_Wenchang	CRA004775	0.833046091
ENSGALG00010020963	CCDC93	2	0.449111891	0.200241254	2.242853973	0.024906233	0.03972264	1.966195855	49.14036679	1	CRA004775;Yang2025_Silkie_Wenchang	PRJNA827465	0.269934231
ENSGALG00010020963	CCDC93	2	0.018946482	0.106437846	0.178005127	0.858718949	0.007622255	1.277678743	21.73306432	0.5	CRA004775;PRJNA827465	Yang2025_Silkie_Wenchang	0.964449906
ENSGALG00010020965	DHX37	2	0.125675933	0.124807462	1.00695849	0.313954723	0.025119128	5.006727298	80.02687304	1	PRJNA827465;Yang2025_Silkie_Wenchang	CRA004775	0.796531448
ENSGALG00010020965	DHX37	2	0.053635314	0.251478134	0.213280227	0.831108395	0.097583657	3.91498829	74.45713943	0.5	CRA004775;Yang2025_Silkie_Wenchang	PRJNA827465	0.957470197
ENSGALG00010020965	DHX37	2	-0.015409532	0.079084567	-0.194848787	0.845511328	0.003408142	1.110786612	9.97370791	0.5	CRA004775;PRJNA827465	Yang2025_Silkie_Wenchang	0.961048404
ENSGALG00010020966	PAPLN	2	-0.087728251	0.129850142	-0.675611511	0.499287331	0	0.225253238	0	1	PRJNA827465;Yang2025_Silkie_Wenchang	CRA004775	0.8618512
ENSGALG00010020966	PAPLN	2	-0.056980407	0.255519841	-0.222997975	0.823537083	0.026921459	1.140715071	12.33568963	0.5	CRA004775;Yang2025_Silkie_Wenchang	PRJNA827465	0.955934625
ENSGALG00010020966	PAPLN	2	-0.008768485	0.15166211	-0.057815927	0.953895252	0	0.787751895	0	0.5	CRA004775;PRJNA827465	Yang2025_Silkie_Wenchang	0.987838152
ENSGALG00010020967	VPS51	2	0.087569438	0.056679565	1.544991357	0.122348354	0	0.832669213	0	0.5	PRJNA827465;Yang2025_Silkie_Wenchang	CRA004775	0.586636252
ENSGALG00010020967	VPS51	2	-0.272009493	0.223741166	-1.215732885	0.224086693	0.059257705	2.419848108	58.67509218	1	CRA004775;Yang2025_Silkie_Wenchang	PRJNA827465	0.737452676
ENSGALG00010020967	VPS51	2	-0.168227589	0.308824069	-0.544736004	0.585505099	0.16631899	7.082209867	85.88011343	0.5	CRA004775;PRJNA827465	Yang2025_Silkie_Wenchang	0.89138793
ENSGALG00010020969	RBP3	2	-0.094418745	0.614257211	-0.153712066	0.877836771	0.565432222	3.801394177	73.69386195	0.5	PRJNA827465;Yang2025_Silkie_Wenchang	CRA004775	0.970941655
ENSGALG00010020969	RBP3	2	-0.533514584	0.472078788	-1.130138862	0.258417717	0	0.871078455	0	0.5	CRA004775;Yang2025_Silkie_Wenchang	PRJNA827465	0.765668415
ENSGALG00010020969	RBP3	2	0.420678127	0.30379032	1.384764753	0.166124412	0	0.055544781	0	1	CRA004775;PRJNA827465	Yang2025_Silkie_Wenchang	0.66529196
ENSGALG00010020970	ARMH3	2	-0.189575866	0.136860326	-1.385177659	0.16599815	0.034348615	11.89929062	91.59613769	1	PRJNA827465;Yang2025_Silkie_Wenchang	CRA004775	0.66529196
ENSGALG00010020970	ARMH3	2	-0.32601994	0.063178196	-5.160323642	2.46523E-07	0	0.065620619	0	1	CRA004775;Yang2025_Silkie_Wenchang	PRJNA827465	7.23968E-05
ENSGALG00010020970	ARMH3	2	-0.065449272	0.043273812	-1.51244526	0.130420628	0	0.929385127	0	1	CRA004775;PRJNA827465	Yang2025_Silkie_Wenchang	0.605614597
ENSGALG00010020971	TMEM51	2	0.317774863	0.187099457	1.698427505	0.089427104	0	0.643965377	0	1	PRJNA827465;Yang2025_Silkie_Wenchang	CRA004775	0.513235693
ENSGALG00010020971	TMEM51	2	-0.02307904	0.262296787	-0.087988268	0.929886001	0	0.198616599	0	0.5	CRA004775;Yang2025_Silkie_Wenchang	PRJNA827465	0.983813577
ENSGALG00010020971	TMEM51	2	0.223603024	0.267669988	0.835387033	0.403499829	0.058402451	1.567588047	36.20772996	0.5	CRA004775;PRJNA827465	Yang2025_Silkie_Wenchang	0.83306585
ENSGALG00010020973	GNB5	2	0.075928596	0.142786259	0.531764025	0.594889442	0.017481716	1.716316856	41.73570011	0.5	PRJNA827465;Yang2025_Silkie_Wenchang	CRA004775	0.893382718
ENSGALG00010020973	GNB5	2	-0.040974343	0.153922671	-0.266200832	0.790084542	0	0.35043159	0	0.5	CRA004775;Yang2025_Silkie_Wenchang	PRJNA827465	0.947390191
ENSGALG00010020973	GNB5	2	0.185464741	0.122119345	1.51871712	0.128833716	0	0.050511532	0	1	CRA004775;PRJNA827465	Yang2025_Silkie_Wenchang	0.602158583
ENSGALG00010020975	HCCS	2	-0.20925827	0.090225215	-2.319288123	0.020379419	0.007662788	1.808513803	44.70597912	1	PRJNA827465;Yang2025_Silkie_Wenchang	CRA004775	0.240387846
ENSGALG00010020975	HCCS	2	-0.052160869	0.103968826	-0.501697196	0.615880537	0	0.765432144	0	0.5	CRA004775;Yang2025_Silkie_Wenchang	PRJNA827465	0.89901403
ENSGALG00010020975	HCCS	2	-0.134152398	0.199530301	-0.672340979	0.501366647	0.05576788	2.76815343	63.87483478	0.5	CRA004775;PRJNA827465	Yang2025_Silkie_Wenchang	0.862658912
ENSGALG00010020976	GDF10	2	-0.167362307	0.223412952	-0.749116402	0.45378705	0	0.0014208	0	1	PRJNA827465;Yang2025_Silkie_Wenchang	CRA004775	0.849904791
ENSGALG00010020976	GDF10	2	-0.15381613	0.30185613	-0.509567687	0.610354367	0	0.00010856	0	1	CRA004775;Yang2025_Silkie_Wenchang	PRJNA827465	0.897438028
ENSGALG00010020976	GDF10	2	-0.168153426	0.245146668	-0.685929885	0.492757324	0	0.001710287	0	1	CRA004775;PRJNA827465	Yang2025_Silkie_Wenchang	0.859474364
ENSGALG00010020978	BCL2L10	2	-0.120783277	0.116380964	-1.037826741	0.299350752	0	0.600023987	0	1	PRJNA827465;Yang2025_Silkie_Wenchang	CRA004775	0.789858093
ENSGALG00010020978	BCL2L10	2	-0.00410135	0.338638684	-0.012111287	0.990336827	0.134656069	2.410402304	58.51314952	0.5	CRA004775;Yang2025_Silkie_Wenchang	PRJNA827465	0.997468525
ENSGALG00010020978	BCL2L10	2	0.03071916	0.190040879	0.16164501	0.871585411	0.032311953	1.516708502	34.06775273	0.5	CRA004775;PRJNA827465	Yang2025_Silkie_Wenchang	0.969425739
ENSGALG00010020979	ENSGALG00010020979	2	0.046911151	0.157320745	0.298187952	0.765589717	0	0.077362402	0	0.5	PRJNA827465;Yang2025_Silkie_Wenchang	CRA004775	0.940892259
ENSGALG00010020979	ENSGALG00010020979	2	-0.107227898	0.192338671	-0.557495261	0.577189097	0	0.229382565	0	1	CRA004775;Yang2025_Silkie_Wenchang	PRJNA827465	0.887145528
ENSGALG00010020979	ENSGALG00010020979	2	-0.014852271	0.157206336	-0.09447629	0.924730816	0	0.691541064	0	0.5	CRA004775;PRJNA827465	Yang2025_Silkie_Wenchang	0.982493782
ENSGALG00010020981	CIT	2	0.210030823	0.261063833	0.804519034	0.421097281	0.120808936	8.629523174	88.41187421	0.5	PRJNA827465;Yang2025_Silkie_Wenchang	CRA004775	0.839269089
ENSGALG00010020981	CIT	2	0.020831333	0.636762898	0.032714426	0.97390292	0.597720565	3.00731439	66.74774	0.5	CRA004775;Yang2025_Silkie_Wenchang	PRJNA827465	0.992472082
ENSGALG00010020981	CIT	2	-0.100451941	0.216197935	-0.464629509	0.642196797	0.041518068	1.142069524	12.48563304	1	CRA004775;PRJNA827465	Yang2025_Silkie_Wenchang	0.906645523
ENSGALG00010020982	TLCD4	2	-0.060288596	0.092839429	-0.649385683	0.516089116	0	0.108800614	0	1	PRJNA827465;Yang2025_Silkie_Wenchang	CRA004775	0.867498525
ENSGALG00010020982	TLCD4	2	-0.137827314	0.161028257	-0.855920054	0.392043001	0	0.210445478	0	1	CRA004775;Yang2025_Silkie_Wenchang	PRJNA827465	0.829251057
ENSGALG00010020982	TLCD4	2	-0.05773189	0.106637484	-0.541384586	0.588242529	0	0.395433507	0	1	CRA004775;PRJNA827465	Yang2025_Silkie_Wenchang	0.89186036
ENSGALG00010020984	PSEN1	2	-0.063902643	0.17834329	-0.358312575	0.720109408	0.055630555	7.764173877	87.12032966	0.5	PRJNA827465;Yang2025_Silkie_Wenchang	CRA004775	0.928564173
ENSGALG00010020984	PSEN1	2	-0.181091692	0.130920332	-1.383220536	0.166597255	0.010239197	1.273214331	21.45862831	0.5	CRA004775;Yang2025_Silkie_Wenchang	PRJNA827465	0.665626909
ENSGALG00010020984	PSEN1	2	0.100676441	0.06338122	1.588427012	0.112189813	0	0.034981378	0	1	CRA004775;PRJNA827465	Yang2025_Silkie_Wenchang	0.5658803
ENSGALG00010020987	NADSYN1	2	-0.061566147	0.101875402	-0.604327588	0.545625669	0.010229749	1.898924744	47.33861871	0.5	PRJNA827465;Yang2025_Silkie_Wenchang	CRA004775	0.837802934
ENSGALG00010020987	NADSYN1	2	-0.215406874	0.28955723	-0.743918135	0.456926004	0.145696386	7.548783593	86.75283259	0.5	CRA004775;Yang2025_Silkie_Wenchang	PRJNA827465	0.84954048
ENSGALG00010020987	NADSYN1	2	-0.301458336	0.183518011	-1.642663487	0.100452577	0.051112149	3.846579215	74.00287518	1	CRA004775;PRJNA827465	Yang2025_Silkie_Wenchang	0.541278811
ENSGALG00010020989	MAPK4	2	0.077029438	0.035529909	2.168016755	0.030157412	0	0.476795783	0	0.5	PRJNA827465;Yang2025_Silkie_Wenchang	CRA004775	0.295973612
ENSGALG00010020989	MAPK4	2	0.026518102	0.114059065	0.232494075	0.816133978	0	0.711047047	0	0.5	CRA004775;Yang2025_Silkie_Wenchang	PRJNA827465	0.95448249
ENSGALG00010020989	MAPK4	2	0.086958674	0.037086521	2.344751445	0.019039768	0	0.44413981	0	1	CRA004775;PRJNA827465	Yang2025_Silkie_Wenchang	0.231177011
ENSGALG00010020990	UBB	2	-0.010985403	0.126719222	-0.086690897	0.930917212	0.019299326	1.921589425	47.95974691	0.5	PRJNA827465;Yang2025_Silkie_Wenchang	CRA004775	0.983943003
ENSGALG00010020990	UBB	2	-0.26191922	0.143596929	-1.823989007	0.068151734	0	0.111749162	0	1	CRA004775;Yang2025_Silkie_Wenchang	PRJNA827465	0.450926316
ENSGALG00010020990	UBB	2	-0.069014264	0.182150444	-0.378886059	0.704772477	0.048852715	3.097440881	67.71528373	0.5	CRA004775;PRJNA827465	Yang2025_Silkie_Wenchang	0.924152671
ENSGALG00010020991	ALG14	2	0.013218952	0.091456059	0.144538833	0.885074994	0	0.170469199	0	0.5	PRJNA827465;Yang2025_Silkie_Wenchang	CRA004775	0.972221982
ENSGALG00010020991	ALG14	2	0.033519629	0.154398293	0.217098443	0.828131627	0	0.237202484	0	0.5	CRA004775;Yang2025_Silkie_Wenchang	PRJNA827465	0.95664866
ENSGALG00010020991	ALG14	2	-0.01887123	0.101102423	-0.186654858	0.851931147	0	0.074065825	0	1	CRA004775;PRJNA827465	Yang2025_Silkie_Wenchang	0.962255491
ENSGALG00010020992	KCNH7	2	0.165579042	0.41885248	0.395315892	0.692609773	0.191180772	2.193942305	54.41994997	0.5	PRJNA827465;Yang2025_Silkie_Wenchang	CRA004775	0.920559211
ENSGALG00010020992	KCNH7	2	0.554013719	0.337214405	1.642912377	0.100401064	0	0.035044911	0	1	CRA004775;Yang2025_Silkie_Wenchang	PRJNA827465	0.541223839
ENSGALG00010020992	KCNH7	2	-0.018312492	0.326867048	-0.056024282	0.955322463	0.007662806	1.031814716	3.083374861	0.5	CRA004775;PRJNA827465	Yang2025_Silkie_Wenchang	0.988131974
ENSGALG00010020994	DHCR7	2	-0.028617768	0.082769234	-0.345755169	0.729527831	0	0.620113214	0	0.5	PRJNA827465;Yang2025_Silkie_Wenchang	CRA004775	0.9323457
ENSGALG00010020994	DHCR7	2	-0.113771749	0.140824186	-0.807899216	0.419148603	0	0.067519812	0	1	CRA004775;Yang2025_Silkie_Wenchang	PRJNA827465	0.838481271
ENSGALG00010020994	DHCR7	2	0.012633995	0.096265972	0.131240506	0.895585054	0	0.005330569	0	0.5	CRA004775;PRJNA827465	Yang2025_Silkie_Wenchang	0.974927462
ENSGALG00010020998	SYT17	2	0.009945985	0.133433038	0.074539147	0.940581393	0.007493871	1.239278954	19.3079172	0.5	PRJNA827465;Yang2025_Silkie_Wenchang	CRA004775	0.984559481
ENSGALG00010020998	SYT17	2	0.265048618	0.177038846	1.497121253	0.134361711	0	0.516022279	0	1	CRA004775;Yang2025_Silkie_Wenchang	PRJNA827465	0.61409585
ENSGALG00010020998	SYT17	2	0.106996158	0.270069587	0.39617996	0.691972274	0.091316722	2.306393383	56.642262	0.5	CRA004775;PRJNA827465	Yang2025_Silkie_Wenchang	0.920348122
ENSGALG00010020999	IFIH1	2	-0.141062792	0.165556441	-0.852052579	0.39418491	0.015494802	1.340005058	25.373416	0.5	PRJNA827465;Yang2025_Silkie_Wenchang	CRA004775	0.830486298
ENSGALG00010020999	IFIH1	2	-0.037079152	0.214544463	-0.172827152	0.862787137	0	0.840807785	0	0.5	CRA004775;Yang2025_Silkie_Wenchang	PRJNA827465	0.964099097
ENSGALG00010020999	IFIH1	2	-0.271668971	0.149449148	-1.817802068	0.06909439	0	0.037393439	0	1	CRA004775;PRJNA827465	Yang2025_Silkie_Wenchang	0.452678251
ENSGALG00010021000	NFKB2	2	0.017521105	0.091851221	0.19075528	0.848717326	0.008116821	1.880512661	46.82301157	0.5	PRJNA827465;Yang2025_Silkie_Wenchang	CRA004775	0.961364896
ENSGALG00010021000	NFKB2	2	-0.121631989	0.277199183	-0.438789131	0.660814337	1.126022417	5.250936948	80.9537797	0.5	CRA004775;Yang2025_Silkie_Wenchang	PRJNA827465	0.910392683
ENSGALG00010021000	NFKB2	2	-0.191325116	0.177581785	-1.077391557	0.281305375	0.04229228	2.577106621	61.19679365	1	CRA004775;PRJNA827465	Yang2025_Silkie_Wenchang	0.779040666
ENSGALG00010021002	SLC44A3	2	-0.339414491	0.316015707	-1.074043103	0.282803363	0.176233552	8.482852839	88.21151305	1	PRJNA827465;Yang2025_Silkie_Wenchang	CRA004775	0.779532565
ENSGALG00010021002	SLC44A3	2	-0.400746863	0.324356718	-1.235512758	0.216639731	0.145933863	2.94624811	66.05852723	0.5	CRA004775;Yang2025_Silkie_Wenchang	PRJNA827465	0.730172415
ENSGALG00010021002	SLC44A3	2	-0.02391217	0.131975721	-0.181186127	0.856221483	0	0.007404198	0	0.5	CRA004775;PRJNA827465	Yang2025_Silkie_Wenchang	0.963499334
ENSGALG00010021003	KAZN	2	0.055430059	0.073566238	0.753471435	0.45116667	0	0.147971154	0	1	PRJNA827465;Yang2025_Silkie_Wenchang	CRA004775	0.847847413
ENSGALG00010021003	KAZN	2	0.002506777	0.177965159	0.014085776	0.988761548	0.019716222	1.307180872	23.49949259	0.5	CRA004775;Yang2025_Silkie_Wenchang	PRJNA827465	0.996891127
ENSGALG00010021003	KAZN	2	0.01156484	0.093971551	0.123067454	0.902053683	0.002504515	1.044396203	4.250896663	0.5	CRA004775;PRJNA827465	Yang2025_Silkie_Wenchang	0.976893417
ENSGALG00010021004	PSD	2	0.357851515	0.222134403	1.610968445	0.1071866	0.07264561	3.697598959	72.95542294	1	PRJNA827465;Yang2025_Silkie_Wenchang	CRA004775	0.554473355
ENSGALG00010021004	PSD	2	0.433879309	0.242687051	1.787814004	0.073806021	0.050846829	1.608825028	37.84283667	1	CRA004775;Yang2025_Silkie_Wenchang	PRJNA827465	0.469352297
ENSGALG00010021004	PSD	2	0.147262518	0.124315094	1.184590816	0.23617927	0	0.035095217	0	1	CRA004775;PRJNA827465	Yang2025_Silkie_Wenchang	0.748669015
ENSGALG00010021010	LEO1	2	0.267392846	0.317632955	0.841829671	0.399883301	0.184848812	11.29179928	91.14401545	0.5	PRJNA827465;Yang2025_Silkie_Wenchang	CRA004775	0.832246851
ENSGALG00010021010	LEO1	2	0.226464156	0.380066734	0.595853664	0.55127299	0.258006787	9.349076214	89.30375604	0.5	CRA004775;Yang2025_Silkie_Wenchang	PRJNA827465	0.878879109
ENSGALG00010021010	LEO1	2	-0.041553483	0.056069624	-0.741105079	0.45862973	0	0.461472077	0	1	CRA004775;PRJNA827465	Yang2025_Silkie_Wenchang	0.850089963
ENSGALG00010021011	SCARB1	2	-0.164405945	0.068062885	-2.415500665	0.015713594	0.001935334	1.148673547	12.94306353	1	PRJNA827465;Yang2025_Silkie_Wenchang	CRA004775	0.207334357
ENSGALG00010021011	SCARB1	2	-0.130415692	0.22583129	-0.577491684	0.563607355	0.058961079	2.174507697	54.01257943	0.5	CRA004775;Yang2025_Silkie_Wenchang	PRJNA827465	0.882532874
ENSGALG00010021011	SCARB1	2	-0.110444227	0.075465345	-1.463509201	0.143328098	0.00233322	1.056937332	5.387011123	0.5	CRA004775;PRJNA827465	Yang2025_Silkie_Wenchang	0.630396624
ENSGALG00010021013	FAP	2	-0.208324607	0.23756558	-0.876914101	0.380533297	0.088473318	4.565767854	78.097879	0.5	PRJNA827465;Yang2025_Silkie_Wenchang	CRA004775	0.824392515
ENSGALG00010021013	FAP	2	-0.427695851	0.169206978	-2.527649013	0.011482908	0	0.362011288	0	1	CRA004775;Yang2025_Silkie_Wenchang	PRJNA827465	0.171081405
ENSGALG00010021013	FAP	2	0.008122527	0.130313854	0.062330491	0.950299647	0	0.05159542	0	0.5	CRA004775;PRJNA827465	Yang2025_Silkie_Wenchang	0.987277827
ENSGALG00010021014	FBXL15	2	-0.056409196	0.066218514	-0.851864424	0.394289344	0	0.79325121	0	1	PRJNA827465;Yang2025_Silkie_Wenchang	CRA004775	0.830486298
ENSGALG00010021014	FBXL15	2	-0.162493611	0.140420911	-1.157189553	0.247194925	0	0.100896852	0	1	CRA004775;Yang2025_Silkie_Wenchang	PRJNA827465	0.75766218
ENSGALG00010021014	FBXL15	2	-0.03349471	0.070231203	-0.47692064	0.633418634	0	0.036392771	0	1	CRA004775;PRJNA827465	Yang2025_Silkie_Wenchang	0.903343828
ENSGALG00010021015	RBM25	2	0.388830613	0.417795767	0.930671501	0.352023516	0.326278069	14.50714793	93.10684633	0.5	PRJNA827465;Yang2025_Silkie_Wenchang	CRA004775	0.810678834
ENSGALG00010021015	RBM25	2	0.500284829	0.339500371	1.473591404	0.140591637	0.18035753	4.589032884	78.20891623	1	CRA004775;Yang2025_Silkie_Wenchang	PRJNA827465	0.624864086
ENSGALG00010021015	RBM25	2	0.003133822	0.049712453	0.06303897	0.949735472	0	0.435405923	0	0.5	CRA004775;PRJNA827465	Yang2025_Silkie_Wenchang	0.98723759
ENSGALG00010021016	FHIT	2	-0.021830365	0.115850222	-0.188436105	0.850534799	0	0.163068774	0	0.5	PRJNA827465;Yang2025_Silkie_Wenchang	CRA004775	0.961985357
ENSGALG00010021016	FHIT	2	-0.03159117	0.175274287	-0.180238472	0.856965357	0	0.352837192	0	0.5	CRA004775;Yang2025_Silkie_Wenchang	PRJNA827465	0.963837729
ENSGALG00010021016	FHIT	2	0.031221273	0.133285324	0.234243895	0.81479564	0	0.145553829	0	1	CRA004775;PRJNA827465	Yang2025_Silkie_Wenchang	0.953885608
ENSGALG00010021017	COQ7	2	0.253840793	0.149668932	1.696015259	0.08988299	0.033575257	3.92342177	74.51204437	1	PRJNA827465;Yang2025_Silkie_Wenchang	CRA004775	0.514366798
ENSGALG00010021017	COQ7	2	0.388253849	0.111397022	3.48531624	0.000491556	0	0.294130046	0	1	CRA004775;Yang2025_Silkie_Wenchang	PRJNA827465	0.023248634
ENSGALG00010021017	COQ7	2	0.12866257	0.085324156	1.507926663	0.131573298	0	0.23210896	0	1	CRA004775;PRJNA827465	Yang2025_Silkie_Wenchang	0.607498118
ENSGALG00010021018	F3	2	-0.462404568	0.102730577	-4.501138607	6.75904E-06	0	0.675200468	0	1	PRJNA827465;Yang2025_Silkie_Wenchang	CRA004775	0.001025678
ENSGALG00010021018	F3	2	0.188690544	0.825073735	0.228695371	0.819105693	1.242941454	10.95163307	90.8689417	0.5	CRA004775;Yang2025_Silkie_Wenchang	PRJNA827465	0.955256039
ENSGALG00010021018	F3	2	0.26603435	0.73421744	0.36233728	0.717100003	0.970612596	9.299991756	89.2473023	0.5	CRA004775;PRJNA827465	Yang2025_Silkie_Wenchang	0.92775671
ENSGALG00010021019	VSIG10	2	0.079862003	0.111031003	0.719276606	0.471970507	0.012514465	1.662301013	39.84242371	0.5	PRJNA827465;Yang2025_Silkie_Wenchang	CRA004775	0.853998373
ENSGALG00010021019	VSIG10	2	-0.193019422	0.152564474	-1.265166241	0.205811741	0	0.785605822	0	1	CRA004775;Yang2025_Silkie_Wenchang	PRJNA827465	0.71832154
ENSGALG00010021019	VSIG10	2	-0.058945271	0.26407493	-0.223214188	0.82336881	0.107847212	3.669482867	72.74820359	0.5	CRA004775;PRJNA827465	Yang2025_Silkie_Wenchang	0.95592309
ENSGALG00010021020	ZDHHC13	2	-0.058003227	0.095826256	-0.605295762	0.544982497	0.00810697	1.731889653	42.25960075	0.5	PRJNA827465;Yang2025_Silkie_Wenchang	CRA004775	0.877354247
ENSGALG00010021020	ZDHHC13	2	-0.207141788	0.118158871	-1.7530786	0.079588516	0	0.89022269	0	1	CRA004775;Yang2025_Silkie_Wenchang	PRJNA827465	0.48542321
ENSGALG00010021020	ZDHHC13	2	-0.144922395	0.276238578	-0.524627646	0.599842064	0.096295879	1.953556931	48.8113203	0.5	CRA004775;PRJNA827465	Yang2025_Silkie_Wenchang	0.895092498
ENSGALG00010021021	GCG	2	-0.383950002	0.28888994	-1.329052865	0.183830529	0	0.082467847	0	1	PRJNA827465;Yang2025_Silkie_Wenchang	CRA004775	0.692470323
ENSGALG00010021021	GCG	2	0.365535291	0.916042057	0.399037673	0.689865445	1.338139118	4.879950691	79.50798966	0.5	CRA004775;Yang2025_Silkie_Wenchang	PRJNA827465	0.919979266
ENSGALG00010021021	GCG	2	0.409130396	0.823597883	0.496759892	0.619358382	1.094984046	4.922246507	79.68407314	0.5	CRA004775;PRJNA827465	Yang2025_Silkie_Wenchang	0.899181533
ENSGALG00010021022	ZFYVE1	2	-0.10423558	0.19760607	-0.527491792	0.597852115	0.069717036	8.969886198	88.85158654	0.5	PRJNA827465;Yang2025_Silkie_Wenchang	CRA004775	0.894645663
ENSGALG00010021022	ZFYVE1	2	-0.116679256	0.241215395	-0.48371397	0.628588877	0.0837414	3.266746531	69.3885035	0.5	CRA004775;Yang2025_Silkie_Wenchang	PRJNA827465	0.901974673
ENSGALG00010021022	ZFYVE1	2	0.085348028	0.056496986	1.51066516	0.130873783	0	0.143400663	0	1	CRA004775;PRJNA827465	Yang2025_Silkie_Wenchang	0.606627305
ENSGALG00010021023	MGEA5	2	0.082345327	0.135049602	0.609741351	0.542033158	0.028635817	4.394412217	77.24382806	0.5	PRJNA827465;Yang2025_Silkie_Wenchang	CRA004775	0.876685104
ENSGALG00010021023	MGEA5	2	0.266123428	0.101680241	2.617258028	0.008863931	0	0.33026265	0	1	CRA004775;Yang2025_Silkie_Wenchang	PRJNA827465	0.147555728
ENSGALG00010021023	MGEA5	2	0.11695662	0.200378232	0.583679272	0.559436116	0.059977088	3.292393284	69.62695785	0.5	CRA004775;PRJNA827465	Yang2025_Silkie_Wenchang	0.881086662
ENSGALG00010021024	ENSGALG00010021024	2	0.115062777	0.104056711	1.105769879	0.268826136	0.010951772	1.933481711	48.27983143	1	PRJNA827465;Yang2025_Silkie_Wenchang	CRA004775	0.771398294
ENSGALG00010021024	ENSGALG00010021024	2	-0.03817413	0.325535892	-0.117265502	0.906649661	0.169167631	4.592526258	78.22549194	0.5	CRA004775;Yang2025_Silkie_Wenchang	PRJNA827465	0.978196186
ENSGALG00010021024	ENSGALG00010021024	2	-0.111576485	0.207624901	-0.537394525	0.590995124	0.056602139	2.339411615	57.25420899	0.5	CRA004775;PRJNA827465	Yang2025_Silkie_Wenchang	0.892766566
ENSGALG00010021025	CUEDC2	2	0.135840776	0.06691721	2.029982653	0.042358303	0	0.444974295	0	1	PRJNA827465;Yang2025_Silkie_Wenchang	CRA004775	0.356238117
ENSGALG00010021025	CUEDC2	2	0.18947913	0.126883661	1.493329625	0.135350925	0	0.233693865	0	1	CRA004775;Yang2025_Silkie_Wenchang	PRJNA827465	0.616776509
ENSGALG00010021025	CUEDC2	2	0.108125214	0.074000841	1.461134935	0.143978415	0	0.032029905	0	1	CRA004775;PRJNA827465	Yang2025_Silkie_Wenchang	0.631067652
ENSGALG00010021026	ADGRA2	2	-0.040060584	0.054510552	-0.734914302	0.462391687	0	0.331772877	0	1	PRJNA827465;Yang2025_Silkie_Wenchang	CRA004775	0.850823289
ENSGALG00010021026	ADGRA2	2	-0.08727516	0.092738218	-0.941091617	0.346657911	0	0.006278641	0	1	CRA004775;Yang2025_Silkie_Wenchang	PRJNA827465	0.808232887
ENSGALG00010021026	ADGRA2	2	-0.021295626	0.065288536	-0.326177117	0.744290353	0	0.066222243	0	1	CRA004775;PRJNA827465	Yang2025_Silkie_Wenchang	0.935821071
ENSGALG00010021027	PLA2G4A	2	0.179462405	0.265935179	0.674835145	0.499780509	0.13182014	14.64831806	93.17327767	0.5	PRJNA827465;Yang2025_Silkie_Wenchang	CRA004775	0.862078151
ENSGALG00010021027	PLA2G4A	2	0.242863713	0.322820822	0.752317376	0.451860221	0.136139941	2.082546574	51.98186621	0.5	CRA004775;Yang2025_Silkie_Wenchang	PRJNA827465	0.848106301
ENSGALG00010021027	PLA2G4A	2	-0.099630058	0.106511651	-0.935391168	0.349586747	0	0.143704722	0	1	CRA004775;PRJNA827465	Yang2025_Silkie_Wenchang	0.809903518
ENSGALG00010021028	ENSGALG00010021028	2	0.466068483	0.531083907	0.877579751	0.380171822	0.465507183	5.689488812	82.42372851	0.5	PRJNA827465;Yang2025_Silkie_Wenchang	CRA004775	0.824392515
ENSGALG00010021028	ENSGALG00010021028	2	0.192405507	0.899965281	0.213792144	0.830709152	1.369175713	6.195392327	83.85897217	0.5	CRA004775;Yang2025_Silkie_Wenchang	PRJNA827465	0.95733143
ENSGALG00010021028	ENSGALG00010021028	2	-0.198391154	0.299597356	-0.662192606	0.507847792	0.032911913	1.134862724	11.88361565	1	CRA004775;PRJNA827465	Yang2025_Silkie_Wenchang	0.864401731
ENSGALG00010021029	PRDM2	2	0.24060821	0.219472147	1.096304079	0.272945725	0.075464638	3.872841459	74.17916508	1	PRJNA827465;Yang2025_Silkie_Wenchang	CRA004775	0.773799118
ENSGALG00010021029	PRDM2	2	0.400288204	0.148786606	2.690351067	0.007137689	0	0.503490147	0	1	CRA004775;Yang2025_Silkie_Wenchang	PRJNA827465	0.129996561
ENSGALG00010021029	PRDM2	2	0.108657566	0.090572912	1.199669561	0.230267699	0.007100567	1.332156415	24.93373983	1	CRA004775;PRJNA827465	Yang2025_Silkie_Wenchang	0.742860818
ENSGALG00010021030	CSRP3	2	0.312742449	0.762541749	0.410131576	0.681709431	0.984153569	6.365656808	84.29070196	0.5	PRJNA827465;Yang2025_Silkie_Wenchang	CRA004775	0.918368413
ENSGALG00010021030	CSRP3	2	0.161356608	1.018951035	0.158355605	0.874176594	1.709766914	5.580769584	82.08132436	0.5	CRA004775;Yang2025_Silkie_Wenchang	PRJNA827465	0.970092404
ENSGALG00010021030	CSRP3	2	-0.497231995	0.299569399	-1.659822388	0.096950188	0	0.438975619	0	1	CRA004775;PRJNA827465	Yang2025_Silkie_Wenchang	0.532111212
ENSGALG00010021031	DDX54	2	0.00576266	0.062939862	0.09155819	0.927049072	0.000153932	1.016170488	1.591316397	0.5	PRJNA827465;Yang2025_Silkie_Wenchang	CRA004775	0.982928581
ENSGALG00010021031	DDX54	2	-0.109782802	0.235338983	-0.466487958	0.640866265	0.087835611	4.679524957	78.6303095	0.5	CRA004775;Yang2025_Silkie_Wenchang	PRJNA827465	0.906429868
ENSGALG00010021031	DDX54	2	-0.157962017	0.161142719	-0.980261582	0.326957013	0.03587967	2.816083623	64.48969086	1	CRA004775;PRJNA827465	Yang2025_Silkie_Wenchang	0.800028239
ENSGALG00010021032	TMC7	2	-0.292157465	0.128205844	-2.27881551	0.022678035	0.023589002	3.408513647	70.6616988	1	PRJNA827465;Yang2025_Silkie_Wenchang	CRA004775	0.256272223
ENSGALG00010021032	TMC7	2	-0.409899417	0.1138707	-3.599691739	0.000318595	0	0.789678744	0	1	CRA004775;Yang2025_Silkie_Wenchang	PRJNA827465	0.017636233
ENSGALG00010021032	TMC7	2	-0.174785591	0.074196441	-2.355713942	0.018487148	0	0.104008476	0	1	CRA004775;PRJNA827465	Yang2025_Silkie_Wenchang	0.227601507
ENSGALG00010021033	RNF122	2	-0.153958871	0.308432416	-0.499165662	0.617662683	0.151977607	4.428415118	77.41855781	0.5	PRJNA827465;Yang2025_Silkie_Wenchang	CRA004775	0.899121942
ENSGALG00010021033	RNF122	2	-0.4242818	0.221515174	-1.915362246	0.055446322	0	0.322940161	0	1	CRA004775;Yang2025_Silkie_Wenchang	PRJNA827465	0.407862026
ENSGALG00010021033	RNF122	2	0.07846625	0.095825632	0.818844061	0.412875389	0	0.961362495	0	0.5	CRA004775;PRJNA827465	Yang2025_Silkie_Wenchang	0.835870654
ENSGALG00010021034	NPM3	2	0.375155818	0.355099368	1.056481233	0.29074841	0.215515843	6.501213693	84.61825672	1	PRJNA827465;Yang2025_Silkie_Wenchang	CRA004775	0.784646441
ENSGALG00010021034	NPM3	2	0.189298558	0.619404029	0.305614024	0.759898542	0.653417106	6.557656694	84.75065032	0.5	CRA004775;Yang2025_Silkie_Wenchang	PRJNA827465	0.93917677
ENSGALG00010021034	NPM3	2	-0.061904895	0.21833299	-0.283534316	0.77676728	0.047086125	1.509958754	33.77302542	0.5	CRA004775;PRJNA827465	Yang2025_Silkie_Wenchang	0.944156005
ENSGALG00010021036	E2F8	2	-0.107515288	0.130384519	-0.824601639	0.40959779	0.017088383	1.861384569	46.27655044	0.5	PRJNA827465;Yang2025_Silkie_Wenchang	CRA004775	0.834547535
ENSGALG00010021036	E2F8	2	0.048179759	0.163343358	0.294960015	0.768024425	0	0.095680994	0	0.5	CRA004775;Yang2025_Silkie_Wenchang	PRJNA827465	0.941444269
ENSGALG00010021036	E2F8	2	-0.200557477	0.095215208	-2.1063597	0.035173119	0	0.055777309	0	1	CRA004775;PRJNA827465	Yang2025_Silkie_Wenchang	0.32344621
ENSGALG00010021038	PTGS2	2	0.525000921	0.672434322	0.780746764	0.43495145	0.87040346	26.65098252	96.24779312	0.5	PRJNA827465;Yang2025_Silkie_Wenchang	CRA004775	0.842762754

ENSGALG00010021038	PTGS2	2	1.147920171	0.17303608	6.633993176	3.26725E-11	0	0.675963348	0	1	CRA004775;Yang2025_Silkie_Wenchang	PRJNA827465	2.46948E-08
ENSGALG00010021038	PTGS2	2	0.15547192	0.419782851	0.370362723	0.711112244	0.23381243	2.486831471	59.78818783	0.5	CRA004775;PRJNA827465	Yang2025_Silkie_Wenchang	0.925588272
ENSGALG00010021041	GLTP	2	0.236588874	0.259075263	0.913205189	0.361134636	0.084149635	2.494165657	59.90643215	0.5	PRJNA827465;Yang2025_Silkie_Wenchang	CRA004775	0.815240214
ENSGALG00010021041	GLTP	2	-0.25601226	0.227695853	-1.124360662	0.260860053	0	0.86654793	0	1	CRA004775;Yang2025_Silkie_Wenchang	PRJNA827465	0.76792161
ENSGALG00010021041	GLTP	2	0.018125317	0.478638956	0.037868454	0.969792565	0.385261659	5.891541113	83.02651241	0.5	CRA004775;PRJNA827465	Yang2025_Silkie_Wenchang	0.991572914
ENSGALG00010021042	LRRC38	2	-0.302141612	0.071033667	-4.253498712	2.10456E-05	0.001478036	1.169478061	14.49176916	1	PRJNA827465;Yang2025_Silkie_Wenchang	CRA004775	0.00255295
ENSGALG00010021042	LRRC38	2	-0.361110547	0.094389302	-3.825757144	0.000130371	0	0.45517645	0	1	CRA004775;Yang2025_Silkie_Wenchang	PRJNA827465	0.009419048
ENSGALG00010021042	LRRC38	2	-0.232288822	0.084458329	-2.750336463	0.00595341	0	0.061727089	0	1	CRA004775;PRJNA827465	Yang2025_Silkie_Wenchang	0.116701682
ENSGALG00010021043	PTPRG	2	-0.049253947	0.093686311	-0.525732594	0.599074014	0.006976052	1.495726732	33.1428677	0.5	PRJNA827465;Yang2025_Silkie_Wenchang	CRA004775	0.894908299
ENSGALG00010021043	PTPRG	2	0.138094799	0.134160229	1.029327395	0.303325854	0	0.397004995	0	1	CRA004775;Yang2025_Silkie_Wenchang	PRJNA827465	0.792084776
ENSGALG00010021043	PTPRG	2	-0.001956391	0.181975067	-0.010750874	0.991422209	0.039301291	1.843606473	45.75848944	0.5	CRA004775;PRJNA827465	Yang2025_Silkie_Wenchang	0.997563866
ENSGALG00010021044	EIF4G2	2	-0.124428437	0.098348436	-1.265179627	0.205806944	0.016988556	7.689783821	86.99573326	1	PRJNA827465;Yang2025_Silkie_Wenchang	CRA004775	0.71832154
ENSGALG00010021044	EIF4G2	2	-0.070152344	0.230854421	-0.303881312	0.761218316	0.07532111	2.678447923	62.6649452	0.5	CRA004775;Yang2025_Silkie_Wenchang	PRJNA827465	0.939618592
ENSGALG00010021044	EIF4G2	2	-0.032980124	0.025360157	-1.300470008	0.193439929	0	0.996387664	0	0.5	CRA004775;PRJNA827465	Yang2025_Silkie_Wenchang	0.703373997
ENSGALG00010021045	DPP4	2	0.138852394	0.247358545	0.561340601	0.574565368	0.106999651	7.821032188	87.21396389	0.5	PRJNA827465;Yang2025_Silkie_Wenchang	CRA004775	0.886130225
ENSGALG00010021045	DPP4	2	0.424891425	0.142425716	2.983249342	0.002852055	0	0.415331571	0	1	CRA004775;Yang2025_Silkie_Wenchang	PRJNA827465	0.072978082
ENSGALG00010021045	DPP4	2	0.169879414	0.393289678	0.431944757	0.665781565	0.216044736	2.511854182	60.18877181	0.5	CRA004775;PRJNA827465	Yang2025_Silkie_Wenchang	0.913532078
ENSGALG00010021046	ENSGALG00010021046	2	0.217453106	0.125999317	1.725827655	0.084378423	0.005703756	1.207660039	17.19523974	1	PRJNA827465;Yang2025_Silkie_Wenchang	CRA004775	0.500017819
ENSGALG00010021046	ENSGALG00010021046	2	-0.00665347	0.186100369	-0.035752053	0.971480065	0.005024047	1.031580662	3.061385639	0.5	CRA004775;Yang2025_Silkie_Wenchang	PRJNA827465	0.991955348
ENSGALG00010021046	ENSGALG00010021046	2	0.062852239	0.383800879	0.163762625	0.869918016	0.192616918	2.265337475	55.85646681	0.5	CRA004775;PRJNA827465	Yang2025_Silkie_Wenchang	0.968621105
ENSGALG00010021047	TMC5	2	0.212107292	0.338540878	0.626533767	0.530964896	0.004526936	1.020142903	1.974517791	0.5	PRJNA827465;Yang2025_Silkie_Wenchang	CRA004775	0.872840748
ENSGALG00010021047	TMC5	2	0.760620532	0.387056037	1.965143184	0.049397668	0	0.644574117	0	1	CRA004775;Yang2025_Silkie_Wenchang	PRJNA827465	0.384595411
ENSGALG00010021047	TMC5	2	0.451703875	0.664980907	0.67927345	0.496964616	0.554116596	2.603115154	61.58448855	0.5	CRA004775;PRJNA827465	Yang2025_Silkie_Wenchang	0.860939846
ENSGALG00010021048	API5	2	-0.264629807	0.273645016	-0.967055095	0.333516492	0.145221055	32.34555907	96.90838548	0.5	PRJNA827465;Yang2025_Silkie_Wenchang	CRA004775	0.802691373
ENSGALG00010021048	API5	2	-0.29658629	0.265493729	-1.117112224	0.263946344	0.123922257	8.013659795	87.52130705	1	CRA004775;Yang2025_Silkie_Wenchang	PRJNA827465	0.768643821
ENSGALG00010021048	API5	2	0.001844007	0.03021298	0.061033595	0.951332454	0	0.00838017	0	0.5	CRA004775;PRJNA827465	Yang2025_Silkie_Wenchang	0.987695991
ENSGALG00010021050	CTR9	2	0.127367156	0.143389415	0.88826052	0.374400631	0.035289476	6.886936543	85.47975586	0.5	PRJNA827465;Yang2025_Silkie_Wenchang	CRA004775	0.822805106
ENSGALG00010021050	CTR9	2	0.192184448	0.143145092	1.34258496	0.179406403	0.019625538	1.615069442	38.08315765	0.5	CRA004775;Yang2025_Silkie_Wenchang	PRJNA827465	0.686254155
ENSGALG00010021050	CTR9	2	-0.009233687	0.056599087	-0.163141972	0.870406654	0	0.019363735	0	1	CRA004775;PRJNA827465	Yang2025_Silkie_Wenchang	0.968890696
ENSGALG00010021051	GDE1	2	0.258250988	0.236342118	1.092699814	0.274525621	0.099848145	9.358832059	89.31490603	1	PRJNA827465;Yang2025_Silkie_Wenchang	CRA004775	0.774528691
ENSGALG00010021051	GDE1	2	0.233167373	0.326365504	0.714436328	0.474957415	0.16330861	3.807193136	73.73393037	0.5	CRA004775;Yang2025_Silkie_Wenchang	PRJNA827465	0.854462864
ENSGALG00010021051	GDE1	2	0.012393136	0.090869451	0.136383966	0.891517749	0	0.336267213	0	0.5	CRA004775;PRJNA827465	Yang2025_Silkie_Wenchang	0.973846899
ENSGALG00010021052	CEP15	2	-0.274550143	0.260733966	-1.052989553	0.29234578	0.114468642	6.189585037	83.84382808	1	PRJNA827465;Yang2025_Silkie_Wenchang	CRA004775	0.785753005
ENSGALG00010021052	CEP15	2	-0.384523629	0.224740648	-1.710966092	0.087087372	0.047937273	1.774979638	43.66132553	1	CRA004775;Yang2025_Silkie_Wenchang	PRJNA827465	0.507587624
ENSGALG00010021052	CEP15	2	-0.036791644	0.107865917	-0.341086836	0.733038211	0	0.027399658	0	1	CRA004775;PRJNA827465	Yang2025_Silkie_Wenchang	0.932985777
ENSGALG00010021055	IRAG1	2	0.396974326	0.252268732	1.573616843	0.115576057	0.106843743	6.108345167	83.62895395	1	PRJNA827465;Yang2025_Silkie_Wenchang	CRA004775	0.572716099
ENSGALG00010021055	IRAG1	2	0.656747331	0.154925588	4.239114647	2.24403E-05	0	0.015103869	0	1	CRA004775;Yang2025_Silkie_Wenchang	PRJNA827465	0.002680764
ENSGALG00010021055	IRAG1	2	0.241201994	0.174265717	1.384104681	0.166326404	0.023924344	1.301971412	23.19339807	1	CRA004775;PRJNA827465	Yang2025_Silkie_Wenchang	0.665303687
ENSGALG00010021057	ZDHHC8	2	0.099758418	0.216909121	0.459908822	0.645581667	0.083705814	8.518533343	88.26088999	0.5	PRJNA827465;Yang2025_Silkie_Wenchang	CRA004775	0.907052464
ENSGALG00010021057	ZDHHC8	2	0.305707676	0.129622554	2.358445086	0.018351675	0	0.355875571	0	1	CRA004775;Yang2025_Silkie_Wenchang	PRJNA827465	0.22688766
ENSGALG00010021057	ZDHHC8	2	-0.094050006	0.051911568	-1.81173502	0.07002715	0	0.362622642	0	0.5	CRA004775;PRJNA827465	Yang2025_Silkie_Wenchang	0.45646282
ENSGALG00010021058	CCP110	2	0.078319542	0.087952545	0.890474994	0.37321089	0.008476599	1.847997256	45.88736553	1	PRJNA827465;Yang2025_Silkie_Wenchang	CRA004775	0.822187352
ENSGALG00010021058	CCP110	2	0.233218037	0.122041584	1.910971894	0.056008193	0	0.157851644	0	1	CRA004775;Yang2025_Silkie_Wenchang	PRJNA827465	0.409427048
ENSGALG00010021058	CCP110	2	0.034848787	0.066225529	0.52621379	0.598739674	0.002028871	1.038825364	3.73742931	1	CRA004775;PRJNA827465	Yang2025_Silkie_Wenchang	0.894762943
ENSGALG00010021062	MYO9A	2	0.040848909	0.060771832	0.672168456	0.50147646	0	0.406061101	0	1	PRJNA827465;Yang2025_Silkie_Wenchang	CRA004775	0.862674719
ENSGALG00010021062	MYO9A	2	0.122207247	0.138025781	0.885394358	0.375943974	0	0.031225274	0	1	CRA004775;Yang2025_Silkie_Wenchang	PRJNA827465	0.822974013
ENSGALG00010021062	MYO9A	2	0.02897724	0.063403716	0.457027471	0.647651301	0	0.04927849	0	1	CRA004775;PRJNA827465	Yang2025_Silkie_Wenchang	0.907376718
ENSGALG00010021064	LYVE1	2	0.136243589	0.181413304	0.751012112	0.452645367	0	0.098785038	0	1	PRJNA827465;Yang2025_Silkie_Wenchang	CRA004775	0.848370093
ENSGALG00010021064	LYVE1	2	0.161153111	0.271257535	0.594096347	0.552447674	0	0.36919904	0	1	CRA004775;Yang2025_Silkie_Wenchang	PRJNA827465	0.879228216
ENSGALG00010021064	LYVE1	2	0.213273524	0.19926581	1.070296623	0.284485814	0	0.190503067	0	1	CRA004775;PRJNA827465	Yang2025_Silkie_Wenchang	0.780265906
ENSGALG00010021065	CNNM2	2	0.064075029	0.063771501	1.004759622	0.315012611	0	0.064359117	0	1	PRJNA827465;Yang2025_Silkie_Wenchang	CRA004775	0.796611803
ENSGALG00010021065	CNNM2	2	-0.055083306	0.117523333	-0.468701025	0.639283354	0	0.812825135	0	0.5	CRA004775;Yang2025_Silkie_Wenchang	PRJNA827465	0.905968982
ENSGALG00010021065	CNNM2	2	0.000750023	0.115613682	0.006487317	0.994823906	0.013434978	1.664567121	39.92432102	0.5	CRA004775;PRJNA827465	Yang2025_Silkie_Wenchang	0.998242335
ENSGALG00010021069	PGAM5	2	-0.055256793	0.045898459	-1.20389213	0.228631274	0	0.670487978	0	1	PRJNA827465;Yang2025_Silkie_Wenchang	CRA004775	0.741349308
ENSGALG00010021069	PGAM5	2	-0.187386004	0.105653359	-1.773592495	0.07613058	0	0.368830483	0	1	CRA004775;Yang2025_Silkie_Wenchang	PRJNA827465	0.476155344
ENSGALG00010021069	PGAM5	2	-0.089101621	0.100208438	-0.889162851	0.373915565	0.009989855	1.447831146	30.93117225	1	CRA004775;PRJNA827465	Yang2025_Silkie_Wenchang	0.822579781
ENSGALG00010021070	RNF141	2	-0.147832629	0.081099711	-1.822850254	0.068326074	0.008873952	2.999643254	66.66270235	1	PRJNA827465;Yang2025_Silkie_Wenchang	CRA004775	0.450487609
ENSGALG00010021070	RNF141	2	-0.118153406	0.153754625	-0.768454317	0.442217321	0.032187371	2.785085213	64.09445587	0.5	CRA004775;Yang2025_Silkie_Wenchang	PRJNA827465	0.845369538
ENSGALG00010021070	RNF141	2	-0.061258472	0.052160344	-1.174426158	0.240224373	0	0.712729355	0	0.5	CRA004775;PRJNA827465	Yang2025_Silkie_Wenchang	0.752613674
ENSGALG00010021071	HIF1AN	2	-0.232228587	0.16827881	-1.380022752	0.16757964	0.050080074	8.408648476	88.1074824	1	PRJNA827465;Yang2025_Silkie_Wenchang	CRA004775	0.667448579
ENSGALG00010021071	HIF1AN	2	-0.385995731	0.096089765	-4.017032747	5.89355E-05	0	0.665187204	0	1	CRA004775;Yang2025_Silkie_Wenchang	PRJNA827465	0.005412017
ENSGALG00010021071	HIF1AN	2	-0.07587454	0.056959165	-1.332086603	0.182831732	0	0.041648991	0	1	CRA004775;PRJNA827465	Yang2025_Silkie_Wenchang	0.691266551
ENSGALG00010021072	ENSGALG00010021072	2	-0.401082098	0.298533969	-1.34350573	0.179108272	0.173177142	34.93286483	97.13736619	1	PRJNA827465;Yang2025_Silkie_Wenchang	CRA004775	0.685776173
ENSGALG00010021072	ENSGALG00010021072	2	-0.080573041	0.627480529	-0.128407237	0.897826704	0.774601849	61.06013857	98.36227034	0.5	CRA004775;Yang2025_Silkie_Wenchang	PRJNA827465	0.975696709
ENSGALG00010021072	ENSGALG00010021072	2	0.210346478	0.328717412	0.639900628	0.522237205	0.206013258	20.85151205	95.20418472	0.5	CRA004775;PRJNA827465	Yang2025_Silkie_Wenchang	0.869568211
ENSGALG00010021073	NDUFB8	2	0.10522455	0.157981779	0.666054981	0.505375958	0.034393336	2.795144257	64.22367119	0.5	PRJNA827465;Yang2025_Silkie_Wenchang	CRA004775	0.863862536
ENSGALG00010021073	NDUFB8	2	0.135197671	0.246337879	0.548830214	0.583121974	0.058650597	1.763583006	43.29725357	0.5	CRA004775;Yang2025_Silkie_Wenchang	PRJNA827465	0.890299155
ENSGALG00010021073	NDUFB8	2	-0.023772947	0.07063703	-0.336550767	0.736455575	0	0.297016999	0	1	CRA004775;PRJNA827465	Yang2025_Silkie_Wenchang	0.933661344
ENSGALG00010021074	TTC17	2	0.010212932	0.036116289	0.282779109	0.77734617	0	0.289607047	0	1	PRJNA827465;Yang2025_Silkie_Wenchang	CRA004775	0.944260471
ENSGALG00010021074	TTC17	2	0.054833544	0.089175185	0.61489689	0.538622817	0	0.010438624	0	1	CRA004775;Yang2025_Silkie_Wenchang	PRJNA827465	0.87481821
ENSGALG00010021074	TTC17	2	0.003358132	0.038325127	0.087622193	0.930176963	0	0.019135619	0	1	CRA004775;PRJNA827465	Yang2025_Silkie_Wenchang	0.983813577
ENSGALG00010021075	DHRS3	2	-0.095994999	0.181201738	-0.529768645	0.596272347	0.051201718	4.452729859	77.54186686	0.5	PRJNA827465;Yang2025_Silkie_Wenchang	CRA004775	0.894064255
ENSGALG00010021075	DHRS3	2	-0.274203927	0.125355225	-2.187415211	0.028712227	0	0.071748381	0	1	CRA004775;Yang2025_Silkie_Wenchang	PRJNA827465	0.288695306
ENSGALG00010021075	DHRS3	2	0.040345749	0.093821673	0.430025895	0.667176805	0	0.898784017	0	0.5	CRA004775;PRJNA827465	Yang2025_Silkie_Wenchang	0.913783154
ENSGALG00010021077	SEC31B	2	0.128461773	0.241093189	0.53283037	0.594151009	0.097258882	5.760532257	82.64049301	0.5	PRJNA827465;Yang2025_Silkie_Wenchang	CRA004775	0.893274007
ENSGALG00010021077	SEC31B	2	0.099060997	0.307676193	0.321965103	0.747479131	0.150502346	4.860054287	79.42409815	0.5	CRA004775;Yang2025_Silkie_Wenchang	PRJNA827465	0.93680487
ENSGALG00010021077	SEC31B	2	-0.105947781	0.079772762	-1.328119753	0.184138549	0	0.327502037	0	1	CRA004775;PRJNA827465	Yang2025_Silkie_Wenchang	0.692545576
ENSGALG00010021078	ENSGALG00010021078	2	-0.777814076	0.689708229	-1.127743651	0.259428197	0.859019859	10.29872065	90.29005608	1	PRJNA827465;Yang2025_Silkie_Wenchang	CRA004775	0.766754882
ENSGALG00010021078	ENSGALG00010021078	2	-0.214457312	0.259361805	-0.826865434	0.408313337	0	0.680838615	0	1	CRA004775;Yang2025_Silkie_Wenchang	PRJNA827465	0.834134074
ENSGALG00010021078	ENSGALG00010021078	2	-1.121619529	0.433182104	-2.589256389	0.009618345	0.213787944	2.187662103	54.28910166	1	CRA004775;PRJNA827465	Yang2025_Silkie_Wenchang	0.154613423
ENSGALG00010021080	TTLL7	2	0.301448345	0.351650858	0.85723762	0.391313576	0.223482906	10.0718114	90.07129939	0.5	PRJNA827465;Yang2025_Silkie_Wenchang	CRA004775	0.829251057
ENSGALG00010021080	TTLL7	2	0.620917116	0.177359546	3.500894823	0.000463699	0	0.38259009	0	1	CRA004775;Yang2025_Silkie_Wenchang	PRJNA827465	0.022279651
ENSGALG00010021080	TTLL7	2	-0.004158116	0.102760584	-0.040464113	0.967723118	0.000731092	1.008216053	0.814909933	0.5	CRA004775;PRJNA827465	Yang2025_Silkie_Wenchang	0.991065147
ENSGALG00010021081	KCNH6	2	0.189016869	0.22332511	0.846375356	0.397343382	0.072591463	3.249382445	69.22492144	1	PRJNA827465;Yang2025_Silkie_Wenchang	CRA004775	0.8315648
ENSGALG00010021081	KCNH6	2	0.405899745	0.214413675	1.893068364	0.058348779	0	0.415225902	0	1	CRA004775;Yang2025_Silkie_Wenchang	PRJNA827465	0.418650753
ENSGALG00010021081	KCNH6	2	0.013575527	0.093882432	0.144601358	0.885025625	0	0.029522266	0	1	CRA004775;PRJNA827465	Yang2025_Silkie_Wenchang	0.972194923
ENSGALG00010021082	SENP8	2	-0.01919605	0.080431849	-0.238662302	0.81136745	0.002451874	1.212380327	17.51763222	0.5	PRJNA827465;Yang2025_Silkie_Wenchang	CRA004775	0.953196081
ENSGALG00010021082	SENP8	2	0.075146489	0.116612445	0.644412258	0.519308138	0	0.084495661	0	0.5	CRA004775;Yang2025_Silkie_Wenchang	PRJNA827465	0.869086345
ENSGALG00010021082	SENP8	2	-0.076033207	0.084971133	-0.894812201	0.370887487	0	0.03060311	0	1	CRA004775;PRJNA827465	Yang2025_Silkie_Wenchang	0.8214684
ENSGALG00010021083	SCD	2	0.954191105	0.219476417	4.347579201	1.37648E-05	0.003228576	1.034673788	3.351180642	1	PRJNA827465;Yang2025_Silkie_Wenchang	CRA004775	0.001827701
ENSGALG00010021083	SCD	2	0.737545806	0.268303253	2.748926066	0.005979087	0	0.000651134	0	1	CRA004775;Yang2025_Silkie_Wenchang	PRJNA827465	0.116888359
ENSGALG00010021083	SCD	2	1.076099015	0.267416972	4.024049056	5.7206E-05	0	0.440685445	0	1	CRA004775;PRJNA827465	Yang2025_Silkie_Wenchang	0.005336024
ENSGALG00010021085	AMPD3	2	-0.118618066	0.088696368	-1.33734975	0.181108499	0	0.019258117	0	1	PRJNA827465;Yang2025_Silkie_Wenchang	CRA004775	0.688594651
ENSGALG00010021085	AMPD3	2	-0.120099825	0.141333804	-0.84976008	0.395458488	0	0.135738289	0	0.5	CRA004775;Yang2025_Silkie_Wenchang	PRJNA827465	0.830946177
ENSGALG00010021085	AMPD3	2	-0.101674183	0.108781281	-0.93466617	0.349960366	0	0.106368166	0	0.5	CRA004775;PRJNA827465	Yang2025_Silkie_Wenchang	0.809993068
ENSGALG00010021086	PSMD14	2	-0.022617872	0.054566904	-0.414497982	0.678509445	0	0.064090616	0	1	PRJNA827465;Yang2025_Silkie_Wenchang	CRA004775	0.917701868
ENSGALG00010021086	PSMD14	2	-0.079392837	0.101483771	-0.782320522	0.434026229	0	0.570527484	0	1	CRA004775;Yang2025_Silkie_Wenchang	PRJNA827465	0.842762754
ENSGALG00010021086	PSMD14	2	-0.028288724	0.06104173	-0.463432538	0.643054361	0	0.842193782	0	1	CRA004775;PRJNA827465	Yang2025_Silkie_Wenchang	0.906634523
ENSGALG00010021087	THBS1	2	0.40439952	0.25608189	1.579180472	0.114294668	0	0.068754168	0	1	PRJNA827465;Yang2025_Silkie_Wenchang	CRA004775	0.57019364
ENSGALG00010021087	THBS1	2	0.469308647	0.300895153	1.559708232	0.118828846	0	0.003114832	0	1	CRA004775;Yang2025_Silkie_Wenchang	PRJNA827465	0.579158002
ENSGALG00010021087	THBS1	2	0.382790899	0.271572121	1.409536802	0.158676499	0	0.031983975	0	1	CRA004775;PRJNA827465	Yang2025_Silkie_Wenchang	0.65275931
ENSGALG00010021088	ANKLE2	2	0.025071775	0.040738013	0.615439313	0.538264635	0.000379493	1.048194398	4.597849194	1	PRJNA827465;Yang2025_Silkie_Wenchang	CRA004775	0.87481821
ENSGALG00010021088	ANKLE2	2	0.171956768	0.102044252	1.685119594	0.0919655	0	0.28290387	0	1	CRA004775;Yang2025_Silkie_Wenchang	PRJNA827465	0.519402219
ENSGALG00010021088	ANKLE2	2	0.061865778	0.101210253	0.611259986	0.541027477	0.011389325	1.567757936	36.21464279	1	CRA004775;PRJNA827465	Yang2025_Silkie_Wenchang	0.875829389
ENSGALG00010021090	ADM	2	-0.132129288	0.348380894	-0.379266747	0.70448979	0.195070892	5.04552599	80.18046083	0.5	PRJNA827465;Yang2025_Silkie_Wenchang	CRA004775	0.924152671
ENSGALG00010021090	ADM	2	0.307356988	0.216749633	1.418027724	0.156182671	0	0.428382758	0	1	CRA004775;Yang2025_Silkie_Wenchang	PRJNA827465	0.649132948
ENSGALG00010021090	ADM	2	-0.030157968	0.514230537	-0.058646785	0.953233446	0.416277147	4.254915779	76.49777218	0.5	CRA004775;PRJNA827465	Yang2025_Silkie_Wenchang	0.987758913
ENSGALG00010021091	TANK	2	-0.095299289	0.087251498	-1.092236707	0.274729072	0.009292886	2.433812909	58.91220742	1	PRJNA827465;Yang2025_Silkie_Wenchang	CRA004775	0.774706274
ENSGALG00010021091	TANK	2	-0.169875141	0.084806187	-2.003098443	0.045166723	0	0.425115551	0	1	CRA004775;Yang2025_Silkie_Wenchang	PRJNA827465	0.366320206
ENSGALG00010021091	TANK	2	-0.028010749	0.056306831	-0.497466263	0.618860288	0	0.066611663	0	1	CRA004775;PRJNA827465	Yang2025_Silkie_Wenchang	0.899168321
ENSGALG00010021092	VPS13D	2	-0.143507107	0.063078678	-2.275049358	0.022902969	0	0.637602815	0	1	PRJNA827465;Yang2025_Silkie_Wenchang	CRA004775	0.258146084
ENSGALG00010021092	VPS13D	2	0.067084794	0.307737501	0.21799356	0.827434129	0.165911367	7.926871684	87.38468289	0.5	CRA004775;Yang2025_Silkie_Wenchang	PRJNA827465	0.956550752
ENSGALG00010021092	VPS13D	2	0.111165555	0.249998548	0.444664802	0.65656201	0.107153145	6.530739615	84.68779864	0.5	CRA004775;PRJNA827465	Yang2025_Silkie_Wenchang	0.910509456
ENSGALG00010021093	GRAMD2	2	0.090453683	0.121050295	0.747240501	0.454918401	0	0.018257088	0	1	PRJNA827465;Yang2025_Silkie_Wenchang	CRA004775	0.849072665
ENSGALG00010021093	GRAMD2	2	0.182075653	0.269957978	0.674459239	0.500019391	0.019698958	1.083291724	7.688762146	1	CRA004775;Yang2025_Silkie_Wenchang	PRJNA827465	0.862130386
ENSGALG00010021093	GRAMD2	2	0.151436852	0.180818305	0.837508415	0.402306849	0.016787973	1.079304214	7.347716543	1	CRA004775;PRJNA827465	Yang2025_Silkie_Wenchang	0.832879175
ENSGALG00010021094	ENSGALG00010021094	2	-0.094607623	0.349636401	-0.270588597	0.786707467	0.194758483	4.909551855	79.63154215	0.5	PRJNA827465;Yang2025_Silkie_Wenchang	CRA004775	0.946722706
ENSGALG00010021094	ENSGALG00010021094	2	0.393651902	0.211796625	1.858631606	0.063079374	0.007980357	1.077290547	7.174531274	1	CRA004775;Yang2025_Silkie_Wenchang	PRJNA827465	0.432579363
ENSGALG00010021094	ENSGALG00010021094	2	0.102046844	0.583745466	0.174813939	0.861225836	0.58694457	6.944513261	85.6001427	0.5	CRA004775;PRJNA827465	Yang2025_Silkie_Wenchang	0.965343309
ENSGALG00010021095	NT5C2	2	-0.036346112	0.171604473	-0.21180166	0.832261774	0.047847929	5.089157279	80.35038131	0.5	PRJNA827465;Yang2025_Silkie_Wenchang	CRA004775	0.957808251
ENSGALG00010021095	NT5C2	2	0.140759842	0.103497894	1.360026145	0.17382165	0	0.025282406	0	1	CRA004775;Yang2025_Silkie_Wenchang	PRJNA827465	0.677480397
ENSGALG00010021095	NT5C2	2	-0.068962287	0.152041248	-0.453576171	0.650133908	0.032502772	3.027653117	66.97111719	0.5	CRA004775;PRJNA827465	Yang2025_Silkie_Wenchang	0.908335059
ENSGALG00010021096	ZFAND4	2	0.081697044	0.063472282	1.287129449	0.198049173	0	0.885253584	0	1	PRJNA827465;Yang2025_Silkie_Wenchang	CRA004775	0.710234722
ENSGALG00010021096	ZFAND4	2	0.026411046	0.095775247	0.275760664	0.782731898	0	0.291732066	0	1	CRA004775;Yang2025_Silkie_Wenchang	PRJNA827465	0.945490372
ENSGALG00010021096	ZFAND4	2	0.126393257	0.074104281	1.705613419	0.088080079	0	0.000500415	0	1	CRA004775;PRJNA827465	Yang2025_Silkie_Wenchang	0.509839669
ENSGALG00010021097	KATNBL1	2	-0.084704856	0.054730315	-1.547677127	0.121700052	0	0.875972175	0	1	PRJNA827465;Yang2025_Silkie_Wenchang	CRA004775	0.5849268
ENSGALG00010021097	KATNBL1	2	0.032644548	0.213726299	0.152739966	0.878603342	0.070763517	4.052054772	75.32116281	0.5	CRA004775;Yang2025_Silkie_Wenchang	PRJNA827465	0.971079508
ENSGALG00010021097	KATNBL1	2	0.069558207	0.156075649	0.445669828	0.655835762	0.032148402	2.470000185	59.51417307	0.5	CRA004775;PRJNA827465	Yang2025_Silkie_Wenchang	0.910411938
ENSGALG00010021098	SDK1	2	-0.297216053	0.108987801	-2.727057994	0.006390181	0.014085108	2.42532891	58.76847896	1	PRJNA827465;Yang2025_Silkie_Wenchang	CRA004775	0.121506179
ENSGALG00010021098	SDK1	2	-0.039577345	0.493211854	-0.080244108	0.93604311	0.373534969	3.512109963	71.52708741	0.5	CRA004775;Yang2025_Silkie_Wenchang	PRJNA827465	0.984822128
ENSGALG00010021098	SDK1	2	0.030663933	0.362411863	0.084610731	0.932570867	0.175995175	2.201759147	54.58177152	0.5	CRA004775;PRJNA827465	Yang2025_Silkie_Wenchang	0.984224753
ENSGALG00010021099	HMCN1	2	0.112353656	0.151486485	0.741674455	0.458284601	0.030529805	2.686531483	62.77728341	0.5	PRJNA827465;Yang2025_Silkie_Wenchang	CRA004775	0.850026426
ENSGALG00010021099	HMCN1	2	0.018745251	0.413436511	0.045340097	0.963836228	0.223391742	2.341482083	57.29200718	0.5	CRA004775;Yang2025_Silkie_Wenchang	PRJNA827465	0.990168281
ENSGALG00010021099	HMCN1	2	-0.032363312	0.11854741	-0.272998896	0.784854064	0.008149436	1.052579777	4.995324667	1	CRA004775;PRJNA827465	Yang2025_Silkie_Wenchang	0.946230653
ENSGALG00010021100	EMC7	2	-0.264422453	0.193264747	-1.36818772	0.171253324	0.065557874	7.826451666	87.22281766	1	PRJNA827465;Yang2025_Silkie_Wenchang	CRA004775	0.673243267
ENSGALG00010021100	EMC7	2	-0.436185339	0.105949352	-4.11692313	3.83964E-05	0	0.326264514	0	1	CRA004775;Yang2025_Silkie_Wenchang	PRJNA827465	0.004008225
ENSGALG00010021100	EMC7	2	-0.138131853	0.102317357	-1.350033438	0.177005257	0.009485851	1.425390073	29.84376566	1	CRA004775;PRJNA827465	Yang2025_Silkie_Wenchang	0.682685058
ENSGALG00010021102	GOLGA3	2	0.002600472	0.041453703	0.062731964	0.949979944	0	0.850242506	0	0.5	PRJNA827465;Yang2025_Silkie_Wenchang	CRA004775	0.987277827
ENSGALG00010021102	GOLGA3	2	0.060055535	0.063601797	0.944242741	0.345045619	0	0.581167175	0	1	CRA004775;Yang2025_Silkie_Wenchang	PRJNA827465	0.806961063
ENSGALG00010021102	GOLGA3	2	-0.009194276	0.074552349	-0.123326442	0.901848603	0.003062103	1.071869792	6.705086086	0.5	CRA004775;PRJNA827465	Yang2025_Silkie_Wenchang	0.976725148
ENSGALG00010021103	RBMS1	2	0.041292355	0.031719467	1.301798498	0.192985277	0	0.385486003	0	0.5	PRJNA827465;Yang2025_Silkie_Wenchang	CRA004775	0.702958174
ENSGALG00010021103	RBMS1	2	0.126993503	0.164770248	0.770730788	0.440866519	0.037575127	3.232192334	69.06124709	0.5	CRA004775;Yang2025_Silkie_Wenchang	PRJNA827465	0.844888742
ENSGALG00010021103	RBMS1	2	0.13700611	0.121084207	1.131494463	0.257847033	0.02173616	3.135096766	68.10305791	1	CRA004775;PRJNA827465	Yang2025_Silkie_Wenchang	0.765151037
ENSGALG00010021104	INA	2	-0.018372531	0.167290404	-0.109824177	0.912548816	0	0.265397845	0	0.5	PRJNA827465;Yang2025_Silkie_Wenchang	CRA004775	0.978967245
ENSGALG00010021104	INA	2	0.196737935	0.239593645	0.821131693	0.41157126	0	0.609137974	0	1	CRA004775;Yang2025_Silkie_Wenchang	PRJNA827465	0.831528673
ENSGALG00010021104	INA	2	0.063290364	0.264316796	0.239448894	0.81075752	0.047164051	1.325655937	24.56564542	0.5	CRA004775;PRJNA827465	Yang2025_Silkie_Wenchang	0.953020781
ENSGALG00010021106	ITGB6	2	0.09632533	0.311003437	0.309724327	0.756770602	0.166307723	6.966838	85.64628602	0.5	PRJNA827465;Yang2025_Silkie_Wenchang	CRA004775	0.938740241
ENSGALG00010021106	ITGB6	2	-0.199294114	0.194518442	-1.024551259	0.305574973	0	0.626649729	0	0.5	CRA004775;Yang2025_Silkie_Wenchang	PRJNA827465	0.79225794
ENSGALG00010021106	ITGB6	2	0.390543411	0.124950904	3.125574909	0.001774579	0	0.009694646	0	1	CRA004775;PRJNA827465	Yang2025_Silkie_Wenchang	0.028220599
ENSGALG00010021108	PCGF6	2	0.012912969	0.101832591	0.126805854	0.899094062	0.012085813	2.188593384	54.30855237	0.5	PRJNA827465;Yang2025_Silkie_Wenchang	CRA004775	0.975990605
ENSGALG00010021108	PCGF6	2	-0.18528268	0.106760261	-1.735502306	0.082651873	0	0.928985223	0	1	CRA004775;Yang2025_Silkie_Wenchang	PRJNA827465	0.495638233
ENSGALG00010021108	PCGF6	2	-0.088015447	0.215535203	-0.408355413	0.683011126	0.07096454	4.356268925	77.04457605	0.5	CRA004775;PRJNA827465	Yang2025_Silkie_Wenchang	0.918800744
ENSGALG00010021110	PKLR	2	-0.261369872	0.099052225	-2.638707733	0.008322269	0	0.214259351	0	1	PRJNA827465;Yang2025_Silkie_Wenchang	CRA004775	0.142213112
ENSGALG00010021110	PKLR	2	-0.20197307	0.137411074	-1.46984566	0.14160356	0	0.151571241	0	0.5	CRA004775;Yang2025_Silkie_Wenchang	PRJNA827465	0.627088401
ENSGALG00010021110	PKLR	2	-0.289294121	0.135241406	-2.139094301	0.032428032	0	0.292129092	0	0.5	CRA004775;PRJNA827465	Yang2025_Silkie_Wenchang	0.309308824
ENSGALG00010021111	AVEN	2	-0.121711122	0.061359655	-1.983569185	0.047303887	0	0.53771749	0	1	PRJNA827465;Yang2025_Silkie_Wenchang	CRA004775	0.37135651
ENSGALG00010021111	AVEN	2	-0.016244756	0.116770896	-0.139116479	0.889358106	0.003555972	1.051920795	4.935808362	0.5	CRA004775;Yang2025_Silkie_Wenchang	PRJNA827465	0.973135755
ENSGALG00010021111	AVEN	2	-0.04056904	0.2019729	-0.200863779	0.840805091	0.047817408	1.730949811	42.22824985	0.5	CRA004775;PRJNA827465	Yang2025_Silkie_Wenchang	0.95980036
ENSGALG00010021112	TAF5	2	-0.080572942	0.044729927	-1.801320672	0.071652351	0	0.020697107	0	1	PRJNA827465;Yang2025_Silkie_Wenchang	CRA004775	0.461891482
ENSGALG00010021112	TAF5	2	-0.08962271	0.084631716	-1.058970566	0.289620021	0	0.004833533	0	1	CRA004775;Yang2025_Silkie_Wenchang	PRJNA827465	0.769014188
ENSGALG00010021112	TAF5	2	-0.077023821	0.049493607	-1.556237793	0.119651557	0	2.44893E-06	0	1	CRA004775;PRJNA827465	Yang2025_Silkie_Wenchang	0.580639051
ENSGALG00010021113	SYP	2	0.523284036	0.178822062	2.926283423	0.00343038	0	0.688065824	0	1	PRJNA827465;Yang2025_Silkie_Wenchang	CRA004775	0.082562356
ENSGALG00010021113	SYP	2	1.275344601	0.897385095	1.421178721	0.155264809	1.465710221	10.62445866	90.55775574	1	CRA004775;Yang2025_Silkie_Wenchang	PRJNA827465	0.634574911
ENSGALG00010021113	SYP	2	1.459978355	0.727576155	2.006633046	0.04478875	0.869269115	5.485315197	81.76950705	1	CRA004775;PRJNA827465	Yang2025_Silkie_Wenchang	0.364835697
ENSGALG00010021114	SBF2	2	-0.046115369	0.064913048	-0.710417555	0.47744524	0	0.009114007	0	1	PRJNA827465;Yang2025_Silkie_Wenchang	CRA004775	0.855104347
ENSGALG00010021114	SBF2	2	0.024007919	0.128188887	0.18728549	0.851436799	0.001485131	1.02662217	2.59318051	0.5	CRA004775;Yang2025_Silkie_Wenchang	PRJNA827465	0.962139388
ENSGALG00010021114	SBF2	2	0.009236572	0.125572985	0.073555277	0.981342869	0.013052063	1.264647858	20.92660467	0.5	CRA004775;PRJNA827465	Yang2025_Silkie_Wenchang	0.994962782
ENSGALG00010021115	ALPL	2	0.073891697	0.198190754	0.372831202	0.709274079	0	0.33703826	0	0.5	PRJNA827465;Yang2025_Silkie_Wenchang	CRA004775	0.925298042
ENSGALG00010021115	ALPL	2	-0.057251186	0.248445999	-0.23043714	0.817752104	0	0.001548681	0	1	CRA004775;Yang2025_Silkie_Wenchang	PRJNA827465	0.954967428
ENSGALG00010021115	ALPL	2	0.116918613	0.218959335	0.50833676	0.611508699	0	0.19117925	0	0.5	CRA004775;PRJNA827465	Yang2025_Silkie_Wenchang	0.893817943
ENSGALG00010021116	FAM21C	2	0.255440242	0.202033362	1.26434684	0.206105564	0.075942425	14.07057593	92.89297037	1	PRJNA827465;Yang2025_Silkie_Wenchang	CRA004775	0.718403416
ENSGALG00010021116	FAM21C	2	0.247331243	0.249051016	0.993094696	0.320663809	0.100620435	4.922569218	79.68540501	0.5	CRA004775;Yang2025_Silkie_Wenchang	PRJNA827465	0.798348216
ENSGALG00010021116	FAM21C	2	0.055034093	0.050459624	1.090656029	0.275424265	0	0.213728485	0	0.5	CRA004775;PRJNA827465	Yang2025_Silkie_Wenchang	0.775135547
ENSGALG00010021118	USMG5	2	-0.20742319	0.188550844	-1.100091548	0.271292236	0.048452662	2.801220043	64.30126928	1	PRJNA827465;Yang2025_Silkie_Wenchang	CRA004775	0.772743863
ENSGALG00010021118	USMG5	2	-0.127801588	0.34322709	-0.372352857	0.709630149	0.175295907	3.850755366	74.0310691	0.5	CRA004775;Yang2025_Silkie_Wenchang	PRJNA827465	0.925298042
ENSGALG00010021118	USMG5	2	-0.024755282	0.095892469	-0.258156685	0.796285986	0.001391719	1.031923009	3.093545577	0.5	CRA004775;PRJNA827465	Yang2025_Silkie_Wenchang	0.949325935
ENSGALG00010021120	PDCD11	2	0.077170853	0.081182146	0.950588981	0.341813064	0.007026881	2.126009694	52.96352585	1	PRJNA827465;Yang2025_Silkie_Wenchang	CRA004775	0.805823616
ENSGALG00010021120	PDCD11	2	0.002390216	0.224246618	0.010658871	0.991495612	0.069478952	2.663543077	62.456023	0.5	CRA004775;Yang2025_Silkie_Wenchang	PRJNA827465	0.997563866
ENSGALG00010021120	PDCD11	2	-0.037058253	0.102969872	-0.35989414	0.718926299	0.006980904	1.171539845	14.66225448	0.5	CRA004775;PRJNA827465	Yang2025_Silkie_Wenchang	0.928449671
ENSGALG00010021121	PLA2R1	2	-0.246728912	0.072274183	-3.413790417	0.000640658	0	0.729085885	0	1	PRJNA827465;Yang2025_Silkie_Wenchang	CRA004775	0.027822979
ENSGALG00010021121	PLA2R1	2	-0.16203597	0.10353554	-1.565027517	0.117576457	0	0.283197086	0	0.5	CRA004775;Yang2025_Silkie_Wenchang	PRJNA827465	0.577165052
ENSGALG00010021121	PLA2R1	2	-0.280782744	0.094045185	-2.985615298	0.002830084	0	0.745896184	0	0.5	CRA004775;PRJNA827465	Yang2025_Silkie_Wenchang	0.072604962
ENSGALG00010021123	RYR3	2	0.12512034	0.314238561	0.398169913	0.690504943	0.168361286	6.712334765	85.10205413	0.5	PRJNA827465;Yang2025_Silkie_Wenchang	CRA004775	0.919979266
ENSGALG00010021123	RYR3	2	0.490530875	0.185712709	2.641342524	0.008257819	0	0.443960995	0	1	CRA004775;Yang2025_Silkie_Wenchang	PRJNA827465	0.141469904
ENSGALG00010021123	RYR3	2	0.18108479	0.505303728	0.358368205	0.720067782	0.361816752	2.701907676	62.98911288	0.5	CRA004775;PRJNA827465	Yang2025_Silkie_Wenchang	0.928564173
ENSGALG00010021124	CALHM2	2	-0.240304974	0.214475073	-1.120433113	0.262529241	0.079125053	7.010073179	85.73481368	1	PRJNA827465;Yang2025_Silkie_Wenchang	CRA004775	0.768418419
ENSGALG00010021124	CALHM2	2	-0.441436168	0.126378766	-3.492961532	0.000477695	0	0.278748409	0	1	CRA004775;Yang2025_Silkie_Wenchang	PRJNA827465	0.022813698

ENSGALG00010021124	CALHM2	2	-0.051613517	0.086570906	-0.596199337	0.551042068	0	0.40680841	0	1	CRA004775;PRJNA827465	Yang2025_Silkie_Wenchang	0.878879109
ENSGALG00010021125	LY75	2	0.203150704	0.310656533	0.653939905	0.513150523	0.181583904	16.86717079	94.07132345	0.5	PRJNA827465;Yang2025_Silkie_Wenchang	CRA004775	0.866263853
ENSGALG00010021125	LY75	2	0.487576377	0.109774447	4.441619967	8.92841E-06	0	0.850634696	0	1	CRA004775;Yang2025_Silkie_Wenchang	PRJNA827465	0.001271426
ENSGALG00010021125	LY75	2	-0.091333628	0.096408193	-0.947363761	0.343451451	0	0.326944508	0	0.5	CRA004775;PRJNA827465	Yang2025_Silkie_Wenchang	0.806276913
ENSGALG00010021126	ECE1	2	-0.078334421	0.081460996	-0.961618742	0.336241153	0.009292509	3.188378774	68.63609781	1	PRJNA827465;Yang2025_Silkie_Wenchang	CRA004775	0.803727281
ENSGALG00010021126	ECE1	2	-0.149219654	0.074892677	-1.992446535	0.046322086	0	0.977309363	0	0.5	CRA004775;Yang2025_Silkie_Wenchang	PRJNA827465	0.371247337
ENSGALG00010021126	ECE1	2	-0.004174294	0.047561268	-0.087766678	0.930062123	0	0.142653203	0	0.5	CRA004775;PRJNA827465	Yang2025_Silkie_Wenchang	0.993813577
ENSGALG00010021127	SWAP70	2	-0.158584666	0.044539607	-3.560531333	0.000370105	0	0.161667362	0	1	PRJNA827465;Yang2025_Silkie_Wenchang	CRA004775	0.01934739
ENSGALG00010021127	SWAP70	2	0.033828692	0.27121833	0.124728636	0.900738386	0.113532754	3.589445639	72.14054479	0.5	CRA004775;Yang2025_Silkie_Wenchang	PRJNA827465	0.976497577
ENSGALG00010021127	SWAP70	2	0.047526353	0.251337116	0.189094048	0.850019107	0.093684538	3.12075885	67.9565116	0.5	CRA004775;PRJNA827465	Yang2025_Silkie_Wenchang	0.961869125
ENSGALG00010021129	TXNRD3	2	-0.039063456	0.06529663	-0.59824615	0.549675715	0	0.754201579	0	1	PRJNA827465;Yang2025_Silkie_Wenchang	CRA004775	0.878554604
ENSGALG00010021129	TXNRD3	2	-0.15435973	0.106553372	-1.448661148	0.147432235	0	0.207795123	0	1	CRA004775;Yang2025_Silkie_Wenchang	PRJNA827465	0.636411768
ENSGALG00010021129	TXNRD3	2	-0.032614265	0.079208639	-0.41175136	0.680521676	0.001157902	1.041661571	3.999530415	1	CRA004775;PRJNA827465	Yang2025_Silkie_Wenchang	0.917851751
ENSGALG00010021130	ENSGALG00010021130	2	-1.727358975	2.148886183	-0.803839211	0.421489841	7.960116154	7.223857126	86.15697982	0.5	PRJNA827465;Yang2025_Silkie_Wenchang	CRA004775	0.839352211
ENSGALG00010021130	ENSGALG00010021130	2	-1.685807465	2.04784865	-0.823209012	0.410389145	7.71336018	12.16372448	91.77883385	0.5	CRA004775;Yang2025_Silkie_Wenchang	PRJNA827465	0.834820782
ENSGALG00010021130	ENSGALG00010021130	2	0.306747972	0.509135901	0.602487412	0.54684974	0	0.021966472	0	1	CRA004775;PRJNA827465	Yang2025_Silkie_Wenchang	0.878275987
ENSGALG00010021131	EDC3	2	-0.202493399	0.100481085	-2.015238988	0.043879614	0.016342935	5.193653982	80.74573309	1	PRJNA827465;Yang2025_Silkie_Wenchang	CRA004775	0.361804689
ENSGALG00010021131	EDC3	2	-0.313247395	0.064497229	-4.856757359	1.19324E-06	0	0.039408752	0	1	CRA004775;Yang2025_Silkie_Wenchang	PRJNA827465	0.000253501
ENSGALG00010021131	EDC3	2	-0.171771441	0.105874489	-1.622406334	0.104716374	0.012516767	1.798981388	44.41298801	1	CRA004775;PRJNA827465	Yang2025_Silkie_Wenchang	0.549804368
ENSGALG00010021133	ENSGALG00010021133	2	0.120125131	0.038213491	3.143526708	0.001669252	0	0.06145979	0	1	PRJNA827465;Yang2025_Silkie_Wenchang	CRA004775	0.051618099
ENSGALG00010021133	ENSGALG00010021133	2	0.091479949	0.077335777	1.182892996	0.236851558	0	0.410899223	0	0.5	CRA004775;Yang2025_Silkie_Wenchang	PRJNA827465	0.749092844
ENSGALG00010021133	ENSGALG00010021133	2	0.121409656	0.04325769	2.806660628	0.005005795	0	0.514257589	0	0.5	CRA004775;PRJNA827465	Yang2025_Silkie_Wenchang	0.105492094
ENSGALG00010021134	CHFR	2	-0.050897463	0.068468945	-0.743365675	0.45726032	0.005056014	2.146477276	53.41203882	0.5	PRJNA827465;Yang2025_Silkie_Wenchang	CRA004775	0.849541335
ENSGALG00010021134	CHFR	2	-0.119791335	0.071132794	-1.684052156	0.092171589	0	0.159929651	0	0.5	CRA004775;Yang2025_Silkie_Wenchang	PRJNA827465	0.519893797
ENSGALG00010021134	CHFR	2	0.010894613	0.058031919	0.187734844	0.851084514	0	0.00072813	0	1	CRA004775;PRJNA827465	Yang2025_Silkie_Wenchang	0.962071965
ENSGALG00010021135	CLK3	2	0.193923065	0.250390706	0.774481882	0.438645879	0.117394452	15.56927188	93.57709206	0.5	PRJNA827465;Yang2025_Silkie_Wenchang	CRA004775	0.844002503
ENSGALG00010021135	CLK3	2	0.217735799	0.275731307	0.789666583	0.429722512	0.119102597	4.176554279	76.05681782	0.5	CRA004775;Yang2025_Silkie_Wenchang	PRJNA827465	0.842012722
ENSGALG00010021135	CLK3	2	-0.055473358	0.069899801	-0.793612532	0.427421035	0	0.049670514	0	1	CRA004775;PRJNA827465	Yang2025_Silkie_Wenchang	0.84125685
ENSGALG00010021136	WEE1	2	-0.223963333	0.310102925	-0.715773096	0.474131467	0.180167996	15.23412478	93.43578962	0.5	PRJNA827465;Yang2025_Silkie_Wenchang	CRA004775	0.85444425
ENSGALG00010021136	WEE1	2	-0.052501602	0.536521429	-0.097855555	0.922046993	0.511160925	8.500021506	88.23532388	0.5	CRA004775;Yang2025_Silkie_Wenchang	PRJNA827465	0.981720024
ENSGALG00010021136	WEE1	2	0.178591002	0.192336083	0.928536128	0.353129536	0.04533264	1.776815181	43.71952632	1	CRA004775;PRJNA827465	Yang2025_Silkie_Wenchang	0.81107437
ENSGALG00010021137	PLAU	2	0.235345043	0.126520811	1.860129111	0.062867261	0	0.210209905	0	1	PRJNA827465;Yang2025_Silkie_Wenchang	CRA004775	0.431819639
ENSGALG00010021137	PLAU	2	0.236569484	0.195953859	1.207271369	0.227327653	0	0.829917124	0	1	CRA004775;Yang2025_Silkie_Wenchang	PRJNA827465	0.740605003
ENSGALG00010021137	PLAU	2	0.31209174	0.148532163	2.101172786	0.035625801	0	0.498086486	0	1	CRA004775;PRJNA827465	Yang2025_Silkie_Wenchang	0.325599715
ENSGALG00010021138	DDX18	2	0.061467415	0.089412369	0.687459865	0.491792984	0.008037763	1.823173783	45.15059347	0.5	PRJNA827465;Yang2025_Silkie_Wenchang	CRA004775	0.858983177
ENSGALG00010021138	DDX18	2	-0.037098331	0.107398176	-0.345427937	0.729772677	0	0.171351355	0	0.5	CRA004775;Yang2025_Silkie_Wenchang	PRJNA827465	0.932444623
ENSGALG00010021138	DDX18	2	0.118592351	0.061215559	1.937290979	0.052709784	0	0.150597131	0	1	CRA004775;PRJNA827465	Yang2025_Silkie_Wenchang	0.398907772
ENSGALG00010021139	ARHGAP29	2	0.094015619	0.142992704	0.657485426	0.510868839	0.0345557	6.254629258	84.01184213	0.5	PRJNA827465;Yang2025_Silkie_Wenchang	CRA004775	0.865436876
ENSGALG00010021139	ARHGAP29	2	0.273886107	0.08559544	3.199774509	0.001375351	0	0.274396187	0	1	CRA004775;Yang2025_Silkie_Wenchang	PRJNA827465	0.04550121
ENSGALG00010021139	ARHGAP29	2	0.123234789	0.192579633	0.639915999	0.522227212	0.059792373	4.587366873	78.20100228	0.5	CRA004775;PRJNA827465	Yang2025_Silkie_Wenchang	0.869568211
ENSGALG00010021140	SLC11A1	2	-0.122727854	0.207803678	-0.590595195	0.554791685	0	0.034106801	0	1	PRJNA827465;Yang2025_Silkie_Wenchang	CRA004775	0.880223402
ENSGALG00010021140	SLC11A1	2	-0.406832195	0.368579326	-1.103784632	0.269686573	0.10777694	1.508748417	33.71989733	1	CRA004775;Yang2025_Silkie_Wenchang	PRJNA827465	0.77189992
ENSGALG00010021140	SLC11A1	2	-0.380083854	0.414102333	-0.917850069	0.358697365	0.173789535	1.826936863	45.26357095	1	CRA004775;PRJNA827465	Yang2025_Silkie_Wenchang	0.814232788
ENSGALG00010021141	SGSM1	2	-0.011846757	0.122312239	-0.096856679	0.922840212	0	0.635934474	0	0.5	PRJNA827465;Yang2025_Silkie_Wenchang	CRA004775	0.981819849
ENSGALG00010021141	SGSM1	2	-0.068404587	0.180758649	-0.378430507	0.705110809	0	0.655190458	0	0.5	CRA004775;Yang2025_Silkie_Wenchang	PRJNA827465	0.924245236
ENSGALG00010021141	SGSM1	2	0.084137174	0.144870599	0.580774669	0.561392329	0	0.151988488	0	1	CRA004775;PRJNA827465	Yang2025_Silkie_Wenchang	0.881623955
ENSGALG00010021142	WNT6	2	0.103011791	0.464871723	0.221591863	0.824631617	0	0.245493511	0	0.5	PRJNA827465;Yang2025_Silkie_Wenchang	CRA004775	0.95661211
ENSGALG00010021142	WNT6	2	0.112395983	0.519702307	0.216269933	0.828777344	0	0.270575011	0	0.5	CRA004775;Yang2025_Silkie_Wenchang	PRJNA827465	0.956840134
ENSGALG00010021142	WNT6	2	-0.10141223	0.452958024	-0.223888804	0.822843828	0	0.005069094	0	1	CRA004775;PRJNA827465	Yang2025_Silkie_Wenchang	0.955822987
ENSGALG00010021144	FOXK1	2	-0.209287327	0.064059331	-3.26708576	0.001086608	0	0.000665147	0	1	PRJNA827465;Yang2025_Silkie_Wenchang	CRA004775	0.039875759
ENSGALG00010021144	FOXK1	2	-0.041927894	0.19756147	-0.212227078	0.831929882	0.050535393	2.727518282	63.33663438	0.5	CRA004775;Yang2025_Silkie_Wenchang	PRJNA827465	0.957762335
ENSGALG00010021144	FOXK1	2	-0.055629408	0.192588443	-0.288851226	0.772695229	0.055061621	3.375758322	70.3770263	0.5	CRA004775;PRJNA827465	Yang2025_Silkie_Wenchang	0.943066211
ENSGALG00010021145	EIF4G3	2	0.116590382	0.254757051	0.457653211	0.647201608	0.11432444	7.855520724	87.27009914	0.5	PRJNA827465;Yang2025_Silkie_Wenchang	CRA004775	0.907350807
ENSGALG00010021145	EIF4G3	2	0.376641428	0.152690894	2.466692138	0.013636755	0	0.070347486	0	1	CRA004775;Yang2025_Silkie_Wenchang	PRJNA827465	0.191338116
ENSGALG00010021145	EIF4G3	2	-0.044976709	0.154384125	-0.291329882	0.770799036	0.024571647	1.41046131	29.10121017	0.5	CRA004775;PRJNA827465	Yang2025_Silkie_Wenchang	0.942491331
ENSGALG00010021146	AP5Z1	2	-0.21003668	0.054154661	-3.878459855	0.00010512	0	0.382694734	0	1	PRJNA827465;Yang2025_Silkie_Wenchang	CRA004775	0.008069387
ENSGALG00010021146	AP5Z1	2	-0.306526008	0.105925051	-2.893800906	0.003806094	0	0.193156315	0	1	CRA004775;Yang2025_Silkie_Wenchang	PRJNA827465	0.088515335
ENSGALG00010021146	AP5Z1	2	-0.208675138	0.057844547	-3.607536159	0.000309142	0	0.78698012	0	1	CRA004775;PRJNA827465	Yang2025_Silkie_Wenchang	0.017332695
ENSGALG00010021147	ARID3B	2	0.051464263	0.14046842	0.366376036	0.714084499	0.024817217	2.352025376	57.48345191	0.5	PRJNA827465;Yang2025_Silkie_Wenchang	CRA004775	0.926442961
ENSGALG00010021147	ARID3B	2	0.066522332	0.208802899	0.318589123	0.750038103	0.043189377	1.923488855	48.01113627	0.5	CRA004775;Yang2025_Silkie_Wenchang	PRJNA827465	0.937374353
ENSGALG00010021147	ARID3B	2	-0.063275982	0.072273526	-0.875507053	0.381298074	0	0.249023393	0	1	CRA004775;PRJNA827465	Yang2025_Silkie_Wenchang	0.823492515
ENSGALG00010021148	GPATCH8	2	0.356195872	0.323060226	1.102568013	0.270214807	0.195737244	15.51932769	93.5564219	1	PRJNA827465;Yang2025_Silkie_Wenchang	CRA004775	0.772430311
ENSGALG00010021148	GPATCH8	2	0.345659427	0.347831694	0.993754833	0.32034224	0.21961025	10.81757754	90.75578616	0.5	CRA004775;Yang2025_Silkie_Wenchang	PRJNA827465	0.798348216
ENSGALG00010021148	GPATCH8	2	0.041496482	0.054358068	0.763391418	0.445229997	0	0.096659028	0	0.5	CRA004775;PRJNA827465	Yang2025_Silkie_Wenchang	0.846013379
ENSGALG00010021150	ENSGALG00010021150	2	0.396596896	0.364347564	1.088512552	0.276368899	0.214775639	5.231672604	80.88565406	1	PRJNA827465;Yang2025_Silkie_Wenchang	CRA004775	0.775851484
ENSGALG00010021150	ENSGALG00010021150	2	-0.092804417	0.954456375	-0.097232435	0.922541811	1.593066467	7.377866537	86.4459462	0.5	CRA004775;Yang2025_Silkie_Wenchang	PRJNA827465	0.981804675
ENSGALG00010021150	ENSGALG00010021150	2	-0.392234999	0.571615977	-0.686186206	0.492595695	0.462451732	2.873175845	65.19530813	0.5	CRA004775;PRJNA827465	Yang2025_Silkie_Wenchang	0.859320399
ENSGALG00010021152	STK11IP	2	0.030785631	0.053494814	0.575488139	0.56496121	0	0.391712955	0	1	PRJNA827465;Yang2025_Silkie_Wenchang	CRA004775	0.883416369
ENSGALG00010021152	STK11IP	2	-0.286802243	0.421886116	-0.679809627	0.49662501	0.302438984	5.945123319	83.17949105	0.5	CRA004775;Yang2025_Silkie_Wenchang	PRJNA827465	0.860597846
ENSGALG00010021152	STK11IP	2	-0.313956522	0.384618317	-0.816280735	0.414339591	0.24661097	5.175821061	80.67939389	0.5	CRA004775;PRJNA827465	Yang2025_Silkie_Wenchang	0.836962991
ENSGALG00010021153	PTPRF	2	0.158828341	0.131400575	1.208733991	0.226765059	0.016611603	1.882038062	46.86611179	1	PRJNA827465;Yang2025_Silkie_Wenchang	CRA004775	0.74036776
ENSGALG00010021153	PTPRF	2	0.4622409	0.222745187	2.075200396	0.037967966	0.047373781	1.688551112	40.77378077	1	CRA004775;Yang2025_Silkie_Wenchang	PRJNA827465	0.33607781
ENSGALG00010021153	PTPRF	2	0.354288915	0.368129141	0.962403884	0.335846763	0.216760501	4.42759714	77.41438599	1	CRA004775;PRJNA827465	Yang2025_Silkie_Wenchang	0.803504387
ENSGALG00010021154	CHST13	2	-0.043210828	0.201474704	-0.21447272	0.830178438	0.062241618	4.24707478	76.45438209	0.5	PRJNA827465;Yang2025_Silkie_Wenchang	CRA004775	0.957215045
ENSGALG00010021154	CHST13	2	0.083838019	0.408484939	0.205241394	0.83738353	0.258617635	3.983704474	74.89773635	0.5	CRA004775;Yang2025_Silkie_Wenchang	PRJNA827465	0.958828842
ENSGALG00010021154	CHST13	2	-0.203755375	0.144064185	-1.414335474	0.157262839	0.008993902	1.10916641	9.842203043	1	CRA004775;PRJNA827465	Yang2025_Silkie_Wenchang	0.650687662
ENSGALG00010021155	RADIL	2	0.290507874	0.148054471	1.96216887	0.049742827	0	0.681409584	0	1	PRJNA827465;Yang2025_Silkie_Wenchang	CRA004775	0.386162809
ENSGALG00010021155	RADIL	2	0.058939858	0.403707004	0.145996619	0.883924058	0.257279533	4.733426133	78.87365363	0.5	CRA004775;Yang2025_Silkie_Wenchang	PRJNA827465	0.972001076
ENSGALG00010021155	RADIL	2	-0.039877527	0.274105023	-0.145482655	0.884329809	0.097249038	2.734860971	63.43506998	0.5	CRA004775;PRJNA827465	Yang2025_Silkie_Wenchang	0.972001076
ENSGALG00010021156	DDOST	2	-0.095161626	0.216116038	-0.440326536	0.659700624	0.077006639	4.972787056	79.89055255	0.5	PRJNA827465;Yang2025_Silkie_Wenchang	CRA004775	0.911617103
ENSGALG00010021156	DDOST	2	-0.367212282	0.140926164	-2.60570693	0.009168487	0	0.011784965	0	1	CRA004775;Yang2025_Silkie_Wenchang	PRJNA827465	0.150270666
ENSGALG00010021156	DDOST	2	-0.105081267	0.230735746	-0.455418239	0.648806376	0.087227738	4.77150967	79.04227238	0.5	CRA004775;PRJNA827465	Yang2025_Silkie_Wenchang	0.907735854
ENSGALG00010021158	ACTG1L	2	-0.025552359	0.166848592	-0.153146985	0.878282364	0.043865068	4.165711074	75.97603989	0.5	PRJNA827465;Yang2025_Silkie_Wenchang	CRA004775	0.971037793
ENSGALG00010021158	ACTG1L	2	-0.266959148	0.132088063	-2.021069439	0.043272577	0	0.212146548	0	1	CRA004775;Yang2025_Silkie_Wenchang	PRJNA827465	0.359703576
ENSGALG00010021158	ACTG1L	2	-0.065072234	0.229030608	-0.284120253	0.776318227	0.08093696	3.611957814	72.31418385	0.5	CRA004775;PRJNA827465	Yang2025_Silkie_Wenchang	0.944109792
ENSGALG00010021159	MMD2	2	-0.136249333	0.177050282	-0.769551629	0.441565908	0	0.252176563	0	1	PRJNA827465;Yang2025_Silkie_Wenchang	CRA004775	0.845053556
ENSGALG00010021159	MMD2	2	-0.112331332	0.264400513	-0.424852926	0.670943892	0	0.52884904	0	1	CRA004775;Yang2025_Silkie_Wenchang	PRJNA827465	0.914552186
ENSGALG00010021159	MMD2	2	-0.239260516	0.207898299	-1.150853648	0.24979245	0	0.221707766	0	1	CRA004775;PRJNA827465	Yang2025_Silkie_Wenchang	0.759757983
ENSGALG00010021160	ZNF143	2	0.02729432	0.049286919	0.553784274	0.579726492	0	0.001129522	0	1	PRJNA827465;Yang2025_Silkie_Wenchang	CRA004775	0.888013685
ENSGALG00010021160	ZNF143	2	0.027192844	0.107827986	0.252187258	0.800896327	0	0.004973835	0	1	CRA004775;Yang2025_Silkie_Wenchang	PRJNA827465	0.950158029
ENSGALG00010021160	ZNF143	2	0.028713928	0.052983321	0.541942773	0.587857929	0	0.003515405	0	1	CRA004775;PRJNA827465	Yang2025_Silkie_Wenchang	0.891758856
ENSGALG00010021161	ENSGALG00010021161	2	0.119034362	0.155740422	0.764312566	0.444680999	0.040148498	5.543282462	81.96014713	0.5	PRJNA827465;Yang2025_Silkie_Wenchang	CRA004775	0.846013379
ENSGALG00010021161	ENSGALG00010021161	2	0.145858178	0.225067992	0.648062733	0.516944377	0.058045639	1.905823613	47.52924702	0.5	CRA004775;Yang2025_Silkie_Wenchang	PRJNA827465	0.867952013
ENSGALG00010021161	ENSGALG00010021161	2	-0.02818704	0.062259083	-0.452737794	0.650737561	0	0.277098131	0	1	CRA004775;PRJNA827465	Yang2025_Silkie_Wenchang	0.908769703
ENSGALG00010021162	WIPI2	2	-0.015909451	0.137527029	-0.115682356	0.90790429	0.029269864	3.861308768	74.10204519	0.5	PRJNA827465;Yang2025_Silkie_Wenchang	CRA004775	0.978196186
ENSGALG00010021162	WIPI2	2	-0.135892261	0.353573155	-0.38433987	0.700726561	0.190427627	3.730006511	73.19039532	0.5	CRA004775;Yang2025_Silkie_Wenchang	PRJNA827465	0.922472203
ENSGALG00010021162	WIPI2	2	-0.212484775	0.173746996	-1.222955102	0.221346655	0.035302712	1.57284698	36.42102423	1	CRA004775;PRJNA827465	Yang2025_Silkie_Wenchang	0.734689158
ENSGALG00010021163	INHA	2	0.252075683	0.315504508	0.798960638	0.424313236	0.122329821	2.585063966	61.31623769	0.5	PRJNA827465;Yang2025_Silkie_Wenchang	CRA004775	0.840617373
ENSGALG00010021163	INHA	2	0.001761598	0.719934878	0.002446886	0.99804767	0.754610491	3.112387897	67.87032873	0.5	CRA004775;Yang2025_Silkie_Wenchang	PRJNA827465	0.999313737
ENSGALG00010021163	INHA	2	-0.182196558	0.280242095	-0.650139866	0.51560188	0.003006348	1.008205385	0.813860451	1	CRA004775;PRJNA827465	Yang2025_Silkie_Wenchang	0.867300757
ENSGALG00010021164	SLC41A3	2	-0.041069315	0.129745783	-0.3165368	0.751595098	0.014886978	1.788956105	44.10147921	0.5	PRJNA827465;Yang2025_Silkie_Wenchang	CRA004775	0.937716403
ENSGALG00010021164	SLC41A3	2	-0.177912144	0.139369828	-1.276547056	0.201762204	0	6.90443E-05	0	1	CRA004775;Yang2025_Silkie_Wenchang	PRJNA827465	0.714788164
ENSGALG00010021164	SLC41A3	2	0.069546383	0.127764945	0.544330708	0.586213919	0	0.195532267	0	0.5	CRA004775;PRJNA827465	Yang2025_Silkie_Wenchang	0.891482799
ENSGALG00010021165	PTRF	2	-0.024739696	0.110968774	-0.222942859	0.823579979	0.014535079	2.415767338	58.60528518	0.5	PRJNA827465;Yang2025_Silkie_Wenchang	CRA004775	0.955934625
ENSGALG00010021165	PTRF	2	0.093564032	0.107026072	0.87421719	0.381999986	0	0.006334202	0	1	CRA004775;Yang2025_Silkie_Wenchang	PRJNA827465	0.824983626
ENSGALG00010021165	PTRF	2	-0.117513852	0.088271294	-1.331280498	0.183096733	0	0.214704808	0	0.5	CRA004775;PRJNA827465	Yang2025_Silkie_Wenchang	0.691392093
ENSGALG00010021166	NEURL1	2	0.582187612	0.138928304	4.190561583	2.78265E-05	0	0.717978476	0	1	PRJNA827465;Yang2025_Silkie_Wenchang	CRA004775	0.00309935
ENSGALG00010021166	NEURL1	2	0.458886048	0.442871668	1.036160318	0.300127356	0.224577215	2.030662037	50.75497636	0.5	CRA004775;Yang2025_Silkie_Wenchang	PRJNA827465	0.790292475
ENSGALG00010021166	NEURL1	2	0.420000557	0.228770224	1.835905699	0.066371605	0.033388026	1.169587264	14.499753	0.5	CRA004775;PRJNA827465	Yang2025_Silkie_Wenchang	0.443651804
ENSGALG00010021167	KIAA1671	2	-0.086107721	0.16349219	-0.526677884	0.598417296	0.043564022	5.245196251	80.93493642	0.5	PRJNA827465;Yang2025_Silkie_Wenchang	CRA004775	0.894645663
ENSGALG00010021167	KIAA1671	2	-0.240262158	0.115484117	-2.080477935	0.037481717	0	0.401660133	0	1	CRA004775;Yang2025_Silkie_Wenchang	PRJNA827465	0.333894819
ENSGALG00010021167	KIAA1671	2	0.061437721	0.074785861	0.821515187	0.411352877	0	0.038804327	0	0.5	CRA004775;PRJNA827465	Yang2025_Silkie_Wenchang	0.835128673
ENSGALG00010021168	IVNS1ABP	2	0.122013178	0.233346943	0.522883122	0.601055591	0.102186305	16.17535983	93.81775731	0.5	PRJNA827465;Yang2025_Silkie_Wenchang	CRA004775	0.895230519
ENSGALG00010021168	IVNS1ABP	2	0.10049469	0.270623655	0.371344809	0.710380727	0.131609696	9.656525135	89.64430801	0.5	CRA004775;Yang2025_Silkie_Wenchang	PRJNA827465	0.925364369
ENSGALG00010021168	IVNS1ABP	2	-0.122809341	0.065514143	-1.874547008	0.060855069	0	0.204751946	0	1	CRA004775;PRJNA827465	Yang2025_Silkie_Wenchang	0.427127227
ENSGALG00010021169	CAMK2G	2	-0.11146421	0.046470886	-2.398581556	0.016458711	0	0.571490493	0	1	PRJNA827465;Yang2025_Silkie_Wenchang	CRA004775	0.212299545
ENSGALG00010021169	CAMK2G	2	0.139403884	0.244607386	0.569908727	0.568739606	0.085603626	2.998141924	66.64600859	0.5	CRA004775;Yang2025_Silkie_Wenchang	PRJNA827465	0.884293565
ENSGALG00010021169	CAMK2G	2	0.101734741	0.288286622	0.352894423	0.724167592	0.134382967	4.391824575	77.23042023	0.5	CRA004775;PRJNA827465	Yang2025_Silkie_Wenchang	0.930377915
ENSGALG00010021170	UBL7	2	-0.16288348	0.164694425	-0.989004214	0.32266108	0.041855983	4.298948893	76.73849992	1	PRJNA827465;Yang2025_Silkie_Wenchang	CRA004775	0.798652022
ENSGALG00010021170	UBL7	2	-0.357631585	0.116625838	-3.066486738	0.002165904	0	0.09837092	0	1	CRA004775;Yang2025_Silkie_Wenchang	PRJNA827465	0.061198081
ENSGALG00010021170	UBL7	2	-0.145927693	0.198054896	-0.736804271	0.461241386	0.049056709	2.196700164	54.4771737	1	CRA004775;PRJNA827465	Yang2025_Silkie_Wenchang	0.85014477
ENSGALG00010021171	ENSGALG00010021171	2	0.226617558	0.292338913	0.775187796	0.438228698	0.159214917	14.571875	93.13746515	0.5	PRJNA827465;Yang2025_Silkie_Wenchang	CRA004775	0.843979791
ENSGALG00010021171	ENSGALG00010021171	2	0.495412	0.107092593	4.626015536	3.72767E-06	0	0.327809608	0	1	CRA004775;Yang2025_Silkie_Wenchang	PRJNA827465	0.000665949
ENSGALG00010021171	ENSGALG00010021171	2	0.073215749	0.19569761	0.374126944	0.708309873	0.04690544	2.159474613	53.6924401	0.5	CRA004775;PRJNA827465	Yang2025_Silkie_Wenchang	0.925061251
ENSGALG00010021172	SLC29A4	2	-0.279428398	0.255645115	-1.093032415	0.274379568	0.068653881	1.872248498	46.58828671	0.5	PRJNA827465;Yang2025_Silkie_Wenchang	CRA004775	0.774526342
ENSGALG00010021172	SLC29A4	2	-0.386657103	0.469416262	-0.823697715	0.410111338	0.312931933	3.449146363	71.00731907	0.5	CRA004775;Yang2025_Silkie_Wenchang	PRJNA827465	0.834652129
ENSGALG00010021172	SLC29A4	2	-0.533679611	0.154399011	-3.456496297	0.000547247	0.000154823	1.001979022	0.197511353	1	CRA004775;PRJNA827465	Yang2025_Silkie_Wenchang	0.024894026
ENSGALG00010021173	KLF15	2	-0.378591993	0.198619306	-1.906118803	0.056634794	0.063903696	5.235307089	80.89892373	1	PRJNA827465;Yang2025_Silkie_Wenchang	CRA004775	0.411670741
ENSGALG00010021173	KLF15	2	-0.164469492	0.525263837	-0.313117867	0.754191116	0.434228874	3.977434152	74.85816328	0.5	CRA004775;Yang2025_Silkie_Wenchang	PRJNA827465	0.938385954
ENSGALG00010021173	KLF15	2	-0.039241994	0.280105942	-0.140096972	0.888583373	0.087811949	1.613499033	38.02289435	0.5	CRA004775;PRJNA827465	Yang2025_Silkie_Wenchang	0.973135755
ENSGALG00010021175	IPO7	2	-0.232900887	0.18024091	-1.292164398	0.196300218	0.059355209	11.40168387	91.22936567	1	PRJNA827465;Yang2025_Silkie_Wenchang	CRA004775	0.707685514
ENSGALG00010021175	IPO7	2	-0.149402569	0.287083789	-0.520414507	0.602774701	0.147484873	9.223680371	89.15834071	0.5	CRA004775;Yang2025_Silkie_Wenchang	PRJNA827465	0.895538966
ENSGALG00010021175	IPO7	2	-0.008956555	0.090814514	-0.098624705	0.921436254	0.007454326	1.479620816	32.41511684	0.5	CRA004775;PRJNA827465	Yang2025_Silkie_Wenchang	0.981548382
ENSGALG00010021176	ENSGALG00010021176	2	-0.192931702	0.129545957	-1.48929158	0.136410604	0.025507056	3.967657517	74.79621172	1	PRJNA827465;Yang2025_Silkie_Wenchang	CRA004775	0.61829693
ENSGALG00010021176	ENSGALG00010021176	2	-0.231931077	0.176585529	-1.313420635	0.189041248	0.029964267	1.614697011	38.06887651	0.5	CRA004775;Yang2025_Silkie_Wenchang	PRJNA827465	0.698162702
ENSGALG00010021176	ENSGALG00010021176	2	-0.072173389	0.064058548	-1.126678505	0.259878434	0	0.206779965	0	0.5	CRA004775;PRJNA827465	Yang2025_Silkie_Wenchang	0.767047318
ENSGALG00010021177	OBSL1	2	0.257890544	0.224083753	1.150866764	0.249787053	0.048655977	1.902321423	47.43264792	1	PRJNA827465;Yang2025_Silkie_Wenchang	CRA004775	0.759757983
ENSGALG00010021177	OBSL1	2	0.144621487	0.50149609	0.288380089	0.773055807	0.321506239	2.414732923	58.58755266	0.5	CRA004775;Yang2025_Silkie_Wenchang	PRJNA827465	0.943100418
ENSGALG00010021177	OBSL1	2	0.009532595	0.188342217	0.050613163	0.959633774	0	0.832514765	0	0.5	CRA004775;PRJNA827465	Yang2025_Silkie_Wenchang	0.989259322
ENSGALG00010021178	NDST2	2	-0.06568901	0.084838331	-0.774284557	0.438762534	0.009612865	2.718543151	63.21559216	0.5	PRJNA827465;Yang2025_Silkie_Wenchang	CRA004775	0.844002503
ENSGALG00010021178	NDST2	2	-0.173170772	0.088417812	-1.958550738	0.050165424	0	1.59394E-05	0	1	CRA004775;Yang2025_Silkie_Wenchang	PRJNA827465	0.387738769
ENSGALG00010021178	NDST2	2	-0.005001292	0.044782445	-0.111679743	0.911077342	0	0.536662987	0	0.5	CRA004775;PRJNA827465	Yang2025_Silkie_Wenchang	0.978845851
ENSGALG00010021180	TMEM198	2	0.05996159	0.124075064	0.48326866	0.62890499	0.018364705	2.444351837	59.0893592	0.5	PRJNA827465;Yang2025_Silkie_Wenchang	CRA004775	0.902084638
ENSGALG00010021180	TMEM198	2	0.037963111	0.230318055	0.164829071	0.869078522	0.062515095	2.059848964	51.45275128	0.5	CRA004775;Yang2025_Silkie_Wenchang	PRJNA827465	0.968461055
ENSGALG00010021180	TMEM198	2	-0.073780718	0.094174276	-0.783448744	0.433363642	0	0.530152189	0	1	CRA004775;PRJNA827465	Yang2025_Silkie_Wenchang	0.842762754
ENSGALG00010021181	DAGLA	2	0.048489752	0.070008635	0.692625303	0.488544727	0	0.446839834	0	0.5	PRJNA827465;Yang2025_Silkie_Wenchang	CRA004775	0.857850753
ENSGALG00010021181	DAGLA	2	-0.238522577	0.288550007	-0.826624748	0.408449786	0.117649084	2.932233744	65.8963068	1	CRA004775;Yang2025_Silkie_Wenchang	PRJNA827465	0.834154426
ENSGALG00010021181	DAGLA	2	-0.194856954	0.340857023	-0.571667711	0.567547114	0.185339564	4.23491784	76.38679101	0.5	CRA004775;PRJNA827465	Yang2025_Silkie_Wenchang	0.883717772
ENSGALG00010021182	ZSWIM8	2	-0.068048024	0.062855141	-1.082616693	0.278978595	0	0.96113462	0	1	PRJNA827465;Yang2025_Silkie_Wenchang	CRA004775	0.777307224
ENSGALG00010021182	ZSWIM8	2	-0.151169207	0.102456234	-1.475451525	0.140091192	0	0.041131152	0	1	CRA004775;Yang2025_Silkie_Wenchang	PRJNA827465	0.623792706
ENSGALG00010021182	ZSWIM8	2	-0.035004865	0.071693016	-0.488260458	0.625365366	0	0.109846156	0	1	CRA004775;PRJNA827465	Yang2025_Silkie_Wenchang	0.901367193
ENSGALG00010021183	CFAP100	2	0.479077582	0.324839357	1.474813849	0.140262597	0.071988252	1.366038086	26.79559886	1	PRJNA827465;Yang2025_Silkie_Wenchang	CRA004775	0.624073416
ENSGALG00010021183	CFAP100	2	0.354688569	0.761044539	0.466054943	0.641176174	0.694533144	2.425577928	58.77271191	0.5	CRA004775;Yang2025_Silkie_Wenchang	PRJNA827465	0.906429868
ENSGALG00010021183	CFAP100	2	0.202198457	0.255066935	0.792727042	0.427936872	0	0.891665141	0	0.5	CRA004775;PRJNA827465	Yang2025_Silkie_Wenchang	0.84125685
ENSGALG00010021184	DRAXIN	2	0.018000987	0.250507711	0.071858016	0.942714902	0	0.442440919	0	0.5	PRJNA827465;Yang2025_Silkie_Wenchang	CRA004775	0.985827325
ENSGALG00010021184	DRAXIN	2	-0.162693304	0.276519066	-0.588361974	0.556289356	0	0.006316399	0	1	CRA004775;Yang2025_Silkie_Wenchang	PRJNA827465	0.880324407
ENSGALG00010021184	DRAXIN	2	0.032878073	0.248510641	0.132300463	0.894746641	0	0.343280304	0	0.5	CRA004775;PRJNA827465	Yang2025_Silkie_Wenchang	0.974565659
ENSGALG00010021186	SWT1	2	0.216810449	0.212941354	1.018169768	0.308597284	0.078688078	7.325055392	86.3482261	1	PRJNA827465;Yang2025_Silkie_Wenchang	CRA004775	0.793561833
ENSGALG00010021186	SWT1	2	0.332214005	0.131722812	2.522068875	0.011666686	0.011319008	1.467447261	31.85445047	1	CRA004775;Yang2025_Silkie_Wenchang	PRJNA827465	0.172836702
ENSGALG00010021186	SWT1	2	0.045565784	0.071143612	0.640476116	0.521863112	0	0.718978596	0	1	CRA004775;PRJNA827465	Yang2025_Silkie_Wenchang	0.86956507
ENSGALG00010021187	SEMA7A	2	-0.035822812	0.099725594	-0.359213828	0.719435133	0.011962628	2.491837705	59.8689755	0.5	PRJNA827465;Yang2025_Silkie_Wenchang	CRA004775	0.928449671
ENSGALG00010021187	SEMA7A	2	0.068864929	0.08023201	0.858322374	0.390714478	0	0.564471139	0	1	CRA004775;Yang2025_Silkie_Wenchang	PRJNA827465	0.829251057
ENSGALG00010021187	SEMA7A	2	-0.043906555	0.205762735	-0.213384384	0.831027159	0.042696059	1.441671659	30.63607834	0.5	CRA004775;PRJNA827465	Yang2025_Silkie_Wenchang	0.957460805
ENSGALG00010021188	TNRC18	2	-0.150308378	0.057753317	-2.602593004	0.009252172	0	0.005838256	0	1	PRJNA827465;Yang2025_Silkie_Wenchang	CRA004775	0.15088718
ENSGALG00010021188	TNRC18	2	-0.142114616	0.096106781	-1.478715795	0.139216289	0	0.200963502	0	1	CRA004775;Yang2025_Silkie_Wenchang	PRJNA827465	0.622954765
ENSGALG00010021188	TNRC18	2	-0.140364804	0.068397319	-2.052197471	0.040150475	0	0.18504275	0	1	CRA004775;PRJNA827465	Yang2025_Silkie_Wenchang	0.347067015
ENSGALG00010021189	ENSGALG00010021189	2	-0.027606043	0.343172588	-0.080443613	0.935884441	0	0.067686332	0	0.5	PRJNA827465;Yang2025_Silkie_Wenchang	CRA004775	0.984821049
ENSGALG00010021189	ENSGALG00010021189	2	-0.028228855	0.35843665	-0.078755494	0.937227105	0	0.074593291	0	0.5	CRA004775;Yang2025_Silkie_Wenchang	PRJNA827465	0.985114195
ENSGALG00010021189	ENSGALG00010021189	2	-0.119438305	0.347243628	-0.343961113	0.730875531	0	0.000592574	0	1	CRA004775;PRJNA827465	Yang2025_Silkie_Wenchang	0.932769533
ENSGALG00010021190	ENSGALG00010021190	2	-0.144255191	0.075732976	-1.904787036	0.05680776	0.007646612	2.999357982	66.65953161	1	PRJNA827465;Yang2025_Silkie_Wenchang	CRA004775	0.412396635
ENSGALG00010021190	ENSGALG00010021190	2	-0.204533096	0.059971411	-3.410509966	0.000648415	0	0.908652825	0	0.5	CRA004775;Yang2025_Silkie_Wenchang	PRJNA827465	0.028097751
ENSGALG00010021190	ENSGALG00010021190	2	-0.063866997	0.059316114	-1.076722549	0.281604235	0	0.094001969	0	0.5	CRA004775;PRJNA827465	Yang2025_Silkie_Wenchang	0.779295548
ENSGALG00010021191	MYRF	2	0.133259303	0.275596543	0.483530385	0.628719191	0.111045113	3.24736117	69.20576592	0.5	PRJNA827465;Yang2025_Silkie_Wenchang	CRA004775	0.902042431
ENSGALG00010021191	MYRF	2	0.238739834	0.319733316	0.746684259	0.455254174	0.074900348	1.515575232	34.01845196	0.5	CRA004775;Yang2025_Silkie_Wenchang	PRJNA827465	0.849072665
ENSGALG00010021191	MYRF	2	-0.090154733	0.108008862	-0.83469756	0.403888016	0	0.043552603	0	1	CRA004775;PRJNA827465	Yang2025_Silkie_Wenchang	0.833112102
ENSGALG00010021192	ENSGALG00010021192	2	-0.017701306	0.119215825	-0.148481175	0.881963041	0.019288673	3.110868709	67.85463826	0.5	PRJNA827465;Yang2025_Silkie_Wenchang	CRA004775	0.971928118
ENSGALG00010021192	ENSGALG00010021192	2	0.088381947	0.093520092	0.945058381	0.344629072	0	0.324548079	0	0.5	CRA004775;Yang2025_Silkie_Wenchang	PRJNA827465	0.806903268
ENSGALG00010021192	ENSGALG00010021192	2	-0.134635113	0.091475854	-1.471810386	0.141072088	0	0.00344123	0	1	CRA004775;PRJNA827465	Yang2025_Silkie_Wenchang	0.625584596
ENSGALG00010021193	USP37	2	-0.022462986	0.047291335	-0.474991585	0.634792971	0	0.009480079	0	1	PRJNA827465;Yang2025_Silkie_Wenchang	CRA004775	0.903958991
ENSGALG00010021193	USP37	2	-0.028297673	0.083440978	-0.339134008	0.734508781	0	0.002296051	0	1	CRA004775;Yang2025_Silkie_Wenchang	PRJNA827465	0.933520234
ENSGALG00010021193	USP37	2	-0.01957988	0.053755527	-0.364239386	0.715679258	0	1.71443E-05	0	1	CRA004775;PRJNA827465	Yang2025_Silkie_Wenchang	0.927131632
ENSGALG00010021194	CYP11A1	2	0.325635179	0.191203206	1.703084308	0.088552292	0	0.022762064	0	1	PRJNA827465;Yang2025_Silkie_Wenchang	CRA004775	0.511076251
ENSGALG00010021194	CYP11A1	2	-0.067614694	0.335426634	-0.201578191	0.840246497	0.074601089	1.495714678	33.1423289	0.5	CRA004775;Yang2025_Silkie_Wenchang	PRJNA827465	0.959662809
ENSGALG00010021194	CYP11A1	2	0.039008949	0.363339095	0.107362378	0.9145015	0.175743248	2.808195011	64.38993745	0.5	CRA004775;PRJNA827465	Yang2025_Silkie_Wenchang	0.979711523
ENSGALG00010021195	GCLM	2	-0.126908288	0.250725744	-0.506163773	0.612741684	0.113100365	9.783383903	89.77858776	0.5	PRJNA827465;Yang2025_Silkie_Wenchang	CRA004775	0.898034557
ENSGALG00010021195	GCLM	2	-0.275347192	0.170263307	-1.617184569	0.105838446	0.023431244	1.52635117	34.48427729	1	CRA004775;Yang2025_Silkie_Wenchang	PRJNA827465	0.552010594
ENSGALG00010021195	GCLM	2	0.100438557	0.08114535	1.237761092	0.215804659	0	0.228414863	0	0.5	CRA004775;PRJNA827465	Yang2025_Silkie_Wenchang	0.729213578

ENSGALG00010021196	ATP6V0A1	2	0.281712792	0.075912388	3.7110253	0.000206421	0	0.467158806	0	1	PRJNA827465;Yang2025_Silkie_Wenchang	CRA004775	0.013022445
ENSGALG00010021196	ATP6V0A1	2	-0.010801839	0.379304166	-0.02847804	0.977280883	0.251401463	7.46619293	86.60629307	0.5	CRA004775;Yang2025_Silkie_Wenchang	PRJNA827465	0.993487352
ENSGALG00010021196	ATP6V0A1	2	-0.056759888	0.326444677	-0.173872916	0.861965339	0.177916509	5.600953863	82.14589828	0.5	CRA004775;PRJNA827465	Yang2025_Silkie_Wenchang	0.96567952
ENSGALG00010021197	CHCHD1	2	0.314203062	0.077729548	4.042260234	5.29384E-05	0	0.666920551	0	1	PRJNA827465;Yang2025_Silkie_Wenchang	CRA004775	0.005037867
ENSGALG00010021197	CHCHD1	2	0.348581877	0.20650189	1.688032381	0.091405013	0	0.984087118	0	1	CRA004775;Yang2025_Silkie_Wenchang	PRJNA827465	0.518286636
ENSGALG00010021197	CHCHD1	2	0.284314728	0.078379747	3.627400419	0.000286289	0	0.304983989	0	1	CRA004775;PRJNA827465	Yang2025_Silkie_Wenchang	0.016522793
ENSGALG00010021199	TMEM258	2	-0.077889363	0.086272921	-0.902825147	0.366618702	0	0.724336881	0	1	PRJNA827465;Yang2025_Silkie_Wenchang	CRA004775	0.819403745
ENSGALG00010021199	TMEM258	2	-0.323895049	0.226543885	-1.429723203	0.152796478	0	0.06962484	0	1	CRA004775;Yang2025_Silkie_Wenchang	PRJNA827465	0.644767015
ENSGALG00010021199	TMEM258	2	-0.082474552	0.084094932	-0.980731544	0.326725144	0	0.58855201	0	1	CRA004775;PRJNA827465	Yang2025_Silkie_Wenchang	0.800028239
ENSGALG00010021200	FTO	2	-0.000999278	0.144267829	-0.006926546	0.99447346	0	0.045537948	0	0.5	PRJNA827465;Yang2025_Silkie_Wenchang	CRA004775	0.99823774
ENSGALG00010021200	FTO	2	0.02075237	0.190298927	0.109051427	0.913161701	0	0.014888706	0	0.5	CRA004775;Yang2025_Silkie_Wenchang	PRJNA827465	0.979129568
ENSGALG00010021200	FTO	2	-0.031716241	0.187020366	-0.169587095	0.865334872	0	5.20324E-05	0	1	CRA004775;PRJNA827465	Yang2025_Silkie_Wenchang	0.967024637
ENSGALG00010021201	TRMT1L	2	0.073020796	0.136842187	0.533613187	0.593609183	0.028323939	3.542125007	71.76835945	0.5	PRJNA827465;Yang2025_Silkie_Wenchang	CRA004775	0.892935613
ENSGALG00010021201	TRMT1L	2	0.253895316	0.117728163	2.156623435	0.031035017	0	0.012116782	0	1	CRA004775;Yang2025_Silkie_Wenchang	PRJNA827465	0.301028942
ENSGALG00010021201	TRMT1L	2	0.049798276	0.138242132	0.360225032	0.718678857	0.024197944	2.027937006	50.6888036	0.5	CRA004775;PRJNA827465	Yang2025_Silkie_Wenchang	0.928327969
ENSGALG00010021203	FUT11	2	-0.127806607	0.059495359	-2.148177776	0.031699635	0	0.002286671	0	1	PRJNA827465;Yang2025_Silkie_Wenchang	CRA004775	0.304916978
ENSGALG00010021203	FUT11	2	0.01884591	0.194386046	0.09695094	0.922765355	0.047783785	2.463970843	59.41510417	0.5	CRA004775;Yang2025_Silkie_Wenchang	PRJNA827465	0.981804675
ENSGALG00010021203	FUT11	2	0.011094837	0.187938383	0.059034437	0.952924679	0.049250923	2.719851929	63.23329262	0.5	CRA004775;PRJNA827465	Yang2025_Silkie_Wenchang	0.987758913
ENSGALG00010021204	NAGLU	2	0.017871604	0.060166932	0.297033655	0.766440815	0	0.298829162	0	1	PRJNA827465;Yang2025_Silkie_Wenchang	CRA004775	0.941152991
ENSGALG00010021204	NAGLU	2	-0.049878423	0.159631655	-0.312459475	0.754691357	0.015781052	1.445981936	30.84284282	0.5	CRA004775;Yang2025_Silkie_Wenchang	PRJNA827465	0.93838723
ENSGALG00010021204	NAGLU	2	-0.028208587	0.07804403	-0.361444517	0.717767175	0.003356288	1.185589436	15.65376939	0.5	CRA004775;PRJNA827465	Yang2025_Silkie_Wenchang	0.927927766
ENSGALG00010021207	MAD2L2	2	-0.020733798	0.060445209	-0.34301805	0.731584881	0	0.213461784	0	0.5	PRJNA827465;Yang2025_Silkie_Wenchang	CRA004775	0.932851968
ENSGALG00010021207	MAD2L2	2	-0.090746785	0.187844306	-0.483095744	0.629027756	0.033399986	1.665923924	39.97324936	0.5	CRA004775;Yang2025_Silkie_Wenchang	PRJNA827465	0.902155813
ENSGALG00010021207	MAD2L2	2	-0.090262177	0.125338895	-0.720144992	0.471435728	0.014100775	1.321449539	24.32552508	1	CRA004775;PRJNA827465	Yang2025_Silkie_Wenchang	0.853998373
ENSGALG00010021210	ENSGALG00010021210	2	-0.032940451	0.06219495	-0.529632245	0.596366933	0.003433485	1.531515295	34.70519012	0.5	PRJNA827465;Yang2025_Silkie_Wenchang	CRA004775	0.894064255
ENSGALG00010021210	ENSGALG00010021210	2	-0.118304009	0.095091606	-1.24410569	0.213460667	0	0.155707066	0	1	CRA004775;Yang2025_Silkie_Wenchang	PRJNA827465	0.726080554
ENSGALG00010021210	ENSGALG00010021210	2	0.002972889	0.042119629	0.070582039	0.943730406	0	0.037585948	0	0.5	CRA004775;PRJNA827465	Yang2025_Silkie_Wenchang	0.985827325
ENSGALG00010021211	FEN1	2	0.034231197	0.069231665	0.494444224	0.620992488	0	0.005201941	0	1	PRJNA827465;Yang2025_Silkie_Wenchang	CRA004775	0.899578487
ENSGALG00010021211	FEN1	2	-0.174835432	0.263042284	-0.664666646	0.506263732	0.092896747	2.668520355	62.52604938	0.5	CRA004775;Yang2025_Silkie_Wenchang	PRJNA827465	0.863929781
ENSGALG00010021211	FEN1	2	-0.161689485	0.266623103	-0.606434638	0.544226173	0.104008928	3.053518334	67.25089256	0.5	CRA004775;PRJNA827465	Yang2025_Silkie_Wenchang	0.877023301
ENSGALG00010021212	MYO18B	2	0.093890318	0.305554668	0.307278296	0.758631554	0.179467564	25.71997723	96.11197167	0.5	PRJNA827465;Yang2025_Silkie_Wenchang	CRA004775	0.938954369
ENSGALG00010021212	MYO18B	2	0.378614815	0.083661067	4.525579571	6.02302E-06	0	0.922170848	0	1	CRA004775;Yang2025_Silkie_Wenchang	PRJNA827465	0.000950694
ENSGALG00010021212	MYO18B	2	-0.193763753	0.081249845	-2.384789206	0.01708891	0	0.711503613	0	0.5	CRA004775;PRJNA827465	Yang2025_Silkie_Wenchang	0.217240225
ENSGALG00010021213	FBXO44	2	0.149375561	0.194233355	0.769052058	0.441862407	0.030003616	1.546638413	35.3436465	0.5	PRJNA827465;Yang2025_Silkie_Wenchang	CRA004775	0.845135451
ENSGALG00010021213	FBXO44	2	-0.306832405	0.164097819	-1.869814032	0.061509647	0	0.585421764	0	1	CRA004775;Yang2025_Silkie_Wenchang	PRJNA827465	0.428485228
ENSGALG00010021213	FBXO44	2	-0.043769391	0.340059383	-0.128711021	0.897586314	0.198270988	6.972210235	85.65734586	0.5	CRA004775;PRJNA827465	Yang2025_Silkie_Wenchang	0.975608116
ENSGALG00010021214	FBXL18	2	-0.1178566	0.155013938	-0.760296794	0.447077198	0.036976697	4.274692192	76.60650276	0.5	PRJNA827465;Yang2025_Silkie_Wenchang	CRA004775	0.84650962
ENSGALG00010021214	FBXL18	2	-0.296824789	0.110588094	-2.68405738	0.007273462	0	0.079755116	0	1	CRA004775;Yang2025_Silkie_Wenchang	PRJNA827465	0.131077397
ENSGALG00010021214	FBXL18	2	-0.082091919	0.177352134	-0.462875282	0.643453766	0.035300508	1.81144832	44.79555452	0.5	CRA004775;PRJNA827465	Yang2025_Silkie_Wenchang	0.906634523
ENSGALG00010021215	SH3PXD2A	2	0.155222306	0.102793325	1.510042664	0.131032538	0.007480358	1.415879963	29.37254386	1	PRJNA827465;Yang2025_Silkie_Wenchang	CRA004775	0.606767161
ENSGALG00010021215	SH3PXD2A	2	0.286667442	0.147172412	1.947834095	0.051434818	0	0.114325743	0	1	CRA004775;Yang2025_Silkie_Wenchang	PRJNA827465	0.393141348
ENSGALG00010021215	SH3PXD2A	2	0.097279896	0.083116668	1.170401774	0.241839322	0	0.121803102	0	1	CRA004775;PRJNA827465	Yang2025_Silkie_Wenchang	0.75406671
ENSGALG00010021217	FBXO2	2	-0.007193044	0.16679401	-0.043125312	0.965601642	0	0.25881127	0	0.5	PRJNA827465;Yang2025_Silkie_Wenchang	CRA004775	0.990668014
ENSGALG00010021217	FBXO2	2	-0.053366991	0.223492636	-0.23878635	0.811271255	0	0.718175164	0	0.5	CRA004775;Yang2025_Silkie_Wenchang	PRJNA827465	0.953179464
ENSGALG00010021217	FBXO2	2	-0.11486917	0.170229807	-0.674788813	0.499809949	0	0.256441877	0	1	CRA004775;PRJNA827465	Yang2025_Silkie_Wenchang	0.862078151
ENSGALG00010021218	DPF3	2	-0.104161587	0.13144623	-0.792427345	0.42811154	0	0.648103451	0	0.5	PRJNA827465;Yang2025_Silkie_Wenchang	CRA004775	0.84125685
ENSGALG00010021218	DPF3	2	0.384704061	0.333864476	1.152276114	0.249207629	0.138673281	2.61810968	61.80450317	1	CRA004775;Yang2025_Silkie_Wenchang	PRJNA827465	0.759411487
ENSGALG00010021218	DPF3	2	0.246100512	0.454169706	0.541869061	0.587908711	0.352983764	6.563482678	84.76418619	0.5	CRA004775;PRJNA827465	Yang2025_Silkie_Wenchang	0.891761457
ENSGALG00010021220	SYNPO2L	2	-0.082311903	0.168234858	-0.489267827	0.624652095	0.029674182	2.052559621	51.28034334	0.5	PRJNA827465;Yang2025_Silkie_Wenchang	CRA004775	0.90097623
ENSGALG00010021220	SYNPO2L	2	0.141696196	0.184315363	0.768770405	0.442029621	0	0.300464094	0	1	CRA004775;Yang2025_Silkie_Wenchang	PRJNA827465	0.845159086
ENSGALG00010021220	SYNPO2L	2	-0.164410139	0.203813483	-0.806669593	0.419856867	0.025844156	1.109605936	9.877915434	0.5	CRA004775;PRJNA827465	Yang2025_Silkie_Wenchang	0.838582908
ENSGALG00010021222	ENSGALG00010021222	2	-0.343660889	0.213842952	-1.607071384	0.10803871	0	0.800830503	0	1	PRJNA827465;Yang2025_Silkie_Wenchang	CRA004775	0.556189254
ENSGALG00010021222	ENSGALG00010021222	2	-0.211667745	0.407051628	-0.520002208	0.603062036	0.185465179	2.238290222	55.32304122	0.5	CRA004775;Yang2025_Silkie_Wenchang	PRJNA827465	0.895538966
ENSGALG00010021222	ENSGALG00010021222	2	-0.0618909	0.232713147	-0.265953602	0.790274943	0	0.700299134	0	0.5	CRA004775;PRJNA827465	Yang2025_Silkie_Wenchang	0.947390191
ENSGALG00010021223	DFFB	2	0.072380437	0.166620982	0.434401692	0.663996769	0.044923652	5.189128571	80.72894155	0.5	PRJNA827465;Yang2025_Silkie_Wenchang	CRA004775	0.91312035
ENSGALG00010021223	DFFB	2	0.161637635	0.152346083	1.060989766	0.288694552	0.017163659	1.367963451	26.89863175	0.5	CRA004775;Yang2025_Silkie_Wenchang	PRJNA827465	0.783385942
ENSGALG00010021223	DFFB	2	-0.088777352	0.085827451	-1.034370133	0.300963161	0	0.006338313	0	1	CRA004775;PRJNA827465	Yang2025_Silkie_Wenchang	0.791009438
ENSGALG00010021224	MYOZ1	2	-0.203370727	0.12374641	-1.643447485	0.100290382	0.011480143	1.5827651	36.81943074	1	PRJNA827465;Yang2025_Silkie_Wenchang	CRA004775	0.540998201
ENSGALG00010021224	MYOZ1	2	-0.073079875	0.15143946	-0.48256825	0.629402327	0	0.136529318	0	1	CRA004775;Yang2025_Silkie_Wenchang	PRJNA827465	0.902390168
ENSGALG00010021224	MYOZ1	2	-0.312642633	0.123087771	-2.539997513	0.011085326	0	0.002758187	0	1	CRA004775;PRJNA827465	Yang2025_Silkie_Wenchang	0.167383943
ENSGALG00010021227	CHCHD4	2	-0.171021702	0.215818978	-0.792431246	0.428109266	0.076495429	5.008757074	80.03496705	0.5	PRJNA827465;Yang2025_Silkie_Wenchang	CRA004775	0.84125685
ENSGALG00010021227	CHCHD4	2	-0.359603913	0.153682481	-2.339914811	0.019288139	0	0.393513568	0	1	CRA004775;Yang2025_Silkie_Wenchang	PRJNA827465	0.232624497
ENSGALG00010021227	CHCHD4	2	0.001676174	0.060664369	0.02763028	0.977957031	0	0.65783257	0	0.5	CRA004775;PRJNA827465	Yang2025_Silkie_Wenchang	0.993759894
ENSGALG00010021228	ESRP2	2	-0.664530003	0.828530632	-0.802058459	0.422519143	0.412407987	1.427865678	29.96540114	0.5	PRJNA827465;Yang2025_Silkie_Wenchang	CRA004775	0.839709737
ENSGALG00010021228	ESRP2	2	0.41398677	1.881992411	0.219972603	0.825892491	6.308729313	9.049529171	88.94970135	0.5	CRA004775;Yang2025_Silkie_Wenchang	PRJNA827465	0.956393839
ENSGALG00010021228	ESRP2	2	1.263144927	1.050128886	1.202847521	0.229035332	1.504364548	3.101678204	67.75938914	1	CRA004775;PRJNA827465	Yang2025_Silkie_Wenchang	0.741680452
ENSGALG00010021229	GLO1	2	-0.278009579	0.251610033	-1.104922468	0.269193186	0.10799891	6.54744809	84.72687395	1	PRJNA827465;Yang2025_Silkie_Wenchang	CRA004775	0.77176306
ENSGALG00010021229	GLO1	2	-0.167312571	0.455259878	-0.367510028	0.713238613	0.324667997	4.181094783	76.08281917	0.5	CRA004775;Yang2025_Silkie_Wenchang	PRJNA827465	0.926216079
ENSGALG00010021229	GLO1	2	-0.025513605	0.092741219	-0.275105343	0.783235307	0	0.957500606	0	0.5	CRA004775;PRJNA827465	Yang2025_Silkie_Wenchang	0.945729563
ENSGALG00010021230	C1orf174	2	-0.171633542	0.289246331	-0.593381915	0.552925589	0.162097841	31.58719144	96.83415981	0.5	PRJNA827465;Yang2025_Silkie_Wenchang	CRA004775	0.87928478
ENSGALG00010021230	C1orf174	2	-0.306579804	0.193778079	-1.582118088	0.113622615	0.054510642	3.337127879	70.03411208	1	CRA004775;Yang2025_Silkie_Wenchang	PRJNA827465	0.568794964
ENSGALG00010021230	C1orf174	2	0.103109693	0.042154023	2.446022609	0.014444194	0	0.851748691	0	0.5	CRA004775;PRJNA827465	Yang2025_Silkie_Wenchang	0.197724547
ENSGALG00010021231	STN1	2	-0.068034219	0.353174209	-0.192636431	0.847243712	0.225916251	10.50106299	90.47715454	0.5	PRJNA827465;Yang2025_Silkie_Wenchang	CRA004775	0.961252712
ENSGALG00010021231	STN1	2	-0.804307911	0.441923857	-1.820014687	0.068756768	0.302475889	4.032520239	75.20161238	1	CRA004775;Yang2025_Silkie_Wenchang	PRJNA827465	0.451745023
ENSGALG00010021231	STN1	2	-0.479842862	0.800389289	-0.599511848	0.548831612	1.193571932	13.95073863	92.83192076	0.5	CRA004775;PRJNA827465	Yang2025_Silkie_Wenchang	0.878281266
ENSGALG00010021232	ACTB	2	0.055473596	0.064315811	0.862518796	0.388402094	0	0.358628691	0	1	PRJNA827465;Yang2025_Silkie_Wenchang	CRA004775	0.828154121
ENSGALG00010021232	ACTB	2	-0.089314541	0.276424567	-0.32310638	0.746614678	0.104957918	2.945599638	66.05105504	0.5	CRA004775;Yang2025_Silkie_Wenchang	PRJNA827465	0.93667635
ENSGALG00010021232	ACTB	2	-0.108237727	0.215214072	-0.50293053	0.615013116	0.062467464	2.386814324	58.10315072	0.5	CRA004775;PRJNA827465	Yang2025_Silkie_Wenchang	0.898782646
ENSGALG00010021233	COASY	2	-0.004511219	0.038720275	-0.116507929	0.907250002	0	0.215482973	0	1	PRJNA827465;Yang2025_Silkie_Wenchang	CRA004775	0.978196186
ENSGALG00010021233	COASY	2	-0.095180165	0.11895007	-0.800169048	0.42361286	0	0.013913451	0	1	CRA004775;Yang2025_Silkie_Wenchang	PRJNA827465	0.84030043
ENSGALG00010021233	COASY	2	-0.006109619	0.038696	-0.157887613	0.874545359	0	0.400302184	0	1	CRA004775;PRJNA827465	Yang2025_Silkie_Wenchang	0.970145533
ENSGALG00010021234	ISLR2	2	0.034061819	0.587886438	0.057939454	0.953796856	0.627294757	10.80852538	90.74804412	0.5	PRJNA827465;Yang2025_Silkie_Wenchang	CRA004775	0.987838152
ENSGALG00010021234	ISLR2	2	-0.316602671	1.079946585	-0.29316512	0.769395943	1.985862267	6.087508561	83.57291838	0.5	CRA004775;Yang2025_Silkie_Wenchang	PRJNA827465	0.941790377
ENSGALG00010021234	ISLR2	2	-0.727186337	0.382712369	-1.900085796	0.057421861	0.118261433	1.306414675	23.45462595	1	CRA004775;PRJNA827465	Yang2025_Silkie_Wenchang	0.415091304
ENSGALG00010021235	XRCC5	2	-0.172915955	0.116333177	-1.486385573	0.137177164	0.019208316	3.441860972	70.94595022	1	PRJNA827465;Yang2025_Silkie_Wenchang	CRA004775	0.619615281
ENSGALG00010021235	XRCC5	2	-0.281020782	0.088804574	-3.164485458	0.001553574	0	0.238014172	0	1	CRA004775;Yang2025_Silkie_Wenchang	PRJNA827465	0.049561793
ENSGALG00010021235	XRCC5	2	-0.060361502	0.084016066	-0.718451898	0.472478697	0	0.009544201	0	1	CRA004775;PRJNA827465	Yang2025_Silkie_Wenchang	0.853998373
ENSGALG00010021236	BTBD9	2	-0.21877575	0.195786405	-1.117420537	0.263814558	0.069479654	10.46638179	90.44559983	1	PRJNA827465;Yang2025_Silkie_Wenchang	CRA004775	0.768643821
ENSGALG00010021236	BTBD9	2	-0.110996051	0.327708474	-0.338703632	0.734833004	0.194298254	10.26592591	90.25903743	0.5	CRA004775;Yang2025_Silkie_Wenchang	PRJNA827465	0.933537081
ENSGALG00010021236	BTBD9	2	0.048915812	0.121600422	0.402266798	0.687487687	0.017374124	1.991535194	49.78748039	0.5	CRA004775;PRJNA827465	Yang2025_Silkie_Wenchang	0.91949273
ENSGALG00010021237	FSCN1	2	-0.061962297	0.118518576	-0.522806632	0.601108824	0.014392701	1.837605546	45.5813571	0.5	PRJNA827465;Yang2025_Silkie_Wenchang	CRA004775	0.895230519
ENSGALG00010021237	FSCN1	2	-0.322697282	0.205022792	-1.573958093	0.115497138	0.023080235	1.185119768	15.62034258	1	CRA004775;Yang2025_Silkie_Wenchang	PRJNA827465	0.572716099
ENSGALG00010021237	FSCN1	2	-0.240269765	0.373744801	-0.642871191	0.520307684	0.201752423	2.766799093	63.85715166	0.5	CRA004775;PRJNA827465	Yang2025_Silkie_Wenchang	0.869331539
ENSGALG00010021238	NDUFS8	2	-0.066483646	0.183673357	-0.361966737	0.717376887	0.046654767	2.659625198	62.40071719	0.5	PRJNA827465;Yang2025_Silkie_Wenchang	CRA004775	0.9277944
ENSGALG00010021238	NDUFS8	2	0.140292263	0.196482626	0.714018665	0.475215638	0	0.171514666	0	0.5	CRA004775;Yang2025_Silkie_Wenchang	PRJNA827465	0.854462864
ENSGALG00010021238	NDUFS8	2	-0.193500461	0.067940294	-2.84809572	0.004398169	0	0.248016531	0	1	CRA004775;PRJNA827465	Yang2025_Silkie_Wenchang	0.097277013
ENSGALG00010021239	USP54	2	0.034374068	0.104340444	0.329441458	0.741822036	0.013854797	2.304334913	56.60353041	0.5	PRJNA827465;Yang2025_Silkie_Wenchang	CRA004775	0.93543545
ENSGALG00010021239	USP54	2	0.261721683	0.130587188	2.004191116	0.045049592	0.004689977	1.122443351	10.90864415	1	CRA004775;Yang2025_Silkie_Wenchang	PRJNA827465	0.3659545
ENSGALG00010021239	USP54	2	0.163951334	0.251555364	0.651750501	0.514562138	0.102180537	4.386914811	77.20493688	0.5	CRA004775;PRJNA827465	Yang2025_Silkie_Wenchang	0.866869697
ENSGALG00010021240	SLK	2	0.116564448	0.161755725	0.720620233	0.471143202	0.03707285	2.873491652	65.19913328	0.5	PRJNA827465;Yang2025_Silkie_Wenchang	CRA004775	0.853923969
ENSGALG00010021240	SLK	2	0.367049818	0.161676851	2.270268233	0.023191313	0	0.12110416	0	1	CRA004775;Yang2025_Silkie_Wenchang	PRJNA827465	0.259609281
ENSGALG00010021240	SLK	2	0.127404251	0.20922915	0.608922091	0.542576081	0.057425841	2.144895835	53.37768932	0.5	CRA004775;PRJNA827465	Yang2025_Silkie_Wenchang	0.876817086
ENSGALG00010021241	TMEM43	2	-0.18638654	0.069208775	-2.693105604	0.007078984	0	0.796253424	0	1	PRJNA827465;Yang2025_Silkie_Wenchang	CRA004775	0.129226809
ENSGALG00010021241	TMEM43	2	-0.277718304	0.100999105	-2.749710547	0.005964793	0	0.175417854	0	1	CRA004775;Yang2025_Silkie_Wenchang	PRJNA827465	0.116866528
ENSGALG00010021241	TMEM43	2	-0.159321273	0.085392395	-1.865754819	0.062075676	0	0.743532084	0	1	CRA004775;PRJNA827465	Yang2025_Silkie_Wenchang	0.42991371
ENSGALG00010021242	BCAR3	2	0.212251028	0.135859469	1.56228366	0.11822118	0	0.038994057	0	1	PRJNA827465;Yang2025_Silkie_Wenchang	CRA004775	0.578003524
ENSGALG00010021242	BCAR3	2	0.325642	0.210681251	1.545661983	0.122186223	0	0.997551756	0	1	CRA004775;Yang2025_Silkie_Wenchang	PRJNA827465	0.586288015
ENSGALG00010021242	BCAR3	2	0.301411973	0.254538373	1.184151408	0.236353133	0.047692832	1.240797807	19.40669186	1	CRA004775;PRJNA827465	Yang2025_Silkie_Wenchang	0.748669015
ENSGALG00010021243	DYL1	2	-0.263351257	0.157967273	-1.667125424	0.095489468	0.032057928	2.21390339	54.83091067	1	PRJNA827465;Yang2025_Silkie_Wenchang	CRA004775	0.529041803
ENSGALG00010021243	DYL1	2	-0.404261693	0.161989418	-2.495605568	0.012574233	0	0.404534556	0	1	CRA004775;Yang2025_Silkie_Wenchang	PRJNA827465	0.181574995
ENSGALG00010021243	DYL1	2	-0.16677547	0.061401208	-2.716159427	0.006604411	0	0.300943951	0	1	CRA004775;PRJNA827465	Yang2025_Silkie_Wenchang	0.124138283
ENSGALG00010021244	PPP3CB	2	0.02707512	0.037693903	0.718289124	0.472579035	0	0.702843733	0	1	PRJNA827465;Yang2025_Silkie_Wenchang	CRA004775	0.853998373
ENSGALG00010021244	PPP3CB	2	0.163751943	0.086574874	1.891448809	0.058564458	0.001231374	1.077382458	7.182450195	1	CRA004775;Yang2025_Silkie_Wenchang	PRJNA827465	0.419187562
ENSGALG00010021244	PPP3CB	2	0.114669221	0.132474303	0.865595957	0.386711773	0.026004829	3.199127681	68.74147894	1	CRA004775;PRJNA827465	Yang2025_Silkie_Wenchang	0.827062786
ENSGALG00010021245	ZFAND3	2	-0.187274652	0.081709363	-2.291960742	0.021907909	0.009759976	3.675345	72.7916699	1	PRJNA827465;Yang2025_Silkie_Wenchang	CRA004775	0.250960808
ENSGALG00010021245	ZFAND3	2	-0.003278073	0.296713925	-0.011047925	0.991185211	0.157042311	8.671305035	88.4677105	0.5	CRA004775;Yang2025_Silkie_Wenchang	PRJNA827465	0.99755571
ENSGALG00010021245	ZFAND3	2	0.066455	0.212505248	0.312721687	0.754492117	0.073738177	4.760105707	78.99206317	0.5	CRA004775;PRJNA827465	Yang2025_Silkie_Wenchang	0.93838723
ENSGALG00010021246	PECR	2	0.215306224	0.230405847	0.934465108	0.350064026	0.093870814	8.604228168	88.37780704	0.5	PRJNA827465;Yang2025_Silkie_Wenchang	CRA004775	0.810057397
ENSGALG00010021246	PECR	2	0.121303268	0.375474482	0.323066609	0.746644797	0.234785828	5.504394724	81.83269823	0.5	CRA004775;Yang2025_Silkie_Wenchang	PRJNA827465	0.936683091
ENSGALG00010021246	PECR	2	-0.040943945	0.095878179	-0.427041332	0.669349223	0	0.879399154	0	1	CRA004775;PRJNA827465	Yang2025_Silkie_Wenchang	0.914377769
ENSGALG00010021247	GNAO1	2	0.298548708	0.169232447	1.764133961	0.077709428	0.01895102	1.394887687	28.30964032	1	PRJNA827465;Yang2025_Silkie_Wenchang	CRA004775	0.480343171
ENSGALG00010021247	GNAO1	2	-0.487757876	1.103867422	-0.441862733	0.65858854	2.224132445	11.02176084	90.92703957	0.5	CRA004775;Yang2025_Silkie_Wenchang	PRJNA827465	0.911105434
ENSGALG00010021247	GNAO1	2	-0.643621467	0.918133983	-0.701010397	0.483296527	1.50720112	8.670341157	88.46642846	0.5	CRA004775;PRJNA827465	Yang2025_Silkie_Wenchang	0.85691916
ENSGALG00010021248	ANXA7	2	-0.028025002	0.072271735	-0.387772651	0.698184286	0	0.048725412	0	1	PRJNA827465;Yang2025_Silkie_Wenchang	CRA004775	0.921603258
ENSGALG00010021248	ANXA7	2	-0.023127404	0.127887329	-0.180842028	0.856491573	0	0.222942327	0	0.5	CRA004775;Yang2025_Silkie_Wenchang	PRJNA827465	0.96353738
ENSGALG00010021248	ANXA7	2	-0.009479711	0.079520681	-0.119210642	0.905108476	0	0.145334533	0	0.5	CRA004775;PRJNA827465	Yang2025_Silkie_Wenchang	0.977938157
ENSGALG00010021250	ISLR	2	-0.386196277	0.313364761	-1.232417695	0.217793094	0.173299607	8.428939888	88.13611186	1	PRJNA827465;Yang2025_Silkie_Wenchang	CRA004775	0.731179806
ENSGALG00010021250	ISLR	2	-0.388402835	0.362264309	-1.072153194	0.28365123	0.204911137	4.348389445	77.00297978	0.5	CRA004775;Yang2025_Silkie_Wenchang	PRJNA827465	0.779713546
ENSGALG00010021250	ISLR	2	-0.067835097	0.118522996	-0.572337005	0.567093684	0	0.096367256	0	0.5	CRA004775;PRJNA827465	Yang2025_Silkie_Wenchang	0.883487517
ENSGALG00010021251	RNF216	2	0.001381041	0.048687073	0.02836566	0.977370513	0	0.846013833	0	0.5	PRJNA827465;Yang2025_Silkie_Wenchang	CRA004775	0.993511271
ENSGALG00010021251	RNF216	2	-0.057089755	0.181751773	-0.314108378	0.753438727	0.045017344	3.118616981	67.9345041	0.5	CRA004775;Yang2025_Silkie_Wenchang	PRJNA827465	0.938087082
ENSGALG00010021251	RNF216	2	-0.085131946	0.106248909	-0.801250073	0.422986888	0.013291524	1.962697808	49.04972147	1	CRA004775;PRJNA827465	Yang2025_Silkie_Wenchang	0.840012882
ENSGALG00010021252	XPC	2	0.21941169	0.198148715	1.107308162	0.268160717	0.070506335	9.531468471	89.5084372	1	PRJNA827465;Yang2025_Silkie_Wenchang	CRA004775	0.771398294
ENSGALG00010021252	XPC	2	0.277895109	0.197151489	1.409551152	0.158672259	0.049025527	2.426706889	58.79189182	1	CRA004775;Yang2025_Silkie_Wenchang	PRJNA827465	0.65275931
ENSGALG00010021252	XPC	2	0.031276263	0.060191146	0.519615672	0.603331472	0	0.002194052	0	1	CRA004775;PRJNA827465	Yang2025_Silkie_Wenchang	0.895538966
ENSGALG00010021253	TMEM169	2	0.027235188	0.064408049	0.422853797	0.672401934	0	0.392246428	0	0.5	PRJNA827465;Yang2025_Silkie_Wenchang	CRA004775	0.914742762
ENSGALG00010021253	TMEM169	2	-0.03091434	0.174264359	-0.177399096	0.859194918	0.031352115	1.749138487	42.82899795	0.5	CRA004775;Yang2025_Silkie_Wenchang	PRJNA827465	0.964605083
ENSGALG00010021253	TMEM169	2	-0.042036177	0.100862446	-0.416767379	0.676848565	0.005269238	1.134238748	11.83514036	1	CRA004775;PRJNA827465	Yang2025_Silkie_Wenchang	0.917000315
ENSGALG00010021254	FNBP1L	2	-0.029250562	0.114176727	-0.256186726	0.797806658	0.016805118	2.200077903	54.54706406	0.5	PRJNA827465;Yang2025_Silkie_Wenchang	CRA004775	0.949922217
ENSGALG00010021254	FNBP1L	2	0.328817958	0.203100779	1.61898915	0.105449598	0.049501206	2.469085282	59.49917132	1	CRA004775;Yang2025_Silkie_Wenchang	PRJNA827465	0.551018754
ENSGALG00010021254	FNBP1L	2	0.193355365	0.326694639	0.591853499	0.553948695	0.193643975	9.958278318	89.95810352	0.5	CRA004775;PRJNA827465	Yang2025_Silkie_Wenchang	0.880026093
ENSGALG00010021255	EIF3F	2	-0.329139918	0.276809631	-1.189047926	0.234420808	0.142446562	13.75143431	92.72803129	1	PRJNA827465;Yang2025_Silkie_Wenchang	CRA004775	0.747509756
ENSGALG00010021255	EIF3F	2	-0.444623179	0.203613037	-2.183667531	0.028986687	0.053463302	2.706448219	63.05120516	1	CRA004775;Yang2025_Silkie_Wenchang	PRJNA827465	0.289878916
ENSGALG00010021255	EIF3F	2	-0.076469351	0.057886208	-1.321028858	0.186491742	0	0.426715363	0	1	CRA004775;PRJNA827465	Yang2025_Silkie_Wenchang	0.695457578
ENSGALG00010021256	ENSGALG00010021256	2	0.052833966	0.099506467	0.530960119	0.595446416	0	0.663579567	0	0.5	PRJNA827465;Yang2025_Silkie_Wenchang	CRA004775	0.893794893
ENSGALG00010021256	ENSGALG00010021256	2	0.088547798	0.145448685	0.608790641	0.542663218	0	0.9250853	0	0.5	CRA004775;Yang2025_Silkie_Wenchang	PRJNA827465	0.876913595
ENSGALG00010021256	ENSGALG00010021256	2	-0.034102951	0.117930627	-0.289178069	0.772445113	0	0.280078288	0	1	CRA004775;PRJNA827465	Yang2025_Silkie_Wenchang	0.943066211
ENSGALG00010021257	RGS6	2	0.390123775	0.185173333	2.106803219	0.03513464	0	0.72566434	0	1	PRJNA827465;Yang2025_Silkie_Wenchang	CRA004775	0.323167978
ENSGALG00010021257	RGS6	2	-0.080286324	0.773305837	-0.103822214	0.917310438	0.916531696	3.698748147	72.96382559	0.5	CRA004775;Yang2025_Silkie_Wenchang	PRJNA827465	0.98082192
ENSGALG00010021257	RGS6	2	-0.198317439	0.597531773	-0.331894382	0.739969013	0.461488217	2.342803272	57.31609172	0.5	CRA004775;PRJNA827465	Yang2025_Silkie_Wenchang	0.935072094
ENSGALG00010021258	LSM3	2	-0.507491534	0.406739645	-1.247706094	0.212138709	0.309535181	14.97316688	93.32138613	1	PRJNA827465;Yang2025_Silkie_Wenchang	CRA004775	0.72461062
ENSGALG00010021258	LSM3	2	-0.730055517	0.252157938	-2.895231148	0.003788794	0.066968209	2.018188329	50.45061031	1	CRA004775;Yang2025_Silkie_Wenchang	PRJNA827465	0.088217406
ENSGALG00010021258	LSM3	2	-0.137787837	0.073470038	-1.875428949	0.060733736	0	0.907174338	0	1	CRA004775;PRJNA827465	Yang2025_Silkie_Wenchang	0.42686302
ENSGALG00010021259	CEP104	2	0.047956925	0.053220845	0.901092878	0.367538939	0	0.954025093	0	0.5	PRJNA827465;Yang2025_Silkie_Wenchang	CRA004775	0.819975483
ENSGALG00010021259	CEP104	2	-0.032129528	0.095073991	-0.337942352	0.735406631	0	0.016992038	0	0.5	CRA004775;Yang2025_Silkie_Wenchang	PRJNA827465	0.933592889
ENSGALG00010021259	CEP104	2	0.075590833	0.06074328	1.244431211	0.213340902	0	0.086187392	0	1	CRA004775;PRJNA827465	Yang2025_Silkie_Wenchang	0.725928657
ENSGALG00010021260	KDM4A	2	-0.026785747	0.038742187	-0.691384497	0.489323944	0	0.513813411	0	1	PRJNA827465;Yang2025_Silkie_Wenchang	CRA004775	0.858337006
ENSGALG00010021260	KDM4A	2	-0.083533089	0.069082526	-1.209178273	0.226594363	0	0.232613262	0	1	CRA004775;Yang2025_Silkie_Wenchang	PRJNA827465	0.740117864
ENSGALG00010021260	KDM4A	2	-0.017493448	0.044499923	-0.393111868	0.694236859	0	0.635922724	0	1	CRA004775;PRJNA827465	Yang2025_Silkie_Wenchang	0.920728119
ENSGALG00010021261	JUP	2	0.130863313	0.159534277	0.820283357	0.412054592	0.043307075	6.462705837	84.52660503	0.5	PRJNA827465;Yang2025_Silkie_Wenchang	CRA004775	0.835532508
ENSGALG00010021261	JUP	2	0.277578757	0.104601158	2.653687239	0.007961758	0	0.617923473	0	1	CRA004775;Yang2025_Silkie_Wenchang	PRJNA827465	0.138706114
ENSGALG00010021261	JUP	2	-0.015174171	0.060682446	-0.250058667	0.802541979	0	0.010686027	0	0.5	CRA004775;PRJNA827465	Yang2025_Silkie_Wenchang	0.950688375
ENSGALG00010021262	MDGA1	2	0.064081149	0.162688678	0.393888194	0.693663587	0	0.108069637	0	0.5	PRJNA827465;Yang2025_Silkie_Wenchang	CRA004775	0.920721644
ENSGALG00010021262	MDGA1	2	0.066817074	0.248592743	0.268781273	0.788098005	0	0.505165432	0	0.5	CRA004775;Yang2025_Silkie_Wenchang	PRJNA827465	0.946919116
ENSGALG00010021262	MDGA1	2	0.139649539	0.194843824	0.71672551	0.473543482	0	0.325807803	0	1	CRA004775;PRJNA827465	Yang2025_Silkie_Wenchang	0.854373829
ENSGALG00010021263	STOML1	2	0.185270577	0.170847518	1.084420649	0.278178332	0.038001605	2.745894355	63.58199294	1	PRJNA827465;Yang2025_Silkie_Wenchang	CRA004775	0.776763074
ENSGALG00010021263	STOML1	2	0.09815789	0.313098483	0.313504839	0.753897145	0.149143585	4.076017967	75.46625142	0.5	CRA004775;Yang2025_Silkie_Wenchang	PRJNA827465	0.938265341
ENSGALG00010021263	STOML1	2	-0.008283385	0.10535224	-0.078625621	0.937330408	0.000661453	1.016800584	1.652298779	0.5	CRA004775;PRJNA827465	Yang2025_Silkie_Wenchang	0.985114195
ENSGALG00010021264	CBFB	2	-0.09786047	0.110304684	-0.887183266	0.37498024	0.010575969	1.649392797	39.37162805	0.5	PRJNA827465;Yang2025_Silkie_Wenchang	CRA004775	0.822916144
ENSGALG00010021264	CBFB	2	-0.194985272	0.30406366	-0.641264633	0.521350763	0.129714398	3.065293485	67.3766964	0.5	CRA004775;Yang2025_Silkie_Wenchang	PRJNA827465	0.869518515
ENSGALG00010021264	CBFB	2	-0.252896291	0.153156583	-1.651227037	0.098692224	0.020990233	1.384670697	27.78066279	1	CRA004775;PRJNA827465	Yang2025_Silkie_Wenchang	0.536091342
ENSGALG00010021265	SIPA1L1	2	0.003655705	0.112177519	0.032588572	0.974002683	0.013675633	1.926363986	48.08873051	0.5	PRJNA827465;Yang2025_Silkie_Wenchang	CRA004775	0.992492038
ENSGALG00010021265	SIPA1L1	2	0.201108373	0.131917239	1.524504112	0.12738283	0	0.203639422	0	1	CRA004775;Yang2025_Silkie_Wenchang	PRJNA827465	0.5992253
ENSGALG00010021265	SIPA1L1	2	0.034890031	0.169160157	0.206254428	0.836592175	0.035605386	2.08301907	51.99275829	0.5	CRA004775;PRJNA827465	Yang2025_Silkie_Wenchang	0.958399882
ENSGALG00010021266	COQ5	2	0.070553444	0.074273593	0.949912899	0.342156512	0.00118955	1.075409831	7.012194651	0.5	PRJNA827465;Yang2025_Silkie_Wenchang	CRA004775	0.806005903
ENSGALG00010021266	COQ5	2	-0.197153019	0.158824494	-1.241326285	0.214485238	0.014098193	1.351210334	25.99227711	1	CRA004775;Yang2025_Silkie_Wenchang	PRJNA827465	0.727205473
ENSGALG00010021266	COQ5	2	-0.101135967	0.252357436	-0.400764759	0.688593327	0.101759958	4.379472346	77.16619901	0.5	CRA004775;PRJNA827465	Yang2025_Silkie_Wenchang	0.919798626
ENSGALG00010021267	LRRC47	2	-0.014925145	0.095262295	-0.156674209	0.875501615	0.011505266	2.524082638	60.38164579	0.5	PRJNA827465;Yang2025_Silkie_Wenchang	CRA004775	0.970347777
ENSGALG00010021267	LRRC47	2	-0.05823609	0.20172192	-0.288694903	0.772814863	0.055294881	2.84169689	64.80975845	0.5	CRA004775;Yang2025_Silkie_Wenchang	PRJNA827465	0.943066211
ENSGALG00010021267	LRRC47	2	-0.105572384	0.056343404	-1.87373104	0.060967505	0	0.917320662	0	1	CRA004775;PRJNA827465	Yang2025_Silkie_Wenchang	0.427185264
ENSGALG00010021269	NUDT13	2	0.261185569	0.30072073	0.868531972	0.385103177	0.167001264	12.90540717	92.25130996	0.5	PRJNA827465;Yang2025_Silkie_Wenchang	CRA004775	0.826444827
ENSGALG00010021269	NUDT13	2	0.518798331	0.127459375	4.070303429	4.69519E-05	0	0.851302314	0	1	CRA004775;Yang2025_Silkie_Wenchang	PRJNA827465	0.004633453
ENSGALG00010021269	NUDT13	2	-0.007780903	0.088936851	-0.087487958	0.930283657	0	0.757400191	0	0.5	CRA004775;PRJNA827465	Yang2025_Silkie_Wenchang	0.983813577
ENSGALG00010021271	ENSGALG00010021271	2	-0.086833708	0.219554794	-0.395499031	0.692474637	0.086053834	9.261046026	89.20208368	0.5	PRJNA827465;Yang2025_Silkie_Wenchang	CRA004775	0.920559211
ENSGALG00010021271	ENSGALG00010021271	2	-0.034854886	0.314590771	-0.110794371	0.911779409	0.162929414	5.26560261	81.00882132	0.5	CRA004775;Yang2025_Silkie_Wenchang	PRJNA827465	0.978895589
ENSGALG00010021271	ENSGALG00010021271	2	0.148957808	0.084154025	1.770061598	0.07671688	0	0.531723506	0	1	CRA004775;PRJNA827465	Yang2025_Silkie_Wenchang	0.477904868
ENSGALG00010021272	ENSGALG00010021272	2	0.018787476	0.102128552	0.183959093	0.854045546	0	0.900928595	0	0.5	PRJNA827465;Yang2025_Silkie_Wenchang	CRA004775	0.962975689

ENSGALG00010021272	ENSGALG00010021272	2	-0.173541831	0.154211525	-1.125349294	0.260441049	0	0.707220551	0	1	CRA004775;Yang2025_Silkie_Wenchang	PRJNA827465	0.767694434
ENSGALG00010021272	ENSGALG00010021272	2	-0.072769761	0.246604339	-0.295087108	0.767927338	0.071410705	1.994857842	49.87111466	0.5	CRA004775;PRJNA827465	Yang2025_Silkie_Wenchang	0.941444269
ENSGALG00010021275	MAP3K2	2	-0.004538128	0.124892505	-0.036336275	0.971014226	0.022627326	3.348850432	70.13900679	0.5	PRJNA827465;Yang2025_Silkie_Wenchang	CRA004775	0.991885126
ENSGALG00010021275	MAP3K2	2	0.12725581	0.107249255	1.186542609	0.235408085	0	0.083739133	0	1	CRA004775;Yang2025_Silkie_Wenchang	PRJNA827465	0.748510012
ENSGALG00010021275	MAP3K2	2	-0.094211476	0.058986607	-1.597167245	0.110228432	0	0.691833252	0	0.5	CRA004775;PRJNA827465	Yang2025_Silkie_Wenchang	0.562335482
ENSGALG00010021277	ENSGALG00010021277	2	0.127888069	0.133477348	0.958125638	0.337999409	0	0.678281001	0	1	PRJNA827465;Yang2025_Silkie_Wenchang	CRA004775	0.80415385
ENSGALG00010021277	ENSGALG00010021277	2	0.406022436	0.199514949	2.035047694	0.041846074	0	0.389225784	0	1	CRA004775;Yang2025_Silkie_Wenchang	PRJNA827465	0.354227064
ENSGALG00010021277	ENSGALG00010021277	2	0.233618449	0.238627671	0.97900821	0.327557923	0.062419903	1.982228434	49.5517276	1	CRA004775;PRJNA827465	Yang2025_Silkie_Wenchang	0.800119103
ENSGALG00010021287	COL17A1	2	-0.810837719	0.917334348	-0.88390642	0.376746731	1.328861438	4.741630159	78.91020669	0.5	PRJNA827465;Yang2025_Silkie_Wenchang	CRA004775	0.822974013
ENSGALG00010021287	COL17A1	2	-0.301131723	1.481217506	-0.203300138	0.838900448	3.899525098	8.950095268	88.82693457	0.5	CRA004775;Yang2025_Silkie_Wenchang	PRJNA827465	0.959283372
ENSGALG00010021287	COL17A1	2	0.528221417	0.551032765	0.958602557	0.337759004	0.186866538	1.415992469	29.37815547	1	CRA004775;PRJNA827465	Yang2025_Silkie_Wenchang	0.80415385
ENSGALG00010021290	ERCC3	2	0.032456754	0.065528787	0.495305272	0.62038465	0.004962898	1.960740384	48.99885735	0.5	PRJNA827465;Yang2025_Silkie_Wenchang	CRA004775	0.899395593
ENSGALG00010021290	ERCC3	2	0.08950948	0.079879229	1.120560145	0.262475138	0	0.640797228	0	0.5	CRA004775;Yang2025_Silkie_Wenchang	PRJNA827465	0.768418419
ENSGALG00010021290	ERCC3	2	-0.010801959	0.033909123	-0.318556113	0.750063138	0	0.000348296	0	1	CRA004775;PRJNA827465	Yang2025_Silkie_Wenchang	0.937374353
ENSGALG00010021293	PCNX1	2	-0.113303223	0.116951387	-0.968806148	0.332641919	0.022509568	5.342469652	81.28206494	1	PRJNA827465;Yang2025_Silkie_Wenchang	CRA004775	0.802178578
ENSGALG00010021293	PCNX1	2	-0.221725946	0.086650883	-2.55884231	0.010502137	0	0.674076979	0	0.5	CRA004775;Yang2025_Silkie_Wenchang	PRJNA827465	0.1632418
ENSGALG00010021293	PCNX1	2	-0.008275647	0.045522976	-0.181790542	0.855747107	0	0.002401795	0	0.5	CRA004775;PRJNA827465	Yang2025_Silkie_Wenchang	0.963486168
ENSGALG00010021296	GATC	2	-0.063517166	0.062628211	-1.014194167	0.310490112	0	0.050701114	0	1	PRJNA827465;Yang2025_Silkie_Wenchang	CRA004775	0.794899969
ENSGALG00010021296	GATC	2	-0.081133129	0.132546844	-0.612109095	0.540465581	0	0.036757174	0	1	CRA004775;Yang2025_Silkie_Wenchang	PRJNA827465	0.875506901
ENSGALG00010021296	GATC	2	-0.055536328	0.067380152	-0.824223839	0.409812384	0	0.007908674	0	1	CRA004775;PRJNA827465	Yang2025_Silkie_Wenchang	0.834652129
ENSGALG00010021298	RPGRIP1L	2	0.118010552	0.103251054	1.142947672	0.253060318	0	0.000620065	0	1	PRJNA827465;Yang2025_Silkie_Wenchang	CRA004775	0.762731469
ENSGALG00010021298	RPGRIP1L	2	-0.041781963	0.244902007	-0.170606862	0.864532902	0.062260341	1.789041926	44.10416069	0.5	CRA004775;Yang2025_Silkie_Wenchang	PRJNA827465	0.966723659
ENSGALG00010021298	RPGRIP1L	2	-0.038493082	0.23950489	-0.160719398	0.872314411	0.062081729	1.813155817	44.84754201	0.5	CRA004775;PRJNA827465	Yang2025_Silkie_Wenchang	0.969626388
ENSGALG00010021301	SMURF1	2	0.008805456	0.140297276	0.062762844	0.949955354	0.031370298	4.516332087	77.8581384	0.5	PRJNA827465;Yang2025_Silkie_Wenchang	CRA004775	0.987277827
ENSGALG00010021301	SMURF1	2	-0.139313343	0.111561481	-1.248758453	0.211734434	0	0.090995293	0	1	CRA004775;Yang2025_Silkie_Wenchang	PRJNA827465	0.724064961
ENSGALG00010021301	SMURF1	2	0.121955724	0.052069199	2.342185531	0.019171182	0	0.503102997	0	0.5	CRA004775;PRJNA827465	Yang2025_Silkie_Wenchang	0.231912888
ENSGALG00010021302	P4HA1	2	-0.00601464	0.040197192	-0.149628356	0.881057835	0	0.208820435	0	0.5	PRJNA827465;Yang2025_Silkie_Wenchang	CRA004775	0.971685305
ENSGALG00010021302	P4HA1	2	0.170936109	0.248454397	0.687997923	0.49145409	0.099351172	4.701001973	78.72793916	0.5	CRA004775;Yang2025_Silkie_Wenchang	PRJNA827465	0.85895707
ENSGALG00010021302	P4HA1	2	0.183480196	0.223014276	0.822728481	0.410662415	0.080284349	4.38496245	77.19478761	1	CRA004775;PRJNA827465	Yang2025_Silkie_Wenchang	0.816028074
ENSGALG00010021307	MAP3K9	2	-0.019181924	0.164827007	-0.1163761	0.907354476	0.009108583	1.177603056	15.08174209	0.5	PRJNA827465;Yang2025_Silkie_Wenchang	CRA004775	0.978196186
ENSGALG00010021307	MAP3K9	2	-0.007685444	0.278081158	-0.027637414	0.977951341	0.054140822	1.491693837	32.96211493	0.5	CRA004775;Yang2025_Silkie_Wenchang	PRJNA827465	0.993759894
ENSGALG00010021307	MAP3K9	2	-0.177784849	0.162708797	-1.092656651	0.27454458	0	0.279711578	0	1	CRA004775;PRJNA827465	Yang2025_Silkie_Wenchang	0.774528691
ENSGALG00010021309	ST3GAL3	2	-0.231706911	0.259332107	-0.893475604	0.371602531	0.118963589	8.181192933	87.77684369	0.5	PRJNA827465;Yang2025_Silkie_Wenchang	CRA004775	0.82156974
ENSGALG00010021309	ST3GAL3	2	-0.468989071	0.155305404	-3.019785907	0.002529534	0	0.493566826	0	1	CRA004775;Yang2025_Silkie_Wenchang	PRJNA827465	0.067541822
ENSGALG00010021309	ST3GAL3	2	-0.000165161	0.068204214	-0.00242157	0.998067869	0	0.275299114	0	0.5	CRA004775;PRJNA827465	Yang2025_Silkie_Wenchang	0.999313737
ENSGALG00010021311	TRIAP1	2	-0.31615343	0.15174566	-2.083442958	0.037210804	0.031043237	2.85557948	64.98083814	1	PRJNA827465;Yang2025_Silkie_Wenchang	CRA004775	0.332764183
ENSGALG00010021311	TRIAP1	2	-0.11044868	0.138067044	-0.799964106	0.423731594	0	0.070815601	0	1	CRA004775;Yang2025_Silkie_Wenchang	PRJNA827465	0.84030043
ENSGALG00010021311	TRIAP1	2	-0.328238342	0.177872046	-1.845362153	0.064984875	0.036774175	1.911154253	47.67559978	1	CRA004775;PRJNA827465	Yang2025_Silkie_Wenchang	0.439528572
ENSGALG00010021314	ZP3	2	0.128317155	0.345485845	0.371410746	0.710331623	0.188244163	4.724461341	78.83356582	0.5	PRJNA827465;Yang2025_Silkie_Wenchang	CRA004775	0.925364369
ENSGALG00010021314	ZP3	2	1.839941309	1.424575799	1.291571365	0.196505626	3.825463032	16.80684805	94.05004438	1	CRA004775;Yang2025_Silkie_Wenchang	PRJNA827465	0.707841684
ENSGALG00010021314	ZP3	2	1.50611029	1.77080106	0.850524841	0.395033356	6.039266239	26.37008277	96.20782381	0.5	CRA004775;PRJNA827465	Yang2025_Silkie_Wenchang	0.830643015
ENSGALG00010021315	COX6A1	2	-0.136832424	0.095262639	-1.436370277	0.150897006	0	0.033680437	0	1	PRJNA827465;Yang2025_Silkie_Wenchang	CRA004775	0.642051813
ENSGALG00010021315	COX6A1	2	0.042490308	0.170877779	0.248659063	0.803624518	0.007363706	1.132288997	11.68383244	0.5	CRA004775;Yang2025_Silkie_Wenchang	PRJNA827465	0.951064358
ENSGALG00010021315	COX6A1	2	-0.025857004	0.143670087	-0.17997486	0.857172305	0.021166804	1.864810623	46.37525186	0.5	CRA004775;PRJNA827465	Yang2025_Silkie_Wenchang	0.963878155
ENSGALG00010021316	PLA2G12B	2	0.973544963	0.396994915	2.452285723	0.01419519	0	0.217116453	0	1	PRJNA827465;Yang2025_Silkie_Wenchang	CRA004775	0.19521731
ENSGALG00010021316	PLA2G12B	2	1.380195216	0.608956793	2.266491205	0.023421325	0.423368346	2.282264329	56.18386585	1	CRA004775;Yang2025_Silkie_Wenchang	PRJNA827465	0.261142791
ENSGALG00010021316	PLA2G12B	2	1.677301269	0.477861261	3.54009045	0.000399384	0	0.772794704	0	1	CRA004775;PRJNA827465	Yang2025_Silkie_Wenchang	0.020176083
ENSGALG00010021318	TP73	2	0.248647795	0.133054454	1.868767163	0.061655215	0	0.283233222	0	1	PRJNA827465;Yang2025_Silkie_Wenchang	CRA004775	0.428734062
ENSGALG00010021318	TP73	2	0.048588196	0.220345417	0.220509218	0.825474594	0.042438396	1.593266839	37.23587441	0.5	CRA004775;Yang2025_Silkie_Wenchang	PRJNA827465	0.956386623
ENSGALG00010021318	TP73	2	0.0835233	0.307917592	0.271252121	0.786197129	0.113015798	2.374279835	57.8819655	0.5	CRA004775;PRJNA827465	Yang2025_Silkie_Wenchang	0.946639719
ENSGALG00010021320	SLC6A6	2	-0.147350265	0.160985347	-0.915302343	0.360032929	0.034196298	2.845517826	64.85701158	1	PRJNA827465;Yang2025_Silkie_Wenchang	CRA004775	0.814880744
ENSGALG00010021320	SLC6A6	2	-0.328225851	0.153589104	-2.137038646	0.032594848	0	0.003817098	0	1	CRA004775;Yang2025_Silkie_Wenchang	PRJNA827465	0.310037591
ENSGALG00010021320	SLC6A6	2	-0.015874141	0.10616751	-0.149519764	0.881143515	0	0.240695574	0	1	CRA004775;PRJNA827465	Yang2025_Silkie_Wenchang	0.971705487
ENSGALG00010021325	ARPC1A	2	-0.158505647	0.114398258	-1.385559972	0.165883308	0.018503544	2.818673655	64.5223207	1	PRJNA827465;Yang2025_Silkie_Wenchang	CRA004775	0.66029196
ENSGALG00010021325	ARPC1A	2	-0.180571028	0.154230569	-1.17078624	0.24168471	0.022844644	1.886345852	46.98745203	1	CRA004775;Yang2025_Silkie_Wenchang	PRJNA827465	0.75406671
ENSGALG00010021325	ARPC1A	2	-0.070587774	0.040210821	-1.755442236	0.079183693	0	0.151237665	0	1	CRA004775;PRJNA827465	Yang2025_Silkie_Wenchang	0.484279839
ENSGALG00010021329	PSMC3IP	2	0.174049816	0.217872255	0.798861772	0.424370568	0.070481495	3.41398243	70.70869518	0.5	PRJNA827465;Yang2025_Silkie_Wenchang	CRA004775	0.840628469
ENSGALG00010021329	PSMC3IP	2	0.104702569	0.38404078	0.272633986	0.785134584	0.213026173	3.474849464	71.22177492	0.5	CRA004775;Yang2025_Silkie_Wenchang	PRJNA827465	0.946325593
ENSGALG00010021329	PSMC3IP	2	-0.019449424	0.083808849	-0.232068854	0.816484536	0	0.830739202	0	1	CRA004775;PRJNA827465	Yang2025_Silkie_Wenchang	0.95460349
ENSGALG00010021330	COMMD4	2	0.250611228	0.306503302	0.817646092	0.4135593	0.154325535	5.125714692	80.49052552	0.5	PRJNA827465;Yang2025_Silkie_Wenchang	CRA004775	0.836246395
ENSGALG00010021330	COMMD4	2	0.190003918	0.41218795	0.460969313	0.644824218	0.276797364	5.393089842	81.45775373	0.5	CRA004775;Yang2025_Silkie_Wenchang	PRJNA827465	0.909053008
ENSGALG00010021330	COMMD4	2	-0.044526721	0.093775929	-0.474820363	0.634915018	0	0.586702128	0	1	CRA004775;PRJNA827465	Yang2025_Silkie_Wenchang	0.904034523
ENSGALG00010021332	OIT3	2	0.981435737	0.217918805	4.5036762	6.67879E-06	0.072650272	4.237634956	76.40193149	1	PRJNA827465;Yang2025_Silkie_Wenchang	CRA004775	0.001019574
ENSGALG00010021332	OIT3	2	0.773579932	0.466817512	1.657135632	0.097492057	0.38004519	7.529858234	86.71953749	1	CRA004775;Yang2025_Silkie_Wenchang	PRJNA827465	0.533219726
ENSGALG00010021332	OIT3	2	0.592332272	0.239616112	2.472005185	0.013435757	0.070125863	2.276584638	56.07455205	1	CRA004775;PRJNA827465	Yang2025_Silkie_Wenchang	0.189678717
ENSGALG00010021335	NFATC3	2	-0.048906274	0.050848876	-0.961796575	0.336151799	0	0.003020506	0	1	PRJNA827465;Yang2025_Silkie_Wenchang	CRA004775	0.803727281
ENSGALG00010021335	NFATC3	2	0.168596844	0.216508793	0.778706686	0.436152517	0.042320106	1.819082871	45.02724334	0.5	CRA004775;Yang2025_Silkie_Wenchang	PRJNA827465	0.8431095
ENSGALG00010021335	NFATC3	2	0.113168739	0.214635819	0.527259335	0.598013509	0.069819701	3.362354223	70.25893366	0.5	CRA004775;PRJNA827465	Yang2025_Silkie_Wenchang	0.894645663
ENSGALG00010021338	PDIA5	2	-0.082179108	0.059396637	-1.383565008	0.166491689	0	0.472794194	0	1	PRJNA827465;Yang2025_Silkie_Wenchang	CRA004775	0.66540806
ENSGALG00010021338	PDIA5	2	0.131715092	0.27197173	0.484296997	0.628175107	0.133951914	10.41707881	90.40037982	0.5	CRA004775;Yang2025_Silkie_Wenchang	PRJNA827465	0.901904489
ENSGALG00010021338	PDIA5	2	0.168753043	0.230401108	0.732431558	0.463905203	0.093467133	8.100551566	87.6551616	0.5	CRA004775;PRJNA827465	Yang2025_Silkie_Wenchang	0.852159649
ENSGALG00010021341	L3MBTL1	2	0.320315446	0.097516765	3.284721835	0.001020831	0	0.929120709	0	1	PRJNA827465;Yang2025_Silkie_Wenchang	CRA004775	0.038357286
ENSGALG00010021341	L3MBTL1	2	-0.18238763	0.67995038	-0.268236676	0.788517145	0.840073888	10.50705002	90.48258076	0.5	CRA004775;Yang2025_Silkie_Wenchang	PRJNA827465	0.947048735
ENSGALG00010021341	L3MBTL1	2	-0.268682138	0.578216016	-0.464674326	0.642164697	0.594435468	8.354683297	88.03066538	0.5	CRA004775;PRJNA827465	Yang2025_Silkie_Wenchang	0.906634523
ENSGALG00010021342	SFR1	2	-0.556763287	0.37625825	-1.479737086	0.138943424	0.251039213	8.603055286	88.37622255	1	PRJNA827465;Yang2025_Silkie_Wenchang	CRA004775	0.622711797
ENSGALG00010021342	SFR1	2	-0.960806825	0.213568166	-4.498829787	6.83285E-06	0	0.009376325	0	1	CRA004775;Yang2025_Silkie_Wenchang	PRJNA827465	0.001032891
ENSGALG00010021342	SFR1	2	-0.248960674	0.193536979	-1.286372637	0.198313043	0.022847486	1.070373325	6.574652312	1	CRA004775;PRJNA827465	Yang2025_Silkie_Wenchang	0.710296638
ENSGALG00010021345	NEIL1	2	0.014978721	0.119948874	0.124875877	0.900621816	0	0.585019249	0	0.5	PRJNA827465;Yang2025_Silkie_Wenchang	CRA004775	0.976497577
ENSGALG00010021345	NEIL1	2	0.09184512	0.157669153	0.582518004	0.560217818	0	0.132681516	0	1	CRA004775;Yang2025_Silkie_Wenchang	PRJNA827465	0.881203589
ENSGALG00010021345	NEIL1	2	-0.037046244	0.130502074	-0.283874756	0.776506362	0	0.0557883	0	0.5	CRA004775;PRJNA827465	Yang2025_Silkie_Wenchang	0.944156005
ENSGALG00010021346	RPS21	2	-0.782349832	0.594811481	-1.315290401	0.188412323	0.659379898	13.99335744	92.85375219	1	PRJNA827465;Yang2025_Silkie_Wenchang	CRA004775	0.697745174
ENSGALG00010021346	RPS21	2	-0.753781526	0.65474814	-1.15125417	0.249627686	0.771167139	9.938228383	89.93784444	1	CRA004775;Yang2025_Silkie_Wenchang	PRJNA827465	0.759668364
ENSGALG00010021346	RPS21	2	-0.210759971	0.087135131	-2.418771505	0.015573019	0	0.157943977	0	1	CRA004775;PRJNA827465	Yang2025_Silkie_Wenchang	0.206527548
ENSGALG00010021348	SLC4A3	2	-0.182927542	0.06770328	-2.701900729	0.006894434	0	0.34225062	0	1	PRJNA827465;Yang2025_Silkie_Wenchang	CRA004775	0.127631755
ENSGALG00010021348	SLC4A3	2	-0.245262723	0.101423295	-2.418208982	0.015597116	0	0.264857652	0	1	CRA004775;Yang2025_Silkie_Wenchang	PRJNA827465	0.206610535
ENSGALG00010021348	SLC4A3	2	-0.164915125	0.085826831	-1.921486825	0.054670362	0	0.538087723	0	1	CRA004775;PRJNA827465	Yang2025_Silkie_Wenchang	0.404653339
ENSGALG00010021350	CABLES2	2	-0.137690755	0.161123917	-0.854564346	0.392792373	0.042176762	5.275897502	81.04587893	0.5	PRJNA827465;Yang2025_Silkie_Wenchang	CRA004775	0.830040251
ENSGALG00010021350	CABLES2	2	-0.150517823	0.241549871	-0.623133526	0.53319678	0.070390807	2.0066058	50.16460134	0.5	CRA004775;Yang2025_Silkie_Wenchang	PRJNA827465	0.873379507
ENSGALG00010021350	CABLES2	2	0.027240917	0.083511659	0.326192975	0.744278356	0	0.318092663	0	1	CRA004775;PRJNA827465	Yang2025_Silkie_Wenchang	0.935821071
ENSGALG00010021352	CFAP43	2	0.009241329	0.323729617	0.028546442	0.977226328	0.16561433	4.708541448	78.7620007	0.5	PRJNA827465;Yang2025_Silkie_Wenchang	CRA004775	0.993487352
ENSGALG00010021352	CFAP43	2	0.480141476	0.268538877	1.787977523	0.073779634	0.032058338	1.155523817	13.45916154	1	CRA004775;Yang2025_Silkie_Wenchang	PRJNA827465	0.469350366
ENSGALG00010021352	CFAP43	2	0.25202719	0.658390022	0.38279315	0.701873141	0.701792562	4.612631427	78.32040093	0.5	CRA004775;PRJNA827465	Yang2025_Silkie_Wenchang	0.922907998
ENSGALG00010021353	IPO13	2	-0.173269435	0.064070717	-2.704346724	0.006843884	0	0.216904553	0	1	PRJNA827465;Yang2025_Silkie_Wenchang	CRA004775	0.126999602
ENSGALG00010021353	IPO13	2	-0.233428968	0.103545063	-2.25437082	0.024172849	0	0.282522784	0	1	CRA004775;Yang2025_Silkie_Wenchang	PRJNA827465	0.265307316
ENSGALG00010021353	IPO13	2	-0.165682313	0.076720867	-2.159546914	0.030807762	0	0.542077562	0	1	CRA004775;PRJNA827465	Yang2025_Silkie_Wenchang	0.299786447
ENSGALG00010021354	ENSGALG00010021354	2	-0.129946801	0.11639537	-1.116425862	0.264239889	0.022077712	5.189454772	80.7301529	1	PRJNA827465;Yang2025_Silkie_Wenchang	CRA004775	0.768790489
ENSGALG00010021354	ENSGALG00010021354	2	-0.238863697	0.083840639	-2.84902046	0.004385406	0	0.428061252	0	1	CRA004775;Yang2025_Silkie_Wenchang	PRJNA827465	0.097063291
ENSGALG00010021354	ENSGALG00010021354	2	-0.026537289	0.050408818	-0.526441408	0.598581552	0	0.050733523	0	1	CRA004775;PRJNA827465	Yang2025_Silkie_Wenchang	0.894762943
ENSGALG00010021358	MMP15	2	-0.014382764	0.046369572	-0.310176767	0.756426537	0	0.007659218	0	1	PRJNA827465;Yang2025_Silkie_Wenchang	CRA004775	0.938640387
ENSGALG00010021358	MMP15	2	-0.084792272	0.149730902	-0.566297742	0.571191395	0.014870351	1.198648675	16.57271889	1	CRA004775;Yang2025_Silkie_Wenchang	PRJNA827465	0.885354083
ENSGALG00010021358	MMP15	2	-0.07311512	0.150518029	-0.485756561	0.627139776	0.023688572	1.336072604	25.15376808	1	CRA004775;PRJNA827465	Yang2025_Silkie_Wenchang	0.901583369
ENSGALG00010021360	PTPRN	2	0.236408476	0.426001821	0.554947102	0.578930843	0.307210309	6.4741942	84.55406234	0.5	PRJNA827465;Yang2025_Silkie_Wenchang	CRA004775	0.887888423
ENSGALG00010021360	PTPRN	2	0.124544951	0.624028258	0.19958223	0.841807327	0.626078071	4.803709173	79.18275308	0.5	CRA004775;Yang2025_Silkie_Wenchang	PRJNA827465	0.960031145
ENSGALG00010021360	PTPRN	2	-0.234989318	0.194906766	-1.205649874	0.22795252	0	0.536486879	0	1	CRA004775;PRJNA827465	Yang2025_Silkie_Wenchang	0.740853612
ENSGALG00010021361	WRAP73	2	0.042257131	0.084668878	0.499086932	0.617718143	0	0.216399666	0	0.5	PRJNA827465;Yang2025_Silkie_Wenchang	CRA004775	0.899121942
ENSGALG00010021361	WRAP73	2	-0.031632054	0.139309513	-0.227063127	0.820374656	0	0.003238363	0	1	CRA004775;Yang2025_Silkie_Wenchang	PRJNA827465	0.955325326
ENSGALG00010021361	WRAP73	2	0.050035246	0.090714476	0.551568492	0.581244029	0	0.168625096	0	0.5	CRA004775;PRJNA827465	Yang2025_Silkie_Wenchang	0.88890219
ENSGALG00010021362	ARPC1B	2	0.057345539	0.101505817	0.564948306	0.572108926	0.008216721	1.274521888	21.53920543	0.5	PRJNA827465;Yang2025_Silkie_Wenchang	CRA004775	0.885538639
ENSGALG00010021362	ARPC1B	2	-0.281193345	0.182266273	-1.542761265	0.122888711	0	0.505006963	0	1	CRA004775;Yang2025_Silkie_Wenchang	PRJNA827465	0.587721466
ENSGALG00010021362	ARPC1B	2	-0.096009646	0.260213758	-0.368964527	0.712154166	0.102777896	3.385594751	70.4630922	0.5	CRA004775;PRJNA827465	Yang2025_Silkie_Wenchang	0.92597752
ENSGALG00010021363	ENSGALG00010021363	2	-0.159764856	0.248969174	-0.64170537	0.521064502	0.109757245	8.059188829	87.59180333	0.5	PRJNA827465;Yang2025_Silkie_Wenchang	CRA004775	0.869518515
ENSGALG00010021363	ENSGALG00010021363	2	-0.441401717	0.139820944	-3.156907003	0.001594522	0	0.003393927	0	1	CRA004775;Yang2025_Silkie_Wenchang	PRJNA827465	0.050539915
ENSGALG00010021363	ENSGALG00010021363	2	-0.136723947	0.252589637	-0.541288821	0.588308524	0.101759647	4.132047036	75.79892022	0.5	CRA004775;PRJNA827465	Yang2025_Silkie_Wenchang	0.891874519
ENSGALG00010021367	GRIP2	2	0.012535108	0.105766351	0.118516978	0.905658044	0.009568552	1.653064624	39.50629725	0.5	PRJNA827465;Yang2025_Silkie_Wenchang	CRA004775	0.978182365
ENSGALG00010021367	GRIP2	2	-0.267632316	0.549469116	-0.487074356	0.626205638	0.457484215	3.401301909	70.59949318	0.5	CRA004775;Yang2025_Silkie_Wenchang	PRJNA827465	0.901504244
ENSGALG00010021367	GRIP2	2	-0.334779103	0.417321608	-0.802208889	0.422432135	0.237836073	2.293809776	56.40440587	1	CRA004775;PRJNA827465	Yang2025_Silkie_Wenchang	0.839704389
ENSGALG00010021373	CEP250	2	0.408331778	0.516246529	0.790962758	0.428965724	0.508678895	21.29846946	95.30482694	0.5	PRJNA827465;Yang2025_Silkie_Wenchang	CRA004775	0.841898328
ENSGALG00010021373	CEP250	2	0.495208836	0.471449121	1.0503972	0.293535533	0.378928024	6.681731905	85.03382036	0.5	CRA004775;Yang2025_Silkie_Wenchang	PRJNA827465	0.786979185
ENSGALG00010021373	CEP250	2	-0.084128109	0.071874274	-1.170489866	0.24180389	0	0.084306452	0	1	CRA004775;PRJNA827465	Yang2025_Silkie_Wenchang	0.75406671
ENSGALG00010021375	TUBG1	2	0.056826273	0.047347751	1.20018947	0.230065764	0	0.005653869	0	1	PRJNA827465;Yang2025_Silkie_Wenchang	CRA004775	0.742845187
ENSGALG00010021375	TUBG1	2	-0.165824359	0.240008498	-0.690910365	0.489621872	0.095658374	5.853875999	82.91730128	0.5	CRA004775;Yang2025_Silkie_Wenchang	PRJNA827465	0.858425426
ENSGALG00010021375	TUBG1	2	-0.157057348	0.234021278	-0.671124222	0.502141397	0.097264079	8.313776627	87.9717721	0.5	CRA004775;PRJNA827465	Yang2025_Silkie_Wenchang	0.862860181
ENSGALG00010021378	KIF11	2	-0.083401355	0.09451211	-0.882440939	0.377538405	0	0.000517308	0	1	PRJNA827465;Yang2025_Silkie_Wenchang	CRA004775	0.82325159
ENSGALG00010021378	KIF11	2	0.170851946	0.396874452	0.430493687	0.666836558	0.207494705	2.206887894	54.68732223	0.5	CRA004775;Yang2025_Silkie_Wenchang	PRJNA827465	0.913648
ENSGALG00010021378	KIF11	2	0.175169515	0.395457768	0.442953785	0.657799163	0.201080228	2.151897055	53.52937551	0.5	CRA004775;PRJNA827465	Yang2025_Silkie_Wenchang	0.910774485
ENSGALG00010021381	DNAJB2	2	0.070269584	0.06240277	1.126065149	0.260137944	0	0.241621641	0	1	PRJNA827465;Yang2025_Silkie_Wenchang	CRA004775	0.76744615
ENSGALG00010021381	DNAJB2	2	0.132535243	0.169758077	0.780730119	0.434961242	0	0.100100237	0	1	CRA004775;Yang2025_Silkie_Wenchang	PRJNA827465	0.842762754
ENSGALG00010021381	DNAJB2	2	0.058113512	0.065478379	0.887522147	0.374797848	0	0.013363386	0	1	CRA004775;PRJNA827465	Yang2025_Silkie_Wenchang	0.822805106
ENSGALG00010021382	TPRG1L	2	-0.120284518	0.193197798	-0.622597768	0.533548878	0.067433904	10.03848598	90.03833843	0.5	PRJNA827465;Yang2025_Silkie_Wenchang	CRA004775	0.873379507
ENSGALG00010021382	TPRG1L	2	-0.304424078	0.101842489	-2.989165715	0.002797404	0	0.30589199	0	1	CRA004775;Yang2025_Silkie_Wenchang	PRJNA827465	0.072072769
ENSGALG00010021382	TPRG1L	2	0.054909488	0.055472318	0.989853855	0.322245557	0	0.482234878	0	0.5	CRA004775;PRJNA827465	Yang2025_Silkie_Wenchang	0.798513866
ENSGALG00010021383	MSI1	2	0.035575912	0.053924541	0.659735106	0.509423834	0	0.459838345	0	0.5	PRJNA827465;Yang2025_Silkie_Wenchang	CRA004775	0.865257661
ENSGALG00010021383	MSI1	2	-0.01338144	0.088288582	-0.151564788	0.879530208	0	0.004715952	0	0.5	CRA004775;Yang2025_Silkie_Wenchang	PRJNA827465	0.971343592
ENSGALG00010021383	MSI1	2	0.059338897	0.064889091	0.91446646	0.360471794	0	0.03206865	0	1	CRA004775;PRJNA827465	Yang2025_Silkie_Wenchang	0.814880744
ENSGALG00010021384	ARTN	2	-0.004645526	0.262429876	-0.017701972	0.985876608	0.028982636	1.265795124	20.99827364	0.5	PRJNA827465;Yang2025_Silkie_Wenchang	CRA004775	0.996013375
ENSGALG00010021384	ARTN	2	0.305976619	0.287094767	1.065768711	0.286528208	0	0.039367217	0	1	CRA004775;Yang2025_Silkie_Wenchang	PRJNA827465	0.782018115
ENSGALG00010021384	ARTN	2	-0.072524176	0.292769034	-0.247718057	0.80435256	0.016391437	1.08368349	7.722133917	0.5	CRA004775;PRJNA827465	Yang2025_Silkie_Wenchang	0.951602591
ENSGALG00010021387	SLC2A14	2	-0.033981591	0.052501513	-0.647249741	0.517470323	0.001384452	1.242330326	19.50611049	1	PRJNA827465;Yang2025_Silkie_Wenchang	CRA004775	0.867952013
ENSGALG00010021387	SLC2A14	2	-0.146829618	0.091170209	-1.610499965	0.107288753	0	0.836502727	0	1	CRA004775;Yang2025_Silkie_Wenchang	PRJNA827465	0.554473355
ENSGALG00010021387	SLC2A14	2	-0.09726412	0.175783256	-0.553318457	0.580045365	0.037509489	1.734014528	42.33035629	1	CRA004775;PRJNA827465	Yang2025_Silkie_Wenchang	0.888203646
ENSGALG00010021388	DPH2	2	0.120407147	0.116276898	1.035520802	0.300425757	0.001080003	1.018803718	1.84566644	0.5	PRJNA827465;Yang2025_Silkie_Wenchang	CRA004775	0.790548575
ENSGALG00010021388	DPH2	2	-0.4631434	0.245394102	-1.887345282	0.0591139	0.042265832	1.520796782	34.24499502	1	CRA004775;Yang2025_Silkie_Wenchang	PRJNA827465	0.421162606
ENSGALG00010021388	DPH2	2	-0.225184208	0.418511977	-0.538059173	0.590536197	0.313663276	9.202451693	89.13333062	0.5	CRA004775;PRJNA827465	Yang2025_Silkie_Wenchang	0.892617651
ENSGALG00010021390	GSTO2	2	0.977208509	0.468435359	2.086111757	0.036968501	0.348437727	4.677622441	78.62161787	1	PRJNA827465;Yang2025_Silkie_Wenchang	CRA004775	0.331653274
ENSGALG00010021390	GSTO2	2	0.37984023	0.32730847	1.160496181	0.245846848	0	0.204835218	0	1	CRA004775;Yang2025_Silkie_Wenchang	PRJNA827465	0.75661406
ENSGALG00010021390	GSTO2	2	0.916666183	0.626876339	1.462275931	0.143665612	0.577016826	3.202372013	68.77314703	1	CRA004775;PRJNA827465	Yang2025_Silkie_Wenchang	0.630564924
ENSGALG00010021391	ENSGALG00010021391	2	0.354379766	0.249074273	1.422787513	0.154797762	0	0.519972405	0	1	PRJNA827465;Yang2025_Silkie_Wenchang	CRA004775	0.647505492
ENSGALG00010021391	ENSGALG00010021391	2	0.195473537	0.316847332	0.616932879	0.537278999	0.087883496	1.72382442	41.98945156	0.5	CRA004775;Yang2025_Silkie_Wenchang	PRJNA827465	0.87481821
ENSGALG00010021391	ENSGALG00010021391	2	0.059616334	0.210193766	0.283625606	0.776697312	0	0.42774952	0	0.5	CRA004775;PRJNA827465	Yang2025_Silkie_Wenchang	0.944156005
ENSGALG00010021393	CCDC174	2	0.181660881	0.162600031	1.117225374	0.263897974	0.044000526	5.650013499	82.30092724	1	PRJNA827465;Yang2025_Silkie_Wenchang	CRA004775	0.768643821
ENSGALG00010021393	CCDC174	2	0.261091361	0.13624698	1.91630935	0.055325731	0.013742844	1.513364929	33.92208443	1	CRA004775;Yang2025_Silkie_Wenchang	PRJNA827465	0.407328965
ENSGALG00010021393	CCDC174	2	0.039139533	0.059284328	0.660200342	0.509125273	0	0.042338462	0	1	CRA004775;PRJNA827465	Yang2025_Silkie_Wenchang	0.865193604
ENSGALG00010021394	MAN2C1	2	0.073468264	0.077616149	0.946558996	0.343863547	0	0.377974477	0	1	PRJNA827465;Yang2025_Silkie_Wenchang	CRA004775	0.806413705
ENSGALG00010021394	MAN2C1	2	-0.150893006	0.31921778	-0.472696119	0.636429996	0.173150818	6.637604303	84.93432337	0.5	CRA004775;Yang2025_Silkie_Wenchang	PRJNA827465	0.904566695
ENSGALG00010021394	MAN2C1	2	-0.18856016	0.26065989	-0.723395379	0.469437009	0.116133948	6.51922964	84.6607643	0.5	CRA004775;PRJNA827465	Yang2025_Silkie_Wenchang	0.853485658
ENSGALG00010021396	MED6	2	-0.067029415	0.172089581	-0.389503039	0.696904063	0.050186485	6.164889813	83.77910992	0.5	PRJNA827465;Yang2025_Silkie_Wenchang	CRA004775	0.921086208
ENSGALG00010021396	MED6	2	-0.244055495	0.106192695	-2.298232432	0.021548564	0	0.043223075	0	1	CRA004775;Yang2025_Silkie_Wenchang	PRJNA827465	0.248218992
ENSGALG00010021396	MED6	2	-0.003432166	0.137254146	-0.025005917	0.980050244	0.023776438	2.154892032	53.59396271	0.5	CRA004775;PRJNA827465	Yang2025_Silkie_Wenchang	0.994658414
ENSGALG00010021400	B4GALT2	2	-0.094806992	0.055532027	-1.707248909	0.087775798	0	0.39172713	0	1	PRJNA827465;Yang2025_Silkie_Wenchang	CRA004775	0.509353637
ENSGALG00010021400	B4GALT2	2	-0.161065919	0.092113845	-1.748552773	0.080368356	0	0.197881641	0	1	CRA004775;Yang2025_Silkie_Wenchang	PRJNA827465	0.487926636
ENSGALG00010021400	B4GALT2	2	-0.083381191	0.065146886	-1.279895257	0.200581976	0	0.542275638	0	1	CRA004775;PRJNA827465	Yang2025_Silkie_Wenchang	0.712970384
ENSGALG00010021401	PDAP1	2	0.065936826	0.178279831	0.369850173	0.711494129	0.054515633	6.637874651	84.93493697	0.5	PRJNA827465;Yang2025_Silkie_Wenchang	CRA004775	0.925894105
ENSGALG00010021401	PDAP1	2	0.232203948	0.105518837	2.200592368	0.027764894	0	0.191902883	0	1	CRA004775;Yang2025_Silkie_Wenchang	PRJNA827465	0.284281094
ENSGALG00010021401	PDAP1	2	-0.031640256	0.111327609	-0.284208525	0.776250583	0.013116218	1.653172806	39.5102559	0.5	CRA004775;PRJNA827465	Yang2025_Silkie_Wenchang	0.944082704
ENSGALG00010021402	ENSGALG00010021402	2	-0.122088758	0.070367616	-1.735013412	0.08273843	0	0.047695542	0	1	PRJNA827465;Yang2025_Silkie_Wenchang	CRA004775	0.495704284
ENSGALG00010021402	ENSGALG00010021402	2	-0.09088286	0.10209176	-0.890207598	0.373354425	0.00175702	1.052868842	5.02140816	0.5	CRA004775;Yang2025_Silkie_Wenchang	PRJNA827465	0.822208157
ENSGALG00010021402	ENSGALG00010021402	2	-0.07551015	0.08806026	-0.857482711	0.391178166	0	0.846476595	0	0.5	CRA004775;PRJNA827465	Yang2025_Silkie_Wenchang	0.829251057
ENSGALG00010021404	UQCC1	2	-0.051111942	0.056636124	-0.902461866	0.36681157	0	0.023643188	0	1	PRJNA827465;Yang2025_Silkie_Wenchang	CRA004775	0.819577857
ENSGALG00010021404	UQCC1	2	-0.082353008	0.092930075	-0.886182523	0.37551918	0	0.197993614	0	1	CRA004775;Yang2025_Silkie_Wenchang	PRJNA827465	0.822974013
ENSGALG00010021404	UQCC1	2	-0.055836601	0.065401257	-0.853754252	0.393241167	0	0.296841869	0	1	CRA004775;PRJNA827465	Yang2025_Silkie_Wenchang	0.830346712
ENSGALG00010021405	IDE	2	-0.107907972	0.082994675	-1.300179468	0.193539466	0.009107989	2.562359109	60.97346401	1	PRJNA827465;Yang2025_Silkie_Wenchang	CRA004775	0.703540661
ENSGALG00010021405	IDE	2	0.035379869	0.322052816	0.10985735	0.912522507	0.158141633	3.580551184	72.07133906	0.5	CRA004775;Yang2025_Silkie_Wenchang	PRJNA827465	0.978967245
ENSGALG00010021405	IDE	2	0.08596396	0.217030693	0.396091255	0.692037709	0.062742531	2.098492101	52.34673509	0.5	CRA004775;PRJNA827465	Yang2025_Silkie_Wenchang	0.920348122
ENSGALG00010021407	SIN3A	2	-0.007666198	0.070719912	-0.108402254	0.913676614	0.004342201	1.668811253	40.07710589	0.5	PRJNA827465;Yang2025_Silkie_Wenchang	CRA004775	0.979364626
ENSGALG00010021407	SIN3A	2	-0.066945941	0.189485726	-0.353303343	0.723861039	0.049904238	2.981636674	66.46137308	0.5	CRA004775;Yang2025_Silkie_Wenchang	PRJNA827465	0.930264404
ENSGALG00010021407	SIN3A	2	-0.103256988	0.091593628	-1.127338112	0.259599554	0.006695836	1.301873465	23.18761949	1	CRA004775;PRJNA827465	Yang2025_Silkie_Wenchang	0.76691144
ENSGALG00010021408	TTC9	2	0.10657182	0.126310099	0.843731581	0.398819416	0	0.115163866	0	1	PRJNA827465;Yang2025_Silkie_Wenchang	CRA004775	0.832054308
ENSGALG00010021408	TTC9	2	0.1301656	0.156730743	0.83050458	0.40625356	0	0.052338818	0	1	CRA004775;Yang2025_Silkie_Wenchang	PRJNA827465	0.833523891
ENSGALG00010021408	TTC9	2	0.055931541	0.172104617	0.324985707	0.745191889	0	0.001188472	0	1	CRA004775;PRJNA827465	Yang2025_Silkie_Wenchang	0.93597621
ENSGALG00010021409	MEGF6	2	0.590641426	0.35192899	1.678297161	0.093289102	0.190331896	4.278380594	76.62667035	1	PRJNA827465;Yang2025_Silkie_Wenchang	CRA004775	0.523343073
ENSGALG00010021409	MEGF6	2	0.373541108	0.620021602	0.602464666	0.546864876	0.659064271	6.886658482	85.47916958	0.5	CRA004775;Yang2025_Silkie_Wenchang	PRJNA827465	0.878275987
ENSGALG00010021409	MEGF6	2	0.088574935	0.249011652	0.355705984	0.722060763	0.045209602	1.456859151	31.35918463	0.5	CRA004775;PRJNA827465	Yang2025_Silkie_Wenchang	0.929404251
ENSGALG00010021411	MCU	2	-0.162123535	0.062034671	-2.613434251	0.008963733	0	0.191603387	0	1	PRJNA827465;Yang2025_Silkie_Wenchang	CRA004775	0.148337513
ENSGALG00010021411	MCU	2	-0.087447371	0.095891652	-0.911939358	0.361800642	0.004196249	1.174761207	14.87631747	0.5	CRA004775;Yang2025_Silkie_Wenchang	PRJNA827465	0.815545459
ENSGALG00010021411	MCU	2	-0.100410421	0.134833313	-0.744700392	0.456452862	0.018244485	1.749181345	42.83039876	0.5	CRA004775;PRJNA827465	Yang2025_Silkie_Wenchang	0.849515887
ENSGALG00010021412	ZNF319	2	-0.013088187	0.05126314	-0.255313789	0.79848075	0	0.604040824	0	0.5	PRJNA827465;Yang2025_Silkie_Wenchang	CRA004775	0.949922217
ENSGALG00010021412	ZNF319	2	-0.106326683	0.106050218	-1.002606919	0.316050556	0.003787761	1.058320738	5.510686526	1	CRA004775;Yang2025_Silkie_Wenchang	PRJNA827465	0.796883862

ENSGALG00010021412	ZNF319	2	-0.086634938	0.189019793	-0.458337914	0.646709689	0.041946911	1.670535933	40.13897097	0.5	CRA004775;PRJNA827465	Yang2025_Silkie_Wenchang	0.907350807
ENSGALG00010021413	SYNJ2BP	2	0.217855236	0.153694012	1.417460795	0.156348249	0.010949542	1.283181684	22.06871308	1	PRJNA827465;Yang2025_Silkie_Wenchang	CRA004775	0.649484056
ENSGALG00010021413	SYNJ2BP	2	-0.000124713	0.40483569	-0.000308057	0.999754206	0.282414458	7.217769911	86.1453051	0.5	CRA004775;Yang2025_Silkie_Wenchang	PRJNA827465	0.999889665
ENSGALG00010021413	SYNJ2BP	2	-0.138920944	0.246985147	-0.562466796	0.573798021	0.087803977	3.53973154	71.74927	0.5	CRA004775;PRJNA827465	Yang2025_Silkie_Wenchang	0.886032852
ENSGALG00010021414	BUD31	2	-0.034089866	0.083994853	-0.405856601	0.684847967	0.005902647	1.42565364	29.85673576	0.5	PRJNA827465;Yang2025_Silkie_Wenchang	CRA004775	0.919003112
ENSGALG00010021414	BUD31	2	0.093788168	0.137471271	0.682238308	0.495088275	0	0.110357046	0	1	CRA004775;Yang2025_Silkie_Wenchang	PRJNA827465	0.860536639
ENSGALG00010021414	BUD31	2	-0.076904688	0.056253451	-1.367110584	0.171590646	0	0.09316283	0	0.5	CRA004775;PRJNA827465	Yang2025_Silkie_Wenchang	0.673998318
ENSGALG00010021415	SIRT4	2	0.048314617	0.22431769	0.215384781	0.829467334	0.080813558	4.917559112	79.6647081	0.5	PRJNA827465;Yang2025_Silkie_Wenchang	CRA004775	0.95692599
ENSGALG00010021415	SIRT4	2	-0.047599557	0.390022388	-0.12204314	0.902864853	0.238863575	4.371549825	77.12481751	0.5	CRA004775;Yang2025_Silkie_Wenchang	PRJNA827465	0.97693511
ENSGALG00010021415	SIRT4	2	-0.188393633	0.10248778	-1.838205816	0.066032083	0	0.916567141	0	1	CRA004775;PRJNA827465	Yang2025_Silkie_Wenchang	0.442444044
ENSGALG00010021416	PLEKHH3	2	0.174858195	0.187897681	0.930603262	0.352058826	0.054976795	4.377250578	77.15460922	1	PRJNA827465;Yang2025_Silkie_Wenchang	CRA004775	0.810678834
ENSGALG00010021416	PLEKHH3	2	0.214526678	0.258512624	0.829849911	0.406623648	0.071674571	1.804052126	44.56922915	0.5	CRA004775;Yang2025_Silkie_Wenchang	PRJNA827465	0.833618122
ENSGALG00010021416	PLEKHH3	2	-0.006885183	0.094629657	-0.072759248	0.9419977	0	0.218269311	0	0.5	CRA004775;PRJNA827465	Yang2025_Silkie_Wenchang	0.985827325
ENSGALG00010021417	STK16	2	0.021911623	0.079320487	0.276241658	0.782362463	0	0.005114778	0	1	PRJNA827465;Yang2025_Silkie_Wenchang	CRA004775	0.945372683
ENSGALG00010021417	STK16	2	-0.298656653	0.350245328	-0.85270703	0.393821792	0.177626121	3.470649198	71.18694679	0.5	CRA004775;Yang2025_Silkie_Wenchang	PRJNA827465	0.830486298
ENSGALG00010021417	STK16	2	-0.26962658	0.354707177	-0.760138497	0.447171804	0.204775301	4.667888099	78.57703572	0.5	CRA004775;PRJNA827465	Yang2025_Silkie_Wenchang	0.84650962
ENSGALG00010021419	MANSC1	2	0.097549474	0.117828269	0.827895332	0.407729779	0.012104233	1.771395116	43.54732093	0.5	PRJNA827465;Yang2025_Silkie_Wenchang	CRA004775	0.833982683
ENSGALG00010021419	MANSC1	2	-0.272292972	0.316456656	-0.860443181	0.389544792	0.150744662	3.564799819	71.94793394	1	CRA004775;Yang2025_Silkie_Wenchang	PRJNA827465	0.828971734
ENSGALG00010021419	MANSC1	2	-0.173550236	0.438851352	-0.39546474	0.692499939	0.330275328	6.535423441	84.69877264	0.5	CRA004775;PRJNA827465	Yang2025_Silkie_Wenchang	0.920559211
ENSGALG00010021420	PLXNB3	2	-0.117908245	0.063644165	-1.852616742	0.063937311	0	0.238622018	0	1	PRJNA827465;Yang2025_Silkie_Wenchang	CRA004775	0.435667153
ENSGALG00010021420	PLXNB3	2	-0.132678122	0.148969676	-0.890638449	0.373123165	0	0.642839589	0	0.5	CRA004775;Yang2025_Silkie_Wenchang	PRJNA827465	0.822187352
ENSGALG00010021420	PLXNB3	2	-0.096393068	0.067371061	-1.430778537	0.152493696	0	0.381285876	0	0.5	CRA004775;PRJNA827465	Yang2025_Silkie_Wenchang	0.64411174
ENSGALG00010021421	GLB1L	2	0.537105995	0.115989644	4.630637503	3.64542E-06	0	0.408089848	0	1	PRJNA827465;Yang2025_Silkie_Wenchang	CRA004775	0.00065654
ENSGALG00010021421	GLB1L	2	0.142981132	0.499595085	0.286194033	0.774729513	0.452549004	10.64302393	90.60417409	0.5	CRA004775;Yang2025_Silkie_Wenchang	PRJNA827465	0.943713409
ENSGALG00010021421	GLB1L	2	0.076705996	0.423046308	0.181318203	0.856117819	0.318481175	8.880936101	88.73992574	0.5	CRA004775;PRJNA827465	Yang2025_Silkie_Wenchang	0.963499334
ENSGALG00010021424	PTCD1	2	-0.022468743	0.048329832	-0.464904219	0.642000049	0	0.064861516	0	1	PRJNA827465;Yang2025_Silkie_Wenchang	CRA004775	0.906634523
ENSGALG00010021424	PTCD1	2	0.007029017	0.084415595	0.083266806	0.933639394	0	0.097163357	0	0.5	CRA004775;Yang2025_Silkie_Wenchang	PRJNA827465	0.984262625
ENSGALG00010021424	PTCD1	2	-0.0243953	0.055595631	-0.438798865	0.660807283	0	0.190311931	0	0.5	CRA004775;PRJNA827465	Yang2025_Silkie_Wenchang	0.91192683
ENSGALG00010021425	PTPN9	2	0.115575991	0.065340761	1.768819187	0.076924054	0.000350017	1.037414871	3.606548563	1	PRJNA827465;Yang2025_Silkie_Wenchang	CRA004775	0.478074966
ENSGALG00010021425	PTPN9	2	0.024023432	0.105909721	0.226829342	0.820556447	0	0.003384499	0	1	CRA004775;Yang2025_Silkie_Wenchang	PRJNA827465	0.955383907
ENSGALG00010021425	PTPN9	2	0.153953851	0.075068514	2.05084453	0.040282089	0	0.143185676	0	1	CRA004775;PRJNA827465	Yang2025_Silkie_Wenchang	0.347442284
ENSGALG00010021426	SLC38A7	2	0.005624127	0.058521972	0.096102832	0.923438902	0	0.520831812	0	0.5	PRJNA827465;Yang2025_Silkie_Wenchang	CRA004775	0.98201108
ENSGALG00010021426	SLC38A7	2	-0.075431709	0.105345878	-0.716038543	0.47396755	0	0.201717322	0	1	CRA004775;Yang2025_Silkie_Wenchang	PRJNA827465	0.85444425
ENSGALG00010021426	SLC38A7	2	0.023264961	0.067989179	0.342186236	0.732210742	0	0.501259226	0	0.5	CRA004775;PRJNA827465	Yang2025_Silkie_Wenchang	0.932899339
ENSGALG00010021427	RPLP0	2	-0.058397898	0.22120935	-0.263993803	0.791784694	0.081252539	5.434389632	81.59866944	0.5	PRJNA827465;Yang2025_Silkie_Wenchang	CRA004775	0.948201801
ENSGALG00010021427	RPLP0	2	-0.349123822	0.159880658	-2.183652646	0.028987782	0	0.253540519	0	1	CRA004775;Yang2025_Silkie_Wenchang	PRJNA827465	0.289878916
ENSGALG00010021427	RPLP0	2	-0.089648215	0.310574932	-0.288652449	0.772847355	0.1418714	2.978743728	66.42880048	0.5	CRA004775;PRJNA827465	Yang2025_Silkie_Wenchang	0.943066211
ENSGALG00010021429	MED8	2	-0.127522403	0.068492145	-1.86185442	0.062623613	0	0.330565638	0	1	PRJNA827465;Yang2025_Silkie_Wenchang	CRA004775	0.431125569
ENSGALG00010021429	MED8	2	-0.055519455	0.107689168	-0.515552823	0.606166767	0	0.008011491	0	1	CRA004775;Yang2025_Silkie_Wenchang	PRJNA827465	0.896709756
ENSGALG00010021429	MED8	2	-0.134138309	0.073476536	-1.825593808	0.067911469	0	0.29343617	0	1	CRA004775;PRJNA827465	Yang2025_Silkie_Wenchang	0.44927192
ENSGALG00010021431	CPSF4	2	-0.01253761	0.052352263	-0.239485542	0.810729106	0	0.340859989	0	0.5	PRJNA827465;Yang2025_Silkie_Wenchang	CRA004775	0.953020781
ENSGALG00010021431	CPSF4	2	-0.111260174	0.086473211	-1.286643257	0.198218659	0	0.574677943	0	1	CRA004775;Yang2025_Silkie_Wenchang	PRJNA827465	0.710234722
ENSGALG00010021431	CPSF4	2	-0.054413319	0.094845063	-0.573707449	0.566165787	0.008882361	1.66575285	39.96708457	0.5	CRA004775;PRJNA827465	Yang2025_Silkie_Wenchang	0.883487517
ENSGALG00010021432	SYT9	2	-0.250348001	0.217524876	-1.150893662	0.249775985	0.037106556	1.645021823	39.2105329	1	PRJNA827465;Yang2025_Silkie_Wenchang	CRA004775	0.759757983
ENSGALG00010021432	SYT9	2	0.209017526	0.393740594	0.530850842	0.595522145	0.155928592	1.66659714	39.99749692	0.5	CRA004775;Yang2025_Silkie_Wenchang	PRJNA827465	0.893794893
ENSGALG00010021432	SYT9	2	0.057394547	0.645294816	0.08894314	0.929127099	0.634741905	3.713874078	73.07393899	0.5	CRA004775;PRJNA827465	Yang2025_Silkie_Wenchang	0.983566083
ENSGALG00010021433	ENSGALG00010021433	2	0.009175306	0.097256038	0.094341758	0.924837679	0	0.075453431	0	0.5	PRJNA827465;Yang2025_Silkie_Wenchang	CRA004775	0.982493782
ENSGALG00010021433	ENSGALG00010021433	2	0.027932195	0.144753392	0.192964011	0.846987154	0	0.184803035	0	0.5	CRA004775;Yang2025_Silkie_Wenchang	PRJNA827465	0.961252712
ENSGALG00010021433	ENSGALG00010021433	2	-0.021965331	0.127816331	-0.171850739	0.863554875	0	0.132610211	0	1	CRA004775;PRJNA827465	Yang2025_Silkie_Wenchang	0.966271807
ENSGALG00010021434	ANKZF1	2	0.20577093	0.187805169	1.095661696	0.273226851	0.056228525	4.549512841	78.01962463	1	PRJNA827465;Yang2025_Silkie_Wenchang	CRA004775	0.773904763
ENSGALG00010021434	ANKZF1	2	-0.000705951	0.445416982	-0.001584921	0.998735416	0.351791983	8.652682537	88.44289045	0.5	CRA004775;Yang2025_Silkie_Wenchang	PRJNA827465	0.999676516
ENSGALG00010021434	ANKZF1	2	-0.153733215	0.249288016	-0.61668915	0.537439779	0.095732028	3.698554999	72.96241369	0.5	CRA004775;PRJNA827465	Yang2025_Silkie_Wenchang	0.87481821
ENSGALG00010021435	GCN1	2	-0.092164815	0.039439182	-2.336884529	0.019445189	0	0.013188424	0	1	PRJNA827465;Yang2025_Silkie_Wenchang	CRA004775	0.233679423
ENSGALG00010021435	GCN1	2	-0.071770309	0.079577164	-0.901895785	0.367112231	0	0.833319952	0	0.5	CRA004775;Yang2025_Silkie_Wenchang	PRJNA827465	0.819739734
ENSGALG00010021435	GCN1	2	-0.09067078	0.04500128	-2.014848895	0.043920484	0	0.891009889	0	0.5	CRA004775;PRJNA827465	Yang2025_Silkie_Wenchang	0.361888215
ENSGALG00010021436	ENSGALG00010021436	2	-0.39162572	0.390764822	-1.002203109	0.316245507	0	0.953475571	0	0.5	PRJNA827465;Yang2025_Silkie_Wenchang	CRA004775	0.796883862
ENSGALG00010021436	ENSGALG00010021436	2	0.032698211	0.46965219	0.069622183	0.94449438	0	0.000243323	0	1	CRA004775;Yang2025_Silkie_Wenchang	PRJNA827465	0.985935636
ENSGALG00010021436	ENSGALG00010021436	2	-0.501290437	0.440874139	-1.137037518	0.255522602	0	0.665671727	0	0.5	CRA004775;PRJNA827465	Yang2025_Silkie_Wenchang	0.764467569
ENSGALG00010021438	GOT2	2	-0.16448302	0.068652545	-2.395876503	0.016580676	0	0.345382244	0	1	PRJNA827465;Yang2025_Silkie_Wenchang	CRA004775	0.213382547
ENSGALG00010021438	GOT2	2	-0.252513385	0.185460009	-1.361551673	0.173339416	0	0.084765934	0	1	CRA004775;Yang2025_Silkie_Wenchang	PRJNA827465	0.676868485
ENSGALG00010021438	GOT2	2	-0.149976747	0.071762127	-2.089915025	0.036625434	0	0.000434209	0	1	CRA004775;PRJNA827465	Yang2025_Silkie_Wenchang	0.330158124
ENSGALG00010021439	ITPRIP	2	-0.261230579	0.277396523	-0.941722615	0.346334674	0.141731257	12.50262256	92.00167809	0.5	PRJNA827465;Yang2025_Silkie_Wenchang	CRA004775	0.807757292
ENSGALG00010021439	ITPRIP	2	-0.510294347	0.125219588	-4.075195867	4.59756E-05	0	0.907292462	0	1	CRA004775;Yang2025_Silkie_Wenchang	PRJNA827465	0.004574413
ENSGALG00010021439	ITPRIP	2	0.001981943	0.084442283	0.023470977	0.981274589	0	0.101062206	0	0.5	CRA004775;PRJNA827465	Yang2025_Silkie_Wenchang	0.994962782
ENSGALG00010021441	ENSGALG00010021441	2	0.32474186	0.16344477	1.986859898	0.046937925	0	0.801412023	0	1	PRJNA827465;Yang2025_Silkie_Wenchang	CRA004775	0.374003609
ENSGALG00010021441	ENSGALG00010021441	2	-0.143078061	0.707207562	-0.2023141	0.839671178	0.846693035	6.183047683	83.82674611	0.5	CRA004775;Yang2025_Silkie_Wenchang	PRJNA827465	0.959493354
ENSGALG00010021441	ENSGALG00010021441	2	-0.264914104	0.553954971	-0.478223173	0.632491371	0.478067169	4.099188333	75.60492666	0.5	CRA004775;PRJNA827465	Yang2025_Silkie_Wenchang	0.903058174
ENSGALG00010021443	SNUPN	2	-0.097274841	0.064940274	-1.49791239	0.134156014	0	0.326780626	0	1	PRJNA827465;Yang2025_Silkie_Wenchang	CRA004775	0.61346525
ENSGALG00010021443	SNUPN	2	-0.32672078	0.163969559	-1.992569731	0.046308582	0	0.303547277	0	1	CRA004775;Yang2025_Silkie_Wenchang	PRJNA827465	0.371214811
ENSGALG00010021443	SNUPN	2	-0.178401802	0.145864374	-1.223066316	0.22130465	0.025106082	1.917119095	47.83839969	1	CRA004775;PRJNA827465	Yang2025_Silkie_Wenchang	0.734685079
ENSGALG00010021444	ATG9A	2	-0.022859718	0.059220466	-0.386010445	0.699488933	0.003180348	1.809652792	44.74078097	0.5	PRJNA827465;Yang2025_Silkie_Wenchang	CRA004775	0.922022134
ENSGALG00010021444	ATG9A	2	0.035826472	0.067962006	0.527154421	0.598086357	0	0.475708603	0	0.5	CRA004775;Yang2025_Silkie_Wenchang	PRJNA827465	0.894645663
ENSGALG00010021444	ATG9A	2	-0.078644489	0.055264265	-1.423062253	0.15471811	0	0.166201331	0	1	CRA004775;PRJNA827465	Yang2025_Silkie_Wenchang	0.647505492
ENSGALG00010021446	HOXB8	2	0.341083692	0.761373405	0.447984773	0.654164186	0.959630887	5.560841851	82.01711132	0.5	PRJNA827465;Yang2025_Silkie_Wenchang	CRA004775	0.909981418
ENSGALG00010021446	HOXB8	2	0.851984122	0.452885358	1.88123574	0.059939858	0	0.913936385	0	1	CRA004775;Yang2025_Silkie_Wenchang	PRJNA827465	0.423882851
ENSGALG00010021446	HOXB8	2	-0.257108826	0.289897904	-0.886894392	0.375135761	0	0.597178053	0	0.5	CRA004775;PRJNA827465	Yang2025_Silkie_Wenchang	0.822941424
ENSGALG00010021448	SZT2	2	0.014207012	0.051271467	0.277093929	0.781707983	0	0.34982026	0	0.5	PRJNA827465;Yang2025_Silkie_Wenchang	CRA004775	0.945265125
ENSGALG00010021448	SZT2	2	-0.166350535	0.271038868	-0.613751585	0.539379495	0.118723746	4.961091334	79.84314473	0.5	CRA004775;Yang2025_Silkie_Wenchang	PRJNA827465	0.87498895
ENSGALG00010021448	SZT2	2	-0.188796519	0.229584112	-0.822341397	0.410882623	0.08489746	4.433253058	77.44320058	1	CRA004775;PRJNA827465	Yang2025_Silkie_Wenchang	0.835087378
ENSGALG00010021450	OLFML1	2	-0.424219941	0.134766801	-3.147807448	0.001645	0	0.163199109	0	1	PRJNA827465;Yang2025_Silkie_Wenchang	CRA004775	0.051226717
ENSGALG00010021450	OLFML1	2	0.342631742	0.916153076	0.373989621	0.708412038	1.495722967	8.676129892	88.47412369	0.5	CRA004775;Yang2025_Silkie_Wenchang	PRJNA827465	0.9250668
ENSGALG00010021450	OLFML1	2	0.387498125	0.857368413	0.451962212	0.651296205	1.304057417	8.158338139	87.7426017	0.5	CRA004775;PRJNA827465	Yang2025_Silkie_Wenchang	0.908887045
ENSGALG00010021452	SLC25A1	2	0.204524755	0.154850027	1.320792506	0.186570559	0.015439096	1.438650081	30.49039421	1	PRJNA827465;Yang2025_Silkie_Wenchang	CRA004775	0.695457578
ENSGALG00010021452	SLC25A1	2	-0.173084464	0.154813219	-1.118021222	0.26355793	0.010209215	1.261898252	20.75430817	0.5	CRA004775;Yang2025_Silkie_Wenchang	PRJNA827465	0.768643821
ENSGALG00010021452	SLC25A1	2	0.017086804	0.315888666	0.054091221	0.956862487	0.172308208	7.303611328	86.30814326	0.5	CRA004775;PRJNA827465	Yang2025_Silkie_Wenchang	0.988540618
ENSGALG00010021454	ATP5J2	2	-0.142211377	0.088271369	-1.611070256	0.10716441	0	0.446313309	0	1	PRJNA827465;Yang2025_Silkie_Wenchang	CRA004775	0.554473355
ENSGALG00010021454	ATP5J2	2	-0.149585025	0.245358758	-0.609658386	0.542088126	0	0.778214229	0	0.5	CRA004775;Yang2025_Silkie_Wenchang	PRJNA827465	0.876685104
ENSGALG00010021454	ATP5J2	2	-0.114214072	0.088249778	-1.294213706	0.195591614	0	0.289704204	0	0.5	CRA004775;PRJNA827465	Yang2025_Silkie_Wenchang	0.706675375
ENSGALG00010021457	USB1	2	-0.014848583	0.068295258	-0.217417476	0.827883013	0	0.046301006	0	0.5	PRJNA827465;Yang2025_Silkie_Wenchang	CRA004775	0.956550752
ENSGALG00010021457	USB1	2	0.068494117	0.202298913	0.338578769	0.734927079	0	0.098326237	0	1	CRA004775;Yang2025_Silkie_Wenchang	PRJNA827465	0.933545748
ENSGALG00010021457	USB1	2	-0.013540254	0.070415299	-0.192291361	0.847513985	0	0.2294289	0	0.5	CRA004775;PRJNA827465	Yang2025_Silkie_Wenchang	0.961252712
ENSGALG00010021458	USP30	2	0.121530314	0.087403963	1.390443977	0.1643941	0.007320089	1.835988101	45.53341606	1	PRJNA827465;Yang2025_Silkie_Wenchang	CRA004775	0.663501882
ENSGALG00010021458	USP30	2	0.240927165	0.09536716	2.526311621	0.011526719	0	0.029467492	0	1	CRA004775;Yang2025_Silkie_Wenchang	PRJNA827465	0.171604025
ENSGALG00010021458	USP30	2	0.092116355	0.084247369	1.093403346	0.274216745	0.003543819	1.17813839	15.12032808	1	CRA004775;PRJNA827465	Yang2025_Silkie_Wenchang	0.774526342
ENSGALG00010021459	ARHGEF16	2	0.230815045	0.152875785	1.509820835	0.131089147	0.037533781	5.004564823	80.01824264	1	PRJNA827465;Yang2025_Silkie_Wenchang	CRA004775	0.606767161
ENSGALG00010021459	ARHGEF16	2	0.3892749	0.0988123	3.939538893	8.16384E-05	0	0.00590566	0	1	CRA004775;Yang2025_Silkie_Wenchang	PRJNA827465	0.006769267
ENSGALG00010021459	ARHGEF16	2	0.155269251	0.122407918	1.268457578	0.204634591	0.013085138	1.464836277	31.73298506	1	CRA004775;PRJNA827465	Yang2025_Silkie_Wenchang	0.71730346
ENSGALG00010021461	SNX33	2	0.085444048	0.044159757	1.934884938	0.053004417	0	0.409555687	0	1	PRJNA827465;Yang2025_Silkie_Wenchang	CRA004775	0.399631766
ENSGALG00010021461	SNX33	2	0.001251404	0.201874908	0.00619891	0.995054017	0.050559234	2.382988917	58.0358938	0.5	CRA004775;Yang2025_Silkie_Wenchang	PRJNA827465	0.998242335
ENSGALG00010021461	SNX33	2	-0.008797301	0.144583881	-0.060845658	0.951482128	0.025552336	1.838883491	45.61917571	0.5	CRA004775;PRJNA827465	Yang2025_Silkie_Wenchang	0.987713746
ENSGALG00010021462	SETD5	2	-0.036235817	0.043731929	-0.828589504	0.407336729	0	0.736538634	0	1	PRJNA827465;Yang2025_Silkie_Wenchang	CRA004775	0.833863243
ENSGALG00010021462	SETD5	2	-0.113443803	0.08251417	-1.374840264	0.169180972	0	0.188967687	0	1	CRA004775;Yang2025_Silkie_Wenchang	PRJNA827465	0.670497101
ENSGALG00010021462	SETD5	2	-0.022292856	0.049552498	-0.449883599	0.652794374	0	0.618535785	0	1	CRA004775;PRJNA827465	Yang2025_Silkie_Wenchang	0.909572411
ENSGALG00010021465	ABCB6	2	0.193230288	0.082684719	2.336952831	0.019441837	0	0.795814408	0	1	PRJNA827465;Yang2025_Silkie_Wenchang	CRA004775	0.233679423
ENSGALG00010021465	ABCB6	2	0.156230549	0.268713778	0.584831353	0.55866112	0.071059798	1.875609647	46.66400211	0.5	CRA004775;Yang2025_Silkie_Wenchang	PRJNA827465	0.880854415
ENSGALG00010021465	ABCB6	2	0.13890155	0.087209179	1.592740029	0.111218524	0	0.96104472	0	0.5	CRA004775;PRJNA827465	Yang2025_Silkie_Wenchang	0.564247016
ENSGALG00010021466	HES4	2	0.068458456	0.086979564	0.787063681	0.431244589	0	0.64568095	0	1	PRJNA827465;Yang2025_Silkie_Wenchang	CRA004775	0.842503929
ENSGALG00010021466	HES4	2	0.114440678	0.139376681	0.821089131	0.4115955	0	0.90146544	0	0.5	CRA004775;Yang2025_Silkie_Wenchang	PRJNA827465	0.835128673
ENSGALG00010021466	HES4	2	0.00102339	0.102895236	0.009945943	0.992064416	0	0.370869152	0	0.5	CRA004775;PRJNA827465	Yang2025_Silkie_Wenchang	0.997699848
ENSGALG00010021467	LHFPL4	2	0.366996732	0.434805021	0.844048975	0.398642039	0.333353169	8.438463024	88.14950072	0.5	PRJNA827465;Yang2025_Silkie_Wenchang	CRA004775	0.831980453
ENSGALG00010021467	LHFPL4	2	0.164459698	0.673759092	0.244092733	0.807159001	0.817577195	9.853373138	89.8511912	0.5	CRA004775;Yang2025_Silkie_Wenchang	PRJNA827465	0.95234753
ENSGALG00010021467	LHFPL4	2	-0.203006079	0.218195508	-0.930386154	0.352171185	0.027186511	1.310003035	23.66429901	1	CRA004775;PRJNA827465	Yang2025_Silkie_Wenchang	0.810517746
ENSGALG00010021469	CSPG4	2	0.140868318	0.065733161	2.143032754	0.032110468	0	0.27384738	0	1	PRJNA827465;Yang2025_Silkie_Wenchang	CRA004775	0.30721464
ENSGALG00010021469	CSPG4	2	0.250099364	0.128905755	1.940172209	0.052358765	0	0.930274029	0	1	CRA004775;Yang2025_Silkie_Wenchang	PRJNA827465	0.397324397
ENSGALG00010021469	CSPG4	2	0.211454199	0.182841469	1.156489278	0.247481083	0.035806377	1.499631017	33.31693006	1	CRA004775;PRJNA827465	Yang2025_Silkie_Wenchang	0.757947282
ENSGALG00010021473	SLC8A3	2	0.067921428	0.13436576	0.505496552	0.613210019	0.026883945	3.805244405	73.72047907	0.5	PRJNA827465;Yang2025_Silkie_Wenchang	CRA004775	0.898218445
ENSGALG00010021473	SLC8A3	2	0.206161183	0.110053562	1.873280428	0.061029671	0	0.095264614	0	1	CRA004775;Yang2025_Silkie_Wenchang	PRJNA827465	0.42722323
ENSGALG00010021473	SLC8A3	2	-0.04797916	0.075707473	-0.633744039	0.526247879	0	0.140944828	0	0.5	CRA004775;PRJNA827465	Yang2025_Silkie_Wenchang	0.870537576
ENSGALG00010021474	PPFIBP2	2	0.267235871	0.301068193	0.88762572	0.374742114	0.151427148	5.752807014	82.61718153	0.5	PRJNA827465;Yang2025_Silkie_Wenchang	CRA004775	0.822805106
ENSGALG00010021474	PPFIBP2	2	0.158902603	0.199759081	2.950565258	0.00317191	0	0.013410689	0	1	CRA004775;Yang2025_Silkie_Wenchang	PRJNA827465	0.073892601
ENSGALG00010021474	PPFIBP2	2	0.115802767	0.226759888	0.51068453	0.609571975	0.053037335	1.541778109	35.1398237	0.5	CRA004775;PRJNA827465	Yang2025_Silkie_Wenchang	0.897238578
ENSGALG00010021476	ALKBH2	2	-0.231027993	0.102182363	-2.260938048	0.023763094	0	0.648792174	0	1	PRJNA827465;Yang2025_Silkie_Wenchang	CRA004775	0.263367861
ENSGALG00010021476	ALKBH2	2	-0.057211835	0.165043984	-0.346645865	0.728857185	0	0.014266894	0	1	CRA004775;Yang2025_Silkie_Wenchang	PRJNA827465	0.93253378
ENSGALG00010021476	ALKBH2	2	-0.23227346	0.104832003	-2.215673203	0.026713891	0	0.75553183	0	1	CRA004775;PRJNA827465	Yang2025_Silkie_Wenchang	0.278912632
ENSGALG00010021477	ZFAND2B	2	0.31587183	0.25495419	1.238935633	0.215369336	0.104436589	4.630624746	78.40464182	1	PRJNA827465;Yang2025_Silkie_Wenchang	CRA004775	0.728757551
ENSGALG00010021477	ZFAND2B	2	0.272326595	0.375462718	0.725309284	0.468262312	0.208261929	3.732828682	73.21066448	0.5	CRA004775;Yang2025_Silkie_Wenchang	PRJNA827465	0.853198008
ENSGALG00010021477	ZFAND2B	2	0.080384642	0.086774681	0.926360561	0.354258633	0	0.510289262	0	0.5	CRA004775;PRJNA827465	Yang2025_Silkie_Wenchang	0.831861636
ENSGALG00010021478	SLC13A3	2	0.344017655	0.159074691	2.162617154	0.030570638	0	0.079810738	0	1	PRJNA827465;Yang2025_Silkie_Wenchang	CRA004775	0.298217368
ENSGALG00010021478	SLC13A3	2	0.160232327	0.306139419	0.523396583	0.60069191	0.091519154	1.833859649	45.47019993	0.5	CRA004775;Yang2025_Silkie_Wenchang	PRJNA827465	0.895230519
ENSGALG00010021478	SLC13A3	2	0.139386596	0.249848783	0.55788383	0.576923714	0.043963953	1.42267873	29.7100618	0.5	CRA004775;PRJNA827465	Yang2025_Silkie_Wenchang	0.887113945
ENSGALG00010021479	SKAP1	2	-0.342868395	0.552052807	-0.656769566	0.511329096	0.34326488	2.518266666	60.29017819	0.5	PRJNA827465;Yang2025_Silkie_Wenchang	CRA004775	0.865658376
ENSGALG00010021479	SKAP1	2	-0.800353196	0.375377747	-2.132127445	0.032996371	0	0.171076619	0	1	CRA004775;Yang2025_Silkie_Wenchang	PRJNA827465	0.312002211
ENSGALG00010021479	SKAP1	2	-0.208491608	0.365570424	-0.570318587	0.568461636	0.10864017	1.625514575	38.48101917	0.5	CRA004775;PRJNA827465	Yang2025_Silkie_Wenchang	0.884151198
ENSGALG00010021480	ENSGALG00010021480	2	0.048813016	0.142916744	0.341548616	0.732699613	0.029996219	3.191175455	68.66358845	0.5	PRJNA827465;Yang2025_Silkie_Wenchang	CRA004775	0.932985777
ENSGALG00010021480	ENSGALG00010021480	2	-0.202745816	0.12560463	-1.614158772	0.106492992	0	0.483376863	0	1	CRA004775;Yang2025_Silkie_Wenchang	PRJNA827465	0.553422082
ENSGALG00010021480	ENSGALG00010021480	2	-0.036867687	0.234670337	-0.157104165	0.875162755	0.090856315	4.962171285	79.8475316	0.5	CRA004775;PRJNA827465	Yang2025_Silkie_Wenchang	0.970310073
ENSGALG00010021481	MICU1	2	0.490828234	0.302664658	1.621689947	0.104869752	0.168770346	12.60512772	92.0667206	1	PRJNA827465;Yang2025_Silkie_Wenchang	CRA004775	0.550048644
ENSGALG00010021481	MICU1	2	0.558249426	0.290344947	1.922711011	0.054516353	0.124227319	3.488549003	71.33725194	1	CRA004775;Yang2025_Silkie_Wenchang	PRJNA827465	0.404997277
ENSGALG00010021481	MICU1	2	0.19691805	0.095317349	2.065920342	0.038836002	0	0.002580844	0	1	CRA004775;PRJNA827465	Yang2025_Silkie_Wenchang	0.340024811
ENSGALG00010021483	UNG	2	-0.14826168	0.263729987	-0.562172248	0.573998667	0.103277757	3.221979507	68.96317938	0.5	PRJNA827465;Yang2025_Silkie_Wenchang	CRA004775	0.886081433
ENSGALG00010021483	UNG	2	-0.430007298	0.235916523	-1.822709546	0.06834759	0	0.136797896	0	1	CRA004775;Yang2025_Silkie_Wenchang	PRJNA827465	0.458457609
ENSGALG00010021483	UNG	2	0.037742768	0.079242353	0.476295404	0.633863939	0	0.818028554	0	0.5	CRA004775;PRJNA827465	Yang2025_Silkie_Wenchang	0.903596555
ENSGALG00010021484	MARCHF5	2	-0.232049948	0.13585732	-1.708041545	0.087628634	0.030297595	5.506504574	81.83965914	1	PRJNA827465;Yang2025_Silkie_Wenchang	CRA004775	0.508950525
ENSGALG00010021484	MARCHF5	2	-0.201580294	0.220648043	-0.913582967	0.36093602	0.070189472	3.137905077	68.13160451	0.5	CRA004775;Yang2025_Silkie_Wenchang	PRJNA827465	0.815139251
ENSGALG00010021484	MARCHF5	2	-0.089312244	0.066148563	-1.350203231	0.17693247	0	0.533783159	0	0.5	CRA004775;PRJNA827465	Yang2025_Silkie_Wenchang	0.662355682
ENSGALG00010021485	SMOC1	2	0.321157756	0.170267262	1.886197923	0.059268288	0	0.334204146	0	1	PRJNA827465;Yang2025_Silkie_Wenchang	CRA004775	0.421843813
ENSGALG00010021485	SMOC1	2	0.229453234	0.241000233	0.952081189	0.341052766	0	0.074556682	0	1	CRA004775;Yang2025_Silkie_Wenchang	PRJNA827465	0.805646109
ENSGALG00010021485	SMOC1	2	0.38009235	0.189128655	2.009702598	0.044462675	0	0.018195331	0	1	CRA004775;PRJNA827465	Yang2025_Silkie_Wenchang	0.363948415
ENSGALG00010021487	KLHL17	2	0.060889848	0.149172956	0.408183287	0.683139441	0.034092361	4.116569447	75.70674807	0.5	PRJNA827465;Yang2025_Silkie_Wenchang	CRA004775	0.918080744
ENSGALG00010021487	KLHL17	2	-0.261453687	0.211431063	-1.236590703	0.216239073	0.05324578	2.192197866	54.3836797	0.5	CRA004775;Yang2025_Silkie_Wenchang	PRJNA827465	0.72989044
ENSGALG00010021487	KLHL17	2	-0.131187967	0.368521455	-0.35598461	0.721852091	0.235949847	6.929821728	85.56961435	0.5	CRA004775;PRJNA827465	Yang2025_Silkie_Wenchang	0.929372448
ENSGALG00010021488	HYI	2	0.062362227	0.276880011	0.225934292	0.82123697	0.126893108	5.57368558	82.03853021	0.5	PRJNA827465;Yang2025_Silkie_Wenchang	CRA004775	0.933598716
ENSGALG00010021488	HYI	2	0.014824339	0.388648267	0.038143329	0.969573405	0.232555197	4.202821398	76.20645977	0.5	CRA004775;Yang2025_Silkie_Wenchang	PRJNA827465	0.991572914
ENSGALG00010021488	HYI	2	-0.215878002	0.10921509	-1.97663163	0.048083284	0	0.434206645	0	1	CRA004775;PRJNA827465	Yang2025_Silkie_Wenchang	0.379219854
ENSGALG00010021489	CNPPD1	2	-0.012202045	0.050357236	-0.24230966	0.808540229	0	0.308749754	0	0.5	PRJNA827465;Yang2025_Silkie_Wenchang	CRA004775	0.952496413
ENSGALG00010021489	CNPPD1	2	-0.088531345	0.08134786	-1.088305761	0.276460148	0	0.933435703	0	1	CRA004775;Yang2025_Silkie_Wenchang	PRJNA827465	0.775851484
ENSGALG00010021489	CNPPD1	2	-0.062265958	0.117854916	-0.528327198	0.597272257	0.014956461	1.69648586	41.05462217	0.5	CRA004775;PRJNA827465	Yang2025_Silkie_Wenchang	0.894372368
ENSGALG00010021490	ACACB	2	-0.086227223	0.206199759	-0.41817325	0.675820452	0.050210627	2.428625414	58.82444471	0.5	PRJNA827465;Yang2025_Silkie_Wenchang	CRA004775	0.916522765
ENSGALG00010021490	ACACB	2	0.076951617	0.167743512	0.458745713	0.646416783	0	0.531065128	0	0.5	CRA004775;Yang2025_Silkie_Wenchang	PRJNA827465	0.907220702
ENSGALG00010021490	ACACB	2	-0.313603658	0.192860504	-1.626064702	0.103935892	0	0.020845249	0	1	CRA004775;PRJNA827465	Yang2025_Silkie_Wenchang	0.548175896
ENSGALG00010021492	PARP6	2	0.051855536	0.152565651	0.339889978	0.733939384	0.038276819	5.581317686	82.08308403	0.5	PRJNA827465;Yang2025_Silkie_Wenchang	CRA004775	0.933391141
ENSGALG00010021492	PARP6	2	0.214400066	0.09675127	2.215992271	0.026692031	0	0.013785974	0	1	CRA004775;Yang2025_Silkie_Wenchang	PRJNA827465	0.278826072
ENSGALG00010021492	PARP6	2	-0.064625556	0.097132047	-0.66533712	0.505834894	0.003897153	1.069964457	6.538951525	0.5	CRA004775;PRJNA827465	Yang2025_Silkie_Wenchang	0.863927652
ENSGALG00010021493	UNC93B1	2	0.027940619	0.079680428	0.350658493	0.725844569	0.000217526	1.01415706	1.395943538	0.5	PRJNA827465;Yang2025_Silkie_Wenchang	CRA004775	0.931029023
ENSGALG00010021493	UNC93B1	2	-0.07557132	0.278499264	-0.271351956	0.78612035	0.108281352	3.056437782	67.28217385	0.5	CRA004775;Yang2025_Silkie_Wenchang	PRJNA827465	0.946639719
ENSGALG00010021493	UNC93B1	2	-0.122331028	0.170518555	-0.717405963	0.473123641	0.03041241	1.661856691	39.82633968	1	CRA004775;PRJNA827465	Yang2025_Silkie_Wenchang	0.854341771
ENSGALG00010021494	SLC2A10	2	-0.089993566	0.259838351	-0.346344433	0.72908388	0.097803407	3.301116313	69.70721704	0.5	PRJNA827465;Yang2025_Silkie_Wenchang	CRA004775	0.932323844
ENSGALG00010021494	SLC2A10	2	-0.492447331	0.225563386	-2.183188234	0.029021951	0	0.395027258	0	1	CRA004775;Yang2025_Silkie_Wenchang	PRJNA827465	0.289904262
ENSGALG00010021494	SLC2A10	2	-0.199065434	0.412094244	-0.483058031	0.629054532	0.258582101	3.539095767	71.74419496	0.5	CRA004775;PRJNA827465	Yang2025_Silkie_Wenchang	0.902161295
ENSGALG00010021496	MTMR14	2	0.156334387	0.067290304	2.323282532	0.02016398	0	0.062201946	0	1	PRJNA827465;Yang2025_Silkie_Wenchang	CRA004775	0.238922186
ENSGALG00010021496	MTMR14	2	-0.009689676	0.209733727	-0.046199896	0.963150925	0.042532491	1.669920503	40.11690985	0.5	CRA004775;Yang2025_Silkie_Wenchang	PRJNA827465	0.989998461
ENSGALG00010021496	MTMR14	2	0.016557016	0.228790799	0.07236749	0.942309456	0.06865064	2.213945838	54.83177669	0.5	CRA004775;PRJNA827465	Yang2025_Silkie_Wenchang	0.985827325
ENSGALG00010021498	B3GALT6	2	-0.119975342	0.176733939	-0.678847212	0.497234676	0.053540384	6.891954585	85.49032807	0.5	PRJNA827465;Yang2025_Silkie_Wenchang	CRA004775	0.86098656
ENSGALG00010021498	B3GALT6	2	-0.225951742	0.150957275	-1.49679267	0.134447214	0.016936338	1.340399268	25.3953636	0.5	CRA004775;Yang2025_Silkie_Wenchang	PRJNA827465	0.614225138
ENSGALG00010021498	B3GALT6	2	0.048823845	0.074604269	0.65443769	0.512829859	0	0.001369828	0	1	CRA004775;PRJNA827465	Yang2025_Silkie_Wenchang	0.866026915
ENSGALG00010021502	DNAJB12	2	0.035456826	0.042143145	0.841342673	0.400155991	0	0.014300396	0	1	PRJNA827465;Yang2025_Silkie_Wenchang	CRA004775	0.832246851
ENSGALG00010021502	DNAJB12	2	0.020658121	0.081532931	0.253371504	0.799981146	0	0.044982795	0	0.5	CRA004775;Yang2025_Silkie_Wenchang	PRJNA827465	0.949956462
ENSGALG00010021502	DNAJB12	2	0.035901882	0.047494745	0.755912721	0.449701527	0	0.070400716	0	0.5	CRA004775;PRJNA827465	Yang2025_Silkie_Wenchang	0.847309577
ENSGALG00010021503	KCNS1	2	0.299677795	0.376588165	0.795770613	0.426165375	0.15438182	2.110835489	52.62539382	0.5	PRJNA827465;Yang2025_Silkie_Wenchang	CRA004775	0.84125685
ENSGALG00010021503	KCNS1	2	0.328035193	0.483355928	0.678661777	0.49735219	0.221324835	1.871682827	46.5721443	0.5	CRA004775;Yang2025_Silkie_Wenchang	PRJNA827465	0.86098656
ENSGALG00010021503	KCNS1	2	-0.058174242	0.259443689	-0.224226853	0.82258079	0	0.108867221	0	1	CRA004775;PRJNA827465	Yang2025_Silkie_Wenchang	0.955822987
ENSGALG00010021504	ODR4	2	0.038541705	0.050272952	0.766648929	0.443290276	0	0.458974045	0	0.5	PRJNA827465;Yang2025_Silkie_Wenchang	CRA004775	0.845733906
ENSGALG00010021504	ODR4	2	-0.032744231	0.099304581	-0.32973535	0.74159994	0	0.09221546	0	1	CRA004775;Yang2025_Silkie_Wenchang	PRJNA827465	0.93543545
ENSGALG00010021504	ODR4	2	0.051870627	0.05655873	0.917110887	0.359084537	0	0.306993255	0	0.5	CRA004775;PRJNA827465	Yang2025_Silkie_Wenchang	0.814241185
ENSGALG00010021505	IHH	2	1.250504245	0.481879926	2.595053617	0.009457616	0	0.001656548	0	1	PRJNA827465;Yang2025_Silkie_Wenchang	CRA004775	0.152962732
ENSGALG00010021505	IHH	2	0.83491926	0.405328545	2.059858032	0.039412115	0	0.361326286	0	1	CRA004775;Yang2025_Silkie_Wenchang	PRJNA827465	0.343326573
ENSGALG00010021505	IHH	2	0.900546362	0.378174218	2.381300253	0.017251644	0	0.555883358	0	1	CRA004775;PRJNA827465	Yang2025_Silkie_Wenchang	0.218160028

ENSGALG00010021506	MAX	2	0.469028405	0.457563079	1.025057366	0.305336121	0.389711601	13.93033271	92.82142056	1	PRJNA827465;Yang2025_Silkie_Wenchang	CRA004775	0.79225794
ENSGALG00010021506	MAX	2	0.334073163	0.613537944	0.544502856	0.586095484	0.705051695	15.74157021	93.64739358	0.5	CRA004775;Yang2025_Silkie_Wenchang	PRJNA827465	0.891402106
ENSGALG00010021506	MAX	2	-0.063900037	0.144567724	-0.442007629	0.658483685	0.022166307	1.848839449	45.91201519	0.5	CRA004775;PRJNA827465	Yang2025_Silkie_Wenchang	0.911105434
ENSGALG00010021509	DDIT4	2	0.101310449	0.314646495	0.321981814	0.747466471	0.168882144	6.209569442	83.89582387	0.5	PRJNA827465;Yang2025_Silkie_Wenchang	CRA004775	0.93680487
ENSGALG00010021509	DDIT4	2	0.392310986	0.217821439	1.801066911	0.071692334	0	0.416560855	0	1	CRA004775;Yang2025_Silkie_Wenchang	PRJNA827465	0.461921937
ENSGALG00010021509	DDIT4	2	-0.168488807	0.080593136	-2.090609889	0.036563049	0	0.280212854	0	0.5	CRA004775;PRJNA827465	Yang2025_Silkie_Wenchang	0.329671369
ENSGALG00010021510	NTSR1	2	-0.131106072	0.255319496	-0.513498084	0.607602951	0.003473998	1.025478199	2.484518813	0.5	PRJNA827465;Yang2025_Silkie_Wenchang	CRA004775	0.897160522
ENSGALG00010021510	NTSR1	2	-0.537273022	0.343552829	-1.56387308	0.117847378	0	0.097520594	0	1	CRA004775;Yang2025_Silkie_Wenchang	PRJNA827465	0.577620253
ENSGALG00010021510	NTSR1	2	-0.135553837	0.334571373	-0.40515671	0.685362323	0.051984353	1.219885609	18.02510064	0.5	CRA004775;PRJNA827465	Yang2025_Silkie_Wenchang	0.919235539
ENSGALG00010021512	CPNE9	2	-0.079323498	0.441550131	-0.179647773	0.857429097	0.271027088	3.213760478	68.88380429	0.5	PRJNA827465;Yang2025_Silkie_Wenchang	CRA004775	0.963964719
ENSGALG00010021512	CPNE9	2	-0.449561503	0.903264211	-0.497707645	0.618690119	1.388529118	6.598596019	84.84526107	0.5	CRA004775;Yang2025_Silkie_Wenchang	PRJNA827465	0.899168321
ENSGALG00010021512	CPNE9	2	-0.805743367	0.440467465	-1.829291448	0.067355959	0.214336474	2.016844425	50.41759356	1	CRA004775;PRJNA827465	Yang2025_Silkie_Wenchang	0.447253128
ENSGALG00010021513	SDF4	2	-0.160671981	0.156106372	-1.02924678	0.303363725	0.046334015	20.25089519	95.06194669	1	PRJNA827465;Yang2025_Silkie_Wenchang	CRA004775	0.792084776
ENSGALG00010021513	SDF4	2	-0.115894038	0.216815968	-0.534527226	0.592976818	0.084860195	9.66866709	89.65731273	0.5	CRA004775;Yang2025_Silkie_Wenchang	PRJNA827465	0.892776493
ENSGALG00010021513	SDF4	2	0.013237485	0.048780493	0.271368417	0.78610769	0	0.744655431	0	0.5	CRA004775;PRJNA827465	Yang2025_Silkie_Wenchang	0.946639719
ENSGALG00010021514	TPR	2	0.195084471	0.319442753	0.610702447	0.541396586	0.186189604	10.8698364	90.80022952	0.5	PRJNA827465;Yang2025_Silkie_Wenchang	CRA004775	0.876108291
ENSGALG00010021514	TPR	2	0.406272797	0.159995382	2.539278265	0.011108143	0.009814247	1.225834713	18.42293345	1	CRA004775;Yang2025_Silkie_Wenchang	PRJNA827465	0.167465807
ENSGALG00010021514	TPR	2	-0.024460303	0.136058764	-0.179777486	0.857327259	0.019915838	1.677918905	40.40236407	0.5	CRA004775;PRJNA827465	Yang2025_Silkie_Wenchang	0.963936745
ENSGALG00010021516	SRSF5	2	0.555503553	0.416322299	1.334311311	0.182101852	0.337749678	38.85971829	97.42664115	1	PRJNA827465;Yang2025_Silkie_Wenchang	CRA004775	0.690033956
ENSGALG00010021516	SRSF5	2	0.274002025	0.742517163	0.369017767	0.712114482	1.031276227	14.74840724	93.21960681	0.5	CRA004775;Yang2025_Silkie_Wenchang	PRJNA827465	0.92597752
ENSGALG00010021516	SRSF5	2	-0.083966696	0.311454739	-0.26959518	0.787471709	0.142148624	2.967904523	66.30619374	0.5	CRA004775;PRJNA827465	Yang2025_Silkie_Wenchang	0.946919116
ENSGALG00010021518	CPEB3	2	-0.250252538	0.138829846	-1.802584568	0.071453482	0.018036797	1.788678125	44.09279198	1	PRJNA827465;Yang2025_Silkie_Wenchang	CRA004775	0.461559554
ENSGALG00010021518	CPEB3	2	0.186917146	0.307736772	0.60739295	0.543590166	0.129510696	2.981093081	66.45525742	0.5	CRA004775;Yang2025_Silkie_Wenchang	PRJNA827465	0.876968972
ENSGALG00010021518	CPEB3	2	0.049558635	0.45277087	0.109456324	0.912840562	0.358812786	7.459276505	86.59387409	0.5	CRA004775;PRJNA827465	Yang2025_Silkie_Wenchang	0.979052329
ENSGALG00010021519	FNTB	2	0.117927309	0.103519733	1.139177098	0.254629296	0.010728414	1.570082791	36.30909111	1	PRJNA827465;Yang2025_Silkie_Wenchang	CRA004775	0.764296259
ENSGALG00010021519	FNTB	2	0.082929577	0.2916838	0.284313275	0.776170313	0.099282663	2.172673516	53.97375663	0.5	CRA004775;Yang2025_Silkie_Wenchang	PRJNA827465	0.944043505
ENSGALG00010021519	FNTB	2	0.051318515	0.057044975	0.899614992	0.368325176	0	0.954723829	0	0.5	CRA004775;PRJNA827465	Yang2025_Silkie_Wenchang	0.820213684
ENSGALG00010021521	ENSGALG00010021521	2	0.230387144	0.271884171	0.847372405	0.396787581	0.095185187	2.741026625	63.517319	0.5	PRJNA827465;Yang2025_Silkie_Wenchang	CRA004775	0.831375233
ENSGALG00010021521	ENSGALG00010021521	2	0.570500116	0.220134647	2.59159621	0.009553183	0	0.050868086	0	1	CRA004775;Yang2025_Silkie_Wenchang	PRJNA827465	0.153870904
ENSGALG00010021521	ENSGALG00010021521	2	0.230291658	0.315448024	0.730046284	0.465361894	0.120725437	2.30302212	56.57879309	0.5	CRA004775;PRJNA827465	Yang2025_Silkie_Wenchang	0.852411124
ENSGALG00010021523	CELF6	2	0.201612736	0.351139382	0.574167259	0.565854623	0.223595228	10.35913276	90.34668226	0.5	PRJNA827465;Yang2025_Silkie_Wenchang	CRA004775	0.883487517
ENSGALG00010021523	CELF6	2	0.318101299	0.315647965	1.007772372	0.313563752	0.126635585	2.556037855	60.87694875	0.5	CRA004775;Yang2025_Silkie_Wenchang	PRJNA827465	0.796531448
ENSGALG00010021523	CELF6	2	-0.125099917	0.091912666	-1.36107375	0.173490384	0	0.025737088	0	1	CRA004775;PRJNA827465	Yang2025_Silkie_Wenchang	0.676927685
ENSGALG00010021525	ENSGALG00010021525	2	-0.172284519	0.085719531	-2.009863067	0.044445684	0	0.024897893	0	1	PRJNA827465;Yang2025_Silkie_Wenchang	CRA004775	0.363948415
ENSGALG00010021525	ENSGALG00010021525	2	-0.116504463	0.132359962	-0.880209252	0.378745962	0	0.715269855	0	0.5	CRA004775;Yang2025_Silkie_Wenchang	PRJNA827465	0.823876439
ENSGALG00010021525	ENSGALG00010021525	2	-0.156885566	0.104830773	-1.496560226	0.134507726	0	0.865088129	0	0.5	CRA004775;PRJNA827465	Yang2025_Silkie_Wenchang	0.614358762
ENSGALG00010021526	KCNJ12	2	-0.104819779	0.207280067	-0.505691552	0.613073199	0.06381123	3.819321575	73.81733954	0.5	PRJNA827465;Yang2025_Silkie_Wenchang	CRA004775	0.898130964
ENSGALG00010021526	KCNJ12	2	-0.041278293	0.420024735	-0.098275863	0.921713244	0.229763762	2.303423434	56.58635815	0.5	CRA004775;Yang2025_Silkie_Wenchang	PRJNA827465	0.981649741
ENSGALG00010021526	KCNJ12	2	0.109878201	0.123336514	0.890881353	0.372992825	0	0.694460696	0	1	CRA004775;PRJNA827465	Yang2025_Silkie_Wenchang	0.822162024
ENSGALG00010021528	BRPF1	2	-0.078794399	0.051608424	-1.526773998	0.126817219	0.000227561	1.036069236	3.481353855	1	PRJNA827465;Yang2025_Silkie_Wenchang	CRA004775	0.597676535
ENSGALG00010021528	BRPF1	2	-0.128132713	0.087517032	-1.464088868	0.143169669	0	0.719228564	0	0.5	CRA004775;Yang2025_Silkie_Wenchang	PRJNA827465	0.630019027
ENSGALG00010021528	BRPF1	2	-0.04022034	0.057083951	-0.704582273	0.481070233	0	0.150927302	0	0.5	CRA004775;PRJNA827465	Yang2025_Silkie_Wenchang	0.856783731
ENSGALG00010021529	BTAF1	2	-0.018975025	0.044778812	-0.423750083	0.671748086	0	0.679827451	0	0.5	PRJNA827465;Yang2025_Silkie_Wenchang	CRA004775	0.914674175
ENSGALG00010021529	BTAF1	2	0.104354089	0.124492763	0.838234182	0.401899193	0	0.054792758	0	1	CRA004775;Yang2025_Silkie_Wenchang	PRJNA827465	0.832806663
ENSGALG00010021529	BTAF1	2	-0.026049931	0.046619761	-0.558774437	0.576315671	0	0.473912838	0	0.5	CRA004775;PRJNA827465	Yang2025_Silkie_Wenchang	0.887048016
ENSGALG00010021530	MLF2	2	0.01305281	0.042519131	0.306986756	0.758853453	0	0.000228077	0	1	PRJNA827465;Yang2025_Silkie_Wenchang	CRA004775	0.938996879
ENSGALG00010021530	MLF2	2	-0.15091229	0.173165977	-0.871489265	0.383487065	0.046472089	4.382555716	77.18226385	0.5	CRA004775;Yang2025_Silkie_Wenchang	PRJNA827465	0.825509017
ENSGALG00010021530	MLF2	2	-0.139026015	0.172953276	-0.803835686	0.421491877	0.050923797	6.161330548	83.76973947	0.5	CRA004775;PRJNA827465	Yang2025_Silkie_Wenchang	0.839352211
ENSGALG00010021531	RAB15	2	0.573372583	0.490901747	1.16799866	0.242807305	0.392997079	5.411936782	81.52232666	1	PRJNA827465;Yang2025_Silkie_Wenchang	CRA004775	0.7550024
ENSGALG00010021531	RAB15	2	0.445669567	0.609555417	0.731138719	0.464694426	0.663430396	9.324891763	89.27601494	0.5	CRA004775;Yang2025_Silkie_Wenchang	PRJNA827465	0.85217126
ENSGALG00010021531	RAB15	2	-0.05621928	0.207068338	-0.27150109	0.786005661	0	0.324235169	0	0.5	CRA004775;PRJNA827465	Yang2025_Silkie_Wenchang	0.946639719
ENSGALG00010021532	DPP10	2	0.041667093	0.11326461	0.367873892	0.712967268	0	0.671536317	0	0.5	PRJNA827465;Yang2025_Silkie_Wenchang	CRA004775	0.926124617
ENSGALG00010021532	DPP10	2	0.21193046	0.135207616	1.567444696	0.117010784	0	0.916011719	0	1	CRA004775;Yang2025_Silkie_Wenchang	PRJNA827465	0.57615266
ENSGALG00010021532	DPP10	2	0.14220569	0.234183039	0.607241627	0.543690571	0.066778394	2.433947263	58.91447547	0.5	CRA004775;PRJNA827465	Yang2025_Silkie_Wenchang	0.876968972
ENSGALG00010021534	KIFC3	2	0.210576422	0.329593659	0.638897068	0.522889896	0.206968223	21.10227822	95.26117517	0.5	PRJNA827465;Yang2025_Silkie_Wenchang	CRA004775	0.869568211
ENSGALG00010021534	KIFC3	2	0.28995057	0.315312208	0.919566584	0.357799295	0.151752509	3.618543469	72.36457131	0.5	CRA004775;Yang2025_Silkie_Wenchang	PRJNA827465	0.813618706
ENSGALG00010021534	KIFC3	2	-0.11808896	0.097155265	-1.215466401	0.224188256	0	0.001153131	0	1	CRA004775;PRJNA827465	Yang2025_Silkie_Wenchang	0.737455024
ENSGALG00010021535	SUSD6	2	-0.00721037	0.122565168	-0.058828869	0.953088414	0.026698241	8.943173192	88.81828655	0.5	PRJNA827465;Yang2025_Silkie_Wenchang	CRA004775	0.987758913
ENSGALG00010021535	SUSD6	2	-0.135191353	0.061635509	-2.193400439	0.028278541	0	0.030190644	0	1	CRA004775;Yang2025_Silkie_Wenchang	PRJNA827465	0.286525261
ENSGALG00010021535	SUSD6	2	0.075508841	0.097123004	0.777455777	0.436889916	0.008121745	1.237124026	19.16736082	0.5	CRA004775;PRJNA827465	Yang2025_Silkie_Wenchang	0.843387647
ENSGALG00010021538	TNFRSF18	2	0.510779142	0.278919684	1.831276783	0.067059241	0	0.333767255	0	1	PRJNA827465;Yang2025_Silkie_Wenchang	CRA004775	0.446111942
ENSGALG00010021538	TNFRSF18	2	0.257296409	0.36560285	0.703759308	0.481582679	0	0.00206025	0	1	CRA004775;Yang2025_Silkie_Wenchang	PRJNA827465	0.85691916
ENSGALG00010021538	TNFRSF18	2	0.51292552	0.285574148	1.796120287	0.072475389	0	0.358573854	0	1	CRA004775;PRJNA827465	Yang2025_Silkie_Wenchang	0.464985342
ENSGALG00010021539	HEXA	2	-0.018474578	0.182116991	-0.101443464	0.919198435	0.054184342	5.353056881	81.31908511	0.5	PRJNA827465;Yang2025_Silkie_Wenchang	CRA004775	0.981159015
ENSGALG00010021539	HEXA	2	-0.215485933	0.126405851	-1.704714861	0.088247617	0	0.00552934	0	1	CRA004775;Yang2025_Silkie_Wenchang	PRJNA827465	0.510208309
ENSGALG00010021539	HEXA	2	0.140969718	0.088167853	1.598878875	0.109847519	0	0.552633029	0	0.5	CRA004775;PRJNA827465	Yang2025_Silkie_Wenchang	0.561203305
ENSGALG00010021540	STK4	2	0.028292232	0.099770683	0.283572603	0.776737935	0.014861126	3.907814238	74.41024729	0.5	PRJNA827465;Yang2025_Silkie_Wenchang	CRA004775	0.944156005
ENSGALG00010021540	STK4	2	-0.092381734	0.077413037	-1.193361449	0.232727846	0	0.935074545	0	1	CRA004775;Yang2025_Silkie_Wenchang	PRJNA827465	0.74577273
ENSGALG00010021540	STK4	2	-0.043076496	0.26383371	-0.163271389	0.87030476	0.093068067	2.185018512	54.23379736	0.5	CRA004775;PRJNA827465	Yang2025_Silkie_Wenchang	0.968832152
ENSGALG00010021541	CYP27A1	2	-0.027268791	0.249879786	-0.109127638	0.913101254	0.07785106	2.528933607	60.45764123	0.5	PRJNA827465;Yang2025_Silkie_Wenchang	CRA004775	0.979129568
ENSGALG00010021541	CYP27A1	2	-0.222373421	0.161889822	-1.373609644	0.169562901	0	0.090695113	0	1	CRA004775;Yang2025_Silkie_Wenchang	PRJNA827465	0.671059781
ENSGALG00010021541	CYP27A1	2	0.209340257	0.244283038	0.85695781	0.391468202	0	0.2751993	0	0.5	CRA004775;PRJNA827465	Yang2025_Silkie_Wenchang	0.829251057
ENSGALG00010021543	PLEKHD1	2	0.613537031	0.530892415	1.155671119	0.247815706	0.500945442	8.955742698	88.83398023	1	PRJNA827465;Yang2025_Silkie_Wenchang	CRA004775	0.7580874
ENSGALG00010021543	PLEKHD1	2	0.318919229	0.87905447	0.362798029	0.716755764	1.385358261	9.200674483	89.13123161	0.5	CRA004775;Yang2025_Silkie_Wenchang	PRJNA827465	0.92761444
ENSGALG00010021543	PLEKHD1	2	-0.127336067	0.316310454	-0.402566735	0.687266985	0.065757844	1.365258054	26.75377398	0.5	CRA004775;PRJNA827465	Yang2025_Silkie_Wenchang	0.919451777
ENSGALG00010021545	CYB5R2	2	0.301858786	0.28215007	1.069851892	0.284685981	0.129227769	5.211476036	80.81157827	1	PRJNA827465;Yang2025_Silkie_Wenchang	CRA004775	0.780265906
ENSGALG00010021545	CYB5R2	2	0.552937636	0.179046554	3.088233899	0.002013499	0	0.276716303	0	1	CRA004775;Yang2025_Silkie_Wenchang	PRJNA827465	0.0584465
ENSGALG00010021545	CYB5R2	2	0.079122487	0.133102494	0.594447816	0.552212636	0	0.695688583	0	1	CRA004775;PRJNA827465	Yang2025_Silkie_Wenchang	0.879151505
ENSGALG00010021546	OGG1	2	0.34594237	0.193213256	1.790469131	0.073378525	0.024554349	1.35175745	26.0222313	1	PRJNA827465;Yang2025_Silkie_Wenchang	CRA004775	0.467957919
ENSGALG00010021546	OGG1	2	-0.122589847	0.238513546	-0.513974508	0.607260918	0	0.412838519	0	0.5	CRA004775;Yang2025_Silkie_Wenchang	PRJNA827465	0.896998866
ENSGALG00010021546	OGG1	2	0.152805896	0.365677863	0.417870239	0.676041993	0.203813787	3.883885622	74.25258885	0.5	CRA004775;PRJNA827465	Yang2025_Silkie_Wenchang	0.91657106
ENSGALG00010021549	ANAPC16	2	0.129920022	0.1650948	0.786941941	0.431315854	0.039343899	3.268816655	69.40878896	0.5	PRJNA827465;Yang2025_Silkie_Wenchang	CRA004775	0.842503929
ENSGALG00010021549	ANAPC16	2	0.03056245	0.358825072	0.085173675	0.932123319	0.190718401	3.545533515	71.79390893	0.5	CRA004775;Yang2025_Silkie_Wenchang	PRJNA827465	0.984183398
ENSGALG00010021549	ANAPC16	2	-0.054737504	0.129976499	-0.42113385	0.673657345	0.013076735	1.204863309	17.00303326	1	CRA004775;PRJNA827465	Yang2025_Silkie_Wenchang	0.915485448
ENSGALG00010021550	MAP2K3	2	0.118597348	0.068660003	1.727313457	0.084111384	0	0.228842951	0	1	PRJNA827465;Yang2025_Silkie_Wenchang	CRA004775	0.499714459
ENSGALG00010021550	MAP2K3	2	0.145848793	0.120163052	1.213757394	0.22484038	0	0.265428944	0	0.5	CRA004775;Yang2025_Silkie_Wenchang	PRJNA827465	0.737885283
ENSGALG00010021550	MAP2K3	2	0.091841593	0.078317583	1.17268166	0.240923489	0	0.098979403	0	0.5	CRA004775;PRJNA827465	Yang2025_Silkie_Wenchang	0.753482604
ENSGALG00010021552	ENSGALG00010021552	2	-0.0375932	0.263344552	-0.142752905	0.886485332	0.118070595	6.490732442	84.59341825	0.5	PRJNA827465;Yang2025_Silkie_Wenchang	CRA004775	0.972729743
ENSGALG00010021552	ENSGALG00010021552	2	0.26813483	0.165891364	1.61632784	0.106023451	0	0.084911487	0	0.5	CRA004775;Yang2025_Silkie_Wenchang	PRJNA827465	0.552458367
ENSGALG00010021552	ENSGALG00010021552	2	-0.07649097	0.305863974	-0.250081659	0.8025242	0.124860425	2.323462137	56.96077918	0.5	CRA004775;PRJNA827465	Yang2025_Silkie_Wenchang	0.950688375
ENSGALG00010021553	SLC39A9	2	-0.300494947	0.332351524	-0.902880418	0.336607905	0.18851101	28.8428788	96.5329397	0.5	PRJNA827465;Yang2025_Silkie_Wenchang	CRA004775	0.803537951
ENSGALG00010021553	SLC39A9	2	-0.477986049	0.22450604	-2.129056522	0.033249584	0.05880953	1.927924205	48.13074097	1	CRA004775;Yang2025_Silkie_Wenchang	PRJNA827465	0.313333153
ENSGALG00010021553	SLC39A9	2	0.001748805	0.035525443	0.049226843	0.96073852	0	0.147353427	0	0.5	CRA004775;PRJNA827465	Yang2025_Silkie_Wenchang	0.989520168
ENSGALG00010021554	SNX11	2	-0.01729656	0.080509106	-0.214839795	0.829892226	0.000797586	1.022016891	2.154259053	0.5	PRJNA827465;Yang2025_Silkie_Wenchang	CRA004775	0.957079054
ENSGALG00010021554	SNX11	2	-0.372364381	0.17177802	-2.167706795	0.030181002	0	0.326887993	0	1	CRA004775;Yang2025_Silkie_Wenchang	PRJNA827465	0.295973612
ENSGALG00010021554	SNX11	2	-0.175784381	0.229457379	-0.76608729	0.443624365	0.080445594	3.694983742	72.93628146	0.5	CRA004775;PRJNA827465	Yang2025_Silkie_Wenchang	0.845827655
ENSGALG00010021556	CAMK1	2	-0.0824512	0.054198986	-1.521268316	0.128192518	0	0.193614269	0	0.5	PRJNA827465;Yang2025_Silkie_Wenchang	CRA004775	0.606096128
ENSGALG00010021556	CAMK1	2	-0.152991304	0.195538512	-0.782410087	0.433973608	0.021788113	1.371797265	27.11029092	0.5	CRA004775;Yang2025_Silkie_Wenchang	PRJNA827465	0.842762754
ENSGALG00010021556	CAMK1	2	-0.124860709	0.098800465	-1.263766411	0.206331881	0.007875986	1.198092372	16.53398159	1	CRA004775;PRJNA827465	Yang2025_Silkie_Wenchang	0.718451597
ENSGALG00010021558	CHURC1	2	0.08787946	0.281913443	0.311724972	0.755249548	0.124761739	4.17911512	76.07148951	0.5	PRJNA827465;Yang2025_Silkie_Wenchang	CRA004775	0.938459928
ENSGALG00010021558	CHURC1	2	0.297538115	0.223880375	1.329009603	0.183846352	0	0.899496074	0	0.5	CRA004775;Yang2025_Silkie_Wenchang	PRJNA827465	0.692470523
ENSGALG00010021558	CHURC1	2	-0.143495004	0.099527047	-1.441768926	0.149367572	0	0.040798599	0	1	CRA004775;PRJNA827465	Yang2025_Silkie_Wenchang	0.639246608
ENSGALG00010021561	TGIF2	2	-0.278457466	0.291448665	-0.955425433	0.339362598	0.156676022	12.38386736	91.92497811	1	PRJNA827465;Yang2025_Silkie_Wenchang	CRA004775	0.804824608
ENSGALG00010021561	TGIF2	2	-0.275342866	0.32372324	-0.850550197	0.395019265	0.178462296	6.67768888	85.02475905	0.5	CRA004775;Yang2025_Silkie_Wenchang	PRJNA827465	0.830643015
ENSGALG00010021561	TGIF2	2	0.002781772	0.060549926	0.045941759	0.963356646	0	0.094514015	0	0.5	CRA004775;PRJNA827465	Yang2025_Silkie_Wenchang	0.999032587
ENSGALG00010021562	LRP6	2	0.012337341	0.089823303	0.137351225	0.890753183	0	0.926716731	0	0.5	PRJNA827465;Yang2025_Silkie_Wenchang	CRA004775	0.973477891
ENSGALG00010021562	LRP6	2	0.125939588	0.17580567	0.716356806	0.473771059	0	0.375738705	0	0.5	CRA004775;Yang2025_Silkie_Wenchang	PRJNA827465	0.85444425
ENSGALG00010021562	LRP6	2	-0.03407347	0.097024651	-0.351183639	0.725450583	0	0.012135766	0	1	CRA004775;PRJNA827465	Yang2025_Silkie_Wenchang	0.930910808
ENSGALG00010021564	PRKAG3	2	0.642057826	0.641590467	1.000724839	0.316950115	0.779808581	18.76050586	94.66965333	1	PRJNA827465;Yang2025_Silkie_Wenchang	CRA004775	0.79096787
ENSGALG00010021564	PRKAG3	2	0.763435074	0.670572087	1.138483227	0.254918759	0.673183359	3.468938723	71.17273956	0.5	CRA004775;Yang2025_Silkie_Wenchang	PRJNA827465	0.764444699
ENSGALG00010021564	PRKAG3	2	0.010600749	0.153860762	0.068898326	0.945070551	0	0.016635152	0	0.5	CRA004775;PRJNA827465	Yang2025_Silkie_Wenchang	0.986215824
ENSGALG00010021565	CBX1	2	0.000707463	0.053002337	0.013347775	0.989350533	0	0.021155979	0	0.5	PRJNA827465;Yang2025_Silkie_Wenchang	CRA004775	0.997119019
ENSGALG00010021565	CBX1	2	-0.02551793	0.08180287	-0.311944242	0.755082899	0	0.538759194	0	0.5	CRA004775;Yang2025_Silkie_Wenchang	PRJNA827465	0.93838723
ENSGALG00010021565	CBX1	2	-0.021322579	0.058517172	-0.364381572	0.715573095	0	0.491333327	0	1	CRA004775;PRJNA827465	Yang2025_Silkie_Wenchang	0.927131632
ENSGALG00010021566	ENSGALG00010021566	2	0.061662668	0.183451538	0.336125106	0.736776527	0.058611082	7.511458496	86.68700625	0.5	PRJNA827465;Yang2025_Silkie_Wenchang	CRA004775	0.937877134
ENSGALG00010021566	ENSGALG00010021566	2	0.207844429	0.125200415	1.66009377	0.09689559	0.00464483	1.075618327	7.030219284	0.5	CRA004775;Yang2025_Silkie_Wenchang	PRJNA827465	0.532106661
ENSGALG00010021566	ENSGALG00010021566	2	-0.109719926	0.065547554	-1.673898091	0.094150629	0	0.00015648	0	1	CRA004775;PRJNA827465	Yang2025_Silkie_Wenchang	0.525712768
ENSGALG00010021568	TADA3	2	-0.059538832	0.066089735	-0.900878658	0.36765284	0	0.364100609	0	0.5	PRJNA827465;Yang2025_Silkie_Wenchang	CRA004775	0.819975483
ENSGALG00010021568	TADA3	2	-0.054567539	0.185205027	-0.294633144	0.76827414	0.001264902	1.015640846	1.539997703	0.5	CRA004775;Yang2025_Silkie_Wenchang	PRJNA827465	0.941444269
ENSGALG00010021568	TADA3	2	-0.083025218	0.067937455	-1.222083147	0.221676186	0	0.600114487	0	1	CRA004775;PRJNA827465	Yang2025_Silkie_Wenchang	0.734987272
ENSGALG00010021570	KATNB1	2	0.082812542	0.065748509	1.2595349	0.207837193	0	0.589124176	0	1	PRJNA827465;Yang2025_Silkie_Wenchang	CRA004775	0.720210297
ENSGALG00010021570	KATNB1	2	-0.219198026	0.385826979	-0.568125191	0.569949964	0.265935134	8.862558087	88.71657607	0.5	CRA004775;Yang2025_Silkie_Wenchang	PRJNA827465	0.884923673
ENSGALG00010021570	KATNB1	2	-0.264257943	0.334524635	-0.78995062	0.429556607	0.193247223	6.793120921	85.27922568	0.5	CRA004775;PRJNA827465	Yang2025_Silkie_Wenchang	0.842012722
ENSGALG00010021572	MYL9	2	-0.102404177	0.180470205	-0.567429827	0.57042219	0.052777881	5.220188075	80.84360208	0.5	PRJNA827465;Yang2025_Silkie_Wenchang	CRA004775	0.8851128
ENSGALG00010021572	MYL9	2	-0.199792055	0.172924501	-1.155371582	0.247938294	0.022747245	1.353071888	26.09409678	0.5	CRA004775;Yang2025_Silkie_Wenchang	PRJNA827465	0.758107886
ENSGALG00010021572	MYL9	2	0.074073505	0.093301885	0.793912201	0.427246546	0	0.025748273	0	1	CRA004775;PRJNA827465	Yang2025_Silkie_Wenchang	0.84125685
ENSGALG00010021573	NATD1	2	-0.046105262	0.116571831	-0.39550946	0.692466941	0.007575032	1.306544352	23.46222322	0.5	PRJNA827465;Yang2025_Silkie_Wenchang	CRA004775	0.920559211
ENSGALG00010021573	NATD1	2	-0.171310333	0.140347809	-1.220612808	0.222232655	0	0.153703467	0	1	CRA004775;Yang2025_Silkie_Wenchang	PRJNA827465	0.73559121
ENSGALG00010021573	NATD1	2	-0.001640907	0.096050606	-0.017083775	0.986369783	0	0.388701933	0	0.5	CRA004775;PRJNA827465	Yang2025_Silkie_Wenchang	0.99618774
ENSGALG00010021574	TTLL4	2	-0.019943968	0.098279172	-0.202931784	0.839188351	0.009464894	1.828421316	45.30801018	0.5	PRJNA827465;Yang2025_Silkie_Wenchang	CRA004775	0.959413439
ENSGALG00010021574	TTLL4	2	-0.134957568	0.120217042	-1.122615947	0.26160064	0	0.085737992	0	1	CRA004775;Yang2025_Silkie_Wenchang	PRJNA827465	0.768310285
ENSGALG00010021574	TTLL4	2	0.047913057	0.073217349	0.654394853	0.51285745	0	0.119352839	0	0.5	CRA004775;PRJNA827465	Yang2025_Silkie_Wenchang	0.866029489
ENSGALG00010021575	ADGRL2	2	-0.086570965	0.11183322	-0.774107778	0.438867058	0.016389081	2.326269826	57.01272532	0.5	PRJNA827465;Yang2025_Silkie_Wenchang	CRA004775	0.844002503
ENSGALG00010021575	ADGRL2	2	0.102260608	0.135693666	0.753613719	0.451081204	0	0.153955678	0	1	CRA004775;Yang2025_Silkie_Wenchang	PRJNA827465	0.847847413
ENSGALG00010021575	ADGRL2	2	-0.10213915	0.138647107	-0.736684327	0.46131434	0.020960105	1.422627767	29.70754381	0.5	CRA004775;PRJNA827465	Yang2025_Silkie_Wenchang	0.85014477
ENSGALG00010021576	ERH	2	-0.044708897	0.04343467	-1.029336641	0.303321511	0	0.043812071	0	1	PRJNA827465;Yang2025_Silkie_Wenchang	CRA004775	0.792084776
ENSGALG00010021576	ERH	2	-0.195894919	0.236665372	-0.827729541	0.407823685	0.066718501	2.24486694	55.45392994	1	CRA004775;Yang2025_Silkie_Wenchang	PRJNA827465	0.833982683
ENSGALG00010021576	ERH	2	-0.185796088	0.210294276	-0.883505208	0.37696337	0.061288072	2.404972804	58.41948823	1	CRA004775;PRJNA827465	Yang2025_Silkie_Wenchang	0.823064945
ENSGALG00010021577	ARIH1	2	0.027228319	0.096641387	0.281745942	0.778138324	0.01233627	2.578795783	61.22221051	0.5	PRJNA827465;Yang2025_Silkie_Wenchang	CRA004775	0.944509772
ENSGALG00010021577	ARIH1	2	0.113800247	0.098740714	1.152515936	0.249109125	0	0.477341312	0	1	CRA004775;Yang2025_Silkie_Wenchang	PRJNA827465	0.759411487
ENSGALG00010021577	ARIH1	2	-0.043241526	0.047185221	-0.916420965	0.359446144	0	0.054187785	0	0.5	CRA004775;PRJNA827465	Yang2025_Silkie_Wenchang	0.814395099
ENSGALG00010021579	C20H20ORF24	2	-0.131900161	0.15585324	-0.846310036	0.397379811	0.042745488	7.925084258	87.38183762	0.5	PRJNA827465;Yang2025_Silkie_Wenchang	CRA004775	0.831571329
ENSGALG00010021579	C20H20ORF24	2	-0.224987548	0.130354475	-1.7259672	0.084353314	0.01257745	1.391515039	28.13588271	1	CRA004775;Yang2025_Silkie_Wenchang	PRJNA827465	0.500017819
ENSGALG00010021579	C20H20ORF24	2	0.010983868	0.045051021	0.24380952	0.807378347	0	0.003383868	0	0.5	CRA004775;PRJNA827465	Yang2025_Silkie_Wenchang	0.95234753
ENSGALG00010021580	KCTD10	2	-0.010682149	0.054494441	-0.196022723	0.84459238	0.002253381	1.569575711	36.2885146	0.5	PRJNA827465;Yang2025_Silkie_Wenchang	CRA004775	0.960769014
ENSGALG00010021580	KCTD10	2	-0.122596215	0.101412291	-1.208889114	0.226705449	0.007018785	1.227257466	18.51750528	1	CRA004775;Yang2025_Silkie_Wenchang	PRJNA827465	0.740342522
ENSGALG00010021580	KCTD10	2	-0.090350142	0.18041837	-0.500781276	0.616525064	0.045207378	2.527839691	60.44052938	0.5	CRA004775;PRJNA827465	Yang2025_Silkie_Wenchang	0.899111936
ENSGALG00010021582	STK36	2	0.090186011	0.074194538	1.215534364	0.22416235	0	0.892634989	0	1	PRJNA827465;Yang2025_Silkie_Wenchang	CRA004775	0.737452676
ENSGALG00010021582	STK36	2	0.133023061	0.107895698	1.232885675	0.21761842	0	0.87443826	0	0.5	CRA004775;Yang2025_Silkie_Wenchang	PRJNA827465	0.731013651
ENSGALG00010021582	STK36	2	0.018146345	0.090486344	0.20054236	0.841056432	0	0.220184324	0	0.5	CRA004775;PRJNA827465	Yang2025_Silkie_Wenchang	0.959871101
ENSGALG00010021583	NFE2L1	2	-0.024316986	0.045930385	-0.529431355	0.596506252	0	0.373149198	0	0.5	PRJNA827465;Yang2025_Silkie_Wenchang	CRA004775	0.894064255
ENSGALG00010021583	NFE2L1	2	0.008822106	0.076913464	0.11470171	0.908681558	0	0.115814665	0	0.5	CRA004775;Yang2025_Silkie_Wenchang	PRJNA827465	0.978382908
ENSGALG00010021583	NFE2L1	2	-0.045969578	0.055688481	-0.825477321	0.409100653	0	0.029512668	0	1	CRA004775;PRJNA827465	Yang2025_Silkie_Wenchang	0.834316328
ENSGALG00010021584	OVCH2	2	0.216829369	0.303481728	0.714472564	0.474935016	0	0.239163195	0	1	PRJNA827465;Yang2025_Silkie_Wenchang	CRA004775	0.854462864
ENSGALG00010021584	OVCH2	2	0.494092202	0.32820807	1.505423683	0.132215184	0	0.326764065	0	1	CRA004775;Yang2025_Silkie_Wenchang	PRJNA827465	0.608983169
ENSGALG00010021584	OVCH2	2	0.333407317	0.346865391	0.961200872	0.336451178	0	0.948863076	0	1	CRA004775;PRJNA827465	Yang2025_Silkie_Wenchang	0.803806405
ENSGALG00010021586	BBS4	2	-0.081434598	0.122972957	-0.662215505	0.507833119	0.015904812	1.813610662	44.861374	0.5	PRJNA827465;Yang2025_Silkie_Wenchang	CRA004775	0.864401731
ENSGALG00010021586	BBS4	2	-0.320028843	0.148781376	-2.151000694	0.031476145	0	0.269201979	0	1	CRA004775;Yang2025_Silkie_Wenchang	PRJNA827465	0.30351004
ENSGALG00010021586	BBS4	2	-0.143860272	0.203196847	-0.707984769	0.478954716	0.056219841	2.54801845	60.75381636	0.5	CRA004775;PRJNA827465	Yang2025_Silkie_Wenchang	0.855875059
ENSGALG00010021588	RNF25	2	0.146775309	0.162743278	0.901882471	0.367119304	0.043632843	5.400552321	81.48337539	1	PRJNA827465;Yang2025_Silkie_Wenchang	CRA004775	0.819739734
ENSGALG00010021588	RNF25	2	-0.03130745	0.390306313	-0.080212512	0.936068239	0.265530255	7.425359919	86.53263935	0.5	CRA004775;Yang2025_Silkie_Wenchang	PRJNA827465	0.984822128
ENSGALG00010021588	RNF25	2	-0.159071755	0.217427061	-0.731609737	0.464406804	0.068267668	2.908791277	65.62145906	0.5	CRA004775;PRJNA827465	Yang2025_Silkie_Wenchang	0.852159649
ENSGALG00010021593	DCAF7	2	-0.297615885	0.339131275	-0.877583128	0.380169989	0.220555597	23.68713633	95.7782993	0.5	PRJNA827465;Yang2025_Silkie_Wenchang	CRA004775	0.824392515
ENSGALG00010021593	DCAF7	2	-0.445503585	0.224162154	-1.987416594	0.04687625	0.073948329	3.682873431	72.84728844	1	CRA004775;Yang2025_Silkie_Wenchang	PRJNA827465	0.37376485
ENSGALG00010021593	DCAF7	2	-0.005433851	0.082962824	-0.065497427	0.947777955	0.006138256	1.308957266	23.60331187	0.5	CRA004775;PRJNA827465	Yang2025_Silkie_Wenchang	0.986619597
ENSGALG00010021594	ADPGK	2	-0.179032413	0.238703897	-0.750018813	0.453243374	0.105393729	12.76970141	92.16896333	0.5	PRJNA827465;Yang2025_Silkie_Wenchang	CRA004775	0.848823429
ENSGALG00010021594	ADPGK	2	-0.412788705	0.112459939	-3.670539988	0.000242039	0	0.125107298	0	1	CRA004775;Yang2025_Silkie_Wenchang	PRJNA827465	0.014523425
ENSGALG00010021594	ADPGK	2	-0.057396443	0.168247667	-0.341142582	0.732996246	0.036741732	2.070931849	51.71255875	0.5	CRA004775;PRJNA827465	Yang2025_Silkie_Wenchang	0.932985777
ENSGALG00010021595	UBE3B	2	0.001770506	0.074750689	0.02368548	0.981103488	0.004110123	1.568555366	36.24707031	0.5	PRJNA827465;Yang2025_Silkie_Wenchang	CRA004775	0.994928779
ENSGALG00010021595	UBE3B	2	-0.135203959	0.107690631	-1.255484885	0.209302792	0.005774668	1.183387914	15.49685544	1	CRA004775;Yang2025_Silkie_Wenchang	PRJNA827465	0.721187439
ENSGALG00010021595	UBE3B	2	-0.086507187	0.203904371	-0.424253715	0.671380791	0.05958837	2.977988626	66.42028814	0.5	CRA004775;PRJNA827465	Yang2025_Silkie_Wenchang	0.914674175
ENSGALG00010021597	GGT7	2	-0.086222866	0.211592796	-0.407494338	0.683644951	0.076945498	7.062482788	85.84067346	0.5	PRJNA827465;Yang2025_Silkie_Wenchang	CRA004775	0.918800744
ENSGALG00010021597	GGT7	2	-0.280074001	0.11732929	-2.387076575	0.016982954	0	0.332863175	0	1	CRA004775;Yang2025_Silkie_Wenchang	PRJNA827465	0.216433535
ENSGALG00010021597	GGT7	2	0.102133212	0.095003973	1.075041488	0.282356154	0	0.359373557	0	0.5	CRA004775;PRJNA827465	Yang2025_Silkie_Wenchang	0.779532565
ENSGALG00010021599	ARPC4	2	0.141496192	0.051317806	2.757253352	0.005828917	0	0.005040221	0	1	PRJNA827465;Yang2025_Silkie_Wenchang	CRA004775	0.115022409
ENSGALG00010021599	ARPC4	2	-0.20555468	0.327981303	-0.626726823	0.530838318	0.169541767	4.715411522	78.7929432	0.5	CRA004775;Yang2025_Silkie_Wenchang	PRJNA827465	0.872805322
ENSGALG00010021599	ARPC4	2	-0.163669127	0.334678789	-0.489033463	0.624818006	0.202885413	9.847396244	89.84503136	0.5	CRA004775;PRJNA827465	Yang2025_Silkie_Wenchang	0.901043264
ENSGALG00010021600	FMN1	2	-0.040741147	0.07170754	-0.568157087	0.569928307	0	0.97940731	0	0.5	PRJNA827465;Yang2025_Silkie_Wenchang	CRA004775	0.884923673
ENSGALG00010021600	FMN1	2	0.195334093	0.212981644	0.917140506	0.359069018	0	0.018164443	0	1	CRA004775;Yang2025_Silkie_Wenchang	PRJNA827465	0.814241185
ENSGALG00010021600	FMN1	2	-0.050452477	0.072682335	-0.694150468	0.487587852	0	0.386638426	0	0.5	CRA004775;PRJNA827465	Yang2025_Silkie_Wenchang	0.85768885
ENSGALG00010021602	PLEKHG4	2	-0.093365364	0.129495785	-0.720991527	0.470914728	0.023519915	3.198710849	68.73740556	0.5	PRJNA827465;Yang2025_Silkie_Wenchang	CRA004775	0.853785476
ENSGALG00010021602	PLEKHG4	2	-0.232175262	0.115867779	-2.003794874	0.045092038	0	0.051843041	0	1	CRA004775;Yang2025_Silkie_Wenchang	PRJNA827465	0.3659545
ENSGALG00010021602	PLEKHG4	2	0.008742041	0.074501582	0.117340343	0.906590356	0	0.322082351	0	0.5	CRA004775;PRJNA827465	Yang2025_Silkie_Wenchang	0.978196186
ENSGALG00010021604	SPTB	2	0.022362167	0.232707702	0.096095517	0.923444712	0.099252229	11.95289196	91.63382382	0.5	PRJNA827465;Yang2025_Silkie_Wenchang	CRA004775	0.98201108
ENSGALG00010021604	SPTB	2	0.029228061	0.264912069	0.11033118	0.91214673	0.112101452	4.563030589	78.08474038	0.5	CRA004775;Yang2025_Silkie_Wenchang	PRJNA827465	0.978967245
ENSGALG00010021604	SPTB	2	-0.217692289	0.083896141	-2.594783102	0.009465063	0	0.081220811	0	1	CRA004775;PRJNA827465	Yang2025_Silkie_Wenchang	0.152962732
ENSGALG00010021605	MAP3K3	2	0.014328195	0.047976248	0.298651838	0.765205708	0	0.000181249	0	1	PRJNA827465;Yang2025_Silkie_Wenchang	CRA004775	0.940779391

ENSGALG00010021605	MAP3K3	2	-0.005313869	0.10334092	-0.051420767	0.958990237	0	0.237459588	0	0.5	CRA004775;Yang2025_Silkie_Wenchang	PRJNA827465	0.989102387
ENSGALG00010021605	MAP3K3	2	0.009864494	0.052509053	0.187862734	0.850984256	0	0.266247759	0	0.5	CRA004775;PRJNA827465	Yang2025_Silkie_Wenchang	0.962056252
ENSGALG00010021606	RNF8	2	-0.084151117	0.059189897	-1.421714198	0.155109237	0	0.025810893	0	1	PRJNA827465;Yang2025_Silkie_Wenchang	CRA004775	0.647765412
ENSGALG00010021606	RNF8	2	-0.000309047	0.116249626	-0.002658476	0.997878846	0.008654984	1.339458684	25.34297535	0.5	CRA004775;Yang2025_Silkie_Wenchang	PRJNA827465	0.999290321
ENSGALG00010021606	RNF8	2	-0.013990902	0.125503948	-0.11147778	0.911237485	0.016288243	1.700323763	41.18767132	0.5	CRA004775;PRJNA827465	Yang2025_Silkie_Wenchang	0.978845851
ENSGALG00010021607	SLC9A5	2	0.465910146	0.113846326	4.092447805	4.26843E-05	0	0.000167886	0	1	PRJNA827465;Yang2025_Silkie_Wenchang	CRA004775	0.004380215
ENSGALG00010021607	SLC9A5	2	0.092251165	0.390183491	0.23643021	0.813098856	0.242295591	4.849298311	79.37845981	0.5	CRA004775;Yang2025_Silkie_Wenchang	PRJNA827465	0.953526116
ENSGALG00010021607	SLC9A5	2	0.112932248	0.39043703	0.28924574	0.772393332	0.259883501	6.419313729	84.42201079	0.5	CRA004775;PRJNA827465	Yang2025_Silkie_Wenchang	0.943066211
ENSGALG00010021609	ENSGALG00010021609	2	-0.029806853	0.327595596	-0.090986732	0.927503134	0.18811029	7.60669697	86.85368953	0.5	PRJNA827465;Yang2025_Silkie_Wenchang	CRA004775	0.982993195
ENSGALG00010021609	ENSGALG00010021609	2	0.338529425	0.203288257	1.66526798	0.095859304	0	0.008494743	0	1	CRA004775;Yang2025_Silkie_Wenchang	PRJNA827465	0.5301492
ENSGALG00010021609	ENSGALG00010021609	2	-0.153184322	0.305612473	-0.50123714	0.616204239	0.115906546	1.852638042	46.02291557	0.5	CRA004775;PRJNA827465	Yang2025_Silkie_Wenchang	0.899111936
ENSGALG00010021610	TMEM11	2	0.123274611	0.063524701	1.940577582	0.052309535	0	0.164925655	0	1	PRJNA827465;Yang2025_Silkie_Wenchang	CRA004775	0.397126071
ENSGALG00010021610	TMEM11	2	-0.072794434	0.202239984	-0.359940862	0.718891359	0.038353812	1.702929525	41.27766385	0.5	CRA004775;Yang2025_Silkie_Wenchang	PRJNA827465	0.928449671
ENSGALG00010021610	TMEM11	2	-0.031695773	0.235215864	-0.134751849	0.892808077	0.07931296	2.70952086	63.09310569	0.5	CRA004775;PRJNA827465	Yang2025_Silkie_Wenchang	0.974381724
ENSGALG00010021611	GREM1	2	0.072995567	0.275769763	0.264697501	0.791242503	0	0.020399993	0	1	PRJNA827465;Yang2025_Silkie_Wenchang	CRA004775	0.947937032
ENSGALG00010021611	GREM1	2	-0.426102694	0.601860197	-0.707976198	0.478960039	0.478116722	2.887001975	65.36199114	0.5	CRA004775;Yang2025_Silkie_Wenchang	PRJNA827465	0.855875059
ENSGALG00010021611	GREM1	2	-0.43929187	0.558265732	-0.786886684	0.431348203	0.410200117	2.799880102	64.28418491	0.5	CRA004775;PRJNA827465	Yang2025_Silkie_Wenchang	0.842503929
ENSGALG00010021612	BCS1L	2	0.190605969	0.078163528	2.43855381	0.014746163	0	0.016419369	0	1	PRJNA827465;Yang2025_Silkie_Wenchang	CRA004775	0.200553277
ENSGALG00010021612	BCS1L	2	0.045497005	0.235920526	0.192848863	0.847077335	0.047350779	1.610175996	37.89498773	0.5	CRA004775;Yang2025_Silkie_Wenchang	PRJNA827465	0.961252712
ENSGALG00010021612	BCS1L	2	0.067407198	0.205108302	0.328641975	0.742426317	0.048813029	1.773435366	43.61226694	0.5	CRA004775;PRJNA827465	Yang2025_Silkie_Wenchang	0.935510459
ENSGALG00010021613	ADGRL4	2	-0.253904594	0.24592214	-1.032459273	0.301857017	0.115431156	21.88465927	95.43058913	1	PRJNA827465;Yang2025_Silkie_Wenchang	CRA004775	0.791453392
ENSGALG00010021613	ADGRL4	2	-0.079556487	0.493016229	-0.161366873	0.871804457	0.414584117	5.959291599	83.21948199	0.5	CRA004775;Yang2025_Silkie_Wenchang	PRJNA827465	0.969462602
ENSGALG00010021613	ADGRL4	2	0.089805685	0.200794201	0.447252385	0.654692842	0.045173273	1.544179645	35.24069539	0.5	CRA004775;PRJNA827465	Yang2025_Silkie_Wenchang	0.910222014
ENSGALG00010021615	FLVCR1	2	0.094998901	0.113660443	0.835813218	0.403259989	0	0.474288227	0	0.5	PRJNA827465;Yang2025_Silkie_Wenchang	CRA004775	0.83306585
ENSGALG00010021615	FLVCR1	2	-0.061135514	0.196384651	-0.311304495	0.755568005	0	0.001999434	0	1	CRA004775;Yang2025_Silkie_Wenchang	PRJNA827465	0.938501652
ENSGALG00010021615	FLVCR1	2	0.108274592	0.119478136	0.906229335	0.364814479	0	0.351395214	0	0.5	CRA004775;PRJNA827465	Yang2025_Silkie_Wenchang	0.817649605
ENSGALG00010021616	ENSGALG00010021616	2	2.43286904	0.518022956	4.696450244	2.64722E-06	0	0.207407624	0	1	PRJNA827465;Yang2025_Silkie_Wenchang	CRA004775	0.000510017
ENSGALG00010021616	ENSGALG00010021616	2	1.35226947	0.716302179	1.887847766	0.05904639	0.578165439	2.240842393	55.37392531	1	CRA004775;Yang2025_Silkie_Wenchang	PRJNA827465	0.420797876
ENSGALG00010021616	ENSGALG00010021616	2	1.644057299	0.959038781	1.71427614	0.086470023	1.436364688	4.534475316	77.94673186	1	CRA004775;PRJNA827465	Yang2025_Silkie_Wenchang	0.509030629
ENSGALG00010021617	SORBS1	2	0.263060906	0.1437781	1.829631258	0.067305096	0.026780347	2.445241381	59.10424191	1	PRJNA827465;Yang2025_Silkie_Wenchang	CRA004775	0.447066453
ENSGALG00010021617	SORBS1	2	0.414274996	0.152236926	2.72125171	0.006503522	0	0.219353292	0	1	CRA004775;Yang2025_Silkie_Wenchang	PRJNA827465	0.123006701
ENSGALG00010021617	SORBS1	2	0.165150951	0.069509689	2.375941448	0.01750424	0	0.222527776	0	1	CRA004775;PRJNA827465	Yang2025_Silkie_Wenchang	0.220220599
ENSGALG00010021618	NEO1	2	-0.086468214	0.073374285	-1.178453925	0.23861569	0	0.692353129	0	0.5	PRJNA827465;Yang2025_Silkie_Wenchang	CRA004775	0.750694914
ENSGALG00010021618	NEO1	2	0.122846719	0.15728211	0.781059708	0.434767378	0	0.154007801	0	1	CRA004775;Yang2025_Silkie_Wenchang	PRJNA827465	0.842762754
ENSGALG00010021618	NEO1	2	-0.045342497	0.125339823	-0.361756509	0.717533995	0.013707528	1.407905043	28.97248256	0.5	CRA004775;PRJNA827465	Yang2025_Silkie_Wenchang	0.9277944
ENSGALG00010021619	FHOD1	2	0.011897452	0.154039285	0.077236481	0.938435421	0.030850931	2.774316721	63.95508875	0.5	PRJNA827465;Yang2025_Silkie_Wenchang	CRA004775	0.985349045
ENSGALG00010021619	FHOD1	2	0.146854569	0.144250503	1.018052384	0.308653062	0	0.591416826	0	0.5	CRA004775;Yang2025_Silkie_Wenchang	PRJNA827465	0.79365334
ENSGALG00010021619	FHOD1	2	-0.125761234	0.102874398	-1.222473585	0.221528587	0	0.006868106	0	1	CRA004775;PRJNA827465	Yang2025_Silkie_Wenchang	0.734941806
ENSGALG00010021621	ZNF518A	2	-0.073816999	0.077136297	-0.956968401	0.338583204	0	0.000876843	0	1	PRJNA827465;Yang2025_Silkie_Wenchang	CRA004775	0.804408854
ENSGALG00010021621	ZNF518A	2	-0.009390569	0.1376643	-0.068213535	0.945615653	0	0.374784162	0	0.5	CRA004775;Yang2025_Silkie_Wenchang	PRJNA827465	0.986321197
ENSGALG00010021621	ZNF518A	2	-0.053338585	0.081438652	-0.654954168	0.512497263	0	0.530062461	0	0.5	CRA004775;PRJNA827465	Yang2025_Silkie_Wenchang	0.865809047
ENSGALG00010021622	PTHLH	2	-0.243029092	0.112461387	-2.161000313	0.030695315	0	0.001182852	0	1	PRJNA827465;Yang2025_Silkie_Wenchang	CRA004775	0.298914265
ENSGALG00010021622	PTHLH	2	-0.140116182	0.247472495	-0.566188909	0.571265369	0.025912168	1.130708134	11.51598473	0.5	CRA004775;Yang2025_Silkie_Wenchang	PRJNA827465	0.885354083
ENSGALG00010021622	PTHLH	2	-0.161437663	0.214689984	-0.751957123	0.452076844	0.035106594	1.191590385	16.07854408	0.5	CRA004775;PRJNA827465	Yang2025_Silkie_Wenchang	0.848275385
ENSGALG00010021623	FAR1	2	0.063235762	0.050551916	1.250907322	0.210968292	0	0.899748589	0	0.5	PRJNA827465;Yang2025_Silkie_Wenchang	CRA004775	0.722839054
ENSGALG00010021623	FAR1	2	0.275390925	0.338952411	0.812476666	0.41651817	0.192298847	5.618318065	82.20107889	0.5	CRA004775;Yang2025_Silkie_Wenchang	PRJNA827465	0.837984974
ENSGALG00010021623	FAR1	2	0.316108321	0.279736347	1.13002234	0.258466811	0.125755352	4.299964009	76.74599139	1	CRA004775;PRJNA827465	Yang2025_Silkie_Wenchang	0.765698431
ENSGALG00010021624	HSD11B2	2	-0.342717634	0.236417796	-1.449627055	0.147162547	0	0.052950082	0	1	PRJNA827465;Yang2025_Silkie_Wenchang	CRA004775	0.635975583
ENSGALG00010021624	HSD11B2	2	0.338662833	0.639766599	0.529353726	0.596560092	0.62925795	4.291010822	76.69546777	0.5	CRA004775;Yang2025_Silkie_Wenchang	PRJNA827465	0.894064255
ENSGALG00010021624	HSD11B2	2	0.261829534	0.695053566	0.376704109	0.706393509	0.812068598	6.097282585	83.59925121	0.5	CRA004775;PRJNA827465	Yang2025_Silkie_Wenchang	0.924548793
ENSGALG00010021626	ZNF142	2	-0.062931337	0.05906493	-1.065450282	0.28666769	0	0.002895606	0	1	PRJNA827465;Yang2025_Silkie_Wenchang	CRA004775	0.782018115
ENSGALG00010021626	ZNF142	2	-0.068140095	0.095320255	-0.714854305	0.474699075	0	0.139548643	0	1	CRA004775;Yang2025_Silkie_Wenchang	PRJNA827465	0.854462864
ENSGALG00010021626	ZNF142	2	-0.07050447	0.072340148	-0.974624346	0.329746632	0	0.130756595	0	1	CRA004775;PRJNA827465	Yang2025_Silkie_Wenchang	0.801177491
ENSGALG00010021627	NCOA6	2	0.233022601	0.233314913	0.998799833	0.317912596	0.089808402	5.029937749	80.11686412	1	PRJNA827465;Yang2025_Silkie_Wenchang	CRA004775	0.8214684
ENSGALG00010021627	NCOA6	2	0.390831915	0.159046866	2.457338037	0.013997091	0	0.55971498	0	1	CRA004775;Yang2025_Silkie_Wenchang	PRJNA827465	0.193842584
ENSGALG00010021627	NCOA6	2	0.022944322	0.055841538	0.410882708	0.681158549	0	0.766719244	0	1	CRA004775;PRJNA827465	Yang2025_Silkie_Wenchang	0.91820222
ENSGALG00010021628	REC114	2	-0.178465037	0.180948242	-0.986276712	0.323997344	0	0.018642891	0	1	PRJNA827465;Yang2025_Silkie_Wenchang	CRA004775	0.799600434
ENSGALG00010021628	REC114	2	-0.041847573	0.268753555	-0.075672419	0.939667972	0.018137634	1.126808828	11.19642789	0.5	CRA004775;Yang2025_Silkie_Wenchang	PRJNA827465	0.983425793
ENSGALG00010021628	REC114	2	-0.019582699	0.21721109	-0.192658549	0.847226389	0.009824789	1.079090479	7.329364957	0.5	CRA004775;PRJNA827465	Yang2025_Silkie_Wenchang	0.961252712
ENSGALG00010021629	DHRS7B	2	-0.131807448	0.070347015	-1.873675052	0.060975227	0	0.124110707	0	1	PRJNA827465;Yang2025_Silkie_Wenchang	CRA004775	0.427185264
ENSGALG00010021629	DHRS7B	2	-0.255554097	0.127098917	-2.010650928	0.044360277	0.006427427	1.204965867	17.01009738	1	CRA004775;Yang2025_Silkie_Wenchang	PRJNA827465	0.363642337
ENSGALG00010021629	DHRS7B	2	-0.221795666	0.153852574	-1.441611666	0.149411955	0.027897269	2.058099638	51.41148748	1	CRA004775;PRJNA827465	Yang2025_Silkie_Wenchang	0.639246608
ENSGALG00010021632	LIMD2	2	0.048366981	0.09700316	0.498612428	0.618052448	0	0.004051149	0	1	PRJNA827465;Yang2025_Silkie_Wenchang	CRA004775	0.899121942
ENSGALG00010021632	LIMD2	2	-0.281312319	0.465895262	-0.603810216	0.545969827	0.291580545	2.566926549	61.04290555	0.5	CRA004775;Yang2025_Silkie_Wenchang	PRJNA827465	0.877958239
ENSGALG00010021632	LIMD2	2	-0.271003591	0.45204752	-0.599502441	0.548837883	0.29209355	2.70590654	63.04380861	0.5	CRA004775;PRJNA827465	Yang2025_Silkie_Wenchang	0.878281266
ENSGALG00010021633	CCNJ	2	0.096316505	0.067126045	1.434860431	0.151326873	0	0.46066801	0	1	PRJNA827465;Yang2025_Silkie_Wenchang	CRA004775	0.64289984
ENSGALG00010021633	CCNJ	2	0.012456546	0.118060823	0.105509569	0.915971478	0	0.122414616	0	0.5	CRA004775;Yang2025_Silkie_Wenchang	PRJNA827465	0.980377086
ENSGALG00010021633	CCNJ	2	0.115847291	0.078225284	1.480944336	0.138621407	0	0.37432639	0	0.5	CRA004775;PRJNA827465	Yang2025_Silkie_Wenchang	0.622371164
ENSGALG00010021634	PLCD4	2	0.405604525	0.42818915	0.947255495	0.343508603	0.342824296	15.36115613	93.49007333	0.5	PRJNA827465;Yang2025_Silkie_Wenchang	CRA004775	0.806276913
ENSGALG00010021634	PLCD4	2	0.446852411	0.474383212	0.941965059	0.346210529	0.350778	3.983853741	74.89867688	0.5	CRA004775;Yang2025_Silkie_Wenchang	PRJNA827465	0.807744676
ENSGALG00010021634	PLCD4	2	-0.032016447	0.143001377	-0.223889082	0.822843612	0	0.053368203	0	1	CRA004775;PRJNA827465	Yang2025_Silkie_Wenchang	0.955822987
ENSGALG00010021635	FOXJ2	2	-0.04494935	0.046046624	-0.976170362	0.328980048	0	0.093101683	0	1	PRJNA827465;Yang2025_Silkie_Wenchang	CRA004775	0.800761292
ENSGALG00010021635	FOXJ2	2	0.003027934	0.085834337	0.03527649	0.97185927	0	0.291825837	0	0.5	CRA004775;Yang2025_Silkie_Wenchang	PRJNA827465	0.991955348
ENSGALG00010021635	FOXJ2	2	-0.042602901	0.050794455	-0.838731335	0.40162009	0	0.552402517	0	0.5	CRA004775;PRJNA827465	Yang2025_Silkie_Wenchang	0.832623399
ENSGALG00010021636	C1orf159	2	-0.04437531	0.168846757	-0.262814111	0.792693859	0.044029018	4.241750149	76.42482549	0.5	PRJNA827465;Yang2025_Silkie_Wenchang	CRA004775	0.948435155
ENSGALG00010021636	C1orf159	2	-0.214572046	0.126054863	-1.702211572	0.088715714	0	0.079251528	0	1	CRA004775;Yang2025_Silkie_Wenchang	PRJNA827465	0.511635175
ENSGALG00010021636	C1orf159	2	0.087989589	0.084318743	1.043535357	0.296700414	0	0.644122021	0	0.5	CRA004775;PRJNA827465	Yang2025_Silkie_Wenchang	0.788458494
ENSGALG00010021637	ATP6V0D1	2	0.067856107	0.060150121	1.128112552	0.259272389	0.000334242	1.029224651	2.839482177	0.5	PRJNA827465;Yang2025_Silkie_Wenchang	CRA004775	0.766467295
ENSGALG00010021637	ATP6V0D1	2	-0.153475102	0.096586931	-1.588984149	0.112063971	0	0.999302241	0	1	CRA004775;Yang2025_Silkie_Wenchang	PRJNA827465	0.56547057
ENSGALG00010021637	ATP6V0D1	2	-0.055478815	0.172014416	-0.322524219	0.747055593	0.048694424	5.289509537	81.09465551	0.5	CRA004775;PRJNA827465	Yang2025_Silkie_Wenchang	0.936729365
ENSGALG00010021638	SRC	2	0.0794687	0.057166447	1.390128367	0.164489901	0	0.359944763	0	1	PRJNA827465;Yang2025_Silkie_Wenchang	CRA004775	0.663752214
ENSGALG00010021638	SRC	2	0.029993439	0.159046176	0.188583212	0.850419491	0.025410151	1.617391207	38.17203928	0.5	CRA004775;Yang2025_Silkie_Wenchang	PRJNA827465	0.961985357
ENSGALG00010021638	SRC	2	0.021668379	0.090104906	0.24047946	0.809958585	0.003776929	1.095546082	8.721320245	0.5	CRA004775;PRJNA827465	Yang2025_Silkie_Wenchang	0.952851096
ENSGALG00010021639	SCG5	2	0.377270879	0.59914949	0.629677376	0.528905688	0.603344183	6.018155931	83.38361432	0.5	PRJNA827465;Yang2025_Silkie_Wenchang	CRA004775	0.871809618
ENSGALG00010021639	SCG5	2	0.7463291	0.318814045	2.340954274	0.019234523	0	0.989410222	0	1	CRA004775;Yang2025_Silkie_Wenchang	PRJNA827465	0.232464467
ENSGALG00010021639	SCG5	2	-0.039563621	0.238918988	-0.165594291	0.86847624	0.017932598	1.126463751	11.2266152	0.5	CRA004775;PRJNA827465	Yang2025_Silkie_Wenchang	0.968167735
ENSGALG00010021640	PLEKHG3	2	0.230299671	0.277105524	0.831090148	0.405922705	0.143141093	14.67837813	93.18725822	0.5	PRJNA827465;Yang2025_Silkie_Wenchang	CRA004775	0.833416425
ENSGALG00010021640	PLEKHG3	2	0.361608695	0.196422155	1.840977127	0.065624911	0.04836451	2.39712621	58.28338133	1	CRA004775;Yang2025_Silkie_Wenchang	PRJNA827465	0.441590298
ENSGALG00010021640	PLEKHG3	2	-0.024143272	0.085013395	-0.283993742	0.776415177	0	0.332544167	0	0.5	CRA004775;PRJNA827465	Yang2025_Silkie_Wenchang	0.944136806
ENSGALG00010021642	NPTN	2	-0.068064475	0.084448183	-0.805990999	0.420248039	0.00903036	2.32516388	56.99227875	0.5	PRJNA827465;Yang2025_Silkie_Wenchang	CRA004775	0.838687527
ENSGALG00010021642	NPTN	2	0.075752219	0.099175136	0.763822692	0.444972912	0	0.358676829	0	1	CRA004775;Yang2025_Silkie_Wenchang	PRJNA827465	0.846013379
ENSGALG00010021642	NPTN	2	-0.044728438	0.145880738	-0.306609623	0.759140528	0.026566223	1.880077029	46.81068996	0.5	CRA004775;PRJNA827465	Yang2025_Silkie_Wenchang	0.939081587
ENSGALG00010021643	ENSGALG00010021643	2	-0.141128536	0.075640405	-1.865782395	0.062071816	0	0.171923027	0	1	PRJNA827465;Yang2025_Silkie_Wenchang	CRA004775	0.42991371
ENSGALG00010021643	ENSGALG00010021643	2	-0.362489757	0.202811392	-1.787324437	0.073885067	0.054085102	2.809099086	64.40139813	1	CRA004775;Yang2025_Silkie_Wenchang	PRJNA827465	0.469467289
ENSGALG00010021643	ENSGALG00010021643	2	-0.323062131	0.235942201	-1.369242676	0.17092343	0.08864464	4.523689939	77.89415248	1	CRA004775;PRJNA827465	Yang2025_Silkie_Wenchang	0.672991742
ENSGALG00010021644	TARDBP	2	0.039217843	0.062703258	0.62545144	0.53167481	0.004088772	1.861771652	46.28772014	0.5	PRJNA827465;Yang2025_Silkie_Wenchang	CRA004775	0.873042777
ENSGALG00010021644	TARDBP	2	0.115091946	0.081802452	1.406949832	0.159442265	0	0.162149655	0	1	CRA004775;Yang2025_Silkie_Wenchang	PRJNA827465	0.655006453
ENSGALG00010021644	TARDBP	2	-0.004069523	0.041910653	-0.097099955	0.922647016	0	0.02163306	0	0.5	CRA004775;PRJNA827465	Yang2025_Silkie_Wenchang	0.981804675
ENSGALG00010021645	ASCC1	2	0.231392645	0.064083151	3.610818782	0.000305232	0	0.06662134	0	1	PRJNA827465;Yang2025_Silkie_Wenchang	CRA004775	0.017197164
ENSGALG00010021645	ASCC1	2	0.0062266	0.311165544	0.020010569	0.984034941	0.141447483	3.43070733	70.85149202	0.5	CRA004775;Yang2025_Silkie_Wenchang	PRJNA827465	0.995637155
ENSGALG00010021645	ASCC1	2	0.004309152	0.28449919	0.015146448	0.987915345	0.124145452	3.540103764	71.75224042	0.5	CRA004775;PRJNA827465	Yang2025_Silkie_Wenchang	0.996800355
ENSGALG00010021646	RPUSD3	2	0.013419601	0.368300551	0.036436549	0.970934271	0.241665477	9.002086294	88.89146396	0.5	PRJNA827465;Yang2025_Silkie_Wenchang	CRA004775	0.991880791
ENSGALG00010021646	RPUSD3	2	0.05018461	0.398040015	0.12607931	0.899669145	0.237450889	3.794913901	73.64894103	0.5	CRA004775;Yang2025_Silkie_Wenchang	PRJNA827465	0.976220878
ENSGALG00010021646	RPUSD3	2	-0.34632621	0.124637157	-2.778675454	0.005458103	0	0.029719402	0	1	CRA004775;PRJNA827465	Yang2025_Silkie_Wenchang	0.110944465
ENSGALG00010021647	ARHGAP11B	2	-0.278671526	0.167554791	-1.663166565	0.096279098	0.044691762	4.896618388	79.57774283	1	PRJNA827465;Yang2025_Silkie_Wenchang	CRA004775	0.530723339
ENSGALG00010021647	ARHGAP11B	2	-0.339059496	0.207369271	-1.635051779	0.102038194	0.043321962	1.484371385	32.63141486	0.5	CRA004775;Yang2025_Silkie_Wenchang	PRJNA827465	0.544448428
ENSGALG00010021647	ARHGAP11B	2	-0.096395683	0.107009741	-0.900812229	0.367688164	0	0.179773699	0	0.5	CRA004775;PRJNA827465	Yang2025_Silkie_Wenchang	0.819975483
ENSGALG00010021648	BRE	2	-0.172431936	0.082475533	-2.09070411	0.036554596	0.009276327	3.126017464	68.01041544	1	PRJNA827465;Yang2025_Silkie_Wenchang	CRA004775	0.329671369
ENSGALG00010021648	BRE	2	-0.235826944	0.066875733	-3.526345578	0.000421337	0	0.937178303	0	1	CRA004775;Yang2025_Silkie_Wenchang	PRJNA827465	0.020751629
ENSGALG00010021648	BRE	2	-0.090798294	0.057908517	-1.567960946	0.116890247	0	0.033130555	0	1	CRA004775;PRJNA827465	Yang2025_Silkie_Wenchang	0.575778591
ENSGALG00010021649	PXN	2	-0.027043339	0.044332817	-0.610007227	0.54185702	0	0.060684882	0	1	PRJNA827465;Yang2025_Silkie_Wenchang	CRA004775	0.876475696
ENSGALG00010021649	PXN	2	-0.024827658	0.066256947	-0.374717812	0.707870344	0	0.293999886	0	1	CRA004775;Yang2025_Silkie_Wenchang	PRJNA827465	0.925061251
ENSGALG00010021649	PXN	2	-0.042581176	0.055962332	-0.76088994	0.446722808	0	0.207114616	0	1	CRA004775;PRJNA827465	Yang2025_Silkie_Wenchang	0.84648396
ENSGALG00010021650	CD276	2	-0.004015511	0.050701283	-0.079199396	0.936874025	0	0.247826264	0	0.5	PRJNA827465;Yang2025_Silkie_Wenchang	CRA004775	0.985038972
ENSGALG00010021650	CD276	2	-0.012721682	0.077819565	-0.163476646	0.87014316	0	0.6631643	0	0.5	CRA004775;Yang2025_Silkie_Wenchang	PRJNA827465	0.968789456
ENSGALG00010021650	CD276	2	0.026243795	0.061325557	0.427942219	0.668693188	0	0.36801892	0	1	CRA004775;PRJNA827465	Yang2025_Silkie_Wenchang	0.914377769
ENSGALG00010021651	RNF113B	2	0.146639939	0.199671125	0.734407333	0.462700519	0.071665467	9.833182548	89.83035253	0.5	PRJNA827465;Yang2025_Silkie_Wenchang	CRA004775	0.851103033
ENSGALG00010021651	RNF113B	2	0.214544309	0.166551392	1.288156808	0.197691384	0.036404042	2.707440828	63.06475143	1	CRA004775;Yang2025_Silkie_Wenchang	PRJNA827465	0.709468779
ENSGALG00010021651	RNF113B	2	-0.038897833	0.072643718	-0.53546038	0.592331547	0	0.091287338	0	0.5	CRA004775;PRJNA827465	Yang2025_Silkie_Wenchang	0.892776493
ENSGALG00010021652	BORCS5	2	0.051976411	0.181701415	0.286053967	0.774836788	0.056886845	6.911737818	85.53185861	0.5	PRJNA827465;Yang2025_Silkie_Wenchang	CRA004775	0.943713409
ENSGALG00010021652	BORCS5	2	-0.247928993	0.1316255	-1.883593931	0.059619923	0.012479189	1.510187647	33.78306319	1	CRA004775;Yang2025_Silkie_Wenchang	PRJNA827465	0.423044205
ENSGALG00010021652	BORCS5	2	-0.076088003	0.317407015	-0.239717458	0.8105493	0.183763839	10.76569091	90.71123248	0.5	CRA004775;PRJNA827465	Yang2025_Silkie_Wenchang	0.952997426
ENSGALG00010021655	USP22	2	0.109324375	0.164606076	0.66415759	0.506589454	0.044272119	4.783732505	79.09582112	0.5	PRJNA827465;Yang2025_Silkie_Wenchang	CRA004775	0.863929781
ENSGALG00010021655	USP22	2	0.129998275	0.219791206	0.591462583	0.554210518	0.057761045	2.325203572	56.99301291	0.5	CRA004775;Yang2025_Silkie_Wenchang	PRJNA827465	0.880144209
ENSGALG00010021655	USP22	2	-0.031060071	0.040848559	-0.760371266	0.447032694	0	0.198988237	0	1	CRA004775;PRJNA827465	Yang2025_Silkie_Wenchang	0.84650962
ENSGALG00010021656	SETD6	2	0.291686408	0.073360269	3.976081511	7.00601E-05	0.004541639	1.671332532	40.16750222	1	PRJNA827465;Yang2025_Silkie_Wenchang	CRA004775	0.006081423
ENSGALG00010021656	SETD6	2	-0.071702273	0.491620437	-0.145848846	0.884040715	0.439526026	10.40206394	90.38652323	0.5	CRA004775;Yang2025_Silkie_Wenchang	PRJNA827465	0.972001076
ENSGALG00010021656	SETD6	2	-0.137083442	0.415153187	-0.330199661	0.741249103	0.305011872	7.88991192	87.32558728	0.5	CRA004775;PRJNA827465	Yang2025_Silkie_Wenchang	0.93538952
ENSGALG00010021657	JAGN1	2	0.102016279	0.094813839	1.075964014	0.281943351	0	0.937223352	0	1	PRJNA827465;Yang2025_Silkie_Wenchang	CRA004775	0.779434446
ENSGALG00010021657	JAGN1	2	-0.039141865	0.354723403	-0.110344749	0.912135969	0.184586242	3.633375301	72.47738213	0.5	CRA004775;Yang2025_Silkie_Wenchang	PRJNA827465	0.978967245
ENSGALG00010021657	JAGN1	2	-0.101920092	0.228112069	-0.446798333	0.655020675	0.06504717	2.190706158	54.35261839	0.5	CRA004775;PRJNA827465	Yang2025_Silkie_Wenchang	0.910335134
ENSGALG00010021658	STRADA	2	0.003284922	0.055336852	0.059362293	0.952663546	0	0.919126605	0	0.5	PRJNA827465;Yang2025_Silkie_Wenchang	CRA004775	0.987758913
ENSGALG00010021658	STRADA	2	-0.110058408	0.084387323	-1.304205469	0.192163536	0.000925328	1.046506745	4.443998631	1	CRA004775;Yang2025_Silkie_Wenchang	PRJNA827465	0.701849591
ENSGALG00010021658	STRADA	2	-0.070016345	0.150974124	-0.463763878	0.642816926	0.030965327	2.697131437	62.92357183	0.5	CRA004775;PRJNA827465	Yang2025_Silkie_Wenchang	0.906634523
ENSGALG00010021659	SPOCK2	2	0.142501525	0.220786294	0.645427405	0.518650248	0.076437909	4.521701374	77.88443072	0.5	PRJNA827465;Yang2025_Silkie_Wenchang	CRA004775	0.868645788
ENSGALG00010021659	SPOCK2	2	0.30912318	0.145486375	2.1247569	0.033606903	0	0.623899373	0	1	CRA004775;Yang2025_Silkie_Wenchang	PRJNA827465	0.314638424
ENSGALG00010021659	SPOCK2	2	-0.031341278	0.107554112	-0.291400088	0.770745347	0	0.408566432	0	0.5	CRA004775;PRJNA827465	Yang2025_Silkie_Wenchang	0.942491331
ENSGALG00010021660	RNF223	2	0.508710056	0.191694462	2.653754582	0.00796017	0	0.141452125	0	1	PRJNA827465;Yang2025_Silkie_Wenchang	CRA004775	0.138706114
ENSGALG00010021660	RNF223	2	0.881207015	0.400853189	2.198328565	0.0279257	0.188943547	2.215650222	54.86652224	1	CRA004775;Yang2025_Silkie_Wenchang	PRJNA827465	0.284609505
ENSGALG00010021660	RNF223	2	0.832767184	0.480311281	1.733807257	0.082952288	0.311895813	2.909504703	65.62988886	1	CRA004775;PRJNA827465	Yang2025_Silkie_Wenchang	0.495859127
ENSGALG00010021661	DUSP16	2	-0.156722831	0.099834855	-1.569820788	0.11645681	0.011108395	2.182655701	54.18425364	1	PRJNA827465;Yang2025_Silkie_Wenchang	CRA004775	0.574631355
ENSGALG00010021661	DUSP16	2	0.04057162	0.373048631	0.108756919	0.913395295	0.225826748	4.737170909	78.8903542	0.5	CRA004775;Yang2025_Silkie_Wenchang	PRJNA827465	0.979193041
ENSGALG00010021661	DUSP16	2	0.112664082	0.261323104	0.431129436	0.666374258	0.096525052	2.692907774	62.86541968	0.5	CRA004775;PRJNA827465	Yang2025_Silkie_Wenchang	0.913579507
ENSGALG00010021662	RASGRP1	2	-0.096120855	0.105371701	-0.912207492	0.361659501	0	0.441400727	0	1	PRJNA827465;Yang2025_Silkie_Wenchang	CRA004775	0.815545459
ENSGALG00010021662	RASGRP1	2	-0.195381681	0.178199792	-1.096419239	0.272895348	0	0.012482329	0	1	CRA004775;Yang2025_Silkie_Wenchang	PRJNA827465	0.773799118
ENSGALG00010021662	RASGRP1	2	-0.05731424	0.121278374	-0.472584171	0.636509878	0	0.041495123	0	1	CRA004775;PRJNA827465	Yang2025_Silkie_Wenchang	0.904566695
ENSGALG00010021663	CNOT9	2	-0.029446821	0.048810149	-0.603293001	0.546313788	0	0.636375473	0	1	PRJNA827465;Yang2025_Silkie_Wenchang	CRA004775	0.878083295
ENSGALG00010021663	CNOT9	2	-0.102431323	0.103433973	-0.990306381	0.322024388	0	0.059408122	0	1	CRA004775;Yang2025_Silkie_Wenchang	PRJNA827465	0.798513866
ENSGALG00010021663	CNOT9	2	-0.015498242	0.051543003	-0.300685665	0.763654201	0	0.055094627	0	1	CRA004775;PRJNA827465	Yang2025_Silkie_Wenchang	0.940361611
ENSGALG00010021664	TYSND1	2	0.40043504	0.408562224	0.980107843	0.327032888	0.306478878	12.09491804	91.73206469	1	PRJNA827465;Yang2025_Silkie_Wenchang	CRA004775	0.800028239
ENSGALG00010021664	TYSND1	2	0.095421471	0.775686433	0.123015522	0.902094807	1.090510505	10.21339126	90.20893281	0.5	CRA004775;Yang2025_Silkie_Wenchang	PRJNA827465	0.976899171
ENSGALG00010021664	TYSND1	2	-0.24266546	0.347602496	-0.698111961	0.485107179	0.162820142	2.492226141	59.87523028	0.5	CRA004775;PRJNA827465	Yang2025_Silkie_Wenchang	0.857004733
ENSGALG00010021665	CNOT1	2	-0.056200115	0.048755152	-1.152701059	0.249033107	0	0.037380715	0	1	PRJNA827465;Yang2025_Silkie_Wenchang	CRA004775	0.759411487
ENSGALG00010021665	CNOT1	2	-0.068118682	0.09775909	-0.696801519	0.485927015	0	0.018930225	0	1	CRA004775;Yang2025_Silkie_Wenchang	PRJNA827465	0.857391223
ENSGALG00010021665	CNOT1	2	-0.051198547	0.052102567	-0.982649213	0.325780115	0	0.001145021	0	1	CRA004775;PRJNA827465	Yang2025_Silkie_Wenchang	0.800023788
ENSGALG00010021666	CCDC47	2	-0.207670984	0.061414566	-3.381461411	0.000721014	0	0.977815603	0	1	PRJNA827465;Yang2025_Silkie_Wenchang	CRA004775	0.0300828
ENSGALG00010021666	CCDC47	2	-0.124812526	0.100014345	-1.247946238	0.212050746	0	0.052713195	0	1	CRA004775;Yang2025_Silkie_Wenchang	PRJNA827465	0.724432597
ENSGALG00010021666	CCDC47	2	-0.237181235	0.068531211	-3.460922857	0.000538327	0	0.137067991	0	1	CRA004775;PRJNA827465	Yang2025_Silkie_Wenchang	0.024602172
ENSGALG00010021667	TBX15	2	-0.133025066	0.075606168	-1.759447259	0.078501571	0	0.021513043	0	1	PRJNA827465;Yang2025_Silkie_Wenchang	CRA004775	0.481915278
ENSGALG00010021667	TBX15	2	-0.108967229	0.099363412	-1.096653456	0.27279291	0	0.613958189	0	0.5	CRA004775;Yang2025_Silkie_Wenchang	PRJNA827465	0.773730226
ENSGALG00010021667	TBX15	2	-0.1266553	0.111660989	-1.134284235	0.25667534	0	0.671918392	0	0.5	CRA004775;PRJNA827465	Yang2025_Silkie_Wenchang	0.764677554
ENSGALG00010021668	PYGO1	2	-0.179401777	0.095410162	-1.880321487	0.060064277	0	0.703107583	0	1	PRJNA827465;Yang2025_Silkie_Wenchang	CRA004775	0.424205978
ENSGALG00010021668	PYGO1	2	-0.28346577	0.160164417	-1.769842363	0.076753405	0	0.10460659	0	1	CRA004775;Yang2025_Silkie_Wenchang	PRJNA827465	0.477904868
ENSGALG00010021668	PYGO1	2	-0.1401668	0.104047826	-1.347138194	0.177935729	0	0.042855197	0	1	CRA004775;PRJNA827465	Yang2025_Silkie_Wenchang	0.683951879
ENSGALG00010021669	ENSGALG00010021669	2	-0.008212039	0.1081218	-0.075951741	0.939457492	0.014785601	2.19111582	54.36115285	0.5	PRJNA827465;Yang2025_Silkie_Wenchang	CRA004775	0.985425793
ENSGALG00010021669	ENSGALG00010021669	2	-0.238855462	0.11700874	-2.041347174	0.041216331	0	0.555019624	0	1	CRA004775;Yang2025_Silkie_Wenchang	PRJNA827465	0.351928866
ENSGALG00010021669	ENSGALG00010021669	2	-0.101613099	0.20198099	-0.503082488	0.614906279	0.066237593	4.585805267	78.19357906	0.5	CRA004775;PRJNA827465	Yang2025_Silkie_Wenchang	0.898781283
ENSGALG00010021670	BLCAP	2	-0.308449108	0.271176428	-1.137448082	0.255351017	0.132518002	9.573111552	89.55407555	1	PRJNA827465;Yang2025_Silkie_Wenchang	CRA004775	0.764467569
ENSGALG00010021670	BLCAP	2	-0.533505426	0.148558186	-3.591222001	0.000329131	0	0.940932865	0	1	CRA004775;Yang2025_Silkie_Wenchang	PRJNA827465	0.017941527
ENSGALG00010021670	BLCAP	2	-0.063049208	0.061492855	-1.025309495	0.305217177	0	0.235583024	0	1	CRA004775;PRJNA827465	Yang2025_Silkie_Wenchang	0.79225794
ENSGALG00010021671	TNFRSF13B	2	-0.520326506	0.30574385	-1.701838014	0.088785738	0	0.807835072	0	1	PRJNA827465;Yang2025_Silkie_Wenchang	CRA004775	0.5117008
ENSGALG00010021671	TNFRSF13B	2	-0.383737619	0.577229921	-0.664791629	0.506183778	0.331541514	1.988737989	49.71685534	0.5	CRA004775;Yang2025_Silkie_Wenchang	PRJNA827465	0.863929781
ENSGALG00010021671	TNFRSF13B	2	-0.192270899	0.310362122	-0.619505041	0.535583702	0	0.627353759	0	0.5	CRA004775;PRJNA827465	Yang2025_Silkie_Wenchang	0.874171355
ENSGALG00010021674	ING4	2	0.219231587	0.162678793	1.347634706	0.177775902	0.043988095	5.68174359	82.39976894	1	PRJNA827465;Yang2025_Silkie_Wenchang	CRA004775	0.683951879
ENSGALG00010021674	ING4	2	0.124375117	0.319951651	0.388730974	0.697475167	0.162746549	4.454222105	77.54939075	0.5	CRA004775;Yang2025_Silkie_Wenchang	PRJNA827465	0.921342994
ENSGALG00010021674	ING4	2	0.027851033	0.109448821	0.254466269	0.79913536	0.0093682	1.222064145	18.17123482	0.5	CRA004775;PRJNA827465	Yang2025_Silkie_Wenchang	0.949922217
ENSGALG00010021675	DDX42	2	0.019848592	0.155718654	0.127464444	0.898572813	0.043155785	8.983575637	88.86857483	0.5	PRJNA827465;Yang2025_Silkie_Wenchang	CRA004775	0.975785574
ENSGALG00010021675	DDX42	2	-0.08662764	0.29225796	-0.296408144	0.766918406	0.150053939	7.801298855	87.18162169	0.5	CRA004775;Yang2025_Silkie_Wenchang	PRJNA827465	0.941375309
ENSGALG00010021675	DDX42	2	-0.207262317	0.123485543	-1.678433857	0.093262433	0.017542138	1.864604283	46.36931767	1	CRA004775;PRJNA827465	Yang2025_Silkie_Wenchang	0.523343073
ENSGALG00010021676	TP53INP2	2	0.37898029	0.24871417	1.523758334	0.127569091	0.093326615	4.043326084	75.26788641	1	PRJNA827465;Yang2025_Silkie_Wenchang	CRA004775	0.599599139
ENSGALG00010021676	TP53INP2	2	0.559763776	0.190057013	2.945241359	0.003227028	0.006004694	1.044344232	4.246131737	1	CRA004775;Yang2025_Silkie_Wenchang	PRJNA827465	0.078974898
ENSGALG00010021676	TP53INP2	2	0.13847694	0.14982336	0.924268017	0.35534679	0	0.004418409	0	1	CRA004775;PRJNA827465	Yang2025_Silkie_Wenchang	0.812301302
ENSGALG00010021677	ENSGALG00010021677	2	0.101684768	0.142694771	0.712603319	0.476091257	0.029786212	3.157136355	68.32572662	0.5	PRJNA827465;Yang2025_Silkie_Wenchang	CRA004775	0.854810196
ENSGALG00010021677	ENSGALG00010021677	2	0.228942676	0.121170324	1.88942861	0.05883442	0	0.329205353	0	1	CRA004775;Yang2025_Silkie_Wenchang	PRJNA827465	0.42013793
ENSGALG00010021677	ENSGALG00010021677	2	0.002501821	0.049408974	0.050634957	0.959616407	0	0.621206177	0	0.5	CRA004775;PRJNA827465	Yang2025_Silkie_Wenchang	0.989259322
ENSGALG00010021678	DNAAF4	2	0.23987548	0.37992294	0.631379301	0.527792547	0.252896064	7.754714943	87.10461948	0.5	PRJNA827465;Yang2025_Silkie_Wenchang	CRA004775	0.871296643
ENSGALG00010021678	DNAAF4	2	0.550730872	0.21637073	2.545311341	0.010918034	0	0.780582076	0	1	CRA004775;Yang2025_Silkie_Wenchang	PRJNA827465	0.166322288

ENSGALG00010021678	DNAAF4	2	-0.093160962	0.12047876	-0.773256309	0.439370707	0	0.363398271	0	0.5	CRA004775;PRJNA827465	Yang2025_Silkie_Wenchang	0.844199375
ENSGALG00010021680	PIGT	2	0.114643931	0.185169681	0.619129066	0.535831337	0.057731907	5.750840761	82.61123823	0.5	PRJNA827465;Yang2025_Silkie_Wenchang	CRA004775	0.874321378
ENSGALG00010021680	PIGT	2	0.09749673	0.252206065	0.386575675	0.699070369	0.094633691	3.807953169	73.73917284	0.5	CRA004775;Yang2025_Silkie_Wenchang	PRJNA827465	0.92185597
ENSGALG00010021680	PIGT	2	-0.053831586	0.049511549	-1.087253111	0.276924965	0	0.365123424	0	1	CRA004775;PRJNA827465	Yang2025_Silkie_Wenchang	0.776084088
ENSGALG00010021681	BTBD10	2	-0.127314654	0.16781074	-0.758680009	0.448043998	0.046452839	5.33290834	81.24850576	0.5	PRJNA827465;Yang2025_Silkie_Wenchang	CRA004775	0.846601815
ENSGALG00010021681	BTBD10	2	-0.087062329	0.269076472	-0.323559799	0.746271328	0.107588705	3.636037099	72.49753034	0.5	CRA004775;Yang2025_Silkie_Wenchang	PRJNA827465	0.936574448
ENSGALG00010021681	BTBD10	2	0.032527142	0.059425652	0.547358612	0.584132384	0	0.631108525	0	1	CRA004775;PRJNA827465	Yang2025_Silkie_Wenchang	0.890817751
ENSGALG00010021682	MYL4	2	0.233398226	0.14594604	1.599209037	0.109774163	0.019366581	1.552604569	35.5920999	1	PRJNA827465;Yang2025_Silkie_Wenchang	CRA004775	0.560974377
ENSGALG00010021682	MYL4	2	1.018616114	0.728109691	1.398987168	0.161816831	0.765055099	2.968726025	66.31551744	1	CRA004775;Yang2025_Silkie_Wenchang	PRJNA827465	0.658987349
ENSGALG00010021682	MYL4	2	0.878512976	0.904441223	0.971332303	0.331382828	1.345912846	4.705175665	78.74680838	1	CRA004775;PRJNA827465	Yang2025_Silkie_Wenchang	0.801798188
ENSGALG00010021684	BIN1	2	-0.179145316	0.078379253	-2.285621628	0.022276404	0	0.772433372	0	1	PRJNA827465;Yang2025_Silkie_Wenchang	CRA004775	0.253189559
ENSGALG00010021684	BIN1	2	-0.037881952	0.130178903	-0.290999168	0.771051956	0	0.813899441	0	0.5	CRA004775;Yang2025_Silkie_Wenchang	PRJNA827465	0.942646827
ENSGALG00010021684	BIN1	2	-0.103883068	0.221371395	-0.469270513	0.638876289	0.05501405	1.677690293	40.39424297	0.5	CRA004775;PRJNA827465	Yang2025_Silkie_Wenchang	0.905769959
ENSGALG00010021685	DBNDD2	2	0.016015807	0.140506081	0.113986576	0.909248435	0.017271018	1.776719969	43.71651034	0.5	PRJNA827465;Yang2025_Silkie_Wenchang	CRA004775	0.978607662
ENSGALG00010021685	DBNDD2	2	0.146056525	0.145141917	1.006301472	0.314270573	0	0.090972645	0	1	CRA004775;Yang2025_Silkie_Wenchang	PRJNA827465	0.796531448
ENSGALG00010021685	DBNDD2	2	-0.110207299	0.139581178	-0.789557019	0.429786518	0	0.066420759	0	0.5	CRA004775;PRJNA827465	Yang2025_Silkie_Wenchang	0.842012722
ENSGALG00010021686	CCPG1	2	0.007924492	0.115610997	0.068544449	0.945352238	0.011413575	1.551686575	35.55399548	0.5	PRJNA827465;Yang2025_Silkie_Wenchang	CRA004775	0.986321197
ENSGALG00010021686	CCPG1	2	0.152151578	0.173131737	0.878819682	0.379499053	0	0.305778546	0	0.5	CRA004775;Yang2025_Silkie_Wenchang	PRJNA827465	0.824165982
ENSGALG00010021686	CCPG1	2	-0.06829404	0.08571986	-0.796711981	0.425618324	0	0.008579087	0	1	CRA004775;PRJNA827465	Yang2025_Silkie_Wenchang	0.84125685
ENSGALG00010021687	ZNF384	2	-0.057757022	0.046804409	-1.234008152	0.217199867	0.000816662	1.150983062	13.11774842	0.5	PRJNA827465;Yang2025_Silkie_Wenchang	CRA004775	0.730748463
ENSGALG00010021687	ZNF384	2	-0.151436086	0.196390061	-0.771098522	0.440648537	0.062113543	4.975508967	79.90155366	0.5	CRA004775;Yang2025_Silkie_Wenchang	PRJNA827465	0.844819973
ENSGALG00010021687	ZNF384	2	-0.188409974	0.136663472	-1.378641788	0.168005226	0.027729273	3.2662905	69.3842296	1	CRA004775;PRJNA827465	Yang2025_Silkie_Wenchang	0.668466226
ENSGALG00010021688	ITPR1	2	0.068273844	0.071304172	0.957501385	0.338314248	0	0.336466366	0	1	PRJNA827465;Yang2025_Silkie_Wenchang	CRA004775	0.804154375
ENSGALG00010021688	ITPR1	2	0.151901955	0.125808931	1.207402001	0.227277365	0	0.000129397	0	1	CRA004775;Yang2025_Silkie_Wenchang	PRJNA827465	0.740605003
ENSGALG00010021688	ITPR1	2	0.061452366	0.074353414	0.826490184	0.408526084	0	0.232214941	0	1	CRA004775;PRJNA827465	Yang2025_Silkie_Wenchang	0.834164004
ENSGALG00010021689	MPRIP	2	0.039815641	0.145476547	0.273691135	0.784321991	0.032559875	3.857472247	74.07628789	0.5	PRJNA827465;Yang2025_Silkie_Wenchang	CRA004775	0.946083234
ENSGALG00010021689	MPRIP	2	0.189567632	0.126829351	1.494666893	0.135001399	0	0.193838745	0	1	CRA004775;Yang2025_Silkie_Wenchang	PRJNA827465	0.615826367
ENSGALG00010021689	MPRIP	2	-0.074745129	0.053272967	-1.403059255	0.16059917	0	0.27071338	0	0.5	CRA004775;PRJNA827465	Yang2025_Silkie_Wenchang	0.65630165
ENSGALG00010021691	PIGB	2	0.040149438	0.128687456	0.311991858	0.755046711	0	0.524954279	0	0.5	PRJNA827465;Yang2025_Silkie_Wenchang	CRA004775	0.93838723
ENSGALG00010021691	PIGB	2	-0.159902123	0.333752239	-0.479104271	0.631864451	0.163570305	3.572245019	72.00639948	0.5	CRA004775;Yang2025_Silkie_Wenchang	PRJNA827465	0.903058174
ENSGALG00010021691	PIGB	2	-0.237557204	0.235594039	-1.008332827	0.313294709	0.054104613	1.836239168	45.54086321	1	CRA004775;PRJNA827465	Yang2025_Silkie_Wenchang	0.796531448
ENSGALG00010021693	PPA1	2	-0.002675339	0.117819227	-0.022707149	0.981883873	0.016961931	2.017866648	50.44271132	0.5	PRJNA827465;Yang2025_Silkie_Wenchang	CRA004775	0.995055866
ENSGALG00010021693	PPA1	2	0.067723677	0.14654937	0.462121922	0.64399389	0.003633169	1.086083557	7.926052913	0.5	CRA004775;Yang2025_Silkie_Wenchang	PRJNA827465	0.906826466
ENSGALG00010021693	PPA1	2	-0.080218593	0.050006019	-1.604178747	0.108674658	0	0.033518285	0	1	CRA004775;PRJNA827465	Yang2025_Silkie_Wenchang	0.558186106
ENSGALG00010021695	FNBP4	2	0.504597099	0.440023995	1.146749052	0.251485351	0.365050768	16.77474657	94.03865808	1	PRJNA827465;Yang2025_Silkie_Wenchang	CRA004775	0.761706068
ENSGALG00010021695	FNBP4	2	0.356209208	0.604147273	0.589606581	0.555454439	0.695659179	21.21394308	95.28611915	0.5	CRA004775;Yang2025_Silkie_Wenchang	PRJNA827465	0.880255849
ENSGALG00010021695	FNBP4	2	-0.043341404	0.159951706	-0.270965561	0.78641752	0.0383316	3.519977613	71.59072841	0.5	CRA004775;PRJNA827465	Yang2025_Silkie_Wenchang	0.946651901
ENSGALG00010021697	PIANP	2	0.086806791	0.127765764	0.679421376	0.49687091	0	0.593510471	0	1	PRJNA827465;Yang2025_Silkie_Wenchang	CRA004775	0.860939846
ENSGALG00010021697	PIANP	2	0.079902488	0.21389773	0.373554635	0.70873569	0.014614661	1.175436275	14.9252051	0.5	CRA004775;Yang2025_Silkie_Wenchang	PRJNA827465	0.925245427
ENSGALG00010021697	PIANP	2	-0.013279731	0.136951247	-0.096966852	0.922752719	0	0.380771266	0	0.5	CRA004775;PRJNA827465	Yang2025_Silkie_Wenchang	0.981804675
ENSGALG00010021698	GYPC	2	-0.179331486	0.078663491	-2.279729572	0.022623732	0	0.201657814	0	1	PRJNA827465;Yang2025_Silkie_Wenchang	CRA004775	0.255732111
ENSGALG00010021698	GYPC	2	-0.00419237	0.283531978	-0.014786232	0.988202724	0.112440138	2.983515201	66.4824902	0.5	CRA004775;Yang2025_Silkie_Wenchang	PRJNA827465	0.996891127
ENSGALG00010021698	GYPC	2	0.014074219	0.238671336	0.058969037	0.95297677	0.076363545	2.476919761	59.62727514	0.5	CRA004775;PRJNA827465	Yang2025_Silkie_Wenchang	0.987758913
ENSGALG00010021699	SYS1	2	-0.032072778	0.088816431	-0.361113115	0.71801489	0	0.03247839	0	1	PRJNA827465;Yang2025_Silkie_Wenchang	CRA004775	0.927927766
ENSGALG00010021699	SYS1	2	-0.157425796	0.174807458	-0.900566818	0.367818684	0	0.144993042	0	1	CRA004775;Yang2025_Silkie_Wenchang	PRJNA827465	0.820142704
ENSGALG00010021699	SYS1	2	-0.053586963	0.086918512	-0.616519571	0.537551659	0	0.572419022	0	1	CRA004775;PRJNA827465	Yang2025_Silkie_Wenchang	0.87481821
ENSGALG00010021700	FLCN	2	-0.036150696	0.049413455	-0.731596201	0.464415068	0	0.748071085	0	1	PRJNA827465;Yang2025_Silkie_Wenchang	CRA004775	0.852159649
ENSGALG00010021700	FLCN	2	-0.140971809	0.092663668	-1.521327744	0.128177611	0.001695377	1.051749689	4.920342712	1	CRA004775;Yang2025_Silkie_Wenchang	PRJNA827465	0.600696128
ENSGALG00010021700	FLCN	2	-0.111603735	0.16154362	-0.690858199	0.489654657	0.033237293	2.096688816	52.30575027	1	CRA004775;PRJNA827465	Yang2025_Silkie_Wenchang	0.858425426
ENSGALG00010021701	DR1	2	0.007299287	0.058321845	0.125155283	0.900400618	0.002173422	1.346185587	25.71603723	0.5	PRJNA827465;Yang2025_Silkie_Wenchang	CRA004775	0.976475201
ENSGALG00010021701	DR1	2	-0.065417821	0.091563686	-0.71445159	0.474947981	0	0.271044917	0	0.5	CRA004775;Yang2025_Silkie_Wenchang	PRJNA827465	0.854462864
ENSGALG00010021701	DR1	2	0.043555698	0.049250392	0.884372601	0.376495109	0	1.20165E-05	0	1	CRA004775;PRJNA827465	Yang2025_Silkie_Wenchang	0.822974013
ENSGALG00010021702	PLD6	2	0.281892242	0.281340521	1.001961041	0.316362409	0.056938825	1.561451481	35.9570238	1	PRJNA827465;Yang2025_Silkie_Wenchang	CRA004775	0.796883862
ENSGALG00010021702	PLD6	2	-0.199739349	0.785323092	-0.254340348	0.799232631	1.102225815	9.359003717	89.31510201	0.5	CRA004775;Yang2025_Silkie_Wenchang	PRJNA827465	0.949922217
ENSGALG00010021702	PLD6	2	-0.468559973	0.503233175	-0.931099133	0.351802287	0.379191625	3.936751943	74.59834873	0.5	CRA004775;PRJNA827465	Yang2025_Silkie_Wenchang	0.810678834
ENSGALG00010021703	CD82	2	-0.09607671	0.063370754	-1.516104876	0.129492835	0	0.466395219	0	1	PRJNA827465;Yang2025_Silkie_Wenchang	CRA004775	0.603529077
ENSGALG00010021703	CD82	2	-0.15651983	0.12927093	-1.21078907	0.225976255	0	0.220806437	0	1	CRA004775;Yang2025_Silkie_Wenchang	PRJNA827465	0.739327792
ENSGALG00010021703	CD82	2	-0.071130571	0.069766078	-1.019558102	0.307938084	0	0.038845684	0	1	CRA004775;PRJNA827465	Yang2025_Silkie_Wenchang	0.793278812
ENSGALG00010021704	COPS7A	2	-0.137161838	0.147143057	-0.932166562	0.351250453	0.036730117	6.330422645	84.20326642	1	PRJNA827465;Yang2025_Silkie_Wenchang	CRA004775	0.810306777
ENSGALG00010021704	COPS7A	2	-0.270472721	0.096490828	-2.80309255	0.005061514	0	0.55700857	0	1	CRA004775;Yang2025_Silkie_Wenchang	PRJNA827465	0.106040882
ENSGALG00010021704	COPS7A	2	-0.005163256	0.055096092	-0.093713649	0.925336628	0	0.073857056	0	1	CRA004775;PRJNA827465	Yang2025_Silkie_Wenchang	0.982594411
ENSGALG00010021705	CCDC18	2	0.661943876	0.376051325	1.760248752	0.078365639	0.261074788	12.99424627	92.30428622	1	PRJNA827465;Yang2025_Silkie_Wenchang	CRA004775	0.481746691
ENSGALG00010021705	CCDC18	2	0.697299078	0.449059771	1.552797919	0.12047142	0.295798484	3.153094091	68.28512023	1	CRA004775;Yang2025_Silkie_Wenchang	PRJNA827465	0.581896058
ENSGALG00010021705	CCDC18	2	0.275239183	0.135804705	2.026727893	0.042690248	0	0.117775057	0	1	CRA004775;PRJNA827465	Yang2025_Silkie_Wenchang	0.357446704
ENSGALG00010021706	ENSGALG00010021706	2	0.022965884	0.164192958	0.139871308	0.888761671	0.027963404	2.044469611	51.08755861	0.5	PRJNA827465;Yang2025_Silkie_Wenchang	CRA004775	0.973135755
ENSGALG00010021706	ENSGALG00010021706	2	0.124230667	0.15832664	0.784647908	0.432660033	0	0.848247046	0	0.5	CRA004775;Yang2025_Silkie_Wenchang	PRJNA827465	0.842762754
ENSGALG00010021706	ENSGALG00010021706	2	-0.12316829	0.129313882	-0.952475394	0.340855939	0	0.000251584	0	1	CRA004775;PRJNA827465	Yang2025_Silkie_Wenchang	0.805635165
ENSGALG00010021710	ENSGALG00010021710	2	0.534780416	0.180102489	2.969311632	0.002984677	0	0.806192733	0	1	PRJNA827465;Yang2025_Silkie_Wenchang	CRA004775	0.075060946
ENSGALG00010021710	ENSGALG00010021710	2	0.34309746	0.246015693	1.394616152	0.163131622	0	0.000615785	0	1	CRA004775;Yang2025_Silkie_Wenchang	PRJNA827465	0.661599642
ENSGALG00010021710	ENSGALG00010021710	2	0.612628852	0.212025743	2.889407877	0.003859681	0	0.323245098	0	1	CRA004775;PRJNA827465	Yang2025_Silkie_Wenchang	0.08923355
ENSGALG00010021713	RAB27A	2	0.095778831	0.232628614	0.411724203	0.680541583	0.100572672	14.02536971	92.87006318	0.5	PRJNA827465;Yang2025_Silkie_Wenchang	CRA004775	0.917851751
ENSGALG00010021713	RAB27A	2	-0.001682345	0.161078226	-0.010444276	0.991666825	0.035460317	3.040546851	67.11117937	0.5	CRA004775;Yang2025_Silkie_Wenchang	PRJNA827465	0.997575757
ENSGALG00010021713	RAB27A	2	0.298562634	0.065137458	4.583578232	4.57086E-06	0	0.665537492	0	1	CRA004775;PRJNA827465	Yang2025_Silkie_Wenchang	0.00075801
ENSGALG00010021714	TSPAN18	2	-0.318694528	0.274648435	-1.160372633	0.245897124	0.142458461	17.94902252	94.42866597	1	PRJNA827465;Yang2025_Silkie_Wenchang	CRA004775	0.75661406
ENSGALG00010021714	TSPAN18	2	-0.545091584	0.134476948	-4.053420256	5.04742E-05	0.012077661	1.155137276	13.43020252	1	CRA004775;Yang2025_Silkie_Wenchang	PRJNA827465	0.004874172
ENSGALG00010021714	TSPAN18	2	-0.050710141	0.089599918	-0.565961918	0.571419669	0	0.100562318	0	1	CRA004775;PRJNA827465	Yang2025_Silkie_Wenchang	0.885354083
ENSGALG00010021715	MMAB	2	0.136090675	0.243916245	0.557940185	0.57688523	0.102779386	7.245835465	86.19896898	0.5	PRJNA827465;Yang2025_Silkie_Wenchang	CRA004775	0.887113945
ENSGALG00010021715	MMAB	2	0.121830408	0.313470293	0.388650569	0.697534653	0.14903053	3.86474499	74.12507157	0.5	CRA004775;Yang2025_Silkie_Wenchang	PRJNA827465	0.921342994
ENSGALG00010021715	MMAB	2	-0.114295363	0.098706	-1.157937342	0.246889607	0	0.227939282	0	1	CRA004775;PRJNA827465	Yang2025_Silkie_Wenchang	0.757435192
ENSGALG00010021716	TFCP2L1	2	0.023235669	0.322544389	0.07203867	0.942571133	0.124876929	2.500879862	60.01407283	0.5	PRJNA827465;Yang2025_Silkie_Wenchang	CRA004775	0.985827325
ENSGALG00010021716	TFCP2L1	2	-0.344234813	0.272599879	-1.262784172	0.206666754	0	0.177059881	0	1	CRA004775;Yang2025_Silkie_Wenchang	PRJNA827465	0.71868063
ENSGALG00010021716	TFCP2L1	2	0.11090929	0.421383457	0.263202762	0.792394302	0.149919221	1.433430326	30.23727892	0.5	CRA004775;PRJNA827465	Yang2025_Silkie_Wenchang	0.948368502
ENSGALG00010021717	SDC4	2	-0.338743561	0.109105146	-3.104744137	0.001904437	0.008454595	1.489351359	32.85667659	1	PRJNA827465;Yang2025_Silkie_Wenchang	CRA004775	0.056193756
ENSGALG00010021717	SDC4	2	-0.160350127	0.371529355	-0.431594771	0.66603596	0.217944281	4.426362716	77.40808731	0.5	CRA004775;Yang2025_Silkie_Wenchang	PRJNA827465	0.913562201
ENSGALG00010021717	SDC4	2	-0.08305138	0.246362005	-0.337111155	0.736033109	0.080478821	2.422588385	58.72183627	0.5	CRA004775;PRJNA827465	Yang2025_Silkie_Wenchang	0.933592889
ENSGALG00010021719	BHLHE40	2	0.072227709	0.079906636	0.903901262	0.36604776	0.000892402	1.05915208	5.584852352	1	PRJNA827465;Yang2025_Silkie_Wenchang	CRA004775	0.819012587
ENSGALG00010021719	BHLHE40	2	-0.050230694	0.339260525	-0.148059353	0.882295928	0.156302899	2.646850268	62.21924556	0.5	CRA004775;Yang2025_Silkie_Wenchang	PRJNA827465	0.971967286
ENSGALG00010021719	BHLHE40	2	-0.091531558	0.218082154	-0.419711363	0.674696323	0.052728167	1.6008933	37.53487504	0.5	CRA004775;PRJNA827465	Yang2025_Silkie_Wenchang	0.915970608
ENSGALG00010021720	CACNB1	2	-0.08661895	0.104539092	-0.828579511	0.407342386	0.012907677	2.437381406	58.97236282	0.5	PRJNA827465;Yang2025_Silkie_Wenchang	CRA004775	0.833863243
ENSGALG00010021720	CACNB1	2	-0.005784094	0.115734112	-0.049977438	0.960140368	0.004360466	1.034526051	3.337378598	0.5	CRA004775;Yang2025_Silkie_Wenchang	PRJNA827465	0.989338243
ENSGALG00010021720	CACNB1	2	-0.197122708	0.088844328	-2.218742752	0.026504231	0	0.363343311	0	1	CRA004775;PRJNA827465	Yang2025_Silkie_Wenchang	0.277711485
ENSGALG00010021721	TP53I11	2	-0.10618848	0.112013317	-0.947998704	0.343130114	0	0.46993914	0	1	PRJNA827465;Yang2025_Silkie_Wenchang	CRA004775	0.806218116
ENSGALG00010021721	TP53I11	2	-0.00560297	0.235151946	-0.02382702	0.980990587	0.061495587	2.208543863	54.72129774	0.5	CRA004775;Yang2025_Silkie_Wenchang	PRJNA827465	0.994928779
ENSGALG00010021721	TP53I11	2	0.031573254	0.137621169	0.229421495	0.818541331	0.006673667	1.158678946	13.6948157	0.5	CRA004775;PRJNA827465	Yang2025_Silkie_Wenchang	0.955256039
ENSGALG00010021722	AARS1	2	-0.080606301	0.049948629	-1.61378405	0.106574275	0	0.177321601	0	1	PRJNA827465;Yang2025_Silkie_Wenchang	CRA004775	0.553677869
ENSGALG00010021722	AARS1	2	-0.049117417	0.099561473	-0.493337586	0.621774075	0	0.04461295	0	1	CRA004775;Yang2025_Silkie_Wenchang	PRJNA827465	0.899796991
ENSGALG00010021722	AARS1	2	-0.091945346	0.055051749	-1.670162131	0.09488729	0	0.000878351	0	1	CRA004775;PRJNA827465	Yang2025_Silkie_Wenchang	0.527384122
ENSGALG00010021723	COPS3	2	0.131210289	0.045535866	2.881471255	0.003958233	0	0.000440671	0	1	PRJNA827465;Yang2025_Silkie_Wenchang	CRA004775	0.09071163
ENSGALG00010021723	COPS3	2	0.141730602	0.097588748	1.452325244	0.146411191	0	0.048803534	0	1	CRA004775;Yang2025_Silkie_Wenchang	PRJNA827465	0.6352709
ENSGALG00010021723	COPS3	2	0.133044677	0.049645935	2.679870528	0.007365064	0	0.058784876	0	1	CRA004775;PRJNA827465	Yang2025_Silkie_Wenchang	0.132057078
ENSGALG00010021725	SH2D6	2	0.11675644	0.269365388	0.433450046	0.66468785	0.10342327	2.990171147	66.55709821	0.5	PRJNA827465;Yang2025_Silkie_Wenchang	CRA004775	0.913382053
ENSGALG00010021725	SH2D6	2	0.390519176	0.259155354	1.506892179	0.131838297	0	0.237266531	0	1	CRA004775;Yang2025_Silkie_Wenchang	PRJNA827465	0.608454531
ENSGALG00010021725	SH2D6	2	-0.077763172	0.10703951	-0.726490362	0.467538214	0	0.285062014	0	0.5	CRA004775;PRJNA827465	Yang2025_Silkie_Wenchang	0.852885838
ENSGALG00010021726	ARNTL	2	0.087022701	0.128314932	0.678196209	0.497647296	0.022564775	2.647027996	62.22178226	0.5	PRJNA827465;Yang2025_Silkie_Wenchang	CRA004775	0.86098656
ENSGALG00010021726	ARNTL	2	0.282540574	0.14776147	1.912139708	0.055858277	0	0.139905061	0	1	CRA004775;Yang2025_Silkie_Wenchang	PRJNA827465	0.409031746
ENSGALG00010021726	ARNTL	2	0.004265992	0.069034204	0.061795337	0.950725817	0.002186273	1.022904567	2.239169537	0.5	CRA004775;PRJNA827465	Yang2025_Silkie_Wenchang	0.98727979
ENSGALG00010021727	MVK	2	-0.174054356	0.225916997	-0.770434976	0.441041912	0.086423775	5.969467521	83.2480871	0.5	PRJNA827465;Yang2025_Silkie_Wenchang	CRA004775	0.844888742
ENSGALG00010021727	MVK	2	-0.484254911	0.128785566	-3.760164474	0.000169802	0	0.196503863	0	1	CRA004775;Yang2025_Silkie_Wenchang	PRJNA827465	0.011330586
ENSGALG00010021727	MVK	2	-0.232770128	0.283707462	-0.820458249	0.411954921	0.142615909	8.154482355	87.73680589	0.5	CRA004775;PRJNA827465	Yang2025_Silkie_Wenchang	0.835413801
ENSGALG00010021728	TMED5	2	0.020712453	0.149180321	0.138841721	0.889575225	0.038115516	6.885935724	85.47764545	0.5	PRJNA827465;Yang2025_Silkie_Wenchang	CRA004775	0.973153999
ENSGALG00010021728	TMED5	2	0.174046118	0.079637539	2.185478344	0.028853792	0	0.002759637	0	1	CRA004775;Yang2025_Silkie_Wenchang	PRJNA827465	0.289410648
ENSGALG00010021728	TMED5	2	-0.014935847	0.139580584	-0.107005187	0.914784865	0.026424096	2.726625155	63.32462501	0.5	CRA004775;PRJNA827465	Yang2025_Silkie_Wenchang	0.97982748
ENSGALG00010021729	RBPJL	2	0.882573844	0.500592884	1.763057113	0.077890858	0.218054556	1.733811651	42.32360826	1	PRJNA827465;Yang2025_Silkie_Wenchang	CRA004775	0.480724545
ENSGALG00010021729	RBPJL	2	1.613237781	0.447850914	3.60217593	0.000315565	0	0.10700036	0	1	CRA004775;Yang2025_Silkie_Wenchang	PRJNA827465	0.017542629
ENSGALG00010021729	RBPJL	2	1.03274794	0.649285883	1.590590473	0.11170177	0.538878433	2.699178264	62.95168743	1	CRA004775;PRJNA827465	Yang2025_Silkie_Wenchang	0.565091513
ENSGALG00010021730	ARL8B	2	-0.114091979	0.051811397	-2.202063371	0.027660832	0.002420221	1.720043813	41.86194603	1	PRJNA827465;Yang2025_Silkie_Wenchang	CRA004775	0.283778046
ENSGALG00010021730	ARL8B	2	-0.185021303	0.068673068	-2.694233809	0.007055066	0	0.041358086	0	1	CRA004775;Yang2025_Silkie_Wenchang	PRJNA827465	0.128969879
ENSGALG00010021730	ARL8B	2	-0.077331019	0.042309367	-1.827751748	0.067586818	0	0.261659276	0	1	CRA004775;PRJNA827465	Yang2025_Silkie_Wenchang	0.44825594
ENSGALG00010021731	SRM	2	0.307531243	0.15254348	2.01602352	0.043797516	0.037763878	5.098822906	80.38763028	1	PRJNA827465;Yang2025_Silkie_Wenchang	CRA004775	0.361804689
ENSGALG00010021731	SRM	2	0.265546761	0.246142986	1.078831317	0.280662931	0.090955417	3.732188667	73.2060705	0.5	CRA004775;Yang2025_Silkie_Wenchang	PRJNA827465	0.778536485
ENSGALG00010021731	SRM	2	0.153462945	0.06554376	2.341381458	0.019212526	0	0.640836386	0	0.5	CRA004775;PRJNA827465	Yang2025_Silkie_Wenchang	0.232311767
ENSGALG00010021732	CASR	2	0.261621231	0.178466933	1.465936724	0.142665526	0	0.447877139	0	1	PRJNA827465;Yang2025_Silkie_Wenchang	CRA004775	0.628960535
ENSGALG00010021732	CASR	2	-0.338698578	0.882156188	-0.383944003	0.701019952	1.253548291	4.59541059	78.23915882	0.5	CRA004775;Yang2025_Silkie_Wenchang	PRJNA827465	0.922547052
ENSGALG00010021732	CASR	2	-0.428686076	0.751701598	-0.570287568	0.568482672	0.860180982	3.554385408	71.86574091	0.5	CRA004775;PRJNA827465	Yang2025_Silkie_Wenchang	0.884151198
ENSGALG00010021733	ENSGALG00010021733	2	-0.120611721	0.094317988	-1.278777501	0.200975419	0	0.735533696	0	1	PRJNA827465;Yang2025_Silkie_Wenchang	CRA004775	0.713544434
ENSGALG00010021733	ENSGALG00010021733	2	-0.177567495	0.116215163	-1.527920204	0.126532351	0	0.05739623	0	1	CRA004775;Yang2025_Silkie_Wenchang	PRJNA827465	0.597082602
ENSGALG00010021733	ENSGALG00010021733	2	-0.027218921	0.141498483	-0.192361929	0.847458711	0	0.019612538	0	1	CRA004775;PRJNA827465	Yang2025_Silkie_Wenchang	0.961252712
ENSGALG00010021734	PARD3B	2	-0.118326184	0.08154912	-1.450980515	0.146785286	0	0.063883104	0	1	PRJNA827465;Yang2025_Silkie_Wenchang	CRA004775	0.635558525
ENSGALG00010021734	PARD3B	2	-0.127543596	0.164070521	-0.777370581	0.436940164	0	0.133816096	0	1	CRA004775;Yang2025_Silkie_Wenchang	PRJNA827465	0.843387647
ENSGALG00010021734	PARD3B	2	-0.10244417	0.087384558	-1.172337216	0.241061696	0	0.064085857	0	1	CRA004775;PRJNA827465	Yang2025_Silkie_Wenchang	0.753615003
ENSGALG00010021735	PIGBOS1	2	0.340950828	0.523783057	0.650939015	0.515085855	0.470743559	6.307893945	84.14684824	0.5	PRJNA827465;Yang2025_Silkie_Wenchang	CRA004775	0.867111635
ENSGALG00010021735	PIGBOS1	2	0.461803603	0.47567063	0.970847417	0.331624266	0.291722255	2.813457853	64.45654947	0.5	CRA004775;Yang2025_Silkie_Wenchang	PRJNA827465	0.801846521
ENSGALG00010021735	PIGBOS1	2	-0.107530192	0.102202773	-1.052125964	0.292741761	0	0.067835544	0	1	CRA004775;PRJNA827465	Yang2025_Silkie_Wenchang	0.786440837
ENSGALG00010021736	EDEM1	2	-0.06771618	0.16856169	-0.40172936	0.687883215	0.04992651	7.883626621	87.31548248	0.5	PRJNA827465;Yang2025_Silkie_Wenchang	CRA004775	0.919631238
ENSGALG00010021736	EDEM1	2	-0.246265442	0.102408167	-2.404744161	0.016183791	0	0.011624024	0	1	CRA004775;Yang2025_Silkie_Wenchang	PRJNA827465	0.210262467
ENSGALG00010021736	EDEM1	2	0.079775718	0.051790619	1.540350749	0.12347488	0	0.91045732	0	0.5	CRA004775;PRJNA827465	Yang2025_Silkie_Wenchang	0.589091186
ENSGALG00010021738	PRDM11	2	-0.186011582	0.170838138	-1.088817663	0.276234301	0.051926054	8.488979518	88.22002105	1	PRJNA827465;Yang2025_Silkie_Wenchang	CRA004775	0.77570997
ENSGALG00010021738	PRDM11	2	-0.37278053	0.101689011	-3.665888036	0.000246482	0	8.55541E-05	0	1	CRA004775;Yang2025_Silkie_Wenchang	PRJNA827465	0.014719311
ENSGALG00010021738	PRDM11	2	-0.107148337	0.142489673	-0.751972652	0.452067505	0.024669634	1.740589635	42.54820438	1	CRA004775;PRJNA827465	Yang2025_Silkie_Wenchang	0.848275385
ENSGALG00010021739	EXOSC10	2	0.066962549	0.100793688	0.664352604	0.50646466	0.016150033	4.757623002	78.98110044	0.5	PRJNA827465;Yang2025_Silkie_Wenchang	CRA004775	0.863929781
ENSGALG00010021739	EXOSC10	2	0.098275108	0.143139386	0.6865693	0.49235418	0.020624352	1.549453162	35.46110171	0.5	CRA004775;Yang2025_Silkie_Wenchang	PRJNA827465	0.859228604
ENSGALG00010021739	EXOSC10	2	-0.03127828	0.050723689	-0.616640469	0.537471895	0	0.268362735	0	1	CRA004775;PRJNA827465	Yang2025_Silkie_Wenchang	0.87481821
ENSGALG00010021740	PDLIM1	2	0.073897466	0.098954413	0.746782927	0.455194603	0	0.878866604	0	1	PRJNA827465;Yang2025_Silkie_Wenchang	CRA004775	0.849072665
ENSGALG00010021740	PDLIM1	2	0.179312744	0.131294894	1.365725197	0.172025232	0	0.031028911	0	1	CRA004775;Yang2025_Silkie_Wenchang	PRJNA827465	0.674672141
ENSGALG00010021740	PDLIM1	2	0.038988479	0.107896846	0.361349568	0.717838144	0	0.33098486	0	1	CRA004775;PRJNA827465	Yang2025_Silkie_Wenchang	0.927927766
ENSGALG00010021741	RPL19	2	-0.04747163	0.0659635	-0.719665122	0.471731205	0	0.284685432	0	0.5	PRJNA827465;Yang2025_Silkie_Wenchang	CRA004775	0.853998373
ENSGALG00010021741	RPL19	2	-0.063545476	0.155409525	-0.408890483	0.682620033	0	0.682241104	0	0.5	CRA004775;Yang2025_Silkie_Wenchang	PRJNA827465	0.918716001
ENSGALG00010021741	RPL19	2	-0.070605253	0.066001845	-1.069746646	0.284733364	0	0.337022113	0	1	CRA004775;PRJNA827465	Yang2025_Silkie_Wenchang	0.780278244
ENSGALG00010021742	MATN4	2	0.882571559	0.482315921	1.829861966	0.067270582	0.233445158	1.994312971	49.85741885	1	PRJNA827465;Yang2025_Silkie_Wenchang	CRA004775	0.446912726
ENSGALG00010021742	MATN4	2	0.176481879	1.246615657	0.141568797	0.887420615	2.791118594	9.786740344	89.78209327	0.5	CRA004775;Yang2025_Silkie_Wenchang	PRJNA827465	0.973030895
ENSGALG00010021742	MATN4	2	-0.271340798	0.760961907	-0.35657606	0.721409202	0.883654695	4.151731319	75.91366292	0.5	CRA004775;PRJNA827465	Yang2025_Silkie_Wenchang	0.929045705
ENSGALG00010021744	LITAF	2	-0.009031521	0.216940738	-0.04163128	0.966792637	0.019965529	1.25374805	20.23915812	0.5	PRJNA827465;Yang2025_Silkie_Wenchang	CRA004775	0.990893423
ENSGALG00010021744	LITAF	2	-0.191865636	0.233536092	-0.821567383	0.411323159	0	0.166467019	0	1	CRA004775;Yang2025_Silkie_Wenchang	PRJNA827465	0.835128673
ENSGALG00010021744	LITAF	2	0.083680669	0.197303947	0.424120603	0.671477861	0	0.359355032	0	0.5	CRA004775;PRJNA827465	Yang2025_Silkie_Wenchang	0.914674175
ENSGALG00010021745	RSL24D1	2	-0.105458481	0.057747351	-1.826204658	0.06781944	0	0.31066474	0	1	PRJNA827465;Yang2025_Silkie_Wenchang	CRA004775	0.449101706
ENSGALG00010021745	RSL24D1	2	-0.027831581	0.115491294	-0.240984233	0.809567337	0	0.143828946	0	0.5	CRA004775;Yang2025_Silkie_Wenchang	PRJNA827465	0.952770549
ENSGALG00010021745	RSL24D1	2	-0.114286942	0.064024844	-1.785040526	0.074254744	0	0.40153339	0	0.5	CRA004775;PRJNA827465	Yang2025_Silkie_Wenchang	0.470563398
ENSGALG00010021746	MTOR	2	-0.101316695	0.059450733	-1.704212715	0.088341355	0	0.265870953	0	1	PRJNA827465;Yang2025_Silkie_Wenchang	CRA004775	0.510441217
ENSGALG00010021746	MTOR	2	-0.041256819	0.101833805	-0.405138733	0.685375537	0	0.054994504	0	0.5	CRA004775;Yang2025_Silkie_Wenchang	PRJNA827465	0.919235539
ENSGALG00010021746	MTOR	2	-0.110415506	0.067363168	-1.639107971	0.101190775	0	0.268128894	0	0.5	CRA004775;PRJNA827465	Yang2025_Silkie_Wenchang	0.542850216
ENSGALG00010021747	AKTIP	2	0.081526099	0.058882	1.384567421	0.166184779	0	0.527932352	0	0.5	PRJNA827465;Yang2025_Silkie_Wenchang	CRA004775	0.66529196
ENSGALG00010021747	AKTIP	2	-0.034305218	0.118898208	-0.288525946	0.772944172	0	0.385785968	0	1	CRA004775;Yang2025_Silkie_Wenchang	PRJNA827465	0.943096979
ENSGALG00010021747	AKTIP	2	0.087417705	0.068248845	1.280867171	0.200240326	0.0005423	1.011548388	1.141654531	0.5	CRA004775;PRJNA827465	Yang2025_Silkie_Wenchang	0.712517205
ENSGALG00010021748	CDCA7	2	-0.179044416	0.164771785	-1.086620602	0.277204516	0.037667069	2.960601709	66.22308239	1	PRJNA827465;Yang2025_Silkie_Wenchang	CRA004775	0.776216095
ENSGALG00010021748	CDCA7	2	-0.409133492	0.168610412	-2.426501948	0.015245168	0	0.225192877	0	1	CRA004775;Yang2025_Silkie_Wenchang	PRJNA827465	0.204219788
ENSGALG00010021748	CDCA7	2	-0.108219424	0.184788718	-0.585638698	0.558118338	0.030879658	1.196270977	16.40689947	1	CRA004775;PRJNA827465	Yang2025_Silkie_Wenchang	0.880678789
ENSGALG00010021749	CLASP1	2	8.95356E-05	0.147725207	0.000606095	0.999516406	0.033071986	3.705672705	73.01434639	0.5	PRJNA827465;Yang2025_Silkie_Wenchang	CRA004775	0.999889665
ENSGALG00010021749	CLASP1	2	0.184043486	0.127161235	1.447323826	0.147806249	0	0.00478777	0	1	CRA004775;Yang2025_Silkie_Wenchang	PRJNA827465	0.637081344
ENSGALG00010021749	CLASP1	2	-0.066621466	0.121245203	-0.549477129	0.582678056	0.014153096	1.375365996	27.29208057	0.5	CRA004775;PRJNA827465	Yang2025_Silkie_Wenchang	0.890043325
ENSGALG00010021750	HELLS	2	-0.063968472	0.065731849	-0.973173176	0.33046724	0	0.88342233	0	0.5	PRJNA827465;Yang2025_Silkie_Wenchang	CRA004775	0.80156474
ENSGALG00010021750	HELLS	2	0.023834717	0.101823443	0.234078872	0.814923748	0	0.280419986	0	1	CRA004775;Yang2025_Silkie_Wenchang	PRJNA827465	0.953897709
ENSGALG00010021750	HELLS	2	-0.10241699	0.083396363	-1.228075017	0.219418809	0	0.630778416	0	0.5	CRA004775;PRJNA827465	Yang2025_Silkie_Wenchang	0.732888275
ENSGALG00010021751	MTCH2	2	-0.01997676	0.060480226	-0.330302337	0.741171527	0	0.25084139	0	0.5	PRJNA827465;Yang2025_Silkie_Wenchang	CRA004775	0.935384591
ENSGALG00010021751	MTCH2	2	-0.119151	0.202162193	-0.589383197	0.555604246	0.041310002	2.009974348	50.24812127	0.5	CRA004775;Yang2025_Silkie_Wenchang	PRJNA827465	0.880272248
ENSGALG00010021751	MTCH2	2	-0.113692395	0.136481073	-0.833026826	0.4048296	0.02147863	1.838570196	45.60990916	1	CRA004775;PRJNA827465	Yang2025_Silkie_Wenchang	0.833112102
ENSGALG00010021752	CSTB	2	-0.268082968	0.075676599	-3.542481711	0.000396381	0	0.649557502	0	1	PRJNA827465;Yang2025_Silkie_Wenchang	CRA004775	0.020084189
ENSGALG00010021752	CSTB	2	-0.393778337	0.13148965	-2.994747769	0.002746719	0	0.000930639	0	1	CRA004775;Yang2025_Silkie_Wenchang	PRJNA827465	0.071540283
ENSGALG00010021752	CSTB	2	-0.260527842	0.078324353	-3.326268688	0.00088017	0	0.514210025	0	1	CRA004775;PRJNA827465	Yang2025_Silkie_Wenchang	0.034801769
ENSGALG00010021753	MTF2	2	0.097380326	0.044350617	2.195692718	0.028113945	0	0.502530924	0	1	PRJNA827465;Yang2025_Silkie_Wenchang	CRA004775	0.285731308
ENSGALG00010021753	MTF2	2	0.122249121	0.069216824	1.766176403	0.077366259	0	0.840746605	0	0.5	CRA004775;Yang2025_Silkie_Wenchang	PRJNA827465	0.479060101
ENSGALG00010021753	MTF2	2	0.064881872	0.055888377	1.160918886	0.245674888	0	0.521564264	0	0.5	CRA004775;PRJNA827465	Yang2025_Silkie_Wenchang	0.75661406
ENSGALG00010021754	SLC17A9	2	0.489832019	0.31358466	1.562040754	0.118278388	0	0.374160775	0	1	PRJNA827465;Yang2025_Silkie_Wenchang	CRA004775	0.578052163
ENSGALG00010021754	SLC17A9	2	0.918745232	0.617622105	1.487552379	0.13686898	0.563842341	3.819144177	73.81612336	1	CRA004775;Yang2025_Silkie_Wenchang	PRJNA827465	0.619028943
ENSGALG00010021754	SLC17A9	2	1.134286384	0.424417069	2.672574849	0.007527158	0.133253611	1.587003908	36.98818289	1	CRA004775;PRJNA827465	Yang2025_Silkie_Wenchang	0.133864204
ENSGALG00010021755	ENSGALG00010021755	2	-0.114323814	0.076976372	-1.485180592	0.137495992	0	0.569253444	0	1	PRJNA827465;Yang2025_Silkie_Wenchang	CRA004775	0.620332372
ENSGALG00010021755	ENSGALG00010021755	2	-0.008746788	0.12699482	-0.068875155	0.945088995	0	0.363082786	0	0.5	CRA004775;Yang2025_Silkie_Wenchang	PRJNA827465	0.986215824
ENSGALG00010021755	ENSGALG00010021755	2	-0.137326747	0.092186638	-1.489659992	0.136313659	0	0.80420333	0	0.5	CRA004775;PRJNA827465	Yang2025_Silkie_Wenchang	0.618139317

ENSGALG00010021756	USP42	2	0.586716463	0.460700743	1.273530533	0.202829845	0.376513927	8.136101536	87.70910127	1	PRJNA827465;Yang2025_Silkie_Wenchang	CRA004775	0.7160533
ENSGALG00010021756	USP42	2	0.685315794	0.408841468	1.676238462	0.093691493	0.240135655	3.548888142	71.82216064	1	CRA004775;Yang2025_Silkie_Wenchang	PRJNA827465	0.524189933
ENSGALG00010021756	USP42	2	0.180621233	0.08289762	2.17884702	0.029343035	0	0.121162363	0	1	CRA004775;PRJNA827465	Yang2025_Silkie_Wenchang	0.291796413
ENSGALG00010021758	RASSF10	2	0.631194799	0.506544083	1.246080689	0.212734774	0.257381641	1.863705275	46.34344747	1	PRJNA827465;Yang2025_Silkie_Wenchang	CRA004775	0.725541376
ENSGALG00010021758	RASSF10	2	0.434549682	0.826643621	0.525679593	0.599110845	0.987184891	3.578246277	72.05334897	0.5	CRA004775;Yang2025_Silkie_Wenchang	PRJNA827465	0.894908299
ENSGALG00010021758	RASSF10	2	0.059884392	0.314981826	0.19012015	0.849214981	0	0.787023846	0	0.5	CRA004775;PRJNA827465	Yang2025_Silkie_Wenchang	0.961476534
ENSGALG00010021759	NIFK	2	0.021763145	0.047329161	0.459825279	0.645641637	0	0.870584623	0	0.5	PRJNA827465;Yang2025_Silkie_Wenchang	CRA004775	0.907052464
ENSGALG00010021759	NIFK	2	-0.154561637	0.105183462	-1.46944808	0.141711295	0	0.376415122	0	1	CRA004775;Yang2025_Silkie_Wenchang	PRJNA827465	0.627129189
ENSGALG00010021759	NIFK	2	-0.051923111	0.126494727	-0.410476483	0.681456452	0.021522551	2.495375835	59.92587626	0.5	CRA004775;PRJNA827465	Yang2025_Silkie_Wenchang	0.918242009
ENSGALG00010021760	CSTA	2	-0.287614846	0.203537489	-1.413080449	0.157632071	0	0.019275407	0	1	PRJNA827465;Yang2025_Silkie_Wenchang	CRA004775	0.650983849
ENSGALG00010021760	CSTA	2	0.335529498	0.821852734	0.408259879	0.683082891	0.942986251	2.698142394	62.93746386	0.5	CRA004775;Yang2025_Silkie_Wenchang	PRJNA827465	0.918800744
ENSGALG00010021760	CSTA	2	0.314490608	0.853846081	0.368322365	0.712632878	1.044883198	2.889885598	65.39655408	0.5	CRA004775;PRJNA827465	Yang2025_Silkie_Wenchang	0.926026913
ENSGALG00010021761	MAFB	2	0.315563263	0.444486144	0.70995073	0.47773469	0.335344316	5.848760841	82.90236125	0.5	PRJNA827465;Yang2025_Silkie_Wenchang	CRA004775	0.855300256
ENSGALG00010021761	MAFB	2	0.319330414	0.550342123	0.580239819	0.561752905	0.436762983	3.542913595	71.7746433	0.5	CRA004775;Yang2025_Silkie_Wenchang	PRJNA827465	0.881802301
ENSGALG00010021761	MAFB	2	-0.07274195	0.085653424	-0.849259102	0.395737133	0	0.188358352	0	1	CRA004775;PRJNA827465	Yang2025_Silkie_Wenchang	0.83121294
ENSGALG00010021762	LAMB2	2	-0.016226382	0.204625645	-0.07929789	0.936795685	0.066157983	4.750765252	78.95075957	0.5	PRJNA827465;Yang2025_Silkie_Wenchang	CRA004775	0.985038972
ENSGALG00010021762	LAMB2	2	-0.101436493	0.369159829	-0.274776628	0.783487856	0.203588467	3.456343393	71.06768957	0.5	CRA004775;Yang2025_Silkie_Wenchang	PRJNA827465	0.945815798
ENSGALG00010021762	LAMB2	2	-0.249164829	0.118467925	-2.103226082	0.035446011	0	0.747727619	0	1	CRA004775;PRJNA827465	Yang2025_Silkie_Wenchang	0.325043067
ENSGALG00010021765	EIF2AK1	2	0.188582454	0.102569412	1.838583738	0.065976435	0.016271352	4.371457583	77.12433483	1	PRJNA827465;Yang2025_Silkie_Wenchang	CRA004775	0.44241327
ENSGALG00010021765	EIF2AK1	2	0.098717784	0.229065747	0.430958297	0.666498694	0.085713193	4.9883309	79.95321441	0.5	CRA004775;Yang2025_Silkie_Wenchang	PRJNA827465	0.913579507
ENSGALG00010021765	EIF2AK1	2	0.027066	0.11139497	0.242973269	0.808026105	0.012787773	1.626903174	38.53352701	0.5	CRA004775;PRJNA827465	Yang2025_Silkie_Wenchang	0.952496413
ENSGALG00010021766	AGRN	2	-0.11003123	0.071697772	-1.534653406	0.124868987	0	0.003348921	0	1	PRJNA827465;Yang2025_Silkie_Wenchang	CRA004775	0.592935337
ENSGALG00010021766	AGRN	2	-0.08978319	0.112398487	-0.79879358	0.424410114	0	0.678955293	0	0.5	CRA004775;Yang2025_Silkie_Wenchang	PRJNA827465	0.840628469
ENSGALG00010021766	AGRN	2	-0.091186539	0.08887995	-1.025951741	0.304914332	0	0.665636768	0	0.5	CRA004775;PRJNA827465	Yang2025_Silkie_Wenchang	0.79225794
ENSGALG00010021767	CCDC58	2	0.019849609	0.112995872	0.17566667	0.860555822	0.007746259	1.317403313	24.0931012	0.5	PRJNA827465;Yang2025_Silkie_Wenchang	CRA004775	0.965059076
ENSGALG00010021767	CCDC58	2	0.157786615	0.164561333	0.958831656	0.337643559	0	0.180517048	0	1	CRA004775;Yang2025_Silkie_Wenchang	PRJNA827465	0.80415385
ENSGALG00010021767	CCDC58	2	-0.039278494	0.094031035	-0.417718411	0.67615301	0	0.105186148	0	0.5	CRA004775;PRJNA827465	Yang2025_Silkie_Wenchang	0.916658333
ENSGALG00010021768	DIPK1A	2	-0.190575751	0.220496057	-0.864304576	0.387420597	0.08267874	6.648903277	84.9599256	0.5	PRJNA827465;Yang2025_Silkie_Wenchang	CRA004775	0.827812968
ENSGALG00010021768	DIPK1A	2	-0.30980419	0.153814611	-2.014140188	0.043984817	0.01784588	1.498831502	33.28135961	1	CRA004775;Yang2025_Silkie_Wenchang	PRJNA827465	0.36242712
ENSGALG00010021768	DIPK1A	2	0.003310816	0.098712123	0.033540118	0.973243874	0	0.195715321	0	0.5	CRA004775;PRJNA827465	Yang2025_Silkie_Wenchang	0.992392211
ENSGALG00010021769	ENSGALG00010021769	2	0.353873651	0.422475815	0.837618719	0.402244876	0.335130293	15.87642741	93.70135375	0.5	PRJNA827465;Yang2025_Silkie_Wenchang	CRA004775	0.832879175
ENSGALG00010021769	ENSGALG00010021769	2	0.285658532	0.522968304	0.546225325	0.58491106	0.495014704	10.45718733	90.43719914	0.5	CRA004775;Yang2025_Silkie_Wenchang	PRJNA827465	0.891037186
ENSGALG00010021769	ENSGALG00010021769	2	-0.069239523	0.078860983	-0.877994671	0.379946611	0	0.548172373	0	1	CRA004775;PRJNA827465	Yang2025_Silkie_Wenchang	0.824392515
ENSGALG00010021770	RALGAPB	2	-0.023035082	0.0375265	-0.613835065	0.539324323	0	0.363714434	0	1	PRJNA827465;Yang2025_Silkie_Wenchang	CRA004775	0.874983893
ENSGALG00010021770	RALGAPB	2	-0.063984405	0.107415599	-0.595671446	0.551394737	0	0.306853905	0	0.5	CRA004775;Yang2025_Silkie_Wenchang	PRJNA827465	0.878888037
ENSGALG00010021770	RALGAPB	2	-0.013521176	0.039183668	-0.345071736	0.730040441	0	0.104023224	0	0.5	CRA004775;PRJNA827465	Yang2025_Silkie_Wenchang	0.932587945
ENSGALG00010021771	ANKRD61	2	0.12448507	0.061144911	2.035902371	0.04176016	0	0.541736412	0	1	PRJNA827465;Yang2025_Silkie_Wenchang	CRA004775	0.354014899
ENSGALG00010021771	ANKRD61	2	0.171625149	0.11810641	1.453140006	0.146184885	0	0.782215776	0	0.5	CRA004775;Yang2025_Silkie_Wenchang	PRJNA827465	0.634916196
ENSGALG00010021771	ANKRD61	2	0.090923532	0.069346582	1.311146559	0.189808253	0	0.431324452	0	0.5	CRA004775;PRJNA827465	Yang2025_Silkie_Wenchang	0.699160501
ENSGALG00010021772	TSN	2	-0.123151213	0.119850731	-1.027538268	0.304167076	0.018613294	2.280298384	56.14609004	1	PRJNA827465;Yang2025_Silkie_Wenchang	CRA004775	0.79225794
ENSGALG00010021772	TSN	2	-0.248762566	0.126844428	-1.961162741	0.049860042	0	0.23833312	0	1	CRA004775;Yang2025_Silkie_Wenchang	PRJNA827465	0.38629001
ENSGALG00010021772	TSN	2	-0.088818746	0.047873807	-1.019738123	0.307852676	0	0.398055482	0	1	CRA004775;PRJNA827465	Yang2025_Silkie_Wenchang	0.793203994
ENSGALG00010021774	FBXL20	2	-0.172965549	0.04878461	-3.54549138	0.000391978	0	0.225174651	0	1	PRJNA827465;Yang2025_Silkie_Wenchang	CRA004775	0.019929928
ENSGALG00010021774	FBXL20	2	-0.21191753	0.073453582	-2.885053733	0.003913469	0	0.113188883	0	1	CRA004775;Yang2025_Silkie_Wenchang	PRJNA827465	0.090106067
ENSGALG00010021774	FBXL20	2	-0.165063762	0.059405272	-2.778604616	0.005459293	0	0.324175483	0	1	CRA004775;PRJNA827465	Yang2025_Silkie_Wenchang	0.110944465
ENSGALG00010021775	CCDC36	2	0.074686052	0.199997669	0.373434613	0.708825002	0	0.453127545	0	0.5	PRJNA827465;Yang2025_Silkie_Wenchang	CRA004775	0.925298042
ENSGALG00010021775	CCDC36	2	-0.193295307	0.238567827	-0.810232082	0.417806802	0	0.78391245	0	1	CRA004775;Yang2025_Silkie_Wenchang	PRJNA827465	0.838112595
ENSGALG00010021775	CCDC36	2	-0.078053799	0.35937006	-0.217196164	0.828055474	0.131720389	1.944058863	48.56122831	0.5	CRA004775;PRJNA827465	Yang2025_Silkie_Wenchang	0.956645041
ENSGALG00010021776	PMS2	2	-0.102768729	0.12823375	-0.801417168	0.42289018	0.023290478	3.373252393	70.35501992	0.5	PRJNA827465;Yang2025_Silkie_Wenchang	CRA004775	0.839988514
ENSGALG00010021776	PMS2	2	0.144075877	0.412655199	0.349143492	0.726981587	0.306714377	9.667515539	89.65608076	0.5	CRA004775;Yang2025_Silkie_Wenchang	PRJNA827465	0.93142315
ENSGALG00010021776	PMS2	2	0.255535064	0.281371343	0.908177291	0.363785456	0.129657438	4.963850834	79.85435031	1	CRA004775;PRJNA827465	Yang2025_Silkie_Wenchang	0.817042253
ENSGALG00010021778	RBL2	2	-0.139771679	0.040099531	-3.485618811	0.000491	0.000409505	1.097465493	8.880961944	1	PRJNA827465;Yang2025_Silkie_Wenchang	CRA004775	0.023248634
ENSGALG00010021778	RBL2	2	-0.030328701	0.223247232	-0.135852527	0.891937866	0.076909663	3.8946266	74.32259754	0.5	CRA004775;Yang2025_Silkie_Wenchang	PRJNA827465	0.974016669
ENSGALG00010021778	RBL2	2	-0.001044427	0.168276816	-0.006206598	0.995047883	0.040369556	2.679720792	62.68267937	0.5	CRA004775;PRJNA827465	Yang2025_Silkie_Wenchang	0.999042335
ENSGALG00010021779	UNC13C	2	0.358426858	0.279769674	1.281149786	0.20014106	0.10938075	3.305854459	69.75063445	1	PRJNA827465;Yang2025_Silkie_Wenchang	CRA004775	0.712448516
ENSGALG00010021779	UNC13C	2	0.579275035	0.215906135	2.682994786	0.007296613	0	0.680504068	0	1	CRA004775;Yang2025_Silkie_Wenchang	PRJNA827465	0.131225376
ENSGALG00010021779	UNC13C	2	0.094589811	0.191041184	0.495127851	0.620509876	0	0.004541892	0	1	CRA004775;PRJNA827465	Yang2025_Silkie_Wenchang	0.899395593
ENSGALG00010021781	RAPGEF4	2	0.165917049	0.177853321	0.932886989	0.350878321	0	0.562690387	0	1	PRJNA827465;Yang2025_Silkie_Wenchang	CRA004775	0.810146221
ENSGALG00010021781	RAPGEF4	2	0.109263936	0.281846902	0.387671234	0.698259346	0.05172791	1.400558139	28.59989372	0.5	CRA004775;Yang2025_Silkie_Wenchang	PRJNA827465	0.921624968
ENSGALG00010021781	RAPGEF4	2	-0.042180901	0.214762563	-0.19640714	0.844291508	0	0.44211698	0	0.5	CRA004775;PRJNA827465	Yang2025_Silkie_Wenchang	0.960723102
ENSGALG00010021782	ADAM11	2	0.130443169	0.45753399	0.28510099	0.775567148	0.348782918	5.880295296	82.99405133	0.5	PRJNA827465;Yang2025_Silkie_Wenchang	CRA004775	0.938815265
ENSGALG00010021782	ADAM11	2	0.337152208	0.416299206	0.809879536	0.418009414	0.154366309	1.617580088	38.1792588	0.5	CRA004775;Yang2025_Silkie_Wenchang	PRJNA827465	0.838112595
ENSGALG00010021782	ADAM11	2	-0.298092226	0.202656417	-1.470924196	0.141311618	0	0.000686401	0	1	CRA004775;PRJNA827465	Yang2025_Silkie_Wenchang	0.626293475
ENSGALG00010021783	GIT2	2	-0.078789683	0.038140605	-2.065769077	0.038850289	0	0.590427435	0	1	PRJNA827465;Yang2025_Silkie_Wenchang	CRA004775	0.340074147
ENSGALG00010021783	GIT2	2	-0.148698484	0.090653448	-1.640295947	0.100943648	0	0.000469901	0	1	CRA004775;Yang2025_Silkie_Wenchang	PRJNA827465	0.542348734
ENSGALG00010021783	GIT2	2	-0.069364734	0.040553962	-1.710430503	0.087186294	0	0.123421454	0	1	CRA004775;PRJNA827465	Yang2025_Silkie_Wenchang	0.507807192
ENSGALG00010021785	CCDC71	2	-0.018118216	0.052939843	-0.342241589	0.732169089	0.002137231	1.445586248	30.82391302	0.5	PRJNA827465;Yang2025_Silkie_Wenchang	CRA004775	0.93287651
ENSGALG00010021785	CCDC71	2	-0.119712153	0.080721636	-1.48302437	0.138067936	0	0.218200124	0	1	CRA004775;Yang2025_Silkie_Wenchang	PRJNA827465	0.621163472
ENSGALG00010021785	CCDC71	2	-0.009629251	0.069109565	-0.139333117	0.889186922	0.003620701	1.178557877	15.15053956	0.5	CRA004775;PRJNA827465	Yang2025_Silkie_Wenchang	0.973135755
ENSGALG00010021786	FAM162A	2	0.273899139	0.256887109	1.066223759	0.286322504	0.096586555	3.408631413	70.66271243	1	PRJNA827465;Yang2025_Silkie_Wenchang	CRA004775	0.781944441
ENSGALG00010021786	FAM162A	2	0.454476387	0.216041912	2.103649161	0.035409062	0	0.89067329	0	1	CRA004775;Yang2025_Silkie_Wenchang	PRJNA827465	0.324780017
ENSGALG00010021786	FAM162A	2	0.066967776	0.116258951	0.576022542	0.564599945	0	0.029317518	0	1	CRA004775;PRJNA827465	Yang2025_Silkie_Wenchang	0.883173142
ENSGALG00010021788	RPL5	2	-0.159160395	0.143806141	-1.1067705	0.268393166	0.030854847	3.537563234	71.73195407	1	PRJNA827465;Yang2025_Silkie_Wenchang	CRA004775	0.771398294
ENSGALG00010021788	RPL5	2	-0.239287891	0.138485997	-1.727885104	0.084008825	0.007663483	1.211047317	17.42684321	1	CRA004775;Yang2025_Silkie_Wenchang	PRJNA827465	0.499515714
ENSGALG00010021788	RPL5	2	-0.041044669	0.058960941	-0.696133202	0.486345415	0	0.000190448	0	1	CRA004775;PRJNA827465	Yang2025_Silkie_Wenchang	0.857603818
ENSGALG00010021789	CHD9	2	0.207314784	0.257188224	0.806081944	0.420195602	0.115781135	7.303274803	86.30751236	0.5	PRJNA827465;Yang2025_Silkie_Wenchang	CRA004775	0.838662794
ENSGALG00010021789	CHD9	2	0.472308018	0.156607778	3.015865637	0.002562468	0	0.062199575	0	1	CRA004775;Yang2025_Silkie_Wenchang	PRJNA827465	0.068279778
ENSGALG00010021789	CHD9	2	0.096507812	0.192082763	0.502428278	0.615366292	0.048168622	2.075545094	51.81988563	0.5	CRA004775;PRJNA827465	Yang2025_Silkie_Wenchang	0.898864379
ENSGALG00010021790	PHF21A	2	0.070996546	0.177588732	0.399780689	0.689318055	0.052007685	5.105705256	80.41406721	0.5	PRJNA827465;Yang2025_Silkie_Wenchang	CRA004775	0.91986176
ENSGALG00010021790	PHF21A	2	0.356434141	0.114011353	3.12630393	0.001770185	0	0.607825491	0	1	CRA004775;Yang2025_Silkie_Wenchang	PRJNA827465	0.053477003
ENSGALG00010021790	PHF21A	2	0.163360003	0.268351565	0.60875368	0.54268772	0.129008425	8.898113821	88.7616632	0.5	CRA004775;PRJNA827465	Yang2025_Silkie_Wenchang	0.876917135
ENSGALG00010021791	PLEKHM2	2	0.018766335	0.170708633	0.109931962	0.912463334	0.047500763	5.305551089	81.15181659	0.5	PRJNA827465;Yang2025_Silkie_Wenchang	CRA004775	0.978967245
ENSGALG00010021791	PLEKHM2	2	-0.03365357	0.334593504	-0.100580465	0.919883505	0.152168369	2.505043271	60.08052989	0.5	CRA004775;Yang2025_Silkie_Wenchang	PRJNA827465	0.981237238
ENSGALG00010021791	PLEKHM2	2	-0.154806227	0.083293134	-1.858571289	0.06308793	0	0.702029058	0	1	CRA004775;PRJNA827465	Yang2025_Silkie_Wenchang	0.432579363
ENSGALG00010021792	KLHDC8B	2	0.180131676	0.1952473	0.922582162	0.356224995	0.027031941	1.468810211	31.91768463	1	PRJNA827465;Yang2025_Silkie_Wenchang	CRA004775	0.812521698
ENSGALG00010021792	KLHDC8B	2	-0.319279649	0.82099183	-0.388895038	0.697353793	1.182811839	7.927133392	87.38509937	0.5	CRA004775;Yang2025_Silkie_Wenchang	PRJNA827465	0.92131151
ENSGALG00010021792	KLHDC8B	2	-0.485874742	0.610036892	-0.7964678	0.425760184	0.617094474	5.285396308	81.07994289	0.5	CRA004775;PRJNA827465	Yang2025_Silkie_Wenchang	0.84125685
ENSGALG00010021793	ENSGALG00010021793	2	-0.295643347	0.102978341	-2.870927461	0.004092694	0	0.09097716	0	1	PRJNA827465;Yang2025_Silkie_Wenchang	CRA004775	0.092818906
ENSGALG00010021793	ENSGALG00010021793	2	-0.211357328	0.148975826	-1.418735734	0.155976075	0	0.671274061	0	0.5	CRA004775;Yang2025_Silkie_Wenchang	PRJNA827465	0.649132948
ENSGALG00010021793	ENSGALG00010021793	2	-0.280720158	0.127974575	-2.193561949	0.028266916	0	0.962333422	0	0.5	CRA004775;PRJNA827465	Yang2025_Silkie_Wenchang	0.286481335
ENSGALG00010021794	ENSGALG00010021794	2	0.255956445	0.311557698	0.82153786	0.411339968	0.171203133	8.391848778	88.08367469	0.5	PRJNA827465;Yang2025_Silkie_Wenchang	CRA004775	0.835128673
ENSGALG00010021794	ENSGALG00010021794	2	0.515025028	0.159402392	3.230974269	0.00123369	0	0.910679949	0	1	CRA004775;Yang2025_Silkie_Wenchang	PRJNA827465	0.042833689
ENSGALG00010021794	ENSGALG00010021794	2	-0.028044654	0.122723416	-0.228519181	0.819242646	0	0.201446716	0	0.5	CRA004775;PRJNA827465	Yang2025_Silkie_Wenchang	0.955256039
ENSGALG00010021795	NDRG3	2	-0.117961603	0.053216387	-2.216640579	0.026647662	0	0.025905959	0	1	PRJNA827465;Yang2025_Silkie_Wenchang	CRA004775	0.278694271
ENSGALG00010021795	NDRG3	2	-0.19379896	0.106648061	-1.817182222	0.069189216	0.005992215	1.252551169	20.16294229	1	CRA004775;Yang2025_Silkie_Wenchang	PRJNA827465	0.453013819
ENSGALG00010021795	NDRG3	2	-0.178639364	0.114050099	-1.566323615	0.117272875	0.013341569	1.632509038	38.7445964	1	CRA004775;PRJNA827465	Yang2025_Silkie_Wenchang	0.576651547
ENSGALG00010021797	SLC25A34	2	0.654418995	0.600354432	1.090054407	0.275689178	0.664451267	12.31801804	91.88181088	1	PRJNA827465;Yang2025_Silkie_Wenchang	CRA004775	0.775427142
ENSGALG00010021797	SLC25A34	2	0.775662738	0.550704433	1.408491909	0.158985461	0.467952938	4.30751823	76.78477614	1	CRA004775;Yang2025_Silkie_Wenchang	PRJNA827465	0.653803724
ENSGALG00010021797	SLC25A34	2	0.095821307	0.127559168	0.751191065	0.452537676	0	0.048310837	0	1	CRA004775;PRJNA827465	Yang2025_Silkie_Wenchang	0.848321192
ENSGALG00010021798	RANBP10	2	-0.060263974	0.063059274	-0.955671869	0.339238039	0	0.61245013	0	0.5	PRJNA827465;Yang2025_Silkie_Wenchang	CRA004775	0.804824608
ENSGALG00010021798	RANBP10	2	-0.050790139	0.167213795	-0.303743716	0.76132315	0.014507538	1.250369895	20.02366626	0.5	CRA004775;Yang2025_Silkie_Wenchang	PRJNA827465	0.939618592
ENSGALG00010021798	RANBP10	2	-0.094910157	0.067507096	-1.4059286	0.159745328	0	0.638999253	0	1	CRA004775;PRJNA827465	Yang2025_Silkie_Wenchang	0.655404348
ENSGALG00010021799	ENSGALG00010021799	2	0.385069498	0.127099506	3.029669509	0.002448215	0	0.059928236	0	1	PRJNA827465;Yang2025_Silkie_Wenchang	CRA004775	0.066177569
ENSGALG00010021799	ENSGALG00010021799	2	0.02968594	0.416136656	0.071336998	0.94312955	0.273868485	4.776680496	79.06495942	0.5	CRA004775;Yang2025_Silkie_Wenchang	PRJNA827465	0.985827325
ENSGALG00010021799	ENSGALG00010021799	2	0.024738002	0.377748178	0.065488078	0.947785398	0.239251844	5.90355012	83.06103989	0.5	CRA004775;PRJNA827465	Yang2025_Silkie_Wenchang	0.986619597
ENSGALG00010021800	TMEM82	2	0.47463567	0.326264898	1.454755548	0.145736949	0.138593868	2.84241881	64.81869609	1	PRJNA827465;Yang2025_Silkie_Wenchang	CRA004775	0.63445938
ENSGALG00010021800	TMEM82	2	0.79934827	0.212861403	3.755252291	0.000173167	0	0.023186544	0	1	CRA004775;Yang2025_Silkie_Wenchang	PRJNA827465	0.011507452
ENSGALG00010021800	TMEM82	2	0.431567815	0.35918849	1.201507918	0.229554237	0.133004996	2.008439833	50.21010917	1	CRA004775;PRJNA827465	Yang2025_Silkie_Wenchang	0.742629165
ENSGALG00010021801	VAMP1	2	0.184126606	0.202320099	0.910075704	0.362782586	0.032734126	1.652740679	39.4944402	1	PRJNA827465;Yang2025_Silkie_Wenchang	CRA004775	0.816073947
ENSGALG00010021801	VAMP1	2	0.149989403	0.383225529	0.391386773	0.695511368	0.155265442	1.840697567	45.67277006	0.5	CRA004775;Yang2025_Silkie_Wenchang	PRJNA827465	0.921038437
ENSGALG00010021801	VAMP1	2	-0.045628412	0.190235887	-0.239851759	0.81044518	0	0.496379585	0	0.5	CRA004775;PRJNA827465	Yang2025_Silkie_Wenchang	0.952997426
ENSGALG00010021803	TLL2	2	-0.094184199	0.109850639	-0.857384177	0.391232601	0.007360619	1.429459345	30.04348089	0.5	PRJNA827465;Yang2025_Silkie_Wenchang	CRA004775	0.829251057
ENSGALG00010021803	TLL2	2	0.16808248	0.494536629	0.339878727	0.733947857	0.373790308	3.565657024	71.95467782	0.5	CRA004775;Yang2025_Silkie_Wenchang	PRJNA827465	0.933391141
ENSGALG00010021803	TLL2	2	0.240839106	0.364993964	0.659844078	0.509353894	0.175653047	2.230949433	55.17603468	1	CRA004775;PRJNA827465	Yang2025_Silkie_Wenchang	0.865257661
ENSGALG00010021805	FUT8	2	-0.149756633	0.192889206	-0.776386797	0.437520638	0.064886925	7.797875082	87.17599359	0.5	PRJNA827465;Yang2025_Silkie_Wenchang	CRA004775	0.843803603
ENSGALG00010021805	FUT8	2	-0.171876702	0.272589151	-0.630533905	0.528345327	0.093801721	2.07957986	51.9133639	0.5	CRA004775;Yang2025_Silkie_Wenchang	PRJNA827465	0.871701504
ENSGALG00010021805	FUT8	2	0.050502415	0.08905839	0.56707083	0.57066606	0	0.270555038	0	1	CRA004775;PRJNA827465	Yang2025_Silkie_Wenchang	0.885152854
ENSGALG00010021807	CDC42	2	-0.393653139	0.2966484	-1.327002402	0.184507888	0.173220387	62.89144861	98.40995871	1	PRJNA827465;Yang2025_Silkie_Wenchang	CRA004775	0.692816808
ENSGALG00010021807	CDC42	2	-0.410514647	0.301267174	-1.362626541	0.17300024	0.166159034	11.3282879	91.17254074	1	CRA004775;Yang2025_Silkie_Wenchang	PRJNA827465	0.676043418
ENSGALG00010021807	CDC42	2	-0.099679985	0.03089091	-3.226838758	0.00125166	0	0.003791337	0	1	CRA004775;PRJNA827465	Yang2025_Silkie_Wenchang	0.042994223
ENSGALG00010021810	ANKRD13A	2	-0.118300665	0.145397731	-0.813634876	0.415854152	0.038093332	9.910887725	89.91008649	0.5	PRJNA827465;Yang2025_Silkie_Wenchang	CRA004775	0.837742614
ENSGALG00010021810	ANKRD13A	2	-0.276388982	0.076547933	-3.610665514	0.000305412	0	0.104232969	0	1	CRA004775;Yang2025_Silkie_Wenchang	PRJNA827465	0.017197164
ENSGALG00010021810	ANKRD13A	2	-0.047941386	0.150747297	-0.318024844	0.750466092	0.025076192	1.456802551	31.35651778	0.5	CRA004775;PRJNA827465	Yang2025_Silkie_Wenchang	0.937393444
ENSGALG00010021812	TEAD1	2	-0.197983273	0.054301534	-3.645997817	0.000266356	0	0.257041817	0	1	PRJNA827465;Yang2025_Silkie_Wenchang	CRA004775	0.015624775
ENSGALG00010021812	TEAD1	2	-0.108120908	0.131465018	-0.822431015	0.410831634	0	0.34643471	0	0.5	CRA004775;Yang2025_Silkie_Wenchang	PRJNA827465	0.83504692
ENSGALG00010021812	TEAD1	2	-0.203254737	0.058470934	-3.476167076	0.000508635	0	0.62770454	0	0.5	CRA004775;PRJNA827465	Yang2025_Silkie_Wenchang	0.023657862
ENSGALG00010021817	EVI5	2	0.066014649	0.093057451	0.709396704	0.478078333	0.009470642	2.07495459	51.80617423	0.5	PRJNA827465;Yang2025_Silkie_Wenchang	CRA004775	0.855366076
ENSGALG00010021817	EVI5	2	0.215516696	0.09583754	2.248771167	0.024527059	0	0.267697293	0	1	CRA004775;Yang2025_Silkie_Wenchang	PRJNA827465	0.267551204
ENSGALG00010021817	EVI5	2	0.097955766	0.143317627	0.683487212	0.494299028	0.02707793	2.508601785	60.13715664	0.5	CRA004775;PRJNA827465	Yang2025_Silkie_Wenchang	0.860175061
ENSGALG00010021821	PPP1R19L	2	0.124709529	0.180697807	0.690155186	0.490096602	0.049955673	3.840423476	73.9612049	0.5	PRJNA827465;Yang2025_Silkie_Wenchang	CRA004775	0.858529056
ENSGALG00010021821	PPP1R19L	2	0.306969628	0.14714893	2.086115255	0.036968184	0	0.148554922	0	1	CRA004775;Yang2025_Silkie_Wenchang	PRJNA827465	0.331653274
ENSGALG00010021821	PPP1R19L	2	-0.010429224	0.070389267	-0.148164979	0.88221257	0	0.690096693	0	0.5	CRA004775;PRJNA827465	Yang2025_Silkie_Wenchang	0.971942333
ENSGALG00010021823	ENSGALG00010021823	2	0.410609096	0.491109068	0.836085348	0.403106891	0.434732452	10.0901639	90.08935821	0.5	PRJNA827465;Yang2025_Silkie_Wenchang	CRA004775	0.833046091
ENSGALG00010021823	ENSGALG00010021823	2	0.162986476	0.743017397	0.219357551	0.826371535	1.054908678	22.39901967	95.5355189	0.5	CRA004775;Yang2025_Silkie_Wenchang	PRJNA827465	0.956393839
ENSGALG00010021823	ENSGALG00010021823	2	-0.319997994	0.251818228	-1.270749922	0.203817629	0.086883091	3.174314431	68.49713468	1	CRA004775;PRJNA827465	Yang2025_Silkie_Wenchang	0.717254253
ENSGALG00010021826	FBXO40	2	-0.250505141	0.1407149	-1.780231813	0.075038031	0	0.933264282	0	1	PRJNA827465;Yang2025_Silkie_Wenchang	CRA004775	0.47268425
ENSGALG00010021826	FBXO40	2	-0.095233216	0.483403131	-0.197005791	0.843823006	0.293348785	2.2605223	55.76243597	0.5	CRA004775;Yang2025_Silkie_Wenchang	PRJNA827465	0.96057435
ENSGALG00010021826	FBXO40	2	-0.036554975	0.267903058	-0.136448516	0.891466722	0.054589584	1.248805891	19.92350397	0.5	CRA004775;PRJNA827465	Yang2025_Silkie_Wenchang	0.973846899
ENSGALG00010021827	CCZ1	2	-0.079572983	0.106402035	-0.747852078	0.454549386	0.018037444	4.648235448	78.48645984	0.5	PRJNA827465;Yang2025_Silkie_Wenchang	CRA004775	0.849072665
ENSGALG00010021827	CCZ1	2	-0.16862863	0.082053595	-2.05510351	0.039869008	0	0.96890687	0	0.5	CRA004775;Yang2025_Silkie_Wenchang	PRJNA827465	0.345604681
ENSGALG00010021827	CCZ1	2	0.015646891	0.045475748	0.344071101	0.730792816	0	0.012632294	0	1	CRA004775;PRJNA827465	Yang2025_Silkie_Wenchang	0.932769533
ENSGALG00010021829	ERCC6	2	0.152398709	0.126571491	1.204052411	0.228569322	0.023495309	3.290224846	69.60694035	1	PRJNA827465;Yang2025_Silkie_Wenchang	CRA004775	0.741270737
ENSGALG00010021829	ERCC6	2	0.183344388	0.164481936	1.114677956	0.264988451	0.025634859	1.803697294	44.55832455	0.5	CRA004775;Yang2025_Silkie_Wenchang	PRJNA827465	0.769274038
ENSGALG00010021829	ERCC6	2	0.0474215	0.050086992	0.946782748	0.343749494	0	0.129715625	0	0.5	CRA004775;PRJNA827465	Yang2025_Silkie_Wenchang	0.806388726
ENSGALG00010021831	ENSGALG00010021831	2	0.127004085	0.213077837	0.596045496	0.551144834	0	0.873214668	0	0.5	PRJNA827465;Yang2025_Silkie_Wenchang	CRA004775	0.878879109
ENSGALG00010021831	ENSGALG00010021831	2	0.121707986	0.257553744	0.472553744	0.63653159	0.018865759	1.134677742	11.86925037	0.5	CRA004775;Yang2025_Silkie_Wenchang	PRJNA827465	0.904566695
ENSGALG00010021831	ENSGALG00010021831	2	-0.173931511	0.270598018	-0.642767127	0.520375217	0	0.077424614	0	1	CRA004775;PRJNA827465	Yang2025_Silkie_Wenchang	0.869331539
ENSGALG00010021835	NUDT8	2	-0.143130184	0.104118627	-1.374683734	0.169229516	0	0.000173126	0	1	PRJNA827465;Yang2025_Silkie_Wenchang	CRA004775	0.670621867
ENSGALG00010021835	NUDT8	2	-0.133187633	0.181890161	-0.732242095	0.464020815	0	0.014449729	0	1	CRA004775;Yang2025_Silkie_Wenchang	PRJNA827465	0.852159649
ENSGALG00010021835	NUDT8	2	-0.137780276	0.114262171	-1.205825828	0.227884654	0	0.015158445	0	1	CRA004775;PRJNA827465	Yang2025_Silkie_Wenchang	0.740853612
ENSGALG00010021837	ENSGALG00010021837	2	0.362797435	0.52056048	0.696936185	0.485842732	0.424850355	4.616224651	78.33727612	0.5	PRJNA827465;Yang2025_Silkie_Wenchang	CRA004775	0.857391223
ENSGALG00010021837	ENSGALG00010021837	2	-0.115157147	1.053210111	-0.109339196	0.912933459	2.00231109	10.11458633	90.1132882	0.5	CRA004775;Yang2025_Silkie_Wenchang	PRJNA827465	0.979071811
ENSGALG00010021837	ENSGALG00010021837	2	-0.581852126	0.524622782	-1.109086655	0.267392801	0.363273033	2.767351868	63.86437115	1	CRA004775;PRJNA827465	Yang2025_Silkie_Wenchang	0.771398294
ENSGALG00010021839	C1orf50	2	-0.039944911	0.081224872	-0.491781765	0.622873629	0	0.55800991	0	1	PRJNA827465;Yang2025_Silkie_Wenchang	CRA004775	0.900261179
ENSGALG00010021839	C1orf50	2	-0.33597414	0.199213167	-1.686505696	0.091698439	0.042308117	2.119944738	52.82895906	1	CRA004775;Yang2025_Silkie_Wenchang	PRJNA827465	0.518914432
ENSGALG00010021839	C1orf50	2	-0.247702833	0.271457294	-0.912492824	0.361509345	0.122764482	5.565832055	82.03323438	1	CRA004775;PRJNA827465	Yang2025_Silkie_Wenchang	0.81550034
ENSGALG00010021840	ENSGALG00010021840	2	0.591888569	0.675128477	0.876705085	0.380646844	0.811661112	9.114333543	89.02827074	0.5	PRJNA827465;Yang2025_Silkie_Wenchang	CRA004775	0.824392515
ENSGALG00010021840	ENSGALG00010021840	2	0.776445302	0.552289387	1.405866781	0.159763687	0.443228805	3.554222396	71.86445054	1	CRA004775;Yang2025_Silkie_Wenchang	PRJNA827465	0.655404348
ENSGALG00010021840	ENSGALG00010021840	2	-0.010231854	0.256405648	-0.039904948	0.968168906	0	0.174774596	0	0.5	CRA004775;PRJNA827465	Yang2025_Silkie_Wenchang	0.991182909
ENSGALG00010021841	CENPT	2	0.040584977	0.133223154	0.304639064	0.760641064	0.023896401	3.010542838	66.78339908	0.5	PRJNA827465;Yang2025_Silkie_Wenchang	CRA004775	0.939384641
ENSGALG00010021841	CENPT	2	-0.167284456	0.450149	-0.371620189	0.710175656	0.312563802	3.675944233	72.79610525	0.5	CRA004775;Yang2025_Silkie_Wenchang	PRJNA827465	0.925364369
ENSGALG00010021841	CENPT	2	-0.251645577	0.288600305	-0.871951875	0.383234632	0.103639471	1.912231627	47.70507997	1	CRA004775;PRJNA827465	Yang2025_Silkie_Wenchang	0.825476201
ENSGALG00010021843	ENSGALG00010021843	2	-0.089688231	0.223910884	-0.400553246	0.688749074	0	2.02335E-07	0	1	PRJNA827465;Yang2025_Silkie_Wenchang	CRA004775	0.919799842
ENSGALG00010021843	ENSGALG00010021843	2	-0.499814961	0.464901867	-1.075097771	0.282330957	0.261035453	2.466257488	59.45273334	1	CRA004775;Yang2025_Silkie_Wenchang	PRJNA827465	0.779519046
ENSGALG00010021843	ENSGALG00010021843	2	-0.47911348	0.461713517	-1.037685625	0.29941644	0.280322209	2.768042433	63.87338618	1	CRA004775;PRJNA827465	Yang2025_Silkie_Wenchang	0.789910488
ENSGALG00010021847	P3H1	2	0.176512254	0.069565886	2.537339262	0.011169865	0	0.306861289	0	1	PRJNA827465;Yang2025_Silkie_Wenchang	CRA004775	0.167907012
ENSGALG00010021847	P3H1	2	-0.059900806	0.204299553	-0.293200868	0.76936862	0.048650949	2.267611712	55.90073932	0.5	CRA004775;Yang2025_Silkie_Wenchang	PRJNA827465	0.941790377
ENSGALG00010021847	P3H1	2	-0.007621681	0.249463085	-0.030552339	0.975626553	0.098096623	4.150407055	75.90597773	0.5	CRA004775;PRJNA827465	Yang2025_Silkie_Wenchang	0.992754467
ENSGALG00010021848	LARGE2	2	-0.042726529	0.071426775	-0.598186443	0.549715536	0	0.742299204	0	0.5	PRJNA827465;Yang2025_Silkie_Wenchang	CRA004775	0.878556837
ENSGALG00010021848	LARGE2	2	0.0051735	0.094584506	0.054697117	0.956379766	0	0.149720899	0	0.5	CRA004775;Yang2025_Silkie_Wenchang	PRJNA827465	0.988540618
ENSGALG00010021848	LARGE2	2	-0.110045501	0.100361456	-1.096491673	0.272863666	0	0.003592351	0	1	CRA004775;PRJNA827465	Yang2025_Silkie_Wenchang	0.773799118
ENSGALG00010021851	ENSGALG00010021851	2	0.933578896	1.024518137	0.911237061	0.362170481	1.977161735	16.95734158	94.10284923	0.5	PRJNA827465;Yang2025_Silkie_Wenchang	CRA004775	0.815579273
ENSGALG00010021851	ENSGALG00010021851	2	1.098923844	0.865445052	1.269778875	0.204163408	1.355023498	10.40366159	90.38799954	1	CRA004775;Yang2025_Silkie_Wenchang	PRJNA827465	0.717271785
ENSGALG00010021851	ENSGALG00010021851	2	0.058889915	0.197288474	0.29849648	0.765324262	0	0.612275858	0	0.5	CRA004775;PRJNA827465	Yang2025_Silkie_Wenchang	0.940779391
ENSGALG00010021854	TAPBPL	2	0.470234839	0.437527211	1.074755642	0.282484145	0.355010568	13.41950915	92.54816261	1	PRJNA827465;Yang2025_Silkie_Wenchang	CRA004775	0.779532565
ENSGALG00010021854	TAPBPL	2	0.451100292	0.52697325	0.856021235	0.391986034	0.459141094	5.502828476	81.82752734	0.5	CRA004775;Yang2025_Silkie_Wenchang	PRJNA827465	0.829251057
ENSGALG00010021854	TAPBPL	2	0.03876565	0.10475544	0.370058583	0.711338841	0	0.197570598	0	0.5	CRA004775;PRJNA827465	Yang2025_Silkie_Wenchang	0.925814638
ENSGALG00010021860	ENSGALG00010021860	2	0.147516983	0.233260081	0.632414181	0.52711627	0.063979422	2.402811624	58.38208913	0.5	PRJNA827465;Yang2025_Silkie_Wenchang	CRA004775	0.870985065
ENSGALG00010021860	ENSGALG00010021860	2	-0.359460992	0.296315097	-1.213103871	0.22509011	0.097196128	2.174414125	54.01060044	1	CRA004775;Yang2025_Silkie_Wenchang	PRJNA827465	0.738087485
ENSGALG00010021860	ENSGALG00010021860	2	-0.137244408	0.532911087	-0.257537159	0.796764133	0.496814432	7.750746856	87.09801754	0.5	CRA004775;PRJNA827465	Yang2025_Silkie_Wenchang	0.949622804
ENSGALG00010021862	PDK1	2	-0.172977331	0.134211624	-1.288840161	0.197453661	0.0305439	6.295463538	84.11554615	1	PRJNA827465;Yang2025_Silkie_Wenchang	CRA004775	0.708952382
ENSGALG00010021862	PDK1	2	-0.294906469	0.090314587	-3.26532489	0.001093386	0	0.734835506	0	1	CRA004775;Yang2025_Silkie_Wenchang	PRJNA827465	0.039938053
ENSGALG00010021862	PDK1	2	-0.050277791	0.049718383	-1.011251542	0.311896053	0	8.81773E-05	0	1	CRA004775;PRJNA827465	Yang2025_Silkie_Wenchang	0.795993742
ENSGALG00010021863	CLDN19	2	0.078445329	0.147273748	0.532649773	0.594276041	0	0.561429065	0	0.5	PRJNA827465;Yang2025_Silkie_Wenchang	CRA004775	0.893334186
ENSGALG00010021863	CLDN19	2	0.482193536	0.435322848	1.107668797	0.26800488	0.249139329	2.428160094	58.81655404	1	CRA004775;Yang2025_Silkie_Wenchang	PRJNA827465	0.771398294
ENSGALG00010021863	CLDN19	2	0.406096881	0.560868368	0.724050248	0.469034887	0.472759275	3.596169122	72.19263149	0.5	CRA004775;PRJNA827465	Yang2025_Silkie_Wenchang	0.85345437
ENSGALG00010021866	SCNN1A	2	0.197488938	0.175010277	1.128441953	0.259133319	0.015323587	1.332279051	24.94064971	1	PRJNA827465;Yang2025_Silkie_Wenchang	CRA004775	0.766314541

ENSGALG00010021866	SCNN1A	2	0.314537141	0.194134724	1.620200318	0.105189253	0	0.466504448	0	0.5	CRA004775;Yang2025_Silkie_Wenchang	PRJNA827465	0.550557098
ENSGALG00010021866	SCNN1A	2	0.004638515	0.206887028	0.02242052	0.982112512	0	0.009629906	0	0.5	CRA004775;PRJNA827465	Yang2025_Silkie_Wenchang	0.995105937
ENSGALG00010021867	CXCL12	2	-0.237879243	0.241966334	-0.983108847	0.32555387	0.106474217	10.92839595	90.84952628	0.5	PRJNA827465;Yang2025_Silkie_Wenchang	CRA004775	0.799987949
ENSGALG00010021867	CXCL12	2	-0.129976752	0.412340797	-0.315216815	0.75259704	0.280286091	4.920207026	79.67565197	0.5	CRA004775;Yang2025_Silkie_Wenchang	PRJNA827465	0.938087082
ENSGALG00010021867	CXCL12	2	0.044952981	0.113376869	0.396491646	0.69174237	0	0.838790625	0	1	CRA004775;PRJNA827465	Yang2025_Silkie_Wenchang	0.920348122
ENSGALG00010021869	GJC3	2	-0.045033307	0.058606294	-0.768403949	0.442247235	0	0.006862218	0	1	PRJNA827465;Yang2025_Silkie_Wenchang	CRA004775	0.845369538
ENSGALG00010021869	GJC3	2	-0.048702429	0.092307303	-0.527611877	0.597768748	0	0.010251682	0	1	CRA004775;Yang2025_Silkie_Wenchang	PRJNA827465	0.894645663
ENSGALG00010021869	GJC3	2	-0.040121904	0.072393565	-0.554219201	0.579428839	0	0.00549785	0	1	CRA004775;PRJNA827465	Yang2025_Silkie_Wenchang	0.887951482
ENSGALG00010021870	ENSGALG00010021870	2	0.017534429	0.135939296	0.128987202	0.897367776	0	0.800431985	0	0.5	PRJNA827465;Yang2025_Silkie_Wenchang	CRA004775	0.97548008
ENSGALG00010021870	ENSGALG00010021870	2	0.069906484	0.148857338	0.469620676	0.63862605	0	0.269830411	0	0.5	CRA004775;Yang2025_Silkie_Wenchang	PRJNA827465	0.905713124
ENSGALG00010021870	ENSGALG00010021870	2	-0.095607247	0.165424957	-0.577949356	0.563298311	0	0.052845228	0	1	CRA004775;PRJNA827465	Yang2025_Silkie_Wenchang	0.882393222
ENSGALG00010021871	ENSGALG00010021871	2	0.161715231	0.345790861	0.467667741	0.640022214	0.216952611	10.74875772	90.69659931	0.5	PRJNA827465;Yang2025_Silkie_Wenchang	CRA004775	0.906064918
ENSGALG00010021871	ENSGALG00010021871	2	0.224834297	0.491540329	0.45740763	0.647378081	0.298603232	1.889913789	47.08753352	0.5	CRA004775;Yang2025_Silkie_Wenchang	PRJNA827465	0.907350807
ENSGALG00010021871	ENSGALG00010021871	2	-0.190458605	0.133506995	-1.426581469	0.153700569	0	0.284661921	0	1	CRA004775;PRJNA827465	Yang2025_Silkie_Wenchang	0.646153969
ENSGALG00010021874	TMCC1	2	-0.06259539	0.058365654	-1.072469619	0.283509153	0	0.385616649	0	1	PRJNA827465;Yang2025_Silkie_Wenchang	CRA004775	0.779568117
ENSGALG00010021874	TMCC1	2	-0.140988447	0.100664209	-1.400581684	0.161339201	0	0.259950703	0	1	CRA004775;Yang2025_Silkie_Wenchang	PRJNA827465	0.657655531
ENSGALG00010021874	TMCC1	2	-0.054153542	0.066849571	-0.810080621	0.417893841	0	0.686152324	0	1	CRA004775;PRJNA827465	Yang2025_Silkie_Wenchang	0.838112595
ENSGALG00010021877	PLEKHN1	2	-0.203950978	0.101988111	-1.999752489	0.045526997	0	0.963121526	0	1	PRJNA827465;Yang2025_Silkie_Wenchang	CRA004775	0.367868629
ENSGALG00010021877	PLEKHN1	2	-0.793110397	0.841043934	-0.943007095	0.345677274	1.219828159	6.578796409	84.79965122	1	CRA004775;Yang2025_Silkie_Wenchang	PRJNA827465	0.807376412
ENSGALG00010021877	PLEKHN1	2	-0.879186307	0.727967655	-1.207727157	0.227152228	0.885051774	5.228457123	80.87389881	1	CRA004775;PRJNA827465	Yang2025_Silkie_Wenchang	0.740605003
ENSGALG00010021879	CREBL2	2	-0.164693081	0.149437375	-1.10208762	0.270423581	0.033652092	3.988573871	74.92838211	1	PRJNA827465;Yang2025_Silkie_Wenchang	CRA004775	0.772529292
ENSGALG00010021879	CREBL2	2	-0.196805094	0.182556685	-1.078049233	0.281011785	0.035337452	1.889698835	47.08151472	0.5	CRA004775;Yang2025_Silkie_Wenchang	PRJNA827465	0.77866654
ENSGALG00010021879	CREBL2	2	-0.016458529	0.083983291	-0.195973852	0.844630632	0	0.105814053	0	0.5	CRA004775;PRJNA827465	Yang2025_Silkie_Wenchang	0.960769014
ENSGALG00010021881	PHF20	2	0.032042275	0.096446749	0.332227635	0.739717378	0.012075348	2.242190208	55.40075072	0.5	PRJNA827465;Yang2025_Silkie_Wenchang	CRA004775	0.934936834
ENSGALG00010021881	PHF20	2	0.220513679	0.110095907	2.002923501	0.0451855	0	0.327687815	0	1	CRA004775;Yang2025_Silkie_Wenchang	PRJNA827465	0.366396888
ENSGALG00010021881	PHF20	2	0.08734997	0.163480078	0.534315685	0.593123142	0.039686306	3.076280442	67.49321075	0.5	CRA004775;PRJNA827465	Yang2025_Silkie_Wenchang	0.892776493
ENSGALG00010021887	NUTF2	2	-0.104040423	0.144191617	-0.721542801	0.470575617	0.030083823	2.934089591	65.91787779	0.5	PRJNA827465;Yang2025_Silkie_Wenchang	CRA004775	0.853744788
ENSGALG00010021887	NUTF2	2	-0.22539012	0.137070347	-1.644339018	0.100106194	0	0.538641865	0	1	CRA004775;Yang2025_Silkie_Wenchang	PRJNA827465	0.54044969
ENSGALG00010021887	NUTF2	2	-0.00128624	0.043357196	-0.029666127	0.976333327	0	0.154455336	0	0.5	CRA004775;PRJNA827465	Yang2025_Silkie_Wenchang	0.99308563
ENSGALG00010021892	NDUFV1	2	-0.051089579	0.067719398	-0.7544305	0.450590763	0	0.139860613	0	1	PRJNA827465;Yang2025_Silkie_Wenchang	CRA004775	0.84760941
ENSGALG00010021892	NDUFV1	2	-0.130230343	0.196695448	-0.662091288	0.507912719	0	0.002733692	0	1	CRA004775;Yang2025_Silkie_Wenchang	PRJNA827465	0.864401731
ENSGALG00010021892	NDUFV1	2	-0.046620404	0.06910559	-0.674625648	0.499913634	0	0.042710307	0	1	CRA004775;PRJNA827465	Yang2025_Silkie_Wenchang	0.862078151
ENSGALG00010021893	FAM43B	2	0.318394747	0.184834251	1.72259603	0.084961603	0	0.111798192	0	1	PRJNA827465;Yang2025_Silkie_Wenchang	CRA004775	0.501162525
ENSGALG00010021893	FAM43B	2	0.186072644	0.219630919	0.847206053	0.39688028	0	0.348198866	0	0.5	CRA004775;Yang2025_Silkie_Wenchang	PRJNA827465	0.831375233
ENSGALG00010021893	FAM43B	2	0.268561743	0.230494315	1.165155606	0.243956012	0	0.671719821	0	0.5	CRA004775;PRJNA827465	Yang2025_Silkie_Wenchang	0.755518239
ENSGALG00010021897	ENSGALG00010021897	2	0.070775505	0.119398223	0.592768493	0.553336096	0	0.670659549	0	0.5	PRJNA827465;Yang2025_Silkie_Wenchang	CRA004775	0.879487916
ENSGALG00010021897	ENSGALG00010021897	2	-0.135772438	0.192709036	-0.704546295	0.48109263	0	0.188924296	0	1	CRA004775;Yang2025_Silkie_Wenchang	PRJNA827465	0.856784795
ENSGALG00010021897	ENSGALG00010021897	2	0.075693879	0.140521643	0.538663493	0.590119068	0.00307043	1.038515377	3.708695894	0.5	CRA004775;PRJNA827465	Yang2025_Silkie_Wenchang	0.892433327
ENSGALG00010021902	LMTK2	2	-0.069929906	0.107463758	-0.650730133	0.515220708	0.016963357	3.301069202	69.70678471	0.5	PRJNA827465;Yang2025_Silkie_Wenchang	CRA004775	0.8671413
ENSGALG00010021902	LMTK2	2	0.258368182	0.504125387	0.512507779	0.608295677	0.456102603	9.17669621	89.10283203	0.5	CRA004775;Yang2025_Silkie_Wenchang	PRJNA827465	0.897204334
ENSGALG00010021902	LMTK2	2	0.349909203	0.390839671	0.895275553	0.370639806	0.262707167	6.223728177	83.93246023	1	CRA004775;PRJNA827465	Yang2025_Silkie_Wenchang	0.821427961
ENSGALG00010021903	MORC2	2	0.106296854	0.167197945	0.635754547	0.524936417	0.04546352	4.719233813	78.81011962	0.5	PRJNA827465;Yang2025_Silkie_Wenchang	CRA004775	0.869907984
ENSGALG00010021903	MORC2	2	0.248406089	0.112974103	2.19878788	0.027893009	0	0.345316021	0	1	CRA004775;Yang2025_Silkie_Wenchang	PRJNA827465	0.284557362
ENSGALG00010021903	MORC2	2	0.001752319	0.077676031	0.022559327	0.982001788	0.005193643	1.326073531	24.58940048	0.5	CRA004775;PRJNA827465	Yang2025_Silkie_Wenchang	0.995091431
ENSGALG00010021905	RBSN	2	0.053810297	0.075663669	0.711177483	0.47697426	0.007010175	2.264180758	55.83391491	0.5	PRJNA827465;Yang2025_Silkie_Wenchang	CRA004775	0.854986476
ENSGALG00010021905	RBSN	2	0.196820644	0.077244707	2.548014619	0.010833793	0	0.490793165	0	1	CRA004775;Yang2025_Silkie_Wenchang	PRJNA827465	0.165874784
ENSGALG00010021905	RBSN	2	0.106329501	0.132915322	0.799979256	0.423722816	0.027562896	3.96980076	74.80981892	0.5	CRA004775;PRJNA827465	Yang2025_Silkie_Wenchang	0.84030043
ENSGALG00010021908	EDC4	2	-0.063633942	0.043159649	-1.474385063	0.140377944	0	0.118821469	0	1	PRJNA827465;Yang2025_Silkie_Wenchang	CRA004775	0.624408593
ENSGALG00010021908	EDC4	2	-0.082407202	0.057741473	-1.427175273	0.15352938	0	0.023239608	0	1	CRA004775;Yang2025_Silkie_Wenchang	PRJNA827465	0.645911732
ENSGALG00010021908	EDC4	2	-0.056195728	0.056232222	-0.999351009	0.317624683	0	0.107838101	0	1	CRA004775;PRJNA827465	Yang2025_Silkie_Wenchang	0.797215846
ENSGALG00010021913	ENSGALG00010021913	2	-0.129627689	0.097479607	-1.329792891	0.183586519	0	0.009317505	0	1	PRJNA827465;Yang2025_Silkie_Wenchang	CRA004775	0.692102743
ENSGALG00010021913	ENSGALG00010021913	2	0.159931627	0.360936273	0.443102118	0.657691873	0.194870752	3.634318354	72.48452385	0.5	CRA004775;Yang2025_Silkie_Wenchang	PRJNA827465	0.910774485
ENSGALG00010021913	ENSGALG00010021913	2	0.159569961	0.348921814	0.45732297	0.647438922	0.186654105	3.746258004	73.30669701	0.5	CRA004775;PRJNA827465	Yang2025_Silkie_Wenchang	0.907350807
ENSGALG00010021916	MRPS25	2	-0.231637706	0.088055142	-2.630598301	0.008523472	0.005930761	1.359818315	26.460764	1	PRJNA827465;Yang2025_Silkie_Wenchang	CRA004775	0.144023217
ENSGALG00010021916	MRPS25	2	-0.149426923	0.282327644	-0.52926777	0.59661971	0.111712296	3.207739861	68.82540221	0.5	CRA004775;Yang2025_Silkie_Wenchang	PRJNA827465	0.894070734
ENSGALG00010021916	MRPS25	2	-0.10081175	0.151548391	-0.665211618	0.50591515	0.025935722	1.679675915	40.46470564	0.5	CRA004775;PRJNA827465	Yang2025_Silkie_Wenchang	0.863927652
ENSGALG00010021919	PARVA	2	-0.270306262	0.264510818	-1.021910045	0.306823478	0.129731891	13.64299836	92.67023294	1	PRJNA827465;Yang2025_Silkie_Wenchang	CRA004775	0.79266003
ENSGALG00010021919	PARVA	2	-0.188103034	0.397620414	-0.473071872	0.636161903	0.269030852	6.190983585	83.84747777	0.5	CRA004775;Yang2025_Silkie_Wenchang	PRJNA827465	0.904466477
ENSGALG00010021919	PARVA	2	0.009132463	0.082376953	0.110861871	0.911725882	0	0.75608944	0	0.5	CRA004775;PRJNA827465	Yang2025_Silkie_Wenchang	0.978895589
ENSGALG00010021922	MRPL51	2	-0.324725107	0.329267751	-0.986203798	0.324033116	0.187399944	6.67027984	85.00812524	1	PRJNA827465;Yang2025_Silkie_Wenchang	CRA004775	0.799600434
ENSGALG00010021922	MRPL51	2	-0.390845837	0.296630374	-1.317619067	0.1876312	0.121817749	3.238155114	69.11821809	1	CRA004775;Yang2025_Silkie_Wenchang	PRJNA827465	0.696888021
ENSGALG00010021922	MRPL51	2	-0.041704531	0.066155058	-0.630405781	0.528429129	0	0.094301793	0	1	CRA004775;PRJNA827465	Yang2025_Silkie_Wenchang	0.871701504
ENSGALG00010021924	ENSGALG00010021924	2	0.988537581	0.450229916	2.195628379	0.028118553	0	0.124718089	0	1	PRJNA827465;Yang2025_Silkie_Wenchang	CRA004775	0.285731308
ENSGALG00010021924	ENSGALG00010021924	2	0.868720891	0.500357886	1.736199058	0.082528644	0	0.443712281	0	1	CRA004775;Yang2025_Silkie_Wenchang	PRJNA827465	0.495285278
ENSGALG00010021924	ENSGALG00010021924	2	0.703953238	0.47525909	1.481198892	0.138553581	0	0.131547573	0	1	CRA004775;PRJNA827465	Yang2025_Silkie_Wenchang	0.622371164
ENSGALG00010021931	ITGA6	2	-0.106594241	0.091747301	-1.16182428	0.24530685	0.010565119	2.684979355	62.75576577	1	PRJNA827465;Yang2025_Silkie_Wenchang	CRA004775	0.75661406
ENSGALG00010021931	ITGA6	2	0.391046196	0.64433573	0.606898202	0.543918471	0.756559442	10.4192342	90.40236566	0.5	CRA004775;Yang2025_Silkie_Wenchang	PRJNA827465	0.876968972
ENSGALG00010021931	ITGA6	2	0.473293721	0.551100295	0.858815946	0.390442069	0.5360516	7.678223096	86.97615337	0.5	CRA004775;PRJNA827465	Yang2025_Silkie_Wenchang	0.829251057
ENSGALG00010021932	RPAP2	2	-0.014741184	0.109460825	-0.134670863	0.892872111	0.017596	3.489967489	71.34643795	0.5	PRJNA827465;Yang2025_Silkie_Wenchang	CRA004775	0.974381724
ENSGALG00010021932	RPAP2	2	0.103592569	0.100323281	1.032587528	0.301796967	0	0.112760904	0	1	CRA004775;Yang2025_Silkie_Wenchang	PRJNA827465	0.791401535
ENSGALG00010021932	RPAP2	2	-0.103867764	0.053190064	-1.95276629	0.050847295	0	0.122934866	0	0.5	CRA004775;PRJNA827465	Yang2025_Silkie_Wenchang	0.390049394
ENSGALG00010021933	PRR14L	2	0.133565449	0.156568133	0.853081956	0.393613857	0.037485987	3.810200949	73.75466508	1	PRJNA827465;Yang2025_Silkie_Wenchang	CRA004775	0.83043598
ENSGALG00010021933	PRR14L	2	0.277553604	0.125829147	2.205797401	0.027398188	0	0.291820221	0	1	CRA004775;Yang2025_Silkie_Wenchang	PRJNA827465	0.282558645
ENSGALG00010021933	PRR14L	2	0.015913676	0.059331675	0.268215528	0.788533423	0	0.534939175	0	1	CRA004775;PRJNA827465	Yang2025_Silkie_Wenchang	0.947048735
ENSGALG00010021934	EFTUD2	2	0.045068473	0.04404907	1.023142454	0.306240497	0	0.25171303	0	1	PRJNA827465;Yang2025_Silkie_Wenchang	CRA004775	0.792485409
ENSGALG00010021934	EFTUD2	2	-0.116030165	0.211748598	-0.547961904	0.583718062	0.076536475	6.61256831	84.87728288	0.5	CRA004775;Yang2025_Silkie_Wenchang	PRJNA827465	0.89056601
ENSGALG00010021934	EFTUD2	2	-0.132928981	0.186244197	-0.713734888	0.475391129	0.058961622	6.119729593	83.65940873	0.5	CRA004775;PRJNA827465	Yang2025_Silkie_Wenchang	0.854462864
ENSGALG00010021935	CETP	2	0.1235821	0.172670616	0.715710076	0.474170388	0	0.018080839	0	1	PRJNA827465;Yang2025_Silkie_Wenchang	CRA004775	0.85444425
ENSGALG00010021935	CETP	2	0.013527839	0.280755829	0.048183644	0.961569885	0	0.323956542	0	0.5	CRA004775;Yang2025_Silkie_Wenchang	PRJNA827465	0.98973612
ENSGALG00010021935	CETP	2	0.097031655	0.198143803	0.489703204	0.624343934	0	0.452768332	0	0.5	CRA004775;PRJNA827465	Yang2025_Silkie_Wenchang	0.900886179
ENSGALG00010021938	CABP2	2	0.707157776	0.270933584	2.610077957	0.009052159	0.123502075	6.270831007	84.05315023	1	PRJNA827465;Yang2025_Silkie_Wenchang	CRA004775	0.149037013
ENSGALG00010021938	CABP2	2	0.418421639	0.582736121	0.71802935	0.47273919	0.634103396	14.9278658	93.30111877	0.5	CRA004775;Yang2025_Silkie_Wenchang	PRJNA827465	0.854086678
ENSGALG00010021938	CABP2	2	0.167495853	0.309871487	0.540533288	0.588829311	0.153278942	4.713927993	78.78626909	0.5	CRA004775;PRJNA827465	Yang2025_Silkie_Wenchang	0.892171138
ENSGALG00010021943	OCM3	2	-0.138767141	0.178468043	-0.777546156	0.436836614	0	0.400551688	0	1	PRJNA827465;Yang2025_Silkie_Wenchang	CRA004775	0.843387647
ENSGALG00010021943	OCM3	2	-0.009033655	0.257598011	-0.035068808	0.972024874	0	0.004552762	0	0.5	CRA004775;Yang2025_Silkie_Wenchang	PRJNA827465	0.991955348
ENSGALG00010021943	OCM3	2	-0.224791319	0.2338869	-0.961111196	0.336496261	0	0.081956371	0	0.5	CRA004775;PRJNA827465	Yang2025_Silkie_Wenchang	0.803806405
ENSGALG00010021945	C1QTNF4	2	-0.055494882	0.107128333	-0.518022456	0.604442601	0	0.150742624	0	1	PRJNA827465;Yang2025_Silkie_Wenchang	CRA004775	0.896163852
ENSGALG00010021945	C1QTNF4	2	-0.076041045	0.170722612	-0.445406993	0.656025659	0	0.421327561	0	0.5	CRA004775;Yang2025_Silkie_Wenchang	PRJNA827465	0.910411938
ENSGALG00010021945	C1QTNF4	2	-0.008644706	0.130066427	-0.066463777	0.947008596	0	0.263159552	0	0.5	CRA004775;PRJNA827465	Yang2025_Silkie_Wenchang	0.986445196
ENSGALG00010021947	CDK2AP2	2	0.078325884	0.201727855	0.388275005	0.697812532	0.046850664	1.959562292	48.96819539	0.5	PRJNA827465;Yang2025_Silkie_Wenchang	CRA004775	0.921422004
ENSGALG00010021947	CDK2AP2	2	0.234806406	0.188496959	1.245677422	0.212882846	0	0.428479486	0	1	CRA004775;Yang2025_Silkie_Wenchang	PRJNA827465	0.725659838
ENSGALG00010021947	CDK2AP2	2	-0.025309907	0.099573396	-0.254183428	0.799353853	0	0.482034513	0	0.5	CRA004775;PRJNA827465	Yang2025_Silkie_Wenchang	0.949922217
ENSGALG00010021949	ENSGALG00010021949	2	-0.245431138	0.156014164	-1.573133695	0.115687864	0	0.23991966	0	1	PRJNA827465;Yang2025_Silkie_Wenchang	CRA004775	0.572716099
ENSGALG00010021949	ENSGALG00010021949	2	0.272785638	0.631357965	0.432061766	0.665696522	0.705259428	8.475625629	88.20146094	0.5	CRA004775;Yang2025_Silkie_Wenchang	PRJNA827465	0.913532078
ENSGALG00010021949	ENSGALG00010021949	2	0.342726172	0.554300623	0.618303782	0.536375109	0.525520761	6.69116171	85.05491209	0.5	CRA004775;PRJNA827465	Yang2025_Silkie_Wenchang	0.874599474
ENSGALG00010021951	PSKH1	2	-0.258621356	0.22375705	-1.155813218	0.247757565	0.091904239	11.90258779	91.59846566	1	PRJNA827465;Yang2025_Silkie_Wenchang	CRA004775	0.7580874
ENSGALG00010021951	PSKH1	2	-0.512642942	0.106020962	-4.835297953	1.32946E-06	0	0.24207886	0	1	CRA004775;Yang2025_Silkie_Wenchang	PRJNA827465	0.000277936
ENSGALG00010021951	PSKH1	2	-0.277463398	0.293847625	-0.944242439	0.345045773	0.13660268	3.994804774	74.96748761	1	CRA004775;PRJNA827465	Yang2025_Silkie_Wenchang	0.806961063
ENSGALG00010021953	PITPNM1	2	0.17817018	0.145000246	1.228757774	0.219162638	0.031052588	3.819068972	73.81560775	1	PRJNA827465;Yang2025_Silkie_Wenchang	CRA004775	0.73285529
ENSGALG00010021953	PITPNM1	2	0.047743556	0.339944048	0.140445337	0.888308139	0.181206938	4.058328243	75.35931201	0.5	CRA004775;Yang2025_Silkie_Wenchang	PRJNA827465	0.973135755
ENSGALG00010021953	PITPNM1	2	-0.046034239	0.162451999	-0.283371332	0.776892202	0.022984385	1.39364278	28.24560107	0.5	CRA004775;PRJNA827465	Yang2025_Silkie_Wenchang	0.944200431
ENSGALG00010021958	BHLHA15	2	-0.01060071	0.471495382	-0.022483169	0.982062538	0.266652083	2.362136601	57.66544578	0.5	PRJNA827465;Yang2025_Silkie_Wenchang	CRA004775	0.995105937
ENSGALG00010021958	BHLHA15	2	0.550239124	0.450552362	1.221254553	0.221989655	0	0.00245976	0	1	CRA004775;Yang2025_Silkie_Wenchang	PRJNA827465	0.735359501
ENSGALG00010021958	BHLHA15	2	-0.265015949	0.321071415	-0.825411224	0.409138165	0.01549082	1.038645924	3.720798738	0.5	CRA004775;PRJNA827465	Yang2025_Silkie_Wenchang	0.834316328
ENSGALG00010021960	CDKN1B	2	0.066441929	0.051737434	1.284213848	0.199067141	0	0.379492892	0	1	PRJNA827465;Yang2025_Silkie_Wenchang	CRA004775	0.71096127
ENSGALG00010021960	CDKN1B	2	0.023944591	0.086601679	0.276491069	0.782170918	0	0.023400076	0	1	CRA004775;Yang2025_Silkie_Wenchang	PRJNA827465	0.945372683
ENSGALG00010021960	CDKN1B	2	0.085683844	0.060101507	1.425652191	0.153968763	0	0.015934139	0	1	CRA004775;PRJNA827465	Yang2025_Silkie_Wenchang	0.646534044
ENSGALG00010021965	PEX16	2	-0.077953339	0.061235165	-1.2730159	0.2030124	0	0.075508375	0	1	PRJNA827465;Yang2025_Silkie_Wenchang	CRA004775	0.716504701
ENSGALG00010021965	PEX16	2	-0.152506673	0.158951781	-0.95945243	0.337330873	0.005574033	1.115271033	10.33569682	1	CRA004775;Yang2025_Silkie_Wenchang	PRJNA827465	0.80415385
ENSGALG00010021965	PEX16	2	-0.115645887	0.087842223	-1.31651822	0.188000167	0.004528149	1.137016322	12.05051494	1	CRA004775;PRJNA827465	Yang2025_Silkie_Wenchang	0.697335841
ENSGALG00010021966	ADGRA1	2	-0.072152399	0.076674105	-0.941026952	0.346691047	0	0.490210249	0	1	PRJNA827465;Yang2025_Silkie_Wenchang	CRA004775	0.808232887
ENSGALG00010021966	ADGRA1	2	-0.130304588	0.13567912	-0.960387917	0.336860017	0	0.315305896	0	0.5	CRA004775;Yang2025_Silkie_Wenchang	PRJNA827465	0.803904536
ENSGALG00010021966	ADGRA1	2	-0.032597694	0.088343482	-0.368988103	0.712136592	0	0.085827084	0	0.5	CRA004775;PRJNA827465	Yang2025_Silkie_Wenchang	0.92597752
ENSGALG00010021969	CCDC103	2	-0.100855258	0.235187841	-0.428828537	0.668048009	0	0.129597442	0	0.5	PRJNA827465;Yang2025_Silkie_Wenchang	CRA004775	0.914120722
ENSGALG00010021969	CCDC103	2	0.014999598	0.348699876	0.043015782	0.965688954	0	0.004231283	0	0.5	CRA004775;Yang2025_Silkie_Wenchang	PRJNA827465	0.990668014
ENSGALG00010021969	CCDC103	2	-0.128135378	0.250868304	-0.510767507	0.609513864	0	0.047847474	0	1	CRA004775;PRJNA827465	Yang2025_Silkie_Wenchang	0.897238578
ENSGALG00010021970	ERGIC3	2	0.00859002	0.043827998	0.195993902	0.844614939	0	0.202251819	0	0.5	PRJNA827465;Yang2025_Silkie_Wenchang	CRA004775	0.960769014
ENSGALG00010021970	ERGIC3	2	-0.092285045	0.115625823	-0.798135253	0.424792006	0.006596913	1.195879031	16.37950213	1	CRA004775;Yang2025_Silkie_Wenchang	PRJNA827465	0.841099792
ENSGALG00010021970	ERGIC3	2	-0.067502949	0.145909371	-0.462636142	0.643625198	0.02624246	1.883767017	46.91487903	0.5	CRA004775;PRJNA827465	Yang2025_Silkie_Wenchang	0.906643868
ENSGALG00010021971	ICOS	2	-0.135000404	0.668682103	-0.20189026	0.840002516	0.603643536	2.962451454	66.24417259	0.5	PRJNA827465;Yang2025_Silkie_Wenchang	CRA004775	0.95957104
ENSGALG00010021971	ICOS	2	-0.500451063	0.412778149	-1.21239718	0.22536038	0	0.616791927	0	1	CRA004775;Yang2025_Silkie_Wenchang	PRJNA827465	0.7385424
ENSGALG00010021971	ICOS	2	0.175993497	0.33582707	0.524059891	0.600236884	0.003456923	1.014880417	1.466223676	0.5	CRA004775;PRJNA827465	Yang2025_Silkie_Wenchang	0.895230519
ENSGALG00010021972	PISD	2	-0.187345276	0.176976648	-1.058587544	0.289787662	0.055504118	8.334615482	88.00184601	1	PRJNA827465;Yang2025_Silkie_Wenchang	CRA004775	0.784067533
ENSGALG00010021972	PISD	2	-0.381004581	0.110028191	-3.462790553	0.000534604	0	0.002978784	0	1	CRA004775;Yang2025_Silkie_Wenchang	PRJNA827465	0.024517568
ENSGALG00010021972	PISD	2	-0.028288581	0.048325849	-0.585371635	0.558297857	0	0.625571789	0	1	CRA004775;PRJNA827465	Yang2025_Silkie_Wenchang	0.880678789
ENSGALG00010021974	GFAP	2	0.081537671	0.848074988	0.096144412	0.923405879	1.17041258	5.214638818	80.82321643	0.5	PRJNA827465;Yang2025_Silkie_Wenchang	CRA004775	0.98201108
ENSGALG00010021974	GFAP	2	0.282601023	0.686917813	0.411404417	0.680776015	0.593352657	2.683910267	62.7409302	0.5	CRA004775;Yang2025_Silkie_Wenchang	PRJNA827465	0.917851751
ENSGALG00010021974	GFAP	2	-0.588704486	0.324292806	-1.815348587	0.069470357	0	0.227695132	0	1	CRA004775;PRJNA827465	Yang2025_Silkie_Wenchang	0.454157258
ENSGALG00010021978	NR2C2	2	-0.051547019	0.053361671	-0.965993335	0.334047516	0	0.829770776	0	1	PRJNA827465;Yang2025_Silkie_Wenchang	CRA004775	0.80304894
ENSGALG00010021978	NR2C2	2	0.048483123	0.223577167	0.216851854	0.8283238	0.074802037	3.80848476	73.74283835	0.5	CRA004775;Yang2025_Silkie_Wenchang	PRJNA827465	0.956777964
ENSGALG00010021978	NR2C2	2	0.082901509	0.155056614	0.534653161	0.592889716	0.032678271	2.542819134	60.67356948	0.5	CRA004775;PRJNA827465	Yang2025_Silkie_Wenchang	0.892776493
ENSGALG00010021980	GLMN	2	-0.258287863	0.267639575	-0.965058562	0.334515481	0.129448454	10.2956938	90.28720143	0.5	PRJNA827465;Yang2025_Silkie_Wenchang	CRA004775	0.803094614
ENSGALG00010021980	GLMN	2	-0.317215393	0.272635951	-1.163512703	0.244621547	0.103182539	2.925614963	65.81915212	0.5	CRA004775;Yang2025_Silkie_Wenchang	PRJNA827465	0.756093682
ENSGALG00010021980	GLMN	2	0.004411475	0.094462844	0.046700639	0.962751821	0	0.006030408	0	1	CRA004775;PRJNA827465	Yang2025_Silkie_Wenchang	0.989980504
ENSGALG00010021988	FAM180B	2	-0.598022741	0.3759506	-1.590695002	0.111678232	0.24108885	6.796636765	85.28684062	1	PRJNA827465;Yang2025_Silkie_Wenchang	CRA004775	0.565091513
ENSGALG00010021988	FAM180B	2	-0.626868072	0.489768015	-1.279928563	0.200570261	0.318703922	2.454618843	59.26047733	0.5	CRA004775;Yang2025_Silkie_Wenchang	PRJNA827465	0.712970384
ENSGALG00010021988	FAM180B	2	-0.196500319	0.192287077	-1.021911205	0.306822929	0	0.186320304	0	0.5	CRA004775;PRJNA827465	Yang2025_Silkie_Wenchang	0.79266003
ENSGALG00010021989	NCAPD2	2	-0.004556084	0.065873631	-0.069164008	0.944859072	0	0.160411823	0	0.5	PRJNA827465;Yang2025_Silkie_Wenchang	CRA004775	0.986080619
ENSGALG00010021989	NCAPD2	2	-0.068451966	0.121288474	-0.564373217	0.572500162	0	0.278171253	0	1	CRA004775;Yang2025_Silkie_Wenchang	PRJNA827465	0.885678394
ENSGALG00010021989	NCAPD2	2	0.00140433	0.075613659	0.018572438	0.98518219	0	0.475055574	0	0.5	CRA004775;PRJNA827465	Yang2025_Silkie_Wenchang	0.995859331
ENSGALG00010021999	ANKRD22	2	-0.450356914	0.640553934	-0.703074153	0.482009539	0.546324709	2.879060304	65.2664448	0.5	PRJNA827465;Yang2025_Silkie_Wenchang	CRA004775	0.85691916
ENSGALG00010021999	ANKRD22	2	-0.418249012	0.820616046	-0.509676863	0.610277865	0.857824665	2.734657302	63.43234675	0.5	CRA004775;Yang2025_Silkie_Wenchang	PRJNA827465	0.897438028
ENSGALG00010021999	ANKRD22	2	0.186510748	0.365433415	0.510382302	0.609783653	0	0.163050067	0	1	CRA004775;PRJNA827465	Yang2025_Silkie_Wenchang	0.897238578
ENSGALG00010022002	KIF18B	2	-0.021598107	0.145614617	-0.148323754	0.88208727	0.027020298	2.615344576	61.76412053	0.5	PRJNA827465;Yang2025_Silkie_Wenchang	CRA004775	0.971928118
ENSGALG00010022002	KIF18B	2	0.116989256	0.149755035	0.781204157	0.434682429	0	0.632699841	0	0.5	CRA004775;Yang2025_Silkie_Wenchang	PRJNA827465	0.842762754
ENSGALG00010022002	KIF18B	2	-0.148091585	0.09372595	-1.580048906	0.114095667	0	0.070451108	0	1	CRA004775;PRJNA827465	Yang2025_Silkie_Wenchang	0.56994179
ENSGALG00010022005	NLRC5	2	0.135959568	0.128739699	1.056081138	0.290931147	0.00612856	1.211264294	17.44163474	1	PRJNA827465;Yang2025_Silkie_Wenchang	CRA004775	0.78467882
ENSGALG00010022005	NLRC5	2	0.063413323	0.260611336	0.243325267	0.807753432	0.086543889	2.711644215	63.12200567	0.5	CRA004775;Yang2025_Silkie_Wenchang	PRJNA827465	0.952496413
ENSGALG00010022005	NLRC5	2	-0.050396015	0.12488553	-0.226509637	0.820735075	0	0.792077955	0	0.5	CRA004775;PRJNA827465	Yang2025_Silkie_Wenchang	0.958454439
ENSGALG00010022006	SFI1	2	0.403806813	0.368031883	1.097206063	0.272551323	0.254909911	16.83688283	94.06065832	1	PRJNA827465;Yang2025_Silkie_Wenchang	CRA004775	0.773495131
ENSGALG00010022006	SFI1	2	0.435858559	0.38356081	1.136347999	0.25581095	0.243534258	5.485152824	81.76896739	1	CRA004775;Yang2025_Silkie_Wenchang	PRJNA827465	0.764478331
ENSGALG00010022006	SFI1	2	0.039279953	0.097257726	0.403874888	0.686309723	0	0.012965619	0	1	CRA004775;PRJNA827465	Yang2025_Silkie_Wenchang	0.919353539
ENSGALG00010022019	BTBD8	2	0.374309192	0.356550618	1.049806599	0.29380704	0.226397165	8.977082052	88.86052289	1	PRJNA827465;Yang2025_Silkie_Wenchang	CRA004775	0.787150519
ENSGALG00010022019	BTBD8	2	0.658007263	0.175676528	3.745561629	0.000179991	0	0.882951981	0	1	CRA004775;Yang2025_Silkie_Wenchang	PRJNA827465	0.011790288
ENSGALG00010022019	BTBD8	2	0.068735358	0.122468239	0.561250479	0.574626795	0	0.667241498	0	1	CRA004775;PRJNA827465	Yang2025_Silkie_Wenchang	0.88613451
ENSGALG00010022021	TECPR1	2	-0.061113988	0.044275965	-1.380250138	0.169413676	0	0.206935977	0	1	PRJNA827465;Yang2025_Silkie_Wenchang	CRA004775	0.670820905
ENSGALG00010022021	TECPR1	2	-0.011525741	0.073926084	-0.15590899	0.876104759	0	0.659187414	0	0.5	CRA004775;Yang2025_Silkie_Wenchang	PRJNA827465	0.970347777
ENSGALG00010022021	TECPR1	2	-0.058796553	0.06048501	-0.972084707	0.33100841	0.000997126	1.03579111	3.45543706	0.5	CRA004775;PRJNA827465	Yang2025_Silkie_Wenchang	0.801722786
ENSGALG00010022022	STAMBPL1	2	-0.107683827	0.382149074	-0.281784868	0.778108474	0.235126299	5.025708625	80.10230846	0.5	PRJNA827465;Yang2025_Silkie_Wenchang	CRA004775	0.944509772
ENSGALG00010022022	STAMBPL1	2	-0.443331545	0.242430663	-1.828677698	0.06744554	0	0.272939917	0	0.5	CRA004775;Yang2025_Silkie_Wenchang	PRJNA827465	0.447353382
ENSGALG00010022022	STAMBPL1	2	0.181016027	0.179967672	1.005825236	0.314499645	0	0.851058166	0	0.5	CRA004775;PRJNA827465	Yang2025_Silkie_Wenchang	0.796531448
ENSGALG00010022024	MICAL2	2	0.15340825	0.290432265	0.528206636	0.597355924	0.150061335	9.037051541	88.93444399	0.5	PRJNA827465;Yang2025_Silkie_Wenchang	CRA004775	0.894395424
ENSGALG00010022024	MICAL2	2	0.446338769	0.142170805	3.139454466	0.001692627	0	0.002849424	0	1	CRA004775;Yang2025_Silkie_Wenchang	PRJNA827465	0.032054246
ENSGALG00010022024	MICAL2	2	-0.118185061	0.126254598	-0.936085204	0.34922932	0	0.492007509	0	0.5	CRA004775;PRJNA827465	Yang2025_Silkie_Wenchang	0.809731578
ENSGALG00010022027	EIF4ENIF1	2	-0.097024297	0.084398356	-1.149599376	0.250308915	0.01101959	4.220371792	76.30540509	1	PRJNA827465;Yang2025_Silkie_Wenchang	CRA004775	0.759943035
ENSGALG00010022027	EIF4ENIF1	2	-0.169300874	0.067140528	-2.52158985	0.011682584	0	0.859377465	0	1	CRA004775;Yang2025_Silkie_Wenchang	PRJNA827465	0.172876725
ENSGALG00010022027	EIF4ENIF1	2	-0.020889169	0.040836477	-0.511532087	0.608978526	0	0.014460864	0	1	CRA004775;PRJNA827465	Yang2025_Silkie_Wenchang	0.897208691
ENSGALG00010022028	FGD5	2	0.080085943	0.077774536	1.029719326	0.303141781	0	0.113987505	0	1	PRJNA827465;Yang2025_Silkie_Wenchang	CRA004775	0.792084776
ENSGALG00010022028	FGD5	2	0.129594991	0.165496024	0.783070116	0.433585939	0.016673592	1.259457639	20.60074359	1	CRA004775;Yang2025_Silkie_Wenchang	PRJNA827465	0.842762754
ENSGALG00010022028	FGD5	2	0.125800631	0.093241796	1.349181729	0.177276868	0.000387581	1.006496258	0.645432942	1	CRA004775;PRJNA827465	Yang2025_Silkie_Wenchang	0.668290452
ENSGALG00010022029	ENSGALG00010022029	2	1.387793883	0.360784145	3.846604412	0.000119766	0	0.107556045	0	1	PRJNA827465;Yang2025_Silkie_Wenchang	CRA004775	0.008808821
ENSGALG00010022029	ENSGALG00010022029	2	0.667515762	0.808753965	0.825363203	0.409165419	0.681459941	1.879349372	46.79009582	0.5	CRA004775;Yang2025_Silkie_Wenchang	PRJNA827465	0.834316328
ENSGALG00010022029	ENSGALG00010022029	2	0.775970307	0.937634951	0.827582628	0.40790691	1.16254794	2.588206613	61.36320822	0.5	CRA004775;PRJNA827465	Yang2025_Silkie_Wenchang	0.833982683
ENSGALG00010022030	ZNF341	2	0.092354196	0.066043928	1.398375284	0.162000401	0	0.032942472	0	1	PRJNA827465;Yang2025_Silkie_Wenchang	CRA004775	0.659256758
ENSGALG00010022030	ZNF341	2	-0.039786646	0.182860413	-0.21757933	0.827756891	0.042926925	2.597766119	61.50538754	0.5	CRA004775;Yang2025_Silkie_Wenchang	PRJNA827465	0.956550752
ENSGALG00010022030	ZNF341	2	-0.041120681	0.167041552	-0.246170374	0.805550348	0.036793329	2.539155681	60.61682994	0.5	CRA004775;PRJNA827465	Yang2025_Silkie_Wenchang	0.951939184
ENSGALG00010022032	KBTBD4	2	-0.044955182	0.044538844	-1.009347733	0.312807896	0	0.3858486	0	1	PRJNA827465;Yang2025_Silkie_Wenchang	CRA004775	0.796054997
ENSGALG00010022032	KBTBD4	2	-0.238990135	0.160722299	-1.486975593	0.137021257	0.02847938	2.093114248	52.22429923	1	CRA004775;Yang2025_Silkie_Wenchang	PRJNA827465	0.619307551
ENSGALG00010022032	KBTBD4	2	-0.193388407	0.199374993	-0.969973238	0.332059832	0.06299225	4.081299612	75.49800076	1	CRA004775;PRJNA827465	Yang2025_Silkie_Wenchang	0.802002658
ENSGALG00010022033	RAPH1	2	0.224331413	0.356505853	0.629250293	0.529185208	0.222730344	7.377780565	86.44578825	0.5	PRJNA827465;Yang2025_Silkie_Wenchang	CRA004775	0.871837903
ENSGALG00010022033	RAPH1	2	0.238425476	0.417176056	0.571522436	0.567645556	0.258578526	3.824825724	73.85501772	0.5	CRA004775;Yang2025_Silkie_Wenchang	PRJNA827465	0.883717772
ENSGALG00010022033	RAPH1	2	-0.097880165	0.069683553	-1.404637978	0.160128957	0	0.114701444	0	1	CRA004775;PRJNA827465	Yang2025_Silkie_Wenchang	0.655509678
ENSGALG00010022034	ENSGALG00010022034	2	-0.071197833	0.074897142	-0.950608132	0.341803339	0	0.862341311	0	0.5	PRJNA827465;Yang2025_Silkie_Wenchang	CRA004775	0.805823616
ENSGALG00010022034	ENSGALG00010022034	2	0.088752301	0.167173479	0.53089941	0.595488487	0	0.012176304	0	1	CRA004775;Yang2025_Silkie_Wenchang	PRJNA827465	0.893794893
ENSGALG00010022034	ENSGALG00010022034	2	-0.088846929	0.078096993	-1.137648521	0.255267277	0	0.256521937	0	0.5	CRA004775;PRJNA827465	Yang2025_Silkie_Wenchang	0.764467569
ENSGALG00010022037	DRG1	2	-0.134904599	0.191798252	-0.703367194	0.481826945	0.055540127	3.426461956	70.81537712	0.5	PRJNA827465;Yang2025_Silkie_Wenchang	CRA004775	0.85691916
ENSGALG00010022037	DRG1	2	-0.313126071	0.155986139	-2.007396768	0.044707433	0	0.26404685	0	1	CRA004775;Yang2025_Silkie_Wenchang	PRJNA827465	0.364399881

ENSGALG00010022037	DRG1	2	0.000792522	0.056435902	0.014042862	0.988795786	0	0.905416128	0	0.5	CRA004775;PRJNA827465	Yang2025_Silkie_Wenchang	0.996891127
ENSGALG00010022038	GAPDH	2	-0.456406853	0.256686343	-1.77807221	0.075391996	0.104825105	4.627191969	78.38862086	1	PRJNA827465;Yang2025_Silkie_Wenchang	CRA004775	0.474101058
ENSGALG00010022038	GAPDH	2	-0.704674146	0.200843292	-3.508576956	0.000450511	0	0.366442763	0	1	CRA004775;Yang2025_Silkie_Wenchang	PRJNA827465	0.02175237
ENSGALG00010022038	GAPDH	2	-0.232875104	0.111118136	-2.095743428	0.036104957	0	0.035919219	0	1	CRA004775;PRJNA827465	Yang2025_Silkie_Wenchang	0.327796981
ENSGALG00010022041	MAPK8IP1	2	-0.064415886	0.088649864	-0.726632659	0.467451016	0	0.524254853	0	0.5	PRJNA827465;Yang2025_Silkie_Wenchang	CRA004775	0.852885838
ENSGALG00010022041	MAPK8IP1	2	0.030395539	0.174767136	0.173920222	0.86192816	0	0.131315907	0	0.5	CRA004775;Yang2025_Silkie_Wenchang	PRJNA827465	0.96567952
ENSGALG00010022041	MAPK8IP1	2	-0.099724983	0.09811568	-1.016402089	0.309437951	0	0.003027274	0	1	CRA004775;PRJNA827465	Yang2025_Silkie_Wenchang	0.794112948
ENSGALG00010022042	AIP	2	0.187438871	0.102574269	1.827347864	0.067647483	0.011817718	2.080559588	51.93600772	1	PRJNA827465;Yang2025_Silkie_Wenchang	CRA004775	0.448431273
ENSGALG00010022042	AIP	2	0.054602006	0.266928492	0.204556679	0.837918505	0.12630212	8.793475778	88.62793251	0.5	CRA004775;Yang2025_Silkie_Wenchang	PRJNA827465	0.959032758
ENSGALG00010022042	AIP	2	-0.033804011	0.159425437	-0.212036494	0.832078564	0.041407684	5.154996874	80.60134614	0.5	CRA004775;PRJNA827465	Yang2025_Silkie_Wenchang	0.957762335
ENSGALG00010022044	PXMP4	2	0.033831217	0.049204961	0.687557026	0.491731778	0	0.012671481	0	1	PRJNA827465;Yang2025_Silkie_Wenchang	CRA004775	0.85895707
ENSGALG00010022044	PXMP4	2	-0.009840864	0.095570007	-0.102970213	0.917986612	0	0.381348538	0	0.5	CRA004775;Yang2025_Silkie_Wenchang	PRJNA827465	0.981033557
ENSGALG00010022044	PXMP4	2	0.02554403	0.053077619	0.481258035	0.630333119	0	0.558256358	0	0.5	CRA004775;PRJNA827465	Yang2025_Silkie_Wenchang	0.902650914
ENSGALG00010022046	CRY2	2	-0.134246635	0.061392006	-2.186711976	0.028765557	0	0.283408431	0	1	PRJNA827465;Yang2025_Silkie_Wenchang	CRA004775	0.288981102
ENSGALG00010022046	CRY2	2	-0.137900686	0.114922796	-1.199941968	0.230161879	0	0.836191744	0	0.5	CRA004775;Yang2025_Silkie_Wenchang	PRJNA827465	0.742845187
ENSGALG00010022046	CRY2	2	-0.103643212	0.06664912	-1.555057484	0.11993238	0	0.466777791	0	0.5	CRA004775;PRJNA827465	Yang2025_Silkie_Wenchang	0.580870455
ENSGALG00010022049	EPHX4	2	1.450590567	0.309179938	4.691735746	2.70897E-06	0	0.13323102	0	1	PRJNA827465;Yang2025_Silkie_Wenchang	CRA004775	0.00051708
ENSGALG00010022049	EPHX4	2	1.599290232	0.458544377	3.487754358	0.000487095	0	0.008737317	0	1	CRA004775;Yang2025_Silkie_Wenchang	PRJNA827465	0.023132362
ENSGALG00010022049	EPHX4	2	1.38168818	0.389210392	3.549977614	0.000385264	0	0.059056449	0	1	CRA004775;PRJNA827465	Yang2025_Silkie_Wenchang	0.019759415
ENSGALG00010022050	ENSGALG00010022050	2	0.672386407	0.450240798	1.493392893	0.135334372	0.320510865	4.561519879	78.07748236	1	PRJNA827465;Yang2025_Silkie_Wenchang	CRA004775	0.616776509
ENSGALG00010022050	ENSGALG00010022050	2	0.418653094	0.744218025	0.562540921	0.573747531	0.991491914	9.50651469	89.48089776	0.5	CRA004775;Yang2025_Silkie_Wenchang	PRJNA827465	0.886016235
ENSGALG00010022050	ENSGALG00010022050	2	0.024005931	0.287689614	0.083443858	0.933498617	0.098753239	2.392148135	58.1965688	0.5	CRA004775;PRJNA827465	Yang2025_Silkie_Wenchang	0.984260547
ENSGALG00010022051	BAIAP2L1	2	0.340590571	0.175433921	1.941417997	0.052207597	0.046837044	4.172559047	76.03389218	1	PRJNA827465;Yang2025_Silkie_Wenchang	CRA004775	0.396812065
ENSGALG00010022051	BAIAP2L1	2	0.190966138	0.103393466	1.84698459	0.064749374	0	0.185993619	0	1	CRA004775;Yang2025_Silkie_Wenchang	PRJNA827465	0.438766317
ENSGALG00010022051	BAIAP2L1	2	0.469225106	0.113317745	4.140791064	3.4611E-05	0	0.73998459	0	1	CRA004775;PRJNA827465	Yang2025_Silkie_Wenchang	0.003696512
ENSGALG00010022052	IFFO1	2	-0.046112901	0.085661618	-0.538314618	0.590359861	0.008395584	2.182065544	54.1718624	0.5	PRJNA827465;Yang2025_Silkie_Wenchang	CRA004775	0.892490001
ENSGALG00010022052	IFFO1	2	-0.173067791	0.097692331	-1.771559643	0.076467684	0	0.30825764	0	1	CRA004775;Yang2025_Silkie_Wenchang	PRJNA827465	0.477200599
ENSGALG00010022052	IFFO1	2	-0.031544918	0.126159733	-0.250039512	0.802556792	0.015212137	1.324365838	24.49216288	0.5	CRA004775;PRJNA827465	Yang2025_Silkie_Wenchang	0.950688375
ENSGALG00010022054	ENSGALG00010022054	2	0.860171653	0.499039304	1.723652124	0.084770012	0.38299717	4.275082126	76.6086365	1	PRJNA827465;Yang2025_Silkie_Wenchang	CRA004775	0.500958903
ENSGALG00010022054	ENSGALG00010022054	2	0.561835948	0.804160268	0.698661659	0.4847635	1.14975915	8.855854703	88.70803515	0.5	CRA004775;Yang2025_Silkie_Wenchang	PRJNA827465	0.857004733
ENSGALG00010022054	ENSGALG00010022054	2	0.072871465	0.301216617	0.241923789	0.808839216	0.009264879	1.052389286	4.978128026	0.5	CRA004775;PRJNA827465	Yang2025_Silkie_Wenchang	0.952595795
ENSGALG00010022055	E2F1	2	0.010344987	0.093326011	0.110847846	0.911737003	0	0.68062637	0	0.5	PRJNA827465;Yang2025_Silkie_Wenchang	CRA004775	0.978895589
ENSGALG00010022055	E2F1	2	-0.118057291	0.183652055	-0.642831309	0.520333565	0	0.111964081	0	1	CRA004775;Yang2025_Silkie_Wenchang	PRJNA827465	0.869331539
ENSGALG00010022055	E2F1	2	0.035065588	0.096294918	0.364147858	0.715747601	0	0.07134268	0	0.5	CRA004775;PRJNA827465	Yang2025_Silkie_Wenchang	0.927131632
ENSGALG00010022058	NLRP3	2	0.155158622	0.111080358	1.396814207	0.162469449	0.011751895	1.818353453	45.00519147	1	PRJNA827465;Yang2025_Silkie_Wenchang	CRA004775	0.660140262
ENSGALG00010022058	NLRP3	2	0.13980905	0.198316764	0.704978476	0.480823631	0.043308194	2.08621143	52.06621986	0.5	CRA004775;Yang2025_Silkie_Wenchang	PRJNA827465	0.856722567
ENSGALG00010022058	NLRP3	2	0.045785143	0.085053	0.538313091	0.590369015	0	0.476498843	0	0.5	CRA004775;PRJNA827465	Yang2025_Silkie_Wenchang	0.892490001
ENSGALG00010022059	NPTX2	2	-0.217571824	0.102088659	-2.131204648	0.033072286	0	0.202997096	0	1	PRJNA827465;Yang2025_Silkie_Wenchang	CRA004775	0.312461555
ENSGALG00010022059	NPTX2	2	0.065207757	0.451755977	0.144342875	0.885229723	0.293467927	3.208520932	68.83299123	0.5	CRA004775;Yang2025_Silkie_Wenchang	PRJNA827465	0.972280795
ENSGALG00010022059	NPTX2	2	0.087719757	0.386859513	0.226748353	0.820619426	0.214470859	2.82917672	64.6540284	0.5	CRA004775;PRJNA827465	Yang2025_Silkie_Wenchang	0.955383907
ENSGALG00010022062	MITF	2	0.16375132	0.072116768	2.270641391	0.023168696	0	0.246170576	0	1	PRJNA827465;Yang2025_Silkie_Wenchang	CRA004775	0.259577892
ENSGALG00010022062	MITF	2	0.298862301	0.164508206	1.816701478	0.069262835	0.017492612	1.262506347	20.79247743	1	CRA004775;Yang2025_Silkie_Wenchang	PRJNA827465	0.453272383
ENSGALG00010022062	MITF	2	0.270432289	0.216174627	1.252423589	0.210415555	0.05637497	1.905656576	47.52464779	1	CRA004775;PRJNA827465	Yang2025_Silkie_Wenchang	0.721968882
ENSGALG00010022065	PABPC1L	2	0.195737229	0.145726069	1.343186092	0.179211724	0.020127855	1.891167275	47.12260447	1	PRJNA827465;Yang2025_Silkie_Wenchang	CRA004775	0.685873949
ENSGALG00010022065	PABPC1L	2	-0.169311844	0.553012635	-0.306162705	0.759480767	0.55269699	10.06594096	90.06550899	0.5	CRA004775;Yang2025_Silkie_Wenchang	PRJNA827465	0.939163539
ENSGALG00010022065	PABPC1L	2	-0.300137037	0.405058635	-0.740971828	0.458710522	0.274822227	5.758702011	82.63497576	0.5	CRA004775;PRJNA827465	Yang2025_Silkie_Wenchang	0.850089963
ENSGALG00010022066	DAD1	2	-0.128792339	0.256511841	-0.502091203	0.615603368	0.107212809	4.603710067	78.27838883	0.5	PRJNA827465;Yang2025_Silkie_Wenchang	CRA004775	0.898886043
ENSGALG00010022066	DAD1	2	-0.523515201	0.155905601	-3.357898603	0.000785374	0	0.202679973	0	1	CRA004775;Yang2025_Silkie_Wenchang	PRJNA827465	0.03200973
ENSGALG00010022066	DAD1	2	-0.221608619	0.331280578	-0.6689454	0.503530308	0.198738298	9.814668969	89.81116935	0.5	CRA004775;PRJNA827465	Yang2025_Silkie_Wenchang	0.863108263
ENSGALG00010022067	TMEM130	2	-0.002429931	0.114909931	-0.021146395	0.983128876	0	0.059368975	0	0.5	PRJNA827465;Yang2025_Silkie_Wenchang	CRA004775	0.995363468
ENSGALG00010022067	TMEM130	2	0.007055824	0.201150876	0.035077274	0.972018123	0	0.135962209	0	0.5	CRA004775;Yang2025_Silkie_Wenchang	PRJNA827465	0.991995348
ENSGALG00010022067	TMEM130	2	-0.02892476	0.127772494	-0.226377046	0.820908179	0	0.066545076	0	1	CRA004775;PRJNA827465	Yang2025_Silkie_Wenchang	0.955548641
ENSGALG00010022069	NOP2	2	0.053391014	0.078589673	0.679364241	0.496907102	0.005613622	1.722686067	41.95111813	0.5	PRJNA827465;Yang2025_Silkie_Wenchang	CRA004775	0.860939846
ENSGALG00010022069	NOP2	2	0.10888907	0.094689229	1.149962576	0.250159286	0	0.955012846	0	0.5	CRA004775;Yang2025_Silkie_Wenchang	PRJNA827465	0.75993411
ENSGALG00010022069	NOP2	2	-0.01115253	0.060761576	-0.183545766	0.854369813	0	0.080519768	0	1	CRA004775;PRJNA827465	Yang2025_Silkie_Wenchang	0.963178635
ENSGALG00010022071	ACTA2	2	-0.040061775	0.128847361	-0.310924296	0.755858174	0.015053959	1.780838681	43.84668244	0.5	PRJNA827465;Yang2025_Silkie_Wenchang	CRA004775	0.938501783
ENSGALG00010022071	ACTA2	2	-0.081973208	0.210159627	-0.390052118	0.696498009	0.031401019	1.328164759	24.70813628	0.5	CRA004775;Yang2025_Silkie_Wenchang	PRJNA827465	0.921041503
ENSGALG00010022071	ACTA2	2	0.084851742	0.109998805	0.771419898	0.440466849	0	0.327638215	0	1	CRA004775;PRJNA827465	Yang2025_Silkie_Wenchang	0.844733855
ENSGALG00010022072	SMTN	2	-0.04901405	0.133230924	-0.367887942	0.712956791	0.023396516	2.926784441	65.83281003	0.5	PRJNA827465;Yang2025_Silkie_Wenchang	CRA004775	0.926124617
ENSGALG00010022072	SMTN	2	0.082166217	0.109595642	0.749721568	0.453422415	0	0.032536949	0	1	CRA004775;Yang2025_Silkie_Wenchang	PRJNA827465	0.848879567
ENSGALG00010022072	SMTN	2	-0.161566225	0.100384239	-1.609478011	0.107511859	0	0.298326687	0	0.5	CRA004775;PRJNA827465	Yang2025_Silkie_Wenchang	0.554966053
ENSGALG00010022073	PTPMT1	2	-0.168517845	0.089394249	-1.885108351	0.059415211	0	0.220282536	0	1	PRJNA827465;Yang2025_Silkie_Wenchang	CRA004775	0.422430541
ENSGALG00010022073	PTPMT1	2	-0.308000005	0.219835287	-1.401048981	0.161199426	0	0.039718446	0	1	CRA004775;Yang2025_Silkie_Wenchang	PRJNA827465	0.657426695
ENSGALG00010022073	PTPMT1	2	-0.163843029	0.09304414	-1.760917228	0.078252414	0	0.272851297	0	1	CRA004775;PRJNA827465	Yang2025_Silkie_Wenchang	0.481684356
ENSGALG00010022076	YWHAB	2	-0.213037467	0.163077021	-1.306361041	0.191429908	0.04977291	15.25466507	93.44462828	1	PRJNA827465;Yang2025_Silkie_Wenchang	CRA004775	0.700915693
ENSGALG00010022076	YWHAB	2	-0.246682148	0.183795284	-1.342157113	0.179545059	0.046694425	2.712364046	63.13179267	0.5	CRA004775;Yang2025_Silkie_Wenchang	PRJNA827465	0.686445472
ENSGALG00010022076	YWHAB	2	-0.054322744	0.037837804	-1.435673799	0.151095184	0	0.067124921	0	0.5	CRA004775;PRJNA827465	Yang2025_Silkie_Wenchang	0.64242966
ENSGALG00010022079	ABI2	2	-0.01017679	0.052516512	-0.193782678	0.846346051	0	0.510674396	0	0.5	PRJNA827465;Yang2025_Silkie_Wenchang	CRA004775	0.96122283
ENSGALG00010022079	ABI2	2	-0.095141968	0.143358727	-0.663665138	0.506905648	0	0.105666177	0	1	CRA004775;Yang2025_Silkie_Wenchang	PRJNA827465	0.863929781
ENSGALG00010022079	ABI2	2	0.002475697	0.054669637	0.045284671	0.963880405	0	0.000657542	0	0.5	CRA004775;PRJNA827465	Yang2025_Silkie_Wenchang	0.990168281
ENSGALG00010022081	FAS	2	0.040671548	0.258793174	0.157158505	0.87511993	0.101424217	4.114919908	75.69819043	0.5	PRJNA827465;Yang2025_Silkie_Wenchang	CRA004775	0.970289963
ENSGALG00010022081	FAS	2	0.256353281	0.156409267	1.638990387	0.101215262	0	0.218063865	0	1	CRA004775;Yang2025_Silkie_Wenchang	PRJNA827465	0.612858001
ENSGALG00010022081	FAS	2	-0.107724855	0.163424888	-0.659170438	0.509786328	0.007640578	1.135549558	11.93691567	0.5	CRA004775;PRJNA827465	Yang2025_Silkie_Wenchang	0.865257661
ENSGALG00010022082	TGFBR3	2	-0.394426362	0.261052724	-1.510906902	0.130812172	0.123440653	10.55224826	90.52334654	1	PRJNA827465;Yang2025_Silkie_Wenchang	CRA004775	0.60657277
ENSGALG00010022082	TGFBR3	2	-0.152171491	0.54854596	-0.277408825	0.781466206	0.544184833	9.924966657	89.9243994	0.5	CRA004775;Yang2025_Silkie_Wenchang	PRJNA827465	0.945235709
ENSGALG00010022082	TGFBR3	2	0.066743286	0.276734968	0.241181252	0.809414641	0.109023898	2.880796327	65.2873759	0.5	CRA004775;PRJNA827465	Yang2025_Silkie_Wenchang	0.952770549
ENSGALG00010022084	SELENOM	2	0.011381129	0.227679235	0.049987559	0.960132302	0.079954392	3.982349271	74.889194	0.5	PRJNA827465;Yang2025_Silkie_Wenchang	CRA004775	0.989338243
ENSGALG00010022084	SELENOM	2	-0.238837932	0.167086967	-1.429422869	0.152882729	0	0.023743479	0	1	CRA004775;Yang2025_Silkie_Wenchang	PRJNA827465	0.644992477
ENSGALG00010022084	SELENOM	2	0.078059558	0.188453184	0.414211936	0.678718901	0.042505765	2.033087761	50.81373174	0.5	CRA004775;PRJNA827465	Yang2025_Silkie_Wenchang	0.917701868
ENSGALG00010022085	MLLT6	2	0.190439986	0.155058344	1.228182769	0.219375365	0.025174003	3.40644115	70.66438423	1	PRJNA827465;Yang2025_Silkie_Wenchang	CRA004775	0.673288275
ENSGALG00010022085	MLLT6	2	0.079508036	0.315683336	0.251860097	0.801149203	0.166216843	5.975563652	83.26517701	0.5	CRA004775;Yang2025_Silkie_Wenchang	PRJNA827465	0.950158029
ENSGALG00010022085	MLLT6	2	-0.032464779	0.14565214	-0.222892568	0.823619121	0.026767174	2.18053804	54.13975901	0.5	CRA004775;PRJNA827465	Yang2025_Silkie_Wenchang	0.955934625
ENSGALG00010022087	SLC35C1	2	-0.176066554	0.193860902	-0.908210741	0.36376689	0.068604247	11.24945252	91.11067851	0.5	PRJNA827465;Yang2025_Silkie_Wenchang	CRA004775	0.817042253
ENSGALG00010022087	SLC35C1	2	-0.369418985	0.093037563	-3.970643383	7.16788E-05	0	0.064828428	0	1	CRA004775;Yang2025_Silkie_Wenchang	PRJNA827465	0.00612433
ENSGALG00010022087	SLC35C1	2	-0.041901352	0.116852699	-0.358582665	0.719907317	0.012805191	1.345432023	25.67443148	0.5	CRA004775;PRJNA827465	Yang2025_Silkie_Wenchang	0.928564173
ENSGALG00010022088	DKK3	2	-0.153188091	0.09085657	-1.686043071	0.091787504	0	0.013412414	0	1	PRJNA827465;Yang2025_Silkie_Wenchang	CRA004775	0.519068239
ENSGALG00010022088	DKK3	2	-0.189833605	0.117768455	-1.611922347	0.106978839	0	0.381967919	0	1	CRA004775;Yang2025_Silkie_Wenchang	PRJNA827465	0.553983328
ENSGALG00010022088	DKK3	2	-0.173910455	0.123027636	-1.413588531	0.157482751	0	0.451089074	0	1	CRA004775;PRJNA827465	Yang2025_Silkie_Wenchang	0.65076873
ENSGALG00010022090	TRRAP	2	-0.08798879	0.045680454	-1.926180262	0.054081869	0	0.069896966	0	1	PRJNA827465;Yang2025_Silkie_Wenchang	CRA004775	0.403227287
ENSGALG00010022090	TRRAP	2	-0.042231514	0.11682116	-0.361505689	0.717721453	0	0.092275968	0	0.5	CRA004775;Yang2025_Silkie_Wenchang	PRJNA827465	0.927927766
ENSGALG00010022090	TRRAP	2	-0.08927432	0.048381946	-1.84519903	0.065008592	0	0.19336658	0	0.5	CRA004775;PRJNA827465	Yang2025_Silkie_Wenchang	0.439539763
ENSGALG00010022093	CHD4	2	0.073581595	0.138374605	0.531756499	0.594894655	0.030736664	4.499445755	77.77504043	0.5	PRJNA827465;Yang2025_Silkie_Wenchang	CRA004775	0.893382718
ENSGALG00010022093	CHD4	2	0.09539127	0.174664566	0.546139794	0.584969847	0.035361447	2.26111697	55.77407037	0.5	CRA004775;Yang2025_Silkie_Wenchang	PRJNA827465	0.891072617
ENSGALG00010022093	CHD4	2	-0.045486365	0.041793118	-1.088369747	0.276431911	0	0.133238981	0	1	CRA004775;PRJNA827465	Yang2025_Silkie_Wenchang	0.775851484
ENSGALG00010022097	INPP5J	2	-0.495292642	0.257738286	-1.921688272	0.054644994	0.099559728	3.962786411	74.76523092	1	PRJNA827465;Yang2025_Silkie_Wenchang	CRA004775	0.404541758
ENSGALG00010022097	INPP5J	2	-0.215419087	0.591497545	-0.364192698	0.715714119	0.612676452	7.753246918	87.10217783	0.5	CRA004775;Yang2025_Silkie_Wenchang	PRJNA827465	0.927131632
ENSGALG00010022097	INPP5J	2	0.000148088	0.323980154	0.000457089	0.999635296	0.140112493	2.66109784	62.42152449	0.5	CRA004775;PRJNA827465	Yang2025_Silkie_Wenchang	0.999889665
ENSGALG00010022098	CDC7	2	0.051544689	0.08186929	0.629597363	0.52895805	0	0.127369508	0	1	PRJNA827465;Yang2025_Silkie_Wenchang	CRA004775	0.871809618
ENSGALG00010022098	CDC7	2	0.108189468	0.129798155	0.833520845	0.404551047	0	0.41380435	0	1	CRA004775;Yang2025_Silkie_Wenchang	PRJNA827465	0.833112102
ENSGALG00010022098	CDC7	2	0.042511776	0.102180018	0.416047842	0.677374995	0	0.565345968	0	1	CRA004775;PRJNA827465	Yang2025_Silkie_Wenchang	0.917144807
ENSGALG00010022100	ENTPD1	2	-0.307426723	0.291500711	-1.054634558	0.291592492	0.154104765	10.72839623	90.67894232	1	PRJNA827465;Yang2025_Silkie_Wenchang	CRA004775	0.785070539
ENSGALG00010022100	ENTPD1	2	-0.609472847	0.118431703	-5.146196782	2.65821E-07	0	0.070803349	0	1	CRA004775;Yang2025_Silkie_Wenchang	PRJNA827465	7.68202E-05
ENSGALG00010022100	ENTPD1	2	-0.273136319	0.332012739	-0.822668189	0.41069671	0.16053011	3.155025767	68.30453778	1	CRA004775;PRJNA827465	Yang2025_Silkie_Wenchang	0.835028074
ENSGALG00010022101	ENSGALG00010022101	2	0.248179426	0.452295198	0.548711166	0.583203682	0.357476227	7.604238693	86.84943963	0.5	PRJNA827465;Yang2025_Silkie_Wenchang	CRA004775	0.890342871
ENSGALG00010022101	ENSGALG00010022101	2	0.166477284	0.619783048	0.26860574	0.788233095	0.623574491	5.157555067	80.61096805	0.5	CRA004775;Yang2025_Silkie_Wenchang	PRJNA827465	0.946992888
ENSGALG00010022101	ENSGALG00010022101	2	-0.202130334	0.142084284	-1.422608667	0.15484963	0	0.479264997	0	1	CRA004775;PRJNA827465	Yang2025_Silkie_Wenchang	0.647505492
ENSGALG00010022104	EXOC3L1	2	0.023526634	0.096914627	0.242756275	0.80819421	0	0.328569392	0	0.5	PRJNA827465;Yang2025_Silkie_Wenchang	CRA004775	0.952496413
ENSGALG00010022104	EXOC3L1	2	-0.159082497	0.167430986	-0.95013773	0.342042274	0	0.4770827	0	1	CRA004775;Yang2025_Silkie_Wenchang	PRJNA827465	0.805904173
ENSGALG00010022104	EXOC3L1	2	-0.038303237	0.162684154	-0.235445407	0.813863047	0.022767332	1.460985244	31.553039	0.5	CRA004775;PRJNA827465	Yang2025_Silkie_Wenchang	0.953869515
ENSGALG00010022105	DLG5	2	0.300182607	0.243325793	1.233665377	0.21732762	0.100830845	5.952460727	83.20022515	1	PRJNA827465;Yang2025_Silkie_Wenchang	CRA004775	0.730896703
ENSGALG00010022105	DLG5	2	0.572895156	0.163422831	3.505600487	0.000455578	0	0.009550302	0	1	CRA004775;Yang2025_Silkie_Wenchang	PRJNA827465	0.021970063
ENSGALG00010022105	DLG5	2	0.228250771	0.209626841	1.088843253	0.276223014	0.060686846	2.376180623	57.91565716	1	CRA004775;PRJNA827465	Yang2025_Silkie_Wenchang	0.77570997
ENSGALG00010022108	USP47	2	0.025127976	0.076899849	0.326762361	0.74384763	0.008645165	3.55566685	71.87588033	0.5	PRJNA827465;Yang2025_Silkie_Wenchang	CRA004775	0.935821071
ENSGALG00010022108	USP47	2	0.249100165	0.182600941	1.364177883	0.172511587	0.047012603	2.842918946	64.8248853	1	CRA004775;Yang2025_Silkie_Wenchang	PRJNA827465	0.675388277
ENSGALG00010022108	USP47	2	0.184284154	0.264773992	0.696005498	0.486425387	0.119663588	5.995137292	83.31981486	0.5	CRA004775;PRJNA827465	Yang2025_Silkie_Wenchang	0.857603818
ENSGALG00010022111	PCGF2	2	0.193116105	0.326751276	0.591018671	0.554507911	0.170896789	4.345484097	76.98760419	0.5	PRJNA827465;Yang2025_Silkie_Wenchang	CRA004775	0.880223402
ENSGALG00010022111	PCGF2	2	0.475168873	0.243264765	1.953299212	0.050784151	0	0.382770096	0	1	CRA004775;Yang2025_Silkie_Wenchang	PRJNA827465	0.390032349
ENSGALG00010022111	PCGF2	2	-0.058445409	0.086124825	-0.678612799	0.497383231	0	0.75761423	0	0.5	CRA004775;PRJNA827465	Yang2025_Silkie_Wenchang	0.86098656
ENSGALG00010022115	E2F4	2	-0.025019864	0.057806697	-0.432819471	0.665145927	0.001256045	1.114732103	10.29234763	0.5	PRJNA827465;Yang2025_Silkie_Wenchang	CRA004775	0.913399315
ENSGALG00010022115	E2F4	2	0.004596235	0.12036006	0.038187378	0.969538285	0.005539275	1.227669653	18.54486278	0.5	CRA004775;Yang2025_Silkie_Wenchang	PRJNA827465	0.991572914
ENSGALG00010022115	E2F4	2	-0.058089245	0.050069331	-1.160176175	0.245977085	0	0.241186893	0	1	CRA004775;PRJNA827465	Yang2025_Silkie_Wenchang	0.75661406
ENSGALG00010022116	CYP20A1	2	-0.073108795	0.064318764	-1.136663541	0.255678966	0.001020313	1.108637362	9.799179185	1	PRJNA827465;Yang2025_Silkie_Wenchang	CRA004775	0.764478331
ENSGALG00010022116	CYP20A1	2	-0.18098361	0.094455623	-1.91607027	0.055356152	0	0.001760782	0	1	CRA004775;Yang2025_Silkie_Wenchang	PRJNA827465	0.407427498
ENSGALG00010022116	CYP20A1	2	-0.05828769	0.062598024	-0.931142652	0.351779778	0	0.803993691	0	1	CRA004775;PRJNA827465	Yang2025_Silkie_Wenchang	0.810678834
ENSGALG00010022117	FAM20C	2	-0.115290241	0.069057989	-1.669470006	0.09502427	0	0.857824145	0	0.5	PRJNA827465;Yang2025_Silkie_Wenchang	CRA004775	0.527729105
ENSGALG00010022117	FAM20C	2	0.011966494	0.130123185	0.091962816	0.926727583	0	0.022330862	0	1	CRA004775;Yang2025_Silkie_Wenchang	PRJNA827465	0.9828798
ENSGALG00010022117	FAM20C	2	-0.136835484	0.075973205	-1.801101892	0.071686822	0	0.431176567	0	0.5	CRA004775;PRJNA827465	Yang2025_Silkie_Wenchang	0.461921937
ENSGALG00010022122	PSMB3	2	0.041450325	0.055041355	0.75307603	0.451404228	0	0.247212919	0	0.5	PRJNA827465;Yang2025_Silkie_Wenchang	CRA004775	0.847847413
ENSGALG00010022122	PSMB3	2	-0.21828281	0.209528212	-1.041782432	0.297512561	0.04542424	2.014989787	50.3719569	1	CRA004775;Yang2025_Silkie_Wenchang	PRJNA827465	0.788562929
ENSGALG00010022122	PSMB3	2	-0.152923485	0.252052157	-0.606713652	0.544040961	0.101584416	4.237664167	76.40209416	0.5	CRA004775;PRJNA827465	Yang2025_Silkie_Wenchang	0.876968972
ENSGALG00010022124	ENSGALG00010022124	2	0.035533448	0.096539485	0.368071655	0.712819806	0	0.034981138	0	1	PRJNA827465;Yang2025_Silkie_Wenchang	CRA004775	0.926097326
ENSGALG00010022124	ENSGALG00010022124	2	-0.115965848	0.196333557	-0.590657296	0.554750066	0.032325864	1.717192381	41.76540666	0.5	CRA004775;Yang2025_Silkie_Wenchang	PRJNA827465	0.880223402
ENSGALG00010022124	ENSGALG00010022124	2	-0.095810237	0.16683516	-0.574280845	0.565777769	0.030447046	1.981178295	49.52498709	0.5	CRA004775;PRJNA827465	Yang2025_Silkie_Wenchang	0.883487517
ENSGALG00010022125	CBFA2T2	2	-0.066540245	0.122542773	-0.542996073	0.587132506	0.017239048	2.232418957	55.20554075	0.5	PRJNA827465;Yang2025_Silkie_Wenchang	CRA004775	0.891596903
ENSGALG00010022125	CBFA2T2	2	0.094773988	0.125330174	0.756194497	0.449532592	0	0.032460549	0	1	CRA004775;Yang2025_Silkie_Wenchang	PRJNA827465	0.847263197
ENSGALG00010022125	CBFA2T2	2	-0.119985486	0.110103484	-1.089751949	0.275822426	0.006456328	1.161705406	13.91965684	0.5	CRA004775;PRJNA827465	Yang2025_Silkie_Wenchang	0.7754953
ENSGALG00010022129	ELMO3	2	0.364192075	0.289101228	1.259738938	0.207763554	0.147132419	8.325595309	87.98884689	1	PRJNA827465;Yang2025_Silkie_Wenchang	CRA004775	0.720154541
ENSGALG00010022129	ELMO3	2	0.30691068	0.419243282	0.732058672	0.464132758	0.276342363	4.209384607	76.2435583	0.5	CRA004775;Yang2025_Silkie_Wenchang	PRJNA827465	0.852159649
ENSGALG00010022129	ELMO3	2	0.052509397	0.123424108	0.425438739	0.670516871	0	0.449376959	0	0.5	CRA004775;PRJNA827465	Yang2025_Silkie_Wenchang	0.914482883
ENSGALG00010022134	CH25H	2	-0.476116444	0.229751753	-2.07230821	0.038236707	0.064030064	2.46593793	59.44747887	1	PRJNA827465;Yang2025_Silkie_Wenchang	CRA004775	0.337384449
ENSGALG00010022134	CH25H	2	-0.497964371	0.282487984	-1.762780719	0.077937481	0.079952406	1.959678073	48.97121043	1	CRA004775;Yang2025_Silkie_Wenchang	PRJNA827465	0.480724545
ENSGALG00010022134	CH25H	2	-0.254293934	0.150147432	-1.693628255	0.090335946	0	0.084475259	0	1	CRA004775;PRJNA827465	Yang2025_Silkie_Wenchang	0.515982221
ENSGALG00010022135	CHST1	2	0.09241176	0.151005362	0.611976681	0.540553186	0	0.10480076	0	1	PRJNA827465;Yang2025_Silkie_Wenchang	CRA004775	0.875579799
ENSGALG00010022135	CHST1	2	-0.004761268	0.219824763	-0.02165938	0.982719666	0	0.019022743	0	0.5	CRA004775;Yang2025_Silkie_Wenchang	PRJNA827465	0.995331299
ENSGALG00010022135	CHST1	2	0.091280567	0.162016184	0.563404008	0.573159809	0	0.172793504	0	0.5	CRA004775;PRJNA827465	Yang2025_Silkie_Wenchang	0.88575805
ENSGALG00010022137	PDDC1	2	-0.104592039	0.186676928	-0.560283697	0.575285945	0.055047277	4.197536296	76.17650142	0.5	PRJNA827465;Yang2025_Silkie_Wenchang	CRA004775	0.886476261
ENSGALG00010022137	PDDC1	2	-0.322273686	0.145194493	-2.219599926	0.026445937	0	0.006931676	0	1	CRA004775;Yang2025_Silkie_Wenchang	PRJNA827465	0.277470548
ENSGALG00010022137	PDDC1	2	-0.038394142	0.153082803	-0.250806367	0.801963819	0.027591887	1.785505494	43.99345152	0.5	CRA004775;PRJNA827465	Yang2025_Silkie_Wenchang	0.950530276
ENSGALG00010022138	SPAG17	2	0.212090495	0.137462891	1.542892722	0.122856807	0.002517813	1.058329559	5.511474017	1	PRJNA827465;Yang2025_Silkie_Wenchang	CRA004775	0.587708865
ENSGALG00010022138	SPAG17	2	-0.134908655	0.690703751	-0.195320577	0.845141989	0.729581081	3.737888144	73.2469255	0.5	CRA004775;Yang2025_Silkie_Wenchang	PRJNA827465	0.960863849
ENSGALG00010022138	SPAG17	2	-0.229473422	0.516740091	-0.444078999	0.65698547	0.367622108	2.485405641	59.76511908	0.5	CRA004775;PRJNA827465	Yang2025_Silkie_Wenchang	0.910695405
ENSGALG00010022142	TMEM208	2	-0.220943553	0.358685079	-0.615982002	0.537906398	0.211587949	4.893536174	79.56487978	0.5	PRJNA827465;Yang2025_Silkie_Wenchang	CRA004775	0.87481821
ENSGALG00010022142	TMEM208	2	-0.466617186	0.230276311	-2.026336034	0.042730361	0	0.692850278	0	1	CRA004775;Yang2025_Silkie_Wenchang	PRJNA827465	0.357546035
ENSGALG00010022142	TMEM208	2	0.055240522	0.085806638	0.643779123	0.519718674	0.001421502	1.02437363	2.379369157	0.5	CRA004775;PRJNA827465	Yang2025_Silkie_Wenchang	0.869101946
ENSGALG00010022143	MATCAP1	2	-0.00068584	0.079315978	-0.008646937	0.993100828	0	0.068748667	0	0.5	PRJNA827465;Yang2025_Silkie_Wenchang	CRA004775	0.99794341
ENSGALG00010022143	MATCAP1	2	-0.187564225	0.255364139	-0.73449712	0.462645815	0.080930277	2.604423148	61.60378161	0.5	CRA004775;Yang2025_Silkie_Wenchang	PRJNA827465	0.851042238
ENSGALG00010022143	MATCAP1	2	-0.178351035	0.220078936	-0.810395753	0.417712758	0.069363321	3.007386972	66.74854252	1	CRA004775;PRJNA827465	Yang2025_Silkie_Wenchang	0.838112595
ENSGALG00010022144	SNTA1	2	0.104353043	0.264702924	0.394227014	0.693413443	0.121793362	7.340671536	86.37726814	0.5	PRJNA827465;Yang2025_Silkie_Wenchang	CRA004775	0.920721644
ENSGALG00010022144	SNTA1	2	0.046762508	0.425858223	0.10980769	0.912561892	0.26937775	3.445586365	70.97736367	0.5	CRA004775;Yang2025_Silkie_Wenchang	PRJNA827465	0.978967245
ENSGALG00010022144	SNTA1	2	-0.1543532	0.088431512	-1.745454721	0.080905748	0	0.583946881	0	1	CRA004775;PRJNA827465	Yang2025_Silkie_Wenchang	0.489847627
ENSGALG00010022148	TALDO1	2	-0.141043692	0.309952218	-0.455049792	0.649073417	0.173032447	9.583398277	89.56528811	0.5	PRJNA827465;Yang2025_Silkie_Wenchang	CRA004775	0.907783669
ENSGALG00010022148	TALDO1	2	-0.501367043	0.164448514	-3.048778192	0.002297741	0	0.0961351	0	1	CRA004775;Yang2025_Silkie_Wenchang	PRJNA827465	0.063663608
ENSGALG00010022148	TALDO1	2	-0.145379002	0.364114875	-0.399266856	0.689696586	0.212056591	4.206039216	76.22466295	0.5	CRA004775;PRJNA827465	Yang2025_Silkie_Wenchang	0.919979266
ENSGALG00010022149	NBEAL1	2	0.132651117	0.077339147	1.715187231	0.086310905	0.003385157	1.333038015	24.98338468	1	PRJNA827465;Yang2025_Silkie_Wenchang	CRA004775	0.505706248
ENSGALG00010022149	NBEAL1	2	0.236763299	0.11511736	2.056712369	0.039713902	0	0.026151157	0	1	CRA004775;Yang2025_Silkie_Wenchang	PRJNA827465	0.344755224
ENSGALG00010022149	NBEAL1	2	0.082853821	0.072774563	1.138499743	0.254911866	0	0.081573738	0	1	CRA004775;PRJNA827465	Yang2025_Silkie_Wenchang	0.764444699
ENSGALG00010022150	CPNE2	2	-0.044376756	0.07164142	-0.619428755	0.535633943	0	0.129183487	0	1	PRJNA827465;Yang2025_Silkie_Wenchang	CRA004775	0.874215303
ENSGALG00010022150	CPNE2	2	-0.224189166	0.193690794	-1.157459071	0.247084852	0.025613588	1.398407834	28.49010311	1	CRA004775;Yang2025_Silkie_Wenchang	PRJNA827465	0.757459165
ENSGALG00010022150	CPNE2	2	-0.180850459	0.2254105	-0.802316037	0.422370168	0.066636983	2.24377282	55.43220815	1	CRA004775;PRJNA827465	Yang2025_Silkie_Wenchang	0.839704389
ENSGALG00010022151	PIP4K2B	2	-0.12659659	0.063417614	-1.996237039	0.045908128	0	0.381938333	0	1	PRJNA827465;Yang2025_Silkie_Wenchang	CRA004775	0.369489827
ENSGALG00010022151	PIP4K2B	2	-0.194270579	0.103904714	-1.869699378	0.061525576	0	0.034700359	0	1	CRA004775;Yang2025_Silkie_Wenchang	PRJNA827465	0.42852024
ENSGALG00010022151	PIP4K2B	2	-0.112110451	0.072882291	-1.538239936	0.12398996	0	0.272495923	0	1	CRA004775;PRJNA827465	Yang2025_Silkie_Wenchang	0.59047345
ENSGALG00010022152	ZNF644	2	0.047972543	0.053434068	0.897789456	0.369297807	0	0.355355043	0	1	PRJNA827465;Yang2025_Silkie_Wenchang	CRA004775	0.820606801
ENSGALG00010022152	ZNF644	2	0.406010174	0.342822124	1.184317305	0.236287481	0.203299807	6.924570328	85.55867075	1	CRA004775;Yang2025_Silkie_Wenchang	PRJNA827465	0.748669015
ENSGALG00010022152	ZNF644	2	0.370723257	0.377848358	0.981142961	0.326522247	0.256638201	9.142618513	89.06221452	1	CRA004775;PRJNA827465	Yang2025_Silkie_Wenchang	0.800028239
ENSGALG00010022154	CPN1	2	0.344794745	0.295170728	1.16811971	0.242758481	0.12300671	3.148678469	68.24064414	1	PRJNA827465;Yang2025_Silkie_Wenchang	CRA004775	0.7550024
ENSGALG00010022154	CPN1	2	-0.32708994	0.551598423	-0.592985633	0.553190767	0.461773779	3.457117956	71.07417181	0.5	CRA004775;Yang2025_Silkie_Wenchang	PRJNA827465	0.87934426
ENSGALG00010022154	CPN1	2	-0.095287515	0.866924915	-0.109914381	0.912477277	1.293567843	6.857824689	85.4181166	0.5	CRA004775;PRJNA827465	Yang2025_Silkie_Wenchang	0.978967245
ENSGALG00010022157	EPS8L2	2	0.045895846	0.166752811	0.275232815	0.783137377	0	0.195566594	0	0.5	PRJNA827465;Yang2025_Silkie_Wenchang	CRA004775	0.945669422
ENSGALG00010022157	EPS8L2	2	-0.002129923	0.233148382	-0.009135483	0.99271104	0	0.108752772	0	0.5	CRA004775;Yang2025_Silkie_Wenchang	PRJNA827465	0.997865013
ENSGALG00010022157	EPS8L2	2	0.097629386	0.179731039	0.543197138	0.586994077	0	3.04046E-05	0	1	CRA004775;PRJNA827465	Yang2025_Silkie_Wenchang	0.891596903
ENSGALG00010022158	CWC25	2	0.119466705	0.096604441	1.236658525	0.216213882	0.011458039	2.49732469	59.95714919	1	PRJNA827465;Yang2025_Silkie_Wenchang	CRA004775	0.72989044
ENSGALG00010022158	CWC25	2	0.087729009	0.175983422	0.498507236	0.61812657	0.041255232	2.783013859	64.06773194	0.5	CRA004775;Yang2025_Silkie_Wenchang	PRJNA827465	0.899121942
ENSGALG00010022158	CWC25	2	0.017515932	0.064653813	0.270918787	0.786453495	0	0.659959519	0	0.5	CRA004775;PRJNA827465	Yang2025_Silkie_Wenchang	0.946651901
ENSGALG00010022159	KIAA2013	2	-0.097539491	0.12632795	-0.772113306	0.440047322	0.028533948	9.075986211	88.98191363	0.5	PRJNA827465;Yang2025_Silkie_Wenchang	CRA004775	0.844353254
ENSGALG00010022159	KIAA2013	2	-0.215845699	0.072045434	-2.995966373	0.002735766	0	0.539993416	0	1	CRA004775;Yang2025_Silkie_Wenchang	PRJNA827465	0.071349576
ENSGALG00010022159	KIAA2013	2	0.018762163	0.036209286	0.51815888	0.604347422	0	0.088546415	0	0.5	CRA004775;PRJNA827465	Yang2025_Silkie_Wenchang	0.89612415

ENSGALG00010022161	CELF1	2	-0.070463	0.056184556	-1.254134685	0.209793055	0	0.961605274	0	1	PRJNA827465;Yang2025_Silkie_Wenchang	CRA004775	0.721625118
ENSGALG00010022161	CELF1	2	0.160897834	0.3353806	0.479746992	0.631407309	0.208574228	13.69904718	92.70022224	0.5	CRA004775;Yang2025_Silkie_Wenchang	PRJNA827465	0.903058174
ENSGALG00010022161	CELF1	2	0.216245716	0.269978725	0.800973175	0.423147176	0.134432974	12.44960377	91.96761585	0.5	CRA004775;PRJNA827465	Yang2025_Silkie_Wenchang	0.840074427
ENSGALG00010022162	POLE	2	0.053904742	0.073882536	0.729600594	0.465634359	0	0.149206419	0	1	PRJNA827465;Yang2025_Silkie_Wenchang	CRA004775	0.852424459
ENSGALG00010022162	POLE	2	-0.011109228	0.125336787	-0.088635012	0.929371982	0	0.773529409	0	0.5	CRA004775;Yang2025_Silkie_Wenchang	PRJNA827465	0.983656242
ENSGALG00010022162	POLE	2	0.060139915	0.089259898	0.673761867	0.500462722	0	0.987334784	0	0.5	CRA004775;PRJNA827465	Yang2025_Silkie_Wenchang	0.862400704
ENSGALG00010022164	PDGFA	2	-0.073514596	0.055953831	-1.31384384	0.188898761	0	0.157080912	0	1	PRJNA827465;Yang2025_Silkie_Wenchang	CRA004775	0.698162702
ENSGALG00010022164	PDGFA	2	0.216488781	0.307003147	0.705167956	0.48070572	0.153713592	4.857695846	79.41410842	0.5	CRA004775;Yang2025_Silkie_Wenchang	PRJNA827465	0.856722567
ENSGALG00010022164	PDGFA	2	0.19422419	0.330422979	0.587804731	0.556663368	0.185229766	5.869889231	82.96390339	0.5	CRA004775;PRJNA827465	Yang2025_Silkie_Wenchang	0.880358001
ENSGALG00010022165	FAM192A	2	0.023511324	0.086239834	0.272627193	0.785139807	0.009448021	2.350185702	57.45017089	0.5	PRJNA827465;Yang2025_Silkie_Wenchang	CRA004775	0.946325593
ENSGALG00010022165	FAM192A	2	0.155162005	0.098164702	1.580629315	0.113962819	0	0.068555951	0	1	CRA004775;Yang2025_Silkie_Wenchang	PRJNA827465	0.569567735
ENSGALG00010022165	FAM192A	2	-0.009637064	0.079800554	-0.120764378	0.903877666	0.005323958	1.214921254	17.69013863	0.5	CRA004775;PRJNA827465	Yang2025_Silkie_Wenchang	0.977333734
ENSGALG00010022166	SDHB	2	-0.232481174	0.159060078	-1.461593485	0.143852641	0.037633976	3.557582952	71.89102788	1	PRJNA827465;Yang2025_Silkie_Wenchang	CRA004775	0.630832183
ENSGALG00010022166	SDHB	2	-0.359924191	0.149082607	-2.414260115	0.015767202	0.002028293	1.024522438	2.393548204	1	CRA004775;Yang2025_Silkie_Wenchang	PRJNA827465	0.207812987
ENSGALG00010022166	SDHB	2	-0.097163032	0.070473539	-1.378716505	0.167982179	0	0.053068444	0	1	CRA004775;PRJNA827465	Yang2025_Silkie_Wenchang	0.668442195
ENSGALG00010022167	WDR3	2	-0.031226574	0.06319038	-0.494166586	0.621188535	0	0.370805159	0	1	PRJNA827465;Yang2025_Silkie_Wenchang	CRA004775	0.899614409
ENSGALG00010022167	WDR3	2	-0.116714267	0.105325723	-1.108126902	0.267807014	0	0.053036228	0	1	CRA004775;Yang2025_Silkie_Wenchang	PRJNA827465	0.771398294
ENSGALG00010022167	WDR3	2	-0.028817042	0.06739815	-0.427564287	0.668968372	0	0.518052851	0	1	CRA004775;PRJNA827465	Yang2025_Silkie_Wenchang	0.914377769
ENSGALG00010022169	TOX2	2	0.134812809	0.40031205	0.336769299	0.736290818	0.260073903	4.722246596	78.82363871	0.5	PRJNA827465;Yang2025_Silkie_Wenchang	CRA004775	0.933661344
ENSGALG00010022169	TOX2	2	0.140312539	0.521601966	0.269003086	0.787927308	0.36669953	2.998209784	66.6467635	0.5	CRA004775;Yang2025_Silkie_Wenchang	PRJNA827465	0.946919116
ENSGALG00010022169	TOX2	2	-0.215654297	0.115215919	-1.871740463	0.061242519	0	0.219342444	0	1	CRA004775;PRJNA827465	Yang2025_Silkie_Wenchang	0.427802453
ENSGALG00010022170	RSPRY1	2	0.052121103	0.047218297	1.103832763	0.26966569	0	0.913619065	0	0.5	PRJNA827465;Yang2025_Silkie_Wenchang	CRA004775	0.77189992
ENSGALG00010022170	RSPRY1	2	-0.033312982	0.094293342	-0.353290926	0.723870348	0	0.008682059	0	1	CRA004775;Yang2025_Silkie_Wenchang	PRJNA827465	0.930264404
ENSGALG00010022170	RSPRY1	2	0.069595744	0.051301028	1.356614983	0.174903579	0	0.168684918	0	0.5	CRA004775;PRJNA827465	Yang2025_Silkie_Wenchang	0.679636281
ENSGALG00010022171	FOXP1	2	0.154345794	0.208980912	0.738564075	0.460171746	0.06377578	3.161037488	68.36481681	0.5	PRJNA827465;Yang2025_Silkie_Wenchang	CRA004775	0.850111874
ENSGALG00010022171	FOXP1	2	0.216213115	0.245080717	0.88221186	0.37766225	0.050527677	1.694563408	40.9877497	0.5	CRA004775;Yang2025_Silkie_Wenchang	PRJNA827465	0.82325159
ENSGALG00010022171	FOXP1	2	-0.011039692	0.074879547	-0.147432682	0.882790511	0	0.042399563	0	1	CRA004775;PRJNA827465	Yang2025_Silkie_Wenchang	0.972001076
ENSGALG00010022172	GIPC2	2	0.070548197	0.14130123	0.499275182	0.617585536	0.020389223	1.926901502	48.10321135	0.5	PRJNA827465;Yang2025_Silkie_Wenchang	CRA004775	0.899121942
ENSGALG00010022172	GIPC2	2	0.374396128	0.551732262	0.678588299	0.497402124	0.506911055	5.515378836	81.86953664	0.5	CRA004775;Yang2025_Silkie_Wenchang	PRJNA827465	0.860986561
ENSGALG00010022172	GIPC2	2	0.482067012	0.395864794	1.217756719	0.223316436	0.235494747	3.307443139	69.76516427	1	CRA004775;PRJNA827465	Yang2025_Silkie_Wenchang	0.736744216
ENSGALG00010022173	RPL23	2	-0.062621343	0.05683265	-1.101855057	0.27052469	0	0.066085192	0	1	PRJNA827465;Yang2025_Silkie_Wenchang	CRA004775	0.772536273
ENSGALG00010022173	RPL23	2	-0.117899138	0.152672646	-0.77223485	0.439975344	0	0.009385106	0	1	CRA004775;Yang2025_Silkie_Wenchang	PRJNA827465	0.814353254
ENSGALG00010022173	RPL23	2	-0.061681678	0.058329008	-1.057478604	0.290293214	0	0.086637915	0	1	CRA004775;PRJNA827465	Yang2025_Silkie_Wenchang	0.784195688
ENSGALG00010022174	KCNMA1	2	0.336699034	0.440751545	0.763920258	0.444914276	0.368306258	18.92343827	94.71554806	0.5	PRJNA827465;Yang2025_Silkie_Wenchang	CRA004775	0.846013379
ENSGALG00010022174	KCNMA1	2	0.577803812	0.285374283	2.024722783	0.042893838	0.094783115	2.147881652	53.4425	1	CRA004775;Yang2025_Silkie_Wenchang	PRJNA827465	0.358148931
ENSGALG00010022174	KCNMA1	2	-0.092780092	0.215763322	-0.785665287	0.432063605	0	0.575446115	0	0.5	CRA004775;PRJNA827465	Yang2025_Silkie_Wenchang	0.842503929
ENSGALG00010022176	ARL2BPL	2	0.279352966	0.178117629	1.568362254	0.116796615	0	0.303968655	0	1	PRJNA827465;Yang2025_Silkie_Wenchang	CRA004775	0.57540293
ENSGALG00010022176	ARL2BPL	2	0.064715117	0.359580345	0.179974011	0.857172971	0.163268778	2.66076298	62.4167952	0.5	CRA004775;Yang2025_Silkie_Wenchang	PRJNA827465	0.963878155
ENSGALG00010022176	ARL2BPL	2	-0.007483777	0.254781512	-0.029373312	0.976566858	0.046299995	1.503246514	33.47731124	0.5	CRA004775;PRJNA827465	Yang2025_Silkie_Wenchang	0.99314221
ENSGALG00010022178	ENSGALG00010022178	2	-0.158666841	0.192117293	-0.825885263	0.408869181	0	0.010267829	0	1	PRJNA827465;Yang2025_Silkie_Wenchang	CRA004775	0.834316328
ENSGALG00010022178	ENSGALG00010022178	2	-0.287440149	0.292783202	-0.981750824	0.32622262	0	0.850155978	0	1	CRA004775;Yang2025_Silkie_Wenchang	PRJNA827465	0.800023788
ENSGALG00010022178	ENSGALG00010022178	2	-0.25220909	0.216537977	-1.164733752	0.244126782	0	0.853324955	0	1	CRA004775;PRJNA827465	Yang2025_Silkie_Wenchang	0.755564606
ENSGALG00010022180	PLLP	2	0.002011765	0.161891937	0.01242659	0.990085271	0	0.044099364	0	0.5	PRJNA827465;Yang2025_Silkie_Wenchang	CRA004775	0.997368295
ENSGALG00010022180	PLLP	2	0.506154425	0.565058396	0.895755957	0.370383117	0.45192244	3.094332494	67.68285238	1	CRA004775;Yang2025_Silkie_Wenchang	PRJNA827465	0.821139872
ENSGALG00010022180	PLLP	2	0.462556502	0.599999604	0.770928012	0.440749602	0.555435713	3.811290746	73.76216965	0.5	CRA004775;PRJNA827465	Yang2025_Silkie_Wenchang	0.844848889
ENSGALG00010022181	ENSGALG00010022181	2	0.038529153	0.089878019	0.428682718	0.668154139	0	0.21420274	0	0.5	PRJNA827465;Yang2025_Silkie_Wenchang	CRA004775	0.914135432
ENSGALG00010022181	ENSGALG00010022181	2	-0.038382613	0.150898589	-0.254360117	0.799217205	0	0.302737967	0	1	CRA004775;Yang2025_Silkie_Wenchang	PRJNA827465	0.949922217
ENSGALG00010022181	ENSGALG00010022181	2	0.056281156	0.109045382	0.516125989	0.605766418	0	0.466494226	0	0.5	CRA004775;PRJNA827465	Yang2025_Silkie_Wenchang	0.896474603
ENSGALG00010022182	CNTNAP1	2	-0.219178472	0.121853207	-1.798709098	0.072064709	0	0.611115246	0	1	PRJNA827465;Yang2025_Silkie_Wenchang	CRA004775	0.463464923
ENSGALG00010022182	CNTNAP1	2	-0.09041452	0.188067088	-0.480756739	0.6306894	0	5.9234E-05	0	1	CRA004775;Yang2025_Silkie_Wenchang	PRJNA827465	0.902770945
ENSGALG00010022182	CNTNAP1	2	-0.271661347	0.144814671	-1.875924201	0.060665689	0	0.161353299	0	1	CRA004775;PRJNA827465	Yang2025_Silkie_Wenchang	0.426613629
ENSGALG00010022184	PRKAR1B	2	-0.03253162	0.050248884	-0.647409794	0.517366759	0	0.282522675	0	1	PRJNA827465;Yang2025_Silkie_Wenchang	CRA004775	0.867952013
ENSGALG00010022184	PRKAR1B	2	0.072687363	0.178133619	0.408049663	0.683232715	0.044481431	2.933136104	65.90679857	0.5	CRA004775;Yang2025_Silkie_Wenchang	PRJNA827465	0.918800744
ENSGALG00010022184	PRKAR1B	2	0.088389206	0.145677753	0.606748708	0.544020047	0.027040177	2.239842994	55.35401353	0.5	CRA004775;PRJNA827465	Yang2025_Silkie_Wenchang	0.876968972
ENSGALG00010022186	DNAJB4	2	0.096285951	0.147564949	0.65249879	0.514079453	0.03738488	6.737499035	85.15769732	0.5	PRJNA827465;Yang2025_Silkie_Wenchang	CRA004775	0.866855965
ENSGALG00010022186	DNAJB4	2	0.588645775	0.425209696	1.384365834	0.166246465	0.279912783	3.669130351	72.74558535	1	CRA004775;Yang2025_Silkie_Wenchang	PRJNA827465	0.66529196
ENSGALG00010022186	DNAJB4	2	0.464329667	0.580765359	0.799513366	0.4239928	0.587673628	6.821380443	85.3402107	0.5	CRA004775;PRJNA827465	Yang2025_Silkie_Wenchang	0.84031007
ENSGALG00010022190	ACVR1B	2	-0.275473498	0.222583262	-1.237620004	0.215856993	0.085764461	7.087865547	85.89102192	1	PRJNA827465;Yang2025_Silkie_Wenchang	CRA004775	0.729227043
ENSGALG00010022190	ACVR1B	2	-0.447745499	0.134585839	-3.326839603	0.000878369	0.000283544	1.005338461	0.531011341	1	CRA004775;Yang2025_Silkie_Wenchang	PRJNA827465	0.034765905
ENSGALG00010022190	ACVR1B	2	-0.078832402	0.07085674	-1.112560391	0.26589728	0	0.162526761	0	1	CRA004775;PRJNA827465	Yang2025_Silkie_Wenchang	0.770065639
ENSGALG00010022193	GDAP2	2	-0.21392627	0.228354746	-0.93681552	0.348853461	0.093953936	9.690495089	89.68060981	1	PRJNA827465;Yang2025_Silkie_Wenchang	CRA004775	0.809343216
ENSGALG00010022193	GDAP2	2	-0.442295187	0.100545759	-4.398944243	1.08779E-05	0	0.032593465	0	1	CRA004775;Yang2025_Silkie_Wenchang	PRJNA827465	0.001507474
ENSGALG00010022193	GDAP2	2	-0.187796758	0.209276629	-0.897361352	0.369526129	0.075130761	6.549205588	84.73097253	1	CRA004775;PRJNA827465	Yang2025_Silkie_Wenchang	0.820606801
ENSGALG00010022194	ENSGALG00010022194	2	-0.278480935	0.355328148	-0.783728889	0.433199207	0.239401992	19.0827927	94.75967687	0.5	PRJNA827465;Yang2025_Silkie_Wenchang	CRA004775	0.842762754
ENSGALG00010022194	ENSGALG00010022194	2	-0.618020837	0.113900465	-5.425972909	5.76397E-08	0	0.096049098	0	1	CRA004775;Yang2025_Silkie_Wenchang	PRJNA827465	2.19943E-05
ENSGALG00010022194	ENSGALG00010022194	2	-0.221539819	0.316186056	-0.700662836	0.483351454	0.176784631	8.188299306	87.78745179	0.5	CRA004775;PRJNA827465	Yang2025_Silkie_Wenchang	0.85691916
ENSGALG00010022196	PLXNA1	2	-0.032491259	0.073186559	-0.443951177	0.657077883	0	0.05785536	0	1	PRJNA827465;Yang2025_Silkie_Wenchang	CRA004775	0.910695405
ENSGALG00010022196	PLXNA1	2	-0.049692108	0.134724948	-0.368841174	0.712246113	0	0.041033958	0	1	CRA004775;Yang2025_Silkie_Wenchang	PRJNA827465	0.92597752
ENSGALG00010022196	PLXNA1	2	-0.021332352	0.079143343	-0.27004611	0.78712478	0	0.005195878	0	1	CRA004775;PRJNA827465	Yang2025_Silkie_Wenchang	0.946848017
ENSGALG00010022199	RBM39	2	0.214166873	0.198909721	1.076703899	0.28161257	0.07325388	13.03523622	92.32848578	1	PRJNA827465;Yang2025_Silkie_Wenchang	CRA004775	0.779295548
ENSGALG00010022199	RBM39	2	0.263501788	0.18043462	1.460372667	0.144187681	0.048015041	3.662445536	72.6958397	1	CRA004775;Yang2025_Silkie_Wenchang	PRJNA827465	0.631632681
ENSGALG00010022199	RBM39	2	0.027751636	0.043102737	0.643848583	0.519673626	0	0.043864187	0	1	CRA004775;PRJNA827465	Yang2025_Silkie_Wenchang	0.869100572
ENSGALG00010022200	DNMBP	2	-0.124694775	0.074225675	-1.679941291	0.092968739	0.006967891	2.66789609	62.5172808	1	PRJNA827465;Yang2025_Silkie_Wenchang	CRA004775	0.52216376
ENSGALG00010022200	DNMBP	2	-0.235043586	0.066596956	-3.52934426	0.000416591	0	0.826951077	0	1	CRA004775;Yang2025_Silkie_Wenchang	PRJNA827465	0.020595307
ENSGALG00010022200	DNMBP	2	-0.1761158	0.150255094	-1.172112004	0.241152091	0.033358057	3.348341491	70.13446798	1	CRA004775;PRJNA827465	Yang2025_Silkie_Wenchang	0.753758453
ENSGALG00010022202	CX3CL1	2	0.04659658	0.103155993	0.451709872	0.651478006	0	0.319407232	0	0.5	PRJNA827465;Yang2025_Silkie_Wenchang	CRA004775	0.908927523
ENSGALG00010022202	CX3CL1	2	0.101587638	0.151043666	0.672571326	0.501220049	0	0.071537801	0	0.5	CRA004775;Yang2025_Silkie_Wenchang	PRJNA827465	0.862658912
ENSGALG00010022202	CX3CL1	2	-0.003285646	0.129212625	-0.02542821	0.97971341	0	0.000460395	0	1	CRA004775;PRJNA827465	Yang2025_Silkie_Wenchang	0.994474095
ENSGALG00010022203	DNAAF5	2	0.063887896	0.076762404	0.832281073	0.405250312	0	0.092862129	0	1	PRJNA827465;Yang2025_Silkie_Wenchang	CRA004775	0.833112102
ENSGALG00010022203	DNAAF5	2	-0.186656446	0.329469124	-0.566536991	0.571028794	0.171091788	4.381516483	77.17685181	0.5	CRA004775;Yang2025_Silkie_Wenchang	PRJNA827465	0.885354083
ENSGALG00010022203	DNAAF5	2	-0.19971061	0.301844917	-0.661633174	0.508206342	0.143879843	4.159018694	75.95586667	0.5	CRA004775;PRJNA827465	Yang2025_Silkie_Wenchang	0.864714223
ENSGALG00010022204	ENSGALG00010022204	2	0.491990216	0.485463896	1.01344347	0.310848385	0.31654283	3.033853219	67.03861631	1	PRJNA827465;Yang2025_Silkie_Wenchang	CRA004775	0.795188367
ENSGALG00010022204	ENSGALG00010022204	2	0.462844478	0.58609307	0.78971157	0.429696233	0.454732583	2.911323772	65.65136419	0.5	CRA004775;Yang2025_Silkie_Wenchang	PRJNA827465	0.842012722
ENSGALG00010022204	ENSGALG00010022204	2	-0.037435757	0.305850566	-0.122398847	0.902583152	0	0.096524177	0	0.5	CRA004775;PRJNA827465	Yang2025_Silkie_Wenchang	0.976923607
ENSGALG00010022205	GALNT18	2	-0.203307515	0.070494929	-2.884001995	0.003926563	0	0.01054622	0	1	PRJNA827465;Yang2025_Silkie_Wenchang	CRA004775	0.090319952
ENSGALG00010022205	GALNT18	2	0.153243717	0.405787858	0.377644905	0.705694402	0.286348258	7.127234684	85.96931286	0.5	CRA004775;Yang2025_Silkie_Wenchang	PRJNA827465	0.924374044
ENSGALG00010022205	GALNT18	2	0.158165763	0.398182577	0.397219196	0.691205828	0.275814236	7.066939017	85.84960196	0.5	CRA004775;PRJNA827465	Yang2025_Silkie_Wenchang	0.920268407
ENSGALG00010022207	ENSGALG00010022207	2	-0.024585206	0.102557264	-0.239721742	0.810545979	0	0.030368865	0	1	PRJNA827465;Yang2025_Silkie_Wenchang	CRA004775	0.952997426
ENSGALG00010022207	ENSGALG00010022207	2	-0.039171414	0.132925992	-0.294685892	0.768233841	0	0.555108124	0	1	CRA004775;Yang2025_Silkie_Wenchang	PRJNA827465	0.941444269
ENSGALG00010022207	ENSGALG00010022207	2	-0.076423616	0.143462657	-0.532707376	0.594236159	0	0.439142467	0	1	CRA004775;PRJNA827465	Yang2025_Silkie_Wenchang	0.893331693
ENSGALG00010022209	ZFYVE19	2	-0.114476526	0.057005547	-2.008164695	0.044625793	0	0.002197095	0	1	PRJNA827465;Yang2025_Silkie_Wenchang	CRA004775	0.364257243
ENSGALG00010022209	ZFYVE19	2	-0.079519101	0.082766146	-0.960768433	0.336668614	0	0.553912908	0	0.5	CRA004775;Yang2025_Silkie_Wenchang	PRJNA827465	0.803887433
ENSGALG00010022209	ZFYVE19	2	-0.095105095	0.067068183	-1.418035964	0.156180265	0	0.648335228	0	0.5	CRA004775;PRJNA827465	Yang2025_Silkie_Wenchang	0.649132948
ENSGALG00010022210	EZH1	2	0.140893572	0.058491486	2.408787682	0.016005606	0	0.133465156	0	1	PRJNA827465;Yang2025_Silkie_Wenchang	CRA004775	0.209024249
ENSGALG00010022210	EZH1	2	-0.041169024	0.212302559	-0.193916758	0.846241062	0.079796816	8.651897232	88.44184145	0.5	CRA004775;Yang2025_Silkie_Wenchang	PRJNA827465	0.96122283
ENSGALG00010022210	EZH1	2	-0.059871681	0.190665615	-0.314014047	0.75351037	0.063245608	7.568493498	86.78733092	0.5	CRA004775;PRJNA827465	Yang2025_Silkie_Wenchang	0.938087082
ENSGALG00010022211	ENSGALG00010022211	2	0.844215138	0.248600516	3.395870423	0.000684107	0	0.062517341	0	1	PRJNA827465;Yang2025_Silkie_Wenchang	CRA004775	0.028942381
ENSGALG00010022211	ENSGALG00010022211	2	1.169459345	0.360219506	3.246518651	0.001168258	0.106126047	1.616861847	38.15179684	1	CRA004775;Yang2025_Silkie_Wenchang	PRJNA827465	0.041590617
ENSGALG00010022211	ENSGALG00010022211	2	1.154968673	0.434357627	2.659027036	0.007836667	0.178851515	1.881398537	46.8480505	1	CRA004775;PRJNA827465	Yang2025_Silkie_Wenchang	0.137440669
ENSGALG00010022213	IDH1	2	-0.323926809	0.351624826	-0.921228493	0.356931145	0.22841369	12.86876618	92.22924727	0.5	PRJNA827465;Yang2025_Silkie_Wenchang	CRA004775	0.812971699
ENSGALG00010022213	IDH1	2	-0.532340222	0.169664068	-3.137613221	0.001703295	0.024581985	1.737678202	42.45194541	1	CRA004775;Yang2025_Silkie_Wenchang	PRJNA827465	0.052228664
ENSGALG00010022213	IDH1	2	-0.12879754	0.177563518	-0.725360374	0.468230976	0.042417685	2.794289544	64.21272798	0.5	CRA004775;PRJNA827465	Yang2025_Silkie_Wenchang	0.853198008
ENSGALG00010022214	ENSGALG00010022214	2	-0.151131071	0.440488597	-0.343098714	0.731524198	0.329307381	6.423725743	84.43271024	0.5	PRJNA827465;Yang2025_Silkie_Wenchang	CRA004775	0.932851968
ENSGALG00010022214	ENSGALG00010022214	2	-0.348461881	0.382319059	-0.911442611	0.362062211	0.133736905	1.700006843	41.17670738	0.5	CRA004775;Yang2025_Silkie_Wenchang	PRJNA827465	0.815579273
ENSGALG00010022214	ENSGALG00010022214	2	0.251329488	0.172348302	1.45826495	0.144767527	0	0.018258107	0	1	CRA004775;PRJNA827465	Yang2025_Silkie_Wenchang	0.632551209
ENSGALG00010022215	ENSGALG00010022215	2	0.175419272	0.143521785	1.222248398	0.221613707	0.020002419	1.519079116	34.17064396	1	PRJNA827465;Yang2025_Silkie_Wenchang	CRA004775	0.734944791
ENSGALG00010022215	ENSGALG00010022215	2	0.340235051	0.184460016	1.844492144	0.06511145	0	0.271548233	0	1	CRA004775;Yang2025_Silkie_Wenchang	PRJNA827465	0.439870073
ENSGALG00010022215	ENSGALG00010022215	2	0.111237848	0.077850971	1.428856274	0.153045547	0	0.309407083	0	1	CRA004775;PRJNA827465	Yang2025_Silkie_Wenchang	0.645187993
ENSGALG00010022216	ROMO1	2	-0.050386136	0.082837601	-0.608251994	0.543020355	0	0.208945344	0	1	PRJNA827465;Yang2025_Silkie_Wenchang	CRA004775	0.876968972
ENSGALG00010022216	ROMO1	2	-0.12269217	0.217622583	-0.563784183	0.572901017	0	0.091184852	0	1	CRA004775;Yang2025_Silkie_Wenchang	PRJNA827465	0.88573298
ENSGALG00010022216	ROMO1	2	-0.04345222	0.081620523	-0.532368798	0.594470591	0	0.00941228	0	1	CRA004775;PRJNA827465	Yang2025_Silkie_Wenchang	0.89334242
ENSGALG00010022218	ENSGALG00010022218	2	0.249813961	0.458628549	0.544697799	0.585961379	0.377658752	9.717075453	89.70883776	0.5	PRJNA827465;Yang2025_Silkie_Wenchang	CRA004775	0.891393407
ENSGALG00010022218	ENSGALG00010022218	2	0.435089562	0.396427827	1.09752528	0.272411836	0.184237198	2.117068722	52.76487769	0.5	CRA004775;Yang2025_Silkie_Wenchang	PRJNA827465	0.77337492
ENSGALG00010022218	ENSGALG00010022218	2	-0.188139669	0.169407633	-1.110573739	0.266751872	0	0.021840992	0	1	CRA004775;PRJNA827465	Yang2025_Silkie_Wenchang	0.770949983
ENSGALG00010022219	EIF4E3	2	-0.165669085	0.079217351	-2.091323218	0.0364991	0	0.195084329	0	1	PRJNA827465;Yang2025_Silkie_Wenchang	CRA004775	0.329454443
ENSGALG00010022219	EIF4E3	2	-0.254688	0.128997053	-1.974370692	0.048339608	0	0.170918686	0	1	CRA004775;Yang2025_Silkie_Wenchang	PRJNA827465	0.38014722
ENSGALG00010022219	EIF4E3	2	-0.16845082	0.087188698	-1.932025873	0.053356313	0	0.558645078	0	1	CRA004775;PRJNA827465	Yang2025_Silkie_Wenchang	0.400126532
ENSGALG00010022220	PPP1R14D	2	0.094105111	0.225531812	0.417258701	0.676489193	0	0.00546912	0	1	PRJNA827465;Yang2025_Silkie_Wenchang	CRA004775	0.916786558
ENSGALG00010022220	PPP1R14D	2	0.154176093	0.250053075	0.616573475	0.537516095	0	0.188588339	0	1	CRA004775;Yang2025_Silkie_Wenchang	PRJNA827465	0.87481821
ENSGALG00010022220	PPP1R14D	2	0.185858642	0.267007688	0.69607974	0.486378894	0	0.128761379	0	1	CRA004775;PRJNA827465	Yang2025_Silkie_Wenchang	0.857603818
ENSGALG00010022221	CUTC	2	0.07966301	0.083195246	0.957542807	0.338293351	0	0.001108168	0	1	PRJNA827465;Yang2025_Silkie_Wenchang	CRA004775	0.804154375
ENSGALG00010022221	CUTC	2	-0.2529427	0.313481349	-0.806882775	0.419734024	0.129513775	2.926097679	65.82479091	0.5	CRA004775;Yang2025_Silkie_Wenchang	PRJNA827465	0.838527387
ENSGALG00010022221	CUTC	2	-0.196144182	0.315657442	-0.621383043	0.534347626	0.168563249	5.929610786	83.13548669	0.5	CRA004775;PRJNA827465	Yang2025_Silkie_Wenchang	0.873423363
ENSGALG00010022222	ENSGALG00010022222	2	0.10656076	0.127810605	0.833739576	0.404427751	0.001463677	1.032686292	3.165171514	1	PRJNA827465;Yang2025_Silkie_Wenchang	CRA004775	0.833112102
ENSGALG00010022222	ENSGALG00010022222	2	0.148197863	0.176686723	0.838760609	0.401603659	0	0.962644065	0	0.5	CRA004775;Yang2025_Silkie_Wenchang	PRJNA827465	0.832623399
ENSGALG00010022222	ENSGALG00010022222	2	0.02534321	0.120909325	0.209605087	0.833975909	0	0.02605746	0	0.5	CRA004775;PRJNA827465	Yang2025_Silkie_Wenchang	0.957968425
ENSGALG00010022223	DOK4	2	0.368650293	0.236128116	1.561229976	0.118469499	0.090209181	5.177861197	80.68700643	1	PRJNA827465;Yang2025_Silkie_Wenchang	CRA004775	0.578338927
ENSGALG00010022223	DOK4	2	0.180561718	0.482235929	0.374426099	0.708087329	0.395839535	6.357118042	84.26960152	0.5	CRA004775;Yang2025_Silkie_Wenchang	PRJNA827465	0.925061251
ENSGALG00010022223	DOK4	2	0.0013868	0.22472404	0.006171122	0.995076188	0.054989872	1.806160479	44.63393417	0.5	CRA004775;PRJNA827465	Yang2025_Silkie_Wenchang	0.998242335
ENSGALG00010022224	NHEJ1	2	-0.137163006	0.14620428	-0.93815999	0.348162196	0	0.114342077	0	1	PRJNA827465;Yang2025_Silkie_Wenchang	CRA004775	0.808976658
ENSGALG00010022224	NHEJ1	2	-0.251656708	0.211038264	-1.192469569	0.233077176	0.013091039	1.16909392	14.46367286	1	CRA004775;Yang2025_Silkie_Wenchang	PRJNA827465	0.74610134
ENSGALG00010022224	NHEJ1	2	-0.255316266	0.152686306	-1.672162185	0.094492345	0	0.849281171	0	1	CRA004775;PRJNA827465	Yang2025_Silkie_Wenchang	0.526635371
ENSGALG00010022226	FBRSL1	2	0.346366392	0.442111444	0.783436838	0.433370631	0.353386228	9.565266688	89.54550843	0.5	PRJNA827465;Yang2025_Silkie_Wenchang	CRA004775	0.842762754
ENSGALG00010022226	FBRSL1	2	0.607979139	0.200633783	3.030292956	0.002443166	0.011633908	1.166206851	14.25191861	1	CRA004775;Yang2025_Silkie_Wenchang	PRJNA827465	0.066177569
ENSGALG00010022226	FBRSL1	2	0.128129268	0.23480131	0.545692302	0.585277464	0.085618931	3.70218469	72.98892184	0.5	CRA004775;PRJNA827465	Yang2025_Silkie_Wenchang	0.891130641
ENSGALG00010022227	TMEM80	2	-0.404113118	0.383513066	-1.053714081	0.29201384	0.278471962	18.5366905	94.60529376	1	PRJNA827465;Yang2025_Silkie_Wenchang	CRA004775	0.785516679
ENSGALG00010022227	TMEM80	2	-0.611342023	0.22913085	-2.668091286	0.007628353	0.063828435	2.39394794	58.22799723	1	CRA004775;Yang2025_Silkie_Wenchang	PRJNA827465	0.134870514
ENSGALG00010022227	TMEM80	2	-0.079644326	0.111142522	-0.716596351	0.473623197	0.006339021	1.165189144	14.17702392	1	CRA004775;PRJNA827465	Yang2025_Silkie_Wenchang	0.854399987
ENSGALG00010022228	FUBP1	2	0.321771894	0.298590708	1.07763532	0.281196533	0.16787426	16.38823875	93.89806302	1	PRJNA827465;Yang2025_Silkie_Wenchang	CRA004775	0.778899386
ENSGALG00010022228	FUBP1	2	0.396661816	0.281216619	1.410520535	0.158386037	0.116329498	3.530928487	71.67883735	1	CRA004775;Yang2025_Silkie_Wenchang	PRJNA827465	0.652042151
ENSGALG00010022228	FUBP1	2	0.038794716	0.041168411	0.942341823	0.346017662	0	0.010842575	0	1	CRA004775;PRJNA827465	Yang2025_Silkie_Wenchang	0.807669092
ENSGALG00010022229	RAPSN	2	0.271410908	0.209290392	1.296814945	0.194694867	0.076839332	7.996153353	87.49398672	1	PRJNA827465;Yang2025_Silkie_Wenchang	CRA004775	0.70591258
ENSGALG00010022229	RAPSN	2	0.468269099	0.120972954	3.870857779	0.000108453	0	0.743492863	0	0.5	CRA004775;Yang2025_Silkie_Wenchang	PRJNA827465	0.008228823
ENSGALG00010022229	RAPSN	2	0.070742122	0.080472128	0.879088498	0.379353294	0	0.017457175	0	0.5	CRA004775;PRJNA827465	Yang2025_Silkie_Wenchang	0.824165982
ENSGALG00010022230	NFS1	2	0.000703305	0.04702341	0.014956478	0.988066902	0.00042589	1.104094022	9.428003385	0.5	PRJNA827465;Yang2025_Silkie_Wenchang	CRA004775	0.996842505
ENSGALG00010022230	NFS1	2	0.041043437	0.065967544	0.62217622	0.533826001	0	0.502182559	0	0.5	CRA004775;Yang2025_Silkie_Wenchang	PRJNA827465	0.873408008
ENSGALG00010022230	NFS1	2	-0.044510201	0.056789698	-0.783772452	0.433173641	0	0.098948848	0	1	CRA004775;PRJNA827465	Yang2025_Silkie_Wenchang	0.842762754
ENSGALG00010022231	ZNF503	2	0.08720059	0.240463443	0.362635536	0.716877161	0.091141804	4.440379148	77.47940059	0.5	PRJNA827465;Yang2025_Silkie_Wenchang	CRA004775	0.927632716
ENSGALG00010022231	ZNF503	2	0.25491301	0.200296144	1.272680568	0.203131416	0.009641433	1.089857137	8.244854659	0.5	CRA004775;Yang2025_Silkie_Wenchang	PRJNA827465	0.716859255
ENSGALG00010022231	ZNF503	2	-0.123704024	0.102552097	-1.206255426	0.227719019	0	1.11845E-05	0	1	CRA004775;PRJNA827465	Yang2025_Silkie_Wenchang	0.740853612
ENSGALG00010022232	GPR27	2	-0.265195798	0.307214071	-0.863228034	0.388012103	0.180676248	23.1684056	95.68377722	0.5	PRJNA827465;Yang2025_Silkie_Wenchang	CRA004775	0.828154121
ENSGALG00010022232	GPR27	2	-0.011335897	0.593415087	-0.019102813	0.984759088	0.661792152	16.0512069	93.76993888	0.5	CRA004775;Yang2025_Silkie_Wenchang	PRJNA827465	0.995703389
ENSGALG00010022232	GPR27	2	0.260668692	0.279887277	0.931334555	0.351680533	0.124020493	4.065718028	75.40409854	1	CRA004775;PRJNA827465	Yang2025_Silkie_Wenchang	0.810678834
ENSGALG00010022233	FAM46C	2	-0.222248491	0.088077057	-2.52334147	0.011624546	0	0.699026976	0	1	PRJNA827465;Yang2025_Silkie_Wenchang	CRA004775	0.172602771
ENSGALG00010022233	FAM46C	2	-0.073826587	0.145678228	-0.506778453	0.612310276	0	0.650938941	0	0.5	CRA004775;Yang2025_Silkie_Wenchang	PRJNA827465	0.897895209
ENSGALG00010022233	FAM46C	2	-0.175514759	0.205011133	-0.856123063	0.391929713	0.040102277	1.404268985	28.78857182	0.5	CRA004775;PRJNA827465	Yang2025_Silkie_Wenchang	0.829251057
ENSGALG00010022234	COX15	2	-0.199790613	0.084260258	-2.371113238	0.017734597	0	0.045806241	0	1	PRJNA827465;Yang2025_Silkie_Wenchang	CRA004775	0.2222112
ENSGALG00010022234	COX15	2	-0.262035838	0.137019493	-1.912398253	0.055825132	0	0.02584552	0	1	CRA004775;Yang2025_Silkie_Wenchang	PRJNA827465	0.408997088
ENSGALG00010022234	COX15	2	-0.209327761	0.08329968	-2.512947961	0.011972699	0	0.189561013	0	1	CRA004775;PRJNA827465	Yang2025_Silkie_Wenchang	0.175911404
ENSGALG00010022235	RAMP2	2	0.008690487	0.054124687	0.160564194	0.872436658	0	0.336464091	0	0.5	PRJNA827465;Yang2025_Silkie_Wenchang	CRA004775	0.969629321
ENSGALG00010022235	RAMP2	2	0.033912704	0.131113723	0.258651062	0.795904482	0	0.44436794	0	0.5	CRA004775;Yang2025_Silkie_Wenchang	PRJNA827465	0.949130222
ENSGALG00010022235	RAMP2	2	-0.00767233	0.055626364	-0.13792614	0.890298791	0	0.133639844	0	1	CRA004775;PRJNA827465	Yang2025_Silkie_Wenchang	0.973417023
ENSGALG00010022236	SHF	2	-0.369751674	0.186909605	-1.978237948	0.04790187	0.058468928	6.012073556	83.36680364	1	PRJNA827465;Yang2025_Silkie_Wenchang	CRA004775	0.378353533
ENSGALG00010022236	SHF	2	-0.529752037	0.118421646	-4.473439223	7.69714E-06	0	0.771766458	0	1	CRA004775;Yang2025_Silkie_Wenchang	PRJNA827465	0.001128809
ENSGALG00010022236	SHF	2	-0.197378201	0.083179888	-2.372907735	0.017648672	0	0.02607085	0	1	CRA004775;PRJNA827465	Yang2025_Silkie_Wenchang	0.221474498
ENSGALG00010022237	SUN1	2	0.015482352	0.063261842	0.244734444	0.806662056	0.003893735	1.891839569	47.14139526	0.5	PRJNA827465;Yang2025_Silkie_Wenchang	CRA004775	0.952273023
ENSGALG00010022237	SUN1	2	-0.031162083	0.070124698	-0.444380998	0.656767149	0	0.928094546	0	0.5	CRA004775;Yang2025_Silkie_Wenchang	PRJNA827465	0.910563743
ENSGALG00010022237	SUN1	2	0.073332712	0.051654606	1.419674223	0.155702546	0	0.097689108	0	1	CRA004775;PRJNA827465	Yang2025_Silkie_Wenchang	0.648878928
ENSGALG00010022238	COMTD1	2	0.124708686	0.088725363	1.405558475	0.159855274	0	0.022613774	0	1	PRJNA827465;Yang2025_Silkie_Wenchang	CRA004775	0.655404348
ENSGALG00010022238	COMTD1	2	0.098711751	0.148726613	0.663712763	0.506874169	0	0.001678352	0	1	CRA004775;Yang2025_Silkie_Wenchang	PRJNA827465	0.863929781
ENSGALG00010022238	COMTD1	2	0.127704715	0.09733843	1.311966038	0.189531594	0	0.020544114	0	1	CRA004775;PRJNA827465	Yang2025_Silkie_Wenchang	0.698754721
ENSGALG00010022239	DEAF1	2	-0.010468984	0.065085652	-0.160849338	0.872212065	0	0.049976702	0	1	PRJNA827465;Yang2025_Silkie_Wenchang	CRA004775	0.969617087
ENSGALG00010022239	DEAF1	2	-0.105774785	0.113284383	-0.933710209	0.350453397	0.004300997	1.162067042	13.94644508	1	CRA004775;Yang2025_Silkie_Wenchang	PRJNA827465	0.810111037
ENSGALG00010022239	DEAF1	2	-0.081750096	0.126908515	-0.644165568	0.519468076	0.016560787	1.731805539	42.25679631	1	CRA004775;PRJNA827465	Yang2025_Silkie_Wenchang	0.869086345
ENSGALG00010022240	ENSGALG00010022240	2	0.157991217	0.090767988	1.740605029	0.081752835	0	0.557250419	0	1	PRJNA827465;Yang2025_Silkie_Wenchang	CRA004775	0.492811609
ENSGALG00010022240	ENSGALG00010022240	2	0.142257055	0.100540729	1.414919662	0.157092052	0	0.438807884	0	1	CRA004775;Yang2025_Silkie_Wenchang	PRJNA827465	0.650687662
ENSGALG00010022240	ENSGALG00010022240	2	0.250918386	0.120365648	2.08463453	0.037102488	0	0.004544326	0	1	CRA004775;PRJNA827465	Yang2025_Silkie_Wenchang	0.332143015
ENSGALG00010022241	POLR2C	2	-0.017539356	0.072214779	-0.242877659	0.808100173	0.003807244	1.44350287	30.7240726	0.5	PRJNA827465;Yang2025_Silkie_Wenchang	CRA004775	0.952496413
ENSGALG00010022241	POLR2C	2	-0.115106478	0.249976268	-0.460469624	0.64517917	0.092070952	3.47055527	71.18616699	0.5	CRA004775;Yang2025_Silkie_Wenchang	PRJNA827465	0.906966303
ENSGALG00010022241	POLR2C	2	-0.158844688	0.155434418	-1.02194025	0.306809181	0.029325094	1.906864217	47.5578811	1	CRA004775;PRJNA827465	Yang2025_Silkie_Wenchang	0.79266003
ENSGALG00010022242	USP19	2	-0.054434373	0.056260176	-0.96754714	0.333270588	0.003305402	2.086641205	52.07609255	1	PRJNA827465;Yang2025_Silkie_Wenchang	CRA004775	0.802360424
ENSGALG00010022242	USP19	2	-0.251082096	0.177902048	-1.411350228	0.158141371	0.047162192	3.305936038	69.7513809	1	CRA004775;Yang2025_Silkie_Wenchang	PRJNA827465	0.65200722
ENSGALG00010022242	USP19	2	-0.204962134	0.23745292	-0.863169564	0.388044245	0.095269095	5.733728846	82.55934267	1	CRA004775;PRJNA827465	Yang2025_Silkie_Wenchang	0.828154121
ENSGALG00010022243	ENSGALG00010022243	2	0.304796218	0.535546534	0.569131157	0.569267136	0.299858796	2.087696895	52.10032633	0.5	PRJNA827465;Yang2025_Silkie_Wenchang	CRA004775	0.884658821
ENSGALG00010022243	ENSGALG00010022243	2	0.637672202	0.896625562	0.711191191	0.476965766	1.305562735	5.318206729	81.19666927	0.5	CRA004775;Yang2025_Silkie_Wenchang	PRJNA827465	0.854986476
ENSGALG00010022243	ENSGALG00010022243	2	1.134756354	0.364998785	3.108931863	0.00187765	0	0.964270581	0	1	CRA004775;PRJNA827465	Yang2025_Silkie_Wenchang	0.055706168
ENSGALG00010022244	ENTPD7	2	0.192099023	0.090483785	2.123021528	0.033752048	0	0.264948972	0	1	PRJNA827465;Yang2025_Silkie_Wenchang	CRA004775	0.315687663

ENSGALG00010022244	ENTPD7	2	-0.017827847	0.165161852	-0.107941678	0.914041956	0.014869794	1.35240477	26.05764027	0.5	CRA004775;Yang2025_Silkie_Wenchang	PRJNA827465	0.979467654
ENSGALG00010022244	ENTPD7	2	0.047657322	0.215941514	0.220695505	0.825329531	0.065271369	2.996467326	66.62736846	0.5	CRA004775;PRJNA827465	Yang2025_Silkie_Wenchang	0.956381808
ENSGALG00010022245	PIKFYVE	2	0.077515598	0.05172814	1.498518957	0.133998471	0	0.02238095	0	1	PRJNA827465;Yang2025_Silkie_Wenchang	CRA004775	0.61327389
ENSGALG00010022245	PIKFYVE	2	0.094846277	0.089368653	1.061292446	0.288557018	0	0.076312289	0	1	CRA004775;Yang2025_Silkie_Wenchang	PRJNA827465	0.783229038
ENSGALG00010022245	PIKFYVE	2	0.075531136	0.061744269	1.22328982	0.221220251	0	0.104675245	0	1	CRA004775;PRJNA827465	Yang2025_Silkie_Wenchang	0.734591037
ENSGALG00010022246	RHOV	2	-0.438815799	0.333971506	-1.313931853	0.188869138	0	0.805601524	0	1	PRJNA827465;Yang2025_Silkie_Wenchang	CRA004775	0.698162702
ENSGALG00010022246	RHOV	2	0.640549803	0.791136656	0.809657596	0.418136996	0.854141224	3.075416934	67.48408358	0.5	CRA004775;Yang2025_Silkie_Wenchang	PRJNA827465	0.838112595
ENSGALG00010022246	RHOV	2	0.336630842	1.098462407	0.306456406	0.759257167	2.06144605	6.647458796	84.95665741	0.5	CRA004775;PRJNA827465	Yang2025_Silkie_Wenchang	0.939145717
ENSGALG00010022247	GALNT9	2	0.407898338	0.161176919	2.530749072	0.011381924	0	0.190347256	0	1	PRJNA827465;Yang2025_Silkie_Wenchang	CRA004775	0.170092687
ENSGALG00010022247	GALNT9	2	-0.056187899	0.461677757	-0.121703716	0.90313367	0.231641307	1.998950807	49.9737564	0.5	CRA004775;Yang2025_Silkie_Wenchang	PRJNA827465	0.97714757
ENSGALG00010022247	GALNT9	2	0.033759535	0.542404471	0.062240518	0.950371296	0.431938007	3.168969624	68.44400172	0.5	CRA004775;PRJNA827465	Yang2025_Silkie_Wenchang	0.987277827
ENSGALG00010022248	FIGNL2	2	-0.017415489	0.130535668	-0.133415557	0.893864743	0.011592335	1.361049077	26.52726366	0.5	PRJNA827465;Yang2025_Silkie_Wenchang	CRA004775	0.974381724
ENSGALG00010022248	FIGNL2	2	-0.380159094	0.260774107	-1.457809972	0.144892929	0.036582456	1.242058963	19.48852432	1	CRA004775;Yang2025_Silkie_Wenchang	PRJNA827465	0.63274742
ENSGALG00010022248	FIGNL2	2	-0.262100127	0.434864769	-0.602716397	0.546697372	0.281926968	3.158271228	68.33710826	0.5	CRA004775;PRJNA827465	Yang2025_Silkie_Wenchang	0.878275987
ENSGALG00010022250	SLC25A28	2	0.036595983	0.062171207	0.588632341	0.556107934	0	0.045824777	0	1	PRJNA827465;Yang2025_Silkie_Wenchang	CRA004775	0.880324407
ENSGALG00010022250	SLC25A28	2	-0.162540688	0.292514817	-0.555666512	0.578438854	0.115542054	2.738708573	63.48643993	0.5	CRA004775;Yang2025_Silkie_Wenchang	PRJNA827465	0.887855709
ENSGALG00010022250	SLC25A28	2	-0.160272593	0.268370129	-0.597207273	0.550369004	0.103707525	2.794088099	64.21014783	0.5	CRA004775;PRJNA827465	Yang2025_Silkie_Wenchang	0.878702974
ENSGALG00010022251	WNK4	2	0.294843689	0.180858448	1.630245601	0.1030496	0.040404875	2.580635796	61.24985937	1	PRJNA827465;Yang2025_Silkie_Wenchang	CRA004775	0.547244753
ENSGALG00010022251	WNK4	2	0.078894742	0.447045264	0.176480434	0.859916519	0.339764691	6.455491141	84.50931187	0.5	CRA004775;Yang2025_Silkie_Wenchang	PRJNA827465	0.964879591
ENSGALG00010022251	WNK4	2	-0.068136951	0.257708661	-0.264395272	0.791475354	0.086032981	2.528976672	60.45831459	0.5	CRA004775;PRJNA827465	Yang2025_Silkie_Wenchang	0.948059392
ENSGALG00010022252	COQ9	2	-0.153251427	0.080217532	-1.910448048	0.056075549	0	0.248080531	0	1	PRJNA827465;Yang2025_Silkie_Wenchang	CRA004775	0.409653777
ENSGALG00010022252	COQ9	2	-0.229658346	0.194657957	-1.179804562	0.238077955	0	0.067929048	0	1	CRA004775;Yang2025_Silkie_Wenchang	PRJNA827465	0.750014255
ENSGALG00010022252	COQ9	2	-0.135603545	0.085831962	-1.579872363	0.1141361	0	0.005167153	0	1	CRA004775;PRJNA827465	Yang2025_Silkie_Wenchang	0.569998872
ENSGALG00010022253	ALPK3	2	-0.09772931	0.087064884	-1.122488258	0.261654897	0	0.526320991	0	1	PRJNA827465;Yang2025_Silkie_Wenchang	CRA004775	0.768310285
ENSGALG00010022253	ALPK3	2	-0.026894132	0.114519967	-0.234842294	0.814331139	0	0.378876986	0	0.5	CRA004775;Yang2025_Silkie_Wenchang	PRJNA827465	0.953869515
ENSGALG00010022253	ALPK3	2	-0.144420402	0.127313549	-1.134367889	0.256640263	0	0.685039345	0	0.5	CRA004775;PRJNA827465	Yang2025_Silkie_Wenchang	0.764677554
ENSGALG00010022255	NOC4L	2	-0.151853353	0.160349696	-0.947013665	0.343631816	0.041078823	4.927450281	79.70552836	0.5	PRJNA827465;Yang2025_Silkie_Wenchang	CRA004775	0.806314104
ENSGALG00010022255	NOC4L	2	-0.32009889	0.102830421	-3.112881259	0.001852705	0	1.13429E-05	0	1	CRA004775;Yang2025_Silkie_Wenchang	PRJNA827465	0.055248013
ENSGALG00010022255	NOC4L	2	-0.080784426	0.140218299	-0.576132559	0.564525113	0.018335704	1.547182882	35.1767696	0.5	CRA004775;PRJNA827465	Yang2025_Silkie_Wenchang	0.883156092
ENSGALG00010022256	PSMC3	2	0.06623283	0.044735897	1.480529839	0.138731904	0	0.65758422	0	0.5	PRJNA827465;Yang2025_Silkie_Wenchang	CRA004775	0.62244064
ENSGALG00010022256	PSMC3	2	-0.066782152	0.116796679	-0.571781256	0.567470178	0	0.212769088	0	1	CRA004775;Yang2025_Silkie_Wenchang	PRJNA827465	0.883717772
ENSGALG00010022256	PSMC3	2	0.069796901	0.046531091	1.50000567	0.133612934	0	0.992551898	0	0.5	CRA004775;PRJNA827465	Yang2025_Silkie_Wenchang	0.612692701
ENSGALG00010022258	ENSGALG00010022258	2	0.754617915	0.541172791	1.39441215	0.163193181	0	0.00101208	0	1	PRJNA827465;Yang2025_Silkie_Wenchang	CRA004775	0.661668506
ENSGALG00010022258	ENSGALG00010022258	2	-0.193026698	0.852044199	-0.226545404	0.820777249	1.012522109	3.24624465	69.19517449	0.5	CRA004775;Yang2025_Silkie_Wenchang	PRJNA827465	0.955454439
ENSGALG00010022258	ENSGALG00010022258	2	-0.1779384	0.869140397	-0.20472919	0.837783722	1.065716514	3.331630865	69.98466995	0.5	CRA004775;PRJNA827465	Yang2025_Silkie_Wenchang	0.959032758
ENSGALG00010022259	GET4	2	-0.04778598	0.042041913	-1.136625157	0.255694183	0	0.2243806	0	1	PRJNA827465;Yang2025_Silkie_Wenchang	CRA004775	0.574478331
ENSGALG00010022259	GET4	2	0.019788111	0.08364199	0.236581059	0.812981816	0	0.44479058	0	0.5	CRA004775;Yang2025_Silkie_Wenchang	PRJNA827465	0.953526116
ENSGALG00010022259	GET4	2	-0.046517471	0.045632362	-1.019396528	0.308014754	0	0.962824831	0	0.5	CRA004775;PRJNA827465	Yang2025_Silkie_Wenchang	0.793278812
ENSGALG00010022264	SCN8A	2	0.66577785	0.324825833	2.049645632	0.040399024	0.173434797	5.53949304	81.94780654	1	PRJNA827465;Yang2025_Silkie_Wenchang	CRA004775	0.347592566
ENSGALG00010022264	SCN8A	2	0.067536634	1.01040912	0.066840879	0.946708379	1.856972011	10.51279233	90.48777938	0.5	CRA004775;Yang2025_Silkie_Wenchang	PRJNA827465	0.986445196
ENSGALG00010022264	SCN8A	2	-0.215463465	0.67811907	-0.317736921	0.750684503	0.761973051	5.146083891	80.5677478	0.5	CRA004775;PRJNA827465	Yang2025_Silkie_Wenchang	0.937473087
ENSGALG00010022266	COA3	2	0.167744695	0.243161073	0.689850118	0.490288448	0.077250436	2.230404486	55.165083	1	PRJNA827465;Yang2025_Silkie_Wenchang	CRA004775	0.858529056
ENSGALG00010022266	COA3	2	0.113654558	0.416543304	0.272851722	0.784967199	0.23447186	3.081520509	67.55164692	0.5	CRA004775;Yang2025_Silkie_Wenchang	PRJNA827465	0.946308994
ENSGALG00010022266	COA3	2	-0.012518153	0.084024259	-0.148982605	0.881567359	0	0.804842379	0	0.5	CRA004775;PRJNA827465	Yang2025_Silkie_Wenchang	0.971802788
ENSGALG00010022271	SLC18A2	2	-0.285897542	0.195502759	-1.462370881	0.143639605	0	0.076915179	0	1	PRJNA827465;Yang2025_Silkie_Wenchang	CRA004775	0.630564924
ENSGALG00010022271	SLC18A2	2	-0.396835741	0.244286469	-1.624468773	0.1042758	0	0.151552503	0	1	CRA004775;Yang2025_Silkie_Wenchang	PRJNA827465	0.548786638
ENSGALG00010022271	SLC18A2	2	-0.310794728	0.237493533	-1.308645015	0.190654625	0	0.343939616	0	1	CRA004775;PRJNA827465	Yang2025_Silkie_Wenchang	0.699915817
ENSGALG00010022274	CCDC91	2	0.478038092	0.36010819	1.327484643	0.184348417	0.232843956	9.506595129	89.48098676	1	PRJNA827465;Yang2025_Silkie_Wenchang	CRA004775	0.692608786
ENSGALG00010022274	CCDC91	2	0.495351254	0.390236917	1.269360311	0.204312585	0.242542602	4.820200161	79.25397356	1	CRA004775;Yang2025_Silkie_Wenchang	PRJNA827465	0.717281769
ENSGALG00010022274	CCDC91	2	0.130281711	0.104034586	1.252292294	0.210463376	0	0.038258071	0	1	CRA004775;PRJNA827465	Yang2025_Silkie_Wenchang	0.722053252
ENSGALG00010022275	VDAC2	2	-0.468202582	0.363464336	-1.28816654	0.197688011	0.242236814	11.40366647	91.2308905	1	PRJNA827465;Yang2025_Silkie_Wenchang	CRA004775	0.709468779
ENSGALG00010022275	VDAC2	2	-0.321414321	0.552906872	-0.581317285	0.561026633	0.551859318	10.18351722	90.18021005	0.5	CRA004775;Yang2025_Silkie_Wenchang	PRJNA827465	0.881417858
ENSGALG00010022275	VDAC2	2	-0.038111337	0.161670233	-0.235735028	0.813638287	0.029825141	1.719429667	41.84118029	0.5	CRA004775;PRJNA827465	Yang2025_Silkie_Wenchang	0.953782677
ENSGALG00010022276	BECN1	2	0.135067013	0.100566284	1.343064572	0.179251066	0.0163283	5.09567472	80.37551345	1	PRJNA827465;Yang2025_Silkie_Wenchang	CRA004775	0.685921979
ENSGALG00010022276	BECN1	2	0.245227073	0.069350004	3.536078692	0.000406114	0	0.014706336	0	1	CRA004775;Yang2025_Silkie_Wenchang	PRJNA827465	0.020366982
ENSGALG00010022276	BECN1	2	0.053313535	0.051957425	1.026100407	0.304844258	0.000202702	1.007718753	0.765962975	1	CRA004775;PRJNA827465	Yang2025_Silkie_Wenchang	0.79225794
ENSGALG00010022279	EP400	2	0.076504377	0.254766733	0.300291864	0.763954539	0.109872877	5.83119927	82.85086903	0.5	PRJNA827465;Yang2025_Silkie_Wenchang	CRA004775	0.940419232
ENSGALG00010022279	EP400	2	0.284084986	0.162125856	1.752249724	0.079730877	0	0.484921114	0	1	CRA004775;Yang2025_Silkie_Wenchang	PRJNA827465	0.48568857
ENSGALG00010022279	EP400	2	-0.121101113	0.076311931	-1.586922399	0.112530221	0.002865872	1.077827406	7.220767061	0.5	CRA004775;PRJNA827465	Yang2025_Silkie_Wenchang	0.566223663
ENSGALG00010022280	PDZD8	2	-0.177460352	0.153654626	-1.154930096	0.248119054	0.039759148	6.156806066	83.75781226	1	PRJNA827465;Yang2025_Silkie_Wenchang	CRA004775	0.758346566
ENSGALG00010022280	PDZD8	2	-0.092582976	0.29314836	-0.315822937	0.752136908	0.13733876	4.530341686	77.92660975	0.5	CRA004775;Yang2025_Silkie_Wenchang	PRJNA827465	0.937945771
ENSGALG00010022280	PDZD8	2	-0.001006756	0.091974516	-0.010946031	0.991266506	0.005318783	1.149995488	13.04313705	0.5	CRA004775;PRJNA827465	Yang2025_Silkie_Wenchang	0.99755571
ENSGALG00010022282	TMT1A	2	-0.181844693	0.243227588	-0.747631854	0.454682245	0.073970749	2.558975963	60.92186818	0.5	PRJNA827465;Yang2025_Silkie_Wenchang	CRA004775	0.849072665
ENSGALG00010022282	TMT1A	2	-0.530107299	0.237287365	-2.234030875	0.025481042	0	0.370516925	0	1	CRA004775;Yang2025_Silkie_Wenchang	PRJNA827465	0.272213827
ENSGALG00010022282	TMT1A	2	-0.23435864	0.404213583	-0.579789127	0.562056831	0.202700797	2.052854492	51.28734142	0.5	CRA004775;PRJNA827465	Yang2025_Silkie_Wenchang	0.883189276
ENSGALG00010022290	NEXN	2	0.542232925	0.671476962	0.807522753	0.419365371	0.859348846	20.80146735	95.19264683	0.5	PRJNA827465;Yang2025_Silkie_Wenchang	CRA004775	0.838527387
ENSGALG00010022290	NEXN	2	1.186255154	0.246859059	4.805394452	1.54447E-06	0	0.203804754	0	1	CRA004775;Yang2025_Silkie_Wenchang	PRJNA827465	0.000312898
ENSGALG00010022290	NEXN	2	0.247822935	0.495123216	0.500527801	0.616703486	0.357760715	2.897048891	65.48211515	0.5	CRA004775;PRJNA827465	Yang2025_Silkie_Wenchang	0.899111936
ENSGALG00010022293	DNAJC10	2	-0.146454894	0.176754708	-0.828577049	0.407343779	0.057366453	11.96614439	91.64308931	0.5	PRJNA827465;Yang2025_Silkie_Wenchang	CRA004775	0.833863243
ENSGALG00010022293	DNAJC10	2	-0.131799233	0.224249736	-0.587734173	0.556710734	0.082033114	5.107146511	80.41959443	0.5	CRA004775;Yang2025_Silkie_Wenchang	PRJNA827465	0.880358001
ENSGALG00010022293	DNAJC10	2	0.030195732	0.048767655	0.619175392	0.535800821	0	0.276538027	0	1	CRA004775;PRJNA827465	Yang2025_Silkie_Wenchang	0.874321378
ENSGALG00010022296	CIAPIN1	2	-0.007410643	0.153786554	-0.048187844	0.961566538	0.015633265	3.447984368	70.9975483	0.5	PRJNA827465;Yang2025_Silkie_Wenchang	CRA004775	0.98973612
ENSGALG00010022296	CIAPIN1	2	0.085535878	0.153356287	0.557759188	0.577008835	0.007627331	1.162027087	13.94348625	0.5	CRA004775;Yang2025_Silkie_Wenchang	PRJNA827465	0.887134413
ENSGALG00010022296	CIAPIN1	2	-0.128318579	0.049242029	-2.60587515	0.009163986	0	0.002771728	0	1	CRA004775;PRJNA827465	Yang2025_Silkie_Wenchang	0.150259547
ENSGALG00010022297	VPS18	2	-0.219106715	0.202204239	-1.083591107	0.278546135	0.075982339	14.09816509	92.90687835	1	PRJNA827465;Yang2025_Silkie_Wenchang	CRA004775	0.776763074
ENSGALG00010022297	VPS18	2	-0.404431113	0.07803051	-5.182986906	2.1836E-07	0	0.585521459	0	1	CRA004775;Yang2025_Silkie_Wenchang	PRJNA827465	6.60171E-05
ENSGALG00010022297	VPS18	2	-0.033023436	0.067426085	-0.489772402	0.624294962	0	0.565353395	0	1	CRA004775;PRJNA827465	Yang2025_Silkie_Wenchang	0.900886179
ENSGALG00010022299	MRGBP	2	-0.032462328	0.060034146	-0.540731063	0.588692964	0.002476	1.40749278	28.95167816	0.5	PRJNA827465;Yang2025_Silkie_Wenchang	CRA004775	0.89216471
ENSGALG00010022299	MRGBP	2	-0.009773988	0.150033015	-0.06514558	0.94805809	0.026040122	2.159327236	53.68927954	0.5	CRA004775;Yang2025_Silkie_Wenchang	PRJNA827465	0.986697717
ENSGALG00010022299	MRGBP	2	0.01972908	0.049967652	0.394837032	0.692963163	0	0.855752243	0	1	CRA004775;PRJNA827465	Yang2025_Silkie_Wenchang	0.92962328
ENSGALG00010022302	ENSGALG00010022302	2	-0.139936471	0.123494476	-1.133139515	0.25715567	0.00507396	1.165880618	14.22792486	1	PRJNA827465;Yang2025_Silkie_Wenchang	CRA004775	0.764870705
ENSGALG00010022302	ENSGALG00010022302	2	-0.419511993	0.180060322	-2.329841398	0.019814535	0	0.77329689	0	1	CRA004775;Yang2025_Silkie_Wenchang	PRJNA827465	0.236401882
ENSGALG00010022302	ENSGALG00010022302	2	-0.28600463	0.30129841	-0.949239095	0.342437595	0.126126961	2.81420375	64.46597017	1	CRA004775;PRJNA827465	Yang2025_Silkie_Wenchang	0.806005903
ENSGALG00010022303	DUSP13A	2	0.156279182	0.13289703	1.175941871	0.239618105	0	0.000171236	0	1	PRJNA827465;Yang2025_Silkie_Wenchang	CRA004775	0.751865818
ENSGALG00010022303	DUSP13A	2	0.161119186	0.179035105	0.899930693	0.368157135	0	0.002465351	0	1	CRA004775;Yang2025_Silkie_Wenchang	PRJNA827465	0.820213684
ENSGALG00010022303	DUSP13A	2	0.157916865	0.176543804	0.894491121	0.371059178	0	0.003107446	0	1	CRA004775;PRJNA827465	Yang2025_Silkie_Wenchang	0.8214684
ENSGALG00010022304	ADAP1	2	0.969946284	0.148104402	6.549071255	5.7896E-11	0.000826356	1.019126328	1.876737658	1	PRJNA827465;Yang2025_Silkie_Wenchang	CRA004775	4.21386E-08
ENSGALG00010022304	ADAP1	2	0.464118593	0.435492939	1.065731616	0.286544982	0.283396772	3.640445916	72.53083762	0.5	CRA004775;Yang2025_Silkie_Wenchang	PRJNA827465	0.782018115
ENSGALG00010022304	ADAP1	2	0.59124647	0.587872741	1.005738876	0.314541197	0.587090061	6.337312247	84.22043982	0.5	CRA004775;PRJNA827465	Yang2025_Silkie_Wenchang	0.796531448
ENSGALG00010022307	ENSGALG00010022307	2	-0.000210716	0.856119409	-0.000246129	0.999803617	1.371363134	15.50653353	93.55110542	0.5	PRJNA827465;Yang2025_Silkie_Wenchang	CRA004775	0.999889665
ENSGALG00010022307	ENSGALG00010022307	2	0.384373262	0.573641561	0.670058253	0.502820656	0.459092759	3.028833263	66.98398647	0.5	CRA004775;Yang2025_Silkie_Wenchang	PRJNA827465	0.863050365
ENSGALG00010022307	ENSGALG00010022307	2	-0.742753731	0.279382338	-2.658556501	0.007847619	0	0.634056015	0	1	CRA004775;PRJNA827465	Yang2025_Silkie_Wenchang	0.137510019
ENSGALG00010022308	OGFR	2	0.12527281	0.201581812	0.621448975	0.534304257	0.062064254	3.983411329	74.89588904	0.5	PRJNA827465;Yang2025_Silkie_Wenchang	CRA004775	0.873408008
ENSGALG00010022308	OGFR	2	0.008471079	0.379012437	0.022350398	0.982168447	0.232873642	5.126012127	80.49165754	0.5	CRA004775;Yang2025_Silkie_Wenchang	PRJNA827465	0.995105937
ENSGALG00010022308	OGFR	2	-0.126015759	0.145237554	-0.867652721	0.385584475	0.017569335	1.390978896	28.10818317	1	CRA004775;PRJNA827465	Yang2025_Silkie_Wenchang	0.82648313
ENSGALG00010022309	WSCD2	2	0.011934635	0.22696008	0.052584732	0.958062783	0	0.178634881	0	0.5	PRJNA827465;Yang2025_Silkie_Wenchang	CRA004775	0.988864712
ENSGALG00010022309	WSCD2	2	0.499103901	0.632362753	0.789268342	0.429955186	0.630492136	4.724680649	78.83454832	0.5	CRA004775;Yang2025_Silkie_Wenchang	PRJNA827465	0.842012722
ENSGALG00010022309	WSCD2	2	0.558486218	0.526320334	1.061114653	0.288637799	0.435963328	4.568024681	78.10869972	1	CRA004775;PRJNA827465	Yang2025_Silkie_Wenchang	0.783313256
ENSGALG00010022310	DCLRE1A	2	0.197040843	0.062064774	3.174761295	0.001499598	0	0.052054731	0	1	PRJNA827465;Yang2025_Silkie_Wenchang	CRA004775	0.048147232
ENSGALG00010022310	DCLRE1A	2	-0.012080241	0.219669463	-0.054992809	0.956144193	0.068075702	3.180425759	68.55766882	0.5	CRA004775;Yang2025_Silkie_Wenchang	PRJNA827465	0.988513657
ENSGALG00010022310	DCLRE1A	2	0.011253879	0.234676997	0.04795476	0.961752297	0.087579702	4.299079911	76.73920884	0.5	CRA004775;PRJNA827465	Yang2025_Silkie_Wenchang	0.98973612
ENSGALG00010022311	DLL4	2	-0.076299931	0.188677282	-0.40439384	0.68592313	0.06260283	8.23186014	87.85207738	0.5	PRJNA827465;Yang2025_Silkie_Wenchang	CRA004775	0.919235539
ENSGALG00010022311	DLL4	2	0.063557396	0.433213644	0.146711437	0.883359791	0.288582693	3.561847889	71.92468541	0.5	CRA004775;Yang2025_Silkie_Wenchang	PRJNA827465	0.972001076
ENSGALG00010022311	DLL4	2	0.16542912	0.164003199	1.008694469	0.313121185	0.023749771	1.214814944	17.68293555	1	CRA004775;PRJNA827465	Yang2025_Silkie_Wenchang	0.796531448
ENSGALG00010022312	ZNF592	2	0.042411168	0.144304559	0.293900406	0.768834008	0.032263487	3.933442475	74.57697662	0.5	PRJNA827465;Yang2025_Silkie_Wenchang	CRA004775	0.941666147
ENSGALG00010022312	ZNF592	2	0.106002051	0.140314172	0.755462188	0.449971712	0.012852941	1.44979998	31.02496802	0.5	CRA004775;Yang2025_Silkie_Wenchang	PRJNA827465	0.847521838
ENSGALG00010022312	ZNF592	2	-0.075840764	0.049687472	-1.526355855	0.126921265	0	0.00098261	0	1	CRA004775;PRJNA827465	Yang2025_Silkie_Wenchang	0.597841142
ENSGALG00010022313	ENSGALG00010022313	2	0.289210897	0.218192008	1.325488037	0.185009334	0	0.953316334	0	1	PRJNA827465;Yang2025_Silkie_Wenchang	CRA004775	0.693265167
ENSGALG00010022313	ENSGALG00010022313	2	0.371565733	0.2268767	1.637743023	0.101475312	0	0.391069662	0	1	CRA004775;Yang2025_Silkie_Wenchang	PRJNA827465	0.543585141
ENSGALG00010022313	ENSGALG00010022313	2	0.141474958	0.226689799	0.624090537	0.532568128	0	0.096654107	0	1	CRA004775;PRJNA827465	Yang2025_Silkie_Wenchang	0.873183244
ENSGALG00010022314	DUSP13B	2	0.042366228	0.218131737	0.194223129	0.846001174	0	0.018352096	0	1	PRJNA827465;Yang2025_Silkie_Wenchang	CRA004775	0.96122283
ENSGALG00010022314	DUSP13B	2	0.045421314	0.302524851	0.150140768	0.880653556	0	0.083713945	0	0.5	CRA004775;Yang2025_Silkie_Wenchang	PRJNA827465	0.971623434
ENSGALG00010022314	DUSP13B	2	-0.005893096	0.286826046	-0.02054589	0.983607905	0	0.054433864	0	0.5	CRA004775;PRJNA827465	Yang2025_Silkie_Wenchang	0.995461349
ENSGALG00010022316	INO80	2	0.036327947	0.041456146	0.87629822	0.380867932	0	0.401547971	0	1	PRJNA827465;Yang2025_Silkie_Wenchang	CRA004775	0.824392515
ENSGALG00010022316	INO80	2	0.118159502	0.115254528	1.025204855	0.305266538	0	0.001226155	0	1	CRA004775;Yang2025_Silkie_Wenchang	PRJNA827465	0.79225794
ENSGALG00010022316	INO80	2	0.031826353	0.042454934	0.749650295	0.453465351	0	0.163168576	0	1	CRA004775;PRJNA827465	Yang2025_Silkie_Wenchang	0.848879567
ENSGALG00010022317	PSME3	2	-0.049775384	0.190462493	-0.26133956	0.793830662	0.066865057	12.26451352	91.8463949	0.5	PRJNA827465;Yang2025_Silkie_Wenchang	CRA004775	0.948830022
ENSGALG00010022317	PSME3	2	-0.302258779	0.086760336	-3.483835966	0.000494282	0	0.957747998	0	1	CRA004775;Yang2025_Silkie_Wenchang	PRJNA827465	0.023284834
ENSGALG00010022317	PSME3	2	-0.133957728	0.286417559	-0.467700823	0.639998553	0.14995418	10.847743	90.78149251	0.5	CRA004775;PRJNA827465	Yang2025_Silkie_Wenchang	0.906064918
ENSGALG00010022320	COL9A3	2	1.026797111	0.547800646	1.874399235	0.060875419	0.096989983	1.186579661	15.72415801	1	PRJNA827465;Yang2025_Silkie_Wenchang	CRA004775	0.427127227
ENSGALG00010022320	COL9A3	2	-0.499129322	2.157228999	-0.231375214	0.817023319	8.752290022	16.75717256	94.03240614	0.5	CRA004775;Yang2025_Silkie_Wenchang	PRJNA827465	0.954817505
ENSGALG00010022320	COL9A3	2	-1.028126488	1.60189681	-0.641818176	0.520991247	4.678288481	11.30220699	91.15217054	0.5	CRA004775;PRJNA827465	Yang2025_Silkie_Wenchang	0.869518515
ENSGALG00010022322	ENSGALG00010022322	2	0.224125812	0.311964581	0.718433521	0.472490025	0.079635329	1.691916231	40.89541894	0.5	PRJNA827465;Yang2025_Silkie_Wenchang	CRA004775	0.853998373
ENSGALG00010022322	ENSGALG00010022322	2	-0.003134411	0.512084629	-0.006120885	0.995116271	0.435046304	5.821154217	82.82127628	0.5	CRA004775;Yang2025_Silkie_Wenchang	PRJNA827465	0.998242335
ENSGALG00010022322	ENSGALG00010022322	2	-0.347724697	0.209480664	-1.659936961	0.096927135	0	0.852062402	0	1	CRA004775;PRJNA827465	Yang2025_Silkie_Wenchang	0.532111212
ENSGALG00010022323	DUSP29	2	0.671432859	0.236255511	2.841977548	0.004483465	0	0.605775664	0	1	PRJNA827465;Yang2025_Silkie_Wenchang	CRA004775	0.097950873
ENSGALG00010022323	DUSP29	2	0.751561671	0.302942333	2.480873712	0.01310608	0	0.624694715	0	1	CRA004775;Yang2025_Silkie_Wenchang	PRJNA827465	0.186701071
ENSGALG00010022323	DUSP29	2	0.428299828	0.317192134	1.350285151	0.176924534	0	0.139468062	0	1	CRA004775;PRJNA827465	Yang2025_Silkie_Wenchang	0.682556882
ENSGALG00010022325	MIGA1	2	-0.047565302	0.073282775	-0.64906523	0.516296215	0	0.004183346	0	1	PRJNA827465;Yang2025_Silkie_Wenchang	CRA004775	0.867685411
ENSGALG00010022325	MIGA1	2	-0.028838662	0.136964191	-0.21055622	0.833233578	0	0.015267048	0	0.5	CRA004775;Yang2025_Silkie_Wenchang	PRJNA827465	0.957968425
ENSGALG00010022325	MIGA1	2	-0.045854886	0.079252888	-0.578589466	0.562866214	0	0.031116203	0	0.5	CRA004775;PRJNA827465	Yang2025_Silkie_Wenchang	0.882070607
ENSGALG00010022326	COX19	2	-0.173470079	0.22678151	-0.764921615	0.444318222	0.079282926	3.677390464	72.80680391	0.5	PRJNA827465;Yang2025_Silkie_Wenchang	CRA004775	0.846013379
ENSGALG00010022326	COX19	2	-0.467528331	0.183776171	-2.544009536	0.010958809	0	0.001558746	0	1	CRA004775;Yang2025_Silkie_Wenchang	PRJNA827465	0.166620534
ENSGALG00010022326	COX19	2	-0.149756101	0.224866027	-0.665979217	0.505424385	0.070518677	2.552129568	60.81703639	0.5	CRA004775;PRJNA827465	Yang2025_Silkie_Wenchang	0.863907741
ENSGALG00010022328	ENSGALG00010022328	2	0.109923865	0.153257066	0.717251528	0.47321891	0.002965251	1.066019104	6.193050704	0.5	PRJNA827465;Yang2025_Silkie_Wenchang	CRA004775	0.854341771
ENSGALG00010022328	ENSGALG00010022328	2	-0.218269865	0.539992157	-0.404209324	0.686058798	0.494301809	6.394533181	84.36164186	0.5	CRA004775;Yang2025_Silkie_Wenchang	PRJNA827465	0.919235539
ENSGALG00010022328	ENSGALG00010022328	2	-0.349641701	0.381784712	-0.915808543	0.359767322	0.213774072	3.513567627	71.53889988	1	CRA004775;PRJNA827465	Yang2025_Silkie_Wenchang	0.814880744
ENSGALG00010022332	KAT6B	2	0.195514647	0.275937425	0.708547042	0.47860561	0.126054181	5.225679036	80.86373095	0.5	PRJNA827465;Yang2025_Silkie_Wenchang	CRA004775	0.85563306
ENSGALG00010022332	KAT6B	2	0.285338056	0.212576587	1.342283546	0.179504076	0.045392046	1.994038279	49.85051138	1	CRA004775;Yang2025_Silkie_Wenchang	PRJNA827465	0.68642233
ENSGALG00010022332	KAT6B	2	-0.022851707	0.07265422	-0.314526908	0.753120883	0	0.394255993	0	0.5	CRA004775;PRJNA827465	Yang2025_Silkie_Wenchang	0.938087082
ENSGALG00010022333	ENSGALG00010022333	2	-0.087427388	0.08463772	-1.032960101	0.301622571	0	0.749641387	0	1	PRJNA827465;Yang2025_Silkie_Wenchang	CRA004775	0.791273005
ENSGALG00010022333	ENSGALG00010022333	2	-0.067349196	0.177805341	-0.378780503	0.704850867	0.025824937	1.466991029	31.83325731	0.5	CRA004775;Yang2025_Silkie_Wenchang	PRJNA827465	0.924152671
ENSGALG00010022333	ENSGALG00010022333	2	0.004017286	0.104169681	0.038564826	0.969237346	0	0.61123162	0	0.5	CRA004775;PRJNA827465	Yang2025_Silkie_Wenchang	0.991572914
ENSGALG00010022334	PDE1A	2	0.016999649	0.072367471	0.234907319	0.814280669	0	0.235814162	0	1	PRJNA827465;Yang2025_Silkie_Wenchang	CRA004775	0.953869515
ENSGALG00010022334	PDE1A	2	0.161465632	0.211742843	0.762555323	0.445728638	0	0.043907124	0	1	CRA004775;Yang2025_Silkie_Wenchang	PRJNA827465	0.846013379
ENSGALG00010022334	PDE1A	2	0.014296881	0.074253985	0.192540254	0.847319039	0	0.311636542	0	1	CRA004775;PRJNA827465	Yang2025_Silkie_Wenchang	0.961252712
ENSGALG00010022340	GFOD2	2	-0.097578988	0.152552737	-0.639641018	0.522406009	0.036336822	4.102073869	75.62208697	0.5	PRJNA827465;Yang2025_Silkie_Wenchang	CRA004775	0.869568211
ENSGALG00010022340	GFOD2	2	-0.281084846	0.11908877	-2.360296832	0.018260317	0	0.000187976	0	1	CRA004775;Yang2025_Silkie_Wenchang	PRJNA827465	0.225971429
ENSGALG00010022340	GFOD2	2	-0.056185911	0.139981257	-0.40138167	0.688139143	0.023812319	1.939320368	48.43554388	0.5	CRA004775;PRJNA827465	Yang2025_Silkie_Wenchang	0.919679555
ENSGALG00010022343	GATA5	2	0.400600607	0.330565846	1.211863269	0.225564725	0	0.002128227	0	1	PRJNA827465;Yang2025_Silkie_Wenchang	CRA004775	0.73884362
ENSGALG00010022343	GATA5	2	1.040773655	0.62195766	1.673383449	0.094251834	0.583994216	4.078324372	75.48012594	1	CRA004775;Yang2025_Silkie_Wenchang	PRJNA827465	0.525785636
ENSGALG00010022343	GATA5	2	1.045804298	0.636840008	1.642177445	0.100553236	0.59654232	3.763791671	73.43104807	1	CRA004775;PRJNA827465	Yang2025_Silkie_Wenchang	0.541393598
ENSGALG00010022345	USP33	2	0.15253138	0.055775768	2.734724888	0.006243243	0.001994527	1.461519082	31.5780401	1	PRJNA827465;Yang2025_Silkie_Wenchang	CRA004775	0.119872099
ENSGALG00010022345	USP33	2	0.21027236	0.069067404	3.044451471	0.00233105	0	0.087676729	0	1	CRA004775;Yang2025_Silkie_Wenchang	PRJNA827465	0.064063502
ENSGALG00010022345	USP33	2	0.104486203	0.058222048	1.794615739	0.072714944	0	0.010313234	0	1	CRA004775;PRJNA827465	Yang2025_Silkie_Wenchang	0.465709132
ENSGALG00010022347	SEC11A	2	-0.01417917	0.055701392	-0.25455683	0.799065406	0	0.11528925	0	0.5	PRJNA827465;Yang2025_Silkie_Wenchang	CRA004775	0.949922217
ENSGALG00010022347	SEC11A	2	-0.165742447	0.250303779	-0.662165181	0.507865366	0.084387593	2.579751268	61.23657298	0.5	CRA004775;Yang2025_Silkie_Wenchang	PRJNA827465	0.864401731
ENSGALG00010022347	SEC11A	2	-0.172212919	0.221796513	-0.776445564	0.437485951	0.066551106	2.342827821	57.31653896	1	CRA004775;PRJNA827465	Yang2025_Silkie_Wenchang	0.843803603
ENSGALG00010022348	G6PC1	2	0.426613229	0.710264458	0.600639979	0.548079804	0.781108861	4.285783048	76.66704103	0.5	PRJNA827465;Yang2025_Silkie_Wenchang	CRA004775	0.878281266
ENSGALG00010022348	G6PC1	2	0.441080269	0.656611259	0.671752522	0.501741259	0.726598352	6.35516804	84.26477485	0.5	CRA004775;Yang2025_Silkie_Wenchang	PRJNA827465	0.86279037
ENSGALG00010022348	G6PC1	2	1.128819799	0.309759061	3.644186531	0.000268239	0	0.027649219	0	1	CRA004775;PRJNA827465	Yang2025_Silkie_Wenchang	0.01571176
ENSGALG00010022353	SLC39A13	2	-0.160090003	0.286451667	-0.558872651	0.576248635	0.156950688	22.67739397	95.59032223	0.5	PRJNA827465;Yang2025_Silkie_Wenchang	CRA004775	0.887048016
ENSGALG00010022353	SLC39A13	2	-0.375314745	0.121424154	-3.090939761	0.001995241	0.009873958	1.381335452	27.60628864	1	CRA004775;Yang2025_Silkie_Wenchang	PRJNA827465	0.058002189
ENSGALG00010022353	SLC39A13	2	0.028280603	0.140071988	0.201900487	0.839994521	0.024465059	2.100699754	52.39681453	0.5	CRA004775;PRJNA827465	Yang2025_Silkie_Wenchang	0.95957104
ENSGALG00010022354	FAR2	2	-0.100651634	0.058515459	-1.720086212	0.085416772	0	0.031330764	0	1	PRJNA827465;Yang2025_Silkie_Wenchang	CRA004775	0.502621951
ENSGALG00010022354	FAR2	2	-0.086101023	0.128801474	-0.668478553	0.503828168	0	0.155105632	0	1	CRA004775;Yang2025_Silkie_Wenchang	PRJNA827465	0.86324419
ENSGALG00010022354	FAR2	2	-0.090509336	0.059616904	-1.518182437	0.128968414	0	0.115208093	0	1	CRA004775;PRJNA827465	Yang2025_Silkie_Wenchang	0.602501553
ENSGALG00010022356	TCFL5	2	0.270757429	0.367766308	0.736221407	0.461595967	0.2230752	5.6892429	82.42296879	0.5	PRJNA827465;Yang2025_Silkie_Wenchang	CRA004775	0.85042452
ENSGALG00010022356	TCFL5	2	0.308904182	0.460004943	0.671523613	0.501887022	0.272172671	2.414717606	58.58728999	0.5	CRA004775;Yang2025_Silkie_Wenchang	PRJNA827465	0.862795569
ENSGALG00010022356	TCFL5	2	-0.114000692	0.191734641	-0.594575351	0.552127361	0	0.13983609	0	1	CRA004775;PRJNA827465	Yang2025_Silkie_Wenchang	0.879061525
ENSGALG00010022357	ENSGALG00010022357	2	0.205457615	0.324230884	0.63367688	0.526291717	0	0.858840884	0	0.5	PRJNA827465;Yang2025_Silkie_Wenchang	CRA004775	0.870537576
ENSGALG00010022357	ENSGALG00010022357	2	0.413832157	0.429770114	0.962915159	0.335590102	0	0.337769461	0	1	CRA004775;Yang2025_Silkie_Wenchang	PRJNA827465	0.803317552
ENSGALG00010022357	ENSGALG00010022357	2	-0.003879361	0.357587179	-0.010848715	0.991344148	0	0.012873246	0	0.5	CRA004775;PRJNA827465	Yang2025_Silkie_Wenchang	0.99755571
ENSGALG00010022359	ZZZ3	2	-0.038928072	0.046534767	-0.836537377	0.402852658	0	0.513377348	0	0.5	PRJNA827465;Yang2025_Silkie_Wenchang	CRA004775	0.832937979
ENSGALG00010022359	ZZZ3	2	0.068504274	0.112265096	0.610200996	0.54172867	0.010166019	1.40318879	28.73375221	1	CRA004775;Yang2025_Silkie_Wenchang	PRJNA827465	0.876340218
ENSGALG00010022359	ZZZ3	2	0.043991732	0.156639414	0.280847142	0.778827644	0.032716345	2.37748663	57.93877503	0.5	CRA004775;PRJNA827465	Yang2025_Silkie_Wenchang	0.944673731
ENSGALG00010022363	KIF18A	2	-0.119448294	0.0961762	-1.241973523	0.214246331	0.001328661	1.076938528	7.144189379	1	PRJNA827465;Yang2025_Silkie_Wenchang	CRA004775	0.72703536
ENSGALG00010022363	KIF18A	2	-0.224311706	0.132512111	-1.692763808	0.090500434	0	0.001930701	0	1	CRA004775;Yang2025_Silkie_Wenchang	PRJNA827465	0.516242452
ENSGALG00010022363	KIF18A	2	-0.040718639	0.123034129	-0.330954013	0.740679222	0	0.146512204	0	1	CRA004775;PRJNA827465	Yang2025_Silkie_Wenchang	0.935170274
ENSGALG00010022365	PLCG1	2	0.054114741	0.089953245	0.601587425	0.547448801	0.011691533	3.600706376	72.22767158	0.5	PRJNA827465;Yang2025_Silkie_Wenchang	CRA004775	0.878281266
ENSGALG00010022365	PLCG1	2	-0.148090514	0.194920413	-0.759748614	0.447404864	0.045534996	1.794066868	44.26071749	1	CRA004775;Yang2025_Silkie_Wenchang	PRJNA827465	0.84650962
ENSGALG00010022365	PLCG1	2	-0.090662784	0.305585612	-0.296685381	0.766706719	0.143589137	3.514989682	71.55041436	0.5	CRA004775;PRJNA827465	Yang2025_Silkie_Wenchang	0.941271762
ENSGALG00010022366	AARSD1	2	0.168227685	0.191914183	0.87657766	0.380716078	0.064069769	7.317124048	86.33342836	0.5	PRJNA827465;Yang2025_Silkie_Wenchang	CRA004775	0.824392515
ENSGALG00010022366	AARSD1	2	0.09517999	0.306493637	0.310544752	0.756146733	0.157077763	5.896950982	83.0420839	0.5	CRA004775;Yang2025_Silkie_Wenchang	PRJNA827465	0.93854559
ENSGALG00010022366	AARSD1	2	-0.027278842	0.063978164	-0.426377387	0.669832876	0.000717821	1.027943348	2.718374353	1	CRA004775;PRJNA827465	Yang2025_Silkie_Wenchang	0.914377769
ENSGALG00010022373	AK5	2	0.486971578	0.494917878	0.983944205	0.325142947	0.404312242	5.45379204	81.66413401	1	PRJNA827465;Yang2025_Silkie_Wenchang	CRA004775	0.799914637
ENSGALG00010022373	AK5	2	1.878978435	1.003287141	1.872822204	0.061092941	1.576465144	4.165993722	75.99612321	1	CRA004775;Yang2025_Silkie_Wenchang	PRJNA827465	0.427248372

ENSGALG00010022373	AK5	2	1.422123395	1.510010797	0.941796839	0.346296664	4.149155172	10.27854521	90.27099672	1	CRA004775;PRJNA827465	Yang2025_Silkie_Wenchang	0.807744676
ENSGALG00010022375	NMB	2	-0.183854569	0.217672618	-0.84463802	0.398312975	0	0.164106862	0	1	PRJNA827465;Yang2025_Silkie_Wenchang	CRA004775	0.831980453
ENSGALG00010022375	NMB	2	0.053085212	0.36534084	0.145303251	0.884471448	0	0.061984722	0	0.5	CRA004775;Yang2025_Silkie_Wenchang	PRJNA827465	0.972062774
ENSGALG00010022375	NMB	2	-0.171815556	0.227654733	-0.754706715	0.450424976	0	0.388021307	0	0.5	CRA004775;PRJNA827465	Yang2025_Silkie_Wenchang	0.847577105
ENSGALG00010022377	ZHX3	2	-0.027304935	0.057261504	-0.476846277	0.63347159	0	0.557675697	0	0.5	PRJNA827465;Yang2025_Silkie_Wenchang	CRA004775	0.903348345
ENSGALG00010022377	ZHX3	2	0.062117427	0.137901832	0.450446712	0.652388369	0	0.064974088	0	0.5	CRA004775;Yang2025_Silkie_Wenchang	PRJNA827465	0.909377929
ENSGALG00010022377	ZHX3	2	-0.042904298	0.060426403	-0.710025687	0.477688207	0	0.014183004	0	1	CRA004775;PRJNA827465	Yang2025_Silkie_Wenchang	0.855255996
ENSGALG00010022379	METTL15	2	0.18154629	0.311201195	0.583372728	0.559642413	0.173481876	9.313829346	89.26327762	0.5	PRJNA827465;Yang2025_Silkie_Wenchang	CRA004775	0.881203589
ENSGALG00010022379	METTL15	2	0.256328851	0.318155869	0.805670667	0.420432767	0.134118568	2.711089992	63.11446677	0.5	CRA004775;Yang2025_Silkie_Wenchang	PRJNA827465	0.838923138
ENSGALG00010022379	METTL15	2	-0.114099505	0.094291041	-1.210077899	0.226249003	0	0.006038767	0	1	CRA004775;PRJNA827465	Yang2025_Silkie_Wenchang	0.739677001
ENSGALG00010022385	ENSGALG00010022385	2	-0.3027444	0.355027965	-0.852733954	0.393806857	0	0.147065946	0	1	PRJNA827465;Yang2025_Silkie_Wenchang	CRA004775	0.830486298
ENSGALG00010022385	ENSGALG00010022385	2	-0.281679987	0.431264557	-0.653148937	0.513660266	0	0.156678358	0	1	CRA004775;Yang2025_Silkie_Wenchang	PRJNA827465	0.866528862
ENSGALG00010022385	ENSGALG00010022385	2	-0.423877201	0.362290719	-1.169991886	0.242004234	0	0.005710409	0	1	CRA004775;PRJNA827465	Yang2025_Silkie_Wenchang	0.75421685
ENSGALG00010022386	SPI1	2	-0.105792566	0.156029354	-0.678029893	0.49775274	0.03216967	2.864106295	65.08509471	0.5	PRJNA827465;Yang2025_Silkie_Wenchang	CRA004775	0.861055278
ENSGALG00010022386	SPI1	2	-0.30760522	0.148559258	-2.070589371	0.038397186	0	0.385071331	0	1	CRA004775;Yang2025_Silkie_Wenchang	PRJNA827465	0.337838507
ENSGALG00010022386	SPI1	2	-0.074804764	0.248517738	-0.30100372	0.763411658	0.062089863	1.356090167	26.25859074	0.5	CRA004775;PRJNA827465	Yang2025_Silkie_Wenchang	0.940301633
ENSGALG00010022388	CYP2W1	2	0.065804071	0.277943984	0.236752996	0.812848418	0	0.852985623	0	0.5	PRJNA827465;Yang2025_Silkie_Wenchang	CRA004775	0.953526116
ENSGALG00010022388	CYP2W1	2	0.578733344	0.888398887	0.651434117	0.514766293	1.305683121	5.745068709	82.59376779	0.5	CRA004775;Yang2025_Silkie_Wenchang	PRJNA827465	0.866930857
ENSGALG00010022388	CYP2W1	2	0.793738142	0.615690087	1.28918454	0.197333939	0.544706224	3.384637601	70.45473938	1	CRA004775;PRJNA827465	Yang2025_Silkie_Wenchang	0.708876317
ENSGALG00010022391	ENSGALG00010022391	2	0.066086301	0.107307943	0.615856564	0.537989192	0.007453847	1.316709443	24.05310029	1	PRJNA827465;Yang2025_Silkie_Wenchang	CRA004775	0.87481821
ENSGALG00010022391	ENSGALG00010022391	2	0.198292711	0.169987435	1.166513934	0.243406718	0	0.296207282	0	1	CRA004775;Yang2025_Silkie_Wenchang	PRJNA827465	0.755298773
ENSGALG00010022391	ENSGALG00010022391	2	0.007291675	0.085488076	0.085294638	0.932027154	0	0.012935342	0	1	CRA004775;PRJNA827465	Yang2025_Silkie_Wenchang	0.98412915
ENSGALG00010022392	EMILIN3	2	0.298969508	0.130333921	2.293873348	0.021797775	0.014433042	1.685495938	40.67028124	1	PRJNA827465;Yang2025_Silkie_Wenchang	CRA004775	0.250063614
ENSGALG00010022392	EMILIN3	2	0.427606005	0.165215312	2.588174179	0.009648618	0	0.9453118	0	0.5	CRA004775;Yang2025_Silkie_Wenchang	PRJNA827465	0.154906126
ENSGALG00010022392	EMILIN3	2	0.118249208	0.116038768	1.572681977	0.115792475	0	0.447847184	0	0.5	CRA004775;PRJNA827465	Yang2025_Silkie_Wenchang	0.572808488
ENSGALG00010022395	PTGES3L	2	0.194501083	0.229780335	0.846465311	0.397293217	0.089567971	6.345596838	84.24104106	0.5	PRJNA827465;Yang2025_Silkie_Wenchang	CRA004775	0.831504091
ENSGALG00010022395	PTGES3L	2	0.206977361	0.323122409	0.640554029	0.521812475	0.135104226	2.431998044	58.88154588	0.5	CRA004775;Yang2025_Silkie_Wenchang	PRJNA827465	0.86956507
ENSGALG00010022395	PTGES3L	2	-0.027099076	0.087187122	-0.310815126	0.75594117	0	0.273360626	0	1	CRA004775;PRJNA827465	Yang2025_Silkie_Wenchang	0.938501783
ENSGALG00010022396	PPP1R1C	2	0.123232442	0.17902712	0.688345106	0.491253483	0	0.002314505	0	1	PRJNA827465;Yang2025_Silkie_Wenchang	CRA004775	0.85895707
ENSGALG00010022396	PPP1R1C	2	0.742483753	0.729680397	1.017546526	0.308891511	0.778440946	3.400635639	70.59373287	1	CRA004775;Yang2025_Silkie_Wenchang	PRJNA827465	0.793908035
ENSGALG00010022396	PPP1R1C	2	0.709184201	0.73480919	0.965127016	0.334481197	0.845673313	3.955547232	74.71904793	1	CRA004775;PRJNA827465	Yang2025_Silkie_Wenchang	0.803094614
ENSGALG00010022397	ATF1	2	-0.049034277	0.036020488	-1.361288502	0.173422535	0	0.337263125	0	1	PRJNA827465;Yang2025_Silkie_Wenchang	CRA004775	0.676868485
ENSGALG00010022397	ATF1	2	-0.066108254	0.088427319	-0.747599892	0.45470153	0	0.5352751	0	0.5	CRA004775;Yang2025_Silkie_Wenchang	PRJNA827465	0.849072665
ENSGALG00010022397	ATF1	2	-0.037306152	0.037487947	-0.995150565	0.319663041	0	0.219267928	0	0.5	CRA004775;PRJNA827465	Yang2025_Silkie_Wenchang	0.798328994
ENSGALG00010022400	NHLRC2	2	0.164917814	0.066099259	2.495002453	0.012595626	9.41396E-05	1.008821877	0.874473217	1	PRJNA827465;Yang2025_Silkie_Wenchang	CRA004775	0.181734907
ENSGALG00010022400	NHLRC2	2	0.257928162	0.109199979	2.36197997	0.018177624	0	0.043491304	0	1	CRA004775;Yang2025_Silkie_Wenchang	PRJNA827465	0.22514412
ENSGALG00010022400	NHLRC2	2	0.132660313	0.073450966	1.806107115	0.07090161	0	0.142197622	0	1	CRA004775;PRJNA827465	Yang2025_Silkie_Wenchang	0.459463474
ENSGALG00010022401	LCAT	2	0.219152408	0.589491804	0.371764979	0.710067841	0.603287788	7.47159953	86.61598502	0.5	PRJNA827465;Yang2025_Silkie_Wenchang	CRA004775	0.925330621
ENSGALG00010022401	LCAT	2	-0.333320687	0.227967339	-1.462142293	0.143702221	0	0.011766033	0	1	CRA004775;Yang2025_Silkie_Wenchang	PRJNA827465	0.630564924
ENSGALG00010022401	LCAT	2	0.385962903	0.545180161	0.707954783	0.478973338	0.374795157	2.552581871	60.82397938	0.5	CRA004775;PRJNA827465	Yang2025_Silkie_Wenchang	0.855875059
ENSGALG00010022406	ITPRID2	2	-0.011968859	0.141137958	-0.084802552	0.932418364	0.032744534	5.582492609	82.08685492	0.5	PRJNA827465;Yang2025_Silkie_Wenchang	CRA004775	0.984224753
ENSGALG00010022406	ITPRID2	2	-0.146220576	0.09029481	-1.619368559	0.105367987	0	0.351938663	0	0.5	CRA004775;Yang2025_Silkie_Wenchang	PRJNA827465	0.550848365
ENSGALG00010022406	ITPRID2	2	0.123236022	0.074456753	1.655135603	0.097896994	0	0.00547959	0	1	CRA004775;PRJNA827465	Yang2025_Silkie_Wenchang	0.534130658
ENSGALG00010022407	RUNDC1	2	0.015638408	0.038441092	0.406814863	0.684143969	0	0.231040283	0	1	PRJNA827465;Yang2025_Silkie_Wenchang	CRA004775	0.918806497
ENSGALG00010022407	RUNDC1	2	0.021922221	0.086359061	0.253849692	0.799611682	0.001131245	1.030347324	2.945348957	0.5	CRA004775;Yang2025_Silkie_Wenchang	PRJNA827465	0.949922217
ENSGALG00010022407	RUNDC1	2	7.10731E-05	0.042507911	0.001671997	0.99866594	0	0.773540203	0	0.5	CRA004775;PRJNA827465	Yang2025_Silkie_Wenchang	0.999657891
ENSGALG00010022408	RAD51AP1	2	0.051227272	0.101032903	0.507035541	0.612129881	0.029274791	2.692006636	62.85298904	0.5	PRJNA827465;Yang2025_Silkie_Wenchang	CRA004775	0.897833503
ENSGALG00010022408	RAD51AP1	2	0.136569905	0.094437687	1.446137757	0.148138568	0	0.789458674	0	0.5	CRA004775;Yang2025_Silkie_Wenchang	PRJNA827465	0.63772401
ENSGALG00010022408	RAD51AP1	2	-0.044335399	0.072114315	-0.614793325	0.538691218	0	0.071905681	0	1	CRA004775;PRJNA827465	Yang2025_Silkie_Wenchang	0.87481821
ENSGALG00010022409	CARNS1	2	0.15697965	0.451696551	0.347533427	0.728190611	0.333077373	5.432956447	81.59381527	0.5	PRJNA827465;Yang2025_Silkie_Wenchang	CRA004775	0.931987594
ENSGALG00010022409	CARNS1	2	0.501059395	0.261782723	1.914027645	0.055616611	0	0.932853681	0	0.5	CRA004775;Yang2025_Silkie_Wenchang	PRJNA827465	0.408441043
ENSGALG00010022409	CARNS1	2	-0.251098438	0.239478434	-1.048522132	0.294398111	0	0.123815473	0	1	CRA004775;PRJNA827465	Yang2025_Silkie_Wenchang	0.787343482
ENSGALG00010022411	ADRB1	2	-0.207958335	0.288644011	-0.720466482	0.47123783	0.107055725	2.7787336	64.0123832	0.5	PRJNA827465;Yang2025_Silkie_Wenchang	CRA004775	0.85393796
ENSGALG00010022411	ADRB1	2	-0.505406894	0.22372193	-2.259083167	0.023878088	0	0.004189351	0	1	CRA004775;Yang2025_Silkie_Wenchang	PRJNA827465	0.204093469
ENSGALG00010022411	ADRB1	2	-0.096250361	0.248625248	-0.387130277	0.698659764	0.037334178	1.333562532	25.01289023	0.5	CRA004775;PRJNA827465	Yang2025_Silkie_Wenchang	0.921745106
ENSGALG00010022412	CHAC1	2	0.846989276	0.299526584	2.827759942	0.004687494	0.063235752	1.489745283	32.87443088	1	PRJNA827465;Yang2025_Silkie_Wenchang	CRA004775	0.100728587
ENSGALG00010022412	CHAC1	2	0.457903127	0.386366038	1.185153667	0.235956696	0	6.81697E-05	0	1	CRA004775;Yang2025_Silkie_Wenchang	PRJNA827465	0.748669015
ENSGALG00010022412	CHAC1	2	1.027202076	0.269895598	3.805925785	0.000141276	0	0.382232525	0	1	CRA004775;PRJNA827465	Yang2025_Silkie_Wenchang	0.010009417
ENSGALG00010022415	DIP2B	2	0.045064061	0.047315194	0.952422603	0.340882701	0	0.744259533	0	0.5	PRJNA827465;Yang2025_Silkie_Wenchang	CRA004775	0.805635165
ENSGALG00010022415	DIP2B	2	-0.049155314	0.098319397	-0.499955403	0.61716048	0	0.497617192	0	1	CRA004775;Yang2025_Silkie_Wenchang	PRJNA827465	0.899121942
ENSGALG00010022415	DIP2B	2	0.060053542	0.053076408	1.131454521	0.257863835	0	0.917104844	0	0.5	CRA004775;PRJNA827465	Yang2025_Silkie_Wenchang	0.76515037
ENSGALG00010022416	CCDC186	2	-0.057223748	0.051191585	-1.117835048	0.26363745	0	0.934947719	0	1	PRJNA827465;Yang2025_Silkie_Wenchang	CRA004775	0.768643821
ENSGALG00010022416	CCDC186	2	0.047917344	0.241777884	0.198187457	0.842898401	0.088270588	3.856389587	74.06900996	0.5	CRA004775;Yang2025_Silkie_Wenchang	PRJNA827465	0.960411546
ENSGALG00010022416	CCDC186	2	0.084496834	0.169015898	0.499934237	0.617121384	0.039400618	2.559969045	60.93702765	0.5	CRA004775;PRJNA827465	Yang2025_Silkie_Wenchang	0.899121942
ENSGALG00010022421	CMKLR1	2	-0.039491144	0.12814985	-0.308163794	0.757957701	0.010678042	1.451103962	31.08694992	0.5	PRJNA827465;Yang2025_Silkie_Wenchang	CRA004775	0.938954369
ENSGALG00010022421	CMKLR1	2	0.060298872	0.334860074	0.180071848	0.857096163	0.14797357	2.612612856	61.7241415	0.5	CRA004775;Yang2025_Silkie_Wenchang	PRJNA827465	0.963878155
ENSGALG00010022421	CMKLR1	2	0.121077965	0.14581368	0.830360809	0.406334817	0.008021101	1.094843297	8.662728034	1	CRA004775;PRJNA827465	Yang2025_Silkie_Wenchang	0.833580937
ENSGALG00010022422	RPS6KB2	2	0.112757865	0.092910918	1.213612649	0.224895675	0.010352732	2.273510154	56.01515136	1	PRJNA827465;Yang2025_Silkie_Wenchang	CRA004775	0.737888351
ENSGALG00010022422	RPS6KB2	2	-0.003157553	0.327513153	-0.009640997	0.992307717	0.150426573	2.718709197	63.21783877	0.5	CRA004775;Yang2025_Silkie_Wenchang	PRJNA827465	0.997811136
ENSGALG00010022422	RPS6KB2	2	-0.047944941	0.190898186	-0.251154511	0.801694653	0.041019859	1.49552817	33.13399104	0.5	CRA004775;PRJNA827465	Yang2025_Silkie_Wenchang	0.950372222
ENSGALG00010022426	EXD1	2	-0.973747546	0.445079024	-2.187808217	0.028683575	0.229129168	2.335237298	57.1777994	1	PRJNA827465;Yang2025_Silkie_Wenchang	CRA004775	0.288600705
ENSGALG00010022426	EXD1	2	-0.733835688	0.707321968	-1.037484655	0.299516041	0.82653736	5.707863707	82.48031047	1	CRA004775;Yang2025_Silkie_Wenchang	PRJNA827465	0.79004325
ENSGALG00010022426	EXD1	2	-0.321159311	0.26011628	-1.234675932	0.216951137	0.002526677	1.019010394	1.865574123	1	CRA004775;PRJNA827465	Yang2025_Silkie_Wenchang	0.730505165
ENSGALG00010022427	RPL27	2	-0.161707351	0.168240741	-0.96116642	0.336468498	0.03389643	1.836782089	45.5569604	1	PRJNA827465;Yang2025_Silkie_Wenchang	CRA004775	0.803806405
ENSGALG00010022427	RPL27	2	-0.332463467	0.181365198	-1.833116109	0.066785306	0	0.308175548	0	1	CRA004775;Yang2025_Silkie_Wenchang	PRJNA827465	0.445135824
ENSGALG00010022427	RPL27	2	-0.075347024	0.062012613	-1.215027396	0.224355643	0	0.539221198	0	1	CRA004775;PRJNA827465	Yang2025_Silkie_Wenchang	0.737471949
ENSGALG00010022429	ADK	2	-0.00450841	0.058309406	-0.077318747	0.938369977	0	0.023363875	0	0.5	PRJNA827465;Yang2025_Silkie_Wenchang	CRA004775	0.985349045
ENSGALG00010022429	ADK	2	-0.03159756	0.121856565	-0.259301249	0.795402816	0	0.465259528	0	0.5	CRA004775;Yang2025_Silkie_Wenchang	PRJNA827465	0.948935068
ENSGALG00010022429	ADK	2	-0.019241632	0.062153913	-0.309580374	0.756880083	0	0.443271574	0	1	CRA004775;PRJNA827465	Yang2025_Silkie_Wenchang	0.93876729
ENSGALG00010022431	LARP4	2	-0.030468153	0.054565418	-0.558378453	0.576585983	0	0.253589087	0	1	PRJNA827465;Yang2025_Silkie_Wenchang	CRA004775	0.887113945
ENSGALG00010022431	LARP4	2	-0.051240455	0.093979721	-0.545228849	0.585596132	0	0.368689751	0	0.5	CRA004775;Yang2025_Silkie_Wenchang	PRJNA827465	0.891148209
ENSGALG00010022431	LARP4	2	-0.006371078	0.063063164	-0.101026933	0.91952908	0	0.167170613	0	0.5	CRA004775;PRJNA827465	Yang2025_Silkie_Wenchang	0.981192162
ENSGALG00010022433	LNPK	2	-0.189220578	0.069611876	-2.718223236	0.006563366	0.001535034	1.153792907	13.32933371	1	PRJNA827465;Yang2025_Silkie_Wenchang	CRA004775	0.123781174
ENSGALG00010022433	LNPK	2	-0.211047011	0.169751497	-1.243270398	0.213768209	0.030612157	1.963898264	49.08086542	1	CRA004775;Yang2025_Silkie_Wenchang	PRJNA827465	0.726416385
ENSGALG00010022433	LNPK	2	-0.254901243	0.069722864	-3.655920404	0.000256261	0	0.746490859	0	1	CRA004775;PRJNA827465	Yang2025_Silkie_Wenchang	0.015122854
ENSGALG00010022435	PIGK	2	-0.279668303	0.231393852	-1.208624606	0.226807099	0.094403765	8.262130552	87.89658438	1	PRJNA827465;Yang2025_Silkie_Wenchang	CRA004775	0.74038201
ENSGALG00010022435	PIGK	2	-0.213869767	0.367098079	-0.582595713	0.560165492	0.212103638	4.255227047	76.49949136	0.5	CRA004775;Yang2025_Silkie_Wenchang	PRJNA827465	0.881203589
ENSGALG00010022435	PIGK	2	-0.043541816	0.082048194	-0.530685857	0.595636489	0	0.66642248	0	0.5	CRA004775;PRJNA827465	Yang2025_Silkie_Wenchang	0.893802528
ENSGALG00010022436	AFAP1L2	2	0.032469405	0.052911394	0.613656196	0.53944254	0	0.556039649	0	0.5	PRJNA827465;Yang2025_Silkie_Wenchang	CRA004775	0.874993194
ENSGALG00010022436	AFAP1L2	2	-0.028797496	0.090058708	-0.31976359	0.749147551	0	0.008176783	0	1	CRA004775;Yang2025_Silkie_Wenchang	PRJNA827465	0.937362187
ENSGALG00010022436	AFAP1L2	2	0.053826876	0.062295192	0.864061493	0.387554181	0	0.144238114	0	0.5	CRA004775;PRJNA827465	Yang2025_Silkie_Wenchang	0.82800822
ENSGALG00010022438	RPN2	2	-0.00382727	0.11997875	-0.031899668	0.974552143	0.024006886	5.767093903	82.66024419	0.5	PRJNA827465;Yang2025_Silkie_Wenchang	CRA004775	0.992492038
ENSGALG00010022438	RPN2	2	-0.145652808	0.083872003	-1.736608199	0.082456351	0	0.14242054	0	1	CRA004775;Yang2025_Silkie_Wenchang	PRJNA827465	0.495221142
ENSGALG00010022438	RPN2	2	0.03595408	0.139741005	0.257290834	0.796954268	0.022029332	1.558946108	35.85410075	0.5	CRA004775;PRJNA827465	Yang2025_Silkie_Wenchang	0.949622804
ENSGALG00010022439	IFI35	2	0.095155411	0.185414978	0.513202397	0.607809749	0.053790505	4.385022448	77.19509964	0.5	PRJNA827465;Yang2025_Silkie_Wenchang	CRA004775	0.897203929
ENSGALG00010022439	IFI35	2	0.058581082	0.267372261	0.219099324	0.826572677	0.107463179	3.933281279	74.57593472	0.5	CRA004775;Yang2025_Silkie_Wenchang	PRJNA827465	0.956393839
ENSGALG00010022439	IFI35	2	-0.092491293	0.082376211	-1.122791309	0.261526137	0	0.467301189	0	1	CRA004775;PRJNA827465	Yang2025_Silkie_Wenchang	0.768310285
ENSGALG00010022440	SORD	2	-0.011148674	0.1023669	-0.108908971	0.913274691	0	0.005021542	0	0.5	PRJNA827465;Yang2025_Silkie_Wenchang	CRA004775	0.979169142
ENSGALG00010022440	SORD	2	-0.35470219	0.400652437	-0.885311452	0.375988675	0.233703607	3.559434969	71.90565332	0.5	CRA004775;Yang2025_Silkie_Wenchang	PRJNA827465	0.822974013
ENSGALG00010022440	SORD	2	-0.335959144	0.387120484	-0.8678413	0.385481217	0.240176283	4.427343635	77.41309276	1	CRA004775;PRJNA827465	Yang2025_Silkie_Wenchang	0.82648313
ENSGALG00010022441	FICD	2	-0.033896647	0.258136319	-0.131312973	0.89552773	0.114307666	6.887976059	85.48194722	0.5	PRJNA827465;Yang2025_Silkie_Wenchang	CRA004775	0.974927462
ENSGALG00010022441	FICD	2	-0.272564875	0.120558929	-2.260843526	0.023768948	0	0.096492799	0	1	CRA004775;Yang2025_Silkie_Wenchang	PRJNA827465	0.263367861
ENSGALG00010022441	FICD	2	0.010752187	0.219922382	0.048890827	0.961006299	0.076286058	4.560224339	78.07125427	0.5	CRA004775;PRJNA827465	Yang2025_Silkie_Wenchang	0.989599947
ENSGALG00010022448	FERRY3	2	0.5411411	0.476422914	1.135841885	0.256022744	0.407311532	9.389548407	89.34986054	1	PRJNA827465;Yang2025_Silkie_Wenchang	CRA004775	0.764570254
ENSGALG00010022448	FERRY3	2	0.388091069	0.659822859	0.588174634	0.556415082	0.794705942	11.45239848	91.26820463	0.5	CRA004775;Yang2025_Silkie_Wenchang	PRJNA827465	0.880324407
ENSGALG00010022448	FERRY3	2	-0.012499992	0.16612173	-0.075245979	0.940019002	0.019971022	1.428827925	30.01256608	0.5	CRA004775;PRJNA827465	Yang2025_Silkie_Wenchang	0.985444231
ENSGALG00010022449	ENSGALG00010022449	2	0.208147112	0.275829435	0.75462255	0.450475489	0.137956497	10.18863476	90.18514233	0.5	PRJNA827465;Yang2025_Silkie_Wenchang	CRA004775	0.847577105
ENSGALG00010022449	ENSGALG00010022449	2	0.050218463	0.46893761	0.107089859	0.914717692	0.400414894	11.05661271	90.95563871	0.5	CRA004775;Yang2025_Silkie_Wenchang	PRJNA827465	0.97982748
ENSGALG00010022449	ENSGALG00010022449	2	-0.176549431	0.181524537	-0.972592652	0.330755796	0.045485072	2.584380371	61.30600544	1	CRA004775;PRJNA827465	Yang2025_Silkie_Wenchang	0.801609117
ENSGALG00010022451	CHP1	2	-0.039356811	0.277319428	-0.141918692	0.887144229	0.142772065	13.71143109	92.70681526	0.5	PRJNA827465;Yang2025_Silkie_Wenchang	CRA004775	0.972973806
ENSGALG00010022451	CHP1	2	-0.091173436	0.242109722	-0.376578996	0.706486499	0.097396006	5.88714867	83.01384837	0.5	CRA004775;Yang2025_Silkie_Wenchang	PRJNA827465	0.924548793
ENSGALG00010022451	CHP1	2	0.214229233	0.068106075	3.145523092	0.001657901	0	0.171821461	0	1	CRA004775;PRJNA827465	Yang2025_Silkie_Wenchang	0.051428955
ENSGALG00010022452	AP3M1	2	-0.178627388	0.144565928	-1.23561195	0.216602841	0.029117184	3.216184976	68.90726101	1	PRJNA827465;Yang2025_Silkie_Wenchang	CRA004775	0.730172415
ENSGALG00010022452	AP3M1	2	-0.286410011	0.107957275	-2.652994061	0.007978127	0	0.472263251	0	1	CRA004775;Yang2025_Silkie_Wenchang	PRJNA827465	0.138929705
ENSGALG00010022452	AP3M1	2	-0.075230739	0.083586374	-0.900035912	0.368101139	0	0.409964268	0	1	CRA004775;PRJNA827465	Yang2025_Silkie_Wenchang	0.820213684
ENSGALG00010022453	ENSGALG00010022453	2	-0.135585831	0.08676719	-1.562639412	0.118137433	0	0.073561553	0	1	PRJNA827465;Yang2025_Silkie_Wenchang	CRA004775	0.578003524
ENSGALG00010022453	ENSGALG00010022453	2	-0.366969333	0.262675313	-1.397045385	0.162399924	0.098432859	3.100018262	67.74212552	1	CRA004775;Yang2025_Silkie_Wenchang	PRJNA827465	0.659930202
ENSGALG00010022453	ENSGALG00010022453	2	-0.349655741	0.288055074	-1.213850308	0.224804891	0.12413673	3.603031863	72.24559654	1	CRA004775;PRJNA827465	Yang2025_Silkie_Wenchang	0.737885283
ENSGALG00010022454	ABLIM1	2	-0.021714642	0.090576433	-0.239738319	0.810533127	0	0.579698922	0	0.5	PRJNA827465;Yang2025_Silkie_Wenchang	CRA004775	0.952997426
ENSGALG00010022454	ABLIM1	2	0.091739921	0.198072376	0.46316363	0.643247083	0	0.166680088	0	0.5	CRA004775;Yang2025_Silkie_Wenchang	PRJNA827465	0.906634523
ENSGALG00010022454	ABLIM1	2	-0.053558521	0.096912364	-0.552649003	0.58050378	0	0.001650156	0	1	CRA004775;PRJNA827465	Yang2025_Silkie_Wenchang	0.888433837
ENSGALG00010022455	C7orf50	2	0.002469766	0.133138065	0.018550409	0.985199764	0	0.193521986	0	0.5	PRJNA827465;Yang2025_Silkie_Wenchang	CRA004775	0.995859331
ENSGALG00010022455	C7orf50	2	-0.177532717	0.170977623	-1.038338903	0.299112303	0	0.642095278	0	1	CRA004775;Yang2025_Silkie_Wenchang	PRJNA827465	0.789748017
ENSGALG00010022455	C7orf50	2	-0.091735209	0.192345172	-0.476930138	0.63341187	0.028003276	1.521924487	34.29371771	0.5	CRA004775;PRJNA827465	Yang2025_Silkie_Wenchang	0.903343828
ENSGALG00010022456	ATP5G3	2	-0.37821794	0.416007896	-0.909160484	0.363265419	0.303422523	7.486870085	86.64328366	1	PRJNA827465;Yang2025_Silkie_Wenchang	CRA004775	0.816832815
ENSGALG00010022456	ATP5G3	2	-0.669127013	0.232206983	-2.881597292	0.003956651	0	0.973670966	0	1	CRA004775;Yang2025_Silkie_Wenchang	PRJNA827465	0.09071163
ENSGALG00010022456	ATP5G3	2	-0.025449797	0.092178667	-0.276092054	0.782477364	0	0.821617005	0	1	CRA004775;PRJNA827465	Yang2025_Silkie_Wenchang	0.945424324
ENSGALG00010022460	VAT1	2	0.024574953	0.156874529	0.156653558	0.87551789	0.034579378	3.135344637	68.10557958	0.5	PRJNA827465;Yang2025_Silkie_Wenchang	CRA004775	0.970347777
ENSGALG00010022460	VAT1	2	-0.139909322	0.146185746	-0.957065412	0.33853424	0	0.139306223	0	1	CRA004775;Yang2025_Silkie_Wenchang	PRJNA827465	0.804408854
ENSGALG00010022460	VAT1	2	0.148094735	0.083422976	1.775227187	0.075860382	0	0.179049324	0	0.5	CRA004775;PRJNA827465	Yang2025_Silkie_Wenchang	0.47539798
ENSGALG00010022462	ITGA5	2	0.083878356	0.093468832	0.897393853	0.369508791	0	0.000339048	0	1	PRJNA827465;Yang2025_Silkie_Wenchang	CRA004775	0.820606801
ENSGALG00010022462	ITGA5	2	0.056640507	0.139045648	0.407351884	0.683749561	0	0.170470626	0	0.5	CRA004775;Yang2025_Silkie_Wenchang	PRJNA827465	0.918800744
ENSGALG00010022462	ITGA5	2	0.068518341	0.112315559	0.610052089	0.541827303	0	0.190059811	0	0.5	CRA004775;PRJNA827465	Yang2025_Silkie_Wenchang	0.876463699
ENSGALG00010022463	DUSP12	2	-0.011783953	0.080541543	-0.146309006	0.883677457	0	0.385254643	0	0.5	PRJNA827465;Yang2025_Silkie_Wenchang	CRA004775	0.972001076
ENSGALG00010022463	DUSP12	2	-0.18562223	0.155732529	-1.19192972	0.233288804	0	0.173095248	0	1	CRA004775;Yang2025_Silkie_Wenchang	PRJNA827465	0.746535568
ENSGALG00010022463	DUSP12	2	-0.034857056	0.099966097	-0.348688772	0.727322974	0.004299114	1.133870058	11.80647262	0.5	CRA004775;PRJNA827465	Yang2025_Silkie_Wenchang	0.931522817
ENSGALG00010022466	NEDD4	2	0.155011378	0.146540827	1.057803348	0.290145106	0.0309336	3.153226278	68.28644976	1	PRJNA827465;Yang2025_Silkie_Wenchang	CRA004775	0.784195688
ENSGALG00010022466	NEDD4	2	0.072871885	0.301540282	0.241665506	0.80903936	0.137489408	3.920289496	74.49167973	0.5	CRA004775;Yang2025_Silkie_Wenchang	PRJNA827465	0.952710749
ENSGALG00010022466	NEDD4	2	-0.007774149	0.110042224	-0.070646968	0.943678729	0.010232941	1.278370574	21.77542095	0.5	CRA004775;PRJNA827465	Yang2025_Silkie_Wenchang	0.985827325
ENSGALG00010022467	SART3	2	0.083575872	0.086060813	0.971125748	0.331485663	0.011814522	4.777686451	79.06936736	1	PRJNA827465;Yang2025_Silkie_Wenchang	CRA004775	0.801798188
ENSGALG00010022467	SART3	2	0.157161919	0.063040771	2.493020279	0.012666161	0	0.804639906	0	1	CRA004775;Yang2025_Silkie_Wenchang	PRJNA827465	0.182551571
ENSGALG00010022467	SART3	2	0.005116176	0.0406463	0.125870648	0.899834318	0	3.79744E-05	0	1	CRA004775;PRJNA827465	Yang2025_Silkie_Wenchang	0.976319826
ENSGALG00010022468	MYBPC3	2	0.032004692	0.267076432	0.119833457	0.904615078	0.107121358	3.903100848	74.37934506	0.5	PRJNA827465;Yang2025_Silkie_Wenchang	CRA004775	0.977683364
ENSGALG00010022468	MYBPC3	2	-1.137857206	1.574545548	-0.722657536	0.46989031	4.572447499	12.09503645	91.73214562	0.5	CRA004775;Yang2025_Silkie_Wenchang	PRJNA827465	0.853503044
ENSGALG00010022468	MYBPC3	2	-1.377433809	1.302503785	-1.057527682	0.290270828	3.03591236	8.625663205	88.40668855	1	CRA004775;PRJNA827465	Yang2025_Silkie_Wenchang	0.784195688
ENSGALG00010022470	ENSGALG00010022470	2	0.694549922	0.158418889	4.38426205	1.1638E-05	0	0.07147575	0	1	PRJNA827465;Yang2025_Silkie_Wenchang	CRA004775	0.001593752
ENSGALG00010022470	ENSGALG00010022470	2	0.276494482	0.432525563	0.639255818	0.522656526	0.230341084	2.449073248	59.16822819	0.5	CRA004775;Yang2025_Silkie_Wenchang	PRJNA827465	0.869568211
ENSGALG00010022470	ENSGALG00010022470	2	0.337893134	0.476279877	0.70944239	0.47804999	0.338736808	3.491952336	71.36272481	0.5	CRA004775;PRJNA827465	Yang2025_Silkie_Wenchang	0.855366076
ENSGALG00010022471	ST6GALNAC5	2	-0.26981114	0.103354745	-2.610534627	0.009040082	0	0.421461014	0	1	PRJNA827465;Yang2025_Silkie_Wenchang	CRA004775	0.149036217
ENSGALG00010022471	ST6GALNAC5	2	-0.278788291	0.208700746	-1.335827961	0.181605512	0.022739439	1.107497849	9.706370899	0.5	CRA004775;Yang2025_Silkie_Wenchang	PRJNA827465	0.689004635
ENSGALG00010022471	ST6GALNAC5	2	-0.189492909	0.131925793	-1.436359821	0.15089998	0	0.723528507	0	0.5	CRA004775;PRJNA827465	Yang2025_Silkie_Wenchang	0.642051813
ENSGALG00010022472	QSER1	2	-0.016712879	0.190865232	-0.087563769	0.930223399	0.0521221	2.932446721	65.89878367	0.5	PRJNA827465;Yang2025_Silkie_Wenchang	CRA004775	0.983813577
ENSGALG00010022472	QSER1	2	0.305178765	0.177500992	1.71930738	0.085558417	0	0.255466487	0	1	CRA004775;Yang2025_Silkie_Wenchang	PRJNA827465	0.502946823
ENSGALG00010022472	QSER1	2	0.061713325	0.283627874	0.217585544	0.827752049	0.124722519	3.69842541	72.96146632	0.5	CRA004775;PRJNA827465	Yang2025_Silkie_Wenchang	0.956550752
ENSGALG00010022473	ZNF385A	2	0.041182513	0.229521648	0.179427576	0.85760198	0.081298125	4.370030615	77.11686512	0.5	PRJNA827465;Yang2025_Silkie_Wenchang	CRA004775	0.964029591
ENSGALG00010022473	ZNF385A	2	-0.484189831	0.798688842	-0.606230869	0.544361457	1.18960869	14.32711242	93.02022647	0.5	CRA004775;Yang2025_Silkie_Wenchang	PRJNA827465	0.877133419
ENSGALG00010022473	ZNF385A	2	-0.695781452	0.567188105	-1.226720812	0.219927543	0.563219221	7.495146322	86.65803232	1	CRA004775;PRJNA827465	Yang2025_Silkie_Wenchang	0.733521063
ENSGALG00010022474	VCL	2	-0.063007506	0.047741021	-1.319777101	0.186909449	0	0.00827584	0	1	PRJNA827465;Yang2025_Silkie_Wenchang	CRA004775	0.695851291
ENSGALG00010022474	VCL	2	0.393519112	0.470372386	0.83661185	0.402810783	0.41241888	14.26837736	92.99149459	0.5	CRA004775;Yang2025_Silkie_Wenchang	PRJNA827465	0.832937979
ENSGALG00010022474	VCL	2	0.384878044	0.475380863	0.809620398	0.418158381	0.425755267	16.45170305	93.92160194	0.5	CRA004775;PRJNA827465	Yang2025_Silkie_Wenchang	0.838112595
ENSGALG00010022476	OIP5	2	-0.499708213	0.391744836	-1.275596171	0.202098309	0.287165507	15.29249394	93.46084423	1	PRJNA827465;Yang2025_Silkie_Wenchang	CRA004775	0.71497298
ENSGALG00010022476	OIP5	2	-0.606207127	0.314616357	-1.926813765	0.054002843	0.157807351	4.87522816	79.48813948	1	CRA004775;Yang2025_Silkie_Wenchang	PRJNA827465	0.402825653
ENSGALG00010022476	OIP5	2	-0.145742938	0.091593001	-1.591201697	0.111564191	0	0.385676166	0	1	CRA004775;PRJNA827465	Yang2025_Silkie_Wenchang	0.564762672
ENSGALG00010022478	ATP6V1A	2	-0.129038848	0.237420658	-0.543503033	0.586783503	0.103721219	12.09487256	91.7320336	0.5	PRJNA827465;Yang2025_Silkie_Wenchang	CRA004775	0.891596903
ENSGALG00010022478	ATP6V1A	2	-0.384994847	0.11704268	-3.289354352	0.001004175	0	0.015690315	0	1	CRA004775;Yang2025_Silkie_Wenchang	PRJNA827465	0.03802225
ENSGALG00010022478	ATP6V1A	2	-0.053906625	0.235931434	-0.228484286	0.819269771	0.074870592	2.225451098	55.06528986	0.5	CRA004775;PRJNA827465	Yang2025_Silkie_Wenchang	0.955256039
ENSGALG00010022479	ENSGALG00010022479	2	-0.25916046	1.090513314	-0.237649974	0.812152587	2.112963921	8.821149025	88.66360837	0.5	PRJNA827465;Yang2025_Silkie_Wenchang	CRA004775	0.953371387
ENSGALG00010022479	ENSGALG00010022479	2	0.340003853	1.66119081	0.204674774	0.837826231	5.292375012	24.26799075	95.87934572	0.5	CRA004775;Yang2025_Silkie_Wenchang	PRJNA827465	0.959032758
ENSGALG00010022479	ENSGALG00010022479	2	1.488022569	0.568477635	2.617556921	0.008856172	0.316018866	1.948650937	48.68244583	1	CRA004775;PRJNA827465	Yang2025_Silkie_Wenchang	0.147549629
ENSGALG00010022480	PIF1	2	-0.307004564	0.301696906	-1.017592685	0.308871565	0.145293694	4.846742861	79.36758709	1	PRJNA827465;Yang2025_Silkie_Wenchang	CRA004775	0.793903546
ENSGALG00010022480	PIF1	2	-0.299801655	0.501923387	-0.59730561	0.550303359	0.310935699	2.096736304	52.30683048	0.5	CRA004775;Yang2025_Silkie_Wenchang	PRJNA827465	0.878679315
ENSGALG00010022480	PIF1	2	-0.005406356	0.151708572	-0.035636455	0.97157224	0	0.436374472	0	0.5	CRA004775;PRJNA827465	Yang2025_Silkie_Wenchang	0.991955348
ENSGALG00010022481	CORO1B	2	0.016649932	0.11864862	0.140329759	0.888399453	0.019167664	3.130210934	68.05327114	0.5	PRJNA827465;Yang2025_Silkie_Wenchang	CRA004775	0.973135755
ENSGALG00010022481	CORO1B	2	0.094182208	0.085583559	1.100470803	0.271127044	0	0.849619608	0	0.5	CRA004775;Yang2025_Silkie_Wenchang	PRJNA827465	0.772743863
ENSGALG00010022481	CORO1B	2	-0.088984335	0.081614218	-1.090304328	0.275579109	0	0.064317648	0	1	CRA004775;PRJNA827465	Yang2025_Silkie_Wenchang	0.775295118
ENSGALG00010022482	PRTG	2	1.220979815	0.242964164	5.025349396	5.02517E-07	0	0.667893526	0	1	PRJNA827465;Yang2025_Silkie_Wenchang	CRA004775	0.000130797
ENSGALG00010022482	PRTG	2	0.812461761	0.353012787	2.301508024	0.021362932	0	0.073487782	0	1	CRA004775;Yang2025_Silkie_Wenchang	PRJNA827465	0.247158031
ENSGALG00010022482	PRTG	2	1.186070626	0.274800856	4.316109644	1.58803E-05	0.018868316	1.101418269	9.207970435	1	CRA004775;PRJNA827465	Yang2025_Silkie_Wenchang	0.002059881
ENSGALG00010022483	TOP2A	2	-0.21800079	0.084623228	-2.576134171	0.009991187	0	0.726942501	0	1	PRJNA827465;Yang2025_Silkie_Wenchang	CRA004775	0.158404041
ENSGALG00010022483	TOP2A	2	-0.044760586	0.396611953	-0.112857381	0.910143626	0.206605064	2.209989991	54.75092628	0.5	CRA004775;Yang2025_Silkie_Wenchang	PRJNA827465	0.978730675
ENSGALG00010022483	TOP2A	2	-0.010607932	0.289753235	-0.036610229	0.970795787	0.092658451	1.549776353	35.47456068	0.5	CRA004775;PRJNA827465	Yang2025_Silkie_Wenchang	0.991851524
ENSGALG00010022485	ATF2	2	-0.014984716	0.059059673	-0.253721623	0.799710628	0.002795515	1.284044518	22.12108024	0.5	PRJNA827465;Yang2025_Silkie_Wenchang	CRA004775	0.949922217
ENSGALG00010022485	ATF2	2	-0.098544774	0.118384978	-0.832409447	0.405177871	0	0.563368994	0	0.5	CRA004775;Yang2025_Silkie_Wenchang	PRJNA827465	0.833112102
ENSGALG00010022485	ATF2	2	0.012450599	0.036772951	0.338580354	0.734925884	0	0.042226184	0	1	CRA004775;PRJNA827465	Yang2025_Silkie_Wenchang	0.933545748
ENSGALG00010022489	NUSAP1	2	-0.112327167	0.06614056	-1.698309883	0.08944929	0	0.002633038	0	1	PRJNA827465;Yang2025_Silkie_Wenchang	CRA004775	0.513235693
ENSGALG00010022489	NUSAP1	2	-0.055993883	0.186115035	-0.300856313	0.763524065	0.018449038	1.108267966	9.769114456	0.5	CRA004775;Yang2025_Silkie_Wenchang	PRJNA827465	0.940301633
ENSGALG00010022489	NUSAP1	2	-0.067188629	0.156224015	-0.430078752	0.667138356	0.020696017	1.126867443	11.25841766	0.5	CRA004775;PRJNA827465	Yang2025_Silkie_Wenchang	0.913766816

ENSGALG00010022520	TMOD2	2	-0.084107624	0.213605094	-0.393752896	0.693763484	0.048321127	2.092516895	52.21066065	0.5	PRJNA827465;Yang2025_Silkie_Wenchang	CRA004775	0.920721644
ENSGALG00010022520	TMOD2	2	0.287811576	0.66347647	0.433793193	0.664438625	0.760166194	7.038986403	85.79340913	0.5	CRA004775;Yang2025_Silkie_Wenchang	PRJNA827465	0.913219726
ENSGALG00010022520	TMOD2	2	0.465412502	0.441272632	1.054705113	0.291560213	0.290943467	3.531608434	71.68429007	1	CRA004775;PRJNA827465	Yang2025_Silkie_Wenchang	0.785037408
ENSGALG00010022521	RTF1	2	-0.030085678	0.030180772	-0.99684917	0.318837727	0	0.901179457	0	1	PRJNA827465;Yang2025_Silkie_Wenchang	CRA004775	0.797974202
ENSGALG00010022521	RTF1	2	0.074551922	0.192876406	0.386526911	0.699106476	0.05884743	4.292636204	76.7042919	0.5	CRA004775;Yang2025_Silkie_Wenchang	PRJNA827465	0.92185597
ENSGALG00010022521	RTF1	2	0.097406198	0.153048576	0.636439751	0.524489838	0.035084116	3.16934334	68.44772268	0.5	CRA004775;PRJNA827465	Yang2025_Silkie_Wenchang	0.869790047
ENSGALG00010022522	TNS4	2	-0.036165819	0.203740389	-0.177509327	0.85910834	0	0.099026656	0	0.5	PRJNA827465;Yang2025_Silkie_Wenchang	CRA004775	0.964605083
ENSGALG00010022522	TNS4	2	0.516819801	0.68076537	0.759174634	0.447748098	0.756046878	5.326017406	81.22424461	0.5	CRA004775;Yang2025_Silkie_Wenchang	PRJNA827465	0.846601815
ENSGALG00010022522	TNS4	2	0.558739222	0.611664372	0.913473545	0.360993541	0.607583382	5.044487057	80.17637891	1	CRA004775;PRJNA827465	Yang2025_Silkie_Wenchang	0.815181068
ENSGALG00010022524	SOX18	2	0.011193137	0.191747016	0.058374504	0.953450323	0.05629851	4.099378779	75.60605999	0.5	PRJNA827465;Yang2025_Silkie_Wenchang	CRA004775	0.987758913
ENSGALG00010022524	SOX18	2	0.191082614	0.146126905	1.307648401	0.190992592	0	0.233657346	0	1	CRA004775;Yang2025_Silkie_Wenchang	PRJNA827465	0.700443419
ENSGALG00010022524	SOX18	2	-0.143221708	0.095075427	-1.506400898	0.13196429	0	0.357378461	0	0.5	CRA004775;PRJNA827465	Yang2025_Silkie_Wenchang	0.608737092
ENSGALG00010022525	ISCU	2	-0.104848189	0.121392291	-0.863713736	0.387745166	0.016790169	1.700781479	41.20349897	1	PRJNA827465;Yang2025_Silkie_Wenchang	CRA004775	0.828154121
ENSGALG00010022525	ISCU	2	-0.293785842	0.181619313	-1.617591413	0.105750681	0	0.067265626	0	1	CRA004775;Yang2025_Silkie_Wenchang	PRJNA827465	0.551748176
ENSGALG00010022525	ISCU	2	-0.040880497	0.049706126	-0.822443838	0.410824338	0	0.262426543	0	1	CRA004775;PRJNA827465	Yang2025_Silkie_Wenchang	0.83504692
ENSGALG00010022526	PPP1CA	2	-0.101724599	0.055449227	-1.834553942	0.066571808	0	0.000944965	0	1	PRJNA827465;Yang2025_Silkie_Wenchang	CRA004775	0.444449088
ENSGALG00010022526	PPP1CA	2	-0.095115299	0.171303523	-0.555244268	0.578727593	0	0.023162957	0	1	CRA004775;Yang2025_Silkie_Wenchang	PRJNA827465	0.88788081
ENSGALG00010022526	PPP1CA	2	-0.099957147	0.057415227	-1.740951894	0.081692011	0	0.022921574	0	1	CRA004775;PRJNA827465	Yang2025_Silkie_Wenchang	0.492520496
ENSGALG00010022528	SIDT1	2	0.199072933	0.199836081	0.996181129	0.319162147	0	0.76753055	0	1	PRJNA827465;Yang2025_Silkie_Wenchang	CRA004775	0.798079048
ENSGALG00010022528	SIDT1	2	0.02826173	0.26837245	0.105307865	0.916131522	0	0.003084597	0	1	CRA004775;Yang2025_Silkie_Wenchang	PRJNA827465	0.980406784
ENSGALG00010022528	SIDT1	2	0.342882744	0.26732363	1.282650336	0.199614607	0	0.115431128	0	1	CRA004775;PRJNA827465	Yang2025_Silkie_Wenchang	0.711489865
ENSGALG00010022529	CCR7	2	0.218461533	0.346500127	0.630480384	0.528380333	0.146697345	2.496705479	59.9472181	0.5	PRJNA827465;Yang2025_Silkie_Wenchang	CRA004775	0.871701504
ENSGALG00010022529	CCR7	2	-0.211639994	0.326131645	-0.648940382	0.516376912	0	0.041792895	0	1	CRA004775;Yang2025_Silkie_Wenchang	PRJNA827465	0.867685411
ENSGALG00010022529	CCR7	2	0.368643915	0.32991745	1.117382288	0.263830905	0.067352987	1.217039982	17.83343074	0.5	CRA004775;PRJNA827465	Yang2025_Silkie_Wenchang	0.768643821
ENSGALG00010022531	ENSGALG00010022531	2	0.089516728	0.321598956	0.278348938	0.780744513	0.085709401	1.661902044	39.82798183	0.5	PRJNA827465;Yang2025_Silkie_Wenchang	CRA004775	0.945235709
ENSGALG00010022531	ENSGALG00010022531	2	0.3771594	0.301756651	1.249879327	0.211343632	0	0.136381239	0	1	CRA004775;Yang2025_Silkie_Wenchang	PRJNA827465	0.723704057
ENSGALG00010022531	ENSGALG00010022531	2	-0.039634254	0.245115547	-0.161696206	0.871545092	0	0.667092629	0	0.5	CRA004775;PRJNA827465	Yang2025_Silkie_Wenchang	0.969425739
ENSGALG00010022532	CBX5	2	0.054625217	0.053238333	1.02605047	0.304867795	0	0.316033752	0	1	PRJNA827465;Yang2025_Silkie_Wenchang	CRA004775	0.79225794
ENSGALG00010022532	CBX5	2	-0.111048725	0.254910118	-0.435638751	0.66309885	0.102185158	4.577118641	78.15219402	0.5	CRA004775;Yang2025_Silkie_Wenchang	PRJNA827465	0.91293776
ENSGALG00010022532	CBX5	2	-0.131095436	0.210228851	-0.623584422	0.532900544	0.070754241	4.361171998	77.07038382	0.5	CRA004775;PRJNA827465	Yang2025_Silkie_Wenchang	0.873237026
ENSGALG00010022533	TMEM119	2	-0.101964453	0.136325113	-0.747950623	0.454489941	0.020790822	2.168464175	53.88441222	0.5	PRJNA827465;Yang2025_Silkie_Wenchang	CRA004775	0.849072665
ENSGALG00010022533	TMEM119	2	-0.327767379	0.145213491	-2.257141384	0.023999242	0	0.888083119	0	1	CRA004775;Yang2025_Silkie_Wenchang	PRJNA827465	0.264584066
ENSGALG00010022533	TMEM119	2	-0.222046796	0.311008424	-0.713957496	0.475253462	0.140321974	3.003637877	66.70703857	0.5	CRA004775;PRJNA827465	Yang2025_Silkie_Wenchang	0.854462864
ENSGALG00010022536	SMUG1	2	-0.187900844	0.273789097	-0.686297759	0.492525362	0.113962336	3.340928869	70.0682044	0.5	PRJNA827465;Yang2025_Silkie_Wenchang	CRA004775	0.859312128
ENSGALG00010022536	SMUG1	2	-0.388256697	0.21666412	-1.791975044	0.073136964	0	0.573473364	0	1	CRA004775;Yang2025_Silkie_Wenchang	PRJNA827465	0.46748834
ENSGALG00010022536	SMUG1	2	0.00952378	0.055549806	0.171445785	0.863873257	0	0.605996967	0	0.5	CRA004775;PRJNA827465	Yang2025_Silkie_Wenchang	0.966447685
ENSGALG00010022538	WIPF1	2	0.200867827	0.112772147	1.78118297	0.074882565	0	0.714983642	0	1	PRJNA827465;Yang2025_Silkie_Wenchang	CRA004775	0.472257613
ENSGALG00010022538	WIPF1	2	-0.017435726	0.457182483	-0.038137345	0.969578176	0.307055546	3.521927497	71.60645695	0.5	CRA004775;Yang2025_Silkie_Wenchang	PRJNA827465	0.991572914
ENSGALG00010022538	WIPF1	2	-0.079712122	0.32844677	-0.242694188	0.80824231	0.142979092	2.385487085	58.07984012	0.5	CRA004775;PRJNA827465	Yang2025_Silkie_Wenchang	0.952496413
ENSGALG00010022539	LYSMD2	2	-0.108676551	0.184697959	-0.588401472	0.55626285	0.051235305	3.806668239	73.73030858	0.5	PRJNA827465;Yang2025_Silkie_Wenchang	CRA004775	0.880324407
ENSGALG00010022539	LYSMD2	2	-0.124322342	0.217430041	-0.571780887	0.567470427	0.058023325	2.544767087	60.70367283	0.5	CRA004775;Yang2025_Silkie_Wenchang	PRJNA827465	0.883717772
ENSGALG00010022539	LYSMD2	2	0.062741917	0.086513894	0.725223596	0.468314869	0	0.073622547	0	1	CRA004775;PRJNA827465	Yang2025_Silkie_Wenchang	0.853198008
ENSGALG00010022540	GPR146	2	0.017144805	0.147306387	0.11638874	0.907344459	0.010163513	1.298160951	22.96794944	0.5	PRJNA827465;Yang2025_Silkie_Wenchang	CRA004775	0.978196186
ENSGALG00010022540	GPR146	2	0.118299447	0.272677072	0.433844497	0.664401367	0.098965263	2.823930506	64.58836371	0.5	CRA004775;Yang2025_Silkie_Wenchang	PRJNA827465	0.913219726
ENSGALG00010022540	GPR146	2	0.271817922	0.163805635	1.659392993	0.097036627	0	0.542869911	0	1	CRA004775;PRJNA827465	Yang2025_Silkie_Wenchang	0.532286192
ENSGALG00010022541	ITPKA	2	-0.149893119	0.197228967	-0.759995458	0.4472573	0.061646301	4.715089671	78.79149561	0.5	PRJNA827465;Yang2025_Silkie_Wenchang	CRA004775	0.84650962
ENSGALG00010022541	ITPKA	2	-0.207832593	0.206782527	-1.005078116	0.314859237	0.044222403	1.908048983	47.59044402	0.5	CRA004775;Yang2025_Silkie_Wenchang	PRJNA827465	0.796531448
ENSGALG00010022541	ITPKA	2	0.037485981	0.096832843	0.387120525	0.698666984	0	0.014733345	0	1	CRA004775;PRJNA827465	Yang2025_Silkie_Wenchang	0.921745106
ENSGALG00010022544	SPICE1	2	0.005544649	0.054490114	0.101755141	0.918951033	0	0.109704208	0	0.5	PRJNA827465;Yang2025_Silkie_Wenchang	CRA004775	0.981086235
ENSGALG00010022544	SPICE1	2	0.032166621	0.088358154	0.364048129	0.71582207	0	0.001752649	0	1	CRA004775;Yang2025_Silkie_Wenchang	PRJNA827465	0.927197535
ENSGALG00010022544	SPICE1	2	-0.001943945	0.060299737	-0.032238027	0.974282231	0	0.029247549	0	0.5	CRA004775;PRJNA827465	Yang2025_Silkie_Wenchang	0.992492038
ENSGALG00010022545	CSTF3	2	0.102254119	0.155225711	0.658744734	0.510059702	0.040859454	6.395012108	84.36281303	0.5	PRJNA827465;Yang2025_Silkie_Wenchang	CRA004775	0.865290927
ENSGALG00010022545	CSTF3	2	0.162235281	0.139324534	1.16444158	0.244245106	0.019783142	1.904267863	47.48637945	0.5	CRA004775;Yang2025_Silkie_Wenchang	PRJNA827465	0.75569278
ENSGALG00010022545	CSTF3	2	-0.039804803	0.060645379	-0.656353438	0.511596742	0	0.013817875	0	1	CRA004775;PRJNA827465	Yang2025_Silkie_Wenchang	0.865694231
ENSGALG00010022546	MTG1	2	0.203353802	0.124974696	1.627159808	0.103703161	0.009562834	1.259023192	20.57334559	1	PRJNA827465;Yang2025_Silkie_Wenchang	CRA004775	0.547964315
ENSGALG00010022546	MTG1	2	-0.010499952	0.514846059	-0.020394353	0.983728788	0.407288815	4.099823208	75.60870435	0.5	CRA004775;Yang2025_Silkie_Wenchang	PRJNA827465	0.995510523
ENSGALG00010022546	MTG1	2	-0.099447284	0.343126798	-0.28982663	0.771948874	0.163917023	2.564514809	61.00626923	0.5	CRA004775;PRJNA827465	Yang2025_Silkie_Wenchang	0.943066211
ENSGALG00010022547	SMARCE1	2	-0.043127476	0.040198195	-1.072870956	0.283329019	0	0.164736686	0	0.5	PRJNA827465;Yang2025_Silkie_Wenchang	CRA004775	0.779568117
ENSGALG00010022547	SMARCE1	2	-0.002168319	0.089186837	-0.024312098	0.980603663	0	0.002739226	0	0.5	CRA004775;Yang2025_Silkie_Wenchang	PRJNA827465	0.994828884
ENSGALG00010022547	SMARCE1	2	-0.045328029	0.040799192	-1.1110031	0.266567015	0	0.084288491	0	1	CRA004775;PRJNA827465	Yang2025_Silkie_Wenchang	0.770699087
ENSGALG00010022548	ENSGALG00010022548	2	0.173199438	0.241137526	0.718259993	0.472596993	0.10255713	8.286435217	87.9320845	0.5	PRJNA827465;Yang2025_Silkie_Wenchang	CRA004775	0.853998373
ENSGALG00010022548	ENSGALG00010022548	2	0.39058479	0.133186324	2.932619349	0.003361157	0	0.526915582	0	1	CRA004775;Yang2025_Silkie_Wenchang	PRJNA827465	0.081207766
ENSGALG00010022548	ENSGALG00010022548	2	-0.044938431	0.08592758	-0.522980296	0.600987965	0	0.257472664	0	0.5	CRA004775;PRJNA827465	Yang2025_Silkie_Wenchang	0.895230519
ENSGALG00010022550	TRIM45	2	0.082362158	0.258900247	0.318123133	0.750391537	0.097787428	3.499897329	71.42773327	0.5	PRJNA827465;Yang2025_Silkie_Wenchang	CRA004775	0.937393444
ENSGALG00010022550	TRIM45	2	-0.166123103	0.557040189	-0.298224628	0.765531726	0.551565692	8.971280601	88.85331934	0.5	CRA004775;Yang2025_Silkie_Wenchang	PRJNA827465	0.940888005
ENSGALG00010022550	TRIM45	2	-0.386141761	0.29106795	-1.326637855	0.184628507	0.125164356	3.530749829	71.67740428	1	CRA004775;PRJNA827465	Yang2025_Silkie_Wenchang	0.692938714
ENSGALG00010022552	GPR155	2	0.041472666	0.143740065	0.288525445	0.772944556	0.036051166	7.767748174	87.12625619	0.5	PRJNA827465;Yang2025_Silkie_Wenchang	CRA004775	0.943096979
ENSGALG00010022552	GPR155	2	-0.052919331	0.099783515	-0.530341423	0.595875232	0.00610655	1.278817824	21.80277901	0.5	CRA004775;Yang2025_Silkie_Wenchang	PRJNA827465	0.893857407
ENSGALG00010022552	GPR155	2	0.175269069	0.058765386	2.982522227	0.002858839	0	0.063790458	0	1	CRA004775;PRJNA827465	Yang2025_Silkie_Wenchang	0.073104063
ENSGALG00010022553	PRPF6	2	-0.150798765	0.140947838	-1.069890591	0.284668559	0.035947256	10.22838673	90.22328715	1	PRJNA827465;Yang2025_Silkie_Wenchang	CRA004775	0.780265906
ENSGALG00010022553	PRPF6	2	-0.27954021	0.07249934	-3.855762143	0.00011537	0	0.536262817	0	1	CRA004775;Yang2025_Silkie_Wenchang	PRJNA827465	0.008571592
ENSGALG00010022553	PRPF6	2	-0.021908735	0.041589128	-0.526789958	0.598339457	0	0.322744186	0	1	CRA004775;PRJNA827465	Yang2025_Silkie_Wenchang	0.894645663
ENSGALG00010022554	SCG3	2	-0.235586756	0.117343135	-2.007673953	0.04467795	0	0.078118014	0	1	PRJNA827465;Yang2025_Silkie_Wenchang	CRA004775	0.364322144
ENSGALG00010022554	SCG3	2	0.022531897	0.48808747	0.046163646	0.963179818	0.278705603	1.963861364	49.07990867	0.5	CRA004775;Yang2025_Silkie_Wenchang	PRJNA827465	0.989998461
ENSGALG00010022554	SCG3	2	0.022737105	0.423848225	0.05364445	0.957218442	0.219817156	1.822153905	45.11989369	0.5	CRA004775;PRJNA827465	Yang2025_Silkie_Wenchang	0.988541306
ENSGALG00010022557	WNT1	2	0.21843016	0.196423414	1.112037283	0.26612212	0	0.023585786	0	1	PRJNA827465;Yang2025_Silkie_Wenchang	CRA004775	0.770092601
ENSGALG00010022557	WNT1	2	-0.02074834	0.285074227	-0.072782237	0.941979406	0.038525597	1.310660594	23.70259663	0.5	CRA004775;Yang2025_Silkie_Wenchang	PRJNA827465	0.985827325
ENSGALG00010022557	WNT1	2	0.013384154	0.243259371	0.055020098	0.956122452	0.037140334	1.410763218	29.11638274	0.5	CRA004775;PRJNA827465	Yang2025_Silkie_Wenchang	0.988513657
ENSGALG00010022559	SSH1	2	0.036793086	0.05530068	0.665327908	0.505840784	0	0.149525327	0	1	PRJNA827465;Yang2025_Silkie_Wenchang	CRA004775	0.863927652
ENSGALG00010022559	SSH1	2	-0.010644446	0.085372784	-0.124681956	0.900775343	0	0.487176766	0	0.5	CRA004775;Yang2025_Silkie_Wenchang	PRJNA827465	0.976497577
ENSGALG00010022559	SSH1	2	0.038034003	0.067809347	0.56089616	0.574868328	0	0.758881435	0	0.5	CRA004775;PRJNA827465	Yang2025_Silkie_Wenchang	0.886211989
ENSGALG00010022564	VTCN1	2	-0.661906815	0.240963953	-2.746912173	0.006015924	0	0.159134026	0	1	PRJNA827465;Yang2025_Silkie_Wenchang	CRA004775	0.117225517
ENSGALG00010022564	VTCN1	2	-0.846466837	0.301965232	-2.803193039	0.005059937	0	0.140785624	0	1	CRA004775;Yang2025_Silkie_Wenchang	PRJNA827465	0.106040882
ENSGALG00010022564	VTCN1	2	-0.691070217	0.276738089	-2.497199502	0.012517851	0	0.483923997	0	1	CRA004775;PRJNA827465	Yang2025_Silkie_Wenchang	0.181211453
ENSGALG00010022565	ARF3	2	-0.105681713	0.076685093	-1.378125912	0.168164417	0	0.005144967	0	1	PRJNA827465;Yang2025_Silkie_Wenchang	CRA004775	0.668761111
ENSGALG00010022565	ARF3	2	-0.165265504	0.137525327	-1.201709587	0.229476065	0	0.759984041	0	1	CRA004775;Yang2025_Silkie_Wenchang	PRJNA827465	0.74255704
ENSGALG00010022565	ARF3	2	-0.123158933	0.086691033	-1.420665189	0.155414117	0	0.904625421	0	1	CRA004775;PRJNA827465	Yang2025_Silkie_Wenchang	0.648167852
ENSGALG00010022567	PAOX	2	-0.348680806	0.230795098	-1.510780817	0.130844304	0.072031461	2.936515905	65.94603835	1	PRJNA827465;Yang2025_Silkie_Wenchang	CRA004775	0.606578636
ENSGALG00010022567	PAOX	2	-0.121790321	0.17213709	-0.707519342	0.479243797	0	0.076591153	0	1	CRA004775;Yang2025_Silkie_Wenchang	PRJNA827465	0.856035423
ENSGALG00010022567	PAOX	2	-0.420478113	0.175680074	-2.393430873	0.016691626	0.026497141	1.622115448	38.35210675	1	CRA004775;PRJNA827465	Yang2025_Silkie_Wenchang	0.214179233
ENSGALG00010022568	SCRN3	2	0.015009275	0.314510952	0.047722582	0.961937337	0.182915187	13.26024021	92.45865849	0.5	PRJNA827465;Yang2025_Silkie_Wenchang	CRA004775	0.989842209
ENSGALG00010022568	SCRN3	2	0.339006679	0.116342891	2.91385812	0.003569921	0	0.085054779	0	1	CRA004775;Yang2025_Silkie_Wenchang	PRJNA827465	0.084829873
ENSGALG00010022568	SCRN3	2	-0.026953069	0.362943378	-0.074262463	0.940801544	0.191059617	3.024224311	66.93366969	0.5	CRA004775;PRJNA827465	Yang2025_Silkie_Wenchang	0.985565295
ENSGALG00010022569	KIAA1549L	2	0.046668212	0.127732856	0.365357931	0.71484424	0	0.23089553	0	1	PRJNA827465;Yang2025_Silkie_Wenchang	CRA004775	0.926755613
ENSGALG00010022569	KIAA1549L	2	0.159321534	0.358464731	0.444455256	0.656713472	0	0.208965626	0	0.5	CRA004775;Yang2025_Silkie_Wenchang	PRJNA827465	0.910530553
ENSGALG00010022569	KIAA1549L	2	0.019678406	0.134392912	0.146424434	0.88358634	0	0.088136882	0	0.5	CRA004775;PRJNA827465	Yang2025_Silkie_Wenchang	0.972001076
ENSGALG00010022571	RIPOR1	2	0.054942208	0.05152419	1.066338127	0.28627082	0	0.002269686	0	1	PRJNA827465;Yang2025_Silkie_Wenchang	CRA004775	0.781944441
ENSGALG00010022571	RIPOR1	2	0.041974479	0.098317402	0.426928283	0.669431565	0	0.099333263	0	0.5	CRA004775;Yang2025_Silkie_Wenchang	PRJNA827465	0.914377769
ENSGALG00010022571	RIPOR1	2	0.048500059	0.055515307	0.873633982	0.382317614	0	0.101932323	0	0.5	CRA004775;PRJNA827465	Yang2025_Silkie_Wenchang	0.825080207
ENSGALG00010022573	AP4E1	2	-0.058283555	0.061766248	-0.943614958	0.345366446	0	0.877337226	0	1	PRJNA827465;Yang2025_Silkie_Wenchang	CRA004775	0.807251705
ENSGALG00010022573	AP4E1	2	6.627E-06	0.182130708	3.6386E-05	0.999970968	0.042050119	2.541996104	60.66083664	0.5	CRA004775;Yang2025_Silkie_Wenchang	PRJNA827465	0.999970968
ENSGALG00010022573	AP4E1	2	0.028231303	0.098165845	0.287587836	0.773662257	0.006624576	1.281589448	21.97189191	0.5	CRA004775;PRJNA827465	Yang2025_Silkie_Wenchang	0.943248868
ENSGALG00010022574	ITPRIPL2	2	-0.036987634	0.089570364	-0.412944998	0.679646912	0	0.191518418	0	1	PRJNA827465;Yang2025_Silkie_Wenchang	CRA004775	0.917701868
ENSGALG00010022574	ITPRIPL2	2	-0.126674696	0.166000573	-0.763097944	0.445404986	0	0.023979104	0	1	CRA004775;Yang2025_Silkie_Wenchang	PRJNA827465	0.846013379
ENSGALG00010022574	ITPRIPL2	2	-0.029795425	0.096682946	-0.308176637	0.757947929	0	0.20173279	0	1	CRA004775;PRJNA827465	Yang2025_Silkie_Wenchang	0.938954369
ENSGALG00010022575	TMEM41B	2	-0.063534314	0.205509316	-0.309155398	0.75720332	0.07909126	15.64527128	93.60829236	0.5	PRJNA827465;Yang2025_Silkie_Wenchang	CRA004775	0.938795135
ENSGALG00010022575	TMEM41B	2	0.0293702	0.338326368	0.086810258	0.930822333	0.198781792	6.999037043	85.71232022	0.5	CRA004775;Yang2025_Silkie_Wenchang	PRJNA827465	0.983943003
ENSGALG00010022575	TMEM41B	2	0.170829058	0.087569337	1.950786247	0.051082479	0.004719589	1.148767784	12.95020505	1	CRA004775;PRJNA827465	Yang2025_Silkie_Wenchang	0.391439785
ENSGALG00010022578	ZNF512B	2	0.085353704	0.065823057	1.296714379	0.19472948	0.004740117	2.196499313	54.47301103	1	PRJNA827465;Yang2025_Silkie_Wenchang	CRA004775	0.705946169
ENSGALG00010022578	ZNF512B	2	0.138738711	0.060684794	2.286218699	0.022241467	0	0.336954019	0	1	CRA004775;Yang2025_Silkie_Wenchang	PRJNA827465	0.252865604
ENSGALG00010022578	ZNF512B	2	0.029729106	0.05385666	0.552004265	0.580945431	0	0.073944964	0	1	CRA004775;PRJNA827465	Yang2025_Silkie_Wenchang	0.888785453
ENSGALG00010022579	TNFAIP8L3	2	-0.13434224	0.085270539	-1.575482479	0.11514512	0	0.000940976	0	1	PRJNA827465;Yang2025_Silkie_Wenchang	CRA004775	0.572141484

ENSGALG00010022593	MANBAL	2	-0.177178269	0.223161858	-0.793945124	0.427227379	0.057077058	2.178729469	54.10169027	1	CRA004775;Yang2025_Silkie_Wenchang	PRJNA827465	0.84125685
ENSGALG00010022593	MANBAL	2	-0.142561284	0.247052818	-0.577047793	0.56390717	0.089169923	3.176660333	68.52039893	0.5	CRA004775;PRJNA827465	Yang2025_Silkie_Wenchang	0.882721184
ENSGALG00010022597	SLCO4A1	2	0.440172019	0.270602352	1.626637816	0.103814043	0.080360881	2.209775816	54.74654067	1	PRJNA827465;Yang2025_Silkie_Wenchang	CRA004775	0.548157801
ENSGALG00010022597	SLCO4A1	2	0.27582374	0.527648724	0.522741225	0.601154346	0.397347186	3.118535794	67.93366932	0.5	CRA004775;Yang2025_Silkie_Wenchang	PRJNA827465	0.895230519
ENSGALG00010022597	SLCO4A1	2	0.054430268	0.244101643	0.222981981	0.823549531	0	0.748622729	0	0.5	CRA004775;PRJNA827465	Yang2025_Silkie_Wenchang	0.955934625
ENSGALG00010022599	RNLS	2	0.042152448	0.151166115	0.278848527	0.780361071	0	0.160855282	0	0.5	PRJNA827465;Yang2025_Silkie_Wenchang	CRA004775	0.945041817
ENSGALG00010022599	RNLS	2	-0.184967783	0.209029762	-0.884887306	0.376217415	0.008038063	1.090532509	8.301679092	1	CRA004775;Yang2025_Silkie_Wenchang	PRJNA827465	0.822974013
ENSGALG00010022599	RNLS	2	-0.119274414	0.272766833	-0.437276091	0.661911133	0.08099618	2.035154308	50.86367672	0.5	CRA004775;PRJNA827465	Yang2025_Silkie_Wenchang	0.912556664
ENSGALG00010022603	ATRNL1	2	-0.002229026	0.110647168	-0.02014535	0.983927424	0	0.493943454	0	0.5	PRJNA827465;Yang2025_Silkie_Wenchang	CRA004775	0.995579629
ENSGALG00010022603	ATRNL1	2	0.166838995	0.19107039	0.873180794	0.382564542	0	0.105122347	0	1	CRA004775;Yang2025_Silkie_Wenchang	PRJNA827465	0.825121999
ENSGALG00010022603	ATRNL1	2	-0.015369551	0.121475307	-0.12652408	0.899317089	0	0.632207375	0	0.5	CRA004775;PRJNA827465	Yang2025_Silkie_Wenchang	0.976081354
ENSGALG00010022608	P3H4	2	0.209311458	0.067159265	3.116643056	0.001829229	0	0.035163734	0	1	PRJNA827465;Yang2025_Silkie_Wenchang	CRA004775	0.054630843
ENSGALG00010022608	P3H4	2	-0.04127837	0.35668403	-0.115728115	0.907868024	0.181150786	2.996036219	66.62256638	0.5	CRA004775;Yang2025_Silkie_Wenchang	PRJNA827465	0.978196186
ENSGALG00010022608	P3H4	2	-0.041273161	0.335918145	-0.122866721	0.902212639	0.166773105	3.017352378	66.85836208	0.5	CRA004775;PRJNA827465	Yang2025_Silkie_Wenchang	0.976923607
ENSGALG00010022614	DAO	2	-0.461786583	0.091012091	-5.073903658	3.89736E-07	0	0.966544116	0	1	PRJNA827465;Yang2025_Silkie_Wenchang	CRA004775	0.000104917
ENSGALG00010022614	DAO	2	-0.083284252	0.582982908	-0.142858822	0.88640168	0.584610562	6.689066563	85.05023099	0.5	CRA004775;Yang2025_Silkie_Wenchang	PRJNA827465	0.972729743
ENSGALG00010022614	DAO	2	-0.005385373	0.478471891	-0.01125536	0.991019712	0.378784408	5.06009048	80.23750753	0.5	CRA004775;PRJNA827465	Yang2025_Silkie_Wenchang	0.99755571
ENSGALG00010022616	GLDN	2	0.11970179	0.127395158	0.939610205	0.34741754	0	0.632754771	0	0.5	PRJNA827465;Yang2025_Silkie_Wenchang	CRA004775	0.808705451
ENSGALG00010022616	GLDN	2	1.013541051	0.858525536	1.180560168	0.237777495	1.363469591	12.76138722	92.16386132	1	CRA004775;Yang2025_Silkie_Wenchang	PRJNA827465	0.749548355
ENSGALG00010022616	GLDN	2	0.919139925	0.961993622	0.955453242	0.33934854	1.732814618	15.2365404	93.43683032	0.5	CRA004775;PRJNA827465	Yang2025_Silkie_Wenchang	0.804824608
ENSGALG00010022619	MADD	2	0.186369026	0.137731825	1.353129719	0.176014193	0.027059932	3.35765798	70.21733584	1	PRJNA827465;Yang2025_Silkie_Wenchang	CRA004775	0.68110013
ENSGALG00010022619	MADD	2	0.142618063	0.26146443	0.545458757	0.585438039	0.092026108	2.678773404	62.66948154	0.5	CRA004775;Yang2025_Silkie_Wenchang	PRJNA827465	0.891135366
ENSGALG00010022619	MADD	2	0.047190489	0.078954185	0.597694587	0.550043738	0	0.698298343	0	0.5	CRA004775;PRJNA827465	Yang2025_Silkie_Wenchang	0.878652984
ENSGALG00010022622	MPPED2	2	-0.077422338	0.125632427	-0.616260786	0.537722416	0.020197149	2.523767497	60.37669868	0.5	PRJNA827465;Yang2025_Silkie_Wenchang	CRA004775	0.87481821
ENSGALG00010022622	MPPED2	2	-0.067260996	0.20567904	-0.327019204	0.743653361	0.050072961	2.331497855	57.10911774	0.5	CRA004775;Yang2025_Silkie_Wenchang	PRJNA827465	0.935821071
ENSGALG00010022622	MPPED2	2	0.035547065	0.069557116	0.511048568	0.609317049	0	0.432017117	0	1	CRA004775;PRJNA827465	Yang2025_Silkie_Wenchang	0.897238578
ENSGALG00010022623	TOP1	2	0.117351851	0.198647407	0.59075451	0.554684919	0.064643828	4.951221283	79.80296289	0.5	PRJNA827465;Yang2025_Silkie_Wenchang	CRA004775	0.880223402
ENSGALG00010022623	TOP1	2	0.256335955	0.139881151	1.832526781	0.066872975	0	0.913601027	0	1	CRA004775;Yang2025_Silkie_Wenchang	PRJNA827465	0.445476026
ENSGALG00010022623	TOP1	2	-0.045172782	0.056467192	-0.799982793	0.423720767	0	0.227644566	0	0.5	CRA004775;PRJNA827465	Yang2025_Silkie_Wenchang	0.84030043
ENSGALG00010022625	ENSGALG00010022625	2	-0.336432772	0.082287836	-4.088487296	4.34195E-05	0	0.586298444	0	1	PRJNA827465;Yang2025_Silkie_Wenchang	CRA004775	0.004409607
ENSGALG00010022625	ENSGALG00010022625	2	-0.41036333	0.097618708	-4.203736522	2.62545E-05	0	0.199327963	0	1	CRA004775;Yang2025_Silkie_Wenchang	PRJNA827465	0.002991336
ENSGALG00010022625	ENSGALG00010022625	2	-0.318834192	0.11125693	-2.865746813	0.004160268	0	0.84374831	0	1	CRA004775;PRJNA827465	Yang2025_Silkie_Wenchang	0.093756322
ENSGALG00010022627	CTCF	2	-0.075762096	0.085010172	-0.891212129	0.372815379	0.010486519	3.40787718	70.65621949	1	PRJNA827465;Yang2025_Silkie_Wenchang	CRA004775	0.822162024
ENSGALG00010022627	CTCF	2	-0.122011312	0.078706183	-1.550212548	0.121090509	0.002336648	1.195877422	16.37938958	1	CRA004775;Yang2025_Silkie_Wenchang	PRJNA827465	0.583289761
ENSGALG00010022627	CTCF	2	-0.003895305	0.04188553	-0.092998812	0.925904505	0	0.000597785	0	1	CRA004775;PRJNA827465	Yang2025_Silkie_Wenchang	0.982765528
ENSGALG00010022630	ENSGALG00010022630	2	0.492488564	0.417316803	1.180131163	0.237948052	0.277118017	4.668240209	78.57865159	1	PRJNA827465;Yang2025_Silkie_Wenchang	CRA004775	0.74978532
ENSGALG00010022630	ENSGALG00010022630	2	0.913991726	0.318822989	2.866768575	0.004146861	0	0.155251471	0	1	CRA004775;Yang2025_Silkie_Wenchang	PRJNA827465	0.093615215
ENSGALG00010022630	ENSGALG00010022630	2	0.136679536	0.186876859	0.731388232	0.464542051	0	0.213356134	0	1	CRA004775;PRJNA827465	Yang2025_Silkie_Wenchang	0.85217126
ENSGALG00010022637	DMXL2	2	0.123136859	0.139088566	0.885312592	0.375988061	0.022786972	2.258063215	55.71426019	1	PRJNA827465;Yang2025_Silkie_Wenchang	CRA004775	0.822974013
ENSGALG00010022637	DMXL2	2	-0.014856202	0.406802332	-0.036519461	0.970868162	0.236609402	3.023520953	66.92597751	0.5	CRA004775;Yang2025_Silkie_Wenchang	PRJNA827465	0.991868977
ENSGALG00010022637	DMXL2	2	-0.085983871	0.212733163	-0.404186494	0.686075585	0.046516568	1.433011947	30.21691117	0.5	CRA004775;PRJNA827465	Yang2025_Silkie_Wenchang	0.919235539
ENSGALG00010022642	SEC13	2	0.059351436	0.038841879	1.528026896	0.12650586	0	0.244453215	0	1	PRJNA827465;Yang2025_Silkie_Wenchang	CRA004775	0.597082602
ENSGALG00010022642	SEC13	2	0.008777851	0.078143549	0.112329819	0.910561899	0	0.106684084	0	0.5	CRA004775;Yang2025_Silkie_Wenchang	PRJNA827465	0.97880407
ENSGALG00010022642	SEC13	2	0.062769938	0.042241119	1.485991371	0.137281403	0	0.372690729	0	0.5	CRA004775;PRJNA827465	Yang2025_Silkie_Wenchang	0.619818823
ENSGALG00010022643	SP3	2	0.120198463	0.182646423	0.658093712	0.510477916	0.052823432	4.003848883	75.02403239	0.5	PRJNA827465;Yang2025_Silkie_Wenchang	CRA004775	0.865365179
ENSGALG00010022643	SP3	2	0.297786202	0.134380336	2.215995377	0.026691819	0	0.179485753	0	1	CRA004775;Yang2025_Silkie_Wenchang	PRJNA827465	0.278826072
ENSGALG00010022643	SP3	2	0.034384154	0.109108087	0.315138459	0.752656529	0.013901311	1.688678966	40.7821131	0.5	CRA004775;PRJNA827465	Yang2025_Silkie_Wenchang	0.938087082
ENSGALG00010022644	ARMC8	2	-0.034799481	0.042856052	-0.812008543	0.416786729	0.000643357	1.165397253	14.19234964	1	PRJNA827465;Yang2025_Silkie_Wenchang	CRA004775	0.837984974
ENSGALG00010022644	ARMC8	2	-0.106878141	0.071620949	-1.492274853	0.135627106	0	0.019000463	0	1	CRA004775;Yang2025_Silkie_Wenchang	PRJNA827465	0.617196137
ENSGALG00010022644	ARMC8	2	-0.01351273	0.042777361	-0.315885066	0.752089747	0	0.364726232	0	1	CRA004775;PRJNA827465	Yang2025_Silkie_Wenchang	0.937945771
ENSGALG00010022647	SVIP	2	-0.346913013	0.262570001	-1.32122105	0.18642767	0.130779097	19.2101569	94.79442044	1	PRJNA827465;Yang2025_Silkie_Wenchang	CRA004775	0.695457578
ENSGALG00010022647	SVIP	2	-0.437372897	0.257363104	-1.699439003	0.089236498	0.087631873	2.379960709	57.98249961	1	CRA004775;Yang2025_Silkie_Wenchang	PRJNA827465	0.512891552
ENSGALG00010022647	SVIP	2	-0.08957806	0.061965804	-1.445604736	0.148283098	0	0.004981363	0	1	CRA004775;PRJNA827465	Yang2025_Silkie_Wenchang	0.638004061
ENSGALG00010022649	PATZ1	2	-0.042607781	0.050165016	-0.849335482	0.395685185	0	0.413035144	0	1	PRJNA827465;Yang2025_Silkie_Wenchang	CRA004775	0.831148247
ENSGALG00010022649	PATZ1	2	-0.10589421	0.104283345	-1.015446995	0.309892801	0	0.000966957	0	1	CRA004775;Yang2025_Silkie_Wenchang	PRJNA827465	0.794395438
ENSGALG00010022649	PATZ1	2	-0.02816874	0.055780501	-0.504992607	0.613564028	0	0.063770909	0	1	CRA004775;PRJNA827465	Yang2025_Silkie_Wenchang	0.898256044
ENSGALG00010022650	NR1H3	2	-0.214039145	0.152158762	-1.406683009	0.159521406	0.043784022	18.2820178	94.53014426	1	PRJNA827465;Yang2025_Silkie_Wenchang	CRA004775	0.655069463
ENSGALG00010022650	NR1H3	2	-0.360181528	0.056210874	-6.407684217	1.47747E-10	0	0.496962361	0	1	CRA004775;Yang2025_Silkie_Wenchang	PRJNA827465	1.00119E-07
ENSGALG00010022650	NR1H3	2	-0.06714863	0.04142865	-1.620825906	0.105054981	0	0.176045422	0	1	CRA004775;PRJNA827465	Yang2025_Silkie_Wenchang	0.550236662
ENSGALG00010022652	CARMIL2	2	0.512252965	0.407831165	1.256041736	0.209100839	0.275647145	5.79826818	82.75347105	1	PRJNA827465;Yang2025_Silkie_Wenchang	CRA004775	0.721011841
ENSGALG00010022652	CARMIL2	2	0.665910698	0.374675708	1.777288832	0.075519086	0.143329686	1.847219626	45.86458557	1	CRA004775;Yang2025_Silkie_Wenchang	PRJNA827465	0.474354409
ENSGALG00010022652	CARMIL2	2	0.122711735	0.19692052	0.623153621	0.533183576	0	0.002090867	0	1	CRA004775;PRJNA827465	Yang2025_Silkie_Wenchang	0.873379507
ENSGALG00010022655	NRIP3	2	1.315227032	0.794638969	1.655125262	0.097899092	0.693148889	1.934350102	48.30305025	1	PRJNA827465;Yang2025_Silkie_Wenchang	CRA004775	0.534130658
ENSGALG00010022655	NRIP3	2	1.001432879	1.27160832	0.7875325	0.430970211	2.492850816	4.117279597	75.71211825	0.5	CRA004775;Yang2025_Silkie_Wenchang	PRJNA827465	0.842503929
ENSGALG00010022655	NRIP3	2	0.391821713	0.430942616	0.909220156	0.363233907	0.077484328	1.259946478	20.37877271	0.5	CRA004775;PRJNA827465	Yang2025_Silkie_Wenchang	0.816808732
ENSGALG00010022656	FKBP10	2	-0.060839403	0.072808006	-0.835614183	0.403371987	0	0.15310884	0	1	PRJNA827465;Yang2025_Silkie_Wenchang	CRA004775	0.83306585
ENSGALG00010022656	FKBP10	2	-0.137820567	0.123346004	-1.117349262	0.26384502	0	0.919288102	0	1	CRA004775;Yang2025_Silkie_Wenchang	PRJNA827465	0.768643821
ENSGALG00010022656	FKBP10	2	-0.106861419	0.167063127	-0.639646946	0.522402154	0.024414796	1.253581094	20.2285353	1	CRA004775;PRJNA827465	Yang2025_Silkie_Wenchang	0.869568211
ENSGALG00010022657	MRPS35	2	-0.001853087	0.056866537	-0.032586606	0.974004251	0	0.887808375	0	0.5	PRJNA827465;Yang2025_Silkie_Wenchang	CRA004775	0.992492038
ENSGALG00010022657	MRPS35	2	-0.139702026	0.139029904	-1.004834369	0.314976611	0	0.001359439	0	1	CRA004775;Yang2025_Silkie_Wenchang	PRJNA827465	0.796572019
ENSGALG00010022657	MRPS35	2	0.013300297	0.060057662	0.221458784	0.824735225	0	0.273867793	0	0.5	CRA004775;PRJNA827465	Yang2025_Silkie_Wenchang	0.95619211
ENSGALG00010022659	TIE1	2	-0.193704667	0.203414922	-0.952263802	0.340963211	0.074843844	10.30665622	90.29753259	0.5	PRJNA827465;Yang2025_Silkie_Wenchang	CRA004775	0.805639763
ENSGALG00010022659	TIE1	2	-0.165385299	0.301950856	-0.547722571	0.583882412	0.137430372	3.511855974	71.52502815	0.5	CRA004775;Yang2025_Silkie_Wenchang	PRJNA827465	0.890763231
ENSGALG00010022659	TIE1	2	0.011622665	0.067326432	0.172631526	0.86294107	0	0.440130065	0	1	CRA004775;PRJNA827465	Yang2025_Silkie_Wenchang	0.966099097
ENSGALG00010022660	METAP1D	2	0.28715335	0.15005424	1.913677015	0.055661438	0	0.001552792	0	1	PRJNA827465;Yang2025_Silkie_Wenchang	CRA004775	0.40857092
ENSGALG00010022660	METAP1D	2	0.103103951	0.215425223	0.478606682	0.632218464	0	0.74903835	0	0.5	CRA004775;Yang2025_Silkie_Wenchang	PRJNA827465	0.903058174
ENSGALG00010022660	METAP1D	2	0.186445193	0.171745802	1.085588067	0.277661281	0.009959664	1.153162833	13.28197792	0.5	CRA004775;PRJNA827465	Yang2025_Silkie_Wenchang	0.776763074
ENSGALG00010022661	ENSGALG00010022661	2	0.154729394	0.282639853	0.547443657	0.584073969	0	0.066528074	0	1	PRJNA827465;Yang2025_Silkie_Wenchang	CRA004775	0.890763231
ENSGALG00010022661	ENSGALG00010022661	2	0.107145681	0.352985473	0.303541335	0.761477352	0	0.414180253	0	0.5	CRA004775;Yang2025_Silkie_Wenchang	PRJNA827465	0.939694947
ENSGALG00010022661	ENSGALG00010022661	2	-0.006823806	0.35327393	-0.019315905	0.984589096	0	0.223295931	0	0.5	CRA004775;PRJNA827465	Yang2025_Silkie_Wenchang	0.995703389
ENSGALG00010022662	GFRA1	2	0.109107465	0.14846571	0.734900095	0.46240034	0	0.205739491	0	1	PRJNA827465;Yang2025_Silkie_Wenchang	CRA004775	0.850823289
ENSGALG00010022662	GFRA1	2	0.733635782	0.757293001	0.968760811	0.332664544	1.014468651	8.27959008	87.92210737	1	CRA004775;Yang2025_Silkie_Wenchang	PRJNA827465	0.802178578
ENSGALG00010022662	GFRA1	2	0.791666272	0.688268823	1.150228291	0.250049857	0.823115643	7.158484579	86.03056291	1	CRA004775;PRJNA827465	Yang2025_Silkie_Wenchang	0.75993411
ENSGALG00010022664	TRPC4AP	2	-0.090437342	0.040475708	-2.234360966	0.025459332	0	0.1294033	0	1	PRJNA827465;Yang2025_Silkie_Wenchang	CRA004775	0.272131662
ENSGALG00010022664	TRPC4AP	2	-0.033656799	0.078769346	-0.42728296	0.669173243	0	0.65308152	0	0.5	CRA004775;Yang2025_Silkie_Wenchang	PRJNA827465	0.914377769
ENSGALG00010022664	TRPC4AP	2	-0.079149389	0.059125471	-1.338668212	0.180678709	0.001760704	1.093254276	8.529971275	0.5	CRA004775;PRJNA827465	Yang2025_Silkie_Wenchang	0.688369068
ENSGALG00010022666	ENSGALG00010022666	2	0.116167303	0.094793175	1.225481722	0.220393773	0.012233362	3.075573569	67.48573957	1	PRJNA827465;Yang2025_Silkie_Wenchang	CRA004775	0.734053249
ENSGALG00010022666	ENSGALG00010022666	2	-0.016388214	0.250178021	-0.065506209	0.947770963	0.110987235	8.611316761	88.38737411	0.5	CRA004775;Yang2025_Silkie_Wenchang	PRJNA827465	0.986619597
ENSGALG00010022666	ENSGALG00010022666	2	-0.097614392	0.152828658	-0.638717854	0.523006496	0.035375554	3.742051863	73.2766932	0.5	CRA004775;PRJNA827465	Yang2025_Silkie_Wenchang	0.869568211
ENSGALG00010022667	PTEN	2	-0.072143399	0.047783206	-1.509806588	0.131092784	0	0.802911174	0	1	PRJNA827465;Yang2025_Silkie_Wenchang	CRA004775	0.606767161
ENSGALG00010022667	PTEN	2	0.040113399	0.240535806	0.166766852	0.867553502	0.0906057	4.548467371	78.01457242	0.5	CRA004775;Yang2025_Silkie_Wenchang	PRJNA827465	0.96788231
ENSGALG00010022667	PTEN	2	0.077212708	0.170640234	0.45248829	0.650917255	0.044281547	3.518875779	71.58183287	0.5	CRA004775;PRJNA827465	Yang2025_Silkie_Wenchang	0.908769703
ENSGALG00010022671	DBR1	2	-0.079113819	0.110675939	-0.714824019	0.474717792	0.019839383	5.02883137	80.11466429	0.5	PRJNA827465;Yang2025_Silkie_Wenchang	CRA004775	0.854462864
ENSGALG00010022671	DBR1	2	-0.201232978	0.078929121	-2.549540352	0.010786503	0	0.000361479	0	1	CRA004775;Yang2025_Silkie_Wenchang	PRJNA827465	0.165420139
ENSGALG00010022671	DBR1	2	-0.009201305	0.075039237	-0.122619916	0.902408083	0.004160744	1.211014229	17.4245871	0.5	CRA004775;PRJNA827465	Yang2025_Silkie_Wenchang	0.976923607
ENSGALG00010022673	KLHL42	2	-0.018918994	0.203432523	-0.092998866	0.925904462	0.055552203	2.886590238	65.35705045	0.5	PRJNA827465;Yang2025_Silkie_Wenchang	CRA004775	0.982765528
ENSGALG00010022673	KLHL42	2	-0.17934972	0.170202587	-1.053742621	0.29200077	0	0.43941701	0	1	CRA004775;Yang2025_Silkie_Wenchang	PRJNA827465	0.785516679
ENSGALG00010022673	KLHL42	2	0.127770534	0.115363046	1.107551671	0.268055485	0	0.286527834	0	0.5	CRA004775;PRJNA827465	Yang2025_Silkie_Wenchang	0.771398294
ENSGALG00010022674	ENSGALG00010022674	2	0.211568566	0.460365273	0.459566736	0.64582724	0.28101527	2.866934722	65.11954066	0.5	PRJNA827465;Yang2025_Silkie_Wenchang	CRA004775	0.907098003
ENSGALG00010022674	ENSGALG00010022674	2	1.008890539	0.278862636	3.617876364	0.00029703	0	0.563357671	0	1	CRA004775;Yang2025_Silkie_Wenchang	PRJNA827465	0.016889382
ENSGALG00010022674	ENSGALG00010022674	2	0.478337874	0.680722436	0.702691506	0.482248022	0.8184313	8.536415802	88.2854816	0.5	CRA004775;PRJNA827465	Yang2025_Silkie_Wenchang	0.85691916
ENSGALG00010022681	PPP2R3A	2	-0.208951149	0.079756874	-2.619851306	0.008796811	0.000502889	1.039500992	3.799995649	1	PRJNA827465;Yang2025_Silkie_Wenchang	CRA004775	0.147123863
ENSGALG00010022681	PPP2R3A	2	-0.073576196	0.309453105	-0.237762021	0.812065677	0.134583777	2.845620601	64.85828084	0.5	CRA004775;Yang2025_Silkie_Wenchang	PRJNA827465	0.953371387
ENSGALG00010022681	PPP2R3A	2	-0.027837752	0.205770013	-0.135285757	0.892385946	0.045613292	1.648666485	39.34491849	0.5	CRA004775;PRJNA827465	Yang2025_Silkie_Wenchang	0.974316485
ENSGALG00010022682	EIF1	2	-0.479965792	0.381730334	-1.257342551	0.208629622	0.26943006	12.46630551	91.97837724	1	PRJNA827465;Yang2025_Silkie_Wenchang	CRA004775	0.721011841
ENSGALG00010022682	EIF1	2	-0.607116969	0.335353742	-1.810377798	0.07023722	0.148073931	2.774810259	63.96149983	1	CRA004775;Yang2025_Silkie_Wenchang	PRJNA827465	0.457193351
ENSGALG00010022682	EIF1	2	-0.126331655	0.050412449	-2.505961498	0.012211892	0	0.057724293	0	1	CRA004775;PRJNA827465	Yang2025_Silkie_Wenchang	0.178358966
ENSGALG00010022684	ENSGALG00010022684	2	0.491573344	0.606198078	0.810912078	0.417416164	0.645202657	8.188435987	87.78765564	0.5	PRJNA827465;Yang2025_Silkie_Wenchang	CRA004775	0.838112595
ENSGALG00010022684	ENSGALG00010022684	2	0.561529861	0.657882038	0.853541864	0.393358882	0.630857707	3.313894921	69.8240281	0.5	CRA004775;Yang2025_Silkie_Wenchang	PRJNA827465	0.830346712
ENSGALG00010022684	ENSGALG00010022684	2	-0.138412155	0.276603572	-0.500399014	0.616794147	0	0.031633377	0	1	CRA004775;PRJNA827465	Yang2025_Silkie_Wenchang	0.899111936
ENSGALG00010022685	ATAD1	2	0.033825795	0.105552888	0.320462998	0.748617376	0.014011882	2.542938169	60.67541035	0.5	PRJNA827465;Yang2025_Silkie_Wenchang	CRA004775	0.937249951
ENSGALG00010022685	ATAD1	2	-0.043653032	0.218797091	-0.199513769	0.841860874	0.07651574	4.897592691	79.58180553	0.5	CRA004775;Yang2025_Silkie_Wenchang	PRJNA827465	0.960041272
ENSGALG00010022685	ATAD1	2	-0.118119461	0.101358118	-1.165367547	0.243870248	0.009693751	1.633346545	38.77600542	1	CRA004775;PRJNA827465	Yang2025_Silkie_Wenchang	0.755365463
ENSGALG00010022689	MPL	2	0.231044157	0.266498332	0.866962864	0.385962357	0.065491317	1.845233786	45.80632505	0.5	PRJNA827465;Yang2025_Silkie_Wenchang	CRA004775	0.826629531
ENSGALG00010022689	MPL	2	0.132859933	0.453842387	0.292744654	0.769717335	0.266824491	2.714595511	63.16209925	0.5	CRA004775;Yang2025_Silkie_Wenchang	PRJNA827465	0.941878652
ENSGALG00010022689	MPL	2	-0.102717798	0.225502847	-0.455505548	0.648745577	0	0.512885647	0	1	CRA004775;PRJNA827465	Yang2025_Silkie_Wenchang	0.907730933
ENSGALG00010022691	ASIC2	2	0.468255686	0.338820192	1.382018241	0.1669661	0	0.060121828	0	1	PRJNA827465;Yang2025_Silkie_Wenchang	CRA004775	0.666022689
ENSGALG00010022691	ASIC2	2	0.572639922	0.610952523	0.937290379	0.34860921	0	0.02199408	0	1	CRA004775;Yang2025_Silkie_Wenchang	PRJNA827465	0.809295875
ENSGALG00010022691	ASIC2	2	0.440561872	0.338599104	1.301131241	0.193213536	0	0.002786414	0	1	CRA004775;PRJNA827465	Yang2025_Silkie_Wenchang	0.703208363
ENSGALG00010022695	PIK3IP1	2	-0.05755082	0.114620513	-0.50209878	0.615598039	0.008075543	1.386462698	27.87400616	0.5	PRJNA827465;Yang2025_Silkie_Wenchang	CRA004775	0.898886043
ENSGALG00010022695	PIK3IP1	2	-0.170863226	0.159333925	-1.07235936	0.283558654	0	0.440558296	0	0.5	CRA004775;Yang2025_Silkie_Wenchang	PRJNA827465	0.779568117
ENSGALG00010022695	PIK3IP1	2	0.029899106	0.106186425	0.281571833	0.77827184	0	0.018514741	0	1	CRA004775;PRJNA827465	Yang2025_Silkie_Wenchang	0.944556432
ENSGALG00010022698	SEMA6D	2	0.097893543	0.198106681	0.494145591	0.621203362	0.06460901	5.22883087	80.87526591	0.5	PRJNA827465;Yang2025_Silkie_Wenchang	CRA004775	0.899614409
ENSGALG00010022698	SEMA6D	2	0.409041527	0.161511547	2.5325838	0.01132253	0.007457054	1.107365402	9.695571259	1	CRA004775;Yang2025_Silkie_Wenchang	PRJNA827465	0.169656655
ENSGALG00010022698	SEMA6D	2	0.251991392	0.39000631	0.646121319	0.518200789	0.254670813	5.323831325	81.21653488	0.5	CRA004775;PRJNA827465	Yang2025_Silkie_Wenchang	0.868374078
ENSGALG00010022702	CDC20	2	-0.245044977	0.270710932	-0.905190549	0.365364448	0.124754695	6.575467212	84.79195519	0.5	PRJNA827465;Yang2025_Silkie_Wenchang	CRA004775	0.818457479
ENSGALG00010022702	CDC20	2	-0.128988578	0.478991056	-0.269292247	0.787704798	0.361216805	4.252339278	76.48353213	0.5	CRA004775;Yang2025_Silkie_Wenchang	PRJNA827465	0.946919116
ENSGALG00010022702	CDC20	2	0.037403098	0.111448203	0.335609703	0.737165206	0	0.904187747	0	1	CRA004775;PRJNA827465	Yang2025_Silkie_Wenchang	0.933895493
ENSGALG00010022708	SPRED1	2	-0.261562039	0.234152375	-1.11705909	0.263969061	0.104362862	20.5153324	95.12559689	1	PRJNA827465;Yang2025_Silkie_Wenchang	CRA004775	0.768643821
ENSGALG00010022708	SPRED1	2	-0.246578573	0.291712487	-0.845279458	0.397954828	0.142033295	5.53037132	81.9180315	0.5	CRA004775;Yang2025_Silkie_Wenchang	PRJNA827465	0.831825697
ENSGALG00010022708	SPRED1	2	-0.026650839	0.052229546	-0.510263665	0.609866755	0	0.251218567	0	0.5	CRA004775;PRJNA827465	Yang2025_Silkie_Wenchang	0.897238578
ENSGALG00010022709	RNF5	2	0.211961273	0.203427292	1.041951015	0.29743439	0.07129937	6.611583058	84.8750293	1	PRJNA827465;Yang2025_Silkie_Wenchang	CRA004775	0.788562929
ENSGALG00010022709	RNF5	2	0.149175959	0.293939798	0.507505143	0.611800429	0.149120837	7.296314748	86.2944509	0.5	CRA004775;Yang2025_Silkie_Wenchang	PRJNA827465	0.897777844
ENSGALG00010022709	RNF5	2	0.004592616	0.064160571	0.071580033	0.942936131	0.002362618	1.175288083	14.914478	0.5	CRA004775;PRJNA827465	Yang2025_Silkie_Wenchang	0.985827325
ENSGALG00010022711	LRRC49	2	0.128902873	0.086072119	1.497614733	0.134233377	0	0.039483071	0	1	PRJNA827465;Yang2025_Silkie_Wenchang	CRA004775	0.613747604
ENSGALG00010022711	LRRC49	2	0.070657645	0.139304487	0.507217293	0.612002363	0	0.267362443	0	0.5	CRA004775;Yang2025_Silkie_Wenchang	PRJNA827465	0.897777844
ENSGALG00010022711	LRRC49	2	0.120458601	0.099321123	1.212819561	0.225198815	0	0.420375111	0	0.5	CRA004775;PRJNA827465	Yang2025_Silkie_Wenchang	0.738276881
ENSGALG00010022712	DDX23	2	0.063500475	0.10371241	0.612274608	0.540356087	0.01820804	6.432561859	84.45409431	0.5	PRJNA827465;Yang2025_Silkie_Wenchang	CRA004775	0.875453039
ENSGALG00010022712	DDX23	2	-0.001618318	0.221103342	-0.007319281	0.994160111	0.074733402	3.593481206	72.17183164	0.5	CRA004775;Yang2025_Silkie_Wenchang	PRJNA827465	0.998198315
ENSGALG00010022712	DDX23	2	-0.056268604	0.068656215	-0.819570425	0.412461039	0.002802978	1.100799325	9.156920999	1	CRA004775;PRJNA827465	Yang2025_Silkie_Wenchang	0.835702455
ENSGALG00010022717	ELOVL1	2	0.026537201	0.13178876	0.201361642	0.840415806	0.01848792	1.699076452	41.14449654	0.5	PRJNA827465;Yang2025_Silkie_Wenchang	CRA004775	0.959662809
ENSGALG00010022717	ELOVL1	2	-0.194160746	0.196902014	-0.986078013	0.324094832	0	3.93767E-05	0	1	CRA004775;Yang2025_Silkie_Wenchang	PRJNA827465	0.799600434
ENSGALG00010022717	ELOVL1	2	0.097879176	0.07013413	1.395599772	0.162835051	0	0.420353952	0	0.5	CRA004775;PRJNA827465	Yang2025_Silkie_Wenchang	0.660874227
ENSGALG00010022718	C12orf43	2	0.082118315	0.172600551	0.475770874	0.634237622	0.035137532	2.270621059	55.95918589	0.5	PRJNA827465;Yang2025_Silkie_Wenchang	CRA004775	0.903815945
ENSGALG00010022718	C12orf43	2	-0.117283614	0.433507403	-0.270545815	0.786740375	0.319675445	6.630714182	84.9186683	0.5	CRA004775;Yang2025_Silkie_Wenchang	PRJNA827465	0.94673332
ENSGALG00010022718	C12orf43	2	-0.254942979	0.250312563	-1.018498535	0.308441096	0.089171386	3.094329832	67.68282457	1	CRA004775;PRJNA827465	Yang2025_Silkie_Wenchang	0.793457188
ENSGALG00010022719	MSL2	2	-0.052801735	0.085715922	-0.616008487	0.537888919	0.008441876	2.133966694	53.13891248	0.5	PRJNA827465;Yang2025_Silkie_Wenchang	CRA004775	0.87481821
ENSGALG00010022719	MSL2	2	-0.12780763	0.099553283	-1.283811303	0.199207988	0	0.787513088	0	0.5	CRA004775;Yang2025_Silkie_Wenchang	PRJNA827465	0.71096127
ENSGALG00010022719	MSL2	2	0.014895348	0.05427713	0.274431387	0.783753125	0	0.060655128	0	1	CRA004775;PRJNA827465	Yang2025_Silkie_Wenchang	0.94583177
ENSGALG00010022724	LUZP2	2	0.168679933	0.243958187	0.691429686	0.489295554	0.037269902	1.420072098	29.58104019	0.5	PRJNA827465;Yang2025_Silkie_Wenchang	CRA004775	0.858337006
ENSGALG00010022724	LUZP2	2	-0.123071188	0.304276137	-0.404472035	0.685865638	0	0.012913464	0	1	CRA004775;Yang2025_Silkie_Wenchang	PRJNA827465	0.919235539
ENSGALG00010022724	LUZP2	2	0.309658683	0.227808344	1.359294735	0.174053212	0	0.36093037	0	0.5	CRA004775;PRJNA827465	Yang2025_Silkie_Wenchang	0.67800817
ENSGALG00010022727	ACP2	2	0.171900389	0.078265042	2.196387875	0.028064193	0	0.394854049	0	1	PRJNA827465;Yang2025_Silkie_Wenchang	CRA004775	0.285514467
ENSGALG00010022727	ACP2	2	0.015230635	0.125691187	0.121175045	0.90355239	0.009623156	1.395964485	28.36493975	0.5	CRA004775;Yang2025_Silkie_Wenchang	PRJNA827465	0.97732491
ENSGALG00010022727	ACP2	2	0.063245639	0.177650322	0.356011959	0.721831609	0.043474635	3.005344244	66.72594156	0.5	CRA004775;PRJNA827465	Yang2025_Silkie_Wenchang	0.929372448
ENSGALG00010022729	ENSGALG00010022729	2	-0.132618495	0.083416979	-1.589826161	0.111873995	0.002988445	1.227348263	18.52353321	1	PRJNA827465;Yang2025_Silkie_Wenchang	CRA004775	0.565455714
ENSGALG00010022729	ENSGALG00010022729	2	-0.309017757	0.123659297	-2.498944801	0.012456371	0	0.741021774	0	1	CRA004775;Yang2025_Silkie_Wenchang	PRJNA827465	0.180654153
ENSGALG00010022729	ENSGALG00010022729	2	-0.221282751	0.208824692	-1.059657981	0.289300226	0.057522531	2.37841612	57.95521264	1	CRA004775;PRJNA827465	Yang2025_Silkie_Wenchang	0.78357099
ENSGALG00010022731	LRRC8B	2	-0.067720681	0.101210062	-0.669110162	0.503425208	0	0.692543576	0	0.5	PRJNA827465;Yang2025_Silkie_Wenchang	CRA004775	0.863108263
ENSGALG00010022731	LRRC8B	2	-0.109753923	0.146400981	-0.749680243	0.45344731	0	0.969804818	0	0.5	CRA004775;Yang2025_Silkie_Wenchang	PRJNA827465	0.848879567
ENSGALG00010022731	LRRC8B	2	0.027304583	0.124569941	0.219190784	0.826501434	0	0.344156671	0	1	CRA004775;PRJNA827465	Yang2025_Silkie_Wenchang	0.956393839
ENSGALG00010022733	PCCB	2	0.047439724	0.06188278	0.766606224	0.443315674	0	0.802900868	0	0.5	PRJNA827465;Yang2025_Silkie_Wenchang	CRA004775	0.845733906
ENSGALG00010022733	PCCB	2	-0.026159306	0.135221466	-0.193455277	0.846602428	0	0.459765383	0	0.5	CRA004775;Yang2025_Silkie_Wenchang	PRJNA827465	0.961252712
ENSGALG00010022733	PCCB	2	0.073233671	0.06457243	1.134132178	0.256739108	0	0.027193429	0	1	CRA004775;PRJNA827465	Yang2025_Silkie_Wenchang	0.764730364
ENSGALG00010022734	ENSGALG00010022734	2	-0.231162415	0.22635866	-1.021221875	0.307149331	0.067617575	2.182537123	54.18176445	1	PRJNA827465;Yang2025_Silkie_Wenchang	CRA004775	0.79266003
ENSGALG00010022734	ENSGALG00010022734	2	-0.248512205	0.357804489	-0.694547477	0.487338938	0.132370737	2.063214073	51.53193199	0.5	CRA004775;Yang2025_Silkie_Wenchang	PRJNA827465	0.85768885
ENSGALG00010022734	ENSGALG00010022734	2	-0.080345674	0.065314506	-1.230135211	0.218646477	0	0.328770865	0	0.5	CRA004775;PRJNA827465	Yang2025_Silkie_Wenchang	0.73229743
ENSGALG00010022737	CYLD	2	-0.049321718	0.049788942	-0.990615906	0.321873168	0	0.0416253	0	1	PRJNA827465;Yang2025_Silkie_Wenchang	CRA004775	0.798513866
ENSGALG00010022737	CYLD	2	-0.046843008	0.070194752	-0.667329207	0.504561874	0	0.292772213	0	0.5	CRA004775;Yang2025_Silkie_Wenchang	PRJNA827465	0.863512504
ENSGALG00010022737	CYLD	2	-0.030877207	0.06349393	-0.48630172	0.62675326	0	0.204979828	0	0.5	CRA004775;PRJNA827465	Yang2025_Silkie_Wenchang	0.901506107
ENSGALG00010022741	HNF1A	2	1.292619932	0.334657578	3.862515052	0.000112226	0	0.922345235	0	1	PRJNA827465;Yang2025_Silkie_Wenchang	CRA004775	0.008421586
ENSGALG00010022741	HNF1A	2	1.635334005	0.394835639	4.14180951	3.44576E-05	0	0.01634813	0	1	CRA004775;Yang2025_Silkie_Wenchang	PRJNA827465	0.003690161
ENSGALG00010022741	HNF1A	2	1.215193735	0.352521967	3.447143291	0.000566548	0	0.552902153	0	1	CRA004775;PRJNA827465	Yang2025_Silkie_Wenchang	0.025506342
ENSGALG00010022742	IFT46	2	0.118923193	0.075939813	1.566019044	0.117344159	0	0.125882506	0	1	PRJNA827465;Yang2025_Silkie_Wenchang	CRA004775	0.57688585
ENSGALG00010022742	IFT46	2	-0.128509623	0.305333992	-0.420882137	0.67384115	0.156776031	6.228680784	83.945236	0.5	CRA004775;Yang2025_Silkie_Wenchang	PRJNA827465	0.915590641
ENSGALG00010022742	IFT46	2	-0.144584474	0.273401765	-0.528835191	0.596919783	0.12833457	6.671727016	85.01137715	0.5	CRA004775;PRJNA827465	Yang2025_Silkie_Wenchang	0.894172266
ENSGALG00010022743	ZNF326	2	0.375242963	0.351645729	1.067105135	0.285924363	0.228889814	12.78691092	92.17950288	1	PRJNA827465;Yang2025_Silkie_Wenchang	CRA004775	0.781752017
ENSGALG00010022743	ZNF326	2	0.381869978	0.400943908	0.952427436	0.340880251	0.261068625	5.157120642	80.60933476	0.5	CRA004775;Yang2025_Silkie_Wenchang	PRJNA827465	0.805635165
ENSGALG00010022743	ZNF326	2	0.041340381	0.059357971	0.696458788	0.486141558	0	0.106510787	0	0.5	CRA004775;PRJNA827465	Yang2025_Silkie_Wenchang	0.857577274
ENSGALG00010022745	PAPSS2	2	0.219064024	0.170169108	1.287331331	0.197978828	0.012606852	1.265286215	20.96649846	1	PRJNA827465;Yang2025_Silkie_Wenchang	CRA004775	0.710154409
ENSGALG00010022745	PAPSS2	2	0.466312682	0.48506613	0.961338367	0.336382063	0.382262401	5.2288817	80.87545182	1	CRA004775;Yang2025_Silkie_Wenchang	PRJNA827465	0.803806405
ENSGALG00010022745	PAPSS2	2	0.603391022	0.305142017	1.977410483	0.04799525	0.116762375	2.480692624	59.68867766	1	CRA004775;PRJNA827465	Yang2025_Silkie_Wenchang	0.378786609
ENSGALG00010022747	NT5C3B	2	-0.075508487	0.142311367	-0.530586477	0.595705368	0.026683165	2.782869052	64.0658622	0.5	PRJNA827465;Yang2025_Silkie_Wenchang	CRA004775	0.893802528
ENSGALG00010022747	NT5C3B	2	-0.337644317	0.116570207	-2.896488961	0.003773639	0.00053619	1.019196728	1.883515459	1	CRA004775;Yang2025_Silkie_Wenchang	PRJNA827465	0.088100594
ENSGALG00010022747	NT5C3B	2	-0.194145115	0.262463788	-0.739702478	0.459480544	0.11795688	6.609779163	84.8709015	0.5	CRA004775;PRJNA827465	Yang2025_Silkie_Wenchang	0.850098539
ENSGALG00010022748	ENSGALG00010022748	2	0.169875604	0.4683302	0.362726137	0.716809473	0.330092994	3.979995139	74.87434117	0.5	PRJNA827465;Yang2025_Silkie_Wenchang	CRA004775	0.927632716
ENSGALG00010022748	ENSGALG00010022748	2	0.233484455	0.524777444	0.444920905	0.656376915	0.326252645	2.346774381	57.38831956	0.5	CRA004775;Yang2025_Silkie_Wenchang	PRJNA827465	0.910509456

ENSGALG00010022748	ENSGALG00010022748	2	-0.290500379	0.254219516	-1.142714706	0.253157062	0	0.038377307	0	1	CRA004775;PRJNA827465	Yang2025_Silkie_Wenchang	0.762731469
ENSGALG00010022749	HSPA12A	2	-0.334344604	0.137607272	-2.429701567	0.01511126	0.02714397	3.527950236	71.65492898	1	PRJNA827465;Yang2025_Silkie_Wenchang	CRA004775	0.203100811
ENSGALG00010022749	HSPA12A	2	-0.197370448	0.382686062	-0.515750291	0.606028825	0.210508766	2.790222469	64.16056386	0.5	CRA004775;Yang2025_Silkie_Wenchang	PRJNA827465	0.896709756
ENSGALG00010022749	HSPA12A	2	-0.126873513	0.177728757	-0.71386035	0.475313537	0.024806045	1.20985934	17.34576351	0.5	CRA004775;PRJNA827465	Yang2025_Silkie_Wenchang	0.854462864
ENSGALG00010022751	SLC25A12	2	-0.02520268	0.076295389	-0.330330318	0.741150387	0	0.166441858	0	0.5	PRJNA827465;Yang2025_Silkie_Wenchang	CRA004775	0.935384591
ENSGALG00010022751	SLC25A12	2	0.022898473	0.132357844	0.173004275	0.862648067	0	0.002845553	0	1	CRA004775;Yang2025_Silkie_Wenchang	PRJNA827465	0.966099097
ENSGALG00010022751	SLC25A12	2	-0.043314253	0.09059048	-0.4781325	0.632555902	0	0.032193404	0	0.5	CRA004775;PRJNA827465	Yang2025_Silkie_Wenchang	0.903058174
ENSGALG00010022754	SNX20	2	-0.372480289	0.275983869	-1.349645144	0.177129836	0.11574016	4.15535104	75.9346445	1	PRJNA827465;Yang2025_Silkie_Wenchang	CRA004775	0.682961725
ENSGALG00010022754	SNX20	2	-0.598603494	0.172185075	-3.476512084	0.000507981	0	0.552889457	0	1	CRA004775;Yang2025_Silkie_Wenchang	PRJNA827465	0.023657862
ENSGALG00010022754	SNX20	2	-0.105883783	0.18805602	-0.563043836	0.573405035	0	0.052028407	0	1	CRA004775;PRJNA827465	Yang2025_Silkie_Wenchang	0.885850914
ENSGALG00010022755	MINPP1	2	0.088825543	0.069625029	1.275770282	0.202036736	0	0.239604756	0	1	PRJNA827465;Yang2025_Silkie_Wenchang	CRA004775	0.71497298
ENSGALG00010022755	MINPP1	2	-0.318464593	0.403797339	-0.788674322	0.430302379	0.271777507	5.530709337	81.91913661	0.5	CRA004775;Yang2025_Silkie_Wenchang	PRJNA827465	0.842040417
ENSGALG00010022755	MINPP1	2	-0.279916079	0.441483296	-0.634035493	0.526057659	0.340620083	7.184606088	86.08135244	0.5	CRA004775;PRJNA827465	Yang2025_Silkie_Wenchang	0.870509645
ENSGALG00010022759	THSD4	2	-0.106980582	0.095422883	-1.121120828	0.262236434	0	0.596019439	0	1	PRJNA827465;Yang2025_Silkie_Wenchang	CRA004775	0.768363052
ENSGALG00010022759	THSD4	2	-0.18125214	0.179977381	-1.007082883	0.313894946	0	0.473412754	0	0.5	CRA004775;Yang2025_Silkie_Wenchang	PRJNA827465	0.796531448
ENSGALG00010022759	THSD4	2	-0.058564623	0.104406855	-0.560926991	0.574847309	0	0.09835475	0	0.5	CRA004775;PRJNA827465	Yang2025_Silkie_Wenchang	0.886211989
ENSGALG00010022764	MFAP5	2	-0.427809851	0.376165894	-1.137290377	0.255416916	0.259239735	11.90147705	91.59768157	1	PRJNA827465;Yang2025_Silkie_Wenchang	CRA004775	0.764467569
ENSGALG00010022764	MFAP5	2	-0.492067987	0.439167948	-1.120455145	0.262519857	0.266001567	2.622323991	61.86588677	0.5	CRA004775;Yang2025_Silkie_Wenchang	PRJNA827465	0.768418419
ENSGALG00010022764	MFAP5	2	-0.043034144	0.141661992	-0.303780452	0.7612916	0	0.094673518	0	0.5	CRA004775;PRJNA827465	Yang2025_Silkie_Wenchang	0.939618592
ENSGALG00010022767	CACNB3	2	0.48901386	0.386353773	1.265715246	0.205615048	0.225200633	4.005905259	75.03685346	1	PRJNA827465;Yang2025_Silkie_Wenchang	CRA004775	0.71825498
ENSGALG00010022767	CACNB3	2	0.304664974	0.701433869	0.43434597	0.664037227	0.755118067	3.982477135	74.89000021	0.5	CRA004775;Yang2025_Silkie_Wenchang	PRJNA827465	0.91312035
ENSGALG00010022767	CACNB3	2	0.055670866	0.216870186	0.256701335	0.797409341	0	0.899832004	0	0.5	CRA004775;PRJNA827465	Yang2025_Silkie_Wenchang	0.949715736
ENSGALG00010022769	FXYD6	2	-0.043474999	0.083919446	-0.518056319	0.604418976	0	0.227179942	0	0.5	PRJNA827465;Yang2025_Silkie_Wenchang	CRA004775	0.89616263
ENSGALG00010022769	FXYD6	2	-0.040050282	0.164514917	-0.243444686	0.80766093	0	0.759267416	0	0.5	CRA004775;Yang2025_Silkie_Wenchang	PRJNA827465	0.952496413
ENSGALG00010022769	FXYD6	2	-0.080169493	0.090845711	-0.882479088	0.377517784	0	0.461803088	0	1	CRA004775;PRJNA827465	Yang2025_Silkie_Wenchang	0.82325159
ENSGALG00010022770	HAT1	2	-0.032385341	0.099352338	-0.325964555	0.744451172	0.011671648	2.222868676	55.01308689	0.5	PRJNA827465;Yang2025_Silkie_Wenchang	CRA004775	0.935846381
ENSGALG00010022770	HAT1	2	-0.170132332	0.109177071	-1.558315596	0.119158454	0	0.017953	0	1	CRA004775;Yang2025_Silkie_Wenchang	PRJNA827465	0.579471011
ENSGALG00010022770	HAT1	2	0.027544515	0.060757514	0.453351585	0.650295593	0	0.973600136	0	0.5	CRA004775;PRJNA827465	Yang2025_Silkie_Wenchang	0.908490659
ENSGALG00010022771	SGMS1	2	-0.023994976	0.093312244	-0.257147138	0.79706519	0.010168943	2.344057035	57.338922	0.5	PRJNA827465;Yang2025_Silkie_Wenchang	CRA004775	0.949962804
ENSGALG00010022771	SGMS1	2	-0.12782183	0.097683443	-1.30853117	0.190693209	0	0.022044323	0	1	CRA004775;Yang2025_Silkie_Wenchang	PRJNA827465	0.699992081
ENSGALG00010022771	SGMS1	2	0.055006595	0.071197735	0.772589112	0.43976559	0	0.055727109	0	0.5	CRA004775;PRJNA827465	Yang2025_Silkie_Wenchang	0.844353254
ENSGALG00010022776	CDH4	2	-0.095435218	0.190312263	-0.501466463	0.616042875	0.03463493	1.892742505	47.16661155	0.5	PRJNA827465;Yang2025_Silkie_Wenchang	CRA004775	0.899057762
ENSGALG00010022776	CDH4	2	0.143044975	0.1957934	0.730591406	0.465028763	0	0.067108245	0	1	CRA004775;Yang2025_Silkie_Wenchang	PRJNA827465	0.852263402
ENSGALG00010022776	CDH4	2	-0.175678406	0.194206613	-0.904595388	0.36567978	0.015397017	1.132828938	11.72541885	0.5	CRA004775;PRJNA827465	Yang2025_Silkie_Wenchang	0.818968857
ENSGALG00010022778	GDF11	2	0.246974248	0.156105354	1.582099789	0.113626792	0	0.012287697	0	1	PRJNA827465;Yang2025_Silkie_Wenchang	CRA004775	0.568794964
ENSGALG00010022778	GDF11	2	0.233983827	0.219877889	1.064153508	0.287259165	0	0.005284113	0	1	CRA004775;Yang2025_Silkie_Wenchang	PRJNA827465	0.782840067
ENSGALG00010022778	GDF11	2	0.261128391	0.182870251	1.427943522	0.153308115	0	2.33682E-05	0	1	CRA004775;PRJNA827465	Yang2025_Silkie_Wenchang	0.645602272
ENSGALG00010022780	DYNC1I2	2	-0.029504772	0.035646534	-0.827703808	0.407838262	0	0.242119781	0	0.5	PRJNA827465;Yang2025_Silkie_Wenchang	CRA004775	0.833982683
ENSGALG00010022780	DYNC1I2	2	-0.01212142	0.082795395	-0.146402098	0.883603971	0	0.435703181	0	0.5	CRA004775;Yang2025_Silkie_Wenchang	PRJNA827465	0.972001076
ENSGALG00010022780	DYNC1I2	2	-0.04044702	0.038182958	-1.059295104	0.289465404	0	0.231518699	0	1	CRA004775;PRJNA827465	Yang2025_Silkie_Wenchang	0.783908136
ENSGALG00010022782	TMPRSS13	2	0.063437861	0.249142234	0.254625078	0.799012688	0.046152906	1.54367947	35.21971243	0.5	PRJNA827465;Yang2025_Silkie_Wenchang	CRA004775	0.949922217
ENSGALG00010022782	TMPRSS13	2	-0.476222225	0.233686657	-2.037866564	0.041563279	0	0.838548318	0	1	CRA004775;Yang2025_Silkie_Wenchang	PRJNA827465	0.353355943
ENSGALG00010022782	TMPRSS13	2	-0.183395102	0.469502672	-0.390615672	0.696083134	0.362810282	5.568026143	82.04031421	0.5	CRA004775;PRJNA827465	Yang2025_Silkie_Wenchang	0.921038437
ENSGALG00010022783	STAG1	2	-0.036803864	0.044693767	-0.823467492	0.410242196	0	0.071571642	0	1	PRJNA827465;Yang2025_Silkie_Wenchang	CRA004775	0.834737228
ENSGALG00010022783	STAG1	2	0.010096033	0.098312526	0.102693254	0.918206428	0.00238697	1.129555407	11.46959293	0.5	CRA004775;Yang2025_Silkie_Wenchang	PRJNA827465	0.981033557
ENSGALG00010022783	STAG1	2	-0.009376145	0.057587027	-0.162816968	0.870662548	0.001449296	1.11004668	9.913698397	0.5	CRA004775;PRJNA827465	Yang2025_Silkie_Wenchang	0.969038318
ENSGALG00010022786	KRT40	2	-0.165653517	0.253393833	-0.653739339	0.51327975	0	0.308362986	0	0.5	PRJNA827465;Yang2025_Silkie_Wenchang	CRA004775	0.88629566
ENSGALG00010022786	KRT40	2	1.038774499	1.134438861	0.915672527	0.359838678	2.177370905	6.162263301	83.77219617	1	CRA004775;Yang2025_Silkie_Wenchang	PRJNA827465	0.814880744
ENSGALG00010022786	KRT40	2	0.895195339	1.279532389	0.699626948	0.484160309	2.907863185	8.428792429	88.1359043	0.5	CRA004775;PRJNA827465	Yang2025_Silkie_Wenchang	0.857004733
ENSGALG00010022787	ENO4	2	0.41981511	0.238395797	1.761000469	0.078238324	0.004450362	1.037427376	3.607710497	1	PRJNA827465;Yang2025_Silkie_Wenchang	CRA004775	0.481684356
ENSGALG00010022787	ENO4	2	0.741691099	0.360487249	2.057468333	0.039641198	0	0.000650244	0	1	CRA004775;Yang2025_Silkie_Wenchang	PRJNA827465	0.344465621
ENSGALG00010022787	ENO4	2	0.288332878	0.276945054	1.041119435	0.297820121	0	0.292638891	0	1	CRA004775;PRJNA827465	Yang2025_Silkie_Wenchang	0.788858094
ENSGALG00010022788	CYBRD1	2	-0.196212061	0.204590965	-0.959045582	0.337535783	0.066155335	4.758919305	78.98682587	0.5	PRJNA827465;Yang2025_Silkie_Wenchang	CRA004775	0.80415385
ENSGALG00010022788	CYBRD1	2	-0.187663324	0.285169804	-0.608631497	0.542766723	0.115604826	3.135498441	68.10714409	0.5	CRA004775;Yang2025_Silkie_Wenchang	PRJNA827465	0.876968972
ENSGALG00010022788	CYBRD1	2	0.030092299	0.115726251	0.260030015	0.794840621	0	0.287321652	0	1	CRA004775;PRJNA827465	Yang2025_Silkie_Wenchang	0.948905846
ENSGALG00010022789	ENSGALG00010022789	2	-0.110731273	0.415910838	-0.266238009	0.790055912	0.249674121	3.379453906	70.40942035	0.5	PRJNA827465;Yang2025_Silkie_Wenchang	CRA004775	0.947390191
ENSGALG00010022789	ENSGALG00010022789	2	-0.709403829	0.31247524	-2.270272133	0.023191077	0	0.199813871	0	1	CRA004775;Yang2025_Silkie_Wenchang	PRJNA827465	0.259609281
ENSGALG00010022789	ENSGALG00010022789	2	-0.230852313	0.557182997	-0.414320455	0.678639435	0.486943478	4.252558953	76.48474693	0.5	CRA004775;PRJNA827465	Yang2025_Silkie_Wenchang	0.917701868
ENSGALG00010022792	DDB2	2	-0.118923129	0.136294017	-0.872548421	0.382909265	0.01507426	1.658703739	39.71195841	1	PRJNA827465;Yang2025_Silkie_Wenchang	CRA004775	0.825217023
ENSGALG00010022792	DDB2	2	-0.180667542	0.209548003	-0.862177351	0.388589931	0.058753144	2.883120425	65.31535793	1	CRA004775;Yang2025_Silkie_Wenchang	PRJNA827465	0.828338548
ENSGALG00010022792	DDB2	2	-0.334264631	0.132007476	-2.532164685	0.011336085	0	0.306840891	0	1	CRA004775;PRJNA827465	Yang2025_Silkie_Wenchang	0.169700306
ENSGALG00010022795	TAF4	2	-0.028566507	0.056688498	-0.503920686	0.614317114	0	0.013343005	0	1	PRJNA827465;Yang2025_Silkie_Wenchang	CRA004775	0.898500503
ENSGALG00010022795	TAF4	2	-0.023724729	0.117357832	-0.202157186	0.839793843	0	0.083142267	0	0.5	CRA004775;Yang2025_Silkie_Wenchang	PRJNA827465	0.959507805
ENSGALG00010022795	TAF4	2	-0.021576528	0.060503488	-0.35661627	0.721379096	0	0.062897041	0	0.5	CRA004775;PRJNA827465	Yang2025_Silkie_Wenchang	0.929037374
ENSGALG00010022796	RIMKLB	2	0.140596446	0.047687336	2.948297326	0.003195296	0	0.441704402	0	1	PRJNA827465;Yang2025_Silkie_Wenchang	CRA004775	0.07839254
ENSGALG00010022796	RIMKLB	2	-0.059322874	0.219673483	-0.270050226	0.787121614	0.058859803	2.035428289	50.87029077	0.5	CRA004775;Yang2025_Silkie_Wenchang	PRJNA827465	0.946848017
ENSGALG00010022796	RIMKLB	2	-0.02900812	0.262696609	-0.110424418	0.912072789	0.101054174	2.889944493	65.39725928	0.5	CRA004775;PRJNA827465	Yang2025_Silkie_Wenchang	0.978967245
ENSGALG00010022797	EFHD1	2	-0.139626279	0.211137976	-0.661303486	0.508417706	0.084243358	17.65623133	94.3362772	0.5	PRJNA827465;Yang2025_Silkie_Wenchang	CRA004775	0.864902608
ENSGALG00010022797	EFHD1	2	-0.335136854	0.087872743	-3.813888627	0.000136797	0	0.649234245	0	1	CRA004775;Yang2025_Silkie_Wenchang	PRJNA827465	0.009757782
ENSGALG00010022797	EFHD1	2	0.059436968	0.038200438	1.555923721	0.119726232	0	0.488776747	0	0.5	CRA004775;PRJNA827465	Yang2025_Silkie_Wenchang	0.580639051
ENSGALG00010022799	DCAF17	2	0.157654415	0.073323348	2.150125717	0.031545272	0	0.156392645	0	1	PRJNA827465;Yang2025_Silkie_Wenchang	CRA004775	0.303832392
ENSGALG00010022799	DCAF17	2	0.058991094	0.12920707	0.456563231	0.647985588	0	0.570582566	0	0.5	CRA004775;Yang2025_Silkie_Wenchang	PRJNA827465	0.907955391
ENSGALG00010022799	DCAF17	2	0.141446239	0.095726549	1.477607211	0.139512943	0.003036909	1.067981636	6.365431193	0.5	CRA004775;PRJNA827465	Yang2025_Silkie_Wenchang	0.623241328
ENSGALG00010022800	KLHL10	2	-0.214855806	0.233560504	-0.919914977	0.357617192	0	0.478105912	0	1	PRJNA827465;Yang2025_Silkie_Wenchang	CRA004775	0.813498486
ENSGALG00010022800	KLHL10	2	-0.586392786	0.308130593	-1.903065776	0.057031963	0	0.629746245	0	1	CRA004775;Yang2025_Silkie_Wenchang	PRJNA827465	0.413453375
ENSGALG00010022800	KLHL10	2	-0.393434842	0.40991786	-0.95978946	0.337161188	0.173265396	1.905104191	47.50943257	1	CRA004775;PRJNA827465	Yang2025_Silkie_Wenchang	0.804086136
ENSGALG00010022801	LSM14B	2	0.064863597	0.12216846	0.530935699	0.595463338	0.024422835	5.499888344	81.8178127	0.5	PRJNA827465;Yang2025_Silkie_Wenchang	CRA004775	0.893794893
ENSGALG00010022801	LSM14B	2	0.076412818	0.159610486	0.4787456	0.632119621	0.032284266	2.250283098	55.56114692	0.5	CRA004775;Yang2025_Silkie_Wenchang	PRJNA827465	0.903058174
ENSGALG00010022801	LSM14B	2	-0.067427828	0.069666128	-0.967871042	0.333108778	0	0.180580327	0	1	CRA004775;PRJNA827465	Yang2025_Silkie_Wenchang	0.802178578
ENSGALG00010022803	ENSGALG00010022803	2	-0.185625839	0.184462906	-1.006320691	0.314261331	0.00756022	1.272975935	21.44391953	1	PRJNA827465;Yang2025_Silkie_Wenchang	CRA004775	0.79653148
ENSGALG00010022803	ENSGALG00010022803	2	-0.533851828	0.265514832	-2.01062903	0.044364655	0	0.143008804	0	1	CRA004775;Yang2025_Silkie_Wenchang	PRJNA827465	0.363642137
ENSGALG00010022803	ENSGALG00010022803	2	-0.204952612	0.260424239	-0.786995145	0.431284708	0.056333603	1.399595304	28.55077484	1	CRA004775;PRJNA827465	Yang2025_Silkie_Wenchang	0.842503929
ENSGALG00010022804	SNX19	2	0.017471724	0.073390867	0.238060412	0.811831447	0	0.957589246	0	0.5	PRJNA827465;Yang2025_Silkie_Wenchang	CRA004775	0.953371387
ENSGALG00010022804	SNX19	2	-0.306147797	0.229188018	-1.335793205	0.181616875	0.074807168	3.37472439	70.36795055	1	CRA004775;Yang2025_Silkie_Wenchang	PRJNA827465	0.689004635
ENSGALG00010022804	SNX19	2	-0.221667061	0.310122785	-0.714771928	0.474749984	0.168778855	7.694062186	87.00296442	0.5	CRA004775;PRJNA827465	Yang2025_Silkie_Wenchang	0.854462864
ENSGALG00010022805	ENSGALG00010022805	2	0.646519504	0.670948759	0.963589984	0.33525153	0.869948421	29.49377689	96.60945425	0.5	PRJNA827465;Yang2025_Silkie_Wenchang	CRA004775	0.803198384
ENSGALG00010022805	ENSGALG00010022805	2	0.403631268	0.96806696	0.416945609	0.676718192	1.74587293	14.13948921	92.92760874	0.5	CRA004775;Yang2025_Silkie_Wenchang	PRJNA827465	0.91685529
ENSGALG00010022805	ENSGALG00010022805	2	-0.142882993	0.243901385	-0.585822802	0.557994599	0.056020606	1.459782	31.49662073	1	CRA004775;PRJNA827465	Yang2025_Silkie_Wenchang	0.880678789
ENSGALG00010022806	SPPL3	2	-0.104450255	0.082963233	-1.258994513	0.208032314	0.011232533	5.390206141	81.44783383	1	PRJNA827465;Yang2025_Silkie_Wenchang	CRA004775	0.720568788
ENSGALG00010022806	SPPL3	2	-0.179295903	0.053858779	-3.329000599	0.000871582	0	0.55609297	0	1	CRA004775;Yang2025_Silkie_Wenchang	PRJNA827465	0.034665833
ENSGALG00010022806	SPPL3	2	-0.025830884	0.043222373	-0.597627607	0.550068439	0	0.007484024	0	1	CRA004775;PRJNA827465	Yang2025_Silkie_Wenchang	0.878652984
ENSGALG00010022807	ADCY6	2	0.290397105	0.249789588	1.162566894	0.245005269	0.116510094	14.96013476	93.31556823	1	PRJNA827465;Yang2025_Silkie_Wenchang	CRA004775	0.756475101
ENSGALG00010022807	ADCY6	2	0.228003181	0.355543203	0.641281227	0.521339984	0.216316872	6.45358365	84.50473327	0.5	CRA004775;Yang2025_Silkie_Wenchang	PRJNA827465	0.869518515
ENSGALG00010022807	ADCY6	2	0.030261553	0.071145433	0.425347799	0.670583153	0	0.632986299	0	0.5	CRA004775;PRJNA827465	Yang2025_Silkie_Wenchang	0.91482883
ENSGALG00010022808	PSMA7	2	-0.065634681	0.139470394	-0.470599378	0.637926852	0.024952332	2.213794856	54.8286962	0.5	PRJNA827465;Yang2025_Silkie_Wenchang	CRA004775	0.905365679
ENSGALG00010022808	PSMA7	2	-0.22429764	0.15562031	-1.441313218	0.149496215	0	0.180255699	0	1	CRA004775;Yang2025_Silkie_Wenchang	PRJNA827465	0.639246608
ENSGALG00010022808	PSMA7	2	0.020228876	0.05542142	0.365001042	0.715110628	0	0.383084831	0	0.5	CRA004775;PRJNA827465	Yang2025_Silkie_Wenchang	0.926907257
ENSGALG00010022809	MYO1E	2	0.293208601	0.311319849	0.941832807	0.346282598	0.176439993	10.56970009	90.53859362	1	PRJNA827465;Yang2025_Silkie_Wenchang	CRA004775	0.80774676
ENSGALG00010022809	MYO1E	2	0.606651221	0.152095982	3.988607811	6.64622E-05	0	0.04846794	0	1	CRA004775;Yang2025_Silkie_Wenchang	PRJNA827465	0.005883252
ENSGALG00010022809	MYO1E	2	0.210935241	0.266731061	0.790816189	0.429051261	0.109818059	3.628616023	72.44128358	1	CRA004775;PRJNA827465	Yang2025_Silkie_Wenchang	0.841924927
ENSGALG00010022811	GIGYF2	2	0.133508721	0.222432522	0.600221226	0.548358809	0.085255089	6.521520863	84.66615348	0.5	PRJNA827465;Yang2025_Silkie_Wenchang	CRA004775	0.878281266
ENSGALG00010022811	GIGYF2	2	0.147688054	0.251839865	0.586436361	0.557582316	0.092202811	3.637959389	72.51206259	0.5	CRA004775;Yang2025_Silkie_Wenchang	PRJNA827465	0.880678789
ENSGALG00010022811	GIGYF2	2	-0.064155804	0.04561992	-1.406311207	0.159631734	0	0.071026842	0	1	CRA004775;PRJNA827465	Yang2025_Silkie_Wenchang	0.655304681
ENSGALG00010022813	USP24	2	-0.07540666	0.106845758	-0.705752489	0.480342073	0.014742272	2.757109334	63.73012896	0.5	PRJNA827465;Yang2025_Silkie_Wenchang	CRA004775	0.856585676
ENSGALG00010022813	USP24	2	0.064281696	0.101734391	0.631858071	0.52747962	0	0.585235531	0	1	CRA004775;Yang2025_Silkie_Wenchang	PRJNA827465	0.871149047
ENSGALG00010022813	USP24	2	-0.052065854	0.223456942	-0.233001729	0.815760063	0.058252454	1.667290639	40.02245459	0.5	CRA004775;PRJNA827465	Yang2025_Silkie_Wenchang	0.954210034
ENSGALG00010022815	LRRC8D	2	0.114588245	0.095092815	1.205014756	0.228197605	0	2.68873E-06	0	1	PRJNA827465;Yang2025_Silkie_Wenchang	CRA004775	0.741040754
ENSGALG00010022815	LRRC8D	2	-0.172627081	0.423525774	-0.407595277	0.683570832	0.238912607	2.350307961	57.45238426	0.5	CRA004775;Yang2025_Silkie_Wenchang	PRJNA827465	0.918800744
ENSGALG00010022815	LRRC8D	2	-0.168210184	0.421974661	-0.398626267	0.690160604	0.243141354	2.405489923	58.42542698	0.5	CRA004775;PRJNA827465	Yang2025_Silkie_Wenchang	0.919797266
ENSGALG00010022816	METTL8	2	0.29544008	0.142535075	2.072753527	0.038195223	0	0.029699527	0	1	PRJNA827465;Yang2025_Silkie_Wenchang	CRA004775	0.33719788
ENSGALG00010022816	METTL8	2	0.272462895	0.185772741	1.466664254	0.14247231	0	0.134179238	0	1	CRA004775;Yang2025_Silkie_Wenchang	PRJNA827465	0.628884683
ENSGALG00010022816	METTL8	2	0.254079876	0.150849454	1.684321455	0.092133403	0	0.065268973	0	1	CRA004775;PRJNA827465	Yang2025_Silkie_Wenchang	0.513962986
ENSGALG00010022819	PACSIN3	2	-0.051388419	0.215175567	-0.238820882	0.811244477	0.079577002	7.098218351	85.91195775	0.5	PRJNA827465;Yang2025_Silkie_Wenchang	CRA004775	0.953179464
ENSGALG00010022819	PACSIN3	2	0.061316794	0.192394796	0.318702975	0.749951759	0.033685578	1.482111986	32.52871515	0.5	CRA004775;Yang2025_Silkie_Wenchang	PRJNA827465	0.937362187
ENSGALG00010022819	PACSIN3	2	-0.265126427	0.102717743	-2.58111616	0.009848144	0	0.004458662	0	1	CRA004775;PRJNA827465	Yang2025_Silkie_Wenchang	0.15667223
ENSGALG00010022820	SHTN1	2	0.135681789	0.127255989	1.066211423	0.286328079	0	0.121095853	0	1	PRJNA827465;Yang2025_Silkie_Wenchang	CRA004775	0.781944441
ENSGALG00010022820	SHTN1	2	0.087206667	0.210278748	0.414719354	0.678347262	0	0.053882528	0	0.5	CRA004775;Yang2025_Silkie_Wenchang	PRJNA827465	0.917670684
ENSGALG00010022820	SHTN1	2	0.171286711	0.154134704	1.111279335	0.266448131	0	0.015471493	0	1	CRA004775;PRJNA827465	Yang2025_Silkie_Wenchang	0.770695533
ENSGALG00010022823	IGHMBP2	2	-0.050053295	0.049905902	-1.002953422	0.315883335	0	0.01164113	0	1	PRJNA827465;Yang2025_Silkie_Wenchang	CRA004775	0.796883862
ENSGALG00010022823	IGHMBP2	2	-0.064503012	0.103142444	-0.625377963	0.531723022	0	0.379573058	0	0.5	CRA004775;Yang2025_Silkie_Wenchang	PRJNA827465	0.873042777
ENSGALG00010022823	IGHMBP2	2	-0.059343017	0.054787308	-1.083152635	0.278740679	0	0.360315305	0	1	CRA004775;PRJNA827465	Yang2025_Silkie_Wenchang	0.776992195
ENSGALG00010022824	PUS1	2	-0.00487377	0.04791451	-0.101718046	0.918980478	0	0.202472045	0	0.5	PRJNA827465;Yang2025_Silkie_Wenchang	CRA004775	0.981086235
ENSGALG00010022824	PUS1	2	-0.011850747	0.079848162	-0.148416025	0.882014454	0	0.435615738	0	0.5	CRA004775;Yang2025_Silkie_Wenchang	PRJNA827465	0.971928118
ENSGALG00010022824	PUS1	2	0.016539876	0.053868452	0.307041979	0.75881142	0	0.199385533	0	1	CRA004775;PRJNA827465	Yang2025_Silkie_Wenchang	0.938996879
ENSGALG00010022825	SS18L1	2	0.445160467	0.46165999	0.964260443	0.334915368	0.36555751	6.866559631	85.4366662	1	PRJNA827465;Yang2025_Silkie_Wenchang	CRA004775	0.803101658
ENSGALG00010022825	SS18L1	2	0.277587396	0.719722672	0.385686608	0.699728782	0.863032489	5.763242015	82.64865509	0.5	CRA004775;Yang2025_Silkie_Wenchang	PRJNA827465	0.922124474
ENSGALG00010022825	SS18L1	2	-0.049534153	0.178522173	-0.27746779	0.781420935	0	0.872855128	0	0.5	CRA004775;PRJNA827465	Yang2025_Silkie_Wenchang	0.945235709
ENSGALG00010022826	SNORC	2	1.673186979	1.431220789	1.169062797	0.242378331	3.236332716	4.739060048	78.89876917	1	PRJNA827465;Yang2025_Silkie_Wenchang	CRA004775	0.754698112
ENSGALG00010022826	SNORC	2	0.449889771	2.614276552	0.172089586	0.863367099	12.89915702	17.75429015	94.36755854	0.5	CRA004775;Yang2025_Silkie_Wenchang	PRJNA827465	0.966256632
ENSGALG00010022826	SNORC	2	-1.032026793	1.181490652	-0.873495521	0.382393047	1.880661089	3.056598786	67.28389723	0.5	CRA004775;PRJNA827465	Yang2025_Silkie_Wenchang	0.825099858
ENSGALG00010022827	CMTR2	2	-0.31672114	0.278848711	-1.13581712	0.256033111	0.146904142	17.74073253	94.36325418	1	PRJNA827465;Yang2025_Silkie_Wenchang	CRA004775	0.764570254
ENSGALG00010022827	CMTR2	2	-0.588474206	0.108161138	-5.440717606	5.30664E-08	0	0.12375065	0	1	CRA004775;Yang2025_Silkie_Wenchang	PRJNA827465	2.06502E-05
ENSGALG00010022827	CMTR2	2	-0.194750401	0.21247892	-0.916563396	0.359371473	0.063336248	2.610247845	61.68946172	1	CRA004775;PRJNA827465	Yang2025_Silkie_Wenchang	0.814335519
ENSGALG00010022828	PRKG1	2	-0.307563182	0.087450569	-3.516994655	0.000436463	0	0.010413954	0	1	PRJNA827465;Yang2025_Silkie_Wenchang	CRA004775	0.02125169
ENSGALG00010022828	PRKG1	2	0.159589604	0.523048702	0.305114234	0.760279151	0.480473455	7.792931422	87.16785833	0.5	CRA004775;Yang2025_Silkie_Wenchang	PRJNA827465	0.939327609
ENSGALG00010022828	PRKG1	2	0.16237665	0.512853325	0.316614209	0.751536353	0.466742855	8.220425683	87.83517985	0.5	CRA004775;PRJNA827465	Yang2025_Silkie_Wenchang	0.937716403
ENSGALG00010022830	MTG2	2	0.137881388	0.089590267	1.539021967	0.123798934	0	0.909963447	0	1	PRJNA827465;Yang2025_Silkie_Wenchang	CRA004775	0.590064211
ENSGALG00010022830	MTG2	2	0.110242409	0.216403623	0.50942959	0.61045114	0.04525796	1.809839354	44.74647719	0.5	CRA004775;Yang2025_Silkie_Wenchang	PRJNA827465	0.897438028
ENSGALG00010022830	MTG2	2	0.053441706	0.099337279	0.537982383	0.590589211	0	0.714749166	0	0.5	CRA004775;PRJNA827465	Yang2025_Silkie_Wenchang	0.892642928
ENSGALG00010022831	LRRC8C	2	-0.051445947	0.066004016	-0.779436622	0.435722558	0.000570559	1.031324125	3.03727255	0.5	PRJNA827465;Yang2025_Silkie_Wenchang	CRA004775	0.842762754
ENSGALG00010022831	LRRC8C	2	0.068670159	0.149708508	0.458692426	0.646455054	0	0.238714902	0	0.5	CRA004775;Yang2025_Silkie_Wenchang	PRJNA827465	0.907220702
ENSGALG00010022831	LRRC8C	2	-0.075484188	0.064876983	-1.163497209	0.24462783	0	0.014600143	0	1	CRA004775;PRJNA827465	Yang2025_Silkie_Wenchang	0.756093682
ENSGALG00010022834	CHRNA7	2	0.0452034	0.229404239	0.197046927	0.843790815	0.047158368	1.810211093	44.75782389	0.5	PRJNA827465;Yang2025_Silkie_Wenchang	CRA004775	0.96057435
ENSGALG00010022834	CHRNA7	2	0.461088884	0.805519309	0.572411956	0.567042918	1.013457251	3.959718621	74.7456803	0.5	CRA004775;Yang2025_Silkie_Wenchang	PRJNA827465	0.883487517
ENSGALG00010022834	CHRNA7	2	0.621831503	0.543495631	1.144133398	0.252568319	0.366302188	2.080537331	51.93549353	1	CRA004775;PRJNA827465	Yang2025_Silkie_Wenchang	0.762655223
ENSGALG00010022837	ITGB3	2	-0.037622363	0.220356773	-0.170733861	0.864433036	0.078840119	5.251284395	80.95703975	0.5	PRJNA827465;Yang2025_Silkie_Wenchang	CRA004775	0.966676482
ENSGALG00010022837	ITGB3	2	0.06006649	0.37150591	0.161683806	0.871554857	0.223229503	4.937169546	79.74547986	0.5	CRA004775;Yang2025_Silkie_Wenchang	PRJNA827465	0.969425739
ENSGALG00010022837	ITGB3	2	0.211308441	0.108005159	1.956466179	0.050410263	0	0.91339178	0	1	CRA004775;PRJNA827465	Yang2025_Silkie_Wenchang	0.38897029
ENSGALG00010022838	ARFGAP2	2	-0.03915666	0.060527969	-0.646918449	0.517684725	0.003221194	1.567037118	36.18530226	0.5	PRJNA827465;Yang2025_Silkie_Wenchang	CRA004775	0.867962259
ENSGALG00010022838	ARFGAP2	2	-0.16780716	0.252571765	-0.66439398	0.506438185	0.104286728	5.134075478	80.52229648	0.5	CRA004775;Yang2025_Silkie_Wenchang	PRJNA827465	0.863929781
ENSGALG00010022838	ARFGAP2	2	-0.213520407	0.180398518	-1.183603993	0.236569859	0.049023455	3.282058488	69.5313169	1	CRA004775;PRJNA827465	Yang2025_Silkie_Wenchang	0.7487803
ENSGALG00010022839	SYT12	2	0.600925094	0.158630215	3.788213318	0.000151735	0	0.781834168	0	1	PRJNA827465;Yang2025_Silkie_Wenchang	CRA004775	0.010480884
ENSGALG00010022839	SYT12	2	0.283188651	0.488603106	0.579588315	0.562192275	0.406239095	6.661289609	84.98789185	0.5	CRA004775;Yang2025_Silkie_Wenchang	PRJNA827465	0.881955027
ENSGALG00010022839	SYT12	2	0.156039496	0.346620822	0.450173462	0.65258537	0.175357638	3.627286016	72.43117869	0.5	CRA004775;PRJNA827465	Yang2025_Silkie_Wenchang	0.909491252
ENSGALG00010022840	MARVELD1	2	0.10555046	0.182500529	0.578357008	0.563023113	0.037237049	2.011044972	50.27460777	0.5	PRJNA827465;Yang2025_Silkie_Wenchang	CRA004775	0.882210957
ENSGALG00010022840	MARVELD1	2	-0.091432274	0.468099142	-0.195326728	0.845137174	0.361716778	5.687721211	82.41826625	0.5	CRA004775;Yang2025_Silkie_Wenchang	PRJNA827465	0.960863849
ENSGALG00010022840	MARVELD1	2	-0.228777403	0.266155821	-0.8595619	0.390030585	0.098689695	2.838989363	64.77619772	1	CRA004775;PRJNA827465	Yang2025_Silkie_Wenchang	0.829204867
ENSGALG00010022842	PHC1	2	0.206513054	0.172611831	1.196401505	0.231539914	0.04548614	3.491646581	71.36021711	1	PRJNA827465;Yang2025_Silkie_Wenchang	CRA004775	0.743924694
ENSGALG00010022842	PHC1	2	0.105993914	0.324895749	0.326239768	0.744242955	0.17640262	6.081885135	83.55772959	0.5	CRA004775;Yang2025_Silkie_Wenchang	PRJNA827465	0.935821071
ENSGALG00010022842	PHC1	2	-0.016605221	0.135146664	-0.12286815	0.902211507	0.024405485	2.323220557	56.95630375	0.5	CRA004775;PRJNA827465	Yang2025_Silkie_Wenchang	0.976923607
ENSGALG00010022845	HYDIN	2	0.122090504	0.391297447	0.31201457	0.755029451	0.215843727	3.386777489	70.47340715	0.5	PRJNA827465;Yang2025_Silkie_Wenchang	CRA004775	0.93838723
ENSGALG00010022845	HYDIN	2	-0.633341263	1.185838506	-0.534087281	0.593281149	2.638378293	15.95312261	93.73163471	0.5	CRA004775;Yang2025_Silkie_Wenchang	PRJNA827465	0.892783052
ENSGALG00010022845	HYDIN	2	-1.005186588	0.793171993	-1.267299649	0.205048166	1.091544601	7.325916176	86.34983016	1	CRA004775;PRJNA827465	Yang2025_Silkie_Wenchang	0.717759892
ENSGALG00010022850	ULK1	2	0.107076234	0.159036165	0.673282298	0.500767712	0.037149515	3.426216511	70.81328641	0.5	PRJNA827465;Yang2025_Silkie_Wenchang	CRA004775	0.862421093
ENSGALG00010022850	ULK1	2	0.338995672	0.147699448	2.29517224	0.021723255	0	0.452649234	0	1	CRA004775;Yang2025_Silkie_Wenchang	PRJNA827465	0.249645938
ENSGALG00010022850	ULK1	2	0.156195188	0.279801838	0.558235032	0.576683902	0.105460348	2.267162973	55.89201077	0.5	CRA004775;PRJNA827465	Yang2025_Silkie_Wenchang	0.887113945
ENSGALG00010022858	HERC4	2	-0.08511161	0.060715896	-1.401801104	0.160974649	0.003357796	1.767869283	43.43473188	1	PRJNA827465;Yang2025_Silkie_Wenchang	CRA004775	0.657055417
ENSGALG00010022858	HERC4	2	0.37243376	0.439799734	0.846825796	0.397092228	0.33330991	6.440086241	84.47225763	0.5	CRA004775;Yang2025_Silkie_Wenchang	PRJNA827465	0.831438925
ENSGALG00010022858	HERC4	2	0.316253795	0.50321805	0.628462741	0.529700847	0.453163944	8.602653702	88.37567994	0.5	CRA004775;PRJNA827465	Yang2025_Silkie_Wenchang	0.871944741
ENSGALG00010022860	MOB3C	2	0.025540304	0.063168595	0.404319652	0.685977676	0	0.104385848	0	0.5	PRJNA827465;Yang2025_Silkie_Wenchang	CRA004775	0.919235539
ENSGALG00010022860	MOB3C	2	-0.214575739	0.272804222	-0.786555784	0.431541951	0.102959995	2.792387256	64.18834824	1	CRA004775;Yang2025_Silkie_Wenchang	PRJNA827465	0.842503929
ENSGALG00010022860	MOB3C	2	-0.190205468	0.295987701	-0.642612741	0.520475414	0.134249083	3.532436554	71.69092821	0.5	CRA004775;PRJNA827465	Yang2025_Silkie_Wenchang	0.869331539
ENSGALG00010022862	ACADS	2	0.014277708	0.125453302	0.113808945	0.909389247	0.023878728	3.81297333	73.77374785	0.5	PRJNA827465;Yang2025_Silkie_Wenchang	CRA004775	0.978607662
ENSGALG00010022862	ACADS	2	0.122283853	0.1078834	1.133481637	0.257012048	0	0.732402538	0	0.5	CRA004775;Yang2025_Silkie_Wenchang	PRJNA827465	0.764848386
ENSGALG00010022862	ACADS	2	-0.091728243	0.054697292	-1.677016157	0.093539322	0	9.26497E-05	0	1	CRA004775;PRJNA827465	Yang2025_Silkie_Wenchang	0.523998859
ENSGALG00010022863	FAM81A	2	-0.119501688	0.50128232	-0.238391987	0.811577082	0.443939442	8.569188185	88.33028312	0.5	PRJNA827465;Yang2025_Silkie_Wenchang	CRA004775	0.953328374
ENSGALG00010022863	FAM81A	2	0.348013143	0.226481411	1.536607978	0.124389338	0	0.231360672	0	0.5	CRA004775;Yang2025_Silkie_Wenchang	PRJNA827465	0.591443762
ENSGALG00010022863	FAM81A	2	-0.577289487	0.238778292	-2.417679936	0.015619809	0	0.439918369	0	1	CRA004775;PRJNA827465	Yang2025_Silkie_Wenchang	0.206771761
ENSGALG00010022864	CSTPP1	2	-0.045839851	0.086446936	-0.530265763	0.595927681	0	0.755590617	0	0.5	PRJNA827465;Yang2025_Silkie_Wenchang	CRA004775	0.893857407
ENSGALG00010022864	CSTPP1	2	-0.117470044	0.235083185	-0.499695644	0.6172894	0.063226573	1.998096481	49.95236668	0.5	CRA004775;Yang2025_Silkie_Wenchang	PRJNA827465	0.899121942
ENSGALG00010022864	CSTPP1	2	-0.146759052	0.109480886	-1.340499309	0.180083069	0.000823153	1.013599836	1.341736247	1	CRA004775;PRJNA827465	Yang2025_Silkie_Wenchang	0.68716552
ENSGALG00010022866	KLHL11	2	-0.179480445	0.217117908	-0.826649661	0.408435661	0.085429926	10.31377185	90.30422609	0.5	PRJNA827465;Yang2025_Silkie_Wenchang	CRA004775	0.834154426
ENSGALG00010022866	KLHL11	2	-0.408579965	0.11493919	-3.554748949	0.00037834	0	0.000783589	0	1	CRA004775;Yang2025_Silkie_Wenchang	PRJNA827465	0.019514308
ENSGALG00010022866	KLHL11	2	-0.036639964	0.149832407	-0.244539647	0.8068129	0.02263541	1.335961845	25.14756288	0.5	CRA004775;PRJNA827465	Yang2025_Silkie_Wenchang	0.95228484
ENSGALG00010022867	ATPAF1	2	0.155946662	0.074468251	2.094136244	0.036247845	0	0.002868718	0	1	PRJNA827465;Yang2025_Silkie_Wenchang	CRA004775	0.328411494
ENSGALG00010022867	ATPAF1	2	0.124144566	0.138921416	0.893631592	0.371519037	0	0.335500259	0	0.5	CRA004775;Yang2025_Silkie_Wenchang	PRJNA827465	0.821526338
ENSGALG00010022867	ATPAF1	2	0.140733235	0.082126891	1.713607241	0.086600883	0	0.352056039	0	0.5	CRA004775;PRJNA827465	Yang2025_Silkie_Wenchang	0.506122601

ENSGALG00010022868	VAC14	2	0.174413097	0.082707194	2.108802014	0.034961675	0.009452294	3.084323966	67.57798432	1	PRJNA827465;Yang2025_Silkie_Wenchang	CRA004775	0.322104712
ENSGALG00010022868	VAC14	2	-0.13060133	0.415769751	-0.314119364	0.753430383	0.327074577	18.1190958	94.48096195	0.5	CRA004775;Yang2025_Silkie_Wenchang	PRJNA827465	0.938087082
ENSGALG00010022868	VAC14	2	-0.20733625	0.331689945	-0.62509055	0.53191163	0.203961084	12.97232307	92.29128049	0.5	CRA004775;PRJNA827465	Yang2025_Silkie_Wenchang	0.873082041
ENSGALG00010022872	KRT13	2	0.141909338	0.391870318	0.362133419	0.717252332	0.13572367	1.791152856	44.17003572	0.5	PRJNA827465;Yang2025_Silkie_Wenchang	CRA004775	0.9277944
ENSGALG00010022872	KRT13	2	-0.167708165	0.388818013	-0.431328178	0.666229765	0	0.304898223	0	0.5	CRA004775;Yang2025_Silkie_Wenchang	PRJNA827465	0.913579507
ENSGALG00010022872	KRT13	2	0.498629182	0.375878798	1.326569055	0.184651278	0	0.030079453	0	1	CRA004775;PRJNA827465	Yang2025_Silkie_Wenchang	0.692958004
ENSGALG00010022873	NGEF	2	0.125520615	0.233814501	0.536838454	0.591379207	0	0.106514705	0	1	PRJNA827465;Yang2025_Silkie_Wenchang	CRA004775	0.892776493
ENSGALG00010022873	NGEF	2	0.006289797	0.305115265	0.020614496	0.983553177	0.019816478	1.097240531	8.86228029	0.5	CRA004775;Yang2025_Silkie_Wenchang	PRJNA827465	0.995457747
ENSGALG00010022873	NGEF	2	-0.085598093	0.279772923	-0.3059556	0.759638451	0	0.06288703	0	0.5	CRA004775;PRJNA827465	Yang2025_Silkie_Wenchang	0.939163539
ENSGALG00010022874	EFCAB14	2	-0.172539225	0.099020364	-1.742462034	0.081427629	0.015681381	4.970545543	79.88148401	1	PRJNA827465;Yang2025_Silkie_Wenchang	CRA004775	0.491466018
ENSGALG00010022874	EFCAB14	2	-0.240278664	0.064593954	-3.719832126	0.000199355	0.001651409	1.118552607	10.5987511	1	CRA004775;Yang2025_Silkie_Wenchang	PRJNA827465	0.012761008
ENSGALG00010022874	EFCAB14	2	-0.073649673	0.062170935	-1.184631899	0.236163019	0	0.01603838	0	1	CRA004775;PRJNA827465	Yang2025_Silkie_Wenchang	0.748669015
ENSGALG00010022875	SPON1	2	-0.138831554	0.231240956	-0.600376147	0.548255581	0	0.006049791	0	1	PRJNA827465;Yang2025_Silkie_Wenchang	CRA004775	0.878281266
ENSGALG00010022875	SPON1	2	-0.262495065	0.289008614	-0.908260151	0.363740685	0	0.484375448	0	1	CRA004775;Yang2025_Silkie_Wenchang	PRJNA827465	0.817042253
ENSGALG00010022875	SPON1	2	-0.26052304	0.259095305	-1.005510463	0.314651114	0	0.445920373	0	1	CRA004775;PRJNA827465	Yang2025_Silkie_Wenchang	0.796531448
ENSGALG00010022877	KYAT3	2	-0.206034696	0.183421959	-1.123282602	0.261317491	0.046068987	3.089364093	67.63087905	1	PRJNA827465;Yang2025_Silkie_Wenchang	CRA004775	0.768082306
ENSGALG00010022877	KYAT3	2	-0.213300955	0.237452273	-0.898289802	0.369031068	0.065883523	2.299013055	56.50307431	0.5	CRA004775;Yang2025_Silkie_Wenchang	PRJNA827465	0.820553374
ENSGALG00010022877	KYAT3	2	-0.022731993	0.109923053	-0.20679914	0.836166727	0	0.149932943	0	0.5	CRA004775;PRJNA827465	Yang2025_Silkie_Wenchang	0.958399882
ENSGALG00010022879	ENSGALG00010022879	2	-0.324863903	0.180547822	-1.799323299	0.071967555	0	0.002822123	0	1	PRJNA827465;Yang2025_Silkie_Wenchang	CRA004775	0.463163714
ENSGALG00010022879	ENSGALG00010022879	2	-0.398589796	0.242823451	-1.641479824	0.100697852	0	0.206685211	0	1	CRA004775;Yang2025_Silkie_Wenchang	PRJNA827465	0.541412814
ENSGALG00010022879	ENSGALG00010022879	2	-0.368628086	0.213509122	-1.72652148	0.084253638	0	0.256397033	0	1	CRA004775;PRJNA827465	Yang2025_Silkie_Wenchang	0.49990641
ENSGALG00010022881	CCNB1	2	-0.21696679	0.256472344	-0.845965636	0.397571915	0.112665106	6.348801259	84.24899506	0.5	PRJNA827465;Yang2025_Silkie_Wenchang	CRA004775	0.831688789
ENSGALG00010022881	CCNB1	2	-0.501140676	0.184630464	-2.714290297	0.006641794	0	0.013497844	0	1	CRA004775;Yang2025_Silkie_Wenchang	PRJNA827465	0.124602584
ENSGALG00010022881	CCNB1	2	0.003160712	0.063000495	0.050169637	0.959987208	0	0.611698693	0	0.5	CRA004775;PRJNA827465	Yang2025_Silkie_Wenchang	0.98933784
ENSGALG00010022885	M6PR	2	-0.144283449	0.157409899	-0.916609754	0.359347171	0.042650361	6.555470583	84.74556499	1	PRJNA827465;Yang2025_Silkie_Wenchang	CRA004775	0.814335519
ENSGALG00010022885	M6PR	2	-0.300903724	0.092204731	-3.263430424	0.001100722	0	0.086713782	0	1	CRA004775;Yang2025_Silkie_Wenchang	PRJNA827465	0.040098299
ENSGALG00010022885	M6PR	2	-0.097038907	0.124818837	-0.777437997	0.436900402	0.022551546	2.93474438	65.92548207	1	CRA004775;PRJNA827465	Yang2025_Silkie_Wenchang	0.843387647
ENSGALG00010022886	PLA2G15	2	-0.477194006	0.234226789	-2.03731609	0.041618376	0.089268548	5.340206659	81.27413293	1	PRJNA827465;Yang2025_Silkie_Wenchang	CRA004775	0.353517841
ENSGALG00010022886	PLA2G15	2	-0.488596731	0.372663604	-1.311093237	0.189826265	0.167899525	1.923715049	48.17597193	0.5	CRA004775;Yang2025_Silkie_Wenchang	PRJNA827465	0.699160501
ENSGALG00010022886	PLA2G15	2	-0.232631194	0.127405475	-1.825912063	0.067863509	0	0.378073633	0	0.5	CRA004775;PRJNA827465	Yang2025_Silkie_Wenchang	0.449111762
ENSGALG00010022888	OSBPL2	2	-0.066048968	0.053573035	-1.232877101	0.21762162	0	0.184314754	0	1	PRJNA827465;Yang2025_Silkie_Wenchang	CRA004775	0.731013651
ENSGALG00010022888	OSBPL2	2	-0.018268568	0.102236311	-0.178689622	0.858181419	0.008329487	1.60168501	37.56575144	0.5	CRA004775;Yang2025_Silkie_Wenchang	PRJNA827465	0.964291891
ENSGALG00010022888	OSBPL2	2	-0.014522029	0.066755286	-0.217541254	0.827786561	0.00136985	1.116731744	10.45297987	0.5	CRA004775;PRJNA827465	Yang2025_Silkie_Wenchang	0.956550752
ENSGALG00010022890	MYPN	2	0.006315659	0.433808844	0.014558621	0.988384311	0.344827236	11.69361325	91.4483233	0.5	PRJNA827465;Yang2025_Silkie_Wenchang	CRA004775	0.996891127
ENSGALG00010022890	MYPN	2	0.464431422	0.207874281	2.234193765	0.025470327	0	0.005384528	0	1	CRA004775;Yang2025_Silkie_Wenchang	PRJNA827465	0.272175161
ENSGALG00010022890	MYPN	2	-0.160845112	0.40775188	-0.394468107	0.693235469	0.211900008	2.013074141	50.32473074	0.5	CRA004775;PRJNA827465	Yang2025_Silkie_Wenchang	0.920721644
ENSGALG00010022891	NTM	2	-0.201421513	0.117709041	-1.711181329	0.087047644	0	0.148014773	0	1	PRJNA827465;Yang2025_Silkie_Wenchang	CRA004775	0.507587624
ENSGALG00010022891	NTM	2	0.124222129	0.381653698	0.325483887	0.744814875	0.193950387	2.52119554	60.33627761	0.5	CRA004775;Yang2025_Silkie_Wenchang	PRJNA827465	0.935893304
ENSGALG00010022891	NTM	2	0.089885283	0.432024673	0.208055902	0.835185319	0.272075752	3.156140427	68.31573172	0.5	CRA004775;PRJNA827465	Yang2025_Silkie_Wenchang	0.958305532
ENSGALG00010022900	MMP17	2	-0.214746593	0.328439048	-0.653840018	0.513214882	0.202776883	16.46775656	93.92752743	0.5	PRJNA827465;Yang2025_Silkie_Wenchang	CRA004775	0.866295966
ENSGALG00010022900	MMP17	2	-0.521034392	0.131938191	-3.949079399	7.84523E-05	0	0.758906978	0	1	CRA004775;Yang2025_Silkie_Wenchang	PRJNA827465	0.006546522
ENSGALG00010022900	MMP17	2	0.099859976	0.085776793	1.16418407	0.244349424	0	0.142628681	0	0.5	CRA004775;PRJNA827465	Yang2025_Silkie_Wenchang	0.755896531
ENSGALG00010022911	SMPD3	2	-0.285903642	0.400448375	-0.713958803	0.475252654	0	0.507796137	0	1	PRJNA827465;Yang2025_Silkie_Wenchang	CRA004775	0.854462864
ENSGALG00010022911	SMPD3	2	-0.816436277	0.368900139	-2.213163375	0.026886384	0	0.207818781	0	1	CRA004775;Yang2025_Silkie_Wenchang	PRJNA827465	0.279388398
ENSGALG00010022911	SMPD3	2	-0.527025491	0.458380316	-1.14975594	0.250244407	0.167799926	1.656968493	39.64882227	1	CRA004775;PRJNA827465	Yang2025_Silkie_Wenchang	0.75993411
ENSGALG00010022917	CYP4B7	2	0.097880492	0.46900345	0.208698875	0.834683319	0.275058981	2.667478733	62.5114162	0.5	PRJNA827465;Yang2025_Silkie_Wenchang	CRA004775	0.958133448
ENSGALG00010022917	CYP4B7	2	0.203676647	0.575856235	0.353693557	0.723368543	0.495761474	3.958447649	74.73757168	0.5	CRA004775;Yang2025_Silkie_Wenchang	PRJNA827465	0.930032096
ENSGALG00010022917	CYP4B7	2	0.678714599	0.294677687	2.303243951	0.021265121	0	0.131466632	0	1	CRA004775;PRJNA827465	Yang2025_Silkie_Wenchang	0.246616423
ENSGALG00010022919	ACLY	2	0.019481168	0.057459075	0.339044223	0.734576416	0	0.638314262	0	0.5	PRJNA827465;Yang2025_Silkie_Wenchang	CRA004775	0.933520234
ENSGALG00010022919	ACLY	2	-0.115819695	0.155927874	-0.742777367	0.45761648	0.021187331	1.368266539	26.91482462	1	CRA004775;Yang2025_Silkie_Wenchang	PRJNA827465	0.849573219
ENSGALG00010022919	ACLY	2	-0.088371218	0.22341909	-0.39554014	0.692444304	0.065168415	2.169294224	53.90205769	0.5	CRA004775;PRJNA827465	Yang2025_Silkie_Wenchang	0.920959211
ENSGALG00010022921	ENSGALG00010022921	2	0.237353781	0.273326811	0.868388214	0.385181845	0.117682557	4.348790248	77.00509928	1	PRJNA827465;Yang2025_Silkie_Wenchang	CRA004775	0.826444827
ENSGALG00010022921	ENSGALG00010022921	2	0.207142571	0.348743822	0.593967716	0.552533706	0.184707079	4.155294938	75.93431959	0.5	CRA004775;Yang2025_Silkie_Wenchang	PRJNA827465	0.879228216
ENSGALG00010022921	ENSGALG00010022921	2	-0.022725786	0.100950924	-0.225120792	0.821886531	0	0.29287932	0	0.5	CRA004775;PRJNA827465	Yang2025_Silkie_Wenchang	0.95563486
ENSGALG00010022923	CD63	2	0.119693749	0.134106177	0.892529726	0.372109067	0.018974198	1.560999948	35.93849882	1	PRJNA827465;Yang2025_Silkie_Wenchang	CRA004775	0.821620523
ENSGALG00010022923	CD63	2	0.117702156	0.233160788	0.504811107	0.613691514	0.056370182	2.05709745	51.38781589	0.5	CRA004775;Yang2025_Silkie_Wenchang	PRJNA827465	0.898256044
ENSGALG00010022923	CD63	2	0.036699025	0.060604116	0.605553336	0.544831397	0	0.437425127	0	0.5	CRA004775;PRJNA827465	Yang2025_Silkie_Wenchang	0.877345822
ENSGALG00010022928	RNF111	2	0.253776443	0.30850785	0.822593146	0.410779697	0.162282348	5.905790203	83.06746488	0.5	PRJNA827465;Yang2025_Silkie_Wenchang	CRA004775	0.835028074
ENSGALG00010022928	RNF111	2	0.331911131	0.255798488	1.297549226	0.194442279	0.081983117	2.627854183	61.94613816	1	CRA004775;Yang2025_Silkie_Wenchang	PRJNA827465	0.705148332
ENSGALG00010022928	RNF111	2	-0.003504844	0.041425414	-0.084606121	0.932574533	0	0.320472912	0	0.5	CRA004775;PRJNA827465	Yang2025_Silkie_Wenchang	0.984224753
ENSGALG00010022929	LRP4	2	-0.333047751	0.093619354	-3.557466859	0.000374448	0	0.030069695	0	1	PRJNA827465;Yang2025_Silkie_Wenchang	CRA004775	0.019374277
ENSGALG00010022929	LRP4	2	-0.374080655	0.168209166	-2.223901733	0.026155056	0	0.03742328	0	1	CRA004775;Yang2025_Silkie_Wenchang	PRJNA827465	0.275965687
ENSGALG00010022929	LRP4	2	-0.331041986	0.105672997	-3.132701767	0.001732053	0	0.082283046	0	1	CRA004775;PRJNA827465	Yang2025_Silkie_Wenchang	0.052667801
ENSGALG00010022930	GTF2B	2	-0.009852733	0.114368832	-0.08614876	0.931348163	0.018255918	3.14056535	68.15859921	0.5	PRJNA827465;Yang2025_Silkie_Wenchang	CRA004775	0.984076328
ENSGALG00010022930	GTF2B	2	-0.068220485	0.128193334	-0.532168744	0.594609129	0.009444844	1.279475408	21.84296833	0.5	CRA004775;Yang2025_Silkie_Wenchang	PRJNA827465	0.893379204
ENSGALG00010022930	GTF2B	2	0.09288872	0.064097228	1.449184662	0.14728602	0	0.066008849	0	1	CRA004775;PRJNA827465	Yang2025_Silkie_Wenchang	0.636129938
ENSGALG00010022931	RDH5	2	-0.044322824	0.112670987	-0.393382763	0.694036799	0	0.227266879	0	0.5	PRJNA827465;Yang2025_Silkie_Wenchang	CRA004775	0.920728119
ENSGALG00010022931	RDH5	2	-0.093578901	0.224943944	-0.416009868	0.677402782	0.021372279	1.204528315	16.97995077	0.5	CRA004775;Yang2025_Silkie_Wenchang	PRJNA827465	0.917144807
ENSGALG00010022931	RDH5	2	-0.1128838	0.124503627	-0.906670773	0.364580923	0	0.811164694	0	1	CRA004775;PRJNA827465	Yang2025_Silkie_Wenchang	0.814749251
ENSGALG00010022932	OPCML	2	-0.040386675	0.098491754	-0.410051337	0.681768289	0	0.000257583	0	1	PRJNA827465;Yang2025_Silkie_Wenchang	CRA004775	0.918378828
ENSGALG00010022932	OPCML	2	0.012850862	0.17728395	0.072487437	0.942214003	0	0.696285369	0	0.5	CRA004775;Yang2025_Silkie_Wenchang	PRJNA827465	0.985827325
ENSGALG00010022932	OPCML	2	-0.020526657	0.113261972	-0.181231675	0.856185733	0	0.755601149	0	0.5	CRA004775;PRJNA827465	Yang2025_Silkie_Wenchang	0.963499334
ENSGALG00010022933	UNC119B	2	-0.092427718	0.193349798	-0.478033693	0.632626224	0.067923105	10.72067231	90.67222679	0.5	PRJNA827465;Yang2025_Silkie_Wenchang	CRA004775	0.903058174
ENSGALG00010022933	UNC119B	2	-0.151211106	0.168743448	-0.896100605	0.370199034	0.038723961	2.958253161	66.196267	0.5	CRA004775;Yang2025_Silkie_Wenchang	PRJNA827465	0.821045695
ENSGALG00010022933	UNC119B	2	0.087595545	0.057470241	1.524189638	0.127461346	0	0.061378057	0	1	CRA004775;PRJNA827465	Yang2025_Silkie_Wenchang	0.599451151
ENSGALG00010022937	BLOC1S1	2	0.159438988	0.262902087	0.606457675	0.54421088	0.116451949	6.221915061	83.92787618	0.5	PRJNA827465;Yang2025_Silkie_Wenchang	CRA004775	0.877023301
ENSGALG00010022937	BLOC1S1	2	0.442440065	0.155746504	2.840770434	0.00450047	0	0.001905107	0	1	CRA004775;Yang2025_Silkie_Wenchang	PRJNA827465	0.09815869
ENSGALG00010022937	BLOC1S1	2	0.091166442	0.25541965	0.356928068	0.721145657	0.08346587	2.300288423	56.52719068	0.5	CRA004775;PRJNA827465	Yang2025_Silkie_Wenchang	0.928950734
ENSGALG00010022942	NEU2	2	0.079157086	0.360280237	0.219709763	0.8260972	0.168866698	2.746546276	63.59063713	0.5	PRJNA827465;Yang2025_Silkie_Wenchang	CRA004775	0.956393839
ENSGALG00010022942	NEU2	2	-0.633625497	0.393581344	-1.609897182	0.107420304	0.104826321	1.451452651	31.10350524	1	CRA004775;Yang2025_Silkie_Wenchang	PRJNA827465	0.554861377
ENSGALG00010022942	NEU2	2	-0.298444772	0.769937927	-0.387621862	0.698295588	1.008448997	6.240024078	83.97442081	0.5	CRA004775;PRJNA827465	Yang2025_Silkie_Wenchang	0.921624968
ENSGALG00010022946	KRT17	2	0.245854474	0.292259851	0.841218776	0.400225384	0	0.104486374	0	1	PRJNA827465;Yang2025_Silkie_Wenchang	CRA004775	0.832246851
ENSGALG00010022946	KRT17	2	0.563041968	0.337844479	1.666571466	0.095599647	0	0.830089679	0	1	CRA004775;Yang2025_Silkie_Wenchang	PRJNA827465	0.529544732
ENSGALG00010022946	KRT17	2	0.472679472	0.407250819	1.160659353	0.245780458	0.09973373	1.396357939	28.38512445	1	CRA004775;PRJNA827465	Yang2025_Silkie_Wenchang	0.75661406
ENSGALG00010022950	ITGA7	2	0.276124673	0.150632755	1.83309847	0.066787929	0.026483232	2.388578729	58.13409925	1	PRJNA827465;Yang2025_Silkie_Wenchang	CRA004775	0.445135824
ENSGALG00010022950	ITGA7	2	0.168266187	0.413158746	0.407267638	0.683811429	0.220700874	2.170264154	53.92265968	0.5	CRA004775;Yang2025_Silkie_Wenchang	PRJNA827465	0.918800744
ENSGALG00010022950	ITGA7	2	0.10818693	0.124652793	0.867906181	0.385445695	0	0.978375411	0	0.5	CRA004775;PRJNA827465	Yang2025_Silkie_Wenchang	0.82648313
ENSGALG00010022954	ADRM1	2	0.012388371	0.059340418	0.208767843	0.834629476	0	0.083668042	0	1	PRJNA827465;Yang2025_Silkie_Wenchang	CRA004775	0.958133448
ENSGALG00010022954	ADRM1	2	-0.199749031	0.306269758	-0.652199655	0.514272382	0.137015753	3.296622925	69.66592714	0.5	CRA004775;Yang2025_Silkie_Wenchang	PRJNA827465	0.866866841
ENSGALG00010022954	ADRM1	2	-0.205068014	0.280064438	-0.732217252	0.464035976	0.117626518	3.212770256	68.87421383	0.5	CRA004775;PRJNA827465	Yang2025_Silkie_Wenchang	0.852159649
ENSGALG00010022956	PBLD	2	-0.122803402	0.166053575	-0.739540851	0.459573669	0	0.886473765	0	0.5	PRJNA827465;Yang2025_Silkie_Wenchang	CRA004775	0.850098539
ENSGALG00010022956	PBLD	2	-0.20055334	0.174507938	-1.149250527	0.250452692	0	0.204899608	0	1	CRA004775;Yang2025_Silkie_Wenchang	PRJNA827465	0.759943035
ENSGALG00010022956	PBLD	2	-0.016805395	0.185298806	-0.090693487	0.927736146	0	0.166939792	0	0.5	CRA004775;PRJNA827465	Yang2025_Silkie_Wenchang	0.982993195
ENSGALG00010022957	SLTM	2	0.418775554	0.488805661	0.856732209	0.391592898	0.446838637	14.62972263	93.1650697	0.5	PRJNA827465;Yang2025_Silkie_Wenchang	CRA004775	0.829251057
ENSGALG00010022957	SLTM	2	0.705977784	0.248315474	2.843068021	0.004468153	0.053718353	1.765650868	43.36366164	1	CRA004775;Yang2025_Silkie_Wenchang	PRJNA827465	0.097752851
ENSGALG00010022957	SLTM	2	0.121977128	0.22798547	0.5350215	0.59263499	0.074902054	2.822629869	64.57204641	0.5	CRA004775;PRJNA827465	Yang2025_Silkie_Wenchang	0.892776493
ENSGALG00010022958	SFSWAP	2	0.496770084	0.456937047	1.087174016	0.276959912	0.400111388	23.46588068	95.73849363	0.5	PRJNA827465;Yang2025_Silkie_Wenchang	CRA004775	0.776084088
ENSGALG00010022958	SFSWAP	2	0.555056886	0.407229451	1.363007721	0.172880077	0.305748845	12.77550067	92.17251812	1	CRA004775;Yang2025_Silkie_Wenchang	PRJNA827465	0.676043418
ENSGALG00010022958	SFSWAP	2	0.073628164	0.068379728	1.076754269	0.28159006	0	0.353318397	0	1	CRA004775;PRJNA827465	Yang2025_Silkie_Wenchang	0.779295548
ENSGALG00010022959	ADAMTS8	2	-0.060021972	0.212812464	-0.282041619	0.777911598	0.074391115	5.206799297	80.79434326	0.5	PRJNA827465;Yang2025_Silkie_Wenchang	CRA004775	0.944432731
ENSGALG00010022959	ADAMTS8	2	-0.048681302	0.402480847	-0.120953191	0.903728391	0.196708393	1.974210119	49.34683038	0.5	CRA004775;Yang2025_Silkie_Wenchang	PRJNA827465	0.977383374
ENSGALG00010022959	ADAMTS8	2	0.13443765	0.076503161	1.757282339	0.078869697	0	0.565456456	0	1	CRA004775;PRJNA827465	Yang2025_Silkie_Wenchang	0.482993365
ENSGALG00010022967	ADAMTS15	2	-0.172529869	0.235556638	-0.732434755	0.463903252	0.10353974	14.92216914	93.29856142	0.5	PRJNA827465;Yang2025_Silkie_Wenchang	CRA004775	0.852159649
ENSGALG00010022967	ADAMTS15	2	-0.122907312	0.373164408	-0.329365046	0.741879786	0.213025095	3.454840415	71.05510299	0.5	CRA004775;Yang2025_Silkie_Wenchang	PRJNA827465	0.93543545
ENSGALG00010022967	ADAMTS15	2	0.078135423	0.087404043	0.893956616	0.371345104	0	0.526313912	0	0.5	CRA004775;PRJNA827465	Yang2025_Silkie_Wenchang	0.8214684
ENSGALG00010022969	TTN	2	0.604441321	0.959372439	0.630038237	0.528669567	1.814337587	69.35297814	98.55810085	0.5	PRJNA827465;Yang2025_Silkie_Wenchang	CRA004775	0.871792397
ENSGALG00010022969	TTN	2	1.51256268	0.188831602	8.010114139	1.14602E-15	0	0.890173238	0	1	CRA004775;Yang2025_Silkie_Wenchang	PRJNA827465	1.28692E-12
ENSGALG00010022969	TTN	2	0.172018985	0.642398381	0.267776181	0.788871606	0.669689059	4.471387729	77.63557847	0.5	CRA004775;PRJNA827465	Yang2025_Silkie_Wenchang	0.947144026
ENSGALG00010022971	CKAP5	2	-0.085905521	0.085469214	-1.005104848	0.314846366	0.011522262	4.542654288	77.9864384	1	PRJNA827465;Yang2025_Silkie_Wenchang	CRA004775	0.79651448
ENSGALG00010022971	CKAP5	2	-0.167336295	0.067091821	-2.494138507	0.012626326	0	0.593876208	0	0.5	CRA004775;Yang2025_Silkie_Wenchang	PRJNA827465	0.182044207
ENSGALG00010022971	CKAP5	2	-0.008121358	0.040647369	-0.199800344	0.841636732	0	0.020611842	0	0.5	CRA004775;PRJNA827465	Yang2025_Silkie_Wenchang	0.959922457
ENSGALG00010022972	ENSGALG00010022972	2	-1.141266378	1.844784498	-0.618644822	0.536150367	6.083134082	9.402326182	89.3643341	0.5	PRJNA827465;Yang2025_Silkie_Wenchang	CRA004775	0.874599474
ENSGALG00010022972	ENSGALG00010022972	2	-0.725414391	2.22381881	-0.326202111	0.744271444	9.317526908	17.23507447	94.19787828	0.5	CRA004775;Yang2025_Silkie_Wenchang	PRJNA827465	0.935821071
ENSGALG00010022972	ENSGALG00010022972	2	1.189517652	0.550280895	2.161655369	0.03064475	0	0.448399384	0	1	CRA004775;PRJNA827465	Yang2025_Silkie_Wenchang	0.298717911
ENSGALG00010022974	INPP5D	2	0.065313264	0.123539	0.528685391	0.597023713	0.018280391	2.407328916	58.46018409	0.5	PRJNA827465;Yang2025_Silkie_Wenchang	CRA004775	0.894204603
ENSGALG00010022974	INPP5D	2	-0.080873679	0.385649244	-0.209707864	0.833899688	0.215319636	3.019327781	66.88804508	0.5	CRA004775;Yang2025_Silkie_Wenchang	PRJNA827465	0.957968425
ENSGALG00010022974	INPP5D	2	-0.147060157	0.218021651	-0.674520887	0.499980211	0.050747703	1.498506963	33.26691004	1	CRA004775;PRJNA827465	Yang2025_Silkie_Wenchang	0.862106651
ENSGALG00010022976	MINDY2	2	-0.22197656	0.22148217	-1.002232191	0.316231464	0.095411022	36.28822514	97.24428517	1	PRJNA827465;Yang2025_Silkie_Wenchang	CRA004775	0.796883862
ENSGALG00010022976	MINDY2	2	-0.228407416	0.278154519	-0.821152992	0.411559129	0.120896805	3.769533687	73.47151974	0.5	CRA004775;Yang2025_Silkie_Wenchang	PRJNA827465	0.835128673
ENSGALG00010022976	MINDY2	2	0.000711555	0.043447988	0.016377166	0.986933496	0	0.199168596	0	0.5	CRA004775;PRJNA827465	Yang2025_Silkie_Wenchang	0.996351319
ENSGALG00010022978	ARL14EP	2	-0.191720588	0.141820948	-1.351849581	0.176423439	0.028825776	3.046488187	67.17531996	1	PRJNA827465;Yang2025_Silkie_Wenchang	CRA004775	0.681691731
ENSGALG00010022978	ARL14EP	2	-0.163586709	0.214688085	-0.761973858	0.446075607	0.065898465	3.504956481	71.46897528	0.5	CRA004775;Yang2025_Silkie_Wenchang	PRJNA827465	0.846013379
ENSGALG00010022978	ARL14EP	2	-0.067196162	0.054690903	-1.228653371	0.219201796	0	0.598800587	0	0.5	CRA004775;PRJNA827465	Yang2025_Silkie_Wenchang	0.73285529
ENSGALG00010022979	LAMA5	2	-0.105166041	0.081206748	-1.295040655	0.195306205	0	0.762908501	0	1	PRJNA827465;Yang2025_Silkie_Wenchang	CRA004775	0.70643959
ENSGALG00010022979	LAMA5	2	-0.021964641	0.109073552	-0.20137458	0.840405691	0	0.15393818	0	0.5	CRA004775;Yang2025_Silkie_Wenchang	PRJNA827465	0.959662809
ENSGALG00010022979	LAMA5	2	-0.150967606	0.110346613	-1.36812179	0.171273957	0	0.573217106	0	0.5	CRA004775;PRJNA827465	Yang2025_Silkie_Wenchang	0.673243267
ENSGALG00010022981	HNRNPH3	2	0.180665108	0.277013702	0.652188346	0.514279677	0.138285702	9.394036158	89.35494836	0.5	PRJNA827465;Yang2025_Silkie_Wenchang	CRA004775	0.866866841
ENSGALG00010022981	HNRNPH3	2	0.294529802	0.230377725	1.278464755	0.201085604	0.058463669	2.129795617	53.04713785	1	CRA004775;Yang2025_Silkie_Wenchang	PRJNA827465	0.713804636
ENSGALG00010022981	HNRNPH3	2	-0.069416184	0.047835682	-1.451138155	0.146741394	0	0.100221104	0	0.5	CRA004775;PRJNA827465	Yang2025_Silkie_Wenchang	0.635558525
ENSGALG00010022983	PKN2	2	-0.080064622	0.04055874	-1.974041137	0.048377066	0	0.121295671	0	1	PRJNA827465;Yang2025_Silkie_Wenchang	CRA004775	0.380196722
ENSGALG00010022983	PKN2	2	-0.091957029	0.096902924	-0.948960312	0.342640798	0	0.320684242	0	0.5	CRA004775;Yang2025_Silkie_Wenchang	PRJNA827465	0.806011702
ENSGALG00010022983	PKN2	2	-0.070818826	0.043448946	-1.629931979	0.103115874	0	0.20601697	0	0.5	CRA004775;PRJNA827465	Yang2025_Silkie_Wenchang	0.5473214
ENSGALG00010022986	ALG9	2	-0.487709731	0.364298722	-1.338763221	0.180647768	0.255146728	25.60326062	96.09424747	1	PRJNA827465;Yang2025_Silkie_Wenchang	CRA004775	0.68836533
ENSGALG00010022986	ALG9	2	-0.580720048	0.280317232	-2.071653051	0.038297809	0.140635031	9.50748575	89.48197214	1	CRA004775;Yang2025_Silkie_Wenchang	PRJNA827465	0.337506202
ENSGALG00010022986	ALG9	2	-0.177439987	0.075619128	-2.346496083	0.018950865	0.002482581	1.213263894	17.57770053	1	CRA004775;PRJNA827465	Yang2025_Silkie_Wenchang	0.230667657
ENSGALG00010022987	F2	2	-0.047217073	1.237379494	-0.038158926	0.969560969	2.63523774	7.086075899	85.88781698	0.5	PRJNA827465;Yang2025_Silkie_Wenchang	CRA004775	0.991572914
ENSGALG00010022987	F2	2	0.029493487	1.24187028	0.023749249	0.981052622	2.833867967	12.27698891	91.85468027	0.5	CRA004775;Yang2025_Silkie_Wenchang	PRJNA827465	0.994928779
ENSGALG00010022987	F2	2	1.251238464	0.388515934	3.220558939	0.001279409	0	6.01713E-05	0	1	CRA004775;PRJNA827465	Yang2025_Silkie_Wenchang	0.043386199
ENSGALG00010022990	SLC25A22	2	0.033546418	0.112162865	0.299086676	0.764873912	0.010515106	1.593670819	37.25178447	0.5	PRJNA827465;Yang2025_Silkie_Wenchang	CRA004775	0.940711384
ENSGALG00010022990	SLC25A22	2	-0.175363501	0.146568489	-1.19646114	0.231516655	0	0.469282735	0	1	CRA004775;Yang2025_Silkie_Wenchang	PRJNA827465	0.743924694
ENSGALG00010022990	SLC25A22	2	-0.030827372	0.222058326	-0.138825564	0.889587993	0.061642751	2.088118128	52.10998906	0.5	CRA004775;PRJNA827465	Yang2025_Silkie_Wenchang	0.973153999
ENSGALG00010022991	TAL1	2	0.041633934	0.153034458	0.272055941	0.785579006	0.019185706	1.674124722	40.26729391	0.5	PRJNA827465;Yang2025_Silkie_Wenchang	CRA004775	0.946523963
ENSGALG00010022991	TAL1	2	-0.165293312	0.17464508	-0.946452727	0.343917723	0	0.253884551	0	1	CRA004775;Yang2025_Silkie_Wenchang	PRJNA827465	0.806445383
ENSGALG00010022991	TAL1	2	0.086251573	0.209551454	0.411600927	0.680631952	0.029687785	1.214793337	17.68147144	0.5	CRA004775;PRJNA827465	Yang2025_Silkie_Wenchang	0.917851751
ENSGALG00010022992	SLC7A6	2	0.090338161	0.071002426	1.272324986	0.203257675	0	0.22412495	0	1	PRJNA827465;Yang2025_Silkie_Wenchang	CRA004775	0.717112781
ENSGALG00010022992	SLC7A6	2	-0.194170398	0.306779441	-0.632931585	0.526778321	0.120546088	2.365319714	57.72241722	0.5	CRA004775;Yang2025_Silkie_Wenchang	PRJNA827465	0.870719503
ENSGALG00010022992	SLC7A6	2	-0.153890291	0.349147284	-0.440760385	0.659386476	0.184168933	3.296660812	69.66627576	0.5	CRA004775;PRJNA827465	Yang2025_Silkie_Wenchang	0.911449691
ENSGALG00010022997	ARHGAP1	2	-0.172835832	0.082434716	-2.096638903	0.036025553	0.007664599	2.203156497	54.61057799	1	PRJNA827465;Yang2025_Silkie_Wenchang	CRA004775	0.327574606
ENSGALG00010022997	ARHGAP1	2	-0.284133111	0.086982815	-3.26654306	0.001088692	0	0.122556201	0	1	CRA004775;Yang2025_Silkie_Wenchang	PRJNA827465	0.039877802
ENSGALG00010022997	ARHGAP1	2	-0.136728672	0.085061625	-1.607407236	0.107965064	0.004434913	1.159971688	13.79099933	1	CRA004775;PRJNA827465	Yang2025_Silkie_Wenchang	0.556183889
ENSGALG00010023002	ATG16L1	2	0.013018291	0.049383741	0.263614915	0.792076665	0	4.97825E-05	0	1	PRJNA827465;Yang2025_Silkie_Wenchang	CRA004775	0.948314332
ENSGALG00010023002	ATG16L1	2	-0.027584953	0.125025053	-0.220635401	0.825376334	0	0.579254639	0	0.5	CRA004775;Yang2025_Silkie_Wenchang	PRJNA827465	0.956381808
ENSGALG00010023002	ATG16L1	2	0.006168374	0.0523387	0.117854939	0.906182598	0	0.667558989	0	0.5	CRA004775;PRJNA827465	Yang2025_Silkie_Wenchang	0.978196186
ENSGALG00010023003	ENSGALG00010023003	2	0.167444619	0.468641517	0.357297876	0.72086882	0	0.529505353	0	0.5	PRJNA827465;Yang2025_Silkie_Wenchang	CRA004775	0.928919926
ENSGALG00010023003	ENSGALG00010023003	2	-0.203946304	0.617153836	-0.330462669	0.741050395	0	0.000321773	0	1	CRA004775;Yang2025_Silkie_Wenchang	PRJNA827465	0.935384591
ENSGALG00010023003	ENSGALG00010023003	2	0.362182708	0.582579008	0.621688566	0.534146671	0	0.213408946	0	0.5	CRA004775;PRJNA827465	Yang2025_Silkie_Wenchang	0.873408008
ENSGALG00010023005	IRF7	2	-0.004660299	0.118241337	-0.039413451	0.968560756	0	0.009941365	0	0.5	PRJNA827465;Yang2025_Silkie_Wenchang	CRA004775	0.991215679
ENSGALG00010023005	IRF7	2	-0.263467871	0.290545184	-0.906805155	0.364509843	0.115088012	2.970137483	66.33152487	1	CRA004775;Yang2025_Silkie_Wenchang	PRJNA827465	0.817496251
ENSGALG00010023005	IRF7	2	-0.250477641	0.302326523	-0.828500385	0.407387177	0.131149381	3.318996026	69.87040683	0.5	CRA004775;PRJNA827465	Yang2025_Silkie_Wenchang	0.833863243
ENSGALG00010023007	LMO4	2	0.014366346	0.138987886	0.103364018	0.917674069	0.028152189	3.622957106	72.39823794	0.5	PRJNA827465;Yang2025_Silkie_Wenchang	CRA004775	0.980974192
ENSGALG00010023007	LMO4	2	0.175267385	0.10868143	1.612670943	0.106816017	0	0.051893515	0	1	CRA004775;Yang2025_Silkie_Wenchang	PRJNA827465	0.553925396
ENSGALG00010023007	LMO4	2	-0.043642248	0.139873502	-0.312012267	0.7550312	0.016884	1.384342201	27.76352558	0.5	CRA004775;PRJNA827465	Yang2025_Silkie_Wenchang	0.93838723
ENSGALG00010023015	METTL2B	2	-0.20886311	0.179664795	-1.162515501	0.245026132	0.054256313	5.926548742	83.12677338	1	PRJNA827465;Yang2025_Silkie_Wenchang	CRA004775	0.756475101
ENSGALG00010023015	METTL2B	2	-0.294409643	0.140054554	-2.102106885	0.035543914	0.014777542	1.565199298	36.11037257	1	CRA004775;Yang2025_Silkie_Wenchang	PRJNA827465	0.325417923
ENSGALG00010023015	METTL2B	2	-0.055235236	0.061208116	-0.902416871	0.366835462	0	0.146282134	0	1	CRA004775;PRJNA827465	Yang2025_Silkie_Wenchang	0.819577857
ENSGALG00010023017	BUB1B	2	-0.094965402	0.07657925	-1.24009313	0.21494095	0	0.165028699	0	1	PRJNA827465;Yang2025_Silkie_Wenchang	CRA004775	0.728256227
ENSGALG00010023017	BUB1B	2	0.018272924	0.275163858	0.066407429	0.947053456	0.084718649	1.618129987	38.2002677	0.5	CRA004775;Yang2025_Silkie_Wenchang	PRJNA827465	0.986445196
ENSGALG00010023017	BUB1B	2	0.029382203	0.219187429	0.134050584	0.893362575	0.045604382	1.33374137	25.02294506	0.5	CRA004775;PRJNA827465	Yang2025_Silkie_Wenchang	0.974381724
ENSGALG00010023019	CABP1	2	-0.064686143	0.202880479	-0.318838673	0.749848851	0	0.363243375	0	0.5	PRJNA827465;Yang2025_Silkie_Wenchang	CRA004775	0.937362187
ENSGALG00010023019	CABP1	2	-0.210518472	0.411038148	-0.512162856	0.608537037	0.146516691	1.688740572	40.78427339	0.5	CRA004775;Yang2025_Silkie_Wenchang	PRJNA827465	0.897208691
ENSGALG00010023019	CABP1	2	-0.249887851	0.225670638	-1.107312203	0.26815897	0	0.954029476	0	1	CRA004775;PRJNA827465	Yang2025_Silkie_Wenchang	0.771398294
ENSGALG00010023024	PRMT7	2	-0.031848703	0.065854889	-0.483619418	0.628655991	0	0.611688533	0	1	PRJNA827465;Yang2025_Silkie_Wenchang	CRA004775	0.901991009
ENSGALG00010023024	PRMT7	2	-0.222058563	0.117115523	-1.8960643	0.057951543	0	0.575944134	0	1	CRA004775;Yang2025_Silkie_Wenchang	PRJNA827465	0.417384914
ENSGALG00010023024	PRMT7	2	-0.123932307	0.151556095	-0.817732255	0.413510088	0.031143877	2.713924565	63.15299206	1	CRA004775;PRJNA827465	Yang2025_Silkie_Wenchang	0.836246395
ENSGALG00010023026	PHRF1	2	0.037735019	0.118861513	0.317470461	0.75088665	0.021452225	3.674751693	72.78727698	0.5	PRJNA827465;Yang2025_Silkie_Wenchang	CRA004775	0.937567095
ENSGALG00010023026	PHRF1	2	0.158906885	0.100600356	1.579585712	0.114201773	0	0.159273055	0	1	CRA004775;Yang2025_Silkie_Wenchang	PRJNA827465	0.570149069
ENSGALG00010023026	PHRF1	2	-0.050911847	0.043880343	-1.160242699	0.245950007	0	0.608977006	0	0.5	CRA004775;PRJNA827465	Yang2025_Silkie_Wenchang	0.75661406
ENSGALG00010023028	TMEM61	2	-0.063489397	0.219946627	-0.288658199	0.772842954	0.02521301	1.236841507	19.14889705	0.5	PRJNA827465;Yang2025_Silkie_Wenchang	CRA004775	0.943066211
ENSGALG00010023028	TMEM61	2	0.300346983	0.288041557	1.042721009	0.297077525	0	0.006776575	0	1	CRA004775;Yang2025_Silkie_Wenchang	PRJNA827465	0.788466926
ENSGALG00010023028	TMEM61	2	-0.0836425	0.194146545	-0.430821469	0.666598188	0.009919234	1.100690195	9.147914267	0.5	CRA004775;PRJNA827465	Yang2025_Silkie_Wenchang	0.913579507
ENSGALG00010023030	HS2ST1	2	-0.135416698	0.119806187	-1.130298044	0.258350658	0.02520706	7.96884138	87.45112429	1	PRJNA827465;Yang2025_Silkie_Wenchang	CRA004775	0.765653331
ENSGALG00010023030	HS2ST1	2	-0.100541051	0.191332479	-0.525478226	0.599250786	0.057585411	4.273798922	76.60161327	0.5	CRA004775;Yang2025_Silkie_Wenchang	PRJNA827465	0.894908299
ENSGALG00010023030	HS2ST1	2	-0.01467816	0.040977797	-0.358197873	0.720195239	0	0.691972582	0	0.5	CRA004775;PRJNA827465	Yang2025_Silkie_Wenchang	0.928564173
ENSGALG00010023041	CD151	2	-0.031572621	0.166180099	-0.189990386	0.849316664	0.050127986	10.8140352	90.75275804	0.5	PRJNA827465;Yang2025_Silkie_Wenchang	CRA004775	0.96156396
ENSGALG00010023041	CD151	2	-0.186246242	0.07230322	-2.575905238	0.009997804	0	0.469459349	0	0.5	CRA004775;Yang2025_Silkie_Wenchang	PRJNA827465	0.15844504
ENSGALG00010023041	CD151	2	0.126216013	0.06620022	1.90657995	0.056575004	0	0.195277003	0	1	CRA004775;PRJNA827465	Yang2025_Silkie_Wenchang	0.411552605
ENSGALG00010023042	ENSGALG00010023042	2	-0.574713265	0.509679621	-1.127597105	0.25949011	0	0.273281079	0	1	PRJNA827465;Yang2025_Silkie_Wenchang	CRA004775	0.766880201

ENSGALG00010023042	ENSGALG00010023042	2	0.145577069	1.034612572	0.140706843	0.888101539	1.500618178	3.325554263	69.92982459	0.5	CRA004775;Yang2025_Silkie_Wenchang	PRJNA827465	0.973135755
ENSGALG00010023042	ENSGALG00010023042	2	0.327784766	0.754409948	0.434491574	0.663931513	0.673359168	2.434446165	58.92289531	0.5	CRA004775;PRJNA827465	Yang2025_Silkie_Wenchang	0.913119244
ENSGALG00010023043	CNP	2	0.10688589	0.298626055	0.357925533	0.720399042	0.159819725	9.371269994	89.32908773	0.5	PRJNA827465;Yang2025_Silkie_Wenchang	CRA004775	0.928749051
ENSGALG00010023043	CNP	2	0.292326133	0.157388001	1.857359713	0.063259994	0.012689567	1.328430707	24.72320952	1	CRA004775;Yang2025_Silkie_Wenchang	PRJNA827465	0.433102794
ENSGALG00010023043	CNP	2	-0.106997703	0.119752769	-0.893488339	0.371595714	0.009920886	1.346133465	25.713161	0.5	CRA004775;PRJNA827465	Yang2025_Silkie_Wenchang	0.82156974
ENSGALG00010023046	SLC7A6OS	2	-0.009080738	0.060421307	-0.15029032	0.88053557	0.001551445	1.221055757	18.10365787	0.5	PRJNA827465;Yang2025_Silkie_Wenchang	CRA004775	0.971623434
ENSGALG00010023046	SLC7A6OS	2	-0.151463811	0.082928339	-1.826442121	0.067783692	0	0.778683249	0	1	CRA004775;Yang2025_Silkie_Wenchang	PRJNA827465	0.449101706
ENSGALG00010023046	SLC7A6OS	2	-0.082332779	0.139993688	-0.588117795	0.556453229	0.028765488	3.362211913	70.25767483	0.5	CRA004775;PRJNA827465	Yang2025_Silkie_Wenchang	0.880324407
ENSGALG00010023048	ARFGAP1	2	-0.079632614	0.14143606	-0.563029076	0.573415085	0.03428068	6.787030389	85.26601558	0.5	PRJNA827465;Yang2025_Silkie_Wenchang	CRA004775	0.885850914
ENSGALG00010023048	ARFGAP1	2	-0.147446107	0.138506075	-1.064546135	0.287081366	0.017486751	1.557365583	35.78900095	0.5	CRA004775;Yang2025_Silkie_Wenchang	PRJNA827465	0.782574486
ENSGALG00010023048	ARFGAP1	2	0.053832175	0.054278057	0.991785227	0.321302305	0	0.01476054	0	1	CRA004775;PRJNA827465	Yang2025_Silkie_Wenchang	0.798513866
ENSGALG00010023050	RUFY2	2	0.208673237	0.180486229	1.156172624	0.247610555	0.052128582	4.642618819	78.46043281	1	PRJNA827465;Yang2025_Silkie_Wenchang	CRA004775	0.757947282
ENSGALG00010023050	RUFY2	2	0.414446296	0.126112588	3.286319802	0.001015057	0	0.000458619	0	1	CRA004775;Yang2025_Silkie_Wenchang	PRJNA827465	0.038315518
ENSGALG00010023050	RUFY2	2	0.170033266	0.168637924	1.008274192	0.313322849	0.037361584	2.384145209	58.05624607	1	CRA004775;PRJNA827465	Yang2025_Silkie_Wenchang	0.796531448
ENSGALG00010023052	RAD54L	2	-0.036196796	0.130486029	-0.277399785	0.781473147	0.016417545	1.71603577	41.72615645	0.5	PRJNA827465;Yang2025_Silkie_Wenchang	CRA004775	0.945235709
ENSGALG00010023052	RAD54L	2	-0.17857672	0.176591223	-1.011243466	0.311899917	0	0.393991924	0	0.5	CRA004775;Yang2025_Silkie_Wenchang	PRJNA827465	0.795993742
ENSGALG00010023052	RAD54L	2	0.053392303	0.090174251	0.59210143	0.553782669	0	0.001625499	0	1	CRA004775;PRJNA827465	Yang2025_Silkie_Wenchang	0.879833465
ENSGALG00010023053	MAN1A2	2	-0.173822226	0.168528069	-1.031414095	0.302346674	0.050487141	8.55674054	88.35413746	1	PRJNA827465;Yang2025_Silkie_Wenchang	CRA004775	0.791733715
ENSGALG00010023053	MAN1A2	2	-0.306595132	0.119863128	-2.557876949	0.010531335	0.005742213	1.109657556	9.882107785	1	CRA004775;Yang2025_Silkie_Wenchang	PRJNA827465	0.163408236
ENSGALG00010023053	MAN1A2	2	-0.017733254	0.046950309	-0.377702616	0.705651524	0	8.42911E-05	0	1	CRA004775;PRJNA827465	Yang2025_Silkie_Wenchang	0.924374044
ENSGALG00010023054	AQP9	2	0.315158848	0.166497117	1.892878713	0.058374001	0	0.420405779	0	1	PRJNA827465;Yang2025_Silkie_Wenchang	CRA004775	0.418650753
ENSGALG00010023054	AQP9	2	0.178300036	0.188399327	0.946394229	0.343947548	0	0.75045095	0	0.5	CRA004775;Yang2025_Silkie_Wenchang	PRJNA827465	0.806445383
ENSGALG00010023054	AQP9	2	0.244563737	0.334331521	0.731500685	0.464473386	0.077096542	1.426371704	29.89204726	0.5	CRA004775;PRJNA827465	Yang2025_Silkie_Wenchang	0.852159649
ENSGALG00010023056	SAG	2	0.315112816	0.303633084	1.037807911	0.299359493	0	0.814565991	0	1	PRJNA827465;Yang2025_Silkie_Wenchang	CRA004775	0.789858093
ENSGALG00010023056	SAG	2	0.393098112	0.362313458	1.084966908	0.277936312	0	0.702137122	0	0.5	CRA004775;Yang2025_Silkie_Wenchang	PRJNA827465	0.776763074
ENSGALG00010023056	SAG	2	0.024853701	0.349638044	0.071084086	0.943330834	0	0.017052859	0	0.5	CRA004775;PRJNA827465	Yang2025_Silkie_Wenchang	0.985827325
ENSGALG00010023057	PAFAH1B2	2	-0.293860677	0.343005805	-0.856722169	0.391598448	0.227568956	29.80236522	96.64456162	0.5	PRJNA827465;Yang2025_Silkie_Wenchang	CRA004775	0.829251057
ENSGALG00010023057	PAFAH1B2	2	-0.443706019	0.22229722	-1.996003456	0.045933547	0.076240015	4.248472524	76.46212858	1	CRA004775;Yang2025_Silkie_Wenchang	PRJNA827465	0.369565237
ENSGALG00010023057	PAFAH1B2	2	-0.01468226	0.100715246	-0.145779919	0.884095129	0.011470539	1.664788446	39.93230777	0.5	CRA004775;PRJNA827465	Yang2025_Silkie_Wenchang	0.972001076
ENSGALG00010023058	PPARA	2	0.085907455	0.104943486	0.81860683	0.413010769	0	0.002386342	0	1	PRJNA827465;Yang2025_Silkie_Wenchang	CRA004775	0.835888542
ENSGALG00010023058	PPARA	2	0.120230788	0.149339906	0.805081452	0.420772679	0	0.271026842	0	1	CRA004775;Yang2025_Silkie_Wenchang	PRJNA827465	0.839211146
ENSGALG00010023058	PPARA	2	0.104951179	0.132949008	0.789409272	0.429872838	0	0.303023936	0	1	CRA004775;PRJNA827465	Yang2025_Silkie_Wenchang	0.842012722
ENSGALG00010023059	SELENOF	2	-0.138782638	0.296063044	-0.468760424	0.639240891	0.158946315	10.04209812	90.04192164	0.5	PRJNA827465;Yang2025_Silkie_Wenchang	CRA004775	0.905968982
ENSGALG00010023059	SELENOF	2	-0.371876601	0.127855838	-2.908561766	0.003630954	0	0.494692614	0	1	CRA004775;Yang2025_Silkie_Wenchang	PRJNA827465	0.08576166
ENSGALG00010023059	SELENOF	2	-0.037026773	0.203913523	-0.181580764	0.855911746	0.067789231	4.758408838	78.98457165	0.5	CRA004775;PRJNA827465	Yang2025_Silkie_Wenchang	0.963486168
ENSGALG00010023060	ALDH1A2	2	-0.215546783	0.10016905	-2.151830166	0.031410733	0.006582314	1.487076936	32.7539836	1	PRJNA827465;Yang2025_Silkie_Wenchang	CRA004775	0.303102391
ENSGALG00010023060	ALDH1A2	2	-0.318377442	0.113213253	-2.812192328	0.004920507	0	0.000970007	0	1	CRA004775;Yang2025_Silkie_Wenchang	PRJNA827465	0.104558504
ENSGALG00010023060	ALDH1A2	2	-0.136469503	0.10761973	-1.268071412	0.20477245	0	0.257336113	0	1	CRA004775;PRJNA827465	Yang2025_Silkie_Wenchang	0.717318293
ENSGALG00010023062	STX2	2	0.101412801	0.179276162	0.565679228	0.571611858	0.048375121	3.59130408	72.1549616	0.5	PRJNA827465;Yang2025_Silkie_Wenchang	CRA004775	0.885429878
ENSGALG00010023062	STX2	2	0.434755539	0.134579797	3.230466601	0.001235883	0	0.902554924	0	1	CRA004775;Yang2025_Silkie_Wenchang	PRJNA827465	0.042841786
ENSGALG00010023062	STX2	2	0.238382951	0.310555897	0.767600786	0.442724395	0.171413638	8.401033128	88.0967021	0.5	CRA004775;PRJNA827465	Yang2025_Silkie_Wenchang	0.845623604
ENSGALG00010023063	ENSGALG00010023063	2	-0.027635285	0.036719055	-0.752614264	0.451681743	0	0.190347507	0	1	PRJNA827465;Yang2025_Silkie_Wenchang	CRA004775	0.848006549
ENSGALG00010023063	ENSGALG00010023063	2	-0.054051983	0.06721644	-0.804148254	0.421311359	0	0.006005775	0	1	CRA004775;Yang2025_Silkie_Wenchang	PRJNA827465	0.839352211
ENSGALG00010023063	ENSGALG00010023063	2	-0.018564186	0.043071728	-0.431006308	0.666463784	0	0.034572719	0	1	CRA004775;PRJNA827465	Yang2025_Silkie_Wenchang	0.913579507
ENSGALG00010023064	ATG13	2	-0.291087756	0.26392422	-1.102921725	0.270061159	0.134990249	31.91053522	96.86623871	1	PRJNA827465;Yang2025_Silkie_Wenchang	CRA004775	0.772336005
ENSGALG00010023064	ATG13	2	-0.445510197	0.152637967	-2.918737756	0.003514517	0.02948268	2.39595138	58.26292602	1	CRA004775;Yang2025_Silkie_Wenchang	PRJNA827465	0.083890916
ENSGALG00010023064	ATG13	2	-0.056937505	0.06803408	-0.836896817	0.40265057	0.00340417	1.182969422	15.46696124	1	CRA004775;PRJNA827465	Yang2025_Silkie_Wenchang	0.832937979
ENSGALG00010023065	DNA2	2	-0.09558402	0.088141797	-1.084434664	0.278172121	0	0.15861167	0	1	PRJNA827465;Yang2025_Silkie_Wenchang	CRA004775	0.776763074
ENSGALG00010023065	DNA2	2	-0.048843748	0.213187512	-0.229111677	0.818782118	0.030766082	1.24177984	19.47042721	0.5	CRA004775;Yang2025_Silkie_Wenchang	PRJNA827465	0.955256039
ENSGALG00010023065	DNA2	2	-0.048468569	0.104342072	-0.464516064	0.642278053	0	0.981108199	0	0.5	CRA004775;PRJNA827465	Yang2025_Silkie_Wenchang	0.906634523
ENSGALG00010023066	TLR21	2	-0.176895851	0.13680799	-1.293022807	0.196003171	0	0.910751765	0	1	PRJNA827465;Yang2025_Silkie_Wenchang	CRA004775	0.707301271
ENSGALG00010023066	TLR21	2	-0.185970477	0.188109556	-0.988628549	0.322844913	0.017326979	1.307300692	23.50650418	1	CRA004775;Yang2025_Silkie_Wenchang	PRJNA827465	0.79874391
ENSGALG00010023066	TLR21	2	-0.334098884	0.151639296	-2.203247403	0.027577316	0	0.140425462	0	1	CRA004775;PRJNA827465	Yang2025_Silkie_Wenchang	0.283513274
ENSGALG00010023068	CDPF1	2	-0.018836285	0.094265114	-0.19982244	0.841619451	0.010807824	2.471824326	59.54405055	0.5	PRJNA827465;Yang2025_Silkie_Wenchang	CRA004775	0.959922457
ENSGALG00010023068	CDPF1	2	-0.142521319	0.088709257	-1.606611571	0.108139603	0	0.102355401	0	1	CRA004775;Yang2025_Silkie_Wenchang	PRJNA827465	0.556321269
ENSGALG00010023068	CDPF1	2	-0.004614107	0.11391304	-0.040505524	0.967690103	0.01278224	1.606973776	37.77123095	0.5	CRA004775;PRJNA827465	Yang2025_Silkie_Wenchang	0.991065147
ENSGALG00010023069	ENSGALG00010023069	2	0.240044485	0.190147089	1.262414727	0.206799593	0	0.105551841	0	1	PRJNA827465;Yang2025_Silkie_Wenchang	CRA004775	0.71868063
ENSGALG00010023069	ENSGALG00010023069	2	0.096802128	0.242184745	0.399703655	0.6893748	0	0.606714899	0	0.5	CRA004775;Yang2025_Silkie_Wenchang	PRJNA827465	0.91986176
ENSGALG00010023069	ENSGALG00010023069	2	0.194657484	0.248596969	0.783024365	0.433612804	0	0.926519206	0	0.5	CRA004775;PRJNA827465	Yang2025_Silkie_Wenchang	0.842762754
ENSGALG00010023070	SIDT2	2	0.152523319	0.162234044	0.940143725	0.347143843	0.046211209	7.973108381	87.45784013	0.5	PRJNA827465;Yang2025_Silkie_Wenchang	CRA004775	0.808616989
ENSGALG00010023070	SIDT2	2	0.007183861	0.358895586	0.020016577	0.984030149	0.219963632	6.304318151	84.13785637	0.5	CRA004775;Yang2025_Silkie_Wenchang	PRJNA827465	0.995637155
ENSGALG00010023070	SIDT2	2	-0.111522438	0.1787201	-0.624006133	0.532623557	0.040977618	2.071864763	51.7343015	1	CRA004775;PRJNA827465	Yang2025_Silkie_Wenchang	0.873183244
ENSGALG00010023071	POLR2M	2	0.028340984	0.118341904	0.239483926	0.810730359	0.021013329	3.849842368	74.02491052	0.5	PRJNA827465;Yang2025_Silkie_Wenchang	CRA004775	0.953020781
ENSGALG00010023071	POLR2M	2	0.128159796	0.094640352	1.354177089	0.175679887	0	0.646734953	0	0.5	CRA004775;Yang2025_Silkie_Wenchang	PRJNA827465	0.6803934
ENSGALG00010023071	POLR2M	2	-0.075032737	0.061833775	-1.213458785	0.224954463	0	0.014819135	0	1	CRA004775;PRJNA827465	Yang2025_Silkie_Wenchang	0.737917946
ENSGALG00010023073	POMGNT1	2	-0.337675848	0.258161627	-1.308001706	0.190872731	0.117404446	7.8629383	87.28210801	1	PRJNA827465;Yang2025_Silkie_Wenchang	CRA004775	0.700389407
ENSGALG00010023073	POMGNT1	2	-0.540144492	0.143562908	-3.76242372	0.000168275	0	0.715614918	0	1	CRA004775;Yang2025_Silkie_Wenchang	PRJNA827465	0.011305467
ENSGALG00010023073	POMGNT1	2	-0.118650311	0.063647599	-1.864175773	0.062297024	0	0.929338073	0	1	CRA004775;PRJNA827465	Yang2025_Silkie_Wenchang	0.430309306
ENSGALG00010023074	CCNT1	2	0.202687959	0.229594638	0.882807893	0.377340075	0.093555305	8.26166226	87.89589832	0.5	PRJNA827465;Yang2025_Silkie_Wenchang	CRA004775	0.82325159
ENSGALG00010023074	CCNT1	2	0.2330079	0.23009777	1.012647365	0.311228627	0.079353045	3.978147515	74.86267173	0.5	CRA004775;Yang2025_Silkie_Wenchang	PRJNA827465	0.795416404
ENSGALG00010023074	CCNT1	2	-0.005444234	0.048111977	-0.113157568	0.909905636	0	2.80851E-05	0	1	CRA004775;PRJNA827465	Yang2025_Silkie_Wenchang	0.978730675
ENSGALG00010023075	PKDREJ	2	0.147284273	0.150334434	0.979710828	0.32722888	0	0.150613674	0	1	PRJNA827465;Yang2025_Silkie_Wenchang	CRA004775	0.800028239
ENSGALG00010023075	PKDREJ	2	0.148157138	0.213302946	0.694585518	0.48731509	0	0.611464679	0	0.5	CRA004775;Yang2025_Silkie_Wenchang	PRJNA827465	0.85768885
ENSGALG00010023075	PKDREJ	2	0.057662146	0.187993472	0.306724195	0.759053312	0	0.362709343	0	0.5	CRA004775;PRJNA827465	Yang2025_Silkie_Wenchang	0.939081587
ENSGALG00010023076	DNAJC7	2	0.110223932	0.268286	0.410844891	0.681186274	0.134168012	14.39868253	93.05492014	0.5	PRJNA827465;Yang2025_Silkie_Wenchang	CRA004775	0.91820222
ENSGALG00010023076	DNAJC7	2	0.341408422	0.111427596	3.063948556	0.002184365	0	0.693779315	0	1	CRA004775;Yang2025_Silkie_Wenchang	PRJNA827465	0.061542714
ENSGALG00010023076	DNAJC7	2	-0.081163896	0.12707433	-0.638711971	0.523010324	0.016269583	1.514802286	33.98478408	0.5	CRA004775;PRJNA827465	Yang2025_Silkie_Wenchang	0.869568211
ENSGALG00010023077	KANSL2	2	0.251754995	0.291780542	0.862823109	0.388234732	0.162713648	22.45732879	95.54711066	0.5	PRJNA827465;Yang2025_Silkie_Wenchang	CRA004775	0.828154121
ENSGALG00010023077	KANSL2	2	0.260920186	0.31086178	0.839344695	0.401275909	0.168883154	7.567882534	86.78626425	0.5	CRA004775;Yang2025_Silkie_Wenchang	PRJNA827465	0.83248071
ENSGALG00010023077	KANSL2	2	-0.041207901	0.069824491	-0.590163997	0.555080707	0	0.034250308	0	1	CRA004775;PRJNA827465	Yang2025_Silkie_Wenchang	0.880223402
ENSGALG00010023078	TAGLN	2	-0.013518908	0.158134178	-0.085490108	0.93187176	0	0.481945788	0	0.5	PRJNA827465;Yang2025_Silkie_Wenchang	CRA004775	0.98412915
ENSGALG00010023078	TAGLN	2	0.2211663	0.213656126	1.035150754	0.300598513	0	0.836787224	0	1	CRA004775;Yang2025_Silkie_Wenchang	PRJNA827465	0.790579721
ENSGALG00010023078	TAGLN	2	0.122670344	0.324501119	0.378027491	0.705410171	0.11494216	1.945735706	48.60555845	0.5	CRA004775;PRJNA827465	Yang2025_Silkie_Wenchang	0.924281103
ENSGALG00010023079	SLC25A16	2	0.106555435	0.062984909	1.691761358	0.090691484	0	0.412135783	0	1	PRJNA827465;Yang2025_Silkie_Wenchang	CRA004775	0.516759185
ENSGALG00010023079	SLC25A16	2	0.071333196	0.085597889	0.833352289	0.404646075	0	0.049730487	0	1	CRA004775;Yang2025_Silkie_Wenchang	PRJNA827465	0.833112102
ENSGALG00010023079	SLC25A16	2	0.143460107	0.082834235	1.731893915	0.083292452	0	0.00542007	0	1	CRA004775;PRJNA827465	Yang2025_Silkie_Wenchang	0.497287446
ENSGALG00010023080	MCRS1	2	-0.02263908	0.038431579	-0.589074939	0.555811004	0	0.132920484	0	1	PRJNA827465;Yang2025_Silkie_Wenchang	CRA004775	0.880324407
ENSGALG00010023080	MCRS1	2	-0.102316585	0.12762161	-0.80171834	0.422715904	0	0.420562232	0	1	CRA004775;Yang2025_Silkie_Wenchang	PRJNA827465	0.839872304
ENSGALG00010023080	MCRS1	2	-0.022281772	0.039794132	-0.559926078	0.57552986	0	0.698344505	0	1	CRA004775;PRJNA827465	Yang2025_Silkie_Wenchang	0.886655396
ENSGALG00010023081	PRLHR2	2	0.376547646	0.225995347	1.666174334	0.095678696	0.036671678	1.447479901	30.91441205	1	PRJNA827465;Yang2025_Silkie_Wenchang	CRA004775	0.529791464
ENSGALG00010023081	PRLHR2	2	-0.056653477	0.329099199	-0.172147112	0.863321875	0	0.54785181	0	0.5	CRA004775;Yang2025_Silkie_Wenchang	PRJNA827465	0.966256632
ENSGALG00010023081	PRLHR2	2	0.243111978	0.507458217	0.479077823	0.631883266	0.301688734	1.751333133	42.9006406	0.5	CRA004775;PRJNA827465	Yang2025_Silkie_Wenchang	0.903058174
ENSGALG00010023083	HARBI1	2	0.02624342	0.096442252	0.272115379	0.785533305	0	0.002141826	0	1	PRJNA827465;Yang2025_Silkie_Wenchang	CRA004775	0.946497915
ENSGALG00010023083	HARBI1	2	-0.14742176	0.236917953	-0.622248157	0.533778705	0.055193873	1.843419487	45.75298748	0.5	CRA004775;Yang2025_Silkie_Wenchang	PRJNA827465	0.873408008
ENSGALG00010023083	HARBI1	2	-0.129454228	0.224066067	-0.577750259	0.563432742	0.060742971	2.103638317	52.46331121	0.5	CRA004775;PRJNA827465	Yang2025_Silkie_Wenchang	0.882407972
ENSGALG00010023084	ENSGALG00010023084	2	0.346364916	0.618904342	0.559642084	0.575723594	0.721353329	17.01181373	94.12173202	0.5	PRJNA827465;Yang2025_Silkie_Wenchang	CRA004775	0.886802963
ENSGALG00010023084	ENSGALG00010023084	2	0.025416907	0.977194432	0.026010082	0.979249297	1.80169518	17.47461789	94.27741421	0.5	CRA004775;Yang2025_Silkie_Wenchang	PRJNA827465	0.994378895
ENSGALG00010023084	ENSGALG00010023084	2	-0.528808235	0.347074197	-1.523617255	0.12760435	0.163298773	2.742518909	63.53717027	1	CRA004775;PRJNA827465	Yang2025_Silkie_Wenchang	0.599693145
ENSGALG00010023086	PCSK7	2	0.097192264	0.066222272	1.467667315	0.142194613	0	0.000398152	0	1	PRJNA827465;Yang2025_Silkie_Wenchang	CRA004775	0.628152735
ENSGALG00010023086	PCSK7	2	-0.165306324	0.265773843	-0.621981162	0.533954255	0.122070773	7.357100729	86.40768916	0.5	CRA004775;Yang2025_Silkie_Wenchang	PRJNA827465	0.873408008
ENSGALG00010023086	PCSK7	2	-0.154556131	0.263885269	-0.585694424	0.558080882	0.126919378	10.99378702	90.90395332	0.5	CRA004775;PRJNA827465	Yang2025_Silkie_Wenchang	0.880678789
ENSGALG00010023087	SH3GLB1	2	0.066792681	0.138924635	0.480783565	0.630670332	0.030258143	4.292047877	76.70109867	0.5	PRJNA827465;Yang2025_Silkie_Wenchang	CRA004775	0.902770945
ENSGALG00010023087	SH3GLB1	2	0.245397391	0.111591804	2.199062845	0.027873454	0	0.121927284	0	1	CRA004775;Yang2025_Silkie_Wenchang	PRJNA827465	0.284557362
ENSGALG00010023087	SH3GLB1	2	0.047961951	0.169350129	0.283211775	0.777014502	0.035478899	1.918865388	47.88587015	0.5	CRA004775;PRJNA827465	Yang2025_Silkie_Wenchang	0.944218792
ENSGALG00010023090	LRRC41	2	-0.063280555	0.112266649	-0.563662993	0.572983507	0.018368679	3.562299454	71.9282443	0.5	PRJNA827465;Yang2025_Silkie_Wenchang	CRA004775	0.88573298
ENSGALG00010023090	LRRC41	2	-0.231312237	0.085690509	-2.699391567	0.006946639	0	0.65234381	0	1	CRA004775;Yang2025_Silkie_Wenchang	PRJNA827465	0.12805992
ENSGALG00010023090	LRRC41	2	-0.124074047	0.188503589	-0.658205221	0.51040627	0.057171939	4.645036936	78.47164589	0.5	CRA004775;PRJNA827465	Yang2025_Silkie_Wenchang	0.865365179
ENSGALG00010023091	ENSGALG00010023091	2	0.043073879	0.145803752	0.295423665	0.767670257	0.036354962	6.607837372	84.86645564	0.5	PRJNA827465;Yang2025_Silkie_Wenchang	CRA004775	0.941444269
ENSGALG00010023091	ENSGALG00010023091	2	-0.091740306	0.305214372	-0.300576626	0.763737357	0.169300146	10.79679601	90.73799302	0.5	CRA004775;Yang2025_Silkie_Wenchang	PRJNA827465	0.940361611
ENSGALG00010023091	ENSGALG00010023091	2	-0.215765592	0.155420662	-1.38826839	0.165055334	0.037038355	3.728114459	73.17678921	1	CRA004775;PRJNA827465	Yang2025_Silkie_Wenchang	0.664377375
ENSGALG00010023093	POP5	2	0.134215567	0.299215092	0.448558813	0.653749951	0.147952271	5.420204761	81.5505125	0.5	PRJNA827465;Yang2025_Silkie_Wenchang	CRA004775	0.909846738
ENSGALG00010023093	POP5	2	0.001499693	0.494543897	0.003032476	0.997580438	0.409680705	6.062536618	83.50525427	0.5	CRA004775;Yang2025_Silkie_Wenchang	PRJNA827465	0.999290321
ENSGALG00010023093	POP5	2	-0.204439727	0.150767101	-1.355996937	0.175100143	0.015135088	1.251366771	20.08737779	1	CRA004775;PRJNA827465	Yang2025_Silkie_Wenchang	0.680094025
ENSGALG00010023094	CCDC32	2	-0.41559355	0.198507601	-2.093590109	0.036296509	0.070173265	9.115897558	89.03015316	1	PRJNA827465;Yang2025_Silkie_Wenchang	CRA004775	0.328473799
ENSGALG00010023094	CCDC32	2	-0.460090011	0.187587741	-2.452665661	0.014180207	0.050080197	3.207022285	68.81842684	1	CRA004775;Yang2025_Silkie_Wenchang	PRJNA827465	0.195210042
ENSGALG00010023094	CCDC32	2	-0.221984086	0.080038922	-2.773451732	0.005546507	0	0.005525877	0	1	CRA004775;PRJNA827465	Yang2025_Silkie_Wenchang	0.111717753
ENSGALG00010023096	SPATS2	2	0.158045618	0.186409675	0.847840211	0.396526966	0.060228969	7.260663555	86.22715414	0.5	PRJNA827465;Yang2025_Silkie_Wenchang	CRA004775	0.831365249
ENSGALG00010023096	SPATS2	2	0.15010207	0.222398798	0.674923025	0.499724671	0.078184511	4.692143106	78.68777705	0.5	CRA004775;Yang2025_Silkie_Wenchang	PRJNA827465	0.862078151
ENSGALG00010023096	SPATS2	2	-0.025918328	0.063177496	-0.410246214	0.681625343	0	0.15991537	0	1	CRA004775;PRJNA827465	Yang2025_Silkie_Wenchang	0.918322769
ENSGALG00010023097	LURAP1	2	0.008159894	0.109952891	0.074212641	0.940841188	0	0.395341072	0	0.5	PRJNA827465;Yang2025_Silkie_Wenchang	CRA004775	0.985565295
ENSGALG00010023097	LURAP1	2	0.004444363	0.203028668	0.021890324	0.982535443	0	0.889120581	0	0.5	CRA004775;Yang2025_Silkie_Wenchang	PRJNA827465	0.995221651
ENSGALG00010023097	LURAP1	2	0.067250177	0.115509447	0.582204998	0.560428607	0	0.385489068	0	1	CRA004775;PRJNA827465	Yang2025_Silkie_Wenchang	0.881203589
ENSGALG00010023102	RNF10	2	-0.195968829	0.166909734	-1.17410066	0.240354709	0.051311885	12.61316457	92.07177553	1	PRJNA827465;Yang2025_Silkie_Wenchang	CRA004775	0.752843928
ENSGALG00010023102	RNF10	2	-0.171470445	0.227641921	-0.753246344	0.451301895	0.084175936	4.800269128	79.16783469	0.5	CRA004775;Yang2025_Silkie_Wenchang	PRJNA827465	0.847847413
ENSGALG00010023102	RNF10	2	-0.020856446	0.057270949	-0.364171471	0.715729969	0	0.380094364	0	0.5	CRA004775;PRJNA827465	Yang2025_Silkie_Wenchang	0.927131632
ENSGALG00010023103	DGKD	2	0.01312798	0.057418732	0.22863583	0.819151974	0	0.043956318	0	0.5	PRJNA827465;Yang2025_Silkie_Wenchang	CRA004775	0.955256039
ENSGALG00010023103	DGKD	2	0.011164371	0.099729504	0.111946523	0.910865808	0	0.337277332	0	0.5	CRA004775;Yang2025_Silkie_Wenchang	PRJNA827465	0.97880407
ENSGALG00010023103	DGKD	2	0.027738676	0.067855974	0.408787529	0.682695593	0	0.274399327	0	1	CRA004775;PRJNA827465	Yang2025_Silkie_Wenchang	0.918738538
ENSGALG00010023104	ENSGALG00010023104	2	0.21264206	0.093598976	2.271841736	0.023096071	0	0.017338586	0	1	PRJNA827465;Yang2025_Silkie_Wenchang	CRA004775	0.259429803
ENSGALG00010023104	ENSGALG00010023104	2	0.117614559	0.183986726	0.639255677	0.522656617	0.027156408	1.440023858	30.55670608	0.5	CRA004775;Yang2025_Silkie_Wenchang	PRJNA827465	0.869568211
ENSGALG00010023104	ENSGALG00010023104	2	0.113648034	0.164346011	0.691516839	0.489240802	0.017842182	1.292821893	22.64982472	0.5	CRA004775;PRJNA827465	Yang2025_Silkie_Wenchang	0.858337006
ENSGALG00010023107	ZC3HAV1	2	-0.145539718	0.115287334	-1.262408566	0.206801808	0.011747579	1.775023117	43.66270553	1	PRJNA827465;Yang2025_Silkie_Wenchang	CRA004775	0.71868063
ENSGALG00010023107	ZC3HAV1	2	-0.239378452	0.10612497	-2.255627979	0.024093939	0	0.094043926	0	1	CRA004775;Yang2025_Silkie_Wenchang	PRJNA827465	0.265011806
ENSGALG00010023107	ZC3HAV1	2	-0.029082751	0.126875552	-0.229222659	0.818695862	0	0.074559145	0	1	CRA004775;PRJNA827465	Yang2025_Silkie_Wenchang	0.955256039
ENSGALG00010023108	TET1	2	0.190285306	0.324372272	0.586626301	0.557454715	0.178435449	6.168758444	83.78928257	0.5	PRJNA827465;Yang2025_Silkie_Wenchang	CRA004775	0.880678789
ENSGALG00010023108	TET1	2	0.437285588	0.198775687	2.199894735	0.027814364	0	0.724922888	0	1	CRA004775;Yang2025_Silkie_Wenchang	PRJNA827465	0.284423997
ENSGALG00010023108	TET1	2	-0.076580374	0.101993376	-0.750836738	0.452750916	0	0.608262278	0	0.5	CRA004775;PRJNA827465	Yang2025_Silkie_Wenchang	0.848446539
ENSGALG00010023110	LIMA1	2	0.058995471	0.097136885	0.607343661	0.543622869	0.016163182	6.96182951	85.63595965	0.5	PRJNA827465;Yang2025_Silkie_Wenchang	CRA004775	0.876968972
ENSGALG00010023110	LIMA1	2	-0.03580481	0.053113177	-0.674122918	0.50023317	0	0.096839261	0	0.5	CRA004775;Yang2025_Silkie_Wenchang	PRJNA827465	0.862271438
ENSGALG00010023110	LIMA1	2	0.150515816	0.048718821	3.089479841	0.002005073	0	0.203308101	0	1	CRA004775;PRJNA827465	Yang2025_Silkie_Wenchang	0.058244929
ENSGALG00010023112	PCMT1L	2	-0.136341949	0.089522316	-1.522993983	0.127760211	0	0.833695672	0	1	PRJNA827465;Yang2025_Silkie_Wenchang	CRA004775	0.600026204
ENSGALG00010023112	PCMT1L	2	-0.195015901	0.154772114	-1.260019621	0.207662284	0	0.642302624	0	1	CRA004775;Yang2025_Silkie_Wenchang	PRJNA827465	0.720024358
ENSGALG00010023112	PCMT1L	2	-0.094078968	0.089867852	-1.046858981	0.295164623	0	0.016482424	0	1	CRA004775;PRJNA827465	Yang2025_Silkie_Wenchang	0.787628307
ENSGALG00010023113	MYZAP	2	0.330571648	0.328535102	1.006198871	0.314319915	0.187513477	7.155837223	86.02539481	1	PRJNA827465;Yang2025_Silkie_Wenchang	CRA004775	0.796531448
ENSGALG00010023113	MYZAP	2	0.599676786	0.203221606	2.950851534	0.003168992	0	0.825837931	0	1	CRA004775;Yang2025_Silkie_Wenchang	PRJNA827465	0.078088334
ENSGALG00010023113	MYZAP	2	0.041503923	0.093055403	0.446013041	0.655587826	0	0.192603645	0	1	CRA004775;PRJNA827465	Yang2025_Silkie_Wenchang	0.910411938
ENSGALG00010023114	UQCRHL	2	-0.133023955	0.163536575	-0.813420211	0.415977176	0.02762739	1.556991578	35.77357682	1	PRJNA827465;Yang2025_Silkie_Wenchang	CRA004775	0.837742614
ENSGALG00010023114	UQCRHL	2	-0.284714659	0.239434064	-1.18911509	0.234394381	0	0.673778162	0	1	CRA004775;Yang2025_Silkie_Wenchang	PRJNA827465	0.747509756
ENSGALG00010023114	UQCRHL	2	-0.044954283	0.080567792	-0.557968416	0.576865952	0	0.000906981	0	1	CRA004775;PRJNA827465	Yang2025_Silkie_Wenchang	0.887113945
ENSGALG00010023115	RPLP2	2	-0.197187428	0.260146176	-0.757987034	0.448458745	0.100768485	3.125437851	68.00448297	1	PRJNA827465;Yang2025_Silkie_Wenchang	CRA004775	0.846878812
ENSGALG00010023115	RPLP2	2	-0.384239727	0.191151465	-2.010132261	0.044417192	0	0.50119865	0	1	CRA004775;Yang2025_Silkie_Wenchang	PRJNA827465	0.363895527
ENSGALG00010023115	RPLP2	2	-0.032585142	0.081389048	-0.400362739	0.688889364	0.003567267	1.109993107	9.90935048	1	CRA004775;PRJNA827465	Yang2025_Silkie_Wenchang	0.919854035
ENSGALG00010023116	PROC	2	0.032891854	1.160244524	0.02834907	0.977383744	2.009461944	3.940452354	74.62220298	0.5	PRJNA827465;Yang2025_Silkie_Wenchang	CRA004775	0.993511271
ENSGALG00010023116	PROC	2	-0.328159926	0.653640693	-0.502049412	0.615632764	0.371442882	1.706874033	41.41336848	0.5	CRA004775;Yang2025_Silkie_Wenchang	PRJNA827465	0.898886043
ENSGALG00010023116	PROC	2	0.527876026	0.467576602	1.128961594	0.258914036	0	0.98612052	0	1	CRA004775;PRJNA827465	Yang2025_Silkie_Wenchang	0.765984068
ENSGALG00010023117	RPUSD2	2	-0.061480953	0.052068633	-1.18076757	0.23769507	0	0.131147501	0	1	PRJNA827465;Yang2025_Silkie_Wenchang	CRA004775	0.749512172
ENSGALG00010023117	RPUSD2	2	-0.021883734	0.091866174	-0.238213188	0.811715749	0	0.040449158	0	0.5	CRA004775;Yang2025_Silkie_Wenchang	PRJNA827465	0.953348795
ENSGALG00010023117	RPUSD2	2	-0.066509448	0.058672785	-1.133565539	0.256976835	0	0.157737696	0	0.5	CRA004775;PRJNA827465	Yang2025_Silkie_Wenchang	0.764848386
ENSGALG00010023118	PIDD1	2	0.014640615	0.099780784	0.146727797	0.883346877	4.37427E-05	1.0020046	0.200058948	0.5	PRJNA827465;Yang2025_Silkie_Wenchang	CRA004775	0.972001076
ENSGALG00010023118	PIDD1	2	-0.070607694	0.150369608	-0.469560934	0.638668741	0	0.44495478	0	0.5	CRA004775;Yang2025_Silkie_Wenchang	PRJNA827465	0.905737083
ENSGALG00010023118	PIDD1	2	0.092826507	0.115956057	0.800531765	0.423402767	0	0.01289141	0	1	CRA004775;PRJNA827465	Yang2025_Silkie_Wenchang	0.840219758
ENSGALG00010023119	NDRG4	2	0.271540767	0.296725374	0.915124863	0.360126084	0.146528946	5.725668002	82.53478896	1	PRJNA827465;Yang2025_Silkie_Wenchang	CRA004775	0.814880744
ENSGALG00010023119	NDRG4	2	0.61179489	0.181508421	3.370614355	0.000750008	0	0.016947315	0	1	CRA004775;Yang2025_Silkie_Wenchang	PRJNA827465	0.03080204
ENSGALG00010023119	NDRG4	2	0.250791529	0.315302948	0.795398617	0.426381664	0.147080539	3.352089074	70.16785718	1	CRA004775;PRJNA827465	Yang2025_Silkie_Wenchang	0.84125685
ENSGALG00010023120	CHRM4	2	-0.265047304	0.136222715	-1.945690944	0.051691875	0	5.36208E-05	0	1	PRJNA827465;Yang2025_Silkie_Wenchang	CRA004775	0.394188158
ENSGALG00010023120	CHRM4	2	-0.304747414	0.223125404	-1.365812265	0.171997896	0	0.109220367	0	1	CRA004775;Yang2025_Silkie_Wenchang	PRJNA827465	0.674672141
ENSGALG00010023120	CHRM4	2	-0.28454261	0.151811724	-1.87431248	0.060887368	0	0.124364224	0	1	CRA004775;PRJNA827465	Yang2025_Silkie_Wenchang	0.427127227
ENSGALG00010023122	ENSGALG00010023122	2	-0.011338469	0.262098207	-0.043260384	0.965493971	0.103341753	4.028223619	75.17516169	0.5	PRJNA827465;Yang2025_Silkie_Wenchang	CRA004775	0.990668014
ENSGALG00010023122	ENSGALG00010023122	2	-0.176662142	0.161632157	-1.092988824	0.274398707	0	0.943585103	0	0.5	CRA004775;Yang2025_Silkie_Wenchang	PRJNA827465	0.774526342
ENSGALG00010023122	ENSGALG00010023122	2	0.196843215	0.150898997	1.304470001	0.192073381	0	0.281828596	0	1	CRA004775;PRJNA827465	Yang2025_Silkie_Wenchang	0.701846421
ENSGALG00010023123	RNF214	2	0.144256106	0.1946534	0.741092148	0.45863757	0.067403585	8.713455787	88.52349717	0.5	PRJNA827465;Yang2025_Silkie_Wenchang	CRA004775	0.850089963
ENSGALG00010023123	RNF214	2	0.299968863	0.102277889	2.932880844	0.003358328	0	0.791874102	0	1	CRA004775;Yang2025_Silkie_Wenchang	PRJNA827465	0.081207766
ENSGALG00010023123	RNF214	2	-0.023391363	0.056717398	-0.412419544	0.680031941	0	0.726039481	0	0.5	CRA004775;PRJNA827465	Yang2025_Silkie_Wenchang	0.917701868
ENSGALG00010023124	NSUN4	2	0.027841412	0.151892607	0.183296689	0.854565232	0.023084833	1.718563325	41.81186197	0.5	PRJNA827465;Yang2025_Silkie_Wenchang	CRA004775	0.963225297
ENSGALG00010023124	NSUN4	2	-0.130582926	0.177249911	-0.736716454	0.461294798	0	0.24406872	0	1	CRA004775;Yang2025_Silkie_Wenchang	PRJNA827465	0.85014477
ENSGALG00010023124	NSUN4	2	0.108636887	0.09283135	1.170260766	0.241896045	0	0.28656122	0	0.5	CRA004775;PRJNA827465	Yang2025_Silkie_Wenchang	0.754123921
ENSGALG00010023125	COL20A1	2	0.020225641	0.089692448	0.225499933	0.821590377	0	0.002271587	0	1	PRJNA827465;Yang2025_Silkie_Wenchang	CRA004775	0.955598716
ENSGALG00010023125	COL20A1	2	0.212734529	0.365972107	0.581286182	0.561047592	0.154215162	1.61686052	38.15174608	1	CRA004775;Yang2025_Silkie_Wenchang	PRJNA827465	0.881417858
ENSGALG00010023125	COL20A1	2	0.211907431	0.373624781	0.567166424	0.570601118	0.161051314	1.641894941	39.09476331	1	CRA004775;PRJNA827465	Yang2025_Silkie_Wenchang	0.885152854
ENSGALG00010023126	CGNL1	2	0.023119482	0.102558645	0.225426948	0.821647149	0.00265938	1.126493577	11.22896564	0.5	PRJNA827465;Yang2025_Silkie_Wenchang	CRA004775	0.955598716
ENSGALG00010023126	CGNL1	2	0.176522581	0.161176937	1.095209925	0.273424679	0	0.0227284	0	1	CRA004775;Yang2025_Silkie_Wenchang	PRJNA827465	0.774237042
ENSGALG00010023126	CGNL1	2	-0.034695363	0.111637417	-0.310786151	0.755963199	0	0.332329606	0	0.5	CRA004775;PRJNA827465	Yang2025_Silkie_Wenchang	0.938501783
ENSGALG00010023127	CERS5	2	-0.041842625	0.200959197	-0.208214531	0.835061463	0.07150324	8.161678231	87.74761793	0.5	PRJNA827465;Yang2025_Silkie_Wenchang	CRA004775	0.95823248
ENSGALG00010023127	CERS5	2	-0.264001121	0.121557759	-2.171816292	0.029869522	0	0.000645334	0	1	CRA004775;Yang2025_Silkie_Wenchang	PRJNA827465	0.295113583
ENSGALG00010023127	CERS5	2	0.042812539	0.175593197	0.243816615	0.807372852	0.038151098	1.815991961	44.93367693	0.5	CRA004775;PRJNA827465	Yang2025_Silkie_Wenchang	0.95234753
ENSGALG00010023128	IWS1	2	0.380894786	0.398612021	0.955552685	0.339298276	0.300977313	18.1198469	94.48118957	1	PRJNA827465;Yang2025_Silkie_Wenchang	CRA004775	0.804824608
ENSGALG00010023128	IWS1	2	0.542687382	0.268879762	2.018327363	0.043557179	0.105047737	3.636675385	72.5023574	1	CRA004775;Yang2025_Silkie_Wenchang	PRJNA827465	0.360709607

ENSGALG00010023128	IWS1	2	0.045768293	0.097716067	0.468380421	0.639512567	0.009640094	1.399503955	28.54611119	1	CRA004775;PRJNA827465	Yang2025_Silkie_Wenchang	0.906064918
ENSGALG00010023131	SOX6	2	0.002122614	0.100983332	0.021019445	0.983230144	0.005629731	1.215356658	17.7196263	0.5	PRJNA827465;Yang2025_Silkie_Wenchang	CRA004775	0.995363468
ENSGALG00010023131	SOX6	2	0.187594651	0.18639917	1.006413552	0.314216677	0	0.024548741	0	1	CRA004775;Yang2025_Silkie_Wenchang	PRJNA827465	0.796531448
ENSGALG00010023131	SOX6	2	-0.040243408	0.082511724	-0.48772958	0.625741397	0	0.211517903	0	0.5	CRA004775;PRJNA827465	Yang2025_Silkie_Wenchang	0.901504244
ENSGALG00010023134	KNL1	2	-0.057125806	0.068371812	-0.835516923	0.403426723	0	0.111440869	0	1	PRJNA827465;Yang2025_Silkie_Wenchang	CRA004775	0.83306585
ENSGALG00010023134	KNL1	2	-0.031157609	0.098048786	-0.317776587	0.750654412	0	0.030902504	0	0.5	CRA004775;Yang2025_Silkie_Wenchang	PRJNA827465	0.937473087
ENSGALG00010023134	KNL1	2	-0.076374273	0.094032	-0.812215767	0.416667834	0	0.054416773	0	0.5	CRA004775;PRJNA827465	Yang2025_Silkie_Wenchang	0.837984974
ENSGALG00010023135	GINS3	2	-0.099304467	0.177496806	-0.559478156	0.575839734	0.045291919	3.085936681	67.59492811	0.5	PRJNA827465;Yang2025_Silkie_Wenchang	CRA004775	0.886924804
ENSGALG00010023135	GINS3	2	-0.334367126	0.150180187	-2.226439668	0.025984746	0	0.003466146	0	1	CRA004775;Yang2025_Silkie_Wenchang	PRJNA827465	0.274758802
ENSGALG00010023135	GINS3	2	-0.087926901	0.180674459	-0.486659276	0.626499809	0.04341145	2.430729555	58.86008799	0.5	CRA004775;PRJNA827465	Yang2025_Silkie_Wenchang	0.901504244
ENSGALG00010023136	MDK	2	-0.084723242	0.139473427	-0.607450779	0.543551798	0.019865502	2.013787765	50.34233412	0.5	PRJNA827465;Yang2025_Silkie_Wenchang	CRA004775	0.876968972
ENSGALG00010023136	MDK	2	0.048015705	0.151213876	0.317535045	0.750837652	0	0.421730764	0	0.5	CRA004775;Yang2025_Silkie_Wenchang	PRJNA827465	0.937567095
ENSGALG00010023136	MDK	2	-0.213597839	0.119876378	-1.781817588	0.074778983	0	0.04721286	0	1	CRA004775;PRJNA827465	Yang2025_Silkie_Wenchang	0.472210536
ENSGALG00010023137	ETV6	2	0.095298806	0.130466005	0.730449328	0.465115576	0.022160543	2.183648815	54.20509044	1	PRJNA827465;Yang2025_Silkie_Wenchang	CRA004775	0.852354058
ENSGALG00010023137	ETV6	2	-0.034652009	0.324701779	-0.106719493	0.915011517	0.179815897	6.775426738	85.24078204	0.5	CRA004775;Yang2025_Silkie_Wenchang	PRJNA827465	0.979990126
ENSGALG00010023137	ETV6	2	-0.13685165	0.178562132	-0.766409142	0.443432895	0.051687345	4.586356314	78.19619908	0.5	CRA004775;PRJNA827465	Yang2025_Silkie_Wenchang	0.845733906
ENSGALG00010023140	CCAR1	2	0.250998039	0.287071869	0.87433868	0.381933841	0.149862881	10.58490126	90.55258074	0.5	PRJNA827465;Yang2025_Silkie_Wenchang	CRA004775	0.824944152
ENSGALG00010023140	CCAR1	2	0.429297802	0.143701093	2.987435891	0.002813283	0.010749397	1.345378382	25.67146809	1	CRA004775;Yang2025_Silkie_Wenchang	PRJNA827465	0.07222107
ENSGALG00010023140	CCAR1	2	0.070544015	0.132308325	0.533178958	0.593909706	0.020345865	1.990872714	49.77077175	0.5	CRA004775;PRJNA827465	Yang2025_Silkie_Wenchang	0.893216744
ENSGALG00010023142	TLK2	2	-0.066393849	0.052472736	-1.265301822	0.205763154	0.001938554	1.374677163	27.25564756	0.5	PRJNA827465;Yang2025_Silkie_Wenchang	CRA004775	0.71832154
ENSGALG00010023142	TLK2	2	0.096468345	0.138108603	0.698496276	0.484866886	0.016129613	1.422457672	29.69913836	1	CRA004775;Yang2025_Silkie_Wenchang	PRJNA827465	0.857004733
ENSGALG00010023142	TLK2	2	0.053192006	0.21203211	0.25086769	0.801916405	0.065929524	2.894472909	65.45139542	0.5	CRA004775;PRJNA827465	Yang2025_Silkie_Wenchang	0.950530276
ENSGALG00010023144	C11orf58	2	-0.03605438	0.044861938	-0.803674165	0.421585178	0	0.185796309	0	1	PRJNA827465;Yang2025_Silkie_Wenchang	CRA004775	0.839352211
ENSGALG00010023144	C11orf58	2	-0.108765455	0.111104436	-0.978947907	0.32760572	0	0.038683669	0	1	CRA004775;Yang2025_Silkie_Wenchang	PRJNA827465	0.80013317
ENSGALG00010023144	C11orf58	2	-0.036054076	0.045953171	-0.784582993	0.432698105	0	0.320189217	0	1	CRA004775;PRJNA827465	Yang2025_Silkie_Wenchang	0.842762754
ENSGALG00010023147	RAD51	2	-0.139993076	0.08184399	-1.710486952	0.087175864	0	0.535623917	0	1	PRJNA827465;Yang2025_Silkie_Wenchang	CRA004775	0.507807192
ENSGALG00010023147	RAD51	2	-0.349871735	0.195928753	-1.785708983	0.07414639	0	0.102656399	0	1	CRA004775;Yang2025_Silkie_Wenchang	PRJNA827465	0.470243057
ENSGALG00010023147	RAD51	2	-0.138141232	0.084307315	-1.638543853	0.101308294	0	0.838322353	0	1	CRA004775;PRJNA827465	Yang2025_Silkie_Wenchang	0.543134617
ENSGALG00010023149	ADGRD1	2	-0.448167534	0.176163068	-2.544049329	0.010957561	0.040950798	2.9177731	65.57728702	1	PRJNA827465;Yang2025_Silkie_Wenchang	CRA004775	0.166620534
ENSGALG00010023149	ADGRD1	2	-0.6194102	0.151890665	-4.078000452	4.54247E-05	0	0.122585412	0	1	CRA004775;Yang2025_Silkie_Wenchang	PRJNA827465	0.004554403
ENSGALG00010023149	ADGRD1	2	-0.292296559	0.128274724	-2.278676195	0.022686321	0	0.089933415	0	1	CRA004775;PRJNA827465	Yang2025_Silkie_Wenchang	0.256292172
ENSGALG00010023160	ENSGALG00010023160	2	0.201284558	0.237408966	0.847838907	0.396527692	0.106400439	17.52284242	94.29316331	0.5	PRJNA827465;Yang2025_Silkie_Wenchang	CRA004775	0.831365249
ENSGALG00010023160	ENSGALG00010023160	2	0.225404812	0.262915354	0.857328446	0.391263392	0.107920964	4.123706015	75.74996868	0.5	CRA004775;Yang2025_Silkie_Wenchang	PRJNA827465	0.829251057
ENSGALG00010023160	ENSGALG00010023160	2	-0.031400785	0.052168287	-0.601913288	0.547231859	0	0.05571846	0	1	CRA004775;PRJNA827465	Yang2025_Silkie_Wenchang	0.878281266
ENSGALG00010023162	CHRNA4	2	-0.248330908	0.157216304	-1.579549329	0.114210111	0	7.453E-06	0	1	PRJNA827465;Yang2025_Silkie_Wenchang	CRA004775	0.570149069
ENSGALG00010023162	CHRNA4	2	-0.994931196	0.854141723	-1.164832514	0.2440872	1.229921853	5.606039349	82.16209452	1	CRA004775;Yang2025_Silkie_Wenchang	PRJNA827465	0.755564606
ENSGALG00010023162	CHRNA4	2	-0.987176387	0.853564344	-1.156534237	0.247462704	1.235162748	5.894553838	83.03518761	1	CRA004775;PRJNA827465	Yang2025_Silkie_Wenchang	0.757947282
ENSGALG00010023163	CRTAC1	2	0.217374799	0.204478967	1.063066786	0.287751666	0.063683496	4.127817383	75.77412208	1	PRJNA827465;Yang2025_Silkie_Wenchang	CRA004775	0.782840067
ENSGALG00010023163	CRTAC1	2	0.135278754	0.364718491	0.370912793	0.710702487	0.197670298	3.531149896	71.68061313	0.5	CRA004775;Yang2025_Silkie_Wenchang	PRJNA827465	0.925476769
ENSGALG00010023163	CRTAC1	2	-0.006349052	0.113908314	-0.055738266	0.955550315	0	0.766923101	0	0.5	CRA004775;PRJNA827465	Yang2025_Silkie_Wenchang	0.988263618
ENSGALG00010023164	NKIRAS2	2	-0.01469533	0.062206309	-0.236235358	0.813250044	0	0.832382407	0	0.5	PRJNA827465;Yang2025_Silkie_Wenchang	CRA004775	0.953555086
ENSGALG00010023164	NKIRAS2	2	-0.181428357	0.148200384	-1.224209763	0.220873107	0	0.108143427	0	1	CRA004775;Yang2025_Silkie_Wenchang	PRJNA827465	0.734182657
ENSGALG00010023164	NKIRAS2	2	-0.003724509	0.065617716	-0.05676073	0.954735796	0	0.744501405	0	0.5	CRA004775;PRJNA827465	Yang2025_Silkie_Wenchang	0.987987175
ENSGALG00010023165	CSNK2A2	2	-0.087108465	0.091791636	-0.948980423	0.342630569	0.010755756	2.299149364	56.50565311	1	PRJNA827465;Yang2025_Silkie_Wenchang	CRA004775	0.806011702
ENSGALG00010023165	CSNK2A2	2	-0.247126333	0.095551009	-2.586328872	0.009700433	0	0.15466963	0	1	CRA004775;Yang2025_Silkie_Wenchang	PRJNA827465	0.155237398
ENSGALG00010023165	CSNK2A2	2	-0.118141609	0.130041346	-0.908492668	0.363617986	0.02420814	2.872360581	65.18542948	1	CRA004775;PRJNA827465	Yang2025_Silkie_Wenchang	0.817042253
ENSGALG00010023166	KDM2A	2	2.36138E-05	0.046482906	0.000508011	0.999594666	0	0.931231108	0	0.5	PRJNA827465;Yang2025_Silkie_Wenchang	CRA004775	0.999889665
ENSGALG00010023166	KDM2A	2	-0.099406585	0.220213053	-0.451410957	0.651693388	0.07378618	3.928522223	74.54338614	0.5	CRA004775;Yang2025_Silkie_Wenchang	PRJNA827465	0.909021727
ENSGALG00010023166	KDM2A	2	-0.132557048	0.156294431	-0.848123931	0.396368956	0.033974594	2.620272918	61.83603651	1	CRA004775;PRJNA827465	Yang2025_Silkie_Wenchang	0.831365249
ENSGALG00010023167	GPD1	2	-0.752127119	0.18851393	-3.989769449	6.61375E-05	0	0.445280284	0	1	PRJNA827465;Yang2025_Silkie_Wenchang	CRA004775	0.005867729
ENSGALG00010023167	GPD1	2	-0.79981012	0.238562817	-3.352618533	0.00080051	0	0.634321404	0	1	CRA004775;Yang2025_Silkie_Wenchang	PRJNA827465	0.032402087
ENSGALG00010023167	GPD1	2	-0.562284213	0.266414613	-2.110560705	0.034810088	0	0.221591769	0	1	CRA004775;PRJNA827465	Yang2025_Silkie_Wenchang	0.321263903
ENSGALG00010023169	RAN	2	-0.15497952	0.211550978	-0.732587114	0.463810293	0.069819783	3.776001812	73.51696186	0.5	PRJNA827465;Yang2025_Silkie_Wenchang	CRA004775	0.852159649
ENSGALG00010023169	RAN	2	-0.366834413	0.150967678	-2.429887092	0.015103527	0	0.135309819	0	1	CRA004775;Yang2025_Silkie_Wenchang	PRJNA827465	0.203100811
ENSGALG00010023169	RAN	2	-0.08836983	0.149470578	-0.591218895	0.554373764	0.029776985	2.299535445	56.5129556	0.5	CRA004775;PRJNA827465	Yang2025_Silkie_Wenchang	0.880177968
ENSGALG00010023170	TCF12	2	-0.247840793	0.206854908	-1.198138324	0.230863171	0.079477136	13.78734056	92.74696962	1	PRJNA827465;Yang2025_Silkie_Wenchang	CRA004775	0.743374303
ENSGALG00010023170	TCF12	2	-0.199617926	0.287566027	-0.694163802	0.48757949	0.142401299	6.8519315	85.40557506	0.5	CRA004775;Yang2025_Silkie_Wenchang	PRJNA827465	0.85768885
ENSGALG00010023170	TCF12	2	-0.036530126	0.053464456	-0.683260047	0.494442536	0	0.630305223	0	0.5	CRA004775;PRJNA827465	Yang2025_Silkie_Wenchang	0.860175061
ENSGALG00010023171	ENSGALG00010023171	2	0.08033987	0.102086186	0.786980816	0.431293096	0.009489005	1.517732176	34.11222244	0.5	PRJNA827465;Yang2025_Silkie_Wenchang	CRA004775	0.842503929
ENSGALG00010023171	ENSGALG00010023171	2	-0.195530421	0.138272557	-1.414094203	0.157334245	0	0.744351308	0	1	CRA004775;Yang2025_Silkie_Wenchang	PRJNA827465	0.650687662
ENSGALG00010023171	ENSGALG00010023171	2	-0.055671144	0.235174178	-0.236723029	0.812871668	0.088891101	4.462471971	77.59089566	0.5	CRA004775;PRJNA827465	Yang2025_Silkie_Wenchang	0.953526116
ENSGALG00010023172	DHX58	2	-0.223965335	0.229170636	-0.977286355	0.328427406	0.051556624	1.860253129	46.24387486	1	PRJNA827465;Yang2025_Silkie_Wenchang	CRA004775	0.800761292
ENSGALG00010023172	DHX58	2	-0.531466083	0.2138359	-2.485392229	0.012940876	0	0.005202278	0	1	CRA004775;Yang2025_Silkie_Wenchang	PRJNA827465	0.184914415
ENSGALG00010023172	DHX58	2	-0.233014046	0.243482874	-0.957003842	0.338565316	0.061648324	1.949827698	48.71341703	1	CRA004775;PRJNA827465	Yang2025_Silkie_Wenchang	0.804408854
ENSGALG00010023175	GRK2	2	0.14676095	0.148752902	0.986608989	0.323834362	0.03898182	8.376288654	88.06153845	1	PRJNA827465;Yang2025_Silkie_Wenchang	CRA004775	0.799600434
ENSGALG00010023175	GRK2	2	0.100433933	0.233211173	0.43065661	0.666718073	0.087396674	4.580786351	78.16968696	0.5	CRA004775;Yang2025_Silkie_Wenchang	PRJNA827465	0.913617955
ENSGALG00010023175	GRK2	2	-0.016484401	0.064224424	-0.256668719	0.797434522	0	0.648866954	0	0.5	CRA004775;PRJNA827465	Yang2025_Silkie_Wenchang	0.949715736
ENSGALG00010023177	LZTS2	2	0.037118585	0.158543187	0.234122867	0.814889594	0.037750352	3.663153373	72.70111573	0.5	PRJNA827465;Yang2025_Silkie_Wenchang	CRA004775	0.953897709
ENSGALG00010023177	LZTS2	2	0.175490179	0.14391381	1.219411669	0.222687983	0	0.971979616	0	0.5	CRA004775;Yang2025_Silkie_Wenchang	PRJNA827465	0.726491702
ENSGALG00010023177	LZTS2	2	-0.098482249	0.069116769	-1.424867669	0.154195457	0	0.078865961	0	1	CRA004775;PRJNA827465	Yang2025_Silkie_Wenchang	0.646830576
ENSGALG00010023178	CFAP20	2	0.04345651	0.045860747	0.947575265	0.343345723	0	0.348234262	0	1	PRJNA827465;Yang2025_Silkie_Wenchang	CRA004775	0.806220394
ENSGALG00010023178	CFAP20	2	-0.033430048	0.152477229	-0.219246167	0.826458294	0.029143042	2.656251613	62.35296404	0.5	CRA004775;Yang2025_Silkie_Wenchang	PRJNA827465	0.956393839
ENSGALG00010023178	CFAP20	2	-0.042266029	0.107427597	-0.393437344	0.693996492	0.014617304	2.248594295	55.52277118	0.5	CRA004775;PRJNA827465	Yang2025_Silkie_Wenchang	0.920728119
ENSGALG00010023179	KAT2A	2	0.094598482	0.064239002	1.472601986	0.140858389	0	0.030521916	0	1	PRJNA827465;Yang2025_Silkie_Wenchang	CRA004775	0.625201272
ENSGALG00010023179	KAT2A	2	-0.264939541	0.3530684	-0.75039154	0.453018925	0.232549115	14.75997265	93.2249197	0.5	CRA004775;Yang2025_Silkie_Wenchang	PRJNA827465	0.848663482
ENSGALG00010023179	KAT2A	2	-0.250485546	0.364607296	-0.687000914	0.492082153	0.25148592	18.18323035	94.50042715	0.5	CRA004775;PRJNA827465	Yang2025_Silkie_Wenchang	0.859158569
ENSGALG00010023180	EEF1A2	2	-0.24744448	0.096896757	-2.553692072	0.010658747	0	0.103668412	0	1	PRJNA827465;Yang2025_Silkie_Wenchang	CRA004775	0.164605401
ENSGALG00010023180	EEF1A2	2	-0.283418251	0.145761233	-1.944400752	0.051847143	0	0.00020069	0	1	CRA004775;Yang2025_Silkie_Wenchang	PRJNA827465	0.394912455
ENSGALG00010023180	EEF1A2	2	-0.222348134	0.125613667	-1.770095079	0.076711303	0	0.005320095	0	1	CRA004775;PRJNA827465	Yang2025_Silkie_Wenchang	0.477904868
ENSGALG00010023181	PLEKHA7	2	-0.020374438	0.075598191	-0.269509601	0.787537555	0	0.659331841	0	0.5	PRJNA827465;Yang2025_Silkie_Wenchang	CRA004775	0.946919116
ENSGALG00010023181	PLEKHA7	2	0.096317405	0.141462864	0.680867068	0.495955608	0	0.01734668	0	1	CRA004775;Yang2025_Silkie_Wenchang	PRJNA827465	0.860911322
ENSGALG00010023181	PLEKHA7	2	-0.044342516	0.084399931	-0.525385691	0.599315099	0	0.276462835	0	0.5	CRA004775;PRJNA827465	Yang2025_Silkie_Wenchang	0.894908299
ENSGALG00010023182	EPHB6	2	-0.090229739	0.077800306	-1.15976072	0.246146241	0.002442678	1.168276267	14.40180772	1	PRJNA827465;Yang2025_Silkie_Wenchang	CRA004775	0.756727496
ENSGALG00010023182	EPHB6	2	-0.232866252	0.144105733	-1.615940235	0.106107235	0	0.00059455	0	1	CRA004775;Yang2025_Silkie_Wenchang	PRJNA827465	0.55265474
ENSGALG00010023182	EPHB6	2	-0.053333348	0.072654268	-0.734070411	0.462905826	0	0.212331518	0	1	CRA004775;PRJNA827465	Yang2025_Silkie_Wenchang	0.851299641
ENSGALG00010023184	SMARCD1	2	0.041011072	0.039555202	1.036806017	0.299826271	0	0.319211639	0	1	PRJNA827465;Yang2025_Silkie_Wenchang	CRA004775	0.790081926
ENSGALG00010023184	SMARCD1	2	-0.163792672	0.182438242	-0.897797908	0.3692933	0.057239206	6.949591409	85.61064414	0.5	CRA004775;Yang2025_Silkie_Wenchang	PRJNA827465	0.820606801
ENSGALG00010023184	SMARCD1	2	-0.1372815	0.206709847	-0.664126564	0.50660931	0.077723042	10.5475697	90.519143	0.5	CRA004775;PRJNA827465	Yang2025_Silkie_Wenchang	0.863929781
ENSGALG00010023185	FBXW4	2	-0.231725255	0.276635081	-0.837656791	0.402223487	0.125139909	5.030058765	80.11951655	0.5	PRJNA827465;Yang2025_Silkie_Wenchang	CRA004775	0.832879175
ENSGALG00010023185	FBXW4	2	-0.537519775	0.204349039	-2.630400089	0.008528438	0	0.015404977	0	1	CRA004775;Yang2025_Silkie_Wenchang	PRJNA827465	0.144045206
ENSGALG00010023185	FBXW4	2	-0.054004475	0.164401086	-0.328492202	0.742539539	0.021973355	1.216245221	17.77573859	0.5	CRA004775;PRJNA827465	Yang2025_Silkie_Wenchang	0.935535551
ENSGALG00010023186	MIER1	2	0.065789008	0.056364985	1.167196415	0.243131058	0.002605357	1.451116148	31.08752863	1	PRJNA827465;Yang2025_Silkie_Wenchang	CRA004775	0.7550024
ENSGALG00010023186	MIER1	2	0.15951568	0.092370966	1.72690281	0.08418512	0	0.005909573	0	1	CRA004775;Yang2025_Silkie_Wenchang	PRJNA827465	0.499744053
ENSGALG00010023186	MIER1	2	0.035722732	0.040137317	0.890012844	0.373455936	0	0.186504414	0	1	CRA004775;PRJNA827465	Yang2025_Silkie_Wenchang	0.822254022
ENSGALG00010023187	ENSGALG00010023187	2	0.53482349	0.805831347	0.663691593	0.506887721	1.237499294	21.16087933	95.27429846	0.5	PRJNA827465;Yang2025_Silkie_Wenchang	CRA004775	0.863929781
ENSGALG00010023187	ENSGALG00010023187	2	1.346129342	0.256936869	5.239144327	1.61323E-07	0	0.001152703	0	1	CRA004775;Yang2025_Silkie_Wenchang	PRJNA827465	5.28373E-05
ENSGALG00010023187	ENSGALG00010023187	2	0.378615535	0.778027835	0.486634947	0.626517053	0.968819383	4.365301136	77.09207294	0.5	CRA004775;PRJNA827465	Yang2025_Silkie_Wenchang	0.901504244
ENSGALG00010023188	KIF1BP	2	-0.076282099	0.035239396	-2.164949496	0.030395705	0	0.466930439	0	1	PRJNA827465;Yang2025_Silkie_Wenchang	CRA004775	0.324168275
ENSGALG00010023188	KIF1BP	2	-0.115662578	0.055255441	-2.093234169	0.036328256	0	0.212202458	0	1	CRA004775;Yang2025_Silkie_Wenchang	PRJNA827465	0.3286583
ENSGALG00010023188	KIF1BP	2	-0.065547409	0.04477105	-1.464057905	0.143178128	0	0.558921323	0	1	CRA004775;PRJNA827465	Yang2025_Silkie_Wenchang	0.630019027
ENSGALG00010023189	RMDN3	2	-0.069849074	0.092901183	-0.751864203	0.452132727	0.012598664	3.688303206	72.88726159	0.5	PRJNA827465;Yang2025_Silkie_Wenchang	CRA004775	0.848321192
ENSGALG00010023189	RMDN3	2	-0.16814654	0.069804648	-2.408815811	0.016004373	0	0.000719963	0	1	CRA004775;Yang2025_Silkie_Wenchang	PRJNA827465	0.209024249
ENSGALG00010023189	RMDN3	2	0.005871934	0.061447044	0.095560882	0.923869334	0	0.708720551	0	0.5	CRA004775;PRJNA827465	Yang2025_Silkie_Wenchang	0.982219519
ENSGALG00010023190	SF3B3	2	-0.194319401	0.186008761	-1.044678753	0.296171468	0.060812518	7.804067379	87.18616907	1	PRJNA827465;Yang2025_Silkie_Wenchang	CRA004775	0.788267543
ENSGALG00010023190	SF3B3	2	-0.379768569	0.100591332	-3.775360757	0.000159776	0	0.063854701	0	1	CRA004775;Yang2025_Silkie_Wenchang	PRJNA827465	0.01094022
ENSGALG00010023190	SF3B3	2	-0.140475046	0.153729977	-0.913777833	0.360833596	0.034423172	3.071198112	67.43941733	1	CRA004775;PRJNA827465	Yang2025_Silkie_Wenchang	0.815095283
ENSGALG00010023193	PPDPF	2	-0.127848712	0.100579035	-1.271126851	0.203683524	0	0.316571772	0	1	PRJNA827465;Yang2025_Silkie_Wenchang	CRA004775	0.717254253
ENSGALG00010023193	PPDPF	2	0.037367334	0.189712519	0.196968205	0.843852419	0	0.700757584	0	0.5	CRA004775;Yang2025_Silkie_Wenchang	PRJNA827465	0.96057435
ENSGALG00010023193	PPDPF	2	-0.070205797	0.210860407	-0.333233717	0.738957869	0.039139	1.294705475	22.76235641	0.5	CRA004775;PRJNA827465	Yang2025_Silkie_Wenchang	0.934613258
ENSGALG00010023194	MRC2	2	-0.017491706	0.075686488	-0.231107382	0.81723138	0	0.341640887	0	0.5	PRJNA827465;Yang2025_Silkie_Wenchang	CRA004775	0.954833525
ENSGALG00010023194	MRC2	2	-0.128797125	0.145090827	-0.887699089	0.374702152	0	0.374702152	0	1	CRA004775;Yang2025_Silkie_Wenchang	PRJNA827465	0.822805106
ENSGALG00010023194	MRC2	2	-0.009783302	0.085205372	-0.092521184	0.926283961	0	0.805571227	0	0.5	CRA004775;PRJNA827465	Yang2025_Silkie_Wenchang	0.98280842
ENSGALG00010023195	ENSGALG00010023195	2	0.70221506	0.474460539	1.480028373	0.138865675	0.407725557	10.47279879	90.45145409	1	PRJNA827465;Yang2025_Silkie_Wenchang	CRA004775	0.622497285
ENSGALG00010023195	ENSGALG00010023195	2	1.13150479	0.214644428	5.27153116	1.3529E-07	0	0.304338199	0	1	CRA004775;Yang2025_Silkie_Wenchang	PRJNA827465	4.58389E-05
ENSGALG00010023195	ENSGALG00010023195	2	0.432912472	0.298704601	1.449299641	0.147253921	0.097524363	1.805937848	44.62710878	1	CRA004775;PRJNA827465	Yang2025_Silkie_Wenchang	0.636077613
ENSGALG00010023196	DGKZ	2	-0.166095452	0.042853438	-3.875895589	0.000106233	0	0.529405005	0	1	PRJNA827465;Yang2025_Silkie_Wenchang	CRA004775	0.008107349
ENSGALG00010023196	DGKZ	2	-0.227833073	0.066081428	-3.447762563	0.000565251	0	0.535194475	0	1	CRA004775;Yang2025_Silkie_Wenchang	PRJNA827465	0.02547712
ENSGALG00010023196	DGKZ	2	-0.176909025	0.075607975	-2.339819635	0.019293055	0.004017934	1.246467513	19.77328015	1	CRA004775;PRJNA827465	Yang2025_Silkie_Wenchang	0.232624497
ENSGALG00010023199	RPS13	2	-0.094597475	0.06831891	-1.384645562	0.166160872	0	0.129981921	0	1	PRJNA827465;Yang2025_Silkie_Wenchang	CRA004775	0.66529196
ENSGALG00010023199	RPS13	2	-0.132258978	0.175638455	-0.753018341	0.451438893	0	0.076079568	0	1	CRA004775;Yang2025_Silkie_Wenchang	PRJNA827465	0.847847413
ENSGALG00010023199	RPS13	2	-0.088573738	0.067409259	-1.313969906	0.188856332	0	0.000225647	0	1	CRA004775;PRJNA827465	Yang2025_Silkie_Wenchang	0.698162702
ENSGALG00010023200	VPS26A	2	-0.178375268	0.187884056	-0.949390128	0.342422229	0.065872636	14.39233731	93.05185823	0.5	PRJNA827465;Yang2025_Silkie_Wenchang	CRA004775	0.806005903
ENSGALG00010023200	VPS26A	2	-0.319906341	0.110369409	-2.898505515	0.003749457	0.006767682	1.222956179	18.22051996	1	CRA004775;Yang2025_Silkie_Wenchang	PRJNA827465	0.08783761
ENSGALG00010023200	VPS26A	2	-0.002421555	0.034892195	-0.069401057	0.944670387	0	0.201874182	0	0.5	CRA004775;PRJNA827465	Yang2025_Silkie_Wenchang	0.986014725
ENSGALG00010023201	ZNF280D	2	0.087475142	0.114153653	0.766292974	0.443501998	0.017678942	2.796236223	64.23764231	0.5	PRJNA827465;Yang2025_Silkie_Wenchang	CRA004775	0.845761624
ENSGALG00010023201	ZNF280D	2	0.24086403	0.109136129	2.207005434	0.02731368	0	0.019289394	0	1	CRA004775;Yang2025_Silkie_Wenchang	PRJNA827465	0.28183501
ENSGALG00010023201	ZNF280D	2	0.054281497	0.110000533	0.493465761	0.621683527	0.011855326	1.48734127	32.76593473	0.5	CRA004775;PRJNA827465	Yang2025_Silkie_Wenchang	0.899796991
ENSGALG00010023202	PID1	2	-0.192594991	0.123982485	-1.553404825	0.120326451	0.024634912	4.926165845	79.70023683	1	PRJNA827465;Yang2025_Silkie_Wenchang	CRA004775	0.58173287
ENSGALG00010023202	PID1	2	-0.274626458	0.11649103	-2.357490169	0.018398942	0.007671418	1.182346333	15.42241286	1	CRA004775;Yang2025_Silkie_Wenchang	PRJNA827465	0.227043528
ENSGALG00010023202	PID1	2	-0.074030081	0.061697358	-1.199890608	0.230181828	0	0.056013056	0	1	CRA004775;PRJNA827465	Yang2025_Silkie_Wenchang	0.742845187
ENSGALG00010023203	COG4	2	0.030054013	0.038628616	0.778024571	0.436554528	0	0.154814613	0	0.5	PRJNA827465;Yang2025_Silkie_Wenchang	CRA004775	0.84322305
ENSGALG00010023203	COG4	2	-0.191504022	0.204920443	-0.934528637	0.350031271	0.068548557	5.267328654	81.01504452	1	CRA004775;Yang2025_Silkie_Wenchang	PRJNA827465	0.810057397
ENSGALG00010023203	COG4	2	-0.165134397	0.224653563	-0.735062444	0.462301465	0.089502454	8.118312549	87.68216924	0.5	CRA004775;PRJNA827465	Yang2025_Silkie_Wenchang	0.850823289
ENSGALG00010023204	SRGN	2	0.182974468	0.453069396	0.403855281	0.686319142	0.152744004	1.496242086	33.16589545	0.5	PRJNA827465;Yang2025_Silkie_Wenchang	CRA004775	0.919235539
ENSGALG00010023204	SRGN	2	-0.74447937	0.43059071	-1.728972208	0.083814069	0	0.305653987	0	1	CRA004775;Yang2025_Silkie_Wenchang	PRJNA827465	0.498734953
ENSGALG00010023204	SRGN	2	-0.164215923	0.72050242	-0.227918627	0.819709501	0.810554672	4.47240235	77.64065212	0.5	CRA004775;PRJNA827465	Yang2025_Silkie_Wenchang	0.955256039
ENSGALG00010023205	DNAJC17	2	0.363854049	0.369325351	0.985185687	0.32453287	0.257994745	18.13797846	94.48670643	1	PRJNA827465;Yang2025_Silkie_Wenchang	CRA004775	0.799859262
ENSGALG00010023205	DNAJC17	2	0.006476863	0.759115236	0.008532121	0.993192435	1.097595705	20.65729857	95.15909597	0.5	CRA004775;Yang2025_Silkie_Wenchang	PRJNA827465	0.99794341
ENSGALG00010023205	DNAJC17	2	-0.332171849	0.386403531	-0.859650138	0.389981928	0.256449028	6.383520548	84.3346631	0.5	CRA004775;PRJNA827465	Yang2025_Silkie_Wenchang	0.829204867
ENSGALG00010023206	HSPB9	2	-0.036234699	0.253964138	-0.142676438	0.886545726	0.066670491	1.900464044	47.38127231	0.5	PRJNA827465;Yang2025_Silkie_Wenchang	CRA004775	0.972729743
ENSGALG00010023206	HSPB9	2	0.737508921	0.574465268	1.283818121	0.199205602	0.401961572	2.283500975	56.20759478	1	CRA004775;Yang2025_Silkie_Wenchang	PRJNA827465	0.71096127
ENSGALG00010023206	HSPB9	2	0.499755965	0.852030586	0.586546978	0.557508002	1.217709735	5.467430438	81.70987246	0.5	CRA004775;PRJNA827465	Yang2025_Silkie_Wenchang	0.880678789
ENSGALG00010023207	MNS1	2	0.38752465	0.347085129	1.116511822	0.264203113	0.213096161	8.088194678	87.63630155	1	PRJNA827465;Yang2025_Silkie_Wenchang	CRA004775	0.768790489
ENSGALG00010023207	MNS1	2	0.671239907	0.197427057	3.399938767	0.000674009	0	0.674065249	0	1	CRA004775;Yang2025_Silkie_Wenchang	PRJNA827465	0.028731562
ENSGALG00010023207	MNS1	2	0.087078204	0.080656641	1.079616049	0.280313192	0	0.725535345	0	1	CRA004775;PRJNA827465	Yang2025_Silkie_Wenchang	0.778461864
ENSGALG00010023208	BACE1	2	-0.099743426	0.070314466	-1.418533515	0.156035061	0.00701255	3.427316302	70.82265213	1	PRJNA827465;Yang2025_Silkie_Wenchang	CRA004775	0.649132948
ENSGALG00010023208	BACE1	2	-0.175607549	0.054829135	-3.202814515	0.001360916	0	0.034727087	0	1	CRA004775;Yang2025_Silkie_Wenchang	PRJNA827465	0.045175746
ENSGALG00010023208	BACE1	2	-0.042676068	0.048734356	-0.87568755	0.381199915	0	0.760847223	0	1	CRA004775;PRJNA827465	Yang2025_Silkie_Wenchang	0.824392515
ENSGALG00010023209	PIK3C2A	2	0.112285381	0.103773482	1.082023832	0.279241938	0.014261213	2.559369603	60.92787854	1	PRJNA827465;Yang2025_Silkie_Wenchang	CRA004775	0.777442633
ENSGALG00010023209	PIK3C2A	2	0.229028365	0.119424315	1.917769971	0.055140185	0	0.323483615	0	1	CRA004775;Yang2025_Silkie_Wenchang	PRJNA827465	0.406523108
ENSGALG00010023209	PIK3C2A	2	0.034418195	0.049896227	0.689795539	0.490322774	0	0.001529719	0	1	CRA004775;PRJNA827465	Yang2025_Silkie_Wenchang	0.858529056
ENSGALG00010023210	ZNHIT6	2	-0.290280238	0.153110255	-1.895890246	0.057974559	0.024874278	1.931228976	48.21950102	1	PRJNA827465;Yang2025_Silkie_Wenchang	CRA004775	0.417400582
ENSGALG00010023210	ZNHIT6	2	-0.287369083	0.234062938	-1.227742781	0.219543541	0.057442779	2.079614603	51.91416724	1	CRA004775;Yang2025_Silkie_Wenchang	PRJNA827465	0.732986732
ENSGALG00010023210	ZNHIT6	2	-0.156070776	0.097424611	-1.601964586	0.109163441	0	0.295823793	0	1	CRA004775;PRJNA827465	Yang2025_Silkie_Wenchang	0.559234974
ENSGALG00010023211	TLK1	2	-9.79999E-05	0.044487978	-0.00220284	0.998242389	0	0.442912725	0	0.5	PRJNA827465;Yang2025_Silkie_Wenchang	CRA004775	0.999412096
ENSGALG00010023211	TLK1	2	0.174182742	0.137731133	1.264657732	0.205994048	0.0107609	1.323025402	24.41566136	1	CRA004775;Yang2025_Silkie_Wenchang	PRJNA827465	0.718403416
ENSGALG00010023211	TLK1	2	0.122474093	0.184607716	0.663428898	0.507055903	0.04962426	2.908720594	65.62062365	0.5	CRA004775;PRJNA827465	Yang2025_Silkie_Wenchang	0.864016158
ENSGALG00010023212	RIMBP2	2	0.322622565	0.491430604	0.656496691	0.511504596	0.423419119	7.790062078	87.16313182	0.5	PRJNA827465;Yang2025_Silkie_Wenchang	CRA004775	0.865694231
ENSGALG00010023212	RIMBP2	2	0.343677904	0.561202543	0.612395486	0.540276128	0.47498589	3.95141016	74.69257912	0.5	CRA004775;Yang2025_Silkie_Wenchang	PRJNA827465	0.875359578
ENSGALG00010023212	RIMBP2	2	-0.147682926	0.151739575	-0.973265711	0.33042126	0	0.081236523	0	1	CRA004775;PRJNA827465	Yang2025_Silkie_Wenchang	0.80156474
ENSGALG00010023213	FCSK	2	0.323624039	0.076496519	4.23057212	2.33098E-05	0.000105564	1.003624766	0.361167468	1	PRJNA827465;Yang2025_Silkie_Wenchang	CRA004775	0.002741334
ENSGALG00010023213	FCSK	2	-0.102814889	0.650257691	-0.158114069	0.874366915	0.781879885	13.22326699	92.43757234	0.5	CRA004775;Yang2025_Silkie_Wenchang	PRJNA827465	0.970135804
ENSGALG00010023213	FCSK	2	-0.200186523	0.528412246	-0.378845352	0.704802707	0.519270749	13.56283078	92.62690794	0.5	CRA004775;PRJNA827465	Yang2025_Silkie_Wenchang	0.924152671
ENSGALG00010023214	SUPV3L1	2	-0.027038279	0.051632138	-0.52367149	0.600507048	0.000549615	1.092636393	8.478245235	0.5	PRJNA827465;Yang2025_Silkie_Wenchang	CRA004775	0.895230519
ENSGALG00010023214	SUPV3L1	2	0.03054943	0.086356335	0.353760148	0.723518642	0	0.451046113	0	0.5	CRA004775;Yang2025_Silkie_Wenchang	PRJNA827465	0.930032096
ENSGALG00010023214	SUPV3L1	2	-0.063008572	0.054962698	-1.146387897	0.251634688	0	0.050685345	0	1	CRA004775;PRJNA827465	Yang2025_Silkie_Wenchang	0.761706574
ENSGALG00010023215	TRIP12	2	-0.070553421	0.033965372	-2.077216181	0.037781611	0	0.421048227	0	1	PRJNA827465;Yang2025_Silkie_Wenchang	CRA004775	0.335429998
ENSGALG00010023215	TRIP12	2	0.027549748	0.094524081	0.291457454	0.770701479	0	0.744595794	0	0.5	CRA004775;Yang2025_Silkie_Wenchang	PRJNA827465	0.942491331
ENSGALG00010023215	TRIP12	2	-0.025203681	0.113407939	-0.222239123	0.82412774	0.014381674	1.491383281	32.9481554	0.5	CRA004775;PRJNA827465	Yang2025_Silkie_Wenchang	0.95619211
ENSGALG00010023216	SLC35D1	2	0.141009307	0.075731701	1.861958787	0.0626089	0	0.501811245	0	1	PRJNA827465;Yang2025_Silkie_Wenchang	CRA004775	0.431124947
ENSGALG00010023216	SLC35D1	2	-0.180430602	0.251654296	-0.716978034	0.473387651	0.083178569	2.799064899	64.27378299	0.5	CRA004775;Yang2025_Silkie_Wenchang	PRJNA827465	0.854354854
ENSGALG00010023216	SLC35D1	2	-0.106474999	0.317072983	-0.335805965	0.737017192	0.168966071	5.669731176	82.36247947	0.5	CRA004775;PRJNA827465	Yang2025_Silkie_Wenchang	0.93387966
ENSGALG00010023217	CREB3L1	2	-0.008899185	0.098243112	-0.090583298	0.927823703	0	0.275667721	0	0.5	PRJNA827465;Yang2025_Silkie_Wenchang	CRA004775	0.982993195
ENSGALG00010023217	CREB3L1	2	-0.122009786	0.282839456	-0.4313747	0.666195943	0.067185638	1.699128408	41.14629624	0.5	CRA004775;Yang2025_Silkie_Wenchang	PRJNA827465	0.913579507
ENSGALG00010023217	CREB3L1	2	-0.109688758	0.165245756	-0.663791679	0.506823652	0.022685668	1.347952025	25.81338343	1	CRA004775;PRJNA827465	Yang2025_Silkie_Wenchang	0.863929781
ENSGALG00010023218	SSH3	2	0.345112627	0.264446019	1.305039979	0.191879235	0.116067721	5.572216714	82.05382074	1	PRJNA827465;Yang2025_Silkie_Wenchang	CRA004775	0.701581425
ENSGALG00010023218	SSH3	2	0.13536212	0.509755082	0.265543443	0.790590848	0.47337288	11.20260942	91.0735083	0.5	CRA004775;Yang2025_Silkie_Wenchang	PRJNA827465	0.947584465
ENSGALG00010023218	SSH3	2	-0.093398528	0.240008107	-0.389147388	0.69716712	0.087518988	3.787110982	73.59464762	0.5	CRA004775;PRJNA827465	Yang2025_Silkie_Wenchang	0.921126814
ENSGALG00010023219	TEX9	2	0.356854952	0.318079174	1.121906057	0.261902385	0.17875414	8.453372555	88.17040189	1	PRJNA827465;Yang2025_Silkie_Wenchang	CRA004775	0.768363052
ENSGALG00010023219	TEX9	2	0.030030695	0.707643865	0.042437583	0.966149869	0.90237417	9.659816527	89.64783651	0.5	CRA004775;Yang2025_Silkie_Wenchang	PRJNA827465	0.990863074
ENSGALG00010023219	TEX9	2	-0.236066599	0.37735196	-0.625587315	0.531585661	0.210501937	3.208170509	68.82958692	0.5	CRA004775;PRJNA827465	Yang2025_Silkie_Wenchang	0.873042777
ENSGALG00010023220	ST3GAL2	2	0.01432741	0.052427283	0.273281565	0.784636785	0	0.043303384	0	1	PRJNA827465;Yang2025_Silkie_Wenchang	CRA004775	0.946117405
ENSGALG00010023220	ST3GAL2	2	-0.056154479	0.133177021	-0.421652914	0.673278378	0.015400286	1.583846664	36.86257499	0.5	CRA004775;Yang2025_Silkie_Wenchang	PRJNA827465	0.915223605
ENSGALG00010023220	ST3GAL2	2	-0.051162995	0.11000786	-0.465084899	0.64187066	0.011794175	1.508618326	33.71418187	0.5	CRA004775;PRJNA827465	Yang2025_Silkie_Wenchang	0.906634523
ENSGALG00010023221	AMY1A	2	-0.07732198	0.159229683	-0.485600288	0.627250592	0.026777037	2.104262636	52.477415	0.5	PRJNA827465;Yang2025_Silkie_Wenchang	CRA004775	0.901612726
ENSGALG00010023221	AMY1A	2	0.129567312	0.148418056	0.872988874	0.382669142	0	0.191190541	0	1	CRA004775;Yang2025_Silkie_Wenchang	PRJNA827465	0.825121999
ENSGALG00010023221	AMY1A	2	-0.070017606	0.222803185	-0.314257653	0.753325358	0.053266872	1.888321964	47.04292916	0.5	CRA004775;PRJNA827465	Yang2025_Silkie_Wenchang	0.938087082

ENSGALG00010023223	CEP164	2	0.278577458	0.331351163	0.840731793	0.400498205	0.189234811	6.746940572	85.17846735	0.5	PRJNA827465;Yang2025_Silkie_Wenchang	CRA004775	0.832251175
ENSGALG00010023223	CEP164	2	0.358426495	0.349264851	1.026231222	0.304782608	0.148846603	2.432918773	58.89710699	0.5	CRA004775;Yang2025_Silkie_Wenchang	PRJNA827465	0.79225794
ENSGALG00010023223	CEP164	2	-0.021324942	0.092195744	-0.231300719	0.817081188	0	0.012311118	0	1	CRA004775;PRJNA827465	Yang2025_Silkie_Wenchang	0.954833525
ENSGALG00010023225	PDPR	2	-0.066167661	0.065955809	-1.003212025	0.315758572	0	0.12615357	0	1	PRJNA827465;Yang2025_Silkie_Wenchang	CRA004775	0.796883862
ENSGALG00010023225	PDPR	2	-0.283261629	0.290642641	-0.97460451	0.329756476	0.13369034	4.514304831	77.84819507	1	CRA004775;Yang2025_Silkie_Wenchang	PRJNA827465	0.801177491
ENSGALG00010023225	PDPR	2	-0.296396223	0.261770552	-1.13227489	0.257518884	0.109235489	4.316913714	76.83530257	1	CRA004775;PRJNA827465	Yang2025_Silkie_Wenchang	0.7650601
ENSGALG00010023226	ENSGALG00010023226	2	0.109139499	0.286858006	0.380465235	0.703600098	0.094359894	2.31701961	56.84110761	0.5	PRJNA827465;Yang2025_Silkie_Wenchang	CRA004775	0.923639641
ENSGALG00010023226	ENSGALG00010023226	2	0.373169614	0.260441421	1.432835118	0.151904963	0	0.134319594	0	1	CRA004775;Yang2025_Silkie_Wenchang	PRJNA827465	0.643294424
ENSGALG00010023226	ENSGALG00010023226	2	-0.098637124	0.218008928	-0.452445341	0.650948189	0	0.329761175	0	0.5	CRA004775;PRJNA827465	Yang2025_Silkie_Wenchang	0.908769703
ENSGALG00010023227	RNPC3	2	0.481650612	0.438218754	1.099109994	0.271720091	0.363552174	18.46902787	94.58552985	1	PRJNA827465;Yang2025_Silkie_Wenchang	CRA004775	0.773086433
ENSGALG00010023227	RNPC3	2	0.63208788	0.309415147	2.042847242	0.041067562	0.154350733	5.137384708	80.53484298	1	CRA004775;Yang2025_Silkie_Wenchang	PRJNA827465	0.351115597
ENSGALG00010023227	RNPC3	2	0.120151003	0.111788445	1.074806998	0.282461147	0.006479864	1.242590521	19.5229657	1	CRA004775;PRJNA827465	Yang2025_Silkie_Wenchang	0.779532565
ENSGALG00010023228	GORASP2	2	0.020254003	0.041425869	0.488921612	0.624897194	0.0009075	1.167407332	14.340096	1	PRJNA827465;Yang2025_Silkie_Wenchang	CRA004775	0.90106521
ENSGALG00010023228	GORASP2	2	0.095248316	0.086164891	1.105419093	0.268978034	0	0.163723274	0	1	CRA004775;Yang2025_Silkie_Wenchang	PRJNA827465	0.771398294
ENSGALG00010023228	GORASP2	2	0.003108774	0.033167745	0.093728841	0.925324559	0	0.027919716	0	1	CRA004775;PRJNA827465	Yang2025_Silkie_Wenchang	0.982594411
ENSGALG00010023230	ENSGALG00010023230	2	-0.231477688	0.19471715	-1.188789418	0.234522543	0.066269446	7.854128969	87.2678434	1	PRJNA827465;Yang2025_Silkie_Wenchang	CRA004775	0.747615801
ENSGALG00010023230	ENSGALG00010023230	2	-0.247054147	0.240984252	-1.025187932	0.305274522	0.080370964	2.846494697	64.86907209	0.5	CRA004775;Yang2025_Silkie_Wenchang	PRJNA827465	0.79225794
ENSGALG00010023230	ENSGALG00010023230	2	-0.034948637	0.078932246	-0.442767549	0.657933878	0	0.143650021	0	0.5	CRA004775;PRJNA827465	Yang2025_Silkie_Wenchang	0.910841476
ENSGALG00010023231	ASIC1	2	-0.045300289	0.10801372	-0.419393842	0.674928324	0	0.017512965	0	1	PRJNA827465;Yang2025_Silkie_Wenchang	CRA004775	0.91599715
ENSGALG00010023231	ASIC1	2	0.320205176	0.430311605	0.74412396	0.456801485	0.292928126	4.609458776	78.30547905	0.5	CRA004775;Yang2025_Silkie_Wenchang	PRJNA827465	0.849515887
ENSGALG00010023231	ASIC1	2	0.317989818	0.411734437	0.77231776	0.439926249	0.279398209	5.154228047	80.59845255	0.5	CRA004775;PRJNA827465	Yang2025_Silkie_Wenchang	0.844353254
ENSGALG00010023234	ENSGALG00010023234	2	0.085797843	0.210104265	0.408358406	0.683010565	0.061614898	2.595928715	61.47814097	0.5	PRJNA827465;Yang2025_Silkie_Wenchang	CRA004775	0.918800744
ENSGALG00010023234	ENSGALG00010023234	2	0.315487795	0.217716036	1.449079274	0.147315445	0	0.19773292	0	1	CRA004775;Yang2025_Silkie_Wenchang	PRJNA827465	0.636173932
ENSGALG00010023234	ENSGALG00010023234	2	-0.053693266	0.062494127	-0.859172991	0.390245082	0	0.433482937	0	0.5	CRA004775;PRJNA827465	Yang2025_Silkie_Wenchang	0.82923632
ENSGALG00010023235	ENSGALG00010023235	2	0.21817731	0.203526351	1.071985566	0.283726515	0	0.603607582	0	1	PRJNA827465;Yang2025_Silkie_Wenchang	CRA004775	0.779756886
ENSGALG00010023235	ENSGALG00010023235	2	0.229443756	0.233921712	0.980857029	0.326663251	0	0.647287394	0	1	CRA004775;Yang2025_Silkie_Wenchang	PRJNA827465	0.800028239
ENSGALG00010023235	ENSGALG00010023235	2	0.090194951	0.194425842	0.463904128	0.642716435	0	0.0118028	0	1	CRA004775;PRJNA827465	Yang2025_Silkie_Wenchang	0.906634523
ENSGALG00010023238	NUCB2	2	0.042079886	0.061878186	0.680043957	0.496476628	0	6.19713E-05	0	1	PRJNA827465;Yang2025_Silkie_Wenchang	CRA004775	0.860939846
ENSGALG00010023238	NUCB2	2	0.006821712	0.142258476	0.047952937	0.96175375	0	0.282125677	0	0.5	CRA004775;Yang2025_Silkie_Wenchang	PRJNA827465	0.98973612
ENSGALG00010023238	NUCB2	2	0.03486065	0.066153575	0.526965478	0.598217562	0	0.331601738	0	0.5	CRA004775;PRJNA827465	Yang2025_Silkie_Wenchang	0.894645663
ENSGALG00010023239	RBPMS2	2	0.196901786	0.399120474	0.493339227	0.621772915	0.26518217	5.464623626	81.70047805	0.5	PRJNA827465;Yang2025_Silkie_Wenchang	CRA004775	0.899796991
ENSGALG00010023239	RBPMS2	2	0.347026608	0.264201773	1.313490838	0.189017606	0.060845423	1.742111793	42.59840246	1	CRA004775;Yang2025_Silkie_Wenchang	PRJNA827465	0.698162702
ENSGALG00010023239	RBPMS2	2	-0.096442588	0.110579012	-0.872159971	0.383121113	0	0.970013283	0	0.5	CRA004775;PRJNA827465	Yang2025_Silkie_Wenchang	0.825405345
ENSGALG00010023243	HK1	2	-0.009867596	0.194234711	-0.050802432	0.959482953	0.065792854	7.579998068	86.8073845	0.5	PRJNA827465;Yang2025_Silkie_Wenchang	CRA004775	0.989259322
ENSGALG00010023243	HK1	2	0.184849626	0.110094183	1.679013556	0.093149402	0	0.057051122	0	1	CRA004775;Yang2025_Silkie_Wenchang	PRJNA827465	0.522932573
ENSGALG00010023243	HK1	2	-0.118644413	0.135018307	-0.878728344	0.379548587	0.018603402	1.554499998	35.40569633	0.5	CRA004775;PRJNA827465	Yang2025_Silkie_Wenchang	0.82419806
ENSGALG00010023244	PPIB	2	0.126462861	0.053212612	2.376558035	0.017475012	0	0.001672333	0	1	PRJNA827465;Yang2025_Silkie_Wenchang	CRA004775	0.220134745
ENSGALG00010023244	PPIB	2	-0.091121016	0.220816173	-0.412655533	0.679859008	0.064320438	2.925648126	65.81953957	0.5	CRA004775;Yang2025_Silkie_Wenchang	PRJNA827465	0.917701868
ENSGALG00010023244	PPIB	2	-0.05890612	0.221364287	-0.266104891	0.790158428	0.08049139	4.927104622	79.70410461	0.5	CRA004775;PRJNA827465	Yang2025_Silkie_Wenchang	0.947390191
ENSGALG00010023245	TANC2	2	0.023110576	0.117279527	0.197055503	0.843784104	0.012004046	1.489178491	32.8488824	0.5	PRJNA827465;Yang2025_Silkie_Wenchang	CRA004775	0.96057435
ENSGALG00010023245	TANC2	2	-0.317268234	0.589844728	-0.537884325	0.59065691	0.5997973	6.946651617	85.60457534	0.5	CRA004775;Yang2025_Silkie_Wenchang	PRJNA827465	0.892697118
ENSGALG00010023245	TANC2	2	-0.418009252	0.449005772	-0.930966322	0.351870985	0.334482842	5.044863215	80.17785701	1	CRA004775;PRJNA827465	Yang2025_Silkie_Wenchang	0.810678834
ENSGALG00010023248	PRDM10	2	0.109545399	0.156181582	0.70139768	0.48305487	0.036842877	3.482623405	71.28601391	0.5	PRJNA827465;Yang2025_Silkie_Wenchang	CRA004775	0.85691916
ENSGALG00010023248	PRDM10	2	0.212403563	0.158282879	1.341923801	0.179620704	0.007183427	1.1270132	11.26989465	0.5	CRA004775;Yang2025_Silkie_Wenchang	PRJNA827465	0.68659821
ENSGALG00010023248	PRDM10	2	-0.014186689	0.050258671	-0.282273462	0.777733833	0	0.014247687	0	1	CRA004775;PRJNA827465	Yang2025_Silkie_Wenchang	0.944388838
ENSGALG00010023249	IL12RB2	2	-0.180871935	0.162285817	-1.114527061	0.265053142	0	0.273848774	0	1	PRJNA827465;Yang2025_Silkie_Wenchang	CRA004775	0.769274038
ENSGALG00010023249	IL12RB2	2	0.025885778	0.390624025	0.066267757	0.947164653	0.17309271	2.278505113	56.11157534	0.5	CRA004775;Yang2025_Silkie_Wenchang	PRJNA827465	0.986445196
ENSGALG00010023249	IL12RB2	2	0.052258555	0.273290208	0.191220005	0.848353231	0.073029449	1.720748703	41.88576182	0.5	CRA004775;PRJNA827465	Yang2025_Silkie_Wenchang	0.961364896
ENSGALG00010023250	FZD10	2	-0.200109812	0.369743641	-0.541212316	0.58836125	0	0.167175544	0	1	PRJNA827465;Yang2025_Silkie_Wenchang	CRA004775	0.891874519
ENSGALG00010023250	FZD10	2	-0.577530499	0.546217534	-1.057326914	0.290362414	0	0.198108056	0	1	CRA004775;Yang2025_Silkie_Wenchang	PRJNA827465	0.784195688
ENSGALG00010023250	FZD10	2	-0.238999823	0.409789118	-0.583226378	0.559740976	0	0.607047026	0	1	CRA004775;PRJNA827465	Yang2025_Silkie_Wenchang	0.881203589
ENSGALG00010023252	GLG1	2	-0.068979199	0.075878635	-0.909072737	0.363311732	0.005746733	1.69459659	40.9889052	0.5	PRJNA827465;Yang2025_Silkie_Wenchang	CRA004775	0.816880132
ENSGALG00010023252	GLG1	2	0.007488759	0.103131791	0.072613483	0.942113697	0	0.510426097	0	0.5	CRA004775;Yang2025_Silkie_Wenchang	PRJNA827465	0.985827325
ENSGALG00010023252	GLG1	2	-0.117449954	0.047158807	-2.490414029	0.012759436	0	0.000138529	0	1	CRA004775;PRJNA827465	Yang2025_Silkie_Wenchang	0.133357994
ENSGALG00010023255	SNX22	2	0.181551913	0.346698357	0.523659572	0.600515339	0.152730774	2.623126892	61.87755907	0.5	PRJNA827465;Yang2025_Silkie_Wenchang	CRA004775	0.895230519
ENSGALG00010023255	SNX22	2	-0.083705023	0.675871721	-0.1238475	0.901436021	0.77614164	6.645598119	84.95244548	0.5	CRA004775;Yang2025_Silkie_Wenchang	PRJNA827465	0.976655087
ENSGALG00010023255	SNX22	2	-0.370806069	0.317619882	-1.167452323	0.243027752	0.113051128	2.157772466	53.65591062	1	CRA004775;PRJNA827465	Yang2025_Silkie_Wenchang	0.7550024
ENSGALG00010023257	PAK6	2	0.228250192	0.201713294	1.113313055	0.257820514	0	0.657639706	0	1	PRJNA827465;Yang2025_Silkie_Wenchang	CRA004775	0.761511037
ENSGALG00010023257	PAK6	2	0.413621071	0.252220022	1.639921639	0.101021461	0	0.000112526	0	1	CRA004775;Yang2025_Silkie_Wenchang	PRJNA827465	0.542487465
ENSGALG00010023257	PAK6	2	0.171984612	0.229312656	0.750000522	0.453254391	0	0.391834396	0	1	CRA004775;PRJNA827465	Yang2025_Silkie_Wenchang	0.848823429
ENSGALG00010023258	ENSGALG00010023258	2	-0.12468793	0.235380498	-0.537291455	0.591066307	0	0.022525606	0	1	PRJNA827465;Yang2025_Silkie_Wenchang	CRA004775	0.892776493
ENSGALG00010023258	ENSGALG00010023258	2	0.053917612	0.354872045	0.151935361	0.879237918	0	0.144936293	0	0.5	CRA004775;Yang2025_Silkie_Wenchang	PRJNA827465	0.971257564
ENSGALG00010023258	ENSGALG00010023258	2	-0.074720985	0.24226412	-0.308427782	0.757756844	0	0.346339385	0	0.5	CRA004775;PRJNA827465	Yang2025_Silkie_Wenchang	0.938954369
ENSGALG00010023259	TSPAN15	2	-0.269205418	0.146308238	-1.839988111	0.065769983	0	0.398704338	0	1	PRJNA827465;Yang2025_Silkie_Wenchang	CRA004775	0.441899536
ENSGALG00010023259	TSPAN15	2	-0.346274404	0.196071107	-1.766065433	0.077384872	0	0.071968778	0	1	CRA004775;Yang2025_Silkie_Wenchang	PRJNA827465	0.47906101
ENSGALG00010023259	TSPAN15	2	-0.196902762	0.17805128	-1.105876701	0.26877989	0	0.014360801	0	1	CRA004775;PRJNA827465	Yang2025_Silkie_Wenchang	0.771398294
ENSGALG00010023260	ENSGALG00010023260	2	-0.403213657	0.230690242	-1.747857442	0.080488716	0	0.782837499	0	1	PRJNA827465;Yang2025_Silkie_Wenchang	CRA004775	0.488261768
ENSGALG00010023260	ENSGALG00010023260	2	-0.291235126	0.584766357	-0.498036734	0.618458151	0.342475813	1.758799351	43.14299877	0.5	CRA004775;Yang2025_Silkie_Wenchang	PRJNA827465	0.899168321
ENSGALG00010023260	ENSGALG00010023260	2	-0.175333316	0.269993553	-0.649393143	0.516084166	0	0.831522837	0	0.5	CRA004775;PRJNA827465	Yang2025_Silkie_Wenchang	0.867498525
ENSGALG00010023261	SNX1	2	0.107507708	0.045871388	2.343676812	0.019094709	0	0.051365398	0	1	PRJNA827465;Yang2025_Silkie_Wenchang	CRA004775	0.231442015
ENSGALG00010023261	SNX1	2	-0.046812504	0.172198619	-0.271851796	0.785719977	0.03853797	2.583160998	61.28774007	0.5	CRA004775;Yang2025_Silkie_Wenchang	PRJNA827465	0.946626034
ENSGALG00010023261	SNX1	2	-0.028401766	0.183524031	-0.154757348	0.877012302	0.050991501	3.425136512	70.80408339	0.5	CRA004775;PRJNA827465	Yang2025_Silkie_Wenchang	0.970499043
ENSGALG00010023262	POLD4	2	-0.072965416	0.274097542	-0.266203373	0.790083356	0	0.00033647	0	1	PRJNA827465;Yang2025_Silkie_Wenchang	CRA004775	0.947390191
ENSGALG00010023262	POLD4	2	0.054247263	0.309347097	0.175360504	0.860796373	0	0.113654987	0	0.5	CRA004775;Yang2025_Silkie_Wenchang	PRJNA827465	0.96519114
ENSGALG00010023262	POLD4	2	0.014198296	0.250389215	0.056704903	0.954780268	0	0.161443132	0	0.5	CRA004775;PRJNA827465	Yang2025_Silkie_Wenchang	0.987987175
ENSGALG00010023263	SRP14	2	-0.061598414	0.272767143	-0.225825737	0.821335326	0.140104409	16.78099626	94.04370713	0.5	PRJNA827465;Yang2025_Silkie_Wenchang	CRA004775	0.955598716
ENSGALG00010023263	SRP14	2	-0.252036847	0.130208034	-1.935647434	0.052910897	0.011417894	1.426060398	29.87674286	1	CRA004775;Yang2025_Silkie_Wenchang	PRJNA827465	0.399377178
ENSGALG00010023263	SRP14	2	0.133843782	0.117110107	1.142888395	0.253084932	0.014579578	1.671706833	40.18089894	0.5	CRA004775;PRJNA827465	Yang2025_Silkie_Wenchang	0.762731469
ENSGALG00010023264	RACGAP1	2	0.137973692	0.106817836	1.291672782	0.196470487	0.009140966	1.495253601	33.12171265	1	PRJNA827465;Yang2025_Silkie_Wenchang	CRA004775	0.707837424
ENSGALG00010023264	RACGAP1	2	0.305301877	0.163615865	1.865992447	0.062041375	0	0.000303729	0	1	CRA004775;Yang2025_Silkie_Wenchang	PRJNA827465	0.42491371
ENSGALG00010023264	RACGAP1	2	0.075938553	0.082381371	0.921792787	0.356636671	0	0.22959822	0	1	CRA004775;PRJNA827465	Yang2025_Silkie_Wenchang	0.812714737
ENSGALG00010023265	RAB5C	2	-0.090031435	0.146606849	-0.614101153	0.539148487	0.038270748	8.535976391	88.28487856	0.5	PRJNA827465;Yang2025_Silkie_Wenchang	CRA004775	0.874983893
ENSGALG00010023265	RAB5C	2	-0.220772218	0.08199319	-2.692567316	0.007090941	0	0.386762109	0	1	CRA004775;Yang2025_Silkie_Wenchang	PRJNA827465	0.129970351
ENSGALG00010023265	RAB5C	2	0.025594803	0.053794602	0.475787572	0.634225725	0.00210607	1.154027094	13.34692179	0.5	CRA004775;PRJNA827465	Yang2025_Silkie_Wenchang	0.903815945
ENSGALG00010023266	RFWD3	2	0.230267445	0.076402095	3.013889152	0.00257922	0	0.776959809	0	1	PRJNA827465;Yang2025_Silkie_Wenchang	CRA004775	0.068626229
ENSGALG00010023266	RFWD3	2	-0.105754145	0.495975985	-0.213224327	0.831151995	0.438177825	8.939569508	88.81377902	0.5	CRA004775;Yang2025_Silkie_Wenchang	PRJNA827465	0.95749236
ENSGALG00010023266	RFWD3	2	-0.171265909	0.41017206	-0.417546487	0.676378729	0.296197864	7.685397582	86.98831141	0.5	CRA004775;PRJNA827465	Yang2025_Silkie_Wenchang	0.91671756
ENSGALG00010023268	KCNJ11	2	-0.091628812	0.077606483	-1.180685015	0.237727877	0	0.200498447	0	1	PRJNA827465;Yang2025_Silkie_Wenchang	CRA004775	0.749512172
ENSGALG00010023268	KCNJ11	2	-0.064632907	0.142937745	-0.452175228	0.651142753	0	0.570261245	0	1	CRA004775;Yang2025_Silkie_Wenchang	PRJNA827465	0.908769703
ENSGALG00010023268	KCNJ11	2	-0.122513378	0.089835421	-1.363753584	0.172645133	0	0.397215088	0	1	CRA004775;PRJNA827465	Yang2025_Silkie_Wenchang	0.675574638
ENSGALG00010023271	PATL2	2	-0.254229637	0.179791027	-1.414025502	0.157353534	0.020516479	1.442420041	30.67206698	1	PRJNA827465;Yang2025_Silkie_Wenchang	CRA004775	0.650687662
ENSGALG00010023271	PATL2	2	-0.411984451	0.46742038	-0.881400274	0.37810121	0.299819623	2.883570904	65.32077645	1	CRA004775;Yang2025_Silkie_Wenchang	PRJNA827465	0.823285408
ENSGALG00010023271	PATL2	2	-0.509391831	0.22684602	-2.245540084	0.024733482	0.028510002	1.19403123	16.25009676	1	CRA004775;PRJNA827465	Yang2025_Silkie_Wenchang	0.268758652
ENSGALG00010023273	HDAC7	2	-0.146614523	0.082554166	-1.775979694	0.075736264	0.001083755	1.085973236	7.899737547	1	PRJNA827465;Yang2025_Silkie_Wenchang	CRA004775	0.487510429
ENSGALG00010023273	HDAC7	2	-0.240604189	0.107216657	-2.244093373	0.024826394	0	0.009099391	0	1	CRA004775;Yang2025_Silkie_Wenchang	PRJNA827465	0.268876212
ENSGALG00010023273	HDAC7	2	-0.097641124	0.099552925	-0.980796136	0.326693284	0	0.455868301	0	1	CRA004775;PRJNA827465	Yang2025_Silkie_Wenchang	0.800028239
ENSGALG00010023274	ZBTB38	2	-0.028282592	0.195522649	-0.144651231	0.884986246	0.057290099	3.665292703	72.71704933	0.5	PRJNA827465;Yang2025_Silkie_Wenchang	CRA004775	0.972194923
ENSGALG00010023274	ZBTB38	2	0.304408548	0.151591512	2.008084386	0.044634325	0	0.83137183	0	1	CRA004775;Yang2025_Silkie_Wenchang	PRJNA827465	0.364257243
ENSGALG00010023274	ZBTB38	2	0.108538615	0.341282476	0.31803161	0.750460959	0.199232578	6.331037694	84.20480104	0.5	CRA004775;PRJNA827465	Yang2025_Silkie_Wenchang	0.937393444
ENSGALG00010023276	ABCC8	2	0.06173237	0.06852269	0.900904067	0.367639329	0	0.82582702	0	1	PRJNA827465;Yang2025_Silkie_Wenchang	CRA004775	0.819975483
ENSGALG00010023276	ABCC8	2	0.128024983	0.103004031	1.242912252	0.213900134	0	0.085850411	0	1	CRA004775;Yang2025_Silkie_Wenchang	PRJNA827465	0.726593954
ENSGALG00010023276	ABCC8	2	0.011503937	0.086127848	0.133568147	0.893744073	0	0.002683525	0	1	CRA004775;PRJNA827465	Yang2025_Silkie_Wenchang	0.974381724
ENSGALG00010023277	KCNH4	2	-0.159768101	0.274616856	-0.581785486	0.560711181	0	0.052433344	0	1	PRJNA827465;Yang2025_Silkie_Wenchang	CRA004775	0.88130295
ENSGALG00010023277	KCNH4	2	-0.463017194	0.244776484	-1.891591816	0.058545387	0	0.709100074	0	1	CRA004775;Yang2025_Silkie_Wenchang	PRJNA827465	0.419127387
ENSGALG00010023277	KCNH4	2	-0.408585721	0.20737601	-1.970265133	0.048807992	0	0.77856473	0	1	CRA004775;PRJNA827465	Yang2025_Silkie_Wenchang	0.381827333
ENSGALG00010023278	ENSGALG00010023278	2	0.375684596	0.608224477	0.617674248	0.536790091	0.686899894	13.8330696	92.77094652	0.5	PRJNA827465;Yang2025_Silkie_Wenchang	CRA004775	0.874651173
ENSGALG00010023278	ENSGALG00010023278	2	0.437136601	0.630496954	0.693320718	0.488108304	0.641443394	4.919568524	79.6730141	0.5	CRA004775;Yang2025_Silkie_Wenchang	PRJNA827465	0.85768885
ENSGALG00010023278	ENSGALG00010023278	2	-0.222201138	0.171600771	-1.294872608	0.195364179	0	0.009436399	0	1	CRA004775;PRJNA827465	Yang2025_Silkie_Wenchang	0.70643959
ENSGALG00010023279	FA2H	2	0.393060802	0.303587845	1.294718509	0.195417352	0	0.055056438	0	1	PRJNA827465;Yang2025_Silkie_Wenchang	CRA004775	0.70644667
ENSGALG00010023279	FA2H	2	1.172834126	0.822522728	1.425898745	0.153897572	1.177740763	7.493510665	86.65512008	1	CRA004775;Yang2025_Silkie_Wenchang	PRJNA827465	0.646463179
ENSGALG00010023279	FA2H	2	1.209392171	0.748935775	1.614814262	0.106350924	1.007178226	9.746162356	89.73955118	1	CRA004775;PRJNA827465	Yang2025_Silkie_Wenchang	0.553237654
ENSGALG00010023281	B2M	2	0.125082995	0.065070245	1.922276389	0.054570989	0	0.521268188	0	1	PRJNA827465;Yang2025_Silkie_Wenchang	CRA004775	0.404298509
ENSGALG00010023281	B2M	2	-0.07295226	0.340845747	-0.214033066	0.830521271	0.190765801	5.541672473	81.95490612	0.5	CRA004775;Yang2025_Silkie_Wenchang	PRJNA827465	0.957295395
ENSGALG00010023281	B2M	2	-0.117334165	0.253938361	-0.462057661	0.644039971	0.112045078	7.096863904	85.90926903	0.5	CRA004775;PRJNA827465	Yang2025_Silkie_Wenchang	0.906826466
ENSGALG00010023282	SERBP1	2	-0.093228216	0.040113963	-2.324083895	0.020120998	0	0.4030606	0	1	PRJNA827465;Yang2025_Silkie_Wenchang	CRA004775	0.238628722
ENSGALG00010023282	SERBP1	2	-0.140223859	0.060901446	-2.302471755	0.021308582	0	0.160146392	0	1	CRA004775;Yang2025_Silkie_Wenchang	PRJNA827465	0.246901892
ENSGALG00010023282	SERBP1	2	-0.087986197	0.046687309	-1.884584903	0.059485902	0	0.620323609	0	1	CRA004775;PRJNA827465	Yang2025_Silkie_Wenchang	0.422595132
ENSGALG00010023285	SLC48A1	2	-0.039420714	0.112810889	-0.349440678	0.726758499	0.013226603	1.892082569	47.1481839	0.5	PRJNA827465;Yang2025_Silkie_Wenchang	CRA004775	0.93132668
ENSGALG00010023285	SLC48A1	2	-0.157875132	0.134311083	-1.175443818	0.239817202	0	0.24374981	0	1	CRA004775;Yang2025_Silkie_Wenchang	PRJNA827465	0.752057407
ENSGALG00010023285	SLC48A1	2	0.03715187	0.07630571	0.48688191	0.626342019	0	0.072753754	0	0.5	CRA004775;PRJNA827465	Yang2025_Silkie_Wenchang	0.901504244
ENSGALG00010023287	RGS19	2	-0.006463723	0.092489722	-0.069885856	0.944284511	0	0.797554403	0	0.5	PRJNA827465;Yang2025_Silkie_Wenchang	CRA004775	0.985873665
ENSGALG00010023287	RGS19	2	-0.129832847	0.353032676	-0.36776439	0.713048924	0.156023876	2.154631816	53.58835823	0.5	CRA004775;Yang2025_Silkie_Wenchang	PRJNA827465	0.926138858
ENSGALG00010023287	RGS19	2	-0.164168461	0.219083477	-0.749342046	0.453651071	0.044428995	1.343542265	25.56988894	1	CRA004775;PRJNA827465	Yang2025_Silkie_Wenchang	0.849044791
ENSGALG00010023289	NFRKB	2	-0.11276488	0.04915338	-2.294142972	0.021782288	0	0.458724623	0	1	PRJNA827465;Yang2025_Silkie_Wenchang	CRA004775	0.249990082
ENSGALG00010023289	NFRKB	2	-0.180163349	0.086940079	-2.072270366	0.038240234	0	0.085850793	0	1	CRA004775;Yang2025_Silkie_Wenchang	PRJNA827465	0.337384449
ENSGALG00010023289	NFRKB	2	-0.102934808	0.055214147	-1.864283239	0.062281939	0	0.438217858	0	1	CRA004775;PRJNA827465	Yang2025_Silkie_Wenchang	0.430309306
ENSGALG00010023293	COL11A1	2	0.649452117	0.459524599	1.413313058	0.157563696	0.29481726	3.277610006	69.48996378	1	PRJNA827465;Yang2025_Silkie_Wenchang	CRA004775	0.650796198
ENSGALG00010023293	COL11A1	2	0.163442518	1.033459844	0.158150816	0.874337959	1.86580585	7.739238473	87.07883206	0.5	CRA004775;Yang2025_Silkie_Wenchang	PRJNA827465	0.970135804
ENSGALG00010023293	COL11A1	2	-0.226206052	0.559965279	-0.403964426	0.686238879	0.41182674	2.659123433	62.39362236	0.5	CRA004775;PRJNA827465	Yang2025_Silkie_Wenchang	0.919235539
ENSGALG00010023295	ENSGALG00010023295	2	0.279358628	0.426015254	0.655747948	0.511986313	0.096032181	1.358280247	26.37749079	0.5	PRJNA827465;Yang2025_Silkie_Wenchang	CRA004775	0.865694231
ENSGALG00010023295	ENSGALG00010023295	2	1.521522766	0.915388517	1.662160642	0.096480569	1.293515828	4.273381273	76.59932648	1	CRA004775;Yang2025_Silkie_Wenchang	PRJNA827465	0.530885356
ENSGALG00010023295	ENSGALG00010023295	2	1.127645444	1.344704463	0.838582362	0.401703712	3.205834443	8.723247127	88.53637888	0.5	CRA004775;PRJNA827465	Yang2025_Silkie_Wenchang	0.832689704
ENSGALG00010023299	PASK	2	-0.177507353	0.085307256	-2.080800179	0.0374522	0	0.741058181	0	1	PRJNA827465;Yang2025_Silkie_Wenchang	CRA004775	0.333858881
ENSGALG00010023299	PASK	2	-0.239863638	0.153667038	-1.560930963	0.118540041	0	0.852909422	0	0.5	CRA004775;Yang2025_Silkie_Wenchang	PRJNA827465	0.578415575
ENSGALG00010023299	PASK	2	-0.120407761	0.095962894	-1.254732482	0.20957589	0	0.306288084	0	0.5	CRA004775;PRJNA827465	Yang2025_Silkie_Wenchang	0.721401401
ENSGALG00010023304	ENSGALG00010023304	2	0.040243965	0.080549147	0.499620004	0.617342669	0	0.000421718	0	1	PRJNA827465;Yang2025_Silkie_Wenchang	CRA004775	0.899121942
ENSGALG00010023304	ENSGALG00010023304	2	-0.29716821	0.392846025	-0.756449579	0.449379692	0.238384991	4.042785677	75.26458042	0.5	CRA004775;Yang2025_Silkie_Wenchang	PRJNA827465	0.847263197
ENSGALG00010023304	ENSGALG00010023304	2	-0.283451526	0.392752693	-0.721704855	0.470475956	0.251159913	4.656500018	78.52464305	0.5	CRA004775;PRJNA827465	Yang2025_Silkie_Wenchang	0.853744788
ENSGALG00010023305	PLCB2	2	-0.153947202	0.339755766	-0.453111374	0.650468546	0.206999681	9.548483744	89.52713303	0.5	PRJNA827465;Yang2025_Silkie_Wenchang	CRA004775	0.908618725
ENSGALG00010023305	PLCB2	2	0.145038477	0.16240504	0.893066359	0.371821637	0	0.43243072	0	0.5	CRA004775;Yang2025_Silkie_Wenchang	PRJNA827465	0.821597118
ENSGALG00010023305	PLCB2	2	-0.409826508	0.156334236	-2.621476385	0.008754983	0.014212976	1.197278465	16.47724154	1	CRA004775;PRJNA827465	Yang2025_Silkie_Wenchang	0.146797439
ENSGALG00010023309	PIK3R3	2	-0.182873113	0.216448759	-0.84487947	0.398178138	0.083373534	9.064666576	88.96815463	0.5	PRJNA827465;Yang2025_Silkie_Wenchang	CRA004775	0.831980453
ENSGALG00010023309	PIK3R3	2	-0.395705372	0.102762737	-3.850669857	0.000117795	0	0.044497082	0	1	CRA004775;Yang2025_Silkie_Wenchang	PRJNA827465	0.008702453
ENSGALG00010023309	PIK3R3	2	0.010426431	0.093517507	0.111491758	0.911226401	0	0.779172266	0	0.5	CRA004775;PRJNA827465	Yang2025_Silkie_Wenchang	0.978845851
ENSGALG00010023311	ETS1	2	-0.053044263	0.125021523	-0.424281048	0.671360859	0.025757825	5.674509951	82.37733287	0.5	PRJNA827465;Yang2025_Silkie_Wenchang	CRA004775	0.914674175
ENSGALG00010023311	ETS1	2	-0.181590567	0.069200826	-2.624109806	0.008687577	0	0.121319345	0	1	CRA004775;Yang2025_Silkie_Wenchang	PRJNA827465	0.14605031
ENSGALG00010023311	ETS1	2	0.021307103	0.12769167	0.166863687	0.867477306	0.013082729	1.248480051	19.90260485	0.5	CRA004775;PRJNA827465	Yang2025_Silkie_Wenchang	0.967862205
ENSGALG00010023312	UBR3	2	-0.071766007	0.043174034	-1.662249293	0.0964628	0	0.701334228	0	1	PRJNA827465;Yang2025_Silkie_Wenchang	CRA004775	0.530885356
ENSGALG00010023312	UBR3	2	0.189443922	0.269807427	0.702144948	0.482588774	0.099687284	2.541467027	60.65264709	0.5	CRA004775;Yang2025_Silkie_Wenchang	PRJNA827465	0.85691916
ENSGALG00010023312	UBR3	2	0.152571211	0.319389101	0.477696986	0.63286589	0.158544059	3.587450269	72.12504913	0.5	CRA004775;PRJNA827465	Yang2025_Silkie_Wenchang	0.903112631
ENSGALG00010023317	RAPGEF3	2	0.210391887	0.071014093	2.962677937	0.003049755	0	0.09016036	0	1	PRJNA827465;Yang2025_Silkie_Wenchang	CRA004775	0.076007935
ENSGALG00010023317	RAPGEF3	2	0.132154365	0.142147867	0.929696429	0.352528284	0	0.391643409	0	0.5	CRA004775;Yang2025_Silkie_Wenchang	PRJNA827465	0.810970286
ENSGALG00010023317	RAPGEF3	2	0.20786716	0.078199983	2.658148413	0.007857128	0	0.641686436	0	0.5	CRA004775;PRJNA827465	Yang2025_Silkie_Wenchang	0.137569534
ENSGALG00010023319	EIF2AK4	2	0.137933063	0.100592321	1.371208671	0.170309916	0.011392726	2.104502943	52.48284147	1	PRJNA827465;Yang2025_Silkie_Wenchang	CRA004775	0.672114378
ENSGALG00010023319	EIF2AK4	2	0.166234663	0.139243506	1.193841401	0.232540013	0.014032138	1.494423421	33.08456052	0.5	CRA004775;Yang2025_Silkie_Wenchang	PRJNA827465	0.745353136
ENSGALG00010023319	EIF2AK4	2	0.052354784	0.064708651	0.809084776	0.418466384	0	0.151110038	0	0.5	CRA004775;PRJNA827465	Yang2025_Silkie_Wenchang	0.838158675
ENSGALG00010023320	ENSGALG00010023320	2	-0.235615021	0.224027523	-1.051723545	0.292926405	0.093079446	13.48863476	92.58635127	1	PRJNA827465;Yang2025_Silkie_Wenchang	CRA004775	0.786774237
ENSGALG00010023320	ENSGALG00010023320	2	-0.374086514	0.162122895	-2.30742558	0.021031107	0.023813047	1.522528209	34.31977194	1	CRA004775;Yang2025_Silkie_Wenchang	PRJNA827465	0.245277621
ENSGALG00010023320	ENSGALG00010023320	2	-0.023428616	0.05785608	-0.404946492	0.685516843	0	0.06918555	0	1	CRA004775;PRJNA827465	Yang2025_Silkie_Wenchang	0.919235539
ENSGALG00010023322	PRKAB1	2	-0.015718159	0.053462589	-0.294002948	0.768755651	0	0.526195542	0	0.5	PRJNA827465;Yang2025_Silkie_Wenchang	CRA004775	0.941623403
ENSGALG00010023322	PRKAB1	2	-0.126148228	0.095906635	-1.31532326	0.188401284	0	0.605088467	0	1	CRA004775;Yang2025_Silkie_Wenchang	PRJNA827465	0.697745174
ENSGALG00010023322	PRKAB1	2	-0.060982091	0.115884593	-0.526231223	0.598727563	0.014160048	1.65487494	39.57247306	0.5	CRA004775;PRJNA827465	Yang2025_Silkie_Wenchang	0.894762943
ENSGALG00010023328	TCEA2	2	0.198950886	0.056501017	3.521191269	0.000429613	0	0.650692002	0	1	PRJNA827465;Yang2025_Silkie_Wenchang	CRA004775	0.021075798
ENSGALG00010023328	TCEA2	2	0.063133957	0.114933345	0.549309316	0.582793195	0	0.675666685	0	0.5	CRA004775;Yang2025_Silkie_Wenchang	PRJNA827465	0.890122448
ENSGALG00010023328	TCEA2	2	0.137940433	0.152201608	0.906300757	0.364776685	0.026185338	1.67486627	40.29374057	0.5	CRA004775;PRJNA827465	Yang2025_Silkie_Wenchang	0.817649605
ENSGALG00010023331	CD4	2	-0.304384852	0.285864465	-1.064787301	0.286972193	0	0.655588188	0	1	PRJNA827465;Yang2025_Silkie_Wenchang	CRA004775	0.782548262
ENSGALG00010023331	CD4	2	-0.598926625	0.350016921	-1.711136203	0.087055972	0	0.070201419	0	1	CRA004775;Yang2025_Silkie_Wenchang	PRJNA827465	0.507587624
ENSGALG00010023331	CD4	2	-0.254145779	0.342959655	-0.741036956	0.458671033	0	0.738695434	0	1	CRA004775;PRJNA827465	Yang2025_Silkie_Wenchang	0.850089963
ENSGALG00010023332	PPP1R7	2	-0.082480519	0.049298871	-1.673071148	0.094313291	0	0.648012495	0	1	PRJNA827465;Yang2025_Silkie_Wenchang	CRA004775	0.525935763
ENSGALG00010023332	PPP1R7	2	0.01380947	0.098856538	0.139692025	0.888903327	0	0.105071427	0	0.5	CRA004775;Yang2025_Silkie_Wenchang	PRJNA827465	0.973135755
ENSGALG00010023332	PPP1R7	2	-0.091447723	0.053359144	-1.713815416	0.086562631	0	0.633588894	0	0.5	CRA004775;PRJNA827465	Yang2025_Silkie_Wenchang	0.506111562
ENSGALG00010023333	ENDOU	2	0.292002893	0.547131579	0.53369775	0.593550666	0.367974762	2.5782323	61.21373548	0.5	PRJNA827465;Yang2025_Silkie_Wenchang	CRA004775	0.892915939
ENSGALG00010023333	ENDOU	2	-0.301515006	0.431085964	-0.699431276	0.484282548	0	0.001279048	0	1	CRA004775;Yang2025_Silkie_Wenchang	PRJNA827465	0.857004733
ENSGALG00010023333	ENDOU	2	0.415313793	0.537127468	0.773212724	0.439396497	0.24571351	1.626250388	38.50885401	0.5	CRA004775;PRJNA827465	Yang2025_Silkie_Wenchang	0.844199375
ENSGALG00010023334	RPS17	2	0.318713775	0.393504998	0.809935774	0.41797709	0.2937844	18.92414239	94.71574468	0.5	PRJNA827465;Yang2025_Silkie_Wenchang	CRA004775	0.838112595
ENSGALG00010023334	RPS17	2	0.218855024	0.522875551	0.418560446	0.675537402	0.504061793	12.68975933	92.1196299	0.5	CRA004775;Yang2025_Silkie_Wenchang	PRJNA827465	0.91647357
ENSGALG00010023334	RPS17	2	-0.08540957	0.077540671	-1.101480921	0.270687404	0.00252307	1.081687417	7.551850559	1	CRA004775;PRJNA827465	Yang2025_Silkie_Wenchang	0.772667394
ENSGALG00010023335	WDR59	2	-0.093047653	0.049814956	-1.867865805	0.061780778	0.000345861	1.05963421	5.627810977	1	PRJNA827465;Yang2025_Silkie_Wenchang	CRA004775	0.429075295
ENSGALG00010023335	WDR59	2	-0.268632984	0.265841041	-1.010502303	0.312254697	0.127371315	9.968808076	89.96871048	1	CRA004775;Yang2025_Silkie_Wenchang	PRJNA827465	0.796054997
ENSGALG00010023335	WDR59	2	-0.314843987	0.209678042	-1.501559172	0.133210994	0.077219568	7.711352696	87.03210657	1	CRA004775;PRJNA827465	Yang2025_Silkie_Wenchang	0.611633901
ENSGALG00010023336	GPR162	2	0.036338934	0.077860128	0.466720711	0.64069971	0	0.019609401	0	1	PRJNA827465;Yang2025_Silkie_Wenchang	CRA004775	0.906328128
ENSGALG00010023336	GPR162	2	-0.009009518	0.125880989	-0.071571716	0.94294275	0	0.115463072	0	0.5	CRA004775;Yang2025_Silkie_Wenchang	PRJNA827465	0.985827325
ENSGALG00010023336	GPR162	2	0.027230836	0.084914067	0.320686987	0.74844761	0	0.224752083	0	0.5	CRA004775;PRJNA827465	Yang2025_Silkie_Wenchang	0.937249951
ENSGALG00010023337	FLI1	2	0.047264127	0.059489558	0.794494498	0.426907611	0	0.362291293	0	1	PRJNA827465;Yang2025_Silkie_Wenchang	CRA004775	0.84125685
ENSGALG00010023337	FLI1	2	0.085829362	0.128129465	0.669864357	0.502944263	0	0.679998299	0	0.5	CRA004775;Yang2025_Silkie_Wenchang	PRJNA827465	0.863050365
ENSGALG00010023337	FLI1	2	0.022942401	0.065487867	0.350330567	0.726090628	0	0.411793589	0	0.5	CRA004775;PRJNA827465	Yang2025_Silkie_Wenchang	0.931029023
ENSGALG00010023338	HDLBP	2	-0.163154491	0.126818128	-1.28652341	0.198260454	0.029170662	10.70181779	90.65579307	1	PRJNA827465;Yang2025_Silkie_Wenchang	CRA004775	0.710234722

ENSGALG00010023338	HDLBP	2	-0.16505522	0.155285015	-1.062917891	0.287819189	0.036713494	3.732941223	73.21147213	0.5	CRA004775;Yang2025_Silkie_Wenchang	PRJNA827465	0.782840067
ENSGALG00010023338	HDLBP	2	-0.032852156	0.046578928	-0.705300817	0.480623052	0	0.154849827	0	0.5	CRA004775;PRJNA827465	Yang2025_Silkie_Wenchang	0.856722567
ENSGALG00010023340	P3H3	2	0.388248082	0.311687513	1.245632456	0.212899361	0.18480642	20.40753105	95.0998482	1	PRJNA827465;Yang2025_Silkie_Wenchang	CRA004775	0.725659838
ENSGALG00010023340	P3H3	2	0.328584401	0.440712473	0.745575452	0.455923917	0.323107203	5.281502659	81.06599457	0.5	CRA004775;Yang2025_Silkie_Wenchang	PRJNA827465	0.84907427
ENSGALG00010023340	P3H3	2	0.067382969	0.082095823	0.820784377	0.411769099	0	0.496939785	0	0.5	CRA004775;PRJNA827465	Yang2025_Silkie_Wenchang	0.835204671
ENSGALG00010023342	FITM2	2	-0.185679866	0.075479085	-2.460017456	0.013893026	0	0.158835202	0	1	PRJNA827465;Yang2025_Silkie_Wenchang	CRA004775	0.19354185
ENSGALG00010023342	FITM2	2	-0.211768817	0.124824658	-1.696530325	0.089785492	0	0.106051161	0	1	CRA004775;Yang2025_Silkie_Wenchang	PRJNA827465	0.514108275
ENSGALG00010023342	FITM2	2	-0.163427253	0.084744168	-1.928477875	0.053795714	0	0.012856362	0	1	CRA004775;PRJNA827465	Yang2025_Silkie_Wenchang	0.401964436
ENSGALG00010023343	TMEM233	2	-0.109585797	0.213748662	-0.512685302	0.608171473	0.046663864	1.792135058	44.20063401	0.5	PRJNA827465;Yang2025_Silkie_Wenchang	CRA004775	0.897204334
ENSGALG00010023343	TMEM233	2	0.0533489	0.138060099	0.38641795	0.699187158	0	0.267609855	0	1	CRA004775;Yang2025_Silkie_Wenchang	PRJNA827465	0.92185597
ENSGALG00010023343	TMEM233	2	-0.195663508	0.351359254	-0.556875921	0.577612207	0.077780897	1.356245776	26.26705147	0.5	CRA004775;PRJNA827465	Yang2025_Silkie_Wenchang	0.887312588
ENSGALG00010023347	STAT5A	2	-0.150010223	0.067508277	-2.222101206	0.026276467	0	0.082835868	0	1	PRJNA827465;Yang2025_Silkie_Wenchang	CRA004775	0.276356429
ENSGALG00010023347	STAT5A	2	-0.155846826	0.134467521	-1.158992339	0.246459308	0	0.443541379	0	1	CRA004775;Yang2025_Silkie_Wenchang	PRJNA827465	0.756843363
ENSGALG00010023347	STAT5A	2	-0.170164684	0.073331176	-2.32049578	0.020314073	0	0.320562972	0	1	CRA004775;PRJNA827465	Yang2025_Silkie_Wenchang	0.240053022
ENSGALG00010023349	ENSGALG00010023349	2	0.715802385	0.904986869	0.790953338	0.428971221	1.346249021	5.341811512	81.27975879	0.5	PRJNA827465;Yang2025_Silkie_Wenchang	CRA004775	0.841898328
ENSGALG00010023349	ENSGALG00010023349	2	1.92709388	0.52158086	3.694717401	0.000220131	0	0.19916797	0	1	CRA004775;Yang2025_Silkie_Wenchang	PRJNA827465	0.013550335
ENSGALG00010023349	ENSGALG00010023349	2	0.940143408	1.139692302	0.824909852	0.409422773	2.241856166	6.924895766	85.55934943	0.5	CRA004775;PRJNA827465	Yang2025_Silkie_Wenchang	0.83440722
ENSGALG00010023350	ZNRF1	2	0.110093675	0.094243696	1.168180783	0.24273385	0.007174268	1.601126659	37.54397916	1	PRJNA827465;Yang2025_Silkie_Wenchang	CRA004775	0.7550024
ENSGALG00010023350	ZNRF1	2	0.289532377	0.131126718	2.208034957	0.027241836	0.003298255	1.052354132	4.974953854	1	CRA004775;Yang2025_Silkie_Wenchang	PRJNA827465	0.281669893
ENSGALG00010023350	ZNRF1	2	0.226849436	0.264518974	0.857592303	0.391117627	0.098663588	2.70318549	63.00660818	1	CRA004775;PRJNA827465	Yang2025_Silkie_Wenchang	0.829251057
ENSGALG00010023351	GNB3	2	0.473568234	0.323562943	1.463604669	0.143301996	0.113757452	2.127217685	52.99023663	1	PRJNA827465;Yang2025_Silkie_Wenchang	CRA004775	0.630352362
ENSGALG00010023351	GNB3	2	-0.156202269	1.042902801	-0.14977644	0.880940998	1.970758133	10.52352089	90.49747693	0.5	CRA004775;Yang2025_Silkie_Wenchang	PRJNA827465	0.971685305
ENSGALG00010023351	GNB3	2	-0.444429664	0.712013126	-0.624187459	0.532504482	0.851656296	5.916170049	83.0971728	0.5	CRA004775;PRJNA827465	Yang2025_Silkie_Wenchang	0.873183244
ENSGALG00010023354	SEPTIN2	2	-0.260311398	0.325250398	-0.80034152	0.423512953	0.198543416	16.19982471	93.8270937	0.5	PRJNA827465;Yang2025_Silkie_Wenchang	CRA004775	0.84030043
ENSGALG00010023354	SEPTIN2	2	-0.583782292	0.121339422	-4.811151064	1.50064E-06	0	0.037529867	0	1	CRA004775;Yang2025_Silkie_Wenchang	PRJNA827465	0.000307185
ENSGALG00010023354	SEPTIN2	2	0.021858571	0.141209294	0.154795552	0.876982498	0.01093607	1.07870879	7.29657443	0.5	CRA004775;PRJNA827465	Yang2025_Silkie_Wenchang	0.970493387
ENSGALG00010023356	ENSGALG00010023356	2	0.046067264	0.120530112	0.382205437	0.70230899	0	0.262109057	0	0.5	PRJNA827465;Yang2025_Silkie_Wenchang	CRA004775	0.923141374
ENSGALG00010023356	ENSGALG00010023356	2	0.13651983	0.164361176	0.830608744	0.406194694	0	0.025256992	0	1	CRA004775;Yang2025_Silkie_Wenchang	PRJNA827465	0.833477131
ENSGALG00010023356	ENSGALG00010023356	2	0.029896547	0.14069842	0.212486727	0.831727331	0	0.263510646	0	0.5	CRA004775;PRJNA827465	Yang2025_Silkie_Wenchang	0.957712669
ENSGALG00010023359	CDCA3	2	-0.037852427	0.076858229	-0.492496738	0.622368227	0	0.119903028	0	1	PRJNA827465;Yang2025_Silkie_Wenchang	CRA004775	0.900093208
ENSGALG00010023359	CDCA3	2	0.052476495	0.268228947	0.195640687	0.844891412	0.047208012	1.35255546	26.06587772	0.5	CRA004775;Yang2025_Silkie_Wenchang	PRJNA827465	0.960769014
ENSGALG00010023359	CDCA3	2	0.028739778	0.162177613	0.177211748	0.859342068	0.022888125	1.205579288	17.05232415	0.5	CRA004775;PRJNA827465	Yang2025_Silkie_Wenchang	0.894606195
ENSGALG00010023362	GADD45A	2	0.080334709	0.103590472	0.775502876	0.438042566	0.004682073	1.260107529	20.6416931	0.5	PRJNA827465;Yang2025_Silkie_Wenchang	CRA004775	0.843965988
ENSGALG00010023362	GADD45A	2	-0.064202923	0.140382073	-0.45734417	0.647423687	0	0.050035657	0	1	CRA004775;Yang2025_Silkie_Wenchang	PRJNA827465	0.907350807
ENSGALG00010023362	GADD45A	2	0.135129675	0.107672456	1.2550069	0.209476255	0	0.636315601	0	0.5	CRA004775;PRJNA827465	Yang2025_Silkie_Wenchang	0.721386257
ENSGALG00010023363	CPEB1	2	0.226346125	0.123113062	1.838522419	0.065985461	0	0.424354612	0	1	PRJNA827465;Yang2025_Silkie_Wenchang	CRA004775	0.44241327
ENSGALG00010023363	CPEB1	2	0.053857689	0.392707066	0.13714469	0.890919429	0.189528733	2.476526662	59.62086678	0.5	CRA004775;Yang2025_Silkie_Wenchang	PRJNA827465	0.973604571
ENSGALG00010023363	CPEB1	2	0.022910187	0.266376522	0.086006781	0.931461027	0.078292768	1.774147451	43.63489915	0.5	CRA004775;PRJNA827465	Yang2025_Silkie_Wenchang	0.984076328
ENSGALG00010023366	GDAP1L1	2	0.710455618	0.527212722	1.347569185	0.177796987	0.509770515	12.0446649	91.69756894	1	PRJNA827465;Yang2025_Silkie_Wenchang	CRA004775	0.683951879
ENSGALG00010023366	GDAP1L1	2	0.425086573	0.915912748	0.464112519	0.642567133	1.451278349	6.753397232	85.19263764	0.5	CRA004775;Yang2025_Silkie_Wenchang	PRJNA827465	0.906634523
ENSGALG00010023366	GDAP1L1	2	0.044855695	0.297908285	0.150568807	0.880315869	0.057609326	1.225451462	18.3974208	0.5	CRA004775;PRJNA827465	Yang2025_Silkie_Wenchang	0.971592092
ENSGALG00010023368	ENSGALG00010023368	2	1.136747048	0.683211457	1.663829019	0.096146603	0.807067686	7.358347343	86.4099919	1	PRJNA827465;Yang2025_Silkie_Wenchang	CRA004775	0.530364903
ENSGALG00010023368	ENSGALG00010023368	2	-0.138818153	2.021699797	-0.068664078	0.945257012	7.835979392	23.82538094	95.80279534	0.5	CRA004775;Yang2025_Silkie_Wenchang	PRJNA827465	0.986321197
ENSGALG00010023368	ENSGALG00010023368	2	-0.789861785	1.336467172	-0.591007248	0.554515565	3.262372448	11.10103678	90.99183239	0.5	CRA004775;PRJNA827465	Yang2025_Silkie_Wenchang	0.880223402
ENSGALG00010023369	ACE	2	-0.219039152	0.210230152	-1.041901696	0.297457258	0.064888613	3.748879002	73.32535941	1	PRJNA827465;Yang2025_Silkie_Wenchang	CRA004775	0.788562929
ENSGALG00010023369	ACE	2	-0.189467124	0.347410785	-0.545369148	0.585499655	0.155121188	2.343613661	57.33085122	0.5	CRA004775;Yang2025_Silkie_Wenchang	PRJNA827465	0.891139408
ENSGALG00010023369	ACE	2	0.013087313	0.136859164	0.095626137	0.923817506	0	0.440923248	0	0.5	CRA004775;PRJNA827465	Yang2025_Silkie_Wenchang	0.982219519
ENSGALG00010023374	ENSGALG00010023374	2	0.153435269	0.26282886	0.583783949	0.559365679	0.071234771	1.989434951	49.7344711	0.5	PRJNA827465;Yang2025_Silkie_Wenchang	CRA004775	0.881050436
ENSGALG00010023374	ENSGALG00010023374	2	0.342709426	0.271237005	1.263501226	0.206409106	0	0.652610564	0	0.5	CRA004775;Yang2025_Silkie_Wenchang	PRJNA827465	0.718451597
ENSGALG00010023374	ENSGALG00010023374	2	-0.059275056	0.197304447	-0.300424326	0.76385351	0	0.00337199	0	1	CRA004775;PRJNA827465	Yang2025_Silkie_Wenchang	0.940361611
ENSGALG00010023375	ST14	2	0.053696685	0.081095393	0.662124234	0.507880071	0	0.088534012	0	1	PRJNA827465;Yang2025_Silkie_Wenchang	CRA004775	0.864401731
ENSGALG00010023375	ST14	2	0.147333356	0.180729295	0.815215686	0.41494886	0.03339919	1.747249647	42.76719405	1	CRA004775;Yang2025_Silkie_Wenchang	PRJNA827465	0.837458421
ENSGALG00010023375	ST14	2	0.160451315	0.147742974	1.086016546	0.277471672	0.015455489	1.345515129	25.67902221	1	CRA004775;PRJNA827465	Yang2025_Silkie_Wenchang	0.776687495
ENSGALG00010023377	OSER1	2	0.030698089	0.053621456	0.572496368	0.566998745	0	0.087026748	0	1	PRJNA827465;Yang2025_Silkie_Wenchang	CRA004775	0.883487517
ENSGALG00010023377	OSER1	2	0.085079225	0.122674598	0.693535795	0.48797337	0	0.109842839	0	1	CRA004775;Yang2025_Silkie_Wenchang	PRJNA827465	0.85768885
ENSGALG00010023377	OSER1	2	0.029328115	0.057422569	0.510741951	0.609531761	0	0.246525547	0	1	CRA004775;PRJNA827465	Yang2025_Silkie_Wenchang	0.897238578
ENSGALG00010023380	USP5	2	0.057973735	0.055189888	1.050441248	0.29351529	0	0.14399978	0	1	PRJNA827465;Yang2025_Silkie_Wenchang	CRA004775	0.786979185
ENSGALG00010023380	USP5	2	-0.177440605	0.225769558	-0.785936806	0.43190451	0.068406382	2.856503495	64.99216606	0.5	CRA004775;Yang2025_Silkie_Wenchang	PRJNA827465	0.843250929
ENSGALG00010023380	USP5	2	-0.139725848	0.252474186	-0.553426272	0.579971553	0.102972373	4.480593663	77.68152894	0.5	CRA004775;PRJNA827465	Yang2025_Silkie_Wenchang	0.888194434
ENSGALG00010023381	HOXB7	2	0.100778336	0.083574409	1.205851612	0.227874711	0	0.165438016	0	1	PRJNA827465;Yang2025_Silkie_Wenchang	CRA004775	0.740853612
ENSGALG00010023381	HOXB7	2	0.126052412	0.126502764	0.996438966	0.319036411	0	0.118783206	0	1	CRA004775;Yang2025_Silkie_Wenchang	PRJNA827465	0.798079048
ENSGALG00010023381	HOXB7	2	0.070548153	0.09883357	0.713807593	0.475346164	0	0.01905396	0	1	CRA004775;PRJNA827465	Yang2025_Silkie_Wenchang	0.854462864
ENSGALG00010023383	HOXB6	2	0.229693238	0.133565738	1.719701792	0.085486662	0.022659351	2.710771333	63.11013076	1	PRJNA827465;Yang2025_Silkie_Wenchang	CRA004775	0.502675386
ENSGALG00010023383	HOXB6	2	0.3528401	0.119887982	2.943081475	0.003249629	0	0.268351771	0	1	CRA004775;Yang2025_Silkie_Wenchang	PRJNA827465	0.079280051
ENSGALG00010023383	HOXB6	2	0.111080491	0.098281049	1.130233064	0.258378011	0	0.030558062	0	1	CRA004775;PRJNA827465	Yang2025_Silkie_Wenchang	0.765653331
ENSGALG00010023384	AP3B2	2	0.323068415	0.26018602	1.241682449	0.214353748	0.094619679	3.087342597	67.60968475	1	PRJNA827465;Yang2025_Silkie_Wenchang	CRA004775	0.727148745
ENSGALG00010023384	AP3B2	2	0.237881489	0.400465311	0.59401272	0.552503605	0.247752799	4.393385181	77.23850838	0.5	CRA004775;Yang2025_Silkie_Wenchang	PRJNA827465	0.879228216
ENSGALG00010023384	AP3B2	2	0.049116391	0.125927279	0.390037734	0.696508644	0	0.77398225	0	0.5	CRA004775;PRJNA827465	Yang2025_Silkie_Wenchang	0.921041503
ENSGALG00010023387	ENSGALG00010023387	2	-0.126844441	0.072556988	-2.972621197	0.002952686	0	0.003006356	0	1	PRJNA827465;Yang2025_Silkie_Wenchang	CRA004775	0.07952399
ENSGALG00010023387	ENSGALG00010023387	2	-0.213132715	0.110440554	-1.929841046	0.053626536	0	0.101141469	0	1	CRA004775;Yang2025_Silkie_Wenchang	PRJNA827465	0.401616567
ENSGALG00010023387	ENSGALG00010023387	2	-0.207372971	0.093359885	-2.221221369	0.026335972	0	0.093231415	0	1	CRA004775;PRJNA827465	Yang2025_Silkie_Wenchang	0.276760077
ENSGALG00010023388	FARP2	2	-0.090240519	0.112401355	-0.802841919	0.42206611	0.015776294	2.527009312	60.42753007	0.5	PRJNA827465;Yang2025_Silkie_Wenchang	CRA004775	0.839649223
ENSGALG00010023388	FARP2	2	-0.211153335	0.114183357	-1.849247918	0.064422022	0	0.096818152	0	1	CRA004775;Yang2025_Silkie_Wenchang	PRJNA827465	0.437453135
ENSGALG00010023388	FARP2	2	-0.003134021	0.07310724	-0.042868812	0.96580611	0	0.145425757	0	0.5	CRA004775;PRJNA827465	Yang2025_Silkie_Wenchang	0.990731137
ENSGALG00010023389	GNG12	2	0.001489512	0.129584879	0.011494493	0.990828924	0.030159071	9.578327143	89.55976357	0.5	PRJNA827465;Yang2025_Silkie_Wenchang	CRA004775	0.997555603
ENSGALG00010023389	GNG12	2	-0.126268385	0.069213354	-1.824335359	0.068101353	0	0.162098536	0	1	CRA004775;Yang2025_Silkie_Wenchang	PRJNA827465	0.450073563
ENSGALG00010023389	GNG12	2	0.11936198	0.041414592	2.882123784	0.003950045	0	0.471688002	0	0.5	CRA004775;PRJNA827465	Yang2025_Silkie_Wenchang	0.090629672
ENSGALG00010023390	HSPB8	2	-0.07691817	0.105632016	-0.728170989	0.466508922	0.002280155	1.112102295	10.08021437	0.5	PRJNA827465;Yang2025_Silkie_Wenchang	CRA004775	0.852792382
ENSGALG00010023390	HSPB8	2	0.289545914	0.415767261	0.696413453	0.48616994	0.211300414	1.896871159	47.2816066	1	CRA004775;Yang2025_Silkie_Wenchang	PRJNA827465	0.85758885
ENSGALG00010023390	HSPB8	2	0.222241158	0.548410436	0.405246042	0.685296665	0.43762796	2.878428254	65.25881795	0.5	CRA004775;PRJNA827465	Yang2025_Silkie_Wenchang	0.919325539
ENSGALG00010023392	TPI1	2	-0.327127565	0.208472008	-1.569167816	0.116608841	0.061613553	3.226263997	69.00439639	1	PRJNA827465;Yang2025_Silkie_Wenchang	CRA004775	0.57477217
ENSGALG00010023392	TPI1	2	-0.509218542	0.194352812	-2.620072936	0.008791096	0	0.910536404	0	0.5	CRA004775;Yang2025_Silkie_Wenchang	PRJNA827465	0.147090871
ENSGALG00010023392	TPI1	2	-0.143829031	0.11256379	-1.277755749	0.201335558	0	0.088762377	0	0.5	CRA004775;PRJNA827465	Yang2025_Silkie_Wenchang	0.714177928
ENSGALG00010023394	HOXB5	2	0.347070559	0.416214675	0.833873911	0.40435204	0.320456804	13.30781465	92.48561821	0.5	PRJNA827465;Yang2025_Silkie_Wenchang	CRA004775	0.833112102
ENSGALG00010023394	HOXB5	2	0.647597214	0.214375416	3.020855783	0.002520614	0.031222018	1.324653515	24.50856101	1	CRA004775;Yang2025_Silkie_Wenchang	PRJNA827465	0.067541822
ENSGALG00010023394	HOXB5	2	-0.025031013	0.142350002	-0.175841325	0.860418603	0	0.575695082	0	0.5	CRA004775;PRJNA827465	Yang2025_Silkie_Wenchang	0.964999892
ENSGALG00010023396	ENSGALG00010023396	2	0.304821824	0.142668906	2.136568034	0.032631342	0	0.874072665	0	1	PRJNA827465;Yang2025_Silkie_Wenchang	CRA004775	0.310251664
ENSGALG00010023396	ENSGALG00010023396	2	0.439523157	0.203501237	2.159805822	0.030787705	0	0.107253606	0	1	CRA004775;Yang2025_Silkie_Wenchang	PRJNA827465	0.299739698
ENSGALG00010023396	ENSGALG00010023396	2	0.219217883	0.166180891	1.319152166	0.187118246	0	0.065409672	0	1	CRA004775;PRJNA827465	Yang2025_Silkie_Wenchang	0.695933761
ENSGALG00010023397	ENSGALG00010023397	2	0.334166757	0.646815814	0.516633561	0.605411983	0.784107697	15.89130772	93.70725168	0.5	PRJNA827465;Yang2025_Silkie_Wenchang	CRA004775	0.896474603
ENSGALG00010023397	ENSGALG00010023397	2	0.661217744	0.391543936	1.68874469	0.091268367	0.200762514	2.711208266	63.11607587	1	CRA004775;Yang2025_Silkie_Wenchang	PRJNA827465	0.51792097
ENSGALG00010023397	ENSGALG00010023397	2	-0.19170471	0.210999147	-0.908556802	0.363584118	0.009969923	1.088181786	8.103589607	0.5	CRA004775;PRJNA827465	Yang2025_Silkie_Wenchang	0.817042253
ENSGALG00010023401	LRRC23	2	0.125008947	0.222168413	0.56267651	0.573655182	0	0.561443837	0	0.5	PRJNA827465;Yang2025_Silkie_Wenchang	CRA004775	0.886012875
ENSGALG00010023401	LRRC23	2	0.203404313	0.263362008	0.772337343	0.439914654	0	0.280778027	0	1	CRA004775;Yang2025_Silkie_Wenchang	PRJNA827465	0.844353254
ENSGALG00010023401	LRRC23	2	-0.005062838	0.24236441	-0.020889363	0.983333912	0	0.009133354	0	0.5	CRA004775;PRJNA827465	Yang2025_Silkie_Wenchang	0.995363468
ENSGALG00010023405	DNAJA2	2	-0.028376038	0.041137789	-0.689780333	0.490332338	0	0.082655421	0	1	PRJNA827465;Yang2025_Silkie_Wenchang	CRA004775	0.858529056
ENSGALG00010023405	DNAJA2	2	-0.060147126	0.085820614	-0.700847072	0.483398458	0	0.050616902	0	1	CRA004775;Yang2025_Silkie_Wenchang	PRJNA827465	0.85691916
ENSGALG00010023405	DNAJA2	2	-0.025856238	0.04499396	-0.574660186	0.565521139	0	0.134428422	0	1	CRA004775;PRJNA827465	Yang2025_Silkie_Wenchang	0.883487517
ENSGALG00010023408	ZBTB44	2	-0.036979935	0.064436295	-0.573899148	0.56603605	0	0.036173999	0	1	PRJNA827465;Yang2025_Silkie_Wenchang	CRA004775	0.883487517
ENSGALG00010023408	ZBTB44	2	-0.008899565	0.133279583	-0.066773654	0.946761898	0	0.004519927	0	0.5	CRA004775;Yang2025_Silkie_Wenchang	PRJNA827465	0.986445196
ENSGALG00010023408	ZBTB44	2	-0.040891832	0.070636157	-0.578907945	0.562651289	0	0.024194948	0	0.5	CRA004775;PRJNA827465	Yang2025_Silkie_Wenchang	0.882015141
ENSGALG00010023411	ENSGALG00010023411	2	0.292196935	0.441238079	0.662220578	0.507829868	0.346964594	8.775230699	88.6042882	0.5	PRJNA827465;Yang2025_Silkie_Wenchang	CRA004775	0.864401731
ENSGALG00010023411	ENSGALG00010023411	2	0.455787134	0.406963624	1.119970206	0.262726459	0.18901348	2.137940789	53.22601985	0.5	CRA004775;Yang2025_Silkie_Wenchang	PRJNA827465	0.768569343
ENSGALG00010023411	ENSGALG00010023411	2	-0.114720308	0.119913744	-0.956690236	0.338723628	0	0.006520313	0	1	CRA004775;PRJNA827465	Yang2025_Silkie_Wenchang	0.804450436
ENSGALG00010023412	GSS	2	0.098552021	0.056476979	1.744994542	0.080985819	0	0.008429116	0	1	PRJNA827465;Yang2025_Silkie_Wenchang	CRA004775	0.489916214
ENSGALG00010023412	GSS	2	-0.150362447	0.217609392	-0.690974069	0.489581837	0.064693911	3.127367215	68.02422193	0.5	CRA004775;Yang2025_Silkie_Wenchang	PRJNA827465	0.858419004
ENSGALG00010023412	GSS	2	-0.104241068	0.226098619	-0.461042478	0.644768129	0.089403856	7.44028999	86.55966365	0.5	CRA004775;PRJNA827465	Yang2025_Silkie_Wenchang	0.906953008
ENSGALG00010023414	SUDS3	2	-0.162212775	0.191896901	-0.845312114	0.397936599	0.071046603	28.16550514	96.44955773	0.5	PRJNA827465;Yang2025_Silkie_Wenchang	CRA004775	0.831825697
ENSGALG00010023414	SUDS3	2	-0.273753976	0.10899917	-2.511523486	0.012021129	0.015161184	2.454953667	59.26603367	1	CRA004775;Yang2025_Silkie_Wenchang	PRJNA827465	0.176332839
ENSGALG00010023414	SUDS3	2	-0.002160889	0.061323963	-0.035237277	0.971890538	0.002985847	1.316344969	24.0320719	0.5	CRA004775;PRJNA827465	Yang2025_Silkie_Wenchang	0.991955348
ENSGALG00010023415	HOXB4	2	-0.108950742	0.069142655	-1.575738486	0.115086084	0	0.484754494	0	1	PRJNA827465;Yang2025_Silkie_Wenchang	CRA004775	0.572141484
ENSGALG00010023415	HOXB4	2	-0.063106193	0.088061895	-0.716611804	0.473613659	0	0.056808381	0	0.5	CRA004775;Yang2025_Silkie_Wenchang	PRJNA827465	0.854399987
ENSGALG00010023415	HOXB4	2	-0.151499632	0.102875386	-1.472651901	0.140844922	0	0.236631355	0	0.5	CRA004775;PRJNA827465	Yang2025_Silkie_Wenchang	0.625201272
ENSGALG00010023421	OLFM3	2	-0.09942298	0.124195219	-0.800537899	0.423399215	0.019432429	2.643859582	62.17650865	0.5	PRJNA827465;Yang2025_Silkie_Wenchang	CRA004775	0.840219758
ENSGALG00010023421	OLFM3	2	0.023806817	0.118588687	0.200751163	0.840893152	0	0.194816054	0	0.5	CRA004775;Yang2025_Silkie_Wenchang	PRJNA827465	0.95980036
ENSGALG00010023421	OLFM3	2	-0.207199875	0.088708684	-2.335733831	0.019505118	0	0.022835558	0	1	CRA004775;PRJNA827465	Yang2025_Silkie_Wenchang	0.234012699
ENSGALG00010023422	WLS	2	-0.190530809	0.137358131	-1.387109795	0.165408286	0.023984695	2.617807479	61.80009385	1	PRJNA827465;Yang2025_Silkie_Wenchang	CRA004775	0.664874006
ENSGALG00010023422	WLS	2	-0.319797963	0.113012948	-2.829746223	0.004658494	0	0.12965368	0	1	CRA004775;Yang2025_Silkie_Wenchang	PRJNA827465	0.100408405
ENSGALG00010023422	WLS	2	-0.115009997	0.081413201	-1.412670122	0.15775274	0	0.948908591	0	1	CRA004775;PRJNA827465	Yang2025_Silkie_Wenchang	0.651232233
ENSGALG00010023428	HOXB3	2	0.357245072	0.377982436	0.945136699	0.344589091	0.242851401	6.35058609	84.25342188	1	PRJNA827465;Yang2025_Silkie_Wenchang	CRA004775	0.806886906
ENSGALG00010023428	HOXB3	2	0.337306437	0.46533651	0.724865618	0.468534476	0.333464185	4.282275797	76.64793097	0.5	CRA004775;Yang2025_Silkie_Wenchang	PRJNA827465	0.853198008
ENSGALG00010023428	HOXB3	2	-0.00858203	0.127487672	-0.067316545	0.946329706	0	0.201409591	0	0.5	CRA004775;PRJNA827465	Yang2025_Silkie_Wenchang	0.986436718
ENSGALG00010023429	BMF	2	-0.028151252	0.289407678	-0.097271958	0.922510424	0.146046988	7.514887521	86.69308094	0.5	PRJNA827465;Yang2025_Silkie_Wenchang	CRA004775	0.981804675
ENSGALG00010023429	BMF	2	-0.271273509	0.156683791	-1.731343788	0.083390466	0	0.451219073	0	1	CRA004775;Yang2025_Silkie_Wenchang	PRJNA827465	0.497419257
ENSGALG00010023429	BMF	2	0.167583868	0.142180072	1.178673392	0.238528255	0.015737524	1.343684254	25.57775406	0.5	CRA004775;PRJNA827465	Yang2025_Silkie_Wenchang	0.750652917
ENSGALG00010023431	BCAR1	2	0.116012177	0.111595995	1.039572942	0.298538353	0.015933997	2.678736099	62.66896166	1	PRJNA827465;Yang2025_Silkie_Wenchang	CRA004775	0.789493533
ENSGALG00010023431	BCAR1	2	0.04076062	0.281345426	0.144877492	0.884807597	0.107690665	2.59549469	61.47169925	0.5	CRA004775;Yang2025_Silkie_Wenchang	PRJNA827465	0.972138605
ENSGALG00010023431	BCAR1	2	-0.005550443	0.092233777	-0.060177989	0.952013876	0.003266179	1.051140752	4.865262049	0.5	CRA004775;PRJNA827465	Yang2025_Silkie_Wenchang	0.987725077
ENSGALG00010023432	TMOD3	2	-0.21652512	0.188452627	-1.14896313	0.250571184	0.064065576	10.10796996	90.10681666	1	PRJNA827465;Yang2025_Silkie_Wenchang	CRA004775	0.760126525
ENSGALG00010023432	TMOD3	2	-0.069568839	0.369291117	-0.188384815	0.850575002	0.243688219	8.926745881	88.79770956	0.5	CRA004775;Yang2025_Silkie_Wenchang	PRJNA827465	0.961985357
ENSGALG00010023432	TMOD3	2	0.082774396	0.16980031	0.487480832	0.625917623	0.03766139	2.321505767	56.92450934	0.5	CRA004775;PRJNA827465	Yang2025_Silkie_Wenchang	0.901504244
ENSGALG00010023434	ATN1	2	0.166058004	0.393422941	0.422085208	0.672962825	0.265474084	6.384054721	84.33597387	0.5	PRJNA827465;Yang2025_Silkie_Wenchang	CRA004775	0.915153756
ENSGALG00010023434	ATN1	2	0.206525694	0.401787957	0.514016636	0.60724036	0.237520399	3.781025698	73.55215013	0.5	CRA004775;Yang2025_Silkie_Wenchang	PRJNA827465	0.896998866
ENSGALG00010023434	ATN1	2	-0.180584774	0.088991976	-2.029225361	0.042435342	0	0.001159458	0	1	CRA004775;PRJNA827465	Yang2025_Silkie_Wenchang	0.356451428
ENSGALG00010023437	KCNJ5	2	0.104817252	0.124255435	0.843562712	0.398913808	0	0.087222753	0	1	PRJNA827465;Yang2025_Silkie_Wenchang	CRA004775	0.832054308
ENSGALG00010023437	KCNJ5	2	0.135246953	0.197579685	0.684518518	0.4936478	0	0.052857552	0	1	CRA004775;Yang2025_Silkie_Wenchang	PRJNA827465	0.860048738
ENSGALG00010023437	KCNJ5	2	0.077657992	0.139327967	0.557375477	0.577270917	0	0.003704085	0	1	CRA004775;PRJNA827465	Yang2025_Silkie_Wenchang	0.887145528
ENSGALG00010023438	HOXB2	2	0.261055649	0.190358177	1.371391831	0.170252843	0.045515522	2.643325527	62.16886684	1	PRJNA827465;Yang2025_Silkie_Wenchang	CRA004775	0.672034495
ENSGALG00010023438	HOXB2	2	0.386167025	0.166307707	2.322003182	0.020232764	0	0.986736991	0	1	CRA004775;Yang2025_Silkie_Wenchang	PRJNA827465	0.23937638
ENSGALG00010023438	HOXB2	2	0.085988586	0.132677405	0.648102715	0.516918518	0	0.003245734	0	1	CRA004775;PRJNA827465	Yang2025_Silkie_Wenchang	0.867952013
ENSGALG00010023439	ACSS2	2	-0.00143344	0.155511321	-0.00921759	0.992645531	0.035448904	3.506243762	71.47945015	0.5	PRJNA827465;Yang2025_Silkie_Wenchang	CRA004775	0.997852036
ENSGALG00010023439	ACSS2	2	-0.318531934	0.524825917	-0.606928744	0.543898202	0.502824773	11.17290454	91.04977585	0.5	CRA004775;Yang2025_Silkie_Wenchang	PRJNA827465	0.876968972
ENSGALG00010023439	ACSS2	2	-0.454217228	0.364872225	-1.244866552	0.213180808	0.229228096	6.506106492	84.62982429	1	CRA004775;PRJNA827465	Yang2025_Silkie_Wenchang	0.72579791
ENSGALG00010023441	ENSGALG00010023441	2	-0.020702439	0.185359576	-0.111687992	0.911070801	0.035544918	1.96299195	49.05735605	0.5	PRJNA827465;Yang2025_Silkie_Wenchang	CRA004775	0.978845851
ENSGALG00010023441	ENSGALG00010023441	2	0.164902644	0.209768778	0.786116246	0.431799387	0	0.24564228	0	0.5	CRA004775;Yang2025_Silkie_Wenchang	PRJNA827465	0.842503929
ENSGALG00010023441	ENSGALG00010023441	2	-0.159698836	0.137506079	-1.161394733	0.24548141	0	0.042528307	0	1	CRA004775;PRJNA827465	Yang2025_Silkie_Wenchang	0.75661406
ENSGALG00010023442	STK25	2	-0.139495205	0.118381763	-1.178350456	0.238656921	0.024630192	7.773285921	87.13542754	1	PRJNA827465;Yang2025_Silkie_Wenchang	CRA004775	0.750694914
ENSGALG00010023442	STK25	2	-0.079566325	0.226965901	-0.350565107	0.725914638	0.081277766	4.238850746	76.40869991	0.5	CRA004775;Yang2025_Silkie_Wenchang	PRJNA827465	0.931029023
ENSGALG00010023442	STK25	2	-0.015804995	0.057143692	-0.276583374	0.782100033	0.002470237	1.10978607	9.892543501	0.5	CRA004775;PRJNA827465	Yang2025_Silkie_Wenchang	0.945372683
ENSGALG00010023443	ENSGALG00010023443	2	0.048951921	0.427772035	0.114434597	0.908893289	0.327592582	9.0383431	88.93602523	0.5	PRJNA827465;Yang2025_Silkie_Wenchang	CRA004775	0.978396455
ENSGALG00010023443	ENSGALG00010023443	2	0.08412648	0.418363314	0.201084744	0.840632311	0.288257255	5.64968916	82.29991117	0.5	CRA004775;Yang2025_Silkie_Wenchang	PRJNA827465	0.95974936
ENSGALG00010023443	ENSGALG00010023443	2	-0.34210069	0.096326004	-3.551488434	0.000383059	0	0.007019478	0	1	CRA004775;PRJNA827465	Yang2025_Silkie_Wenchang	0.019680211
ENSGALG00010023447	C12orf57	2	-0.288202705	0.260944408	-1.104460168	0.269393573	0.097569919	3.117401866	67.92200548	1	PRJNA827465;Yang2025_Silkie_Wenchang	CRA004775	0.77189992
ENSGALG00010023447	C12orf57	2	-0.387707685	0.290192728	-1.336035151	0.181537784	0.058096824	1.485202553	32.66911653	1	CRA004775;Yang2025_Silkie_Wenchang	PRJNA827465	0.689004635
ENSGALG00010023447	C12orf57	2	-0.076672701	0.110948944	-0.691062917	0.489526003	0	0.020952287	0	1	CRA004775;PRJNA827465	Yang2025_Silkie_Wenchang	0.858419004
ENSGALG00010023448	FAM219B	2	-0.005606842	0.212638813	-0.026367913	0.978963887	0.080238218	8.822290384	88.66507498	0.5	PRJNA827465;Yang2025_Silkie_Wenchang	CRA004775	0.994259988
ENSGALG00010023448	FAM219B	2	-0.025230703	0.266306056	-0.094743255	0.92451876	0.114899875	4.999396522	79.9975858	0.5	CRA004775;Yang2025_Silkie_Wenchang	PRJNA827465	0.982382364
ENSGALG00010023448	FAM219B	2	-0.229054633	0.081619164	-2.806382981	0.005010111	0	0.232294163	0	1	CRA004775;PRJNA827465	Yang2025_Silkie_Wenchang	0.105526462
ENSGALG00010023453	ARHGAP32	2	0.154252214	0.220282225	0.700248123	0.483772363	0.066472163	2.710792692	63.11042143	0.5	PRJNA827465;Yang2025_Silkie_Wenchang	CRA004775	0.85691916
ENSGALG00010023453	ARHGAP32	2	0.292740663	0.258829317	1.131018181	0.258047439	0.02273793	1.15227431	13.21510938	0.5	CRA004775;Yang2025_Silkie_Wenchang	PRJNA827465	0.765205863
ENSGALG00010023453	ARHGAP32	2	-0.015865674	0.094674479	-0.167581319	0.866912669	0	0.068008026	0	1	CRA004775;PRJNA827465	Yang2025_Silkie_Wenchang	0.967687266
ENSGALG00010023454	HOXB1	2	-0.211420364	0.393611017	-0.537130199	0.591177682	0.22112977	3.340577574	70.06505678	0.5	PRJNA827465;Yang2025_Silkie_Wenchang	CRA004775	0.892776493
ENSGALG00010023454	HOXB1	2	-0.55245946	0.284973364	-1.938635428	0.052545746	0	0.215885189	0	1	CRA004775;Yang2025_Silkie_Wenchang	PRJNA827465	0.398073529
ENSGALG00010023454	HOXB1	2	0.010078057	0.221829106	0.045431625	0.963763274	0.012238833	1.097454499	8.88004918	0.5	CRA004775;PRJNA827465	Yang2025_Silkie_Wenchang	0.990167386
ENSGALG00010023455	CFDP1	2	0.257232131	0.229933617	1.118723456	0.263258135	0.096996865	11.73464199	91.47822319	1	PRJNA827465;Yang2025_Silkie_Wenchang	CRA004775	0.768643821
ENSGALG00010023455	CFDP1	2	0.263655811	0.25712161	1.02541288	0.305168413	0.107166328	5.116163155	80.45410262	0.5	CRA004775;Yang2025_Silkie_Wenchang	PRJNA827465	0.79225794
ENSGALG00010023455	CFDP1	2	0.034401343	0.056238055	0.611709342	0.54073008	0	0.07567005	0	0.5	CRA004775;PRJNA827465	Yang2025_Silkie_Wenchang	0.875630422
ENSGALG00010023456	MPI	2	-0.020533777	0.065592101	-0.313052594	0.754240705	0	0.003132713	0	1	PRJNA827465;Yang2025_Silkie_Wenchang	CRA004775	0.93838723
ENSGALG00010023456	MPI	2	-0.034191136	0.120860054	-0.282898568	0.777254593	0	0.28398411	0	1	CRA004775;Yang2025_Silkie_Wenchang	PRJNA827465	0.944218792
ENSGALG00010023456	MPI	2	-0.030164618	0.075444025	-0.399827791	0.68928336	0	0.279012257	0	1	CRA004775;PRJNA827465	Yang2025_Silkie_Wenchang	0.91986176
ENSGALG00010023466	CLCF1	2	0.279764773	0.358635317	0.780081492	0.43534291	0.133301913	1.985186009	49.62688658	0.5	PRJNA827465;Yang2025_Silkie_Wenchang	CRA004775	0.842762754
ENSGALG00010023466	CLCF1	2	-0.192320006	0.930658954	-0.206649283	0.836283768	1.510875905	7.807453377	87.19172627	0.5	CRA004775;Yang2025_Silkie_Wenchang	PRJNA827465	0.958399882
ENSGALG00010023466	CLCF1	2	-0.49888793	0.559091386	-0.892319113	0.372221912	0.470269388	3.821365801	73.83134585	1	CRA004775;PRJNA827465	Yang2025_Silkie_Wenchang	0.821693879
ENSGALG00010023469	ENSGALG00010023469	2	0.052163035	0.321464598	0.162266811	0.871095749	0.136599459	2.949127962	66.0916714	0.5	PRJNA827465;Yang2025_Silkie_Wenchang	CRA004775	0.969259951
ENSGALG00010023469	ENSGALG00010023469	2	0.05851373	0.35069648	0.166850062	0.867488027	0.152190143	2.578480933	61.21747549	0.5	CRA004775;Yang2025_Silkie_Wenchang	PRJNA827465	0.967862205
ENSGALG00010023469	ENSGALG00010023469	2	-0.29065193	0.210239423	-1.382480626	0.166824175	0	0.020222846	0	1	CRA004775;PRJNA827465	Yang2025_Silkie_Wenchang	0.665789049
ENSGALG00010023472	PTPN6	2	-0.030590535	0.142218465	-0.215095384	0.829692953	0.02865647	3.401172829	70.59837738	0.5	PRJNA827465;Yang2025_Silkie_Wenchang	CRA004775	0.957045817
ENSGALG00010023472	PTPN6	2	-0.277136402	0.219343211	-1.263482926	0.206415679	0.0429743	1.31612861	24.01958346	1	CRA004775;Yang2025_Silkie_Wenchang	PRJNA827465	0.718451597
ENSGALG00010023472	PTPN6	2	-0.197591631	0.418633188	-0.471992275	0.636932303	0.256375149	2.919450192	65.74697515	0.5	CRA004775;PRJNA827465	Yang2025_Silkie_Wenchang	0.904841694
ENSGALG00010023473	BOK	2	0.573867606	0.231954411	2.474053426	0.013358972	0.083985504	4.195558352	76.16527013	1	PRJNA827465;Yang2025_Silkie_Wenchang	CRA004775	0.188936172
ENSGALG00010023473	BOK	2	0.378927004	0.47626768	0.795617715	0.426254267	0.400785069	8.550199631	88.30436664	0.5	CRA004775;Yang2025_Silkie_Wenchang	PRJNA827465	0.84125685
ENSGALG00010023473	BOK	2	0.189445046	0.235114761	0.805755645	0.420383758	0.08076147	3.246449175	69.19711518	0.5	CRA004775;PRJNA827465	Yang2025_Silkie_Wenchang	0.838867934
ENSGALG00010023476	SCAMP2	2	-0.070570751	0.074438739	-0.94803797	0.343110125	0.004263515	1.470323426	31.98775303	1	PRJNA827465;Yang2025_Silkie_Wenchang	CRA004775	0.806218116
ENSGALG00010023476	SCAMP2	2	-0.315205197	0.151952055	-2.074372715	0.038044709	0.025319097	2.148155052	53.44842548	1	CRA004775;Yang2025_Silkie_Wenchang	PRJNA827465	0.336310939
ENSGALG00010023476	SCAMP2	2	-0.230416688	0.233917207	-0.985035222	0.32460677	0.094374404	6.691995682	85.05677458	1	CRA004775;PRJNA827465	Yang2025_Silkie_Wenchang	0.799859262
ENSGALG00010023478	TAOK3	2	0.168144649	0.411262414	0.408850027	0.682649724	0.313540829	12.99420814	92.30426365	0.5	PRJNA827465;Yang2025_Silkie_Wenchang	CRA004775	0.918716001
ENSGALG00010023478	TAOK3	2	0.48529449	0.172572371	2.812121591	0.004921589	0	0.809756046	0	1	CRA004775;Yang2025_Silkie_Wenchang	PRJNA827465	0.104558504

ENSGALG00010023478	TAOK3	2	-0.038842834	0.241166515	-0.161062304	0.87204433	0.085787964	3.074728839	67.47680682	0.5	CRA004775;PRJNA827465	Yang2025_Silkie_Wenchang	0.969560463
ENSGALG00010023479	MCCC2L	2	-0.234548326	0.097232323	-2.412246448	0.015854561	0	0.212852866	0	1	PRJNA827465;Yang2025_Silkie_Wenchang	CRA004775	0.208335606
ENSGALG00010023479	MCCC2L	2	-0.550000858	0.460054186	-1.195513213	0.231886577	0.327678189	3.99920992	74.99506102	1	CRA004775;Yang2025_Silkie_Wenchang	PRJNA827465	0.744189487
ENSGALG00010023479	MCCC2L	2	-0.578323631	0.40521623	-1.427197599	0.153522946	0.249877523	3.500764007	71.43480686	1	CRA004775;PRJNA827465	Yang2025_Silkie_Wenchang	0.645911732
ENSGALG00010023482	MTCL2	2	0.077744267	0.068328251	1.137805602	0.255201665	0	0.230301916	0	1	PRJNA827465;Yang2025_Silkie_Wenchang	CRA004775	0.764467569
ENSGALG00010023482	MTCL2	2	0.081983611	0.109657034	0.747636594	0.454679386	0.001297149	1.020332524	1.99273502	0.5	CRA004775;Yang2025_Silkie_Wenchang	PRJNA827465	0.849072665
ENSGALG00010023482	MTCL2	2	0.033650563	0.084437794	0.398524889	0.690243316	0	0.699610581	0	0.5	CRA004775;PRJNA827465	Yang2025_Silkie_Wenchang	0.919979296
ENSGALG00010023485	BARX2	2	0.132571452	0.135141029	0.98098596	0.326599665	0.016593922	1.832306106	45.42396618	0.5	PRJNA827465;Yang2025_Silkie_Wenchang	CRA004775	0.800028239
ENSGALG00010023485	BARX2	2	0.028143505	0.135343542	0.207941247	0.835274843	0	0.71257984	0	0.5	CRA004775;Yang2025_Silkie_Wenchang	PRJNA827465	0.958309459
ENSGALG00010023485	BARX2	2	0.282453128	0.138094603	2.04535964	0.040819415	0	0.13350958	0	1	CRA004775;PRJNA827465	Yang2025_Silkie_Wenchang	0.349602411
ENSGALG00010023487	GRN	2	0.160112087	0.069737742	2.295917272	0.021680612	0	0.079954644	0	1	PRJNA827465;Yang2025_Silkie_Wenchang	CRA004775	0.246941453
ENSGALG00010023487	GRN	2	0.141191019	0.214651826	0.657767615	0.510687467	0	0.265814171	0	1	CRA004775;Yang2025_Silkie_Wenchang	PRJNA827465	0.865386785
ENSGALG00010023487	GRN	2	0.148627562	0.070213981	2.116780148	0.034278511	0	0.176396463	0	1	CRA004775;PRJNA827465	Yang2025_Silkie_Wenchang	0.318385282
ENSGALG00010023489	ETV5	2	-0.090555395	0.170891129	-0.529901085	0.596180514	0.049690794	6.673683383	85.01577101	0.5	PRJNA827465;Yang2025_Silkie_Wenchang	CRA004775	0.894064255
ENSGALG00010023489	ETV5	2	0.086011554	0.097940522	0.878201909	0.379834156	0	0.001582845	0	1	CRA004775;Yang2025_Silkie_Wenchang	PRJNA827465	0.824375826
ENSGALG00010023489	ETV5	2	-0.239153239	0.082730013	-2.890767565	0.003843022	0	0.882955025	0	0.5	CRA004775;PRJNA827465	Yang2025_Silkie_Wenchang	0.089057965
ENSGALG00010023490	PHB2	2	-0.120203346	0.120706498	-0.995831608	0.31933197	0.019208296	2.350306556	57.45235882	1	PRJNA827465;Yang2025_Silkie_Wenchang	CRA004775	0.798079048
ENSGALG00010023490	PHB2	2	-0.211410058	0.126316293	-1.673656283	0.09419817	0	0.695255216	0	1	CRA004775;Yang2025_Silkie_Wenchang	PRJNA827465	0.525712768
ENSGALG00010023490	PHB2	2	-0.03947201	0.046122354	-0.855810826	0.392102426	0	0.042481642	0	1	CRA004775;PRJNA827465	Yang2025_Silkie_Wenchang	0.829251057
ENSGALG00010023496	PLEKHA3	2	-0.020874931	0.186446533	-0.111962022	0.910853519	0.046707933	2.766432487	63.85236203	0.5	PRJNA827465;Yang2025_Silkie_Wenchang	CRA004775	0.97880407
ENSGALG00010023496	PLEKHA3	2	-0.290547516	0.174796707	-1.662202455	0.096472188	0	0.105592986	0	1	CRA004775;Yang2025_Silkie_Wenchang	PRJNA827465	0.530885356
ENSGALG00010023496	PLEKHA3	2	-0.030873997	0.235543905	-0.131075338	0.89571571	0.070499672	2.200988515	54.56586924	0.5	CRA004775;PRJNA827465	Yang2025_Silkie_Wenchang	0.974927462
ENSGALG00010023498	DISP2	2	-0.324180681	0.218460151	-1.483935077	0.137826145	0	0.336580828	0	1	PRJNA827465;Yang2025_Silkie_Wenchang	CRA004775	0.62064915
ENSGALG00010023498	DISP2	2	-0.109363512	0.270607187	-0.404141197	0.686108892	0	0.461506612	0	0.5	CRA004775;Yang2025_Silkie_Wenchang	PRJNA827465	0.919235539
ENSGALG00010023498	DISP2	2	-0.284308093	0.304078971	-0.934981106	0.349798036	0.022637322	1.098834146	8.969602457	0.5	CRA004775;PRJNA827465	Yang2025_Silkie_Wenchang	0.949909518
ENSGALG00010023499	PEBP1	2	-0.007822645	0.057465602	-0.136127444	0.891720532	0	0.038047709	0	0.5	PRJNA827465;Yang2025_Silkie_Wenchang	CRA004775	0.973985832
ENSGALG00010023499	PEBP1	2	-0.032883333	0.176829034	-0.185961165	0.852475216	0	0.570060011	0	0.5	CRA004775;Yang2025_Silkie_Wenchang	PRJNA827465	0.962607579
ENSGALG00010023499	PEBP1	2	-0.017457507	0.059551015	-0.293152134	0.769405869	0	0.532557437	0	1	CRA004775;PRJNA827465	Yang2025_Silkie_Wenchang	0.941790377
ENSGALG00010023510	ASPHD2	2	-0.134652063	0.140444791	-0.958754409	0.337682482	0	0.011786368	0	1	PRJNA827465;Yang2025_Silkie_Wenchang	CRA004775	0.80441385
ENSGALG00010023510	ASPHD2	2	-0.208916051	0.191511682	-1.090878889	0.275326177	0	0.645268628	0	1	CRA004775;Yang2025_Silkie_Wenchang	PRJNA827465	0.775135547
ENSGALG00010023510	ASPHD2	2	-0.176094374	0.181853153	-0.96833281	0.332878184	0	0.743015747	0	1	CRA004775;PRJNA827465	Yang2025_Silkie_Wenchang	0.802178578
ENSGALG00010023513	CDCA9	2	-0.171174917	0.146288085	-1.170122077	0.241951845	0.017986096	1.683780934	40.60905132	0.5	PRJNA827465;Yang2025_Silkie_Wenchang	CRA004775	0.754178236
ENSGALG00010023513	CDCA9	2	0.048024734	0.169374954	0.283540945	0.776762199	0	0.323669294	0	1	CRA004775;Yang2025_Silkie_Wenchang	PRJNA827465	0.944156005
ENSGALG00010023513	CDCA9	2	-0.152987325	0.241213344	-0.634240722	0.525923736	0.059520912	1.625587521	38.48377973	0.5	CRA004775;PRJNA827465	Yang2025_Silkie_Wenchang	0.870414822
ENSGALG00010023514	ULK3	2	0.098834103	0.080182419	1.232615627	0.217719204	0	0.120997873	0	1	PRJNA827465;Yang2025_Silkie_Wenchang	CRA004775	0.731070128
ENSGALG00010023514	ULK3	2	-0.113719023	0.294697771	-0.385885552	0.699582913	0.131442028	3.782766916	73.56432415	0.5	CRA004775;Yang2025_Silkie_Wenchang	PRJNA827465	0.922065803
ENSGALG00010023514	ULK3	2	-0.130604689	0.262807494	-0.496959532	0.619217589	0.102168423	3.341421229	70.07261487	0.5	CRA004775;PRJNA827465	Yang2025_Silkie_Wenchang	0.899168321
ENSGALG00010023518	METTL5	2	-0.095670385	0.048533948	-1.971205505	0.048700375	0	0.314256849	0	1	PRJNA827465;Yang2025_Silkie_Wenchang	CRA004775	0.381461853
ENSGALG00010023518	METTL5	2	-0.127165941	0.115060409	-1.105210229	0.269068506	0	0.425992774	0	0.5	CRA004775;Yang2025_Silkie_Wenchang	PRJNA827465	0.771461882
ENSGALG00010023518	METTL5	2	-0.080118218	0.051678772	-1.550311951	0.12106666	0	0.189097855	0	0.5	CRA004775;PRJNA827465	Yang2025_Silkie_Wenchang	0.583289761
ENSGALG00010023519	DGKG	2	0.150907077	0.302252165	0.49927542	0.617585368	0.154428764	6.404966284	84.38171532	0.5	PRJNA827465;Yang2025_Silkie_Wenchang	CRA004775	0.899121942
ENSGALG00010023519	DGKG	2	0.388459019	0.161653674	2.40303242	0.016259746	0	0.610441805	0	1	CRA004775;Yang2025_Silkie_Wenchang	PRJNA827465	0.210770941
ENSGALG00010023519	DGKG	2	-0.081600173	0.132142426	-0.617516841	0.536893876	0	0.872437649	0	0.5	CRA004775;PRJNA827465	Yang2025_Silkie_Wenchang	0.874713452
ENSGALG00010023522	EMG1	2	-0.195033359	0.130446639	-1.495119844	0.134883168	0.014578555	1.483496663	32.59169198	1	PRJNA827465;Yang2025_Silkie_Wenchang	CRA004775	0.615501352
ENSGALG00010023522	EMG1	2	-0.466855084	0.173669768	-2.688177042	0.007184329	0	0.11039932	0	1	CRA004775;Yang2025_Silkie_Wenchang	PRJNA827465	0.130302584
ENSGALG00010023522	EMG1	2	-0.250318111	0.193501084	-1.293626405	0.195794497	0.046510264	2.162178714	53.75035405	1	CRA004775;PRJNA827465	Yang2025_Silkie_Wenchang	0.706834861
ENSGALG00010023526	KNSTRN	2	0.122187212	0.08707962	1.403166576	0.160565172	0	0.299575752	0	1	PRJNA827465;Yang2025_Silkie_Wenchang	CRA004775	0.65630165
ENSGALG00010023526	KNSTRN	2	0.212466917	0.187008528	1.136134908	0.255900107	0	0.020628361	0	1	CRA004775;Yang2025_Silkie_Wenchang	PRJNA827465	0.764548987
ENSGALG00010023526	KNSTRN	2	0.102921941	0.093579259	1.099837096	0.271403106	0	0.016849248	0	1	CRA004775;PRJNA827465	Yang2025_Silkie_Wenchang	0.772743863
ENSGALG00010023527	TMEM98	2	0.01898157	0.103996966	0.182520421	0.85517433	0.009150023	1.655201734	39.58440354	0.5	PRJNA827465;Yang2025_Silkie_Wenchang	CRA004775	0.963353229
ENSGALG00010023527	TMEM98	2	0.17042027	0.134694414	1.265236359	0.205786612	0	0.165728051	0	1	CRA004775;Yang2025_Silkie_Wenchang	PRJNA827465	0.71832154
ENSGALG00010023527	TMEM98	2	-0.046247499	0.088382418	-0.523265826	0.600789279	0	0.763715817	0	0.5	CRA004775;PRJNA827465	Yang2025_Silkie_Wenchang	0.895230519
ENSGALG00010023533	FKBP7	2	0.073098961	0.048783648	1.498431626	0.134021145	0	0.222677182	0	1	PRJNA827465;Yang2025_Silkie_Wenchang	CRA004775	0.61327389
ENSGALG00010023533	FKBP7	2	0.012011254	0.110566922	0.108633341	0.913493315	0	0.139930127	0	0.5	CRA004775;Yang2025_Silkie_Wenchang	PRJNA827465	0.979269146
ENSGALG00010023533	FKBP7	2	0.080028203	0.05338144	1.499176541	0.133827839	0	0.285709683	0	0.5	CRA004775;PRJNA827465	Yang2025_Silkie_Wenchang	0.613104446
ENSGALG00010023536	ENSGALG00010023536	2	0.29790315	0.150947219	1.973558386	0.048431979	0	0.085288978	0	1	PRJNA827465;Yang2025_Silkie_Wenchang	CRA004775	0.380432851
ENSGALG00010023536	ENSGALG00010023536	2	-0.112746764	0.479894782	-0.234940593	0.814254843	0.365289383	4.805447253	79.19028246	0.5	CRA004775;Yang2025_Silkie_Wenchang	PRJNA827465	0.953869515
ENSGALG00010023536	ENSGALG00010023536	2	-0.120087767	0.424173278	-0.283110165	0.77709239	0.308187439	6.774527707	85.23882338	0.5	CRA004775;PRJNA827465	Yang2025_Silkie_Wenchang	0.94421879
ENSGALG00010023544	SSB	2	0.235976526	0.334071728	0.706364849	0.479961275	0.206995434	13.23287025	92.44306049	0.5	PRJNA827465;Yang2025_Silkie_Wenchang	CRA004775	0.856321884
ENSGALG00010023544	SSB	2	0.317254073	0.315826334	1.004520645	0.315127725	0.144292329	3.418891486	70.75075345	0.5	CRA004775;Yang2025_Silkie_Wenchang	PRJNA827465	0.796635323
ENSGALG00010023544	SSB	2	-0.018396247	0.064219934	-1.220746506	0.222182089	0	0.00991897	0	1	CRA004775;PRJNA827465	Yang2025_Silkie_Wenchang	0.73229121
ENSGALG00010023545	PTPRT	2	0.142303067	0.203753323	0.698408567	0.48492172	0.062223169	3.794883594	73.64873058	0.5	PRJNA827465;Yang2025_Silkie_Wenchang	CRA004775	0.857004733
ENSGALG00010023545	PTPRT	2	0.123859363	0.417942059	0.296355344	0.766958725	0.204300145	1.83693026	45.5613519	0.5	CRA004775;Yang2025_Silkie_Wenchang	PRJNA827465	0.941375309
ENSGALG00010023545	PTPRT	2	-0.04910072	0.104431116	-0.470173275	0.638231226	0	0.61633019	0	1	CRA004775;PRJNA827465	Yang2025_Silkie_Wenchang	0.905508695
ENSGALG00010023546	SLC11A2	2	-0.235594928	0.074925745	-3.144378847	0.001664398	0	0.545131286	0	1	PRJNA827465;Yang2025_Silkie_Wenchang	CRA004775	0.051549125
ENSGALG00010023546	SLC11A2	2	-0.34444218	0.123616037	-2.786387503	0.005329911	0	0.372494911	0	1	CRA004775;Yang2025_Silkie_Wenchang	PRJNA827465	0.109048157
ENSGALG00010023546	SLC11A2	2	-0.21945833	0.088037583	-2.492780051	0.012674733	0	0.919650212	0	1	CRA004775;PRJNA827465	Yang2025_Silkie_Wenchang	0.182608155
ENSGALG00010023551	ENSGALG00010023551	2	0.165069532	0.213581102	0.772865814	0.439601798	0.081007198	8.860135937	88.71349145	0.5	PRJNA827465;Yang2025_Silkie_Wenchang	CRA004775	0.844278492
ENSGALG00010023551	ENSGALG00010023551	2	0.047541625	0.426208536	0.111545916	0.911183457	0.282564072	3.800805303	73.68978623	0.5	CRA004775;Yang2025_Silkie_Wenchang	PRJNA827465	0.978280851
ENSGALG00010023551	ENSGALG00010023551	2	-0.064472912	0.097213243	-0.663211201	0.507195299	0.002330403	1.023733052	2.318285193	1	CRA004775;PRJNA827465	Yang2025_Silkie_Wenchang	0.864048203
ENSGALG00010023552	SGPP2	2	-0.312197282	0.364341712	-0.856880428	0.391510971	0.154335634	2.246789936	55.49205629	1	PRJNA827465;Yang2025_Silkie_Wenchang	CRA004775	0.829251057
ENSGALG00010023552	SGPP2	2	-0.55307989	0.36014926	-1.491070644	0.135942949	0	0.970706452	0	0.5	CRA004775;Yang2025_Silkie_Wenchang	PRJNA827465	0.617541114
ENSGALG00010023552	SGPP2	2	-0.014328903	0.237372017	-0.060364754	0.95186513	0	0.00240666	0	0.5	CRA004775;PRJNA827465	Yang2025_Silkie_Wenchang	0.987719213
ENSGALG00010023555	HPS4	2	-0.038801746	0.123120904	-0.315151568	0.752646576	0.022778642	3.945186434	74.65265542	0.5	PRJNA827465;Yang2025_Silkie_Wenchang	CRA004775	0.938087082
ENSGALG00010023555	HPS4	2	-0.297145137	0.166587474	-1.783718368	0.07446944	0.033186973	2.227491245	55.10644533	1	CRA004775;Yang2025_Silkie_Wenchang	PRJNA827465	0.471164262
ENSGALG00010023555	HPS4	2	-0.189720793	0.295261854	-0.642550977	0.520515502	0.151875238	7.175006684	86.06273075	0.5	CRA004775;PRJNA827465	Yang2025_Silkie_Wenchang	0.869331539
ENSGALG00010023556	IVD	2	0.085819062	0.112634533	0.761924956	0.446104794	0.015723428	2.534913667	60.55092475	0.5	PRJNA827465;Yang2025_Silkie_Wenchang	CRA004775	0.846013379
ENSGALG00010023556	IVD	2	0.178685794	0.11142647	1.603620708	0.108797683	0	0.836006573	0	0.5	CRA004775;Yang2025_Silkie_Wenchang	PRJNA827465	0.558526038
ENSGALG00010023556	IVD	2	-0.015712222	0.077079676	-0.203843906	0.838475481	0	0.062092078	0	1	CRA004775;PRJNA827465	Yang2025_Silkie_Wenchang	0.959197438
ENSGALG00010023569	LPCAT3	2	-0.048231137	0.067797183	-0.711402359	0.47681438	0	0.116432212	0	1	PRJNA827465;Yang2025_Silkie_Wenchang	CRA004775	0.854986476
ENSGALG00010023569	LPCAT3	2	-0.207213474	0.163955799	-1.263837421	0.206288387	0.026464535	1.83192982	45.41275605	1	CRA004775;Yang2025_Silkie_Wenchang	PRJNA827465	0.718451597
ENSGALG00010023569	LPCAT3	2	-0.176415644	0.188788966	-0.934459506	0.350066915	0.04961287	2.819158654	64.52842415	1	CRA004775;PRJNA827465	Yang2025_Silkie_Wenchang	0.810057397
ENSGALG00010023571	APLP2	2	-0.039101806	0.071571759	-0.546330104	0.584839067	0.005097718	1.980656748	49.51679363	0.5	PRJNA827465;Yang2025_Silkie_Wenchang	CRA004775	0.891016154
ENSGALG00010023571	APLP2	2	0.06361898	0.224515449	0.283361257	0.776899925	0.076699649	3.621525097	72.38732376	0.5	CRA004775;Yang2025_Silkie_Wenchang	PRJNA827465	0.944200431
ENSGALG00010023571	APLP2	2	0.110138054	0.139040214	0.79213093	0.428284337	0.021656069	1.758772018	43.14214749	1	CRA004775;PRJNA827465	Yang2025_Silkie_Wenchang	0.841329286
ENSGALG00010023572	MYBL2	2	0.022527032	0.076278783	0.295324998	0.767745622	0	0.404886477	0	0.5	PRJNA827465;Yang2025_Silkie_Wenchang	CRA004775	0.941444269
ENSGALG00010023572	MYBL2	2	-0.069566494	0.151365409	-0.459593077	0.645808329	0	0.002954498	0	1	CRA004775;Yang2025_Silkie_Wenchang	PRJNA827465	0.907098003
ENSGALG00010023572	MYBL2	2	0.044254076	0.085389758	0.51825977	0.604277037	0	0.08811321	0	0.5	CRA004775;PRJNA827465	Yang2025_Silkie_Wenchang	0.89612415
ENSGALG00010023574	DEPDC1	2	-0.093786918	0.076631705	-1.223865728	0.221002884	0	0.203057844	0	1	PRJNA827465;Yang2025_Silkie_Wenchang	CRA004775	0.734231106
ENSGALG00010023574	DEPDC1	2	-0.084206337	0.202473569	-0.415888047	0.677491926	0.024217946	1.111239051	10.01036197	0.5	CRA004775;Yang2025_Silkie_Wenchang	PRJNA827465	0.917144807
ENSGALG00010023574	DEPDC1	2	-0.060340843	0.089410206	-0.674876462	0.499754256	0	0.905732795	0	0.5	CRA004775;PRJNA827465	Yang2025_Silkie_Wenchang	0.862078151
ENSGALG00010023578	SRRD	2	-0.029070707	0.161932183	-0.179523964	0.857526303	0.030967831	2.017872981	50.44286686	0.5	PRJNA827465;Yang2025_Silkie_Wenchang	CRA004775	0.963999665
ENSGALG00010023578	SRRD	2	0.001803797	0.226372808	0.00796826	0.993642316	0.04199487	1.688581746	40.77870364	0.5	CRA004775;Yang2025_Silkie_Wenchang	PRJNA827465	0.997979742
ENSGALG00010023578	SRRD	2	-0.147007722	0.079601907	-1.846786423	0.0647781	0	0.142442599	0	1	CRA004775;PRJNA827465	Yang2025_Silkie_Wenchang	0.438869713
ENSGALG00010023580	RDM1	2	-0.059673892	0.0916843	-0.650862708	0.515135116	0	0.434869591	0	0.5	PRJNA827465;Yang2025_Silkie_Wenchang	CRA004775	0.8671413
ENSGALG00010023580	RDM1	2	-0.131224085	0.256468251	-0.511658203	0.608890244	0.061778328	1.738829247	42.49004024	0.5	CRA004775;Yang2025_Silkie_Wenchang	PRJNA827465	0.897208691
ENSGALG00010023580	RDM1	2	-0.137794374	0.126614874	-1.088295317	0.276464757	0.007440601	1.106894495	9.657153028	1	CRA004775;PRJNA827465	Yang2025_Silkie_Wenchang	0.775851484
ENSGALG00010023585	TFIP11	2	-0.239697799	0.159921283	-1.498848643	0.133912902	0.042133443	5.161237081	80.62480014	1	PRJNA827465;Yang2025_Silkie_Wenchang	CRA004775	0.613104446
ENSGALG00010023585	TFIP11	2	-0.263104431	0.172080229	-1.528963743	0.126273432	0.038048574	2.777952096	64.00225902	1	CRA004775;Yang2025_Silkie_Wenchang	PRJNA827465	0.596720538
ENSGALG00010023585	TFIP11	2	-0.099639212	0.047785074	-2.08515344	0.037055375	0	0.016800907	0	1	CRA004775;PRJNA827465	Yang2025_Silkie_Wenchang	0.332064293
ENSGALG00010023588	FARSB	2	0.034402571	0.105370686	0.326490911	0.744052965	0.015260929	2.974021202	66.3754919	0.5	PRJNA827465;Yang2025_Silkie_Wenchang	CRA004775	0.935821071
ENSGALG00010023588	FARSB	2	0.122222175	0.101357067	1.205857454	0.227872458	0	0.900516023	0	0.5	CRA004775;Yang2025_Silkie_Wenchang	PRJNA827465	0.740853612
ENSGALG00010023588	FARSB	2	-0.055788675	0.05761835	-0.968244926	0.332922063	0	0.048705052	0	1	CRA004775;PRJNA827465	Yang2025_Silkie_Wenchang	0.802178578
ENSGALG00010023590	S1PR1	2	-0.024750874	0.048661447	-0.50863415	0.611008687	0	0.204810876	0	0.5	PRJNA827465;Yang2025_Silkie_Wenchang	CRA004775	0.897536045
ENSGALG00010023590	S1PR1	2	0.054231781	0.095010856	0.570795627	0.568138187	0	0.95343285	0	1	CRA004775;Yang2025_Silkie_Wenchang	PRJNA827465	0.883987933
ENSGALG00010023590	S1PR1	2	0.027855266	0.127582783	0.218330919	0.827171286	0.017378404	1.528814569	34.58984363	0.5	CRA004775;PRJNA827465	Yang2025_Silkie_Wenchang	0.956550752
ENSGALG00010023592	BAHD1	2	-0.147706083	0.218667194	-0.675483507	0.499368626	0.090348747	17.82154417	94.38881395	0.5	PRJNA827465;Yang2025_Silkie_Wenchang	CRA004775	0.861953672
ENSGALG00010023592	BAHD1	2	-0.359544891	0.086130266	-4.174431436	2.98731E-05	0	0.212086785	0	1	CRA004775;Yang2025_Silkie_Wenchang	PRJNA827465	0.003288804
ENSGALG00010023592	BAHD1	2	0.020595716	0.107623365	0.191368446	0.84823694	0.011049653	1.302990533	23.25347159	0.5	CRA004775;PRJNA827465	Yang2025_Silkie_Wenchang	0.961363811
ENSGALG00010023593	CSK	2	0.086294939	0.044883192	1.922655992	0.054523267	0	0.042408669	0	1	PRJNA827465;Yang2025_Silkie_Wenchang	CRA004775	0.404097297
ENSGALG00010023593	CSK	2	0.074611617	0.096531988	0.772921173	0.439569033	0	0.304998902	0	0.5	CRA004775;Yang2025_Silkie_Wenchang	PRJNA827465	0.844278492
ENSGALG00010023593	CSK	2	0.076092704	0.047189375	1.612496543	0.106853932	0	0.243709089	0	0.5	CRA004775;PRJNA827465	Yang2025_Silkie_Wenchang	0.553927143
ENSGALG00010023597	MOGAT1	2	-0.208598359	0.687435129	-0.303444427	0.761551193	0.865110511	11.71607279	91.46471674	0.5	PRJNA827465;Yang2025_Silkie_Wenchang	CRA004775	0.939738254
ENSGALG00010023597	MOGAT1	2	-0.929990863	0.215327111	-4.318967812	1.56761E-05	0	0.323739553	0	1	CRA004775;Yang2025_Silkie_Wenchang	PRJNA827465	0.00204012
ENSGALG00010023597	MOGAT1	2	-0.302906711	0.855067332	-0.354248958	0.723152316	1.265833116	7.243510547	86.19453933	0.5	CRA004775;PRJNA827465	Yang2025_Silkie_Wenchang	0.930032096
ENSGALG00010023600	CHST14	2	-0.197586023	0.180297578	-1.095888394	0.273127619	0.057544157	8.493847804	88.2267728	1	PRJNA827465;Yang2025_Silkie_Wenchang	CRA004775	0.773904763
ENSGALG00010023600	CHST14	2	-0.148601531	0.246995396	-0.601636845	0.547415898	0.107135506	8.149455191	87.72924108	0.5	CRA004775;Yang2025_Silkie_Wenchang	PRJNA827465	0.878281266
ENSGALG00010023600	CHST14	2	-0.003714959	0.057320893	-0.064809855	0.948325395	0	0.783330753	0	0.5	CRA004775;PRJNA827465	Yang2025_Silkie_Wenchang	0.986763555
ENSGALG00010023601	TPST2	2	-0.117069938	0.235585759	-0.496931302	0.619237497	0.097894702	7.97415786	87.4594908	0.5	PRJNA827465;Yang2025_Silkie_Wenchang	CRA004775	0.899168321
ENSGALG00010023601	TPST2	2	-0.317573785	0.131482653	-2.415328399	0.015721028	0	0.477726006	0	1	CRA004775;Yang2025_Silkie_Wenchang	PRJNA827465	0.207343482
ENSGALG00010023601	TPST2	2	0.065499248	0.088188466	0.742718988	0.457651831	0.004996245	1.155876386	13.48555852	0.5	CRA004775;PRJNA827465	Yang2025_Silkie_Wenchang	0.849573219
ENSGALG00010023603	C1S	2	-0.255819288	0.384713251	-0.664960949	0.506075471	0.246202919	5.868543513	82.95999684	0.5	PRJNA827465;Yang2025_Silkie_Wenchang	CRA004775	0.863929781
ENSGALG00010023603	C1S	2	-0.270572729	0.448902306	-0.602743014	0.546679662	0.28798635	3.340123614	70.06098829	0.5	CRA004775;Yang2025_Silkie_Wenchang	PRJNA827465	0.878275987
ENSGALG00010023603	C1S	2	0.132241457	0.172251979	0.767720973	0.442652974	0	0.085979538	0	1	CRA004775;PRJNA827465	Yang2025_Silkie_Wenchang	0.845623604
ENSGALG00010023604	ENSGALG00010023604	2	0.653837535	0.239293002	2.732372149	0.006288007	0	0.181533727	0	1	PRJNA827465;Yang2025_Silkie_Wenchang	CRA004775	0.120477095
ENSGALG00010023604	ENSGALG00010023604	2	-0.075851543	0.917987855	-0.082628046	0.9341473	1.379938376	5.329529955	81.23661921	0.5	CRA004775;Yang2025_Silkie_Wenchang	PRJNA827465	0.98432801
ENSGALG00010023604	ENSGALG00010023604	2	-0.116349754	0.765166316	-0.152058123	0.879141093	1.056306201	10.03356361	90.03345134	0.5	CRA004775;PRJNA827465	Yang2025_Silkie_Wenchang	0.971214053
ENSGALG00010023605	INSYN1	2	-0.226656564	0.216715538	-1.04587131	0.295620452	0	0.759786356	0	1	PRJNA827465;Yang2025_Silkie_Wenchang	CRA004775	0.787628307
ENSGALG00010023605	INSYN1	2	-0.117339375	0.488480218	-0.240213157	0.810165015	0.240764877	1.958923845	48.95156326	0.5	CRA004775;Yang2025_Silkie_Wenchang	PRJNA827465	0.952951325
ENSGALG00010023605	INSYN1	2	-0.014823545	0.234044721	-0.063336378	0.949498649	0	0.815839172	0	0.5	CRA004775;PRJNA827465	Yang2025_Silkie_Wenchang	0.98723759
ENSGALG00010023612	GPT2	2	0.098136784	0.236782447	0.414459707	0.67853747	0.06075734	2.170291652	53.92324349	0.5	PRJNA827465;Yang2025_Silkie_Wenchang	CRA004775	0.917701868
ENSGALG00010023612	GPT2	2	0.220243647	0.431051916	0.510944595	0.609389853	0.267392208	3.404125072	70.62387607	0.5	CRA004775;Yang2025_Silkie_Wenchang	PRJNA827465	0.897238578
ENSGALG00010023612	GPT2	2	0.403364358	0.186273683	2.165439322	0.030354057	0	0.756251341	0	1	CRA004775;PRJNA827465	Yang2025_Silkie_Wenchang	0.296841384
ENSGALG00010023613	COPB1	2	-0.23538622	0.267172434	-0.881027345	0.37830302	0.139039046	38.01723189	97.36961386	0.5	PRJNA827465;Yang2025_Silkie_Wenchang	CRA004775	0.823345221
ENSGALG00010023613	COPB1	2	-0.372169278	0.169943665	-2.189956763	0.028527373	0.041041262	3.057847233	67.29725445	1	CRA004775;Yang2025_Silkie_Wenchang	PRJNA827465	0.287785248
ENSGALG00010023613	COPB1	2	0.019486595	0.038960118	0.500167771	0.61695695	0	0.96435076	0	0.5	CRA004775;PRJNA827465	Yang2025_Silkie_Wenchang	0.899111936
ENSGALG00010023617	ENSGALG00010023617	2	-0.060706918	0.445093039	-0.136391525	0.891511773	0.314277447	4.757982435	78.98268828	0.5	PRJNA827465;Yang2025_Silkie_Wenchang	CRA004775	0.973846899
ENSGALG00010023617	ENSGALG00010023617	2	0.055336139	0.383759316	0.144194909	0.885346561	0.166637983	2.289687541	56.32591861	0.5	CRA004775;Yang2025_Silkie_Wenchang	PRJNA827465	0.972280795
ENSGALG00010023617	ENSGALG00010023617	2	-0.443350963	0.203900302	-2.174351677	0.029678734	0	0.073095051	0	1	CRA004775;PRJNA827465	Yang2025_Silkie_Wenchang	0.29397955
ENSGALG00010023620	IRS1	2	-0.079095029	0.061250913	-1.29132815	0.196589914	0	0.492967937	0	1	PRJNA827465;Yang2025_Silkie_Wenchang	CRA004775	0.707950648
ENSGALG00010023620	IRS1	2	0.093108617	0.218044621	0.427016343	0.669367424	0.052695952	1.655031978	39.57820675	0.5	CRA004775;Yang2025_Silkie_Wenchang	PRJNA827465	0.914377769
ENSGALG00010023620	IRS1	2	0.066872932	0.276775822	0.241614068	0.80907922	0.103222178	2.307140643	56.65630516	0.5	CRA004775;PRJNA827465	Yang2025_Silkie_Wenchang	0.952716213
ENSGALG00010023621	CD79B	2	0.586168379	0.363242561	1.613710621	0.106590209	0.043387356	1.185139094	15.6217186	1	PRJNA827465;Yang2025_Silkie_Wenchang	CRA004775	0.553677869
ENSGALG00010023621	CD79B	2	1.031551374	0.404495491	2.550217236	0.010765581	0	0.000826689	0	1	CRA004775;Yang2025_Silkie_Wenchang	PRJNA827465	0.165305655
ENSGALG00010023621	CD79B	2	0.528138745	0.342187338	1.543419895	0.12272893	0	0.969394439	0	1	CRA004775;PRJNA827465	Yang2025_Silkie_Wenchang	0.587672408
ENSGALG00010023623	OSBPL6	2	0.006001674	0.128322638	0.04677019	0.962696388	0	0.000801245	0	1	PRJNA827465;Yang2025_Silkie_Wenchang	CRA004775	0.989980504
ENSGALG00010023623	OSBPL6	2	0.261069013	0.33754323	0.773438749	0.439262765	0.134015226	2.221563789	54.98666278	1	CRA004775;Yang2025_Silkie_Wenchang	PRJNA827465	0.844161867
ENSGALG00010023623	OSBPL6	2	0.247711446	0.328529906	0.753999685	0.450849411	0.139894437	2.419847714	58.67508546	1	CRA004775;PRJNA827465	Yang2025_Silkie_Wenchang	0.847631399
ENSGALG00010023624	PSMA1	2	-0.097604685	0.075047099	-1.300579049	0.193402582	0.004709917	1.27767375	21.73275845	1	PRJNA827465;Yang2025_Silkie_Wenchang	CRA004775	0.703339473
ENSGALG00010023624	PSMA1	2	-0.299598059	0.144223689	-2.077315189	0.037772478	0	0.059349078	0	1	CRA004775;Yang2025_Silkie_Wenchang	PRJNA827465	0.335429998
ENSGALG00010023624	PSMA1	2	-0.104490201	0.097160625	-1.075437723	0.2821788	0.00900554	1.293136065	22.66861727	1	CRA004775;PRJNA827465	Yang2025_Silkie_Wenchang	0.779519046
ENSGALG00010023626	LOXL1	2	-0.053107894	0.068711127	-0.772915477	0.439572405	0	0.182169705	0	1	PRJNA827465;Yang2025_Silkie_Wenchang	CRA004775	0.844278492
ENSGALG00010023626	LOXL1	2	0.031875553	0.11189446	0.284871592	0.775742521	0	0.703809927	0	0.5	CRA004775;Yang2025_Silkie_Wenchang	PRJNA827465	0.943815265
ENSGALG00010023626	LOXL1	2	-0.024121483	0.114268016	-0.211095663	0.832812624	0.008554215	1.206658157	17.12648735	0.5	CRA004775;PRJNA827465	Yang2025_Silkie_Wenchang	0.957861248
ENSGALG00010023627	IFT52	2	0.056821583	0.053829952	1.055575578	0.291162163	0	0.131630389	0	1	PRJNA827465;Yang2025_Silkie_Wenchang	CRA004775	0.78467882
ENSGALG00010023627	IFT52	2	-0.044251585	0.121301712	-0.364805941	0.71525627	0	0.419656899	0	0.5	CRA004775;Yang2025_Silkie_Wenchang	PRJNA827465	0.926953446
ENSGALG00010023627	IFT52	2	0.049541285	0.056562376	0.875869945	0.38110074	6.87823E-05	1.002664591	0.265750944	0.5	CRA004775;PRJNA827465	Yang2025_Silkie_Wenchang	0.824392515
ENSGALG00010023631	ENSGALG00010023631	2	-0.405747831	0.234312377	-1.73165343	0.083335287	0.075348214	2.920631266	65.76082672	1	PRJNA827465;Yang2025_Silkie_Wenchang	CRA004775	0.497316548
ENSGALG00010023631	ENSGALG00010023631	2	-0.380942508	0.359994844	-1.05818879	0.289969381	0.16495592	2.660558703	62.41390957	0.5	CRA004775;Yang2025_Silkie_Wenchang	PRJNA827465	0.784195688
ENSGALG00010023631	ENSGALG00010023631	2	-0.189356203	0.119118943	-1.589639711	0.11191604	0	0.40600296	0	0.5	CRA004775;PRJNA827465	Yang2025_Silkie_Wenchang	0.56547057
ENSGALG00010023635	SCN4A	2	-0.459673997	0.106198346	-4.328447817	1.50164E-05	0	0.583025299	0	1	PRJNA827465;Yang2025_Silkie_Wenchang	CRA004775	0.001967297
ENSGALG00010023635	SCN4A	2	-0.38460868	0.124660958	-3.085237646	0.002033896	0	0.363611206	0	1	CRA004775;Yang2025_Silkie_Wenchang	PRJNA827465	0.058631999
ENSGALG00010023635	SCN4A	2	-0.517594444	0.175679483	-2.94624298	0.003216596	0	0.827573576	0	1	CRA004775;PRJNA827465	Yang2025_Silkie_Wenchang	0.078816632
ENSGALG00010023636	PDE3B	2	-0.15861297	0.089378692	-1.774617261	0.075961105	0.010043397	2.670749504	62.55732713	1	PRJNA827465;Yang2025_Silkie_Wenchang	CRA004775	0.47539798
ENSGALG00010023636	PDE3B	2	-0.238645516	0.0783919	-3.04426241	0.002332516	0	0.25390078	0	1	CRA004775;Yang2025_Silkie_Wenchang	PRJNA827465	0.064063502
ENSGALG00010023636	PDE3B	2	-0.081636466	0.066414453	-1.229197307	0.218997838	0	0.084321338	0	1	CRA004775;PRJNA827465	Yang2025_Silkie_Wenchang	0.73259614
ENSGALG00010023637	RHBDD1	2	-0.000267013	0.091863801	-0.002906617	0.997680859	0.005348534	1.444981226	30.79494861	0.5	PRJNA827465;Yang2025_Silkie_Wenchang	CRA004775	0.999290321
ENSGALG00010023637	RHBDD1	2	0.058083349	0.100490518	0.577998304	0.563265265	0	0.526871492	0	0.5	CRA004775;Yang2025_Silkie_Wenchang	PRJNA827465	0.882393222
ENSGALG00010023637	RHBDD1	2	-0.070901709	0.083931772	-0.844754109	0.398248142	0	0.023122532	0	1	CRA004775;PRJNA827465	Yang2025_Silkie_Wenchang	0.831980453
ENSGALG00010023638	DCTN2	2	0.051014222	0.17868641	0.285495814	0.775264311	0.055667295	7.401389855	86.48902409	0.5	PRJNA827465;Yang2025_Silkie_Wenchang	CRA004775	0.943815265
ENSGALG00010023638	DCTN2	2	0.214223727	0.111972176	1.913187137	0.0557241	0	0.552343499	0	0.5	CRA004775;Yang2025_Silkie_Wenchang	PRJNA827465	0.408696004
ENSGALG00010023638	DCTN2	2	-0.109156348	0.053862017	-2.026592296	0.042704125	0	0.123068621	0	1	CRA004775;PRJNA827465	Yang2025_Silkie_Wenchang	0.357486737
ENSGALG00010023639	KLHL23	2	0.259660904	0.143231155	1.812880055	0.069850323	0	0.98282924	0	1	PRJNA827465;Yang2025_Silkie_Wenchang	CRA004775	0.455777165
ENSGALG00010023639	KLHL23	2	0.061194558	0.451813979	0.135441931	0.892262473	0.28111619	2.790029103	64.15807996	0.5	CRA004775;Yang2025_Silkie_Wenchang	PRJNA827465	0.974208739
ENSGALG00010023639	KLHL23	2	-0.025279331	0.273161897	-0.0925434	0.926266311	0.056982565	1.366383392	26.81409875	0.5	CRA004775;PRJNA827465	Yang2025_Silkie_Wenchang	0.98280842
ENSGALG00010023641	LRRC40	2	-0.212885145	0.0804932	-2.644759355	0.008174903	0.007095008	2.122595522	52.88786819	1	PRJNA827465;Yang2025_Silkie_Wenchang	CRA004775	0.140550403
ENSGALG00010023641	LRRC40	2	-0.325110238	0.089843663	-3.618621813	0.000296176	0	0.319574873	0	1	CRA004775;Yang2025_Silkie_Wenchang	PRJNA827465	0.016889382
ENSGALG00010023641	LRRC40	2	-0.18525569	0.110075143	-1.682992946	0.092376457	0.009805272	1.192431245	16.13772249	1	CRA004775;PRJNA827465	Yang2025_Silkie_Wenchang	0.520517459
ENSGALG00010023643	SCN4B	2	-0.07929867	0.119377333	-0.664269073	0.506518112	0	0.216537482	0	1	PRJNA827465;Yang2025_Silkie_Wenchang	CRA004775	0.863929781
ENSGALG00010023643	SCN4B	2	-0.157677006	0.184556428	-0.854356623	0.392907422	0	0.00267008	0	1	CRA004775;Yang2025_Silkie_Wenchang	PRJNA827465	0.830060225
ENSGALG00010023643	SCN4B	2	-0.056824439	0.132435084	-0.429073912	0.667869437	0	0.068884782	0	1	CRA004775;PRJNA827465	Yang2025_Silkie_Wenchang	0.914120722
ENSGALG00010023646	DPH5	2	-0.012885363	0.0693243	-0.185870797	0.852546083	0	0.546783753	0	0.5	PRJNA827465;Yang2025_Silkie_Wenchang	CRA004775	0.962607579
ENSGALG00010023646	DPH5	2	0.030976654	0.12188953	0.254137119	0.799389628	0	0.539723839	0	0.5	CRA004775;Yang2025_Silkie_Wenchang	PRJNA827465	0.949922217
ENSGALG00010023646	DPH5	2	-0.052445915	0.078069965	-0.671780946	0.50172316	0	0.158168465	0	1	CRA004775;PRJNA827465	Yang2025_Silkie_Wenchang	0.86279037
ENSGALG00010023648	SCN2B	2	0.317218827	0.349921894	0.906541808	0.364649146	0.229684588	16.10001185	93.78882445	0.5	PRJNA827465;Yang2025_Silkie_Wenchang	CRA004775	0.817512144
ENSGALG00010023648	SCN2B	2	0.086021076	0.688003489	0.12503	0.9004998	0.795127143	5.424985067	81.56676954	0.5	CRA004775;Yang2025_Silkie_Wenchang	PRJNA827465	0.976475201
ENSGALG00010023648	SCN2B	2	-0.146308033	0.25589335	-0.571753946	0.567488682	0.061740297	1.341529502	25.45821774	1	CRA004775;PRJNA827465	Yang2025_Silkie_Wenchang	0.883717772

ENSGALG00010023652	SLC30A7	2	-0.267422031	0.361693901	-0.739360078	0.459688377	0.249622278	21.69263397	95.39014026	0.5	PRJNA827465;Yang2025_Silkie_Wenchang	CRA004775	0.850098539
ENSGALG00010023652	SLC30A7	2	-0.612996243	0.117047107	-5.237175506	1.63053E-07	0	0.262530002	0	1	CRA004775;Yang2025_Silkie_Wenchang	PRJNA827465	5.29625E-05
ENSGALG00010023652	SLC30A7	2	-0.060698235	0.23856326	-0.254432453	0.799161482	0.07075812	2.025644139	50.63298726	0.5	CRA004775;PRJNA827465	Yang2025_Silkie_Wenchang	0.949922217
ENSGALG00010023655	C1R	2	-0.124128448	0.182346834	-0.680727189	0.496044129	0.055979479	6.129599596	83.68572067	0.5	PRJNA827465;Yang2025_Silkie_Wenchang	CRA004775	0.860911322
ENSGALG00010023655	C1R	2	-0.29068828	0.118448898	-2.454123976	0.014122828	0	0.459137735	0	1	CRA004775;Yang2025_Silkie_Wenchang	PRJNA827465	0.194761234
ENSGALG00010023655	C1R	2	0.038727516	0.074226951	0.521744667	0.60184812	0	0.1280234	0	0.5	CRA004775;PRJNA827465	Yang2025_Silkie_Wenchang	0.895289227
ENSGALG00010023663	PHOSPHO2	2	-0.082195238	0.083830915	-0.980488383	0.326845101	0	0.906666392	0	1	PRJNA827465;Yang2025_Silkie_Wenchang	CRA004775	0.800028239
ENSGALG00010023663	PHOSPHO2	2	-0.068246835	0.188873923	-0.361335404	0.717848731	0.030716179	1.723694941	41.98509398	0.5	CRA004775;Yang2025_Silkie_Wenchang	PRJNA827465	0.927927766
ENSGALG00010023663	PHOSPHO2	2	-0.011214026	0.08821857	-0.127116392	0.898848278	0	0.584817599	0	0.5	CRA004775;PRJNA827465	Yang2025_Silkie_Wenchang	0.975893962
ENSGALG00010023664	SLC6A9	2	0.009292026	0.07237389	0.128389196	0.897840981	0	0.405982068	0	0.5	PRJNA827465;Yang2025_Silkie_Wenchang	CRA004775	0.975696709
ENSGALG00010023664	SLC6A9	2	-0.492275679	0.510347299	-0.964589564	0.334750429	0.437663671	5.590479509	82.11244673	1	CRA004775;Yang2025_Silkie_Wenchang	PRJNA827465	0.803101658
ENSGALG00010023664	SLC6A9	2	-0.44743823	0.559741614	-0.79936567	0.424078411	0.545798123	6.943130046	85.59727395	0.5	CRA004775;PRJNA827465	Yang2025_Silkie_Wenchang	0.840437362
ENSGALG00010023665	C16orf87	2	-0.140872526	0.089212381	-1.57906924	0.114320176	0	0.001616214	0	1	PRJNA827465;Yang2025_Silkie_Wenchang	CRA004775	0.57019364
ENSGALG00010023665	C16orf87	2	-0.144735145	0.127505515	-1.135128507	0.256321479	0	4.10073E-05	0	1	CRA004775;Yang2025_Silkie_Wenchang	PRJNA827465	0.764677554
ENSGALG00010023665	C16orf87	2	-0.138300236	0.111278701	-1.242827558	0.213931385	0	0.000176806	0	1	CRA004775;PRJNA827465	Yang2025_Silkie_Wenchang	0.726593954
ENSGALG00010023666	ENSGALG00010023666	2	-0.013255493	0.131184365	-0.101044759	0.919514929	0	0.155474598	0	0.5	PRJNA827465;Yang2025_Silkie_Wenchang	CRA004775	0.981192162
ENSGALG00010023666	ENSGALG00010023666	2	-0.208784088	0.21294095	-0.980478803	0.326849828	0.004260249	1.035182295	3.398656935	1	CRA004775;Yang2025_Silkie_Wenchang	PRJNA827465	0.800028239
ENSGALG00010023666	ENSGALG00010023666	2	-0.152332742	0.276130919	-0.551668543	0.581175466	0.081480307	1.768615903	43.45861085	0.5	CRA004775;PRJNA827465	Yang2025_Silkie_Wenchang	0.88890219
ENSGALG00010023668	RCN1	2	-0.06955761	0.10939297	-0.635850826	0.524873656	0.01599898	2.798767331	64.26998454	0.5	PRJNA827465;Yang2025_Silkie_Wenchang	CRA004775	0.869892816
ENSGALG00010023668	RCN1	2	-0.155547002	0.106358958	-1.462472038	0.143611901	0	0.912720916	0	0.5	CRA004775;Yang2025_Silkie_Wenchang	PRJNA827465	0.630564924
ENSGALG00010023668	RCN1	2	0.022596235	0.061379072	0.368142339	0.712767103	0	0.026033693	0	1	CRA004775;PRJNA827465	Yang2025_Silkie_Wenchang	0.926097326
ENSGALG00010023670	EXTL2	2	-0.217858844	0.055576743	-3.919964243	8.85621E-05	0	0.568173505	0	1	PRJNA827465;Yang2025_Silkie_Wenchang	CRA004775	0.007095294
ENSGALG00010023670	EXTL2	2	-0.234770849	0.121725572	-1.928689629	0.053769405	0.005097487	1.117989373	10.55371152	0.5	CRA004775;Yang2025_Silkie_Wenchang	PRJNA827465	0.401964436
ENSGALG00010023670	EXTL2	2	-0.183612097	0.061837322	-2.969276317	0.00298502	0	0.581247423	0	0.5	CRA004775;PRJNA827465	Yang2025_Silkie_Wenchang	0.075060946
ENSGALG00010023671	ENSGALG00010023671	2	0.246778836	0.236605989	1.070131947	0.284559921	0	0.020718462	0	1	PRJNA827465;Yang2025_Silkie_Wenchang	CRA004775	0.780265906
ENSGALG00010023671	ENSGALG00010023671	2	-0.134239102	0.381107354	-0.352234352	0.724662517	0.122837718	1.715827079	41.71906878	0.5	CRA004775;Yang2025_Silkie_Wenchang	PRJNA827465	0.930460989
ENSGALG00010023671	ENSGALG00010023671	2	-0.073416996	0.411656676	-0.17834521	0.858451876	0.202838577	2.387159089	58.10920165	0.5	CRA004775;PRJNA827465	Yang2025_Silkie_Wenchang	0.964378153
ENSGALG00010023676	ENSGALG00010023676	2	0.819453362	0.589586755	1.389877494	0.164566081	0.635256688	11.34005605	91.18170143	1	PRJNA827465;Yang2025_Silkie_Wenchang	CRA004775	0.663855145
ENSGALG00010023676	ENSGALG00010023676	2	0.862042345	0.640075467	1.346782354	0.178050339	0.646574027	4.545532453	78.08037707	1	CRA004775;Yang2025_Silkie_Wenchang	PRJNA827465	0.684819679
ENSGALG00010023676	ENSGALG00010023676	2	0.245842347	0.163004462	1.508193971	0.13150489	0	0.038185201	0	1	CRA004775;PRJNA827465	Yang2025_Silkie_Wenchang	0.607348611
ENSGALG00010023677	MN1	2	-0.052268558	0.085081901	-0.614332285	0.538995772	0.002976941	1.242755247	19.53363285	0.5	PRJNA827465;Yang2025_Silkie_Wenchang	CRA004775	0.87481821
ENSGALG00010023677	MN1	2	0.126132145	0.147302763	0.856278204	0.391843914	0.01115124	1.188857601	15.88563681	1	CRA004775;Yang2025_Silkie_Wenchang	PRJNA827465	0.829251057
ENSGALG00010023677	MN1	2	0.069428165	0.261098294	0.265908266	0.790309859	0.095215408	2.711353332	63.11804928	0.5	CRA004775;PRJNA827465	Yang2025_Silkie_Wenchang	0.947390191
ENSGALG00010023686	MYLK3	2	-0.379629177	0.15006748	-2.52972314	0.011415256	0	0.661810755	0	1	PRJNA827465;Yang2025_Silkie_Wenchang	CRA004775	0.17044842
ENSGALG00010023686	MYLK3	2	-0.179704794	0.384231894	-0.467698794	0.640000004	0.213591871	3.535258838	71.71352804	0.5	CRA004775;Yang2025_Silkie_Wenchang	PRJNA827465	0.906064918
ENSGALG00010023686	MYLK3	2	-0.090459503	0.251277397	-0.359998566	0.718848206	0.061781964	1.827591625	45.27719257	0.5	CRA004775;PRJNA827465	Yang2025_Silkie_Wenchang	0.928449671
ENSGALG00010023689	WT1	2	-0.182592416	0.143219161	-1.274916115	0.202338935	0	0.049757635	0	1	PRJNA827465;Yang2025_Silkie_Wenchang	CRA004775	0.715289846
ENSGALG00010023689	WT1	2	0.669219202	0.946404915	0.707117209	0.479493642	1.638259237	11.26830258	91.12554892	0.5	CRA004775;Yang2025_Silkie_Wenchang	PRJNA827465	0.856126448
ENSGALG00010023689	WT1	2	0.694942196	0.913208139	0.760989928	0.446663083	1.525492273	11.13473422	91.01909412	0.5	CRA004775;PRJNA827465	Yang2025_Silkie_Wenchang	0.84648396
ENSGALG00010023693	SRSF11	2	0.397930305	0.351733707	1.131339696	0.257912143	0.227207153	11.53493473	91.3306835	1	PRJNA827465;Yang2025_Silkie_Wenchang	CRA004775	0.765151037
ENSGALG00010023693	SRSF11	2	0.345108843	0.42526059	0.811523218	0.417065265	0.327121452	10.44181864	90.42312423	0.5	CRA004775;Yang2025_Silkie_Wenchang	PRJNA827465	0.838112595
ENSGALG00010023693	SRSF11	2	0.056499273	0.05449275	1.036821831	0.299818899	0	0.643841101	0	0.5	CRA004775;PRJNA827465	Yang2025_Silkie_Wenchang	0.790081926
ENSGALG00010023694	PPCDC	2	-0.237587666	0.138273482	-1.718244618	0.085752006	0.015214626	1.548357127	35.4154166	1	PRJNA827465;Yang2025_Silkie_Wenchang	CRA004775	0.503632861
ENSGALG00010023694	PPCDC	2	-0.231940133	0.193652001	-1.197716173	0.231027531	0.035832273	1.912496702	47.71232813	1	CRA004775;Yang2025_Silkie_Wenchang	PRJNA827465	0.743719801
ENSGALG00010023694	PPCDC	2	-0.114135067	0.099300944	-1.149385509	0.250397053	0	0.178172196	0	1	CRA004775;PRJNA827465	Yang2025_Silkie_Wenchang	0.759943035
ENSGALG00010023695	CLSTN3	2	0.001959221	0.061496883	0.031858874	0.974584596	0	0.077975438	0	0.5	PRJNA827465;Yang2025_Silkie_Wenchang	CRA004775	0.992492038
ENSGALG00010023695	CLSTN3	2	-0.06543555	0.105905154	-0.617869362	0.536664457	0	0.836441502	0	1	CRA004775;Yang2025_Silkie_Wenchang	PRJNA827465	0.874599474
ENSGALG00010023695	CLSTN3	2	-0.029061076	0.106171928	-0.273717133	0.78430201	0.00772352	1.19034816	15.99096518	0.5	CRA004775;PRJNA827465	Yang2025_Silkie_Wenchang	0.946083234
ENSGALG00010023696	LRRC4C	2	0.195132386	0.240663346	0.810810576	0.41747446	0.078053199	3.003326872	66.70359096	0.5	PRJNA827465;Yang2025_Silkie_Wenchang	CRA004775	0.838112595
ENSGALG00010023696	LRRC4C	2	0.482169344	0.213829765	2.254921549	0.024138253	0	0.087716901	0	1	CRA004775;Yang2025_Silkie_Wenchang	PRJNA827465	0.265075657
ENSGALG00010023696	LRRC4C	2	0.015972941	0.160379302	0.099594775	0.920666041	0	0.984173094	0	0.5	CRA004775;PRJNA827465	Yang2025_Silkie_Wenchang	0.981469977
ENSGALG00010023698	ENSGALG00010023698	2	0.040352484	0.172702301	0.233653426	0.815254047	0.026192474	1.707820162	41.44582538	0.5	PRJNA827465;Yang2025_Silkie_Wenchang	CRA004775	0.953933933
ENSGALG00010023698	ENSGALG00010023698	2	-0.058658599	0.418792549	-0.140066004	0.888607841	0.22509373	2.477017297	59.62886487	0.5	CRA004775;Yang2025_Silkie_Wenchang	PRJNA827465	0.973135755
ENSGALG00010023698	ENSGALG00010023698	2	-0.136468288	0.142734177	-0.958581854	0.337801347	0	0.95887557	0	1	CRA004775;PRJNA827465	Yang2025_Silkie_Wenchang	0.80415385
ENSGALG00010023699	PPIG	2	0.420288734	0.588203458	0.71452952	0.474899809	0.629825384	10.34518839	90.33367047	0.5	PRJNA827465;Yang2025_Silkie_Wenchang	CRA004775	0.854462864
ENSGALG00010023699	PPIG	2	0.694542266	0.323850793	2.144636592	0.031981914	0.111965475	2.106868791	52.53619949	1	CRA004775;Yang2025_Silkie_Wenchang	PRJNA827465	0.306656545
ENSGALG00010023699	PPIG	2	0.081170287	0.256136793	0.3169021	0.751317889	0.09876039	3.4308361	70.85258605	0.5	CRA004775;PRJNA827465	Yang2025_Silkie_Wenchang	0.937684688
ENSGALG00010023702	COL4A4	2	-0.074893314	0.129581132	-0.577966658	0.563287981	0	0.39405691	0	1	PRJNA827465;Yang2025_Silkie_Wenchang	CRA004775	0.582393222
ENSGALG00010023702	COL4A4	2	0.376668775	0.534884481	0.70420584	0.481304595	0.412300877	2.887215748	65.36455578	0.5	CRA004775;Yang2025_Silkie_Wenchang	PRJNA827465	0.85682839
ENSGALG00010023702	COL4A4	2	0.322246582	0.630755058	0.510890206	0.609427939	0.606765485	3.654079027	72.63332313	0.5	CRA004775;PRJNA827465	Yang2025_Silkie_Wenchang	0.897238578
ENSGALG00010023703	PITPNB	2	0.29718304	0.326614106	0.909881649	0.362884928	0.208414106	42.75997268	97.66136427	0.5	PRJNA827465;Yang2025_Silkie_Wenchang	CRA004775	0.816210733
ENSGALG00010023703	PITPNB	2	0.227714263	0.427710319	0.532403014	0.594446898	0.335556791	11.53844514	91.33332102	0.5	CRA004775;Yang2025_Silkie_Wenchang	PRJNA827465	0.893340972
ENSGALG00010023703	PITPNB	2	-0.031071639	0.041939964	-0.740859939	0.458778368	0	0.725110774	0	1	CRA004775;PRJNA827465	Yang2025_Silkie_Wenchang	0.850089963
ENSGALG00010023704	ANKRD13C	2	-0.09561972	0.071190265	-1.343157242	0.179221064	0.007969685	4.595129463	78.23782751	1	PRJNA827465;Yang2025_Silkie_Wenchang	CRA004775	0.685873949
ENSGALG00010023704	ANKRD13C	2	-0.164619022	0.053200267	-3.094326982	0.0019726	0	0.530245882	0	0.5	CRA004775;Yang2025_Silkie_Wenchang	PRJNA827465	0.057514153
ENSGALG00010023704	ANKRD13C	2	-0.0280333	0.038380205	-0.730410366	0.465139384	0	0.055977224	0	0.5	CRA004775;PRJNA827465	Yang2025_Silkie_Wenchang	0.852357945
ENSGALG00010023706	CDK5RAP3	2	0.230416505	0.253006699	0.91071306	0.362446581	0.117172376	11.44883965	91.26549039	0.5	PRJNA827465;Yang2025_Silkie_Wenchang	CRA004775	0.815644888
ENSGALG00010023706	CDK5RAP3	2	-0.052721418	0.551630321	-0.095573821	0.923859058	0.588710098	30.60184532	96.73222321	0.5	CRA004775;Yang2025_Silkie_Wenchang	PRJNA827465	0.982219519
ENSGALG00010023706	CDK5RAP3	2	-0.293218838	0.297914629	-0.984241839	0.324971157	0.164854161	13.6084279	92.65161261	1	CRA004775;PRJNA827465	Yang2025_Silkie_Wenchang	0.799914637
ENSGALG00010023707	MPZL2	2	-0.138498114	0.388636454	-0.356369332	0.721563993	0.137107662	1.813003957	44.84292237	0.5	PRJNA827465;Yang2025_Silkie_Wenchang	CRA004775	0.929153713
ENSGALG00010023707	MPZL2	2	-0.694552354	0.359780693	-1.930488118	0.053546386	0	0.205346596	0	1	CRA004775;Yang2025_Silkie_Wenchang	PRJNA827465	0.401322198
ENSGALG00010023707	MPZL2	2	-0.251046386	0.55183321	-0.454931639	0.649158421	0.381619362	2.541961428	60.6603	0.5	CRA004775;PRJNA827465	Yang2025_Silkie_Wenchang	0.950702768
ENSGALG00010023710	CDC14A	2	0.005252991	0.056905685	0.092310478	0.926451364	0	0.096342659	0	0.5	PRJNA827465;Yang2025_Silkie_Wenchang	CRA004775	0.98280842
ENSGALG00010023710	CDC14A	2	6.03034E-05	0.102970936	0.000585636	0.99953273	0	0.283846707	0	0.5	CRA004775;Yang2025_Silkie_Wenchang	PRJNA827465	0.999889665
ENSGALG00010023710	CDC14A	2	0.022417746	0.063573354	0.352628023	0.724367126	0	0.165720797	0	1	CRA004775;PRJNA827465	Yang2025_Silkie_Wenchang	0.930387861
ENSGALG00010023711	ENSGALG00010023711	2	-0.41657942	0.320188188	-1.30108556	0.19324286	0.106615755	2.083216033	51.99729724	1	PRJNA827465;Yang2025_Silkie_Wenchang	CRA004775	0.703216219
ENSGALG00010023711	ENSGALG00010023711	2	-0.430721906	0.47293063	-0.910750707	0.36242674	0.237013934	1.88984168	47.0855146	1	CRA004775;Yang2025_Silkie_Wenchang	PRJNA827465	0.815644888
ENSGALG00010023711	ENSGALG00010023711	2	-0.803890586	0.283301322	-2.83758149	0.004545674	0	0.247311903	0	1	CRA004775;PRJNA827465	Yang2025_Silkie_Wenchang	0.098687396
ENSGALG00010023712	ARHGAP40	2	-0.008645902	0.245862768	-0.035165558	0.971947726	0.109640519	10.5413419	90.51354173	0.5	PRJNA827465;Yang2025_Silkie_Wenchang	CRA004775	0.991955348
ENSGALG00010023712	ARHGAP40	2	-0.249837123	0.125434381	-1.991775471	0.046395699	0	0.227667263	0	1	CRA004775;Yang2025_Silkie_Wenchang	PRJNA827465	0.371687521
ENSGALG00010023712	ARHGAP40	2	0.220475579	0.077510697	2.844453553	0.004448767	0	0.248441676	0	0.5	CRA004775;PRJNA827465	Yang2025_Silkie_Wenchang	0.097660808
ENSGALG00010023714	EIF3M	2	-0.116533105	0.12977743	-0.897945858	0.369214415	0.022114912	2.71245255	63.13299563	1	PRJNA827465;Yang2025_Silkie_Wenchang	CRA004775	0.820606801
ENSGALG00010023714	EIF3M	2	-0.168498404	0.110185109	-1.52923027	0.126207369	0.000964528	1.17747411	15.07248599	1	CRA004775;Yang2025_Silkie_Wenchang	PRJNA827465	0.596623553
ENSGALG00010023714	EIF3M	2	-0.019815521	0.068723191	-0.288338193	0.773087874	0	0.070776231	0	1	CRA004775;PRJNA827465	Yang2025_Silkie_Wenchang	0.943100418
ENSGALG00010023715	CD3E	2	-0.104951245	0.418872435	-0.25055658	0.802156955	0.16367094	1.644526375	39.19221878	0.5	PRJNA827465;Yang2025_Silkie_Wenchang	CRA004775	0.950630607
ENSGALG00010023715	CD3E	2	-0.744735748	0.377900013	-1.970721679	0.048755719	0	1.56902E-07	0	1	CRA004775;Yang2025_Silkie_Wenchang	PRJNA827465	0.381722319
ENSGALG00010023715	CD3E	2	-0.217114549	0.451622599	-0.480743324	0.630698936	0.266299687	2.756593166	63.72172338	0.5	CRA004775;PRJNA827465	Yang2025_Silkie_Wenchang	0.902770945
ENSGALG00010023716	FASTKD1	2	-0.169685804	0.073301228	-2.314910801	0.020617814	0	0.533796835	0	1	PRJNA827465;Yang2025_Silkie_Wenchang	CRA004775	0.242254693
ENSGALG00010023716	FASTKD1	2	-0.235564458	0.110213397	-2.137348669	0.032569643	0	0.013248681	0	1	CRA004775;Yang2025_Silkie_Wenchang	PRJNA827465	0.310037591
ENSGALG00010023716	FASTKD1	2	-0.139471673	0.085673294	-1.627948067	0.103535898	0	0.091525224	0	1	CRA004775;PRJNA827465	Yang2025_Silkie_Wenchang	0.547964315
ENSGALG00010023720	SCAMP5	2	0.07213491	0.073834864	0.976976259	0.328580096	0	0.108228981	0	1	PRJNA827465;Yang2025_Silkie_Wenchang	CRA004775	0.800761292
ENSGALG00010023720	SCAMP5	2	-0.127887189	0.254706339	-0.502096607	0.615599567	0.103209126	4.777803226	79.06987892	0.5	CRA004775;Yang2025_Silkie_Wenchang	PRJNA827465	0.898886043
ENSGALG00010023720	SCAMP5	2	-0.141657353	0.227096265	-0.623776672	0.532774262	0.082517125	4.637345133	78.45393756	0.5	CRA004775;PRJNA827465	Yang2025_Silkie_Wenchang	0.873183244
ENSGALG00010023724	CYP2R1	2	-0.282061862	0.297240502	-0.948938419	0.342653764	0.16149086	11.52965748	91.32596343	0.5	PRJNA827465;Yang2025_Silkie_Wenchang	CRA004775	0.800011702
ENSGALG00010023724	CYP2R1	2	-0.129196381	0.49224389	-0.262464164	0.79296361	0.432135441	8.860051448	88.71338382	0.5	CRA004775;Yang2025_Silkie_Wenchang	PRJNA827465	0.948501088
ENSGALG00010023724	CYP2R1	2	0.102510919	0.168148966	0.609643469	0.54209801	0.026687682	1.530811013	34.67514988	1	CRA004775;PRJNA827465	Yang2025_Silkie_Wenchang	0.876685104
ENSGALG00010023726	PNPO	2	0.386616503	0.252937607	1.528505423	0.126387098	0.107889279	6.376818031	84.31819765	1	PRJNA827465;Yang2025_Silkie_Wenchang	CRA004775	0.59697057
ENSGALG00010023726	PNPO	2	0.242250466	0.47152385	0.51376079	0.607419245	0.360165328	4.689707642	78.67670916	0.5	CRA004775;Yang2025_Silkie_Wenchang	PRJNA827465	0.897023476
ENSGALG00010023726	PNPO	2	0.080308842	0.145858939	0.550592526	0.581913033	0.004155118	1.042602193	4.086140732	0.5	CRA004775;PRJNA827465	Yang2025_Silkie_Wenchang	0.889192798
ENSGALG00010023730	SP2	2	-0.003319793	0.093962146	-0.035331174	0.971815666	0.006264607	1.319744352	24.22774921	0.5	PRJNA827465;Yang2025_Silkie_Wenchang	CRA004775	0.991955348
ENSGALG00010023730	SP2	2	0.002850778	0.171673913	0.016605774	0.986751118	0.024752305	1.723929748	41.99299588	0.5	CRA004775;Yang2025_Silkie_Wenchang	PRJNA827465	0.996282251
ENSGALG00010023730	SP2	2	-0.067854002	0.066234364	-1.024453143	0.305621292	0	0.342370634	0	1	CRA004775;PRJNA827465	Yang2025_Silkie_Wenchang	0.79225794
ENSGALG00010023731	ACTR5	2	-0.209812689	0.068015635	-3.084771452	0.002037086	0	0.051689643	0	1	PRJNA827465;Yang2025_Silkie_Wenchang	CRA004775	0.058631999
ENSGALG00010023731	ACTR5	2	-0.225934344	0.109510258	-2.063134067	0.039099892	0	0.017182622	0	1	CRA004775;Yang2025_Silkie_Wenchang	PRJNA827465	0.341557454
ENSGALG00010023731	ACTR5	2	-0.201399157	0.07294304	-2.761046923	0.00576164	0	0.000548219	0	1	CRA004775;PRJNA827465	Yang2025_Silkie_Wenchang	0.114426341
ENSGALG00010023732	TM2D1	2	-0.011203559	0.082107145	-0.13645048	0.891465169	0	0.578767423	0	0.5	PRJNA827465;Yang2025_Silkie_Wenchang	CRA004775	0.973846899
ENSGALG00010023732	TM2D1	2	0.043342263	0.130872181	0.331180108	0.740508445	0	0.304921523	0	0.5	CRA004775;Yang2025_Silkie_Wenchang	PRJNA827465	0.935128797
ENSGALG00010023732	TM2D1	2	-0.054505607	0.091037446	-0.598716349	0.549362053	0	0.009409812	0	1	CRA004775;PRJNA827465	Yang2025_Silkie_Wenchang	0.878554604
ENSGALG00010023733	ORC6	2	-0.019783733	0.095385732	-0.207407681	0.835691484	0	0.050164989	0	0.5	PRJNA827465;Yang2025_Silkie_Wenchang	CRA004775	0.958339923
ENSGALG00010023733	ORC6	2	-0.085433764	0.203819975	-0.419162862	0.675097112	0.010573939	1.093768585	8.572982133	0.5	CRA004775;Yang2025_Silkie_Wenchang	PRJNA827465	0.916111653
ENSGALG00010023733	ORC6	2	-0.060309958	0.110394841	-0.546311382	0.584851913	0.001456518	1.014952363	1.473208326	1	CRA004775;PRJNA827465	Yang2025_Silkie_Wenchang	0.891016154
ENSGALG00010023738	ENSGALG00010023738	2	-0.04170992	0.110754875	-0.376596701	0.70647334	0	0.001822069	0	1	PRJNA827465;Yang2025_Silkie_Wenchang	CRA004775	0.924548793
ENSGALG00010023738	ENSGALG00010023738	2	-0.107290748	0.178008032	-0.602729812	0.546688446	0	0.507669029	0	1	CRA004775;Yang2025_Silkie_Wenchang	PRJNA827465	0.878275987
ENSGALG00010023738	ENSGALG00010023738	2	-0.078563896	0.123412447	-0.636596211	0.524387894	0	0.553459944	0	1	CRA004775;PRJNA827465	Yang2025_Silkie_Wenchang	0.869790047
ENSGALG00010023740	PPP1R16B	2	-0.131871341	0.232102604	-0.568159678	0.569926548	0.093076748	7.192810848	86.09722929	0.5	PRJNA827465;Yang2025_Silkie_Wenchang	CRA004775	0.884923673
ENSGALG00010023740	PPP1R16B	2	-0.346354837	0.135425937	-2.557522178	0.010542084	0	0.335336101	0	1	CRA004775;Yang2025_Silkie_Wenchang	PRJNA827465	0.163510462
ENSGALG00010023740	PPP1R16B	2	0.072327558	0.090650865	0.797869472	0.424946241	0	0.402740072	0	0.5	CRA004775;PRJNA827465	Yang2025_Silkie_Wenchang	0.841139484
ENSGALG00010023743	CD3D	2	0.574318531	0.251094069	2.287264425	0.022180394	0.086913965	3.149766757	68.25161743	1	PRJNA827465;Yang2025_Silkie_Wenchang	CRA004775	0.252609681
ENSGALG00010023743	CD3D	2	0.07428904	0.811287708	0.09156929	0.927040252	1.209401753	12.05672443	91.7058733	0.5	CRA004775;Yang2025_Silkie_Wenchang	PRJNA827465	0.982928581
ENSGALG00010023743	CD3D	2	-0.15114325	0.556296866	-0.271695311	0.785856307	0.52974082	6.473652054	84.5527688	0.5	CRA004775;PRJNA827465	Yang2025_Silkie_Wenchang	0.946639719
ENSGALG00010023744	MT4	2	-0.020279875	0.223510394	-0.090733478	0.927704369	0.043531258	1.732508114	42.28021262	0.5	PRJNA827465;Yang2025_Silkie_Wenchang	CRA004775	0.982993195
ENSGALG00010023744	MT4	2	-0.136229655	0.221319818	-0.615533017	0.538202771	0	0.798823997	0	0.5	CRA004775;Yang2025_Silkie_Wenchang	PRJNA827465	0.87481821
ENSGALG00010023744	MT4	2	0.157417909	0.17840143	0.882380309	0.377571181	0	0.010459742	0	1	CRA004775;PRJNA827465	Yang2025_Silkie_Wenchang	0.82325159
ENSGALG00010023746	TTC28	2	0.003733574	0.066360878	0.056261675	0.95513335	0	0.229385672	0	0.5	PRJNA827465;Yang2025_Silkie_Wenchang	CRA004775	0.988118398
ENSGALG00010023746	TTC28	2	-0.056345945	0.13194816	-0.427030926	0.669356802	0	0.00050724	0	1	CRA004775;Yang2025_Silkie_Wenchang	PRJNA827465	0.914377769
ENSGALG00010023746	TTC28	2	0.017845187	0.074035712	0.241034856	0.809528102	0	0.045227352	0	0.5	CRA004775;PRJNA827465	Yang2025_Silkie_Wenchang	0.952770549
ENSGALG00010023748	ENSGALG00010023748	2	-0.574983952	0.360528315	-1.594837154	0.110748656	0.232102513	9.08431679	88.99201753	1	PRJNA827465;Yang2025_Silkie_Wenchang	CRA004775	0.563098892
ENSGALG00010023748	ENSGALG00010023748	2	-0.856420512	0.164826504	-5.195890791	2.03742E-07	0	0.611951735	0	1	CRA004775;Yang2025_Silkie_Wenchang	PRJNA827465	6.25599E-05
ENSGALG00010023748	ENSGALG00010023748	2	-0.398755277	0.218979274	-1.820972691	0.068611008	0.061123627	2.423774386	58.7420345	1	CRA004775;PRJNA827465	Yang2025_Silkie_Wenchang	0.45121266
ENSGALG00010023749	RPP25	2	0.225592889	0.111923132	2.015605583	0.043841236	0	0.079853872	0	1	PRJNA827465;Yang2025_Silkie_Wenchang	CRA004775	0.361804689
ENSGALG00010023749	RPP25	2	0.287867365	0.152153825	1.891949579	0.058497699	0	0.250206531	0	1	CRA004775;Yang2025_Silkie_Wenchang	PRJNA827465	0.418938606
ENSGALG00010023749	RPP25	2	0.229146473	0.145076439	1.579487851	0.114224201	0	0.412803233	0	1	CRA004775;PRJNA827465	Yang2025_Silkie_Wenchang	0.570149069
ENSGALG00010023750	ENSGALG00010023750	2	-0.128041278	0.110608066	-1.157612481	0.247022214	0.010731474	1.72064779	41.88235352	1	PRJNA827465;Yang2025_Silkie_Wenchang	CRA004775	0.757459165
ENSGALG00010023750	ENSGALG00010023750	2	-0.199321427	0.122486729	-1.627289976	0.103675526	0	0.816013441	0	1	CRA004775;Yang2025_Silkie_Wenchang	PRJNA827465	0.547964315
ENSGALG00010023750	ENSGALG00010023750	2	-0.035304564	0.090071386	-0.391962038	0.695086263	0	0.009548601	0	1	CRA004775;PRJNA827465	Yang2025_Silkie_Wenchang	0.92097163
ENSGALG00010023751	PRRG4	2	-0.042068673	0.213233388	-0.197289334	0.843601126	0.027736446	1.434819408	30.30481782	0.5	PRJNA827465;Yang2025_Silkie_Wenchang	CRA004775	0.96057435
ENSGALG00010023751	PRRG4	2	0.109166358	0.202730152	0.538481115	0.590244939	0	0.212264502	0	0.5	CRA004775;Yang2025_Silkie_Wenchang	PRJNA827465	0.892451983
ENSGALG00010023751	PRRG4	2	-0.151224487	0.189032613	-0.799991514	0.423715714	0	0.370535399	0	1	CRA004775;PRJNA827465	Yang2025_Silkie_Wenchang	0.84030043
ENSGALG00010023752	C1QL1	2	0.025637021	0.213786038	0.11991906	0.904547266	0.028168329	1.424836485	29.81650802	0.5	PRJNA827465;Yang2025_Silkie_Wenchang	CRA004775	0.977669174
ENSGALG00010023752	C1QL1	2	0.785275641	0.579687094	1.354654345	0.17552771	0.505894924	3.814593343	73.78488583	1	CRA004775;Yang2025_Silkie_Wenchang	PRJNA827465	0.680297576
ENSGALG00010023752	C1QL1	2	0.579633298	0.799730592	0.724785701	0.46858351	1.126391594	7.921918125	87.37679456	0.5	CRA004775;PRJNA827465	Yang2025_Silkie_Wenchang	0.853198008
ENSGALG00010023759	CALCA	2	0.491539934	0.289639678	1.697073887	0.089682692	0.070143218	1.673129986	40.23178064	1	PRJNA827465;Yang2025_Silkie_Wenchang	CRA004775	0.513744186
ENSGALG00010023759	CALCA	2	0.010222488	0.947084363	0.010793641	0.991388088	1.454261907	4.942028976	79.76539585	0.5	CRA004775;Yang2025_Silkie_Wenchang	PRJNA827465	0.99755571
ENSGALG00010023759	CALCA	2	-0.205253021	0.628244438	-0.326708854	0.743888103	0.534850092	2.596191473	61.48203973	0.5	CRA004775;PRJNA827465	Yang2025_Silkie_Wenchang	0.935821071
ENSGALG00010023760	ZW10	2	0.143977767	0.074832851	1.923991476	0.054355652	0.00619409	2.190456714	54.34742016	1	PRJNA827465;Yang2025_Silkie_Wenchang	CRA004775	0.40381596
ENSGALG00010023760	ZW10	2	0.091182673	0.178685505	0.510296976	0.609843421	0.044329625	2.860078621	65.03592619	0.5	CRA004775;Yang2025_Silkie_Wenchang	PRJNA827465	0.897238578
ENSGALG00010023760	ZW10	2	0.054659398	0.067979049	0.804062413	0.421360931	0.001482196	1.066369279	6.223855134	0.5	CRA004775;PRJNA827465	Yang2025_Silkie_Wenchang	0.839352211
ENSGALG00010023762	BBS5	2	-0.102816205	0.102647224	-1.001646227	0.316514486	0.005837041	1.376569095	27.35562613	0.5	PRJNA827465;Yang2025_Silkie_Wenchang	CRA004775	0.796883862
ENSGALG00010023762	BBS5	2	0.279558049	0.395608465	0.706653355	0.479781923	0.210227651	2.372878216	57.85708709	1	CRA004775;Yang2025_Silkie_Wenchang	PRJNA827465	0.856274132
ENSGALG00010023762	BBS5	2	0.204971895	0.513333436	0.39929582	0.689675247	0.4100461	3.719995648	73.11824812	0.5	CRA004775;PRJNA827465	Yang2025_Silkie_Wenchang	0.919979266
ENSGALG00010023764	COX5A	2	-0.559095552	0.537338891	-1.040489645	0.298112472	0.554742604	24.77457061	95.9636031	1	PRJNA827465;Yang2025_Silkie_Wenchang	CRA004775	0.789110621
ENSGALG00010023764	COX5A	2	-0.458782925	0.680070886	-0.674610447	0.499923294	0.849932646	12.13585484	91.75995417	0.5	CRA004775;Yang2025_Silkie_Wenchang	PRJNA827465	0.862078151
ENSGALG00010023764	COX5A	2	-0.027618907	0.06615516	-0.417486815	0.676322366	0	0.709816033	0	0.5	CRA004775;PRJNA827465	Yang2025_Silkie_Wenchang	0.916729823
ENSGALG00010023766	ENSGALG00010023766	2	-0.174988288	0.313849997	-0.557553895	0.577149047	0.079641283	1.678216293	40.41292506	0.5	PRJNA827465;Yang2025_Silkie_Wenchang	CRA004775	0.887145528
ENSGALG00010023766	ENSGALG00010023766	2	0.087482308	0.283288831	0.308809591	0.757466373	0	0.062234192	0	0.5	CRA004775;Yang2025_Silkie_Wenchang	PRJNA827465	0.938842663
ENSGALG00010023766	ENSGALG00010023766	2	-0.349001471	0.289396184	-1.205964314	0.227831251	0	0.568915425	0	1	CRA004775;PRJNA827465	Yang2025_Silkie_Wenchang	0.740853612
ENSGALG00010023767	ENSGALG00010023767	2	0.15621692	0.223588188	0.698681451	0.484751129	0.05512881	2.216908925	54.89214785	0.5	PRJNA827465;Yang2025_Silkie_Wenchang	CRA004775	0.857004733
ENSGALG00010023767	ENSGALG00010023767	2	-0.246409785	0.275387529	-0.894774668	0.370907555	0.074684845	1.772511726	43.58288382	1	CRA004775;Yang2025_Silkie_Wenchang	PRJNA827465	0.8214684
ENSGALG00010023767	ENSGALG00010023767	2	-0.07182859	0.514406307	-0.13963396	0.888949207	0.430784906	5.166842063	80.64581832	0.5	CRA004775;PRJNA827465	Yang2025_Silkie_Wenchang	0.973135755
ENSGALG00010023770	SCRN2	2	-0.004415512	0.225739669	-0.019560195	0.984394217	0.071440347	3.102213383	67.76495113	0.5	PRJNA827465;Yang2025_Silkie_Wenchang	CRA004775	0.995703389
ENSGALG00010023770	SCRN2	2	0.076865506	0.238684368	0.322038292	0.747423686	0.039928853	1.499158226	33.29590015	0.5	CRA004775;Yang2025_Silkie_Wenchang	PRJNA827465	0.93680487
ENSGALG00010023770	SCRN2	2	-0.195841619	0.113859059	-1.720035463	0.085425996	0	0.007682608	0	1	CRA004775;PRJNA827465	Yang2025_Silkie_Wenchang	0.502621951
ENSGALG00010023773	OVOA	2	0.226409432	0.214047667	1.057752397	0.290168341	0	0.036035633	0	1	PRJNA827465;Yang2025_Silkie_Wenchang	CRA004775	0.784195688
ENSGALG00010023773	OVOA	2	0.386542231	0.344565611	1.121824752	0.26193696	0	0.952927135	0	1	CRA004775;Yang2025_Silkie_Wenchang	PRJNA827465	0.768363052
ENSGALG00010023773	OVOA	2	0.33071445	0.390101937	0.847764183	0.396569314	0.099530114	1.141313759	12.38167487	1	CRA004775;PRJNA827465	Yang2025_Silkie_Wenchang	0.831365249
ENSGALG00010023775	ENSGALG00010023775	2	-0.231279135	0.235940293	-0.980244331	0.326965527	0.083931512	3.953578006	74.70645581	1	PRJNA827465;Yang2025_Silkie_Wenchang	CRA004775	0.800028239
ENSGALG00010023775	ENSGALG00010023775	2	-0.230172941	0.300829433	-0.765127728	0.44419549	0.119337407	2.824423995	64.5945509	0.5	CRA004775;Yang2025_Silkie_Wenchang	PRJNA827465	0.846013379
ENSGALG00010023775	ENSGALG00010023775	2	0.004926902	0.121952546	0.040400157	0.967774105	0	0.16360925	0	0.5	CRA004775;PRJNA827465	Yang2025_Silkie_Wenchang	0.991065147
ENSGALG00010023777	OAZ2	2	-0.413487212	0.333929781	-1.238245987	0.215624865	0.198893832	8.484379714	88.21363454	1	PRJNA827465;Yang2025_Silkie_Wenchang	CRA004775	0.729132243
ENSGALG00010023777	OAZ2	2	-0.548998416	0.268868086	-2.041887623	0.04116268	0.077553733	2.113934865	52.69485278	1	CRA004775;Yang2025_Silkie_Wenchang	PRJNA827465	0.351699305
ENSGALG00010023777	OAZ2	2	-0.117023071	0.054483013	-2.147881767	0.031723149	0	0.180356842	0	1	CRA004775;PRJNA827465	Yang2025_Silkie_Wenchang	0.305037635
ENSGALG00010023778	FANCF	2	-0.050255114	0.149133164	-0.336981481	0.736130861	0	0.002234387	0	1	PRJNA827465;Yang2025_Silkie_Wenchang	CRA004775	0.933656616
ENSGALG00010023778	FANCF	2	-0.236102996	0.341635047	-0.691097117	0.489504512	0	0.379806143	0	1	CRA004775;Yang2025_Silkie_Wenchang	PRJNA827465	0.858419004
ENSGALG00010023778	FANCF	2	-0.08255237	0.152519788	-0.541256788	0.588330601	0	0.629185244	0	1	CRA004775;PRJNA827465	Yang2025_Silkie_Wenchang	0.891874519
ENSGALG00010023781	COL4A3	2	0.109565646	0.12890761	0.84995483	0.3953502	0	0.129924366	0	1	PRJNA827465;Yang2025_Silkie_Wenchang	CRA004775	0.830888664
ENSGALG00010023781	COL4A3	2	0.047247619	0.313906109	0.15051513	0.880358215	0.076172654	1.179289984	15.20321433	0.5	CRA004775;Yang2025_Silkie_Wenchang	PRJNA827465	0.971592092
ENSGALG00010023781	COL4A3	2	0.030653585	0.175373108	0.174790681	0.861244111	0	0.97337583	0	0.5	CRA004775;PRJNA827465	Yang2025_Silkie_Wenchang	0.965343309
ENSGALG00010023782	TMPRSS5	2	1.542629608	0.349109834	4.418751513	9.92727E-06	0	0.359754758	0	1	PRJNA827465;Yang2025_Silkie_Wenchang	CRA004775	0.001403495
ENSGALG00010023782	TMPRSS5	2	0.779819899	1.02343663	0.761962076	0.446082639	1.777585391	6.598890989	84.84593848	0.5	CRA004775;Yang2025_Silkie_Wenchang	PRJNA827465	0.846013379
ENSGALG00010023782	TMPRSS5	2	0.597129024	0.809781361	0.737395368	0.460881952	1.041408043	4.809142064	79.20627033	0.5	CRA004775;PRJNA827465	Yang2025_Silkie_Wenchang	0.85014477
ENSGALG00010023783	VPS35	2	-0.130415572	0.094978822	-1.373101602	0.169720763	0.016428341	11.14761056	91.02946775	1	PRJNA827465;Yang2025_Silkie_Wenchang	CRA004775	0.671232541
ENSGALG00010023783	VPS35	2	-0.220412453	0.042337397	-5.206093599	1.92857E-07	0	0.321830915	0	1	CRA004775;Yang2025_Silkie_Wenchang	PRJNA827465	6.01577E-05
ENSGALG00010023783	VPS35	2	-0.041786841	0.03527916	-1.184462468	0.236230045	0	0.348236475	0	1	CRA004775;PRJNA827465	Yang2025_Silkie_Wenchang	0.748669015
ENSGALG00010023786	ENSGALG00010023786	2	-0.16166457	0.231153463	-0.699381992	0.484313339	0.067445927	2.565438507	61.02030912	0.5	PRJNA827465;Yang2025_Silkie_Wenchang	CRA004775	0.857004733
ENSGALG00010023786	ENSGALG00010023786	2	-0.45380134	0.236312983	-1.920340281	0.054814931	0	0.027489993	0	1	CRA004775;Yang2025_Silkie_Wenchang	PRJNA827465	0.405341722
ENSGALG00010023786	ENSGALG00010023786	2	0.003516796	0.147998738	0.023762335	0.981042184	0	0.698924284	0	0.5	CRA004775;PRJNA827465	Yang2025_Silkie_Wenchang	0.994928779
ENSGALG00010023788	RTCA	2	0.250378502	0.260379149	0.961591983	0.3362546	0.12817071	18.23826806	94.51702324	0.5	PRJNA827465;Yang2025_Silkie_Wenchang	CRA004775	0.803727281

ENSGALG00010023788	RTCA	2	0.310355863	0.244629261	1.268678413	0.204555785	0.092293388	3.915779533	74.46230076	1	CRA004775;Yang2025_Silkie_Wenchang	PRJNA827465	0.71729696
ENSGALG00010023788	RTCA	2	-0.005297918	0.074590389	-0.071026816	0.943376413	0	0.008615333	0	0.5	CRA004775;PRJNA827465	Yang2025_Silkie_Wenchang	0.985827325
ENSGALG00010023791	VTI1B	2	0.056715094	0.049500623	1.145745048	0.251900659	0	0.550831408	0	1	PRJNA827465;Yang2025_Silkie_Wenchang	CRA004775	0.761871849
ENSGALG00010023791	VTI1B	2	0.105549967	0.094353873	1.11866067	0.26328493	0	0.185541588	0	1	CRA004775;Yang2025_Silkie_Wenchang	PRJNA827465	0.768643821
ENSGALG00010023791	VTI1B	2	0.036281351	0.054214105	0.669223456	0.503352946	0	0.003755106	0	1	CRA004775;PRJNA827465	Yang2025_Silkie_Wenchang	0.863108263
ENSGALG00010023792	ZNF609	2	0.041154641	0.099572374	0.413313843	0.679376689	0.012392423	2.373315453	57.8648511	0.5	PRJNA827465;Yang2025_Silkie_Wenchang	CRA004775	0.917701868
ENSGALG00010023792	ZNF609	2	0.165263416	0.110982792	1.489090442	0.136463554	0	0.022409125	0	1	CRA004775;Yang2025_Silkie_Wenchang	PRJNA827465	0.618333765
ENSGALG00010023792	ZNF609	2	-0.027254345	0.055305722	-0.492794303	0.622157937	0	0.351858336	0	0.5	CRA004775;PRJNA827465	Yang2025_Silkie_Wenchang	0.900054232
ENSGALG00010023793	RBM45	2	0.018925502	0.047563553	0.397899238	0.690704462	0	0.909436403	0	0.5	PRJNA827465;Yang2025_Silkie_Wenchang	CRA004775	0.920083812
ENSGALG00010023793	RBM45	2	-0.075908768	0.079515092	-0.954646046	0.339756723	0	0.265513762	0	1	CRA004775;Yang2025_Silkie_Wenchang	PRJNA827465	0.805078728
ENSGALG00010023793	RBM45	2	0.010789939	0.077074788	0.139993102	0.88866544	0.004123947	1.260193359	20.64709807	0.5	CRA004775;PRJNA827465	Yang2025_Silkie_Wenchang	0.973135755
ENSGALG00010023798	ARCN1	2	0.012210449	0.061994542	0.196960059	0.843858794	0.005053988	2.69460234	62.88877268	0.5	PRJNA827465;Yang2025_Silkie_Wenchang	CRA004775	0.96057435
ENSGALG00010023798	ARCN1	2	-0.078604262	0.063021798	-1.247255154	0.212303955	0	0.293499791	0	1	CRA004775;Yang2025_Silkie_Wenchang	PRJNA827465	0.724950682
ENSGALG00010023798	ARCN1	2	0.008276498	0.095607651	0.086567322	0.931015442	0.010761659	1.762322633	43.25670107	0.5	CRA004775;PRJNA827465	Yang2025_Silkie_Wenchang	0.983943003
ENSGALG00010023799	NMT1	2	-0.018646673	0.072001403	-0.258976523	0.795653354	0.002929736	1.315213161	23.96669761	0.5	PRJNA827465;Yang2025_Silkie_Wenchang	CRA004775	0.94908992
ENSGALG00010023799	NMT1	2	-0.26990981	0.391089754	-0.690147996	0.490101123	0.268425008	7.779330906	87.14542405	0.5	CRA004775;Yang2025_Silkie_Wenchang	PRJNA827465	0.858529056
ENSGALG00010023799	NMT1	2	-0.332417518	0.310541239	-1.07044565	0.284418761	0.162688182	5.670696141	82.3654808	1	CRA004775;PRJNA827465	Yang2025_Silkie_Wenchang	0.780265906
ENSGALG00010023800	PEX5	2	-0.115023258	0.045118135	-2.549379739	0.010791472	0	0.375096549	0	1	PRJNA827465;Yang2025_Silkie_Wenchang	CRA004775	0.165420139
ENSGALG00010023800	PEX5	2	-0.166590824	0.11656637	-1.42914997	0.152961134	0	0.150316885	0	1	CRA004775;Yang2025_Silkie_Wenchang	PRJNA827465	0.645068129
ENSGALG00010023800	PEX5	2	-0.10451566	0.046905113	-2.228235967	0.025864784	0	0.004974727	0	1	CRA004775;PRJNA827465	Yang2025_Silkie_Wenchang	0.274154152
ENSGALG00010023802	ENSGALG00010023802	2	-0.145874365	0.172444997	-0.845918218	0.397598369	0	0.675698389	0	0.5	PRJNA827465;Yang2025_Silkie_Wenchang	CRA004775	0.831689959
ENSGALG00010023802	ENSGALG00010023802	2	0.069462674	0.209439659	0.331659602	0.740146308	0	0.137538054	0	1	CRA004775;Yang2025_Silkie_Wenchang	PRJNA827465	0.93508234
ENSGALG00010023802	ENSGALG00010023802	2	-0.129811941	0.208885168	-0.621451211	0.534302786	0.010536923	1.116955578	10.4709245	0.5	CRA004775;PRJNA827465	Yang2025_Silkie_Wenchang	0.873408008
ENSGALG00010023805	GALNT6	2	0.242960183	0.342908212	0.70852833	0.478617226	0.116353195	1.935037169	48.32140611	0.5	PRJNA827465;Yang2025_Silkie_Wenchang	CRA004775	0.85563306
ENSGALG00010023805	GALNT6	2	0.486327294	0.314833585	1.544712233	0.122415885	0	0.394990587	0	1	CRA004775;Yang2025_Silkie_Wenchang	PRJNA827465	0.63688845
ENSGALG00010023805	GALNT6	2	0.021993086	0.255704539	0.086009758	0.93145866	0	0.239350548	0	0.5	CRA004775;PRJNA827465	Yang2025_Silkie_Wenchang	0.984076328
ENSGALG00010023806	PATJ	2	0.109945815	0.112779705	0.974872348	0.329623584	0.008815166	1.398351345	28.48721437	1	PRJNA827465;Yang2025_Silkie_Wenchang	CRA004775	0.801177491
ENSGALG00010023806	PATJ	2	0.270623566	0.168374184	1.607274696	0.107994123	0	0.038305997	0	1	CRA004775;Yang2025_Silkie_Wenchang	PRJNA827465	0.556183889
ENSGALG00010023806	PATJ	2	0.047711864	0.092440214	0.516137537	0.605758353	0	0.17656504	0	1	CRA004775;PRJNA827465	Yang2025_Silkie_Wenchang	0.896474603
ENSGALG00010023807	MRPL10	2	0.276827335	0.077053908	3.592644956	0.000327338	0	0.12433413	0	1	PRJNA827465;Yang2025_Silkie_Wenchang	CRA004775	0.017894809
ENSGALG00010023807	MRPL10	2	0.018208774	0.226564336	0.080369111	0.935943693	0.003614142	1.033041255	3.19844484	0.5	CRA004775;Yang2025_Silkie_Wenchang	PRJNA827465	0.984822128
ENSGALG00010023807	MRPL10	2	0.115694137	0.262352771	0.440986907	0.659222475	0.090973371	2.212972392	54.81190801	0.5	CRA004775;PRJNA827465	Yang2025_Silkie_Wenchang	0.911449691
ENSGALG00010023808	DHX35	2	0.043368361	0.06648114	0.652340813	0.514181337	0	0.168958376	0	0.5	PRJNA827465;Yang2025_Silkie_Wenchang	CRA004775	0.866866841
ENSGALG00010023808	DHX35	2	-0.055309064	0.120121227	-0.460443718	0.645197761	0	0.872591822	0	1	CRA004775;Yang2025_Silkie_Wenchang	PRJNA827465	0.906966303
ENSGALG00010023808	DHX35	2	-0.01300351	0.143739198	-0.090466001	0.92791691	0.019968565	1.452010564	31.12997767	0.5	CRA004775;PRJNA827465	Yang2025_Silkie_Wenchang	0.982993195
ENSGALG00010023809	CDON	2	-0.028962431	0.05103202	-0.567534482	0.570351106	0	0.799612068	0	1	PRJNA827465;Yang2025_Silkie_Wenchang	CRA004775	0.8851128
ENSGALG00010023809	CDON	2	-0.098544417	0.092491774	-1.065439793	0.286676957	0	0.017739677	0	1	CRA004775;Yang2025_Silkie_Wenchang	PRJNA827465	0.782018115
ENSGALG00010023809	CDON	2	-0.002427328	0.059112218	-0.041063059	0.967245625	0	0.029666908	0	1	CRA004775;PRJNA827465	Yang2025_Silkie_Wenchang	0.990894822
ENSGALG00010023811	MRPL44	2	-0.072910601	0.059322042	-1.229064265	0.219047712	0	0.047058204	0	1	PRJNA827465;Yang2025_Silkie_Wenchang	CRA004775	0.732700615
ENSGALG00010023811	MRPL44	2	0.009348467	0.199889249	0.046768235	0.962697946	0	0.057094409	0	0.5	CRA004775;Yang2025_Silkie_Wenchang	PRJNA827465	0.989980504
ENSGALG00010023811	MRPL44	2	-0.071789081	0.060357107	-1.189405591	0.234280102	0	0.184787124	0	0.5	CRA004775;PRJNA827465	Yang2025_Silkie_Wenchang	0.74745603
ENSGALG00010023815	PLCD3	2	0.05066757	0.253974282	0.199498823	0.841872564	0.113052544	7.853970229	87.26758606	0.5	PRJNA827465;Yang2025_Silkie_Wenchang	CRA004775	0.960041272
ENSGALG00010023815	PLCD3	2	0.24150069	0.162278023	1.488190983	0.136700534	0.007886559	1.104212656	9.438472406	0.5	CRA004775;Yang2025_Silkie_Wenchang	PRJNA827465	0.618582531
ENSGALG00010023815	PLCD3	2	-0.181342768	0.08735951	-2.075821721	0.037910443	0	0.074616704	0	1	CRA004775;PRJNA827465	Yang2025_Silkie_Wenchang	0.335670361
ENSGALG00010023817	BBS2	2	0.039522467	0.065700006	0.601559561	0.547467354	0	0.581547548	0	1	PRJNA827465;Yang2025_Silkie_Wenchang	CRA004775	0.878281266
ENSGALG00010023817	BBS2	2	0.03379794	0.170998551	0.197650446	0.843318563	0.020811152	1.318908242	24.17971408	0.5	CRA004775;Yang2025_Silkie_Wenchang	PRJNA827465	0.96057435
ENSGALG00010023817	BBS2	2	-0.004930536	0.074547131	-0.066139852	0.947266483	0	0.755253099	0	0.5	CRA004775;PRJNA827465	Yang2025_Silkie_Wenchang	0.986445196
ENSGALG00010023818	ALOX5	2	-0.130474268	0.142629719	-0.914776172	0.360309146	0	0.331660646	0	0.5	PRJNA827465;Yang2025_Silkie_Wenchang	CRA004775	0.814880744
ENSGALG00010023818	ALOX5	2	-0.174732151	0.198581339	-0.879902169	0.37891231	0	0.910195001	0	0.5	CRA004775;Yang2025_Silkie_Wenchang	PRJNA827465	0.823876439
ENSGALG00010023818	ALOX5	2	-0.229797597	0.144993598	-1.58488099	0.112993376	0	0.340775999	0	1	CRA004775;PRJNA827465	Yang2025_Silkie_Wenchang	0.56739219
ENSGALG00010023819	BIN2	2	0.011091759	0.165594669	0.066981375	0.946596531	0	0.152066253	0	0.5	PRJNA827465;Yang2025_Silkie_Wenchang	CRA004775	0.986445196
ENSGALG00010023819	BIN2	2	-0.290928852	0.471494144	-0.617035982	0.537210993	0.277840435	2.614260844	61.74826998	0.5	CRA004775;Yang2025_Silkie_Wenchang	PRJNA827465	0.87481821
ENSGALG00010023819	BIN2	2	-0.30308306	0.379043338	-0.799600019	0.423942576	0.186568906	2.455120767	59.2688061	1	CRA004775;PRJNA827465	Yang2025_Silkie_Wenchang	0.84030043
ENSGALG00010023821	KANK4	2	0.228598547	0.055939009	4.086567678	4.37802E-05	0	0.02917763	0	1	PRJNA827465;Yang2025_Silkie_Wenchang	CRA004775	0.004434774
ENSGALG00010023821	KANK4	2	0.252794967	0.08012022	3.155195633	0.001603905	0	0.444277528	0	1	CRA004775;Yang2025_Silkie_Wenchang	PRJNA827465	0.050669962
ENSGALG00010023821	KANK4	2	0.232580072	0.073868227	3.14858068	0.001640654	0	0.530071932	0	1	CRA004775;PRJNA827465	Yang2025_Silkie_Wenchang	0.051226717
ENSGALG00010023822	CHEK2	2	0.220480228	0.125239143	1.760473788	0.078327508	0.016217216	2.030098535	50.74130724	1	PRJNA827465;Yang2025_Silkie_Wenchang	CRA004775	0.481707034
ENSGALG00010023822	CHEK2	2	-0.000157893	0.41050511	-0.000384616	0.999691091	0.28213326	5.747639311	82.60155264	0.5	CRA004775;Yang2025_Silkie_Wenchang	PRJNA827465	0.999906103
ENSGALG00010023822	CHEK2	2	-0.099441962	0.276550646	-0.359579568	0.719161565	0.109476101	2.987573315	66.52801808	0.5	CRA004775;PRJNA827465	Yang2025_Silkie_Wenchang	0.928449671
ENSGALG00010023823	MUC2	2	0.051801857	0.336199217	0.154080837	0.877545998	0.03170151	1.147990904	12.89129588	0.5	PRJNA827465;Yang2025_Silkie_Wenchang	CRA004775	0.970706998
ENSGALG00010023823	MUC2	2	1.490640655	1.035551871	1.439449379	0.150018825	1.765514871	5.599478437	82.14119384	1	CRA004775;Yang2025_Silkie_Wenchang	PRJNA827465	0.650194343
ENSGALG00010023823	MUC2	2	1.126967102	1.385973636	0.813123044	0.416147516	3.535345101	12.25989772	91.8433251	0.5	CRA004775;PRJNA827465	Yang2025_Silkie_Wenchang	0.837742614
ENSGALG00010023826	DBT	2	-0.135504852	0.133934146	-1.011727449	0.311668387	0.029610794	5.484596315	81.76711753	1	PRJNA827465;Yang2025_Silkie_Wenchang	CRA004775	0.795887377
ENSGALG00010023826	DBT	2	-0.167386824	0.200961256	-0.832930819	0.404883747	0.043666622	1.678360448	40.41804302	0.5	CRA004775;Yang2025_Silkie_Wenchang	PRJNA827465	0.833112102
ENSGALG00010023826	DBT	2	-0.00892222	0.05505121	-0.16203128	0.871249722	0	0.316400821	0	0.5	CRA004775;PRJNA827465	Yang2025_Silkie_Wenchang	0.99079598
ENSGALG00010023828	CHEK1	2	0.128441775	0.219890648	0.58411659	0.559141874	0.078756083	5.11641543	80.45506637	0.5	PRJNA827465;Yang2025_Silkie_Wenchang	CRA004775	0.881014886
ENSGALG00010023828	CHEK1	2	-0.124792691	0.605449757	-0.206115684	0.836700548	0.585474513	4.335384931	76.93399742	0.5	CRA004775;Yang2025_Silkie_Wenchang	PRJNA827465	0.958399882
ENSGALG00010023828	CHEK1	2	-0.266404033	0.342695352	-0.777378601	0.436935434	0.148259472	1.899816076	47.36332572	1	CRA004775;PRJNA827465	Yang2025_Silkie_Wenchang	0.843387647
ENSGALG00010023829	USP1	2	0.07341017	0.10821142	0.678395772	0.497520789	0.018762012	4.71823989	78.80565585	0.5	PRJNA827465;Yang2025_Silkie_Wenchang	CRA004775	0.86098656
ENSGALG00010023829	USP1	2	0.201298217	0.084469526	2.383086851	0.017168143	0	0.024650191	0	1	CRA004775;Yang2025_Silkie_Wenchang	PRJNA827465	0.217734604
ENSGALG00010023829	USP1	2	-0.00503835	0.063692062	-0.079104837	0.936949236	0.002331653	1.074489606	6.932557168	0.5	CRA004775;PRJNA827465	Yang2025_Silkie_Wenchang	0.985068196
ENSGALG00010023830	AGFG1	2	0.12946758	0.146146687	0.885874209	0.375685315	0.033425219	3.880819558	74.23224695	1	PRJNA827465;Yang2025_Silkie_Wenchang	CRA004775	0.822974013
ENSGALG00010023830	AGFG1	2	0.177921642	0.120541328	1.476021912	0.13993801	0.011110065	1.605325938	37.70735423	1	CRA004775;Yang2025_Silkie_Wenchang	PRJNA827465	0.623792706
ENSGALG00010023830	AGFG1	2	0.018342406	0.035815282	0.51213909	0.608553667	0	0.201610948	0	1	CRA004775;PRJNA827465	Yang2025_Silkie_Wenchang	0.897208691
ENSGALG00010023831	SATB2	2	-0.472460839	0.607084126	-0.778246077	0.436423958	0.357084215	1.931321708	48.22198726	0.5	PRJNA827465;Yang2025_Silkie_Wenchang	CRA004775	0.843136591
ENSGALG00010023831	SATB2	2	-0.527376521	0.607712576	-0.867805837	0.385500634	0.289505471	1.644111222	39.17686428	0.5	CRA004775;Yang2025_Silkie_Wenchang	PRJNA827465	0.82648313
ENSGALG00010023831	SATB2	2	0.094273898	0.440666449	0.213934823	0.830597884	0	1.98213E-06	0	1	CRA004775;PRJNA827465	Yang2025_Silkie_Wenchang	0.957295959
ENSGALG00010023832	ENSGALG00010023832	2	-0.20960877	0.218775435	-0.958100115	0.338012278	0.072860939	4.105722884	75.64375317	1	PRJNA827465;Yang2025_Silkie_Wenchang	CRA004775	0.80415385
ENSGALG00010023832	ENSGALG00010023832	2	-0.191116145	0.304711787	-0.627202993	0.530526182	0.126944447	2.963492565	66.25603141	0.5	CRA004775;Yang2025_Silkie_Wenchang	PRJNA827465	0.872584136
ENSGALG00010023832	ENSGALG00010023832	2	0.017650666	0.116937801	0.150940638	0.880022543	0	0.284192868	0	0.5	CRA004775;PRJNA827465	Yang2025_Silkie_Wenchang	0.971546618
ENSGALG00010023833	PIP4K2C	2	0.262713135	0.141170207	1.860967271	0.062748799	0.030198842	3.861267354	74.10176742	1	PRJNA827465;Yang2025_Silkie_Wenchang	CRA004775	0.431609387
ENSGALG00010023833	PIP4K2C	2	0.090229206	0.345103548	0.261455459	0.793741294	0.215658094	10.50422422	90.48002043	0.5	CRA004775;Yang2025_Silkie_Wenchang	PRJNA827465	0.948796049
ENSGALG00010023833	PIP4K2C	2	-0.031376544	0.200239336	-0.156695204	0.875485067	0.065337204	4.923947164	79.69108996	0.5	CRA004775;PRJNA827465	Yang2025_Silkie_Wenchang	0.970347777
ENSGALG00010023835	INAFM2	2	0.007379663	0.087653322	0.084191485	0.932904188	0	0.081691576	0	0.5	PRJNA827465;Yang2025_Silkie_Wenchang	CRA004775	0.984224753
ENSGALG00010023835	INAFM2	2	-0.156767061	0.304134959	-0.51545229	0.606237001	0	0.032668512	0	1	CRA004775;Yang2025_Silkie_Wenchang	PRJNA827465	0.896709756
ENSGALG00010023835	INAFM2	2	0.004184002	0.088022451	0.047533353	0.962088149	0	0.248422238	0	0.5	CRA004775;PRJNA827465	Yang2025_Silkie_Wenchang	0.989880527
ENSGALG00010023836	NDUFS3	2	-0.116467844	0.070627203	-1.64905078	0.099137232	0	0.3385428	0	1	PRJNA827465;Yang2025_Silkie_Wenchang	CRA004775	0.537214481
ENSGALG00010023836	NDUFS3	2	-0.189406809	0.21603111	-0.876757101	0.380618585	0	0.261826366	0	1	CRA004775;Yang2025_Silkie_Wenchang	PRJNA827465	0.824392515
ENSGALG00010023836	NDUFS3	2	-0.101789594	0.072291668	-1.408040475	0.159119085	0	0.047647345	0	1	CRA004775;PRJNA827465	Yang2025_Silkie_Wenchang	0.654100764
ENSGALG00010023837	STT3A	2	0.023535307	0.089430878	0.263167571	0.792421425	0.007167192	1.655927902	39.61089739	0.5	PRJNA827465;Yang2025_Silkie_Wenchang	CRA004775	0.948372084
ENSGALG00010023837	STT3A	2	-0.011316499	0.172679113	-0.065534845	0.947748163	0.039089644	2.881721692	65.29852266	0.5	CRA004775;Yang2025_Silkie_Wenchang	PRJNA827465	0.986619597
ENSGALG00010023837	STT3A	2	-0.064592444	0.063152063	-1.022808127	0.306398575	0	0.830439977	0	1	CRA004775;PRJNA827465	Yang2025_Silkie_Wenchang	0.792485409
ENSGALG00010023840	SMAGP	2	-0.478902905	0.467582368	-1.024210787	0.305735725	0.397644807	10.9171335	90.84008636	1	PRJNA827465;Yang2025_Silkie_Wenchang	CRA004775	0.79225794
ENSGALG00010023840	SMAGP	2	-0.481330665	0.495093779	-0.972200996	0.330950565	0.421788862	7.124730255	85.96438091	0.5	CRA004775;Yang2025_Silkie_Wenchang	PRJNA827465	0.801685675
ENSGALG00010023840	SMAGP	2	-0.013523418	0.140079603	-0.096540948	0.923090954	0	0.027717674	0	0.5	CRA004775;PRJNA827465	Yang2025_Silkie_Wenchang	0.981836718
ENSGALG00010023841	OSBPL7	2	0.114724726	0.187279387	0.612585975	0.540150134	0.056526573	4.996892256	79.98756129	0.5	PRJNA827465;Yang2025_Silkie_Wenchang	CRA004775	0.87529516
ENSGALG00010023841	OSBPL7	2	-0.035293667	0.359282875	-0.098233648	0.921746765	0.234289679	10.78974623	90.73194143	0.5	CRA004775;Yang2025_Silkie_Wenchang	PRJNA827465	0.981649741
ENSGALG00010023841	OSBPL7	2	-0.199872131	0.168946966	-1.183046587	0.236790684	0.040860165	3.288046552	69.58680529	1	CRA004775;PRJNA827465	Yang2025_Silkie_Wenchang	0.749020867
ENSGALG00010023847	PIK3AP1	2	-0.203911606	0.28709059	-0.71026921	0.477537209	0.136882054	5.795955582	82.74658966	0.5	PRJNA827465;Yang2025_Silkie_Wenchang	CRA004775	0.855180432
ENSGALG00010023847	PIK3AP1	2	-0.05291714	0.532333361	-0.099406019	0.920815904	0.452637994	4.537678192	77.96229795	0.5	CRA004775;Yang2025_Silkie_Wenchang	PRJNA827465	0.981469977
ENSGALG00010023847	PIK3AP1	2	0.119960276	0.154741878	0.775228255	0.438204795	0.008052345	1.068157847	6.38087779	1	CRA004775;PRJNA827465	Yang2025_Silkie_Wenchang	0.843979791
ENSGALG00010023850	DAZAP2	2	0.142109524	0.239516112	0.593319268	0.552967507	0.100320044	7.905090139	87.34992287	0.5	PRJNA827465;Yang2025_Silkie_Wenchang	CRA004775	0.87928478
ENSGALG00010023850	DAZAP2	2	0.074353858	0.346866915	0.214358461	0.830267532	0.201349085	5.846856646	82.89679292	0.5	CRA004775;Yang2025_Silkie_Wenchang	PRJNA827465	0.957231
ENSGALG00010023850	DAZAP2	2	-0.121387255	0.096738703	-1.254795144	0.209553136	0	0.624542939	0	1	CRA004775;PRJNA827465	Yang2025_Silkie_Wenchang	0.721386257
ENSGALG00010023851	HSCB	2	0.161222454	0.30480148	0.528942491	0.596845345	0.155761498	5.527728928	81.90938787	0.5	PRJNA827465;Yang2025_Silkie_Wenchang	CRA004775	0.894172266
ENSGALG00010023851	HSCB	2	0.431300867	0.215004913	2.006004701	0.044855746	0	0.425567433	0	1	CRA004775;Yang2025_Silkie_Wenchang	PRJNA827465	0.365078774
ENSGALG00010023851	HSCB	2	-0.090978065	0.073791568	-1.232905967	0.217610849	0	0.506439544	0	0.5	CRA004775;PRJNA827465	Yang2025_Silkie_Wenchang	0.731013651
ENSGALG00010023852	LRRC39	2	-0.110804098	0.130869963	-0.84667326	0.397177267	0.02026876	2.424950398	58.76204311	0.5	PRJNA827465;Yang2025_Silkie_Wenchang	CRA004775	0.831438925
ENSGALG00010023852	LRRC39	2	0.027837275	0.12740187	0.218499738	0.827039762	0	0.015135921	0	0.5	CRA004775;Yang2025_Silkie_Wenchang	PRJNA827465	0.956540044
ENSGALG00010023852	LRRC39	2	-0.224668653	0.10471326	-2.145560675	0.031908046	0	0.109903885	0	1	CRA004775;PRJNA827465	Yang2025_Silkie_Wenchang	0.306172344
ENSGALG00010023854	SHCBP1	2	-0.556777116	0.128771115	-4.32377335	1.53383E-05	0	0.087002559	0	1	PRJNA827465;Yang2025_Silkie_Wenchang	CRA004775	0.002002793
ENSGALG00010023854	SHCBP1	2	-0.256103775	0.3613571	-0.708727668	0.478493492	0.158820825	2.189521374	54.32791788	0.5	CRA004775;Yang2025_Silkie_Wenchang	PRJNA827465	0.855528601
ENSGALG00010023854	SHCBP1	2	-0.288175763	0.403543016	-0.714114114	0.475156619	0.224649932	2.738897057	63.4889527	0.5	CRA004775;PRJNA827465	Yang2025_Silkie_Wenchang	0.854462864
ENSGALG00010023857	ACBD4	2	0.554527305	0.32689973	1.696322309	0.089824858	0.180835954	6.368289571	84.29719646	1	PRJNA827465;Yang2025_Silkie_Wenchang	CRA004775	0.514211987
ENSGALG00010023857	ACBD4	2	0.408372499	0.497059581	0.821576557	0.411317936	0.443963514	9.844414752	89.84195582	0.5	CRA004775;Yang2025_Silkie_Wenchang	PRJNA827465	0.835128673
ENSGALG00010023857	ACBD4	2	0.126080266	0.162424529	0.776239073	0.437607839	0.020366823	1.548417642	35.41794067	0.5	CRA004775;PRJNA827465	Yang2025_Silkie_Wenchang	0.843827727
ENSGALG00010023859	ENSGALG00010023859	2	0.767343921	0.2947152	2.603679484	0.009222896	0	0.545589264	0	1	PRJNA827465;Yang2025_Silkie_Wenchang	CRA004775	0.150534672
ENSGALG00010023859	ENSGALG00010023859	2	0.594059318	0.519244086	1.1440849	0.252588429	0.2484935	1.759752405	43.17382392	0.5	CRA004775;Yang2025_Silkie_Wenchang	PRJNA827465	0.762655223
ENSGALG00010023859	ENSGALG00010023859	2	0.394185901	0.350232799	1.125496821	0.260378564	0	0.636599799	0	0.5	CRA004775;PRJNA827465	Yang2025_Silkie_Wenchang	0.767694434
ENSGALG00010023860	TRIP4	2	0.162682606	0.099776839	1.630464622	0.103003336	0.014566574	3.588846257	72.13589192	1	PRJNA827465;Yang2025_Silkie_Wenchang	CRA004775	0.54714693
ENSGALG00010023860	TRIP4	2	0.003606849	0.299206498	0.012054715	0.990381962	0.155189813	7.070901767	85.85753228	0.5	CRA004775;Yang2025_Silkie_Wenchang	PRJNA827465	0.997468525
ENSGALG00010023860	TRIP4	2	-0.076256588	0.193848893	-0.3933816	0.694037657	0.056940905	3.457011096	71.07327769	0.5	CRA004775;PRJNA827465	Yang2025_Silkie_Wenchang	0.920728119
ENSGALG00010023861	ENSGALG00010023861	2	0.524296776	0.094402082	5.553868766	2.79415E-08	0	0.039918996	0	1	PRJNA827465;Yang2025_Silkie_Wenchang	CRA004775	1.22021E-05
ENSGALG00010023861	ENSGALG00010023861	2	0.137669017	0.423193047	0.325310206	0.744946307	0.323829074	10.35481421	90.34265628	0.5	CRA004775;Yang2025_Silkie_Wenchang	PRJNA827465	0.935927651
ENSGALG00010023861	ENSGALG00010023861	2	0.125410573	0.403225914	0.311018137	0.755786833	0.296819081	11.2094337	91.07894273	0.5	CRA004775;PRJNA827465	Yang2025_Silkie_Wenchang	0.938501783
ENSGALG00010023862	TRMT13	2	-0.110648788	0.165095366	-0.670211349	0.50272307	0.035982849	2.911329997	65.65143762	0.5	PRJNA827465;Yang2025_Silkie_Wenchang	CRA004775	0.863050365
ENSGALG00010023862	TRMT13	2	-0.000532785	0.149201018	-0.003570922	0.997150822	0.0039759	1.053151581	5.046907058	0.5	CRA004775;Yang2025_Silkie_Wenchang	PRJNA827465	0.999108213
ENSGALG00010023862	TRMT13	2	-0.270911131	0.115114526	-2.353405257	0.018602348	0	0.030633449	0	1	CRA004775;PRJNA827465	Yang2025_Silkie_Wenchang	0.228192282
ENSGALG00010023863	EYA2	2	0.099332144	0.10479112	0.947906118	0.34317725	0.003553411	1.176357069	14.99179744	1	PRJNA827465;Yang2025_Silkie_Wenchang	CRA004775	0.806218116
ENSGALG00010023863	EYA2	2	0.147303808	0.243414555	0.60515612	0.545075269	0.040849534	1.17211489	14.68413132	0.5	CRA004775;Yang2025_Silkie_Wenchang	PRJNA827465	0.877354247
ENSGALG00010023863	EYA2	2	0.004265926	0.115407675	0.036963971	0.970513733	0	0.604058396	0	0.5	CRA004775;PRJNA827465	Yang2025_Silkie_Wenchang	0.991760517
ENSGALG00010023866	AMBRA1	2	-0.065927741	0.049902407	-1.321133487	0.186456859	0.000461697	1.083332779	7.692260464	1	PRJNA827465;Yang2025_Silkie_Wenchang	CRA004775	0.695457578
ENSGALG00010023866	AMBRA1	2	-0.159603924	0.074952423	-2.129403127	0.033220922	0	0.110672725	0	1	CRA004775;Yang2025_Silkie_Wenchang	PRJNA827465	0.313263411
ENSGALG00010023866	AMBRA1	2	-0.06786135	0.066569813	-1.019401238	0.308012519	0.002531176	1.231803695	18.81823344	1	CRA004775;PRJNA827465	Yang2025_Silkie_Wenchang	0.793278812
ENSGALG00010023869	DOCK7	2	0.081256845	0.111072464	0.731566066	0.464433467	0.018175766	3.490394681	71.34994488	0.5	PRJNA827465;Yang2025_Silkie_Wenchang	CRA004775	0.852159649
ENSGALG00010023869	DOCK7	2	0.213008408	0.098691986	2.158315145	0.030903338	0	0.000590392	0	1	CRA004775;Yang2025_Silkie_Wenchang	PRJNA827465	0.300270431
ENSGALG00010023869	DOCK7	2	-0.001124672	0.051490244	-0.021842424	0.982573653	0	0.825725264	0	0.5	CRA004775;PRJNA827465	Yang2025_Silkie_Wenchang	0.995234705
ENSGALG00010023871	UQCRFS1	2	-0.221981441	0.258331592	-0.859288787	0.390181209	0.111338069	5.434221743	81.59810094	0.5	PRJNA827465;Yang2025_Silkie_Wenchang	CRA004775	0.829234633
ENSGALG00010023871	UQCRFS1	2	-0.30833276	0.294390429	-1.047360001	0.294933572	0.086953407	1.828635026	45.31440196	0.5	CRA004775;Yang2025_Silkie_Wenchang	PRJNA827465	0.787569357
ENSGALG00010023871	UQCRFS1	2	0.003475388	0.069363573	0.05010393	0.960039568	0	0.055610042	0	1	CRA004775;PRJNA827465	Yang2025_Silkie_Wenchang	0.989338243
ENSGALG00010023874	HEXIM1	2	0.231066915	0.279380245	0.827069627	0.408197598	0.148398895	19.88487564	94.97105228	0.5	PRJNA827465;Yang2025_Silkie_Wenchang	CRA004775	0.834134074
ENSGALG00010023874	HEXIM1	2	0.496082351	0.106946245	4.638614037	3.50753E-06	0	0.423586153	0	1	CRA004775;Yang2025_Silkie_Wenchang	PRJNA827465	0.000647214
ENSGALG00010023874	HEXIM1	2	-0.031415959	0.054169181	-0.579960013	0.561941583	0	0.907340282	0	0.5	CRA004775;PRJNA827465	Yang2025_Silkie_Wenchang	0.881836663
ENSGALG00010023876	POU6F1	2	0.61977369	0.500943081	1.237213794	0.216007722	0.361923747	3.541581992	71.76403081	1	PRJNA827465;Yang2025_Silkie_Wenchang	CRA004775	0.729548121
ENSGALG00010023876	POU6F1	2	0.632913918	0.567999471	1.114286106	0.265156466	0.419661576	2.826168102	64.61640059	1	CRA004775;Yang2025_Silkie_Wenchang	PRJNA827465	0.769274038
ENSGALG00010023876	POU6F1	2	0.115207631	0.278856175	0.413143552	0.679501442	0	0.047062997	0	1	CRA004775;PRJNA827465	Yang2025_Silkie_Wenchang	0.917701868
ENSGALG00010023879	EI24	2	-0.119646811	0.056576737	-2.114770436	0.034449518	0.001264291	1.176471954	15.00009867	1	PRJNA827465;Yang2025_Silkie_Wenchang	CRA004775	0.319633946
ENSGALG00010023879	EI24	2	-0.222051076	0.09918992	-2.238645579	0.025178986	0	0.021913417	0	1	CRA004775;Yang2025_Silkie_Wenchang	PRJNA827465	0.270237491
ENSGALG00010023879	EI24	2	-0.093204912	0.053992978	-1.726241372	0.084303998	0	0.380467124	0	1	CRA004775;PRJNA827465	Yang2025_Silkie_Wenchang	0.499984914
ENSGALG00010023880	ENSGALG00010023880	2	-0.047619465	0.121260534	-0.392703737	0.694538311	0.002218304	1.022815412	2.230648019	0.5	PRJNA827465;Yang2025_Silkie_Wenchang	CRA004775	0.920728119
ENSGALG00010023880	ENSGALG00010023880	2	0.023001564	0.346742326	0.066336187	0.947110174	0.054805371	1.295029643	22.78169036	0.5	CRA004775;Yang2025_Silkie_Wenchang	PRJNA827465	0.986445196
ENSGALG00010023880	ENSGALG00010023880	2	-0.100141566	0.113333074	-0.883604071	0.376909981	0	0.299778449	0	1	CRA004775;PRJNA827465	Yang2025_Silkie_Wenchang	0.823064945
ENSGALG00010023881	VSTM2B	2	-0.068152147	0.092143169	-0.739633202	0.45952259	0	0.616706243	0	1	PRJNA827465;Yang2025_Silkie_Wenchang	CRA004775	0.850098539
ENSGALG00010023881	VSTM2B	2	-0.030656345	0.229322975	-0.133681962	0.89365407	0.047362717	1.456477162	31.34118229	0.5	CRA004775;Yang2025_Silkie_Wenchang	PRJNA827465	0.974381724
ENSGALG00010023881	VSTM2B	2	0.021888178	0.116100031	0.188528614	0.850462287	0	0.805296563	0	0.5	CRA004775;PRJNA827465	Yang2025_Silkie_Wenchang	0.961985357
ENSGALG00010023883	TBX21	2	-0.108945204	0.219262323	-0.496871521	0.619279656	0.063444604	2.919552239	65.74817238	0.5	PRJNA827465;Yang2025_Silkie_Wenchang	CRA004775	0.899168321
ENSGALG00010023883	TBX21	2	0.050014815	0.175200457	0.285471942	0.775282597	0	0.670924436	0	0.5	CRA004775;Yang2025_Silkie_Wenchang	PRJNA827465	0.943815265
ENSGALG00010023883	TBX21	2	-0.301910008	0.151681453	-1.990421389	0.046544536	0	0.04300969	0	1	CRA004775;PRJNA827465	Yang2025_Silkie_Wenchang	0.372346812
ENSGALG00010023884	C2orf69	2	-0.272913078	0.251799808	-1.083849431	0.278431564	0.11550957	10.90583042	90.8305928	1	PRJNA827465;Yang2025_Silkie_Wenchang	CRA004775	0.776763074
ENSGALG00010023884	C2orf69	2	-0.031656041	0.529875634	-0.059742399	0.952360803	0.519260719	12.97036419	92.29011626	0.5	CRA004775;Yang2025_Silkie_Wenchang	PRJNA827465	0.987758913
ENSGALG00010023884	C2orf69	2	0.186992369	0.272333968	0.686628886	0.492316621	0.118254585	4.233233257	76.37739432	0.5	CRA004775;PRJNA827465	Yang2025_Silkie_Wenchang	0.859228604
ENSGALG00010023887	RASA2	2	-0.028912611	0.070664128	-0.409155425	0.682425604	0.000872003	1.082128559	7.58953808	0.5	PRJNA827465;Yang2025_Silkie_Wenchang	CRA004775	0.918625039
ENSGALG00010023887	RASA2	2	-0.036846467	0.170455248	-0.21616505	0.828859095	0.023358894	1.411696245	29.16323159	0.5	CRA004775;Yang2025_Silkie_Wenchang	PRJNA827465	0.956840134
ENSGALG00010023887	RASA2	2	0.034750472	0.077879829	0.446206322	0.655448217	0	0.587916537	0	1	CRA004775;PRJNA827465	Yang2025_Silkie_Wenchang	0.910411938
ENSGALG00010023888	CTH	2	-0.000911524	0.221213877	-0.004120553	0.996712283	0.077788081	4.810383661	79.21163736	0.5	PRJNA827465;Yang2025_Silkie_Wenchang	CRA004775	0.99895145
ENSGALG00010023888	CTH	2	-0.184794472	0.110924162	-1.665953278	0.095722721	0	0.256474779	0	1	CRA004775;Yang2025_Silkie_Wenchang	PRJNA827465	0.529885929
ENSGALG00010023888	CTH	2	0.158539912	0.139261665	1.138431823	0.254940212	0	0.836369768	0	0.5	CRA004775;PRJNA827465	Yang2025_Silkie_Wenchang	0.764444699
ENSGALG00010023889	SAMD8	2	-0.159125198	0.117385478	-1.355578225	0.175233404	0.01773286	2.701759413	62.98708186	1	PRJNA827465;Yang2025_Silkie_Wenchang	CRA004775	0.680258136
ENSGALG00010023889	SAMD8	2	0.078417369	0.452403807	0.173334901	0.862388192	0.329606262	4.470115253	77.62921215	0.5	CRA004775;Yang2025_Silkie_Wenchang	PRJNA827465	0.965970249
ENSGALG00010023889	SAMD8	2	0.160797779	0.319005598	0.504059428	0.614219616	0.143185667	2.575316818	61.16982606	0.5	CRA004775;PRJNA827465	Yang2025_Silkie_Wenchang	0.898391336
ENSGALG00010023890	SASS6	2	0.065322169	0.123555232	0.528688002	0.597021902	0.022322307	3.638572683	72.51669577	0.5	PRJNA827465;Yang2025_Silkie_Wenchang	CRA004775	0.894204603
ENSGALG00010023890	SASS6	2	0.08975762	0.159216911	0.563744263	0.572928189	0.026393601	1.844295452	45.77875258	0.5	CRA004775;Yang2025_Silkie_Wenchang	PRJNA827465	0.88573298
ENSGALG00010023890	SASS6	2	-0.05667012	0.071527222	-0.79228745	0.428193088	0	0.14680329	0	1	CRA004775;PRJNA827465	Yang2025_Silkie_Wenchang	0.84125685
ENSGALG00010023891	ENSGALG00010023891	2	-0.650362421	0.277056718	-2.347398132	0.018905042	0	0.883206138	0	1	PRJNA827465;Yang2025_Silkie_Wenchang	CRA004775	0.230466768
ENSGALG00010023891	ENSGALG00010023891	2	-0.354850488	0.547852894	-0.647711259	0.517171721	0.467581763	4.475661184	77.65693249	0.5	CRA004775;Yang2025_Silkie_Wenchang	PRJNA827465	0.867952013
ENSGALG00010023891	ENSGALG00010023891	2	-0.088797509	0.285240891	-0.311307081	0.755567185	0.043706751	1.35983661	26.46175339	0.5	CRA004775;PRJNA827465	Yang2025_Silkie_Wenchang	0.938501652
ENSGALG00010023896	POP4	2	0.117314757	0.084602516	1.386658025	0.165546066	0	0.552714686	0	1	PRJNA827465;Yang2025_Silkie_Wenchang	CRA004775	0.664874006
ENSGALG00010023896	POP4	2	-0.078698169	0.321638606	-0.244678866	0.806705093	0.168649367	5.369899417	81.37767727	0.5	CRA004775;Yang2025_Silkie_Wenchang	PRJNA827465	0.952273023
ENSGALG00010023896	POP4	2	-0.127772542	0.2450327	-0.521450984	0.602052643	0.094773232	4.362622777	77.078009	0.5	CRA004775;PRJNA827465	Yang2025_Silkie_Wenchang	0.89532296
ENSGALG00010023902	PTGER3	2	-0.313758013	0.283006838	-1.108658769	0.267577415	0.123300625	4.195677464	76.16594677	1	PRJNA827465;Yang2025_Silkie_Wenchang	CRA004775	0.771398294
ENSGALG00010023902	PTGER3	2	-0.174326621	0.486700748	-0.358180302	0.720208388	0.386990319	5.338598369	81.26849164	0.5	CRA004775;Yang2025_Silkie_Wenchang	PRJNA827465	0.928564173
ENSGALG00010023902	PTGER3	2	0.020657972	0.160987049	0.128320705	0.897895181	0.010164506	1.139977259	12.27895189	0.5	CRA004775;PRJNA827465	Yang2025_Silkie_Wenchang	0.975696709
ENSGALG00010023903	TBKBP1	2	0.620737942	0.220417598	2.816190489	0.004859684	0	0.071628853	0	1	PRJNA827465;Yang2025_Silkie_Wenchang	CRA004775	0.103579269
ENSGALG00010023903	TBKBP1	2	0.186100597	0.489498213	0.380186469	0.703807002	0.363555045	4.104666343	75.63748387	0.5	CRA004775;Yang2025_Silkie_Wenchang	PRJNA827465	0.92372025
ENSGALG00010023903	TBKBP1	2	0.160718461	0.427794297	0.375690986	0.70714664	0.284073506	4.458092602	77.56888227	0.5	CRA004775;PRJNA827465	Yang2025_Silkie_Wenchang	0.924856272
ENSGALG00010023909	FOXRED1	2	-0.125870076	0.0980358	-1.283919518	0.199170117	0	0.268535586	0	1	PRJNA827465;Yang2025_Silkie_Wenchang	CRA004775	0.71096127
ENSGALG00010023909	FOXRED1	2	-0.226099315	0.175179767	-1.290670259	0.196818042	0	0.000640708	0	1	CRA004775;Yang2025_Silkie_Wenchang	PRJNA827465	0.707994211

ENSGALG00010023909	FOXRED1	2	-0.116425058	0.101867447	-1.142907395	0.253077042	0	0.154787783	0	1	CRA004775;PRJNA827465	Yang2025_Silkie_Wenchang	0.762731469
ENSGALG00010023910	RNF7	2	-0.203755035	0.193447907	-1.053281151	0.292212155	0.068215306	11.15635066	91.03649544	1	PRJNA827465;Yang2025_Silkie_Wenchang	CRA004775	0.785617484
ENSGALG00010023910	RNF7	2	-0.074273724	0.366150327	-0.202850356	0.839251998	0.233464005	7.203572807	86.11799968	0.5	CRA004775;Yang2025_Silkie_Wenchang	PRJNA827465	0.959427611
ENSGALG00010023910	RNF7	2	0.069261924	0.150499249	0.460214418	0.645362323	0.025905065	1.738641816	42.48384049	0.5	CRA004775;PRJNA827465	Yang2025_Silkie_Wenchang	0.906966468
ENSGALG00010023911	TFCP2	2	0.031925529	0.198224769	0.161057214	0.872048338	0.071372936	10.66179616	90.62071732	0.5	PRJNA827465;Yang2025_Silkie_Wenchang	CRA004775	0.969560463
ENSGALG00010023911	TFCP2	2	-0.205677146	0.451930178	-0.455108235	0.649031374	0.391509198	23.94916369	95.82448885	0.5	CRA004775;Yang2025_Silkie_Wenchang	PRJNA827465	0.907783669
ENSGALG00010023911	TFCP2	2	-0.391651228	0.252815784	-1.549156552	0.121344091	0.115200921	9.62379947	89.60909355	1	CRA004775;PRJNA827465	Yang2025_Silkie_Wenchang	0.583884665
ENSGALG00010023913	ZMYND8	2	0.103231185	0.189738936	0.544069588	0.586393587	0.056639568	4.027483573	75.17060015	0.5	PRJNA827465;Yang2025_Silkie_Wenchang	CRA004775	0.891596903
ENSGALG00010023913	ZMYND8	2	0.214935598	0.161744239	1.328860921	0.183893859	0.006776227	1.136904206	12.04184179	1	CRA004775;Yang2025_Silkie_Wenchang	PRJNA827465	0.692470323
ENSGALG00010023913	ZMYND8	2	-0.047743855	0.052062844	-0.917042778	0.359120225	0	0.041337423	0	0.5	CRA004775;PRJNA827465	Yang2025_Silkie_Wenchang	0.814241185
ENSGALG00010023914	CCDC117	2	-0.268124174	0.374501335	-0.715949848	0.474022317	0.272457906	34.40404947	97.09336542	0.5	PRJNA827465;Yang2025_Silkie_Wenchang	CRA004775	0.85444425
ENSGALG00010023914	CCDC117	2	-0.429703135	0.25184328	-1.706232284	0.08796484	0.098275699	4.187430088	76.11900428	1	CRA004775;Yang2025_Silkie_Wenchang	PRJNA827465	0.509638136
ENSGALG00010023914	CCDC117	2	0.072562278	0.07528403	0.963846895	0.335122692	0.003810943	1.151047203	13.12258982	0.5	CRA004775;PRJNA827465	Yang2025_Silkie_Wenchang	0.803129704
ENSGALG00010023917	ZRANB2	2	-0.054300402	0.055917044	-0.971088548	0.331504186	0.0012008	1.208151829	17.22894622	0.5	PRJNA827465;Yang2025_Silkie_Wenchang	CRA004775	0.801798188
ENSGALG00010023917	ZRANB2	2	-0.034177671	0.092474115	-0.369591766	0.711686687	0.003893075	1.225522649	18.40216085	0.5	CRA004775;Yang2025_Silkie_Wenchang	PRJNA827465	0.92597752
ENSGALG00010023917	ZRANB2	2	-0.104714264	0.055847455	-1.87500511	0.060792021	0	0.255496173	0	1	CRA004775;PRJNA827465	Yang2025_Silkie_Wenchang	0.42696726
ENSGALG00010023918	MFSD14A	2	-0.261560987	0.258107799	-1.01337886	0.310879233	0.122085609	11.35290499	91.19168177	1	PRJNA827465;Yang2025_Silkie_Wenchang	CRA004775	0.795215522
ENSGALG00010023918	MFSD14A	2	-0.46489893	0.131551797	-3.533961078	0.000409381	0.001632984	1.035301249	3.409756259	1	CRA004775;Yang2025_Silkie_Wenchang	PRJNA827465	0.020366982
ENSGALG00010023918	MFSD14A	2	-0.02785645	0.049573868	-0.561918023	0.574171873	0	0.584264512	0	1	CRA004775;PRJNA827465	Yang2025_Silkie_Wenchang	0.886081433
ENSGALG00010023921	GRK7	2	0.309786715	0.162947139	1.901148541	0.057282559	0.016592815	1.45398651	31.223571	1	PRJNA827465;Yang2025_Silkie_Wenchang	CRA004775	0.414473866
ENSGALG00010023921	GRK7	2	0.477241111	0.174611853	2.733154158	0.006273096	0	5.80389E-07	0	1	CRA004775;Yang2025_Silkie_Wenchang	PRJNA827465	0.120327725
ENSGALG00010023921	GRK7	2	0.211567276	0.169174169	1.250588533	0.211084636	0	0.561288897	0	1	CRA004775;PRJNA827465	Yang2025_Silkie_Wenchang	0.72317464
ENSGALG00010023923	CSNK1G1	2	-0.018002699	0.059544716	-0.302339156	0.762393532	0	0.373603794	0	0.5	PRJNA827465;Yang2025_Silkie_Wenchang	CRA004775	0.940082621
ENSGALG00010023923	CSNK1G1	2	-0.006463654	0.12703605	-0.050880472	0.959420767	0.007109021	1.208464324	17.25034988	0.5	CRA004775;Yang2025_Silkie_Wenchang	PRJNA827465	0.989259322
ENSGALG00010023923	CSNK1G1	2	0.018507152	0.065406624	0.28295531	0.777211095	0	0.701412956	0	1	CRA004775;PRJNA827465	Yang2025_Silkie_Wenchang	0.944218792
ENSGALG00010023925	NCOA3	2	0.129098842	0.204324177	0.631833415	0.527495736	0.065312598	4.007205261	75.04495191	0.5	PRJNA827465;Yang2025_Silkie_Wenchang	CRA004775	0.871149047
ENSGALG00010023925	NCOA3	2	0.31528098	0.172651917	1.826107607	0.067834055	0	0.439749593	0	1	CRA004775;Yang2025_Silkie_Wenchang	PRJNA827465	0.449101706
ENSGALG00010023925	NCOA3	2	-0.032588864	0.063586302	-0.512513906	0.60829139	0	0.174328972	0	0.5	CRA004775;PRJNA827465	Yang2025_Silkie_Wenchang	0.897204334
ENSGALG00010023926	ZYX	2	0.095199875	0.145335749	0.655034122	0.512445785	0.026198272	2.147805422	53.4408476	0.5	PRJNA827465;Yang2025_Silkie_Wenchang	CRA004775	0.865809047
ENSGALG00010023926	ZYX	2	0.069406057	0.288517366	0.240561107	0.809895297	0.100063189	2.390128471	58.16124479	0.5	CRA004775;Yang2025_Silkie_Wenchang	PRJNA827465	0.952818762
ENSGALG00010023926	ZYX	2	-0.014838162	0.066330346	-0.223700965	0.822989995	0	0.695636386	0	1	CRA004775;PRJNA827465	Yang2025_Silkie_Wenchang	0.955822987
ENSGALG00010023927	KPNB1	2	-0.002993193	0.054972267	-0.054449153	0.956577318	0.001292496	1.264168066	20.89659384	0.5	PRJNA827465;Yang2025_Silkie_Wenchang	CRA004775	0.988540618
ENSGALG00010023927	KPNB1	2	0.103853169	0.088568429	1.172575486	0.240966084	0.003851186	1.177615507	15.08263996	1	CRA004775;Yang2025_Silkie_Wenchang	PRJNA827465	0.753551568
ENSGALG00010023927	KPNB1	2	0.068611315	0.161082083	0.42594008	0.670151509	0.03610548	2.740203901	63.50636536	0.5	CRA004775;PRJNA827465	Yang2025_Silkie_Wenchang	0.914452132
ENSGALG00010023930	SRPRA	2	-0.149391075	0.153267886	-0.974705652	0.329706289	0.041371844	7.79084207	87.164417	1	PRJNA827465;Yang2025_Silkie_Wenchang	CRA004775	0.801177491
ENSGALG00010023930	SRPRA	2	-0.222731317	0.140078075	-1.59005124	0.111823256	0.01800779	1.666198741	39.98314996	1	CRA004775;Yang2025_Silkie_Wenchang	PRJNA827465	0.565344663
ENSGALG00010023930	SRPRA	2	-0.011098586	0.035643527	-0.311377321	0.755513793	0	0.001429833	0	1	CRA004775;PRJNA827465	Yang2025_Silkie_Wenchang	0.938501652
ENSGALG00010023931	CSRNP2	2	0.296161791	0.231952323	1.276821834	0.201665155	0.083445958	4.302117185	76.75563084	1	PRJNA827465;Yang2025_Silkie_Wenchang	CRA004775	0.714508754
ENSGALG00010023931	CSRNP2	2	0.361612429	0.212985982	1.697822668	0.089541236	0.04568397	1.991106317	49.7766648	1	CRA004775;Yang2025_Silkie_Wenchang	PRJNA827465	0.513242777
ENSGALG00010023931	CSRNP2	2	0.094298502	0.106631004	0.884344123	0.376510477	0	0.02042408	0	1	CRA004775;PRJNA827465	Yang2025_Silkie_Wenchang	0.822974013
ENSGALG00010023937	XBP1	2	0.142045139	0.257190075	0.552296349	0.580745332	0.109791109	5.271740609	81.03093316	0.5	PRJNA827465;Yang2025_Silkie_Wenchang	CRA004775	0.888579914
ENSGALG00010023937	XBP1	2	-0.155043667	0.155105213	-0.999603197	0.317502575	0	0.000452603	0	1	CRA004775;Yang2025_Silkie_Wenchang	PRJNA827465	0.797125693
ENSGALG00010023937	XBP1	2	0.149070249	0.253233448	0.588667295	0.556084481	0.104725848	4.828918289	79.29142843	0.5	CRA004775;PRJNA827465	Yang2025_Silkie_Wenchang	0.880324407
ENSGALG00010023940	ATP1B3	2	-0.055612285	0.171160539	-0.324913003	0.745246915	0.05321833	10.47094688	90.44976532	0.5	PRJNA827465;Yang2025_Silkie_Wenchang	CRA004775	0.93597621
ENSGALG00010023940	ATP1B3	2	-0.12372777	0.177967053	-0.695228515	0.486912105	0.035358614	1.859576399	46.22431214	0.5	CRA004775;Yang2025_Silkie_Wenchang	PRJNA827465	0.857631005
ENSGALG00010023940	ATP1B3	2	0.10599643	0.042114169	2.516882835	0.011839819	0	0.030982856	0	1	CRA004775;PRJNA827465	Yang2025_Silkie_Wenchang	0.174611791
ENSGALG00010023942	NEGR1	2	-0.159130273	0.20961952	-0.759138618	0.44776964	0.074563435	6.573017525	84.78628733	0.5	PRJNA827465;Yang2025_Silkie_Wenchang	CRA004775	0.846601815
ENSGALG00010023942	NEGR1	2	-0.340102789	0.119355701	-2.849489274	0.004378948	0	0.799630417	0	1	CRA004775;Yang2025_Silkie_Wenchang	PRJNA827465	0.097063291
ENSGALG00010023942	NEGR1	2	0.04141109	0.101466169	0.408127074	0.683180385	0	0.017155751	0	0.5	CRA004775;PRJNA827465	Yang2025_Silkie_Wenchang	0.918800744
ENSGALG00010023945	LETMD1	2	0.223670743	0.370329207	0.603978133	0.545858181	0.248418164	10.22826399	90.22316983	0.5	PRJNA827465;Yang2025_Silkie_Wenchang	CRA004775	0.877954577
ENSGALG00010023945	LETMD1	2	0.099251643	0.545509897	0.181942882	0.855627551	0.522365452	8.025950151	87.54041601	0.5	CRA004775;Yang2025_Silkie_Wenchang	PRJNA827465	0.963486168
ENSGALG00010023945	LETMD1	2	-0.164267762	0.111194922	-1.477295536	0.139596434	0.004033205	1.070332286	6.57107019	1	CRA004775;PRJNA827465	Yang2025_Silkie_Wenchang	0.623410749
ENSGALG00010023946	ZNRF3	2	-0.031074059	0.072676176	-0.427568714	0.668965147	0	0.649423849	0	0.5	PRJNA827465;Yang2025_Silkie_Wenchang	CRA004775	0.914377769
ENSGALG00010023946	ZNRF3	2	0.053774879	0.145482969	0.369630066	0.711658146	0	0.198395973	0	0.5	CRA004775;Yang2025_Silkie_Wenchang	PRJNA827465	0.92597752
ENSGALG00010023946	ZNRF3	2	-0.061159804	0.078773905	-0.776396753	0.437514761	0	0.001968078	0	1	CRA004775;PRJNA827465	Yang2025_Silkie_Wenchang	0.843803603
ENSGALG00010023948	SULF2	2	-0.045945927	0.107976785	-0.42551672	0.670460036	0	0.398804521	0	0.5	PRJNA827465;Yang2025_Silkie_Wenchang	CRA004775	0.914482883
ENSGALG00010023948	SULF2	2	0.073933584	0.162657346	0.454535785	0.649443241	0	0.157070348	0	1	CRA004775;Yang2025_Silkie_Wenchang	PRJNA827465	0.908043243
ENSGALG00010023948	SULF2	2	-0.063853837	0.127837676	-0.499491532	0.617433151	0	0.571853046	0	0.5	CRA004775;PRJNA827465	Yang2025_Silkie_Wenchang	0.899121942
ENSGALG00010023953	SLC35A3	2	0.022563182	0.13736378	0.164258598	0.869527573	0.023569504	2.65102683	62.27876729	0.5	PRJNA827465;Yang2025_Silkie_Wenchang	CRA004775	0.968578601
ENSGALG00010023953	SLC35A3	2	-0.137075749	0.104587344	-1.310634191	0.189981382	0	0.655556825	0	1	CRA004775;Yang2025_Silkie_Wenchang	PRJNA827465	0.699277793
ENSGALG00010023953	SLC35A3	2	-0.028915843	0.262897085	-0.109989212	0.912417931	0.091383519	2.550185383	60.78716447	0.5	CRA004775;PRJNA827465	Yang2025_Silkie_Wenchang	0.978967245
ENSGALG00010023954	PLEKHF1	2	0.117502522	0.122911934	0.955989534	0.339077522	0.023459406	4.358053841	77.05397784	1	PRJNA827465;Yang2025_Silkie_Wenchang	CRA004775	0.804767595
ENSGALG00010023954	PLEKHF1	2	0.251807725	0.08711878	2.890395448	0.003847575	0	1.6543E-05	0	1	CRA004775;Yang2025_Silkie_Wenchang	PRJNA827465	0.089058443
ENSGALG00010023954	PLEKHF1	2	0.065279061	0.106351051	0.613807389	0.539342614	0.01062505	1.530337037	34.65491744	1	CRA004775;PRJNA827465	Yang2025_Silkie_Wenchang	0.874983893
ENSGALG00010023955	C1QL4	2	0.56101281	0.377474745	1.486226078	0.137219332	0	0.718494329	0	1	PRJNA827465;Yang2025_Silkie_Wenchang	CRA004775	0.619615281
ENSGALG00010023955	C1QL4	2	1.079965792	0.62601905	1.725132472	0.084503602	0	0.095462809	0	1	CRA004775;Yang2025_Silkie_Wenchang	PRJNA827465	0.500072565
ENSGALG00010023955	C1QL4	2	0.518722177	0.368585729	1.407331146	0.159329218	0	0.277157758	0	1	CRA004775;PRJNA827465	Yang2025_Silkie_Wenchang	0.654827591
ENSGALG00010023956	KREMEN1	2	0.007364921	0.101812393	0.072338158	0.942332798	0	0.003975468	0	1	PRJNA827465;Yang2025_Silkie_Wenchang	CRA004775	0.985827325
ENSGALG00010023956	KREMEN1	2	0.013475897	0.136779655	0.098522668	0.921517273	0	0.040639702	0	1	CRA004775;Yang2025_Silkie_Wenchang	PRJNA827465	0.981565735
ENSGALG00010023956	KREMEN1	2	0.022345687	0.126045922	0.177282106	0.859286805	0	0.028118951	0	1	CRA004775;PRJNA827465	Yang2025_Silkie_Wenchang	0.964605083
ENSGALG00010023957	YBX3	2	-0.148473937	0.065679722	-2.260575008	0.023785587	0	0.763319117	0	1	PRJNA827465;Yang2025_Silkie_Wenchang	CRA004775	0.263410797
ENSGALG00010023957	YBX3	2	0.009744623	0.135316506	0.07201356	0.942591116	0	0.474678983	0	0.5	CRA004775;Yang2025_Silkie_Wenchang	PRJNA827465	0.985827325
ENSGALG00010023957	YBX3	2	-0.111198276	0.146701759	-0.757988707	0.448457743	0.020459887	1.358762859	26.40364037	0.5	CRA004775;PRJNA827465	Yang2025_Silkie_Wenchang	0.846878812
ENSGALG00010023962	FPGT	2	-0.2971612	0.139707319	-2.127026711	0.033417864	0.026963506	3.083518357	67.56951364	1	PRJNA827465;Yang2025_Silkie_Wenchang	CRA004775	0.314027665
ENSGALG00010023962	FPGT	2	-0.270153036	0.241089241	-1.12055202	0.262478598	0.075054478	2.557551804	60.90010774	0.5	CRA004775;Yang2025_Silkie_Wenchang	PRJNA827465	0.768418419
ENSGALG00010023962	FPGT	2	-0.158907057	0.08041067	-1.976193675	0.048132846	0	0.541146363	0	0.5	CRA004775;PRJNA827465	Yang2025_Silkie_Wenchang	0.379424312
ENSGALG00010023966	PEAK1	2	0.087148166	0.0667249	1.306081635	0.191524797	0	0.119893658	0	1	PRJNA827465;Yang2025_Silkie_Wenchang	CRA004775	0.701145596
ENSGALG00010023966	PEAK1	2	0.197011855	0.181889794	1.083138587	0.278746914	0	0.244924928	0	1	CRA004775;Yang2025_Silkie_Wenchang	PRJNA827465	0.776992195
ENSGALG00010023966	PEAK1	2	0.087576926	0.069684192	1.256768909	0.208837327	0	0.515925745	0	1	CRA004775;PRJNA827465	Yang2025_Silkie_Wenchang	0.721011841
ENSGALG00010023967	PRPH	2	1.146668468	0.590182488	1.942904934	0.052027646	0.568688883	5.334791876	81.25512629	1	PRJNA827465;Yang2025_Silkie_Wenchang	CRA004775	0.395759125
ENSGALG00010023967	PRPH	2	1.055561768	0.758167649	1.392253771	0.163845565	0.918626961	4.932334872	79.72562638	1	CRA004775;Yang2025_Silkie_Wenchang	PRJNA827465	0.662308161
ENSGALG00010023967	PRPH	2	0.520208062	0.254457882	2.044377866	0.040916232	0	0.301695524	0	1	CRA004775;PRJNA827465	Yang2025_Silkie_Wenchang	0.350050212
ENSGALG00010023968	TYW5	2	-0.013936566	0.101286149	-0.137595975	0.890559737	0	0.051343009	0	1	PRJNA827465;Yang2025_Silkie_Wenchang	CRA004775	0.973458375
ENSGALG00010023968	TYW5	2	-0.00169464	0.15205569	-0.011144861	0.991107871	0	0.126221599	0	0.5	CRA004775;Yang2025_Silkie_Wenchang	PRJNA827465	0.99755571
ENSGALG00010023968	TYW5	2	0.009780646	0.100292784	0.097520932	0.922312712	0	0.047196766	0	0.5	CRA004775;PRJNA827465	Yang2025_Silkie_Wenchang	0.981804675
ENSGALG00010023972	ENSGALG00010023972	2	0.285028531	0.234285187	1.216587935	0.223761035	0	0.384071469	0	1	PRJNA827465;Yang2025_Silkie_Wenchang	CRA004775	0.737297114
ENSGALG00010023972	ENSGALG00010023972	2	-0.031331025	0.550486328	-0.056915174	0.954612767	0.409804115	3.083692576	67.57134586	0.5	CRA004775;Yang2025_Silkie_Wenchang	PRJNA827465	0.987987175
ENSGALG00010023972	ENSGALG00010023972	2	-0.11847774	0.383424235	-0.30899909	0.757322219	0.162916717	2.11915385	52.81135439	0.5	CRA004775;PRJNA827465	Yang2025_Silkie_Wenchang	0.938838283
ENSGALG00010023974	PREX1	2	0.096039498	0.073702555	1.303068771	0.192551284	0.002217811	1.139551801	12.24620076	1	PRJNA827465;Yang2025_Silkie_Wenchang	CRA004775	0.702467122
ENSGALG00010023974	PREX1	2	0.197804224	0.153775013	1.286322267	0.198330614	0	0.775816574	0	0.5	CRA004775;Yang2025_Silkie_Wenchang	PRJNA827465	0.710296638
ENSGALG00010023974	PREX1	2	0.055980529	0.065780275	0.851023026	0.394756562	0	0.236005297	0	0.5	CRA004775;PRJNA827465	Yang2025_Silkie_Wenchang	0.830643015
ENSGALG00010023978	TFDP2	2	0.0044584	0.125845621	0.035427537	0.971738827	0.017162836	1.921326439	47.95262378	0.5	PRJNA827465;Yang2025_Silkie_Wenchang	CRA004775	0.991955348
ENSGALG00010023978	TFDP2	2	0.383755656	0.240503557	1.595634011	0.110570529	0.067135548	2.278907195	56.11931884	1	CRA004775;Yang2025_Silkie_Wenchang	PRJNA827465	0.562832796
ENSGALG00010023978	TFDP2	2	0.249644659	0.375689121	0.66449797	0.506371648	0.245935147	7.094202846	85.90398355	0.5	CRA004775;PRJNA827465	Yang2025_Silkie_Wenchang	0.863929781
ENSGALG00010023979	FAM118B	2	0.004172225	0.063010637	0.066214615	0.947206961	0	0.028660996	0	0.5	PRJNA827465;Yang2025_Silkie_Wenchang	CRA004775	0.986445196
ENSGALG00010023979	FAM118B	2	-0.071347958	0.110139988	-0.647793409	0.517118579	0	0.151155492	0	1	CRA004775;Yang2025_Silkie_Wenchang	PRJNA827465	0.867952013
ENSGALG00010023979	FAM118B	2	-0.012371546	0.061634635	-0.20072393	0.840914447	0	0.528683025	0	0.5	CRA004775;PRJNA827465	Yang2025_Silkie_Wenchang	0.95980036
ENSGALG00010023981	ENSGALG00010023981	2	0.114629298	0.327042008	0.350503286	0.725961025	0.144658557	2.94901382	66.09035898	0.5	PRJNA827465;Yang2025_Silkie_Wenchang	CRA004775	0.931029023
ENSGALG00010023981	ENSGALG00010023981	2	0.041298761	0.435432175	0.094845452	0.924437584	0.288507456	4.158081309	75.95044623	0.5	CRA004775;Yang2025_Silkie_Wenchang	PRJNA827465	0.982375838
ENSGALG00010023981	ENSGALG00010023981	2	-0.240533977	0.163331733	-1.472671432	0.140839653	0	0.377636291	0	1	CRA004775;PRJNA827465	Yang2025_Silkie_Wenchang	0.625201272
ENSGALG00010023984	FMNL1	2	0.133976398	0.108612422	1.233527397	0.217379062	0	0.006459789	0	1	PRJNA827465;Yang2025_Silkie_Wenchang	CRA004775	0.730896703
ENSGALG00010023984	FMNL1	2	-0.143105163	0.345041011	-0.414748272	0.678326191	0.170571768	3.266120419	69.38263531	0.5	CRA004775;Yang2025_Silkie_Wenchang	PRJNA827465	0.917670684
ENSGALG00010023984	FMNL1	2	-0.141076996	0.333627508	-0.422857806	0.672399009	0.16450432	3.389640962	70.49835038	0.5	CRA004775;PRJNA827465	Yang2025_Silkie_Wenchang	0.914742762
ENSGALG00010023986	SYCE3	2	0.585064655	0.381636837	1.533040312	0.125265923	0.189489896	2.858771118	65.01993484	1	PRJNA827465;Yang2025_Silkie_Wenchang	CRA004775	0.594102397
ENSGALG00010023986	SYCE3	2	0.821820442	0.277250415	2.964181107	0.003034896	0	0.998700169	0	1	CRA004775;Yang2025_Silkie_Wenchang	PRJNA827465	0.075885251
ENSGALG00010023986	SYCE3	2	0.208978552	0.291427398	0.717086155	0.473320938	0	0.008391754	0	1	CRA004775;PRJNA827465	Yang2025_Silkie_Wenchang	0.854343014
ENSGALG00010023987	TNNI3K	2	0.316937557	0.237973859	1.331816688	0.182920433	0.034983254	1.446251787	30.85574661	1	PRJNA827465;Yang2025_Silkie_Wenchang	CRA004775	0.691347415
ENSGALG00010023987	TNNI3K	2	0.57203921	0.261730207	2.185606384	0.028844415	0	0.095633049	0	1	CRA004775;Yang2025_Silkie_Wenchang	PRJNA827465	0.289410648
ENSGALG00010023987	TNNI3K	2	0.141600773	0.272902338	0.518869768	0.603851561	0	0.658175424	0	1	CRA004775;PRJNA827465	Yang2025_Silkie_Wenchang	0.895892991
ENSGALG00010023989	ENSGALG00010023989	2	0.104465764	0.09166785	1.139611809	0.254448063	0.007518457	1.573400802	36.44340343	1	PRJNA827465;Yang2025_Silkie_Wenchang	CRA004775	0.764087626
ENSGALG00010023989	ENSGALG00010023989	2	-0.097652343	0.358671572	-0.272261173	0.785421208	0.227345329	8.526214182	88.27146517	0.5	CRA004775;Yang2025_Silkie_Wenchang	PRJNA827465	0.946449894
ENSGALG00010023989	ENSGALG00010023989	2	-0.177437509	0.255136229	-0.695461832	0.486765923	0.111071844	6.208655915	83.89345434	0.5	CRA004775;PRJNA827465	Yang2025_Silkie_Wenchang	0.857603818
ENSGALG00010023991	TSPAN3	2	-0.216640748	0.123475728	-1.75452092	0.079341288	0.023606653	4.142814057	75.86181793	1	PRJNA827465;Yang2025_Silkie_Wenchang	CRA004775	0.484805397
ENSGALG00010023991	TSPAN3	2	-0.313992579	0.092179438	-3.406319101	0.000658452	0	0.592865203	0	1	CRA004775;Yang2025_Silkie_Wenchang	PRJNA827465	0.028283199
ENSGALG00010023991	TSPAN3	2	-0.118376881	0.052967617	-2.234891569	0.025424468	0	0.262599305	0	1	CRA004775;PRJNA827465	Yang2025_Silkie_Wenchang	0.271950723
ENSGALG00010023992	FAM131B	2	0.244468561	0.104551003	2.338270824	0.019373204	0.005587476	1.312359301	23.80135539	1	PRJNA827465;Yang2025_Silkie_Wenchang	CRA004775	0.23313687
ENSGALG00010023992	FAM131B	2	0.183472864	0.283250915	0.647739705	0.517153319	0.095289865	2.097762194	52.33015435	0.5	CRA004775;Yang2025_Silkie_Wenchang	PRJNA827465	0.867952013
ENSGALG00010023992	FAM131B	2	0.13639555	0.104306003	1.307648134	0.190992683	0	0.927552834	0	0.5	CRA004775;PRJNA827465	Yang2025_Silkie_Wenchang	0.700443419
ENSGALG00010023993	C19orf12	2	-0.124307194	0.179186897	-0.693729266	0.487852009	0.050212963	4.282827656	76.65093998	0.5	PRJNA827465;Yang2025_Silkie_Wenchang	CRA004775	0.85768885
ENSGALG00010023993	C19orf12	2	-0.336607624	0.127547705	-2.639072366	0.008313323	0	0.004223142	0	1	CRA004775;Yang2025_Silkie_Wenchang	PRJNA827465	0.142122029
ENSGALG00010023993	C19orf12	2	-0.102730226	0.181373972	-0.566400047	0.571121863	0.044754167	2.641715165	62.14580537	0.5	CRA004775;PRJNA827465	Yang2025_Silkie_Wenchang	0.885354083
ENSGALG00010023996	MYO7B	2	0.2738497	0.290344663	0.94318834	0.345584576	0	0.11372183	0	1	PRJNA827465;Yang2025_Silkie_Wenchang	CRA004775	0.807376412
ENSGALG00010023996	MYO7B	2	1.119206907	0.709099777	1.578348977	0.11448546	0.808195578	5.085765634	80.33727718	1	CRA004775;Yang2025_Silkie_Wenchang	PRJNA827465	0.570438899
ENSGALG00010023996	MYO7B	2	0.992774483	0.809552187	1.226325492	0.220076212	1.152687222	8.277937158	87.91969568	1	CRA004775;PRJNA827465	Yang2025_Silkie_Wenchang	0.733691086
ENSGALG00010023997	ARFGEF2	2	0.119534374	0.105277846	1.135418124	0.256200169	0.012799798	2.209500245	54.74089661	1	PRJNA827465;Yang2025_Silkie_Wenchang	CRA004775	0.764613396
ENSGALG00010023997	ARFGEF2	2	0.210380093	0.118848102	1.770159472	0.076700579	0	0.982748816	0	0.5	CRA004775;Yang2025_Silkie_Wenchang	PRJNA827465	0.477904868
ENSGALG00010023997	ARFGEF2	2	0.029774995	0.070996328	0.419387817	0.674932727	0	0.187628131	0	0.5	CRA004775;PRJNA827465	Yang2025_Silkie_Wenchang	0.91599715
ENSGALG00010023999	DGCR14	2	0.009931256	0.049839846	0.199263376	0.842056726	0	0.301220556	0	0.5	PRJNA827465;Yang2025_Silkie_Wenchang	CRA004775	0.960064308
ENSGALG00010023999	DGCR14	2	-0.1417013	0.130717026	-1.084030931	0.278351085	0.015787513	1.755916746	43.0496917	1	CRA004775;Yang2025_Silkie_Wenchang	PRJNA827465	0.776763074
ENSGALG00010023999	DGCR14	2	-0.103530317	0.163514738	-0.633155875	0.526631858	0.03993077	3.373926529	70.36094321	0.5	CRA004775;PRJNA827465	Yang2025_Silkie_Wenchang	0.87066367
ENSGALG00010024001	ENSGALG00010024001	2	0.194397152	0.369561194	0.526021548	0.598873235	0	0.242009227	0	1	PRJNA827465;Yang2025_Silkie_Wenchang	CRA004775	0.894792426
ENSGALG00010024001	ENSGALG00010024001	2	0.648490403	0.612098255	1.059454749	0.289392727	0.508509052	3.109958267	67.84522768	1	CRA004775;Yang2025_Silkie_Wenchang	PRJNA827465	0.783765322
ENSGALG00010024001	ENSGALG00010024001	2	0.860752344	0.427959591	2.011293498	0.044294465	0.106247925	1.403726767	28.76106492	1	CRA004775;PRJNA827465	Yang2025_Silkie_Wenchang	0.363642137
ENSGALG00010024002	DGCR2	2	-0.056476066	0.037636426	-1.500569286	0.133467001	0	0.149006247	0	1	PRJNA827465;Yang2025_Silkie_Wenchang	CRA004775	0.612342914
ENSGALG00010024002	DGCR2	2	-0.081386688	0.078065206	-1.042547526	0.297157903	0	0.027866728	0	1	CRA004775;Yang2025_Silkie_Wenchang	PRJNA827465	0.788466926
ENSGALG00010024002	DGCR2	2	-0.051547199	0.038987917	-1.322132682	0.186123973	0	0.009488943	0	1	CRA004775;PRJNA827465	Yang2025_Silkie_Wenchang	0.69509982
ENSGALG00010024003	ERICH3	2	0.731840741	0.637507283	1.147972361	0.250979974	0.749685446	12.5214991	92.0137358	1	PRJNA827465;Yang2025_Silkie_Wenchang	CRA004775	0.760779417
ENSGALG00010024003	ERICH3	2	0.278176988	1.118848123	0.248628015	0.803648536	2.408146235	26.20847681	96.1844406	0.5	CRA004775;Yang2025_Silkie_Wenchang	PRJNA827465	0.951064358
ENSGALG00010024003	ERICH3	2	-0.32431784	0.479548694	-0.676298036	0.49885144	0.405993368	8.256471676	87.88828886	0.5	CRA004775;PRJNA827465	Yang2025_Silkie_Wenchang	0.861571318
ENSGALG00010024004	EIF4B	2	0.017110898	0.092873099	0.18423955	0.853825534	0.012054233	3.167533745	68.42969703	0.5	PRJNA827465;Yang2025_Silkie_Wenchang	CRA004775	0.962975689
ENSGALG00010024004	EIF4B	2	-0.067906836	0.245724597	-0.276353435	0.782276618	0.089291205	3.298252862	69.68091769	0.5	CRA004775;Yang2025_Silkie_Wenchang	PRJNA827465	0.945372683
ENSGALG00010024004	EIF4B	2	-0.118002198	0.120193259	-0.981770522	0.326212914	0.014419093	1.397031756	28.41966581	1	CRA004775;PRJNA827465	Yang2025_Silkie_Wenchang	0.800023788
ENSGALG00010024005	STYK1	2	-0.001739432	0.147910393	-0.011760037	0.990617064	0	0.314639366	0	0.5	PRJNA827465;Yang2025_Silkie_Wenchang	CRA004775	0.997520496
ENSGALG00010024005	STYK1	2	0.198405831	0.403793981	0.491354107	0.623176018	0.19718659	2.366327352	57.74042002	0.5	CRA004775;Yang2025_Silkie_Wenchang	PRJNA827465	0.900348922
ENSGALG00010024005	STYK1	2	0.23285168	0.289514796	0.804282486	0.42123385	0.084628335	1.681603638	40.53295455	1	CRA004775;PRJNA827465	Yang2025_Silkie_Wenchang	0.839352211
ENSGALG00010024008	CCNE1	2	0.037027579	0.073395776	0.504491971	0.613915703	0	0.019585944	0	1	PRJNA827465;Yang2025_Silkie_Wenchang	CRA004775	0.898256044
ENSGALG00010024008	CCNE1	2	0.079524121	0.10648167	0.746833899	0.45516383	0	0.179472096	0	1	CRA004775;Yang2025_Silkie_Wenchang	PRJNA827465	0.849072665
ENSGALG00010024008	CCNE1	2	0.050215951	0.082456765	0.608997342	0.542526201	0	0.306956488	0	1	CRA004775;PRJNA827465	Yang2025_Silkie_Wenchang	0.876817086
ENSGALG00010024009	CSE1L	2	-0.2096003	0.148040649	-1.415829382	0.156825465	0.03948507	9.743201071	89.73643269	1	PRJNA827465;Yang2025_Silkie_Wenchang	CRA004775	0.650212649
ENSGALG00010024009	CSE1L	2	-0.186213747	0.194111731	-0.959312174	0.337401504	0.063163068	6.024790525	83.40191255	0.5	CRA004775;Yang2025_Silkie_Wenchang	PRJNA827465	0.80415385
ENSGALG00010024009	CSE1L	2	-0.062062352	0.040322892	-1.539134438	0.123771479	0	0.434986097	0	0.5	CRA004775;PRJNA827465	Yang2025_Silkie_Wenchang	0.590004905
ENSGALG00010024010	ENSGALG00010024010	2	-0.227466481	0.055255686	-4.116616727	3.84475E-05	0	0.501937994	0	1	PRJNA827465;Yang2025_Silkie_Wenchang	CRA004775	0.004008225
ENSGALG00010024010	ENSGALG00010024010	2	-0.055660119	0.166005679	-0.33529045	0.737405997	0.03274603	2.129963839	53.05084614	0.5	CRA004775;Yang2025_Silkie_Wenchang	PRJNA827465	0.933968189
ENSGALG00010024010	ENSGALG00010024010	2	-0.090588421	0.210616402	-0.430110952	0.667114933	0.067055677	3.490014511	71.346824	0.5	CRA004775;PRJNA827465	Yang2025_Silkie_Wenchang	0.913766816
ENSGALG00010024011	AGPS	2	-0.211583562	0.16189793	-1.30689479	0.191248446	0.047557818	10.71587232	90.66804857	1	PRJNA827465;Yang2025_Silkie_Wenchang	CRA004775	0.700590704
ENSGALG00010024011	AGPS	2	-0.205683328	0.202911535	-1.013660108	0.310744965	0.06451212	4.202981301	76.207365	0.5	CRA004775;Yang2025_Silkie_Wenchang	PRJNA827465	0.795107883
ENSGALG00010024011	AGPS	2	-0.045152797	0.057235679	-0.78889248	0.430174851	0	0.203227308	0	0.5	CRA004775;PRJNA827465	Yang2025_Silkie_Wenchang	0.842032082
ENSGALG00010024013	PTPRJ	2	0.100181468	0.073203212	1.368539243	0.171143347	0	0.353828234	0	1	PRJNA827465;Yang2025_Silkie_Wenchang	CRA004775	0.673243267
ENSGALG00010024013	PTPRJ	2	0.161998917	0.166246711	0.974448851	0.329833724	0	0.294019222	0	0.5	CRA004775;Yang2025_Silkie_Wenchang	PRJNA827465	0.801177491
ENSGALG00010024013	PTPRJ	2	0.07610093	0.079096686	0.962125392	0.33598662	0	0.105137348	0	0.5	CRA004775;PRJNA827465	Yang2025_Silkie_Wenchang	0.803504387
ENSGALG00010024016	CRYZ	2	0.011198972	0.082805382	0.135244491	0.892418571	0.005152022	1.442933668	30.69674495	0.5	PRJNA827465;Yang2025_Silkie_Wenchang	CRA004775	0.97432504
ENSGALG00010024016	CRYZ	2	-0.149690722	0.322290619	-0.464458825	0.642319053	0.170136529	5.287465651	81.08734759	0.5	CRA004775;Yang2025_Silkie_Wenchang	PRJNA827465	0.906634523
ENSGALG00010024016	CRYZ	2	-0.213139694	0.224306215	-0.950217514	0.342001741	0.075413699	3.32013632	69.88075477	1	CRA004775;PRJNA827465	Yang2025_Silkie_Wenchang	0.805904173
ENSGALG00010024017	TYW3	2	0.088421397	0.262128404	0.337320932	0.735874982	0.109115642	4.834095718	79.31360779	0.5	PRJNA827465;Yang2025_Silkie_Wenchang	CRA004775	0.933592889
ENSGALG00010024017	TYW3	2	0.194848984	0.191840713	1.015681087	0.309781277	0.030850074	1.69327193	40.94274036	0.5	CRA004775;Yang2025_Silkie_Wenchang	PRJNA827465	0.794371602
ENSGALG00010024017	TYW3	2	-0.124342449	0.129376722	-0.961088265	0.33650779	0	0.232842865	0	1	CRA004775;PRJNA827465	Yang2025_Silkie_Wenchang	0.803806405
ENSGALG00010024018	KRT18	2	-0.444771593	0.756092479	-0.588250255	0.55636433	0	0.916951803	0	0.5	PRJNA827465;Yang2025_Silkie_Wenchang	CRA004775	0.880324407
ENSGALG00010024018	KRT18	2	0.800233198	1.898255408	0.421562449	0.67334442	6.35482423	8.357652074	88.03491709	0.5	CRA004775;Yang2025_Silkie_Wenchang	PRJNA827465	0.915250069
ENSGALG00010024018	KRT18	2	1.566517937	1.169716269	1.33922899	0.180496138	1.912912105	3.245543146	69.18851622	1	CRA004775;PRJNA827465	Yang2025_Silkie_Wenchang	0.68814043
ENSGALG00010024019	MAP3K14	2	-0.055086439	0.051895935	-1.061478875	0.288472328	0	0.04810014	0	1	PRJNA827465;Yang2025_Silkie_Wenchang	CRA004775	0.783088502
ENSGALG00010024019	MAP3K14	2	-0.154861864	0.127567526	-1.213959926	0.224763027	0.017754913	1.896816035	47.28007452	1	CRA004775;Yang2025_Silkie_Wenchang	PRJNA827465	0.737885283
ENSGALG00010024019	MAP3K14	2	-0.145148003	0.140414706	-1.033709411	0.301272032	0.024926143	2.300322422	56.5278332	1	CRA004775;PRJNA827465	Yang2025_Silkie_Wenchang	0.791222412
ENSGALG00010024020	MAIP1	2	-0.257818178	0.269181303	-0.957786353	0.338170502	0.134434672	13.09978078	92.36628447	1	PRJNA827465;Yang2025_Silkie_Wenchang	CRA004775	0.80415385
ENSGALG00010024020	MAIP1	2	-0.296929217	0.30266706	-0.981042392	0.326571837	0.132625494	3.292812909	69.63082848	0.5	CRA004775;Yang2025_Silkie_Wenchang	PRJNA827465	0.800028239
ENSGALG00010024020	MAIP1	2	-0.004528057	0.039050649	-0.115953441	0.907689442	0	0.062478643	0	0.5	CRA004775;PRJNA827465	Yang2025_Silkie_Wenchang	0.978196186
ENSGALG00010024024	AGL	2	-0.183391387	0.08061504	-2.274902877	0.022911757	0	0.15492888	0	1	PRJNA827465;Yang2025_Silkie_Wenchang	CRA004775	0.258171094
ENSGALG00010024024	AGL	2	0.019402983	0.272131729	0.071299964	0.943159024	0.081224672	1.719464481	41.84235783	0.5	CRA004775;Yang2025_Silkie_Wenchang	PRJNA827465	0.985827325
ENSGALG00010024024	AGL	2	-0.005232235	0.313622956	-0.016683201	0.986689349	0.128958197	2.186082291	54.25606785	0.5	CRA004775;PRJNA827465	Yang2025_Silkie_Wenchang	0.996259695
ENSGALG00010024025	CLCN1	2	0.010016646	0.321156638	0.031189285	0.975118585	0.157723446	4.245114482	76.44350926	0.5	PRJNA827465;Yang2025_Silkie_Wenchang	CRA004775	0.992672134
ENSGALG00010024025	CLCN1	2	0.313069723	0.197559546	1.584685373	0.113037837	0	0.02759028	0	1	CRA004775;Yang2025_Silkie_Wenchang	PRJNA827465	0.567470443
ENSGALG00010024025	CLCN1	2	-0.227972449	0.208190578	-1.095018089	0.273508712	0	0.934771194	0	0.5	CRA004775;PRJNA827465	Yang2025_Silkie_Wenchang	0.774252172
ENSGALG00010024026	TIRAP	2	0.03833843	0.06919758	0.55404293	0.579549466	0	0.00346852	0	1	PRJNA827465;Yang2025_Silkie_Wenchang	CRA004775	0.887969027
ENSGALG00010024026	TIRAP	2	-0.202966823	0.259414851	-0.78240248	0.433978077	0.084389185	2.621596818	61.8553092	0.5	CRA004775;Yang2025_Silkie_Wenchang	PRJNA827465	0.842762754
ENSGALG00010024026	TIRAP	2	-0.168921104	0.260747175	-0.647834837	0.51709178	0.10608955	3.890976655	74.2995117	0.5	CRA004775;PRJNA827465	Yang2025_Silkie_Wenchang	0.867952013
ENSGALG00010024029	SCAPER	2	0.010762415	0.150182585	0.071662202	0.942870737	0.031443887	3.115095957	67.89826016	0.5	PRJNA827465;Yang2025_Silkie_Wenchang	CRA004775	0.985827325
ENSGALG00010024029	SCAPER	2	0.052256832	0.194942469	0.268062843	0.788650947	0.033951793	1.644180637	39.17943214	0.5	CRA004775;Yang2025_Silkie_Wenchang	PRJNA827465	0.947060654
ENSGALG00010024029	SCAPER	2	-0.126071978	0.08247992	-1.528517221	0.126384171	0	0.138547958	0	1	CRA004775;PRJNA827465	Yang2025_Silkie_Wenchang	0.59697057

ENSGALG00010024030	STAU1	2	0.059570225	0.075430468	0.789736923	0.429681423	0.006494685	2.156683266	53.63250525	1	PRJNA827465;Yang2025_Silkie_Wenchang	CRA004775	0.842012722
ENSGALG00010024030	STAU1	2	0.136984823	0.084967917	1.612194672	0.106919584	0	0.277922073	0	1	CRA004775;Yang2025_Silkie_Wenchang	PRJNA827465	0.553983328
ENSGALG00010024030	STAU1	2	0.002695773	0.049959613	0.053959039	0.956967799	0	0.023057742	0	1	CRA004775;PRJNA827465	Yang2025_Silkie_Wenchang	0.988540618
ENSGALG00010024031	LIMS2	2	-0.234793916	0.240703229	-0.975449796	0.329337193	0.106034333	11.37312629	91.20734287	1	PRJNA827465;Yang2025_Silkie_Wenchang	CRA004775	0.800981213
ENSGALG00010024031	LIMS2	2	-0.173217613	0.359096117	-0.482371165	0.629542301	0.212227917	5.260450069	80.99021972	0.5	CRA004775;Yang2025_Silkie_Wenchang	PRJNA827465	0.902457014
ENSGALG00010024031	LIMS2	2	0.002520464	0.058525191	0.043066312	0.965648673	0	0.676923344	0	0.5	CRA004775;PRJNA827465	Yang2025_Silkie_Wenchang	0.990668014
ENSGALG00010024034	LRP2	2	-0.252607859	0.150408553	-1.679478022	0.093058599	0	0.303012303	0	1	PRJNA827465;Yang2025_Silkie_Wenchang	CRA004775	0.52249924
ENSGALG00010024034	LRP2	2	-0.178384554	0.25004074	-0.713421956	0.475584691	0	0.21724601	0	1	CRA004775;Yang2025_Silkie_Wenchang	PRJNA827465	0.854526154
ENSGALG00010024034	LRP2	2	-0.317765037	0.173013779	-1.836645832	0.066262197	0	0.044173758	0	1	CRA004775;PRJNA827465	Yang2025_Silkie_Wenchang	0.443285637
ENSGALG00010024035	OSBPL5	2	0.074944896	0.063225259	1.185363195	0.235873877	0	0.010355237	0	1	PRJNA827465;Yang2025_Silkie_Wenchang	CRA004775	0.748669015
ENSGALG00010024035	OSBPL5	2	0.026489854	0.139052634	0.190502357	0.848915497	0	0.685871743	0	0.5	CRA004775;Yang2025_Silkie_Wenchang	PRJNA827465	0.961364896
ENSGALG00010024035	OSBPL5	2	0.058388272	0.067052046	0.870790322	0.38386865	0	0.739263318	0	0.5	CRA004775;PRJNA827465	Yang2025_Silkie_Wenchang	0.825601288
ENSGALG00010024038	NKD1	2	0.167101265	0.151966701	1.099591316	0.271510227	0.015347502	1.428723559	30.00745358	1	PRJNA827465;Yang2025_Silkie_Wenchang	CRA004775	0.772805786
ENSGALG00010024038	NKD1	2	0.091892258	0.435406964	0.211049124	0.83284894	0.219032497	1.942040244	48.50776121	0.5	CRA004775;Yang2025_Silkie_Wenchang	PRJNA827465	0.957874985
ENSGALG00010024038	NKD1	2	0.028048446	0.137703637	0.203687039	0.838598071	0	0.91765609	0	0.5	CRA004775;PRJNA827465	Yang2025_Silkie_Wenchang	0.959226718
ENSGALG00010024039	MRPL45	2	-0.058465054	0.057720957	-1.012891288	0.31111209	0	0.020058778	0	1	PRJNA827465;Yang2025_Silkie_Wenchang	CRA004775	0.79529356
ENSGALG00010024039	MRPL45	2	-0.102441723	0.11190083	-0.91546884	0.359945554	0	0.733320011	0	1	CRA004775;Yang2025_Silkie_Wenchang	PRJNA827465	0.814880744
ENSGALG00010024039	MRPL45	2	-0.078882575	0.061289083	-1.287057512	0.198074243	0	0.764333665	0	1	CRA004775;PRJNA827465	Yang2025_Silkie_Wenchang	0.710234722
ENSGALG00010024042	DCPS	2	-0.310212234	0.105971764	-2.927310284	0.003419076	0.012125789	2.014816861	50.36769746	1	PRJNA827465;Yang2025_Silkie_Wenchang	CRA004775	0.082340648
ENSGALG00010024042	DCPS	2	-0.352287782	0.132075822	-2.667314706	0.007646004	0.008486637	1.282307065	22.01555873	1	CRA004775;Yang2025_Silkie_Wenchang	PRJNA827465	0.135061073
ENSGALG00010024042	DCPS	2	-0.221927073	0.070916026	-3.129434722	0.00175143	0	0.071162152	0	1	CRA004775;PRJNA827465	Yang2025_Silkie_Wenchang	0.05307359
ENSGALG00010024045	PSTPIP1	2	-0.296727282	0.117527785	-2.524741548	0.011578341	0	0.644189247	0	1	PRJNA827465;Yang2025_Silkie_Wenchang	CRA004775	0.172111769
ENSGALG00010024045	PSTPIP1	2	-0.610411262	0.335113213	-1.821512484	0.068528991	0.117058603	1.667308824	40.02310876	1	CRA004775;Yang2025_Silkie_Wenchang	PRJNA827465	0.451003838
ENSGALG00010024045	PSTPIP1	2	-0.545189451	0.453510967	-1.202152737	0.229304357	0.288598454	2.700917534	62.97554888	1	CRA004775;PRJNA827465	Yang2025_Silkie_Wenchang	0.742284374
ENSGALG00010024049	RCN2	2	-0.211943162	0.17491201	-1.211713028	0.225622225	0.056436649	12.86209816	92.22521872	1	PRJNA827465;Yang2025_Silkie_Wenchang	CRA004775	0.73884362
ENSGALG00010024049	RCN2	2	-0.313891095	0.138955464	-2.258933085	0.023887548	0.019527032	1.542724037	35.17959298	1	CRA004775;Yang2025_Silkie_Wenchang	PRJNA827465	0.264093469
ENSGALG00010024049	RCN2	2	-0.03972603	0.062531255	-0.635298779	0.52523357	0	0.003920254	0	1	CRA004775;PRJNA827465	Yang2025_Silkie_Wenchang	0.870179017
ENSGALG00010024051	ENSGALG00010024051	2	0.186696868	0.457156068	0.41276247	0.67978065	0	0.17943794	0	1	PRJNA827465;Yang2025_Silkie_Wenchang	CRA004775	0.917701868
ENSGALG00010024051	ENSGALG00010024051	2	1.125521827	1.128042077	0.996731232	0.318849986	2.132602572	6.119815749	83.65903877	1	CRA004775;Yang2025_Silkie_Wenchang	PRJNA827465	0.798009017
ENSGALG00010024051	ENSGALG00010024051	2	1.328185576	0.935095913	1.42037363	0.155498936	1.312309153	4.006415908	75.04003521	1	CRA004775;PRJNA827465	Yang2025_Silkie_Wenchang	0.648167852
ENSGALG00010024052	ENSGALG00010024052	2	0.567923556	0.338951199	1.675533927	0.093829912	0.209355308	11.23241296	91.09719342	1	PRJNA827465;Yang2025_Silkie_Wenchang	CRA004775	0.524786069
ENSGALG00010024052	ENSGALG00010024052	2	0.516100925	0.430950252	1.197588172	0.231077383	0.318465361	6.711779729	85.10082213	1	CRA004775;Yang2025_Silkie_Wenchang	PRJNA827465	0.743758446
ENSGALG00010024052	ENSGALG00010024052	2	0.202429841	0.120671578	1.677527079	0.093439459	0	0.332087308	0	1	CRA004775;PRJNA827465	Yang2025_Silkie_Wenchang	0.52381273
ENSGALG00010024053	ISL2	2	-0.147618884	0.900395249	-0.163948982	0.869771307	1.442736709	9.070099708	88.97476288	0.5	PRJNA827465;Yang2025_Silkie_Wenchang	CRA004775	0.968610705
ENSGALG00010024053	ISL2	2	0.387557701	0.478658936	0.809674012	0.418127558	0.176642948	1.567285291	36.19540706	0.5	CRA004775;Yang2025_Silkie_Wenchang	PRJNA827465	0.838112595
ENSGALG00010024053	ISL2	2	-0.798053318	0.372269157	-2.143753526	0.032052641	0.031027566	1.103644673	9.391127018	1	CRA004775;PRJNA827465	Yang2025_Silkie_Wenchang	0.309978889
ENSGALG00010024058	SLC44A5	2	-0.126200992	0.081321423	-1.551878792	0.120691228	0.006281152	1.731587912	42.2495391	1	PRJNA827465;Yang2025_Silkie_Wenchang	CRA004775	0.582493642
ENSGALG00010024058	SLC44A5	2	0.128422642	0.38158722	0.336548592	0.736457215	0.26860043	12.71903046	92.13776551	0.5	CRA004775;Yang2025_Silkie_Wenchang	PRJNA827465	0.933661344
ENSGALG00010024058	SLC44A5	2	0.202696216	0.294572044	0.688104051	0.49138726	0.156739806	9.716685178	89.70842441	0.5	CRA004775;PRJNA827465	Yang2025_Silkie_Wenchang	0.85895707
ENSGALG00010024060	FRRS1	2	-0.206576045	0.323435819	-0.638692574	0.523022967	0.192492414	12.4092332	91.94148434	0.5	PRJNA827465;Yang2025_Silkie_Wenchang	CRA004775	0.869568211
ENSGALG00010024060	FRRS1	2	-0.129533974	0.438479224	-0.295416447	0.767675771	0.337235538	7.860804191	87.27865527	0.5	CRA004775;Yang2025_Silkie_Wenchang	PRJNA827465	0.941444269
ENSGALG00010024060	FRRS1	2	0.13881302	0.099591586	1.393822769	0.163371131	0	0.615902478	0	1	CRA004775;PRJNA827465	Yang2025_Silkie_Wenchang	0.661683383
ENSGALG00010024062	ST3GAL4	2	0.156934899	0.074149726	2.116459464	0.034305749	0	0.005460777	0	1	PRJNA827465;Yang2025_Silkie_Wenchang	CRA004775	0.318385282
ENSGALG00010024062	ST3GAL4	2	0.102448421	0.119966397	0.853975979	0.393118299	0	0.719715115	0	0.5	CRA004775;Yang2025_Silkie_Wenchang	PRJNA827465	0.830282579
ENSGALG00010024062	ST3GAL4	2	0.13596378	0.085644271	1.587540859	0.112390201	0	0.864030564	0	0.5	CRA004775;PRJNA827465	Yang2025_Silkie_Wenchang	0.56602666
ENSGALG00010024063	BRD7	2	0.088913727	0.180720984	0.49199448	0.622723246	0.055216052	5.824318533	82.83060938	0.5	PRJNA827465;Yang2025_Silkie_Wenchang	CRA004775	0.900255255
ENSGALG00010024063	BRD7	2	0.28988905	0.125202517	2.315361191	0.020593174	0	0.010730921	0	1	CRA004775;Yang2025_Silkie_Wenchang	PRJNA827465	0.232182376
ENSGALG00010024063	BRD7	2	0.009269254	0.14101315	0.065733257	0.947590194	0.02382806	1.736675727	42.41872651	0.5	CRA004775;PRJNA827465	Yang2025_Silkie_Wenchang	0.986560458
ENSGALG00010024064	GPR17	2	0.274471898	0.280018726	0.980191224	0.326991736	0.128251121	5.110158652	80.43113594	1	PRJNA827465;Yang2025_Silkie_Wenchang	CRA004775	0.800028239
ENSGALG00010024064	GPR17	2	0.320626947	0.338848376	0.94622542	0.344033624	0.137374725	2.32931132	57.06885587	0.5	CRA004775;Yang2025_Silkie_Wenchang	PRJNA827465	0.806445383
ENSGALG00010024064	GPR17	2	0.02005201	0.100093313	0.200333168	0.841220024	0	0.100637985	0	0.5	CRA004775;PRJNA827465	Yang2025_Silkie_Wenchang	0.95989279
ENSGALG00010024068	CASP2	2	-0.0057949	0.111058774	-0.052178678	0.958386322	0.014852537	2.343696511	57.33235957	0.5	PRJNA827465;Yang2025_Silkie_Wenchang	CRA004775	0.989036566
ENSGALG00010024068	CASP2	2	-0.200518221	0.147973645	-1.355094153	0.175387561	0.008319398	1.09092399	8.334585277	1	CRA004775;Yang2025_Silkie_Wenchang	PRJNA827465	0.680258136
ENSGALG00010024068	CASP2	2	-0.134798242	0.315414161	-0.427369024	0.669110565	0.141591152	2.649571479	62.25804783	0.5	CRA004775;PRJNA827465	Yang2025_Silkie_Wenchang	0.914579769
ENSGALG00010024073	RABGGTB	2	-0.115791573	0.124433503	-0.930549812	0.352086486	0.021594349	3.187233218	68.62482499	1	PRJNA827465;Yang2025_Silkie_Wenchang	CRA004775	0.810678834
ENSGALG00010024073	RABGGTB	2	-0.182297225	0.139211759	-1.309495881	0.190366432	0.011126658	1.249107023	19.94280861	0.5	CRA004775;Yang2025_Silkie_Wenchang	PRJNA827465	0.699411386
ENSGALG00010024073	RABGGTB	2	-0.000131204	0.074015219	-0.001772663	0.998585621	0	0.084992233	0	0.5	CRA004775;PRJNA827465	Yang2025_Silkie_Wenchang	0.99962841
ENSGALG00010024074	ADCY7	2	-0.005511438	0.111896746	-0.049254674	0.960716341	0	0.003064454	0	1	PRJNA827465;Yang2025_Silkie_Wenchang	CRA004775	0.989520168
ENSGALG00010024074	ADCY7	2	-0.055445145	0.198071932	-0.279924292	0.779535589	0	0.339121067	0	1	CRA004775;Yang2025_Silkie_Wenchang	PRJNA827465	0.944807046
ENSGALG00010024074	ADCY7	2	-0.019310353	0.129230672	-0.149425463	0.881217921	0	0.389953987	0	1	CRA004775;PRJNA827465	Yang2025_Silkie_Wenchang	0.971760275
ENSGALG00010024075	SPATS2L	2	0.084747173	0.125970495	0.672917335	0.500999083	0.019317909	2.221632263	54.98805015	0.5	PRJNA827465;Yang2025_Silkie_Wenchang	CRA004775	0.884241093
ENSGALG00010024075	SPATS2L	2	0.235784866	0.138307073	1.704792541	0.088233123	0	0.067841968	0	0.5	CRA004775;Yang2025_Silkie_Wenchang	PRJNA827465	0.510208309
ENSGALG00010024075	SPATS2L	2	0.00599101	0.067541158	0.088701617	0.929319048	0	0.475520078	0	0.5	CRA004775;PRJNA827465	Yang2025_Silkie_Wenchang	0.983653628
ENSGALG00010024077	SFT2D3	2	0.2435004	0.13523109	1.800624397	0.071762101	0.027969041	4.131788792	75.7974076	1	PRJNA827465;Yang2025_Silkie_Wenchang	CRA004775	0.462169374
ENSGALG00010024077	SFT2D3	2	0.256296938	0.222979603	1.149418757	0.250383349	0.055762766	1.794107127	44.26196825	0.5	CRA004775;Yang2025_Silkie_Wenchang	PRJNA827465	0.759943035
ENSGALG00010024077	SFT2D3	2	0.11098181	0.072419432	1.532486624	0.125402396	0	0.397246773	0	0.5	CRA004775;PRJNA827465	Yang2025_Silkie_Wenchang	0.594562606
ENSGALG00010024078	KIRREL3	2	0.107855507	0.138394694	0.779332675	0.435783772	0	0.678709807	0	1	PRJNA827465;Yang2025_Silkie_Wenchang	CRA004775	0.842811218
ENSGALG00010024078	KIRREL3	2	-0.060234257	0.458621134	-0.131337727	0.895508149	0.266644586	2.492291999	59.87629056	0.5	CRA004775;Yang2025_Silkie_Wenchang	PRJNA827465	0.974927462
ENSGALG00010024078	KIRREL3	2	-0.112926902	0.285136302	-0.396045335	0.692071584	0.078960296	1.510527538	33.79796298	0.5	CRA004775;PRJNA827465	Yang2025_Silkie_Wenchang	0.920348122
ENSGALG00010024080	TENT4B	2	-0.062854143	0.061529672	-1.02152572	0.30700543	0	0.120613117	0	1	PRJNA827465;Yang2025_Silkie_Wenchang	CRA004775	0.79266003
ENSGALG00010024080	TENT4B	2	0.297300126	0.332399608	0.894405764	0.371104829	0.190564368	7.15278312	86.01942792	0.5	CRA004775;Yang2025_Silkie_Wenchang	PRJNA827465	0.8214684
ENSGALG00010024080	TENT4B	2	0.260818516	0.359080871	0.72635035	0.46762402	0.235846465	11.08703097	90.98045272	0.5	CRA004775;PRJNA827465	Yang2025_Silkie_Wenchang	0.852885838
ENSGALG00010024081	CARS	2	0.072736662	0.106193498	0.684944585	0.49337889	0.016048409	2.899641513	65.51297823	0.5	PRJNA827465;Yang2025_Silkie_Wenchang	CRA004775	0.859868321
ENSGALG00010024081	CARS	2	0.176308492	0.103618912	1.701508813	0.088847484	0	0.317655646	0	1	CRA004775;Yang2025_Silkie_Wenchang	PRJNA827465	0.511771357
ENSGALG00010024081	CARS	2	-0.002866549	0.038544726	-0.074369415	0.940716444	0	0.236632488	0	0.5	CRA004775;PRJNA827465	Yang2025_Silkie_Wenchang	0.985565295
ENSGALG00010024083	PALMD	2	0.119012139	0.103123609	1.154072673	0.248470376	0.006568715	1.346255557	25.71989806	1	PRJNA827465;Yang2025_Silkie_Wenchang	CRA004775	0.758731348
ENSGALG00010024083	PALMD	2	0.337619072	0.157288657	2.146493456	0.03183363	0	0.754699203	0	1	CRA004775;Yang2025_Silkie_Wenchang	PRJNA827465	0.305575589
ENSGALG00010024083	PALMD	2	0.22942036	0.273637221	0.838410646	0.401800112	0.097750876	2.190605443	54.35051971	1	CRA004775;PRJNA827465	Yang2025_Silkie_Wenchang	0.832733063
ENSGALG00010024086	GABARAPL1	2	0.007093569	0.04342838	0.163339486	0.870251146	0.000677544	1.201561966	16.77499554	0.5	PRJNA827465;Yang2025_Silkie_Wenchang	CRA004775	0.96881514
ENSGALG00010024086	GABARAPL1	2	0.069790777	0.065666515	1.062806168	0.287869862	0	0.438649148	0	1	CRA004775;Yang2025_Silkie_Wenchang	PRJNA827465	0.782840067
ENSGALG00010024086	GABARAPL1	2	-0.022029039	0.047941332	-0.459499946	0.645875191	0	0.73570129	0	0.5	CRA004775;PRJNA827465	Yang2025_Silkie_Wenchang	0.907098003
ENSGALG00010024091	ETFA	2	0.001214151	0.118611011	0.010236414	0.991832666	0.013000986	1.543151599	35.19755283	0.5	PRJNA827465;Yang2025_Silkie_Wenchang	CRA004775	0.997575757
ENSGALG00010024091	ETFA	2	-0.139066475	0.363558124	-0.382515109	0.702079324	0.207524002	4.592486384	78.22530289	0.5	CRA004775;Yang2025_Silkie_Wenchang	PRJNA827465	0.923083392
ENSGALG00010024091	ETFA	2	-0.222568739	0.215949523	-1.03065168	0.302704192	0.064509787	2.623418243	61.88179286	1	CRA004775;PRJNA827465	Yang2025_Silkie_Wenchang	0.791852471
ENSGALG00010024095	RBM12	2	-0.069973948	0.035707526	-1.959641446	0.050037713	0	0.779036766	0	1	PRJNA827465;Yang2025_Silkie_Wenchang	CRA004775	0.387208555
ENSGALG00010024095	RBM12	2	-0.155889767	0.093213764	-1.67239	0.094447443	0	0.001399835	0	1	CRA004775;Yang2025_Silkie_Wenchang	PRJNA827465	0.52645977
ENSGALG00010024095	RBM12	2	-0.061040203	0.037601415	-1.623348596	0.104514907	0	0.206276962	0	1	CRA004775;PRJNA827465	Yang2025_Silkie_Wenchang	0.549447931
ENSGALG00010024096	MYO1D	2	0.288790585	0.18043856	1.600492628	0.109489341	0.042666666	2.898104667	65.49468998	1	PRJNA827465;Yang2025_Silkie_Wenchang	CRA004775	0.560028576
ENSGALG00010024096	MYO1D	2	0.052987922	0.448392814	0.118172996	0.905930586	0.35368074	8.053477172	87.58300323	0.5	CRA004775;Yang2025_Silkie_Wenchang	PRJNA827465	0.978196186
ENSGALG00010024096	MYO1D	2	-0.105296225	0.263899262	-0.399001589	0.689892034	0.095812664	2.964729985	66.27011549	0.5	CRA004775;PRJNA827465	Yang2025_Silkie_Wenchang	0.919979266
ENSGALG00010024101	CNEP1R1	2	-0.039724029	0.095512925	-0.415902135	0.677481617	0.012533615	3.024557699	66.93731449	0.5	PRJNA827465;Yang2025_Silkie_Wenchang	CRA004775	0.917144807
ENSGALG00010024101	CNEP1R1	2	-0.133998455	0.085635054	-1.564761736	0.117638787	0	0.174141527	0	1	CRA004775;Yang2025_Silkie_Wenchang	PRJNA827465	0.577223126
ENSGALG00010024101	CNEP1R1	2	0.033121635	0.054202229	0.611075148	0.541149832	0	0.275746031	0	0.5	CRA004775;PRJNA827465	Yang2025_Silkie_Wenchang	0.875908568
ENSGALG00010024102	SGF29	2	0.176767906	0.095258367	1.855668029	0.063500891	0.01373478	4.063332725	75.38965997	1	PRJNA827465;Yang2025_Silkie_Wenchang	CRA004775	0.433896992
ENSGALG00010024102	SGF29	2	-0.051266618	0.35504524	-0.144394608	0.885188874	0.228945247	10.31079188	90.30142387	0.5	CRA004775;Yang2025_Silkie_Wenchang	PRJNA827465	0.972280795
ENSGALG00010024102	SGF29	2	-0.13478137	0.25773092	-0.522953822	0.601006389	0.112367225	5.78986944	82.72845337	0.5	CRA004775;PRJNA827465	Yang2025_Silkie_Wenchang	0.895230519
ENSGALG00010024104	PLPPR5	2	0.034092757	0.076362001	0.446462328	0.655263319	0	0.166468164	0	1	PRJNA827465;Yang2025_Silkie_Wenchang	CRA004775	0.910411938
ENSGALG00010024104	PLPPR5	2	0.052674402	0.126001051	0.418047322	0.675912519	0	0.512034004	0	0.5	CRA004775;Yang2025_Silkie_Wenchang	PRJNA827465	0.916522765
ENSGALG00010024104	PLPPR5	2	0.000855456	0.092342038	0.009263994	0.992608508	0	0.351531306	0	0.5	CRA004775;PRJNA827465	Yang2025_Silkie_Wenchang	0.997852036
ENSGALG00010024106	WDR33	2	-0.085349263	0.03280128	-2.602010129	0.009267911	0	0.340034078	0	1	PRJNA827465;Yang2025_Silkie_Wenchang	CRA004775	0.150955958
ENSGALG00010024106	WDR33	2	-0.102546862	0.064087487	-1.600107402	0.109574759	0	0.646178153	0	0.5	CRA004775;Yang2025_Silkie_Wenchang	PRJNA827465	0.560246749
ENSGALG00010024106	WDR33	2	-0.070522471	0.036882531	-1.91208325	0.055865517	0	0.376714137	0	0.5	CRA004775;PRJNA827465	Yang2025_Silkie_Wenchang	0.409031746
ENSGALG00010024107	NFE2L2	2	-0.106020187	0.099704886	-1.063339934	0.287627822	0.01694005	6.603920061	84.85747873	1	PRJNA827465;Yang2025_Silkie_Wenchang	CRA004775	0.782840067
ENSGALG00010024107	NFE2L2	2	-0.205490392	0.063460847	-3.238065715	0.001203431	0	0.173668493	0	1	CRA004775;Yang2025_Silkie_Wenchang	PRJNA827465	0.042344175
ENSGALG00010024107	NFE2L2	2	-0.01483263	0.041312903	-0.359031421	0.719571584	0	0.121437828	0	1	CRA004775;PRJNA827465	Yang2025_Silkie_Wenchang	0.928449671
ENSGALG00010024110	CPNE1	2	0.019303985	0.07420849	0.260131755	0.794762144	0.006093834	2.237740195	55.31205981	0.5	PRJNA827465;Yang2025_Silkie_Wenchang	CRA004775	0.948905846
ENSGALG00010024110	CPNE1	2	-0.113478625	0.104203477	-1.089009965	0.276149491	0.009020602	1.42040717	29.59765192	1	CRA004775;Yang2025_Silkie_Wenchang	PRJNA827465	0.775682505
ENSGALG00010024110	CPNE1	2	-0.063620995	0.193190097	-0.329318097	0.741915267	0.056879378	3.674965103	72.78885726	0.5	CRA004775;PRJNA827465	Yang2025_Silkie_Wenchang	0.93543545
ENSGALG00010024112	NAP1L4	2	0.024227988	0.134126804	0.180634944	0.856654125	0.032696568	10.66609728	90.62449954	0.5	PRJNA827465;Yang2025_Silkie_Wenchang	CRA004775	0.963625561
ENSGALG00010024112	NAP1L4	2	0.164091307	0.070660486	2.322249901	0.020219483	0	0.007892432	0	1	CRA004775;Yang2025_Silkie_Wenchang	PRJNA827465	0.239291285
ENSGALG00010024112	NAP1L4	2	-0.099268813	0.038310049	-2.591195151	0.009564324	0	0.609042945	0	0.5	CRA004775;PRJNA827465	Yang2025_Silkie_Wenchang	0.153870904
ENSGALG00010024113	ENSGALG00010024113	2	0.162188617	0.540550511	0.300043408	0.764144046	0.547412819	15.72119334	93.63915971	0.5	PRJNA827465;Yang2025_Silkie_Wenchang	CRA004775	0.940483053
ENSGALG00010024113	ENSGALG00010024113	2	0.627542113	0.188062964	3.336872386	0.000847268	0	0.568616139	0	1	CRA004775;Yang2025_Silkie_Wenchang	PRJNA827465	0.033876084
ENSGALG00010024113	ENSGALG00010024113	2	-0.057975371	0.37379174	-0.155100728	0.876741908	0.209843944	3.6427993	72.54858373	0.5	CRA004775;PRJNA827465	Yang2025_Silkie_Wenchang	0.97046577
ENSGALG00010024115	POLR2D	2	-0.04943592	0.075863178	-0.651645775	0.514629711	0.003138009	1.094699831	8.650757822	1	PRJNA827465;Yang2025_Silkie_Wenchang	CRA004775	0.866869697
ENSGALG00010024115	POLR2D	2	-0.368412347	0.164199987	-2.243680724	0.024852951	0	0.147540602	0	1	CRA004775;Yang2025_Silkie_Wenchang	PRJNA827465	0.268931467
ENSGALG00010024115	POLR2D	2	-0.16913945	0.190556429	-0.887608208	0.374751537	0.053625622	3.134898535	68.10104095	1	CRA004775;PRJNA827465	Yang2025_Silkie_Wenchang	0.822805106
ENSGALG00010024116	MCAM	2	0.11431228	0.202176723	0.56540772	0.571796475	0.057118282	3.307154877	69.7625289	0.5	PRJNA827465;Yang2025_Silkie_Wenchang	CRA004775	0.885470687
ENSGALG00010024116	MCAM	2	0.275711613	0.158823694	1.73596021	0.08257087	0	0.874903125	0	0.5	CRA004775;Yang2025_Silkie_Wenchang	PRJNA827465	0.495344636
ENSGALG00010024116	MCAM	2	-0.087530904	0.141089566	-0.620392468	0.53499943	0	0.033021692	0	1	CRA004775;PRJNA827465	Yang2025_Silkie_Wenchang	0.87393405
ENSGALG00010024118	TMEM266	2	0.141048148	0.084590087	1.667431171	0.0954287	0.002642702	1.181978826	15.39611557	1	PRJNA827465;Yang2025_Silkie_Wenchang	CRA004775	0.528960129
ENSGALG00010024118	TMEM266	2	0.125731575	0.189885315	0.662144805	0.507878424	0.035773276	1.861809673	46.28881704	0.5	CRA004775;Yang2025_Silkie_Wenchang	PRJNA827465	0.864401731
ENSGALG00010024118	TMEM266	2	0.06352424	0.081872802	0.775889407	0.437814288	0	0.626014794	0	0.5	CRA004775;PRJNA827465	Yang2025_Silkie_Wenchang	0.843899392
ENSGALG00010024119	AMMECR1L	2	-0.147999414	0.172850672	-0.856227012	0.391872224	0.052378722	7.689337936	86.99497917	0.5	PRJNA827465;Yang2025_Silkie_Wenchang	CRA004775	0.829251057
ENSGALG00010024119	AMMECR1L	2	-0.297824036	0.105251002	-2.829655109	0.004659821	0	0.757736047	0	1	CRA004775;Yang2025_Silkie_Wenchang	PRJNA827465	0.100408405
ENSGALG00010024119	AMMECR1L	2	0.007284324	0.05011312	0.145357618	0.884428525	0	0.111104252	0	0.5	CRA004775;PRJNA827465	Yang2025_Silkie_Wenchang	0.97205521
ENSGALG00010024121	NRG4	2	0.071245185	0.100441222	0.70932217	0.478124574	0	0.086547459	0	1	PRJNA827465;Yang2025_Silkie_Wenchang	CRA004775	0.855366076
ENSGALG00010024121	NRG4	2	0.110297143	0.138247153	0.797825785	0.424971596	0.000940671	1.017109556	1.682174342	1	CRA004775;Yang2025_Silkie_Wenchang	PRJNA827465	0.841147134
ENSGALG00010024121	NRG4	2	0.143467751	0.121107426	1.184632151	0.23616292	0	0.724984527	0	1	CRA004775;PRJNA827465	Yang2025_Silkie_Wenchang	0.748669015
ENSGALG00010024122	SOCS7	2	0.08224027	0.114706254	0.716964133	0.473396228	0.020283616	4.300108451	76.74477257	0.5	PRJNA827465;Yang2025_Silkie_Wenchang	CRA004775	0.854354854
ENSGALG00010024122	SOCS7	2	0.15224097	0.126792917	1.200705633	0.229865408	0.010947351	1.205275908	17.0314454	0.5	CRA004775;Yang2025_Silkie_Wenchang	PRJNA827465	0.742767343
ENSGALG00010024122	SOCS7	2	-0.030626255	0.064927978	-0.471695812	0.637143927	0	0.160371663	0	1	CRA004775;PRJNA827465	Yang2025_Silkie_Wenchang	0.905076903
ENSGALG00010024123	PLPPR4	2	-0.397773716	0.415524237	-0.957281623	0.338425128	0.220878556	2.583765735	61.29680077	0.5	PRJNA827465;Yang2025_Silkie_Wenchang	CRA004775	0.804276383
ENSGALG00010024123	PLPPR4	2	-0.538566902	0.849907632	-0.63367698	0.526291651	0.934311616	2.520730008	60.32895245	0.5	CRA004775;Yang2025_Silkie_Wenchang	PRJNA827465	0.870537576
ENSGALG00010024123	PLPPR4	2	-0.791071188	0.248750472	-3.180179647	0.001471838	0	0.767308485	0	1	CRA004775;PRJNA827465	Yang2025_Silkie_Wenchang	0.047532982
ENSGALG00010024124	DIDO1	2	0.340434511	0.379141515	0.897908823	0.36923416	0.272472742	18.41578976	94.56987719	0.5	PRJNA827465;Yang2025_Silkie_Wenchang	CRA004775	0.820606801
ENSGALG00010024124	DIDO1	2	0.340565303	0.413744007	0.82313048	0.410433797	0.299804679	7.948787664	87.41946518	0.5	CRA004775;Yang2025_Silkie_Wenchang	PRJNA827465	0.834820782
ENSGALG00010024124	DIDO1	2	-0.024120201	0.046683346	-0.51667678	0.605381808	0	0.080180557	0	1	CRA004775;PRJNA827465	Yang2025_Silkie_Wenchang	0.896474603
ENSGALG00010024126	CDK5R1	2	-0.059833871	0.131191249	-0.45608127	0.648331538	0.005118239	1.172637476	14.72215238	0.5	PRJNA827465;Yang2025_Silkie_Wenchang	CRA004775	0.90762535
ENSGALG00010024126	CDK5R1	2	-0.207853129	0.348996772	-0.595573214	0.551460375	0.174524801	3.278081566	69.49435272	0.5	CRA004775;Yang2025_Silkie_Wenchang	PRJNA827465	0.878888037
ENSGALG00010024126	CDK5R1	2	-0.295558368	0.195894951	-1.508759497	0.131360255	0.025866836	1.353585407	26.12213498	1	CRA004775;PRJNA827465	Yang2025_Silkie_Wenchang	0.607072924
ENSGALG00010024128	PHLDA2	2	0.910967091	0.694973961	1.310793125	0.189927666	0.937425829	33.6796521	97.03084819	1	PRJNA827465;Yang2025_Silkie_Wenchang	CRA004775	0.699271855
ENSGALG00010024128	PHLDA2	2	0.979542329	0.663838073	1.475574193	0.140058237	0.803377357	11.0709394	90.96734284	1	CRA004775;Yang2025_Silkie_Wenchang	PRJNA827465	0.623792706
ENSGALG00010024128	PHLDA2	2	0.231843564	0.123017508	1.884638764	0.059478625	0	0.027214624	0	1	CRA004775;PRJNA827465	Yang2025_Silkie_Wenchang	0.422595132
ENSGALG00010024129	GID8	2	-0.404504169	0.393477732	-1.028023028	0.303938996	0.304945072	65.61167337	98.47588097	1	PRJNA827465;Yang2025_Silkie_Wenchang	CRA004775	0.792208658
ENSGALG00010024129	GID8	2	-0.207761163	0.606203218	-0.342725273	0.731805148	0.712229942	31.84382253	96.85967349	0.5	CRA004775;Yang2025_Silkie_Wenchang	PRJNA827465	0.932851968
ENSGALG00010024129	GID8	2	0.155927891	0.208442623	0.748061452	0.454423092	0.06970501	4.33258375	76.91908437	0.5	CRA004775;PRJNA827465	Yang2025_Silkie_Wenchang	0.849072665
ENSGALG00010024132	ENSGALG00010024132	2	0.024190245	0.181128225	0.133553151	0.893755932	0.049715209	4.123852566	75.75083047	0.5	PRJNA827465;Yang2025_Silkie_Wenchang	CRA004775	0.974381724
ENSGALG00010024132	ENSGALG00010024132	2	-0.166595523	0.112667614	-1.478646048	0.139234939	0	0.011274826	0	1	CRA004775;Yang2025_Silkie_Wenchang	PRJNA827465	0.622954765
ENSGALG00010024132	ENSGALG00010024132	2	0.053786201	0.189432187	0.283933803	0.776461111	0.043108078	2.309327388	56.69734811	0.5	CRA004775;PRJNA827465	Yang2025_Silkie_Wenchang	0.944156005
ENSGALG00010024133	PSMD11	2	0.027109817	0.092543036	0.292942811	0.769565865	0.011713892	3.158885696	68.34326733	0.5	PRJNA827465;Yang2025_Silkie_Wenchang	CRA004775	0.941843401
ENSGALG00010024133	PSMD11	2	-0.038819742	0.238301575	-0.162901745	0.870595797	0.076176421	2.292158934	56.37300777	0.5	CRA004775;Yang2025_Silkie_Wenchang	PRJNA827465	0.969018906
ENSGALG00010024133	PSMD11	2	-0.084379854	0.074682099	-1.129853816	0.258537827	0	0.943149559	0	1	CRA004775;PRJNA827465	Yang2025_Silkie_Wenchang	0.765790021
ENSGALG00010024135	FBXO22	2	0.021754693	0.067480176	0.322386433	0.747159961	0.003703411	1.594962435	37.30259861	0.5	PRJNA827465;Yang2025_Silkie_Wenchang	CRA004775	0.93678921
ENSGALG00010024135	FBXO22	2	-0.052593931	0.090097169	-0.583746767	0.559390697	0	0.278475021	0	0.5	CRA004775;Yang2025_Silkie_Wenchang	PRJNA827465	0.881050436
ENSGALG00010024135	FBXO22	2	0.072523027	0.056814506	1.276487852	0.201783118	0	0.002303583	0	1	CRA004775;PRJNA827465	Yang2025_Silkie_Wenchang	0.714797828
ENSGALG00010024136	SAP130	2	0.192783904	0.3200969	0.602267326	0.546996206	0.176933363	6.673883872	85.01622115	0.5	PRJNA827465;Yang2025_Silkie_Wenchang	CRA004775	0.878281266
ENSGALG00010024136	SAP130	2	0.161682186	0.397318363	0.406933585	0.684056768	0.255851656	5.271354325	81.0295431	0.5	CRA004775;Yang2025_Silkie_Wenchang	PRJNA827465	0.918806497
ENSGALG00010024136	SAP130	2	-0.101682017	0.070547405	-1.441328953	0.149491771	0	0.327556643	0	1	CRA004775;PRJNA827465	Yang2025_Silkie_Wenchang	0.639246608
ENSGALG00010024137	ENSGALG00010024137	2	0.362099815	0.265819258	1.362203089	0.173133801	0	0.006864632	0	1	PRJNA827465;Yang2025_Silkie_Wenchang	CRA004775	0.676207671
ENSGALG00010024137	ENSGALG00010024137	2	0.605184778	0.311935586	1.940095345	0.052368104	0	0.286368856	0	1	CRA004775;Yang2025_Silkie_Wenchang	PRJNA827465	0.397324397
ENSGALG00010024137	ENSGALG00010024137	2	0.497418672	0.247055072	2.013391863	0.044073421	0	0.590677717	0	1	CRA004775;PRJNA827465	Yang2025_Silkie_Wenchang	0.36277554
ENSGALG00010024138	CBL	2	-0.07281809	0.063312294	-1.150141389	0.250085642	0	0.422966245	0	1	PRJNA827465;Yang2025_Silkie_Wenchang	CRA004775	0.75993411
ENSGALG00010024138	CBL	2	-0.249651424	0.166015802	-1.503781093	0.132637741	0	0.877288956	0	1	CRA004775;Yang2025_Silkie_Wenchang	PRJNA827465	0.610000132
ENSGALG00010024138	CBL	2	-0.179037385	0.229404075	-0.780445531	0.435128677	0.062849251	1.692285702	40.90832304	1	CRA004775;PRJNA827465	Yang2025_Silkie_Wenchang	0.842762754
ENSGALG00010024141	YTHDF1	2	-0.11499481	0.110749861	-1.038329164	0.299116835	0.019322901	4.265856954	76.55805127	1	PRJNA827465;Yang2025_Silkie_Wenchang	CRA004775	0.789748017
ENSGALG00010024141	YTHDF1	2	-0.011205378	0.254841898	-0.043969919	0.964928382	0.109999846	6.301484373	84.13072316	0.5	CRA004775;Yang2025_Silkie_Wenchang	PRJNA827465	0.990508259
ENSGALG00010024141	YTHDF1	2	0.070132388	0.133684695	0.524610449	0.599854022	0.024576148	2.487641971	59.80128927	0.5	CRA004775;PRJNA827465	Yang2025_Silkie_Wenchang	0.895092498
ENSGALG00010024142	DNAJC6	2	-0.026017599	0.234395224	-0.110998846	0.911617262	0.033867151	1.339699674	25.35640491	0.5	PRJNA827465;Yang2025_Silkie_Wenchang	CRA004775	0.978895589
ENSGALG00010024142	DNAJC6	2	-0.178557246	0.526454232	-0.339169551	0.734482006	0.3913856	3.400950441	70.59645481	0.5	CRA004775;Yang2025_Silkie_Wenchang	PRJNA827465	0.933520234
ENSGALG00010024142	DNAJC6	2	-0.325604776	0.240661535	-1.352957277	0.176069279	0.036956586	1.346128521	25.71288815	1	CRA004775;PRJNA827465	Yang2025_Silkie_Wenchang	0.681107371
ENSGALG00010024143	UBE2Q2	2	-0.138409916	0.104498414	-1.324516901	0.185331434	0.014771034	3.081802492	67.55145722	1	PRJNA827465;Yang2025_Silkie_Wenchang	CRA004775	0.693795574
ENSGALG00010024143	UBE2Q2	2	0.075243496	0.367194289	0.204914669	0.837638794	0.230566158	6.263129245	84.03354041	0.5	CRA004775;Yang2025_Silkie_Wenchang	PRJNA827465	0.959001319
ENSGALG00010024143	UBE2Q2	2	0.159283448	0.25670011	0.620504014	0.534926012	0.098280234	3.283118992	69.54115881	0.5	CRA004775;PRJNA827465	Yang2025_Silkie_Wenchang	0.873896294
ENSGALG00010024144	HNRNPA3	2	0.12081719	0.220940635	0.546831007	0.584494839	0.091414293	15.19712229	93.41980685	0.5	PRJNA827465;Yang2025_Silkie_Wenchang	CRA004775	0.891016154
ENSGALG00010024144	HNRNPA3	2	0.075441683	0.311246517	0.242385628	0.808481369	0.162566171	5.809160232	82.78580793	0.5	CRA004775;Yang2025_Silkie_Wenchang	PRJNA827465	0.952496413
ENSGALG00010024144	HNRNPA3	2	-0.095370255	0.041317373	-2.308236192	0.020986003	0	0.58313095	0	1	CRA004775;PRJNA827465	Yang2025_Silkie_Wenchang	0.245277621
ENSGALG00010024145	RAP1GAP3	2	0.087519898	0.133863516	0.653799488	0.513240997	0.01814238	2.024853053	50.61370017	0.5	PRJNA827465;Yang2025_Silkie_Wenchang	CRA004775	0.866295966
ENSGALG00010024145	RAP1GAP3	2	0.00902378	0.255375597	0.035335325	0.971812356	0.092888619	3.230612773	69.04612003	0.5	CRA004775;Yang2025_Silkie_Wenchang	PRJNA827465	0.991955348
ENSGALG00010024145	RAP1GAP3	2	-0.097170133	0.116758161	-0.832234185	0.405276772	0	0.764684755	0	1	CRA004775;PRJNA827465	Yang2025_Silkie_Wenchang	0.833112102
ENSGALG00010024146	CDKN1C	2	0.129332689	0.196634621	0.657731017	0.510710988	0.056110206	3.494273968	71.38175172	0.5	PRJNA827465;Yang2025_Silkie_Wenchang	CRA004775	0.865386785
ENSGALG00010024146	CDKN1C	2	0.294395523	0.169419335	1.737673701	0.082268325	0	0.897419859	0	0.5	CRA004775;Yang2025_Silkie_Wenchang	PRJNA827465	0.494402444
ENSGALG00010024146	CDKN1C	2	-0.048639269	0.108986028	-0.446289033	0.655388477	0	0.040940879	0	1	CRA004775;PRJNA827465	Yang2025_Silkie_Wenchang	0.910411938
ENSGALG00010024147	ENSGALG00010024147	2	0.010599495	0.264414918	0.040086599	0.968024086	0.096563026	2.992172627	66.57946835	0.5	PRJNA827465;Yang2025_Silkie_Wenchang	CRA004775	0.991128245
ENSGALG00010024147	ENSGALG00010024147	2	-0.106170155	0.435041576	-0.244045997	0.807195196	0.304346405	5.102581719	80.40207771	0.5	CRA004775;Yang2025_Silkie_Wenchang	PRJNA827465	0.95234753
ENSGALG00010024147	ENSGALG00010024147	2	-0.295875843	0.146489615	-2.019773506	0.043406887	0.00823833	1.176364564	14.99233907	1	CRA004775;PRJNA827465	Yang2025_Silkie_Wenchang	0.360031851
ENSGALG00010024148	UGGT1	2	-0.083462522	0.134817836	-0.619076264	0.535866119	0.031624106	7.579141239	86.80589306	0.5	PRJNA827465;Yang2025_Silkie_Wenchang	CRA004775	0.874341833

ENSGALG00010024148	UGGT1	2	-0.21649083	0.078092988	-2.772218544	0.005567564	0	0.166642117	0	1	CRA004775;Yang2025_Silkie_Wenchang	PRJNA827465	0.111986684
ENSGALG00010024148	UGGT1	2	0.040950336	0.054890339	0.746039039	0.455643832	0	0.189391364	0	0.5	CRA004775;PRJNA827465	Yang2025_Silkie_Wenchang	0.84907427
ENSGALG00010024149	TCIRG1	2	0.061657274	0.122214326	0.504501202	0.613909218	0.020422215	3.158801244	68.34242098	0.5	PRJNA827465;Yang2025_Silkie_Wenchang	CRA004775	0.898256044
ENSGALG00010024149	TCIRG1	2	0.174507814	0.097600436	1.787981906	0.073778927	0	0.286197293	0	0.5	CRA004775;Yang2025_Silkie_Wenchang	PRJNA827465	0.469350366
ENSGALG00010024149	TCIRG1	2	-0.058515528	0.092477762	-0.632752419	0.526895333	0	3.82583E-05	0	1	CRA004775;PRJNA827465	Yang2025_Silkie_Wenchang	0.870839667
ENSGALG00010024150	NHERF4	2	-0.002087398	0.233055435	-0.008956659	0.992853716	0	0.06479803	0	0.5	PRJNA827465;Yang2025_Silkie_Wenchang	CRA004775	0.997880823
ENSGALG00010024150	NHERF4	2	0.036264056	0.255793726	0.141770703	0.887261126	0	0.143636396	0	0.5	CRA004775;Yang2025_Silkie_Wenchang	PRJNA827465	0.972987228
ENSGALG00010024150	NHERF4	2	0.067860281	0.20998236	0.323171342	0.746565483	0	0.02912137	0	1	CRA004775;PRJNA827465	Yang2025_Silkie_Wenchang	0.936668296
ENSGALG00010024151	LEPROT	2	-0.177098344	0.149302358	-1.18617245	0.235554204	0.039249153	8.101021813	87.6558782	1	PRJNA827465;Yang2025_Silkie_Wenchang	CRA004775	0.748604097
ENSGALG00010024151	LEPROT	2	-0.311041219	0.088064506	-3.531970296	0.000412476	0	0.906307134	0	1	CRA004775;Yang2025_Silkie_Wenchang	PRJNA827465	0.020443294
ENSGALG00010024151	LEPROT	2	-0.037278069	0.05008951	-0.744229056	0.456737912	0	2.57805E-05	0	1	CRA004775;PRJNA827465	Yang2025_Silkie_Wenchang	0.849515887
ENSGALG00010024152	ZNF423	2	-0.138634903	0.074565751	-1.859230286	0.062994504	0.005353775	1.767445876	43.42118119	1	PRJNA827465;Yang2025_Silkie_Wenchang	CRA004775	0.432176808
ENSGALG00010024152	ZNF423	2	-0.229229917	0.090046255	-2.545690734	0.010906176	0	0.057386842	0	1	CRA004775;Yang2025_Silkie_Wenchang	PRJNA827465	0.166322288
ENSGALG00010024152	ZNF423	2	-0.094179741	0.053284959	-1.76747328	0.077149	0	0.326360123	0	1	CRA004775;PRJNA827465	Yang2025_Silkie_Wenchang	0.478533748
ENSGALG00010024153	KCNQ1	2	-0.163722775	0.153957406	-1.06342903	0.287587434	0	0.731888052	0	1	PRJNA827465;Yang2025_Silkie_Wenchang	CRA004775	0.782840067
ENSGALG00010024153	KCNQ1	2	-0.03297312	0.361806288	-0.091134734	0.927385534	0.156129534	2.298652849	56.49625821	0.5	CRA004775;Yang2025_Silkie_Wenchang	PRJNA827465	0.982993195
ENSGALG00010024153	KCNQ1	2	0.035802286	0.184939328	0.193589359	0.846497431	0.001297095	1.011566038	1.143379398	0.5	CRA004775;PRJNA827465	Yang2025_Silkie_Wenchang	0.96122283
ENSGALG00010024155	SNX7	2	-0.001109737	0.042485166	-0.026120573	0.979161167	0	0.297828556	0	0.5	PRJNA827465;Yang2025_Silkie_Wenchang	CRA004775	0.994324552
ENSGALG00010024155	SNX7	2	0.020097805	0.06286317	0.31970715	0.749190339	0	0.121091207	0	0.5	CRA004775;Yang2025_Silkie_Wenchang	PRJNA827465	0.937362187
ENSGALG00010024155	SNX7	2	-0.023298955	0.054944278	-0.424046969	0.671531559	0	0.029770962	0	1	CRA004775;PRJNA827465	Yang2025_Silkie_Wenchang	0.914674175
ENSGALG00010024158	ENSGALG00010024158	2	-0.074409283	0.247226933	-0.300975634	0.763433075	0.074370499	2.48577273	59.77106081	0.5	PRJNA827465;Yang2025_Silkie_Wenchang	CRA004775	0.940301633
ENSGALG00010024158	ENSGALG00010024158	2	-0.341816165	0.160521619	-2.12940891	0.033220444	0	0.333496227	0	1	CRA004775;Yang2025_Silkie_Wenchang	PRJNA827465	0.313263411
ENSGALG00010024158	ENSGALG00010024158	2	-0.120263604	0.35782178	-0.336099171	0.736796083	0.169292864	2.909015305	65.62410661	0.5	CRA004775;PRJNA827465	Yang2025_Silkie_Wenchang	0.933777134
ENSGALG00010024159	NLRX1	2	0.10893545	0.084421814	1.290370869	0.196921922	0	0.188080347	0	1	PRJNA827465;Yang2025_Silkie_Wenchang	CRA004775	0.707994211
ENSGALG00010024159	NLRX1	2	-0.042730341	0.249800126	-0.171058125	0.864170862	0.07980314	2.677460164	62.65117168	0.5	CRA004775;Yang2025_Silkie_Wenchang	PRJNA827465	0.966583863
ENSGALG00010024159	NLRX1	2	-0.054237561	0.198580301	-0.273126594	0.784755904	0.050335458	2.340629357	57.27644802	0.5	CRA004775;PRJNA827465	Yang2025_Silkie_Wenchang	0.946214033
ENSGALG00010024162	SRCIN1	2	0.48588551	0.449803395	1.080217525	0.280045326	0.366706487	10.58234998	90.55030308	1	PRJNA827465;Yang2025_Silkie_Wenchang	CRA004775	0.778098827
ENSGALG00010024162	SRCIN1	2	0.970107806	0.21036328	4.611583385	3.99613E-06	0	0.082356443	0	1	CRA004775;Yang2025_Silkie_Wenchang	PRJNA827465	0.000694016
ENSGALG00010024162	SRCIN1	2	0.425297445	0.518915087	0.819589671	0.412450064	0.384354336	2.817416624	64.50649181	1	CRA004775;PRJNA827465	Yang2025_Silkie_Wenchang	0.843570455
ENSGALG00010024163	HS6ST1	2	0.013992662	0.060941409	0.229608434	0.818396053	0.002022419	1.328183453	24.70919603	0.5	PRJNA827465;Yang2025_Silkie_Wenchang	CRA004775	0.955256039
ENSGALG00010024163	HS6ST1	2	0.117306725	0.084302008	1.391505704	0.164072131	0	0.255903294	0	1	CRA004775;Yang2025_Silkie_Wenchang	PRJNA827465	0.662814983
ENSGALG00010024163	HS6ST1	2	0.013644401	0.092832864	0.146978132	0.883149281	0.006895621	1.317269967	24.08541717	0.5	CRA004775;PRJNA827465	Yang2025_Silkie_Wenchang	0.972001076
ENSGALG00010024164	SNAP29	2	-0.321248193	0.316797991	-1.014047441	0.310560116	0.194416985	31.4508225	96.82043292	1	PRJNA827465;Yang2025_Silkie_Wenchang	CRA004775	0.794899969
ENSGALG00010024164	SNAP29	2	-0.036778904	0.627417403	-0.058619515	0.953255167	0.752496655	22.12783897	95.48080587	0.5	CRA004775;Yang2025_Silkie_Wenchang	PRJNA827465	0.987758913
ENSGALG00010024164	SNAP29	2	0.253164685	0.307302714	0.823828342	0.410037101	0.161674114	6.113587184	83.6429911	0.5	CRA004775;PRJNA827465	Yang2025_Silkie_Wenchang	0.834652129
ENSGALG00010024165	LEPR	2	0.229673604	0.289065059	0.794539487	0.42688143	0.081970643	1.956957207	48.90026229	0.5	PRJNA827465;Yang2025_Silkie_Wenchang	CRA004775	0.84125685
ENSGALG00010024165	LEPR	2	0.386703407	0.25667464	1.506589852	0.13191582	0	0.759216356	0	1	CRA004775;Yang2025_Silkie_Wenchang	PRJNA827465	0.608602827
ENSGALG00010024165	LEPR	2	-0.019330494	0.237481416	-0.081397925	0.9351255	0	0.030049357	0	0.5	CRA004775;PRJNA827465	Yang2025_Silkie_Wenchang	0.984512821
ENSGALG00010024169	FAM168B	2	-0.075572811	0.040818053	-1.851455535	0.064104046	0	0.321416823	0	1	PRJNA827465;Yang2025_Silkie_Wenchang	CRA004775	0.436198289
ENSGALG00010024169	FAM168B	2	-0.113296031	0.101772805	-1.113225001	0.265611808	0	0.205645412	0	1	CRA004775;Yang2025_Silkie_Wenchang	PRJNA827465	0.769794389
ENSGALG00010024169	FAM168B	2	-0.064886888	0.043091688	-1.505786647	0.132121952	0	0.044848759	0	1	CRA004775;PRJNA827465	Yang2025_Silkie_Wenchang	0.608786928
ENSGALG00010024170	NUP160	2	-0.19646954	0.066598469	-2.950060907	0.003177113	0.004272175	1.904425092	47.49071496	1	PRJNA827465;Yang2025_Silkie_Wenchang	CRA004775	0.078092601
ENSGALG00010024170	NUP160	2	-0.294533719	0.071195381	-4.136977922	3.5191E-05	0	0.744875031	0	1	CRA004775;Yang2025_Silkie_Wenchang	PRJNA827465	0.00374827
ENSGALG00010024170	NUP160	2	-0.239531684	0.149458184	-1.602666892	0.109008217	0.029123822	2.305103338	56.61799697	1	CRA004775;PRJNA827465	Yang2025_Silkie_Wenchang	0.55900318
ENSGALG00010024172	ABCG4	2	-0.219567657	0.267298477	-0.821432503	0.411399956	0.081170805	2.206104186	54.67122512	0.5	PRJNA827465;Yang2025_Silkie_Wenchang	CRA004775	0.835128673
ENSGALG00010024172	ABCG4	2	0.225789643	0.28412022	0.794697549	0.426789459	0	0.940174176	0	1	CRA004775;Yang2025_Silkie_Wenchang	PRJNA827465	0.84125685
ENSGALG00010024172	ABCG4	2	0.029225457	0.622984193	0.046912036	0.962583335	0.569034619	3.092136418	67.65990032	0.5	CRA004775;PRJNA827465	Yang2025_Silkie_Wenchang	0.989980504
ENSGALG00010024173	CBLN1	2	0.473447577	0.957293023	0.494569129	0.620904298	1.654228539	10.21638028	90.2117974	0.5	PRJNA827465;Yang2025_Silkie_Wenchang	CRA004775	0.899565085
ENSGALG00010024173	CBLN1	2	0.6005702	0.934790665	0.64246491	0.520571366	1.37898262	4.591849642	78.22228344	0.5	CRA004775;Yang2025_Silkie_Wenchang	PRJNA827465	0.869331539
ENSGALG00010024173	CBLN1	2	-0.455352162	0.33291289	-1.367781712	0.171380414	0	0.002030429	0	1	CRA004775;PRJNA827465	Yang2025_Silkie_Wenchang	0.673441752
ENSGALG00010024174	PLEKHB2	2	0.173492206	0.063041851	2.752016375	0.005922956	0.002959819	1.546059813	35.31944937	1	PRJNA827465;Yang2025_Silkie_Wenchang	CRA004775	0.116522637
ENSGALG00010024174	PLEKHB2	2	0.240101962	0.081049395	2.962415212	0.003052359	0	0.21792656	0	1	CRA004775;Yang2025_Silkie_Wenchang	PRJNA827465	0.076024621
ENSGALG00010024174	PLEKHB2	2	0.123707546	0.057747892	2.142200201	0.032177375	0	0.000357095	0	1	CRA004775;PRJNA827465	Yang2025_Silkie_Wenchang	0.30763011
ENSGALG00010024175	DHRS9	2	-0.169116665	0.288358963	-0.586479654	0.557587523	0	0.176715533	0	1	PRJNA827465;Yang2025_Silkie_Wenchang	CRA004775	0.880678789
ENSGALG00010024175	DHRS9	2	0.067355665	0.340833913	0.197620197	0.843342231	0	0.136600134	0	0.5	CRA004775;Yang2025_Silkie_Wenchang	PRJNA827465	0.96057435
ENSGALG00010024175	DHRS9	2	-0.110582963	0.31542554	-0.350583414	0.725900902	0	0.562330332	0	0.5	CRA004775;PRJNA827465	Yang2025_Silkie_Wenchang	0.931029023
ENSGALG00010024178	N4BP1	2	-0.109513472	0.056170577	-1.949659	0.051216776	0	0.532439231	0	1	PRJNA827465;Yang2025_Silkie_Wenchang	CRA004775	0.392010312
ENSGALG00010024178	N4BP1	2	0.079271974	0.284287312	0.278848571	0.780364107	0.132989442	5.232681819	80.8893406	0.5	CRA004775;Yang2025_Silkie_Wenchang	PRJNA827465	0.945041817
ENSGALG00010024178	N4BP1	2	0.111027549	0.237385692	0.467709522	0.639992331	0.088957213	4.101959322	75.62140622	0.5	CRA004775;PRJNA827465	Yang2025_Silkie_Wenchang	0.906064918
ENSGALG00010024179	ATG4C	2	0.065350798	0.055325146	1.181213287	0.237518004	0.000551052	1.073463532	6.843598279	0.5	PRJNA827465;Yang2025_Silkie_Wenchang	CRA004775	0.749400455
ENSGALG00010024179	ATG4C	2	-0.055624612	0.095234396	-0.584081115	0.559165734	0	0.29486558	0	1	CRA004775;Yang2025_Silkie_Wenchang	PRJNA827465	0.855101486
ENSGALG00010024179	ATG4C	2	0.049436476	0.099781163	0.495448983	0.620283226	0.008730463	1.369319145	26.97100572	0.5	CRA004775;PRJNA827465	Yang2025_Silkie_Wenchang	0.893995593
ENSGALG00010024180	HINFP	2	0.0612274	0.077845692	0.786522656	0.431561352	0	0.472629267	0	1	PRJNA827465;Yang2025_Silkie_Wenchang	CRA004775	0.842503929
ENSGALG00010024180	HINFP	2	-0.021825338	0.209551954	-0.104152393	0.917052958	0.050897459	2.195263701	54.447386	0.5	CRA004775;Yang2025_Silkie_Wenchang	PRJNA827465	0.980728718
ENSGALG00010024180	HINFP	2	-0.043190471	0.130435097	-0.331126145	0.740549203	0.012944817	1.348207825	25.82745914	0.5	CRA004775;PRJNA827465	Yang2025_Silkie_Wenchang	0.935128797
ENSGALG00010024181	FOXD3	2	0.005349727	0.172765538	0.030965244	0.975297258	0.034258171	2.314746922	56.79873293	0.5	PRJNA827465;Yang2025_Silkie_Wenchang	CRA004775	0.992750665
ENSGALG00010024181	FOXD3	2	0.30338033	0.266525131	1.138280391	0.255003419	0.050268961	1.202891944	16.8670133	1	CRA004775;Yang2025_Silkie_Wenchang	PRJNA827465	0.764467569
ENSGALG00010024181	FOXD3	2	0.210907257	0.523467237	0.402904408	0.687018548	0.384842449	2.592553525	61.4279902	0.5	CRA004775;PRJNA827465	Yang2025_Silkie_Wenchang	0.919369765
ENSGALG00010024183	MTX2	2	-0.384740127	0.270661093	-1.421482943	0.155176409	0.118856155	4.676339039	78.61575066	1	PRJNA827465;Yang2025_Silkie_Wenchang	CRA004775	0.647854089
ENSGALG00010024183	MTX2	2	-0.412482666	0.312025584	-1.321951426	0.186184326	0.125791641	2.809862363	64.4110682	1	CRA004775;Yang2025_Silkie_Wenchang	PRJNA827465	0.695193101
ENSGALG00010024183	MTX2	2	-0.150093816	0.073274153	-2.089329047	0.036678113	0	0.065670412	0	1	CRA004775;PRJNA827465	Yang2025_Silkie_Wenchang	0.330557187
ENSGALG00010024184	ENSGALG00010024184	2	-0.044249458	0.164458297	-0.25082017	0.801955147	0.037932401	3.305077989	69.74152789	0.5	PRJNA827465;Yang2025_Silkie_Wenchang	CRA004775	0.959530276
ENSGALG00010024184	ENSGALG00010024184	2	-0.186240439	0.138499581	-1.344700372	0.178722015	0	0.802239661	0	0.5	CRA004775;Yang2025_Silkie_Wenchang	PRJNA827465	0.685472119
ENSGALG00010024184	ENSGALG00010024184	2	0.118619294	0.10937006	1.084568239	0.278112928	0	0.104092234	0	1	CRA004775;PRJNA827465	Yang2025_Silkie_Wenchang	0.776763074
ENSGALG00010024185	CHRNA5	2	-0.037902896	0.185326698	-0.204519351	0.837947672	0.051478527	3.893474921	74.31600253	0.5	PRJNA827465;Yang2025_Silkie_Wenchang	CRA004775	0.959032758
ENSGALG00010024185	CHRNA5	2	0.172707577	0.145191929	1.189512244	0.234238156	0	0.018565131	0	1	CRA004775;Yang2025_Silkie_Wenchang	PRJNA827465	0.747435122
ENSGALG00010024185	CHRNA5	2	-0.171646634	0.118409447	-1.449602531	0.14716939	0.003806618	1.04256266	4.082503821	0.5	CRA004775;PRJNA827465	Yang2025_Silkie_Wenchang	0.635975583
ENSGALG00010024188	TSSC4	2	0.147915925	0.051570957	2.868202043	0.004128118	0.001584495	1.352722789	26.07502379	1	PRJNA827465;Yang2025_Silkie_Wenchang	CRA004775	0.093485158
ENSGALG00010024188	TSSC4	2	0.098972429	0.189585113	0.52204747	0.601637279	0.04563647	2.077981629	51.87637918	0.5	CRA004775;Yang2025_Silkie_Wenchang	PRJNA827465	0.895230519
ENSGALG00010024188	TSSC4	2	0.081238419	0.092061724	0.882434252	0.37754202	0.00709088	1.180915909	15.31996544	0.5	CRA004775;PRJNA827465	Yang2025_Silkie_Wenchang	0.82325159
ENSGALG00010024189	KAT7	2	-0.162246062	0.144637811	-1.121740303	0.261972875	0.028285854	11.57629621	91.3616585	1	PRJNA827465;Yang2025_Silkie_Wenchang	CRA004775	0.768363052
ENSGALG00010024189	KAT7	2	-0.292223925	0.067035953	-4.359211934	1.30532E-05	0	0.535757367	0	1	CRA004775;Yang2025_Silkie_Wenchang	PRJNA827465	0.001744995
ENSGALG00010024189	KAT7	2	-0.032457686	0.042919316	-0.756248926	0.449489963	0	0.715232365	0	1	CRA004775;PRJNA827465	Yang2025_Silkie_Wenchang	0.881853197
ENSGALG00010024190	WARS2	2	-0.078453258	0.078004562	-1.005752179	0.314534796	0.001500283	1.10344798	9.374975669	0.5	PRJNA827465;Yang2025_Silkie_Wenchang	CRA004775	0.796531448
ENSGALG00010024190	WARS2	2	-0.070441178	0.183761778	-0.38332878	0.701476003	0.027113213	1.533131442	34.77402051	0.5	CRA004775;Yang2025_Silkie_Wenchang	PRJNA827465	0.922848915
ENSGALG00010024190	WARS2	2	-0.140865855	0.078441752	-1.795802002	0.072526012	0	0.542155882	0	1	CRA004775;PRJNA827465	Yang2025_Silkie_Wenchang	0.465234186
ENSGALG00010024191	DPYD	2	-0.151558288	0.096945245	-1.563339956	0.117972867	0	0.140341234	0	1	PRJNA827465;Yang2025_Silkie_Wenchang	CRA004775	0.577760513
ENSGALG00010024191	DPYD	2	-0.149884252	0.09741841	-1.538561875	0.123911292	0	0.226474579	0	0.5	CRA004775;Yang2025_Silkie_Wenchang	PRJNA827465	0.590384957
ENSGALG00010024191	DPYD	2	0.012528951	0.281580023	0.04449517	0.964509702	0	0.009532679	0	0.5	CRA004775;PRJNA827465	Yang2025_Silkie_Wenchang	0.990364463
ENSGALG00010024192	ALG6	2	0.228100259	0.382067598	0.597015451	0.550497065	0.264937054	10.76604232	90.71153567	0.5	PRJNA827465;Yang2025_Silkie_Wenchang	CRA004775	0.878769592
ENSGALG00010024192	ALG6	2	0.201088021	0.4930827	0.407818042	0.683407267	0.383874797	4.30009649	76.74995255	0.5	CRA004775;Yang2025_Silkie_Wenchang	PRJNA827465	0.918300744
ENSGALG00010024192	ALG6	2	-0.169276281	0.138892877	-1.218754231	0.222937488	0	0.251726252	0	1	CRA004775;PRJNA827465	Yang2025_Silkie_Wenchang	0.736458649
ENSGALG00010024193	GSTK1	2	-0.042127093	0.090888187	-0.463504605	0.643002715	0	0.004451708	0	1	PRJNA827465;Yang2025_Silkie_Wenchang	CRA004775	0.906634523
ENSGALG00010024193	GSTK1	2	-0.120230837	0.157353827	-0.764079522	0.444819855	0	0.432579862	0	1	CRA004775;Yang2025_Silkie_Wenchang	PRJNA827465	0.846013379
ENSGALG00010024193	GSTK1	2	-0.066334089	0.097775576	-0.678432098	0.497497763	0	0.615294719	0	0.5	CRA004775;PRJNA827465	Yang2025_Silkie_Wenchang	0.86098656
ENSGALG00010024194	NKAIN4	2	-0.004830263	0.071116878	-0.067920061	0.945849269	0	0.034900708	0	0.5	PRJNA827465;Yang2025_Silkie_Wenchang	CRA004775	0.986321197
ENSGALG00010024194	NKAIN4	2	0.088822574	0.210056828	0.422850213	0.672404549	0.035399359	1.416209004	29.38895341	0.5	CRA004775;Yang2025_Silkie_Wenchang	PRJNA827465	0.914742762
ENSGALG00010024194	NKAIN4	2	0.078237195	0.17054978	0.458735241	0.646424304	0.028356676	1.368663957	26.9360463	1	CRA004775;PRJNA827465	Yang2025_Silkie_Wenchang	0.907220702
ENSGALG00010024195	PSMA4	2	-0.000942928	0.05540191	-0.017019774	0.986420841	0.00051009	1.064506743	6.059777764	0.5	PRJNA827465;Yang2025_Silkie_Wenchang	CRA004775	0.99618774
ENSGALG00010024195	PSMA4	2	0.056518268	0.09911309	0.570240198	0.568514795	0	0.687899292	0	0.5	CRA004775;Yang2025_Silkie_Wenchang	PRJNA827465	0.884153886
ENSGALG00010024195	PSMA4	2	-0.037138163	0.057365019	-0.647400864	0.517372537	0	0.108706826	0	1	CRA004775;PRJNA827465	Yang2025_Silkie_Wenchang	0.867952013
ENSGALG00010024196	MRPL21	2	-0.109878776	0.202745266	-0.54195483	0.587849623	0.059923861	3.13381532	68.09001496	0.5	PRJNA827465;Yang2025_Silkie_Wenchang	CRA004775	0.891758856
ENSGALG00010024196	MRPL21	2	-0.122804195	0.279624314	-0.439175669	0.660534253	0.090360738	2.330909054	57.09828325	0.5	CRA004775;Yang2025_Silkie_Wenchang	PRJNA827465	0.91192683
ENSGALG00010024196	MRPL21	2	0.054740502	0.071868512	0.761675738	0.446253559	0	0.225821648	0	1	CRA004775;PRJNA827465	Yang2025_Silkie_Wenchang	0.846054146
ENSGALG00010024197	KRT7	2	-0.250796305	0.183008129	-1.370410735	0.170558723	0	0.076033523	0	1	PRJNA827465;Yang2025_Silkie_Wenchang	CRA004775	0.672201941
ENSGALG00010024197	KRT7	2	-0.186717356	0.25321172	-0.737396186	0.460881454	0.022141917	1.090424978	8.292636372	0.5	CRA004775;Yang2025_Silkie_Wenchang	PRJNA827465	0.85014477
ENSGALG00010024197	KRT7	2	-0.073963276	0.276818536	-0.267190476	0.789322509	0	0.738222886	0	0.5	CRA004775;PRJNA827465	Yang2025_Silkie_Wenchang	0.947379026
ENSGALG00010024198	PI4KA	2	-0.000282062	0.041135992	-0.006856821	0.994529091	0	0.479830623	0	0.5	PRJNA827465;Yang2025_Silkie_Wenchang	CRA004775	0.99823774
ENSGALG00010024198	PI4KA	2	0.046758285	0.073408352	0.636961378	0.524150001	0	0.007260257	0	1	CRA004775;Yang2025_Silkie_Wenchang	PRJNA827465	0.869776968
ENSGALG00010024198	PI4KA	2	-0.015050944	0.047728672	-0.315343874	0.752500576	0	0.116385962	0	0.5	CRA004775;PRJNA827465	Yang2025_Silkie_Wenchang	0.938087082
ENSGALG00010024199	SLC35B1	2	0.005195762	0.117276726	0.044303437	0.964662532	0.016032472	2.14345969	53.34645179	0.5	PRJNA827465;Yang2025_Silkie_Wenchang	CRA004775	0.990394578
ENSGALG00010024199	SLC35B1	2	-0.214766215	0.112656249	-1.906385291	0.056600236	0	0.377798027	0	1	CRA004775;Yang2025_Silkie_Wenchang	PRJNA827465	0.411574294
ENSGALG00010024199	SLC35B1	2	-0.069762112	0.189866932	-0.367426342	0.713301026	0.056975307	4.35641481	77.04534477	0.5	CRA004775;PRJNA827465	Yang2025_Silkie_Wenchang	0.926216079
ENSGALG00010024201	IREB2	2	-0.103073043	0.067221991	-1.533323284	0.12519622	0.004746219	1.936135598	48.35072497	1	PRJNA827465;Yang2025_Silkie_Wenchang	CRA004775	0.593887252
ENSGALG00010024201	IREB2	2	-0.068133617	0.157193241	-0.433438594	0.664696168	0.032432264	2.648028667	62.23605837	0.5	CRA004775;Yang2025_Silkie_Wenchang	PRJNA827465	0.913382053
ENSGALG00010024201	IREB2	2	-0.040294558	0.046349295	-0.869367223	0.384646305	0	0.986912551	0	0.5	CRA004775;PRJNA827465	Yang2025_Silkie_Wenchang	0.826332535
ENSGALG00010024203	SPOP	2	-0.004695711	0.044481713	-0.105564988	0.915927505	0	0.534085272	0	0.5	PRJNA827465;Yang2025_Silkie_Wenchang	CRA004775	0.980356719
ENSGALG00010024203	SPOP	2	-0.008115259	0.094978224	-0.085443361	0.931908922	0.003212361	1.200768291	16.71998606	0.5	CRA004775;Yang2025_Silkie_Wenchang	PRJNA827465	0.98412915
ENSGALG00010024203	SPOP	2	-0.031180789	0.04638498	-0.672217373	0.501445322	0	0.528691416	0	1	CRA004775;PRJNA827465	Yang2025_Silkie_Wenchang	0.862658912
ENSGALG00010024204	PTBP2	2	-0.199766635	0.098974324	-2.018368263	0.043552922	0.007656049	1.510644237	33.80307716	1	PRJNA827465;Yang2025_Silkie_Wenchang	CRA004775	0.360709607
ENSGALG00010024204	PTBP2	2	-0.212262558	0.189483039	-1.120219303	0.26262032	0.030283452	1.595017693	37.30477072	0.5	CRA004775;Yang2025_Silkie_Wenchang	PRJNA827465	0.768434743
ENSGALG00010024204	PTBP2	2	-0.11755142	0.078415754	-1.499079121	0.133853107	0	0.424425255	0	0.5	CRA004775;PRJNA827465	Yang2025_Silkie_Wenchang	0.613104446
ENSGALG00010024205	ANKRD13D	2	0.306593106	0.353979589	0.86613216	0.386417691	0.238515752	20.61887913	95.15007584	0.5	PRJNA827465;Yang2025_Silkie_Wenchang	CRA004775	0.826928809
ENSGALG00010024205	ANKRD13D	2	0.228899897	0.481473142	0.475415712	0.634490698	0.406998167	7.686223972	86.98971038	0.5	CRA004775;Yang2025_Silkie_Wenchang	PRJNA827465	0.903872221
ENSGALG00010024205	ANKRD13D	2	-0.059733332	0.086054885	-0.694130638	0.487600286	0	0.59303377	0	1	CRA004775;PRJNA827465	Yang2025_Silkie_Wenchang	0.85768885
ENSGALG00010024206	KRT80	2	-0.070765547	0.23896533	-0.296133111	0.767128429	0	0.157436001	0	0.5	PRJNA827465;Yang2025_Silkie_Wenchang	CRA004775	0.941432527
ENSGALG00010024206	KRT80	2	-1.235816722	1.276745293	-0.967943041	0.333072817	2.691277695	5.067058012	80.26468224	1	CRA004775;Yang2025_Silkie_Wenchang	PRJNA827465	0.802178578
ENSGALG00010024206	KRT80	2	-1.159462058	1.376138066	-0.84254777	0.399481413	3.191415731	5.739733949	82.57758968	0.5	CRA004775;PRJNA827465	Yang2025_Silkie_Wenchang	0.832246851
ENSGALG00010024207	KCTD18	2	-0.132843398	0.090854807	-1.462150461	0.143699983	0.008945715	2.17835994	54.09390425	1	PRJNA827465;Yang2025_Silkie_Wenchang	CRA004775	0.630564924
ENSGALG00010024207	KCTD18	2	-0.223100907	0.08643808	-2.581048845	0.009850064	0	0.024432878	0	1	CRA004775;Yang2025_Silkie_Wenchang	PRJNA827465	0.15667223
ENSGALG00010024207	KCTD18	2	-0.056881407	0.079624293	-0.714372522	0.474996858	0	0.221141821	0	1	CRA004775;PRJNA827465	Yang2025_Silkie_Wenchang	0.854462864
ENSGALG00010024208	SERPIND1	2	-0.109462245	1.099783539	-0.099530718	0.9207169	2.004300756	5.691319319	82.42938159	0.5	PRJNA827465;Yang2025_Silkie_Wenchang	CRA004775	0.981469977
ENSGALG00010024208	SERPIND1	2	-0.116697365	1.015120647	-0.114959109	0.908477533	1.810487514	8.22878167	87.84753272	0.5	CRA004775;Yang2025_Silkie_Wenchang	PRJNA827465	0.978291088
ENSGALG00010024208	SERPIND1	2	0.948262728	0.419009383	2.26310619	0.023629144	0	0.036051168	0	1	CRA004775;PRJNA827465	Yang2025_Silkie_Wenchang	0.262343574
ENSGALG00010024209	RWDD3	2	-0.158379619	0.099812456	-1.586772078	0.112564275	0	0.20083697	0	1	PRJNA827465;Yang2025_Silkie_Wenchang	CRA004775	0.566223663
ENSGALG00010024209	RWDD3	2	-0.182514826	0.138793935	-1.315005773	0.188507962	0	0.239713132	0	0.5	CRA004775;Yang2025_Silkie_Wenchang	PRJNA827465	0.697920084
ENSGALG00010024209	RWDD3	2	-0.105141157	0.128571795	-0.817762222	0.413492974	0	0.08132067	0	0.5	CRA004775;PRJNA827465	Yang2025_Silkie_Wenchang	0.836246395
ENSGALG00010024211	TRPM5	2	0.619097104	0.274312081	2.256907903	0.02401383	0	0.122766746	0	1	PRJNA827465;Yang2025_Silkie_Wenchang	CRA004775	0.264653015
ENSGALG00010024211	TRPM5	2	1.615628906	0.95555748	1.690771033	0.090880542	1.492855267	5.307404984	81.15840033	1	CRA004775;Yang2025_Silkie_Wenchang	PRJNA827465	0.517213717
ENSGALG00010024211	TRPM5	2	1.509282428	1.05352846	1.43259768	0.151972845	1.922287986	7.13053084	85.97579868	1	CRA004775;PRJNA827465	Yang2025_Silkie_Wenchang	0.643294424
ENSGALG00010024212	RIMS4	2	-0.327263675	0.348911615	-0.937955806	0.348267122	0.194827958	4.8694969	79.46399761	1	PRJNA827465;Yang2025_Silkie_Wenchang	CRA004775	0.80902788
ENSGALG00010024212	RIMS4	2	-0.599487112	0.240852852	-2.48901811	0.012809644	0	0.898837916	0	1	CRA004775;Yang2025_Silkie_Wenchang	PRJNA827465	0.183676556
ENSGALG00010024212	RIMS4	2	-0.015312374	0.165506954	-0.09251801	0.926286483	0	0.032005149	0	1	CRA004775;PRJNA827465	Yang2025_Silkie_Wenchang	0.98280842
ENSGALG00010024213	ENSGALG00010024213	2	0.496441087	0.55515501	0.894238686	0.371194198	0.584068843	19.00639605	94.73861327	0.5	PRJNA827465;Yang2025_Silkie_Wenchang	CRA004775	0.8214684
ENSGALG00010024213	ENSGALG00010024213	2	0.978802614	0.182222615	5.371466182	7.8099E-08	0	0.857672889	0	1	CRA004775;Yang2025_Silkie_Wenchang	PRJNA827465	2.89578E-05
ENSGALG00010024213	ENSGALG00010024213	2	0.171197373	0.317469443	0.539256224	0.58971007	0.126143179	2.236802144	55.29331896	0.5	CRA004775;PRJNA827465	Yang2025_Silkie_Wenchang	0.892320726
ENSGALG00010024215	ENSGALG00010024215	2	0.496734392	0.534027874	0.930165664	0.352285317	0.487707408	6.857922879	85.41832538	0.5	PRJNA827465;Yang2025_Silkie_Wenchang	CRA004775	0.810786136
ENSGALG00010024215	ENSGALG00010024215	2	0.279386289	0.868014559	0.321868205	0.747552541	1.242949586	5.342460547	81.28203304	0.5	CRA004775;Yang2025_Silkie_Wenchang	PRJNA827465	0.93680487
ENSGALG00010024215	ENSGALG00010024215	2	-0.102740083	0.238781278	-0.430268587	0.667000275	0	0.863702968	0	1	CRA004775;PRJNA827465	Yang2025_Silkie_Wenchang	0.913766816
ENSGALG00010024218	NGFR	2	-0.134205119	0.120124984	-1.11721238	0.263903529	0.015941084	2.151070599	53.51152116	0.5	PRJNA827465;Yang2025_Silkie_Wenchang	CRA004775	0.768643821
ENSGALG00010024218	NGFR	2	-0.237107994	0.091061012	-2.603836581	0.00921867	0	0.019988087	0	1	CRA004775;Yang2025_Silkie_Wenchang	PRJNA827465	0.150534672
ENSGALG00010024218	NGFR	2	-0.013589278	0.133105745	-0.102093851	0.918682181	0	0.378908167	0	0.5	CRA004775;PRJNA827465	Yang2025_Silkie_Wenchang	0.981033557
ENSGALG00010024220	SIAH1	2	-0.169345064	0.05320814	-3.182690931	0.001459133	0.000607472	1.057913546	5.474317453	1	PRJNA827465;Yang2025_Silkie_Wenchang	CRA004775	0.047278067
ENSGALG00010024220	SIAH1	2	0.220639183	0.562544531	0.392216386	0.694898338	0.56507612	8.800354795	88.63682177	0.5	CRA004775;Yang2025_Silkie_Wenchang	PRJNA827465	0.92087205
ENSGALG00010024220	SIAH1	2	0.279910612	0.486007615	0.575938737	0.564656591	0.41571975	7.427661717	86.53681282	0.5	CRA004775;PRJNA827465	Yang2025_Silkie_Wenchang	0.883221157
ENSGALG00010024222	ENSGALG00010024222	2	-0.018589838	0.134684465	-0.138025108	0.890220574	0	0.127062062	0	0.5	PRJNA827465;Yang2025_Silkie_Wenchang	CRA004775	0.973412509
ENSGALG00010024222	ENSGALG00010024222	2	-0.244003662	0.239165649	-1.020228711	0.307620004	0	0.558406858	0	1	CRA004775;Yang2025_Silkie_Wenchang	PRJNA827465	0.792968388
ENSGALG00010024222	ENSGALG00010024222	2	-0.098398638	0.201419183	-0.488526646	0.625176856	0.028592989	1.307100407	23.49478319	0.5	CRA004775;PRJNA827465	Yang2025_Silkie_Wenchang	0.901331406
ENSGALG00010024223	ENSGALG00010024223	2	-0.026878453	0.19248977	-0.13963575	0.888947793	0	0.001031893	0	1	PRJNA827465;Yang2025_Silkie_Wenchang	CRA004775	0.973135755
ENSGALG00010024223	ENSGALG00010024223	2	0.021381261	0.303415555	0.070468573	0.943820714	0	0.100090313	0	0.5	CRA004775;Yang2025_Silkie_Wenchang	PRJNA827465	0.985827325
ENSGALG00010024223	ENSGALG00010024223	2	-0.007931819	0.227616093	-0.034847354	0.972201461	0	0.118137286	0	0.5	CRA004775;PRJNA827465	Yang2025_Silkie_Wenchang	0.99196841
ENSGALG00010024225	BCDIN3D	2	-0.104798036	0.09527812	-1.099917132	0.27136823	0	0.285164253	0	1	PRJNA827465;Yang2025_Silkie_Wenchang	CRA004775	0.772743863
ENSGALG00010024225	BCDIN3D	2	-0.128049468	0.172197656	-0.743619113	0.457106937	0	0.402044707	0	0.5	CRA004775;Yang2025_Silkie_Wenchang	PRJNA827465	0.849541335
ENSGALG00010024225	BCDIN3D	2	-0.067061865	0.100539563	-0.667019661	0.504759575	0	0.110603458	0	0.5	CRA004775;PRJNA827465	Yang2025_Silkie_Wenchang	0.863512504
ENSGALG00010024230	LONP2	2	0.011751487	0.091110651	0.128980382	0.897373172	0.009537075	2.226007012	55.07651167	0.5	PRJNA827465;Yang2025_Silkie_Wenchang	CRA004775	0.97548008
ENSGALG00010024230	LONP2	2	0.09267772	0.100503244	0.922136605	0.356457326	0	0.805790144	0	0.5	CRA004775;Yang2025_Silkie_Wenchang	PRJNA827465	0.812521698
ENSGALG00010024230	LONP2	2	-0.066990355	0.063885374	-1.048602365	0.294361167	0	0.115442328	0	1	CRA004775;PRJNA827465	Yang2025_Silkie_Wenchang	0.787343482
ENSGALG00010024233	FAIM2	2	0.215990055	0.203677911	1.060449089	0.28894034	0.019101214	1.230470244	18.7302574	0.5	PRJNA827465;Yang2025_Silkie_Wenchang	CRA004775	0.783530627
ENSGALG00010024233	FAIM2	2	0.34797126	0.590308496	0.589473576	0.555543633	0.428840505	2.356388631	57.56217855	0.5	CRA004775;Yang2025_Silkie_Wenchang	PRJNA827465	0.880255849
ENSGALG00010024233	FAIM2	2	0.425477684	0.23629778	1.800599583	0.071766015	0.023577953	1.086641595	7.973336864	1	CRA004775;PRJNA827465	Yang2025_Silkie_Wenchang	0.462169374
ENSGALG00010024234	ENSGALG00010024234	2	-0.102721318	0.201746373	-0.509160667	0.61063961	0.053455582	2.807794737	64.38486094	0.5	PRJNA827465;Yang2025_Silkie_Wenchang	CRA004775	0.897438028
ENSGALG00010024234	ENSGALG00010024234	2	0.141122064	0.179702102	0.785311148	0.432271161	0	0.015204276	0	1	CRA004775;Yang2025_Silkie_Wenchang	PRJNA827465	0.842762754
ENSGALG00010024234	ENSGALG00010024234	2	-0.190092328	0.181182681	-1.049174938	0.294097611	0.02258794	1.269412726	21.22341464	0.5	CRA004775;PRJNA827465	Yang2025_Silkie_Wenchang	0.787324026
ENSGALG00010024238	ATF7	2	-0.078184117	0.077520146	-1.008565136	0.313183235	0.001967397	1.16509289	14.16993371	0.5	PRJNA827465;Yang2025_Silkie_Wenchang	CRA004775	0.796531448
ENSGALG00010024238	ATF7	2	0.001280062	0.116022534	0.011032876	0.991197217	0	0.333742191	0	0.5	CRA004775;Yang2025_Silkie_Wenchang	PRJNA827465	0.99755571
ENSGALG00010024238	ATF7	2	-0.130806203	0.078579057	-1.664644611	0.09598368	0	3.73043E-05	0	1	CRA004775;PRJNA827465	Yang2025_Silkie_Wenchang	0.5301492
ENSGALG00010024240	WDR61	2	-0.180522511	0.279614902	-0.645611193	0.518531187	0.136837508	7.516024204	86.69509341	0.5	PRJNA827465;Yang2025_Silkie_Wenchang	CRA004775	0.868525042
ENSGALG00010024240	WDR61	2	-0.418177349	0.131087385	-3.190065544	0.001422405	0	0.260049409	0	1	CRA004775;Yang2025_Silkie_Wenchang	PRJNA827465	0.046588307
ENSGALG00010024240	WDR61	2	-0.109238822	0.211665656	-0.516091387	0.605790583	0.072349379	4.761721752	78.9991929	0.5	CRA004775;PRJNA827465	Yang2025_Silkie_Wenchang	0.896474603
ENSGALG00010024242	KMT2A	2	-0.028768002	0.174108311	-0.165230491	0.868762567	0.043007038	2.965703965	66.28119287	0.5	PRJNA827465;Yang2025_Silkie_Wenchang	CRA004775	0.968349597
ENSGALG00010024242	KMT2A	2	0.169409153	0.177377831	0.955075121	0.339539709	0	0.112067107	0	1	CRA004775;Yang2025_Silkie_Wenchang	PRJNA827465	0.80490319
ENSGALG00010024242	KMT2A	2	-0.156621418	0.071417887	-2.193027886	0.02830537	0	0.240664749	0	0.5	CRA004775;PRJNA827465	Yang2025_Silkie_Wenchang	0.286649306
ENSGALG00010024243	MPP3	2	0.398825543	0.406842083	0.980295696	0.326940179	0.293596193	8.782845322	88.61416815	0.5	PRJNA827465;Yang2025_Silkie_Wenchang	CRA004775	0.800028239
ENSGALG00010024243	MPP3	2	0.338729205	0.507386834	0.667595574	0.504391784	0.436629781	6.178459401	83.81473544	0.5	CRA004775;Yang2025_Silkie_Wenchang	PRJNA827465	0.863512504
ENSGALG00010024243	MPP3	2	-0.072076697	0.188166887	-0.383046657	0.70168517	0	0.227448536	0	1	CRA004775;PRJNA827465	Yang2025_Silkie_Wenchang	0.922904951
ENSGALG00010024245	TARBP2	2	0.1508737	0.188129488	0.801967311	0.422571868	0.042843171	2.21877776	54.93014136	1	PRJNA827465;Yang2025_Silkie_Wenchang	CRA004775	0.8397382
ENSGALG00010024245	TARBP2	2	0.198618437	0.269095339	0.738096905	0.460455564	0.057551705	1.596756767	37.37305389	0.5	CRA004775;Yang2025_Silkie_Wenchang	PRJNA827465	0.85014477

ENSGALG00010024245	TARBP2	2	0.000855356	0.102657915	0.008332103	0.99335202	0	0.176016331	0	0.5	CRA004775;PRJNA827465	Yang2025_Silkie_Wenchang	0.997979742
ENSGALG00010024250	AP2A2	2	-0.099367896	0.051091992	-1.944882023	0.051789179	0.003168169	2.415382695	58.5986932	1	PRJNA827465;Yang2025_Silkie_Wenchang	CRA004775	0.394831847
ENSGALG00010024250	AP2A2	2	-0.162169494	0.055163856	-2.939778093	0.003284474	0	0.00694128	0	1	CRA004775;Yang2025_Silkie_Wenchang	PRJNA827465	0.079946251
ENSGALG00010024250	AP2A2	2	-0.059989218	0.034732283	-1.727189018	0.084133723	0	0.296650519	0	1	CRA004775;PRJNA827465	Yang2025_Silkie_Wenchang	0.499714459
ENSGALG00010024251	ENSGALG00010024251	2	-0.103991463	0.130847272	-0.794754536	0.426756302	0.000799806	1.02387902	2.332211058	0.5	PRJNA827465;Yang2025_Silkie_Wenchang	CRA004775	0.84125685
ENSGALG00010024251	ENSGALG00010024251	2	0.06639028	0.173305964	0.383081333	0.70165946	0	0.246077603	0	1	CRA004775;Yang2025_Silkie_Wenchang	PRJNA827465	0.922904951
ENSGALG00010024251	ENSGALG00010024251	2	-0.156048116	0.17971426	-0.868312375	0.385223349	0.005582872	1.044589508	4.268615322	0.5	CRA004775;PRJNA827465	Yang2025_Silkie_Wenchang	0.826444827
ENSGALG00010024254	ENSGALG00010024254	2	-0.366034153	0.159335035	-2.297260943	0.021603888	0	0.104475837	0	1	PRJNA827465;Yang2025_Silkie_Wenchang	CRA004775	0.248710494
ENSGALG00010024254	ENSGALG00010024254	2	-0.44935811	0.253764064	-1.770771256	0.076598748	0.006163729	1.040963198	3.935124495	1	CRA004775;Yang2025_Silkie_Wenchang	PRJNA827465	0.47762877
ENSGALG00010024254	ENSGALG00010024254	2	-0.464049918	0.176324605	-2.631793324	0.008493551	0	0.791732936	0	1	CRA004775;PRJNA827465	Yang2025_Silkie_Wenchang	0.14369705
ENSGALG00010024255	CFAP97D1	2	0.978081914	0.347711508	2.812912115	0.004909507	0.109019777	1.821002977	45.08520785	1	PRJNA827465;Yang2025_Silkie_Wenchang	CRA004775	0.104414688
ENSGALG00010024255	CFAP97D1	2	-0.100735176	1.498558096	-0.067221402	0.946405448	4.145875499	12.62371211	92.07839983	0.5	CRA004775;Yang2025_Silkie_Wenchang	PRJNA827465	0.986445196
ENSGALG00010024255	CFAP97D1	2	-0.426157672	1.148614108	-0.371019012	0.710623371	2.300171633	7.437309883	86.55427815	0.5	CRA004775;PRJNA827465	Yang2025_Silkie_Wenchang	0.925404854
ENSGALG00010024258	DNAJA4	2	0.093061001	0.167542052	0.555448615	0.578587848	0.038009795	3.072943911	67.45791563	0.5	PRJNA827465;Yang2025_Silkie_Wenchang	CRA004775	0.88788081
ENSGALG00010024258	DNAJA4	2	0.559854661	0.385622198	1.451821663	0.146551199	0.210381608	2.868736439	65.14144742	1	CRA004775;Yang2025_Silkie_Wenchang	PRJNA827465	0.635517732
ENSGALG00010024258	DNAJA4	2	0.422324106	0.563657936	0.749256029	0.453702905	0.539924969	5.945328479	83.18007149	0.5	CRA004775;PRJNA827465	Yang2025_Silkie_Wenchang	0.849044791
ENSGALG00010024259	ENSGALG00010024259	2	-0.273598394	0.312379273	-0.875853225	0.381109831	0.138205251	2.565620105	61.02306814	1	PRJNA827465;Yang2025_Silkie_Wenchang	CRA004775	0.824392515
ENSGALG00010024259	ENSGALG00010024259	2	-0.322748314	0.382877582	-0.842954327	0.399253988	0.144255363	1.958085658	48.92971123	0.5	CRA004775;Yang2025_Silkie_Wenchang	PRJNA827465	0.83210929
ENSGALG00010024259	ENSGALG00010024259	2	-0.05932536	0.062696082	-0.946237115	0.34402766	0	0.067812708	0	0.5	CRA004775;PRJNA827465	Yang2025_Silkie_Wenchang	0.806445383
ENSGALG00010024263	PHKB	2	-0.13679942	0.122955589	-1.112592126	0.265883644	0.015238373	1.81947064	45.03895923	0.5	PRJNA827465;Yang2025_Silkie_Wenchang	CRA004775	0.770065639
ENSGALG00010024263	PHKB	2	0.074150512	0.162093684	0.457454666	0.64734428	0	0.441344908	0	1	CRA004775;Yang2025_Silkie_Wenchang	PRJNA827465	0.907350807
ENSGALG00010024263	PHKB	2	-0.111571108	0.234494864	-0.475793396	0.634221575	0.06016726	1.502062683	33.42488225	0.5	CRA004775;PRJNA827465	Yang2025_Silkie_Wenchang	0.903815945
ENSGALG00010024264	AMER3	2	-0.174218032	0.139578052	-1.248176413	0.21196646	0.020994519	1.856684605	46.14055628	1	PRJNA827465;Yang2025_Silkie_Wenchang	CRA004775	0.724364644
ENSGALG00010024264	AMER3	2	0.33202886	0.788130998	0.421286386	0.67354597	1.0895522	7.610419312	86.86011954	0.5	CRA004775;Yang2025_Silkie_Wenchang	PRJNA827465	0.915386909
ENSGALG00010024264	AMER3	2	0.451131222	0.631710535	0.714145713	0.475139184	0.674489999	5.570435142	82.04808108	0.5	CRA004775;PRJNA827465	Yang2025_Silkie_Wenchang	0.854462864
ENSGALG00010024266	ENSGALG00010024266	2	-0.252322394	0.209359006	-1.205213946	0.22812072	0	0.0157224	0	1	PRJNA827465;Yang2025_Silkie_Wenchang	CRA004775	0.740938585
ENSGALG00010024266	ENSGALG00010024266	2	-0.202981851	0.271932949	-0.746440811	0.455401174	0	0.015305076	0	1	CRA004775;Yang2025_Silkie_Wenchang	PRJNA827465	0.84907427
ENSGALG00010024266	ENSGALG00010024266	2	-0.247852437	0.248212633	-0.998548843	0.318013292	0	0.046036337	0	1	CRA004775;PRJNA827465	Yang2025_Silkie_Wenchang	0.797439961
ENSGALG00010024267	MAP3K12	2	0.010048118	0.168484244	0.059638326	0.952434693	0.023631941	1.646126156	39.25131458	0.5	PRJNA827465;Yang2025_Silkie_Wenchang	CRA004775	0.987758913
ENSGALG00010024267	MAP3K12	2	0.177526465	0.177451685	1.000421408	0.317106614	0	0.115709504	0	1	CRA004775;Yang2025_Silkie_Wenchang	PRJNA827465	0.797007794
ENSGALG00010024267	MAP3K12	2	-0.074764515	0.132246032	-0.56534411	0.571839732	0	0.474284459	0	0.5	CRA004775;PRJNA827465	Yang2025_Silkie_Wenchang	0.885470687
ENSGALG00010024270	RAB18L	2	-0.317621728	0.276253367	-1.149747897	0.250247721	0.003269894	1.02094872	2.051887572	0.5	PRJNA827465;Yang2025_Silkie_Wenchang	CRA004775	0.75993411
ENSGALG00010024270	RAB18L	2	-0.905710067	0.456001232	-1.986200922	0.047011018	0.216952165	1.983914386	49.59459008	1	CRA004775;Yang2025_Silkie_Wenchang	PRJNA827465	0.374081275
ENSGALG00010024270	RAB18L	2	-0.684359422	0.7487274	-0.914030155	0.360701001	0.871629198	4.441852726	77.48687177	0.5	CRA004775;PRJNA827465	Yang2025_Silkie_Wenchang	0.815038264
ENSGALG00010024274	ACSBG1	2	-0.084877934	0.09860011	-0.860830019	0.389331669	0	0.998383766	0	1	PRJNA827465;Yang2025_Silkie_Wenchang	CRA004775	0.828877233
ENSGALG00010024274	ACSBG1	2	0.138606712	0.381666054	0.363219916	0.71644061	0.237541201	5.111926272	80.43790253	0.5	CRA004775;Yang2025_Silkie_Wenchang	PRJNA827465	0.947487786
ENSGALG00010024274	ACSBG1	2	0.217001882	0.276587203	0.784569496	0.432706021	0.107249327	2.964673818	66.26947646	0.5	CRA004775;PRJNA827465	Yang2025_Silkie_Wenchang	0.842762754
ENSGALG00010024277	ITGB3BP	2	-0.402284227	0.10848489	-3.708205152	0.000208734	0	0.057908931	0	1	PRJNA827465;Yang2025_Silkie_Wenchang	CRA004775	0.013147201
ENSGALG00010024277	ITGB3BP	2	-0.420261123	0.142811411	-2.942769901	0.003252901	0	0.0278369	0	1	CRA004775;Yang2025_Silkie_Wenchang	PRJNA827465	0.079310649
ENSGALG00010024277	ITGB3BP	2	-0.370459294	0.154768937	-2.393628216	0.016682649	0	0.006362038	0	1	CRA004775;PRJNA827465	Yang2025_Silkie_Wenchang	0.214133955
ENSGALG00010024280	ENSGALG00010024280	2	-0.007632728	0.091523485	-0.083396393	0.933536357	0.007512127	1.752121271	42.926325	0.5	PRJNA827465;Yang2025_Silkie_Wenchang	CRA004775	0.984262625
ENSGALG00010024280	ENSGALG00010024280	2	0.087132042	0.111646049	0.780431039	0.435137204	0	0.28781496	0	0.5	CRA004775;Yang2025_Silkie_Wenchang	PRJNA827465	0.842762754
ENSGALG00010024280	ENSGALG00010024280	2	-0.083047036	0.078172674	-1.062353785	0.288075106	0	0.005491075	0	1	CRA004775;PRJNA827465	Yang2025_Silkie_Wenchang	0.782840067
ENSGALG00010024282	HIC2	2	-0.111241225	0.163495631	-0.680392645	0.496255877	0.038248426	3.374028782	70.36184145	0.5	PRJNA827465;Yang2025_Silkie_Wenchang	CRA004775	0.860939846
ENSGALG00010024282	HIC2	2	-0.303811468	0.142853775	-2.126730419	0.033442489	0	0.015789415	0	1	CRA004775;Yang2025_Silkie_Wenchang	PRJNA827465	0.31414678
ENSGALG00010024282	HIC2	2	0.013338876	0.093127901	0.14323179	0.886107124	0	0.80705632	0	0.5	CRA004775;PRJNA827465	Yang2025_Silkie_Wenchang	0.972567463
ENSGALG00010024283	UBE4A	2	-0.063492884	0.076348326	-0.831621164	0.405622812	0.007421208	2.69180344	62.85018493	0.5	PRJNA827465;Yang2025_Silkie_Wenchang	CRA004775	0.833257341
ENSGALG00010024283	UBE4A	2	-0.166111131	0.070251081	-2.364534889	0.018052727	0	0.375978255	0	1	CRA004775;Yang2025_Silkie_Wenchang	PRJNA827465	0.224108125
ENSGALG00010024283	UBE4A	2	-0.075605916	0.12635222	-0.598374256	0.54959024	0.01935565	1.987678594	49.69005537	0.5	CRA004775;PRJNA827465	Yang2025_Silkie_Wenchang	0.878554604
ENSGALG00010024285	CCND2	2	-0.104977005	0.13425857	-0.781901711	0.43427234	0.028072536	4.231740319	76.36906037	0.5	PRJNA827465;Yang2025_Silkie_Wenchang	CRA004775	0.842762754
ENSGALG00010024285	CCND2	2	-0.238935123	0.103792734	-2.302040949	0.021332863	0	0.138407883	0	1	CRA004775;Yang2025_Silkie_Wenchang	PRJNA827465	0.247110379
ENSGALG00010024285	CCND2	2	-0.000281096	0.057799284	-0.004863315	0.996119651	0	0.59789605	0	0.5	CRA004775;PRJNA827465	Yang2025_Silkie_Wenchang	0.998737007
ENSGALG00010024286	ADA	2	-0.281387838	0.163958609	-1.716212641	0.086123213	0.040297373	3.945947991	74.65754738	1	PRJNA827465;Yang2025_Silkie_Wenchang	CRA004775	0.504895124
ENSGALG00010024286	ADA	2	-0.141792663	0.327481659	-0.432978064	0.665030067	0.188882203	8.271261367	87.90994559	0.5	CRA004775;Yang2025_Silkie_Wenchang	PRJNA827465	0.913099315
ENSGALG00010024286	ADA	2	-0.001516798	0.15909826	-0.009533717	0.99239331	0.030855433	2.36538824	57.72364202	0.5	CRA004775;PRJNA827465	Yang2025_Silkie_Wenchang	0.997813736
ENSGALG00010024288	CHID1	2	0.144778893	0.102639531	1.410556851	0.158375322	0.010806946	2.022678956	50.56061679	1	PRJNA827465;Yang2025_Silkie_Wenchang	CRA004775	0.652042151
ENSGALG00010024288	CHID1	2	0.250216415	0.108852574	2.298672468	0.021523545	0	0.083238058	0	1	CRA004775;Yang2025_Silkie_Wenchang	PRJNA827465	0.248109211
ENSGALG00010024288	CHID1	2	0.059916798	0.086738577	0.690774511	0.489707256	0	0.077476254	0	1	CRA004775;PRJNA827465	Yang2025_Silkie_Wenchang	0.858425426
ENSGALG00010024290	SOST	2	0.092201063	0.309859226	0.297557906	0.766040606	0	0.5272035	0	0.5	PRJNA827465;Yang2025_Silkie_Wenchang	CRA004775	0.941071295
ENSGALG00010024290	SOST	2	-0.899895535	0.853632891	-1.054195011	0.291793644	1.020526927	3.14061165	68.15906863	1	CRA004775;Yang2025_Silkie_Wenchang	PRJNA827465	0.78534312
ENSGALG00010024290	SOST	2	-0.685239369	1.086238277	-0.630837068	0.528147064	1.949919443	5.407809121	81.50784691	0.5	CRA004775;PRJNA827465	Yang2025_Silkie_Wenchang	0.871698822
ENSGALG00010024291	ENSGALG00010024291	2	0.277875442	0.171383959	1.62136202	0.104940021	0	0.000169563	0	1	PRJNA827465;Yang2025_Silkie_Wenchang	CRA004775	0.550147746
ENSGALG00010024291	ENSGALG00010024291	2	-0.15916377	0.575639453	-0.276499063	0.782164779	0.475584493	3.005433827	66.72693336	0.5	CRA004775;Yang2025_Silkie_Wenchang	PRJNA827465	0.945372683
ENSGALG00010024291	ENSGALG00010024291	2	-0.171973745	0.576069057	-0.298529738	0.765298883	0.456897865	2.824756182	64.59871453	0.5	CRA004775;PRJNA827465	Yang2025_Silkie_Wenchang	0.940779391
ENSGALG00010024300	MEOX1	2	0.206903515	0.120936272	1.710847374	0.087109273	0	0.043319153	0	1	PRJNA827465;Yang2025_Silkie_Wenchang	CRA004775	0.507558762
ENSGALG00010024300	MEOX1	2	0.243268397	0.201735369	1.205878761	0.227864241	0	8.43112E-05	0	1	CRA004775;Yang2025_Silkie_Wenchang	PRJNA827465	0.740853612
ENSGALG00010024300	MEOX1	2	0.191702937	0.144731812	1.32453905	0.185324083	0	0.00686973	0	1	CRA004775;PRJNA827465	Yang2025_Silkie_Wenchang	0.693795574
ENSGALG00010024304	PKIG	2	-0.031322223	0.08010294	-0.391024629	0.695779031	0	0.007254744	0	1	PRJNA827465;Yang2025_Silkie_Wenchang	CRA004775	0.921038437
ENSGALG00010024304	PKIG	2	0.004875807	0.109755901	0.044424096	0.964564355	0	0.575786987	0	0.5	CRA004775;Yang2025_Silkie_Wenchang	PRJNA827465	0.990364463
ENSGALG00010024304	PKIG	2	0.005708353	0.095587767	0.059718444	0.952379882	0	0.533250073	0	0.5	CRA004775;PRJNA827465	Yang2025_Silkie_Wenchang	0.987758913
ENSGALG00010024305	ENSGALG00010024305	2	-0.796704643	0.266203466	-2.992840982	0.002763937	0	0.075236256	0	1	PRJNA827465;Yang2025_Silkie_Wenchang	CRA004775	0.071732358
ENSGALG00010024305	ENSGALG00010024305	2	-0.764698592	0.361278439	-2.116608084	0.034289896	0	0.075871517	0	1	CRA004775;Yang2025_Silkie_Wenchang	PRJNA827465	0.318385282
ENSGALG00010024305	ENSGALG00010024305	2	-0.874611557	0.317669576	-2.753211586	0.005901374	0	0.011589936	0	1	CRA004775;PRJNA827465	Yang2025_Silkie_Wenchang	0.116086942
ENSGALG00010024309	ABCB11	2	0.401964446	0.184198994	2.18222932	0.029092612	0	0.585895976	0	1	PRJNA827465;Yang2025_Silkie_Wenchang	CRA004775	0.290337858
ENSGALG00010024309	ABCB11	2	0.559297126	0.209447624	2.670343614	0.007577366	0	0.17509624	0	1	CRA004775;Yang2025_Silkie_Wenchang	PRJNA827465	0.134392245
ENSGALG00010024309	ABCB11	2	0.37334463	0.248439639	1.502757899	0.132901486	0	0.857175705	0	1	CRA004775;PRJNA827465	Yang2025_Silkie_Wenchang	0.610855703
ENSGALG00010024311	KLHL6	2	-0.074135069	0.204196281	-0.363057881	0.716561647	0.066872901	4.976085882	79.90388382	0.5	PRJNA827465;Yang2025_Silkie_Wenchang	CRA004775	0.927487786
ENSGALG00010024311	KLHL6	2	-0.276971131	0.14173174	-1.954192702	0.050678432	0	0.112238578	0	1	CRA004775;Yang2025_Silkie_Wenchang	PRJNA827465	0.389748565
ENSGALG00010024311	KLHL6	2	0.10774407	0.10622912	1.014261156	0.310458154	0	0.206675709	0	0.5	CRA004775;PRJNA827465	Yang2025_Silkie_Wenchang	0.794899969
ENSGALG00010024313	SERINC3	2	-0.218404824	0.161946181	-1.348625963	0.177457137	0.040218144	3.889770864	74.2915448	1	PRJNA827465;Yang2025_Silkie_Wenchang	CRA004775	0.683574458
ENSGALG00010024313	SERINC3	2	-0.303402605	0.113746407	-2.667359908	0.007644975	0.001255786	1.050887268	4.84231464	1	CRA004775;Yang2025_Silkie_Wenchang	PRJNA827465	0.135061073
ENSGALG00010024313	SERINC3	2	-0.097524033	0.061170558	-1.594296938	0.110869543	0	0.33903613	0	1	CRA004775;PRJNA827465	Yang2025_Silkie_Wenchang	0.563494848
ENSGALG00010024314	ITFG1	2	0.008832231	0.055469425	0.159227016	0.87349002	0	0.895532514	0	0.5	PRJNA827465;Yang2025_Silkie_Wenchang	CRA004775	0.969960396
ENSGALG00010024314	ITFG1	2	0.158850116	0.111394391	1.426015383	0.153863902	0	0.827673612	0	1	CRA004775;Yang2025_Silkie_Wenchang	PRJNA827465	0.646425755
ENSGALG00010024314	ITFG1	2	0.096070157	0.179625823	0.534834887	0.592764036	0.041363296	2.102179215	52.43031646	0.5	CRA004775;PRJNA827465	Yang2025_Silkie_Wenchang	0.892776493
ENSGALG00010024321	UBE2L3	2	-0.330441636	0.336211873	-0.982837496	0.325687424	0.219788881	35.3602803	97.17196812	1	PRJNA827465;Yang2025_Silkie_Wenchang	CRA004775	0.800023788
ENSGALG00010024321	UBE2L3	2	-0.366778872	0.328035765	-1.11810635	0.263521575	0.190702821	8.499500888	88.23460326	1	CRA004775;Yang2025_Silkie_Wenchang	PRJNA827465	0.768643821
ENSGALG00010024321	UBE2L3	2	-0.002050504	0.037270369	-0.055016997	0.956124922	0	0.005582216	0	1	CRA004775;PRJNA827465	Yang2025_Silkie_Wenchang	0.988513657
ENSGALG00010024322	IDH3A	2	0.046299507	0.076310864	0.606722352	0.544035186	0	0.10052	0	1	PRJNA827465;Yang2025_Silkie_Wenchang	CRA004775	0.876968972
ENSGALG00010024322	IDH3A	2	0.082685326	0.11627916	0.711093249	0.477026453	0	0.014816334	0	1	CRA004775;Yang2025_Silkie_Wenchang	PRJNA827465	0.854986476
ENSGALG00010024322	IDH3A	2	0.034778608	0.092830446	0.374646567	0.707923336	0	0.072697435	0	1	CRA004775;PRJNA827465	Yang2025_Silkie_Wenchang	0.925061251
ENSGALG00010024323	TTPAL	2	-0.093109356	0.151930957	-0.612839926	0.539982188	0.035847166	4.151831913	75.9142465	0.5	PRJNA827465;Yang2025_Silkie_Wenchang	CRA004775	0.87529516
ENSGALG00010024323	TTPAL	2	-0.327351585	0.119991042	-2.728133532	0.006369382	0	0.823449511	0	1	CRA004775;Yang2025_Silkie_Wenchang	PRJNA827465	0.121286733
ENSGALG00010024323	TTPAL	2	-0.193884226	0.280210616	-0.691923199	0.488985561	0.127778999	4.644394044	78.46866587	0.5	CRA004775;PRJNA827465	Yang2025_Silkie_Wenchang	0.858232954
ENSGALG00010024325	PCBP2	2	-0.070494294	0.052170165	-1.351237694	0.176619303	0	0.799258297	0	1	PRJNA827465;Yang2025_Silkie_Wenchang	CRA004775	0.682034027
ENSGALG00010024325	PCBP2	2	-0.017354439	0.087540307	-0.198245121	0.842853286	0	0.296383215	0	1	CRA004775;Yang2025_Silkie_Wenchang	PRJNA827465	0.960387984
ENSGALG00010024325	PCBP2	2	-0.106254465	0.062461349	-1.701123438	0.088919811	0	0.063382014	0	1	CRA004775;PRJNA827465	Yang2025_Silkie_Wenchang	0.51183587
ENSGALG00010024326	KLHL24	2	-0.085905907	0.063207168	-1.359116526	0.174109667	0	0.079696804	0	1	PRJNA827465;Yang2025_Silkie_Wenchang	CRA004775	0.67813222
ENSGALG00010024326	KLHL24	2	0.101271994	0.291404789	0.347530302	0.728192958	0.104304866	1.849107098	45.91984416	0.5	CRA004775;Yang2025_Silkie_Wenchang	PRJNA827465	0.931987594
ENSGALG00010024326	KLHL24	2	0.090927262	0.315932743	0.287805756	0.773495431	0.129783422	2.0625165	51.5155394	0.5	CRA004775;PRJNA827465	Yang2025_Silkie_Wenchang	0.943248868
ENSGALG00010024327	PPP1R1B	2	0.218002455	0.424962368	0.512992376	0.607956653	0.271924166	4.041897974	75.25914789	0.5	PRJNA827465;Yang2025_Silkie_Wenchang	CRA004775	0.897204334
ENSGALG00010024327	PPP1R1B	2	0.186906642	0.570033585	0.32788707	0.74299705	0.443166828	2.80787946	64.38593557	0.5	CRA004775;Yang2025_Silkie_Wenchang	PRJNA827465	0.935735651
ENSGALG00010024327	PPP1R1B	2	-0.27686815	0.279078819	-0.99207869	0.321159141	0	0.207254645	0	1	CRA004775;PRJNA827465	Yang2025_Silkie_Wenchang	0.798513866
ENSGALG00010024328	YDJC	2	-0.046848853	0.084295039	-0.555772359	0.578366483	0.005426687	1.455684338	31.30378794	0.5	PRJNA827465;Yang2025_Silkie_Wenchang	CRA004775	0.887855709
ENSGALG00010024328	YDJC	2	-0.172801086	0.124406899	-1.388999225	0.164832986	0	0.011743313	0	1	CRA004775;Yang2025_Silkie_Wenchang	PRJNA827465	0.664067023
ENSGALG00010024328	YDJC	2	-0.001598101	0.065580018	-0.024368722	0.980558497	0	0.278720007	0	0.5	CRA004775;PRJNA827465	Yang2025_Silkie_Wenchang	0.994808741
ENSGALG00010024330	SDF2L1	2	-0.151516976	0.339825924	-0.445866443	0.655693724	0.219541102	19.70458331	94.92503858	0.5	PRJNA827465;Yang2025_Silkie_Wenchang	CRA004775	0.910411938
ENSGALG00010024330	SDF2L1	2	-0.475587836	0.132695261	-3.584060456	0.000338294	0	0.305527549	0	1	CRA004775;Yang2025_Silkie_Wenchang	PRJNA827465	0.018263679
ENSGALG00010024330	SDF2L1	2	0.08559977	0.18353776	0.466387791	0.640937949	0.038655466	1.563820217	36.05403043	0.5	CRA004775;PRJNA827465	Yang2025_Silkie_Wenchang	0.906429868
ENSGALG00010024332	EFCAB7	2	-0.131364012	0.10712768	-1.226237821	0.220109193	0	0.186139903	0	1	PRJNA827465;Yang2025_Silkie_Wenchang	CRA004775	0.733691086
ENSGALG00010024332	EFCAB7	2	-0.074906187	0.164559683	-0.455191607	0.648971398	0.008533854	1.166147545	14.24755777	0.5	CRA004775;Yang2025_Silkie_Wenchang	PRJNA827465	0.907783669
ENSGALG00010024332	EFCAB7	2	-0.047251996	0.118696741	-0.398090087	0.690563781	0	0.709270638	0	0.5	CRA004775;PRJNA827465	Yang2025_Silkie_Wenchang	0.919979266
ENSGALG00010024333	ETV4	2	0.079496471	0.128976844	0.616362351	0.537655396	0.017805602	2.030291893	50.74599846	0.5	PRJNA827465;Yang2025_Silkie_Wenchang	CRA004775	0.87481821
ENSGALG00010024333	ETV4	2	0.247076616	0.149696817	1.650513497	0.098837955	0	0.008675438	0	1	CRA004775;Yang2025_Silkie_Wenchang	PRJNA827465	0.536752215
ENSGALG00010024333	ETV4	2	-0.008960233	0.094229936	-0.09508903	0.924244111	0	0.460251337	0	0.5	CRA004775;PRJNA827465	Yang2025_Silkie_Wenchang	0.98232425
ENSGALG00010024334	USP28	2	-0.115010433	0.124941325	-0.92051556	0.357303406	0.02221427	3.348454792	70.13547854	0.5	PRJNA827465;Yang2025_Silkie_Wenchang	CRA004775	0.813292764
ENSGALG00010024334	USP28	2	0.010903869	0.110222998	0.098925535	0.921197394	0	0.204479495	0	0.5	CRA004775;Yang2025_Silkie_Wenchang	PRJNA827465	0.981519273
ENSGALG00010024334	USP28	2	-0.223173844	0.073900995	-3.019903093	0.002528556	0	0.032902058	0	1	CRA004775;PRJNA827465	Yang2025_Silkie_Wenchang	0.067541822
ENSGALG00010024335	AMHR2	2	0.059178903	0.286170882	0.206795682	0.836169429	0	0.390557797	0	0.5	PRJNA827465;Yang2025_Silkie_Wenchang	CRA004775	0.958399882
ENSGALG00010024335	AMHR2	2	-0.155406582	0.350781249	-0.4430299	0.657744108	0	0.043664123	0	1	CRA004775;Yang2025_Silkie_Wenchang	PRJNA827465	0.910774485
ENSGALG00010024335	AMHR2	2	0.115299879	0.357448203	0.32256388	0.747025552	0	0.402840439	0	0.5	CRA004775;PRJNA827465	Yang2025_Silkie_Wenchang	0.936729365
ENSGALG00010024336	TSPAN4	2	0.067400914	0.250386003	0.269188028	0.787784993	0.104647589	5.569457181	82.04492884	0.5	PRJNA827465;Yang2025_Silkie_Wenchang	CRA004775	0.946919116
ENSGALG00010024336	TSPAN4	2	0.269931321	0.167697628	1.609631123	0.10747841	0	0.603328526	0	1	CRA004775;Yang2025_Silkie_Wenchang	PRJNA827465	0.554966053
ENSGALG00010024336	TSPAN4	2	-0.139074182	0.076883682	-1.808890765	0.070467975	0	0.455430612	0	0.5	CRA004775;PRJNA827465	Yang2025_Silkie_Wenchang	0.458021454
ENSGALG00010024338	NDUFA9	2	-0.057060465	0.055882602	-1.021077452	0.307217745	0	0.00403265	0	1	PRJNA827465;Yang2025_Silkie_Wenchang	CRA004775	0.79266003
ENSGALG00010024338	NDUFA9	2	-0.042067307	0.126008357	-0.333845373	0.738496243	0	0.01282935	0	1	CRA004775;Yang2025_Silkie_Wenchang	PRJNA827465	0.934485814
ENSGALG00010024338	NDUFA9	2	-0.056663519	0.059670409	-0.949608359	0.342311289	0	0.024198835	0	1	CRA004775;PRJNA827465	Yang2025_Silkie_Wenchang	0.806005903
ENSGALG00010024339	TOP3B	2	0.141087956	0.144542475	0.976100316	0.329014755	0.037494023	9.679766131	89.66917189	1	PRJNA827465;Yang2025_Silkie_Wenchang	CRA004775	0.800761292
ENSGALG00010024339	TOP3B	2	0.158655877	0.171413487	0.925574056	0.354667381	0.041079311	2.859977545	65.03469051	0.5	CRA004775;Yang2025_Silkie_Wenchang	PRJNA827465	0.812016118
ENSGALG00010024339	TOP3B	2	-0.004806273	0.054562324	-0.088087753	0.92980693	0	0.104533355	0	0.5	CRA004775;PRJNA827465	Yang2025_Silkie_Wenchang	0.983813577
ENSGALG00010024340	NETO2	2	-0.100772363	0.160270245	-0.628765265	0.529502743	0	0.034968908	0	1	PRJNA827465;Yang2025_Silkie_Wenchang	CRA004775	0.871944741
ENSGALG00010024340	NETO2	2	0.316873141	0.534713655	0.592603421	0.553446589	0.400167222	3.212688584	68.87342256	0.5	CRA004775;Yang2025_Silkie_Wenchang	PRJNA827465	0.879514268
ENSGALG00010024340	NETO2	2	0.310551594	0.491825374	0.63142654	0.527761667	0.363851788	3.530194228	71.67294671	0.5	CRA004775;PRJNA827465	Yang2025_Silkie_Wenchang	0.871296643
ENSGALG00010024343	POLR2L	2	0.119267082	0.072414622	1.647002754	0.09955748	0	0.348623741	0	0.5	PRJNA827465;Yang2025_Silkie_Wenchang	CRA004775	0.538819558
ENSGALG00010024343	POLR2L	2	-0.151040758	0.240395586	-0.628300878	0.529806857	0	0.101000382	0	1	CRA004775;Yang2025_Silkie_Wenchang	PRJNA827465	0.871944741
ENSGALG00010024343	POLR2L	2	0.106328544	0.086436698	1.230131957	0.218647695	0.002473148	1.040365614	3.879945079	0.5	CRA004775;PRJNA827465	Yang2025_Silkie_Wenchang	0.73229743
ENSGALG00010024344	YEATS2	2	-0.006735952	0.113595753	-0.05929757	0.952715096	0.019191922	3.44850921	71.00196232	0.5	PRJNA827465;Yang2025_Silkie_Wenchang	CRA004775	0.987758913
ENSGALG00010024344	YEATS2	2	0.109550563	0.105818785	1.035265743	0.300544823	0	0.255208013	0	0.5	CRA004775;Yang2025_Silkie_Wenchang	PRJNA827465	0.790579721
ENSGALG00010024344	YEATS2	2	-0.095344905	0.044991142	-2.119192791	0.034074177	0	0.12883254	0	1	CRA004775;PRJNA827465	Yang2025_Silkie_Wenchang	0.317399999
ENSGALG00010024345	STARD3	2	-0.061812917	0.141045511	-0.438248026	0.661206498	0.026280358	2.309451374	56.69967287	0.5	PRJNA827465;Yang2025_Silkie_Wenchang	CRA004775	0.91217832
ENSGALG00010024345	STARD3	2	-0.156295576	0.152379576	-1.025698984	0.305033493	0	0.925956976	0	0.5	CRA004775;Yang2025_Silkie_Wenchang	PRJNA827465	0.79225794
ENSGALG00010024345	STARD3	2	0.033034151	0.050205109	0.657983861	0.510548502	0	0.000100566	0	1	CRA004775;PRJNA827465	Yang2025_Silkie_Wenchang	0.865365179
ENSGALG00010024346	PPM1F	2	0.024388441	0.072139567	0.338073023	0.73530816	0	0.006513031	0	1	PRJNA827465;Yang2025_Silkie_Wenchang	CRA004775	0.933592889
ENSGALG00010024346	PPM1F	2	-0.008443561	0.146252014	-0.057732955	0.953961343	0	0.77919813	0	0.5	CRA004775;Yang2025_Silkie_Wenchang	PRJNA827465	0.987846193
ENSGALG00010024346	PPM1F	2	0.008532145	0.080301929	0.106250804	0.915383364	0	0.786932042	0	0.5	CRA004775;PRJNA827465	Yang2025_Silkie_Wenchang	0.980163067
ENSGALG00010024347	OTOG	2	0.746386187	0.594648653	1.255171744	0.209416421	0.61608304	7.705868082	87.02287673	1	PRJNA827465;Yang2025_Silkie_Wenchang	CRA004775	0.721294679
ENSGALG00010024347	OTOG	2	0.927409298	0.681290373	1.361254075	0.173433411	0.537028997	1.902572936	47.43959712	0.5	CRA004775;Yang2025_Silkie_Wenchang	PRJNA827465	0.676868485
ENSGALG00010024347	OTOG	2	0.151705573	0.254573919	0.595919541	0.551228979	0	0.086587647	0	0.5	CRA004775;PRJNA827465	Yang2025_Silkie_Wenchang	0.878879109
ENSGALG00010024349	R3HDML	2	-0.178923378	0.331503203	-0.539733483	0.589380846	0	0.257610719	0	1	PRJNA827465;Yang2025_Silkie_Wenchang	CRA004775	0.892320726
ENSGALG00010024349	R3HDML	2	-0.459749296	0.51081419	-0.900032351	0.368103035	0	0.072829253	0	1	CRA004775;Yang2025_Silkie_Wenchang	PRJNA827465	0.820213684
ENSGALG00010024349	R3HDML	2	-0.117134081	0.396077637	-0.295735155	0.767432348	0	0.278766759	0	1	CRA004775;PRJNA827465	Yang2025_Silkie_Wenchang	0.941444269
ENSGALG00010024350	PGM1	2	-0.276186296	0.110072817	-2.509123538	0.012103115	0	0.04392637	0	1	PRJNA827465;Yang2025_Silkie_Wenchang	CRA004775	0.17736343
ENSGALG00010024350	PGM1	2	-0.274995177	0.163080627	-1.686252878	0.091747103	0	0.37578523	0	0.5	CRA004775;Yang2025_Silkie_Wenchang	PRJNA827465	0.518914432
ENSGALG00010024350	PGM1	2	-0.238036599	0.140756282	-1.691125931	0.090812754	0	0.295528139	0	0.5	CRA004775;PRJNA827465	Yang2025_Silkie_Wenchang	0.517052536
ENSGALG00010024351	SP1	2	0.131511113	0.206697095	0.63625042	0.524613215	0.067914921	4.465730113	77.60724507	0.5	PRJNA827465;Yang2025_Silkie_Wenchang	CRA004775	0.869811145
ENSGALG00010024351	SP1	2	0.321773426	0.147803219	2.177039361	0.029477631	0	0.232810628	0	1	CRA004775;Yang2025_Silkie_Wenchang	PRJNA827465	0.292694292
ENSGALG00010024351	SP1	2	-0.020690523	0.077685181	-0.266338091	0.78997884	0.000495142	1.014015534	1.382181395	0.5	CRA004775;PRJNA827465	Yang2025_Silkie_Wenchang	0.947390191
ENSGALG00010024352	MAP6D1	2	0.078616738	0.280074942	0.280698936	0.778941325	0	0.110587453	0	0.5	PRJNA827465;Yang2025_Silkie_Wenchang	CRA004775	0.944714533
ENSGALG00010024352	MAP6D1	2	-0.076319824	0.40087401	-0.190383567	0.849008574	0	0.013993417	0	1	CRA004775;Yang2025_Silkie_Wenchang	PRJNA827465	0.961364896
ENSGALG00010024352	MAP6D1	2	0.095877297	0.31716419	0.302295468	0.762426833	0	0.128156779	0	0.5	CRA004775;PRJNA827465	Yang2025_Silkie_Wenchang	0.940082621
ENSGALG00010024354	C2CD2L	2	-0.026092502	0.090051767	-0.289750024	0.772007483	0.006956201	1.644745646	39.20032544	0.5	PRJNA827465;Yang2025_Silkie_Wenchang	CRA004775	0.943066211
ENSGALG00010024354	C2CD2L	2	-0.169417315	0.110128587	-1.538359113	0.123960834	0	0.119603874	0	1	CRA004775;Yang2025_Silkie_Wenchang	PRJNA827465	0.59047345
ENSGALG00010024354	C2CD2L	2	-0.017806456	0.112994384	-0.157587088	0.874782179	0.010580122	1.379452956	27.50749523	0.5	CRA004775;PRJNA827465	Yang2025_Silkie_Wenchang	0.970159044
ENSGALG00010024356	PARL	2	-0.131700906	0.09523963	-1.382837222	0.166714783	0.007658599	1.397373293	28.437161	1	PRJNA827465;Yang2025_Silkie_Wenchang	CRA004775	0.665690452
ENSGALG00010024356	PARL	2	-0.253839103	0.155343393	-1.634051494	0.102248039	0	0.344240233	0	1	CRA004775;Yang2025_Silkie_Wenchang	PRJNA827465	0.54496402
ENSGALG00010024356	PARL	2	-0.083536042	0.060871598	-1.372332008	0.169960106	0	0.013786588	0	1	CRA004775;PRJNA827465	Yang2025_Silkie_Wenchang	0.67148593
ENSGALG00010024358	MAPK3	2	-0.309089222	0.239172494	-1.29232763	0.196243707	0.111746634	42.45476639	97.64455187	1	PRJNA827465;Yang2025_Silkie_Wenchang	CRA004775	0.707666824
ENSGALG00010024358	MAPK3	2	-0.294278787	0.286230808	-1.028117096	0.30389475	0.143900608	7.631527329	86.8964631	0.5	CRA004775;Yang2025_Silkie_Wenchang	PRJNA827465	0.792208658
ENSGALG00010024358	MAPK3	2	-0.072390435	0.025957449	-2.788811576	0.005290183	0	0.239333509	0	0.5	CRA004775;PRJNA827465	Yang2025_Silkie_Wenchang	0.108631224
ENSGALG00010024360	FTL	2	0.204962935	0.138669705	1.478065699	0.139390194	0.005514383	1.164769416	14.14609739	1	PRJNA827465;Yang2025_Silkie_Wenchang	CRA004775	0.623009874
ENSGALG00010024360	FTL	2	-0.127123183	0.222359622	-0.571700841	0.567524665	0.042544157	1.688793931	40.78614438	0.5	CRA004775;Yang2025_Silkie_Wenchang	PRJNA827465	0.883717772
ENSGALG00010024360	FTL	2	-0.001133139	0.365970174	-0.003096261	0.997529545	0.213574756	4.730526417	78.86070361	0.5	CRA004775;PRJNA827465	Yang2025_Silkie_Wenchang	0.999290321
ENSGALG00010024361	H2AX	2	0.200801235	0.147065681	1.365384735	0.172132159	0	0.456955755	0	1	PRJNA827465;Yang2025_Silkie_Wenchang	CRA004775	0.674809614
ENSGALG00010024361	H2AX	2	0.025177305	0.42698603	0.058965172	0.952979849	0.215340166	1.992116373	49.80212937	0.5	CRA004775;Yang2025_Silkie_Wenchang	PRJNA827465	0.987758913
ENSGALG00010024361	H2AX	2	-0.020836705	0.277569092	-0.075068535	0.940160183	0.052456028	1.244476396	19.64492032	0.5	CRA004775;PRJNA827465	Yang2025_Silkie_Wenchang	0.985494335
ENSGALG00010024362	DHX8	2	-0.084573708	0.035464308	-2.384755604	0.017090471	0	0.982272931	0	1	PRJNA827465;Yang2025_Silkie_Wenchang	CRA004775	0.217240225
ENSGALG00010024362	DHX8	2	-0.062060356	0.097930518	-0.633718248	0.526264714	0.005391864	1.23800218	19.22469795	1	CRA004775;Yang2025_Silkie_Wenchang	PRJNA827465	0.870537576
ENSGALG00010024362	DHX8	2	-0.10680613	0.038464585	-2.776739403	0.005490718	0	0.532460857	0	1	CRA004775;PRJNA827465	Yang2025_Silkie_Wenchang	0.111278432
ENSGALG00010024363	EDEM2	2	-0.018442935	0.079547228	-0.231848876	0.816655394	0.000602345	1.041835305	4.015539199	0.5	PRJNA827465;Yang2025_Silkie_Wenchang	CRA004775	0.954671394
ENSGALG00010024363	EDEM2	2	-0.216661683	0.112734566	-1.92187446	0.054621556	0	0.785716534	0	1	CRA004775;Yang2025_Silkie_Wenchang	PRJNA827465	0.404444428
ENSGALG00010024363	EDEM2	2	-0.12239386	0.186838859	-0.65507711	0.512418109	0.050875514	3.401236265	70.59892574	0.5	CRA004775;PRJNA827465	Yang2025_Silkie_Wenchang	0.865809047
ENSGALG00010024365	ENSGALG00010024365	2	0.338896807	0.294087413	1.152367603	0.249170048	0.152004789	7.912480632	87.36173842	1	PRJNA827465;Yang2025_Silkie_Wenchang	CRA004775	0.759411487
ENSGALG00010024365	ENSGALG00010024365	2	0.186591638	0.484619421	0.385027157	0.700217295	0.420294668	9.428143465	89.39345796	0.5	CRA004775;Yang2025_Silkie_Wenchang	PRJNA827465	0.922299095
ENSGALG00010024365	ENSGALG00010024365	2	-0.049970676	0.175138805	-0.285320413	0.775398676	0.035576241	1.973442745	49.32713388	0.5	CRA004775;PRJNA827465	Yang2025_Silkie_Wenchang	0.943815265
ENSGALG00010024366	PARD6A	2	0.413362326	0.25801638	1.602077845	0.109138396	0	0.066008691	0	1	PRJNA827465;Yang2025_Silkie_Wenchang	CRA004775	0.559179559
ENSGALG00010024366	PARD6A	2	0.095652367	0.323869409	0.295342396	0.767732333	0	0.87685334	0	0.5	CRA004775;Yang2025_Silkie_Wenchang	PRJNA827465	0.941444269
ENSGALG00010024366	PARD6A	2	0.196784457	0.364327789	0.540130241	0.589107217	0.09560222	1.492051106	32.97816702	0.5	CRA004775;PRJNA827465	Yang2025_Silkie_Wenchang	0.892171138

ENSGALG00010024367	ENSGALG00010024367	2	0.246104349	0.253941349	0.969138543	0.332476069	0.093279391	3.217291431	68.91795408	1	PRJNA827465;Yang2025_Silkie_Wenchang	CRA004775	0.802178578
ENSGALG00010024367	ENSGALG00010024367	2	0.422938486	0.268293384	1.576402966	0.114932965	0.020071589	1.103308814	9.363546673	0.5	CRA004775;Yang2025_Silkie_Wenchang	PRJNA827465	0.571871165
ENSGALG00010024367	ENSGALG00010024367	2	0.037249056	0.110471617	0.337182136	0.735979603	0	0.054832324	0	0.5	CRA004775;PRJNA827465	Yang2025_Silkie_Wenchang	0.933592889
ENSGALG00010024368	DPAGT1	2	0.015174932	0.043855667	0.346019857	0.729327792	0	0.010584328	0	1	PRJNA827465;Yang2025_Silkie_Wenchang	CRA004775	0.9323457
ENSGALG00010024368	DPAGT1	2	0.039160486	0.07667625	0.510725092	0.609543568	0	0.337028614	0	1	CRA004775;Yang2025_Silkie_Wenchang	PRJNA827465	0.897238578
ENSGALG00010024368	DPAGT1	2	0.027968822	0.046179354	0.605656431	0.544742922	0	0.352043494	0	1	CRA004775;PRJNA827465	Yang2025_Silkie_Wenchang	0.877345822
ENSGALG00010024369	MYOD1	2	-0.137556981	0.086769912	-1.585307373	0.112896515	0	0.065336601	0	1	PRJNA827465;Yang2025_Silkie_Wenchang	CRA004775	0.566980581
ENSGALG00010024369	MYOD1	2	0.353045699	0.605670271	0.582900822	0.559960067	0.551974679	3.291167013	69.61564102	0.5	CRA004775;Yang2025_Silkie_Wenchang	PRJNA827465	0.881203589
ENSGALG00010024369	MYOD1	2	0.330301322	0.630875653	0.523560103	0.600584537	0.620128296	3.647176685	72.58153124	0.5	CRA004775;PRJNA827465	Yang2025_Silkie_Wenchang	0.895230519
ENSGALG00010024370	HERPUD1	2	0.071735833	0.211130625	0.339769906	0.734025812	0.080835678	10.19240944	90.18877719	0.5	PRJNA827465;Yang2025_Silkie_Wenchang	CRA004775	0.933401505
ENSGALG00010024370	HERPUD1	2	-0.258170492	0.173422319	-1.48868089	0.13657142	0.025302827	1.478518517	32.36472939	1	CRA004775;Yang2025_Silkie_Wenchang	PRJNA827465	0.618470274
ENSGALG00010024370	HERPUD1	2	-0.087583628	0.405851672	-0.215802062	0.829142041	0.288890432	7.23443294	86.17721654	0.5	CRA004775;PRJNA827465	Yang2025_Silkie_Wenchang	0.956860127
ENSGALG00010024371	YPEL1	2	-0.051599597	0.075648091	-0.682100455	0.495175433	0.002582698	1.233830926	18.95161814	1	PRJNA827465;Yang2025_Silkie_Wenchang	CRA004775	0.860619541
ENSGALG00010024371	YPEL1	2	-0.123970701	0.118517673	-1.046010256	0.295556297	0	0.761306215	0	0.5	CRA004775;Yang2025_Silkie_Wenchang	PRJNA827465	0.787628307
ENSGALG00010024371	YPEL1	2	0.004928688	0.072656677	0.067835309	0.945916736	0	0.157609594	0	0.5	CRA004775;PRJNA827465	Yang2025_Silkie_Wenchang	0.986321197
ENSGALG00010024373	TCAP	2	0.637486837	0.498500986	1.278807575	0.200964826	0.260299904	1.978532823	49.45749757	1	PRJNA827465;Yang2025_Silkie_Wenchang	CRA004775	0.713544434
ENSGALG00010024373	TCAP	2	0.97705813	0.492502946	1.98386251	0.047271169	0	0.587284788	0	1	CRA004775;Yang2025_Silkie_Wenchang	PRJNA827465	0.375330384
ENSGALG00010024373	TCAP	2	0.290424585	0.340778581	0.85223838	0.394081799	0	0.078397514	0	1	CRA004775;PRJNA827465	Yang2025_Silkie_Wenchang	0.830486298
ENSGALG00010024374	ENSGALG00010024374	2	-0.343065711	0.471926728	-0.726946983	0.467258434	0	0.846362507	0	0.5	PRJNA827465;Yang2025_Silkie_Wenchang	CRA004775	0.852885838
ENSGALG00010024374	ENSGALG00010024374	2	-0.182889396	0.538606143	-0.339560547	0.734187494	0.175078051	1.421256987	29.63974782	0.5	CRA004775;Yang2025_Silkie_Wenchang	PRJNA827465	0.933481207
ENSGALG00010024374	ENSGALG00010024374	2	0.360167094	0.531154697	0.678083233	0.497718921	0	0.024996883	0	1	CRA004775;PRJNA827465	Yang2025_Silkie_Wenchang	0.861055278
ENSGALG00010024377	CIB2	2	-0.199722253	0.135600827	-1.472868989	0.140786365	0	0.789834899	0	1	PRJNA827465;Yang2025_Silkie_Wenchang	CRA004775	0.625201272
ENSGALG00010024377	CIB2	2	-0.462181519	0.212670222	-2.173230999	0.029762936	0	0.427057451	0	1	CRA004775;Yang2025_Silkie_Wenchang	PRJNA827465	0.294469727
ENSGALG00010024377	CIB2	2	-0.279040511	0.25953756	-1.075145005	0.282309813	0.066224233	1.640260397	39.03407035	1	CRA004775;PRJNA827465	Yang2025_Silkie_Wenchang	0.779519046
ENSGALG00010024378	ZDHHC1	2	-0.051397046	0.059323523	-0.866385601	0.386278738	0	0.176795628	0	1	PRJNA827465;Yang2025_Silkie_Wenchang	CRA004775	0.826894808
ENSGALG00010024378	ZDHHC1	2	-0.005374055	0.104594534	-0.05137976	0.959022817	0	0.025870976	0	0.5	CRA004775;Yang2025_Silkie_Wenchang	PRJNA827465	0.989102387
ENSGALG00010024378	ZDHHC1	2	-0.061512191	0.068195309	-0.902000337	0.367056689	0	0.120567488	0	0.5	CRA004775;PRJNA827465	Yang2025_Silkie_Wenchang	0.819739734
ENSGALG00010024383	DUSP28	2	0.186148699	0.129154612	1.441285729	0.149503977	0	0.37061998	0	1	PRJNA827465;Yang2025_Silkie_Wenchang	CRA004775	0.639246608
ENSGALG00010024383	DUSP28	2	0.250550124	0.18403903	1.36139667	0.173388366	0	0.141083956	0	1	CRA004775;Yang2025_Silkie_Wenchang	PRJNA827465	0.675868485
ENSGALG00010024383	DUSP28	2	0.112187409	0.163382882	0.686653383	0.49230118	0	0.009541294	0	1	CRA004775;PRJNA827465	Yang2025_Silkie_Wenchang	0.859228604
ENSGALG00010024386	TPPP3	2	0.377384227	0.177371224	2.127651933	0.033365953	0	0.4533274	0	1	PRJNA827465;Yang2025_Silkie_Wenchang	CRA004775	0.313802836
ENSGALG00010024386	TPPP3	2	0.279992618	0.376899299	0.742884422	0.457551658	0.124948461	1.526752319	34.50149135	0.5	CRA004775;Yang2025_Silkie_Wenchang	PRJNA827465	0.849573219
ENSGALG00010024386	TPPP3	2	0.191925868	0.22486077	0.853532025	0.393364336	0	0.80682737	0	0.5	CRA004775;PRJNA827465	Yang2025_Silkie_Wenchang	0.830346712
ENSGALG00010024387	PPIL2	2	-0.024302944	0.070932116	-0.342622571	0.73188242	0.006593465	2.788946285	64.14416422	0.5	PRJNA827465;Yang2025_Silkie_Wenchang	CRA004775	0.932851968
ENSGALG00010024387	PPIL2	2	-0.100136594	0.067112133	-1.492078841	0.135678478	0	0.058204745	0	1	CRA004775;Yang2025_Silkie_Wenchang	PRJNA827465	0.617196137
ENSGALG00010024387	PPIL2	2	0.031312919	0.045425897	0.689318666	0.490625758	0	0.246228956	0	0.5	CRA004775;PRJNA827465	Yang2025_Silkie_Wenchang	0.858798485
ENSGALG00010024389	AAAS	2	-0.010443125	0.065267906	-0.160003987	0.872877933	0	0.004577752	0	0.5	PRJNA827465;Yang2025_Silkie_Wenchang	CRA004775	0.969664257
ENSGALG00010024389	AAAS	2	-0.090845001	0.129959643	-0.470797936	0.637785039	0	0.495628669	0	0.5	CRA004775;Yang2025_Silkie_Wenchang	PRJNA827465	0.905279419
ENSGALG00010024389	AAAS	2	-0.02245034	0.066560415	-0.337292669	0.735896286	0	0.632827958	0	1	CRA004775;PRJNA827465	Yang2025_Silkie_Wenchang	0.933592889
ENSGALG00010024391	TBC1D2B	2	-0.111828505	0.154096051	-0.725706491	0.468018722	0.035069072	3.686523698	72.8741741	0.5	PRJNA827465;Yang2025_Silkie_Wenchang	CRA004775	0.853145337
ENSGALG00010024391	TBC1D2B	2	-0.321227995	0.130245242	-2.466331898	0.013650478	0	0.831671039	0	1	CRA004775;Yang2025_Silkie_Wenchang	PRJNA827465	0.191338116
ENSGALG00010024391	TBC1D2B	2	-0.212194605	0.343179847	-0.618318956	0.536365109	0.166739162	2.634421749	62.04100576	0.5	CRA004775;PRJNA827465	Yang2025_Silkie_Wenchang	0.874599474
ENSGALG00010024392	CHMP4B	2	0.23204051	0.308812572	0.751395928	0.452414412	0.184096976	28.26617725	96.4622029	0.5	PRJNA827465;Yang2025_Silkie_Wenchang	CRA004775	0.848321192
ENSGALG00010024392	CHMP4B	2	0.435907642	0.148546412	2.934487851	0.003340986	0.023269763	1.957346302	48.91042024	1	CRA004775;Yang2025_Silkie_Wenchang	PRJNA827465	0.080906214
ENSGALG00010024392	CHMP4B	2	0.028753715	0.143888533	0.199833262	0.841610987	0.028295574	2.447141003	59.13598772	0.5	CRA004775;PRJNA827465	Yang2025_Silkie_Wenchang	0.959922457
ENSGALG00010024393	OGFOD1	2	0.058015127	0.06435106	0.901541134	0.367300673	0	0.674850569	0	1	PRJNA827465;Yang2025_Silkie_Wenchang	CRA004775	0.819739734
ENSGALG00010024393	OGFOD1	2	-0.083684792	0.25494286	-0.328249211	0.742722243	0.100186827	4.069838158	75.42599837	0.5	CRA004775;Yang2025_Silkie_Wenchang	PRJNA827465	0.935621057
ENSGALG00010024393	OGFOD1	2	-0.121019093	0.192330589	-0.629224363	0.529202181	0.051524223	2.775113143	63.96543317	0.5	CRA004775;PRJNA827465	Yang2025_Silkie_Wenchang	0.871837903
ENSGALG00010024396	ENSGALG00010024396	2	0.384084896	0.453341778	0.847230312	0.396866761	0.347217158	6.420652154	84.42525812	0.5	PRJNA827465;Yang2025_Silkie_Wenchang	CRA004775	0.831375233
ENSGALG00010024396	ENSGALG00010024396	2	0.408204398	0.621287922	0.657029348	0.511162046	0.505881918	2.399418448	58.3232345	0.5	CRA004775;Yang2025_Silkie_Wenchang	PRJNA827465	0.865649044
ENSGALG00010024396	ENSGALG00010024396	2	-0.090197012	0.223738347	-0.403136134	0.686848098	0	0.239179121	0	1	CRA004775;PRJNA827465	Yang2025_Silkie_Wenchang	0.919235539
ENSGALG00010024398	PHB	2	-0.041634125	0.057701199	-0.721546973	0.470573051	0	0.030698462	0	1	PRJNA827465;Yang2025_Silkie_Wenchang	CRA004775	0.853744788
ENSGALG00010024398	PHB	2	-0.079533088	0.172774998	-0.46032753	0.645281144	0	0.001159547	0	1	CRA004775;Yang2025_Silkie_Wenchang	PRJNA827465	0.906966303
ENSGALG00010024398	PHB	2	-0.04149903	0.057313561	-0.724070006	0.469022758	0	0.021579167	0	1	CRA004775;PRJNA827465	Yang2025_Silkie_Wenchang	0.85345437
ENSGALG00010024399	ROR1	2	-0.026267832	0.078187709	-0.335958586	0.736902097	0	0.971922027	0	0.5	PRJNA827465;Yang2025_Silkie_Wenchang	CRA004775	0.93386802
ENSGALG00010024399	ROR1	2	0.509782411	0.39323116	1.296393733	0.19483987	0.22403198	3.436375108	70.89956805	1	CRA004775;Yang2025_Silkie_Wenchang	PRJNA827465	0.706046493
ENSGALG00010024399	ROR1	2	0.390271691	0.506834179	0.770018493	0.441288923	0.449430405	7.194807262	86.10108703	0.5	CRA004775;PRJNA827465	Yang2025_Silkie_Wenchang	0.844907543
ENSGALG00010024401	KCNC1	2	-0.250193963	0.133348053	-1.876247588	0.060621229	0.014923145	1.70417913	41.32072285	1	PRJNA827465;Yang2025_Silkie_Wenchang	CRA004775	0.426454013
ENSGALG00010024401	KCNC1	2	-0.051296933	0.151319558	-0.338997373	0.734611709	0	0.831606071	0	0.5	CRA004775;Yang2025_Silkie_Wenchang	PRJNA827465	0.933520234
ENSGALG00010024401	KCNC1	2	-0.148222274	0.329608713	-0.449691615	0.652932818	0.140199117	2.221150002	54.97827705	0.5	CRA004775;PRJNA827465	Yang2025_Silkie_Wenchang	0.909572411
ENSGALG00010024402	GPC1	2	-0.00513771	0.074438273	-0.06901974	0.944971906	0	0.007155619	0	1	PRJNA827465;Yang2025_Silkie_Wenchang	CRA004775	0.986174277
ENSGALG00010024402	GPC1	2	0.019261944	0.112379671	0.171400613	0.863908773	0	0.448572335	0	0.5	CRA004775;Yang2025_Silkie_Wenchang	PRJNA827465	0.966447685
ENSGALG00010024402	GPC1	2	0.018924652	0.088911265	0.212848754	0.831444935	0	0.42054157	0	0.5	CRA004775;PRJNA827465	Yang2025_Silkie_Wenchang	0.957633345
ENSGALG00010024403	HMBS	2	0.040148535	0.059315331	0.676866063	0.498490938	0	0.565001368	0	0.5	PRJNA827465;Yang2025_Silkie_Wenchang	CRA004775	0.861478809
ENSGALG00010024403	HMBS	2	-0.252588392	0.230901137	-1.093924418	0.273988128	0.057681993	2.020051752	50.49631777	1	CRA004775;Yang2025_Silkie_Wenchang	PRJNA827465	0.774526342
ENSGALG00010024403	HMBS	2	-0.180799664	0.297826746	-0.607063221	0.543808957	0.141384651	4.139964866	75.84520564	0.5	CRA004775;PRJNA827465	Yang2025_Silkie_Wenchang	0.876968972
ENSGALG00010024406	NUDT21	2	-0.067103212	0.05984511	-1.121281462	0.262168074	0	0.000844809	0	1	PRJNA827465;Yang2025_Silkie_Wenchang	CRA004775	0.768363052
ENSGALG00010024406	NUDT21	2	-0.206136142	0.144264377	-1.428877637	0.153039406	0	0.848846685	0	1	CRA004775;Yang2025_Silkie_Wenchang	PRJNA827465	0.645187993
ENSGALG00010024406	NUDT21	2	-0.131707335	0.112162669	-1.174252863	0.240293758	0.012801918	1.606880903	37.76763431	1	CRA004775;PRJNA827465	Yang2025_Silkie_Wenchang	0.752771048
ENSGALG00010024407	HIRA	2	0.071345204	0.143064937	0.498691056	0.617997047	0.032499873	4.703220033	78.73797116	0.5	PRJNA827465;Yang2025_Silkie_Wenchang	CRA004775	0.899121942
ENSGALG00010024407	HIRA	2	0.09564966	0.17520512	0.545929595	0.585114334	0.035702157	2.165439735	53.82000322	0.5	CRA004775;Yang2025_Silkie_Wenchang	PRJNA827465	0.891086887
ENSGALG00010024407	HIRA	2	-0.066449477	0.067495563	-0.984501412	0.324869038	0	0.110054483	0	1	CRA004775;PRJNA827465	Yang2025_Silkie_Wenchang	0.799914637
ENSGALG00010024410	MYG1	2	0.054586483	0.064824141	0.842070287	0.399748612	0	0.085262823	0	0.5	PRJNA827465;Yang2025_Silkie_Wenchang	CRA004775	0.832246851
ENSGALG00010024410	MYG1	2	-0.210126092	0.202635137	-1.0369677	0.299750911	0	0.735012194	0	1	CRA004775;Yang2025_Silkie_Wenchang	PRJNA827465	0.79004225
ENSGALG00010024410	MYG1	2	-0.073493915	0.200304437	-0.366911068	0.713685355	0.053226734	2.279842198	56.13731508	0.5	CRA004775;PRJNA827465	Yang2025_Silkie_Wenchang	0.926292039
ENSGALG00010024412	PIGU	2	0.09886562	0.18518296	0.533880765	0.593424031	0.051016532	3.438895918	70.92089951	0.5	PRJNA827465;Yang2025_Silkie_Wenchang	CRA004775	0.892896317
ENSGALG00010024412	PIGU	2	-0.152582417	0.478159878	-0.319103345	0.749648147	0.424539835	13.957729	92.8355107	0.5	CRA004775;Yang2025_Silkie_Wenchang	PRJNA827465	0.937362187
ENSGALG00010024412	PIGU	2	-0.317861537	0.287793929	-1.104476175	0.269386633	0.149251143	9.656090279	89.64384165	1	CRA004775;PRJNA827465	Yang2025_Silkie_Wenchang	0.77189992
ENSGALG00010024413	AMFR	2	-0.250167968	0.221290124	-1.130497662	0.258266583	0.088000153	9.314455039	89.26399885	1	PRJNA827465;Yang2025_Silkie_Wenchang	CRA004775	0.765554168
ENSGALG00010024413	AMFR	2	-0.436986235	0.120348387	-3.631010332	0.000282314	0	0.693198372	0	1	CRA004775;Yang2025_Silkie_Wenchang	PRJNA827465	0.016365461
ENSGALG00010024413	AMFR	2	-0.053798806	0.050075606	-1.07435156	0.282665144	0	0.595494209	0	1	CRA004775;PRJNA827465	Yang2025_Silkie_Wenchang	0.779532565
ENSGALG00010024415	SIK2	2	-0.015377869	0.039768205	-0.386687529	0.69898755	0	0.080283157	0	1	PRJNA827465;Yang2025_Silkie_Wenchang	CRA004775	0.92185597
ENSGALG00010024415	SIK2	2	-0.043950964	0.112292349	-0.391397676	0.69550331	0	0.008919197	0	1	CRA004775;Yang2025_Silkie_Wenchang	PRJNA827465	0.921038437
ENSGALG00010024415	SIK2	2	-0.012023057	0.041252222	-0.291452348	0.770705383	0	0.002510157	0	1	CRA004775;PRJNA827465	Yang2025_Silkie_Wenchang	0.942491331
ENSGALG00010024416	VDR	2	0.206278172	0.240487175	0.857751238	0.39102984	0	0.070895642	0	1	PRJNA827465;Yang2025_Silkie_Wenchang	CRA004775	0.829251057
ENSGALG00010024416	VDR	2	-0.028011387	0.300123709	-0.093332803	0.925639173	0.042573242	1.308723368	23.58965813	0.5	CRA004775;Yang2025_Silkie_Wenchang	PRJNA827465	0.982594411
ENSGALG00010024416	VDR	2	-0.045072426	0.235052396	-0.191754802	0.847934276	0	0.976329124	0	0.5	CRA004775;PRJNA827465	Yang2025_Silkie_Wenchang	0.961328089
ENSGALG00010024419	PGAP3	2	0.056175251	0.104799209	0.536027432	0.591939591	0.010412989	1.739715305	42.51933078	0.5	PRJNA827465;Yang2025_Silkie_Wenchang	CRA004775	0.892776493
ENSGALG00010024419	PGAP3	2	-0.131596617	0.345946494	-0.380395867	0.703651582	0.209265718	7.885955936	87.31922917	0.5	CRA004775;Yang2025_Silkie_Wenchang	PRJNA827465	0.923639641
ENSGALG00010024419	PGAP3	2	-0.219825464	0.23294392	-0.943684057	0.345331124	0.088397146	4.923931377	79.69102484	1	CRA004775;PRJNA827465	Yang2025_Silkie_Wenchang	0.807217149
ENSGALG00010024420	TMEM106C	2	-0.104359076	0.174605754	-0.59768406	0.550050764	0.035790808	2.183816109	54.20859861	0.5	PRJNA827465;Yang2025_Silkie_Wenchang	CRA004775	0.878652984
ENSGALG00010024420	TMEM106C	2	-0.496481513	0.181457147	-2.736081325	0.006217566	0.010091661	1.177697309	15.08853828	1	CRA004775;Yang2025_Silkie_Wenchang	PRJNA827465	0.1195543
ENSGALG00010024420	TMEM106C	2	-0.301053693	0.362738293	-0.829947371	0.40656854	0.227279953	6.898526746	85.50415129	0.5	CRA004775;PRJNA827465	Yang2025_Silkie_Wenchang	0.833618122
ENSGALG00010024421	DAPK2	2	0.195181798	0.082577515	2.363619173	0.018097405	0.002389441	1.205530826	17.04898968	1	PRJNA827465;Yang2025_Silkie_Wenchang	CRA004775	0.224591825
ENSGALG00010024421	DAPK2	2	0.293227949	0.114941807	2.551099176	0.010738376	0	0.000512347	0	1	CRA004775;Yang2025_Silkie_Wenchang	PRJNA827465	0.165236469
ENSGALG00010024421	DAPK2	2	0.131452925	0.09487656	1.385515302	0.165894957	0	0.120136189	0	1	CRA004775;PRJNA827465	Yang2025_Silkie_Wenchang	0.66529196
ENSGALG00010024423	SERGEF	2	-0.127222637	0.079749189	-1.595284396	0.110648653	0	0.552348038	0	1	PRJNA827465;Yang2025_Silkie_Wenchang	CRA004775	0.562832796
ENSGALG00010024423	SERGEF	2	-0.191586129	0.109474284	-1.75005601	0.080108649	0	0.001082166	0	1	CRA004775;Yang2025_Silkie_Wenchang	PRJNA827465	0.487008546
ENSGALG00010024423	SERGEF	2	-0.088299716	0.099949138	-0.8834465	0.376995077	0	0.136563026	0	1	CRA004775;PRJNA827465	Yang2025_Silkie_Wenchang	0.823064945
ENSGALG00010024424	SAMHD1	2	-0.091711117	0.053850267	-1.703076349	0.088553781	0	0.149457403	0	1	PRJNA827465;Yang2025_Silkie_Wenchang	CRA004775	0.511076251
ENSGALG00010024424	SAMHD1	2	-0.097575443	0.072098527	-1.353362497	0.175939852	0	0.512008323	0	0.5	CRA004775;Yang2025_Silkie_Wenchang	PRJNA827465	0.681008865
ENSGALG00010024424	SAMHD1	2	-0.058036822	0.074630319	-0.777657432	0.436770993	0	0.320590469	0	0.5	CRA004775;PRJNA827465	Yang2025_Silkie_Wenchang	0.843375996
ENSGALG00010024425	MRPL40	2	-0.085376936	0.110298638	-0.774052495	0.438899748	0.011035754	1.683579351	40.60274025	0.5	PRJNA827465;Yang2025_Silkie_Wenchang	CRA004775	0.844002503
ENSGALG00010024425	MRPL40	2	0.030655397	0.131344514	0.233396858	0.815453253	0	0.19053252	0	0.5	CRA004775;Yang2025_Silkie_Wenchang	PRJNA827465	0.954009864
ENSGALG00010024425	MRPL40	2	-0.151564013	0.080579124	-1.880933989	0.059980899	0	0.187742351	0	1	CRA004775;PRJNA827465	Yang2025_Silkie_Wenchang	0.424074343
ENSGALG00010024426	PHAF1	2	-0.163775332	0.061950776	-2.64363648	0.008202069	0	0.161105049	0	1	PRJNA827465;Yang2025_Silkie_Wenchang	CRA004775	0.140757277
ENSGALG00010024426	PHAF1	2	-0.183776347	0.09286199	-1.979026579	0.047813014	0	0.144469128	0	1	CRA004775;Yang2025_Silkie_Wenchang	PRJNA827465	0.378107622
ENSGALG00010024426	PHAF1	2	-0.13842831	0.078966437	-1.753001842	0.079601691	0	0.06037388	0	1	CRA004775;PRJNA827465	Yang2025_Silkie_Wenchang	0.48542321
ENSGALG00010024427	C22orf39	2	-0.131923447	0.090883403	-1.451568086	0.146621738	0	0.24939807	0	0.5	PRJNA827465;Yang2025_Silkie_Wenchang	CRA004775	0.635558525
ENSGALG00010024427	C22orf39	2	-0.175278595	0.238262796	-0.735652391	0.46194227	0	0.66347272	0	0.5	CRA004775;Yang2025_Silkie_Wenchang	PRJNA827465	0.850747865
ENSGALG00010024427	C22orf39	2	-0.160236545	0.090214184	-1.776179076	0.075703406	0	0.381260946	0	1	CRA004775;PRJNA827465	Yang2025_Silkie_Wenchang	0.475071205
ENSGALG00010024428	SAAL1	2	-0.145175619	0.191676251	-0.757400137	0.448810174	0.068466654	14.20714275	92.96128703	0.5	PRJNA827465;Yang2025_Silkie_Wenchang	CRA004775	0.847038957
ENSGALG00010024428	SAAL1	2	-0.331145562	0.08576022	-3.861295601	0.000112787	0	0.167027349	0	1	CRA004775;Yang2025_Silkie_Wenchang	PRJNA827465	0.008443583
ENSGALG00010024428	SAAL1	2	-0.02828561	0.120806035	-0.234140704	0.814875747	0.017284607	1.717235697	41.76687558	0.5	CRA004775;PRJNA827465	Yang2025_Silkie_Wenchang	0.953897709
ENSGALG00010024430	ZNF652	2	-0.023034214	0.03046022	-0.756206424	0.449525442	0	0.943758179	0	0.5	PRJNA827465;Yang2025_Silkie_Wenchang	CRA004775	0.847263197
ENSGALG00010024430	ZNF652	2	0.170214679	0.125531705	1.355949708	0.17511517	0	0.07926701	0	1	CRA004775;Yang2025_Silkie_Wenchang	PRJNA827465	0.680094025
ENSGALG00010024430	ZNF652	2	0.008792547	0.070899144	0.124014856	0.901303512	0.005325085	1.505988565	33.59843339	0.5	CRA004775;PRJNA827465	Yang2025_Silkie_Wenchang	0.976619209
ENSGALG00010024431	FEZ1	2	0.136352044	0.067965262	2.006201998	0.044834701	0	0.269414179	0	1	PRJNA827465;Yang2025_Silkie_Wenchang	CRA004775	0.36505868
ENSGALG00010024431	FEZ1	2	0.132122625	0.143660422	0.919687018	0.357736338	0.003129603	1.053614393	5.088616204	0.5	CRA004775;Yang2025_Silkie_Wenchang	PRJNA827465	0.813616765
ENSGALG00010024431	FEZ1	2	0.103470749	0.07374513	1.403085853	0.160591239	0	0.693199206	0	0.5	CRA004775;PRJNA827465	Yang2025_Silkie_Wenchang	0.65630165
ENSGALG00010024433	ENSGALG00010024433	2	-0.161093118	0.070360994	-2.289523052	0.02204898	0	0.091488809	0	1	PRJNA827465;Yang2025_Silkie_Wenchang	CRA004775	0.25198926
ENSGALG00010024433	ENSGALG00010024433	2	-0.146491394	0.123531245	-1.185865119	0.23567557	0	0.215074588	0	1	CRA004775;Yang2025_Silkie_Wenchang	PRJNA827465	0.748626601
ENSGALG00010024433	ENSGALG00010024433	2	-0.181009141	0.082013605	-2.207062375	0.027309702	0	0.132419148	0	1	CRA004775;PRJNA827465	Yang2025_Silkie_Wenchang	0.28183501
ENSGALG00010024434	B3GNT9	2	0.040675576	0.106473715	0.382024575	0.702443137	0.014050451	2.601630208	61.56256193	0.5	PRJNA827465;Yang2025_Silkie_Wenchang	CRA004775	0.923191989
ENSGALG00010024434	B3GNT9	2	-0.075580685	0.099487612	-0.759699462	0.44743425	0	0.000838293	0	1	CRA004775;Yang2025_Silkie_Wenchang	PRJNA827465	0.84650962
ENSGALG00010024434	B3GNT9	2	0.128610266	0.081295869	1.582002476	0.113649005	0	0.332792108	0	0.5	CRA004775;PRJNA827465	Yang2025_Silkie_Wenchang	0.568794964
ENSGALG00010024437	HERC1	2	-0.095594497	0.037156517	-2.572751844	0.010089352	0	0.099622073	0	1	PRJNA827465;Yang2025_Silkie_Wenchang	CRA004775	0.159574167
ENSGALG00010024437	HERC1	2	-0.044839515	0.11473639	-0.390804656	0.695941633	0	0.104304913	0	0.5	CRA004775;Yang2025_Silkie_Wenchang	PRJNA827465	0.921038437
ENSGALG00010024437	HERC1	2	-0.097173626	0.038717453	-2.509814569	0.012079457	0	0.217021772	0	0.5	CRA004775;PRJNA827465	Yang2025_Silkie_Wenchang	0.177082773
ENSGALG00010024438	RBL1	2	0.011337261	0.045488078	0.249235887	0.803178321	0	0.146049188	0	1	PRJNA827465;Yang2025_Silkie_Wenchang	CRA004775	0.950951274
ENSGALG00010024438	RBL1	2	-0.158299022	0.327519788	-0.48332659	0.628863863	0.138881545	2.053626588	51.30565578	0.5	CRA004775;Yang2025_Silkie_Wenchang	PRJNA827465	0.902084638
ENSGALG00010024438	RBL1	2	-0.164636352	0.296483194	-0.555297419	0.578691244	0.112767999	1.874717987	46.65864376	0.5	CRA004775;PRJNA827465	Yang2025_Silkie_Wenchang	0.88788081
ENSGALG00010024439	UFD1	2	-0.027899209	0.1150264	-0.242546134	0.808357014	0.017946692	2.525747431	60.40775939	0.5	PRJNA827465;Yang2025_Silkie_Wenchang	CRA004775	0.952496413
ENSGALG00010024439	UFD1	2	0.092012878	0.134661465	0.683290334	0.494423401	0	0.517566819	0	0.5	CRA004775;Yang2025_Silkie_Wenchang	PRJNA827465	0.860175061
ENSGALG00010024439	UFD1	2	-0.109831702	0.043681188	-2.514393669	0.011923725	0	0.003672868	0	1	CRA004775;PRJNA827465	Yang2025_Silkie_Wenchang	0.175322923
ENSGALG00010024440	PKNOX2	2	-0.082553297	0.083311606	-0.990897916	0.32173543	0	0.110665696	0	1	PRJNA827465;Yang2025_Silkie_Wenchang	CRA004775	0.798513866
ENSGALG00010024440	PKNOX2	2	-0.075167613	0.154400648	-0.486834831	0.626375385	0	0.523353143	0	1	CRA004775;Yang2025_Silkie_Wenchang	PRJNA827465	0.901504244
ENSGALG00010024440	PKNOX2	2	-0.111151746	0.094718516	-1.173495436	0.240597186	0	0.381083153	0	1	CRA004775;PRJNA827465	Yang2025_Silkie_Wenchang	0.753158164
ENSGALG00010024441	SAA	2	0.316459604	0.297240288	1.06465919	0.287030184	0	0.117260552	0	1	PRJNA827465;Yang2025_Silkie_Wenchang	CRA004775	0.782566585
ENSGALG00010024441	SAA	2	-0.535817186	0.723083299	-0.741017233	0.458682991	0.781876092	3.96183453	74.75916794	0.5	CRA004775;Yang2025_Silkie_Wenchang	PRJNA827465	0.850089963
ENSGALG00010024441	SAA	2	-0.395856204	0.828617761	-0.477730773	0.632841839	1.170156273	6.662536907	84.99070228	0.5	CRA004775;PRJNA827465	Yang2025_Silkie_Wenchang	0.903112631
ENSGALG00010024446	GRM4	2	0.509271331	0.760218203	0.669901521	0.50292057	0.973731013	6.315965165	84.1671071	0.5	PRJNA827465;Yang2025_Silkie_Wenchang	CRA004775	0.863050365
ENSGALG00010024446	GRM4	2	1.137436558	0.40684753	2.795731755	0.005178236	0	0.460363193	0	1	CRA004775;Yang2025_Silkie_Wenchang	PRJNA827465	0.107436917
ENSGALG00010024446	GRM4	2	-0.073417608	0.349546448	-0.210036772	0.833638973	0	0.91363022	0	0.5	CRA004775;PRJNA827465	Yang2025_Silkie_Wenchang	0.957968425
ENSGALG00010024449	CACHD1	2	-0.040747435	0.125154005	-0.325578354	0.744743391	0.018682583	2.295113321	56.42916666	0.5	PRJNA827465;Yang2025_Silkie_Wenchang	CRA004775	0.935893304
ENSGALG00010024449	CACHD1	2	0.111799058	0.12122458	0.922247431	0.356399528	0	0.004789553	0	1	CRA004775;Yang2025_Silkie_Wenchang	PRJNA827465	0.812521698
ENSGALG00010024449	CACHD1	2	-0.097222206	0.091383752	-1.063889401	0.287378805	0.003397621	1.134319658	11.84142911	0.5	CRA004775;PRJNA827465	Yang2025_Silkie_Wenchang	0.782840067
ENSGALG00010024451	PHOSPHO1	2	0.092962988	0.229283642	0.405449717	0.685146972	0.039863126	1.603062065	37.61938345	0.5	PRJNA827465;Yang2025_Silkie_Wenchang	CRA004775	0.919196979
ENSGALG00010024451	PHOSPHO1	2	-0.173636948	0.174813852	-0.993267671	0.320579528	0	0.122670319	0	1	CRA004775;Yang2025_Silkie_Wenchang	PRJNA827465	0.798348216
ENSGALG00010024451	PHOSPHO1	2	0.043910298	0.29147913	0.150646456	0.880254613	0.099229464	2.401225186	58.35459306	0.5	CRA004775;PRJNA827465	Yang2025_Silkie_Wenchang	0.971588195
ENSGALG00010024452	SLC16A1	2	-0.043043001	0.21852447	-0.19697108	0.843850169	0.07279099	3.971351368	74.81965438	0.5	PRJNA827465;Yang2025_Silkie_Wenchang	CRA004775	0.96057435
ENSGALG00010024452	SLC16A1	2	-0.18770015	0.164254206	-1.142741818	0.253145802	0.000495969	1.007675415	0.761695176	0.5	CRA004775;Yang2025_Silkie_Wenchang	PRJNA827465	0.762731469
ENSGALG00010024452	SLC16A1	2	0.138822401	0.100403834	1.38264044	0.166775143	0	0.062569623	0	1	CRA004775;PRJNA827465	Yang2025_Silkie_Wenchang	0.665766006
ENSGALG00010024453	HTR3A	2	0.499896283	0.780563798	0.640429756	0.521893243	1.043122395	6.85078881	85.40314075	0.5	PRJNA827465;Yang2025_Silkie_Wenchang	CRA004775	0.86956507
ENSGALG00010024453	HTR3A	2	0.09377445	1.223414562	0.076649774	0.938902161	2.699607173	9.839374573	89.8367524	0.5	CRA004775;Yang2025_Silkie_Wenchang	PRJNA827465	0.985349045
ENSGALG00010024453	HTR3A	2	-0.620563491	0.420629847	-1.47531968	0.140126618	0.030381322	1.083371457	7.695555952	1	CRA004775;PRJNA827465	Yang2025_Silkie_Wenchang	0.623855512
ENSGALG00010024454	HPS5	2	-0.096062692	0.113924478	-0.843213806	0.399108878	0.018041047	3.021533533	66.904223	1	PRJNA827465;Yang2025_Silkie_Wenchang	CRA004775	0.832054308
ENSGALG00010024454	HPS5	2	-0.213223312	0.110236794	-1.934229984	0.053084857	0	0.230715262	0	1	CRA004775;Yang2025_Silkie_Wenchang	PRJNA827465	0.399631766
ENSGALG00010024454	HPS5	2	-0.005679792	0.059653022	-0.095213814	0.924144998	0	0.070633295	0	1	CRA004775;PRJNA827465	Yang2025_Silkie_Wenchang	0.98230395
ENSGALG00010024457	CHD6	2	-0.044838459	0.045865219	-0.977613529	0.328265504	0	0.471005967	0	1	PRJNA827465;Yang2025_Silkie_Wenchang	CRA004775	0.800761292
ENSGALG00010024457	CHD6	2	-0.118104813	0.113189101	-1.043429196	0.296749558	0	0.015550401	0	1	CRA004775;Yang2025_Silkie_Wenchang	PRJNA827465	0.788458494
ENSGALG00010024457	CHD6	2	-0.034306342	0.048462848	-0.707889511	0.479013874	0	0.042776805	0	1	CRA004775;PRJNA827465	Yang2025_Silkie_Wenchang	0.855875059
ENSGALG00010024459	AHCYL1	2	-0.010689725	0.057127239	-0.18712134	0.851565497	0	0.020736491	0	0.5	PRJNA827465;Yang2025_Silkie_Wenchang	CRA004775	0.96214473
ENSGALG00010024459	AHCYL1	2	-0.02830771	0.105831542	-0.26747895	0.78910042	0	0.226222231	0	0.5	CRA004775;Yang2025_Silkie_Wenchang	PRJNA827465	0.947224171
ENSGALG00010024459	AHCYL1	2	-0.02382269	0.060493544	-0.393805496	0.693724647	0	0.19435336	0	1	CRA004775;PRJNA827465	Yang2025_Silkie_Wenchang	0.920721644
ENSGALG00010024460	IMP4	2	-0.062343612	0.087104422	-0.715734184	0.474155499	0	0.110051103	0	1	PRJNA827465;Yang2025_Silkie_Wenchang	CRA004775	0.85444425
ENSGALG00010024460	IMP4	2	-0.209432077	0.184647637	-1.134225597	0.256699929	0	0.123663853	0	1	CRA004775;Yang2025_Silkie_Wenchang	PRJNA827465	0.764677554
ENSGALG00010024460	IMP4	2	-0.076922964	0.087061786	-0.883544525	0.376942137	0	0.644507709	0	1	CRA004775;PRJNA827465	Yang2025_Silkie_Wenchang	0.823064945
ENSGALG00010024462	ZBTB32	2	-0.609319038	0.387455701	-1.57261601	0.115807758	0.255779456	6.703031746	85.08137753	1	PRJNA827465;Yang2025_Silkie_Wenchang	CRA004775	0.572808488
ENSGALG00010024462	ZBTB32	2	-0.204211366	0.219668932	-0.929632442	0.352561424	0	0.001686372	0	1	CRA004775;Yang2025_Silkie_Wenchang	PRJNA827465	0.810998575
ENSGALG00010024462	ZBTB32	2	-0.788229636	0.343998932	-2.291372336	0.021941888	0.122239822	1.613076761	38.00666997	1	CRA004775;PRJNA827465	Yang2025_Silkie_Wenchang	0.25105736
ENSGALG00010024463	FBXL22	2	0.023331613	0.123915729	0.188286128	0.850652359	0	0.070962305	0	0.5	PRJNA827465;Yang2025_Silkie_Wenchang	CRA004775	0.961985357
ENSGALG00010024463	FBXL22	2	-0.030126325	0.194801519	-0.154651386	0.877096157	0.006520894	1.056954876	5.388581596	0.5	CRA004775;Yang2025_Silkie_Wenchang	PRJNA827465	0.970509861
ENSGALG00010024463	FBXL22	2	-0.051538164	0.147757811	-0.348801623	0.727238245	0	0.843281051	0	1	CRA004775;PRJNA827465	Yang2025_Silkie_Wenchang	0.931522817
ENSGALG00010024465	SENP1	2	0.21285294	0.148145882	1.436779326	0.150780707	0.028976434	2.662369751	62.4394771	1	PRJNA827465;Yang2025_Silkie_Wenchang	CRA004775	0.642051813
ENSGALG00010024465	SENP1	2	0.111947502	0.299427416	0.373871917	0.708499611	0.144762607	5.160956829	80.62374802	0.5	CRA004775;Yang2025_Silkie_Wenchang	PRJNA827465	0.9250668
ENSGALG00010024465	SENP1	2	0.007598305	0.134999854	0.056283804	0.955115721	0.019402762	1.808720372	44.71229408	0.5	CRA004775;PRJNA827465	Yang2025_Silkie_Wenchang	0.988118398
ENSGALG00010024467	ENSGALG00010024467	2	-0.035287406	0.17354323	-0.203334961	0.838873231	0	0.614418951	0	0.5	PRJNA827465;Yang2025_Silkie_Wenchang	CRA004775	0.959283372
ENSGALG00010024467	ENSGALG00010024467	2	0.039085652	0.212011879	0.184355953	0.853734222	0	0.272017736	0	0.5	CRA004775;Yang2025_Silkie_Wenchang	PRJNA827465	0.962975689
ENSGALG00010024467	ENSGALG00010024467	2	-0.137931815	0.191182288	-0.721467538	0.470621906	0	0.012229016	0	1	CRA004775;PRJNA827465	Yang2025_Silkie_Wenchang	0.853744788
ENSGALG00010024469	GTF2H1	2	0.222917049	0.366407389	0.608385792	0.542931632	0.255729229	20.6137458	95.14886809	0.5	PRJNA827465;Yang2025_Silkie_Wenchang	CRA004775	0.876968972
ENSGALG00010024469	GTF2H1	2	0.39320493	0.252306866	1.55843928	0.119129151	0.085408197	2.825090266	64.60290093	1	CRA004775;Yang2025_Silkie_Wenchang	PRJNA827465	0.579471011
ENSGALG00010024469	GTF2H1	2	-0.118915465	0.066422604	-1.790286101	0.073407929	0	0.623330229	0	0.5	CRA004775;PRJNA827465	Yang2025_Silkie_Wenchang	0.468064869
ENSGALG00010024471	CCDC61	2	0.072318655	0.248219814	0.291349242	0.770784231	0.107401127	7.641920055	86.91428342	0.5	PRJNA827465;Yang2025_Silkie_Wenchang	CRA004775	0.942491331
ENSGALG00010024471	CCDC61	2	0.050470898	0.305939267	0.164970316	0.868967346	0.151299516	5.091251797	80.35846507	0.5	CRA004775;Yang2025_Silkie_Wenchang	PRJNA827465	0.9684491
ENSGALG00010024471	CCDC61	2	-0.18155277	0.090075451	-2.015563275	0.043845663	0	0.222903439	0	1	CRA004775;PRJNA827465	Yang2025_Silkie_Wenchang	0.361804689
ENSGALG00010024472	LDHA	2	-0.387135982	0.180962331	-2.139318059	0.032409918	0	0.005781113	0	1	PRJNA827465;Yang2025_Silkie_Wenchang	CRA004775	0.309308824

ENSGALG00000024488	ADGRG5	2	0.294875378	0.296317863	0.995131968	0.319672085	0	0.022018921	0	1	PRJNA827465;Yang2025_Silkie_Wenchang	CRA004775	0.798328994
ENSGALG00000024488	ADGRG5	2	-0.344565053	0.386828997	-0.890742566	0.373067294	0.110729795	1.476122231	32.2549326	0.5	CRA004775;Yang2025_Silkie_Wenchang	PRJNA827465	0.822162024
ENSGALG00000024488	ADGRG5	2	-0.148971016	0.465267434	-0.320183631	0.748829131	0.319857437	3.827639038	73.87423448	0.5	CRA004775;PRJNA827465	Yang2025_Silkie_Wenchang	0.937249951
ENSGALG00000024490	TGM3	2	0.207351905	0.690243987	0.300403783	0.763869178	0.599550104	2.696187861	62.9105963	0.5	PRJNA827465;Yang2025_Silkie_Wenchang	CRA004775	0.940361611
ENSGALG00000024490	TGM3	2	-0.272283657	0.430753873	-0.632109596	0.527315265	0	0.267368481	0	1	CRA004775;Yang2025_Silkie_Wenchang	PRJNA827465	0.871149047
ENSGALG00000024490	TGM3	2	0.440531031	0.467151035	0.94301628	0.345672576	0.077771435	1.216388038	17.78939216	0.5	CRA004775;PRJNA827465	Yang2025_Silkie_Wenchang	0.807376412
ENSGALG00000024493	JAK1	2	-0.041218224	0.092942345	-0.443481645	0.657417392	0.01168426	2.989042869	66.54447447	0.5	PRJNA827465;Yang2025_Silkie_Wenchang	CRA004775	0.910711339
ENSGALG00000024493	JAK1	2	0.089224786	0.084147702	1.060335381	0.288992048	0	0.607169202	0	1	CRA004775;Yang2025_Silkie_Wenchang	PRJNA827465	0.783530627
ENSGALG00000024493	JAK1	2	0.00117396	0.180027417	0.006521007	0.994797026	0.044190278	2.47433187	59.58504952	0.5	CRA004775;PRJNA827465	Yang2025_Silkie_Wenchang	0.998242335
ENSGALG00000024494	DDAH1	2	0.25660765	0.65073709	0.394333832	0.693334588	0.768151188	10.73854745	90.68775359	0.5	PRJNA827465;Yang2025_Silkie_Wenchang	CRA004775	0.920721644
ENSGALG00000024494	DDAH1	2	0.789957369	0.260493663	3.03253968	0.002425052	0	0.752703445	0	1	CRA004775;Yang2025_Silkie_Wenchang	PRJNA827465	0.06593704
ENSGALG00000024494	DDAH1	2	-0.146763969	0.355852538	-0.412429176	0.680024882	0.115031541	1.640359457	39.03775202	0.5	CRA004775;PRJNA827465	Yang2025_Silkie_Wenchang	0.917701868
ENSGALG00000024495	CLDN5	2	0.275831184	0.245718259	1.12255062	0.261628397	0.098754941	5.330258948	81.23918538	1	PRJNA827465;Yang2025_Silkie_Wenchang	CRA004775	0.768310285
ENSGALG00000024495	CLDN5	2	0.567313343	0.165085218	3.436487832	0.000589309	0	0.113522128	0	1	CRA004775;Yang2025_Silkie_Wenchang	PRJNA827465	0.026171305
ENSGALG00000024495	CLDN5	2	0.253278916	0.29899663	0.847096222	0.396941491	0.118070495	2.338299923	57.23388646	1	CRA004775;PRJNA827465	Yang2025_Silkie_Wenchang	0.831432073
ENSGALG00000024496	UEVLD	2	-0.228626386	0.135861952	-1.682784492	0.092416818	0.026147783	3.328725675	69.95847368	1	PRJNA827465;Yang2025_Silkie_Wenchang	CRA004775	0.520543885
ENSGALG00000024496	UEVLD	2	0.359614928	0.775453136	0.463748112	0.642828223	1.133701868	16.8395688	94.06160566	0.5	CRA004775;Yang2025_Silkie_Wenchang	PRJNA827465	0.906634523
ENSGALG00000024496	UEVLD	2	0.48534348	0.637851124	0.760904013	0.446714401	0.750727922	12.13324951	91.75818482	0.5	CRA004775;PRJNA827465	Yang2025_Silkie_Wenchang	0.84648396
ENSGALG00000024497	ABI3	2	-0.021487877	0.169897495	-0.126475536	0.899355513	0	0.678167435	0	0.5	PRJNA827465;Yang2025_Silkie_Wenchang	CRA004775	0.976081354
ENSGALG00000024497	ABI3	2	0.20979552	0.201411327	1.041627217	0.297584545	0	0.65122705	0	1	CRA004775;Yang2025_Silkie_Wenchang	PRJNA827465	0.788562929
ENSGALG00000024497	ABI3	2	0.084879122	0.316221535	0.26841664	0.788378632	0.103422858	1.968871204	49.20947608	0.5	CRA004775;PRJNA827465	Yang2025_Silkie_Wenchang	0.94704362
ENSGALG00000024498	CCDC102A	2	0.325591511	0.211118729	1.54221993	0.123020159	0.083496091	15.66782232	93.61749208	1	PRJNA827465;Yang2025_Silkie_Wenchang	CRA004775	0.58801364
ENSGALG00000024498	CCDC102A	2	0.263486993	0.317231599	0.830582432	0.406209563	0.170245267	5.929364718	83.13478682	0.5	CRA004775;Yang2025_Silkie_Wenchang	PRJNA827465	0.833477131
ENSGALG00000024498	CCDC102A	2	0.106978635	0.058769313	1.820314532	0.06871112	0	0.728527822	0	0.5	CRA004775;PRJNA827465	Yang2025_Silkie_Wenchang	0.451522009
ENSGALG00000024499	CA12	2	0.065819892	0.579449921	0.113590303	0.909562575	0	0.091664291	0	0.5	PRJNA827465;Yang2025_Silkie_Wenchang	CRA004775	0.978607662
ENSGALG00000024499	CA12	2	-0.829077972	1.061458843	-0.781074064	0.434758935	1.31656131	2.371297864	57.82900093	0.5	CRA004775;Yang2025_Silkie_Wenchang	PRJNA827465	0.842762754
ENSGALG00000024499	CA12	2	-0.840967373	0.868757834	-0.968011268	0.333038742	0.921941966	2.539529079	60.62262062	1	CRA004775;PRJNA827465	Yang2025_Silkie_Wenchang	0.802178578
ENSGALG00000024500	GNGT2	2	-0.399004675	0.206771845	-1.929685708	0.053645792	0	0.77839216	0	1	PRJNA827465;Yang2025_Silkie_Wenchang	CRA004775	0.401684237
ENSGALG00000024500	GNGT2	2	-0.115690531	0.231914564	-0.498849788	0.61788521	0	0.050159037	0	1	CRA004775;Yang2025_Silkie_Wenchang	PRJNA827465	0.899121942
ENSGALG00000024500	GNGT2	2	-0.341008704	0.236483867	-1.441995636	0.149303605	0.027358403	1.313883935	23.88977647	1	CRA004775;PRJNA827465	Yang2025_Silkie_Wenchang	0.639246608
ENSGALG00000024501	ARL8A	2	0.008249931	0.061910165	0.133256487	0.893990539	0	0.014916621	0	0.5	PRJNA827465;Yang2025_Silkie_Wenchang	CRA004775	0.974381724
ENSGALG00000024501	ARL8A	2	-0.035791171	0.124109347	-0.288384168	0.773052685	0	0.087277879	0	1	CRA004775;Yang2025_Silkie_Wenchang	PRJNA827465	0.943100418
ENSGALG00000024501	ARL8A	2	0.002425004	0.064376916	0.037668847	0.969951715	0	0.194931042	0	0.5	CRA004775;PRJNA827465	Yang2025_Silkie_Wenchang	0.991572914
ENSGALG00000024502	VPS8	2	-0.119201707	0.03599914	-3.311237608	0.000928843	0	0.553194662	0	1	PRJNA827465;Yang2025_Silkie_Wenchang	CRA004775	0.035966816
ENSGALG00000024502	VPS8	2	-0.14703139	0.05198625	-2.828274568	0.004679965	0	0.014418996	0	1	CRA004775;Yang2025_Silkie_Wenchang	PRJNA827465	0.10067688
ENSGALG00000024502	VPS8	2	-0.095728936	0.047777862	-2.003625358	0.045110207	0	0.010840229	0	1	CRA004775;PRJNA827465	Yang2025_Silkie_Wenchang	0.366012899
ENSGALG00000024504	CASC3	2	0.121560022	0.154577347	0.786402567	0.43163168	0.042687001	9.235431284	89.17213534	0.5	PRJNA827465;Yang2025_Silkie_Wenchang	CRA004775	0.842503929
ENSGALG00000024504	CASC3	2	0.201022118	0.143822203	1.397712686	0.162199363	0.020186982	1.562010265	35.97993415	0.5	CRA004775;Yang2025_Silkie_Wenchang	PRJNA827465	0.659645143
ENSGALG00000024504	CASC3	2	-0.027951636	0.055233664	-0.506061583	0.612813419	0	0.009626219	0	1	CRA004775;PRJNA827465	Yang2025_Silkie_Wenchang	0.898034557
ENSGALG00000024505	DNASE2B	2	-0.495835996	0.297910789	-1.664377437	0.096037026	0.160489142	10.36542936	90.35254629	1	PRJNA827465;Yang2025_Silkie_Wenchang	CRA004775	0.530281432
ENSGALG00000024505	DNASE2B	2	-0.813313935	0.143338649	-5.674072827	1.39442E-08	0	0.105066887	0	1	CRA004775;Yang2025_Silkie_Wenchang	PRJNA827465	6.73616E-06
ENSGALG00000024505	DNASE2B	2	-0.389705996	0.334975647	-1.163386055	0.244672905	0.133623966	1.736513506	42.41334739	1	CRA004775;PRJNA827465	Yang2025_Silkie_Wenchang	0.756093682
ENSGALG00000024506	RAB8B	2	-0.170503697	0.158013588	-1.079044524	0.28056788	0.04530642	10.67735722	90.63438659	1	PRJNA827465;Yang2025_Silkie_Wenchang	CRA004775	0.778536485
ENSGALG00000024506	RAB8B	2	-0.255055448	0.118585711	-2.15081097	0.031491123	0.013956947	1.727177755	42.10207969	1	CRA004775;Yang2025_Silkie_Wenchang	PRJNA827465	0.303579986
ENSGALG00000024506	RAB8B	2	-0.022618716	0.052987677	-0.426867481	0.669475852	0	0.097957717	0	1	CRA004775;PRJNA827465	Yang2025_Silkie_Wenchang	0.914377769
ENSGALG00000024508	RPS27L	2	-0.229865431	0.093361001	-2.462114037	0.013812074	0	0.59013888	0	1	PRJNA827465;Yang2025_Silkie_Wenchang	CRA004775	0.192817251
ENSGALG00000024508	RPS27L	2	-0.160234499	0.308713991	-0.519038669	0.603733776	0.093503834	1.895197867	47.23506091	0.5	CRA004775;Yang2025_Silkie_Wenchang	PRJNA827465	0.895856405
ENSGALG00000024508	RPS27L	2	-0.152447813	0.134886031	-1.130197186	0.258393145	0.010611093	1.139053932	12.20784444	0.5	CRA004775;PRJNA827465	Yang2025_Silkie_Wenchang	0.765653331
ENSGALG00000024509	SEPTIN5	2	-0.131983735	0.067102036	-1.966911017	0.04919347	0	6.82498E-05	0	1	PRJNA827465;Yang2025_Silkie_Wenchang	CRA004775	0.383545122
ENSGALG00000024509	SEPTIN5	2	-0.144621902	0.096438281	-1.499631684	0.133709836	0	0.222076223	0	1	CRA004775;Yang2025_Silkie_Wenchang	PRJNA827465	0.612847937
ENSGALG00000024509	SEPTIN5	2	-0.141594192	0.088166585	-1.605984773	0.108277256	0	0.227361903	0	1	CRA004775;PRJNA827465	Yang2025_Silkie_Wenchang	0.556717702
ENSGALG00000024510	CNN3	2	-0.213274193	0.193422317	-1.102634877	0.270185758	0.072200474	28.39750879	96.47856434	1	PRJNA827465;Yang2025_Silkie_Wenchang	CRA004775	0.772430311
ENSGALG00000024510	CNN3	2	-0.085344416	0.354620114	-0.240664341	0.809815278	0.22701282	9.537627752	89.51521252	0.5	CRA004775;Yang2025_Silkie_Wenchang	PRJNA827465	0.952818762
ENSGALG00000024510	CNN3	2	0.066796101	0.145032052	0.460560962	0.645113625	0.027246278	2.06153929	51.4925568	0.5	CRA004775;PRJNA827465	Yang2025_Silkie_Wenchang	0.906966303
ENSGALG00000024511	ENSGALG00000024511	2	0.001943262	0.071125181	0.027321709	0.978203142	0	0.974190433	0	0.5	PRJNA827465;Yang2025_Silkie_Wenchang	CRA004775	0.993932889
ENSGALG00000024511	ENSGALG00000024511	2	0.094930494	0.144532231	0.65681193	0.511301852	0	0.97273746	0	0.5	CRA004775;Yang2025_Silkie_Wenchang	PRJNA827465	0.865658376
ENSGALG00000024511	ENSGALG00000024511	2	-0.044179158	0.080146664	-0.551228907	0.581476767	0	0.52418491	0	1	CRA004775;PRJNA827465	Yang2025_Silkie_Wenchang	0.889045686
ENSGALG00000024512	CCNDBP1	2	-0.092222651	0.191768893	-0.480905163	0.630583903	0.058416682	4.343282663	76.97594014	0.5	PRJNA827465;Yang2025_Silkie_Wenchang	CRA004775	0.902756437
ENSGALG00000024512	CCNDBP1	2	-0.334501612	0.147287443	-2.271080314	0.023142116	0	0.019397232	0	1	CRA004775;Yang2025_Silkie_Wenchang	PRJNA827465	0.259577892
ENSGALG00000024512	CCNDBP1	2	-0.069220315	0.202135287	-0.34244548	0.732015667	0.055640951	2.435026077	58.93267799	0.5	CRA004775;PRJNA827465	Yang2025_Silkie_Wenchang	0.93287651
ENSGALG00000024513	SPTY2D1	2	0.187083024	0.16032881	1.166870906	0.243262507	0.039661759	3.944707843	74.64958015	1	PRJNA827465;Yang2025_Silkie_Wenchang	CRA004775	0.755238542
ENSGALG00000024513	SPTY2D1	2	0.333525239	0.133961569	2.489708362	0.012784795	0	0.414012315	0	1	CRA004775;Yang2025_Silkie_Wenchang	PRJNA827465	0.183473076
ENSGALG00000024513	SPTY2D1	2	0.058839158	0.061074871	0.963393886	0.335349893	0	0.163769918	0	1	CRA004775;PRJNA827465	Yang2025_Silkie_Wenchang	0.803198384
ENSGALG00000024514	FAF1	2	-0.155070403	0.186283033	-0.832445126	0.40515774	0.064989242	15.5006405	93.54865368	0.5	PRJNA827465;Yang2025_Silkie_Wenchang	CRA004775	0.833112102
ENSGALG00000024514	FAF1	2	-0.270230175	0.133627756	-2.022260821	0.043149414	0.017134106	1.561061798	35.94103697	1	CRA004775;Yang2025_Silkie_Wenchang	PRJNA827465	0.359301147
ENSGALG00000024514	FAF1	2	0.022861978	0.047006776	0.486354944	0.62671553	0	0.072143206	0	0.5	CRA004775;PRJNA827465	Yang2025_Silkie_Wenchang	0.901504244
ENSGALG00000024515	C3orf70	2	-0.082139101	0.063436369	-1.294826648	0.195380037	0	0.872843453	0	1	PRJNA827465;Yang2025_Silkie_Wenchang	CRA004775	0.70643959
ENSGALG00000024515	C3orf70	2	-0.134193554	0.08859835	-1.514628146	0.1298666	0	0.196722427	0	0.5	CRA004775;Yang2025_Silkie_Wenchang	PRJNA827465	0.604541867
ENSGALG00000024515	C3orf70	2	-0.020869426	0.087007242	-0.23985849	0.810439962	0	0.029517752	0	0.5	CRA004775;PRJNA827465	Yang2025_Silkie_Wenchang	0.952997426
ENSGALG00000024516	EHHADH	2	-0.040200252	0.420976636	-0.095492834	0.923923382	0.308086803	7.562583119	86.77700484	0.5	PRJNA827465;Yang2025_Silkie_Wenchang	CRA004775	0.98222777
ENSGALG00000024516	EHHADH	2	-0.282174585	0.226708567	-1.244657797	0.213257565	0.045226097	1.650355932	39.40701028	0.5	CRA004775;Yang2025_Silkie_Wenchang	PRJNA827465	0.725873568
ENSGALG00000024516	EHHADH	2	0.265411769	0.197406289	1.344494999	0.178788373	0	0.786610589	0	1	CRA004775;PRJNA827465	Yang2025_Silkie_Wenchang	0.68554543
ENSGALG00000024517	LACTB	2	-0.065714844	0.109827903	-0.598343794	0.549610561	0.016019777	2.721498926	63.2555431	0.5	PRJNA827465;Yang2025_Silkie_Wenchang	CRA004775	0.878554604
ENSGALG00000024517	LACTB	2	0.112416626	0.101840164	1.103853548	0.269656672	0	0.379547245	0	1	CRA004775;Yang2025_Silkie_Wenchang	PRJNA827465	0.77189992
ENSGALG00000024517	LACTB	2	-0.015253	0.175912288	-0.086707984	0.93090363	0.046332663	3.417220075	70.73644723	0.5	CRA004775;PRJNA827465	Yang2025_Silkie_Wenchang	0.983943003
ENSGALG00000024519	MIEN1	2	0.131016774	0.133488862	0.981480942	0.326355628	0.018485566	1.932773018	48.26086711	1	PRJNA827465;Yang2025_Silkie_Wenchang	CRA004775	0.800028239
ENSGALG00000024519	MIEN1	2	0.06864508	0.270404627	0.253860598	0.799603256	0.099491836	3.019743987	66.88460994	0.5	CRA004775;Yang2025_Silkie_Wenchang	PRJNA827465	0.949922217
ENSGALG00000024519	MIEN1	2	-0.004197447	0.092247468	-0.04550203	0.963707157	0	0.921655917	0	0.5	CRA004775;PRJNA827465	Yang2025_Silkie_Wenchang	0.990159788
ENSGALG00000024520	MAP3K13	2	-0.072767096	0.119502999	-0.60891439	0.542581186	0	0.071441919	0	1	PRJNA827465;Yang2025_Silkie_Wenchang	CRA004775	0.876817086
ENSGALG00000024520	MAP3K13	2	0.001372577	0.165068946	0.008315171	0.99336553	0	0.072911759	0	0.5	CRA004775;Yang2025_Silkie_Wenchang	PRJNA827465	0.997979742
ENSGALG00000024520	MAP3K13	2	-0.062587206	0.132217265	-0.47336636	0.635951826	0	0.247795009	0	0.5	CRA004775;PRJNA827465	Yang2025_Silkie_Wenchang	0.904429535
ENSGALG00000024521	HEPACAM	2	0.822378455	0.612710964	1.342196408	0.179532321	0.595012536	4.458014573	77.56848966	1	PRJNA827465;Yang2025_Silkie_Wenchang	CRA004775	0.686445472
ENSGALG00000024521	HEPACAM	2	1.309293469	0.352447904	3.714856732	0.000203319	0	0.310477718	0	1	CRA004775;Yang2025_Silkie_Wenchang	PRJNA827465	0.012909598
ENSGALG00000024521	HEPACAM	2	0.619348525	0.410478215	1.508846275	0.131338072	0.214438559	2.524182072	60.38320646	1	CRA004775;PRJNA827465	Yang2025_Silkie_Wenchang	0.607072924
ENSGALG00000024522	DNTTIP2	2	-0.108502639	0.055853148	-1.942641411	0.0520595	0	0.363658356	0	1	PRJNA827465;Yang2025_Silkie_Wenchang	CRA004775	0.395839528
ENSGALG00000024522	DNTTIP2	2	-0.027496957	0.092021678	-0.298809558	0.765085357	0	0.328435873	0	0.5	CRA004775;Yang2025_Silkie_Wenchang	PRJNA827465	0.940779391
ENSGALG00000024522	DNTTIP2	2	-0.109316305	0.062703295	-1.743390128	0.081265492	0	0.948605313	0	0.5	CRA004775;PRJNA827465	Yang2025_Silkie_Wenchang	0.491003477
ENSGALG00000024523	TOX3	2	0.417505165	0.356382506	1.171508586	0.241394409	0.186363941	3.3569294	70.21087188	1	PRJNA827465;Yang2025_Silkie_Wenchang	CRA004775	0.753935509
ENSGALG00000024523	TOX3	2	0.900327682	0.324151664	2.777489006	0.005478069	0	0.063541999	0	1	CRA004775;Yang2025_Silkie_Wenchang	PRJNA827465	0.111211032
ENSGALG00000024523	TOX3	2	0.34351197	0.391091862	0.878340878	0.379758758	0.17505263	1.687583818	40.74368399	1	CRA004775;PRJNA827465	Yang2025_Silkie_Wenchang	0.824361963
ENSGALG00000024524	TMEM41A	2	0.112649659	0.091269487	1.234253229	0.217108558	0	0.015629162	0	1	PRJNA827465;Yang2025_Silkie_Wenchang	CRA004775	0.730610701
ENSGALG00000024524	TMEM41A	2	0.076584529	0.164964691	0.464248006	0.642470071	0	0.444553625	0	0.5	CRA004775;Yang2025_Silkie_Wenchang	PRJNA827465	0.906634523
ENSGALG00000024524	TMEM41A	2	0.087277739	0.100746463	0.866310702	0.386319799	0	0.420883833	0	0.5	CRA004775;PRJNA827465	Yang2025_Silkie_Wenchang	0.826894808
ENSGALG00000024525	GP1BB	2	0.623627493	0.330446357	1.887227621	0.059129717	0.163999032	4.011271028	75.07024599	1	PRJNA827465;Yang2025_Silkie_Wenchang	CRA004775	0.421162606
ENSGALG00000024525	GP1BB	2	0.382255426	0.631125846	0.605672273	0.5447324	0.673811362	6.221588353	83.92693403	0.5	CRA004775;Yang2025_Silkie_Wenchang	PRJNA827465	0.877345822
ENSGALG00000024525	GP1BB	2	0.119048385	0.276985036	0.429800782	0.667340564	0.05861811	1.468569236	31.90651311	0.5	CRA004775;PRJNA827465	Yang2025_Silkie_Wenchang	0.913852694
ENSGALG00000024527	ATP6V0B	2	-0.282308478	0.365347693	-0.772711812	0.439692955	0.232518067	6.994092373	85.70221915	0.5	PRJNA827465;Yang2025_Silkie_Wenchang	CRA004775	0.844305853
ENSGALG00000024527	ATP6V0B	2	-0.632630871	0.183451565	-3.448489908	0.000563731	0	0.105775003	0	1	CRA004775;Yang2025_Silkie_Wenchang	PRJNA827465	0.025467013
ENSGALG00000024527	ATP6V0B	2	-0.222171031	0.304867549	-0.728746078	0.466157001	0.15691118	5.700546624	82.45782263	0.5	CRA004775;PRJNA827465	Yang2025_Silkie_Wenchang	0.852631007

ENSGALG00010024554	ST6GALNAC3	2	-0.075895723	0.083675554	-0.907023845	0.364394188	0	0.273447257	0	1	CRA004775;PRJNA827465	Yang2025_Silkie_Wenchang	0.817496251
ENSGALG00010024555	EPHB1	2	-0.091835152	0.066947119	-1.371756588	0.170139226	0.003182606	1.505861971	33.5928512	1	PRJNA827465;Yang2025_Silkie_Wenchang	CRA004775	0.671788425
ENSGALG00010024555	EPHB1	2	-0.108930985	0.108809303	-1.001118305	0.316769617	0.009025259	1.154894982	13.41204045	0.5	CRA004775;Yang2025_Silkie_Wenchang	PRJNA827465	0.796959041
ENSGALG00010024555	EPHB1	2	0.00905467	0.088848239	0.101911638	0.918826812	0	0.434421619	0	0.5	CRA004775;PRJNA827465	Yang2025_Silkie_Wenchang	0.981033557
ENSGALG00010024556	ENSGALG00010024556	2	0.087672957	0.130281361	0.672950884	0.500978539	0	0.988643494	0	1	PRJNA827465;Yang2025_Silkie_Wenchang	CRA004775	0.862421093
ENSGALG00010024556	ENSGALG00010024556	2	0.267770706	0.250428127	1.069251722	0.284956258	0	0.301890079	0	1	CRA004775;Yang2025_Silkie_Wenchang	PRJNA827465	0.780497609
ENSGALG00010024556	ENSGALG00010024556	2	0.034935703	0.135269205	0.258267969	0.796200105	0	0.017574731	0	1	CRA004775;PRJNA827465	Yang2025_Silkie_Wenchang	0.949325935
ENSGALG00010024557	TSPAN32	2	-0.078626505	0.28084256	-0.279966489	0.779503215	0.076477444	1.868820097	46.49030148	0.5	PRJNA827465;Yang2025_Silkie_Wenchang	CRA004775	0.944807046
ENSGALG00010024557	TSPAN32	2	-0.34881898	0.296694479	-1.175684095	0.239721137	0	0.167504658	0	1	CRA004775;Yang2025_Silkie_Wenchang	PRJNA827465	0.751936141
ENSGALG00010024557	TSPAN32	2	0.106089291	0.216134014	0.490849584	0.623532837	0	0.242370069	0	0.5	CRA004775;PRJNA827465	Yang2025_Silkie_Wenchang	0.900444194
ENSGALG00010024558	PFDN5	2	-0.147704734	0.044799693	-3.297003272	0.000977224	0	0.00029664	0	1	PRJNA827465;Yang2025_Silkie_Wenchang	CRA004775	0.037252978
ENSGALG00010024558	PFDN5	2	-0.214314498	0.195062167	-1.098698439	0.271899624	0	0.221994043	0	1	CRA004775;Yang2025_Silkie_Wenchang	PRJNA827465	0.773184343
ENSGALG00010024558	PFDN5	2	-0.150994165	0.045238407	-3.337742767	0.000844619	0	0.333039925	0	1	CRA004775;PRJNA827465	Yang2025_Silkie_Wenchang	0.033804543
ENSGALG00010024561	ROBO3	2	0.710723706	0.328844515	2.161275838	0.030674038	0.098799277	1.838583886	45.61031413	1	PRJNA827465;Yang2025_Silkie_Wenchang	CRA004775	0.298814255
ENSGALG00010024561	ROBO3	2	0.444069151	0.669888522	0.662900075	0.507394554	0.692968595	4.256847899	76.50843949	0.5	CRA004775;Yang2025_Silkie_Wenchang	PRJNA827465	0.864149944
ENSGALG00010024561	ROBO3	2	0.188855288	0.314914642	0.599703104	0.548704119	0.032264394	1.157997809	13.6440508	0.5	CRA004775;PRJNA827465	Yang2025_Silkie_Wenchang	0.878281266
ENSGALG00010024563	ESPL1	2	-0.036860777	0.099112529	-0.371908345	0.709961091	0	0.000168427	0	1	PRJNA827465;Yang2025_Silkie_Wenchang	CRA004775	0.925298042
ENSGALG00010024563	ESPL1	2	-0.125628811	0.193912436	-0.647863613	0.517073167	0	0.889793165	0	1	CRA004775;Yang2025_Silkie_Wenchang	PRJNA827465	0.867952013
ENSGALG00010024563	ESPL1	2	-0.071424718	0.124779692	-0.572406592	0.567046551	0.004318718	1.039437447	3.794114533	1	CRA004775;PRJNA827465	Yang2025_Silkie_Wenchang	0.883487517
ENSGALG00010024564	PPP1R3D	2	-0.127018394	0.097708793	-1.299968918	0.193611622	0	0.251289553	0	1	PRJNA827465;Yang2025_Silkie_Wenchang	CRA004775	0.703672794
ENSGALG00010024564	PPP1R3D	2	-0.041237555	0.162707138	-0.253446501	0.799923198	0	0.043381983	0	0.5	CRA004775;Yang2025_Silkie_Wenchang	PRJNA827465	0.949922217
ENSGALG00010024564	PPP1R3D	2	-0.1467453	0.114000551	-1.287233253	0.198013	0	0.1974114	0	0.5	CRA004775;PRJNA827465	Yang2025_Silkie_Wenchang	0.710212169
ENSGALG00010024565	FAM217B	2	-0.076756097	0.112984752	-0.679349165	0.496916652	0.015287342	2.13564173	53.17566679	0.5	PRJNA827465;Yang2025_Silkie_Wenchang	CRA004775	0.860939846
ENSGALG00010024565	FAM217B	2	0.129410947	0.110523271	1.170893197	0.24164171	0	0.196162613	0	1	CRA004775;Yang2025_Silkie_Wenchang	PRJNA827465	0.75404876
ENSGALG00010024565	FAM217B	2	-0.020327129	0.165730007	-0.122652073	0.902382617	0.042176602	3.831216839	73.89863216	0.5	CRA004775;PRJNA827465	Yang2025_Silkie_Wenchang	0.976923607
ENSGALG00010024566	MTSS2	2	0.317435171	0.288373875	1.100776455	0.270993962	0.128302993	3.93913407	74.61370996	1	PRJNA827465;Yang2025_Silkie_Wenchang	CRA004775	0.772743863
ENSGALG00010024566	MTSS2	2	0.247558164	0.453465745	0.545924729	0.585117679	0.304055899	3.758123003	73.39097206	0.5	CRA004775;Yang2025_Silkie_Wenchang	PRJNA827465	0.891086887
ENSGALG00010024566	MTSS2	2	0.051682763	0.107368455	0.481358914	0.630261432	0	0.662822906	0	0.5	CRA004775;PRJNA827465	Yang2025_Silkie_Wenchang	0.902650914
ENSGALG00010024568	DZIP1L	2	0.294496992	0.27152719	1.084594851	0.278101136	0.093577159	2.645353387	62.19786721	1	PRJNA827465;Yang2025_Silkie_Wenchang	CRA004775	0.776763074
ENSGALG00010024568	DZIP1L	2	0.51823984	0.236698908	2.189447535	0.028564328	0	0.360653512	0	1	CRA004775;Yang2025_Silkie_Wenchang	PRJNA827465	0.287842636
ENSGALG00010024568	DZIP1L	2	0.094428996	0.173803902	0.54330769	0.58691797	0	0.231934582	0	1	CRA004775;PRJNA827465	Yang2025_Silkie_Wenchang	0.891596903
ENSGALG00010024569	ASCL2	2	-0.094814645	0.170752228	-0.555276183	0.578705767	0	0.990456392	0	0.5	PRJNA827465;Yang2025_Silkie_Wenchang	CRA004775	0.88788081
ENSGALG00010024569	ASCL2	2	-0.262556697	0.245381595	-1.06999344	0.284622262	0	0.1604406	0	1	CRA004775;Yang2025_Silkie_Wenchang	PRJNA827465	0.780265906
ENSGALG00010024569	ASCL2	2	0.014989471	0.196962154	0.076103304	0.939336911	0	0.052163783	0	0.5	CRA004775;PRJNA827465	Yang2025_Silkie_Wenchang	0.985393654
ENSGALG00010024570	TPM1	2	0.068799507	0.213130557	0.322804521	0.746843289	0.074894074	5.686492629	82.41446766	0.5	PRJNA827465;Yang2025_Silkie_Wenchang	CRA004775	0.936729365
ENSGALG00010024570	TPM1	2	0.275437409	0.129568627	2.125803253	0.033519645	0	0.14191021	0	1	CRA004775;Yang2025_Silkie_Wenchang	PRJNA827465	0.314546348
ENSGALG00010024570	TPM1	2	-0.134616273	0.11815445	-1.139324616	0.254567785	0	0.076940474	0	0.5	CRA004775;PRJNA827465	Yang2025_Silkie_Wenchang	0.764286735
ENSGALG00010024571	KCNA3	2	0.539381096	0.440088769	1.22561886	0.220342137	0.045082919	1.121868354	10.86298174	1	PRJNA827465;Yang2025_Silkie_Wenchang	CRA004775	0.734031787
ENSGALG00010024571	KCNA3	2	0.27984259	0.75244919	0.371908952	0.70996064	0.782441439	3.163967344	68.39411121	0.5	CRA004775;Yang2025_Silkie_Wenchang	PRJNA827465	0.925298042
ENSGALG00010024571	KCNA3	2	-0.109210826	0.356486656	-0.306353196	0.75933574	0	0.711136725	0	0.5	CRA004775;PRJNA827465	Yang2025_Silkie_Wenchang	0.939145717
ENSGALG00010024573	ENSGALG00010024573	2	0.577166205	0.791123185	0.729552889	0.465663528	1.191877343	20.87290011	95.2090989	0.5	PRJNA827465;Yang2025_Silkie_Wenchang	CRA004775	0.852424459
ENSGALG00010024573	ENSGALG00010024573	2	0.384656698	1.016713979	0.378333244	0.705183053	1.944108509	16.59161458	93.97285903	0.5	CRA004775;Yang2025_Silkie_Wenchang	PRJNA827465	0.924245236
ENSGALG00010024573	ENSGALG00010024573	2	-0.303961175	0.195654274	-1.553562662	0.120288771	0	0.899456774	0	1	CRA004775;PRJNA827465	Yang2025_Silkie_Wenchang	0.581657183
ENSGALG00010024575	TXNRD2	2	0.188873342	0.101881555	1.853852181	0.063760311	0.009477527	1.685460269	40.66902563	1	PRJNA827465;Yang2025_Silkie_Wenchang	CRA004775	0.434988977
ENSGALG00010024575	TXNRD2	2	-0.061721377	0.42348975	-0.145744678	0.88412295	0.313215785	7.619644587	86.87602829	0.5	CRA004775;Yang2025_Silkie_Wenchang	PRJNA827465	0.972001076
ENSGALG00010024575	TXNRD2	2	-0.147152593	0.312185274	-0.471363017	0.637381521	0.160896832	5.061422442	80.24270822	0.5	CRA004775;PRJNA827465	Yang2025_Silkie_Wenchang	0.905152693
ENSGALG00010024576	CSAD	2	0.249536442	0.238563961	1.045993874	0.295563861	0.077743052	3.099465754	67.73637526	0.5	PRJNA827465;Yang2025_Silkie_Wenchang	CRA004775	0.787628307
ENSGALG00010024576	CSAD	2	-0.105689912	0.17864613	-0.591616016	0.554107747	0	0.567341885	0	1	CRA004775;Yang2025_Silkie_Wenchang	PRJNA827465	0.8801009
ENSGALG00010024576	CSAD	2	0.130662431	0.380111074	0.343748025	0.731035791	0.231224792	4.728086812	78.84979613	0.5	CRA004775;PRJNA827465	Yang2025_Silkie_Wenchang	0.932769533
ENSGALG00010024579	ZNF740	2	0.041792737	0.131768228	0.31716854	0.75111572	0.024064217	2.906642275	65.59604157	0.5	PRJNA827465;Yang2025_Silkie_Wenchang	CRA004775	0.937683866
ENSGALG00010024579	ZNF740	2	0.010134833	0.222017477	0.045648806	0.963590168	0.0675525	3.092459667	67.66328077	0.5	CRA004775;Yang2025_Silkie_Wenchang	PRJNA827465	0.990117239
ENSGALG00010024579	ZNF740	2	-0.077650446	0.061496607	-1.262678544	0.206704728	0	0.650453844	0	1	CRA004775;PRJNA827465	Yang2025_Silkie_Wenchang	0.71868063
ENSGALG00010024580	IGF2	2	0.086081993	0.100243	0.858733216	0.39048772	0	0.154865283	0	1	PRJNA827465;Yang2025_Silkie_Wenchang	CRA004775	0.829251057
ENSGALG00010024580	IGF2	2	0.057249548	0.172031126	0.332785985	0.739295838	0.004062217	1.047946491	4.575280479	0.5	CRA004775;Yang2025_Silkie_Wenchang	PRJNA827465	0.93477525
ENSGALG00010024580	IGF2	2	0.028206801	0.113971775	0.247489358	0.804529527	0	0.747928367	0	0.5	CRA004775;PRJNA827465	Yang2025_Silkie_Wenchang	0.951665484
ENSGALG00010024581	RARA	2	0.108831712	0.13016521	0.836104453	0.403096143	0.026723011	4.587816031	78.20313646	0.5	PRJNA827465;Yang2025_Silkie_Wenchang	CRA004775	0.833046091
ENSGALG00010024581	RARA	2	0.171524434	0.144395387	1.18788029	0.234880576	0.016673455	1.403055251	28.72696931	0.5	CRA004775;Yang2025_Silkie_Wenchang	PRJNA827465	0.747791642
ENSGALG00010024581	RARA	2	-0.01442028	0.063884267	-0.225725059	0.821415267	0	0.082180262	0	1	CRA004775;PRJNA827465	Yang2025_Silkie_Wenchang	0.955598716
ENSGALG00010024582	TLN2	2	-0.035075088	0.059800552	-0.586534522	0.557516369	0	0.873007581	0	0.5	PRJNA827465;Yang2025_Silkie_Wenchang	CRA004775	0.880678789
ENSGALG00010024582	TLN2	2	0.166239048	0.358848552	0.463256844	0.643180276	0.197936209	3.672686438	72.77197449	0.5	CRA004775;Yang2025_Silkie_Wenchang	PRJNA827465	0.906634523
ENSGALG00010024582	TLN2	2	0.204850193	0.289640434	0.707256892	0.47940685	0.119504393	2.683512315	62.73540485	1	CRA004775;PRJNA827465	Yang2025_Silkie_Wenchang	0.85611011
ENSGALG00010024585	TBCCD1	2	-0.003640003	0.077761843	-0.046809625	0.962664957	0	0.029758518	0	0.5	PRJNA827465;Yang2025_Silkie_Wenchang	CRA004775	0.989980504
ENSGALG00010024585	TBCCD1	2	-0.29097525	0.315145399	-0.923304771	0.355848402	0.158471715	4.551585024	78.02963155	1	CRA004775;Yang2025_Silkie_Wenchang	PRJNA827465	0.812301302
ENSGALG00010024585	TBCCD1	2	-0.276163599	0.328722638	-0.84011129	0.400845991	0.178271547	5.191222477	80.73671463	0.5	CRA004775;PRJNA827465	Yang2025_Silkie_Wenchang	0.832353691
ENSGALG00010024586	DLGAP4	2	0.015741791	0.045673742	0.344657343	0.730351991	0	0.458992741	0	1	PRJNA827465;Yang2025_Silkie_Wenchang	CRA004775	0.932617298
ENSGALG00010024586	DLGAP4	2	0.13164165	0.105728274	1.245094093	0.213097165	0	0.314916704	0	1	CRA004775;Yang2025_Silkie_Wenchang	PRJNA827465	0.72579791
ENSGALG00010024586	DLGAP4	2	0.031404101	0.073637286	0.426470108	0.669765325	0.00378831	1.18037801	15.28137667	1	CRA004775;PRJNA827465	Yang2025_Silkie_Wenchang	0.914377769
ENSGALG00010024587	ENSGALG00010024587	2	-0.166013892	0.26995855	-0.614960672	0.538580693	0.122634055	5.924537193	83.12104444	0.5	PRJNA827465;Yang2025_Silkie_Wenchang	CRA004775	0.87481821
ENSGALG00010024587	ENSGALG00010024587	2	-0.248192352	0.243684893	-1.018497082	0.308441787	0.066346913	2.234445032	55.24615795	0.5	CRA004775;Yang2025_Silkie_Wenchang	PRJNA827465	0.793457188
ENSGALG00010024587	ENSGALG00010024587	2	0.069594791	0.087661009	0.793908167	0.427248895	0	0.037586449	0	1	CRA004775;PRJNA827465	Yang2025_Silkie_Wenchang	0.84125685
ENSGALG00010024588	TTC39A	2	0.789435584	0.305241718	2.58626373	0.009702266	0	0.480045157	0	1	PRJNA827465;Yang2025_Silkie_Wenchang	CRA004775	0.155237398
ENSGALG00010024588	TTC39A	2	0.982864739	0.36988124	2.657244091	0.007878238	0	0.029246771	0	1	CRA004775;Yang2025_Silkie_Wenchang	PRJNA827465	0.137800793
ENSGALG00010024588	TTC39A	2	0.693283673	0.33897479	2.045236675	0.04083153	0	0.159929667	0	1	CRA004775;PRJNA827465	Yang2025_Silkie_Wenchang	0.349629987
ENSGALG00010024589	ADORA1	2	0.001130966	0.188372258	0.006003889	0.995209618	0	0.004990093	0	0.5	PRJNA827465;Yang2025_Silkie_Wenchang	CRA004775	0.998242335
ENSGALG00010024589	ADORA1	2	0.297099378	0.367491969	0.808451349	0.418830802	0.165840447	2.47016648	59.51689863	0.5	CRA004775;Yang2025_Silkie_Wenchang	PRJNA827465	0.838250071
ENSGALG00010024589	ADORA1	2	0.318897087	0.355338507	0.897445903	0.369481028	0.138408965	2.147594134	53.43626693	1	CRA004775;PRJNA827465	Yang2025_Silkie_Wenchang	0.820606801
ENSGALG00010024590	MRPL23	2	-0.23207308	0.339620974	-0.683329645	0.494398566	0.186842579	4.659548302	78.53869227	0.5	PRJNA827465;Yang2025_Silkie_Wenchang	CRA004775	0.860175061
ENSGALG00010024590	MRPL23	2	-0.343014277	0.259362165	-1.322530126	0.185991684	0.061225912	1.811610247	44.80048886	1	CRA004775;Yang2025_Silkie_Wenchang	PRJNA827465	0.695002013
ENSGALG00010024590	MRPL23	2	0.029121973	0.090918195	0.320309627	0.748733626	0	0.409875997	0	0.5	CRA004775;PRJNA827465	Yang2025_Silkie_Wenchang	0.937249951
ENSGALG00010024592	COMT	2	-0.122375055	0.122863211	-0.996026837	0.319237106	0.013936305	1.704831179	41.3431657	1	PRJNA827465;Yang2025_Silkie_Wenchang	CRA004775	0.798079048
ENSGALG00010024592	COMT	2	-0.342059093	0.154793308	-2.209779587	0.027120463	0	0.342632746	0	1	CRA004775;Yang2025_Silkie_Wenchang	PRJNA827465	0.280945645
ENSGALG00010024592	COMT	2	-0.168750858	0.215106432	-0.784499359	0.432747158	0.055413764	1.935138721	48.3241181	1	CRA004775;PRJNA827465	Yang2025_Silkie_Wenchang	0.842762754
ENSGALG00010024593	SRSF6	2	0.127391242	0.140431696	0.907140238	0.364332642	0.036202111	11.92081321	91.61131055	0.5	PRJNA827465;Yang2025_Silkie_Wenchang	CRA004775	0.817496251
ENSGALG00010024593	SRSF6	2	0.00739453	0.285850363	0.025868536	0.979362197	0.147794578	10.00375732	90.0037559	0.5	CRA004775;Yang2025_Silkie_Wenchang	PRJNA827465	0.994390773
ENSGALG00010024593	SRSF6	2	-0.108155762	0.138906741	-0.77862141	0.436202763	0.027923115	2.900340914	65.52129458	1	CRA004775;PRJNA827465	Yang2025_Silkie_Wenchang	0.843122822
ENSGALG00010024594	TNNI2	2	-0.162618927	0.108135552	-1.503843324	0.132621713	0	0.133547671	0	1	PRJNA827465;Yang2025_Silkie_Wenchang	CRA004775	0.609997798
ENSGALG00010024594	TNNI2	2	-0.092803518	0.18700361	-0.496265917	0.61970681	0	0.057414728	0	0.5	CRA004775;Yang2025_Silkie_Wenchang	PRJNA827465	0.899325257
ENSGALG00010024594	TNNI2	2	-0.181501108	0.128665731	-1.410640627	0.158350606	0	0.125584579	0	0.5	CRA004775;PRJNA827465	Yang2025_Silkie_Wenchang	0.652042151
ENSGALG00010024595	MYBPHL	2	0.209598529	0.394517221	0.53127853	0.595225781	0.268108025	7.123864743	85.96267565	0.5	PRJNA827465;Yang2025_Silkie_Wenchang	CRA004775	0.893657226
ENSGALG00010024595	MYBPHL	2	0.550577409	0.227213835	2.423168506	0.015385788	0	0.868241436	0	0.5	CRA004775;Yang2025_Silkie_Wenchang	PRJNA827465	0.205165677
ENSGALG00010024595	MYBPHL	2	-0.16295176	0.16853239	-0.966886899	0.333600576	0	0.014901438	0	1	CRA004775;PRJNA827465	Yang2025_Silkie_Wenchang	0.802691373
ENSGALG00010024596	EPS15	2	-0.052321232	0.044476359	-1.176383004	0.23944186	0	0.002290859	0	1	PRJNA827465;Yang2025_Silkie_Wenchang	CRA004775	0.751706259
ENSGALG00010024596	EPS15	2	-0.09497431	0.111456869	-0.852117154	0.394149072	0	0.667011237	0	1	CRA004775;Yang2025_Silkie_Wenchang	PRJNA827465	0.830486298
ENSGALG00010024596	EPS15	2	-0.061320922	0.046604959	-1.315759617	0.188254737	0	0.774449003	0	1	CRA004775;PRJNA827465	Yang2025_Silkie_Wenchang	0.697615303
ENSGALG00010024597	DNAJB11	2	0.09456677	0.182083344	0.519359802	0.603509858	0.050719359	3.990575819	74.94095977	0.5	PRJNA827465;Yang2025_Silkie_Wenchang	CRA004775	0.895659402
ENSGALG00010024597	DNAJB11	2	-0.22761723	0.159078622	-1.430847377	0.152473961	0.011271464	1.246024147	19.74473348	1	CRA004775;Yang2025_Silkie_Wenchang	PRJNA827465	0.644106525
ENSGALG00010024597	DNAJB11	2	-0.059674109	0.350525907	-0.170241651	0.864820097	0.212895601	6.88370881	85.4729474	0.5	CRA004775;PRJNA827465	Yang2025_Silkie_Wenchang	0.966769016
ENSGALG00010024598	LRRC3C	2	0.118705457	0.177070904	0.670383756	0.502613187	0.049394562	4.584123941	78.18558108	0.5	PRJNA827465;Yang2025_Silkie_Wenchang	CRA004775	0.863050365
ENSGALG00010024598	LRRC3C	2	0.268164094	0.129546819	2.070016816	0.03845077	0	0.779246363	0	0.5	CRA004775;Yang2025_Silkie_Wenchang	PRJNA827465	0.338234244
ENSGALG00010024598	LRRC3C	2	-0.043035751	0.088601312	-0.485723633	0.627163125	0	0.000975053	0	1	CRA004775;PRJNA827465	Yang2025_Silkie_Wenchang	0.901583369
ENSGALG00010024599	ENSGALG00010024599	2	0.215971115	0.276228105	0.781857861	0.434298112	0.061642737	1.676200799	40.3412765	0.5	PRJNA827465;Yang2025_Silkie_Wenchang	CRA004775	0.842762754
ENSGALG00010024599	ENSGALG00010024599	2	0.078867083	0.473592599	0.166529382	0.867740362	0.306958278	3.084562484	67.58049139	0.5	CRA004775;Yang2025_Silkie_Wenchang	PRJNA827465	0.967946362
ENSGALG00010024599	ENSGALG00010024599	2	-0.171395299	0.244475888	-0.701072409	0.483257828	0	0.565264237	0	1	CRA004775;PRJNA827465	Yang2025_Silkie_Wenchang	0.85691916
ENSGALG00010024600	CD9	2	-0.03334267	0.322822247	-0.103284921	0.917736843	0.178656663	6.849225622	85.39980933	0.5	PRJNA827465;Yang2025_Silkie_Wenchang	CRA004775	0.98098528
ENSGALG00010024600	CD9	2	0.932932948	1.333115302	0.699814147	0.484043379	3.448318273	33.15946013	96.98426936	0.5	CRA004775;Yang2025_Silkie_Wenchang	PRJNA827465	0.857004733
ENSGALG00010024600	CD9	2	1.242378174	1.00919784	1.231055126	0.218302247	1.945325113	21.58151234	95.36640443	1	CRA004775;PRJNA827465	Yang2025_Silkie_Wenchang	0.731951308
ENSGALG00010024601	ENSGALG00010024601	2	-0.060261417	0.158533214	-0.380118557	0.703857411	0	0.864591356	0	0.5	PRJNA827465;Yang2025_Silkie_Wenchang	CRA004775	0.923724717
ENSGALG00010024601	ENSGALG00010024601	2	0.534686489	1.009144755	0.529841221	0.596222023	1.664650254	5.001711197	80.00684244	0.5	CRA004775;Yang2025_Silkie_Wenchang	PRJNA827465	0.894064255
ENSGALG00010024601	ENSGALG00010024601	2	0.656912984	0.817306097	0.803753926	0.421539104	1.04366123	3.781513221	73.55555987	1	CRA004775;PRJNA827465	Yang2025_Silkie_Wenchang	0.839352211
ENSGALG00010024602	PROCR	2	-0.109492306	0.121140537	-0.903845314	0.36607743	0	0.187209276	0	1	PRJNA827465;Yang2025_Silkie_Wenchang	CRA004775	0.819032346
ENSGALG00010024602	PROCR	2	0.034930644	0.190434155	0.183426359	0.854463495	0	0.435020801	0	0.5	CRA004775;Yang2025_Silkie_Wenchang	PRJNA827465	0.96322236
ENSGALG00010024602	PROCR	2	-0.096905957	0.137604592	-0.704234907	0.481286496	0	0.93247156	0	0.5	CRA004775;PRJNA827465	Yang2025_Silkie_Wenchang	0.85682839
ENSGALG00010024604	MFSD5	2	0.062214246	0.059055268	1.053491896	0.292115607	0	0.78347478	0	1	PRJNA827465;Yang2025_Silkie_Wenchang	CRA004775	0.785516679
ENSGALG00010024604	MFSD5	2	0.173747081	0.179129197	0.969953998	0.332069423	0	0.382281968	0	0.5	CRA004775;Yang2025_Silkie_Wenchang	PRJNA827465	0.802002658
ENSGALG00010024604	MFSD5	2	0.044775813	0.060730732	0.737284254	0.460949506	0	0.031823417	0	0.5	CRA004775;PRJNA827465	Yang2025_Silkie_Wenchang	0.85014477
ENSGALG00010024605	LSP1P1	2	-0.14582822	0.202438144	-0.7203594	0.47130374	0.068392303	6.00941829	83.35945425	0.5	PRJNA827465;Yang2025_Silkie_Wenchang	CRA004775	0.853939282
ENSGALG00010024605	LSP1P1	2	-0.388862818	0.119820409	-3.245380489	0.001172938	0	0.610883841	0	1	CRA004775;Yang2025_Silkie_Wenchang	PRJNA827465	0.041644062
ENSGALG00010024605	LSP1P1	2	-0.217761864	0.338927195	-0.642503368	0.520546403	0.175671301	3.623792307	72.40459951	0.5	CRA004775;PRJNA827465	Yang2025_Silkie_Wenchang	0.869331539
ENSGALG00010024607	RARG	2	-0.040712932	0.083917256	-0.485155662	0.627565931	0.004332699	1.228168159	18.57792494	1	PRJNA827465;Yang2025_Silkie_Wenchang	CRA004775	0.901840054
ENSGALG00010024607	RARG	2	-0.272334343	0.138849678	-1.961361002	0.049836926	0	0.215437907	0	1	CRA004775;Yang2025_Silkie_Wenchang	PRJNA827465	0.386284807
ENSGALG00010024607	RARG	2	-0.114893201	0.162789579	-0.705777372	0.480326596	0.035231733	2.425676148	58.77438128	1	CRA004775;PRJNA827465	Yang2025_Silkie_Wenchang	0.856585676
ENSGALG00010024608	DENND2D	2	0.106947096	0.230368637	0.464243299	0.642473443	0	0.121210299	0	1	PRJNA827465;Yang2025_Silkie_Wenchang	CRA004775	0.906634523
ENSGALG00010024608	DENND2D	2	0.033120254	0.284958625	0.116228292	0.907471615	0	0.542462495	0	0.5	CRA004775;Yang2025_Silkie_Wenchang	PRJNA827465	0.978196186
ENSGALG00010024608	DENND2D	2	-0.036103095	0.245155856	-0.147265888	0.882922156	0	0.241292651	0	0.5	CRA004775;PRJNA827465	Yang2025_Silkie_Wenchang	0.972001076
ENSGALG00010024609	PRR33	2	-0.034933538	0.366889731	-0.095215361	0.924143769	0.141148995	2.098286239	52.34205985	0.5	PRJNA827465;Yang2025_Silkie_Wenchang	CRA004775	0.98230395
ENSGALG00010024609	PRR33	2	0.312289885	0.309410418	1.009306301	0.312827759	0	0.001133821	0	1	CRA004775;Yang2025_Silkie_Wenchang	PRJNA827465	0.796054997
ENSGALG00010024609	PRR33	2	-0.266722792	0.331474396	-0.804655791	0.421018338	0	0.762720924	0	0.5	CRA004775;PRJNA827465	Yang2025_Silkie_Wenchang	0.839269089
ENSGALG00010024610	PFKL	2	0.170428138	0.080466768	2.117994085	0.034175569	0	0.048112778	0	1	PRJNA827465;Yang2025_Silkie_Wenchang	CRA004775	0.317969834
ENSGALG00010024610	PFKL	2	-0.034261419	0.275422696	-0.124395772	0.901001921	0.109095786	3.289321376	69.59859237	0.5	CRA004775;Yang2025_Silkie_Wenchang	PRJNA827465	0.976551823
ENSGALG00010024610	PFKL	2	-0.039953423	0.253431582	-0.157649742	0.874732806	0.093739906	3.194670026	68.69786263	0.5	CRA004775;PRJNA827465	Yang2025_Silkie_Wenchang	0.970159044
ENSGALG00010024611	ENSGALG00010024611	2	-0.017868253	0.555746463	-0.032151808	0.974350988	0.468730327	3.775127039	73.51082521	0.5	PRJNA827465;Yang2025_Silkie_Wenchang	CRA004775	0.992492038
ENSGALG00010024611	ENSGALG00010024611	2	0.00676604	0.567613375	0.011920157	0.990489316	0.449931266	3.208548583	68.83325984	0.5	CRA004775;Yang2025_Silkie_Wenchang	PRJNA827465	0.997468525
ENSGALG00010024611	ENSGALG00010024611	2	-0.487043905	0.197080784	-2.471290687	0.013462634	0	0.001091944	0	1	CRA004775;PRJNA827465	Yang2025_Silkie_Wenchang	0.189853567
ENSGALG00010024613	ENSGALG00010024613	2	-0.128314101	0.115971338	-1.106429422	0.268540696	0.020860656	4.320928913	76.85682824	1	PRJNA827465;Yang2025_Silkie_Wenchang	CRA004775	0.771398294
ENSGALG00010024613	ENSGALG00010024613	2	-0.263485643	0.085360638	-3.086734677	0.002023681	0	0.037288491	0	1	CRA004775;Yang2025_Silkie_Wenchang	PRJNA827465	0.058569034
ENSGALG00010024613	ENSGALG00010024613	2	-0.093099753	0.120276848	-0.774045501	0.438903884	0.015690428	1.698934471	41.13957796	1	CRA004775;PRJNA827465	Yang2025_Silkie_Wenchang	0.844002503
ENSGALG00010024616	ARVCF	2	-0.108876969	0.06169915	-1.764642914	0.077623798	0	0.313558262	0	1	PRJNA827465;Yang2025_Silkie_Wenchang	CRA004775	0.479994989
ENSGALG00010024616	ARVCF	2	-0.109986769	0.161510032	-0.680990324	0.495877613	0.006843465	1.094009586	8.593122697	0.5	CRA004775;Yang2025_Silkie_Wenchang	PRJNA827465	0.860911322
ENSGALG00010024616	ARVCF	2	-0.08374236	0.065564655	-1.277248546	0.201514513	0	0.732102836	0	0.5	CRA004775;PRJNA827465	Yang2025_Silkie_Wenchang	0.714377024
ENSGALG00010024618	VPS13C	2	-0.178506525	0.083248049	-2.144272765	0.032011038	0	0.70031156	0	1	PRJNA827465;Yang2025_Silkie_Wenchang	CRA004775	0.306786105
ENSGALG00010024618	VPS13C	2	-0.031267719	0.161800464	-0.193248634	0.846764252	0	0.018058574	0	0.5	CRA004775;Yang2025_Silkie_Wenchang	PRJNA827465	0.961252712
ENSGALG00010024618	VPS13C	2	-0.199230774	0.090374355	-2.20450561	0.027488806	0	0.384742144	0	0.5	CRA004775;PRJNA827465	Yang2025_Silkie_Wenchang	0.282973425
ENSGALG00010024619	ENSGALG00010024619	2	-0.300660273	0.21877874	-1.374266408	0.169358991	0	0.109519316	0	1	PRJNA827465;Yang2025_Silkie_Wenchang	CRA004775	0.670729184
ENSGALG00010024619	ENSGALG00010024619	2	-0.225321643	0.231580185	-0.97297462	0.330565916	0	0.360322762	0	1	CRA004775;Yang2025_Silkie_Wenchang	PRJNA827465	0.80156474
ENSGALG00010024619	ENSGALG00010024619	2	-0.187479535	0.199524235	-0.939632899	0.347405895	0	0.110451409	0	1	CRA004775;PRJNA827465	Yang2025_Silkie_Wenchang	0.808705451
ENSGALG00010024620	MSANTD2	2	0.026276961	0.052512126	0.500397974	0.61679488	0	0.843616739	0	0.5	PRJNA827465;Yang2025_Silkie_Wenchang	CRA004775	0.899111936
ENSGALG00010024620	MSANTD2	2	-0.02098174	0.149625592	-0.140228285	0.888479625	0.027571603	2.384294891	58.05887922	0.5	CRA004775;Yang2025_Silkie_Wenchang	PRJNA827465	0.973135755
ENSGALG00010024620	MSANTD2	2	-0.043069099	0.078085571	-0.55156284	0.581247902	0.003334891	1.188755327	15.8784001	1	CRA004775;PRJNA827465	Yang2025_Silkie_Wenchang	0.88890219
ENSGALG00010024621	THRA	2	-0.061655563	0.062507064	-0.986377519	0.323947893	0	0.744957462	0	0.5	PRJNA827465;Yang2025_Silkie_Wenchang	CRA004775	0.799600434
ENSGALG00010024621	THRA	2	0.006933937	0.122934197	0.056403644	0.955020254	0	0.346709748	0	0.5	CRA004775;Yang2025_Silkie_Wenchang	PRJNA827465	0.988093796
ENSGALG00010024621	THRA	2	-0.091006646	0.06733255	-1.351599578	0.176503445	0	0.018571594	0	1	CRA004775;PRJNA827465	Yang2025_Silkie_Wenchang	0.681914372
ENSGALG00010024622	PRMT8	2	0.34764327	0.165331468	2.102704792	0.035491583	0.03726854	3.14162491	68.1693382	1	PRJNA827465;Yang2025_Silkie_Wenchang	CRA004775	0.325309162
ENSGALG00010024622	PRMT8	2	0.50692288	0.127696533	3.969746632	7.19491E-05	0	0.033272665	0	1	CRA004775;Yang2025_Silkie_Wenchang	PRJNA827465	0.006128778
ENSGALG00010024622	PRMT8	2	0.1921493	0.129742378	1.481006465	0.138604851	0	0.133681191	0	1	CRA004775;PRJNA827465	Yang2025_Silkie_Wenchang	0.622371164
ENSGALG00010024623	NRDC	2	-0.081617925	0.127732691	-0.638974446	0.522839556	0.029153221	9.281093267	89.22540727	0.5	PRJNA827465;Yang2025_Silkie_Wenchang	CRA004775	0.869568211
ENSGALG00010024623	NRDC	2	0.050426187	0.065374104	0.77134804	0.440500665	0	0.000333124	0	1	CRA004775;Yang2025_Silkie_Wenchang	PRJNA827465	0.844733855
ENSGALG00010024623	NRDC	2	-0.168660705	0.088244104	-1.911297145	0.055966406	0.006751705	1.271525421	21.35430536	0.5	CRA004775;PRJNA827465	Yang2025_Silkie_Wenchang	0.409290467
ENSGALG00010024624	CTSD	2	-0.099716033	0.182121986	-0.547523311	0.58401926	0.053257574	4.686275729	78.66109342	0.5	PRJNA827465;Yang2025_Silkie_Wenchang	CRA004775	0.890763231
ENSGALG00010024624	CTSD	2	-0.290527883	0.134981176	-2.152358509	0.031369129	0	0.063875437	0	1	CRA004775;Yang2025_Silkie_Wenchang	PRJNA827465	0.302998494
ENSGALG00010024624	CTSD	2	0.035434728	0.07764476	0.456369862	0.648124034	0.001720638	1.043142968	4.135863343	0.5	CRA004775;PRJNA827465	Yang2025_Silkie_Wenchang	0.90762535
ENSGALG00010024625	SPRYD3	2	0.383483589	0.376960059	1.017305629	0.30900806	0.27019994	19.79314297	94.94774528	1	PRJNA827465;Yang2025_Silkie_Wenchang	CRA004775	0.794014496
ENSGALG00010024625	SPRYD3	2	0.166815667	0.621982846	0.268199788	0.788545538	0.731339265	18.08492438	94.47053259	0.5	CRA004775;Yang2025_Silkie_Wenchang	PRJNA827465	0.947048735
ENSGALG00010024625	SPRYD3	2	-0.166856891	0.237966067	-0.701179344	0.483191099	0.087580521	3.70222456	72.98921273	0.5	CRA004775;PRJNA827465	Yang2025_Silkie_Wenchang	0.85691916
ENSGALG00010024626	TSHZ3	2	0.043590108	0.080598165	0.540832504	0.588623037	0	0.641421745	0	0.5	PRJNA827465;Yang2025_Silkie_Wenchang	CRA004775	0.89212762
ENSGALG00010024626	TSHZ3	2	-0.058580011	0.152360182	-0.384483732	0.700619951	0	0.048584113	0	0.5	CRA004775;Yang2025_Silkie_Wenchang	PRJNA827465	0.922371071
ENSGALG00010024626	TSHZ3	2	0.075372917	0.089252355	0.844492195	0.398394423	0	0.027888539	0	1	CRA004775;PRJNA827465	Yang2025_Silkie_Wenchang	0.831980453
ENSGALG00010024627	CEPT1	2	-0.123450056	0.135097327	-0.913786072	0.360829266	0.029539081	4.897955793	79.5833192	1	PRJNA827465;Yang2025_Silkie_Wenchang	CRA004775	0.815095283
ENSGALG00010024627	CEPT1	2	-0.259591199	0.101774681	-2.550646159	0.010752343	0	0.155503618	0	1	CRA004775;Yang2025_Silkie_Wenchang	PRJNA827465	0.165236469
ENSGALG00010024627	CEPT1	2	-0.011593786	0.051812574	-0.223763947	0.822940985	0	0.399157886	0	1	CRA004775;PRJNA827465	Yang2025_Silkie_Wenchang	0.955822987
ENSGALG00010024628	TANGO2	2	-0.068934296	0.062030921	-1.11128925	0.266443865	0	0.346772086	0	1	PRJNA827465;Yang2025_Silkie_Wenchang	CRA004775	0.770695533
ENSGALG00010024628	TANGO2	2	-0.108626558	0.095626449	-1.13594679	0.255978834	0	0.054339564	0	1	CRA004775;Yang2025_Silkie_Wenchang	PRJNA827465	0.764570254
ENSGALG00010024628	TANGO2	2	-0.043488137	0.073337395	-0.592987209	0.553189712	0	0.004063281	0	1	CRA004775;PRJNA827465	Yang2025_Silkie_Wenchang	0.87934426
ENSGALG00010024629	ENSGALG00010024629	2	-0.054301117	0.119845778	-0.453091615	0.650482773	0.017808327	2.434797383	58.92882065	0.5	PRJNA827465;Yang2025_Silkie_Wenchang	CRA004775	0.908618725
ENSGALG00010024629	ENSGALG00010024629	2	-0.135218284	0.118284712	-1.14315943	0.252972403	0	0.935373661	0	0.5	CRA004775;Yang2025_Silkie_Wenchang	PRJNA827465	0.762731469
ENSGALG00010024629	ENSGALG00010024629	2	0.04249578	0.071634188	0.593233221	0.553025082	0	0.002591837	0	1	CRA004775;PRJNA827465	Yang2025_Silkie_Wenchang	0.879340756
ENSGALG00010024630	CFAP410	2	-0.207243544	0.327646444	-0.632521877	0.527045917	0.186942317	7.02819257	85.77159077	0.5	PRJNA827465;Yang2025_Silkie_Wenchang	CRA004775	0.87095077
ENSGALG00010024630	CFAP410	2	-0.39962975	0.160580689	-2.488653848	0.012822774	0	0.995606637	0	1	CRA004775;Yang2025_Silkie_Wenchang	PRJNA827465	0.183797777
ENSGALG00010024630	CFAP410	2	-0.023898784	0.157003016	-0.152218628	0.879014502	0.031585219	2.236915058	55.29557565	0.5	CRA004775;PRJNA827465	Yang2025_Silkie_Wenchang	0.971128798
ENSGALG00010024631	EPB41L1	2	-0.027972574	0.097116764	-0.288030332	0.773323521	0.009559425	1.634639804	38.82444332	0.5	PRJNA827465;Yang2025_Silkie_Wenchang	CRA004775	0.943245453
ENSGALG00010024631	EPB41L1	2	0.090813874	0.143741197	0.631787381	0.52752582	0	0.332906795	0	0.5	CRA004775;Yang2025_Silkie_Wenchang	PRJNA827465	0.871149047
ENSGALG00010024631	EPB41L1	2	-0.084371171	0.055192036	-1.528683794	0.126342852	0	0.007386359	0	1	CRA004775;PRJNA827465	Yang2025_Silkie_Wenchang	0.596957212
ENSGALG00010024632	IFITM10	2	-0.084718398	0.226289081	-0.374381291	0.708120661	0	0.765397994	0	0.5	PRJNA827465;Yang2025_Silkie_Wenchang	CRA004775	0.925061251
ENSGALG00010024632	IFITM10	2	-0.031190865	0.267004547	-0.116817728	0.907004495	0	0.713974199	0	0.5	CRA004775;Yang2025_Silkie_Wenchang	PRJNA827465	0.978196186
ENSGALG00010024632	IFITM10	2	-0.336405753	0.284913778	-1.180728274	0.237710686	0	0.040745035	0	1	CRA004775;PRJNA827465	Yang2025_Silkie_Wenchang	0.749512172
ENSGALG00010024633	NRGN	2	-1.163358797	0.511438998	-2.274677531	0.022925281	0.391737126	3.933566927	74.57778097	1	PRJNA827465;Yang2025_Silkie_Wenchang	CRA004775	0.258244821
ENSGALG00010024633	NRGN	2	-0.710387505	1.046692587	-0.678697369	0.497329634	1.924783269	8.094671939	87.64619484	0.5	CRA004775;Yang2025_Silkie_Wenchang	PRJNA827465	0.86098656
ENSGALG00010024633	NRGN	2	-0.276801878	0.519705868	-0.532612571	0.594301798	0.335470232	2.410587331	58.51633387	0.5	CRA004775;PRJNA827465	Yang2025_Silkie_Wenchang	0.893334186

ENSGALG00010024634	ENSGALG00010024634	2	0.727425766	0.678868202	1.071527233	0.283932432	0.874720021	18.7322335	94.66160829	1	PRJNA827465;Yang2025_Silkie_Wenchang	CRA004775	0.77978499
ENSGALG00010024634	ENSGALG00010024634	2	0.768711312	0.66728665	1.151995641	0.249322864	0.801892906	10.04002443	90.03986487	1	CRA004775;Yang2025_Silkie_Wenchang	PRJNA827465	0.759540418
ENSGALG00010024634	ENSGALG00010024634	2	0.082671917	0.053578112	1.543016622	0.122826743	0	0.00728106	0	1	CRA004775;PRJNA827465	Yang2025_Silkie_Wenchang	0.587706885
ENSGALG00010024635	ENSGALG00010024635	2	-0.150898392	0.168133115	-0.897493584	0.369455596	0	0.652717394	0	0.5	PRJNA827465;Yang2025_Silkie_Wenchang	CRA004775	0.820606801
ENSGALG00010024635	ENSGALG00010024635	2	0.067691455	0.270610763	0.250143247	0.802476573	0	2.45443E-12	0	1	CRA004775;Yang2025_Silkie_Wenchang	PRJNA827465	0.950688375
ENSGALG00010024635	ENSGALG00010024635	2	-0.196032975	0.184677209	-1.061489811	0.288467361	0	0.303710015	0	0.5	CRA004775;PRJNA827465	Yang2025_Silkie_Wenchang	0.783088502
ENSGALG00010024636	PANX3	2	0.204278382	0.143491653	1.423625534	0.1545549	0	0.13398188	0	1	PRJNA827465;Yang2025_Silkie_Wenchang	CRA004775	0.647391159
ENSGALG00010024636	PANX3	2	-0.296665224	0.476852067	-0.622132617	0.533854669	0.403356029	8.73726262	88.55476774	0.5	CRA004775;Yang2025_Silkie_Wenchang	PRJNA827465	0.873408008
ENSGALG00010024636	PANX3	2	-0.253265819	0.53083849	-0.477105228	0.633287192	0.502701274	9.237620509	89.17470144	0.5	CRA004775;PRJNA827465	Yang2025_Silkie_Wenchang	0.9032522
ENSGALG00010024637	DUSP8	2	0.021636386	0.205793121	0.105136586	0.916267428	0.059634529	3.315105322	69.83504586	0.5	PRJNA827465;Yang2025_Silkie_Wenchang	CRA004775	0.98043852
ENSGALG00010024637	DUSP8	2	0.271657219	0.17824892	1.524032907	0.127500491	0	0.367523991	0	1	CRA004775;Yang2025_Silkie_Wenchang	PRJNA827465	0.599452995
ENSGALG00010024637	DUSP8	2	-0.051421078	0.302164993	-0.170175498	0.86487212	0.086450295	1.264560931	20.92116913	0.5	CRA004775;PRJNA827465	Yang2025_Silkie_Wenchang	0.966782393
ENSGALG00010024638	ZNF507	2	0.007194353	0.071243392	0.100982737	0.919564164	0	0.063385955	0	0.5	PRJNA827465;Yang2025_Silkie_Wenchang	CRA004775	0.981192162
ENSGALG00010024638	ZNF507	2	0.102814146	0.157748327	0.651760609	0.514555616	0	0.660207684	0	1	CRA004775;Yang2025_Silkie_Wenchang	PRJNA827465	0.866869697
ENSGALG00010024638	ZNF507	2	0.014148643	0.076747728	0.184352604	0.853736849	0	0.977728569	0	0.5	CRA004775;PRJNA827465	Yang2025_Silkie_Wenchang	0.962975689
ENSGALG00010024639	AAR2	2	-0.297990408	0.320953386	-0.928453854	0.353172194	0.191466125	13.42712061	92.55238685	0.5	PRJNA827465;Yang2025_Silkie_Wenchang	CRA004775	0.81107437
ENSGALG00010024639	AAR2	2	-0.323711626	0.298488618	-1.084502414	0.278142097	0.160606199	9.825163025	89.82205183	1	CRA004775;Yang2025_Silkie_Wenchang	PRJNA827465	0.776763074
ENSGALG00010024639	AAR2	2	-0.006811291	0.040919456	-0.16645606	0.86779806	0	0.20746439	0	0.5	CRA004775;PRJNA827465	Yang2025_Silkie_Wenchang	0.967946362
ENSGALG00010024640	GSDMA	2	-0.239690766	0.143057962	-1.675480083	0.093840076	0	0.361571461	0	1	PRJNA827465;Yang2025_Silkie_Wenchang	CRA004775	0.524786069
ENSGALG00010024640	GSDMA	2	-0.329609841	0.187769495	-1.755396113	0.079191576	0.022126731	1.456114829	31.32409751	1	CRA004775;Yang2025_Silkie_Wenchang	PRJNA827465	0.484279839
ENSGALG00010024640	GSDMA	2	-0.395070867	0.139509582	-2.831854706	0.004627887	0	0.503424143	0	1	CRA004775;PRJNA827465	Yang2025_Silkie_Wenchang	0.099994421
ENSGALG00010024641	TRA2B	2	0.070928118	0.04267442	1.66207571	0.096497595	0	0.033287507	0	1	PRJNA827465;Yang2025_Silkie_Wenchang	CRA004775	0.530885356
ENSGALG00010024641	TRA2B	2	-0.062115949	0.210531834	-0.295043025	0.767961012	0.058976128	2.39442941	58.2363967	0.5	CRA004775;Yang2025_Silkie_Wenchang	PRJNA827465	0.941444269
ENSGALG00010024641	TRA2B	2	-0.062970116	0.197951509	-0.318108794	0.750402413	0.053317873	2.323490271	56.9613003	0.5	CRA004775;PRJNA827465	Yang2025_Silkie_Wenchang	0.937393444
ENSGALG00010024642	RAB3B	2	-0.038160974	0.311760267	-0.122404866	0.902578385	0.151109186	4.457258215	77.56468323	0.5	PRJNA827465;Yang2025_Silkie_Wenchang	CRA004775	0.979692607
ENSGALG00010024642	RAB3B	2	0.088501588	0.509412873	0.173732531	0.86207567	0.412229343	4.620850596	78.35896272	0.5	CRA004775;Yang2025_Silkie_Wenchang	PRJNA827465	0.965763853
ENSGALG00010024642	RAB3B	2	0.325603417	0.169904553	1.916390179	0.05531545	0	0.769859939	0	1	CRA004775;PRJNA827465	Yang2025_Silkie_Wenchang	0.407328965
ENSGALG00010024643	ETV7	2	0.469072296	0.315841947	1.485148824	0.137504405	0	0.464016686	0	1	PRJNA827465;Yang2025_Silkie_Wenchang	CRA004775	0.620332372
ENSGALG00010024643	ETV7	2	0.231638041	0.514414466	0.450294054	0.652498852	0.262629803	1.984503453	49.6095611	0.5	CRA004775;Yang2025_Silkie_Wenchang	PRJNA827465	0.909401809
ENSGALG00010024643	ETV7	2	0.087665609	0.311976991	0.28100024	0.778710216	0	0.815679539	0	0.5	CRA004775;PRJNA827465	Yang2025_Silkie_Wenchang	0.944673731
ENSGALG00010024644	EIF6	2	-0.013584796	0.042383206	-0.320523089	0.748571831	0	0.569898681	0	1	PRJNA827465;Yang2025_Silkie_Wenchang	CRA004775	0.937249951
ENSGALG00010024644	EIF6	2	-0.203649308	0.122182862	-1.66675837	0.095562461	0.015284235	2.03464373	50.85134635	1	CRA004775;Yang2025_Silkie_Wenchang	PRJNA827465	0.529371587
ENSGALG00010024644	EIF6	2	-0.145790106	0.166412916	-0.876074459	0.380989556	0.046737893	5.837446569	82.86922221	1	CRA004775;PRJNA827465	Yang2025_Silkie_Wenchang	0.824392515
ENSGALG00010024645	PARP11	2	-0.125017321	0.087938837	-1.421639468	0.155130941	0.00907733	2.420162987	58.68046883	1	PRJNA827465;Yang2025_Silkie_Wenchang	CRA004775	0.647765412
ENSGALG00010024645	PARP11	2	-0.199520549	0.075676643	-2.63648785	0.00837692	0	0.326100504	0	1	CRA004775;Yang2025_Silkie_Wenchang	PRJNA827465	0.142771619
ENSGALG00010024645	PARP11	2	-0.036865579	0.077902554	-0.473226841	0.63605135	0	0.00161792	0	1	CRA004775;PRJNA827465	Yang2025_Silkie_Wenchang	0.904440167
ENSGALG00010024646	RORA	2	0.267279261	0.363381159	0.735534176	0.462014234	0.219502675	5.61937277	82.20441959	0.5	PRJNA827465;Yang2025_Silkie_Wenchang	CRA004775	0.850747865
ENSGALG00010024646	RORA	2	0.708721073	0.244209684	2.902100614	0.003706695	0	0.090101078	0	1	CRA004775;Yang2025_Silkie_Wenchang	PRJNA827465	0.087131711
ENSGALG00010024646	RORA	2	0.250308003	0.431609065	0.579941488	0.561954076	0.262927834	2.731684272	63.39254831	0.5	CRA004775;PRJNA827465	Yang2025_Silkie_Wenchang	0.881836663
ENSGALG00010024647	RPUSD4	2	0.246066485	0.155526173	1.582154822	0.113614231	0.025024991	1.950086343	48.7202193	1	PRJNA827465;Yang2025_Silkie_Wenchang	CRA004775	0.568794964
ENSGALG00010024647	RPUSD4	2	0.107800354	0.327236842	0.329426091	0.74183365	0.182042791	6.633058504	84.92399849	0.5	CRA004775;Yang2025_Silkie_Wenchang	PRJNA827465	0.93543545
ENSGALG00010024647	RPUSD4	2	-0.024782904	0.166166743	-0.149144791	0.881439383	0.035933883	2.80314628	64.32580037	0.5	CRA004775;PRJNA827465	Yang2025_Silkie_Wenchang	0.971790895
ENSGALG00010024648	MMP24	2	-0.046522013	0.049667417	-0.936670662	0.348927992	0	0.447307006	0	0.5	PRJNA827465;Yang2025_Silkie_Wenchang	CRA004775	0.809415141
ENSGALG00010024648	MMP24	2	0.160087085	0.221475304	0.722821381	0.46978963	0.063273374	2.287198522	56.27839077	1	CRA004775;Yang2025_Silkie_Wenchang	PRJNA827465	0.853485658
ENSGALG00010024648	MMP24	2	0.129191718	0.262958833	0.491300166	0.623214162	0.104204029	3.248562886	69.2171574	0.5	CRA004775;PRJNA827465	Yang2025_Silkie_Wenchang	0.900348922
ENSGALG00010024649	NCOR2	2	0.135692642	0.248858721	0.545259741	0.585574888	0.102417174	5.436510029	81.60584649	0.5	PRJNA827465;Yang2025_Silkie_Wenchang	CRA004775	0.891148209
ENSGALG00010024649	NCOR2	2	0.333448591	0.158449467	2.097900526	0.035913934	0	0.544198289	0	1	CRA004775;Yang2025_Silkie_Wenchang	PRJNA827465	0.132696036
ENSGALG00010024649	NCOR2	2	-0.060830385	0.08861028	-0.686493545	0.492401933	0	0.727161643	0	0.5	CRA004775;PRJNA827465	Yang2025_Silkie_Wenchang	0.859249353
ENSGALG00010024650	PRKAG1	2	0.010692146	0.239064205	0.044724996	0.96432651	0.102040845	8.852245444	88.70343116	0.5	PRJNA827465;Yang2025_Silkie_Wenchang	CRA004775	0.990299069
ENSGALG00010024650	PRKAG1	2	0.21081186	0.135915327	1.551052883	0.120889011	0	0.935428828	0	0.5	CRA004775;Yang2025_Silkie_Wenchang	PRJNA827465	0.582767179
ENSGALG00010024650	PRKAG1	2	-0.205759731	0.060728609	-3.388184508	0.000703569	0	0.141284341	0	1	CRA004775;PRJNA827465	Yang2025_Silkie_Wenchang	0.029606402
ENSGALG00010024651	PSMD3	2	-0.102211459	0.227874238	-0.448543284	0.653761156	0.080514146	3.75699947	73.38301461	0.5	PRJNA827465;Yang2025_Silkie_Wenchang	CRA004775	0.909846738
ENSGALG00010024651	PSMD3	2	-0.427362921	0.195456014	-2.18649154	0.028779663	0	0.082665989	0	1	CRA004775;Yang2025_Silkie_Wenchang	PRJNA827465	0.289069025
ENSGALG00010024651	PSMD3	2	-0.113177409	0.276447008	-0.409400013	0.68224613	0.109079957	2.662595267	62.4426584	0.5	CRA004775;PRJNA827465	Yang2025_Silkie_Wenchang	0.918550276
ENSGALG00010024652	PUS3	2	-0.046709582	0.072928431	-0.640485221	0.521857194	0	0.284092484	0	1	PRJNA827465;Yang2025_Silkie_Wenchang	CRA004775	0.86956507
ENSGALG00010024652	PUS3	2	-0.192184348	0.134380366	-1.430151985	0.152673403	0	0.172758413	0	1	CRA004775;Yang2025_Silkie_Wenchang	PRJNA827465	0.644378159
ENSGALG00010024652	PUS3	2	-0.069220094	0.083994216	-0.824105479	0.409879627	0.002300191	1.105158888	9.515273271	1	CRA004775;PRJNA827465	Yang2025_Silkie_Wenchang	0.834652129
ENSGALG00010024653	RFLNA	2	0.148605356	0.099834775	1.488532959	0.136615667	0	0.000247247	0	1	PRJNA827465;Yang2025_Silkie_Wenchang	CRA004775	0.618470274
ENSGALG00010024653	RFLNA	2	0.107185733	0.149801941	0.715516318	0.474290061	0	0.397649223	0	0.5	CRA004775;Yang2025_Silkie_Wenchang	PRJNA827465	0.854462864
ENSGALG00010024653	RFLNA	2	0.124062398	0.120403147	1.030391654	0.30282619	0	0.432488356	0	0.5	CRA004775;PRJNA827465	Yang2025_Silkie_Wenchang	0.792039511
ENSGALG00010024654	CCDC92	2	0.012601652	0.132818091	0.094878034	0.92441091	0	0.784803086	0	0.5	PRJNA827465;Yang2025_Silkie_Wenchang	CRA004775	0.982374054
ENSGALG00010024654	CCDC92	2	-0.157620631	0.198854443	-0.792643244	0.427985706	0	0.093874247	0	1	CRA004775;Yang2025_Silkie_Wenchang	PRJNA827465	0.84125685
ENSGALG00010024654	CCDC92	2	0.070812906	0.162722016	0.435177166	0.663433835	0	0.526084776	0	0.5	CRA004775;PRJNA827465	Yang2025_Silkie_Wenchang	0.913099491
ENSGALG00010024655	IGF2BP2	2	0.074864649	0.094602054	0.791363884	0.428731679	0.01179029	2.91155877	65.65413653	0.5	PRJNA827465;Yang2025_Silkie_Wenchang	CRA004775	0.841764471
ENSGALG00010024655	IGF2BP2	2	0.151303952	0.080359457	1.882839403	0.059722135	0	0.762160596	0	0.5	CRA004775;Yang2025_Silkie_Wenchang	PRJNA827465	0.423249156
ENSGALG00010024655	IGF2BP2	2	-0.017592208	0.068914205	-0.255276945	0.798509206	0	0.025275477	0	1	CRA004775;PRJNA827465	Yang2025_Silkie_Wenchang	0.949922217
ENSGALG00010024656	HYLS1	2	-0.196942567	0.203042811	-0.969955874	0.332068487	0.058924358	3.24201811	69.15501507	1	PRJNA827465;Yang2025_Silkie_Wenchang	CRA004775	0.802002658
ENSGALG00010024656	HYLS1	2	-0.41644872	0.16433742	-2.534107684	0.011273408	0	0.039483969	0	1	CRA004775;Yang2025_Silkie_Wenchang	PRJNA827465	0.169049509
ENSGALG00010024656	HYLS1	2	-0.103971412	0.143039935	-0.72686982	0.467305707	0.015321801	1.34839841	25.83794281	1	CRA004775;PRJNA827465	Yang2025_Silkie_Wenchang	0.852388538
ENSGALG00010024657	DPY19L3	2	0.084694005	0.05563084	1.522429005	0.127901622	0	0.010244047	0	1	PRJNA827465;Yang2025_Silkie_Wenchang	CRA004775	0.60032911
ENSGALG00010024657	DPY19L3	2	0.11653563	0.164509306	0.708383208	0.478707317	0	0.317615233	0	1	CRA004775;Yang2025_Silkie_Wenchang	PRJNA827465	0.8557308
ENSGALG00010024657	DPY19L3	2	0.091996071	0.057470473	1.600766632	0.109429942	0	0.330506129	0	1	CRA004775;PRJNA827465	Yang2025_Silkie_Wenchang	0.559801483
ENSGALG00010024659	BEND5	2	0.127604604	0.285826863	0.446440205	0.655279296	0.154391319	17.89364408	94.41142343	0.5	PRJNA827465;Yang2025_Silkie_Wenchang	CRA004775	0.910411938
ENSGALG00010024659	BEND5	2	0.414135295	0.109098151	3.795988194	0.000147056	0	0.030053395	0	1	CRA004775;Yang2025_Silkie_Wenchang	PRJNA827465	0.010268856
ENSGALG00010024659	BEND5	2	0.031608106	0.247044784	0.127944841	0.898192625	0.089626355	3.021971825	66.90902306	0.5	CRA004775;PRJNA827465	Yang2025_Silkie_Wenchang	0.975750151
ENSGALG00010024660	KMT2D	2	-0.02282526	0.141093961	-0.161773473	0.871484243	0.025520986	2.525991649	60.40688497	0.5	PRJNA827465;Yang2025_Silkie_Wenchang	CRA004775	0.969403821
ENSGALG00010024660	KMT2D	2	0.124326422	0.149808634	0.829901577	0.406594433	0	0.247207655	0	0.5	CRA004775;Yang2025_Silkie_Wenchang	PRJNA827465	0.833618122
ENSGALG00010024660	KMT2D	2	-0.129142013	0.079272819	-1.62908313	0.103295422	0	0.05651315	0	1	CRA004775;PRJNA827465	Yang2025_Silkie_Wenchang	0.547576788
ENSGALG00010024662	MOB2	2	-0.022111589	0.039352085	-0.561891159	0.574190178	0	0.03629604	0	1	PRJNA827465;Yang2025_Silkie_Wenchang	CRA004775	0.886081433
ENSGALG00010024662	MOB2	2	0.03107046	0.098230079	0.316302913	0.751772602	0	0.404628061	0	0.5	CRA004775;Yang2025_Silkie_Wenchang	PRJNA827465	0.937802658
ENSGALG00010024662	MOB2	2	-0.018183002	0.041237686	-0.440931678	0.659262459	0	0.661441858	0	0.5	CRA004775;PRJNA827465	Yang2025_Silkie_Wenchang	0.911449691
ENSGALG00010024663	ICE2	2	0.004123198	0.112936686	0.036508938	0.970876552	0.014783471	2.304096303	56.5990363	0.5	PRJNA827465;Yang2025_Silkie_Wenchang	CRA004775	0.991868977
ENSGALG00010024663	ICE2	2	0.127654298	0.112408122	1.135632338	0.256110469	0	0.025068378	0	1	CRA004775;Yang2025_Silkie_Wenchang	PRJNA827465	0.764570254
ENSGALG00010024663	ICE2	2	-0.075845649	0.081629189	-0.929148635	0.352812062	0	0.416938245	0	0.5	CRA004775;PRJNA827465	Yang2025_Silkie_Wenchang	0.811035834
ENSGALG00010024665	MAP1LC3A	2	-0.368864063	0.075839442	-4.86374969	1.15183E-06	0	0.852764645	0	1	PRJNA827465;Yang2025_Silkie_Wenchang	CRA004775	0.000248737
ENSGALG00010024665	MAP1LC3A	2	-0.440266456	0.132619528	-3.319770954	0.000900913	0	0.665166108	0	1	CRA004775;Yang2025_Silkie_Wenchang	PRJNA827465	0.015302688
ENSGALG00010024665	MAP1LC3A	2	-0.314710626	0.087914034	-3.579774115	0.000343879	0	0.219874918	0	1	CRA004775;PRJNA827465	Yang2025_Silkie_Wenchang	0.018390471
ENSGALG00010024666	MED24	2	0.067072996	0.04963078	1.351439472	0.176554697	0	0.089704375	0	1	PRJNA827465;Yang2025_Silkie_Wenchang	CRA004775	0.682016071
ENSGALG00010024666	MED24	2	-0.172801164	0.268170059	-0.644371578	0.519334511	0.132574688	12.65575713	92.09845772	0.5	CRA004775;Yang2025_Silkie_Wenchang	PRJNA827465	0.869086345
ENSGALG00010024666	MED24	2	-0.184536829	0.252322314	-0.731353585	0.464563208	0.117986209	13.26672215	92.46234308	0.5	CRA004775;PRJNA827465	Yang2025_Silkie_Wenchang	0.85217126
ENSGALG00010024667	RBM7	2	0.324195751	0.301181328	1.076413841	0.281742215	0.172202031	19.56660879	94.88925234	1	PRJNA827465;Yang2025_Silkie_Wenchang	CRA004775	0.779295548
ENSGALG00010024667	RBM7	2	0.386591073	0.299657492	1.290109821	0.197012531	0.136926072	3.721810139	73.13135376	1	CRA004775;Yang2025_Silkie_Wenchang	PRJNA827465	0.708095167
ENSGALG00010024667	RBM7	2	0.027670048	0.075379275	0.367077658	0.713561092	0	0.000953917	0	1	CRA004775;PRJNA827465	Yang2025_Silkie_Wenchang	0.926253108
ENSGALG00010024668	ENSGALG00010024668	2	0.479952958	0.545221033	0.880290614	0.378701896	0.565540519	20.3495536	95.08598431	0.5	PRJNA827465;Yang2025_Silkie_Wenchang	CRA004775	0.878874716
ENSGALG00010024668	ENSGALG00010024668	2	0.974257729	0.178060507	5.471498134	4.46247E-08	0	0.409162674	0	1	CRA004775;Yang2025_Silkie_Wenchang	PRJNA827465	1.82224E-05
ENSGALG00010024668	ENSGALG00010024668	2	0.280726738	0.402764897	0.696999018	0.485803408	0.257221563	4.286663536	76.67183366	0.5	CRA004775;PRJNA827465	Yang2025_Silkie_Wenchang	0.857391223
ENSGALG00010024669	STIL	2	0.048847876	0.071984059	0.678592958	0.497395806	0	0.041524191	0	1	PRJNA827465;Yang2025_Silkie_Wenchang	CRA004775	0.86098656
ENSGALG00010024669	STIL	2	0.05268289	0.114354691	0.460697237	0.645015837	0	0.235828616	0	0.5	CRA004775;Yang2025_Silkie_Wenchang	PRJNA827465	0.906966303
ENSGALG00010024669	STIL	2	0.029721617	0.088588306	0.335502716	0.737245896	0	0.179376984	0	0.5	CRA004775;PRJNA827465	Yang2025_Silkie_Wenchang	0.933895493
ENSGALG00010024670	SENP2	2	-0.071579988	0.082165714	-0.871166142	0.383663444	0.005736725	1.630411284	38.66578268	0.5	PRJNA827465;Yang2025_Silkie_Wenchang	CRA004775	0.825509017
ENSGALG00010024670	SENP2	2	0.021130571	0.101618697	0.209710983	0.833892253	0	0.112817983	0	0.5	CRA004775;Yang2025_Silkie_Wenchang	PRJNA827465	0.957586425
ENSGALG00010024670	SENP2	2	-0.122969386	0.064812426	-1.897311896	0.057786786	0	0.176467983	0	1	CRA004775;PRJNA827465	Yang2025_Silkie_Wenchang	0.416579979
ENSGALG00010024671	KCTD20	2	-0.185244287	0.152930948	-1.211293652	0.225782881	0.041367672	8.465088643	88.18677462	1	PRJNA827465;Yang2025_Silkie_Wenchang	CRA004775	0.73906426
ENSGALG00010024671	KCTD20	2	-0.167391618	0.310206616	-0.539613307	0.589463737	0.120286505	1.883028634	46.89406301	0.5	CRA004775;Yang2025_Silkie_Wenchang	PRJNA827465	0.892320726
ENSGALG00010024671	KCTD20	2	-0.035602816	0.054820911	-0.649436694	0.516054918	0	0.62841089	0	0.5	CRA004775;PRJNA827465	Yang2025_Silkie_Wenchang	0.867498525
ENSGALG00010024672	DYNLRB1	2	-0.275454471	0.198757334	-1.385883306	0.165782539	0.074312563	16.50390352	93.9408274	1	PRJNA827465;Yang2025_Silkie_Wenchang	CRA004775	0.66529196
ENSGALG00010024672	DYNLRB1	2	-0.295530514	0.235610556	-1.254317799	0.209726517	0.08245878	3.358676751	70.22636967	0.5	CRA004775;Yang2025_Silkie_Wenchang	PRJNA827465	0.721625118
ENSGALG00010024672	DYNLRB1	2	-0.080797691	0.044302907	-1.823755975	0.068188972	0	0.11712928	0	0.5	CRA004775;PRJNA827465	Yang2025_Silkie_Wenchang	0.450274056
ENSGALG00010024673	REXO2	2	-0.015957997	0.043822121	-0.364153921	0.715743074	0.000335738	1.01715185	1.682718451	1	PRJNA827465;Yang2025_Silkie_Wenchang	CRA004775	0.927131632
ENSGALG00010024673	REXO2	2	-0.102364279	0.152255507	-0.672319061	0.501380598	0	0.741855274	0	0.5	CRA004775;Yang2025_Silkie_Wenchang	PRJNA827465	0.862658912
ENSGALG00010024673	REXO2	2	-0.003933191	0.040515255	-0.097079265	0.922663447	0	0.078820708	0	0.5	CRA004775;PRJNA827465	Yang2025_Silkie_Wenchang	0.981804675
ENSGALG00010024674	DHH	2	0.090736758	0.169845984	0.534228638	0.593182666	0	0.041744693	0	1	PRJNA827465;Yang2025_Silkie_Wenchang	CRA004775	0.892776493
ENSGALG00010024674	DHH	2	0.291284832	0.32091889	0.907658729	0.364058555	0.056596517	1.356907404	26.30300365	1	CRA004775;Yang2025_Silkie_Wenchang	PRJNA827465	0.817213628
ENSGALG00010024674	DHH	2	0.256300498	0.250252761	1.024166514	0.305756632	0.045151644	1.373678046	27.20273844	1	CRA004775;PRJNA827465	Yang2025_Silkie_Wenchang	0.79225794
ENSGALG00010024675	NR1D1	2	0.443822209	0.292547616	1.517093917	0.129242974	0	0.808599072	0	1	PRJNA827465;Yang2025_Silkie_Wenchang	CRA004775	0.603386934
ENSGALG00010024675	NR1D1	2	0.237286672	0.516975458	0.458990206	0.646241199	0.312321505	2.352005763	57.48309737	0.5	CRA004775;Yang2025_Silkie_Wenchang	PRJNA827465	0.907220702
ENSGALG00010024675	NR1D1	2	-0.029138164	0.336378613	-0.086623118	0.930971089	0	0.551431946	0	0.5	CRA004775;PRJNA827465	Yang2025_Silkie_Wenchang	0.983943003
ENSGALG00010024676	ENSGALG00010024676	2	0.144488944	0.065939688	2.191228802	0.028435239	0	0.477725	0	1	PRJNA827465;Yang2025_Silkie_Wenchang	CRA004775	0.287193179
ENSGALG00010024676	ENSGALG00010024676	2	0.542138656	0.416422613	1.301895332	0.192952169	0.278552136	4.383427837	77.18680364	1	CRA004775;Yang2025_Silkie_Wenchang	PRJNA827465	0.702958174
ENSGALG00010024676	ENSGALG00010024676	2	0.501684842	0.465017382	1.078851807	0.280653796	0.364175069	5.531585682	81.92200108	1	CRA004775;PRJNA827465	Yang2025_Silkie_Wenchang	0.778536485
ENSGALG00010024677	LMBR1L	2	0.09444569	0.198748037	0.475203133	0.634642194	0.034398163	1.721637466	41.91576218	0.5	PRJNA827465;Yang2025_Silkie_Wenchang	CRA004775	0.903872221
ENSGALG00010024677	LMBR1L	2	-0.288176748	0.703122384	-0.4098529	0.681913858	0.820639488	5.505102585	81.83503423	0.5	CRA004775;Yang2025_Silkie_Wenchang	PRJNA827465	0.918480478
ENSGALG00010024677	LMBR1L	2	-0.441492647	0.487715737	-0.905225346	0.365346017	0.344819549	3.073451475	67.46328978	1	CRA004775;PRJNA827465	Yang2025_Silkie_Wenchang	0.818457479
ENSGALG00010024678	AMOTL2	2	-0.086832231	0.066807653	-1.299734784	0.193691884	0	0.007314708	0	1	PRJNA827465;Yang2025_Silkie_Wenchang	CRA004775	0.70389941
ENSGALG00010024678	AMOTL2	2	-0.061132443	0.090471877	-0.675706588	0.499226952	0	0.521026203	0	0.5	CRA004775;Yang2025_Silkie_Wenchang	PRJNA827465	0.861784825
ENSGALG00010024678	AMOTL2	2	-0.07168569	0.090249378	-0.794306742	0.42701688	0	0.573531906	0	0.5	CRA004775;PRJNA827465	Yang2025_Silkie_Wenchang	0.84125685
ENSGALG00010024679	MSL1	2	0.03648878	0.166904439	0.21862079	0.826945457	0.048613394	7.220198255	86.1499648	0.5	PRJNA827465;Yang2025_Silkie_Wenchang	CRA004775	0.956540044
ENSGALG00010024679	MSL1	2	0.166259365	0.104316705	1.593794248	0.110982126	0	0.993047009	0	0.5	CRA004775;Yang2025_Silkie_Wenchang	PRJNA827465	0.563777127
ENSGALG00010024679	MSL1	2	-0.109868181	0.037694272	-2.914718217	0.003560098	0	0.165831108	0	1	CRA004775;PRJNA827465	Yang2025_Silkie_Wenchang	0.084750176
ENSGALG00010024680	BRSK1	2	-0.31242418	0.28288701	-1.104413311	0.269413889	0.088377703	2.18481128	54.22945637	0.5	PRJNA827465;Yang2025_Silkie_Wenchang	CRA004775	0.77189992
ENSGALG00010024680	BRSK1	2	0.251374894	0.392795877	0.639963168	0.522196545	0.115547319	1.421732927	29.66330165	1	CRA004775;Yang2025_Silkie_Wenchang	PRJNA827465	0.869568211
ENSGALG00010024680	BRSK1	2	0.029932012	0.713714612	0.041938349	0.966547845	0.80424208	4.189304804	76.12969104	0.5	CRA004775;PRJNA827465	Yang2025_Silkie_Wenchang	0.990881287
ENSGALG00010024681	ATP6V0A2	2	-0.002961937	0.152457486	-0.019427956	0.984499709	0.03860635	5.669569488	82.36197647	0.5	PRJNA827465;Yang2025_Silkie_Wenchang	CRA004775	0.995703389
ENSGALG00010024681	ATP6V0A2	2	-0.16007388	0.090466839	-1.769420498	0.076823727	0	0.022546728	0	1	CRA004775;Yang2025_Silkie_Wenchang	PRJNA827465	0.477904868
ENSGALG00010024681	ATP6V0A2	2	0.032677802	0.134261071	0.243389997	0.807703292	0.024739085	2.792251609	64.18660852	0.5	CRA004775;PRJNA827465	Yang2025_Silkie_Wenchang	0.952496413
ENSGALG00010024682	ENSGALG00010024682	2	-0.298029537	0.359425405	-0.829183282	0.407000703	0.203271927	4.533259689	77.94081812	0.5	PRJNA827465;Yang2025_Silkie_Wenchang	CRA004775	0.833707648
ENSGALG00010024682	ENSGALG00010024682	2	-0.850801797	0.237464238	-3.582862846	0.000339849	0	0.93440038	0	1	CRA004775;Yang2025_Silkie_Wenchang	PRJNA827465	0.01832248
ENSGALG00010024682	ENSGALG00010024682	2	-0.519683609	0.604592956	-0.859559483	0.390031918	0.633380823	7.044166344	85.80385597	0.5	CRA004775;PRJNA827465	Yang2025_Silkie_Wenchang	0.829204867
ENSGALG00010024683	SHISA4	2	0.373931388	0.559963505	0.667778141	0.504275222	0.578195663	12.12407259	91.75194645	0.5	PRJNA827465;Yang2025_Silkie_Wenchang	CRA004775	0.863512504
ENSGALG00010024683	SHISA4	2	0.500835845	0.521849842	0.959731718	0.337190256	0.400680441	3.686623982	72.87491198	0.5	CRA004775;Yang2025_Silkie_Wenchang	PRJNA827465	0.804086136
ENSGALG00010024683	SHISA4	2	-0.145465596	0.088275488	-1.647859431	0.09938152	0	0.02602022	0	1	CRA004775;PRJNA827465	Yang2025_Silkie_Wenchang	0.538163666
ENSGALG00010024684	ENSGALG00010024684	2	-0.501374549	0.26187753	-1.914538251	0.055551416	0	0.00156373	0	1	PRJNA827465;Yang2025_Silkie_Wenchang	CRA004775	0.408228819
ENSGALG00010024684	ENSGALG00010024684	2	-0.415949225	0.343926381	-1.20941355	0.226504006	0	0.308528637	0	1	CRA004775;Yang2025_Silkie_Wenchang	PRJNA827465	0.740007226
ENSGALG00010024684	ENSGALG00010024684	2	-0.41018555	0.323239877	-1.26898189	0.204447523	0	0.29051709	0	1	CRA004775;PRJNA827465	Yang2025_Silkie_Wenchang	0.71729696
ENSGALG00010024685	ENSGALG00010024685	2	-0.061020127	0.248674792	-0.245381234	0.806161258	0.075291849	2.298252689	56.48868357	0.5	PRJNA827465;Yang2025_Silkie_Wenchang	CRA004775	0.952109208
ENSGALG00010024685	ENSGALG00010024685	2	-0.110844681	0.34222697	-0.323892301	0.746019573	0.165686139	3.340592674	70.06519209	0.5	CRA004775;Yang2025_Silkie_Wenchang	PRJNA827465	0.936484152
ENSGALG00010024685	ENSGALG00010024685	2	-0.304239641	0.123137712	-2.470726761	0.01348388	0	0.41968879	0	1	CRA004775;PRJNA827465	Yang2025_Silkie_Wenchang	0.19001683
ENSGALG00010024686	CMPK1	2	-0.257468751	0.402294461	-0.640000735	0.522172122	0.310724885	24.79014245	95.96613855	0.5	PRJNA827465;Yang2025_Silkie_Wenchang	CRA004775	0.869568211
ENSGALG00010024686	CMPK1	2	-0.522718642	0.200231598	-2.610570193	0.009039142	0.042051832	1.893989759	47.20140406	1	CRA004775;Yang2025_Silkie_Wenchang	PRJNA827465	0.149036217
ENSGALG00010024686	CMPK1	2	0.035172381	0.169440621	0.207579392	0.835557397	0.031965779	1.775355663	43.67325822	0.5	CRA004775;PRJNA827465	Yang2025_Silkie_Wenchang	0.958339923
ENSGALG00010024687	RYK	2	-0.18050879	0.045675253	-3.952004149	7.74994E-05	0	0.001421207	0	1	PRJNA827465;Yang2025_Silkie_Wenchang	CRA004775	0.006494582
ENSGALG00010024687	RYK	2	-0.081777848	0.144660759	-0.565307751	0.571864457	0.023861538	1.942263491	48.51367981	0.5	CRA004775;Yang2025_Silkie_Wenchang	PRJNA827465	0.885474088
ENSGALG00010024687	RYK	2	-0.087989331	0.144758829	-0.607833949	0.543297611	0.026628841	2.123004513	52.89694421	0.5	CRA004775;PRJNA827465	Yang2025_Silkie_Wenchang	0.876968972
ENSGALG00010024688	CADM1	2	-0.045559345	0.092740738	-0.491254935	0.623246149	0.005719821	1.498078148	33.24780813	0.5	PRJNA827465;Yang2025_Silkie_Wenchang	CRA004775	0.900348922
ENSGALG00010024688	CADM1	2	0.030243219	0.088063434	0.343425391	0.73127846	0	0.082142148	0	0.5	CRA004775;Yang2025_Silkie_Wenchang	PRJNA827465	0.932851968
ENSGALG00010024688	CADM1	2	-0.093998593	0.087138348	-1.078728196	0.280708912	0	0.506128819	0	1	CRA004775;PRJNA827465	Yang2025_Silkie_Wenchang	0.778536485
ENSGALG00010024689	FOXD2	2	0.154637306	0.160435374	0.963860413	0.335115913	0.030036218	2.391625119	58.18742695	1	PRJNA827465;Yang2025_Silkie_Wenchang	CRA004775	0.803129704
ENSGALG00010024689	FOXD2	2	0.29053049	0.148604852	1.95505387	0.050576712	0	0.500514397	0	0.5	CRA004775;Yang2025_Silkie_Wenchang	PRJNA827465	0.389309933
ENSGALG00010024689	FOXD2	2	-0.000877761	0.132408399	-0.006629196	0.994710706	0	0.007787188	0	0.5	CRA004775;PRJNA827465	Yang2025_Silkie_Wenchang	0.998242335
ENSGALG00010024690	TUBA1C	2	0.390757957	0.647530677	0.603458602	0.546203648	0.731593412	7.837397935	87.24066319	0.5	PRJNA827465;Yang2025_Silkie_Wenchang	CRA004775	0.878012469
ENSGALG00010024690	TUBA1C	2	1.024566931	0.291503305	3.514769522	0.000440136	0	0.00827966	0	1	CRA004775;Yang2025_Silkie_Wenchang	PRJNA827465	0.021345626
ENSGALG00010024690	TUBA1C	2	0.23025534	0.601225325	0.382976782	0.701736979	0.491553252	2.863965922	65.08338342	0.5	CRA004775;PRJNA827465	Yang2025_Silkie_Wenchang	0.922904951
ENSGALG00010024691	TCTN2	2	-0.156363557	0.283675963	-0.551204816	0.581493279	0.144641834	9.562850515	89.54286697	0.5	PRJNA827465;Yang2025_Silkie_Wenchang	CRA004775	0.889045686
ENSGALG00010024691	TCTN2	2	-0.41240244	0.137920918	-2.990136996	0.002788524	0	0.269312949	0	1	CRA004775;Yang2025_Silkie_Wenchang	PRJNA827465	0.0719461
ENSGALG00010024691	TCTN2	2	-0.022015435	0.191832344	-0.114763936	0.908632235	0.047269829	2.284652087	56.2296594	0.5	CRA004775;PRJNA827465	Yang2025_Silkie_Wenchang	0.978356603
ENSGALG00010024692	DRAM2	2	-0.091242188	0.058651684	-1.55566187	0.119788518	0	0.515426598	0	1	PRJNA827465;Yang2025_Silkie_Wenchang	CRA004775	0.580639051
ENSGALG00010024692	DRAM2	2	-0.031898386	0.102649453	-0.310750669	0.755990174	0	0.069026761	0	1	CRA004775;Yang2025_Silkie_Wenchang	PRJNA827465	0.938501783
ENSGALG00010024692	DRAM2	2	-0.110067444	0.063205065	-1.741433911	0.081607548	0	0.038975018	0	1	CRA004775;PRJNA827465	Yang2025_Silkie_Wenchang	0.492162264
ENSGALG00010024693	ANXA2	2	-0.181765113	0.126053893	-1.441963504	0.14931267	0.01576973	1.984592553	49.61182341	1	PRJNA827465;Yang2025_Silkie_Wenchang	CRA004775	0.639246608
ENSGALG00010024693	ANXA2	2	-0.319225448	0.121960846	-2.617442052	0.008859153	0	0.056002078	0	1	CRA004775;Yang2025_Silkie_Wenchang	PRJNA827465	0.147549629
ENSGALG00010024693	ANXA2	2	-0.090013052	0.118739028	-0.758074692	0.448406269	0	0.7287845	0	1	CRA004775;PRJNA827465	Yang2025_Silkie_Wenchang	0.846878812
ENSGALG00010024694	GTF2H3	2	-0.147434796	0.144935101	-1.017246995	0.309035945	0.030683954	3.478955006	71.25573633	1	PRJNA827465;Yang2025_Silkie_Wenchang	CRA004775	0.794014496
ENSGALG00010024694	GTF2H3	2	-0.310406309	0.112232485	-2.765743895	0.005679312	0	0.006837208	0	1	CRA004775;Yang2025_Silkie_Wenchang	PRJNA827465	0.113196293
ENSGALG00010024694	GTF2H3	2	-0.103342772	0.124421211	-0.83058806	0.406206382	0.016182856	1.75891987	43.14692686	1	CRA004775;PRJNA827465	Yang2025_Silkie_Wenchang	0.833477131
ENSGALG00010024695	TRABD2B	2	0.259171938	0.137712958	1.881972056	0.05983981	0.005892846	1.13856809	12.17038238	1	PRJNA827465;Yang2025_Silkie_Wenchang	CRA004775	0.423839259
ENSGALG00010024695	TRABD2B	2	0.428266429	0.231142929	1.852820811	0.063908046	0	0.298354588	0	1	CRA004775;Yang2025_Silkie_Wenchang	PRJNA827465	0.435607433
ENSGALG00010024695	TRABD2B	2	0.17485348	0.136879588	1.277425528	0.201452057	0	0.001046361	0	1	CRA004775;PRJNA827465	Yang2025_Silkie_Wenchang	0.714377024
ENSGALG00010024696	TRADD	2	0.110241868	0.054324688	2.029314322	0.042426285	0	0.059304249	0	1	PRJNA827465;Yang2025_Silkie_Wenchang	CRA004775	0.356451428
ENSGALG00010024696	TRADD	2	-0.241336442	0.399677946	-0.603827268	0.545958489	0.260119355	4.68399516	78.65070382	0.5	CRA004775;Yang2025_Silkie_Wenchang	PRJNA827465	0.877958239
ENSGALG00010024696	TRADD	2	-0.226350744	0.414152057	-0.546540191	0.584694668	0.286558332	5.219066921	80.83948692	0.5	CRA004775;PRJNA827465	Yang2025_Silkie_Wenchang	0.891016154
ENSGALG00010024697	IGF2BP1	2	-0.112874928	0.099145061	-1.138482611	0.254919016	0.013028332	2.901798592	65.53861448	1	PRJNA827465;Yang2025_Silkie_Wenchang	CRA004775	0.764444699
ENSGALG00010024697	IGF2BP1	2	0.00318845	0.09358836	0.034068869	0.972822233	0	0.192905762	0	0.5	CRA004775;Yang2025_Silkie_Wenchang	PRJNA827465	0.992213869
ENSGALG00010024697	IGF2BP1	2	-0.196768761	0.068634142	-2.866922428	0.004144846	0	0.55059818	0	0.5	CRA004775;PRJNA827465	Yang2025_Silkie_Wenchang	0.093615215
ENSGALG00010024698	ENSGALG00010024698	2	-0.054081129	0.115231313	-0.46932667	0.638836154	0	0.337950638	0	0.5	PRJNA827465;Yang2025_Silkie_Wenchang	CRA004775	0.905768875
ENSGALG00010024698	ENSGALG00010024698	2	-0.05007364	0.173665612	-0.288333654	0.773091349	0	0.934401271	0	0.5	CRA004775;Yang2025_Silkie_Wenchang	PRJNA827465	0.943100418
ENSGALG00010024698	ENSGALG00010024698	2	-0.136559115	0.13299743	-1.026780108	0.304524018	0	0.445446155	0	1	CRA004775;PRJNA827465	Yang2025_Silkie_Wenchang	0.79225794
ENSGALG00010024699	FBXL8	2	0.062504207	0.05681827	1.100072331	0.271300608	0	0.371493084	0	1	PRJNA827465;Yang2025_Silkie_Wenchang	CRA004775	0.772743863

ENSGALG00010024699	FBXL8	2	-0.300176361	0.335778069	-0.893972504	0.371336603	0.200638118	8.718701924	88.53040271	0.5	CRA004775;Yang2025_Silkie_Wenchang	PRJNA827465	0.8214684
ENSGALG00010024699	FBXL8	2	-0.262120039	0.372760627	-0.70318596	0.481939868	0.255726959	11.92005163	91.61077459	0.5	CRA004775;PRJNA827465	Yang2025_Silkie_Wenchang	0.85691916
ENSGALG00010024700	TOLLIP	2	-0.242910488	0.210970766	-1.151394063	0.249570155	0.084750559	20.83245344	95.19979726	1	PRJNA827465;Yang2025_Silkie_Wenchang	CRA004775	0.759622031
ENSGALG00010024700	TOLLIP	2	-0.439115337	0.068953433	-6.368288237	1.91149E-10	0	0.972435142	0	1	CRA004775;Yang2025_Silkie_Wenchang	PRJNA827465	1.27335E-07
ENSGALG00010024700	TOLLIP	2	-0.038780024	0.058048202	-0.66806589	0.504091534	0	0.181706773	0	1	CRA004775;PRJNA827465	Yang2025_Silkie_Wenchang	0.863448292
ENSGALG00010024701	EIF2B1	2	-0.184720306	0.253322517	-0.729190235	0.465885304	0.106356328	5.252481549	80.96138005	0.5	PRJNA827465;Yang2025_Silkie_Wenchang	CRA004775	0.852511753
ENSGALG00010024701	EIF2B1	2	-0.358678292	0.145920639	-2.458036741	0.013969888	0	0.621332912	0	1	CRA004775;Yang2025_Silkie_Wenchang	PRJNA827465	0.193808158
ENSGALG00010024701	EIF2B1	2	-0.045724385	0.126418669	-0.361690131	0.717583603	0.017045943	1.748372911	42.80396397	0.5	CRA004775;PRJNA827465	Yang2025_Silkie_Wenchang	0.92782802
ENSGALG00010024702	INAVA	2	0.665590591	0.416658444	1.597448942	0.11016567	0.247669463	3.488163467	71.33161879	1	PRJNA827465;Yang2025_Silkie_Wenchang	CRA004775	0.562244038
ENSGALG00010024702	INAVA	2	0.878006034	0.243198996	3.610237079	0.000305917	0.001293018	1.010719223	1.060553942	1	CRA004775;Yang2025_Silkie_Wenchang	PRJNA827465	0.017200953
ENSGALG00010024702	INAVA	2	0.389068653	0.241745156	1.609416542	0.10752529	0	0.478030783	0	1	CRA004775;PRJNA827465	Yang2025_Silkie_Wenchang	0.554966053
ENSGALG00010024705	SLCO2A1	2	-0.049320052	0.185816803	-0.265422992	0.790683626	0	0.002858735	0	1	PRJNA827465;Yang2025_Silkie_Wenchang	CRA004775	0.947644914
ENSGALG00010024705	SLCO2A1	2	0.104105356	0.210585868	0.4943606	0.621051535	0	0.576596634	0	0.5	CRA004775;Yang2025_Silkie_Wenchang	PRJNA827465	0.899579438
ENSGALG00010024705	SLCO2A1	2	0.07132089	0.194946954	0.365847675	0.714478742	0	0.72406882	0	0.5	CRA004775;PRJNA827465	Yang2025_Silkie_Wenchang	0.926596201
ENSGALG00010024706	ENSGALG00010024706	2	0.340772048	0.693782382	0.491180025	0.623299126	0.838505069	7.503346932	86.67261411	0.5	PRJNA827465;Yang2025_Silkie_Wenchang	CRA004775	0.900348922
ENSGALG00010024706	ENSGALG00010024706	2	0.954660383	0.355367767	2.686401161	0.007222631	0	0.241090203	0	1	CRA004775;Yang2025_Silkie_Wenchang	PRJNA827465	0.130635564
ENSGALG00010024706	ENSGALG00010024706	2	0.065935153	0.493626958	0.133572837	0.893740365	0.320924317	2.553338914	60.83559473	0.5	CRA004775;PRJNA827465	Yang2025_Silkie_Wenchang	0.974381724
ENSGALG00010024707	ENSGALG00010024707	2	0.414766809	0.869013328	0.47728475	0.633159369	1.453325982	26.2674762	96.19301073	0.5	PRJNA827465;Yang2025_Silkie_Wenchang	CRA004775	0.903249582
ENSGALG00010024707	ENSGALG00010024707	2	0.739673491	0.570884202	1.295662919	0.195091642	0.554812074	6.704272842	85.08413927	1	CRA004775;Yang2025_Silkie_Wenchang	PRJNA827465	0.706113528
ENSGALG00010024707	ENSGALG00010024707	2	-0.207465688	0.290607856	-0.713902545	0.475287444	0.107325923	2.523113035	60.36642093	0.5	CRA004775;PRJNA827465	Yang2025_Silkie_Wenchang	0.854462864
ENSGALG00010024708	BNIP2	2	0.219502638	0.297174954	0.738631015	0.460131086	0.164104583	13.35440299	92.51183298	0.5	PRJNA827465;Yang2025_Silkie_Wenchang	CRA004775	0.850104868
ENSGALG00010024708	BNIP2	2	0.282462292	0.245199872	1.151967536	0.249334414	0.099920392	5.877069656	82.98471758	1	CRA004775;Yang2025_Silkie_Wenchang	PRJNA827465	0.759540418
ENSGALG00010024708	BNIP2	2	-0.048980972	0.038774585	-1.263223629	0.206508823	0	0.620754421	0	0.5	CRA004775;PRJNA827465	Yang2025_Silkie_Wenchang	0.718584885
ENSGALG00010024709	TEAD4	2	0.02889425	0.060888021	0.474547357	0.635109636	0	0.08718708	0	1	PRJNA827465;Yang2025_Silkie_Wenchang	CRA004775	0.904051292
ENSGALG00010024709	TEAD4	2	0.048864222	0.095265366	0.512923455	0.608002068	0	0.013357242	0	1	CRA004775;Yang2025_Silkie_Wenchang	PRJNA827465	0.897204334
ENSGALG00010024709	TEAD4	2	0.014449403	0.075833279	0.190541719	0.848884655	0	0.000394806	0	1	CRA004775;PRJNA827465	Yang2025_Silkie_Wenchang	0.961364896
ENSGALG00010024710	HSDL1	2	0.044557759	0.105600472	0.421946591	0.673064002	0	0.519182836	0	0.5	PRJNA827465;Yang2025_Silkie_Wenchang	CRA004775	0.915153756
ENSGALG00010024710	HSDL1	2	-0.202876	0.192248454	-1.055280265	0.291297164	0	0.669870498	0	1	CRA004775;Yang2025_Silkie_Wenchang	PRJNA827465	0.784705445
ENSGALG00010024710	HSDL1	2	-0.076221756	0.243957591	-0.312438552	0.754707256	0.071068433	2.01312907	50.32608615	0.5	CRA004775;PRJNA827465	Yang2025_Silkie_Wenchang	0.93813723
ENSGALG00010024711	DDX55	2	0.183010047	0.096764844	1.891286542	0.058586104	0.005532945	1.401544296	28.65013237	1	PRJNA827465;Yang2025_Silkie_Wenchang	CRA004775	0.419266142
ENSGALG00010024711	DDX55	2	0.259811226	0.111178211	2.336889791	0.019444915	0	0.311196723	0	1	CRA004775;Yang2025_Silkie_Wenchang	PRJNA827465	0.233679423
ENSGALG00010024711	DDX55	2	0.112933008	0.091840929	1.229658806	0.218824899	0	0.06194689	0	1	CRA004775;PRJNA827465	Yang2025_Silkie_Wenchang	0.73259614
ENSGALG00010024712	LRIF1	2	0.030858197	0.058187365	0.530324696	0.595886827	0.002056246	1.228485787	18.59897683	1	PRJNA827465;Yang2025_Silkie_Wenchang	CRA004775	0.893857407
ENSGALG00010024712	LRIF1	2	0.170770866	0.109579198	1.558424132	0.11913274	0	0.095957209	0	1	CRA004775;Yang2025_Silkie_Wenchang	PRJNA827465	0.579471011
ENSGALG00010024712	LRIF1	2	0.013236192	0.04764059	0.277834351	0.781139521	0.000261448	1.010184426	1.008174904	1	CRA004775;PRJNA827465	Yang2025_Silkie_Wenchang	0.945235709
ENSGALG00010024713	RND2	2	0.190720199	0.319920389	0.596154533	0.551071996	0.071412104	1.497867409	33.23841657	0.5	PRJNA827465;Yang2025_Silkie_Wenchang	CRA004775	0.851087109
ENSGALG00010024713	RND2	2	0.525830921	0.386251961	1.361367642	0.173397536	0	0.099684769	0	1	CRA004775;Yang2025_Silkie_Wenchang	PRJNA827465	0.676868485
ENSGALG00010024713	RND2	2	-0.021829587	0.291350989	-0.074925392	0.940274074	0	0.163084153	0	0.5	CRA004775;PRJNA827465	Yang2025_Silkie_Wenchang	0.985508732
ENSGALG00010024714	SRPRB	2	-0.165059508	0.290545745	-0.56810162	0.569965968	0.151617105	8.988876052	88.87513863	0.5	PRJNA827465;Yang2025_Silkie_Wenchang	CRA004775	0.884923673
ENSGALG00010024714	SRPRB	2	-0.515531307	0.141970211	-3.631263939	0.000282037	0	0.051085165	0	1	CRA004775;Yang2025_Silkie_Wenchang	PRJNA827465	0.016365461
ENSGALG00010024714	SRPRB	2	-0.193377835	0.322592001	-0.599450187	0.548872719	0.186927928	8.965383167	88.84598704	0.5	CRA004775;PRJNA827465	Yang2025_Silkie_Wenchang	0.878281266
ENSGALG00010024715	DNAAF1	2	0.223469908	0.160164628	1.395251322	0.162940065	0.036340672	3.402654015	70.611176	1	PRJNA827465;Yang2025_Silkie_Wenchang	CRA004775	0.660959169
ENSGALG00010024715	DNAAF1	2	0.240197092	0.276577956	0.868460724	0.385142165	0.081796878	1.620072033	38.27434955	0.5	CRA004775;Yang2025_Silkie_Wenchang	PRJNA827465	0.826444827
ENSGALG00010024715	DNAAF1	2	0.056207358	0.108026745	0.520309647	0.602847773	0	0.429527838	0	0.5	CRA004775;PRJNA827465	Yang2025_Silkie_Wenchang	0.895538966
ENSGALG00010024716	GTF2A2	2	-0.062295195	0.055359631	-1.125292813	0.260464974	0	0.512748634	0	1	PRJNA827465;Yang2025_Silkie_Wenchang	CRA004775	0.767694434
ENSGALG00010024716	GTF2A2	2	-0.168845376	0.137939922	-1.224050109	0.220933325	0	0.032298677	0	1	CRA004775;Yang2025_Silkie_Wenchang	PRJNA827465	0.73419654
ENSGALG00010024716	GTF2A2	2	-0.057288272	0.055614734	-1.030091625	0.302966997	0	0.195378286	0	1	CRA004775;PRJNA827465	Yang2025_Silkie_Wenchang	0.792084776
ENSGALG00010024717	TF	2	-0.488144436	1.280818992	-0.381118987	0.703114959	2.786409542	6.558580162	84.75279748	0.5	PRJNA827465;Yang2025_Silkie_Wenchang	CRA004775	0.923459557
ENSGALG00010024717	TF	2	-1.230546805	0.457709755	-2.688487174	0.007177659	0.088778212	1.267947294	21.13236843	1	CRA004775;Yang2025_Silkie_Wenchang	PRJNA827465	0.130253231
ENSGALG00010024717	TF	2	-0.075119485	0.817326828	-0.091908747	0.926770542	0.877629579	2.829550027	64.65869165	0.5	CRA004775;PRJNA827465	Yang2025_Silkie_Wenchang	0.982888437
ENSGALG00010024718	ENSGALG00010024718	2	0.278632134	0.159306664	1.749029993	0.080285835	0	0.035827397	0	1	PRJNA827465;Yang2025_Silkie_Wenchang	CRA004775	0.487575691
ENSGALG00010024718	ENSGALG00010024718	2	0.099659259	0.272453565	0.365784383	0.714525974	0	0.742056312	0	0.5	CRA004775;Yang2025_Silkie_Wenchang	PRJNA827465	0.926596201
ENSGALG00010024718	ENSGALG00010024718	2	0.19673161	0.157870005	1.246162057	0.212704906	0	0.880193945	0	0.5	CRA004775;PRJNA827465	Yang2025_Silkie_Wenchang	0.725541376
ENSGALG00010024719	PYM1	2	0.424259439	0.330358708	1.284238705	0.199058446	0.201869537	13.18201331	92.4139054	1	PRJNA827465;Yang2025_Silkie_Wenchang	CRA004775	0.71096127
ENSGALG00010024719	PYM1	2	0.199791037	0.578478671	0.345373213	0.729813813	0.630224254	16.96487413	94.10546761	0.5	CRA004775;Yang2025_Silkie_Wenchang	PRJNA827465	0.932444623
ENSGALG00010024719	PYM1	2	-0.099122103	0.243849392	-0.40648903	0.684383315	0.089746228	3.683987056	72.85549638	0.5	CRA004775;PRJNA827465	Yang2025_Silkie_Wenchang	0.91887806
ENSGALG00010024720	BUD13	2	0.021143457	0.100574189	0.210227469	0.833490142	0.010013655	1.871246678	46.55969138	0.5	PRJNA827465;Yang2025_Silkie_Wenchang	CRA004775	0.957968425
ENSGALG00010024720	BUD13	2	-0.282468792	0.189364971	-1.491663181	0.135787413	0.049626674	3.199561557	68.74571774	1	CRA004775;Yang2025_Silkie_Wenchang	PRJNA827465	0.617196137
ENSGALG00010024720	BUD13	2	-0.175263954	0.293079404	-0.598008429	0.549834309	0.155179476	9.958613682	89.95844169	0.5	CRA004775;PRJNA827465	Yang2025_Silkie_Wenchang	0.878603751
ENSGALG00010024722	BRCA1	2	0.12301611	0.088898275	1.383785109	0.166424264	0.006121535	1.629165894	38.61889673	1	PRJNA827465;Yang2025_Silkie_Wenchang	CRA004775	0.665350942
ENSGALG00010024722	BRCA1	2	0.202690785	0.093450641	2.168969583	0.030085654	0	0.141499224	0	0.5	CRA004775;Yang2025_Silkie_Wenchang	PRJNA827465	0.295973612
ENSGALG00010024722	BRCA1	2	0.02732404	0.101817455	0.268363025	0.788419898	0	0.017249836	0	0.5	CRA004775;PRJNA827465	Yang2025_Silkie_Wenchang	0.94704362
ENSGALG00010024723	ITCH	2	0.025197496	0.040397704	0.623735853	0.532801073	0	0.217806387	0	0.5	PRJNA827465;Yang2025_Silkie_Wenchang	CRA004775	0.873183244
ENSGALG00010024723	ITCH	2	0.011385023	0.081565672	0.139581057	0.888991008	0	0.714886884	0	0.5	CRA004775;Yang2025_Silkie_Wenchang	PRJNA827465	0.971335755
ENSGALG00010024723	ITCH	2	0.040063601	0.045240847	0.898823138	0.368745008	0	0.506453008	0	1	CRA004775;PRJNA827465	Yang2025_Silkie_Wenchang	0.820425502
ENSGALG00010024724	HOXD3	2	-0.027768807	0.151981956	-0.182711207	0.855024622	0.024391208	2.096844448	52.30929022	0.5	PRJNA827465;Yang2025_Silkie_Wenchang	CRA004775	0.963317625
ENSGALG00010024724	HOXD3	2	0.144364084	0.152289302	0.947959457	0.343150094	0	0.00638363	0	0.5	CRA004775;Yang2025_Silkie_Wenchang	PRJNA827465	0.806218116
ENSGALG00010024724	HOXD3	2	-0.13440755	0.126963121	-1.058634581	0.289766232	0	0.602957087	0	0.5	CRA004775;PRJNA827465	Yang2025_Silkie_Wenchang	0.784067533
ENSGALG00010024726	TMED2	2	-0.171375316	0.222879284	-0.768915413	0.441943527	0.096037559	29.58447577	96.61984884	0.5	PRJNA827465;Yang2025_Silkie_Wenchang	CRA004775	0.84515555
ENSGALG00010024726	TMED2	2	-0.326283692	0.106834206	-3.054112584	0.002257274	0.011202752	1.740338878	42.53992542	1	CRA004775;Yang2025_Silkie_Wenchang	PRJNA827465	0.062920299
ENSGALG00010024726	TMED2	2	-0.007064925	0.093505448	-0.075556297	0.939772107	0.010436341	1.804604915	44.58620878	0.5	CRA004775;PRJNA827465	Yang2025_Silkie_Wenchang	0.985429356
ENSGALG00010024728	SLC5A9	2	0.559946013	0.245926138	2.276889049	0.022792977	0	0.593589562	0	1	PRJNA827465;Yang2025_Silkie_Wenchang	CRA004775	0.23727524
ENSGALG00010024728	SLC5A9	2	1.391622371	0.631222437	2.204646556	0.027478906	0.528652118	2.891641464	65.41756603	1	CRA004775;Yang2025_Silkie_Wenchang	PRJNA827465	0.282945624
ENSGALG00010024728	SLC5A9	2	1.179847674	0.833061265	1.416279598	0.156693659	1.170028478	6.034476803	83.42855507	1	CRA004775;PRJNA827465	Yang2025_Silkie_Wenchang	0.649910897
ENSGALG00010024729	FOXM1	2	0.015281956	0.046202607	0.330759603	0.740826077	0	0.164715031	0	0.5	PRJNA827465;Yang2025_Silkie_Wenchang	CRA004775	0.935265557
ENSGALG00010024729	FOXM1	2	0.115646255	0.230234301	0.502298115	0.615451585	0.059608121	1.849733418	45.93815573	0.5	CRA004775;Yang2025_Silkie_Wenchang	PRJNA827465	0.898864379
ENSGALG00010024729	FOXM1	2	0.116217147	0.183947554	0.631795011	0.527520834	0.040022088	1.624604622	38.44656194	1	CRA004775;PRJNA827465	Yang2025_Silkie_Wenchang	0.871149047
ENSGALG00010024731	AOX1	2	0.024083739	0.404112602	0.059596604	0.952476923	0.276967814	6.477457919	84.5618449	0.5	PRJNA827465;Yang2025_Silkie_Wenchang	CRA004775	0.987758913
ENSGALG00010024731	AOX1	2	0.446030498	0.238449058	1.870548378	0.061407706	0	0.001571506	0	1	CRA004775;Yang2025_Silkie_Wenchang	PRJNA827465	0.427938152
ENSGALG00010024731	AOX1	2	-0.16892215	0.336021861	-0.502711786	0.615160922	0.115885156	1.605930491	37.73080433	0.5	CRA004775;PRJNA827465	Yang2025_Silkie_Wenchang	0.8988191
ENSGALG00010024732	AHCY	2	-0.16887235	0.209872322	-0.804643264	0.421025569	0.066068747	3.955419856	74.71823381	0.5	PRJNA827465;Yang2025_Silkie_Wenchang	CRA004775	0.839269089
ENSGALG00010024732	AHCY	2	-0.338923398	0.13358006	-2.537230459	0.011173337	0	0.283975488	0	1	CRA004775;Yang2025_Silkie_Wenchang	PRJNA827465	0.167907012
ENSGALG00010024732	AHCY	2	-0.04682512	0.121726216	-0.384675723	0.700477684	0.003550871	1.106190296	9.599640885	0.5	CRA004775;PRJNA827465	Yang2025_Silkie_Wenchang	0.922303014
ENSGALG00010024733	MBTPS1	2	-0.198551358	0.18115103	-1.09605426	0.27305503	0.060575537	12.59172347	92.0582754	1	PRJNA827465;Yang2025_Silkie_Wenchang	CRA004775	0.773904763
ENSGALG00010024733	MBTPS1	2	-0.236658137	0.169393079	-1.397094485	0.16238516	0.043379506	3.962147948	74.76116457	1	CRA004775;Yang2025_Silkie_Wenchang	PRJNA827465	0.659930202
ENSGALG00010024733	MBTPS1	2	-0.028224259	0.041537959	-0.679481114	0.49683307	0	0.021632997	0	1	CRA004775;PRJNA827465	Yang2025_Silkie_Wenchang	0.860939846
ENSGALG00010024734	ZPR1	2	-0.027204767	0.058522697	-0.464858393	0.642032868	0	0.769153464	0	0.5	PRJNA827465;Yang2025_Silkie_Wenchang	CRA004775	0.906634523
ENSGALG00010024734	ZPR1	2	-0.141411732	0.224298135	-0.630463254	0.528391537	0.079190867	4.629852213	78.40103844	0.5	CRA004775;Yang2025_Silkie_Wenchang	PRJNA827465	0.871701504
ENSGALG00010024734	ZPR1	2	-0.180901516	0.158682531	-1.140021621	0.254277294	0.03677148	3.264976893	69.37191187	1	CRA004775;PRJNA827465	Yang2025_Silkie_Wenchang	0.764021784
ENSGALG00010024735	OTUD7A	2	-0.053907315	0.22362617	-0.241059957	0.809508648	0.083123954	5.876981049	82.98446104	0.5	PRJNA827465;Yang2025_Silkie_Wenchang	CRA004775	0.952770549
ENSGALG00010024735	OTUD7A	2	0.200087126	0.136098554	1.4701635	0.141517478	0	0.192317646	0	1	CRA004775;Yang2025_Silkie_Wenchang	PRJNA827465	0.626852409
ENSGALG00010024735	OTUD7A	2	-0.068191335	0.295170391	-0.231023629	0.817296445	0.114299547	2.312328383	56.75354732	0.5	CRA004775;PRJNA827465	Yang2025_Silkie_Wenchang	0.954854362
ENSGALG00010024736	DGKA	2	-0.041800726	0.079029226	-0.528927437	0.596855788	0	0.01655501	0	1	PRJNA827465;Yang2025_Silkie_Wenchang	CRA004775	0.894172266
ENSGALG00010024736	DGKA	2	-0.147829713	0.143878906	-1.027459254	0.304204262	0	0.713454518	0	1	CRA004775;Yang2025_Silkie_Wenchang	PRJNA827465	0.79225794
ENSGALG00010024736	DGKA	2	-0.082287182	0.104107084	-0.79040905	0.429288918	0.005096474	1.145461026	12.69890662	1	CRA004775;PRJNA827465	Yang2025_Silkie_Wenchang	0.842008086
ENSGALG00010024737	STK38	2	-0.106247622	0.042987198	-2.471610785	0.013450587	0	0.10262841	0	1	PRJNA827465;Yang2025_Silkie_Wenchang	CRA004775	0.189751764
ENSGALG00010024737	STK38	2	-0.133981995	0.077286773	-1.733569525	0.082994492	0	0.078960335	0	1	CRA004775;Yang2025_Silkie_Wenchang	PRJNA827465	0.495960545
ENSGALG00010024737	STK38	2	-0.100967029	0.050140442	-2.013684458	0.044042673	0	0.152957296	0	1	CRA004775;PRJNA827465	Yang2025_Silkie_Wenchang	0.362678958
ENSGALG00010024738	NBR1	2	0.063465084	0.040093533	1.582925708	0.113438399	0	0.033132939	0	1	PRJNA827465;Yang2025_Silkie_Wenchang	CRA004775	0.568537283
ENSGALG00010024738	NBR1	2	0.081679416	0.066384612	1.230396823	0.218548543	0	0.073901905	0	1	CRA004775;Yang2025_Silkie_Wenchang	PRJNA827465	0.732214932
ENSGALG00010024738	NBR1	2	0.063301605	0.046609742	1.358119611	0.174425733	0	0.136741128	0	1	CRA004775;PRJNA827465	Yang2025_Silkie_Wenchang	0.678690684
ENSGALG00010024741	RILPL2	2	0.033456778	0.076353118	0.438184833	0.661252302	0	0.155357017	0	0.5	PRJNA827465;Yang2025_Silkie_Wenchang	CRA004775	0.912189788
ENSGALG00010024741	RILPL2	2	-0.018354699	0.156433156	-0.11733254	0.90659654	0	0.017496078	0	0.5	CRA004775;Yang2025_Silkie_Wenchang	PRJNA827465	0.978196186
ENSGALG00010024741	RILPL2	2	0.046462758	0.082279172	0.564696467	0.572280238	0	0.005432952	0	1	CRA004775;PRJNA827465	Yang2025_Silkie_Wenchang	0.885651577
ENSGALG00010024742	PAK2	2	-0.02574359	0.042406815	-0.607062582	0.543809381	0	0.244392074	0	1	PRJNA827465;Yang2025_Silkie_Wenchang	CRA004775	0.876968972
ENSGALG00010024742	PAK2	2	-0.056348394	0.081930771	-0.687756181	0.491606335	0	0.05724998	0	0.5	CRA004775;Yang2025_Silkie_Wenchang	PRJNA827465	0.85895707
ENSGALG00010024742	PAK2	2	-0.013457725	0.047741299	-0.281888534	0.778028982	0	0.003205814	0	0.5	CRA004775;PRJNA827465	Yang2025_Silkie_Wenchang	0.944480563
ENSGALG00010024744	KLF13	2	0.099634204	0.248228164	0.401381545	0.688139235	0.094416616	3.897456127	74.34223844	0.5	PRJNA827465;Yang2025_Silkie_Wenchang	CRA004775	0.919679555
ENSGALG00010024744	KLF13	2	0.315215283	0.170083795	1.853294032	0.063840226	0	0.24952043	0	1	CRA004775;Yang2025_Silkie_Wenchang	PRJNA827465	0.435307496
ENSGALG00010024744	KLF13	2	-0.018482466	0.148353299	-0.124584125	0.900852797	0.019484106	1.538140666	34.98644029	0.5	CRA004775;PRJNA827465	Yang2025_Silkie_Wenchang	0.976507735
ENSGALG00010024745	PMEL	2	9.10018767	0.30661005	29.68000448	1.3912E-193	0	0.098071077	0	1	PRJNA827465;Yang2025_Silkie_Wenchang	CRA004775	5.468E-189
ENSGALG00010024745	PMEL	2	8.735420883	0.322850845	27.05714115	3.1476E-161	0	0.379288851	0	1	CRA004775;Yang2025_Silkie_Wenchang	PRJNA827465	3.0928E-157
ENSGALG00010024745	PMEL	2	8.894520075	0.300883727	29.56131977	4.6979E-192	0.019485711	1.119833704	10.70102672	1	CRA004775;PRJNA827465	Yang2025_Silkie_Wenchang	9.2322E-188
ENSGALG00010024747	TULP3	2	-0.182771248	0.217586559	-0.839993284	0.400912152	0.089530301	18.07471115	94.46740813	0.5	PRJNA827465;Yang2025_Silkie_Wenchang	CRA004775	0.832395176
ENSGALG00010024747	TULP3	2	-0.28350678	0.165965414	-1.708228074	0.087594031	0.034700726	2.369378976	57.79484792	1	CRA004775;Yang2025_Silkie_Wenchang	PRJNA827465	0.508938499
ENSGALG00010024747	TULP3	2	0.023638656	0.047568949	0.496934584	0.619235182	0	0.175498207	0	0.5	CRA004775;PRJNA827465	Yang2025_Silkie_Wenchang	0.899168321
ENSGALG00010024749	APOA5	2	-0.02099857	0.631643377	-0.033244344	0.973479736	0.595130533	3.934059948	74.58096691	0.5	PRJNA827465;Yang2025_Silkie_Wenchang	CRA004775	0.992392211
ENSGALG00010024749	APOA5	2	-0.595300428	0.271861246	-2.189721544	0.028544438	0	0.027494641	0	1	CRA004775;Yang2025_Silkie_Wenchang	PRJNA827465	0.287842636
ENSGALG00010024749	APOA5	2	-0.012932003	0.583520141	-0.02216205	0.98231869	0.526577204	4.354226142	77.03380653	0.5	CRA004775;PRJNA827465	Yang2025_Silkie_Wenchang	0.99513702
ENSGALG00010024751	MTMR10	2	-0.020276367	0.058593658	-0.346050537	0.729304735	0.002075863	1.37670717	27.36291191	0.5	PRJNA827465;Yang2025_Silkie_Wenchang	CRA004775	0.9323457
ENSGALG00010024751	MTMR10	2	-0.091159163	0.084857681	-1.074259422	0.282706426	0	0.144852685	0	0.5	CRA004775;Yang2025_Silkie_Wenchang	PRJNA827465	0.779532565
ENSGALG00010024751	MTMR10	2	0.022138062	0.055337255	0.400057097	0.689114463	0	0.000363908	0	1	CRA004775;PRJNA827465	Yang2025_Silkie_Wenchang	0.919854035
ENSGALG00010024752	MPP2	2	0.004926749	0.114303493	0.043102348	0.965619947	0	0.719792735	0	0.5	PRJNA827465;Yang2025_Silkie_Wenchang	CRA004775	0.990668014
ENSGALG00010024752	MPP2	2	0.062445779	0.152767498	0.408763509	0.682713222	0	0.441855569	0	0.5	CRA004775;Yang2025_Silkie_Wenchang	PRJNA827465	0.918738538
ENSGALG00010024752	MPP2	2	-0.094730258	0.14217794	-0.666279576	0.505232418	0	0.030051439	0	1	CRA004775;PRJNA827465	Yang2025_Silkie_Wenchang	0.863862536
ENSGALG00010024753	HOXD8	2	-0.090139281	0.108338922	-0.832011975	0.405402185	0.01269767	2.097404251	52.32201902	0.5	PRJNA827465;Yang2025_Silkie_Wenchang	CRA004775	0.833112102
ENSGALG00010024753	HOXD8	2	0.053330342	0.11750416	0.453859181	0.649930185	0	0.083356281	0	1	CRA004775;Yang2025_Silkie_Wenchang	PRJNA827465	0.908335059
ENSGALG00010024753	HOXD8	2	-0.159423069	0.081921714	-1.94604168	0.051649733	0	0.932416731	0	0.5	CRA004775;PRJNA827465	Yang2025_Silkie_Wenchang	0.394019694
ENSGALG00010024754	EIF2S2	2	0.299518466	0.339552827	0.882096812	0.377724456	0.223876325	33.79087519	97.04062119	0.5	PRJNA827465;Yang2025_Silkie_Wenchang	CRA004775	0.82325159
ENSGALG00010024754	EIF2S2	2	0.541574294	0.194341673	2.786712115	0.005324576	0.036904397	1.519642087	34.19503129	1	CRA004775;Yang2025_Silkie_Wenchang	PRJNA827465	0.109021737
ENSGALG00010024754	EIF2S2	2	-0.029792338	0.041625604	-0.715721449	0.474163364	0	0.349415347	0	0.5	CRA004775;PRJNA827465	Yang2025_Silkie_Wenchang	0.85444425
ENSGALG00010024755	PIGX	2	-0.052216501	0.098988715	-0.527499527	0.597846745	0	0.286528607	0	1	PRJNA827465;Yang2025_Silkie_Wenchang	CRA004775	0.894645663
ENSGALG00010024755	PIGX	2	-0.326063719	0.204227216	-1.596573297	0.110360855	0.007858	1.096135611	8.770412162	1	CRA004775;Yang2025_Silkie_Wenchang	PRJNA827465	0.562436811
ENSGALG00010024755	PIGX	2	-0.228099084	0.27007272	-0.844583946	0.398343176	0.101045799	2.726536313	63.32342998	1	CRA004775;PRJNA827465	Yang2025_Silkie_Wenchang	0.831980453
ENSGALG00010024756	CDK2	2	0.080612612	0.074383985	1.083736122	0.278481815	0	0.556734536	0	1	PRJNA827465;Yang2025_Silkie_Wenchang	CRA004775	0.776763074
ENSGALG00010024756	CDK2	2	0.018915544	0.26359912	0.071758751	0.9427939	0.070188094	1.850981254	45.97460142	0.5	CRA004775;Yang2025_Silkie_Wenchang	PRJNA827465	0.985827325
ENSGALG00010024756	CDK2	2	0.011682994	0.126608079	0.092276841	0.926478088	0.011591902	1.172085087	14.68196198	0.5	CRA004775;PRJNA827465	Yang2025_Silkie_Wenchang	0.98280842
ENSGALG00010024757	PPFIA1	2	0.085152507	0.114212469	0.745562267	0.455931884	0.018041038	2.548468705	60.76075024	1	PRJNA827465;Yang2025_Silkie_Wenchang	CRA004775	0.84907427
ENSGALG00010024757	PPFIA1	2	0.289453682	0.125392427	2.308382487	0.020977872	0	0.255326383	0	1	CRA004775;Yang2025_Silkie_Wenchang	PRJNA827465	0.245277621
ENSGALG00010024757	PPFIA1	2	0.127669494	0.178763247	0.714182001	0.475114645	0.045512259	2.612308086	61.71967597	1	CRA004775;PRJNA827465	Yang2025_Silkie_Wenchang	0.854462864
ENSGALG00010024758	SPATA6	2	-0.100352622	0.118114103	-0.84962438	0.395533953	0.017339371	2.339616795	57.25795771	1	PRJNA827465;Yang2025_Silkie_Wenchang	CRA004775	0.83100823
ENSGALG00010024758	SPATA6	2	-0.237580878	0.134944664	-1.760580014	0.078309514	0	0.128113269	0	1	CRA004775;Yang2025_Silkie_Wenchang	PRJNA827465	0.481707034
ENSGALG00010024758	SPATA6	2	-0.015743799	0.065529855	-0.240253828	0.810133487	0	0.098181768	0	1	CRA004775;PRJNA827465	Yang2025_Silkie_Wenchang	0.95294276
ENSGALG00010024759	APOC3	2	0.030331196	0.925724921	0.032764804	0.973862145	1.151072423	2.98238521	66.4697908	0.5	PRJNA827465;Yang2025_Silkie_Wenchang	CRA004775	0.992472082
ENSGALG00010024759	APOC3	2	0.19067378	1.004472221	0.189824841	0.849446389	1.596938522	4.792149608	79.13253797	0.5	CRA004775;Yang2025_Silkie_Wenchang	PRJNA827465	0.961655426
ENSGALG00010024759	APOC3	2	1.148929918	0.526296861	2.183045354	0.02903247	0	0.018349873	0	1	CRA004775;PRJNA827465	Yang2025_Silkie_Wenchang	0.289904262
ENSGALG00010024760	ENSGALG00010024760	2	-0.058209732	0.111071293	-0.524075397	0.6002261	0	0.031342016	0	1	PRJNA827465;Yang2025_Silkie_Wenchang	CRA004775	0.895230519
ENSGALG00010024760	ENSGALG00010024760	2	-0.109081885	0.146968383	-0.742213271	0.457958129	0	0.045541594	0	1	CRA004775;Yang2025_Silkie_Wenchang	PRJNA827465	0.849857328
ENSGALG00010024760	ENSGALG00010024760	2	-0.070107509	0.119790513	-0.585250932	0.558379003	0	0.146447916	0	1	CRA004775;PRJNA827465	Yang2025_Silkie_Wenchang	0.880678789
ENSGALG00010024761	NECAB2	2	-0.022385764	0.162225647	-0.137991522	0.890247118	0.023228624	1.638039107	38.95139647	0.5	PRJNA827465;Yang2025_Silkie_Wenchang	CRA004775	0.973414452
ENSGALG00010024761	NECAB2	2	-0.287731783	0.221098567	-1.301373353	0.19313069	0	0.141139187	0	1	CRA004775;Yang2025_Silkie_Wenchang	PRJNA827465	0.703200102
ENSGALG00010024761	NECAB2	2	0.065556675	0.123593944	0.530419804	0.595820898	0	0.936137284	0	0.5	CRA004775;PRJNA827465	Yang2025_Silkie_Wenchang	0.893857407
ENSGALG00010024762	FAN1	2	0.123741717	0.068608407	1.803594097	0.071294962	0	0.573432812	0	1	PRJNA827465;Yang2025_Silkie_Wenchang	CRA004775	0.461027938
ENSGALG00010024762	FAN1	2	0.138235022	0.109843538	1.25847205	0.208221089	0	0.902382427	0	0.5	CRA004775;Yang2025_Silkie_Wenchang	PRJNA827465	0.720917211
ENSGALG00010024762	FAN1	2	0.077286485	0.073834593	1.04675169	0.295214117	0	0.252508233	0	0.5	CRA004775;PRJNA827465	Yang2025_Silkie_Wenchang	0.787628307
ENSGALG00010024763	HOXD9	2	0.138186323	0.167763774	0.823695844	0.410112401	0.011253213	1.182193526	15.41148063	1	PRJNA827465;Yang2025_Silkie_Wenchang	CRA004775	0.834652129
ENSGALG00010024763	HOXD9	2	-0.051250569	0.483140466	-0.106077989	0.915520475	0.359463333	4.339444228	76.95557432	0.5	CRA004775;Yang2025_Silkie_Wenchang	PRJNA827465	0.980165711
ENSGALG00010024763	HOXD9	2	-0.175126533	0.281456243	-0.622215843	0.53379995	0.098069075	2.349116343	57.43080145	0.5	CRA004775;PRJNA827465	Yang2025_Silkie_Wenchang	0.873408008
ENSGALG00010024764	ENSGALG00010024764	2	0.073242179	0.153436738	0.477344477	0.633116845	0	0.00169478	0	1	PRJNA827465;Yang2025_Silkie_Wenchang	CRA004775	0.903241183
ENSGALG00010024764	ENSGALG00010024764	2	0.010248591	0.243817323	0.042033891	0.966471681	0	0.431078765	0	0.5	CRA004775;Yang2025_Silkie_Wenchang	PRJNA827465	0.990881287
ENSGALG00010024764	ENSGALG00010024764	2	0.045694634	0.185229803	0.246691587	0.805146918	0	0.475221013	0	0.5	CRA004775;PRJNA827465	Yang2025_Silkie_Wenchang	0.951863117
ENSGALG00010024765	SNRNP35	2	0.151887592	0.05351916	2.838004042	0.004539661	0	0.171225567	0	1	PRJNA827465;Yang2025_Silkie_Wenchang	CRA004775	0.098668611
ENSGALG00010024765	SNRNP35	2	0.17894988	0.119879542	1.492747439	0.135503311	0	0.337366269	0	0.5	CRA004775;Yang2025_Silkie_Wenchang	PRJNA827465	0.616997992
ENSGALG00010024765	SNRNP35	2	0.137660687	0.058762618	2.342657463	0.019146953	0	0.221806385	0	0.5	CRA004775;PRJNA827465	Yang2025_Silkie_Wenchang	0.231833851
ENSGALG00010024767	CTTN	2	0.2500158	0.246491322	1.014298589	0.310440297	0.110247497	10.20539848	90.20126454	1	PRJNA827465;Yang2025_Silkie_Wenchang	CRA004775	0.794899969
ENSGALG00010024767	CTTN	2	0.382450304	0.14080487	2.716172414	0.006604152	0.016393312	1.701510854	41.22870285	1	CRA004775;Yang2025_Silkie_Wenchang	PRJNA827465	0.124138283
ENSGALG00010024767	CTTN	2	0.074198984	0.091672987	0.809387663	0.418292196	0.008493972	1.60384332	37.64976993	1	CRA004775;PRJNA827465	Yang2025_Silkie_Wenchang	0.838112595
ENSGALG00010024769	RALY	2	-0.188726399	0.141064308	-1.337874911	0.180937217	0.023578308	2.42390959	58.74433584	1	PRJNA827465;Yang2025_Silkie_Wenchang	CRA004775	0.688486342
ENSGALG00010024769	RALY	2	-0.041047297	0.137325799	-0.298904485	0.765012925	0	0.03530381	0	1	CRA004775;Yang2025_Silkie_Wenchang	PRJNA827465	0.940771616
ENSGALG00010024769	RALY	2	-0.309269507	0.110884194	-2.789121652	0.005285121	0	0.114107216	0	1	CRA004775;PRJNA827465	Yang2025_Silkie_Wenchang	0.108631224
ENSGALG00010024770	CDC27	2	0.050658422	0.210126471	0.241085391	0.809488935	0.07921978	8.986336211	88.87199436	0.5	PRJNA827465;Yang2025_Silkie_Wenchang	CRA004775	0.952770549
ENSGALG00010024770	CDC27	2	0.248209907	0.113971659	2.177821306	0.029419343	0	0.208064834	0	1	CRA004775;Yang2025_Silkie_Wenchang	PRJNA827465	0.292356116
ENSGALG00010024770	CDC27	2	-0.053899377	0.138465814	-0.389261256	0.697082894	0.025291808	2.131298319	53.08024264	0.5	CRA004775;PRJNA827465	Yang2025_Silkie_Wenchang	0.921116491
ENSGALG00010024771	AOX2	2	-0.069675469	0.307042538	-0.226924482	0.820482465	0.129022574	3.162384705	68.37829381	0.5	PRJNA827465;Yang2025_Silkie_Wenchang	CRA004775	0.955381125
ENSGALG00010024771	AOX2	2	0.23593271	0.225230991	1.047514413	0.294862388	0	0.006953558	0	1	CRA004775;Yang2025_Silkie_Wenchang	PRJNA827465	0.78756211
ENSGALG00010024771	AOX2	2	-0.241652145	0.236751751	-1.020698449	0.30739733	0.017596347	1.122076509	10.87951741	0.5	CRA004775;PRJNA827465	Yang2025_Silkie_Wenchang	0.792675978
ENSGALG00010024772	ELAPOR1	2	0.386356238	0.243118872	1.58916597	0.112022927	0.097538113	5.707962079	82.48061241	1	PRJNA827465;Yang2025_Silkie_Wenchang	CRA004775	0.56547057
ENSGALG00010024772	ELAPOR1	2	0.303029167	0.374832336	0.808439235	0.418837773	0.227163848	4.90417198	79.60919796	0.5	CRA004775;Yang2025_Silkie_Wenchang	PRJNA827465	0.838250071
ENSGALG00010024772	ELAPOR1	2	0.103337887	0.12421752	0.831910728	0.405459336	0	0.647237116	0	0.5	CRA004775;PRJNA827465	Yang2025_Silkie_Wenchang	0.833112102
ENSGALG00010024773	APOA1	2	0.093666296	0.23720802	0.394869853	0.69293894	0	0.221161462	0	1	PRJNA827465;Yang2025_Silkie_Wenchang	CRA004775	0.92062328
ENSGALG00010024773	APOA1	2	-0.100790355	0.244558359	-0.41213212	0.680242587	0	0.672055684	0	0.5	CRA004775;Yang2025_Silkie_Wenchang	PRJNA827465	0.917768536
ENSGALG00010024773	APOA1	2	-0.055940656	0.346839159	-0.161287025	0.871867342	0.037860361	1.184961905	15.60910136	0.5	CRA004775;PRJNA827465	Yang2025_Silkie_Wenchang	0.969462602
ENSGALG00010024775	ITFG2	2	0.036375122	0.09313512	0.390562891	0.69612036	0.012991564	3.922172451	74.50392576	0.5	PRJNA827465;Yang2025_Silkie_Wenchang	CRA004775	0.921038437
ENSGALG00010024775	ITFG2	2	0.148657733	0.072505693	2.050290482	0.040336093	0	0.343711561	0	1	CRA004775;Yang2025_Silkie_Wenchang	PRJNA827465	0.347442284
ENSGALG00010024775	ITFG2	2	0.028507257	0.145311222	0.1961807	0.844468734	0.024130402	1.67092849	40.15303433	0.5	CRA004775;PRJNA827465	Yang2025_Silkie_Wenchang	0.960752464
ENSGALG00010024776	MPHOSPH10	2	0.383041697	0.417566878	0.917318199	0.358975924	0.325967876	14.64465091	93.1715682	0.5	PRJNA827465;Yang2025_Silkie_Wenchang	CRA004775	0.814241185
ENSGALG00010024776	MPHOSPH10	2	0.537119838	0.318001506	1.689048093	0.091210214	0.140249966	3.191531594	68.66708129	1	CRA004775;Yang2025_Silkie_Wenchang	PRJNA827465	0.517862098

ENSGALG00010024776	MPHOSPH10	2	-0.002496571	0.061618277	-0.040516729	0.96768117	0	0.441775183	0	0.5	CRA004775;PRJNA827465	Yang2025_Silkie_Wenchang	0.991065147
ENSGALG00010024778	RAB5B	2	0.036451402	0.122482807	0.297604236	0.766005241	0.018315188	2.012979612	50.32239801	0.5	PRJNA827465;Yang2025_Silkie_Wenchang	CRA004775	0.941057264
ENSGALG00010024778	RAB5B	2	0.031719365	0.214832694	0.147646823	0.8826215	0.051300345	2.228136757	55.1194514	0.5	CRA004775;Yang2025_Silkie_Wenchang	PRJNA827465	0.972001076
ENSGALG00010024778	RAB5B	2	-0.050536603	0.05174501	-0.976646889	0.328743997	0	0.492644257	0	1	CRA004775;PRJNA827465	Yang2025_Silkie_Wenchang	0.800761292
ENSGALG00010024781	CEP19	2	-0.043465732	0.112262625	-0.387179015	0.698623685	0	0.160246597	0	1	PRJNA827465;Yang2025_Silkie_Wenchang	CRA004775	0.921745106
ENSGALG00010024781	CEP19	2	-0.121550576	0.194141094	-0.626094012	0.53125328	0	0.001590879	0	1	CRA004775;Yang2025_Silkie_Wenchang	PRJNA827465	0.872939824
ENSGALG00010024781	CEP19	2	-0.030587699	0.119323128	-0.256343422	0.797685671	0	0.063452124	0	1	CRA004775;PRJNA827465	Yang2025_Silkie_Wenchang	0.949842152
ENSGALG00010024783	UBE2T	2	-0.046123739	0.08616024	-0.535325099	0.592425074	0	0.031832432	0	1	PRJNA827465;Yang2025_Silkie_Wenchang	CRA004775	0.892776493
ENSGALG00010024783	UBE2T	2	0.013202936	0.180201181	0.073267756	0.94159305	0	0.058089203	0	0.5	CRA004775;Yang2025_Silkie_Wenchang	PRJNA827465	0.985827325
ENSGALG00010024783	UBE2T	2	-0.043865977	0.091436444	-0.47974281	0.631410283	0	0.144880917	0	0.5	CRA004775;PRJNA827465	Yang2025_Silkie_Wenchang	0.903058174
ENSGALG00010024784	ENSGALG00010024784	2	0.035533954	0.085912107	0.413608227	0.679161047	0	0.924725298	0	0.5	PRJNA827465;Yang2025_Silkie_Wenchang	CRA004775	0.917701868
ENSGALG00010024784	ENSGALG00010024784	2	0.148184503	0.139229028	1.0643219	0.2871829	0	0.055001915	0	1	CRA004775;Yang2025_Silkie_Wenchang	PRJNA827465	0.782742686
ENSGALG00010024784	ENSGALG00010024784	2	-0.002833041	0.094973114	-0.029829928	0.976202691	0	0.141853868	0	0.5	CRA004775;PRJNA827465	Yang2025_Silkie_Wenchang	0.992992231
ENSGALG00010024785	MCEE	2	-0.166590948	0.166561411	-1.00017733	0.317224698	0.043386796	4.321606588	76.86045734	1	PRJNA827465;Yang2025_Silkie_Wenchang	CRA004775	0.797082395
ENSGALG00010024785	MCEE	2	-0.347920214	0.133676516	-2.602702584	0.009249215	0	0.028941903	0	1	CRA004775;Yang2025_Silkie_Wenchang	PRJNA827465	0.15088718
ENSGALG00010024785	MCEE	2	-0.030815997	0.0739524	-0.416700428	0.676897541	0	0.483218707	0	1	CRA004775;PRJNA827465	Yang2025_Silkie_Wenchang	0.917035058
ENSGALG00010024786	PDCD5	2	-0.165348816	0.054131537	-3.054574589	0.0022538	0	0.304236258	0	1	PRJNA827465;Yang2025_Silkie_Wenchang	CRA004775	0.062868053
ENSGALG00010024786	PDCD5	2	0.080343376	0.335937123	0.239161945	0.81098001	0.188671246	5.591998161	82.11730456	0.5	CRA004775;Yang2025_Silkie_Wenchang	PRJNA827465	0.953118313
ENSGALG00010024786	PDCD5	2	0.103450814	0.300539939	0.344216527	0.730683454	0.148931866	4.937563765	79.747097	0.5	CRA004775;PRJNA827465	Yang2025_Silkie_Wenchang	0.932769533
ENSGALG00010024787	SARS1	2	-0.021414378	0.06336887	-0.337932144	0.735414323	0	0.000243627	0	1	PRJNA827465;Yang2025_Silkie_Wenchang	CRA004775	0.933592889
ENSGALG00010024787	SARS1	2	-0.128250084	0.141517026	-0.906251972	0.3648025	0.009656272	1.282957392	22.05508881	1	CRA004775;Yang2025_Silkie_Wenchang	PRJNA827465	0.817649605
ENSGALG00010024787	SARS1	2	-0.099868392	0.131629504	-0.758708258	0.448027096	0.018610417	1.717875966	41.78857963	1	CRA004775;PRJNA827465	Yang2025_Silkie_Wenchang	0.846601815
ENSGALG00010024788	SIK3	2	0.409827654	0.320230498	1.279789578	0.20061915	0.180360067	7.753582888	87.1027367	1	PRJNA827465;Yang2025_Silkie_Wenchang	CRA004775	0.712970384
ENSGALG00010024788	SIK3	2	0.552473322	0.252002135	2.192335879	0.028355263	0.060912979	1.867059364	46.43983908	1	CRA004775;Yang2025_Silkie_Wenchang	PRJNA827465	0.286858918
ENSGALG00010024788	SIK3	2	0.127988365	0.077988593	1.641116466	0.100773241	0	0.172346705	0	1	CRA004775;PRJNA827465	Yang2025_Silkie_Wenchang	0.541717388
ENSGALG00010024789	SUOX	2	-0.036878455	0.075948928	-0.485569132	0.627272686	0	0.852019252	0	1	PRJNA827465;Yang2025_Silkie_Wenchang	CRA004775	0.901612726
ENSGALG00010024789	SUOX	2	-0.181533089	0.153036329	-1.18620912	0.235539725	0	0.006766857	0	1	CRA004775;Yang2025_Silkie_Wenchang	PRJNA827465	0.748604097
ENSGALG00010024789	SUOX	2	-0.017377247	0.080128035	-0.2168685	0.828310827	0	0.285381495	0	1	CRA004775;PRJNA827465	Yang2025_Silkie_Wenchang	0.956777964
ENSGALG00010024792	BZW1	2	-0.165883999	0.137675113	-1.204894586	0.228243999	0.031747684	5.704343558	82.46949908	1	PRJNA827465;Yang2025_Silkie_Wenchang	CRA004775	0.741040754
ENSGALG00010024792	BZW1	2	-0.345867086	0.089591927	-3.860471539	0.000113168	0	0.167143398	0	1	CRA004775;Yang2025_Silkie_Wenchang	PRJNA827465	0.008456005
ENSGALG00010024792	BZW1	2	-0.188522302	0.175277193	-1.075566645	0.282121112	0.049533469	4.470068681	77.62897907	1	CRA004775;PRJNA827465	Yang2025_Silkie_Wenchang	0.779519046
ENSGALG00010024793	FKBP4	2	0.007745625	0.040669578	0.190452544	0.848954527	0	0.597253587	0	0.5	PRJNA827465;Yang2025_Silkie_Wenchang	CRA004775	0.961364896
ENSGALG00010024793	FKBP4	2	0.054211562	0.097708773	0.554827989	0.579012321	0	0.342930916	0	0.5	CRA004775;Yang2025_Silkie_Wenchang	PRJNA827465	0.887888423
ENSGALG00010024793	FKBP4	2	-0.004977478	0.041873978	-0.118868042	0.905379901	0	0.016846766	0	1	CRA004775;PRJNA827465	Yang2025_Silkie_Wenchang	0.978016333
ENSGALG00010024794	DDX6	2	-0.221278235	0.132117608	-1.674858022	0.093962091	0.030307416	7.193373964	86.09831763	1	PRJNA827465;Yang2025_Silkie_Wenchang	CRA004775	0.525133608
ENSGALG00010024794	DDX6	2	-0.254596703	0.169774611	-1.499615886	0.13371393	0.033141845	1.93737754	48.38383437	0.5	CRA004775;Yang2025_Silkie_Wenchang	PRJNA827465	0.612847937
ENSGALG00010024794	DDX6	2	-0.09790965	0.040612564	-2.410821711	0.015916627	0	0.170760847	0	0.5	CRA004775;PRJNA827465	Yang2025_Silkie_Wenchang	0.208471156
ENSGALG00010024795	NRROS	2	0.048971567	0.109276795	0.448142413	0.65405042	0	0.02400678	0	1	PRJNA827465;Yang2025_Silkie_Wenchang	CRA004775	0.909919778
ENSGALG00010024795	NRROS	2	-0.186668772	0.26229073	-0.711686501	0.476658929	0.07122256	1.968205769	49.19230419	0.5	CRA004775;Yang2025_Silkie_Wenchang	PRJNA827465	0.854986476
ENSGALG00010024795	NRROS	2	-0.15060464	0.277136899	-0.543430486	0.586833441	0.10426329	2.700484348	62.96960578	0.5	CRA004775;PRJNA827465	Yang2025_Silkie_Wenchang	0.891596903
ENSGALG00010024797	IKZF4	2	-0.014163597	0.103422593	-0.136948774	0.891071286	0.009093823	1.554594431	35.67454121	0.5	PRJNA827465;Yang2025_Silkie_Wenchang	CRA004775	0.973638442
ENSGALG00010024797	IKZF4	2	0.062250987	0.13763331	0.452295936	0.651055803	0	0.870687336	0	0.5	CRA004775;Yang2025_Silkie_Wenchang	PRJNA827465	0.908769703
ENSGALG00010024797	IKZF4	2	-0.086749714	0.075133896	-1.154601575	0.248253621	0	0.046341814	0	1	CRA004775;PRJNA827465	Yang2025_Silkie_Wenchang	0.758364066
ENSGALG00010024798	FBXO45	2	-0.155820947	0.057583276	-2.706010441	0.00680969	0.00347537	2.087778616	52.10220125	1	PRJNA827465;Yang2025_Silkie_Wenchang	CRA004775	0.126544334
ENSGALG00010024798	FBXO45	2	-0.211180065	0.058084249	-3.635754385	0.000277168	0	0.140093238	0	1	CRA004775;Yang2025_Silkie_Wenchang	PRJNA827465	0.016138588
ENSGALG00010024798	FBXO45	2	-0.10504548	0.050098162	-2.096793093	0.036011895	0	0.03751636	0	1	CRA004775;PRJNA827465	Yang2025_Silkie_Wenchang	0.327557399
ENSGALG00010024799	ENSGALG00010024799	2	0.457240784	0.55946263	0.817285658	0.413765201	0.600340064	24.05396407	95.84268108	0.5	PRJNA827465;Yang2025_Silkie_Wenchang	CRA004775	0.836533627
ENSGALG00010024799	ENSGALG00010024799	2	0.814924389	0.271916984	2.996960235	0.002726863	0.078963806	2.026102547	50.64415662	1	CRA004775;Yang2025_Silkie_Wenchang	PRJNA827465	0.071211886
ENSGALG00010024799	ENSGALG00010024799	2	0.101100246	0.265320792	0.381049088	0.703166824	0.095823613	2.559392641	60.92823023	0.5	CRA004775;PRJNA827465	Yang2025_Silkie_Wenchang	0.923496815
ENSGALG00010024800	ENSGALG00010024800	2	0.190053856	0.146252697	1.299489581	0.193775967	0.031160422	3.432923065	70.87030554	1	PRJNA827465;Yang2025_Silkie_Wenchang	CRA004775	0.70398689
ENSGALG00010024800	ENSGALG00010024800	2	0.333986215	0.117310077	2.847037726	0.004412813	0	0.131406185	0	1	CRA004775;Yang2025_Silkie_Wenchang	PRJNA827465	0.097436404
ENSGALG00010024800	ENSGALG00010024800	2	0.089238918	0.070358845	1.268339714	0.20467666	0	0.847396889	0	1	CRA004775;PRJNA827465	Yang2025_Silkie_Wenchang	0.71730346
ENSGALG00010024802	CD59	2	-0.126956305	0.160686886	-0.79008504	0.429478106	0.039217615	4.08506006	75.52055575	0.5	PRJNA827465;Yang2025_Silkie_Wenchang	CRA004775	0.842012722
ENSGALG00010024802	CD59	2	-0.299745822	0.120402001	-2.489541871	0.012790785	0	6.58503E-05	0	1	CRA004775;Yang2025_Silkie_Wenchang	PRJNA827465	0.183473076
ENSGALG00010024802	CD59	2	-0.000628152	0.090589587	-0.006934042	0.994467479	0	0.82329673	0	0.5	CRA004775;PRJNA827465	Yang2025_Silkie_Wenchang	0.99823774
ENSGALG00010024803	CLK1	2	0.263382599	0.230283806	1.143730442	0.252735445	0.084084846	4.527401416	77.91227444	1	PRJNA827465;Yang2025_Silkie_Wenchang	CRA004775	0.762731469
ENSGALG00010024803	CLK1	2	0.420553844	0.145645231	2.887522253	0.003882891	0	0.664237165	0	1	CRA004775;Yang2025_Silkie_Wenchang	PRJNA827465	0.089664677
ENSGALG00010024803	CLK1	2	0.099399265	0.089694121	1.10820268	0.267774293	0	0.882365757	0	1	CRA004775;PRJNA827465	Yang2025_Silkie_Wenchang	0.771398294
ENSGALG00010024804	WDR53	2	-0.026881554	0.070138001	-0.383266611	0.701522093	0.001331588	1.104238008	9.439813469	0.5	PRJNA827465;Yang2025_Silkie_Wenchang	CRA004775	0.922874082
ENSGALG00010024804	WDR53	2	0.035592435	0.120765388	0.294723811	0.768204872	0	0.770459732	0	0.5	CRA004775;Yang2025_Silkie_Wenchang	PRJNA827465	0.941444269
ENSGALG00010024804	WDR53	2	-0.070626164	0.067537879	-1.045726703	0.29568723	0	0.078004239	0	1	CRA004775;PRJNA827465	Yang2025_Silkie_Wenchang	0.787730985
ENSGALG00010024805	ENSGALG00010024805	2	0.461952528	0.55504861	0.832274002	0.405254302	0.553674021	9.849870546	89.84758231	0.5	PRJNA827465;Yang2025_Silkie_Wenchang	CRA004775	0.833112102
ENSGALG00010024805	ENSGALG00010024805	2	0.652702313	0.490116852	1.331727955	0.1829496	0.308032185	2.46052507	59.35826819	1	CRA004775;Yang2025_Silkie_Wenchang	PRJNA827465	0.691391164
ENSGALG00010024805	ENSGALG00010024805	2	-0.074387392	0.219107779	-0.339501374	0.734232062	0	0.020486078	0	0.5	CRA004775;PRJNA827465	Yang2025_Silkie_Wenchang	0.933492903
ENSGALG00010024806	ANKRD27	2	0.049517611	0.134885912	0.367107359	0.713538939	0.023288525	2.51799569	60.13964964	0.5	PRJNA827465;Yang2025_Silkie_Wenchang	CRA004775	0.926253108
ENSGALG00010024806	ANKRD27	2	0.192668175	0.132477779	1.454343338	0.145851141	0	0.12238066	0	1	CRA004775;Yang2025_Silkie_Wenchang	PRJNA827465	0.634745585
ENSGALG00010024806	ANKRD27	2	-0.039634677	0.073633582	-0.538269029	0.59039133	0	0.473379205	0	0.5	CRA004775;PRJNA827465	Yang2025_Silkie_Wenchang	0.892501652
ENSGALG00010024807	PPIL3	2	0.056899066	0.121858623	0.466926872	0.640552199	0.011263457	1.533452489	34.78767638	0.5	PRJNA827465;Yang2025_Silkie_Wenchang	CRA004775	0.90623926
ENSGALG00010024807	PPIL3	2	-0.263100111	0.200387521	-1.312956564	0.189197585	0.034116098	1.665147808	39.99452721	1	CRA004775;Yang2025_Silkie_Wenchang	PRJNA827465	0.698184834
ENSGALG00010024807	PPIL3	2	-0.141155779	0.330811473	-0.426695537	0.669601102	0.180978676	5.31125955	81.17207433	0.5	CRA004775;PRJNA827465	Yang2025_Silkie_Wenchang	0.914377769
ENSGALG00010024808	CELSR2	2	0.074886837	0.288689296	0.259402888	0.795324402	0.098681141	2.406196238	58.44062989	0.5	PRJNA827465;Yang2025_Silkie_Wenchang	CRA004775	0.948935068
ENSGALG00010024808	CELSR2	2	0.580829267	0.235926364	2.461909215	0.013819968	0	0.988848419	0	1	CRA004775;Yang2025_Silkie_Wenchang	PRJNA827465	0.193817251
ENSGALG00010024808	CELSR2	2	0.304437777	0.530583311	0.573779406	0.566117086	0.467814611	5.709220746	82.48447417	0.5	CRA004775;PRJNA827465	Yang2025_Silkie_Wenchang	0.883487517
ENSGALG00010024809	KANSL1	2	0.044909278	0.183466887	0.24478138	0.806625712	0.055244344	5.040267044	80.15978139	0.5	PRJNA827465;Yang2025_Silkie_Wenchang	CRA004775	0.952273023
ENSGALG00010024809	KANSL1	2	0.124339658	0.164513712	0.755801179	0.44976841	0.020978831	1.592909244	37.22178438	0.5	CRA004775;Yang2025_Silkie_Wenchang	PRJNA827465	0.847341953
ENSGALG00010024809	KANSL1	2	-0.110189511	0.054564466	-2.019437188	0.0434418	0	0.025435676	0	1	CRA004775;PRJNA827465	Yang2025_Silkie_Wenchang	0.360133527
ENSGALG00010024810	ADIPOR1	2	-0.055871266	0.127428089	-0.4384533	0.661057716	0.021324789	2.366030282	57.73511406	0.5	PRJNA827465;Yang2025_Silkie_Wenchang	CRA004775	0.912081423
ENSGALG00010024810	ADIPOR1	2	-0.270362202	0.138325304	-1.954538998	0.050637508	0	0.105708336	0	1	CRA004775;Yang2025_Silkie_Wenchang	PRJNA827465	0.389701578
ENSGALG00010024810	ADIPOR1	2	-0.075292195	0.167496503	-0.449515027	0.65306017	0.036963416	2.207390093	54.69763125	0.5	CRA004775;PRJNA827465	Yang2025_Silkie_Wenchang	0.909572411
ENSGALG00010024811	NIF3L1	2	-0.121585076	0.069516395	-1.749012963	0.080285779	0	0.108625251	0	1	PRJNA827465;Yang2025_Silkie_Wenchang	CRA004775	0.487575691
ENSGALG00010024811	NIF3L1	2	-0.173581402	0.138696541	-1.251519328	0.210745066	0	0.002900059	0	1	CRA004775;Yang2025_Silkie_Wenchang	PRJNA827465	0.722326096
ENSGALG00010024811	NIF3L1	2	-0.116066739	0.074286322	-1.562424083	0.118188117	0	0.070091106	0	1	CRA004775;PRJNA827465	Yang2025_Silkie_Wenchang	0.578003524
ENSGALG00010024812	SBNO1	2	0.087101822	0.08398951	1.037055963	0.299709977	0.01024002	3.189526111	68.64738005	1	PRJNA827465;Yang2025_Silkie_Wenchang	CRA004775	0.76990225
ENSGALG00010024812	SBNO1	2	0.208083018	0.081749702	2.545367303	0.010916285	0	0.143679854	0	1	CRA004775;Yang2025_Silkie_Wenchang	PRJNA827465	0.166322288
ENSGALG00010024812	SBNO1	2	0.076627129	0.104992014	0.729837687	0.465489405	0.012930763	1.674413766	40.27760521	1	CRA004775;PRJNA827465	Yang2025_Silkie_Wenchang	0.852424459
ENSGALG00010024813	RNF168	2	0.021437586	0.253444873	0.084584809	0.932591476	0.10484455	5.30904763	81.16423002	0.5	PRJNA827465;Yang2025_Silkie_Wenchang	CRA004775	0.984224753
ENSGALG00010024813	RNF168	2	0.28364516	0.133786272	2.120136515	0.033994534	0	0.007211135	0	1	CRA004775;Yang2025_Silkie_Wenchang	PRJNA827465	0.31717041
ENSGALG00010024813	RNF168	2	0.005012091	0.241588051	0.020746434	0.983447928	0.091513768	4.472543743	77.64135898	0.5	CRA004775;PRJNA827465	Yang2025_Silkie_Wenchang	0.995439878
ENSGALG00010024814	APBA2	2	0.156084041	0.227679922	0.685541525	0.493002267	0.078374118	3.746230793	73.30650312	0.5	PRJNA827465;Yang2025_Silkie_Wenchang	CRA004775	0.859609959
ENSGALG00010024814	APBA2	2	0.351294396	0.216455312	1.622941901	0.104601825	0.005522212	1.023685653	2.313762357	0.5	CRA004775;Yang2025_Silkie_Wenchang	PRJNA827465	0.549694549
ENSGALG00010024814	APBA2	2	-0.040185418	0.098522228	-0.407881748	0.683360493	0	0.12162986	0	1	CRA004775;PRJNA827465	Yang2025_Silkie_Wenchang	0.918800744
ENSGALG00010024816	NFIA	2	0.180171081	0.209799094	0.85877912	0.390462389	0.064137223	3.061291669	67.33405019	1	PRJNA827465;Yang2025_Silkie_Wenchang	CRA004775	0.829251057
ENSGALG00010024816	NFIA	2	0.175724764	0.299853108	0.58603616	0.557851216	0.112528231	2.656893954	62.36206574	0.5	CRA004775;Yang2025_Silkie_Wenchang	PRJNA827465	0.880678789
ENSGALG00010024816	NFIA	2	0.012392259	0.067839536	0.182670158	0.855056832	0	0.323643564	0	0.5	CRA004775;PRJNA827465	Yang2025_Silkie_Wenchang	0.963317625
ENSGALG00010024817	UCKL1	2	0.103495481	0.153083065	0.676074002	0.498993662	0.03693111	4.692929503	78.69134835	0.5	PRJNA827465;Yang2025_Silkie_Wenchang	CRA004775	0.861571318
ENSGALG00010024817	UCKL1	2	0.232842056	0.103983626	2.239218466	0.025141705	0	0.958764164	0	0.5	CRA004775;Yang2025_Silkie_Wenchang	PRJNA827465	0.269968973
ENSGALG00010024817	UCKL1	2	-0.048285728	0.088441927	-0.545959697	0.585091642	0	0.038096509	0	1	CRA004775;PRJNA827465	Yang2025_Silkie_Wenchang	0.891086887
ENSGALG00010024818	FBXO3	2	-0.146743587	0.175468729	-0.836294806	0.402989075	0.057538301	15.13677673	93.39357369	0.5	PRJNA827465;Yang2025_Silkie_Wenchang	CRA004775	0.833046091
ENSGALG00010024818	FBXO3	2	-0.222775892	0.151739024	-1.468151613	0.142063044	0.02827799	2.135377223	53.16986671	0.5	CRA004775;Yang2025_Silkie_Wenchang	PRJNA827465	0.627783206
ENSGALG00010024818	FBXO3	2	0.023469869	0.050569822	0.464108203	0.642570225	0	0.013143451	0	1	CRA004775;PRJNA827465	Yang2025_Silkie_Wenchang	0.906634523
ENSGALG00010024820	NUDT19	2	0.031510651	0.133200378	0.236565779	0.812999671	0.025907334	3.663228337	72.70167437	0.5	PRJNA827465;Yang2025_Silkie_Wenchang	CRA004775	0.953526116
ENSGALG00010024820	NUDT19	2	-0.065509217	0.106032229	-0.617823632	0.536691604	0.001236854	1.029415687	2.857512995	0.5	CRA004775;Yang2025_Silkie_Wenchang	PRJNA827465	0.874599474
ENSGALG00010024820	NUDT19	2	0.158862823	0.081981407	1.937790886	0.05264874	0	0.009351792	0	1	CRA004775;PRJNA827465	Yang2025_Silkie_Wenchang	0.398623278
ENSGALG00010024821	NECAP1	2	-0.169962214	0.166214443	-1.022547807	0.306521698	0.050331592	10.90270852	90.82796721	1	PRJNA827465;Yang2025_Silkie_Wenchang	CRA004775	0.792500392
ENSGALG00010024821	NECAP1	2	-0.39169179	0.098969205	-3.957711384	7.56705E-05	0.003884564	1.132470654	11.69749108	1	CRA004775;Yang2025_Silkie_Wenchang	PRJNA827465	0.006362144
ENSGALG00010024821	NECAP1	2	-0.266712507	0.292445916	-0.912006264	0.36176542	0.148069429	6.619555863	84.8932463	1	CRA004775;PRJNA827465	Yang2025_Silkie_Wenchang	0.815545459
ENSGALG00010024822	RPS26	2	-0.075236644	0.050227445	-1.497919008	0.134154295	0	0.069736258	0	1	PRJNA827465;Yang2025_Silkie_Wenchang	CRA004775	0.61346525
ENSGALG00010024822	RPS26	2	-0.028486715	0.155078972	-0.18369167	0.854255344	0	0.000560528	0	1	CRA004775;Yang2025_Silkie_Wenchang	PRJNA827465	0.963091083
ENSGALG00010024822	RPS26	2	-0.077700877	0.051713803	-1.502517179	0.132963395	0	0.030697514	0	1	CRA004775;PRJNA827465	Yang2025_Silkie_Wenchang	0.610998265
ENSGALG00010024823	ASB10	2	-0.314529937	0.238667483	-1.317858355	0.187551069	0	0.953487835	0	1	PRJNA827465;Yang2025_Silkie_Wenchang	CRA004775	0.696676054
ENSGALG00010024823	ASB10	2	-0.151856533	0.283417404	-0.535805251	0.592093153	0	0.067778013	0	1	CRA004775;Yang2025_Silkie_Wenchang	PRJNA827465	0.892776493
ENSGALG00010024823	ASB10	2	-0.525539025	0.324103603	-1.621535528	0.10490723	0	0.132435998	0	1	CRA004775;PRJNA827465	Yang2025_Silkie_Wenchang	0.550686644
ENSGALG00010024824	DNAJC5	2	-0.128857409	0.072324832	-1.781648232	0.074806614	0	0.064504383	0	1	PRJNA827465;Yang2025_Silkie_Wenchang	CRA004775	0.472248889
ENSGALG00010024824	DNAJC5	2	-0.058263407	0.120240491	-0.48455729	0.627990416	0	0.642470086	0	0.5	CRA004775;Yang2025_Silkie_Wenchang	PRJNA827465	0.901904489
ENSGALG00010024824	DNAJC5	2	-0.117407053	0.083131086	-1.41231227	0.157858035	0	0.942706012	0	0.5	CRA004775;PRJNA827465	Yang2025_Silkie_Wenchang	0.651369486
ENSGALG00010024825	UBXN7	2	-0.045941301	0.234700807	-0.195744112	0.844810456	0.099936088	10.33820624	90.32714209	0.5	PRJNA827465;Yang2025_Silkie_Wenchang	CRA004775	0.960760914
ENSGALG00010024825	UBXN7	2	-0.255433603	0.120982851	-2.111320735	0.034744753	0	0.514841353	0	1	CRA004775;Yang2025_Silkie_Wenchang	PRJNA827465	0.321084652
ENSGALG00010024825	UBXN7	2	0.163674464	0.057897968	2.826946618	0.004699416	0	0.721255369	0	1	CRA004775;PRJNA827465	Yang2025_Silkie_Wenchang	0.100929591
ENSGALG00010024826	RBM33	2	0.475507674	0.556922006	0.853813763	0.393208187	0.57361323	12.50750027	92.00479729	0.5	PRJNA827465;Yang2025_Silkie_Wenchang	CRA004775	0.830338566
ENSGALG00010024826	RBM33	2	0.606832441	0.461910975	1.313743925	0.188932394	0.337014067	4.754924302	78.96917098	1	CRA004775;Yang2025_Silkie_Wenchang	PRJNA827465	0.698162702
ENSGALG00010024826	RBM33	2	-0.031304249	0.072679243	-0.430717869	0.666673524	0	0.408104495	0	0.5	CRA004775;PRJNA827465	Yang2025_Silkie_Wenchang	0.913607724
ENSGALG00010024828	TIMM17B	2	0.01249114	0.195358503	0.063939577	0.949018339	0.048976776	2.267434464	55.89729203	0.5	PRJNA827465;Yang2025_Silkie_Wenchang	CRA004775	0.987161201
ENSGALG00010024828	TIMM17B	2	0.078164799	0.239532766	0.326322766	0.744180166	0.035140288	1.436214164	30.3725012	0.5	CRA004775;Yang2025_Silkie_Wenchang	PRJNA827465	0.935821071
ENSGALG00010024828	TIMM17B	2	-0.126255976	0.08163885	-1.546518309	0.121979442	0	0.039672608	0	1	CRA004775;PRJNA827465	Yang2025_Silkie_Wenchang	0.585707157
ENSGALG00010024829	ERBB3	2	0.126150308	0.158384452	0.796481635	0.425752146	0.0366615	3.676057362	72.79694244	0.5	PRJNA827465;Yang2025_Silkie_Wenchang	CRA004775	0.84125685
ENSGALG00010024829	ERBB3	2	0.596613849	0.39213862	1.521436089	0.128150438	0.228202079	3.264181471	69.36444837	1	CRA004775;Yang2025_Silkie_Wenchang	PRJNA827465	0.600696128
ENSGALG00010024829	ERBB3	2	0.464388559	0.558962137	0.830661915	0.406164647	0.539093475	6.518174619	84.65828152	0.5	CRA004775;PRJNA827465	Yang2025_Silkie_Wenchang	0.833477131
ENSGALG00010024830	ORC2	2	0.127613734	0.070105305	1.820314938	0.068711058	0	0.001786699	0	1	PRJNA827465;Yang2025_Silkie_Wenchang	CRA004775	0.451522009
ENSGALG00010024830	ORC2	2	0.024700423	0.186982855	0.132099936	0.894905247	0.031999639	1.515783677	34.02752551	0.5	CRA004775;Yang2025_Silkie_Wenchang	PRJNA827465	0.974641371
ENSGALG00010024830	ORC2	2	0.031325918	0.177144227	0.176838493	0.859635253	0.032789445	1.559936134	35.89481147	0.5	CRA004775;PRJNA827465	Yang2025_Silkie_Wenchang	0.964839196
ENSGALG00010024831	SLC7A9	2	0.582451754	0.315586519	1.845616712	0.064947878	0	0.789656072	0	1	PRJNA827465;Yang2025_Silkie_Wenchang	CRA004775	0.439429584
ENSGALG00010024831	SLC7A9	2	1.423073164	0.695140124	2.047174542	0.04064095	0.563087628	2.210585925	54.76312461	1	CRA004775;Yang2025_Silkie_Wenchang	PRJNA827465	0.348841826
ENSGALG00010024831	SLC7A9	2	1.184991801	0.989177419	1.197956786	0.230933841	1.540130122	4.406697353	77.30726846	1	CRA004775;PRJNA827465	Yang2025_Silkie_Wenchang	0.743479092
ENSGALG00010024833	MAPT	2	0.049900378	0.392339788	0.127186636	0.898792682	0.267171201	2.531799362	86.73379436	0.5	PRJNA827465;Yang2025_Silkie_Wenchang	CRA004775	0.997588294
ENSGALG00010024833	MAPT	2	0.336426309	0.191421994	1.757511255	0.078830705	0.001727097	1.018756591	1.841125852	1	CRA004775;Yang2025_Silkie_Wenchang	PRJNA827465	0.482974778
ENSGALG00010024833	MAPT	2	-0.263617795	0.163993258	-1.607491666	0.107946556	0	0.730079376	0	0.5	CRA004775;PRJNA827465	Yang2025_Silkie_Wenchang	0.556183889
ENSGALG00010024834	INSIG1	2	0.299266056	0.14301034	2.092618313	0.03638324	0	0.049871976	0	1	PRJNA827465;Yang2025_Silkie_Wenchang	CRA004775	0.32890494
ENSGALG00010024834	INSIG1	2	0.380217864	0.178909188	2.125200129	0.033569901	0	0.28211751	0	1	CRA004775;Yang2025_Silkie_Wenchang	PRJNA827465	0.314626202
ENSGALG00010024834	INSIG1	2	0.330595947	0.179946527	1.83718993	0.066181862	0	0.456903938	0	1	CRA004775;PRJNA827465	Yang2025_Silkie_Wenchang	0.442974406
ENSGALG00010024835	ENTREP2	2	0.101595944	0.144898108	0.701154388	0.483206671	0.027240083	2.7486879	63.61900527	0.5	PRJNA827465;Yang2025_Silkie_Wenchang	CRA004775	0.85691916
ENSGALG00010024835	ENTREP2	2	0.188376291	0.15359193	1.226472585	0.220020886	0.008442539	1.136258212	11.99183516	0.5	CRA004775;Yang2025_Silkie_Wenchang	PRJNA827465	0.733691086
ENSGALG00010024835	ENTREP2	2	-0.031491495	0.093811994	-0.3356873	0.737106684	0	0.060913251	0	1	CRA004775;PRJNA827465	Yang2025_Silkie_Wenchang	0.93387966
ENSGALG00010024836	TXNDC12	2	-0.175332339	0.175430805	-0.999438718	0.317582212	0.054580939	8.169877852	87.75991492	1	PRJNA827465;Yang2025_Silkie_Wenchang	CRA004775	0.797211066
ENSGALG00010024836	TXNDC12	2	-0.345608128	0.09998312	-3.456664476	0.000546905	0	0.146189462	0	1	CRA004775;Yang2025_Silkie_Wenchang	PRJNA827465	0.024894026
ENSGALG00010024836	TXNDC12	2	-0.087078373	0.117753386	-0.73949783	0.45960457	0.017499475	1.953289634	48.85045142	1	CRA004775;PRJNA827465	Yang2025_Silkie_Wenchang	0.850098539
ENSGALG00010024837	TNFRSF1A	2	-0.282401381	0.080351492	-3.514575445	0.000440458	0.00183145	1.12762613	11.31812453	1	PRJNA827465;Yang2025_Silkie_Wenchang	CRA004775	0.021345626
ENSGALG00010024837	TNFRSF1A	2	-0.344039145	0.122827252	-2.80100009	0.00509445	0	0.722873959	0	1	CRA004775;Yang2025_Silkie_Wenchang	PRJNA827465	0.10649726
ENSGALG00010024837	TNFRSF1A	2	-0.226981923	0.078729026	-2.883077996	0.003938099	0	0.039585342	0	1	CRA004775;PRJNA827465	Yang2025_Silkie_Wenchang	0.090514101
ENSGALG00010024838	EN2	2	1.169569303	0.695822146	1.680851185	0.092792989	0.39730299	1.556400587	35.74918897	1	PRJNA827465;Yang2025_Silkie_Wenchang	CRA004775	0.52167685
ENSGALG00010024838	EN2	2	-0.306539337	2.523229133	-0.121487994	0.903311652	11.44220299	9.843567951	89.84108196	0.5	CRA004775;Yang2025_Silkie_Wenchang	PRJNA827465	0.977253688
ENSGALG00010024838	EN2	2	-0.924729004	1.771080999	-0.522126885	0.601581989	5.429095623	7.047915316	85.81140727	0.5	CRA004775;PRJNA827465	Yang2025_Silkie_Wenchang	0.895230519
ENSGALG00010024839	PA2G4	2	0.114651089	0.172425986	0.664930794	0.506097518	0.044980689	3.53196567	71.68715403	0.5	PRJNA827465;Yang2025_Silkie_Wenchang	CRA004775	0.863659781
ENSGALG00010024839	PA2G4	2	0.136216837	0.189444194	0.719034108	0.472119905	0.042216674	2.423992387	58.74574502	0.5	CRA004775;Yang2025_Silkie_Wenchang	PRJNA827465	0.853998373
ENSGALG00010024839	PA2G4	2	-0.024721195	0.055040695	-0.449143954	0.653327814	0	0.02021669	0	1	CRA004775;PRJNA827465	Yang2025_Silkie_Wenchang	0.909597225
ENSGALG00010024840	BTF3L4	2	-0.19709147	0.163222239	-1.20750378	0.22723819	0.04871492	11.42649465	91.24840967	1	PRJNA827465;Yang2025_Silkie_Wenchang	CRA004775	0.740605003
ENSGALG00010024840	BTF3L4	2	-0.341126763	0.073790169	-4.622929665	3.78358E-06	0	0.445094598	0	1	CRA004775;Yang2025_Silkie_Wenchang	PRJNA827465	0.000669847
ENSGALG00010024840	BTF3L4	2	-0.093164024	0.090481793	-1.029643885	0.303177207	0.008601522	1.650102191	39.39769273	1	CRA004775;PRJNA827465	Yang2025_Silkie_Wenchang	0.792084776
ENSGALG00010024841	ENSGALG00010024841	2	-0.133377292	0.110999077	-1.201607219	0.229515743	0	0.141464922	0	1	PRJNA827465;Yang2025_Silkie_Wenchang	CRA004775	0.742624291
ENSGALG00010024841	ENSGALG00010024841	2	-0.068208298	0.18553841	-0.367623596	0.713153918	0	0.001673054	0	1	CRA004775;Yang2025_Silkie_Wenchang	PRJNA827465	0.926214012
ENSGALG00010024841	ENSGALG00010024841	2	-0.149629377	0.12185656	-1.227904027	0.219479293	0	0.040099285	0	1	CRA004775;PRJNA827465	Yang2025_Silkie_Wenchang	0.732888275
ENSGALG00010024842	ZFYVE9	2	0.022707028	0.050364232	0.450856227	0.652093174	0.000650399	1.086546445	7.965278016	0.5	PRJNA827465;Yang2025_Silkie_Wenchang	CRA004775	0.909322729
ENSGALG00010024842	ZFYVE9	2	-0.053570606	0.102584746	-0.522208304	0.601525305	0	0.359931918	0	0.5	CRA004775;Yang2025_Silkie_Wenchang	PRJNA827465	0.895230519
ENSGALG00010024842	ZFYVE9	2	0.048738843	0.048977699	0.995123174	0.319676361	0	0.022372716	0	1	CRA004775;PRJNA827465	Yang2025_Silkie_Wenchang	0.798328994
ENSGALG00010024843	BCL9L	2	0.055056511	0.100974784	0.545250097	0.585581521	0.008547303	1.544254443	35.24383209	0.5	PRJNA827465;Yang2025_Silkie_Wenchang	CRA004775	0.891148209
ENSGALG00010024843	BCL9L	2	0.17152017	0.142666987	1.202241537	0.229269961	0	0.281076625	0	1	CRA004775;Yang2025_Silkie_Wenchang	PRJNA827465	0.742256776
ENSGALG00010024843	BCL9L	2	-0.009442325	0.075465567	-0.125120938	0.900427807	0	0.008516353	0	0.5	CRA004775;PRJNA827465	Yang2025_Silkie_Wenchang	0.976475201
ENSGALG00010024845	HYCC2	2	0.029887249	0.092120944	0.324434484	0.745608806	0	0.14981784	0	1	PRJNA827465;Yang2025_Silkie_Wenchang	CRA004775	0.936221299
ENSGALG00010024845	HYCC2	2	0.074970652	0.133210299	0.562799217	0.573571614	0	0.002676824	0	1	CRA004775;Yang2025_Silkie_Wenchang	PRJNA827465	0.885953434
ENSGALG00010024845	HYCC2	2	0.014419818	0.10340483	0.139450141	0.889094453	0	0.047585917	0	1	CRA004775;PRJNA827465	Yang2025_Silkie_Wenchang	0.973135755
ENSGALG00010024846	LMO2	2	0.082743215	0.157063198	0.526814784	0.598323215	0.042541376	7.120982115	85.95683547	0.5	PRJNA827465;Yang2025_Silkie_Wenchang	CRA004775	0.894665663
ENSGALG00010024846	LMO2	2	0.232031573	0.095570118	2.427867393	0.015187895	0	0.489425662	0	0.5	CRA004775;Yang2025_Silkie_Wenchang	PRJNA827465	0.203660816
ENSGALG00010024846	LMO2	2	-0.064956632	0.063872071	-1.016980207	0.309162845	0	0.006309873	0	1	CRA004775;PRJNA827465	Yang2025_Silkie_Wenchang	0.794043289
ENSGALG00010024847	PPIL1	2	0.06184899	0.08663189	0.713928663	0.475271292	0	0.1129882	0	0.5	PRJNA827465;Yang2025_Silkie_Wenchang	CRA004775	0.854462864
ENSGALG00010024847	PPIL1	2	-0.074631178	0.248314532	-0.300550986	0.763756911	0	0.003737534	0	1	CRA004775;Yang2025_Silkie_Wenchang	PRJNA827465	0.940361611
ENSGALG00010024847	PPIL1	2	0.058623304	0.086185265	0.680201005	0.496377196	0	0.20401597	0	0.5	CRA004775;PRJNA827465	Yang2025_Silkie_Wenchang	0.860939846
ENSGALG00010024848	ESYT1	2	0.026343513	0.084536776	0.311621926	0.755327869	0.007308141	1.939503182	48.44040426	0.5	PRJNA827465;Yang2025_Silkie_Wenchang	CRA004775	0.938464456
ENSGALG00010024848	ESYT1	2	-0.060626747	0.216827056	-0.279608771	0.779777677	0.073312323	4.345813043	76.98934606	0.5	CRA004775;Yang2025_Silkie_Wenchang	PRJNA827465	0.944967824
ENSGALG00010024848	ESYT1	2	-0.118134886	0.119825458	-0.985891382	0.324186417	0.016054267	1.87011542	46.52736459	1	CRA004775;PRJNA827465	Yang2025_Silkie_Wenchang	0.799686366
ENSGALG00010024849	CENATAC	2	0.340287511	0.257412327	1.32195499	0.186183139	0.110919173	6.023996245	83.39972405	1	PRJNA827465;Yang2025_Silkie_Wenchang	CRA004775	0.695193101
ENSGALG00010024849	CENATAC	2	0.420134818	0.200327319	2.097241756	0.03597218	0.047523113	2.447225015	59.13739056	1	CRA004775;Yang2025_Silkie_Wenchang	PRJNA827465	0.32742348
ENSGALG00010024849	CENATAC	2	0.130423385	0.100597027	1.296493457	0.194805533	0	0.264951194	0	1	CRA004775;PRJNA827465	Yang2025_Silkie_Wenchang	0.706046493
ENSGALG00010024850	CDK2AP1	2	-0.109396235	0.066550736	-1.643802032	0.100217101	0	0.286043734	0	1	PRJNA827465;Yang2025_Silkie_Wenchang	CRA004775	0.540825585
ENSGALG00010024850	CDK2AP1	2	-0.066841754	0.096518637	-0.692526911	0.488606492	0	0.000166394	0	1	CRA004775;Yang2025_Silkie_Wenchang	PRJNA827465	0.857920879
ENSGALG00010024850	CDK2AP1	2	-0.131577662	0.081682214	-1.61084838	0.107212773	0	0.066921358	0	1	CRA004775;PRJNA827465	Yang2025_Silkie_Wenchang	0.554473355
ENSGALG00010024852	CEP89	2	0.25933685	0.358503736	0.723386743	0.469442314	0.241276079	15.85751558	93.69384192	0.5	PRJNA827465;Yang2025_Silkie_Wenchang	CRA004775	0.853485658
ENSGALG00010024852	CEP89	2	0.578100785	0.15410326	3.751385825	0.00017586	0	0.889462808	0	1	CRA004775;Yang2025_Silkie_Wenchang	PRJNA827465	0.01153893
ENSGALG00010024852	CEP89	2	-0.076543307	0.071728819	-1.067120691	0.285917339	0	0.407834118	0	0.5	CRA004775;PRJNA827465	Yang2025_Silkie_Wenchang	0.781752017

ENSGALG00010024853	TJP1	2	-0.034700001	0.050383223	-0.688721344	0.490998642	0	0.09293408	0	0.5	PRJNA827465;Yang2025_Silkie_Wenchang	CRA004775	0.85895707
ENSGALG00010024853	TJP1	2	0.209570794	0.246864323	0.84893107	0.395919649	0.076355397	2.491344284	59.86102739	1	CRA004775;Yang2025_Silkie_Wenchang	PRJNA827465	0.831296327
ENSGALG00010024853	TJP1	2	0.169943576	0.269256129	0.631159547	0.527936209	0.113717665	3.810354768	73.75572458	0.5	CRA004775;PRJNA827465	Yang2025_Silkie_Wenchang	0.8714972
ENSGALG00010024854	C12orf65	2	0.148280528	0.263075206	0.56364311	0.572997041	0.119991594	7.173398103	86.05960542	0.5	PRJNA827465;Yang2025_Silkie_Wenchang	CRA004775	0.88573298
ENSGALG00010024854	C12orf65	2	0.370579725	0.149106655	2.485333243	0.012943021	0	0.454300459	0	1	CRA004775;Yang2025_Silkie_Wenchang	PRJNA827465	0.184914415
ENSGALG00010024854	C12orf65	2	-0.052701383	0.103878796	-0.507335329	0.611919554	0.004874255	1.115723365	10.3720482	0.5	CRA004775;PRJNA827465	Yang2025_Silkie_Wenchang	0.897777844
ENSGALG00010024855	CAPRIN1	2	-0.090135246	0.037004775	-2.435773421	0.014859989	0	0.956152811	0	0.5	PRJNA827465;Yang2025_Silkie_Wenchang	CRA004775	0.201463318
ENSGALG00010024855	CAPRIN1	2	0.047016696	0.097947067	0.480021476	0.631212122	0	0.212763899	0	1	CRA004775;Yang2025_Silkie_Wenchang	PRJNA827465	0.903058174
ENSGALG00010024855	CAPRIN1	2	-0.063614302	0.083569822	-0.761211406	0.446530806	0.006953993	1.406412827	28.89712174	0.5	CRA004775;PRJNA827465	Yang2025_Silkie_Wenchang	0.846394998
ENSGALG00010024856	SORT1	2	0.014382477	0.097367067	0.147713978	0.8825685	0.012023577	2.705698437	63.04096619	0.5	PRJNA827465;Yang2025_Silkie_Wenchang	CRA004775	0.972001076
ENSGALG00010024856	SORT1	2	0.115648482	0.089621545	1.290409371	0.19690856	0	0.031310211	0	1	CRA004775;Yang2025_Silkie_Wenchang	PRJNA827465	0.707994211
ENSGALG00010024856	SORT1	2	-0.071032247	0.07333212	-0.968637567	0.332726053	0	0.10766566	0	0.5	CRA004775;PRJNA827465	Yang2025_Silkie_Wenchang	0.802178578
ENSGALG00010024857	SLC37A4	2	-0.102095477	0.063460965	-1.608791738	0.107661888	0	0.959604729	0	1	PRJNA827465;Yang2025_Silkie_Wenchang	CRA004775	0.55523359
ENSGALG00010024857	SLC37A4	2	-0.208227	0.155524028	-1.338873507	0.180611856	0	0.451340783	0	1	CRA004775;Yang2025_Silkie_Wenchang	PRJNA827465	0.688337781
ENSGALG00010024857	SLC37A4	2	-0.090270139	0.062157632	-1.452277638	0.146424423	0	0.040340026	0	1	CRA004775;PRJNA827465	Yang2025_Silkie_Wenchang	0.6352709
ENSGALG00010024858	SLC25A39	2	-0.065896908	0.084584967	-0.779061694	0.435943374	0.003832627	1.120904393	10.78632522	1	PRJNA827465;Yang2025_Silkie_Wenchang	CRA004775	0.842995445
ENSGALG00010024858	SLC25A39	2	-0.352117333	0.159703324	-2.204821562	0.027466618	0	0.074040294	0	1	CRA004775;Yang2025_Silkie_Wenchang	PRJNA827465	0.282945624
ENSGALG00010024858	SLC25A39	2	-0.155498702	0.167646956	-0.927536689	0.353647952	0.038237819	2.558422228	60.91341026	1	CRA004775;PRJNA827465	Yang2025_Silkie_Wenchang	0.811318534
ENSGALG00010024859	TARSL2	2	-0.019712779	0.056604254	-0.348256135	0.727647834	0	0.291808206	0	1	PRJNA827465;Yang2025_Silkie_Wenchang	CRA004775	0.931639802
ENSGALG00010024859	TARSL2	2	-0.116293563	0.092697911	-1.254543509	0.209644522	0	0.58155408	0	1	CRA004775;Yang2025_Silkie_Wenchang	PRJNA827465	0.721511264
ENSGALG00010024859	TARSL2	2	-0.058175193	0.09764635	-0.595774374	0.551325966	0.008405118	1.498835224	33.28152529	1	CRA004775;PRJNA827465	Yang2025_Silkie_Wenchang	0.878879109
ENSGALG00010024861	ORC1	2	0.054510643	0.074551613	0.73117993	0.464669257	0.007122125	2.703621737	63.01257731	0.5	PRJNA827465;Yang2025_Silkie_Wenchang	CRA004775	0.85217126
ENSGALG00010024861	ORC1	2	0.112920307	0.069741429	1.619128088	0.105419707	0	0.907449579	0	0.5	CRA004775;Yang2025_Silkie_Wenchang	PRJNA827465	0.551018754
ENSGALG00010024861	ORC1	2	-0.014065563	0.050332341	-0.279453774	0.779896608	0	0.048411115	0	1	CRA004775;PRJNA827465	Yang2025_Silkie_Wenchang	0.944978771
ENSGALG00010024862	METTL16	2	-0.145740345	0.087098812	-1.673275916	0.094272991	0.00767723	1.89282068	47.16879362	1	PRJNA827465;Yang2025_Silkie_Wenchang	CRA004775	0.525785636
ENSGALG00010024862	METTL16	2	-0.013904548	0.103348174	-0.134540822	0.892974933	0	0.152492534	0	0.5	CRA004775;Yang2025_Silkie_Wenchang	PRJNA827465	0.974381724
ENSGALG00010024862	METTL16	2	-0.170960068	0.101219395	-1.689005039	0.091218464	0.007607112	1.223915849	18.29503629	0.5	CRA004775;PRJNA827465	Yang2025_Silkie_Wenchang	0.517862098
ENSGALG00010024863	LMOD1	2	0.0537484	0.359267235	0.149605628	0.881075767	0.218113189	6.383317204	84.33416407	0.5	PRJNA827465;Yang2025_Silkie_Wenchang	CRA004775	0.971685305
ENSGALG00010024863	LMOD1	2	0.431120876	0.197812939	2.179437188	0.029299206	0	0.000390456	0	1	CRA004775;Yang2025_Silkie_Wenchang	PRJNA827465	0.291530809
ENSGALG00010024863	LMOD1	2	-0.016477972	0.352777063	-0.046709308	0.962744912	0.170100788	2.788211381	64.13471349	0.5	CRA004775;PRJNA827465	Yang2025_Silkie_Wenchang	0.989980504
ENSGALG00010024864	MPHOSPH9	2	-0.005069642	0.07234298	-0.070077871	0.94413168	0	0.434602726	0	0.5	PRJNA827465;Yang2025_Silkie_Wenchang	CRA004775	0.985827325
ENSGALG00010024864	MPHOSPH9	2	-0.167786144	0.316277918	-0.530502241	0.595763756	0.139002285	2.845029717	64.85098226	0.5	CRA004775;Yang2025_Silkie_Wenchang	PRJNA827465	0.893850317
ENSGALG00010024864	MPHOSPH9	2	-0.193425552	0.248572685	-0.778144838	0.436483632	0.0802966	2.165876944	53.82932521	1	CRA004775;PRJNA827465	Yang2025_Silkie_Wenchang	0.843168986
ENSGALG00010024865	SNAP47	2	-0.03847181	0.139406074	-0.275969397	0.782571572	0.028277231	3.571415273	71.99989574	0.5	PRJNA827465;Yang2025_Silkie_Wenchang	CRA004775	0.945446706
ENSGALG00010024865	SNAP47	2	-0.189371232	0.118553032	-1.597354615	0.110186683	0	0.012947036	0	1	CRA004775;Yang2025_Silkie_Wenchang	PRJNA827465	0.562278266
ENSGALG00010024865	SNAP47	2	0.080650499	0.082420463	0.978525189	0.327814639	0	0.21677449	0	0.5	CRA004775;PRJNA827465	Yang2025_Silkie_Wenchang	0.800354004
ENSGALG00010024866	TRAPPC4	2	-0.08521626	0.124594038	-0.683951346	0.49400589	0.01795174	1.785837443	44.00386195	1	PRJNA827465;Yang2025_Silkie_Wenchang	CRA004775	0.860175061
ENSGALG00010024866	TRAPPC4	2	-0.305310597	0.171733723	-1.777813884	0.075434427	0	0.004183215	0	1	CRA004775;Yang2025_Silkie_Wenchang	PRJNA827465	0.474292002
ENSGALG00010024866	TRAPPC4	2	-0.021395778	0.053062829	-0.403215922	0.686789387	0	0.980131891	0	1	CRA004775;PRJNA827465	Yang2025_Silkie_Wenchang	0.919235539
ENSGALG00010024867	TM2D3	2	-0.032639614	0.176259947	-0.185178846	0.85308876	0.04602139	3.520106085	71.59176525	0.5	PRJNA827465;Yang2025_Silkie_Wenchang	CRA004775	0.962724663
ENSGALG00010024867	TM2D3	2	-0.250661048	0.132151248	-1.89677397	0.057857776	0	0.002839668	0	1	CRA004775;Yang2025_Silkie_Wenchang	PRJNA827465	0.416938793
ENSGALG00010024867	TM2D3	2	-0.025266316	0.180323019	-0.140116975	0.888567569	0.045364067	2.879196406	65.26808669	0.5	CRA004775;PRJNA827465	Yang2025_Silkie_Wenchang	0.973135755
ENSGALG00010024868	FAAP24	2	0.074702053	0.199638922	0.374185813	0.708266078	0.045901267	2.227963226	55.11595575	0.5	PRJNA827465;Yang2025_Silkie_Wenchang	CRA004775	0.925061251
ENSGALG00010024868	FAAP24	2	0.303395451	0.212714124	1.426306093	0.153780007	0	0.033098482	0	1	CRA004775;Yang2025_Silkie_Wenchang	PRJNA827465	0.646349653
ENSGALG00010024868	FAAP24	2	-0.065430415	0.136778755	-0.478366801	0.632389159	0	0.328878786	0	0.5	CRA004775;PRJNA827465	Yang2025_Silkie_Wenchang	0.903058174
ENSGALG00010024869	NDUFB3	2	-0.124635051	0.210376533	-0.59243799	0.553557333	0.056988335	2.31442098	56.79264884	0.5	PRJNA827465;Yang2025_Silkie_Wenchang	CRA004775	0.879610907
ENSGALG00010024869	NDUFB3	2	-0.195356744	0.269640891	-0.724507113	0.468754462	0.047878322	1.473074484	32.11477009	0.5	CRA004775;Yang2025_Silkie_Wenchang	PRJNA827465	0.853293345
ENSGALG00010024869	NDUFB3	2	0.028995136	0.091165828	0.318048294	0.750448304	0	0.07203109	0	1	CRA004775;PRJNA827465	Yang2025_Silkie_Wenchang	0.937393444
ENSGALG00010024870	TPD52L2	2	-0.038083063	0.054184647	-0.702838632	0.482156319	0.001823179	1.167474015	14.34498868	1	PRJNA827465;Yang2025_Silkie_Wenchang	CRA004775	0.85691916
ENSGALG00010024870	TPD52L2	2	-0.102463865	0.103833256	-0.986811633	0.323734991	0	0.577839873	0	1	CRA004775;Yang2025_Silkie_Wenchang	PRJNA827465	0.799600434
ENSGALG00010024870	TPD52L2	2	-0.016913921	0.038275973	-0.441893954	0.658565946	0	6.01711E-05	0	1	CRA004775;PRJNA827465	Yang2025_Silkie_Wenchang	0.911105434
ENSGALG00010024871	POLQ	2	-0.087457367	0.129245667	-0.676675426	0.498611911	0.018251639	2.177074305	54.06679516	0.5	PRJNA827465;Yang2025_Silkie_Wenchang	CRA004775	0.861478809
ENSGALG00010024871	POLQ	2	0.021366028	0.108131263	0.197593437	0.84336317	0	0.162713271	0	0.5	CRA004775;Yang2025_Silkie_Wenchang	PRJNA827465	0.96057435
ENSGALG00010024871	POLQ	2	-0.230050106	0.13420982	-1.714107848	0.086508921	0	0.000303406	0	1	CRA004775;PRJNA827465	Yang2025_Silkie_Wenchang	0.506036629
ENSGALG00010024872	RUNDC3A	2	0.241009954	0.258464551	0.932468118	0.351094656	0.06379906	1.859541284	46.22329666	1	PRJNA827465;Yang2025_Silkie_Wenchang	CRA004775	0.810161472
ENSGALG00010024872	RUNDC3A	2	0.024766048	0.537001312	0.046119157	0.963215277	0.466063705	5.201523415	80.77486305	0.5	CRA004775;Yang2025_Silkie_Wenchang	PRJNA827465	0.989998461
ENSGALG00010024872	RUNDC3A	2	-0.182245636	0.266143634	-0.684764215	0.49349272	0.064369471	1.74640189	42.73941147	0.5	CRA004775;PRJNA827465	Yang2025_Silkie_Wenchang	0.859868321
ENSGALG00010024873	HYOU1	2	0.061663453	0.066476969	0.927591222	0.353619653	0.004359268	1.912912606	47.72369649	0.5	PRJNA827465;Yang2025_Silkie_Wenchang	CRA004775	0.811318534
ENSGALG00010024873	HYOU1	2	-0.034841112	0.077358907	-0.450382679	0.652434532	0	0.645449727	0	1	CRA004775;Yang2025_Silkie_Wenchang	PRJNA827465	0.909401809
ENSGALG00010024873	HYOU1	2	0.037223587	0.15543876	0.239474293	0.810737828	0.027407276	1.599529922	37.48163218	0.5	CRA004775;PRJNA827465	Yang2025_Silkie_Wenchang	0.953020781
ENSGALG00010024874	JMJD4	2	-0.153670803	0.080626458	-1.905959995	0.056655396	0.005856774	1.818432552	45.00758367	1	PRJNA827465;Yang2025_Silkie_Wenchang	CRA004775	0.411670741
ENSGALG00010024874	JMJD4	2	-0.226592311	0.082098561	-2.760003453	0.005780075	0	0.128049012	0	1	CRA004775;Yang2025_Silkie_Wenchang	PRJNA827465	0.114502839
ENSGALG00010024874	JMJD4	2	-0.08181515	0.078184836	-1.046432462	0.295361412	0	0.062529307	0	1	CRA004775;PRJNA827465	Yang2025_Silkie_Wenchang	0.787628307
ENSGALG00010024876	CFLAR	2	-0.034044233	0.057017773	-0.597081067	0.550453258	0.000909744	1.137329921	12.07476554	0.5	PRJNA827465;Yang2025_Silkie_Wenchang	CRA004775	0.878740242
ENSGALG00010024876	CFLAR	2	-0.060695799	0.115522736	-0.525401332	0.599304228	0.007232007	1.208110632	17.22612373	0.5	CRA004775;Yang2025_Silkie_Wenchang	PRJNA827465	0.894908299
ENSGALG00010024876	CFLAR	2	0.014636596	0.060046935	0.243752583	0.807422446	0	0.436357231	0	1	CRA004775;PRJNA827465	Yang2025_Silkie_Wenchang	0.95234753
ENSGALG00010024877	PRPF38A	2	0.031388292	0.047884174	0.65550451	0.512142984	0	0.026254034	0	1	PRJNA827465;Yang2025_Silkie_Wenchang	CRA004775	0.865694231
ENSGALG00010024877	PRPF38A	2	-0.079073063	0.176398108	-0.4482648	0.653962102	0.034265032	1.983506365	49.58423036	0.5	CRA004775;Yang2025_Silkie_Wenchang	PRJNA827465	0.909861322
ENSGALG00010024877	PRPF38A	2	-0.07125727	0.158098801	-0.450713539	0.652196023	0.032026987	2.078104831	51.87923223	0.5	CRA004775;PRJNA827465	Yang2025_Silkie_Wenchang	0.909376064
ENSGALG00010024878	RHPN2	2	-0.13666653	0.154390266	-0.88520173	0.37604784	0.006381746	1.154092053	13.35179918	0.5	PRJNA827465;Yang2025_Silkie_Wenchang	CRA004775	0.822974013
ENSGALG00010024878	RHPN2	2	0.434451356	0.76911784	0.564869691	0.572162401	1.067841934	9.833441597	89.83062044	0.5	CRA004775;Yang2025_Silkie_Wenchang	PRJNA827465	0.885543355
ENSGALG00010024878	RHPN2	2	0.57769553	0.612978088	0.942440752	0.345967031	0.636473888	6.161044496	83.76898591	1	CRA004775;PRJNA827465	Yang2025_Silkie_Wenchang	0.807598873
ENSGALG00010024879	D2HGDH	2	-0.282282789	0.139895134	-2.017817068	0.043610317	0.027998193	3.251611928	69.24602252	1	PRJNA827465;Yang2025_Silkie_Wenchang	CRA004775	0.360845532
ENSGALG00010024879	D2HGDH	2	-0.466847006	0.118546117	-3.938104566	8.21278E-05	0	0.051330206	0	1	CRA004775;Yang2025_Silkie_Wenchang	PRJNA827465	0.006795514
ENSGALG00010024879	D2HGDH	2	-0.273368603	0.159542305	-1.713455268	0.086628816	0.031878905	2.170379884	53.92511663	1	CRA004775;PRJNA827465	Yang2025_Silkie_Wenchang	0.506210579
ENSGALG00010024880	NAT10	2	-0.032035535	0.06024927	-0.531716566	0.594922317	0	0.583625277	0	0.5	PRJNA827465;Yang2025_Silkie_Wenchang	CRA004775	0.893382718
ENSGALG00010024880	NAT10	2	-0.169233129	0.160316711	-1.055617523	0.291142992	0.027913787	1.712738915	41.61398499	1	CRA004775;Yang2025_Silkie_Wenchang	PRJNA827465	0.78467882
ENSGALG00010024880	NAT10	2	-0.142124231	0.21852541	-0.650378515	0.515447752	0.065429723	2.646868798	62.21951006	0.5	CRA004775;PRJNA827465	Yang2025_Silkie_Wenchang	0.867199306
ENSGALG00010024881	CASP10	2	0.038904078	0.121965877	0.3189751	0.749745395	0	0.669206034	0	0.5	PRJNA827465;Yang2025_Silkie_Wenchang	CRA004775	0.937362187
ENSGALG00010024881	CASP10	2	-0.003742401	0.194808437	-0.019210672	0.984673044	0.028145279	1.551157338	35.53200727	0.5	CRA004775;Yang2025_Silkie_Wenchang	PRJNA827465	0.995703389
ENSGALG00010024881	CASP10	2	-0.101785814	0.136824787	-0.743913552	0.456928776	0	0.413394383	0	1	CRA004775;PRJNA827465	Yang2025_Silkie_Wenchang	0.84954048
ENSGALG00010024882	MYL6	2	0.812700454	0.563661163	1.441824464	0.1493519	0.624322252	56.9956138	98.24547902	1	PRJNA827465;Yang2025_Silkie_Wenchang	CRA004775	0.639246608
ENSGALG00010024882	MYL6	2	0.637492091	0.758703925	0.840238294	0.400774792	1.112103281	29.06420128	96.5593412	0.5	CRA004775;Yang2025_Silkie_Wenchang	PRJNA827465	0.832353691
ENSGALG00010024882	MYL6	2	0.131482307	0.18103025	0.726300198	0.467654758	0.04184442	2.218396564	54.92239681	0.5	CRA004775;PRJNA827465	Yang2025_Silkie_Wenchang	0.852885838
ENSGALG00010024883	ING5	2	-0.162754392	0.162827527	-0.999550847	0.31752792	0.046494446	7.744057219	87.08687227	1	PRJNA827465;Yang2025_Silkie_Wenchang	CRA004775	0.797125693
ENSGALG00010024883	ING5	2	-0.301078966	0.092782177	-3.245008641	0.001174471	0	0.552376891	0	1	CRA004775;Yang2025_Silkie_Wenchang	PRJNA827465	0.041660846
ENSGALG00010024883	ING5	2	-0.02346333	0.048284855	-0.485935589	0.627012834	0	0.702496583	0	1	CRA004775;PRJNA827465	Yang2025_Silkie_Wenchang	0.901573209
ENSGALG00010024884	ZBTB46	2	-0.059575617	0.172297324	-0.345772156	0.729513952	0.051181856	7.00943328	85.7335114	0.5	PRJNA827465;Yang2025_Silkie_Wenchang	CRA004775	0.9323457
ENSGALG00010024884	ZBTB46	2	-0.23387583	0.105132139	-2.224589282	0.026108823	0	0.067314675	0	1	CRA004775;Yang2025_Silkie_Wenchang	PRJNA827465	0.275774002
ENSGALG00010024884	ZBTB46	2	0.086716993	0.062411403	1.389441487	0.164698542	0	0.895314866	0	0.5	CRA004775;PRJNA827465	Yang2025_Silkie_Wenchang	0.663901457
ENSGALG00010024885	SLC4A1	2	0.074375048	0.289676601	0.256752003	0.797370224	0.13662031	5.367239478	81.36844827	0.5	PRJNA827465;Yang2025_Silkie_Wenchang	CRA004775	0.949715736
ENSGALG00010024885	SLC4A1	2	-0.217682355	0.168222218	-1.294016676	0.195659661	0	0.00235026	0	1	CRA004775;Yang2025_Silkie_Wenchang	PRJNA827465	0.706714537
ENSGALG00010024885	SLC4A1	2	0.187554169	0.257180728	0.72926992	0.465836568	0.067488972	1.76200389	43.24643632	0.5	CRA004775;PRJNA827465	Yang2025_Silkie_Wenchang	0.852511753
ENSGALG00010024886	ABTB2	2	0.422642694	0.092185173	4.584714437	4.54607E-06	0	0.923741086	0	1	PRJNA827465;Yang2025_Silkie_Wenchang	CRA004775	0.000757095
ENSGALG00010024886	ABTB2	2	0.578977966	0.177911272	3.25430738	0.001136692	0	0.017469425	0	1	CRA004775;Yang2025_Silkie_Wenchang	PRJNA827465	0.040911549
ENSGALG00010024886	ABTB2	2	0.385071122	0.101089203	3.809221055	0.000139405	0	0.131320301	0	1	CRA004775;PRJNA827465	Yang2025_Silkie_Wenchang	0.009907859
ENSGALG00010024887	CASP18	2	-0.046897231	0.171350044	-0.273692552	0.784320902	0	0.419172062	0	0.5	PRJNA827465;Yang2025_Silkie_Wenchang	CRA004775	0.946083234
ENSGALG00010024887	CASP18	2	-0.224706019	0.237135128	-0.947586385	0.34334006	0	0.208774	0	1	CRA004775;Yang2025_Silkie_Wenchang	PRJNA827465	0.806220394
ENSGALG00010024887	CASP18	2	-0.02119745	0.210616513	-0.100644765	0.91983246	0	0.697532465	0	0.5	CRA004775;PRJNA827465	Yang2025_Silkie_Wenchang	0.981237238
ENSGALG00010024888	PITPNM2	2	0.12967552	0.295513846	0.438813687	0.660796542	0.152336896	7.381620226	86.45283868	0.5	PRJNA827465;Yang2025_Silkie_Wenchang	CRA004775	0.91192683
ENSGALG00010024888	PITPNM2	2	0.197989204	0.344287702	0.575069056	0.565244595	0.143850018	2.257000347	55.69340514	0.5	CRA004775;Yang2025_Silkie_Wenchang	PRJNA827465	0.883427937
ENSGALG00010024888	PITPNM2	2	-0.143985685	0.084043098	-1.713236287	0.086669078	0	0.079907269	0	1	CRA004775;PRJNA827465	Yang2025_Silkie_Wenchang	0.506300423
ENSGALG00010024889	SLC2A4RG	2	-0.218777794	0.287749402	-0.760306684	0.447071288	0.089099004	2.043767787	51.07076223	0.5	PRJNA827465;Yang2025_Silkie_Wenchang	CRA004775	0.84650962
ENSGALG00010024889	SLC2A4RG	2	0.129236676	0.217516868	0.594145534	0.552414778	0	0.002497827	0	1	CRA004775;Yang2025_Silkie_Wenchang	PRJNA827465	0.879228216
ENSGALG00010024889	SLC2A4RG	2	-0.18998711	0.282901907	-0.671565322	0.501860461	0.100335228	2.640959739	62.13497748	0.5	CRA004775;PRJNA827465	Yang2025_Silkie_Wenchang	0.862795569
ENSGALG00010024890	SMARCC2	2	0.087566654	0.222669511	0.393258393	0.694128645	0.091746443	13.17416271	92.40938478	0.5	PRJNA827465;Yang2025_Silkie_Wenchang	CRA004775	0.920728119
ENSGALG00010024890	SMARCC2	2	0.079998877	0.246517646	0.324515825	0.745547543	0.106482042	8.002190662	87.50342197	0.5	CRA004775;Yang2025_Silkie_Wenchang	PRJNA827465	0.936204195
ENSGALG00010024890	SMARCC2	2	-0.135824389	0.05816627	-2.335105714	0.019537899	0	0.096789579	0	1	CRA004775;PRJNA827465	Yang2025_Silkie_Wenchang	0.234180956
ENSGALG00010024891	PKP1	2	0.029716678	0.335387401	0.088604037	0.9293966	0	0.19108564	0	0.5	PRJNA827465;Yang2025_Silkie_Wenchang	CRA004775	0.983656242
ENSGALG00010024891	PKP1	2	0.298132699	0.425690535	0.700350782	0.483708265	0	0.185484735	0	1	CRA004775;Yang2025_Silkie_Wenchang	PRJNA827465	0.85691916
ENSGALG00010024891	PKP1	2	0.058861683	0.399775755	0.147236751	0.882945154	0	0.561877581	0	0.5	CRA004775;PRJNA827465	Yang2025_Silkie_Wenchang	0.972001076
ENSGALG00010024893	ARL6IP4	2	0.234411919	0.25638057	0.914312338	0.360552749	0.122052039	13.5430795	92.61615499	0.5	PRJNA827465;Yang2025_Silkie_Wenchang	CRA004775	0.814882387
ENSGALG00010024893	ARL6IP4	2	0.347655527	0.181582322	1.914589054	0.055544932	0.041111724	2.560195334	60.94048033	1	CRA004775;Yang2025_Silkie_Wenchang	PRJNA827465	0.408228819
ENSGALG00010024893	ARL6IP4	2	0.00226512	0.054471439	0.041583628	0.966830625	0	0.537420593	0	0.5	CRA004775;PRJNA827465	Yang2025_Silkie_Wenchang	0.990893423
ENSGALG00010024894	TUT4	2	0.112616611	0.164796446	0.68336796	0.494374361	0.043492679	4.517337706	77.86306747	0.5	PRJNA827465;Yang2025_Silkie_Wenchang	CRA004775	0.860175061
ENSGALG00010024894	TUT4	2	0.304613333	0.126367142	2.410542246	0.015928826	0	0.001472279	0	1	CRA004775;Yang2025_Silkie_Wenchang	PRJNA827465	0.208471156
ENSGALG00010024894	TUT4	2	0.045466614	0.134623895	0.337730641	0.735566182	0.020252664	1.633256865	38.77264372	0.5	CRA004775;PRJNA827465	Yang2025_Silkie_Wenchang	0.933592889
ENSGALG00010024896	RNF41	2	0.108907528	0.098306992	1.107830951	0.26793483	0.012152109	2.226390107	55.08424167	1	PRJNA827465;Yang2025_Silkie_Wenchang	CRA004775	0.771398294
ENSGALG00010024896	RNF41	2	0.020318869	0.306754163	0.066238284	0.947188117	0.129591033	2.742702451	63.53961036	0.5	CRA004775;Yang2025_Silkie_Wenchang	PRJNA827465	0.986445196
ENSGALG00010024896	RNF41	2	-0.023095103	0.152700707	-0.151244247	0.879783048	0.025260852	1.379302903	27.49960883	0.5	CRA004775;PRJNA827465	Yang2025_Silkie_Wenchang	0.971407853
ENSGALG00010024897	ADAL	2	-0.325621592	0.237908089	-1.368686511	0.171097288	0.106989988	18.13771981	94.48662781	1	PRJNA827465;Yang2025_Silkie_Wenchang	CRA004775	0.673238496
ENSGALG00010024897	ADAL	2	-0.401388437	0.260122338	-1.543075614	0.122812431	0.087127585	2.128305032	53.01425383	0.5	CRA004775;Yang2025_Silkie_Wenchang	PRJNA827465	0.587706885
ENSGALG00010024897	ADAL	2	-0.088906605	0.064827016	-1.371443738	0.170236671	0	0.063035099	0	0.5	CRA004775;PRJNA827465	Yang2025_Silkie_Wenchang	0.672034495
ENSGALG00010024898	NABP2	2	0.225278443	0.151160123	1.490329851	0.136137527	0.033211222	3.545055467	71.79169665	1	PRJNA827465;Yang2025_Silkie_Wenchang	CRA004775	0.617782384
ENSGALG00010024898	NABP2	2	0.251388028	0.179734646	1.398662052	0.161914349	0.034531096	2.028587992	50.70462784	1	CRA004775;Yang2025_Silkie_Wenchang	PRJNA827465	0.659179579
ENSGALG00010024898	NABP2	2	0.080891558	0.082711749	0.977993553	0.328077513	0	0.07393333	0	1	CRA004775;PRJNA827465	Yang2025_Silkie_Wenchang	0.800597943
ENSGALG00010024899	OGFOD2	2	0.216110069	0.16293462	1.326360655	0.184720265	0.041383457	4.423030086	77.39106494	1	PRJNA827465;Yang2025_Silkie_Wenchang	CRA004775	0.69308359
ENSGALG00010024899	OGFOD2	2	-0.073388712	0.500694525	-0.146573825	0.883468415	0.451971799	9.70978555	89.70111137	0.5	CRA004775;Yang2025_Silkie_Wenchang	PRJNA827465	0.972001076
ENSGALG00010024899	OGFOD2	2	-0.213266882	0.333266375	-0.639929192	0.522218635	0.181651815	4.832495925	79.30675958	0.5	CRA004775;PRJNA827465	Yang2025_Silkie_Wenchang	0.869568211
ENSGALG00010024900	DTYMK	2	-0.113652598	0.151686742	-0.749258611	0.453701349	0.029925008	2.425402674	58.76973293	1	PRJNA827465;Yang2025_Silkie_Wenchang	CRA004775	0.849044791
ENSGALG00010024900	DTYMK	2	-0.36469769	0.166058742	-2.196196878	0.028077855	0	0.191218634	0	1	CRA004775;Yang2025_Silkie_Wenchang	PRJNA827465	0.285522361
ENSGALG00010024900	DTYMK	2	-0.139199726	0.218198879	-0.637948861	0.52350697	0.060802786	2.05489329	51.33567252	1	CRA004775;PRJNA827465	Yang2025_Silkie_Wenchang	0.869661505
ENSGALG00010024901	ZGPAT	2	-0.08854612	0.126280858	-0.701184026	0.483188177	0.019999788	2.107281264	52.54548992	1	PRJNA827465;Yang2025_Silkie_Wenchang	CRA004775	0.85691916
ENSGALG00010024901	ZGPAT	2	-0.274940507	0.111912382	-2.456747867	0.014020105	0	0.002411608	0	1	CRA004775;Yang2025_Silkie_Wenchang	PRJNA827465	0.193842584
ENSGALG00010024901	ZGPAT	2	-0.105700438	0.128170331	-0.824687256	0.409549168	0.023602494	3.071857618	67.44640787	1	CRA004775;PRJNA827465	Yang2025_Silkie_Wenchang	0.834491729
ENSGALG00010024902	LARP6	2	-0.1118556	0.082483776	-1.356092135	0.175069855	0	0.407847615	0	0.5	PRJNA827465;Yang2025_Silkie_Wenchang	CRA004775	0.680094025
ENSGALG00010024902	LARP6	2	0.036625432	0.166282129	0.220260781	0.825668063	0	0.01767658	0	1	CRA004775;Yang2025_Silkie_Wenchang	PRJNA827465	0.956388903
ENSGALG00010024902	LARP6	2	-0.112091193	0.08408532	-1.333064954	0.182510489	0	0.52291884	0	0.5	CRA004775;PRJNA827465	Yang2025_Silkie_Wenchang	0.690661444
ENSGALG00010024903	CASP8	2	-0.16714092	0.176264575	-0.94823886	0.343007868	0.040158777	2.749459925	63.62922075	1	PRJNA827465;Yang2025_Silkie_Wenchang	CRA004775	0.806218116
ENSGALG00010024903	CASP8	2	-0.363264353	0.160871314	-2.258105217	0.0239391	0	0.002636815	0	1	CRA004775;Yang2025_Silkie_Wenchang	PRJNA827465	0.264351316
ENSGALG00010024903	CASP8	2	-0.039971463	0.117558491	-0.340013412	0.733846428	0	0.625213298	0	1	CRA004775;PRJNA827465	Yang2025_Silkie_Wenchang	0.933391141
ENSGALG00010024904	ALS2CL	2	0.208203463	0.078923631	2.638037054	0.008338747	0	0.067247648	0	1	PRJNA827465;Yang2025_Silkie_Wenchang	CRA004775	0.142432756
ENSGALG00010024904	ALS2CL	2	0.065994855	0.281287927	0.234616733	0.814506221	0.088534262	1.806324411	44.63895886	0.5	CRA004775;Yang2025_Silkie_Wenchang	PRJNA827465	0.953869515
ENSGALG00010024904	ALS2CL	2	0.064493111	0.243438039	0.264926185	0.791066326	0.067304651	1.647501861	39.30204125	0.5	CRA004775;PRJNA827465	Yang2025_Silkie_Wenchang	0.947840178
ENSGALG00010024905	GPX7	2	-0.286836587	0.304188225	-0.942957561	0.345702611	0.178873315	29.83603362	96.64834806	0.5	PRJNA827465;Yang2025_Silkie_Wenchang	CRA004775	0.807376412
ENSGALG00010024905	GPX7	2	-0.588167999	0.086997052	-6.760780827	1.3725E-11	0	0.097604128	0	1	CRA004775;Yang2025_Silkie_Wenchang	PRJNA827465	1.12382E-08
ENSGALG00010024905	GPX7	2	-0.032789372	0.14183549	-0.231178893	0.817175827	0.017850879	1.190794811	16.02247588	0.5	CRA004775;PRJNA827465	Yang2025_Silkie_Wenchang	0.954833525
ENSGALG00010024907	PHLDB1	2	0.082266035	0.133720287	0.615209831	0.538416156	0.028591298	4.762499097	79.00262069	0.5	PRJNA827465;Yang2025_Silkie_Wenchang	CRA004775	0.87481821
ENSGALG00010024907	PHLDB1	2	0.11993093	0.204037336	0.587789138	0.556673836	0.04252474	1.566652536	36.16963705	0.5	CRA004775;Yang2025_Silkie_Wenchang	PRJNA827465	0.880358001
ENSGALG00010024907	PHLDB1	2	-0.043182065	0.059737893	-0.722858853	0.469766605	0	0.327682329	0	1	CRA004775;PRJNA827465	Yang2025_Silkie_Wenchang	0.853485658
ENSGALG00010024908	ENSGALG00010024908	2	-0.317495942	0.122689528	-2.587799848	0.009659109	0.018545123	2.141886481	53.31218489	1	PRJNA827465;Yang2025_Silkie_Wenchang	CRA004775	0.154951817
ENSGALG00010024908	ENSGALG00010024908	2	-0.320642269	0.228076291	-1.405855324	0.15976709	0.054386794	1.96348732	49.0702084	1	CRA004775;Yang2025_Silkie_Wenchang	PRJNA827465	0.655404348
ENSGALG00010024908	ENSGALG00010024908	2	-0.225740871	0.057812145	-3.904730966	9.43303E-05	0	0.490649126	0	1	CRA004775;PRJNA827465	Yang2025_Silkie_Wenchang	0.007444704
ENSGALG00010024910	UBTF	2	0.064414195	0.043181431	1.491710512	0.135775052	0	0.06598746	0	1	PRJNA827465;Yang2025_Silkie_Wenchang	CRA004775	0.617196137
ENSGALG00010024910	UBTF	2	-0.073751996	0.178986684	-0.412052978	0.680300593	0.045145909	3.350735368	70.15580492	0.5	CRA004775;Yang2025_Silkie_Wenchang	PRJNA827465	0.917768536
ENSGALG00010024910	UBTF	2	-0.069582146	0.159242528	-0.436957051	0.662142496	0.040079587	4.135136135	75.81699931	0.5	CRA004775;PRJNA827465	Yang2025_Silkie_Wenchang	0.912691648
ENSGALG00010024911	CAT	2	-0.301063026	0.150417423	-2.001516976	0.045336706	0.036940488	5.397034016	81.47130448	1	PRJNA827465;Yang2025_Silkie_Wenchang	CRA004775	0.367105422
ENSGALG00010024911	CAT	2	-0.178821016	0.355097886	-0.503582315	0.614554923	0.193812646	3.651936545	72.61726791	0.5	CRA004775;Yang2025_Silkie_Wenchang	PRJNA827465	0.898614239
ENSGALG00010024911	CAT	2	-0.093107824	0.153065964	-0.608285611	0.542998063	0.021170808	1.297957494	22.95587457	0.5	CRA004775;PRJNA827465	Yang2025_Silkie_Wenchang	0.876968972
ENSGALG00010024915	STXBP5L	2	-0.336397954	0.141088867	-2.384298356	0.017111723	0	0.00476344	0	1	PRJNA827465;Yang2025_Silkie_Wenchang	CRA004775	0.217369762
ENSGALG00010024915	STXBP5L	2	0.048971746	0.524729365	0.093327626	0.925643286	0.3633149	2.59387968	61.44771064	0.5	CRA004775;Yang2025_Silkie_Wenchang	PRJNA827465	0.982594411
ENSGALG00010024915	STXBP5L	2	0.035544069	0.505999207	0.070245306	0.943998415	0.36261644	2.768009967	63.87296245	0.5	CRA004775;PRJNA827465	Yang2025_Silkie_Wenchang	0.985827325
ENSGALG00010024917	TRAK2	2	-0.048875376	0.117428126	-0.416215245	0.677252505	0.019892191	3.323719407	69.91322439	0.5	PRJNA827465;Yang2025_Silkie_Wenchang	CRA004775	0.917144807
ENSGALG00010024917	TRAK2	2	-0.065281583	0.158220897	-0.412597732	0.679901363	0.026806254	2.0176455	50.43727949	0.5	CRA004775;Yang2025_Silkie_Wenchang	PRJNA827465	0.917701868
ENSGALG00010024917	TRAK2	2	0.056815111	0.057226	0.992819891	0.320797735	0	0.179186116	0	1	CRA004775;PRJNA827465	Yang2025_Silkie_Wenchang	0.798348216
ENSGALG00010024918	ATG4B	2	-0.099329843	0.163718358	-0.606711701	0.544042256	0.045093022	5.980219388	83.27820544	0.5	PRJNA827465;Yang2025_Silkie_Wenchang	CRA004775	0.876968972
ENSGALG00010024918	ATG4B	2	-0.301309431	0.094121065	-3.201296454	0.001368107	0	0.078751302	0	1	CRA004775;Yang2025_Silkie_Wenchang	PRJNA827465	0.045337865
ENSGALG00010024918	ATG4B	2	-0.117802036	0.190002959	-0.620001062	0.535257088	0.060203448	5.541704727	81.95501115	0.5	CRA004775;PRJNA827465	Yang2025_Silkie_Wenchang	0.874073846
ENSGALG00010024919	ANKRD52	2	-0.076821493	0.102993844	-0.74588432	0.455737298	0.01191983	2.189539106	54.32828775	0.5	PRJNA827465;Yang2025_Silkie_Wenchang	CRA004775	0.84907427
ENSGALG00010024919	ANKRD52	2	-0.170997665	0.110182043	-1.551955838	0.120672791	0	0.468923229	0	0.5	CRA004775;Yang2025_Silkie_Wenchang	PRJNA827465	0.582493642
ENSGALG00010024919	ANKRD52	2	0.009652289	0.075216253	0.128327176	0.897890059	0	0.001595071	0	1	CRA004775;PRJNA827465	Yang2025_Silkie_Wenchang	0.975696709
ENSGALG00010024920	VIPR2	2	-0.159331192	0.178865093	-0.890789752	0.373041974	0.026549296	1.704511908	41.33217872	0.5	PRJNA827465;Yang2025_Silkie_Wenchang	CRA004775	0.822162024
ENSGALG00010024920	VIPR2	2	0.102894157	0.533706003	0.192791831	0.847122002	0.436302586	3.72313201	73.14089328	0.5	CRA004775;Yang2025_Silkie_Wenchang	PRJNA827465	0.961252712
ENSGALG00010024920	VIPR2	2	0.230970546	0.326725144	0.706926142	0.479612377	0.105046233	1.639682196	39.01257194	1	CRA004775;PRJNA827465	Yang2025_Silkie_Wenchang	0.856184215
ENSGALG00010024921	STRADB	2	-0.097517507	0.090838007	-1.073532	0.283032489	0.005515899	1.340128287	25.38027816	0.5	PRJNA827465;Yang2025_Silkie_Wenchang	CRA004775	0.779568117
ENSGALG00010024921	STRADB	2	0.022840163	0.131906563	0.173154108	0.862530295	0	0.12939224	0	0.5	CRA004775;Yang2025_Silkie_Wenchang	PRJNA827465	0.966088008
ENSGALG00010024921	STRADB	2	-0.139572978	0.070665028	-1.9751351	0.048252818	0	0.217151896	0	1	CRA004775;PRJNA827465	Yang2025_Silkie_Wenchang	0.379964424
ENSGALG00010024924	MNX1	2	1.425449851	0.89405923	1.594357289	0.110856033	1.304803431	5.439741684	81.61677414	1	PRJNA827465;Yang2025_Silkie_Wenchang	CRA004775	0.563494848
ENSGALG00010024924	MNX1	2	1.346647363	0.989810278	1.360510587	0.173668405	1.632680045	5.989865742	83.30513499	1	CRA004775;Yang2025_Silkie_Wenchang	PRJNA827465	0.677420535
ENSGALG00010024924	MNX1	2	0.447800702	0.399688794	1.120373423	0.262554666	0	0.056905649	0	1	CRA004775;PRJNA827465	Yang2025_Silkie_Wenchang	0.768418419
ENSGALG00010024925	GTF2E1	2	0.02405207	0.051111002	0.47058498	0.637937136	0	0.291858143	0	1	PRJNA827465;Yang2025_Silkie_Wenchang	CRA004775	0.905365679
ENSGALG00010024925	GTF2E1	2	-0.163891044	0.246329949	-0.665331378	0.505838565	0.105215488	7.343161983	86.38188832	0.5	CRA004775;Yang2025_Silkie_Wenchang	PRJNA827465	0.863927652
ENSGALG00010024925	GTF2E1	2	-0.185965555	0.214865265	-0.865498456	0.386765263	0.079637066	6.770441979	85.22991552	0.5	CRA004775;PRJNA827465	Yang2025_Silkie_Wenchang	0.827124608
ENSGALG00010024927	NOM1	2	-0.166806493	0.210316897	-0.793119791	0.427708033	0.075084115	6.496317177	84.60666293	0.5	PRJNA827465;Yang2025_Silkie_Wenchang	CRA004775	0.84125685
ENSGALG00010024927	NOM1	2	-0.287007248	0.14180545	-2.023950765	0.042975218	0.011930929	1.364368597	26.70602342	1	CRA004775;Yang2025_Silkie_Wenchang	PRJNA827465	0.358504015
ENSGALG00010024927	NOM1	2	0.012895761	0.085614495	0.150625912	0.88027082	0	0.269637929	0	0.5	CRA004775;PRJNA827465	Yang2025_Silkie_Wenchang	0.971588195
ENSGALG00010024928	THAP4	2	-0.002386876	0.057947287	-0.041190471	0.96714405	0	0.110359428	0	0.5	PRJNA827465;Yang2025_Silkie_Wenchang	CRA004775	0.990893423

ENSGALG00010024928	THAP4	2	-0.06283775	0.117144471	-0.536412426	0.591673545	0	0.260986844	0	1	CRA004775;Yang2025_Silkie_Wenchang	PRJNA827465	0.892776493
ENSGALG00010024928	THAP4	2	-0.001225175	0.06398935	-0.019146552	0.984724195	0	0.463453185	0	0.5	CRA004775;PRJNA827465	Yang2025_Silkie_Wenchang	0.995703389
ENSGALG00010024930	TMEM237	2	0.012401423	0.102038673	0.121536502	0.903266104	0.01376229	2.856159224	64.98794634	0.5	PRJNA827465;Yang2025_Silkie_Wenchang	CRA004775	0.977253688
ENSGALG00010024930	TMEM237	2	-0.063761934	0.092307911	-0.69075265	0.489720996	0	0.984157228	0	0.5	CRA004775;Yang2025_Silkie_Wenchang	PRJNA827465	0.858425426
ENSGALG00010024930	TMEM237	2	0.105560492	0.065587567	1.609458879	0.10751604	0	0.033123042	0	1	CRA004775;PRJNA827465	Yang2025_Silkie_Wenchang	0.554966053
ENSGALG00010024931	UBE3C	2	-0.20736861	0.182018433	-1.139272578	0.254589482	0.059976632	10.16030876	90.15777942	1	PRJNA827465;Yang2025_Silkie_Wenchang	CRA004775	0.764293494
ENSGALG00010024931	UBE3C	2	-0.103681709	0.323625395	-0.320375689	0.748683552	0.182527366	7.421603118	86.5258222	0.5	CRA004775;Yang2025_Silkie_Wenchang	PRJNA827465	0.937249951
ENSGALG00010024931	UBE3C	2	0.029123398	0.119892643	0.242912302	0.808073336	0.016306712	1.672611699	40.21326047	0.5	CRA004775;PRJNA827465	Yang2025_Silkie_Wenchang	0.952496413
ENSGALG00010024933	CS	2	-0.161166446	0.072032021	-2.237427807	0.025258393	0	0.053320703	0	1	PRJNA827465;Yang2025_Silkie_Wenchang	CRA004775	0.270438551
ENSGALG00010024933	CS	2	-0.134870747	0.161187639	-0.836731327	0.402743606	0	0.021543956	0	1	CRA004775;Yang2025_Silkie_Wenchang	PRJNA827465	0.832937979
ENSGALG00010024933	CS	2	-0.169023284	0.07673844	-2.20258953	0.027623693	0	0.001342589	0	1	CRA004775;PRJNA827465	Yang2025_Silkie_Wenchang	0.283693229
ENSGALG00010024934	G6PC3	2	0.135068742	0.138583979	0.974634606	0.329741542	0.017353729	1.723997294	41.99526859	1	PRJNA827465;Yang2025_Silkie_Wenchang	CRA004775	0.801177491
ENSGALG00010024934	G6PC3	2	0.039702351	0.324567744	0.122323771	0.902642607	0.14728126	3.144510956	68.19855252	0.5	CRA004775;Yang2025_Silkie_Wenchang	PRJNA827465	0.976923607
ENSGALG00010024934	G6PC3	2	-0.034450868	0.139824289	-0.246386862	0.805382776	0.010638996	1.184357244	15.56601647	0.5	CRA004775;PRJNA827465	Yang2025_Silkie_Wenchang	0.951870339
ENSGALG00010024935	KLHL30	2	-0.03814498	0.102178925	-0.373315537	0.708913614	0	0.076490039	0	1	PRJNA827465;Yang2025_Silkie_Wenchang	CRA004775	0.925298042
ENSGALG00010024935	KLHL30	2	-0.028445578	0.144664457	-0.196631422	0.844115979	0	0.409516063	0	1	CRA004775;Yang2025_Silkie_Wenchang	PRJNA827465	0.960723102
ENSGALG00010024935	KLHL30	2	-0.081048288	0.138385013	-0.585672443	0.558095656	0	0.314352046	0	1	CRA004775;PRJNA827465	Yang2025_Silkie_Wenchang	0.880678789
ENSGALG00010024937	ILKAP	2	0.308824234	0.324976209	0.950297979	0.341960866	0.204402853	30.78581634	96.75175091	0.5	PRJNA827465;Yang2025_Silkie_Wenchang	CRA004775	0.805904173
ENSGALG00010024937	ILKAP	2	0.138000784	0.522132357	0.264302302	0.791546987	0.513277515	16.58661306	93.97104161	0.5	CRA004775;Yang2025_Silkie_Wenchang	PRJNA827465	0.948076163
ENSGALG00010024937	ILKAP	2	-0.14299478	0.186243901	-0.767782351	0.442616501	0.048270799	2.619478166	61.82445752	1	CRA004775;PRJNA827465	Yang2025_Silkie_Wenchang	0.845623
ENSGALG00010024938	ENSGALG00010024938	2	0.355563354	0.254259286	1.398428194	0.161984522	0.096894698	3.929291994	74.55012248	1	PRJNA827465;Yang2025_Silkie_Wenchang	CRA004775	0.659256758
ENSGALG00010024938	ENSGALG00010024938	2	0.219176405	0.444643844	0.492925761	0.622065044	0.32311163	5.310563882	81.16960793	0.5	CRA004775;Yang2025_Silkie_Wenchang	PRJNA827465	0.899986101
ENSGALG00010024938	ENSGALG00010024938	2	0.037342475	0.156740517	0.238243917	0.811691917	0.007544075	1.115040035	10.31712147	0.5	CRA004775;PRJNA827465	Yang2025_Silkie_Wenchang	0.953348795
ENSGALG00010024939	COQ10A	2	0.103877172	0.123110039	0.843774989	0.398795154	0.004961195	1.192154854	16.11827974	0.5	PRJNA827465;Yang2025_Silkie_Wenchang	CRA004775	0.832054308
ENSGALG00010024939	COQ10A	2	0.058258679	0.282473551	0.206244721	0.836599756	0.084391299	1.799535019	44.43008948	0.5	CRA004775;Yang2025_Silkie_Wenchang	PRJNA827465	0.958399882
ENSGALG00010024939	COQ10A	2	-0.044736591	0.140145628	-0.319215033	0.749563458	0	0.666282547	0	1	CRA004775;PRJNA827465	Yang2025_Silkie_Wenchang	0.937362187
ENSGALG00010024940	RABL3	2	-0.081067385	0.069552399	-1.16555843	0.243793024	0.001563982	1.167398366	14.33943809	1	PRJNA827465;Yang2025_Silkie_Wenchang	CRA004775	0.755365463
ENSGALG00010024940	RABL3	2	-0.237687702	0.092593107	-2.567012908	0.010257879	0	0.757397154	0	1	CRA004775;Yang2025_Silkie_Wenchang	PRJNA827465	0.160880061
ENSGALG00010024940	RABL3	2	-0.160240136	0.155862825	-1.028084381	0.303910137	0.035051694	3.274189948	69.45809449	1	CRA004775;PRJNA827465	Yang2025_Silkie_Wenchang	0.792208658
ENSGALG00010024941	PDHX	2	0.079621766	0.097468566	0.816896866	0.413987369	0.014888032	4.496236505	77.75917706	0.5	PRJNA827465;Yang2025_Silkie_Wenchang	CRA004775	0.836724075
ENSGALG00010024941	PDHX	2	0.113515063	0.158968551	0.71407245	0.475182381	0.024595903	1.383958784	27.74351292	0.5	CRA004775;Yang2025_Silkie_Wenchang	PRJNA827465	0.854462864
ENSGALG00010024941	PDHX	2	-0.014345343	0.050321528	-0.285073677	0.775587696	0	0.407167487	0	1	CRA004775;PRJNA827465	Yang2025_Silkie_Wenchang	0.943815265
ENSGALG00010024942	DNAJB6	2	0.060472873	0.069226846	0.873546556	0.382365242	0.005408244	1.936304625	48.35523361	1	PRJNA827465;Yang2025_Silkie_Wenchang	CRA004775	0.82508516
ENSGALG00010024942	DNAJB6	2	0.154434551	0.082417652	1.873804302	0.060957403	0	0.025775677	0	1	CRA004775;Yang2025_Silkie_Wenchang	PRJNA827465	0.427185264
ENSGALG00010024942	DNAJB6	2	0.022231751	0.037960136	0.585660474	0.558103701	0	0.647411979	0	1	CRA004775;PRJNA827465	Yang2025_Silkie_Wenchang	0.880678789
ENSGALG00010024943	ABCB9	2	-0.031619794	0.115802999	-0.273048148	0.784816204	0	0.00241364	0	1	PRJNA827465;Yang2025_Silkie_Wenchang	CRA004775	0.946214033
ENSGALG00010024943	ABCB9	2	-0.026369292	0.181550848	-0.145244667	0.884517701	0	0.043521133	0	0.5	CRA004775;Yang2025_Silkie_Wenchang	PRJNA827465	0.972062774
ENSGALG00010024943	ABCB9	2	-0.020352974	0.140853341	-0.144497633	0.885107525	0	0.037309703	0	0.5	CRA004775;PRJNA827465	Yang2025_Silkie_Wenchang	0.972230543
ENSGALG00010024944	ENSGALG00010024944	2	-0.017092845	0.064232053	-0.266110831	0.790153854	0	0.084818698	0	0.5	PRJNA827465;Yang2025_Silkie_Wenchang	CRA004775	0.947390191
ENSGALG00010024944	ENSGALG00010024944	2	-0.27328404	0.356432313	-0.766720721	0.443247581	0.178391187	3.261408588	69.3384017	0.5	CRA004775;Yang2025_Silkie_Wenchang	PRJNA827465	0.845733906
ENSGALG00010024944	ENSGALG00010024944	2	-0.267926258	0.314742573	-0.851255219	0.394627595	0.154709877	3.762189688	73.4197347	1	CRA004775;PRJNA827465	Yang2025_Silkie_Wenchang	0.830643015
ENSGALG00010024945	APIP	2	0.005898167	0.071191209	0.082849655	0.933971086	0	0.448310919	0	0.5	PRJNA827465;Yang2025_Silkie_Wenchang	CRA004775	0.98432801
ENSGALG00010024945	APIP	2	-0.074429613	0.110086084	-0.676103736	0.498974785	0	0.00816629	0	1	CRA004775;Yang2025_Silkie_Wenchang	PRJNA827465	0.861571318
ENSGALG00010024945	APIP	2	0.017752096	0.078134791	0.227198361	0.820269502	0	0.332898847	0	0.5	CRA004775;PRJNA827465	Yang2025_Silkie_Wenchang	0.955308792
ENSGALG00010024946	KIF1A	2	0.130879662	0.307762236	0.425262254	0.670645506	0.079052465	1.506263027	33.61053267	0.5	PRJNA827465;Yang2025_Silkie_Wenchang	CRA004775	0.914482883
ENSGALG00010024946	KIF1A	2	0.268860764	0.447369077	0.600982004	0.547851973	0.067131848	1.189484771	15.92998717	0.5	CRA004775;Yang2025_Silkie_Wenchang	PRJNA827465	0.878281266
ENSGALG00010024946	KIF1A	2	-0.085060024	0.216606857	-0.392693126	0.694546148	0	0.107605058	0	1	CRA004775;PRJNA827465	Yang2025_Silkie_Wenchang	0.920728119
ENSGALG00010024947	MPP4	2	0.757574339	0.755120729	1.003249295	0.315740594	1.101761	29.49072654	96.60910355	1	PRJNA827465;Yang2025_Silkie_Wenchang	CRA004775	0.796883862
ENSGALG00010024947	MPP4	2	1.32938431	0.32664007	4.069875174	4.70383E-05	0.098279366	1.517707407	34.11114716	1	CRA004775;Yang2025_Silkie_Wenchang	PRJNA827465	0.004633453
ENSGALG00010024947	MPP4	2	0.179717298	0.317111044	0.566733015	0.570895587	0.096714156	1.521644429	34.28162447	1	CRA004775;PRJNA827465	Yang2025_Silkie_Wenchang	0.88529924
ENSGALG00010024949	PAN2	2	0.032783202	0.050390677	0.650580696	0.515317195	0.000690894	1.081418764	7.528883936	1	PRJNA827465;Yang2025_Silkie_Wenchang	CRA004775	0.867165256
ENSGALG00010024949	PAN2	2	-0.203641123	0.374364811	-0.54396438	0.586465984	0.247630707	8.282964076	87.9270272	0.5	CRA004775;Yang2025_Silkie_Wenchang	PRJNA827465	0.891596903
ENSGALG00010024949	PAN2	2	-0.255718283	0.304706453	-0.839228313	0.401341202	0.161265386	6.786566952	85.26500944	0.5	CRA004775;PRJNA827465	Yang2025_Silkie_Wenchang	0.832528277
ENSGALG00010024950	NCAM1	2	-0.150269142	0.063209936	-2.377302548	0.017439777	0	0.044531438	0	1	PRJNA827465;Yang2025_Silkie_Wenchang	CRA004775	0.21990233
ENSGALG00010024950	NCAM1	2	-0.305589576	0.269501943	-1.13390491	0.256834437	0.091900118	2.087204763	52.08903228	1	CRA004775;Yang2025_Silkie_Wenchang	PRJNA827465	0.764750282
ENSGALG00010024950	NCAM1	2	-0.309833635	0.249451046	-1.242061878	0.214213732	0.077932015	1.943606075	48.54924499	1	CRA004775;PRJNA827465	Yang2025_Silkie_Wenchang	0.72702531
ENSGALG00010024953	NCAPG2	2	-0.227787354	0.114229104	-1.994127119	0.046138164	0	0.107966346	0	1	PRJNA827465;Yang2025_Silkie_Wenchang	CRA004775	0.370377506
ENSGALG00010024953	NCAPG2	2	-0.259260212	0.174475983	-1.485936383	0.137295949	0	0.129133245	0	0.5	CRA004775;Yang2025_Silkie_Wenchang	PRJNA827465	0.619818823
ENSGALG00010024953	NCAPG2	2	-0.189390332	0.146258608	-1.294900407	0.195354588	0	0.073171923	0	0.5	CRA004775;PRJNA827465	Yang2025_Silkie_Wenchang	0.70643959
ENSGALG00010024956	VPS37B	2	-0.27902712	0.221654669	-1.258837096	0.208089178	0.092380134	16.29999734	93.86502967	1	PRJNA827465;Yang2025_Silkie_Wenchang	CRA004775	0.720702237
ENSGALG00010024956	VPS37B	2	0.06947794	0.600329482	0.115733013	0.907864142	0.68588416	20.14044695	95.03486689	0.5	CRA004775;Yang2025_Silkie_Wenchang	PRJNA827465	0.978196186
ENSGALG00010024956	VPS37B	2	0.273522311	0.376838587	0.725834138	0.467940456	0.255196857	8.997371046	88.88564232	0.5	CRA004775;PRJNA827465	Yang2025_Silkie_Wenchang	0.853115491
ENSGALG00010024960	ARFRP1	2	-0.1920463	0.259245293	-0.740789922	0.458820827	0.123082302	11.26758424	91.12498315	0.5	PRJNA827465;Yang2025_Silkie_Wenchang	CRA004775	0.850089963
ENSGALG00010024960	ARFRP1	2	-0.32558973	0.160508458	-2.028489552	0.042510309	0.025233856	1.939110694	48.42996829	1	CRA004775;Yang2025_Silkie_Wenchang	PRJNA827465	0.356625761
ENSGALG00010024960	ARFRP1	2	0.026852773	0.06312944	0.425360551	0.670573859	0.001917505	1.109730527	9.888033562	0.5	CRA004775;PRJNA827465	Yang2025_Silkie_Wenchang	0.914482883
ENSGALG00010024961	LSM12	2	0.062748581	0.246470694	0.254588406	0.799041015	0.10589899	7.465152346	86.60442609	0.5	PRJNA827465;Yang2025_Silkie_Wenchang	CRA004775	0.949922217
ENSGALG00010024961	LSM12	2	0.359831931	0.128682923	2.796267931	0.005169652	0	0.093852499	0	1	CRA004775;Yang2025_Silkie_Wenchang	PRJNA827465	0.107382666
ENSGALG00010024961	LSM12	2	0.090023193	0.286212647	0.314532549	0.7531166	0.140221256	6.425197563	84.43627624	0.5	CRA004775;PRJNA827465	Yang2025_Silkie_Wenchang	0.938087082
ENSGALG00010024963	TNFRSF6B	2	-0.574589013	0.363756233	-1.579599087	0.114198708	0.168706597	2.749383279	63.62820681	1	PRJNA827465;Yang2025_Silkie_Wenchang	CRA004775	0.570149069
ENSGALG00010024963	TNFRSF6B	2	-0.446074047	0.637099011	-0.7001644	0.483824641	0.556478026	2.872095827	65.18222022	0.5	CRA004775;Yang2025_Silkie_Wenchang	PRJNA827465	0.85691916
ENSGALG00010024963	TNFRSF6B	2	-0.140500507	0.268779126	-0.522735934	0.601158029	0	0.58575791	0	0.5	CRA004775;PRJNA827465	Yang2025_Silkie_Wenchang	0.895230519
ENSGALG00010024964	ITM2C	2	-0.060942917	0.186620468	-0.326560736	0.744000145	0.028376631	1.680476935	40.49308388	0.5	PRJNA827465;Yang2025_Silkie_Wenchang	CRA004775	0.935821071
ENSGALG00010024964	ITM2C	2	0.300628532	0.587527316	0.511684348	0.608871942	0.615355151	9.070675791	88.9754631	0.5	CRA004775;Yang2025_Silkie_Wenchang	PRJNA827465	0.897208691
ENSGALG00010024964	ITM2C	2	0.469468385	0.398538849	1.17797958	0.238806989	0.250580409	4.539311889	77.97022931	1	CRA004775;PRJNA827465	Yang2025_Silkie_Wenchang	0.750806421
ENSGALG00010024965	STAT2	2	0.238221955	0.222622419	1.07007172	0.284587027	0.08976284	10.56953179	90.53884297	1	PRJNA827465;Yang2025_Silkie_Wenchang	CRA004775	0.780265906
ENSGALG00010024965	STAT2	2	0.006859731	0.474259811	0.014464078	0.988459738	0.423191368	16.52910853	93.95006695	0.5	CRA004775;Yang2025_Silkie_Wenchang	PRJNA827465	0.996891127
ENSGALG00010024965	STAT2	2	-0.197610899	0.249419153	-0.792284379	0.428194878	0.101141873	4.921126049	79.67944755	0.5	CRA004775;PRJNA827465	Yang2025_Silkie_Wenchang	0.84125685
ENSGALG00010024966	AGAP3	2	0.150571632	0.102497759	1.469023648	0.141826375	0.017557274	5.810937685	82.79107342	1	PRJNA827465;Yang2025_Silkie_Wenchang	CRA004775	0.627301601
ENSGALG00010024966	AGAP3	2	0.014267671	0.291870986	0.048883486	0.96101215	0.141582843	5.277890456	81.05303609	0.5	CRA004775;Yang2025_Silkie_Wenchang	PRJNA827465	0.989599947
ENSGALG00010024966	AGAP3	2	-0.058621634	0.176728664	-0.331704167	0.740112654	0.043096702	2.387435429	58.11405041	0.5	CRA004775;PRJNA827465	Yang2025_Silkie_Wenchang	0.93508234
ENSGALG00010024968	TMEM101	2	-0.053236589	0.070477412	-0.755370935	0.450026447	0	0.081570607	0	1	PRJNA827465;Yang2025_Silkie_Wenchang	CRA004775	0.847526417
ENSGALG00010024968	TMEM101	2	-0.134240756	0.186061384	-0.721486387	0.470610313	0	0.008696747	0	1	CRA004775;Yang2025_Silkie_Wenchang	PRJNA827465	0.853744788
ENSGALG00010024968	TMEM101	2	-0.053697152	0.071365322	-0.752426392	0.45179468	0	0.13034586	0	1	CRA004775;PRJNA827465	Yang2025_Silkie_Wenchang	0.848029338
ENSGALG00010024970	CAB39	2	-0.074333165	0.095273158	-0.780210992	0.435266694	0.013414141	3.281755799	69.52850665	0.5	PRJNA827465;Yang2025_Silkie_Wenchang	CRA004775	0.842762754
ENSGALG00010024970	CAB39	2	0.032944924	0.251485376	0.131001349	0.89577424	0.105283979	5.725143926	82.53319021	0.5	CRA004775;Yang2025_Silkie_Wenchang	PRJNA827465	0.974955414
ENSGALG00010024970	CAB39	2	0.10121476	0.144899101	0.698518892	0.484852748	0.030045385	2.707725836	63.06863912	1	CRA004775;PRJNA827465	Yang2025_Silkie_Wenchang	0.857004733
ENSGALG00010024973	RTEL1	2	0.063772815	0.067406341	0.946095189	0.344100038	0	0.09941873	0	1	PRJNA827465;Yang2025_Silkie_Wenchang	CRA004775	0.806449838
ENSGALG00010024973	RTEL1	2	0.055258468	0.135021759	0.409256026	0.682351783	0	0.609679891	0	1	CRA004775;Yang2025_Silkie_Wenchang	PRJNA827465	0.918625039
ENSGALG00010024973	RTEL1	2	0.083762246	0.07588906	1.103746004	0.269703334	0	0.490617526	0	1	CRA004775;PRJNA827465	Yang2025_Silkie_Wenchang	0.77189992
ENSGALG00010024974	CD44	2	0.06229236	0.091008023	0.684471086	0.493677741	0	0.08001897	0	1	PRJNA827465;Yang2025_Silkie_Wenchang	CRA004775	0.860054554
ENSGALG00010024974	CD44	2	0.055693855	0.139748284	0.398529795	0.6902397	0	0.559544026	0	0.5	CRA004775;Yang2025_Silkie_Wenchang	PRJNA827465	0.919979266
ENSGALG00010024974	CD44	2	0.021950401	0.109931854	0.199672798	0.841736489	0	0.406199633	0	0.5	CRA004775;PRJNA827465	Yang2025_Silkie_Wenchang	0.960006072
ENSGALG00010024977	LRP3	2	-0.006631546	0.062518823	-0.106072788	0.915524601	0	0.932047532	0	0.5	PRJNA827465;Yang2025_Silkie_Wenchang	CRA004775	0.980165711
ENSGALG00010024977	LRP3	2	-0.119954511	0.123443456	-0.971736498	0.331181655	0	0.018365813	0	1	CRA004775;Yang2025_Silkie_Wenchang	PRJNA827465	0.801725552
ENSGALG00010024977	LRP3	2	0.021734854	0.070628115	0.307736573	0.758282788	0	0.208574561	0	0.5	CRA004775;PRJNA827465	Yang2025_Silkie_Wenchang	0.938954369
ENSGALG00010024986	PTS	2	0.115239092	0.08484096	1.358295468	0.174369948	0	0.097886901	0	1	PRJNA827465;Yang2025_Silkie_Wenchang	CRA004775	0.678675189
ENSGALG00010024986	PTS	2	0.09899883	0.131294955	0.754018537	0.450838091	0	0.11271753	0	1	CRA004775;Yang2025_Silkie_Wenchang	PRJNA827465	0.847631399
ENSGALG00010024986	PTS	2	0.140477398	0.099278803	1.41497876	0.157074724	0	0.032778491	0	1	CRA004775;PRJNA827465	Yang2025_Silkie_Wenchang	0.650687662
ENSGALG00010024989	TIMELESS	2	0.215368281	0.288909854	0.745451492	0.455998825	0.143333248	6.921767218	85.55282244	0.5	PRJNA827465;Yang2025_Silkie_Wenchang	CRA004775	0.84907427
ENSGALG00010024989	TIMELESS	2	0.05107166	0.54063305	0.094466405	0.924738668	0.479937056	5.144535433	80.56189887	0.5	CRA004775;Yang2025_Silkie_Wenchang	PRJNA827465	0.982493782
ENSGALG00010024989	TIMELESS	2	-0.134428977	0.183832545	-0.731257772	0.464621719	0.025650723	1.242503619	19.517337	1	CRA004775;PRJNA827465	Yang2025_Silkie_Wenchang	0.85217126
ENSGALG00010024994	NDUFB4	2	0.011766415	0.223793373	0.052577138	0.958068833	0.078355213	4.116232383	75.70593915	0.5	PRJNA827465;Yang2025_Silkie_Wenchang	CRA004775	0.988864712
ENSGALG00010024994	NDUFB4	2	0.239884141	0.181718897	1.320083624	0.1868071	0	0.151019762	0	1	CRA004775;Yang2025_Silkie_Wenchang	PRJNA827465	0.69566794
ENSGALG00010024994	NDUFB4	2	-0.16140307	0.079166387	-2.038782829	0.041471707	0	0.596935322	0	0.5	CRA004775;PRJNA827465	Yang2025_Silkie_Wenchang	0.353111459
ENSGALG00010024996	COA7	2	-0.036751616	0.08791274	-0.418046534	0.675913094	0	0.265575405	0	0.5	PRJNA827465;Yang2025_Silkie_Wenchang	CRA004775	0.916522765
ENSGALG00010024996	COA7	2	0.022464796	0.155838249	0.14415457	0.885378414	0	0.078727941	0	0.5	CRA004775;Yang2025_Silkie_Wenchang	PRJNA827465	0.972280795
ENSGALG00010024996	COA7	2	-0.052348447	0.088939719	-0.588583453	0.556140737	0	0.017404232	0	1	CRA004775;PRJNA827465	Yang2025_Silkie_Wenchang	0.880324407
ENSGALG00010025003	ENSGALG00010025003	2	0.098792043	0.338118045	0.292182109	0.77014739	0.187834457	5.474425432	81.73324283	0.5	PRJNA827465;Yang2025_Silkie_Wenchang	CRA004775	0.942170227
ENSGALG00010025003	ENSGALG00010025003	2	0.445561586	0.227333582	1.959946182	0.050002081	0	0.040115854	0	1	CRA004775;Yang2025_Silkie_Wenchang	PRJNA827465	0.387009017
ENSGALG00010025003	ENSGALG00010025003	2	-0.187362012	0.155673679	-1.203556142	0.22876118	0	0.598965581	0	0.5	CRA004775;PRJNA827465	Yang2025_Silkie_Wenchang	0.741525826
ENSGALG00010025005	HIP1R	2	0.346323241	0.261153189	1.326130625	0.184796434	0.126564335	13.85484722	92.78230944	1	PRJNA827465;Yang2025_Silkie_Wenchang	CRA004775	0.69308359
ENSGALG00010025005	HIP1R	2	0.236193096	0.389724987	0.606050688	0.544481095	0.280341706	12.68405198	92.11608403	0.5	CRA004775;Yang2025_Silkie_Wenchang	PRJNA827465	0.877225185
ENSGALG00010025005	HIP1R	2	0.013106043	0.116667638	0.112336579	0.910556539	0.010295462	1.448637692	30.96962717	0.5	CRA004775;PRJNA827465	Yang2025_Silkie_Wenchang	0.97880407
ENSGALG00010025007	ZYG11B	2	0.021816667	0.054579606	0.399721963	0.689361314	0	0.717939073	0	0.5	PRJNA827465;Yang2025_Silkie_Wenchang	CRA004775	0.91986176
ENSGALG00010025007	ZYG11B	2	-0.052738609	0.085292537	-0.618326183	0.536360346	0	0.013401265	0	1	CRA004775;Yang2025_Silkie_Wenchang	PRJNA827465	0.874599474
ENSGALG00010025007	ZYG11B	2	0.037344732	0.061439905	0.607825353	0.543303312	0	0.441022546	0	0.5	CRA004775;PRJNA827465	Yang2025_Silkie_Wenchang	0.876968972
ENSGALG00010025008	ENSGALG00010025008	2	-0.559226825	1.100000848	-0.508387631	0.611181526	2.073296629	6.882655904	85.47072505	0.5	PRJNA827465;Yang2025_Silkie_Wenchang	CRA004775	0.897609796
ENSGALG00010025008	ENSGALG00010025008	2	-0.904596268	1.002901893	-0.901978821	0.367068118	1.190684894	2.30171295	56.55409594	0.5	CRA004775;Yang2025_Silkie_Wenchang	PRJNA827465	0.819739734
ENSGALG00010025008	ENSGALG00010025008	2	0.466367127	0.447198243	1.042864399	0.2970111	0	0.014230655	0	1	CRA004775;PRJNA827465	Yang2025_Silkie_Wenchang	0.788466926
ENSGALG00010025026	DENR	2	-0.083607707	0.061774828	-1.353426778	0.175919328	0	0.9955059	0	1	PRJNA827465;Yang2025_Silkie_Wenchang	CRA004775	0.680996487
ENSGALG00010025026	DENR	2	-0.173770785	0.135884614	-1.278811339	0.2009635	0	0.550864695	0	0.5	CRA004775;Yang2025_Silkie_Wenchang	PRJNA827465	0.713544434
ENSGALG00010025026	DENR	2	-0.051147985	0.066562345	-0.76842222	0.442236384	0	0.101613065	0	0.5	CRA004775;PRJNA827465	Yang2025_Silkie_Wenchang	0.845369538
ENSGALG00010025028	HES6	2	0.215224891	0.24655696	0.87292158	0.382705823	0.104101896	6.620860882	84.89622395	0.5	PRJNA827465;Yang2025_Silkie_Wenchang	CRA004775	0.825121999
ENSGALG00010025028	HES6	2	0.37495814	0.202754407	1.849321776	0.064411363	0.0235407	1.254089286	20.2608609	1	CRA004775;Yang2025_Silkie_Wenchang	PRJNA827465	0.437453135
ENSGALG00010025028	HES6	2	-0.008978131	0.083051316	-0.10810342	0.913913655	0	0.001065843	0	0.5	CRA004775;PRJNA827465	Yang2025_Silkie_Wenchang	0.979374752
ENSGALG00010025031	ECHDC2	2	-0.150215406	0.088733808	-1.692876804	0.090478919	0	0.18428692	0	1	PRJNA827465;Yang2025_Silkie_Wenchang	CRA004775	0.516198718
ENSGALG00010025031	ECHDC2	2	-0.158344693	0.140938134	-1.123504962	0.261223094	0	0.522478412	0	0.5	CRA004775;Yang2025_Silkie_Wenchang	PRJNA827465	0.768039422
ENSGALG00010025031	ECHDC2	2	-0.106191485	0.103183676	-1.029150048	0.303409171	0	0.280533541	0	0.5	CRA004775;PRJNA827465	Yang2025_Silkie_Wenchang	0.792088386
ENSGALG00010025032	FSTL1	2	-0.27526064	0.302164256	-0.910963605	0.362314551	0.172683474	18.35449489	94.55174329	0.5	PRJNA827465;Yang2025_Silkie_Wenchang	CRA004775	0.815644888
ENSGALG00010025032	FSTL1	2	-0.277445992	0.369418972	-0.751034416	0.452632546	0.216518454	4.230145711	76.36015238	0.5	CRA004775;Yang2025_Silkie_Wenchang	PRJNA827465	0.818370093
ENSGALG00010025032	FSTL1	2	0.032079503	0.08518057	0.376605868	0.706466526	0	0.170593771	0	1	CRA004775;PRJNA827465	Yang2025_Silkie_Wenchang	0.924548793
ENSGALG00010025039	CEBPA	2	-0.017062165	0.155257936	-0.109895608	0.912492166	0	0.012994047	0	0.5	PRJNA827465;Yang2025_Silkie_Wenchang	CRA004775	0.978967245
ENSGALG00010025039	CEBPA	2	-0.633963745	0.696407111	-0.910334968	0.362654883	0.796684278	5.080143395	80.31551627	1	CRA004775;Yang2025_Silkie_Wenchang	PRJNA827465	0.815941879
ENSGALG00010025039	CEBPA	2	-0.61925553	0.714733608	-0.866414455	0.386262692	0.84656211	5.322122347	81.21050335	1	CRA004775;PRJNA827465	Yang2025_Silkie_Wenchang	0.826894808
ENSGALG00010025044	TMEM9	2	0.573737407	0.142546123	4.024924679	5.69936E-05	0	0.982977015	0	1	PRJNA827465;Yang2025_Silkie_Wenchang	CRA004775	0.005333375
ENSGALG00010025044	TMEM9	2	-0.248882936	1.019635449	-0.244090117	0.807161027	1.986619784	22.24390638	95.50438676	0.5	CRA004775;Yang2025_Silkie_Wenchang	PRJNA827465	0.95234753
ENSGALG00010025044	TMEM9	2	-0.385040781	0.874442593	-0.440327111	0.659700208	1.446914891	18.21091241	94.50878694	0.5	CRA004775;PRJNA827465	Yang2025_Silkie_Wenchang	0.911617103
ENSGALG00010025047	PER2	2	0.41273186	0.283742553	1.454599797	0.145780087	0.141337751	7.714440787	87.03729761	1	PRJNA827465;Yang2025_Silkie_Wenchang	CRA004775	0.438470894
ENSGALG00010025047	PER2	2	0.673571652	0.159746217	3.991153374	6.57527E-05	0	0.713027286	0	1	CRA004775;Yang2025_Silkie_Wenchang	PRJNA827465	0.005846787
ENSGALG00010025047	PER2	2	0.171754106	0.075791177	2.266149099	0.023442256	0	0.790449225	0	1	CRA004775;PRJNA827465	Yang2025_Silkie_Wenchang	0.261227952
ENSGALG00010025048	ESYT2	2	-0.098159451	0.076868379	-1.276980884	0.201608996	0	0.070809619	0	1	PRJNA827465;Yang2025_Silkie_Wenchang	CRA004775	0.714501332
ENSGALG00010025048	ESYT2	2	-0.065494546	0.14003085	-0.467715124	0.639988324	0	0.00666602	0	0.5	CRA004775;Yang2025_Silkie_Wenchang	PRJNA827465	0.906064918
ENSGALG00010025048	ESYT2	2	-0.108891517	0.087668404	-1.242083946	0.214205591	0	0.006952138	0	1	CRA004775;PRJNA827465	Yang2025_Silkie_Wenchang	0.72702531
ENSGALG00010025055	ALS2	2	-0.011431698	0.087715226	-0.130327403	0.8963074	0.008585632	1.881067149	46.83868672	0.5	PRJNA827465;Yang2025_Silkie_Wenchang	CRA004775	0.975115773
ENSGALG00010025055	ALS2	2	0.121176065	0.119332647	1.015447731	0.309892458	0	0.000771474	0	1	CRA004775;Yang2025_Silkie_Wenchang	PRJNA827465	0.679435438
ENSGALG00010025055	ALS2	2	-0.063820841	0.048322571	-1.320725278	0.186592982	0	0.379488938	0	0.5	CRA004775;PRJNA827465	Yang2025_Silkie_Wenchang	0.695457578
ENSGALG00010025056	RBMS2	2	0.064730674	0.12350302	0.524122196	0.600193552	0.020205007	2.96604697	65.5889939	0.5	PRJNA827465;Yang2025_Silkie_Wenchang	CRA004775	0.895230519
ENSGALG00010025056	RBMS2	2	-0.095395495	0.313165319	-0.304617049	0.760657833	0.172329681	8.061366267	87.59515488	0.5	CRA004775;Yang2025_Silkie_Wenchang	PRJNA827465	0.939384641
ENSGALG00010025056	RBMS2	2	-0.200250451	0.185995937	-1.076638643	0.281641604	0.050474247	3.3623627	70.2578903	1	CRA004775;PRJNA827465	Yang2025_Silkie_Wenchang	0.712979548
ENSGALG00010025057	SCP2	2	-0.043302543	0.062348612	-0.694522967	0.487354303	0	0.248876374	0	1	PRJNA827465;Yang2025_Silkie_Wenchang	CRA004775	0.85768885
ENSGALG00010025057	SCP2	2	-0.046816391	0.082509335	-0.567407206	0.570437555	0	0.639078219	0	0.5	CRA004775;Yang2025_Silkie_Wenchang	PRJNA827465	0.8851128
ENSGALG00010025057	SCP2	2	0.004098745	0.079615915	0.05148148	0.958941859	0	0.275940033	0	0.5	CRA004775;PRJNA827465	Yang2025_Silkie_Wenchang	0.989102387
ENSGALG00010025060	GPR156	2	-0.064495465	0.113426658	-0.568609405	0.569621241	0	0.069123702	0	1	PRJNA827465;Yang2025_Silkie_Wenchang	CRA004775	0.88485924
ENSGALG00010025060	GPR156	2	-0.055604205	0.180201772	-0.308566362	0.757651413	0	0.278878291	0	0.5	CRA004775;Yang2025_Silkie_Wenchang	PRJNA827465	0.938953568
ENSGALG00010025060	GPR156	2	-0.026019611	0.126777964	-0.205237645	0.837386459	0	0.16126207	0	0.5	CRA004775;PRJNA827465	Yang2025_Silkie_Wenchang	0.958828842
ENSGALG00010025064	GPN3	2	-0.086769294	0.090543012	-0.958321168	0.337900833	0.008068733	1.77084334	43.52773085	1	PRJNA827465;Yang2025_Silkie_Wenchang	CRA004775	0.80415385
ENSGALG00010025064	GPN3	2	-0.205247408	0.115277037	-1.780470885	0.07499893	0	0.03173373	0	1	CRA004775;Yang2025_Silkie_Wenchang	PRJNA827465	0.47268425
ENSGALG00010025064	GPN3	2	-0.031877639	0.0625654	-0.509509068	0.610395444	0	0.294999056	0	1	CRA004775;PRJNA827465	Yang2025_Silkie_Wenchang	0.897438028
ENSGALG00010025067	SLC1A2	2	0.182131978	0.142604274	1.277184565	0.201537095	0	0.130334226	0	1	PRJNA827465;Yang2025_Silkie_Wenchang	CRA004775	0.714377024
ENSGALG00010025067	SLC1A2	2	-0.118103605	0.472120154	-0.250155822	0.802468684	0.298103154	2.764136283	63.82233372	0.5	CRA004775;Yang2025_Silkie_Wenchang	PRJNA827465	0.916522765
ENSGALG00010025067	SLC1A2	2	-0.137249415	0.402928924	-0.340629345	0.733382635	0.215468608	2.448608043	59.16047067	0.5	CRA004775;PRJNA827465	Yang2025_Silkie_Wenchang	0.933182392
ENSGALG00010025069	PODN	2	-0.232318619	0.348084036	-0.667421066	0.504503214	0.205499479	6.569635966	84.77845644	0.5	PRJNA827465;Yang2025_Silkie_Wenchang	CRA004775	0.863512504
ENSGALG00010025069	PODN	2	-0.10344747	0.516447257	-0.200307417	0.841241598	0.458967272	6.971235193	85.6553398	0.5	CRA004775;Yang2025_Silkie_Wenchang	PRJNA827465	0.959989279
ENSGALG00010025069	PODN	2	0.186190694	0.159298345	1.168817502	0.242477167	0	0.789905052	0	1	CRA004775;PRJNA827465	Yang2025_Silkie_Wenchang	0.754738268
ENSGALG00010025071	CDK15	2	1.083344103	0.216709782	4.999054921	5.7612E-07	0.009341512	1.103244996	9.358301797	1	PRJNA827465;Yang2025_Silkie_Wenchang	CRA004775	0.00014241
ENSGALG00010025071	CDK15	2	0.420105041	0.426709972	0.984521265	0.324859281	0.215990964	2.421625156	58.70541741	0.5	CRA004775;Yang2025_Silkie_Wenchang	PRJNA827465	0.799914637
ENSGALG00010025071	CDK15	2	0.647525911	0.650610738	0.994838961	0.319814594	0.723350279	6.66551544	84.99740929	0.5	CRA004775;PRJNA827465	Yang2025_Silkie_Wenchang	0.779384216
ENSGALG00010025073	IL18	2	0.434751361	0.197527835	2.20096252	0.027738677	0	0.733390577	0	1	PRJNA827465;Yang2025_Silkie_Wenchang	CRA004775	0.284131674
ENSGALG00010025073	IL18	2	0.233795696	0.476547512	0.49060312	0.623707179	0.301971796	2.928748862	65.85572723	0.5	CRA004775;Yang2025_Silkie_Wenchang	PRJNA827465	0.900519204
ENSGALG00010025073	IL18	2	0.129931451	0.277580604	0.457279793	0.647469952	0.051051901	1.391105032	28.11470185	0.5	CRA004775;PRJNA827465	Yang2025_Silkie_Wenchang	0.897350807
ENSGALG00010025080	BAZ2A	2	0.173727542	0.105831345	1.641550919	0.100683106	0	0.811689783	0	1	PRJNA827465;Yang2025_Silkie_Wenchang	CRA004775	0.541412814
ENSGALG00010025080	BAZ2A	2	0.024099687	0.308389286	0.078146967	0.937711147	0.141418989	3.798277546	73.67227677	0.5	CRA004775;Yang2025_Silkie_Wenchang	PRJNA827465	0.985239694
ENSGALG00010025080	BAZ2A	2	-0.043952608	0.200437841	-0.219282985	0.826429615	0.044930926	2.044536874	51.0891678	0.5	CRA004775;PRJNA827465	Yang2025_Silkie_Wenchang	0.956393839
ENSGALG00010025092	COL1A1	2	-0.327588643	0.126339272	-2.592928065	0.009516267	0	0.063751798	0	1	PRJNA827465;Yang2025_Silkie_Wenchang	CRA004775	0.153474704
ENSGALG00010025092	COL1A1	2	-0.320012644	0.207123646	-1.545022571	0.122338544	0	0.331958042	0	1	CRA004775;Yang2025_Silkie_Wenchang	PRJNA827465	0.586636252
ENSGALG00010025092	COL1A1	2	-0.367123999	0.151775191	-2.418866988	0.015568932	0	0.249888472	0	1	CRA004775;PRJNA827465	Yang2025_Silkie_Wenchang	0.206527548
ENSGALG00010025097	LMBR1	2	-0.084751751	0.274920624	-0.308349915	0.757816088	0.144674032	23.26392625	95.70149944	0.5	PRJNA827465;Yang2025_Silkie_Wenchang	CRA004775	0.938954369
ENSGALG00010025097	LMBR1	2	-0.348712942	0.082700594	-4.216571216	2.48045E-05	0	0.20262464	0	1	CRA004775;Yang2025_Silkie_Wenchang	PRJNA827465	0.002867327
ENSGALG00010025097	LMBR1	2	0.037442947	0.205629704	0.182089192	0.85551273	0.05964378	2.784775522	64.09046287	0.5	CRA004775;PRJNA827465	Yang2025_Silkie_Wenchang	0.963463555
ENSGALG00010025098	ATP5B	2	-0.228929785	0.105291209	-2.174253548	0.029686099	0.007896081	1.400709472	28.60760777	1	PRJNA827465;Yang2025_Silkie_Wenchang	CRA004775	0.29397955
ENSGALG00010025098	ATP5B	2	-0.332876241	0.163892551	-2.031063889	0.042248514	0	0.846967212	0	0.5	CRA004775;Yang2025_Silkie_Wenchang	PRJNA827465	0.355831985
ENSGALG00010025098	ATP5B	2	-0.156041947	0.084545769	-1.845650569	0.064942959	0	0.178451186	0	0.5	CRA004775;PRJNA827465	Yang2025_Silkie_Wenchang	0.439429584
ENSGALG00010025102	ARPC3	2	-0.032292326	0.135990863	-0.237459524	0.812300316	0.026633546	3.31124482	69.79987726	0.5	PRJNA827465;Yang2025_Silkie_Wenchang	CRA004775	0.953371387
ENSGALG00010025102	ARPC3	2	-0.215428382	0.125709963	-1.713693783	0.086584979	0	0.180541258	0	1	CRA004775;Yang2025_Silkie_Wenchang	PRJNA827465	0.506111562
ENSGALG00010025102	ARPC3	2	-0.003010793	0.171991665	-0.017505461	0.986033376	0.03190599	1.512076549	33.86578209	0.5	CRA004775;PRJNA827465	Yang2025_Silkie_Wenchang	0.996120544
ENSGALG00010025103	GSK3B	2	-0.019363137	0.151086449	-0.128159322	0.898022891	0.032916036	3.031451229	67.01249914	0.5	PRJNA827465;Yang2025_Silkie_Wenchang	CRA004775	0.975733636
ENSGALG00010025103	GSK3B	2	0.138598774	0.119494493	1.159875823	0.246099368	0	0.108900245	0	1	CRA004775;Yang2025_Silkie_Wenchang	PRJNA827465	0.756727496

ENSGALG00010025103	GSK3B	2	-0.069927761	0.104740374	-0.667629475	0.504370138	0.011977113	1.771333736	43.54536475	0.5	CRA004775;PRJNA827465	Yang2025_Silkie_Wenchang	0.863512504
ENSGALG00010025113	SDHD	2	-0.186175844	0.141998971	-1.311106991	0.189821619	0.028957091	3.125514547	68.0052681	1	PRJNA827465;Yang2025_Silkie_Wenchang	CRA004775	0.699160501
ENSGALG00010025113	SDHD	2	-0.28596494	0.154516157	-1.850712224	0.064210965	0.006806746	1.104516231	9.462625182	0.5	CRA004775;Yang2025_Silkie_Wenchang	PRJNA827465	0.43662345
ENSGALG00010025113	SDHD	2	-0.07080279	0.06079466	-1.164621871	0.244172087	0	0.069142297	0	0.5	CRA004775;PRJNA827465	Yang2025_Silkie_Wenchang	0.755585822
ENSGALG00010025114	CEBPG	2	-0.249436115	0.139296097	-1.790689913	0.073343069	0.034574265	9.159211418	89.08202951	1	PRJNA827465;Yang2025_Silkie_Wenchang	CRA004775	0.467957919
ENSGALG00010025114	CEBPG	2	-0.145926109	0.288968273	-0.504990072	0.613565809	0.138551179	5.17467801	80.6751261	0.5	CRA004775;Yang2025_Silkie_Wenchang	PRJNA827465	0.898256044
ENSGALG00010025114	CEBPG	2	-0.052129233	0.121518362	-0.428982353	0.667936067	0.014514356	1.443161381	30.70768013	0.5	CRA004775;PRJNA827465	Yang2025_Silkie_Wenchang	0.914120722
ENSGALG00010025115	MGAT4F	2	0.231525413	0.207030971	1.118312934	0.263433365	0.056594759	2.834429251	64.71952865	0.5	PRJNA827465;Yang2025_Silkie_Wenchang	CRA004775	0.768643821
ENSGALG00010025115	MGAT4F	2	-0.009182121	0.18654892	-0.049220982	0.96074319	0	0.000613303	0	1	CRA004775;Yang2025_Silkie_Wenchang	PRJNA827465	0.989520168
ENSGALG00010025115	MGAT4F	2	0.37111704	0.129779549	2.859595704	0.004241814	0	0.929351426	0	0.5	CRA004775;PRJNA827465	Yang2025_Silkie_Wenchang	0.095157544
ENSGALG00010025116	ANAPC7	2	-0.097309839	0.044699767	-2.176965232	0.029483162	0	0.930614748	0	1	PRJNA827465;Yang2025_Silkie_Wenchang	CRA004775	0.292694292
ENSGALG00010025116	ANAPC7	2	-0.040196094	0.07617586	-0.527674956	0.597724958	0	0.100316817	0	1	CRA004775;Yang2025_Silkie_Wenchang	PRJNA827465	0.894645663
ENSGALG00010025116	ANAPC7	2	-0.12258034	0.051016563	-2.402755736	0.016272052	0	0.023110523	0	1	CRA004775;PRJNA827465	Yang2025_Silkie_Wenchang	0.210791192
ENSGALG00010025121	DYNC2I1	2	0.381668304	0.451760423	0.844846703	0.398196435	0.36490203	8.716628063	88.52767386	0.5	PRJNA827465;Yang2025_Silkie_Wenchang	CRA004775	0.831980453
ENSGALG00010025121	DYNC2I1	2	0.467431797	0.433707633	1.077757828	0.281141844	0.270717849	3.5446667	71.78860286	1	CRA004775;Yang2025_Silkie_Wenchang	PRJNA827465	0.778862191
ENSGALG00010025121	DYNC2I1	2	-0.025518058	0.079217449	-0.322126737	0.747356684	0	0.011873977	0	0.5	CRA004775;PRJNA827465	Yang2025_Silkie_Wenchang	0.93680487
ENSGALG00010025125	PIK3CB	2	0.119180534	0.064934011	1.835410001	0.066444963	0	0.662624263	0	1	PRJNA827465;Yang2025_Silkie_Wenchang	CRA004775	0.443979324
ENSGALG00010025125	PIK3CB	2	0.04157875	0.134723215	0.308623501	0.757607942	0	0.339607362	0	1	CRA004775;Yang2025_Silkie_Wenchang	PRJNA827465	0.938948211
ENSGALG00010025125	PIK3CB	2	0.150330143	0.07222282	2.081477057	0.037390262	0	0.103678884	0	1	CRA004775;PRJNA827465	Yang2025_Silkie_Wenchang	0.333556507
ENSGALG00010025126	ENSGALG00010025126	2	0.48450741	0.364766532	1.328267173	0.18408986	0.243279992	11.14795707	91.02974658	1	PRJNA827465;Yang2025_Silkie_Wenchang	CRA004775	0.692545576
ENSGALG00010025126	ENSGALG00010025126	2	0.532976583	0.34909436	1.526740743	0.126825492	0.196687238	5.168802419	80.65315872	1	CRA004775;Yang2025_Silkie_Wenchang	PRJNA827465	0.597676535
ENSGALG00010025126	ENSGALG00010025126	2	0.146448692	0.076019175	1.926470404	0.054045664	0	0.018024995	0	1	CRA004775;PRJNA827465	Yang2025_Silkie_Wenchang	0.403065793
ENSGALG00010025127	PAMR1	2	-0.048644092	0.118027015	-0.412143713	0.68023409	0	0.011766599	0	1	PRJNA827465;Yang2025_Silkie_Wenchang	CRA004775	0.917768536
ENSGALG00010025127	PAMR1	2	-0.076007236	0.204568025	-0.371549931	0.710227973	0	0.006368967	0	1	CRA004775;Yang2025_Silkie_Wenchang	PRJNA827465	0.925364369
ENSGALG00010025127	PAMR1	2	-0.045505372	0.13460264	-0.338071915	0.735308995	0	0.018586939	0	1	CRA004775;PRJNA827465	Yang2025_Silkie_Wenchang	0.933592889
ENSGALG00010025130	ATP2A2	2	0.173000694	0.092938247	1.861458548	0.062679449	0	0.04603423	0	1	PRJNA827465;Yang2025_Silkie_Wenchang	CRA004775	0.43135885
ENSGALG00010025130	ATP2A2	2	0.093308589	0.132410613	0.704691164	0.481002451	0	0.890103508	0	0.5	CRA004775;Yang2025_Silkie_Wenchang	PRJNA827465	0.856783731
ENSGALG00010025130	ATP2A2	2	0.122314707	0.144160248	0.84846349	0.396179897	0.010658696	1.177578043	15.07993837	0.5	CRA004775;PRJNA827465	Yang2025_Silkie_Wenchang	0.831365249
ENSGALG00010025131	SUMO1	2	-0.308240421	0.312271931	-0.987089744	0.323598645	0.180339287	12.60184355	92.06465311	1	PRJNA827465;Yang2025_Silkie_Wenchang	CRA004775	0.799600434
ENSGALG00010025131	SUMO1	2	-0.511947986	0.144808778	-3.535338069	0.000407254	0.009361117	1.283047081	22.0605374	1	CRA004775;Yang2025_Silkie_Wenchang	PRJNA827465	0.020366982
ENSGALG00010025131	SUMO1	2	-0.130877836	0.158882019	-0.823742276	0.410086012	0.035850734	2.781379178	64.04661371	1	CRA004775;PRJNA827465	Yang2025_Silkie_Wenchang	0.834652129
ENSGALG00010025138	CFAP91	2	0.920549057	0.171710417	5.361055377	8.27371E-08	0	0.06625572	0	1	PRJNA827465;Yang2025_Silkie_Wenchang	CRA004775	3.03908E-05
ENSGALG00010025138	CFAP91	2	0.349179854	0.614662369	0.568083995	0.569977935	0.536064813	3.150506664	68.25907365	0.5	CRA004775;Yang2025_Silkie_Wenchang	PRJNA827465	0.884923673
ENSGALG00010025138	CFAP91	2	0.408514971	0.661316636	0.617729765	0.536753488	0.687943873	4.0953439	75.58202621	0.5	CRA004775;PRJNA827465	Yang2025_Silkie_Wenchang	0.874651173
ENSGALG00010025139	COPB2	2	0.014330197	0.044887303	0.319248351	0.749538195	0.001106339	1.336027798	25.15125796	0.5	PRJNA827465;Yang2025_Silkie_Wenchang	CRA004775	0.937362187
ENSGALG00010025139	COPB2	2	-0.054869763	0.06251854	-0.877655854	0.380130509	0	0.115568294	0	1	CRA004775;Yang2025_Silkie_Wenchang	PRJNA827465	0.824392515
ENSGALG00010025139	COPB2	2	0.036958962	0.043343017	0.852708561	0.393820942	0	0.816987467	0	0.5	CRA004775;PRJNA827465	Yang2025_Silkie_Wenchang	0.830486298
ENSGALG00010025144	PIH1D2	2	0.314039407	0.105553145	2.975178104	0.002928185	0.003929138	1.202164083	16.81667969	1	PRJNA827465;Yang2025_Silkie_Wenchang	CRA004775	0.074041237
ENSGALG00010025144	PIH1D2	2	0.378688011	0.138050809	2.743106061	0.006086102	0	0.822976657	0	1	CRA004775;Yang2025_Silkie_Wenchang	PRJNA827465	0.118095213
ENSGALG00010025144	PIH1D2	2	0.216944359	0.111113563	1.952456146	0.050884073	0	0.072955591	0	1	CRA004775;PRJNA827465	Yang2025_Silkie_Wenchang	0.390223748
ENSGALG00010025145	CPT2	2	0.025573293	0.06430424	0.397692172	0.690857108	0	0.9881709	0	0.5	PRJNA827465;Yang2025_Silkie_Wenchang	CRA004775	0.920151714
ENSGALG00010025145	CPT2	2	-0.179888203	0.181715284	-0.989945369	0.322200822	0.03418183	1.740388987	42.54158081	1	CRA004775;Yang2025_Silkie_Wenchang	PRJNA827465	0.798513866
ENSGALG00010025145	CPT2	2	-0.12800113	0.259042422	-0.494131924	0.621213013	0.100534168	3.329515313	69.96559842	0.5	CRA004775;PRJNA827465	Yang2025_Silkie_Wenchang	0.899614409
ENSGALG00010025147	COX17	2	-0.054252614	0.10177728	-0.533052313	0.593997368	0.00527334	1.119442304	10.66980438	1	PRJNA827465;Yang2025_Silkie_Wenchang	CRA004775	0.893224663
ENSGALG00010025147	COX17	2	-0.257585335	0.215161736	-1.197170745	0.23124001	0	0.152789232	0	1	CRA004775;Yang2025_Silkie_Wenchang	PRJNA827465	0.743891332
ENSGALG00010025147	COX17	2	-0.025152271	0.078179558	-0.321724397	0.747661494	0	0.185143198	0	1	CRA004775;PRJNA827465	Yang2025_Silkie_Wenchang	0.936853271
ENSGALG00010025149	IFT81	2	-0.063223281	0.057493127	-1.099666769	0.271477339	0	0.546178645	0	1	PRJNA827465;Yang2025_Silkie_Wenchang	CRA004775	0.772805786
ENSGALG00010025149	IFT81	2	0.0423836	0.242404095	0.17484688	0.861199951	0.075929811	2.235956581	55.27641241	0.5	CRA004775;Yang2025_Silkie_Wenchang	PRJNA827465	0.965343309
ENSGALG00010025149	IFT81	2	0.061747511	0.178633482	0.345665945	0.72959378	0.035447771	1.5967852	37.37416906	0.5	CRA004775;PRJNA827465	Yang2025_Silkie_Wenchang	0.9323457
ENSGALG00010025154	TMEM183A	2	-0.007160662	0.048691518	-0.147061788	0.88308325	0	0.073799916	0	0.5	PRJNA827465;Yang2025_Silkie_Wenchang	CRA004775	0.972001076
ENSGALG00010025154	TMEM183A	2	0.033776129	0.113144846	0.298521149	0.765305437	0	0.064823848	0	1	CRA004775;Yang2025_Silkie_Wenchang	PRJNA827465	0.940779391
ENSGALG00010025154	TMEM183A	2	-0.009680183	0.052476752	-0.184466125	0.8536478	0	0.143554571	0	0.5	CRA004775;PRJNA827465	Yang2025_Silkie_Wenchang	0.962972345
ENSGALG00010025155	CZIB	2	-0.43624445	0.3644602	-1.196960463	0.231321966	0.23482507	8.106457759	87.66415579	1	PRJNA827465;Yang2025_Silkie_Wenchang	CRA004775	0.743891332
ENSGALG00010025155	CZIB	2	-0.325345232	0.520700117	-0.624822659	0.532087457	0.473959859	7.916351359	87.36791794	0.5	CRA004775;Yang2025_Silkie_Wenchang	PRJNA827465	0.873104119
ENSGALG00010025155	CZIB	2	-0.063230382	0.104146677	-0.607128177	0.543765852	0.003734932	1.084470344	7.789087487	0.5	CRA004775;PRJNA827465	Yang2025_Silkie_Wenchang	0.876968972
ENSGALG00010025156	DLAT	2	-0.184918236	0.069510351	-2.660297844	0.007807158	0	0.106029447	0	1	PRJNA827465;Yang2025_Silkie_Wenchang	CRA004775	0.13708394
ENSGALG00010025156	DLAT	2	-0.188135501	0.105656921	-1.78062638	0.074973507	0	0.238324568	0	1	CRA004775;Yang2025_Silkie_Wenchang	PRJNA827465	0.47268425
ENSGALG00010025156	DLAT	2	-0.159369535	0.078845741	-2.021282727	0.043250506	0	0.097737036	0	1	CRA004775;PRJNA827465	Yang2025_Silkie_Wenchang	0.359703576
ENSGALG00010025157	NOP58	2	0.052538511	0.050918259	1.031820659	0.302156139	0	0.381051662	0	0.5	PRJNA827465;Yang2025_Silkie_Wenchang	CRA004775	0.791603969
ENSGALG00010025157	NOP58	2	0.020334423	0.113220917	0.179599523	0.857466979	0	0.426606928	0	0.5	CRA004775;Yang2025_Silkie_Wenchang	PRJNA827465	0.963964719
ENSGALG00010025157	NOP58	2	0.070600909	0.053829383	1.311568228	0.189665859	0	0.131035021	0	1	CRA004775;PRJNA827465	Yang2025_Silkie_Wenchang	0.698962706
ENSGALG00010025160	MRPS22	2	-0.117729943	0.062982107	-1.86926017	0.061586627	0	0.534572293	0	0.5	PRJNA827465;Yang2025_Silkie_Wenchang	CRA004775	0.428647789
ENSGALG00010025160	MRPS22	2	0.009462402	0.161810878	0.05847816	0.953367758	0	0.000791721	0	1	CRA004775;Yang2025_Silkie_Wenchang	PRJNA827465	0.987758913
ENSGALG00010025160	MRPS22	2	-0.128213141	0.065370273	-1.961337089	0.049839713	0	0.177719808	0	0.5	CRA004775;PRJNA827465	Yang2025_Silkie_Wenchang	0.386284807
ENSGALG00010025161	ENSGALG00010025161	2	0.02825103	0.068329233	0.413454514	0.679273642	0	0.048924944	0	0.5	PRJNA827465;Yang2025_Silkie_Wenchang	CRA004775	0.917701868
ENSGALG00010025161	ENSGALG00010025161	2	-0.161885277	0.186047052	-0.870130836	0.384228908	0.037500033	2.127153721	52.98882305	1	CRA004775;Yang2025_Silkie_Wenchang	PRJNA827465	0.825842107
ENSGALG00010025161	ENSGALG00010025161	2	-0.125179541	0.201719454	-0.620562563	0.534887478	0.060772773	3.451099906	71.02373078	0.5	CRA004775;PRJNA827465	Yang2025_Silkie_Wenchang	0.873896294
ENSGALG00010025163	LDLRAD3	2	0.040415122	0.072381887	0.55835961	0.576598848	0	0.804013065	0	1	PRJNA827465;Yang2025_Silkie_Wenchang	CRA004775	0.887113945
ENSGALG00010025163	LDLRAD3	2	0.148921529	0.149092151	0.998855597	0.317864649	0	0.112478305	0	1	CRA004775;Yang2025_Silkie_Wenchang	PRJNA827465	0.797439961
ENSGALG00010025163	LDLRAD3	2	0.008176528	0.0795304	0.102810099	0.91811369	0	0.001353831	0	1	CRA004775;PRJNA827465	Yang2025_Silkie_Wenchang	0.981033557
ENSGALG00010025164	C11orf52	2	-0.463442672	0.247740281	-1.870679531	0.061389514	0.109240774	8.671293176	88.46769473	1	PRJNA827465;Yang2025_Silkie_Wenchang	CRA004775	0.427938152
ENSGALG00010025164	C11orf52	2	-0.725668049	0.145600991	-4.983949924	6.22992E-07	0	0.011329492	0	1	CRA004775;Yang2025_Silkie_Wenchang	PRJNA827465	0.000152084
ENSGALG00010025164	C11orf52	2	-0.244515262	0.068058731	-3.592709664	0.000327257	0	0.983286911	0	1	CRA004775;PRJNA827465	Yang2025_Silkie_Wenchang	0.017894809
ENSGALG00010025167	PTGES3	2	-0.050315964	0.140595695	-0.357876993	0.720435369	0.032423627	5.332538732	81.24720606	0.5	PRJNA827465;Yang2025_Silkie_Wenchang	CRA004775	0.928755937
ENSGALG00010025167	PTGES3	2	-0.178845719	0.096339954	-1.856402368	0.063396227	0	0.33781146	0	1	CRA004775;Yang2025_Silkie_Wenchang	PRJNA827465	0.433558714
ENSGALG00010025167	PTGES3	2	0.068185055	0.058180359	1.171960041	0.2412131	0	0.34460296	0	0.5	CRA004775;PRJNA827465	Yang2025_Silkie_Wenchang	0.753788539
ENSGALG00010025168	POPDC2	2	-0.053140387	0.131834853	-0.403082993	0.686887171	0.013400852	1.627364152	38.55093841	0.5	PRJNA827465;Yang2025_Silkie_Wenchang	CRA004775	0.919256554
ENSGALG00010025168	POPDC2	2	0.044066315	0.142246431	0.309788548	0.756721761	0	0.976373142	0	0.5	CRA004775;Yang2025_Silkie_Wenchang	PRJNA827465	0.938740241
ENSGALG00010025168	POPDC2	2	-0.203839441	0.141374503	-1.441840198	0.14934746	0	0.304668871	0	1	CRA004775;PRJNA827465	Yang2025_Silkie_Wenchang	0.639246608
ENSGALG00010025170	COMMD9	2	-0.117532281	0.281803351	-0.417071976	0.676625762	0.145485149	11.30079735	91.15106687	0.5	PRJNA827465;Yang2025_Silkie_Wenchang	CRA004775	0.916786558
ENSGALG00010025170	COMMD9	2	-0.340406134	0.122731848	-2.77357621	0.005544385	0	0.615920059	0	1	CRA004775;Yang2025_Silkie_Wenchang	PRJNA827465	0.111717753
ENSGALG00010025170	COMMD9	2	0.00729202	0.178418503	0.040870313	0.967399284	0.047899648	3.384499812	70.45353655	0.5	CRA004775;PRJNA827465	Yang2025_Silkie_Wenchang	0.990999924
ENSGALG00010025182	PLA1A	2	0.32385902	0.111820682	2.896235433	0.003776689	0	0.221467003	0	1	PRJNA827465;Yang2025_Silkie_Wenchang	CRA004775	0.088100594
ENSGALG00010025182	PLA1A	2	-0.035395371	0.376227153	-0.094079788	0.925045776	0.236116608	5.595531082	82.12859539	0.5	CRA004775;Yang2025_Silkie_Wenchang	PRJNA827465	0.982594411
ENSGALG00010025182	PLA1A	2	-0.002332231	0.443618293	-0.005257293	0.995805306	0.320633771	5.366806537	81.36694526	0.5	CRA004775;PRJNA827465	Yang2025_Silkie_Wenchang	0.998609527
ENSGALG00010025186	TMEM120B	2	-0.130221915	0.082647881	-1.575623144	0.115112679	0	0.016151021	0	1	PRJNA827465;Yang2025_Silkie_Wenchang	CRA004775	0.572141484
ENSGALG00010025186	TMEM120B	2	-0.210166138	0.156820942	-1.34016628	0.180191292	0	0.278661096	0	1	CRA004775;Yang2025_Silkie_Wenchang	PRJNA827465	0.687244865
ENSGALG00010025186	TMEM120B	2	-0.145717405	0.086871545	-1.677389364	0.093466368	0	0.49658757	0	1	CRA004775;PRJNA827465	Yang2025_Silkie_Wenchang	0.523888855
ENSGALG00010025188	PPFIA4	2	0.285265369	0.285647364	0.998662704	0.317958114	0.025958625	1.15440409	13.37522027	1	PRJNA827465;Yang2025_Silkie_Wenchang	CRA004775	0.797439961
ENSGALG00010025188	PPFIA4	2	0.175677224	0.562392117	0.312374976	0.754755566	0.368174079	2.389374575	58.14804382	0.5	CRA004775;Yang2025_Silkie_Wenchang	PRJNA827465	0.93838723
ENSGALG00010025188	PPFIA4	2	-0.023402102	0.262232282	-0.089241881	0.928889682	0	0.672561313	0	0.5	CRA004775;PRJNA827465	Yang2025_Silkie_Wenchang	0.983490508
ENSGALG00010025190	PRR5L	2	-0.208415645	0.278617273	-0.748035621	0.454438672	0.121970601	4.656750747	78.52579933	0.5	PRJNA827465;Yang2025_Silkie_Wenchang	CRA004775	0.849072665
ENSGALG00010025190	PRR5L	2	-0.514194158	0.180786972	-2.844199181	0.004452321	0	0.11267982	0	1	CRA004775;Yang2025_Silkie_Wenchang	PRJNA827465	0.097660808
ENSGALG00010025190	PRR5L	2	-0.131675224	0.330272353	-0.398686791	0.690124002	0.117450064	1.708865268	41.48163587	0.5	CRA004775;PRJNA827465	Yang2025_Silkie_Wenchang	0.919979266
ENSGALG00010025192	CRYAB	2	0.066624431	0.085653905	0.777832969	0.436667489	0	0.263556771	0	1	PRJNA827465;Yang2025_Silkie_Wenchang	CRA004775	0.843275468
ENSGALG00010025192	CRYAB	2	0.183779235	0.225521118	0.814909207	0.415124282	0	0.000108084	0	1	CRA004775;Yang2025_Silkie_Wenchang	PRJNA827465	0.837489728
ENSGALG00010025192	CRYAB	2	0.052970859	0.090596179	0.584691973	0.558754853	0	0.049692496	0	1	CRA004775;PRJNA827465	Yang2025_Silkie_Wenchang	0.880871906
ENSGALG00010025195	P2RX7	2	0.281292307	0.172714852	1.62865153	0.103386809	0	0.622142224	0	1	PRJNA827465;Yang2025_Silkie_Wenchang	CRA004775	0.547576788
ENSGALG00010025195	P2RX7	2	0.193808595	0.357310996	0.542408707	0.587536983	0.118081755	1.643517105	39.15487725	0.5	CRA004775;Yang2025_Silkie_Wenchang	PRJNA827465	0.89167336
ENSGALG00010025195	P2RX7	2	0.079150968	0.215407903	0.367446908	0.713285688	0	0.710180409	0	0.5	CRA004775;PRJNA827465	Yang2025_Silkie_Wenchang	0.926216079
ENSGALG00010025200	PEPD	2	-0.057857259	0.158705729	-0.364556837	0.715442239	0.038425997	3.880990699	74.23338324	0.5	PRJNA827465;Yang2025_Silkie_Wenchang	CRA004775	0.927131632
ENSGALG00010025200	PEPD	2	-0.272395826	0.112109884	-2.429721762	0.015110418	0	0.080644896	0	1	CRA004775;Yang2025_Silkie_Wenchang	PRJNA827465	0.203100811
ENSGALG00010025200	PEPD	2	-0.084322471	0.190404278	-0.442860168	0.65786688	0.057313281	4.321568231	76.86025196	0.5	CRA004775;PRJNA827465	Yang2025_Silkie_Wenchang	0.910812384
ENSGALG00010025204	TRAF6	2	-0.204502135	0.195500567	-1.04604369	0.295540861	0.071175129	14.3745611	93.04326586	1	PRJNA827465;Yang2025_Silkie_Wenchang	CRA004775	0.787628307
ENSGALG00010025204	TRAF6	2	-0.01095505	0.410376979	-0.02669509	0.978702929	0.316575023	16.23853296	93.84180823	0.5	CRA004775;Yang2025_Silkie_Wenchang	PRJNA827465	0.994183692
ENSGALG00010025204	TRAF6	2	0.164997475	0.212109177	0.777889371	0.436634235	0.073853089	4.94780357	79.78901171	0.5	CRA004775;PRJNA827465	Yang2025_Silkie_Wenchang	0.843275468
ENSGALG00010025207	BMPR2	2	0.075540668	0.218994658	0.34494297	0.730137245	0.077599361	4.615010764	78.33157817	0.5	PRJNA827465;Yang2025_Silkie_Wenchang	CRA004775	0.932614156
ENSGALG00010025207	BMPR2	2	0.355066204	0.172638366	2.056705084	0.039714603	0	0.03208348	0	1	CRA004775;Yang2025_Silkie_Wenchang	PRJNA827465	0.344755224
ENSGALG00010025207	BMPR2	2	0.034361111	0.230828081	0.148860186	0.881663958	0.069555722	2.079151473	51.90345614	0.5	CRA004775;PRJNA827465	Yang2025_Silkie_Wenchang	0.971870384
ENSGALG00010025210	HSPB2	2	0.284756954	0.131259041	2.169427361	0.030050254	0	0.690881238	0	1	PRJNA827465;Yang2025_Silkie_Wenchang	CRA004775	0.295709844
ENSGALG00010025210	HSPB2	2	0.395658434	0.239140245	1.65450376	0.0980252	0	0.702632125	0	0.5	CRA004775;Yang2025_Silkie_Wenchang	PRJNA827465	0.534130658
ENSGALG00010025210	HSPB2	2	0.201527414	0.150366771	1.340239026	0.180167648	0	0.293012681	0	0.5	CRA004775;PRJNA827465	Yang2025_Silkie_Wenchang	0.687225928
ENSGALG00010025212	P2RX4	2	0.062852818	0.324117979	0.193919566	0.846238863	0.183032142	7.757489348	87.10923141	0.5	PRJNA827465;Yang2025_Silkie_Wenchang	CRA004775	0.96122283
ENSGALG00010025212	P2RX4	2	0.210277056	0.248681636	0.845567287	0.397794181	0.065234811	1.903718589	47.47122786	0.5	CRA004775;Yang2025_Silkie_Wenchang	PRJNA827465	0.831711071
ENSGALG00010025212	P2RX4	2	-0.236366287	0.144382024	-1.637089446	0.101611781	0	0.109011671	0	1	CRA004775;PRJNA827465	Yang2025_Silkie_Wenchang	0.543686036
ENSGALG00010025214	LRP8	2	0.67862564	0.509109785	1.332965227	0.182543215	0.4536877	7.535465123	86.72941904	1	PRJNA827465;Yang2025_Silkie_Wenchang	CRA004775	0.690718782
ENSGALG00010025214	LRP8	2	0.858606519	0.50135305	1.712578628	0.086790088	0.276660532	2.041501425	51.01644369	1	CRA004775;Yang2025_Silkie_Wenchang	PRJNA827465	0.506700954
ENSGALG00010025214	LRP8	2	0.214941133	0.137190261	1.566737549	0.11717605	0	0.000800384	0	1	CRA004775;PRJNA827465	Yang2025_Silkie_Wenchang	0.57646425
ENSGALG00010025215	MYOG	2	0.104542797	0.107913148	0.96876793	0.332660991	0	0.254831306	0	1	PRJNA827465;Yang2025_Silkie_Wenchang	CRA004775	0.802178578
ENSGALG00010025215	MYOG	2	-0.005260039	0.184708608	-0.028477496	0.977281316	0	0.384190952	0	0.5	CRA004775;Yang2025_Silkie_Wenchang	PRJNA827465	0.993487352
ENSGALG00010025215	MYOG	2	0.123465643	0.128691608	0.959391567	0.337361522	0	0.623755778	0	0.5	CRA004775;PRJNA827465	Yang2025_Silkie_Wenchang	0.80415385
ENSGALG00010025219	NACA	2	0.19523807	0.248258777	0.78642968	0.431615801	0.113509858	12.22082187	91.81724429	0.5	PRJNA827465;Yang2025_Silkie_Wenchang	CRA004775	0.842503929
ENSGALG00010025219	NACA	2	0.147109051	0.329028642	0.447101048	0.654802102	0.187678824	7.341633999	86.37905403	0.5	CRA004775;Yang2025_Silkie_Wenchang	PRJNA827465	0.910222014
ENSGALG00010025219	NACA	2	-0.052089155	0.056372107	-0.924023557	0.355474051	0	0.572363112	0	1	CRA004775;PRJNA827465	Yang2025_Silkie_Wenchang	0.812301302
ENSGALG00010025222	FAM117B	2	0.058859208	0.115085266	0.511439997	0.609042994	0	0.015254472	0	1	PRJNA827465;Yang2025_Silkie_Wenchang	CRA004775	0.897212042
ENSGALG00010025222	FAM117B	2	-0.116118022	0.338554179	-0.342982095	0.73161193	0.101868766	1.515130058	33.99906531	0.5	CRA004775;Yang2025_Silkie_Wenchang	PRJNA827465	0.932851968
ENSGALG00010025222	FAM117B	2	-0.100715719	0.298810996	-0.337054927	0.736075494	0.094476951	1.523878722	34.37798	0.5	CRA004775;PRJNA827465	Yang2025_Silkie_Wenchang	0.933616521
ENSGALG00010025228	FDXACB1	2	-0.07120502	0.079465951	-0.89604439	0.370229056	0	0.006055596	0	1	PRJNA827465;Yang2025_Silkie_Wenchang	CRA004775	0.821045695
ENSGALG00010025228	FDXACB1	2	-0.333993784	0.292880002	-1.140377569	0.254129034	0.141948246	5.630716408	82.24027055	1	CRA004775;Yang2025_Silkie_Wenchang	PRJNA827465	0.763894203
ENSGALG00010025228	FDXACB1	2	-0.333585927	0.285817076	-1.167130854	0.24315753	0.138119391	6.110827775	83.63560491	1	CRA004775;PRJNA827465	Yang2025_Silkie_Wenchang	0.7550024
ENSGALG00010025229	ENSGALG00010025229	2	-0.128270528	0.193361508	-0.663371575	0.507092606	0.065180386	7.625592912	86.88626561	0.5	PRJNA827465;Yang2025_Silkie_Wenchang	CRA004775	0.864016158
ENSGALG00010025229	ENSGALG00010025229	2	-0.319381775	0.112531648	-2.838150701	0.004537575	0	0.128615069	0	1	CRA004775;Yang2025_Silkie_Wenchang	PRJNA827465	0.098668611
ENSGALG00010025229	ENSGALG00010025229	2	0.043610118	0.073141365	0.596244246	0.551012071	0	0.490790725	0	0.5	CRA004775;PRJNA827465	Yang2025_Silkie_Wenchang	0.878879109
ENSGALG00010025232	PRIM1	2	0.022717406	0.135193135	0.168036683	0.866554421	0.021556811	1.990346232	49.75748522	0.5	PRJNA827465;Yang2025_Silkie_Wenchang	CRA004775	0.967507198
ENSGALG00010025232	PRIM1	2	-0.031635284	0.253574492	-0.12475736	0.900715646	0.09285737	3.592607371	72.16506296	0.5	CRA004775;Yang2025_Silkie_Wenchang	PRJNA827465	0.976497577
ENSGALG00010025232	PRIM1	2	-0.106772158	0.08355839	-1.277814929	0.201314689	0.003979616	1.212916963	17.55412525	1	CRA004775;PRJNA827465	Yang2025_Silkie_Wenchang	0.714168358
ENSGALG00010025233	WDR12	2	0.00822751	0.060743638	0.135446449	0.892258902	0	0.004619391	0	1	PRJNA827465;Yang2025_Silkie_Wenchang	CRA004775	0.974208739
ENSGALG00010025233	WDR12	2	-0.182434757	0.206500143	-0.883460683	0.376987417	0.06025279	3.29389554	69.64081016	0.5	CRA004775;Yang2025_Silkie_Wenchang	PRJNA827465	0.823064945
ENSGALG00010025233	WDR12	2	-0.165043631	0.209581577	-0.787491119	0.430994425	0.069935953	4.378186299	77.15949181	0.5	CRA004775;PRJNA827465	Yang2025_Silkie_Wenchang	0.842503929
ENSGALG00010025236	ENSGALG00010025236	2	0.206908097	0.211049361	0.980377743	0.326908692	0	0.331217744	0	1	PRJNA827465;Yang2025_Silkie_Wenchang	CRA004775	0.800028239
ENSGALG00010025236	ENSGALG00010025236	2	0.296407377	0.229294768	1.292691413	0.196117809	0	0.994391099	0	1	CRA004775;Yang2025_Silkie_Wenchang	PRJNA827465	0.70748217
ENSGALG00010025236	ENSGALG00010025236	2	0.39851086	0.208959137	1.907123399	0.05650461	0	0.248693513	0	1	CRA004775;PRJNA827465	Yang2025_Silkie_Wenchang	0.411523553
ENSGALG00010025238	IGSF9B	2	0.01890806	0.182798169	0.103436814	0.917616295	0.007999326	1.128286958	11.37006475	0.5	PRJNA827465;Yang2025_Silkie_Wenchang	CRA004775	0.980974192
ENSGALG00010025238	IGSF9B	2	0.202902189	0.259760872	0.78111144	0.434736954	0	0.195170132	0	0.5	CRA004775;Yang2025_Silkie_Wenchang	PRJNA827465	0.842762754
ENSGALG00010025238	IGSF9B	2	-0.123203501	0.206411406	-0.596883205	0.550585361	0	0.003004616	0	1	CRA004775;PRJNA827465	Yang2025_Silkie_Wenchang	0.878839153
ENSGALG00010025242	CLSTN2	2	0.264891561	0.14146601	1.872474954	0.061140924	0	0.513022551	0	1	PRJNA827465;Yang2025_Silkie_Wenchang	CRA004775	0.427371325
ENSGALG00010025242	CLSTN2	2	0.389341756	0.269145857	1.44658276	0.148013818	0	0.237834068	0	1	CRA004775;Yang2025_Silkie_Wenchang	PRJNA827465	0.637591744
ENSGALG00010025242	CLSTN2	2	0.205554275	0.15473642	1.328415606	0.184040847	0	0.017516614	0	1	CRA004775;PRJNA827465	Yang2025_Silkie_Wenchang	0.692524875
ENSGALG00010025243	PAXIP1	2	0.195001773	0.212583263	0.917295983	0.358987562	0.068520964	3.511696404	71.52373426	1	PRJNA827465;Yang2025_Silkie_Wenchang	CRA004775	0.814241185
ENSGALG00010025243	PAXIP1	2	0.241289849	0.236940727	1.018355318	0.308509128	0.056532754	2.008265851	50.20579574	0.5	CRA004775;Yang2025_Silkie_Wenchang	PRJNA827465	0.793490889
ENSGALG00010025243	PAXIP1	2	0.025701159	0.064755749	0.396893859	0.691445733	0	0.020377483	0	0.5	CRA004775;PRJNA827465	Yang2025_Silkie_Wenchang	0.920268407
ENSGALG00010025245	ENSGALG00010025245	2	0.23262353	0.256421172	0.907193145	0.364304668	0.121102445	12.06032903	91.70835226	0.5	PRJNA827465;Yang2025_Silkie_Wenchang	CRA004775	0.817496251
ENSGALG00010025245	ENSGALG00010025245	2	0.321184005	0.208711441	1.538890268	0.123831088	0.058309876	2.937837927	65.96136258	1	CRA004775;Yang2025_Silkie_Wenchang	PRJNA827465	0.5901459
ENSGALG00010025245	ENSGALG00010025245	2	-0.002491646	0.047998204	-0.051911244	0.958599415	0	0.202241115	0	0.5	CRA004775;PRJNA827465	Yang2025_Silkie_Wenchang	0.989070013
ENSGALG00010025247	SGCA	2	-0.089185348	0.084236209	-1.0587531	0.289712238	0	0.214370472	0	1	PRJNA827465;Yang2025_Silkie_Wenchang	CRA004775	0.784049259
ENSGALG00010025247	SGCA	2	-0.038543641	0.146237145	-0.263569431	0.792111716	0	0.038272967	0	1	CRA004775;Yang2025_Silkie_Wenchang	PRJNA827465	0.948314332
ENSGALG00010025247	SGCA	2	-0.116360104	0.099957793	-1.164092371	0.24438658	0	0.003175419	0	1	CRA004775;PRJNA827465	Yang2025_Silkie_Wenchang	0.755929523
ENSGALG00010025248	C11orf74	2	-0.082385227	0.181885863	-0.452950141	0.650584644	0.038460489	2.261229238	55.77626616	0.5	PRJNA827465;Yang2025_Silkie_Wenchang	CRA004775	0.908636451
ENSGALG00010025248	C11orf74	2	-0.388594255	0.177899828	-2.184343062	0.028937049	0	0.54407164	0	1	CRA004775;Yang2025_Silkie_Wenchang	PRJNA827465	0.289687424
ENSGALG00010025248	C11orf74	2	-0.193944181	0.323091841	-0.600275701	0.54832251	0.155307146	3.445039588	70.97275737	0.5	CRA004775;PRJNA827465	Yang2025_Silkie_Wenchang	0.878281266
ENSGALG00010025250	GLIS1	2	0.144540257	0.296589368	0.487341329	0.626016464	0	0.760992459	0	0.5	PRJNA827465;Yang2025_Silkie_Wenchang	CRA004775	0.901504244
ENSGALG00010025250	GLIS1	2	-0.406764285	0.998719019	-0.407286011	0.683797936	1.546366167	4.236347574	76.39476028	0.5	CRA004775;Yang2025_Silkie_Wenchang	PRJNA827465	0.918800744
ENSGALG00010025250	GLIS1	2	-0.598537834	0.712294819	-0.840295084	0.400742957	0.660786346	2.532808625	60.51813823	1	CRA004775;PRJNA827465	Yang2025_Silkie_Wenchang	0.832353691
ENSGALG00010025252	PPP1R9B	2	0.324435786	0.244422085	1.327358717	0.184390049	0.114413863	23.52885494	95.74989942	1	PRJNA827465;Yang2025_Silkie_Wenchang	CRA004775	0.692639023
ENSGALG00010025252	PPP1R9B	2	0.054312456	0.539987293	0.100580988	0.919883089	0.552364414	18.28539125	94.53115339	0.5	CRA004775;Yang2025_Silkie_Wenchang	PRJNA827465	0.981237238
ENSGALG00010025252	PPP1R9B	2	-0.168683307	0.292588567	-0.576520501	0.564263417	0.144364239	5.661145189	82.33572949	0.5	CRA004775;PRJNA827465	Yang2025_Silkie_Wenchang	0.883027875
ENSGALG00010025256	ANAPC5	2	-0.101403625	0.361915886	-0.280185615	0.779335102	0.245598015	15.76032249	93.65495217	0.5	PRJNA827465;Yang2025_Silkie_Wenchang	CRA004775	0.944727706
ENSGALG00010025256	ANAPC5	2	-0.441255872	0.137497299	-3.209196654	0.001331064	0	0.186383743	0	1	CRA004775;Yang2025_Silkie_Wenchang	PRJNA827465	0.044565154
ENSGALG00010025256	ANAPC5	2	0.014478362	0.287255738	0.050402341	0.959801771	0.129334154	4.038712939	75.23963661	0.5	CRA004775;PRJNA827465	Yang2025_Silkie_Wenchang	0.989302378
ENSGALG00010025257	LPCAT2	2	0.014849496	0.106812404	0.139024083	0.889431118	0.002920189	1.143711521	12.56536444	0.5	PRJNA827465;Yang2025_Silkie_Wenchang	CRA004775	0.973135755
ENSGALG00010025257	LPCAT2	2	-0.353832378	0.370107235	-0.956026645	0.339058773	0.195645516	2.900540281	65.52366445	1	CRA004775;Yang2025_Silkie_Wenchang	PRJNA827465	0.804767595
ENSGALG00010025257	LPCAT2	2	-0.273411626	0.487059885	-0.561351148	0.57455818	0.382356449	4.60460382	78.282605	0.5	CRA004775;PRJNA827465	Yang2025_Silkie_Wenchang	0.886130225
ENSGALG00010025262	CARF	2	0.113060732	0.08191635	1.380197373	0.167525883	0	0.427060507	0	1	PRJNA827465;Yang2025_Silkie_Wenchang	CRA004775	0.667369731
ENSGALG00010025262	CARF	2	0.003912898	0.117044963	0.033430723	0.97333111	0	0.881616498	0	0.5	CRA004775;Yang2025_Silkie_Wenchang	PRJNA827465	0.992392211
ENSGALG00010025262	CARF	2	0.04621983	0.185114875	0.249681877	0.802833374	0.035327511	1.680358554	40.48889164	0.5	CRA004775;PRJNA827465	Yang2025_Silkie_Wenchang	0.950786757
ENSGALG00010025263	SAMD14	2	-0.066658311	0.259163804	-0.257205326	0.797020273	0	0.289620637	0	0.5	PRJNA827465;Yang2025_Silkie_Wenchang	CRA004775	0.949622804
ENSGALG00010025263	SAMD14	2	0.1845722	0.383173231	0.48169388	0.630023424	0	0.005256513	0	1	CRA004775;Yang2025_Silkie_Wenchang	PRJNA827465	0.902533841
ENSGALG00010025263	SAMD14	2	-0.076698239	0.271111738	-0.282902686	0.777251435	0	0.301607811	0	0.5	CRA004775;PRJNA827465	Yang2025_Silkie_Wenchang	0.944218792
ENSGALG00010025268	ZBTB39	2	0.077232898	0.216493447	0.356744739	0.721282909	0.078630527	6.110833834	83.63562114	0.5	PRJNA827465;Yang2025_Silkie_Wenchang	CRA004775	0.928950734
ENSGALG00010025268	ZBTB39	2	-0.236339827	0.115223869	-2.051136018	0.040253703	1.47719E-05	1.00050809	0.050783191	1	CRA004775;Yang2025_Silkie_Wenchang	PRJNA827465	0.347442284
ENSGALG00010025268	ZBTB39	2	-0.034845379	0.336801435	-0.10345971	0.917598125	0.20372096	9.498159781	89.47164479	0.5	CRA004775;PRJNA827465	Yang2025_Silkie_Wenchang	0.980974192
ENSGALG00010025270	NDC1	2	-0.039094673	0.159010478	-0.245862244	0.805788872	0.036067062	3.366593345	70.2963828	0.5	PRJNA827465;Yang2025_Silkie_Wenchang	CRA004775	0.952057827
ENSGALG00010025270	NDC1	2	-0.246033874	0.136300666	-1.805081965	0.071061857	0	0.269502683	0	1	CRA004775;Yang2025_Silkie_Wenchang	PRJNA827465	0.460426007
ENSGALG00010025270	NDC1	2	-0.038321435	0.233636979	-0.164021277	0.869714394	0.065151587	1.867787354	46.46071473	0.5	CRA004775;PRJNA827465	Yang2025_Silkie_Wenchang	0.968586462
ENSGALG00010025271	RNF34	2	-0.075838507	0.113017801	-0.67103152	0.50220045	0.021038019	5.453586446	81.66344277	0.5	PRJNA827465;Yang2025_Silkie_Wenchang	CRA004775	0.862860181
ENSGALG00010025271	RNF34	2	-0.037880619	0.181925217	-0.208220829	0.835056546	0.052207174	4.504664086	77.80078645	0.5	CRA004775;Yang2025_Silkie_Wenchang	PRJNA827465	0.95823248
ENSGALG00010025271	RNF34	2	0.040415551	0.046289554	0.873103041	0.382606917	0	0.774399959	0	1	CRA004775;PRJNA827465	Yang2025_Silkie_Wenchang	0.825121999

ENSGALG00010025272	NCAPD3	2	-0.200798237	0.169514518	-1.184548906	0.236195849	0.042240504	3.591506607	72.1565318	1	PRJNA827465;Yang2025_Silkie_Wenchang	CRA004775	0.748669015
ENSGALG00010025272	NCAPD3	2	-0.201307977	0.292121474	-0.689124199	0.490745113	0.098642552	1.962429593	49.04275785	0.5	CRA004775;Yang2025_Silkie_Wenchang	PRJNA827465	0.858798485
ENSGALG00010025272	NCAPD3	2	-0.039891861	0.089522665	-0.445606277	0.655881676	0	0.450995873	0	0.5	CRA004775;PRJNA827465	Yang2025_Silkie_Wenchang	0.910411938
ENSGALG00010025274	PDK2	2	-0.125652899	0.27247803	-0.461148737	0.644691898	0.117919122	4.730803356	78.8619411	0.5	PRJNA827465;Yang2025_Silkie_Wenchang	CRA004775	0.906953008
ENSGALG00010025274	PDK2	2	0.106213877	0.193665379	0.548440187	0.583389689	0	0.6314042	0	0.5	CRA004775;Yang2025_Silkie_Wenchang	PRJNA827465	0.890479822
ENSGALG00010025274	PDK2	2	-0.368225457	0.133415012	-2.760000184	0.005780133	0	0.048278312	0	1	CRA004775;PRJNA827465	Yang2025_Silkie_Wenchang	0.114502839
ENSGALG00010025280	HSPB11	2	0.088963483	0.086464302	1.028904196	0.303524697	0	0.103945532	0	1	PRJNA827465;Yang2025_Silkie_Wenchang	CRA004775	0.792205992
ENSGALG00010025280	HSPB11	2	0.044383358	0.142569514	0.311310301	0.755564737	0	0.004178828	0	1	CRA004775;Yang2025_Silkie_Wenchang	PRJNA827465	0.938501652
ENSGALG00010025280	HSPB11	2	0.10198509	0.100964261	1.010110795	0.312442211	0	0.047318972	0	1	CRA004775;PRJNA827465	Yang2025_Silkie_Wenchang	0.796054997
ENSGALG00010025286	ITGA3	2	0.257708951	0.376099596	0.685214644	0.493208484	0.26822583	19.19402164	94.79004443	0.5	PRJNA827465;Yang2025_Silkie_Wenchang	CRA004775	0.859740678
ENSGALG00010025286	ITGA3	2	0.222238961	0.43325828	0.512947984	0.607987707	0.342020223	10.76882965	90.71393984	0.5	CRA004775;Yang2025_Silkie_Wenchang	PRJNA827465	0.897204334
ENSGALG00010025286	ITGA3	2	-0.152347765	0.120230124	-1.267134721	0.205107122	0	0.171212684	0	1	CRA004775;PRJNA827465	Yang2025_Silkie_Wenchang	0.717838399
ENSGALG00010025289	KDM2B	2	-0.048378159	0.0437324	-1.106231502	0.268626331	0.001018221	1.285005989	22.17935105	1	PRJNA827465;Yang2025_Silkie_Wenchang	CRA004775	0.771398294
ENSGALG00010025289	KDM2B	2	-0.105222478	0.070580716	-1.490810583	0.136011232	0	0.242666653	0	0.5	CRA004775;Yang2025_Silkie_Wenchang	PRJNA827465	0.617575642
ENSGALG00010025289	KDM2B	2	-0.019228439	0.040614336	-0.473439688	0.635899521	0	0.013544486	0	0.5	CRA004775;PRJNA827465	Yang2025_Silkie_Wenchang	0.904387873
ENSGALG00010025291	DUS2	2	0.09541097	0.063105887	1.511918688	0.130554549	0	0.414185706	0	1	PRJNA827465;Yang2025_Silkie_Wenchang	CRA004775	0.606115935
ENSGALG00010025291	DUS2	2	-0.024185257	0.116929178	-0.206836803	0.836137313	0	0.786427811	0	0.5	CRA004775;Yang2025_Silkie_Wenchang	PRJNA827465	0.958399882
ENSGALG00010025291	DUS2	2	0.0336195	0.15513529	0.216710793	0.828433736	0.025989481	1.617837239	38.18908503	0.5	CRA004775;PRJNA827465	Yang2025_Silkie_Wenchang	0.956777964
ENSGALG00010025292	ATP1A1	2	-0.090402944	0.132446251	-0.682563249	0.494882863	0.026925713	3.87879849	74.21882053	0.5	PRJNA827465;Yang2025_Silkie_Wenchang	CRA004775	0.860445712
ENSGALG00010025292	ATP1A1	2	-0.098390473	0.188291415	-0.522543596	0.601291901	0.042359139	2.322502625	56.94299808	0.5	CRA004775;Yang2025_Silkie_Wenchang	PRJNA827465	0.895230519
ENSGALG00010025292	ATP1A1	2	0.02616933	0.051172658	0.511392834	0.609076012	0	0.266009887	0	1	CRA004775;PRJNA827465	Yang2025_Silkie_Wenchang	0.897212042
ENSGALG00010025297	SPSB4	2	-0.123339324	0.262317611	-0.470190786	0.638218717	0.128047691	14.36167212	93.03702249	0.5	PRJNA827465;Yang2025_Silkie_Wenchang	CRA004775	0.905508695
ENSGALG00010025297	SPSB4	2	0.267640067	0.71280614	0.375473852	0.707308089	0.927058109	10.629233	90.59198345	0.5	CRA004775;Yang2025_Silkie_Wenchang	PRJNA827465	0.924856272
ENSGALG00010025297	SPSB4	2	0.494060717	0.442759392	1.115867277	0.264478951	0.315273553	4.314775926	76.82382545	1	CRA004775;PRJNA827465	Yang2025_Silkie_Wenchang	0.768790489
ENSGALG00010025298	ENSGALG00010025298	2	0.034286335	0.126485407	0.27106949	0.786315787	0	0.140648312	0	1	PRJNA827465;Yang2025_Silkie_Wenchang	CRA004775	0.786439719
ENSGALG00010025298	ENSGALG00010025298	2	-0.328500089	0.591359187	-0.555500102	0.578552641	0.397766989	2.296648995	56.45830068	0.5	CRA004775;Yang2025_Silkie_Wenchang	PRJNA827465	0.88788081
ENSGALG00010025298	ENSGALG00010025298	2	-0.299570025	0.465075168	-0.644132488	0.519489525	0.302462105	2.575573515	61.1736961	0.5	CRA004775;PRJNA827465	Yang2025_Silkie_Wenchang	0.869086345
ENSGALG00010025301	MORN3	2	-0.525369127	0.432018518	-1.21608011	0.223934407	0.310990178	5.992384339	83.31215184	1	PRJNA827465;Yang2025_Silkie_Wenchang	CRA004775	0.737452676
ENSGALG00010025301	MORN3	2	0.12753129	0.318407752	0.400052822	0.688767503	0.089304251	1.630481613	38.85782874	0.5	CRA004775;Yang2025_Silkie_Wenchang	PRJNA827465	0.919799842
ENSGALG00010025301	MORN3	2	-0.238172924	0.769619007	-0.309468609	0.756965087	1.048008845	8.391454326	88.08311455	0.5	CRA004775;PRJNA827465	Yang2025_Silkie_Wenchang	0.938795135
ENSGALG00010025303	TMCC2	2	-0.022050394	0.072303513	-0.304969892	0.760389084	0	0.16301707	0	0.5	PRJNA827465;Yang2025_Silkie_Wenchang	CRA004775	0.939327609
ENSGALG00010025303	TMCC2	2	-0.263084893	0.339713803	-0.77443098	0.438675969	0.178421235	3.803494567	73.7083889	0.5	CRA004775;Yang2025_Silkie_Wenchang	PRJNA827465	0.884002503
ENSGALG00010025303	TMCC2	2	-0.284051371	0.307497149	-0.923752861	0.355615003	0.140276182	3.236328912	69.10079206	1	CRA004775;PRJNA827465	Yang2025_Silkie_Wenchang	0.812301302
ENSGALG00010025304	DDX28	2	-0.043457801	0.070785888	-0.613933122	0.539259522	0	0.090189351	0	1	PRJNA827465;Yang2025_Silkie_Wenchang	CRA004775	0.874983893
ENSGALG00010025304	DDX28	2	-0.147827687	0.140947349	-1.048814952	0.294263295	0	0.275419523	0	1	CRA004775;Yang2025_Silkie_Wenchang	PRJNA827465	0.787343482
ENSGALG00010025304	DDX28	2	-0.054962813	0.073373269	-0.749084973	0.453805991	0	0.737962892	0	1	CRA004775;PRJNA827465	Yang2025_Silkie_Wenchang	0.854904791
ENSGALG00010025305	GPR182	2	-0.194158159	0.22231122	-0.873361943	0.382465827	0	0.005830177	0	1	PRJNA827465;Yang2025_Silkie_Wenchang	CRA004775	0.825121002
ENSGALG00010025305	GPR182	2	0.493355657	0.721634979	0.683663725	0.494187535	0.92180855	8.658170819	88.45021632	0.5	CRA004775;Yang2025_Silkie_Wenchang	PRJNA827465	0.860175061
ENSGALG00010025305	GPR182	2	0.513482358	0.704706771	0.728646834	0.466217722	0.868277394	7.923999745	87.38011065	0.5	CRA004775;PRJNA827465	Yang2025_Silkie_Wenchang	0.852664268
ENSGALG00010025312	PLEKHM3	2	-0.027247037	0.134917392	-0.201953478	0.839953094	0.026194572	3.462883741	71.12233402	0.5	PRJNA827465;Yang2025_Silkie_Wenchang	CRA004775	0.95957104
ENSGALG00010025312	PLEKHM3	2	-0.053668123	0.186960575	-0.287055828	0.774069574	0.035703179	1.763804004	43.30435821	0.5	CRA004775;Yang2025_Silkie_Wenchang	PRJNA827465	0.94329829
ENSGALG00010025312	PLEKHM3	2	0.104391478	0.078475609	1.330241069	0.183438857	0	0.211918426	0	1	CRA004775;PRJNA827465	Yang2025_Silkie_Wenchang	0.691847354
ENSGALG00010025315	LRRC42	2	0.251151482	0.120215028	2.089185415	0.036691036	0.01614753	2.160579444	53.71611986	1	PRJNA827465;Yang2025_Silkie_Wenchang	CRA004775	0.330558668
ENSGALG00010025315	LRRC42	2	0.340747539	0.121690935	2.800106168	0.00510858	0	0.650714606	0	1	CRA004775;Yang2025_Silkie_Wenchang	PRJNA827465	0.106629064
ENSGALG00010025315	LRRC42	2	0.154926467	0.083968644	1.845051437	0.065030057	0	0.00556511	0	1	CRA004775;PRJNA827465	Yang2025_Silkie_Wenchang	0.439607209
ENSGALG00010025316	SLC25A36	2	-0.025171694	0.12499057	-0.201388746	0.840394614	0.021027351	2.726792955	63.32688193	0.5	PRJNA827465;Yang2025_Silkie_Wenchang	CRA004775	0.959662809
ENSGALG00010025316	SLC25A36	2	-0.006025009	0.197165436	-0.030558514	0.975621926	0.05077812	2.834849309	64.72475637	0.5	CRA004775;Yang2025_Silkie_Wenchang	PRJNA827465	0.992754467
ENSGALG00010025316	SLC25A36	2	0.086890729	0.06066703	1.432256181	0.152070518	0	0.488510195	0	1	CRA004775;PRJNA827465	Yang2025_Silkie_Wenchang	0.643569246
ENSGALG00010025318	NUAK2	2	0.160058179	0.312630945	0.511971645	0.608670855	0	0.770103831	0	0.5	PRJNA827465;Yang2025_Silkie_Wenchang	CRA004775	0.897208691
ENSGALG00010025318	NUAK2	2	0.274529557	0.525317884	0.522597012	0.60125472	0.235126255	1.741757142	42.58671454	0.5	CRA004775;Yang2025_Silkie_Wenchang	PRJNA827465	0.895230519
ENSGALG00010025318	NUAK2	2	0.488524458	0.315829915	1.546796027	0.121912439	0	0.453509925	0	1	CRA004775;PRJNA827465	Yang2025_Silkie_Wenchang	0.585546203
ENSGALG00010025319	LDLRAD1	2	0.307089685	0.424682861	0.723103552	0.469616267	0.282572846	4.577217946	78.15266802	0.5	PRJNA827465;Yang2025_Silkie_Wenchang	CRA004775	0.853485658
ENSGALG00010025319	LDLRAD1	2	0.252997135	0.594515567	0.425551741	0.670434511	0.506826503	3.288129405	69.58757163	0.5	CRA004775;Yang2025_Silkie_Wenchang	PRJNA827465	0.914482883
ENSGALG00010025319	LDLRAD1	2	-0.151122663	0.228407424	-0.661636386	0.508204283	0	0.311696536	0	1	CRA004775;PRJNA827465	Yang2025_Silkie_Wenchang	0.864714223
ENSGALG00010025320	JAM3	2	-0.093239725	0.219962251	-0.423889665	0.671646282	0.092164221	21.02034242	95.24270357	0.5	PRJNA827465;Yang2025_Silkie_Wenchang	CRA004775	0.914674175
ENSGALG00010025320	JAM3	2	-0.077000979	0.305848781	-0.251761601	0.80122534	0.144754438	3.641016448	72.53514192	0.5	CRA004775;Yang2025_Silkie_Wenchang	PRJNA827465	0.950158029
ENSGALG00010025320	JAM3	2	0.133591867	0.064251678	2.079196552	0.037599288	0	0.337611577	0	1	CRA004775;PRJNA827465	Yang2025_Silkie_Wenchang	0.334407867
ENSGALG00010025323	KLHDC8A	2	0.188752099	0.16883013	1.118000076	0.263566961	0.027316845	1.893349401	47.18354683	0.5	PRJNA827465;Yang2025_Silkie_Wenchang	CRA004775	0.768643821
ENSGALG00010025323	KLHDC8A	2	0.033245665	0.177635297	0.187156865	0.851537644	0	0.205816351	0	0.5	CRA004775;Yang2025_Silkie_Wenchang	PRJNA827465	0.96214473
ENSGALG00010025323	KLHDC8A	2	0.32451579	0.144462735	2.246363322	0.024680746	0	0.069736976	0	1	CRA004775;PRJNA827465	Yang2025_Silkie_Wenchang	0.268408231
ENSGALG00010025325	DPEP2	2	0.043444231	0.121782002	0.35673909	0.721288185	0	0.892965903	0	0.5	PRJNA827465;Yang2025_Silkie_Wenchang	CRA004775	0.929850734
ENSGALG00010025325	DPEP2	2	-0.05593051	0.166992739	-0.334927799	0.737679552	0	0.157218211	0	0.5	CRA004775;Yang2025_Silkie_Wenchang	PRJNA827465	0.934083553
ENSGALG00010025325	DPEP2	2	0.138727774	0.15160088	0.915085549	0.360146721	0	0.014983075	0	1	CRA004775;PRJNA827465	Yang2025_Silkie_Wenchang	0.814880744
ENSGALG00010025327	TMEM59	2	-0.216766353	0.27754778	-0.78100554	0.434799237	0.143974558	14.84050834	93.26168634	0.5	PRJNA827465;Yang2025_Silkie_Wenchang	CRA004775	0.842762754
ENSGALG00010025327	TMEM59	2	-0.292549451	0.221468266	-1.320954269	0.186516613	0.077732397	4.798569622	79.16045658	1	CRA004775;Yang2025_Silkie_Wenchang	PRJNA827465	0.699457578
ENSGALG00010025327	TMEM59	2	0.034163064	0.054991405	0.621243701	0.53443929	0	0.46767252	0	0.5	CRA004775;PRJNA827465	Yang2025_Silkie_Wenchang	0.873427894
ENSGALG00010025329	MYO1A	2	0.100625659	0.085879076	1.171713339	0.241312166	0	0.040561921	0	1	PRJNA827465;Yang2025_Silkie_Wenchang	CRA004775	0.753847713
ENSGALG00010025329	MYO1A	2	0.112107448	0.144623953	0.77516515	0.438242078	0	0.868144805	0	1	CRA004775;Yang2025_Silkie_Wenchang	PRJNA827465	0.843979791
ENSGALG00010025329	MYO1A	2	0.12984179	0.103026202	1.260279304	0.207568623	0	0.780149117	0	1	CRA004775;PRJNA827465	Yang2025_Silkie_Wenchang	0.71978733
ENSGALG00010025330	USH1C	2	0.613185153	0.190190608	3.224055913	0.001263887	0	0.130799541	0	1	PRJNA827465;Yang2025_Silkie_Wenchang	CRA004775	0.043232862
ENSGALG00010025330	USH1C	2	1.449569222	0.842158123	1.721255407	0.085204187	1.132160208	4.614990848	78.33148465	1	CRA004775;Yang2025_Silkie_Wenchang	PRJNA827465	0.502163128
ENSGALG00010025330	USH1C	2	1.36745165	0.916026823	1.492807433	0.135487601	1.427436386	6.072102977	83.53124019	1	CRA004775;PRJNA827465	Yang2025_Silkie_Wenchang	0.616997992
ENSGALG00010025331	ENSGALG00010025331	2	0.186244377	0.355067103	0.524532899	0.599907944	0	0.272871304	0	0.5	PRJNA827465;Yang2025_Silkie_Wenchang	CRA004775	0.895092498
ENSGALG00010025331	ENSGALG00010025331	2	-0.159776986	0.459957521	-0.347373351	0.728310851	0.249902808	2.432453304	58.88924164	0.5	CRA004775;Yang2025_Silkie_Wenchang	PRJNA827465	0.932099951
ENSGALG00010025331	ENSGALG00010025331	2	-0.433491929	0.325039945	-1.333657402	0.182316161	0	0.572227386	0	1	CRA004775;PRJNA827465	Yang2025_Silkie_Wenchang	0.690258365
ENSGALG00010025333	FZD5	2	-0.335069234	0.259849096	-1.289470235	0.197232573	0.128337546	19.908345	94.97698076	1	PRJNA827465;Yang2025_Silkie_Wenchang	CRA004775	0.708706512
ENSGALG00010025333	FZD5	2	-0.150849655	0.481802459	-0.313094407	0.754208938	0.42501016	11.27850503	91.13357668	0.5	CRA004775;Yang2025_Silkie_Wenchang	PRJNA827465	0.938385954
ENSGALG00010025333	FZD5	2	0.063915174	0.209053474	0.305736007	0.759805655	0.061396584	2.614637906	61.75378633	0.5	CRA004775;PRJNA827465	Yang2025_Silkie_Wenchang	0.939165042
ENSGALG00010025334	RHOF	2	0.337653452	0.17349467	1.946189191	0.051632018	0	0.776210899	0	1	PRJNA827465;Yang2025_Silkie_Wenchang	CRA004775	0.393961017
ENSGALG00010025334	RHOF	2	0.084550633	0.557487838	0.151663636	0.879452524	0.435533901	3.058531829	67.30457435	0.5	CRA004775;Yang2025_Silkie_Wenchang	PRJNA827465	0.971291607
ENSGALG00010025334	RHOF	2	-0.008747265	0.368831326	-0.023716167	0.98107901	0.142263767	1.734642815	42.35124421	0.5	CRA004775;PRJNA827465	Yang2025_Silkie_Wenchang	0.994928779
ENSGALG00010025336	ADAM10	2	0.146230194	0.120242563	1.216126717	0.223936654	0.018003172	2.116984039	52.76298821	1	PRJNA827465;Yang2025_Silkie_Wenchang	CRA004775	0.737452676
ENSGALG00010025336	ADAM10	2	0.307800356	0.151528094	2.031308829	0.042223676	0	0.06205122	0	1	CRA004775;Yang2025_Silkie_Wenchang	PRJNA827465	0.285737867
ENSGALG00010025336	ADAM10	2	0.071062017	0.0521455	1.362764136	0.172956857	0	0.270330375	0	1	CRA004775;PRJNA827465	Yang2025_Silkie_Wenchang	0.676043418
ENSGALG00010025337	NEMP1	2	0.061059489	0.095475912	0.639527682	0.522479711	0	0.101265043	0	1	PRJNA827465;Yang2025_Silkie_Wenchang	CRA004775	0.869568211
ENSGALG00010025337	NEMP1	2	-0.186297	0.29308823	-0.635634532	0.525014656	0.135863427	4.74160239	78.91008318	0.5	CRA004775;Yang2025_Silkie_Wenchang	PRJNA827465	0.86996294
ENSGALG00010025337	NEMP1	2	-0.193783705	0.255290838	-0.759070344	0.447810478	0.11100352	6.582709581	84.80868725	0.5	CRA004775;PRJNA827465	Yang2025_Silkie_Wenchang	0.846601815
ENSGALG00010025338	FADD	2	6.25203E-05	0.06387827	0.000978741	0.999219078	0.001525235	1.211970419	17.48973534	0.5	PRJNA827465;Yang2025_Silkie_Wenchang	CRA004775	0.999889665
ENSGALG00010025338	FADD	2	0.073930604	0.094843654	0.779499745	0.435685388	0	0.193114984	0	0.5	CRA004775;Yang2025_Silkie_Wenchang	PRJNA827465	0.842762754
ENSGALG00010025338	FADD	2	-0.050939414	0.069773049	-0.730072933	0.465345605	0	0.024583665	0	1	CRA004775;PRJNA827465	Yang2025_Silkie_Wenchang	0.852411124
ENSGALG00010025340	METTL21A	2	-0.207429696	0.126878088	-1.634874067	0.10207545	0.016091108	1.706110166	41.39400798	1	PRJNA827465;Yang2025_Silkie_Wenchang	CRA004775	0.544451388
ENSGALG00010025340	METTL21A	2	-0.368383544	0.167577401	-2.198288916	0.027928524	0	0.12149221	0	1	CRA004775;Yang2025_Silkie_Wenchang	PRJNA827465	0.284609505
ENSGALG00010025340	METTL21A	2	-0.134989331	0.079107856	-1.706396015	0.087934372	0	0.204605237	0	1	CRA004775;PRJNA827465	Yang2025_Silkie_Wenchang	0.509638136
ENSGALG00010025341	VPS26B	2	-0.126213665	0.13624279	-0.926383548	0.354244475	0.028806882	4.294012022	76.71175593	1	PRJNA827465;Yang2025_Silkie_Wenchang	CRA004775	0.831861636
ENSGALG00010025341	VPS26B	2	-0.264448369	0.101948484	-2.593941172	0.009488272	0	0.076281144	0	1	CRA004775;Yang2025_Silkie_Wenchang	PRJNA827465	0.153228591
ENSGALG00010025341	VPS26B	2	-0.019491801	0.066092283	-0.294917953	0.768056557	0	0.667056906	0	1	CRA004775;PRJNA827465	Yang2025_Silkie_Wenchang	0.941444269
ENSGALG00010025342	TCEANC2	2	-0.014839348	0.129291557	-0.114774302	0.908624018	0.020167548	2.340997793	57.28317203	0.5	PRJNA827465;Yang2025_Silkie_Wenchang	CRA004775	0.978356603
ENSGALG00010025342	TCEANC2	2	-0.229204207	0.129661028	-1.767718578	0.077107963	0	0.439434663	0	1	CRA004775;Yang2025_Silkie_Wenchang	PRJNA827465	0.478533748
ENSGALG00010025342	TCEANC2	2	-0.081059172	0.221640498	-0.365723651	0.714571296	0.070558057	3.015299702	66.83580078	0.5	CRA004775;PRJNA827465	Yang2025_Silkie_Wenchang	0.926596201
ENSGALG00010025343	NAB2	2	0.203322681	0.116577123	1.744104468	0.081140875	0	0.597327901	0	1	PRJNA827465;Yang2025_Silkie_Wenchang	CRA004775	0.490552194
ENSGALG00010025343	NAB2	2	0.139071191	0.291664308	0.476819367	0.633490753	0.080369522	1.716785974	41.74890663	0.5	CRA004775;Yang2025_Silkie_Wenchang	PRJNA827465	0.930400348
ENSGALG00010025343	NAB2	2	0.104261901	0.131637573	0.792037556	0.428338779	0	0.905753085	0	1	CRA004775;PRJNA827465	Yang2025_Silkie_Wenchang	0.841329286
ENSGALG00010025345	CCM2	2	0.01484649	0.055293885	0.268501481	0.788313335	0	0.022358473	0	1	PRJNA827465;Yang2025_Silkie_Wenchang	CRA004775	0.947024357
ENSGALG00010025345	CCM2	2	0.029272398	0.124268461	0.235564568	0.813770571	0	0.0055711	0	1	CRA004775;Yang2025_Silkie_Wenchang	PRJNA827465	0.953869515
ENSGALG00010025345	CCM2	2	0.011183066	0.059042142	0.189408211	0.849727289	0	1.52303E-05	0	1	CRA004775;PRJNA827465	Yang2025_Silkie_Wenchang	0.96185718
ENSGALG00010025346	PXYLP1	2	0.23557246	0.118878962	1.981616058	0.047522229	0	0.146119591	0	1	PRJNA827465;Yang2025_Silkie_Wenchang	CRA004775	0.376717658
ENSGALG00010025346	PXYLP1	2	0.045167272	0.265692341	0.169998397	0.865011397	0.068709207	1.561136327	35.94409515	0.5	CRA004775;Yang2025_Silkie_Wenchang	PRJNA827465	0.966855585
ENSGALG00010025346	PXYLP1	2	0.064800898	0.329715849	0.196535586	0.844190982	0.125561433	1.991822382	49.79472021	0.5	CRA004775;PRJNA827465	Yang2025_Silkie_Wenchang	0.960723102
ENSGALG00010025348	HIPK1	2	-0.036427923	0.063334994	-0.575162655	0.565181297	0	0.036432861	0	1	PRJNA827465;Yang2025_Silkie_Wenchang	CRA004775	0.883427937
ENSGALG00010025348	HIPK1	2	-0.069645749	0.160824518	-0.433054298	0.664975324	0	0.001067776	0	1	CRA004775;Yang2025_Silkie_Wenchang	PRJNA827465	0.913399315
ENSGALG00010025348	HIPK1	2	-0.03302887	0.067086103	-0.492335503	0.622482186	0	0.014340152	0	1	CRA004775;PRJNA827465	Yang2025_Silkie_Wenchang	0.900102128
ENSGALG00010025349	SETD1A	2	0.101179247	0.15783089	0.641061117	0.521482975	0.031952598	2.16923118	53.90071794	0.5	PRJNA827465;Yang2025_Silkie_Wenchang	CRA004775	0.869518515
ENSGALG00010025349	SETD1A	2	0.044096004	0.336929675	0.130875988	0.89587341	0.151328905	2.911909968	65.6582789	0.5	CRA004775;Yang2025_Silkie_Wenchang	PRJNA827465	0.975002393
ENSGALG00010025349	SETD1A	2	-0.014644379	0.063289428	-0.23138744	0.817013822	0.000512435	1.009237981	0.915342196	1	CRA004775;PRJNA827465	Yang2025_Silkie_Wenchang	0.954817505
ENSGALG00010025351	CREB1	2	-0.17308508	0.198792533	-0.870681998	0.383927811	0.072519512	11.61811199	91.39274952	0.5	PRJNA827465;Yang2025_Silkie_Wenchang	CRA004775	0.825601288
ENSGALG00010025351	CREB1	2	-0.315896501	0.145765147	-2.167160722	0.030222601	0.014182339	1.248696971	19.91651914	1	CRA004775;Yang2025_Silkie_Wenchang	PRJNA827465	0.295973612
ENSGALG00010025351	CREB1	2	0.013229391	0.042641952	0.31024356	0.756375748	0	0.004231245	0	0.5	CRA004775;PRJNA827465	Yang2025_Silkie_Wenchang	0.938618977
ENSGALG00010025353	SLC12A4	2	0.075741759	0.039041549	1.940029573	0.052376096	0	0.141572715	0	1	PRJNA827465;Yang2025_Silkie_Wenchang	CRA004775	0.397324397
ENSGALG00010025353	SLC12A4	2	0.108494246	0.069988721	1.55016757	0.121101301	0	0.268292388	0	1	CRA004775;Yang2025_Silkie_Wenchang	PRJNA827465	0.583289761
ENSGALG00010025353	SLC12A4	2	0.071444838	0.04566373	1.56458611	0.117679988	0	0.41899684	0	1	CRA004775;PRJNA827465	Yang2025_Silkie_Wenchang	0.577281149
ENSGALG00010025355	STAT6	2	0.142080378	0.168403882	0.843688259	0.39884363	0.052718203	14.08646829	92.90098853	0.5	PRJNA827465;Yang2025_Silkie_Wenchang	CRA004775	0.832054308
ENSGALG00010025355	STAT6	2	0.048590886	0.307481336	0.158028733	0.874434157	0.159824836	5.792731417	82.73698661	0.5	CRA004775;Yang2025_Silkie_Wenchang	PRJNA827465	0.970135804
ENSGALG00010025355	STAT6	2	-0.062044351	0.098252747	-0.631477011	0.527728676	0.008409513	1.261885818	20.7535273	1	CRA004775;PRJNA827465	Yang2025_Silkie_Wenchang	0.871296643
ENSGALG00010025357	OLFML3	2	-0.135265308	0.177402763	-0.762475769	0.445776099	0.046926387	3.304635383	69.73947549	0.5	PRJNA827465;Yang2025_Silkie_Wenchang	CRA004775	0.846013379
ENSGALG00010025357	OLFML3	2	-0.166755322	0.277948657	-0.59995009	0.548539499	0.079214341	1.847613254	45.87611894	0.5	CRA004775;Yang2025_Silkie_Wenchang	PRJNA827465	0.878281266
ENSGALG00010025357	OLFML3	2	0.006268638	0.056515011	0.110919871	0.911679888	0	0.319267883	0	1	CRA004775;PRJNA827465	Yang2025_Silkie_Wenchang	0.978895589
ENSGALG00010025358	INCENP	2	-0.02359026	0.084190395	-0.280201318	0.779323056	0	0.309558053	0	0.5	PRJNA827465;Yang2025_Silkie_Wenchang	CRA004775	0.944727706
ENSGALG00010025358	INCENP	2	0.086980824	0.176198351	0.493652883	0.621551346	0	0.068668279	0	1	CRA004775;Yang2025_Silkie_Wenchang	PRJNA827465	0.899772838
ENSGALG00010025358	INCENP	2	-0.038444802	0.092589279	-0.415218724	0.677981793	0	0.251462473	0	0.5	CRA004775;PRJNA827465	Yang2025_Silkie_Wenchang	0.917534751
ENSGALG00010025359	CDCP2	2	-0.015122936	0.485836107	-0.031127649	0.97516774	0.336329273	3.42161775	70.77405856	0.5	PRJNA827465;Yang2025_Silkie_Wenchang	CRA004775	0.992684416
ENSGALG00010025359	CDCP2	2	-0.058580474	0.480100845	-0.122017018	0.90288554	0.29375612	2.623303687	61.88012829	0.5	CRA004775;Yang2025_Silkie_Wenchang	PRJNA827465	0.97693511
ENSGALG00010025359	CDCP2	2	0.511756986	0.325224061	1.573552045	0.115591047	0	5.93106E-07	0	1	CRA004775;PRJNA827465	Yang2025_Silkie_Wenchang	0.572716099
ENSGALG00010025360	THYN1	2	0.179162792	0.058955493	3.038949952	0.002374043	0	0.432735684	0	1	PRJNA827465;Yang2025_Silkie_Wenchang	CRA004775	0.065022305
ENSGALG00010025360	THYN1	2	0.076541159	0.199563071	0.3835437	0.701316675	0.054914213	3.156673996	68.32108728	0.5	CRA004775;Yang2025_Silkie_Wenchang	PRJNA827465	0.922671124
ENSGALG00010025360	THYN1	2	0.05603564	0.143893759	0.389423704	0.69696274	0.027211917	2.454475103	59.25809152	0.5	CRA004775;PRJNA827465	Yang2025_Silkie_Wenchang	0.921086208
ENSGALG00010025362	G6PC2	2	-0.007558423	0.105802616	-0.071438908	0.943048445	0	0.304617184	0	0.5	PRJNA827465;Yang2025_Silkie_Wenchang	CRA004775	0.985827325
ENSGALG00010025362	G6PC2	2	-0.10642014	0.283002108	-0.376040097	0.706887087	0.081934095	1.621693874	38.3360808	0.5	CRA004775;Yang2025_Silkie_Wenchang	PRJNA827465	0.92461376
ENSGALG00010025362	G6PC2	2	-0.125181671	0.174687807	-0.716602224	0.473619572	0.014305817	1.110400299	9.942387345	1	CRA004775;PRJNA827465	Yang2025_Silkie_Wenchang	0.854399987
ENSGALG00010025364	EPHA4	2	-0.031714694	0.083309671	-0.38068443	0.703437424	0	0.753862309	0	0.5	PRJNA827465;Yang2025_Silkie_Wenchang	CRA004775	0.923639641
ENSGALG00010025364	EPHA4	2	-0.152261809	0.128192235	-1.18776156	0.234927363	0	0.814062035	0	1	CRA004775;Yang2025_Silkie_Wenchang	PRJNA827465	0.747791642
ENSGALG00010025364	EPHA4	2	-0.087042017	0.238026176	-0.365682543	0.714601974	0.058122389	1.449906878	31.03005338	0.5	CRA004775;PRJNA827465	Yang2025_Silkie_Wenchang	0.926596201
ENSGALG00010025365	SIKE1	2	-0.135831204	0.093303381	-1.455801523	0.145447495	0.009460186	1.745899852	42.72294605	1	PRJNA827465;Yang2025_Silkie_Wenchang	CRA004775	0.633959706
ENSGALG00010025365	SIKE1	2	-0.253791405	0.133585866	-1.899837252	0.057454481	0	0.254043284	0	1	CRA004775;Yang2025_Silkie_Wenchang	PRJNA827465	0.41517438
ENSGALG00010025365	SIKE1	2	-0.080673602	0.04872896	-1.655557628	0.097811437	0	0.028055439	0	1	CRA004775;PRJNA827465	Yang2025_Silkie_Wenchang	0.534130658
ENSGALG00010025368	LRP1	2	-0.077967834	0.151660512	-0.51409449	0.607185929	0.022682387	1.858979884	46.20705643	0.5	PRJNA827465;Yang2025_Silkie_Wenchang	CRA004775	0.896998866
ENSGALG00010025368	LRP1	2	0.037850401	0.175062406	0.216210905	0.828823354	0	0.795826641	0	0.5	CRA004775;Yang2025_Silkie_Wenchang	PRJNA827465	0.956840134
ENSGALG00010025368	LRP1	2	-0.201573284	0.113417433	-1.777268974	0.075523996	0	0.031425435	0	1	CRA004775;PRJNA827465	Yang2025_Silkie_Wenchang	0.474458409
ENSGALG00010025369	ENSGALG00010025369	2	0.122009723	0.823551306	0.148150725	0.882223818	0.933965095	3.209767709	68.84509751	0.5	PRJNA827465;Yang2025_Silkie_Wenchang	CRA004775	0.971942333
ENSGALG00010025369	ENSGALG00010025369	2	0.380100237	1.059030464	0.358913412	0.719659866	1.864618954	5.921561888	83.11256356	0.5	CRA004775;Yang2025_Silkie_Wenchang	PRJNA827465	0.928466114
ENSGALG00010025369	ENSGALG00010025369	2	1.215887457	0.440451994	2.760544787	0.005770505	0	0.282398115	0	1	CRA004775;PRJNA827465	Yang2025_Silkie_Wenchang	0.114486693
ENSGALG00010025370	HPD	2	0.270982642	0.308832723	0.877441482	0.38024689	0	0.468838083	0	1	PRJNA827465;Yang2025_Silkie_Wenchang	CRA004775	0.824392515
ENSGALG00010025370	HPD	2	-0.435678986	0.591312558	-0.73679982	0.461244093	0.536728838	4.27960671	76.63336686	0.5	CRA004775;Yang2025_Silkie_Wenchang	PRJNA827465	0.85014477
ENSGALG00010025370	HPD	2	-0.26682457	0.821756926	-0.324700117	0.745408046	1.098583731	5.308337523	81.16171032	0.5	CRA004775;PRJNA827465	Yang2025_Silkie_Wenchang	0.936118751
ENSGALG00010025373	CYB5RL	2	-0.36429991	0.215980744	-1.686724024	0.09165643	0.070881952	3.617223615	72.35448768	1	PRJNA827465;Yang2025_Silkie_Wenchang	CRA004775	0.518914432
ENSGALG00010025373	CYB5RL	2	-0.497105242	0.157341051	-3.15941223	0.001580877	0	0.789530231	0	1	CRA004775;Yang2025_Silkie_Wenchang	PRJNA827465	0.050188378
ENSGALG00010025373	CYB5RL	2	-0.207785067	0.071545993	-2.904216689	0.003681732	0	0.47174518	0	1	CRA004775;PRJNA827465	Yang2025_Silkie_Wenchang	0.086700498
ENSGALG00010025377	MYO1G	2	0.091529793	0.147555715	0.620306659	0.535055911	0	0.42849407	0	1	PRJNA827465;Yang2025_Silkie_Wenchang	CRA004775	0.873963199
ENSGALG00010025377	MYO1G	2	-0.074252332	0.369915323	-0.200727918	0.840911329	0.162648478	2.17301245	53.98093555	0.5	CRA004775;Yang2025_Silkie_Wenchang	PRJNA827465	0.95980036
ENSGALG00010025377	MYO1G	2	-0.125911275	0.241478709	-0.52141771	0.602075817	0.034545549	1.255735097	20.36536983	0.5	CRA004775;PRJNA827465	Yang2025_Silkie_Wenchang	0.89532296
ENSGALG00010025378	PAX3	2	0.253840096	0.149244845	1.700829901	0.088974933	0	0.163645467	0	1	PRJNA827465;Yang2025_Silkie_Wenchang	CRA004775	0.511998468
ENSGALG00010025378	PAX3	2	0.372075189	0.195603549	1.902190374	0.057146271	0	0.594386418	0	1	CRA004775;Yang2025_Silkie_Wenchang	PRJNA827465	0.414005744
ENSGALG00010025378	PAX3	2	0.268674887	0.197605671	1.359651704	0.173940168	0	0.956046656	0	1	CRA004775;PRJNA827465	Yang2025_Silkie_Wenchang	0.677807894
ENSGALG00010025379	ACAD8	2	-0.270523814	0.12991759	-2.082272407	0.037317595	0.020983865	2.40290403	58.38368958	1	PRJNA827465;Yang2025_Silkie_Wenchang	CRA004775	0.333247689
ENSGALG00010025379	ACAD8	2	-0.264865727	0.258618486	-1.024156208	0.305761499	0.072911767	1.869688733	46.51516147	0.5	CRA004775;Yang2025_Silkie_Wenchang	PRJNA827465	0.79225794
ENSGALG00010025379	ACAD8	2	-0.157650692	0.076481506	-2.061291687	0.039275221	0	0.567820058	0	0.5	CRA004775;PRJNA827465	Yang2025_Silkie_Wenchang	0.342649058
ENSGALG00010025380	SPC25	2	-0.119277647	0.086305503	-1.382039883	0.166959455	0	0.334180862	0	1	PRJNA827465;Yang2025_Silkie_Wenchang	CRA004775	0.666022689
ENSGALG00010025380	SPC25	2	-0.051238608	0.155433004	-0.32965076	0.741663864	0	0.058955302	0	1	CRA004775;Yang2025_Silkie_Wenchang	PRJNA827465	0.93543545
ENSGALG00010025380	SPC25	2	-0.151259905	0.099896411	-1.514167562	0.129983347	0	0.001667919	0	1	CRA004775;PRJNA827465	Yang2025_Silkie_Wenchang	0.604798801
ENSGALG00010025381	MRPL37	2	-0.126517284	0.070975335	-1.782552836	0.074659123	0	0.058326097	0	1	PRJNA827465;Yang2025_Silkie_Wenchang	CRA004775	0.471681002
ENSGALG00010025381	MRPL37	2	-0.106358182	0.159404721	-0.667221029	0.504630961	0	0.515139585	0	0.5	CRA004775;Yang2025_Silkie_Wenchang	PRJNA827465	0.863512504
ENSGALG00010025381	MRPL37	2	-0.108023309	0.075336829	-1.43387119	0.151609025	0	0.430665557	0	0.5	CRA004775;PRJNA827465	Yang2025_Silkie_Wenchang	0.643070311
ENSGALG00010025382	MAB21L3	2	0.929880797	0.232105265	4.00628912	6.16801E-05	0	0.029864497	0	1	PRJNA827465;Yang2025_Silkie_Wenchang	CRA004775	0.005572907
ENSGALG00010025382	MAB21L3	2	0.48226658	0.416209813	1.158710259	0.246574308	0.129407424	1.589926669	37.10401748	1	CRA004775;Yang2025_Silkie_Wenchang	PRJNA827465	0.756999688
ENSGALG00010025382	MAB21L3	2	0.586279299	0.452621033	1.295298398	0.195217312	0.265798293	2.646964138	62.22087086	1	CRA004775;PRJNA827465	Yang2025_Silkie_Wenchang	0.706438265
ENSGALG00010025386	TPCN2	2	-0.065374168	0.229129158	-0.285315797	0.775402212	0.090704123	7.308309979	86.31694601	0.5	PRJNA827465;Yang2025_Silkie_Wenchang	CRA004775	0.943815265
ENSGALG00010025386	TPCN2	2	-0.221453139	0.14882482	-1.488012145	0.136747691	0.011158536	1.210892805	17.41630674	0.5	CRA004775;Yang2025_Silkie_Wenchang	PRJNA827465	0.618582531
ENSGALG00010025386	TPCN2	2	0.145931396	0.100641893	1.450006463	0.147056717	0	0.101008092	0	1	CRA004775;PRJNA827465	Yang2025_Silkie_Wenchang	0.635768359
ENSGALG00010025387	PSMD9	2	-0.066543405	0.053764238	-1.23768898	0.215831407	0	0.56891872	0	0.5	PRJNA827465;Yang2025_Silkie_Wenchang	CRA004775	0.729213578
ENSGALG00010025387	PSMD9	2	-0.056665395	0.126511387	-0.447907467	0.654219979	0.006416083	1.175902961	14.95896912	0.5	CRA004775;Yang2025_Silkie_Wenchang	PRJNA827465	0.910008212
ENSGALG00010025387	PSMD9	2	-0.097540543	0.058130966	-1.677944644	0.093357906	0	0.587914517	0	1	CRA004775;PRJNA827465	Yang2025_Silkie_Wenchang	0.523579592
ENSGALG00010025389	SNED1	2	0.180983438	0.064909605	2.788238141	0.005299557	0.001537597	1.182799763	15.45483593	1	PRJNA827465;Yang2025_Silkie_Wenchang	CRA004775	0.108766838
ENSGALG00010025389	SNED1	2	0.198819485	0.154346245	1.288139437	0.197697429	0.017965068	1.338294715	25.27804308	0.5	CRA004775;Yang2025_Silkie_Wenchang	PRJNA827465	0.709468779
ENSGALG00010025389	SNED1	2	0.127209329	0.065665448	1.937233854	0.052716764	0	0.565648135	0	0.5	CRA004775;PRJNA827465	Yang2025_Silkie_Wenchang	0.398907772
ENSGALG00010025390	BCL7A	2	-0.2761791	0.149339041	-1.849342939	0.064408309	0.038425248	7.13849533	85.99144562	1	PRJNA827465;Yang2025_Silkie_Wenchang	CRA004775	0.437453135
ENSGALG00010025390	BCL7A	2	-0.04903877	0.415813679	-0.117934479	0.906119574	0.311405082	9.474976512	89.44588413	0.5	CRA004775;Yang2025_Silkie_Wenchang	PRJNA827465	0.978196186
ENSGALG00010025390	BCL7A	2	0.077765254	0.261626669	0.29723749	0.766285202	0.108532962	4.114692525	75.69684748	0.5	CRA004775;PRJNA827465	Yang2025_Silkie_Wenchang	0.941123716
ENSGALG00010025392	SLC6A2	2	-0.368801835	0.168577087	-2.187734063	0.028688979	0.007867834	1.157556076	13.61109663	1	PRJNA827465;Yang2025_Silkie_Wenchang	CRA004775	0.288600705

ENSGALG00010025392	SLC6A2	2	-0.189885082	0.409133607	-0.464115093	0.642565289	0.247557898	3.736924538	73.24002693	0.5	CRA004775;Yang2025_Silkie_Wenchang	PRJNA827465	0.906634523
ENSGALG00010025392	SLC6A2	2	-0.063722941	0.225980303	-0.281984492	0.777955401	0.033282994	1.401142551	28.62967448	0.5	CRA004775;PRJNA827465	Yang2025_Silkie_Wenchang	0.944432731
ENSGALG00010025398	TSPAN2	2	0.244843035	0.185550426	1.319549838	0.18698536	0.032874922	1.648128748	39.32512851	1	PRJNA827465;Yang2025_Silkie_Wenchang	CRA004775	0.695858627
ENSGALG00010025398	TSPAN2	2	0.362225286	0.226830584	1.596897914	0.110288465	0	0.869827286	0	1	CRA004775;Yang2025_Silkie_Wenchang	PRJNA827465	0.562335482
ENSGALG00010025398	TSPAN2	2	0.123767137	0.118011059	1.048775753	0.294281339	0	0.003621615	0	1	CRA004775;PRJNA827465	Yang2025_Silkie_Wenchang	0.787343482
ENSGALG00010025400	SLC22A15	2	0.438836201	0.166682165	2.632772386	0.008469108	0	0.109467391	0	1	PRJNA827465;Yang2025_Silkie_Wenchang	CRA004775	0.143536595
ENSGALG00010025400	SLC22A15	2	-0.075176147	0.502200451	-0.149693508	0.88100643	0.355303849	3.223705993	68.97980144	0.5	CRA004775;Yang2025_Silkie_Wenchang	PRJNA827465	0.971685305
ENSGALG00010025400	SLC22A15	2	-0.004228798	0.55995431	-0.007552041	0.9939744	0.504056222	4.665171693	78.5645617	0.5	CRA004775;PRJNA827465	Yang2025_Silkie_Wenchang	0.998139346
ENSGALG00010025403	ENSGALG00010025403	2	0.356817472	0.289485264	1.232592867	0.2177277	0.145582203	6.939307685	85.58934053	1	PRJNA827465;Yang2025_Silkie_Wenchang	CRA004775	0.731070128
ENSGALG00010025403	ENSGALG00010025403	2	0.300349889	0.41780415	0.718877226	0.47221657	0.27644964	4.673353165	78.60208795	0.5	CRA004775;Yang2025_Silkie_Wenchang	PRJNA827465	0.853998373
ENSGALG00010025403	ENSGALG00010025403	2	0.091296593	0.061553	1.483219228	0.138016174	0	0.596700007	0	0.5	CRA004775;PRJNA827465	Yang2025_Silkie_Wenchang	0.621022596
ENSGALG00010025404	CERS6	2	-0.105053358	0.210950333	-0.498000438	0.618483733	0.073589346	5.775196135	82.68457077	0.5	PRJNA827465;Yang2025_Silkie_Wenchang	CRA004775	0.899168321
ENSGALG00010025404	CERS6	2	-0.337923349	0.122228348	-2.764688825	0.005697712	0	0.959457755	0	1	CRA004775;Yang2025_Silkie_Wenchang	PRJNA827465	0.113443351
ENSGALG00010025404	CERS6	2	-0.240319194	0.510693617	-0.47057411	0.6379449	0.365245629	2.526211181	60.41502756	0.5	CRA004775;PRJNA827465	Yang2025_Silkie_Wenchang	0.905365679
ENSGALG00010025405	TAMALIN	2	-0.04193257	0.120568382	-0.347790765	0.727997326	0	0.217913302	0	0.5	PRJNA827465;Yang2025_Silkie_Wenchang	CRA004775	0.931881152
ENSGALG00010025405	TAMALIN	2	0.115075393	0.218391461	0.526922584	0.59824735	0	0.01364689	0	1	CRA004775;Yang2025_Silkie_Wenchang	PRJNA827465	0.894645663
ENSGALG00010025405	TAMALIN	2	-0.034707221	0.120790332	-0.287334427	0.773856265	0	0.376231538	0	0.5	CRA004775;PRJNA827465	Yang2025_Silkie_Wenchang	0.943248868
ENSGALG00010025406	ENSGALG00010025406	2	-0.074091284	0.057869062	-1.280326322	0.200430394	0	0.010420574	0	1	PRJNA827465;Yang2025_Silkie_Wenchang	CRA004775	0.712703862
ENSGALG00010025406	ENSGALG00010025406	2	-0.034568522	0.092635456	-0.373167293	0.709023937	0	0.853196193	0	0.5	CRA004775;Yang2025_Silkie_Wenchang	PRJNA827465	0.925298042
ENSGALG00010025406	ENSGALG00010025406	2	-0.060119511	0.069058047	-0.870564883	0.383991778	0	0.9910411	0	0.5	CRA004775;PRJNA827465	Yang2025_Silkie_Wenchang	0.825648495
ENSGALG00010025407	MLXIP	2	-0.188958783	0.074821251	-2.525469446	0.011554383	0	0.5044831	0	1	PRJNA827465;Yang2025_Silkie_Wenchang	CRA004775	0.171919086
ENSGALG00010025407	MLXIP	2	0.108325278	0.262943881	0.411971091	0.680360612	0.077210801	2.016647985	50.41276379	0.5	CRA004775;Yang2025_Silkie_Wenchang	PRJNA827465	0.91777228
ENSGALG00010025407	MLXIP	2	0.04298189	0.332672834	0.129201684	0.897198063	0.170448827	3.608913222	72.29082723	0.5	CRA004775;PRJNA827465	Yang2025_Silkie_Wenchang	0.975346564
ENSGALG00010025413	NECAB3	2	0.125612333	0.135238564	0.928820368	0.352982187	0.02843723	4.425090522	77.40159224	0.5	PRJNA827465;Yang2025_Silkie_Wenchang	CRA004775	0.811035834
ENSGALG00010025413	NECAB3	2	-0.001816562	0.317269266	-0.005725615	0.995431645	0.163874009	4.876847732	79.49495135	0.5	CRA004775;Yang2025_Silkie_Wenchang	PRJNA827465	0.998378287
ENSGALG00010025413	NECAB3	2	-0.094916283	0.161349062	-0.58826672	0.55635328	0.028991323	1.727721817	42.12031182	1	CRA004775;PRJNA827465	Yang2025_Silkie_Wenchang	0.880324407
ENSGALG00010025414	MMP2	2	-0.222450548	0.187919728	-1.183753031	0.23651084	0.05987456	6.364709603	84.28836408	1	PRJNA827465;Yang2025_Silkie_Wenchang	CRA004775	0.7487803
ENSGALG00010025414	MMP2	2	0.015538687	0.496900253	0.031271239	0.975053227	0.424609123	6.561279495	84.75907023	0.5	CRA004775;Yang2025_Silkie_Wenchang	PRJNA827465	0.99263131
ENSGALG00010025414	MMP2	2	0.159285888	0.294011098	0.541768285	0.587978142	0.123741804	2.743598301	63.55151555	0.5	CRA004775;PRJNA827465	Yang2025_Silkie_Wenchang	0.891761457
ENSGALG00010025415	ENSGALG00010025415	2	0.591728475	0.166858174	3.546295998	0.000390687	0	0.904892886	0	1	PRJNA827465;Yang2025_Silkie_Wenchang	CRA004775	0.019924928
ENSGALG00010025415	ENSGALG00010025415	2	0.253465761	0.562538477	0.450574976	0.652295905	0.514272371	5.099181111	80.389008	0.5	CRA004775;Yang2025_Silkie_Wenchang	PRJNA827465	0.909377929
ENSGALG00010025415	ENSGALG00010025415	2	0.121547897	0.397432556	0.305832765	0.75973198	0.208542533	2.70934861	63.0907593	0.5	CRA004775;PRJNA827465	Yang2025_Silkie_Wenchang	0.939163539
ENSGALG00010025417	B3GAT1	2	-0.110006185	0.078878191	-1.394633726	0.163126319	0	0.055227951	0	1	PRJNA827465;Yang2025_Silkie_Wenchang	CRA004775	0.661599642
ENSGALG00010025417	B3GAT1	2	-0.109790598	0.129977332	-0.844690352	0.398283748	0	0.615928683	0	0.5	CRA004775;Yang2025_Silkie_Wenchang	PRJNA827465	0.831980453
ENSGALG00010025417	B3GAT1	2	-0.083780029	0.095963052	-0.873044645	0.382638744	0	0.524267635	0	0.5	CRA004775;PRJNA827465	Yang2025_Silkie_Wenchang	0.825121999
ENSGALG00010025418	CASQ2	2	-0.128253568	0.072045261	-1.780180492	0.075046427	0	0.233273195	0	1	PRJNA827465;Yang2025_Silkie_Wenchang	CRA004775	0.47268425
ENSGALG00010025418	CASQ2	2	-0.09189148	0.097817329	-0.939419225	0.347515546	0	0.08102275	0	0.5	CRA004775;Yang2025_Silkie_Wenchang	PRJNA827465	0.808715941
ENSGALG00010025418	CASQ2	2	-0.15968837	0.104306937	-1.530946778	0.125782547	0	0.143643028	0	0.5	CRA004775;PRJNA827465	Yang2025_Silkie_Wenchang	0.595474757
ENSGALG00010025419	CCND1	2	0.195777151	0.226112702	0.865838802	0.386578567	0.094444151	12.65997436	92.10108985	0.5	PRJNA827465;Yang2025_Silkie_Wenchang	CRA004775	0.827047925
ENSGALG00010025419	CCND1	2	0.252586381	0.236461398	1.068192876	0.285433518	0.075363097	2.696707559	62.91774402	0.5	CRA004775;Yang2025_Silkie_Wenchang	PRJNA827465	0.781170778
ENSGALG00010025419	CCND1	2	-0.019854391	0.050555363	-0.392725711	0.694522079	0	0.017174108	0	1	CRA004775;PRJNA827465	Yang2025_Silkie_Wenchang	0.920728119
ENSGALG00010025420	STK39	2	-0.086874746	0.093992406	-0.924274091	0.355343629	0	0.40442093	0	1	PRJNA827465;Yang2025_Silkie_Wenchang	CRA004775	0.812301302
ENSGALG00010025420	STK39	2	-0.208345113	0.160570308	-1.297532005	0.1944482	0	0.274909118	0	1	CRA004775;Yang2025_Silkie_Wenchang	PRJNA827465	0.705148332
ENSGALG00010025420	STK39	2	-0.068863129	0.109430255	-0.629287842	0.52916063	0	0.660313586	0	1	CRA004775;PRJNA827465	Yang2025_Silkie_Wenchang	0.871837903
ENSGALG00010025421	NR4A1	2	0.043258009	0.13218172	0.327261656	0.743469991	0	0.081675968	0	1	PRJNA827465;Yang2025_Silkie_Wenchang	CRA004775	0.935816088
ENSGALG00010025421	NR4A1	2	0.058416841	0.186835683	0.312664263	0.754535749	0	0.220375913	0	0.5	CRA004775;Yang2025_Silkie_Wenchang	PRJNA827465	0.93838723
ENSGALG00010025421	NR4A1	2	-0.008720617	0.173776395	-0.050182976	0.959976577	0	0.131115447	0	0.5	CRA004775;PRJNA827465	Yang2025_Silkie_Wenchang	0.98933784
ENSGALG00010025422	SSBP3	2	-0.029056	0.048418138	-0.600105682	0.548435806	0	0.583387275	0	1	PRJNA827465;Yang2025_Silkie_Wenchang	CRA004775	0.878281266
ENSGALG00010025422	SSBP3	2	-0.112932223	0.083874275	-1.34644649	0.178158567	0	0.14455659	0	1	CRA004775;Yang2025_Silkie_Wenchang	PRJNA827465	0.684540634
ENSGALG00010025422	SSBP3	2	-0.024443401	0.05276214	-0.463275396	0.643166979	0	0.840089866	0	1	CRA004775;PRJNA827465	Yang2025_Silkie_Wenchang	0.906634523
ENSGALG00010025423	NGF	2	0.002535736	0.220135241	0.011518993	0.990809376	0.042741435	1.742317036	42.60516432	0.5	PRJNA827465;Yang2025_Silkie_Wenchang	CRA004775	0.997555603
ENSGALG00010025423	NGF	2	0.312794573	0.237353547	1.317842422	0.187556404	0	0.102995893	0	1	CRA004775;Yang2025_Silkie_Wenchang	PRJNA827465	0.696676054
ENSGALG00010025423	NGF	2	-0.012642302	0.275868219	-0.045827322	0.963447882	0.065068646	1.507455395	33.66304547	0.5	CRA004775;PRJNA827465	Yang2025_Silkie_Wenchang	0.99005635
ENSGALG00010025424	IRX6	2	0.357696243	0.269042805	1.32951425	0.183678368	0	0.051828294	0	1	PRJNA827465;Yang2025_Silkie_Wenchang	CRA004775	0.692148694
ENSGALG00010025424	IRX6	2	0.311523969	0.294128222	1.059143412	0.289534472	0	0.303247908	0	1	CRA004775;Yang2025_Silkie_Wenchang	PRJNA827465	0.78398714
ENSGALG00010025424	IRX6	2	0.189051088	0.353239457	0.535192442	0.592516792	0	0.113255544	0	1	CRA004775;PRJNA827465	Yang2025_Silkie_Wenchang	0.892776493
ENSGALG00010025425	ENSGALG00010025425	2	0.171607782	0.192075569	0.893438884	0.371622188	0.066709813	10.27229414	90.26507627	0.5	PRJNA827465;Yang2025_Silkie_Wenchang	CRA004775	0.82156974
ENSGALG00010025425	ENSGALG00010025425	2	0.040222368	0.375791181	0.107033826	0.914762145	0.24083114	6.22250698	83.9293069	0.5	CRA004775;Yang2025_Silkie_Wenchang	PRJNA827465	0.97982748
ENSGALG00010025425	ENSGALG00010025425	2	-0.095164275	0.154656599	-0.615326313	0.538339244	0.026443248	1.612717906	37.9928755	1	CRA004775;PRJNA827465	Yang2025_Silkie_Wenchang	0.87481821
ENSGALG00010025426	ENSGALG00010025426	2	0.015863893	0.099491025	0.159450491	0.873313961	0.01308483	2.897088284	65.48258451	0.5	PRJNA827465;Yang2025_Silkie_Wenchang	CRA004775	0.969874502
ENSGALG00010025426	ENSGALG00010025426	2	0.115230494	0.091697677	1.256634814	0.208885902	0	0.25269969	0	0.5	CRA004775;Yang2025_Silkie_Wenchang	PRJNA827465	0.721011841
ENSGALG00010025426	ENSGALG00010025426	2	-0.074962486	0.06956685	-1.077560456	0.281229957	0	0.001826238	0	1	CRA004775;PRJNA827465	Yang2025_Silkie_Wenchang	0.778899386
ENSGALG00010025427	SLC35G2	2	0.072472125	0.18698044	0.387592015	0.698317979	0.057197099	5.258505434	80.98318976	0.5	PRJNA827465;Yang2025_Silkie_Wenchang	CRA004775	0.921624968
ENSGALG00010025427	SLC35G2	2	-0.172662412	0.13444361	-1.284273845	0.199046155	0	0.612448501	0	1	CRA004775;Yang2025_Silkie_Wenchang	PRJNA827465	0.71096127
ENSGALG00010025427	SLC35G2	2	-0.00477057	0.335147874	-0.014234224	0.988643116	0.166656504	3.084888416	67.58391665	0.5	CRA004775;PRJNA827465	Yang2025_Silkie_Wenchang	0.996891127
ENSGALG00010025428	VANGL1	2	-0.077225632	0.075608283	-1.021391168	0.307069148	0	0.21636434	0	1	PRJNA827465;Yang2025_Silkie_Wenchang	CRA004775	0.79266003
ENSGALG00010025428	VANGL1	2	-0.027941641	0.130217678	-0.214576403	0.830097593	0	0.013258119	0	1	CRA004775;Yang2025_Silkie_Wenchang	PRJNA827465	0.957212554
ENSGALG00010025428	VANGL1	2	-0.096969247	0.086339034	-1.123121741	0.261385794	0	0.011226986	0	1	CRA004775;PRJNA827465	Yang2025_Silkie_Wenchang	0.76815058
ENSGALG00010025432	IRX3	2	0.540087368	0.867325062	0.622704672	0.533478612	1.236342155	5.580755446	82.08127896	0.5	PRJNA827465;Yang2025_Silkie_Wenchang	CRA004775	0.873379507
ENSGALG00010025432	IRX3	2	0.648600814	0.797712679	0.813075724	0.416174644	0.937474994	3.79528099	73.65148977	0.5	CRA004775;Yang2025_Silkie_Wenchang	PRJNA827465	0.837742614
ENSGALG00010025432	IRX3	2	-0.244355155	0.366852437	-0.66608568	0.505356337	0	0.034538302	0	1	CRA004775;PRJNA827465	Yang2025_Silkie_Wenchang	0.863862536
ENSGALG00010025433	CHPF2	2	-0.017190411	0.051266147	-0.335317019	0.737385957	0	1.75478E-07	0	1	PRJNA827465;Yang2025_Silkie_Wenchang	CRA004775	0.933968189
ENSGALG00010025433	CHPF2	2	-0.200367201	0.22254162	-0.900358329	0.36792959	0.070108563	3.140643967	68.15939628	1	CRA004775;Yang2025_Silkie_Wenchang	PRJNA827465	0.820188115
ENSGALG00010025433	CHPF2	2	-0.189700965	0.220215599	-0.861432912	0.388999656	0.074961385	3.684617534	72.86014109	1	CRA004775;PRJNA827465	Yang2025_Silkie_Wenchang	0.828615121
ENSGALG00010025434	SYT6	2	-0.267438768	0.352063396	-0.759632416	0.447474337	0.230826669	14.47418645	93.09114883	0.5	PRJNA827465;Yang2025_Silkie_Wenchang	CRA004775	0.84650962
ENSGALG00010025434	SYT6	2	-0.53807131	0.227109382	-2.369216572	0.017825812	0.03814065	1.197538311	16.49536462	0.5	CRA004775;Yang2025_Silkie_Wenchang	PRJNA827465	0.222839658
ENSGALG00010025434	SYT6	2	0.078323434	0.113117285	0.692409068	0.488680473	0	0.001545376	0	1	CRA004775;PRJNA827465	Yang2025_Silkie_Wenchang	0.857953638
ENSGALG00010025435	SCLY	2	-0.078513913	0.065002203	-1.207865421	0.227099032	0	0.115421449	0	1	PRJNA827465;Yang2025_Silkie_Wenchang	CRA004775	0.740605003
ENSGALG00010025435	SCLY	2	-0.27585461	0.16623298	-1.659445742	0.097026005	0	0.385089788	0	1	CRA004775;Yang2025_Silkie_Wenchang	PRJNA827465	0.532286192
ENSGALG00010025435	SCLY	2	-0.143868825	0.126424598	-1.137981272	0.255128303	0.016535886	1.610035422	37.88956527	1	CRA004775;PRJNA827465	Yang2025_Silkie_Wenchang	0.764467569
ENSGALG00010025438	EXOSC6	2	0.530656351	0.519975192	1.02054167	0.307471637	0.477495974	7.904630993	87.34918808	1	PRJNA827465;Yang2025_Silkie_Wenchang	CRA004775	0.792741915
ENSGALG00010025438	EXOSC6	2	0.414657704	0.707938528	0.585725579	0.558059942	0.855648345	6.814672684	85.32578091	0.5	CRA004775;Yang2025_Silkie_Wenchang	PRJNA827465	0.880678789
ENSGALG00010025438	EXOSC6	2	0.036855825	0.107257204	0.343620974	0.731131349	0	0.760032022	0	0.5	CRA004775;PRJNA827465	Yang2025_Silkie_Wenchang	0.932769533
ENSGALG00010025442	ABCF2	2	0.011180268	0.051352882	0.217714511	0.827651557	0	0.671355237	0	0.5	PRJNA827465;Yang2025_Silkie_Wenchang	CRA004775	0.956550752
ENSGALG00010025442	ABCF2	2	-0.055051096	0.109005131	-0.505032156	0.613536251	0	0.260216517	0	0.5	CRA004775;Yang2025_Silkie_Wenchang	PRJNA827465	0.898256044
ENSGALG00010025442	ABCF2	2	0.03455861	0.056803148	0.608392512	0.542927176	0	0.060302354	0	1	CRA004775;PRJNA827465	Yang2025_Silkie_Wenchang	0.876968972
ENSGALG00010025445	B3GNT4	2	0.554779652	0.330243389	1.679911456	0.092974545	0.189561257	7.636513293	86.90501854	1	PRJNA827465;Yang2025_Silkie_Wenchang	CRA004775	0.52216376
ENSGALG00010025445	B3GNT4	2	0.752307107	0.237835382	3.163142083	0.001560761	0.045114786	1.411873103	29.17210491	1	CRA004775;Yang2025_Silkie_Wenchang	PRJNA827465	0.049710376
ENSGALG00010025445	B3GNT4	2	0.239113941	0.1555209	1.537503587	0.124170037	0	0.050083435	0	1	CRA004775;PRJNA827465	Yang2025_Silkie_Wenchang	0.59073738
ENSGALG00010025448	CHD1L	2	0.219265739	0.196415414	1.11633672	0.264278029	0.0630353	5.462881961	81.69464383	1	PRJNA827465;Yang2025_Silkie_Wenchang	CRA004775	0.768790489
ENSGALG00010025448	CHD1L	2	0.048894525	0.414944273	0.117833957	0.906199223	0.292895767	6.182125819	83.82433439	0.5	CRA004775;Yang2025_Silkie_Wenchang	PRJNA827465	0.978196186
ENSGALG00010025448	CHD1L	2	-0.10214067	0.199841753	-0.511107758	0.609275604	0.042013851	1.748017975	42.7923503	0.5	CRA004775;PRJNA827465	Yang2025_Silkie_Wenchang	0.897238578
ENSGALG00010025449	ENSGALG00010025449	2	0.141846345	0.157207799	0.902285678	0.366905132	0.042102572	6.733713254	85.14935278	0.5	PRJNA827465;Yang2025_Silkie_Wenchang	CRA004775	0.819577857
ENSGALG00010025449	ENSGALG00010025449	2	0.098439882	0.260967316	0.377211535	0.706016409	0.10127925	3.327634701	69.94862447	0.5	CRA004775;Yang2025_Silkie_Wenchang	PRJNA827465	0.924548793
ENSGALG00010025449	ENSGALG00010025449	2	-0.029348307	0.077084655	-0.380728264	0.703404894	0	0.587186922	0	1	CRA004775;PRJNA827465	Yang2025_Silkie_Wenchang	0.923639641
ENSGALG00010025451	LRRC43	2	0.88974115	0.752378068	1.182571885	0.236978861	1.027030792	10.77010838	90.71504237	1	PRJNA827465;Yang2025_Silkie_Wenchang	CRA004775	0.749355649
ENSGALG00010025451	LRRC43	2	0.548216521	1.146405621	0.478204669	0.63250454	2.406832086	11.59857217	91.3782491	0.5	CRA004775;Yang2025_Silkie_Wenchang	PRJNA827465	0.903058174
ENSGALG00010025451	LRRC43	2	-0.101960579	0.363838766	-0.280235611	0.779296747	0.08766299	1.390101866	28.06282588	0.5	CRA004775;PRJNA827465	Yang2025_Silkie_Wenchang	0.944727706
ENSGALG00010025452	TRIM33	2	0.014599561	0.043293348	0.337224124	0.735947953	0	0.002092844	0	1	PRJNA827465;Yang2025_Silkie_Wenchang	CRA004775	0.933592889
ENSGALG00010025452	TRIM33	2	0.04860935	0.104282913	0.46612958	0.641122752	0	0.738570067	0	1	CRA004775;Yang2025_Silkie_Wenchang	PRJNA827465	0.906429868
ENSGALG00010025452	TRIM33	2	0.022826398	0.046095947	0.495193175	0.620463768	0	0.811593208	0	1	CRA004775;PRJNA827465	Yang2025_Silkie_Wenchang	0.899395593
ENSGALG00010025458	BCAS2	2	0.073111912	0.067382107	1.08503452	0.277906366	0	0.136792369	0	1	PRJNA827465;Yang2025_Silkie_Wenchang	CRA004775	0.776763074
ENSGALG00010025458	BCAS2	2	-0.070686242	0.255179378	-0.277006093	0.781775428	0.069780362	2.084774477	52.033181	0.5	CRA004775;Yang2025_Silkie_Wenchang	PRJNA827465	0.945265125
ENSGALG00010025458	BCAS2	2	-0.061004596	0.197992043	-0.308116404	0.757993759	0.049824752	2.080573181	51.93632172	0.5	CRA004775;PRJNA827465	Yang2025_Silkie_Wenchang	0.938954369
ENSGALG00010025459	DIABLO	2	-0.042964697	0.072100248	-0.595902202	0.551240562	0.005360476	1.952645883	48.78743715	0.5	PRJNA827465;Yang2025_Silkie_Wenchang	CRA004775	0.878879109
ENSGALG00010025459	DIABLO	2	-0.107501555	0.083558103	-1.286548545	0.198251688	0	0.600918608	0	0.5	CRA004775;Yang2025_Silkie_Wenchang	PRJNA827465	0.710234722
ENSGALG00010025459	DIABLO	2	0.016104127	0.053791928	0.29937813	0.764651547	0	0.024426956	0	1	CRA004775;PRJNA827465	Yang2025_Silkie_Wenchang	0.940711384
ENSGALG00010025464	VPS33A	2	0.006176972	0.049476507	0.124846557	0.900645029	0	0.598519958	0	0.5	PRJNA827465;Yang2025_Silkie_Wenchang	CRA004775	0.976497577
ENSGALG00010025464	VPS33A	2	-0.139291418	0.104895825	-1.327902404	0.184210351	0.00432004	1.203589398	16.91518707	1	CRA004775;Yang2025_Silkie_Wenchang	PRJNA827465	0.692545576
ENSGALG00010025464	VPS33A	2	-0.09110022	0.154162368	-0.590936826	0.55456275	0.034347857	3.03165135	67.01467667	0.5	CRA004775;PRJNA827465	Yang2025_Silkie_Wenchang	0.880223402
ENSGALG00010025465	ENSGALG00010025465	2	-0.059688283	0.182692098	-0.326715184	0.743883315	0.054954938	5.131527718	80.51262597	0.5	PRJNA827465;Yang2025_Silkie_Wenchang	CRA004775	0.935821071
ENSGALG00010025465	ENSGALG00010025465	2	-0.28131566	0.134489636	-2.092349734	0.036407242	0	0.016751974	0	1	CRA004775;Yang2025_Silkie_Wenchang	PRJNA827465	0.32890494
ENSGALG00010025465	ENSGALG00010025465	2	-0.01544127	0.18351686	-0.084140882	0.932944421	0.043292887	2.052467668	51.27816063	0.5	CRA004775;PRJNA827465	Yang2025_Silkie_Wenchang	0.984224753
ENSGALG00010025470	ORAOV1	2	0.200800191	0.179757478	1.117061684	0.263967952	0.049124788	4.068503659	75.42093891	1	PRJNA827465;Yang2025_Silkie_Wenchang	CRA004775	0.768643821
ENSGALG00010025470	ORAOV1	2	0.335993898	0.094147962	3.568785656	0.000358664	0	0.618725133	0	1	CRA004775;Yang2025_Silkie_Wenchang	PRJNA827465	0.018920289
ENSGALG00010025470	ORAOV1	2	0.040482298	0.127791323	0.316823528	0.75137751	0	0.239772384	0	1	CRA004775;PRJNA827465	Yang2025_Silkie_Wenchang	0.917684688
ENSGALG00010025472	ZCCHC8	2	0.293060708	0.31702646	0.924404567	0.355275718	0.18422139	11.58052151	91.36481031	0.5	PRJNA827465;Yang2025_Silkie_Wenchang	CRA004775	0.812301302
ENSGALG00010025472	ZCCHC8	2	0.29210916	0.350230101	0.83404927	0.40425322	0.207015446	6.34597648	84.24198383	0.5	CRA004775;Yang2025_Silkie_Wenchang	PRJNA827465	0.833112102
ENSGALG00010025472	ZCCHC8	2	-0.014288762	0.074276969	-0.192371365	0.847451321	0	0.079163807	0	1	CRA004775;PRJNA827465	Yang2025_Silkie_Wenchang	0.961252712
ENSGALG00010025473	PARS2	2	0.021157887	0.06343853	0.333801664	0.738529228	0	0.41377092	0	1	PRJNA827465;Yang2025_Silkie_Wenchang	CRA004775	0.934485814
ENSGALG00010025473	PARS2	2	0.018640551	0.126448692	0.147415924	0.882803737	0.000271409	1.007901149	0.783921004	0.5	CRA004775;Yang2025_Silkie_Wenchang	PRJNA827465	0.972001076
ENSGALG00010025473	PARS2	2	-0.011829269	0.065809037	-0.179751445	0.857547704	0	0.477295133	0	0.5	CRA004775;PRJNA827465	Yang2025_Silkie_Wenchang	0.963936745
ENSGALG00010025477	ENSGALG00010025477	2	0.140825935	0.152354734	0.924325917	0.355314935	0.036076596	3.687488775	72.8380792	1	PRJNA827465;Yang2025_Silkie_Wenchang	CRA004775	0.812301302
ENSGALG00010025477	ENSGALG00010025477	2	0.212112365	0.113237657	1.873161019	0.061046153	0.002747212	1.118617562	10.60394241	1	CRA004775;Yang2025_Silkie_Wenchang	PRJNA827465	0.42722323
ENSGALG00010025477	ENSGALG00010025477	2	0.027080997	0.032075147	0.844298447	0.398502654	0	0.349924696	0	1	CRA004775;PRJNA827465	Yang2025_Silkie_Wenchang	0.831980453
ENSGALG00010025481	TMEM39A	2	0.093901457	0.045193701	2.077755439	0.03771189	0	0.484815202	0	1	PRJNA827465;Yang2025_Silkie_Wenchang	CRA004775	0.335363291
ENSGALG00010025481	TMEM39A	2	-0.076944374	0.230423547	-0.333925341	0.738435528	0.095294335	9.668222621	89.65583726	0.5	CRA004775;Yang2025_Silkie_Wenchang	PRJNA827465	0.934485814
ENSGALG00010025481	TMEM39A	2	-0.104988165	0.194233272	-0.540526161	0.588834225	0.067390199	9.933263293	88.80588238	0.5	CRA004775;PRJNA827465	Yang2025_Silkie_Wenchang	0.892171138
ENSGALG00010025483	FGF4	2	0.345926021	0.400711532	0.863279425	0.387983854	0.287235344	9.381089716	89.34025758	1	PRJNA827465;Yang2025_Silkie_Wenchang	CRA004775	0.828154121
ENSGALG00010025483	FGF4	2	0.796779975	0.203662985	3.912247366	9.14412E-05	0	0.360467578	0	1	CRA004775;Yang2025_Silkie_Wenchang	PRJNA827465	0.00724579
ENSGALG00010025483	FGF4	2	0.388291438	0.593544494	0.654190953	0.51298879	0.513578659	2.922997973	65.78854965	0.5	CRA004775;PRJNA827465	Yang2025_Silkie_Wenchang	0.866156104
ENSGALG00010025485	DENND2C	2	0.326741377	0.072238642	4.523083034	6.09453E-06	0.001328406	1.143220976	12.52784713	1	PRJNA827465;Yang2025_Silkie_Wenchang	CRA004775	0.000954316
ENSGALG00010025485	DENND2C	2	0.31011894	0.152336835	2.035744939	0.041775974	0.022317067	1.666927496	40.00938839	1	CRA004775;Yang2025_Silkie_Wenchang	PRJNA827465	0.354014899
ENSGALG00010025485	DENND2C	2	0.239769879	0.0834449	2.87339164	0.004060904	0	0.552181663	0	1	CRA004775;PRJNA827465	Yang2025_Silkie_Wenchang	0.092471433
ENSGALG00010025486	B3GALT1	2	-0.119599434	0.173772372	-0.688253449	0.491293191	0.036070332	2.428093538	58.81542517	0.5	PRJNA827465;Yang2025_Silkie_Wenchang	CRA004775	0.85895707
ENSGALG00010025486	B3GALT1	2	0.149839581	0.210240453	0.712705754	0.476027855	0.016660869	1.077513129	7.193706253	1	CRA004775;Yang2025_Silkie_Wenchang	PRJNA827465	0.854810196
ENSGALG00010025486	B3GALT1	2	0.058296853	0.481439559	0.121088622	0.903620841	0.323917837	2.56200737	60.96810604	0.5	CRA004775;PRJNA827465	Yang2025_Silkie_Wenchang	0.97732491
ENSGALG00010025488	SLC4A8	2	0.033425288	0.183178656	0.182473704	0.855210989	0.036335808	2.101147756	52.40696437	0.5	PRJNA827465;Yang2025_Silkie_Wenchang	CRA004775	0.963532279
ENSGALG00010025488	SLC4A8	2	-0.390854158	0.298018429	-1.311510025	0.189685509	0.085585399	1.725082873	42.03177044	1	CRA004775;Yang2025_Silkie_Wenchang	PRJNA827465	0.698969582
ENSGALG00010025488	SLC4A8	2	-0.234535476	0.498153548	-0.470809607	0.637776703	0.404758221	4.828316781	79.28884857	0.5	CRA004775;PRJNA827465	Yang2025_Silkie_Wenchang	0.905279419
ENSGALG00010025490	ENSGALG00010025490	2	0.849656767	1.414362498	0.600731855	0.548016668	3.677173993	12.32795505	91.88440474	0.5	PRJNA827465;Yang2025_Silkie_Wenchang	CRA004775	0.878256266
ENSGALG00010025490	ENSGALG00010025490	2	1.12926824	1.198084836	0.942610064	0.34588039	2.412356529	6.081584971	83.55691806	0.5	CRA004775;Yang2025_Silkie_Wenchang	PRJNA827465	0.807492544
ENSGALG00010025490	ENSGALG00010025490	2	-0.434423187	0.49998815	-0.868866967	0.384919899	0	0.167702761	0	1	CRA004775;PRJNA827465	Yang2025_Silkie_Wenchang	0.826444827
ENSGALG00010025491	ZMYND11	2	0.014129074	0.096655715	0.146179396	0.883779771	0.010041877	1.825651781	45.22504178	0.5	PRJNA827465;Yang2025_Silkie_Wenchang	CRA004775	0.972001076
ENSGALG00010025491	ZMYND11	2	0.204270992	0.111204902	1.83688838	0.066225375	0	0.212367168	0	1	CRA004775;Yang2025_Silkie_Wenchang	PRJNA827465	0.44312142
ENSGALG00010025491	ZMYND11	2	0.065673336	0.151602235	0.433195041	0.664873081	0.032877134	2.968863145	66.31707319	0.5	CRA004775;PRJNA827465	Yang2025_Silkie_Wenchang	0.913399315
ENSGALG00010025496	ATG101	2	-0.003324074	0.067331779	-0.049368569	0.960625576	0	0.133433121	0	0.5	PRJNA827465;Yang2025_Silkie_Wenchang	CRA004775	0.989511782
ENSGALG00010025496	ATG101	2	-0.103451529	0.154464738	-0.669742039	0.503022248	0	0.07972779	0	1	CRA004775;Yang2025_Silkie_Wenchang	PRJNA827465	0.863050365
ENSGALG00010025496	ATG101	2	-0.036054668	0.069058602	-0.09471185	0.92454706	0	0.396260587	0	0.5	CRA004775;PRJNA827465	Yang2025_Silkie_Wenchang	0.982382364
ENSGALG00010025499	STAC3	2	0.494146561	0.5083853	0.971992228	0.331054415	0.497004413	25.91494913	96.14122337	0.5	PRJNA827465;Yang2025_Silkie_Wenchang	CRA004775	0.801722786
ENSGALG00010025499	STAC3	2	0.481768326	0.661826539	0.727937453	0.466651875	0.689190318	3.847614886	74.00987288	0.5	CRA004775;Yang2025_Silkie_Wenchang	PRJNA827465	0.852842649
ENSGALG00010025499	STAC3	2	-0.036123779	0.152778348	-0.236445758	0.813086793	0	0.245362016	0	1	CRA004775;PRJNA827465	Yang2025_Silkie_Wenchang	0.959352616
ENSGALG00010025504	AMPD1	2	-0.191370334	0.097838884	-1.956038246	0.050460649	0	0.352611597	0	0.5	PRJNA827465;Yang2025_Silkie_Wenchang	CRA004775	0.389039127
ENSGALG00010025504	AMPD1	2	-0.127025398	0.149767059	-0.848153119	0.396352702	0	0.010215535	0	1	CRA004775;Yang2025_Silkie_Wenchang	PRJNA827465	0.831365249
ENSGALG00010025504	AMPD1	2	-0.24196863	0.127853038	-1.892552842	0.05841736	0	0.002159596	0	1	CRA004775;PRJNA827465	Yang2025_Silkie_Wenchang	0.418744757
ENSGALG00010025505	ENSGALG00010025505	2	-0.244946389	0.270034568	-0.907092917	0.364337772	0.054275002	1.582409958	36.80489166	1	PRJNA827465;Yang2025_Silkie_Wenchang	CRA004775	0.831749625
ENSGALG00010025505	ENSGALG00010025505	2	0.086132369	0.264211452	0.325997863	0.744425972	0	0.984270029	0	0.5	CRA004775;Yang2025_Silkie_Wenchang	PRJNA827465	0.935846381
ENSGALG00010025505	ENSGALG00010025505	2	-0.04467834	0.675092912	-0.066181024	0.947233704	0.597407989	2.499696162	59.995136	0.5	CRA004775;PRJNA827465	Yang2025_Silkie_Wenchang	0.986445196
ENSGALG00010025506	R3HDM2	2	0.284895558	0.31840292	0.894764276	0.370913111	0.182346317	9.5255427	89.50191048	0.5	PRJNA827465;Yang2025_Silkie_Wenchang	CRA004775	0.8214684
ENSGALG00010025506	R3HDM2	2	0.264374978	0.378792466	0.697946561	0.485250785	0.238592466	5.872920307	82.9726959	0.5	CRA004775;Yang2025_Silkie_Wenchang	PRJNA827465	0.857504733
ENSGALG00010025506	R3HDM2	2	-0.024773052	0.08086308	-0.306358008	0.759332077	0	0.20652731	0	1	CRA004775;PRJNA827465	Yang2025_Silkie_Wenchang	0.939145717
ENSGALG00010025508	ENSGALG00010025508	2	-0.06991561	0.0569841	-1.226931891	0.219848191	0	0.257809833	0	1	PRJNA827465;Yang2025_Silkie_Wenchang	CRA004775	0.733380873
ENSGALG00010025508	ENSGALG00010025508	2	-0.118439199	0.114434703	-1.209439862	0.226402286	0	0.109529834	0	1	CRA004775;Yang2025_Silkie_Wenchang	PRJNA827465	0.739815009
ENSGALG00010025508	ENSGALG00010025508	2	-0.061359891	0.063298676	-0.969370838	0.332360197	0	0.304708137	0	1	CRA004775;PRJNA827465	Yang2025_Silkie_Wenchang	0.802178578
ENSGALG00010025512	NRAS	2	0.060517836	0.038297119	1.580219048	0.114056711	0	0.787042502	0	1	PRJNA827465;Yang2025_Silkie_Wenchang	CRA004775	0.569819615
ENSGALG00010025512	NRAS	2	-0.017186039	0.065642693	-0.261811917	0.793464451	0	0.570915077	0	1	CRA004775;Yang2025_Silkie_Wenchang	PRJNA827465	0.948740761
ENSGALG00010025512	NRAS	2	0.032865366	0.087348981	0.376253563	0.706728398	0.007976944	1.61037093	37.99792605	0.5	CRA004775;PRJNA827465	Yang2025_Silkie_Wenchang	0.932453846
ENSGALG00010025513	RSRC2	2	0.288402407	0.265037369	1.088157524	0.276525573	0.131344432	14.73247611	93.21227475	1	PRJNA827465;Yang2025_Silkie_Wenchang	CRA004775	0.775851484
ENSGALG00010025513	RSRC2	2	0.427012233	0.162388169	2.629576758	0.008549123	0.029085008	2.185622648	54.09483838	1	CRA004775;Yang2025_Silkie_Wenchang	PRJNA827465	0.14427058
ENSGALG00010025513	RSRC2	2	0.064430803	0.068903133	0.935090594	0.34974863	0.003597948	1.211708079	17.47187157	1	CRA004775;PRJNA827465	Yang2025_Silkie_Wenchang	0.809803518
ENSGALG00010025520	ACOT11	2	0.26431951	0.199581902	1.324366123	0.18538148	0.05189296	2.564028792	60.99887789	1	PRJNA827465;Yang2025_Silkie_Wenchang	CRA004775	0.693843281
ENSGALG00010025520	ACOT11	2	0.454713803	0.215174304	2.113324686	0.034580686	0	0.552893788	0	1	CRA004775;Yang2025_Silkie_Wenchang	PRJNA827465	0.320322559
ENSGALG00010025520	ACOT11	2	0.108848421	0.106294489	1.02402695	0.305822546	0	0.003393101	0	1	CRA004775;PRJNA827465	Yang2025_Silkie_Wenchang	0.79225794
ENSGALG00010025522	SUMO3	2	-0.098881282	0.051581556	-1.916989128	0.055239313	0	0.433357295	0	1	PRJNA827465;Yang2025_Silkie_Wenchang	CRA004775	0.407036233
ENSGALG00010025522	SUMO3	2	-0.154318195	0.094940557	-1.62541911	0.104073287	0	0.032485638	0	1	CRA004775;Yang2025_Silkie_Wenchang	PRJNA827465	0.548383482
ENSGALG00010025522	SUMO3	2	-0.086334164	0.054869911	-1.573433642	0.115618442	0	0.066094979	0	1	CRA004775;PRJNA827465	Yang2025_Silkie_Wenchang	0.572716099
ENSGALG00010025523	B4GALT4	2	0.064338195	0.098050268	0.656175618	0.511711135	0	0.493667483	0	1	PRJNA827465;Yang2025_Silkie_Wenchang	CRA004775	0.865694231
ENSGALG00010025523	B4GALT4	2	0.205108827	0.147725297	1.388447549	0.165000806	0	0.386124659	0	1	CRA004775;Yang2025_Silkie_Wenchang	PRJNA827465	0.664342845
ENSGALG00010025523	B4GALT4	2	0.076980296	0.139498973	0.551834145	0.581061991	0.008666243	1.143807484	12.57270006	1	CRA004775;PRJNA827465	Yang2025_Silkie_Wenchang	0.888894576
ENSGALG00010025527	KNTC1	2	-0.057952286	0.096297665	-0.601803643	0.54730485	0	0.013458522	0	1	PRJNA827465;Yang2025_Silkie_Wenchang	CRA004775	0.878281266
ENSGALG00010025527	KNTC1	2	-0.064385512	0.142280631	-0.304226319	0.760955475	0	0.007084374	0	1	CRA004775;Yang2025_Silkie_Wenchang	PRJNA827465	0.939493404
ENSGALG00010025527	KNTC1	2	-0.065630269	0.127469344	-0.514871004	0.606643164	0	0.012566501	0	0.5	CRA004775;PRJNA827465	Yang2025_Silkie_Wenchang	0.896799223
ENSGALG00010025531	ENSGALG00010025531	2	0.054895432	0.117295722	0.468008819	0.639778283	0.016491728	2.365769772	57.73046001	0.5	PRJNA827465;Yang2025_Silkie_Wenchang	CRA004775	0.906064918
ENSGALG00010025531	ENSGALG00010025531	2	-0.089495055	0.107677771	-0.831137701	0.405895844	0	0.000646341	0	1	CRA004775;Yang2025_Silkie_Wenchang	PRJNA827465	0.833416425
ENSGALG00010025531	ENSGALG00010025531	2	0.085380431	0.108692402	0.78552346	0.432146721	0.009587229	1.470466775	31.9943832	0.5	CRA004775;PRJNA827465	Yang2025_Silkie_Wenchang	0.842618573
ENSGALG00010025533	INHBC	2	0.351019856	0.399981165	0.877590964	0.380165735	0.099378477	1.445071346	30.7992645	0.5	PRJNA827465;Yang2025_Silkie_Wenchang	CRA004775	0.824392515
ENSGALG00010025533	INHBC	2	-0.121365322	0.290347297	-0.418000523	0.675946735	0	0.105409439	0	1	CRA004775;Yang2025_Silkie_Wenchang	PRJNA827465	0.916530594

ENSGALG00010025533	INHBC	2	0.240530971	0.493993068	0.486911632	0.626320956	0.292500988	2.455290752	59.27162601	0.5	CRA004775;PRJNA827465	Yang2025_Silkie_Wenchang	0.901504244
ENSGALG00010025538	ANO1	2	0.058767349	0.203359855	0.288982056	0.772595109	0.049367496	2.469354859	59.50359275	0.5	PRJNA827465;Yang2025_Silkie_Wenchang	CRA004775	0.943066211
ENSGALG00010025538	ANO1	2	0.256180555	0.15240631	1.680905177	0.092781334	0	0.004392074	0	1	CRA004775;Yang2025_Silkie_Wenchang	PRJNA827465	0.52167685
ENSGALG00010025538	ANO1	2	-0.030640987	0.196360749	-0.156044357	0.875998058	0.016123925	1.208138174	17.22801072	0.5	CRA004775;PRJNA827465	Yang2025_Silkie_Wenchang	0.970347777
ENSGALG00010025541	TTC21B	2	0.138592634	0.140311178	0.987751907	0.323274166	0.030587811	4.35049627	77.0141166	1	PRJNA827465;Yang2025_Silkie_Wenchang	CRA004775	0.79919767
ENSGALG00010025541	TTC21B	2	0.219440782	0.133205774	1.647381904	0.099479573	0.010277868	1.259858632	20.6260151	0.5	CRA004775;Yang2025_Silkie_Wenchang	PRJNA827465	0.538620422
ENSGALG00010025541	TTC21B	2	0.008194963	0.070904429	0.115577592	0.907987323	0	0.015973013	0	0.5	CRA004775;PRJNA827465	Yang2025_Silkie_Wenchang	0.978196186
ENSGALG00010025544	CSDE1	2	0.043843783	0.127726445	0.343263159	0.731400494	0.027249991	5.493669241	81.79722957	0.5	PRJNA827465;Yang2025_Silkie_Wenchang	CRA004775	0.932851968
ENSGALG00010025544	CSDE1	2	0.2196299	0.090617902	2.423692166	0.015363622	0	0.302116054	0	1	CRA004775;Yang2025_Silkie_Wenchang	PRJNA827465	0.205049211
ENSGALG00010025544	CSDE1	2	0.076980072	0.182027086	0.422904493	0.672364945	0.052493728	3.99064186	74.94137447	0.5	CRA004775;PRJNA827465	Yang2025_Silkie_Wenchang	0.914742762
ENSGALG00010025548	ENSGALG00010025548	2	0.051627022	0.109624477	0.470944299	0.637680512	0	0.52899998	0	0.5	PRJNA827465;Yang2025_Silkie_Wenchang	CRA004775	0.905279419
ENSGALG00010025548	ENSGALG00010025548	2	0.558734466	0.664171313	0.841250525	0.400207601	0.775588657	7.753967852	87.10337702	0.5	CRA004775;Yang2025_Silkie_Wenchang	PRJNA827465	0.832246851
ENSGALG00010025548	ENSGALG00010025548	2	0.627084108	0.581056128	1.079214345	0.280492186	0.576811282	6.233460267	83.95754593	1	CRA004775;PRJNA827465	Yang2025_Silkie_Wenchang	0.778536485
ENSGALG00010025549	DHCR24	2	0.242134171	0.171181887	1.414484766	0.157219617	0	0.097213449	0	1	PRJNA827465;Yang2025_Silkie_Wenchang	CRA004775	0.650687662
ENSGALG00010025549	DHCR24	2	0.112193314	0.257973913	0.434901781	0.663633722	0	0.142077831	0	0.5	CRA004775;Yang2025_Silkie_Wenchang	PRJNA827465	0.913119244
ENSGALG00010025549	DHCR24	2	0.233254057	0.194929868	1.196605009	0.231460547	0	0.361548864	0	0.5	CRA004775;PRJNA827465	Yang2025_Silkie_Wenchang	0.743924694
ENSGALG00010025553	UBE2G2	2	-0.152687289	0.18434194	-0.828282965	0.407510268	0.050028238	3.231682775	69.05636878	1	PRJNA827465;Yang2025_Silkie_Wenchang	CRA004775	0.833925651
ENSGALG00010025553	UBE2G2	2	-0.348511967	0.134022541	-2.60039814	0.009311566	0	0.077876369	0	1	CRA004775;Yang2025_Silkie_Wenchang	PRJNA827465	0.151478671
ENSGALG00010025553	UBE2G2	2	-0.116675116	0.145683164	-0.800882634	0.423199594	0.028268302	2.53980915	60.62696286	1	CRA004775;PRJNA827465	Yang2025_Silkie_Wenchang	0.840095671
ENSGALG00010025554	DIP2C	2	-0.012489809	0.115002586	-0.108604593	0.913516118	0.017718339	2.791731441	64.1799356	0.5	PRJNA827465;Yang2025_Silkie_Wenchang	CRA004775	0.979269146
ENSGALG00010025554	DIP2C	2	0.106062509	0.315966144	0.335676814	0.737114592	0.151939786	3.743134006	73.28441893	0.5	CRA004775;Yang2025_Silkie_Wenchang	PRJNA827465	0.93387966
ENSGALG00010025554	DIP2C	2	0.17417935	0.171563587	1.01524661	0.309988287	0.033885111	1.681249683	40.52043487	1	CRA004775;PRJNA827465	Yang2025_Silkie_Wenchang	0.794395438
ENSGALG00010025557	VPS29	2	-0.529299611	0.532920317	-0.993205914	0.320609617	0.553315213	38.04136522	97.37128257	1	PRJNA827465;Yang2025_Silkie_Wenchang	CRA004775	0.798348216
ENSGALG00010025557	VPS29	2	-0.659717205	0.420411162	-1.56921905	0.116596907	0.321145084	10.90026005	90.82590694	1	CRA004775;Yang2025_Silkie_Wenchang	PRJNA827465	0.57477217
ENSGALG00010025557	VPS29	2	-0.042167142	0.081423027	-0.517877348	0.604543848	0.004701257	1.227747271	18.55001243	1	CRA004775;PRJNA827465	Yang2025_Silkie_Wenchang	0.896276948
ENSGALG00010025558	GALNT3	2	0.952285581	0.808458554	1.177902783	0.238835366	1.233332857	17.6215405	94.32512725	1	PRJNA827465;Yang2025_Silkie_Wenchang	CRA004775	0.750835576
ENSGALG00010025558	GALNT3	2	0.908602086	0.968877823	0.937788093	0.348353321	1.592112167	6.142329722	83.7195324	0.5	CRA004775;Yang2025_Silkie_Wenchang	PRJNA827465	0.809042603
ENSGALG00010025558	GALNT3	2	0.125530013	0.220599879	0.569039356	0.569329432	0	0.191959958	0	0.5	CRA004775;PRJNA827465	Yang2025_Silkie_Wenchang	0.88472065
ENSGALG00010025562	GRAMD1B	2	-0.133923488	0.083574727	-1.602440021	0.109058341	0	0.020099814	0	1	PRJNA827465;Yang2025_Silkie_Wenchang	CRA004775	0.559060907
ENSGALG00010025562	GRAMD1B	2	-0.167446239	0.150354984	-1.113672681	0.265419633	0	0.077981488	0	1	CRA004775;Yang2025_Silkie_Wenchang	PRJNA827465	0.769476127
ENSGALG00010025562	GRAMD1B	2	-0.133602136	0.096135682	-1.389724743	0.164612478	0	0.118699987	0	1	CRA004775;PRJNA827465	Yang2025_Silkie_Wenchang	0.663901457
ENSGALG00010025563	TSPEAR	2	0.266451841	0.18576184	1.434373393	0.151465736	0	0.013067987	0	1	PRJNA827465;Yang2025_Silkie_Wenchang	CRA004775	0.643006634
ENSGALG00010025563	TSPEAR	2	0.260493133	0.242122278	1.07587429	0.281983482	0	0.03258253	0	1	CRA004775;Yang2025_Silkie_Wenchang	PRJNA827465	0.77949056
ENSGALG00010025563	TSPEAR	2	0.238248776	0.201374959	1.183110238	0.236765461	0	0.010145573	0	1	CRA004775;PRJNA827465	Yang2025_Silkie_Wenchang	0.749020867
ENSGALG00010025565	ENSGALG00010025565	2	-0.061390257	0.277446477	-0.221268829	0.824883119	0.133919197	7.63105602	86.89565379	0.5	PRJNA827465;Yang2025_Silkie_Wenchang	CRA004775	0.956260685
ENSGALG00010025565	ENSGALG00010025565	2	-0.359235483	0.149255485	-2.406849458	0.016090801	0	0.043499616	0	1	CRA004775;Yang2025_Silkie_Wenchang	PRJNA827465	0.209635104
ENSGALG00010025565	ENSGALG00010025565	2	0.016904875	0.296583959	0.056998616	0.954546298	0.108975273	2.048642521	51.1871891	0.5	CRA004775;PRJNA827465	Yang2025_Silkie_Wenchang	0.987987175
ENSGALG00010025569	PPTC7	2	0.134122281	0.042607321	3.147869375	0.001644652	0	0.252354286	0	1	PRJNA827465;Yang2025_Silkie_Wenchang	CRA004775	0.051226717
ENSGALG00010025569	PPTC7	2	0.367244796	0.308812891	1.189214592	0.234355234	0.135320449	2.583097847	61.28679364	1	CRA004775;Yang2025_Silkie_Wenchang	PRJNA827465	0.747486154
ENSGALG00010025569	PPTC7	2	0.352724141	0.334324613	1.055034919	0.291409355	0.165149067	2.939700833	65.98293307	1	CRA004775;PRJNA827465	Yang2025_Silkie_Wenchang	0.784817029
ENSGALG00010025571	SRSF3	2	0.172231403	0.129576565	1.329186359	0.183786494	0.027022007	4.732060687	78.86755758	1	PRJNA827465;Yang2025_Silkie_Wenchang	CRA004775	0.692423369
ENSGALG00010025571	SRSF3	2	-0.09243713	0.436641798	-0.211700141	0.832340978	0.349997722	11.8043958	91.52857955	0.5	CRA004775;Yang2025_Silkie_Wenchang	PRJNA827465	0.957808251
ENSGALG00010025571	SRSF3	2	-0.205506853	0.303610472	-0.676876696	0.498484191	0.160170056	6.811925882	85.31986376	0.5	CRA004775;PRJNA827465	Yang2025_Silkie_Wenchang	0.861478809
ENSGALG00010025572	ENSGALG00010025572	2	0.079943929	0.051386707	1.555731707	0.119771904	0.00083309	1.074694529	6.950303297	1	PRJNA827465;Yang2025_Silkie_Wenchang	CRA004775	0.580639051
ENSGALG00010025572	ENSGALG00010025572	2	-0.048497911	0.269379784	-0.18003545	0.857124738	0.122016091	6.263992487	84.03574075	0.5	CRA004775;Yang2025_Silkie_Wenchang	PRJNA827465	0.963878155
ENSGALG00010025572	ENSGALG00010025572	2	-0.100280081	0.189453506	-0.529312352	0.596588788	0.05953201	5.188151391	80.72531188	0.5	CRA004775;PRJNA827465	Yang2025_Silkie_Wenchang	0.894064255
ENSGALG00010025573	CLMP	2	-0.258078493	0.196107131	-1.316007691	0.188171461	0.068571888	8.754419229	88.57719771	1	PRJNA827465;Yang2025_Silkie_Wenchang	CRA004775	0.697483156
ENSGALG00010025573	CLMP	2	-0.452550788	0.118632923	-3.81471499	0.00013634	0	0.331776978	0	1	CRA004775;Yang2025_Silkie_Wenchang	PRJNA827465	0.009757782
ENSGALG00010025573	CLMP	2	-0.0785381	0.050961693	-1.541120322	0.123287505	0	0.062659029	0	1	CRA004775;PRJNA827465	Yang2025_Silkie_Wenchang	0.588697461
ENSGALG00010025574	ARHGAP31	2	-0.153555336	0.184082059	-0.834167852	0.404186404	0.057304259	6.339243862	84.22524797	0.5	PRJNA827465;Yang2025_Silkie_Wenchang	CRA004775	0.833112102
ENSGALG00010025574	ARHGAP31	2	-0.26252883	0.182462633	-1.438808739	0.15020472	0.025881135	1.306403701	23.45398292	0.5	CRA004775;Yang2025_Silkie_Wenchang	PRJNA827465	0.640639837
ENSGALG00010025574	ARHGAP31	2	0.023467168	0.078917242	0.297364268	0.766188421	0	0.063454269	0	1	CRA004775;PRJNA827465	Yang2025_Silkie_Wenchang	0.941123716
ENSGALG00010025575	TRPM2	2	0.579118996	0.321071472	1.803707419	0.071277186	0	0.248284526	0	1	PRJNA827465;Yang2025_Silkie_Wenchang	CRA004775	0.461027938
ENSGALG00010025575	TRPM2	2	0.103337738	0.794188381	0.130117414	0.896473533	0.815509955	2.747887289	63.60840549	0.5	CRA004775;Yang2025_Silkie_Wenchang	PRJNA827465	0.975115773
ENSGALG00010025575	TRPM2	2	0.020110219	0.60328079	0.033334758	0.973407636	0.405480331	2.032951007	50.81042306	0.5	CRA004775;PRJNA827465	Yang2025_Silkie_Wenchang	0.992392211
ENSGALG00010025577	PTH2R	2	0.203630006	0.181654897	1.120971738	0.262299893	0	0.332773887	0	1	PRJNA827465;Yang2025_Silkie_Wenchang	CRA004775	0.768366452
ENSGALG00010025577	PTH2R	2	0.065932936	0.260293387	0.253302387	0.800034551	0	0.011094099	0	1	CRA004775;Yang2025_Silkie_Wenchang	PRJNA827465	0.949991177
ENSGALG00010025577	PTH2R	2	0.25755461	0.22231707	1.158501277	0.246659531	0	0.172007776	0	1	CRA004775;PRJNA827465	Yang2025_Silkie_Wenchang	0.757083915
ENSGALG00010025578	ORMDL2	2	0.402597095	0.37222185	1.081605218	0.279427985	0.261759515	17.7108567	94.35374574	1	PRJNA827465;Yang2025_Silkie_Wenchang	CRA004775	0.777457036
ENSGALG00010025578	ORMDL2	2	0.172671155	0.62049605	0.278279216	0.780798029	0.738224144	24.1673497	95.86218591	0.5	CRA004775;Yang2025_Silkie_Wenchang	PRJNA827465	0.945235709
ENSGALG00010025578	ORMDL2	2	-0.172515678	0.245920969	-0.701508613	0.482985661	0.100722175	5.503674861	81.83032201	0.5	CRA004775;PRJNA827465	Yang2025_Silkie_Wenchang	0.85691916
ENSGALG00010025580	RASSF7	2	-0.203429958	0.111300083	-1.827761058	0.06758542	0	0.120589556	0	1	PRJNA827465;Yang2025_Silkie_Wenchang	CRA004775	0.44825594
ENSGALG00010025580	RASSF7	2	-0.297461431	0.165756249	-1.79457144	0.072722007	0	0.523524043	0	1	CRA004775;Yang2025_Silkie_Wenchang	PRJNA827465	0.465709132
ENSGALG00010025580	RASSF7	2	-0.210410462	0.136507408	-1.541384941	0.123223127	0	0.816928935	0	1	CRA004775;PRJNA827465	Yang2025_Silkie_Wenchang	0.588575077
ENSGALG00010025582	DMAP1	2	-0.096943235	0.04365102	-2.220869894	0.026359775	0	0.246545473	0	1	PRJNA827465;Yang2025_Silkie_Wenchang	CRA004775	0.276862168
ENSGALG00010025582	DMAP1	2	-0.152951054	0.142523398	-1.073164523	0.283197305	0.022325255	1.715786836	41.71770182	1	CRA004775;Yang2025_Silkie_Wenchang	PRJNA827465	0.779568117
ENSGALG00010025582	DMAP1	2	-0.159345057	0.103588315	-1.5382532	0.123986718	0.009665331	1.319196141	24.19626099	1	CRA004775;PRJNA827465	Yang2025_Silkie_Wenchang	0.59047345
ENSGALG00010025585	SARNP	2	-0.008132869	0.082491544	-0.098590339	0.921463541	0.007635909	2.209128953	54.73328986	0.5	PRJNA827465;Yang2025_Silkie_Wenchang	CRA004775	0.981550846
ENSGALG00010025585	SARNP	2	-0.097652808	0.220946928	-0.441974049	0.658507985	0.074955039	3.929791168	74.5533552	0.5	CRA004775;Yang2025_Silkie_Wenchang	PRJNA827465	0.911105434
ENSGALG00010025585	SARNP	2	-0.150796367	0.122897674	-1.227007492	0.219819775	0.016185237	1.691638499	40.88571521	1	CRA004775;PRJNA827465	Yang2025_Silkie_Wenchang	0.733380873
ENSGALG00010025586	TCTN1	2	-0.112880072	0.070075003	-1.61084649	0.107213185	0.004755498	1.784305788	43.95579466	1	PRJNA827465;Yang2025_Silkie_Wenchang	CRA004775	0.554473355
ENSGALG00010025586	TCTN1	2	0.195119004	0.438387242	0.445083673	0.656259288	0.345956686	9.438221814	89.40478387	0.5	CRA004775;Yang2025_Silkie_Wenchang	PRJNA827465	0.910446833
ENSGALG00010025586	TCTN1	2	0.256959478	0.363195319	0.707496667	0.479257884	0.230656284	7.11606623	85.94729212	0.5	CRA004775;PRJNA827465	Yang2025_Silkie_Wenchang	0.856035423
ENSGALG00010025588	ENSGALG00010025588	2	-0.212002461	0.194680905	-1.088974087	0.276165313	0	0.018034456	0	1	PRJNA827465;Yang2025_Silkie_Wenchang	CRA004775	0.775682505
ENSGALG00010025588	ENSGALG00010025588	2	-0.225982983	0.237387317	-0.951958958	0.341117797	0	0.066665539	0	1	CRA004775;Yang2025_Silkie_Wenchang	PRJNA827465	0.805646109
ENSGALG00010025588	ENSGALG00010025588	2	-0.258167411	0.222228748	-1.161719236	0.245349529	0	0.025268493	0	1	CRA004775;PRJNA827465	Yang2025_Silkie_Wenchang	0.75661406
ENSGALG00010025590	HRAS	2	0.014249875	0.156694185	0.090940677	0.927539728	0.038484983	4.567694353	78.10711657	0.5	PRJNA827465;Yang2025_Silkie_Wenchang	CRA004775	0.982993195
ENSGALG00010025590	HRAS	2	0.158006251	0.10936288	1.444788677	0.148517253	0	0.295217719	0	0.5	CRA004775;Yang2025_Silkie_Wenchang	PRJNA827465	0.63857057
ENSGALG00010025590	HRAS	2	-0.123506239	0.085601921	-1.442797515	0.149077519	0	0.1697868	0	1	CRA004775;PRJNA827465	Yang2025_Silkie_Wenchang	0.639246608
ENSGALG00010025591	OTOS	2	0.076559167	0.257569652	0.297236753	0.766285764	0.051380333	1.62969006	38.63863907	0.5	PRJNA827465;Yang2025_Silkie_Wenchang	CRA004775	0.941123716
ENSGALG00010025591	OTOS	2	0.306908285	0.247287261	1.241100264	0.214568712	0	0.058757765	0	1	CRA004775;Yang2025_Silkie_Wenchang	PRJNA827465	0.727312987
ENSGALG00010025591	OTOS	2	-0.064237171	0.234167039	-0.274322001	0.783837178	0	0.536753315	0	0.5	CRA004775;PRJNA827465	Yang2025_Silkie_Wenchang	0.945884474
ENSGALG00010025592	HSPA8	2	0.26204422	0.116911827	2.241383326	0.025001256	0	0.728151349	0	1	PRJNA827465;Yang2025_Silkie_Wenchang	CRA004775	0.269433603
ENSGALG00010025592	HSPA8	2	0.13196153	0.16066358	0.821353102	0.411445169	0	0.001741668	0	1	CRA004775;Yang2025_Silkie_Wenchang	PRJNA827465	0.835128673
ENSGALG00010025592	HSPA8	2	0.299246116	0.134220092	2.229518031	0.025779457	0	0.413572466	0	1	CRA004775;PRJNA827465	Yang2025_Silkie_Wenchang	0.273678181
ENSGALG00010025595	ERI3	2	-0.017066825	0.062161808	-0.274554833	0.783658271	0	0.68955535	0	0.5	PRJNA827465;Yang2025_Silkie_Wenchang	CRA004775	0.945815798
ENSGALG00010025595	ERI3	2	0.012186906	0.085787165	0.14205978	0.887032787	0	0.84996936	0	0.5	CRA004775;Yang2025_Silkie_Wenchang	PRJNA827465	0.972937943
ENSGALG00010025595	ERI3	2	-0.080857235	0.082503801	-0.980042541	0.32706512	0	0.339052937	0	1	CRA004775;PRJNA827465	Yang2025_Silkie_Wenchang	0.800028239
ENSGALG00010025598	ENSGALG00010025598	2	0.610299903	0.438456003	1.391929633	0.163943707	0.232796052	2.473400711	59.56983455	1	PRJNA827465;Yang2025_Silkie_Wenchang	CRA004775	0.662500466
ENSGALG00010025598	ENSGALG00010025598	2	0.344462857	0.736761655	0.467536353	0.640116189	0.918918362	6.464803194	84.53162502	0.5	CRA004775;Yang2025_Silkie_Wenchang	PRJNA827465	0.906122334
ENSGALG00010025598	ENSGALG00010025598	2	-0.053592451	0.294709157	-0.181848611	0.855701534	0.055795849	1.470840943	32.01168319	0.5	CRA004775;PRJNA827465	Yang2025_Silkie_Wenchang	0.963486168
ENSGALG00010025599	COPS9	2	0.114231087	0.264978239	0.431096107	0.666398491	0.11938776	6.224057112	83.93330938	0.5	PRJNA827465;Yang2025_Silkie_Wenchang	CRA004775	0.913579507
ENSGALG00010025599	COPS9	2	0.31334094	0.168978287	1.854326644	0.063692442	0	0.920860547	0	1	CRA004775;Yang2025_Silkie_Wenchang	PRJNA827465	0.434844802
ENSGALG00010025599	COPS9	2	-0.112418129	0.079904659	-1.406903298	0.159456066	0	0.243240917	0	0.5	CRA004775;PRJNA827465	Yang2025_Silkie_Wenchang	0.655006453
ENSGALG00010025600	ENSGALG00010025600	2	0.063251136	0.040374479	1.566611832	0.11720545	0	0.923724357	0	1	PRJNA827465;Yang2025_Silkie_Wenchang	CRA004775	0.57646425
ENSGALG00010025600	ENSGALG00010025600	2	-0.030946381	0.123297931	-0.25098865	0.801822884	0.011692925	1.142117973	12.44337068	0.5	CRA004775;Yang2025_Silkie_Wenchang	PRJNA827465	0.950449221
ENSGALG00010025600	ENSGALG00010025600	2	-0.015659569	0.208490771	-0.075109171	0.940127851	0.052270278	1.645380669	39.22379067	0.5	CRA004775;PRJNA827465	Yang2025_Silkie_Wenchang	0.985486876
ENSGALG00010025604	HVCN1	2	-0.187089086	0.211079118	-0.886345783	0.375431225	0.065139266	3.454935354	71.05589837	1	PRJNA827465;Yang2025_Silkie_Wenchang	CRA004775	0.822974013
ENSGALG00010025604	HVCN1	2	0.092826679	0.554346464	0.167452459	0.867014051	0.546141466	8.833571398	88.67955038	0.5	CRA004775;Yang2025_Silkie_Wenchang	PRJNA827465	0.967699
ENSGALG00010025604	HVCN1	2	0.268663399	0.334879287	0.802269383	0.422397149	0.177930282	4.271662687	76.58991186	0.5	CRA004775;PRJNA827465	Yang2025_Silkie_Wenchang	0.839704389
ENSGALG00010025606	CCDC12	2	-0.058238528	0.054264276	-1.073238833	0.283163972	0	0.012558752	0	1	PRJNA827465;Yang2025_Silkie_Wenchang	CRA004775	0.779568117
ENSGALG00010025606	CCDC12	2	-0.090510391	0.126279994	-0.716743702	0.473532255	0	0.079475134	0	1	CRA004775;Yang2025_Silkie_Wenchang	PRJNA827465	0.854373829
ENSGALG00010025606	CCDC12	2	-0.060083406	0.057569949	-1.043659187	0.296643099	0	0.134919203	0	1	CRA004775;PRJNA827465	Yang2025_Silkie_Wenchang	0.788458494
ENSGALG00010025608	ENSGALG00010025608	2	-1.017687179	0.425753424	-2.390320599	0.016833671	0	0.002923284	0	1	PRJNA827465;Yang2025_Silkie_Wenchang	CRA004775	0.215439197
ENSGALG00010025608	ENSGALG00010025608	2	-0.184943758	0.666967325	-0.277290583	0.78155699	0.505724639	2.223933804	55.03463286	0.5	CRA004775;Yang2025_Silkie_Wenchang	PRJNA827465	0.945249331
ENSGALG00010025608	ENSGALG00010025608	2	-0.325306247	0.702611748	-0.462995741	0.64336742	0.730591869	3.845079047	73.99273233	0.5	CRA004775;PRJNA827465	Yang2025_Silkie_Wenchang	0.906634523
ENSGALG00010025610	RNH1	2	-0.170025172	0.236723836	-0.718242723	0.47260764	0.092170577	5.090152585	80.35422351	0.5	PRJNA827465;Yang2025_Silkie_Wenchang	CRA004775	0.853998373
ENSGALG00010025610	RNH1	2	-0.338566723	0.135213658	-2.503938794	0.012281928	0	0.531585483	0	1	CRA004775;Yang2025_Silkie_Wenchang	PRJNA827465	0.179115626
ENSGALG00010025610	RNH1	2	-0.056523601	0.131041946	-0.43133976	0.666221344	0.020053547	2.030646837	50.75460776	0.5	CRA004775;PRJNA827465	Yang2025_Silkie_Wenchang	0.913579507
ENSGALG00010025612	TMEM53	2	-0.019980423	0.174465017	-0.114523953	0.90882246	0.040187789	2.616430944	61.77999643	0.5	PRJNA827465;Yang2025_Silkie_Wenchang	CRA004775	0.978387968
ENSGALG00010025612	TMEM53	2	-0.333340981	0.167422571	-1.991015779	0.046479153	0	0.573052982	0	1	CRA004775;Yang2025_Silkie_Wenchang	PRJNA827465	0.372278405
ENSGALG00010025612	TMEM53	2	-0.139041684	0.309784365	-0.44883377	0.653551576	0.151958287	4.184041973	76.09966615	0.5	CRA004775;PRJNA827465	Yang2025_Silkie_Wenchang	0.90967658
ENSGALG00010025613	CDKN1A	2	0.330585239	0.168984919	1.956300249	0.050429795	0.016594925	1.333682197	25.01961847	1	PRJNA827465;Yang2025_Silkie_Wenchang	CRA004775	0.38897029
ENSGALG00010025613	CDKN1A	2	0.102332126	0.240862009	0.424857892	0.670940272	0	0.028739683	0	1	CRA004775;Yang2025_Silkie_Wenchang	PRJNA827465	0.914552186
ENSGALG00010025613	CDKN1A	2	0.421711363	0.151113103	2.790700168	0.005259417	0	0.245509594	0	1	CRA004775;PRJNA827465	Yang2025_Silkie_Wenchang	0.108331963
ENSGALG00010025618	ADIPOQ	2	-0.093635604	0.112509061	-0.832249451	0.405268157	0	0.017476819	0	1	PRJNA827465;Yang2025_Silkie_Wenchang	CRA004775	0.833112102
ENSGALG00010025618	ADIPOQ	2	-0.040405413	0.176536386	-0.22887867	0.81896322	0	0.870540057	0	0.5	CRA004775;Yang2025_Silkie_Wenchang	PRJNA827465	0.955256039
ENSGALG00010025618	ADIPOQ	2	-0.044772231	0.131644474	-0.340099583	0.733781535	0	0.818018572	0	0.5	CRA004775;PRJNA827465	Yang2025_Silkie_Wenchang	0.933391141
ENSGALG00010025621	PPP1CC	2	-0.21210334	0.1381145	-1.535706539	0.124610371	0.03483001	10.96714716	90.88185847	1	PRJNA827465;Yang2025_Silkie_Wenchang	CRA004775	0.592140711
ENSGALG00010025621	PPP1CC	2	-0.344728286	0.075838227	-4.545574147	5.47857E-06	0	0.713748883	0	1	CRA004775;Yang2025_Silkie_Wenchang	PRJNA827465	0.000878875
ENSGALG00010025621	PPP1CC	2	-0.082900371	0.030165925	-2.748146178	0.005993328	0	0.000895415	0	1	CRA004775;PRJNA827465	Yang2025_Silkie_Wenchang	0.117017277
ENSGALG00010025623	ENSGALG00010025623	2	0.327666548	0.285213006	1.148848548	0.250618437	0	0.408524082	0	1	PRJNA827465;Yang2025_Silkie_Wenchang	CRA004775	0.760152524
ENSGALG00010025623	ENSGALG00010025623	2	-0.03258904	0.59298439	-0.054957669	0.956172188	0.487331069	3.243957245	69.17345315	0.5	CRA004775;Yang2025_Silkie_Wenchang	PRJNA827465	0.98851389
ENSGALG00010025623	ENSGALG00010025623	2	-0.178503788	0.40472197	-0.441052873	0.65917472	0.140307169	1.718716223	41.8170384	0.5	CRA004775;PRJNA827465	Yang2025_Silkie_Wenchang	0.911449691
ENSGALG00010025624	ARMH1	2	0.468825795	0.196280545	2.388549485	0.01691503	0	0.68282113	0	1	PRJNA827465;Yang2025_Silkie_Wenchang	CRA004775	0.216007777
ENSGALG00010025624	ARMH1	2	0.16188242	0.523573987	0.309187285	0.757179065	0.427149379	4.462901897	77.59305441	0.5	CRA004775;Yang2025_Silkie_Wenchang	PRJNA827465	0.938795135
ENSGALG00010025624	ARMH1	2	0.027264759	0.355706809	0.076649529	0.938902357	0.14740615	2.299579192	56.51378289	0.5	CRA004775;PRJNA827465	Yang2025_Silkie_Wenchang	0.985349045
ENSGALG00010025629	ST6GAL1	2	-0.034591015	0.061193636	-0.565271454	0.571889142	0	0.118858468	0	1	PRJNA827465;Yang2025_Silkie_Wenchang	CRA004775	0.885477425
ENSGALG00010025629	ST6GAL1	2	0.111726699	0.218759071	0.510729448	0.609540518	0.067841462	3.070291721	67.42980502	0.5	CRA004775;Yang2025_Silkie_Wenchang	PRJNA827465	0.897238578
ENSGALG00010025629	ST6GAL1	2	0.122489867	0.192884243	0.635043408	0.525400105	0.051412565	2.692003335	62.8529435	0.5	CRA004775;PRJNA827465	Yang2025_Silkie_Wenchang	0.870215879
ENSGALG00010025635	CHKA	2	0.096901109	0.074758073	1.296195917	0.194907996	0	0.038018425	0	1	PRJNA827465;Yang2025_Silkie_Wenchang	CRA004775	0.706054964
ENSGALG00010025635	CHKA	2	0.14162998	0.121328933	1.167322386	0.243080202	0	0.683346383	0	1	CRA004775;Yang2025_Silkie_Wenchang	PRJNA827465	0.7550024
ENSGALG00010025635	CHKA	2	0.131171811	0.080290718	1.633710772	0.102319595	0	0.618543304	0	1	CRA004775;PRJNA827465	Yang2025_Silkie_Wenchang	0.545029796
ENSGALG00010025637	C6orf89	2	-0.188312993	0.189663434	-0.992879804	0.320768533	0.067030638	14.50929753	93.10786757	1	PRJNA827465;Yang2025_Silkie_Wenchang	CRA004775	0.798348216
ENSGALG00010025637	C6orf89	2	-0.387152345	0.078091443	-4.957679456	7.13401E-07	0	0.024554348	0	1	CRA004775;Yang2025_Silkie_Wenchang	PRJNA827465	0.000169932
ENSGALG00010025637	C6orf89	2	-0.148150863	0.199291292	-0.743388545	0.457246477	0.057542352	2.897449785	65.48689109	1	CRA004775;PRJNA827465	Yang2025_Silkie_Wenchang	0.849541335
ENSGALG00010025642	RPS8	2	-0.175156657	0.105207555	-1.664867665	0.09593916	0.011043697	1.618574358	38.21723452	1	PRJNA827465;Yang2025_Silkie_Wenchang	CRA004775	0.5301492
ENSGALG00010025642	RPS8	2	-0.339729045	0.152201888	-2.232094822	0.025608697	0	0.021874979	0	1	CRA004775;Yang2025_Silkie_Wenchang	PRJNA827465	0.27294819
ENSGALG00010025642	RPS8	2	-0.121363153	0.060893783	-1.993030259	0.046258134	0	0.40499347	0	1	CRA004775;PRJNA827465	Yang2025_Silkie_Wenchang	0.370886053
ENSGALG00010025644	ENSGALG00010025644	2	0.230417192	0.234923405	0.980818374	0.326682316	0	0.095534099	0	1	PRJNA827465;Yang2025_Silkie_Wenchang	CRA004775	0.800028239
ENSGALG00010025644	ENSGALG00010025644	2	0.287056938	0.409628276	0.700774226	0.483443925	0	0.073335341	0	1	CRA004775;Yang2025_Silkie_Wenchang	PRJNA827465	0.85691916
ENSGALG00010025644	ENSGALG00010025644	2	0.187085068	0.244822763	0.764165334	0.444768722	0	0.004584724	0	1	CRA004775;PRJNA827465	Yang2025_Silkie_Wenchang	0.846013379
ENSGALG00010025645	NBEAL2	2	0.053050662	0.069547721	0.762795119	0.445585593	0	0.014648079	0	1	PRJNA827465;Yang2025_Silkie_Wenchang	CRA004775	0.846013379
ENSGALG00010025645	NBEAL2	2	-0.203943292	0.348375849	-0.585411681	0.558270936	0.177826306	3.195528764	68.70627449	0.5	CRA004775;Yang2025_Silkie_Wenchang	PRJNA827465	0.880678789
ENSGALG00010025645	NBEAL2	2	-0.203961069	0.336250128	-0.606575439	0.544132704	0.169508743	3.224881702	68.9911106	0.5	CRA004775;PRJNA827465	Yang2025_Silkie_Wenchang	0.877010098
ENSGALG00010025650	PPP1R1A	2	-0.04265331	0.244556019	-0.174411205	0.861542309	0.04097908	1.503095745	33.47063864	0.5	PRJNA827465;Yang2025_Silkie_Wenchang	CRA004775	0.965479094
ENSGALG00010025650	PPP1R1A	2	0.212782385	0.28526831	0.745902641	0.45572623	0	0.046721381	0	1	CRA004775;Yang2025_Silkie_Wenchang	PRJNA827465	0.84907427
ENSGALG00010025650	PPP1R1A	2	-0.210164078	0.234440689	-0.896448815	0.370013106	0	0.226775283	0	0.5	CRA004775;PRJNA827465	Yang2025_Silkie_Wenchang	0.820855964
ENSGALG00010025652	KMT5B	2	0.058504611	0.095993698	0.609463033	0.542217568	0.009730906	1.858651859	46.19756277	0.5	PRJNA827465;Yang2025_Silkie_Wenchang	CRA004775	0.876721948
ENSGALG00010025652	KMT5B	2	0.240478179	0.118534044	2.02876887	0.042481837	0	0.391859138	0	1	CRA004775;Yang2025_Silkie_Wenchang	PRJNA827465	0.35661334
ENSGALG00010025652	KMT5B	2	0.111286475	0.174985481	0.635975475	0.524792407	0.04092438	2.371923639	57.84012674	0.5	CRA004775;PRJNA827465	Yang2025_Silkie_Wenchang	0.869876873
ENSGALG00010025653	IGSF11	2	-0.03009275	0.088556987	-0.339812262	0.733997913	0	0.81089493	0	0.5	PRJNA827465;Yang2025_Silkie_Wenchang	CRA004775	0.933391141
ENSGALG00010025653	IGSF11	2	-0.12838169	0.125006988	-1.026996106	0.304422298	0	0.528236738	0	1	CRA004775;Yang2025_Silkie_Wenchang	PRJNA827465	0.79225794
ENSGALG00010025653	IGSF11	2	0.027299831	0.121702129	0.224316787	0.822510815	0	0.901661647	0	0.5	CRA004775;PRJNA827465	Yang2025_Silkie_Wenchang	0.955822987
ENSGALG00010025658	PDE1B	2	0.472480012	0.410242182	1.151709973	0.249440273	0.213547353	2.725345251	63.30740116	1	PRJNA827465;Yang2025_Silkie_Wenchang	CRA004775	0.759622031
ENSGALG00010025658	PDE1B	2	0.660335551	0.276717056	2.386320379	0.017017918	0	0.931207969	0	1	CRA004775;Yang2025_Silkie_Wenchang	PRJNA827465	0.216668366
ENSGALG00010025658	PDE1B	2	0.191507321	0.257102626	0.74486723	0.456351987	0	0.291374072	0	1	CRA004775;PRJNA827465	Yang2025_Silkie_Wenchang	0.849483857
ENSGALG00010025659	COBLL1	2	0.229196893	0.248221367	0.923356822	0.355821285	0.10227578	5.528884582	81.91316919	1	PRJNA827465;Yang2025_Silkie_Wenchang	CRA004775	0.812301302
ENSGALG00010025659	COBLL1	2	0.470468533	0.175514436	2.680511897	0.007350965	0	0.228113785	0	1	CRA004775;Yang2025_Silkie_Wenchang	PRJNA827465	0.131918938
ENSGALG00010025659	COBLL1	2	0.019489435	0.089217349	0.218448937	0.82707934	0	0.381315979	0	1	CRA004775;PRJNA827465	Yang2025_Silkie_Wenchang	0.956550752
ENSGALG00010025660	KIF2C	2	-0.025296268	0.087432542	-0.289323266	0.77233401	0	0.192905234	0	0.5	PRJNA827465;Yang2025_Silkie_Wenchang	CRA004775	0.943066211
ENSGALG00010025660	KIF2C	2	0.331850067	0.433280242	0.765901683	0.443734804	0.266263057	2.755961986	63.71502926	1	CRA004775;Yang2025_Silkie_Wenchang	PRJNA827465	0.845840387
ENSGALG00010025660	KIF2C	2	0.301577861	0.47681537	0.632483514	0.527070978	0.343294827	3.305223622	69.74486103	0.5	CRA004775;PRJNA827465	Yang2025_Silkie_Wenchang	0.87095077
ENSGALG00010025661	SNRPE	2	-0.129620543	0.202128761	-0.641277086	0.521342674	0.065650891	4.816462547	79.23787447	0.5	PRJNA827465;Yang2025_Silkie_Wenchang	CRA004775	0.869518515
ENSGALG00010025661	SNRPE	2	-0.020367634	0.171969479	-0.11843749	0.905721022	0.016612302	1.324074604	24.47555469	0.5	CRA004775;Yang2025_Silkie_Wenchang	PRJNA827465	0.978196186
ENSGALG00010025661	SNRPE	2	-0.305793674	0.082500465	-3.706569083	0.000210086	0	0.017964893	0	1	CRA004775;PRJNA827465	Yang2025_Silkie_Wenchang	0.013167626
ENSGALG00010025685	NCKAP1L	2	0.009277497	0.080067069	0.115871573	0.907754325	0	0.767683939	0	0.5	PRJNA827465;Yang2025_Silkie_Wenchang	CRA004775	0.978196186
ENSGALG00010025685	NCKAP1L	2	-0.181523157	0.219741938	-0.826074251	0.408761973	0.047624403	1.499215834	33.2984633	1	CRA004775;Yang2025_Silkie_Wenchang	PRJNA827465	0.834305842
ENSGALG00010025685	NCKAP1L	2	-0.141436203	0.31492883	-0.44910529	0.653355705	0.133783091	2.41711977	58.62844644	0.5	CRA004775;PRJNA827465	Yang2025_Silkie_Wenchang	0.909597225
ENSGALG00010025688	BEST4	2	0.445400563	0.312498613	1.425288131	0.15407393	0.01216364	1.063592454	5.979024563	1	PRJNA827465;Yang2025_Silkie_Wenchang	CRA004775	0.646788679
ENSGALG00010025688	BEST4	2	-0.052433859	0.376509671	-0.139262982	0.889242341	0	0.147039383	0	0.5	CRA004775;Yang2025_Silkie_Wenchang	PRJNA827465	0.973135755
ENSGALG00010025688	BEST4	2	0.348120482	0.452751432	0.768899791	0.441952802	0.178604558	1.693320276	40.9444265	0.5	CRA004775;PRJNA827465	Yang2025_Silkie_Wenchang	0.84513555
ENSGALG00010025691	ENSGALG00010025691	2	0.042513387	0.111655008	0.380756647	0.703383831	0	0.033450458	0	1	PRJNA827465;Yang2025_Silkie_Wenchang	CRA004775	0.923639641
ENSGALG00010025691	ENSGALG00010025691	2	0.09285041	0.245793559	0.377757701	0.705610599	0	0.000114977	0	1	CRA004775;Yang2025_Silkie_Wenchang	PRJNA827465	0.924358822
ENSGALG00010025691	ENSGALG00010025691	2	0.037911823	0.117214143	0.323440686	0.746361521	0	0.017064247	0	1	CRA004775;PRJNA827465	Yang2025_Silkie_Wenchang	0.936627825
ENSGALG00010025696	MTCH1	2	0.10412968	0.12206217	0.85308724	0.393610927	0.022896427	4.265784878	76.55765519	0.5	PRJNA827465;Yang2025_Silkie_Wenchang	CRA004775	0.83043598
ENSGALG00010025696	MTCH1	2	0.034033639	0.254324777	0.133819596	0.893545231	0.095770594	3.31993156	69.87889714	0.5	CRA004775;Yang2025_Silkie_Wenchang	PRJNA827465	0.974381724
ENSGALG00010025696	MTCH1	2	-0.029162016	0.069725668	-0.418239324	0.675772147	0	0.985794675	0	1	CRA004775;PRJNA827465	Yang2025_Silkie_Wenchang	0.916522765
ENSGALG00010025702	GTSF2	2	0.793112885	0.601228851	1.31915307	0.187117944	0.667866205	13.0412382	92.33201645	1	PRJNA827465;Yang2025_Silkie_Wenchang	CRA004775	0.695933761
ENSGALG00010025702	GTSF2	2	0.588504532	0.836894754	0.703200169	0.481931014	1.300376966	13.87395184	92.7922483	0.5	CRA004775;Yang2025_Silkie_Wenchang	PRJNA827465	0.85691916
ENSGALG00010025702	GTSF2	2	0.066420555	0.214973259	0.308971241	0.757343403	0.026642855	1.315470478	23.98157036	0.5	CRA004775;PRJNA827465	Yang2025_Silkie_Wenchang	0.938838283
ENSGALG00010025703	UBASH3B	2	-0.369066982	0.193795382	-1.904415774	0.056856056	0.068501056	11.35291887	91.19169254	1	PRJNA827465;Yang2025_Silkie_Wenchang	CRA004775	0.412594827
ENSGALG00010025703	UBASH3B	2	-0.554835461	0.083427828	-6.650484321	2.9213E-11	0	0.396435344	0	1	CRA004775;Yang2025_Silkie_Wenchang	PRJNA827465	2.25129E-08
ENSGALG00010025703	UBASH3B	2	-0.179096384	0.076700199	-2.335018513	0.019542454	0	0.034240644	0	1	CRA004775;PRJNA827465	Yang2025_Silkie_Wenchang	0.234180956
ENSGALG00010025705	ACSL3	2	-0.033837342	0.058826022	-0.575210445	0.56514898	0	0.643009036	0	0.5	PRJNA827465;Yang2025_Silkie_Wenchang	CRA004775	0.883427937
ENSGALG00010025705	ACSL3	2	-0.083501004	0.082178036	-1.016098807	0.309582337	0	0.006169752	0	1	CRA004775;Yang2025_Silkie_Wenchang	PRJNA827465	0.794246638
ENSGALG00010025705	ACSL3	2	0.00137508	0.075291289	0.01826347	0.98542867	0	0.089639787	0	0.5	CRA004775;PRJNA827465	Yang2025_Silkie_Wenchang	0.99592834

ENSGALG00010025708	HOXC8	2	0.764372707	0.360225115	2.121930636	0.033843563	0.194492665	3.968456115	74.80128365	1	PRJNA827465;Yang2025_Silkie_Wenchang	CRA004775	0.316060879
ENSGALG00010025708	HOXC8	2	0.840849605	0.421198538	1.996326028	0.045898447	0.190448145	1.919751288	47.90991904	1	CRA004775;Yang2025_Silkie_Wenchang	PRJNA827465	0.369489827
ENSGALG00010025708	HOXC8	2	0.395808996	0.216186665	1.830866844	0.06712042	0	0.074248096	0	1	CRA004775;PRJNA827465	Yang2025_Silkie_Wenchang	0.446292315
ENSGALG00010025720	PHETA1	2	0.167610549	0.16559865	1.01214925	0.311466697	0	0.313911531	0	1	PRJNA827465;Yang2025_Silkie_Wenchang	CRA004775	0.795682523
ENSGALG00010025720	PHETA1	2	-0.098289206	0.268528454	-0.366029017	0.714343423	0	0.331645574	0	0.5	CRA004775;Yang2025_Silkie_Wenchang	PRJNA827465	0.92653974
ENSGALG00010025720	PHETA1	2	0.120360561	0.211661482	0.568646501	0.569596061	0.018278934	1.141961732	12.43139138	0.5	CRA004775;PRJNA827465	Yang2025_Silkie_Wenchang	0.88485924
ENSGALG00010025723	SETD2	2	-0.074363308	0.049459688	-1.503513497	0.132706679	0	0.178564011	0	1	PRJNA827465;Yang2025_Silkie_Wenchang	CRA004775	0.61017438
ENSGALG00010025723	SETD2	2	0.308506252	0.359314849	0.858595887	0.390563508	0.218963658	6.31110732	84.15492006	0.5	CRA004775;Yang2025_Silkie_Wenchang	PRJNA827465	0.829251057
ENSGALG00010025723	SETD2	2	0.270059219	0.389624047	0.6931277	0.488229416	0.273293787	9.1916725	89.12058714	0.5	CRA004775;PRJNA827465	Yang2025_Silkie_Wenchang	0.85768885
ENSGALG00010025727	GLI1	2	0.22435801	0.12402796	1.808930908	0.070461738	0.016240425	2.112730416	52.66788453	1	PRJNA827465;Yang2025_Silkie_Wenchang	CRA004775	0.458021454
ENSGALG00010025727	GLI1	2	0.329642719	0.12006684	2.74549343	0.006041998	0	0.373507792	0	1	CRA004775;Yang2025_Silkie_Wenchang	PRJNA827465	0.117500562
ENSGALG00010025727	GLI1	2	0.106629799	0.109587168	0.973013542	0.330546572	0	0.000258516	0	1	CRA004775;PRJNA827465	Yang2025_Silkie_Wenchang	0.80156474
ENSGALG00010025734	PLK3	2	0.388751231	0.240421228	1.616958842	0.105887166	0.086844867	3.745282737	73.2997461	1	PRJNA827465;Yang2025_Silkie_Wenchang	CRA004775	0.552086395
ENSGALG00010025734	PLK3	2	0.327932463	0.38424308	0.853450539	0.393409505	0.214601027	3.534156142	71.70470235	0.5	CRA004775;Yang2025_Silkie_Wenchang	PRJNA827465	0.830352025
ENSGALG00010025734	PLK3	2	0.154722919	0.108943685	1.420210073	0.155546532	0	0.621364449	0	0.5	CRA004775;PRJNA827465	Yang2025_Silkie_Wenchang	0.648297492
ENSGALG00010025736	KIF9	2	-0.098876468	0.177965016	-0.555594972	0.578487769	0	0.763117092	0	0.5	PRJNA827465;Yang2025_Silkie_Wenchang	CRA004775	0.88785945
ENSGALG00010025736	KIF9	2	0.100698545	0.209801243	0.479971156	0.631247903	0	0.186995059	0	1	CRA004775;Yang2025_Silkie_Wenchang	PRJNA827465	0.903058174
ENSGALG00010025736	KIF9	2	-0.103325943	0.240004761	-0.43051622	0.66682017	0.015939138	1.134633072	11.86578066	0.5	CRA004775;PRJNA827465	Yang2025_Silkie_Wenchang	0.913648
ENSGALG00010025737	ENSGALG00010025737	2	0.147821193	0.073329643	2.015844984	0.043816188	0	0.008665943	0	1	PRJNA827465;Yang2025_Silkie_Wenchang	CRA004775	0.361804689
ENSGALG00010025737	ENSGALG00010025737	2	0.064703213	0.148396549	0.436015616	0.6628254	0.020809666	1.579541955	36.69050722	0.5	CRA004775;Yang2025_Silkie_Wenchang	PRJNA827465	0.912821987
ENSGALG00010025737	ENSGALG00010025737	2	0.054904507	0.142049352	0.386517122	0.699113724	0.014201931	1.37437854	27.2398418	0.5	CRA004775;PRJNA827465	Yang2025_Silkie_Wenchang	0.92185597
ENSGALG00010025738	SOX13	2	7.05892E-05	0.182082965	0.000387676	0.999690679	0.05677089	6.829059498	85.35669516	0.5	PRJNA827465;Yang2025_Silkie_Wenchang	CRA004775	0.999889665
ENSGALG00010025738	SOX13	2	0.138430943	0.129085541	1.07396966	0.28354177	0.00618123	1.10392974	9.414524882	0.5	CRA004775;Yang2025_Silkie_Wenchang	PRJNA827465	0.779568117
ENSGALG00010025738	SOX13	2	-0.172117312	0.075797731	-2.270744895	0.023162426	0	4.94202E-05	0	1	CRA004775;PRJNA827465	Yang2025_Silkie_Wenchang	0.259577892
ENSGALG00010025743	SH2B3	2	0.073126439	0.051818211	1.411211195	0.15818235	0	0.036511976	0	1	PRJNA827465;Yang2025_Silkie_Wenchang	CRA004775	0.65200722
ENSGALG00010025743	SH2B3	2	0.047422904	0.090028986	0.526751512	0.598366159	0	0.180552241	0	0.5	CRA004775;Yang2025_Silkie_Wenchang	PRJNA827465	0.894645663
ENSGALG00010025743	SH2B3	2	0.073985856	0.061162633	1.20965781	0.226410225	0	0.247754885	0	0.5	CRA004775;PRJNA827465	Yang2025_Silkie_Wenchang	0.739871509
ENSGALG00010025745	COX14	2	-0.174846847	0.177535178	-0.984857472	0.324690486	0	0.052573262	0	1	PRJNA827465;Yang2025_Silkie_Wenchang	CRA004775	0.799890413
ENSGALG00010025745	COX14	2	-0.298375095	0.27455039	-1.086777168	0.277135301	0	0.0507335	0	1	CRA004775;Yang2025_Silkie_Wenchang	PRJNA827465	0.77638179
ENSGALG00010025745	COX14	2	-0.192774917	0.184242886	-1.046308608	0.295418572	0	0.21686947	0	1	CRA004775;PRJNA827465	Yang2025_Silkie_Wenchang	0.787628307
ENSGALG00010025748	ETNK2	2	-0.259346834	0.085468252	-3.034423041	0.002409962	0	0.551581675	0	1	PRJNA827465;Yang2025_Silkie_Wenchang	CRA004775	0.06582262
ENSGALG00010025748	ETNK2	2	-0.258101075	0.143939585	-1.793120883	0.072953597	0.010968051	1.144398026	12.61781499	0.5	CRA004775;Yang2025_Silkie_Wenchang	PRJNA827465	0.466530301
ENSGALG00010025748	ETNK2	2	-0.157137342	0.121606376	-1.292180126	0.196294772	0	0.537938164	0	0.5	CRA004775;PRJNA827465	Yang2025_Silkie_Wenchang	0.707685514
ENSGALG00010025753	SORL1	2	0.073324343	0.099572446	0.736391903	0.461492231	0	0.361020805	0	0.5	PRJNA827465;Yang2025_Silkie_Wenchang	CRA004775	0.850392852
ENSGALG00010025753	SORL1	2	-0.21470714	0.314013999	-0.683750217	0.494132907	0.100818636	1.68557553	40.67308274	1	CRA004775;Yang2025_Silkie_Wenchang	PRJNA827465	0.860175061
ENSGALG00010025753	SORL1	2	-0.160934823	0.391927495	-0.410623969	0.681348286	0.213690512	2.565738833	61.02487178	0.5	CRA004775;PRJNA827465	Yang2025_Silkie_Wenchang	0.918242009
ENSGALG00010025755	ENSGALG00010025755	2	0.488726386	0.442089894	1.105490969	0.268946905	0.247341668	2.498734829	59.97974702	1	PRJNA827465;Yang2025_Silkie_Wenchang	CRA004775	0.771398294
ENSGALG00010025755	ENSGALG00010025755	2	0.98444411	0.321158331	3.065292145	0.002174575	0	0.024649213	0	1	CRA004775;Yang2025_Silkie_Wenchang	PRJNA827465	0.061310839
ENSGALG00010025755	ENSGALG00010025755	2	0.478508994	0.397713071	1.20315129	0.228917781	0.203017422	2.67626526	62.63449611	1	CRA004775;PRJNA827465	Yang2025_Silkie_Wenchang	0.741605303
ENSGALG00010025787	KCNE4	2	-0.182077117	0.103154789	-1.765086415	0.077549243	0.008745867	1.695102279	41.00650961	1	PRJNA827465;Yang2025_Silkie_Wenchang	CRA004775	0.479609428
ENSGALG00010025787	KCNE4	2	-0.117219766	0.245667994	-0.477147082	0.63325739	0.075436126	2.133819674	53.13568375	0.5	CRA004775;Yang2025_Silkie_Wenchang	PRJNA827465	0.9032522
ENSGALG00010025787	KCNE4	2	-0.041205175	0.110499196	-0.372900225	0.709222705	0	0.788505706	0	0.5	CRA004775;PRJNA827465	Yang2025_Silkie_Wenchang	0.925298042
ENSGALG00010025790	LRP5	2	0.065827295	0.065625673	0.88795966	0.374562451	0	0.745665552	0	1	PRJNA827465;Yang2025_Silkie_Wenchang	CRA004775	0.822805106
ENSGALG00010025790	LRP5	2	0.115067113	0.161354464	0.713132501	0.475763769	0	0.724331273	0	0.5	CRA004775;Yang2025_Silkie_Wenchang	PRJNA827465	0.854613502
ENSGALG00010025790	LRP5	2	0.033778745	0.06646552	0.508214552	0.611302886	0	0.108820127	0	0.5	CRA004775;PRJNA827465	Yang2025_Silkie_Wenchang	0.897609796
ENSGALG00010025791	FIGN	2	-0.089595947	0.209531265	-0.427601803	0.668941053	0.055813729	2.512043687	60.19177512	0.5	PRJNA827465;Yang2025_Silkie_Wenchang	CRA004775	0.914377769
ENSGALG00010025791	FIGN	2	0.421275133	0.343524875	1.226330797	0.220074216	0.120230408	1.839997165	45.65209017	1	CRA004775;Yang2025_Silkie_Wenchang	PRJNA827465	0.733691086
ENSGALG00010025791	FIGN	2	0.231027189	0.570857718	0.404701875	0.685696664	0.546857932	5.490791443	81.78768925	0.5	CRA004775;PRJNA827465	Yang2025_Silkie_Wenchang	0.919235539
ENSGALG00010025799	SC5D	2	0.51559052	0.488946558	1.054492586	0.291657453	0.41259978	6.52538978	84.67524495	1	PRJNA827465;Yang2025_Silkie_Wenchang	CRA004775	0.785137868
ENSGALG00010025799	SC5D	2	0.411574895	0.64021944	0.642865352	0.520311474	0.71097453	7.464069134	86.60248208	0.5	CRA004775;Yang2025_Silkie_Wenchang	PRJNA827465	0.869313539
ENSGALG00010025799	SC5D	2	0.062532303	0.085004045	0.735639148	0.461950931	4.18515E-06	1.000096725	0.009671585	0.5	CRA004775;PRJNA827465	Yang2025_Silkie_Wenchang	0.850747865
ENSGALG00010025800	ARL2BP	2	0.035350949	0.167418986	0.211152569	0.83276822	0.019078877	1.457374604	31.38346194	0.5	PRJNA827465;Yang2025_Silkie_Wenchang	CRA004775	0.957861248
ENSGALG00010025800	ARL2BP	2	-0.386910958	0.216539332	-1.786791328	0.073971222	0.027915271	1.419693919	29.56228192	1	CRA004775;Yang2025_Silkie_Wenchang	PRJNA827465	0.469669846
ENSGALG00010025800	ARL2BP	2	-0.198930685	0.390352267	-0.509618367	0.610318853	0.257206721	6.191064343	83.84768846	0.5	CRA004775;PRJNA827465	Yang2025_Silkie_Wenchang	0.897438028
ENSGALG00010025801	PTPN7	2	0.324079976	0.339149407	0.955566983	0.339291049	0	0.066715303	0	1	PRJNA827465;Yang2025_Silkie_Wenchang	CRA004775	0.804824608
ENSGALG00010025801	PTPN7	2	-0.161862352	0.555905244	-0.29116896	0.770922101	0.389963056	2.698003635	62.93555772	0.5	CRA004775;Yang2025_Silkie_Wenchang	PRJNA827465	0.942531226
ENSGALG00010025801	PTPN7	2	-0.226001714	0.468376836	-0.482521116	0.629483802	0.236606652	2.170294736	53.92330897	0.5	CRA004775;PRJNA827465	Yang2025_Silkie_Wenchang	0.902390168
ENSGALG00010025805	ATXN2	2	0.093197171	0.280225513	0.332579078	0.739481963	0.129872412	4.959948463	79.83606098	0.5	PRJNA827465;Yang2025_Silkie_Wenchang	CRA004775	0.934873078
ENSGALG00010025805	ATXN2	2	0.269981688	0.158889304	1.699181011	0.089285083	0	0.689472009	0	1	CRA004775;Yang2025_Silkie_Wenchang	PRJNA827465	0.512891552
ENSGALG00010025805	ATXN2	2	-0.054077744	0.13549703	-0.399106485	0.689814744	0.023006476	2.030036088	50.73979197	0.5	CRA004775;PRJNA827465	Yang2025_Silkie_Wenchang	0.919979266
ENSGALG00010025809	ENSGALG00010025809	2	0.15036136	0.189585212	0.793103007	0.427715481	0.047523931	2.86955445	65.15138439	0.5	PRJNA827465;Yang2025_Silkie_Wenchang	CRA004775	0.84125685
ENSGALG00010025809	ENSGALG00010025809	2	0.263222185	0.177974918	1.478984719	0.139144399	0.007693105	1.098437859	8.961622962	0.5	CRA004775;Yang2025_Silkie_Wenchang	PRJNA827465	0.622869284
ENSGALG00010025809	ENSGALG00010025809	2	-0.021491424	0.120987289	-0.177633737	0.859010627	0	0.007568177	0	1	CRA004775;PRJNA827465	Yang2025_Silkie_Wenchang	0.964605083
ENSGALG00010025822	KLF7	2	0.201935349	0.138078555	1.462467143	0.143631242	0.020681965	1.970186115	49.24337389	1	PRJNA827465;Yang2025_Silkie_Wenchang	CRA004775	0.630564924
ENSGALG00010025822	KLF7	2	0.225826246	0.278207215	0.811719586	0.416952553	0.070560766	1.540811072	35.09911642	0.5	CRA004775;Yang2025_Silkie_Wenchang	PRJNA827465	0.830063117
ENSGALG00010025822	KLF7	2	0.088467645	0.090882028	0.973433886	0.330337704	0	0.503352305	0	0.5	CRA004775;PRJNA827465	Yang2025_Silkie_Wenchang	0.80153493
ENSGALG00010025829	ENSGALG00010025829	2	0.032955069	0.162692447	0.202560535	0.839478539	0.041755916	4.649795861	78.49367951	0.5	PRJNA827465;Yang2025_Silkie_Wenchang	CRA004775	0.959493354
ENSGALG00010025829	ENSGALG00010025829	2	-0.278268607	0.270637614	-1.028196353	0.303857474	0.076795665	1.514630647	33.97730317	1	CRA004775;Yang2025_Silkie_Wenchang	PRJNA827465	0.792208658
ENSGALG00010025829	ENSGALG00010025829	2	-0.176603301	0.479811869	-0.368067803	0.712822677	0.353006613	3.425930494	70.81084973	0.5	CRA004775;PRJNA827465	Yang2025_Silkie_Wenchang	0.92609326
ENSGALG00010025830	TBCEL	2	0.21270662	0.110751713	1.920571828	0.05478571	0	0.251709313	0	1	PRJNA827465;Yang2025_Silkie_Wenchang	CRA004775	0.405278138
ENSGALG00010025830	TBCEL	2	-0.291541556	0.681034623	-0.428086248	0.668538328	0.773574605	5.53966587	81.98436975	0.5	CRA004775;Yang2025_Silkie_Wenchang	PRJNA827465	0.914377769
ENSGALG00010025830	TBCEL	2	-0.333527316	0.614121715	-0.543096437	0.587063405	0.623399013	5.003902823	80.01559003	0.5	CRA004775;PRJNA827465	Yang2025_Silkie_Wenchang	0.891596903
ENSGALG00010025831	BRAP	2	-0.106040183	0.085825355	-1.235534455	0.216631662	0.010687	3.379083442	70.4061762	1	PRJNA827465;Yang2025_Silkie_Wenchang	CRA004775	0.730172415
ENSGALG00010025831	BRAP	2	-0.174166823	0.073894069	-2.356979749	0.018424252	0	0.703878929	0	1	CRA004775;Yang2025_Silkie_Wenchang	PRJNA827465	0.227213166
ENSGALG00010025831	BRAP	2	-0.035871116	0.041805702	-0.858043636	0.390868369	0	0.028645674	0	1	CRA004775;PRJNA827465	Yang2025_Silkie_Wenchang	0.829251057
ENSGALG00010025832	ENKD1	2	-0.296234022	0.207595224	-1.426978693	0.153585992	0.051580236	2.205793163	54.70179846	1	PRJNA827465;Yang2025_Silkie_Wenchang	CRA004775	0.645948495
ENSGALG00010025832	ENKD1	2	-0.290749537	0.365974745	-0.794452462	0.426932073	0.145531923	2.048235466	51.1774883	0.5	CRA004775;Yang2025_Silkie_Wenchang	PRJNA827465	0.84125685
ENSGALG00010025832	ENKD1	2	-0.121067157	0.118540148	-1.021317744	0.307103922	0	0.460101764	0	0.5	CRA004775;PRJNA827465	Yang2025_Silkie_Wenchang	0.79266003
ENSGALG00010025835	PPP6R3	2	0.097536149	0.036603986	2.664631918	0.007707263	0	0.11750171	0	1	PRJNA827465;Yang2025_Silkie_Wenchang	CRA004775	0.13583792
ENSGALG00010025835	PPP6R3	2	0.123905464	0.104148641	1.189698327	0.234164983	0	0.058778124	0	1	CRA004775;Yang2025_Silkie_Wenchang	PRJNA827465	0.747392101
ENSGALG00010025835	PPP6R3	2	0.092575946	0.038393145	2.411262357	0.015897408	0	0.013900296	0	1	CRA004775;PRJNA827465	Yang2025_Silkie_Wenchang	0.208471156
ENSGALG00010025836	ENSGALG00010025836	2	0.21877113	0.14337767	1.525838232	0.127050157	0.033380596	5.223367803	80.85526354	1	PRJNA827465;Yang2025_Silkie_Wenchang	CRA004775	0.598304854
ENSGALG00010025836	ENSGALG00010025836	2	0.362516185	0.096541655	3.755015754	0.000173313	0	0.067893827	0	1	CRA004775;Yang2025_Silkie_Wenchang	PRJNA827465	0.001507452
ENSGALG00010025836	ENSGALG00010025836	2	0.097237662	0.070209154	1.384971279	0.16606125	0	0.514008294	0	1	CRA004775;PRJNA827465	Yang2025_Silkie_Wenchang	0.66529196
ENSGALG00010025837	ENSGALG00010025837	2	-0.164009588	0.143919892	-1.13958943	0.254457391	0	0.103077487	0	1	PRJNA827465;Yang2025_Silkie_Wenchang	CRA004775	0.764087626
ENSGALG00010025837	ENSGALG00010025837	2	-0.301346294	0.199221494	-1.512619388	0.130376366	0	0.189950079	0	1	CRA004775;Yang2025_Silkie_Wenchang	PRJNA827465	0.605608015
ENSGALG00010025837	ENSGALG00010025837	2	-0.196332248	0.152851453	-1.284452051	0.198997819	0	0.58289242	0	1	CRA004775;PRJNA827465	Yang2025_Silkie_Wenchang	0.71096127
ENSGALG00010025846	GRIK4	2	-0.078890237	0.170519299	-0.462646967	0.643617437	0	0.004005172	0	1	PRJNA827465;Yang2025_Silkie_Wenchang	CRA004775	0.906643868
ENSGALG00010025846	GRIK4	2	-0.048454066	0.22466072	-0.215676624	0.829239824	0	0.112531746	0	0.5	CRA004775;Yang2025_Silkie_Wenchang	PRJNA827465	0.956860127
ENSGALG00010025846	GRIK4	2	-0.038779779	0.203232399	-0.190814946	0.848670579	0	0.091421359	0	0.5	CRA004775;PRJNA827465	Yang2025_Silkie_Wenchang	0.961364896
ENSGALG00010025847	DEF6	2	0.251417296	0.395393839	0.640923736	0.521572223	0.295280338	17.62891258	94.32750037	0.5	PRJNA827465;Yang2025_Silkie_Wenchang	CRA004775	0.869918515
ENSGALG00010025847	DEF6	2	0.160611192	0.547207371	0.293510652	0.769131859	0.522932473	7.502814658	86.67166863	0.5	CRA004775;Yang2025_Silkie_Wenchang	PRJNA827465	0.941790377
ENSGALG00010025847	DEF6	2	-0.144279139	0.081928037	-1.761047184	0.078230417	0	0.67691091	0	1	CRA004775;PRJNA827465	Yang2025_Silkie_Wenchang	0.481684356
ENSGALG00010025849	OBSCN	2	0.231509764	0.515572535	0.449034323	0.653406897	0.500227107	16.71979767	94.01906638	0.5	PRJNA827465;Yang2025_Silkie_Wenchang	CRA004775	0.909626907
ENSGALG00010025849	OBSCN	2	0.773604752	0.203653772	3.798627165	0.0001455	0	0.026671465	0	1	CRA004775;Yang2025_Silkie_Wenchang	PRJNA827465	0.010211774
ENSGALG00010025849	OBSCN	2	0.144137227	0.546980686	0.26351429	0.792154211	0.453632459	3.357306116	70.21421445	0.5	CRA004775;PRJNA827465	Yang2025_Silkie_Wenchang	0.948314332
ENSGALG00010025852	BTBD19	2	-0.101721307	0.109967704	-0.925010734	0.354960324	0	0.028743542	0	1	PRJNA827465;Yang2025_Silkie_Wenchang	CRA004775	0.812191047
ENSGALG00010025852	BTBD19	2	0.189691863	0.323205325	0.586908222	0.557265347	0.141870174	2.893737443	65.44261462	0.5	CRA004775;Yang2025_Silkie_Wenchang	PRJNA827465	0.880678789
ENSGALG00010025852	BTBD19	2	0.163184572	0.341601973	0.477703832	0.632861016	0.175581005	3.599605713	72.21917955	0.5	CRA004775;PRJNA827465	Yang2025_Silkie_Wenchang	0.903112631
ENSGALG00010025862	PTCH2	2	0.371670809	0.255999501	1.451841927	0.146545563	0.077660565	2.453015287	59.23384558	1	PRJNA827465;Yang2025_Silkie_Wenchang	CRA004775	0.635517732
ENSGALG00010025862	PTCH2	2	0.650950825	0.206006552	3.159854955	0.001578477	0	0.086118691	0	1	CRA004775;Yang2025_Silkie_Wenchang	PRJNA827465	0.050152693
ENSGALG00010025862	PTCH2	2	0.305253208	0.304578802	1.002214224	0.31624014	0.07472294	1.49394543	33.06315078	1	CRA004775;PRJNA827465	Yang2025_Silkie_Wenchang	0.796883862
ENSGALG00010025869	FASTKD2	2	0.06725006	0.054704891	1.229324451	0.218950183	0	0.195487082	0	1	PRJNA827465;Yang2025_Silkie_Wenchang	CRA004775	0.73259614
ENSGALG00010025869	FASTKD2	2	-0.089789532	0.131942033	-0.680522573	0.496173634	0.009547167	1.346224506	25.71818478	0.5	CRA004775;Yang2025_Silkie_Wenchang	PRJNA827465	0.860939846
ENSGALG00010025869	FASTKD2	2	-0.041823492	0.161634764	-0.25875307	0.795825769	0.036891354	2.826226852	64.61713612	0.5	CRA004775;PRJNA827465	Yang2025_Silkie_Wenchang	0.949107318
ENSGALG00010025870	ENSGALG00010025870	2	-0.165960833	0.182038358	-0.911680566	0.361936897	0.034154082	1.904561082	47.49446424	0.5	PRJNA827465;Yang2025_Silkie_Wenchang	CRA004775	0.815579273
ENSGALG00010025870	ENSGALG00010025870	2	-0.029059401	0.208186273	-0.139583654	0.888988956	0	0.769485938	0	0.5	CRA004775;Yang2025_Silkie_Wenchang	PRJNA827465	0.973135755
ENSGALG00010025870	ENSGALG00010025870	2	-0.306371092	0.126458484	-2.42270096	0.015405602	0	0.009181795	0	1	CRA004775;PRJNA827465	Yang2025_Silkie_Wenchang	0.205241414
ENSGALG00010025881	GAL	2	-0.306169902	0.534714874	-0.572585347	0.566925483	0	0.075463845	0	1	PRJNA827465;Yang2025_Silkie_Wenchang	CRA004775	0.883487517
ENSGALG00010025881	GAL	2	-0.462708945	0.618202465	-0.748474766	0.45417384	0	0.000252095	0	1	CRA004775;Yang2025_Silkie_Wenchang	PRJNA827465	0.849072665
ENSGALG00010025881	GAL	2	-0.267293814	0.61120197	-0.437324858	0.661875771	0	0.059024259	0	1	CRA004775;PRJNA827465	Yang2025_Silkie_Wenchang	0.912556664
ENSGALG00010025882	LDHD	2	0.108814729	0.29971117	0.363065312	0.716556096	0.148546655	5.388014427	81.44028726	0.5	PRJNA827465;Yang2025_Silkie_Wenchang	CRA004775	0.927487786
ENSGALG00010025882	LDHD	2	0.185761272	0.337117276	0.551028634	0.581614046	0.126111817	2.088188514	52.11160326	0.5	CRA004775;Yang2025_Silkie_Wenchang	PRJNA827465	0.889045686
ENSGALG00010025882	LDHD	2	-0.161751494	0.103775178	-1.558672282	0.119073965	0	0.044981255	0	1	CRA004775;PRJNA827465	Yang2025_Silkie_Wenchang	0.579471011
ENSGALG00010025886	ALDH2	2	-0.013223068	0.090889002	-0.145485896	0.88432725	0.008303165	1.819012484	45.02511617	0.5	PRJNA827465;Yang2025_Silkie_Wenchang	CRA004775	0.972001076
ENSGALG00010025886	ALDH2	2	0.0001733	0.204192914	0.000848705	0.999322831	0.045319355	1.90049624	47.38216373	0.5	CRA004775;Yang2025_Silkie_Wenchang	PRJNA827465	0.999889665
ENSGALG00010025886	ALDH2	2	0.063397206	0.063171723	1.003569362	0.315586229	0	0.678083884	0	1	CRA004775;PRJNA827465	Yang2025_Silkie_Wenchang	0.796883862
ENSGALG00010025887	ZNF76	2	0.034204524	0.328607589	0.104089271	0.917098505	0.202630345	16.19155743	93.82394186	0.5	PRJNA827465;Yang2025_Silkie_Wenchang	CRA004775	0.980728718
ENSGALG00010025887	ZNF76	2	-0.087199597	0.453770072	-0.19216692	0.847611457	0.394795369	24.1300131	95.85578343	0.5	CRA004775;Yang2025_Silkie_Wenchang	PRJNA827465	0.961267114
ENSGALG00010025887	ZNF76	2	-0.405878084	0.124297859	-3.265366659	0.001093225	0.013497343	1.756302186	43.06219008	1	CRA004775;PRJNA827465	Yang2025_Silkie_Wenchang	0.039938053
ENSGALG00010025890	SCG2	2	-0.364977083	0.853781982	-0.427482765	0.669027736	1.283932427	8.341429222	88.01164677	0.5	PRJNA827465;Yang2025_Silkie_Wenchang	CRA004775	0.914377769
ENSGALG00010025890	SCG2	2	0.40053064	0.333186344	1.202122017	0.229316257	0	0.120398086	0	1	CRA004775;Yang2025_Silkie_Wenchang	PRJNA827465	0.742284374
ENSGALG00010025890	SCG2	2	-0.49139028	0.737604039	-0.666197925	0.505284599	0.904563785	5.872705457	82.97207297	0.5	CRA004775;PRJNA827465	Yang2025_Silkie_Wenchang	0.863862536
ENSGALG00010025891	NUP93	2	-0.22446396	0.088154328	-2.546261386	0.010888362	0.006116257	1.604615252	37.67976475	1	PRJNA827465;Yang2025_Silkie_Wenchang	CRA004775	0.166322288
ENSGALG00010025891	NUP93	2	-0.333843705	0.107879259	-3.094605099	0.001970751	0	0.000282901	0	1	CRA004775;Yang2025_Silkie_Wenchang	PRJNA827465	0.057502915
ENSGALG00010025891	NUP93	2	-0.166498817	0.079215174	-2.101855102	0.035565971	0	0.371315903	0	1	CRA004775;PRJNA827465	Yang2025_Silkie_Wenchang	0.325417923
ENSGALG00010025892	ZC3H11A	2	0.248645003	0.224794967	1.106096841	0.268684606	0.096131703	20.41980005	95.1027924	1	PRJNA827465;Yang2025_Silkie_Wenchang	CRA004775	0.771398294
ENSGALG00010025892	ZC3H11A	2	0.310668464	0.190967623	1.626812225	0.103776984	0.057672537	4.443058664	77.4929823	1	CRA004775;Yang2025_Silkie_Wenchang	PRJNA827465	0.548071327
ENSGALG00010025892	ZC3H11A	2	0.034252435	0.056839888	0.602612637	0.546766412	0	0.131685574	0	1	CRA004775;PRJNA827465	Yang2025_Silkie_Wenchang	0.878275987
ENSGALG00010025897	MAPKAPK5	2	0.026352087	0.055022157	0.478935914	0.631984221	0	0.154084175	0	1	PRJNA827465;Yang2025_Silkie_Wenchang	CRA004775	0.903058174
ENSGALG00010025897	MAPKAPK5	2	0.145690048	0.153639834	0.948257004	0.342998634	0.023208453	1.599669178	37.48707458	1	CRA004775;Yang2025_Silkie_Wenchang	PRJNA827465	0.806218116
ENSGALG00010025897	MAPKAPK5	2	0.128027306	0.181314371	0.706106775	0.480121739	0.042033879	2.142820392	53.33253297	1	CRA004775;PRJNA827465	Yang2025_Silkie_Wenchang	0.856413938
ENSGALG00010025899	ENSGALG00010025899	2	0.021748871	0.113965586	0.190837176	0.848653162	0	0.648797984	0	0.5	PRJNA827465;Yang2025_Silkie_Wenchang	CRA004775	0.961364896
ENSGALG00010025899	ENSGALG00010025899	2	0.073966349	0.16820995	0.43972636	0.660135308	0	0.593228769	0	0.5	CRA004775;Yang2025_Silkie_Wenchang	PRJNA827465	0.911833064
ENSGALG00010025899	ENSGALG00010025899	2	-0.060978256	0.130194437	-0.46836299	0.63952503	0	0.086534583	0	1	CRA004775;PRJNA827465	Yang2025_Silkie_Wenchang	0.906064918
ENSGALG00010025904	RPL10A	2	-0.092435139	0.064349724	-1.436449652	0.150874433	0	0.453370856	0	1	PRJNA827465;Yang2025_Silkie_Wenchang	CRA004775	0.642051813
ENSGALG00010025904	RPL10A	2	-0.288128926	0.17551724	-1.641599007	0.100673134	0	0.026917167	0	1	CRA004775;Yang2025_Silkie_Wenchang	PRJNA827465	0.541412814
ENSGALG00010025904	RPL10A	2	-0.09748114	0.064151598	-1.519543452	0.128625759	0	0.868619492	0	1	CRA004775;PRJNA827465	Yang2025_Silkie_Wenchang	0.60183074
ENSGALG00010025905	CPT1A	2	-0.047073899	0.07843068	-0.600197521	0.548374605	0	0.03241223	0	1	PRJNA827465;Yang2025_Silkie_Wenchang	CRA004775	0.878281266
ENSGALG00010025905	CPT1A	2	0.226081792	0.396964291	0.569526774	0.568998706	0.227881533	2.873892527	65.2039876	0.5	CRA004775;Yang2025_Silkie_Wenchang	PRJNA827465	0.884492059
ENSGALG00010025905	CPT1A	2	0.23591081	0.380564474	0.61989709	0.535325541	0.204433797	2.677877622	62.65699404	0.5	CRA004775;PRJNA827465	Yang2025_Silkie_Wenchang	0.874112994
ENSGALG00010025907	ARHGEF12	2	0.011834915	0.134758447	0.087823178	0.930017216	0.029115298	4.654175123	78.51391549	0.5	PRJNA827465;Yang2025_Silkie_Wenchang	CRA004775	0.983813577
ENSGALG00010025907	ARHGEF12	2	0.170579023	0.105824044	1.611911779	0.106981139	0	0.004426655	0	1	CRA004775;Yang2025_Silkie_Wenchang	PRJNA827465	0.553983328
ENSGALG00010025907	ARHGEF12	2	-0.076517884	0.08630463	-0.886602311	0.375293049	0.005600766	1.151631183	13.16664442	0.5	CRA004775;PRJNA827465	Yang2025_Silkie_Wenchang	0.822941424
ENSGALG00010025910	HEATR3	2	-0.084149578	0.083483274	-1.007981293	0.313463443	0.006454018	1.767292028	43.41625582	1	PRJNA827465;Yang2025_Silkie_Wenchang	CRA004775	0.796531448
ENSGALG00010025910	HEATR3	2	-0.176263129	0.096470913	-1.827111653	0.067682984	0	0.086677581	0	1	CRA004775;Yang2025_Silkie_Wenchang	PRJNA827465	0.448590945
ENSGALG00010025910	HEATR3	2	-0.03050264	0.064858905	-0.470292243	0.638146239	0	0.247088766	0	1	CRA004775;PRJNA827465	Yang2025_Silkie_Wenchang	0.905453488
ENSGALG00010025913	EIF2B3	2	-0.046714517	0.048732838	-0.958583967	0.337768373	0	0.013067311	0	1	PRJNA827465;Yang2025_Silkie_Wenchang	CRA004775	0.80415385
ENSGALG00010025913	EIF2B3	2	-0.069872185	0.102689703	-0.68042056	0.496238206	0	0.480518124	0	1	CRA004775;Yang2025_Silkie_Wenchang	PRJNA827465	0.860939846
ENSGALG00010025913	EIF2B3	2	-0.057739578	0.05247528	-1.100319576	0.271192906	0	0.478904338	0	1	CRA004775;PRJNA827465	Yang2025_Silkie_Wenchang	0.772743863
ENSGALG00010025917	SCUBE3	2	0.245559506	0.210657091	1.165683552	0.243742413	0	0.27075002	0	1	PRJNA827465;Yang2025_Silkie_Wenchang	CRA004775	0.755365463
ENSGALG00010025917	SCUBE3	2	1.125563851	0.652208754	1.725772375	0.084388372	0.64831398	4.198512246	76.18203922	1	CRA004775;Yang2025_Silkie_Wenchang	PRJNA827465	0.500017819
ENSGALG00010025917	SCUBE3	2	0.936386907	0.787162778	1.189572136	0.234214603	1.120891117	10.19690203	90.19309985	1	CRA004775;PRJNA827465	Yang2025_Silkie_Wenchang	0.74742908
ENSGALG00010025919	RGS9BP	2	0.608244221	0.241495152	2.518660168	0.011780229	0	0.183351658	0	1	PRJNA827465;Yang2025_Silkie_Wenchang	CRA004775	0.173863443
ENSGALG00010025919	RGS9BP	2	0.579705619	0.326695415	1.774452875	0.07598827	0	0.579005361	0	1	CRA004775;Yang2025_Silkie_Wenchang	PRJNA827465	0.475492277
ENSGALG00010025919	RGS9BP	2	0.456732166	0.275495848	1.657854992	0.097346739	0	0.243479657	0	1	CRA004775;PRJNA827465	Yang2025_Silkie_Wenchang	0.532942995
ENSGALG00010025920	GTSF1	2	0.256081705	0.389349643	0.657716553	0.510720284	0.100493344	1.493845761	33.05868474	0.5	PRJNA827465;Yang2025_Silkie_Wenchang	CRA004775	0.865386785
ENSGALG00010025920	GTSF1	2	-0.417676178	0.374212445	-1.116147213	0.264359126	0	0.881269781	0	1	CRA004775;Yang2025_Silkie_Wenchang	PRJNA827465	0.768790489
ENSGALG00010025920	GTSF1	2	-0.063410464	0.752713041	-0.084242548	0.93286359	0.853901045	3.964619012	74.77689541	0.5	CRA004775;PRJNA827465	Yang2025_Silkie_Wenchang	0.984224753
ENSGALG00010025924	TMEM116	2	0.224359967	0.168085579	1.334796048	0.181943107	0.043556492	4.347930957	77.00055475	1	PRJNA827465;Yang2025_Silkie_Wenchang	CRA004775	0.689745682
ENSGALG00010025924	TMEM116	2	0.066026713	0.101581962	0.649984616	0.515702159	0	0.022396336	0	1	CRA004775;Yang2025_Silkie_Wenchang	PRJNA827465	0.867330307
ENSGALG00010025924	TMEM116	2	0.370952337	0.115845458	3.202131025	0.001364149	0	0.507867762	0	1	CRA004775;PRJNA827465	Yang2025_Silkie_Wenchang	0.045244862
ENSGALG00010025925	KLHL18	2	-0.015919153	0.057100742	-0.278790642	0.780405496	0.00173828	1.31555917	23.98669531	0.5	PRJNA827465;Yang2025_Silkie_Wenchang	CRA004775	0.945041817
ENSGALG00010025925	KLHL18	2	0.03598209	0.083914535	0.428794489	0.66807279	0	0.983269649	0	0.5	CRA004775;Yang2025_Silkie_Wenchang	PRJNA827465	0.914120722
ENSGALG00010025925	KLHL18	2	-0.063887025	0.05548043	-1.1515236	0.249516892	0	0.339935041	0	1	CRA004775;PRJNA827465	Yang2025_Silkie_Wenchang	0.759622031
ENSGALG00010025929	NELFE	2	0.032788194	0.058749676	0.558099992	0.576776106	0	0.928742284	0	1	PRJNA827465;Yang2025_Silkie_Wenchang	CRA004775	0.887113945
ENSGALG00010025929	NELFE	2	-0.066369673	0.279654211	-0.237327637	0.812402623	0.114064371	3.627181387	72.43038345	0.5	CRA004775;Yang2025_Silkie_Wenchang	PRJNA827465	0.953371387
ENSGALG00010025929	NELFE	2	-0.110176028	0.178595687	-0.61690195	0.537299401	0.043063506	2.428752165	58.82659357	0.5	CRA004775;PRJNA827465	Yang2025_Silkie_Wenchang	0.87481821
ENSGALG00010025931	TMEM136	2	0.044306579	0.102849837	0.43078901	0.666621792	0	0.00022687	0	1	PRJNA827465;Yang2025_Silkie_Wenchang	CRA004775	0.913579507
ENSGALG00010025931	TMEM136	2	-0.022334244	0.153494157	-0.145505499	0.884311774	0	0.94147381	0	0.5	CRA004775;Yang2025_Silkie_Wenchang	PRJNA827465	0.972001076
ENSGALG00010025931	TMEM136	2	-4.05021E-05	0.122196538	-0.00033145	0.999735541	0	0.998200686	0	0.5	CRA004775;PRJNA827465	Yang2025_Silkie_Wenchang	0.999889665
ENSGALG00010025932	AP1S3	2	0.187713527	0.254565814	0.73738702	0.460887026	0	0.412495215	0	0.5	PRJNA827465;Yang2025_Silkie_Wenchang	CRA004775	0.85014477
ENSGALG00010025932	AP1S3	2	-0.262233365	0.298917311	-0.877277278	0.380336051	0	0.92073948	0	1	CRA004775;Yang2025_Silkie_Wenchang	PRJNA827465	0.824392515
ENSGALG00010025932	AP1S3	2	-0.079989538	0.452451986	-0.176791219	0.859672387	0.258003237	2.645073897	62.19387288	0.5	CRA004775;PRJNA827465	Yang2025_Silkie_Wenchang	0.964839196
ENSGALG00010025933	SKIC2	2	0.23312814	0.299472872	0.778461628	0.436296919	0.163555371	11.07011007	90.96666615	0.5	PRJNA827465;Yang2025_Silkie_Wenchang	CRA004775	0.843122822
ENSGALG00010025933	SKIC2	2	0.219626892	0.353064038	0.622059649	0.533902647	0.20719954	5.766981622	82.65990659	0.5	CRA004775;Yang2025_Silkie_Wenchang	PRJNA827465	0.873408008
ENSGALG00010025933	SKIC2	2	-0.063710769	0.081588818	-0.780876232	0.434875293	0	0.169354554	0	1	CRA004775;PRJNA827465	Yang2025_Silkie_Wenchang	0.842762754
ENSGALG00010025935	CATH3	2	0.196991901	0.223621532	0.88091652	0.378363005	0	0.016662074	0	1	PRJNA827465;Yang2025_Silkie_Wenchang	CRA004775	0.823410919
ENSGALG00010025935	CATH3	2	0.34518664	0.262950452	1.312744042	0.189269212	0	0.374680781	0	1	CRA004775;Yang2025_Silkie_Wenchang	PRJNA827465	0.698221121
ENSGALG00010025935	CATH3	2	0.297408528	0.2531428	1.174864652	0.24004887	0	0.534001318	0	1	CRA004775;PRJNA827465	Yang2025_Silkie_Wenchang	0.752483707
ENSGALG00010025936	GOSR2	2	-0.078372398	0.194230725	-0.403501544	0.686579299	0.071561475	19.37170525	94.83783184	0.5	PRJNA827465;Yang2025_Silkie_Wenchang	CRA004775	0.919235539
ENSGALG00010025936	GOSR2	2	-0.161336155	0.161607559	-0.998320597	0.318123923	0.034330687	2.37858129	57.95813227	0.5	CRA004775;Yang2025_Silkie_Wenchang	PRJNA827465	0.797501246
ENSGALG00010025936	GOSR2	2	0.111237064	0.055062126	2.020210127	0.043361596	0	0.040424383	0	1	CRA004775;PRJNA827465	Yang2025_Silkie_Wenchang	0.360023385
ENSGALG00010025938	ERP29	2	-0.208976455	0.346170257	-0.60368114	0.546055656	0.228619248	21.16832293	95.2759602	0.5	PRJNA827465;Yang2025_Silkie_Wenchang	CRA004775	0.877958239
ENSGALG00010025938	ERP29	2	-0.3896676	0.229775799	-1.695860062	0.089912385	0.064726974	2.348288223	57.4157895	1	CRA004775;Yang2025_Silkie_Wenchang	PRJNA827465	0.514454377
ENSGALG00010025938	ERP29	2	0.117090294	0.054030183	2.16712746	0.030225137	0	0.598007021	0	0.5	CRA004775;PRJNA827465	Yang2025_Silkie_Wenchang	0.295973612
ENSGALG00010025940	HECTD3	2	-0.097971782	0.045859159	-2.136362386	0.032649887	0	0.003553436	0	1	PRJNA827465;Yang2025_Silkie_Wenchang	CRA004775	0.310329141

ENSGALG00010025940	HECTD3	2	-0.246688751	0.166813537	-1.478829329	0.139185935	0.031172846	2.262744796	55.80588664	1	CRA004775;Yang2025_Silkie_Wenchang	PRJNA827465	0.622913323
ENSGALG00010025940	HECTD3	2	-0.221819851	0.1576433	-1.407099766	0.159397807	0.037632909	3.474908316	71.22226231	1	CRA004775;PRJNA827465	Yang2025_Silkie_Wenchang	0.654972506
ENSGALG00010025943	CATH1	2	0.306869608	0.296865422	1.033699397	0.301276715	0	0.75985023	0	1	PRJNA827465;Yang2025_Silkie_Wenchang	CRA004775	0.791222412
ENSGALG00010025943	CATH1	2	1.132860743	0.644197538	1.758561117	0.078652082	0.516102783	2.525049323	60.3968132	1	CRA004775;Yang2025_Silkie_Wenchang	PRJNA827465	0.482554155
ENSGALG00010025943	CATH1	2	0.895828648	0.909226059	0.985265039	0.324493901	1.346372188	5.17489768	80.67594643	1	CRA004775;PRJNA827465	Yang2025_Silkie_Wenchang	0.799859262
ENSGALG00010025949	GPATCH1	2	0.170084795	0.149562677	1.137214163	0.255448768	0.036927326	5.695520372	82.44234179	1	PRJNA827465;Yang2025_Silkie_Wenchang	CRA004775	0.764467569
ENSGALG00010025949	GPATCH1	2	0.310592655	0.090113079	3.446698933	0.000567481	0	0.158491359	0	1	CRA004775;Yang2025_Silkie_Wenchang	PRJNA827465	0.025519096
ENSGALG00010025949	GPATCH1	2	0.044467011	0.075520903	0.58880402	0.555992749	0	0.582732279	0	1	CRA004775;PRJNA827465	Yang2025_Silkie_Wenchang	0.880324407
ENSGALG00010025951	TBRG4	2	-0.00378724	0.076029483	-0.049812781	0.960271581	0.003681086	1.316155703	24.02114752	0.5	PRJNA827465;Yang2025_Silkie_Wenchang	CRA004775	0.989338243
ENSGALG00010025951	TBRG4	2	0.014161876	0.166835289	0.084885373	0.93235252	0.021357265	1.494229082	33.07585749	0.5	CRA004775;Yang2025_Silkie_Wenchang	PRJNA827465	0.984224753
ENSGALG00010025951	TBRG4	2	-0.058417661	0.06179763	-0.945305854	0.344502751	0	0.476772293	0	1	CRA004775;PRJNA827465	Yang2025_Silkie_Wenchang	0.806768255
ENSGALG00010025954	TUBA1A	2	0.068809274	0.128311969	0.536265433	0.591775117	0.017835894	1.967358463	49.17042222	0.5	PRJNA827465;Yang2025_Silkie_Wenchang	CRA004775	0.892776493
ENSGALG00010025954	TUBA1A	2	0.182776343	0.151598498	1.205660649	0.227948364	0	0.5864253	0	0.5	CRA004775;Yang2025_Silkie_Wenchang	PRJNA827465	0.740853612
ENSGALG00010025954	TUBA1A	2	-0.025693658	0.083244522	-0.308652843	0.757585619	0	0.001085116	0	1	CRA004775;PRJNA827465	Yang2025_Silkie_Wenchang	0.938948211
ENSGALG00010025957	ENSGALG00010025957	2	-0.046469285	0.153748695	-0.302241816	0.76246773	0.040375237	6.759140513	85.20521954	0.5	PRJNA827465;Yang2025_Silkie_Wenchang	CRA004775	0.940082621
ENSGALG00010025957	ENSGALG00010025957	2	-0.203822778	0.093681654	-2.175695771	0.029578017	0	0.030881582	0	1	CRA004775;Yang2025_Silkie_Wenchang	PRJNA827465	0.293445145
ENSGALG00010025957	ENSGALG00010025957	2	0.093405366	0.067880965	1.37601705	0.168816355	0	0.331302645	0	0.5	CRA004775;PRJNA827465	Yang2025_Silkie_Wenchang	0.669862614
ENSGALG00010025959	UROD	2	-0.209413554	0.121529964	-1.723143385	0.084862599	0.014640554	1.526313444	34.48265795	1	PRJNA827465;Yang2025_Silkie_Wenchang	CRA004775	0.501029702
ENSGALG00010025959	UROD	2	-0.514088012	0.176256274	-2.916707595	0.003537472	0	0.306305393	0	1	CRA004775;Yang2025_Silkie_Wenchang	PRJNA827465	0.08431369
ENSGALG00010025959	UROD	2	-0.313744074	0.233516853	-1.343560735	0.179090474	0.078918099	2.89456031	65.45243861	1	CRA004775;PRJNA827465	Yang2025_Silkie_Wenchang	0.685774834
ENSGALG00010025960	CMKLR2	2	-0.266186961	0.157167368	-1.693652856	0.090331268	0.041830735	6.399233485	84.3731284	1	PRJNA827465;Yang2025_Silkie_Wenchang	CRA004775	0.515982221
ENSGALG00010025960	CMKLR2	2	-0.168371366	0.299528711	-0.562120957	0.57403361	0.149046283	5.494994024	81.80161806	0.5	CRA004775;Yang2025_Silkie_Wenchang	PRJNA827465	0.886081433
ENSGALG00010025960	CMKLR2	2	-0.061708312	0.114472693	-0.539065783	0.589841464	0.011482708	1.38007281	27.54005491	0.5	CRA004775;PRJNA827465	Yang2025_Silkie_Wenchang	0.892320726
ENSGALG00010025962	ENSGALG00010025962	2	0.017922054	0.112728227	0.158984616	0.873680994	0	0.82668125	0	0.5	PRJNA827465;Yang2025_Silkie_Wenchang	CRA004775	0.9699681
ENSGALG00010025962	ENSGALG00010025962	2	0.19222241	0.40359095	0.476264466	0.633857131	0.228695916	3.041562137	67.11999575	0.5	CRA004775;Yang2025_Silkie_Wenchang	PRJNA827465	0.903596555
ENSGALG00010025962	ENSGALG00010025962	2	0.252809162	0.269648227	0.937551731	0.348474827	0.081699761	1.809295244	44.72986079	1	CRA004775;PRJNA827465	Yang2025_Silkie_Wenchang	0.809147894
ENSGALG00010025963	OAF	2	0.005031155	0.290181962	0.01733793	0.986167026	0.151093364	9.205285449	89.13667582	0.5	PRJNA827465;Yang2025_Silkie_Wenchang	CRA004775	0.99618277
ENSGALG00010025963	OAF	2	-0.265481395	0.169183331	-1.569193568	0.116602842	0	0.566152056	0	0.5	CRA004775;Yang2025_Silkie_Wenchang	PRJNA827465	0.57477217
ENSGALG00010025963	OAF	2	0.269759403	0.068541003	3.935737594	8.29416E-05	0	0.106154422	0	1	CRA004775;PRJNA827465	Yang2025_Silkie_Wenchang	0.006805537
ENSGALG00010025968	FADS2	2	0.075414772	0.103730331	0.727027204	0.467209291	0	0.214909017	0	0.5	PRJNA827465;Yang2025_Silkie_Wenchang	CRA004775	0.852885838
ENSGALG00010025968	FADS2	2	0.011363568	0.180804151	0.062850152	0.949885829	0	0.035948616	0	0.5	CRA004775;Yang2025_Silkie_Wenchang	PRJNA827465	0.987277827
ENSGALG00010025968	FADS2	2	0.100312654	0.113807004	0.879670419	0.379037879	0	0.006551648	0	1	CRA004775;PRJNA827465	Yang2025_Silkie_Wenchang	0.823921561
ENSGALG00010025970	KLHL12	2	0.013355917	0.052426112	0.254756959	0.79891082	0	0.279703649	0	1	PRJNA827465;Yang2025_Silkie_Wenchang	CRA004775	0.949922217
ENSGALG00010025970	KLHL12	2	-0.095927858	0.181837674	-0.527546665	0.597814019	0.046192389	3.271413129	69.43217012	0.5	CRA004775;Yang2025_Silkie_Wenchang	PRJNA827465	0.894645663
ENSGALG00010025970	KLHL12	2	-0.108258016	0.141414044	-0.765539353	0.443950442	0.028431457	2.971394382	66.34576662	0.5	CRA004775;PRJNA827465	Yang2025_Silkie_Wenchang	0.846013379
ENSGALG00010025971	TRAFD1	2	0.165796217	0.062421741	2.656065273	0.007905831	0	0.058195395	0	1	PRJNA827465;Yang2025_Silkie_Wenchang	CRA004775	0.138064133
ENSGALG00010025971	TRAFD1	2	-0.036999604	0.284802746	-0.129913088	0.896635189	0.120252424	3.294283491	69.64438541	0.5	CRA004775;Yang2025_Silkie_Wenchang	PRJNA827465	0.975115773
ENSGALG00010025971	TRAFD1	2	-0.045233311	0.266822233	-0.169526019	0.865382908	0.10437335	3.061155263	67.33259459	0.5	CRA004775;PRJNA827465	Yang2025_Silkie_Wenchang	0.967050821
ENSGALG00010025974	EEF1B2	2	0.192135852	0.25922008	0.741207441	0.458567672	0.121874247	10.33769831	90.32666683	0.5	PRJNA827465;Yang2025_Silkie_Wenchang	CRA004775	0.850089963
ENSGALG00010025974	EEF1B2	2	0.268750416	0.242484275	1.108321007	0.267723206	0.077039951	2.708452497	63.07854758	0.5	CRA004775;Yang2025_Silkie_Wenchang	PRJNA827465	0.771398294
ENSGALG00010025974	EEF1B2	2	-0.049661447	0.065307784	-0.760421558	0.447002641	0	0.008705194	0	1	CRA004775;PRJNA827465	Yang2025_Silkie_Wenchang	0.84650962
ENSGALG00010025976	NAA25	2	0.081882982	0.04811112	1.701955439	0.088763722	0	0.042946463	0	1	PRJNA827465;Yang2025_Silkie_Wenchang	CRA004775	0.511686794
ENSGALG00010025976	NAA25	2	0.050550616	0.096441463	0.589613622	0.55549077	0	0.026490855	0	1	CRA004775;Yang2025_Silkie_Wenchang	PRJNA827465	0.880255849
ENSGALG00010025976	NAA25	2	0.08431971	0.053270817	1.582849956	0.113455667	0	0.067360185	0	1	CRA004775;PRJNA827465	Yang2025_Silkie_Wenchang	0.568551332
ENSGALG00010025980	HECTD4	2	-0.0972055	0.083443931	-1.16491994	0.244051401	0.006991226	1.616615701	38.14237981	0.5	PRJNA827465;Yang2025_Silkie_Wenchang	CRA004775	0.755564606
ENSGALG00010025980	HECTD4	2	0.000116542	0.129939056	0.000896901	0.999284377	0	0.59776179	0	0.5	CRA004775;Yang2025_Silkie_Wenchang	PRJNA827465	0.999889665
ENSGALG00010025980	HECTD4	2	-0.147775856	0.047955894	-3.081532739	0.002059378	0	0.057291788	0	1	CRA004775;PRJNA827465	Yang2025_Silkie_Wenchang	0.059037013
ENSGALG00010025982	SERPINE2	2	-0.075794535	0.255173268	-0.297031641	0.766442352	0.122752191	17.40666087	94.25507277	0.5	PRJNA827465;Yang2025_Silkie_Wenchang	CRA004775	0.941152991
ENSGALG00010025982	SERPINE2	2	-0.314733198	0.088953773	-3.538165814	0.000402917	0	0.609020582	0	1	CRA004775;Yang2025_Silkie_Wenchang	PRJNA827465	0.020302365
ENSGALG00010025982	SERPINE2	2	0.166153037	0.07864388	2.112726836	0.034624155	0	0.394388363	0	0.5	CRA004775;PRJNA827465	Yang2025_Silkie_Wenchang	0.320497684
ENSGALG00010025983	RNF26	2	-0.072212457	0.057778634	-1.249812469	0.21136806	0	0.552515601	0	1	PRJNA827465;Yang2025_Silkie_Wenchang	CRA004775	0.723794057
ENSGALG00010025983	RNF26	2	-0.348986234	0.197651323	-1.765666062	0.07745189	0.039471357	1.952324504	48.77900688	1	CRA004775;Yang2025_Silkie_Wenchang	PRJNA827465	0.479280283
ENSGALG00010025983	RNF26	2	-0.272155032	0.261134702	-1.042201707	0.297318172	0.11114392	4.687297777	78.66574629	1	CRA004775;PRJNA827465	Yang2025_Silkie_Wenchang	0.788535199
ENSGALG00010025984	NDUFS1	2	-0.010205756	0.193142794	-0.052840547	0.957858916	0.067909051	11.13429722	91.01874164	0.5	PRJNA827465;Yang2025_Silkie_Wenchang	CRA004775	0.988864712
ENSGALG00010025984	NDUFS1	2	0.177942771	0.080800702	2.202242894	0.027648156	0	0.066398061	0	1	CRA004775;Yang2025_Silkie_Wenchang	PRJNA827465	0.28376061
ENSGALG00010025984	NDUFS1	2	-0.180909474	0.078397247	-2.307599835	0.021021404	0.000263177	1.005612979	0.558164976	0.5	CRA004775;PRJNA827465	Yang2025_Silkie_Wenchang	0.245277621
ENSGALG00010025985	CHCHD10	2	0.219338051	0.207141202	1.058881812	0.289653609	0.033078208	1.502561828	33.44699823	1	PRJNA827465;Yang2025_Silkie_Wenchang	CRA004775	0.784039654
ENSGALG00010025985	CHCHD10	2	0.500642235	0.280024714	1.791432292	0.073225715	0	0.018766218	0	1	CRA004775;Yang2025_Silkie_Wenchang	PRJNA827465	0.467793099
ENSGALG00010025985	CHCHD10	2	0.106265966	0.165680118	0.641392387	0.521267778	0	0.336615481	0	1	CRA004775;PRJNA827465	Yang2025_Silkie_Wenchang	0.869518515
ENSGALG00010025986	RAB3IL1	2	-0.158365163	0.23813446	-0.665024133	0.506035058	0.102508397	9.84128327	89.83872354	0.5	PRJNA827465;Yang2025_Silkie_Wenchang	CRA004775	0.863929781
ENSGALG00010025986	RAB3IL1	2	-0.44641057	0.114951948	-3.883403133	0.000102983	0	0.130005738	0	1	CRA004775;Yang2025_Silkie_Wenchang	PRJNA827465	0.007067611
ENSGALG00010025986	RAB3IL1	2	-0.189572233	0.280527293	-0.675771085	0.499185996	0.139221232	7.943455357	87.41102008	0.5	CRA004775;PRJNA827465	Yang2025_Silkie_Wenchang	0.861784825
ENSGALG00010025988	NSF	2	-0.038353917	0.042947697	-0.893037798	0.371836931	0	0.158033631	0	1	PRJNA827465;Yang2025_Silkie_Wenchang	CRA004775	0.821597118
ENSGALG00010025988	NSF	2	-0.133304667	0.09518717	-1.400447846	0.161379251	0	0.619873393	0	1	CRA004775;Yang2025_Silkie_Wenchang	PRJNA827465	0.657750564
ENSGALG00010025988	NSF	2	-0.075080306	0.08499857	-0.883312275	0.377067412	0.00698221	1.466700404	31.81975016	1	CRA004775;PRJNA827465	Yang2025_Silkie_Wenchang	0.823766945
ENSGALG00010025990	FAM124B	2	0.03253583	0.165686496	0.196369835	0.844320705	0	0.052268918	0	0.5	PRJNA827465;Yang2025_Silkie_Wenchang	CRA004775	0.960723102
ENSGALG00010025990	FAM124B	2	0.073446987	0.224371434	0.327345535	0.743406556	0	0.486622204	0	0.5	CRA004775;Yang2025_Silkie_Wenchang	PRJNA827465	0.935816088
ENSGALG00010025990	FAM124B	2	0.117229828	0.197339706	0.594050887	0.552478078	0	0.327331151	0	1	CRA004775;PRJNA827465	Yang2025_Silkie_Wenchang	0.879228216
ENSGALG00010025992	MMP11	2	0.11956982	0.353420981	0.338321228	0.735121129	0.206482174	5.731044472	82.55117362	0.5	PRJNA827465;Yang2025_Silkie_Wenchang	CRA004775	0.931592889
ENSGALG00010025992	MMP11	2	0.439566547	0.205059271	2.143607288	0.032064366	0	0.203952106	0	1	CRA004775;Yang2025_Silkie_Wenchang	PRJNA827465	0.306978889
ENSGALG00010025992	MMP11	2	-0.154340106	0.172829313	-0.893020421	0.371846237	0	0.918390459	0	0.5	CRA004775;PRJNA827465	Yang2025_Silkie_Wenchang	0.821597118
ENSGALG00010025994	INO80D	2	0.060495455	0.127176403	0.475683144	0.634301345	0.013287224	1.433247542	30.22838199	0.5	PRJNA827465;Yang2025_Silkie_Wenchang	CRA004775	0.903815945
ENSGALG00010025994	INO80D	2	0.140695418	0.200247475	0.702607702	0.482300261	0.007645043	1.093803056	8.575863398	0.5	CRA004775;Yang2025_Silkie_Wenchang	PRJNA827465	0.85691916
ENSGALG00010025994	INO80D	2	-0.020192293	0.086751503	-0.232760145	0.815947662	0	0.134201673	0	1	CRA004775;PRJNA827465	Yang2025_Silkie_Wenchang	0.954326597
ENSGALG00010025996	CUL3	2	-0.171762841	0.052555248	-3.268233828	0.001082209	0	0.007419736	0	1	PRJNA827465;Yang2025_Silkie_Wenchang	CRA004775	0.039806134
ENSGALG00010025996	CUL3	2	0.089709095	0.305614185	0.295473473	0.767632214	0.149760369	4.728968087	78.85373761	0.5	CRA004775;Yang2025_Silkie_Wenchang	PRJNA827465	0.941444269
ENSGALG00010025996	CUL3	2	0.083109613	0.308070777	0.269774413	0.787333808	0.157293763	5.087184487	80.34276126	0.5	CRA004775;PRJNA827465	Yang2025_Silkie_Wenchang	0.946919116
ENSGALG00010025997	CCDC43	2	0.236875325	0.243387775	0.973242494	0.330432796	0.109711841	13.29523295	92.47850712	1	PRJNA827465;Yang2025_Silkie_Wenchang	CRA004775	0.80156474
ENSGALG00010025997	CCDC43	2	0.191915247	0.33188463	0.578258917	0.563089327	0.185346888	5.929167413	83.13422559	0.5	CRA004775;Yang2025_Silkie_Wenchang	PRJNA827465	0.882244362
ENSGALG00010025997	CCDC43	2	-0.010154151	0.065236397	-0.155651621	0.876307633	0	0.490773512	0	0.5	CRA004775;PRJNA827465	Yang2025_Silkie_Wenchang	0.970347777
ENSGALG00010025998	SMARCB1	2	-0.016558658	0.068337551	-0.242306867	0.808542393	0.004481507	1.590528552	37.12781838	0.5	PRJNA827465;Yang2025_Silkie_Wenchang	CRA004775	0.952496413
ENSGALG00010025998	SMARCB1	2	-0.112334957	0.09393133	-1.195926401	0.231725282	0	0.078920772	0	1	CRA004775;Yang2025_Silkie_Wenchang	PRJNA827465	0.744150932
ENSGALG00010025998	SMARCB1	2	0.017592802	0.041543592	0.423478118	0.67194646	0	0.35493964	0	0.5	CRA004775;PRJNA827465	Yang2025_Silkie_Wenchang	0.914714609
ENSGALG00010025999	DBF4B	2	-0.070679027	0.165481862	-0.427110418	0.669298905	0.03109961	2.272550748	55.99658221	0.5	PRJNA827465;Yang2025_Silkie_Wenchang	CRA004775	0.914377769
ENSGALG00010025999	DBF4B	2	-0.079245786	0.238215121	-0.3326648	0.739387323	0.063726507	2.171091075	53.94020953	0.5	CRA004775;Yang2025_Silkie_Wenchang	PRJNA827465	0.934830469
ENSGALG00010025999	DBF4B	2	-0.246264984	0.120638509	-2.041346379	0.04121641	0	0.243863424	0	1	CRA004775;PRJNA827465	Yang2025_Silkie_Wenchang	0.351928866
ENSGALG00010026001	ANKS1A	2	0.062860584	0.085123443	0.738463832	0.460232638	0.004899796	1.336617445	25.18427741	1	PRJNA827465;Yang2025_Silkie_Wenchang	CRA004775	0.85014477
ENSGALG00010026001	ANKS1A	2	0.1553066	0.130391844	1.191076031	0.233623739	0	0.424904797	0	1	CRA004775;Yang2025_Silkie_Wenchang	PRJNA827465	0.746938404
ENSGALG00010026001	ANKS1A	2	0.01500105	0.066042054	0.227143906	0.820311844	0	0.002467966	0	1	CRA004775;PRJNA827465	Yang2025_Silkie_Wenchang	0.955308792
ENSGALG00010026002	FTH1	2	-0.136675039	0.270633666	-0.505018615	0.613545762	0.116559252	4.287060572	76.67399415	0.5	PRJNA827465;Yang2025_Silkie_Wenchang	CRA004775	0.898256044
ENSGALG00010026002	FTH1	2	-0.082360138	0.394196765	-0.208931543	0.83450168	0.237321173	4.218463852	76.29468842	0.5	CRA004775;Yang2025_Silkie_Wenchang	PRJNA827465	0.958093639
ENSGALG00010026002	FTH1	2	0.103545468	0.077390263	1.337965057	0.180907828	0	0.629674443	0	1	CRA004775;PRJNA827465	Yang2025_Silkie_Wenchang	0.688484814
ENSGALG00010026003	NECTIN1	2	0.111200901	0.070220592	1.583593904	0.113286162	0	0.546257821	0	1	PRJNA827465;Yang2025_Silkie_Wenchang	CRA004775	0.568136536
ENSGALG00010026003	NECTIN1	2	-0.018572083	0.15935837	-0.116542877	0.907222307	0.015373945	1.154221805	13.3615397	0.5	CRA004775;Yang2025_Silkie_Wenchang	PRJNA827465	0.978196186
ENSGALG00010026003	NECTIN1	2	0.007606902	0.251928121	0.030194731	0.975911751	0.076076199	1.786772663	44.03317106	0.5	CRA004775;PRJNA827465	Yang2025_Silkie_Wenchang	0.992868007
ENSGALG00010026005	DERL3	2	0.086081413	0.101083098	0.851590568	0.394441377	0.005666252	1.278136682	21.76110628	0.5	PRJNA827465;Yang2025_Silkie_Wenchang	CRA004775	0.830577521
ENSGALG00010026005	DERL3	2	-0.162319186	0.139258803	-1.165593721	0.243778748	0	0.507068903	0	1	CRA004775;Yang2025_Silkie_Wenchang	PRJNA827465	0.755365463
ENSGALG00010026005	DERL3	2	-0.021605783	0.21016115	-0.102805787	0.918117112	0.064387861	3.279662924	69.50906165	0.5	CRA004775;PRJNA827465	Yang2025_Silkie_Wenchang	0.981033557
ENSGALG00010026006	ITGA2B	2	0.500270097	0.179384698	2.788811441	0.005290186	0.02775369	1.75659392	43.0716463	1	PRJNA827465;Yang2025_Silkie_Wenchang	CRA004775	0.108631224
ENSGALG00010026006	ITGA2B	2	0.114022915	0.664143707	0.1716841	0.863685886	0.729342016	5.224200564	80.8583153	0.5	CRA004775;Yang2025_Silkie_Wenchang	PRJNA827465	0.966335868
ENSGALG00010026006	ITGA2B	2	-0.026869228	0.472275709	-0.056893098	0.954630352	0.313857745	2.841770253	64.81066692	0.5	CRA004775;PRJNA827465	Yang2025_Silkie_Wenchang	0.987987175
ENSGALG00010026008	NRP2	2	-0.108691625	0.079117202	-1.373805214	0.169502162	7.89418E-05	1.005820375	0.578669424	1	PRJNA827465;Yang2025_Silkie_Wenchang	CRA004775	0.670890582
ENSGALG00010026008	NRP2	2	-0.035258178	0.12267686	-0.287406919	0.773800764	0	0.4445994	0	1	CRA004775;Yang2025_Silkie_Wenchang	PRJNA827465	0.943248868
ENSGALG00010026008	NRP2	2	-0.173420338	0.094128534	-1.842377977	0.065419881	0	0.043143651	0	1	CRA004775;PRJNA827465	Yang2025_Silkie_Wenchang	0.440801916
ENSGALG00010026009	ENSGALG00010026009	2	0.309316293	0.266320414	1.161444173	0.245461314	0.11022404	4.479596202	77.67655934	1	PRJNA827465;Yang2025_Silkie_Wenchang	CRA004775	0.75661406
ENSGALG00010026009	ENSGALG00010026009	2	0.225121414	0.4231741	0.531982969	0.594737791	0.271142171	3.766526349	73.4503384	0.5	CRA004775;Yang2025_Silkie_Wenchang	PRJNA827465	0.893382718
ENSGALG00010026009	ENSGALG00010026009	2	-0.001880074	0.156957753	-0.011978218	0.990442994	0	0.558792549	0	0.5	CRA004775;PRJNA827465	Yang2025_Silkie_Wenchang	0.997468525
ENSGALG00010026010	ZSWIM5	2	0.061479752	0.223766702	0.274749331	0.783508829	0.092776595	13.58825198	92.64070168	0.5	PRJNA827465;Yang2025_Silkie_Wenchang	CRA004775	0.945815798
ENSGALG00010026010	ZSWIM5	2	0.270974797	0.084745792	3.19750149	0.001386237	0	0.308884093	0	1	CRA004775;Yang2025_Silkie_Wenchang	PRJNA827465	0.045707442
ENSGALG00010026010	ZSWIM5	2	-0.129331064	0.090365124	-1.43120552	0.152371322	0.002396956	1.060491859	5.704132344	0.5	CRA004775;PRJNA827465	Yang2025_Silkie_Wenchang	0.643940868
ENSGALG00010026011	GJC2	2	-0.025560107	0.061826672	-0.413415541	0.67930219	0	0.44095758	0	0.5	PRJNA827465;Yang2025_Silkie_Wenchang	CRA004775	0.917701868
ENSGALG00010026011	GJC2	2	0.019543015	0.169769044	0.115115303	0.908353731	0.027918443	1.628841686	38.60667929	0.5	CRA004775;Yang2025_Silkie_Wenchang	PRJNA827465	0.978271131
ENSGALG00010026011	GJC2	2	0.027176437	0.080822321	0.33624915	0.736682992	0.001476809	1.035750074	3.451612048	1	CRA004775;PRJNA827465	Yang2025_Silkie_Wenchang	0.933754245
ENSGALG00010026013	RABIF	2	0.264142353	0.326305592	0.809493798	0.418231169	0.199868467	15.80895274	93.67447031	0.5	PRJNA827465;Yang2025_Silkie_Wenchang	CRA004775	0.838112595
ENSGALG00010026013	RABIF	2	0.279785255	0.370938681	0.75426282	0.450691423	0.220564415	4.714380307	78.7883044	0.5	CRA004775;Yang2025_Silkie_Wenchang	PRJNA827465	0.847631399
ENSGALG00010026013	RABIF	2	-0.051981055	0.062668169	-0.829465036	0.406841314	0	0.090311512	0	1	CRA004775;PRJNA827465	Yang2025_Silkie_Wenchang	0.833618122
ENSGALG00010026016	SLC2A11	2	0.591356495	0.264984629	2.23166339	0.025637219	0	0.010279242	0	1	PRJNA827465;Yang2025_Silkie_Wenchang	CRA004775	0.27294819
ENSGALG00010026016	SLC2A11	2	-0.03301405	0.691271571	-0.047758437	0.961908761	0.671515468	3.192398269	68.67558757	0.5	CRA004775;Yang2025_Silkie_Wenchang	PRJNA827465	0.989838719
ENSGALG00010026016	SLC2A11	2	0.00174324	0.717458634	0.002429743	0.998061348	0.765526335	3.645044371	72.56549171	0.5	CRA004775;PRJNA827465	Yang2025_Silkie_Wenchang	0.999313737
ENSGALG00010026017	PLEKHM1	2	-0.062312148	0.061977576	-1.005398277	0.314705109	0	0.485243848	0	0.5	PRJNA827465;Yang2025_Silkie_Wenchang	CRA004775	0.796531448
ENSGALG00010026017	PLEKHM1	2	-0.24321698	0.261951153	-0.92848219	0.353157502	0.116631473	6.520826203	84.66451997	0.5	CRA004775;Yang2025_Silkie_Wenchang	PRJNA827465	0.81107437
ENSGALG00010026017	PLEKHM1	2	-0.278367578	0.213675614	-1.302757826	0.192657453	0.075321543	5.290956326	81.09982509	1	CRA004775;PRJNA827465	Yang2025_Silkie_Wenchang	0.702608879
ENSGALG00010026023	THY1	2	0.153927121	0.230680609	0.667273776	0.504597274	0.04958768	1.773898325	43.62698324	0.5	PRJNA827465;Yang2025_Silkie_Wenchang	CRA004775	0.863512504
ENSGALG00010026023	THY1	2	0.324394821	0.232429835	1.395667733	0.162814575	0	0.423527333	0	1	CRA004775;Yang2025_Silkie_Wenchang	PRJNA827465	0.660874227
ENSGALG00010026023	THY1	2	0.012214915	0.166712654	0.073269274	0.941591842	0	0.175517393	0	0.5	CRA004775;PRJNA827465	Yang2025_Silkie_Wenchang	0.985827325
ENSGALG00010026030	IBA57	2	-0.159411053	0.113775993	-1.401095691	0.16118546	0.012050889	1.836370888	45.5447695	1	PRJNA827465;Yang2025_Silkie_Wenchang	CRA004775	0.657426695
ENSGALG00010026030	IBA57	2	-0.198494937	0.096891828	-2.048624117	0.040498885	0	0.967600171	0	1	CRA004775;Yang2025_Silkie_Wenchang	PRJNA827465	0.348223071
ENSGALG00010026030	IBA57	2	-0.075159946	0.082523289	-0.910772548	0.362415229	0	0.051948099	0	1	CRA004775;PRJNA827465	Yang2025_Silkie_Wenchang	0.815644888
ENSGALG00010026036	TUB5A	2	0.056000742	0.063508247	0.881786931	0.37789204	0	0.313880259	0	1	PRJNA827465;Yang2025_Silkie_Wenchang	CRA004775	0.82325159
ENSGALG00010026036	TUB5A	2	0.10830768	0.152741451	0.709091601	0.478267635	0	0.423151473	0	0.5	CRA004775;Yang2025_Silkie_Wenchang	PRJNA827465	0.855397171
ENSGALG00010026036	TUB5A	2	0.036163952	0.068671596	0.526621685	0.59845633	0	0.242793321	0	0.5	CRA004775;PRJNA827465	Yang2025_Silkie_Wenchang	0.894645663
ENSGALG00010026040	GUK1	2	-0.091771447	0.110386964	-0.831361272	0.40576957	0.009426611	1.5755633	36.53063638	1	PRJNA827465;Yang2025_Silkie_Wenchang	CRA004775	0.833357445
ENSGALG00010026040	GUK1	2	-0.226178643	0.134165119	-1.685823002	0.091829896	0	0.007960164	0	1	CRA004775;Yang2025_Silkie_Wenchang	PRJNA827465	0.519097275
ENSGALG00010026040	GUK1	2	-0.025073249	0.096149084	-0.2607747	0.794266256	0	0.385208168	0	1	CRA004775;PRJNA827465	Yang2025_Silkie_Wenchang	0.948905846
ENSGALG00010026044	CERKL	2	-0.279690468	0.335157078	-0.834505628	0.403996117	0.17043241	4.056898517	75.35062817	0.5	PRJNA827465;Yang2025_Silkie_Wenchang	CRA004775	0.833112102
ENSGALG00010026044	CERKL	2	-0.578506277	0.251558223	-2.299691379	0.021465711	0	0.383592469	0	1	CRA004775;Yang2025_Silkie_Wenchang	PRJNA827465	0.247834011
ENSGALG00010026044	CERKL	2	0.010150307	0.184916024	0.054891441	0.956224951	0	0.118843353	0	0.5	CRA004775;PRJNA827465	Yang2025_Silkie_Wenchang	0.988519141
ENSGALG00010026051	TAF11	2	-0.126556092	0.187569305	-0.674716431	0.499855943	0.050991638	3.019171532	66.87833104	0.5	PRJNA827465;Yang2025_Silkie_Wenchang	CRA004775	0.862078151
ENSGALG00010026051	TAF11	2	-0.400744338	0.16397329	-2.443961071	0.014526994	0	0.031250273	0	1	CRA004775;Yang2025_Silkie_Wenchang	PRJNA827465	0.198277074
ENSGALG00010026051	TAF11	2	-0.143574889	0.214516944	-0.66929393	0.503307998	0.066916685	2.969309109	66.32213208	0.5	CRA004775;PRJNA827465	Yang2025_Silkie_Wenchang	0.863108263
ENSGALG00010026061	MPEG1	2	-0.167091935	0.114942343	-1.453702184	0.146028892	0.002502135	1.098437968	8.961631924	1	PRJNA827465;Yang2025_Silkie_Wenchang	CRA004775	0.634897819
ENSGALG00010026061	MPEG1	2	-0.405349877	0.164114704	-2.46991809	0.013514399	0.003651761	1.052354913	4.975024336	1	CRA004775;Yang2025_Silkie_Wenchang	PRJNA827465	0.190294637
ENSGALG00010026061	MPEG1	2	-0.3144605	0.301347133	-1.04351582	0.296709458	0.128578469	3.031738568	67.0156256	1	CRA004775;PRJNA827465	Yang2025_Silkie_Wenchang	0.788458494
ENSGALG00010026062	ITGA4	2	0.093843412	0.084109222	1.115732737	0.264536553	0	0.483743803	0	1	PRJNA827465;Yang2025_Silkie_Wenchang	CRA004775	0.768855521
ENSGALG00010026062	ITGA4	2	0.149636396	0.148563823	1.007219606	0.313829253	0	0.626723642	0	0.5	CRA004775;Yang2025_Silkie_Wenchang	PRJNA827465	0.796531448
ENSGALG00010026062	ITGA4	2	0.045302856	0.098395027	0.460418149	0.64521611	0	0.325971407	0	0.5	CRA004775;PRJNA827465	Yang2025_Silkie_Wenchang	0.906966303
ENSGALG00010026064	ENSGALG00010026064	2	-0.030793741	0.161880707	-0.190224899	0.849132901	0.030004255	1.831913312	45.41226414	0.5	PRJNA827465;Yang2025_Silkie_Wenchang	CRA004775	0.961438996
ENSGALG00010026064	ENSGALG00010026064	2	-0.228871817	0.200461546	-1.141724297	0.253568638	0	0.169676083	0	1	CRA004775;Yang2025_Silkie_Wenchang	PRJNA827465	0.763346521
ENSGALG00010026064	ENSGALG00010026064	2	0.057525635	0.075431232	0.762623562	0.445687928	0	0.348248848	0	0.5	CRA004775;PRJNA827465	Yang2025_Silkie_Wenchang	0.846013379
ENSGALG00010026066	SELENOP2	2	0.613358616	0.294879823	2.080029114	0.037522863	0	0.199322594	0	1	PRJNA827465;Yang2025_Silkie_Wenchang	CRA004775	0.334034216
ENSGALG00010026066	SELENOP2	2	0.982709243	0.544480577	1.80485638	0.071097159	0.379876251	2.660042095	62.40660996	1	CRA004775;Yang2025_Silkie_Wenchang	PRJNA827465	0.460518035
ENSGALG00010026066	SELENOP2	2	1.170127538	0.41020266	2.852559604	0.004336868	0.046708248	1.159322053	13.7426915	1	CRA004775;PRJNA827465	Yang2025_Silkie_Wenchang	0.096523938
ENSGALG00010026068	BLTP3A	2	-0.140187201	0.047616832	-2.944068187	0.003239286	0	0.051172289	0	1	PRJNA827465;Yang2025_Silkie_Wenchang	CRA004775	0.079076812
ENSGALG00010026068	BLTP3A	2	-0.109064806	0.125644648	-0.868041797	0.385371452	0	0.000170736	0	1	CRA004775;Yang2025_Silkie_Wenchang	PRJNA827465	0.82648313
ENSGALG00010026068	BLTP3A	2	-0.142730512	0.049679614	-2.873019726	0.004065687	0	0.019244701	0	1	CRA004775;PRJNA827465	Yang2025_Silkie_Wenchang	0.092526756
ENSGALG00010026073	HPDL	2	0.062225771	0.186633785	0.333411074	0.738824005	0	0.297624632	0	1	PRJNA827465;Yang2025_Silkie_Wenchang	CRA004775	0.934613258
ENSGALG00010026073	HPDL	2	-0.197453098	0.474657681	-0.415990526	0.677416936	0.268825167	2.473142242	59.56560917	0.5	CRA004775;Yang2025_Silkie_Wenchang	PRJNA827465	0.917144807
ENSGALG00010026073	HPDL	2	-0.229480833	0.332201632	-0.690787794	0.489698907	0.123274108	2.008124248	50.20228449	0.5	CRA004775;PRJNA827465	Yang2025_Silkie_Wenchang	0.858425426
ENSGALG00010026076	C1orf35	2	0.194119337	0.254270013	0.763437789	0.44520235	0.108949472	6.230979392	83.9511586	0.5	PRJNA827465;Yang2025_Silkie_Wenchang	CRA004775	0.846013379
ENSGALG00010026076	C1orf35	2	0.221900864	0.254526445	0.871818502	0.383307399	0.094845617	3.707055825	73.02441487	0.5	CRA004775;Yang2025_Silkie_Wenchang	PRJNA827465	0.825486615
ENSGALG00010026076	C1orf35	2	-0.045320614	0.099507921	-0.455447299	0.648787474	0	7.77731E-07	0	1	CRA004775;PRJNA827465	Yang2025_Silkie_Wenchang	0.907735854
ENSGALG00010026078	UBE2E3	2	-0.422530435	0.347103521	-1.217303799	0.22348865	0.236302334	51.54430664	98.05992152	1	PRJNA827465;Yang2025_Silkie_Wenchang	CRA004775	0.736893825
ENSGALG00010026078	UBE2E3	2	-0.379016887	0.412987676	-0.917743819	0.358753001	0.318726566	14.74178105	93.21655914	0.5	CRA004775;Yang2025_Silkie_Wenchang	PRJNA827465	0.814232788
ENSGALG00010026078	UBE2E3	2	-0.06971164	0.050501918	-1.380376083	0.16747088	0	0.414558568	0	0.5	CRA004775;PRJNA827465	Yang2025_Silkie_Wenchang	0.667218247
ENSGALG00010026081	KPNA1	2	-0.032190167	0.094969983	-0.33895096	0.734646673	0.010025956	1.77601812	43.69426816	0.5	PRJNA827465;Yang2025_Silkie_Wenchang	CRA004775	0.933520234
ENSGALG00010026081	KPNA1	2	-0.038635069	0.19849504	-0.194639972	0.845674808	0.038429356	1.823664474	45.16535171	0.5	CRA004775;Yang2025_Silkie_Wenchang	PRJNA827465	0.961178629
ENSGALG00010026081	KPNA1	2	0.03173794	0.047272034	0.671389346	0.501972531	0	0.545442083	0	1	CRA004775;PRJNA827465	Yang2025_Silkie_Wenchang	0.862795569
ENSGALG00010026083	MUTYH	2	0.161193292	0.136422908	1.18157056	0.237376141	0	0.000373535	0	1	PRJNA827465;Yang2025_Silkie_Wenchang	CRA004775	0.749366344
ENSGALG00010026083	MUTYH	2	-0.222772191	0.371008695	-0.600450053	0.548206338	0.186793434	3.095086099	67.69072109	0.5	CRA004775;Yang2025_Silkie_Wenchang	PRJNA827465	0.878281266
ENSGALG00010026083	MUTYH	2	-0.172098565	0.366266524	-0.469872494	0.638446117	0.221674427	5.504037983	81.83152073	0.5	CRA004775;PRJNA827465	Yang2025_Silkie_Wenchang	0.90561743
ENSGALG00010026088	SNRPC	2	-0.234676714	0.194031987	-1.209474362	0.226480655	0.058460246	3.89199527	74.30623804	1	PRJNA827465;Yang2025_Silkie_Wenchang	CRA004775	0.73999245
ENSGALG00010026088	SNRPC	2	-0.383341959	0.155232838	-2.469464352	0.01353155	0	0.66339112	0	1	CRA004775;Yang2025_Silkie_Wenchang	PRJNA827465	0.190294637
ENSGALG00010026088	SNRPC	2	-0.084228868	0.060890886	-1.383275455	0.166580421	0	0.225300406	0	1	CRA004775;PRJNA827465	Yang2025_Silkie_Wenchang	0.665626909
ENSGALG00010026090	USP2	2	-0.144433888	0.066566837	-2.169757407	0.030025229	0	0.476922362	0	1	PRJNA827465;Yang2025_Silkie_Wenchang	CRA004775	0.295537581
ENSGALG00010026090	USP2	2	-0.107549216	0.085628017	-1.256004983	0.209114164	0	0.009506671	0	1	CRA004775;Yang2025_Silkie_Wenchang	PRJNA827465	0.721011841
ENSGALG00010026090	USP2	2	-0.199525328	0.103371588	-1.930175701	0.053585071	0	0.001479947	0	1	CRA004775;PRJNA827465	Yang2025_Silkie_Wenchang	0.401444454
ENSGALG00010026091	DTX4	2	-0.125435545	0.055673802	-2.25304437	0.024256352	0	0.579607986	0	1	PRJNA827465;Yang2025_Silkie_Wenchang	CRA004775	0.266149465
ENSGALG00010026091	DTX4	2	-0.228420806	0.113725134	-2.008534061	0.04458657	0	0.211461275	0	1	CRA004775;Yang2025_Silkie_Wenchang	PRJNA827465	0.364257243
ENSGALG00010026091	DTX4	2	-0.113607121	0.061359766	-1.851492106	0.064098789	0	0.656086036	0	1	CRA004775;PRJNA827465	Yang2025_Silkie_Wenchang	0.436198289
ENSGALG00010026093	TOE1	2	0.005632484	0.061432058	0.091686395	0.926947207	0.001459184	1.213388587	17.58617059	0.5	PRJNA827465;Yang2025_Silkie_Wenchang	CRA004775	0.982896295
ENSGALG00010026093	TOE1	2	-0.088706081	0.090111964	-0.984398489	0.324919621	0	0.0921857	0	1	CRA004775;Yang2025_Silkie_Wenchang	PRJNA827465	0.799914637
ENSGALG00010026093	TOE1	2	0.037028043	0.063734691	0.580971564	0.561259618	0	0.660768139	0	0.5	CRA004775;PRJNA827465	Yang2025_Silkie_Wenchang	0.881506202
ENSGALG00010026094	C6orf106	2	-0.270369709	0.183407614	-1.474146591	0.140442126	0.060646861	9.713713778	89.70527624	1	PRJNA827465;Yang2025_Silkie_Wenchang	CRA004775	0.624550824
ENSGALG00010026094	C6orf106	2	-0.227464903	0.270373966	-0.841297356	0.400181372	0.118830503	5.02576253	80.10252187	0.5	CRA004775;Yang2025_Silkie_Wenchang	PRJNA827465	0.832246851
ENSGALG00010026094	C6orf106	2	-0.091379276	0.046207218	-1.977597426	0.04797414	0	0.631684998	0	0.5	CRA004775;PRJNA827465	Yang2025_Silkie_Wenchang	0.378772122
ENSGALG00010026095	MIF	2	0.348705163	0.111809224	3.118751312	0.001816192	0	0.001801015	0	1	PRJNA827465;Yang2025_Silkie_Wenchang	CRA004775	0.054422978
ENSGALG00010026095	MIF	2	-0.030177107	0.380403104	-0.079329286	0.936770713	0.225922447	4.546071805	78.00298713	0.5	CRA004775;Yang2025_Silkie_Wenchang	PRJNA827465	0.985038972

ENSGALG00010026095	MIF	2	0.000995118	0.384556321	0.002587705	0.997935312	0.254450149	6.795450418	85.284272	0.5	CRA004775;PRJNA827465	Yang2025_Silkie_Wenchang	0.999290321
ENSGALG00010026097	ARF1	2	-0.24640936	0.284795479	-0.865215141	0.386920717	0.156136481	25.90919547	96.14036645	0.5	PRJNA827465;Yang2025_Silkie_Wenchang	CRA004775	0.827149575
ENSGALG00010026097	ARF1	2	-0.29972379	0.250890137	-1.194641582	0.232227096	0.109398122	7.557271514	86.7677111	1	CRA004775;Yang2025_Silkie_Wenchang	PRJNA827465	0.744836098
ENSGALG00010026097	ARF1	2	0.026596994	0.026851046	0.990538464	0.321910999	0	0.205699368	0	0.5	CRA004775;PRJNA827465	Yang2025_Silkie_Wenchang	0.798513866
ENSGALG00010026098	STAT3	2	-0.083438951	0.099156635	-0.841486312	0.40007555	0.012998161	2.360769165	57.6409242	1	PRJNA827465;Yang2025_Silkie_Wenchang	CRA004775	0.832246851
ENSGALG00010026098	STAT3	2	-0.293546361	0.114882316	-2.555191878	0.010612928	0	0.983241274	0	1	CRA004775;Yang2025_Silkie_Wenchang	PRJNA827465	0.16428511
ENSGALG00010026098	STAT3	2	-0.199014908	0.23138861	-0.860089475	0.389739723	0.085828364	4.174284309	76.04379755	1	CRA004775;PRJNA827465	Yang2025_Silkie_Wenchang	0.829068422
ENSGALG00010026102	IPO9	2	-0.06144974	0.043145305	-1.424250908	0.154373853	0.001963111	2.114673618	52.71137866	1	PRJNA827465;Yang2025_Silkie_Wenchang	CRA004775	0.646989113
ENSGALG00010026102	IPO9	2	-0.108053599	0.041659022	-2.593762293	0.00949321	0	0.076186433	0	1	CRA004775;Yang2025_Silkie_Wenchang	PRJNA827465	0.153228591
ENSGALG00010026102	IPO9	2	-0.025656175	0.040163441	-0.638794252	0.522956788	0	0.537879029	0	1	CRA004775;PRJNA827465	Yang2025_Silkie_Wenchang	0.869568211
ENSGALG00010026104	DDX51	2	0.207363083	0.114055833	1.818083985	0.069051297	0.01576201	2.532446572	60.51249369	1	PRJNA827465;Yang2025_Silkie_Wenchang	CRA004775	0.452607154
ENSGALG00010026104	DDX51	2	0.031370465	0.312873905	0.10026552	0.92013353	0.173150584	8.403010897	88.09950371	0.5	CRA004775;Yang2025_Silkie_Wenchang	PRJNA827465	0.981364549
ENSGALG00010026104	DDX51	2	-0.069375526	0.196631033	-0.352820837	0.724222762	0.056983397	3.532111846	71.68832575	0.5	CRA004775;PRJNA827465	Yang2025_Silkie_Wenchang	0.930387861
ENSGALG00010026105	CNTF	2	0.066665622	0.181141876	0.368029877	0.712850957	0	0.46917067	0	1	PRJNA827465;Yang2025_Silkie_Wenchang	CRA004775	0.926097326
ENSGALG00010026105	CNTF	2	-0.250300832	0.577630662	-0.433323313	0.664779904	0.488282708	3.713866617	73.0738849	0.5	CRA004775;Yang2025_Silkie_Wenchang	PRJNA827465	0.913393112
ENSGALG00010026105	CNTF	2	-0.327947598	0.415115303	-0.790015681	0.429518611	0.239937704	2.9515876	66.11992813	0.5	CRA004775;PRJNA827465	Yang2025_Silkie_Wenchang	0.842012722
ENSGALG00010026106	TESK2	2	-0.070877182	0.094513771	-0.749913805	0.45330662	0.007244344	1.666578712	39.99683348	0.5	PRJNA827465;Yang2025_Silkie_Wenchang	CRA004775	0.848869458
ENSGALG00010026106	TESK2	2	-0.145288351	0.107814275	-1.347579909	0.177793536	0	0.747632795	0	0.5	CRA004775;Yang2025_Silkie_Wenchang	PRJNA827465	0.683951879
ENSGALG00010026106	TESK2	2	0.021529266	0.090085884	0.238986011	0.81111643	0	0.108998647	0	1	CRA004775;PRJNA827465	Yang2025_Silkie_Wenchang	0.953179464
ENSGALG00010026111	ENSGALG00010026111	2	-0.147075278	0.37429337	-0.392941178	0.694362927	0.267473829	21.91796724	95.4375331	0.5	PRJNA827465;Yang2025_Silkie_Wenchang	CRA004775	0.920728119
ENSGALG00010026111	ENSGALG00010026111	2	-0.268821755	0.289452469	-0.928725038	0.353031602	0.133397998	4.672735881	78.59926121	0.5	CRA004775;Yang2025_Silkie_Wenchang	PRJNA827465	0.811035834
ENSGALG00010026111	ENSGALG00010026111	2	0.199581014	0.085281309	2.340266763	0.01926997	0	0.432184091	0	1	CRA004775;PRJNA827465	Yang2025_Silkie_Wenchang	0.232624497
ENSGALG00010026112	ZFP91	2	0.017757444	0.03189594	0.556730552	0.57771154	0	0.002157131	0	1	PRJNA827465;Yang2025_Silkie_Wenchang	CRA004775	0.887389782
ENSGALG00010026112	ZFP91	2	0.024128105	0.054725854	0.440890419	0.65929233	0	0.080207852	0	1	CRA004775;Yang2025_Silkie_Wenchang	PRJNA827465	0.911449691
ENSGALG00010026112	ZFP91	2	0.018869739	0.037942974	0.497313188	0.618964547	0	0.092108288	0	1	CRA004775;PRJNA827465	Yang2025_Silkie_Wenchang	0.899168321
ENSGALG00010026113	MFRP	2	-0.150185049	0.309843064	-0.484713285	0.627879742	0.076042724	1.655750906	39.60444193	0.5	PRJNA827465;Yang2025_Silkie_Wenchang	CRA004775	0.901891583
ENSGALG00010026113	MFRP	2	0.07440199	0.313351478	0.237439409	0.81231592	0	0.409981988	0	0.5	CRA004775;Yang2025_Silkie_Wenchang	PRJNA827465	0.953371387
ENSGALG00010026113	MFRP	2	-0.450898928	0.313661385	-1.437534073	0.150566299	0	0.004932429	0	1	CRA004775;PRJNA827465	Yang2025_Silkie_Wenchang	0.64169456
ENSGALG00010026114	CWC22	2	0.057163284	0.048120291	1.187924745	0.23486306	0	0.027964335	0	1	PRJNA827465;Yang2025_Silkie_Wenchang	CRA004775	0.74779162
ENSGALG00010026114	CWC22	2	0.137957651	0.113765132	1.212653195	0.225262442	0	0.605072207	0	1	CRA004775;Yang2025_Silkie_Wenchang	PRJNA827465	0.738344571
ENSGALG00010026114	CWC22	2	0.071473388	0.05864132	1.218822966	0.222911393	0.001097562	1.049483909	4.715070769	1	CRA004775;PRJNA827465	Yang2025_Silkie_Wenchang	0.736458649
ENSGALG00010026115	MMACHC	2	0.088414524	0.345959466	0.255563245	0.798288103	0.20239873	6.304397371	84.13805569	0.5	PRJNA827465;Yang2025_Silkie_Wenchang	CRA004775	0.949992217
ENSGALG00010026115	MMACHC	2	-0.270443251	0.185385761	-1.458813497	0.144616447	0	0.007259401	0	1	CRA004775;Yang2025_Silkie_Wenchang	PRJNA827465	0.632135862
ENSGALG00010026115	MMACHC	2	0.133617028	0.326192858	0.409625854	0.682080428	0.161997951	3.869197251	74.15484569	0.5	CRA004775;PRJNA827465	Yang2025_Silkie_Wenchang	0.918515969
ENSGALG00010026116	DLX3	2	0.447998621	0.376726427	1.189188198	0.234365618	0	0.063328089	0	1	PRJNA827465;Yang2025_Silkie_Wenchang	CRA004775	0.747486154
ENSGALG00010026116	DLX3	2	0.215596188	0.431370101	0.499794	0.61722013	0	0.93976582	0	0.5	CRA004775;Yang2025_Silkie_Wenchang	PRJNA827465	0.899121942
ENSGALG00010026116	DLX3	2	0.120997521	0.413189925	0.292837541	0.769646332	0	0.599354085	0	0.5	CRA004775;PRJNA827465	Yang2025_Silkie_Wenchang	0.941850415
ENSGALG00010026117	LZTR1	2	-0.079720553	0.039638214	-2.011204488	0.044303862	0	0.773672941	0	1	PRJNA827465;Yang2025_Silkie_Wenchang	CRA004775	0.363642137
ENSGALG00010026117	LZTR1	2	-0.256363804	0.132249779	-1.93848191	0.052564456	0.022071273	2.557226389	60.89513215	1	CRA004775;Yang2025_Silkie_Wenchang	PRJNA827465	0.398138524
ENSGALG00010026117	LZTR1	2	-0.212150213	0.175226625	-1.210719054	0.226003097	0.051368143	5.478786621	81.74778342	1	CRA004775;PRJNA827465	Yang2025_Silkie_Wenchang	0.739354064
ENSGALG00010026120	C1QTNF5	2	0.060374711	0.108643368	0.555714653	0.578405938	0	0.002110503	0	1	PRJNA827465;Yang2025_Silkie_Wenchang	CRA004775	0.887855709
ENSGALG00010026120	C1QTNF5	2	-0.007036893	0.221127675	-0.031822762	0.974613394	0	0.132702632	0	0.5	CRA004775;Yang2025_Silkie_Wenchang	PRJNA827465	0.992492038
ENSGALG00010026120	C1QTNF5	2	0.046634416	0.113501349	0.410871028	0.681167108	0	0.209559058	0	0.5	CRA004775;PRJNA827465	Yang2025_Silkie_Wenchang	0.91820222
ENSGALG00010026121	ENSGALG00010026121	2	0.397930344	0.212528659	1.87236087	0.061156695	0.036992032	1.663589799	39.88902789	1	PRJNA827465;Yang2025_Silkie_Wenchang	CRA004775	0.427390038
ENSGALG00010026121	ENSGALG00010026121	2	-0.424081718	0.597801605	-0.709402107	0.478074981	0.613923507	7.007168079	85.72889948	0.5	CRA004775;Yang2025_Silkie_Wenchang	PRJNA827465	0.855366076
ENSGALG00010026121	ENSGALG00010026121	2	-0.206552412	0.813215075	-0.253994814	0.799499564	1.236988848	15.17294671	93.4093224	0.5	CRA004775;PRJNA827465	Yang2025_Silkie_Wenchang	0.949922217
ENSGALG00010026124	PACSIN1	2	0.323359031	0.136573072	2.367663153	0.017900825	0	0.79287897	0	1	PRJNA827465;Yang2025_Silkie_Wenchang	CRA004775	0.223309996
ENSGALG00010026124	PACSIN1	2	0.602809645	0.327319891	1.841652958	0.065525929	0	0.044170288	0	1	CRA004775;Yang2025_Silkie_Wenchang	PRJNA827465	0.441389365
ENSGALG00010026124	PACSIN1	2	0.289311693	0.141927385	2.0384487	0.04150508	0	0.119369835	0	1	CRA004775;PRJNA827465	Yang2025_Silkie_Wenchang	0.353189581
ENSGALG00010026127	ENSGALG00010026127	2	-1.704314826	0.867269684	-1.965149776	0.049396905	1.27151349	6.333889046	84.21191163	1	PRJNA827465;Yang2025_Silkie_Wenchang	CRA004775	0.384595411
ENSGALG00010026127	ENSGALG00010026127	2	-1.366343943	1.286027478	-1.062453149	0.288030077	2.910475011	8.300369718	87.95234388	1	CRA004775;Yang2025_Silkie_Wenchang	PRJNA827465	0.782840067
ENSGALG00010026127	ENSGALG00010026127	2	-0.662851306	0.370647658	-1.788359623	0.073718006	0.041634344	1.135681763	11.94716402	1	CRA004775;PRJNA827465	Yang2025_Silkie_Wenchang	0.469290648
ENSGALG00010026128	PRDX1	2	-0.286592441	0.400040957	-0.716407747	0.473739613	0.473739613	10.84649859	90.78043488	0.5	PRJNA827465;Yang2025_Silkie_Wenchang	CRA004775	0.85444425
ENSGALG00010026128	PRDX1	2	-0.338865657	0.366855655	-0.923730294	0.355641001	0.224413886	5.955347994	83.20837001	0.5	CRA004775;Yang2025_Silkie_Wenchang	PRJNA827465	0.812301302
ENSGALG00010026128	PRDX1	2	0.076414834	0.044971683	1.699176638	0.089285907	0	0.132764331	0	1	CRA004775;PRJNA827465	Yang2025_Silkie_Wenchang	0.512891552
ENSGALG00010026132	DDTL	2	-0.30086654	0.51965302	-0.578975833	0.56260548	0.508388983	16.67694676	94.00369855	0.5	PRJNA827465;Yang2025_Silkie_Wenchang	CRA004775	0.881978509
ENSGALG00010026132	DDTL	2	-0.726050863	0.168667463	-4.304629054	1.67266E-05	0	0.542478923	0	1	CRA004775;Yang2025_Silkie_Wenchang	PRJNA827465	0.002141383
ENSGALG00010026132	DDTL	2	-0.16393929	0.394070986	-0.416014615	0.677399908	0.275250081	8.37639093	88.06168303	0.5	CRA004775;PRJNA827465	Yang2025_Silkie_Wenchang	0.917144807
ENSGALG00010026137	LPXN	2	0.698922754	0.22232927	3.143638052	0.001668617	0	0.023156897	0	1	PRJNA827465;Yang2025_Silkie_Wenchang	CRA004775	0.051618099
ENSGALG00010026137	LPXN	2	0.073540355	0.669420753	0.1098567	0.912523023	0.782225844	7.849896682	87.26097883	0.5	CRA004775;Yang2025_Silkie_Wenchang	PRJNA827465	0.978967245
ENSGALG00010026137	LPXN	2	0.042397737	0.635525672	0.066712863	0.946810294	0.696686389	7.254621799	86.21569415	0.5	CRA004775;PRJNA827465	Yang2025_Silkie_Wenchang	0.986645196
ENSGALG00010026143	RNPEP	2	-0.047162068	0.084584152	-0.557575707	0.577134149	0.00421858	1.149030294	12.97009271	1	PRJNA827465;Yang2025_Silkie_Wenchang	CRA004775	0.887145528
ENSGALG00010026143	RNPEP	2	-0.188711671	0.123256416	-1.531049471	0.125757167	0	0.176955678	0	1	CRA004775;Yang2025_Silkie_Wenchang	PRJNA827465	0.595474757
ENSGALG00010026143	RNPEP	2	-0.037097149	0.057831403	-0.641471407	0.521216452	0	0.780939508	0	1	CRA004775;PRJNA827465	Yang2025_Silkie_Wenchang	0.869518515
ENSGALG00010026148	RPS10	2	0.062531799	0.148522477	0.421025827	0.673736223	0.028454499	2.361390544	57.65207062	0.5	PRJNA827465;Yang2025_Silkie_Wenchang	CRA004775	0.915497675
ENSGALG00010026148	RPS10	2	0.005465151	0.307325652	0.01778293	0.985812022	0.127871353	2.96321117	66.252827	0.5	CRA004775;Yang2025_Silkie_Wenchang	PRJNA827465	0.996013375
ENSGALG00010026148	RPS10	2	-0.058481835	0.067718136	-0.863606678	0.387803994	0	0.961349203	0	1	CRA004775;PRJNA827465	Yang2025_Silkie_Wenchang	0.828154121
ENSGALG00010026149	LASP1	2	0.072454828	0.113707412	0.637204093	0.523991912	0.021413197	5.575120954	82.06316942	0.5	PRJNA827465;Yang2025_Silkie_Wenchang	CRA004775	0.869661505
ENSGALG00010026149	LASP1	2	0.114788208	0.149831439	0.766115635	0.443607501	0.022137169	1.545983203	35.30996759	0.5	CRA004775;Yang2025_Silkie_Wenchang	PRJNA827465	0.844927655
ENSGALG00010026149	LASP1	2	-0.03457056	0.046273867	-0.747086047	0.455011622	0	0.207312199	0	1	CRA004775;PRJNA827465	Yang2025_Silkie_Wenchang	0.849072665
ENSGALG00010026154	ZNF385B	2	0.139730669	0.113106352	1.235391883	0.216684692	0	0.78560425	0	1	PRJNA827465;Yang2025_Silkie_Wenchang	CRA004775	0.730172415
ENSGALG00010026154	ZNF385B	2	-0.304963585	0.655924943	-0.464934711	0.641979578	0.741757658	6.82868475	85.35589156	0.5	CRA004775;Yang2025_Silkie_Wenchang	PRJNA827465	0.906654523
ENSGALG00010026154	ZNF385B	2	-0.390158744	0.543771386	-0.717505103	0.473062489	0.491013198	5.232969365	80.89039071	0.5	CRA004775;PRJNA827465	Yang2025_Silkie_Wenchang	0.854341771
ENSGALG00010026162	MED1	2	-0.122866777	0.037543142	-3.27268233	0.001065321	0	0.274313628	0	1	PRJNA827465;Yang2025_Silkie_Wenchang	CRA004775	0.039425919
ENSGALG00010026162	MED1	2	-0.042621363	0.135756437	-0.31395464	0.75355549	0	0.002189228	0	1	CRA004775;Yang2025_Silkie_Wenchang	PRJNA827465	0.938087082
ENSGALG00010026162	MED1	2	-0.12630841	0.038433088	-3.286449728	0.001014589	0	0.102789872	0	1	CRA004775;PRJNA827465	Yang2025_Silkie_Wenchang	0.038315518
ENSGALG00010026163	CTNND1	2	-0.06729014	0.068153598	-0.987330711	0.32348054	0.006777861	3.543851033	71.78210961	1	PRJNA827465;Yang2025_Silkie_Wenchang	CRA004775	0.799456433
ENSGALG00010026163	CTNND1	2	-0.137722161	0.060117617	-2.290878568	0.021970438	0	0.196473132	0	1	CRA004775;Yang2025_Silkie_Wenchang	PRJNA827465	0.251237742
ENSGALG00010026163	CTNND1	2	-0.008053853	0.037778586	-0.213185661	0.831182152	0	0.014777189	0	1	CRA004775;PRJNA827465	Yang2025_Silkie_Wenchang	0.957499036
ENSGALG00010026166	WNT9A	2	-0.270121173	0.116883785	-2.311032495	0.020831557	0	0.190029625	0	1	PRJNA827465;Yang2025_Silkie_Wenchang	CRA004775	0.243976022
ENSGALG00010026166	WNT9A	2	-0.335960193	0.208310241	-1.61278769	0.106790641	0	0.045098892	0	1	CRA004775;Yang2025_Silkie_Wenchang	PRJNA827465	0.553925396
ENSGALG00010026166	WNT9A	2	-0.241332541	0.12948828	-1.863740422	0.062358166	0	0.000309035	0	1	CRA004775;PRJNA827465	Yang2025_Silkie_Wenchang	0.430504654
ENSGALG00010026167	CABIN1	2	0.054334264	0.14890842	0.364883757	0.715198619	0.032396576	3.136690621	68.11926578	0.5	PRJNA827465;Yang2025_Silkie_Wenchang	CRA004775	0.926939292
ENSGALG00010026167	CABIN1	2	0.240430461	0.13392941	1.795202863	0.072621384	0	0.006980741	0	1	CRA004775;Yang2025_Silkie_Wenchang	PRJNA827465	0.465466124
ENSGALG00010026167	CABIN1	2	-0.002814705	0.113970626	-0.024696754	0.980296844	0.013432701	1.494617939	33.09326928	0.5	CRA004775;PRJNA827465	Yang2025_Silkie_Wenchang	0.994676009
ENSGALG00010026173	CDK12	2	-0.048364265	0.045023164	-1.074208497	0.282729245	0	0.527494292	0	1	PRJNA827465;Yang2025_Silkie_Wenchang	CRA004775	0.779532565
ENSGALG00010026173	CDK12	2	0.025488536	0.111335305	0.22893489	0.818919522	0	0.062539859	0	1	CRA004775;Yang2025_Silkie_Wenchang	PRJNA827465	0.955525039
ENSGALG00010026173	CDK12	2	-0.0571186	0.046288917	-1.233958449	0.217218388	0	0.054010156	0	1	CRA004775;PRJNA827465	Yang2025_Silkie_Wenchang	0.730748463
ENSGALG00010026183	SESTD1	2	0.108590864	0.19460251	0.558013686	0.576835039	0.068990539	10.60743381	90.57264916	0.5	PRJNA827465;Yang2025_Silkie_Wenchang	CRA004775	0.887113945
ENSGALG00010026183	SESTD1	2	0.270197906	0.098288974	2.749015436	0.005977457	0	0.715486983	0	1	CRA004775;Yang2025_Silkie_Wenchang	PRJNA827465	0.116888359
ENSGALG00010026183	SESTD1	2	-0.064643435	0.037376005	-1.729543705	0.08371832	0	0.99213808	0	0.5	CRA004775;PRJNA827465	Yang2025_Silkie_Wenchang	0.4985005
ENSGALG00010026184	NUDT3	2	-0.232499748	0.143722071	-1.61770386	0.105726434	0.030638266	3.619321522	72.37051215	1	PRJNA827465;Yang2025_Silkie_Wenchang	CRA004775	0.551748176
ENSGALG00010026184	NUDT3	2	-0.355434918	0.122879982	-2.892537192	0.003821439	0	0.641906493	0	1	CRA004775;Yang2025_Silkie_Wenchang	PRJNA827465	0.088674731
ENSGALG00010026184	NUDT3	2	-0.11136655	0.069503301	-1.602320005	0.109084798	0	0.010624595	0	1	CRA004775;PRJNA827465	Yang2025_Silkie_Wenchang	0.559123605
ENSGALG00010026192	SMIM29	2	-0.089220611	0.140619303	-0.634483582	0.525765409	0	0.936566797	0	0.5	PRJNA827465;Yang2025_Silkie_Wenchang	CRA004775	0.87039964
ENSGALG00010026192	SMIM29	2	0.04685265	0.212148423	0.220848471	0.825210421	0	0.231654724	0	0.5	CRA004775;Yang2025_Silkie_Wenchang	PRJNA827465	0.956356178
ENSGALG00010026192	SMIM29	2	-0.173073985	0.157173001	-1.101168675	0.270823253	0	0.020229142	0	1	CRA004775;PRJNA827465	Yang2025_Silkie_Wenchang	0.772743863
ENSGALG00010026199	TBX6	2	0.383513014	0.320711684	1.195818654	0.231767335	0	0.319753133	0	1	PRJNA827465;Yang2025_Silkie_Wenchang	CRA004775	0.744150932
ENSGALG00010026199	TBX6	2	0.592527401	0.407133638	1.455363415	0.145568679	0	6.57865E-05	0	1	CRA004775;Yang2025_Silkie_Wenchang	PRJNA827465	0.634178701
ENSGALG00010026199	TBX6	2	0.316837845	0.369995193	0.856329625	0.391815479	0	0.189320408	0	1	CRA004775;PRJNA827465	Yang2025_Silkie_Wenchang	0.829251057
ENSGALG00010026203	TMX2	2	0.057418823	0.064481532	0.890469279	0.373215957	0	0.00076662	0	1	PRJNA827465;Yang2025_Silkie_Wenchang	CRA004775	0.822187352
ENSGALG00010026203	TMX2	2	-0.31216369	0.377029217	-0.827956232	0.407695288	0.247493114	7.721370965	87.04893206	0.5	CRA004775;Yang2025_Silkie_Wenchang	PRJNA827465	0.833982683
ENSGALG00010026203	TMX2	2	-0.294770065	0.373555698	-0.789092675	0.430057843	0.258392842	12.89244549	92.24351966	0.5	CRA004775;PRJNA827465	Yang2025_Silkie_Wenchang	0.842012722
ENSGALG00010026205	ENSGALG00010026205	2	-0.149279569	0.636356036	-0.234584982	0.814530867	0.478721021	2.311844246	56.74449082	0.5	PRJNA827465;Yang2025_Silkie_Wenchang	CRA004775	0.953869515
ENSGALG00010026205	ENSGALG00010026205	2	0.439799521	1.137097061	0.386773049	0.698923356	2.325927392	9.887394805	89.88611155	0.5	CRA004775;Yang2025_Silkie_Wenchang	PRJNA827465	0.92185597
ENSGALG00010026205	ENSGALG00010026205	2	1.223829431	0.476386924	2.568981995	0.010199776	0.045818253	1.106445635	9.620502914	1	CRA004775;PRJNA827465	Yang2025_Silkie_Wenchang	0.160364698
ENSGALG00010026209	MED19	2	-0.066378415	0.071769806	-0.924879397	0.355026645	0	0.785215177	0	0.5	PRJNA827465;Yang2025_Silkie_Wenchang	CRA004775	0.812276513
ENSGALG00010026209	MED19	2	0.010559479	0.14082124	0.074984988	0.940226656	0	0.488944573	0	0.5	CRA004775;Yang2025_Silkie_Wenchang	PRJNA827465	0.985508732
ENSGALG00010026209	MED19	2	-0.105540833	0.078918514	-1.33733934	0.181111895	0	0.095744217	0	1	CRA004775;PRJNA827465	Yang2025_Silkie_Wenchang	0.688594651
ENSGALG00010026213	AKR1A1	2	-0.02632367	0.200439504	-0.131329749	0.895514459	0.072443005	9.900658324	89.89966154	0.5	PRJNA827465;Yang2025_Silkie_Wenchang	CRA004775	0.974927462
ENSGALG00010026213	AKR1A1	2	-0.279924914	0.101209602	-2.765794024	0.005678439	0	0.806985314	0	1	CRA004775;Yang2025_Silkie_Wenchang	PRJNA827465	0.113196293
ENSGALG00010026213	AKR1A1	2	-0.12025616	0.317055749	-0.359308677	0.719364184	0.173751688	6.626865437	84.91396154	0.5	CRA004775;PRJNA827465	Yang2025_Silkie_Wenchang	0.928449671
ENSGALG00010026218	FTSJ3	2	0.332071068	0.133880413	2.480355873	0.013125131	0.028000505	4.52595878	77.90523404	1	PRJNA827465;Yang2025_Silkie_Wenchang	CRA004775	0.186904724
ENSGALG00010026218	FTSJ3	2	0.0811078	0.430651255	0.188337545	0.850612056	0.329243367	8.31797634	87.9778451	0.5	CRA004775;Yang2025_Silkie_Wenchang	PRJNA827465	0.961985357
ENSGALG00010026218	FTSJ3	2	-0.031593432	0.29199338	-0.108199139	0.913837728	0.135067654	4.129846831	75.78602692	0.5	CRA004775;PRJNA827465	Yang2025_Silkie_Wenchang	0.979364626
ENSGALG00010026220	ENSGALG00010026220	2	-0.156793782	0.146807748	-1.068023116	0.285510085	0.031207307	3.414399775	70.71227549	1	PRJNA827465;Yang2025_Silkie_Wenchang	CRA004775	0.781226409
ENSGALG00010026220	ENSGALG00010026220	2	-0.417982295	0.149294843	-2.799710412	0.005114847	0.010722701	1.231589677	18.80412622	1	CRA004775;Yang2025_Silkie_Wenchang	PRJNA827465	0.106703201
ENSGALG00010026220	ENSGALG00010026220	2	-0.295742942	0.313016663	-0.944815331	0.344753164	0.161922139	5.092555325	80.36349266	1	CRA004775;PRJNA827465	Yang2025_Silkie_Wenchang	0.806945375
ENSGALG00010026221	CRKL	2	-0.117701722	0.114309585	-1.029674999	0.303162596	0.017590585	2.806293482	64.36580826	1	PRJNA827465;Yang2025_Silkie_Wenchang	CRA004775	0.792084776
ENSGALG00010026221	CRKL	2	-0.219577206	0.110608679	-1.98517157	0.047125386	0	0.483739302	0	1	CRA004775;Yang2025_Silkie_Wenchang	PRJNA827465	0.374478176
ENSGALG00010026221	CRKL	2	-0.028103105	0.06135272	-0.458058008	0.646910766	0	0.031400876	0	1	CRA004775;PRJNA827465	Yang2025_Silkie_Wenchang	0.907350807
ENSGALG00010026229	CRHR2	2	-0.224558361	0.178158596	-1.260440786	0.207510396	0.045515006	3.502494265	71.44891827	1	PRJNA827465;Yang2025_Silkie_Wenchang	CRA004775	0.71964891
ENSGALG00010026229	CRHR2	2	-0.37149947	0.142044989	-2.615336428	0.008913963	0	0.999309363	0	1	CRA004775;Yang2025_Silkie_Wenchang	PRJNA827465	0.147825104
ENSGALG00010026229	CRHR2	2	-0.047651811	0.11679543	-0.407993796	0.68327823	0	0.102234131	0	1	CRA004775;PRJNA827465	Yang2025_Silkie_Wenchang	0.918800744
ENSGALG00010026232	PSMC5	2	0.019094859	0.059180878	0.322652507	0.746958424	0	0.292803526	0	1	PRJNA827465;Yang2025_Silkie_Wenchang	CRA004775	0.936729365
ENSGALG00010026232	PSMC5	2	-0.153967389	0.183258509	-0.840165018	0.40081587	0	0.124683283	0	1	CRA004775;Yang2025_Silkie_Wenchang	PRJNA827465	0.832353691
ENSGALG00010026232	PSMC5	2	0.016705193	0.059917534	0.278803088	0.780309282	0	0.759798827	0	0.5	CRA004775;PRJNA827465	Yang2025_Silkie_Wenchang	0.945041817
ENSGALG00010026234	ZDHHC5	2	-0.020466172	0.088655012	-0.230851831	0.817429913	0.007284589	1.823956521	45.17413171	0.5	PRJNA827465;Yang2025_Silkie_Wenchang	CRA004775	0.954888783
ENSGALG00010026234	ZDHHC5	2	0.052626213	0.098508424	0.534230589	0.593182008	0	0.564753049	0	0.5	CRA004775;Yang2025_Silkie_Wenchang	PRJNA827465	0.892776493
ENSGALG00010026234	ZDHHC5	2	-0.098506359	0.075696015	-1.301341406	0.19314102	0	0.011303523	0	1	CRA004775;PRJNA827465	Yang2025_Silkie_Wenchang	0.703200102
ENSGALG00010026237	KLHL22	2	-0.066086727	0.084108879	-0.785728302	0.432026679	0.008206568	2.30115282	56.54352065	0.5	PRJNA827465;Yang2025_Silkie_Wenchang	CRA004775	0.842503929
ENSGALG00010026237	KLHL22	2	-0.0546486	0.16544025	-0.330322274	0.741156465	0.030891424	1.924238713	48.03139584	0.5	CRA004775;Yang2025_Silkie_Wenchang	PRJNA827465	0.935384591
ENSGALG00010026237	KLHL22	2	0.017704135	0.061216392	0.289205786	0.772423904	0	0.569504722	0	1	CRA004775;PRJNA827465	Yang2025_Silkie_Wenchang	0.943066211
ENSGALG00010026242	CCDC141	2	0.306025174	0.303124074	1.009570669	0.312701026	0.149434957	4.918752246	79.6696408	1	PRJNA827465;Yang2025_Silkie_Wenchang	CRA004775	0.796054997
ENSGALG00010026242	CCDC141	2	0.171650071	0.577519165	0.297219696	0.766298786	0.503152709	3.722144143	73.1337648	0.5	CRA004775;Yang2025_Silkie_Wenchang	PRJNA827465	0.941123716
ENSGALG00010026242	CCDC141	2	0.023103019	0.104989319	0.220051138	0.825831328	0	0.993431582	0	0.5	CRA004775;PRJNA827465	Yang2025_Silkie_Wenchang	0.956393839
ENSGALG00010026244	CLP1	2	0.043049949	0.06846993	0.628742424	0.529517699	0	0.586487188	0	0.5	PRJNA827465;Yang2025_Silkie_Wenchang	CRA004775	0.871944741
ENSGALG00010026244	CLP1	2	-0.232892252	0.131054807	-1.777059977	0.075558372	0	0.713965424	0	1	CRA004775;Yang2025_Silkie_Wenchang	PRJNA827465	0.474539901
ENSGALG00010026244	CLP1	2	-0.101342742	0.191974301	-0.527897442	0.59757052	0.058211903	4.336171584	76.93818197	0.5	CRA004775;PRJNA827465	Yang2025_Silkie_Wenchang	0.894645663
ENSGALG00010026252	ENO2	2	0.480867086	0.503295125	0.955437598	0.339356448	0.381055404	3.703091579	72.99553687	1	PRJNA827465;Yang2025_Silkie_Wenchang	CRA004775	0.804824608
ENSGALG00010026252	ENO2	2	0.252882866	0.919547625	0.275007906	0.783310165	1.29827446	4.150326761	75.9055116	0.5	CRA004775;Yang2025_Silkie_Wenchang	PRJNA827465	0.94576184
ENSGALG00010026252	ENO2	2	-0.028547106	0.252966671	-0.112849276	0.910150051	0.020172391	1.062568173	5.888391427	0.5	CRA004775;PRJNA827465	Yang2025_Silkie_Wenchang	0.978730675
ENSGALG00010026259	MED15	2	0.441887792	0.478055931	0.924343291	0.35530761	0.414870649	9.957396268	89.95721398	1	PRJNA827465;Yang2025_Silkie_Wenchang	CRA004775	0.812301302
ENSGALG00010026259	MED15	2	0.448197827	0.49143081	0.912026308	0.361754869	0.421244847	7.662329512	86.94913866	0.5	CRA004775;Yang2025_Silkie_Wenchang	PRJNA827465	0.815545459
ENSGALG00010026259	MED15	2	0.005342727	0.050744911	0.105285973	0.916148893	0	0.01456894	0	0.5	CRA004775;PRJNA827465	Yang2025_Silkie_Wenchang	0.980406784
ENSGALG00010026261	IFT70A	2	-0.068139264	0.067271448	-1.012900222	0.311107822	0	0.619598084	0	1	PRJNA827465;Yang2025_Silkie_Wenchang	CRA004775	0.79529356
ENSGALG00010026261	IFT70A	2	-0.165713578	0.104473101	-1.586184163	0.112697539	0	0.362245237	0	1	CRA004775;Yang2025_Silkie_Wenchang	PRJNA827465	0.566553488
ENSGALG00010026261	IFT70A	2	-0.056196452	0.084398608	-0.665845714	0.505509722	0.000860995	1.021105467	2.06692328	1	CRA004775;PRJNA827465	Yang2025_Silkie_Wenchang	0.863927652
ENSGALG00010026262	NAV1	2	-0.020854325	0.080203159	-0.260018752	0.794849309	0	0.748980403	0	0.5	PRJNA827465;Yang2025_Silkie_Wenchang	CRA004775	0.948905846
ENSGALG00010026262	NAV1	2	0.037531007	0.130270329	0.288100962	0.773269456	0	0.659091424	0	0.5	CRA004775;Yang2025_Silkie_Wenchang	PRJNA827465	0.943230857
ENSGALG00010026262	NAV1	2	-0.081093887	0.094714524	-0.856192725	0.391891186	0	0.202292199	0	1	CRA004775;PRJNA827465	Yang2025_Silkie_Wenchang	0.829251057
ENSGALG00010026264	FZD7	2	-0.134067211	0.070069459	-1.913347308	0.055703605	0	0.072414623	0	1	PRJNA827465;Yang2025_Silkie_Wenchang	CRA004775	0.408696004
ENSGALG00010026264	FZD7	2	-0.157681769	0.154720109	-1.019142049	0.308135534	0	0.047932479	0	1	CRA004775;Yang2025_Silkie_Wenchang	PRJNA827465	0.793353295
ENSGALG00010026264	FZD7	2	-0.125176834	0.072826607	-1.718833801	0.085644639	0	0.004141994	0	1	CRA004775;PRJNA827465	Yang2025_Silkie_Wenchang	0.503152652
ENSGALG00010026268	GARS1	2	-0.303552728	0.285570717	-1.062968678	0.287796156	0.152865729	15.21322556	93.42677202	1	PRJNA827465;Yang2025_Silkie_Wenchang	CRA004775	0.782840067
ENSGALG00010026268	GARS1	2	-0.490282788	0.157168068	-3.119480907	0.0018117	0.01804623	1.498243579	33.25517868	1	CRA004775;Yang2025_Silkie_Wenchang	PRJNA827465	0.05435515
ENSGALG00010026268	GARS1	2	-0.049989504	0.060810359	-0.822055732	0.411045178	0.002430628	1.090026958	8.259149643	1	CRA004775;PRJNA827465	Yang2025_Silkie_Wenchang	0.835128673
ENSGALG00010026272	LRRC58	2	-0.112152336	0.086035164	-1.303563918	0.19238231	0.008116436	2.148728647	53.46085223	1	PRJNA827465;Yang2025_Silkie_Wenchang	CRA004775	0.702137035
ENSGALG00010026272	LRRC58	2	-0.182918408	0.089705432	-2.039100677	0.041439981	0	0.64109497	0	1	CRA004775;Yang2025_Silkie_Wenchang	PRJNA827465	0.352975427
ENSGALG00010026272	LRRC58	2	-0.03660065	0.065555562	-0.558314947	0.57662934	0	0.012651656	0	1	CRA004775;PRJNA827465	Yang2025_Silkie_Wenchang	0.887113945
ENSGALG00010026277	SMARCD2	2	-0.034198603	0.064219918	-0.532523297	0.59436361	0.00228903	1.381389956	27.60914502	0.5	PRJNA827465;Yang2025_Silkie_Wenchang	CRA004775	0.893337382
ENSGALG00010026277	SMARCD2	2	-0.155257521	0.090588049	-1.713885246	0.086549803	0.004322162	1.244748078	19.66245879	1	CRA004775;Yang2025_Silkie_Wenchang	PRJNA827465	0.506111562
ENSGALG00010026277	SMARCD2	2	-0.109197602	0.165679667	-0.659088736	0.509838788	0.040206038	3.35585663	70.20134916	0.5	CRA004775;PRJNA827465	Yang2025_Silkie_Wenchang	0.865257661
ENSGALG00010026287	NASP	2	0.257319168	0.274638536	0.936937589	0.348790662	0.139016055	12.38149925	91.92343367	0.5	PRJNA827465;Yang2025_Silkie_Wenchang	CRA004775	0.809333419
ENSGALG00010026287	NASP	2	0.243490669	0.344377442	0.707045932	0.479537934	0.190361986	4.74515115	78.92585571	0.5	CRA004775;Yang2025_Silkie_Wenchang	PRJNA827465	0.856149697
ENSGALG00010026287	NASP	2	-0.011702184	0.066382643	-0.176283785	0.860071	0	0.241511332	0	1	CRA004775;PRJNA827465	Yang2025_Silkie_Wenchang	0.964928366
ENSGALG00010026291	SERPING1	2	0.075405987	0.077556374	0.972273239	0.330914634	0.000866185	1.074296638	6.915840105	1	PRJNA827465;Yang2025_Silkie_Wenchang	CRA004775	0.801678056
ENSGALG00010026291	SERPING1	2	-0.12657167	0.352604623	-0.358962026	0.719623497	0.198296088	4.430326391	77.42829959	0.5	CRA004775;Yang2025_Silkie_Wenchang	PRJNA827465	0.928449671
ENSGALG00010026291	SERPING1	2	-0.183920148	0.266127686	-0.691097386	0.489504343	0.100038489	2.808436027	64.39299345	0.5	CRA004775;PRJNA827465	Yang2025_Silkie_Wenchang	0.858419004
ENSGALG00010026295	SMPD4	2	0.015765027	0.05648079	0.279121937	0.780151249	0	0.023814556	0	1	PRJNA827465;Yang2025_Silkie_Wenchang	CRA004775	0.945041817
ENSGALG00010026295	SMPD4	2	-0.050484357	0.094485522	-0.534307853	0.59312856	0.001080928	1.046045999	4.401909608	0.5	CRA004775;Yang2025_Silkie_Wenchang	PRJNA827465	0.892776493
ENSGALG00010026295	SMPD4	2	-0.032888114	0.100624628	-0.326839604	0.743789203	0.008524938	1.417303727	29.44349322	0.5	CRA004775;PRJNA827465	Yang2025_Silkie_Wenchang	0.935821071
ENSGALG00010026298	SNF8	2	0.077560994	0.167734156	0.462404292	0.643791422	0.04366127	4.050544846	75.31196325	0.5	PRJNA827465;Yang2025_Silkie_Wenchang	CRA004775	0.906687722
ENSGALG00010026298	SNF8	2	0.197652951	0.133196278	1.483922478	0.137829488	0	0.967085795	0	0.5	CRA004775;Yang2025_Silkie_Wenchang	PRJNA827465	0.62064915
ENSGALG00010026298	SNF8	2	-0.060712564	0.064038522	-0.94806317	0.343097296	0	0.02867755	0	1	CRA004775;PRJNA827465	Yang2025_Silkie_Wenchang	0.806218116
ENSGALG00010026300	HMGA1	2	0.032197103	0.059883081	0.5376661	0.590807587	0	0.418708946	0	1	PRJNA827465;Yang2025_Silkie_Wenchang	CRA004775	0.892752146
ENSGALG00010026300	HMGA1	2	-0.159339491	0.278038582	-0.573084103	0.56658775	0.128251109	5.580712801	82.08114204	0.5	CRA004775;Yang2025_Silkie_Wenchang	PRJNA827465	0.883487517
ENSGALG00010026300	HMGA1	2	-0.189300605	0.233615443	-0.81030861	0.417762828	0.087955765	4.581849686	78.17475324	0.5	CRA004775;PRJNA827465	Yang2025_Silkie_Wenchang	0.838112595
ENSGALG00010026305	ATP5MC1	2	-0.075745709	0.132067001	-0.573540009	0.566279118	0.010695051	1.117504516	10.51490298	1	PRJNA827465;Yang2025_Silkie_Wenchang	CRA004775	0.883487517
ENSGALG00010026305	ATP5MC1	2	-0.227818649	0.296911432	-0.767294975	0.442906156	0	0.769655532	0	0.5	CRA004775;Yang2025_Silkie_Wenchang	PRJNA827465	0.845720426
ENSGALG00010026305	ATP5MC1	2	-0.029834566	0.090444476	-0.329866092	0.741501146	0	0.02608993	0	0.5	CRA004775;PRJNA827465	Yang2025_Silkie_Wenchang	0.93543545
ENSGALG00010026315	SYT2	2	-0.004806334	0.229205097	-0.020969579	0.983269923	0.017196808	1.15821093	13.65994103	0.5	PRJNA827465;Yang2025_Silkie_Wenchang	CRA004775	0.995363468
ENSGALG00010026315	SYT2	2	0.485125445	0.347356646	1.396620594	0.162527694	0	0.478378451	0	1	CRA004775;Yang2025_Silkie_Wenchang	PRJNA827465	0.660240409
ENSGALG00010026315	SYT2	2	0.171592003	0.488881208	0.350989156	0.725596484	0.287298808	2.046301105	51.13133656	0.5	CRA004775;PRJNA827465	Yang2025_Silkie_Wenchang	0.930960683
ENSGALG00010026316	ENSGALG00010026316	2	-0.113224168	0.135746493	-0.834085405	0.404232859	0.002634437	1.063867324	6.003316624	1	PRJNA827465;Yang2025_Silkie_Wenchang	CRA004775	0.833112102
ENSGALG00010026316	ENSGALG00010026316	2	-0.346733042	0.18217006	-1.903348118	0.056995136	0	0.026583214	0	1	CRA004775;Yang2025_Silkie_Wenchang	PRJNA827465	0.413451427
ENSGALG00010026316	ENSGALG00010026316	2	-0.128784434	0.162122671	-0.794364124	0.426983483	0.01349809	1.27036928	21.2827313	1	CRA004775;PRJNA827465	Yang2025_Silkie_Wenchang	0.84125685

ENSGALG00010026320	ENSGALG00010026320	2	-0.014571101	0.060434566	-0.241105408	0.809473422	0	0.053913075	0	1	PRJNA827465;Yang2025_Silkie_Wenchang	CRA004775	0.952770549
ENSGALG00010026320	ENSGALG00010026320	2	-0.096183953	0.146159374	-0.658075841	0.510489399	0	0.466142345	0	1	CRA004775;Yang2025_Silkie_Wenchang	PRJNA827465	0.865365179
ENSGALG00010026320	ENSGALG00010026320	2	-0.019299998	0.064032383	-0.301409963	0.763101898	0	0.733672077	0	1	CRA004775;PRJNA827465	Yang2025_Silkie_Wenchang	0.940184187
ENSGALG00010026321	GPBP1L1	2	-0.116976472	0.089435607	-1.307940719	0.190893417	0.013217787	5.560824899	82.01705649	1	PRJNA827465;Yang2025_Silkie_Wenchang	CRA004775	0.700399923
ENSGALG00010026321	GPBP1L1	2	-0.140222993	0.113573688	-1.234643299	0.216963287	0.014127152	1.849503272	45.93142845	0.5	CRA004775;Yang2025_Silkie_Wenchang	PRJNA827465	0.730505165
ENSGALG00010026321	GPBP1L1	2	-0.031500292	0.037961708	-0.829791227	0.406656832	0	0.153808478	0	0.5	CRA004775;PRJNA827465	Yang2025_Silkie_Wenchang	0.833618122
ENSGALG00010026325	TMEM69	2	-0.069636644	0.071041976	-0.980218293	0.326978377	0.002557504	1.26485242	20.9393931	1	PRJNA827465;Yang2025_Silkie_Wenchang	CRA004775	0.800028239
ENSGALG00010026325	TMEM69	2	-0.181608439	0.104313416	-1.740988325	0.081685625	0	0.000416164	0	1	CRA004775;Yang2025_Silkie_Wenchang	PRJNA827465	0.492520496
ENSGALG00010026325	TMEM69	2	-0.040014461	0.064150044	-0.623763582	0.53278286	0	0.563259549	0	1	CRA004775;PRJNA827465	Yang2025_Silkie_Wenchang	0.873183244
ENSGALG00010026329	ENSGALG00010026329	2	-0.437571394	0.333863296	-1.310630426	0.189982654	0.168653668	4.091828517	75.56104818	1	PRJNA827465;Yang2025_Silkie_Wenchang	CRA004775	0.699277793
ENSGALG00010026329	ENSGALG00010026329	2	-0.065350763	0.229319725	-0.284976631	0.775662046	0	0.071858824	0	0.5	CRA004775;Yang2025_Silkie_Wenchang	PRJNA827465	0.943815265
ENSGALG00010026329	ENSGALG00010026329	2	-0.51401119	0.381376564	-1.347778649	0.177729587	0.159192414	1.83191964	45.4124527	0.5	CRA004775;PRJNA827465	Yang2025_Silkie_Wenchang	0.683951879
ENSGALG00010026334	ENSGALG00010026334	2	-0.078511552	0.109233637	-0.718748857	0.472295675	0.011473675	1.924226206	48.03105805	0.5	PRJNA827465;Yang2025_Silkie_Wenchang	CRA004775	0.853998373
ENSGALG00010026334	ENSGALG00010026334	2	-0.058341026	0.147012842	-0.396843063	0.691483193	0.026110051	2.487290299	59.79560568	0.5	CRA004775;Yang2025_Silkie_Wenchang	PRJNA827465	0.920268407
ENSGALG00010026334	ENSGALG00010026334	2	0.054004732	0.087246311	0.61899158	0.535921906	0	0.176214995	0	1	CRA004775;PRJNA827465	Yang2025_Silkie_Wenchang	0.874396558
ENSGALG00010026335	IPP	2	0.082048349	0.058763771	1.396240371	0.162642123	0	0.126097228	0	1	PRJNA827465;Yang2025_Silkie_Wenchang	CRA004775	0.660469716
ENSGALG00010026335	IPP	2	-0.168511269	0.267495824	-0.629958505	0.528721733	0.111051896	3.997302792	74.98313108	0.5	CRA004775;Yang2025_Silkie_Wenchang	PRJNA827465	0.871809618
ENSGALG00010026335	IPP	2	-0.146502256	0.289689065	-0.505722422	0.613051526	0.13808006	4.969855617	79.87869111	0.5	CRA004775;PRJNA827465	Yang2025_Silkie_Wenchang	0.898130964
ENSGALG00010026344	TIMM10	2	-0.01027877	0.111276529	-0.092371409	0.926402955	0	0.008986591	0	1	PRJNA827465;Yang2025_Silkie_Wenchang	CRA004775	0.98280842
ENSGALG00010026344	TIMM10	2	-0.163070042	0.215896924	-0.755314336	0.450060399	0	0.431934341	0	1	CRA004775;Yang2025_Silkie_Wenchang	PRJNA827465	0.847526417
ENSGALG00010026344	TIMM10	2	-0.044368062	0.112898639	-0.39299023	0.694326697	0	0.835266026	0	1	CRA004775;PRJNA827465	Yang2025_Silkie_Wenchang	0.920728119
ENSGALG00010026346	FZD2	2	-0.126361943	0.078719816	-1.60521137	0.108447296	0	0.008140197	0	1	PRJNA827465;Yang2025_Silkie_Wenchang	CRA004775	0.557236773
ENSGALG00010026346	FZD2	2	-0.079656587	0.14795228	-0.538393782	0.590305218	0	0.422453382	0	0.5	CRA004775;Yang2025_Silkie_Wenchang	PRJNA827465	0.892474457
ENSGALG00010026346	FZD2	2	-0.117320698	0.089061076	-1.317306085	0.187736046	0	0.50774538	0	0.5	CRA004775;PRJNA827465	Yang2025_Silkie_Wenchang	0.696930043
ENSGALG00010026354	ENSGALG00010026354	2	-0.337862851	0.171762943	-1.966913495	0.049191184	0.027347998	1.863879134	46.34845244	1	PRJNA827465;Yang2025_Silkie_Wenchang	CRA004775	0.383545122
ENSGALG00010026354	ENSGALG00010026354	2	-0.138119153	0.167978139	-0.822244814	0.410937578	0	0.215757346	0	0.5	CRA004775;Yang2025_Silkie_Wenchang	PRJNA827465	0.835128673
ENSGALG00010026354	ENSGALG00010026354	2	-0.40073275	0.228934348	-1.750426505	0.080044746	0.032645484	1.229607893	18.67326118	0.5	CRA004775;PRJNA827465	Yang2025_Silkie_Wenchang	0.486695335
ENSGALG00010026355	PPP1R12B	2	-0.07952596	0.114636225	-0.693724515	0.487834989	0.019756344	3.991166845	74.9446706	0.5	PRJNA827465;Yang2025_Silkie_Wenchang	CRA004775	0.85768885
ENSGALG00010026355	PPP1R12B	2	0.033113138	0.070627771	0.469097879	0.638999675	0	0.093840066	0	1	CRA004775;Yang2025_Silkie_Wenchang	PRJNA827465	0.905879535
ENSGALG00010026355	PPP1R12B	2	-0.182124125	0.087261484	-2.087107804	0.036878391	0	0.798365459	0	0.5	CRA004775;PRJNA827465	Yang2025_Silkie_Wenchang	0.331459091
ENSGALG00010026360	CTDSPL	2	-0.010778943	0.050397117	-0.213880159	0.830640513	0	0.309513172	0	0.5	PRJNA827465;Yang2025_Silkie_Wenchang	CRA004775	0.957295959
ENSGALG00010026360	CTDSPL	2	0.073159502	0.098910786	0.739651404	0.459511542	0	0.92518603	0	1	CRA004775;Yang2025_Silkie_Wenchang	PRJNA827465	0.850098539
ENSGALG00010026360	CTDSPL	2	0.043160018	0.145444123	0.296746387	0.76666014	0.022655962	1.4776391	32.32447625	0.5	CRA004775;PRJNA827465	Yang2025_Silkie_Wenchang	0.941271762
ENSGALG00010026362	ENSGALG00010026362	2	-0.050756925	0.201720725	-0.251619783	0.801334966	0	0.423880355	0	0.5	PRJNA827465;Yang2025_Silkie_Wenchang	CRA004775	0.950158029
ENSGALG00010026362	ENSGALG00010026362	2	0.383882717	0.613825819	0.625393565	0.531712785	0.630361175	6.092712932	83.58695033	0.5	CRA004775;Yang2025_Silkie_Wenchang	PRJNA827465	0.873042777
ENSGALG00010026362	ENSGALG00010026362	2	0.494856534	0.478047346	1.035162181	0.300593177	0.354374427	4.345168788	76.98699354	1	CRA004775;PRJNA827465	Yang2025_Silkie_Wenchang	0.790579721
ENSGALG00010026367	RBP	2	0.153473792	0.5402978	0.284054075	0.776368941	0.273338263	1.869058888	46.49713786	0.5	PRJNA827465;Yang2025_Silkie_Wenchang	CRA004775	0.944109792
ENSGALG00010026367	RBP	2	0.297558808	0.76635588	0.388277582	0.697810625	0.758625394	2.812328905	64.44228134	0.5	CRA004775;Yang2025_Silkie_Wenchang	PRJNA827465	0.921422004
ENSGALG00010026367	RBP	2	0.812564455	0.412750117	1.968659536	0.048992201	0	0.273784447	0	1	CRA004775;PRJNA827465	Yang2025_Silkie_Wenchang	0.382604056
ENSGALG00010026377	ENSGALG00010026377	2	-0.07778498	0.135937997	-0.572209255	0.567180218	0.007865537	1.204581534	16.95836165	0.5	PRJNA827465;Yang2025_Silkie_Wenchang	CRA004775	0.883581756
ENSGALG00010026377	ENSGALG00010026377	2	-0.416687422	0.638674482	-0.65242535	0.514126815	0.661643766	4.925393955	79.69705552	0.5	CRA004775;Yang2025_Silkie_Wenchang	PRJNA827465	0.866855965
ENSGALG00010026377	ENSGALG00010026377	2	-0.521483829	0.474022435	-1.100124785	0.271277756	0.336604032	3.278340486	69.49676203	1	CRA004775;PRJNA827465	Yang2025_Silkie_Wenchang	0.772743863
ENSGALG00010026388	SNRPD3	2	-0.051535099	0.088863389	-0.579934656	0.560881642	0.008717801	2.022341565	50.55236874	0.5	PRJNA827465;Yang2025_Silkie_Wenchang	CRA004775	0.881354866
ENSGALG00010026388	SNRPD3	2	-0.017237835	0.135694315	-0.127034318	0.898913237	0.012163752	1.364855363	26.73216321	0.5	CRA004775;Yang2025_Silkie_Wenchang	PRJNA827465	0.975912573
ENSGALG00010026388	SNRPD3	2	-0.125303845	0.057958206	-2.16196901	0.030620565	0	0.236742001	0	1	CRA004775;PRJNA827465	Yang2025_Silkie_Wenchang	0.298556206
ENSGALG00010026393	SLC43A3	2	0.047372028	0.100687958	0.47048355	0.638009584	0	0.912123082	0	0.5	PRJNA827465;Yang2025_Silkie_Wenchang	CRA004775	0.905365679
ENSGALG00010026393	SLC43A3	2	-0.094570286	0.312442819	-0.302680299	0.762133513	0.144315959	3.687369295	72.88039466	0.5	CRA004775;Yang2025_Silkie_Wenchang	PRJNA827465	0.939973436
ENSGALG00010026393	SLC43A3	2	-0.160966181	0.199982516	-0.804901269	0.420876656	0.044365333	1.960321762	48.98796618	1	CRA004775;PRJNA827465	Yang2025_Silkie_Wenchang	0.839239169
ENSGALG00010026402	TFEB	2	-0.010577073	0.177393253	-0.059625003	0.952454305	0.052571557	6.031249178	83.41968686	0.5	PRJNA827465;Yang2025_Silkie_Wenchang	CRA004775	0.987758913
ENSGALG00010026402	TFEB	2	0.09342414	0.126855735	0.736459733	0.461450964	0.009742373	1.32705397	24.64511445	0.5	CRA004775;Yang2025_Silkie_Wenchang	PRJNA827465	0.850356679
ENSGALG00010026402	TFEB	2	-0.170667067	0.083320034	-2.048331704	0.040527508	0	0.099182007	0	1	CRA004775;PRJNA827465	Yang2025_Silkie_Wenchang	0.348354009
ENSGALG00010026403	GUCD1	2	-0.241893938	0.059900707	-4.038248484	5.38518E-05	0.000221335	1.029724285	2.886625598	1	PRJNA827465;Yang2025_Silkie_Wenchang	CRA004775	0.005100089
ENSGALG00010026403	GUCD1	2	-0.016328552	0.343629856	-0.047517849	0.962100505	0.200598811	6.105327619	83.62086259	0.5	CRA004775;Yang2025_Silkie_Wenchang	PRJNA827465	0.989880527
ENSGALG00010026403	GUCD1	2	0.032777645	0.278849172	0.117546145	0.906427279	0.12443114	4.33141118	76.09283606	0.5	CRA004775;PRJNA827465	Yang2025_Silkie_Wenchang	0.979196186
ENSGALG00010026409	VILL	2	-0.10334093	0.216388405	-0.477571479	0.632955235	0.05663219	2.521410426	60.33965793	0.5	PRJNA827465;Yang2025_Silkie_Wenchang	CRA004775	0.903207334
ENSGALG00010026409	VILL	2	-0.306075192	0.175334343	-1.74566595	0.080869015	0	0.00663332	0	1	CRA004775;Yang2025_Silkie_Wenchang	PRJNA827465	0.489847627
ENSGALG00010026409	VILL	2	0.094060113	0.19587775	0.480198044	0.631086577	0	0.264571685	0	0.5	CRA004775;PRJNA827465	Yang2025_Silkie_Wenchang	0.903058174
ENSGALG00010026410	ENSGALG00010026410	2	0.613200977	1.623228168	0.377765964	0.705604175	4.943621805	15.29910357	93.46166932	0.5	PRJNA827465;Yang2025_Silkie_Wenchang	CRA004775	0.923190822
ENSGALG00010026410	ENSGALG00010026410	2	0.458538457	1.876367294	0.244373721	0.806941393	6.387109876	10.75989541	90.70622936	0.5	CRA004775;Yang2025_Silkie_Wenchang	PRJNA827465	0.95234753
ENSGALG00010026410	ENSGALG00010026410	2	-0.931819885	0.151028855	-6.169813616	6.83705E-10	0	0.376489906	0	1	CRA004775;PRJNA827465	Yang2025_Silkie_Wenchang	4.1987E-07
ENSGALG00010026427	PGC	2	0.167592657	0.261300322	0.641373451	0.52127618	0	0.004213887	0	1	PRJNA827465;Yang2025_Silkie_Wenchang	CRA004775	0.869518515
ENSGALG00010026427	PGC	2	0.005396165	0.322491736	0.016732722	0.986649843	0	0.245832601	0	0.5	CRA004775;Yang2025_Silkie_Wenchang	PRJNA827465	0.996259695
ENSGALG00010026427	PGC	2	0.069006507	0.273339408	0.252457221	0.800687676	0	0.367619616	0	0.5	CRA004775;PRJNA827465	Yang2025_Silkie_Wenchang	0.950158029
ENSGALG00010026433	VPS11	2	0.060386163	0.111989637	0.539212065	0.589740536	0.02060457	5.497575415	81.81016313	0.5	PRJNA827465;Yang2025_Silkie_Wenchang	CRA004775	0.892320726
ENSGALG00010026433	VPS11	2	-0.044185027	0.256862015	-0.172018532	0.86342296	0.109942732	5.485560171	81.77032119	0.5	CRA004775;Yang2025_Silkie_Wenchang	PRJNA827465	0.966256632
ENSGALG00010026433	VPS11	2	-0.123979488	0.130523533	-0.949863098	0.34218182	0.020351004	1.888540556	47.04905876	1	CRA004775;PRJNA827465	Yang2025_Silkie_Wenchang	0.806005903
ENSGALG00010026434	ENSGALG00010026434	2	0.255703909	0.101693944	2.514445795	0.011921963	0	0.517311345	0	1	PRJNA827465;Yang2025_Silkie_Wenchang	CRA004775	0.175322923
ENSGALG00010026434	ENSGALG00010026434	2	-0.210784416	0.336159197	-0.62703748	0.530634668	0.186515748	5.720743954	82.51975603	0.5	CRA004775;Yang2025_Silkie_Wenchang	PRJNA827465	0.872665695
ENSGALG00010026434	ENSGALG00010026434	2	-0.108984198	0.420564316	-0.259138006	0.795528761	0.328558815	13.84207135	92.7756477	0.5	CRA004775;PRJNA827465	Yang2025_Silkie_Wenchang	0.949027709
ENSGALG00010026436	PLCD1	2	-0.249554998	0.225363487	-1.107344411	0.26814505	0.096678707	20.71629655	95.17288239	1	PRJNA827465;Yang2025_Silkie_Wenchang	CRA004775	0.771398294
ENSGALG00010026436	PLCD1	2	-0.237005481	0.31028761	-0.763825151	0.444971447	0.148021306	3.56302388	71.93395179	0.5	CRA004775;Yang2025_Silkie_Wenchang	PRJNA827465	0.846013379
ENSGALG00010026436	PLCD1	2	-0.018703573	0.06364336	-0.293880973	0.768848858	0	0.318974308	0	0.5	CRA004775;PRJNA827465	Yang2025_Silkie_Wenchang	0.941666147
ENSGALG00010026438	TOMM6	2	-0.350628265	0.283466997	-1.236927998	0.216113815	0.122405168	3.3683936	70.31225805	1	PRJNA827465;Yang2025_Silkie_Wenchang	CRA004775	0.729718321
ENSGALG00010026438	TOMM6	2	-0.516137126	0.229899879	-2.245051753	0.02476481	0	0.948236049	0	1	CRA004775;Yang2025_Silkie_Wenchang	PRJNA827465	0.268761753
ENSGALG00010026438	TOMM6	2	-0.141578097	0.057596221	-2.458114328	0.01396687	0	0.173405249	0	1	CRA004775;PRJNA827465	Yang2025_Silkie_Wenchang	0.193808158
ENSGALG00010026443	UPB1	2	0.499019767	0.263319168	1.895113718	0.058077338	0	0.025313154	0	1	PRJNA827465;Yang2025_Silkie_Wenchang	CRA004775	0.417588463
ENSGALG00010026443	UPB1	2	0.580271641	0.264784257	2.191488449	0.028416465	0	0.263343712	0	1	CRA004775;Yang2025_Silkie_Wenchang	PRJNA827465	0.28718239
ENSGALG00010026443	UPB1	2	0.640350798	0.283514189	2.258619928	0.023907037	0	0.112206101	0	1	CRA004775;PRJNA827465	Yang2025_Silkie_Wenchang	0.264234612
ENSGALG00010026448	ADORA2A	2	0.008502881	0.17988614	0.04726813	0.962299528	0.055284085	6.717049083	85.11251016	0.5	PRJNA827465;Yang2025_Silkie_Wenchang	CRA004775	0.989950484
ENSGALG00010026448	ADORA2A	2	-0.174410543	0.110237136	-1.582139632	0.113617698	0	0.046500835	0	1	CRA004775;Yang2025_Silkie_Wenchang	PRJNA827465	0.568794964
ENSGALG00010026448	ADORA2A	2	0.164381642	0.074029134	2.220499322	0.026384892	0	0.662638395	0	0.5	CRA004775;PRJNA827465	Yang2025_Silkie_Wenchang	0.276903978
ENSGALG00010026457	SSRP1	2	0.096087854	0.088801582	1.082051152	0.279229799	0.013034745	5.747209569	82.60025169	1	PRJNA827465;Yang2025_Silkie_Wenchang	CRA004775	0.777442633
ENSGALG00010026457	SSRP1	2	-0.071994307	0.288156167	-0.249844756	0.802707406	0.147011136	8.017789057	87.52773373	0.5	CRA004775;Yang2025_Silkie_Wenchang	PRJNA827465	0.950704475
ENSGALG00010026457	SSRP1	2	-0.146286945	0.195796146	-0.747139041	0.454979636	0.060204278	3.926805752	74.53400898	0.5	CRA004775;PRJNA827465	Yang2025_Silkie_Wenchang	0.849072665
ENSGALG00010026458	DLEC1	2	0.100174249	0.113875047	0.879685685	0.379029606	0.005608384	1.231656529	18.80853335	0.5	PRJNA827465;Yang2025_Silkie_Wenchang	CRA004775	0.823921561
ENSGALG00010026458	DLEC1	2	-0.010448364	0.173451467	-0.060237969	0.951966105	0	0.63153866	0	0.5	CRA004775;Yang2025_Silkie_Wenchang	PRJNA827465	0.987725077
ENSGALG00010026458	DLEC1	2	0.187230463	0.112511095	1.66410666	0.096091116	0	0.102853159	0	1	CRA004775;PRJNA827465	Yang2025_Silkie_Wenchang	0.530362009
ENSGALG00010026480	USP49	2	0.171340207	0.236127745	0.725625053	0.468068658	0.075285154	3.073857056	67.46758285	0.5	PRJNA827465;Yang2025_Silkie_Wenchang	CRA004775	0.853145513
ENSGALG00010026480	USP49	2	0.200140469	0.291564177	0.686437103	0.492437514	0.091410722	1.955632069	48.86563706	0.5	CRA004775;Yang2025_Silkie_Wenchang	PRJNA827465	0.859260507
ENSGALG00010026480	USP49	2	-0.083842337	0.165527001	-0.506517586	0.612493347	0	0.104506462	0	1	CRA004775;PRJNA827465	Yang2025_Silkie_Wenchang	0.897938231
ENSGALG00010026481	DIXDC1	2	-0.210515364	0.069118391	-3.04572141	0.002321228	0.000688677	1.077668689	7.207102679	1	PRJNA827465;Yang2025_Silkie_Wenchang	CRA004775	0.063887413
ENSGALG00010026481	DIXDC1	2	-0.266458251	0.090090116	-2.957685755	0.003099579	0	0.237470947	0	1	CRA004775;Yang2025_Silkie_Wenchang	PRJNA827465	0.076908312
ENSGALG00010026481	DIXDC1	2	-0.138596344	0.090929201	-1.524222606	0.127453113	0	0.006747086	0	1	CRA004775;PRJNA827465	Yang2025_Silkie_Wenchang	0.599451151
ENSGALG00010026484	TNKS1BP1	2	0.06062338	0.089790835	0.675162226	0.499572703	0.007262771	1.694337817	40.97989255	0.5	PRJNA827465;Yang2025_Silkie_Wenchang	CRA004775	0.862078151
ENSGALG00010026484	TNKS1BP1	2	0.011787121	0.213054631	0.055324407	0.955880018	0.05893288	2.625501172	61.91203377	0.5	CRA004775;Yang2025_Silkie_Wenchang	PRJNA827465	0.988406776
ENSGALG00010026484	TNKS1BP1	2	-0.02584672	0.068640586	-0.376551559	0.706506892	0	0.99454437	0	1	CRA004775;PRJNA827465	Yang2025_Silkie_Wenchang	0.924548793
ENSGALG00010026485	ACAA1	2	-0.165892993	0.269076013	-0.61652836	0.53754586	0.133871726	13.23919221	92.44666907	0.5	PRJNA827465;Yang2025_Silkie_Wenchang	CRA004775	0.87481821
ENSGALG00010026485	ACAA1	2	-0.347017032	0.098182645	-3.534402965	0.000408697	0.005820221	1.427379834	29.94156313	1	CRA004775;Yang2025_Silkie_Wenchang	PRJNA827465	0.020366982
ENSGALG00010026485	ACAA1	2	-0.061160359	0.17031366	-0.359104253	0.7195171	0.045441417	4.579290381	78.1625554	0.5	CRA004775;PRJNA827465	Yang2025_Silkie_Wenchang	0.928449671
ENSGALG00010026492	SPECC1L	2	-0.01104237	0.067294998	-0.164089015	0.86966107	0	0.534179279	0	0.5	PRJNA827465;Yang2025_Silkie_Wenchang	CRA004775	0.968586462
ENSGALG00010026492	SPECC1L	2	0.179799079	0.166289391	1.081242034	0.279589465	0	0.244899852	0	1	CRA004775;Yang2025_Silkie_Wenchang	PRJNA827465	0.777720009
ENSGALG00010026492	SPECC1L	2	0.008377484	0.104931047	0.079837988	0.936366111	0.007273154	1.150080259	13.04954654	0.5	CRA004775;PRJNA827465	Yang2025_Silkie_Wenchang	0.98495871
ENSGALG00010026498	ENSGALG00010026498	2	-0.09013024	0.051263471	-1.758176705	0.078717448	0	0.150820564	0	1	PRJNA827465;Yang2025_Silkie_Wenchang	CRA004775	0.482600227
ENSGALG00010026498	ENSGALG00010026498	2	-0.18450277	0.154634238	-1.193156009	0.23280828	0	0.063755092	0	1	CRA004775;Yang2025_Silkie_Wenchang	PRJNA827465	0.745799878
ENSGALG00010026498	ENSGALG00010026498	2	-0.089870866	0.052436715	-1.713891991	0.086548564	0	0.30861927	0	1	CRA004775;PRJNA827465	Yang2025_Silkie_Wenchang	0.506111562
ENSGALG00010026507	APLNR	2	0.064863821	0.089490587	0.724811664	0.468567579	0.00163289	1.093834831	8.578519164	1	PRJNA827465;Yang2025_Silkie_Wenchang	CRA004775	0.853198008
ENSGALG00010026507	APLNR	2	-0.04181187	0.419736793	-0.099614498	0.920650383	0.210284006	1.814739435	44.89567037	0.5	CRA004775;Yang2025_Silkie_Wenchang	PRJNA827465	0.981469977
ENSGALG00010026507	APLNR	2	-0.06874653	0.228619648	-0.300702635	0.763641259	0.048053378	1.19181379	16.09427513	0.5	CRA004775;PRJNA827465	Yang2025_Silkie_Wenchang	0.940361611
ENSGALG00010026508	CSRP1	2	0.121168333	0.159711254	0.758671224	0.448049255	0.022259224	1.720771837	41.8865431	0.5	PRJNA827465;Yang2025_Silkie_Wenchang	CRA004775	0.846601815
ENSGALG00010026508	CSRP1	2	0.314373324	0.167827671	1.873191243	0.061041981	0	0.000434088	0	1	CRA004775;Yang2025_Silkie_Wenchang	PRJNA827465	0.42722323
ENSGALG00010026508	CSRP1	2	0.050576966	0.128256569	0.39434211	0.693328477	0	0.940643061	0	0.5	CRA004775;PRJNA827465	Yang2025_Silkie_Wenchang	0.920721644
ENSGALG00010026516	ENSGALG00010026516	2	-0.087606849	0.056805141	-1.54223453	0.123016612	0	0.345097736	0	1	PRJNA827465;Yang2025_Silkie_Wenchang	CRA004775	0.58801364
ENSGALG00010026516	ENSGALG00010026516	2	-0.031317842	0.181532984	-0.172518744	0.863029727	0.034068793	1.606632671	37.75801911	0.5	CRA004775;Yang2025_Silkie_Wenchang	PRJNA827465	0.966099097
ENSGALG00010026516	ENSGALG00010026516	2	-0.024229917	0.11106026	-0.218169097	0.827297362	0.008924977	1.165365499	14.19001158	0.5	CRA004775;PRJNA827465	Yang2025_Silkie_Wenchang	0.956550752
ENSGALG00010026522	MED20	2	-0.171826784	0.084835739	-2.025405646	0.042825728	0	0.906857622	0	1	PRJNA827465;Yang2025_Silkie_Wenchang	CRA004775	0.357943462
ENSGALG00010026522	MED20	2	-0.395094328	0.185217479	-2.133137382	0.032913458	0	0.007304091	0	1	CRA004775;Yang2025_Silkie_Wenchang	PRJNA827465	0.311661412
ENSGALG00010026522	MED20	2	-0.164292323	0.086887607	-1.890860253	0.058643001	0	0.778578489	0	1	CRA004775;PRJNA827465	Yang2025_Silkie_Wenchang	0.419491742
ENSGALG00010026536	BCR	2	-0.045174382	0.049671753	-0.909458174	0.363108326	0	0.262557266	0	1	PRJNA827465;Yang2025_Silkie_Wenchang	CRA004775	0.816619738
ENSGALG00010026536	BCR	2	-0.148278363	0.145799314	-1.017003163	0.309151925	0	0.166111394	0	1	CRA004775;Yang2025_Silkie_Wenchang	PRJNA827465	0.794043289
ENSGALG00010026536	BCR	2	-0.041755777	0.051768015	-0.806594132	0.419900356	0	0.450581057	0	1	CRA004775;PRJNA827465	Yang2025_Silkie_Wenchang	0.838584536
ENSGALG00010026537	FJX1	2	0.101661253	0.120199519	0.845770882	0.397680572	0.005899279	1.087048242	8.007762538	1	PRJNA827465;Yang2025_Silkie_Wenchang	CRA004775	0.831701623
ENSGALG00010026537	FJX1	2	0.125967436	0.372857499	0.337843376	0.735481221	0.114461496	1.66974647	40.11066843	0.5	CRA004775;Yang2025_Silkie_Wenchang	PRJNA827465	0.933592889
ENSGALG00010026537	FJX1	2	0.042431047	0.099333406	0.427157879	0.669264338	0	0.608527827	0	0.5	CRA004775;PRJNA827465	Yang2025_Silkie_Wenchang	0.914377769
ENSGALG00010026550	MYD88	2	-0.307500973	0.403040209	-0.762953587	0.445491076	0.309358624	20.18846629	95.04667672	0.5	PRJNA827465;Yang2025_Silkie_Wenchang	CRA004775	0.846013379
ENSGALG00010026550	MYD88	2	-0.608685748	0.153106069	-3.975582099	7.02073E-05	0.007653277	1.181508217	15.3624168	1	CRA004775;Yang2025_Silkie_Wenchang	PRJNA827465	0.006081423
ENSGALG00010026550	MYD88	2	-0.113943233	0.239792714	-0.475173877	0.634663045	0.092409135	4.275221383	76.60939843	0.5	CRA004775;PRJNA827465	Yang2025_Silkie_Wenchang	0.903872221
ENSGALG00010026553	PLPP3	2	-0.160318624	0.055264105	-2.900953964	0.003720285	0	0.025762533	0	1	PRJNA827465;Yang2025_Silkie_Wenchang	CRA004775	0.087190443
ENSGALG00010026553	PLPP3	2	0.101880075	0.306077489	0.332857131	0.73924213	0.144616252	3.829754985	73.88866902	0.5	CRA004775;Yang2025_Silkie_Wenchang	PRJNA827465	0.934767179
ENSGALG00010026553	PLPP3	2	0.089084227	0.315473675	0.282382442	0.777650277	0.160280056	4.345332276	76.98680017	0.5	CRA004775;PRJNA827465	Yang2025_Silkie_Wenchang	0.944388838
ENSGALG00010026560	TNNI1	2	0.131281024	0.125728535	1.044162521	0.296410204	0	0.253709819	0	1	PRJNA827465;Yang2025_Silkie_Wenchang	CRA004775	0.788267543
ENSGALG00010026560	TNNI1	2	0.069629489	0.24961044	0.278952629	0.780281179	0	0.341521902	0	1	CRA004775;Yang2025_Silkie_Wenchang	PRJNA827465	0.945041817
ENSGALG00010026560	TNNI1	2	0.176368058	0.139245243	1.266600238	0.205298267	0	0.155207473	0	1	CRA004775;PRJNA827465	Yang2025_Silkie_Wenchang	0.718123692
ENSGALG00010026566	ENSGALG00010026566	2	0.084860739	0.113838926	0.74544571	0.45600232	0.007510063	1.334405399	25.06025522	1	PRJNA827465;Yang2025_Silkie_Wenchang	CRA004775	0.84907427
ENSGALG00010026566	ENSGALG00010026566	2	0.198031551	0.171930597	1.151810986	0.249398753	0	0.751546331	0	0.5	CRA004775;Yang2025_Silkie_Wenchang	PRJNA827465	0.759618659
ENSGALG00010026566	ENSGALG00010026566	2	-0.002035534	0.104625564	-0.019455413	0.984477806	0	0.181552823	0	0.5	CRA004775;PRJNA827465	Yang2025_Silkie_Wenchang	0.995703389
ENSGALG00010026568	BYSL	2	0.010060516	0.069387042	0.14499128	0.884717757	0	0.185221444	0	1	PRJNA827465;Yang2025_Silkie_Wenchang	CRA004775	0.972074082
ENSGALG00010026568	BYSL	2	-0.21295045	0.348340606	-0.61132824	0.540982299	0.169022249	3.232434366	69.06356365	0.5	CRA004775;Yang2025_Silkie_Wenchang	PRJNA827465	0.875817741
ENSGALG00010026568	BYSL	2	-0.219120887	0.287335566	-0.76259577	0.445704508	0.125568619	3.428458509	70.8323727	0.5	CRA004775;PRJNA827465	Yang2025_Silkie_Wenchang	0.846013379
ENSGALG00010026569	GNAZ	2	-0.192295982	0.091797673	-2.094780577	0.036190503	0	0.328403778	0	1	PRJNA827465;Yang2025_Silkie_Wenchang	CRA004775	0.328194582
ENSGALG00010026569	GNAZ	2	0.093575743	0.348019536	0.26888072	0.788021474	0.130973097	1.681383285	40.52516111	0.5	CRA004775;Yang2025_Silkie_Wenchang	PRJNA827465	0.946919116
ENSGALG00010026569	GNAZ	2	0.050239814	0.421703388	0.119135428	0.905168063	0.241845808	2.32294541	56.95120533	0.5	CRA004775;PRJNA827465	Yang2025_Silkie_Wenchang	0.977975655
ENSGALG00010026581	DNER	2	-0.244405277	0.365332047	-0.668994904	0.503498729	0.216799051	5.139215463	80.5417771	0.5	PRJNA827465;Yang2025_Silkie_Wenchang	CRA004775	0.863108263
ENSGALG00010026581	DNER	2	0.188079738	0.262212064	0.717281024	0.473200713	0	0.126331364	0	1	CRA004775;Yang2025_Silkie_Wenchang	PRJNA827465	0.854341771
ENSGALG00010026581	DNER	2	-0.358371447	0.423303591	-0.846606206	0.397214654	0.19951557	1.591718084	37.17480438	0.5	CRA004775;PRJNA827465	Yang2025_Silkie_Wenchang	0.831438925
ENSGALG00010026584	ENSGALG00010026584	2	-0.069911608	0.056045429	-1.247409621	0.212247341	0.000541056	1.078249818	7.257113942	1	PRJNA827465;Yang2025_Silkie_Wenchang	CRA004775	0.724883318
ENSGALG00010026584	ENSGALG00010026584	2	0.107548903	0.29022834	0.370566509	0.710960431	0.143391177	6.360894738	84.27894123	0.5	CRA004775;Yang2025_Silkie_Wenchang	PRJNA827465	0.925563881
ENSGALG00010026584	ENSGALG00010026584	2	0.154532405	0.226566574	0.682061799	0.495199875	0.082573106	4.470925516	77.63326639	0.5	CRA004775;PRJNA827465	Yang2025_Silkie_Wenchang	0.860619541
ENSGALG00010026588	CCND3	2	-0.001908887	0.072689447	-0.026260858	0.979049275	0	0.506696083	0	0.5	PRJNA827465;Yang2025_Silkie_Wenchang	CRA004775	0.994278537
ENSGALG00010026588	CCND3	2	-0.101703588	0.108746146	-0.93523855	0.349665376	0	0.018270209	0	1	CRA004775;Yang2025_Silkie_Wenchang	PRJNA827465	0.809903518
ENSGALG00010026588	CCND3	2	-0.001051714	0.075492907	-0.013931301	0.988884789	0	0.616917594	0	0.5	CRA004775;PRJNA827465	Yang2025_Silkie_Wenchang	0.996891127
ENSGALG00010026595	PRKAA2	2	-0.129250191	0.188381729	-0.686107893	0.492645074	0.052763327	3.888460355	74.2828804	0.5	PRJNA827465;Yang2025_Silkie_Wenchang	CRA004775	0.859368397
ENSGALG00010026595	PRKAA2	2	-0.306291978	0.138987645	-2.203735293	0.027542966	0	0.300010213	0	0.5	CRA004775;Yang2025_Silkie_Wenchang	PRJNA827465	0.283382507
ENSGALG00010026595	PRKAA2	2	0.053510672	0.125250895	0.427227863	0.669213369	0	0.000340865	0	1	CRA004775;PRJNA827465	Yang2025_Silkie_Wenchang	0.914377769
ENSGALG00010026612	MFF	2	-0.08199638	0.055592758	-1.474947138	0.140226756	0	0.391352445	0	1	PRJNA827465;Yang2025_Silkie_Wenchang	CRA004775	0.624052921
ENSGALG00010026612	MFF	2	-0.130574295	0.090743526	-1.438937851	0.150168132	0	0.00849142	0	1	CRA004775;Yang2025_Silkie_Wenchang	PRJNA827465	0.640555393
ENSGALG00010026612	MFF	2	-0.064066931	0.063458828	-1.009582642	0.312695289	0	0.061783347	0	1	CRA004775;PRJNA827465	Yang2025_Silkie_Wenchang	0.796054997
ENSGALG00010026630	XIRP1	2	0.030924759	0.296744128	0.10421355	0.916999882	0.151366864	6.884770351	85.47518728	0.5	PRJNA827465;Yang2025_Silkie_Wenchang	CRA004775	0.980728718
ENSGALG00010026630	XIRP1	2	0.870744313	0.741976656	1.173546777	0.24057661	0.782259875	2.687336959	62.78844019	1	CRA004775;Yang2025_Silkie_Wenchang	PRJNA827465	0.753158164
ENSGALG00010026630	XIRP1	2	0.640808448	1.067991638	0.600012608	0.548497833	1.911907981	5.244256621	80.93152047	0.5	CRA004775;PRJNA827465	Yang2025_Silkie_Wenchang	0.878281266
ENSGALG00010026633	SLC5A1	2	0.4110724	0.249165484	1.649796726	0.098984519	0	0.81965045	0	1	PRJNA827465;Yang2025_Silkie_Wenchang	CRA004775	0.536907794
ENSGALG00010026633	SLC5A1	2	1.04908284	1.021289524	1.027213944	0.304319734	1.675754296	4.62680347	78.38680622	1	CRA004775;Yang2025_Silkie_Wenchang	PRJNA827465	0.79225794
ENSGALG00010026633	SLC5A1	2	1.226762756	0.780630668	1.571502128	0.116066061	0.849123295	2.858254862	65.01361676	1	CRA004775;PRJNA827465	Yang2025_Silkie_Wenchang	0.573371591
ENSGALG00010026638	LAD1	2	0.573375777	0.235985962	2.429702899	0.015111204	0	0.006664459	0	1	PRJNA827465;Yang2025_Silkie_Wenchang	CRA004775	0.203100811
ENSGALG00010026638	LAD1	2	1.36011613	0.930339989	1.461956001	0.143753267	1.405321259	4.883744323	79.5239076	1	CRA004775;Yang2025_Silkie_Wenchang	PRJNA827465	0.63068235
ENSGALG00010026638	LAD1	2	1.377746721	0.910624114	1.51296973	0.130287348	1.331453102	4.657753711	78.53042342	1	CRA004775;PRJNA827465	Yang2025_Silkie_Wenchang	0.605608015
ENSGALG00010026643	YWHAH	2	0.028526603	0.060718855	0.469814581	0.638487496	0	0.857083298	0	0.5	PRJNA827465;Yang2025_Silkie_Wenchang	CRA004775	0.905621386
ENSGALG00010026643	YWHAH	2	0.086947234	0.247958634	0.350652175	0.725849309	0.070379697	1.801018819	44.47587168	0.5	CRA004775;Yang2025_Silkie_Wenchang	PRJNA827465	0.931029023
ENSGALG00010026643	YWHAH	2	0.102349835	0.124272073	0.823594817	0.410169822	0.011820813	1.140976378	12.35576656	1	CRA004775;PRJNA827465	Yang2025_Silkie_Wenchang	0.834717782
ENSGALG00010026659	LIPH	2	0.80020903	0.198725317	4.026709037	5.6563E-05	0.038564144	1.928651401	48.15029821	1	PRJNA827465;Yang2025_Silkie_Wenchang	CRA004775	0.005305713
ENSGALG00010026659	LIPH	2	0.315239277	0.74929949	0.420711986	0.673965407	1.012642574	9.901594728	89.90061674	0.5	CRA004775;Yang2025_Silkie_Wenchang	PRJNA827465	0.915650814
ENSGALG00010026659	LIPH	2	0.138899447	0.546426838	0.254195873	0.799344239	0.501436773	5.805759625	82.77572506	0.5	CRA004775;PRJNA827465	Yang2025_Silkie_Wenchang	0.949922217
ENSGALG00010026671	TAF8	2	-0.00782832	0.058766077	-0.13321154	0.894026085	0	0.062198922	0	0.5	PRJNA827465;Yang2025_Silkie_Wenchang	CRA004775	0.974381724
ENSGALG00010026671	TAF8	2	-0.302551932	0.352628442	-0.857990721	0.390897587	0.21332123	6.604822062	84.8595467	0.5	CRA004775;Yang2025_Silkie_Wenchang	PRJNA827465	0.829251057
ENSGALG00010026671	TAF8	2	-0.311666786	0.335474199	-0.92903355	0.3528717	0.194469172	6.645586408	84.95241896	1	CRA004775;PRJNA827465	Yang2025_Silkie_Wenchang	0.811035834
ENSGALG00010026673	DEPDC5	2	0.02124613	0.066545561	0.319271936	0.749520312	0.004332089	1.927369232	48.11580557	0.5	PRJNA827465;Yang2025_Silkie_Wenchang	CRA004775	0.937362187
ENSGALG00010026673	DEPDC5	2	-0.122369851	0.237816178	-0.514556459	0.606862998	0.09881883	7.57005949	86.79006418	0.5	CRA004775;Yang2025_Silkie_Wenchang	PRJNA827465	0.896799223
ENSGALG00010026673	DEPDC5	2	-0.178554341	0.168819371	-1.057665005	0.290208195	0.044936776	4.234962845	76.38704195	1	CRA004775;PRJNA827465	Yang2025_Silkie_Wenchang	0.784195688
ENSGALG00010026681	PLXDC1	2	-0.01160533	0.18656452	-0.062205448	0.950399224	0.050984638	3.714784409	73.08053739	0.5	PRJNA827465;Yang2025_Silkie_Wenchang	CRA004775	0.987277827
ENSGALG00010026681	PLXDC1	2	-0.243767571	0.142285248	-1.713231519	0.086669955	0	0.748712851	0	1	CRA004775;Yang2025_Silkie_Wenchang	PRJNA827465	0.506300423
ENSGALG00010026681	PLXDC1	2	-0.103924966	0.386751152	-0.268712751	0.788150739	0.20715094	2.569146222	61.07656343	0.5	CRA004775;PRJNA827465	Yang2025_Silkie_Wenchang	0.946941439
ENSGALG00010026686	ENSGALG00010026686	2	1.099314648	0.519252444	2.117110207	0.034250496	0.41823036	4.446888155	77.51236449	1	PRJNA827465;Yang2025_Silkie_Wenchang	CRA004775	0.318313367
ENSGALG00010026686	ENSGALG00010026686	2	1.343459944	0.336501275	3.992436418	6.53979E-05	0.061651585	1.361872124	26.57166688	1	CRA004775;Yang2025_Silkie_Wenchang	PRJNA827465	0.005828418
ENSGALG00010026686	ENSGALG00010026686	2	0.715597475	0.26640362	2.686140206	0.007228275	0	0.406669636	0	1	CRA004775;PRJNA827465	Yang2025_Silkie_Wenchang	0.130677504
ENSGALG00010026690	KRT14	2	0.164278732	0.26969642	0.609124629	0.542441834	0	0.026982251	0	1	PRJNA827465;Yang2025_Silkie_Wenchang	CRA004775	0.876817086
ENSGALG00010026690	KRT14	2	0.602382367	0.55854993	1.078475414	0.280821648	0.406785273	2.753130698	63.67771421	1	CRA004775;Yang2025_Silkie_Wenchang	PRJNA827465	0.778581075
ENSGALG00010026690	KRT14	2	0.650835777	0.514187932	1.265754673	0.205600928	0.298422119	2.223695298	55.02981002	1	CRA004775;PRJNA827465	Yang2025_Silkie_Wenchang	0.71825498
ENSGALG00010026696	CSRNP1	2	-0.151851196	0.135425188	-1.12129212	0.262163539	0.027466605	3.976693001	74.85347751	1	PRJNA827465;Yang2025_Silkie_Wenchang	CRA004775	0.768363052

ENSGALG00010026696	CSRNP1	2	0.142480397	0.485757014	0.293316191	0.769280479	0.409902657	6.86734924	85.43834069	0.5	CRA004775;Yang2025_Silkie_Wenchang	PRJNA827465	0.941790377
ENSGALG00010026696	CSRNP1	2	0.254996397	0.34384086	0.741611677	0.458322647	0.180520206	3.557854095	71.89317006	0.5	CRA004775;PRJNA827465	Yang2025_Silkie_Wenchang	0.850026426
ENSGALG00010026697	CHN1	2	-0.150241744	0.224710474	-0.668601432	0.503749759	0.095065173	16.92997074	94.09331525	0.5	PRJNA827465;Yang2025_Silkie_Wenchang	CRA004775	0.86322274
ENSGALG00010026697	CHN1	2	-0.17083038	0.250724218	-0.681347744	0.49565148	0.09841042	4.117554219	75.71373813	0.5	CRA004775;Yang2025_Silkie_Wenchang	PRJNA827465	0.860911322
ENSGALG00010026697	CHN1	2	0.073833466	0.060181637	1.226843766	0.219881318	0	0.061760549	0	1	CRA004775;PRJNA827465	Yang2025_Silkie_Wenchang	0.733429129
ENSGALG00010026704	WDFY1	2	-0.020726797	0.184681824	-0.11222976	0.910641233	0.063669996	14.81151848	93.24849777	0.5	PRJNA827465;Yang2025_Silkie_Wenchang	CRA004775	0.97880407
ENSGALG00010026704	WDFY1	2	-0.208842431	0.076482584	-2.730588076	0.006322144	0	0.006417404	0	1	CRA004775;Yang2025_Silkie_Wenchang	PRJNA827465	0.12091446
ENSGALG00010026704	WDFY1	2	0.043458269	0.164797253	0.263707486	0.792005327	0.037854273	2.587364583	61.35063429	0.5	CRA004775;PRJNA827465	Yang2025_Silkie_Wenchang	0.948314332
ENSGALG00010026706	KRT24	2	-0.322928794	0.298648667	-1.081299967	0.279563702	0	0.113621069	0	1	PRJNA827465;Yang2025_Silkie_Wenchang	CRA004775	0.777720009
ENSGALG00010026706	KRT24	2	0.841052938	1.331438236	0.631687535	0.527591075	3.209730826	10.48724032	90.46460299	0.5	CRA004775;Yang2025_Silkie_Wenchang	PRJNA827465	0.871220201
ENSGALG00010026706	KRT24	2	0.921745345	1.226104191	0.751767551	0.452190859	2.726078808	10.47497902	90.4534415	0.5	CRA004775;PRJNA827465	Yang2025_Silkie_Wenchang	0.848321192
ENSGALG00010026707	GORASP1	2	-0.147983884	0.118667295	-1.247048597	0.212379679	0.02192897	4.509622863	77.82519669	1	PRJNA827465;Yang2025_Silkie_Wenchang	CRA004775	0.725020284
ENSGALG00010026707	GORASP1	2	-0.244233417	0.07833075	-3.117976239	0.001820975	0	0.862937933	0	1	CRA004775;Yang2025_Silkie_Wenchang	PRJNA827465	0.054467104
ENSGALG00010026707	GORASP1	2	-0.031945949	0.071663975	-0.445774171	0.655760381	0	1.07415E-05	0	1	CRA004775;PRJNA827465	Yang2025_Silkie_Wenchang	0.910411938
ENSGALG00010026715	WDR48	2	-0.11773724	0.169800722	-0.693384802	0.488068097	0.050380192	7.430620401	86.54217352	0.5	PRJNA827465;Yang2025_Silkie_Wenchang	CRA004775	0.85768885
ENSGALG00010026715	WDR48	2	-0.152350828	0.189166443	-0.805379779	0.420600557	0.045571148	2.552439709	60.82179742	0.5	CRA004775;Yang2025_Silkie_Wenchang	PRJNA827465	0.839130136
ENSGALG00010026715	WDR48	2	0.038394382	0.045561619	0.842691355	0.399401084	0	0.044434177	0	1	CRA004775;PRJNA827465	Yang2025_Silkie_Wenchang	0.832237344
ENSGALG00010026724	OLA1	2	-0.04408198	0.183212549	-0.240605682	0.809860746	0.056135168	5.760784606	82.64125343	0.5	PRJNA827465;Yang2025_Silkie_Wenchang	CRA004775	0.952818762
ENSGALG00010026724	OLA1	2	0.161446729	0.1245942	1.29578046	0.195051132	0	0.000490524	0	1	CRA004775;Yang2025_Silkie_Wenchang	PRJNA827465	0.706113528
ENSGALG00010026724	OLA1	2	-0.124356596	0.156588925	-0.794159586	0.427102533	0.027140862	1.625206019	38.46933938	0.5	CRA004775;PRJNA827465	Yang2025_Silkie_Wenchang	0.84125685
ENSGALG00010026740	DAB1	2	0.072594768	0.138860783	0.522788118	0.601121709	0.019084452	1.79726184	44.35980457	0.5	PRJNA827465;Yang2025_Silkie_Wenchang	CRA004775	0.895230519
ENSGALG00010026740	DAB1	2	0.692444375	0.504707416	1.371971864	0.170072197	0.398398737	4.165527171	75.99343471	1	CRA004775;Yang2025_Silkie_Wenchang	PRJNA827465	0.671658717
ENSGALG00010026740	DAB1	2	0.55441806	0.654089792	0.847617663	0.396650935	0.755631198	7.74625015	87.09052792	0.5	CRA004775;PRJNA827465	Yang2025_Silkie_Wenchang	0.831375233
ENSGALG00010026743	TNNT2	2	0.904129329	0.472196341	1.914731754	0.055526722	0.39999176	9.631877108	89.61780774	1	PRJNA827465;Yang2025_Silkie_Wenchang	CRA004775	0.408228819
ENSGALG00010026743	TNNT2	2	0.505193579	1.034916483	0.488149129	0.625444215	1.795310503	5.494784185	81.80092309	0.5	CRA004775;Yang2025_Silkie_Wenchang	PRJNA827465	0.901367193
ENSGALG00010026743	TNNT2	2	0.177523814	0.486393193	0.364980055	0.715126295	0.275396221	1.713101	41.62632558	0.5	CRA004775;PRJNA827465	Yang2025_Silkie_Wenchang	0.926907257
ENSGALG00010026755	ENSGALG00010026755	2	-0.265826888	0.573703864	-0.463352096	0.64311201	0.518998207	4.702212534	78.73341554	0.5	PRJNA827465;Yang2025_Silkie_Wenchang	CRA004775	0.906634523
ENSGALG00010026755	ENSGALG00010026755	2	-0.505984718	0.319572058	-1.583319646	0.113348627	0.084170681	1.700362436	41.18900896	1	CRA004775;Yang2025_Silkie_Wenchang	PRJNA827465	0.568377278
ENSGALG00010026755	ENSGALG00010026755	2	0.039999056	0.266396151	0.150148774	0.88064724	0	0.894894676	0	0.5	CRA004775;PRJNA827465	Yang2025_Silkie_Wenchang	0.971623434
ENSGALG00010026759	OMA1	2	0.028834795	0.056280842	0.512337658	0.608414714	0	0.00717662	0	1	PRJNA827465;Yang2025_Silkie_Wenchang	CRA004775	0.897208691
ENSGALG00010026759	OMA1	2	-0.107209358	0.208059481	-0.515282254	0.606355798	0.053955283	2.188030294	54.29679367	0.5	CRA004775;Yang2025_Silkie_Wenchang	PRJNA827465	0.896731537
ENSGALG00010026759	OMA1	2	-0.104610979	0.199387935	-0.524660524	0.599819205	0.052002842	2.212745572	54.80727596	0.5	CRA004775;PRJNA827465	Yang2025_Silkie_Wenchang	0.895092498
ENSGALG00010026775	BTG2	2	-0.228298413	0.2569598	-0.888459647	0.374293553	0.116493544	8.350088746	88.02407938	0.5	PRJNA827465;Yang2025_Silkie_Wenchang	CRA004775	0.822805106
ENSGALG00010026775	BTG2	2	-0.103129097	0.531697669	-0.193961913	0.846205704	0.416225925	3.06896883	67.41576551	0.5	CRA004775;Yang2025_Silkie_Wenchang	PRJNA827465	0.96122283
ENSGALG00010026775	BTG2	2	0.031812048	0.097234496	0.327168333	0.743540571	0	0.912973654	0	1	CRA004775;PRJNA827465	Yang2025_Silkie_Wenchang	0.935816088
ENSGALG00010026780	MYSM1	2	0.115335807	0.06462961	1.784566029	0.074331736	0	0.548772807	0	1	PRJNA827465;Yang2025_Silkie_Wenchang	CRA004775	0.470803516
ENSGALG00010026780	MYSM1	2	0.136430475	0.088823859	1.535966532	0.124546589	0	0.583207849	0	1	CRA004775;Yang2025_Silkie_Wenchang	PRJNA827465	0.591976609
ENSGALG00010026780	MYSM1	2	0.064121278	0.077101923	0.831643047	0.405610456	0	0.103332069	0	1	CRA004775;PRJNA827465	Yang2025_Silkie_Wenchang	0.833257341
ENSGALG00010026782	HOXB9	2	1.277109346	0.698276959	1.828943844	0.067408021	0.708521379	3.607391639	72.27913961	1	PRJNA827465;Yang2025_Silkie_Wenchang	CRA004775	0.44752322
ENSGALG00010026782	HOXB9	2	0.798912106	1.233313495	0.647776992	0.517129198	2.688409503	8.599567691	88.37150848	0.5	CRA004775;Yang2025_Silkie_Wenchang	PRJNA827465	0.867952013
ENSGALG00010026782	HOXB9	2	0.171074835	0.52765467	0.324217419	0.745773435	0.285448083	2.007861972	50.19577969	0.5	CRA004775;PRJNA827465	Yang2025_Silkie_Wenchang	0.936338274
ENSGALG00010026796	DCAKD	2	0.078307683	0.149139538	0.5250632	0.599539257	0	0.000920949	0	1	PRJNA827465;Yang2025_Silkie_Wenchang	CRA004775	0.895010988
ENSGALG00010026796	DCAKD	2	-0.071659287	0.195821568	-0.365941749	0.714408542	0	0.938682376	0	0.5	CRA004775;Yang2025_Silkie_Wenchang	PRJNA827465	0.926588092
ENSGALG00010026796	DCAKD	2	-0.032768532	0.170385651	-0.192319787	0.84749172	0.00665332	1.103217483	9.356041264	0.5	CRA004775;PRJNA827465	Yang2025_Silkie_Wenchang	0.961252712
ENSGALG00010026801	FMOD	2	0.869727166	0.68602223	1.267782774	0.204875535	0.831347857	8.554393259	88.31010021	1	PRJNA827465;Yang2025_Silkie_Wenchang	CRA004775	0.717411187
ENSGALG00010026801	FMOD	2	1.42150052	0.293934225	4.83611774	1.324E-06	0	0.578033305	0	1	CRA004775;Yang2025_Silkie_Wenchang	PRJNA827465	0.000277936
ENSGALG00010026801	FMOD	2	0.518394143	0.42488488	1.220081408	0.222434016	0.188546313	1.950941415	48.74269457	1	CRA004775;PRJNA827465	Yang2025_Silkie_Wenchang	0.736006309
ENSGALG00010026822	PRELP	2	-0.068294339	0.09592071	-0.711987425	0.476472563	0	0.989850806	0	1	PRJNA827465;Yang2025_Silkie_Wenchang	CRA004775	0.854986476
ENSGALG00010026822	PRELP	2	-0.261479425	0.208983652	-1.251195594	0.210863125	0	0.090126337	0	1	CRA004775;Yang2025_Silkie_Wenchang	PRJNA827465	0.72260471
ENSGALG00010026822	PRELP	2	-0.041509602	0.099176102	-0.418544404	0.675549128	0	0.154303594	0	1	CRA004775;PRJNA827465	Yang2025_Silkie_Wenchang	0.91647357
ENSGALG00010026825	ADAM23	2	-0.006626554	0.268885101	-0.024644557	0.980338479	0.128696082	9.051806514	88.95248149	0.5	PRJNA827465;Yang2025_Silkie_Wenchang	CRA004775	0.994676009
ENSGALG00010026825	ADAM23	2	0.293396121	0.131005282	2.239574754	0.025118543	0	0.271131326	0	1	CRA004775;Yang2025_Silkie_Wenchang	PRJNA827465	0.269931537
ENSGALG00010026825	ADAM23	2	0.016046861	0.364525226	0.044021263	0.964887455	0.201697193	3.509948548	71.50955388	0.5	CRA004775;PRJNA827465	Yang2025_Silkie_Wenchang	0.990492116
ENSGALG00010026827	FAM117A	2	-0.139275541	0.10583776	-1.315934322	0.188196088	0.014637618	2.877852233	65.25186427	1	PRJNA827465;Yang2025_Silkie_Wenchang	CRA004775	0.697483156
ENSGALG00010026827	FAM117A	2	-0.255236358	0.089001064	-2.867789973	0.004133498	0	0.056816212	0	1	CRA004775;Yang2025_Silkie_Wenchang	PRJNA827465	0.093485158
ENSGALG00010026827	FAM117A	2	-0.056190752	0.080795724	-0.695466912	0.48676274	0	0.789516447	0	1	CRA004775;PRJNA827465	Yang2025_Silkie_Wenchang	0.857603818
ENSGALG00010026839	FGGY	2	-0.327385041	0.096552297	-3.390753534	0.000697008	0	0.009672417	0	1	PRJNA827465;Yang2025_Silkie_Wenchang	CRA004775	0.029424798
ENSGALG00010026839	FGGY	2	-0.135426552	0.266828179	-0.507542166	0.611774458	0.085017714	2.263431465	55.81929405	0.5	CRA004775;Yang2025_Silkie_Wenchang	PRJNA827465	0.897777844
ENSGALG00010026839	FGGY	2	-0.141685073	0.251284637	-0.563842957	0.572861014	0.081339076	2.355421334	57.54475067	0.5	CRA004775;PRJNA827465	Yang2025_Silkie_Wenchang	0.88573298
ENSGALG00010026846	MICAL1	2	0.146238645	0.131374511	1.113143206	0.26564693	0	0.275248538	0	1	PRJNA827465;Yang2025_Silkie_Wenchang	CRA004775	0.769794389
ENSGALG00010026846	MICAL1	2	-0.123740308	0.251162635	-0.49267005	0.622245743	0.04529673	1.466032262	31.78867711	0.5	CRA004775;Yang2025_Silkie_Wenchang	PRJNA827465	0.900093208
ENSGALG00010026846	MICAL1	2	-0.054381865	0.327548885	-0.166026713	0.868135928	0.143873055	2.672095235	62.57618416	0.5	CRA004775;PRJNA827465	Yang2025_Silkie_Wenchang	0.968042774
ENSGALG00010026854	SMPD2	2	0.291611269	0.132113149	2.207284215	0.027294209	0.021516365	2.474957743	59.59526975	1	PRJNA827465;Yang2025_Silkie_Wenchang	CRA004775	0.281782062
ENSGALG00010026854	SMPD2	2	0.098011687	0.3805139	0.257577153	0.796733264	0.247827507	6.702218544	85.07956741	0.5	CRA004775;Yang2025_Silkie_Wenchang	PRJNA827465	0.949622804
ENSGALG00010026854	SMPD2	2	-0.007181093	0.240284786	-0.029885757	0.976158165	0.085401528	3.315072369	69.83474601	0.5	CRA004775;PRJNA827465	Yang2025_Silkie_Wenchang	0.992992231
ENSGALG00010026856	UBE2Z	2	-0.236079019	0.310231802	-0.760976204	0.446671281	0.180046139	14.78199806	93.23501467	0.5	PRJNA827465;Yang2025_Silkie_Wenchang	CRA004775	0.84648396
ENSGALG00010026856	UBE2Z	2	-0.38402436	0.181905631	-2.11111859	0.03476212	0.04161683	2.694021667	62.88077367	1	CRA004775;Yang2025_Silkie_Wenchang	PRJNA827465	0.321095119
ENSGALG00010026856	UBE2Z	2	-0.027161884	0.120836939	-0.2247813	0.822149414	0.019586718	2.439865759	59.01413853	0.5	CRA004775;PRJNA827465	Yang2025_Silkie_Wenchang	0.955636543
ENSGALG00010026874	TEAD3	2	0.005743763	0.069045568	0.083188011	0.933702045	0.003554272	1.527742372	34.54393764	0.5	PRJNA827465;Yang2025_Silkie_Wenchang	CRA004775	0.984262625
ENSGALG00010026874	TEAD3	2	0.081515309	0.095370108	0.854725982	0.392702863	0	0.555430817	0	0.5	CRA004775;Yang2025_Silkie_Wenchang	PRJNA827465	0.829914191
ENSGALG00010026874	TEAD3	2	-0.050375246	0.062236079	-0.809421914	0.418272501	0	0.193770082	0	1	CRA004775;PRJNA827465	Yang2025_Silkie_Wenchang	0.838112595
ENSGALG00010026885	TULP1	2	0.201074763	0.559410289	0.359440589	0.719265515	0.438490124	3.317855238	69.86004728	0.5	PRJNA827465;Yang2025_Silkie_Wenchang	CRA004775	0.928449671
ENSGALG00010026885	TULP1	2	0.596797785	0.394912647	1.511214667	0.130733767	0	0.566256645	0	1	CRA004775;Yang2025_Silkie_Wenchang	PRJNA827465	0.60646754
ENSGALG00010026885	TULP1	2	-0.220601498	0.346152856	-0.63729504	0.523932681	0	0.329175718	0	0.5	CRA004775;PRJNA827465	Yang2025_Silkie_Wenchang	0.869661505
ENSGALG00010026886	JUN	2	-0.245762244	0.212694105	-1.155472757	0.247896883	0.081973215	10.53900586	90.511439	1	PRJNA827465;Yang2025_Silkie_Wenchang	CRA004775	0.75810588
ENSGALG00010026886	JUN	2	-0.066397174	0.468142063	-0.141831249	0.8872133	0.366795338	5.4263704	81.57147548	0.5	CRA004775;Yang2025_Silkie_Wenchang	PRJNA827465	0.972987228
ENSGALG00010026886	JUN	2	0.079639165	0.219369116	0.363037269	0.716577044	0.056597714	1.707251895	41.42633535	0.5	CRA004775;PRJNA827465	Yang2025_Silkie_Wenchang	0.927487786
ENSGALG00010026889	ENSGALG00010026889	2	-0.109349396	0.179081596	-0.610612138	0.541456385	0	0.066678449	0	1	PRJNA827465;Yang2025_Silkie_Wenchang	CRA004775	0.87615218
ENSGALG00010026889	ENSGALG00010026889	2	-0.168750826	0.251956962	-0.669760521	0.503010464	0	0.004198248	0	1	CRA004775;Yang2025_Silkie_Wenchang	PRJNA827465	0.863050365
ENSGALG00010026889	ENSGALG00010026889	2	-0.084495013	0.224387699	-0.376558134	0.706502006	0	0.038726333	0	1	CRA004775;PRJNA827465	Yang2025_Silkie_Wenchang	0.924548793
ENSGALG00010026893	FKBP5	2	0.225366761	0.564612166	0.399153215	0.689780314	0.607856707	21.44163174	95.33617585	0.5	PRJNA827465;Yang2025_Silkie_Wenchang	CRA004775	0.919979266
ENSGALG00010026893	FKBP5	2	0.666881167	0.290556466	2.295186121	0.02172246	0.067121089	1.269534365	21.23096249	0.5	CRA004775;Yang2025_Silkie_Wenchang	PRJNA827465	0.249645938
ENSGALG00010026893	FKBP5	2	-0.318307748	0.157612909	-2.019553794	0.043429692	0	0.227267674	0	1	CRA004775;PRJNA827465	Yang2025_Silkie_Wenchang	0.360109112
ENSGALG00010026894	IP6K1	2	0.0314085	0.122289579	0.256837093	0.797304534	0.020314683	2.516924245	60.2689671	0.5	PRJNA827465;Yang2025_Silkie_Wenchang	CRA004775	0.949715736
ENSGALG00010026894	IP6K1	2	-0.18523642	0.102105391	-1.814168858	0.069651733	0	0.131001512	0	1	CRA004775;Yang2025_Silkie_Wenchang	PRJNA827465	0.455191564
ENSGALG00010026894	IP6K1	2	-0.02572265	0.165524457	-0.155400901	0.876505274	0.045877083	5.561322971	82.01866705	0.5	CRA004775;PRJNA827465	Yang2025_Silkie_Wenchang	0.970347777
ENSGALG00010026897	ENSGALG00010026897	2	0.374060357	0.386026101	0.969002759	0.332543813	0.244053119	5.506959457	81.84115921	0.5	PRJNA827465;Yang2025_Silkie_Wenchang	CRA004775	0.802178578
ENSGALG00010026897	ENSGALG00010026897	2	0.501209111	0.337923764	1.483201728	0.138020822	0.124850522	2.076911478	51.85158297	1	CRA004775;Yang2025_Silkie_Wenchang	PRJNA827465	0.621022596
ENSGALG00010026897	ENSGALG00010026897	2	0.014093821	0.191967802	0.07341763	0.941473789	0	0.028193389	0	0.5	CRA004775;PRJNA827465	Yang2025_Silkie_Wenchang	0.985827325
ENSGALG00010026898	PNPLA2	2	-0.244861381	0.166323158	-1.472202571	0.140966183	0.038185461	3.227644712	69.01765562	1	PRJNA827465;Yang2025_Silkie_Wenchang	CRA004775	0.625397212
ENSGALG00010026898	PNPLA2	2	-0.380819535	0.127324098	-2.990946264	0.002781144	0	0.89224428	0	0.5	CRA004775;Yang2025_Silkie_Wenchang	PRJNA827465	0.071912707
ENSGALG00010026898	PNPLA2	2	-0.068737407	0.126420671	-0.543719678	0.586634389	0	0.100886318	0	0.5	CRA004775;PRJNA827465	Yang2025_Silkie_Wenchang	0.891596903
ENSGALG00010026899	HOOK1	2	0.304325562	0.222706021	1.36649005	0.171785202	0.07268407	3.659303133	72.67239243	1	PRJNA827465;Yang2025_Silkie_Wenchang	CRA004775	0.674398921
ENSGALG00010026899	HOOK1	2	0.380787265	0.229414729	1.659820475	0.096950574	0.044269449	1.616632666	38.14302894	1	CRA004775;Yang2025_Silkie_Wenchang	PRJNA827465	0.532111212
ENSGALG00010026899	HOOK1	2	0.095919603	0.124953443	0.767642735	0.442699466	0	0.009509566	0	1	CRA004775;PRJNA827465	Yang2025_Silkie_Wenchang	0.845623604
ENSGALG00010026901	FAM212A	2	0.106077391	0.087857717	1.207377038	0.227286974	0	0.000486139	0	1	PRJNA827465;Yang2025_Silkie_Wenchang	CRA004775	0.740605003
ENSGALG00010026901	FAM212A	2	0.14894902	0.180718168	0.824206123	0.409822449	0	0.213806689	0	1	CRA004775;Yang2025_Silkie_Wenchang	PRJNA827465	0.834652129
ENSGALG00010026901	FAM212A	2	0.116219806	0.095411089	1.218095367	0.223187734	0	0.258486903	0	1	CRA004775;PRJNA827465	Yang2025_Silkie_Wenchang	0.736744216
ENSGALG00010026907	ENSGALG00010026907	2	0.293030236	0.115427123	2.538660132	0.011127787	0.007708017	1.243012341	19.5502758	1	PRJNA827465;Yang2025_Silkie_Wenchang	CRA004775	0.167633344
ENSGALG00010026907	ENSGALG00010026907	2	0.178753844	0.328401076	0.544315647	0.586224281	0.165650498	4.305085054	76.77165521	0.5	CRA004775;Yang2025_Silkie_Wenchang	PRJNA827465	0.891482799
ENSGALG00010026907	ENSGALG00010026907	2	0.093433571	0.181578852	0.514561963	0.606859151	0.043056166	2.555892806	60.87472849	0.5	CRA004775;PRJNA827465	Yang2025_Silkie_Wenchang	0.896799223
ENSGALG00010026909	ENSGALG00010026909	2	-0.276175011	0.069807336	-3.956246258	7.61367E-05	0	0.331200006	0	1	PRJNA827465;Yang2025_Silkie_Wenchang	CRA004775	0.006407708
ENSGALG00010026909	ENSGALG00010026909	2	-0.294797425	0.103617078	-2.84506599	0.004440222	0	0.619942734	0	1	CRA004775;Yang2025_Silkie_Wenchang	PRJNA827465	0.097602941
ENSGALG00010026909	ENSGALG00010026909	2	-0.229415816	0.085929196	-2.669823827	0.007589105	0	0.287877821	0	1	CRA004775;PRJNA827465	Yang2025_Silkie_Wenchang	0.134479085
ENSGALG00010026916	ENSGALG00010026916	2	0.349604216	0.527503256	0.662752717	0.507488941	0.490796597	8.203432443	87.80998068	0.5	PRJNA827465;Yang2025_Silkie_Wenchang	CRA004775	0.86416697
ENSGALG00010026916	ENSGALG00010026916	2	0.396474473	0.537604534	0.7374835	0.460828374	0.450942289	4.522351761	77.8876113	0.5	CRA004775;Yang2025_Silkie_Wenchang	PRJNA827465	0.85014477
ENSGALG00010026916	ENSGALG00010026916	2	-0.14755247	0.161299474	-0.914773411	0.360310596	0	0.00193172	0	1	CRA004775;PRJNA827465	Yang2025_Silkie_Wenchang	0.814880744
ENSGALG00010026917	AMT	2	0.325024031	0.132141165	2.459672806	0.013906373	0	0.564437619	0	1	PRJNA827465;Yang2025_Silkie_Wenchang	CRA004775	0.19354185
ENSGALG00010026917	AMT	2	0.014865092	0.239955391	0.061949398	0.950603129	0.064301244	2.205329656	54.65530529	0.5	CRA004775;Yang2025_Silkie_Wenchang	PRJNA827465	0.987277827
ENSGALG00010026917	AMT	2	0.105790986	0.340819726	0.310401592	0.756255583	0.181672589	4.487755723	77.71714724	0.5	CRA004775;PRJNA827465	Yang2025_Silkie_Wenchang	0.938571038
ENSGALG00010026919	NICN1	2	0.167939773	0.139375923	1.204941063	0.228226055	0.016022511	1.47672904	32.28277001	1	PRJNA827465;Yang2025_Silkie_Wenchang	CRA004775	0.741040754
ENSGALG00010026919	NICN1	2	0.127425453	0.279350475	0.456149049	0.648282802	0.09498843	2.551768718	60.81149546	0.5	CRA004775;Yang2025_Silkie_Wenchang	PRJNA827465	0.90762535
ENSGALG00010026919	NICN1	2	0.050277716	0.095060687	0.528901246	0.596873958	0	0.780846844	0	0.5	CRA004775;PRJNA827465	Yang2025_Silkie_Wenchang	0.894172266
ENSGALG00010026920	GATA2	2	0.237325273	0.272521745	0.87084894	0.383836639	0.111747679	3.796856581	73.66242368	1	PRJNA827465;Yang2025_Silkie_Wenchang	CRA004775	0.825601288
ENSGALG00010026920	GATA2	2	0.000956849	0.64335087	0.00148729	0.998813315	0.656256265	4.430275703	77.42804134	0.5	CRA004775;Yang2025_Silkie_Wenchang	PRJNA827465	0.999703568
ENSGALG00010026920	GATA2	2	-0.172372609	0.316946224	-0.543854433	0.586541648	0.112718608	1.665412015	39.95479852	0.5	CRA004775;PRJNA827465	Yang2025_Silkie_Wenchang	0.891596903
ENSGALG00010026924	TPH1	2	-0.171500686	0.164210426	-1.044395844	0.296302286	0	0.68874286	0	1	PRJNA827465;Yang2025_Silkie_Wenchang	CRA004775	0.788267543
ENSGALG00010026924	TPH1	2	-0.30957023	0.229253429	-1.350340677	0.17690673	0	0.038562522	0	1	CRA004775;Yang2025_Silkie_Wenchang	PRJNA827465	0.682556882
ENSGALG00010026924	TPH1	2	-0.075222713	0.201582885	-0.373160215	0.709029205	0	0.069496001	0	1	CRA004775;PRJNA827465	Yang2025_Silkie_Wenchang	0.925298042
ENSGALG00010026942	CDK18	2	0.088746072	0.06966971	1.273811415	0.202730259	0	0.411044275	0	1	PRJNA827465;Yang2025_Silkie_Wenchang	CRA004775	0.715766022
ENSGALG00010026942	CDK18	2	0.074012199	0.223021399	0.331861423	0.739993901	0.031257801	1.292692075	22.64205686	0.5	CRA004775;Yang2025_Silkie_Wenchang	PRJNA827465	0.935072094
ENSGALG00010026942	CDK18	2	0.058295886	0.074121882	0.786486861	0.431582314	0	0.854464172	0	0.5	CRA004775;PRJNA827465	Yang2025_Silkie_Wenchang	0.842503929
ENSGALG00010026943	EEFSEC	2	-0.0104382	0.050888853	-0.205117609	0.837480239	0	0.889467111	0	0.5	PRJNA827465;Yang2025_Silkie_Wenchang	CRA004775	0.958908286
ENSGALG00010026943	EEFSEC	2	-0.027342201	0.104896879	-0.260657905	0.794356331	0.005380858	1.220576549	18.07150474	0.5	CRA004775;Yang2025_Silkie_Wenchang	PRJNA827465	0.948905846
ENSGALG00010026943	EEFSEC	2	0.031099503	0.057492962	0.540927142	0.588557802	0	0.436668975	0	1	CRA004775;PRJNA827465	Yang2025_Silkie_Wenchang	0.892097466
ENSGALG00010026950	LHFPL5	2	0.252023066	0.269586138	0.934851727	0.349864717	0.089810148	2.429175301	58.83376554	1	PRJNA827465;Yang2025_Silkie_Wenchang	CRA004775	0.809953147
ENSGALG00010026950	LHFPL5	2	0.463742528	0.250089091	1.854309301	0.063694922	0	0.463705647	0	1	CRA004775;Yang2025_Silkie_Wenchang	PRJNA827465	0.434844802
ENSGALG00010026950	LHFPL5	2	0.063824063	0.159042703	0.401301419	0.688198219	0	0.183558698	0	1	CRA004775;PRJNA827465	Yang2025_Silkie_Wenchang	0.919719653
ENSGALG00010026951	RUVBL1	2	-0.076645985	0.04462334	-1.717620986	0.08586577	0	0.304662215	0	1	PRJNA827465;Yang2025_Silkie_Wenchang	CRA004775	0.503999754
ENSGALG00010026951	RUVBL1	2	-0.060307313	0.069522782	-0.867446777	0.385697262	0	0.435402787	0	1	CRA004775;Yang2025_Silkie_Wenchang	PRJNA827465	0.82648313
ENSGALG00010026951	RUVBL1	2	-0.101774397	0.054225229	-1.876882759	0.060534164	0	0.195079797	0	1	CRA004775;PRJNA827465	Yang2025_Silkie_Wenchang	0.426049285
ENSGALG00010026954	MFSD4A	2	-0.23868182	0.203899209	-1.170587276	0.241764714	0.076630016	12.71016594	92.1322821	1	PRJNA827465;Yang2025_Silkie_Wenchang	CRA004775	0.75406671
ENSGALG00010026954	MFSD4A	2	-0.033203864	0.441986276	-0.075124197	0.940115896	0.357861349	11.32542541	91.1703096	0.5	CRA004775;Yang2025_Silkie_Wenchang	PRJNA827465	0.985486876
ENSGALG00010026954	MFSD4A	2	0.142060794	0.231740987	0.613015398	0.539866159	0.081080141	3.439115977	70.92276019	0.5	CRA004775;PRJNA827465	Yang2025_Silkie_Wenchang	0.87529516
ENSGALG00010026958	CLTCL1	2	0.008017641	0.049463895	0.162090778	0.871234369	0	0.925930121	0	0.5	PRJNA827465;Yang2025_Silkie_Wenchang	CRA004775	0.969278598
ENSGALG00010026958	CLTCL1	2	0.104691203	0.105392673	0.993344224	0.320542233	0	0.005618779	0	1	CRA004775;Yang2025_Silkie_Wenchang	PRJNA827465	0.798348216
ENSGALG00010026958	CLTCL1	2	-0.010302227	0.053591624	-0.192235776	0.847557523	0	0.145313045	0	0.5	CRA004775;PRJNA827465	Yang2025_Silkie_Wenchang	0.961258308
ENSGALG00010026960	SEC61A1	2	-0.214259273	0.295368402	-0.725396731	0.468208678	0.167501229	24.91080826	95.98567823	0.5	PRJNA827465;Yang2025_Silkie_Wenchang	CRA004775	0.853198008
ENSGALG00010026960	SEC61A1	2	-0.230636888	0.305823223	-0.754150997	0.450758559	0.164271969	7.848689869	87.25902008	0.5	CRA004775;Yang2025_Silkie_Wenchang	PRJNA827465	0.847631399
ENSGALG00010026960	SEC61A1	2	0.080473685	0.066977356	1.201505847	0.22955504	0	0.011825026	0	1	CRA004775;PRJNA827465	Yang2025_Silkie_Wenchang	0.742629165
ENSGALG00010026964	SRPK1	2	0.031285251	0.040079491	0.780580044	0.435049532	0.000526782	1.176554689	15.00607583	0.5	PRJNA827465;Yang2025_Silkie_Wenchang	CRA004775	0.842762754
ENSGALG00010026964	SRPK1	2	-0.026262671	0.061157239	-0.429428657	0.667611303	0	0.044819113	0	1	CRA004775;Yang2025_Silkie_Wenchang	PRJNA827465	0.913995707
ENSGALG00010026964	SRPK1	2	0.056413286	0.043536174	1.295779597	0.195051429	0	0.316197282	0	0.5	CRA004775;PRJNA827465	Yang2025_Silkie_Wenchang	0.706113528
ENSGALG00010026972	TNNT3	2	0.147630247	0.378983924	0.389542242	0.696875069	0.270594372	17.16053261	94.17267504	0.5	PRJNA827465;Yang2025_Silkie_Wenchang	CRA004775	0.921086208
ENSGALG00010026972	TNNT3	2	0.521620087	0.144190253	3.617582172	0.000297368	0	0.168016303	0	1	CRA004775;Yang2025_Silkie_Wenchang	PRJNA827465	0.016889382
ENSGALG00010026972	TNNT3	2	-0.216159486	0.108085154	-1.999899881	0.045511076	0	0.328318631	0	0.5	CRA004775;PRJNA827465	Yang2025_Silkie_Wenchang	0.367822707
ENSGALG00010026973	MGLL	2	-0.114389044	0.148744886	-0.769028413	0.441876444	0.028296403	2.703770229	63.01460866	0.5	PRJNA827465;Yang2025_Silkie_Wenchang	CRA004775	0.845135451
ENSGALG00010026973	MGLL	2	-0.163839179	0.140544999	-1.165741791	0.243718858	0.011761265	1.406850569	28.91924543	0.5	CRA004775;Yang2025_Silkie_Wenchang	PRJNA827465	0.755365463
ENSGALG00010026973	MGLL	2	0.013913627	0.092319381	0.150711873	0.880203007	0	0.004464867	0	1	CRA004775;PRJNA827465	Yang2025_Silkie_Wenchang	0.971588195
ENSGALG00010026977	ELK4	2	-0.041077117	0.081470003	-0.504199284	0.614121344	0	0.063975638	0	1	PRJNA827465;Yang2025_Silkie_Wenchang	CRA004775	0.898282836
ENSGALG00010026977	ELK4	2	-0.132615608	0.194227932	-0.682783405	0.494743718	0	0.14353812	0	1	CRA004775;Yang2025_Silkie_Wenchang	PRJNA827465	0.860356282
ENSGALG00010026977	ELK4	2	-0.043251465	0.085720017	-0.504566692	0.613863209	0	0.318479906	0	1	CRA004775;PRJNA827465	Yang2025_Silkie_Wenchang	0.898256044
ENSGALG00010026980	LIMK2	2	0.000611524	0.054599936	0.011200087	0.99106381	0	0.322636695	0	0.5	PRJNA827465;Yang2025_Silkie_Wenchang	CRA004775	0.99755571
ENSGALG00010026980	LIMK2	2	0.038724495	0.081331274	0.476132897	0.633979702	0	5.80834E-05	0	1	CRA004775;Yang2025_Silkie_Wenchang	PRJNA827465	0.903714339
ENSGALG00010026980	LIMK2	2	-0.019782038	0.067804119	-0.291752742	0.77047568	0	0.065388038	0	0.5	CRA004775;PRJNA827465	Yang2025_Silkie_Wenchang	0.942454504
ENSGALG00010026982	ABTB1	2	-0.283149473	0.28422661	-0.99621029	0.319147981	0.151439383	15.51757353	93.5556935	1	PRJNA827465;Yang2025_Silkie_Wenchang	CRA004775	0.798079048
ENSGALG00010026982	ABTB1	2	-0.400129102	0.220170474	-1.817360411	0.069161945	0.063382842	2.68558414	62.76415305	1	CRA004775;Yang2025_Silkie_Wenchang	PRJNA827465	0.452969829
ENSGALG00010026982	ABTB1	2	-0.01766386	0.057040573	-0.309671858	0.756810505	0	0.2334671	0	1	CRA004775;PRJNA827465	Yang2025_Silkie_Wenchang	0.938740241
ENSGALG00010026999	MLEC	2	-0.092638322	0.060450628	-1.532462517	0.125408341	0.001084587	1.093345403	8.537595023	1	PRJNA827465;Yang2025_Silkie_Wenchang	CRA004775	0.594562606
ENSGALG00010026999	MLEC	2	-0.269886018	0.123941587	-2.17752591	0.029441351	0	0.458610473	0	1	CRA004775;Yang2025_Silkie_Wenchang	PRJNA827465	0.292500862
ENSGALG00010026999	MLEC	2	-0.155532039	0.156104915	-0.996330186	0.319089742	0.029246583	1.827093295	45.26825735	1	CRA004775;PRJNA827465	Yang2025_Silkie_Wenchang	0.798079048
ENSGALG00010027001	SLC26A8	2	-0.011605433	0.254597366	-0.045583475	0.963642241	0.095599508	3.785775102	73.58533	0.5	PRJNA827465;Yang2025_Silkie_Wenchang	CRA004775	0.990144859
ENSGALG00010027001	SLC26A8	2	0.180733982	0.180517083	1.001201545	0.31672938	0	0.714239717	0	0.5	CRA004775;Yang2025_Silkie_Wenchang	PRJNA827465	0.796959041
ENSGALG00010027001	SLC26A8	2	-0.237260843	0.155804677	-1.522809502	0.127806372	0	0.075271403	0	1	CRA004775;PRJNA827465	Yang2025_Silkie_Wenchang	0.600058027
ENSGALG00010027003	SEPTIN2L	2	0.025177112	0.032929906	0.764566757	0.444529571	0	0.416593599	0	1	PRJNA827465;Yang2025_Silkie_Wenchang	CRA004775	0.846013379
ENSGALG00010027003	SEPTIN2L	2	0.01427391	0.086196315	0.165597684	0.86847357	0.004168286	1.327082442	24.64673119	0.5	CRA004775;Yang2025_Silkie_Wenchang	PRJNA827465	0.968167735
ENSGALG00010027003	SEPTIN2L	2	0.007834992	0.034712441	0.225711344	0.821425934	0	0.792786658	0	0.5	CRA004775;PRJNA827465	Yang2025_Silkie_Wenchang	0.955598716
ENSGALG00010027012	RILPL1	2	0.12323323	0.264198201	0.466442352	0.640898902	0.119708698	6.693078102	85.05919123	0.5	PRJNA827465;Yang2025_Silkie_Wenchang	CRA004775	0.906429868
ENSGALG00010027012	RILPL1	2	0.484851608	0.15595709	3.10887826	0.001877991	0	0.563305375	0	1	CRA004775;Yang2025_Silkie_Wenchang	PRJNA827465	0.055706168
ENSGALG00010027012	RILPL1	2	0.227714402	0.392807376	0.579710097	0.562110133	0.265576689	6.529600686	84.6851278	0.5	CRA004775;PRJNA827465	Yang2025_Silkie_Wenchang	0.881940701
ENSGALG00010027025	ORAI1	2	-0.014273127	0.122446035	-0.116566676	0.907203447	0.013224269	1.488630861	32.82417918	0.5	PRJNA827465;Yang2025_Silkie_Wenchang	CRA004775	0.978196186
ENSGALG00010027025	ORAI1	2	0.049856064	0.150672268	0.33089078	0.740726987	0	0.904466118	0	0.5	CRA004775;Yang2025_Silkie_Wenchang	PRJNA827465	0.935170497
ENSGALG00010027025	ORAI1	2	-0.084830746	0.07615602	-1.113907291	0.265318961	0	0.000178294	0	1	CRA004775;PRJNA827465	Yang2025_Silkie_Wenchang	0.76929776
ENSGALG00010027026	SLC45A3	2	-0.025252776	0.470960949	-0.053619681	0.957238176	0.374001054	6.326220499	84.19277355	0.5	PRJNA827465;Yang2025_Silkie_Wenchang	CRA004775	0.988541306
ENSGALG00010027026	SLC45A3	2	-0.471071734	0.263939312	-1.784772907	0.07429816	0	0.10829142	0	1	CRA004775;Yang2025_Silkie_Wenchang	PRJNA827465	0.470762626
ENSGALG00010027026	SLC45A3	2	0.274296452	0.296447668	0.925277818	0.354821414	0.057936826	1.283813993	22.1070961	0.5	CRA004775;PRJNA827465	Yang2025_Silkie_Wenchang	0.812016118
ENSGALG00010027027	ARHGEF3	2	-0.050765978	0.074918264	-0.67761819	0.49801381	0	0.049462987	0	1	PRJNA827465;Yang2025_Silkie_Wenchang	CRA004775	0.861203659
ENSGALG00010027027	ARHGEF3	2	-0.089238713	0.106629251	-0.8369065	0.402645126	0	0.35852888	0	1	CRA004775;Yang2025_Silkie_Wenchang	PRJNA827465	0.832937979
ENSGALG00010027027	ARHGEF3	2	-0.054827186	0.097139702	-0.564415835	0.572471164	0	0.481959997	0	1	CRA004775;PRJNA827465	Yang2025_Silkie_Wenchang	0.885678394
ENSGALG00010027036	NUCKS1	2	-0.088124473	0.03915706	-2.250538542	0.02441478	0	0.97878513	0	0.5	PRJNA827465;Yang2025_Silkie_Wenchang	CRA004775	0.267184364
ENSGALG00010027036	NUCKS1	2	0.037042691	0.121103436	0.305876465	0.759698706	0	0.017901517	0	1	CRA004775;Yang2025_Silkie_Wenchang	PRJNA827465	0.939163539
ENSGALG00010027036	NUCKS1	2	-0.095937405	0.040119401	-2.391297042	0.016788964	0	0.218150568	0	0.5	CRA004775;PRJNA827465	Yang2025_Silkie_Wenchang	0.215077129
ENSGALG00010027040	CLIP1	2	0.29872812	0.453988258	0.658008472	0.510532687	0.397549503	27.51610557	96.36576478	0.5	PRJNA827465;Yang2025_Silkie_Wenchang	CRA004775	0.865365179
ENSGALG00010027040	CLIP1	2	0.479463827	0.323129821	1.483811754	0.137858868	0.159698378	4.059444439	75.36608728	1	CRA004775;Yang2025_Silkie_Wenchang	PRJNA827465	0.62064915
ENSGALG00010027040	CLIP1	2	-0.132092522	0.056238637	-2.348785958	0.01883473	0	0.817569885	0	0.5	CRA004775;PRJNA827465	Yang2025_Silkie_Wenchang	0.229831303
ENSGALG00010027041	MAPK14	2	-0.267992672	0.104220883	-2.57139129	0.010129081	0.015500751	3.389652137	70.49844764	1	PRJNA827465;Yang2025_Silkie_Wenchang	CRA004775	0.159816649
ENSGALG00010027041	MAPK14	2	-0.390418896	0.087786834	-4.447351366	8.69356E-06	0	0.031358202	0	1	CRA004775;Yang2025_Silkie_Wenchang	PRJNA827465	0.001256959

ENSGALG00010027041	MAPK14	2	-0.197245531	0.072578235	-2.717695326	0.006573834	0.00169947	1.054963364	5.209978484	1	CRA004775;PRJNA827465	Yang2025_Silkie_Wenchang	0.123859732
ENSGALG00010027043	IL17RD	2	-0.140745899	0.167364617	-0.840953728	0.400373857	0.047753048	6.717004755	85.11241191	0.5	PRJNA827465;Yang2025_Silkie_Wenchang	CRA004775	0.832251175
ENSGALG00010027043	IL17RD	2	-0.199407748	0.234380919	-0.850784907	0.394888847	0.057706739	1.491718125	32.96320643	0.5	CRA004775;Yang2025_Silkie_Wenchang	PRJNA827465	0.830643015
ENSGALG00010027043	IL17RD	2	0.027151373	0.077560777	0.350065771	0.726289338	0	0.286117206	0	1	CRA004775;PRJNA827465	Yang2025_Silkie_Wenchang	0.931029023
ENSGALG00010027045	MAPK13	2	0.125920194	0.136511068	0.922417468	0.356310862	0.010571009	1.373608025	27.19902755	1	PRJNA827465;Yang2025_Silkie_Wenchang	CRA004775	0.812521698
ENSGALG00010027045	MAPK13	2	0.275975121	0.179451626	1.537880301	0.124077885	0	0.077999857	0	1	CRA004775;Yang2025_Silkie_Wenchang	PRJNA827465	0.590534404
ENSGALG00010027045	MAPK13	2	0.022451659	0.138181351	0.162479662	0.870928143	0	0.044600351	0	1	CRA004775;PRJNA827465	Yang2025_Silkie_Wenchang	0.969196692
ENSGALG00010027050	GSTT1	2	-0.196498713	0.09500379	-2.068324985	0.038609474	0	0.181318224	0	1	PRJNA827465;Yang2025_Silkie_Wenchang	CRA004775	0.338965227
ENSGALG00010027050	GSTT1	2	-0.460701301	0.318937027	-1.444489856	0.148601231	0.139813353	2.632157745	62.00835601	1	CRA004775;Yang2025_Silkie_Wenchang	PRJNA827465	0.638722024
ENSGALG00010027050	GSTT1	2	-0.435675217	0.366357886	-1.189206604	0.234358376	0.194996145	3.185898012	68.61167569	1	CRA004775;PRJNA827465	Yang2025_Silkie_Wenchang	0.747486154
ENSGALG00010027052	APPL1	2	0.070763982	0.066098402	1.070585369	0.284355905	0	0.055382142	0	1	PRJNA827465;Yang2025_Silkie_Wenchang	CRA004775	0.780265906
ENSGALG00010027052	APPL1	2	0.123354563	0.096290227	1.281070441	0.200168925	0	0.495878296	0	1	CRA004775;Yang2025_Silkie_Wenchang	PRJNA827465	0.712483179
ENSGALG00010027052	APPL1	2	0.084427208	0.078583351	1.07436508	0.282659087	0	0.750861039	0	1	CRA004775;PRJNA827465	Yang2025_Silkie_Wenchang	0.779532565
ENSGALG00010027059	ASB14	2	-0.203499632	0.082966032	-2.452806623	0.014174652	0	0.029322229	0	1	PRJNA827465;Yang2025_Silkie_Wenchang	CRA004775	0.195201938
ENSGALG00010027059	ASB14	2	-0.165785095	0.135557477	-1.222987464	0.221334431	0	0.236176928	0	0.5	CRA004775;Yang2025_Silkie_Wenchang	PRJNA827465	0.734689158
ENSGALG00010027059	ASB14	2	-0.202624345	0.100694401	-2.012270226	0.044191459	0	0.304584264	0	0.5	CRA004775;PRJNA827465	Yang2025_Silkie_Wenchang	0.363282309
ENSGALG00010027063	RAB7L1	2	-0.093515716	0.22282398	-0.419684254	0.67471613	0.091302707	12.23307533	91.82544068	0.5	PRJNA827465;Yang2025_Silkie_Wenchang	CRA004775	0.915970608
ENSGALG00010027063	RAB7L1	2	-0.306419839	0.09951614	-3.079096909	0.002076291	0	0.180813384	0	1	CRA004775;Yang2025_Silkie_Wenchang	PRJNA827465	0.059305579
ENSGALG00010027063	RAB7L1	2	0.047175079	0.137512874	0.343059363	0.731553802	0.019882147	1.580782715	36.74019899	0.5	CRA004775;PRJNA827465	Yang2025_Silkie_Wenchang	0.932851968
ENSGALG00010027074	BRPF3	2	0.06089361	0.034885504	1.745527585	0.080893075	0	0.011059763	0	1	PRJNA827465;Yang2025_Silkie_Wenchang	CRA004775	0.489847627
ENSGALG00010027074	BRPF3	2	0.034956959	0.059874448	0.583837683	0.559329523	0	0.739803218	0	0.5	CRA004775;Yang2025_Silkie_Wenchang	PRJNA827465	0.881050436
ENSGALG00010027074	BRPF3	2	0.053625196	0.040243267	1.332525912	0.182687433	0	0.890274488	0	0.5	CRA004775;PRJNA827465	Yang2025_Silkie_Wenchang	0.6911231
ENSGALG00010027075	SLC41A1	2	-0.032717694	0.034132603	-0.958546707	0.337787151	0	0.355712125	0	1	PRJNA827465;Yang2025_Silkie_Wenchang	CRA004775	0.80415385
ENSGALG00010027075	SLC41A1	2	-0.105794555	0.102021203	-1.036985968	0.299742397	0.007619459	1.198527861	16.56430923	1	CRA004775;Yang2025_Silkie_Wenchang	PRJNA827465	0.79004225
ENSGALG00010027075	SLC41A1	2	-0.093640527	0.14218722	-0.658572037	0.510170624	0.023990159	1.648324133	39.33232062	1	CRA004775;PRJNA827465	Yang2025_Silkie_Wenchang	0.865365179
ENSGALG00010027076	VDHAP	2	0.209380427	0.088861462	2.356256833	0.018460149	0	0.360277976	0	1	PRJNA827465;Yang2025_Silkie_Wenchang	CRA004775	0.227370496
ENSGALG00010027076	VDHAP	2	0.116932826	0.194098373	0.602441038	0.5468806	0	0.087866236	0	1	CRA004775;Yang2025_Silkie_Wenchang	PRJNA827465	0.878275987
ENSGALG00010027076	VDHAP	2	0.227315452	0.091527602	2.483572694	0.013007178	0	0.012242063	0	1	CRA004775;PRJNA827465	Yang2025_Silkie_Wenchang	0.185626401
ENSGALG00010027077	ARF4	2	-0.081485149	0.150929006	-0.539890586	0.58927249	0.041514645	11.14352928	91.02618233	0.5	PRJNA827465;Yang2025_Silkie_Wenchang	CRA004775	0.892217301
ENSGALG00010027077	ARF4	2	-0.244322292	0.069243849	-3.528433151	0.000418028	0	0.06124578	0	1	CRA004775;Yang2025_Silkie_Wenchang	PRJNA827465	0.020640371
ENSGALG00010027077	ARF4	2	-0.066099887	0.169838301	-0.389193053	0.697133342	0.043321328	3.352447715	70.17104859	0.5	CRA004775;PRJNA827465	Yang2025_Silkie_Wenchang	0.921116491
ENSGALG00010027078	PDE12	2	-0.094028922	0.17431181	-0.539429442	0.589590571	0.039936975	2.69241944	62.85868445	0.5	PRJNA827465;Yang2025_Silkie_Wenchang	CRA004775	0.892320726
ENSGALG00010027078	PDE12	2	-0.058831868	0.300842669	-0.195556926	0.844956977	0.116461664	2.648232735	62.2389684	0.5	CRA004775;Yang2025_Silkie_Wenchang	PRJNA827465	0.960779519
ENSGALG00010027078	PDE12	2	0.070018439	0.097009706	0.721767351	0.470437526	0	0.567648646	0	1	CRA004775;PRJNA827465	Yang2025_Silkie_Wenchang	0.853744788
ENSGALG00010027079	PM20D1	2	-0.084578126	0.111158238	-0.760880408	0.446728501	0.013644995	2.034097133	50.83813925	1	PRJNA827465;Yang2025_Silkie_Wenchang	CRA004775	0.84648396
ENSGALG00010027079	PM20D1	2	-0.311611734	0.117669454	-2.648195632	0.008092268	0	0.893921812	0	1	CRA004775;Yang2025_Silkie_Wenchang	PRJNA827465	0.139660521
ENSGALG00010027079	PM20D1	2	-0.196800893	0.230714206	-0.853007261	0.393655278	0.08579191	4.631831933	78.41027018	1	CRA004775;PRJNA827465	Yang2025_Silkie_Wenchang	0.83043598
ENSGALG00010027080	TBC1D22B	2	-0.018799048	0.046848033	-0.401277212	0.688216039	0	0.674983236	0	0.5	PRJNA827465;Yang2025_Silkie_Wenchang	CRA004775	0.919719653
ENSGALG00010027080	TBC1D22B	2	-0.097083141	0.207128301	-0.468710168	0.639276818	0.055714982	2.436987634	58.96573351	0.5	CRA004775;Yang2025_Silkie_Wenchang	PRJNA827465	0.905968982
ENSGALG00010027080	TBC1D22B	2	-0.115023888	0.141980205	-0.810140313	0.417859537	0.023129844	1.658813242	39.71593821	1	CRA004775;PRJNA827465	Yang2025_Silkie_Wenchang	0.838812595
ENSGALG00010027081	PDE4B	2	0.328577177	0.121682498	2.70028297	0.006928052	0	0.115629874	0	1	PRJNA827465;Yang2025_Silkie_Wenchang	CRA004775	0.127897246
ENSGALG00010027081	PDE4B	2	0.322223422	0.183957162	1.751622051	0.079838818	0	0.814668027	0	0.5	CRA004775;Yang2025_Silkie_Wenchang	PRJNA827465	0.486023928
ENSGALG00010027081	PDE4B	2	0.263014935	0.15000903	1.753327357	0.079545832	0	0.599535268	0	0.5	CRA004775;PRJNA827465	Yang2025_Silkie_Wenchang	0.485313543
ENSGALG00010027082	SLC26A9	2	-0.155152722	0.36119516	-0.429553713	0.667520314	0.051821632	1.247398181	19.83313627	0.5	PRJNA827465;Yang2025_Silkie_Wenchang	CRA004775	0.913973577
ENSGALG00010027082	SLC26A9	2	0.394715247	0.376775613	1.047613576	0.29481668	0	0.396039248	0	1	CRA004775;Yang2025_Silkie_Wenchang	PRJNA827465	0.787551963
ENSGALG00010027082	SLC26A9	2	0.023223592	0.603895942	0.038456281	0.969323888	0.442000847	2.467212651	59.46843092	0.5	CRA004775;PRJNA827465	Yang2025_Silkie_Wenchang	0.991572914
ENSGALG00010027083	DENND6A	2	-0.058784343	0.046893318	-1.253576125	0.209996114	0	0.218380015	0	1	PRJNA827465;Yang2025_Silkie_Wenchang	CRA004775	0.507285135
ENSGALG00010027083	DENND6A	2	0.012607303	0.160796303	0.07840543	0.937505555	0.029917913	1.868489508	46.48083408	0.5	CRA004775;Yang2025_Silkie_Wenchang	PRJNA827465	0.985139256
ENSGALG00010027083	DENND6A	2	0.019933858	0.124743512	0.159798759	0.873039601	0.015882769	1.477736853	32.32895304	0.5	CRA004775;PRJNA827465	Yang2025_Silkie_Wenchang	0.9697477
ENSGALG00010027084	OSBPL9	2	-0.088964411	0.032122917	-2.769499784	0.005614244	0	0.121307122	0	1	PRJNA827465;Yang2025_Silkie_Wenchang	CRA004775	0.112465157
ENSGALG00010027084	OSBPL9	2	-0.106440017	0.065715813	-1.61970174	0.105296362	0	0.031877832	0	1	CRA004775;Yang2025_Silkie_Wenchang	PRJNA827465	0.550766958
ENSGALG00010027084	OSBPL9	2	-0.08274687	0.035735379	-2.315544755	0.020583138	0	0.003287155	0	1	CRA004775;PRJNA827465	Yang2025_Silkie_Wenchang	0.242156628
ENSGALG00010027085	PIM1	2	0.015228036	0.243048479	0.062654315	0.950041777	0.091980818	4.283971301	76.65717322	0.5	PRJNA827465;Yang2025_Silkie_Wenchang	CRA004775	0.987277827
ENSGALG00010027085	PIM1	2	-0.532382815	0.313221804	-1.699699091	0.089181541	0.129369506	2.829389389	64.65668515	1	CRA004775;Yang2025_Silkie_Wenchang	PRJNA827465	0.653176171
ENSGALG00010027085	PIM1	2	-0.293390171	0.559943779	-0.523963622	0.600303842	0.574129853	11.28176978	91.13614247	0.5	CRA004775;PRJNA827465	Yang2025_Silkie_Wenchang	0.895230519
ENSGALG00010027086	CACNA1S	2	-0.238913114	0.097702033	-2.445323865	0.014472212	0	0.483742353	0	1	PRJNA827465;Yang2025_Silkie_Wenchang	CRA004775	0.197843946
ENSGALG00010027086	CACNA1S	2	-0.183592816	0.135614068	-1.353788871	0.175803745	0	0.204503338	0	1	CRA004775;Yang2025_Silkie_Wenchang	PRJNA827465	0.680750207
ENSGALG00010027086	CACNA1S	2	-0.313428863	0.134639785	-2.327906743	0.019917056	0	0.066957186	0	1	CRA004775;PRJNA827465	Yang2025_Silkie_Wenchang	0.237140278
ENSGALG00010027087	RAB7B	2	-0.088318509	0.143309808	-0.616276794	0.537711852	0	0.541033474	0	0.5	PRJNA827465;Yang2025_Silkie_Wenchang	CRA004775	0.87481821
ENSGALG00010027087	RAB7B	2	0.589704686	0.547264241	1.077550189	0.281234542	0.448855958	3.835457845	73.92749339	1	CRA004775;Yang2025_Silkie_Wenchang	PRJNA827465	0.778899386
ENSGALG00010027087	RAB7B	2	0.454306299	0.671395608	0.676659624	0.49862194	0.784963952	7.144773168	86.0037544	0.5	CRA004775;PRJNA827465	Yang2025_Silkie_Wenchang	0.843478809
ENSGALG00010027088	AGBL4	2	0.144339026	0.180090057	0.80148248	0.422852383	0	0.617940996	0	0.5	PRJNA827465;Yang2025_Silkie_Wenchang	CRA004775	0.839988514
ENSGALG00010027088	AGBL4	2	0.313139732	0.27317208	1.146309435	0.25166714	0	0.029048467	0	1	CRA004775;Yang2025_Silkie_Wenchang	PRJNA827465	0.761746139
ENSGALG00010027088	AGBL4	2	0.058650212	0.211528565	0.27726852	0.78157393	0	0.067443146	0	0.5	CRA004775;PRJNA827465	Yang2025_Silkie_Wenchang	0.945249331
ENSGALG00010027092	ENSGALG00010027092	2	-0.120584083	0.179853913	-0.67045571	0.502567331	0.055016031	6.55099145	84.73513501	0.5	PRJNA827465;Yang2025_Silkie_Wenchang	CRA004775	0.863040321
ENSGALG00010027092	ENSGALG00010027092	2	-0.1668529	0.22223471	-0.750795856	0.452775524	0.056527488	1.91781322	47.85727883	0.5	CRA004775;Yang2025_Silkie_Wenchang	PRJNA827465	0.848452198
ENSGALG00010027092	ENSGALG00010027092	2	0.05538219	0.075586928	0.732695339	0.463744267	0	0.136873142	0	1	CRA004775;PRJNA827465	Yang2025_Silkie_Wenchang	0.852159649
ENSGALG00010027093	SLMAP	2	0.116442624	0.321397528	0.362300931	0.717127162	0.196154672	19.49828707	94.87134436	0.5	PRJNA827465;Yang2025_Silkie_Wenchang	CRA004775	0.92775671
ENSGALG00010027093	SLMAP	2	0.373341199	0.122016709	3.059752452	0.0022152	0.003864826	1.113250409	10.72695016	1	CRA004775;Yang2025_Silkie_Wenchang	PRJNA827465	0.022717545
ENSGALG00010027093	SLMAP	2	-0.076298485	0.172184292	-0.443121053	0.657678178	0.039222614	2.387417612	58.11373783	0.5	CRA004775;PRJNA827465	Yang2025_Silkie_Wenchang	0.910774485
ENSGALG00010027095	SCN5A	2	0.253926598	0.155686743	1.631009766	0.102858259	0.025118701	2.074784568	51.80222489	1	PRJNA827465;Yang2025_Silkie_Wenchang	CRA004775	0.546797836
ENSGALG00010027095	SCN5A	2	0.395059348	0.153586925	2.572219924	0.010104866	0	0.511434458	0	1	CRA004775;Yang2025_Silkie_Wenchang	PRJNA827465	0.159642386
ENSGALG00010027095	SCN5A	2	0.091648287	0.148591037	0.616782068	0.537378481	0	0.16567162	0	0.5	CRA004775;PRJNA827465	Yang2025_Silkie_Wenchang	0.87481821
ENSGALG00010027096	YIPF1	2	0.078644344	0.054775796	1.435749916	0.151073516	0	0.692833131	0	0.5	PRJNA827465;Yang2025_Silkie_Wenchang	CRA004775	0.64242966
ENSGALG00010027096	YIPF1	2	-0.197770816	0.17824091	-1.109570278	0.267184245	0.031784421	1.964418404	49.09434783	1	CRA004775;Yang2025_Silkie_Wenchang	PRJNA827465	0.771292132
ENSGALG00010027096	YIPF1	2	-0.114686556	0.246018277	-0.466170879	0.641093192	0.101412855	5.48685519	81.7746238	0.5	CRA004775;PRJNA827465	Yang2025_Silkie_Wenchang	0.906429868
ENSGALG00010027097	ENSGALG00010027097	2	-0.051284761	0.062376941	-0.822174987	0.410977313	0.00221103	1.300887151	23.1293814	0.5	PRJNA827465;Yang2025_Silkie_Wenchang	CRA004775	0.835128673
ENSGALG00010027097	ENSGALG00010027097	2	0.017632825	0.09869855	0.178653331	0.858209916	0	0.521531324	0	0.5	CRA004775;Yang2025_Silkie_Wenchang	PRJNA827465	0.964291891
ENSGALG00010027097	ENSGALG00010027097	2	-0.093504637	0.05543189	-1.686838336	0.091634442	0	0.05472856	0	1	CRA004775;PRJNA827465	Yang2025_Silkie_Wenchang	0.518914432
ENSGALG00010027098	EXOG	2	-0.48180949	0.369337017	-1.304525321	0.192054532	0.24146427	8.650206256	88.43958201	1	PRJNA827465;Yang2025_Silkie_Wenchang	CRA004775	0.701884421
ENSGALG00010027098	EXOG	2	-0.436199599	0.449191083	-0.971078046	0.331509415	0.343540613	6.612172483	84.87637758	0.5	CRA004775;Yang2025_Silkie_Wenchang	PRJNA827465	0.801798188
ENSGALG00010027098	EXOG	2	-0.086536204	0.136576266	-0.633610851	0.526334818	0	0.240742746	0	0.5	CRA004775;PRJNA827465	Yang2025_Silkie_Wenchang	0.870546826
ENSGALG00010027099	GNA11	2	-0.100928011	0.05732684	-1.760571693	0.078310993	0	0.25899649	0	1	PRJNA827465;Yang2025_Silkie_Wenchang	CRA004775	0.481707034
ENSGALG00010027099	GNA11	2	0.026200283	0.192772456	0.135913002	0.891890056	0.05532196	3.87999785	74.22678984	0.5	CRA004775;Yang2025_Silkie_Wenchang	PRJNA827465	0.974015927
ENSGALG00010027099	GNA11	2	0.041812105	0.154650784	0.270364647	0.786879735	0.035974997	3.631732248	72.46493046	0.5	CRA004775;PRJNA827465	Yang2025_Silkie_Wenchang	0.946784559
ENSGALG00010027100	MIER2	2	0.078667089	0.058815513	1.337522779	0.181052052	0	0.950392391	0	0.5	PRJNA827465;Yang2025_Silkie_Wenchang	CRA004775	0.688594651
ENSGALG00010027100	MIER2	2	-0.2291509	0.188079633	-1.218371687	0.223082759	0.044001552	2.62293152	61.87471947	1	CRA004775;Yang2025_Silkie_Wenchang	PRJNA827465	0.726667929
ENSGALG00010027100	MIER2	2	-0.137621729	0.266040517	-0.517296128	0.604949457	0.12477777	7.871256267	87.29554767	0.5	CRA004775;PRJNA827465	Yang2025_Silkie_Wenchang	0.896474603
ENSGALG00010027101	RNF220	2	-0.032873956	0.042832976	-0.767491767	0.442789186	0.00046212	1.141357665	12.38504542	0.5	PRJNA827465;Yang2025_Silkie_Wenchang	CRA004775	0.845624071
ENSGALG00010027101	RNF220	2	0.011555152	0.058341486	0.198060642	0.842997618	0	0.054138521	0	0.5	CRA004775;Yang2025_Silkie_Wenchang	PRJNA827465	0.960416186
ENSGALG00010027101	RNF220	2	-0.067724888	0.050914931	-1.330157697	0.183469139	0	0.039327347	0	1	CRA004775;PRJNA827465	Yang2025_Silkie_Wenchang	0.691847354
ENSGALG00010027102	DUS3L	2	0.144434036	0.06550434	2.204935642	0.027457345	0	0.897732744	0	1	PRJNA827465;Yang2025_Silkie_Wenchang	CRA004775	0.282945624
ENSGALG00010027102	DUS3L	2	0.163630435	0.112555608	1.453774159	0.14600893	0.004981458	1.157346362	13.59544276	0.5	CRA004775;Yang2025_Silkie_Wenchang	PRJNA827465	0.634897819
ENSGALG00010027102	DUS3L	2	0.079146174	0.078121848	1.013111913	0.311006709	0	0.346078182	0	0.5	CRA004775;PRJNA827465	Yang2025_Silkie_Wenchang	0.795218958
ENSGALG00010027103	ACVR2B	2	-0.136197487	0.115068263	-1.183623384	0.23656218	0	0.16034923	0	1	PRJNA827465;Yang2025_Silkie_Wenchang	CRA004775	0.7487803
ENSGALG00010027103	ACVR2B	2	-0.040804934	0.173530668	-0.235145375	0.814095902	0	0.304567764	0	0.5	CRA004775;Yang2025_Silkie_Wenchang	PRJNA827465	0.953869515
ENSGALG00010027103	ACVR2B	2	-0.1404281	0.140166	-1.001869927	0.316406419	0	0.568283258	0	0.5	CRA004775;PRJNA827465	Yang2025_Silkie_Wenchang	0.796883862
ENSGALG00010027105	CHERP	2	-0.051692598	0.039894422	-1.295734972	0.195066808	0	0.014458353	0	1	PRJNA827465;Yang2025_Silkie_Wenchang	CRA004775	0.706113528
ENSGALG00010027105	CHERP	2	-0.068063782	0.074460897	-0.914087587	0.360670825	0	0.177461578	0	1	CRA004775;Yang2025_Silkie_Wenchang	PRJNA827465	0.815038264
ENSGALG00010027105	CHERP	2	-0.060291434	0.040728689	-1.48031856	0.138788252	0	0.171505831	0	1	CRA004775;PRJNA827465	Yang2025_Silkie_Wenchang	0.622497285
ENSGALG00010027106	CYP2J23	2	0.17429594	0.157889509	1.103909509	0.269632394	0.034308342	3.167154212	68.42591383	1	PRJNA827465;Yang2025_Silkie_Wenchang	CRA004775	0.77189992
ENSGALG00010027106	CYP2J23	2	0.319843186	0.13264034	2.411356793	0.015893292	0	0.304875249	0	1	CRA004775;Yang2025_Silkie_Wenchang	PRJNA827465	0.208471156
ENSGALG00010027106	CYP2J23	2	0.032668844	0.106249137	0.307473972	0.758482631	0	0.029873159	0	1	CRA004775;PRJNA827465	Yang2025_Silkie_Wenchang	0.938954369
ENSGALG00010027107	KIF21B	2	0.151507309	0.086473429	1.752067776	0.079762154	0	0.618394409	0	1	PRJNA827465;Yang2025_Silkie_Wenchang	CRA004775	0.485803806
ENSGALG00010027107	KIF21B	2	-0.055383645	0.434127609	-0.127574575	0.898485653	0.255299834	2.407918784	58.4703601	0.5	CRA004775;Yang2025_Silkie_Wenchang	PRJNA827465	0.97575249
ENSGALG00010027107	KIF21B	2	-0.09006006	0.33803729	-0.266420488	0.789915388	0.137918167	1.777516469	43.74173081	0.5	CRA004775;PRJNA827465	Yang2025_Silkie_Wenchang	0.947390191
ENSGALG00010027108	ENSGALG00010027108	2	0.000241479	0.103126605	0.002341574	0.998131696	0.003347665	1.184604543	15.58364304	0.5	PRJNA827465;Yang2025_Silkie_Wenchang	CRA004775	0.999326728
ENSGALG00010027108	ENSGALG00010027108	2	0.135281848	0.280834126	0.48171442	0.630008831	0.11502825	3.321752294	69.89540726	0.5	CRA004775;Yang2025_Silkie_Wenchang	PRJNA827465	0.902533841
ENSGALG00010027108	ENSGALG00010027108	2	0.211668928	0.161929075	1.30717061	0.191154775	0.0154604	1.299177421	23.02821897	0.5	CRA004775;PRJNA827465	Yang2025_Silkie_Wenchang	0.700443419
ENSGALG00010027109	C19orf44	2	-0.021479097	0.127070284	-0.169033205	0.865770524	0.016363405	1.969949082	49.23726662	0.5	PRJNA827465;Yang2025_Silkie_Wenchang	CRA004775	0.967098441
ENSGALG00010027109	C19orf44	2	0.133489738	0.132988089	1.003772134	0.315488459	0	0.004022319	0	1	CRA004775;Yang2025_Silkie_Wenchang	PRJNA827465	0.796883862
ENSGALG00010027109	C19orf44	2	-0.095463028	0.099669789	-0.957793024	0.338167128	0	0.820967403	0	0.5	CRA004775;PRJNA827465	Yang2025_Silkie_Wenchang	0.80415385
ENSGALG00010027110	IKBKE	2	0.310274757	0.210345581	1.475071431	0.14019334	0.027131724	1.411853891	29.17114113	1	PRJNA827465;Yang2025_Silkie_Wenchang	CRA004775	0.624011195
ENSGALG00010027110	IKBKE	2	-0.247956221	0.886373977	-0.279742217	0.779675286	1.373996533	7.655589561	86.93764873	0.5	CRA004775;Yang2025_Silkie_Wenchang	PRJNA827465	0.944885381
ENSGALG00010027110	IKBKE	2	-0.426751515	0.66515299	-0.641583998	0.521143325	0.720707224	4.875962052	79.49122677	0.5	CRA004775;PRJNA827465	Yang2025_Silkie_Wenchang	0.869518515
ENSGALG00010027112	XYLB	2	0.125361996	0.246555171	0.508454133	0.611134897	0.085447964	3.364035948	70.27380161	0.5	PRJNA827465;Yang2025_Silkie_Wenchang	CRA004775	0.897609796
ENSGALG00010027112	XYLB	2	-0.174960019	0.16419385	-1.065569871	0.286618125	0	0.403658703	0	1	CRA004775;Yang2025_Silkie_Wenchang	PRJNA827465	0.782018115
ENSGALG00010027112	XYLB	2	0.058379051	0.367142328	0.159009317	0.873661534	0.19732517	3.519403782	71.58609634	0.5	CRA004775;PRJNA827465	Yang2025_Silkie_Wenchang	0.9699681
ENSGALG00010027113	PIAS4	2	0.148052112	0.055312666	2.67664032	0.007436442	0	0.847554383	0	1	PRJNA827465;Yang2025_Silkie_Wenchang	CRA004775	0.132925083
ENSGALG00010027113	PIAS4	2	0.084283146	0.203189294	0.414801118	0.678287501	0.052754974	2.505873397	60.09375409	0.5	CRA004775;Yang2025_Silkie_Wenchang	PRJNA827465	0.917670684
ENSGALG00010027113	PIAS4	2	0.060558474	0.119313019	0.507559651	0.611762194	0.01390332	1.460557002	31.53297007	0.5	CRA004775;PRJNA827465	Yang2025_Silkie_Wenchang	0.897777844
ENSGALG00010027114	FLNB	2	-0.078735725	0.060328041	-1.305126517	0.191849771	0	0.17619156	0	1	PRJNA827465;Yang2025_Silkie_Wenchang	CRA004775	0.701581425
ENSGALG00010027114	FLNB	2	-0.115232096	0.116056855	-0.992893493	0.320761861	0	0.046798939	0	1	CRA004775;Yang2025_Silkie_Wenchang	PRJNA827465	0.798348216
ENSGALG00010027114	FLNB	2	-0.063329148	0.068443455	-0.925276899	0.354821892	0	0.005742462	0	1	CRA004775;PRJNA827465	Yang2025_Silkie_Wenchang	0.812016118
ENSGALG00010027116	CCDC94	2	0.149086272	0.234711962	0.635188216	0.525305668	0.101266789	12.10029299	91.7357373	0.5	PRJNA827465;Yang2025_Silkie_Wenchang	CRA004775	0.870188344
ENSGALG00010027116	CCDC94	2	-0.094834495	0.507420829	-0.186895157	0.851742837	0.4827639	15.68090628	93.62281757	0.5	CRA004775;Yang2025_Silkie_Wenchang	PRJNA827465	0.962215236
ENSGALG00010027116	CCDC94	2	-0.304878108	0.269330209	-1.131986304	0.257640193	0.120804038	5.304295583	81.1473553	1	CRA004775;PRJNA827465	Yang2025_Silkie_Wenchang	0.7650601
ENSGALG00010027117	RASSF5	2	-0.060758007	0.179037512	-0.339359093	0.734339231	0	0.110493348	0	1	PRJNA827465;Yang2025_Silkie_Wenchang	CRA004775	0.933501228
ENSGALG00010027117	RASSF5	2	-0.486134504	0.458412012	-1.060475055	0.288928533	0.224528554	1.953239606	48.80300416	1	CRA004775;Yang2025_Silkie_Wenchang	PRJNA827465	0.783530627
ENSGALG00010027117	RASSF5	2	-0.414452687	0.522618208	-0.793031473	0.427759486	0.381636044	2.826175841	64.61649748	1	CRA004775;PRJNA827465	Yang2025_Silkie_Wenchang	0.84125685
ENSGALG00010027118	ABHD6	2	-0.007858466	0.109599832	-0.071701439	0.942839511	0	0.668692176	0	0.5	PRJNA827465;Yang2025_Silkie_Wenchang	CRA004775	0.985827325
ENSGALG00010027118	ABHD6	2	0.192489183	0.123595073	1.557417934	0.119371291	0	0.2473703	0	1	CRA004775;Yang2025_Silkie_Wenchang	PRJNA827465	0.579860319
ENSGALG00010027118	ABHD6	2	0.073731495	0.157096651	0.469338425	0.638827754	0.027617156	2.249988727	55.55533289	0.5	CRA004775;PRJNA827465	Yang2025_Silkie_Wenchang	0.905768875
ENSGALG00010027119	NUB1	2	-0.080342022	0.080082576	-1.003239725	0.315745211	0.006487467	1.94651669	48.626179	0.5	PRJNA827465;Yang2025_Silkie_Wenchang	CRA004775	0.796883862
ENSGALG00010027119	NUB1	2	-0.002239493	0.093019692	-0.024075474	0.980792406	0	0.467850595	0	0.5	CRA004775;Yang2025_Silkie_Wenchang	PRJNA827465	0.994928779
ENSGALG00010027119	NUB1	2	-0.148519388	0.063719373	-2.330835665	0.019762027	0	0.037721822	0	1	CRA004775;PRJNA827465	Yang2025_Silkie_Wenchang	0.2360094
ENSGALG00010027120	ENSGALG00010027120	2	0.62639716	1.16884245	0.535912398	0.592019096	2.427830753	8.946724731	88.8227253	0.5	PRJNA827465;Yang2025_Silkie_Wenchang	CRA004775	0.892776493
ENSGALG00010027120	ENSGALG00010027120	2	0.994693626	0.907788746	1.095732492	0.273195859	1.176376427	3.303173298	69.72608126	0.5	CRA004775;Yang2025_Silkie_Wenchang	PRJNA827465	0.773904763
ENSGALG00010027120	ENSGALG00010027120	2	-0.405483381	0.493452265	-0.821727672	0.411231906	0	0.223482909	0	1	CRA004775;PRJNA827465	Yang2025_Silkie_Wenchang	0.835128673
ENSGALG00010027122	DNASE1L3	2	-0.00347221	0.144509514	-0.024027552	0.980830631	0	0.165430878	0	0.5	PRJNA827465;Yang2025_Silkie_Wenchang	CRA004775	0.994928779
ENSGALG00010027122	DNASE1L3	2	0.160890489	0.317478884	0.506775402	0.612312418	0.096500638	1.834037836	45.47549781	0.5	CRA004775;Yang2025_Silkie_Wenchang	PRJNA827465	0.897895209
ENSGALG00010027122	DNASE1L3	2	0.170522449	0.229118337	0.744254919	0.456722269	0.040617276	1.420331568	29.59390451	1	CRA004775;PRJNA827465	Yang2025_Silkie_Wenchang	0.849515887
ENSGALG00010027123	WDR86	2	-0.19713844	0.068780306	-2.86620476	0.004154255	0	0.830541184	0	1	PRJNA827465;Yang2025_Silkie_Wenchang	CRA004775	0.093728281
ENSGALG00010027123	WDR86	2	-0.537290776	0.271426775	-1.979505435	0.047759128	0.097297033	2.684602919	62.75054338	1	CRA004775;Yang2025_Silkie_Wenchang	PRJNA827465	0.377909608
ENSGALG00010027123	WDR86	2	-0.460976587	0.351007037	-1.313297279	0.189082795	0.204627326	5.164478117	80.6369593	1	CRA004775;PRJNA827465	Yang2025_Silkie_Wenchang	0.698162702
ENSGALG00010027124	IP6K2	2	-0.199025195	0.199810725	-0.996068628	0.319216801	0.070548272	8.136781188	87.71012791	1	PRJNA827465;Yang2025_Silkie_Wenchang	CRA004775	0.798079048
ENSGALG00010027124	IP6K2	2	-0.016187924	0.410818856	-0.039404044	0.968568256	0.312411169	13.32268388	92.49400489	0.5	CRA004775;Yang2025_Silkie_Wenchang	PRJNA827465	0.991215679
ENSGALG00010027124	IP6K2	2	0.15807259	0.207176207	0.762986215	0.445471617	0.070051617	4.787758635	79.1133999	0.5	CRA004775;PRJNA827465	Yang2025_Silkie_Wenchang	0.846013379
ENSGALG00010027126	ENSGALG00010027126	2	-0.41149473	0.339624469	-1.211616854	0.22565908	0.099523643	1.758702227	43.13989119	1	PRJNA827465;Yang2025_Silkie_Wenchang	CRA004775	0.73884362
ENSGALG00010027126	ENSGALG00010027126	2	-0.968105836	0.289457259	-3.344555388	0.000824146	0	0.942266116	0	1	CRA004775;Yang2025_Silkie_Wenchang	PRJNA827465	0.03312004
ENSGALG00010027126	ENSGALG00010027126	2	-0.66627079	0.629645899	-1.058167442	0.289979112	0.617450771	4.460063387	77.57879399	1	CRA004775;PRJNA827465	Yang2025_Silkie_Wenchang	0.784195688
ENSGALG00010027128	EIF2D	2	0.124796615	0.162344298	0.768715727	0.442062086	0.044025494	5.872224573	82.97067853	0.5	PRJNA827465;Yang2025_Silkie_Wenchang	CRA004775	0.845180045
ENSGALG00010027128	EIF2D	2	0.105074324	0.22944901	0.457941938	0.646994156	0.077652405	3.562337169	71.9285415	0.5	CRA004775;Yang2025_Silkie_Wenchang	PRJNA827465	0.907350807
ENSGALG00010027128	EIF2D	2	-0.037479373	0.065543865	-0.571821226	0.567443096	0	0.371106573	0	1	CRA004775;PRJNA827465	Yang2025_Silkie_Wenchang	0.883717772
ENSGALG00010027129	NCLN	2	0.034655502	0.067343795	0.514605725	0.606828564	0.000216819	1.023904539	2.334645262	0.5	PRJNA827465;Yang2025_Silkie_Wenchang	CRA004775	0.896799223
ENSGALG00010027129	NCLN	2	-0.072021527	0.0934568	-0.770639769	0.440920482	0	0.450909905	0	1	CRA004775;Yang2025_Silkie_Wenchang	PRJNA827465	0.844888742
ENSGALG00010027129	NCLN	2	0.013365089	0.13346634	0.100138275	0.920234549	0.016406613	1.548092918	35.4043941	0.5	CRA004775;PRJNA827465	Yang2025_Silkie_Wenchang	0.981364549
ENSGALG00010027130	NCKIPSD	2	0.028961174	0.109874133	0.263585009	0.792099711	0.011230791	1.850792505	45.96909178	0.5	PRJNA827465;Yang2025_Silkie_Wenchang	CRA004775	0.948314332
ENSGALG00010027130	NCKIPSD	2	0.015764671	0.16307465	0.096671498	0.922987275	0.029158925	2.128198234	53.01189598	0.5	CRA004775;Yang2025_Silkie_Wenchang	PRJNA827465	0.981836718
ENSGALG00010027130	NCKIPSD	2	-0.094758387	0.090297958	-1.04939678	0.29399554	0	0.26376314	0	1	CRA004775;PRJNA827465	Yang2025_Silkie_Wenchang	0.787324026
ENSGALG00010027131	UBA52	2	-0.051536005	0.058549957	-0.880205692	0.37874789	0	0.468758444	0	0.5	PRJNA827465;Yang2025_Silkie_Wenchang	CRA004775	0.823876439
ENSGALG00010027131	UBA52	2	0.070107845	0.177493557	0.394988115	0.692851659	0	0.047589233	0	1	CRA004775;Yang2025_Silkie_Wenchang	PRJNA827465	0.92062328
ENSGALG00010027131	UBA52	2	-0.057872298	0.059053832	-0.979992254	0.327089942	0	0.095985361	0	0.5	CRA004775;PRJNA827465	Yang2025_Silkie_Wenchang	0.800028239
ENSGALG00010027133	CELF5	2	0.172410586	0.337966404	0.510141197	0.609952545	0.188899819	5.563189509	82.02470007	0.5	PRJNA827465;Yang2025_Silkie_Wenchang	CRA004775	0.897258959
ENSGALG00010027133	CELF5	2	0.147623414	0.510450815	0.289202033	0.772426776	0.354184514	2.751119987	63.65116736	0.5	CRA004775;Yang2025_Silkie_Wenchang	PRJNA827465	0.943066211
ENSGALG00010027133	CELF5	2	-0.157961144	0.139825687	-1.129700462	0.258602462	0	0.389804645	0	1	CRA004775;PRJNA827465	Yang2025_Silkie_Wenchang	0.765798911
ENSGALG00010027134	DYRK3	2	-0.183021122	0.067888687	-2.695900147	0.007019871	0.001558481	1.203471673	16.90705963	1	PRJNA827465;Yang2025_Silkie_Wenchang	CRA004775	0.12854434
ENSGALG00010027134	DYRK3	2	-0.230945403	0.084530267	-2.732103075	0.006293145	0	0.740079231	0	0.5	CRA004775;Yang2025_Silkie_Wenchang	PRJNA827465	0.120477095
ENSGALG00010027134	DYRK3	2	-0.105055635	0.084112477	-1.248989908	0.211668765	0	0.198078457	0	0.5	CRA004775;PRJNA827465	Yang2025_Silkie_Wenchang	0.724013667
ENSGALG00010027135	PRKAG2	2	-0.060326475	0.150960844	-0.399616702	0.689438853	0.040553628	8.786765665	88.6192481	0.5	PRJNA827465;Yang2025_Silkie_Wenchang	CRA004775	0.919914966
ENSGALG00010027135	PRKAG2	2	-0.148604688	0.123782038	-1.200535156	0.229931568	0.012482618	1.449544848	31.01282781	0.5	CRA004775;Yang2025_Silkie_Wenchang	PRJNA827465	0.742776697
ENSGALG00010027135	PRKAG2	2	0.080490418	0.04695091	1.714352658	0.086463978	0	0.007108075	0	1	CRA004775;PRJNA827465	Yang2025_Silkie_Wenchang	0.506036629
ENSGALG00010027136	MAPKAPK2	2	-0.154404692	0.061044707	-2.529370681	0.011426727	0	0.253785697	0	1	PRJNA827465;Yang2025_Silkie_Wenchang	CRA004775	0.17044842
ENSGALG00010027136	MAPKAPK2	2	-0.098192805	0.08995698	-1.091552931	0.275029654	0	0.215773731	0	0.5	CRA004775;Yang2025_Silkie_Wenchang	PRJNA827465	0.77498498
ENSGALG00010027136	MAPKAPK2	2	-0.160452322	0.074007383	-2.168058322	0.030154249	0	0.543764086	0	0.5	CRA004775;PRJNA827465	Yang2025_Silkie_Wenchang	0.295973612
ENSGALG00010027137	FKBP8	2	-0.06788625	0.051507242	-1.317994285	0.187505561	0	0.60812184	0	0.5	PRJNA827465;Yang2025_Silkie_Wenchang	CRA004775	0.696631608
ENSGALG00010027137	FKBP8	2	-0.091100041	0.194221835	-0.469051489	0.639032833	0.032590002	1.619557375	38.25473458	0.5	CRA004775;Yang2025_Silkie_Wenchang	PRJNA827465	0.905893866
ENSGALG00010027137	FKBP8	2	-0.09220731	0.053669614	-1.718054272	0.085786717	0	0.963557905	0	1	CRA004775;PRJNA827465	Yang2025_Silkie_Wenchang	0.503725202
ENSGALG00010027139	HSD11B1L	2	-0.071365503	0.109097943	-0.6541416	0.513020582	0	0.000183391	0	1	PRJNA827465;Yang2025_Silkie_Wenchang	CRA004775	0.866156104
ENSGALG00010027139	HSD11B1L	2	-0.095745448	0.13969839	-0.685372594	0.493108833	0	0.055698789	0	1	CRA004775;Yang2025_Silkie_Wenchang	PRJNA827465	0.859667262
ENSGALG00010027139	HSD11B1L	2	-0.088548471	0.123480293	-0.717106099	0.473308633	0	0.066759898	0	1	CRA004775;PRJNA827465	Yang2025_Silkie_Wenchang	0.854343014
ENSGALG00010027143	GALNT11	2	-0.022629686	0.164530053	-0.137541356	0.890602906	0.044306296	5.394375128	81.46217168	0.5	PRJNA827465;Yang2025_Silkie_Wenchang	CRA004775	0.973477891
ENSGALG00010027143	GALNT11	2	-0.196725795	0.110503763	-1.780263314	0.075032878	0	0.002477606	0	1	CRA004775;Yang2025_Silkie_Wenchang	PRJNA827465	0.47268425
ENSGALG00010027143	GALNT11	2	0.113719687	0.077775163	1.462159415	0.14369753	0	0.849083041	0	0.5	CRA004775;PRJNA827465	Yang2025_Silkie_Wenchang	0.630564924
ENSGALG00010027145	CELSR3	2	0.167259384	0.118530217	1.411111771	0.15821166	0.010415063	1.484933802	32.65693067	1	PRJNA827465;Yang2025_Silkie_Wenchang	CRA004775	0.65200722
ENSGALG00010027145	CELSR3	2	0.070216942	0.299134726	0.234733501	0.814415584	0.128225909	3.41579091	70.72420337	0.5	CRA004775;Yang2025_Silkie_Wenchang	PRJNA827465	0.953869515
ENSGALG00010027145	CELSR3	2	0.00011468	0.149623951	0.000766458	0.999388455	0.019035796	1.452383016	31.1476388	0.5	CRA004775;PRJNA827465	Yang2025_Silkie_Wenchang	0.999889665

ENSGALG00010027146	KXD1	2	-0.168843891	0.138961764	-1.21503848	0.224351415	0.023296282	2.236395945	55.28519884	1	PRJNA827465;Yang2025_Silkie_Wenchang	CRA004775	0.737471949
ENSGALG00010027146	KXD1	2	-0.336585364	0.151156559	-2.226733434	0.025965095	0	0.045831948	0	1	CRA004775;Yang2025_Silkie_Wenchang	PRJNA827465	0.274758802
ENSGALG00010027146	KXD1	2	-0.081104308	0.078685611	-1.030738734	0.302663355	0	0.503268438	0	1	CRA004775;PRJNA827465	Yang2025_Silkie_Wenchang	0.791852471
ENSGALG00010027147	KMT2C	2	0.131507858	0.226345096	0.581005998	0.561236411	0.078326589	3.515855893	71.55742356	0.5	PRJNA827465;Yang2025_Silkie_Wenchang	CRA004775	0.881506202
ENSGALG00010027147	KMT2C	2	0.4445207	0.196074392	2.267102269	0.023383979	0	0.019502569	0	1	CRA004775;Yang2025_Silkie_Wenchang	PRJNA827465	0.260874405
ENSGALG00010027147	KMT2C	2	0.117056611	0.241955971	0.483793025	0.628532766	0.080793798	2.411224059	58.52728841	0.5	CRA004775;PRJNA827465	Yang2025_Silkie_Wenchang	0.901942802
ENSGALG00010027148	ELL	2	0.031707133	0.224269225	0.141379775	0.887569931	0.085135021	6.176707385	83.81014451	0.5	PRJNA827465;Yang2025_Silkie_Wenchang	CRA004775	0.973038314
ENSGALG00010027148	ELL	2	0.329703258	0.149916563	2.199245039	0.027860503	0	0.637495459	0	1	CRA004775;Yang2025_Silkie_Wenchang	PRJNA827465	0.284557362
ENSGALG00010027148	ELL	2	0.130353624	0.382022181	0.34122004	0.732937938	0.230589987	3.963324278	74.76865556	0.5	CRA004775;PRJNA827465	Yang2025_Silkie_Wenchang	0.932985777
ENSGALG00010027149	C1orf116	2	0.304201145	0.156072958	1.949095785	0.051283987	0	0.019467687	0	1	PRJNA827465;Yang2025_Silkie_Wenchang	CRA004775	0.392371918
ENSGALG00010027149	C1orf116	2	0.204226297	0.214296342	0.953008789	0.340585618	0.01380585	1.123673709	11.00619405	0.5	CRA004775;Yang2025_Silkie_Wenchang	PRJNA827465	0.805524125
ENSGALG00010027149	C1orf116	2	0.179145157	0.198760505	0.90131164	0.367422647	0	0.919396794	0	0.5	CRA004775;PRJNA827465	Yang2025_Silkie_Wenchang	0.819941647
ENSGALG00010027150	YOD1	2	-0.105394525	0.142623671	-0.738969371	0.459925596	0.034476261	6.536597355	84.7015206	0.5	PRJNA827465;Yang2025_Silkie_Wenchang	CRA004775	0.850104868
ENSGALG00010027150	YOD1	2	-0.26035363	0.079064653	-3.292920664	0.000991524	0	0.094230703	0	1	CRA004775;Yang2025_Silkie_Wenchang	PRJNA827465	0.037652065
ENSGALG00010027150	YOD1	2	-0.082483551	0.168114764	-0.490638352	0.623682256	0.036530994	2.30852749	56.68234386	0.5	CRA004775;PRJNA827465	Yang2025_Silkie_Wenchang	0.900519204
ENSGALG00010027151	MICOS13	2	-0.117583732	0.096554605	-1.217795175	0.223301818	0	0.829461468	0	0.5	PRJNA827465;Yang2025_Silkie_Wenchang	CRA004775	0.736744216
ENSGALG00010027151	MICOS13	2	0.132586404	0.249294791	0.531845867	0.594832753	0	0.012871747	0	1	CRA004775;Yang2025_Silkie_Wenchang	PRJNA827465	0.893382718
ENSGALG00010027151	MICOS13	2	-0.132147564	0.098522728	-1.341290147	0.179826268	0	0.325829351	0	0.5	CRA004775;PRJNA827465	Yang2025_Silkie_Wenchang	0.68659821
ENSGALG00010027152	C4BPS	2	0.602068147	0.186549214	3.227395789	0.001249225	0	0.086854073	0	1	PRJNA827465;Yang2025_Silkie_Wenchang	CRA004775	0.042993261
ENSGALG00010027152	C4BPS	2	-0.073775783	0.847783601	-0.087021951	0.930654064	1.182837257	5.289859464	81.0959061	0.5	CRA004775;Yang2025_Silkie_Wenchang	PRJNA827465	0.983927281
ENSGALG00010027152	C4BPS	2	-0.109307668	0.786367255	-0.139003332	0.889447516	1.015751333	5.027511243	80.10944279	0.5	CRA004775;PRJNA827465	Yang2025_Silkie_Wenchang	0.973135755
ENSGALG00010027153	ENSGALG00010027153	2	-0.251024203	0.252205982	-0.995314234	0.319583457	0.111434684	7.981722638	87.47137623	0.5	PRJNA827465;Yang2025_Silkie_Wenchang	CRA004775	0.798328994
ENSGALG00010027153	ENSGALG00010027153	2	-0.14447686	0.409288789	-0.352994911	0.724092255	0.278457267	5.282759076	81.07049772	0.5	CRA004775;Yang2025_Silkie_Wenchang	PRJNA827465	0.930355448
ENSGALG00010027153	ENSGALG00010027153	2	0.059003046	0.132908238	0.443938217	0.657087253	0	0.752182328	0	1	CRA004775;PRJNA827465	Yang2025_Silkie_Wenchang	0.910695405
ENSGALG00010027154	PLPP2	2	0.162310886	0.23342518	0.695344376	0.486839511	0.09560054	7.845417546	87.25370582	0.5	PRJNA827465;Yang2025_Silkie_Wenchang	CRA004775	0.857631005
ENSGALG00010027154	PLPP2	2	0.257895349	0.201100338	1.282421261	0.19969491	0.042658663	1.977422406	49.42911556	0.5	CRA004775;Yang2025_Silkie_Wenchang	PRJNA827465	0.71156927
ENSGALG00010027154	PLPP2	2	-0.050173661	0.073644569	-0.681294791	0.495684978	0	0.038951422	0	1	CRA004775;PRJNA827465	Yang2025_Silkie_Wenchang	0.860911322
ENSGALG00010027155	ENSGALG00010027155	2	-0.03108903	0.33294697	-0.09337532	0.925605397	0.184516619	5.936872038	83.15611329	0.5	PRJNA827465;Yang2025_Silkie_Wenchang	CRA004775	0.982594411
ENSGALG00010027155	ENSGALG00010027155	2	-0.126320941	0.356841215	-0.353997621	0.723340666	0.144797844	1.994833051	49.87049169	0.5	CRA004775;Yang2025_Silkie_Wenchang	PRJNA827465	0.930032096
ENSGALG00010027155	ENSGALG00010027155	2	0.303063173	0.165920245	1.826559336	0.067766052	0	0.033020275	0	1	CRA004775;PRJNA827465	Yang2025_Silkie_Wenchang	0.449065781
ENSGALG00010027156	ISYNA1	2	-0.019393983	0.064205145	-0.302062762	0.762604219	0	0.292471464	0	0.5	PRJNA827465;Yang2025_Silkie_Wenchang	CRA004775	0.940082621
ENSGALG00010027156	ISYNA1	2	0.092152355	0.126840467	0.726521722	0.467518996	0.004206252	1.075468696	7.017284319	1	CRA004775;Yang2025_Silkie_Wenchang	PRJNA827465	0.852885838
ENSGALG00010027156	ISYNA1	2	0.063995204	0.182891285	0.349908437	0.726407415	0.038744042	1.758028393	43.11809731	0.5	CRA004775;PRJNA827465	Yang2025_Silkie_Wenchang	0.93109166
ENSGALG00010027157	ENSGALG00010027157	2	-0.236775657	0.471825303	-0.50182908	0.615787756	0.402644732	10.3448216	90.33332774	0.5	PRJNA827465;Yang2025_Silkie_Wenchang	CRA004775	0.898956845
ENSGALG00010027157	ENSGALG00010027157	2	-0.286174763	0.439099862	-0.651730479	0.514575057	0.327080538	6.587508928	84.81975492	0.5	CRA004775;Yang2025_Silkie_Wenchang	PRJNA827465	0.866869697
ENSGALG00010027157	ENSGALG00010027157	2	0.198271694	0.138269244	1.433953698	0.151585476	0	0.049799898	0	1	CRA004775;PRJNA827465	Yang2025_Silkie_Wenchang	0.643051067
ENSGALG00010027158	XRCC2	2	0.015772433	0.143763293	0.109711126	0.912638476	0.029169431	3.290001013	69.60487258	0.5	PRJNA827465;Yang2025_Silkie_Wenchang	CRA004775	0.978995879
ENSGALG00010027158	XRCC2	2	-0.178403032	0.117061983	-1.524004873	0.127507494	0	0.269244536	0	1	CRA004775;Yang2025_Silkie_Wenchang	PRJNA827465	0.599452995
ENSGALG00010027158	XRCC2	2	-0.012497918	0.208841133	-0.059844141	0.952279769	0.060144156	2.734288129	63.42740952	0.5	CRA004775;PRJNA827465	Yang2025_Silkie_Wenchang	0.987758913
ENSGALG00010027159	TMUB1	2	0.371804523	0.446898743	0.831965918	0.405428182	0.382884917	23.60833253	95.76420741	0.5	PRJNA827465;Yang2025_Silkie_Wenchang	CRA004775	0.833112102
ENSGALG00010027159	TMUB1	2	0.349350683	0.52975728	0.659454237	0.509604124	0.48501406	6.988684726	85.69115593	0.5	CRA004775;Yang2025_Silkie_Wenchang	PRJNA827465	0.865257661
ENSGALG00010027159	TMUB1	2	-0.067092889	0.066344993	-1.011272829	0.311885867	0	0.209957652	0	1	CRA004775;PRJNA827465	Yang2025_Silkie_Wenchang	0.795993742
ENSGALG00010027160	FASTK	2	-0.043025069	0.053358067	-0.80634611	0.42004331	0	0.218717454	0	0.5	PRJNA827465;Yang2025_Silkie_Wenchang	CRA004775	0.838603917
ENSGALG00010027160	FASTK	2	3.81483E-05	0.10254302	0.000372023	0.999703169	0	1.15871E-05	0	0.5	CRA004775;Yang2025_Silkie_Wenchang	PRJNA827465	0.999889665
ENSGALG00010027160	FASTK	2	-0.055972201	0.060817761	-0.920326573	0.357402127	0	0.021918906	0	1	CRA004775;PRJNA827465	Yang2025_Silkie_Wenchang	0.813374394
ENSGALG00010027161	C4BPG	2	0.070161737	0.168451641	0.416509669	0.677037094	0.042547231	3.995086835	74.96925496	0.5	PRJNA827465;Yang2025_Silkie_Wenchang	CRA004775	0.917109899
ENSGALG00010027161	C4BPG	2	-0.219274173	0.194588089	-1.126863282	0.25980029	0.037887736	1.667473443	40.0290299	1	CRA004775;Yang2025_Silkie_Wenchang	PRJNA827465	0.767047318
ENSGALG00010027161	C4BPG	2	-0.092961309	0.382040229	-0.243328587	0.80775086	0.241462117	5.27176092	81.03100624	0.5	CRA004775;PRJNA827465	Yang2025_Silkie_Wenchang	0.952496413
ENSGALG00010027162	SLC4A2	2	0.120172765	0.074140048	1.620888683	0.105041514	0	0.061333426	0	1	PRJNA827465;Yang2025_Silkie_Wenchang	CRA004775	0.550236662
ENSGALG00010027162	SLC4A2	2	-0.013484092	0.1919322	-0.070254456	0.943991132	0.046338523	2.542233978	60.66451756	0.5	CRA004775;Yang2025_Silkie_Wenchang	PRJNA827465	0.985827325
ENSGALG00010027162	SLC4A2	2	-0.018496476	0.168642854	-0.109678386	0.912664442	0.035638018	2.347364065	57.39902409	0.5	CRA004775;PRJNA827465	Yang2025_Silkie_Wenchang	0.978997014
ENSGALG00010027163	ENSGALG00010027163	2	0.509543172	0.554808266	0.918413087	0.358402642	0.594424141	28.21102007	96.45528592	0.5	PRJNA827465;Yang2025_Silkie_Wenchang	CRA004775	0.814005058
ENSGALG00010027163	ENSGALG00010027163	2	0.596867322	0.479322078	1.245232276	0.213046382	0.423904406	12.88209116	92.23728517	1	CRA004775;Yang2025_Silkie_Wenchang	PRJNA827465	0.72579791
ENSGALG00010027163	ENSGALG00010027163	2	-0.018401505	0.042382783	-0.434174069	0.664162042	0	0.72864787	0	0.5	CRA004775;PRJNA827465	Yang2025_Silkie_Wenchang	0.91312035
ENSGALG00010027164	ENSGALG00010027164	2	-0.237894344	0.306885795	-0.775188516	0.438228273	0	0.861486246	0	0.5	PRJNA827465;Yang2025_Silkie_Wenchang	CRA004775	0.843979791
ENSGALG00010027164	ENSGALG00010027164	2	1.008159629	1.60451967	0.62832488	0.529791136	4.814624375	15.27270745	93.45237245	0.5	CRA004775;Yang2025_Silkie_Wenchang	PRJNA827465	0.871944741
ENSGALG00010027164	ENSGALG00010027164	2	1.278095233	1.317015166	0.970448379	0.331823044	3.160180951	11.03098612	90.93462734	1	CRA004775;PRJNA827465	Yang2025_Silkie_Wenchang	0.802002658
ENSGALG00010027165	ENSGALG00010027165	2	0.575302808	0.333559844	1.724736413	0.084574986	0.205554291	13.01460966	92.31632737	1	PRJNA827465;Yang2025_Silkie_Wenchang	CRA004775	0.500108811
ENSGALG00010027165	ENSGALG00010027165	2	0.328333717	0.613345768	0.53531586	0.592431461	0.700400716	14.15935589	92.93753185	0.5	CRA004775;Yang2025_Silkie_Wenchang	PRJNA827465	0.892776493
ENSGALG00010027165	ENSGALG00010027165	2	0.034227795	0.272673727	0.125526562	0.900106698	0.109341094	3.293789482	69.63983262	0.5	CRA004775;PRJNA827465	Yang2025_Silkie_Wenchang	0.976466979
ENSGALG00010027166	CDK5	2	0.033629295	0.187031874	0.179805154	0.857305538	0.057166597	5.18336399	80.7075096	0.5	PRJNA827465;Yang2025_Silkie_Wenchang	CRA004775	0.963936745
ENSGALG00010027166	CDK5	2	-0.023096697	0.286050427	-0.080743445	0.935645985	0.130945215	4.898255675	79.58456915	0.5	CRA004775;Yang2025_Silkie_Wenchang	PRJNA827465	0.984800337
ENSGALG00010027166	CDK5	2	-0.155988257	0.071821841	-2.171877725	0.029864887	0	0.7558772	0	1	CRA004775;PRJNA827465	Yang2025_Silkie_Wenchang	0.295113583
ENSGALG00010027167	ENSGALG00010027167	2	0.356712295	0.518009033	0.688621765	0.491061321	0.395561497	3.709711965	73.04372929	0.5	PRJNA827465;Yang2025_Silkie_Wenchang	CRA004775	0.85895707
ENSGALG00010027167	ENSGALG00010027167	2	-0.027153134	0.998930837	-0.027182196	0.978314416	1.709836542	6.89194387	85.49030551	0.5	CRA004775;Yang2025_Silkie_Wenchang	PRJNA827465	0.993994558
ENSGALG00010027167	ENSGALG00010027167	2	-0.443593132	0.455483804	-0.973894414	0.330108967	0.22131031	1.926668664	48.09693962	1	CRA004775;PRJNA827465	Yang2025_Silkie_Wenchang	0.801237299
ENSGALG00010027168	SSBP4	2	-0.017182132	0.079473192	-0.216200347	0.828831583	0	0.688457745	0	0.5	PRJNA827465;Yang2025_Silkie_Wenchang	CRA004775	0.956840134
ENSGALG00010027168	SSBP4	2	0.039388911	0.146112932	0.269578538	0.787484513	0	0.476110019	0	0.5	CRA004775;Yang2025_Silkie_Wenchang	PRJNA827465	0.946919116
ENSGALG00010027168	SSBP4	2	-0.042575442	0.077592887	-0.548702903	0.583209353	0	0.000359852	0	1	CRA004775;PRJNA827465	Yang2025_Silkie_Wenchang	0.890342871
ENSGALG00010027169	ABCB8	2	-0.086914205	0.079583419	-1.092114494	0.27478278	0	0.623655724	0	0.5	PRJNA827465;Yang2025_Silkie_Wenchang	CRA004775	0.774719194
ENSGALG00010027169	ABCB8	2	-0.023917604	0.190971639	-0.125241653	0.900332243	0	0.811508294	0	0.5	CRA004775;Yang2025_Silkie_Wenchang	PRJNA827465	0.976475201
ENSGALG00010027169	ABCB8	2	-0.121238523	0.083271331	-1.455945544	0.145407674	0	0.289085406	0	1	CRA004775;PRJNA827465	Yang2025_Silkie_Wenchang	0.633907279
ENSGALG00010027170	TLE2	2	0.45430863	0.299059746	1.519123306	0.128731461	0.161730839	10.39019719	90.37554359	1	PRJNA827465;Yang2025_Silkie_Wenchang	CRA004775	0.602157487
ENSGALG00010027170	TLE2	2	0.189442456	0.602645159	0.314351577	0.753254029	0.667452666	11.92111163	91.61152054	0.5	CRA004775;Yang2025_Silkie_Wenchang	PRJNA827465	0.938087082
ENSGALG00010027170	TLE2	2	-0.070808049	0.295774013	-0.239399156	0.810796084	0.128043654	3.234052503	69.0790425	0.5	CRA004775;PRJNA827465	Yang2025_Silkie_Wenchang	0.953037608
ENSGALG00010027171	ATG9B	2	-0.170293401	0.205475958	-0.828775311	0.407231561	0.055183008	2.885345431	65.34210465	0.5	PRJNA827465;Yang2025_Silkie_Wenchang	CRA004775	0.833863243
ENSGALG00010027171	ATG9B	2	0.001419042	0.162736718	0.008719865	0.993042642	0	0.548819834	0	0.5	CRA004775;Yang2025_Silkie_Wenchang	PRJNA827465	0.99794341
ENSGALG00010027171	ATG9B	2	-0.383867423	0.167989329	-2.285070288	0.022308707	0	0.015698669	0	1	CRA004775;PRJNA827465	Yang2025_Silkie_Wenchang	0.253336926
ENSGALG00010027172	ENSGALG00010027172	2	-0.275767513	0.48941576	-0.563462674	0.57311987	0.353968862	3.829137427	73.88445782	0.5	PRJNA827465;Yang2025_Silkie_Wenchang	CRA004775	0.885753405
ENSGALG00010027172	ENSGALG00010027172	2	-0.219562407	0.566738063	-0.38741426	0.698449548	0.384201427	2.243743419	55.43162416	0.5	CRA004775;Yang2025_Silkie_Wenchang	PRJNA827465	0.921666253
ENSGALG00010027172	ENSGALG00010027172	2	-0.809893582	0.319817476	-2.532361873	0.0113297	0	0.063029983	0	1	CRA004775;PRJNA827465	Yang2025_Silkie_Wenchang	0.169699387
ENSGALG00010027173	ENSGALG00010027173	2	0.064597872	0.189383197	0.34109611	0.733031229	0.061905346	7.007148047	85.72885869	0.5	PRJNA827465;Yang2025_Silkie_Wenchang	CRA004775	0.932985777
ENSGALG00010027173	ENSGALG00010027173	2	0.280506839	0.113193043	2.478127903	0.013207379	0	0.032234636	0	1	CRA004775;Yang2025_Silkie_Wenchang	PRJNA827465	0.187667975
ENSGALG00010027173	ENSGALG00010027173	2	0.038731508	0.204540415	0.189358704	0.849811688	0.059515559	2.80543868	64.35495072	0.5	CRA004775;PRJNA827465	Yang2025_Silkie_Wenchang	0.961856718
ENSGALG00010027174	SHISA5	2	0.012553211	0.099887607	0.125673361	0.89999049	0.01530748	4.248736578	76.46359143	0.5	PRJNA827465;Yang2025_Silkie_Wenchang	CRA004775	0.976407823
ENSGALG00010027174	SHISA5	2	-0.150346637	0.129211036	-1.163574266	0.244596585	0.01411968	1.301502322	23.16571523	1	CRA004775;Yang2025_Silkie_Wenchang	PRJNA827465	0.756093682
ENSGALG00010027174	SHISA5	2	-0.090999791	0.263695813	-0.345093804	0.730023851	0.1051355	3.300705975	69.70345109	0.5	CRA004775;PRJNA827465	Yang2025_Silkie_Wenchang	0.932582945
ENSGALG00010027175	NOS3	2	0.319174065	0.27173384	1.174583426	0.240161417	0.135514539	12.09191485	91.73001123	1	PRJNA827465;Yang2025_Silkie_Wenchang	CRA004775	0.75252297
ENSGALG00010027175	NOS3	2	0.523428456	0.165555858	3.16164261	0.00156882	0.017667373	1.21168354	17.47020021	1	CRA004775;Yang2025_Silkie_Wenchang	PRJNA827465	0.049926578
ENSGALG00010027175	NOS3	2	0.058031459	0.094127623	0.616518903	0.5375521	0	0.054586828	0	1	CRA004775;PRJNA827465	Yang2025_Silkie_Wenchang	0.87481821
ENSGALG00010027176	ENSGALG00010027176	2	0.47826873	0.458202152	1.043794159	0.296580635	0.404298615	26.22727241	96.187175	1	PRJNA827465;Yang2025_Silkie_Wenchang	CRA004775	0.788400993
ENSGALG00010027176	ENSGALG00010027176	2	0.622185021	0.346354898	1.796380025	0.072434099	0.1988676	5.771136415	82.67239018	1	CRA004775;Yang2025_Silkie_Wenchang	PRJNA827465	0.464872204
ENSGALG00010027176	ENSGALG00010027176	2	0.044844264	0.050150393	0.894195665	0.371217211	0	0.880844654	0	1	CRA004775;PRJNA827465	Yang2025_Silkie_Wenchang	0.8214684
ENSGALG00010027178	C4BPM	2	0.116863223	0.117233267	0.996843527	0.318840467	0.020820728	4.020227904	75.12578829	0.5	PRJNA827465;Yang2025_Silkie_Wenchang	CRA004775	0.797974202
ENSGALG00010027178	C4BPM	2	-0.118811973	0.198806151	-0.597627249	0.550088678	0.041633989	1.582396408	36.80470993	1	CRA004775;Yang2025_Silkie_Wenchang	PRJNA827465	0.878652984
ENSGALG00010027178	C4BPM	2	-0.040781943	0.343741613	-0.118641273	0.905559565	0.183841805	3.665224331	72.71654039	0.5	CRA004775;PRJNA827465	Yang2025_Silkie_Wenchang	0.978182365
ENSGALG00010027183	CD34	2	0.327022368	0.336314225	0.972371503	0.330865763	0.197853286	7.408760517	86.50246559	1	PRJNA827465;Yang2025_Silkie_Wenchang	CRA004775	0.801677893
ENSGALG00010027183	CD34	2	0.463427417	0.347815898	1.332392851	0.18273113	0.122403863	1.780842808	43.84681256	0.5	CRA004775;Yang2025_Silkie_Wenchang	PRJNA827465	0.691163661
ENSGALG00010027183	CD34	2	0.022437565	0.082018933	0.273565674	0.784418416	0	0.018905938	0	0.5	CRA004775;PRJNA827465	Yang2025_Silkie_Wenchang	0.946111735
ENSGALG00010027184	MPND	2	0.68852498	0.507331095	1.357151152	0.174733188	0.47618097	13.30600154	92.48459429	1	PRJNA827465;Yang2025_Silkie_Wenchang	CRA004775	0.679348946
ENSGALG00010027184	MPND	2	1.117655291	0.192251473	5.813507037	6.11774E-09	0	0.801262005	0	1	CRA004775;Yang2025_Silkie_Wenchang	PRJNA827465	3.12267E-06
ENSGALG00010027184	MPND	2	0.346807507	0.259333743	1.3373019	0.181124111	0.061040098	1.560417931	35.91460466	1	CRA004775;PRJNA827465	Yang2025_Silkie_Wenchang	0.688594651
ENSGALG00010027185	ATRIP	2	0.172709295	0.066296725	2.605095433	0.009184867	0.004667511	2.131883853	53.09312942	1	PRJNA827465;Yang2025_Silkie_Wenchang	CRA004775	0.150281327
ENSGALG00010027185	ATRIP	2	0.070357528	0.228912645	0.30735536	0.758572902	0.076602757	2.988447389	66.53780811	0.5	CRA004775;Yang2025_Silkie_Wenchang	PRJNA827465	0.938954369
ENSGALG00010027185	ATRIP	2	0.031274051	0.142588636	0.219330601	0.826392526	0.021690453	1.561923233	35.97636691	0.5	CRA004775;PRJNA827465	Yang2025_Silkie_Wenchang	0.956393839
ENSGALG00010027186	PLXNA2	2	-0.085757761	0.088281153	-0.971416411	0.33134096	0	0.513472678	0	1	PRJNA827465;Yang2025_Silkie_Wenchang	CRA004775	0.801798188
ENSGALG00010027186	PLXNA2	2	-0.135478488	0.133444424	-1.015242778	0.309990114	0	0.416920752	0	0.5	CRA004775;Yang2025_Silkie_Wenchang	PRJNA827465	0.794395438
ENSGALG00010027186	PLXNA2	2	-0.024152129	0.109485126	-0.22059735	0.825405965	0	0.130015367	0	0.5	CRA004775;PRJNA827465	Yang2025_Silkie_Wenchang	0.956381808
ENSGALG00010027187	ENSGALG00010027187	2	-0.000587637	0.211334894	-0.002780597	0.997781408	0.063285174	3.06164506	67.33782067	0.5	PRJNA827465;Yang2025_Silkie_Wenchang	CRA004775	0.999290321
ENSGALG00010027187	ENSGALG00010027187	2	-0.216158471	0.168567995	-1.282322133	0.199729667	0	0.105420586	0	1	CRA004775;Yang2025_Silkie_Wenchang	PRJNA827465	0.711587863
ENSGALG00010027187	ENSGALG00010027187	2	0.088762379	0.139087629	0.638175944	0.523359155	0.015836925	1.433934432	30.26180434	0.5	CRA004775;PRJNA827465	Yang2025_Silkie_Wenchang	0.869661505
ENSGALG00010027188	KCNH2	2	0.179565766	0.322044097	0.557581297	0.577130331	0.180734376	7.744510199	87.08762757	0.5	PRJNA827465;Yang2025_Silkie_Wenchang	CRA004775	0.887145528
ENSGALG00010027188	KCNH2	2	-0.150604533	0.726667012	-0.207253846	0.835811617	0.921966724	7.179028637	86.07053892	0.5	CRA004775;Yang2025_Silkie_Wenchang	PRJNA827465	0.958339923
ENSGALG00010027188	KCNH2	2	-0.410193854	0.381506191	-1.075195801	0.282287075	0.181809696	2.18121441	54.15397976	1	CRA004775;PRJNA827465	Yang2025_Silkie_Wenchang	0.779519046
ENSGALG00010027189	ENSGALG00010027189	2	0.774123386	0.683753605	1.132167173	0.257564158	0.662000779	3.390756779	70.50805866	1	PRJNA827465;Yang2025_Silkie_Wenchang	CRA004775	0.7650601
ENSGALG00010027189	ENSGALG00010027189	2	1.32694177	0.420658903	3.154436438	0.001608084	0	0.194042334	0	1	CRA004775;Yang2025_Silkie_Wenchang	PRJNA827465	0.050669962
ENSGALG00010027189	ENSGALG00010027189	2	0.554595955	0.493515986	1.123764926	0.261112765	0.204702965	1.694936098	41.00072555	1	CRA004775;PRJNA827465	Yang2025_Silkie_Wenchang	0.767929536
ENSGALG00010027190	IPPK	2	0.029637723	0.055464877	0.534351187	0.593098584	0	0.558094175	0	0.5	PRJNA827465;Yang2025_Silkie_Wenchang	CRA004775	0.892776493
ENSGALG00010027190	IPPK	2	0.27709682	0.313746992	0.883185583	0.37713601	0.179386721	10.94180388	90.86073913	0.5	CRA004775;Yang2025_Silkie_Wenchang	PRJNA827465	0.823064945
ENSGALG00010027190	IPPK	2	0.314500184	0.271164982	1.159811203	0.246125682	0.131128164	8.780788627	88.61150128	1	CRA004775;PRJNA827465	Yang2025_Silkie_Wenchang	0.756727496
ENSGALG00010027191	AOC1	2	-0.096192682	0.931343848	-0.103283747	0.917737775	0.788973296	1.828341833	45.30563256	0.5	PRJNA827465;Yang2025_Silkie_Wenchang	CRA004775	0.98098528
ENSGALG00010027191	AOC1	2	0.781747762	1.827092621	0.427864331	0.668749897	5.770276669	7.343444291	86.38241184	0.5	CRA004775;Yang2025_Silkie_Wenchang	PRJNA827465	0.914377769
ENSGALG00010027191	AOC1	2	1.697669952	0.894109859	1.898726353	0.057600465	0.797929082	1.994822028	49.8702147	1	CRA004775;PRJNA827465	Yang2025_Silkie_Wenchang	0.415770628
ENSGALG00010027192	SH3GL1	2	-0.116187296	0.06540127	-1.776529649	0.07564566	0.004812727	2.267508101	55.89872426	1	PRJNA827465;Yang2025_Silkie_Wenchang	CRA004775	0.474784631
ENSGALG00010027192	SH3GL1	2	-0.185219714	0.063563472	-2.913933249	0.003569062	0	0.007492653	0	1	CRA004775;Yang2025_Silkie_Wenchang	PRJNA827465	0.084829873
ENSGALG00010027192	SH3GL1	2	-0.063828511	0.053738736	-1.18775608	0.234929522	0	0.290554993	0	1	CRA004775;PRJNA827465	Yang2025_Silkie_Wenchang	0.747791642
ENSGALG00010027193	CAMK1G	2	-0.160579576	0.279025829	-0.575500758	0.564952678	0	0.823991313	0	0.5	PRJNA827465;Yang2025_Silkie_Wenchang	CRA004775	0.883416369
ENSGALG00010027193	CAMK1G	2	0.13312201	0.372174293	0.35768728	0.720577353	0	0.000648874	0	1	CRA004775;Yang2025_Silkie_Wenchang	PRJNA827465	0.928774793
ENSGALG00010027193	CAMK1G	2	-0.284976667	0.335467657	-0.849490736	0.395608283	0	0.379002957	0	0.5	CRA004775;PRJNA827465	Yang2025_Silkie_Wenchang	0.831031125
ENSGALG00010027194	ENSGALG00010027194	2	0.185517903	0.260837784	0.711238611	0.476936386	0	0.543486353	0	0.5	PRJNA827465;Yang2025_Silkie_Wenchang	CRA004775	0.854986476
ENSGALG00010027194	ENSGALG00010027194	2	-0.103866889	0.303107883	-0.342673006	0.731844473	0	0.008880205	0	1	CRA004775;Yang2025_Silkie_Wenchang	PRJNA827465	0.932851968
ENSGALG00010027194	ENSGALG00010027194	2	0.147570438	0.235107802	0.627671376	0.53021924	0	0.627847967	0	0.5	CRA004775;PRJNA827465	Yang2025_Silkie_Wenchang	0.872246963
ENSGALG00010027195	CENPP	2	-0.063200167	0.127368891	-0.496197829	0.619754843	0	0.069560356	0	1	PRJNA827465;Yang2025_Silkie_Wenchang	CRA004775	0.899325257
ENSGALG00010027195	CENPP	2	0.323533742	0.453579695	0.713289738	0.475666486	0.272160365	2.555077754	60.8622478	0.5	CRA004775;Yang2025_Silkie_Wenchang	PRJNA827465	0.854555922
ENSGALG00010027195	CENPP	2	0.285311556	0.489895217	0.582393022	0.560301981	0.35424483	3.175215748	68.50607709	0.5	CRA004775;PRJNA827465	Yang2025_Silkie_Wenchang	0.881203589
ENSGALG00010027196	ENSGALG00010027196	2	-0.727170321	0.304128732	-2.390995141	0.016802775	0.032875527	1.216093285	17.7694662	1	PRJNA827465;Yang2025_Silkie_Wenchang	CRA004775	0.215137283
ENSGALG00010027196	ENSGALG00010027196	2	-0.75811674	0.289542153	-2.618329432	0.008836145	0.006140224	1.037930397	3.6544259	1	CRA004775;Yang2025_Silkie_Wenchang	PRJNA827465	0.147467951
ENSGALG00010027196	ENSGALG00010027196	2	-0.439825848	0.282165662	-1.558750433	0.11905546	0	0.002559321	0	1	CRA004775;PRJNA827465	Yang2025_Silkie_Wenchang	0.579471011
ENSGALG00010027197	ENSGALG00010027197	2	0.185489316	0.287576696	0.645008162	0.518921897	0	0.46238484	0	1	PRJNA827465;Yang2025_Silkie_Wenchang	CRA004775	0.868878597
ENSGALG00010027197	ENSGALG00010027197	2	-1.041258049	1.552096636	-0.670871919	0.502302127	4.406902189	11.54990902	91.34192314	0.5	CRA004775;Yang2025_Silkie_Wenchang	PRJNA827465	0.862860181
ENSGALG00010027197	ENSGALG00010027197	2	-1.217142675	1.344783429	-0.905084529	0.365420608	3.270693041	10.05028321	90.05003164	0.5	CRA004775;PRJNA827465	Yang2025_Silkie_Wenchang	0.81849468
ENSGALG00010027198	ECM2	2	-0.274140605	0.092753226	-2.955591058	0.003120706	0	0.340235158	0	1	PRJNA827465;Yang2025_Silkie_Wenchang	CRA004775	0.077237464
ENSGALG00010027198	ECM2	2	-0.046897862	0.209151167	-0.224229504	0.822578727	0	0.746264537	0	0.5	CRA004775;Yang2025_Silkie_Wenchang	PRJNA827465	0.955822987
ENSGALG00010027198	ECM2	2	-0.16168999	0.245177146	-0.659482309	0.509586103	0.068278389	1.684842597	40.64727459	0.5	CRA004775;PRJNA827465	Yang2025_Silkie_Wenchang	0.865257661
ENSGALG00010027199	LAMB3	2	-0.53636613	0.33483447	-1.60188445	0.109181164	0.125147171	2.259738537	55.74709269	1	PRJNA827465;Yang2025_Silkie_Wenchang	CRA004775	0.559252871
ENSGALG00010027199	LAMB3	2	-0.552246144	0.457739544	-1.206463701	0.227638747	0.233815285	2.121264689	52.85831111	1	CRA004775;Yang2025_Silkie_Wenchang	PRJNA827465	0.740853612
ENSGALG00010027199	LAMB3	2	-0.921665951	0.26598361	-3.465123104	0.000529989	0	0.182175271	0	1	CRA004775;PRJNA827465	Yang2025_Silkie_Wenchang	0.024386237
ENSGALG00010027200	G0S2	2	-0.270417196	0.145321377	-1.86082187	0.062769336	0	0.809630719	0	1	PRJNA827465;Yang2025_Silkie_Wenchang	CRA004775	0.431675103
ENSGALG00010027200	G0S2	2	-0.200082531	0.193901335	-1.031878048	0.302129251	0	0.542120028	0	1	CRA004775;Yang2025_Silkie_Wenchang	PRJNA827465	0.791603969
ENSGALG00010027200	G0S2	2	-0.392346542	0.169780715	-2.310901696	0.020838285	0	0.019551327	0	1	CRA004775;PRJNA827465	Yang2025_Silkie_Wenchang	0.243976022
ENSGALG00010027201	ASPN	2	0.011425624	0.311558819	0.036672445	0.970746179	0.102803332	2.115358225	52.72668298	0.5	PRJNA827465;Yang2025_Silkie_Wenchang	CRA004775	0.991843322
ENSGALG00010027201	ASPN	2	0.43316598	0.260614025	1.662097731	0.09649318	0	0.214272085	0	1	CRA004775;Yang2025_Silkie_Wenchang	PRJNA827465	0.530885356
ENSGALG00010027201	ASPN	2	0.093603676	0.433692047	0.215829818	0.829120405	0.248532501	2.827896293	64.6380243	0.5	CRA004775;PRJNA827465	Yang2025_Silkie_Wenchang	0.956860127
ENSGALG00010027202	CHAF1A	2	0.036978749	0.045676098	0.809586413	0.41817792	0	0.104228845	0	1	PRJNA827465;Yang2025_Silkie_Wenchang	CRA004775	0.838112595
ENSGALG00010027202	CHAF1A	2	0.01584972	0.06756998	0.234567477	0.814544455	0	0.072811334	0	0.5	CRA004775;Yang2025_Silkie_Wenchang	PRJNA827465	0.953869515
ENSGALG00010027202	CHAF1A	2	0.045378387	0.059443576	0.763385891	0.445233292	0	0.13671249	0	0.5	CRA004775;PRJNA827465	Yang2025_Silkie_Wenchang	0.846013379
ENSGALG00010027203	ENSGALG00010027203	2	-0.190062622	0.199573486	-0.952344048	0.340922525	0	0.17063858	0	1	PRJNA827465;Yang2025_Silkie_Wenchang	CRA004775	0.805639763
ENSGALG00010027203	ENSGALG00010027203	2	-0.188747819	0.252450947	-0.747661362	0.454664442	0	0.338501386	0	1	CRA004775;Yang2025_Silkie_Wenchang	PRJNA827465	0.849072665
ENSGALG00010027203	ENSGALG00010027203	2	-0.305369	0.237600887	-1.285218269	0.198716021	0	0.089271017	0	1	CRA004775;PRJNA827465	Yang2025_Silkie_Wenchang	0.71096127
ENSGALG00010027205	OGN	2	0.024562112	0.098639201	0.24900964	0.803353325	0	0.778933264	0	0.5	PRJNA827465;Yang2025_Silkie_Wenchang	CRA004775	0.951001347
ENSGALG00010027205	OGN	2	0.370421162	0.265958163	1.392779818	0.163686383	0	0.368182028	0	1	CRA004775;Yang2025_Silkie_Wenchang	PRJNA827465	0.662073265
ENSGALG00010027205	OGN	2	0.112310256	0.252934255	0.444029441	0.657021299	0.066911826	1.439471885	30.53007769	0.5	CRA004775;PRJNA827465	Yang2025_Silkie_Wenchang	0.910695405
ENSGALG00010027206	ENSGALG00010027206	2	-0.053972776	0.837203546	-0.064467925	0.948597646	1.036737088	3.839635616	73.95586196	0.5	PRJNA827465;Yang2025_Silkie_Wenchang	CRA004775	0.9869162
ENSGALG00010027206	ENSGALG00010027206	2	0.25253847	1.136284263	0.222249357	0.824119773	2.235686798	7.446741569	86.57130786	0.5	CRA004775;Yang2025_Silkie_Wenchang	PRJNA827465	0.95619211
ENSGALG00010027206	ENSGALG00010027206	2	1.095819899	0.413282566	2.651502843	0.008013444	0	0.522343044	0	1	CRA004775;PRJNA827465	Yang2025_Silkie_Wenchang	0.139297234
ENSGALG00010027207	NOL8	2	0.182648856	0.200363141	0.911589105	0.36198506	0.070053234	7.365309156	86.4228374	1	PRJNA827465;Yang2025_Silkie_Wenchang	CRA004775	0.815579273
ENSGALG00010027207	NOL8	2	0.370990078	0.120269026	3.084668522	0.002037791	0	0.239472002	0	1	CRA004775;Yang2025_Silkie_Wenchang	PRJNA827465	0.058631999
ENSGALG00010027207	NOL8	2	0.021737018	0.071102009	0.305715948	0.759820929	0.002372875	1.073232411	6.82353703	1	CRA004775;PRJNA827465	Yang2025_Silkie_Wenchang	0.939165042
ENSGALG00010027208	UBXN6	2	-0.159963014	0.090341286	-1.770652405	0.076618522	0.00628826	1.566156574	36.14942357	1	PRJNA827465;Yang2025_Silkie_Wenchang	CRA004775	0.47762877
ENSGALG00010027208	UBXN6	2	-0.249326044	0.105116698	-2.371897614	0.017696994	0	0.167219004	0	1	CRA004775;Yang2025_Silkie_Wenchang	PRJNA827465	0.222006049
ENSGALG00010027208	UBXN6	2	-0.103385385	0.076032428	-1.359753818	0.173907841	0	0.181710378	0	1	CRA004775;PRJNA827465	Yang2025_Silkie_Wenchang	0.677749121
ENSGALG00010027209	TRAF3IP3	2	0.353959087	0.173660427	2.038225363	0.0415274	0	0.072015842	0	1	PRJNA827465;Yang2025_Silkie_Wenchang	CRA004775	0.353203075
ENSGALG00010027209	TRAF3IP3	2	0.087755561	0.403683664	0.21738695	0.8279068	0.195883849	2.319473976	56.88677648	0.5	CRA004775;Yang2025_Silkie_Wenchang	PRJNA827465	0.956550752
ENSGALG00010027209	TRAF3IP3	2	0.063321945	0.348369717	0.181766501	0.855765975	0.129915569	1.922109794	47.97383567	0.5	CRA004775;PRJNA827465	Yang2025_Silkie_Wenchang	0.963486168
ENSGALG00010027210	ENSGALG00010027210	2	-0.015620287	0.210528099	-0.074195735	0.940854639	0.039468216	1.797482084	44.36662213	0.5	PRJNA827465;Yang2025_Silkie_Wenchang	CRA004775	0.985565295
ENSGALG00010027210	ENSGALG00010027210	2	-0.039167373	0.349783063	-0.111976184	0.91084229	0.128572158	1.874883125	46.66334203	0.5	CRA004775;Yang2025_Silkie_Wenchang	PRJNA827465	0.97880407
ENSGALG00010027210	ENSGALG00010027210	2	-0.263970359	0.193138336	-1.366742433	0.171706053	0	0.362619445	0	1	CRA004775;PRJNA827465	Yang2025_Silkie_Wenchang	0.674249474
ENSGALG00010027211	HDGFRP2	2	-0.011743706	0.062129815	-0.189018842	0.850078049	0.004488383	2.332066731	57.11958038	0.5	PRJNA827465;Yang2025_Silkie_Wenchang	CRA004775	0.96189951
ENSGALG00010027211	HDGFRP2	2	0.068833027	0.059942476	1.148318045	0.250837292	0	0.112879282	0	1	CRA004775;Yang2025_Silkie_Wenchang	PRJNA827465	0.760522881
ENSGALG00010027211	HDGFRP2	2	-0.025707248	0.074359394	-0.34571621	0.729556001	0.004833746	1.444753691	30.78404949	0.5	CRA004775;PRJNA827465	Yang2025_Silkie_Wenchang	0.9323457
ENSGALG00010027212	IRF6	2	0.050143036	0.357923761	0.14009418	0.888585579	0.010176427	1.040355704	3.879029476	0.5	PRJNA827465;Yang2025_Silkie_Wenchang	CRA004775	0.973135755
ENSGALG00010027212	IRF6	2	0.029407659	0.389187958	0.075561584	0.9397679	0.016649327	1.058133438	5.493960977	0.5	CRA004775;Yang2025_Silkie_Wenchang	PRJNA827465	0.985429356
ENSGALG00010027212	IRF6	2	0.380296259	0.347529142	1.094285955	0.273829583	0	0.005956029	0	1	CRA004775;PRJNA827465	Yang2025_Silkie_Wenchang	0.774526342
ENSGALG00010027213	KCNC4	2	-0.054583371	0.317465455	-0.171934836	0.863488759	0.138440649	3.139583464	68.14864101	0.5	PRJNA827465;Yang2025_Silkie_Wenchang	CRA004775	0.96626958
ENSGALG00010027213	KCNC4	2	-0.326388902	0.250714881	-1.301832986	0.192973485	0	0.284729544	0	1	CRA004775;Yang2025_Silkie_Wenchang	PRJNA827465	0.702958174
ENSGALG00010027213	KCNC4	2	0.187133507	0.199339518	0.938767732	0.347850009	0	0.338265217	0	0.5	CRA004775;PRJNA827465	Yang2025_Silkie_Wenchang	0.808847403
ENSGALG00010027214	PLIN4	2	0.154882341	0.196543619	0.788030373	0.43067894	0.059056481	4.040160429	75.24850764	0.5	PRJNA827465;Yang2025_Silkie_Wenchang	CRA004775	0.842473342

ENSGALG00010027214	PLIN4	2	0.201772401	0.219651525	0.918602323	0.358303617	0.049976163	1.97326648	49.32260747	0.5	CRA004775;Yang2025_Silkie_Wenchang	PRJNA827465	0.813964918
ENSGALG00010027214	PLIN4	2	-0.024734856	0.092262414	-0.268092437	0.788628167	0	0.035063401	0	1	CRA004775;PRJNA827465	Yang2025_Silkie_Wenchang	0.947060654
ENSGALG00010027215	ENSGALG00010027215	2	0.351882832	0.159454096	2.206797068	0.02732824	0.016114172	1.453665703	31.20839281	1	PRJNA827465;Yang2025_Silkie_Wenchang	CRA004775	0.281911238
ENSGALG00010027215	ENSGALG00010027215	2	0.442879302	0.177071494	2.501132694	0.012379678	0	0.760582696	0	1	CRA004775;Yang2025_Silkie_Wenchang	PRJNA827465	0.180073464
ENSGALG00010027215	ENSGALG00010027215	2	0.204423155	0.1537447	1.329627328	0.18364109	0	0.007807739	0	1	CRA004775;PRJNA827465	Yang2025_Silkie_Wenchang	0.692148694
ENSGALG00010027216	ENSGALG00010027216	2	0.383115128	0.534495893	0.716778433	0.473510821	0.442338773	4.272002275	76.59177277	0.5	PRJNA827465;Yang2025_Silkie_Wenchang	CRA004775	0.854373829
ENSGALG00010027216	ENSGALG00010027216	2	0.337608968	0.698954170	0.483020176	0.62908141	0.701217098	3.469810006	71.17997821	0.5	CRA004775;Yang2025_Silkie_Wenchang	PRJNA827465	0.902166922
ENSGALG00010027216	ENSGALG00010027216	2	-0.154179759	0.250667473	-0.615076845	0.538503973	0	0.241189715	0	1	CRA004775;PRJNA827465	Yang2025_Silkie_Wenchang	0.87481821
ENSGALG00010027217	SEMA6B	2	0.174981149	0.176067683	0.993828884	0.320306181	0.0492727	4.865451659	79.44692353	1	PRJNA827465;Yang2025_Silkie_Wenchang	CRA004775	0.798348216
ENSGALG00010027217	SEMA6B	2	0.238650044	0.248442848	0.960583273	0.336761743	0.061224214	1.408825882	29.01890769	0.5	CRA004775;Yang2025_Silkie_Wenchang	PRJNA827465	0.803887433
ENSGALG00010027217	SEMA6B	2	-0.009591396	0.105727547	-0.090718039	0.927716636	0	0.297500402	0	0.5	CRA004775;PRJNA827465	Yang2025_Silkie_Wenchang	0.982993195
ENSGALG00010027218	IARS1	2	-0.107361332	0.044985232	-2.386590578	0.017005418	0	0.002780046	0	1	PRJNA827465;Yang2025_Silkie_Wenchang	CRA004775	0.216579369
ENSGALG00010027218	IARS1	2	-0.09976555	0.08551403	-1.166657094	0.243348877	0	0.092868589	0	1	CRA004775;Yang2025_Silkie_Wenchang	PRJNA827465	0.755238542
ENSGALG00010027218	IARS1	2	-0.102365091	0.049694303	-2.05989959	0.039408494	0	0.089141479	0	1	CRA004775;PRJNA827465	Yang2025_Silkie_Wenchang	0.343326573
ENSGALG00010027219	ENSGALG00010027219	2	-0.079857426	0.054275073	-1.471346254	0.1411975	0	0.665529398	0	0.5	PRJNA827465;Yang2025_Silkie_Wenchang	CRA004775	0.625999476
ENSGALG00010027219	ENSGALG00010027219	2	-0.090875497	0.142896814	-0.635951881	0.524807786	0.015019968	1.515290113	34.00603676	0.5	CRA004775;Yang2025_Silkie_Wenchang	PRJNA827465	0.869876873
ENSGALG00010027219	ENSGALG00010027219	2	-0.114427379	0.057264788	-1.998215376	0.045693315	0	0.723985956	0	1	CRA004775;PRJNA827465	Yang2025_Silkie_Wenchang	0.368613377
ENSGALG00010027221	LRRC61	2	-0.286167772	0.287664048	-0.994798528	0.319834262	0.122105584	3.602765782	72.32153134	1	PRJNA827465;Yang2025_Silkie_Wenchang	CRA004775	0.798348216
ENSGALG00010027221	LRRC61	2	-0.199788531	0.416801257	-0.47933764	0.631698449	0.278007249	5.00303167	80.01211933	0.5	CRA004775;Yang2025_Silkie_Wenchang	PRJNA827465	0.903058174
ENSGALG00010027221	LRRC61	2	0.025637422	0.1299894	0.197227023	0.843649884	0	0.705737056	0	0.5	CRA004775;PRJNA827465	Yang2025_Silkie_Wenchang	0.96057435
ENSGALG00010027223	SIRT6	2	-0.016895943	0.054863286	-0.307964471	0.758109367	0	0.136393934	0	1	PRJNA827465;Yang2025_Silkie_Wenchang	CRA004775	0.938954369
ENSGALG00010027223	SIRT6	2	-0.156424053	0.109162026	-1.432953008	0.151871267	0.002928569	1.136803656	12.03406195	1	CRA004775;Yang2025_Silkie_Wenchang	PRJNA827465	0.643294424
ENSGALG00010027223	SIRT6	2	-0.10645678	0.131828516	-0.807539853	0.419355524	0.023496066	2.647472494	62.22812504	1	CRA004775;PRJNA827465	Yang2025_Silkie_Wenchang	0.838527387
ENSGALG00010027224	RARRES2	2	0.219441345	0.156032833	1.406379287	0.159611528	0.023557046	1.553694707	35.63729118	1	PRJNA827465;Yang2025_Silkie_Wenchang	CRA004775	0.655302608
ENSGALG00010027224	RARRES2	2	0.285726894	0.155317622	1.849930171	0.064323485	0	0.820275383	0	1	CRA004775;Yang2025_Silkie_Wenchang	PRJNA827465	0.643237276
ENSGALG00010027224	RARRES2	2	0.142909867	0.083227517	1.717098773	0.085961126	0	0.084527813	0	1	CRA004775;PRJNA827465	Yang2025_Silkie_Wenchang	0.504285479
ENSGALG00010027225	CREB3L3	2	-0.394589646	0.397142428	-0.993572124	0.320431221	0	0.790380534	0	1	PRJNA827465;Yang2025_Silkie_Wenchang	CRA004775	0.798348216
ENSGALG00010027225	CREB3L3	2	0.750723013	1.487481381	0.504694057	0.613773736	4.065579186	12.26396759	91.84603194	0.5	CRA004775;Yang2025_Silkie_Wenchang	PRJNA827465	0.898256044
ENSGALG00010027225	CREB3L3	2	1.133074445	1.132397326	0.982936306	0.325638788	2.169881812	6.487183809	84.58499051	0.5	CRA004775;PRJNA827465	Yang2025_Silkie_Wenchang	0.806037788
ENSGALG00010027227	ENSGALG00010027227	2	0.351703138	0.325165583	1.081612436	0.279424776	0.20030025	18.5004731	94.59473282	1	PRJNA827465;Yang2025_Silkie_Wenchang	CRA004775	0.777457036
ENSGALG00010027227	ENSGALG00010027227	2	0.299443447	0.449743019	0.665810106	0.505532485	0.334813557	5.283999675	81.07494206	0.5	CRA004775;Yang2025_Silkie_Wenchang	PRJNA827465	0.863927652
ENSGALG00010027227	ENSGALG00010027227	2	0.031311103	0.060343107	0.518884498	0.603841289	0	0.472598869	0	0.5	CRA004775;PRJNA827465	Yang2025_Silkie_Wenchang	0.893892991
ENSGALG00010027229	MDM4	2	0.058742785	0.073879593	0.795115172	0.4265461	0	0.214792298	0	1	PRJNA827465;Yang2025_Silkie_Wenchang	CRA004775	0.84125685
ENSGALG00010027229	MDM4	2	-0.036041709	0.189230607	-0.190464479	0.848945175	0.039795129	2.067596357	51.63466037	0.5	CRA004775;Yang2025_Silkie_Wenchang	PRJNA827465	0.961364896
ENSGALG00010027229	MDM4	2	-0.04705272	0.139569144	-0.337128385	0.736020121	0.018114663	1.550019564	35.48468526	0.5	CRA004775;PRJNA827465	Yang2025_Silkie_Wenchang	0.933592889
ENSGALG00010027230	MAP2K2	2	0.087155108	0.067755168	1.286619319	0.198227007	0	0.719342943	0	1	PRJNA827465;Yang2025_Silkie_Wenchang	CRA004775	0.710234722
ENSGALG00010027230	MAP2K2	2	-0.006540566	0.214099773	-0.03054915	0.975629096	0.064259335	3.219827428	68.94243489	0.5	CRA004775;Yang2025_Silkie_Wenchang	PRJNA827465	0.992754467
ENSGALG00010027230	MAP2K2	2	-0.041694372	0.139901957	-0.29802565	0.765683587	0.022106565	1.937172963	48.3783834	0.5	CRA004775;PRJNA827465	Yang2025_Silkie_Wenchang	0.940926806
ENSGALG00010027231	ENSGALG00010027231	2	0.409236294	0.466632909	0.876998355	0.380487531	0.409759582	16.79864454	94.04713876	0.5	PRJNA827465;Yang2025_Silkie_Wenchang	CRA004775	0.824392515
ENSGALG00010027231	ENSGALG00010027231	2	0.852445904	0.172277106	4.948109038	7.49379E-07	0	0.199275426	0	1	CRA004775;Yang2025_Silkie_Wenchang	PRJNA827465	0.000177427
ENSGALG00010027231	ENSGALG00010027231	2	0.198180944	0.342752283	0.578204591	0.563125999	0.161277038	2.600558143	61.54671632	0.5	CRA004775;PRJNA827465	Yang2025_Silkie_Wenchang	0.882266649
ENSGALG00010027232	ARRDC2	2	-0.011309569	0.186313009	-0.060701984	0.951596552	0.04378889	2.429143252	58.8332224	0.5	PRJNA827465;Yang2025_Silkie_Wenchang	CRA004775	0.987713746
ENSGALG00010027232	ARRDC2	2	-0.233164236	0.208982033	-1.115714267	0.264544462	0	0.070894495	0	1	CRA004775;Yang2025_Silkie_Wenchang	PRJNA827465	0.768855521
ENSGALG00010027232	ARRDC2	2	0.124709314	0.104803641	1.189933028	0.234072716	0	0.131997456	0	0.5	CRA004775;PRJNA827465	Yang2025_Silkie_Wenchang	0.747279664
ENSGALG00010027233	ENSGALG00010027233	2	0.208741772	0.461608229	0.452205483	0.651120959	0.389137002	11.47765324	91.28741757	0.5	PRJNA827465;Yang2025_Silkie_Wenchang	CRA004775	0.908769703
ENSGALG00010027233	ENSGALG00010027233	2	0.048802158	0.690114238	0.070716608	0.94362374	0.82796955	7.238028384	86.18408292	0.5	CRA004775;Yang2025_Silkie_Wenchang	PRJNA827465	0.985827325
ENSGALG00010027233	ENSGALG00010027233	2	-0.301011698	0.163060675	-1.846010107	0.0648907	0	0.8512615	0	1	CRA004775;PRJNA827465	Yang2025_Silkie_Wenchang	0.438420946
ENSGALG00010027236	PEX11G	2	0.036672402	0.098528681	0.37220027	0.709743745	0.006795862	1.39895699	28.51817409	0.5	PRJNA827465;Yang2025_Silkie_Wenchang	CRA004775	0.925298042
ENSGALG00010027236	PEX11G	2	-0.110079044	0.159496238	-0.690167026	0.490089157	0	0.047566463	0	0.5	CRA004775;Yang2025_Silkie_Wenchang	PRJNA827465	0.858529056
ENSGALG00010027236	PEX11G	2	0.097428928	0.081594145	1.194067641	0.23245151	0	0.016752241	0	1	CRA004775;PRJNA827465	Yang2025_Silkie_Wenchang	0.745130226
ENSGALG00010027237	CTC-575C13.4	2	-0.158612567	0.10793147	-1.469569228	0.141678459	0	0.397037139	0	1	PRJNA827465;Yang2025_Silkie_Wenchang	CRA004775	0.627088401
ENSGALG00010027237	CTC-575C13.4	2	0.0370525	0.320804562	0.115498668	0.908049876	0.11442782	2.002170441	50.05420219	0.5	CRA004775;Yang2025_Silkie_Wenchang	PRJNA827465	0.978196186
ENSGALG00010027237	CTC-575C13.4	2	0.016719859	0.205794302	0.081245489	0.935246724	0.036188852	1.357497823	26.33505682	0.5	CRA004775;PRJNA827465	Yang2025_Silkie_Wenchang	0.984597077
ENSGALG00010027239	ENSGALG00010027239	2	0.034380833	0.038255872	0.898703734	0.368808569	0	0.136652436	0	1	PRJNA827465;Yang2025_Silkie_Wenchang	CRA004775	0.826856397
ENSGALG00010027239	ENSGALG00010027239	2	-0.123434669	0.203834992	-0.605561723	0.544805826	0.068895532	5.603691042	82.1546193	0.5	CRA004775;Yang2025_Silkie_Wenchang	PRJNA827465	0.877345822
ENSGALG00010027239	ENSGALG00010027239	2	-0.13216295	0.185285267	-0.713294438	0.475663579	0.057838023	5.660706505	82.33436058	0.5	CRA004775;PRJNA827465	Yang2025_Silkie_Wenchang	0.854555922
ENSGALG00010027240	SLC6A1	2	0.221293775	0.25421025	0.870514759	0.384019157	0	0.890020606	0	1	PRJNA827465;Yang2025_Silkie_Wenchang	CRA004775	0.825662196
ENSGALG00010027240	SLC6A1	2	0.551802055	0.387717597	1.423206114	0.154676414	0	0.021054156	0	1	CRA004775;Yang2025_Silkie_Wenchang	PRJNA827465	0.647505492
ENSGALG00010027240	SLC6A1	2	0.128486199	0.278457436	0.461421324	0.644496356	0	0.277474987	0	1	CRA004775;PRJNA827465	Yang2025_Silkie_Wenchang	0.906953008
ENSGALG00010027241	LRRN2	2	0.047641206	0.0634471	0.75088075	0.452724426	0	0.048606348	0	1	PRJNA827465;Yang2025_Silkie_Wenchang	CRA004775	0.848446539
ENSGALG00010027241	LRRN2	2	0.001224017	0.095207727	0.012856276	0.989742459	0	0.271804915	0	0.5	CRA004775;Yang2025_Silkie_Wenchang	PRJNA827465	0.997311131
ENSGALG00010027241	LRRN2	2	0.037974831	0.072821144	0.521480834	0.602031854	0	0.476564061	0	0.5	CRA004775;PRJNA827465	Yang2025_Silkie_Wenchang	0.89532296
ENSGALG00010027242	ENSGALG00010027242	2	0.193641896	0.265130663	0.730364017	0.465167707	0.125885157	9.552419166	89.53144766	0.5	PRJNA827465;Yang2025_Silkie_Wenchang	CRA004775	0.852370105
ENSGALG00010027242	ENSGALG00010027242	2	0.43496889	0.11640849	3.736573579	0.000186545	0	0.266516349	0	1	CRA004775;Yang2025_Silkie_Wenchang	PRJNA827465	0.012118634
ENSGALG00010027242	ENSGALG00010027242	2	0.021188727	0.158121156	0.134003115	0.893400111	0.019854295	1.414641256	29.31169948	0.5	CRA004775;PRJNA827465	Yang2025_Silkie_Wenchang	0.974381724
ENSGALG00010027243	HRH1	2	0.092096851	0.108753766	0.846838268	0.397085275	0	0.304705054	0	1	PRJNA827465;Yang2025_Silkie_Wenchang	CRA004775	0.831438925
ENSGALG00010027243	HRH1	2	0.173407414	0.189737086	0.913935261	0.360750864	0	0.035768445	0	1	CRA004775;Yang2025_Silkie_Wenchang	PRJNA827465	0.815095283
ENSGALG00010027243	HRH1	2	0.05666802	0.124095589	0.456648144	0.647923969	0	0.003995146	0	1	CRA004775;PRJNA827465	Yang2025_Silkie_Wenchang	0.907532279
ENSGALG00010027244	ARHGEF18	2	-0.014575997	0.041637294	-0.350070699	0.726028564	0	0.9306578	0	0.5	PRJNA827465;Yang2025_Silkie_Wenchang	CRA004775	0.931029023
ENSGALG00010027244	ARHGEF18	2	0.082587884	0.107201049	0.77040182	0.441061574	0	0.0027492	0	1	CRA004775;Yang2025_Silkie_Wenchang	PRJNA827465	0.844888742
ENSGALG00010027244	ARHGEF18	2	-0.030083874	0.044748009	-0.672295259	0.501395747	0	0.038786156	0	0.5	CRA004775;PRJNA827465	Yang2025_Silkie_Wenchang	0.862658912
ENSGALG00010027245	LRIG2	2	-0.152709027	0.081279989	-1.878806264	0.060271498	0.00795145	2.321406352	56.92266462	1	PRJNA827465;Yang2025_Silkie_Wenchang	CRA004775	0.425316544
ENSGALG00010027245	LRIG2	2	-0.222646128	0.0898732	-2.477336159	0.013236717	0	0.919538157	0	0.5	CRA004775;Yang2025_Silkie_Wenchang	PRJNA827465	0.187881067
ENSGALG00010027245	LRIG2	2	-0.084765451	0.051488881	-1.646286519	0.099704784	0	0.124630473	0	0.5	CRA004775;PRJNA827465	Yang2025_Silkie_Wenchang	0.539319724
ENSGALG00010027246	ZNF767	2	-0.034815589	0.062281202	-0.559006386	0.576157362	0	0.153443956	0	0.5	PRJNA827465;Yang2025_Silkie_Wenchang	CRA004775	0.887048016
ENSGALG00010027246	ZNF767	2	-0.119944094	0.151605243	-0.79116059	0.428850286	0.024752112	2.068288139	51.65083718	0.5	CRA004775;Yang2025_Silkie_Wenchang	PRJNA827465	0.841871174
ENSGALG00010027246	ZNF767	2	-0.124565265	0.116067361	-1.073215281	0.283174536	0.013541431	1.70731421	41.42847321	1	CRA004775;PRJNA827465	Yang2025_Silkie_Wenchang	0.779568117
ENSGALG00010027247	ATG7	2	0.035645895	0.060724728	0.587007904	0.557198398	0.001237741	1.19650703	16.4233912	0.5	PRJNA827465;Yang2025_Silkie_Wenchang	CRA004775	0.880678789
ENSGALG00010027247	ATG7	2	-0.059682197	0.08199731	-0.727855553	0.466702014	0	0.918275218	0	1	CRA004775;Yang2025_Silkie_Wenchang	PRJNA827465	0.852842649
ENSGALG00010027247	ATG7	2	-0.023239167	0.177725662	-0.130758634	0.895966247	0.03877535	1.987063511	49.67448224	0.5	CRA004775;PRJNA827465	Yang2025_Silkie_Wenchang	0.975902393
ENSGALG00010027250	ENSGALG00010027250	2	0.159587048	0.118715423	1.344282347	0.178857102	0.012858153	1.670894321	40.15181046	1	PRJNA827465;Yang2025_Silkie_Wenchang	CRA004775	0.685601971
ENSGALG00010027250	ENSGALG00010027250	2	0.13481978	0.23593725	0.56575203	0.57156236	0.064006122	2.252453967	55.60397617	0.5	CRA004775;Yang2025_Silkie_Wenchang	PRJNA827465	0.885429878
ENSGALG00010027250	ENSGALG00010027250	2	0.053993933	0.08523361	0.633075996	0.526684017	0	0.64231119	0	0.5	CRA004775;PRJNA827465	Yang2025_Silkie_Wenchang	0.87306637
ENSGALG00010027251	ENSGALG00010027251	2	-0.105367087	0.129323501	-0.814755913	0.415212041	0.018216558	2.03503073	50.8606929	1	PRJNA827465;Yang2025_Silkie_Wenchang	CRA004775	0.837489728
ENSGALG00010027251	ENSGALG00010027251	2	-0.201944887	0.139098909	-1.451807842	0.146555043	0	0.688166313	0	1	CRA004775;Yang2025_Silkie_Wenchang	PRJNA827465	0.635517732
ENSGALG00010027251	ENSGALG00010027251	2	-0.007044459	0.086533076	-0.081407707	0.93511772	0	0.001324368	0	1	CRA004775;PRJNA827465	Yang2025_Silkie_Wenchang	0.984512821
ENSGALG00010027252	MAGI3	2	-0.022932575	0.055058249	-0.416331171	0.677166625	0	0.014957384	0	1	PRJNA827465;Yang2025_Silkie_Wenchang	CRA004775	0.971144807
ENSGALG00010027252	MAGI3	2	0.012636325	0.115046747	0.109836444	0.912539088	0	0.61730205	0	0.5	CRA004775;Yang2025_Silkie_Wenchang	PRJNA827465	0.978967245
ENSGALG00010027252	MAGI3	2	-0.00783239	0.058730212	-0.133362202	0.893906936	0	0.637095514	0	0.5	CRA004775;PRJNA827465	Yang2025_Silkie_Wenchang	0.974381724
ENSGALG00010027253	VGLL4	2	-0.009538077	0.073476316	-0.129811579	0.896715502	0.005390303	1.799844217	44.43963592	0.5	PRJNA827465;Yang2025_Silkie_Wenchang	CRA004775	0.975115773
ENSGALG00010027253	VGLL4	2	-0.116128142	0.098549913	-1.178399169	0.238640961	0	0.008075013	0	1	CRA004775;Yang2025_Silkie_Wenchang	PRJNA827465	0.750694914
ENSGALG00010027253	VGLL4	2	0.037609063	0.051455052	0.730910999	0.464833517	0	0.336008524	0	0.5	CRA004775;PRJNA827465	Yang2025_Silkie_Wenchang	0.85217126
ENSGALG00010027254	INSR	2	-0.403356913	0.169057554	-2.385914757	0.017036699	0.047979885	6.127346628	83.67972206	1	PRJNA827465;Yang2025_Silkie_Wenchang	CRA004775	0.216696893
ENSGALG00010027254	INSR	2	-0.433353336	0.252524633	-1.716083422	0.086146775	0.073346237	1.78521028	43.98418992	1	CRA004775;Yang2025_Silkie_Wenchang	PRJNA827465	0.504893124
ENSGALG00010027254	INSR	2	-0.235261238	0.077227378	-3.046344996	0.002316419	0	0.316188832	0	1	CRA004775;PRJNA827465	Yang2025_Silkie_Wenchang	0.063799725
ENSGALG00010027255	ENSGALG00010027255	2	0.431440572	0.407151464	1.059656199	0.289301036	0.282513547	6.443691021	84.48094428	1	PRJNA827465;Yang2025_Silkie_Wenchang	CRA004775	0.78357099
ENSGALG00010027255	ENSGALG00010027255	2	0.253887247	0.627838626	0.404382967	0.685931123	0.701096952	9.031752771	88.92795202	0.5	CRA004775;Yang2025_Silkie_Wenchang	PRJNA827465	0.919235539
ENSGALG00010027255	ENSGALG00010027255	2	-0.077722448	0.203474561	-0.38197624	0.702478989	0.039539803	1.70192638	41.24505189	0.5	CRA004775;PRJNA827465	Yang2025_Silkie_Wenchang	0.923191989
ENSGALG00010027256	PHTF1	2	0.162092532	0.157581708	1.028625301	0.303655784	0.041410537	5.813008436	82.7972037	1	PRJNA827465;Yang2025_Silkie_Wenchang	CRA004775	0.792205992
ENSGALG00010027256	PHTF1	2	-0.230791431	0.615908269	-0.374717214	0.707870789	0.684939124	9.606701175	89.5905995	0.5	CRA004775;Yang2025_Silkie_Wenchang	PRJNA827465	0.925061251
ENSGALG00010027256	PHTF1	2	-0.363815462	0.453625527	-0.806426096	0.419997205	0.348053905	5.616367268	82.19489659	0.5	CRA004775;PRJNA827465	Yang2025_Silkie_Wenchang	0.838603917
ENSGALG00010027257	TAFA3	2	-0.349720025	0.199759748	-1.750703174	0.079997052	0	0.121444994	0	1	PRJNA827465;Yang2025_Silkie_Wenchang	CRA004775	0.486631195
ENSGALG00010027257	TAFA3	2	-0.247575023	0.2635268	-0.939468101	0.347490463	0	0.004486081	0	1	CRA004775;Yang2025_Silkie_Wenchang	PRJNA827465	0.808705451
ENSGALG00010027257	TAFA3	2	-0.360457774	0.220654871	-1.63358177	0.102346698	0	0.122606752	0	1	CRA004775;PRJNA827465	Yang2025_Silkie_Wenchang	0.545058575
ENSGALG00010027258	ENSGALG00010027258	2	0.082997873	0.117929283	0.703793587	0.481561328	0	0.609336846	0	0.5	PRJNA827465;Yang2025_Silkie_Wenchang	CRA004775	0.85691916
ENSGALG00010027258	ENSGALG00010027258	2	-0.070171116	0.184730346	-0.379857004	0.704051565	0	0.038253439	0	1	CRA004775;Yang2025_Silkie_Wenchang	PRJNA827465	0.92388697
ENSGALG00010027258	ENSGALG00010027258	2	0.113534673	0.134577325	0.84363895	0.398871192	0	0.454676848	0	0.5	CRA004775;PRJNA827465	Yang2025_Silkie_Wenchang	0.832054308
ENSGALG00010027259	ENSGALG00010027259	2	-0.031208215	0.043587067	-0.715997133	0.473993119	0	0.174059643	0	1	PRJNA827465;Yang2025_Silkie_Wenchang	CRA004775	0.85444425
ENSGALG00010027259	ENSGALG00010027259	2	-0.122218887	0.154757996	-0.789741988	0.429678465	0.031513072	2.63072972	61.98773319	1	CRA004775;Yang2025_Silkie_Wenchang	PRJNA827465	0.842012722
ENSGALG00010027259	ENSGALG00010027259	2	-0.128973517	0.128743412	-1.001787318	0.316446323	0.021511974	2.260287134	55.75783337	1	CRA004775;PRJNA827465	Yang2025_Silkie_Wenchang	0.796883862
ENSGALG00010027260	LEMD2	2	0.01044863	0.039176386	0.26670735	0.789694495	0	0.311076939	0	0.5	PRJNA827465;Yang2025_Silkie_Wenchang	CRA004775	0.947390191
ENSGALG00010027260	LEMD2	2	0.025934194	0.05936196	0.436882378	0.662196653	0	0.690010461	0	0.5	CRA004775;Yang2025_Silkie_Wenchang	PRJNA827465	0.912691648
ENSGALG00010027260	LEMD2	2	-0.014177781	0.050528364	-0.280590533	0.779024478	0	0.469010522	0	1	CRA004775;PRJNA827465	Yang2025_Silkie_Wenchang	0.944714533
ENSGALG00010027261	ENSGALG00010027261	2	0.142400357	0.244981907	0.581268872	0.561059257	0.106183895	8.360963213	88.03965554	0.5	PRJNA827465;Yang2025_Silkie_Wenchang	CRA004775	0.881417858
ENSGALG00010027261	ENSGALG00010027261	2	0.039147569	0.401773382	0.097436941	0.922379409	0.272469549	6.119488503	83.65876495	0.5	CRA004775;Yang2025_Silkie_Wenchang	PRJNA827465	0.981804675
ENSGALG00010027261	ENSGALG00010027261	2	-0.12136085	0.099378264	-1.22120114	0.222009873	0.005029459	1.112063044	10.0770406	1	CRA004775;PRJNA827465	Yang2025_Silkie_Wenchang	0.735359501
ENSGALG00010027262	USE1	2	0.305073813	0.283274476	1.076954825	0.281500448	0.151062202	16.81282993	94.05216133	1	PRJNA827465;Yang2025_Silkie_Wenchang	CRA004775	0.779251452
ENSGALG00010027262	USE1	2	0.167793596	0.442384467	0.379293598	0.704469853	0.365357716	14.81755033	93.25124614	0.5	CRA004775;Yang2025_Silkie_Wenchang	PRJNA827465	0.924152671
ENSGALG00010027262	USE1	2	-0.076544059	0.150126977	-0.509862123	0.610148059	0.028767383	2.311526656	56.73854777	0.5	CRA004775;PRJNA827465	Yang2025_Silkie_Wenchang	0.897438028
ENSGALG00010027263	TAMM41	2	0.110528637	0.109101382	1.013081917	0.311021035	0.007951306	1.465858367	31.78058519	0.5	PRJNA827465;Yang2025_Silkie_Wenchang	CRA004775	0.795218958
ENSGALG00010027263	TAMM41	2	-0.025905397	0.123080995	-0.210474391	0.833297437	0	2.32326E-07	0	1	CRA004775;Yang2025_Silkie_Wenchang	PRJNA827465	0.957968425
ENSGALG00010027263	TAMM41	2	0.152883457	0.096121153	1.590528743	0.111715672	0	0.867732019	0	0.5	CRA004775;PRJNA827465	Yang2025_Silkie_Wenchang	0.565091513
ENSGALG00010027264	ENSGALG00010027264	2	0.546444817	0.437388607	1.249334821	0.211542637	0.363355161	19.24446965	94.80370196	1	PRJNA827465;Yang2025_Silkie_Wenchang	CRA004775	0.724013667
ENSGALG00010027264	ENSGALG00010027264	2	0.319577004	0.694540364	0.460127331	0.645424828	0.912731602	18.4431671	94.57793775	0.5	CRA004775;Yang2025_Silkie_Wenchang	PRJNA827465	0.906966468
ENSGALG00010027264	ENSGALG00010027264	2	-0.067038225	0.249014571	-0.269214067	0.787764956	0.0954756	3.615764553	72.34333195	0.5	CRA004775;PRJNA827465	Yang2025_Silkie_Wenchang	0.946919116
ENSGALG00010027265	IP6K3	2	0.149852078	0.115134509	1.301539205	0.193073954	0.012434154	1.851698158	45.99551791	1	PRJNA827465;Yang2025_Silkie_Wenchang	CRA004775	0.703149149
ENSGALG00010027265	IP6K3	2	0.268347678	0.132494617	2.025347785	0.042831665	0	0.242245178	0	0.5	CRA004775;Yang2025_Silkie_Wenchang	PRJNA827465	0.357943462
ENSGALG00010027265	IP6K3	2	0.047458688	0.102764374	0.461820433	0.644210097	0	0.018296325	0	0.5	CRA004775;PRJNA827465	Yang2025_Silkie_Wenchang	0.906873169
ENSGALG00010027267	MYO9B	2	0.110955934	0.048798634	2.273750828	0.022980972	0	0.410685943	0	1	PRJNA827465;Yang2025_Silkie_Wenchang	CRA004775	0.258432375
ENSGALG00010027267	MYO9B	2	0.151974332	0.115414264	1.316772528	0.187914884	0	0.685316115	0	0.5	CRA004775;Yang2025_Silkie_Wenchang	PRJNA827465	0.697228375
ENSGALG00010027267	MYO9B	2	0.092482548	0.052833267	1.750460522	0.080038881	0	0.412430162	0	0.5	CRA004775;PRJNA827465	Yang2025_Silkie_Wenchang	0.486695335
ENSGALG00010027268	UQCRC1	2	-0.115553	0.072092407	-1.602845632	0.108968739	0	0.942938009	0	0.5	PRJNA827465;Yang2025_Silkie_Wenchang	CRA004775	0.558893171
ENSGALG00010027268	UQCRC1	2	0.020137596	0.150731243	0.133599349	0.893719399	0	0.025654018	0	0.5	CRA004775;Yang2025_Silkie_Wenchang	PRJNA827465	0.974381724
ENSGALG00010027268	UQCRC1	2	-0.142415983	0.077657117	-1.833907675	0.0666677	0	0.119442709	0	1	CRA004775;PRJNA827465	Yang2025_Silkie_Wenchang	0.444787068
ENSGALG00010027269	CDC25A	2	-0.201872772	0.070851998	-2.84921776	0.004382687	0	0.366415295	0	1	PRJNA827465;Yang2025_Silkie_Wenchang	CRA004775	0.097063291
ENSGALG00010027269	CDC25A	2	-0.274617369	0.199061613	-1.379559651	0.167722268	0.042319119	1.846525883	45.84424679	1	CRA004775;Yang2025_Silkie_Wenchang	PRJNA827465	0.667779227
ENSGALG00010027269	CDC25A	2	-0.286949903	0.124900914	-2.29742036	0.021594801	0.011153818	1.241972382	19.48291165	1	CRA004775;PRJNA827465	Yang2025_Silkie_Wenchang	0.248678723
ENSGALG00010027270	ENSGALG00010027270	2	0.056308237	0.306054966	0.183980798	0.854028518	0.118796042	2.733149632	63.41217516	0.5	PRJNA827465;Yang2025_Silkie_Wenchang	CRA004775	0.962975689
ENSGALG00010027270	ENSGALG00010027270	2	0.346753397	0.241712752	1.434568075	0.151410217	0	0.024597995	0	1	CRA004775;Yang2025_Silkie_Wenchang	PRJNA827465	0.642990359
ENSGALG00010027270	ENSGALG00010027270	2	-0.173889502	0.241131961	-0.721138341	0.470824404	0	0.559180309	0	0.5	CRA004775;PRJNA827465	Yang2025_Silkie_Wenchang	0.853762555
ENSGALG00010027271	INKA2	2	-0.276371962	0.082220617	-3.361346244	0.000775635	0	0.49468801	0	1	PRJNA827465;Yang2025_Silkie_Wenchang	CRA004775	0.031688974
ENSGALG00010027271	INKA2	2	0.16618755	0.549915563	0.302205576	0.762495354	0.536297409	8.165221618	87.75293499	0.5	CRA004775;Yang2025_Silkie_Wenchang	PRJNA827465	0.940082621
ENSGALG00010027271	INKA2	2	0.218511616	0.490931182	0.445096225	0.656250217	0.413905959	6.461581366	84.52391228	0.5	CRA004775;PRJNA827465	Yang2025_Silkie_Wenchang	0.910446833
ENSGALG00010027272	DDX20	2	-0.042579397	0.051712233	-0.823391192	0.41028557	0	0.433929011	0	1	PRJNA827465;Yang2025_Silkie_Wenchang	CRA004775	0.834737228
ENSGALG00010027272	DDX20	2	-0.054235611	0.092873653	-0.583971978	0.559239165	0.000583176	1.021249738	2.080758268	0.5	CRA004775;Yang2025_Silkie_Wenchang	PRJNA827465	0.881014886
ENSGALG00010027272	DDX20	2	-0.010093969	0.058034944	-0.173929153	0.861921141	0	0.525820112	0	0.5	CRA004775;PRJNA827465	Yang2025_Silkie_Wenchang	0.96567952
ENSGALG00010027273	HAUS8	2	0.049452959	0.067772312	0.729692662	0.465578067	0	0.021898245	0	1	PRJNA827465;Yang2025_Silkie_Wenchang	CRA004775	0.852424459
ENSGALG00010027273	HAUS8	2	0.015260276	0.099671741	0.153105338	0.878315206	0	0.6702973	0	0.5	CRA004775;Yang2025_Silkie_Wenchang	PRJNA827465	0.971037793
ENSGALG00010027273	HAUS8	2	0.039712398	0.087225502	0.455284255	0.648904752	0	0.772416748	0	0.5	CRA004775;PRJNA827465	Yang2025_Silkie_Wenchang	0.907783669
ENSGALG00010027274	COPG1	2	-0.130325641	0.116343229	-1.120182428	0.26263603	0.021338079	4.181674711	76.08613608	1	PRJNA827465;Yang2025_Silkie_Wenchang	CRA004775	0.768434743
ENSGALG00010027274	COPG1	2	-0.213989268	0.078710314	-2.718694129	0.006554018	0	0.562727271	0	1	CRA004775;Yang2025_Silkie_Wenchang	PRJNA827465	0.123728698
ENSGALG00010027274	COPG1	2	-0.046208855	0.03646241	-1.267301146	0.205047631	0	0.979749029	0	1	CRA004775;PRJNA827465	Yang2025_Silkie_Wenchang	0.717759892
ENSGALG00010027275	CPAMD8	2	-0.304866101	0.135840428	-2.244295795	0.024813376	0.002454522	1.070407101	6.577600355	1	PRJNA827465;Yang2025_Silkie_Wenchang	CRA004775	0.268809293
ENSGALG00010027275	CPAMD8	2	0.057769243	0.584596055	0.098819077	0.921281921	0.566725791	5.416743629	81.53872384	0.5	CRA004775;Yang2025_Silkie_Wenchang	PRJNA827465	0.981519273
ENSGALG00010027275	CPAMD8	2	0.167137242	0.441006348	0.378990559	0.704694875	0.285863843	3.298011827	69.67870182	0.5	CRA004775;PRJNA827465	Yang2025_Silkie_Wenchang	0.924152671
ENSGALG00010027276	ENSGALG00010027276	2	0.096212936	0.230402874	0.417585661	0.676250083	0.079809643	3.369444667	70.32151886	0.5	PRJNA827465;Yang2025_Silkie_Wenchang	CRA004775	0.91671756
ENSGALG00010027276	ENSGALG00010027276	2	0.251799773	0.189528163	1.32856125	0.183992764	0	0.770530386	0	1	CRA004775;Yang2025_Silkie_Wenchang	PRJNA827465	0.692470323
ENSGALG00010027276	ENSGALG00010027276	2	-0.073888104	0.067769653	-1.09028306	0.275588475	0	0.219222218	0	0.5	CRA004775;PRJNA827465	Yang2025_Silkie_Wenchang	0.775295118
ENSGALG00010027277	KCND3	2	0.008976749	0.790880416	0.011350324	0.990943946	1.129400848	10.14957721	90.14737285	0.5	PRJNA827465;Yang2025_Silkie_Wenchang	CRA004775	0.997555603
ENSGALG00010027277	KCND3	2	0.740134058	0.360812342	2.051299169	0.040237821	0	0.19870892	0	1	CRA004775;Yang2025_Silkie_Wenchang	PRJNA827465	0.347442284
ENSGALG00010027277	KCND3	2	-0.340560273	0.572189525	-0.595187885	0.551717888	0.421480676	2.37234796	57.8476675	0.5	CRA004775;PRJNA827465	Yang2025_Silkie_Wenchang	0.879061525
ENSGALG00010027278	H1-10	2	-0.239740108	0.146510041	-1.63633909	0.101768639	0.023341394	1.664746423	39.93079149	1	PRJNA827465;Yang2025_Silkie_Wenchang	CRA004775	0.543700483
ENSGALG00010027278	H1-10	2	-0.388886789	0.184127073	-2.112056546	0.0346816	0	0.38556651	0	1	CRA004775;Yang2025_Silkie_Wenchang	PRJNA827465	0.320651825
ENSGALG00010027278	H1-10	2	-0.164348107	0.068262221	-2.407599768	0.016057774	0	0.169197493	0	1	CRA004775;PRJNA827465	Yang2025_Silkie_Wenchang	0.209326266
ENSGALG00010027279	F2RL3	2	-0.221372124	0.139089975	-1.591574973	0.111480237	0.002058728	1.050044948	4.765981461	1	PRJNA827465;Yang2025_Silkie_Wenchang	CRA004775	0.564762672
ENSGALG00010027279	F2RL3	2	0.048642137	0.551403702	0.088215108	0.929705709	0.464658645	3.876675144	74.20469957	0.5	CRA004775;Yang2025_Silkie_Wenchang	PRJNA827465	0.983767889
ENSGALG00010027279	F2RL3	2	0.144821763	0.385926817	0.37525706	0.707469298	0.192872851	2.306925516	56.65226324	0.5	CRA004775;PRJNA827465	Yang2025_Silkie_Wenchang	0.92489559
ENSGALG00010027280	HMCES	2	0.162643521	0.066203867	2.456707255	0.01402169	0	0.341822953	0	1	PRJNA827465;Yang2025_Silkie_Wenchang	CRA004775	0.193842584
ENSGALG00010027280	HMCES	2	0.029794185	0.126819642	0.234933518	0.814260334	0	0.167612186	0	0.5	CRA004775;Yang2025_Silkie_Wenchang	PRJNA827465	0.953869515
ENSGALG00010027280	HMCES	2	0.153030334	0.069893382	2.189482473	0.028561791	0.000372558	1.016995082	1.671107611	0.5	CRA004775;PRJNA827465	Yang2025_Silkie_Wenchang	0.287842636
ENSGALG00010027281	ENSGALG00010027281	2	0.435812579	0.770576367	0.56556702	0.571688153	1.134666961	22.35585853	95.52689959	0.5	PRJNA827465;Yang2025_Silkie_Wenchang	CRA004775	0.885445282
ENSGALG00010027281	ENSGALG00010027281	2	0.708324754	0.500878424	1.414165036	0.157313451	0.440250215	8.143652836	87.72049816	1	CRA004775;Yang2025_Silkie_Wenchang	PRJNA827465	0.650687662
ENSGALG00010027281	ENSGALG00010027281	2	-0.07032982	0.269249884	-0.261206499	0.793933267	0.100115954	3.206593389	68.81425617	0.5	CRA004775;PRJNA827465	Yang2025_Silkie_Wenchang	0.948872932
ENSGALG00010027282	ENSGALG00010027282	2	0.08079246	0.070051802	1.153324503	0.248777217	0	0.801906552	0	0.5	PRJNA827465;Yang2025_Silkie_Wenchang	CRA004775	0.759196439
ENSGALG00010027282	ENSGALG00010027282	2	-0.221290103	0.236601999	-0.935284162	0.349641875	0	0.042628216	0	1	CRA004775;Yang2025_Silkie_Wenchang	PRJNA827465	0.809903518
ENSGALG00010027282	ENSGALG00010027282	2	0.082637627	0.070755666	1.16792946	0.24283522	0	0.956312784	0	0.5	CRA004775;PRJNA827465	Yang2025_Silkie_Wenchang	0.7550024
ENSGALG00010027283	RAB7A	2	-0.025047726	0.032800422	-0.763640354	0.445081594	0	0.537507314	0	1	PRJNA827465;Yang2025_Silkie_Wenchang	CRA004775	0.846013379
ENSGALG00010027283	RAB7A	2	-0.102277866	0.091954171	-1.112270012	0.266022074	0	0.085929693	0	1	CRA004775;Yang2025_Silkie_Wenchang	PRJNA827465	0.770081145
ENSGALG00010027283	RAB7A	2	-0.019087638	0.034535482	-0.552696444	0.580471288	0	0.345074571	0	1	CRA004775;PRJNA827465	Yang2025_Silkie_Wenchang	0.888433837
ENSGALG00010027284	CTTNBP2NL	2	0.031454653	0.122065157	0.257687403	0.796648169	0.023670985	4.569525294	78.11588873	0.5	PRJNA827465;Yang2025_Silkie_Wenchang	CRA004775	0.949584902
ENSGALG00010027284	CTTNBP2NL	2	0.178083177	0.096475793	1.845884566	0.064908968	0	0.048171527	0	1	CRA004775;Yang2025_Silkie_Wenchang	PRJNA827465	0.439424217
ENSGALG00010027284	CTTNBP2NL	2	-0.04335316	0.095252261	-0.455140479	0.649008178	0.007395816	1.178907149	15.17567768	0.5	CRA004775;PRJNA827465	Yang2025_Silkie_Wenchang	0.907783669
ENSGALG00010027285	PTPRS	2	-0.052033827	0.051542689	-1.009528751	0.31272112	0	0.300656943	0	0.5	PRJNA827465;Yang2025_Silkie_Wenchang	CRA004775	0.796054997
ENSGALG00010027285	PTPRS	2	0.028290622	0.112245977	0.252041302	0.801009139	0	0.199750051	0	1	CRA004775;Yang2025_Silkie_Wenchang	PRJNA827465	0.950158029
ENSGALG00010027285	PTPRS	2	-0.057737989	0.055962054	-1.031734631	0.302196449	0	0.509810052	0	0.5	CRA004775;PRJNA827465	Yang2025_Silkie_Wenchang	0.791647454
ENSGALG00010027286	RPN1	2	-0.076129008	0.080986007	-0.940026698	0.347203866	0.008159296	2.312705103	56.7605918	1	PRJNA827465;Yang2025_Silkie_Wenchang	CRA004775	0.808660951
ENSGALG00010027286	RPN1	2	-0.166876517	0.085570308	-1.950168482	0.051156041	0	0.116071233	0	1	CRA004775;Yang2025_Silkie_Wenchang	PRJNA827465	0.391850688

ENSGALG00010027286	RPN1	2	-0.024327793	0.042632978	-0.570633224	0.568248292	0	0.426406333	0	1	CRA004775;PRJNA827465	Yang2025_Silkie_Wenchang	0.884054254
ENSGALG00010027287	DHX30	2	-0.090342789	0.160666902	-0.562298692	0.57391253	0.04008422	3.837332075	73.94022773	0.5	PRJNA827465;Yang2025_Silkie_Wenchang	CRA004775	0.886081433
ENSGALG00010027287	DHX30	2	-0.239347048	0.1044129	-2.292313001	0.021887588	0	0.176636962	0	1	CRA004775;Yang2025_Silkie_Wenchang	PRJNA827465	0.25080113
ENSGALG00010027287	DHX30	2	-0.047503148	0.115174988	-0.412443265	0.680014557	0.018344432	2.740000495	63.50365623	0.5	CRA004775;PRJNA827465	Yang2025_Silkie_Wenchang	0.917701868
ENSGALG00010027288	DAG1	2	-0.004886485	0.112949028	-0.043262741	0.965492091	0.010515219	1.541192407	35.11517475	0.5	PRJNA827465;Yang2025_Silkie_Wenchang	CRA004775	0.990668014
ENSGALG00010027288	DAG1	2	0.171178708	0.154365916	1.108915181	0.267466774	0	0.006192706	0	1	CRA004775;Yang2025_Silkie_Wenchang	PRJNA827465	0.771398294
ENSGALG00010027288	DAG1	2	-0.060772019	0.08573621	-0.708825579	0.478432722	0	0.671267396	0	0.5	CRA004775;PRJNA827465	Yang2025_Silkie_Wenchang	0.855469318
ENSGALG00010027289	SMARCC1	2	0.036344854	0.087148132	0.417046855	0.676644136	0.006679908	1.606594646	37.75654597	0.5	PRJNA827465;Yang2025_Silkie_Wenchang	CRA004775	0.916786558
ENSGALG00010027289	SMARCC1	2	0.139094448	0.112432835	1.237133686	0.216037456	0	0.141622788	0	1	CRA004775;Yang2025_Silkie_Wenchang	PRJNA827465	0.729551847
ENSGALG00010027289	SMARCC1	2	-0.013912308	0.063000576	-0.220828269	0.825226151	0	0.18229446	0	0.5	CRA004775;PRJNA827465	Yang2025_Silkie_Wenchang	0.956356178
ENSGALG00010027290	CSPG5	2	0.278760343	0.296698715	0.939540112	0.347453508	0.127213884	3.402220916	70.60743483	1	PRJNA827465;Yang2025_Silkie_Wenchang	CRA004775	0.808705451
ENSGALG00010027290	CSPG5	2	0.663745598	0.259963936	2.553221841	0.010673149	0	0.123991802	0	1	CRA004775;Yang2025_Silkie_Wenchang	PRJNA827465	0.164714096
ENSGALG00010027290	CSPG5	2	0.2124039	0.353589009	0.600708435	0.5480342	0.134795839	1.591056566	37.14868337	1	CRA004775;PRJNA827465	Yang2025_Silkie_Wenchang	0.878281266
ENSGALG00010027291	MST1	2	0.043473954	0.151265544	0.287401562	0.773804865	0.018356491	1.669685627	40.10848607	0.5	PRJNA827465;Yang2025_Silkie_Wenchang	CRA004775	0.943248868
ENSGALG00010027291	MST1	2	-0.092679254	0.353267335	-0.262348778	0.793052559	0.179591738	3.207920152	68.82715427	0.5	CRA004775;Yang2025_Silkie_Wenchang	PRJNA827465	0.948571413
ENSGALG00010027291	MST1	2	-0.1907909	0.166044571	-1.149034255	0.250541856	0.006341859	1.077531571	7.195294646	1	CRA004775;PRJNA827465	Yang2025_Silkie_Wenchang	0.760096222
ENSGALG00010027292	KDM4B	2	-0.076731614	0.079143562	-0.969524399	0.332283613	0.008937638	3.347303213	70.12520419	1	PRJNA827465;Yang2025_Silkie_Wenchang	CRA004775	0.802178578
ENSGALG00010027292	KDM4B	2	-0.143857468	0.069301884	-2.075808905	0.037911628	0	0.797372503	0	0.5	CRA004775;Yang2025_Silkie_Wenchang	PRJNA827465	0.335670361
ENSGALG00010027292	KDM4B	2	-0.005833117	0.045059122	-0.129454734	0.89699784	0	0.020447487	0	0.5	CRA004775;PRJNA827465	Yang2025_Silkie_Wenchang	0.975287875
ENSGALG00010027293	ELP6	2	0.224048338	0.13895256	1.612408851	0.106873	0	0.013042505	0	1	PRJNA827465;Yang2025_Silkie_Wenchang	CRA004775	0.553927143
ENSGALG00010027293	ELP6	2	-0.160113335	0.368933219	-0.433990019	0.664295689	0.217892509	5.006736491	80.02690971	0.5	CRA004775;Yang2025_Silkie_Wenchang	PRJNA827465	0.913214881
ENSGALG00010027293	ELP6	2	-0.131352802	0.384909968	-0.341255911	0.732910935	0.2522764	6.650823587	84.96426816	0.5	CRA004775;PRJNA827465	Yang2025_Silkie_Wenchang	0.932985777
ENSGALG00010027295	ENSGALG00010027295	2	0.506281194	0.423690234	1.194932413	0.232113438	0.314014284	7.826185664	87.22238338	1	PRJNA827465;Yang2025_Silkie_Wenchang	CRA004775	0.74465386
ENSGALG00010027295	ENSGALG00010027295	2	0.396794658	0.620566374	0.639407281	0.522558014	0.629364325	5.190840888	80.73529855	0.5	CRA004775;Yang2025_Silkie_Wenchang	PRJNA827465	0.869568211
ENSGALG00010027295	ENSGALG00010027295	2	0.062907651	0.155979967	0.403305962	0.686723156	0	0.60951427	0	0.5	CRA004775;PRJNA827465	Yang2025_Silkie_Wenchang	0.929325539
ENSGALG00010027299	PGPEP1	2	-0.130872225	0.116675822	-1.121673904	0.262001116	0.012884315	1.707384681	41.43089071	1	PRJNA827465;Yang2025_Silkie_Wenchang	CRA004775	0.768363052
ENSGALG00010027299	PGPEP1	2	-0.279849946	0.142973892	-1.957349986	0.050306334	0	0.044455311	0	1	CRA004775;Yang2025_Silkie_Wenchang	PRJNA827465	0.388598636
ENSGALG00010027299	PGPEP1	2	-0.067791185	0.080908381	-0.837875927	0.40210039	0	0.446640478	0	1	CRA004775;PRJNA827465	Yang2025_Silkie_Wenchang	0.832879175
ENSGALG00010027301	SCAP	2	-0.0342586	0.046832807	-0.731508578	0.464468567	0	0.036044271	0	1	PRJNA827465;Yang2025_Silkie_Wenchang	CRA004775	0.853159649
ENSGALG00010027301	SCAP	2	-0.107425744	0.112733262	-0.952919686	0.340630765	0.014992382	2.150018531	53.48877298	1	CRA004775;Yang2025_Silkie_Wenchang	PRJNA827465	0.805524125
ENSGALG00010027301	SCAP	2	-0.112961246	0.102119027	-1.10617237	0.268651919	0.011031865	1.85227203	46.01224961	1	CRA004775;PRJNA827465	Yang2025_Silkie_Wenchang	0.771398294
ENSGALG00010027303	APEH	2	-0.00092252	0.061038579	-0.015113719	0.987941456	0	0.034318891	0	0.5	PRJNA827465;Yang2025_Silkie_Wenchang	CRA004775	0.996800355
ENSGALG00010027303	APEH	2	-0.163511687	0.211788079	-0.77205331	0.440082854	0.053919211	2.100709429	52.39703378	1	CRA004775;Yang2025_Silkie_Wenchang	PRJNA827465	0.844353254
ENSGALG00010027303	APEH	2	-0.148352097	0.223797158	-0.66288642	0.5074033	0.068720181	2.506799267	60.10849321	0.5	CRA004775;PRJNA827465	Yang2025_Silkie_Wenchang	0.864149944
ENSGALG00010027304	PTPN23	2	0.020457205	0.0627907	0.325799929	0.744575732	0.001806548	1.286341898	22.26017037	0.5	PRJNA827465;Yang2025_Silkie_Wenchang	CRA004775	0.935893304
ENSGALG00010027304	PTPN23	2	0.068561767	0.08275212	0.82752512	0.407793949	0	0.855862525	0	0.5	CRA004775;Yang2025_Silkie_Wenchang	PRJNA827465	0.843982683
ENSGALG00010027304	PTPN23	2	-0.041113605	0.067767396	-0.606687104	0.544058582	0	0.21153711	0	1	CRA004775;PRJNA827465	Yang2025_Silkie_Wenchang	0.876968972
ENSGALG00010027305	LSM4	2	0.050357004	0.06019692	0.836537885	0.402852373	0	0.554117802	0	0.5	PRJNA827465;Yang2025_Silkie_Wenchang	CRA004775	0.832937979
ENSGALG00010027305	LSM4	2	-0.279662701	0.268824932	-1.040315341	0.298193419	0.123980947	6.926209488	85.56208844	1	CRA004775;Yang2025_Silkie_Wenchang	PRJNA827465	0.789118382
ENSGALG00010027305	LSM4	2	-0.224328371	0.319348805	-0.702455647	0.482395052	0.188049268	12.34158315	91.89731181	0.5	CRA004775;PRJNA827465	Yang2025_Silkie_Wenchang	0.85691916
ENSGALG00010027306	ENSGALG00010027306	2	-0.483661847	0.252695246	-1.91401245	0.055618565	0.035547027	1.385591136	27.82863761	1	PRJNA827465;Yang2025_Silkie_Wenchang	CRA004775	0.408441043
ENSGALG00010027306	ENSGALG00010027306	2	-0.347742491	0.438633448	-0.792786077	0.42790247	0.252238203	2.835881214	64.73759214	0.5	CRA004775;Yang2025_Silkie_Wenchang	PRJNA827465	0.84125685
ENSGALG00010027306	ENSGALG00010027306	2	-0.108764364	0.24547062	-0.44308506	0.657704211	0	0.525021964	0	0.5	CRA004775;PRJNA827465	Yang2025_Silkie_Wenchang	0.910774485
ENSGALG00010027307	JUND	2	-0.086432806	0.226238954	-0.382042102	0.702430136	0.074085915	3.199161236	68.7418068	0.5	PRJNA827465;Yang2025_Silkie_Wenchang	CRA004775	0.923191989
ENSGALG00010027307	JUND	2	-0.125991895	0.381588067	-0.330177764	0.741265647	0.147596652	1.719438331	41.84147336	0.5	CRA004775;Yang2025_Silkie_Wenchang	PRJNA827465	0.93538952
ENSGALG00010027307	JUND	2	0.103622714	0.096428961	1.074601588	0.282553141	0	0.389820464	0	1	CRA004775;PRJNA827465	Yang2025_Silkie_Wenchang	0.779532565
ENSGALG00010027310	ARHGEF2	2	-0.008591324	0.059310125	-0.144852438	0.88482595	0	0.536058221	0	0.5	PRJNA827465;Yang2025_Silkie_Wenchang	CRA004775	0.972138605
ENSGALG00010027310	ARHGEF2	2	-0.097603489	0.201694914	-0.483916462	0.628445157	0.054193711	2.948107806	66.07993785	0.5	CRA004775;Yang2025_Silkie_Wenchang	PRJNA827465	0.901912657
ENSGALG00010027310	ARHGEF2	2	-0.119967977	0.13474702	-0.890320079	0.373294043	0.022305411	2.133133657	53.12061218	1	CRA004775;PRJNA827465	Yang2025_Silkie_Wenchang	0.822187352
ENSGALG00010027311	NFASC	2	0.321890033	0.431895777	0.745295626	0.456093025	0.309541043	5.492028647	81.79179199	0.5	PRJNA827465;Yang2025_Silkie_Wenchang	CRA004775	0.849162679
ENSGALG00010027311	NFASC	2	0.427260781	0.448406487	0.95284255	0.340669852	0.232573634	2.299974808	56.35045847	0.5	CRA004775;Yang2025_Silkie_Wenchang	PRJNA827465	0.805526077
ENSGALG00010027311	NFASC	2	-0.064628632	0.147254456	-0.438890842	0.660740633	0	0.005501796	0	1	CRA004775;PRJNA827465	Yang2025_Silkie_Wenchang	0.91192683
ENSGALG00010027313	BSN	2	0.007862662	0.355757965	0.022101154	0.982362766	0.21240569	6.054471792	83.48328253	0.5	PRJNA827465;Yang2025_Silkie_Wenchang	CRA004775	0.99513702
ENSGALG00010027313	BSN	2	-0.152298417	0.564681046	-0.269706975	0.787385694	0.552779492	7.428466095	86.53826133	0.5	CRA004775;Yang2025_Silkie_Wenchang	PRJNA827465	0.934691916
ENSGALG00010027313	BSN	2	-0.426318532	0.179833211	-2.370632927	0.017757657	0.019127393	1.282149052	22.00594788	1	CRA004775;PRJNA827465	Yang2025_Silkie_Wenchang	0.22222937
ENSGALG00010027314	ENSGALG00010027314	2	0.472734484	0.377370514	1.252706469	0.210312551	0.233697885	5.442323682	81.62549568	1	PRJNA827465;Yang2025_Silkie_Wenchang	CRA004775	0.721900195
ENSGALG00010027314	ENSGALG00010027314	2	0.554377615	0.42516292	1.303918072	0.192261518	0.212484458	2.204509421	54.63843382	0.5	CRA004775;Yang2025_Silkie_Wenchang	PRJNA827465	0.702082087
ENSGALG00010027314	ENSGALG00010027314	2	0.108807899	0.169371404	0.642427901	0.520599283	0	0.050324091	0	0.5	CRA004775;PRJNA827465	Yang2025_Silkie_Wenchang	0.869931539
ENSGALG00010027315	RAB3A	2	0.225176326	0.493677236	0.456120537	0.648303303	0.463317288	20.18895625	95.04679693	0.5	PRJNA827465;Yang2025_Silkie_Wenchang	CRA004775	0.90762535
ENSGALG00010027315	RAB3A	2	0.558482068	0.239843046	2.328531419	0.019883903	0.056629238	1.747177373	42.76482653	1	CRA004775;Yang2025_Silkie_Wenchang	PRJNA827465	0.236817284
ENSGALG00010027315	RAB3A	2	-0.13764885	0.209619531	-0.656660423	0.511399288	0.039643501	1.549796375	35.47539429	0.5	CRA004775;PRJNA827465	Yang2025_Silkie_Wenchang	0.865687234
ENSGALG00010027316	TRAIP	2	0.018412168	0.113248991	0.162581296	0.870848115	0	0.019161757	0	0.5	PRJNA827465;Yang2025_Silkie_Wenchang	CRA004775	0.969164319
ENSGALG00010027316	TRAIP	2	0.060350249	0.260158332	0.231975078	0.816557371	0	0.435492313	0	0.5	CRA004775;Yang2025_Silkie_Wenchang	PRJNA827465	0.954613592
ENSGALG00010027316	TRAIP	2	0.040507498	0.121017329	0.334724773	0.737832713	0	0.414993396	0	1	CRA004775;PRJNA827465	Yang2025_Silkie_Wenchang	0.934161348
ENSGALG00010027317	MPV17L2	2	-0.044077127	0.093907648	-0.469366745	0.638807514	0	0.819660713	0	0.5	PRJNA827465;Yang2025_Silkie_Wenchang	CRA004775	0.905768875
ENSGALG00010027317	MPV17L2	2	-0.17188737	0.155450303	-1.105738402	0.268839763	0	0.038328276	0	1	CRA004775;Yang2025_Silkie_Wenchang	PRJNA827465	0.771398294
ENSGALG00010027317	MPV17L2	2	-0.013703801	0.100456288	-0.13641556	0.891492773	0	0.205759676	0	0.5	CRA004775;PRJNA827465	Yang2025_Silkie_Wenchang	0.973846899
ENSGALG00010027319	MST1R	2	0.048499429	0.333512906	0.145419948	0.884379316	0.115809119	2.079426077	51.90980766	0.5	PRJNA827465;Yang2025_Silkie_Wenchang	CRA004775	0.972028308
ENSGALG00010027319	MST1R	2	-0.050834407	0.539802832	-0.094172175	0.924972388	0.375725505	2.662417046	62.44014432	0.5	CRA004775;Yang2025_Silkie_Wenchang	PRJNA827465	0.982572225
ENSGALG00010027319	MST1R	2	-0.363240178	0.272282584	-1.334055864	0.182185549	0	0.429889669	0	1	CRA004775;PRJNA827465	Yang2025_Silkie_Wenchang	0.690033956
ENSGALG00010027320	MON1A	2	0.06661175	0.071347596	0.933622911	0.350498443	0	0.575523945	0	1	PRJNA827465;Yang2025_Silkie_Wenchang	CRA004775	0.810111037
ENSGALG00010027320	MON1A	2	-0.022336837	0.249208014	-0.089631293	0.928580216	0.075261428	2.26110408	55.77381825	0.5	CRA004775;Yang2025_Silkie_Wenchang	PRJNA827465	0.98332179
ENSGALG00010027320	MON1A	2	-0.043187302	0.157794821	-0.273692773	0.784320732	0.024604315	1.471391677	32.03713085	0.5	CRA004775;PRJNA827465	Yang2025_Silkie_Wenchang	0.946083234
ENSGALG00010027321	IFI30	2	0.490244201	0.178307598	2.749429679	0.005969907	0.037203132	2.133702949	53.13312002	1	PRJNA827465;Yang2025_Silkie_Wenchang	CRA004775	0.116888359
ENSGALG00010027321	IFI30	2	0.182700112	0.592545149	0.308331125	0.757830385	0.60107073	6.735440035	85.15316008	0.5	CRA004775;Yang2025_Silkie_Wenchang	PRJNA827465	0.938954369
ENSGALG00010027321	IFI30	2	0.041306037	0.397250374	0.103979856	0.917185335	0.247679616	3.963437025	74.76937331	0.5	CRA004775;PRJNA827465	Yang2025_Silkie_Wenchang	0.9807682
ENSGALG00010027322	CRABP2	2	0.140468067	0.106366951	1.320598786	0.186635178	0	0.934751731	0	1	PRJNA827465;Yang2025_Silkie_Wenchang	CRA004775	0.695457578
ENSGALG00010027322	CRABP2	2	0.344672545	0.19410688	1.775684329	0.075784962	0	0.490299382	0	1	CRA004775;Yang2025_Silkie_Wenchang	PRJNA827465	0.47520363
ENSGALG00010027322	CRABP2	2	0.121801158	0.178150864	0.683696703	0.494166706	0.018105599	1.069171566	6.469641403	1	CRA004775;PRJNA827465	Yang2025_Silkie_Wenchang	0.860175061
ENSGALG00010027323	ISG20L2	2	0.027437861	0.069782351	0.393191987	0.694177688	0	0.088186074	0	1	PRJNA827465;Yang2025_Silkie_Wenchang	CRA004775	0.920728119
ENSGALG00010027323	ISG20L2	2	-0.126744332	0.198321733	-0.639084433	0.522768006	0.053211223	3.089527287	67.63258883	0.5	CRA004775;Yang2025_Silkie_Wenchang	PRJNA827465	0.869568211
ENSGALG00010027323	ISG20L2	2	-0.125688913	0.169843029	-0.74002986	0.459281876	0.043151705	3.647905655	72.58701034	0.5	CRA004775;PRJNA827465	Yang2025_Silkie_Wenchang	0.850098539
ENSGALG00010027324	PIK3R2	2	-0.092850922	0.07234492	-1.283447711	0.199335268	0.006933111	2.929629357	65.86598924	1	PRJNA827465;Yang2025_Silkie_Wenchang	CRA004775	0.711181159
ENSGALG00010027324	PIK3R2	2	-0.167481693	0.06353866	-2.635902185	0.008391392	0	0.032457209	0	1	CRA004775;Yang2025_Silkie_Wenchang	PRJNA827465	0.142771619
ENSGALG00010027324	PIK3R2	2	-0.030968072	0.051614064	-0.599992888	0.548510975	0	0.191550228	0	1	CRA004775;PRJNA827465	Yang2025_Silkie_Wenchang	0.878281266
ENSGALG00010027325	METTL25B	2	0.164008125	0.137315591	1.194388227	0.23232614	0	0.790453742	0	1	PRJNA827465;Yang2025_Silkie_Wenchang	CRA004775	0.745032171
ENSGALG00010027325	METTL25B	2	-0.148981533	0.518346136	-0.28741708	0.773792985	0.465442743	7.471377041	86.61558646	0.5	CRA004775;Yang2025_Silkie_Wenchang	PRJNA827465	0.943248868
ENSGALG00010027325	METTL25B	2	-0.257336658	0.377993517	-0.680796485	0.496000274	0.239167802	5.939492522	83.16354476	0.5	CRA004775;PRJNA827465	Yang2025_Silkie_Wenchang	0.860911322
ENSGALG00010027326	MRPL24	2	-0.04818918	0.064751354	-0.744218879	0.456744068	0	0.520083313	0	0.5	PRJNA827465;Yang2025_Silkie_Wenchang	CRA004775	0.849515887
ENSGALG00010027326	MRPL24	2	-0.002526074	0.199263817	-0.012677034	0.989885461	0	0.784775263	0	0.5	CRA004775;Yang2025_Silkie_Wenchang	PRJNA827465	0.997311131
ENSGALG00010027326	MRPL24	2	-0.068072376	0.065961595	-1.032000154	0.302072045	0	0.300169611	0	1	CRA004775;PRJNA827465	Yang2025_Silkie_Wenchang	0.791603969
ENSGALG00010027327	RBM6	2	0.183478275	0.241260123	0.760499798	0.44695589	0.103321297	8.324523779	87.98730082	0.5	PRJNA827465;Yang2025_Silkie_Wenchang	CRA004775	0.84650962
ENSGALG00010027327	RBM6	2	0.231091192	0.235179465	0.982616367	0.325796286	0.077472304	3.288965145	69.59529956	0.5	CRA004775;Yang2025_Silkie_Wenchang	PRJNA827465	0.800023788
ENSGALG00010027327	RBM6	2	-0.035418611	0.054487545	-0.650031333	0.515671983	0	0.00335834	0	1	CRA004775;PRJNA827465	Yang2025_Silkie_Wenchang	0.867316669
ENSGALG00010027328	RBM15	2	0.012177784	0.196389574	0.062008303	0.95055622	0.065846051	6.555862889	84.74647782	0.5	PRJNA827465;Yang2025_Silkie_Wenchang	CRA004775	0.987277827
ENSGALG00010027328	RBM15	2	-0.154017496	0.119503433	-1.28881232	0.197463342	0	0.546672519	0	0.5	CRA004775;Yang2025_Silkie_Wenchang	PRJNA827465	0.708952382
ENSGALG00010027328	RBM15	2	0.178481227	0.069658095	2.562229829	0.010400247	0	0.490497532	0	1	CRA004775;PRJNA827465	Yang2025_Silkie_Wenchang	0.162400043
ENSGALG00010027329	MINDY4	2	0.06831802	0.078100374	0.874746395	0.38171191	0	0.544172286	0	0.5	PRJNA827465;Yang2025_Silkie_Wenchang	CRA004775	0.824699008
ENSGALG00010027329	MINDY4	2	-0.060459347	0.141812108	-0.426334168	0.669864364	0	0.219707427	0	1	CRA004775;Yang2025_Silkie_Wenchang	PRJNA827465	0.914377769
ENSGALG00010027329	MINDY4	2	0.082129969	0.087441305	0.939258266	0.34759816	0	0.750305013	0	0.5	CRA004775;PRJNA827465	Yang2025_Silkie_Wenchang	0.808764533
ENSGALG00010027330	ENSGALG00010027330	2	0.10720209	0.095866736	1.118260743	0.263464188	0	0.240596466	0	1	PRJNA827465;Yang2025_Silkie_Wenchang	CRA004775	0.768643821
ENSGALG00010027330	ENSGALG00010027330	2	0.16871337	0.111211912	1.517044062	0.12925556	0	0.85442052	0	1	CRA004775;Yang2025_Silkie_Wenchang	PRJNA827465	0.603386934
ENSGALG00010027330	ENSGALG00010027330	2	0.1462686	0.202669801	0.721708905	0.470473466	0.023601187	1.248543421	19.90667017	1	CRA004775;PRJNA827465	Yang2025_Silkie_Wenchang	0.853744788
ENSGALG00010027331	SLC16A4	2	0.098738678	0.093807797	1.052563657	0.292541021	0	0.025363146	0	1	PRJNA827465;Yang2025_Silkie_Wenchang	CRA004775	0.786116487
ENSGALG00010027331	SLC16A4	2	0.100509553	0.134725179	0.746033916	0.455649557	0	0.053054036	0	1	CRA004775;Yang2025_Silkie_Wenchang	PRJNA827465	0.84907427
ENSGALG00010027331	SLC16A4	2	0.080980162	0.107579732	0.752745521	0.45160285	0	0.018989228	0	1	CRA004775;PRJNA827465	Yang2025_Silkie_Wenchang	0.848006549
ENSGALG00010027332	HDGF	2	-0.079359744	0.043423885	-1.827559747	0.067615652	0	0.62599501	0	1	PRJNA827465;Yang2025_Silkie_Wenchang	CRA004775	0.448330927
ENSGALG00010027332	HDGF	2	-0.092140137	0.092377418	-0.997431401	0.318555156	0.00334741	1.138089277	12.13343102	0.5	CRA004775;Yang2025_Silkie_Wenchang	PRJNA827465	0.797754958
ENSGALG00010027332	HDGF	2	-0.04976832	0.049044515	-1.014758121	0.310221141	0	0.56483982	0	0.5	CRA004775;PRJNA827465	Yang2025_Silkie_Wenchang	0.794813537
ENSGALG00010027333	PROK1	2	0.552324114	0.291414931	1.895318516	0.058050217	0	0.472651752	0	1	PRJNA827465;Yang2025_Silkie_Wenchang	CRA004775	0.417532916
ENSGALG00010027333	PROK1	2	-0.141340974	0.386514794	-0.365680631	0.714603401	0	0.619026471	0	0.5	CRA004775;Yang2025_Silkie_Wenchang	PRJNA827465	0.926596201
ENSGALG00010027333	PROK1	2	0.178413616	0.533712385	0.334892565	0.708030036	0.39053842	3.064815577	67.36805313	0.5	CRA004775;PRJNA827465	Yang2025_Silkie_Wenchang	0.927061251
ENSGALG00010027334	PRCC	2	-0.198378413	0.178011154	-1.114415632	0.26510092	0.059432827	15.93964808	93.72633577	1	PRJNA827465;Yang2025_Silkie_Wenchang	CRA004775	0.769274038
ENSGALG00010027334	PRCC	2	-0.291139834	0.119853951	-2.429121715	0.01513545	0.016903254	2.17793281	54.08490128	1	CRA004775;Yang2025_Silkie_Wenchang	PRJNA827465	0.203234917
ENSGALG00010027334	PRCC	2	-0.034766343	0.047394967	-0.733545039	0.463226069	0	0.437801697	0	1	CRA004775;PRJNA827465	Yang2025_Silkie_Wenchang	0.851591476
ENSGALG00010027335	MAST3	2	0.257670646	0.204114306	1.262384057	0.206810625	0.065435213	4.170315758	76.02100037	1	PRJNA827465;Yang2025_Silkie_Wenchang	CRA004775	0.671868063
ENSGALG00010027335	MAST3	2	-0.209762686	0.735003458	-0.285390067	0.775345317	1.011104383	15.35175709	93.48608766	0.5	CRA004775;Yang2025_Silkie_Wenchang	PRJNA827465	0.943815265
ENSGALG00010027335	MAST3	2	-0.393448095	0.525808864	-0.748272086	0.454296058	0.502334624	10.12229909	90.12082145	0.5	CRA004775;PRJNA827465	Yang2025_Silkie_Wenchang	0.849072665
ENSGALG00010027336	RAD54L2	2	-0.101545431	0.069963318	-1.451409607	0.146665836	0.005439605	2.199191794	54.52874994	1	PRJNA827465;Yang2025_Silkie_Wenchang	CRA004775	0.635558525
ENSGALG00010027336	RAD54L2	2	-0.119255782	0.164918431	-0.723129792	0.469606291	0.025213389	1.256083583	20.38746356	0.5	CRA004775;Yang2025_Silkie_Wenchang	PRJNA827465	0.854585608
ENSGALG00010027336	RAD54L2	2	-0.034742371	0.055877228	-0.621762611	0.534097975	0	0.652948842	0	0.5	CRA004775;PRJNA827465	Yang2025_Silkie_Wenchang	0.873408008
ENSGALG00010027337	AQP1	2	0.080288167	0.077976375	1.02964733	0.303157588	0	0.55959248	0	1	PRJNA827465;Yang2025_Silkie_Wenchang	CRA004775	0.792084776
ENSGALG00010027337	AQP1	2	0.027495725	0.124726388	0.220448342	0.825521979	0	0.421112895	0	0.5	CRA004775;Yang2025_Silkie_Wenchang	PRJNA827465	0.956386623
ENSGALG00010027337	AQP1	2	0.131871075	0.094431031	1.396480302	0.162569908	0	0.139029834	0	1	CRA004775;PRJNA827465	Yang2025_Silkie_Wenchang	0.660343645
ENSGALG00010027339	LAMTOR5	2	0.035983443	0.064495486	0.557921879	0.576897731	0	0.053934144	0	0.5	PRJNA827465;Yang2025_Silkie_Wenchang	CRA004775	0.887113945
ENSGALG00010027339	LAMTOR5	2	-0.040516652	0.151012192	-0.268300538	0.788467992	0	0.142485134	0	1	CRA004775;Yang2025_Silkie_Wenchang	PRJNA827465	0.947048735
ENSGALG00010027339	LAMTOR5	2	0.031714373	0.06698186	0.473477047	0.635872873	0	0.354882024	0	0.5	CRA004775;PRJNA827465	Yang2025_Silkie_Wenchang	0.904387873
ENSGALG00010027346	SIN3B	2	-0.223485817	0.177814556	-1.256847706	0.208808787	0.060068239	19.91027837	94.97746851	1	PRJNA827465;Yang2025_Silkie_Wenchang	CRA004775	0.721011841
ENSGALG00010027346	SIN3B	2	-0.200864122	0.240165913	-0.836355666	0.402954846	0.094781438	4.955643017	79.82098395	0.5	CRA004775;Yang2025_Silkie_Wenchang	PRJNA827465	0.833046091
ENSGALG00010027346	SIN3B	2	-0.040839694	0.048034863	-0.850209447	0.39520865	0	0.373330894	0	0.5	CRA004775;PRJNA827465	Yang2025_Silkie_Wenchang	0.83072444
ENSGALG00010027348	ENSGALG00010027348	2	0.145113242	0.099246489	1.462149886	0.143700141	0	0.114698567	0	1	PRJNA827465;Yang2025_Silkie_Wenchang	CRA004775	0.630564924
ENSGALG00010027348	ENSGALG00010027348	2	0.030682838	0.172768006	0.177595602	0.859040579	0	0.060796478	0	0.5	CRA004775;Yang2025_Silkie_Wenchang	PRJNA827465	0.964605083
ENSGALG00010027348	ENSGALG00010027348	2	0.132554692	0.099977941	1.325839393	0.184892901	0	0.408529495	0	0.5	CRA004775;PRJNA827465	Yang2025_Silkie_Wenchang	0.693265167
ENSGALG00010027350	INSRR	2	0.335002468	0.227925605	1.469788654	0.141619003	0.040164348	1.62365639	38.4106141	1	PRJNA827465;Yang2025_Silkie_Wenchang	CRA004775	0.627088401
ENSGALG00010027350	INSRR	2	0.142459116	0.465781971	0.305849357	0.759719347	0.337316879	4.439868397	77.47680988	0.5	CRA004775;Yang2025_Silkie_Wenchang	PRJNA827465	0.939163539
ENSGALG00010027350	INSRR	2	-0.042588223	0.227157676	-0.187483088	0.851281882	0.023490536	1.261825082	20.74971292	0.5	CRA004775;PRJNA827465	Yang2025_Silkie_Wenchang	0.962071965
ENSGALG00010027351	TMEM38A	2	-0.3191377	0.088203367	-3.618203156	0.000296655	0	0.465097133	0	1	PRJNA827465;Yang2025_Silkie_Wenchang	CRA004775	0.016889382
ENSGALG00010027351	TMEM38A	2	-0.141229711	0.35766354	-0.394867508	0.69294067	0.169456718	2.31051376	56.71958258	0.5	CRA004775;Yang2025_Silkie_Wenchang	PRJNA827465	0.92062328
ENSGALG00010027351	TMEM38A	2	-0.108825001	0.275886598	-0.394455553	0.693244736	0.084154617	1.653332597	39.51610208	0.5	CRA004775;PRJNA827465	Yang2025_Silkie_Wenchang	0.920721644
ENSGALG00010027353	ENSGALG00010027353	2	0.167249591	0.291205607	0.574335066	0.565741084	0	0.221274137	0	1	PRJNA827465;Yang2025_Silkie_Wenchang	CRA004775	0.883487517
ENSGALG00010027353	ENSGALG00010027353	2	-0.015653819	0.310403617	-0.050430531	0.959779307	0	0.002799132	0	0.5	CRA004775;Yang2025_Silkie_Wenchang	PRJNA827465	0.989302378
ENSGALG00010027353	ENSGALG00010027353	2	0.142500737	0.282133242	0.505083115	0.61350046	0	0.301328463	0	0.5	CRA004775;PRJNA827465	Yang2025_Silkie_Wenchang	0.898256044
ENSGALG00010027355	ADCYAP1R1	2	0.061805307	0.107881252	0.572901278	0.566711539	0	0.083914761	0	1	PRJNA827465;Yang2025_Silkie_Wenchang	CRA004775	0.883487517
ENSGALG00010027355	ADCYAP1R1	2	0.068084347	0.217724325	0.312708957	0.75450179	0	0.984165642	0	0.5	CRA004775;Yang2025_Silkie_Wenchang	PRJNA827465	0.93838723
ENSGALG00010027355	ADCYAP1R1	2	0.029911849	0.122160202	0.24485756	0.806566724	0	0.871993315	0	0.5	CRA004775;PRJNA827465	Yang2025_Silkie_Wenchang	0.952270941
ENSGALG00010027356	ENSGALG00010027356	2	0.40881311	0.475709276	0.859375947	0.390133136	0.439304338	34.03352715	97.06172094	0.5	PRJNA827465;Yang2025_Silkie_Wenchang	CRA004775	0.829204867
ENSGALG00010027356	ENSGALG00010027356	2	0.339809755	0.578403004	0.587496526	0.556870283	0.617762097	12.4451171	91.96472004	0.5	CRA004775;Yang2025_Silkie_Wenchang	PRJNA827465	0.880511839
ENSGALG00010027356	ENSGALG00010027356	2	-0.095976155	0.112971312	-0.849562182	0.395568545	0	0.389233083	0	1	CRA004775;PRJNA827465	Yang2025_Silkie_Wenchang	0.831031125
ENSGALG00010027358	RNF126	2	0.031981366	0.042945209	0.744701602	0.456452131	0	0.91563128	0	1	PRJNA827465;Yang2025_Silkie_Wenchang	CRA004775	0.849515887
ENSGALG00010027358	RNF126	2	0.023396277	0.145895892	0.160362823	0.872595273	0.02031302	1.76152621	43.23104621	0.5	CRA004775;Yang2025_Silkie_Wenchang	PRJNA827465	0.969629321
ENSGALG00010027358	RNF126	2	0.005083147	0.045405087	0.111951039	0.910862227	0	0.848513353	0	0.5	CRA004775;PRJNA827465	Yang2025_Silkie_Wenchang	0.97880407
ENSGALG00010027359	ENSGALG00010027359	2	-0.047014066	0.067829628	-0.693119918	0.488234299	0	0.849749086	0	0.5	PRJNA827465;Yang2025_Silkie_Wenchang	CRA004775	0.85768885
ENSGALG00010027359	ENSGALG00010027359	2	0.069759243	0.250615232	0.278351967	0.780742188	0	0.70273181	0	0.5	CRA004775;Yang2025_Silkie_Wenchang	PRJNA827465	0.945235709
ENSGALG00010027359	ENSGALG00010027359	2	-0.063573695	0.068165029	-0.932643847	0.351003887	0	0.081659982	0	1	CRA004775;PRJNA827465	Yang2025_Silkie_Wenchang	0.810146221
ENSGALG00010027360	SMIM7	2	-0.125308595	0.074034698	-1.692565755	0.090538154	0	0.010321907	0	1	PRJNA827465;Yang2025_Silkie_Wenchang	CRA004775	0.516311821
ENSGALG00010027360	SMIM7	2	-0.0787865	0.149946927	-0.525429245	0.599284828	0	0.416493731	0	0.5	CRA004775;Yang2025_Silkie_Wenchang	PRJNA827465	0.894908299
ENSGALG00010027360	SMIM7	2	-0.106446519	0.077969843	-1.365226799	0.172181778	0	0.447440261	0	0.5	CRA004775;PRJNA827465	Yang2025_Silkie_Wenchang	0.674903801
ENSGALG00010027361	FANCD2	2	-0.035568054	0.062004499	-0.573636672	0.56621369	0	0.346393012	0	1	PRJNA827465;Yang2025_Silkie_Wenchang	CRA004775	0.883487517
ENSGALG00010027361	FANCD2	2	-0.047346875	0.225098961	-0.210338044	0.833403845	0	0.766223398	0	0.5	CRA004775;Yang2025_Silkie_Wenchang	PRJNA827465	0.957968425
ENSGALG00010027361	FANCD2	2	-0.020602314	0.06262289	-0.328990145	0.742163137	0	0.398546151	0	0.5	CRA004775;PRJNA827465	Yang2025_Silkie_Wenchang	0.935481151
ENSGALG00010027362	ENSGALG00010027362	2	-0.332537687	0.314386027	-1.057736851	0.29017543	0.167145618	5.77415939	82.6814618	1	PRJNA827465;Yang2025_Silkie_Wenchang	CRA004775	0.784195688
ENSGALG00010027362	ENSGALG00010027362	2	-0.351775481	0.334684759	-1.051065135	0.293228676	0.166637783	3.891093777	74.30028529	1	CRA004775;Yang2025_Silkie_Wenchang	PRJNA827465	0.786834618
ENSGALG00010027362	ENSGALG00010027362	2	-0.058907229	0.066936587	-0.880045309	0.378834765	0	0.020840011	0	1	CRA004775;PRJNA827465	Yang2025_Silkie_Wenchang	0.823876439
ENSGALG00010027363	VIPR1	2	-0.008247452	0.136248397	-0.060532467	0.951731558	0	0.137822439	0	0.5	PRJNA827465;Yang2025_Silkie_Wenchang	CRA004775	0.987713746
ENSGALG00010027363	VIPR1	2	0.213332099	0.214610532	0.99404301	0.320201929	0.037155476	1.618871398	38.22857076	1	CRA004775;Yang2025_Silkie_Wenchang	PRJNA827465	0.798348216
ENSGALG00010027363	VIPR1	2	0.170887539	0.267724223	0.638296891	0.523280435	0.087566331	2.475303456	59.60091286	0.5	CRA004775;PRJNA827465	Yang2025_Silkie_Wenchang	0.869661505
ENSGALG00010027364	FSTL3	2	-0.137054186	0.071412326	-1.91919511	0.054959646	0	0.558446996	0	1	PRJNA827465;Yang2025_Silkie_Wenchang	CRA004775	0.405768983
ENSGALG00010027364	FSTL3	2	-0.187826145	0.119268452	-1.57481833	0.115298384	0	0.592740133	0	0.5	CRA004775;Yang2025_Silkie_Wenchang	PRJNA827465	0.572240484
ENSGALG00010027364	FSTL3	2	-0.090369756	0.086033071	-1.050407188	0.293530942	0	0.294944373	0	0.5	CRA004775;PRJNA827465	Yang2025_Silkie_Wenchang	0.786979185
ENSGALG00010027365	PLEKHA6	2	0.353670007	0.174374642	2.02821926	0.042537875	0.035522107	2.113661883	52.68874326	1	PRJNA827465;Yang2025_Silkie_Wenchang	CRA004775	0.356625761
ENSGALG00010027365	PLEKHA6	2	0.509444515	0.211062128	2.413718271	0.015790667	0	0.860735973	0	1	CRA004775;Yang2025_Silkie_Wenchang	PRJNA827465	0.20791309
ENSGALG00010027365	PLEKHA6	2	0.221303227	0.101940002	2.170916451	0.029937489	0	0.096849517	0	1	CRA004775;PRJNA827465	Yang2025_Silkie_Wenchang	0.295167651
ENSGALG00010027366	USP4	2	-0.060273847	0.042110734	-1.431317885	0.15233913	0	0.185077747	0	1	PRJNA827465;Yang2025_Silkie_Wenchang	CRA004775	0.643874056
ENSGALG00010027366	USP4	2	-0.127279283	0.086934833	-1.464076929	0.14317293	0	0.353316042	0	1	CRA004775;Yang2025_Silkie_Wenchang	PRJNA827465	0.630019027
ENSGALG00010027366	USP4	2	-0.062325674	0.045041981	-1.383724073	0.16644296	0	0.826957471	0	1	CRA004775;PRJNA827465	Yang2025_Silkie_Wenchang	0.665350942
ENSGALG00010027367	SEC22C	2	-0.234401156	0.278765092	-0.840855484	0.4004289	0.141283939	10.58246818	90.55040863	0.5	PRJNA827465;Yang2025_Silkie_Wenchang	CRA004775	0.832251175
ENSGALG00010027367	SEC22C	2	-0.279950097	0.258861666	-1.081466025	0.279489866	0.105625167	4.704237429	78.74256954	1	CRA004775;Yang2025_Silkie_Wenchang	PRJNA827465	0.777574163
ENSGALG00010027367	SEC22C	2	0.022054186	0.065858323	0.334873182	0.737720753	0	0.044032943	0	0.5	CRA004775;PRJNA827465	Yang2025_Silkie_Wenchang	0.93410563
ENSGALG00010027368	EMC3	2	0.061757002	0.054973545	1.123394928	0.261269803	0	0.07960725	0	1	PRJNA827465;Yang2025_Silkie_Wenchang	CRA004775	0.768039422
ENSGALG00010027368	EMC3	2	-0.06449516	0.140961625	-0.457537009	0.647285108	0	0.262183298	0	0.5	CRA004775;Yang2025_Silkie_Wenchang	PRJNA827465	0.907350807
ENSGALG00010027368	EMC3	2	0.05077776	0.054973164	0.923682691	0.355651546	0	0.956981892	0	0.5	CRA004775;PRJNA827465	Yang2025_Silkie_Wenchang	0.812301302
ENSGALG00010027369	ENSGALG00010027369	2	-0.090546205	0.11030859	-0.820844552	0.411734818	0.017973115	3.64079871	72.53349938	0.5	PRJNA827465;Yang2025_Silkie_Wenchang	CRA004775	0.835183193
ENSGALG00010027369	ENSGALG00010027369	2	0.238334806	0.478037808	0.498568946	0.618083087	0.425492652	14.02944075	92.87213213	0.5	CRA004775;Yang2025_Silkie_Wenchang	PRJNA827465	0.899121942
ENSGALG00010027369	ENSGALG00010027369	2	0.337997958	0.36561952	0.924452715	0.355250659	0.240489006	9.195854754	89.12553507	1	CRA004775;PRJNA827465	Yang2025_Silkie_Wenchang	0.812301302
ENSGALG00010027370	NUP210L	2	0.726283281	0.226723851	3.203382792	0.001358233	0	0.104415118	0	1	PRJNA827465;Yang2025_Silkie_Wenchang	CRA004775	0.045124799
ENSGALG00010027370	NUP210L	2	0.368568996	0.456512263	0.807358368	0.419460045	0.287100151	3.201349087	68.76316912	0.5	CRA004775;Yang2025_Silkie_Wenchang	PRJNA827465	0.838527387
ENSGALG00010027370	NUP210L	2	0.310996893	0.381741487	0.814679315	0.415255897	0.172953515	2.430154424	58.85035165	0.5	CRA004775;PRJNA827465	Yang2025_Silkie_Wenchang	0.837489728

ENSGALG00010027372	PIK3C2B	2	0.054606424	0.174558385	0.31282613	0.754412763	0.055263761	10.72895722	90.67942971	0.5	PRJNA827465;Yang2025_Silkie_Wenchang	CRA004775	0.93838723
ENSGALG00010027372	PIK3C2B	2	0.151608694	0.179290532	0.845603461	0.397773994	0.032712875	1.359740591	26.45656041	0.5	CRA004775;Yang2025_Silkie_Wenchang	PRJNA827465	0.831711071
ENSGALG00010027372	PIK3C2B	2	-0.126091122	0.076764796	-1.642564416	0.100473088	0	0.120153635	0	1	CRA004775;PRJNA827465	Yang2025_Silkie_Wenchang	0.54128455
ENSGALG00010027374	NKTR	2	0.205496546	0.185066726	1.110391642	0.266830298	0.052575458	4.012836266	75.07977004	1	PRJNA827465;Yang2025_Silkie_Wenchang	CRA004775	0.771006559
ENSGALG00010027374	NKTR	2	0.415023174	0.142855406	2.905197549	0.003670214	0	0.003688958	0	1	CRA004775;Yang2025_Silkie_Wenchang	PRJNA827465	0.086556316
ENSGALG00010027374	NKTR	2	0.124999891	0.147547708	0.847182868	0.396893201	0.020898976	1.497972338	33.24309306	1	CRA004775;PRJNA827465	Yang2025_Silkie_Wenchang	0.831375233
ENSGALG00010027375	FUT7	2	0.052466493	0.258272033	0.203144307	0.839022242	0	0.017452458	0	1	PRJNA827465;Yang2025_Silkie_Wenchang	CRA004775	0.959360287
ENSGALG00010027375	FUT7	2	-0.649580312	0.722453987	-0.899130358	0.36858323	0.807920921	4.315853547	76.82961229	0.5	CRA004775;Yang2025_Silkie_Wenchang	PRJNA827465	0.82033738
ENSGALG00010027375	FUT7	2	-0.606172223	0.756205605	-0.801597104	0.422786053	0.928048976	5.111292975	80.43547875	0.5	CRA004775;PRJNA827465	Yang2025_Silkie_Wenchang	0.839951486
ENSGALG00010027376	DSTYK	2	-0.140572372	0.058723088	-2.39381778	0.01667403	0.002821074	1.538663611	35.00853644	1	PRJNA827465;Yang2025_Silkie_Wenchang	CRA004775	0.214133955
ENSGALG00010027376	DSTYK	2	-0.215012624	0.084290144	-2.550863154	0.010745651	0	0.144746386	0	1	CRA004775;Yang2025_Silkie_Wenchang	PRJNA827465	0.165236469
ENSGALG00010027376	DSTYK	2	-0.10269278	0.045673269	-2.248421924	0.024549298	0	0.024420112	0	1	CRA004775;PRJNA827465	Yang2025_Silkie_Wenchang	0.2677195
ENSGALG00010027378	ZBTB47	2	0.075018672	0.068021492	1.102867199	0.27008484	0	0.015618243	0	1	PRJNA827465;Yang2025_Silkie_Wenchang	CRA004775	0.772347532
ENSGALG00010027378	ZBTB47	2	0.043013451	0.138822607	0.309844714	0.756679047	0	0.069490423	0	0.5	CRA004775;Yang2025_Silkie_Wenchang	PRJNA827465	0.938740241
ENSGALG00010027378	ZBTB47	2	0.074025081	0.074622437	0.991994956	0.321199986	0	0.113237767	0	0.5	CRA004775;PRJNA827465	Yang2025_Silkie_Wenchang	0.798513866
ENSGALG00010027379	OPTC	2	0.065022577	0.27177545	0.239251107	0.810910876	0	0.18930685	0	0.5	PRJNA827465;Yang2025_Silkie_Wenchang	CRA004775	0.953115528
ENSGALG00010027379	OPTC	2	-1.182900816	1.455049154	-0.812962788	0.416239394	3.872615421	11.47334868	91.28414879	0.5	CRA004775;Yang2025_Silkie_Wenchang	PRJNA827465	0.837742614
ENSGALG00010027379	OPTC	2	-1.286265731	1.33325148	-0.964758525	0.334665774	3.229497674	10.53588889	90.50863187	1	CRA004775;PRJNA827465	Yang2025_Silkie_Wenchang	0.803101658
ENSGALG00010027380	GPX1	2	-0.186072936	0.16747626	-1.111040673	0.266550843	0.035599221	2.10393723	52.47006489	1	PRJNA827465;Yang2025_Silkie_Wenchang	CRA004775	0.770699087
ENSGALG00010027380	GPX1	2	-0.503284836	0.206885914	-2.432668454	0.014988018	0	0.179784734	0	1	CRA004775;Yang2025_Silkie_Wenchang	PRJNA827465	0.20236141
ENSGALG00010027380	GPX1	2	-0.232286912	0.250299634	-0.928035364	0.353389226	0.082275708	2.09387056	52.24155594	1	CRA004775;PRJNA827465	Yang2025_Silkie_Wenchang	0.811193595
ENSGALG00010027381	KLHL40	2	-0.09424415	0.066655186	-1.413905732	0.157389583	0	0.557945529	0	1	PRJNA827465;Yang2025_Silkie_Wenchang	CRA004775	0.650687662
ENSGALG00010027381	KLHL40	2	0.32390447	0.598980308	0.540759798	0.588673156	0.478402544	2.086234622	52.06675273	0.5	CRA004775;Yang2025_Silkie_Wenchang	PRJNA827465	0.89216471
ENSGALG00010027381	KLHL40	2	0.294611059	0.665141217	0.44293009	0.657816302	0.621191756	2.41465203	58.58616532	0.5	CRA004775;PRJNA827465	Yang2025_Silkie_Wenchang	0.910774485
ENSGALG00010027382	PALM	2	0.26845858	0.299255204	0.897089095	0.369671377	0.166006805	13.43088386	92.55447363	0.5	PRJNA827465;Yang2025_Silkie_Wenchang	CRA004775	0.820606801
ENSGALG00010027382	PALM	2	0.401200567	0.261957679	1.53154727	0.125634193	0.080200763	2.020622704	50.51030566	1	CRA004775;Yang2025_Silkie_Wenchang	PRJNA827465	0.595034397
ENSGALG00010027382	PALM	2	-0.018114463	0.0793919	-0.228165127	0.819517871	0	0.008726702	0	0.5	CRA004775;PRJNA827465	Yang2025_Silkie_Wenchang	0.955256039
ENSGALG00010027383	ASS1	2	0.03714491	0.146833981	0.252972166	0.800209722	0	0.245010567	0	0.5	PRJNA827465;Yang2025_Silkie_Wenchang	CRA004775	0.950064546
ENSGALG00010027383	ASS1	2	-0.390126092	0.553902957	-0.704322099	0.481232209	0.516344515	6.057566331	83.49172018	0.5	CRA004775;Yang2025_Silkie_Wenchang	PRJNA827465	0.85682839
ENSGALG00010027383	ASS1	2	-0.451556449	0.47926106	-0.942193069	0.346093802	0.368649071	4.758205463	78.98367341	1	CRA004775;PRJNA827465	Yang2025_Silkie_Wenchang	0.807702909
ENSGALG00010027384	HHATL	2	-0.110298014	0.084181991	-1.310232904	0.190117058	0	0.131643976	0	1	PRJNA827465;Yang2025_Silkie_Wenchang	CRA004775	0.699403589
ENSGALG00010027384	HHATL	2	-0.087006219	0.118026134	-0.73717757	0.461014372	0	0.106265658	0	1	CRA004775;Yang2025_Silkie_Wenchang	PRJNA827465	0.85014477
ENSGALG00010027384	HHATL	2	-0.145309302	0.115233084	-1.261003318	0.207307652	0	0.049611859	0	1	CRA004775;PRJNA827465	Yang2025_Silkie_Wenchang	0.719377239
ENSGALG00010027385	CNTN2	2	-0.286243122	0.730388513	-0.391905291	0.695128194	0.575059244	2.153084255	53.55499917	0.5	PRJNA827465;Yang2025_Silkie_Wenchang	CRA004775	0.92097163
ENSGALG00010027385	CNTN2	2	1.130319472	0.842750866	1.341226117	0.179847049	0.848232284	2.425063341	58.76396368	1	CRA004775;Yang2025_Silkie_Wenchang	PRJNA827465	0.68659821
ENSGALG00010027385	CNTN2	2	0.47433119	1.581977456	0.299834155	0.764303509	4.326250149	7.353159759	86.40118102	0.5	CRA004775;PRJNA827465	Yang2025_Silkie_Wenchang	0.940495329
ENSGALG00010027386	RHOA	2	-0.05661619	0.068365925	-0.828134637	0.407594256	0.005939652	2.38509371	58.0729262	1	PRJNA827465;Yang2025_Silkie_Wenchang	CRA004775	0.833982683
ENSGALG00010027386	RHOA	2	-0.117620195	0.074639437	-1.575845145	0.115061495	0	0.577192588	0	1	CRA004775;Yang2025_Silkie_Wenchang	PRJNA827465	0.572141484
ENSGALG00010027386	RHOA	2	-0.006568003	0.034948912	-0.187931546	0.850930313	0	0.003551964	0	1	CRA004775;PRJNA827465	Yang2025_Silkie_Wenchang	0.962056252
ENSGALG00010027388	URM1	2	-0.443016621	0.238276438	-1.859254841	0.062991025	0.095569426	6.006057142	83.35014176	1	PRJNA827465;Yang2025_Silkie_Wenchang	CRA004775	0.432176808
ENSGALG00010027388	URM1	2	-0.522299947	0.229449101	-2.276321609	0.022826768	0.056026105	2.026852496	50.66241861	1	CRA004775;Yang2025_Silkie_Wenchang	PRJNA827465	0.25743485
ENSGALG00010027388	URM1	2	-0.227900715	0.084318021	-2.702870789	0.006874346	0	0.000850845	0	1	CRA004775;PRJNA827465	Yang2025_Silkie_Wenchang	0.127384458
ENSGALG00010027391	TPM3	2	0.261951946	0.239514598	1.093678417	0.274096043	0.107964858	16.90375938	94.08415621	1	PRJNA827465;Yang2025_Silkie_Wenchang	CRA004775	0.774526342
ENSGALG00010027391	TPM3	2	0.123468098	0.423387113	0.291619878	0.770577275	0.316229241	7.83472335	87.23630746	0.5	CRA004775;Yang2025_Silkie_Wenchang	PRJNA827465	0.942491331
ENSGALG00010027391	TPM3	2	-0.054683869	0.153071596	-0.357243735	0.720909347	0.024548801	1.548073773	35.40359528	0.5	CRA004775;PRJNA827465	Yang2025_Silkie_Wenchang	0.928919926
ENSGALG00010027392	RBBP5	2	-0.032588284	0.040557902	-0.803500247	0.421683654	0	0.674426914	0	1	PRJNA827465;Yang2025_Silkie_Wenchang	CRA004775	0.839352211
ENSGALG00010027392	RBBP5	2	-0.116622905	0.078228297	-1.49080232	0.136013402	0	0.035904445	0	1	CRA004775;Yang2025_Silkie_Wenchang	PRJNA827465	0.617575642
ENSGALG00010027392	RBBP5	2	-0.029800223	0.042194417	-0.706259851	0.480026556	0	0.743551018	0	1	CRA004775;PRJNA827465	Yang2025_Silkie_Wenchang	0.856321884
ENSGALG00010027393	TEX264	2	0.075180977	0.159321912	0.471880962	0.637011758	0.03886113	3.878066853	74.21395665	0.5	PRJNA827465;Yang2025_Silkie_Wenchang	CRA004775	0.904921861
ENSGALG00010027393	TEX264	2	-0.00892381	0.303203739	-0.029431728	0.976520269	0.144454205	4.499351366	77.77457419	0.5	CRA004775;Yang2025_Silkie_Wenchang	PRJNA827465	0.993126926
ENSGALG00010027393	TEX264	2	-0.104134832	0.104822609	-0.993435816	0.320497615	0.008723366	1.271447384	21.34947837	1	CRA004775;PRJNA827465	Yang2025_Silkie_Wenchang	0.79834216
ENSGALG00010027394	SLC27A4	2	-0.131687019	0.149976085	-0.878053449	0.379914713	0.032270861	3.429401604	70.84039388	0.5	PRJNA827465;Yang2025_Silkie_Wenchang	CRA004775	0.824392515
ENSGALG00010027394	SLC27A4	2	-0.34179275	0.103732722	-3.294936657	0.000984439	0	0.235289467	0	1	CRA004775;Yang2025_Silkie_Wenchang	PRJNA827465	0.037419142
ENSGALG00010027394	SLC27A4	2	-0.177620224	0.200791636	-0.884599718	0.376372559	0.064520677	4.777113193	79.06685566	0.5	CRA004775;PRJNA827465	Yang2025_Silkie_Wenchang	0.832974013
ENSGALG00010027395	BCL2L15	2	0.441658363	0.300738054	1.468581566	0.141946318	0	0.121460869	0	1	PRJNA827465;Yang2025_Silkie_Wenchang	CRA004775	0.62757683
ENSGALG00010027395	BCL2L15	2	0.357438592	0.208362986	1.715461072	0.086260727	0	0.260922512	0	1	CRA004775;Yang2025_Silkie_Wenchang	PRJNA827465	0.505487601
ENSGALG00010027395	BCL2L15	2	0.305908998	0.209310759	1.461506325	0.143876541	0	0.005961805	0	1	CRA004775;PRJNA827465	Yang2025_Silkie_Wenchang	0.630832183
ENSGALG00010027397	RFX5	2	0.164255806	0.124211218	1.32239107	0.186037961	0.014843098	1.857406141	46.16147874	1	PRJNA827465;Yang2025_Silkie_Wenchang	CRA004775	0.69509982
ENSGALG00010027397	RFX5	2	-0.037072502	0.381755414	-0.097110613	0.922638552	0.246997798	6.347323999	84.24532921	0.5	CRA004775;Yang2025_Silkie_Wenchang	PRJNA827465	0.981804675
ENSGALG00010027397	RFX5	2	-0.136759109	0.249794679	-0.547486078	0.584044833	0.091422986	3.301059445	69.70669518	0.5	CRA004775;PRJNA827465	Yang2025_Silkie_Wenchang	0.890763231
ENSGALG00010027399	MAPKAP1	2	-0.075328478	0.046447969	-1.62178195	0.104850045	0	0.025990683	0	1	PRJNA827465;Yang2025_Silkie_Wenchang	CRA004775	0.550048644
ENSGALG00010027399	MAPKAP1	2	-0.010511966	0.089501223	-0.117450524	0.906503049	0	0.716234235	0	0.5	CRA004775;Yang2025_Silkie_Wenchang	PRJNA827465	0.978196186
ENSGALG00010027399	MAPKAP1	2	-0.052172195	0.065040896	-0.802144469	0.422469394	0.002286419	1.154640374	13.39294705	0.5	CRA004775;PRJNA827465	Yang2025_Silkie_Wenchang	0.839704389
ENSGALG00010027401	ENSGALG00010027401	2	0.123863395	0.148644872	0.833284011	0.404684572	0	0.988478618	0	0.5	PRJNA827465;Yang2025_Silkie_Wenchang	CRA004775	0.833112102
ENSGALG00010027401	ENSGALG00010027401	2	0.252733053	0.161513614	1.56477864	0.117634822	0	0.021432367	0	1	CRA004775;Yang2025_Silkie_Wenchang	PRJNA827465	0.577223126
ENSGALG00010027401	ENSGALG00010027401	2	0.078590443	0.160916746	0.488391946	0.625272246	0	0.572679618	0	0.5	CRA004775;PRJNA827465	Yang2025_Silkie_Wenchang	0.901367193
ENSGALG00010027402	AP4B1	2	-0.02857419	0.077782568	-0.367359819	0.71335064	0	0.018176742	0	1	PRJNA827465;Yang2025_Silkie_Wenchang	CRA004775	0.926216079
ENSGALG00010027402	AP4B1	2	-0.264868378	0.25311453	-1.04643688	0.295359373	0.103803788	5.265241663	81.00751943	1	CRA004775;Yang2025_Silkie_Wenchang	PRJNA827465	0.787628307
ENSGALG00010027402	AP4B1	2	-0.259262835	0.240298024	-1.078922045	0.28062248	0.100328179	7.401879486	86.48991784	1	CRA004775;PRJNA827465	Yang2025_Silkie_Wenchang	0.778536485
ENSGALG00010027403	ACKR2	2	0.210236064	0.566075807	0.371392066	0.710345534	0.498338006	4.445595864	77.50582755	0.5	PRJNA827465;Yang2025_Silkie_Wenchang	CRA004775	0.925364369
ENSGALG00010027403	ACKR2	2	-0.413194699	0.280901977	-1.470956893	0.141302775	0	0.270382548	0	1	CRA004775;Yang2025_Silkie_Wenchang	PRJNA827465	0.626293475
ENSGALG00010027403	ACKR2	2	0.116038767	0.730598001	0.158827108	0.873805091	0.833444237	4.516527946	77.85909858	0.5	CRA004775;PRJNA827465	Yang2025_Silkie_Wenchang	0.969974876
ENSGALG00010027405	DCLRE1B	2	-0.068862515	0.075893521	-0.907356976	0.364218053	0	0.016785668	0	1	PRJNA827465;Yang2025_Silkie_Wenchang	CRA004775	0.817449342
ENSGALG00010027405	DCLRE1B	2	-0.618703031	0.645950491	-0.957818037	0.338154523	0.675071409	4.527815123	77.91429259	1	CRA004775;Yang2025_Silkie_Wenchang	PRJNA827465	0.80415385
ENSGALG00010027405	DCLRE1B	2	-0.602668886	0.656999907	-0.91730437	0.358983168	0.714883914	4.91556456	79.6564568	1	CRA004775;PRJNA827465	Yang2025_Silkie_Wenchang	0.814241185
ENSGALG00010027410	ALAS1	2	-0.037985953	0.062107094	-0.611620193	0.540789075	0	0.448627967	0	0.5	PRJNA827465;Yang2025_Silkie_Wenchang	CRA004775	0.875657957
ENSGALG00010027410	ALAS1	2	-0.078244129	0.152151711	-0.514250738	0.607076699	0.017750912	1.604697401	37.68295508	0.5	CRA004775;Yang2025_Silkie_Wenchang	PRJNA827465	0.89694739
ENSGALG00010027410	ALAS1	2	-0.079065829	0.063980107	-1.235787709	0.216537485	0	0.949389111	0	1	CRA004775;PRJNA827465	Yang2025_Silkie_Wenchang	0.730172415
ENSGALG00010027412	ENSGALG00010027412	2	0.426396019	0.41058208	1.038515901	0.299029936	0.278585388	5.698966202	82.45295788	1	PRJNA827465;Yang2025_Silkie_Wenchang	CRA004775	0.789748017
ENSGALG00010027412	ENSGALG00010027412	2	0.411598203	0.473702206	0.86889653	0.384903728	0.347345062	4.364752796	77.08919504	0.5	CRA004775;Yang2025_Silkie_Wenchang	PRJNA827465	0.826444827
ENSGALG00010027412	ENSGALG00010027412	2	0.002572697	0.181167662	0.01420064	0.988669909	0	0.092354399	0	0.5	CRA004775;PRJNA827465	Yang2025_Silkie_Wenchang	0.996891127
ENSGALG00010027414	PBX3	2	0.038282567	0.053013055	0.722134702	0.470211664	0	0.06647607	0	1	PRJNA827465;Yang2025_Silkie_Wenchang	CRA004775	0.853726346
ENSGALG00010027414	PBX3	2	-0.068414607	0.147057418	-0.465223776	0.641771214	0.026857822	2.585689932	61.3256026	0.5	CRA004775;Yang2025_Silkie_Wenchang	PRJNA827465	0.906634523
ENSGALG00010027414	PBX3	2	-0.066637998	0.127238612	-0.523724651	0.600470067	0.022009144	2.727446279	63.33566649	0.5	CRA004775;PRJNA827465	Yang2025_Silkie_Wenchang	0.895230519
ENSGALG00010027417	WNT2B	2	-0.302391388	0.243854858	-1.240046601	0.214958158	0	0.754193261	0	1	PRJNA827465;Yang2025_Silkie_Wenchang	CRA004775	0.728256227
ENSGALG00010027417	WNT2B	2	-0.282962489	0.373621115	-0.757351441	0.44883934	0.068611541	1.291998887	22.60055253	0.5	CRA004775;Yang2025_Silkie_Wenchang	PRJNA827465	0.847053326
ENSGALG00010027417	WNT2B	2	-0.047976062	0.275074526	-0.174411142	0.861542358	0	0.286346988	0	0.5	CRA004775;PRJNA827465	Yang2025_Silkie_Wenchang	0.965479094
ENSGALG00010027418	ENSGALG00010027418	2	-0.212460523	0.690784356	-0.307564178	0.758413982	0	0.143902921	0	0.5	PRJNA827465;Yang2025_Silkie_Wenchang	CRA004775	0.938954369
ENSGALG00010027418	ENSGALG00010027418	2	0.461822812	0.542936562	0.85060179	0.394990595	0	0.279663013	0	1	CRA004775;Yang2025_Silkie_Wenchang	PRJNA827465	0.830643015
ENSGALG00010027418	ENSGALG00010027418	2	0.309909554	0.548150243	0.565373377	0.571819829	0	0.918286984	0	0.5	CRA004775;PRJNA827465	Yang2025_Silkie_Wenchang	0.885470687
ENSGALG00010027419	PTBP1	2	-0.139984475	0.132163462	-1.059176815	0.289519262	0.031436084	9.985502625	89.98548158	1	PRJNA827465;Yang2025_Silkie_Wenchang	CRA004775	0.78398714
ENSGALG00010027419	PTBP1	2	-0.273860557	0.057768183	-4.740681477	2.13001E-06	0	0.064309806	0	1	CRA004775;Yang2025_Silkie_Wenchang	PRJNA827465	0.000420681
ENSGALG00010027419	PTBP1	2	-0.015104038	0.059321528	-0.254613104	0.799021937	0	0.786999364	0	1	CRA004775;PRJNA827465	Yang2025_Silkie_Wenchang	0.949922217
ENSGALG00010027421	PLPPR3	2	0.206871057	0.269070046	0.768837185	0.441989971	0.126874257	8.008832651	87.5137858	0.5	PRJNA827465;Yang2025_Silkie_Wenchang	CRA004775	0.845159086
ENSGALG00010027421	PLPPR3	2	0.317555073	0.268340687	1.183402627	0.236649617	0.077039116	1.763341792	43.28949699	0.5	CRA004775;Yang2025_Silkie_Wenchang	PRJNA827465	0.748936298
ENSGALG00010027421	PLPPR3	2	-0.05685161	0.110117631	-0.516280722	0.605658358	0	0.017625891	0	1	CRA004775;PRJNA827465	Yang2025_Silkie_Wenchang	0.896474603
ENSGALG00010027423	PEX11B	2	0.243584355	0.222723816	1.093661015	0.274103678	0.074527627	3.90598453	74.3982601	1	PRJNA827465;Yang2025_Silkie_Wenchang	CRA004775	0.774526342
ENSGALG00010027423	PEX11B	2	0.27039529	0.252336361	1.071566891	0.283914611	0.076137486	2.415286501	58.59704431	0.5	CRA004775;Yang2025_Silkie_Wenchang	PRJNA827465	0.77978499
ENSGALG00010027423	PEX11B	2	0.030527733	0.113932025	0.267946904	0.788740189	0	0.04394265	0	0.5	CRA004775;PRJNA827465	Yang2025_Silkie_Wenchang	0.947138883
ENSGALG00010027425	CFD	2	0.499727905	0.100129114	4.990835162	6.01188E-07	0	0.500617819	0	1	PRJNA827465;Yang2025_Silkie_Wenchang	CRA004775	0.000147678
ENSGALG00010027425	CFD	2	0.31232079	0.172249174	1.813191799	0.069802244	0	0.820175325	0	0.5	CRA004775;Yang2025_Silkie_Wenchang	PRJNA827465	0.455644844
ENSGALG00010027425	CFD	2	0.395254639	0.248494209	1.590598994	0.111699851	0.06988525	1.780842009	43.84678736	0.5	CRA004775;PRJNA827465	Yang2025_Silkie_Wenchang	0.565091513
ENSGALG00010027427	UBAC1	2	-0.10560615	0.046609378	-2.265770427	0.023465443	0	0.079103831	0	1	PRJNA827465;Yang2025_Silkie_Wenchang	CRA004775	0.261412218
ENSGALG00010027427	UBAC1	2	0.0902803	0.295850528	0.30515511	0.76024802	0.126991095	2.898947283	65.5047194	0.5	CRA004775;Yang2025_Silkie_Wenchang	PRJNA827465	0.939327609
ENSGALG00010027427	UBAC1	2	0.096933816	0.278598596	0.347933613	0.727890041	0.111691467	2.711368241	63.11825208	0.5	CRA004775;PRJNA827465	Yang2025_Silkie_Wenchang	0.931804517
ENSGALG00010027438	ST7L	2	-0.072382813	0.098821282	-0.732461785	0.463886759	0.011860312	2.183914299	54.21065743	1	PRJNA827465;Yang2025_Silkie_Wenchang	CRA004775	0.852159649
ENSGALG00010027438	ST7L	2	-0.349245677	0.139255713	-2.507945066	0.012143554	0.021257372	2.212626036	54.80483445	1	CRA004775;Yang2025_Silkie_Wenchang	PRJNA827465	0.177757215
ENSGALG00010027438	ST7L	2	-0.23049662	0.243364443	-0.947125295	0.343574937	0.108562596	11.47454491	91.28505742	1	CRA004775;PRJNA827465	Yang2025_Silkie_Wenchang	0.806276913
ENSGALG00010027440	TWF2	2	0.076956458	0.070562648	1.090611827	0.275443723	0.004228697	1.629094801	38.61621806	1	PRJNA827465;Yang2025_Silkie_Wenchang	CRA004775	0.775135547
ENSGALG00010027440	TWF2	2	-0.140069118	0.442466245	-0.316564529	0.751574054	0.283912494	2.817781566	64.51108873	0.5	CRA004775;Yang2025_Silkie_Wenchang	PRJNA827465	0.937716403
ENSGALG00010027440	TWF2	2	-0.180714234	0.346880786	-0.52096928	0.602388171	0.159316796	2.042037517	51.02930324	0.5	CRA004775;PRJNA827465	Yang2025_Silkie_Wenchang	0.895451466
ENSGALG00010027443	MED16	2	-0.014549009	0.132106017	-0.110131315	0.912305233	0.0228219	2.624001536	61.89026621	0.5	PRJNA827465;Yang2025_Silkie_Wenchang	CRA004775	0.978967245
ENSGALG00010027443	MED16	2	-0.261902485	0.113258338	-2.312434475	0.020753752	0	0.659322331	0	1	CRA004775;Yang2025_Silkie_Wenchang	PRJNA827465	0.243560684
ENSGALG00010027443	MED16	2	-0.112333838	0.227081191	-0.494685786	0.620821936	0.086913825	5.933884021	83.14763153	0.5	CRA004775;PRJNA827465	Yang2025_Silkie_Wenchang	0.899512075
ENSGALG00010027444	SELENBP1	2	-0.1609455	0.116947445	-1.376220743	0.168753302	0.015840761	2.287713683	56.28823628	1	PRJNA827465;Yang2025_Silkie_Wenchang	CRA004775	0.669861707
ENSGALG00010027444	SELENBP1	2	-0.197217753	0.141427074	-1.39448373	0.163171579	0.013435285	1.434270881	30.2781634	1	CRA004775;Yang2025_Silkie_Wenchang	PRJNA827465	0.661668506
ENSGALG00010027444	SELENBP1	2	-0.053406043	0.080570567	-0.662848046	0.507427879	0	0.070149562	0	1	CRA004775;PRJNA827465	Yang2025_Silkie_Wenchang	0.864149944
ENSGALG00010027445	PPM1M	2	-0.005149296	0.084287137	-0.061092313	0.951285691	0	0.452251172	0	0.5	PRJNA827465;Yang2025_Silkie_Wenchang	CRA004775	0.987687454
ENSGALG00010027445	PPM1M	2	-0.156417682	0.165148962	-0.94713088	0.343572091	0	0.588198618	0	1	CRA004775;Yang2025_Silkie_Wenchang	PRJNA827465	0.806276913
ENSGALG00010027445	PPM1M	2	-0.02985157	0.161020546	-0.18538982	0.852923292	0.019804469	1.16998007	14.52845856	0.5	CRA004775;PRJNA827465	Yang2025_Silkie_Wenchang	0.962708451
ENSGALG00010027448	ADAMTSL5	2	-0.050438954	0.303919179	-0.165961734	0.868187065	0.156260662	6.348811813	84.24902124	0.5	PRJNA827465;Yang2025_Silkie_Wenchang	CRA004775	0.968042774
ENSGALG00010027448	ADAMTSL5	2	0.282425349	0.19119814	1.477134395	0.139639615	0	0.018851261	0	1	CRA004775;Yang2025_Silkie_Wenchang	PRJNA827465	0.623452891
ENSGALG00010027448	ADAMTSL5	2	-0.222254149	0.2566466	-0.865992963	0.386494021	0.061251019	1.341134387	25.43625685	0.5	CRA004775;PRJNA827465	Yang2025_Silkie_Wenchang	0.826981729
ENSGALG00010027450	CAMSAP1	2	-0.091359255	0.047913696	-1.906746132	0.05655347	0.001222688	1.158806827	13.70433994	0.5	PRJNA827465;Yang2025_Silkie_Wenchang	CRA004775	0.411552605
ENSGALG00010027450	CAMSAP1	2	0.136727929	0.176182823	0.776057091	0.437715277	0.030296336	1.690020241	40.82911107	1	CRA004775;Yang2025_Silkie_Wenchang	PRJNA827465	0.843889118
ENSGALG00010027450	CAMSAP1	2	0.078001706	0.249843725	0.312201983	0.754887025	0.096893927	3.570767618	71.99481717	0.5	CRA004775;PRJNA827465	Yang2025_Silkie_Wenchang	0.93838723
ENSGALG00010027455	R3HDM4	2	0.022127193	0.087750995	0.252158883	0.800918258	0	0.466445313	0	0.5	PRJNA827465;Yang2025_Silkie_Wenchang	CRA004775	0.950158029
ENSGALG00010027455	R3HDM4	2	-0.268370545	0.192596932	-1.393431051	0.163489481	0	0.466947834	0	1	CRA004775;Yang2025_Silkie_Wenchang	PRJNA827465	0.661791656
ENSGALG00010027455	R3HDM4	2	-0.104584975	0.212847766	-0.491360455	0.623117528	0.05853389	2.313018127	56.76644346	0.5	CRA004775;PRJNA827465	Yang2025_Silkie_Wenchang	0.900348922
ENSGALG00010027457	CAPZA1	2	-0.113237344	0.126209522	-0.897217122	0.36960307	0.026573234	5.575744686	82.06517593	1	PRJNA827465;Yang2025_Silkie_Wenchang	CRA004775	0.820606801
ENSGALG00010027457	CAPZA1	2	-0.216138698	0.090098356	-2.398919451	0.016443531	0	0.909302777	0	1	CRA004775;Yang2025_Silkie_Wenchang	PRJNA827465	0.212234878
ENSGALG00010027457	CAPZA1	2	-0.003326764	0.040379721	-0.082386987	0.934338983	0	0.021774868	0	1	CRA004775;PRJNA827465	Yang2025_Silkie_Wenchang	0.98432801
ENSGALG00010027460	WDR82	2	-0.209631997	0.21745056	-0.96404441	0.335023661	0.087648617	13.1811533	92.41341044	1	PRJNA827465;Yang2025_Silkie_Wenchang	CRA004775	0.803101658
ENSGALG00010027460	WDR82	2	-0.35258181	0.108087568	-3.262001517	0.001106286	0.006872481	1.388968662	28.00413519	1	CRA004775;Yang2025_Silkie_Wenchang	PRJNA827465	0.040185157
ENSGALG00010027460	WDR82	2	-0.061634067	0.095143724	-0.647799607	0.51711457	0.0103298	1.822073046	45.11745827	1	CRA004775;PRJNA827465	Yang2025_Silkie_Wenchang	0.867952013
ENSGALG00010027467	GLYCTK	2	0.13454497	0.123308307	1.091126565	0.275217195	0.011169812	1.578348843	36.64265005	1	PRJNA827465;Yang2025_Silkie_Wenchang	CRA004775	0.775068889
ENSGALG00010027467	GLYCTK	2	-0.022551384	0.128647359	-0.175296129	0.860846954	0	0.17880491	0	0.5	CRA004775;Yang2025_Silkie_Wenchang	PRJNA827465	0.965220318
ENSGALG00010027467	GLYCTK	2	0.143677683	0.172080446	0.834944852	0.403748759	0.023515482	1.476446606	32.26981618	0.5	CRA004775;PRJNA827465	Yang2025_Silkie_Wenchang	0.833112102
ENSGALG00010027468	BSG	2	0.010098044	0.055728713	0.181200013	0.856210584	0	0.006187646	0	1	PRJNA827465;Yang2025_Silkie_Wenchang	CRA004775	0.963499334
ENSGALG00010027468	BSG	2	0.038862139	0.086904592	0.44718165	0.654743909	0	0.354781596	0	1	CRA004775;Yang2025_Silkie_Wenchang	PRJNA827465	0.910222014
ENSGALG00010027468	BSG	2	0.028522835	0.060263243	0.473304023	0.635996292	0	0.368109471	0	1	CRA004775;PRJNA827465	Yang2025_Silkie_Wenchang	0.90443615
ENSGALG00010027471	ENSGALG00010027471	2	-0.110306518	0.072309592	-1.525475581	0.12714052	0	0.931429558	0	0.5	PRJNA827465;Yang2025_Silkie_Wenchang	CRA004775	0.598452543
ENSGALG00010027471	ENSGALG00010027471	2	0.032576463	0.144114577	0.226045582	0.821165968	0	0.020161146	0	1	CRA004775;Yang2025_Silkie_Wenchang	PRJNA827465	0.955598716
ENSGALG00010027471	ENSGALG00010027471	2	-0.135944233	0.079571824	-1.708446867	0.087553457	0	0.364651438	0	0.5	CRA004775;PRJNA827465	Yang2025_Silkie_Wenchang	0.508888073
ENSGALG00010027475	ARID3A	2	0.425173219	0.269866986	1.575491783	0.115142974	0.132592866	11.02924745	90.93319826	1	PRJNA827465;Yang2025_Silkie_Wenchang	CRA004775	0.572141484
ENSGALG00010027475	ARID3A	2	0.250892178	0.517471516	0.484842489	0.627788081	0.455120744	6.061315638	83.5019316	0.5	CRA004775;Yang2025_Silkie_Wenchang	PRJNA827465	0.901878949
ENSGALG00010027475	ARID3A	2	0.064170924	0.20089756	0.319421124	0.749407194	0.041896027	1.492111721	32.9808897	0.5	CRA004775;PRJNA827465	Yang2025_Silkie_Wenchang	0.937362187
ENSGALG00010027479	HCN2	2	-0.227041256	0.145738699	-1.557865259	0.119265192	0	0.019191692	0	1	PRJNA827465;Yang2025_Silkie_Wenchang	CRA004775	0.579671051
ENSGALG00010027479	HCN2	2	-0.210813952	0.202276657	-1.04220603	0.297316168	0	0.006644246	0	1	CRA004775;Yang2025_Silkie_Wenchang	PRJNA827465	0.788535199
ENSGALG00010027479	HCN2	2	-0.239549353	0.158090783	-1.515264518	0.12970543	0	0.000472821	0	1	CRA004775;PRJNA827465	Yang2025_Silkie_Wenchang	0.60409121
ENSGALG00010027487	MPZ	2	0.07232699	0.312738132	0.231270134	0.817104947	0.159909813	5.474300638	81.73282642	0.5	PRJNA827465;Yang2025_Silkie_Wenchang	CRA004775	0.954833525
ENSGALG00010027487	MPZ	2	1.186321832	0.919579528	1.290069859	0.197026404	1.461726971	6.637493721	84.93407238	1	CRA004775;Yang2025_Silkie_Wenchang	PRJNA827465	0.708095167
ENSGALG00010027487	MPZ	2	0.905426742	1.237127081	0.731878523	0.464242716	2.815152113	11.74261925	91.48401239	0.5	CRA004775;PRJNA827465	Yang2025_Silkie_Wenchang	0.852159649
ENSGALG00010027490	ATP2B4	2	-0.114047371	0.119074526	-0.957781438	0.338172981	0.020478969	3.284236073	69.55151889	1	PRJNA827465;Yang2025_Silkie_Wenchang	CRA004775	0.80415385
ENSGALG00010027490	ATP2B4	2	-0.172827416	0.150330774	-1.149647616	0.250289038	0.015350045	1.336837974	25.19661922	0.5	CRA004775;Yang2025_Silkie_Wenchang	PRJNA827465	0.759943035
ENSGALG00010027490	ATP2B4	2	-0.011614539	0.056314225	-0.206245211	0.836599374	0	0.141787559	0	0.5	CRA004775;PRJNA827465	Yang2025_Silkie_Wenchang	0.958399882
ENSGALG00010027492	PCP4L1	2	0.106219754	0.610269871	0.174053741	0.861823227	0.398777426	2.134382578	53.14804335	0.5	PRJNA827465;Yang2025_Silkie_Wenchang	CRA004775	0.965635503
ENSGALG00010027492	PCP4L1	2	0.30246826	0.803500905	0.376437983	0.706591313	0.930380218	3.545221516	71.79301786	0.5	CRA004775;Yang2025_Silkie_Wenchang	PRJNA827465	0.924548793
ENSGALG00010027492	PCP4L1	2	0.957134186	0.463176149	2.066458275	0.038785228	0	0.172473283	0	1	CRA004775;PRJNA827465	Yang2025_Silkie_Wenchang	0.339943161
ENSGALG00010027497	KCNT1	2	0.063072052	0.129645843	0.486494981	0.626616263	0.004523054	1.153008574	13.27037612	0.5	PRJNA827465;Yang2025_Silkie_Wenchang	CRA004775	0.901504244
ENSGALG00010027497	KCNT1	2	0.160964887	0.173622157	0.927098761	0.353875259	0	0.674312247	0	0.5	CRA004775;Yang2025_Silkie_Wenchang	PRJNA827465	0.811456203
ENSGALG00010027497	KCNT1	2	-0.068905274	0.153425852	-0.449111235	0.653351416	0	0.137910483	0	1	CRA004775;PRJNA827465	Yang2025_Silkie_Wenchang	0.909597225
ENSGALG00010027499	MOV10	2	0.005489316	0.147295056	0.037267485	0.970271731	0	0.586632409	0	0.5	PRJNA827465;Yang2025_Silkie_Wenchang	CRA004775	0.991681174
ENSGALG00010027499	MOV10	2	-0.264862117	0.250864305	-1.055798338	0.291060358	0.05067865	1.520082809	34.21411032	1	CRA004775;Yang2025_Silkie_Wenchang	PRJNA827465	0.78467882
ENSGALG00010027499	MOV10	2	-0.18740412	0.37899693	-0.494473979	0.620971479	0.195885333	2.937216958	65.95416633	0.5	CRA004775;PRJNA827465	Yang2025_Silkie_Wenchang	0.899578487
ENSGALG00010027501	BAP1	2	0.06358695	0.123736883	0.513888413	0.607330009	0.020917277	2.557710732	60.90253728	0.5	PRJNA827465;Yang2025_Silkie_Wenchang	CRA004775	0.896998866
ENSGALG00010027501	BAP1	2	-0.03308641	0.25928445	-0.12760661	0.8984603	0.115860642	7.145401335	86.00498484	0.5	CRA004775;Yang2025_Silkie_Wenchang	PRJNA827465	0.97575249
ENSGALG00010027501	BAP1	2	-0.13168403	0.126741127	-1.03899999	0.298804741	0.025473948	4.376701399	77.15174263	1	CRA004775;PRJNA827465	Yang2025_Silkie_Wenchang	0.78974322
ENSGALG00010027502	THOP1	2	-0.03529783	0.057733952	-0.611387731	0.540942923	0	0.999759479	0	1	PRJNA827465;Yang2025_Silkie_Wenchang	CRA004775	0.875790068
ENSGALG00010027502	THOP1	2	-0.222180072	0.124806436	-1.780197231	0.075043688	0	0.454451374	0	1	CRA004775;Yang2025_Silkie_Wenchang	PRJNA827465	0.47268425
ENSGALG00010027502	THOP1	2	-0.109090285	0.15667211	-0.696296773	0.486242994	0.030304584	2.00536483	50.13376193	1	CRA004775;PRJNA827465	Yang2025_Silkie_Wenchang	0.857603818
ENSGALG00010027510	RHOC	2	-0.099706068	0.129950773	-0.767260293	0.442926772	0	0.867664284	0	0.5	PRJNA827465;Yang2025_Silkie_Wenchang	CRA004775	0.845720426
ENSGALG00010027510	RHOC	2	-0.033006508	0.211117297	-0.156342035	0.875763424	0	0.911606341	0	0.5	CRA004775;Yang2025_Silkie_Wenchang	PRJNA827465	0.970347777
ENSGALG00010027510	RHOC	2	-0.192430457	0.139570723	-1.378730817	0.167977764	0	0.146624201	0	1	CRA004775;PRJNA827465	Yang2025_Silkie_Wenchang	0.668442195
ENSGALG00010027511	POLRMT	2	-0.002698107	0.045438109	-0.059379821	0.952649585	0	0.549169559	0	0.5	PRJNA827465;Yang2025_Silkie_Wenchang	CRA004775	0.987758913
ENSGALG00010027511	POLRMT	2	0.02651566	0.078752881	0.336694469	0.736347233	0	0.642243385	0	0.5	CRA004775;Yang2025_Silkie_Wenchang	PRJNA827465	0.933661344
ENSGALG00010027511	POLRMT	2	-0.030825954	0.052643392	-0.585561706	0.558170089	0	0.268052529	0	1	CRA004775;PRJNA827465	Yang2025_Silkie_Wenchang	0.880678789
ENSGALG00010027513	SEMA3G	2	0.132268108	0.103156041	1.282213876	0.19976763	0.008520136	1.554480951	35.66984534	1	PRJNA827465;Yang2025_Silkie_Wenchang	CRA004775	0.711634837
ENSGALG00010027513	SEMA3G	2	0.294653991	0.140732893	2.093710892	0.036285742	0	0.084050239	0	1	CRA004775;Yang2025_Silkie_Wenchang	PRJNA827465	0.328451985
ENSGALG00010027513	SEMA3G	2	0.076738516	0.084161581	0.911799838	0.361874096	0	0.78775268	0	1	CRA004775;PRJNA827465	Yang2025_Silkie_Wenchang	0.815579273
ENSGALG00010027516	PPM1J	2	-0.24136892	0.16856198	-1.431929787	0.152163916	0	0.004926579	0	1	PRJNA827465;Yang2025_Silkie_Wenchang	CRA004775	0.643687268
ENSGALG00010027516	PPM1J	2	-0.327052641	0.247342272	-1.322267472	0.1860791	0	0.294454469	0	1	CRA004775;Yang2025_Silkie_Wenchang	PRJNA827465	0.69509982
ENSGALG00010027516	PPM1J	2	-0.280036395	0.196676377	-1.423843572	0.15449176	0	0.374475575	0	1	CRA004775;PRJNA827465	Yang2025_Silkie_Wenchang	0.647288693
ENSGALG00010027520	FANCE	2	-0.144352603	0.12232888	-1.180036987	0.237985505	0	0.008878337	0	1	PRJNA827465;Yang2025_Silkie_Wenchang	CRA004775	0.749843217
ENSGALG00010027520	FANCE	2	-0.205058373	0.266456154	-0.76957642	0.441551197	0	0.268704768	0	1	CRA004775;Yang2025_Silkie_Wenchang	PRJNA827465	0.845053556
ENSGALG00010027520	FANCE	2	-0.167419928	0.128145757	-1.306480463	0.191389219	0	0.281987288	0	1	CRA004775;PRJNA827465	Yang2025_Silkie_Wenchang	0.7008451
ENSGALG00010027522	PSMD5	2	-0.141038486	0.079556431	-1.772810632	0.076260091	0.006176716	1.848222993	45.8939747	1	PRJNA827465;Yang2025_Silkie_Wenchang	CRA004775	0.47652731

ENSGALG00010027522	PSMD5	2	-0.214509518	0.094186741	-2.27749166	0.022756882	0	0.482061588	0	1	CRA004775;Yang2025_Silkie_Wenchang	PRJNA827465	0.257015441
ENSGALG00010027522	PSMD5	2	-0.078050989	0.061079265	-1.27786392	0.201297412	0	0.003402505	0	1	CRA004775;PRJNA827465	Yang2025_Silkie_Wenchang	0.714168358
ENSGALG00010027524	ENSGALG00010027524	2	-0.188439928	0.069607103	-2.707193949	0.00678546	0	0.053295912	0	1	PRJNA827465;Yang2025_Silkie_Wenchang	CRA004775	0.126452796
ENSGALG00010027524	ENSGALG00010027524	2	-0.226608978	0.188200602	-1.204082108	0.228557845	0	0.00674433	0	1	CRA004775;Yang2025_Silkie_Wenchang	PRJNA827465	0.741270737
ENSGALG00010027524	ENSGALG00010027524	2	-0.183248671	0.072579538	-2.524798001	0.011576481	0	0.00103118	0	1	CRA004775;PRJNA827465	Yang2025_Silkie_Wenchang	0.172111769
ENSGALG00010027525	ENSGALG00010027525	2	0.047451299	0.152834587	0.310474876	0.756199862	0	0.120403424	0	1	PRJNA827465;Yang2025_Silkie_Wenchang	CRA004775	0.93854559
ENSGALG00010027525	ENSGALG00010027525	2	0.043776155	0.253039788	0.173001074	0.862650583	0	0.41091884	0	0.5	CRA004775;Yang2025_Silkie_Wenchang	PRJNA827465	0.966099097
ENSGALG00010027525	ENSGALG00010027525	2	-0.013639654	0.170610348	-0.079946232	0.93628002	0	0.23021683	0	0.5	CRA004775;PRJNA827465	Yang2025_Silkie_Wenchang	0.984894511
ENSGALG00010027527	ENSGALG00010027527	2	-0.027607015	0.354544525	-0.077866145	0.93793453	0	0.499660349	0	0.5	PRJNA827465;Yang2025_Silkie_Wenchang	CRA004775	0.985349045
ENSGALG00010027527	ENSGALG00010027527	2	0.397859306	0.412062054	0.965532502	0.334278165	0	0.076330539	0	1	CRA004775;Yang2025_Silkie_Wenchang	PRJNA827465	0.803094614
ENSGALG00010027527	ENSGALG00010027527	2	0.045669537	0.360177551	0.126797288	0.899100842	0	0.986810301	0	0.5	CRA004775;PRJNA827465	Yang2025_Silkie_Wenchang	0.975990605
ENSGALG00010027529	ACY1	2	-0.046206551	0.083836349	-0.551151754	0.58152965	0	0.114630843	0	0.5	PRJNA827465;Yang2025_Silkie_Wenchang	CRA004775	0.889045686
ENSGALG00010027529	ACY1	2	-0.020589943	0.201392244	-0.102238012	0.918567757	0	0.284931967	0	0.5	CRA004775;Yang2025_Silkie_Wenchang	PRJNA827465	0.981033557
ENSGALG00010027529	ACY1	2	-0.064389689	0.089765744	-0.717308032	0.473184053	0	0.179957107	0	1	CRA004775;PRJNA827465	Yang2025_Silkie_Wenchang	0.854341771
ENSGALG00010027530	PHF19	2	0.01790315	0.085739792	0.208807948	0.834598167	0	0.000128971	0	1	PRJNA827465;Yang2025_Silkie_Wenchang	CRA004775	0.958133448
ENSGALG00010027530	PHF19	2	-0.311203361	0.38951641	-0.798948011	0.424320558	0.229090651	3.740169926	73.26324686	0.5	CRA004775;Yang2025_Silkie_Wenchang	PRJNA827465	0.840617373
ENSGALG00010027530	PHF19	2	-0.297614218	0.388288382	-0.766472267	0.443392373	0.240914901	4.274652397	76.60628498	0.5	CRA004775;PRJNA827465	Yang2025_Silkie_Wenchang	0.845733906
ENSGALG00010027531	UFC1	2	0.050623399	0.086505239	0.583856223	0.559317048	0	0.386337855	0	1	PRJNA827465;Yang2025_Silkie_Wenchang	CRA004775	0.881050436
ENSGALG00010027531	UFC1	2	0.064441191	0.17579961	0.366560491	0.713946883	0	0.789946251	0	0.5	CRA004775;Yang2025_Silkie_Wenchang	PRJNA827465	0.926399535
ENSGALG00010027531	UFC1	2	0.010433426	0.09076175	0.114954002	0.908481581	0	0.341447387	0	0.5	CRA004775;PRJNA827465	Yang2025_Silkie_Wenchang	0.978291088
ENSGALG00010027533	WDR18	2	-0.078029055	0.059087562	-1.320566486	0.186645954	0	0.509150507	0	1	PRJNA827465;Yang2025_Silkie_Wenchang	CRA004775	0.695457578
ENSGALG00010027533	WDR18	2	-0.257625417	0.126664686	-2.033916678	0.041959997	0	0.152307691	0	1	CRA004775;Yang2025_Silkie_Wenchang	PRJNA827465	0.354529664
ENSGALG00010027533	WDR18	2	-0.129557756	0.106796997	-1.213121718	0.225083288	0.011829884	1.702651247	41.26806639	1	CRA004775;PRJNA827465	Yang2025_Silkie_Wenchang	0.738087485
ENSGALG00010027534	KCNN3	2	-0.062563826	0.161253409	-0.387984519	0.69802749	0.03253365	2.547438565	60.74488258	0.5	PRJNA827465;Yang2025_Silkie_Wenchang	CRA004775	0.921516414
ENSGALG00010027534	KCNN3	2	0.312687128	0.254259771	1.22976953	0.218774273	0.068268096	1.908372609	47.59933174	1	CRA004775;Yang2025_Silkie_Wenchang	PRJNA827465	0.73259614
ENSGALG00010027534	KCNN3	2	0.170354038	0.426846858	0.399098729	0.689820459	0.306231703	5.637369802	82.26123112	0.5	CRA004775;PRJNA827465	Yang2025_Silkie_Wenchang	0.919979266
ENSGALG00010027536	USP21	2	-0.004066971	0.109362649	-0.03718793	0.970335163	0	0.831524114	0	0.5	PRJNA827465;Yang2025_Silkie_Wenchang	CRA004775	0.991681174
ENSGALG00010027536	USP21	2	-0.147075735	0.153055117	-0.960933141	0.336585786	0	0.017253615	0	1	CRA004775;Yang2025_Silkie_Wenchang	PRJNA827465	0.803810565
ENSGALG00010027536	USP21	2	0.025935621	0.124354379	0.208562181	0.834790037	0	0.613499293	0	0.5	CRA004775;PRJNA827465	Yang2025_Silkie_Wenchang	0.958133448
ENSGALG00010027537	SGTA	2	-0.054024876	0.100463703	-0.537755174	0.590746083	0.013775434	2.653576988	62.31504679	0.5	PRJNA827465;Yang2025_Silkie_Wenchang	CRA004775	0.892708037
ENSGALG00010027537	SGTA	2	0.082186314	0.099344753	0.827283898	0.408076168	0	1.70757E-06	0	1	CRA004775;Yang2025_Silkie_Wenchang	PRJNA827465	0.834084853
ENSGALG00010027537	SGTA	2	-0.088510527	0.082325532	-1.075128512	0.282317196	0.006535146	1.418146924	29.48544445	0.5	CRA004775;PRJNA827465	Yang2025_Silkie_Wenchang	0.779519046
ENSGALG00010027538	ABHD14A	2	0.155470055	0.108973175	1.42668188	0.153671611	0.000229599	1.007432447	0.737761287	1	PRJNA827465;Yang2025_Silkie_Wenchang	CRA004775	0.646153969
ENSGALG00010027538	ABHD14A	2	0.200683755	0.18988151	1.056889397	0.290562068	0.009684072	1.139831884	12.26776384	0.5	CRA004775;Yang2025_Silkie_Wenchang	PRJNA827465	0.784499619
ENSGALG00010027538	ABHD14A	2	0.069922234	0.11599546	0.602801475	0.546640765	0	0.202009081	0	0.5	CRA004775;PRJNA827465	Yang2025_Silkie_Wenchang	0.878275987
ENSGALG00010027539	DDR2L	2	-0.288091798	0.155808855	-1.849011486	0.064456154	0.038422721	4.71809051	78.80498482	1	PRJNA827465;Yang2025_Silkie_Wenchang	CRA004775	0.437533718
ENSGALG00010027539	DDR2L	2	-0.261800139	0.270058242	-0.96942103	0.332335164	0.09651046	2.446889174	59.13178208	0.5	CRA004775;Yang2025_Silkie_Wenchang	PRJNA827465	0.802178578
ENSGALG00010027539	DDR2L	2	-0.127424077	0.082303302	-1.548225572	0.121567997	0	0.528066532	0	0.5	CRA004775;PRJNA827465	Yang2025_Silkie_Wenchang	0.584749355
ENSGALG00010027540	PPOX	2	0.098549312	0.101319204	0.972661735	0.330721449	0	0.303070338	0	1	PRJNA827465;Yang2025_Silkie_Wenchang	CRA004775	0.801575303
ENSGALG00010027540	PPOX	2	0.010804709	0.248134745	0.043543716	0.965268118	0.037974944	1.441705379	30.63770072	0.5	CRA004775;Yang2025_Silkie_Wenchang	PRJNA827465	0.990650011
ENSGALG00010027540	PPOX	2	0.034768582	0.110579345	0.314422026	0.753200529	0.002100148	1.038948479	3.748836411	0.5	CRA004775;PRJNA827465	Yang2025_Silkie_Wenchang	0.938087082
ENSGALG00010027543	TUBB4B	2	0.07186692	0.061547996	1.167656537	0.242945335	0	0.253198809	0	0.5	PRJNA827465;Yang2025_Silkie_Wenchang	CRA004775	0.7550024
ENSGALG00010027543	TUBB4B	2	-0.040286157	0.140488957	-0.486060675	0.626924148	0	0.469648594	0	1	CRA004775;Yang2025_Silkie_Wenchang	PRJNA827465	0.901573209
ENSGALG00010027543	TUBB4B	2	0.037421329	0.109197857	0.342692884	0.731829517	0.009723756	1.271107346	21.32843828	0.5	CRA004775;PRJNA827465	Yang2025_Silkie_Wenchang	0.932851968
ENSGALG00010027545	PPARD	2	-0.103092321	0.086685101	-1.189273818	0.234331935	0.008070152	2.141011893	53.29311326	1	PRJNA827465;Yang2025_Silkie_Wenchang	CRA004775	0.747486154
ENSGALG00010027545	PPARD	2	-0.000187306	0.254216482	-0.000736797	0.999412121	0.095235864	3.244190182	69.17566653	0.5	CRA004775;Yang2025_Silkie_Wenchang	PRJNA827465	0.999889665
ENSGALG00010027545	PPARD	2	0.050876734	0.14345895	0.354643148	0.722856947	0.020376143	1.495519381	33.13359808	0.5	CRA004775;PRJNA827465	Yang2025_Silkie_Wenchang	0.930032096
ENSGALG00010027547	B4GALT3	2	0.075263849	0.165742934	0.454099894	0.649756929	0.043159159	4.541934244	77.98294853	0.5	PRJNA827465;Yang2025_Silkie_Wenchang	CRA004775	0.90820125
ENSGALG00010027547	B4GALT3	2	0.215490619	0.121410337	1.774895153	0.075915201	0	0.750105958	0	0.5	CRA004775;Yang2025_Silkie_Wenchang	PRJNA827465	0.47539798
ENSGALG00010027547	B4GALT3	2	-0.075787508	0.083715369	-0.905299817	0.365306573	0	0.002683405	0	1	CRA004775;PRJNA827465	Yang2025_Silkie_Wenchang	0.818452533
ENSGALG00010027548	PCBP4	2	-0.038361579	0.067640525	-0.567138984	0.570619759	0	0.033696558	0	1	PRJNA827465;Yang2025_Silkie_Wenchang	CRA004775	0.883152854
ENSGALG00010027548	PCBP4	2	-0.019315099	0.114820317	-0.168220222	0.866410033	0	0.552823186	0	0.5	CRA004775;Yang2025_Silkie_Wenchang	PRJNA827465	0.967428436
ENSGALG00010027548	PCBP4	2	-0.015186428	0.077055674	-0.197083839	0.84376193	0	0.475612862	0	0.5	CRA004775;PRJNA827465	Yang2025_Silkie_Wenchang	0.96057435
ENSGALG00010027553	FGF22	2	0.046438828	0.273251699	0.16994891	0.865050316	0.071912264	1.876154852	46.69949556	0.5	PRJNA827465;Yang2025_Silkie_Wenchang	CRA004775	0.96068159
ENSGALG00010027553	FGF22	2	-0.280590256	0.286990278	-0.977699516	0.328222961	0	6.36471E-05	0	1	CRA004775;Yang2025_Silkie_Wenchang	PRJNA827465	0.800761292
ENSGALG00010027553	FGF22	2	0.196974539	0.218020378	0.903468478	0.366277311	0	0.797305671	0	0.5	CRA004775;PRJNA827465	Yang2025_Silkie_Wenchang	0.819159863
ENSGALG00010027554	ENSGALG00010027554	2	0.064847176	0.099944942	0.648828993	0.516448915	0	0.118235023	0	1	PRJNA827465;Yang2025_Silkie_Wenchang	CRA004775	0.867747143
ENSGALG00010027554	ENSGALG00010027554	2	-0.099152736	0.239324082	-0.382441077	0.702134327	0.071078292	2.507950797	60.12680946	0.5	CRA004775;Yang2025_Silkie_Wenchang	PRJNA827465	0.923083392
ENSGALG00010027554	ENSGALG00010027554	2	-0.107326032	0.198111174	-0.541746483	0.587993163	0.044265941	2.05348684	51.30234191	0.5	CRA004775;PRJNA827465	Yang2025_Silkie_Wenchang	0.891761457
ENSGALG00010027555	ENSGALG00010027555	2	0.313879572	0.46874363	0.669618854	0.503100792	0.366788235	5.596122513	82.13048414	0.5	PRJNA827465;Yang2025_Silkie_Wenchang	CRA004775	0.863052661
ENSGALG00010027555	ENSGALG00010027555	2	0.130711314	0.700204024	0.186674039	0.851914644	0.865918469	8.522452831	88.26628883	0.5	CRA004775;Yang2025_Silkie_Wenchang	PRJNA827465	0.962254991
ENSGALG00010027555	ENSGALG00010027555	2	-0.249205336	0.214243165	-1.163189203	0.244752746	0.045209119	1.771488905	43.55030974	1	CRA004775;PRJNA827465	Yang2025_Silkie_Wenchang	0.756114592
ENSGALG00010027557	C6orf132	2	0.849361041	0.668491898	1.270562955	0.203884173	0	0.67528503	0	0.5	PRJNA827465;Yang2025_Silkie_Wenchang	CRA004775	0.717254253
ENSGALG00010027557	C6orf132	2	-0.48083536	0.637980253	-0.753683767	0.451019132	0	0.187183762	0	1	CRA004775;Yang2025_Silkie_Wenchang	PRJNA827465	0.847847413
ENSGALG00010027557	C6orf132	2	0.255864559	0.921164189	0.277762164	0.781194937	1.109105128	2.88359068	65.32101428	0.5	CRA004775;PRJNA827465	Yang2025_Silkie_Wenchang	0.945255709
ENSGALG00010027559	ENSGALG00010027559	2	-0.021007726	0.276686275	-0.075926158	0.939477846	0.105845641	3.219992565	68.94402767	0.5	PRJNA827465;Yang2025_Silkie_Wenchang	CRA004775	0.985425793
ENSGALG00010027559	ENSGALG00010027559	2	-0.309405697	0.189645835	-1.631492181	0.102786508	0	0.000469336	0	1	CRA004775;Yang2025_Silkie_Wenchang	PRJNA827465	0.546438271
ENSGALG00010027559	ENSGALG00010027559	2	0.025721428	0.26678124	0.09641393	0.923191829	0.078096371	2.104571769	52.48439542	0.5	CRA004775;PRJNA827465	Yang2025_Silkie_Wenchang	0.981874993
ENSGALG00010027561	ADAMTS4	2	-0.055164298	0.18472479	-0.29862964	0.765222648	0.035035793	1.698855514	41.13684231	0.5	PRJNA827465;Yang2025_Silkie_Wenchang	CRA004775	0.940779391
ENSGALG00010027561	ADAMTS4	2	-0.390768724	0.247544818	-1.578577676	0.114432958	0	0.051765629	0	1	CRA004775;Yang2025_Silkie_Wenchang	PRJNA827465	0.570394238
ENSGALG00010027561	ADAMTS4	2	-0.031631602	0.200895094	-0.157453331	0.874887585	0.036442057	1.341435134	25.45297384	0.5	CRA004775;PRJNA827465	Yang2025_Silkie_Wenchang	0.970159044
ENSGALG00010027562	SLC39A3	2	-0.043640952	0.176777411	-0.246869504	0.805009218	0.03295176	1.700844096	41.20566356	0.5	PRJNA827465;Yang2025_Silkie_Wenchang	CRA004775	0.951863117
ENSGALG00010027562	SLC39A3	2	-0.203098715	0.15935825	-1.274478826	0.202493772	0	0.30417165	0	1	CRA004775;Yang2025_Silkie_Wenchang	PRJNA827465	0.715289846
ENSGALG00010027562	SLC39A3	2	0.019172785	0.087288625	0.219648147	0.826145191	0	0.943896645	0	0.5	CRA004775;PRJNA827465	Yang2025_Silkie_Wenchang	0.956393839
ENSGALG00010027563	WDR77	2	-0.034543924	0.068370631	-0.505245072	0.613386717	0	0.830624715	0	1	PRJNA827465;Yang2025_Silkie_Wenchang	CRA004775	0.898256044
ENSGALG00010027563	WDR77	2	-0.293453276	0.169121924	-1.735158098	0.082712806	0	0.005182736	0	1	CRA004775;Yang2025_Silkie_Wenchang	PRJNA827465	0.495704284
ENSGALG00010027563	WDR77	2	-0.088806146	0.122621408	-0.724230357	0.468924324	0.014986177	1.589126395	37.07234347	1	CRA004775;PRJNA827465	Yang2025_Silkie_Wenchang	0.85345437
ENSGALG00010027567	PARP3	2	0.242816497	0.090969533	2.669206804	0.007603062	0	0.620101237	0	1	PRJNA827465;Yang2025_Silkie_Wenchang	CRA004775	0.134538115
ENSGALG00010027567	PARP3	2	-0.1305951	0.251394405	-0.519482922	0.603424019	0.068766387	2.158280266	53.66681446	0.5	CRA004775;Yang2025_Silkie_Wenchang	PRJNA827465	0.895599646
ENSGALG00010027567	PARP3	2	-0.021605906	0.341993238	-0.063176412	0.949626028	0.196003932	5.641401204	82.27390742	0.5	CRA004775;PRJNA827465	Yang2025_Silkie_Wenchang	0.98723759
ENSGALG00010027568	NELFB	2	-0.132486892	0.178625757	-0.741700938	0.458268551	0.055252024	7.247482795	86.20210591	0.5	PRJNA827465;Yang2025_Silkie_Wenchang	CRA004775	0.850026426
ENSGALG00010027568	NELFB	2	-0.364311752	0.091402035	-3.98581663	6.72484E-05	0	0.35375518	0	1	CRA004775;Yang2025_Silkie_Wenchang	PRJNA827465	0.005912897
ENSGALG00010027568	NELFB	2	-0.181295383	0.234765115	-0.77224158	0.439971359	0.097158837	8.019903847	87.53102258	0.5	CRA004775;PRJNA827465	Yang2025_Silkie_Wenchang	0.844353254
ENSGALG00010027569	ATP5PB	2	-0.056404336	0.056078562	-1.005809239	0.314507342	0	0.016415528	0	1	PRJNA827465;Yang2025_Silkie_Wenchang	CRA004775	0.796531448
ENSGALG00010027569	ATP5PB	2	0.060556941	0.185655782	0.326178588	0.744289241	0	0.631425726	0	0.5	CRA004775;Yang2025_Silkie_Wenchang	PRJNA827465	0.935821071
ENSGALG00010027569	ATP5PB	2	-0.042781441	0.056033869	-0.763492537	0.445169712	0	0.954262241	0	0.5	CRA004775;PRJNA827465	Yang2025_Silkie_Wenchang	0.846013379
ENSGALG00010027570	NDUFS2	2	-0.16470162	0.071826205	-2.29305752	0.021844694	0	0.174475735	0	1	PRJNA827465;Yang2025_Silkie_Wenchang	CRA004775	0.25052874
ENSGALG00010027570	NDUFS2	2	-0.074303024	0.167257843	-0.444242388	0.656867349	0	0.027327072	0	1	CRA004775;Yang2025_Silkie_Wenchang	PRJNA827465	0.910647529
ENSGALG00010027570	NDUFS2	2	-0.169222058	0.075454671	-2.242698224	0.024916281	0	0.189927492	0	1	CRA004775;PRJNA827465	Yang2025_Silkie_Wenchang	0.269034231
ENSGALG00010027571	RBM10	2	0.256577862	0.192800108	1.330797294	0.183255718	0.058908844	4.398019349	77.26249203	1	PRJNA827465;Yang2025_Silkie_Wenchang	CRA004775	0.691694865
ENSGALG00010027571	RBM10	2	0.070034911	0.404080658	0.173319138	0.862400582	0.304164656	14.4940061	93.10059625	0.5	CRA004775;Yang2025_Silkie_Wenchang	PRJNA827465	0.965970249
ENSGALG00010027571	RBM10	2	-0.104302817	0.208547564	-0.500139225	0.616977048	0.076793907	8.313316704	87.97110665	0.5	CRA004775;PRJNA827465	Yang2025_Silkie_Wenchang	0.899111936
ENSGALG00010027574	GNG7	2	-0.169407078	0.209418133	-0.808941782	0.418548634	0.064953282	3.089283033	67.63002971	1	PRJNA827465;Yang2025_Silkie_Wenchang	CRA004775	0.838184907
ENSGALG00010027574	GNG7	2	-0.217714408	0.198203921	-1.098436436	0.272013959	0.035308607	1.774239078	43.63781002	1	CRA004775;Yang2025_Silkie_Wenchang	PRJNA827465	0.773184343
ENSGALG00010027574	GNG7	2	-0.017721279	0.050761845	-0.349106272	0.727009528	0	0.037411705	0	1	CRA004775;PRJNA827465	Yang2025_Silkie_Wenchang	0.93142315
ENSGALG00010027575	FCER1G	2	-0.476066292	0.212187853	-2.243607659	0.024857656	0	0.561548847	0	1	PRJNA827465;Yang2025_Silkie_Wenchang	CRA004775	0.268931467
ENSGALG00010027575	FCER1G	2	-0.18240191	0.301415703	-0.605150654	0.5450789	0	0.036083556	0	1	CRA004775;Yang2025_Silkie_Wenchang	PRJNA827465	0.877354247
ENSGALG00010027575	FCER1G	2	-0.475380497	0.22277569	-2.133897541	0.032851169	0	0.754152222	0	1	CRA004775;PRJNA827465	Yang2025_Silkie_Wenchang	0.311420523
ENSGALG00010027576	GADD45B	2	-0.051086736	0.117614845	-0.434356187	0.664029809	0.005927346	1.182295113	15.41874873	0.5	PRJNA827465;Yang2025_Silkie_Wenchang	CRA004775	0.91312035
ENSGALG00010027576	GADD45B	2	0.147535881	0.217687464	0.677741744	0.497935454	0	0.032865504	0	1	CRA004775;Yang2025_Silkie_Wenchang	PRJNA827465	0.861171456
ENSGALG00010027576	GADD45B	2	-0.112493856	0.108842537	-1.0335468	0.301348081	0	0.046558133	0	0.5	CRA004775;PRJNA827465	Yang2025_Silkie_Wenchang	0.791222412
ENSGALG00010027578	ENSGALG00010027578	2	0.159080144	0.162441885	0.979304959	0.327429321	0	0.911196368	0	0.5	PRJNA827465;Yang2025_Silkie_Wenchang	CRA004775	0.800077397
ENSGALG00010027578	ENSGALG00010027578	2	-0.099950588	0.212970329	-0.469316961	0.638843094	0	0.785319456	0	1	CRA004775;Yang2025_Silkie_Wenchang	PRJNA827465	0.905768875
ENSGALG00010027578	ENSGALG00010027578	2	0.026707235	0.375448477	0.071134222	0.943290932	0.168485586	2.20397231	54.62737915	0.5	CRA004775;PRJNA827465	Yang2025_Silkie_Wenchang	0.985827325
ENSGALG00010027579	C26H6ORF125	2	-0.058294028	0.077388436	-0.75326536	0.45129047	0	0.200616674	0	1	PRJNA827465;Yang2025_Silkie_Wenchang	CRA004775	0.847847413
ENSGALG00010027579	C26H6ORF125	2	-0.146354728	0.178445597	-0.820164415	0.412122385	0	0.00757211	0	1	CRA004775;Yang2025_Silkie_Wenchang	PRJNA827465	0.835532508
ENSGALG00010027579	C26H6ORF125	2	-0.053672409	0.078114662	-0.687097752	0.492021131	0	0.088429755	0	1	CRA004775;PRJNA827465	Yang2025_Silkie_Wenchang	0.859157033
ENSGALG00010027583	GLE1	2	0.090462709	0.047331947	1.911239973	0.055973749	0	0.051400772	0	1	PRJNA827465;Yang2025_Silkie_Wenchang	CRA004775	0.409290467
ENSGALG00010027583	GLE1	2	0.054844885	0.087561333	0.626359633	0.531079081	0	0.173646239	0	0.5	CRA004775;Yang2025_Silkie_Wenchang	PRJNA827465	0.872848946
ENSGALG00010027583	GLE1	2	0.088958294	0.052647637	1.68969206	0.091086883	0	0.311770445	0	0.5	CRA004775;PRJNA827465	Yang2025_Silkie_Wenchang	0.517711803
ENSGALG00010027586	HJV	2	-0.178744364	0.206201971	-0.866841201	0.386029024	0.074737325	8.221880744	87.83733271	0.5	PRJNA827465;Yang2025_Silkie_Wenchang	CRA004775	0.826727263
ENSGALG00010027586	HJV	2	0.021163662	0.090126442	0.234821892	0.814346975	0	0.01015128	0	0.5	CRA004775;Yang2025_Silkie_Wenchang	PRJNA827465	0.953869515
ENSGALG00010027586	HJV	2	-0.376503409	0.109062886	-3.45216805	0.000556101	0	0.384364413	0	1	CRA004775;PRJNA827465	Yang2025_Silkie_Wenchang	0.025180239
ENSGALG00010027590	SEMA3F	2	0.022570244	0.227917356	0.099028194	0.921115884	0.089840072	7.334079475	86.36502368	0.5	PRJNA827465;Yang2025_Silkie_Wenchang	CRA004775	0.981519273
ENSGALG00010027590	SEMA3F	2	0.235769027	0.097538896	2.417179574	0.015641299	0	0.046845805	0	1	CRA004775;Yang2025_Silkie_Wenchang	PRJNA827465	0.20696121
ENSGALG00010027590	SEMA3F	2	-0.131619162	0.157212358	-0.837206208	0.402476668	0.013558563	1.226056782	18.43770903	0.5	CRA004775;PRJNA827465	Yang2025_Silkie_Wenchang	0.832905052
ENSGALG00010027592	MAP1S	2	-0.017003638	0.056928528	-0.29868395	0.765181204	0	0.358229981	0	0.5	PRJNA827465;Yang2025_Silkie_Wenchang	CRA004775	0.940779391
ENSGALG00010027592	MAP1S	2	0.017140085	0.074686376	0.229494138	0.818484876	0	0.00112258	0	1	CRA004775;Yang2025_Silkie_Wenchang	PRJNA827465	0.955256039
ENSGALG00010027592	MAP1S	2	-0.042591115	0.076352083	-0.55782519	0.57696376	0	0.106792635	0	0.5	CRA004775;PRJNA827465	Yang2025_Silkie_Wenchang	0.887113945
ENSGALG00010027593	ENSGALG00010027593	2	0.733552272	0.718016872	1.021636539	0.306952957	0.948993812	12.55230652	92.03333667	1	PRJNA827465;Yang2025_Silkie_Wenchang	CRA004775	0.79266003
ENSGALG00010027593	ENSGALG00010027593	2	1.229224293	0.38167251	3.220625698	0.001279111	0.108294995	1.40273682	28.7107898	1	CRA004775;Yang2025_Silkie_Wenchang	PRJNA827465	0.043386199
ENSGALG00010027593	ENSGALG00010027593	2	0.101828299	0.253994039	0.400908223	0.688487695	0	0.614419839	0	1	CRA004775;PRJNA827465	Yang2025_Silkie_Wenchang	0.919798626
ENSGALG00010027594	ITPR3	2	-0.018491859	0.07868736	-0.235004188	0.814205482	0	0.125070079	0	0.5	PRJNA827465;Yang2025_Silkie_Wenchang	CRA004775	0.953869515
ENSGALG00010027594	ITPR3	2	0.027229647	0.123774511	0.219993977	0.825875845	0	0.073015332	0	1	CRA004775;Yang2025_Silkie_Wenchang	PRJNA827465	0.956393839
ENSGALG00010027594	ITPR3	2	-0.03131669	0.097007558	-0.322827324	0.746826018	0	0.160245577	0	0.5	CRA004775;PRJNA827465	Yang2025_Silkie_Wenchang	0.936729365
ENSGALG00010027595	GNAT1	2	-0.314474489	0.493425761	-0.637328883	0.523910641	0.427001784	8.006159078	87.50961616	0.5	PRJNA827465;Yang2025_Silkie_Wenchang	CRA004775	0.869661505
ENSGALG00010027595	GNAT1	2	-0.434223537	0.407014922	-1.066849184	0.286039945	0.239853452	3.615954852	72.34478745	1	CRA004775;Yang2025_Silkie_Wenchang	PRJNA827465	0.781850473
ENSGALG00010027595	GNAT1	2	0.108595932	0.167156639	0.649665684	0.515908195	0	0.216800236	0	0.5	CRA004775;PRJNA827465	Yang2025_Silkie_Wenchang	0.867498525
ENSGALG00010027597	EXOSC2	2	-0.090568289	0.181435544	-0.499176112	0.617655322	0.051758155	4.394974472	77.24673929	0.5	PRJNA827465;Yang2025_Silkie_Wenchang	CRA004775	0.899121942
ENSGALG00010027597	EXOSC2	2	-0.297317407	0.124761204	-2.383091843	0.017167911	0	2.18018E-05	0	1	CRA004775;Yang2025_Silkie_Wenchang	PRJNA827465	0.217734604
ENSGALG00010027597	EXOSC2	2	-0.062577911	0.174997863	-0.357592428	0.720648346	0.041352225	2.640559328	62.12923567	0.5	CRA004775;PRJNA827465	Yang2025_Silkie_Wenchang	0.928796259
ENSGALG00010027600	KIRREL1	2	-0.102434621	0.061816706	-1.657070196	0.097505284	0	0.288232402	0	1	PRJNA827465;Yang2025_Silkie_Wenchang	CRA004775	0.533219726
ENSGALG00010027600	KIRREL1	2	-0.143611326	0.099242649	-1.447072676	0.14787657	0	0.016712863	0	1	CRA004775;Yang2025_Silkie_Wenchang	PRJNA827465	0.637279915
ENSGALG00010027600	KIRREL1	2	-0.080064818	0.074050809	-1.081214629	0.279601652	0	0.008575158	0	1	CRA004775;PRJNA827465	Yang2025_Silkie_Wenchang	0.777720009
ENSGALG00010027605	FCHO1	2	0.26980473	0.294710869	0.91548958	0.35993467	0.097030376	2.198506342	54.51457289	1	PRJNA827465;Yang2025_Silkie_Wenchang	CRA004775	0.814880744
ENSGALG00010027605	FCHO1	2	0.078208637	0.540741142	0.144632304	0.885001191	0.471328048	5.152089572	80.59039956	0.5	CRA004775;Yang2025_Silkie_Wenchang	PRJNA827465	0.972194923
ENSGALG00010027605	FCHO1	2	-0.157548143	0.232957386	-0.676295977	0.498852746	0.031642081	1.368305365	26.91689838	0.5	CRA004775;PRJNA827465	Yang2025_Silkie_Wenchang	0.861571318
ENSGALG00010027607	ABL1	2	-0.26570545	0.241526955	-1.100106818	0.271285583	0.107616586	12.33640356	91.8939098	1	PRJNA827465;Yang2025_Silkie_Wenchang	CRA004775	0.772743863
ENSGALG00010027607	ABL1	2	-0.366565143	0.209641273	-1.74853519	0.080371398	0.051602435	2.166890985	53.85093173	1	CRA004775;Yang2025_Silkie_Wenchang	PRJNA827465	0.487926636
ENSGALG00010027607	ABL1	2	-0.03998835	0.047430204	-0.843098838	0.399173168	0	0.028784232	0	1	CRA004775;PRJNA827465	Yang2025_Silkie_Wenchang	0.832054308
ENSGALG00010027608	GNAI2	2	0.300289564	0.289598547	1.036916678	0.29977469	0.156263603	13.92895675	92.82071143	1	PRJNA827465;Yang2025_Silkie_Wenchang	CRA004775	0.790051945
ENSGALG00010027608	GNAI2	2	0.272659968	0.357215211	0.763293274	0.445288512	0.217763931	6.661209672	84.9877117	0.5	CRA004775;Yang2025_Silkie_Wenchang	PRJNA827465	0.846013379
ENSGALG00010027608	GNAI2	2	0.023125434	0.042945128	0.538487943	0.590240226	0	0.325020899	0	0.5	CRA004775;PRJNA827465	Yang2025_Silkie_Wenchang	0.892451983
ENSGALG00010027609	RPL36	2	0.594938404	0.523066086	1.137405809	0.25536868	0.524978203	24.00846079	95.8348017	1	PRJNA827465;Yang2025_Silkie_Wenchang	CRA004775	0.764467569
ENSGALG00010027609	RPL36	2	0.459053733	0.686816253	0.668379251	0.503891537	0.888494386	17.10956066	94.15531456	0.5	CRA004775;Yang2025_Silkie_Wenchang	PRJNA827465	0.863315129
ENSGALG00010027609	RPL36	2	0.02420849	0.130277317	0.185822752	0.852583761	0.016448467	1.444029739	30.74934863	0.5	CRA004775;PRJNA827465	Yang2025_Silkie_Wenchang	0.962607579
ENSGALG00010027612	BAK1	2	0.086736193	0.060208667	1.44059315	0.149699657	0	0.032117219	0	1	PRJNA827465;Yang2025_Silkie_Wenchang	CRA004775	0.639457191
ENSGALG00010027612	BAK1	2	-0.280499975	0.371050856	-0.755960995	0.449672582	0.238353115	7.202503827	86.11593935	0.5	CRA004775;Yang2025_Silkie_Wenchang	PRJNA827465	0.847309577
ENSGALG00010027612	BAK1	2	-0.258707447	0.384042522	-0.673642715	0.500538489	0.266015053	9.473606469	89.44435782	0.5	CRA004775;PRJNA827465	Yang2025_Silkie_Wenchang	0.862400704
ENSGALG00010027614	LONP1	2	-0.046539511	0.041592014	-1.118953041	0.263160173	0	0.317565768	0	0.5	PRJNA827465;Yang2025_Silkie_Wenchang	CRA004775	0.768643821
ENSGALG00010027614	LONP1	2	0.038793816	0.086997295	0.445919792	0.655655185	0	0.772604391	0	1	CRA004775;Yang2025_Silkie_Wenchang	PRJNA827465	0.910411938
ENSGALG00010027614	LONP1	2	-0.004677327	0.104127662	-0.044919157	0.964171747	0.011533938	1.534489396	34.83174256	0.5	CRA004775;PRJNA827465	Yang2025_Silkie_Wenchang	0.990248829
ENSGALG00010027616	SLC38A3	2	0.13515261	0.251859369	0.536619348	0.591530577	0.065236973	1.954241354	48.82924784	0.5	PRJNA827465;Yang2025_Silkie_Wenchang	CRA004775	0.892776493
ENSGALG00010027616	SLC38A3	2	0.315606923	0.181523475	1.73865624	0.082095248	0	0.073644297	0	1	CRA004775;Yang2025_Silkie_Wenchang	PRJNA827465	0.493815353
ENSGALG00010027616	SLC38A3	2	-0.070041823	0.263066484	-0.266251412	0.79004559	0	0.424806051	0	0.5	CRA004775;PRJNA827465	Yang2025_Silkie_Wenchang	0.947390191
ENSGALG00010027618	PPP1R26	2	-0.095118282	0.068952403	-1.379477399	0.16774761	0.00285424	1.358112057	26.36837329	1	PRJNA827465;Yang2025_Silkie_Wenchang	CRA004775	0.667779227
ENSGALG00010027618	PPP1R26	2	-0.070244025	0.170751744	-0.411381008	0.680793177	0.032810527	2.036737779	50.90187797	0.5	CRA004775;Yang2025_Silkie_Wenchang	PRJNA827465	0.917851751
ENSGALG00010027618	PPP1R26	2	-0.031245967	0.062294753	-0.501582643	0.615961131	0	0.805540528	0	0.5	CRA004775;PRJNA827465	Yang2025_Silkie_Wenchang	0.899050535
ENSGALG00010027619	JAK3	2	0.136463789	0.232104317	0.587941625	0.556571476	0	0.442047848	0	0.5	PRJNA827465;Yang2025_Silkie_Wenchang	CRA004775	0.880358001
ENSGALG00010027619	JAK3	2	-0.233832062	0.266188913	-0.878444031	0.379702798	0.001429039	1.009600444	0.950915231	1	CRA004775;Yang2025_Silkie_Wenchang	PRJNA827465	0.824318332
ENSGALG00010027619	JAK3	2	-0.097113173	0.425940006	-0.227997304	0.819648336	0.223539875	2.553896816	60.84415025	0.5	CRA004775;PRJNA827465	Yang2025_Silkie_Wenchang	0.955256039
ENSGALG00010027621	TSPO2	2	0.2871055	0.257599023	1.114544212	0.265045788	0.108219444	5.410398029	81.51707149	1	PRJNA827465;Yang2025_Silkie_Wenchang	CRA004775	0.769274038
ENSGALG00010027621	TSPO2	2	-0.044464725	0.183436766	-0.242398106	0.808471702	0.011549774	1.081179873	7.508452113	0.5	CRA004775;Yang2025_Silkie_Wenchang	PRJNA827465	0.952496413
ENSGALG00010027621	TSPO2	2	0.115218607	0.523530193	0.220080156	0.825808729	0.432480573	4.09124672	75.55757283	0.5	CRA004775;PRJNA827465	Yang2025_Silkie_Wenchang	0.956393839
ENSGALG00010027623	APOBEC2	2	-0.046086778	0.127112595	-0.362566576	0.716928682	0.0132826	1.6737412	40.25360673	0.5	PRJNA827465;Yang2025_Silkie_Wenchang	CRA004775	0.927632716
ENSGALG00010027623	APOBEC2	2	0.101837927	0.150896127	0.674887615	0.499747169	0	0.00060457	0	1	CRA004775;Yang2025_Silkie_Wenchang	PRJNA827465	0.862078151
ENSGALG00010027623	APOBEC2	2	-0.144273252	0.119667745	-1.2056152	0.227965896	0	0.213902237	0	0.5	CRA004775;PRJNA827465	Yang2025_Silkie_Wenchang	0.740853612
ENSGALG00010027626	SEMA3B	2	0.102588822	0.271313619	0.378118956	0.705342227	0.136596628	13.82482228	92.76663396	0.5	PRJNA827465;Yang2025_Silkie_Wenchang	CRA004775	0.924281103
ENSGALG00010027626	SEMA3B	2	0.347299298	0.106788166	3.25222645	0.001145048	0	0.771114136	0	1	CRA004775;Yang2025_Silkie_Wenchang	PRJNA827465	0.041064434
ENSGALG00010027626	SEMA3B	2	-0.150364795	0.09017905	-1.667402735	0.09543435	0	0.376103346	0	0.5	CRA004775;PRJNA827465	Yang2025_Silkie_Wenchang	0.528960129
ENSGALG00010027627	OARD1	2	-0.217672784	0.160338328	-1.357584222	0.174595652	0.045172253	8.220053316	87.83462878	1	PRJNA827465;Yang2025_Silkie_Wenchang	CRA004775	0.679015726
ENSGALG00010027627	OARD1	2	-0.317008972	0.149572111	-2.119439044	0.03405338	0.019358346	1.288289702	22.37770758	0.5	CRA004775;Yang2025_Silkie_Wenchang	PRJNA827465	0.31730678
ENSGALG00010027627	OARD1	2	-0.055838852	0.07242655	-0.770972134	0.440723449	0	0.068335095	0	0.5	CRA004775;PRJNA827465	Yang2025_Silkie_Wenchang	0.844848889
ENSGALG00010027628	PIERCE1	2	-0.061713289	0.280627266	-0.219911951	0.825939729	0.116301136	3.676309633	72.79880914	0.5	PRJNA827465;Yang2025_Silkie_Wenchang	CRA004775	0.956393839
ENSGALG00010027628	PIERCE1	2	0.221484696	0.213520587	1.037299024	0.299596521	0	0.064661379	0	1	CRA004775;Yang2025_Silkie_Wenchang	PRJNA827465	0.79004225
ENSGALG00010027628	PIERCE1	2	-0.252487052	0.149728991	-1.686293684	0.091739247	0.001334659	1.013794372	1.36066769	0.5	CRA004775;PRJNA827465	Yang2025_Silkie_Wenchang	0.518914432
ENSGALG00010027632	LAMC3	2	0.508233844	0.463418391	1.096706246	0.272769825	0.193676649	1.799800057	44.43827268	0.5	PRJNA827465;Yang2025_Silkie_Wenchang	CRA004775	0.773720586
ENSGALG00010027632	LAMC3	2	0.684050471	0.326328528	2.096201869	0.036064287	0	0.662680004	0	1	CRA004775;Yang2025_Silkie_Wenchang	PRJNA827465	0.327683285
ENSGALG00010027632	LAMC3	2	0.203545261	0.364848808	0.557889341	0.576919951	0	0.295140273	0	0.5	CRA004775;PRJNA827465	Yang2025_Silkie_Wenchang	0.887113945
ENSGALG00010027634	OASL	2	0.193478356	0.167333403	1.156244673	0.247581092	0.020321126	1.566252017	36.15331446	1	PRJNA827465;Yang2025_Silkie_Wenchang	CRA004775	0.757947282
ENSGALG00010027634	OASL	2	0.092766209	0.369189739	0.251271099	0.801604518	0.174580983	2.451954053	59.21620151	0.5	CRA004775;Yang2025_Silkie_Wenchang	PRJNA827465	0.950313523
ENSGALG00010027634	OASL	2	-0.024108491	0.167214016	-0.144177456	0.885360342	0	0.800928568	0	0.5	CRA004775;PRJNA827465	Yang2025_Silkie_Wenchang	0.972280795
ENSGALG00010027635	CD48	2	0.020886318	0.100089249	0.20867694	0.834700443	0	0.958347693	0	0.5	PRJNA827465;Yang2025_Silkie_Wenchang	CRA004775	0.958133448
ENSGALG00010027635	CD48	2	-0.126051583	0.372074603	-0.338780401	0.734775167	0.18757548	2.646102412	62.20856777	0.5	CRA004775;Yang2025_Silkie_Wenchang	PRJNA827465	0.933533809
ENSGALG00010027635	CD48	2	-0.177963601	0.23665734	-0.751988513	0.452057967	0.055998647	1.518890465	34.16246772	1	CRA004775;PRJNA827465	Yang2025_Silkie_Wenchang	0.848275385
ENSGALG00010027636	SLC1A6	2	-0.721923227	0.330614364	-2.183580953	0.028993054	0	0.659682674	0	1	PRJNA827465;Yang2025_Silkie_Wenchang	CRA004775	0.289878916
ENSGALG00010027636	SLC1A6	2	-1.210076703	0.377188954	-3.20814459	0.001335943	0	0.370255077	0	1	CRA004775;Yang2025_Silkie_Wenchang	PRJNA827465	0.044663156

ENSGALG00010027636	SLC1A6	2	-0.908715789	0.497679363	-1.825906107	0.067864406	0.233670091	1.858433169	46.1912316	1	CRA004775;PRJNA827465	Yang2025_Silkie_Wenchang	0.449111762
ENSGALG00010027638	MRPS2	2	-0.111221187	0.070249859	-1.583222913	0.113370666	0	0.002036548	0	1	PRJNA827465;Yang2025_Silkie_Wenchang	CRA004775	0.568380061
ENSGALG00010027638	MRPS2	2	-0.155530751	0.141795416	-1.096867268	0.272699419	0	0.408720817	0	1	CRA004775;Yang2025_Silkie_Wenchang	PRJNA827465	0.773576706
ENSGALG00010027638	MRPS2	2	-0.126520473	0.075215497	-1.682106449	0.0925482	0	0.463719777	0	1	CRA004775;PRJNA827465	Yang2025_Silkie_Wenchang	0.520896735
ENSGALG00010027641	HYAL3	2	0.125472355	0.232288972	0.540156312	0.589089238	0.082757624	3.875382306	74.19609419	0.5	PRJNA827465;Yang2025_Silkie_Wenchang	CRA004775	0.892171138
ENSGALG00010027641	HYAL3	2	-0.16386707	0.587119057	-0.279103646	0.780165285	0.618512499	9.631627821	89.61753902	0.5	CRA004775;Yang2025_Silkie_Wenchang	PRJNA827465	0.945041817
ENSGALG00010027641	HYAL3	2	-0.358380099	0.345941725	-1.035955113	0.300223084	0.195244888	4.787125054	79.11063553	1	CRA004775;PRJNA827465	Yang2025_Silkie_Wenchang	0.790332744
ENSGALG00010027642	DIPK1B	2	0.005849245	0.086253907	0.067814267	0.945933487	0	0.78308902	0	0.5	PRJNA827465;Yang2025_Silkie_Wenchang	CRA004775	0.986321197
ENSGALG00010027642	DIPK1B	2	-0.123006302	0.161325799	-0.762471361	0.445778729	0	0.043963734	0	1	CRA004775;Yang2025_Silkie_Wenchang	PRJNA827465	0.846013379
ENSGALG00010027642	DIPK1B	2	0.03430337	0.09224467	0.371873739	0.709986858	0	0.125955897	0	0.5	CRA004775;PRJNA827465	Yang2025_Silkie_Wenchang	0.925298042
ENSGALG00010027644	NAA80	2	0.062606817	0.144099355	0.434469792	0.663947326	0	0.649897919	0	1	PRJNA827465;Yang2025_Silkie_Wenchang	CRA004775	0.913119244
ENSGALG00010027644	NAA80	2	0.011466968	0.302266189	0.037936655	0.969738188	0.069842176	1.605853997	37.72783815	0.5	CRA004775;Yang2025_Silkie_Wenchang	PRJNA827465	0.991572914
ENSGALG00010027644	NAA80	2	-0.052790414	0.149265044	-0.353668964	0.723586984	0	0.661024125	0	0.5	CRA004775;PRJNA827465	Yang2025_Silkie_Wenchang	0.930032096
ENSGALG00010027648	NFYA	2	-0.128081453	0.189176017	-0.677049107	0.498379798	0.066658517	14.26547049	92.99006646	1	PRJNA827465;Yang2025_Silkie_Wenchang	CRA004775	0.861478809
ENSGALG00010027648	NFYA	2	-0.304887444	0.084084526	-3.625963746	0.000287886	0	0.532519841	0	1	CRA004775;Yang2025_Silkie_Wenchang	PRJNA827465	0.016559912
ENSGALG00010027648	NFYA	2	0.048937262	0.046612726	1.049869123	0.293778289	0	0.373731976	0	0.5	CRA004775;PRJNA827465	Yang2025_Silkie_Wenchang	0.787150519
ENSGALG00010027649	FBLN2	2	-0.132229693	0.158323633	-0.83518607	0.403612951	0.038531927	4.30383898	76.76492999	0.5	PRJNA827465;Yang2025_Silkie_Wenchang	CRA004775	0.83306585
ENSGALG00010027649	FBLN2	2	0.074198673	0.413402287	0.179482976	0.857558484	0.290087182	5.959703167	83.22064083	0.5	CRA004775;Yang2025_Silkie_Wenchang	PRJNA827465	0.964008268
ENSGALG00010027649	FBLN2	2	0.200436195	0.242780148	0.825587249	0.409038271	0.07396848	2.227646165	55.1095674	1	CRA004775;PRJNA827465	Yang2025_Silkie_Wenchang	0.834316328
ENSGALG00010027650	CCDC124	2	0.095071471	0.089187869	1.06596863	0.286437823	0.010223913	2.634624981	62.04393388	1	PRJNA827465;Yang2025_Silkie_Wenchang	CRA004775	0.782018115
ENSGALG00010027650	CCDC124	2	0.008958118	0.280583226	0.031926776	0.974530445	0.10695135	2.463236942	59.40301223	0.5	CRA004775;Yang2025_Silkie_Wenchang	PRJNA827465	0.992492038
ENSGALG00010027650	CCDC124	2	-0.028394036	0.135239091	-0.209954356	0.833703298	0.017555813	1.250694395	20.04441663	0.5	CRA004775;PRJNA827465	Yang2025_Silkie_Wenchang	0.957968425
ENSGALG00010027654	AIF1L	2	-0.325449718	0.264111153	-1.232245267	0.217857478	0.126468514	10.22526809	90.22030531	1	PRJNA827465;Yang2025_Silkie_Wenchang	CRA004775	0.731333487
ENSGALG00010027654	AIF1L	2	-0.453783946	0.181834976	-2.495581189	0.012575097	0.033605416	1.980696216	49.51270204	1	CRA004775;Yang2025_Silkie_Wenchang	PRJNA827465	0.181574995
ENSGALG00010027654	AIF1L	2	-0.092186664	0.060839118	-1.51525314	0.12970831	0	0.527393629	0	1	CRA004775;PRJNA827465	Yang2025_Silkie_Wenchang	0.60409121
ENSGALG00010027663	COPA	2	-0.016655983	0.030432357	-0.54731162	0.584164662	0	0.015766195	0	1	PRJNA827465;Yang2025_Silkie_Wenchang	CRA004775	0.89083241
ENSGALG00010027663	COPA	2	-0.110766815	0.130219842	-0.850613957	0.394983834	0.023650003	2.873256294	65.19628263	1	CRA004775;Yang2025_Silkie_Wenchang	PRJNA827465	0.830643015
ENSGALG00010027663	COPA	2	-0.109904004	0.124268978	-0.884404183	0.376478066	0.022428004	2.93713786	65.95324946	1	CRA004775;PRJNA827465	Yang2025_Silkie_Wenchang	0.822974013
ENSGALG00010027664	TREMB1	2	0.007419177	0.436054797	0.017014322	0.986425219	0.274132149	3.413156605	70.70160805	0.5	PRJNA827465;Yang2025_Silkie_Wenchang	CRA004775	0.99618774
ENSGALG00010027664	TREMB1	2	-0.192051997	0.364886369	-0.526333712	0.598656364	0.056926147	1.259870402	20.62675663	0.5	CRA004775;Yang2025_Silkie_Wenchang	PRJNA827465	0.894762943
ENSGALG00010027664	TREMB1	2	0.361787144	0.221436688	1.633817534	0.102297169	0	0.057309662	0	1	CRA004775;PRJNA827465	Yang2025_Silkie_Wenchang	0.545029796
ENSGALG00010027665	TRAF1	2	0.345261642	0.106780221	3.233385721	0.001223323	0	0.001462939	0	1	PRJNA827465;Yang2025_Silkie_Wenchang	CRA004775	0.043262437
ENSGALG00010027665	TRAF1	2	-0.103298922	0.486159896	-0.212479316	0.831733113	0.411726732	7.513261814	86.69020161	0.5	CRA004775;Yang2025_Silkie_Wenchang	PRJNA827465	0.957712669
ENSGALG00010027665	TRAF1	2	-0.100788832	0.481411541	-0.209361064	0.834166386	0.40975295	8.189881751	87.7898115	0.5	CRA004775;PRJNA827465	Yang2025_Silkie_Wenchang	0.957968425
ENSGALG00010027667	PTPDC1	2	0.018997316	0.086922721	0.218554092	0.826997417	0	0.138851725	0	0.5	PRJNA827465;Yang2025_Silkie_Wenchang	CRA004775	0.956540044
ENSGALG00010027667	PTPDC1	2	-0.03396594	0.134841324	-0.251895963	0.801127138	0	0.019660505	0	1	CRA004775;Yang2025_Silkie_Wenchang	PRJNA827465	0.950158029
ENSGALG00010027667	PTPDC1	2	0.031184722	0.102224295	0.305061744	0.760319127	0	0.117029379	0	0.5	CRA004775;PRJNA827465	Yang2025_Silkie_Wenchang	0.939327609
ENSGALG00010027668	RANBP3	2	-0.081787455	0.052309174	-1.563539419	0.117925771	0.001916832	1.387100566	27.90717383	1	PRJNA827465;Yang2025_Silkie_Wenchang	CRA004775	0.577693705
ENSGALG00010027668	RANBP3	2	-0.156031976	0.071612003	-2.178852296	0.029342643	0	0.039788311	0	1	CRA004775;Yang2025_Silkie_Wenchang	PRJNA827465	0.291796413
ENSGALG00010027668	RANBP3	2	-0.059756638	0.040495474	-1.475637431	0.140041251	0	0.478794866	0	1	CRA004775;PRJNA827465	Yang2025_Silkie_Wenchang	0.623792706
ENSGALG00010027672	CNTRL	2	0.558011323	0.367939462	1.516584603	0.129371595	0.250846788	13.38422671	92.52851867	1	PRJNA827465;Yang2025_Silkie_Wenchang	CRA004775	0.603517352
ENSGALG00010027672	CNTRL	2	0.471469342	0.512189715	0.92049748	0.357312849	0.45128739	6.80733578	85.30996513	0.5	CRA004775;Yang2025_Silkie_Wenchang	PRJNA827465	0.813292764
ENSGALG00010027672	CNTRL	2	0.180163253	0.098904851	1.82158156	0.068518501	0	0.627400856	0	0.5	CRA004775;PRJNA827465	Yang2025_Silkie_Wenchang	0.451003838
ENSGALG00010027674	TREM2	2	0.165384043	0.126543083	1.306938622	0.191233558	0.004678187	1.169698278	14.50786763	1	PRJNA827465;Yang2025_Silkie_Wenchang	CRA004775	0.700590704
ENSGALG00010027674	TREM2	2	-0.062078128	0.261149923	-0.237710688	0.812105494	0.046072379	1.14708783	12.82271734	0.5	CRA004775;Yang2025_Silkie_Wenchang	PRJNA827465	0.953371387
ENSGALG00010027674	TREM2	2	-0.009643974	0.485978335	-0.019844452	0.984167457	0.296258528	1.953911189	48.82060117	0.5	CRA004775;PRJNA827465	Yang2025_Silkie_Wenchang	0.995669738
ENSGALG00010027685	ENSGALG00010027685	2	-0.124161261	0.240870821	-0.515468264	0.606222585	0	0.479516847	0	0.5	PRJNA827465;Yang2025_Silkie_Wenchang	CRA004775	0.896709756
ENSGALG00010027685	ENSGALG00010027685	2	-0.537334312	0.272555122	-1.971470238	0.048670114	0	0.640056666	0	1	CRA004775;Yang2025_Silkie_Wenchang	PRJNA827465	0.381461853
ENSGALG00010027685	ENSGALG00010027685	2	-0.338526057	0.386425779	-0.876044184	0.381006015	0.175667039	2.39821184	58.30226573	0.5	CRA004775;PRJNA827465	Yang2025_Silkie_Wenchang	0.824392515
ENSGALG00010027692	PHF2	2	-0.03458775	0.154457298	-0.223930824	0.822811131	0.033041367	2.652966023	62.30633971	0.5	PRJNA827465;Yang2025_Silkie_Wenchang	CRA004775	0.955822987
ENSGALG00010027692	PHF2	2	0.259813353	0.135152245	1.922360852	0.054560368	0	0.345743175	0	1	CRA004775;Yang2025_Silkie_Wenchang	PRJNA827465	0.40429603
ENSGALG00010027692	PHF2	2	0.062300956	0.239889092	0.259707333	0.795089536	0.097681545	5.947625513	83.18656752	0.5	CRA004775;PRJNA827465	Yang2025_Silkie_Wenchang	0.948933254
ENSGALG00010027693	RAB14	2	-0.037180288	0.154412544	-0.240785412	0.809721437	0.044623645	15.30509689	93.46622888	0.5	PRJNA827465;Yang2025_Silkie_Wenchang	CRA004775	0.952818762
ENSGALG00010027693	RAB14	2	-0.06757439	0.156914857	-0.430643672	0.666727481	0.036611075	3.528006405	71.65538025	0.5	CRA004775;Yang2025_Silkie_Wenchang	PRJNA827465	0.913617955
ENSGALG00010027693	RAB14	2	0.112616569	0.037293022	3.019775919	0.002529618	0	0.004423956	0	1	CRA004775;PRJNA827465	Yang2025_Silkie_Wenchang	0.067541822
ENSGALG00010027695	TREMB2	2	0.000697413	0.226291903	0.00308192	0.997540988	0.035986074	1.527579156	34.5369439	0.5	PRJNA827465;Yang2025_Silkie_Wenchang	CRA004775	0.999290321
ENSGALG00010027695	TREMB2	2	-0.33796806	0.270578523	-1.249057229	0.211644143	0	0.753770277	0	1	CRA004775;Yang2025_Silkie_Wenchang	PRJNA827465	0.724013667
ENSGALG00010027695	TREMB2	2	-0.149623337	0.551820653	-0.271144866	0.786279615	0.371533175	1.990783696	49.76852574	0.5	CRA004775;PRJNA827465	Yang2025_Silkie_Wenchang	0.946639719
ENSGALG00010027697	CATH2	2	-0.614968272	0.415602445	-1.479703208	0.138952469	0	0.204694015	0	1	PRJNA827465;Yang2025_Silkie_Wenchang	CRA004775	0.622711797
ENSGALG00010027697	CATH2	2	0.31174672	1.149959207	0.27109372	0.786318951	2.206077657	5.935566949	83.15240973	0.5	CRA004775;Yang2025_Silkie_Wenchang	PRJNA827465	0.946639719
ENSGALG00010027697	CATH2	2	0.517884803	0.960201932	0.539349887	0.589645453	1.346849128	3.683993227	72.85554184	0.5	CRA004775;PRJNA827465	Yang2025_Silkie_Wenchang	0.892320726
ENSGALG00010027698	FAM120A	2	-0.083721447	0.071129988	-1.177020403	0.239187363	0.006304216	2.198801709	54.52068297	1	PRJNA827465;Yang2025_Silkie_Wenchang	CRA004775	0.751401241
ENSGALG00010027698	FAM120A	2	-0.163448455	0.090627682	-1.803515778	0.07130725	0	0.4747944	0	0.5	CRA004775;Yang2025_Silkie_Wenchang	PRJNA827465	0.461027938
ENSGALG00010027698	FAM120A	2	-0.033887058	0.033950079	-0.998143708	0.318209678	0	0.022435874	0	0.5	CRA004775;PRJNA827465	Yang2025_Silkie_Wenchang	0.797512752
ENSGALG00010027699	VANGL2	2	-0.129211003	0.108437145	-1.191575104	0.233427892	0.015996121	2.965599066	66.28000018	1	PRJNA827465;Yang2025_Silkie_Wenchang	CRA004775	0.746676685
ENSGALG00010027699	VANGL2	2	-0.223615501	0.102718	-2.176984568	0.029481719	0	0.753681531	0	0.5	CRA004775;Yang2025_Silkie_Wenchang	PRJNA827465	0.292694292
ENSGALG00010027699	VANGL2	2	-0.03439752	0.063874737	-0.538515249	0.590221381	0	0.032006792	0	0.5	CRA004775;PRJNA827465	Yang2025_Silkie_Wenchang	0.892451983
ENSGALG00010027700	TCF3	2	-0.029300949	0.050020988	-0.585773091	0.558028009	0	0.259366295	0	0.5	PRJNA827465;Yang2025_Silkie_Wenchang	CRA004775	0.880678789
ENSGALG00010027700	TCF3	2	-0.000549532	0.0846631	-0.006490812	0.994821117	0	0.101617254	0	0.5	CRA004775;Yang2025_Silkie_Wenchang	PRJNA827465	0.998242335
ENSGALG00010027700	TCF3	2	-0.048341851	0.059074775	-0.818316298	0.413176602	0	0.017634878	0	1	CRA004775;PRJNA827465	Yang2025_Silkie_Wenchang	0.836055586
ENSGALG00010027701	NHLH1	2	0.40711408	0.501127433	0.812396316	0.416564259	0.320259233	2.631810866	62.00334861	0.5	PRJNA827465;Yang2025_Silkie_Wenchang	CRA004775	0.837984974
ENSGALG00010027701	NHLH1	2	-0.063569032	1.080825914	-0.058815237	0.953099272	2.016746513	7.297495253	86.29666803	0.5	CRA004775;Yang2025_Silkie_Wenchang	PRJNA827465	0.987758913
ENSGALG00010027701	NHLH1	2	-0.481179066	0.562458785	-0.855492133	0.392278758	0.421730735	2.79905901	64.27370783	1	CRA004775;PRJNA827465	Yang2025_Silkie_Wenchang	0.82944545
ENSGALG00010027702	FOXP4	2	0.236519034	0.27323187	0.865634868	0.386690428	0.119455841	4.890833521	79.55358743	0.5	PRJNA827465;Yang2025_Silkie_Wenchang	CRA004775	0.827062786
ENSGALG00010027702	FOXP4	2	-0.279661237	0.881502109	-0.317255324	0.751049874	1.392397865	9.067371665	88.97144578	0.5	CRA004775;Yang2025_Silkie_Wenchang	PRJNA827465	0.937631446
ENSGALG00010027702	FOXP4	2	-0.511302116	0.599425286	-0.852987232	0.393666385	0.584301556	4.621943003	78.36407763	1	CRA004775;PRJNA827465	Yang2025_Silkie_Wenchang	0.83043598
ENSGALG00010027703	NCSTN	2	-0.166764187	0.16944227	-0.984194714	0.325019784	0.046415781	4.884642264	79.52767171	1	PRJNA827465;Yang2025_Silkie_Wenchang	CRA004775	0.799914637
ENSGALG00010027703	NCSTN	2	-0.285696026	0.116970039	-2.442471836	0.014587068	0	0.953084591	0	1	CRA004775;Yang2025_Silkie_Wenchang	PRJNA827465	0.198860743
ENSGALG00010027703	NCSTN	2	-0.02570125	0.064255215	-0.399986991	0.689166099	0	0.161551308	0	1	CRA004775;PRJNA827465	Yang2025_Silkie_Wenchang	0.919854035
ENSGALG00010027704	MDFI	2	0.194033264	0.127732296	1.519061893	0.128746917	0.024168066	3.734764286	73.2245485	1	PRJNA827465;Yang2025_Silkie_Wenchang	CRA004775	0.602157487
ENSGALG00010027704	MDFI	2	0.162113956	0.310229318	0.522561688	0.601279307	0.114187862	1.760941636	43.21220081	0.5	CRA004775;Yang2025_Silkie_Wenchang	PRJNA827465	0.895230519
ENSGALG00010027704	MDFI	2	0.070715479	0.072289109	0.978231435	0.327959872	0	0.753857207	0	0.5	CRA004775;PRJNA827465	Yang2025_Silkie_Wenchang	0.800530298
ENSGALG00010027705	S100A11	2	0.243159059	0.116308563	2.090637636	0.036560559	0	0.015335028	0	1	PRJNA827465;Yang2025_Silkie_Wenchang	CRA004775	0.329671369
ENSGALG00010027705	S100A11	2	-0.1695382	0.468331759	-0.362004491	0.717348674	0.335485698	3.873541623	74.18383233	0.5	CRA004775;Yang2025_Silkie_Wenchang	PRJNA827465	0.9277944
ENSGALG00010027705	S100A11	2	-0.148051313	0.482498667	-0.306842947	0.758962917	0.373391853	4.458039822	77.5686167	0.5	CRA004775;PRJNA827465	Yang2025_Silkie_Wenchang	0.939031852
ENSGALG00010027706	GSN	2	-0.144825199	0.332213005	-0.435940788	0.662879691	0.202357681	11.80904085	91.53191176	0.5	PRJNA827465;Yang2025_Silkie_Wenchang	CRA004775	0.91282818
ENSGALG00010027706	GSN	2	-0.218113271	0.35390681	-0.616301424	0.537695599	0.171947401	2.788790578	64.14216227	0.5	CRA004775;Yang2025_Silkie_Wenchang	PRJNA827465	0.87481821
ENSGALG00010027706	GSN	2	0.178292695	0.096874434	1.840451471	0.065701983	0	0.025901159	0	1	CRA004775;PRJNA827465	Yang2025_Silkie_Wenchang	0.441718274
ENSGALG00010027707	HIGD1C	2	0.279865227	0.149125777	1.876705908	0.060558412	0.020888701	1.804681333	44.58855525	1	PRJNA827465;Yang2025_Silkie_Wenchang	CRA004775	0.426087949
ENSGALG00010027707	HIGD1C	2	-0.06581968	0.220187767	-0.298925237	0.764997091	0.037014811	1.52178876	34.2878574	0.5	CRA004775;Yang2025_Silkie_Wenchang	PRJNA827465	0.940771616
ENSGALG00010027707	HIGD1C	2	0.071465482	0.380932873	0.187606499	0.851185131	0.23663115	4.917749285	79.66549448	0.5	CRA004775;PRJNA827465	Yang2025_Silkie_Wenchang	0.962071965
ENSGALG00010027708	ENSGALG00010027708	2	-0.121502026	0.261236443	-0.46510366	0.641857225	0	0.001769201	0	1	PRJNA827465;Yang2025_Silkie_Wenchang	CRA004775	0.906634523
ENSGALG00010027708	ENSGALG00010027708	2	-0.405861497	0.384576878	-1.055345551	0.291267316	0.087492871	1.358517953	26.39037286	1	CRA004775;Yang2025_Silkie_Wenchang	PRJNA827465	0.78467882
ENSGALG00010027708	ENSGALG00010027708	2	-0.399947223	0.397867844	-1.005226305	0.314787892	0.104324329	1.425212663	29.83503264	1	CRA004775;PRJNA827465	Yang2025_Silkie_Wenchang	0.796531448
ENSGALG00010027709	PSMA5	2	-0.076925094	0.069011111	-1.114676933	0.264988889	0	0.503786164	0	0.5	PRJNA827465;Yang2025_Silkie_Wenchang	CRA004775	0.769274038
ENSGALG00010027709	PSMA5	2	-0.095685522	0.205755885	-0.465043914	0.641900009	0.02450816	1.366890928	26.84127316	0.5	CRA004775;Yang2025_Silkie_Wenchang	PRJNA827465	0.906634523
ENSGALG00010027709	PSMA5	2	-0.109518569	0.071210035	-1.537965389	0.124057078	0	0.7675599	0	1	CRA004775;PRJNA827465	Yang2025_Silkie_Wenchang	0.590534404
ENSGALG00010027710	ENSGALG00010027710	2	0.238655307	0.177511845	1.344447224	0.178803812	0.002431585	1.039696013	3.818040359	1	PRJNA827465;Yang2025_Silkie_Wenchang	CRA004775	0.68554543
ENSGALG00010027710	ENSGALG00010027710	2	0.035863077	0.221450978	0.161945894	0.871348461	0	0.3180882	0	0.5	CRA004775;Yang2025_Silkie_Wenchang	PRJNA827465	0.96930765
ENSGALG00010027710	ENSGALG00010027710	2	0.315616171	0.278115187	1.134839758	0.256442465	0.023916851	1.088823266	8.157730311	0.5	CRA004775;PRJNA827465	Yang2025_Silkie_Wenchang	0.764677554
ENSGALG00010027711	WNK2	2	0.006278085	0.167083628	0.037574509	0.970026932	0.036409403	2.663910467	62.46120085	0.5	PRJNA827465;Yang2025_Silkie_Wenchang	CRA004775	0.991624015
ENSGALG00010027711	WNK2	2	0.217651995	0.17153248	1.268867531	0.204488314	0	0.007085516	0	1	CRA004775;Yang2025_Silkie_Wenchang	PRJNA827465	0.71729696
ENSGALG00010027711	WNK2	2	-0.113173354	0.097442691	-1.161435029	0.245465031	0	0.628604748	0	0.5	CRA004775;PRJNA827465	Yang2025_Silkie_Wenchang	0.75661406
ENSGALG00010027712	STOM	2	-0.497759208	0.120830089	-4.11949715	3.797E-05	0.001646263	1.059026513	5.573657753	1	PRJNA827465;Yang2025_Silkie_Wenchang	CRA004775	0.004000873
ENSGALG00010027712	STOM	2	-0.434466906	0.191899005	-2.264039385	0.023571693	0.041618296	2.263714128	55.82481076	1	CRA004775;Yang2025_Silkie_Wenchang	PRJNA827465	0.262001762
ENSGALG00010027712	STOM	2	-0.30171376	0.133007429	-2.268397804	0.023304972	0	0.283686287	0	1	CRA004775;PRJNA827465	Yang2025_Silkie_Wenchang	0.260268986
ENSGALG00010027713	ENSGALG00010027713	2	0.038621359	0.092719813	0.416538361	0.677016103	0	0.046421622	0	1	PRJNA827465;Yang2025_Silkie_Wenchang	CRA004775	0.917109899
ENSGALG00010027713	ENSGALG00010027713	2	0.165399789	0.261172236	0.633297748	0.526539224	0	0.048400073	0	1	CRA004775;Yang2025_Silkie_Wenchang	PRJNA827465	0.87066367
ENSGALG00010027713	ENSGALG00010027713	2	0.044590719	0.093202464	0.478428546	0.63234522	0	0.239840027	0	1	CRA004775;PRJNA827465	Yang2025_Silkie_Wenchang	0.903058174
ENSGALG00010027714	SYPL2	2	-0.123880073	0.107763889	-1.149550881	0.250328899	0	0.015285434	0	1	PRJNA827465;Yang2025_Silkie_Wenchang	CRA004775	0.759943035
ENSGALG00010027714	SYPL2	2	-0.211186322	0.231730441	-0.911344755	0.362113752	0	0.606050912	0	1	CRA004775;Yang2025_Silkie_Wenchang	PRJNA827465	0.815579273
ENSGALG00010027714	SYPL2	2	-0.133954089	0.118495036	-1.130461609	0.258281766	0	0.731432985	0	1	CRA004775;PRJNA827465	Yang2025_Silkie_Wenchang	0.765554168
ENSGALG00010027715	NINJ1	2	-0.09163591	0.171352366	-0.534780537	0.592801623	0.048111949	5.518820467	81.88018606	0.5	PRJNA827465;Yang2025_Silkie_Wenchang	CRA004775	0.892776493
ENSGALG00010027715	NINJ1	2	-0.457104043	0.316052195	-1.446292891	0.148095069	0.123832501	1.934533163	48.30794225	1	CRA004775;Yang2025_Silkie_Wenchang	PRJNA827465	0.63772401
ENSGALG00010027715	NINJ1	2	-0.33877885	0.516751471	-0.655593393	0.512085778	0.429496581	4.2771227	76.6197963	0.5	CRA004775;PRJNA827465	Yang2025_Silkie_Wenchang	0.865694231
ENSGALG00010027717	ATXN7L2	2	0.131290535	0.175781766	0.746895075	0.455126899	0.043838166	3.440836917	70.93730322	0.5	PRJNA827465;Yang2025_Silkie_Wenchang	CRA004775	0.849072665
ENSGALG00010027717	ATXN7L2	2	0.265403157	0.124495267	2.131833306	0.033020553	0	0.709748636	0	1	CRA004775;Yang2025_Silkie_Wenchang	PRJNA827465	0.312122842
ENSGALG00010027717	ATXN7L2	2	-0.03834664	0.124628755	-0.307686935	0.758320562	0	0.015728559	0	0.5	CRA004775;PRJNA827465	Yang2025_Silkie_Wenchang	0.938954369
ENSGALG00010027718	ENSGALG00010027718	2	-0.010994521	0.359769975	-0.030559864	0.975620551	0.165555167	2.666114463	62.49223303	0.5	PRJNA827465;Yang2025_Silkie_Wenchang	CRA004775	0.992754467
ENSGALG00010027718	ENSGALG00010027718	2	-0.531523282	0.306496444	-1.734190694	0.082884254	0	0.226879666	0	1	CRA004775;Yang2025_Silkie_Wenchang	PRJNA827465	0.495859127
ENSGALG00010027718	ENSGALG00010027718	2	-0.111684384	0.507875003	-0.219905258	0.825944941	0.366410616	3.109752616	67.84310126	0.5	CRA004775;PRJNA827465	Yang2025_Silkie_Wenchang	0.956393839
ENSGALG00010027720	DAB2IP	2	-0.044044595	0.124276738	-0.354407393	0.723033595	0.020586938	2.594698039	61.45986989	0.5	PRJNA827465;Yang2025_Silkie_Wenchang	CRA004775	0.930032096
ENSGALG00010027720	DAB2IP	2	0.14013866	0.133566944	1.049201662	0.294085314	0	0.078901154	0	1	CRA004775;Yang2025_Silkie_Wenchang	PRJNA827465	0.787324026
ENSGALG00010027720	DAB2IP	2	-0.083265593	0.127134757	-0.654939643	0.512506615	0.015524329	1.332069628	24.92884916	0.5	CRA004775;PRJNA827465	Yang2025_Silkie_Wenchang	0.865809047
ENSGALG00010027721	SUSD3	2	-0.010083276	0.133943193	-0.075280243	0.939991741	0	0.037127887	0	0.5	PRJNA827465;Yang2025_Silkie_Wenchang	CRA004775	0.985444231
ENSGALG00010027721	SUSD3	2	-0.002396842	0.219699689	-0.010909628	0.991295549	0	0.053276432	0	0.5	CRA004775;Yang2025_Silkie_Wenchang	PRJNA827465	0.99755571
ENSGALG00010027721	SUSD3	2	-0.031008189	0.140717934	-0.220357049	0.825593093	0	0.012549587	0	1	CRA004775;PRJNA827465	Yang2025_Silkie_Wenchang	0.956386623
ENSGALG00010027722	GNAI3	2	-0.029285987	0.038510484	-0.760467893	0.446974954	0	0.321450419	0	1	PRJNA827465;Yang2025_Silkie_Wenchang	CRA004775	0.84650962
ENSGALG00010027722	GNAI3	2	-0.153837664	0.147343352	-1.044076044	0.296450209	0.020630199	1.506106594	33.60363709	1	CRA004775;Yang2025_Silkie_Wenchang	PRJNA827465	0.788267543
ENSGALG00010027722	GNAI3	2	-0.132184943	0.182100073	-0.725891764	0.467905126	0.044241881	2.166935805	53.85188627	1	CRA004775;PRJNA827465	Yang2025_Silkie_Wenchang	0.853115491
ENSGALG00010027723	ENSGALG00010027723	2	0.62317485	0.731135281	0.852338638	0.394026167	0.542512038	2.030201887	50.74381487	0.5	PRJNA827465;Yang2025_Silkie_Wenchang	CRA004775	0.830486298
ENSGALG00010027723	ENSGALG00010027723	2	-0.548936529	0.524317878	-1.046953674	0.295120945	0	0.780503483	0	1	CRA004775;Yang2025_Silkie_Wenchang	PRJNA827465	0.787628307
ENSGALG00010027723	ENSGALG00010027723	2	0.170654232	1.194817769	0.142828669	0.886425495	2.302916468	5.168389892	80.65161451	0.5	CRA004775;PRJNA827465	Yang2025_Silkie_Wenchang	0.972729743
ENSGALG00010027724	TTLL11	2	-0.215856525	0.160793198	-1.342448113	0.179450744	0.033163974	2.546897449	60.7365424	1	PRJNA827465;Yang2025_Silkie_Wenchang	CRA004775	0.686323221
ENSGALG00010027724	TTLL11	2	-0.226858368	0.224347804	-1.011190497	0.311925263	0.053289772	2.070295824	51.69772415	0.5	CRA004775;Yang2025_Silkie_Wenchang	PRJNA827465	0.795993742
ENSGALG00010027724	TTLL11	2	-0.073941705	0.088383616	-0.836599672	0.40281763	0	0.205178402	0	0.5	CRA004775;PRJNA827465	Yang2025_Silkie_Wenchang	0.832937979
ENSGALG00010027725	BTN1A1	2	-0.361439563	0.316672456	-1.141367228	0.253717137	0.02745486	1.132966463	11.73613405	1	PRJNA827465;Yang2025_Silkie_Wenchang	CRA004775	0.763418915
ENSGALG00010027725	BTN1A1	2	-0.862951619	0.34964183	-2.468101773	0.013583169	0	0.00533152	0	1	CRA004775;Yang2025_Silkie_Wenchang	PRJNA827465	0.190732158
ENSGALG00010027725	BTN1A1	2	-0.454878834	0.362713953	-1.254097973	0.209806397	0.107239322	1.655963864	39.61220884	1	CRA004775;PRJNA827465	Yang2025_Silkie_Wenchang	0.721625118
ENSGALG00010027727	GSTM2	2	0.045262268	0.240848451	0.187928417	0.850932766	0.049741565	1.548211155	35.40932729	0.5	PRJNA827465;Yang2025_Silkie_Wenchang	CRA004775	0.962056252
ENSGALG00010027727	GSTM2	2	-0.188924399	0.27362858	-0.690441032	0.489916881	0	0.268551837	0	1	CRA004775;Yang2025_Silkie_Wenchang	PRJNA827465	0.858512413
ENSGALG00010027727	GSTM2	2	0.156269336	0.162656638	0.960731376	0.336687251	0	0.333247673	0	0.5	CRA004775;PRJNA827465	Yang2025_Silkie_Wenchang	0.803887433
ENSGALG00010027729	EPS8L3	2	0.080838956	0.176718003	0.457446071	0.647350456	0	0.019131113	0	1	PRJNA827465;Yang2025_Silkie_Wenchang	CRA004775	0.907350807
ENSGALG00010027729	EPS8L3	2	0.69310985	0.594550186	1.165771816	0.243706715	0.556569346	4.670918136	78.59093286	1	CRA004775;Yang2025_Silkie_Wenchang	PRJNA827465	0.755365463
ENSGALG00010027729	EPS8L3	2	0.63389213	0.6205938	1.021428397	0.307051517	0.665106152	6.971535831	85.6559584	1	CRA004775;PRJNA827465	Yang2025_Silkie_Wenchang	0.79266003
ENSGALG00010027730	ENSGALG00010027730	2	0.120791646	0.078218172	1.544291332	0.122517771	0	0.038816789	0	1	PRJNA827465;Yang2025_Silkie_Wenchang	CRA004775	0.587278379
ENSGALG00010027730	ENSGALG00010027730	2	0.045037886	0.123419617	0.364916752	0.715173549	0	0.596291194	0	0.5	CRA004775;Yang2025_Silkie_Wenchang	PRJNA827465	0.926937937
ENSGALG00010027730	ENSGALG00010027730	2	0.100183898	0.088218247	1.135636923	0.256108549	0	0.903536317	0	0.5	CRA004775;PRJNA827465	Yang2025_Silkie_Wenchang	0.764570254
ENSGALG00010027731	OLFML2A	2	-0.020252024	0.216726222	-0.093445196	0.925549886	0.081428798	7.507269057	86.67957692	0.5	PRJNA827465;Yang2025_Silkie_Wenchang	CRA004775	0.982594411
ENSGALG00010027731	OLFML2A	2	0.198441719	0.101064933	1.963507162	0.049587273	0	0.004497358	0	1	CRA004775;Yang2025_Silkie_Wenchang	PRJNA827465	0.385697328
ENSGALG00010027731	OLFML2A	2	-0.069966493	0.218472431	-0.320253192	0.748776403	0.063016887	2.607744759	61.65268873	0.5	CRA004775;PRJNA827465	Yang2025_Silkie_Wenchang	0.937249951
ENSGALG00010027732	EEF2	2	-0.360812267	0.252532127	-1.428777683	0.153068142	0.118230566	13.39127757	92.5324526	1	PRJNA827465;Yang2025_Silkie_Wenchang	CRA004775	0.645187993
ENSGALG00010027732	EEF2	2	-0.49486156	0.176953961	-2.796555428	0.005165055	0.033119724	1.923702003	48.01689667	1	CRA004775;Yang2025_Silkie_Wenchang	PRJNA827465	0.107367602
ENSGALG00010027732	EEF2	2	-0.127854097	0.062224886	-2.054710022	0.039907021	0	0.295084368	0	1	CRA004775;PRJNA827465	Yang2025_Silkie_Wenchang	0.345781671
ENSGALG00010027733	RPL35	2	-0.135630481	0.178388673	-0.760308818	0.447070012	0.035073183	1.591537007	37.16765645	1	PRJNA827465;Yang2025_Silkie_Wenchang	CRA004775	0.84650962
ENSGALG00010027733	RPL35	2	-0.366965739	0.204895071	-1.790993498	0.073294338	0	0.169489413	0	1	CRA004775;Yang2025_Silkie_Wenchang	PRJNA827465	0.467947918
ENSGALG00010027733	RPL35	2	-0.061963663	0.070986388	-0.872895005	0.382720309	0	0.934943656	0	1	CRA004775;PRJNA827465	Yang2025_Silkie_Wenchang	0.825121999
ENSGALG00010027734	CSF1	2	0.122915446	0.117329439	1.047609598	0.294818513	0.015713385	2.275117675	56.04622956	1	PRJNA827465;Yang2025_Silkie_Wenchang	CRA004775	0.787551963
ENSGALG00010027734	CSF1	2	0.238083107	0.117003547	2.034836671	0.04186731	0	0.152582172	0	1	CRA004775;Yang2025_Silkie_Wenchang	PRJNA827465	0.354330508
ENSGALG00010027734	CSF1	2	0.029618898	0.088513486	0.334625818	0.737907366	0	0.116750978	0	1	CRA004775;PRJNA827465	Yang2025_Silkie_Wenchang	0.934161348
ENSGALG00010027738	ENSGALG00010027738	2	0.648131012	0.173751188	3.730224926	0.000191309	0	0.211768012	0	1	PRJNA827465;Yang2025_Silkie_Wenchang	CRA004775	0.012366799
ENSGALG00010027738	ENSGALG00010027738	2	-0.03859815	0.77372982	-0.049885824	0.960213374	1.127950391	17.25095815	94.20322053	0.5	CRA004775;Yang2025_Silkie_Wenchang	PRJNA827465	0.989338243
ENSGALG00010027738	ENSGALG00010027738	2	-0.116757004	0.693720653	-0.168305504	0.866342944	0.894975368	14.23829948	92.97668938	0.5	CRA004775;PRJNA827465	Yang2025_Silkie_Wenchang	0.967381009
ENSGALG00010027739	FGD3	2	0.054714071	0.045856981	1.193145945	0.23281222	0	0.880209142	0	0.5	PRJNA827465;Yang2025_Silkie_Wenchang	CRA004775	0.745799878
ENSGALG00010027739	FGD3	2	-0.031162062	0.0879256	-0.354413982	0.723028657	0	0.231642095	0	1	CRA004775;Yang2025_Silkie_Wenchang	PRJNA827465	0.930032096
ENSGALG00010027739	FGD3	2	0.072977926	0.052348967	1.394066201	0.163297614	0	0.627838134	0	0.5	CRA004775;PRJNA827465	Yang2025_Silkie_Wenchang	0.661668506
ENSGALG00010027740	MARCHF2	2	-0.204825079	0.277965812	-0.736871479	0.46120051	0.14523656	16.42121702	93.91031737	0.5	PRJNA827465;Yang2025_Silkie_Wenchang	CRA004775	0.85014477
ENSGALG00010027740	MARCHF2	2	-0.376769502	0.144115591	-2.614356294	0.008939576	0.019964171	1.835503041	45.51902244	1	CRA004775;Yang2025_Silkie_Wenchang	PRJNA827465	0.148099201
ENSGALG00010027740	MARCHF2	2	-0.008946699	0.117461432	-0.07616712	0.939286141	0.014289706	1.732579959	42.28260606	0.5	CRA004775;PRJNA827465	Yang2025_Silkie_Wenchang	0.985393654
ENSGALG00010027741	RAB11B	2	-0.107631969	0.160617947	-0.670111722	0.502786573	0.045549025	8.046951178	87.57293318	0.5	PRJNA827465;Yang2025_Silkie_Wenchang	CRA004775	0.863050365
ENSGALG00010027741	RAB11B	2	-0.231818776	0.073928888	-3.135699473	0.001714448	0	0.469385439	0	1	CRA004775;Yang2025_Silkie_Wenchang	PRJNA827465	0.052438091
ENSGALG00010027741	RAB11B	2	-0.051196144	0.108745995	-0.470786474	0.637793224	0.018188061	3.896153444	74.33365974	0.5	CRA004775;PRJNA827465	Yang2025_Silkie_Wenchang	0.905279419
ENSGALG00010027743	C1orf74	2	-0.111207321	0.16690873	-0.666276243	0.505234547	0	0.644091538	0	0.5	PRJNA827465;Yang2025_Silkie_Wenchang	CRA004775	0.863862536
ENSGALG00010027743	C1orf74	2	0.00987066	0.288296269	0.034237906	0.97268744	0	0.384264874	0	0.5	CRA004775;Yang2025_Silkie_Wenchang	PRJNA827465	0.992213869
ENSGALG00010027743	C1orf74	2	-0.182878901	0.172836502	-1.058103462	0.290008276	0	0.003907039	0	1	CRA004775;PRJNA827465	Yang2025_Silkie_Wenchang	0.784195688
ENSGALG00010027745	BICD2	2	0.086634498	0.124527183	0.695707524	0.486612012	0.022357447	3.134223798	68.09417373	0.5	PRJNA827465;Yang2025_Silkie_Wenchang	CRA004775	0.857603818
ENSGALG00010027745	BICD2	2	0.258641084	0.124015833	2.085548899	0.037019504	0	0.066663507	0	1	CRA004775;Yang2025_Silkie_Wenchang	PRJNA827465	0.331959293
ENSGALG00010027745	BICD2	2	0.021194335	0.100769082	0.21032577	0.833413424	0.008465103	1.166165508	14.24887865	0.5	CRA004775;PRJNA827465	Yang2025_Silkie_Wenchang	0.957968425
ENSGALG00010027746	UQCR11	2	-0.093464378	0.112939183	-0.827563783	0.407917586	0	0.094818181	0	0.5	PRJNA827465;Yang2025_Silkie_Wenchang	CRA004775	0.833982683
ENSGALG00010027746	UQCR11	2	-0.007076359	0.287022806	-0.024654346	0.980330671	0	0.006252794	0	0.5	CRA004775;Yang2025_Silkie_Wenchang	PRJNA827465	0.994676009
ENSGALG00010027746	UQCR11	2	-0.101698665	0.115998133	-0.876726742	0.380635078	0	0.016599933	0	1	CRA004775;PRJNA827465	Yang2025_Silkie_Wenchang	0.824392515
ENSGALG00010027751	ANGPTL4	2	0.278635568	0.118858296	2.344266897	0.019064523	0	0.457498717	0	1	PRJNA827465;Yang2025_Silkie_Wenchang	CRA004775	0.231290606
ENSGALG00010027751	ANGPTL4	2	0.392613522	0.20750069	1.892107064	0.058476717	0	0.03985245	0	1	CRA004775;Yang2025_Silkie_Wenchang	PRJNA827465	0.418938606
ENSGALG00010027751	ANGPTL4	2	0.237018179	0.132702981	1.786080289	0.074086259	0	0.026310188	0	1	CRA004775;PRJNA827465	Yang2025_Silkie_Wenchang	0.470177983
ENSGALG00010027752	SCAI	2	0.040287115	0.059180591	0.680748772	0.49603047	0	0.064339862	0	1	PRJNA827465;Yang2025_Silkie_Wenchang	CRA004775	0.860911322
ENSGALG00010027752	SCAI	2	0.10321187	0.106099365	0.972784988	0.330660175	0	0.253859883	0	1	CRA004775;Yang2025_Silkie_Wenchang	PRJNA827465	0.801575303
ENSGALG00010027752	SCAI	2	0.048487402	0.063354315	0.765337022	0.444070884	0	0.552718771	0	1	CRA004775;PRJNA827465	Yang2025_Silkie_Wenchang	0.846013379

ENSGALG00010027775	MVB12B	2	-0.112056863	0.041048485	-2.729865986	0.006336008	0	0.005038394	0	1	PRJNA827465;Yang2025_Silkie_Wenchang	CRA004775	0.121049048
ENSGALG00010027775	MVB12B	2	-0.068671099	0.087158274	-0.787889612	0.430761278	0	0.9852658	0	0.5	CRA004775;Yang2025_Silkie_Wenchang	PRJNA827465	0.842503929
ENSGALG00010027775	MVB12B	2	-0.088671917	0.061266085	-1.447324688	0.147806008	0.002219803	1.136829991	12.03609972	0.5	CRA004775;PRJNA827465	Yang2025_Silkie_Wenchang	0.637081344
ENSGALG00010027776	TKT	2	0.001601991	0.207053773	0.007737079	0.993826766	0.074673127	7.744692298	87.08793117	0.5	PRJNA827465;Yang2025_Silkie_Wenchang	CRA004775	0.998042093
ENSGALG00010027776	TKT	2	-0.169033935	0.11069013	-1.527091303	0.126738309	0.002848567	1.040782077	3.918406944	0.5	CRA004775;Yang2025_Silkie_Wenchang	PRJNA827465	0.597552276
ENSGALG00010027776	TKT	2	0.206973979	0.102692174	2.015479572	0.043854424	0	0.007659034	0	1	CRA004775;PRJNA827465	Yang2025_Silkie_Wenchang	0.361804689
ENSGALG00010027784	PEAR1	2	0.132078028	0.068260689	1.934906179	0.05300181	0	0.104388615	0	1	PRJNA827465;Yang2025_Silkie_Wenchang	CRA004775	0.399631766
ENSGALG00010027784	PEAR1	2	0.129091848	0.089000056	1.4504693	0.146927694	0	0.760462986	0	0.5	CRA004775;Yang2025_Silkie_Wenchang	PRJNA827465	0.635630068
ENSGALG00010027784	PEAR1	2	0.085481746	0.095627163	0.89390654	0.371371898	0	0.532372558	0	0.5	CRA004775;PRJNA827465	Yang2025_Silkie_Wenchang	0.821478485
ENSGALG00010027787	DCP1A	2	-0.04730199	0.074824747	-0.632170398	0.527275538	0	0.006837328	0	1	PRJNA827465;Yang2025_Silkie_Wenchang	CRA004775	0.871138813
ENSGALG00010027787	DCP1A	2	0.32582236	0.435016089	0.748989217	0.453863705	0.308158321	5.068838709	80.2716153	0.5	CRA004775;Yang2025_Silkie_Wenchang	PRJNA827465	0.849063008
ENSGALG00010027787	DCP1A	2	0.319296074	0.424989246	0.751303891	0.452469788	0.305975607	5.772944959	82.67781857	0.5	CRA004775;PRJNA827465	Yang2025_Silkie_Wenchang	0.848321192
ENSGALG00010027789	UAP1L1	2	-0.215914959	0.30065417	-0.718150553	0.472664463	0.174659883	29.49913316	96.61006988	0.5	PRJNA827465;Yang2025_Silkie_Wenchang	CRA004775	0.85404245
ENSGALG00010027789	UAP1L1	2	-0.507261641	0.081084228	-6.255984086	3.95017E-10	0	0.450003988	0	1	CRA004775;Yang2025_Silkie_Wenchang	PRJNA827465	2.54514E-07
ENSGALG00010027789	UAP1L1	2	0.074778957	0.072690165	1.028735552	0.303603959	0	0.499023228	0	0.5	CRA004775;PRJNA827465	Yang2025_Silkie_Wenchang	0.792205992
ENSGALG00010027801	MBD3	2	-0.007927294	0.04343505	-0.182509157	0.855183168	0	0.37086295	0	0.5	PRJNA827465;Yang2025_Silkie_Wenchang	CRA004775	0.963353229
ENSGALG00010027801	MBD3	2	-0.046570073	0.148071087	-0.314511588	0.753132517	0.021589495	1.563124435	36.02556666	0.5	CRA004775;Yang2025_Silkie_Wenchang	PRJNA827465	0.938087082
ENSGALG00010027801	MBD3	2	-0.049507022	0.082130418	-0.602785469	0.546651415	0.004875039	1.135568849	11.93778817	1	CRA004775;PRJNA827465	Yang2025_Silkie_Wenchang	0.878275987
ENSGALG00010027803	SAPCD2	2	-0.180617746	0.135102715	-1.336892056	0.181257874	0	0.328711867	0	1	PRJNA827465;Yang2025_Silkie_Wenchang	CRA004775	0.688706325
ENSGALG00010027803	SAPCD2	2	-0.180903643	0.22537589	-0.802675225	0.422162477	0	0.845070709	0	0.5	CRA004775;Yang2025_Silkie_Wenchang	PRJNA827465	0.839649223
ENSGALG00010027803	SAPCD2	2	-0.101016007	0.146728894	-0.688453402	0.491167305	0	0.390193629	0	0.5	CRA004775;PRJNA827465	Yang2025_Silkie_Wenchang	0.85895707
ENSGALG00010027811	MEX3D	2	0.185380202	0.221529873	0.836818076	0.402694836	0.088264781	9.705966619	89.69705915	0.5	PRJNA827465;Yang2025_Silkie_Wenchang	CRA004775	0.832937979
ENSGALG00010027811	MEX3D	2	0.267721831	0.1837687	1.456841291	0.145160195	0.041361836	2.432552614	58.89092001	1	CRA004775;Yang2025_Silkie_Wenchang	PRJNA827465	0.633281291
ENSGALG00010027811	MEX3D	2	-0.017775398	0.067822693	-0.262086291	0.793254916	0	0.099168841	0	0.5	CRA004775;PRJNA827465	Yang2025_Silkie_Wenchang	0.948644003
ENSGALG00010027813	LRRC71	2	0.720323577	0.31329345	2.299197688	0.021493716	0.103223984	2.08029964	51.93000176	1	PRJNA827465;Yang2025_Silkie_Wenchang	CRA004775	0.24802335
ENSGALG00010027813	LRRC71	2	-0.081181727	1.202054516	-0.067535811	0.946155154	2.65410905	12.01232014	91.67521354	0.5	CRA004775;Yang2025_Silkie_Wenchang	PRJNA827465	0.986370359
ENSGALG00010027813	LRRC71	2	-0.365768197	0.884101546	-0.413717404	0.67908108	1.357834735	7.204230942	86.11926786	0.5	CRA004775;PRJNA827465	Yang2025_Silkie_Wenchang	0.917701868
ENSGALG00010027814	ENSGALG00010027814	2	0.218121625	0.274298258	0.795198723	0.426497914	0.145389852	29.56820673	96.61798901	0.5	PRJNA827465;Yang2025_Silkie_Wenchang	CRA004775	0.84125685
ENSGALG00010027814	ENSGALG00010027814	2	0.13484089	0.38515536	0.350094804	0.726267551	0.271112611	10.95957961	90.87556242	0.5	CRA004775;Yang2025_Silkie_Wenchang	PRJNA827465	0.931029023
ENSGALG00010027814	ENSGALG00010027814	2	-0.077196045	0.068034491	-1.134660429	0.256517623	0	0.919747811	0	1	CRA004775;PRJNA827465	Yang2025_Silkie_Wenchang	0.764677554
ENSGALG00010027816	ZMYND19	2	-0.034019086	0.063944772	-0.532007311	0.594720932	0	0.087928235	0	1	PRJNA827465;Yang2025_Silkie_Wenchang	CRA004775	0.893382718
ENSGALG00010027816	ZMYND19	2	-0.122560227	0.141478945	-0.866278921	0.386337223	0	0.050780777	0	1	CRA004775;Yang2025_Silkie_Wenchang	PRJNA827465	0.826894808
ENSGALG00010027816	ZMYND19	2	-0.040648138	0.063899384	-0.636127223	0.524693503	0	0.361584827	0	1	CRA004775;PRJNA827465	Yang2025_Silkie_Wenchang	0.869834181
ENSGALG00010027821	NMRK2	2	0.025070835	0.108892491	0.23023475	0.817909361	0	0.019417899	0	1	PRJNA827465;Yang2025_Silkie_Wenchang	CRA004775	0.955109707
ENSGALG00010027821	NMRK2	2	-0.198103449	0.355656543	-0.557007746	0.577522137	0.128447317	1.591675001	37.17310386	0.5	CRA004775;Yang2025_Silkie_Wenchang	PRJNA827465	0.88731056
ENSGALG00010027821	NMRK2	2	-0.177672206	0.37235788	-0.477154413	0.63325217	0.166798698	1.80753129	44.67592314	0.5	CRA004775;PRJNA827465	Yang2025_Silkie_Wenchang	0.9032522
ENSGALG00010027822	DPH7	2	0.239550241	0.077643465	3.08525952	0.002033746	0	0.019446057	0	1	PRJNA827465;Yang2025_Silkie_Wenchang	CRA004775	0.058631999
ENSGALG00010027822	DPH7	2	0.186262602	0.112232242	1.659617576	0.09699141	0	0.506851153	0	1	CRA004775;Yang2025_Silkie_Wenchang	PRJNA827465	0.532209736
ENSGALG00010027822	DPH7	2	0.218466019	0.092144704	2.370901513	0.017744759	0	0.675427173	0	1	CRA004775;PRJNA827465	Yang2025_Silkie_Wenchang	0.2222112
ENSGALG00010027826	ARHGEF11	2	0.015954979	0.072152872	0.22112743	0.824993213	0.005176755	1.941357487	48.48965187	0.5	PRJNA827465;Yang2025_Silkie_Wenchang	CRA004775	0.956283606
ENSGALG00010027826	ARHGEF11	2	0.080193691	0.081235853	0.987171154	0.32355874	0	0.712984854	0	0.5	CRA004775;Yang2025_Silkie_Wenchang	PRJNA827465	0.79959942
ENSGALG00010027826	ARHGEF11	2	-0.050338571	0.060417767	-0.833174958	0.404746063	0	0.130751028	0	1	CRA004775;PRJNA827465	Yang2025_Silkie_Wenchang	0.833112102
ENSGALG00010027831	ATCAY	2	-0.015204941	0.093869389	-0.161979763	0.87132179	0	0.246826124	0	0.5	PRJNA827465;Yang2025_Silkie_Wenchang	CRA004775	0.969305415
ENSGALG00010027831	ATCAY	2	0.028963194	0.163216686	0.17745241	0.859153043	0	0.250334839	0	0.5	CRA004775;Yang2025_Silkie_Wenchang	PRJNA827465	0.964605083
ENSGALG00010027831	ATCAY	2	-0.053195948	0.109335601	-0.486538208	0.626585623	0	0.102534851	0	1	CRA004775;PRJNA827465	Yang2025_Silkie_Wenchang	0.901504244
ENSGALG00010027838	ENSGALG00010027838	2	-0.033012093	0.256060071	-0.128923235	0.897483391	0	0.910101295	0	0.5	PRJNA827465;Yang2025_Silkie_Wenchang	CRA004775	0.975502255
ENSGALG00010027838	ENSGALG00010027838	2	0.849488336	1.184080218	0.71742665	0.473112114	2.569662638	11.80612704	91.52982179	0.5	CRA004775;Yang2025_Silkie_Wenchang	PRJNA827465	0.85341771
ENSGALG00010027838	ENSGALG00010027838	2	1.078930207	0.938265069	1.149920468	0.25017663	1.538090553	7.717749729	87.0428553	1	CRA004775;PRJNA827465	Yang2025_Silkie_Wenchang	0.75993411
ENSGALG00010027842	SLAMF8	2	-0.146883456	0.150124897	-0.978408374	0.327872388	0	0.977162416	0	0.5	PRJNA827465;Yang2025_Silkie_Wenchang	CRA004775	0.80043771
ENSGALG00010027842	SLAMF8	2	0.378090613	0.368478525	1.026085858	0.304851103	0.134284624	1.859731459	46.22879581	1	CRA004775;Yang2025_Silkie_Wenchang	PRJNA827465	0.79225794
ENSGALG00010027842	SLAMF8	2	0.218115069	0.539718516	0.404127453	0.686118998	0.468454374	4.584044898	78.18520494	0.5	CRA004775;PRJNA827465	Yang2025_Silkie_Wenchang	0.919235539
ENSGALG00010027847	NRTN	2	1.113743222	0.513880797	2.167318233	0.030210597	0	0.107719637	0	1	PRJNA827465;Yang2025_Silkie_Wenchang	CRA004775	0.295973612
ENSGALG00010027847	NRTN	2	0.40738929	0.856646238	0.475563041	0.634385711	0.474470126	1.449710362	31.02070413	0.5	CRA004775;Yang2025_Silkie_Wenchang	PRJNA827465	0.903815945
ENSGALG00010027847	NRTN	2	0.528680585	0.641811047	0.823732448	0.410091598	0.336744163	1.626467086	38.51704665	0.5	CRA004775;PRJNA827465	Yang2025_Silkie_Wenchang	0.834652129
ENSGALG00010027860	ENSGALG00010027860	2	0.337883916	0.246523032	1.370597762	0.170500381	0	0.596727016	0	1	PRJNA827465;Yang2025_Silkie_Wenchang	CRA004775	0.672201941
ENSGALG00010027860	ENSGALG00010027860	2	0.387231733	0.27875097	1.389167305	0.164781881	0	0.454136974	0	1	CRA004775;Yang2025_Silkie_Wenchang	PRJNA827465	0.663999614
ENSGALG00010027860	ENSGALG00010027860	2	0.142709073	0.279500141	0.510586768	0.609640443	0	0.000353557	0	1	CRA004775;PRJNA827465	Yang2025_Silkie_Wenchang	0.897238578
ENSGALG00010027861	PNPLA7	2	-0.077908181	0.069318853	-1.123947931	0.261051032	0.004340244	1.803230397	44.54196943	1	PRJNA827465;Yang2025_Silkie_Wenchang	CRA004775	0.761979536
ENSGALG00010027861	PNPLA7	2	-0.154565407	0.074514867	-2.074289501	0.038052432	0	0.001736712	0	1	CRA004775;Yang2025_Silkie_Wenchang	PRJNA827465	0.336310939
ENSGALG00010027861	PNPLA7	2	-0.029925692	0.06179493	-0.484274232	0.628191261	0	0.419130795	0	1	CRA004775;PRJNA827465	Yang2025_Silkie_Wenchang	0.901904489
ENSGALG00010027863	CHDH	2	-0.144222543	0.105039371	-1.373033203	0.169742025	0.013219195	2.380388846	57.99095688	1	PRJNA827465;Yang2025_Silkie_Wenchang	CRA004775	0.67232541
ENSGALG00010027863	CHDH	2	-0.064274519	0.323735265	-0.19854037	0.842622302	0.133505616	2.093018008	52.22210242	0.5	CRA004775;Yang2025_Silkie_Wenchang	PRJNA827465	0.96029183
ENSGALG00010027863	CHDH	2	-0.030237556	0.111832038	-0.270383666	0.786865105	0.007699341	1.065211482	6.121928164	0.5	CRA004775;PRJNA827465	Yang2025_Silkie_Wenchang	0.946784559
ENSGALG00010027864	ACTR8	2	0.03849967	0.057201647	0.673051777	0.500914351	0	0.042937712	0	1	PRJNA827465;Yang2025_Silkie_Wenchang	CRA004775	0.862421093
ENSGALG00010027864	ACTR8	2	-0.070831097	0.119099837	-0.594720356	0.552030412	0	0.969292541	0	0.5	CRA004775;Yang2025_Silkie_Wenchang	PRJNA827465	0.879061525
ENSGALG00010027864	ACTR8	2	-0.027858653	0.122859711	-0.226751737	0.820616794	0.016324675	1.690468673	40.84480738	0.5	CRA004775;PRJNA827465	Yang2025_Silkie_Wenchang	0.955383907
ENSGALG00010027865	SELENOK	2	0.188902146	0.178896971	1.055927025	0.291001556	0.046996371	3.491846285	71.36185507	1	PRJNA827465;Yang2025_Silkie_Wenchang	CRA004775	0.78467882
ENSGALG00010027865	SELENOK	2	-0.023485875	0.447364908	-0.052498251	0.958131689	0.350229404	7.844547267	87.25229174	0.5	CRA004775;Yang2025_Silkie_Wenchang	PRJNA827465	0.98887765
ENSGALG00010027865	SELENOK	2	-0.168083953	0.259550564	-0.647596178	0.51724617	0.101943395	3.539905799	71.7506607	0.5	CRA004775;PRJNA827465	Yang2025_Silkie_Wenchang	0.867952013
ENSGALG00010027866	KCNA2	2	-0.022963189	0.08260637	-0.277983277	0.781025196	0	0.764706874	0	0.5	PRJNA827465;Yang2025_Silkie_Wenchang	CRA004775	0.945235709
ENSGALG00010027866	KCNA2	2	-0.044480395	0.1878066	-0.236841491	0.812779762	0.021837491	1.174201881	14.8357692	0.5	CRA004775;Yang2025_Silkie_Wenchang	PRJNA827465	0.95348724
ENSGALG00010027866	KCNA2	2	0.047969329	0.100703118	0.47634403	0.633829302	0	0.633734336	0	1	CRA004775;PRJNA827465	Yang2025_Silkie_Wenchang	0.903596555
ENSGALG00010027867	UBAP2L	2	0.260121197	0.383819986	0.677716653	0.497951365	0.252699588	6.282979236	84.08398369	0.5	PRJNA827465;Yang2025_Silkie_Wenchang	CRA004775	0.861171456
ENSGALG00010027867	UBAP2L	2	0.106257727	0.570866878	0.186133985	0.852339692	0.590941923	10.52697003	90.50059042	0.5	CRA004775;Yang2025_Silkie_Wenchang	PRJNA827465	0.96254653
ENSGALG00010027867	UBAP2L	2	-0.21834042	0.179640806	-1.215427751	0.224202989	0.047594835	3.387975391	70.483847	1	CRA004775;PRJNA827465	Yang2025_Silkie_Wenchang	0.737455024
ENSGALG00010027869	NSMF	2	-0.05441307	0.088747977	-0.613118987	0.539797667	0.004994927	1.390035207	28.05937612	0.5	PRJNA827465;Yang2025_Silkie_Wenchang	CRA004775	0.875269925
ENSGALG00010027869	NSMF	2	-0.014546147	0.240988562	-0.060360321	0.95186866	0.066857952	2.011921779	50.29627838	0.5	CRA004775;Yang2025_Silkie_Wenchang	PRJNA827465	0.987719213
ENSGALG00010027869	NSMF	2	0.026350009	0.079910495	0.329744031	0.74159338	0	0.890213054	0	1	CRA004775;PRJNA827465	Yang2025_Silkie_Wenchang	0.93543545
ENSGALG00010027870	HAX1	2	-0.059555728	0.100913742	-0.590164694	0.55508024	0.008114522	1.24117781	19.43136658	1	PRJNA827465;Yang2025_Silkie_Wenchang	CRA004775	0.880223402
ENSGALG00010027870	HAX1	2	-0.221928658	0.215426954	-1.030180553	0.302925257	0	0.427350266	0	0.5	CRA004775;Yang2025_Silkie_Wenchang	PRJNA827465	0.792084776
ENSGALG00010027870	HAX1	2	-0.017874062	0.060630639	-0.294802464	0.768144784	0	0.004578226	0	0.5	CRA004775;PRJNA827465	Yang2025_Silkie_Wenchang	0.941444269
ENSGALG00010027872	KDM5B	2	-0.131167204	0.042773585	-3.06654692	0.002165468	0	0.394816364	0	1	PRJNA827465;Yang2025_Silkie_Wenchang	CRA004775	0.061198081
ENSGALG00010027872	KDM5B	2	-0.183088564	0.088181247	-2.076275524	0.037868476	0	0.016016645	0	1	CRA004775;Yang2025_Silkie_Wenchang	PRJNA827465	0.335620339
ENSGALG00010027872	KDM5B	2	-0.121960921	0.045414523	-2.685504842	0.007242033	0	0.064273702	0	1	CRA004775;PRJNA827465	Yang2025_Silkie_Wenchang	0.130866025
ENSGALG00010027873	RFX2	2	0.050309428	0.109465302	0.459592467	0.645808767	0.004307492	1.209211422	17.30147586	0.5	PRJNA827465;Yang2025_Silkie_Wenchang	CRA004775	0.907098003
ENSGALG00010027873	RFX2	2	-0.211239815	0.212759998	-0.992854939	0.320780652	0.036348142	1.458314915	31.42770536	1	CRA004775;Yang2025_Silkie_Wenchang	PRJNA827465	0.798348216
ENSGALG00010027873	RFX2	2	-0.12834861	0.342026485	-0.375259274	0.707467652	0.173381381	3.327490731	69.94732425	0.5	CRA004775;PRJNA827465	Yang2025_Silkie_Wenchang	0.92489559
ENSGALG00010027874	FRS3	2	0.03021493	0.049073134	0.615712252	0.53808445	0	0.011235398	0	1	PRJNA827465;Yang2025_Silkie_Wenchang	CRA004775	0.87481821
ENSGALG00010027874	FRS3	2	-0.004147123	0.096868012	-0.042812104	0.965851315	0	0.410120645	0	0.5	CRA004775;Yang2025_Silkie_Wenchang	PRJNA827465	0.990731137
ENSGALG00010027874	FRS3	2	0.024684668	0.054418725	0.453606152	0.650112325	0	0.519373974	0	0.5	CRA004775;PRJNA827465	Yang2025_Silkie_Wenchang	0.908335059
ENSGALG00010027875	WNT5A	2	-0.443144263	0.119213869	-3.717220699	0.000201426	0.007932697	1.382817196	27.68386141	1	PRJNA827465;Yang2025_Silkie_Wenchang	CRA004775	0.012862829
ENSGALG00010027875	WNT5A	2	-0.2119619	0.389217125	-0.544585236	0.586038811	0.249033721	5.186587809	80.71950121	0.5	CRA004775;Yang2025_Silkie_Wenchang	PRJNA827465	0.891402106
ENSGALG00010027875	WNT5A	2	-0.110622836	0.26367544	-0.419541677	0.674820302	0.087069993	2.403114859	58.38734065	0.5	CRA004775;PRJNA827465	Yang2025_Silkie_Wenchang	0.915989029
ENSGALG00010027876	ANAPC2	2	0.120964953	0.069205278	1.747915139	0.080478723	0	0.178196504	0	1	PRJNA827465;Yang2025_Silkie_Wenchang	CRA004775	0.488261768
ENSGALG00010027876	ANAPC2	2	-0.154974679	0.336514604	-0.460528836	0.645136678	0.200018835	8.405442966	88.10294705	0.5	CRA004775;Yang2025_Silkie_Wenchang	PRJNA827465	0.906966303
ENSGALG00010027876	ANAPC2	2	-0.177681501	0.304006807	-0.584465533	0.558907148	0.16366254	8.27678535	87.91801457	0.5	CRA004775;PRJNA827465	Yang2025_Silkie_Wenchang	0.880926437
ENSGALG00010027877	OAZ1	2	-0.340917784	0.25543502	-1.334655616	0.181989086	0.114164933	7.246243832	86.19974675	1	PRJNA827465;Yang2025_Silkie_Wenchang	CRA004775	0.689817443
ENSGALG00010027877	OAZ1	2	-0.511442784	0.142951702	-3.577731346	0.000346589	2.34567E-05	1.000553661	0.055335461	1	CRA004775;Yang2025_Silkie_Wenchang	PRJNA827465	0.018458
ENSGALG00010027877	OAZ1	2	-0.124200773	0.046054492	-2.696822104	0.007000466	0	0.949535027	0	1	CRA004775;PRJNA827465	Yang2025_Silkie_Wenchang	0.12854434
ENSGALG00010027879	ENSGALG00010027879	2	0.448736995	0.730257604	0.614491369	0.538890675	1.044120269	47.24415353	97.88333598	0.5	PRJNA827465;Yang2025_Silkie_Wenchang	CRA004775	0.87481821
ENSGALG00010027879	ENSGALG00010027879	2	0.66300962	0.606139064	1.093824273	0.274032056	0.607525514	5.25640057	80.9755747	0.5	CRA004775;Yang2025_Silkie_Wenchang	PRJNA827465	0.774526342
ENSGALG00010027879	ENSGALG00010027879	2	-0.263893264	0.098453911	-2.680373594	0.007354003	0	0.213209592	0	1	CRA004775;PRJNA827465	Yang2025_Silkie_Wenchang	0.131918938
ENSGALG00010027880	AMH	2	-0.318175927	0.096895531	-3.283700729	0.001024537	0	0.395400646	0	1	PRJNA827465;Yang2025_Silkie_Wenchang	CRA004775	0.038424195
ENSGALG00010027880	AMH	2	-0.360233418	0.178024931	-2.023499828	0.043021641	0	0.752718004	0	0.5	CRA004775;Yang2025_Silkie_Wenchang	PRJNA827465	0.358693162
ENSGALG00010027880	AMH	2	-0.266458849	0.109282963	-2.438246933	0.014758688	0	0.390865395	0	0.5	CRA004775;PRJNA827465	Yang2025_Silkie_Wenchang	0.200535277
ENSGALG00010027881	ENSGALG00010027881	2	0.048771024	0.184371706	0.264525532	0.791374993	0	0.401580665	0	0.5	PRJNA827465;Yang2025_Silkie_Wenchang	CRA004775	0.948014611
ENSGALG00010027881	ENSGALG00010027881	2	-0.080320847	0.288013421	-0.278878834	0.780337812	0.070662429	1.740887433	42.55803212	0.5	CRA004775;Yang2025_Silkie_Wenchang	PRJNA827465	0.945041817
ENSGALG00010027881	ENSGALG00010027881	2	-0.159636588	0.187723134	-0.850383139	0.395112107	0	0.726096162	0	1	CRA004775;PRJNA827465	Yang2025_Silkie_Wenchang	0.830643015
ENSGALG00010027883	ATP8B2	2	-0.027953275	0.041533843	-0.673024042	0.500931996	0	0.097168648	0	1	PRJNA827465;Yang2025_Silkie_Wenchang	CRA004775	0.862421093
ENSGALG00010027883	ATP8B2	2	-0.220139147	0.205277383	-1.072398446	0.283541106	0.065452186	4.034160865	75.21169746	1	CRA004775;Yang2025_Silkie_Wenchang	PRJNA827465	0.779568117
ENSGALG00010027883	ATP8B2	2	-0.203988344	0.219571922	-0.929027458	0.352874857	0.079720997	5.043195256	80.17130115	1	CRA004775;PRJNA827465	Yang2025_Silkie_Wenchang	0.811035834
ENSGALG00010027884	SF3A2	2	0.050458646	0.152224969	0.331474177	0.740286344	0.031008116	2.432185591	58.88471654	0.5	PRJNA827465;Yang2025_Silkie_Wenchang	CRA004775	0.935124836
ENSGALG00010027884	SF3A2	2	0.028017785	0.2187867	0.128059819	0.898101634	0.066011367	3.156278159	68.31711434	0.5	CRA004775;Yang2025_Silkie_Wenchang	PRJNA827465	0.975733636
ENSGALG00010027884	SF3A2	2	-0.071496583	0.054267704	-1.317479417	0.187677976	0	0.534859776	0	1	CRA004775;PRJNA827465	Yang2025_Silkie_Wenchang	0.696930043
ENSGALG00010027885	PLEKHJ1	2	0.035942476	0.047157168	0.762184783	0.445949727	0	0.135692913	0	0.5	PRJNA827465;Yang2025_Silkie_Wenchang	CRA004775	0.846013379
ENSGALG00010027885	PLEKHJ1	2	-0.052783926	0.082691388	-0.638324341	0.52326257	0	0.38041502	0	1	CRA004775;Yang2025_Silkie_Wenchang	PRJNA827465	0.869661505
ENSGALG00010027885	PLEKHJ1	2	0.007468604	0.063830001	0.117007733	0.906853925	0.00271959	1.326365952	24.60602609	0.5	CRA004775;PRJNA827465	Yang2025_Silkie_Wenchang	0.978196186
ENSGALG00010027886	IL6R	2	-0.230942807	0.111445689	-2.072245311	0.03824257	0	0.018037402	0	1	PRJNA827465;Yang2025_Silkie_Wenchang	CRA004775	0.337384449
ENSGALG00010027886	IL6R	2	-0.17138975	0.181381706	-0.944911994	0.344703808	0	0.024419211	0	1	CRA004775;Yang2025_Silkie_Wenchang	PRJNA827465	0.806945375
ENSGALG00010027886	IL6R	2	-0.220172827	0.111366177	-1.977017021	0.048039706	0	0.11161371	0	1	CRA004775;PRJNA827465	Yang2025_Silkie_Wenchang	0.379037942
ENSGALG00010027888	PFKFB2	2	0.010296112	0.092456459	0.111361742	0.911329496	0	0.887435791	0	0.5	PRJNA827465;Yang2025_Silkie_Wenchang	CRA004775	0.978845851
ENSGALG00010027888	PFKFB2	2	-0.194958681	0.355720843	-0.548066511	0.583646234	0.201455379	4.559208313	78.06636742	0.5	CRA004775;Yang2025_Silkie_Wenchang	PRJNA827465	0.890558522
ENSGALG00010027888	PFKFB2	2	-0.261393236	0.260991402	-1.001539648	0.316565982	0.093089432	2.740061022	63.50446242	1	CRA004775;PRJNA827465	Yang2025_Silkie_Wenchang	0.796883862
ENSGALG00010027889	RBM8A	2	0.316252237	0.215365947	1.468441236	0.141984408	0.075268157	5.067730035	80.2672993	1	PRJNA827465;Yang2025_Silkie_Wenchang	CRA004775	0.62757683
ENSGALG00010027889	RBM8A	2	0.134276089	0.433075323	0.310052506	0.756521028	0.336528649	9.654986914	89.64265815	0.5	CRA004775;Yang2025_Silkie_Wenchang	PRJNA827465	0.938659623
ENSGALG00010027889	RBM8A	2	-0.047543159	0.211556843	-0.22472995	0.822189363	0.064768102	3.220695057	68.95080154	0.5	CRA004775;PRJNA827465	Yang2025_Silkie_Wenchang	0.955654716
ENSGALG00010027890	ENSGALG00010027890	2	-0.20484596	0.491180852	-0.417047934	0.676643347	0.355220192	3.789681836	73.61256055	0.5	PRJNA827465;Yang2025_Silkie_Wenchang	CRA004775	0.916786558
ENSGALG00010027890	ENSGALG00010027890	2	-0.329510115	0.49643005	-0.663759406	0.506844311	0.252410173	1.889912049	47.08748481	0.5	CRA004775;Yang2025_Silkie_Wenchang	PRJNA827465	0.863929781
ENSGALG00010027890	ENSGALG00010027890	2	0.293761949	0.309847427	0.948085808	0.343085773	0	0.00502979	0	1	CRA004775;PRJNA827465	Yang2025_Silkie_Wenchang	0.806218116
ENSGALG00010027891	CCDC66	2	0.478180087	0.345220907	1.385142315	0.166008955	0.218668371	11.86776404	91.57381292	1	PRJNA827465;Yang2025_Silkie_Wenchang	CRA004775	0.66529196
ENSGALG00010027891	CCDC66	2	0.677970569	0.212852247	3.185169886	0.001446691	0.040756047	1.711545856	41.57328613	1	CRA004775;Yang2025_Silkie_Wenchang	PRJNA827465	0.047151552
ENSGALG00010027891	CCDC66	2	0.173574868	0.094283162	1.8409954	0.065622233	0	0.654420722	0	1	CRA004775;PRJNA827465	Yang2025_Silkie_Wenchang	0.441590298
ENSGALG00010027892	SHE	2	-0.052276974	0.061658803	-0.847842835	0.396525504	0	0.017566951	0	1	PRJNA827465;Yang2025_Silkie_Wenchang	CRA004775	0.831365249
ENSGALG00010027892	SHE	2	0.017247787	0.191640731	0.090000634	0.928286711	0	0.072485597	0	0.5	CRA004775;Yang2025_Silkie_Wenchang	PRJNA827465	0.983169921
ENSGALG00010027892	SHE	2	-0.049048518	0.06256333	-0.78398189	0.433050737	0	0.186192941	0	0.5	CRA004775;PRJNA827465	Yang2025_Silkie_Wenchang	0.842762754
ENSGALG00010027893	DOT1L	2	0.115042832	0.225727989	0.509652493	0.610294941	0.079817449	4.220940655	76.30859845	0.5	PRJNA827465;Yang2025_Silkie_Wenchang	CRA004775	0.897438028
ENSGALG00010027893	DOT1L	2	0.280524096	0.167872035	1.671059128	0.094709999	0	0.745836659	0	1	CRA004775;Yang2025_Silkie_Wenchang	PRJNA827465	0.526919621
ENSGALG00010027893	DOT1L	2	-0.064642314	0.085618553	-0.755003571	0.45024684	0	0.226436589	0	0.5	CRA004775;PRJNA827465	Yang2025_Silkie_Wenchang	0.847564661
ENSGALG00010027894	RSBN1	2	-0.20143911	0.203624717	-0.989266497	0.322532771	0.077663038	15.30893739	93.46786799	1	PRJNA827465;Yang2025_Silkie_Wenchang	CRA004775	0.798652022
ENSGALG00010027894	RSBN1	2	-0.089543615	0.335223871	-0.267115867	0.789379952	0.20874031	13.85789596	92.78389734	0.5	CRA004775;Yang2025_Silkie_Wenchang	PRJNA827465	0.947386107
ENSGALG00010027894	RSBN1	2	0.083810569	0.124989957	0.670538431	0.502514616	0.022082072	2.761428575	63.78685985	0.5	CRA004775;PRJNA827465	Yang2025_Silkie_Wenchang	0.862987501
ENSGALG00010027895	LIX1L	2	0.269249374	0.117745868	2.286699139	0.02221339	0.011760402	1.708206359	41.4590635	1	PRJNA827465;Yang2025_Silkie_Wenchang	CRA004775	0.252849587
ENSGALG00010027895	LIX1L	2	-0.026367786	0.446498737	-0.059054559	0.952908652	0.365619599	11.91300229	91.60581039	0.5	CRA004775;Yang2025_Silkie_Wenchang	PRJNA827465	0.987758913
ENSGALG00010027895	LIX1L	2	-0.133824232	0.326613483	-0.409732722	0.682002024	0.1854341	7.339935258	86.37590163	0.5	CRA004775;PRJNA827465	Yang2025_Silkie_Wenchang	0.918515969
ENSGALG00010027896	TOR4A	2	-0.186767777	0.325117703	-0.574462034	0.565655185	0.179425606	6.225864822	83.93797442	0.5	PRJNA827465;Yang2025_Silkie_Wenchang	CRA004775	0.883487517
ENSGALG00010027896	TOR4A	2	-0.441780618	0.19000369	-2.325115986	0.020065759	0	0.545846376	0	1	CRA004775;Yang2025_Silkie_Wenchang	PRJNA827465	0.238261187
ENSGALG00010027896	TOR4A	2	0.045226107	0.13517894	0.334564738	0.737953449	0.010056837	1.18117005	15.33818519	0.5	CRA004775;PRJNA827465	Yang2025_Silkie_Wenchang	0.934189596
ENSGALG00010027897	AP3D1	2	-0.007766616	0.038185533	-0.203391585	0.838828977	0	0.113981968	0	0.5	PRJNA827465;Yang2025_Silkie_Wenchang	CRA004775	0.959283372
ENSGALG00010027897	AP3D1	2	0.010098957	0.096656048	0.104483443	0.916785707	0	0.091596952	0	0.5	CRA004775;Yang2025_Silkie_Wenchang	PRJNA827465	0.980687732
ENSGALG00010027897	AP3D1	2	-0.013462613	0.039519215	-0.340659918	0.733359616	0	0.016358851	0	1	CRA004775;PRJNA827465	Yang2025_Silkie_Wenchang	0.933182392
ENSGALG00010027898	TASOR	2	0.008899447	0.042572675	0.209041292	0.834416004	0	0.082364861	0	1	PRJNA827465;Yang2025_Silkie_Wenchang	CRA004775	0.958051246
ENSGALG00010027898	TASOR	2	-0.028017295	0.122955236	-0.227865811	0.819750562	0.008661184	1.236256516	19.11063869	0.5	CRA004775;Yang2025_Silkie_Wenchang	PRJNA827465	0.955256039
ENSGALG00010027898	TASOR	2	-0.018250703	0.073019881	-0.249941558	0.802632544	0.003607327	1.110999046	9.990921785	0.5	CRA004775;PRJNA827465	Yang2025_Silkie_Wenchang	0.950704475
ENSGALG00010027899	PMVK	2	1.023234929	0.848271138	1.206259276	0.227717535	1.412002171	52.99174668	98.11291368	1	PRJNA827465;Yang2025_Silkie_Wenchang	CRA004775	0.740853612
ENSGALG00010027899	PMVK	2	0.727975979	1.156444095	0.629495176	0.529024926	2.620285205	48.99116322	97.95881556	0.5	CRA004775;Yang2025_Silkie_Wenchang	PRJNA827465	0.871809618
ENSGALG00010027899	PMVK	2	-0.084692328	0.304965385	-0.277711281	0.781234	0.142213521	3.977789546	74.86040957	0.5	CRA004775;PRJNA827465	Yang2025_Silkie_Wenchang	0.945235709
ENSGALG00010027901	MOB3A	2	-0.06648858	0.175009859	-0.379913341	0.704009744	0.049589364	4.80739168	79.19869928	0.5	PRJNA827465;Yang2025_Silkie_Wenchang	CRA004775	0.923862937
ENSGALG00010027901	MOB3A	2	-0.310540736	0.125532257	-2.473792345	0.013368738	0	0.260108978	0	1	CRA004775;Yang2025_Silkie_Wenchang	PRJNA827465	0.188936172
ENSGALG00010027901	MOB3A	2	-0.112190688	0.242107276	-0.463392466	0.643083078	0.093457511	4.230203562	76.36047567	0.5	CRA004775;PRJNA827465	Yang2025_Silkie_Wenchang	0.906634523
ENSGALG00010027902	UBE2Q1	2	-0.011008269	0.059329357	-0.185545055	0.852801545	0.002973318	1.407120073	28.93285945	0.5	PRJNA827465;Yang2025_Silkie_Wenchang	CRA004775	0.962680849
ENSGALG00010027902	UBE2Q1	2	-0.074361696	0.083435115	-0.891251794	0.372794104	0	0.385941053	0	1	CRA004775;Yang2025_Silkie_Wenchang	PRJNA827465	0.822162024
ENSGALG00010027902	UBE2Q1	2	0.016754699	0.037314627	0.449011566	0.653423313	0	0.09484955	0	0.5	CRA004775;PRJNA827465	Yang2025_Silkie_Wenchang	0.909626907
ENSGALG00010027903	NDUFA8	2	-0.03192602	0.078697336	-0.405681083	0.684976944	0	0.007228009	0	1	PRJNA827465;Yang2025_Silkie_Wenchang	CRA004775	0.919109925
ENSGALG00010027903	NDUFA8	2	-0.020639773	0.217477187	-0.09490546	0.924389919	0	0.040839224	0	0.5	CRA004775;Yang2025_Silkie_Wenchang	PRJNA827465	0.982374054
ENSGALG00010027903	NDUFA8	2	-0.026578343	0.080088964	-0.331860247	0.739994789	0	0.033041459	0	0.5	CRA004775;PRJNA827465	Yang2025_Silkie_Wenchang	0.935072094
ENSGALG00010027905	SYN2	2	-0.144458704	0.205631468	-0.702512634	0.482359526	0	0.784627272	0	0.5	PRJNA827465;Yang2025_Silkie_Wenchang	CRA004775	0.85691916
ENSGALG00010027905	SYN2	2	0.098148184	0.291704903	0.336463951	0.736521032	0	0.054442744	0	1	CRA004775;Yang2025_Silkie_Wenchang	PRJNA827465	0.933661344
ENSGALG00010027905	SYN2	2	-0.184465567	0.207931451	-0.887146057	0.37500027	0	0.331661356	0	0.5	CRA004775;PRJNA827465	Yang2025_Silkie_Wenchang	0.822916144
ENSGALG00010027906	LHX6	2	-0.175433801	0.090628992	-1.935735995	0.052900044	0	0.310479762	0	1	PRJNA827465;Yang2025_Silkie_Wenchang	CRA004775	0.399377178
ENSGALG00010027906	LHX6	2	-0.212361331	0.124620765	-1.704060566	0.088369773	0	0.1973744	0	0.5	CRA004775;Yang2025_Silkie_Wenchang	PRJNA827465	0.510464018
ENSGALG00010027906	LHX6	2	-0.117298851	0.124172218	-0.944646501	0.344839378	0	0.064798515	0	0.5	CRA004775;PRJNA827465	Yang2025_Silkie_Wenchang	0.806961063
ENSGALG00010027907	MKNK2	2	-0.179256228	0.068885878	-2.602220278	0.009262234	0	0.261545686	0	1	PRJNA827465;Yang2025_Silkie_Wenchang	CRA004775	0.150926028
ENSGALG00010027907	MKNK2	2	-0.215032283	0.099918752	-2.152071353	0.031391735	0	0.031023037	0	1	CRA004775;Yang2025_Silkie_Wenchang	PRJNA827465	0.303067885
ENSGALG00010027907	MKNK2	2	-0.154136663	0.082799605	-1.86156279	0.062664742	0	0.010467667	0	1	CRA004775;PRJNA827465	Yang2025_Silkie_Wenchang	0.431333164
ENSGALG00010027908	PBXIP1	2	0.324351647	0.539727374	0.600954598	0.547870227	0.564952527	32.83595137	96.95455756	0.5	PRJNA827465;Yang2025_Silkie_Wenchang	CRA004775	0.878281266
ENSGALG00010027908	PBXIP1	2	0.295335885	0.603857625	0.489081983	0.624783656	0.670839101	12.15233302	91.77112742	0.5	CRA004775;Yang2025_Silkie_Wenchang	PRJNA827465	0.901043264
ENSGALG00010027908	PBXIP1	2	-0.221803679	0.097782881	-2.268328321	0.023309203	0	0.158331127	0	1	CRA004775;PRJNA827465	Yang2025_Silkie_Wenchang	0.260268986
ENSGALG00010027909	TIMP4	2	-0.177838175	0.29648374	-0.599824378	0.548623285	0	0.043875887	0	1	PRJNA827465;Yang2025_Silkie_Wenchang	CRA004775	0.878281266
ENSGALG00010027909	TIMP4	2	-0.232835133	0.238591274	-0.975874469	0.329126675	0	0.004806285	0	1	CRA004775;Yang2025_Silkie_Wenchang	PRJNA827465	0.800761292
ENSGALG00010027909	TIMP4	2	-0.191810042	0.217499983	-0.88188532	0.377838826	0	0.040036167	0	1	CRA004775;PRJNA827465	Yang2025_Silkie_Wenchang	0.82325159
ENSGALG00010027910	CHRNB2	2	-0.018165874	0.118752898	-0.152972052	0.878420315	0.004805313	1.163619142	14.06122809	0.5	PRJNA827465;Yang2025_Silkie_Wenchang	CRA004775	0.971037793
ENSGALG00010027910	CHRNB2	2	0.101413226	0.190811121	0.531484882	0.595082815	0	0.597249067	0	0.5	CRA004775;Yang2025_Silkie_Wenchang	PRJNA827465	0.893476711
ENSGALG00010027910	CHRNB2	2	-0.102930909	0.118807584	-0.86636649	0.386289215	0	0.077657981	0	1	CRA004775;PRJNA827465	Yang2025_Silkie_Wenchang	0.826894808
ENSGALG00010027911	PTPN22	2	0.546321536	0.342771978	1.593833716	0.110973284	0	0.10206874	0	1	PRJNA827465;Yang2025_Silkie_Wenchang	CRA004775	0.563777127
ENSGALG00010027911	PTPN22	2	-0.108452155	0.755975688	-0.143459845	0.885927022	0.919184919	5.102863614	80.40316035	0.5	CRA004775;Yang2025_Silkie_Wenchang	PRJNA827465	0.97244495
ENSGALG00010027911	PTPN22	2	-0.21600983	0.646459542	-0.334142844	0.738271771	0.616789895	3.814129807	73.78169987	0.5	CRA004775;PRJNA827465	Yang2025_Silkie_Wenchang	0.934442079
ENSGALG00010027912	RGS3	2	0.059931268	0.100858079	0.594213859	0.552369084	0.006806735	1.376190966	27.33566602	0.5	PRJNA827465;Yang2025_Silkie_Wenchang	CRA004775	0.879228216

ENSGALG00010027912	RGS3	2	0.14630761	0.139491575	1.048863411	0.294240988	0	0.572752036	0	0.5	CRA004775;Yang2025_Silkie_Wenchang	PRJNA827465	0.787343482
ENSGALG00010027912	RGS3	2	-0.004205404	0.081389255	-0.05167026	0.958791435	0	9.81836E-05	0	1	CRA004775;PRJNA827465	Yang2025_Silkie_Wenchang	0.989102387
ENSGALG00010027913	PYGO2	2	-0.039221418	0.112697666	-0.34802334	0.727822655	0.014237664	2.248459506	55.5251052	0.5	PRJNA827465;Yang2025_Silkie_Wenchang	CRA004775	0.931748602
ENSGALG00010027913	PYGO2	2	-0.123906181	0.102586906	-1.207816722	0.227117768	0	0.464167421	0	1	CRA004775;Yang2025_Silkie_Wenchang	PRJNA827465	0.740605003
ENSGALG00010027913	PYGO2	2	0.052264768	0.086036512	0.607471951	0.543537751	0	0.076499573	0	0.5	CRA004775;PRJNA827465	Yang2025_Silkie_Wenchang	0.876968972
ENSGALG00010027914	BTBD2	2	-0.27450345	0.079335241	-3.460044302	0.000540087	0	0.661417435	0	1	PRJNA827465;Yang2025_Silkie_Wenchang	CRA004775	0.024653915
ENSGALG00010027914	BTBD2	2	-0.37311253	0.153211958	-2.435353637	0.014877242	0	0.102461496	0	1	CRA004775;Yang2025_Silkie_Wenchang	PRJNA827465	0.201558166
ENSGALG00010027914	BTBD2	2	-0.242085413	0.087065736	-2.780490064	0.005427692	0	0.006336667	0	1	CRA004775;PRJNA827465	Yang2025_Silkie_Wenchang	0.110473631
ENSGALG00010027915	GSTM3	2	-0.034544345	0.138653171	-0.249142122	0.803250848	0.02311278	2.174228603	54.00667628	0.5	PRJNA827465;Yang2025_Silkie_Wenchang	CRA004775	0.950951274
ENSGALG00010027915	GSTM3	2	-0.215386374	0.160024453	-1.345959132	0.178315697	0	0.01884753	0	1	CRA004775;Yang2025_Silkie_Wenchang	PRJNA827465	0.684689586
ENSGALG00010027915	GSTM3	2	0.052656975	0.075704215	0.695562004	0.486703168	0	0.495731642	0	0.5	CRA004775;PRJNA827465	Yang2025_Silkie_Wenchang	0.857603818
ENSGALG00010027916	ADAR	2	0.129350484	0.037272716	3.470379864	0.000519723	0	0.636128653	0	1	PRJNA827465;Yang2025_Silkie_Wenchang	CRA004775	0.024059675
ENSGALG00010027916	ADAR	2	-0.162291094	0.365719156	-0.4437588	0.657216979	0.244243975	10.89205817	90.81899872	0.5	CRA004775;Yang2025_Silkie_Wenchang	PRJNA827465	0.910711339
ENSGALG00010027916	ADAR	2	-0.190557107	0.331885326	-0.57416551	0.565855806	0.199196437	9.614990317	89.59957351	0.5	CRA004775;PRJNA827465	Yang2025_Silkie_Wenchang	0.863487517
ENSGALG00010027917	PPARG	2	-0.473508663	0.293067659	-1.61569743	0.106159747	0.117157376	3.143907539	68.1924488	1	PRJNA827465;Yang2025_Silkie_Wenchang	CRA004775	0.552781735
ENSGALG00010027917	PPARG	2	-0.529378343	0.353386881	-1.498013567	0.134129726	0.126931156	1.754788005	43.0130593	0.5	CRA004775;Yang2025_Silkie_Wenchang	PRJNA827465	0.61346525
ENSGALG00010027917	PPARG	2	-0.146408661	0.217534676	-0.673035968	0.500924409	0	0.097312773	0	0.5	CRA004775;PRJNA827465	Yang2025_Silkie_Wenchang	0.862421093
ENSGALG00010027918	CSNK1G2	2	0.096865162	0.124279889	0.779411639	0.43573727	0.025954883	5.97927759	83.27557159	0.5	PRJNA827465;Yang2025_Silkie_Wenchang	CRA004775	0.842762754
ENSGALG00010027918	CSNK1G2	2	0.101375367	0.182673784	0.554953011	0.578926801	0.042667335	2.361451585	57.65316526	0.5	CRA004775;Yang2025_Silkie_Wenchang	PRJNA827465	0.887888423
ENSGALG00010027918	CSNK1G2	2	-0.021625539	0.046752463	-0.462554012	0.643684079	0	0.321083065	0	1	CRA004775;PRJNA827465	Yang2025_Silkie_Wenchang	0.906643868
ENSGALG00010027919	TSEN2	2	0.059922545	0.102943654	0.582090715	0.560505579	0	0.675819655	0	0.5	PRJNA827465;Yang2025_Silkie_Wenchang	CRA004775	0.881203589
ENSGALG00010027919	TSEN2	2	-0.046569053	0.158263853	-0.294249456	0.768567292	0	0.106883331	0	0.5	CRA004775;Yang2025_Silkie_Wenchang	PRJNA827465	0.941493634
ENSGALG00010027919	TSEN2	2	0.087869464	0.10539681	0.833701355	0.404449294	0	0.130541819	0	1	CRA004775;PRJNA827465	Yang2025_Silkie_Wenchang	0.833112102
ENSGALG00010027920	SCAMP4	2	-0.150057696	0.194993395	-0.769552713	0.441565264	0.067325615	8.448288235	88.16328264	0.5	PRJNA827465;Yang2025_Silkie_Wenchang	CRA004775	0.845053556
ENSGALG00010027920	SCAMP4	2	-0.081350608	0.094185567	-0.048926298	5.1452E-05	0	0.105724666	0	1	CRA004775;Yang2025_Silkie_Wenchang	PRJNA827465	0.004949403
ENSGALG00010027920	SCAMP4	2	-0.172302761	0.226112667	-0.762021712	0.446047046	0.089055859	7.278847713	86.26156173	0.5	CRA004775;PRJNA827465	Yang2025_Silkie_Wenchang	0.846013379
ENSGALG00010027921	MKRN2OS	2	-0.597134921	0.367929264	-1.622961205	0.104597698	0.225758866	5.814903151	82.80280902	1	PRJNA827465;Yang2025_Silkie_Wenchang	CRA004775	0.549694549
ENSGALG00010027921	MKRN2OS	2	-0.45109989	0.538682974	-0.837412563	0.402360707	0.518738645	9.403551896	89.36572041	0.5	CRA004775;Yang2025_Silkie_Wenchang	PRJNA827465	0.832879175
ENSGALG00010027921	MKRN2OS	2	-0.13301914	0.163763829	-0.812262026	0.416641295	0.018516413	1.475348963	32.21942569	0.5	CRA004775;PRJNA827465	Yang2025_Silkie_Wenchang	0.837984974
ENSGALG00010027922	SHC1	2	-0.058331156	0.047449288	-1.229336802	0.218945555	0	0.941334189	0	1	PRJNA827465;Yang2025_Silkie_Wenchang	CRA004775	0.73259614
ENSGALG00010027922	SHC1	2	-0.181047067	0.113190397	-1.599491401	0.109711458	0	0.044014271	0	1	CRA004775;Yang2025_Silkie_Wenchang	PRJNA827465	0.560799769
ENSGALG00010027922	SHC1	2	-0.053413422	0.047588766	-1.122395617	0.261694067	0	0.438321247	0	1	CRA004775;PRJNA827465	Yang2025_Silkie_Wenchang	0.768310285
ENSGALG00010027923	AMPD2	2	0.207020598	0.349308358	0.592658588	0.553409661	0.225705181	13.03900738	92.33070455	0.5	PRJNA827465;Yang2025_Silkie_Wenchang	CRA004775	0.879514268
ENSGALG00010027923	AMPD2	2	0.104576322	0.484110985	0.216017247	0.828974303	0.423148363	10.15535233	90.15297582	0.5	CRA004775;Yang2025_Silkie_Wenchang	PRJNA827465	0.956840134
ENSGALG00010027923	AMPD2	2	-0.161053093	0.088330006	-1.823331242	0.068256265	0.000277665	1.007695411	0.763664431	1	CRA004775;PRJNA827465	Yang2025_Silkie_Wenchang	0.450415709
ENSGALG00010027924	ENSGALG00010027924	2	1.201731151	0.345739767	3.475824798	0.000509285	0	0.348844957	0	1	PRJNA827465;Yang2025_Silkie_Wenchang	CRA004775	0.023660075
ENSGALG00010027924	ENSGALG00010027924	2	0.743643996	0.645379398	1.152258653	0.249214802	0.616856928	3.840831786	73.96397302	1	CRA004775;Yang2025_Silkie_Wenchang	PRJNA827465	0.759411487
ENSGALG00010027924	ENSGALG00010027924	2	0.536332584	0.440768306	1.216812953	0.223675391	0.178971327	1.84886956	45.91289609	1	CRA004775;PRJNA827465	Yang2025_Silkie_Wenchang	0.737076707
ENSGALG00010027925	MKRN2	2	0.070507893	0.070303879	1.002901883	0.315908204	0.006595728	2.739975886	63.50332843	1	PRJNA827465;Yang2025_Silkie_Wenchang	CRA004775	0.796883862
ENSGALG00010027925	MKRN2	2	0.136586394	0.072261839	1.890159393	0.058736646	0	0.559602435	0	1	CRA004775;Yang2025_Silkie_Wenchang	PRJNA827465	0.419728393
ENSGALG00010027925	MKRN2	2	0.013820918	0.038085294	0.36289382	0.716684204	0	0.003490357	0	1	CRA004775;PRJNA827465	Yang2025_Silkie_Wenchang	0.927580573
ENSGALG00010027926	CKS1B	2	0.016443755	0.196097789	0.083854872	0.93317182	0.041621056	1.66038509	37.51503895	0.5	PRJNA827465;Yang2025_Silkie_Wenchang	CRA004775	0.984259239
ENSGALG00010027926	CKS1B	2	-0.572052863	0.350201654	-1.633495605	0.102364804	0.041380627	1.166950338	13.82655794	1	CRA004775;Yang2025_Silkie_Wenchang	PRJNA827465	0.545081141
ENSGALG00010027926	CKS1B	2	-0.349342721	0.603639728	-0.578727185	0.56277327	0.578215475	3.943282738	74.64041849	0.5	CRA004775;PRJNA827465	Yang2025_Silkie_Wenchang	0.882065633
ENSGALG00010027927	ENSGALG00010027927	2	-0.266263428	0.092261173	-2.885974896	0.003902033	0	0.069603916	0	1	PRJNA827465;Yang2025_Silkie_Wenchang	CRA004775	0.089895425
ENSGALG00010027927	ENSGALG00010027927	2	-0.26067179	0.125511498	-2.076875781	0.037813026	0	0.390847363	0	1	CRA004775;Yang2025_Silkie_Wenchang	PRJNA827465	0.335608236
ENSGALG00010027927	ENSGALG00010027927	2	-0.221621942	0.117978573	-1.878493153	0.060313733	0	0.244699739	0	1	CRA004775;PRJNA827465	Yang2025_Silkie_Wenchang	0.425432635
ENSGALG00010027928	FLAD1	2	0.149619687	0.074992936	1.995117076	0.046030112	0	0.324556292	0	1	PRJNA827465;Yang2025_Silkie_Wenchang	CRA004775	0.370039171
ENSGALG00010027928	FLAD1	2	0.170019531	0.17002515	0.999966953	0.317326501	0	0.783928569	0	0.5	CRA004775;Yang2025_Silkie_Wenchang	PRJNA827465	0.797095376
ENSGALG00010027928	FLAD1	2	0.119801938	0.079371677	1.509378946	0.13120197	0	0.43060019	0	0.5	CRA004775;PRJNA827465	Yang2025_Silkie_Wenchang	0.606878893
ENSGALG00010027929	CDK5RAP2	2	0.276623996	0.232796264	1.188266475	0.234728442	0.093742206	7.161756925	86.03694581	1	PRJNA827465;Yang2025_Silkie_Wenchang	CRA004775	0.747791642
ENSGALG00010027929	CDK5RAP2	2	0.494592044	0.139476455	3.546061181	0.000391035	0	0.287604551	0	1	CRA004775;Yang2025_Silkie_Wenchang	PRJNA827465	0.019924928
ENSGALG00010027929	CDK5RAP2	2	0.074693279	0.083295878	0.896722392	0.369867069	0	0.519904626	0	1	CRA004775;PRJNA827465	Yang2025_Silkie_Wenchang	0.820829216
ENSGALG00010027930	RAF1	2	-0.064168519	0.044744015	-1.434125193	0.15153454	0	0.000567163	0	1	PRJNA827465;Yang2025_Silkie_Wenchang	CRA004775	0.643402891
ENSGALG00010027930	RAF1	2	-0.061329565	0.06657429	-0.921219971	0.356935594	0	0.012840333	0	1	CRA004775;Yang2025_Silkie_Wenchang	PRJNA827465	0.812971699
ENSGALG00010027930	RAF1	2	-0.063443608	0.057800926	-1.097622699	0.272369277	0	0.014447335	0	1	CRA004775;PRJNA827465	Yang2025_Silkie_Wenchang	0.773337076
ENSGALG00010027931	ABHD17A	2	-0.04473735	0.055929801	-0.799883955	0.423778035	0	0.499477441	0	0.5	PRJNA827465;Yang2025_Silkie_Wenchang	CRA004775	0.84030043
ENSGALG00010027931	ABHD17A	2	-0.000557162	0.134673269	-0.004137139	0.996699395	0	0.882426442	0	0.5	CRA004775;Yang2025_Silkie_Wenchang	PRJNA827465	0.99989145
ENSGALG00010027931	ABHD17A	2	-0.068203362	0.059803787	-1.140452221	0.254097947	0	0.485365676	0	1	CRA004775;PRJNA827465	Yang2025_Silkie_Wenchang	0.763894203
ENSGALG00010027932	POLE3	2	-0.02035969	0.047159943	-0.431715741	0.665940025	0	0.037911191	0	1	PRJNA827465;Yang2025_Silkie_Wenchang	CRA004775	0.913546323
ENSGALG00010027932	POLE3	2	-0.123257337	0.163304119	-0.749260567	0.45370017	0.028319682	1.605607652	37.71828388	1	CRA004775;Yang2025_Silkie_Wenchang	PRJNA827465	0.838964191
ENSGALG00010027932	POLE3	2	-0.114059492	0.175403562	-0.650268963	0.515518502	0.03743269	1.826415378	45.24794239	1	CRA004775;PRJNA827465	Yang2025_Silkie_Wenchang	0.867281212
ENSGALG00010027933	ENSGALG00010027933	2	0.655975196	0.481041188	1.363657027	0.172675535	0.431033022	14.54530297	93.12492836	1	PRJNA827465;Yang2025_Silkie_Wenchang	CRA004775	0.675626336
ENSGALG00010027933	ENSGALG00010027933	2	0.804456441	0.366207652	2.196722098	0.0280403	0.211393808	4.608577862	78.30133221	1	CRA004775;Yang2025_Silkie_Wenchang	PRJNA827465	0.285361962
ENSGALG00010027933	ENSGALG00010027933	2	0.237000984	0.141870813	1.670540822	0.09481241	0	0.481958739	0	1	CRA004775;PRJNA827465	Yang2025_Silkie_Wenchang	0.527148414
ENSGALG00010027934	CNBP	2	-0.058749213	0.041549698	-1.413950406	0.157376465	0	0.345353505	0	0.5	PRJNA827465;Yang2025_Silkie_Wenchang	CRA004775	0.650687662
ENSGALG00010027934	CNBP	2	0.00177656	0.111734559	0.015899827	0.987314308	0	0.021320734	0	0.5	CRA004775;Yang2025_Silkie_Wenchang	PRJNA827465	0.996492495
ENSGALG00010027934	CNBP	2	-0.066613665	0.04355439	-1.529436291	0.126156321	0	0.015609217	0	1	CRA004775;PRJNA827465	Yang2025_Silkie_Wenchang	0.596525734
ENSGALG00010027935	ISY1	2	0.329662902	0.321559523	1.025200245	0.305268712	0.198980928	26.35009281	96.20494695	1	PRJNA827465;Yang2025_Silkie_Wenchang	CRA004775	0.79252794
ENSGALG00010027935	ISY1	2	0.223580824	0.45501592	0.491369234	0.62316532	0.382912089	12.84183709	92.21295214	0.5	CRA004775;Yang2025_Silkie_Wenchang	PRJNA827465	0.900348922
ENSGALG00010027935	ISY1	2	-0.030859028	0.098172015	-0.314336302	0.75326563	0.006074074	1.203293677	16.89476813	0.5	CRA004775;PRJNA827465	Yang2025_Silkie_Wenchang	0.938087082
ENSGALG00010027936	ENSGALG00010027936	2	0.032882419	0.047454989	0.69291806	0.488360975	0	0.49152875	0	1	PRJNA827465;Yang2025_Silkie_Wenchang	CRA004775	0.857796363
ENSGALG00010027936	ENSGALG00010027936	2	0.104584918	0.132277231	0.790649433	0.429148592	0	0.154136513	0	1	CRA004775;Yang2025_Silkie_Wenchang	PRJNA827465	0.841982849
ENSGALG00010027936	ENSGALG00010027936	2	0.022090125	0.048913008	0.451620663	0.651542281	0	0.000100745	0	1	CRA004775;PRJNA827465	Yang2025_Silkie_Wenchang	0.908939988
ENSGALG00010027937	HDHD3	2	-0.063473478	0.049413691	-1.284532218	0.198955798	0	0.011081701	0	1	PRJNA827465;Yang2025_Silkie_Wenchang	CRA004775	0.71096127
ENSGALG00010027937	HDHD3	2	-0.250455711	0.237118756	-1.056245888	0.290855891	0.056194532	1.966269849	49.14228073	1	CRA004775;Yang2025_Silkie_Wenchang	PRJNA827465	0.784646441
ENSGALG00010027937	HDHD3	2	-0.215418442	0.214362732	-1.004908081	0.314941113	0.066066076	2.750475028	63.64264392	1	CRA004775;PRJNA827465	Yang2025_Silkie_Wenchang	0.796572019
ENSGALG00010027938	ENSGALG00010027938	2	0.224505859	0.101404112	2.21397194	0.026830709	0.004584648	1.257887165	20.50161354	1	PRJNA827465;Yang2025_Silkie_Wenchang	CRA004775	0.279212912
ENSGALG00010027938	ENSGALG00010027938	2	0.342374809	0.143327219	2.388763357	0.016905187	0	0.089108309	0	1	CRA004775;Yang2025_Silkie_Wenchang	PRJNA827465	0.216002789
ENSGALG00010027938	ENSGALG00010027938	2	0.155679286	0.102795699	1.514453305	0.129910908	0	0.055256855	0	1	CRA004775;PRJNA827465	Yang2025_Silkie_Wenchang	0.604676508
ENSGALG00010027939	BSPRY	2	0.188840049	0.46905821	0.40259406	0.68724688	0	0.014061167	0	1	PRJNA827465;Yang2025_Silkie_Wenchang	CRA004775	0.919451777
ENSGALG00010027939	BSPRY	2	0.343343364	0.465161394	0.738116638	0.460443574	0	0.020159978	0	1	CRA004775;Yang2025_Silkie_Wenchang	PRJNA827465	0.85014477
ENSGALG00010027939	BSPRY	2	0.271567381	0.420492117	0.645832275	0.518387983	0	0.088578106	0	1	CRA004775;PRJNA827465	Yang2025_Silkie_Wenchang	0.868525042
ENSGALG00010027940	ZBTB7B	2	0.7069989	0.264026248	2.677759904	0.007411633	0	0.175978183	0	1	PRJNA827465;Yang2025_Silkie_Wenchang	CRA004775	0.132770918
ENSGALG00010027940	ZBTB7B	2	0.195597266	0.680526324	0.287420574	0.77379031	0.709522071	4.214173297	76.27055345	0.5	CRA004775;Yang2025_Silkie_Wenchang	PRJNA827465	0.943248868
ENSGALG00010027940	ZBTB7B	2	0.108355048	0.56613477	0.191394441	0.848216575	0.447685983	3.20594208	68.80792057	0.5	CRA004775;PRJNA827465	Yang2025_Silkie_Wenchang	0.961363811
ENSGALG00010027941	KLF2	2	-0.078736828	0.135344171	-0.581752633	0.560733314	0.023725751	2.72630054	63.32025815	0.5	PRJNA827465;Yang2025_Silkie_Wenchang	CRA004775	0.88130295
ENSGALG00010027941	KLF2	2	-0.023365495	0.273789672	-0.085341038	0.931990267	0.10085292	2.713861136	63.15213086	0.5	CRA004775;Yang2025_Silkie_Wenchang	PRJNA827465	0.98412915
ENSGALG00010027941	KLF2	2	0.060037621	0.086782759	0.691815081	0.489053465	0	0.797214716	0	1	CRA004775;PRJNA827465	Yang2025_Silkie_Wenchang	0.858232954
ENSGALG00010027942	KHDC4	2	0.185019867	0.170736337	1.083658403	0.278516285	0.052874947	10.52441108	90.49828068	1	PRJNA827465;Yang2025_Silkie_Wenchang	CRA004775	0.776763074
ENSGALG00010027942	KHDC4	2	0.13073356	0.257466439	0.507769326	0.611615124	0.112374337	6.222252461	83.92864953	0.5	CRA004775;Yang2025_Silkie_Wenchang	PRJNA827465	0.897777844
ENSGALG00010027942	KHDC4	2	0.008684728	0.05134592	0.169141545	0.865685307	0	0.819636568	0	0.5	CRA004775;PRJNA827465	Yang2025_Silkie_Wenchang	0.967088846
ENSGALG00010027943	RAB43	2	-0.107614709	0.164017169	-0.656118564	0.511747841	0.044176975	5.23544119	80.89941299	0.5	PRJNA827465;Yang2025_Silkie_Wenchang	CRA004775	0.865694231
ENSGALG00010027943	RAB43	2	-0.05806835	0.274850661	-0.211272369	0.832674743	0.113633801	3.756530945	73.37969487	0.5	CRA004775;Yang2025_Silkie_Wenchang	PRJNA827465	0.957861248
ENSGALG00010027943	RAB43	2	0.050000614	0.059958625	0.833918622	0.404326843	0	0.738434886	0	1	CRA004775;PRJNA827465	Yang2025_Silkie_Wenchang	0.833112102
ENSGALG00010027944	WDR31	2	0.035750391	0.183979485	0.19431727	0.845927464	0	0.439951804	0	0.5	PRJNA827465;Yang2025_Silkie_Wenchang	CRA004775	0.96122283
ENSGALG00010027944	WDR31	2	0.089223583	0.244381483	0.365099607	0.715037055	0	0.340600636	0	0.5	CRA004775;Yang2025_Silkie_Wenchang	PRJNA827465	0.926852721
ENSGALG00010027944	WDR31	2	-0.059996272	0.19505021	-0.307593988	0.758391295	0	0.005434892	0	1	CRA004775;PRJNA827465	Yang2025_Silkie_Wenchang	0.938954369
ENSGALG00010027946	AP1M1	2	-0.067904261	0.050521079	-1.344077823	0.178923223	0	0.671889533	0	1	PRJNA827465;Yang2025_Silkie_Wenchang	CRA004775	0.685601971
ENSGALG00010027946	AP1M1	2	-0.145112233	0.078179502	-1.856141695	0.063433364	0	0.195375627	0	1	CRA004775;Yang2025_Silkie_Wenchang	PRJNA827465	0.433586349
ENSGALG00010027946	AP1M1	2	-0.05915232	0.057927787	-1.021138952	0.307188611	0	0.917373473	0	1	CRA004775;PRJNA827465	Yang2025_Silkie_Wenchang	0.79266003
ENSGALG00010027947	GP9	2	-0.730182941	0.277789547	-2.628547221	0.008575045	0	0.259387454	0	1	PRJNA827465;Yang2025_Silkie_Wenchang	CRA004775	0.144583866
ENSGALG00010027947	GP9	2	-1.134752718	0.344214581	-3.29664338	0.000978477	0	0.523051853	0	1	CRA004775;Yang2025_Silkie_Wenchang	PRJNA827465	0.037264597
ENSGALG00010027947	GP9	2	-0.925994042	0.386446578	-2.396176069	0.01656713	0.119552808	1.607321017	37.78467465	1	CRA004775;PRJNA827465	Yang2025_Silkie_Wenchang	0.213278058
ENSGALG00010027948	PRPF4	2	0.016249563	0.049013241	0.331534148	0.740241051	0	0.973186331	0	0.5	PRJNA827465;Yang2025_Silkie_Wenchang	CRA004775	0.935124836
ENSGALG00010027948	PRPF4	2	-0.297105995	0.182398224	-1.628886448	0.10333706	0.040996287	2.595124374	61.46620139	1	CRA004775;Yang2025_Silkie_Wenchang	PRJNA827465	0.547576788
ENSGALG00010027948	PRPF4	2	-0.202646524	0.259596674	-0.78062065	0.435025643	0.12022461	8.597523146	88.36874315	0.5	CRA004775;PRJNA827465	Yang2025_Silkie_Wenchang	0.842762754
ENSGALG00010027950	CDC26	2	-0.070320376	0.075194866	-0.935175234	0.349698	0	0.951136544	0	1	PRJNA827465;Yang2025_Silkie_Wenchang	CRA004775	0.809903518
ENSGALG00010027950	CDC26	2	-0.181011186	0.16638249	-1.087922086	0.276629505	0	0.394953277	0	1	CRA004775;Yang2025_Silkie_Wenchang	PRJNA827465	0.775851484
ENSGALG00010027950	CDC26	2	-0.0420948	0.077358474	-0.54415241	0.586336598	0	1.41108E-05	0	1	CRA004775;PRJNA827465	Yang2025_Silkie_Wenchang	0.891584605
ENSGALG00010027951	FAM32A	2	-0.091517996	0.066773914	-1.37056509	0.170510572	0	0.076991758	0	1	PRJNA827465;Yang2025_Silkie_Wenchang	CRA004775	0.672201941
ENSGALG00010027951	FAM32A	2	-0.153316105	0.14393026	-1.065211058	0.28678043	0	0.010291844	0	1	CRA004775;Yang2025_Silkie_Wenchang	PRJNA827465	0.782188151
ENSGALG00010027951	FAM32A	2	-0.092646179	0.06788994	-1.364652535	0.172362285	0	0.136456892	0	1	CRA004775;PRJNA827465	Yang2025_Silkie_Wenchang	0.675240867
ENSGALG00010027952	CIB3	2	-0.303746034	0.30069916	-1.010132635	0.312431749	0.165989534	11.69667592	91.45056247	1	PRJNA827465;Yang2025_Silkie_Wenchang	CRA004775	0.796054997
ENSGALG00010027952	CIB3	2	-0.271645111	0.401095708	-0.677257586	0.498242536	0.257991648	4.718953297	78.80885999	0.5	CRA004775;Yang2025_Silkie_Wenchang	PRJNA827465	0.861454634
ENSGALG00010027952	CIB3	2	-0.012773994	0.06585602	-0.193968514	0.846200535	0	0.365816924	0	0.5	CRA004775;PRJNA827465	Yang2025_Silkie_Wenchang	0.96122283
ENSGALG00010027953	SLC31A1	2	0.092482109	0.217222783	0.425747739	0.670291672	0.073044587	4.2330236	76.37622431	0.5	PRJNA827465;Yang2025_Silkie_Wenchang	CRA004775	0.914482883
ENSGALG00010027953	SLC31A1	2	-0.220239374	0.164955242	-1.33514626	0.181828481	0	0.742745213	0	1	CRA004775;Yang2025_Silkie_Wenchang	PRJNA827465	0.689474655
ENSGALG00010027953	SLC31A1	2	-0.031584195	0.391267955	-0.080722674	0.935662505	0.242358566	4.133307105	75.80629809	0.5	CRA004775;PRJNA827465	Yang2025_Silkie_Wenchang	0.984800337
ENSGALG00010027954	CFAP92	2	0.209806735	0.231790898	0.905155194	0.365383175	0	0.492047672	0	1	PRJNA827465;Yang2025_Silkie_Wenchang	CRA004775	0.818457479
ENSGALG00010027954	CFAP92	2	0.363084947	0.314340689	1.155068242	0.248062482	0	0.071287956	0	1	CRA004775;Yang2025_Silkie_Wenchang	PRJNA827465	0.758257812
ENSGALG00010027954	CFAP92	2	0.115922004	0.260696575	0.444662552	0.656563635	0	0.057929918	0	1	CRA004775;PRJNA827465	Yang2025_Silkie_Wenchang	0.910509456
ENSGALG00010027955	ENSGALG00010027955	2	0.631887245	0.114563102	5.515626182	3.4754E-08	0	0.008550586	0	1	PRJNA827465;Yang2025_Silkie_Wenchang	CRA004775	1.50103E-05
ENSGALG00010027955	ENSGALG00010027955	2	0.1145376	0.498589297	0.229723344	0.818306754	0.445824848	9.680672334	89.67013896	0.5	CRA004775;Yang2025_Silkie_Wenchang	PRJNA827465	0.955256039
ENSGALG00010027955	ENSGALG00010027955	2	0.143545047	0.510516795	0.281175955	0.778575446	0.487596109	15.26212386	93.44783197	0.5	CRA004775;PRJNA827465	Yang2025_Silkie_Wenchang	0.944673731
ENSGALG00010027956	RAB8A	2	0.101790311	0.158637939	0.641651744	0.521099328	0.044606927	8.525175639	88.27003639	0.5	PRJNA827465;Yang2025_Silkie_Wenchang	CRA004775	0.869518515
ENSGALG00010027956	RAB8A	2	-0.021573697	0.332199772	-0.064941939	0.948220229	0.186718866	5.964463542	83.23403282	0.5	CRA004775;Yang2025_Silkie_Wenchang	PRJNA827465	0.98671346
ENSGALG00010027956	RAB8A	2	-0.133021155	0.151174061	-0.879920499	0.378902379	0.027508109	1.799395708	44.42578719	1	CRA004775;PRJNA827465	Yang2025_Silkie_Wenchang	0.823876439
ENSGALG00010027957	ENTREP3	2	0.336351923	0.385103396	0.873406796	0.382441388	0.194425499	2.843174906	64.82805199	0.5	PRJNA827465;Yang2025_Silkie_Wenchang	CRA004775	0.825113569
ENSGALG00010027957	ENTREP3	2	0.607029939	0.289338424	2.097992831	0.035905779	0	0.464167129	0	1	CRA004775;Yang2025_Silkie_Wenchang	PRJNA827465	0.326969036
ENSGALG00010027957	ENTREP3	2	0.08439028	0.234712641	0.359547232	0.719185751	0	0.473234418	0	0.5	CRA004775;PRJNA827465	Yang2025_Silkie_Wenchang	0.928449671
ENSGALG00010027958	ENSGALG00010027958	2	-0.172106657	0.109874604	-1.566391598	0.117256969	0	0.303346359	0	1	PRJNA827465;Yang2025_Silkie_Wenchang	CRA004775	0.576645475
ENSGALG00010027958	ENSGALG00010027958	2	-0.061271397	0.180096039	-0.340215126	0.733694528	0	0.060717142	0	0.5	CRA004775;Yang2025_Silkie_Wenchang	PRJNA827465	0.933377698
ENSGALG00010027958	ENSGALG00010027958	2	-0.191482018	0.126406087	-1.514816435	0.129818897	0	0.29968551	0	0.5	CRA004775;PRJNA827465	Yang2025_Silkie_Wenchang	0.604462992
ENSGALG00010027959	TPM4	2	0.320153861	0.226755877	1.411887818	0.157982995	0.087165991	6.253598637	84.00920721	1	PRJNA827465;Yang2025_Silkie_Wenchang	CRA004775	0.651594473
ENSGALG00010027959	TPM4	2	0.379711859	0.251922579	1.507256162	0.13174501	0.073072512	2.135376716	53.16985557	1	CRA004775;Yang2025_Silkie_Wenchang	PRJNA827465	0.60817173
ENSGALG00010027959	TPM4	2	0.10955519	0.079934956	1.37055421	0.170513965	0	0.043280143	0	1	CRA004775;PRJNA827465	Yang2025_Silkie_Wenchang	0.672201941
ENSGALG00010027960	PPP6C	2	0.05868329	0.115508453	0.508043251	0.611423012	0.020751397	4.211247163	76.25406533	0.5	PRJNA827465;Yang2025_Silkie_Wenchang	CRA004775	0.897609796
ENSGALG00010027960	PPP6C	2	-0.068353558	0.294656961	-0.23197673	0.816556087	0.144139928	5.469854648	81.71797855	0.5	CRA004775;Yang2025_Silkie_Wenchang	PRJNA827465	0.954613592
ENSGALG00010027960	PPP6C	2	-0.150530237	0.165547096	-0.90928951	0.363197326	0.03658232	2.285728705	56.250276	1	CRA004775;PRJNA827465	Yang2025_Silkie_Wenchang	0.81677316
ENSGALG00010027962	SCAMP3	2	-0.07388498	0.089113344	-0.829112416	0.407040799	0	0.001489775	0	1	PRJNA827465;Yang2025_Silkie_Wenchang	CRA004775	0.833746326
ENSGALG00010027962	SCAMP3	2	-0.211660395	0.197647009	-1.070901076	0.284213912	0.030871707	1.511384027	33.83547911	1	CRA004775;Yang2025_Silkie_Wenchang	PRJNA827465	0.780082638
ENSGALG00010027962	SCAMP3	2	-0.192867167	0.194941849	-0.98935743	0.322488295	0.041234274	1.774837829	43.65682409	1	CRA004775;PRJNA827465	Yang2025_Silkie_Wenchang	0.798652022
ENSGALG00010027964	RABEPK	2	-0.033417673	0.069990173	-0.477462356	0.633032921	0	0.000991154	0	1	PRJNA827465;Yang2025_Silkie_Wenchang	CRA004775	0.903225139
ENSGALG00010027964	RABEPK	2	-0.303889674	0.290404358	-1.046436344	0.29535962	0.134590342	4.891888662	79.55799755	1	CRA004775;Yang2025_Silkie_Wenchang	PRJNA827465	0.787628307
ENSGALG00010027964	RABEPK	2	-0.289789804	0.286178513	-1.012619016	0.311242173	0.141001448	6.653552493	84.97043495	1	CRA004775;PRJNA827465	Yang2025_Silkie_Wenchang	0.795416404
ENSGALG00010027965	ACAD9	2	0.075471685	0.204499696	0.369055242	0.71208655	0.070082965	6.160492787	83.76753233	0.5	PRJNA827465;Yang2025_Silkie_Wenchang	CRA004775	0.92597752
ENSGALG00010027965	ACAD9	2	0.245270075	0.113330963	2.164192977	0.030449542	0	0.681503515	0	1	CRA004775;Yang2025_Silkie_Wenchang	PRJNA827465	0.297553047
ENSGALG00010027965	ACAD9	2	-0.108787361	0.103590083	-1.050171583	0.293639232	0	0.195668047	0	0.5	CRA004775;PRJNA827465	Yang2025_Silkie_Wenchang	0.786979185
ENSGALG00010027966	CLK2	2	0.094845817	0.088438285	1.072452024	0.283517052	0.011720355	3.906315346	74.40042825	1	PRJNA827465;Yang2025_Silkie_Wenchang	CRA004775	0.779568117
ENSGALG00010027966	CLK2	2	0.008447609	0.222020344	0.038048805	0.969648769	0.077276273	4.075110041	75.46078536	0.5	CRA004775;Yang2025_Silkie_Wenchang	PRJNA827465	0.991572914
ENSGALG00010027966	CLK2	2	-0.047305933	0.114157888	-0.414390397	0.67858822	0.014018696	1.588755073	37.05763607	0.5	CRA004775;PRJNA827465	Yang2025_Silkie_Wenchang	0.917701868
ENSGALG00010027968	FKBP15	2	0.132215239	0.138938862	0.951607329	0.341296163	0.033561117	7.414888686	86.51362087	1	PRJNA827465;Yang2025_Silkie_Wenchang	CRA004775	0.805665554
ENSGALG00010027968	FKBP15	2	0.042056293	0.287788847	0.146135937	0.883814078	0.132054798	4.354999147	77.03788299	0.5	CRA004775;Yang2025_Silkie_Wenchang	PRJNA827465	0.972001076
ENSGALG00010027968	FKBP15	2	-0.039751793	0.105820441	-0.375653255	0.707174694	0.010434223	1.284445197	22.14537433	0.5	CRA004775;PRJNA827465	Yang2025_Silkie_Wenchang	0.924856272
ENSGALG00010027969	HSPA5	2	0.177091211	0.086264576	2.052884508	0.04008378	0.005672297	1.602575554	37.6004459	1	PRJNA827465;Yang2025_Silkie_Wenchang	CRA004775	0.346798223
ENSGALG00010027969	HSPA5	2	-0.038501392	0.193334832	-0.199143586	0.842150427	0.040301577	1.750258899	42.86559545	0.5	CRA004775;Yang2025_Silkie_Wenchang	PRJNA827465	0.960064308
ENSGALG00010027969	HSPA5	2	0.023158962	0.294773678	0.078565229	0.937378445	0.132456987	3.548778757	71.8212921	0.5	CRA004775;PRJNA827465	Yang2025_Silkie_Wenchang	0.985114195
ENSGALG00010027970	GRIN3B	2	0.321393048	0.484013747	0.664016363	0.506679838	0.452618484	29.25826692	96.58216256	0.5	PRJNA827465;Yang2025_Silkie_Wenchang	CRA004775	0.863929781
ENSGALG00010027970	GRIN3B	2	0.343635815	0.473547863	0.725662265	0.46804584	0.421247729	16.43416304	93.91511452	0.5	CRA004775;Yang2025_Silkie_Wenchang	PRJNA827465	0.853145513
ENSGALG00010027970	GRIN3B	2	-0.150969519	0.084736595	-1.78163307	0.074809088	0	0.010714159	0	1	CRA004775;PRJNA827465	Yang2025_Silkie_Wenchang	0.472248889
ENSGALG00010027971	ENSGALG00010027971	2	-0.239200967	0.208276737	-1.148476638	0.250771852	0.029059304	1.496300762	33.1685163	1	PRJNA827465;Yang2025_Silkie_Wenchang	CRA004775	0.760383127
ENSGALG00010027971	ENSGALG00010027971	2	-0.005144185	0.22484136	-0.022879176	0.981746651	0	0.001015398	0	0.5	CRA004775;Yang2025_Silkie_Wenchang	PRJNA827465	0.99503081
ENSGALG00010027971	ENSGALG00010027971	2	-0.342350746	0.200258096	-1.709547593	0.087349564	0	0.745542445	0	0.5	CRA004775;PRJNA827465	Yang2025_Silkie_Wenchang	0.508203451
ENSGALG00010027972	TMEM259	2	-0.157012485	0.128995455	-1.217193936	0.223530437	0.027609681	5.67868744	82.39029687	1	PRJNA827465;Yang2025_Silkie_Wenchang	CRA004775	0.736969781
ENSGALG00010027972	TMEM259	2	-0.296174415	0.086318185	-3.431193731	0.000600931	0	0.00086363	0	1	CRA004775;Yang2025_Silkie_Wenchang	PRJNA827465	0.02653753
ENSGALG00010027972	TMEM259	2	-0.066103548	0.078858874	-0.83825123	0.40188962	0.003872809	1.139393235	12.23398827	1	CRA004775;PRJNA827465	Yang2025_Silkie_Wenchang	0.832806663
ENSGALG00010027973	GAPVD1	2	0.053862818	0.037901071	1.421142379	0.155275372	0	0.536799023	0	1	PRJNA827465;Yang2025_Silkie_Wenchang	CRA004775	0.647874911
ENSGALG00010027973	GAPVD1	2	0.112826698	0.093443308	1.207434752	0.227264759	0	0.065743596	0	1	CRA004775;Yang2025_Silkie_Wenchang	PRJNA827465	0.740605003
ENSGALG00010027973	GAPVD1	2	0.043320818	0.040158797	1.078737941	0.280704567	0	0.005183448	0	1	CRA004775;PRJNA827465	Yang2025_Silkie_Wenchang	0.778536485
ENSGALG00010027974	ENSGALG00010027974	2	0.238430874	0.422974734	0.563700039	0.572958291	0.095892643	1.366096634	26.79873628	0.5	PRJNA827465;Yang2025_Silkie_Wenchang	CRA004775	0.88573298
ENSGALG00010027974	ENSGALG00010027974	2	-0.352856952	0.996924257	-0.353945597	0.723379654	1.758027585	8.639010252	88.42459992	0.5	CRA004775;Yang2025_Silkie_Wenchang	PRJNA827465	0.930032096
ENSGALG00010027974	ENSGALG00010027974	2	-0.788534181	0.573388054	-1.375219061	0.169063541	0.427052466	2.840513025	64.79509191	1	CRA004775;PRJNA827465	Yang2025_Silkie_Wenchang	0.67030648
ENSGALG00010027975	IQSEC1	2	0.389171788	0.473206825	0.822413726	0.41084147	0.394497923	7.708622004	87.02751284	0.5	PRJNA827465;Yang2025_Silkie_Wenchang	CRA004775	0.83504692
ENSGALG00010027975	IQSEC1	2	0.454092964	0.500465044	0.907342021	0.364225959	0.356658098	3.422151086	70.77861337	0.5	CRA004775;Yang2025_Silkie_Wenchang	PRJNA827465	0.817449342
ENSGALG00010027975	IQSEC1	2	-0.037100881	0.093074959	-0.398612922	0.690178439	0	0.013724578	0	1	CRA004775;PRJNA827465	Yang2025_Silkie_Wenchang	0.919979266
ENSGALG00010027976	LRSAM1	2	0.073353756	0.043057967	1.70360471	0.088454961	0	0.0093769	0	1	PRJNA827465;Yang2025_Silkie_Wenchang	CRA004775	0.510805955
ENSGALG00010027976	LRSAM1	2	-0.122480113	0.197634492	-0.619730451	0.535435264	0.061494743	4.636196921	78.43059695	0.5	CRA004775;Yang2025_Silkie_Wenchang	PRJNA827465	0.874171355
ENSGALG00010027976	LRSAM1	2	-0.104786352	0.202392948	-0.517737171	0.60464166	0.071142485	6.965952732	85.64446188	0.5	CRA004775;PRJNA827465	Yang2025_Silkie_Wenchang	0.896289929
ENSGALG00010027977	BOLA1	2	-0.048966447	0.113152944	-0.432745672	0.665199546	0	0.052256607	0	0.5	PRJNA827465;Yang2025_Silkie_Wenchang	CRA004775	0.913399315
ENSGALG00010027977	BOLA1	2	0.111824174	0.240603369	0.464765619	0.642099311	0	0.239399115	0	1	CRA004775;Yang2025_Silkie_Wenchang	PRJNA827465	0.906634523
ENSGALG00010027977	BOLA1	2	-0.030144457	0.115890657	-0.260111188	0.794778008	0	0.621361675	0	0.5	CRA004775;PRJNA827465	Yang2025_Silkie_Wenchang	0.948905846
ENSGALG00010027979	RPL12	2	-0.076715681	0.071515175	-1.072718924	0.283397247	0	0.708490412	0	0.5	PRJNA827465;Yang2025_Silkie_Wenchang	CRA004775	0.779568117
ENSGALG00010027979	RPL12	2	-0.036164039	0.167618553	-0.215752003	0.829181063	0	0.826943396	0	0.5	CRA004775;Yang2025_Silkie_Wenchang	PRJNA827465	0.956860127
ENSGALG00010027979	RPL12	2	-0.105137926	0.071892079	-1.46244102	0.143620396	0	0.157062272	0	1	CRA004775;PRJNA827465	Yang2025_Silkie_Wenchang	0.630564924
ENSGALG00010027980	NUP210	2	0.211705184	0.286606921	0.738660406	0.460113234	0.143213403	7.401098154	86.48849158	0.5	PRJNA827465;Yang2025_Silkie_Wenchang	CRA004775	0.850104868
ENSGALG00010027980	NUP210	2	-0.165244192	0.755148037	-0.218823574	0.826787483	1.009552613	8.200810472	87.80608327	0.5	CRA004775;Yang2025_Silkie_Wenchang	PRJNA827465	0.956501588

ENSGALG00010027980	NUP210	2	-0.395786668	0.453496448	-0.872744803	0.382802191	0.317149328	3.526477148	71.64308861	1	CRA004775;PRJNA827465	Yang2025_Silkie_Wenchang	0.825121999
ENSGALG00010027981	CNN2	2	0.063801539	0.221846333	0.287593392	0.773658004	0.070895799	3.043714996	67.14541271	0.5	PRJNA827465;Yang2025_Silkie_Wenchang	CRA004775	0.943248868
ENSGALG00010027981	CNN2	2	-0.347786829	0.20396	-1.705171751	0.088162397	0	0.723621397	0	1	CRA004775;Yang2025_Silkie_Wenchang	PRJNA827465	0.510165884
ENSGALG00010027981	CNN2	2	-0.108828933	0.407903981	-0.266800371	0.789622869	0.276547208	5.161097504	80.62427615	0.5	CRA004775;PRJNA827465	Yang2025_Silkie_Wenchang	0.947390191
ENSGALG00010027982	ENTPD2	2	-0.169418695	0.295244569	-0.573824932	0.566086276	0.147267722	6.406443487	84.39071535	0.5	PRJNA827465;Yang2025_Silkie_Wenchang	CRA004775	0.883487517
ENSGALG00010027982	ENTPD2	2	-0.228709478	0.347635672	-0.657899912	0.510602448	0.148349051	2.196661548	54.47637343	0.5	CRA004775;Yang2025_Silkie_Wenchang	PRJNA827465	0.865381975
ENSGALG00010027982	ENTPD2	2	0.130343258	0.14075428	0.926034061	0.35442828	0	0.09141291	0	1	CRA004775;PRJNA827465	Yang2025_Silkie_Wenchang	0.81193713
ENSGALG00010027983	POGZ	2	-0.088129311	0.051800492	-1.701321885	0.088882561	0	0.407292598	0	0.5	PRJNA827465;Yang2025_Silkie_Wenchang	CRA004775	0.511771357
ENSGALG00010027983	POGZ	2	-0.017888653	0.118514719	-0.150940353	0.880022768	0	0.04591859	0	0.5	CRA004775;Yang2025_Silkie_Wenchang	PRJNA827465	0.971546618
ENSGALG00010027983	POGZ	2	-0.096488573	0.053048024	-1.818890998	0.068928061	0	0.061798269	0	1	CRA004775;PRJNA827465	Yang2025_Silkie_Wenchang	0.452044654
ENSGALG00010027984	ARHGAP45	2	0.148255339	0.098015388	1.512572069	0.130388393	0	0.003462315	0	1	PRJNA827465;Yang2025_Silkie_Wenchang	CRA004775	0.605608015
ENSGALG00010027984	ARHGAP45	2	-0.222731269	0.46026402	-0.483920662	0.628442176	0.329027632	3.962808447	74.76537124	0.5	CRA004775;Yang2025_Silkie_Wenchang	PRJNA827465	0.901912657
ENSGALG00010027984	ARHGAP45	2	-0.221650365	0.452703288	-0.489615099	0.62440629	0.323335896	4.062017148	75.38168935	0.5	CRA004775;PRJNA827465	Yang2025_Silkie_Wenchang	0.900886179
ENSGALG00010027985	PSMB4	2	-0.036142679	0.046257651	-0.781334076	0.434606033	0	0.730722078	0	0.5	PRJNA827465;Yang2025_Silkie_Wenchang	CRA004775	0.842762754
ENSGALG00010027985	PSMB4	2	0.065467008	0.168994505	0.387391342	0.698466513	0	0.378540998	0	0.5	CRA004775;Yang2025_Silkie_Wenchang	PRJNA827465	0.921666253
ENSGALG00010027985	PSMB4	2	-0.047028445	0.04711517	-0.998159294	0.318202122	0	0.037056361	0	1	CRA004775;PRJNA827465	Yang2025_Silkie_Wenchang	0.797512752
ENSGALG00010027986	CGN	2	0.786999962	0.50641619	1.554057666	0.120170662	0.461582341	9.991975726	89.99196928	1	PRJNA827465;Yang2025_Silkie_Wenchang	CRA004775	0.581455423
ENSGALG00010027986	CGN	2	1.256630532	0.208133363	6.037621798	1.56402E-09	0	0.164255971	0	1	CRA004775;Yang2025_Silkie_Wenchang	PRJNA827465	9.03981E-07
ENSGALG00010027986	CGN	2	0.535875313	0.367343519	1.458785267	0.144624219	0.148983593	1.939495794	48.44020785	1	CRA004775;PRJNA827465	Yang2025_Silkie_Wenchang	0.632135862
ENSGALG00010027987	TMEM141	2	0.239926749	0.337511326	0.710870217	0.477164663	0.199244171	7.519676308	86.70155524	0.5	PRJNA827465;Yang2025_Silkie_Wenchang	CRA004775	0.854986476
ENSGALG00010027987	TMEM141	2	0.378314729	0.286321011	1.32129573	0.186402777	0.083940255	1.96067595	48.9971813	1	CRA004775;Yang2025_Silkie_Wenchang	PRJNA827465	0.695457578
ENSGALG00010027987	TMEM141	2	-0.061126482	0.092733121	-0.659165588	0.509789442	0	0.064893735	0	0.5	CRA004775;PRJNA827465	Yang2025_Silkie_Wenchang	0.865257661
ENSGALG00010027988	HDAC11	2	0.104989988	0.080146945	1.309968683	0.19020643	0	0.031416328	0	1	PRJNA827465;Yang2025_Silkie_Wenchang	CRA004775	0.699403589
ENSGALG00010027988	HDAC11	2	0.272760076	0.187635857	1.453667121	0.146038618	0.041922287	2.311072059	56.73003807	1	CRA004775;Yang2025_Silkie_Wenchang	PRJNA827465	0.634897819
ENSGALG00010027988	HDAC11	2	0.254953324	0.20172931	1.263838772	0.206267902	0.056058823	2.907308733	65.60392818	1	CRA004775;PRJNA827465	Yang2025_Silkie_Wenchang	0.718451597
ENSGALG00010027989	ENSGALG00010027989	2	-0.125176099	0.096748023	-1.293836243	0.195721991	0	0.778623383	0	1	PRJNA827465;Yang2025_Silkie_Wenchang	CRA004775	0.706808553
ENSGALG00010027989	ENSGALG00010027989	2	-0.279074483	0.206351459	-1.352423113	0.17624	0	0.211973293	0	1	CRA004775;Yang2025_Silkie_Wenchang	PRJNA827465	0.681432435
ENSGALG00010027989	ENSGALG00010027989	2	-0.109063855	0.095461783	-1.142487096	0.253251607	0	0.111124971	0	1	CRA004775;PRJNA827465	Yang2025_Silkie_Wenchang	0.762899356
ENSGALG00010027990	ENSGALG00010027990	2	0.222249404	0.333931802	0.665553273	0.505696682	0.159835142	3.3298356	69.96848733	0.5	PRJNA827465;Yang2025_Silkie_Wenchang	CRA004775	0.863927652
ENSGALG00010027990	ENSGALG00010027990	2	0.097375966	0.526764212	0.184856838	0.853341326	0.438802621	4.768252018	79.02795414	0.5	CRA004775;Yang2025_Silkie_Wenchang	PRJNA827465	0.962916051
ENSGALG00010027990	ENSGALG00010027990	2	-0.1413728	0.162942384	-0.867624473	0.385599944	0	0.878522376	0	1	CRA004775;PRJNA827465	Yang2025_Silkie_Wenchang	0.82648313
ENSGALG00010027991	ENSGALG00010027991	2	-0.215079532	0.14519177	-1.481347963	0.138513874	0	0.275511803	0	1	PRJNA827465;Yang2025_Silkie_Wenchang	CRA004775	0.622343774
ENSGALG00010027991	ENSGALG00010027991	2	-0.186693134	0.171613022	-1.087872768	0.276651279	0	0.881364151	0	0.5	CRA004775;Yang2025_Silkie_Wenchang	PRJNA827465	0.775851484
ENSGALG00010027991	ENSGALG00010027991	2	-0.07783097	0.173133564	-0.449542935	0.653040043	0	0.283748697	0	0.5	CRA004775;PRJNA827465	Yang2025_Silkie_Wenchang	0.909572411
ENSGALG00010027993	CLIC3	2	-0.09951095	0.103145329	-0.964764478	0.334662792	0	0.439134669	0	0.5	PRJNA827465;Yang2025_Silkie_Wenchang	CRA004775	0.803101658
ENSGALG00010027993	CLIC3	2	0.053328312	0.163879866	0.325411007	0.744870026	0	0.277626935	0	1	CRA004775;Yang2025_Silkie_Wenchang	PRJNA827465	0.935893304
ENSGALG00010027993	CLIC3	2	-0.102211189	0.11571161	-0.883326996	0.377059622	0	0.971905635	0	0.5	CRA004775;PRJNA827465	Yang2025_Silkie_Wenchang	0.823064945
ENSGALG00010027994	TUFT1	2	0.308365035	0.146499553	2.104887207	0.035301128	0.032966618	4.290515903	76.69277955	1	PRJNA827465;Yang2025_Silkie_Wenchang	CRA004775	0.324168275
ENSGALG00010027994	TUFT1	2	0.352913845	0.177567159	1.987495027	0.046867566	0.031464859	1.644754994	39.20067101	1	CRA004775;Yang2025_Silkie_Wenchang	PRJNA827465	0.37376485
ENSGALG00010027994	TUFT1	2	0.157722512	0.087470614	1.80314856	0.071364887	0	0.122369474	0	1	CRA004775;PRJNA827465	Yang2025_Silkie_Wenchang	0.46124883
ENSGALG00010027995	POLR2E	2	-0.025749351	0.108344519	-0.237661778	0.812143431	0.012991961	1.682426623	40.56211443	0.5	PRJNA827465;Yang2025_Silkie_Wenchang	CRA004775	0.953371387
ENSGALG00010027995	POLR2E	2	-0.219572845	0.158938818	-1.381492874	0.167127467	0	0.000342094	0	1	CRA004775;Yang2025_Silkie_Wenchang	PRJNA827465	0.666416556
ENSGALG00010027995	POLR2E	2	0.028197879	0.049168908	0.573490046	0.566312937	0	0.752522815	0	0.5	CRA004775;PRJNA827465	Yang2025_Silkie_Wenchang	0.883387517
ENSGALG00010027996	GPX4	2	0.154704267	0.133303101	1.16054515	0.245826922	0.018516449	1.689623454	40.81521549	1	PRJNA827465;Yang2025_Silkie_Wenchang	CRA004775	0.75661406
ENSGALG00010027996	GPX4	2	0.126932975	0.320779446	0.395701709	0.692325095	0.116676033	2.134523022	53.15112604	0.5	CRA004775;Yang2025_Silkie_Wenchang	PRJNA827465	0.920516008
ENSGALG00010027996	GPX4	2	0.061510592	0.074393127	0.826831647	0.40833249	0	0.798964708	0	0.5	CRA004775;PRJNA827465	Yang2025_Silkie_Wenchang	0.834134074
ENSGALG00010027998	SNX27	2	-0.040151592	0.044670196	-0.898845234	0.368735102	0	0.002976982	0	1	PRJNA827465;Yang2025_Silkie_Wenchang	CRA004775	0.820425502
ENSGALG00010027998	SNX27	2	-0.062256129	0.094138974	-0.661321515	0.508406146	0	0.585072718	0	1	CRA004775;Yang2025_Silkie_Wenchang	PRJNA827465	0.864902608
ENSGALG00010027998	SNX27	2	-0.048481468	0.049019579	-0.98902254	0.322652114	0	0.609529387	0	1	CRA004775;PRJNA827465	Yang2025_Silkie_Wenchang	0.798652022
ENSGALG00010027999	ENSGALG00010027999	2	-0.455631129	0.495253991	-0.919994866	0.357575442	0.331644415	3.032946926	67.02876692	0.5	PRJNA827465;Yang2025_Silkie_Wenchang	CRA004775	0.813486201
ENSGALG00010027999	ENSGALG00010027999	2	-0.0209603	0.365522066	-0.057343461	0.954271	0	0.156949413	0	0.5	CRA004775;Yang2025_Silkie_Wenchang	PRJNA827465	0.987981052
ENSGALG00010027999	ENSGALG00010027999	2	-0.740356759	0.298617677	-2.479279752	0.013164801	0	0.984085025	0	1	CRA004775;PRJNA827465	Yang2025_Silkie_Wenchang	0.187266073
ENSGALG00010028000	MRPL9	2	0.345926932	0.446957828	0.773958771	0.438955173	0.375470574	15.9716315	93.73889887	0.5	PRJNA827465;Yang2025_Silkie_Wenchang	CRA004775	0.844002503
ENSGALG00010028000	MRPL9	2	0.495788048	0.375498412	1.320359699	0.186714952	0.198297354	3.180493147	68.55833502	1	CRA004775;Yang2025_Silkie_Wenchang	PRJNA827465	0.695457578
ENSGALG00010028000	MRPL9	2	-0.074415853	0.069434635	-1.071739674	0.283836976	0	0.127579239	0	0.5	CRA004775;PRJNA827465	Yang2025_Silkie_Wenchang	0.77978499
ENSGALG00010028001	SBNO2	2	0.141151177	0.098586995	1.431742368	0.152217566	0.011402389	2.11020395	52.6112156	1	PRJNA827465;Yang2025_Silkie_Wenchang	CRA004775	0.643706371
ENSGALG00010028001	SBNO2	2	-0.245596109	0.587585856	-0.417974848	0.675965507	0.60288525	7.23814747	86.18431022	0.5	CRA004775;Yang2025_Silkie_Wenchang	PRJNA827465	0.916530594
ENSGALG00010028001	SBNO2	2	-0.326212222	0.479184689	-0.684938874	0.493382494	0.365357712	5.295606446	81.11642151	0.5	CRA004775;PRJNA827465	Yang2025_Silkie_Wenchang	0.859868321
ENSGALG00010028002	ABCA2	2	0.081291503	0.217249532	0.374184941	0.708266727	0.083446549	8.356017529	88.03257656	0.5	PRJNA827465;Yang2025_Silkie_Wenchang	CRA004775	0.925061251
ENSGALG00010028002	ABCA2	2	0.013386964	0.319248763	0.041932706	0.966552344	0.174256799	6.732188007	85.14598822	0.5	CRA004775;Yang2025_Silkie_Wenchang	PRJNA827465	0.990881287
ENSGALG00010028002	ABCA2	2	-0.144313105	0.068247291	-2.114561674	0.034467323	0	0.86028423	0	1	CRA004775;PRJNA827465	Yang2025_Silkie_Wenchang	0.319372672
ENSGALG00010028004	ENSGALG00010028004	2	-0.301929163	0.673440299	-0.448338426	0.653908973	0.823137369	10.67147865	90.62922737	0.5	PRJNA827465;Yang2025_Silkie_Wenchang	CRA004775	0.909861322
ENSGALG00010028004	ENSGALG00010028004	2	-0.177983866	0.600129209	-0.29657591	0.766790305	0.572001978	4.821391615	79.25910028	0.5	CRA004775;Yang2025_Silkie_Wenchang	PRJNA827465	0.941315572
ENSGALG00010028004	ENSGALG00010028004	2	-0.918623629	0.201081903	-4.568405285	4.91449E-06	0	0.091851714	0	1	CRA004775;PRJNA827465	Yang2025_Silkie_Wenchang	0.000804809
ENSGALG00010028005	IER5L	2	0.119448819	0.202757467	0.589121677	0.555779653	0.069213338	6.229813644	83.94815548	0.5	PRJNA827465;Yang2025_Silkie_Wenchang	CRA004775	0.880324407
ENSGALG00010028005	IER5L	2	0.320263104	0.120726411	2.652800681	0.007982699	0	0.05551691	0	1	CRA004775;Yang2025_Silkie_Wenchang	PRJNA827465	0.138947756
ENSGALG00010028005	IER5L	2	-0.024218588	0.117135404	-0.206757195	0.836199487	0.007182321	1.151618678	13.16570156	0.5	CRA004775;PRJNA827465	Yang2025_Silkie_Wenchang	0.958399882
ENSGALG00010028007	STK11	2	-0.078538477	0.058202523	-1.349399867	0.177208563	0	0.330630914	0	1	PRJNA827465;Yang2025_Silkie_Wenchang	CRA004775	0.683161174
ENSGALG00010028007	STK11	2	-0.026685892	0.081471508	-0.327548763	0.743252867	0	0.268588124	0	0.5	CRA004775;Yang2025_Silkie_Wenchang	PRJNA827465	0.93574436
ENSGALG00010028007	STK11	2	-0.089870236	0.074838247	-1.200859707	0.229805626	0	0.61949253	0	0.5	CRA004775;PRJNA827465	Yang2025_Silkie_Wenchang	0.742706236
ENSGALG00010028008	NTMT1	2	-0.235812437	0.190340888	-1.23889533	0.215384264	0.054873259	3.539976533	71.75122517	1	PRJNA827465;Yang2025_Silkie_Wenchang	CRA004775	0.728757551
ENSGALG00010028008	NTMT1	2	-0.401373839	0.170325352	-2.356512608	0.018447441	0	0.552838849	0	1	CRA004775;Yang2025_Silkie_Wenchang	PRJNA827465	0.227285201
ENSGALG00010028008	NTMT1	2	-0.088658339	0.062704801	-1.413900332	0.157391169	0	0.089317888	0	1	CRA004775;PRJNA827465	Yang2025_Silkie_Wenchang	0.650687662
ENSGALG00010028009	RRP9	2	0.229394486	0.21624902	1.060788555	0.288786004	0.084373887	9.973143451	89.97307113	1	PRJNA827465;Yang2025_Silkie_Wenchang	CRA004775	0.783530627
ENSGALG00010028009	RRP9	2	-0.058700657	0.530695997	-0.110610702	0.91192506	0.533450136	18.6155717	94.62815316	0.5	CRA004775;Yang2025_Silkie_Wenchang	PRJNA827465	0.978928739
ENSGALG00010028009	RRP9	2	-0.257137585	0.312614772	-0.822538177	0.410770668	0.172216276	7.768081026	87.12680781	0.5	CRA004775;PRJNA827465	Yang2025_Silkie_Wenchang	0.835032562
ENSGALG00010028010	ASB6	2	-0.012109876	0.052485317	-0.230728825	0.817525478	0	0.411582706	0	1	PRJNA827465;Yang2025_Silkie_Wenchang	CRA004775	0.954888783
ENSGALG00010028010	ASB6	2	-0.261577299	0.157459903	-1.661231176	0.096667026	0.013909079	1.38390108	27.74050007	1	CRA004775;Yang2025_Silkie_Wenchang	PRJNA827465	0.531222615
ENSGALG00010028010	ASB6	2	-0.174836866	0.212929915	-0.821100534	0.411589006	0.072330593	4.249427634	76.467419	1	CRA004775;PRJNA827465	Yang2025_Silkie_Wenchang	0.835128673
ENSGALG00010028011	ENSGALG00010028011	2	-0.277915751	0.192499623	-1.443721016	0.148817467	0.05990145	4.983255133	79.93279547	1	PRJNA827465;Yang2025_Silkie_Wenchang	CRA004775	0.63890906
ENSGALG00010028011	ENSGALG00010028011	2	-0.454576668	0.13996307	-3.247832938	0.001162875	0	0.417515883	0	1	CRA004775;Yang2025_Silkie_Wenchang	PRJNA827465	0.041511795
ENSGALG00010028011	ENSGALG00010028011	2	-0.111250766	0.082476796	-1.348873514	0.177377597	0	0.11944518	0	1	CRA004775;PRJNA827465	Yang2025_Silkie_Wenchang	0.683500856
ENSGALG00010028012	PRRX2	2	0.0924091	0.137417003	0.672472092	0.501283201	0	0.305647299	0	1	PRJNA827465;Yang2025_Silkie_Wenchang	CRA004775	0.862658912
ENSGALG00010028012	PRRX2	2	0.016914982	0.278970332	0.060633625	0.951650994	0.056033469	1.422541632	29.7032876	0.5	CRA004775;Yang2025_Silkie_Wenchang	PRJNA827465	0.987713746
ENSGALG00010028012	PRRX2	2	-0.018606211	0.159988516	-0.116297167	0.907417031	0	0.860812061	0	0.5	CRA004775;PRJNA827465	Yang2025_Silkie_Wenchang	0.978196186
ENSGALG00010028013	CBARP	2	0.166765424	0.266292593	0.626248828	0.531151745	0.126309986	8.929268509	88.80087435	0.5	PRJNA827465;Yang2025_Silkie_Wenchang	CRA004775	0.872848946
ENSGALG00010028013	CBARP	2	0.40870736	0.145025852	2.818169005	0.004829838	0	0.592185565	0	1	CRA004775;Yang2025_Silkie_Wenchang	PRJNA827465	0.1030268
ENSGALG00010028013	CBARP	2	-0.078240219	0.088867233	-0.880416953	0.378633476	0	0.15515733	0	0.5	CRA004775;PRJNA827465	Yang2025_Silkie_Wenchang	0.823771464
ENSGALG00010028014	ENSGALG00010028014	2	-0.170239331	0.321498199	-0.52951877	0.596445627	0	0.366009663	0	0.5	PRJNA827465;Yang2025_Silkie_Wenchang	CRA004775	0.894064255
ENSGALG00010028014	ENSGALG00010028014	2	-0.601337853	0.697100347	-0.862627391	0.388342366	0.64905998	2.893561972	65.440519	0.5	CRA004775;Yang2025_Silkie_Wenchang	PRJNA827465	0.828154121
ENSGALG00010028014	ENSGALG00010028014	2	-0.700856668	0.465483814	-1.505652069	0.132156514	0.269396351	2.614914793	61.75783614	1	CRA004775;PRJNA827465	Yang2025_Silkie_Wenchang	0.608786928
ENSGALG00010028015	DUSP7	2	-0.043749954	0.064268707	-0.680734937	0.496039225	0	0.522886567	0	1	PRJNA827465;Yang2025_Silkie_Wenchang	CRA004775	0.860911322
ENSGALG00010028015	DUSP7	2	-0.129967836	0.118112567	-1.100372626	0.2711698	0	0.061999411	0	1	CRA004775;Yang2025_Silkie_Wenchang	PRJNA827465	0.772743863
ENSGALG00010028015	DUSP7	2	-0.023139632	0.073744515	-0.313781055	0.753687334	0	0.274407988	0	1	CRA004775;PRJNA827465	Yang2025_Silkie_Wenchang	0.938112996
ENSGALG00010028019	RPL29	2	-0.254638505	0.301283269	-0.845179707	0.39801051	0.148369131	4.86394935	79.44057538	1	PRJNA827465;Yang2025_Silkie_Wenchang	CRA004775	0.831850795
ENSGALG00010028019	RPL29	2	-0.459237152	0.187648549	-2.44732589	0.014392064	0	0.672046521	0	1	CRA004775;Yang2025_Silkie_Wenchang	PRJNA827465	0.197297279
ENSGALG00010028019	RPL29	2	-0.046352626	0.109020142	-0.425174877	0.670709197	0.006857297	1.174946415	14.88973565	1	CRA004775;PRJNA827465	Yang2025_Silkie_Wenchang	0.914482883
ENSGALG00010028020	ENSGALG00010028020	2	-0.113547157	0.11388679	-0.997017798	0.318755871	0	0.214490819	0	1	PRJNA827465;Yang2025_Silkie_Wenchang	CRA004775	0.79791491
ENSGALG00010028020	ENSGALG00010028020	2	-0.077870582	0.192505931	-0.404510041	0.685837695	0	0.208103322	0	1	CRA004775;Yang2025_Silkie_Wenchang	PRJNA827465	0.919235539
ENSGALG00010028020	ENSGALG00010028020	2	-0.15243751	0.123386954	-1.235442689	0.216665793	0	0.035441797	0	1	CRA004775;PRJNA827465	Yang2025_Silkie_Wenchang	0.730172415
ENSGALG00010028021	ATP5F1D	2	-0.149746594	0.106350245	-1.408051239	0.159115898	0	0.289278558	0	0.5	PRJNA827465;Yang2025_Silkie_Wenchang	CRA004775	0.654100764
ENSGALG00010028021	ATP5F1D	2	-0.050126553	0.245927216	-0.203826783	0.838488862	0	0.089255096	0	0.5	CRA004775;Yang2025_Silkie_Wenchang	PRJNA827465	0.959197438
ENSGALG00010028021	ATP5F1D	2	-0.170159078	0.109813475	-1.549528221	0.121254793	0	0.001569568	0	1	CRA004775;PRJNA827465	Yang2025_Silkie_Wenchang	0.583784676
ENSGALG00010028022	ENSGALG00010028022	2	-0.15287782	0.071318191	-2.143602062	0.032064785	0	0.00027629	0	1	PRJNA827465;Yang2025_Silkie_Wenchang	CRA004775	0.306978889
ENSGALG00010028022	ENSGALG00010028022	2	-0.109081235	0.116909663	-0.933038661	0.350800007	0	0.193788191	0	1	CRA004775;Yang2025_Silkie_Wenchang	PRJNA827465	0.810146221
ENSGALG00010028022	ENSGALG00010028022	2	-0.136273961	0.074275426	-1.834711273	0.066548481	0	0.284003582	0	1	CRA004775;PRJNA827465	Yang2025_Silkie_Wenchang	0.444444342
ENSGALG00010028024	RBM3	2	0.038393187	0.078921272	0.486474507	0.626630775	0.001579564	1.121765838	10.85483562	0.5	PRJNA827465;Yang2025_Silkie_Wenchang	CRA004775	0.901504244
ENSGALG00010028024	RBM3	2	-0.258380912	0.218882442	-1.18045518	0.237819226	0.061498233	2.608839153	61.66877521	1	CRA004775;Yang2025_Silkie_Wenchang	PRJNA827465	0.749619781
ENSGALG00010028024	RBM3	2	-0.176349969	0.305871509	-0.576549185	0.564244035	0.157758628	5.823776455	82.82901125	0.5	CRA004775;PRJNA827465	Yang2025_Silkie_Wenchang	0.883027875
ENSGALG00010028025	TNNC1	2	0.511001975	0.37010019	1.380712542	0.167367363	0.221104728	4.961508033	79.84483763	1	PRJNA827465;Yang2025_Silkie_Wenchang	CRA004775	0.666945317
ENSGALG00010028025	TNNC1	2	0.874914995	0.271419667	3.223476787	0.001266446	0	0.201718621	0	1	CRA004775;Yang2025_Silkie_Wenchang	PRJNA827465	0.043282706
ENSGALG00010028025	TNNC1	2	0.19452911	0.160977656	1.208423051	0.226884577	0	0.239009004	0	1	CRA004775;PRJNA827465	Yang2025_Silkie_Wenchang	0.740444589
ENSGALG00010028026	ANKMY1	2	0.266044646	0.242525496	1.09697599	0.272651888	0.098462362	6.134109642	83.69771559	1	PRJNA827465;Yang2025_Silkie_Wenchang	CRA004775	0.773556489
ENSGALG00010028026	ANKMY1	2	0.294791304	0.262001132	1.125152786	0.260524296	0.095098095	2.997217285	66.63571891	0.5	CRA004775;Yang2025_Silkie_Wenchang	PRJNA827465	0.767694434
ENSGALG00010028026	ANKMY1	2	0.015067283	0.122055953	0.123445707	0.901754165	0	0.025746704	0	0.5	CRA004775;PRJNA827465	Yang2025_Silkie_Wenchang	0.976676696
ENSGALG00010028027	ENSGALG00010028027	2	0.057762977	0.139795394	0.413196565	0.679462605	0	0.105537034	0	1	PRJNA827465;Yang2025_Silkie_Wenchang	CRA004775	0.917701868
ENSGALG00010028027	ENSGALG00010028027	2	-0.165651693	0.352932867	-0.469357513	0.638814112	0.1028106	1.698446405	41.1226638	0.5	CRA004775;Yang2025_Silkie_Wenchang	PRJNA827465	0.905768875
ENSGALG00010028027	ENSGALG00010028027	2	-0.1305593	0.263459206	-0.495557936	0.620206336	0.075183289	1.812727835	44.83452063	0.5	CRA004775;PRJNA827465	Yang2025_Silkie_Wenchang	0.899395593
ENSGALG00010028028	FAM174C	2	0.086360019	0.126210472	0.684253987	0.493814797	0	0.334307357	0	1	PRJNA827465;Yang2025_Silkie_Wenchang	CRA004775	0.860149041
ENSGALG00010028028	FAM174C	2	-0.130506523	0.345669377	-0.377547251	0.705766957	0.172073677	3.559885316	71.90920742	0.5	CRA004775;Yang2025_Silkie_Wenchang	PRJNA827465	0.924409595
ENSGALG00010028028	FAM174C	2	-0.170116464	0.253534753	-0.670978878	0.502233985	0.092032503	3.340357864	70.06308783	0.5	CRA004775;PRJNA827465	Yang2025_Silkie_Wenchang	0.862860181
ENSGALG00010028029	EFNA2	2	-0.080169674	0.094442963	-0.848868688	0.395954364	0	0.036359193	0	1	PRJNA827465;Yang2025_Silkie_Wenchang	CRA004775	0.831296327
ENSGALG00010028029	EFNA2	2	-0.158586079	0.163660957	-0.968991517	0.332549422	0	0.744407118	0	1	CRA004775;Yang2025_Silkie_Wenchang	PRJNA827465	0.802178578
ENSGALG00010028029	EFNA2	2	-0.093209927	0.109584433	-0.850576345	0.395004735	0	0.944923304	0	1	CRA004775;PRJNA827465	Yang2025_Silkie_Wenchang	0.830643015
ENSGALG00010028030	PFDN2	2	0.05083048	0.114471899	0.444043302	0.657011277	0	0.419469362	0	1	PRJNA827465;Yang2025_Silkie_Wenchang	CRA004775	0.910695405
ENSGALG00010028030	PFDN2	2	-0.121780296	0.356619725	-0.341485024	0.732738477	0.155634022	2.558462621	60.91402736	0.5	CRA004775;Yang2025_Silkie_Wenchang	PRJNA827465	0.932985777
ENSGALG00010028030	PFDN2	2	-0.144516645	0.229184454	-0.630569145	0.528322279	0.066332879	2.360182264	57.63039088	0.5	CRA004775;PRJNA827465	Yang2025_Silkie_Wenchang	0.871701504
ENSGALG00010028031	NISCH	2	-0.097057262	0.139524155	-0.695630532	0.48666024	0.033424165	6.877357289	85.45953107	0.5	PRJNA827465;Yang2025_Silkie_Wenchang	CRA004775	0.857603818
ENSGALG00010028031	NISCH	2	-0.01987562	0.243909468	-0.081487694	0.935054111	0.102445018	6.956967976	85.62592205	0.5	CRA004775;Yang2025_Silkie_Wenchang	PRJNA827465	0.984512821
ENSGALG00010028031	NISCH	2	0.083168994	0.089173261	0.932667413	0.350991715	0.007376561	1.506440565	33.61835684	1	CRA004775;PRJNA827465	Yang2025_Silkie_Wenchang	0.810146221
ENSGALG00010028033	ENSGALG00010028033	2	-0.063667048	0.160226588	-0.397356327	0.691104716	0	0.637093104	0	0.5	PRJNA827465;Yang2025_Silkie_Wenchang	CRA004775	0.920268407
ENSGALG00010028033	ENSGALG00010028033	2	-0.183143659	0.210330637	-0.870741711	0.383895198	0	0.051735745	0	1	CRA004775;Yang2025_Silkie_Wenchang	PRJNA827465	0.825601288
ENSGALG00010028033	ENSGALG00010028033	2	0.015075568	0.18801492	0.080182829	0.936091847	0	0.106032894	0	0.5	CRA004775;PRJNA827465	Yang2025_Silkie_Wenchang	0.984822128
ENSGALG00010028034	MUM1L1	2	0.017079699	0.056069181	0.304618307	0.760656875	0.002814606	1.682122243	40.55128845	0.5	PRJNA827465;Yang2025_Silkie_Wenchang	CRA004775	0.939384641
ENSGALG00010028034	MUM1L1	2	0.070379507	0.073994021	0.951151271	0.341527591	0	0.71859465	0	0.5	CRA004775;Yang2025_Silkie_Wenchang	PRJNA827465	0.805758959
ENSGALG00010028034	MUM1L1	2	-0.026889654	0.043701585	-0.61530157	0.538355581	0	0.112945123	0	1	CRA004775;PRJNA827465	Yang2025_Silkie_Wenchang	0.87481821
ENSGALG00010028036	NDUFS7	2	0.132413319	0.124546377	1.063164759	0.287707241	0.015750031	1.683756228	40.60897991	1	PRJNA827465;Yang2025_Silkie_Wenchang	CRA004775	0.782840067
ENSGALG00010028036	NDUFS7	2	0.301493769	0.1812687	1.663242297	0.096263944	0	0.140087007	0	1	CRA004775;Yang2025_Silkie_Wenchang	PRJNA827465	0.530714236
ENSGALG00010028036	NDUFS7	2	0.058707903	0.074212085	0.791082792	0.42889568	0	0.059744994	0	1	CRA004775;PRJNA827465	Yang2025_Silkie_Wenchang	0.841876188
ENSGALG00010028037	ENSGALG00010028037	2	-0.168109866	0.150275266	-1.11867954	0.263276877	0	0.013879211	0	1	PRJNA827465;Yang2025_Silkie_Wenchang	CRA004775	0.768643821
ENSGALG00010028037	ENSGALG00010028037	2	-0.314138339	0.193226179	-1.625754546	0.104001881	0.002125206	1.024433384	2.38506324	1	CRA004775;Yang2025_Silkie_Wenchang	PRJNA827465	0.548383482
ENSGALG00010028037	ENSGALG00010028037	2	-0.291279527	0.210472076	-1.383934308	0.166378571	0.023143278	1.283455965	22.0853674	1	CRA004775;PRJNA827465	Yang2025_Silkie_Wenchang	0.665350942
ENSGALG00010028039	STAB1	2	-0.05774511	0.12521084	-0.461182989	0.644667325	0.019134467	2.50244582	60.03909488	0.5	PRJNA827465;Yang2025_Silkie_Wenchang	CRA004775	0.906953008
ENSGALG00010028039	STAB1	2	-0.199206471	0.122310026	-1.628701078	0.103376314	0	0.000360923	0	1	CRA004775;Yang2025_Silkie_Wenchang	PRJNA827465	0.547576788
ENSGALG00010028039	STAB1	2	0.037899572	0.090389967	0.419289584	0.675004508	0	0.410404264	0	0.5	CRA004775;PRJNA827465	Yang2025_Silkie_Wenchang	0.916045007
ENSGALG00010028042	DPP7	2	0.228895997	0.064482396	3.549743979	0.000385606	0	0.011226477	0	1	PRJNA827465;Yang2025_Silkie_Wenchang	CRA004775	0.019759415
ENSGALG00010028042	DPP7	2	0.226131146	0.143240484	1.578681805	0.11440906	0	0.040791268	0	1	CRA004775;Yang2025_Silkie_Wenchang	PRJNA827465	0.57034745
ENSGALG00010028042	DPP7	2	0.234247193	0.069582018	3.366490382	0.000761312	0	0.027883335	0	1	CRA004775;PRJNA827465	Yang2025_Silkie_Wenchang	0.031201105
ENSGALG00010028043	NT5DC2	2	0.119938596	0.082796161	1.448600921	0.147449064	0.004301698	1.249070514	19.94046863	1	PRJNA827465;Yang2025_Silkie_Wenchang	CRA004775	0.636414513
ENSGALG00010028043	NT5DC2	2	0.262028716	0.15776075	1.660924639	0.096728583	0	0.110726444	0	1	CRA004775;Yang2025_Silkie_Wenchang	PRJNA827465	0.531460189
ENSGALG00010028043	NT5DC2	2	0.080866964	0.064202761	1.259555872	0.207829623	0	0.03469202	0	1	CRA004775;PRJNA827465	Yang2025_Silkie_Wenchang	0.720210297
ENSGALG00010028044	GAMT	2	-0.439848206	0.172680242	-2.547183164	0.010859642	0.021940512	1.579023854	36.66973445	1	PRJNA827465;Yang2025_Silkie_Wenchang	CRA004775	0.166076458
ENSGALG00010028044	GAMT	2	-0.237257742	0.191449547	-1.239270324	0.215245405	0	0.08351082	0	1	CRA004775;Yang2025_Silkie_Wenchang	PRJNA827465	0.728664095
ENSGALG00010028044	GAMT	2	-0.552951839	0.176528327	-3.132368877	0.001734019	0	0.754900982	0	1	CRA004775;PRJNA827465	Yang2025_Silkie_Wenchang	0.052667801
ENSGALG00010028045	ENSGALG00010028045	2	-0.028621991	0.215559765	-0.132779839	0.8943675	0	0.271401578	0	0.5	PRJNA827465;Yang2025_Silkie_Wenchang	CRA004775	0.974381724
ENSGALG00010028045	ENSGALG00010028045	2	-0.377538584	0.350881329	-1.075972282	0.281939653	0	0.474743102	0	1	CRA004775;Yang2025_Silkie_Wenchang	PRJNA827465	0.779434446
ENSGALG00010028045	ENSGALG00010028045	2	-0.13405291	0.32364481	-0.414197619	0.678729385	0.071485285	1.30705885	23.49235076	0.5	CRA004775;PRJNA827465	Yang2025_Silkie_Wenchang	0.917701868
ENSGALG00010028046	SMIM4	2	-0.287287341	0.099181587	-2.896579384	0.003772552	0	0.558143987	0	1	PRJNA827465;Yang2025_Silkie_Wenchang	CRA004775	0.088100594
ENSGALG00010028046	SMIM4	2	-0.378305902	0.215335312	-1.756822413	0.078948084	0	0.434538776	0	1	CRA004775;Yang2025_Silkie_Wenchang	PRJNA827465	0.483166697
ENSGALG00010028046	SMIM4	2	-0.246703761	0.105656513	-2.334960272	0.019545496	0	0.092236415	0	1	CRA004775;PRJNA827465	Yang2025_Silkie_Wenchang	0.234180956
ENSGALG00010028047	ENSGALG00010028047	2	0.03170542	0.321041469	0.09875802	0.921330401	0.084232709	1.63522715	38.84641655	0.5	PRJNA827465;Yang2025_Silkie_Wenchang	CRA004775	0.981519273
ENSGALG00010028047	ENSGALG00010028047	2	-0.176979604	0.343224295	-0.515638335	0.606107031	0	0.596025015	0	0.5	CRA004775;Yang2025_Silkie_Wenchang	PRJNA827465	0.896709756
ENSGALG00010028047	ENSGALG00010028047	2	0.267377417	0.259934742	1.028632861	0.30365223	0	0.024798217	0	1	CRA004775;PRJNA827465	Yang2025_Silkie_Wenchang	0.792205992
ENSGALG00010028048	S100A10	2	0.220191004	0.097645334	2.255007951	0.02413283	0	0.464040351	0	1	PRJNA827465;Yang2025_Silkie_Wenchang	CRA004775	0.265075657
ENSGALG00010028048	S100A10	2	-0.046077306	0.208890522	-0.220581121	0.825418602	0	0.057005671	0	0.5	CRA004775;Yang2025_Silkie_Wenchang	PRJNA827465	0.956381808
ENSGALG00010028048	S100A10	2	0.175063069	0.132368027	1.322547991	0.18598574	0.011419873	1.264740244	20.93238081	0.5	CRA004775;PRJNA827465	Yang2025_Silkie_Wenchang	0.695002013
ENSGALG00010028049	MAN1B1	2	-0.170492326	0.259800809	-0.656242476	0.511668123	0.127614177	17.98181331	94.43882559	0.5	PRJNA827465;Yang2025_Silkie_Wenchang	CRA004775	0.865694231
ENSGALG00010028049	MAN1B1	2	-0.423616605	0.102416357	-4.136220219	3.53073E-05	0	0.172633303	0	1	CRA004775;Yang2025_Silkie_Wenchang	PRJNA827465	0.003750498
ENSGALG00010028049	MAN1B1	2	0.00735323	0.151462183	0.048548289	0.961279281	0.0251654	1.51606586	34.03980485	0.5	CRA004775;PRJNA827465	Yang2025_Silkie_Wenchang	0.98973612
ENSGALG00010028050	PBRM1	2	-0.068480538	0.040358228	-1.696817293	0.089731208	0	0.233382648	0	1	PRJNA827465;Yang2025_Silkie_Wenchang	CRA004775	0.513947199
ENSGALG00010028050	PBRM1	2	-0.096960102	0.116043341	-0.835550759	0.40340768	0	0.322236656	0	0.5	CRA004775;Yang2025_Silkie_Wenchang	PRJNA827465	0.83306585
ENSGALG00010028050	PBRM1	2	-0.059498863	0.0420665	-1.414400147	0.157244447	0	0.150297487	0	0.5	CRA004775;PRJNA827465	Yang2025_Silkie_Wenchang	0.650687662
ENSGALG00010028051	GNL3	2	0.161085126	0.19456418	0.82792797	0.407711294	0.070619109	14.75511517	93.2226893	0.5	PRJNA827465;Yang2025_Silkie_Wenchang	CRA004775	0.833982683
ENSGALG00010028051	GNL3	2	0.113332359	0.283336689	0.399991826	0.689162537	0.134352352	5.592942409	82.12032367	0.5	CRA004775;Yang2025_Silkie_Wenchang	PRJNA827465	0.919854035
ENSGALG00010028051	GNL3	2	-0.040017802	0.055973125	-0.714946716	0.474641969	0	0.608478706	0	1	CRA004775;PRJNA827465	Yang2025_Silkie_Wenchang	0.854462864
ENSGALG00010028052	ENSGALG00010028052	2	0.55138046	0.287209533	1.919784674	0.054885103	0.124133395	4.011800079	75.07353357	1	PRJNA827465;Yang2025_Silkie_Wenchang	CRA004775	0.405555409
ENSGALG00010028052	ENSGALG00010028052	2	0.151286936	0.748053314	0.202240847	0.839728442	1.002272155	9.243938612	89.18210038	0.5	CRA004775;Yang2025_Silkie_Wenchang	PRJNA827465	0.959493354
ENSGALG00010028052	ENSGALG00010028052	2	-0.097671056	0.453829087	-0.215215504	0.829599304	0.313293605	3.7542187	73.36329927	0.5	CRA004775;PRJNA827465	Yang2025_Silkie_Wenchang	0.956965879
ENSGALG00010028054	RPS15	2	-0.199996252	0.199599293	-1.00198878	0.316349012	0.047745751	1.767756421	43.43112049	1	PRJNA827465;Yang2025_Silkie_Wenchang	CRA004775	0.796883862
ENSGALG00010028054	RPS15	2	-0.301245699	0.186803686	-1.612632519	0.106824369	0	0.772504672	0	1	CRA004775;Yang2025_Silkie_Wenchang	PRJNA827465	0.553925396
ENSGALG00010028054	RPS15	2	-0.097572252	0.064321921	-1.516936216	0.129282789	0	0.214967165	0	1	CRA004775;PRJNA827465	Yang2025_Silkie_Wenchang	0.603386934
ENSGALG00010028055	ENSGALG00010028055	2	0.279849094	0.150114898	1.864232652	0.06228904	0.032646644	3.359137844	70.23045655	1	PRJNA827465;Yang2025_Silkie_Wenchang	CRA004775	0.430309306
ENSGALG00010028055	ENSGALG00010028055	2	0.179764389	0.293403107	0.612687406	0.540083051	0.142057582	5.651435197	82.30537969	0.5	CRA004775;Yang2025_Silkie_Wenchang	PRJNA827465	0.87529516
ENSGALG00010028055	ENSGALG00010028055	2	0.071787738	0.128361485	0.559262286	0.57598273	0.017510926	1.78078622	43.84502819	0.5	CRA004775;PRJNA827465	Yang2025_Silkie_Wenchang	0.886945049
ENSGALG00010028056	SLC25A25	2	0.280683802	0.086015204	3.263188244	0.001101663	0	0.054509858	0	1	PRJNA827465;Yang2025_Silkie_Wenchang	CRA004775	0.040098299
ENSGALG00010028056	SLC25A25	2	-0.059583721	0.429541429	-0.138714724	0.889675583	0.261192138	2.787047936	64.11974164	0.5	CRA004775;Yang2025_Silkie_Wenchang	PRJNA827465	0.9731958
ENSGALG00010028056	SLC25A25	2	-0.039496379	0.451919117	-0.087397008	0.930355948	0.305014021	3.159422232	68.34864331	0.5	CRA004775;PRJNA827465	Yang2025_Silkie_Wenchang	0.983813577

ENSGALG00010028057	APC2	2	-0.248197626	0.591662389	-0.419491978	0.674856616	0.498785353	3.317419622	69.85608955	0.5	PRJNA827465;Yang2025_Silkie_Wenchang	CRA004775	0.91599715
ENSGALG00010028057	APC2	2	0.399789104	0.474272331	0.842952621	0.399254942	0	0.012006571	0	1	CRA004775;Yang2025_Silkie_Wenchang	PRJNA827465	0.83210929
ENSGALG00010028057	APC2	2	-0.53360092	0.441269897	-1.209239344	0.226570906	0.131405322	1.290890575	22.53410018	0.5	CRA004775;PRJNA827465	Yang2025_Silkie_Wenchang	0.740117063
ENSGALG00010028058	GLT8D1	2	-0.065405076	0.230571958	-0.283664486	0.776667514	0.096828585	10.86069462	90.7924858	0.5	PRJNA827465;Yang2025_Silkie_Wenchang	CRA004775	0.944156005
ENSGALG00010028058	GLT8D1	2	-0.349654412	0.115399555	-3.029945917	0.002445975	0	0.701151249	0	1	CRA004775;Yang2025_Silkie_Wenchang	PRJNA827465	0.066177569
ENSGALG00010028058	GLT8D1	2	-0.155482292	0.361501766	-0.430101059	0.66712213	0.221780173	5.794108788	82.74109036	0.5	CRA004775;PRJNA827465	Yang2025_Silkie_Wenchang	0.913766816
ENSGALG00010028060	ENSGALG00010028060	2	-0.087082186	0.072810132	-1.196017415	0.231689763	0	0.014379106	0	1	PRJNA827465;Yang2025_Silkie_Wenchang	CRA004775	0.744145032
ENSGALG00010028060	ENSGALG00010028060	2	-0.077387972	0.246731423	-0.313652681	0.753784843	0	0.018980697	0	1	CRA004775;Yang2025_Silkie_Wenchang	PRJNA827465	0.938155284
ENSGALG00010028060	ENSGALG00010028060	2	-0.090216035	0.073521047	-1.227077665	0.219793402	0	0.005857344	0	1	CRA004775;PRJNA827465	Yang2025_Silkie_Wenchang	0.733380873
ENSGALG00010028061	NAIF1	2	-0.211820869	0.110560097	-1.915888959	0.055379231	0.020218913	5.733419688	82.55840224	1	PRJNA827465;Yang2025_Silkie_Wenchang	CRA004775	0.407521049
ENSGALG00010028061	NAIF1	2	-0.195747168	0.147611707	-1.326095148	0.184808183	0.033750024	4.238404544	76.40621631	1	CRA004775;Yang2025_Silkie_Wenchang	PRJNA827465	0.69308359
ENSGALG00010028061	NAIF1	2	-0.09272079	0.051785352	-1.79048296	0.073376304	0	0.306795862	0	1	CRA004775;PRJNA827465	Yang2025_Silkie_Wenchang	0.467957919
ENSGALG00010028062	ENSGALG00010028062	2	0.296689743	0.451195313	0.657563885	0.510818408	0	0.281527365	0	1	PRJNA827465;Yang2025_Silkie_Wenchang	CRA004775	0.865436876
ENSGALG00010028062	ENSGALG00010028062	2	1.535282333	0.857191099	1.791061916	0.07328336	0.742529693	2.021321847	50.52742336	1	CRA004775;Yang2025_Silkie_Wenchang	PRJNA827465	0.467947918
ENSGALG00010028062	ENSGALG00010028062	2	1.171154733	1.119088038	1.046526005	0.295318245	2.018064373	4.985166157	79.94048807	1	CRA004775;PRJNA827465	Yang2025_Silkie_Wenchang	0.787628307
ENSGALG00010028064	SPCS1	2	-0.018853194	0.059613512	-0.316257065	0.751807397	0	0.188513135	0	0.5	PRJNA827465;Yang2025_Silkie_Wenchang	CRA004775	0.937802658
ENSGALG00010028064	SPCS1	2	-0.110411592	0.179244394	-0.615983515	0.5379054	0.027549124	1.740493001	42.54501456	0.5	CRA004775;Yang2025_Silkie_Wenchang	PRJNA827465	0.87481821
ENSGALG00010028064	SPCS1	2	-0.097186782	0.120155409	-0.808842343	0.418605837	0.015208425	1.648895696	39.35335009	1	CRA004775;PRJNA827465	Yang2025_Silkie_Wenchang	0.838193076
ENSGALG00010028065	REEP6	2	0.054812327	0.279892589	0.195833436	0.844740538	0	0.269361356	0	0.5	PRJNA827465;Yang2025_Silkie_Wenchang	CRA004775	0.960769014
ENSGALG00010028065	REEP6	2	0.546407346	0.712733144	0.766636644	0.443297582	0.760304658	3.921799086	74.50149847	0.5	CRA004775;Yang2025_Silkie_Wenchang	PRJNA827465	0.845733906
ENSGALG00010028065	REEP6	2	0.65536942	0.559881952	1.170549286	0.241779992	0.411598563	2.787185822	64.12151669	1	CRA004775;PRJNA827465	Yang2025_Silkie_Wenchang	0.75406671
ENSGALG00010028066	ENSGALG00010028066	2	0.291959895	0.432598683	0.674897789	0.499740705	0.357766788	22.37314312	95.53035532	0.5	PRJNA827465;Yang2025_Silkie_Wenchang	CRA004775	0.862078151
ENSGALG00010028066	ENSGALG00010028066	2	0.500605854	0.305637745	1.637905863	0.101441333	0.122596463	2.579881621	61.23853157	1	CRA004775;Yang2025_Silkie_Wenchang	PRJNA827465	0.54352396
ENSGALG00010028066	ENSGALG00010028066	2	-0.11728865	0.084766519	-1.383667178	0.166460388	0	0.395407016	0	0.5	CRA004775;PRJNA827465	Yang2025_Silkie_Wenchang	0.665350942
ENSGALG00010028067	FAM102A	2	-0.002233574	0.089089005	-0.02507126	0.979998124	0	0.011584656	0	0.5	PRJNA827465;Yang2025_Silkie_Wenchang	CRA004775	0.992465102
ENSGALG00010028067	FAM102A	2	-0.045082986	0.140417269	-0.321064396	0.748161591	0	0.739785502	0	0.5	CRA004775;Yang2025_Silkie_Wenchang	PRJNA827465	0.937091527
ENSGALG00010028067	FAM102A	2	-0.037633194	0.103421587	-0.363881416	0.715946562	0	0.711814978	0	1	CRA004775;PRJNA827465	Yang2025_Silkie_Wenchang	0.927261187
ENSGALG00010028068	POLE4	2	0.002124244	0.101236749	0.020982939	0.983259266	0	0.606458841	0	0.5	PRJNA827465;Yang2025_Silkie_Wenchang	CRA004775	0.995363468
ENSGALG00010028068	POLE4	2	-0.025887439	0.273085436	-0.094794093	0.924501491	0.044155915	1.418058566	29.48105077	0.5	CRA004775;Yang2025_Silkie_Wenchang	PRJNA827465	0.993262364
ENSGALG00010028068	POLE4	2	-0.051577861	0.102157341	-0.504886485	0.613638567	0	0.672942072	0	1	CRA004775;PRJNA827465	Yang2025_Silkie_Wenchang	0.898256044
ENSGALG00010028069	NEK4	2	-0.051155419	0.203246449	-0.251691574	0.801279471	0.073723452	9.275302481	89.21868045	0.5	PRJNA827465;Yang2025_Silkie_Wenchang	CRA004775	0.950158029
ENSGALG00010028069	NEK4	2	0.009293309	0.287126792	0.032366567	0.974179724	0.143404867	7.399323511	86.485251	0.5	CRA004775;Yang2025_Silkie_Wenchang	PRJNA827465	0.992492038
ENSGALG00010028069	NEK4	2	0.180539614	0.079958739	2.257909719	0.023951289	0	0.67219504	0	1	CRA004775;PRJNA827465	Yang2025_Silkie_Wenchang	0.264352006
ENSGALG00010028070	MEX3A	2	0.126378553	0.097666309	1.293983091	0.195671262	0	0.751053129	0	1	PRJNA827465;Yang2025_Silkie_Wenchang	CRA004775	0.706714537
ENSGALG00010028070	MEX3A	2	0.211991174	0.156512629	1.354466891	0.17558747	0	0.293116815	0	0.5	CRA004775;Yang2025_Silkie_Wenchang	PRJNA827465	0.680297576
ENSGALG00010028070	MEX3A	2	0.060371235	0.115933963	0.520738127	0.60254921	0	0.027607089	0	0.5	CRA004775;PRJNA827465	Yang2025_Silkie_Wenchang	0.895518685
ENSGALG00010028071	DPM2	2	-0.030627315	0.085929968	-0.356421816	0.721524694	0	0.070401255	0	0.5	PRJNA827465;Yang2025_Silkie_Wenchang	CRA004775	0.929153713
ENSGALG00010028071	DPM2	2	-0.137308982	0.181216011	-0.757708887	0.448625278	0	0.794495432	0	0.5	CRA004775;Yang2025_Silkie_Wenchang	PRJNA827465	0.846878812
ENSGALG00010028071	DPM2	2	-0.067963402	0.085251218	-0.797213261	0.425327185	0	0.893561474	0	1	CRA004775;PRJNA827465	Yang2025_Silkie_Wenchang	0.84125685
ENSGALG00010028073	ST6GALNAC4	2	-0.150291251	0.107903317	-1.39283254	0.163670436	0.001674007	1.072463737	6.756754085	1	PRJNA827465;Yang2025_Silkie_Wenchang	CRA004775	0.662073265
ENSGALG00010028073	ST6GALNAC4	2	-0.064769494	0.159562008	-0.405920525	0.684800996	0	0.757926174	0	1	CRA004775;Yang2025_Silkie_Wenchang	PRJNA827465	0.919003112
ENSGALG00010028073	ST6GALNAC4	2	-0.249539115	0.125264209	-1.992102269	0.046359838	0	0.173670995	0	1	CRA004775;PRJNA827465	Yang2025_Silkie_Wenchang	0.371474152
ENSGALG00010028074	ENSGALG00010028074	2	-0.669457658	0.298168	-2.245236441	0.024757958	0	0.008652778	0	1	PRJNA827465;Yang2025_Silkie_Wenchang	CRA004775	0.268761753
ENSGALG00010028074	ENSGALG00010028074	2	-0.547260621	0.259342318	-2.110186357	0.034842307	0	0.07275593	0	1	CRA004775;Yang2025_Silkie_Wenchang	PRJNA827465	0.321381648
ENSGALG00010028074	ENSGALG00010028074	2	-0.571664845	0.25489262	-2.242767342	0.024911821	0	0.14716702	0	1	CRA004775;PRJNA827465	Yang2025_Silkie_Wenchang	0.269034231
ENSGALG00010028075	RAB25	2	-0.283510349	0.327240374	-0.86636727	0.386288787	0	0.204310352	0	1	PRJNA827465;Yang2025_Silkie_Wenchang	CRA004775	0.826894808
ENSGALG00010028075	RAB25	2	-0.189997656	0.371097656	-0.511852887	0.608759973	0	0.685123883	0	0.5	CRA004775;Yang2025_Silkie_Wenchang	PRJNA827465	0.897208691
ENSGALG00010028075	RAB25	2	-0.024257973	0.35500623	-0.06833112	0.945522052	0	0.200659566	0	0.5	CRA004775;PRJNA827465	Yang2025_Silkie_Wenchang	0.986321197
ENSGALG00010028076	ST6GALNAC6	2	-0.164333414	0.210961912	-0.778971958	0.435996234	0.073573169	5.765201817	82.65455344	0.5	PRJNA827465;Yang2025_Silkie_Wenchang	CRA004775	0.843014708
ENSGALG00010028076	ST6GALNAC6	2	0.018496784	0.121271762	0.15252342	0.87877412	0	0.6902858	0	0.5	CRA004775;Yang2025_Silkie_Wenchang	PRJNA827465	0.971109264
ENSGALG00010028076	ST6GALNAC6	2	-0.370635789	0.11758418	-3.152088903	0.001621069	0	0.010783155	0	1	CRA004775;PRJNA827465	Yang2025_Silkie_Wenchang	0.010888881
ENSGALG00010028078	LAMTOR2	2	0.232579635	0.444482061	0.523259893	0.600793408	0.372618911	17.32846082	94.229147	0.5	PRJNA827465;Yang2025_Silkie_Wenchang	CRA004775	0.895230519
ENSGALG00010028078	LAMTOR2	2	0.298052933	0.440092567	0.67725055	0.498247	0.313164923	4.939611262	79.75549194	0.5	CRA004775;Yang2025_Silkie_Wenchang	PRJNA827465	0.861454634
ENSGALG00010028078	LAMTOR2	2	-0.19934783	0.103595027	-1.924299724	0.054317088	0	7.18228E-05	0	1	CRA004775;PRJNA827465	Yang2025_Silkie_Wenchang	0.40381596
ENSGALG00010028080	MUSTN1	2	0.168845104	0.427418975	0.39503418	0.692817663	0.268219331	3.422137073	70.77849371	0.5	PRJNA827465;Yang2025_Silkie_Wenchang	CRA004775	0.92062328
ENSGALG00010028080	MUSTN1	2	0.077660981	0.713482026	0.108847845	0.913323175	0.697315221	3.011868899	66.79802363	0.5	CRA004775;Yang2025_Silkie_Wenchang	PRJNA827465	0.979169142
ENSGALG00010028080	MUSTN1	2	-0.224779677	0.190336802	-1.180957518	0.2376196	0	0.600091149	0	1	CRA004775;PRJNA827465	Yang2025_Silkie_Wenchang	0.749411261
ENSGALG00010028081	UBQLN4	2	-0.10076468	0.048174844	-2.091645167	0.036470269	0	0.344854286	0	1	PRJNA827465;Yang2025_Silkie_Wenchang	CRA004775	0.329288072
ENSGALG00010028081	UBQLN4	2	-0.155209176	0.077231283	-2.009667202	0.044466423	0	0.152941675	0	1	CRA004775;Yang2025_Silkie_Wenchang	PRJNA827465	0.363948415
ENSGALG00010028081	UBQLN4	2	-0.093628222	0.055946	-1.673546307	0.094219798	0	0.512106455	0	1	CRA004775;PRJNA827465	Yang2025_Silkie_Wenchang	0.525712768
ENSGALG00010028082	AK1	2	-0.15010277	0.119868792	-1.252225601	0.210487674	0	0.195234349	0	1	PRJNA827465;Yang2025_Silkie_Wenchang	CRA004775	0.72207357
ENSGALG00010028082	AK1	2	-0.050130378	0.196993016	-0.254477946	0.79912634	0	0.166542866	0	0.5	CRA004775;Yang2025_Silkie_Wenchang	PRJNA827465	0.949922217
ENSGALG00010028082	AK1	2	-0.168779249	0.143505065	-1.176120504	0.239546726	0	0.343217018	0	0.5	CRA004775;PRJNA827465	Yang2025_Silkie_Wenchang	0.751810791
ENSGALG00010028083	ELAVL1	2	-0.15379232	0.148113665	-1.038339847	0.299111863	0.040898603	14.26422893	92.98945632	1	PRJNA827465;Yang2025_Silkie_Wenchang	CRA004775	0.789748017
ENSGALG00010028083	ELAVL1	2	-0.206203418	0.127206158	-1.621016191	0.105014038	0.021579336	2.765651398	63.84215304	1	CRA004775;Yang2025_Silkie_Wenchang	PRJNA827465	0.550168852
ENSGALG00010028083	ELAVL1	2	-0.014470405	0.029590456	-0.489022706	0.624825622	0	0.066544527	0	1	CRA004775;PRJNA827465	Yang2025_Silkie_Wenchang	0.901043264
ENSGALG00010028084	ENG	2	0.088863777	0.103170025	0.861333292	0.389054505	0.008825271	1.70803761	41.45327981	0.5	PRJNA827465;Yang2025_Silkie_Wenchang	CRA004775	0.828615121
ENSGALG00010028084	ENG	2	0.186564987	0.102684143	1.81688216	0.069235159	0	0.012103119	0	1	CRA004775;Yang2025_Silkie_Wenchang	PRJNA827465	0.45322276
ENSGALG00010028084	ENG	2	0.010034389	0.103984377	0.096499003	0.923124266	0	0.346992586	0	0.5	CRA004775;PRJNA827465	Yang2025_Silkie_Wenchang	0.981836718
ENSGALG00010028085	ENSGALG00010028085	2	0.622223178	0.660035521	0.942711655	0.34582841	0.855145996	53.82084039	98.14198368	0.5	PRJNA827465;Yang2025_Silkie_Wenchang	CRA004775	0.807479003
ENSGALG00010028085	ENSGALG00010028085	2	0.626622638	0.669954321	0.935321437	0.349622671	0.862509302	25.39187725	96.0617327	0.5	CRA004775;Yang2025_Silkie_Wenchang	PRJNA827465	0.809903518
ENSGALG00010028085	ENSGALG00010028085	2	-0.036712179	0.091638806	-0.400618258	0.688701201	0	0.006785146	0	1	CRA004775;PRJNA827465	Yang2025_Silkie_Wenchang	0.919799842
ENSGALG00010028086	MATK	2	0.559417481	0.253178588	2.209576591	0.027134561	0	0.964877336	0	1	PRJNA827465;Yang2025_Silkie_Wenchang	CRA004775	0.280945645
ENSGALG00010028086	MATK	2	0.579014129	0.29611383	1.955376854	0.050538606	0.011836692	1.067868119	6.355477549	1	CRA004775;Yang2025_Silkie_Wenchang	PRJNA827465	0.389297528
ENSGALG00010028086	MATK	2	0.271620786	0.283635204	0.9576413	0.338243666	0	0.036800049	0	1	CRA004775;PRJNA827465	Yang2025_Silkie_Wenchang	0.804154375
ENSGALG00010028087	STIMATE	2	-0.003255757	0.105873275	-0.030751454	0.975467756	0.016472702	3.64147685	72.53861438	0.5	PRJNA827465;Yang2025_Silkie_Wenchang	CRA004775	0.992754467
ENSGALG00010028087	STIMATE	2	-0.108620507	0.089826329	-1.209227939	0.226575287	0	0.23833407	0	0.5	CRA004775;Yang2025_Silkie_Wenchang	PRJNA827465	0.740117063
ENSGALG00010028087	STIMATE	2	0.089245856	0.059440203	1.501439291	0.133241978	0	0.029854783	0	1	CRA004775;PRJNA827465	Yang2025_Silkie_Wenchang	0.611633901
ENSGALG00010028088	NAXE	2	-0.162873253	0.096189631	-1.69325166	0.090407575	0	0.45268557	0	1	PRJNA827465;Yang2025_Silkie_Wenchang	CRA004775	0.5160164
ENSGALG00010028088	NAXE	2	-0.006626238	0.208073629	-0.03184564	0.974595149	0	0.001086528	0	0.5	CRA004775;Yang2025_Silkie_Wenchang	PRJNA827465	0.992492038
ENSGALG00010028088	NAXE	2	-0.177512228	0.101021939	-1.757165126	0.078889668	0	0.230300697	0	0.5	CRA004775;PRJNA827465	Yang2025_Silkie_Wenchang	0.482993365
ENSGALG00010028090	ENSGALG00010028090	2	0.557479005	0.621382601	0.897159019	0.369634069	0.6550338	5.793901561	82.74047307	1	PRJNA827465;Yang2025_Silkie_Wenchang	CRA004775	0.820606801
ENSGALG00010028090	ENSGALG00010028090	2	0.710761578	0.480890649	1.478010809	0.139404885	0.300567385	2.70081536	62.97414422	1	CRA004775;Yang2025_Silkie_Wenchang	PRJNA827465	0.623009874
ENSGALG00010028090	ENSGALG00010028090	2	0.060515692	0.105255677	0.574939927	0.565331925	0	0.751226735	0	1	CRA004775;PRJNA827465	Yang2025_Silkie_Wenchang	0.883427937
ENSGALG00010028091	FPGS	2	-0.071749528	0.064976717	-1.104234429	0.269491458	0	0.102875405	0	1	PRJNA827465;Yang2025_Silkie_Wenchang	CRA004775	0.77189992
ENSGALG00010028091	FPGS	2	-0.231161217	0.222038907	-1.041084288	0.297836431	0.053656903	2.193267791	54.40593237	1	CRA004775;Yang2025_Silkie_Wenchang	PRJNA827465	0.788858094
ENSGALG00010028091	FPGS	2	-0.211852064	0.178313089	-1.188090367	0.234797809	0.044975439	2.866897879	65.11909241	1	CRA004775;PRJNA827465	Yang2025_Silkie_Wenchang	0.747791642
ENSGALG00010028093	SFMBT1	2	-0.137551907	0.112062643	-1.227455494	0.21965144	0	0.826329077	0	1	PRJNA827465;Yang2025_Silkie_Wenchang	CRA004775	0.733094397
ENSGALG00010028093	SFMBT1	2	-0.276794641	0.17221741	-1.607239602	0.108001818	0	0.010229121	0	1	CRA004775;Yang2025_Silkie_Wenchang	PRJNA827465	0.556183889
ENSGALG00010028093	SFMBT1	2	-0.079679781	0.135723602	-0.587073872	0.557154095	0	0.268683375	0	1	CRA004775;PRJNA827465	Yang2025_Silkie_Wenchang	0.880678789
ENSGALG00010028095	CDK9	2	0.039863195	0.055771928	0.714753754	0.474761216	0	0.005543368	0	1	PRJNA827465;Yang2025_Silkie_Wenchang	CRA004775	0.854462864
ENSGALG00010028095	CDK9	2	-0.192889041	0.219444814	-0.878986553	0.379408567	0.046303341	1.925648542	48.06944371	0.5	CRA004775;Yang2025_Silkie_Wenchang	PRJNA827465	0.824165982
ENSGALG00010028095	CDK9	2	-0.134536353	0.221841394	-0.606452883	0.544214061	0.07668565	3.814106122	73.78153706	0.5	CRA004775;PRJNA827465	Yang2025_Silkie_Wenchang	0.877023301
ENSGALG00010028096	TOR2A	2	0.04502794	0.084306102	0.534100605	0.593271931	0	0.000411587	0	1	PRJNA827465;Yang2025_Silkie_Wenchang	CRA004775	0.892783052
ENSGALG00010028096	TOR2A	2	-0.035122528	0.151782151	-0.231400911	0.817003358	0	0.929595367	0	0.5	CRA004775;Yang2025_Silkie_Wenchang	PRJNA827465	0.954817505
ENSGALG00010028096	TOR2A	2	0.001230694	0.118565062	0.010379903	0.991718184	0.006691333	1.116002764	10.39448719	0.5	CRA004775;PRJNA827465	Yang2025_Silkie_Wenchang	0.997575757
ENSGALG00010028097	TTC24	2	0.377348441	0.324444185	1.163061192	0.244804676	0.165922518	4.526138037	77.9061091	1	PRJNA827465;Yang2025_Silkie_Wenchang	CRA004775	0.756114592
ENSGALG00010028097	TTC24	2	0.177653322	0.679397121	0.261486716	0.793717192	0.706425758	3.814573673	73.78475065	0.5	CRA004775;Yang2025_Silkie_Wenchang	PRJNA827465	0.948796049
ENSGALG00010028097	TTC24	2	-0.000609227	0.236285925	-0.002578346	0.99794278	0.039120829	1.171471194	14.6372523	0.5	CRA004775;PRJNA827465	Yang2025_Silkie_Wenchang	0.999290321
ENSGALG00010028098	RFT1	2	-0.165627471	0.109782774	-1.5086836	0.131379659	0	0.216327764	0	1	PRJNA827465;Yang2025_Silkie_Wenchang	CRA004775	0.607072924
ENSGALG00010028098	RFT1	2	-0.273019126	0.134005369	-2.037374536	0.041612524	0	0.54115152	0	1	CRA004775;Yang2025_Silkie_Wenchang	PRJNA827465	0.353517841
ENSGALG00010028098	RFT1	2	-0.206741599	0.148302016	-1.394057912	0.163300117	0.006263541	1.129881508	11.49514415	1	CRA004775;PRJNA827465	Yang2025_Silkie_Wenchang	0.661668506
ENSGALG00010028099	PCASP2	2	-0.037714436	0.342666312	-0.110061697	0.912360445	0.197696602	6.280434146	84.07753387	0.5	PRJNA827465;Yang2025_Silkie_Wenchang	CRA004775	0.978967245
ENSGALG00010028099	PCASP2	2	0.101300521	0.270384294	0.374653865	0.707917907	0.073997355	1.92835123	48.14222718	0.5	CRA004775;Yang2025_Silkie_Wenchang	PRJNA827465	0.925061251
ENSGALG00010028099	PCASP2	2	-0.341665378	0.152211846	-2.244670083	0.02478932	0	0.120807197	0	1	CRA004775;PRJNA827465	Yang2025_Silkie_Wenchang	0.268761753
ENSGALG00010028101	MRPL54	2	0.066310415	0.209297502	0.316823726	0.75137736	0.053376657	2.097702945	52.32880793	0.5	PRJNA827465;Yang2025_Silkie_Wenchang	CRA004775	0.937684688
ENSGALG00010028101	MRPL54	2	0.239520607	0.231445876	1.034888205	0.300721123	0	0.566124372	0	1	CRA004775;Yang2025_Silkie_Wenchang	PRJNA827465	0.790743448
ENSGALG00010028101	MRPL54	2	-0.068256444	0.097990412	-0.696562475	0.486076647	0	0.058447704	0	0.5	CRA004775;PRJNA827465	Yang2025_Silkie_Wenchang	0.857501254
ENSGALG00010028102	CERCAM	2	-0.079710367	0.054674966	-1.45789512	0.144869454	0	0.124455943	0	1	PRJNA827465;Yang2025_Silkie_Wenchang	CRA004775	0.632715207
ENSGALG00010028102	CERCAM	2	-0.116676538	0.092838383	-1.256770468	0.208836763	0	0.208697554	0	1	CRA004775;Yang2025_Silkie_Wenchang	PRJNA827465	0.721011841
ENSGALG00010028102	CERCAM	2	-0.071955888	0.065998117	-1.090271836	0.275593418	0	0.311189308	0	1	CRA004775;PRJNA827465	Yang2025_Silkie_Wenchang	0.775295118
ENSGALG00010028103	APBA3	2	-0.061935438	0.069032275	-0.897195378	0.369614671	0	0.474246689	0	1	PRJNA827465;Yang2025_Silkie_Wenchang	CRA004775	0.820606801
ENSGALG00010028103	APBA3	2	-0.363031174	0.254888234	-1.424275918	0.154366616	0.092876744	3.3028606	69.72321508	1	CRA004775;Yang2025_Silkie_Wenchang	PRJNA827465	0.646989113
ENSGALG00010028103	APBA3	2	-0.304408346	0.310272212	-0.981100898	0.326542988	0.162614769	5.816869495	82.80862239	1	CRA004775;PRJNA827465	Yang2025_Silkie_Wenchang	0.800028239
ENSGALG00010028104	GOLGA1	2	0.280398113	0.383632813	0.730902321	0.464838818	0.276615156	16.28671864	93.86002778	0.5	PRJNA827465;Yang2025_Silkie_Wenchang	CRA004775	0.85217126
ENSGALG00010028104	GOLGA1	2	0.397854701	0.284653518	1.397680605	0.162209001	0.130928392	5.199991788	80.7692004	1	CRA004775;Yang2025_Silkie_Wenchang	PRJNA827465	0.659645143
ENSGALG00010028104	GOLGA1	2	-0.053067035	0.079494346	-0.667557349	0.504416191	0	0.943707618	0	0.5	CRA004775;PRJNA827465	Yang2025_Silkie_Wenchang	0.863512504
ENSGALG00010028105	PRKCD	2	0.157368465	0.075443715	2.085905568	0.036987178	0	0.058713927	0	1	PRJNA827465;Yang2025_Silkie_Wenchang	CRA004775	0.331745105
ENSGALG00010028105	PRKCD	2	0.163898095	0.113634381	1.442328393	0.149209754	0	0.297203232	0	0.5	CRA004775;Yang2025_Silkie_Wenchang	PRJNA827465	0.639246608
ENSGALG00010028105	PRKCD	2	0.132497721	0.096686982	1.370378076	0.170568912	0	0.224470316	0	0.5	CRA004775;PRJNA827465	Yang2025_Silkie_Wenchang	0.672201941
ENSGALG00010028106	ARPC5L	2	0.148350517	0.194248335	0.763715774	0.445036638	0.053391688	3.097426302	67.71513178	0.5	PRJNA827465;Yang2025_Silkie_Wenchang	CRA004775	0.846013379
ENSGALG00010028106	ARPC5L	2	-0.024058015	0.429274626	-0.056043414	0.955307222	0.312306428	6.492299613	84.59713723	0.5	CRA004775;Yang2025_Silkie_Wenchang	PRJNA827465	0.988131974
ENSGALG00010028106	ARPC5L	2	-0.172199784	0.220860362	-0.779677177	0.435580916	0.066263681	2.634881287	62.04762602	1	CRA004775;PRJNA827465	Yang2025_Silkie_Wenchang	0.842762754
ENSGALG00010028107	NOTCH1	2	0.037718523	0.139932213	0.269548536	0.787507597	0.025977248	2.766512837	63.8534119	0.5	PRJNA827465;Yang2025_Silkie_Wenchang	CRA004775	0.946919116
ENSGALG00010028107	NOTCH1	2	0.165120482	0.140651057	1.173972563	0.240406015	0	0.656906163	0	0.5	CRA004775;Yang2025_Silkie_Wenchang	PRJNA827465	0.752942675
ENSGALG00010028107	NOTCH1	2	-0.079838531	0.080993966	-0.985734308	0.32426351	0	0.022441329	0	1	CRA004775;PRJNA827465	Yang2025_Silkie_Wenchang	0.799731973
ENSGALG00010028108	IQGAP3	2	0.366112035	0.206437861	1.773473302	0.076150312	0.067965076	4.672516276	78.5982554	1	PRJNA827465;Yang2025_Silkie_Wenchang	CRA004775	0.476202977
ENSGALG00010028108	IQGAP3	2	0.588410758	0.158110522	3.721515503	0.000198031	0	0.02868455	0	1	CRA004775;Yang2025_Silkie_Wenchang	PRJNA827465	0.012696902
ENSGALG00010028108	IQGAP3	2	0.19715039	0.088417625	2.229763459	0.02576315	0	0.582183506	0	1	CRA004775;PRJNA827465	Yang2025_Silkie_Wenchang	0.273593381
ENSGALG00010028109	TJP3	2	-0.032394258	0.218989154	-0.147926314	0.882400921	0.061886668	2.813879619	64.461877	0.5	PRJNA827465;Yang2025_Silkie_Wenchang	CRA004775	0.972001076
ENSGALG00010028109	TJP3	2	-0.147524703	0.158012243	-0.933628308	0.350495658	0.003270852	1.055032501	5.216190106	0.5	CRA004775;Yang2025_Silkie_Wenchang	PRJNA827465	0.810111037
ENSGALG00010028109	TJP3	2	0.168523085	0.161473513	1.043657761	0.296643759	0	0.056597586	0	1	CRA004775;PRJNA827465	Yang2025_Silkie_Wenchang	0.788458494
ENSGALG00010028110	COQ4	2	0.185548189	0.179545663	1.033431748	0.301401895	0.04454524	2.82083707	64.54952997	1	PRJNA827465;Yang2025_Silkie_Wenchang	CRA004775	0.791222412
ENSGALG00010028110	COQ4	2	0.06614479	0.369213479	0.179150529	0.857819507	0.218616726	5.005003678	80.0199947	0.5	CRA004775;Yang2025_Silkie_Wenchang	PRJNA827465	0.964081098
ENSGALG00010028110	COQ4	2	-0.054500859	0.164579481	-0.331152212	0.740529515	0.03070244	1.832199236	45.42078284	0.5	CRA004775;PRJNA827465	Yang2025_Silkie_Wenchang	0.935128797
ENSGALG00010028111	TRUB2	2	-0.063355129	0.089943417	-0.704388726	0.481190725	0.005009645	1.241781937	19.47056318	1	PRJNA827465;Yang2025_Silkie_Wenchang	CRA004775	0.85682839
ENSGALG00010028111	TRUB2	2	-0.186269478	0.148006535	-1.258521986	0.208203041	0	0.245235207	0	1	CRA004775;Yang2025_Silkie_Wenchang	PRJNA827465	0.720917211
ENSGALG00010028111	TRUB2	2	-0.025508739	0.067977891	-0.37525052	0.707474161	0	0.098991192	0	1	CRA004775;PRJNA827465	Yang2025_Silkie_Wenchang	0.92489559
ENSGALG00010028112	SWI5	2	0.076266825	0.058133321	1.311929611	0.189543885	0	0.00833822	0	1	PRJNA827465;Yang2025_Silkie_Wenchang	CRA004775	0.698754721
ENSGALG00010028112	SWI5	2	0.081758041	0.176552615	0.463080319	0.643306796	0	0.03081506	0	1	CRA004775;Yang2025_Silkie_Wenchang	PRJNA827465	0.906634523
ENSGALG00010028112	SWI5	2	0.079526656	0.058716105	1.354426625	0.175600308	0	0.021430611	0	1	CRA004775;PRJNA827465	Yang2025_Silkie_Wenchang	0.680297576
ENSGALG00010028113	PIP5K1C	2	0.105472708	0.092476227	1.140538623	0.254061971	0.0133345	4.520822406	77.88013087	1	PRJNA827465;Yang2025_Silkie_Wenchang	CRA004775	0.763894203
ENSGALG00010028113	PIP5K1C	2	-0.03039997	0.261043814	-0.116455431	0.907291607	0.117349157	6.607460468	84.86559239	0.5	CRA004775;Yang2025_Silkie_Wenchang	PRJNA827465	0.978196186
ENSGALG00010028113	PIP5K1C	2	-0.104170175	0.162827444	-0.639758094	0.522329881	0.037713121	2.851375858	64.92921138	0.5	CRA004775;PRJNA827465	Yang2025_Silkie_Wenchang	0.869568211
ENSGALG00010028114	PFKFB4	2	0.124468393	0.205733098	0.604999361	0.545179421	0.077310857	11.31762388	91.16422307	0.5	PRJNA827465;Yang2025_Silkie_Wenchang	CRA004775	0.877408247
ENSGALG00010028114	PFKFB4	2	0.171237139	0.22156924	0.77283805	0.439618232	0.066189283	2.664718259	62.47258049	0.5	CRA004775;Yang2025_Silkie_Wenchang	PRJNA827465	0.844278492
ENSGALG00010028114	PFKFB4	2	-0.075356977	0.060787449	-1.239679878	0.215093823	0	0.032500173	0	1	CRA004775;PRJNA827465	Yang2025_Silkie_Wenchang	0.728473987
ENSGALG00010028115	CACTIN	2	0.129392134	0.220868704	0.585832811	0.557987872	0.092206271	18.14590816	94.48911572	0.5	PRJNA827465;Yang2025_Silkie_Wenchang	CRA004775	0.880678789
ENSGALG00010028115	CACTIN	2	0.228761359	0.172268667	1.327933649	0.184200027	0.038477917	2.406874975	58.4523496	0.5	CRA004775;Yang2025_Silkie_Wenchang	PRJNA827465	0.692545576
ENSGALG00010028115	CACTIN	2	-0.082349786	0.061118844	-1.347371475	0.177860623	0	0.119714192	0	1	CRA004775;PRJNA827465	Yang2025_Silkie_Wenchang	0.683951879
ENSGALG00010028116	HYAL1	2	0.058414004	0.141845825	0.411813346	0.680476239	0.027677316	3.066512353	67.3896634	0.5	PRJNA827465;Yang2025_Silkie_Wenchang	CRA004775	0.917851751
ENSGALG00010028116	HYAL1	2	0.176549686	0.124458836	1.418538789	0.156033522	0	0.55416419	0	0.5	CRA004775;Yang2025_Silkie_Wenchang	PRJNA827465	0.649132948
ENSGALG00010028116	HYAL1	2	-0.059959059	0.083064194	-0.721840012	0.470392846	0	0.031698492	0	1	CRA004775;PRJNA827465	Yang2025_Silkie_Wenchang	0.853744788
ENSGALG00010028117	ENSGALG00010028117	2	0.808146809	0.328610468	2.459285045	0.013921404	0	0.366788361	0	1	PRJNA827465;Yang2025_Silkie_Wenchang	CRA004775	0.193621431
ENSGALG00010028117	ENSGALG00010028117	2	0.29764429	0.732482461	0.406350057	0.68448541	0.838425145	4.522222231	77.88697793	0.5	CRA004775;Yang2025_Silkie_Wenchang	PRJNA827465	0.918983742
ENSGALG00010028117	ENSGALG00010028117	2	0.135755767	0.526529742	0.257831147	0.796537225	0.381154325	3.198829274	68.73856294	0.5	CRA004775;PRJNA827465	Yang2025_Silkie_Wenchang	0.949491531
ENSGALG00010028118	ENSGALG00010028118	2	0.195474865	0.187786641	1.040941272	0.297902805	0.066658822	17.53051795	94.29566198	1	PRJNA827465;Yang2025_Silkie_Wenchang	CRA004775	0.788927563
ENSGALG00010028118	ENSGALG00010028118	2	0.264952788	0.151097614	1.753520654	0.079512677	0.031671418	3.068676146	67.41265769	1	CRA004775;Yang2025_Silkie_Wenchang	PRJNA827465	0.485261919
ENSGALG00010028118	ENSGALG00010028118	2	0.018357413	0.024789109	0.74054349	0.458970283	0	0.202657795	0	1	CRA004775;PRJNA827465	Yang2025_Silkie_Wenchang	0.850089963
ENSGALG00010028119	HYAL2	2	0.187349472	0.172683389	1.084930475	0.277952449	0.052442843	8.214999219	87.82714431	1	PRJNA827465;Yang2025_Silkie_Wenchang	CRA004775	0.776763074
ENSGALG00010028119	HYAL2	2	0.33816305	0.091923008	3.678763976	0.000234367	0	0.873417167	0	1	CRA004775;Yang2025_Silkie_Wenchang	PRJNA827465	0.014149505
ENSGALG00010028119	HYAL2	2	0.023574677	0.070011259	0.336726944	0.73632275	0	0.031148266	0	1	CRA004775;PRJNA827465	Yang2025_Silkie_Wenchang	0.933661344
ENSGALG00010028120	TBXA2R	2	0.319546189	0.325001971	0.983213082	0.325502578	0.191252366	10.45125117	90.4317676	1	PRJNA827465;Yang2025_Silkie_Wenchang	CRA004775	0.799987949
ENSGALG00010028120	TBXA2R	2	-0.00092559	0.690896025	-0.001339695	0.998931078	0.881044152	12.57419097	92.04720206	0.5	CRA004775;Yang2025_Silkie_Wenchang	PRJNA827465	0.999795976
ENSGALG00010028120	TBXA2R	2	-0.285181141	0.358603286	-0.795255236	0.426465047	0.199746671	3.892171623	74.30740222	0.5	CRA004775;PRJNA827465	Yang2025_Silkie_Wenchang	0.84125685
ENSGALG00010028121	TUSC2	2	-0.129002472	0.101763146	-1.267673786	0.20491447	0.010441478	1.761007714	43.21433165	1	PRJNA827465;Yang2025_Silkie_Wenchang	CRA004775	0.7174836
ENSGALG00010028121	TUSC2	2	-0.28214557	0.138744972	-2.033555271	0.041996456	0	0.000795532	0	1	CRA004775;Yang2025_Silkie_Wenchang	PRJNA827465	0.354529664
ENSGALG00010028121	TUSC2	2	-0.068975217	0.066509179	-1.037078158	0.299699434	0	0.419461535	0	1	CRA004775;PRJNA827465	Yang2025_Silkie_Wenchang	0.79004225
ENSGALG00010028122	SEC16A	2	-0.030324525	0.047869408	-0.633484435	0.526417342	0	0.335365108	0	1	PRJNA827465;Yang2025_Silkie_Wenchang	CRA004775	0.870598813
ENSGALG00010028122	SEC16A	2	-0.087948294	0.104282605	-0.843364952	0.399024367	0	0.000775007	0	1	CRA004775;Yang2025_Silkie_Wenchang	PRJNA827465	0.832054308
ENSGALG00010028122	SEC16A	2	-0.019402608	0.052020012	-0.372983536	0.709160698	0	0.048713026	0	1	CRA004775;PRJNA827465	Yang2025_Silkie_Wenchang	0.925298042
ENSGALG00010028123	RASSF1	2	0.228151003	0.086543175	2.636267996	0.00838235	0	0.30364723	0	1	PRJNA827465;Yang2025_Silkie_Wenchang	CRA004775	0.142771619
ENSGALG00010028123	RASSF1	2	0.222877907	0.192358087	1.158661486	0.246594195	0	0.7217436	0	1	CRA004775;Yang2025_Silkie_Wenchang	PRJNA827465	0.757001614
ENSGALG00010028123	RASSF1	2	0.194594397	0.088337402	2.202853975	0.027605042	0	0.348220254	0	1	CRA004775;PRJNA827465	Yang2025_Silkie_Wenchang	0.283613514
ENSGALG00010028124	SUGP2	2	0.12137337	0.082000851	1.480147684	0.138833838	0.004995115	1.491983351	32.97512338	1	PRJNA827465;Yang2025_Silkie_Wenchang	CRA004775	0.622497285
ENSGALG00010028124	SUGP2	2	0.011161092	0.246416869	0.04529354	0.963873337	0.096748489	4.783924461	79.0966599	0.5	CRA004775;Yang2025_Silkie_Wenchang	PRJNA827465	0.990168281
ENSGALG00010028124	SUGP2	2	-0.048684724	0.152888073	-0.318433763	0.750155931	0.030881057	2.50684211	60.10917496	0.5	CRA004775;PRJNA827465	Yang2025_Silkie_Wenchang	0.937374353
ENSGALG00010028125	HMG20B	2	0.090823945	0.084575288	1.073882776	0.282875224	0.005906058	1.59241834	37.20243135	1	PRJNA827465;Yang2025_Silkie_Wenchang	CRA004775	0.779532565
ENSGALG00010028125	HMG20B	2	0.030927907	0.198122889	0.156104663	0.875950523	0.055621827	3.328714104	69.95836924	0.5	CRA004775;Yang2025_Silkie_Wenchang	PRJNA827465	0.970347777
ENSGALG00010028125	HMG20B	2	-0.017457509	0.090462971	-0.192979614	0.846974934	0.005433724	1.287596628	22.33592583	0.5	CRA004775;PRJNA827465	Yang2025_Silkie_Wenchang	0.961252712
ENSGALG00010028126	ZMYND10	2	-0.051423287	0.055912194	-0.919715058	0.357721682	0	0.112353692	0	1	PRJNA827465;Yang2025_Silkie_Wenchang	CRA004775	0.813616765
ENSGALG00010028126	ZMYND10	2	-0.125742477	0.128591053	-0.977847792	0.32814961	0	0.253079265	0	1	CRA004775;Yang2025_Silkie_Wenchang	PRJNA827465	0.800724164
ENSGALG00010028126	ZMYND10	2	-0.051988635	0.059611883	-0.872118644	0.383143656	0	0.509416188	0	1	CRA004775;PRJNA827465	Yang2025_Silkie_Wenchang	0.825405345
ENSGALG00010028127	MFSD12	2	0.338107092	0.104544412	3.234100082	0.001220267	0.004457922	1.241263319	19.43691685	1	PRJNA827465;Yang2025_Silkie_Wenchang	CRA004775	0.042624337
ENSGALG00010028127	MFSD12	2	0.464191069	0.144171365	3.219717512	0.00128317	0	0.006633612	0	1	CRA004775;Yang2025_Silkie_Wenchang	PRJNA827465	0.043419476
ENSGALG00010028127	MFSD12	2	0.266384841	0.112166038	2.374915315	0.017552977	0	0.159555604	0	1	CRA004775;PRJNA827465	Yang2025_Silkie_Wenchang	0.220692466
ENSGALG00010028128	DNM1	2	0.093930486	0.070831496	1.326111852	0.184802651	0	0.897272372	0	1	PRJNA827465;Yang2025_Silkie_Wenchang	CRA004775	0.69308359

ENSGALG00010028128	DNM1	2	-0.062048762	0.397821491	-0.155971368	0.87605559	0.223541276	2.997825401	66.64248692	0.5	CRA004775;Yang2025_Silkie_Wenchang	PRJNA827465	0.970347777
ENSGALG00010028128	DNM1	2	-0.105463772	0.281151396	-0.375113811	0.707575826	0.102207594	2.035137675	50.86327513	0.5	CRA004775;PRJNA827465	Yang2025_Silkie_Wenchang	0.924990943
ENSGALG00010028129	CYB561D2	2	0.352117587	0.351311481	1.002294563	0.316201348	0.223240741	9.733430663	89.72613013	1	PRJNA827465;Yang2025_Silkie_Wenchang	CRA004775	0.796883862
ENSGALG00010028129	CYB561D2	2	0.155306833	0.58812794	0.264069809	0.791726127	0.635613701	12.29323562	91.8654451	0.5	CRA004775;Yang2025_Silkie_Wenchang	PRJNA827465	0.948175147
ENSGALG00010028129	CYB561D2	2	-0.13949422	0.226251503	-0.616544943	0.537534919	0.076706713	3.243954031	69.17342261	0.5	CRA004775;PRJNA827465	Yang2025_Silkie_Wenchang	0.87481821
ENSGALG00010028130	TMEM115	2	0.015835852	0.059428142	0.266470588	0.789876808	0	0.932908048	0	0.5	PRJNA827465;Yang2025_Silkie_Wenchang	CRA004775	0.947390191
ENSGALG00010028130	TMEM115	2	-0.215850227	0.167475272	-1.288848342	0.197450816	0	0.133214789	0	1	CRA004775;Yang2025_Silkie_Wenchang	PRJNA827465	0.708952382
ENSGALG00010028130	TMEM115	2	-0.013909506	0.116834664	-0.119052905	0.905233442	0.012019123	1.269562106	21.23268368	0.5	CRA004775;PRJNA827465	Yang2025_Silkie_Wenchang	0.977980705
ENSGALG00010028131	TMEM161A	2	-0.20967225	0.094186439	-2.226140537	0.026004769	0	0.638761415	0	1	PRJNA827465;Yang2025_Silkie_Wenchang	CRA004775	0.274896572
ENSGALG00010028131	TMEM161A	2	-0.223181298	0.21571209	-1.034625818	0.300843692	0.033761856	1.378314895	27.4476389	1	CRA004775;Yang2025_Silkie_Wenchang	PRJNA827465	0.790854098
ENSGALG00010028131	TMEM161A	2	-0.286554781	0.108865286	-2.63219609	0.008483488	0	0.682477626	0	1	CRA004775;PRJNA827465	Yang2025_Silkie_Wenchang	0.14369705
ENSGALG00010028132	CACNA2D2	2	0.071790467	0.210660613	0.340787326	0.733263692	0.075813656	6.824679433	85.34729712	0.5	PRJNA827465;Yang2025_Silkie_Wenchang	CRA004775	0.933121871
ENSGALG00010028132	CACNA2D2	2	0.197887993	0.158059397	1.251984997	0.210575331	0.017637459	1.357219052	26.31992616	0.5	CRA004775;Yang2025_Silkie_Wenchang	PRJNA827465	0.722116705
ENSGALG00010028132	CACNA2D2	2	-0.126696004	0.096373742	-1.314631989	0.188633614	0	0.032785174	0	1	CRA004775;PRJNA827465	Yang2025_Silkie_Wenchang	0.698039126
ENSGALG00010028133	ENSGALG00010028133	2	0.567995728	0.20721017	2.741157576	0.006122314	0	0.528347559	0	1	PRJNA827465;Yang2025_Silkie_Wenchang	CRA004775	0.118495743
ENSGALG00010028133	ENSGALG00010028133	2	0.622357019	0.244392739	2.546544633	0.01087953	0	0.354326849	0	1	CRA004775;Yang2025_Silkie_Wenchang	PRJNA827465	0.166315892
ENSGALG00010028133	ENSGALG00010028133	2	0.445445317	0.21943887	2.029928958	0.042363761	0	0.000620331	0	1	CRA004775;PRJNA827465	Yang2025_Silkie_Wenchang	0.356238117
ENSGALG00010028134	INPP5E	2	-0.184942405	0.062973236	-2.93684139	0.003315736	0.002490609	1.437692055	30.44407554	1	PRJNA827465;Yang2025_Silkie_Wenchang	CRA004775	0.080463346
ENSGALG00010028134	INPP5E	2	-0.240561353	0.076433199	-3.147341163	0.001647626	0	0.297989373	0	1	CRA004775;Yang2025_Silkie_Wenchang	PRJNA827465	0.051226717
ENSGALG00010028134	INPP5E	2	-0.134378162	0.061383949	-2.189141692	0.028586543	0	0.009988581	0	1	CRA004775;PRJNA827465	Yang2025_Silkie_Wenchang	0.287890952
ENSGALG00010028136	C3orf18	2	0.039765792	0.067196747	0.591781508	0.553996907	0.001707292	1.162463799	13.97581574	1	PRJNA827465;Yang2025_Silkie_Wenchang	CRA004775	0.880067113
ENSGALG00010028136	C3orf18	2	-0.50825266	0.700019745	-0.726054749	0.467805208	0.916943506	14.99177081	93.32967391	0.5	CRA004775;Yang2025_Silkie_Wenchang	PRJNA827465	0.853066769
ENSGALG00010028136	C3orf18	2	-0.577029991	0.621161359	-0.928953455	0.352913208	0.716290176	13.0797821	92.3546127	0.5	CRA004775;PRJNA827465	Yang2025_Silkie_Wenchang	0.811035834
ENSGALG00010028137	NUP214	2	-0.080999421	0.053226202	-1.521796006	0.128060203	0	0.917593448	0	0.5	PRJNA827465;Yang2025_Silkie_Wenchang	CRA004775	0.600542912
ENSGALG00010028137	NUP214	2	-0.033442561	0.118465133	-0.282298768	0.77771443	0	0.947352344	0	0.5	CRA004775;Yang2025_Silkie_Wenchang	PRJNA827465	0.944388838
ENSGALG00010028137	NUP214	2	-0.107916658	0.055078714	-1.95931695	0.05007568	0	0.198002228	0	1	CRA004775;PRJNA827465	Yang2025_Silkie_Wenchang	0.387273603
ENSGALG00010028138	ENSGALG00010028138	2	-0.024331228	0.390063093	-0.062377673	0.950262075	0.22783039	3.973579072	74.83377122	0.5	PRJNA827465;Yang2025_Silkie_Wenchang	CRA004775	0.987277827
ENSGALG00010028138	ENSGALG00010028138	2	0.144413636	0.287298235	0.502661062	0.615202591	0.052096865	1.429625751	30.05162369	0.5	CRA004775;Yang2025_Silkie_Wenchang	PRJNA827465	0.8988191
ENSGALG00010028138	ENSGALG00010028138	2	-0.347409525	0.220332471	-1.576751371	0.114852745	0	0.1601305	0	1	CRA004775;PRJNA827465	Yang2025_Silkie_Wenchang	0.571689138
ENSGALG00010028139	FZR1	2	-0.106729889	0.093071394	-1.146752873	0.251483771	0.010422124	2.471883403	59.54501742	1	PRJNA827465;Yang2025_Silkie_Wenchang	CRA004775	0.761706068
ENSGALG00010028139	FZR1	2	-0.238153127	0.083675475	-2.84615208	0.004425106	0	0.437832228	0	1	CRA004775;Yang2025_Silkie_Wenchang	PRJNA827465	0.097602941
ENSGALG00010028139	FZR1	2	-0.140631091	0.155667679	-0.903405847	0.366310538	0.032844124	2.689953316	62.82463364	1	CRA004775;PRJNA827465	Yang2025_Silkie_Wenchang	0.819159863
ENSGALG00010028140	HEMK1	2	-0.066540442	0.117063347	-0.56841397	0.569753907	0	0.42311652	0	1	PRJNA827465;Yang2025_Silkie_Wenchang	CRA004775	0.884923673
ENSGALG00010028140	HEMK1	2	-0.266321733	0.171875822	-1.549500856	0.121261366	0.00778852	1.120158556	10.72692395	1	CRA004775;Yang2025_Silkie_Wenchang	PRJNA827465	0.583784676
ENSGALG00010028140	HEMK1	2	-0.203058479	0.259178156	-0.783470652	0.433350781	0.083935197	2.406489501	58.44569446	1	CRA004775;PRJNA827465	Yang2025_Silkie_Wenchang	0.842762754
ENSGALG00010028141	CISH	2	-0.036280856	0.060064835	-0.604028236	0.54582487	0	0.035285274	0	1	PRJNA827465;Yang2025_Silkie_Wenchang	CRA004775	0.877943723
ENSGALG00010028141	CISH	2	-0.068103585	0.092747104	-0.734293389	0.462769946	0	0.827043146	0	1	CRA004775;Yang2025_Silkie_Wenchang	PRJNA827465	0.851151069
ENSGALG00010028141	CISH	2	-0.039985986	0.076451345	-0.523025277	0.600956663	0	0.935439604	0	1	CRA004775;PRJNA827465	Yang2025_Silkie_Wenchang	0.895230519
ENSGALG00010028142	PMPCA	2	0.028387256	0.105615429	0.268779444	0.788099412	0.017298011	4.303181913	76.76138215	0.5	PRJNA827465;Yang2025_Silkie_Wenchang	CRA004775	0.946919116
ENSGALG00010028142	PMPCA	2	0.125559073	0.081216796	1.545974222	0.122110794	0	0.429002978	0	0.5	CRA004775;Yang2025_Silkie_Wenchang	PRJNA827465	0.586069181
ENSGALG00010028142	PMPCA	2	-0.064666203	0.053192632	-1.215698503	0.224099794	0	0.040077972	0	1	CRA004775;PRJNA827465	Yang2025_Silkie_Wenchang	0.737452676
ENSGALG00010028144	FAM78A	2	0.036748848	0.160072718	0.229575963	0.818421287	0.03642972	3.391441269	70.51401099	0.5	PRJNA827465;Yang2025_Silkie_Wenchang	CRA004775	0.955256039
ENSGALG00010028144	FAM78A	2	0.218759182	0.128739038	1.699245113	0.08927301	0	0.014253032	0	1	CRA004775;Yang2025_Silkie_Wenchang	PRJNA827465	0.512891552
ENSGALG00010028144	FAM78A	2	-0.051188471	0.133276452	-0.384077385	0.700921092	0.010573271	1.191470761	16.07011828	0.5	CRA004775;PRJNA827465	Yang2025_Silkie_Wenchang	0.922496697
ENSGALG00010028145	ENSGALG00010028145	2	-0.152685731	0.131240973	-1.163399874	0.244667301	0	0.937622658	0	0.5	PRJNA827465;Yang2025_Silkie_Wenchang	CRA004775	0.756093682
ENSGALG00010028145	ENSGALG00010028145	2	0.018810044	0.199286845	0.094386781	0.924801916	0	0.113017603	0	0.5	CRA004775;Yang2025_Silkie_Wenchang	PRJNA827465	0.982493782
ENSGALG00010028145	ENSGALG00010028145	2	-0.184867604	0.132265931	-1.39769631	0.162204283	0	0.277851128	0	1	CRA004775;PRJNA827465	Yang2025_Silkie_Wenchang	0.659645143
ENSGALG00010028146	MAPKAPK3	2	-0.100276384	0.080231825	-1.249833017	0.211360552	0.000593116	1.046840049	4.474422762	1	PRJNA827465;Yang2025_Silkie_Wenchang	CRA004775	0.723704057
ENSGALG00010028146	MAPKAPK3	2	-0.147039975	0.109354757	-1.344614343	0.178749809	0	0.795270157	0	0.5	CRA004775;Yang2025_Silkie_Wenchang	PRJNA827465	0.685472119
ENSGALG00010028146	MAPKAPK3	2	-0.020629427	0.094246409	-0.218888203	0.826737136	0	0.094716848	0	0.5	CRA004775;PRJNA827465	Yang2025_Silkie_Wenchang	0.956499652
ENSGALG00010028147	SLC25A42	2	-0.206544388	0.094996311	-2.174235891	0.029687424	0	0.089162294	0	1	PRJNA827465;Yang2025_Silkie_Wenchang	CRA004775	0.29397955
ENSGALG00010028147	SLC25A42	2	-0.178154573	0.151446976	-1.176349486	0.239455248	0	0.826034153	0	0.5	CRA004775;Yang2025_Silkie_Wenchang	PRJNA827465	0.751706259
ENSGALG00010028147	SLC25A42	2	-0.157931821	0.108239056	-1.459101981	0.144537041	0	0.625865231	0	0.5	CRA004775;PRJNA827465	Yang2025_Silkie_Wenchang	0.631965661
ENSGALG00010028148	ENSGALG00010028148	2	-0.155471743	0.154653754	-1.005289159	0.314757634	0.016421948	1.483624458	32.59749835	0.5	PRJNA827465;Yang2025_Silkie_Wenchang	CRA004775	0.796531448
ENSGALG00010028148	ENSGALG00010028148	2	0.014028259	0.196911349	0.071241494	0.943205558	0	0.095123451	0	0.5	CRA004775;Yang2025_Silkie_Wenchang	PRJNA827465	0.985827325
ENSGALG00010028148	ENSGALG00010028148	2	-0.267375526	0.144526907	-1.850005171	0.064312804	0	0.071047564	0	1	CRA004775;PRJNA827465	Yang2025_Silkie_Wenchang	0.437237276
ENSGALG00010028149	SNAPC4	2	0.344556366	0.233308484	1.476827416	0.139721905	0.098861867	10.595524	90.56205243	1	PRJNA827465;Yang2025_Silkie_Wenchang	CRA004775	0.623597383
ENSGALG00010028149	SNAPC4	2	0.269545546	0.35166813	0.766477036	0.443392511	0.210610059	6.426048003	84.43833598	0.5	CRA004775;Yang2025_Silkie_Wenchang	PRJNA827465	0.845733906
ENSGALG00010028149	SNAPC4	2	0.106586922	0.065351713	1.630973646	0.102895881	0	0.863092029	0	0.5	CRA004775;PRJNA827465	Yang2025_Silkie_Wenchang	0.546797836
ENSGALG00010028150	ARMC6	2	-0.081109584	0.057261175	-1.41648479	0.156633614	0	0.46351374	0	1	PRJNA827465;Yang2025_Silkie_Wenchang	CRA004775	0.64980313
ENSGALG00010028150	ARMC6	2	-0.189293449	0.111426982	-1.6988116	0.089354687	0	0.247444283	0	1	CRA004775;Yang2025_Silkie_Wenchang	PRJNA827465	0.513108919
ENSGALG00010028150	ARMC6	2	-0.077926417	0.061747087	-1.262025797	0.206939504	0	0.911217304	0	1	CRA004775;PRJNA827465	Yang2025_Silkie_Wenchang	0.718911823
ENSGALG00010028151	DOCK3	2	0.613235109	0.533310269	1.149865557	0.250199249	0.502880156	8.602262428	88.37515121	1	PRJNA827465;Yang2025_Silkie_Wenchang	CRA004775	0.75993411
ENSGALG00010028151	DOCK3	2	1.054208573	0.256370211	4.112055648	3.92152E-05	0	0.966250942	0	1	CRA004775;Yang2025_Silkie_Wenchang	PRJNA827465	0.004077444
ENSGALG00010028151	DOCK3	2	0.126239986	0.223176307	0.565651377	0.571630795	0	0.225532266	0	1	CRA004775;PRJNA827465	Yang2025_Silkie_Wenchang	0.885429878
ENSGALG00010028152	ENSGALG00010028152	2	-0.057751683	0.230333624	-0.250730579	0.802022417	0	0.305759471	0	0.5	PRJNA827465;Yang2025_Silkie_Wenchang	CRA004775	0.950571064
ENSGALG00010028152	ENSGALG00010028152	2	-1.134081955	1.437910111	-0.788701565	0.430286453	3.450890054	5.393467973	81.45905371	0.5	CRA004775;Yang2025_Silkie_Wenchang	PRJNA827465	0.842040417
ENSGALG00010028152	ENSGALG00010028152	2	-1.233945027	1.300998086	-0.948460294	0.342895177	2.75027031	4.596466353	78.24415707	1	CRA004775;PRJNA827465	Yang2025_Silkie_Wenchang	0.806218116
ENSGALG00010028153	MANF	2	0.156350381	0.054879526	2.848974702	0.004386037	0	0.449062648	0	1	PRJNA827465;Yang2025_Silkie_Wenchang	CRA004775	0.097063291
ENSGALG00010028153	MANF	2	-0.052283019	0.167073112	-0.31293497	0.754330069	0	0.325039853	0	0.5	CRA004775;Yang2025_Silkie_Wenchang	PRJNA827465	0.93838723
ENSGALG00010028153	MANF	2	0.100326438	0.135288377	0.7415747	0.458345057	0.019423148	1.491220828	32.94085079	0.5	CRA004775;PRJNA827465	Yang2025_Silkie_Wenchang	0.850026426
ENSGALG00010028154	ENSGALG00010028154	2	0.107361091	0.113498243	0.94592734	0.344185648	0	0.037205109	0	1	PRJNA827465;Yang2025_Silkie_Wenchang	CRA004775	0.806554287
ENSGALG00010028154	ENSGALG00010028154	2	-0.005260629	0.11651983	-0.045147932	0.963989396	0	0.270115894	0	0.5	CRA004775;Yang2025_Silkie_Wenchang	PRJNA827465	0.990214803
ENSGALG00010028154	ENSGALG00010028154	2	0.039891044	0.100425473	0.397220369	0.691204963	0	0.740733218	0	0.5	CRA004775;PRJNA827465	Yang2025_Silkie_Wenchang	0.920268407
ENSGALG00010028155	CIZ1	2	0.151829223	0.110947019	1.368484021	0.17116062	0.021070924	6.713486084	85.10460903	1	PRJNA827465;Yang2025_Silkie_Wenchang	CRA004775	0.673243267
ENSGALG00010028155	CIZ1	2	0.13969866	0.165688914	0.843138247	0.39915113	0.039264263	3.11410096	67.88800322	0.5	CRA004775;Yang2025_Silkie_Wenchang	PRJNA827465	0.832054308
ENSGALG00010028155	CIZ1	2	0.042527	0.041523042	1.024178335	0.30575105	0	0.413735626	0	0.5	CRA004775;PRJNA827465	Yang2025_Silkie_Wenchang	0.79225794
ENSGALG00010028156	DOHH	2	0.032721724	0.066356514	0.493119988	0.621927808	0	0.291777027	0	0.5	PRJNA827465;Yang2025_Silkie_Wenchang	CRA004775	0.899853801
ENSGALG00010028156	DOHH	2	-0.234540773	0.219076354	-1.070589176	0.284354193	0	0.212774491	0	1	CRA004775;Yang2025_Silkie_Wenchang	PRJNA827465	0.780265906
ENSGALG00010028156	DOHH	2	-0.026695507	0.143753713	-0.185703079	0.852677614	0.020571517	1.414640867	29.31068064	0.5	CRA004775;PRJNA827465	Yang2025_Silkie_Wenchang	0.962651546
ENSGALG00010028157	PRRC2B	2	-0.076871705	0.059920982	-1.282884606	0.199532507	0	0.099884803	0	1	PRJNA827465;Yang2025_Silkie_Wenchang	CRA004775	0.71137755
ENSGALG00010028157	PRRC2B	2	0.124009368	0.214111468	0.579181346	0.562466816	0.03345023	1.462431475	31.62072771	0.5	CRA004775;Yang2025_Silkie_Wenchang	PRJNA827465	0.881955027
ENSGALG00010028157	PRRC2B	2	0.074717731	0.239661084	0.311764137	0.755219781	0.07842322	2.364206315	57.70250703	0.5	CRA004775;PRJNA827465	Yang2025_Silkie_Wenchang	0.938453928
ENSGALG00010028158	DDX49	2	-0.014007717	0.056522027	-0.247827581	0.804267814	0	0.171702219	0	0.5	PRJNA827465;Yang2025_Silkie_Wenchang	CRA004775	0.95154709
ENSGALG00010028158	DDX49	2	0.005418469	0.08581251	0.063143115	0.949652542	0	0.085807661	0	0.5	CRA004775;Yang2025_Silkie_Wenchang	PRJNA827465	0.98723759
ENSGALG00010028158	DDX49	2	-0.032266229	0.06510358	-0.49561375	0.62016695	0	0.003473286	0	1	CRA004775;PRJNA827465	Yang2025_Silkie_Wenchang	0.899395593
ENSGALG00010028159	DCAF1	2	0.011583496	0.087632376	0.132182836	0.894839677	0.010042975	2.51575813	60.2505508	0.5	PRJNA827465;Yang2025_Silkie_Wenchang	CRA004775	0.974639984
ENSGALG00010028159	DCAF1	2	0.091992506	0.094305566	0.975472709	0.329325832	0	0.553244164	0	0.5	CRA004775;Yang2025_Silkie_Wenchang	PRJNA827465	0.800981213
ENSGALG00010028159	DCAF1	2	-0.053617133	0.043196266	-1.241244635	0.21451539	0	0.00287549	0	1	CRA004775;PRJNA827465	Yang2025_Silkie_Wenchang	0.727205473
ENSGALG00010028160	ENSGALG00010028160	2	0.710714465	0.304527282	2.333828552	0.019604701	0.135075177	3.669644098	72.74940094	1	PRJNA827465;Yang2025_Silkie_Wenchang	CRA004775	0.234756108
ENSGALG00010028160	ENSGALG00010028160	2	0.322102156	0.755819163	0.426162992	0.669989082	1.009169045	8.244430957	87.8706001	0.5	CRA004775;Yang2025_Silkie_Wenchang	PRJNA827465	0.914428878
ENSGALG00010028160	ENSGALG00010028160	2	0.060773728	0.442392954	0.13737499	0.890734398	0.27735041	3.081627802	67.54961779	0.5	CRA004775;PRJNA827465	Yang2025_Silkie_Wenchang	0.973477891
ENSGALG00010028161	ENSGALG00010028161	2	1.831843937	0.505240765	3.625685149	0.000288196	0.327531962	2.781244772	64.04487624	1	PRJNA827465;Yang2025_Silkie_Wenchang	CRA004775	0.016559912
ENSGALG00010028161	ENSGALG00010028161	2	0.394982759	1.974513858	0.20004051	0.841448899	7.548763454	31.32930369	96.80810014	0.5	CRA004775;Yang2025_Silkie_Wenchang	PRJNA827465	0.95989279
ENSGALG00010028161	ENSGALG00010028161	2	-0.099669999	1.468705523	-0.06786248	0.945895107	4.087415288	18.94645788	94.72196858	0.5	CRA004775;PRJNA827465	Yang2025_Silkie_Wenchang	0.986321197
ENSGALG00010028162	CARD9	2	0.273027489	0.20780961	1.313834761	0.188901817	0.067722102	4.57746966	78.1538694	1	PRJNA827465;Yang2025_Silkie_Wenchang	CRA004775	0.698162702
ENSGALG00010028162	CARD9	2	-0.013839269	0.617125467	-0.022425374	0.98210864	0.613915863	4.455947505	77.55808391	0.5	CRA004775;Yang2025_Silkie_Wenchang	PRJNA827465	0.995105937
ENSGALG00010028162	CARD9	2	-0.155684486	0.376392129	-0.413623118	0.67915014	0.181722271	2.052292159	51.27399404	0.5	CRA004775;PRJNA827465	Yang2025_Silkie_Wenchang	0.917701868
ENSGALG00010028163	GBP	2	0.520738902	0.503042067	1.035179632	0.300585029	0.37599881	3.826278673	73.86494593	0.5	PRJNA827465;Yang2025_Silkie_Wenchang	CRA004775	0.790579721
ENSGALG00010028163	GBP	2	0.85222081	0.274459227	3.105090763	0.001902206	0	0.951736807	0	1	CRA004775;Yang2025_Silkie_Wenchang	PRJNA827465	0.056193756
ENSGALG00010028163	GBP	2	0.085804985	0.342859606	0.250262741	0.802384168	0	0.214943405	0	0.5	CRA004775;PRJNA827465	Yang2025_Silkie_Wenchang	0.950688375
ENSGALG00010028164	BBLN	2	-0.193806566	0.081221368	-2.386152449	0.017025692	0	0.230433815	0	1	PRJNA827465;Yang2025_Silkie_Wenchang	CRA004775	0.216673778
ENSGALG00010028164	BBLN	2	-0.390705975	0.168143928	-2.323640112	0.020144791	0	0.259885141	0	1	CRA004775;Yang2025_Silkie_Wenchang	PRJNA827465	0.238838826
ENSGALG00010028164	BBLN	2	-0.246074181	0.12291588	-2.001972249	0.045287716	0.012051767	1.385389456	27.81813116	1	CRA004775;PRJNA827465	Yang2025_Silkie_Wenchang	0.366947722
ENSGALG00010028165	NFIC	2	0.382154627	0.489630276	0.780496316	0.435098795	0.427779012	8.752466893	88.57464973	0.5	PRJNA827465;Yang2025_Silkie_Wenchang	CRA004775	0.842762754
ENSGALG00010028165	NFIC	2	0.510372037	0.383201182	1.331864466	0.182904729	0.214609027	3.683593537	72.85259652	1	CRA004775;Yang2025_Silkie_Wenchang	PRJNA827465	0.691347415
ENSGALG00010028165	NFIC	2	-0.028385874	0.107655559	-0.263673095	0.792031829	0	0.594642232	0	0.5	CRA004775;PRJNA827465	Yang2025_Silkie_Wenchang	0.948314332
ENSGALG00010028167	ENSGALG00010028167	2	-0.128857718	0.091372629	-1.410244179	0.158467595	0.001285035	1.037166935	3.583505566	1	PRJNA827465;Yang2025_Silkie_Wenchang	CRA004775	0.652239905
ENSGALG00010028167	ENSGALG00010028167	2	0.207922424	0.559024835	0.371937723	0.709939218	0.574986547	12.45164295	91.9689313	0.5	CRA004775;Yang2025_Silkie_Wenchang	PRJNA827465	0.925298042
ENSGALG00010028167	ENSGALG00010028167	2	0.313979382	0.424804761	0.739114555	0.459837439	0.337597572	15.06852046	93.36364839	0.5	CRA004775;PRJNA827465	Yang2025_Silkie_Wenchang	0.850098539
ENSGALG00010028168	PTGES2	2	-0.046485624	0.066363128	-0.70047368	0.483631537	0	0.270397225	0	0.5	PRJNA827465;Yang2025_Silkie_Wenchang	CRA004775	0.85691916
ENSGALG00010028168	PTGES2	2	0.045661752	0.132729147	0.344022039	0.730829712	0	0.335368304	0	1	CRA004775;Yang2025_Silkie_Wenchang	PRJNA827465	0.932769533
ENSGALG00010028168	PTGES2	2	-0.053466358	0.073888455	-0.72360909	0.469305759	0	0.657899979	0	0.5	CRA004775;PRJNA827465	Yang2025_Silkie_Wenchang	0.853485658
ENSGALG00010028169	DNLZ	2	0.100431586	0.132646939	0.757134588	0.448969234	0.008207774	1.189657084	15.94216407	1	PRJNA827465;Yang2025_Silkie_Wenchang	CRA004775	0.847095089
ENSGALG00010028169	DNLZ	2	0.109920417	0.292221122	0.376154935	0.706801716	0.071337977	1.674211207	40.27037952	0.5	CRA004775;Yang2025_Silkie_Wenchang	PRJNA827465	0.924594037
ENSGALG00010028169	DNLZ	2	0.00470011	0.113930238	0.041254281	0.967093181	0	0.489693049	0	0.5	CRA004775;PRJNA827465	Yang2025_Silkie_Wenchang	0.990893423
ENSGALG00010028170	COPE	2	-0.005927416	0.203324964	-0.029152425	0.976743024	0.062041594	3.655195111	72.64167932	0.5	PRJNA827465;Yang2025_Silkie_Wenchang	CRA004775	0.993193912
ENSGALG00010028170	COPE	2	-0.366783783	0.146410916	-2.505166922	0.012239362	0	0.800421478	0	1	CRA004775;Yang2025_Silkie_Wenchang	PRJNA827465	0.178627415
ENSGALG00010028170	COPE	2	-0.145038046	0.337140944	-0.430200034	0.667050137	0.200939611	8.140706775	87.7160543	0.5	CRA004775;PRJNA827465	Yang2025_Silkie_Wenchang	0.913766816
ENSGALG00010028171	RNF123	2	-0.195193439	0.057586523	-3.389568065	0.000700028	0	0.495010044	0	1	PRJNA827465;Yang2025_Silkie_Wenchang	CRA004775	0.029488973
ENSGALG00010028171	RNF123	2	-0.246130796	0.088653128	-2.776335141	0.005497551	0	0.001108869	0	1	CRA004775;Yang2025_Silkie_Wenchang	PRJNA827465	0.111278432
ENSGALG00010028171	RNF123	2	-0.166543278	0.072417965	-2.299750885	0.021462337	0	0.070574053	0	1	CRA004775;PRJNA827465	Yang2025_Silkie_Wenchang	0.247834011
ENSGALG00010028172	CERS1	2	-0.186340108	0.17804777	-1.04657367	0.295296251	0.037966241	2.318820641	56.87462919	1	PRJNA827465;Yang2025_Silkie_Wenchang	CRA004775	0.787628307
ENSGALG00010028172	CERS1	2	-0.42369453	0.180549036	-2.346700591	0.018940468	0	0.016883077	0	1	CRA004775;Yang2025_Silkie_Wenchang	PRJNA827465	0.230641818
ENSGALG00010028172	CERS1	2	-0.118137692	0.159450558	-0.740904848	0.458751136	0.017005407	1.239958755	19.3521562	1	CRA004775;PRJNA827465	Yang2025_Silkie_Wenchang	0.850089963
ENSGALG00010028173	GDF3	2	-0.008001874	0.093639466	-0.085454072	0.931900407	0	0.029289587	0	0.5	PRJNA827465;Yang2025_Silkie_Wenchang	CRA004775	0.98412915
ENSGALG00010028173	GDF3	2	-0.00700615	0.167468922	-0.041835526	0.966629815	0	0.094693483	0	0.5	CRA004775;Yang2025_Silkie_Wenchang	PRJNA827465	0.990893423
ENSGALG00010028173	GDF3	2	-0.024359825	0.103200527	-0.236043613	0.813398828	0	0.054647842	0	1	CRA004775;PRJNA827465	Yang2025_Silkie_Wenchang	0.953587297
ENSGALG00010028174	GMPPB	2	0.308514316	0.203626444	1.515099467	0.129747217	0.059342084	2.862600789	65.06673218	1	PRJNA827465;Yang2025_Silkie_Wenchang	CRA004775	0.604200812
ENSGALG00010028174	GMPPB	2	0.078420348	0.506124825	0.154942702	0.876866489	0.454854017	8.913620173	88.78121369	0.5	CRA004775;Yang2025_Silkie_Wenchang	PRJNA827465	0.97046577
ENSGALG00010028174	GMPPB	2	-0.089514573	0.289290671	-0.309427792	0.756996132	0.14120463	5.705786439	82.4739322	0.5	CRA004775;PRJNA827465	Yang2025_Silkie_Wenchang	0.938795135
ENSGALG00010028175	POMT1	2	-0.174515027	0.08503899	-2.052176626	0.0401525	0.002277959	1.139544306	12.24562358	1	PRJNA827465;Yang2025_Silkie_Wenchang	CRA004775	0.347067015
ENSGALG00010028175	POMT1	2	-0.334739735	0.127952083	-2.616133526	0.008893177	0	0.038334872	0	1	CRA004775;Yang2025_Silkie_Wenchang	PRJNA827465	0.147648184
ENSGALG00010028175	POMT1	2	-0.165785797	0.092640254	-1.789565434	0.0735238	0.002815225	1.103057652	9.342907162	1	CRA004775;PRJNA827465	Yang2025_Silkie_Wenchang	0.468547845
ENSGALG00010028176	UPF1	2	-0.105661271	0.038733471	-2.727906079	0.006373775	0	0.219774772	0	1	PRJNA827465;Yang2025_Silkie_Wenchang	CRA004775	0.121311618
ENSGALG00010028176	UPF1	2	-0.151905083	0.084559582	-1.796426601	0.072426696	0	0.124821347	0	1	CRA004775;Yang2025_Silkie_Wenchang	PRJNA827465	0.464872204
ENSGALG00010028176	UPF1	2	-0.100076594	0.042636921	-2.347181558	0.018916035	0	0.271478547	0	1	CRA004775;PRJNA827465	Yang2025_Silkie_Wenchang	0.230529277
ENSGALG00010028177	ENSGALG00010028177	2	0.667847083	0.507169494	1.316812409	0.187901512	0.420549493	5.471561903	81.72368297	1	PRJNA827465;Yang2025_Silkie_Wenchang	CRA004775	0.697228375
ENSGALG00010028177	ENSGALG00010028177	2	0.655669795	0.620910609	1.055980983	0.290976903	0.561576781	3.444017183	70.96414022	0.5	CRA004775;Yang2025_Silkie_Wenchang	PRJNA827465	0.78467882
ENSGALG00010028177	ENSGALG00010028177	2	0.122561145	0.26204201	0.467715636	0.639987958	0	0.13435156	0	0.5	CRA004775;PRJNA827465	Yang2025_Silkie_Wenchang	0.906064918
ENSGALG00010028178	ENSGALG00010028178	2	0.005291465	0.113937695	0.04644174	0.962958169	0.010142724	1.614485512	38.06076347	0.5	PRJNA827465;Yang2025_Silkie_Wenchang	CRA004775	0.989998461
ENSGALG00010028178	ENSGALG00010028178	2	-0.165394006	0.137303824	-1.204584122	0.228363889	0	0.700769362	0	1	CRA004775;Yang2025_Silkie_Wenchang	PRJNA827465	0.741167494
ENSGALG00010028178	ENSGALG00010028178	2	-0.059014788	0.27407765	-0.215321418	0.829516732	0.088880191	1.824694754	45.1963131	0.5	CRA004775;PRJNA827465	Yang2025_Silkie_Wenchang	0.956939641
ENSGALG00010028179	MCM2	2	-0.100942612	0.069997669	-1.442085325	0.149278305	0	0.061894975	0	1	PRJNA827465;Yang2025_Silkie_Wenchang	CRA004775	0.639246608
ENSGALG00010028179	MCM2	2	-0.406038605	0.398141369	-1.019835257	0.307806599	0.189420853	2.00690124	50.1719377	1	CRA004775;Yang2025_Silkie_Wenchang	PRJNA827465	0.793203994
ENSGALG00010028179	MCM2	2	-0.37304438	0.422960016	-0.881984978	0.377784931	0.251036709	2.455634565	59.27732838	1	CRA004775;PRJNA827465	Yang2025_Silkie_Wenchang	0.82325159
ENSGALG00010028180	ODF2	2	0.326789249	0.227775285	1.43470021	0.151372544	0.091527432	7.962871652	87.44171646	1	PRJNA827465;Yang2025_Silkie_Wenchang	CRA004775	0.64289984
ENSGALG00010028180	ODF2	2	0.283108579	0.362553418	0.780874115	0.434876538	0.194847396	3.440222877	70.93211586	0.5	CRA004775;Yang2025_Silkie_Wenchang	PRJNA827465	0.842762754
ENSGALG00010028180	ODF2	2	0.112483549	0.057506225	1.956023863	0.050462343	0	0.574009168	0	0.5	CRA004775;PRJNA827465	Yang2025_Silkie_Wenchang	0.389039127
ENSGALG00010028181	BGLAP	2	-0.074800699	0.249016092	-0.300385001	0.763883503	0	0.217700835	0	0.5	PRJNA827465;Yang2025_Silkie_Wenchang	CRA004775	0.940361611
ENSGALG00010028181	BGLAP	2	-0.027111801	0.270847214	-0.100099982	0.92026495	0	0.104094506	0	0.5	CRA004775;Yang2025_Silkie_Wenchang	PRJNA827465	0.981364549
ENSGALG00010028181	BGLAP	2	-0.151201013	0.251621333	-0.600906972	0.547901949	0	0.01370816	0	1	CRA004775;PRJNA827465	Yang2025_Silkie_Wenchang	0.878281266
ENSGALG00010028182	SMG5	2	0.131494888	0.062666142	2.098340228	0.035875101	0	0.219072602	0	1	PRJNA827465;Yang2025_Silkie_Wenchang	CRA004775	0.326969036
ENSGALG00010028182	SMG5	2	0.015897356	0.213336185	0.074517862	0.940598329	0.057846182	2.454016884	59.25048412	0.5	CRA004775;Yang2025_Silkie_Wenchang	PRJNA827465	0.985559481
ENSGALG00010028182	SMG5	2	0.004989202	0.170392132	0.029280707	0.976640714	0.035412454	2.003483924	50.08694665	0.5	CRA004775;PRJNA827465	Yang2025_Silkie_Wenchang	0.993151507
ENSGALG00010028183	CRTC1	2	0.155970852	0.214955835	0.725594873	0.468087165	0.071676811	3.900188591	74.36021421	0.5	PRJNA827465;Yang2025_Silkie_Wenchang	CRA004775	0.853145513
ENSGALG00010028183	CRTC1	2	0.178003393	0.25196206	0.706469035	0.479896503	0.077801728	2.575884036	61.17837658	0.5	CRA004775;Yang2025_Silkie_Wenchang	PRJNA827465	0.856284208
ENSGALG00010028183	CRTC1	2	-0.024577667	0.068467756	-0.358967025	0.719619758	0	0.071481857	0	1	CRA004775;PRJNA827465	Yang2025_Silkie_Wenchang	0.928449671
ENSGALG00010028184	TMEM79	2	0.03700206	0.098744923	0.374723672	0.707865986	0	0.409122965	0	0.5	PRJNA827465;Yang2025_Silkie_Wenchang	CRA004775	0.925061251
ENSGALG00010028184	TMEM79	2	-0.326715363	0.324243867	-1.007622338	0.313635801	0.155922843	3.639477495	72.52352841	1	CRA004775;Yang2025_Silkie_Wenchang	PRJNA827465	0.796531448
ENSGALG00010028184	TMEM79	2	-0.261386775	0.390767497	-0.668906132	0.503555358	0.256946974	5.840832581	82.87915317	0.5	CRA004775;PRJNA827465	Yang2025_Silkie_Wenchang	0.863108263
ENSGALG00010028185	GLMP	2	0.093778945	0.086794889	1.080466213	0.279934624	0.003135053	1.166926134	14.30477294	1	PRJNA827465;Yang2025_Silkie_Wenchang	CRA004775	0.778040488
ENSGALG00010028185	GLMP	2	0.001732805	0.251541014	0.006888756	0.994503611	0.097391268	4.337422623	76.94483368	0.5	CRA004775;Yang2025_Silkie_Wenchang	PRJNA827465	0.99823774
ENSGALG00010028185	GLMP	2	-0.063864907	0.142556871	-0.447995996	0.654156086	0.026222148	2.548900387	60.76739581	0.5	CRA004775;PRJNA827465	Yang2025_Silkie_Wenchang	0.909981418
ENSGALG00010028186	CCT3	2	0.114329409	0.175092322	0.652966434	0.513777918	0.054166896	8.221609402	87.8369313	0.5	PRJNA827465;Yang2025_Silkie_Wenchang	CRA004775	0.866584405
ENSGALG00010028186	CCT3	2	0.072174673	0.268522075	0.268784876	0.788095232	0.11331502	4.315393126	76.82714018	0.5	CRA004775;Yang2025_Silkie_Wenchang	PRJNA827465	0.946919116
ENSGALG00010028186	CCT3	2	-0.057202411	0.051954685	-1.101005821	0.270894124	0	0.624115948	0	1	CRA004775;PRJNA827465	Yang2025_Silkie_Wenchang	0.772743863
ENSGALG00010028187	TPRA1	2	-0.168087346	0.222028417	-0.757053301	0.44901793	0.085471545	6.856929598	85.41621311	0.5	PRJNA827465;Yang2025_Silkie_Wenchang	CRA004775	0.847106887
ENSGALG00010028187	TPRA1	2	-0.332030656	0.095827905	-3.464863958	0.0005305	0	0.430030279	0	1	CRA004775;Yang2025_Silkie_Wenchang	PRJNA827465	0.024386237
ENSGALG00010028187	TPRA1	2	-0.110749637	0.156450469	-0.707889454	0.479013909	0.041399366	6.033986771	83.42720927	0.5	CRA004775;PRJNA827465	Yang2025_Silkie_Wenchang	0.855875059
ENSGALG00010028188	ENSGALG00010028188	2	0.259744218	0.371589724	0.699008076	0.484546984	0.259598616	16.35528759	93.88576939	0.5	PRJNA827465;Yang2025_Silkie_Wenchang	CRA004775	0.857004733
ENSGALG00010028188	ENSGALG00010028188	2	0.178191545	0.492419641	0.361869289	0.717449711	0.433442092	9.189541655	89.11806445	0.5	CRA004775;Yang2025_Silkie_Wenchang	PRJNA827465	0.9277944
ENSGALG00010028188	ENSGALG00010028188	2	-0.118912307	0.080782398	-1.472007645	0.141018813	0	0.682416254	0	1	CRA004775;PRJNA827465	Yang2025_Silkie_Wenchang	0.625418914
ENSGALG00010028189	GPSM1	2	-0.135811449	0.067916795	-1.999673998	0.045535478	0	0.883709591	0	1	PRJNA827465;Yang2025_Silkie_Wenchang	CRA004775	0.367868629
ENSGALG00010028189	GPSM1	2	-0.217373741	0.0962654	-2.258067194	0.023941471	0	0.159000851	0	1	CRA004775;Yang2025_Silkie_Wenchang	PRJNA827465	0.264351316
ENSGALG00010028189	GPSM1	2	-0.096656734	0.088630195	-1.090562128	0.275465602	0	0.602641375	0	1	CRA004775;PRJNA827465	Yang2025_Silkie_Wenchang	0.775135547
ENSGALG00010028190	ILF2	2	0.025043616	0.042767229	0.585579567	0.558158083	0	0.161269441	0	1	PRJNA827465;Yang2025_Silkie_Wenchang	CRA004775	0.880678789
ENSGALG00010028190	ILF2	2	0.00945803	0.122604459	0.07714263	0.938510081	0.002740382	1.065431707	6.141332837	0.5	CRA004775;Yang2025_Silkie_Wenchang	PRJNA827465	0.985349045
ENSGALG00010028190	ILF2	2	0.011716686	0.044695367	0.262145425	0.793209327	0	0.858829148	0	0.5	CRA004775;PRJNA827465	Yang2025_Silkie_Wenchang	0.94862178
ENSGALG00010028191	KLHL26	2	-0.067096584	0.062479205	-1.073902652	0.282866314	0.002981898	1.592730302	37.21473129	1	PRJNA827465;Yang2025_Silkie_Wenchang	CRA004775	0.779532565
ENSGALG00010028191	KLHL26	2	-0.157444012	0.076026337	-2.070914095	0.038366825	0	0.536390914	0	1	CRA004775;Yang2025_Silkie_Wenchang	PRJNA827465	0.337788438
ENSGALG00010028191	KLHL26	2	-0.078407327	0.130041175	-0.602942315	0.546547065	0.017319492	1.480187591	32.4409956	1	CRA004775;PRJNA827465	Yang2025_Silkie_Wenchang	0.878275987
ENSGALG00010028193	BRINP1	2	-0.127174206	0.142026798	-0.895424016	0.370560467	0	0.051419302	0	1	PRJNA827465;Yang2025_Silkie_Wenchang	CRA004775	0.821334353
ENSGALG00010028193	BRINP1	2	0.028429771	0.305960937	0.092919611	0.925967426	0	0.598940148	0	0.5	CRA004775;Yang2025_Silkie_Wenchang	PRJNA827465	0.982799198
ENSGALG00010028193	BRINP1	2	-0.117244636	0.155174318	-0.75556727	0.449908686	0	0.82149833	0	0.5	CRA004775;PRJNA827465	Yang2025_Silkie_Wenchang	0.84744374
ENSGALG00010028194	ENSGALG00010028194	2	0.131672668	0.073700928	1.786580859	0.074005258	0	0.003194245	0	1	PRJNA827465;Yang2025_Silkie_Wenchang	CRA004775	0.469739768
ENSGALG00010028194	ENSGALG00010028194	2	0.118418325	0.099848356	1.185981716	0.23562952	0	0.099886272	0	1	CRA004775;Yang2025_Silkie_Wenchang	PRJNA827465	0.748626601

ENSGALG00010028194	ENSGALG00010028194	2	0.126003211	0.100383253	1.255221442	0.209398384	0	0.114336155	0	1	CRA004775;PRJNA827465	Yang2025_Silkie_Wenchang	0.721294679
ENSGALG00010028195	ENSGALG00010028195	2	0.004034155	0.188228675	0.021432202	0.982900886	0	0.007683372	0	0.5	PRJNA827465;Yang2025_Silkie_Wenchang	CRA004775	0.995363468
ENSGALG00010028195	ENSGALG00010028195	2	0.064529149	0.219128921	0.294480292	0.768390921	0	0.219014189	0	1	CRA004775;Yang2025_Silkie_Wenchang	PRJNA827465	0.941444269
ENSGALG00010028195	ENSGALG00010028195	2	0.058830035	0.260854955	0.225527765	0.821568729	0	0.252771333	0	0.5	CRA004775;PRJNA827465	Yang2025_Silkie_Wenchang	0.955598716
ENSGALG00010028196	ENSGALG00010028196	2	0.219161608	0.104558219	2.096072507	0.036075759	0.005667551	1.293849626	22.71126568	1	PRJNA827465;Yang2025_Silkie_Wenchang	CRA004775	0.327683285
ENSGALG00010028196	ENSGALG00010028196	2	-0.006774095	0.401262216	-0.016881967	0.986530779	0.275029855	6.699729421	85.07402408	0.5	CRA004775;Yang2025_Silkie_Wenchang	PRJNA827465	0.99618774
ENSGALG00010028196	ENSGALG00010028196	2	-0.093696855	0.286027334	-0.327580074	0.74322919	0.129127737	4.271379217	76.58835825	0.5	CRA004775;PRJNA827465	Yang2025_Silkie_Wenchang	0.93574436
ENSGALG00010028197	INTS3	2	-0.025479903	0.051080942	-0.498814263	0.617910239	0	0.364634715	0	0.5	PRJNA827465;Yang2025_Silkie_Wenchang	CRA004775	0.899121942
ENSGALG00010028197	INTS3	2	-0.109657395	0.136383189	-0.804038942	0.421374485	0.026511085	3.215407163	68.89973962	0.5	CRA004775;Yang2025_Silkie_Wenchang	PRJNA827465	0.839352211
ENSGALG00010028197	INTS3	2	-0.134587273	0.10319477	-1.304206338	0.19216324	0.011005711	1.898947484	47.33924933	1	CRA004775;PRJNA827465	Yang2025_Silkie_Wenchang	0.701849591
ENSGALG00010028198	UCK1	2	-0.130087489	0.115940622	-1.122018215	0.261854695	0.018210558	2.91013526	65.63733604	1	PRJNA827465;Yang2025_Silkie_Wenchang	CRA004775	0.768363052
ENSGALG00010028198	UCK1	2	-0.233506932	0.096346125	-2.423625582	0.015366439	0	0.239838763	0	1	CRA004775;Yang2025_Silkie_Wenchang	PRJNA827465	0.205049211
ENSGALG00010028198	UCK1	2	-0.053786185	0.062815182	-0.856260916	0.391853475	0	0.611362657	0	1	CRA004775;PRJNA827465	Yang2025_Silkie_Wenchang	0.829251057
ENSGALG00010028199	DPP9	2	-0.067433048	0.042852388	-1.573612394	0.115577086	0	0.274891274	0	1	PRJNA827465;Yang2025_Silkie_Wenchang	CRA004775	0.572716099
ENSGALG00010028199	DPP9	2	-0.105564813	0.121593214	-0.868180139	0.385295725	0.014786307	1.467384303	31.85152672	1	CRA004775;Yang2025_Silkie_Wenchang	PRJNA827465	0.826451313
ENSGALG00010028199	DPP9	2	-0.112180107	0.072677977	-1.543522706	0.122704003	0.002097027	1.066271048	6.215215948	1	CRA004775;PRJNA827465	Yang2025_Silkie_Wenchang	0.58762464
ENSGALG00010028201	ENSGALG00010028201	2	0.065210122	0.222654077	0.292876389	0.769616636	0.067283764	2.719271117	63.22543958	0.5	PRJNA827465;Yang2025_Silkie_Wenchang	CRA004775	0.941843401
ENSGALG00010028201	ENSGALG00010028201	2	0.0691296	0.33939691	0.203683645	0.838600723	0.130633736	2.229204471	55.14094768	0.5	CRA004775;Yang2025_Silkie_Wenchang	PRJNA827465	0.959226718
ENSGALG00010028201	ENSGALG00010028201	2	-0.121551979	0.102493938	-1.185943105	0.235644769	0	0.328371991	0	1	CRA004775;PRJNA827465	Yang2025_Silkie_Wenchang	0.748626601
ENSGALG00010028202	QSOX2	2	-0.217722032	0.253796873	-0.857859393	0.390970109	0.121955642	18.25310118	94.5214789	0.5	PRJNA827465;Yang2025_Silkie_Wenchang	CRA004775	0.829251057
ENSGALG00010028202	QSOX2	2	-0.476234248	0.104718067	-4.54777541	5.42159E-06	0	0.025456528	0	1	CRA004775;Yang2025_Silkie_Wenchang	PRJNA827465	0.000873299
ENSGALG00010028202	QSOX2	2	-0.074957608	0.188261543	-0.398156774	0.690514627	0.043689696	1.751588188	42.90895505	0.5	CRA004775;PRJNA827465	Yang2025_Silkie_Wenchang	0.919979266
ENSGALG00010028205	PRRT1B	2	0.263791645	0.243796114	1.082017433	0.279244782	0	0.357952518	0	1	PRJNA827465;Yang2025_Silkie_Wenchang	CRA004775	0.777442633
ENSGALG00010028205	PRRT1B	2	0.330118464	0.317505892	1.039723899	0.298468194	0	0.825898358	0	1	CRA004775;Yang2025_Silkie_Wenchang	PRJNA827465	0.789414228
ENSGALG00010028205	PRRT1B	2	0.432287967	0.24194461	1.786722863	0.073982292	0	0.23212246	0	1	CRA004775;PRJNA827465	Yang2025_Silkie_Wenchang	0.469669846
ENSGALG00010028206	RBM15B	2	-0.088517812	0.053261972	-1.66193267	0.096526275	0.001777622	1.385121769	27.80418138	1	PRJNA827465;Yang2025_Silkie_Wenchang	CRA004775	0.530898652
ENSGALG00010028206	RBM15B	2	-0.039161397	0.160843901	-0.243474552	0.807637796	0.034701284	2.698637717	62.94426652	0.5	CRA004775;Yang2025_Silkie_Wenchang	PRJNA827465	0.952496413
ENSGALG00010028206	RBM15B	2	-0.013673962	0.082297029	-0.166153776	0.868035935	0.005691473	1.311284248	23.73888413	0.5	CRA004775;PRJNA827465	Yang2025_Silkie_Wenchang	0.968006366
ENSGALG00010028208	AMIGO3	2	0.040551878	0.189834848	0.213616615	0.830846042	0	0.204522597	0	0.5	PRJNA827465;Yang2025_Silkie_Wenchang	CRA004775	0.957336479
ENSGALG00010028208	AMIGO3	2	-0.020051829	0.220432034	-0.090966037	0.927519578	0	0.738158084	0	0.5	CRA004775;Yang2025_Silkie_Wenchang	PRJNA827465	0.982993195
ENSGALG00010028208	AMIGO3	2	-0.109532469	0.203877499	-0.537246479	0.591097369	0	0.246195798	0	1	CRA004775;PRJNA827465	Yang2025_Silkie_Wenchang	0.892776493
ENSGALG00010028211	GATAD2B	2	0.089105034	0.18066192	0.493214253	0.621861208	0.044801133	2.76409303	63.82176761	0.5	PRJNA827465;Yang2025_Silkie_Wenchang	CRA004775	0.89982369
ENSGALG00010028211	GATAD2B	2	0.302465264	0.164720946	1.836228312	0.066323897	0	0.033013709	0	1	CRA004775;Yang2025_Silkie_Wenchang	PRJNA827465	0.443593793
ENSGALG00010028211	GATAD2B	2	0.005692383	0.118582002	0.048003771	0.961713237	0.010201765	1.262780753	20.80968943	0.5	CRA004775;PRJNA827465	Yang2025_Silkie_Wenchang	0.98973612
ENSGALG00010028221	TNFAIP8L1	2	-0.163748462	0.220031998	-0.744202951	0.456753703	0.081697971	5.802956493	82.76740484	0.5	PRJNA827465;Yang2025_Silkie_Wenchang	CRA004775	0.849515887
ENSGALG00010028221	TNFAIP8L1	2	-0.452168227	0.166693806	-2.712567664	0.006676415	0	0.445651866	0	1	CRA004775;Yang2025_Silkie_Wenchang	PRJNA827465	0.124953874
ENSGALG00010028221	TNFAIP8L1	2	-0.207406302	0.373289046	-0.555618506	0.578471678	0.191255576	2.264072062	55.83179454	0.5	CRA004775;PRJNA827465	Yang2025_Silkie_Wenchang	0.88785945
ENSGALG00010028232	C9orf69	2	-0.373182074	0.289135457	-1.290682499	0.196813796	0.147608347	7.97422674	87.45959912	1	PRJNA827465;Yang2025_Silkie_Wenchang	CRA004775	0.707994211
ENSGALG00010028232	C9orf69	2	-0.396447213	0.340777043	-1.163362443	0.244682481	0.165856973	3.329391999	69.964486	1	CRA004775;Yang2025_Silkie_Wenchang	PRJNA827465	0.756093682
ENSGALG00010028232	C9orf69	2	-0.105982207	0.068174916	-1.554563076	0.120050163	0	0.105393859	0	1	CRA004775;PRJNA827465	Yang2025_Silkie_Wenchang	0.581146887
ENSGALG00010028242	ENSGALG00010028242	2	0.341301507	0.171628762	1.98860321	0.046745017	0.01656864	1.370091208	27.01215846	1	PRJNA827465;Yang2025_Silkie_Wenchang	CRA004775	0.373490609
ENSGALG00010028242	ENSGALG00010028242	2	0.430437478	0.160389614	2.683699195	0.007281259	0	0.382315025	0	1	CRA004775;Yang2025_Silkie_Wenchang	PRJNA827465	0.131092673
ENSGALG00010028242	ENSGALG00010028242	2	0.165308302	0.197151022	0.838485649	0.401758004	0	0.063700254	0	1	CRA004775;PRJNA827465	Yang2025_Silkie_Wenchang	0.832689704
ENSGALG00010028247	MVP	2	0.050405561	0.088490042	0.569618452	0.56893651	0.003348049	1.200279382	16.68606371	0.5	PRJNA827465;Yang2025_Silkie_Wenchang	CRA004775	0.884492059
ENSGALG00010028247	MVP	2	0.047104185	0.164359243	0.286592859	0.774424083	0.023513604	1.759931021	43.17959125	0.5	CRA004775;Yang2025_Silkie_Wenchang	PRJNA827465	0.943630883
ENSGALG00010028247	MVP	2	-0.024856412	0.077936239	-0.318932654	0.749777582	0	0.390408817	0	1	CRA004775;PRJNA827465	Yang2025_Silkie_Wenchang	0.937362187
ENSGALG00010028262	C1orf43	2	-0.099148369	0.064358735	-1.540558074	0.123424379	0	0.227565186	0	1	PRJNA827465;Yang2025_Silkie_Wenchang	CRA004775	0.588993243
ENSGALG00010028262	C1orf43	2	-0.070980409	0.156803114	-0.452672189	0.650784808	0	0.385851556	0	1	CRA004775;Yang2025_Silkie_Wenchang	PRJNA827465	0.908769703
ENSGALG00010028262	C1orf43	2	-0.117010193	0.067757686	-1.726891816	0.084187095	0	0.181659232	0	1	CRA004775;PRJNA827465	Yang2025_Silkie_Wenchang	0.499744053
ENSGALG00010028270	RAPGEF1	2	-0.081584679	0.094135984	-0.866668366	0.386123743	0.006057009	1.377837794	27.42251632	0.5	PRJNA827465;Yang2025_Silkie_Wenchang	CRA004775	0.826885058
ENSGALG00010028270	RAPGEF1	2	0.09973565	0.14081707	0.708263921	0.478781378	0	0.144719329	0	1	CRA004775;Yang2025_Silkie_Wenchang	PRJNA827465	0.855770817
ENSGALG00010028270	RAPGEF1	2	-0.088642546	0.121063557	-0.732198424	0.464047466	0.011077619	1.242502344	19.51725444	0.5	CRA004775;PRJNA827465	Yang2025_Silkie_Wenchang	0.852159649
ENSGALG00010028283	ENSGALG00010028283	2	-0.167610094	0.147377116	-1.137287108	0.255418282	0.036052367	5.858461672	82.93067266	1	PRJNA827465;Yang2025_Silkie_Wenchang	CRA004775	0.764467569
ENSGALG00010028283	ENSGALG00010028283	2	-0.286592641	0.098944735	-2.896492082	0.003773602	0.004160102	1.04812393	4.591435103	0.5	CRA004775;Yang2025_Silkie_Wenchang	PRJNA827465	0.088100594
ENSGALG00010028283	ENSGALG00010028283	2	-0.009692612	0.090511948	-0.107086547	0.91472032	0	0.097641612	0	0.5	CRA004775;PRJNA827465	Yang2025_Silkie_Wenchang	0.97982748
ENSGALG00010028301	FBN3	2	-0.125382689	0.093278197	-1.344180019	0.178890181	0	0.15358583	0	1	PRJNA827465;Yang2025_Silkie_Wenchang	CRA004775	0.685601971
ENSGALG00010028301	FBN3	2	-0.140110777	0.158008533	-0.88672918	0.375224724	0	0.889862931	0	0.5	CRA004775;Yang2025_Silkie_Wenchang	PRJNA827465	0.822941424
ENSGALG00010028301	FBN3	2	-0.083291708	0.112026764	-0.743498296	0.457180053	0	0.704428008	0	0.5	CRA004775;PRJNA827465	Yang2025_Silkie_Wenchang	0.849541335
ENSGALG00010028311	ENSGALG00010028311	2	0.084582431	0.267570357	0.31611286	0.751916846	0.058004807	1.582211329	36.79731767	0.5	PRJNA827465;Yang2025_Silkie_Wenchang	CRA004775	0.93787965
ENSGALG00010028311	ENSGALG00010028311	2	0.206420555	0.55600862	0.371254234	0.710448182	0.399520645	2.740830457	63.51470783	0.5	CRA004775;Yang2025_Silkie_Wenchang	PRJNA827465	0.925404854
ENSGALG00010028311	ENSGALG00010028311	2	0.360315647	0.21094538	1.708099254	0.087617927	0	0.863001889	0	1	CRA004775;PRJNA827465	Yang2025_Silkie_Wenchang	0.508950525
ENSGALG00010028314	MEF2D	2	0.278452729	0.36499529	0.762894032	0.445526596	0.245028737	11.88807633	91.58821014	0.5	PRJNA827465;Yang2025_Silkie_Wenchang	CRA004775	0.846013379
ENSGALG00010028314	MEF2D	2	0.454374173	0.24005862	1.892763412	0.058389339	0.063486681	2.188788858	54.31263293	1	CRA004775;Yang2025_Silkie_Wenchang	PRJNA827465	0.418650753
ENSGALG00010028314	MEF2D	2	-0.045417735	0.070870916	-0.640851536	0.521619146	0	0.846306391	0	0.5	CRA004775;PRJNA827465	Yang2025_Silkie_Wenchang	0.869542236
ENSGALG00010028316	TIMM44	2	0.11514425	0.307669543	0.374246502	0.70822093	0.177519026	15.56460295	93.57516537	0.5	PRJNA827465;Yang2025_Silkie_Wenchang	CRA004775	0.925061251
ENSGALG00010028316	TIMM44	2	0.156406639	0.308104565	0.507641421	0.611704837	0.152926291	4.962532243	79.84899743	0.5	CRA004775;Yang2025_Silkie_Wenchang	PRJNA827465	0.897777844
ENSGALG00010028316	TIMM44	2	-0.17911812	0.058382361	-3.068017748	0.002154838	0	0.000143381	0	1	CRA004775;PRJNA827465	Yang2025_Silkie_Wenchang	0.061008576
ENSGALG00010028318	NACC2	2	-0.068939021	0.107009051	-0.644235423	0.519422784	0	0.475086532	0	0.5	PRJNA827465;Yang2025_Silkie_Wenchang	CRA004775	0.869086345
ENSGALG00010028318	NACC2	2	-0.367927015	0.463421225	-0.793936478	0.427232412	0.340072383	4.388158391	77.21139688	0.5	CRA004775;Yang2025_Silkie_Wenchang	PRJNA827465	0.84125685
ENSGALG00010028318	NACC2	2	-0.421789427	0.380839219	-1.107526237	0.268066475	0.214738231	3.285731001	69.56537222	1	CRA004775;PRJNA827465	Yang2025_Silkie_Wenchang	0.771398294
ENSGALG00010028321	RHBG	2	-0.126493997	0.133851545	-0.945032028	0.344642525	0	0.280031072	0	1	PRJNA827465;Yang2025_Silkie_Wenchang	CRA004775	0.806903268
ENSGALG00010028321	RHBG	2	-0.057965163	0.189852635	-0.30531661	0.760125027	0	0.058353185	0	1	CRA004775;Yang2025_Silkie_Wenchang	PRJNA827465	0.939294282
ENSGALG00010028321	RHBG	2	-0.164941157	0.145805338	-1.131242236	0.25795315	0	0.022309307	0	1	CRA004775;PRJNA827465	Yang2025_Silkie_Wenchang	0.765157824
ENSGALG00010028322	STRBP	2	0.11485753	0.072654162	1.580880264	0.113905418	0	0.743290006	0	1	PRJNA827465;Yang2025_Silkie_Wenchang	CRA004775	0.569481237
ENSGALG00010028322	STRBP	2	0.175041887	0.098846705	1.770841903	0.076586996	0	0.062959515	0	1	CRA004775;Yang2025_Silkie_Wenchang	PRJNA827465	0.47762877
ENSGALG00010028322	STRBP	2	0.072705682	0.087061737	0.835104884	0.403658657	0	0.08319341	0	1	CRA004775;PRJNA827465	Yang2025_Silkie_Wenchang	0.833112102
ENSGALG00010028326	CRB2	2	0.467094536	0.309617245	1.508619248	0.131396112	0.139834838	3.5039219	71.4605511	1	PRJNA827465;Yang2025_Silkie_Wenchang	CRA004775	0.607072924
ENSGALG00010028326	CRB2	2	0.434724165	0.496706178	0.875213927	0.381457517	0.314866574	2.512122256	60.19302017	0.5	CRA004775;Yang2025_Silkie_Wenchang	PRJNA827465	0.824620471
ENSGALG00010028326	CRB2	2	0.167916744	0.160774484	1.044424086	0.296289225	0	0.437353056	0	0.5	CRA004775;PRJNA827465	Yang2025_Silkie_Wenchang	0.788267543
ENSGALG00010028328	HNRNPM	2	0.221062492	0.241534985	0.915240048	0.360065625	0.109689668	16.26713894	93.85263749	0.5	PRJNA827465;Yang2025_Silkie_Wenchang	CRA004775	0.814880744
ENSGALG00010028328	HNRNPM	2	0.252866079	0.252482386	1.001519684	0.316575629	0.100103306	4.353387915	77.02938448	0.5	CRA004775;Yang2025_Silkie_Wenchang	PRJNA827465	0.796883862
ENSGALG00010028328	HNRNPM	2	-0.012034098	0.047781741	-0.25185558	0.801152695	0	0.013837519	0	1	CRA004775;PRJNA827465	Yang2025_Silkie_Wenchang	0.950158029
ENSGALG00010028329	HAPLN2	2	-0.006386505	0.324447473	-0.019684248	0.984295256	0	0.362464856	0	0.5	PRJNA827465;Yang2025_Silkie_Wenchang	CRA004775	0.995703389
ENSGALG00010028329	HAPLN2	2	0.616711154	0.329130167	1.873760645	0.060963423	0	0.295319698	0	1	CRA004775;Yang2025_Silkie_Wenchang	PRJNA827465	0.427185264
ENSGALG00010028329	HAPLN2	2	0.297966001	0.423905252	0.702907075	0.482113662	0.214046685	2.472457223	59.55440641	0.5	CRA004775;PRJNA827465	Yang2025_Silkie_Wenchang	0.85691916
ENSGALG00010028333	ENSGALG00010028333	2	-0.52842089	0.27288908	-1.936394412	0.052819413	0.043657986	1.346098277	25.7112191	1	PRJNA827465;Yang2025_Silkie_Wenchang	CRA004775	0.399347184
ENSGALG00010028333	ENSGALG00010028333	2	-0.037415185	0.343691932	-0.108863932	0.913312001	0	0.140723718	0	0.5	CRA004775;Yang2025_Silkie_Wenchang	PRJNA827465	0.979169142
ENSGALG00010028333	ENSGALG00010028333	2	-0.443973929	0.397411063	-1.117165501	0.263923568	0.166217696	1.849462848	45.93024666	0.5	CRA004775;PRJNA827465	Yang2025_Silkie_Wenchang	0.768643821
ENSGALG00010028337	ENSGALG00010028337	2	-0.246431733	0.163166951	-1.51030421	0.130965818	0	0.552159793	0	1	PRJNA827465;Yang2025_Silkie_Wenchang	CRA004775	0.606744617
ENSGALG00010028337	ENSGALG00010028337	2	-0.133950454	0.251108268	-0.533437052	0.593731075	0	0.206814477	0	1	CRA004775;Yang2025_Silkie_Wenchang	PRJNA827465	0.893084788
ENSGALG00010028337	ENSGALG00010028337	2	-0.33472218	0.187610429	-1.784134181	0.074401864	0	0.001477077	0	1	CRA004775;PRJNA827465	Yang2025_Silkie_Wenchang	0.470928071
ENSGALG00010028339	BCAN	2	0.200255216	0.209067343	0.95785029	0.338138256	0	0.020276115	0	1	PRJNA827465;Yang2025_Silkie_Wenchang	CRA004775	0.80415385
ENSGALG00010028339	BCAN	2	0.11536356	0.314501564	0.366813948	0.713757803	0	0.177551881	0	0.5	CRA004775;Yang2025_Silkie_Wenchang	PRJNA827465	0.926324888
ENSGALG00010028339	BCAN	2	0.187432947	0.257412504	0.728142355	0.466525448	0	0.243514776	0	0.5	CRA004775;PRJNA827465	Yang2025_Silkie_Wenchang	0.843279284
ENSGALG00010028344	LSM7	2	-0.276209853	0.229308519	-1.204533762	0.228383341	0.090747378	6.730791759	85.14290687	1	PRJNA827465;Yang2025_Silkie_Wenchang	CRA004775	0.741167494
ENSGALG00010028344	LSM7	2	-0.459150557	0.147709796	-3.108463811	0.001880627	0	0.880495926	0	1	CRA004775;Yang2025_Silkie_Wenchang	PRJNA827465	0.055742288
ENSGALG00010028344	LSM7	2	-0.076532582	0.058467676	-1.308972536	0.190543654	0	0.166122594	0	1	CRA004775;PRJNA827465	Yang2025_Silkie_Wenchang	0.699656801
ENSGALG00010028346	DENND1A	2	0.118049932	0.16195714	0.728889932	0.466080094	0.041467799	4.109480663	75.66602493	0.5	PRJNA827465;Yang2025_Silkie_Wenchang	CRA004775	0.853230314
ENSGALG00010028346	DENND1A	2	0.170748871	0.215223321	0.793356733	0.427570011	0.043280747	1.682048582	40.54868507	0.5	CRA004775;Yang2025_Silkie_Wenchang	PRJNA827465	0.84125685
ENSGALG00010028346	DENND1A	2	-0.015771839	0.044324302	-0.355828243	0.721969196	0	0.164525731	0	1	CRA004775;PRJNA827465	Yang2025_Silkie_Wenchang	0.929385887
ENSGALG00010028348	NES	2	0.53342433	0.471584632	1.131131709	0.25799966	0.414383016	14.52880959	93.11712365	1	PRJNA827465;Yang2025_Silkie_Wenchang	CRA004775	0.76517824
ENSGALG00010028348	NES	2	0.956314222	0.19038022	5.023180561	5.08227E-07	0	0.792114458	0	1	CRA004775;Yang2025_Silkie_Wenchang	PRJNA827465	0.000131118
ENSGALG00010028348	NES	2	0.095928163	0.139071116	0.689777757	0.490333958	0	0.45956653	0	1	CRA004775;PRJNA827465	Yang2025_Silkie_Wenchang	0.858529056
ENSGALG00010028355	RBM18	2	-0.307385238	0.187573326	-1.638747068	0.101265947	0.059261144	5.609261509	82.17234125	1	PRJNA827465;Yang2025_Silkie_Wenchang	CRA004775	0.54305574
ENSGALG00010028355	RBM18	2	-0.324012717	0.210743208	-1.537476442	0.12417668	0.06146784	3.236247186	69.10001176	1	CRA004775;Yang2025_Silkie_Wenchang	PRJNA827465	0.59073738
ENSGALG00010028355	RBM18	2	-0.135648302	0.039242918	-3.456516686	0.000220305	0	0.054297321	0	1	CRA004775;PRJNA827465	Yang2025_Silkie_Wenchang	0.013550335
ENSGALG00010028357	SPPL2C	2	-0.249992415	0.202319464	-1.235632053	0.216595365	0.074836388	11.36875297	91.20396052	1	PRJNA827465;Yang2025_Silkie_Wenchang	CRA004775	0.730172415
ENSGALG00010028357	SPPL2C	2	-0.173083111	0.309226197	-0.55972978	0.575663767	0.168084761	7.997555626	87.4961795	0.5	CRA004775;Yang2025_Silkie_Wenchang	PRJNA827465	0.88677096
ENSGALG00010028357	SPPL2C	2	-0.027998834	0.073528022	-0.38079134	0.703350886	0.002960099	1.147286114	12.83778406	0.5	CRA004775;PRJNA827465	Yang2025_Silkie_Wenchang	0.923639641
ENSGALG00010028362	TMPRSS9	2	-0.560898723	0.240642689	-2.330836334	0.019761991	0	0.005082476	0	1	PRJNA827465;Yang2025_Silkie_Wenchang	CRA004775	0.2360094
ENSGALG00010028362	TMPRSS9	2	-0.403306831	0.343834452	-1.172968063	0.240808613	0	0.744115808	0	0.5	CRA004775;Yang2025_Silkie_Wenchang	PRJNA827465	0.753303162
ENSGALG00010028362	TMPRSS9	2	-0.472021188	0.29141131	-1.621035021	0.105010128	0	0.860959109	0	0.5	CRA004775;PRJNA827465	Yang2025_Silkie_Wenchang	0.505168852
ENSGALG00010028364	MHM2	2	-0.367655073	0.338998461	-1.084533161	0.278128472	0	0.016170229	0	1	PRJNA827465;Yang2025_Silkie_Wenchang	CRA004775	0.776763074
ENSGALG00010028364	MHM2	2	-1.037735807	0.885953513	-1.171320833	0.241469841	1.050011777	2.77484586	63.96196219	1	CRA004775;Yang2025_Silkie_Wenchang	PRJNA827465	0.753951804
ENSGALG00010028364	MHM2	2	-1.061508837	0.837081236	-1.268107313	0.20475963	0.914284086	2.591669655	61.41483548	1	CRA004775;PRJNA827465	Yang2025_Silkie_Wenchang	0.717318293
ENSGALG00010028366	MRRF	2	0.167363844	0.216052095	0.774645758	0.438548998	0.084104602	9.821850515	89.81861923	0.5	PRJNA827465;Yang2025_Silkie_Wenchang	CRA004775	0.844002503
ENSGALG00010028366	MRRF	2	0.079020126	0.347178572	0.227606577	0.819952107	0.206090096	6.56492229	84.76752723	0.5	CRA004775;Yang2025_Silkie_Wenchang	PRJNA827465	0.955308792
ENSGALG00010028366	MRRF	2	-0.065488265	0.081811494	-0.800477562	0.423434159	0.00309413	1.096988936	8.841377798	1	CRA004775;PRJNA827465	Yang2025_Silkie_Wenchang	0.840219758
ENSGALG00010028368	TIMM13	2	-0.080132255	0.084055949	-0.953320449	0.340427734	0	0.003476994	0	1	PRJNA827465;Yang2025_Silkie_Wenchang	CRA004775	0.805454073
ENSGALG00010028368	TIMM13	2	-0.120610596	0.267002698	-0.451720513	0.651470338	0	0.004027362	0	1	CRA004775;Yang2025_Silkie_Wenchang	PRJNA827465	0.908927523
ENSGALG00010028368	TIMM13	2	-0.081998775	0.084025471	-0.975879976	0.329123946	0	0.024182381	0	1	CRA004775;PRJNA827465	Yang2025_Silkie_Wenchang	0.800761292
ENSGALG00010028370	S100A12	2	0.097936496	0.34458167	0.284218531	0.776242915	0.080553312	1.51239546	33.87972748	0.5	PRJNA827465;Yang2025_Silkie_Wenchang	CRA004775	0.944082704
ENSGALG00010028370	S100A12	2	-0.722117261	1.199837145	-0.601845917	0.547276707	2.619677146	10.91748735	90.84038325	0.5	CRA004775;Yang2025_Silkie_Wenchang	PRJNA827465	0.878281266
ENSGALG00010028370	S100A12	2	-1.052428123	0.853722784	-1.232751595	0.217668456	1.193044513	5.379044428	81.40933741	1	CRA004775;PRJNA827465	Yang2025_Silkie_Wenchang	0.731070128
ENSGALG00010028372	PTGS1	2	0.008065468	0.148790353	0.054206862	0.956770354	0.027611137	2.590459698	61.39681305	0.5	PRJNA827465;Yang2025_Silkie_Wenchang	CRA004775	0.988540618
ENSGALG00010028372	PTGS1	2	0.145680242	0.139033491	1.047806832	0.294727614	0	0.265096711	0	0.5	CRA004775;Yang2025_Silkie_Wenchang	PRJNA827465	0.787482391
ENSGALG00010028372	PTGS1	2	-0.114188964	0.103638855	-1.101796846	0.270550002	0	0.079681718	0	1	CRA004775;PRJNA827465	Yang2025_Silkie_Wenchang	0.770536273
ENSGALG00010028374	FDPS	2	0.140917441	0.102385662	1.376339593	0.16871652	0	0.009577393	0	1	PRJNA827465;Yang2025_Silkie_Wenchang	CRA004775	0.669861707
ENSGALG00010028374	FDPS	2	0.087801322	0.187598155	0.468028705	0.639764062	0	0.101418759	0	0.5	CRA004775;Yang2025_Silkie_Wenchang	PRJNA827465	0.906064918
ENSGALG00010028374	FDPS	2	0.132134188	0.111004235	1.190352673	0.233907806	0	0.170029783	0	0.5	CRA004775;PRJNA827465	Yang2025_Silkie_Wenchang	0.747279664
ENSGALG00010028375	LMNB2	2	-0.156515064	0.164165029	-0.953400764	0.340385055	0.042891639	4.830727042	79.29918227	1	PRJNA827465;Yang2025_Silkie_Wenchang	CRA004775	0.805432415
ENSGALG00010028375	LMNB2	2	-0.311550715	0.115319263	-2.701636369	0.006899918	0	0.335880161	0	1	CRA004775;Yang2025_Silkie_Wenchang	PRJNA827465	0.127631755
ENSGALG00010028375	LMNB2	2	-0.005073277	0.087514403	-0.057970763	0.953771917	0	0.031473514	0	1	CRA004775;PRJNA827465	Yang2025_Silkie_Wenchang	0.987838152
ENSGALG00010028377	PDCL	2	-0.035808058	0.044110487	-0.811781065	0.416917299	0.000318287	1.06418804	6.02437997	1	PRJNA827465;Yang2025_Silkie_Wenchang	CRA004775	0.818063117
ENSGALG00010028377	PDCL	2	-0.125873414	0.079198345	-1.589343997	0.111982751	0	0.107589856	0	1	CRA004775;Yang2025_Silkie_Wenchang	PRJNA827465	0.56547057
ENSGALG00010028377	PDCL	2	-0.021271229	0.045982575	-0.46259326	0.643655941	0	0.790663595	0	1	CRA004775;PRJNA827465	Yang2025_Silkie_Wenchang	0.906643868
ENSGALG00010028380	RUSC1	2	-0.224802192	0.063144003	-3.560151091	0.000370641	0	0.374470802	0	1	PRJNA827465;Yang2025_Silkie_Wenchang	CRA004775	0.01934739
ENSGALG00010028380	RUSC1	2	-0.30093007	0.084580483	-3.55791384	0.000373812	0	0.960870552	0	1	CRA004775;Yang2025_Silkie_Wenchang	PRJNA827465	0.019374277
ENSGALG00010028380	RUSC1	2	-0.278430079	0.140022261	-1.988470092	0.046759723	0.020183934	1.743945227	42.65874957	1	CRA004775;PRJNA827465	Yang2025_Silkie_Wenchang	0.373490609
ENSGALG00010028391	MED26	2	-0.042622719	0.048634043	-0.87639679	0.380814363	0	0.051293458	0	1	PRJNA827465;Yang2025_Silkie_Wenchang	CRA004775	0.824392515
ENSGALG00010028391	MED26	2	-0.029128542	0.123243457	-0.236349496	0.813161402	0	0.435431509	0	0.5	CRA004775;Yang2025_Silkie_Wenchang	PRJNA827465	0.953526116
ENSGALG00010028391	MED26	2	-0.032431368	0.050910328	-0.637029236	0.5241058	0	0.368525429	0	0.5	CRA004775;PRJNA827465	Yang2025_Silkie_Wenchang	0.869740342
ENSGALG00010028392	ASH1L	2	-0.016644788	0.151110295	-0.110149928	0.912290472	0.035166802	3.939779636	74.61786972	0.5	PRJNA827465;Yang2025_Silkie_Wenchang	CRA004775	0.978967245
ENSGALG00010028392	ASH1L	2	0.260137342	0.155405174	1.673929738	0.094144409	0.013155935	1.2792832	21.83122546	1	CRA004775;Yang2025_Silkie_Wenchang	PRJNA827465	0.525712768
ENSGALG00010028392	ASH1L	2	0.130240038	0.322648688	0.403658915	0.686644515	0.175166975	5.513183665	81.86276106	0.5	CRA004775;PRJNA827465	Yang2025_Silkie_Wenchang	0.919235539
ENSGALG00010028394	ZBTB26	2	-0.080251221	0.085068965	-0.943366608	0.345493417	0.008303807	2.299083723	56.5044113	1	PRJNA827465;Yang2025_Silkie_Wenchang	CRA004775	0.807289734
ENSGALG00010028394	ZBTB26	2	-0.015904502	0.189899292	-0.0837523	0.933253374	0.053658013	3.616435683	72.34846441	0.5	CRA004775;Yang2025_Silkie_Wenchang	PRJNA827465	0.984260547
ENSGALG00010028394	ZBTB26	2	0.035717678	0.083984992	0.42528643	0.670627885	0.004133428	1.221036516	18.10236737	0.5	CRA004775;PRJNA827465	Yang2025_Silkie_Wenchang	0.914482883
ENSGALG00010028399	SLC35E1	2	-0.19521774	0.163071234	-1.197113658	0.231255234	0.050245094	17.91627331	94.41848211	0.5	PRJNA827465;Yang2025_Silkie_Wenchang	CRA004775	0.734891332
ENSGALG00010028399	SLC35E1	2	-0.304945787	0.097990599	-3.11199024	0.001858306	0.008653842	1.518953302	34.16519134	1	CRA004775;Yang2025_Silkie_Wenchang	PRJNA827465	0.055331074
ENSGALG00010028399	SLC35E1	2	-0.041601441	0.039948272	-1.041382746	0.297697947	0	0.334687566	0	1	CRA004775;PRJNA827465	Yang2025_Silkie_Wenchang	0.788810248
ENSGALG00010028405	S100A6	2	-0.219577434	0.261180069	-0.840712624	0.400508234	0.073348583	1.75594541	43.05062564	1	PRJNA827465;Yang2025_Silkie_Wenchang	CRA004775	0.832251175
ENSGALG00010028405	S100A6	2	-0.416672582	0.259243483	-1.607263476	0.107996583	0	0.478416393	0	1	CRA004775;Yang2025_Silkie_Wenchang	PRJNA827465	0.556183889
ENSGALG00010028405	S100A6	2	-0.092589177	0.132718036	-0.697638236	0.485403463	0	0.355970859	0	1	CRA004775;PRJNA827465	Yang2025_Silkie_Wenchang	0.857076964
ENSGALG00010028412	ENSGALG00010028412	2	0.273282466	0.36532752	0.748047851	0.454431296	0.142415878	2.009353165	50.2327407	0.5	PRJNA827465;Yang2025_Silkie_Wenchang	CRA004775	0.849072665
ENSGALG00010028412	ENSGALG00010028412	2	0.614800259	0.326091094	1.885458992	0.059379774	0	0.14647642	0	1	CRA004775;Yang2025_Silkie_Wenchang	PRJNA827465	0.422252444
ENSGALG00010028412	ENSGALG00010028412	2	0.111729495	0.233709508	0.478069957	0.632600414	0	0.857155414	0	0.5	CRA004775;PRJNA827465	Yang2025_Silkie_Wenchang	0.903058174
ENSGALG00010028414	KBTBD12	2	0.099306952	0.149550081	0.664038101	0.506665926	0	0.259998924	0	0.5	PRJNA827465;Yang2025_Silkie_Wenchang	CRA004775	0.863929781
ENSGALG00010028414	KBTBD12	2	-0.111186583	0.299082431	-0.371758993	0.710072298	0.07426216	1.357183959	26.318021	0.5	CRA004775;Yang2025_Silkie_Wenchang	PRJNA827465	0.925330621
ENSGALG00010028414	KBTBD12	2	-0.082743105	0.417163871	-0.198343072	0.842771656	0.193943388	1.892331683	47.15514152	0.5	CRA004775;PRJNA827465	Yang2025_Silkie_Wenchang	0.969035733
ENSGALG00010028415	FSD1	2	0.062808404	0.101772644	0.617144266	0.537139573	0.00703861	1.476641282	32.27874552	0.5	PRJNA827465;Yang2025_Silkie_Wenchang	CRA004775	0.87481821
ENSGALG00010028415	FSD1	2	0.170146359	0.133496871	1.274534434	0.202474077	0	0.229062884	0	0.5	CRA004775;Yang2025_Silkie_Wenchang	PRJNA827465	0.715289846
ENSGALG00010028415	FSD1	2	-0.01881865	0.096988697	-0.194029312	0.84615293	0	0.000646534	0	1	CRA004775;PRJNA827465	Yang2025_Silkie_Wenchang	0.96122283
ENSGALG00010028418	ENSGALG00010028418	2	-0.049491582	0.136685775	-0.362082903	0.71729008	0.026979183	3.498659594	71.41762515	0.5	PRJNA827465;Yang2025_Silkie_Wenchang	CRA004775	0.9277944
ENSGALG00010028418	ENSGALG00010028418	2	-0.086437232	0.17888449	-0.483201377	0.628952758	0.030446796	1.648067443	39.32287153	0.5	CRA004775;Yang2025_Silkie_Wenchang	PRJNA827465	0.902114088
ENSGALG00010028418	ENSGALG00010028418	2	0.083067448	0.079450362	1.045526362	0.295779763	0	0.16352687	0	1	CRA004775;PRJNA827465	Yang2025_Silkie_Wenchang	0.78787069
ENSGALG00010028419	ENSGALG00010028419	2	0.235022585	0.46035921	0.510520001	0.609687206	0.361751588	6.813285259	85.32279272	0.5	PRJNA827465;Yang2025_Silkie_Wenchang	CRA004775	0.897238578
ENSGALG00010028419	ENSGALG00010028419	2	-0.025211911	0.767153923	-0.032864214	0.973782871	1.041692926	8.351991881	88.02680828	0.5	CRA004775;Yang2025_Silkie_Wenchang	PRJNA827465	0.992472082
ENSGALG00010028419	ENSGALG00010028419	2	-0.413235778	0.280708718	-1.472115939	0.140989572	0.041917435	1.281729939	21.98044458	1	CRA004775;PRJNA827465	Yang2025_Silkie_Wenchang	0.625404318
ENSGALG00010028420	ENSGALG00010028420	2	1.312044037	0.333867644	3.929832861	8.50049E-05	0	0.75223714	0	1	PRJNA827465;Yang2025_Silkie_Wenchang	CRA004775	0.006902787
ENSGALG00010028420	ENSGALG00010028420	2	-0.217113238	1.86003046	-0.116725636	0.907077475	6.516166145	16.94557544	94.09875455	0.5	CRA004775;Yang2025_Silkie_Wenchang	PRJNA827465	0.978196186
ENSGALG00010028420	ENSGALG00010028420	2	-0.499748523	1.569902254	-0.31833098	0.750233887	4.519753754	11.85437026	91.56429251	0.5	CRA004775;PRJNA827465	Yang2025_Silkie_Wenchang	0.937386904
ENSGALG00010028421	ZBTB7A	2	0.128293298	0.134620676	0.952998471	0.340590845	0.027435059	3.911790221	74.43625697	1	PRJNA827465;Yang2025_Silkie_Wenchang	CRA004775	0.805524125
ENSGALG00010028421	ZBTB7A	2	0.279471763	0.109398019	2.554632762	0.010629988	0	0.001929657	0	1	CRA004775;Yang2025_Silkie_Wenchang	PRJNA827465	0.164355008
ENSGALG00010028421	ZBTB7A	2	0.024806476	0.0663817	0.373694496	0.70863162	0	0.816431194	0	1	CRA004775;PRJNA827465	Yang2025_Silkie_Wenchang	0.925140295
ENSGALG00010028422	RXRA	2	-0.268141215	0.08083267	-3.317238127	0.000909121	0	0.004749324	0	1	PRJNA827465;Yang2025_Silkie_Wenchang	CRA004775	0.035518073
ENSGALG00010028422	RXRA	2	-0.251530347	0.130701197	-1.924468579	0.054295876	0	0.051115735	0	1	CRA004775;Yang2025_Silkie_Wenchang	PRJNA827465	0.40381596
ENSGALG00010028422	RXRA	2	-0.266618202	0.097831003	-2.725293552	0.006424434	0	0.065599599	0	1	CRA004775;PRJNA827465	Yang2025_Silkie_Wenchang	0.122039404
ENSGALG00010028425	DAP3	2	0.047125868	0.094482679	0.498777857	0.617935889	0.008159036	1.608937696	37.84718933	0.5	PRJNA827465;Yang2025_Silkie_Wenchang	CRA004775	0.899121942
ENSGALG00010028425	DAP3	2	0.150614068	0.135176745	1.114201022	0.265192957	0	0.463031945	0	0.5	CRA004775;Yang2025_Silkie_Wenchang	PRJNA827465	0.769274038
ENSGALG00010028425	DAP3	2	-0.014972316	0.06536996	-0.229039707	0.818838055	0	0.009052275	0	1	CRA004775;PRJNA827465	Yang2025_Silkie_Wenchang	0.955256039
ENSGALG00010028426	GPR21	2	0.135034533	0.091674478	1.472978476	0.14075684	0	0.046595208	0	1	PRJNA827465;Yang2025_Silkie_Wenchang	CRA004775	0.625201272
ENSGALG00010028426	GPR21	2	0.293413606	0.30274074	0.969191019	0.332449891	0.097966824	1.764395195	43.32335507	1	CRA004775;Yang2025_Silkie_Wenchang	PRJNA827465	0.802178578
ENSGALG00010028426	GPR21	2	0.29080588	0.263732382	1.10265519	0.270176933	0.078393321	1.657985927	39.68585719	1	CRA004775;PRJNA827465	Yang2025_Silkie_Wenchang	0.772430311
ENSGALG00010028427	ENSGALG00010028427	2	0.281155968	0.317123157	0.886582898	0.375303504	0.120161829	2.481868389	59.70777482	0.5	PRJNA827465;Yang2025_Silkie_Wenchang	CRA004775	0.822941424
ENSGALG00010028427	ENSGALG00010028427	2	0.408766282	0.19163625	2.133032145	0.032922089	0	0.790660068	0	1	CRA004775;Yang2025_Silkie_Wenchang	PRJNA827465	0.311661412
ENSGALG00010028427	ENSGALG00010028427	2	0.129053938	0.186654482	0.691405515	0.489310739	0	0.598904008	0	0.5	CRA004775;PRJNA827465	Yang2025_Silkie_Wenchang	0.858337006

ENSGALG00010028428	RABGAP1	2	-0.023409576	0.043406559	-0.539309656	0.589673208	0	0.874110076	0	0.5	PRJNA827465;Yang2025_Silkie_Wenchang	CRA004775	0.892320726
ENSGALG00010028428	RABGAP1	2	0.085630693	0.121686126	0.703701368	0.481618768	0	0.026343502	0	1	CRA004775;Yang2025_Silkie_Wenchang	PRJNA827465	0.85691916
ENSGALG00010028428	RABGAP1	2	-0.035540375	0.045368644	-0.783368683	0.433410641	0	0.068890211	0	0.5	CRA004775;PRJNA827465	Yang2025_Silkie_Wenchang	0.842762754
ENSGALG00010028429	REXO1	2	0.191311075	0.201463769	0.949605363	0.342312812	0.071568206	7.750006968	87.0967858	1	PRJNA827465;Yang2025_Silkie_Wenchang	CRA004775	0.806005903
ENSGALG00010028429	REXO1	2	0.208365943	0.22434684	0.928767005	0.353009847	0.073928007	3.706418463	73.0197761	0.5	CRA004775;Yang2025_Silkie_Wenchang	PRJNA827465	0.811035834
ENSGALG00010028429	REXO1	2	0.009025758	0.038619375	0.233710622	0.815209641	0	0.054423571	0	0.5	CRA004775;PRJNA827465	Yang2025_Silkie_Wenchang	0.953933933
ENSGALG00010028430	NR2C2AP	2	0.034042046	0.078158192	0.435553136	0.663160979	0	0.782208734	0	0.5	PRJNA827465;Yang2025_Silkie_Wenchang	CRA004775	0.91293776
ENSGALG00010028430	NR2C2AP	2	-0.195244139	0.184653661	-1.057353197	0.290350423	0	0.033491563	0	1	CRA004775;Yang2025_Silkie_Wenchang	PRJNA827465	0.784195688
ENSGALG00010028430	NR2C2AP	2	0.035510416	0.079446678	0.446971685	0.654895504	0	0.958087768	0	0.5	CRA004775;PRJNA827465	Yang2025_Silkie_Wenchang	0.910225547
ENSGALG00010028431	ENSGALG00010028431	2	0.540276185	0.293703417	1.839529792	0.0658373	0	0.115612121	0	1	PRJNA827465;Yang2025_Silkie_Wenchang	CRA004775	0.442023131
ENSGALG00010028431	ENSGALG00010028431	2	0.161219076	0.413668786	0.389729855	0.696736318	0.181010942	2.005633552	50.1404432	0.5	CRA004775;Yang2025_Silkie_Wenchang	PRJNA827465	0.921084006
ENSGALG00010028431	ENSGALG00010028431	2	0.127595978	0.317653262	0.401683198	0.687917192	0.08986667	1.794493933	44.27398266	0.5	CRA004775;PRJNA827465	Yang2025_Silkie_Wenchang	0.919631238
ENSGALG00010028432	RFXANK	2	-0.078892378	0.149634662	-0.527233312	0.598031578	0.021951925	1.601212519	37.54732817	0.5	PRJNA827465;Yang2025_Silkie_Wenchang	CRA004775	0.894645663
ENSGALG00010028432	RFXANK	2	-0.359879665	0.18651573	-1.92948694	0.05367044	0	0.02483075	0	1	CRA004775;Yang2025_Silkie_Wenchang	PRJNA827465	0.401792247
ENSGALG00010028432	RFXANK	2	-0.10765444	0.182260467	-0.590662595	0.554746515	0.038684575	1.928302951	48.14092882	0.5	CRA004775;PRJNA827465	Yang2025_Silkie_Wenchang	0.880223402
ENSGALG00010028433	MSTO1	2	0.376166095	0.227665494	1.6522754	0.098478422	0.093256978	9.82172513	89.81848925	1	PRJNA827465;Yang2025_Silkie_Wenchang	CRA004775	0.535930498
ENSGALG00010028433	MSTO1	2	0.049469538	0.587108197	0.084259662	0.932849984	0.647662263	16.10450928	93.79055901	0.5	CRA004775;Yang2025_Silkie_Wenchang	PRJNA827465	0.984224753
ENSGALG00010028433	MSTO1	2	-0.157442593	0.356937219	-0.441093235	0.659145501	0.220102516	6.68176975	85.03392131	0.5	CRA004775;PRJNA827465	Yang2025_Silkie_Wenchang	0.911449691
ENSGALG00010028434	COL5A1	2	-0.163414175	0.103801672	-1.574292329	0.115419882	0	0.719790854	0	1	PRJNA827465;Yang2025_Silkie_Wenchang	CRA004775	0.572524855
ENSGALG00010028434	COL5A1	2	-0.002415003	0.18270882	-0.01321777	0.989454052	0	0.048401178	0	0.5	CRA004775;Yang2025_Silkie_Wenchang	PRJNA827465	0.997141349
ENSGALG00010028434	COL5A1	2	-0.19748712	0.117743184	-1.67727009	0.093489679	0	0.414032681	0	0.5	CRA004775;PRJNA827465	Yang2025_Silkie_Wenchang	0.523944793
ENSGALG00010028435	ATP8B3	2	0.422782351	0.607619046	0.695801677	0.486553039	0.636247217	7.217855083	86.14546858	0.5	PRJNA827465;Yang2025_Silkie_Wenchang	CRA004775	0.857603818
ENSGALG00010028435	ATP8B3	2	0.489364865	0.615806032	0.794673713	0.426803328	0.568541423	3.849717641	74.02406895	0.5	CRA004775;Yang2025_Silkie_Wenchang	PRJNA827465	0.84125685
ENSGALG00010028435	ATP8B3	2	-0.181463245	0.262769612	-0.690579262	0.489829984	0	0.001531766	0	1	CRA004775;PRJNA827465	Yang2025_Silkie_Wenchang	0.858512413
ENSGALG00010028436	S100A1	2	0.378889426	0.439309101	0.862466599	0.388430805	0.34992116	10.69543797	90.65021925	0.5	PRJNA827465;Yang2025_Silkie_Wenchang	CRA004775	0.828154121
ENSGALG00010028436	S100A1	2	0.699541584	0.258800993	2.703009655	0.006871475	0.042605164	1.23457885	19.00071833	1	CRA004775;Yang2025_Silkie_Wenchang	PRJNA827465	0.127384458
ENSGALG00010028436	S100A1	2	-0.037492677	0.172658119	-0.217149805	0.828091601	0	0.122423581	0	0.5	CRA004775;PRJNA827465	Yang2025_Silkie_Wenchang	0.95664866
ENSGALG00010028437	ENSGALG00010028437	2	-0.003069877	0.037446899	-0.081979471	0.934663038	0	0.00318026	0	1	PRJNA827465;Yang2025_Silkie_Wenchang	CRA004775	0.984397102
ENSGALG00010028437	ENSGALG00010028437	2	-0.068613631	0.124462224	-0.551320944	0.581413684	0.009756149	1.241860505	19.47565804	1	CRA004775;Yang2025_Silkie_Wenchang	PRJNA827465	0.889045686
ENSGALG00010028437	ENSGALG00010028437	2	-0.055438655	0.119731736	-0.463023894	0.64334724	0.015523334	1.425714784	29.85974395	1	CRA004775;PRJNA827465	Yang2025_Silkie_Wenchang	0.906634523
ENSGALG00010028438	MED27	2	-0.238811292	0.138794583	-1.720609608	0.085321689	0.029229618	3.76769752	73.45859123	1	PRJNA827465;Yang2025_Silkie_Wenchang	CRA004775	0.502590779
ENSGALG00010028438	MED27	2	-0.434054179	0.110603731	-3.924408099	8.69432E-05	0	0.155974549	0	1	CRA004775;Yang2025_Silkie_Wenchang	PRJNA827465	0.003013535
ENSGALG00010028438	MED27	2	-0.263007162	0.1821142	-1.44418811	0.148686069	0.049326454	3.276454662	69.47920532	1	CRA004775;PRJNA827465	Yang2025_Silkie_Wenchang	0.638795624
ENSGALG00010028439	BORCS8	2	-0.127734443	0.150585851	-0.848249961	0.396298779	0.025947434	2.134078944	53.14137732	0.5	PRJNA827465;Yang2025_Silkie_Wenchang	CRA004775	0.831365249
ENSGALG00010028439	BORCS8	2	-0.087118993	0.167659266	-0.519619316	0.603328932	0.016952411	1.429228169	30.03216547	0.5	CRA004775;Yang2025_Silkie_Wenchang	PRJNA827465	0.895538966
ENSGALG00010028439	BORCS8	2	-0.246674267	0.089552708	-2.754514888	0.005877022	0	0.016833337	0	1	CRA004775;PRJNA827465	Yang2025_Silkie_Wenchang	0.131570434
ENSGALG00010028440	PSMB7	2	0.051145591	0.046828667	1.092185494	0.274751577	0	0.135927138	0	1	PRJNA827465;Yang2025_Silkie_Wenchang	CRA004775	0.774706274
ENSGALG00010028440	PSMB7	2	-0.127592423	0.262607879	-0.485866698	0.627061681	0.089887163	2.864532712	65.09029218	0.5	CRA004775;Yang2025_Silkie_Wenchang	PRJNA827465	0.901573209
ENSGALG00010028440	PSMB7	2	-0.119417089	0.216034012	-0.552769852	0.580421015	0.072109146	3.604702611	72.25846046	0.5	CRA004775;PRJNA827465	Yang2025_Silkie_Wenchang	0.888433837
ENSGALG00010028442	CHTOP	2	0.336036102	0.386939417	0.868446292	0.385150062	0.294256302	57.53578124	98.26195112	0.5	PRJNA827465;Yang2025_Silkie_Wenchang	CRA004775	0.826444827
ENSGALG00010028442	CHTOP	2	0.32254698	0.425011952	0.758912728	0.447904765	0.335868866	13.72630202	92.71471662	0.5	CRA004775;Yang2025_Silkie_Wenchang	PRJNA827465	0.846601815
ENSGALG00010028442	CHTOP	2	-0.052745406	0.052695852	-1.000940374	0.316855636	0	0.123697602	0	1	CRA004775;PRJNA827465	Yang2025_Silkie_Wenchang	0.796959041
ENSGALG00010028443	ENSGALG00010028443	2	0.283819342	0.14533645	1.952843503	0.050838142	0	0.101413299	0	1	PRJNA827465;Yang2025_Silkie_Wenchang	CRA004775	0.390049394
ENSGALG00010028443	ENSGALG00010028443	2	0.095307506	0.21305968	0.447327746	0.654638437	0.008672348	1.082262207	7.600949832	0.5	CRA004775;Yang2025_Silkie_Wenchang	PRJNA827465	0.910222014
ENSGALG00010028443	ENSGALG00010028443	2	0.141514077	0.265590313	0.532828459	0.594152332	0.067999832	1.70927825	41.49577459	0.5	CRA004775;PRJNA827465	Yang2025_Silkie_Wenchang	0.893274007
ENSGALG00010028444	MEF2B	2	0.424649583	0.371410397	1.143343285	0.25289609	0.235545903	6.837991314	85.37582231	1	PRJNA827465;Yang2025_Silkie_Wenchang	CRA004775	0.762731469
ENSGALG00010028444	MEF2B	2	0.139867462	0.748423507	0.186882775	0.851752545	0.949869305	5.991297935	83.30912582	0.5	CRA004775;Yang2025_Silkie_Wenchang	PRJNA827465	0.962215236
ENSGALG00010028444	MEF2B	2	-0.135844641	0.330109828	-0.411513471	0.680696066	0.103370387	1.541282439	35.11896493	0.5	CRA004775;PRJNA827465	Yang2025_Silkie_Wenchang	0.917851751
ENSGALG00010028445	SYT11	2	-0.131307681	0.187215589	-0.701371514	0.483071194	0.060066056	6.390582929	84.35197522	0.5	PRJNA827465;Yang2025_Silkie_Wenchang	CRA004775	0.85691916
ENSGALG00010028445	SYT11	2	-0.330704187	0.132125017	-2.502964197	0.0123158	0	0.064122104	0	1	CRA004775;Yang2025_Silkie_Wenchang	PRJNA827465	0.179397068
ENSGALG00010028445	SYT11	2	0.029302265	0.047090584	0.62225316	0.533775416	0	0.537257555	0	0.5	CRA004775;PRJNA827465	Yang2025_Silkie_Wenchang	0.873408008
ENSGALG00010028446	ENSGALG00010028446	2	0.048840548	0.13761621	0.354904034	0.722661486	0	0.026978119	0	1	PRJNA827465;Yang2025_Silkie_Wenchang	CRA004775	0.92995758
ENSGALG00010028446	ENSGALG00010028446	2	0.008029216	0.201341669	0.039878563	0.968189942	0	0.537358559	0	0.5	CRA004775;Yang2025_Silkie_Wenchang	PRJNA827465	0.991182909
ENSGALG00010028446	ENSGALG00010028446	2	-0.009563844	0.164180257	-0.058252998	0.953547823	0	0.445547301	0	0.5	CRA004775;PRJNA827465	Yang2025_Silkie_Wenchang	0.987781987
ENSGALG00010028447	OLFM1	2	-0.174752359	0.223339262	-0.782452477	0.433948704	0.085770254	7.040800385	85.79706929	0.5	PRJNA827465;Yang2025_Silkie_Wenchang	CRA004775	0.842762754
ENSGALG00010028447	OLFM1	2	0.13245066	0.184391177	0.71831344	0.472564045	0.020635591	1.167440385	14.34252125	1	CRA004775;Yang2025_Silkie_Wenchang	PRJNA827465	0.853998373
ENSGALG00010028447	OLFM1	2	-0.007207711	0.479126263	-0.015043449	0.987997517	0.365023163	4.075625107	75.46388655	0.5	CRA004775;PRJNA827465	Yang2025_Silkie_Wenchang	0.996823679
ENSGALG00010028448	RIT1	2	-0.095916967	0.082221973	-1.166559129	0.243387604	0.007048767	1.775338174	43.67270335	1	PRJNA827465;Yang2025_Silkie_Wenchang	CRA004775	0.755105773
ENSGALG00010028448	RIT1	2	-0.205840201	0.10166626	-2.024665814	0.042901691	0	0.049293179	0	1	CRA004775;Yang2025_Silkie_Wenchang	PRJNA827465	0.358148931
ENSGALG00010028448	RIT1	2	-0.052545818	0.048865619	-1.075312631	0.282234783	0	0.523408273	0	1	CRA004775;PRJNA827465	Yang2025_Silkie_Wenchang	0.779519046
ENSGALG00010028449	EFCAB12	2	0.006324417	0.184570364	0.034265614	0.972665314	0.01150281	1.1584026	13.67422692	0.5	PRJNA827465;Yang2025_Silkie_Wenchang	CRA004775	0.992213869
ENSGALG00010028449	EFCAB12	2	-0.212535122	0.591568133	-0.359274122	0.719390031	0.504194929	3.402293422	70.60806121	0.5	CRA004775;Yang2025_Silkie_Wenchang	PRJNA827465	0.928449671
ENSGALG00010028449	EFCAB12	2	-0.326909078	0.35131928	-0.930518468	0.352102707	0.134811609	1.778323707	43.76726825	1	CRA004775;PRJNA827465	Yang2025_Silkie_Wenchang	0.810678834
ENSGALG00010028450	PTGDS	2	0.339665366	0.223631817	1.518859751	0.128797802	0	0.141272673	0	1	PRJNA827465;Yang2025_Silkie_Wenchang	CRA004775	0.602157487
ENSGALG00010028450	PTGDS	2	0.336066055	0.211036746	1.592452788	0.111283003	0	0.132215808	0	1	CRA004775;Yang2025_Silkie_Wenchang	PRJNA827465	0.56444558
ENSGALG00010028450	PTGDS	2	0.239355121	0.243236007	0.984044773	0.325093499	0	0.001660363	0	1	CRA004775;PRJNA827465	Yang2025_Silkie_Wenchang	0.799914637
ENSGALG00010028451	TMEM59L	2	0.302589027	0.134507944	2.249599669	0.024474369	0	0.20806213	0	1	PRJNA827465;Yang2025_Silkie_Wenchang	CRA004775	0.267198929
ENSGALG00010028451	TMEM59L	2	0.334109752	0.21426362	1.55933962	0.118916019	0	0.349423291	0	1	CRA004775;Yang2025_Silkie_Wenchang	PRJNA827465	0.579224536
ENSGALG00010028451	TMEM59L	2	0.240973341	0.156934224	1.535492603	0.124662873	0	0.146693924	0	1	CRA004775;PRJNA827465	Yang2025_Silkie_Wenchang	0.592226514
ENSGALG00010028453	ENSGALG00010028453	2	-0.097050659	0.133568374	-0.726599089	0.467471586	0.02676408	3.861830232	74.1055422	0.5	PRJNA827465;Yang2025_Silkie_Wenchang	CRA004775	0.852885838
ENSGALG00010028453	ENSGALG00010028453	2	-0.216946696	0.094140657	-2.304495241	0.021194859	0	0.191890053	0	1	CRA004775;Yang2025_Silkie_Wenchang	PRJNA827465	0.246205819
ENSGALG00010028453	ENSGALG00010028453	2	-0.00989908	0.069536131	-0.142358796	0.886796607	0.000502345	1.031509934	3.054738784	0.5	CRA004775;PRJNA827465	Yang2025_Silkie_Wenchang	0.972850601
ENSGALG00010028454	CSG	2	0.440761855	0.133072607	3.312190717	0.000925684	0	0.115229952	0	1	PRJNA827465;Yang2025_Silkie_Wenchang	CRA004775	0.035879847
ENSGALG00010028454	CSG	2	0.051651923	0.470277689	0.109832815	0.912541966	0.374089241	6.234765314	83.96090391	0.5	CRA004775;Yang2025_Silkie_Wenchang	PRJNA827465	0.978967245
ENSGALG00010028454	CSG	2	0.008325401	0.424665582	0.019604605	0.98435879	0.290764631	4.958020792	79.83066143	0.5	CRA004775;PRJNA827465	Yang2025_Silkie_Wenchang	0.995703389
ENSGALG00010028455	ENSA	2	-0.212182104	0.105805167	-2.005403984	0.044919876	0.011786159	1.672593804	40.21262081	1	PRJNA827465;Yang2025_Silkie_Wenchang	CRA004775	0.365383522
ENSGALG00010028455	ENSA	2	-0.376799957	0.140547667	-2.680940672	0.007341553	0	0.019390856	0	1	CRA004775;Yang2025_Silkie_Wenchang	PRJNA827465	0.131815925
ENSGALG00010028455	ENSA	2	-0.161872593	0.055567568	-2.913076828	0.003578865	0	0.733135069	0	1	CRA004775;PRJNA827465	Yang2025_Silkie_Wenchang	0.084991025
ENSGALG00010028456	FBXW5	2	-0.206301935	0.171773239	-1.201013241	0.229746065	0.050445949	6.463003299	84.5273172	1	PRJNA827465;Yang2025_Silkie_Wenchang	CRA004775	0.742686486
ENSGALG00010028456	FBXW5	2	-0.372523305	0.113519149	-3.28511641	0.001019412	0	0.222644471	0	1	CRA004775;Yang2025_Silkie_Wenchang	PRJNA827465	0.038353353
ENSGALG00010028456	FBXW5	2	-0.059413023	0.053021299	-1.120550117	0.262479408	0	0.526699085	0	1	CRA004775;PRJNA827465	Yang2025_Silkie_Wenchang	0.768418419
ENSGALG00010028457	KANK3	2	0.058338148	0.06859608	0.85045892	0.39506999	0	0.004737172	0	1	PRJNA827465;Yang2025_Silkie_Wenchang	CRA004775	0.830643015
ENSGALG00010028457	KANK3	2	0.040081062	0.136021818	0.294666418	0.768248719	0	0.2214217	0	0.5	CRA004775;Yang2025_Silkie_Wenchang	PRJNA827465	0.941444269
ENSGALG00010028457	KANK3	2	0.047991468	0.075575209	0.635016015	0.52541797	0	0.21799611	0	0.5	CRA004775;PRJNA827465	Yang2025_Silkie_Wenchang	0.870215879
ENSGALG00010028458	TRAF2	2	-0.193904611	0.25980923	-0.746334573	0.455465332	0.117799491	7.352870252	86.39986882	0.5	PRJNA827465;Yang2025_Silkie_Wenchang	CRA004775	0.84907427
ENSGALG00010028458	TRAF2	2	-0.307069543	0.218531173	-1.405152127	0.159976046	0.045907435	1.839305833	45.63166266	1	CRA004775;Yang2025_Silkie_Wenchang	PRJNA827465	0.655404348
ENSGALG00010028458	TRAF2	2	0.036810668	0.068680349	0.535970896	0.591978665	0	0.065827465	0	0.5	CRA004775;PRJNA827465	Yang2025_Silkie_Wenchang	0.892776493
ENSGALG00010028459	MCL1	2	-0.055386585	0.081592221	-0.678821888	0.497250723	0.004542631	1.229965644	18.69691603	1	PRJNA827465;Yang2025_Silkie_Wenchang	CRA004775	0.86098656
ENSGALG00010028459	MCL1	2	-0.179935682	0.175391761	-1.025907265	0.304935298	0	0.670790443	0	0.5	CRA004775;Yang2025_Silkie_Wenchang	PRJNA827465	0.79225794
ENSGALG00010028459	MCL1	2	-0.016289404	0.05800022	-0.280850727	0.778824894	0	0.160799164	0	0.5	CRA004775;PRJNA827465	Yang2025_Silkie_Wenchang	0.944673731
ENSGALG00010028460	CRLF1	2	0.146179961	0.087467745	1.671244191	0.094673455	0	0.558985766	0	1	PRJNA827465;Yang2025_Silkie_Wenchang	CRA004775	0.526919621
ENSGALG00010028460	CRLF1	2	-0.001368882	0.193785901	-0.007063888	0.99436388	0.024958588	1.165876597	14.22762902	0.5	CRA004775;Yang2025_Silkie_Wenchang	PRJNA827465	0.99823774
ENSGALG00010028460	CRLF1	2	0.02687532	0.310719406	0.086493857	0.931073839	0.114130815	1.769760664	43.4951844	0.5	CRA004775;PRJNA827465	Yang2025_Silkie_Wenchang	0.983943003
ENSGALG00010028461	ENSGALG00010028461	2	0.16449332	0.190969389	0.861359619	0.389040009	0.047655338	2.838313655	64.7678121	0.5	PRJNA827465;Yang2025_Silkie_Wenchang	CRA004775	0.828615121
ENSGALG00010028461	ENSGALG00010028461	2	0.031264358	0.449432616	0.069564061	0.944540643	0.286800768	2.944937853	66.04342605	0.5	CRA004775;Yang2025_Silkie_Wenchang	PRJNA827465	0.985957742
ENSGALG00010028461	ENSGALG00010028461	2	-0.061941795	0.161369359	-0.383851032	0.701088862	0.009737897	1.06926095	6.477459988	1	CRA004775;PRJNA827465	Yang2025_Silkie_Wenchang	0.922547052
ENSGALG00010028462	EDF1	2	-0.039728487	0.038587703	-1.029563411	0.303214998	0	0.225402486	0	1	PRJNA827465;Yang2025_Silkie_Wenchang	CRA004775	0.792084776
ENSGALG00010028462	EDF1	2	-0.060497533	0.113971849	-0.530811188	0.595549627	0	0.37081942	0	0.5	CRA004775;Yang2025_Silkie_Wenchang	PRJNA827465	0.893802007
ENSGALG00010028462	EDF1	2	-0.031210046	0.039827052	-0.78363938	0.433251742	0	0.172525589	0	0.5	CRA004775;PRJNA827465	Yang2025_Silkie_Wenchang	0.842762754
ENSGALG00010028463	RPS28	2	-0.346588226	0.442837801	-0.782652758	0.433831052	0.318145156	4.548953686	78.01692281	0.5	PRJNA827465;Yang2025_Silkie_Wenchang	CRA004775	0.842762754
ENSGALG00010028463	RPS28	2	-0.542705732	0.30417515	-1.784188262	0.074393079	0.041293512	1.281817932	21.98580038	1	CRA004775;Yang2025_Silkie_Wenchang	PRJNA827465	0.470928071
ENSGALG00010028463	RPS28	2	-0.004881895	0.095913733	-0.050898807	0.959406156	0	0.630183961	0	0.5	CRA004775;PRJNA827465	Yang2025_Silkie_Wenchang	0.989259322
ENSGALG00010028464	ENSGALG00010028464	2	0.182840054	0.237036348	0.771358721	0.440494335	0.101883159	10.56007041	90.53036617	0.5	PRJNA827465;Yang2025_Silkie_Wenchang	CRA004775	0.844733855
ENSGALG00010028464	ENSGALG00010028464	2	0.133757194	0.342690177	0.390315228	0.696303465	0.189728143	4.76828651	79.02810584	0.5	CRA004775;Yang2025_Silkie_Wenchang	PRJNA827465	0.921038437
ENSGALG00010028464	ENSGALG00010028464	2	-0.060199904	0.076667935	-0.785203143	0.432334473	0	0.518932524	0	1	CRA004775;PRJNA827465	Yang2025_Silkie_Wenchang	0.842762754
ENSGALG00010028465	ENSGALG00010028465	2	-0.03455033	0.101030446	-0.341979388	0.732366404	0	0.19879906	0	0.5	PRJNA827465;Yang2025_Silkie_Wenchang	CRA004775	0.932985777
ENSGALG00010028465	ENSGALG00010028465	2	0.063787475	0.223145379	0.285856132	0.774988314	0	0.006963805	0	1	CRA004775;Yang2025_Silkie_Wenchang	PRJNA827465	0.943774112
ENSGALG00010028465	ENSGALG00010028465	2	-0.046802868	0.105270767	-0.444595111	0.656612381	0	0.045269311	0	0.5	CRA004775;PRJNA827465	Yang2025_Silkie_Wenchang	0.910509456
ENSGALG00010028467	ENSGALG00010028467	2	0.111200538	0.267093385	0.416335801	0.677164299	0.058416454	1.558830288	35.84933476	0.5	PRJNA827465;Yang2025_Silkie_Wenchang	CRA004775	0.917144807
ENSGALG00010028467	ENSGALG00010028467	2	-0.024968438	0.518754206	-0.048131538	0.961611412	0.379879596	3.398880633	70.578549	0.5	CRA004775;Yang2025_Silkie_Wenchang	PRJNA827465	0.98973612
ENSGALG00010028467	ENSGALG00010028467	2	-0.191548649	0.202827223	-0.944393191	0.344968759	0.008001585	1.079316045	7.348732123	1	CRA004775;PRJNA827465	Yang2025_Silkie_Wenchang	0.806961063
ENSGALG00010028468	PHPT1	2	-0.019163129	0.141989656	-0.134961446	0.892642357	0.019662571	1.495696413	33.14151244	0.5	PRJNA827465;Yang2025_Silkie_Wenchang	CRA004775	0.974381724
ENSGALG00010028468	PHPT1	2	-0.249696133	0.195889284	-1.274679899	0.202422564	0	0.044032372	0	1	CRA004775;Yang2025_Silkie_Wenchang	PRJNA827465	0.715289846
ENSGALG00010028468	PHPT1	2	0.037178506	0.07522295	0.494244187	0.621133737	0	0.792917496	0	0.5	CRA004775;PRJNA827465	Yang2025_Silkie_Wenchang	0.899614409
ENSGALG00010028470	AJM1	2	-0.326079899	0.290120362	-1.123946961	0.261035527	0.029450679	1.208741381	17.26931701	1	PRJNA827465;Yang2025_Silkie_Wenchang	CRA004775	0.767929536
ENSGALG00010028470	AJM1	2	-0.105057909	0.320271228	-0.328027935	0.742890541	0	0.18738196	0	1	CRA004775;Yang2025_Silkie_Wenchang	PRJNA827465	0.935707824
ENSGALG00010028470	AJM1	2	-0.496419132	0.291957737	-1.700311618	0.089072326	0	0.214746955	0	1	CRA004775;PRJNA827465	Yang2025_Silkie_Wenchang	0.512338598
ENSGALG00010028471	TPGS1	2	-0.076042551	0.137061121	-0.554807597	0.57902627	0.016740698	1.412799115	29.2185287	1	PRJNA827465;Yang2025_Silkie_Wenchang	CRA004775	0.887888423
ENSGALG00010028471	TPGS1	2	-0.481658334	0.195437003	-2.464519656	0.013719704	0	0.522144717	0	1	CRA004775;Yang2025_Silkie_Wenchang	PRJNA827465	0.192100292
ENSGALG00010028471	TPGS1	2	-0.258822131	0.305112076	-0.848285435	0.396279027	0.150027415	4.501737924	77.78635681	1	CRA004775;PRJNA827465	Yang2025_Silkie_Wenchang	0.831365249
ENSGALG00010028472	RPRD2	2	-0.083625766	0.057028442	-1.466386994	0.142542888	0	0.006393283	0	1	PRJNA827465;Yang2025_Silkie_Wenchang	CRA004775	0.628893678
ENSGALG00010028472	RPRD2	2	-0.012784776	0.138234255	-0.092486306	0.926311671	0	0.421614578	0	0.5	CRA004775;Yang2025_Silkie_Wenchang	PRJNA827465	0.98280842
ENSGALG00010028472	RPRD2	2	-0.073854813	0.059097682	-1.249707457	0.211406432	0	0.652039514	0	0.5	CRA004775;PRJNA827465	Yang2025_Silkie_Wenchang	0.723738713
ENSGALG00010028473	CDC34	2	-0.154783986	0.060923643	-2.540622616	0.011065529	0	0.020282433	0	1	PRJNA827465;Yang2025_Silkie_Wenchang	CRA004775	0.167336849
ENSGALG00010028473	CDC34	2	-0.232717916	0.153127318	-1.519767466	0.128569429	0	0.82361382	0	1	CRA004775;Yang2025_Silkie_Wenchang	PRJNA827465	0.601638797
ENSGALG00010028473	CDC34	2	-0.172621591	0.092780165	-1.860544125	0.062808582	0.004764499	1.05548278	5.256625781	1	CRA004775;PRJNA827465	Yang2025_Silkie_Wenchang	0.43178313
ENSGALG00010028474	PEX19	2	0.003847631	0.17239108	0.022319199	0.982193334	0.034269337	2.101791628	52.42154423	0.5	PRJNA827465;Yang2025_Silkie_Wenchang	CRA004775	0.995105937
ENSGALG00010028474	PEX19	2	0.147463931	0.197491647	0.746684394	0.455254092	0	0.712003451	0	0.5	CRA004775;Yang2025_Silkie_Wenchang	PRJNA827465	0.849072665
ENSGALG00010028474	PEX19	2	-0.124572359	0.103344878	-1.205404285	0.228047268	0	0.006093645	0	1	CRA004775;PRJNA827465	Yang2025_Silkie_Wenchang	0.740938585
ENSGALG00010028475	AMBP	2	-0.415148564	2.000763162	-0.207495106	0.835623215	6.173073577	4.362936195	77.07965564	0.5	PRJNA827465;Yang2025_Silkie_Wenchang	CRA004775	0.958339923
ENSGALG00010028475	AMBP	2	-0.40157168	1.792177912	-0.224069094	0.822703541	5.437420029	5.926664424	83.12710273	0.5	CRA004775;Yang2025_Silkie_Wenchang	PRJNA827465	0.955822987
ENSGALG00010028475	AMBP	2	1.210443434	0.448046944	2.701599577	0.006900681	0	0.077320126	0	1	CRA004775;PRJNA827465	Yang2025_Silkie_Wenchang	0.127631755
ENSGALG00010028476	ENSGALG00010028476	2	0.109890218	0.198980461	0.552266373	0.580765866	0.076130965	25.90420118	96.13962232	0.5	PRJNA827465;Yang2025_Silkie_Wenchang	CRA004775	0.888579914
ENSGALG00010028476	ENSGALG00010028476	2	0.057450326	0.320880545	0.179039605	0.857906604	0.163211123	3.974873809	74.84196862	0.5	CRA004775;Yang2025_Silkie_Wenchang	PRJNA827465	0.964106119
ENSGALG00010028476	ENSGALG00010028476	2	-0.094771552	0.051460736	-1.841628389	0.065529526	0	0.631674809	0	1	CRA004775;PRJNA827465	Yang2025_Silkie_Wenchang	0.441389365
ENSGALG00010028477	NRARP	2	-0.088770119	0.09154926	-0.969643218	0.332224363	0.006791077	1.470162921	31.98032776	1	PRJNA827465;Yang2025_Silkie_Wenchang	CRA004775	0.802178578
ENSGALG00010028477	NRARP	2	-0.118863257	0.203192207	-0.584979409	0.558561562	0.031554593	1.41565665	29.36140269	0.5	CRA004775;Yang2025_Silkie_Wenchang	PRJNA827465	0.880826892
ENSGALG00010028477	NRARP	2	-0.02387493	0.06731759	-0.354661087	0.722843506	0	0.501158885	0	0.5	CRA004775;PRJNA827465	Yang2025_Silkie_Wenchang	0.930032096
ENSGALG00010028478	PEA15	2	0.340485189	0.072940184	4.668005624	3.04138E-06	0	0.166891027	0	1	PRJNA827465;Yang2025_Silkie_Wenchang	CRA004775	0.000571939
ENSGALG00010028478	PEA15	2	0.339041269	0.106676745	3.1782116	0.001481866	0	0.638328838	0	1	CRA004775;Yang2025_Silkie_Wenchang	PRJNA827465	0.047700052
ENSGALG00010028478	PEA15	2	0.297705149	0.087544659	3.40060895	0.000672359	0	0.361889028	0	1	CRA004775;PRJNA827465	Yang2025_Silkie_Wenchang	0.028692443
ENSGALG00010028479	CASQ1	2	-0.076039056	0.173918651	-0.437210472	0.661958716	0	0.027036545	0	1	PRJNA827465;Yang2025_Silkie_Wenchang	CRA004775	0.912556664
ENSGALG00010028479	CASQ1	2	-0.150326519	0.238354388	-0.630684923	0.528246559	0	0.538408976	0	1	CRA004775;Yang2025_Silkie_Wenchang	PRJNA827465	0.871701504
ENSGALG00010028479	CASQ1	2	-0.096499977	0.237691797	-0.405987831	0.684751542	0	0.628055562	0	1	CRA004775;PRJNA827465	Yang2025_Silkie_Wenchang	0.919003112
ENSGALG00010028480	F11R	2	-0.202073168	0.175311256	-1.152653701	0.249052552	0.036052177	2.37656899	57.92253437	1	PRJNA827465;Yang2025_Silkie_Wenchang	CRA004775	0.759411487
ENSGALG00010028480	F11R	2	-0.139345658	0.272357508	-0.511627747	0.608911562	0.108031172	3.653512241	72.62907761	0.5	CRA004775;Yang2025_Silkie_Wenchang	PRJNA827465	0.897208691
ENSGALG00010028480	F11R	2	0.009555269	0.117317236	0.081448122	0.93508558	0	0.549203753	0	0.5	CRA004775;PRJNA827465	Yang2025_Silkie_Wenchang	0.984512821
ENSGALG00010028481	ENSGALG00010028481	2	0.161323705	0.177536943	0.90867682	0.363520744	0.037457135	2.458875508	59.33100327	0.5	PRJNA827465;Yang2025_Silkie_Wenchang	CRA004775	0.817042253
ENSGALG00010028481	ENSGALG00010028481	2	0.032546314	0.149235944	0.218086292	0.827361878	0	0.532264945	0	0.5	CRA004775;Yang2025_Silkie_Wenchang	PRJNA827465	0.956550752
ENSGALG00010028481	ENSGALG00010028481	2	0.316664349	0.138212719	2.291137545	0.02195546	0	0.057898058	0	1	CRA004775;PRJNA827465	Yang2025_Silkie_Wenchang	0.251139533
ENSGALG00010028482	CD320	2	0.193180957	0.111087275	1.739001673	0.082034469	0	0.282520592	0	1	PRJNA827465;Yang2025_Silkie_Wenchang	CRA004775	0.493676423
ENSGALG00010028482	CD320	2	-0.021878067	0.185194752	-0.11813546	0.905960327	0	0.806211165	0	0.5	CRA004775;Yang2025_Silkie_Wenchang	PRJNA827465	0.978196186
ENSGALG00010028482	CD320	2	0.073444492	0.22920123	0.320436728	0.748637287	0.059726237	1.946605155	48.62851373	0.5	CRA004775;PRJNA827465	Yang2025_Silkie_Wenchang	0.937249951
ENSGALG00010028483	EXD3	2	0.220431058	0.196035555	1.124444275	0.260824598	0.06600658	7.064530291	85.84477723	1	PRJNA827465;Yang2025_Silkie_Wenchang	CRA004775	0.76792161
ENSGALG00010028483	EXD3	2	0.154049719	0.293395946	0.525057422	0.599543274	0.144616214	5.95972823	83.22071139	0.5	CRA004775;Yang2025_Silkie_Wenchang	PRJNA827465	0.895010988
ENSGALG00010028483	EXD3	2	-0.005119569	0.088120359	-0.058097459	0.953670999	0	0.705270283	0	0.5	CRA004775;PRJNA827465	Yang2025_Silkie_Wenchang	0.987834059
ENSGALG00010028485	USF1	2	0.12314832	0.261554836	0.470831746	0.637760892	0.120056566	7.72162698	87.04936146	0.5	PRJNA827465;Yang2025_Silkie_Wenchang	CRA004775	0.905279419
ENSGALG00010028485	USF1	2	0.159869376	0.273300935	0.584957296	0.558576431	0.106682365	3.426173696	70.81292168	0.5	CRA004775;Yang2025_Silkie_Wenchang	PRJNA827465	0.880826892
ENSGALG00010028485	USF1	2	-0.118126997	0.071258571	-1.657723336	0.097373321	0	0.008876637	0	1	CRA004775;PRJNA827465	Yang2025_Silkie_Wenchang	0.532942995
ENSGALG00010028486	ARHGAP30	2	0.269868892	0.124717188	2.163846827	0.030476107	0	0.709139397	0	1	PRJNA827465;Yang2025_Silkie_Wenchang	CRA004775	0.297646478
ENSGALG00010028486	ARHGAP30	2	-0.106700231	0.574431275	-0.185749342	0.852641333	0.541525238	5.101754438	80.39889979	0.5	CRA004775;Yang2025_Silkie_Wenchang	PRJNA827465	0.962638237
ENSGALG00010028486	ARHGAP30	2	-0.189573446	0.462274284	-0.410088671	0.681740903	0.322262513	3.527835667	71.65400845	0.5	CRA004775;PRJNA827465	Yang2025_Silkie_Wenchang	0.918373414
ENSGALG00010028488	PTGES	2	-0.099190654	0.07512401	-1.320358891	0.186715222	0	0.084071961	0	1	PRJNA827465;Yang2025_Silkie_Wenchang	CRA004775	0.695457578
ENSGALG00010028488	PTGES	2	-0.171270028	0.221133406	-0.774509968	0.438629276	0.008120507	1.072728887	6.779801278	1	CRA004775;Yang2025_Silkie_Wenchang	PRJNA827465	0.844002503
ENSGALG00010028488	PTGES	2	-0.124493305	0.085177309	-1.461578274	0.143856811	0.001364487	1.016130224	1.587417017	1	CRA004775;PRJNA827465	Yang2025_Silkie_Wenchang	0.630832183
ENSGALG00010028490	NECTIN4	2	0.602085766	0.607556791	0.990995039	0.321688003	0.62881715	6.73475346	85.15164652	1	PRJNA827465;Yang2025_Silkie_Wenchang	CRA004775	0.798513866
ENSGALG00010028490	NECTIN4	2	0.834822767	0.412183567	2.025366449	0.04282975	0.196062967	2.349617624	57.43988342	1	CRA004775;Yang2025_Silkie_Wenchang	PRJNA827465	0.357943462
ENSGALG00010028490	NECTIN4	2	0.14712739	0.248040896	0.593157792	0.553075557	0	0.57067147	0	1	CRA004775;PRJNA827465	Yang2025_Silkie_Wenchang	0.87934426
ENSGALG00010028491	TOR1B	2	0.060910184	0.050504313	1.206039257	0.227802354	0	0.227117862	0	1	PRJNA827465;Yang2025_Silkie_Wenchang	CRA004775	0.740853612
ENSGALG00010028491	TOR1B	2	-0.111604832	0.138128114	-0.807980568	0.419101768	0.020422965	2.116045468	52.74203625	0.5	CRA004775;Yang2025_Silkie_Wenchang	PRJNA827465	0.838438196
ENSGALG00010028491	TOR1B	2	-0.068371713	0.167477425	-0.408244353	0.683094289	0.0447442	4.427478576	77.41378116	0.5	CRA004775;PRJNA827465	Yang2025_Silkie_Wenchang	0.918800744
ENSGALG00010028492	ENSGALG00010028492	2	0.315047101	0.183039175	1.721200395	0.085214466	0.044988306	2.930880014	65.88055481	1	PRJNA827465;Yang2025_Silkie_Wenchang	CRA004775	0.502163128
ENSGALG00010028492	ENSGALG00010028492	2	0.285015121	0.316149134	0.901521117	0.367311311	0.12495592	2.381408628	58.00804666	0.5	CRA004775;Yang2025_Silkie_Wenchang	PRJNA827465	0.819739734
ENSGALG00010028492	ENSGALG00010028492	2	0.130914691	0.112904138	1.159520755	0.246243981	0	0.507135066	0	0.5	CRA004775;PRJNA827465	Yang2025_Silkie_Wenchang	0.756728888
ENSGALG00010028493	TOR1A	2	0.165394615	0.058281042	2.837880216	0.004541422	0	0.605637472	0	1	PRJNA827465;Yang2025_Silkie_Wenchang	CRA004775	0.098668611
ENSGALG00010028493	TOR1A	2	-0.015607354	0.124980794	-0.12487802	0.900620119	0.013833384	1.754405639	43.00063922	0.5	CRA004775;Yang2025_Silkie_Wenchang	PRJNA827465	0.976497577
ENSGALG00010028493	TOR1A	2	0.041192463	0.176233237	0.233738332	0.815188127	0.049354422	4.473121297	77.64424585	0.5	CRA004775;PRJNA827465	Yang2025_Silkie_Wenchang	0.953933933
ENSGALG00010028494	ENSGALG00010028494	2	0.209941846	0.128994273	1.627528424	0.103624917	0	0.009212556	0	1	PRJNA827465;Yang2025_Silkie_Wenchang	CRA004775	0.547964315
ENSGALG00010028494	ENSGALG00010028494	2	0.107596211	0.217112715	0.495577659	0.620192418	0	0.493319599	0	0.5	CRA004775;Yang2025_Silkie_Wenchang	PRJNA827465	0.899395593
ENSGALG00010028494	ENSGALG00010028494	2	0.160507506	0.133433665	1.202901123	0.229014586	0	0.572919658	0	0.5	CRA004775;PRJNA827465	Yang2025_Silkie_Wenchang	0.741680452
ENSGALG00010028495	LINGO3	2	-0.025234816	0.222839248	-0.113242241	0.909838508	0.038390118	1.594949639	37.30209559	0.5	PRJNA827465;Yang2025_Silkie_Wenchang	CRA004775	0.978730675
ENSGALG00010028495	LINGO3	2	-0.220500793	0.583283042	-0.378033951	0.705405373	0.472244453	2.934983622	65.92825962	0.5	CRA004775;Yang2025_Silkie_Wenchang	PRJNA827465	0.924281103
ENSGALG00010028495	LINGO3	2	-0.339697651	0.282394867	-1.20291723	0.229008352	0.051389176	1.22859753	18.6063804	1	CRA004775;PRJNA827465	Yang2025_Silkie_Wenchang	0.741680452
ENSGALG00010028496	ENSGALG00010028496	2	-0.159596579	0.184507537	-0.86498677	0.387046051	0.045263447	2.980113939	66.44423601	0.5	PRJNA827465;Yang2025_Silkie_Wenchang	CRA004775	0.827206927
ENSGALG00010028496	ENSGALG00010028496	2	-0.330072695	0.125263006	-2.635037304	0.008412804	0	0.038500216	0	1	CRA004775;Yang2025_Silkie_Wenchang	PRJNA827465	0.142952193
ENSGALG00010028496	ENSGALG00010028496	2	-0.10272522	0.154904138	-0.663153491	0.507232255	0.01794328	1.550950384	35.52340485	0.5	CRA004775;PRJNA827465	Yang2025_Silkie_Wenchang	0.864048203
ENSGALG00010028498	C9orf78	2	-0.027528014	0.048512141	-0.567445871	0.570411292	0	0.959233473	0	0.5	PRJNA827465;Yang2025_Silkie_Wenchang	CRA004775	0.8851128

ENSGALG00010028498	C9orf78	2	0.09890548	0.112918091	0.875904648	0.381081873	0	0.007017246	0	1	CRA004775;Yang2025_Silkie_Wenchang	PRJNA827465	0.824392515
ENSGALG00010028498	C9orf78	2	-0.037421105	0.050686986	-0.738278364	0.460345311	0	0.521000175	0	0.5	CRA004775;PRJNA827465	Yang2025_Silkie_Wenchang	0.85014477
ENSGALG00010028500	VPS45	2	-0.042482591	0.049276212	-0.862131829	0.388614978	0	0.729325118	0	1	PRJNA827465;Yang2025_Silkie_Wenchang	CRA004775	0.828338548
ENSGALG00010028500	VPS45	2	-0.122131617	0.089603612	-1.363021135	0.17287585	0	0.006278695	0	1	CRA004775;Yang2025_Silkie_Wenchang	PRJNA827465	0.676043418
ENSGALG00010028500	VPS45	2	-0.028518984	0.054029937	-0.527836699	0.597612683	0	0.343572034	0	1	CRA004775;PRJNA827465	Yang2025_Silkie_Wenchang	0.894645663
ENSGALG00010028501	USP20	2	0.116430146	0.044505254	2.616098893	0.008894079	0	0.03219267	0	1	PRJNA827465;Yang2025_Silkie_Wenchang	CRA004775	0.147648184
ENSGALG00010028501	USP20	2	-0.042654088	0.184083464	-0.231710589	0.816762806	0.055911166	5.608293568	82.16926436	0.5	CRA004775;Yang2025_Silkie_Wenchang	PRJNA827465	0.954684235
ENSGALG00010028501	USP20	2	-0.043826467	0.174156228	-0.251650302	0.801311374	0.051896453	6.44202044	84.47691979	0.5	CRA004775;PRJNA827465	Yang2025_Silkie_Wenchang	0.950158029
ENSGALG00010028502	PLEKHO1	2	-0.03854651	0.061347702	-0.62832851	0.529788759	0	0.511130091	0	0.5	PRJNA827465;Yang2025_Silkie_Wenchang	CRA004775	0.871944741
ENSGALG00010028502	PLEKHO1	2	-0.102114998	0.089251372	-1.144128045	0.252570539	0	0.418009409	0	1	CRA004775;Yang2025_Silkie_Wenchang	PRJNA827465	0.762655223
ENSGALG00010028502	PLEKHO1	2	-0.015331473	0.080290363	-0.190950348	0.848564494	0	0.784783428	0	0.5	CRA004775;PRJNA827465	Yang2025_Silkie_Wenchang	0.961364896
ENSGALG00010028503	NDOR1	2	0.167301052	0.16975393	0.985550392	0.324353791	0.036654802	2.694047794	62.88113365	1	PRJNA827465;Yang2025_Silkie_Wenchang	CRA004775	0.79980407
ENSGALG00010028503	NDOR1	2	-0.115357702	0.481090748	-0.239783664	0.810497972	0.425959944	12.47432912	91.98353683	0.5	CRA004775;Yang2025_Silkie_Wenchang	PRJNA827465	0.952997426
ENSGALG00010028503	NDOR1	2	-0.270789469	0.309461912	-0.87503327	0.381555803	0.161854308	6.234114107	83.95922848	0.5	CRA004775;PRJNA827465	Yang2025_Silkie_Wenchang	0.824696862
ENSGALG00010028507	ANP32E	2	0.017848944	0.04434676	0.402485873	0.687326483	0	0.080244559	0	0.5	PRJNA827465;Yang2025_Silkie_Wenchang	CRA004775	0.919465461
ENSGALG00010028507	ANP32E	2	-0.016149125	0.084562456	-0.190972757	0.848546937	0	0.176776867	0	1	CRA004775;Yang2025_Silkie_Wenchang	PRJNA827465	0.961364896
ENSGALG00010028507	ANP32E	2	0.019743442	0.05016078	0.393603173	0.693874038	0	0.296340473	0	0.5	CRA004775;PRJNA827465	Yang2025_Silkie_Wenchang	0.920728119
ENSGALG00010028510	CILP2	2	0.9533151	0.760190161	1.254048197	0.209824488	0.939264399	5.261825016	80.99518709	1	PRJNA827465;Yang2025_Silkie_Wenchang	CRA004775	0.721625118
ENSGALG00010028510	CILP2	2	0.460208945	1.355890111	0.339414634	0.734297396	3.237350192	8.252387282	87.88229435	0.5	CRA004775;Yang2025_Silkie_Wenchang	PRJNA827465	0.933501228
ENSGALG00010028510	CILP2	2	-0.170123026	0.564680568	-0.301273031	0.763206305	0.3303565	1.870878679	46.54917974	0.5	CRA004775;PRJNA827465	Yang2025_Silkie_Wenchang	0.940204908
ENSGALG00010028511	YJEFN3	2	0.139037928	0.361959117	0.384126056	0.70088502	0.045138745	1.196448419	16.41929697	0.5	PRJNA827465;Yang2025_Silkie_Wenchang	CRA004775	0.922496697
ENSGALG00010028511	YJEFN3	2	0.527291508	0.366204862	1.439882232	0.149901004	0	0.025381447	0	1	CRA004775;Yang2025_Silkie_Wenchang	PRJNA827465	0.63983049
ENSGALG00010028511	YJEFN3	2	0.101556218	0.318248462	0.319109846	0.749643218	0	0.928023573	0	0.5	CRA004775;PRJNA827465	Yang2025_Silkie_Wenchang	0.937362187
ENSGALG00010028512	NDUFA13	2	-0.049331724	0.064649547	-0.763063711	0.4454254	0	0.819277618	0	0.5	PRJNA827465;Yang2025_Silkie_Wenchang	CRA004775	0.846013379
ENSGALG00010028512	NDUFA13	2	0.020482062	0.266078212	0.076977601	0.938641364	0.008847608	1.065452	6.143120479	0.5	CRA004775;Yang2025_Silkie_Wenchang	PRJNA827465	0.985349045
ENSGALG00010028512	NDUFA13	2	-0.066706667	0.064933412	-1.027308819	0.304275071	0	0.31177734	0	1	CRA004775;PRJNA827465	Yang2025_Silkie_Wenchang	0.79225794
ENSGALG00010028514	FNBP1	2	0.051816986	0.249324759	0.207829283	0.835362268	0.11464669	12.33831055	91.89516266	0.5	PRJNA827465;Yang2025_Silkie_Wenchang	CRA004775	0.958339923
ENSGALG00010028514	FNBP1	2	0.098748484	0.229648475	0.42999843	0.667196783	0.082601569	4.562637468	78.08285215	0.5	CRA004775;Yang2025_Silkie_Wenchang	PRJNA827465	0.913783154
ENSGALG00010028514	FNBP1	2	-0.179783457	0.050245729	-3.57808434	0.000346122	0	0.049359698	0	1	CRA004775;PRJNA827465	Yang2025_Silkie_Wenchang	0.018458
ENSGALG00010028516	GATAD2A	2	-0.004950411	0.038666194	-0.128029441	0.898125674	0	0.157458314	0	0.5	PRJNA827465;Yang2025_Silkie_Wenchang	CRA004775	0.975733636
ENSGALG00010028516	GATAD2A	2	-0.142498917	0.138961522	-1.025455933	0.305148108	0.021989302	2.111278175	52.63532718	1	CRA004775;Yang2025_Silkie_Wenchang	PRJNA827465	0.79225794
ENSGALG00010028516	GATAD2A	2	-0.11750321	0.158290303	-0.742327275	0.45788907	0.037570653	3.270585863	69.42443826	0.5	CRA004775;PRJNA827465	Yang2025_Silkie_Wenchang	0.849809422
ENSGALG00010028517	RNF208	2	0.182457701	0.107435203	1.698304617	0.089450284	0	0.519096297	0	1	PRJNA827465;Yang2025_Silkie_Wenchang	CRA004775	0.513235693
ENSGALG00010028517	RNF208	2	-0.341808515	0.741094416	-0.461221279	0.644639856	0.928109811	5.878803979	82.98973731	0.5	CRA004775;Yang2025_Silkie_Wenchang	PRJNA827465	0.906953008
ENSGALG00010028517	RNF208	2	-0.405475007	0.650315007	-0.623505536	0.532952365	0.6979334	4.926759038	79.70268097	0.5	CRA004775;PRJNA827465	Yang2025_Silkie_Wenchang	0.873285534
ENSGALG00010028518	MAU2	2	0.1268529	0.114305131	1.109774321	0.267096288	0.019144172	3.570482182	71.99257834	1	PRJNA827465;Yang2025_Silkie_Wenchang	CRA004775	0.771247817
ENSGALG00010028518	MAU2	2	0.21717964	0.097707992	2.222741825	0.026233214	0.000212128	1.005523853	0.549350746	0.5	CRA004775;Yang2025_Silkie_Wenchang	PRJNA827465	0.276215866
ENSGALG00010028518	MAU2	2	0.024045207	0.061031647	0.393979322	0.693596306	0	0.032029203	0	0.5	CRA004775;PRJNA827465	Yang2025_Silkie_Wenchang	0.920721644
ENSGALG00010028523	SUGP1	2	0.238387504	0.258948669	0.920597523	0.357260596	0.122968955	11.40623437	91.2328647	0.5	PRJNA827465;Yang2025_Silkie_Wenchang	CRA004775	0.813292764
ENSGALG00010028523	SUGP1	2	-0.072410701	0.591475077	-0.122423925	0.902563291	0.675156369	28.5084867	96.4922726	0.5	CRA004775;Yang2025_Silkie_Wenchang	PRJNA827465	0.976923607
ENSGALG00010028523	SUGP1	2	-0.315521842	0.331325406	-0.952301984	0.340943852	0.205297109	14.69450782	93.19473634	1	CRA004775;PRJNA827465	Yang2025_Silkie_Wenchang	0.805639763
ENSGALG00010028528	UHRF1	2	-0.064335131	0.074550845	-0.862969843	0.388154048	0	0.508450364	0	0.5	PRJNA827465;Yang2025_Silkie_Wenchang	CRA004775	0.828154121
ENSGALG00010028528	UHRF1	2	0.035302974	0.137479319	0.256787524	0.797342801	0	0.130891467	0	1	CRA004775;Yang2025_Silkie_Wenchang	PRJNA827465	0.949715736
ENSGALG00010028528	UHRF1	2	-0.088705144	0.086380971	-1.02690608	0.304464691	0	0.347354217	0	0.5	CRA004775;PRJNA827465	Yang2025_Silkie_Wenchang	0.79225794
ENSGALG00010028535	TICAM1	2	-0.126887662	0.074580863	-1.701343444	0.088878515	0.005698334	1.77601942	43.69430937	1	PRJNA827465;Yang2025_Silkie_Wenchang	CRA004775	0.511771357
ENSGALG00010028535	TICAM1	2	-0.203433998	0.104106576	-1.954093636	0.050690145	0	0.821749623	0	0.5	CRA004775;Yang2025_Silkie_Wenchang	PRJNA827465	0.389748565
ENSGALG00010028535	TICAM1	2	-0.073834589	0.047782297	-1.545228962	0.122290891	0	0.176614592	0	0.5	CRA004775;PRJNA827465	Yang2025_Silkie_Wenchang	0.586575409
ENSGALG00010028536	GPR107	2	-0.13581788	0.204262508	-0.664918301	0.50610275	0.078790961	17.73010698	94.35987611	0.5	PRJNA827465;Yang2025_Silkie_Wenchang	CRA004775	0.863929781
ENSGALG00010028536	GPR107	2	-0.333957937	0.078840339	-4.235876465	2.27662E-05	0	0.209291006	0	1	CRA004775;Yang2025_Silkie_Wenchang	PRJNA827465	0.002703262
ENSGALG00010028536	GPR107	2	0.038247641	0.080409777	0.475659087	0.634317273	0.00451055	1.12686559	11.25827175	0.5	CRA004775;PRJNA827465	Yang2025_Silkie_Wenchang	0.903815945
ENSGALG00010028538	FEM1A	2	-0.475703917	0.330739352	-1.438304554	0.15034766	0.205062856	15.88018602	93.70284455	1	PRJNA827465;Yang2025_Silkie_Wenchang	CRA004775	0.6410408
ENSGALG00010028538	FEM1A	2	-0.349311124	0.517591672	-0.674877791	0.499753412	0.464629893	6.966978208	85.64657488	0.5	CRA004775;Yang2025_Silkie_Wenchang	PRJNA827465	0.862078151
ENSGALG00010028538	FEM1A	2	-0.125133266	0.096380244	-1.298328991	0.194174308	0	0.975345007	0	0.5	CRA004775;PRJNA827465	Yang2025_Silkie_Wenchang	0.704870494
ENSGALG00010028541	ENSGALG00010028541	2	0.145757076	0.406887719	0.358224319	0.720175449	0.097292172	1.406563583	28.90474258	0.5	PRJNA827465;Yang2025_Silkie_Wenchang	CRA004775	0.928564173
ENSGALG00010028541	ENSGALG00010028541	2	-0.15324859	0.438954996	-0.349121417	0.726998158	0	0.298747011	0	0.5	CRA004775;Yang2025_Silkie_Wenchang	PRJNA827465	0.93142315
ENSGALG00010028541	ENSGALG00010028541	2	0.429115245	0.385189106	1.114037852	0.265262948	0	0.115781608	0	1	CRA004775;PRJNA827465	Yang2025_Silkie_Wenchang	0.76929776
ENSGALG00010028543	ANP32B	2	0.086964139	0.061433066	1.415591715	0.156895079	0.002350933	1.338506863	25.28988625	1	PRJNA827465;Yang2025_Silkie_Wenchang	CRA004775	0.650302901
ENSGALG00010028543	ANP32B	2	-0.011874178	0.254213341	-0.046709501	0.962744758	0.092290998	3.019827018	66.88552046	0.5	CRA004775;Yang2025_Silkie_Wenchang	PRJNA827465	0.989980504
ENSGALG00010028543	ANP32B	2	-0.046521281	0.167038954	-0.278505579	0.780624283	0.033988631	1.815826362	44.92865503	0.5	CRA004775;PRJNA827465	Yang2025_Silkie_Wenchang	0.94523121
ENSGALG00010028544	ZNF414	2	0.271099483	0.262791767	1.031613302	0.302253306	0.106653791	4.100415983	75.61223046	1	PRJNA827465;Yang2025_Silkie_Wenchang	CRA004775	0.791647454
ENSGALG00010028544	ZNF414	2	0.132141923	0.45654109	0.289441466	0.772243567	0.349784477	6.184859228	83.83148326	0.5	CRA004775;Yang2025_Silkie_Wenchang	PRJNA827465	0.943066211
ENSGALG00010028544	ZNF414	2	-0.060880041	0.170513062	-0.357040336	0.72106161	0.025985723	1.551511651	35.54672957	0.5	CRA004775;PRJNA827465	Yang2025_Silkie_Wenchang	0.928950734
ENSGALG00010028547	ZNF618	2	-0.171554968	0.171357811	-1.001150562	0.316754023	0.04795615	5.214933317	80.82429939	1	PRJNA827465;Yang2025_Silkie_Wenchang	CRA004775	0.796959041
ENSGALG00010028547	ZNF618	2	-0.135334843	0.272722949	-0.496235625	0.619728179	0.108021347	3.344206992	70.09754473	0.5	CRA004775;Yang2025_Silkie_Wenchang	PRJNA827465	0.899325257
ENSGALG00010028547	ZNF618	2	-0.002600225	0.070827325	-0.03671217	0.970714505	0	0.553715376	0	0.5	CRA004775;PRJNA827465	Yang2025_Silkie_Wenchang	0.991843322
ENSGALG00010028548	MYO1F	2	-0.081622105	0.10418538	-0.783431466	0.433373784	0	0.000566049	0	1	PRJNA827465;Yang2025_Silkie_Wenchang	CRA004775	0.842762754
ENSGALG00010028548	MYO1F	2	-0.265902285	0.310788183	-0.855573986	0.392233464	0.10011761	1.645231538	39.21828164	1	CRA004775;Yang2025_Silkie_Wenchang	PRJNA827465	0.829438924
ENSGALG00010028548	MYO1F	2	-0.256260835	0.309653311	-0.827573372	0.407912153	0.1097936	1.736035517	42.39749185	1	CRA004775;PRJNA827465	Yang2025_Silkie_Wenchang	0.833982683
ENSGALG00010028549	ADAMTS10	2	-0.048512426	0.116924674	-0.414903237	0.67821274	0.017684758	2.828181429	64.64158948	0.5	PRJNA827465;Yang2025_Silkie_Wenchang	CRA004775	0.917647993
ENSGALG00010028549	ADAMTS10	2	-0.150562536	0.098571377	-1.527446819	0.126649942	0	0.515606479	0	0.5	CRA004775;Yang2025_Silkie_Wenchang	PRJNA827465	0.597422307
ENSGALG00010028549	ADAMTS10	2	0.069139324	0.092504498	0.747415803	0.454812609	0	0.025792178	0	1	CRA004775;PRJNA827465	Yang2025_Silkie_Wenchang	0.849072665
ENSGALG00010028551	NCS1	2	-0.146344039	0.133282149	-1.098001791	0.272203707	0	0.987197843	0	0.5	PRJNA827465;Yang2025_Silkie_Wenchang	CRA004775	0.773286758
ENSGALG00010028551	NCS1	2	0.005274537	0.211740455	0.024910388	0.980126442	0	0.157024874	0	0.5	CRA004775;Yang2025_Silkie_Wenchang	PRJNA827465	0.994658414
ENSGALG00010028551	NCS1	2	-0.23189593	0.154186762	-1.503993775	0.13258297	0	0.015387686	0	1	CRA004775;PRJNA827465	Yang2025_Silkie_Wenchang	0.609921713
ENSGALG00010028556	SPTAN1	2	0.019144251	0.204710077	0.09351885	0.925491375	0.074012463	8.305741127	87.96013523	0.5	PRJNA827465;Yang2025_Silkie_Wenchang	CRA004775	0.982594411
ENSGALG00010028556	SPTAN1	2	0.249257893	0.114812581	2.17099808	0.029931318	0	0.066259065	0	1	CRA004775;Yang2025_Silkie_Wenchang	PRJNA827465	0.295167651
ENSGALG00010028556	SPTAN1	2	-0.013394309	0.231171586	-0.057940985	0.953795637	0.074093995	2.541101901	60.64699336	0.5	CRA004775;PRJNA827465	Yang2025_Silkie_Wenchang	0.987838152
ENSGALG00010028559	DYNC2I2	2	0.020877475	0.079777633	0.261695848	0.793555942	0	0.00809103	0	1	PRJNA827465;Yang2025_Silkie_Wenchang	CRA004775	0.948743674
ENSGALG00010028559	DYNC2I2	2	-0.23180472	0.253450258	-0.914596502	0.360403496	0.093758806	3.669104849	72.74539592	0.5	CRA004775;Yang2025_Silkie_Wenchang	PRJNA827465	0.814880744
ENSGALG00010028559	DYNC2I2	2	-0.205065712	0.260373749	-0.787582131	0.43094117	0.112374693	5.435834841	81.60356175	0.5	CRA004775;PRJNA827465	Yang2025_Silkie_Wenchang	0.842503929
ENSGALG00010028561	SET	2	0.232103787	0.270929248	0.856695204	0.391613354	0.122902848	5.268359491	81.01875922	1	PRJNA827465;Yang2025_Silkie_Wenchang	CRA004775	0.829251057
ENSGALG00010028561	SET	2	0.230435866	0.313103439	0.735973604	0.461746762	0.148620419	4.109676573	75.66718494	0.5	CRA004775;Yang2025_Silkie_Wenchang	PRJNA827465	0.850622591
ENSGALG00010028561	SET	2	0.003281279	0.038667261	0.084859354	0.932373206	0	0.140320172	0	0.5	CRA004775;PRJNA827465	Yang2025_Silkie_Wenchang	0.984224753
ENSGALG00010028562	ACSBG2	2	0.167028033	0.202142802	0.826287315	0.408641127	0.066505487	5.331749042	81.24442857	0.5	PRJNA827465;Yang2025_Silkie_Wenchang	CRA004775	0.834241753
ENSGALG00010028562	ACSBG2	2	0.248206632	0.182946207	1.356719202	0.174870449	0.031522227	1.719872929	41.85616955	0.5	CRA004775;Yang2025_Silkie_Wenchang	PRJNA827465	0.679636281
ENSGALG00010028562	ACSBG2	2	-0.024607826	0.101192801	-0.243177638	0.807867789	0	0.00337216	0	1	CRA004775;PRJNA827465	Yang2025_Silkie_Wenchang	0.952496413
ENSGALG00010028565	PKN1	2	0.072538162	0.14247747	0.509120227	0.610667954	0.028943014	3.401057429	70.59737976	0.5	PRJNA827465;Yang2025_Silkie_Wenchang	CRA004775	0.897438028
ENSGALG00010028565	PKN1	2	0.234196225	0.119777496	1.955260649	0.050552313	0	0.011014448	0	1	CRA004775;Yang2025_Silkie_Wenchang	PRJNA827465	0.389297528
ENSGALG00010028565	PKN1	2	-0.037863614	0.086940489	-0.435511862	0.663190931	0	0.761076819	0	0.5	CRA004775;PRJNA827465	Yang2025_Silkie_Wenchang	0.91293776
ENSGALG00010028566	ZDHHC12	2	-0.039513941	0.070259079	-0.562403338	0.573841246	0	0.045681341	0	1	PRJNA827465;Yang2025_Silkie_Wenchang	CRA004775	0.886032852
ENSGALG00010028566	ZDHHC12	2	-0.338837972	0.360583554	-0.93969336	0.347374872	0.218455909	5.791775304	82.73413682	1	CRA004775;Yang2025_Silkie_Wenchang	PRJNA827465	0.808705451
ENSGALG00010028566	ZDHHC12	2	-0.348639113	0.344186441	-1.012936802	0.311090348	0.198996146	5.625705718	82.22445236	1	CRA004775;PRJNA827465	Yang2025_Silkie_Wenchang	0.79529356
ENSGALG00010028568	MLLT1	2	-0.322684114	0.310203492	-1.040233661	0.298231356	0.187016659	35.06275711	97.147971	1	PRJNA827465;Yang2025_Silkie_Wenchang	CRA004775	0.789118382
ENSGALG00010028568	MLLT1	2	-0.426859154	0.246696525	-1.730300632	0.083576577	0.096773342	4.469990887	77.62858974	1	CRA004775;Yang2025_Silkie_Wenchang	PRJNA827465	0.498062361
ENSGALG00010028568	MLLT1	2	-0.023188266	0.046321806	-0.500590721	0.616659194	0	0.297861791	0	1	CRA004775;PRJNA827465	Yang2025_Silkie_Wenchang	0.899111936
ENSGALG00010028569	ZER1	2	-0.041368937	0.034802437	-1.188679324	0.23456588	0	0.001860401	0	1	PRJNA827465;Yang2025_Silkie_Wenchang	CRA004775	0.747639509
ENSGALG00010028569	ZER1	2	-0.05624803	0.064597298	-0.870748961	0.383891239	0	0.76312456	0	1	CRA004775;Yang2025_Silkie_Wenchang	PRJNA827465	0.825601288
ENSGALG00010028569	ZER1	2	-0.048795019	0.03992047	-1.222305718	0.221592038	0	0.780701204	0	1	CRA004775;PRJNA827465	Yang2025_Silkie_Wenchang	0.734944791
ENSGALG00010028572	TBC1D13	2	-0.093424057	0.155412556	-0.60113584	0.547749514	0.04020662	5.384767993	81.42909776	0.5	PRJNA827465;Yang2025_Silkie_Wenchang	CRA004775	0.878281266
ENSGALG00010028572	TBC1D13	2	-0.273494558	0.093163756	-2.935632573	0.003328683	0	4.42252E-06	0	1	CRA004775;Yang2025_Silkie_Wenchang	PRJNA827465	0.08065798
ENSGALG00010028572	TBC1D13	2	-0.090092437	0.154714429	-0.582314382	0.56035494	0.039108382	4.855094837	79.40307999	0.5	CRA004775;PRJNA827465	Yang2025_Silkie_Wenchang	0.881203589
ENSGALG00010028573	KCNN1	2	0.299497039	0.229047683	1.307575065	0.191017479	0.096246577	12.06519731	91.71169791	1	PRJNA827465;Yang2025_Silkie_Wenchang	CRA004775	0.700443419
ENSGALG00010028573	KCNN1	2	0.322021512	0.344474511	0.934819565	0.349881295	0.152495527	2.02792946	50.6886201	0.5	CRA004775;Yang2025_Silkie_Wenchang	PRJNA827465	0.809953147
ENSGALG00010028573	KCNN1	2	0.065641293	0.084287537	0.77877816	0.436110405	0	0.343136016	0	0.5	CRA004775;PRJNA827465	Yang2025_Silkie_Wenchang	0.843095975
ENSGALG00010028574	ENDOG	2	0.029594506	0.074883521	0.395207189	0.692689988	0	3.0836E-06	0	1	PRJNA827465;Yang2025_Silkie_Wenchang	CRA004775	0.920579378
ENSGALG00010028574	ENDOG	2	-0.048179584	0.148270706	-0.324943378	0.745223926	0	0.683796768	0	0.5	CRA004775;Yang2025_Silkie_Wenchang	PRJNA827465	0.93597621
ENSGALG00010028574	ENDOG	2	0.007432534	0.079351713	0.093665698	0.92537472	0	0.880936525	0	0.5	CRA004775;PRJNA827465	Yang2025_Silkie_Wenchang	0.982594411
ENSGALG00010028575	SETDB1	2	0.056076403	0.119917387	0.467625291	0.640052576	0.025369783	8.310926888	87.96764773	0.5	PRJNA827465;Yang2025_Silkie_Wenchang	CRA004775	0.906064918
ENSGALG00010028575	SETDB1	2	-0.123581749	0.323801103	-0.381659445	0.702713985	0.193527628	12.49613538	91.99752588	0.5	CRA004775;Yang2025_Silkie_Wenchang	PRJNA827465	0.923272306
ENSGALG00010028575	SETDB1	2	-0.229215586	0.201595256	-1.137008825	0.255534597	0.068102624	5.445469871	81.63611178	1	CRA004775;PRJNA827465	Yang2025_Silkie_Wenchang	0.764467569
ENSGALG00010028577	CERS2	2	0.024604646	0.061974596	0.397011795	0.691358763	0.001740001	1.194274595	16.26716297	0.5	PRJNA827465;Yang2025_Silkie_Wenchang	CRA004775	0.920268407
ENSGALG00010028577	CERS2	2	-0.076061279	0.094020717	-0.808984248	0.418532207	0	0.631597943	0	0.5	CRA004775;Yang2025_Silkie_Wenchang	PRJNA827465	0.831378696
ENSGALG00010028577	CERS2	2	-0.010537251	0.053461695	-0.197099076	0.843750006	0	0.001661867	0	1	CRA004775;PRJNA827465	Yang2025_Silkie_Wenchang	0.96057435
ENSGALG00010028578	SPOUT1	2	0.025895402	0.059880179	0.432453662	0.665411724	0	0.089459032	0	0.5	PRJNA827465;Yang2025_Silkie_Wenchang	CRA004775	0.91350776
ENSGALG00010028578	SPOUT1	2	-0.158747331	0.180185383	-0.88102225	0.378305777	0.033730625	1.989690988	49.74093936	1	CRA004775;Yang2025_Silkie_Wenchang	PRJNA827465	0.823345221
ENSGALG00010028578	SPOUT1	2	-0.120661665	0.200746975	-0.601063428	0.547797741	0.059929678	3.28658943	69.57332149	0.5	CRA004775;PRJNA827465	Yang2025_Silkie_Wenchang	0.878281266
ENSGALG00010028579	OCLN	2	-0.17146593	0.105592408	-1.62384715	0.104408434	0.002459829	1.113390387	10.18424338	1	PRJNA827465;Yang2025_Silkie_Wenchang	CRA004775	0.549118786
ENSGALG00010028579	OCLN	2	0.015775627	0.156094624	0.101064512	0.919499249	0	0.547329681	0	0.5	CRA004775;Yang2025_Silkie_Wenchang	PRJNA827465	0.981192162
ENSGALG00010028579	OCLN	2	-0.117930275	0.235780634	-0.500169473	0.616955752	0.058275353	1.623018505	38.38640799	0.5	CRA004775;PRJNA827465	Yang2025_Silkie_Wenchang	0.899111936
ENSGALG00010028580	PRUNE1	2	0.024655777	0.135219393	0.182335208	0.855332208	0.019166835	1.952288126	48.77805245	0.5	PRJNA827465;Yang2025_Silkie_Wenchang	CRA004775	0.965406774
ENSGALG00010028580	PRUNE1	2	-0.099107299	0.155913082	-0.635657365	0.524999771	0	0.461209606	0	0.5	CRA004775;Yang2025_Silkie_Wenchang	PRJNA827465	0.869962943
ENSGALG00010028580	PRUNE1	2	0.126714009	0.095166693	1.331495349	0.183026074	0	0.002114063	0	1	CRA004775;PRJNA827465	Yang2025_Silkie_Wenchang	0.691392093
ENSGALG00010028581	NR2F6	2	-0.021036562	0.040568109	-0.518549237	0.604075116	0	0.005668628	0	1	PRJNA827465;Yang2025_Silkie_Wenchang	CRA004775	0.896058435
ENSGALG00010028581	NR2F6	2	-0.0447375	0.108467112	-0.412452208	0.680008003	0	0.157569655	0	1	CRA004775;Yang2025_Silkie_Wenchang	PRJNA827465	0.917701868
ENSGALG00010028581	NR2F6	2	-0.022497824	0.042942443	-0.523906475	0.600343591	0	0.199891346	0	1	CRA004775;PRJNA827465	Yang2025_Silkie_Wenchang	0.895230519
ENSGALG00010028582	KYAT1	2	-0.017179673	0.101401411	-0.169422424	0.865464386	0.005576545	1.274261153	21.52315109	0.5	PRJNA827465;Yang2025_Silkie_Wenchang	CRA004775	0.967086879
ENSGALG00010028582	KYAT1	2	-0.19024079	0.162829843	-1.168341051	0.242669223	0	0.010195435	0	1	CRA004775;Yang2025_Silkie_Wenchang	PRJNA827465	0.7550024
ENSGALG00010028582	KYAT1	2	0.030516909	0.090049823	0.338889166	0.734693227	0	0.456432561	0	0.5	CRA004775;PRJNA827465	Yang2025_Silkie_Wenchang	0.933520234
ENSGALG00010028583	USHBP1	2	0.517131131	0.489100317	1.05731097	0.290369688	0.453639687	19.25301634	94.80600867	1	PRJNA827465;Yang2025_Silkie_Wenchang	CRA004775	0.784195688
ENSGALG00010028583	USHBP1	2	0.573971857	0.490956723	1.169088498	0.242367978	0.402492757	5.679082994	82.3915234	1	CRA004775;Yang2025_Silkie_Wenchang	PRJNA827465	0.734698121
ENSGALG00010028583	USHBP1	2	0.031970278	0.131695885	0.242758367	0.808192589	0	0.000641511	0	1	CRA004775;PRJNA827465	Yang2025_Silkie_Wenchang	0.952496413
ENSGALG00010028584	LRRC8A	2	-0.179277944	0.201163619	-0.891204607	0.372819413	0.076442769	17.7637048	94.3705437	0.5	PRJNA827465;Yang2025_Silkie_Wenchang	CRA004775	0.822162024
ENSGALG00010028584	LRRC8A	2	-0.190946642	0.22972725	-0.831188471	0.405867167	0.084052838	4.462635351	77.59171608	0.5	CRA004775;Yang2025_Silkie_Wenchang	PRJNA827465	0.833416425
ENSGALG00010028584	LRRC8A	2	-0.019241302	0.045640093	-0.421587705	0.673325982	0	0.090478515	0	0.5	CRA004775;PRJNA827465	Yang2025_Silkie_Wenchang	0.915250069
ENSGALG00010028585	MINDY1	2	-0.039452596	0.072642648	-0.543105141	0.587057413	0	0.528038307	0	0.5	PRJNA827465;Yang2025_Silkie_Wenchang	CRA004775	0.891596903
ENSGALG00010028585	MINDY1	2	-0.103762446	0.237856782	-0.436239175	0.662663208	0.057996305	1.925430772	48.06357027	0.5	CRA004775;Yang2025_Silkie_Wenchang	PRJNA827465	0.912778072
ENSGALG00010028585	MINDY1	2	-0.11242498	0.121821088	-0.922869608	0.356075161	0.01101842	1.222417694	18.19490141	1	CRA004775;PRJNA827465	Yang2025_Silkie_Wenchang	0.812424361
ENSGALG00010028586	BABAM1	2	-0.077688168	0.066992699	-1.159651126	0.246190822	0.003227364	1.318390244	24.13841592	1	PRJNA827465;Yang2025_Silkie_Wenchang	CRA004775	0.75672496
ENSGALG00010028586	BABAM1	2	-0.201151838	0.124657415	-1.613637169	0.10660615	0	0.011793043	0	1	CRA004775;Yang2025_Silkie_Wenchang	PRJNA827465	0.553677869
ENSGALG00010028586	BABAM1	2	-0.045245861	0.049280795	-0.91812361	0.358554156	0	0.121390268	0	1	CRA004775;PRJNA827465	Yang2025_Silkie_Wenchang	0.814138066
ENSGALG00010028587	ANKLE1	2	0.296964782	0.440169527	0.674660021	0.49989179	0.365821985	17.83617127	94.39341558	0.5	PRJNA827465;Yang2025_Silkie_Wenchang	CRA004775	0.862078151
ENSGALG00010028587	ANKLE1	2	0.044704971	0.717729325	0.062286672	0.950334542	0.975502176	18.54295073	94.60711505	0.5	CRA004775;Yang2025_Silkie_Wenchang	PRJNA827465	0.987277827
ENSGALG00010028587	ANKLE1	2	-0.354991474	0.271049212	-1.309693807	0.19029944	0.103248425	3.019653776	66.88362063	1	CRA004775;PRJNA827465	Yang2025_Silkie_Wenchang	0.699411386
ENSGALG00010028588	ABHD8	2	0.303280315	0.686278635	0.441920088	0.658547033	0.776950173	5.528473666	81.91182485	0.5	PRJNA827465;Yang2025_Silkie_Wenchang	CRA004775	0.911105434
ENSGALG00010028588	ABHD8	2	0.176366852	0.884918758	0.199302874	0.842025831	1.294502669	5.759999799	82.63888828	0.5	CRA004775;Yang2025_Silkie_Wenchang	PRJNA827465	0.969064308
ENSGALG00010028588	ABHD8	2	-0.422343243	0.265380673	-1.591461947	0.111505653	0	0.399353503	0	1	CRA004775;PRJNA827465	Yang2025_Silkie_Wenchang	0.564762672
ENSGALG00010028589	PHYHD1	2	0.187012277	0.205290279	0.910965086	0.36231377	0.042934254	1.943466901	48.54556054	1	PRJNA827465;Yang2025_Silkie_Wenchang	CRA004775	0.815644888
ENSGALG00010028589	PHYHD1	2	-0.144839165	0.220849661	-0.655826976	0.511935458	0	0.363297677	0	1	CRA004775;Yang2025_Silkie_Wenchang	PRJNA827465	0.865694231
ENSGALG00010028589	PHYHD1	2	0.104768023	0.345292479	0.303418203	0.761571175	0.153875469	2.369224071	57.79208847	0.5	CRA004775;PRJNA827465	Yang2025_Silkie_Wenchang	0.939738254
ENSGALG00010028590	ENSGALG00010028590	2	0.04558102	0.152718546	0.298464207	0.76534889	0.028716254	2.081263972	51.95227452	0.5	PRJNA827465;Yang2025_Silkie_Wenchang	CRA004775	0.940780241
ENSGALG00010028590	ENSGALG00010028590	2	0.157062224	0.172512029	0.910442158	0.362589373	0	0.778433167	0	0.5	CRA004775;Yang2025_Silkie_Wenchang	PRJNA827465	0.815907913
ENSGALG00010028590	ENSGALG00010028590	2	-0.053846783	0.066691521	-0.807400733	0.419435645	0	0.004734033	0	0.5	CRA004775;PRJNA827465	Yang2025_Silkie_Wenchang	0.838527387
ENSGALG00010028591	GABPB2	2	0.02152412	0.07987381	0.269476558	0.787562979	1.61217E-06	1.00012201	0.012199471	0.5	PRJNA827465;Yang2025_Silkie_Wenchang	CRA004775	0.946919116
ENSGALG00010028591	GABPB2	2	-0.024969483	0.206523258	-0.120903977	0.903767092	0.04739713	1.947521122	48.65267499	0.5	CRA004775;Yang2025_Silkie_Wenchang	PRJNA827465	0.977333734
ENSGALG00010028591	GABPB2	2	-0.076208563	0.097689329	-0.780111438	0.435325284	0	0.817205086	0	0.5	CRA004775;PRJNA827465	Yang2025_Silkie_Wenchang	0.842762754
ENSGALG00010028592	SEMA6C	2	0.04245312	0.302430411	0.140373182	0.888365146	0.1563281	6.83375828	85.37538808	0.5	PRJNA827465;Yang2025_Silkie_Wenchang	CRA004775	0.971315755
ENSGALG00010028592	SEMA6C	2	0.405146323	0.160629431	2.522242157	0.01166094	0	0.383603124	0	1	CRA004775;Yang2025_Silkie_Wenchang	PRJNA827465	0.172816719
ENSGALG00010028592	SEMA6C	2	0.113686968	0.421155568	0.269940556	0.787205986	0.289072097	4.950881518	79.80157682	0.5	CRA004775;PRJNA827465	Yang2025_Silkie_Wenchang	0.946848352
ENSGALG00010028593	DOLK	2	-0.002957738	0.078003772	-0.037917889	0.96975315	0	0.205909365	0	1	PRJNA827465;Yang2025_Silkie_Wenchang	CRA004775	0.991572914
ENSGALG00010028593	DOLK	2	-0.015993005	0.143765027	-0.111244054	0.911425391	0.000525005	1.008910704	0.883200454	0.5	CRA004775;Yang2025_Silkie_Wenchang	PRJNA827465	0.978945861
ENSGALG00010028593	DOLK	2	-0.043250665	0.085450183	-0.506150645	0.6127509	0	0.683926932	0	1	CRA004775;PRJNA827465	Yang2025_Silkie_Wenchang	0.898034557
ENSGALG00010028594	ENSGALG00010028594	2	-0.14839012	0.121209161	-1.224248391	0.220858539	0.009915617	1.266710875	21.05538688	1	PRJNA827465;Yang2025_Silkie_Wenchang	CRA004775	0.734182657
ENSGALG00010028594	ENSGALG00010028594	2	-0.271937592	0.216961205	-1.253397071	0.210069567	0	0.80942692	0	0.5	CRA004775;Yang2025_Silkie_Wenchang	PRJNA827465	0.721625118
ENSGALG00010028594	ENSGALG00010028594	2	-0.083041429	0.08887145	-0.934399388	0.350097913	0	0.089970245	0	0.5	CRA004775;PRJNA827465	Yang2025_Silkie_Wenchang	0.8100729
ENSGALG00010028595	SCNM1	2	-0.023947708	0.060940499	-0.392968682	0.694342613	0	0.11935163	0	1	PRJNA827465;Yang2025_Silkie_Wenchang	CRA004775	0.920728119
ENSGALG00010028595	SCNM1	2	-0.208221588	0.222827399	-0.934452354	0.350070603	0.056099968	1.915645175	47.79826593	1	CRA004775;Yang2025_Silkie_Wenchang	PRJNA827465	0.810057397
ENSGALG00010028595	SCNM1	2	-0.182779698	0.248898695	-0.734353784	0.462733146	0.086208811	2.536358551	60.57308675	1	CRA004775;PRJNA827465	Yang2025_Silkie_Wenchang	0.851123214
ENSGALG00010028596	DDA1	2	0.054949055	0.033457212	1.642368007	0.100513761	0	0.797168038	0	0.5	PRJNA827465;Yang2025_Silkie_Wenchang	CRA004775	0.541385822
ENSGALG00010028596	DDA1	2	-0.062071672	0.109606403	-0.566314287	0.57118015	0	0.162169458	0	1	CRA004775;Yang2025_Silkie_Wenchang	PRJNA827465	0.885354083
ENSGALG00010028596	DDA1	2	0.060797728	0.03468944	1.752639273	0.079665663	0	0.606346159	0	0.5	CRA004775;PRJNA827465	Yang2025_Silkie_Wenchang	0.48542321
ENSGALG00010028597	NUP188	2	-0.1583007	0.147230541	-1.075189284	0.282289992	0.036327862	5.613025791	82.18429708	1	PRJNA827465;Yang2025_Silkie_Wenchang	CRA004775	0.779519046
ENSGALG00010028597	NUP188	2	-0.396242662	0.139646629	-2.837466719	0.004547309	0.010214694	1.196557634	16.42692574	1	CRA004775;Yang2025_Silkie_Wenchang	PRJNA827465	0.098687396
ENSGALG00010028597	NUP188	2	-0.287397865	0.317834182	-0.904238377	0.365869015	0.165399798	4.608122198	78.29918659	1	CRA004775;PRJNA827465	Yang2025_Silkie_Wenchang	0.818968857
ENSGALG00010028598	TMOD4	2	-0.254956414	0.111974244	-2.276920162	0.022790994	0	0.015824437	0	1	PRJNA827465;Yang2025_Silkie_Wenchang	CRA004775	0.257275242
ENSGALG00010028598	TMOD4	2	-0.236221526	0.136228908	-1.734004405	0.082917302	0	0.057899648	0	1	CRA004775;Yang2025_Silkie_Wenchang	PRJNA827465	0.495859127
ENSGALG00010028598	TMOD4	2	-0.25717264	0.176588728	-1.456336669	0.145299573	0	0.080983812	0	1	CRA004775;PRJNA827465	Yang2025_Silkie_Wenchang	0.633748655
ENSGALG00010028599	VPS72	2	-0.201995628	0.063111543	-3.200613045	0.001371356	0	0.068717889	0	1	PRJNA827465;Yang2025_Silkie_Wenchang	CRA004775	0.045407237
ENSGALG00010028599	VPS72	2	-0.188686375	0.127014758	-1.485546857	0.137399021	0	0.120924588	0	1	CRA004775;Yang2025_Silkie_Wenchang	PRJNA827465	0.620141676
ENSGALG00010028599	VPS72	2	-0.214241442	0.069290573	-3.091927725	0.001988613	0	0.006028098	0	1	CRA004775;PRJNA827465	Yang2025_Silkie_Wenchang	0.057852288
ENSGALG00010028600	PSMD4	2	-0.001640068	0.063276271	-0.025919158	0.979321819	0	0.482087809	0	0.5	PRJNA827465;Yang2025_Silkie_Wenchang	CRA004775	0.994390773
ENSGALG00010028600	PSMD4	2	0.036091726	0.168493146	0.214202934	0.830388808	0	0.780558022	0	0.5	CRA004775;Yang2025_Silkie_Wenchang	PRJNA827465	0.957262428
ENSGALG00010028600	PSMD4	2	-0.024240066	0.065326649	-0.371059387	0.7105933	0	0.326710836	0	1	CRA004775;PRJNA827465	Yang2025_Silkie_Wenchang	0.925404854
ENSGALG00010028601	ANO8	2	0.03943321	0.331738225	0.118868455	0.905379574	0.206154029	15.69688215	93.6293081	0.5	PRJNA827465;Yang2025_Silkie_Wenchang	CRA004775	0.978016333
ENSGALG00010028601	ANO8	2	0.351231252	0.097545605	3.600687615	0.000317377	0	0.622311409	0	0.5	CRA004775;Yang2025_Silkie_Wenchang	PRJNA827465	0.017593592
ENSGALG00010028601	ANO8	2	-0.286761899	0.131268237	-2.18454903	0.028921929	0	0.141696161	0	1	CRA004775;PRJNA827465	Yang2025_Silkie_Wenchang	0.289667055
ENSGALG00010028602	ZNF687	2	0.23258036	0.245807869	0.946187609	0.344052905	0.104114149	6.695100659	85.06370478	1	PRJNA827465;Yang2025_Silkie_Wenchang	CRA004775	0.806445383
ENSGALG00010028602	ZNF687	2	0.17877122	0.333925009	0.535363376	0.59239861	0.187715146	6.310958185	84.15454562	0.5	CRA004775;Yang2025_Silkie_Wenchang	PRJNA827465	0.892776493

ENSGALG00010028689	CFAP45	2	0.553383558	0.590310549	0.937444806	0.348329802	0	0.378472907	0	1	CRA004775;Yang2025_Silkie_Wenchang	PRJNA827465	0.809196124
ENSGALG00010028689	CFAP45	2	-0.79532913	1.628580859	-0.488357164	0.625296878	4.769923804	9.917330167	89.91664104	0.5	CRA004775;PRJNA827465	Yang2025_Silkie_Wenchang	0.901367193
ENSGALG00010028692	RALGPS1	2	0.15171041	0.216600873	0.700414582	0.483668432	0.074655754	4.814361499	79.22881362	0.5	PRJNA827465;Yang2025_Silkie_Wenchang	CRA004775	0.85691916
ENSGALG00010028692	RALGPS1	2	0.378627383	0.140489154	2.695064875	0.007037493	0	0.002988122	0	1	CRA004775;Yang2025_Silkie_Wenchang	PRJNA827465	0.128708505
ENSGALG00010028692	RALGPS1	2	0.070359354	0.189191432	0.371895037	0.709971001	0.037125353	1.75207127	42.9246962	0.5	CRA004775;PRJNA827465	Yang2025_Silkie_Wenchang	0.925298042
ENSGALG00010028700	ANGPTL2	2	-0.000810751	0.129414126	-0.00626478	0.995001461	0.003747374	1.095466857	8.71471886	0.5	PRJNA827465;Yang2025_Silkie_Wenchang	CRA004775	0.998242335
ENSGALG00010028700	ANGPTL2	2	0.31811764	0.21127966	1.505670922	0.132151672	0	0.720398914	0	1	CRA004775;Yang2025_Silkie_Wenchang	PRJNA827465	0.608786928
ENSGALG00010028700	ANGPTL2	2	0.162355091	0.335339581	0.484151293	0.628278501	0.151428823	2.529282	60.46308794	0.5	CRA004775;PRJNA827465	Yang2025_Silkie_Wenchang	0.901904489
ENSGALG00010028712	FBXW2	2	-0.056091326	0.053598472	-1.046509793	0.295325726	0	0.699145071	0	1	PRJNA827465;Yang2025_Silkie_Wenchang	CRA004775	0.787628307
ENSGALG00010028712	FBXW2	2	-0.129534296	0.088744067	-1.459638945	0.14438933	0	0.004929447	0	1	CRA004775;Yang2025_Silkie_Wenchang	PRJNA827465	0.631870428
ENSGALG00010028712	FBXW2	2	-0.038479651	0.060442796	-0.636629239	0.524366375	0	0.310178888	0	1	CRA004775;PRJNA827465	Yang2025_Silkie_Wenchang	0.869790047
ENSGALG00010028726	SLC2A8	2	0.114250121	0.066164803	1.726750719	0.084212443	0	0.459141631	0	1	PRJNA827465;Yang2025_Silkie_Wenchang	CRA004775	0.499819032
ENSGALG00010028726	SLC2A8	2	-0.171290796	0.23227407	-0.737451217	0.460847999	0.071008911	2.77884202	64.01378729	0.5	CRA004775;Yang2025_Silkie_Wenchang	PRJNA827465	0.85014477
ENSGALG00010028726	SLC2A8	2	-0.109145315	0.287731713	-0.379330156	0.704442709	0.137814531	5.325120781	81.22108322	0.5	CRA004775;PRJNA827465	Yang2025_Silkie_Wenchang	0.924152671
ENSGALG00010028727	ENSGALG00010028727	2	0.060750078	0.110385669	0.550343884	0.58208353	0	0.52638975	0	0.5	PRJNA827465;Yang2025_Silkie_Wenchang	CRA004775	0.889349595
ENSGALG00010028727	ENSGALG00010028727	2	-0.146014499	0.19386627	-0.75317124	0.451347019	0	0.000152367	0	1	CRA004775;Yang2025_Silkie_Wenchang	PRJNA827465	0.847847413
ENSGALG00010028727	ENSGALG00010028727	2	0.048894064	0.107372659	0.455367913	0.648844575	0	0.739762513	0	0.5	CRA004775;PRJNA827465	Yang2025_Silkie_Wenchang	0.907754186
ENSGALG00010028734	ENSGALG00010028734	2	0.349164274	0.600203123	0.581743514	0.560739457	0.695141632	28.38215714	96.47665963	0.5	PRJNA827465;Yang2025_Silkie_Wenchang	CRA004775	0.88130295
ENSGALG00010028734	ENSGALG00010028734	2	0.943655627	0.169399537	5.570591541	2.53876E-08	0	0.049775082	0	1	CRA004775;Yang2025_Silkie_Wenchang	PRJNA827465	1.14151E-05
ENSGALG00010028734	ENSGALG00010028734	2	0.088682754	0.489748205	0.181078262	0.856306147	0.332259929	2.511995602	60.19101311	0.5	CRA004775;PRJNA827465	Yang2025_Silkie_Wenchang	0.96353738
ENSGALG00010028735	ENSGALG00010028735	2	0.389790055	0.589471465	0.661253476	0.508449772	0.580447424	6.068750091	83.52214237	0.5	PRJNA827465;Yang2025_Silkie_Wenchang	CRA004775	0.864902608
ENSGALG00010028735	ENSGALG00010028735	2	0.659532108	0.363237066	1.815707068	0.069415319	0.118508738	1.794025576	44.25943458	1	CRA004775;Yang2025_Silkie_Wenchang	PRJNA827465	0.453929672
ENSGALG00010028735	ENSGALG00010028735	2	-0.026898096	0.263164825	-0.102210074	0.918589932	0	0.681338874	0	0.5	CRA004775;PRJNA827465	Yang2025_Silkie_Wenchang	0.981033557
ENSGALG00010028738	MIDN	2	0.333464864	0.302528932	1.102257763	0.270349626	0.164367973	9.49007141	89.46267149	1	PRJNA827465;Yang2025_Silkie_Wenchang	CRA004775	0.772430311
ENSGALG00010028738	MIDN	2	0.642614425	0.167915061	3.82702077	0.000129704	0	0.065402938	0	1	CRA004775;Yang2025_Silkie_Wenchang	PRJNA827465	0.009388102
ENSGALG00010028738	MIDN	2	0.058517048	0.089444597	0.654226749	0.512965731	0	0.527537763	0	1	CRA004775;PRJNA827465	Yang2025_Silkie_Wenchang	0.866156104
ENSGALG00010028739	GARNL3	2	-0.087305738	0.067447965	-1.294416187	0.195521702	0.007273712	4.963867482	79.85441788	1	PRJNA827465;Yang2025_Silkie_Wenchang	CRA004775	0.706563945
ENSGALG00010028739	GARNL3	2	-0.148004657	0.045213313	-3.273475161	0.001062337	0	0.849413993	0	0.5	CRA004775;Yang2025_Silkie_Wenchang	PRJNA827465	0.039389662
ENSGALG00010028739	GARNL3	2	-0.019935406	0.038463302	-0.518296797	0.604251207	0	0.092624951	0	0.5	CRA004775;PRJNA827465	Yang2025_Silkie_Wenchang	0.89612415
ENSGALG00010028740	DAZAP1	2	0.02757102	0.040025232	0.688840977	0.490923346	0	0.1130392	0	1	PRJNA827465;Yang2025_Silkie_Wenchang	CRA004775	0.85895707
ENSGALG00010028740	DAZAP1	2	0.103327784	0.10036976	1.029471273	0.303258272	0.007622317	1.366232543	26.80601813	1	CRA004775;Yang2025_Silkie_Wenchang	PRJNA827465	0.792084776
ENSGALG00010028740	DAZAP1	2	0.089686485	0.118161548	0.759015829	0.447843088	0.016705743	1.869407034	46.50710189	1	CRA004775;PRJNA827465	Yang2025_Silkie_Wenchang	0.846601815
ENSGALG00010028742	ENSGALG00010028742	2	0.144159072	0.178177466	0.809075779	0.418471559	0.025240782	1.538714811	35.01069902	1	PRJNA827465;Yang2025_Silkie_Wenchang	CRA004775	0.838158675
ENSGALG00010028742	ENSGALG00010028742	2	0.051894397	0.328867094	0.157797474	0.874616389	0.157295299	3.635498675	72.49345717	0.5	CRA004775;Yang2025_Silkie_Wenchang	PRJNA827465	0.970159044
ENSGALG00010028742	ENSGALG00010028742	2	-0.073664454	0.131721894	-0.559242294	0.575996373	0.004146278	1.119203561	10.65074894	0.5	CRA004775;PRJNA827465	Yang2025_Silkie_Wenchang	0.886945049
ENSGALG00010028743	ARRDC1	2	0.061375166	0.063820405	0.961685637	0.336207539	0	0.825378827	0	1	PRJNA827465;Yang2025_Silkie_Wenchang	CRA004775	0.803727281
ENSGALG00010028743	ARRDC1	2	0.040265411	0.151253629	0.266211207	0.790076553	0.021238127	1.6237677	38.41483605	0.5	CRA004775;Yang2025_Silkie_Wenchang	PRJNA827465	0.947390191
ENSGALG00010028743	ARRDC1	2	-0.004322011	0.076047494	-0.056833052	0.954678185	0	0.710061072	0	0.5	CRA004775;PRJNA827465	Yang2025_Silkie_Wenchang	0.987987175
ENSGALG00010028744	HOMER3	2	-0.043268902	0.06499272	-0.665749975	0.505570924	0	0.008692645	0	1	PRJNA827465;Yang2025_Silkie_Wenchang	CRA004775	0.863927652
ENSGALG00010028744	HOMER3	2	0.041053891	0.191575616	0.214296013	0.830316227	0.034524683	1.399756374	28.55899653	0.5	CRA004775;Yang2025_Silkie_Wenchang	PRJNA827465	0.9572455
ENSGALG00010028744	HOMER3	2	0.039535423	0.176811551	0.223602038	0.823066978	0.030302698	1.358283991	26.37769374	0.5	CRA004775;PRJNA827465	Yang2025_Silkie_Wenchang	0.955822987
ENSGALG00010028745	ENSGALG00010028745	2	0.055678841	0.064939016	0.857401983	0.391222764	0	0.024488471	0	1	PRJNA827465;Yang2025_Silkie_Wenchang	CRA004775	0.829251057
ENSGALG00010028745	ENSGALG00010028745	2	-0.103521869	0.186838166	-0.554072388	0.579529306	0	0.886397266	0	0.5	CRA004775;Yang2025_Silkie_Wenchang	PRJNA827465	0.887969027
ENSGALG00010028745	ENSGALG00010028745	2	-0.009762818	0.124671946	-0.078308059	0.937583007	0.015072118	1.459061952	31.46281427	0.5	CRA004775;PRJNA827465	Yang2025_Silkie_Wenchang	0.985157731
ENSGALG00010028746	MYDGF	2	-0.106082567	0.31407961	-0.337756937	0.735546364	0.159818757	4.466666899	77.61194146	0.5	PRJNA827465;Yang2025_Silkie_Wenchang	CRA004775	0.933592889
ENSGALG00010028746	MYDGF	2	-0.424271583	0.201099916	-2.109755153	0.034879451	0	0.097760002	0	1	CRA004775;Yang2025_Silkie_Wenchang	PRJNA827465	0.321626212
ENSGALG00010028746	MYDGF	2	-0.04010222	0.248490573	-0.161383266	0.871791546	0.093598438	3.381698801	70.42906365	0.5	CRA004775;PRJNA827465	Yang2025_Silkie_Wenchang	0.969462602
ENSGALG00010028748	PIAS3	2	0.42749639	0.257126971	1.662588674	0.0963948	0.118543161	9.619772974	89.60474428	1	PRJNA827465;Yang2025_Silkie_Wenchang	CRA004775	0.530885356
ENSGALG00010028748	PIAS3	2	0.453431066	0.280824378	1.61464282	0.106388067	0.118323053	3.690706265	72.90491499	1	CRA004775;Yang2025_Silkie_Wenchang	PRJNA827465	0.553237654
ENSGALG00010028748	PIAS3	2	0.168822462	0.096736837	1.745172434	0.080954858	0	0.037362974	0	1	CRA004775;PRJNA827465	Yang2025_Silkie_Wenchang	0.489847627
ENSGALG00010028750	OTUD7B	2	0.118669569	0.074047241	1.602619734	0.109018634	0.004767886	1.70990402	41.51718529	1	PRJNA827465;Yang2025_Silkie_Wenchang	CRA004775	0.55900318
ENSGALG00010028750	OTUD7B	2	0.213790827	0.094019062	2.273909372	0.022971436	0	0.033409532	0	1	CRA004775;Yang2025_Silkie_Wenchang	PRJNA827465	0.258400479
ENSGALG00010028750	OTUD7B	2	0.063024307	0.064858713	0.97171689	0.331191412	0	0.231826057	0	1	CRA004775;PRJNA827465	Yang2025_Silkie_Wenchang	0.801725552
ENSGALG00010028751	EHMT1	2	-0.09761653	0.039481059	-2.472490167	0.01341754	0	0.049400778	0	1	PRJNA827465;Yang2025_Silkie_Wenchang	CRA004775	0.189489613
ENSGALG00010028751	EHMT1	2	-0.168752094	0.116129358	-1.45313895	0.146185179	0.009310242	1.256787489	20.43205326	1	CRA004775;Yang2025_Silkie_Wenchang	PRJNA827465	0.634916196
ENSGALG00010028751	EHMT1	2	-0.156491923	0.128408126	-1.218707321	0.222955298	0.018528642	1.550149414	35.49008948	1	CRA004775;PRJNA827465	Yang2025_Silkie_Wenchang	0.736458649
ENSGALG00010028752	MTMR11	2	0.076247123	0.114357608	0.6667429	0.504936371	0	0.160048506	0	1	PRJNA827465;Yang2025_Silkie_Wenchang	CRA004775	0.863712155
ENSGALG00010028752	MTMR11	2	0.034136587	0.164757311	0.207193156	0.835859012	0	0.041180025	0	1	CRA004775;Yang2025_Silkie_Wenchang	PRJNA827465	0.958339923
ENSGALG00010028752	MTMR11	2	0.105140083	0.127910636	0.821980768	0.411087843	0	0.004661294	0	1	CRA004775;PRJNA827465	Yang2025_Silkie_Wenchang	0.835128673
ENSGALG00010028753	SV2A	2	0.479540676	0.5771207	0.83091921	0.406019271	0.379230775	2.272680171	55.99908809	0.5	PRJNA827465;Yang2025_Silkie_Wenchang	CRA004775	0.833434763
ENSGALG00010028753	SV2A	2	0.498574398	0.669576436	0.744611625	0.456506538	0.469873606	2.095311277	52.27439422	0.5	CRA004775;Yang2025_Silkie_Wenchang	PRJNA827465	0.849515887
ENSGALG00010028753	SV2A	2	-0.082588095	0.382274232	-0.216044108	0.828953366	0	0.045747982	0	1	CRA004775;PRJNA827465	Yang2025_Silkie_Wenchang	0.956840134
ENSGALG00010028760	SSR2	2	-0.13619346	0.145356652	-0.93696063	0.34877881	0.028498978	2.379881385	57.98109913	1	PRJNA827465;Yang2025_Silkie_Wenchang	CRA004775	0.809333419
ENSGALG00010028760	SSR2	2	-0.37760063	0.13370548	-2.824122323	0.004741029	0	0.031721833	0	1	CRA004775;Yang2025_Silkie_Wenchang	PRJNA827465	0.101435303
ENSGALG00010028760	SSR2	2	-0.176301372	0.175595517	-1.004019779	0.31536908	0.047678167	3.70849819	73.03490662	1	CRA004775;PRJNA827465	Yang2025_Silkie_Wenchang	0.796795511
ENSGALG00010028761	CTSS	2	-0.253454098	0.288917939	-0.877252894	0.380349292	0.136717891	5.301981073	81.13912543	0.5	PRJNA827465;Yang2025_Silkie_Wenchang	CRA004775	0.824392515
ENSGALG00010028761	CTSS	2	-0.459553564	0.205237046	-2.23913554	0.025147098	0.0067213	1.059803478	5.642883726	1	CRA004775;Yang2025_Silkie_Wenchang	PRJNA827465	0.269968973
ENSGALG00010028761	CTSS	2	0.001455685	0.119781135	0.012152873	0.990303649	0	0.044396327	0	0.5	CRA004775;PRJNA827465	Yang2025_Silkie_Wenchang	0.997468525
ENSGALG00010028763	CTSK	2	0.432196978	0.12987649	3.327753751	0.000875492	0.013551493	1.534507481	34.83251058	1	PRJNA827465;Yang2025_Silkie_Wenchang	CRA004775	0.034721966
ENSGALG00010028763	CTSK	2	0.635156158	0.191015662	3.325152246	0.000883703	0	0.052515673	0	1	CRA004775;Yang2025_Silkie_Wenchang	PRJNA827465	0.034801769
ENSGALG00010028763	CTSK	2	0.353778359	0.104474038	3.386280125	0.00070847	0	0.405120245	0	1	CRA004775;PRJNA827465	Yang2025_Silkie_Wenchang	0.029780755
ENSGALG00010028764	ARNT	2	-0.071924059	0.040435883	-1.778718639	0.075285902	0	0.248906329	0	1	PRJNA827465;Yang2025_Silkie_Wenchang	CRA004775	0.473509649
ENSGALG00010028764	ARNT	2	-0.059085596	0.066967488	-0.882302715	0.377613129	0	0.443891557	0	1	CRA004775;Yang2025_Silkie_Wenchang	PRJNA827465	0.82325159
ENSGALG00010028764	ARNT	2	-0.091483318	0.047541581	-1.924280095	0.054319484	0	0.222070535	0	1	CRA004775;PRJNA827465	Yang2025_Silkie_Wenchang	0.40381596
ENSGALG00010028768	NTNG2	2	0.319849634	0.271652154	1.177423512	0.23902651	0.118493174	4.928069146	79.70807693	1	PRJNA827465;Yang2025_Silkie_Wenchang	CRA004775	0.751233576
ENSGALG00010028768	NTNG2	2	0.305735773	0.386902645	0.790213706	0.429402973	0.202939632	2.820904363	64.55037564	0.5	CRA004775;Yang2025_Silkie_Wenchang	PRJNA827465	0.842012722
ENSGALG00010028768	NTNG2	2	0.04833623	0.126487612	0.382142007	0.702356035	0	0.310336032	0	0.5	CRA004775;PRJNA827465	Yang2025_Silkie_Wenchang	0.923167672
ENSGALG00010028771	SETX	2	0.181223429	0.100563972	1.802071117	0.071534217	0.011522154	1.958835253	48.94925448	1	PRJNA827465;Yang2025_Silkie_Wenchang	CRA004775	0.46165999
ENSGALG00010028771	SETX	2	0.308701617	0.126458054	2.44113845	0.014641041	0	0.097044594	0	1	CRA004775;Yang2025_Silkie_Wenchang	PRJNA827465	0.199389056
ENSGALG00010028771	SETX	2	0.1189242	0.055181085	2.155162415	0.031149127	0	0.213247021	0	1	CRA004775;PRJNA827465	Yang2025_Silkie_Wenchang	0.30191224
ENSGALG00010028772	TTF1	2	-0.004454737	0.052565024	-0.084747171	0.932462394	0.001050733	1.145049669	12.66754389	0.5	PRJNA827465;Yang2025_Silkie_Wenchang	CRA004775	0.984224753
ENSGALG00010028772	TTF1	2	0.015141545	0.147043562	0.102973195	0.917984245	0.024275657	2.178230123	54.09116836	0.5	CRA004775;Yang2025_Silkie_Wenchang	PRJNA827465	0.981033557
ENSGALG00010028772	TTF1	2	0.035982206	0.047558041	0.756595626	0.449292163	0	0.922487672	0	1	CRA004775;PRJNA827465	Yang2025_Silkie_Wenchang	0.847263197
ENSGALG00010028782	MLLT11	2	-0.665994481	0.425977656	-1.563449332	0.117946944	0.293577315	5.191099378	80.73625783	1	PRJNA827465;Yang2025_Silkie_Wenchang	CRA004775	0.577725418
ENSGALG00010028782	MLLT11	2	-0.885644794	0.317372657	-2.790551656	0.00526183	0.065492477	1.40843288	28.99910147	1	CRA004775;Yang2025_Silkie_Wenchang	PRJNA827465	0.108331963
ENSGALG00010028782	MLLT11	2	-0.29225649	0.209458689	-1.395294184	0.162927145	0	0.113182071	0	1	CRA004775;PRJNA827465	Yang2025_Silkie_Wenchang	0.660959169
ENSGALG00010028783	DDX31	2	-0.286049416	0.174773259	-1.636688684	0.101695535	0.054795463	9.626929964	89.61247247	1	PRJNA827465;Yang2025_Silkie_Wenchang	CRA004775	0.543700483
ENSGALG00010028783	DDX31	2	-0.439695636	0.08407187	-5.229997095	1.69513E-07	0	0.620830439	0	1	CRA004775;Yang2025_Silkie_Wenchang	PRJNA827465	5.46095E-05
ENSGALG00010028783	DDX31	2	-0.12920046	0.064353732	-2.007660732	0.044679356	0	0.444960463	0	1	CRA004775;PRJNA827465	Yang2025_Silkie_Wenchang	0.364322144
ENSGALG00010028786	GTF3C4	2	-0.099390703	0.090530472	-1.097870148	0.272261195	0.010675307	2.484320906	59.74755123	1	PRJNA827465;Yang2025_Silkie_Wenchang	CRA004775	0.773331629
ENSGALG00010028786	GTF3C4	2	-0.233665992	0.103802027	-2.25107351	0.024380882	0	0.102156804	0	1	CRA004775;Yang2025_Silkie_Wenchang	PRJNA827465	0.267068507
ENSGALG00010028786	GTF3C4	2	-0.038822253	0.044818319	-0.866213946	0.386372847	0	0.953431147	0	1	CRA004775;PRJNA827465	Yang2025_Silkie_Wenchang	0.826900117
ENSGALG00010028806	SPACA9	2	0.423108213	0.186259738	2.271603173	0.023110489	0	0.737226976	0	1	PRJNA827465;Yang2025_Silkie_Wenchang	CRA004775	0.259517586
ENSGALG00010028806	SPACA9	2	0.618498787	0.255251463	2.423095955	0.015388861	0	0.061692093	0	1	CRA004775;Yang2025_Silkie_Wenchang	PRJNA827465	0.205165677
ENSGALG00010028806	SPACA9	2	0.330811824	0.240184677	1.377322764	0.16841248	0	0.449195468	0	1	CRA004775;PRJNA827465	Yang2025_Silkie_Wenchang	0.669138264
ENSGALG00010028818	TSC1	2	0.014210053	0.089535461	0.158708661	0.873898414	0.00766274	1.863809875	46.34645875	0.5	PRJNA827465;Yang2025_Silkie_Wenchang	CRA004775	0.970002807
ENSGALG00010028818	TSC1	2	0.0997403	0.099354044	1.003887671	0.31543276	0	0.239787406	0	0.5	CRA004775;Yang2025_Silkie_Wenchang	PRJNA827465	0.796883862
ENSGALG00010028818	TSC1	2	-0.056559198	0.073922532	-0.765114456	0.444203393	0	0.035458566	0	1	CRA004775;PRJNA827465	Yang2025_Silkie_Wenchang	0.846013379
ENSGALG00010028823	GFI1B	2	0.111361668	0.336759823	0.330685732	0.740881881	0.177183414	4.546426493	78.00470322	0.5	PRJNA827465;Yang2025_Silkie_Wenchang	CRA004775	0.935305964
ENSGALG00010028823	GFI1B	2	-0.094463916	0.214701421	-0.439978064	0.659952995	0.00821955	1.082636369	7.63288315	0.5	CRA004775;Yang2025_Silkie_Wenchang	PRJNA827465	0.911773501
ENSGALG00010028823	GFI1B	2	0.387777746	0.178630012	2.170843195	0.029943028	0	0.242657027	0	1	CRA004775;PRJNA827465	Yang2025_Silkie_Wenchang	0.295167651
ENSGALG00010028829	GTF3C5	2	-0.059285659	0.057371403	-1.033366039	0.301432633	0	0.028939364	0	1	PRJNA827465;Yang2025_Silkie_Wenchang	CRA004775	0.791238013
ENSGALG00010028829	GTF3C5	2	-0.078560742	0.103144545	-0.761656778	0.446264878	0	0.001832571	0	1	CRA004775;Yang2025_Silkie_Wenchang	PRJNA827465	0.846054146
ENSGALG00010028829	GTF3C5	2	-0.056279951	0.063517711	-0.886051306	0.375589881	0	0.019859084	0	1	CRA004775;PRJNA827465	Yang2025_Silkie_Wenchang	0.822974013
ENSGALG00010028833	GBGT1	2	-0.126095672	0.076089296	-1.657206453	0.097477742	5.24927E-05	1.004416351	0.439693292	1	PRJNA827465;Yang2025_Silkie_Wenchang	CRA004775	0.533219726
ENSGALG00010028833	GBGT1	2	-0.243515312	0.112538761	-2.163835018	0.030477014	0	0.553998495	0	1	CRA004775;Yang2025_Silkie_Wenchang	PRJNA827465	0.297646478
ENSGALG00010028833	GBGT1	2	-0.14084076	0.169361639	-0.831597762	0.405636026	0.025046857	1.341666208	25.46581305	1	CRA004775;PRJNA827465	Yang2025_Silkie_Wenchang	0.833257341
ENSGALG00010028849	ENSGALG00010028849	2	0.219629977	0.215162301	1.020764216	0.307366162	0	0.069881729	0	1	PRJNA827465;Yang2025_Silkie_Wenchang	CRA004775	0.792675978
ENSGALG00010028849	ENSGALG00010028849	2	0.150571162	0.291529227	0.516487364	0.605514062	0	0.00558292	0	1	CRA004775;Yang2025_Silkie_Wenchang	PRJNA827465	0.896474603
ENSGALG00010028849	ENSGALG00010028849	2	0.251328365	0.276751911	0.90813597	0.363806389	0	0.044404929	0	1	CRA004775;PRJNA827465	Yang2025_Silkie_Wenchang	0.817042253
ENSGALG00010028854	RPL7A	2	-0.114304379	0.064842013	-1.762813562	0.077931939	0	0.139030237	0	1	PRJNA827465;Yang2025_Silkie_Wenchang	CRA004775	0.480724545
ENSGALG00010028854	RPL7A	2	-0.210036807	0.16656245	-1.261009354	0.207305477	0	0.031715027	0	1	CRA004775;Yang2025_Silkie_Wenchang	PRJNA827465	0.719377239
ENSGALG00010028854	RPL7A	2	-0.114484497	0.066289981	-1.727025638	0.084163059	0	0.250097021	0	1	CRA004775;PRJNA827465	Yang2025_Silkie_Wenchang	0.499744053
ENSGALG00010028855	SURF6	2	0.392732229	0.418709189	0.937959422	0.348265264	0.326744668	13.96274251	92.83808321	0.5	PRJNA827465;Yang2025_Silkie_Wenchang	CRA004775	0.80902788
ENSGALG00010028855	SURF6	2	0.409737973	0.438202546	0.935042427	0.349766435	0.328597762	6.904474238	85.51663797	0.5	CRA004775;Yang2025_Silkie_Wenchang	PRJNA827465	0.809903518
ENSGALG00010028855	SURF6	2	-0.003138608	0.05902248	-0.053176491	0.957591286	0	0.020770498	0	1	CRA004775;PRJNA827465	Yang2025_Silkie_Wenchang	0.988770061
ENSGALG00010028856	ETV3	2	0.013606153	0.082135494	0.165654971	0.868428484	0.007539229	2.192057341	54.3807554	0.5	PRJNA827465;Yang2025_Silkie_Wenchang	CRA004775	0.968167735
ENSGALG00010028856	ETV3	2	0.1203894	0.087111572	1.382013857	0.166967447	0	0.097523193	0	1	CRA004775;Yang2025_Silkie_Wenchang	PRJNA827465	0.666022689
ENSGALG00010028856	ETV3	2	-0.041251057	0.061782891	-0.66767768	0.50433936	0	0.915035485	0	0.5	CRA004775;PRJNA827465	Yang2025_Silkie_Wenchang	0.863512504

ENSGALG00010028857	MED22	2	-0.366527169	0.334605833	-1.095399819	0.273341513	0.20965672	15.35770272	93.48860947	1	PRJNA827465;Yang2025_Silkie_Wenchang	CRA004775	0.774113093
ENSGALG00010028857	MED22	2	-0.403615732	0.317759739	-1.270191537	0.204016411	0.174240856	7.267167537	86.23948058	1	CRA004775;Yang2025_Silkie_Wenchang	PRJNA827465	0.717254253
ENSGALG00010028857	MED22	2	-0.04991285	0.070679291	-0.706187757	0.480071383	0	0.030314047	0	1	CRA004775;PRJNA827465	Yang2025_Silkie_Wenchang	0.856362982
ENSGALG00010028858	ENSGALG00010028858	2	0.000316654	0.453241278	0.000698644	0.999442563	0.111389592	1.371382634	27.08089083	0.5	PRJNA827465;Yang2025_Silkie_Wenchang	CRA004775	0.999889665
ENSGALG00010028858	ENSGALG00010028858	2	0.547189111	0.465867828	1.174558701	0.240171314	0	0.063048963	0	1	CRA004775;Yang2025_Silkie_Wenchang	PRJNA827465	0.75252297
ENSGALG00010028858	ENSGALG00010028858	2	-0.037801492	0.55150674	-0.068542212	0.945354019	0.178165805	1.359942898	26.46750082	0.5	CRA004775;PRJNA827465	Yang2025_Silkie_Wenchang	0.986321197
ENSGALG00010028859	SURF1	2	-0.121876074	0.116148904	-1.049308866	0.294035987	0.013964171	1.83379206	45.46819012	1	PRJNA827465;Yang2025_Silkie_Wenchang	CRA004775	0.787324026
ENSGALG00010028859	SURF1	2	-0.209831539	0.132942541	-1.578362634	0.114482324	0	0.640743002	0	1	CRA004775;Yang2025_Silkie_Wenchang	PRJNA827465	0.570438899
ENSGALG00010028859	SURF1	2	-0.04392873	0.073398599	-0.598495491	0.549509367	0	0.011790409	0	1	CRA004775;PRJNA827465	Yang2025_Silkie_Wenchang	0.878554604
ENSGALG00010028860	SURF2	2	0.001009197	0.059537185	0.016950707	0.98647594	0	0.001329122	0	1	PRJNA827465;Yang2025_Silkie_Wenchang	CRA004775	0.99618774
ENSGALG00010028860	SURF2	2	0.0301695	0.145457294	0.207411391	0.835688587	0	0.075517857	0	1	CRA004775;Yang2025_Silkie_Wenchang	PRJNA827465	0.958339923
ENSGALG00010028860	SURF2	2	0.004768209	0.062137888	0.076735933	0.938833619	0	0.111050273	0	1	CRA004775;PRJNA827465	Yang2025_Silkie_Wenchang	0.985349045
ENSGALG00010028861	SURF4	2	-0.206423191	0.197682096	-1.04421794	0.296384569	0.07407913	18.65735274	94.64018281	1	PRJNA827465;Yang2025_Silkie_Wenchang	CRA004775	0.788267543
ENSGALG00010028861	SURF4	2	-0.427175664	0.081885137	-5.216766793	1.82073E-07	0	0.437787326	0	1	CRA004775;Yang2025_Silkie_Wenchang	PRJNA827465	5.81791E-05
ENSGALG00010028861	SURF4	2	-0.243221725	0.292349353	-0.83195575	0.405433922	0.135817507	3.932344055	74.56987522	1	CRA004775;PRJNA827465	Yang2025_Silkie_Wenchang	0.833112102
ENSGALG00010028865	STKLD1	2	0.05059805	0.121318369	0.417068333	0.676628427	0	0.22873885	0	0.5	PRJNA827465;Yang2025_Silkie_Wenchang	CRA004775	0.916786558
ENSGALG00010028865	STKLD1	2	0.030864928	0.1428858	0.216011165	0.828979045	0	0.165775336	0	0.5	CRA004775;Yang2025_Silkie_Wenchang	PRJNA827465	0.956840134
ENSGALG00010028865	STKLD1	2	0.116819967	0.149363747	0.782117277	0.434145654	0	0.002273121	0	1	CRA004775;PRJNA827465	Yang2025_Silkie_Wenchang	0.842762754
ENSGALG00010028867	REXO4	2	0.073566478	0.178205851	0.412817409	0.679740396	0.053000839	5.801212575	82.7622245	0.5	PRJNA827465;Yang2025_Silkie_Wenchang	CRA004775	0.917701868
ENSGALG00010028867	REXO4	2	0.111605257	0.198547881	0.562107518	0.574042767	0.047265435	2.313758083	56.78026985	0.5	CRA004775;Yang2025_Silkie_Wenchang	PRJNA827465	0.886081433
ENSGALG00010028867	REXO4	2	-0.093522072	0.06917039	-1.35205355	0.176358185	0	0.041660216	0	1	CRA004775;PRJNA827465	Yang2025_Silkie_Wenchang	0.681691731
ENSGALG00010028869	ADAMTS13	2	-0.061185809	0.153670364	-0.398162711	0.690510251	0	0.63430317	0	0.5	PRJNA827465;Yang2025_Silkie_Wenchang	CRA004775	0.919979266
ENSGALG00010028869	ADAMTS13	2	-0.055528455	0.163163873	-0.340323222	0.733613131	0	0.869607107	0	0.5	CRA004775;Yang2025_Silkie_Wenchang	PRJNA827465	0.933324601
ENSGALG00010028869	ADAMTS13	2	0.118137589	0.18390697	0.642376897	0.520628497	0	0.039798276	0	1	CRA004775;PRJNA827465	Yang2025_Silkie_Wenchang	0.869331539
ENSGALG00010028870	CACFD1	2	-0.011852347	0.329476642	-0.035973254	0.971303686	0.176595955	5.353936099	81.32215287	0.5	PRJNA827465;Yang2025_Silkie_Wenchang	CRA004775	0.991955348
ENSGALG00010028870	CACFD1	2	-0.409065232	0.187480399	-2.181909335	0.029116225	0	0.460832743	0	1	CRA004775;Yang2025_Silkie_Wenchang	PRJNA827465	0.290337858
ENSGALG00010028870	CACFD1	2	-0.104548618	0.48436437	-0.215847045	0.829106976	0.37143946	4.431484122	77.43419648	0.5	CRA004775;PRJNA827465	Yang2025_Silkie_Wenchang	0.956860127
ENSGALG00010028871	PIP5K1A	2	-0.107238651	0.053959697	-1.987384239	0.046879833	0	0.0197186	0	1	PRJNA827465;Yang2025_Silkie_Wenchang	CRA004775	0.37376485
ENSGALG00010028871	PIP5K1A	2	-0.158858237	0.110853312	-1.433049076	0.151843813	0	0.29674167	0	1	CRA004775;Yang2025_Silkie_Wenchang	PRJNA827465	0.643294424
ENSGALG00010028871	PIP5K1A	2	-0.114539634	0.057405091	-1.995287033	0.046011583	0	0.48465117	0	1	CRA004775;PRJNA827465	Yang2025_Silkie_Wenchang	0.369999825
ENSGALG00010028872	SLC2A6	2	-0.407177999	0.195276061	-2.085140374	0.03705656	0	0.372570153	0	1	PRJNA827465;Yang2025_Silkie_Wenchang	CRA004775	0.332064293
ENSGALG00010028872	SLC2A6	2	-0.416330325	0.308852882	-1.347989122	0.177661881	0	0.648768451	0	1	CRA004775;Yang2025_Silkie_Wenchang	PRJNA827465	0.683951879
ENSGALG00010028872	SLC2A6	2	-0.300454202	0.203620934	-1.475556545	0.140062978	0	0.181213333	0	1	CRA004775;PRJNA827465	Yang2025_Silkie_Wenchang	0.623792706
ENSGALG00010028873	TMEM8C	2	-0.034021232	0.245973683	-0.138312488	0.889993456	0.068129496	2.266561182	55.88029973	0.5	PRJNA827465;Yang2025_Silkie_Wenchang	CRA004775	0.973310644
ENSGALG00010028873	TMEM8C	2	-0.252181298	0.241934314	-1.042354406	0.297247397	0	0.431894958	0	0.5	CRA004775;Yang2025_Silkie_Wenchang	PRJNA827465	0.788466926
ENSGALG00010028873	TMEM8C	2	0.195296656	0.201313083	0.97011408	0.331989632	0	0.005330672	0	1	CRA004775;PRJNA827465	Yang2025_Silkie_Wenchang	0.802002658
ENSGALG00010028874	CRPL1	2	5.846620251	1.047875389	5.579499541	2.41212E-08	1.850969823	6.322319463	84.18302008	1	PRJNA827465;Yang2025_Silkie_Wenchang	CRA004775	1.10236E-05
ENSGALG00010028874	CRPL1	2	6.265810443	0.575836292	10.88123574	1.4163E-27	0.411755349	2.634064333	62.0358551	1	CRA004775;Yang2025_Silkie_Wenchang	PRJNA827465	2.14095E-24
ENSGALG00010028874	CRPL1	2	5.33545918	0.461422419	11.56306882	6.34017E-31	0.117931089	1.359056355	26.41953393	1	CRA004775;PRJNA827465	Yang2025_Silkie_Wenchang	1.08343E-27
ENSGALG00010028875	DUSP23	2	0.265268373	0.280987037	0.944059113	0.345139442	0.126258505	4.576263487	78.14811136	1	PRJNA827465;Yang2025_Silkie_Wenchang	CRA004775	0.806961063
ENSGALG00010028875	DUSP23	2	0.180670839	0.453996208	0.397956715	0.690662093	0.315896491	4.149235935	75.8991772	0.5	CRA004775;Yang2025_Silkie_Wenchang	PRJNA827465	0.920079051
ENSGALG00010028875	DUSP23	2	-0.001090927	0.101023015	-0.010798796	0.991383974	0	0.778116608	0	0.5	CRA004775;PRJNA827465	Yang2025_Silkie_Wenchang	0.99755571
ENSGALG00010028876	ADAMTSL2	2	0.254160207	0.442511265	0.574358528	0.565725076	0.362875454	13.61592646	92.65565951	0.5	PRJNA827465;Yang2025_Silkie_Wenchang	CRA004775	0.883487517
ENSGALG00010028876	ADAMTSL2	2	0.034126507	0.692621766	0.049271491	0.960702939	0.89071613	13.65144399	92.67476759	0.5	CRA004775;Yang2025_Silkie_Wenchang	PRJNA827465	0.989520168
ENSGALG00010028876	ADAMTSL2	2	-0.354243707	0.236555825	-1.497505745	0.134261712	0.056876689	1.826287687	45.24411421	1	CRA004775;PRJNA827465	Yang2025_Silkie_Wenchang	0.613805754
ENSGALG00010028878	IGSF9	2	0.163698402	0.128062963	1.278264989	0.201156008	0.022163699	3.036015746	67.06209442	1	PRJNA827465;Yang2025_Silkie_Wenchang	CRA004775	0.713804636
ENSGALG00010028878	IGSF9	2	0.046868609	0.267552724	0.175175214	0.86094196	0.123642467	7.251060475	86.20891381	0.5	CRA004775;Yang2025_Silkie_Wenchang	PRJNA827465	0.965299305
ENSGALG00010028878	IGSF9	2	-0.060990316	0.135528416	-0.450018661	0.652696985	0.021866562	2.293176534	56.3923673	0.5	CRA004775;PRJNA827465	Yang2025_Silkie_Wenchang	0.909572411
ENSGALG00010028881	SARDH	2	-0.211465227	0.151846571	-1.392624316	0.163733426	0.031126211	3.034624519	67.046994	1	PRJNA827465;Yang2025_Silkie_Wenchang	CRA004775	0.66212726
ENSGALG00010028881	SARDH	2	-0.35790095	0.133957771	-2.671744586	0.007545806	0	0.294422964	0	1	CRA004775;Yang2025_Silkie_Wenchang	PRJNA827465	0.134120107
ENSGALG00010028881	SARDH	2	-0.071503441	0.105262258	-0.679288502	0.496935508	0	5.90623E-06	0	1	CRA004775;PRJNA827465	Yang2025_Silkie_Wenchang	0.860939846
ENSGALG00010028884	VAV2	2	-0.056922528	0.144218765	-0.394695707	0.693067471	0.036591295	8.231754762	87.85192187	0.5	PRJNA827465;Yang2025_Silkie_Wenchang	CRA004775	0.920631027
ENSGALG00010028884	VAV2	2	-0.190085668	0.078847642	-2.410797711	0.015917701	0	0.808058217	0	0.5	CRA004775;Yang2025_Silkie_Wenchang	PRJNA827465	0.208471156
ENSGALG00010028884	VAV2	2	0.08124919	0.058938956	1.412464613	0.157813203	0	0.010151748	0	1	CRA004775;PRJNA827465	Yang2025_Silkie_Wenchang	0.651321256
ENSGALG00010028885	BRD3	2	-0.089758054	0.057762854	-1.553906142	0.120206806	0	0.314933984	0	1	PRJNA827465;Yang2025_Silkie_Wenchang	CRA004775	0.58147546
ENSGALG00010028885	BRD3	2	-0.098703066	0.140357077	-0.703228285	0.481913495	0	0.89292299	0	0.5	CRA004775;Yang2025_Silkie_Wenchang	PRJNA827465	0.85691916
ENSGALG00010028885	BRD3	2	-0.067743423	0.060419038	-1.121226448	0.262191485	0	0.529708559	0	0.5	CRA004775;PRJNA827465	Yang2025_Silkie_Wenchang	0.768363052
ENSGALG00010028887	SF3B4	2	-0.026108319	0.065525605	-0.398444537	0.690302534	0	0.186364997	0	0.5	PRJNA827465;Yang2025_Silkie_Wenchang	CRA004775	0.919979266
ENSGALG00010028887	SF3B4	2	-0.098296093	0.114931837	-0.854472489	0.392843246	0.003858034	1.163556156	14.05657602	0.5	CRA004775;Yang2025_Silkie_Wenchang	PRJNA827465	0.830058498
ENSGALG00010028887	SF3B4	2	-0.072545615	0.064492363	-1.12487141	0.260643529	0.000315066	1.027021133	2.631020116	1	CRA004775;PRJNA827465	Yang2025_Silkie_Wenchang	0.76772293
ENSGALG00010028888	WDR5B	2	-0.148623775	0.044672546	-3.32690013	0.00087799	0	0.497729966	0	1	PRJNA827465;Yang2025_Silkie_Wenchang	CRA004775	0.03476905
ENSGALG00010028888	WDR5B	2	-0.177279341	0.080255907	-2.208925755	0.027179805	0	0.432009484	0	1	CRA004775;Yang2025_Silkie_Wenchang	PRJNA827465	0.281339977
ENSGALG00010028888	WDR5B	2	-0.125989238	0.049577354	-2.541265855	0.01104519	0	0.101152856	0	1	CRA004775;PRJNA827465	Yang2025_Silkie_Wenchang	0.167157912
ENSGALG00010028889	FUBP3	2	0.164647507	0.178257904	0.923647723	0.355669758	0.058002316	11.01819123	90.92410016	0.5	PRJNA827465;Yang2025_Silkie_Wenchang	CRA004775	0.812301302
ENSGALG00010028889	FUBP3	2	0.217142547	0.153840431	1.41147906	0.158103406	0.032747669	3.151773995	68.26982322	1	CRA004775;Yang2025_Silkie_Wenchang	PRJNA827465	0.651971268
ENSGALG00010028889	FUBP3	2	0.000947928	0.041465134	0.022860853	0.981761267	0	0.099591577	0	0.5	CRA004775;PRJNA827465	Yang2025_Silkie_Wenchang	0.99503081
ENSGALG00010028891	SLC31A2	2	0.742791498	0.337448471	2.201199773	0.027721884	0.050866072	1.265956653	21.00835385	1	PRJNA827465;Yang2025_Silkie_Wenchang	CRA004775	0.284073056
ENSGALG00010028891	SLC31A2	2	0.25129162	0.88201417	0.284906557	0.775715732	1.381892393	8.789281044	88.62250513	0.5	CRA004775;Yang2025_Silkie_Wenchang	PRJNA827465	0.943815265
ENSGALG00010028891	SLC31A2	2	-0.068333339	0.53488295	-0.127753818	0.898343798	0.454131423	4.835471024	79.31949142	0.5	CRA004775;PRJNA827465	Yang2025_Silkie_Wenchang	0.97575249
ENSGALG00010028892	NIBAN2	2	0.135548436	0.066765252	2.030224273	0.042333748	0	0.162826609	0	1	PRJNA827465;Yang2025_Silkie_Wenchang	CRA004775	0.356207082
ENSGALG00010028892	NIBAN2	2	0.157389414	0.133089588	1.182582475	0.236974662	0	0.8867262	0	1	CRA004775;Yang2025_Silkie_Wenchang	PRJNA827465	0.749355649
ENSGALG00010028892	NIBAN2	2	0.164874725	0.070668287	2.333079375	0.01964398	0	0.637084237	0	1	CRA004775;PRJNA827465	Yang2025_Silkie_Wenchang	0.219028108
ENSGALG00010028893	STXBP1	2	-0.054509776	0.142652756	-0.382115125	0.702375973	0.022912104	1.994744329	49.86826204	0.5	PRJNA827465;Yang2025_Silkie_Wenchang	CRA004775	0.923167672
ENSGALG00010028893	STXBP1	2	0.003512267	0.126141598	0.027843846	0.977786695	0	0.925331238	0	0.5	CRA004775;Yang2025_Silkie_Wenchang	PRJNA827465	0.993739602
ENSGALG00010028893	STXBP1	2	-0.142566803	0.076675606	-1.85935021	0.062977514	0	0.092798863	0	1	CRA004775;PRJNA827465	Yang2025_Silkie_Wenchang	0.432176808
ENSGALG00010028894	AKNA	2	0.203770502	0.219116067	0.929960044	0.352385866	0.086702223	10.03007037	90.02998021	0.5	PRJNA827465;Yang2025_Silkie_Wenchang	CRA004775	0.810828603
ENSGALG00010028894	AKNA	2	0.040443189	0.453566644	0.089167027	0.928949169	0.346633628	5.758489644	82.63433536	0.5	CRA004775;Yang2025_Silkie_Wenchang	PRJNA827465	0.983500504
ENSGALG00010028894	AKNA	2	-0.109804563	0.19830962	-0.553702655	0.579782358	0.046148335	1.68213978	40.55190823	1	CRA004775;PRJNA827465	Yang2025_Silkie_Wenchang	0.888013685
ENSGALG00010028896	ATP6V1G1	2	-0.053706915	0.092071486	-0.583317565	0.55967954	0.001458908	1.05175489	4.920812879	1	PRJNA827465;Yang2025_Silkie_Wenchang	CRA004775	0.881205589
ENSGALG00010028896	ATP6V1G1	2	-0.120909668	0.168134832	-0.719123326	0.472069077	0	0.943789515	0	0.5	CRA004775;Yang2025_Silkie_Wenchang	PRJNA827465	0.853998373
ENSGALG00010028896	ATP6V1G1	2	-0.00101349	0.088731117	-0.011422038	0.99088673	0	0.105904361	0	1	CRA004775;PRJNA827465	Yang2025_Silkie_Wenchang	0.997555603
ENSGALG00010028897	TMEM268	2	0.03698582	0.13721426	0.269547929	0.787508064	0	0.476468058	0	0.5	PRJNA827465;Yang2025_Silkie_Wenchang	CRA004775	0.946919116
ENSGALG00010028897	TMEM268	2	-0.088251014	0.256277491	-0.344353729	0.730577629	0	0.142033655	0	0.5	CRA004775;Yang2025_Silkie_Wenchang	PRJNA827465	0.932772487
ENSGALG00010028897	TMEM268	2	0.085649675	0.148074834	0.57842155	0.562979548	0	0.000281561	0	1	CRA004775;PRJNA827465	Yang2025_Silkie_Wenchang	0.882177863
ENSGALG00010028898	TNFSF15	2	-0.306950709	0.309591777	-0.991469189	0.32145653	0.124018135	2.739571251	63.49793787	1	PRJNA827465;Yang2025_Silkie_Wenchang	CRA004775	0.798513866
ENSGALG00010028898	TNFSF15	2	-0.07239089	0.602402665	-0.120170269	0.904348269	0.612625699	6.398143135	84.37046532	0.5	CRA004775;Yang2025_Silkie_Wenchang	PRJNA827465	0.977586231
ENSGALG00010028898	TNFSF15	2	0.180328162	0.280774208	0.642253303	0.520708729	0.081385989	1.933588829	48.34392773	0.5	CRA004775;PRJNA827465	Yang2025_Silkie_Wenchang	0.869353369
ENSGALG00010028900	TNC	2	0.701814674	0.728456708	0.963426744	0.33533341	0.872200068	5.580910386	82.08177643	0.5	PRJNA827465;Yang2025_Silkie_Wenchang	CRA004775	0.803198384
ENSGALG00010028900	TNC	2	0.704525185	0.765752653	0.92004276	0.357550414	0.923845728	4.705591107	78.74868476	0.5	CRA004775;Yang2025_Silkie_Wenchang	PRJNA827465	0.813486201
ENSGALG00010028900	TNC	2	-0.02829874	0.312870339	-0.09044878	0.927930595	0	0.0128937	0	1	CRA004775;PRJNA827465	Yang2025_Silkie_Wenchang	0.982993195
ENSGALG00010028901	PAPPA	2	-0.115887395	0.09316004	-1.243960341	0.213514159	0	0.131832103	0	1	PRJNA827465;Yang2025_Silkie_Wenchang	CRA004775	0.726080554
ENSGALG00010028901	PAPPA	2	-0.068776374	0.178248424	-0.385845621	0.699611006	0	0.052189041	0	1	CRA004775;Yang2025_Silkie_Wenchang	PRJNA827465	0.922065803
ENSGALG00010028901	PAPPA	2	-0.137761871	0.106771078	-1.290254569	0.196962285	0	0.015718194	0	1	CRA004775;PRJNA827465	Yang2025_Silkie_Wenchang	0.707994211
ENSGALG00010028903	ASTN2	2	0.550474545	0.413046468	1.3327182	0.182624298	0.285527141	6.111018888	83.63611669	1	PRJNA827465;Yang2025_Silkie_Wenchang	CRA004775	0.690959068
ENSGALG00010028903	ASTN2	2	0.512046658	0.480498827	1.065656417	0.286578986	0.378305034	5.3657633	81.36332253	0.5	CRA004775;Yang2025_Silkie_Wenchang	PRJNA827465	0.782018115
ENSGALG00010028903	ASTN2	2	0.081393903	0.20542293	0.396225987	0.691938322	0	0.100851548	0	0.5	CRA004775;PRJNA827465	Yang2025_Silkie_Wenchang	0.920348122
ENSGALG00010028904	TRIM32	2	-0.032682542	0.05471375	-0.597336905	0.55028247	0.001702702	1.322975943	24.41283565	0.5	PRJNA827465;Yang2025_Silkie_Wenchang	CRA004775	0.878679315
ENSGALG00010028904	TRIM32	2	-0.10810041	0.07775791	-1.390217533	0.164462831	0	0.018532657	0	1	CRA004775;Yang2025_Silkie_Wenchang	PRJNA827465	0.663711125
ENSGALG00010028904	TRIM32	2	-0.004512473	0.049002802	-0.092086013	0.926629701	0	0.304693975	0	0.5	CRA004775;PRJNA827465	Yang2025_Silkie_Wenchang	0.982834319
ENSGALG00010028905	COLGALT1	2	0.126083255	0.164877522	0.764708574	0.444445099	0.046829315	7.186938077	86.08586871	0.5	PRJNA827465;Yang2025_Silkie_Wenchang	CRA004775	0.846013379
ENSGALG00010028905	COLGALT1	2	0.09940029	0.22548513	0.440828582	0.6593371	0.080205296	4.401736279	77.28169212	0.5	CRA004775;Yang2025_Silkie_Wenchang	PRJNA827465	0.911449691
ENSGALG00010028905	COLGALT1	2	-0.052793133	0.073602535	-0.717273296	0.473205481	0	0.35427415	0	1	CRA004775;PRJNA827465	Yang2025_Silkie_Wenchang	0.854341771
ENSGALG00010028909	ENSGALG00010028909	2	-0.025797014	0.112513872	-0.229278522	0.818652446	0.011200861	1.709246926	41.49470243	0.5	PRJNA827465;Yang2025_Silkie_Wenchang	CRA004775	0.955256039
ENSGALG00010028909	ENSGALG00010028909	2	-0.056515976	0.199401493	-0.283428051	0.776848728	0.030330676	1.397107053	28.42352361	0.5	CRA004775;Yang2025_Silkie_Wenchang	PRJNA827465	0.944200431
ENSGALG00010028909	ENSGALG00010028909	2	0.076389391	0.092079466	0.829602883	0.406763346	0	0.378480868	0	1	CRA004775;PRJNA827465	Yang2025_Silkie_Wenchang	0.833618122
ENSGALG00010028912	EXFABP	2	0.062609235	0.326253491	0.191903648	0.84781768	0.125970332	2.415566257	58.60183933	0.5	PRJNA827465;Yang2025_Silkie_Wenchang	CRA004775	0.96132275
ENSGALG00010028912	EXFABP	2	-1.073982341	1.547261496	-0.694118185	0.487608095	4.523885829	17.78955923	94.37872526	0.5	CRA004775;Yang2025_Silkie_Wenchang	PRJNA827465	0.85768885
ENSGALG00010028912	EXFABP	2	-1.379340805	1.218033521	-1.132432549	0.257452627	2.729829424	11.99334862	91.66204509	1	CRA004775;PRJNA827465	Yang2025_Silkie_Wenchang	0.7650601
ENSGALG00010028914	TLR4	2	-0.181891307	0.197975914	-0.918754728	0.358223877	0.062445167	4.865618374	79.44762776	0.5	PRJNA827465;Yang2025_Silkie_Wenchang	CRA004775	0.813921114
ENSGALG00010028914	TLR4	2	-0.42494815	0.137086762	-3.099848188	0.001936199	0	0.837605227	0	1	CRA004775;Yang2025_Silkie_Wenchang	PRJNA827465	0.056832271
ENSGALG00010028914	TLR4	2	-0.293687873	0.419380835	-0.700289209	0.48374671	0.253662413	2.821571197	64.55875361	0.5	CRA004775;PRJNA827465	Yang2025_Silkie_Wenchang	0.85691916
ENSGALG00010028917	NR6A1	2	0.18071657	0.073933808	2.444302203	0.014513264	0	0.106888612	0	1	PRJNA827465;Yang2025_Silkie_Wenchang	CRA004775	0.198198336
ENSGALG00010028917	NR6A1	2	-0.03329003	0.288889328	-0.115234545	0.908259218	0.122651835	3.711943886	73.05993758	0.5	CRA004775;Yang2025_Silkie_Wenchang	PRJNA827465	0.978236585
ENSGALG00010028917	NR6A1	2	-0.037661545	0.252526273	-0.149139115	0.881443862	0.10025839	4.107765955	75.65586718	0.5	CRA004775;PRJNA827465	Yang2025_Silkie_Wenchang	0.971790895
ENSGALG00010028918	AGPAT2	2	-0.243449068	0.133536613	-1.82308853	0.068289984	0.021359074	2.251711856	55.5893443	1	PRJNA827465;Yang2025_Silkie_Wenchang	CRA004775	0.450487609
ENSGALG00010028918	AGPAT2	2	-0.284299409	0.235407961	-1.207688168	0.227167231	0.046254096	1.450624711	31.06418275	0.5	CRA004775;Yang2025_Silkie_Wenchang	PRJNA827465	0.740605003
ENSGALG00010028918	AGPAT2	2	-0.132145856	0.080746449	-1.636553157	0.10172387	0	0.39031782	0	0.5	CRA004775;PRJNA827465	Yang2025_Silkie_Wenchang	0.543700483
ENSGALG00010028919	EGFL7	2	-0.059333714	0.094239528	-0.629605384	0.5289528	0.009915973	1.886077987	46.97992306	0.5	PRJNA827465;Yang2025_Silkie_Wenchang	CRA004775	0.871809618
ENSGALG00010028919	EGFL7	2	-0.214903942	0.125621627	-1.710724084	0.087132059	0	0.037436757	0	1	CRA004775;Yang2025_Silkie_Wenchang	PRJNA827465	0.507641763
ENSGALG00010028919	EGFL7	2	-0.006368624	0.051923706	-0.122653486	0.902381499	0	0.848835266	0	0.5	CRA004775;PRJNA827465	Yang2025_Silkie_Wenchang	0.976923607
ENSGALG00010028921	MRPL41	2	-0.095318908	0.071257432	-1.337669716	0.181004127	0	0.491350343	0	1	PRJNA827465;Yang2025_Silkie_Wenchang	CRA004775	0.688594651
ENSGALG00010028921	MRPL41	2	-0.276368251	0.197047094	-1.402549233	0.160751299	0	0.001035034	0	1	CRA004775;Yang2025_Silkie_Wenchang	PRJNA827465	0.656689359
ENSGALG00010028921	MRPL41	2	-0.094120165	0.07132984	-1.319506183	0.186999944	0	0.418871488	0	1	CRA004775;PRJNA827465	Yang2025_Silkie_Wenchang	0.695858627
ENSGALG00010028923	ENSGALG00010028923	2	0.336657839	0.594379353	0.566402311	0.571120324	0.632798669	9.012712843	88.90456162	0.5	PRJNA827465;Yang2025_Silkie_Wenchang	CRA004775	0.885354083
ENSGALG00010028923	ENSGALG00010028923	2	0.259350543	0.756121509	0.343001144	0.731597599	0.95732859	6.088084025	83.57447112	0.5	CRA004775;Yang2025_Silkie_Wenchang	PRJNA827465	0.932851968
ENSGALG00010028923	ENSGALG00010028923	2	-0.231747601	0.129799895	-1.785422105	0.074192876	0	0.403320293	0	1	CRA004775;PRJNA827465	Yang2025_Silkie_Wenchang	0.470322999
ENSGALG00010028924	TMEM203	2	0.029690271	0.061121447	0.485758633	0.627138307	0	0.044028883	0	1	PRJNA827465;Yang2025_Silkie_Wenchang	CRA004775	0.901583369
ENSGALG00010028924	TMEM203	2	0.039542292	0.204709321	0.193163125	0.846831217	0	0.083810481	0	0.5	CRA004775;Yang2025_Silkie_Wenchang	PRJNA827465	0.961252712
ENSGALG00010028924	TMEM203	2	0.024271703	0.062128646	0.390668474	0.696042305	0	0.039832358	0	0.5	CRA004775;PRJNA827465	Yang2025_Silkie_Wenchang	0.921038437
ENSGALG00010028925	ENSGALG00010028925	2	-0.015866557	0.209713991	-0.075658075	0.939691132	0	0.144792002	0	0.5	PRJNA827465;Yang2025_Silkie_Wenchang	CRA004775	0.985425793
ENSGALG00010028925	ENSGALG00010028925	2	-0.532426459	0.706921952	-0.753161586	0.45135282	0.751523702	3.965234346	74.78080959	0.5	CRA004775;Yang2025_Silkie_Wenchang	PRJNA827465	0.847847413
ENSGALG00010028925	ENSGALG00010028925	2	-0.56821011	0.606375175	-0.93706031	0.348727536	0.566275012	3.933904468	74.57996227	1	CRA004775;PRJNA827465	Yang2025_Silkie_Wenchang	0.809333419
ENSGALG00010028926	ENSGALG00010028926	2	-0.08974301	0.884233885	-0.10149239	0.919159598	1.235435581	4.631123138	78.40696587	0.5	PRJNA827465;Yang2025_Silkie_Wenchang	CRA004775	0.981150999
ENSGALG00010028926	ENSGALG00010028926	2	0.334146208	1.643814733	0.203274859	0.838920205	4.153344972	3.95391299	74.70859873	0.5	CRA004775;Yang2025_Silkie_Wenchang	PRJNA827465	0.959283372
ENSGALG00010028926	ENSGALG00010028926	2	0.845418367	0.432277919	1.955728779	0.050497113	0	0.95803304	0	1	CRA004775;PRJNA827465	Yang2025_Silkie_Wenchang	0.389230834
ENSGALG00010029140	NPDC1	2	0.02215427	0.151044199	0.146674089	0.883389271	0.037197388	5.372144173	81.38545862	0.5	PRJNA827465;Yang2025_Silkie_Wenchang	CRA004775	0.972001076
ENSGALG00010029140	NPDC1	2	0.167255586	0.098081134	1.705277856	0.088142616	0	0.198156717	0	1	CRA004775;Yang2025_Silkie_Wenchang	PRJNA827465	0.510126523
ENSGALG00010029140	NPDC1	2	-0.116378412	0.079495197	-1.463967833	0.143202738	0	0.130787835	0	0.5	CRA004775;PRJNA827465	Yang2025_Silkie_Wenchang	0.630056778
ENSGALG00010029142	ENSGALG00010029142	2	-0.028273179	0.107568989	-0.262837635	0.792675727	0	0.526377438	0	0.5	PRJNA827465;Yang2025_Silkie_Wenchang	CRA004775	0.948435155
ENSGALG00010029142	ENSGALG00010029142	2	-0.125991729	0.179880366	-0.700419574	0.483665316	0	0.11011799	0	1	CRA004775;Yang2025_Silkie_Wenchang	PRJNA827465	0.85691916
ENSGALG00010029142	ENSGALG00010029142	2	0.006879932	0.114150816	0.060270543	0.951940162	0	0.031204523	0	0.5	CRA004775;PRJNA827465	Yang2025_Silkie_Wenchang	0.987725077
ENSGALG00010029143	SH2D3C	2	0.09880816	0.1114126	0.886867016	0.375150502	0.017142246	3.19957985	68.74589644	0.5	PRJNA827465;Yang2025_Silkie_Wenchang	CRA004775	0.822941424
ENSGALG00010029143	SH2D3C	2	0.212425689	0.091925742	2.31084008	0.02084169	0	0.029831956	0	1	CRA004775;Yang2025_Silkie_Wenchang	PRJNA827465	0.243976022
ENSGALG00010029143	SH2D3C	2	0.006824003	0.075487211	0.09039946	0.927969786	0	0.342915811	0	0.5	CRA004775;PRJNA827465	Yang2025_Silkie_Wenchang	0.982993195
ENSGALG00010029144	ENSGALG00010029144	2	-0.346510102	0.106104068	-3.265756996	0.001091719	0	0.02418491	0	1	PRJNA827465;Yang2025_Silkie_Wenchang	CRA004775	0.039938053
ENSGALG00010029144	ENSGALG00010029144	2	-0.289931015	0.188921679	-1.534662494	0.124866753	0	0.150367153	0	1	CRA004775;Yang2025_Silkie_Wenchang	PRJNA827465	0.592935337
ENSGALG00010029144	ENSGALG00010029144	2	-0.342404449	0.120698546	-2.836856457	0.004556009	0	0.227839768	0	1	CRA004775;PRJNA827465	Yang2025_Silkie_Wenchang	0.098821645
ENSGALG00010029145	RC3H2	2	-0.019454796	0.083765771	-0.23225234	0.816342027	0.005752338	1.351954444	26.03301063	0.5	PRJNA827465;Yang2025_Silkie_Wenchang	CRA004775	0.954546835
ENSGALG00010029145	RC3H2	2	0.16937583	0.156162436	1.084613139	0.278093033	0	0.077119733	0	1	CRA004775;Yang2025_Silkie_Wenchang	PRJNA827465	0.776763074
ENSGALG00010029145	RC3H2	2	-0.054123805	0.054147351	-0.999565135	0.317521003	0	0.749928318	0	0.5	CRA004775;PRJNA827465	Yang2025_Silkie_Wenchang	0.797125693
ENSGALG00010029146	NEK6	2	-0.143934184	0.239369317	-0.601305903	0.547636258	0.10722454	15.5434778	93.56643338	0.5	PRJNA827465;Yang2025_Silkie_Wenchang	CRA004775	0.878281266
ENSGALG00010029146	NEK6	2	-0.192038214	0.224045496	-0.857139364	0.391367868	0.079314315	4.382220283	77.18051729	0.5	CRA004775;Yang2025_Silkie_Wenchang	PRJNA827465	0.829251057
ENSGALG00010029146	NEK6	2	0.090650415	0.076613077	1.183223789	0.236720467	0	0.014022241	0	1	CRA004775;PRJNA827465	Yang2025_Silkie_Wenchang	0.749020867
ENSGALG00010029147	RABL6	2	0.016830352	0.045735041	0.367996868	0.712875569	0	0.997207493	0	1	PRJNA827465;Yang2025_Silkie_Wenchang	CRA004775	0.926097326
ENSGALG00010029147	RABL6	2	-0.043923678	0.206579807	-0.212623287	0.831620806	0.060520676	3.409205541	70.66765298	0.5	CRA004775;Yang2025_Silkie_Wenchang	PRJNA827465	0.957712669
ENSGALG00010029147	RABL6	2	-0.078248144	0.121184904	-0.645692174	0.51847873	0.018675948	2.151215746	53.51465785	0.5	CRA004775;PRJNA827465	Yang2025_Silkie_Wenchang	0.868525042
ENSGALG00010029148	RALGDS	2	-0.17367989	0.131864259	-1.317111181	0.18780136	0.027404146	4.715661501	78.79406739	1	PRJNA827465;Yang2025_Silkie_Wenchang	CRA004775	0.697058913
ENSGALG00010029148	RALGDS	2	-0.307202736	0.085367377	-3.598596425	0.000319939	0	0.000405092	0	1	CRA004775;Yang2025_Silkie_Wenchang	PRJNA827465	0.017685754
ENSGALG00010029148	RALGDS	2	-0.056502497	0.081842956	-0.690377041	0.489957111	0	0.5178598	0	1	CRA004775;PRJNA827465	Yang2025_Silkie_Wenchang	0.858512413
ENSGALG00010029149	ENSGALG00010029149	2	0.091853318	0.163611276	0.561411903	0.574516771	0	0.412076823	0	0.5	PRJNA827465;Yang2025_Silkie_Wenchang	CRA004775	0.88612482
ENSGALG00010029149	ENSGALG00010029149	2	0.045421444	0.240060305	0.189208475	0.849929426	0	0.422870644	0	0.5	CRA004775;Yang2025_Silkie_Wenchang	PRJNA827465	0.961869125
ENSGALG00010029149	ENSGALG00010029149	2	0.181252044	0.177971517	1.018432877	0.308472285	0	0.051104851	0	1	CRA004775;PRJNA827465	Yang2025_Silkie_Wenchang	0.793457188
ENSGALG00010029150	NARF	2	-0.162663861	0.280783161	-0.579321995	0.562371927	0.148580229	16.99764413	94.11683177	0.5	PRJNA827465;Yang2025_Silkie_Wenchang	CRA004775	0.881955027
ENSGALG00010029150	NARF	2	-0.1050268	0.390469126	-0.268975939	0.787948198	0.259037745	6.192173275	83.85058112	0.5	CRA004775;Yang2025_Silkie_Wenchang	PRJNA827465	0.946919116
ENSGALG00010029150	NARF	2	0.118186906	0.060448977	1.955151463	0.050565195	0	0.565460321	0	1	CRA004775;PRJNA827465	Yang2025_Silkie_Wenchang	0.389297528
ENSGALG00010029151	CYBC1	2	-0.211992149	0.204294544	-1.037678953	0.299419547	0.072638476	7.493141063	86.65446184	1	PRJNA827465;Yang2025_Silkie_Wenchang	CRA004775	0.789910488
ENSGALG00010029151	CYBC1	2	-0.324509745	0.175987143	-1.843940074	0.065191874	0.025474073	1.47035314	31.98912745	1	CRA004775;Yang2025_Silkie_Wenchang	PRJNA827465	0.439917301
ENSGALG00010029151	CYBC1	2	-0.021161482	0.07354512	-0.287734689	0.773549835	0	0.001103424	0	1	CRA004775;PRJNA827465	Yang2025_Silkie_Wenchang	0.943248868
ENSGALG00010029152	HEXD	2	0.268416233	0.083093023	3.230310109	0.00123656	0	0.66608277	0	1	PRJNA827465;Yang2025_Silkie_Wenchang	CRA004775	0.042841786
ENSGALG00010029152	HEXD	2	-0.219045614	0.59457837	-0.368404949	0.712571308	0.665456613	16.69113776	94.00879668	0.5	CRA004775;Yang2025_Silkie_Wenchang	PRJNA827465	0.92597752
ENSGALG00010029152	HEXD	2	-0.282870173	0.524285157	-0.539534962	0.589517779	0.512356474	14.20971328	92.96256033	0.5	CRA004775;PRJNA827465	Yang2025_Silkie_Wenchang	0.892320726
ENSGALG00010029153	OGFOD3	2	0.105647944	0.074884356	1.410814619	0.158299283	0	0.959440177	0	1	PRJNA827465;Yang2025_Silkie_Wenchang	CRA004775	0.652026484
ENSGALG00010029153	OGFOD3	2	0.038016315	0.114435697	0.332206789	0.739733118	0	0.365008731	0	1	CRA004775;Yang2025_Silkie_Wenchang	PRJNA827465	0.934936834
ENSGALG00010029153	OGFOD3	2	0.163845767	0.088795816	1.845196936	0.065008896	0	0.0132082	0	1	CRA004775;PRJNA827465	Yang2025_Silkie_Wenchang	0.439539763
ENSGALG00010029154	UTS2R	2	0.357405455	0.191889065	1.862562906	0.062523787	0.047330741	2.659862792	62.40407577	1	PRJNA827465;Yang2025_Silkie_Wenchang	CRA004775	0.431065134
ENSGALG00010029154	UTS2R	2	0.515448089	0.183914598	2.802649131	0.005068478	0	0.64123339	0	1	CRA004775;Yang2025_Silkie_Wenchang	PRJNA827465	0.106130197
ENSGALG00010029154	UTS2R	2	0.196574875	0.119883183	1.639720188	0.101063359	0	0.00042597	0	1	CRA004775;PRJNA827465	Yang2025_Silkie_Wenchang	0.542487465
ENSGALG00010029155	ENSGALG00010029155	2	0.120225853	0.219066189	0.548810632	0.583135413	0.072718626	4.065176756	75.40082363	0.5	PRJNA827465;Yang2025_Silkie_Wenchang	CRA004775	0.890299155
ENSGALG00010029155	ENSGALG00010029155	2	0.335307302	0.171591697	1.954099804	0.050689415	0	0.404819335	0	0.5	CRA004775;Yang2025_Silkie_Wenchang	PRJNA827465	0.389748565
ENSGALG00010029155	ENSGALG00010029155	2	-0.087477975	0.129373017	-0.67616862	0.498933593	0	0.03602486	0	1	CRA004775;PRJNA827465	Yang2025_Silkie_Wenchang	0.861571318
ENSGALG00010029158	CD7	2	-0.05632305	0.337350748	-0.166956943	0.867403928	0	0.191577111	0	0.5	PRJNA827465;Yang2025_Silkie_Wenchang	CRA004775	0.9678508
ENSGALG00010029158	CD7	2	-0.701576636	0.436830699	-1.606060743	0.108260564	0	0.405133952	0	1	CRA004775;Yang2025_Silkie_Wenchang	PRJNA827465	0.556717702
ENSGALG00010029158	CD7	2	-0.362425821	0.459837176	-0.788161202	0.43060242	0.213420415	1.955717029	48.86785844	0.5	CRA004775;PRJNA827465	Yang2025_Silkie_Wenchang	0.842407512
ENSGALG00010029160	DUS1L	2	0.168827169	0.166896102	1.011570469	0.311743472	0.048506468	7.436862453	86.5534692	1	PRJNA827465;Yang2025_Silkie_Wenchang	CRA004775	0.795975682
ENSGALG00010029160	DUS1L	2	0.280096448	0.111446815	2.51327458	0.011961619	0.005327522	1.207634422	17.19348322	1	CRA004775;Yang2025_Silkie_Wenchang	PRJNA827465	0.175814334
ENSGALG00010029160	DUS1L	2	0.020809404	0.054499617	0.381826612	0.702589978	0	0.172728632	0	1	CRA004775;PRJNA827465	Yang2025_Silkie_Wenchang	0.923265404
ENSGALG00010029162	ELN	2	0.341147797	0.304204987	1.121440514	0.262100399	0.00327246	1.017842798	1.753001333	1	PRJNA827465;Yang2025_Silkie_Wenchang	CRA004775	0.768363052

ENSGALG00010029162	ELN	2	0.51906387	0.322035974	1.611819521	0.107001219	0	0.158080081	0	1	CRA004775;Yang2025_Silkie_Wenchang	PRJNA827465	0.554007234
ENSGALG00010029162	ELN	2	0.153331708	0.340858202	0.449840159	0.652825699	0	0.252133833	0	1	CRA004775;PRJNA827465	Yang2025_Silkie_Wenchang	0.909572411
ENSGALG00010029163	ANKFY1	2	-0.18827305	0.203535879	-0.925011606	0.354959871	0.074531671	9.534891934	89.51220416	0.5	PRJNA827465;Yang2025_Silkie_Wenchang	CRA004775	0.812191047
ENSGALG00010029163	ANKFY1	2	-0.390415501	0.111820625	-3.491444445	0.000480416	0	0.126832478	0	1	CRA004775;Yang2025_Silkie_Wenchang	PRJNA827465	0.022914811
ENSGALG00010029163	ANKFY1	2	-0.007916573	0.05285903	-0.149767655	0.880947929	0	0.953460099	0	0.5	CRA004775;PRJNA827465	Yang2025_Silkie_Wenchang	0.971685305
ENSGALG00010029164	CYB5D2	2	-0.11013187	0.163605935	-0.673153272	0.500849786	0.033321195	2.109939029	52.60526555	1	PRJNA827465;Yang2025_Silkie_Wenchang	CRA004775	0.862421093
ENSGALG00010029164	CYB5D2	2	-0.344449562	0.185912081	-1.852755136	0.063917463	0	0.009174409	0	1	CRA004775;Yang2025_Silkie_Wenchang	PRJNA827465	0.435607433
ENSGALG00010029164	CYB5D2	2	-0.023704227	0.079996654	-0.296315229	0.766989357	0.001766473	1.035951957	3.470427101	1	CRA004775;PRJNA827465	Yang2025_Silkie_Wenchang	0.941383508
ENSGALG00010029165	RAC3	2	-0.03917675	0.069210983	-0.566048158	0.571361043	0	0.704126297	0	1	PRJNA827465;Yang2025_Silkie_Wenchang	CRA004775	0.885354083
ENSGALG00010029165	RAC3	2	-0.182823503	0.148700882	-1.229471549	0.218895059	0	0.06340119	0	1	CRA004775;Yang2025_Silkie_Wenchang	PRJNA827465	0.73259614
ENSGALG00010029165	RAC3	2	-0.024007168	0.074432254	-0.322534995	0.747047431	0	0.498783359	0	1	CRA004775;PRJNA827465	Yang2025_Silkie_Wenchang	0.936729365
ENSGALG00010029166	ZZEF1	2	-0.066827417	0.099187511	-0.673748299	0.500471349	0.012369479	2.542431565	60.66757455	0.5	PRJNA827465;Yang2025_Silkie_Wenchang	CRA004775	0.862400704
ENSGALG00010029166	ZZEF1	2	-0.000160894	0.242141805	-0.00066446	0.999469837	0.081587546	2.860547184	65.04165337	0.5	CRA004775;Yang2025_Silkie_Wenchang	PRJNA827465	0.999889665
ENSGALG00010029166	ZZEF1	2	0.044872399	0.089300121	0.502489783	0.615323038	0.00464347	1.115971948	10.39201283	1	CRA004775;PRJNA827465	Yang2025_Silkie_Wenchang	0.898864379
ENSGALG00010029167	ENSGALG00010029167	2	-0.032001431	0.110142838	-0.290544818	0.771399469	0.012782634	1.697576783	41.09250254	0.5	PRJNA827465;Yang2025_Silkie_Wenchang	CRA004775	0.94283287
ENSGALG00010029167	ENSGALG00010029167	2	0.180603349	0.15926672	1.133967906	0.256808011	0	0.114437308	0	1	CRA004775;Yang2025_Silkie_Wenchang	PRJNA827465	0.764750282
ENSGALG00010029167	ENSGALG00010029167	2	-0.050935043	0.124968111	-0.407584325	0.683578874	0.014119306	1.24699845	19.80743843	0.5	CRA004775;PRJNA827465	Yang2025_Silkie_Wenchang	0.918800744
ENSGALG00010029168	RFNG	2	-0.172359145	0.158034604	-1.090641799	0.27543053	0.039903983	4.298438847	76.73573975	1	PRJNA827465;Yang2025_Silkie_Wenchang	CRA004775	0.775135547
ENSGALG00010029168	RFNG	2	-0.123349714	0.266695124	-0.462512072	0.643714148	0.10740223	3.969802165	74.80982783	0.5	CRA004775;Yang2025_Silkie_Wenchang	PRJNA827465	0.906643868
ENSGALG00010029168	RFNG	2	-0.035841578	0.041322202	-0.867368538	0.385740115	0	0.880966471	0	0.5	CRA004775;PRJNA827465	Yang2025_Silkie_Wenchang	0.82648313
ENSGALG00010029169	SBDS	2	-0.112698345	0.077627582	-1.45178224	0.146562164	0.00780116	2.740945398	63.51623783	1	PRJNA827465;Yang2025_Silkie_Wenchang	CRA004775	0.635517732
ENSGALG00010029169	SBDS	2	-0.024347181	0.21555931	-0.112948873	0.910071089	0.07178934	3.905745905	74.39669593	0.5	CRA004775;Yang2025_Silkie_Wenchang	PRJNA827465	0.978730675
ENSGALG00010029169	SBDS	2	0.024656381	0.121818252	0.202403015	0.839601672	0.016899876	1.733979191	42.32918104	0.5	CRA004775;PRJNA827465	Yang2025_Silkie_Wenchang	0.959493354
ENSGALG00010029170	DCXR	2	0.053499397	0.104734364	0.510810347	0.609483863	0.00559762	1.224113841	18.30825155	0.5	PRJNA827465;Yang2025_Silkie_Wenchang	CRA004775	0.897238578
ENSGALG00010029170	DCXR	2	0.042465752	0.267973834	0.158469771	0.874080639	0.070738528	1.829714514	45.31678896	0.5	CRA004775;Yang2025_Silkie_Wenchang	PRJNA827465	0.970697439
ENSGALG00010029170	DCXR	2	-0.024097987	0.090424295	-0.266499027	0.789854909	0	0.701840369	0	1	CRA004775;PRJNA827465	Yang2025_Silkie_Wenchang	0.947390191
ENSGALG00010029171	ENSGALG00010029171	2	-0.037366534	0.047768153	-0.782247835	0.434068937	0.000213424	1.035353929	3.414670902	0.5	PRJNA827465;Yang2025_Silkie_Wenchang	CRA004775	0.842762754
ENSGALG00010029171	ENSGALG00010029171	2	-0.029789306	0.126553719	-0.235388622	0.813907116	0.012717631	1.45880874	31.45091797	0.5	CRA004775;Yang2025_Silkie_Wenchang	PRJNA827465	0.953869515
ENSGALG00010029171	ENSGALG00010029171	2	-0.074066921	0.051292776	-1.444005976	0.148738138	0	0.608503287	0	1	CRA004775;PRJNA827465	Yang2025_Silkie_Wenchang	0.618797624
ENSGALG00010029172	LRRC45	2	0.113101182	0.105295763	1.074128516	0.282765085	0.016044353	3.459033053	71.09018663	1	PRJNA827465;Yang2025_Silkie_Wenchang	CRA004775	0.779532565
ENSGALG00010029172	LRRC45	2	-0.005038185	0.253766181	-0.01985365	0.98416012	0.111729813	7.360239423	86.41348545	0.5	CRA004775;Yang2025_Silkie_Wenchang	PRJNA827465	0.995669738
ENSGALG00010029172	LRRC45	2	-0.0898157	0.143256333	-0.626957969	0.530686787	0.029244262	3.005420437	66.72678512	0.5	CRA004775;PRJNA827465	Yang2025_Silkie_Wenchang	0.872665695
ENSGALG00010029173	ZNHIT3	2	0.161584797	0.270914375	0.596442316	0.550879779	0.112779143	3.836513802	73.93466956	0.5	PRJNA827465;Yang2025_Silkie_Wenchang	CRA004775	0.878879109
ENSGALG00010029173	ZNHIT3	2	0.049329309	0.455778	0.108231	0.913812455	0.333760218	5.06548212	80.2585425	0.5	CRA004775;Yang2025_Silkie_Wenchang	PRJNA827465	0.979364626
ENSGALG00010029173	ZNHIT3	2	-0.123071674	0.135936237	-0.905363253	0.365272976	0.012191037	1.232294431	18.85056244	1	CRA004775;PRJNA827465	Yang2025_Silkie_Wenchang	0.818443863
ENSGALG00010029175	CENPX	2	-0.200460036	0.247768031	-0.696002869	0.48605116	0.106212274	2.400889659	58.34877308	0.5	PRJNA827465;Yang2025_Silkie_Wenchang	CRA004775	0.857495134
ENSGALG00010029175	CENPX	2	-0.517898538	0.263198403	-1.96771155	0.049101236	0	0.091493325	0	1	CRA004775;Yang2025_Silkie_Wenchang	PRJNA827465	0.383282201
ENSGALG00010029175	CENPX	2	-0.068742313	0.170100154	-0.40412846	0.686118257	0.014072155	1.166679035	14.28662298	0.5	CRA004775;PRJNA827465	Yang2025_Silkie_Wenchang	0.919235539
ENSGALG00010029177	ASPSCR1	2	-0.204079699	0.191838837	-1.063808052	0.287415663	0.066304998	9.904844551	89.9039304	1	PRJNA827465;Yang2025_Silkie_Wenchang	CRA004775	0.782840067
ENSGALG00010029177	ASPSCR1	2	-0.302203932	0.136768925	-2.209594999	0.027133283	0.018470827	1.834005527	45.47453726	1	CRA004775;Yang2025_Silkie_Wenchang	PRJNA827465	0.280945645
ENSGALG00010029177	ASPSCR1	2	-0.031529423	0.060818396	-0.518419185	0.604165832	0	0.260073038	0	1	CRA004775;PRJNA827465	Yang2025_Silkie_Wenchang	0.89612415
ENSGALG00010029178	NOTUM	2	0.135188626	0.076674506	1.763149616	0.077875259	0	0.391478212	0	1	PRJNA827465;Yang2025_Silkie_Wenchang	CRA004775	0.480724545
ENSGALG00010029178	NOTUM	2	0.095761605	0.116063447	0.825079793	0.409320291	0	0.297574667	0	1	CRA004775;Yang2025_Silkie_Wenchang	PRJNA827465	0.834355341
ENSGALG00010029178	NOTUM	2	0.181764397	0.095904391	1.895266687	0.058057079	0	0.097743732	0	1	CRA004775;PRJNA827465	Yang2025_Silkie_Wenchang	0.417532916
ENSGALG00010029179	PIGW	2	-0.094327618	0.182263848	-0.517533339	0.604783902	0.045964682	3.148480464	68.23864682	0.5	PRJNA827465;Yang2025_Silkie_Wenchang	CRA004775	0.896344437
ENSGALG00010029179	PIGW	2	-0.396823671	0.139522014	-2.844165294	0.004452794	0	0.971610028	0	1	CRA004775;Yang2025_Silkie_Wenchang	PRJNA827465	0.097660808
ENSGALG00010029179	PIGW	2	-0.22758841	0.327491084	-0.694945361	0.487089544	0.180953694	6.048341961	83.46654329	0.5	CRA004775;PRJNA827465	Yang2025_Silkie_Wenchang	0.85768885
ENSGALG00010029180	CA4	2	-0.443383619	0.286614687	-1.546967545	0.121871072	0.074656143	1.820078157	45.05730449	1	PRJNA827465;Yang2025_Silkie_Wenchang	CRA004775	0.585419059
ENSGALG00010029180	CA4	2	0.386281996	0.640176014	0.60339967	0.546242842	0.656906703	4.765780575	79.01707844	0.5	CRA004775;Yang2025_Silkie_Wenchang	PRJNA827465	0.878012469
ENSGALG00010029180	CA4	2	0.130209578	0.931484353	0.139787188	0.888828137	1.549678221	9.171360419	89.09649219	0.5	CRA004775;PRJNA827465	Yang2025_Silkie_Wenchang	0.973135755
ENSGALG00010029181	MYO19	2	0.281402089	0.166598512	1.689103259	0.091199643	0.046811464	6.327686348	84.19643539	1	PRJNA827465;Yang2025_Silkie_Wenchang	CRA004775	0.517862098
ENSGALG00010029181	MYO19	2	0.154075631	0.30992794	0.497133724	0.619094754	0.175469226	11.43359998	91.25384829	0.5	CRA004775;Yang2025_Silkie_Wenchang	PRJNA827465	0.899168321
ENSGALG00010029181	MYO19	2	0.005971965	0.140575615	0.042482225	0.966114281	0.026484209	2.812938126	64.44998235	0.5	CRA004775;PRJNA827465	Yang2025_Silkie_Wenchang	0.990863074
ENSGALG00010029182	HEATR6	2	-0.049609286	0.126888523	-0.390967476	0.695821277	0.023154333	3.407816779	70.65569939	0.5	PRJNA827465;Yang2025_Silkie_Wenchang	CRA004775	0.921038437
ENSGALG00010029182	HEATR6	2	-0.279116304	0.130785533	-2.13415274	0.03283028	0.010331793	1.335679649	25.13174842	1	CRA004775;Yang2025_Silkie_Wenchang	PRJNA827465	0.311372705
ENSGALG00010029182	HEATR6	2	-0.170860376	0.268560559	-0.636207999	0.524640861	0.120830533	5.56660529	82.03573007	0.5	CRA004775;PRJNA827465	Yang2025_Silkie_Wenchang	0.869820288
ENSGALG00010029183	ENSGALG00010029183	2	0.045784561	0.064324324	0.711776788	0.476600009	0.000168888	1.008222496	0.815543807	1	PRJNA827465;Yang2025_Silkie_Wenchang	CRA004775	0.854986476
ENSGALG00010029183	ENSGALG00010029183	2	0.164729962	0.168699902	0.976465665	0.328833755	0	0.467819029	0	0.5	CRA004775;Yang2025_Silkie_Wenchang	PRJNA827465	0.800762292
ENSGALG00010029183	ENSGALG00010029183	2	0.02036441	0.065888946	0.309071716	0.757266974	0	0.041379677	0	0.5	CRA004775;PRJNA827465	Yang2025_Silkie_Wenchang	0.938802759
ENSGALG00010029184	DYNLL2	2	-0.260056293	0.324782009	-0.80071028	0.42329939	0.190440343	9.713046488	89.70456899	0.5	PRJNA827465;Yang2025_Silkie_Wenchang	CRA004775	0.84012238
ENSGALG00010029184	DYNLL2	2	-0.546727634	0.175002658	-3.124104062	0.001783435	0	0.524480639	0	1	CRA004775;Yang2025_Silkie_Wenchang	PRJNA827465	0.0537214
ENSGALG00010029184	DYNLL2	2	0.028709583	0.069875654	0.410866675	0.681170244	0	0.755588454	0	0.5	CRA004775;PRJNA827465	Yang2025_Silkie_Wenchang	0.91820222
ENSGALG00010029186	ZNF302	2	0.254417828	0.123485319	2.060308302	0.039369077	0.017691722	2.244387528	55.44441468	1	PRJNA827465;Yang2025_Silkie_Wenchang	CRA004775	0.34316319
ENSGALG00010029186	ZNF302	2	0.388563553	0.134601885	2.88676166	0.00389229	0	0.124833861	0	1	CRA004775;Yang2025_Silkie_Wenchang	PRJNA827465	0.089776224
ENSGALG00010029186	ZNF302	2	0.166039126	0.084320772	1.905059542	0.056767653	0	0.089922587	0	1	CRA004775;PRJNA827465	Yang2025_Silkie_Wenchang	0.41225771
ENSGALG00010029187	SRSF1	2	0.063041103	0.042039338	1.499574136	0.133724752	0	0.003189239	0	1	PRJNA827465;Yang2025_Silkie_Wenchang	CRA004775	0.612847937
ENSGALG00010029187	SRSF1	2	-0.121260132	0.226589807	-0.535152633	0.592544317	0.075301087	3.46151781	71.11093876	0.5	CRA004775;Yang2025_Silkie_Wenchang	PRJNA827465	0.892776493
ENSGALG00010029187	SRSF1	2	-0.112900855	0.220742662	-0.511459153	0.609020583	0.077271717	4.025171796	75.15633989	0.5	CRA004775;PRJNA827465	Yang2025_Silkie_Wenchang	0.897212042
ENSGALG00010029188	ENSGALG00010029188	2	0.828571377	0.836972003	0.989963074	0.322192168	0.87834492	2.600536015	61.54638912	1	PRJNA827465;Yang2025_Silkie_Wenchang	CRA004775	0.795913866
ENSGALG00010029188	ENSGALG00010029188	2	0.795229947	0.784103484	1.014190043	0.310492079	0.85769656	2.943232244	66.02374815	1	CRA004775;Yang2025_Silkie_Wenchang	PRJNA827465	0.794899969
ENSGALG00010029188	ENSGALG00010029188	2	0.153599976	0.317813814	0.483301762	0.628881489	0	0.012111804	0	1	CRA004775;PRJNA827465	Yang2025_Silkie_Wenchang	0.902084638
ENSGALG00010029189	ENSGALG00010029189	2	1.016601609	0.37464417	2.71354284	0.006658556	0	0.675138491	0	1	PRJNA827465;Yang2025_Silkie_Wenchang	CRA004775	0.125062444
ENSGALG00010029189	ENSGALG00010029189	2	0.905387269	0.494590769	1.830578583	0.067163468	0.140157682	1.378074445	27.43497975	1	CRA004775;Yang2025_Silkie_Wenchang	PRJNA827465	0.446498062
ENSGALG00010029189	ENSGALG00010029189	2	0.538446694	0.427922627	1.258280492	0.208290334	0	0.20083943	0	1	CRA004775;PRJNA827465	Yang2025_Silkie_Wenchang	0.720917211
ENSGALG00010029190	DHRS7C	2	-0.595227912	0.116775512	-5.097198052	3.44718E-07	0	0.057001322	0	1	PRJNA827465;Yang2025_Silkie_Wenchang	CRA004775	9.34375E-05
ENSGALG00010029190	DHRS7C	2	-0.580348657	0.154172818	-3.764273528	0.000167034	0.001163713	1.005658075	0.562624172	0.5	CRA004775;Yang2025_Silkie_Wenchang	PRJNA827465	0.001248673
ENSGALG00010029190	DHRS7C	2	-0.519565038	0.170211275	-3.05247134	0.002269654	0	0.823173988	0	0.5	CRA004775;PRJNA827465	Yang2025_Silkie_Wenchang	0.063175788
ENSGALG00010029191	GSG1L2	2	0.111243036	0.533306175	0.208591315	0.834767291	0.388013828	3.079666812	67.52895488	0.5	PRJNA827465;Yang2025_Silkie_Wenchang	CRA004775	0.958133448
ENSGALG00010029191	GSG1L2	2	0.951948178	0.363761901	2.616954045	0.008871828	0	0.563889848	0	1	CRA004775;Yang2025_Silkie_Wenchang	PRJNA827465	0.147624671
ENSGALG00010029191	GSG1L2	2	0.38968037	0.80862566	0.481904532	0.629873767	1.103605646	6.280002528	84.07643953	0.5	CRA004775;PRJNA827465	Yang2025_Silkie_Wenchang	0.902488805
ENSGALG00010029192	DTX2	2	-0.100471624	0.097652227	-1.028871811	0.303539916	0.007550871	1.452310065	31.14418027	1	PRJNA827465;Yang2025_Silkie_Wenchang	CRA004775	0.792205992
ENSGALG00010029192	DTX2	2	-0.285645514	0.140329114	-2.035539917	0.041796576	0	0.071497853	0	1	CRA004775;Yang2025_Silkie_Wenchang	PRJNA827465	0.354113136
ENSGALG00010029192	DTX2	2	-0.090904491	0.112083526	-0.811042392	0.417341326	0.009535561	1.263145661	20.83256658	1	CRA004775;PRJNA827465	Yang2025_Silkie_Wenchang	0.838112595
ENSGALG00010029194	DHRS11	2	-0.064616649	0.079331516	-0.814514233	0.415350422	0	0.736281016	0	1	PRJNA827465;Yang2025_Silkie_Wenchang	CRA004775	0.837576587
ENSGALG00010029194	DHRS11	2	0.10613273	0.322225683	0.3293739	0.741873093	0.162797807	4.471066846	77.6339734	0.5	CRA004775;Yang2025_Silkie_Wenchang	PRJNA827465	0.93543545
ENSGALG00010029194	DHRS11	2	0.157026578	0.236442106	0.664122736	0.50661176	0.081252961	3.141957136	68.17270393	0.5	CRA004775;PRJNA827465	Yang2025_Silkie_Wenchang	0.863929781
ENSGALG00010029196	MRM1	2	-0.085682914	0.206513124	-0.414902997	0.678212916	0.05358514	2.317061719	56.84189196	0.5	PRJNA827465;Yang2025_Silkie_Wenchang	CRA004775	0.917647993
ENSGALG00010029196	MRM1	2	0.007405254	0.255499867	0.028983397	0.976877832	0.031991111	1.291004378	22.54092884	0.5	CRA004775;Yang2025_Silkie_Wenchang	PRJNA827465	0.993272855
ENSGALG00010029196	MRM1	2	-0.242869603	0.103758433	-2.340721569	0.019246514	0	0.06008002	0	1	CRA004775;PRJNA827465	Yang2025_Silkie_Wenchang	0.23253789
ENSGALG00010029198	PPM1D	2	-0.139396922	0.087608389	-1.591136701	0.111578815	0.0091034	2.452265865	59.22138728	1	PRJNA827465;Yang2025_Silkie_Wenchang	CRA004775	0.564762672
ENSGALG00010029198	PPM1D	2	-0.130317139	0.185231207	-0.703537711	0.481720714	0.037472584	1.617533104	38.17746312	0.5	CRA004775;Yang2025_Silkie_Wenchang	PRJNA827465	0.85691916
ENSGALG00010029198	PPM1D	2	-0.042310913	0.073659133	-0.574415029	0.565686985	0	0.596240642	0	0.5	CRA004775;PRJNA827465	Yang2025_Silkie_Wenchang	0.883487517
ENSGALG00010029199	APPBP2	2	-0.143396305	0.04809938	-2.98125057	0.002870738	0	0.067030192	0	1	PRJNA827465;Yang2025_Silkie_Wenchang	CRA004775	0.073170308
ENSGALG00010029199	APPBP2	2	-0.054615198	0.165225154	-0.33055014	0.740984314	0.028608418	1.842728578	45.7326482	0.5	CRA004775;Yang2025_Silkie_Wenchang	PRJNA827465	0.935375188
ENSGALG00010029199	APPBP2	2	-0.05868075	0.138407777	-0.423970033	0.671587668	0.022433215	1.758575959	43.13580855	0.5	CRA004775;PRJNA827465	Yang2025_Silkie_Wenchang	0.914674175
ENSGALG00010029200	GAS7	2	0.007856462	0.095966634	0.081866597	0.934752797	0	0.126428177	0	0.5	PRJNA827465;Yang2025_Silkie_Wenchang	CRA004775	0.984413039
ENSGALG00010029200	GAS7	2	-0.069853784	0.173103739	-0.403537121	0.686553132	0	0.006846337	0	1	CRA004775;Yang2025_Silkie_Wenchang	PRJNA827465	0.919235539
ENSGALG00010029200	GAS7	2	0.011852334	0.101149843	0.117175999	0.906720586	0	0.131871705	0	0.5	CRA004775;PRJNA827465	Yang2025_Silkie_Wenchang	0.978196186
ENSGALG00010029201	PIGS	2	-0.057633144	0.05722391	-1.007151449	0.313862	0	0.663907204	0	1	PRJNA827465;Yang2025_Silkie_Wenchang	CRA004775	0.796531448
ENSGALG00010029201	PIGS	2	-0.162379588	0.084816429	-1.914482714	0.055558506	0	0.244888522	0	1	CRA004775;Yang2025_Silkie_Wenchang	PRJNA827465	0.408228819
ENSGALG00010029201	PIGS	2	-0.082962778	0.086230073	-0.962109564	0.33599457	0.005862201	1.480672376	32.46311501	1	CRA004775;PRJNA827465	Yang2025_Silkie_Wenchang	0.803504387
ENSGALG00010029202	PSMD12	2	-0.027380203	0.053818872	-0.508747254	0.610929396	0.001814482	1.313384605	23.86084047	0.5	PRJNA827465;Yang2025_Silkie_Wenchang	CRA004775	0.897506931
ENSGALG00010029202	PSMD12	2	0.050941322	0.09355707	0.544494628	0.586101144	0	0.241176129	0	0.5	CRA004775;Yang2025_Silkie_Wenchang	PRJNA827465	0.891402106
ENSGALG00010029202	PSMD12	2	-0.059813914	0.045169283	-1.324216589	0.185431123	0	0.019704055	0	1	CRA004775;PRJNA827465	Yang2025_Silkie_Wenchang	0.693865902
ENSGALG00010029203	ALDOC	2	0.160445772	0.201089241	0.797883423	0.424938144	0.06407974	4.6791886	78.62877338	0.5	PRJNA827465;Yang2025_Silkie_Wenchang	CRA004775	0.841139484
ENSGALG00010029203	ALDOC	2	0.093158548	0.355210861	0.262262668	0.793118942	0.183885403	3.293071996	69.63321783	0.5	CRA004775;Yang2025_Silkie_Wenchang	PRJNA827465	0.948571413
ENSGALG00010029203	ALDOC	2	-0.04652067	0.097814273	-0.475602065	0.634357904	0	0.714002702	0	1	CRA004775;PRJNA827465	Yang2025_Silkie_Wenchang	0.903815945
ENSGALG00010029204	HELZ	2	0.167487071	0.278832024	0.600673728	0.548057321	0.121872787	3.902500571	74.37540413	0.5	PRJNA827465;Yang2025_Silkie_Wenchang	CRA004775	0.878281266
ENSGALG00010029204	HELZ	2	0.424927213	0.202547782	2.097910965	0.035913011	0	0.229995186	0	1	CRA004775;Yang2025_Silkie_Wenchang	PRJNA827465	0.326969036
ENSGALG00010029204	HELZ	2	0.02866743	0.15631145	0.183399421	0.85448463	0.026317188	1.582950667	36.82683732	0.5	CRA004775;PRJNA827465	Yang2025_Silkie_Wenchang	0.96322236
ENSGALG00010029205	CACNG1	2	-0.100008594	0.090763701	-1.101856727	0.270523964	0	0.149032486	0	1	PRJNA827465;Yang2025_Silkie_Wenchang	CRA004775	0.772536273
ENSGALG00010029205	CACNG1	2	-0.173343436	0.165559736	-1.047014452	0.295092913	0	0.25881151	0	1	CRA004775;Yang2025_Silkie_Wenchang	PRJNA827465	0.787628307
ENSGALG00010029205	CACNG1	2	-0.086694158	0.106516384	-0.813904442	0.415699696	0	0.375972574	0	1	CRA004775;PRJNA827465	Yang2025_Silkie_Wenchang	0.837701508
ENSGALG00010029206	CACNG4	2	0.371642011	0.150003699	2.477552314	0.013228701	0.002648119	1.058295481	5.508431447	1	PRJNA827465;Yang2025_Silkie_Wenchang	CRA004775	0.187835134
ENSGALG00010029206	CACNG4	2	0.578536639	0.227543765	2.542529079	0.011005344	0	0.027652942	0	1	CRA004775;Yang2025_Silkie_Wenchang	PRJNA827465	0.166811819
ENSGALG00010029206	CACNG4	2	0.2822957	0.17677762	1.596897273	0.110288608	0	0.347096175	0	1	CRA004775;PRJNA827465	Yang2025_Silkie_Wenchang	0.562335482
ENSGALG00010029207	SPAG5	2	0.190870813	0.363414781	0.525214775	0.599433895	0.249935183	18.52096856	94.60071434	0.5	PRJNA827465;Yang2025_Silkie_Wenchang	CRA004775	0.895010988
ENSGALG00010029207	SPAG5	2	0.538450153	0.130539567	4.124804188	3.7105E-05	0	0.382659451	0	1	CRA004775;Yang2025_Silkie_Wenchang	PRJNA827465	0.003930833
ENSGALG00010029207	SPAG5	2	-0.151963273	0.099546709	-1.526552457	0.126872337	0	0.588839249	0	0.5	CRA004775;PRJNA827465	Yang2025_Silkie_Wenchang	0.597716824
ENSGALG00010029209	PRKCA	2	0.141469485	0.090546507	1.562395831	0.118194769	0.009938081	2.511681181	60.18602968	1	PRJNA827465;Yang2025_Silkie_Wenchang	CRA004775	0.578003524
ENSGALG00010029209	PRKCA	2	0.397800236	0.225260204	1.765958787	0.077402763	0.06771861	2.436809748	58.96273804	1	CRA004775;Yang2025_Silkie_Wenchang	PRJNA827465	0.479080443
ENSGALG00010029209	PRKCA	2	0.32732769	0.324752774	1.007928851	0.31348862	0.172704569	4.774664235	79.05611891	1	CRA004775;PRJNA827465	Yang2025_Silkie_Wenchang	0.796531448
ENSGALG00010029210	RSKR	2	-0.018014161	0.321188553	-0.056085937	0.955273347	0.166022513	5.105611363	80.41370702	0.5	PRJNA827465;Yang2025_Silkie_Wenchang	CRA004775	0.988131974
ENSGALG00010029210	RSKR	2	-0.211134136	0.617463264	-0.341937971	0.732397573	0.60232502	4.158135041	75.950757	0.5	CRA004775;Yang2025_Silkie_Wenchang	PRJNA827465	0.932985777
ENSGALG00010029210	RSKR	2	-0.419530173	0.201011367	-2.087096764	0.036879388	0	0.970425765	0	1	CRA004775;PRJNA827465	Yang2025_Silkie_Wenchang	0.331459091
ENSGALG00010029211	SDF2	2	0.117601546	0.102184143	1.15087862	0.249782175	0.006656883	1.267075204	21.07808625	1	PRJNA827465;Yang2025_Silkie_Wenchang	CRA004775	0.759757983
ENSGALG00010029211	SDF2	2	0.023945037	0.310702102	0.077067508	0.938569841	0.138760801	3.522069366	71.60760064	0.5	CRA004775;Yang2025_Silkie_Wenchang	PRJNA827465	0.985349045
ENSGALG00010029211	SDF2	2	-0.039462969	0.166969444	-0.236348451	0.813162294	0.032264455	1.880655811	46.82705925	0.5	CRA004775;PRJNA827465	Yang2025_Silkie_Wenchang	0.953526116
ENSGALG00010029212	SUPT6H	2	-0.087302126	0.097923522	-0.891533756	0.372642891	0.011323701	1.914836629	47.77622358	0.5	PRJNA827465;Yang2025_Silkie_Wenchang	CRA004775	0.822016521
ENSGALG00010029212	SUPT6H	2	0.044518045	0.127856241	0.348188285	0.727698785	0	0.094769803	0	0.5	CRA004775;Yang2025_Silkie_Wenchang	PRJNA827465	0.931639802
ENSGALG00010029212	SUPT6H	2	-0.144232811	0.044220257	-3.261690921	0.001107498	0	0.281245606	0	1	CRA004775;PRJNA827465	Yang2025_Silkie_Wenchang	0.040192063
ENSGALG00010029213	APOH	2	2.481842133	1.054237576	2.354158293	0.018564704	1.169372925	2.106198625	52.5210971	1	PRJNA827465;Yang2025_Silkie_Wenchang	CRA004775	0.227943938
ENSGALG00010029213	APOH	2	2.236947705	0.527082202	4.244020562	2.1955E-05	0	0.836728574	0	1	CRA004775;Yang2025_Silkie_Wenchang	PRJNA827465	0.002631952
ENSGALG00010029213	APOH	2	2.780188884	0.515573516	5.392419896	6.95151E-08	0	0.727161706	0	1	CRA004775;PRJNA827465	Yang2025_Silkie_Wenchang	2.62707E-05
ENSGALG00010029214	ENSGALG00010029214	2	-0.31051701	0.1885081	-1.647234312	0.099509894	0	0.124820843	0	1	PRJNA827465;Yang2025_Silkie_Wenchang	CRA004775	0.53870846
ENSGALG00010029214	ENSGALG00010029214	2	0.220041786	0.607300479	0.3623277	0.717107161	0.486501331	2.52923155	60.46229931	0.5	CRA004775;Yang2025_Silkie_Wenchang	PRJNA827465	0.92775671
ENSGALG00010029214	ENSGALG00010029214	2	0.161029721	0.679884172	0.236848758	0.812774124	0.679876614	3.225725589	68.99922289	0.5	CRA004775;PRJNA827465	Yang2025_Silkie_Wenchang	0.95348724
ENSGALG00010029215	ENSGALG00010029215	2	-0.257320346	0.136760276	-1.881543047	0.059898085	0	0.122478183	0	1	PRJNA827465;Yang2025_Silkie_Wenchang	CRA004775	0.423882851
ENSGALG00010029215	ENSGALG00010029215	2	-0.104073872	0.280737915	-0.370715413	0.710849509	0	0.178692541	0	0.5	CRA004775;Yang2025_Silkie_Wenchang	PRJNA827465	0.92554556
ENSGALG00010029215	ENSGALG00010029215	2	-0.259100296	0.148458129	-1.745275233	0.080936971	0	0.401651403	0	0.5	CRA004775;PRJNA827465	Yang2025_Silkie_Wenchang	0.489847627
ENSGALG00010029216	RAB34	2	-0.046192337	0.116305821	-0.39716273	0.691247464	0.011712184	1.568168081	36.23132546	0.5	PRJNA827465;Yang2025_Silkie_Wenchang	CRA004775	0.920268407
ENSGALG00010029216	RAB34	2	0.013306427	0.162476589	0.0818975	0.934728222	0.006885657	1.134469644	11.85308433	0.5	CRA004775;Yang2025_Silkie_Wenchang	PRJNA827465	0.984413039
ENSGALG00010029216	RAB34	2	-0.130562495	0.082524988	-1.582096495	0.113627544	0	0.098261409	0	1	CRA004775;PRJNA827465	Yang2025_Silkie_Wenchang	0.568794964
ENSGALG00010029217	CEP112	2	0.241353164	0.253883808	0.950644179	0.341785034	0.102819521	4.834620063	79.31585136	1	PRJNA827465;Yang2025_Silkie_Wenchang	CRA004775	0.805823616
ENSGALG00010029217	CEP112	2	0.203470064	0.344691789	0.590295652	0.554992454	0.179336065	3.952356253	74.69863707	0.5	CRA004775;Yang2025_Silkie_Wenchang	PRJNA827465	0.880223402
ENSGALG00010029217	CEP112	2	-0.025134982	0.119488126	-0.210355479	0.833390238	0	0.330456293	0	0.5	CRA004775;PRJNA827465	Yang2025_Silkie_Wenchang	0.957968425
ENSGALG00010029218	RPL23A	2	-0.320607475	0.26696814	-1.200920363	0.229782095	0.115788372	4.652611675	78.50669538	1	PRJNA827465;Yang2025_Silkie_Wenchang	CRA004775	0.742691255
ENSGALG00010029218	RPL23A	2	-0.437830784	0.217818229	-2.010074115	0.044423345	0.030379235	1.467984804	31.87940383	1	CRA004775;Yang2025_Silkie_Wenchang	PRJNA827465	0.363895527
ENSGALG00010029218	RPL23A	2	-0.10536897	0.066366317	-1.58768747	0.112357028	0	0.149744446	0	1	CRA004775;PRJNA827465	Yang2025_Silkie_Wenchang	0.56602666
ENSGALG00010029219	TLCD1	2	0.041473342	0.106131768	0.390772172	0.695965646	0	0.003513165	0	1	PRJNA827465;Yang2025_Silkie_Wenchang	CRA004775	0.921038437
ENSGALG00010029219	TLCD1	2	-0.288794302	0.363966663	-0.793463609	0.427507763	0.188626453	3.331763635	69.98586606	0.5	CRA004775;Yang2025_Silkie_Wenchang	PRJNA827465	0.84125685
ENSGALG00010029219	TLCD1	2	-0.261839067	0.36826574	-0.711005773	0.477080658	0.21487042	4.281491988	76.64365593	0.5	CRA004775;PRJNA827465	Yang2025_Silkie_Wenchang	0.854986476
ENSGALG00010029220	NEK8	2	0.005132113	0.099841795	0.051402447	0.959004835	0.006708812	1.487371714	32.76731091	0.5	PRJNA827465;Yang2025_Silkie_Wenchang	CRA004775	0.989102387
ENSGALG00010029220	NEK8	2	0.073567233	0.116201766	0.633099098	0.526668931	0	0.674486824	0	0.5	CRA004775;Yang2025_Silkie_Wenchang	PRJNA827465	0.87066367
ENSGALG00010029220	NEK8	2	-0.084138784	0.094819472	-0.887357654	0.374886375	0	0.013790623	0	1	CRA004775;PRJNA827465	Yang2025_Silkie_Wenchang	0.822916144
ENSGALG00010029221	AXIN2	2	0.127408313	0.170203465	0.748564741	0.45411959	0.034993814	2.476687232	59.62348467	0.5	PRJNA827465;Yang2025_Silkie_Wenchang	CRA004775	0.849072665
ENSGALG00010029221	AXIN2	2	0.300280696	0.158455463	1.895047925	0.058086053	0	0.06074463	0	1	CRA004775;Yang2025_Silkie_Wenchang	PRJNA827465	0.417588463
ENSGALG00010029221	AXIN2	2	0.000602668	0.123537843	0.004878409	0.996107608	0	0.35219669	0	0.5	CRA004775;PRJNA827465	Yang2025_Silkie_Wenchang	0.998737007
ENSGALG00010029222	TRAF4	2	0.255031395	0.113712024	2.242783	0.024910811	0.008887899	1.478574382	32.36728484	1	PRJNA827465;Yang2025_Silkie_Wenchang	CRA004775	0.269034231
ENSGALG00010029222	TRAF4	2	0.220450163	0.208617713	1.056718338	0.290640153	0.050734124	2.327657076	57.03834512	0.5	CRA004775;Yang2025_Silkie_Wenchang	PRJNA827465	0.784622961
ENSGALG00010029222	TRAF4	2	0.130688545	0.098423115	1.327823709	0.184236353	0	0.556057574	0	0.5	CRA004775;PRJNA827465	Yang2025_Silkie_Wenchang	0.692545576
ENSGALG00010029223	NME2	2	0.283326412	0.29346454	0.965453652	0.33431764	0.154111033	8.759316858	88.58358459	1	PRJNA827465;Yang2025_Silkie_Wenchang	CRA004775	0.803094614
ENSGALG00010029223	NME2	2	0.25532092	0.358507011	0.712178317	0.476354362	0.215467872	6.17157162	83.79667188	0.5	CRA004775;Yang2025_Silkie_Wenchang	PRJNA827465	0.854973661
ENSGALG00010029223	NME2	2	0.011218998	0.048835706	0.229729414	0.818302037	0	0.332957564	0	0.5	CRA004775;PRJNA827465	Yang2025_Silkie_Wenchang	0.955256039
ENSGALG00010029224	FAM222B	2	0.101504461	0.204071975	0.4973954	0.618910249	0.065045569	4.018273789	75.11369179	0.5	PRJNA827465;Yang2025_Silkie_Wenchang	CRA004775	0.899168321
ENSGALG00010029224	FAM222B	2	0.206803488	0.132071032	1.56585047	0.117383628	0	0.978486541	0	1	CRA004775;Yang2025_Silkie_Wenchang	PRJNA827465	0.57688585
ENSGALG00010029224	FAM222B	2	-0.046437632	0.06359996	-0.730151909	0.465297335	0	0.646745876	0	0.5	CRA004775;PRJNA827465	Yang2025_Silkie_Wenchang	0.852411124
ENSGALG00010029225	ENSGALG00010029225	2	3.322669638	1.414513103	2.348984701	0.01882468	2.322501106	2.370117955	57.80800708	1	PRJNA827465;Yang2025_Silkie_Wenchang	CRA004775	0.229831303
ENSGALG00010029225	ENSGALG00010029225	2	2.473613249	2.052013927	1.205456365	0.228027173	7.508598298	8.96687663	88.84784478	1	CRA004775;Yang2025_Silkie_Wenchang	PRJNA827465	0.740938585
ENSGALG00010029225	ENSGALG00010029225	2	0.775962483	0.600613867	1.291948996	0.196374808	0	0.681657196	0	1	CRA004775;PRJNA827465	Yang2025_Silkie_Wenchang	0.707824568
ENSGALG00010029226	MBTD1	2	-0.064040259	0.069067929	-0.927206878	0.353819133	0.003731175	1.516260541	34.04827383	1	PRJNA827465;Yang2025_Silkie_Wenchang	CRA004775	0.811422183
ENSGALG00010029226	MBTD1	2	-0.093320652	0.135266403	-0.689902667	0.490255399	0.012221205	1.294569579	22.75424851	0.5	CRA004775;Yang2025_Silkie_Wenchang	PRJNA827465	0.858529056
ENSGALG00010029226	MBTD1	2	-0.008779872	0.056053107	-0.156634889	0.875532604	0	0.430047635	0	0.5	CRA004775;PRJNA827465	Yang2025_Silkie_Wenchang	0.970347777
ENSGALG00010029227	UTP18	2	0.06431225	0.052596553	1.222746489	0.221425462	0.001768936	1.326010599	24.58582152	1	PRJNA827465;Yang2025_Silkie_Wenchang	CRA004775	0.734775831
ENSGALG00010029227	UTP18	2	0.101976185	0.090371527	1.128410553	0.259146573	0.001898459	1.093397762	8.541974905	0.5	CRA004775;Yang2025_Silkie_Wenchang	PRJNA827465	0.766314541
ENSGALG00010029227	UTP18	2	0.027861133	0.04304694	0.647226803	0.517485167	0	0.244780177	0	0.5	CRA004775;PRJNA827465	Yang2025_Silkie_Wenchang	0.867952013
ENSGALG00010029228	ERAL1	2	0.118494057	0.053521564	2.213949822	0.026832231	0	0.577333977	0	1	PRJNA827465;Yang2025_Silkie_Wenchang	CRA004775	0.279212912
ENSGALG00010029228	ERAL1	2	0.018837483	0.212447097	0.088669053	0.929344928	0.063514106	3.174945418	68.50339554	0.5	CRA004775;Yang2025_Silkie_Wenchang	PRJNA827465	0.983654531
ENSGALG00010029228	ERAL1	2	-0.005001562	0.152722251	-0.032749397	0.973874432	0.030338701	2.267689717	55.90225627	0.5	CRA004775;PRJNA827465	Yang2025_Silkie_Wenchang	0.992472082
ENSGALG00010029229	CBX4	2	0.287961538	0.282701551	1.018606149	0.308389984	0.149367018	15.19495544	93.41886849	1	PRJNA827465;Yang2025_Silkie_Wenchang	CRA004775	0.793457188
ENSGALG00010029229	CBX4	2	0.288611894	0.323505948	0.892137827	0.372319062	0.17372915	5.502110213	81.82515505	0.5	CRA004775;Yang2025_Silkie_Wenchang	PRJNA827465	0.82179465
ENSGALG00010029229	CBX4	2	0.000887914	0.082975367	0.010700937	0.99146205	0	0.1037066	0	0.5	CRA004775;PRJNA827465	Yang2025_Silkie_Wenchang	0.997563866
ENSGALG00010029230	PPM1E	2	-0.11679928	0.086499522	-1.350288163	0.176923568	0.00360295	1.197806394	16.51405394	0.5	PRJNA827465;Yang2025_Silkie_Wenchang	CRA004775	0.682556882
ENSGALG00010029230	PPM1E	2	0.336024716	0.367456123	0.914462148	0.360474059	0.188590188	2.945275125	66.04731452	1	CRA004775;Yang2025_Silkie_Wenchang	PRJNA827465	0.814880744
ENSGALG00010029230	PPM1E	2	0.23796154	0.475335505	0.500618064	0.616639947	0.380492572	5.491174741	81.78896052	0.5	CRA004775;PRJNA827465	Yang2025_Silkie_Wenchang	0.899111936
ENSGALG00010029231	CBX8	2	0.056506208	0.157265946	0.359303523	0.719368039	0.028366806	1.928538771	48.14727011	0.5	PRJNA827465;Yang2025_Silkie_Wenchang	CRA004775	0.928449671
ENSGALG00010029231	CBX8	2	-0.2520881	0.181498538	-1.388926342	0.16485515	0	0.219435009	0	1	CRA004775;Yang2025_Silkie_Wenchang	PRJNA827465	0.664067023

ENSGALG00010029231	CBX8	2	-0.024769916	0.247749432	-0.099979709	0.920360435	0.088344817	2.940239361	65.98916356	0.5	CRA004775;PRJNA827465	Yang2025_Silkie_Wenchang	0.981413158
ENSGALG00010029233	CBX2	2	-0.234884672	0.252646742	-0.929696027	0.352528492	0.107319298	6.118592362	83.65637158	0.5	PRJNA827465;Yang2025_Silkie_Wenchang	CRA004775	0.810970286
ENSGALG00010029233	CBX2	2	-0.504505166	0.162943728	-3.096192609	0.00196023	0	0.000519455	0	1	CRA004775;Yang2025_Silkie_Wenchang	PRJNA827465	0.057238421
ENSGALG00010029233	CBX2	2	-0.023294063	0.106906601	-0.21789172	0.827513479	0	0.988399494	0	0.5	CRA004775;PRJNA827465	Yang2025_Silkie_Wenchang	0.956550752
ENSGALG00010029234	TOM1L1	2	-0.225178242	0.27749208	-0.811476285	0.417092207	0.103858737	3.019290481	66.87963592	0.5	PRJNA827465;Yang2025_Silkie_Wenchang	CRA004775	0.838112595
ENSGALG00010029234	TOM1L1	2	-0.106457528	0.542028959	-0.19640561	0.844292706	0.406936329	2.869223825	65.14736873	0.5	CRA004775;Yang2025_Silkie_Wenchang	PRJNA827465	0.960723102
ENSGALG00010029234	TOM1L1	2	0.07942123	0.184047551	0.431525603	0.66608624	0	0.771423896	0	1	CRA004775;PRJNA827465	Yang2025_Silkie_Wenchang	0.913579507
ENSGALG00010029235	TRIM37	2	-0.134427883	0.043739292	-3.073389543	0.00211642	0	0.942371994	0	1	PRJNA827465;Yang2025_Silkie_Wenchang	CRA004775	0.060189337
ENSGALG00010029235	TRIM37	2	-0.067597858	0.172439203	-0.3920098	0.695050973	0.041378824	2.944986545	66.04398748	0.5	CRA004775;Yang2025_Silkie_Wenchang	PRJNA827465	0.92097163
ENSGALG00010029235	TRIM37	2	-0.043262697	0.112311365	-0.385203199	0.700086873	0.014293659	1.752144161	42.970706	0.5	CRA004775;PRJNA827465	Yang2025_Silkie_Wenchang	0.922267298
ENSGALG00010029237	COX11	2	0.073409428	0.074125593	0.990338491	0.322008699	0	0.497122661	0	1	PRJNA827465;Yang2025_Silkie_Wenchang	CRA004775	0.798513866
ENSGALG00010029237	COX11	2	0.157523383	0.119937214	1.313382041	0.189054246	0	0.00595334	0	1	CRA004775;Yang2025_Silkie_Wenchang	PRJNA827465	0.698162702
ENSGALG00010029237	COX11	2	0.052969557	0.08408976	0.629916852	0.528748987	0	0.242309628	0	1	CRA004775;PRJNA827465	Yang2025_Silkie_Wenchang	0.871809618
ENSGALG00010029238	CAMKK1	2	0.294484872	0.145247677	2.027466999	0.042614676	0	0.733455088	0	1	PRJNA827465;Yang2025_Silkie_Wenchang	CRA004775	0.357019514
ENSGALG00010029238	CAMKK1	2	0.084891684	0.201074154	0.422190932	0.67288566	0	0.31658068	0	0.5	CRA004775;Yang2025_Silkie_Wenchang	PRJNA827465	0.915153756
ENSGALG00010029238	CAMKK1	2	0.276263826	0.221976458	1.244563628	0.213292197	0.028396503	1.256968518	20.44351264	0.5	CRA004775;PRJNA827465	Yang2025_Silkie_Wenchang	0.725875498
ENSGALG00010029239	RBFOX3	2	0.138431017	0.606646437	0.228190604	0.819498066	0.650854538	8.368827394	88.05089467	0.5	PRJNA827465;Yang2025_Silkie_Wenchang	CRA004775	0.955256039
ENSGALG00010029239	RBFOX3	2	0.346407435	0.492367372	0.703554815	0.481710059	0.299158735	2.55849851	60.91457563	0.5	CRA004775;Yang2025_Silkie_Wenchang	PRJNA827465	0.85691916
ENSGALG00010029239	RBFOX3	2	-0.398496933	0.185854982	-2.144128337	0.032022605	0	0.175752904	0	1	CRA004775;PRJNA827465	Yang2025_Silkie_Wenchang	0.306822147
ENSGALG00010029240	MIS12	2	-0.226262855	0.093127279	-2.42960879	0.015115128	0	0.318040615	0	1	PRJNA827465;Yang2025_Silkie_Wenchang	CRA004775	0.203100811
ENSGALG00010029240	MIS12	2	-0.058045245	0.353464411	-0.164218076	0.869559472	0.154407051	2.446202958	59.1203176	0.5	CRA004775;Yang2025_Silkie_Wenchang	PRJNA827465	0.968578601
ENSGALG00010029240	MIS12	2	-0.041805536	0.260193726	-0.160670805	0.872352685	0.083256056	1.971005851	49.26448343	0.5	CRA004775;PRJNA827465	Yang2025_Silkie_Wenchang	0.96962889
ENSGALG00010029241	STXBP4	2	-0.193458475	0.096630375	-2.002046205	0.045279762	0.007508135	1.575454485	36.52625262	1	PRJNA827465;Yang2025_Silkie_Wenchang	CRA004775	0.366947722
ENSGALG00010029241	STXBP4	2	-0.290065658	0.129521338	-2.239520247	0.025122085	0	0.504516353	0	1	CRA004775;Yang2025_Silkie_Wenchang	PRJNA827465	0.269931537
ENSGALG00010029241	STXBP4	2	-0.119169612	0.080551784	-1.47941618	0.139029118	0	0.037425471	0	1	CRA004775;PRJNA827465	Yang2025_Silkie_Wenchang	0.622849816
ENSGALG00010029242	DERL2	2	-0.120575831	0.182803704	-0.659591836	0.509515795	0.053971279	4.957719915	79.82943738	0.5	PRJNA827465;Yang2025_Silkie_Wenchang	CRA004775	0.865257661
ENSGALG00010029242	DERL2	2	-0.29986984	0.120330087	-2.492060361	0.012700445	0	0.091906199	0	1	CRA004775;Yang2025_Silkie_Wenchang	PRJNA827465	0.182777589
ENSGALG00010029242	DERL2	2	-0.029841889	0.125703929	-0.237398223	0.812347868	0.014793267	1.551080841	35.52882779	0.5	CRA004775;PRJNA827465	Yang2025_Silkie_Wenchang	0.953371387
ENSGALG00010029243	NME1	2	0.487852879	0.177537054	2.747893288	0.005997953	0	0.942341905	0	1	PRJNA827465;Yang2025_Silkie_Wenchang	CRA004775	0.117049421
ENSGALG00010029243	NME1	2	0.06919944	0.697430007	0.099220624	0.9209631	0.789819676	4.955554985	79.82062548	0.5	CRA004775;Yang2025_Silkie_Wenchang	PRJNA827465	0.981469977
ENSGALG00010029243	NME1	2	-0.067472016	0.514166406	-0.13122603	0.895596505	0.369233027	2.919721316	65.75015586	0.5	CRA004775;PRJNA827465	Yang2025_Silkie_Wenchang	0.974927462
ENSGALG00010029244	HLF	2	0.048777002	0.071002353	0.686977257	0.49209706	0	0.133630824	0	1	PRJNA827465;Yang2025_Silkie_Wenchang	CRA004775	0.859158569
ENSGALG00010029244	HLF	2	0.273285301	0.244369563	1.118327902	0.263426974	0.064479579	1.922430195	47.98250659	1	CRA004775;Yang2025_Silkie_Wenchang	PRJNA827465	0.768643821
ENSGALG00010029244	HLF	2	0.234407769	0.276385457	0.848119042	0.396371678	0.10954071	2.810127455	64.41442546	1	CRA004775;PRJNA827465	Yang2025_Silkie_Wenchang	0.831365249
ENSGALG00010029245	SKA2	2	0.003985173	0.117097013	0.034033087	0.972850766	0.019982098	3.322076631	69.8983464	0.5	PRJNA827465;Yang2025_Silkie_Wenchang	CRA004775	0.992213869
ENSGALG00010029245	SKA2	2	0.077803831	0.13261379	0.586694849	0.557408062	0.00809342	1.185553081	15.65118289	0.5	CRA004775;Yang2025_Silkie_Wenchang	PRJNA827465	0.880678789
ENSGALG00010029245	SKA2	2	-0.094537957	0.052254911	-1.809168851	0.070424775	0	0.089871312	0	1	CRA004775;PRJNA827465	Yang2025_Silkie_Wenchang	0.458021454
ENSGALG00010029246	DHX33	2	-0.092971031	0.172178872	-0.539967708	0.589219302	0.046729585	4.535168442	77.95010234	0.5	PRJNA827465;Yang2025_Silkie_Wenchang	CRA004775	0.892171138
ENSGALG00010029246	DHX33	2	-0.264537323	0.117564758	-2.250141337	0.024439975	0	0.068329348	0	1	CRA004775;Yang2025_Silkie_Wenchang	PRJNA827465	0.267184364
ENSGALG00010029246	DHX33	2	-0.004483031	0.118774994	-0.037743894	0.969891879	0.011589252	1.42575447	29.86169631	0.5	CRA004775;PRJNA827465	Yang2025_Silkie_Wenchang	0.991572914
ENSGALG00010029247	CCDC92B	2	-0.031032712	0.482081644	-0.064372316	0.948673773	0.364580111	4.556680768	78.05420105	0.5	PRJNA827465;Yang2025_Silkie_Wenchang	CRA004775	0.986942018
ENSGALG00010029247	CCDC92B	2	0.40843901	0.246968807	1.653808085	0.098166513	0	0.011321425	0	1	CRA004775;Yang2025_Silkie_Wenchang	PRJNA827465	0.534678278
ENSGALG00010029247	CCDC92B	2	-0.298598419	0.398993177	-0.748379762	0.454231126	0.086285549	1.280471894	21.90179154	0.5	CRA004775;PRJNA827465	Yang2025_Silkie_Wenchang	0.849072665
ENSGALG00010029248	C1QBP	2	-0.084872479	0.059408112	-1.428634512	0.153109309	0	0.542266433	0	0.5	PRJNA827465;Yang2025_Silkie_Wenchang	CRA004775	0.645187993
ENSGALG00010029248	C1QBP	2	-0.042196191	0.144752482	-0.29150582	0.770664493	0	0.592720331	0	0.5	CRA004775;Yang2025_Silkie_Wenchang	PRJNA827465	0.942491331
ENSGALG00010029248	C1QBP	2	-0.105957917	0.060921335	-1.739257969	0.081989397	0	0.135524838	0	1	CRA004775;PRJNA827465	Yang2025_Silkie_Wenchang	0.493597945
ENSGALG00010029249	PRR11	2	-0.151562843	0.15153317	-0.97379896	0.33015637	0.024124677	1.7391197	42.50625689	0.5	PRJNA827465;Yang2025_Silkie_Wenchang	CRA004775	0.801276642
ENSGALG00010029249	PRR11	2	0.118548212	0.213800822	0.554479661	0.579250607	0	0.049435636	0	1	CRA004775;Yang2025_Silkie_Wenchang	PRJNA827465	0.887888423
ENSGALG00010029249	PRR11	2	-0.227058308	0.087020344	-2.609255479	0.009073947	0	0.892171852	0	0.5	CRA004775;PRJNA827465	Yang2025_Silkie_Wenchang	0.14921897
ENSGALG00010029250	SPAG9	2	0.145181444	0.162449594	0.893701484	0.371481631	0.045854324	7.28963456	86.2818912	0.5	PRJNA827465;Yang2025_Silkie_Wenchang	CRA004775	0.821526338
ENSGALG00010029250	SPAG9	2	0.311501122	0.10132473	3.074285243	0.002110076	0	0.072852171	0	1	CRA004775;Yang2025_Silkie_Wenchang	PRJNA827465	0.060095876
ENSGALG00010029250	SPAG9	2	0.003729652	0.052821648	0.070608405	0.943709421	0	0.676305555	0	0.5	CRA004775;PRJNA827465	Yang2025_Silkie_Wenchang	0.985827325
ENSGALG00010029251	ENGASE	2	0.102387071	0.141418233	0.724001912	0.469064561	0	0.371636318	0	1	PRJNA827465;Yang2025_Silkie_Wenchang	CRA004775	0.85345437
ENSGALG00010029251	ENGASE	2	-0.015764878	0.288793893	-0.054588431	0.956466157	0.072690461	1.769529167	43.48140422	0.5	CRA004775;Yang2025_Silkie_Wenchang	PRJNA827465	0.988540618
ENSGALG00010029251	ENGASE	2	-0.029500628	0.156410109	-0.188610749	0.850397907	0.005624158	1.087618839	8.056024406	0.5	CRA004775;PRJNA827465	Yang2025_Silkie_Wenchang	0.961985357
ENSGALG00010029252	RPAIN	2	-0.064970705	0.254869465	-0.25491757	0.798786766	0.083585406	2.401611335	58.36128914	0.5	PRJNA827465;Yang2025_Silkie_Wenchang	CRA004775	0.949922217
ENSGALG00010029252	RPAIN	2	-0.264929261	0.227691358	-1.163545528	0.244608238	0	0.432737884	0	1	CRA004775;Yang2025_Silkie_Wenchang	PRJNA827465	0.756093682
ENSGALG00010029252	RPAIN	2	0.089702975	0.117714761	0.762036761	0.446038064	0	0.46241293	0	0.5	CRA004775;PRJNA827465	Yang2025_Silkie_Wenchang	0.846013379
ENSGALG00010029253	NUP88	2	-0.043953957	0.045553371	-0.964889232	0.334600295	0	0.049302537	0	1	PRJNA827465;Yang2025_Silkie_Wenchang	CRA004775	0.803101658
ENSGALG00010029253	NUP88	2	-0.012768442	0.093225879	-0.13696242	0.8910605	0	0.054923879	0	0.5	CRA004775;Yang2025_Silkie_Wenchang	PRJNA827465	0.973638442
ENSGALG00010029253	NUP88	2	-0.044597393	0.049367026	-0.903384234	0.366322695	0	0.136446953	0	0.5	CRA004775;PRJNA827465	Yang2025_Silkie_Wenchang	0.81919863
ENSGALG00010029254	MMD	2	-0.285652105	0.257245505	-1.110426029	0.266815487	0.126047094	20.75378602	95.18160205	1	PRJNA827465;Yang2025_Silkie_Wenchang	CRA004775	0.771006559
ENSGALG00010029254	MMD	2	-0.067795307	0.504072446	-0.134495165	0.893011034	0.476252978	15.45310813	93.51957262	0.5	CRA004775;Yang2025_Silkie_Wenchang	PRJNA827465	0.974381724
ENSGALG00010029254	MMD	2	0.158902621	0.241356139	0.658374059	0.5102978	0.092501923	4.104276918	75.63517229	0.5	CRA004775;PRJNA827465	Yang2025_Silkie_Wenchang	0.865365179
ENSGALG00010029255	RAP1GAP2	2	-0.092949005	0.083483771	-1.113378125	0.265546066	0	0.08445732	0	1	PRJNA827465;Yang2025_Silkie_Wenchang	CRA004775	0.769672347
ENSGALG00010029255	RAP1GAP2	2	-0.090390004	0.167843706	-0.538536749	0.590206541	0	0.40769601	0	1	CRA004775;Yang2025_Silkie_Wenchang	PRJNA827465	0.892451983
ENSGALG00010029255	RAP1GAP2	2	-0.116194672	0.091920237	-1.264081511	0.206200772	0	0.295204081	0	1	CRA004775;PRJNA827465	Yang2025_Silkie_Wenchang	0.718403416
ENSGALG00010029256	RABEP1	2	0.088454685	0.163765174	0.540131233	0.589106533	0.048858456	10.85124259	90.78446554	0.5	PRJNA827465;Yang2025_Silkie_Wenchang	CRA004775	0.892171138
ENSGALG00010029256	RABEP1	2	0.262261046	0.086328661	3.03793714	0.002382036	0	0.000325991	0	1	CRA004775;Yang2025_Silkie_Wenchang	PRJNA827465	0.065395805
ENSGALG00010029256	RABEP1	2	-0.043814545	0.08303772	-0.527646294	0.597744856	0.005544748	1.112073463	10.07788308	0.5	CRA004775;PRJNA827465	Yang2025_Silkie_Wenchang	0.894645663
ENSGALG00010029257	YPEL2	2	-0.021186741	0.104640274	-0.202472143	0.839547634	0.008299803	1.607987991	37.81048081	0.5	PRJNA827465;Yang2025_Silkie_Wenchang	CRA004775	0.959493354
ENSGALG00010029257	YPEL2	2	0.071834053	0.109199237	0.657825599	0.510650203	0	0.067506759	0	0.5	CRA004775;Yang2025_Silkie_Wenchang	PRJNA827465	0.86538785
ENSGALG00010029257	YPEL2	2	-0.12602301	0.117576584	-1.071837656	0.283792956	0	0.028197427	0	1	CRA004775;PRJNA827465	Yang2025_Silkie_Wenchang	0.77978499
ENSGALG00010029258	TMEM100	2	-0.195538986	0.238488339	-0.81991005	0.412267387	0.098826599	7.541070972	86.73928407	0.5	PRJNA827465;Yang2025_Silkie_Wenchang	CRA004775	0.835611629
ENSGALG00010029258	TMEM100	2	-0.418962815	0.133635227	-3.135122549	0.001717823	0	0.341033484	0	1	CRA004775;Yang2025_Silkie_Wenchang	PRJNA827465	0.052459682
ENSGALG00010029258	TMEM100	2	0.022970153	0.100070916	0.229538748	0.818450208	0	0.208415308	0	0.5	CRA004775;PRJNA827465	Yang2025_Silkie_Wenchang	0.955256039
ENSGALG00010029259	PTRH2	2	-0.146641497	0.216167627	-0.678369371	0.497537524	0.061730522	2.554594845	60.85484938	0.5	PRJNA827465;Yang2025_Silkie_Wenchang	CRA004775	0.86098656
ENSGALG00010029259	PTRH2	2	-0.398729934	0.183908805	-2.168085064	0.030152215	0	0.038362286	0	1	CRA004775;Yang2025_Silkie_Wenchang	PRJNA827465	0.295973612
ENSGALG00010029259	PTRH2	2	-0.099951213	0.174181734	-0.57383292	0.566080869	0.032390396	1.826418044	45.2480223	0.5	CRA004775;PRJNA827465	Yang2025_Silkie_Wenchang	0.883487517
ENSGALG00010029260	DHX40	2	0.196459752	0.152126956	1.29141973	0.196558173	0.038024412	5.532770947	81.92587366	1	PRJNA827465;Yang2025_Silkie_Wenchang	CRA004775	0.707901206
ENSGALG00010029260	DHX40	2	0.125873763	0.28578881	0.440443286	0.65961608	0.124174396	3.658281764	72.66476273	0.5	CRA004775;Yang2025_Silkie_Wenchang	PRJNA827465	0.911617103
ENSGALG00010029260	DHX40	2	0.033306257	0.074509428	0.44700728	0.654869803	0	0.884990105	0	0.5	CRA004775;PRJNA827465	Yang2025_Silkie_Wenchang	0.910222014
ENSGALG00010029261	IFT22	2	-0.135187473	0.091985889	-1.469654468	0.141655536	0	0.502674994	0	1	PRJNA827465;Yang2025_Silkie_Wenchang	CRA004775	0.627088401
ENSGALG00010029261	IFT22	2	-0.175236281	0.11467218	-1.528149912	0.126475322	0	0.164360191	0	1	CRA004775;Yang2025_Silkie_Wenchang	PRJNA827465	0.597028533
ENSGALG00010029261	IFT22	2	-0.070608377	0.117748434	-0.599654486	0.548736527	0	0.002216067	0	1	CRA004775;PRJNA827465	Yang2025_Silkie_Wenchang	0.878281266
ENSGALG00010029262	CLTC	2	-0.194233284	0.160829358	-1.207697936	0.227163472	0.047345487	11.51600273	91.31643138	1	PRJNA827465;Yang2025_Silkie_Wenchang	CRA004775	0.740605003
ENSGALG00010029262	CLTC	2	-0.351482961	0.080477174	-4.367486355	1.25685E-05	0	0.272645726	0	1	CRA004775;Yang2025_Silkie_Wenchang	PRJNA827465	0.00169752
ENSGALG00010029262	CLTC	2	-0.044108261	0.044845156	-0.983568003	0.325327964	0	0.248011743	0	1	CRA004775;PRJNA827465	Yang2025_Silkie_Wenchang	0.799987949
ENSGALG00010029263	TOB1	2	0.095142342	0.054162462	1.756610359	0.078984246	0	0.371148424	0	1	PRJNA827465;Yang2025_Silkie_Wenchang	CRA004775	0.483312755
ENSGALG00010029263	TOB1	2	0.157198447	0.092836992	1.693273801	0.090403363	0	0.902773643	0	1	CRA004775;Yang2025_Silkie_Wenchang	PRJNA827465	0.5160164
ENSGALG00010029263	TOB1	2	0.123825649	0.168651893	0.734208477	0.462821688	0.026751723	1.174445477	14.85343342	1	CRA004775;PRJNA827465	Yang2025_Silkie_Wenchang	0.851206402
ENSGALG00010029264	COL26A1	2	-0.182156912	0.258210241	-0.705459672	0.48052422	0.103371839	4.366826026	77.10007236	0.5	PRJNA827465;Yang2025_Silkie_Wenchang	CRA004775	0.85670417
ENSGALG00010029264	COL26A1	2	-0.48178507	0.188212831	-2.559788654	0.010473584	0	0.090304626	0	1	CRA004775;Yang2025_Silkie_Wenchang	PRJNA827465	0.162995658
ENSGALG00010029264	COL26A1	2	-0.098959105	0.28834176	-0.343200737	0.73144745	0.08685831	1.581172338	36.75578709	0.5	CRA004775;PRJNA827465	Yang2025_Silkie_Wenchang	0.932851968
ENSGALG00010029266	CANT1	2	0.124975282	0.068151954	1.833774015	0.066687547	0	0.03564479	0	1	PRJNA827465;Yang2025_Silkie_Wenchang	CRA004775	0.444843965
ENSGALG00010029266	CANT1	2	0.079639488	0.113756479	0.700087486	0.48387267	0	0.53358508	0	0.5	CRA004775;Yang2025_Silkie_Wenchang	PRJNA827465	0.85691916
ENSGALG00010029266	CANT1	2	0.096558513	0.070261386	1.374275661	0.169356119	0	0.508096786	0	0.5	CRA004775;PRJNA827465	Yang2025_Silkie_Wenchang	0.670729184
ENSGALG00010029267	ENSGALG00010029267	2	0.2959732	0.111788799	2.647610507	0.008106286	0	0.566219692	0	1	PRJNA827465;Yang2025_Silkie_Wenchang	CRA004775	0.139734161
ENSGALG00010029267	ENSGALG00010029267	2	-0.03679897	0.437448067	-0.084121916	0.9329595	0.342853966	9.493491258	89.46646736	0.5	CRA004775;Yang2025_Silkie_Wenchang	PRJNA827465	0.984224753
ENSGALG00010029267	ENSGALG00010029267	2	-0.115230113	0.352809807	-0.326606889	0.743965233	0.21097777	6.422602817	84.42998846	0.5	CRA004775;PRJNA827465	Yang2025_Silkie_Wenchang	0.935821071
ENSGALG00010029268	PCTP	2	-0.571528112	0.444895142	-1.284635543	0.198919672	0.359060814	10.67530405	90.63258531	1	PRJNA827465;Yang2025_Silkie_Wenchang	CRA004775	0.71096127
ENSGALG00010029268	PCTP	2	-0.444609747	0.61189451	-0.726611759	0.467463823	0.667779614	9.081245673	88.98829482	0.5	CRA004775;Yang2025_Silkie_Wenchang	PRJNA827465	0.852885838
ENSGALG00010029268	PCTP	2	-0.077916219	0.145990932	-0.533705881	0.59354504	0	0.783678274	0	0.5	CRA004775;PRJNA827465	Yang2025_Silkie_Wenchang	0.892915939
ENSGALG00010029269	WSCD1	2	-0.082379412	0.103750073	-0.794017863	0.427185032	0.011189997	2.045625095	51.11518712	0.5	PRJNA827465;Yang2025_Silkie_Wenchang	CRA004775	0.84125685
ENSGALG00010029269	WSCD1	2	-0.190281785	0.112207929	-1.695796248	0.089924473	0	0.111656501	0	1	CRA004775;Yang2025_Silkie_Wenchang	PRJNA827465	0.514454377
ENSGALG00010029269	WSCD1	2	0.006226372	0.086916829	0.071635982	0.942891604	0	0.019696065	0	0.5	CRA004775;PRJNA827465	Yang2025_Silkie_Wenchang	0.985827325
ENSGALG00010029270	TIMP2	2	0.17867345	0.10194898	1.75257712	0.079674621	0.013856155	2.909209696	65.62640358	1	PRJNA827465;Yang2025_Silkie_Wenchang	CRA004775	0.48542321
ENSGALG00010029270	TIMP2	2	0.291693653	0.09462483	3.08263332	0.002051778	0	0.006743324	0	1	CRA004775;Yang2025_Silkie_Wenchang	PRJNA827465	0.058948122
ENSGALG00010029270	TIMP2	2	0.097000529	0.066461381	1.459502157	0.144426947	0	0.320386326	0	1	CRA004775;PRJNA827465	Yang2025_Silkie_Wenchang	0.631870428
ENSGALG00010029271	MYL10	2	-0.299670096	0.398221087	-0.752521918	0.451737254	0.268728612	6.475876471	84.55807481	0.5	PRJNA827465;Yang2025_Silkie_Wenchang	CRA004775	0.848006549
ENSGALG00010029271	MYL10	2	-0.319557695	0.551497662	-0.579436173	0.562294902	0.399551867	2.22938803	55.1446412	0.5	CRA004775;Yang2025_Silkie_Wenchang	PRJNA827465	0.881955027
ENSGALG00010029271	MYL10	2	0.154860068	0.239702338	0.646051554	0.518245968	0	0.243548968	0	1	CRA004775;PRJNA827465	Yang2025_Silkie_Wenchang	0.868375737
ENSGALG00010029273	SLC13A5	2	0.2265179	0.207920399	1.089445294	0.275957567	0	0.296928243	0	1	PRJNA827465;Yang2025_Silkie_Wenchang	CRA004775	0.775541559
ENSGALG00010029273	SLC13A5	2	0.018275325	0.287618507	0.063540157	0.949336383	0	0.269261908	0	0.5	CRA004775;Yang2025_Silkie_Wenchang	PRJNA827465	0.98723759
ENSGALG00010029273	SLC13A5	2	0.230807676	0.250979277	0.919628419	0.35776697	0	0.74456746	0	0.5	CRA004775;PRJNA827465	Yang2025_Silkie_Wenchang	0.813618706
ENSGALG00010029274	VMP1	2	-0.042467119	0.161148418	-0.263527995	0.792143649	0.045876613	8.360149812	88.03849186	0.5	PRJNA827465;Yang2025_Silkie_Wenchang	CRA004775	0.948314332
ENSGALG00010029274	VMP1	2	-0.247876262	0.088626638	-2.796859585	0.005160195	0	0.877833568	0	1	CRA004775;Yang2025_Silkie_Wenchang	PRJNA827465	0.107364299
ENSGALG00010029274	VMP1	2	-0.123857974	0.274485039	-0.451237613	0.651818304	0.126639751	5.520734057	81.88646673	0.5	CRA004775;PRJNA827465	Yang2025_Silkie_Wenchang	0.909163702
ENSGALG00010029275	PCYT2	2	0.15713388	0.112590315	1.395625198	0.16282739	0.02019604	4.675556551	78.61217185	1	PRJNA827465;Yang2025_Silkie_Wenchang	CRA004775	0.660874227
ENSGALG00010029275	PCYT2	2	0.143049391	0.188815718	0.757613785	0.448682225	0.046927773	2.522895198	60.36299879	0.5	CRA004775;Yang2025_Silkie_Wenchang	PRJNA827465	0.846878812
ENSGALG00010029275	PCYT2	2	0.049119594	0.049326942	0.995796448	0.319349056	0	0.50419251	0	0.5	CRA004775;PRJNA827465	Yang2025_Silkie_Wenchang	0.798079048
ENSGALG00010029276	TUBD1	2	-0.19183225	0.13202157	-1.45303718	0.146213431	0.02325694	2.990278591	66.55829985	1	PRJNA827465;Yang2025_Silkie_Wenchang	CRA004775	0.634916196
ENSGALG00010029276	TUBD1	2	-0.046521953	0.108974575	-0.426906485	0.669447442	0	0.029682941	0	0.5	CRA004775;Yang2025_Silkie_Wenchang	PRJNA827465	0.914377769
ENSGALG00010029276	TUBD1	2	-0.289521117	0.095492386	-3.031876469	0.002430386	0	0.909534448	0	0.5	CRA004775;PRJNA827465	Yang2025_Silkie_Wenchang	0.065967872
ENSGALG00010029277	WFIKKN2	2	-0.124690945	0.442176658	-0.281993505	0.777948491	0.276262034	3.386185907	70.46824872	0.5	PRJNA827465;Yang2025_Silkie_Wenchang	CRA004775	0.944432731
ENSGALG00010029277	WFIKKN2	2	-0.675427295	0.343588019	-1.965805722	0.049321057	0	0.419475673	0	1	CRA004775;Yang2025_Silkie_Wenchang	PRJNA827465	0.384311159
ENSGALG00010029277	WFIKKN2	2	-0.214669604	0.728148165	-0.294815827	0.768134575	0.694693714	2.374753581	57.89036775	0.5	CRA004775;PRJNA827465	Yang2025_Silkie_Wenchang	0.941444269
ENSGALG00010029278	MED31	2	0.046439657	0.155174921	0.299272951	0.764731791	0.040679813	6.230262481	83.94931188	0.5	PRJNA827465;Yang2025_Silkie_Wenchang	CRA004775	0.940711384
ENSGALG00010029278	MED31	2	-0.062179119	0.306260978	-0.203026581	0.839114257	0.158665223	6.135942337	83.70258479	0.5	CRA004775;Yang2025_Silkie_Wenchang	PRJNA827465	0.959403482
ENSGALG00010029278	MED31	2	-0.173259756	0.131911822	-1.313451317	0.189030915	0.019247276	1.710593439	41.54075554	1	CRA004775;PRJNA827465	Yang2025_Silkie_Wenchang	0.698162702
ENSGALG00010029279	ENSGALG00010029279	2	-0.168427183	0.257057312	-0.655212573	0.512330901	0.115202211	7.403067437	86.49208577	0.5	PRJNA827465;Yang2025_Silkie_Wenchang	CRA004775	0.865809047
ENSGALG00010029279	ENSGALG00010029279	2	-0.403243791	0.149970026	-2.68882925	0.007170309	0	0.291957082	0	1	CRA004775;Yang2025_Silkie_Wenchang	PRJNA827465	0.130241134
ENSGALG00010029279	ENSGALG00010029279	2	0.034475594	0.097755033	0.352673335	0.724333352	0.004366055	1.088677759	8.145455181	0.5	CRA004775;PRJNA827465	Yang2025_Silkie_Wenchang	0.930387861
ENSGALG00010029280	USP36	2	0.143038349	0.142310385	1.00511533	0.314841319	0.033083413	5.337429759	81.26439044	1	PRJNA827465;Yang2025_Silkie_Wenchang	CRA004775	0.796531448
ENSGALG00010029280	USP36	2	0.229661095	0.141809173	1.619508036	0.105337999	0.013967435	1.256454512	20.41096667	0.5	CRA004775;Yang2025_Silkie_Wenchang	PRJNA827465	0.550809984
ENSGALG00010029280	USP36	2	0.006643791	0.067173017	0.098905651	0.921213182	0	0.069405477	0	0.5	CRA004775;PRJNA827465	Yang2025_Silkie_Wenchang	0.981519273
ENSGALG00010029281	KIAA0753	2	0.19121099	0.285020367	0.67086781	0.502304744	0.144721799	8.929444629	88.80109524	0.5	PRJNA827465;Yang2025_Silkie_Wenchang	CRA004775	0.862860181
ENSGALG00010029281	KIAA0753	2	0.187835605	0.374511526	0.501548263	0.615985321	0.205789244	3.369209933	70.31945115	0.5	CRA004775;Yang2025_Silkie_Wenchang	PRJNA827465	0.899050535
ENSGALG00010029281	KIAA0753	2	-0.089945472	0.093163612	-0.965457107	0.33431591	0	0.242094754	0	1	CRA004775;PRJNA827465	Yang2025_Silkie_Wenchang	0.803094614
ENSGALG00010029282	LUC7L3	2	0.846696192	0.852479077	0.99321639	0.320604513	1.401420868	27.11232282	96.31164026	1	PRJNA827465;Yang2025_Silkie_Wenchang	CRA004775	0.798348216
ENSGALG00010029282	LUC7L3	2	0.882558103	0.825047291	1.06970608	0.284751629	1.293452399	19.80156188	94.94989332	1	CRA004775;Yang2025_Silkie_Wenchang	PRJNA827465	0.780278244
ENSGALG00010029282	LUC7L3	2	0.028991337	0.064303061	0.450854705	0.652094271	0	0.077219961	0	1	CRA004775;PRJNA827465	Yang2025_Silkie_Wenchang	0.909322729
ENSGALG00010029283	RPS6KB1	2	-0.101907801	0.081862077	-1.244871923	0.213178833	0.008843227	2.503066833	60.0490092	1	PRJNA827465;Yang2025_Silkie_Wenchang	CRA004775	0.72579791
ENSGALG00010029283	RPS6KB1	2	-0.027762069	0.208929192	-0.132877883	0.894289959	0.06783098	4.278118315	76.6252374	0.5	CRA004775;Yang2025_Silkie_Wenchang	PRJNA827465	0.974381724
ENSGALG00010029283	RPS6KB1	2	0.023311383	0.109480691	0.212926889	0.831383989	0.014760935	1.906882226	47.55837636	0.5	CRA004775;PRJNA827465	Yang2025_Silkie_Wenchang	0.957626151
ENSGALG00010029284	CYTH1	2	0.175218712	0.06531107	2.682833288	0.007300137	0	0.05993994	0	1	PRJNA827465;Yang2025_Silkie_Wenchang	CRA004775	0.131225376
ENSGALG00010029284	CYTH1	2	-0.021184271	0.180185579	-0.117569179	0.906409027	0.037570053	2.344615019	57.34907471	0.5	CRA004775;Yang2025_Silkie_Wenchang	PRJNA827465	0.978196186
ENSGALG00010029284	CYTH1	2	0.017754965	0.197926169	0.089704991	0.928521649	0.061212431	4.107571903	75.6547171	0.5	CRA004775;PRJNA827465	Yang2025_Silkie_Wenchang	0.983311758
ENSGALG00010029285	CUX1	2	0.121342447	0.237768588	0.510338424	0.609814388	0.106384276	16.67318303	94.00234497	0.5	PRJNA827465;Yang2025_Silkie_Wenchang	CRA004775	0.897238578
ENSGALG00010029285	CUX1	2	0.239559254	0.157976873	1.516419775	0.129413242	0.030833853	2.432990303	58.89831542	1	CRA004775;Yang2025_Silkie_Wenchang	PRJNA827465	0.603529077
ENSGALG00010029285	CUX1	2	-0.095204824	0.055168318	-1.72571554	0.084398601	0	0.640297942	0	0.5	CRA004775;PRJNA827465	Yang2025_Silkie_Wenchang	0.500017819
ENSGALG00010029286	MAFG	2	-0.031567932	0.073577209	-0.429044982	0.66789049	0.001184287	1.083949547	7.744783598	0.5	PRJNA827465;Yang2025_Silkie_Wenchang	CRA004775	0.914120722
ENSGALG00010029286	MAFG	2	-0.145509972	0.13126691	-1.108504593	0.267643957	0	0.083736174	0	1	CRA004775;Yang2025_Silkie_Wenchang	PRJNA827465	0.771398294
ENSGALG00010029286	MAFG	2	0.002512986	0.073637104	0.034126624	0.972776178	0	0.088466401	0	0.5	CRA004775;PRJNA827465	Yang2025_Silkie_Wenchang	0.992213869
ENSGALG00010029287	ANKRD40	2	0.102198831	0.075141649	1.360082351	0.173803865	0	0.098045154	0	1	PRJNA827465;Yang2025_Silkie_Wenchang	CRA004775	0.677480397
ENSGALG00010029287	ANKRD40	2	0.130448391	0.132850988	0.981915095	0.326141679	0	0.032185474	0	1	CRA004775;Yang2025_Silkie_Wenchang	PRJNA827465	0.800023788
ENSGALG00010029287	ANKRD40	2	0.087708147	0.083992276	1.044240626	0.296374075	0	0.000532086	0	1	CRA004775;PRJNA827465	Yang2025_Silkie_Wenchang	0.788267543
ENSGALG00010029288	PYCR1	2	0.009143009	0.144330479	0.063347733	0.949489607	0.030950521	3.658195193	72.66411585	0.5	PRJNA827465;Yang2025_Silkie_Wenchang	CRA004775	0.98723759
ENSGALG00010029288	PYCR1	2	-0.469187883	0.392000887	-1.196905156	0.231343524	0.232329448	3.529450109	71.66697449	1	CRA004775;Yang2025_Silkie_Wenchang	PRJNA827465	0.743891332
ENSGALG00010029288	PYCR1	2	-0.34193126	0.543644568	-0.628961053	0.529374554	0.516237453	7.034246198	85.78383565	0.5	CRA004775;PRJNA827465	Yang2025_Silkie_Wenchang	0.871944741
ENSGALG00010029289	PITPNM3	2	0.295676659	0.1310772	2.255744396	0.024086643	0	0.404808485	0	1	PRJNA827465;Yang2025_Silkie_Wenchang	CRA004775	0.265011806
ENSGALG00010029289	PITPNM3	2	0.191305743	0.272470935	0.702114313	0.482607877	0.071779195	1.872340399	46.59090833	0.5	CRA004775;Yang2025_Silkie_Wenchang	PRJNA827465	0.85691916
ENSGALG00010029289	PITPNM3	2	0.163714157	0.149857388	1.09246637	0.274628164	0.002526211	1.036868078	3.555715407	0.5	CRA004775;PRJNA827465	Yang2025_Silkie_Wenchang	0.774575583
ENSGALG00010029290	ENSGALG00010029290	2	0.390851672	0.263199163	1.485003473	0.137542904	0.118562113	6.781419917	85.25382571	1	PRJNA827465;Yang2025_Silkie_Wenchang	CRA004775	0.62043484
ENSGALG00010029290	ENSGALG00010029290	2	0.214017164	0.57919037	0.369510915	0.711746939	0.514994243	3.647291776	72.58239644	0.5	CRA004775;Yang2025_Silkie_Wenchang	PRJNA827465	0.92597752
ENSGALG00010029290	ENSGALG00010029290	2	0.073533727	0.203601548	0.361164869	0.717976203	0.034334993	1.184580439	15.58192527	0.5	CRA004775;PRJNA827465	Yang2025_Silkie_Wenchang	0.927927766
ENSGALG00010029291	RNFT1	2	0.056272739	0.052694573	1.067903881	0.285563872	0	0.749421597	0	0.5	PRJNA827465;Yang2025_Silkie_Wenchang	CRA004775	0.781255524
ENSGALG00010029291	RNFT1	2	-0.076065292	0.095600564	-0.795657359	0.426231218	0	0.287001373	0	1	CRA004775;Yang2025_Silkie_Wenchang	PRJNA827465	0.84125685
ENSGALG00010029291	RNFT1	2	0.016021299	0.099856728	0.160442859	0.87253223	0.010442285	1.735420616	42.3770819	0.5	CRA004775;PRJNA827465	Yang2025_Silkie_Wenchang	0.969629321
ENSGALG00010029292	ENSGALG00010029292	2	0.463912286	0.296860069	1.562730505	0.118115996	0.143378787	5.147596527	80.57345803	1	PRJNA827465;Yang2025_Silkie_Wenchang	CRA004775	0.578003524
ENSGALG00010029292	ENSGALG00010029292	2	0.249442467	0.590065369	0.422737006	0.672487153	0.589864924	6.287615227	84.0957189	0.5	CRA004775;Yang2025_Silkie_Wenchang	PRJNA827465	0.914749172
ENSGALG00010029292	ENSGALG00010029292	2	0.031789821	0.26313332	0.120812602	0.903839468	0.078598559	1.834530296	45.49013434	0.5	CRA004775;PRJNA827465	Yang2025_Silkie_Wenchang	0.977333734
ENSGALG00010029293	DNAH17	2	0.049779368	0.179614279	0.277145942	0.781668046	0.041996081	2.812801228	64.44825215	0.5	PRJNA827465;Yang2025_Silkie_Wenchang	CRA004775	0.945265125
ENSGALG00010029293	DNAH17	2	-0.186564978	0.473157658	-0.394297705	0.693361258	0.383248296	6.658681506	84.98201184	0.5	CRA004775;Yang2025_Silkie_Wenchang	PRJNA827465	0.920721644
ENSGALG00010029293	DNAH17	2	-0.331495781	0.284454142	-1.165375123	0.243867182	0.112017158	2.828251231	64.64246213	1	CRA004775;PRJNA827465	Yang2025_Silkie_Wenchang	0.755365463
ENSGALG00010029294	PGS1	2	-0.135601443	0.110384735	-1.228443794	0.219280417	0.016926113	2.986932527	66.52083731	1	PRJNA827465;Yang2025_Silkie_Wenchang	CRA004775	0.73285529
ENSGALG00010029294	PGS1	2	-0.280190169	0.093378458	-3.000586792	0.002694599	0	0.014732804	0	1	CRA004775;Yang2025_Silkie_Wenchang	PRJNA827465	0.070599041
ENSGALG00010029294	PGS1	2	-0.129313755	0.117534239	-1.100221993	0.27123541	0.017468314	2.288054686	56.29475091	1	CRA004775;PRJNA827465	Yang2025_Silkie_Wenchang	0.772743863
ENSGALG00010029295	SOCS3	2	0.249351108	0.111986421	2.22661913	0.025972739	0	0.558547564	0	1	PRJNA827465;Yang2025_Silkie_Wenchang	CRA004775	0.274758802
ENSGALG00010029295	SOCS3	2	-0.363743285	0.551535656	-0.659510008	0.509568322	0.46966622	4.005232121	75.03265804	0.5	CRA004775;Yang2025_Silkie_Wenchang	PRJNA827465	0.865257661
ENSGALG00010029295	SOCS3	2	-0.266936703	0.650265167	-0.410504386	0.681435987	0.724151059	6.214833453	83.90946423	0.5	CRA004775;PRJNA827465	Yang2025_Silkie_Wenchang	0.918242009
ENSGALG00010029296	FBXO39	2	0.311712952	0.136981272	2.275588091	0.022870675	0.020967838	2.242539126	55.40768996	1	PRJNA827465;Yang2025_Silkie_Wenchang	CRA004775	0.257856033
ENSGALG00010029296	FBXO39	2	0.217554012	0.293338809	0.741647561	0.458300899	0.125385338	3.386360705	70.46977309	0.5	CRA004775;Yang2025_Silkie_Wenchang	PRJNA827465	0.850026426
ENSGALG00010029296	FBXO39	2	0.136962891	0.116254655	1.178128225	0.238745491	0.002567992	1.052312579	4.971201539	0.5	CRA004775;PRJNA827465	Yang2025_Silkie_Wenchang	0.75079325
ENSGALG00010029297	SH2B2	2	0.178933255	0.284818306	0.628236496	0.529849025	0.123181172	4.145075572	75.8749875	0.5	PRJNA827465;Yang2025_Silkie_Wenchang	CRA004775	0.871944741
ENSGALG00010029297	SH2B2	2	0.434935876	0.197655495	2.200474494	0.027773248	0	0.309524716	0	1	CRA004775;Yang2025_Silkie_Wenchang	PRJNA827465	0.284281094
ENSGALG00010029297	SH2B2	2	-0.08273711	0.179520049	-0.460879501	0.64488506	0	0.090932541	0	0.5	CRA004775;PRJNA827465	Yang2025_Silkie_Wenchang	0.906953008

ENSGALG00010029298	ENDOV	2	0.018601934	0.0855083	0.217545361	0.82778336	0	0.402591282	0	0.5	PRJNA827465;Yang2025_Silkie_Wenchang	CRA004775	0.956550752
ENSGALG00010029298	ENDOV	2	-0.131855445	0.24573952	-0.536565894	0.591567508	0.090885983	4.040316453	75.24946346	0.5	CRA004775;Yang2025_Silkie_Wenchang	PRJNA827465	0.892776493
ENSGALG00010029298	ENDOV	2	-0.167436694	0.180895177	-0.925600655	0.354653553	0.046901968	3.326561683	69.93893109	1	CRA004775;PRJNA827465	Yang2025_Silkie_Wenchang	0.812016118
ENSGALG00010029299	PRKRIP1	2	-0.094153824	0.074110272	-1.270455781	0.203922324	0.003729387	1.452077096	31.13313315	1	PRJNA827465;Yang2025_Silkie_Wenchang	CRA004775	0.717254253
ENSGALG00010029299	PRKRIP1	2	-0.135557094	0.113083721	-1.198732165	0.230632107	0.004484497	1.131617938	11.63095193	0.5	CRA004775;Yang2025_Silkie_Wenchang	PRJNA827465	0.743374303
ENSGALG00010029299	PRKRIP1	2	-0.029427586	0.066502085	-0.44250622	0.658122931	0	0.24667345	0	0.5	CRA004775;PRJNA827465	Yang2025_Silkie_Wenchang	0.910938006
ENSGALG00010029300	TEKT1	2	0.602279475	0.483654814	1.245267198	0.213033549	0.421928538	10.18904319	90.18553576	1	PRJNA827465;Yang2025_Silkie_Wenchang	CRA004775	0.72579791
ENSGALG00010029300	TEKT1	2	0.854472671	0.340231541	2.511444614	0.012023815	0.123784006	1.856986697	46.14931806	1	CRA004775;Yang2025_Silkie_Wenchang	PRJNA827465	0.176332839
ENSGALG00010029300	TEKT1	2	0.155617912	0.197823492	0.786650314	0.431486597	0	0.190050256	0	1	CRA004775;PRJNA827465	Yang2025_Silkie_Wenchang	0.842503929
ENSGALG00010029301	RNF213	2	0.302080104	0.092931568	3.250565033	0.001151759	0	0.07819105	0	1	PRJNA827465;Yang2025_Silkie_Wenchang	CRA004775	0.041251469
ENSGALG00010029301	RNF213	2	-0.110061313	0.522074019	-0.210815534	0.833031217	0.447264291	5.144886322	80.56322458	0.5	CRA004775;Yang2025_Silkie_Wenchang	PRJNA827465	0.957916437
ENSGALG00010029301	RNF213	2	-0.124628239	0.489760421	-0.254467763	0.799134206	0.397365754	5.090831671	80.35684413	0.5	CRA004775;PRJNA827465	Yang2025_Silkie_Wenchang	0.949922217
ENSGALG00010029303	SLC26A11	2	0.055006118	0.118077343	0.465848203	0.641324159	0.00786171	1.295547984	22.81258492	0.5	PRJNA827465;Yang2025_Silkie_Wenchang	CRA004775	0.906558891
ENSGALG00010029303	SLC26A11	2	0.173960976	0.178765587	0.973123403	0.330491974	0	0.500308932	0	0.5	CRA004775;Yang2025_Silkie_Wenchang	PRJNA827465	0.80156474
ENSGALG00010029303	SLC26A11	2	-0.021756047	0.103193272	-0.21082815	0.833021372	0	0.009025034	0	1	CRA004775;PRJNA827465	Yang2025_Silkie_Wenchang	0.957916437
ENSGALG00010029304	ORAI3	2	0.133025781	0.073419057	1.811869911	0.0700063	0.004276886	1.537754442	34.97011144	1	PRJNA827465;Yang2025_Silkie_Wenchang	CRA004775	0.456446183
ENSGALG00010029304	ORAI3	2	-0.17906686	0.262868488	-0.681203216	0.495742913	0.099774386	3.146831734	68.22200599	0.5	CRA004775;Yang2025_Silkie_Wenchang	PRJNA827465	0.860911322
ENSGALG00010029304	ORAI3	2	-0.109060411	0.344208807	-0.316843755	0.751362161	0.200799624	5.750977216	82.61165081	0.5	CRA004775;PRJNA827465	Yang2025_Silkie_Wenchang	0.937684688
ENSGALG00010029305	SMTNL2	2	0.054763818	0.076596733	0.714962843	0.474632003	0	0.039897024	0	1	PRJNA827465;Yang2025_Silkie_Wenchang	CRA004775	0.854462864
ENSGALG00010029305	SMTNL2	2	0.039979045	0.109962258	0.363570608	0.716178678	0	0.004802948	0	1	CRA004775;Yang2025_Silkie_Wenchang	PRJNA827465	0.927384376
ENSGALG00010029305	SMTNL2	2	0.068918164	0.10143891	0.679405609	0.496880897	0	2.05329E-05	0	1	CRA004775;PRJNA827465	Yang2025_Silkie_Wenchang	0.860939846
ENSGALG00010029306	CEP95	2	-0.103160322	0.168112809	-0.613637488	0.539454905	0.0482822	6.493936186	84.60101899	0.5	PRJNA827465;Yang2025_Silkie_Wenchang	CRA004775	0.874993194
ENSGALG00010029306	CEP95	2	-0.262367384	0.11045873	-2.375252573	0.017536946	0	0.31714411	0	1	CRA004775;Yang2025_Silkie_Wenchang	PRJNA827465	0.220561462
ENSGALG00010029306	CEP95	2	0.042965389	0.055503404	0.77410368	0.433869481	0	0.321188213	0	0.5	CRA004775;PRJNA827465	Yang2025_Silkie_Wenchang	0.844002503
ENSGALG00010029307	SGSH	2	0.186028785	0.218398535	0.851785066	0.394332953	0.07581707	4.859862351	79.42328552	0.5	PRJNA827465;Yang2025_Silkie_Wenchang	CRA004775	0.830486298
ENSGALG00010029307	SGSH	2	0.359375373	0.132042628	2.721661771	0.006495458	0	0.551602027	0	1	CRA004775;Yang2025_Silkie_Wenchang	PRJNA827465	0.123006701
ENSGALG00010029307	SGSH	2	0.00710307	0.119235415	0.05957181	0.952496671	0	0.388306374	0	0.5	CRA004775;PRJNA827465	Yang2025_Silkie_Wenchang	0.987758913
ENSGALG00010029308	GAA	2	0.086568085	0.114386626	0.756802502	0.449168193	0	0.368553753	0	1	PRJNA827465;Yang2025_Silkie_Wenchang	CRA004775	0.847263197
ENSGALG00010029308	GAA	2	0.114135949	0.171692838	0.664768258	0.506198728	0	0.419998267	0	0.5	CRA004775;Yang2025_Silkie_Wenchang	PRJNA827465	0.863929781
ENSGALG00010029308	GAA	2	0.02784233	0.123028334	0.226308271	0.820961666	0	0.06292027	0	0.5	CRA004775;PRJNA827465	Yang2025_Silkie_Wenchang	0.955548641
ENSGALG00010029309	ALKBH4	2	-0.084673867	0.170096668	-0.497798504	0.61862607	0	0.685599202	0	0.5	PRJNA827465;Yang2025_Silkie_Wenchang	CRA004775	0.899168321
ENSGALG00010029309	ALKBH4	2	-0.262642687	0.232032564	-1.131921669	0.257667368	0	0.036844793	0	1	CRA004775;Yang2025_Silkie_Wenchang	PRJNA827465	0.7650601
ENSGALG00010029309	ALKBH4	2	-0.050371224	0.174452887	-0.288738265	0.772781678	0	0.303120299	0	0.5	CRA004775;PRJNA827465	Yang2025_Silkie_Wenchang	0.943066211
ENSGALG00010029310	LRWD1	2	-0.120892969	0.092584248	-1.305761745	0.191633593	0.013486193	4.436343831	77.45891576	1	PRJNA827465;Yang2025_Silkie_Wenchang	CRA004775	0.701282599
ENSGALG00010029310	LRWD1	2	-0.329438006	0.123813472	-2.660760597	0.007796437	0.019999649	2.718920189	63.22069312	1	CRA004775;Yang2025_Silkie_Wenchang	PRJNA827465	0.136979598
ENSGALG00010029310	LRWD1	2	-0.240617815	0.218361626	-1.101923538	0.27049015	0.086504394	10.12629034	90.12471521	1	CRA004775;PRJNA827465	Yang2025_Silkie_Wenchang	0.773236273
ENSGALG00010029311	ENSGALG00010029311	2	0.130213912	0.169894977	0.766437679	0.443415921	0.020373283	1.403744013	28.76194015	1	PRJNA827465;Yang2025_Silkie_Wenchang	CRA004775	0.845733906
ENSGALG00010029311	ENSGALG00010029311	2	0.212516321	0.22735754	0.934722995	0.349931073	0.005671792	1.055745266	5.280181435	0.5	CRA004775;Yang2025_Silkie_Wenchang	PRJNA827465	0.809993068
ENSGALG00010029311	ENSGALG00010029311	2	0.0107517	0.131105439	0.082008042	0.934640319	0	0.053233463	0	0.5	CRA004775;PRJNA827465	Yang2025_Silkie_Wenchang	0.984397102
ENSGALG00010029312	SMURF2	2	-0.038032197	0.053867249	-0.706035628	0.480165982	0	0.044202831	0	1	PRJNA827465;Yang2025_Silkie_Wenchang	CRA004775	0.85643503
ENSGALG00010029312	SMURF2	2	-0.062812823	0.12644457	-0.496761726	0.619357089	0	0.001520287	0	1	CRA004775;Yang2025_Silkie_Wenchang	PRJNA827465	0.899181533
ENSGALG00010029312	SMURF2	2	-0.034002561	0.057291962	-0.593496192	0.552849131	0	0.003927589	0	1	CRA004775;PRJNA827465	Yang2025_Silkie_Wenchang	0.87928478
ENSGALG00010029313	CCDC40	2	0.445820028	0.277939718	1.604016983	0.108710309	0.094077467	2.479930835	59.66653639	1	PRJNA827465;Yang2025_Silkie_Wenchang	CRA004775	0.558296261
ENSGALG00010029313	CCDC40	2	0.717196436	0.270636994	2.650031046	0.008048437	0	0.190595813	0	1	CRA004775;Yang2025_Silkie_Wenchang	PRJNA827465	0.139351426
ENSGALG00010029313	CCDC40	2	0.222438188	0.195102913	1.140106955	0.254241745	0	0.097491525	0	1	CRA004775;PRJNA827465	Yang2025_Silkie_Wenchang	0.764008204
ENSGALG00010029314	MED13	2	-0.016202316	0.183725003	-0.088187866	0.929727361	0.053719283	4.454220736	77.54938385	0.5	PRJNA827465;Yang2025_Silkie_Wenchang	CRA004775	0.983767889
ENSGALG00010029314	MED13	2	0.268995593	0.13313655	2.020488867	0.043336849	0	0.543110175	0	1	CRA004775;Yang2025_Silkie_Wenchang	PRJNA827465	0.359946782
ENSGALG00010029314	MED13	2	0.075621378	0.287114306	0.263384223	0.79225445	0.139558657	5.842072164	82.8827859	0.5	CRA004775;PRJNA827465	Yang2025_Silkie_Wenchang	0.948368502
ENSGALG00010029315	TBC1D16	2	0.006745281	0.070055141	0.096285304	0.923293983	0	0.767989539	0	0.5	PRJNA827465;Yang2025_Silkie_Wenchang	CRA004775	0.981957067
ENSGALG00010029315	TBC1D16	2	-0.064713181	0.099791132	-0.648486297	0.516670471	0	0.000551941	0	1	CRA004775;Yang2025_Silkie_Wenchang	PRJNA827465	0.865952013
ENSGALG00010029315	TBC1D16	2	0.047277544	0.088175105	0.536177916	0.591835594	0	0.19572683	0	0.5	CRA004775;PRJNA827465	Yang2025_Silkie_Wenchang	0.892776493
ENSGALG00010029316	INTS2	2	-0.079926646	0.050621872	-1.578895505	0.114360027	0	0.220480051	0	1	PRJNA827465;Yang2025_Silkie_Wenchang	CRA004775	0.570247672
ENSGALG00010029316	INTS2	2	-0.292268837	0.283805225	-1.029821902	0.303093617	0.132375155	5.008489535	80.03390058	1	CRA004775;Yang2025_Silkie_Wenchang	PRJNA827465	0.792084776
ENSGALG00010029316	INTS2	2	-0.310292486	0.258056443	-1.202420098	0.229200458	0.106708418	4.333456907	76.92370304	1	CRA004775;PRJNA827465	Yang2025_Silkie_Wenchang	0.742154028
ENSGALG00010029317	KPNA2	2	-0.192918761	0.139124909	-1.386658671	0.165545869	0.021621966	2.084697921	52.03141953	1	PRJNA827465;Yang2025_Silkie_Wenchang	CRA004775	0.664874006
ENSGALG00010029317	KPNA2	2	-0.322128281	0.166345022	-1.936506896	0.052805649	0	0.884233627	0	0.5	CRA004775;Yang2025_Silkie_Wenchang	PRJNA827465	0.399347184
ENSGALG00010029317	KPNA2	2	-0.079095373	0.093647831	-0.844604432	0.398331734	0	0.186796686	0	0.5	CRA004775;PRJNA827465	Yang2025_Silkie_Wenchang	0.831980453
ENSGALG00010029318	C17orf58	2	-0.057249967	0.102030057	-0.561108846	0.574723338	0	0.916082374	0	0.5	PRJNA827465;Yang2025_Silkie_Wenchang	CRA004775	0.886144156
ENSGALG00010029318	C17orf58	2	-0.183683246	0.149657314	-1.227358962	0.219687703	0	0.099582442	0	1	CRA004775;Yang2025_Silkie_Wenchang	PRJNA827465	0.733094397
ENSGALG00010029318	C17orf58	2	0.005136036	0.13091758	0.039231064	0.968706167	0	0.455600666	0	0.5	CRA004775;PRJNA827465	Yang2025_Silkie_Wenchang	0.991279382
ENSGALG00010029319	BRIP1	2	-0.019187606	0.126868298	-0.15124015	0.879786122	0.02185478	3.03080024	67.00541373	0.5	PRJNA827465;Yang2025_Silkie_Wenchang	CRA004775	0.970407853
ENSGALG00010029319	BRIP1	2	-0.181022842	0.117483558	-1.540835549	0.123356815	0	0.558881616	0	1	CRA004775;Yang2025_Silkie_Wenchang	PRJNA827465	0.588880549
ENSGALG00010029319	BRIP1	2	-0.027739365	0.258189518	-0.107437999	0.91444151	0.074428941	1.497120057	33.20508963	0.5	CRA004775;PRJNA827465	Yang2025_Silkie_Wenchang	0.979700005
ENSGALG00010029321	TBX2	2	0.304342542	0.171971093	1.769730806	0.076771995	0.046748202	4.660748977	78.54422101	1	PRJNA827465;Yang2025_Silkie_Wenchang	CRA004775	0.477904868
ENSGALG00010029321	TBX2	2	0.443969735	0.12089637	3.672316501	0.000240362	0	0.758712547	0	1	CRA004775;Yang2025_Silkie_Wenchang	PRJNA827465	0.014444858
ENSGALG00010029321	TBX2	2	0.150397894	0.086072314	1.747343444	0.080577781	0	0.036827336	0	1	CRA004775;PRJNA827465	Yang2025_Silkie_Wenchang	0.488651218
ENSGALG00010029322	BPTF	2	0.102094039	0.276223373	0.369606808	0.711675478	0.126290892	5.181584516	80.70088412	0.5	PRJNA827465;Yang2025_Silkie_Wenchang	CRA004775	0.92597752
ENSGALG00010029322	BPTF	2	0.377326542	0.187195189	2.015685039	0.043832921	0	0.119104136	0	1	CRA004775;Yang2025_Silkie_Wenchang	PRJNA827465	0.361804689
ENSGALG00010029322	BPTF	2	-0.028550226	0.183273849	-0.155779047	0.876207187	0.039088856	1.763189463	43.28459755	0.5	CRA004775;PRJNA827465	Yang2025_Silkie_Wenchang	0.970347777
ENSGALG00010029323	NOL11	2	-0.240873827	0.189538784	-1.270841896	0.203784901	0.062151473	6.918675705	85.54636693	1	PRJNA827465;Yang2025_Silkie_Wenchang	CRA004775	0.717254253
ENSGALG00010029323	NOL11	2	-0.109898003	0.359025553	-0.306100785	0.75952791	0.22745418	8.341232735	88.01136437	0.5	CRA004775;Yang2025_Silkie_Wenchang	PRJNA827465	0.939163539
ENSGALG00010029323	NOL11	2	0.037922294	0.157611633	0.240605933	0.809860551	0.033690441	2.442998697	59.06669941	0.5	CRA004775;PRJNA827465	Yang2025_Silkie_Wenchang	0.952818762
ENSGALG00010029324	BCAS3	2	-0.150399222	0.058839335	-2.556099976	0.01058527	0.000669795	1.093988387	8.591351464	1	PRJNA827465;Yang2025_Silkie_Wenchang	CRA004775	0.164050817
ENSGALG00010029324	BCAS3	2	-0.208881356	0.092607659	-2.255551634	0.024098725	0	0.53275392	0	1	CRA004775;Yang2025_Silkie_Wenchang	PRJNA827465	0.265011806
ENSGALG00010029324	BCAS3	2	-0.103895121	0.06521127	-1.593208057	0.111113524	0	0.09196465	0	1	CRA004775;PRJNA827465	Yang2025_Silkie_Wenchang	0.563970481
ENSGALG00010029325	LRRC75A	2	0.164707853	0.14226375	1.15776403	0.246960346	0.020284284	1.658767249	39.71426667	1	PRJNA827465;Yang2025_Silkie_Wenchang	CRA004775	0.757459165
ENSGALG00010029325	LRRC75A	2	0.049528561	0.439431319	0.112710585	0.910260009	0.259992485	2.759946381	63.76741204	0.5	CRA004775;Yang2025_Silkie_Wenchang	PRJNA827465	0.97880407
ENSGALG00010029325	LRRC75A	2	-0.013570788	0.211933265	-0.064033306	0.948943707	0.045719332	1.367335368	26.86505278	0.5	CRA004775;PRJNA827465	Yang2025_Silkie_Wenchang	0.987119459
ENSGALG00010029326	NCBP3	2	-0.028841664	0.039426386	-0.731532019	0.464454255	0	0.57588655	0	1	PRJNA827465;Yang2025_Silkie_Wenchang	CRA004775	0.852159649
ENSGALG00010029326	NCBP3	2	-0.080463465	0.07998069	-1.006036146	0.314398183	0	0.052783332	0	1	CRA004775;Yang2025_Silkie_Wenchang	PRJNA827465	0.796531448
ENSGALG00010029326	NCBP3	2	-0.015694154	0.04272371	-0.367340625	0.713364955	0	0.024175406	0	1	CRA004775;PRJNA827465	Yang2025_Silkie_Wenchang	0.926216079
ENSGALG00010029327	CRK	2	-0.257909662	0.230840728	-1.117262385	0.263882154	0.103487355	34.18677135	97.07489195	1	PRJNA827465;Yang2025_Silkie_Wenchang	CRA004775	0.768643821
ENSGALG00010029327	CRK	2	-0.105538957	0.39501924	-0.267174219	0.789335025	0.301009787	27.88458967	96.4137898	0.5	CRA004775;Yang2025_Silkie_Wenchang	PRJNA827465	0.947379026
ENSGALG00010029327	CRK	2	0.10867023	0.162372646	0.669264392	0.503326837	0.044555274	5.747876322	82.60227006	0.5	CRA004775;PRJNA827465	Yang2025_Silkie_Wenchang	0.863108263
ENSGALG00010029328	ENSGALG00010029328	2	-0.121996742	0.235313309	-0.518443869	0.604148614	0.099336331	9.439276838	89.40596809	0.5	PRJNA827465;Yang2025_Silkie_Wenchang	CRA004775	0.89612415
ENSGALG00010029328	ENSGALG00010029328	2	-0.278961452	0.166681825	-1.673616501	0.094205993	0.018166738	1.306491815	23.45914541	0.5	CRA004775;Yang2025_Silkie_Wenchang	PRJNA827465	0.525712768
ENSGALG00010029328	ENSGALG00010029328	2	0.096251099	0.071747172	1.341531617	0.179747912	0	0.057250586	0	1	CRA004775;PRJNA827465	Yang2025_Silkie_Wenchang	0.68659821
ENSGALG00010029329	ENSGALG00010029329	2	-0.130531136	0.086265986	-1.513124027	0.130248158	0	0.823646151	0	0.5	PRJNA827465;Yang2025_Silkie_Wenchang	CRA004775	0.605600773
ENSGALG00010029329	ENSGALG00010029329	2	-0.158094783	0.247996158	-0.63748884	0.523806477	0.058131211	1.78595387	44.00751237	0.5	CRA004775;Yang2025_Silkie_Wenchang	PRJNA827465	0.869661505
ENSGALG00010029329	ENSGALG00010029329	2	-0.192844071	0.092000967	-2.09610917	0.036072508	0	0.837789161	0	1	CRA004775;PRJNA827465	Yang2025_Silkie_Wenchang	0.327683285
ENSGALG00010029330	AFMID	2	0.360046416	0.225000381	1.600203585	0.109553427	0.069468452	2.957568294	66.18843926	1	PRJNA827465;Yang2025_Silkie_Wenchang	CRA004775	0.560210558
ENSGALG00010029330	AFMID	2	0.528347022	0.207612457	2.54487149	0.010931796	0	0.890503375	0	1	CRA004775;Yang2025_Silkie_Wenchang	PRJNA827465	0.166338515
ENSGALG00010029330	AFMID	2	0.172867267	0.120815231	1.430840011	0.152476073	0	0.001111763	0	1	CRA004775;PRJNA827465	Yang2025_Silkie_Wenchang	0.644106525
ENSGALG00010029331	TK1	2	-0.142261739	0.17098708	-0.832002858	0.405407331	0.034541901	1.920251907	47.92349918	1	PRJNA827465;Yang2025_Silkie_Wenchang	CRA004775	0.833112102
ENSGALG00010029331	TK1	2	-0.279939123	0.24730528	-1.131957729	0.257652206	0.010437721	1.0640495	6.019409787	0.5	CRA004775;Yang2025_Silkie_Wenchang	PRJNA827465	0.7650601
ENSGALG00010029331	TK1	2	-0.029953793	0.077940384	-0.384316728	0.700743711	0	0.166251825	0	0.5	CRA004775;PRJNA827465	Yang2025_Silkie_Wenchang	0.922472203
ENSGALG00010029332	YWHAE	2	0.226018183	0.329210023	0.686547088	0.492368181	0.201471606	13.36356055	92.51696435	0.5	PRJNA827465;Yang2025_Silkie_Wenchang	CRA004775	0.859228604
ENSGALG00010029332	YWHAE	2	0.216482625	0.367774991	0.588627912	0.556110906	0.235163031	7.638828541	86.90898749	0.5	CRA004775;Yang2025_Silkie_Wenchang	PRJNA827465	0.880324407
ENSGALG00010029332	YWHAE	2	-0.084207625	0.037704965	-2.233329905	0.025527197	0	0.139055069	0	1	CRA004775;PRJNA827465	Yang2025_Silkie_Wenchang	0.272486534
ENSGALG00010029333	ENSGALG00010029333	2	-0.503858147	0.356252244	-1.414329748	0.157265106	0.243725543	24.66238464	95.94524206	1	PRJNA827465;Yang2025_Silkie_Wenchang	CRA004775	0.650687662
ENSGALG00010029333	ENSGALG00010029333	2	-0.402417106	0.499936727	-0.804936074	0.42085657	0.451341694	9.892078566	89.89090116	0.5	CRA004775;Yang2025_Silkie_Wenchang	PRJNA827465	0.839239169
ENSGALG00010029333	ENSGALG00010029333	2	-0.14649759	0.056136375	-2.609673155	0.009062877	0	0.957046928	0	0.5	CRA004775;PRJNA827465	Yang2025_Silkie_Wenchang	0.149099306
ENSGALG00010029336	SYNGR2	2	-0.044543553	0.047234818	-0.943023704	0.345668778	0	0.543059345	0	0.5	PRJNA827465;Yang2025_Silkie_Wenchang	CRA004775	0.807376412
ENSGALG00010029336	SYNGR2	2	-0.210202856	0.329567982	-0.63781334	0.523595195	0.156851386	3.101521985	67.75776522	0.5	CRA004775;Yang2025_Silkie_Wenchang	PRJNA827465	0.869661505
ENSGALG00010029336	SYNGR2	2	-0.234152405	0.265684631	-0.881317088	0.37814622	0.099058057	2.471317346	59.53575118	1	CRA004775;PRJNA827465	Yang2025_Silkie_Wenchang	0.823285408
ENSGALG00010029337	MYO1C	2	0.069197967	0.187461444	0.36913173	0.71202954	0.056722694	4.928193456	79.70858878	0.5	PRJNA827465;Yang2025_Silkie_Wenchang	CRA004775	0.92597752
ENSGALG00010029337	MYO1C	2	0.272402261	0.131480482	2.071807584	0.038283389	0	0.00806683	0	1	CRA004775;Yang2025_Silkie_Wenchang	PRJNA827465	0.337506202
ENSGALG00010029337	MYO1C	2	-0.023925211	0.143046297	-0.167255018	0.867169396	0.01948793	1.470395254	31.99107539	0.5	CRA004775;PRJNA827465	Yang2025_Silkie_Wenchang	0.967699
ENSGALG00010029338	INPP5K	2	0.115214702	0.071110512	1.620220402	0.10518494	0	0.342936732	0	1	PRJNA827465;Yang2025_Silkie_Wenchang	CRA004775	0.550557098
ENSGALG00010029338	INPP5K	2	0.069582411	0.093668569	0.742857633	0.457567879	0	0.031798742	0	1	CRA004775;Yang2025_Silkie_Wenchang	PRJNA827465	0.849573219
ENSGALG00010029338	INPP5K	2	0.143666316	0.096930351	1.482160287	0.138297651	0	0.196051519	0	1	CRA004775;PRJNA827465	Yang2025_Silkie_Wenchang	0.621841044
ENSGALG00010029339	PITPNA	2	-0.122612122	0.059784527	-2.050900589	0.040276629	0	0.082952342	0	1	PRJNA827465;Yang2025_Silkie_Wenchang	CRA004775	0.347442284
ENSGALG00010029339	PITPNA	2	0.031537409	0.206073643	0.153039507	0.87836712	0.064704303	3.788740797	73.60600649	0.5	CRA004775;Yang2025_Silkie_Wenchang	PRJNA827465	0.971037793
ENSGALG00010029339	PITPNA	2	0.045609297	0.187854561	0.242790471	0.808167718	0.050739771	3.175987712	68.51373209	0.5	CRA004775;PRJNA827465	Yang2025_Silkie_Wenchang	0.952496413
ENSGALG00010029340	NDEL1	2	0.102212802	0.054499845	1.875469589	0.060728149	0.001651221	1.271887468	21.37669208	1	PRJNA827465;Yang2025_Silkie_Wenchang	CRA004775	0.42686302
ENSGALG00010029340	NDEL1	2	0.000600686	0.09248227	0.006495145	0.994817661	0	0.074781071	0	0.5	CRA004775;Yang2025_Silkie_Wenchang	PRJNA827465	0.998242335
ENSGALG00010029340	NDEL1	2	0.127756737	0.047767532	2.674551754	0.007482923	0	0.572146714	0	0.5	CRA004775;PRJNA827465	Yang2025_Silkie_Wenchang	0.133439794
ENSGALG00010029341	SLC43A2	2	-0.525270837	0.211631019	-2.482012518	0.013064269	0.074575951	5.87869035	82.98940852	1	PRJNA827465;Yang2025_Silkie_Wenchang	CRA004775	0.186172933
ENSGALG00010029341	SLC43A2	2	-0.439699559	0.451901854	-0.972997909	0.330554341	0.279651802	2.470855763	59.52819202	0.5	CRA004775;Yang2025_Silkie_Wenchang	PRJNA827465	0.80156474
ENSGALG00010029341	SLC43A2	2	-0.310226763	0.09964521	-3.113313343	0.001849995	0	0.803630761	0	0.5	CRA004775;PRJNA827465	Yang2025_Silkie_Wenchang	0.055209073
ENSGALG00010029342	TMC6	2	0.570019796	0.618166493	0.922113707	0.356469268	0.746917714	43.92128536	97.7231996	0.5	PRJNA827465;Yang2025_Silkie_Wenchang	CRA004775	0.812521698
ENSGALG00010029342	TMC6	2	0.430408276	0.798439304	0.539061985	0.589844085	1.195928655	15.64364925	93.60762962	0.5	CRA004775;Yang2025_Silkie_Wenchang	PRJNA827465	0.892320726
ENSGALG00010029342	TMC6	2	-0.068371256	0.099346301	-0.688211395	0.491319669	0	0.875472648	0	1	CRA004775;PRJNA827465	Yang2025_Silkie_Wenchang	0.85895707
ENSGALG00010029343	SCARF1	2	-0.026772645	0.067146968	-0.398717115	0.690101656	0.000758433	1.074285814	6.914902238	0.5	PRJNA827465;Yang2025_Silkie_Wenchang	CRA004775	0.919979266
ENSGALG00010029343	SCARF1	2	0.04800697	0.108373398	0.442977437	0.657782055	0	0.301117405	0	0.5	CRA004775;Yang2025_Silkie_Wenchang	PRJNA827465	0.910774485
ENSGALG00010029343	SCARF1	2	-0.069446182	0.071280722	-0.974263159	0.329925892	0	4.50111E-05	0	1	CRA004775;PRJNA827465	Yang2025_Silkie_Wenchang	0.801209935
ENSGALG00010029344	ENSGALG00010029344	2	0.236751932	0.105471457	2.244701434	0.024787306	0	0.278389804	0	1	PRJNA827465;Yang2025_Silkie_Wenchang	CRA004775	0.268761753
ENSGALG00010029344	ENSGALG00010029344	2	-0.011180437	0.418572879	-0.026710849	0.97869036	0.248237416	3.273376831	69.45050779	0.5	CRA004775;Yang2025_Silkie_Wenchang	PRJNA827465	0.994183692
ENSGALG00010029344	ENSGALG00010029344	2	-0.038087698	0.335981056	-0.113362634	0.909743062	0.160702285	2.857787186	65.00789124	0.5	CRA004775;PRJNA827465	Yang2025_Silkie_Wenchang	0.978696684
ENSGALG00010029345	RILP	2	0.258744103	0.316068887	0.818631992	0.412996408	0.181616891	10.95647916	90.8729804	0.5	PRJNA827465;Yang2025_Silkie_Wenchang	CRA004775	0.835888542
ENSGALG00010029345	RILP	2	0.142873768	0.550827693	0.259380146	0.795341947	0.473068683	3.839840523	73.95725176	0.5	CRA004775;Yang2025_Silkie_Wenchang	PRJNA827465	0.948935068
ENSGALG00010029345	RILP	2	-0.074586606	0.118904623	-0.627280956	0.530475084	0	0.76257962	0	1	CRA004775;PRJNA827465	Yang2025_Silkie_Wenchang	0.872536608
ENSGALG00010029346	ARL16	2	-0.233635646	0.101512811	-2.301538539	0.02136121	0	0.023150602	0	1	PRJNA827465;Yang2025_Silkie_Wenchang	CRA004775	0.247158031
ENSGALG00010029346	ARL16	2	-0.196715469	0.191332578	-1.028133692	0.303886944	0	0.006798679	0	1	CRA004775;Yang2025_Silkie_Wenchang	PRJNA827465	0.792208658
ENSGALG00010029346	ARL16	2	-0.236585383	0.111050634	-2.130428029	0.033136292	0	0.030499972	0	1	CRA004775;PRJNA827465	Yang2025_Silkie_Wenchang	0.312915828
ENSGALG00010029347	PRPF8	2	-0.23856396	0.090748616	-2.628844055	0.008567564	0.011948146	3.575799335	72.0342249	1	PRJNA827465;Yang2025_Silkie_Wenchang	CRA004775	0.144519728
ENSGALG00010029347	PRPF8	2	-0.330320078	0.073906539	-4.469429645	7.84285E-06	0	0.085148034	0	1	CRA004775;Yang2025_Silkie_Wenchang	PRJNA827465	0.001145901
ENSGALG00010029347	PRPF8	2	-0.160823663	0.055489353	-2.898279681	0.003752158	0	0.189817527	0	1	CRA004775;PRJNA827465	Yang2025_Silkie_Wenchang	0.087832684
ENSGALG00010029348	HGS	2	-0.009278671	0.122380228	-0.075818386	0.939563588	0.0251288	5.944898099	83.17885381	0.5	PRJNA827465;Yang2025_Silkie_Wenchang	CRA004775	0.985425793
ENSGALG00010029348	HGS	2	-0.054132628	0.21954706	-0.246565031	0.80524487	0.072406451	3.58125906	72.07685947	0.5	CRA004775;Yang2025_Silkie_Wenchang	PRJNA827465	0.951864391
ENSGALG00010029348	HGS	2	-0.130053605	0.046823601	-2.777522487	0.005477505	0	0.810915283	0	1	CRA004775;PRJNA827465	Yang2025_Silkie_Wenchang	0.111211032
ENSGALG00010029349	MYH10	2	0.244926645	0.279171271	0.877334704	0.380304868	0.13326209	6.38279051	84.33287136	0.5	PRJNA827465;Yang2025_Silkie_Wenchang	CRA004775	0.824392515
ENSGALG00010029349	MYH10	2	0.500941833	0.162275256	3.086988398	0.002021955	0	0.198593841	0	1	CRA004775;Yang2025_Silkie_Wenchang	PRJNA827465	0.058563901
ENSGALG00010029349	MYH10	2	0.129655812	0.193312847	0.670704579	0.502408745	0.048732286	2.31963277	56.88972786	0.5	CRA004775;PRJNA827465	Yang2025_Silkie_Wenchang	0.8629188
ENSGALG00010029350	MRPL12	2	0.260296995	0.394922448	0.659109139	0.509825687	0.28258271	9.93232212	89.93186097	0.5	PRJNA827465;Yang2025_Silkie_Wenchang	CRA004775	0.865257661
ENSGALG00010029350	MRPL12	2	0.342093304	0.390736392	0.875509195	0.381296909	0.215604216	3.299164377	69.68929445	0.5	CRA004775;Yang2025_Silkie_Wenchang	PRJNA827465	0.824392515
ENSGALG00010029350	MRPL12	2	-0.102006563	0.068058051	-1.498816984	0.133921117	0	0.000280513	0	1	CRA004775;PRJNA827465	Yang2025_Silkie_Wenchang	0.613104446
ENSGALG00010029351	ENSGALG00010029351	2	0.076573106	0.111396154	0.687394528	0.491834145	0	0.007288755	0	1	PRJNA827465;Yang2025_Silkie_Wenchang	CRA004775	0.858983177
ENSGALG00010029351	ENSGALG00010029351	2	0.058589834	0.149075829	0.393020348	0.694304453	0	0.005052521	0	1	CRA004775;Yang2025_Silkie_Wenchang	PRJNA827465	0.920728119
ENSGALG00010029351	ENSGALG00010029351	2	0.07563492	0.128979569	0.586410085	0.557599969	0	0.018184572	0	1	CRA004775;PRJNA827465	Yang2025_Silkie_Wenchang	0.880678789
ENSGALG00010029352	AATF	2	0.203093132	0.204309689	0.994045521	0.320200706	0.068191108	5.344232495	81.28823922	1	PRJNA827465;Yang2025_Silkie_Wenchang	CRA004775	0.798348216
ENSGALG00010029352	AATF	2	0.017911434	0.416326624	0.043022551	0.965683557	0.31517832	10.91709043	90.84005023	0.5	CRA004775;Yang2025_Silkie_Wenchang	PRJNA827465	0.990668014
ENSGALG00010029352	AATF	2	-0.161230998	0.208270694	-0.774141554	0.438847086	0.063673493	3.452190467	71.0328845	0.5	CRA004775;PRJNA827465	Yang2025_Silkie_Wenchang	0.844002503
ENSGALG00010029353	GCGR	2	-0.234341086	0.501437605	-0.467338475	0.640257734	0.216633911	1.737504867	42.44620438	0.5	PRJNA827465;Yang2025_Silkie_Wenchang	CRA004775	0.906137307
ENSGALG00010029353	GCGR	2	-0.955878407	0.364252619	-2.624218351	0.008684809	0	0.776637569	0	1	CRA004775;Yang2025_Silkie_Wenchang	PRJNA827465	0.14605031
ENSGALG00010029353	GCGR	2	-0.511234205	0.829447022	-0.616355465	0.537659939	1.043494652	4.135070775	75.81661707	0.5	CRA004775;PRJNA827465	Yang2025_Silkie_Wenchang	0.87481821
ENSGALG00010029354	TLCD2	2	0.242700809	0.168708449	1.438581235	0.150269206	0	0.533634166	0	1	PRJNA827465;Yang2025_Silkie_Wenchang	CRA004775	0.640845336
ENSGALG00010029354	TLCD2	2	0.593023975	0.253159726	2.342489401	0.019155578	0	0.150835086	0	1	CRA004775;Yang2025_Silkie_Wenchang	PRJNA827465	0.231866857
ENSGALG00010029354	TLCD2	2	0.34978573	0.237821562	1.470790655	0.141347741	0.049520527	1.642259018	39.10826555	1	CRA004775;PRJNA827465	Yang2025_Silkie_Wenchang	0.626382935
ENSGALG00010029355	MCRIP1	2	0.037194032	0.236327357	0.157383522	0.874942599	0.087937878	4.51540861	77.85361002	0.5	PRJNA827465;Yang2025_Silkie_Wenchang	CRA004775	0.970159044
ENSGALG00010029355	MCRIP1	2	0.314558996	0.17640471	1.783166651	0.074559179	0	0.037349656	0	1	CRA004775;Yang2025_Silkie_Wenchang	PRJNA827465	0.471620961
ENSGALG00010029355	MCRIP1	2	-0.053762066	0.233313737	-0.230428206	0.817759045	0.056115306	1.530403214	34.65774308	0.5	CRA004775;PRJNA827465	Yang2025_Silkie_Wenchang	0.954967428
ENSGALG00010029357	ARHGDIA	2	0.013459845	0.051196081	0.262907722	0.792621704	0	0.014982172	0	1	PRJNA827465;Yang2025_Silkie_Wenchang	CRA004775	0.948435155
ENSGALG00010029357	ARHGDIA	2	-0.070164359	0.105343101	-0.666055569	0.505375583	0.009449661	1.726613454	42.08315719	0.5	CRA004775;Yang2025_Silkie_Wenchang	PRJNA827465	0.863862536
ENSGALG00010029357	ARHGDIA	2	-0.058591841	0.093952218	-0.62363446	0.532867675	0.010029961	2.061001065	51.47988922	0.5	CRA004775;PRJNA827465	Yang2025_Silkie_Wenchang	0.873237026
ENSGALG00010029359	ENSGALG00010029359	2	-0.350013947	0.287106496	-1.219108418	0.222803045	0.067387442	1.678947257	40.43886753	1	PRJNA827465;Yang2025_Silkie_Wenchang	CRA004775	0.736424866
ENSGALG00010029359	ENSGALG00010029359	2	-0.722436142	0.293051595	-2.465218257	0.013692982	0	0.095083783	0	1	CRA004775;Yang2025_Silkie_Wenchang	PRJNA827465	0.191862835
ENSGALG00010029359	ENSGALG00010029359	2	-0.326053718	0.358678443	-0.909041859	0.363328031	0.101778874	1.497744815	33.23295197	1	CRA004775;PRJNA827465	Yang2025_Silkie_Wenchang	0.816880132
ENSGALG00010029360	ACACA	2	-0.072408492	0.050781953	-1.425870569	0.153905706	0	0.11325313	0	1	PRJNA827465;Yang2025_Silkie_Wenchang	CRA004775	0.646463179
ENSGALG00010029360	ACACA	2	-0.093762214	0.091050159	-1.029786387	0.303110293	0	0.03391929	0	1	CRA004775;Yang2025_Silkie_Wenchang	PRJNA827465	0.792084776
ENSGALG00010029360	ACACA	2	-0.062069137	0.056787071	-1.093015293	0.274387085	0	0.00043444	0	1	CRA004775;PRJNA827465	Yang2025_Silkie_Wenchang	0.774526342
ENSGALG00010029361	ALYREF	2	0.217964224	0.221278882	0.985020449	0.324614027	0.091207715	14.28839058	93.00131114	1	PRJNA827465;Yang2025_Silkie_Wenchang	CRA004775	0.799859262
ENSGALG00010029361	ALYREF	2	0.090581728	0.380446172	0.238093415	0.811808642	0.261776437	10.07460109	90.07404868	0.5	CRA004775;Yang2025_Silkie_Wenchang	PRJNA827465	0.953371387
ENSGALG00010029361	ALYREF	2	-0.085983048	0.144183096	-0.596346249	0.550943941	0.025939438	2.033211558	50.81672658	0.5	CRA004775;PRJNA827465	Yang2025_Silkie_Wenchang	0.878879109
ENSGALG00010029362	TADA2A	2	0.045507689	0.062840127	0.724181997	0.468954009	0	0.033763398	0	1	PRJNA827465;Yang2025_Silkie_Wenchang	CRA004775	0.85345437
ENSGALG00010029362	TADA2A	2	-0.370067761	0.467056843	-0.792339876	0.428162526	0.391057632	9.319483881	89.26979205	0.5	CRA004775;Yang2025_Silkie_Wenchang	PRJNA827465	0.84125685
ENSGALG00010029362	TADA2A	2	-0.375608798	0.452114459	-0.830782537	0.406096489	0.371836759	10.30805555	90.29884934	0.5	CRA004775;PRJNA827465	Yang2025_Silkie_Wenchang	0.833477131
ENSGALG00010029363	PIK3R6	2	0.213597524	0.08961437	2.38351868	0.017148014	0.001508135	1.101882125	9.246190953	1	PRJNA827465;Yang2025_Silkie_Wenchang	CRA004775	0.217619761
ENSGALG00010029363	PIK3R6	2	-0.237542555	0.612475602	-0.387840028	0.698134422	0.643537942	6.257558237	84.01932572	0.5	CRA004775;Yang2025_Silkie_Wenchang	PRJNA827465	0.921568388
ENSGALG00010029363	PIK3R6	2	-0.314625543	0.518644048	-0.606630972	0.544095841	0.434286271	4.49050349	77.73078226	0.5	CRA004775;PRJNA827465	Yang2025_Silkie_Wenchang	0.876993063
ENSGALG00010029364	ANAPC11	2	0.020795666	0.093609508	0.222153353	0.824194506	0	0.862515766	0	0.5	PRJNA827465;Yang2025_Silkie_Wenchang	CRA004775	0.95619211
ENSGALG00010029364	ANAPC11	2	-0.295930482	0.269388076	-1.098528513	0.271973774	0	0.0131384	0	1	CRA004775;Yang2025_Silkie_Wenchang	PRJNA827465	0.773184343
ENSGALG00010029364	ANAPC11	2	0.02337127	0.093441212	0.25011737	0.802496583	0	0.633228946	0	0.5	CRA004775;PRJNA827465	Yang2025_Silkie_Wenchang	0.950688375
ENSGALG00010029365	PIK3R5	2	-0.090077386	0.14969548	-0.601737515	0.547348874	0.031373371	3.330723207	69.97649046	0.5	PRJNA827465;Yang2025_Silkie_Wenchang	CRA004775	0.878281266
ENSGALG00010029365	PIK3R5	2	-0.527538038	0.358730643	-1.470568652	0.141407807	0.195500909	3.578942703	72.05878711	1	CRA004775;Yang2025_Silkie_Wenchang	PRJNA827465	0.626437222
ENSGALG00010029365	PIK3R5	2	-0.401102522	0.51452852	-0.779553526	0.43565372	0.461779614	7.161850926	86.03712908	0.5	CRA004775;PRJNA827465	Yang2025_Silkie_Wenchang	0.842762754
ENSGALG00010029366	MGAT5B	2	-0.43153291	0.289017824	-1.493101376	0.135410653	0.015472386	1.101545714	9.218474793	1	PRJNA827465;Yang2025_Silkie_Wenchang	CRA004775	0.616905631

ENSGALG00010029366	MGAT5B	2	-0.207286528	0.606733287	-0.341643573	0.732619142	0.489125106	2.886218259	65.35258563	0.5	CRA004775;Yang2025_Silkie_Wenchang	PRJNA827465	0.932985777
ENSGALG00010029366	MGAT5B	2	0.021531597	0.317603557	0.067793942	0.945949667	0	0.83678388	0	0.5	CRA004775;PRJNA827465	Yang2025_Silkie_Wenchang	0.986321197
ENSGALG00010029367	DUSP14	2	-0.315814744	0.335746911	-0.940633357	0.346892782	0.217398011	27.94409197	96.42142604	0.5	PRJNA827465;Yang2025_Silkie_Wenchang	CRA004775	0.808511356
ENSGALG00010029367	DUSP14	2	-0.324213428	0.367607319	-0.881955857	0.377800679	0.234192597	6.969985516	85.65276789	0.5	CRA004775;Yang2025_Silkie_Wenchang	PRJNA827465	0.82325159
ENSGALG00010029367	DUSP14	2	0.022933527	0.075734237	0.302815845	0.762030207	0	0.061736117	0	1	CRA004775;PRJNA827465	Yang2025_Silkie_Wenchang	0.93993451
ENSGALG00010029368	DDX52	2	-0.015664387	0.05159366	-0.303610702	0.761424497	0	0.160822807	0	1	PRJNA827465;Yang2025_Silkie_Wenchang	CRA004775	0.939659225
ENSGALG00010029368	DDX52	2	-0.060380296	0.085663017	-0.704858381	0.480898373	0	0.155833174	0	1	CRA004775;Yang2025_Silkie_Wenchang	PRJNA827465	0.856722567
ENSGALG00010029368	DDX52	2	-0.011721074	0.059901458	-0.195672601	0.844866431	0	0.363012693	0	1	CRA004775;PRJNA827465	Yang2025_Silkie_Wenchang	0.960769014
ENSGALG00010029369	MFSD11	2	-0.317073109	0.308852527	-1.026616526	0.304601069	0.181311136	19.92648316	94.98155298	1	PRJNA827465;Yang2025_Silkie_Wenchang	CRA004775	0.79225794
ENSGALG00010029369	MFSD11	2	-0.50550402	0.174170213	-2.902356329	0.00370367	0.032524936	1.953918348	48.8207887	1	CRA004775;Yang2025_Silkie_Wenchang	PRJNA827465	0.087112712
ENSGALG00010029369	MFSD11	2	-0.045059655	0.081143682	-0.555306998	0.578684693	0.002479088	1.084064327	7.754551546	1	CRA004775;PRJNA827465	Yang2025_Silkie_Wenchang	0.88788081
ENSGALG00010029370	SRSF2	2	0.285378568	0.235863808	1.209929453	0.226305964	0.104737508	16.68981205	94.00832078	1	PRJNA827465;Yang2025_Silkie_Wenchang	CRA004775	0.739727883
ENSGALG00010029370	SRSF2	2	0.231574163	0.326101479	0.710129141	0.477624057	0.184671449	7.221235948	86.15195505	0.5	CRA004775;Yang2025_Silkie_Wenchang	PRJNA827465	0.855218676
ENSGALG00010029370	SRSF2	2	0.048748388	0.049621353	0.982407472	0.325899147	0	0.651995708	0	0.5	CRA004775;PRJNA827465	Yang2025_Silkie_Wenchang	0.800023788
ENSGALG00010029371	AUTS2	2	0.356319278	0.498163787	0.715265314	0.474445118	0.439633754	7.900262489	87.34219273	0.5	PRJNA827465;Yang2025_Silkie_Wenchang	CRA004775	0.854462864
ENSGALG00010029371	AUTS2	2	0.494372815	0.417423594	1.184343246	0.236277216	0.22990432	2.937859557	65.96161319	1	CRA004775;Yang2025_Silkie_Wenchang	PRJNA827465	0.748669015
ENSGALG00010029371	AUTS2	2	-0.080021208	0.064395601	-1.242650224	0.213996754	0	0.239468192	0	0.5	CRA004775;PRJNA827465	Yang2025_Silkie_Wenchang	0.726753168
ENSGALG00010029372	METTL23	2	0.017285431	0.131102755	0.131846438	0.895105755	0.010099364	1.319151218	24.19367953	0.5	PRJNA827465;Yang2025_Silkie_Wenchang	CRA004775	0.974641371
ENSGALG00010029372	METTL23	2	-0.187951225	0.182319266	-1.03089064	0.302592106	0	3.05431E-07	0	1	CRA004775;Yang2025_Silkie_Wenchang	PRJNA827465	0.791852471
ENSGALG00010029372	METTL23	2	0.066094146	0.111359746	0.593519186	0.552833747	0	0.716733419	0	0.5	CRA004775;PRJNA827465	Yang2025_Silkie_Wenchang	0.87928478
ENSGALG00010029373	GATSL2	2	-0.139077395	0.093898766	-1.481141889	0.138568767	0	0.007686585	0	1	PRJNA827465;Yang2025_Silkie_Wenchang	CRA004775	0.622371164
ENSGALG00010029373	GATSL2	2	-0.112459075	0.164625531	-0.683120499	0.494530703	0	0.618248912	0	0.5	CRA004775;Yang2025_Silkie_Wenchang	PRJNA827465	0.860214217
ENSGALG00010029373	GATSL2	2	-0.116883331	0.109940056	-1.063155099	0.287711621	0	0.600532946	0	0.5	CRA004775;PRJNA827465	Yang2025_Silkie_Wenchang	0.782840067
ENSGALG00010029374	RCC1L	2	-0.180565018	0.130197413	-1.386855649	0.165485784	0.023176858	2.93987874	65.98499161	1	PRJNA827465;Yang2025_Silkie_Wenchang	CRA004775	0.664874006
ENSGALG00010029374	RCC1L	2	-0.316854922	0.111533311	-2.84089944	0.00449865	0	0.072429404	0	1	CRA004775;Yang2025_Silkie_Wenchang	PRJNA827465	0.09815869
ENSGALG00010029374	RCC1L	2	-0.09777604	0.068741716	-1.422368332	0.154919351	0	0.932166847	0	1	CRA004775;PRJNA827465	Yang2025_Silkie_Wenchang	0.647606389
ENSGALG00010029375	JMJD6	2	0.09067164	0.086901326	1.043386143	0.296769489	0	0.582375068	0	0.5	PRJNA827465;Yang2025_Silkie_Wenchang	CRA004775	0.788458494
ENSGALG00010029375	JMJD6	2	0.008811553	0.16919702	0.052078655	0.958466021	0	0.284127608	0	0.5	CRA004775;Yang2025_Silkie_Wenchang	PRJNA827465	0.989066874
ENSGALG00010029375	JMJD6	2	0.127374643	0.093655643	1.360031689	0.173819896	0	0.016848565	0	1	CRA004775;PRJNA827465	Yang2025_Silkie_Wenchang	0.677480397
ENSGALG00010029376	ENSGALG00010029376	2	0.241455532	0.123471859	1.955551126	0.050518055	0.01800153	2.264199919	55.83428868	1	PRJNA827465;Yang2025_Silkie_Wenchang	CRA004775	0.389239584
ENSGALG00010029376	ENSGALG00010029376	2	-0.032269631	0.474681702	-0.067981621	0.945800265	0.388707902	6.862962056	85.42903207	0.5	CRA004775;Yang2025_Silkie_Wenchang	PRJNA827465	0.986321197
ENSGALG00010029376	ENSGALG00010029376	2	-0.131564694	0.342014432	-0.384675855	0.700477586	0.186222693	4.167774142	76.00637736	0.5	CRA004775;PRJNA827465	Yang2025_Silkie_Wenchang	0.922303014
ENSGALG00010029377	NCF1C	2	-0.008246414	0.156751539	-0.052608184	0.958044096	0	0.547925629	0	0.5	PRJNA827465;Yang2025_Silkie_Wenchang	CRA004775	0.988864712
ENSGALG00010029377	NCF1C	2	0.194972512	0.242654564	0.803498227	0.421686821	0	0.121889702	0	1	CRA004775;Yang2025_Silkie_Wenchang	PRJNA827465	0.839352211
ENSGALG00010029377	NCF1C	2	-0.041986627	0.182132096	-0.230528437	0.817681169	0	0.611740606	0	0.5	CRA004775;PRJNA827465	Yang2025_Silkie_Wenchang	0.954961608
ENSGALG00010029378	ST6GALNAC1	2	0.376611341	0.260362626	1.434965349	0.151296972	0	0.051738187	0	1	PRJNA827465;Yang2025_Silkie_Wenchang	CRA004775	0.64289984
ENSGALG00010029378	ST6GALNAC1	2	0.220776007	0.316480034	0.697598532	0.4854283	0	0.219012412	0	0.5	CRA004775;Yang2025_Silkie_Wenchang	PRJNA827465	0.857076964
ENSGALG00010029378	ST6GALNAC1	2	0.310521978	0.310977769	0.99853433	0.318020326	0	0.409818063	0	0.5	CRA004775;PRJNA827465	Yang2025_Silkie_Wenchang	0.797439961
ENSGALG00010029379	GTF2I	2	0.095070661	0.084935303	1.119330339	0.262999237	0.005371362	1.482443504	32.54380372	1	PRJNA827465;Yang2025_Silkie_Wenchang	CRA004775	0.768643821
ENSGALG00010029379	GTF2I	2	0.192426287	0.123176802	1.562195829	0.118241863	0	0.364061266	0	0.5	CRA004775;Yang2025_Silkie_Wenchang	PRJNA827465	0.578003524
ENSGALG00010029379	GTF2I	2	0.034343072	0.071339801	0.481401292	0.630231318	0	0.023522738	0	0.5	CRA004775;PRJNA827465	Yang2025_Silkie_Wenchang	0.902650914
ENSGALG00010029380	RGS9	2	0.139538055	0.129768437	1.075285007	0.282247146	0.0109425	1.432501881	30.19206376	1	PRJNA827465;Yang2025_Silkie_Wenchang	CRA004775	0.779519046
ENSGALG00010029380	RGS9	2	0.015469533	0.346076635	0.044699732	0.964346647	0.171551215	3.309401427	69.78305528	0.5	CRA004775;Yang2025_Silkie_Wenchang	PRJNA827465	0.990299069
ENSGALG00010029380	RGS9	2	-0.061859957	0.183815703	-0.336532496	0.736469351	0.02864929	1.443176378	30.70840023	0.5	CRA004775;PRJNA827465	Yang2025_Silkie_Wenchang	0.933661344
ENSGALG00010029382	GTF2IRD1	2	-0.142723208	0.05560705	-2.566638705	0.010268955	0	0.334959573	0	1	PRJNA827465;Yang2025_Silkie_Wenchang	CRA004775	0.160989519
ENSGALG00010029382	GTF2IRD1	2	-0.116268757	0.0764303	-1.521239045	0.128199861	0	0.124534522	0	1	CRA004775;Yang2025_Silkie_Wenchang	PRJNA827465	0.606696128
ENSGALG00010029382	GTF2IRD1	2	-0.178676441	0.078219477	-2.284296047	0.022354138	0	0.04108164	0	1	CRA004775;PRJNA827465	Yang2025_Silkie_Wenchang	0.223559795
ENSGALG00010029383	GNA13	2	-0.230681032	0.245327271	-0.940299181	0.347064119	0.113810588	17.799742	94.38194104	0.5	PRJNA827465;Yang2025_Silkie_Wenchang	CRA004775	0.808602357
ENSGALG00010029383	GNA13	2	0.017505154	0.544783488	0.032132314	0.974366534	0.539029355	10.27199869	90.26479626	0.5	CRA004775;Yang2025_Silkie_Wenchang	PRJNA827465	0.992492038
ENSGALG00010029383	GNA13	2	0.220046731	0.288521966	0.762668901	0.445660882	0.128006191	3.412797816	70.6985279	1	CRA004775;PRJNA827465	Yang2025_Silkie_Wenchang	0.846013379
ENSGALG00010029384	WDR81	2	-0.035460178	0.179533897	-0.197512437	0.84342655	0.057832775	9.517350222	89.49287379	0.5	PRJNA827465;Yang2025_Silkie_Wenchang	CRA004775	0.96057435
ENSGALG00010029384	WDR81	2	-0.250832059	0.092257407	-2.718828404	0.006551359	0	0.485052126	0	1	CRA004775;Yang2025_Silkie_Wenchang	PRJNA827465	0.123728698
ENSGALG00010029384	WDR81	2	-0.084912355	0.265671898	-0.31961361	0.749261256	0.115526963	4.770440546	79.03757545	0.5	CRA004775;PRJNA827465	Yang2025_Silkie_Wenchang	0.937362187
ENSGALG00010029385	CLIP2	2	-0.077097734	0.06275571	-1.228537366	0.219245311	0	0.264125198	0	1	PRJNA827465;Yang2025_Silkie_Wenchang	CRA004775	0.7328529
ENSGALG00010029385	CLIP2	2	-0.017322007	0.100558746	-0.172257587	0.863235026	0	0.619364607	0	0.5	CRA004775;Yang2025_Silkie_Wenchang	PRJNA827465	0.966235427
ENSGALG00010029385	CLIP2	2	-0.073685696	0.074770996	-0.985485006	0.324385893	0	0.931939886	0	0.5	CRA004775;PRJNA827465	Yang2025_Silkie_Wenchang	0.799833046
ENSGALG00010029386	SLC16A6	2	-0.330383237	0.096129028	-3.436872733	0.000588472	0	0.014665007	0	1	PRJNA827465;Yang2025_Silkie_Wenchang	CRA004775	0.026163707
ENSGALG00010029386	SLC16A6	2	-0.180460906	0.235874122	-0.76507293	0.444231216	0.045636028	1.43116839	30.1270201	0.5	CRA004775;Yang2025_Silkie_Wenchang	PRJNA827465	0.846013379
ENSGALG00010029386	SLC16A6	2	-0.19886232	0.246656783	-0.806230809	0.420109726	0.066524094	1.672897779	40.22348451	0.5	CRA004775;PRJNA827465	Yang2025_Silkie_Wenchang	0.838638439
ENSGALG00010029387	SERPINF1	2	-0.243531509	0.237134453	-1.026976494	0.304431533	0.097668164	7.338041895	86.37238634	1	PRJNA827465;Yang2025_Silkie_Wenchang	CRA004775	0.79225794
ENSGALG00010029387	SERPINF1	2	-0.486624935	0.148134532	-3.285030229	0.00101975	0	0.083038962	0	1	CRA004775;Yang2025_Silkie_Wenchang	PRJNA827465	0.003033353
ENSGALG00010029387	SERPINF1	2	-0.03070882	0.083181548	-0.369178268	0.711994853	0	0.330181808	0	1	CRA004775;PRJNA827465	Yang2025_Silkie_Wenchang	0.92597752
ENSGALG00010029388	ST6GALNAC2	2	1.725267108	0.194595418	8.86591844	7.5876E-19	0.040837897	2.169060159	53.89708322	1	PRJNA827465;Yang2025_Silkie_Wenchang	CRA004775	9.31923E-16
ENSGALG00010029388	ST6GALNAC2	2	0.795057402	1.141964747	0.696218867	0.486291774	2.526631982	31.64014086	96.83945781	0.5	CRA004775;Yang2025_Silkie_Wenchang	PRJNA827465	0.857603818
ENSGALG00010029388	ST6GALNAC2	2	0.603973777	0.947194688	0.63764481	0.523704919	1.71151463	21.35118905	95.31642009	0.5	CRA004775;PRJNA827465	Yang2025_Silkie_Wenchang	0.869061505
ENSGALG00010029389	SMYD4	2	0.095827604	0.064720992	1.480626323	0.138706177	0	0.661528166	0	1	PRJNA827465;Yang2025_Silkie_Wenchang	CRA004775	0.622396264
ENSGALG00010029389	SMYD4	2	0.016740323	0.222033771	0.07539539	0.939900127	0.062808969	2.674264509	62.6065411	0.5	CRA004775;Yang2025_Silkie_Wenchang	PRJNA827465	0.985444231
ENSGALG00010029389	SMYD4	2	-0.008439796	0.135337129	-0.062361279	0.95027513	0.019701924	1.707054629	41.41956661	0.5	CRA004775;PRJNA827465	Yang2025_Silkie_Wenchang	0.987277827
ENSGALG00010029390	RPA1	2	0.135667926	0.065638756	2.066885715	0.038744754	0.003235566	1.588682281	37.0547521	1	PRJNA827465;Yang2025_Silkie_Wenchang	CRA004775	0.339679921
ENSGALG00010029390	RPA1	2	-0.055430885	0.175555074	-0.315746413	0.752194995	0.03811404	2.119850029	52.8268516	0.5	CRA004775;Yang2025_Silkie_Wenchang	PRJNA827465	0.937988447
ENSGALG00010029390	RPA1	2	-0.006108667	0.249866773	-0.024447696	0.980495504	0.098047174	3.968154185	74.79936632	0.5	CRA004775;PRJNA827465	Yang2025_Silkie_Wenchang	0.99477051
ENSGALG00010029391	ARSG	2	-0.058848284	0.074210525	-0.792565549	0.451711027	0	0.102077088	0	1	PRJNA827465;Yang2025_Silkie_Wenchang	CRA004775	0.834000549
ENSGALG00010029391	ARSG	2	-0.353803713	0.290599696	-1.232980344	0.217583098	0.143313944	6.436651517	84.46397172	1	CRA004775;Yang2025_Silkie_Wenchang	PRJNA827465	0.731013651
ENSGALG00010029391	ARSG	2	-0.330441409	0.314976165	-1.049099729	0.294132221	0.176182131	8.561629801	88.31998085	1	CRA004775;PRJNA827465	Yang2025_Silkie_Wenchang	0.787324026
ENSGALG00010029392	RTN4RL1	2	-0.527200479	0.30440521	-1.731903601	0.083290727	0.041438149	1.285622184	22.21665023	1	PRJNA827465;Yang2025_Silkie_Wenchang	CRA004775	0.497287446
ENSGALG00010029392	RTN4RL1	2	-0.118014259	0.305114331	-0.386783507	0.698913897	0	0.443607476	0	0.5	CRA004775;Yang2025_Silkie_Wenchang	PRJNA827465	0.92185597
ENSGALG00010029392	RTN4RL1	2	-0.405136074	0.524665417	-0.772179871	0.440007902	0.319465477	2.312217626	56.75147578	0.5	CRA004775;PRJNA827465	Yang2025_Silkie_Wenchang	0.844353254
ENSGALG00010029394	CYGB	2	-0.026920848	0.126652935	-0.212556054	0.831673252	0.023250461	3.63264414	72.47184251	0.5	PRJNA827465;Yang2025_Silkie_Wenchang	CRA004775	0.957712669
ENSGALG00010029394	CYGB	2	-0.179880058	0.095585142	-1.881883048	0.059851897	0.001074698	1.01590241	1.565348225	1	CRA004775;Yang2025_Silkie_Wenchang	PRJNA827465	0.423848485
ENSGALG00010029394	CYGB	2	-0.117774048	0.297376397	-0.396043697	0.692072792	0.126956373	2.876798888	65.23914119	0.5	CRA004775;PRJNA827465	Yang2025_Silkie_Wenchang	0.920548122
ENSGALG00010029395	DPH1	2	0.406249371	0.227651484	1.784523273	0.074338676	0.091195203	8.207227772	87.8156178	1	PRJNA827465;Yang2025_Silkie_Wenchang	CRA004775	0.470803516
ENSGALG00010029395	DPH1	2	0.157196102	0.492510371	0.319173181	0.749595193	0.462424421	21.30602634	95.30649224	0.5	CRA004775;Yang2025_Silkie_Wenchang	PRJNA827465	0.937362187
ENSGALG00010029395	DPH1	2	-0.095724246	0.263982767	-0.216772659	0.828358521	0.121867682	7.682430949	86.98328684	0.5	CRA004775;PRJNA827465	Yang2025_Silkie_Wenchang	0.958272669
ENSGALG00010029396	WIPI1	2	0.02899093	0.074321504	0.390074591	0.696481391	0.005370217	1.597620752	37.4069222	0.5	PRJNA827465;Yang2025_Silkie_Wenchang	CRA004775	0.921041503
ENSGALG00010029396	WIPI1	2	0.166621722	0.116690702	1.427892014	0.153322943	0	0.06616675	0	1	CRA004775;Yang2025_Silkie_Wenchang	PRJNA827465	0.645602272
ENSGALG00010029396	WIPI1	2	-0.009586128	0.04526503	-0.211777796	0.832280392	0	0.667308733	0	0.5	CRA004775;PRJNA827465	Yang2025_Silkie_Wenchang	0.957808251
ENSGALG00010029397	OVCA2	2	0.301114413	0.134976148	2.230871289	0.025689656	0	0.498742042	0	1	PRJNA827465;Yang2025_Silkie_Wenchang	CRA004775	0.273403886
ENSGALG00010029397	OVCA2	2	-0.014610764	0.470402063	-0.03106016	0.975221562	0.374947603	6.542698129	84.71578575	0.5	CRA004775;Yang2025_Silkie_Wenchang	PRJNA827465	0.992699828
ENSGALG00010029397	OVCA2	2	-0.092718498	0.363667011	-0.254954382	0.798758333	0.217851098	5.476899151	81.74149327	0.5	CRA004775;PRJNA827465	Yang2025_Silkie_Wenchang	0.949922217
ENSGALG00010029398	RHBDF2	2	0.114777651	0.066768427	1.71904081	0.085606941	0.00170466	1.222506774	18.20086223	1	PRJNA827465;Yang2025_Silkie_Wenchang	CRA004775	0.503006371
ENSGALG00010029398	RHBDF2	2	0.00385043	0.227497532	0.016925152	0.986496327	0.079815483	4.036866813	75.22831329	0.5	CRA004775;Yang2025_Silkie_Wenchang	PRJNA827465	0.99618774
ENSGALG00010029398	RHBDF2	2	-0.043038665	0.151332011	-0.28439895	0.776104664	0.028187992	2.16336308	53.77567412	0.5	CRA004775;PRJNA827465	Yang2025_Silkie_Wenchang	0.944001494
ENSGALG00010029399	PRKAR1A	2	-0.228420467	0.199773777	-1.143395646	0.252874359	0.077100747	29.12982256	96.56709203	1	PRJNA827465;Yang2025_Silkie_Wenchang	CRA004775	0.762731469
ENSGALG00010029399	PRKAR1A	2	-0.204029855	0.254165615	-0.802743734	0.422122869	0.112296497	7.072883178	85.86149418	0.5	CRA004775;Yang2025_Silkie_Wenchang	PRJNA827465	0.839649223
ENSGALG00010029399	PRKAR1A	2	-0.027408287	0.036647309	-0.747893589	0.454524345	0	0.364660865	0	0.5	CRA004775;PRJNA827465	Yang2025_Silkie_Wenchang	0.849072665
ENSGALG00010029400	AANAT	2	0.746723653	0.683088334	1.093158257	0.274324321	0.503029368	2.155765959	53.61277528	1	PRJNA827465;Yang2025_Silkie_Wenchang	CRA004775	0.774526342
ENSGALG00010029400	AANAT	2	1.163598493	0.472378616	2.463275122	0.013767423	0	0.360645317	0	1	CRA004775;Yang2025_Silkie_Wenchang	PRJNA827465	0.192562641
ENSGALG00010029400	AANAT	2	0.493693223	0.437417919	1.128653403	0.259044073	0	0.831966191	0	1	CRA004775;PRJNA827465	Yang2025_Silkie_Wenchang	0.766195756
ENSGALG00010029401	HIC1	2	0.065762351	0.068765721	0.956324599	0.338908266	0	0.351178372	0	1	PRJNA827465;Yang2025_Silkie_Wenchang	CRA004775	0.804646102
ENSGALG00010029401	HIC1	2	0.130262736	0.117376551	1.109785004	0.267091683	0	0.027781448	0	1	CRA004775;Yang2025_Silkie_Wenchang	PRJNA827465	0.771247817
ENSGALG00010029401	HIC1	2	0.043106844	0.082080685	0.525176461	0.599460527	0	0.127615071	0	1	CRA004775;PRJNA827465	Yang2025_Silkie_Wenchang	0.895010988
ENSGALG00010029402	SMG6	2	0.022259046	0.083348437	0.267060154	0.789422846	0.01066111	4.154593932	75.93025898	0.5	PRJNA827465;Yang2025_Silkie_Wenchang	CRA004775	0.947390191
ENSGALG00010029402	SMG6	2	0.049225076	0.103228078	0.476857428	0.633463649	0.010116566	1.634003953	38.80063765	0.5	CRA004775;Yang2025_Silkie_Wenchang	PRJNA827465	0.903348345
ENSGALG00010029402	SMG6	2	-0.057031668	0.042591473	-1.339039569	0.180557792	0	0.127204257	0	1	CRA004775;PRJNA827465	Yang2025_Silkie_Wenchang	0.688241964
ENSGALG00010029403	FAM20A	2	-0.129114061	0.202616099	-0.63723496	0.523971809	0.06166501	3.959884269	74.74673672	0.5	PRJNA827465;Yang2025_Silkie_Wenchang	CRA004775	0.869661505
ENSGALG00010029403	FAM20A	2	-0.315798346	0.144655311	-2.183109233	0.029027767	0	0.165331781	0	1	CRA004775;Yang2025_Silkie_Wenchang	PRJNA827465	0.289904262
ENSGALG00010029403	FAM20A	2	0.02526796	0.114629226	0.220432092	0.825534654	0	0.600604281	0	0.5	CRA004775;PRJNA827465	Yang2025_Silkie_Wenchang	0.956386623
ENSGALG00010029404	SRR	2	-0.084910049	0.129450546	-0.655926539	0.511871392	0.012079229	1.541763529	35.13921033	0.5	PRJNA827465;Yang2025_Silkie_Wenchang	CRA004775	0.865694231
ENSGALG00010029404	SRR	2	0.003656586	0.143215081	0.02553213	0.979630521	0	0.554248088	0	0.5	CRA004775;Yang2025_Silkie_Wenchang	PRJNA827465	0.994472942
ENSGALG00010029404	SRR	2	-0.191871201	0.117834424	-1.628311953	0.103458756	0	0.003885614	0	1	CRA004775;PRJNA827465	Yang2025_Silkie_Wenchang	0.547715449
ENSGALG00010029405	POLDIP2	2	-0.038085909	0.037942142	-1.003789111	0.315480274	0	0.373445459	0	1	PRJNA827465;Yang2025_Silkie_Wenchang	CRA004775	0.796883862
ENSGALG00010029405	POLDIP2	2	-0.160868299	0.117816571	-1.365413183	0.172123223	0	0.682807372	0	1	CRA004775;Yang2025_Silkie_Wenchang	PRJNA827465	0.674809614
ENSGALG00010029405	POLDIP2	2	-0.088230261	0.125786403	-0.701429243	0.483035177	0.017520201	1.458512928	31.43701503	1	CRA004775;PRJNA827465	Yang2025_Silkie_Wenchang	0.85691916
ENSGALG00010029406	TMEM199	2	0.136614957	0.076341743	1.789518432	0.073531362	0	0.017485506	0	1	PRJNA827465;Yang2025_Silkie_Wenchang	CRA004775	0.468547845
ENSGALG00010029406	TMEM199	2	-0.129160464	0.270145864	-0.478113793	0.632569215	0.087985103	2.475036921	59.59656233	0.5	CRA004775;Yang2025_Silkie_Wenchang	PRJNA827465	0.903058174
ENSGALG00010029406	TMEM199	2	-0.08191948	0.250045602	-0.327618161	0.743200389	0.102695087	5.088890546	80.34935138	0.5	CRA004775;PRJNA827465	Yang2025_Silkie_Wenchang	0.93573824
ENSGALG00010029407	UBE2O	2	0.017671725	0.09657976	0.182975455	0.854817278	0.013137656	3.199991492	68.74991691	0.5	PRJNA827465;Yang2025_Silkie_Wenchang	CRA004775	0.963248391
ENSGALG00010029407	UBE2O	2	0.135435626	0.089697639	1.509912941	0.13106564	0	0.028097612	0	1	CRA004775;Yang2025_Silkie_Wenchang	PRJNA827465	0.606767161
ENSGALG00010029407	UBE2O	2	-0.056281691	0.054025974	-1.041752465	0.297526458	0	0.70983458	0	0.5	CRA004775;PRJNA827465	Yang2025_Silkie_Wenchang	0.788562929
ENSGALG00010029408	KIF12	2	0.267884099	0.234783381	1.140984074	0.253876551	0	0.791553946	0	1	PRJNA827465;Yang2025_Silkie_Wenchang	CRA004775	0.76368264
ENSGALG00010029408	KIF12	2	0.352338715	0.248483835	1.417954268	0.156204117	0	0.248781986	0	1	CRA004775;Yang2025_Silkie_Wenchang	PRJNA827465	0.649132948
ENSGALG00010029408	KIF12	2	0.08273914	0.280664903	0.294796886	0.768149045	0	0.081905578	0	1	CRA004775;PRJNA827465	Yang2025_Silkie_Wenchang	0.941444269
ENSGALG00010029409	ENSGALG00010029409	2	-0.118230591	0.130392716	-0.906726963	0.364551201	0	0.02463891	0	1	PRJNA827465;Yang2025_Silkie_Wenchang	CRA004775	0.817496251
ENSGALG00010029409	ENSGALG00010029409	2	-0.053101324	0.157274966	-0.33763367	0.735639265	0	0.583302687	0	0.5	CRA004775;Yang2025_Silkie_Wenchang	PRJNA827465	0.933592889
ENSGALG00010029409	ENSGALG00010029409	2	-0.069214603	0.183866482	-0.376439479	0.706590201	0	0.688267523	0	0.5	CRA004775;PRJNA827465	Yang2025_Silkie_Wenchang	0.924548793
ENSGALG00010029410	VTN	2	-0.219772234	0.924842151	-0.237632156	0.812166408	1.287972145	3.916229436	74.46523457	0.5	PRJNA827465;Yang2025_Silkie_Wenchang	CRA004775	0.953371387
ENSGALG00010029410	VTN	2	-0.269989103	0.755757132	-0.357243208	0.720909742	0.922710445	5.17524702	80.67725084	0.5	CRA004775;Yang2025_Silkie_Wenchang	PRJNA827465	0.928919926
ENSGALG00010029410	VTN	2	0.538463562	0.374837489	1.436525369	0.150852903	0	0.150147589	0	1	CRA004775;PRJNA827465	Yang2025_Silkie_Wenchang	0.642051813
ENSGALG00010029411	SARM1	2	0.17350488	0.11372019	1.525717462	0.127080244	0	0.486892703	0	1	PRJNA827465;Yang2025_Silkie_Wenchang	CRA004775	0.598374844
ENSGALG00010029411	SARM1	2	-0.37422363	0.746981807	-0.500980917	0.616384554	0.932850927	5.386359256	81.43458406	0.5	CRA004775;Yang2025_Silkie_Wenchang	PRJNA827465	0.899111936
ENSGALG00010029411	SARM1	2	-0.437757936	0.662913836	-0.660354201	0.509026555	0.706746072	4.363490453	77.08256702	0.5	CRA004775;PRJNA827465	Yang2025_Silkie_Wenchang	0.865193604
ENSGALG00010029412	SLC46A1	2	-0.193905306	0.076407317	-2.537784518	0.011155665	0	0.323693156	0	1	PRJNA827465;Yang2025_Silkie_Wenchang	CRA004775	0.167907012
ENSGALG00010029412	SLC46A1	2	-0.394763743	0.183228866	-2.154484447	0.0312022	0	0.221366535	0	1	CRA004775;Yang2025_Silkie_Wenchang	PRJNA827465	0.302054207
ENSGALG00010029412	SLC46A1	2	-0.220732422	0.107243107	-2.058243451	0.03956677	0.006720111	1.152601131	13.2397173	1	CRA004775;PRJNA827465	Yang2025_Silkie_Wenchang	0.344047067
ENSGALG00010029413	B3GNTL1	2	0.113093028	0.139463932	0.810912369	0.417415997	0	0.548870392	0	0.5	PRJNA827465;Yang2025_Silkie_Wenchang	CRA004775	0.838112595
ENSGALG00010029413	B3GNTL1	2	0.01927835	0.187171395	0.102998379	0.917964258	0	0.075496069	0	0.5	CRA004775;Yang2025_Silkie_Wenchang	PRJNA827465	0.981033557
ENSGALG00010029413	B3GNTL1	2	0.179528262	0.1617483	1.109923643	0.267031931	0	0.054698875	0	1	CRA004775;PRJNA827465	Yang2025_Silkie_Wenchang	0.771247817
ENSGALG00010029417	ENSGALG00010029417	2	-0.072928317	0.09954849	-0.732590891	0.463807989	0	0.44926459	0	0.5	PRJNA827465;Yang2025_Silkie_Wenchang	CRA004775	0.852159649
ENSGALG00010029417	ENSGALG00010029417	2	-0.094581763	0.189598238	-0.498853597	0.617882526	0.020655425	1.278354102	21.77441305	0.5	CRA004775;Yang2025_Silkie_Wenchang	PRJNA827465	0.899121942
ENSGALG00010029417	ENSGALG00010029417	2	-0.15664015	0.117154988	-1.337033562	0.181211682	0	0.644940747	0	1	CRA004775;PRJNA827465	Yang2025_Silkie_Wenchang	0.688663966
ENSGALG00010029418	ABCA5	2	-0.14379193	0.089124544	-1.613381943	0.106661554	0.005234739	1.446334097	30.8596816	1	PRJNA827465;Yang2025_Silkie_Wenchang	CRA004775	0.553853751
ENSGALG00010029418	ABCA5	2	-0.050071642	0.113604858	-0.440752647	0.659392078	0	0.150384072	0	1	CRA004775;Yang2025_Silkie_Wenchang	PRJNA827465	0.911449691
ENSGALG00010029418	ABCA5	2	-0.205381354	0.081886463	-2.508123407	0.012137427	0	0.071190479	0	1	CRA004775;PRJNA827465	Yang2025_Silkie_Wenchang	0.177733715
ENSGALG00010029419	RFC2	2	-0.088192928	0.063199063	-1.395478422	0.162871617	0	0.003036824	0	1	PRJNA827465;Yang2025_Silkie_Wenchang	CRA004775	0.660954381
ENSGALG00010029419	RFC2	2	-0.167094847	0.142657039	-1.171304612	0.241476358	0	0.772241034	0	1	CRA004775;Yang2025_Silkie_Wenchang	PRJNA827465	0.753951804
ENSGALG00010029419	RFC2	2	-0.106476314	0.074781948	-1.423823747	0.1544975	0.001288458	1.028873141	2.806287773	1	CRA004775;PRJNA827465	Yang2025_Silkie_Wenchang	0.647288693
ENSGALG00010029420	LAT2	2	0.245292277	0.306180779	0.801135452	0.423053234	0	0.171220298	0	1	PRJNA827465;Yang2025_Silkie_Wenchang	CRA004775	0.840046095
ENSGALG00010029420	LAT2	2	0.32404964	0.243472484	1.33094974	0.183205549	0	0.314619262	0	1	CRA004775;Yang2025_Silkie_Wenchang	PRJNA827465	0.691626904
ENSGALG00010029420	LAT2	2	0.387044651	0.230902097	1.676228389	0.093693466	0	0.01208915	0	1	CRA004775;PRJNA827465	Yang2025_Silkie_Wenchang	0.524189933
ENSGALG00010029421	TBCD	2	-0.032758029	0.077479462	-0.422796296	0.672443891	0.007350316	2.530790721	60.48665773	0.5	PRJNA827465;Yang2025_Silkie_Wenchang	CRA004775	0.914742762
ENSGALG00010029421	TBCD	2	-0.099680068	0.072578226	-1.373415614	0.169623178	0	0.442425864	0	0.5	CRA004775;Yang2025_Silkie_Wenchang	PRJNA827465	0.671099233
ENSGALG00010029421	TBCD	2	0.035187241	0.056349762	0.624443469	0.532336385	0	0.007264963	0	1	CRA004775;PRJNA827465	Yang2025_Silkie_Wenchang	0.873104119
ENSGALG00010029422	EIF4H	2	-0.094947612	0.057700864	-1.645514557	0.099863743	0.004180496	2.478365908	59.65083296	1	PRJNA827465;Yang2025_Silkie_Wenchang	CRA004775	0.539882352
ENSGALG00010029422	EIF4H	2	-0.077551522	0.122186335	-0.63469882	0.525624863	0.018602707	2.312232245	56.75174922	0.5	CRA004775;Yang2025_Silkie_Wenchang	PRJNA827465	0.870277065
ENSGALG00010029422	EIF4H	2	-0.042079799	0.034634023	-1.214984448	0.224372023	0	0.751229584	0	0.5	CRA004775;PRJNA827465	Yang2025_Silkie_Wenchang	0.737471949
ENSGALG00010029423	MYBBP1A	2	-0.027637913	0.05190594	-0.532461459	0.594406429	0	0.002021431	0	1	PRJNA827465;Yang2025_Silkie_Wenchang	CRA004775	0.893337382
ENSGALG00010029423	MYBBP1A	2	-0.03117203	0.069503292	-0.448497177	0.653794423	0	0.07667948	0	1	CRA004775;Yang2025_Silkie_Wenchang	PRJNA827465	0.909853488
ENSGALG00010029423	MYBBP1A	2	-0.035673668	0.070922375	-0.502995967	0.614967107	0	0.066471994	0	1	CRA004775;PRJNA827465	Yang2025_Silkie_Wenchang	0.898781283
ENSGALG00010029424	MAP2K6	2	-0.141380994	0.074140866	-1.906923965	0.056530434	0	0.400472558	0	1	PRJNA827465;Yang2025_Silkie_Wenchang	CRA004775	0.411523553
ENSGALG00010029424	MAP2K6	2	-0.1960397	0.142692385	-1.373862384	0.169484409	0	0.463817939	0	0.5	CRA004775;Yang2025_Silkie_Wenchang	PRJNA827465	0.670890582
ENSGALG00010029424	MAP2K6	2	-0.107521975	0.084668531	-1.269916629	0.204114329	0	0.251667063	0	0.5	CRA004775;PRJNA827465	Yang2025_Silkie_Wenchang	0.717254253
ENSGALG00010029425	SPNS2	2	0.045800411	0.10972301	0.417418469	0.676372348	0	0.027143732	0	1	PRJNA827465;Yang2025_Silkie_Wenchang	CRA004775	0.916760849
ENSGALG00010029425	SPNS2	2	0.057558833	0.167407202	0.343825312	0.730977664	0	0.025623177	0	1	CRA004775;Yang2025_Silkie_Wenchang	PRJNA827465	0.932769533
ENSGALG00010029425	SPNS2	2	0.02922475	0.128064682	0.228203041	0.819488397	0	0.005539113	0	1	CRA004775;PRJNA827465	Yang2025_Silkie_Wenchang	0.955256039
ENSGALG00010029427	SPNS3	2	-0.174774354	0.209816321	-0.83298741	0.40485183	0	0.695056322	0	1	PRJNA827465;Yang2025_Silkie_Wenchang	CRA004775	0.833112102
ENSGALG00010029427	SPNS3	2	-0.85955345	0.424702092	-2.023897377	0.042980712	0.193101813	2.148042859	53.44599407	1	CRA004775;Yang2025_Silkie_Wenchang	PRJNA827465	0.358504015
ENSGALG00010029427	SPNS3	2	-0.633893958	0.616674008	-1.027923912	0.30398562	0.643308495	6.271760109	84.0555126	1	CRA004775;PRJNA827465	Yang2025_Silkie_Wenchang	0.792225107
ENSGALG00010029428	FN3K	2	0.24930558	0.186609638	1.335973763	0.181557849	0	0.088496505	0	1	PRJNA827465;Yang2025_Silkie_Wenchang	CRA004775	0.689004635
ENSGALG00010029428	FN3K	2	0.115599711	0.220401687	0.524495583	0.599933891	0	0.351496547	0	0.5	CRA004775;Yang2025_Silkie_Wenchang	PRJNA827465	0.895092498
ENSGALG00010029428	FN3K	2	0.189633501	0.226846758	0.835954208	0.403180665	0	0.653874553	0	0.5	CRA004775;PRJNA827465	Yang2025_Silkie_Wenchang	0.83306585
ENSGALG00010029429	UBE2G1	2	-0.087160277	0.042321105	-2.059499069	0.039446454	0	0.605424449	0	1	PRJNA827465;Yang2025_Silkie_Wenchang	CRA004775	0.343380728
ENSGALG00010029429	UBE2G1	2	-0.029402094	0.198144819	-0.148386892	0.882037444	0.045474655	1.835371281	45.51511129	0.5	CRA004775;Yang2025_Silkie_Wenchang	PRJNA827465	0.971928118
ENSGALG00010029429	UBE2G1	2	-0.020042819	0.125657532	-0.159503521	0.873272184	0.015971226	1.310258402	23.67917668	0.5	CRA004775;PRJNA827465	Yang2025_Silkie_Wenchang	0.96985551
ENSGALG00010029430	FN3KRP	2	-0.490770686	0.227458916	-2.157623422	0.030957122	0.087323444	6.08747331	83.57282325	1	PRJNA827465;Yang2025_Silkie_Wenchang	CRA004775	0.300524815
ENSGALG00010029430	FN3KRP	2	-0.37999487	0.375050555	-1.013183064	0.310972729	0.244145462	7.503288894	86.67251103	0.5	CRA004775;Yang2025_Silkie_Wenchang	PRJNA827465	0.795218958
ENSGALG00010029430	FN3KRP	2	-0.207424103	0.129248408	-1.604848415	0.108527168	0.015799705	1.57969805	36.69676302	0.5	CRA004775;PRJNA827465	Yang2025_Silkie_Wenchang	0.557501411
ENSGALG00010029431	ENSGALG00010029431	2	3.39610699	2.298907654	1.477269861	0.139603314	7.857419101	3.76152831	73.41506118	1	PRJNA827465;Yang2025_Silkie_Wenchang	CRA004775	0.623410749
ENSGALG00010029431	ENSGALG00010029431	2	2.853367537	2.760933303	1.033479344	0.301379632	12.7211132	5.929519588	83.13522731	0.5	CRA004775;Yang2025_Silkie_Wenchang	PRJNA827465	0.791222412
ENSGALG00010029431	ENSGALG00010029431	2	0.340659332	1.370061201	0.248645339	0.803635134	0	0.108649574	0	0.5	CRA004775;PRJNA827465	Yang2025_Silkie_Wenchang	0.951064358
ENSGALG00010029432	ENSGALG00010029432	2	-0.495509729	2.058483677	-0.24071589	0.809775323	0	0.07079217	0	0.5	PRJNA827465;Yang2025_Silkie_Wenchang	CRA004775	0.952818762
ENSGALG00010029432	ENSGALG00010029432	2	-0.211272546	1.541493939	-0.137057008	0.890985735	0	0.011313792	0	0.5	CRA004775;Yang2025_Silkie_Wenchang	PRJNA827465	0.973636056
ENSGALG00010029432	ENSGALG00010029432	2	-0.518434192	1.542426091	-0.336116068	0.736783342	0	0.045245372	0	1	CRA004775;PRJNA827465	Yang2025_Silkie_Wenchang	0.933777134
ENSGALG00010029436	WDR45B	2	-0.01281144	0.040761307	-0.314303966	0.753290186	0	0.950177368	0	0.5	PRJNA827465;Yang2025_Silkie_Wenchang	CRA004775	0.938087082
ENSGALG00010029436	WDR45B	2	-0.083902646	0.073564859	-1.140526159	0.254067161	0	0.031021616	0	1	CRA004775;Yang2025_Silkie_Wenchang	PRJNA827465	0.763894203
ENSGALG00010029436	WDR45B	2	0.004332536	0.046301009	0.093573248	0.925448161	0	0.383105228	0	0.5	CRA004775;PRJNA827465	Yang2025_Silkie_Wenchang	0.982594411
ENSGALG00010029437	ENSGALG00010029437	2	1.626708977	0.718279189	2.264730766	0.023529206	0	0.008429582	0	1	PRJNA827465;Yang2025_Silkie_Wenchang	CRA004775	0.261825707
ENSGALG00010029437	ENSGALG00010029437	2	1.125692719	0.892243547	1.26164288	0.207077319	0	0.640589188	0	1	CRA004775;Yang2025_Silkie_Wenchang	PRJNA827465	0.718997292

ENSGALG00010029437	ENSGALG00010029437	2	1.180177367	0.769585614	1.533523165	0.125147003	0	0.617359113	0	1	CRA004775;PRJNA827465	Yang2025_Silkie_Wenchang	0.593753341
ENSGALG00010029438	ENSGALG00010029438	2	-0.057234087	0.146778949	-0.389933892	0.696585432	0	0.442060573	0	0.5	PRJNA827465;Yang2025_Silkie_Wenchang	CRA004775	0.921041503
ENSGALG00010029438	ENSGALG00010029438	2	-0.370094189	0.605537728	-0.611182708	0.54107863	0.520444903	3.414928357	70.71680881	0.5	CRA004775;Yang2025_Silkie_Wenchang	PRJNA827465	0.875829389
ENSGALG00010029438	ENSGALG00010029438	2	-0.425768023	0.441181583	-0.965063003	0.334513256	0.279946676	2.971957032	66.35213803	1	CRA004775;PRJNA827465	Yang2025_Silkie_Wenchang	0.803094614
ENSGALG00010029439	ENSGALG00010029439	2	0.612216601	0.322872735	1.896154533	0.057939613	0.114546038	2.058172699	51.4132123	1	PRJNA827465;Yang2025_Silkie_Wenchang	CRA004775	0.417375481
ENSGALG00010029439	ENSGALG00010029439	2	0.303989165	0.699318386	0.434693512	0.663784908	0.852227603	7.695005811	87.00455822	0.5	CRA004775;Yang2025_Silkie_Wenchang	PRJNA827465	0.913119244
ENSGALG00010029439	ENSGALG00010029439	2	0.022961318	0.365140836	0.062883458	0.949859307	0.19716288	3.782977766	73.56579758	0.5	CRA004775;PRJNA827465	Yang2025_Silkie_Wenchang	0.987277827
ENSGALG00010029440	FOXK2	2	-0.013419719	0.204175909	-0.065726259	0.947595766	0.062897619	3.514945202	71.55005434	0.5	PRJNA827465;Yang2025_Silkie_Wenchang	CRA004775	0.986560458
ENSGALG00010029440	FOXK2	2	0.306177245	0.183512623	1.668426075	0.095231176	0	0.344239614	0	1	CRA004775;Yang2025_Silkie_Wenchang	PRJNA827465	0.528281001
ENSGALG00010029440	FOXK2	2	0.06166008	0.319493308	0.192993339	0.846964185	0.153256266	3.202453102	68.77393772	0.5	CRA004775;PRJNA827465	Yang2025_Silkie_Wenchang	0.961252712
ENSGALG00010029442	ENSGALG00010029442	2	0.534334057	0.542862592	0.984289699	0.324973093	0.390365962	2.961289909	66.2309321	0.5	PRJNA827465;Yang2025_Silkie_Wenchang	CRA004775	0.799914637
ENSGALG00010029442	ENSGALG00010029442	2	0.271241304	0.840765308	0.322612389	0.74698881	1.156933028	5.458190808	81.67891092	0.5	CRA004775;Yang2025_Silkie_Wenchang	PRJNA827465	0.936729365
ENSGALG00010029442	ENSGALG00010029442	2	-0.23880728	0.351047443	-0.680270672	0.496333091	0	0.684963412	0	1	CRA004775;PRJNA827465	Yang2025_Silkie_Wenchang	0.860939846
ENSGALG00010029443	ENSGALG00010029443	2	-0.055940964	0.216572533	-0.258301287	0.796174393	0.013751778	1.143712101	12.56540878	0.5	PRJNA827465;Yang2025_Silkie_Wenchang	CRA004775	0.949325935
ENSGALG00010029443	ENSGALG00010029443	2	-0.828751075	1.146324536	-0.722963741	0.469702161	2.37635743	10.23761681	90.23210169	0.5	CRA004775;Yang2025_Silkie_Wenchang	PRJNA827465	0.853485658
ENSGALG00010029443	ENSGALG00010029443	2	-1.026627911	0.909306798	-1.129022584	0.258888307	1.453430241	7.732482724	87.06754304	1	CRA004775;PRJNA827465	Yang2025_Silkie_Wenchang	0.765965607
ENSGALG00010029445	SPHK1	2	0.306487027	0.098968324	3.096819414	0.00195609	0	0.597176569	0	1	PRJNA827465;Yang2025_Silkie_Wenchang	CRA004775	0.057202533
ENSGALG00010029445	SPHK1	2	0.163877751	0.317369257	0.516363029	0.605600883	0.139025292	3.187636093	68.6287904	0.5	CRA004775;Yang2025_Silkie_Wenchang	PRJNA827465	0.896474603
ENSGALG00010029445	SPHK1	2	0.119333992	0.210279877	0.567500771	0.570374003	0.054034144	2.188170739	54.29972706	0.5	CRA004775;PRJNA827465	Yang2025_Silkie_Wenchang	0.8851128
ENSGALG00010029446	PRPSAP1	2	-0.018400632	0.055803641	-0.329738923	0.74159724	0.000277907	1.029219106	2.838958753	0.5	PRJNA827465;Yang2025_Silkie_Wenchang	CRA004775	0.93543545
ENSGALG00010029446	PRPSAP1	2	-0.173523705	0.10484807	-1.655001423	0.097924209	0	0.417475344	0	1	CRA004775;Yang2025_Silkie_Wenchang	PRJNA827465	0.534130658
ENSGALG00010029446	PRPSAP1	2	-0.075571587	0.131878357	-0.573040101	0.566617542	0.020999003	2.001659508	50.0414533	0.5	CRA004775;PRJNA827465	Yang2025_Silkie_Wenchang	0.883487517
ENSGALG00010029449	UBALD2	2	0.233549385	0.565006931	0.413356672	0.679345315	0.586682084	11.75547921	91.4933285	0.5	PRJNA827465;Yang2025_Silkie_Wenchang	CRA004775	0.917701868
ENSGALG00010029449	UBALD2	2	0.511856004	0.30650295	1.6699872	0.094921896	0.102986527	2.203761207	54.62303281	1	CRA004775;Yang2025_Silkie_Wenchang	PRJNA827465	0.527384122
ENSGALG00010029449	UBALD2	2	-0.096125676	0.252197923	-0.381151736	0.70309066	0.090906033	3.091866377	67.65707575	0.5	CRA004775;PRJNA827465	Yang2025_Silkie_Wenchang	0.923459557
ENSGALG00010029451	ENSGALG00010029451	2	0.390439949	0.114005953	3.424733006	0.000615404	0	0.801876176	0	1	PRJNA827465;Yang2025_Silkie_Wenchang	CRA004775	0.026994664
ENSGALG00010029451	ENSGALG00010029451	2	0.133820298	0.201027263	0.665682337	0.505614166	0	0.742890504	0	0.5	CRA004775;Yang2025_Silkie_Wenchang	PRJNA827465	0.863927652
ENSGALG00010029451	ENSGALG00010029451	2	0.258077473	0.304287074	0.848138141	0.396361043	0.112226707	1.982770039	49.56550783	0.5	CRA004775;PRJNA827465	Yang2025_Silkie_Wenchang	0.831365249
ENSGALG00010029452	ENSGALG00010029452	2	-5.134103545	1.322718209	-3.881479449	0.000103823	0	0.023080197	0	1	PRJNA827465;Yang2025_Silkie_Wenchang	CRA004775	0.008001083
ENSGALG00010029452	ENSGALG00010029452	2	-2.976729514	1.843217131	-1.61496411	0.106318467	4.094110213	2.258405547	55.72097308	1	CRA004775;Yang2025_Silkie_Wenchang	PRJNA827465	0.553168483
ENSGALG00010029452	ENSGALG00010029452	2	-3.086339968	1.691262127	-1.824873815	0.068020074	3.873990666	3.008371287	66.75942214	1	CRA004775;PRJNA827465	Yang2025_Silkie_Wenchang	0.4497039
ENSGALG00010029454	ENSGALG00010029454	2	-0.429405562	0.353516957	-1.214667512	0.224492928	0.113620348	1.481073648	32.48141295	1	PRJNA827465;Yang2025_Silkie_Wenchang	CRA004775	0.737544559
ENSGALG00010029454	ENSGALG00010029454	2	-0.869167974	0.415432339	-2.092201045	0.036420535	0	0.162721431	0	1	CRA004775;Yang2025_Silkie_Wenchang	PRJNA827465	0.328914588
ENSGALG00010029454	ENSGALG00010029454	2	-0.306293034	0.205329041	-1.49171804	0.135773078	0	0.720261594	0	1	CRA004775;PRJNA827465	Yang2025_Silkie_Wenchang	0.617196137
ENSGALG00010029455	ENSGALG00010029455	2	0.31451923	0.510738891	0.61581218	0.538018487	0.442165585	6.5540182	84.74218457	0.5	PRJNA827465;Yang2025_Silkie_Wenchang	CRA004775	0.87481821
ENSGALG00010029455	ENSGALG00010029455	2	-0.130311151	0.996246224	-0.130802153	0.89593182	1.831331634	12.72520816	92.14158238	0.5	CRA004775;Yang2025_Silkie_Wenchang	PRJNA827465	0.975002393
ENSGALG00010029455	ENSGALG00010029455	2	-0.597717328	0.479678508	-1.246079025	0.212735384	0.32240311	3.146667956	68.22035201	1	CRA004775;PRJNA827465	Yang2025_Silkie_Wenchang	0.725541376
ENSGALG00010029456	RNF157	2	0.489192516	0.546834464	0.894589769	0.371006423	0.437649966	3.564021089	71.94180463	1	PRJNA827465;Yang2025_Silkie_Wenchang	CRA004775	0.8214684
ENSGALG00010029456	RNF157	2	1.216338845	0.391837152	3.104194789	0.001907977	0	0.112221363	0	1	CRA004775;Yang2025_Silkie_Wenchang	PRJNA827465	0.05625597
ENSGALG00010029456	RNF157	2	0.583982756	0.677212282	0.862333379	0.388504089	0.697186551	3.845279747	73.99408975	1	CRA004775;PRJNA827465	Yang2025_Silkie_Wenchang	0.82823694
ENSGALG00010029457	ENSGALG00010029457	2	0.187225274	0.142054279	1.317984046	0.187508989	0	0.000470004	0	1	PRJNA827465;Yang2025_Silkie_Wenchang	CRA004775	0.696631608
ENSGALG00010029457	ENSGALG00010029457	2	-0.12243535	0.224973272	-0.544221759	0.58628888	0.032210856	1.396367141	28.38559638	0.5	CRA004775;Yang2025_Silkie_Wenchang	PRJNA827465	0.891546539
ENSGALG00010029457	ENSGALG00010029457	2	-0.027272155	0.23315665	-0.116969234	0.906884434	0.075455738	3.20953115	68.84280123	0.5	CRA004775;PRJNA827465	Yang2025_Silkie_Wenchang	0.978196186
ENSGALG00010029458	ENSGALG00010029458	2	-0.660875046	1.150366239	-0.574490996	0.565635592	0	0.011130796	0	1	PRJNA827465;Yang2025_Silkie_Wenchang	CRA004775	0.883487517
ENSGALG00010029458	ENSGALG00010029458	2	-0.600026073	0.789854074	-0.759666998	0.447453661	0	0.003275406	0	1	CRA004775;Yang2025_Silkie_Wenchang	PRJNA827465	0.84650962
ENSGALG00010029458	ENSGALG00010029458	2	-0.655777766	0.744056526	-0.881354766	0.378125832	0	0.006167412	0	1	CRA004775;PRJNA827465	Yang2025_Silkie_Wenchang	0.823285408
ENSGALG00010029460	ENSGALG00010029460	2	0.037446711	0.125434198	0.2985367	0.76529357	0	0.360542798	0	1	PRJNA827465;Yang2025_Silkie_Wenchang	CRA004775	0.940779391
ENSGALG00010029460	ENSGALG00010029460	2	-0.352418007	0.562053113	-0.627019046	0.53064675	0.536310725	6.57914569	84.80045819	0.5	CRA004775;Yang2025_Silkie_Wenchang	PRJNA827465	0.872665695
ENSGALG00010029460	ENSGALG00010029460	2	-0.412650642	0.451172147	-0.914619054	0.360391652	0.361989478	8.673334932	88.4704095	0.5	CRA004775;PRJNA827465	Yang2025_Silkie_Wenchang	0.814880744
ENSGALG00010029461	ENSGALG00010029461	2	0.35868638	0.528602657	0.678555763	0.49741938	0.513875276	12.19893619	91.80256389	0.5	PRJNA827465;Yang2025_Silkie_Wenchang	CRA004775	0.86098656
ENSGALG00010029461	ENSGALG00010029461	2	-0.201633263	1.136452553	-0.177423389	0.859175837	2.456829861	20.25938307	95.06401554	0.5	CRA004775;Yang2025_Silkie_Wenchang	PRJNA827465	0.964605083
ENSGALG00010029461	ENSGALG00010029461	2	-0.689414475	0.603984057	-1.141444823	0.253684861	0.633751372	7.042954348	85.80141301	1	CRA004775;PRJNA827465	Yang2025_Silkie_Wenchang	0.763418915
ENSGALG00010029462	ENSGALG00010029462	2	-3.253514422	3.441642686	-0.945337654	0.344486521	22.50392419	19.33746858	94.82869231	0.5	PRJNA827465;Yang2025_Silkie_Wenchang	CRA004775	0.806768255
ENSGALG00010029462	ENSGALG00010029462	2	-2.910073474	3.830202575	-0.759770121	0.447392006	27.91222236	20.2656626	95.06554501	0.5	CRA004775;Yang2025_Silkie_Wenchang	PRJNA827465	0.84650962
ENSGALG00010029462	ENSGALG00010029462	2	0.247279368	0.419169421	0.589927021	0.555239578	0	0.652863815	0	1	CRA004775;PRJNA827465	Yang2025_Silkie_Wenchang	0.880255849
ENSGALG00010029464	ENSGALG00010029464	2	1.091904714	0.386093779	2.828081607	0.004682787	0.022250765	1.069150902	6.46783367	1	PRJNA827465;Yang2025_Silkie_Wenchang	CRA004775	0.100682477
ENSGALG00010029464	ENSGALG00010029464	2	0.619268659	1.031622949	0.600285851	0.548315747	1.696035644	4.920927602	79.67862808	0.5	CRA004775;Yang2025_Silkie_Wenchang	PRJNA827465	0.878281266
ENSGALG00010029464	ENSGALG00010029464	2	0.301518417	0.610336072	0.494020313	0.621291834	0.45422657	2.479953349	59.67666084	0.5	CRA004775;PRJNA827465	Yang2025_Silkie_Wenchang	0.899693435
ENSGALG00010029465	ENSGALG00010029465	2	3.351831073	1.376612082	2.434840662	0.014898349	0	0.264731484	0	1	PRJNA827465;Yang2025_Silkie_Wenchang	CRA004775	0.201566194
ENSGALG00010029465	ENSGALG00010029465	2	0.191602292	4.145712258	0.046216978	0.963137311	28.7745087	5.947336984	83.18575183	0.5	CRA004775;Yang2025_Silkie_Wenchang	PRJNA827465	0.989998461
ENSGALG00010029465	ENSGALG00010029465	2	-0.283311275	3.305598241	-0.085706506	0.93169973	19.10272179	7.931394765	87.3918771	0.5	CRA004775;PRJNA827465	Yang2025_Silkie_Wenchang	0.98412915
ENSGALG00010029466	SLC39A11	2	-0.085945323	0.245845847	-0.349590299	0.726646193	0.095844894	4.338883952	76.95259862	0.5	PRJNA827465;Yang2025_Silkie_Wenchang	CRA004775	0.931213124
ENSGALG00010029466	SLC39A11	2	-0.44854554	0.169928924	-2.63960678	0.008300227	0	0.232420867	0	1	CRA004775;Yang2025_Silkie_Wenchang	PRJNA827465	0.141959889
ENSGALG00010029466	SLC39A11	2	-0.165914735	0.329284108	-0.503864995	0.614356251	0.183289132	5.854523754	82.91919135	0.5	CRA004775;PRJNA827465	Yang2025_Silkie_Wenchang	0.898523733
ENSGALG00010029468	ENSGALG00010029468	2	0.184010325	0.201538973	0.913026012	0.361228861	0	0.03031434	0	1	PRJNA827465;Yang2025_Silkie_Wenchang	CRA004775	0.815332104
ENSGALG00010029468	ENSGALG00010029468	2	0.091193225	0.277285308	0.328878676	0.742247393	0	0.427503911	0	0.5	CRA004775;Yang2025_Silkie_Wenchang	PRJNA827465	0.935497348
ENSGALG00010029468	ENSGALG00010029468	2	0.159289339	0.268125799	0.594084343	0.552455702	0	0.529937907	0	0.5	CRA004775;PRJNA827465	Yang2025_Silkie_Wenchang	0.879228216
ENSGALG00010029469	ENSGALG00010029469	2	0.630444231	0.434450379	1.451130581	0.146743502	0.280873443	3.905043846	74.39209291	1	PRJNA827465;Yang2025_Silkie_Wenchang	CRA004775	0.635558525
ENSGALG00010029469	ENSGALG00010029469	2	0.399479712	0.772661313	0.517017878	0.605143679	0.94056701	4.318111207	76.84172658	0.5	CRA004775;Yang2025_Silkie_Wenchang	PRJNA827465	0.896474603
ENSGALG00010029469	ENSGALG00010029469	2	0.067760929	0.289311078	0.234214774	0.814818246	0	0.839065032	0	0.5	CRA004775;PRJNA827465	Yang2025_Silkie_Wenchang	0.953885608
ENSGALG00010029470	SSTR2	2	-0.680157979	0.466716017	-1.457327269	0.145026063	0.270709661	2.639048499	62.10755503	1	PRJNA827465;Yang2025_Silkie_Wenchang	CRA004775	0.632983695
ENSGALG00010029470	SSTR2	2	-2.03986493	0.998886191	-2.042139483	0.041137697	1.658763801	5.646840005	82.29098046	1	CRA004775;Yang2025_Silkie_Wenchang	PRJNA827465	0.351638738
ENSGALG00010029470	SSTR2	2	-1.608278433	1.469314956	-1.094577052	0.273701974	3.959414555	11.79265895	91.52014822	1	CRA004775;PRJNA827465	Yang2025_Silkie_Wenchang	0.774415754
ENSGALG00010029472	ENSGALG00010029472	2	0.801390027	0.361107525	2.219255957	0.026469316	0	0.501095384	0	1	PRJNA827465;Yang2025_Silkie_Wenchang	CRA004775	0.277493606
ENSGALG00010029472	ENSGALG00010029472	2	0.50073136	0.471651388	1.06165565	0.28839204	0	0.032706764	0	1	CRA004775;Yang2025_Silkie_Wenchang	PRJNA827465	0.783088502
ENSGALG00010029472	ENSGALG00010029472	2	0.863813306	0.367493839	2.350551806	0.018745598	0	0.220109626	0	1	CRA004775;PRJNA827465	Yang2025_Silkie_Wenchang	0.229376786
ENSGALG00010029473	COG1	2	-0.099674173	0.086293255	-1.15506332	0.248064497	0.009052744	2.289867685	56.32935446	0.5	PRJNA827465;Yang2025_Silkie_Wenchang	CRA004775	0.758257812
ENSGALG00010029473	COG1	2	-0.165488934	0.256943567	-0.644067239	0.519531834	0.086951847	2.387074631	58.1077195	0.5	CRA004775;Yang2025_Silkie_Wenchang	PRJNA827465	0.869086345
ENSGALG00010029473	COG1	2	-0.197571718	0.104424744	-1.892000971	0.058490851	0.009295268	1.159339971	13.74402465	1	CRA004775;PRJNA827465	Yang2025_Silkie_Wenchang	0.418938606
ENSGALG00010029477	FAM104A	2	-0.111422903	0.195189193	-0.570845657	0.568104271	0.06849782	9.464864633	89.43460854	0.5	PRJNA827465;Yang2025_Silkie_Wenchang	CRA004775	0.883970155
ENSGALG00010029477	FAM104A	2	-0.265517556	0.095004484	-2.794789719	0.005193348	0	0.679881118	0	1	CRA004775;Yang2025_Silkie_Wenchang	PRJNA827465	0.107598403
ENSGALG00010029477	FAM104A	2	0.011529285	0.098943592	0.116523813	0.907237415	0.010628341	1.719245025	41.83493422	0.5	CRA004775;PRJNA827465	Yang2025_Silkie_Wenchang	0.978196186
ENSGALG00010029479	ENSGALG00010029479	2	0.458021899	0.373119482	1.227547531	0.219616868	0	0.914523937	0	1	PRJNA827465;Yang2025_Silkie_Wenchang	CRA004775	0.733094397
ENSGALG00010029479	ENSGALG00010029479	2	-0.041385037	0.893547249	-0.046315443	0.963058831	1.201925434	3.904415999	74.38797504	0.5	CRA004775;Yang2025_Silkie_Wenchang	PRJNA827465	0.989998461
ENSGALG00010029479	ENSGALG00010029479	2	-0.381518447	0.523757489	-0.728425759	0.466353	0.115946779	1.251546057	20.09882542	0.5	CRA004775;PRJNA827465	Yang2025_Silkie_Wenchang	0.852763198
ENSGALG00010029480	ENSGALG00010029480	2	-0.058920441	0.081788792	-0.720397488	0.471280296	0	0.016376119	0	1	PRJNA827465;Yang2025_Silkie_Wenchang	CRA004775	0.853939282
ENSGALG00010029480	ENSGALG00010029480	2	-0.050671142	0.143799616	-0.352373277	0.724558341	0	0.01340048	0	1	CRA004775;Yang2025_Silkie_Wenchang	PRJNA827465	0.930388018
ENSGALG00010029480	ENSGALG00010029480	2	-0.065660335	0.086843959	-0.756072566	0.449605689	0	0.001440755	0	1	CRA004775;PRJNA827465	Yang2025_Silkie_Wenchang	0.847275788
ENSGALG00010029481	ENSGALG00010029481	2	0.337354383	0.258339689	1.305855808	0.191601597	0	0.032371674	0	1	PRJNA827465;Yang2025_Silkie_Wenchang	CRA004775	0.701230801
ENSGALG00010029481	ENSGALG00010029481	2	0.075416325	0.548793472	0.137422052	0.890697201	0	0.72712267	0	0.5	CRA004775;Yang2025_Silkie_Wenchang	PRJNA827465	0.973477891
ENSGALG00010029481	ENSGALG00010029481	2	0.243447891	0.264393215	0.920779646	0.357165484	0	0.805052318	0	0.5	CRA004775;PRJNA827465	Yang2025_Silkie_Wenchang	0.813260191
ENSGALG00010029482	DDX25	2	-0.239281127	0.12288955	-1.947123471	0.051519934	0.010692523	1.524821916	34.41857116	1	PRJNA827465;Yang2025_Silkie_Wenchang	CRA004775	0.393564229
ENSGALG00010029482	DDX25	2	-0.600471144	0.2906473	-2.065978744	0.038830487	0.105492273	2.337198257	57.21372815	1	CRA004775;Yang2025_Silkie_Wenchang	PRJNA827465	0.340024811
ENSGALG00010029482	DDX25	2	-0.495358549	0.422777655	-1.171676277	0.241327051	0.293102573	4.976197023	79.90433266	1	CRA004775;PRJNA827465	Yang2025_Silkie_Wenchang	0.753847713
ENSGALG00010029487	CDC42EP4	2	-0.181855483	0.137204673	-1.325432137	0.185027864	0.023742072	2.505226303	60.0834464	1	PRJNA827465;Yang2025_Silkie_Wenchang	CRA004775	0.693265167
ENSGALG00010029487	CDC42EP4	2	-0.366353886	0.145616798	-2.515876542	0.011873677	0	0.056948572	0	1	CRA004775;Yang2025_Silkie_Wenchang	PRJNA827465	0.174848676
ENSGALG00010029487	CDC42EP4	2	-0.088001471	0.082984445	-1.060457429	0.288936547	0	0.865269763	0	1	CRA004775;PRJNA827465	Yang2025_Silkie_Wenchang	0.783530627
ENSGALG00010029489	ENSGALG00010029489	2	0.468582095	1.425377266	0.328742507	0.742350323	3.510362062	7.346630225	86.38831724	0.5	PRJNA827465;Yang2025_Silkie_Wenchang	CRA004775	0.935510459
ENSGALG00010029489	ENSGALG00010029489	2	2.296087553	0.567519538	4.045829967	5.21381E-05	0	0.634215073	0	1	CRA004775;Yang2025_Silkie_Wenchang	PRJNA827465	0.004985845
ENSGALG00010029489	ENSGALG00010029489	2	0.903099217	1.881061461	0.480100856	0.63115568	6.439511028	11.08142203	90.97588741	0.5	CRA004775;PRJNA827465	Yang2025_Silkie_Wenchang	0.903058174
ENSGALG00010029491	ENSGALG00010029491	2	0.106273909	0.174776753	0.608055171	0.543150883	0	0.187045222	0	1	PRJNA827465;Yang2025_Silkie_Wenchang	CRA004775	0.876968972
ENSGALG00010029491	ENSGALG00010029491	2	0.078148265	0.218926403	0.356961354	0.721120738	0	0.422086293	0	0.5	CRA004775;Yang2025_Silkie_Wenchang	PRJNA827465	0.928950734
ENSGALG00010029491	ENSGALG00010029491	2	0.013320725	0.178196218	0.074753131	0.940411133	0	0.105764945	0	0.5	CRA004775;PRJNA827465	Yang2025_Silkie_Wenchang	0.985539065
ENSGALG00010029492	SDK2	2	0.068712576	0.157648993	0.435858006	0.662939755	0.026211706	2.092994188	52.22155868	0.5	PRJNA827465;Yang2025_Silkie_Wenchang	CRA004775	0.912851529
ENSGALG00010029492	SDK2	2	-0.032303754	0.385119272	-0.083879869	0.933151945	0.195037649	2.428817842	58.82770693	0.5	CRA004775;Yang2025_Silkie_Wenchang	PRJNA827465	0.984259239
ENSGALG00010029492	SDK2	2	-0.110098517	0.132893441	-0.828472167	0.407403151	0	0.940509944	0	1	CRA004775;PRJNA827465	Yang2025_Silkie_Wenchang	0.833863243
ENSGALG00010029494	MYH7	2	0.723037692	0.493851546	1.46407903	0.143172356	0.42140194	7.24558876	86.19849907	1	PRJNA827465;Yang2025_Silkie_Wenchang	CRA004775	0.630019027
ENSGALG00010029494	MYH7	2	1.480367906	0.546062245	2.710987472	0.006708316	0.335832876	1.615510052	38.10004472	1	CRA004775;Yang2025_Silkie_Wenchang	PRJNA827465	0.125312237
ENSGALG00010029494	MYH7	2	1.157104677	1.139738072	1.015237365	0.309992693	2.114454769	4.717683757	78.80315741	1	CRA004775;PRJNA827465	Yang2025_Silkie_Wenchang	0.794395438
ENSGALG00010029495	RPL38	2	-0.830911964	0.651833739	-1.27472991	0.202404856	0.809573691	20.43684094	95.10687585	1	PRJNA827465;Yang2025_Silkie_Wenchang	CRA004775	0.715289846
ENSGALG00010029495	RPL38	2	-0.757592569	0.742284467	-1.02062296	0.307433107	1.040663486	17.91569396	94.41830162	1	CRA004775;Yang2025_Silkie_Wenchang	PRJNA827465	0.792694575
ENSGALG00010029495	RPL38	2	-0.183252057	0.075543628	-2.425777821	0.015275619	0	0.613652715	0	1	CRA004775;PRJNA827465	Yang2025_Silkie_Wenchang	0.204363429
ENSGALG00010029496	TTYH2	2	-0.036703683	0.126973355	-0.289066028	0.77253085	0.023921524	3.871560873	74.17062438	0.5	PRJNA827465;Yang2025_Silkie_Wenchang	CRA004775	0.943066211
ENSGALG00010029496	TTYH2	2	-0.148955647	0.09004317	-1.654269238	0.09807282	0	0.388401552	0	0.5	CRA004775;Yang2025_Silkie_Wenchang	PRJNA827465	0.534242003
ENSGALG00010029496	TTYH2	2	0.084812579	0.085245217	0.994924776	0.319772852	0	0.021836971	0	1	CRA004775;PRJNA827465	Yang2025_Silkie_Wenchang	0.798348216
ENSGALG00010029498	ENSGALG00010029498	2	-0.337959216	0.365819781	-0.923840736	0.355569242	0.25056496	15.32039367	93.47275258	0.5	PRJNA827465;Yang2025_Silkie_Wenchang	CRA004775	0.812301302
ENSGALG00010029498	ENSGALG00010029498	2	-0.403361808	0.379682929	-1.062364875	0.288070073	0.215407852	3.593548683	72.17235418	0.5	CRA004775;Yang2025_Silkie_Wenchang	PRJNA827465	0.782840067
ENSGALG00010029498	ENSGALG00010029498	2	0.015699297	0.081427754	0.192800317	0.847115355	0	0.011255513	0	1	CRA004775;PRJNA827465	Yang2025_Silkie_Wenchang	0.961252712
ENSGALG00010029501	ENSGALG00010029501	2	-0.475006957	0.218348574	-2.175452521	0.029596222	0.02459315	1.230874262	18.75693309	1	PRJNA827465;Yang2025_Silkie_Wenchang	CRA004775	0.293446097
ENSGALG00010029501	ENSGALG00010029501	2	0.021280644	0.207294701	0.102658889	0.918233703	0	0.085947582	0	0.5	CRA004775;Yang2025_Silkie_Wenchang	PRJNA827465	0.981033557
ENSGALG00010029501	ENSGALG00010029501	2	-0.285162596	0.326318565	-0.873877943	0.382184728	0.165885508	4.492244081	77.73941082	0.5	CRA004775;PRJNA827465	Yang2025_Silkie_Wenchang	0.825058022
ENSGALG00010029505	VEZF1	2	0.071819481	0.212990838	0.337195165	0.735969782	0.0676153	3.023260817	66.92313167	0.5	PRJNA827465;Yang2025_Silkie_Wenchang	CRA004775	0.933592889
ENSGALG00010029505	VEZF1	2	0.173577262	0.117889991	1.472366398	0.140921962	0	0.732610143	0	1	CRA004775;Yang2025_Silkie_Wenchang	PRJNA827465	0.625271605
ENSGALG00010029505	VEZF1	2	-0.020614047	0.092698263	-0.222377924	0.824019696	0.01110571	2.182632896	54.18377492	0.5	CRA004775;PRJNA827465	Yang2025_Silkie_Wenchang	0.956170356
ENSGALG00010029506	OXLD1	2	0.110878694	0.107500172	1.031428057	0.30234013	0	0.031133391	0	1	PRJNA827465;Yang2025_Silkie_Wenchang	CRA004775	0.791733715
ENSGALG00010029506	OXLD1	2	-0.166061952	0.249856394	-0.664629588	0.50628744	0.047929084	1.618982522	38.23281065	0.5	CRA004775;Yang2025_Silkie_Wenchang	PRJNA827465	0.863929781
ENSGALG00010029506	OXLD1	2	-0.091652058	0.268103648	-0.341853082	0.73246146	0.102539984	3.06633467	67.38777376	0.5	CRA004775;PRJNA827465	Yang2025_Silkie_Wenchang	0.932985777
ENSGALG00010029507	VPS37D	2	0.043555654	0.13549114	0.321464962	0.747858061	0	0.022443528	0	1	PRJNA827465;Yang2025_Silkie_Wenchang	CRA004775	0.936938957
ENSGALG00010029507	VPS37D	2	-0.013880391	0.235870189	-0.058847586	0.953073506	0	0.017302927	0	0.5	CRA004775;Yang2025_Silkie_Wenchang	PRJNA827465	0.987758913
ENSGALG00010029507	VPS37D	2	0.041424274	0.145369852	0.284957806	0.775676468	0	0.064292497	0	0.5	CRA004775;PRJNA827465	Yang2025_Silkie_Wenchang	0.943815265
ENSGALG00010029508	ENSGALG00010029508	2	0.070438722	0.134873454	0.522257868	0.6014908	0	0.067161202	0	1	PRJNA827465;Yang2025_Silkie_Wenchang	CRA004775	0.895230519
ENSGALG00010029508	ENSGALG00010029508	2	-0.199236592	0.386195093	-0.515896228	0.605926889	0.200644538	2.810661503	64.42118701	0.5	CRA004775;Yang2025_Silkie_Wenchang	PRJNA827465	0.896605719
ENSGALG00010029508	ENSGALG00010029508	2	-0.215122701	0.344237215	-0.624925755	0.532019788	0.155133741	2.516389356	60.26052177	0.5	CRA004775;PRJNA827465	Yang2025_Silkie_Wenchang	0.873104119
ENSGALG00010029509	DNAJC30	2	0.035371378	0.15462574	0.228754784	0.819059512	0.020859262	1.582423389	36.80578745	0.5	PRJNA827465;Yang2025_Silkie_Wenchang	CRA004775	0.955256039
ENSGALG00010029509	DNAJC30	2	-0.243276763	0.559793247	-0.43458324	0.663864962	0.518859816	5.609917948	82.17442734	0.5	CRA004775;Yang2025_Silkie_Wenchang	PRJNA827465	0.913119244
ENSGALG00010029509	DNAJC30	2	-0.362467288	0.381273458	-0.950675376	0.341769192	0.219600051	3.435935166	70.89584198	1	CRA004775;PRJNA827465	Yang2025_Silkie_Wenchang	0.805823616
ENSGALG00010029510	PDE6G	2	-0.088707588	0.224133051	-0.395780934	0.692266643	0	0.088762216	0	1	PRJNA827465;Yang2025_Silkie_Wenchang	CRA004775	0.92046943
ENSGALG00010029510	PDE6G	2	-0.069610316	0.309492766	-0.224917425	0.822043513	0	0.125893647	0	1	CRA004775;Yang2025_Silkie_Wenchang	PRJNA827465	0.955636543
ENSGALG00010029510	PDE6G	2	-0.162856097	0.259917402	-0.626568655	0.53094202	0	0.028866301	0	1	CRA004775;PRJNA827465	Yang2025_Silkie_Wenchang	0.872840748
ENSGALG00010029511	CUEDC1	2	-0.015033967	0.05776259	-0.260271685	0.794654212	0.000899372	1.131648409	11.63333135	0.5	PRJNA827465;Yang2025_Silkie_Wenchang	CRA004775	0.948905846
ENSGALG00010029511	CUEDC1	2	-0.065136161	0.084022535	-0.775222518	0.438208184	0	0.467347662	0	0.5	CRA004775;Yang2025_Silkie_Wenchang	PRJNA827465	0.843979791
ENSGALG00010029511	CUEDC1	2	0.024776098	0.058697256	0.422099773	0.672952194	0	0.00076966	0	1	CRA004775;PRJNA827465	Yang2025_Silkie_Wenchang	0.915153756
ENSGALG00010029512	TSPAN10	2	3.551610911	0.511609542	6.942034154	3.86494E-12	0	0.527332627	0	1	PRJNA827465;Yang2025_Silkie_Wenchang	CRA004775	3.45236E-09
ENSGALG00010029512	TSPAN10	2	1.73012943	2.405963259	0.71910052	0.472078987	10.72419616	13.55651494	92.62347289	0.5	CRA004775;Yang2025_Silkie_Wenchang	PRJNA827465	0.853998373
ENSGALG00010029512	TSPAN10	2	1.383843831	1.98845347	0.69593976	0.486466556	7.358138679	14.2689294	92.99176573	0.5	CRA004775;PRJNA827465	Yang2025_Silkie_Wenchang	0.857603818
ENSGALG00010029513	WBSCR22	2	-0.057480622	0.053015361	-1.084225806	0.278264692	0	0.200806248	0	1	PRJNA827465;Yang2025_Silkie_Wenchang	CRA004775	0.776763074
ENSGALG00010029513	WBSCR22	2	-0.24419833	0.158843923	-1.537347638	0.124208202	0	0.494124071	0	1	CRA004775;Yang2025_Silkie_Wenchang	PRJNA827465	0.590771723
ENSGALG00010029513	WBSCR22	2	-0.125361747	0.135777157	-0.92329041	0.355855884	0.020922835	1.670427124	40.1350717	1	CRA004775;PRJNA827465	Yang2025_Silkie_Wenchang	0.812301302
ENSGALG00010029514	MRPS23	2	0.024822054	0.248852562	0.099746027	0.920545959	0.113709858	11.85357644	91.56372758	0.5	PRJNA827465;Yang2025_Silkie_Wenchang	CRA004775	0.98144788
ENSGALG00010029514	MRPS23	2	0.001753826	0.320005994	0.005480602	0.995627134	0.166808637	5.094558726	80.37121459	0.5	CRA004775;Yang2025_Silkie_Wenchang	PRJNA827465	0.998484999
ENSGALG00010029514	MRPS23	2	-0.220123966	0.061816955	-3.560899537	0.000369586	0	0.310122399	0	1	CRA004775;PRJNA827465	Yang2025_Silkie_Wenchang	0.01934739
ENSGALG00010029515	NPLOC4	2	-0.068896696	0.056343483	-1.222797946	0.221406021	0	0.052317188	0	1	PRJNA827465;Yang2025_Silkie_Wenchang	CRA004775	0.734773356
ENSGALG00010029515	NPLOC4	2	-0.060043785	0.109739091	-0.547150378	0.584275424	0	0.10444622	0	1	CRA004775;Yang2025_Silkie_Wenchang	PRJNA827465	0.890842213
ENSGALG00010029515	NPLOC4	2	-0.079143851	0.06227728	-1.270830259	0.203789041	0	0.054849436	0	1	CRA004775;PRJNA827465	Yang2025_Silkie_Wenchang	0.717254253
ENSGALG00010029516	ENSGALG00010029516	2	0.584555415	0.123692815	4.725863943	2.29139E-06	0	0.436250047	0	1	PRJNA827465;Yang2025_Silkie_Wenchang	CRA004775	0.000450293
ENSGALG00010029516	ENSGALG00010029516	2	0.066419948	0.710644142	0.093464427	0.925534609	0.85931775	6.216794621	83.91454019	0.5	CRA004775;Yang2025_Silkie_Wenchang	PRJNA827465	0.98259441
ENSGALG00010029516	ENSGALG00010029516	2	0.000316708	0.620728433	0.000510219	0.999592904	0.638310285	5.149040318	80.57890523	0.5	CRA004775;PRJNA827465	Yang2025_Silkie_Wenchang	0.999889665
ENSGALG00010029517	MSI2	2	0.231147338	0.285310832	0.8101597	0.417848396	0.148957409	11.2402111	91.10336994	0.5	PRJNA827465;Yang2025_Silkie_Wenchang	CRA004775	0.838112595
ENSGALG00010029517	MSI2	2	0.453249903	0.160351129	2.826608734	0.004704377	0.007283275	1.103483654	9.37790547	1	CRA004775;Yang2025_Silkie_Wenchang	PRJNA827465	0.100980954
ENSGALG00010029517	MSI2	2	-0.030339847	0.061072251	-0.496786131	0.619339876	0	0.264600361	0	0.5	CRA004775;PRJNA827465	Yang2025_Silkie_Wenchang	0.899181533
ENSGALG00010029518	FAAP100	2	0.422953059	0.291655068	1.450182445	0.147007649	0.160731575	18.00861042	94.44710071	1	PRJNA827465;Yang2025_Silkie_Wenchang	CRA004775	0.635668678
ENSGALG00010029518	FAAP100	2	0.022148358	0.724946943	0.030551695	0.975627066	0.999943866	19.98042651	94.99510183	0.5	CRA004775;Yang2025_Silkie_Wenchang	PRJNA827465	0.992754467
ENSGALG00010029518	FAAP100	2	-0.247385452	0.430899251	-0.574114371	0.565890409	0.326725659	7.588609363	86.82235503	0.5	CRA004775;PRJNA827465	Yang2025_Silkie_Wenchang	0.883487517
ENSGALG00010029519	STX1A	2	0.089944563	0.091786384	0.979933612	0.32711889	0	0.041664408	0	1	PRJNA827465;Yang2025_Silkie_Wenchang	CRA004775	0.800028239
ENSGALG00010029519	STX1A	2	-0.021170019	0.18281963	-0.115797298	0.907813192	0.027496864	1.610872583	37.9218437	0.5	CRA004775;Yang2025_Silkie_Wenchang	PRJNA827465	0.978196186
ENSGALG00010029519	STX1A	2	-0.017229222	0.15231519	-0.113115586	0.909938919	0.019209405	1.492236241	32.98648211	0.5	CRA004775;PRJNA827465	Yang2025_Silkie_Wenchang	0.978730675
ENSGALG00010029520	MKS1	2	0.295136587	0.172716021	1.708796816	0.087488593	0.046177558	4.292580523	76.70398972	1	PRJNA827465;Yang2025_Silkie_Wenchang	CRA004775	0.508663337
ENSGALG00010029520	MKS1	2	-0.011001272	0.502003841	-0.021914717	0.982515985	0.478369564	19.60110496	94.8982468	0.5	CRA004775;Yang2025_Silkie_Wenchang	PRJNA827465	0.995221651
ENSGALG00010029520	MKS1	2	-0.172666668	0.328060191	-0.526326182	0.598661595	0.195868081	10.78743891	90.72995909	0.5	CRA004775;PRJNA827465	Yang2025_Silkie_Wenchang	0.894762943
ENSGALG00010029521	ACTG1	2	0.021451613	0.050116377	0.42803598	0.668624925	0	0.394935618	0	1	PRJNA827465;Yang2025_Silkie_Wenchang	CRA004775	0.914377769
ENSGALG00010029521	ACTG1	2	-0.015855566	0.14014842	-0.113134106	0.909924236	0.016797097	1.638611485	38.97272116	0.5	CRA004775;Yang2025_Silkie_Wenchang	PRJNA827465	0.978730675
ENSGALG00010029521	ACTG1	2	-0.015263487	0.064305482	-0.237359028	0.812378272	0.001516623	1.071852746	6.70360239	0.5	CRA004775;PRJNA827465	Yang2025_Silkie_Wenchang	0.953371387
ENSGALG00010029522	AKAP1	2	0.126538385	0.063568339	1.990588181	0.046526181	0.004107143	2.025084188	50.61933691	1	PRJNA827465;Yang2025_Silkie_Wenchang	CRA004775	0.372346812
ENSGALG00010029522	AKAP1	2	0.180754926	0.064315769	2.81042936	0.004947545	0	0.478877709	0	1	CRA004775;Yang2025_Silkie_Wenchang	PRJNA827465	0.104831857
ENSGALG00010029522	AKAP1	2	0.064644326	0.057148747	1.131159105	0.257988131	0	0.020786256	0	1	CRA004775;PRJNA827465	Yang2025_Silkie_Wenchang	0.765157824
ENSGALG00010029524	ENSGALG00010029524	2	0.123288115	0.253331181	0.486667748	0.626493805	0.119794993	14.89700559	93.28724156	0.5	PRJNA827465;Yang2025_Silkie_Wenchang	CRA004775	0.901504244
ENSGALG00010029524	ENSGALG00010029524	2	0.376370168	0.101892701	3.693789282	0.000220937	0	0.0326387	0	1	CRA004775;Yang2025_Silkie_Wenchang	PRJNA827465	0.013567942
ENSGALG00010029524	ENSGALG00010029524	2	-0.023315459	0.186053453	-0.125315917	0.900273451	0.03906054	1.660407564	39.77382291	0.5	CRA004775;PRJNA827465	Yang2025_Silkie_Wenchang	0.976475201
ENSGALG00010029525	SLC38A10	2	0.12724066	0.097124351	1.310079898	0.190168808	0.010795764	2.218721099	54.92899038	1	PRJNA827465;Yang2025_Silkie_Wenchang	CRA004775	0.699403589
ENSGALG00010029525	SLC38A10	2	-0.042588763	0.337073282	-0.126348676	0.899455927	0.184234886	4.800824325	79.17024385	0.5	CRA004775;Yang2025_Silkie_Wenchang	PRJNA827465	0.976081354
ENSGALG00010029525	SLC38A10	2	-0.11216374	0.227709502	-0.492573822	0.622313749	0.072566385	2.629330329	61.96750218	0.5	CRA004775;PRJNA827465	Yang2025_Silkie_Wenchang	0.900093208
ENSGALG00010029526	BCL7B	2	-0.051928941	0.090571857	-0.573345218	0.566410975	0.007056386	1.467729671	31.86756255	1	PRJNA827465;Yang2025_Silkie_Wenchang	CRA004775	0.883487517
ENSGALG00010029526	BCL7B	2	-0.270939474	0.135506898	-1.999451521	0.045559522	0	0.453830187	0	1	CRA004775;Yang2025_Silkie_Wenchang	PRJNA827465	0.367927862
ENSGALG00010029526	BCL7B	2	-0.135438894	0.192399481	-0.703946253	0.481466245	0.051473781	2.599738675	61.5345954	1	CRA004775;PRJNA827465	Yang2025_Silkie_Wenchang	0.85686777
ENSGALG00010029527	NDUFAF8	2	-0.085919369	0.090996531	-0.944204886	0.345064959	0	0.448867608	0	0.5	PRJNA827465;Yang2025_Silkie_Wenchang	CRA004775	0.806961063
ENSGALG00010029527	NDUFAF8	2	-0.02021247	0.215368645	-0.093850568	0.925227861	0	0.485981919	0	0.5	CRA004775;Yang2025_Silkie_Wenchang	PRJNA827465	0.982594411
ENSGALG00010029527	NDUFAF8	2	-0.117689849	0.094897787	-1.240174852	0.214910728	0	0.134391387	0	1	CRA004775;PRJNA827465	Yang2025_Silkie_Wenchang	0.728221083

ENSGALG00010029528	TBL2	2	-0.101416553	0.146671223	-0.691455017	0.48927964	0.021988957	1.516965653	34.07892935	1	PRJNA827465;Yang2025_Silkie_Wenchang	CRA004775		0.858337006
ENSGALG00010029528	TBL2	2	-0.376666721	0.162303559	-2.320754533	0.020300096	0	0.001211086	0	1	CRA004775;Yang2025_Silkie_Wenchang	PRJNA827465		0.240028478
ENSGALG00010029528	TBL2	2	-0.150074975	0.166547575	-0.901093728	0.367538487	0.037972157	2.702094924	62.99167764	1	CRA004775;PRJNA827465		Yang2025_Silkie_Wenchang	0.819975483
ENSGALG00010029529	TEPSIN	2	0.332173627	0.23040723	1.441680571	0.149392507	0.085558705	4.871713202	79.47334011	1	PRJNA827465;Yang2025_Silkie_Wenchang	CRA004775		0.639246608
ENSGALG00010029529	TEPSIN	2	0.256390917	0.346528976	0.739883052	0.459370959	0.197587222	5.610946289	82.1776943	0.5	CRA004775;Yang2025_Silkie_Wenchang	PRJNA827465		0.850098539
ENSGALG00010029529	TEPSIN	2	0.091450405	0.089352573	1.023478134	0.306081833	0	0.870946239	0	0.5	CRA004775;PRJNA827465		Yang2025_Silkie_Wenchang	0.792381392
ENSGALG00010029530	ENSGALG00010029530	2	0.050250425	0.131954367	0.38081669	0.703339274	0	0.711015292	0	0.5	PRJNA827465;Yang2025_Silkie_Wenchang	CRA004775		0.923639641
ENSGALG00010029530	ENSGALG00010029530	2	0.153942679	0.201840981	0.762692876	0.44564658	0	0.631652183	0	0.5	CRA004775;Yang2025_Silkie_Wenchang	PRJNA827465		0.846013379
ENSGALG00010029530	ENSGALG00010029530	2	-0.058763283	0.170495666	-0.344661449	0.730348903	0	0.364741621	0	1	CRA004775;PRJNA827465		Yang2025_Silkie_Wenchang	0.932617298
ENSGALG00010029531	CEP131	2	0.462973733	0.468494321	0.98821632	0.323046717	0.427252695	37.06912572	97.30233724	1	PRJNA827465;Yang2025_Silkie_Wenchang	CRA004775		0.798909393
ENSGALG00010029531	CEP131	2	0.432540187	0.526759746	0.821133715	0.411570107	0.51516742	13.6868684	92.69372679	0.5	CRA004775;Yang2025_Silkie_Wenchang	PRJNA827465		0.835128673
ENSGALG00010029531	CEP131	2	-0.005571221	0.064501617	-0.086373351	0.93116963	0	0.206659232	0	0.5	CRA004775;PRJNA827465		Yang2025_Silkie_Wenchang	0.983943003
ENSGALG00010029532	EVI2A	2	0.109447563	0.204971295	0.533965322	0.593365527	0	0.500572091	0	0.5	PRJNA827465;Yang2025_Silkie_Wenchang	CRA004775		0.89284247
ENSGALG00010029532	EVI2A	2	0.338317339	0.265465046	1.274432714	0.202510105	0	0.150988413	0	1	CRA004775;Yang2025_Silkie_Wenchang	PRJNA827465		0.715289846
ENSGALG00010029532	EVI2A	2	0.099442951	0.240090871	0.414188805	0.67873584	0	0.826136505	0	0.5	CRA004775;PRJNA827465		Yang2025_Silkie_Wenchang	0.917701868
ENSGALG00010029533	MLXIPL	2	-0.098116443	0.230496515	-0.425674303	0.670345189	0	0.484725703	0	0.5	PRJNA827465;Yang2025_Silkie_Wenchang	CRA004775		0.914482883
ENSGALG00010029533	MLXIPL	2	0.6078373	0.892694706	0.680901652	0.495933723	1.454842448	11.44130313	91.25973686	0.5	CRA004775;Yang2025_Silkie_Wenchang	PRJNA827465		0.860911322
ENSGALG00010029533	MLXIPL	2	0.760214974	0.731515302	1.039233181	0.298696302	0.940041419	8.166527164	87.75489287	1	CRA004775;PRJNA827465		Yang2025_Silkie_Wenchang	0.78974322
ENSGALG00010029534	NF1	2	0.172588846	0.227009466	0.760271584	0.447092264	0.085308724	5.208944831	80.80225396	0.5	PRJNA827465;Yang2025_Silkie_Wenchang	CRA004775		0.84650962
ENSGALG00010029534	NF1	2	0.371379979	0.166624977	2.228837395	0.025824726	0	0.501811278	0	1	CRA004775;Yang2025_Silkie_Wenchang	PRJNA827465		0.27387728
ENSGALG00010029534	NF1	2	-0.01562217	0.062086575	-0.251619124	0.801335475	0	0.281129439	0	0.5	CRA004775;PRJNA827465		Yang2025_Silkie_Wenchang	0.950158029
ENSGALG00010029536	BAZ1B	2	0.003117713	0.044334854	0.070290227	0.943962661	0	0.757681905	0	0.5	PRJNA827465;Yang2025_Silkie_Wenchang	CRA004775		0.985827325
ENSGALG00010029536	BAZ1B	2	0.068376316	0.111400181	0.613789987	0.539354115	0	0.4268592	0	0.5	CRA004775;Yang2025_Silkie_Wenchang	PRJNA827465		0.874983893
ENSGALG00010029536	BAZ1B	2	-0.014019198	0.046694786	-0.300230489	0.764001351	0	0.071804186	0	1	CRA004775;PRJNA827465		Yang2025_Silkie_Wenchang	0.940447402
ENSGALG00010029537	MYH1B	2	0.083280559	0.506886637	0.164298195	0.869496403	0.48229462	16.13487645	93.80224569	0.5	PRJNA827465;Yang2025_Silkie_Wenchang	CRA004775		0.906857601
ENSGALG00010029537	MYH1B	2	0.614021571	0.202509617	3.032061283	0.002428899	0	0.043063066	0	1	CRA004775;Yang2025_Silkie_Wenchang	PRJNA827465		0.065967872
ENSGALG00010029537	MYH1B	2	-0.060023148	0.538988482	-0.111362581	0.911328832	0.39455484	2.319283271	56.88323144	0.5	CRA004775;PRJNA827465		Yang2025_Silkie_Wenchang	0.978845851
ENSGALG00010029538	FZD9	2	0.170263012	0.075968322	2.241236964	0.02501073	0	0.696364822	0	1	PRJNA827465;Yang2025_Silkie_Wenchang	CRA004775		0.269461817
ENSGALG00010029538	FZD9	2	-0.001131379	0.385669998	-0.002933542	0.997659375	0.199660852	2.37548421	57.98103963	0.5	CRA004775;Yang2025_Silkie_Wenchang	PRJNA827465		0.99990321
ENSGALG00010029538	FZD9	2	-0.032386192	0.291491554	-0.111105077	0.911533023	0.101291501	1.719517566	41.84415328	0.5	CRA004775;PRJNA827465		Yang2025_Silkie_Wenchang	0.978851978
ENSGALG00010029539	FKBP6	2	0.349995665	0.330008521	1.060565539	0.28888739	0	0.032104671	0	1	PRJNA827465;Yang2025_Silkie_Wenchang	CRA004775		0.783530627
ENSGALG00010029539	FKBP6	2	-0.270787964	0.47872176	-0.565647912	0.571633151	0.011897769	1.026528203	2.584264391	0.5	CRA004775;Yang2025_Silkie_Wenchang	PRJNA827465		0.885429878
ENSGALG00010029539	FKBP6	2	-0.042493653	0.535770375	-0.079313182	0.936783522	0.326361261	2.168166593	53.87808284	0.5	CRA004775;PRJNA827465		Yang2025_Silkie_Wenchang	0.985038972
ENSGALG00010029541	ENSGALG00010029541	2	-0.233957663	0.147362292	-1.587635885	0.112368699	0	0.732122196	0	1	PRJNA827465;Yang2025_Silkie_Wenchang	CRA004775		0.56602666
ENSGALG00010029541	ENSGALG00010029541	2	-0.337218809	0.191481876	-1.761100402	0.078221411	0	0.236306015	0	1	CRA004775;Yang2025_Silkie_Wenchang	PRJNA827465		0.481684356
ENSGALG00010029541	ENSGALG00010029541	2	-0.182688217	0.145577653	-1.254919372	0.209508031	0	0.086521109	0	1	CRA004775;PRJNA827465		Yang2025_Silkie_Wenchang	0.721386257
ENSGALG00010029542	AATK	2	0.097959636	0.077530614	1.263496208	0.206410909	0	0.577513212	0	1	PRJNA827465;Yang2025_Silkie_Wenchang	CRA004775		0.718451597
ENSGALG00010029542	AATK	2	0.183202047	0.103917886	1.7629501	0.077908906	0	0.586677802	0	1	CRA004775;Yang2025_Silkie_Wenchang	PRJNA827465		0.480724545
ENSGALG00010029542	AATK	2	0.105088648	0.144965952	0.724919517	0.468501406	0.013345139	1.221454741	18.13040908	1	CRA004775;PRJNA827465		Yang2025_Silkie_Wenchang	0.853198008
ENSGALG00010029543	NSUN5	2	-0.194161645	0.08058806	-2.409310308	0.015982702	0	0.278991207	0	1	PRJNA827465;Yang2025_Silkie_Wenchang	CRA004775		0.208971439
ENSGALG00010029543	NSUN5	2	-0.195320429	0.178554179	-1.09390007	0.273998808	0	0.533899172	0	1	CRA004775;Yang2025_Silkie_Wenchang	PRJNA827465		0.774526342
ENSGALG00010029543	NSUN5	2	-0.220961897	0.080992312	-2.728183605	0.006368415	0	0.205066049	0	1	CRA004775;PRJNA827465		Yang2025_Silkie_Wenchang	0.121286733
ENSGALG00010029544	ENSGALG00010029544	2	0.107712409	0.227280416	0.473918569	0.635557977	0.082675658	4.483186502	77.68776776	0.5	PRJNA827465;Yang2025_Silkie_Wenchang	CRA004775		0.904244739
ENSGALG00010029544	ENSGALG00010029544	2	0.130478939	0.26795891	0.486936371	0.626303423	0.091041447	2.702138796	62.9922785	0.5	CRA004775;Yang2025_Silkie_Wenchang	PRJNA827465		0.901504244
ENSGALG00010029544	ENSGALG00010029544	2	-0.089549548	0.071563912	-1.251322697	0.210816768	0	0.082441792	0	1	CRA004775;PRJNA827465		Yang2025_Silkie_Wenchang	0.722508844
ENSGALG00010029545	OMG	2	0.389946814	0.496298539	0.785710178	0.432037299	0.381170824	4.295313921	76.71881454	0.5	PRJNA827465;Yang2025_Silkie_Wenchang	CRA004775		0.842503929
ENSGALG00010029545	OMG	2	0.196545088	0.730630674	0.269007441	0.787923957	0.916635078	7.066118858	85.84795954	0.5	CRA004775;Yang2025_Silkie_Wenchang	PRJNA827465		0.946919116
ENSGALG00010029545	OMG	2	-0.218508955	0.219358578	-0.996126786	0.319188547	0.002542382	1.024136222	2.356739392	1	CRA004775;PRJNA827465		Yang2025_Silkie_Wenchang	0.798079048
ENSGALG00010029546	WSB1	2	0.22433355	0.153718044	1.459383325	0.144459633	0.038017616	5.089332555	80.35105804	1	PRJNA827465;Yang2025_Silkie_Wenchang	CRA004775		0.631870428
ENSGALG00010029546	WSB1	2	0.171201672	0.2402761	0.712520605	0.476142457	0.09185019	4.615701628	78.33482143	0.5	CRA004775;Yang2025_Silkie_Wenchang	PRJNA827465		0.854810196
ENSGALG00010029546	WSB1	2	0.047320608	0.080206004	0.589988352	0.555189459	0	0.65615136	0	0.5	CRA004775;PRJNA827465		Yang2025_Silkie_Wenchang	0.880025849
ENSGALG00010029547	HIP1	2	0.222547922	0.337064196	0.660253818	0.509090961	0.220716799	34.91599989	97.13598349	0.5	PRJNA827465;Yang2025_Silkie_Wenchang	CRA004775		0.865193604
ENSGALG00010029547	HIP1	2	0.370040463	0.20562895	1.799554307	0.071931042	0.071700441	6.341496731	84.23085208	1	CRA004775;Yang2025_Silkie_Wenchang	PRJNA827465		0.463004541
ENSGALG00010029547	HIP1	2	-0.010158847	0.1280571	-0.079330601	0.936769867	0.020801055	2.552927023	60.82927593	0.5	CRA004775;PRJNA827465		Yang2025_Silkie_Wenchang	0.995038972
ENSGALG00010029548	BAIAP2	2	-0.001412282	0.166214914	-0.008496721	0.993220679	0.02780911	2.012204216	50.30325491	0.5	PRJNA827465;Yang2025_Silkie_Wenchang	CRA004775		0.99794341
ENSGALG00010029548	BAIAP2	2	0.21850571	0.142113194	1.53754696	0.124159425	0	0.292218989	0	1	CRA004775;Yang2025_Silkie_Wenchang	PRJNA827465		0.59073738
ENSGALG00010029548	BAIAP2	2	0.04465494	0.246827389	0.18091566	0.856437377	0.078200154	2.659055914	62.39266746	0.5	CRA004775;PRJNA827465		Yang2025_Silkie_Wenchang	0.96353738
ENSGALG00010029549	KSR1	2	-0.006959131	0.125661179	-0.055380121	0.955835631	0.016518732	2.021846472	50.54026041	0.5	PRJNA827465;Yang2025_Silkie_Wenchang	CRA004775		0.988406776
ENSGALG00010029549	KSR1	2	0.212117265	0.131410764	1.614154414	0.106493937	0	0.936848762	0	1	CRA004775;Yang2025_Silkie_Wenchang	PRJNA827465		0.553422082
ENSGALG00010029549	KSR1	2	0.109344368	0.27452363	0.39830585	0.690404749	0.113891221	3.580144659	72.06816776	0.5	CRA004775;PRJNA827465		Yang2025_Silkie_Wenchang	0.919979266
ENSGALG00010029550	CHMP6	2	-0.033350122	0.068319197	-0.488131554	0.625442496	0.001436056	1.087471729	8.043586517	0.5	PRJNA827465;Yang2025_Silkie_Wenchang	CRA004775		0.901367193
ENSGALG00010029550	CHMP6	2	0.091692788	0.155552575	0.589464929	0.555549432	0	0.26447809	0	0.5	CRA004775;Yang2025_Silkie_Wenchang	PRJNA827465		0.880255849
ENSGALG00010029550	CHMP6	2	-0.063354166	0.063077801	-1.004381348	0.315194837	0	0.005797026	0	1	CRA004775;PRJNA827465		Yang2025_Silkie_Wenchang	0.796662552
ENSGALG00010029552	NOS2	2	0.445566791	0.258646492	1.722686386	0.084945254	0.115573707	7.249359951	86.20567875	1	PRJNA827465;Yang2025_Silkie_Wenchang	CRA004775		0.501162525
ENSGALG00010029552	NOS2	2	0.462418654	0.312870534	1.477987234	0.139411195	0.137728868	3.062551547	67.34748838	1	CRA004775;Yang2025_Silkie_Wenchang	PRJNA827465		0.462300874
ENSGALG00010029552	NOS2	2	0.185820933	0.105752358	1.757132756	0.078895184	0	0.121954786	0	1	CRA004775;PRJNA827465		Yang2025_Silkie_Wenchang	0.482993365
ENSGALG00010029553	MYH1C	2	0.160115092	0.702144757	0.228037154	0.819617356	0.959008864	36.51079248	97.26108383	0.5	PRJNA827465;Yang2025_Silkie_Wenchang	CRA004775		0.955256039
ENSGALG00010029553	MYH1C	2	0.856368318	0.162779098	5.260923115	1.43334E-07	0	0.034184006	0	1	CRA004775;Yang2025_Silkie_Wenchang	PRJNA827465		4.81492E-05
ENSGALG00010029553	MYH1C	2	-0.148726811	0.582251503	-0.255437966	0.79838794	0.466997372	2.452157602	59.21958691	0.5	CRA004775;PRJNA827465		Yang2025_Silkie_Wenchang	0.969922217
ENSGALG00010029554	ENSGALG00010029554	2	-0.58242699	0.623718532	-0.933797795	0.350408207	0	0.90174444	0	0.5	PRJNA827465;Yang2025_Silkie_Wenchang	CRA004775		0.810111037
ENSGALG00010029554	ENSGALG00010029554	2	0.592719887	0.589845242	1.004873558	0.314957738	0	0.387501904	0	1	CRA004775;Yang2025_Silkie_Wenchang	PRJNA827465		0.796572019
ENSGALG00010029554	ENSGALG00010029554	2	-0.075130622	0.079622934	-0.076692409	0.938867448	1.309382889	3.140947107	68.16246928	0.5	CRA004775;PRJNA827465		Yang2025_Silkie_Wenchang	0.985349045
ENSGALG00010029555	LYRM9	2	0.136403126	0.159242905	0.856572704	0.391681076	0	0.29787835	0	1	PRJNA827465;Yang2025_Silkie_Wenchang	CRA004775		0.829251057
ENSGALG00010029555	LYRM9	2	0.118495867	0.196506149	0.603013531	0.546499688	0	0.533884573	0	0.5	CRA004775;Yang2025_Silkie_Wenchang	PRJNA827465		0.878275987
ENSGALG00010029555	LYRM9	2	0.04076603	0.160676818	0.253714448	0.799716171	0	0.090940888	0	0.5	CRA004775;PRJNA827465		Yang2025_Silkie_Wenchang	0.949922217
ENSGALG00010029556	NPTX1	2	0.2187274	0.238538818	0.938517016	0.347978276	0	0.134783284	0	1	PRJNA827465;Yang2025_Silkie_Wenchang	CRA004775		0.808847403
ENSGALG00010029556	NPTX1	2	0.293883638	0.293384204	1.001702321	0.316487385	0	0.002654415	0	1	CRA004775;Yang2025_Silkie_Wenchang	PRJNA827465		0.796883862
ENSGALG00010029556	NPTX1	2	0.146259165	0.330512152	0.442522806	0.658110931	0	0.023349221	0	1	CRA004775;PRJNA827465		Yang2025_Silkie_Wenchang	0.910938006
ENSGALG00010029558	ENSGALG00010029558	2	0.431930915	0.735215866	0.587488566	0.556875627	0.923102097	6.701451594	85.07785983	0.5	PRJNA827465;Yang2025_Silkie_Wenchang	CRA004775		0.880511839
ENSGALG00010029558	ENSGALG00010029558	2	1.420803692	0.36755919	3.865509915	0.000110857	0	0.470112752	0	1	CRA004775;Yang2025_Silkie_Wenchang	PRJNA827465		0.003785897
ENSGALG00010029558	ENSGALG00010029558	2	0.673308437	0.991426446	0.679131004	0.49705486	1.757938934	9.175927581	89.10191922	0.5	CRA004775;PRJNA827465		Yang2025_Silkie_Wenchang	0.86098489
ENSGALG00010029560	ENSGALG00010029560	2	-0.122597041	0.156007324	-0.785841574	0.431960307	0	0.003471217	0	1	PRJNA827465;Yang2025_Silkie_Wenchang	CRA004775		0.842503929
ENSGALG00010029560	ENSGALG00010029560	2	-0.29068859	0.146312491	-1.986746977	0.046951045	0.000529943	1.012057852	1.191419288	1	CRA004775;Yang2025_Silkie_Wenchang	PRJNA827465		0.157003609
ENSGALG00010029560	ENSGALG00010029560	2	-0.288367311	0.14092336	-2.04627048	0.040729766	0	0.970622046	0	1	CRA004775;PRJNA827465		Yang2025_Silkie_Wenchang	0.349194657
ENSGALG00010029561	SCO1	2	0.178521442	0.044636549	3.999445466	6.34911E-05	0	0.006692089	0	1	PRJNA827465;Yang2025_Silkie_Wenchang	CRA004775		0.005697237
ENSGALG00010029561	SCO1	2	-0.027324748	0.202282513	-0.135082108	0.892546956	0.069611648	6.678904313	85.02748425	0.5	CRA004775;Yang2025_Silkie_Wenchang	PRJNA827465		0.974359592
ENSGALG00010029561	SCO1	2	-0.012889774	0.206555844	-0.062409381	0.950236825	0.07751747	10.47131915	90.4557374	0.5	CRA004775;PRJNA827465		Yang2025_Silkie_Wenchang	0.997027827
ENSGALG00010029563	ADPRM	2	-0.065524203	0.09442163	-0.693953307	0.487711492	0	0.000598009	0	1	PRJNA827465;Yang2025_Silkie_Wenchang	CRA004775		0.85768885
ENSGALG00010029563	ADPRM	2	-0.100625571	0.13945724	-0.721551429	0.47057031	0	0.128178001	0	1	CRA004775;Yang2025_Silkie_Wenchang	PRJNA827465		0.853744788
ENSGALG00010029563	ADPRM	2	-0.082802776	0.105174916	-0.787286348	0.43111426	0	0.164333697	0	1	CRA004775;PRJNA827465		Yang2025_Silkie_Wenchang	0.842503929
ENSGALG00010029564	NLK	2	0.048592681	0.042856814	1.13383792	0.256862542	0	0.651808064	0	1	PRJNA827465;Yang2025_Silkie_Wenchang	CRA004775		0.764750282
ENSGALG00010029564	NLK	2	0.1120025	0.113486119	0.986926868	0.323678492	0	0.324035241	0	0.5	CRA004775;Yang2025_Silkie_Wenchang	PRJNA827465		0.799600434
ENSGALG00010029564	NLK	2	0.034823932	0.04465813	0.779789303	0.435514904	0	0.033842535	0	0.5	CRA004775;PRJNA827465		Yang2025_Silkie_Wenchang	0.842762754
ENSGALG00010029566	TMEM97	2	-0.008002438	0.059548725	-0.134384713	0.893098369	0	0.464221915	0	0.5	PRJNA827465;Yang2025_Silkie_Wenchang	CRA004775		0.974381724
ENSGALG00010029566	TMEM97	2	-0.104084139	0.110316386	-0.9435057	0.345422301	0	0.24753875	0	1	CRA004775;Yang2025_Silkie_Wenchang	PRJNA827465		0.80728624
ENSGALG00010029566	TMEM97	2	0.000451966	0.066357983	0.006811028	0.994565628	0	0.751314251	0	0.5	CRA004775;PRJNA827465		Yang2025_Silkie_Wenchang	0.998242335
ENSGALG00010029567	IFT20	2	-0.07384343	0.058689701	-1.258200815	0.20831914	0	0.424947479	0	0.5	PRJNA827465;Yang2025_Silkie_Wenchang	CRA004775		0.720917211
ENSGALG00010029567	IFT20	2	0.007187703	0.133658132	0.053776769	0.957113018	0	0.008170205	0	0.5	CRA004775;Yang2025_Silkie_Wenchang	PRJNA827465		0.988541306
ENSGALG00010029567	IFT20	2	-0.088694317	0.063209914	-1.40317098	0.160565859	0	0.036109071	0	1	CRA004775;PRJNA827465		Yang2025_Silkie_Wenchang	0.65630165
ENSGALG00010029569	TMEM220	2	0.123566314	0.38593012	0.320177947	0.748833439	0.268922672	10.25972675	90.25315173	0.5	PRJNA827465;Yang2025_Silkie_Wenchang	CRA004775		0.937249951
ENSGALG00010029569	TMEM220	2	0.208991112	0.370911489	0.563452785	0.573126602	0.197822876	3.26556661	69.37744289	0.5	CRA004775;Yang2025_Silkie_Wenchang	PRJNA827465		0.885753405
ENSGALG00010029569	TMEM220	2	-0.253602765	0.143536101	-1.766822169	0.077258015	0	0.001711532	0	1	CRA004775;PRJNA827465		Yang2025_Silkie_Wenchang	0.478793445
ENSGALG00010029570	TNFAIP1	2	-0.190997232	0.195919161	-0.974877757	0.329620901	0.069457956	10.27994065	90.27231738	1	PRJNA827465;Yang2025_Silkie_Wenchang	CRA004775		0.801177491
ENSGALG00010029570	TNFAIP1	2	-0.108141857	0.315415043	-0.342855737	0.731706993	0.171473924	6.890917452	85.48814426	0.5	CRA004775;Yang2025_Silkie_Wenchang	PRJNA827465		0.932851968
ENSGALG00010029570	TNFAIP1	2	0.026672096	0.080543748	0.33115042	0.740530869	0.00361435	1.142025708	12.43629689	0.5	CRA004775;PRJNA827465		Yang2025_Silkie_Wenchang	0.935128797
ENSGALG00010029571	EPX	2	-0.567479866	0.812646133	-0.698311162	0.48498262	1.102915955	6.053155537	83.47969098	0.5	PRJNA827465;Yang2025_Silkie_Wenchang	CRA004775		0.857004733
ENSGALG00010029571	EPX	2	-1.131253768	0.427549635	-2.645900441	0.008147379	0.010957376	1.024458103	2.387418571	1	CRA004775;Yang2025_Silkie_Wenchang	PRJNA827465		0.140261254
ENSGALG00010029571	EPX	2	0.077863007	0.388043042	0.200655594	0.840967884	0	0.523770763	0	0.5	CRA004775;PRJNA827465		Yang2025_Silkie_Wenchang	0.959805361
ENSGALG00010029572	TSPOAP1	2	0.319270359	0.285795857	1.117127317	0.263939892	0.147511358	10.19790513	90.19406449	1	PRJNA827465;Yang2025_Silkie_Wenchang	CRA004775		0.768643821
ENSGALG00010029572	TSPOAP1	2	0.080361354	0.547403751	0.146804537	0.883286303	0.563859374	16.76049697	94.03358981	0.5	CRA004775;Yang2025_Silkie_Wenchang	PRJNA827465		0.972001076
ENSGALG00010029572	TSPOAP1	2	-0.18080259	0.258859126	-0.698459401	0.484889939	0.106665229	4.532220152	77.93575849	0.5	CRA004775;PRJNA827465		Yang2025_Silkie_Wenchang	0.857004733
ENSGALG00010029573	MAP2K4	2	-0.110099309	0.117470492	-0.937250766	0.348629582	0.023406507	6.365109887	84.28935214	1	PRJNA827465;Yang2025_Silkie_Wenchang	CRA004775		0.809295875
ENSGALG00010029573	MAP2K4	2	-0.219622671	0.077000128	-2.852237743	0.004341262	0	0.504433275	0	1	CRA004775;Yang2025_Silkie_Wenchang	PRJNA827465		0.096545833
ENSGALG00010029573	MAP2K4	2	-0.003087607	0.045972093	-0.067162635	0.946452231	0	0.034565394	0	1	CRA004775;PRJNA827465		Yang2025_Silkie_Wenchang	0.986445196
ENSGALG00010029574	SGSM2	2	0.067139928	0.125483242	0.535050952	0.592614625	0.017321415	2.077893976	51.87434915	0.5	PRJNA827465;Yang2025_Silkie_Wenchang	CRA004775		0.892776493
ENSGALG00010029574	SGSM2	2	-0.076783076	0.132531685	-0.579356369	0.562348738	0	0.040086718	0	1	CRA004775;Yang2025_Silkie_Wenchang	PRJNA827465		0.881955027
ENSGALG00010029574	SGSM2	2	0.14359453	0.084917223	1.690994179	0.090837914	0	0.537540638	0	0.5	CRA004775;PRJNA827465		Yang2025_Silkie_Wenchang	0.517120879
ENSGALG00010029576	ATP2A3	2	-0.323610269	0.295947116	-1.093473298	0.274186047	0.163929174	15.44451499	93.52520943	1	PRJNA827465;Yang2025_Silkie_Wenchang	CRA004775		0.774526342
ENSGALG00010029576	ATP2A3	2	-0.609296338	0.120467238	-5.057776277	4.24174E-07	0	0.261594487	0	1	CRA004775;Yang2025_Silkie_Wenchang	PRJNA827465		0.000111888
ENSGALG00010029576	ATP2A3	2	-0.047720019	0.082077729	-0.58140033	0.560970675	0	0.658874459	0	1	CRA004775;PRJNA827465		Yang2025_Silkie_Wenchang	0.881417858
ENSGALG00010029577	NOG	2	0.44732875	0.458245999	0.976176008	0.328977251	0	0.018681095	0	1	PRJNA827465;Yang2025_Silkie_Wenchang	CRA004775		0.800761292
ENSGALG00010029577	NOG	2	0.160245224	0.61798361	0.259303356	0.79540119	0	0.591425936	0	0.5	CRA004775;Yang2025_Silkie_Wenchang	PRJNA827465		0.948935068
ENSGALG00010029577	NOG	2	0.198126849	0.490891986	0.403605793	0.686502625	0	0.543514543	0	0.5	CRA004775;PRJNA827465		Yang2025_Silkie_Wenchang	0.919235539
ENSGALG00010029579	GALNT17	2	0.059950015	0.089865471	0.667108447	0.504702865	0	0.303349241	0	0.5	PRJNA827465;Yang2025_Silkie_Wenchang	CRA004775		0.863512504
ENSGALG00010029579	GALNT17	2	-0.040399824	0.168101654	-0.240329723	0.810074654	0	0.021229072	0	1	CRA004775;Yang2025_Silkie_Wenchang	PRJNA827465		0.952908565
ENSGALG00010029579	GALNT17	2	0.076347093	0.099603313	0.76651158	0.443371964	0	0.190247457	0	0.5	CRA004775;PRJNA827465		Yang2025_Silkie_Wenchang	0.845733906
ENSGALG00010029580	P2RX1	2	0.282697387	0.176889085	1.59816185	0.110006962	0	0.139444062	0	1	PRJNA827465;Yang2025_Silkie_Wenchang	CRA004775		0.561870599
ENSGALG00010029580	P2RX1	2	0.516134555	0.356571793	1.447491263	0.147759382	0.148548496	2.348804676	57.42515287	1	CRA004775;Yang2025_Silkie_Wenchang	PRJNA827465		0.637081344
ENSGALG00010029580	P2RX1	2	0.552507162	0.281974229	1.959424321	0.050063115	0.07266526	1.738327183	42.47343021	1	CRA004775;PRJNA827465		Yang2025_Silkie_Wenchang	0.387273603
ENSGALG00010029581	DGKE	2	-0.027292611	0.04891561	-0.557952985	0.576876489	0	0.948277307	0	1	PRJNA827465;Yang2025_Silkie_Wenchang	CRA004775		0.887113945
ENSGALG00010029581	DGKE	2	-0.127006044	0.130065697	-0.976476093	0.328828589	0	0.267264033	0	0.5	CRA004775;Yang2025_Silkie_Wenchang	PRJNA827465		0.800761292
ENSGALG00010029581	DGKE	2	-0.009772899	0.051117775	-0.19118396	0.84838147	0	0.003324178	0	0.5	CRA004775;PRJNA827465		Yang2025_Silkie_Wenchang	0.961364896
ENSGALG00010029582	SCPEP1	2	-0.330707602	0.19113369	-1.73024234	0.083586987	0.063320463	7.485770276	86.64132129	1	PRJNA827465;Yang2025_Silkie_Wenchang	CRA004775		0.498062361
ENSGALG00010029582	SCPEP1	2	-0.311652745	0.268867031	-1.15913336	0.24640183	0.106844253	3.342160989	70.07923905	0.5	CRA004775;Yang2025_Silkie_Wenchang	PRJNA827465		0.756843363
ENSGALG00010029582	SCPEP1	2	-0.12625734	0.088025605	-1.434325171	0.151479491	0	0.324770115	0	0.5	CRA004775;PRJNA827465		Yang2025_Silkie_Wenchang	0.643006634
ENSGALG00010029583	LIMK1	2	0.189340577	0.248922668	0.760640154	0.446872029	0.109955136	8.678163567	88.47682471	0.5	PRJNA827465;Yang2025_Silkie_Wenchang	CRA004775		0.84650962
ENSGALG00010029583	LIMK1	2	0.401979521	0.134285223	2.993475475	0.002758197	0	0.861011692	0	1	CRA004775;Yang2025_Silkie_Wenchang	PRJNA827465		0.071732358
ENSGALG00010029583	LIMK1	2	-0.039025016	0.085371969	-0.457117442	0.647586634	0	0.136468865	0	0.5	CRA004775;PRJNA827465		Yang2025_Silkie_Wenchang	0.907350807
ENSGALG00010029584	TRIM25	2	-0.099952876	0.272166984	-0.367248352	0.713433776	0.141440192	22.0574275	95.46637975	0.5	PRJNA827465;Yang2025_Silkie_Wenchang	CRA004775		0.926253108
ENSGALG00010029584	TRIM25	2	-0.384391346	0.08527662	-4.507581887	6.55706E-06	0	0.219800392	0	1	CRA004775;Yang2025_Silkie_Wenchang	PRJNA827465		0.001014615
ENSGALG00010029584	TRIM25	2	-0.106622983	0.345964791	-0.308190273	0.757937553	0.190706758	4.140092433	75.84594991	0.5	CRA004775;PRJNA827465		Yang2025_Silkie_Wenchang	0.938954369
ENSGALG00010029585	ENSGALG00010029585	2	0.4404251	0.265049598	1.661670508	0.096578858	0.061218927	1.750452608	42.87191808	1	PRJNA827465;Yang2025_Silkie_Wenchang	CRA004775		0.531035092
ENSGALG00010029585	ENSGALG00010029585	2	0.477385584	0.25389732	1.88023089	0.060076617	0.034543044	1.365207164	26.75104363	1	CRA004775;Yang2025_Silkie_Wenchang	PRJNA827465		0.424216905
ENSGALG00010029585	ENSGALG00010029585	2	0.217956674	0.192717393	1.130965249	0.258069718	0	0.004492969	0	1	CRA004775;PRJNA827465		Yang2025_Silkie_Wenchang	0.765214193
ENSGALG00010029586	COIL	2	0.118989911	0.121655282	0.978090787	0.328029424	0.022882502	4.263195808	76.54341848	1	PRJNA827465;Yang2025_Silkie_Wenchang	CRA004775		0.800530298
ENSGALG00010029586	COIL	2	0.023243686	0.250746384	0.092697991	0.926143493	0.105860182	6.086078924	83.56905961	0.5	CRA004775;Yang2025_Silkie_Wenchang	PRJNA827465		0.98280842
ENSGALG00010029586	COIL	2	-0.067213022	0.11791135	-0.570030131	0.568657262	0.015648134	1.876885721	46.72025105	0.5	CRA004775;PRJNA827465		Yang2025_Silkie_Wenchang	0.884235495
ENSGALG00010029588	RAB11FIP4	2	0.248848642	0.164468481	1.513047611	0.130267566	0.013301026	1.312140254	23.78863488	1	PRJNA827465;Yang2025_Silkie_Wenchang	CRA004775		0.605608015
ENSGALG00010029588	RAB11FIP4	2	0.140648997	0.187995481	0.748150945	0.454369116	0	0.457996147	0	1	CRA004775;Yang2025_Silkie_Wenchang	PRJNA827465		0.849072665
ENSGALG00010029588	RAB11FIP4	2	0.371133527	0.158030569	2.348491999	0.018849604	0	0.028957879	0	1	CRA004775;PRJNA827465		Yang2025_Silkie_Wenchang	0.22993358
ENSGALG00010029590	UTP6	2	0.074985585	0.084999626	0.882187234	0.377675564	0.008286476	2.289457199	56.32152458	1	PRJNA827465;Yang2025_Silkie_Wenchang	CRA004775		0.82325159
ENSGALG00010029590	UTP6	2	0.062535032	0.166915829	0.374650102	0.707920707	0.031498716	1.909388297	47.62720598	0.5	CRA004775;Yang2025_Silkie_Wenchang	PRJNA827465		0.925061251
ENSGALG00010029590	UTP6	2	-0.012432431	0.064460308	-0.192869554	0.84706113	0	0.565080582	0	0.5	CRA004775;PRJNA827465		Yang2025_Silkie_Wenchang	0.961252712
ENSGALG00010029591	SUZ12	2	-0.103628776	0.101283438	-1.023156185	0.306234005	0.016504006	4.951605937	79.80453185	1	PRJNA827465;Yang2025_Silkie_Wenchang	CRA004775		0.792485409
ENSGALG00010029591	SUZ12	2	-0.165415955	0.132944726	-1.244246087	0.213409007	0.014053144	1.200759102	16.71934878	0.5	CRA004775;Yang2025_Silkie_Wenchang	PRJNA827465		0.726074635
ENSGALG00010029591	SUZ12	2	-0.007453004	0.04791265	-0.15555399	0.876384594	0	0.313218543	0	0.5	CRA004775;PRJNA827465		Yang2025_Silkie_Wenchang	0.970347777
ENSGALG00010029592	MNT	2	0.053167721	0.171564939	0.309898521	0.756638128	0.043080692	3.22666109	69.0082109	0.5	PRJNA827465;Yang2025_Silkie_Wenchang	CRA004775		0.938733809
ENSGALG00010029592	MNT	2	0.188919516	0.158674203	1.190612665	0.233805677	0	0.760989454	0	0.5	CRA004775;Yang2025_Silkie_Wenchang	PRJNA827465		0.747094677
ENSGALG00010029592	MNT	2	-0.079548768	0.065137752	-1.221239081	0.221995511	0	0.015849993	0	1	CRA004775;PRJNA827465		Yang2025_Silkie_Wenchang	0.735359501
ENSGALG00010029593	PAFAH1B1	2	-0.116657857	0.09561032	-1.220138761	0.222412277	0.015430588	5.857259087	82.92716806	1	PRJNA827465;Yang2025_Silkie_Wenchang	CRA004775		0.735999809
ENSGALG00010029593	PAFAH1B1	2	-0.081658881	0.181534119	-0.449826631	0.652835454	0.048112452	3.253128499	69.26035967	0.5	CRA004775;Yang2025_Silkie_Wenchang	PRJNA827465		0.909572411
ENSGALG00010029593	PAFAH1B1	2	-0.029524674	0.02655209	-1.111952929	0.26615839	0	0.855677878	0	0.5	CRA004775;PRJNA827465		Yang2025_Silkie_Wenchang	0.770140851
ENSGALG00010029594	CRLF3	2	-0.178186038	0.052355765	-3.403369939	0.000665601	0.001059921	1.227489987	18.53294038	1	PRJNA827465;Yang2025_Silkie_Wenchang	CRA004775		0.028470581
ENSGALG00010029594	CRLF3	2	-0.270639094	0.105890565	-2.555837672	0.010593252	0.006179062	1.072637102	6.771824524	1	CRA004775;Yang2025_Silkie_Wenchang	PRJNA827465		0.164107985
ENSGALG00010029594	CRLF3	2	-0.252127487	0.220602366	-1.142904725	0.25307815	0.058750183	1.700209916	41.18373321	1	CRA004775;PRJNA827465		Yang2025_Silkie_Wenchang	0.762731469
ENSGALG00010029595	CLUH	2	-0.088603861	0.055864918	-1.586037626	0.112730775	0	0.375280507	0	1	PRJNA827465;Yang2025_Silkie_Wenchang	CRA004775		0.566553488
ENSGALG00010029595	CLUH	2	-0.061528074	0.07304253	-0.84235956	0.399586722	0	0.049556226	0	1	CRA004775;Yang2025_Silkie_Wenchang	PRJNA827465		0.832246851
ENSGALG00010029595	CLUH	2	-0.122735567	0.076198464	-1.610735438	0.107237398	0	0.004128631	0	1	CRA004775;PRJNA827465		Yang2025_Silkie_Wenchang	0.554473355
ENSGALG00010029596	ATAD5	2	-0.030635188	0.075213936	-0.407307328	0.683782281	0	0.002834738	0	1	PRJNA827465;Yang2025_Silkie_Wenchang	CRA004775		0.918800744
ENSGALG00010029596	ATAD5	2	0.05601835	0.201468587	0.278050046	0.780973942	0.034483546	1.318261164	24.14249718	0.5	CRA004775;Yang2025_Silkie_Wenchang	PRJNA827465		0.945235709
ENSGALG00010029596	ATAD5	2	0.05241054	0.188797	0.277602611	0.781317428	0.032746466	1.309711169	23.64728774	0.5	CRA004775;PRJNA827465		Yang2025_Silkie_Wenchang	0.945235709
ENSGALG00010029597	ADAP2	2	-0.010550273	0.123610801	-0.085350734	0.931982559	0	0.047832059	0	0.5	PRJNA827465;Yang2025_Silkie_Wenchang	CRA004775		0.98412915
ENSGALG00010029597	ADAP2	2	-0.164656183	0.282715501	-0.582409464	0.560290908	0.075540351	1.673764802	40.25444919	0.5	CRA004775;Yang2025_Silkie_Wenchang	PRJNA827465		0.881203589
ENSGALG00010029597	ADAP2	2	-0.167118143	0.238848251	-0.699683346	0.484125079	0.050578044	1.483738544	32.60268097	1	CRA004775;PRJNA827465		Yang2025_Silkie_Wenchang	0.857004733
ENSGALG00010029598	SUPT4H1	2	0.03060854	0.107439638	0.28489057	0.775727981	0	0.082058655	0	0.5	PRJNA827465;Yang2025_Silkie_Wenchang	CRA004775		0.943815265
ENSGALG00010029598	SUPT4H1	2	-0.319865247	0.158017665	-2.024237276	0.042945744	0	0.813976963	0	1	CRA004775;Yang2025_Silkie_Wenchang	PRJNA827465		0.358364452
ENSGALG00010029598	SUPT4H1	2	-0.156027806	0.218325366	-0.714657251	0.474820859	0.073656318	4.250178984	76.47157911	0.5	CRA004775;PRJNA827465		Yang2025_Silkie_Wenchang	0.854462864
ENSGALG00010029599	RNF43	2	0.533527883	0.27180163	1.962931135	0.049654176	0	0.054441791	0	1	PRJNA827465;Yang2025_Silkie_Wenchang	CRA004775		0.386010596
ENSGALG00010029599	RNF43	2	0.565762003	0.339664502	1.665649485	0.095783249	0	0.030351052	0	1	CRA004775;Yang2025_Silkie_Wenchang	PRJNA827465		0.530071674
ENSGALG00010029599	RNF43	2	0.464752393	0.344676637	1.348372192	0.177538703	0	0.000548803	0	1	CRA004775;PRJNA827465		Yang2025_Silkie_Wenchang	0.683696223
ENSGALG00010029600	RHOT1	2	-0.066121049	0.04732185	-1.39726256	0.16233463	0	0.698769936	0	1	PRJNA827465;Yang2025_Silkie_Wenchang	CRA004775		0.659930202
ENSGALG00010029600	RHOT1	2	-0.008750269	0.141495314	-0.061841403	0.950689131	0.025591982	2.420488854	58.68603161	0.5	CRA004775;Yang2025_Silkie_Wenchang	PRJNA827465		0.98727979
ENSGALG00010029600	RHOT1	2	0.011537135	0.087460275	0.131912861	0.895053216	0.005927163	1.346176235	25.7155212	0.5	CRA004775;PRJNA827465		Yang2025_Silkie_Wenchang	0.974641371
ENSGALG00010029602	MTMR4	2	0.04572324	0.051292744	0.891417325	0.372705327	0	0.633119504	0	0.5	PRJNA827465;Yang2025_Silkie_Wenchang	CRA004775		0.822068435
ENSGALG00010029602	MTMR4	2	-0.239245904	0.259159781	-0.923159846	0.355923911	0.117335109	7.509419019	86.68339059	1	CRA004775;Yang2025_Silkie_Wenchang	PRJNA827465		0.81231505
ENSGALG00010029602	MTMR4	2	-0.199717084	0.300713426	-0.664144219	0.506598011	0.164526274	10.55992629	90.53023693	0.5	CRA004775;PRJNA827465		Yang2025_Silkie_Wenchang	0.863929781
ENSGALG00010029603	RHBDL3	2	0.223278006	0.265601642	0.840649947	0.400544069	0	0.884258287	0	0.5	PRJNA827465;Yang2025_Silkie_Wenchang	CRA004775		0.832280388

ENSGALG00010029603	RHBDL3	2	-0.027930894	0.398092648	-0.070161794	0.944064883	0	0.236136921	0	0.5	CRA004775;Yang2025_Silkie_Wenchang	PRJNA827465	0.985827325
ENSGALG00010029603	RHBDL3	2	0.351843064	0.283166433	1.242530974	0.21404072	0	0.043853232	0	1	CRA004775;PRJNA827465	Yang2025_Silkie_Wenchang	0.726832726
ENSGALG00010029604	ENSGALG00010029604	2	-0.120445957	0.066402411	-1.81387927	0.069696315	0	0.011199729	0	1	PRJNA827465;Yang2025_Silkie_Wenchang	CRA004775	0.455407192
ENSGALG00010029604	ENSGALG00010029604	2	-0.127374081	0.103500762	-1.230658379	0.218450661	0	0.003607799	0	1	CRA004775;Yang2025_Silkie_Wenchang	PRJNA827465	0.732175117
ENSGALG00010029604	ENSGALG00010029604	2	-0.115345891	0.077109458	-1.495872153	0.134686974	0	1.83786E-05	0	1	CRA004775;PRJNA827465	Yang2025_Silkie_Wenchang	0.61510599
ENSGALG00010029605	C17orf75	2	-0.098754566	0.227095523	-0.434859151	0.663664667	0.095629233	13.61423875	92.65474906	0.5	PRJNA827465;Yang2025_Silkie_Wenchang	CRA004775	0.913119244
ENSGALG00010029605	C17orf75	2	-0.335521151	0.094629161	-3.545644372	0.000391654	0	0.012235498	0	1	CRA004775;Yang2025_Silkie_Wenchang	PRJNA827465	0.019924928
ENSGALG00010029605	C17orf75	2	-0.049309224	0.232575429	-0.212013903	0.832096188	0.078694342	2.985224348	66.50168016	0.5	CRA004775;PRJNA827465	Yang2025_Silkie_Wenchang	0.957762335
ENSGALG00010029606	ABHD11	2	0.213058506	0.125158508	1.702309417	0.08869738	0	0.567426527	0	1	PRJNA827465;Yang2025_Silkie_Wenchang	CRA004775	0.51163023
ENSGALG00010029606	ABHD11	2	0.010864707	0.156683995	0.069341524	0.944717774	0	0.602446192	0	0.5	CRA004775;Yang2025_Silkie_Wenchang	PRJNA827465	0.986014725
ENSGALG00010029606	ABHD11	2	0.11723797	0.217745916	0.53841659	0.590289613	0.05131745	2.06609028	51.59940444	0.5	CRA004775;PRJNA827465	Yang2025_Silkie_Wenchang	0.892474457
ENSGALG00010029607	ZNF207	2	0.058358875	0.072753441	0.802145904	0.422468564	0.003890175	1.265862726	21.00249266	1	PRJNA827465;Yang2025_Silkie_Wenchang	CRA004775	0.839704389
ENSGALG00010029607	ZNF207	2	0.10915907	0.119588328	0.912790336	0.361352822	0	0.943915702	0	0.5	CRA004775;Yang2025_Silkie_Wenchang	PRJNA827465	0.815424583
ENSGALG00010029607	ZNF207	2	0.022063609	0.048152262	0.458205034	0.646805143	0	0.049256311	0	0.5	CRA004775;PRJNA827465	Yang2025_Silkie_Wenchang	0.907350807
ENSGALG00010029608	ENSGALG00010029608	2	0.192684447	0.23866362	0.807347374	0.419466377	0.085691939	3.962202757	74.7615137	0.5	PRJNA827465;Yang2025_Silkie_Wenchang	CRA004775	0.838527387
ENSGALG00010029608	ENSGALG00010029608	2	0.098260938	0.397161284	0.247408148	0.804592369	0.244125978	4.218376815	76.29419931	0.5	CRA004775;Yang2025_Silkie_Wenchang	PRJNA827465	0.951665484
ENSGALG00010029608	ENSGALG00010029608	2	-0.075951654	0.130350515	-0.582672449	0.560113824	0	0.771016417	0	1	CRA004775;PRJNA827465	Yang2025_Silkie_Wenchang	0.881203589
ENSGALG00010029609	METTL27	2	0.064996581	0.094764767	0.685872849	0.492793293	0	0.859447149	0	0.5	PRJNA827465;Yang2025_Silkie_Wenchang	CRA004775	0.859474364
ENSGALG00010029609	METTL27	2	0.121963399	0.13336883	0.914482038	0.360463612	0	0.787268972	0	0.5	CRA004775;Yang2025_Silkie_Wenchang	PRJNA827465	0.814880744
ENSGALG00010029609	METTL27	2	-0.033733122	0.122863342	-0.274558073	0.783655782	0	0.246608697	0	1	CRA004775;PRJNA827465	Yang2025_Silkie_Wenchang	0.945815798
ENSGALG00010029610	ERN1	2	0.20966493	0.217405785	0.964394438	0.33484821	0.089408959	18.34395227	94.54861207	0.5	PRJNA827465;Yang2025_Silkie_Wenchang	CRA004775	0.803101658
ENSGALG00010029610	ERN1	2	0.177704427	0.306843149	0.579137671	0.562496283	0.151478577	4.46000644	77.57850771	0.5	CRA004775;Yang2025_Silkie_Wenchang	PRJNA827465	0.881953027
ENSGALG00010029610	ERN1	2	-0.011326831	0.056926169	-0.198974067	0.842283031	0	0.42986367	0	1	CRA004775;PRJNA827465	Yang2025_Silkie_Wenchang	0.96015575
ENSGALG00010029611	YWHAG	2	-0.006816366	0.089173869	-0.076439052	0.939069802	0.00761967	1.723530023	41.97954275	0.5	PRJNA827465;Yang2025_Silkie_Wenchang	CRA004775	0.985349045
ENSGALG00010029611	YWHAG	2	0.096792649	0.11961409	0.809207754	0.418395656	0	0.24282085	0	0.5	CRA004775;Yang2025_Silkie_Wenchang	PRJNA827465	0.838348675
ENSGALG00010029611	YWHAG	2	-0.067393038	0.062989306	-1.069912367	0.284658756	0	0.007722088	0	1	CRA004775;PRJNA827465	Yang2025_Silkie_Wenchang	0.780265906
ENSGALG00010029612	HSPB1	2	0.027893697	0.202014191	0.138077909	0.890178845	0.062605056	4.277710475	76.62300883	0.5	PRJNA827465;Yang2025_Silkie_Wenchang	CRA004775	0.973393961
ENSGALG00010029612	HSPB1	2	0.215594047	0.143132018	1.506260092	0.132000419	0	0.348587046	0	0.5	CRA004775;Yang2025_Silkie_Wenchang	PRJNA827465	0.608737092
ENSGALG00010029612	HSPB1	2	-0.165773779	0.135785596	-1.122185636	0.186106343	0	0.00490017	0	1	CRA004775;PRJNA827465	Yang2025_Silkie_Wenchang	0.69509982
ENSGALG00010029614	MDH2	2	-0.07193337	0.074607985	-0.964151081	0.334970186	0	0.980369358	0	0.5	PRJNA827465;Yang2025_Silkie_Wenchang	CRA004775	0.803101658
ENSGALG00010029614	MDH2	2	0.060911395	0.142807185	0.426528925	0.669722476	0	0.000209889	0	1	CRA004775;Yang2025_Silkie_Wenchang	PRJNA827465	0.914377769
ENSGALG00010029614	MDH2	2	-0.104578185	0.083148244	-1.22573174	0.208488789	0	0.189673487	0	0.5	CRA004775;PRJNA827465	Yang2025_Silkie_Wenchang	0.721011841
ENSGALG00010029615	ENSGALG00010029615	2	-0.00272753	0.21548862	-0.012657422	0.989901108	0	0.255345357	0	0.5	PRJNA827465;Yang2025_Silkie_Wenchang	CRA004775	0.997311131
ENSGALG00010029615	ENSGALG00010029615	2	-0.189470632	0.306813367	-0.617543603	0.53687623	0	0.066928086	0	1	CRA004775;Yang2025_Silkie_Wenchang	PRJNA827465	0.874713452
ENSGALG00010029615	ENSGALG00010029615	2	0.014827267	0.248979349	0.059552197	0.952512292	0	0.36710351	0	0.5	CRA004775;PRJNA827465	Yang2025_Silkie_Wenchang	0.987758913
ENSGALG00010029616	TMEM120A	2	-0.022468381	0.125902221	-0.178458977	0.858362554	0.015804953	1.612352143	37.97880915	0.5	PRJNA827465;Yang2025_Silkie_Wenchang	CRA004775	0.994330358
ENSGALG00010029616	TMEM120A	2	-0.14315134	0.326338378	-0.438799738	0.66080665	0.17275542	5.290700914	81.09891267	0.5	CRA004775;Yang2025_Silkie_Wenchang	PRJNA827465	0.91192683
ENSGALG00010029616	TMEM120A	2	-0.238744249	0.177733168	-1.343273467	0.17918344	0.046374872	3.324270203	69.91820945	1	CRA004775;PRJNA827465	Yang2025_Silkie_Wenchang	0.685873949
ENSGALG00010029617	POR	2	-0.077759518	0.046581287	-1.669329521	0.095052093	0	0.10749088	0	1	PRJNA827465;Yang2025_Silkie_Wenchang	CRA004775	0.527809043
ENSGALG00010029617	POR	2	-0.075697237	0.085111017	-0.889394104	0.373791313	0	0.265342809	0	1	CRA004775;Yang2025_Silkie_Wenchang	PRJNA827465	0.822579781
ENSGALG00010029617	POR	2	-0.064181775	0.048458909	-1.324457705	0.185351081	0	0.120298154	0	1	CRA004775;PRJNA827465	Yang2025_Silkie_Wenchang	0.693795574
ENSGALG00010029618	TEX2	2	0.000311405	0.167753897	0.001856321	0.998518871	0.048822865	7.418523882	86.52022942	0.5	PRJNA827465;Yang2025_Silkie_Wenchang	CRA004775	0.999587051
ENSGALG00010029618	TEX2	2	0.217832445	0.092722445	2.349297288	0.018809059	0	0.890581887	0	1	CRA004775;Yang2025_Silkie_Wenchang	PRJNA827465	0.129831303
ENSGALG00010029618	TEX2	2	0.084588917	0.279275688	0.302886791	0.761976138	0.132648195	6.051653711	83.47559117	0.5	CRA004775;PRJNA827465	Yang2025_Silkie_Wenchang	0.939897315
ENSGALG00010029619	ENSGALG00010029619	2	-0.149918296	0.103234671	-1.45220878	0.146443562	0.007165704	1.331999528	24.92489834	1	PRJNA827465;Yang2025_Silkie_Wenchang	CRA004775	0.63528381
ENSGALG00010029619	ENSGALG00010029619	2	-0.257641871	0.151166055	-1.70436326	0.088313243	0	0.40064399	0	1	CRA004775;Yang2025_Silkie_Wenchang	PRJNA827465	0.510437559
ENSGALG00010029619	ENSGALG00010029619	2	-0.095183444	0.079121878	-1.202997179	0.228977179	0	0.024966652	0	1	CRA004775;PRJNA827465	Yang2025_Silkie_Wenchang	0.741680452
ENSGALG00010029620	ENSGALG00010029620	2	0.234842147	0.237173586	0.990169908	0.322091078	0	0.23279361	0	1	PRJNA827465;Yang2025_Silkie_Wenchang	CRA004775	0.798513866
ENSGALG00010029620	ENSGALG00010029620	2	0.074201976	0.301968227	0.24572776	0.805892982	0.008040174	1.045278413	4.33170842	0.5	CRA004775;Yang2025_Silkie_Wenchang	PRJNA827465	0.952057827
ENSGALG00010029620	ENSGALG00010029620	2	0.044335962	0.227251097	0.195096801	0.845317167	0	0.542377049	0	0.5	CRA004775;PRJNA827465	Yang2025_Silkie_Wenchang	0.960938873
ENSGALG00010029621	TAF15	2	0.168058588	0.240264947	0.699169665	0.484446006	0.109515132	18.98664999	94.73314144	0.5	PRJNA827465;Yang2025_Silkie_Wenchang	CRA004775	0.857804733
ENSGALG00010029621	TAF15	2	0.040360936	0.385814616	0.104612253	0.916683491	0.279616903	16.18487465	93.82139175	0.5	CRA004775;Yang2025_Silkie_Wenchang	PRJNA827465	0.980670715
ENSGALG00010029621	TAF15	2	-0.170486549	0.139317671	-1.223725229	0.221035899	0.026467565	2.661065981	62.42107459	1	CRA004775;PRJNA827465	Yang2025_Silkie_Wenchang	0.734279503
ENSGALG00010029622	MMP28	2	0.085738689	0.088208759	0.971260557	0.331414545	0	0.039711775	0	1	PRJNA827465;Yang2025_Silkie_Wenchang	CRA004775	0.801798188
ENSGALG00010029622	MMP28	2	0.039013123	0.131050799	0.29769466	0.76593622	0	0.164736984	0	0.5	CRA004775;Yang2025_Silkie_Wenchang	PRJNA827465	0.941031299
ENSGALG00010029622	MMP28	2	0.079809249	0.105390448	0.757272125	0.448886847	0	0.278584264	0	0.5	CRA004775;PRJNA827465	Yang2025_Silkie_Wenchang	0.847095089
ENSGALG00010029623	ENSGALG00010029623	2	-0.275952289	0.42687804	-0.646442927	0.517992548	0.180922364	1.972750826	49.30936098	0.5	PRJNA827465;Yang2025_Silkie_Wenchang	CRA004775	0.868202369
ENSGALG00010029623	ENSGALG00010029623	2	0.181302025	0.379871766	0.477271651	0.633168695	0	0.000528375	0	1	CRA004775;Yang2025_Silkie_Wenchang	PRJNA827465	0.903249582
ENSGALG00010029623	ENSGALG00010029623	2	-0.416335008	0.38467746	-1.082296343	0.279120871	0.055977647	1.189610339	15.93886106	0.5	CRA004775;PRJNA827465	Yang2025_Silkie_Wenchang	0.777370468
ENSGALG00010029624	RASL10B	2	0.125142734	0.137264714	0.911689036	0.361923437	0	0.539782653	0	1	PRJNA827465;Yang2025_Silkie_Wenchang	CRA004775	0.815579273
ENSGALG00010029624	RASL10B	2	-0.06411815	0.455192309	-0.140859475	0.887990975	0.257771829	2.17835365	54.09777169	0.5	CRA004775;Yang2025_Silkie_Wenchang	PRJNA827465	0.973135755
ENSGALG00010029624	RASL10B	2	-0.110088312	0.306875597	-0.35873922	0.719790185	0.085775087	1.406125811	28.88260834	0.5	CRA004775;PRJNA827465	Yang2025_Silkie_Wenchang	0.928564173
ENSGALG00010029625	PECAM1	2	0.247816725	0.087522243	2.831471355	0.004633438	0.005825009	1.611086289	37.93007817	1	PRJNA827465;Yang2025_Silkie_Wenchang	CRA004775	0.100059356
ENSGALG00010029625	PECAM1	2	0.381098665	0.115546164	3.298237266	0.000972939	0.00477583	1.077607156	7.201804063	1	CRA004775;Yang2025_Silkie_Wenchang	PRJNA827465	0.037125646
ENSGALG00010029625	PECAM1	2	0.337797311	0.251900124	1.340997001	0.179921426	0.084711186	2.393612855	58.2221495	1	CRA004775;PRJNA827465	Yang2025_Silkie_Wenchang	0.686815444
ENSGALG00010029626	AP2B1	2	-0.187429893	0.115730535	-1.619537084	0.105331754	0.022557015	6.254067856	84.01040694	1	PRJNA827465;Yang2025_Silkie_Wenchang	CRA004775	0.550809984
ENSGALG00010029626	AP2B1	2	-0.320387022	0.071552761	-4.477633262	7.54751E-06	0	0.276675664	0	1	CRA004775;Yang2025_Silkie_Wenchang	PRJNA827465	0.001111011
ENSGALG00010029626	AP2B1	2	-0.182473974	0.16590117	-1.099895647	0.271377592	0.03561846	2.126713453	52.97909087	1	CRA004775;PRJNA827465	Yang2025_Silkie_Wenchang	0.772743863
ENSGALG00010029627	PEX12	2	-0.182138638	0.092395584	-1.971291578	0.048690534	0	0.918159375	0	1	PRJNA827465;Yang2025_Silkie_Wenchang	CRA004775	0.381461853
ENSGALG00010029627	PEX12	2	-0.222948694	0.14513249	-1.53617356	0.124495819	0	0.973964737	0	1	CRA004775;Yang2025_Silkie_Wenchang	PRJNA827465	0.591806866
ENSGALG00010029627	PEX12	2	-0.115208407	0.097317936	-1.183835286	0.236478271	0	0.124535892	0	1	CRA004775;PRJNA827465	Yang2025_Silkie_Wenchang	0.7487803
ENSGALG00010029628	UNC45B	2	-0.096554748	0.133193094	-0.724923082	0.468499219	0.020157803	2.313997118	56.78473442	0.5	PRJNA827465;Yang2025_Silkie_Wenchang	CRA004775	0.853198008
ENSGALG00010029628	UNC45B	2	0.053073516	0.124718007	0.425548139	0.670437137	0	0.309353958	0	1	CRA004775;Yang2025_Silkie_Wenchang	PRJNA827465	0.914482883
ENSGALG00010029628	UNC45B	2	-0.205968496	0.118620564	-1.736364162	0.082499465	0	0.914307687	0	0.5	CRA004775;PRJNA827465	Yang2025_Silkie_Wenchang	0.495285278
ENSGALG00010029629	ENSGALG00010029629	2	0.49089474	0.360312328	1.362414501	0.17306711	0.220706287	6.597961403	84.84380343	1	PRJNA827465;Yang2025_Silkie_Wenchang	CRA004775	0.676043418
ENSGALG00010029629	ENSGALG00010029629	2	0.796762169	0.199831622	3.987167596	6.68668E-05	0	0.462161505	0	1	CRA004775;Yang2025_Silkie_Wenchang	PRJNA827465	0.005898997
ENSGALG00010029629	ENSGALG00010029629	2	0.196256267	0.156702859	1.252410247	0.210420414	0	0.69483746	0	1	CRA004775;PRJNA827465	Yang2025_Silkie_Wenchang	0.721968882
ENSGALG00010029630	RAD51D	2	0.042275571	0.090230291	0.468529701	0.639405836	0	0.925672079	0	1	PRJNA827465;Yang2025_Silkie_Wenchang	CRA004775	0.906063152
ENSGALG00010029630	RAD51D	2	-0.233051136	0.510183332	-0.456798803	0.647815666	0.458256297	8.327820374	87.99205608	0.5	CRA004775;Yang2025_Silkie_Wenchang	PRJNA827465	0.907478507
ENSGALG00010029630	RAD51D	2	-0.331977034	0.371119087	-0.89452967	0.371038562	0.246592218	9.081884724	88.98906967	0.5	CRA004775;PRJNA827465	Yang2025_Silkie_Wenchang	0.8214684
ENSGALG00010029631	LIG3	2	0.039918227	0.045662002	0.874211043	0.382003334	0	0.171469378	0	1	PRJNA827465;Yang2025_Silkie_Wenchang	CRA004775	0.824983626
ENSGALG00010029631	LIG3	2	-0.001871672	0.103883456	-0.018017038	0.985625261	0.010095511	1.576893662	36.58418294	0.5	CRA004775;Yang2025_Silkie_Wenchang	PRJNA827465	0.995939127
ENSGALG00010029631	LIG3	2	-0.008210718	0.071701617	-0.11451231	0.908831688	0.002300746	1.134625412	11.86518567	0.5	CRA004775;PRJNA827465	Yang2025_Silkie_Wenchang	0.978387968
ENSGALG00010029632	TMEM132E	2	-0.122850827	0.137474745	-0.893624688	0.371522732	0	0.066699857	0	1	PRJNA827465;Yang2025_Silkie_Wenchang	CRA004775	0.821526338
ENSGALG00010029632	TMEM132E	2	-0.049352148	0.21757734	-0.226825773	0.820559222	0	0.052864252	0	0.5	CRA004775;Yang2025_Silkie_Wenchang	PRJNA827465	0.955383907
ENSGALG00010029632	TMEM132E	2	-0.128325984	0.1598975	-0.802551536	0.42223399	0	0.143127572	0	0.5	CRA004775;PRJNA827465	Yang2025_Silkie_Wenchang	0.839649223
ENSGALG00010029633	POLG2	2	0.667684332	0.438645326	1.522150796	0.127971301	0.361998381	16.86176136	94.06942146	1	PRJNA827465;Yang2025_Silkie_Wenchang	CRA004775	0.60032911
ENSGALG00010029633	POLG2	2	0.486244356	0.63454772	0.766284931	0.443506783	0.764076948	19.32468971	94.82527267	0.5	CRA004775;Yang2025_Silkie_Wenchang	PRJNA827465	0.845761624
ENSGALG00010029633	POLG2	2	0.080365265	0.190089755	0.42277536	0.672459167	0.034578554	1.817030557	44.96515228	0.5	CRA004775;PRJNA827465	Yang2025_Silkie_Wenchang	0.914742762
ENSGALG00010029634	DDX5	2	0.063611232	0.125407584	0.50723593	0.611989287	0.027828449	8.652100154	88.44211253	0.5	PRJNA827465;Yang2025_Silkie_Wenchang	CRA004775	0.897777844
ENSGALG00010029634	DDX5	2	-0.056910744	0.061363956	-0.927429516	0.353703572	0	0.114377777	0	0.5	CRA004775;Yang2025_Silkie_Wenchang	PRJNA827465	0.811318534
ENSGALG00010029634	DDX5	2	0.171764641	0.052921024	3.245678732	0.00117171	0	0.902487506	0	1	CRA004775;PRJNA827465	Yang2025_Silkie_Wenchang	0.041644062
ENSGALG00010029635	ENSGALG00010029635	2	0.19534167	0.224603374	0.869718322	0.384454357	0	0.261586811	0	1	PRJNA827465;Yang2025_Silkie_Wenchang	CRA004775	0.826099934
ENSGALG00010029635	ENSGALG00010029635	2	-0.508078616	0.596213145	-0.85217614	0.394116337	0.52104633	3.682843244	72.84706587	0.5	CRA004775;Yang2025_Silkie_Wenchang	PRJNA827465	0.830486298
ENSGALG00010029635	ENSGALG00010029635	2	-0.375759933	0.714939356	-0.525582946	0.599178009	0.864781398	6.255263236	84.01346255	0.5	CRA004775;PRJNA827465	Yang2025_Silkie_Wenchang	0.894908299
ENSGALG00010029636	SPATA20	2	-0.163592052	0.113284196	-1.444085392	0.148714957	0.016393564	2.476938557	59.62758151	1	PRJNA827465;Yang2025_Silkie_Wenchang	CRA004775	0.638795624
ENSGALG00010029636	SPATA20	2	-0.066122771	0.112376937	-0.588401617	0.556262752	0	0.449785404	0	1	CRA004775;Yang2025_Silkie_Wenchang	PRJNA827465	0.880324407
ENSGALG00010029636	SPATA20	2	-0.24434227	0.060480114	-4.040043181	5.34414E-05	0	0.11083424	0	1	CRA004775;PRJNA827465	Yang2025_Silkie_Wenchang	0.005073444
ENSGALG00010029638	EPN3	2	0.110238372	0.325778162	0.338384781	0.735073241	0.196036304	13.07157001	92.34980956	0.5	PRJNA827465;Yang2025_Silkie_Wenchang	CRA004775	0.933592889
ENSGALG00010029638	EPN3	2	0.278996536	0.273695908	1.019366849	0.308028838	0.082474818	1.715022983	41.69174351	0.5	CRA004775;Yang2025_Silkie_Wenchang	PRJNA827465	0.793278812
ENSGALG00010029638	EPN3	2	-0.211554029	0.116809156	-1.811108267	0.070124094	0	0.002241655	0	1	CRA004775;PRJNA827465	Yang2025_Silkie_Wenchang	0.456835285
ENSGALG00010029641	ENSGALG00010029641	2	-0.060765415	0.207297814	-0.293130997	0.769422025	0	0.316362047	0	0.5	PRJNA827465;Yang2025_Silkie_Wenchang	CRA004775	0.941790377
ENSGALG00010029641	ENSGALG00010029641	2	-0.129086953	0.337633836	-0.382328247	0.702217905	0	0.950986219	0	0.5	CRA004775;Yang2025_Silkie_Wenchang	PRJNA827465	0.923083392
ENSGALG00010029641	ENSGALG00010029641	2	-0.186501328	0.211690125	-0.881010996	0.378311868	0	0.455028812	0	1	CRA004775;PRJNA827465	Yang2025_Silkie_Wenchang	0.823345221
ENSGALG00010029642	NAT9	2	-0.161071384	0.090372682	-1.782301701	0.074700046	0	0.936137931	0	0.5	PRJNA827465;Yang2025_Silkie_Wenchang	CRA004775	0.471863692
ENSGALG00010029642	NAT9	2	-0.005520132	0.181192385	-0.030465582	0.975695742	0	0.03436043	0	0.5	CRA004775;Yang2025_Silkie_Wenchang	PRJNA827465	0.992765259
ENSGALG00010029642	NAT9	2	-0.200003177	0.099112717	-2.017936568	0.043597868	0	0.071631595	0	1	CRA004775;PRJNA827465	Yang2025_Silkie_Wenchang	0.360845532
ENSGALG00010029644	MYCBPAP	2	0.207336656	0.274727752	0.75469862	0.450429835	0.135593763	9.821954336	89.81872685	0.5	PRJNA827465;Yang2025_Silkie_Wenchang	CRA004775	0.847577105
ENSGALG00010029644	MYCBPAP	2	0.227041339	0.305665623	0.742776817	0.457616814	0.143009903	3.896293064	74.33457947	0.5	CRA004775;Yang2025_Silkie_Wenchang	PRJNA827465	0.849573219
ENSGALG00010029644	MYCBPAP	2	-0.075148474	0.10922652	-0.688005756	0.491449157	0	0.052280582	0	1	CRA004775;PRJNA827465	Yang2025_Silkie_Wenchang	0.85895707
ENSGALG00010029646	MRPS17	2	-0.26745078	0.289447602	-0.92400413	0.355484165	0.152519233	10.65507271	90.61479891	0.5	PRJNA827465;Yang2025_Silkie_Wenchang	CRA004775	0.812301302
ENSGALG00010029646	MRPS17	2	-0.313638274	0.28974306	-1.082470359	0.279043579	0.126229444	3.921433386	74.49912056	0.5	CRA004775;Yang2025_Silkie_Wenchang	PRJNA827465	0.777322971
ENSGALG00010029646	MRPS17	2	0.00334086	0.064774011	0.051577174	0.958865608	0	5.83171E-05	0	1	CRA004775;PRJNA827465	Yang2025_Silkie_Wenchang	0.989102387
ENSGALG00010029647	RSAD1	2	0.562708613	0.619293104	0.908630516	0.363545193	0.710046337	13.2122617	92.43127314	0.5	PRJNA827465;Yang2025_Silkie_Wenchang	CRA004775	0.817042253
ENSGALG00010029647	RSAD1	2	0.55685258	0.639554763	0.870687878	0.383924599	0.743125254	10.89122797	90.81829889	0.5	CRA004775;Yang2025_Silkie_Wenchang	PRJNA827465	0.825601288
ENSGALG00010029647	RSAD1	2	-0.045625584	0.139985562	-0.325930641	0.744476833	0	0.017356021	0	1	CRA004775;PRJNA827465	Yang2025_Silkie_Wenchang	0.935846381
ENSGALG00010029648	NIPSNAP2	2	0.015428125	0.138620185	0.111297824	0.911380181	0.029503918	3.988754098	74.92951494	0.5	PRJNA827465;Yang2025_Silkie_Wenchang	CRA004775	0.978845851
ENSGALG00010029648	NIPSNAP2	2	0.165222735	0.120055166	1.376223452	0.168752464	0	0.096391569	0	1	CRA004775;Yang2025_Silkie_Wenchang	PRJNA827465	0.669861707
ENSGALG00010029648	NIPSNAP2	2	-0.099298408	0.060659127	-1.636990385	0.101632479	0	0.158342785	0	0.5	CRA004775;PRJNA827465	Yang2025_Silkie_Wenchang	0.543686036
ENSGALG00010029649	PSPH	2	-0.026535444	0.103229583	-0.257052713	0.79713808	0	0.021825382	0	1	PRJNA827465;Yang2025_Silkie_Wenchang	CRA004775	0.949622804
ENSGALG00010029649	PSPH	2	0.017261122	0.182734104	0.094460318	0.924743503	0.004762014	1.029890605	2.902308776	0.5	CRA004775;Yang2025_Silkie_Wenchang	PRJNA827465	0.982493782
ENSGALG00010029649	PSPH	2	0.012227196	0.122161454	0.100090458	0.920272511	0	0.968320903	0	0.5	CRA004775;PRJNA827465	Yang2025_Silkie_Wenchang	0.981364549
ENSGALG00010029650	ACSF2	2	0.058787328	0.079665684	0.737925354	0.460559811	0	0.016300786	0	1	PRJNA827465;Yang2025_Silkie_Wenchang	CRA004775	0.85014477
ENSGALG00010029650	ACSF2	2	0.068141649	0.108978588	0.625275573	0.531790209	0	0.00135033	0	1	CRA004775;Yang2025_Silkie_Wenchang	PRJNA827465	0.873042777
ENSGALG00010029650	ACSF2	2	0.050241194	0.102636777	0.489504788	0.624484365	0	0.000675264	0	1	CRA004775;PRJNA827465	Yang2025_Silkie_Wenchang	0.900892482
ENSGALG00010029651	ENSGALG00010029651	2	0.479712434	0.391458418	1.225449276	0.220405991	0.180581018	2.102294256	52.43291955	1	PRJNA827465;Yang2025_Silkie_Wenchang	CRA004775	0.734053249
ENSGALG00010029651	ENSGALG00010029651	2	0.527083796	0.533538095	0.987902834	0.323200237	0.261886266	1.847188005	45.86365886	0.5	CRA004775;Yang2025_Silkie_Wenchang	PRJNA827465	0.799115433
ENSGALG00010029651	ENSGALG00010029651	2	0.172098356	0.207940836	0.82763136	0.407879303	0	0.146229583	0	0.5	CRA004775;PRJNA827465	Yang2025_Silkie_Wenchang	0.833982683
ENSGALG00010029652	LRRC59	2	0.004669061	0.049128643	0.095037448	0.924285083	0	0.336735392	0	0.5	PRJNA827465;Yang2025_Silkie_Wenchang	CRA004775	0.98232425
ENSGALG00010029652	LRRC59	2	-0.045587678	0.103751217	-0.439394154	0.660375962	0	0.052887534	0	1	CRA004775;Yang2025_Silkie_Wenchang	PRJNA827465	0.911923994
ENSGALG00010029652	LRRC59	2	0.015269885	0.05162756	0.295770042	0.767405703	0	0.015943997	0	0.5	CRA004775;PRJNA827465	Yang2025_Silkie_Wenchang	0.941444269
ENSGALG00010029653	CCT6A	2	0.053703262	0.067115095	0.800166679	0.423614232	0.003235688	1.408318707	28.9933454	1	PRJNA827465;Yang2025_Silkie_Wenchang	CRA004775	0.84030043
ENSGALG00010029653	CCT6A	2	0.038088705	0.134223637	0.283770473	0.776586284	0.020348068	2.275446259	56.05257667	0.5	CRA004775;Yang2025_Silkie_Wenchang	PRJNA827465	0.944156005
ENSGALG00010029653	CCT6A	2	-0.004624354	0.051096139	-0.090503012	0.927887501	0	0.651906849	0	0.5	CRA004775;PRJNA827465	Yang2025_Silkie_Wenchang	0.982993195
ENSGALG00010029654	EME1	2	0.080175896	0.106243843	0.754640405	0.450464773	0.01010702	1.717808126	41.78628075	0.5	PRJNA827465;Yang2025_Silkie_Wenchang	CRA004775	0.847577105
ENSGALG00010029654	EME1	2	-0.422419715	0.492591004	-0.857546548	0.391142901	0.354988573	3.018258875	66.86831576	1	CRA004775;Yang2025_Silkie_Wenchang	PRJNA827465	0.829251057
ENSGALG00010029654	EME1	2	-0.334011171	0.611638416	-0.546092531	0.585002334	0.612052243	4.607851886	78.29791354	0.5	CRA004775;PRJNA827465	Yang2025_Silkie_Wenchang	0.891072617
ENSGALG00010029655	PHKG1	2	-0.006289652	0.080763581	-0.07787733	0.937925633	0	0.140986609	0	0.5	PRJNA827465;Yang2025_Silkie_Wenchang	CRA004775	0.985349045
ENSGALG00010029655	PHKG1	2	0.013873945	0.178700614	0.077637926	0.938116073	0	0.612287766	0	0.5	CRA004775;Yang2025_Silkie_Wenchang	PRJNA827465	0.985349045
ENSGALG00010029655	PHKG1	2	-0.029752152	0.088580223	-0.335878036	0.73696284	0	0.466457271	0	1	CRA004775;PRJNA827465	Yang2025_Silkie_Wenchang	0.93386802
ENSGALG00010029656	MRPL27	2	0.027630003	0.076504917	0.361153296	0.717984853	0	0.081003799	0	0.5	PRJNA827465;Yang2025_Silkie_Wenchang	CRA004775	0.927927766
ENSGALG00010029656	MRPL27	2	-0.20801093	0.224306922	-0.927349582	0.353745059	0.019956911	1.225143393	18.37690136	1	CRA004775;Yang2025_Silkie_Wenchang	PRJNA827465	0.811318534
ENSGALG00010029656	MRPL27	2	-0.131166369	0.24883716	-0.527117287	0.598112143	0.082983449	2.332460129	57.12681269	0.5	CRA004775;PRJNA827465	Yang2025_Silkie_Wenchang	0.894645663
ENSGALG00010029657	CHCHD2	2	-0.085237487	0.079551389	-1.071477049	0.283954985	0	0.00364744	0	1	PRJNA827465;Yang2025_Silkie_Wenchang	CRA004775	0.77978499
ENSGALG00010029657	CHCHD2	2	-0.038985594	0.175362955	-0.222313736	0.82406966	0	0.220354231	0	0.5	CRA004775;Yang2025_Silkie_Wenchang	PRJNA827465	0.95619211
ENSGALG00010029657	CHCHD2	2	-0.07839364	0.08580606	-0.913614264	0.360919568	0	0.281171987	0	0.5	CRA004775;PRJNA827465	Yang2025_Silkie_Wenchang	0.815139251
ENSGALG00010029658	XYLT2	2	0.024202274	0.053600982	0.451526697	0.651609988	0	0.204868117	0	0.5	PRJNA827465;Yang2025_Silkie_Wenchang	CRA004775	0.908943868
ENSGALG00010029658	XYLT2	2	-0.176431243	0.159193308	-1.108283039	0.267739598	0.036038685	3.453364018	71.04272834	1	CRA004775;Yang2025_Silkie_Wenchang	PRJNA827465	0.771398294
ENSGALG00010029658	XYLT2	2	-0.135111984	0.187937189	-0.71892096	0.472189621	0.061268849	7.176029175	86.06471663	0.5	CRA004775;PRJNA827465	Yang2025_Silkie_Wenchang	0.853998373
ENSGALG00010029659	VKORC1L1	2	-0.229265839	0.343523684	-0.667394564	0.504520137	0.230134929	39.90615413	97.49412084	0.5	PRJNA827465;Yang2025_Silkie_Wenchang	CRA004775	0.863512504
ENSGALG00010029659	VKORC1L1	2	-0.545895652	0.084814351	-6.436359481	1.22373E-10	0	0.634245979	0	1	CRA004775;Yang2025_Silkie_Wenchang	PRJNA827465	8.58862E-08
ENSGALG00010029659	VKORC1L1	2	-0.106606947	0.251630801	-0.423664142	0.67181077	0.107208033	5.817175691	82.80952728	0.5	CRA004775;PRJNA827465	Yang2025_Silkie_Wenchang	0.914674175
ENSGALG00010029660	CD300LG	2	-0.349787156	0.176087416	-1.9864404	0.046984444	0	0.515157459	0	1	PRJNA827465;Yang2025_Silkie_Wenchang	CRA004775	0.374081275
ENSGALG00010029660	CD300LG	2	-0.445968712	0.234347458	-1.903023468	0.057037483	0	0.227996376	0	1	CRA004775;Yang2025_Silkie_Wenchang	PRJNA827465	0.413453375
ENSGALG00010029660	CD300LG	2	-0.302494802	0.167642627	-1.804402662	0.071168206	0	0.039350874	0	1	CRA004775;PRJNA827465	Yang2025_Silkie_Wenchang	0.460681013
ENSGALG00010029663	GUSB	2	0.038505663	0.056752774	0.678480723	0.497466942	0	0.014762505	0	1	PRJNA827465;Yang2025_Silkie_Wenchang	CRA004775	0.86098656
ENSGALG00010029663	GUSB	2	0.022889786	0.095836642	0.238841695	0.811228338	0	0.078586129	0	0.5	CRA004775;Yang2025_Silkie_Wenchang	PRJNA827465	0.953179464
ENSGALG00010029663	GUSB	2	0.04001862	0.068746998	0.58211444	0.5604896	0	0.099768351	0	0.5	CRA004775;PRJNA827465	Yang2025_Silkie_Wenchang	0.881203589
ENSGALG00010029665	ASL1	2	0.003372499	0.148700346	0.022679831	0.981905664	0	0.703425793	0	0.5	PRJNA827465;Yang2025_Silkie_Wenchang	CRA004775	0.995055866
ENSGALG00010029665	ASL1	2	-0.102729415	0.277708369	-0.369918325	0.711443347	0.087218169	2.243260769	55.42203503	0.5	CRA004775;Yang2025_Silkie_Wenchang	PRJNA827465	0.92587565
ENSGALG00010029665	ASL1	2	-0.207566011	0.168530699	-1.231621375	0.218090553	0	0.724456201	0	1	CRA004775;PRJNA827465	Yang2025_Silkie_Wenchang	0.73160572
ENSGALG00010029666	SLC9A3R1	2	0.024124138	0.173740575	0.138851493	0.889567503	0.039692137	2.802570227	64.31846773	0.5	PRJNA827465;Yang2025_Silkie_Wenchang	CRA004775	0.973153999
ENSGALG00010029666	SLC9A3R1	2	-0.214844475	0.161871793	-1.327250846	0.184425718	0	0.28511342	0	1	CRA004775;Yang2025_Silkie_Wenchang	PRJNA827465	0.692706803
ENSGALG00010029666	SLC9A3R1	2	0.009776291	0.263870421	0.03704959	0.970445466	0.0836376	1.935002465	48.32047928	0.5	CRA004775;PRJNA827465	Yang2025_Silkie_Wenchang	0.99171654
ENSGALG00010029667	TMEM104	2	0.007356338	0.195240778	0.037678286	0.969944189	0.066773895	7.908039953	87.35464153	0.5	PRJNA827465;Yang2025_Silkie_Wenchang	CRA004775	0.991572914
ENSGALG00010029667	TMEM104	2	-0.224822061	0.098980726	-2.271372108	0.023124461	0	0.186615557	0	1	CRA004775;Yang2025_Silkie_Wenchang	PRJNA827465	0.259577892
ENSGALG00010029667	TMEM104	2	-0.020188589	0.241267831	-0.083677087	0.933313175	0.097840092	5.796418728	82.74796824	0.5	CRA004775;PRJNA827465	Yang2025_Silkie_Wenchang	0.984260547
ENSGALG00010029668	CRCP	2	0.056222317	0.087544331	0.642215392	0.52073334	0.002237979	1.161032623	13.86977588	0.5	PRJNA827465;Yang2025_Silkie_Wenchang	CRA004775	0.869353339
ENSGALG00010029668	CRCP	2	0.126076946	0.12119538	1.04027848	0.298210539	0	0.569266953	0	0.5	CRA004775;Yang2025_Silkie_Wenchang	PRJNA827465	0.789118382
ENSGALG00010029668	CRCP	2	-0.020510995	0.096150927	-0.213320824	0.831076732	0	0.047092457	0	1	CRA004775;PRJNA827465	Yang2025_Silkie_Wenchang	0.957470197
ENSGALG00010029669	HID1	2	0.083090287	0.057510017	1.444796764	0.14851498	0	0.116506921	0	1	PRJNA827465;Yang2025_Silkie_Wenchang	CRA004775	0.63857057
ENSGALG00010029669	HID1	2	-0.110627243	0.234376986	-0.472005569	0.636922813	0.093840319	6.522373776	84.66815864	0.5	CRA004775;Yang2025_Silkie_Wenchang	PRJNA827465	0.904841694
ENSGALG00010029669	HID1	2	-0.128208767	0.21443291	-0.597896876	0.549908745	0.076147139	5.486530202	81.77354424	0.5	CRA004775;PRJNA827465	Yang2025_Silkie_Wenchang	0.878621348
ENSGALG00010029670	TPST1	2	0.185832341	0.163714155	1.135102461	0.256332391	0.041040113	3.968173291	74.79948766	1	PRJNA827465;Yang2025_Silkie_Wenchang	CRA004775	0.764677554
ENSGALG00010029670	TPST1	2	0.207130055	0.237767538	0.871145222	0.383674865	0.063099622	1.99459614	49.86453748	0.5	CRA004775;Yang2025_Silkie_Wenchang	PRJNA827465	0.825509017
ENSGALG00010029670	TPST1	2	0.036743985	0.072319007	0.508081994	0.611395842	0	0.267776538	0	0.5	CRA004775;PRJNA827465	Yang2025_Silkie_Wenchang	0.897609796
ENSGALG00010029671	GRIN2C	2	0.003828845	0.088105766	0.043457372	0.965336944	0	0.022811109	0	0.5	PRJNA827465;Yang2025_Silkie_Wenchang	CRA004775	0.990668014
ENSGALG00010029671	GRIN2C	2	0.008294807	0.131491958	0.063082241	0.949701016	0	0.126088322	0	0.5	CRA004775;Yang2025_Silkie_Wenchang	PRJNA827465	0.98723759
ENSGALG00010029671	GRIN2C	2	-0.014813029	0.113909148	-0.130042486	0.896532813	0	0.09705301	0	1	CRA004775;PRJNA827465	Yang2025_Silkie_Wenchang	0.975115773
ENSGALG00010029672	KCTD7	2	-0.051400084	0.132935176	-0.386655252	0.699011448	0	0.005012097	0	1	PRJNA827465;Yang2025_Silkie_Wenchang	CRA004775	0.92185597
ENSGALG00010029672	KCTD7	2	-0.279261054	0.280822627	-0.994439289	0.320009048	0.06688654	1.650034713	39.3952144	1	CRA004775;Yang2025_Silkie_Wenchang	PRJNA827465	0.798348216
ENSGALG00010029672	KCTD7	2	-0.245021607	0.283905899	-0.863038095	0.388116522	0.095069981	2.095069886	52.26889533	1	CRA004775;PRJNA827465	Yang2025_Silkie_Wenchang	0.828154121
ENSGALG00010029673	TMEM248	2	-0.280652398	0.283235144	-0.990881265	0.321743562	0.155988603	35.90024663	97.21450382	1	PRJNA827465;Yang2025_Silkie_Wenchang	CRA004775	0.798513866
ENSGALG00010029673	TMEM248	2	-0.434232954	0.194315953	-2.234674746	0.02543871	0.049300804	2.299551347	56.51325632	1	CRA004775;Yang2025_Silkie_Wenchang	PRJNA827465	0.271985203
ENSGALG00010029673	TMEM248	2	-0.006117011	0.05344455	-0.114455277	0.908876898	0	0.303732666	0	1	CRA004775;PRJNA827465	Yang2025_Silkie_Wenchang	0.978396455
ENSGALG00010029674	FDXR	2	-0.425751754	0.476561722	-0.893382189	0.371652538	0.42618162	16.08668432	93.7836786	0.5	PRJNA827465;Yang2025_Silkie_Wenchang	CRA004775	0.821590623
ENSGALG00010029674	FDXR	2	-0.622077536	0.281095492	-2.213047005	0.026894405	0.125455976	4.831134613	79.30092866	1	CRA004775;Yang2025_Silkie_Wenchang	PRJNA827465	0.279388398

ENSGALG00010029674	FDXR	2	-0.143201158	0.19508254	-0.734054202	0.462915704	0.053362719	3.318453503	69.86548104	0.5	CRA004775;PRJNA827465	Yang2025_Silkie_Wenchang	0.851299641
ENSGALG00010029675	DHRS13	2	0.378871535	0.189878554	1.995336108	0.046006234	0	0.121735795	0	1	PRJNA827465;Yang2025_Silkie_Wenchang	CRA004775	0.369999825
ENSGALG00010029675	DHRS13	2	0.216986633	0.199327791	1.08859197	0.27633386	0.01431403	1.212288368	17.51137549	1	CRA004775;Yang2025_Silkie_Wenchang	PRJNA827465	0.77582325
ENSGALG00010029675	DHRS13	2	0.171354605	0.171509318	0.999097935	0.317747251	0	0.618723706	0	1	CRA004775;PRJNA827465	Yang2025_Silkie_Wenchang	0.797410398
ENSGALG00010029677	PHF12	2	-0.07690472	0.039543971	-1.944789996	0.051800259	0	0.939802534	0	0.5	PRJNA827465;Yang2025_Silkie_Wenchang	CRA004775	0.394831847
ENSGALG00010029677	PHF12	2	0.022426025	0.081308849	0.275812845	0.782691817	0	0.234533248	0	1	CRA004775;Yang2025_Silkie_Wenchang	PRJNA827465	0.945490372
ENSGALG00010029677	PHF12	2	-0.075821634	0.058757926	-1.290406922	0.19690941	0.001908096	1.110302187	9.934429425	0.5	CRA004775;PRJNA827465	Yang2025_Silkie_Wenchang	0.707994211
ENSGALG00010029682	CDR2L	2	-0.052854897	0.147621155	-0.358044193	0.720310242	0.035727283	5.318666574	81.19829498	0.5	PRJNA827465;Yang2025_Silkie_Wenchang	CRA004775	0.92866503
ENSGALG00010029682	CDR2L	2	0.189278203	0.458315082	0.41298707	0.679616087	0.361429191	6.546333417	84.72427333	0.5	CRA004775;Yang2025_Silkie_Wenchang	PRJNA827465	0.917701868
ENSGALG00010029682	CDR2L	2	0.303830649	0.300100907	1.012428292	0.311333316	0.135699089	3.22870555	69.02783532	1	CRA004775;PRJNA827465	Yang2025_Silkie_Wenchang	0.795445189
ENSGALG00010029683	MRPL58	2	0.012585496	0.150817335	0.083448606	0.933494841	0.027142499	2.123679072	52.91190589	0.5	PRJNA827465;Yang2025_Silkie_Wenchang	CRA004775	0.984260547
ENSGALG00010029683	MRPL58	2	0.210777725	0.172607654	1.221137765	0.222033863	0	0.021590303	0	1	CRA004775;Yang2025_Silkie_Wenchang	PRJNA827465	0.735359501
ENSGALG00010029683	MRPL58	2	-0.077595008	0.080478499	-0.96417067	0.334960367	0	0.615625183	0	0.5	CRA004775;PRJNA827465	Yang2025_Silkie_Wenchang	0.803101658
ENSGALG00010029684	ENSGALG00010029684	2	0.414266405	0.299020767	1.385410146	0.16592709	0	0.985170432	0	1	PRJNA827465;Yang2025_Silkie_Wenchang	CRA004775	0.66529196
ENSGALG00010029684	ENSGALG00010029684	2	0.619565226	0.419848932	1.475686083	0.140028184	0	0.499981289	0	1	CRA004775;Yang2025_Silkie_Wenchang	PRJNA827465	0.623792706
ENSGALG00010029684	ENSGALG00010029684	2	0.200545711	0.328604046	0.610295929	0.541665793	0	2.89064E-05	0	1	CRA004775;PRJNA827465	Yang2025_Silkie_Wenchang	0.876288927
ENSGALG00010029685	ATP5PD	2	-0.212900841	0.234284752	-0.908726835	0.363494335	0.084140598	3.590840647	72.15136793	1	PRJNA827465;Yang2025_Silkie_Wenchang	CRA004775	0.817042253
ENSGALG00010029685	ATP5PD	2	-0.325757234	0.229054945	-1.42217944	0.154974166	0.026752819	1.324133032	24.47888728	1	CRA004775;Yang2025_Silkie_Wenchang	PRJNA827465	0.647628882
ENSGALG00010029685	ATP5PD	2	-0.030503736	0.063811794	-0.478026623	0.632631256	0	0.002189156	0	1	CRA004775;PRJNA827465	Yang2025_Silkie_Wenchang	0.903058174
ENSGALG00010029686	KCTD2	2	-0.161010966	0.158567031	-1.015412631	0.309909175	0.046427389	12.76811383	92.16798962	1	PRJNA827465;Yang2025_Silkie_Wenchang	CRA004775	0.794395438
ENSGALG00010029686	KCTD2	2	-0.29147373	0.063362584	-4.600092222	4.22304E-06	0	0.602869909	0	1	CRA004775;Yang2025_Silkie_Wenchang	PRJNA827465	0.000712352
ENSGALG00010029686	KCTD2	2	-0.091474974	0.103802001	-0.881244805	0.378185333	0.015732674	3.260899142	69.33361148	1	CRA004775;PRJNA827465	Yang2025_Silkie_Wenchang	0.823285408
ENSGALG00010029687	SLC16A5	2	-0.019511632	0.30824331	-0.06329945	0.949528054	0.140517835	3.810997756	73.76015249	0.5	PRJNA827465;Yang2025_Silkie_Wenchang	CRA004775	0.98723759
ENSGALG00010029687	SLC16A5	2	-0.004386969	0.460931194	-0.009517622	0.992406151	0.267994017	2.352628052	57.49434343	0.5	CRA004775;Yang2025_Silkie_Wenchang	PRJNA827465	0.997813736
ENSGALG00010029687	SLC16A5	2	0.310993215	0.190011203	1.636709883	0.101691103	0	0.321584942	0	1	CRA004775;PRJNA827465	Yang2025_Silkie_Wenchang	0.543700483
ENSGALG00010029689	ENSGALG00010029689	2	0.311663086	0.533692373	0.583975155	0.559237028	0.55351631	35.11171483	97.15194771	0.5	PRJNA827465;Yang2025_Silkie_Wenchang	CRA004775	0.881014886
ENSGALG00010029689	ENSGALG00010029689	2	0.649180125	0.275122195	2.359606518	0.018294328	0.0935382	2.292852132	56.38619752	1	CRA004775;Yang2025_Silkie_Wenchang	PRJNA827465	0.226321049
ENSGALG00010029689	ENSGALG00010029689	2	-0.083908573	0.217586579	-0.385633036	0.699768463	0.055491415	1.850077252	45.94820302	0.5	CRA004775;PRJNA827465	Yang2025_Silkie_Wenchang	0.922145848
ENSGALG00010029690	ENSGALG00010029690	2	-0.185228169	0.07532221	-2.459144106	0.01392687	0	0.034759163	0	1	PRJNA827465;Yang2025_Silkie_Wenchang	CRA004775	0.193621431
ENSGALG00010029690	ENSGALG00010029690	2	-0.124018941	0.137449668	-0.90228622	0.366904844	0	0.508345237	0	0.5	CRA004775;Yang2025_Silkie_Wenchang	PRJNA827465	0.819577857
ENSGALG00010029690	ENSGALG00010029690	2	-0.177459034	0.08545828	-2.076557536	0.037842416	0	0.681826733	0	0.5	CRA004775;PRJNA827465	Yang2025_Silkie_Wenchang	0.335620339
ENSGALG00010029691	NUFIP2	2	-0.115217139	0.113623994	-1.014021204	0.310572634	0.018086475	3.138483774	68.13748064	1	PRJNA827465;Yang2025_Silkie_Wenchang	CRA004775	0.794899969
ENSGALG00010029691	NUFIP2	2	-0.002045438	0.29833252	-0.006856237	0.994529558	0.137366232	3.925693298	74.52679249	0.5	CRA004775;Yang2025_Silkie_Wenchang	PRJNA827465	0.99823774
ENSGALG00010029691	NUFIP2	2	0.067167187	0.158254304	0.424429451	0.671252646	0.028398374	1.667097021	40.01548876	0.5	CRA004775;PRJNA827465	Yang2025_Silkie_Wenchang	0.914674175
ENSGALG00010029692	CRYBA1	2	-0.065833094	0.229196589	-0.287234178	0.773933019	0	0.143297641	0	1	PRJNA827465;Yang2025_Silkie_Wenchang	CRA004775	0.943248868
ENSGALG00010029692	CRYBA1	2	0.323276686	0.645824455	0.500564332	0.616677777	0.498545136	2.372093771	57.84315055	0.5	CRA004775;Yang2025_Silkie_Wenchang	PRJNA827465	0.899111936
ENSGALG00010029692	CRYBA1	2	0.33983437	0.518874232	0.654945551	0.512502811	0.325323345	2.106380284	52.52519179	0.5	CRA004775;PRJNA827465	Yang2025_Silkie_Wenchang	0.865809047
ENSGALG00010029693	HN1	2	-0.010061606	0.09167497	-0.109753032	0.912605241	0.009913442	2.124381712	52.92748029	0.5	PRJNA827465;Yang2025_Silkie_Wenchang	CRA004775	0.978986947
ENSGALG00010029693	HN1	2	-0.123549929	0.107798967	-1.146114217	0.251747896	0	0.063833224	0	1	CRA004775;Yang2025_Silkie_Wenchang	PRJNA827465	0.761871849
ENSGALG00010029693	HN1	2	0.049387585	0.051743587	0.954467737	0.339849933	0	0.285950714	0	0.5	CRA004775;PRJNA827465	Yang2025_Silkie_Wenchang	0.80512381
ENSGALG00010029694	ARMC7	2	-0.182381698	0.197657006	-0.922718104	0.356154129	0.058615282	3.467709511	71.16252105	1	PRJNA827465;Yang2025_Silkie_Wenchang	CRA004775	0.812471559
ENSGALG00010029694	ARMC7	2	-0.29173033	0.15028682	-1.941157111	0.052239224	0	0.982280253	0	1	CRA004775;Yang2025_Silkie_Wenchang	PRJNA827465	0.396836711
ENSGALG00010029694	ARMC7	2	-0.035662724	0.067818752	-0.525853446	0.598990037	0	0.215862411	0	1	CRA004775;PRJNA827465	Yang2025_Silkie_Wenchang	0.894898902
ENSGALG00010029695	TAOK1	2	-0.051247278	0.044030054	-1.16391586	0.244458112	0.000801828	1.104842553	9.489365986	1	PRJNA827465;Yang2025_Silkie_Wenchang	CRA004775	0.755994741
ENSGALG00010029695	TAOK1	2	-0.122066489	0.109532358	-1.114433142	0.265093412	0	0.944885542	0	0.5	CRA004775;Yang2025_Silkie_Wenchang	PRJNA827465	0.769274038
ENSGALG00010029695	TAOK1	2	-0.031185231	0.037542958	-0.830654602	0.406168779	0	0.365568933	0	0.5	CRA004775;PRJNA827465	Yang2025_Silkie_Wenchang	0.833477131
ENSGALG00010029696	SUMO2	2	-0.239172961	0.259809992	-0.920568757	0.357270562	0.129676972	25.95911863	96.14778909	0.5	PRJNA827465;Yang2025_Silkie_Wenchang	CRA004775	0.813292764
ENSGALG00010029696	SUMO2	2	-0.216791962	0.319733182	-0.678040236	0.497746182	0.177018646	6.97920229	85.67171493	0.5	CRA004775;Yang2025_Silkie_Wenchang	PRJNA827465	0.861055278
ENSGALG00010029696	SUMO2	2	0.019029056	0.043394343	0.438514682	0.661013229	0	0.287338761	0	1	CRA004775;PRJNA827465	Yang2025_Silkie_Wenchang	0.912052061
ENSGALG00010029697	GIT1	2	0.26831736	0.28255134	0.949623384	0.342303652	0.14997691	16.24157442	93.84296144	0.5	PRJNA827465;Yang2025_Silkie_Wenchang	CRA004775	0.806005903
ENSGALG00010029697	GIT1	2	0.280357843	0.378051261	0.741586848	0.458337694	0.206196137	2.931990922	65.89348239	0.5	CRA004775;Yang2025_Silkie_Wenchang	PRJNA827465	0.850026426
ENSGALG00010029697	GIT1	2	-0.011350905	0.069860164	-0.162480368	0.870927587	0	0.250072088	0	1	CRA004775;PRJNA827465	Yang2025_Silkie_Wenchang	0.969196692
ENSGALG00010029698	NUP85	2	0.070129928	0.046044252	1.523098432	0.127734081	0	0.419671226	0	0.5	PRJNA827465;Yang2025_Silkie_Wenchang	CRA004775	0.600026204
ENSGALG00010029698	NUP85	2	-0.222769546	0.215018862	-1.036048653	0.300180436	0.076420855	5.76254159	82.64654607	1	CRA004775;Yang2025_Silkie_Wenchang	PRJNA827465	0.790325453
ENSGALG00010029698	NUP85	2	-0.16489547	0.259073362	-0.636481763	0.524462464	0.125468144	14.78797571	93.23774924	0.5	CRA004775;PRJNA827465	Yang2025_Silkie_Wenchang	0.869790047
ENSGALG00010029699	ANKRD13B	2	0.408720711	0.337005241	1.212802238	0.22520544	0.211831071	14.60387283	93.15250132	1	PRJNA827465;Yang2025_Silkie_Wenchang	CRA004775	0.738276881
ENSGALG00010029699	ANKRD13B	2	0.354740426	0.437579663	0.810687644	0.417545071	0.330276991	6.966851736	85.64631432	0.5	CRA004775;Yang2025_Silkie_Wenchang	PRJNA827465	0.838112595
ENSGALG00010029699	ANKRD13B	2	0.065946532	0.085224708	0.773790933	0.439031478	0	0.435446315	0	0.5	CRA004775;PRJNA827465	Yang2025_Silkie_Wenchang	0.844022511
ENSGALG00010029700	GGA3	2	0.047618059	0.113624554	0.41908247	0.675155862	0.019955306	4.214377114	76.27170106	0.5	PRJNA827465;Yang2025_Silkie_Wenchang	CRA004775	0.916130363
ENSGALG00010029700	GGA3	2	-0.048615033	0.253920012	-0.19145806	0.848166736	0.105312513	5.094423692	80.3706943	0.5	CRA004775;Yang2025_Silkie_Wenchang	PRJNA827465	0.961363811
ENSGALG00010029700	GGA3	2	-0.126353058	0.123687653	-1.02154948	0.306994179	0.017365717	1.764065517	43.31276302	1	CRA004775;PRJNA827465	Yang2025_Silkie_Wenchang	0.79266003
ENSGALG00010029701	MRPS7	2	-0.098452127	0.045358353	-2.170540154	0.02996595	0	0.013332308	0	1	PRJNA827465;Yang2025_Silkie_Wenchang	CRA004775	0.295175875
ENSGALG00010029701	MRPS7	2	-0.113757494	0.076338971	-1.490162784	0.136181439	0	0.044235832	0	1	CRA004775;Yang2025_Silkie_Wenchang	PRJNA827465	0.61791031
ENSGALG00010029701	MRPS7	2	-0.099276959	0.051862014	-1.91425189	0.055587978	0	0.078688669	0	1	CRA004775;PRJNA827465	Yang2025_Silkie_Wenchang	0.408369031
ENSGALG00010029702	CORO6	2	-0.036713383	0.197816802	-0.185592846	0.852764064	0.039452378	1.961893106	49.02882339	0.5	PRJNA827465;Yang2025_Silkie_Wenchang	CRA004775	0.962680849
ENSGALG00010029702	CORO6	2	0.128554717	0.216932615	0.592602068	0.553447495	0	0.645302205	0	0.5	CRA004775;Yang2025_Silkie_Wenchang	PRJNA827465	0.879514268
ENSGALG00010029702	CORO6	2	-0.212170969	0.160227179	-1.324188388	0.185440486	0	0.032238342	0	1	CRA004775;PRJNA827465	Yang2025_Silkie_Wenchang	0.693865902
ENSGALG00010029703	MIF4GD	2	0.003712424	0.077909144	0.047650683	0.961994639	0.005242956	1.6983558	41.11952278	0.5	PRJNA827465;Yang2025_Silkie_Wenchang	CRA004775	0.989875256
ENSGALG00010029703	MIF4GD	2	-0.137049421	0.081301676	-1.685689981	0.091855528	0	0.511099141	0	1	CRA004775;Yang2025_Silkie_Wenchang	PRJNA827465	0.515097275
ENSGALG00010029703	MIF4GD	2	-0.049959977	0.138345621	-0.361124381	0.718006468	0.027888317	3.407044961	70.64905185	0.5	CRA004775;PRJNA827465	Yang2025_Silkie_Wenchang	0.927927766
ENSGALG00010029704	SLC25A19	2	-0.123208817	0.151984467	-0.810667165	0.417558634	0.033313897	3.139383105	68.14660822	1	PRJNA827465;Yang2025_Silkie_Wenchang	CRA004775	0.838112595
ENSGALG00010029704	SLC25A19	2	-0.315843428	0.141206927	-2.236738425	0.025302807	0	1.83485E-05	0	1	CRA004775;Yang2025_Silkie_Wenchang	PRJNA827465	0.279826855
ENSGALG00010029704	SLC25A19	2	-0.043732613	0.104409033	-0.418858524	0.675319531	0.008407683	1.209934751	17.35091506	1	CRA004775;PRJNA827465	Yang2025_Silkie_Wenchang	0.916318563
ENSGALG00010029705	GRB2	2	-0.214313479	0.223080123	-0.960701815	0.336702119	0.096973755	38.61297837	97.41019719	0.5	PRJNA827465;Yang2025_Silkie_Wenchang	CRA004775	0.803887433
ENSGALG00010029705	GRB2	2	-0.367640377	0.126352218	-2.909647199	0.00361837	0.017239047	1.683306724	40.59312032	1	CRA004775;Yang2025_Silkie_Wenchang	PRJNA827465	0.085650848
ENSGALG00010029705	GRB2	2	0.001910114	0.032524154	0.058729105	0.953167877	0	0.504667677	0	0.5	CRA004775;PRJNA827465	Yang2025_Silkie_Wenchang	0.987758913
ENSGALG00010029706	SSH2	2	0.021654297	0.187992258	0.115187175	0.908296764	0.05742752	5.148812901	80.57804742	0.5	PRJNA827465;Yang2025_Silkie_Wenchang	CRA004775	0.978236585
ENSGALG00010029706	SSH2	2	-0.12302078	0.428163287	-0.287322112	0.773865693	0.285920389	3.991548909	74.94706885	0.5	CRA004775;Yang2025_Silkie_Wenchang	PRJNA827465	0.943248868
ENSGALG00010029706	SSH2	2	-0.233154737	0.185289043	-1.258329869	0.208272483	0.033877296	1.380188365	27.54612158	1	CRA004775;PRJNA827465	Yang2025_Silkie_Wenchang	0.720917211
ENSGALG00010029707	NSRP1	2	0.381234877	0.505966873	0.75347794	0.451162763	0.478867284	14.75108334	93.22083689	0.5	PRJNA827465;Yang2025_Silkie_Wenchang	CRA004775	0.84125413
ENSGALG00010029707	NSRP1	2	0.481622348	0.439573708	1.09565777	0.27322857	0.318584968	5.692009517	82.43151216	1	CRA004775;Yang2025_Silkie_Wenchang	PRJNA827465	0.773904763
ENSGALG00010029707	NSRP1	2	-0.087107085	0.070810682	-1.230140456	0.218644513	0	0.234829885	0	0.5	CRA004775;PRJNA827465	Yang2025_Silkie_Wenchang	0.73229743
ENSGALG00010029708	TMEM94	2	-0.043595424	0.042216705	-1.032658101	0.301763909	0	0.899143587	0	1	PRJNA827465;Yang2025_Silkie_Wenchang	CRA004775	0.791367646
ENSGALG00010029708	TMEM94	2	-0.175926942	0.085060513	-2.068256294	0.038615929	0	0.9285003	0	1	CRA004775;Yang2025_Silkie_Wenchang	PRJNA827465	0.338965227
ENSGALG00010029708	TMEM94	2	-0.125396885	0.138657049	-0.904367183	0.365800733	0.027200818	2.778076726	64.00387396	1	CRA004775;PRJNA827465	Yang2025_Silkie_Wenchang	0.818968857
ENSGALG00010029709	SLC6A4	2	0.02946073	0.141934223	0.207566075	0.835567796	0	0.064971113	0	0.5	PRJNA827465;Yang2025_Silkie_Wenchang	CRA004775	0.958339923
ENSGALG00010029709	SLC6A4	2	0.299109606	0.301552719	0.99189822	0.321247177	0.097941037	1.999234954	49.98086652	1	CRA004775;Yang2025_Silkie_Wenchang	PRJNA827465	0.798513866
ENSGALG00010029709	SLC6A4	2	0.264784912	0.339711471	0.779440599	0.435720216	0.150839208	2.615597276	61.7678146	0.5	CRA004775;PRJNA827465	Yang2025_Silkie_Wenchang	0.842762754
ENSGALG00010029710	BLMH	2	0.020422115	0.050000381	0.408439198	0.68295126	0.00089876	1.137668836	12.10095874	0.5	PRJNA827465;Yang2025_Silkie_Wenchang	CRA004775	0.918800744
ENSGALG00010029710	BLMH	2	-0.040981833	0.088823699	-0.461383995	0.644523132	0	0.409559544	0	0.5	CRA004775;Yang2025_Silkie_Wenchang	PRJNA827465	0.906953008
ENSGALG00010029710	BLMH	2	0.046992817	0.046027711	1.020967935	0.307269631	0	0.001332139	0	1	CRA004775;PRJNA827465	Yang2025_Silkie_Wenchang	0.792675978
ENSGALG00010029711	CASKIN2	2	0.052696128	0.044394192	1.187004998	0.235225649	0	0.016227083	0	1	PRJNA827465;Yang2025_Silkie_Wenchang	CRA004775	0.748286012
ENSGALG00010029711	CASKIN2	2	0.062491073	0.09400467	0.664765623	0.506200413	0	0.002492025	0	1	CRA004775;Yang2025_Silkie_Wenchang	PRJNA827465	0.863929781
ENSGALG00010029711	CASKIN2	2	0.049685722	0.049519389	1.003358948	0.315687704	0	0.000530287	0	1	CRA004775;PRJNA827465	Yang2025_Silkie_Wenchang	0.796883862
ENSGALG00010029713	CPD	2	-0.097783364	0.096691803	-1.011289071	0.311878096	0.011582898	2.600228825	61.54184622	1	PRJNA827465;Yang2025_Silkie_Wenchang	CRA004775	0.795993742
ENSGALG00010029713	CPD	2	-0.187751437	0.086357308	-2.174123309	0.029695876	0	0.170117627	0	1	CRA004775;Yang2025_Silkie_Wenchang	PRJNA827465	0.293989171
ENSGALG00010029713	CPD	2	-0.018190195	0.071659205	-0.2538431	0.799616775	0	0.157259798	0	1	CRA004775;PRJNA827465	Yang2025_Silkie_Wenchang	0.949922217
ENSGALG00010029716	GOSR1	2	-0.02390739	0.03708223	-0.644712838	0.519113295	0	3.3295E-05	0	1	PRJNA827465;Yang2025_Silkie_Wenchang	CRA004775	0.868939943
ENSGALG00010029716	GOSR1	2	-0.026132707	0.083747926	-0.312040052	0.755010085	0	0.001528933	0	1	CRA004775;Yang2025_Silkie_Wenchang	PRJNA827465	0.93838723
ENSGALG00010029716	GOSR1	2	-0.024224627	0.039330168	-0.615929915	0.537940777	0	0.002146853	0	1	CRA004775;PRJNA827465	Yang2025_Silkie_Wenchang	0.87481821
ENSGALG00010029717	TRARG1	2	0.193214866	0.264769598	0.729743174	0.465544766	0	0.311212598	0	1	PRJNA827465;Yang2025_Silkie_Wenchang	CRA004775	0.853424459
ENSGALG00010029717	TRARG1	2	0.259728034	0.311446821	0.833940233	0.404314664	0	0.147126484	0	1	CRA004775;Yang2025_Silkie_Wenchang	PRJNA827465	0.833112102
ENSGALG00010029717	TRARG1	2	0.028747169	0.342131792	0.084023672	0.933037611	0	0.000184389	0	1	CRA004775;PRJNA827465	Yang2025_Silkie_Wenchang	0.984247176
ENSGALG00010029718	ABR	2	0.075998028	0.054300501	1.399582446	0.161638393	0	0.128478181	0	1	PRJNA827465;Yang2025_Silkie_Wenchang	CRA004775	0.658328889
ENSGALG00010029718	ABR	2	0.090473897	0.074301666	1.217656368	0.223354584	0	0.052745098	0	1	CRA004775;Yang2025_Silkie_Wenchang	PRJNA827465	0.736744216
ENSGALG00010029718	ABR	2	0.055813753	0.071286533	0.78294946	0.433656792	0	0.004604915	0	1	CRA004775;PRJNA827465	Yang2025_Silkie_Wenchang	0.842762754
ENSGALG00010029720	TIMM22	2	-0.04257334	0.092253656	-0.461481334	0.64445331	0	0.361273919	0	0.5	PRJNA827465;Yang2025_Silkie_Wenchang	CRA004775	0.906953008
ENSGALG00010029720	TIMM22	2	-0.086861334	0.169610825	-0.512121403	0.608566046	0.003996868	1.074603571	6.942427197	0.5	CRA004775;Yang2025_Silkie_Wenchang	PRJNA827465	0.897208691
ENSGALG00010029720	TIMM22	2	-0.096399257	0.091862329	-1.049388341	0.293999422	0	0.556108163	0	1	CRA004775;PRJNA827465	Yang2025_Silkie_Wenchang	0.787324026
ENSGALG00010029721	EXOC7	2	0.001809551	0.038428684	0.047088551	0.962442653	0	0.769741471	0	0.5	PRJNA827465;Yang2025_Silkie_Wenchang	CRA004775	0.98997945
ENSGALG00010029721	EXOC7	2	-0.06592458	0.163750345	-0.402592005	0.687248392	0.037763917	3.126457812	68.01492103	0.5	CRA004775;Yang2025_Silkie_Wenchang	PRJNA827465	0.919451777
ENSGALG00010029721	EXOC7	2	-0.086418473	0.112494969	-0.768198558	0.44236923	0.015934019	2.067453767	51.63132467	1	CRA004775;PRJNA827465	Yang2025_Silkie_Wenchang	0.845479108
ENSGALG00010029722	SPECC1	2	-0.076608361	0.070721242	-1.083243997	0.278700136	0	0.473009589	0	1	PRJNA827465;Yang2025_Silkie_Wenchang	CRA004775	0.776992195
ENSGALG00010029722	SPECC1	2	0.022801292	0.110038411	0.207212119	0.835844204	0	0.189069309	0	0.5	CRA004775;Yang2025_Silkie_Wenchang	PRJNA827465	0.958339923
ENSGALG00010029722	SPECC1	2	-0.081696561	0.079629266	-1.025961494	0.304909735	0	0.823414758	0	0.5	CRA004775;PRJNA827465	Yang2025_Silkie_Wenchang	0.79225794
ENSGALG00010029723	ADORA2B	2	-0.089760578	0.189661138	-0.473268162	0.636021874	0.046499808	2.786650084	64.11461899	0.5	PRJNA827465;Yang2025_Silkie_Wenchang	CRA004775	0.90443615
ENSGALG00010029723	ADORA2B	2	-0.005076852	0.35163931	-0.014437668	0.988480808	0.176494978	3.223282829	68.975729	0.5	CRA004775;Yang2025_Silkie_Wenchang	PRJNA827465	0.996891127
ENSGALG00010029723	ADORA2B	2	0.128391001	0.130027523	0.987414035	0.323439707	0	0.767707011	0	1	CRA004775;PRJNA827465	Yang2025_Silkie_Wenchang	0.799456433
ENSGALG00010029724	CACNA1G	2	0.221806312	0.095048873	2.333602752	0.019616532	0	0.246936336	0	1	PRJNA827465;Yang2025_Silkie_Wenchang	CRA004775	0.234771185
ENSGALG00010029724	CACNA1G	2	0.220919943	0.182905015	1.207837211	0.227108919	0	0.939844411	0	0.5	CRA004775;Yang2025_Silkie_Wenchang	PRJNA827465	0.746605003
ENSGALG00010029724	CACNA1G	2	0.175677357	0.103472694	1.697813705	0.089542928	0	0.599441776	0	0.5	CRA004775;PRJNA827465	Yang2025_Silkie_Wenchang	0.513242777
ENSGALG00010029725	TSEN54	2	0.197116534	0.133488062	1.476660393	0.139760694	0.02826096	4.648119051	78.4859211	1	PRJNA827465;Yang2025_Silkie_Wenchang	CRA004775	0.623597383
ENSGALG00010029725	TSEN54	2	-0.151385445	0.507623528	-0.298223545	0.765532316	0.492574617	22.36775503	95.52927865	0.5	CRA004775;Yang2025_Silkie_Wenchang	PRJNA827465	0.940888005
ENSGALG00010029725	TSEN54	2	-0.276558285	0.373012843	-0.741417594	0.45844028	0.259712061	14.38419184	93.04792364	0.5	CRA004775;PRJNA827465	Yang2025_Silkie_Wenchang	0.850809963
ENSGALG00010029726	SEPTIN9	2	-0.124300814	0.20792274	-0.597822126	0.549958625	0.082516713	21.8303228	95.4192157	0.5	PRJNA827465;Yang2025_Silkie_Wenchang	CRA004775	0.878652984
ENSGALG00010029726	SEPTIN9	2	-0.326989553	0.068436214	-4.778019309	1.7703E-06	0	0.160410787	0	1	CRA004775;Yang2025_Silkie_Wenchang	PRJNA827465	0.000353189
ENSGALG00010029726	SEPTIN9	2	0.016596877	0.126557791	0.131140694	0.89566401	0.017312322	1.545451728	35.29399967	0.5	CRA004775;PRJNA827465	Yang2025_Silkie_Wenchang	0.974974862
ENSGALG00010029727	ZSWIM7	2	-0.017948359	0.143626884	-0.124965179	0.900551118	0.013519249	1.384731497	27.78383374	0.5	PRJNA827465;Yang2025_Silkie_Wenchang	CRA004775	0.976497577
ENSGALG00010029727	ZSWIM7	2	-0.192346911	0.186626192	-1.030653358	0.302703404	0	0.093266887	0	1	CRA004775;Yang2025_Silkie_Wenchang	PRJNA827465	0.791852471
ENSGALG00010029727	ZSWIM7	2	0.049814274	0.117764516	0.422999014	0.67229598	0	0.326916172	0	0.5	CRA004775;PRJNA827465	Yang2025_Silkie_Wenchang	0.914742762
ENSGALG00010029728	TNRC6C	2	0.097944261	0.246394701	0.397509608	0.690991703	0.100857351	5.357423198	81.33431011	0.5	PRJNA827465;Yang2025_Silkie_Wenchang	CRA004775	0.920199347
ENSGALG00010029728	TNRC6C	2	0.342265454	0.169995182	2.013383261	0.044074325	0	0.135843308	0	1	CRA004775;Yang2025_Silkie_Wenchang	PRJNA827465	0.36277554
ENSGALG00010029728	TNRC6C	2	-0.062850188	0.126979322	-0.494963958	0.620625562	0.013606031	1.264407733	20.91158779	0.5	CRA004775;PRJNA827465	Yang2025_Silkie_Wenchang	0.899395593
ENSGALG00010029730	TTC19	2	0.181716724	0.324595577	0.559820656	0.575598799	0.199569953	18.61432659	94.62779384	0.5	PRJNA827465;Yang2025_Silkie_Wenchang	CRA004775	0.886714992
ENSGALG00010029730	TTC19	2	0.232790871	0.347984311	0.668969444	0.50351497	0.18378216	3.667632219	72.73445263	0.5	CRA004775;Yang2025_Silkie_Wenchang	PRJNA827465	0.863108263
ENSGALG00010029730	TTC19	2	-0.136216423	0.070918167	-1.920754985	0.054762604	0	0.030028673	0	1	CRA004775;PRJNA827465	Yang2025_Silkie_Wenchang	0.405183476
ENSGALG00010029731	NCOR1	2	0.096863085	0.246341178	0.393207037	0.694166673	0.100894291	5.264491602	81.00481346	0.5	PRJNA827465;Yang2025_Silkie_Wenchang	CRA004775	0.920728119
ENSGALG00010029731	NCOR1	2	0.380208056	0.188169017	2.020568732	0.043324636	0	0.00458011	0	1	CRA004775;Yang2025_Silkie_Wenchang	PRJNA827465	0.353992143
ENSGALG00010029731	NCOR1	2	-0.077951639	0.122613028	-0.635753311	0.524937223	0.012506818	1.129881019	11.49510582	0.5	CRA004775;PRJNA827465	Yang2025_Silkie_Wenchang	0.869907984
ENSGALG00010029732	PIGL	2	-0.185179633	0.083968009	-1.769479042	0.076813965	0	0.053726906	0	1	PRJNA827465;Yang2025_Silkie_Wenchang	CRA004775	0.477904868
ENSGALG00010029732	PIGL	2	-0.209905234	0.119090858	-1.762545766	0.077979489	0	0.551876675	0	1	CRA004775;Yang2025_Silkie_Wenchang	PRJNA827465	0.480799441
ENSGALG00010029732	PIGL	2	-0.165669079	0.103330956	-1.603286038	0.108871518	0	0.780921109	0	1	CRA004775;PRJNA827465	Yang2025_Silkie_Wenchang	0.558759109
ENSGALG00010029733	ENSGALG00010029733	2	0.003690117	0.110995539	0.033245631	0.97347871	0	0.073801049	0	0.5	PRJNA827465;Yang2025_Silkie_Wenchang	CRA004775	0.992392211
ENSGALG00010029733	ENSGALG00010029733	2	-0.013306445	0.263758882	-0.050450041	0.959763676	0	0.358627157	0	0.5	CRA004775;Yang2025_Silkie_Wenchang	PRJNA827465	0.989302378
ENSGALG00010029733	ENSGALG00010029733	2	-0.021715288	0.115912269	-0.187342445	0.851392146	0	0.260137839	0	1	CRA004775;PRJNA827465	Yang2025_Silkie_Wenchang	0.962139388
ENSGALG00010029734	SEC14L1	2	0.002129118	0.136928489	0.015549125	0.987594093	0.029982491	4.743523803	78.91862587	0.5	PRJNA827465;Yang2025_Silkie_Wenchang	CRA004775	0.996672503
ENSGALG00010029734	SEC14L1	2	-0.166056088	0.104538646	-1.58846603	0.112180997	0	0.15269675	0	1	CRA004775;Yang2025_Silkie_Wenchang	PRJNA827465	0.5658803
ENSGALG00010029734	SEC14L1	2	0.036112828	0.166888344	0.216389156	0.828684418	0.031938985	1.642389875	39.11311708	0.5	CRA004775;PRJNA827465	Yang2025_Silkie_Wenchang	0.956837266
ENSGALG00010029735	SRP68	2	0.151660047	0.190464294	0.796264981	0.425878036	0.069326513	22.22438449	95.50043782	0.5	PRJNA827465;Yang2025_Silkie_Wenchang	CRA004775	0.84125685
ENSGALG00010029735	SRP68	2	0.062050838	0.303491436	0.204456634	0.837996677	0.16812802	10.97781276	90.89071729	0.5	CRA004775;Yang2025_Silkie_Wenchang	PRJNA827465	0.959032758
ENSGALG00010029735	SRP68	2	-0.08822568	0.096599751	-0.91331167	0.361078647	0.010656603	1.703168324	41.28589722	1	CRA004775;PRJNA827465	Yang2025_Silkie_Wenchang	0.815240214
ENSGALG00010029736	ENSGALG00010029736	2	-0.546576537	0.417389779	-1.309511072	0.19036129	0.33394904	23.77525881	95.79394694	1	PRJNA827465;Yang2025_Silkie_Wenchang	CRA004775	0.699411386
ENSGALG00010029736	ENSGALG00010029736	2	-0.569257281	0.490287426	-1.161068489	0.245614048	0.381258222	4.243069633	76.43215675	0.5	CRA004775;Yang2025_Silkie_Wenchang	PRJNA827465	0.75661406
ENSGALG00010029736	ENSGALG00010029736	2	-0.133668211	0.080401114	-1.662516902	0.096409177	0	0.122469149	0	0.5	CRA004775;PRJNA827465	Yang2025_Silkie_Wenchang	0.530885356
ENSGALG00010029737	ENSGALG00010029737	2	0.177004968	0.599607305	0.295201487	0.767839966	0	0.007758082	0	1	PRJNA827465;Yang2025_Silkie_Wenchang	CRA004775	0.941444269
ENSGALG00010029737	ENSGALG00010029737	2	0.861276154	0.599641048	1.436319538	0.150911437	0.14285875	1.234691869	19.0081327	1	CRA004775;Yang2025_Silkie_Wenchang	PRJNA827465	0.642051813
ENSGALG00010029737	ENSGALG00010029737	2	0.887665943	0.550955651	1.611138647	0.107149507	0.061904032	1.109462437	9.866258936	1	CRA004775;PRJNA827465	Yang2025_Silkie_Wenchang	0.554473355
ENSGALG00010029738	TRPV2	2	-0.12598223	0.162806809	-0.773814256	0.439040641	0.045876838	7.412408113	86.50910764	0.5	PRJNA827465;Yang2025_Silkie_Wenchang	CRA004775	0.844022511
ENSGALG00010029738	TRPV2	2	-0.15704753	0.177214155	-0.88620195	0.375508713	0.041431536	2.582524203	61.27819446	0.5	CRA004775;Yang2025_Silkie_Wenchang	PRJNA827465	0.822974013
ENSGALG00010029738	TRPV2	2	0.038898182	0.073464684	0.529481373	0.596471562	0	0.035130314	0	1	CRA004775;PRJNA827465	Yang2025_Silkie_Wenchang	0.894064255
ENSGALG00010029739	P2RX5	2	-0.026649163	0.174773147	-0.152478592	0.878809474	0.048807422	4.954130249	79.81482219	0.5	PRJNA827465;Yang2025_Silkie_Wenchang	CRA004775	0.971120667
ENSGALG00010029739	P2RX5	2	0.180418346	0.111435708	1.619035316	0.105439665	0	0.390437037	0	1	CRA004775;Yang2025_Silkie_Wenchang	PRJNA827465	0.551018754
ENSGALG00010029739	P2RX5	2	0.005603886	0.267949457	0.020913965	0.983314287	0.102026469	2.907607224	65.60745922	0.5	CRA004775;PRJNA827465	Yang2025_Silkie_Wenchang	0.995363468
ENSGALG00010029740	HASPIN	2	0.042699429	0.087640663	0.487210252	0.626109341	0	0.026962374	0	1	PRJNA827465;Yang2025_Silkie_Wenchang	CRA004775	0.901504244
ENSGALG00010029740	HASPIN	2	-0.08946965	0.245941718	-0.36378395	0.716019349	0.039041414	1.206210898	17.09575814	0.5	CRA004775;Yang2025_Silkie_Wenchang	PRJNA827465	0.927269711
ENSGALG00010029740	HASPIN	2	-0.067598005	0.264492933	-0.255575844	0.798278373	0.073906828	1.4152409	29.34065148	0.5	CRA004775;PRJNA827465	Yang2025_Silkie_Wenchang	0.949922217
ENSGALG00010029741	EMC6	2	-0.385925081	0.29965679	-1.287890327	0.197784143	0.165764966	12.40682586	91.93992073	1	PRJNA827465;Yang2025_Silkie_Wenchang	CRA004775	0.709650373
ENSGALG00010029741	EMC6	2	-0.46069888	0.316431889	-1.455917992	0.145415291	0.133974703	2.674892807	62.61532435	1	CRA004775;Yang2025_Silkie_Wenchang	PRJNA827465	0.633907279
ENSGALG00010029741	EMC6	2	-0.103240104	0.056148329	-1.838703041	0.065958876	0	0.020966904	0	1	CRA004775;PRJNA827465	Yang2025_Silkie_Wenchang	0.442385956
ENSGALG00010029742	TAX1BP3	2	-0.090031686	0.060154891	-1.496664439	0.134480594	0.003491301	1.859993066	46.23635872	1	PRJNA827465;Yang2025_Silkie_Wenchang	CRA004775	0.614306227
ENSGALG00010029742	TAX1BP3	2	0.021920033	0.21258924	0.103109797	0.91787583	0.074162272	5.152494186	80.59192376	0.5	CRA004775;Yang2025_Silkie_Wenchang	PRJNA827465	0.981033557
ENSGALG00010029742	TAX1BP3	2	0.066863378	0.146266922	0.457132596	0.647575742	0.030532223	2.870794183	65.16643352	0.5	CRA004775;PRJNA827465	Yang2025_Silkie_Wenchang	0.907350807
ENSGALG00010029743	MYOCD	2	-0.280505883	0.115711627	-2.42418061	0.015342973	0	0.352540381	0	1	PRJNA827465;Yang2025_Silkie_Wenchang	CRA004775	0.20497106
ENSGALG00010029743	MYOCD	2	0.190805968	0.520233251	0.366770036	0.71379056	0.411129935	3.515281708	71.55277776	0.5	CRA004775;Yang2025_Silkie_Wenchang	PRJNA827465	0.926336813
ENSGALG00010029743	MYOCD	2	0.138097078	0.594219244	0.232400885	0.816226662	0.566467574	4.428718848	77.42010649	0.5	CRA004775;PRJNA827465	Yang2025_Silkie_Wenchang	0.95448249
ENSGALG00010029744	CTNS	2	-0.208564643	0.238860109	-0.873166487	0.382572339	0.101048996	8.454846325	88.17246392	0.5	PRJNA827465;Yang2025_Silkie_Wenchang	CRA004775	0.825121999
ENSGALG00010029744	CTNS	2	-0.152748851	0.328790444	-0.464578134	0.642233594	0.183692653	6.534826444	84.69737477	0.5	CRA004775;Yang2025_Silkie_Wenchang	PRJNA827465	0.906634523
ENSGALG00010029744	CTNS	2	0.036173348	0.072890267	0.496271306	0.619703008	0	0.630456761	0	1	CRA004775;PRJNA827465	Yang2025_Silkie_Wenchang	0.899325257
ENSGALG00010029745	SHPK	2	0.097377803	0.230308169	0.422815238	0.672430069	0.069197165	2.768407159	63.87814572	0.5	PRJNA827465;Yang2025_Silkie_Wenchang	CRA004775	0.914742762
ENSGALG00010029745	SHPK	2	0.065617816	0.316378093	0.207403161	0.835695014	0.134169394	3.010322783	66.78097094	0.5	CRA004775;Yang2025_Silkie_Wenchang	PRJNA827465	0.958339923
ENSGALG00010029745	SHPK	2	-0.140621142	0.134626587	-1.044527271	0.296241509	0	0.277301965	0	1	CRA004775;PRJNA827465	Yang2025_Silkie_Wenchang	0.788267543
ENSGALG00010029747	TRPV3	2	0.62209828	0.356745282	1.74381642	0.081191106	0.202925222	4.93122684	79.72107079	1	PRJNA827465;Yang2025_Silkie_Wenchang	CRA004775	0.490780384
ENSGALG00010029747	TRPV3	2	0.964758546	0.211230013	4.567336492	4.93961E-06	0	0.030963037	0	1	CRA004775;Yang2025_Silkie_Wenchang	PRJNA827465	0.000805566
ENSGALG00010029747	TRPV3	2	0.388435033	0.243760452	1.593511289	0.111045538	0.023981632	1.146649686	12.78940617	1	CRA004775;PRJNA827465	Yang2025_Silkie_Wenchang	0.56392513
ENSGALG00010029749	ASPA	2	0.457622594	0.307533323	1.488042299	0.136739739	0.107333746	2.309798683	56.70618364	1	PRJNA827465;Yang2025_Silkie_Wenchang	CRA004775	0.618582531
ENSGALG00010029749	ASPA	2	0.65992505	0.259769322	2.54042719	0.011071714	0	0.662765333	0	1	CRA004775;Yang2025_Silkie_Wenchang	PRJNA827465	0.167365996
ENSGALG00010029749	ASPA	2	0.179720945	0.247805013	0.725251451	0.468297784	0	0.030298483	0	1	CRA004775;PRJNA827465	Yang2025_Silkie_Wenchang	0.853198008

ENSGALG00010029750	TEN1	2	0.135505809	0.164184052	0.825328692	0.409185007	0	0.073327212	0	1	PRJNA827465;Yang2025_Silkie_Wenchang	CRA004775	0.834316328
ENSGALG00010029750	TEN1	2	-0.424654974	0.745941067	-0.569287566	0.569161004	0.867219536	3.998136282	74.98834633	0.5	CRA004775;Yang2025_Silkie_Wenchang	PRJNA827465	0.884646991
ENSGALG00010029750	TEN1	2	-0.453991314	0.696413177	-0.651899374	0.514466089	0.743363074	3.654528932	72.63669221	0.5	CRA004775;PRJNA827465	Yang2025_Silkie_Wenchang	0.866869697
ENSGALG00010029752	ENSGALG00010029752	2	0.011135517	0.087134852	0.127796364	0.898310127	0	0.978367919	0	0.5	PRJNA827465;Yang2025_Silkie_Wenchang	CRA004775	0.97575249
ENSGALG00010029752	ENSGALG00010029752	2	0.303549023	0.294397963	1.031083978	0.30250144	0.102473709	1.936356134	48.35660743	1	CRA004775;Yang2025_Silkie_Wenchang	PRJNA827465	0.791852471
ENSGALG00010029752	ENSGALG00010029752	2	0.241235229	0.397718401	0.60654782	0.544151038	0.235741499	3.218935544	68.93382963	0.5	CRA004775;PRJNA827465	Yang2025_Silkie_Wenchang	0.877010098
ENSGALG00010029753	AKAP10	2	0.010163379	0.039725006	0.255843352	0.798071797	0	0.031352049	0	0.5	PRJNA827465;Yang2025_Silkie_Wenchang	CRA004775	0.949922217
ENSGALG00010029753	AKAP10	2	-0.003952602	0.073075792	-0.054089079	0.956864193	0	0.008749533	0	1	CRA004775;Yang2025_Silkie_Wenchang	PRJNA827465	0.988540618
ENSGALG00010029753	AKAP10	2	0.012805477	0.045336043	0.282456877	0.777593208	0	0.027860812	0	0.5	CRA004775;PRJNA827465	Yang2025_Silkie_Wenchang	0.944388838
ENSGALG00010029754	NLE1	2	-0.171808487	0.115251286	-1.490729462	0.136032538	0	0.233137434	0	1	PRJNA827465;Yang2025_Silkie_Wenchang	CRA004775	0.617591179
ENSGALG00010029754	NLE1	2	-0.367851447	0.377715072	-0.973886071	0.33011311	0.176452313	2.342049355	57.30235156	1	CRA004775;Yang2025_Silkie_Wenchang	PRJNA827465	0.801237299
ENSGALG00010029754	NLE1	2	-0.387543399	0.2947236	-1.314938466	0.188530584	0.101393679	1.869409348	46.50716811	1	CRA004775;PRJNA827465	Yang2025_Silkie_Wenchang	0.697920084
ENSGALG00010029755	ACOX1	2	-0.080209925	0.17646597	-0.454534801	0.64944395	0.05115896	5.599727402	82.14198785	0.5	PRJNA827465;Yang2025_Silkie_Wenchang	CRA004775	0.908043243
ENSGALG00010029755	ACOX1	2	-0.203571052	0.102107952	-1.993684605	0.046186533	0.00226556	1.07749661	7.192283402	1	CRA004775;Yang2025_Silkie_Wenchang	PRJNA827465	0.370491759
ENSGALG00010029755	ACOX1	2	0.077221671	0.095634207	0.807469138	0.419396248	0	0.176500421	0	0.5	CRA004775;PRJNA827465	Yang2025_Silkie_Wenchang	0.838527387
ENSGALG00010029756	ELAC2	2	-0.01158131	0.051873699	-0.223259764	0.823333341	0	0.870267107	0	0.5	PRJNA827465;Yang2025_Silkie_Wenchang	CRA004775	0.955918024
ENSGALG00010029756	ELAC2	2	-0.226307438	0.177912362	-1.272016375	0.203367301	0.047030351	3.610734466	72.30480365	1	CRA004775;Yang2025_Silkie_Wenchang	PRJNA827465	0.717242017
ENSGALG00010029756	ELAC2	2	-0.179058977	0.22908371	-0.781631211	0.434431339	0.089917243	6.46859707	84.54069732	0.5	CRA004775;PRJNA827465	Yang2025_Silkie_Wenchang	0.842762754
ENSGALG00010029757	ULK2	2	0.006254928	0.148147945	0.042220825	0.966322661	0.036682663	5.633884363	82.25025692	0.5	PRJNA827465;Yang2025_Silkie_Wenchang	CRA004775	0.990881287
ENSGALG00010029757	ULK2	2	0.02578217	0.297982424	0.086522453	0.931051108	0.110474008	2.023660056	50.58458574	0.5	CRA004775;Yang2025_Silkie_Wenchang	PRJNA827465	0.983943003
ENSGALG00010029757	ULK2	2	0.140933158	0.047851212	2.945236978	0.003227074	0	0.64121238	0	1	CRA004775;PRJNA827465	Yang2025_Silkie_Wenchang	0.078974898
ENSGALG00010029758	DNAH9	2	-0.227126741	0.210881881	-1.077032981	0.281465532	0	0.330107381	0	1	PRJNA827465;Yang2025_Silkie_Wenchang	CRA004775	0.779251452
ENSGALG00010029758	DNAH9	2	-0.304928221	0.325877146	-0.93571527	0.349419807	0.048788332	1.246470516	19.77347343	1	CRA004775;Yang2025_Silkie_Wenchang	PRJNA827465	0.809903518
ENSGALG00010029758	DNAH9	2	-0.425776629	0.246248086	-1.72905559	0.083799146	0	0.569545561	0	1	CRA004775;PRJNA827465	Yang2025_Silkie_Wenchang	0.498721661
ENSGALG00010029759	ALDH3A2	2	-0.112720107	0.074020792	-1.52281681	0.127804543	0	0.624033014	0	1	PRJNA827465;Yang2025_Silkie_Wenchang	CRA004775	0.600058027
ENSGALG00010029759	ALDH3A2	2	-0.267071213	0.149297006	-1.788858469	0.073637612	0	0.377346207	0	1	CRA004775;Yang2025_Silkie_Wenchang	PRJNA827465	0.468996768
ENSGALG00010029759	ALDH3A2	2	-0.111663573	0.102747207	-1.08677965	0.277134203	0.004478873	1.060791659	5.73078218	1	CRA004775;PRJNA827465	Yang2025_Silkie_Wenchang	0.776188179
ENSGALG00010029761	MRPL38	2	-0.037522451	0.068674784	-0.546378871	0.584805531	0	0.697337401	0	1	PRJNA827465;Yang2025_Silkie_Wenchang	CRA004775	0.891016154
ENSGALG00010029761	MRPL38	2	-0.212781497	0.125622253	-1.693820101	0.090299473	0	0.430615517	0	1	CRA004775;Yang2025_Silkie_Wenchang	PRJNA827465	0.515967344
ENSGALG00010029761	MRPL38	2	-0.104797185	0.145861916	-0.718468451	0.472468494	0.024554582	1.943293898	48.54097979	1	CRA004775;PRJNA827465	Yang2025_Silkie_Wenchang	0.853998373
ENSGALG00010029762	SLC47A2	2	0.34564431	0.224302056	1.540977006	0.123322382	0	0.495727256	0	1	PRJNA827465;Yang2025_Silkie_Wenchang	CRA004775	0.588792468
ENSGALG00010029762	SLC47A2	2	0.530681116	0.358182618	1.481593718	0.138448432	0	0.058075853	0	1	CRA004775;Yang2025_Silkie_Wenchang	PRJNA827465	0.622313717
ENSGALG00010029762	SLC47A2	2	0.231239913	0.270923272	0.85352547	0.393367969	0	0.001240469	0	1	CRA004775;PRJNA827465	Yang2025_Silkie_Wenchang	0.830346712
ENSGALG00010029763	GEMIN4	2	-0.067815918	0.165430826	-0.409935195	0.681853487	0.037535439	2.528951925	60.45792765	0.5	PRJNA827465;Yang2025_Silkie_Wenchang	CRA004775	0.918430638
ENSGALG00010029763	GEMIN4	2	-0.322748011	0.165249734	-1.953092464	0.05080864	0	0.017646033	0	1	CRA004775;Yang2025_Silkie_Wenchang	PRJNA827465	0.390049394
ENSGALG00010029763	GEMIN4	2	-0.069957498	0.181343685	-0.385773003	0.699664791	0.043553183	2.234227878	55.24180815	0.5	CRA004775;PRJNA827465	Yang2025_Silkie_Wenchang	0.922101981
ENSGALG00010029764	NXN	2	-0.124145116	0.065657091	-1.890810476	0.058649648	0	0.589139096	0	1	PRJNA827465;Yang2025_Silkie_Wenchang	CRA004775	0.419491742
ENSGALG00010029764	NXN	2	-0.256638644	0.158756567	-1.616554508	0.105974478	0	0.000826846	0	1	CRA004775;Yang2025_Silkie_Wenchang	PRJNA827465	0.552329257
ENSGALG00010029764	NXN	2	-0.11170686	0.068842276	-1.622649144	0.104664429	0	0.229573173	0	1	CRA004775;PRJNA827465	Yang2025_Silkie_Wenchang	0.549804368
ENSGALG00010029765	KCNJ16	2	0.04838683	0.294463667	0.164321904	0.869477739	0.075292977	1.759340281	43.16051243	0.5	PRJNA827465;Yang2025_Silkie_Wenchang	CRA004775	0.968578601
ENSGALG00010029765	KCNJ16	2	0.927413249	0.669943276	1.384316079	0.166261693	0.650959254	3.364810965	70.28064844	1	CRA004775;Yang2025_Silkie_Wenchang	PRJNA827465	0.66529196
ENSGALG00010029765	KCNJ16	2	0.661382293	0.971681843	0.680657252	0.496088391	1.642758163	7.273478295	86.25141976	0.5	CRA004775;PRJNA827465	Yang2025_Silkie_Wenchang	0.860911322
ENSGALG00010029766	ENSGALG00010029766	2	0.107180779	0.080873501	1.325289229	0.18507524	0	0.031898092	0	1	PRJNA827465;Yang2025_Silkie_Wenchang	CRA004775	0.693279213
ENSGALG00010029766	ENSGALG00010029766	2	0.010835809	0.178882736	0.060574926	0.951697743	0	0.263608582	0	0.5	CRA004775;Yang2025_Silkie_Wenchang	PRJNA827465	0.987713746
ENSGALG00010029766	ENSGALG00010029766	2	0.096740881	0.084302752	1.147541197	0.251158017	0	0.515519103	0	0.5	CRA004775;PRJNA827465	Yang2025_Silkie_Wenchang	0.761142998
ENSGALG00010029767	GLOD4	2	-0.03387139	0.189113451	-0.179106192	0.85785432	0.063140943	8.421802326	88.12605709	0.5	PRJNA827465;Yang2025_Silkie_Wenchang	CRA004775	0.964092655
ENSGALG00010029767	GLOD4	2	-0.099108479	0.296433515	-0.334336279	0.738125817	0.145214324	5.376760396	81.40144015	0.5	CRA004775;Yang2025_Silkie_Wenchang	PRJNA827465	0.93431752
ENSGALG00010029767	GLOD4	2	-0.235517329	0.071097946	-3.312575737	0.000924411	0	0.7913789	0	1	CRA004775;PRJNA827465	Yang2025_Silkie_Wenchang	0.035865867
ENSGALG00010029768	KCNJ2	2	-0.037466633	0.065416978	-0.572735623	0.566823714	0	0.38731621	0	0.5	PRJNA827465;Yang2025_Silkie_Wenchang	CRA004775	0.883487517
ENSGALG00010029768	KCNJ2	2	0.376522471	0.423147011	0.889814797	0.37356534	0.294559056	4.994135264	79.97651351	1	CRA004775;Yang2025_Silkie_Wenchang	PRJNA827465	0.822442223
ENSGALG00010029768	KCNJ2	2	0.337000289	0.467059869	0.721535529	0.470580089	0.374777921	6.294975214	84.11431394	0.5	CRA004775;PRJNA827465	Yang2025_Silkie_Wenchang	0.853744788
ENSGALG00010029770	TLCD3A	2	-0.078250218	0.146333353	-0.534739463	0.592830029	0.013781413	1.413138596	29.23553268	0.5	PRJNA827465;Yang2025_Silkie_Wenchang	CRA004775	0.892776493
ENSGALG00010029770	TLCD3A	2	-0.165737456	0.172539145	-0.960578864	0.33676396	0	0.708842227	0	0.5	CRA004775;Yang2025_Silkie_Wenchang	PRJNA827465	0.803887433
ENSGALG00010029770	TLCD3A	2	0.027490897	0.124087112	0.221545144	0.82466799	0	0.000490952	0	1	CRA004775;PRJNA827465	Yang2025_Silkie_Wenchang	0.95619211
ENSGALG00010029771	SOX9	2	0.380181663	0.336657942	1.129281729	0.258779006	0.145148463	2.731472539	63.38971065	1	PRJNA827465;Yang2025_Silkie_Wenchang	CRA004775	0.765872837
ENSGALG00010029771	SOX9	2	0.765175982	0.250244288	3.057716068	0.002230308	0	0.003660713	0	1	CRA004775;Yang2025_Silkie_Wenchang	PRJNA827465	0.062434324
ENSGALG00010029771	SOX9	2	0.365477048	0.34789762	1.050530465	0.293474292	0.144105392	2.357554703	57.58316874	1	CRA004775;PRJNA827465	Yang2025_Silkie_Wenchang	0.786979185
ENSGALG00010029772	VPS53	2	0.027878433	0.037581776	0.741807228	0.45820414	0	0.011040666	0	1	PRJNA827465;Yang2025_Silkie_Wenchang	CRA004775	0.850026426
ENSGALG00010029772	VPS53	2	-0.147276117	0.217049639	-0.678536569	0.497431546	0.075455211	4.440071834	77.47784186	0.5	CRA004775;Yang2025_Silkie_Wenchang	PRJNA827465	0.86098656
ENSGALG00010029772	VPS53	2	-0.148086442	0.212090185	-0.698223928	0.485037164	0.072905438	4.506136236	77.80803891	0.5	CRA004775;PRJNA827465	Yang2025_Silkie_Wenchang	0.857004733
ENSGALG00010029773	TRIM65	2	0.419234845	0.380960542	1.100467894	0.271128311	0.240667154	5.775389875	82.68515162	1	PRJNA827465;Yang2025_Silkie_Wenchang	CRA004775	0.772743863
ENSGALG00010029773	TRIM65	2	0.255941778	0.557829629	0.458817109	0.646365507	0.564505684	10.7431968	90.69178366	0.5	CRA004775;Yang2025_Silkie_Wenchang	PRJNA827465	0.907220702
ENSGALG00010029773	TRIM65	2	-0.097228027	0.175085755	-0.555316604	0.578678124	0.019570419	1.458822838	31.4515804	0.5	CRA004775;PRJNA827465	Yang2025_Silkie_Wenchang	0.88788081
ENSGALG00010029774	RFLNB	2	-0.03814266	0.051281914	-0.743783868	0.457007241	0	0.014650799	0	1	PRJNA827465;Yang2025_Silkie_Wenchang	CRA004775	0.849541335
ENSGALG00010029774	RFLNB	2	-0.03309904	0.088931197	-0.372187048	0.709753589	0	0.024237262	0	1	CRA004775;Yang2025_Silkie_Wenchang	PRJNA827465	0.925298042
ENSGALG00010029774	RFLNB	2	-0.043667624	0.06009396	-0.726655791	0.467436842	0	0.013203579	0	1	CRA004775;PRJNA827465	Yang2025_Silkie_Wenchang	0.852885838
ENSGALG00010029775	ENSGALG00010029775	2	0.016070929	0.112756516	0.142527716	0.886663189	0	0.194446435	0	0.5	PRJNA827465;Yang2025_Silkie_Wenchang	CRA004775	0.972824613
ENSGALG00010029775	ENSGALG00010029775	2	-0.100387312	0.233629576	-0.429685804	0.667424211	0.054025317	1.852772582	46.02683515	0.5	CRA004775;Yang2025_Silkie_Wenchang	PRJNA827465	0.913903556
ENSGALG00010029775	ENSGALG00010029775	2	-0.119421223	0.165588299	-0.721193611	0.470790402	0.01556272	1.269799573	21.24741406	1	CRA004775;PRJNA827465	Yang2025_Silkie_Wenchang	0.853762555
ENSGALG00010029776	WBP2	2	-0.051137628	0.066487917	-0.769126649	0.44181813	0	0.754873208	0	1	PRJNA827465;Yang2025_Silkie_Wenchang	CRA004775	0.845135451
ENSGALG00010029776	WBP2	2	-0.07426287	0.1376772	-0.539398462	0.589611942	0.00710289	1.159204885	13.7339729	0.5	CRA004775;Yang2025_Silkie_Wenchang	PRJNA827465	0.892320726
ENSGALG00010029776	WBP2	2	-0.003774357	0.073653185	-0.051244991	0.959130301	0	0.456212735	0	0.5	CRA004775;PRJNA827465	Yang2025_Silkie_Wenchang	0.989155754
ENSGALG00010029777	ENSGALG00010029777	2	0.085900102	0.115563451	0.743315478	0.457290703	0.009953536	1.588119471	37.0324451	0.5	PRJNA827465;Yang2025_Silkie_Wenchang	CRA004775	0.849541335
ENSGALG00010029777	ENSGALG00010029777	2	-0.074632791	0.122525637	-0.609119792	0.54244504	0	0.311105484	0	1	CRA004775;Yang2025_Silkie_Wenchang	PRJNA827465	0.876817086
ENSGALG00010029777	ENSGALG00010029777	2	0.063330936	0.186779289	0.339068302	0.734558277	0.036813451	1.859753114	46.22942194	0.5	CRA004775;PRJNA827465	Yang2025_Silkie_Wenchang	0.933520234
ENSGALG00010029778	DOC2B	2	-0.293182289	0.23657167	-1.23929585	0.215235955	0.052140626	1.844187703	45.77558464	1	PRJNA827465;Yang2025_Silkie_Wenchang	CRA004775	0.728664095
ENSGALG00010029778	DOC2B	2	-0.518846831	0.256198959	-2.025171503	0.042849757	0	0.1772487	0	1	CRA004775;Yang2025_Silkie_Wenchang	PRJNA827465	0.357943462
ENSGALG00010029778	DOC2B	2	-0.10191891	0.205910073	-0.494968063	0.620622665	0	0.058346955	0	1	CRA004775;PRJNA827465	Yang2025_Silkie_Wenchang	0.899395593
ENSGALG00010029780	TEX14	2	0.387149697	0.228914195	1.691243732	0.090790262	0.050936273	1.910558203	47.65927578	1	PRJNA827465;Yang2025_Silkie_Wenchang	CRA004775	0.516999371
ENSGALG00010029780	TEX14	2	0.081652224	0.640785307	0.127425245	0.898603837	0.663378919	4.913032795	79.64597344	0.5	CRA004775;Yang2025_Silkie_Wenchang	PRJNA827465	0.975792303
ENSGALG00010029780	TEX14	2	-0.088860747	0.390805282	-0.227378572	0.820129381	0.188420069	2.218381354	54.92208776	0.5	CRA004775;PRJNA827465	Yang2025_Silkie_Wenchang	0.955308792
ENSGALG00010029781	UNC13D	2	0.201834447	0.16976055	1.188936102	0.234464812	0	0.106546436	0	1	PRJNA827465;Yang2025_Silkie_Wenchang	CRA004775	0.747509756
ENSGALG00010029781	UNC13D	2	-0.385006798	0.736432664	-0.522790731	0.601113627	0.898617546	5.539177086	81.9467765	0.5	CRA004775;Yang2025_Silkie_Wenchang	PRJNA827465	0.893230519
ENSGALG00010029781	UNC13D	2	-0.422172305	0.67504241	-0.625401157	0.531707803	0.752569651	5.224697387	80.86013551	0.5	CRA004775;PRJNA827465	Yang2025_Silkie_Wenchang	0.873042777
ENSGALG00010029782	UNK	2	0.180648358	0.117362087	1.539239478	0.123745843	0.018577814	2.541418523	60.65189614	1	PRJNA827465;Yang2025_Silkie_Wenchang	CRA004775	0.590002486
ENSGALG00010029782	UNK	2	0.089880929	0.277961625	0.32335733	0.746424641	0.1198979	4.338754392	76.9519104	0.5	CRA004775;Yang2025_Silkie_Wenchang	PRJNA827465	0.936647222
ENSGALG00010029782	UNK	2	0.050996841	0.135057783	0.13015754	0.8964432	0.020079015	1.842991067	45.73572745	0.5	CRA004775;PRJNA827465	Yang2025_Silkie_Wenchang	0.975157773
ENSGALG00010029783	RAD51C	2	-0.122188785	0.168781243	-0.723947654	0.469097873	0.042381107	3.82205404	73.83605806	0.5	PRJNA827465;Yang2025_Silkie_Wenchang	CRA004775	0.85345437
ENSGALG00010029783	RAD51C	2	-0.307721508	0.397424816	-0.774288609	0.438760138	0.274532102	7.378718381	86.44751096	0.5	CRA004775;Yang2025_Silkie_Wenchang	PRJNA827465	0.844002503
ENSGALG00010029783	RAD51C	2	-0.44493275	0.220753473	-2.015518717	0.043850327	0.066844547	2.754388536	63.6943014	1	CRA004775;PRJNA827465	Yang2025_Silkie_Wenchang	0.361804689
ENSGALG00010029785	ENSGALG00010029785	2	-1.085321826	1.553130268	-0.698863777	0.484639942	3.888509961	5.021342696	80.08500793	0.5	PRJNA827465;Yang2025_Silkie_Wenchang	CRA004775	0.857004733
ENSGALG00010029785	ENSGALG00010029785	2	-1.376153595	1.15567157	-1.190778958	0.233738942	1.917957026	3.265129759	69.37334581	1	CRA004775;Yang2025_Silkie_Wenchang	PRJNA827465	0.747082975
ENSGALG00010029785	ENSGALG00010029785	2	-0.134580457	0.462012367	-0.291293894	0.770828086	0	0.637900822	0	0.5	CRA004775;PRJNA827465	Yang2025_Silkie_Wenchang	0.942491331
ENSGALG00010029787	H3-3B	2	0.006253714	0.044271569	0.141258185	0.887669592	0	0.095454831	0	1	PRJNA827465;Yang2025_Silkie_Wenchang	CRA004775	0.977055616
ENSGALG00010029787	H3-3B	2	-0.011722937	0.116008637	-0.101052279	0.91950896	0	0.699550633	0	0.5	CRA004775;Yang2025_Silkie_Wenchang	PRJNA827465	0.981192162
ENSGALG00010029787	H3-3B	2	-0.005695348	0.045915951	-0.124038551	0.901284751	0	0.58100106	0	0.5	CRA004775;PRJNA827465	Yang2025_Silkie_Wenchang	0.976619209
ENSGALG00010029791	GALK1	2	0.348802263	0.162596532	2.145201118	0.03193677	0.035720342	2.847474792	64.88116408	1	PRJNA827465;Yang2025_Silkie_Wenchang	CRA004775	0.306373171
ENSGALG00010029791	GALK1	2	0.160395704	0.387386383	0.414045798	0.678840567	0.269806451	9.895703086	89.89060184	0.5	CRA004775;Yang2025_Silkie_Wenchang	PRJNA827465	0.917701868
ENSGALG00010029791	GALK1	2	0.019252405	0.220076692	0.087480436	0.930289636	0.078724671	5.063164635	80.24950654	0.5	CRA004775;PRJNA827465	Yang2025_Silkie_Wenchang	0.983813577
ENSGALG00010029804	ITGB4	2	0.029254979	0.117089055	0.249852381	0.80270151	0	0.629888638	0	0.5	PRJNA827465;Yang2025_Silkie_Wenchang	CRA004775	0.950704475
ENSGALG00010029804	ITGB4	2	-0.046994447	0.168404953	-0.279056205	0.780201692	0	0.245660967	0	0.5	CRA004775;Yang2025_Silkie_Wenchang	PRJNA827465	0.945041817
ENSGALG00010029804	ITGB4	2	0.11199909	0.144130401	0.777067777	0.437118784	0	0.009878463	0	1	CRA004775;PRJNA827465	Yang2025_Silkie_Wenchang	0.843453774
ENSGALG00010029812	SAP30BP	2	0.355533478	0.3502914	1.014964909	0.310122555	0.234490875	21.87347228	95.42825214	1	PRJNA827465;Yang2025_Silkie_Wenchang	CRA004775	0.794676411
ENSGALG00010029812	SAP30BP	2	0.204347412	0.527790313	0.387175375	0.698626379	0.52299807	16.15870111	93.81138377	0.5	CRA004775;Yang2025_Silkie_Wenchang	PRJNA827465	0.921745106
ENSGALG00010029812	SAP30BP	2	-0.09429025	0.164895033	-0.571819828	0.567440043	0.037296809	2.452745811	59.22771666	0.5	CRA004775;PRJNA827465	Yang2025_Silkie_Wenchang	0.883717772
ENSGALG00010029835	RECQL5	2	-0.162754471	0.07613815	-2.137620511	0.032547556	0	0.610221885	0	1	PRJNA827465;Yang2025_Silkie_Wenchang	CRA004775	0.310037591
ENSGALG00010029835	RECQL5	2	-0.233872162	0.104294383	-2.242423372	0.024934023	0	0.05757875	0	1	CRA004775;Yang2025_Silkie_Wenchang	PRJNA827465	0.26907795
ENSGALG00010029835	RECQL5	2	-0.125773196	0.099845274	-1.259681016	0.207784456	0	0.355743274	0	1	CRA004775;PRJNA827465	Yang2025_Silkie_Wenchang	0.720154541
ENSGALG00010029841	SMIM5	2	-0.028068674	0.134920049	-0.208039312	0.835198272	0.026147266	3.516263138	71.5607177	0.5	PRJNA827465;Yang2025_Silkie_Wenchang	CRA004775	0.958305532
ENSGALG00010029841	SMIM5	2	-0.002529645	0.257534852	-0.009822536	0.992162876	0.082238743	2.089116764	52.13288136	0.5	CRA004775;Yang2025_Silkie_Wenchang	PRJNA827465	0.997744161
ENSGALG00010029841	SMIM5	2	0.1152679	0.087972094	1.310278	0.190101807	0	0.578951667	0	1	CRA004775;PRJNA827465	Yang2025_Silkie_Wenchang	0.669403589
ENSGALG00010029859	CSNK1D	2	0.016397252	0.041721766	0.393014341	0.694308889	0	0.424023758	0	0.5	PRJNA827465;Yang2025_Silkie_Wenchang	CRA004775	0.920728119
ENSGALG00010029859	CSNK1D	2	-0.013900795	0.082354597	-0.168791978	0.865960268	0	0.314487143	0	0.5	CRA004775;Yang2025_Silkie_Wenchang	PRJNA827465	0.967228499
ENSGALG00010029859	CSNK1D	2	0.033106121	0.045333587	0.730278004	0.46522027	0	0.063665674	0	1	CRA004775;PRJNA827465	Yang2025_Silkie_Wenchang	0.852386942
ENSGALG00010029864	SLC16A3	2	0.076461032	0.126532504	0.604279767	0.545657656	0	0.123824698	0	1	PRJNA827465;Yang2025_Silkie_Wenchang	CRA004775	0.877818462
ENSGALG00010029864	SLC16A3	2	0.101281834	0.186692542	0.542506051	0.58746994	0	0.19181678	0	0.5	CRA004775;Yang2025_Silkie_Wenchang	PRJNA827465	0.89167336
ENSGALG00010029864	SLC16A3	2	0.025984366	0.157984837	0.164473798	0.869358172	0	0.084860651	0	0.5	CRA004775;PRJNA827465	Yang2025_Silkie_Wenchang	0.968558268
ENSGALG00010029879	ENSGALG00010029879	2	0.624616984	0.464992159	1.34328498	0.179179714	0.360199894	5.963570313	83.2315216	1	PRJNA827465;Yang2025_Silkie_Wenchang	CRA004775	0.685873949
ENSGALG00010029879	ENSGALG00010029879	2	0.687064224	0.525570977	1.307272002	0.191120349	0.364397346	2.663588539	62.45666381	1	CRA004775;Yang2025_Silkie_Wenchang	PRJNA827465	0.700443419
ENSGALG00010029879	ENSGALG00010029879	2	0.150431818	0.227383474	0.661577621	0.508241954	0	0.054251719	0	1	CRA004775;PRJNA827465	Yang2025_Silkie_Wenchang	0.864714831
ENSGALG00010029895	FASN	2	-0.035534503	0.112723507	-0.315235963	0.752582502	0.011912772	1.776059519	43.69558062	0.5	PRJNA827465;Yang2025_Silkie_Wenchang	CRA004775	0.938087082
ENSGALG00010029895	FASN	2	-0.193347681	0.139842693	-1.382608395	0.166784974	0	0.039132489	0	1	CRA004775;Yang2025_Silkie_Wenchang	PRJNA827465	0.665766006
ENSGALG00010029895	FASN	2	0.034498921	0.088447523	0.390049604	0.696499867	0	0.624816044	0	0.5	CRA004775;PRJNA827465	Yang2025_Silkie_Wenchang	0.921041503
ENSGALG00010029907	NTN1	2	0.111019211	0.27254223	0.407346821	0.683753279	0.096915782	2.781005088	64.0417774	0.5	PRJNA827465;Yang2025_Silkie_Wenchang	CRA004775	0.918800744
ENSGALG00010029907	NTN1	2	0.317666558	0.223040592	1.424254456	0.154372826	0	0.443839846	0	1	CRA004775;Yang2025_Silkie_Wenchang	PRJNA827465	0.646989113
ENSGALG00010029907	NTN1	2	-0.082920486	0.166149271	-0.499072222	0.617728506	0	0.304709973	0	0.5	CRA004775;PRJNA827465	Yang2025_Silkie_Wenchang	0.899121942
ENSGALG00010029921	STX8	2	0.030711285	0.090492025	0.339381124	0.734322636	0.007667018	1.52698647	34.51153499	0.5	PRJNA827465;Yang2025_Silkie_Wenchang	CRA004775	0.933501228
ENSGALG00010029921	STX8	2	-0.061088003	0.261459496	-0.233642318	0.815262671	0.105536179	4.360575725	77.06724839	0.5	CRA004775;Yang2025_Silkie_Wenchang	PRJNA827465	0.953933933
ENSGALG00010029921	STX8	2	-0.122999116	0.146673915	-0.838588892	0.401700046	0.028827557	2.440255243	59.0206802	1	CRA004775;PRJNA827465	Yang2025_Silkie_Wenchang	0.832689704
ENSGALG00010029925	GGNBP2	2	0.188619398	0.265508578	0.710407926	0.47745121	0.132936266	17.15113697	94.16948275	0.5	PRJNA827465;Yang2025_Silkie_Wenchang	CRA004775	0.855104347
ENSGALG00010029925	GGNBP2	2	0.443122469	0.10187281	4.349761902	1.36285E-05	0	0.148388997	0	1	CRA004775;Yang2025_Silkie_Wenchang	PRJNA827465	0.001815738
ENSGALG00010029925	GGNBP2	2	0.098916178	0.211831271	0.466957394	0.640530361	0.069386485	3.705232697	73.01114176	0.5	CRA004775;PRJNA827465	Yang2025_Silkie_Wenchang	0.90623926
ENSGALG00010029926	BLTP2	2	0.134806494	0.099279407	1.357849513	0.17451144	0.017172556	7.757044127	87.10849154	1	PRJNA827465;Yang2025_Silkie_Wenchang	CRA004775	0.67885473
ENSGALG00010029926	BLTP2	2	0.06947277	0.214941428	0.323217216	0.746530743	0.070656874	3.460251185	71.10036392	0.5	CRA004775;Yang2025_Silkie_Wenchang	PRJNA827465	0.936668296
ENSGALG00010029926	BLTP2	2	0.017004103	0.063825765	0.266414398	0.789920078	0.001694543	1.058691147	5.543745875	0.5	CRA004775;PRJNA827465	Yang2025_Silkie_Wenchang	0.947390191
ENSGALG00010029927	ENSGALG00010029927	2	0.599208787	0.440830827	1.359271517	0.174060566	0	0.260214327	0	1	PRJNA827465;Yang2025_Silkie_Wenchang	CRA004775	0.67800817
ENSGALG00010029927	ENSGALG00010029927	2	0.301599019	0.511394054	0.589758557	0.555352531	0.095400289	1.218619049	17.93990082	0.5	CRA004775;Yang2025_Silkie_Wenchang	PRJNA827465	0.880255849
ENSGALG00010029927	ENSGALG00010029927	2	0.158310304	0.412417402	0.383859418	0.701082646	0	0.473431072	0	0.5	CRA004775;PRJNA827465	Yang2025_Silkie_Wenchang	0.922547052
ENSGALG00010029928	USP43	2	0.283006526	0.343041507	0.824992078	0.409376088	0.205338936	7.626275427	86.88743923	0.5	PRJNA827465;Yang2025_Silkie_Wenchang	CRA004775	0.834355341
ENSGALG00010029928	USP43	2	0.511782144	0.193854289	2.640035184	0.008289742	0.010206303	1.137240449	12.06784801	1	CRA004775;Yang2025_Silkie_Wenchang	PRJNA827465	0.141842286
ENSGALG00010029928	USP43	2	-0.00176985	0.1170994	-0.015114081	0.987941167	0	0.644334875	0	0.5	CRA004775;PRJNA827465	Yang2025_Silkie_Wenchang	0.996800355
ENSGALG00010029929	GDPD1	2	0.029575593	0.032577065	0.907865479	0.363949298	0	0.009496425	0	1	PRJNA827465;Yang2025_Silkie_Wenchang	CRA004775	0.817108378
ENSGALG00010029929	GDPD1	2	0.027549419	0.057381189	0.480112373	0.631147491	0	0.122796762	0	0.5	CRA004775;Yang2025_Silkie_Wenchang	PRJNA827465	0.903058174
ENSGALG00010029929	GDPD1	2	0.024860772	0.037671	0.65994457	0.509289401	0	0.104338181	0	0.5	CRA004775;PRJNA827465	Yang2025_Silkie_Wenchang	0.865257661
ENSGALG00010029930	ARHGAP44	2	0.339934364	0.379394452	0.895991923	0.370257077	0.257576158	9.473148597	89.44384763	0.5	PRJNA827465;Yang2025_Silkie_Wenchang	CRA004775	0.821045695
ENSGALG00010029930	ARHGAP44	2	0.756421095	0.180918453	4.18100577	2.90222E-05	0	0.313535578	0	1	CRA004775;Yang2025_Silkie_Wenchang	PRJNA827465	0.003204105
ENSGALG00010029930	ARHGAP44	2	0.365109695	0.539512947	0.67673945	0.498571282	0.424102982	2.983190806	66.47884547	0.5	CRA004775;PRJNA827465	Yang2025_Silkie_Wenchang	0.861478809
ENSGALG00010029932	RASA4B	2	0.092651473	0.164317169	0.563857528	0.572851097	0	0.1953951	0	1	PRJNA827465;Yang2025_Silkie_Wenchang	CRA004775	0.88573298
ENSGALG00010029932	RASA4B	2	-0.13299134	0.400158779	-0.332346425	0.739627688	0.174152342	2.153728815	53.56889907	0.5	CRA004775;Yang2025_Silkie_Wenchang	PRJNA827465	0.934914078
ENSGALG00010029932	RASA4B	2	-0.145259518	0.294303829	-0.49356992	0.621609948	0.089690376	1.792703386	44.21832368	0.5	CRA004775;PRJNA827465	Yang2025_Silkie_Wenchang	0.899796991
ENSGALG00010029933	TVP23B	2	-0.246565061	0.207579062	-1.187812775	0.23490718	0.074397841	6.630173653	84.91743878	1	PRJNA827465;Yang2025_Silkie_Wenchang	CRA004775	0.747791642
ENSGALG00010029933	TVP23B	2	-0.363750184	0.14922605	-2.437578309	0.014786011	0.011980791	1.343324964	25.55784889	1	CRA004775;Yang2025_Silkie_Wenchang	PRJNA827465	0.200806704
ENSGALG00010029933	TVP23B	2	-0.068801077	0.043830354	-1.569712992	0.116481897	0	0.266687614	0	1	CRA004775;PRJNA827465	Yang2025_Silkie_Wenchang	0.574631355
ENSGALG00010029934	TEKT3	2	0.404603666	0.252112842	1.604851474	0.108526495	0	0.125711126	0	1	PRJNA827465;Yang2025_Silkie_Wenchang	CRA004775	0.557501411
ENSGALG00010029934	TEKT3	2	0.349418423	0.330921488	1.055895237	0.291016081	0	0.586950063	0	0.5	CRA004775;Yang2025_Silkie_Wenchang	PRJNA827465	0.78467882
ENSGALG00010029934	TEKT3	2	0.245806606	0.28997958	0.847668675	0.396622516	0	0.292819795	0	0.5	CRA004775;PRJNA827465	Yang2025_Silkie_Wenchang	0.831375233
ENSGALG00010029935	SYNRG	2	0.078881848	0.141504758	0.557450143	0.577219914	0.027245577	2.907044445	65.60080113	0.5	PRJNA827465;Yang2025_Silkie_Wenchang	CRA004775	0.887145528
ENSGALG00010029935	SYNRG	2	0.155720401	0.155503496	1.001394859	0.316635948	0.010974078	1.217733487	17.88022498	0.5	CRA004775;Yang2025_Silkie_Wenchang	PRJNA827465	0.796935817
ENSGALG00010029935	SYNRG	2	-0.042670597	0.077114283	-0.553342327	0.580029023	0	0.045390103	0	1	CRA004775;PRJNA827465	Yang2025_Silkie_Wenchang	0.888203646
ENSGALG00010029936	SERPINF2	2	-0.043521809	1.12858075	-0.03856331	0.969238555	2.238097714	8.102380109	87.65794758	0.5	PRJNA827465;Yang2025_Silkie_Wenchang	CRA004775	0.991572914
ENSGALG00010029936	SERPINF2	2	-1.028076426	0.30954765	-3.321221874	0.000896243	0	0.085722025	0	1	CRA004775;Yang2025_Silkie_Wenchang	PRJNA827465	0.035189832
ENSGALG00010029936	SERPINF2	2	0.041689805	1.037538805	0.040181442	0.967948473	1.846394959	6.887061344	85.48001898	0.5	CRA004775;PRJNA827465	Yang2025_Silkie_Wenchang	0.991128245
ENSGALG00010029937	PMP22	2	-0.167485871	0.057476779	-2.913974548	0.00356859	0	0.390200702	0	1	PRJNA827465;Yang2025_Silkie_Wenchang	CRA004775	0.084829873
ENSGALG00010029937	PMP22	2	0.005764277	0.301058274	0.019146716	0.984724065	0.128610015	2.706477764	63.0516085	0.5	CRA004775;Yang2025_Silkie_Wenchang	PRJNA827465	0.995703389
ENSGALG00010029937	PMP22	2	0.027131391	0.256022656	0.105972617	0.915604079	0.085777565	2.147973091	53.44448196	0.5	CRA004775;PRJNA827465	Yang2025_Silkie_Wenchang	0.980197399
ENSGALG00010029938	ENSGALG00010029938	2	0.252156583	0.244883201	1.029701431	0.303150183	0.063700667	2.0492608	51.20191632	1	PRJNA827465;Yang2025_Silkie_Wenchang	CRA004775	0.792084776
ENSGALG00010029938	ENSGALG00010029938	2	0.057408547	0.171994152	0.33378197	0.73854409	0	0.007189605	0	1	CRA004775;Yang2025_Silkie_Wenchang	PRJNA827465	0.934485814
ENSGALG00010029938	ENSGALG00010029938	2	0.379367938	0.231111481	1.641493256	0.100695066	0	0.93000423	0	1	CRA004775;PRJNA827465	Yang2025_Silkie_Wenchang	0.541412814
ENSGALG00010029941	COX10	2	-0.044898244	0.070028479	-0.641142641	0.521430012	0	0.002533296	0	1	PRJNA827465;Yang2025_Silkie_Wenchang	CRA004775	0.869518515
ENSGALG00010029941	COX10	2	-0.067264055	0.103240693	-0.651526575	0.514706628	0	0.181747971	0	1	CRA004775;Yang2025_Silkie_Wenchang	PRJNA827465	0.866880125
ENSGALG00010029941	COX10	2	-0.055089476	0.085444398	-0.644740647	0.519095271	0	0.213917605	0	1	CRA004775;PRJNA827465	Yang2025_Silkie_Wenchang	0.868939943
ENSGALG00010029943	HS3ST3A1	2	0.351242309	0.134273163	2.615878719	0.008899816	0	0.003523361	0	1	PRJNA827465;Yang2025_Silkie_Wenchang	CRA004775	0.147652799
ENSGALG00010029943	HS3ST3A1	2	0.245415104	0.235856577	1.040526862	0.298095191	0	0.597310801	0	0.5	CRA004775;Yang2025_Silkie_Wenchang	PRJNA827465	0.789110621
ENSGALG00010029943	HS3ST3A1	2	0.304337331	0.143687851	2.11804497	0.034171259	0	0.690140531	0	0.5	CRA004775;PRJNA827465	Yang2025_Silkie_Wenchang	0.317969834
ENSGALG00010029946	METRNL	2	0.042298103	0.052278982	0.809084284	0.418466667	0	0.087564834	0	1	PRJNA827465;Yang2025_Silkie_Wenchang	CRA004775	0.838158675
ENSGALG00010029946	METRNL	2	-0.014655857	0.091180671	-0.160734256	0.872302708	0	0.826197046	0	0.5	CRA004775;Yang2025_Silkie_Wenchang	PRJNA827465	0.969626388
ENSGALG00010029946	METRNL	2	0.018333629	0.090843578	0.20181536	0.840061073	0.005567735	1.167802916	14.36911261	0.5	CRA004775;PRJNA827465	Yang2025_Silkie_Wenchang	0.95957104
ENSGALG00010029948	MYADML2	2	-0.030670187	0.139231727	-0.220281596	0.825651852	0.024563788	2.584131171	61.302274	0.5	PRJNA827465;Yang2025_Silkie_Wenchang	CRA004775	0.956388903
ENSGALG00010029948	MYADML2	2	-0.218701937	0.145767769	-1.500344957	0.13352507	0	0.299526012	0	1	CRA004775;Yang2025_Silkie_Wenchang	PRJNA827465	0.6124327
ENSGALG00010029948	MYADML2	2	0.044992618	0.151309266	0.297355339	0.766195237	0.016234867	1.096594545	8.808592489	0.5	CRA004775;PRJNA827465	Yang2025_Silkie_Wenchang	0.941123716
ENSGALG00010029950	RFFL	2	-0.154487902	0.169074067	-0.913729141	0.360859188	0.041836098	3.724829891	73.15313641	0.5	PRJNA827465;Yang2025_Silkie_Wenchang	CRA004775	0.815106245
ENSGALG00010029950	RFFL	2	-0.264065276	0.126667223	-2.08471671	0.037095023	0.003591285	1.075826463	7.048205762	1	CRA004775;Yang2025_Silkie_Wenchang	PRJNA827465	0.332143015
ENSGALG00010029950	RFFL	2	0.00870353	0.110126207	0.079032322	0.937006915	0	0.003882524	0	0.5	CRA004775;PRJNA827465	Yang2025_Silkie_Wenchang	0.985068196
ENSGALG00010029956	CCLI5	2	0.435885896	0.126644052	3.44181895	0.000577817	0.002580811	1.083829967	7.73460498	1	PRJNA827465;Yang2025_Silkie_Wenchang	CRA004775	0.025895023
ENSGALG00010029956	CCLI5	2	0.364440978	0.399443581	0.912371599	0.361573135	0.173286423	1.663891294	39.89991994	0.5	CRA004775;Yang2025_Silkie_Wenchang	PRJNA827465	0.81550034
ENSGALG00010029956	CCLI5	2	0.300045331	0.151898449	1.975302135	0.048233871	0	0.891740994	0	0.5	CRA004775;PRJNA827465	Yang2025_Silkie_Wenchang	0.379964424
ENSGALG00010029962	ENSGALG00010029962	2	0.697622918	0.42465554	1.642797167	0.100424907	0	0.877146434	0	1	PRJNA827465;Yang2025_Silkie_Wenchang	CRA004775	0.541278127

ENSGALG00010029962	ENSGALG00010029962	2	0.413354612	0.832382946	0.496591881	0.61947688	0.812433787	2.342208011	57.3052438	0.5	CRA004775;Yang2025_Silkie_Wenchang	PRJNA827465	0.899253917
ENSGALG00010029962	ENSGALG00010029962	2	0.074123546	0.486901648	0.152235151	0.879001471	0	0.681147164	0	0.5	CRA004775;PRJNA827465	Yang2025_Silkie_Wenchang	0.971128798
ENSGALG00010029966	RABGEF1	2	0.09062217	0.079234894	1.143715424	0.252741675	0.008511728	3.080145649	67.53400281	1	PRJNA827465;Yang2025_Silkie_Wenchang	CRA004775	0.762731469
ENSGALG00010029966	RABGEF1	2	0.012210282	0.063036798	0.193700857	0.846410121	0	0.038236309	0	1	CRA004775;Yang2025_Silkie_Wenchang	PRJNA827465	0.96122283
ENSGALG00010029966	RABGEF1	2	0.148313716	0.053661821	2.763859177	0.005712219	0	0.643268375	0	1	CRA004775;PRJNA827465	Yang2025_Silkie_Wenchang	0.113559599
ENSGALG00010030004	FLOT2	2	-0.04076692	0.070566995	-0.577705201	0.563463167	0	0.022031191	0	1	PRJNA827465;Yang2025_Silkie_Wenchang	CRA004775	0.882407972
ENSGALG00010030004	FLOT2	2	-0.122081973	0.099586162	-1.225892938	0.220238967	0.004912066	1.328404266	24.72171118	1	CRA004775;Yang2025_Silkie_Wenchang	PRJNA827465	0.733812488
ENSGALG00010030004	FLOT2	2	-0.114388268	0.085726229	-1.334343881	0.182091183	0.004000585	1.340804608	25.41791742	1	CRA004775;PRJNA827465	Yang2025_Silkie_Wenchang	0.690033956
ENSGALG00010030014	ENSGALG00010030014	2	0.123314062	0.139343898	0.884962053	0.376177098	0.010233859	1.279145007	21.82278049	1	PRJNA827465;Yang2025_Silkie_Wenchang	CRA004775	0.822974013
ENSGALG00010030014	ENSGALG00010030014	2	0.285063103	0.226949709	1.256062872	0.209093176	0	0.654552634	0	0.5	CRA004775;Yang2025_Silkie_Wenchang	PRJNA827465	0.721011841
ENSGALG00010030014	ENSGALG00010030014	2	0.023993729	0.129454156	0.185345371	0.852958154	0	0.20420387	0	0.5	CRA004775;PRJNA827465	Yang2025_Silkie_Wenchang	0.962708451
ENSGALG00010030102	GPS1	2	-0.01196365	0.091493992	-0.130758859	0.895966069	0.010993116	2.732442251	63.40270322	0.5	PRJNA827465;Yang2025_Silkie_Wenchang	CRA004775	0.975002393
ENSGALG00010030102	GPS1	2	-0.011508399	0.167675701	-0.068634865	0.945280266	0.030636817	1.845603851	45.81719152	0.5	CRA004775;Yang2025_Silkie_Wenchang	PRJNA827465	0.986321197
ENSGALG00010030102	GPS1	2	-0.096314263	0.054605345	-1.763824831	0.077761476	0	0.487849822	0	1	CRA004775;PRJNA827465	Yang2025_Silkie_Wenchang	0.480392844
ENSGALG00010030103	C1QTNF1	2	0.136963245	0.112728971	1.21497822	0.224374398	0	0.685034281	0	0.5	PRJNA827465;Yang2025_Silkie_Wenchang	CRA004775	0.737471949
ENSGALG00010030103	C1QTNF1	2	0.015901387	0.185791437	0.085587296	0.931794498	0	0.058696475	0	0.5	CRA004775;Yang2025_Silkie_Wenchang	PRJNA827465	0.98412915
ENSGALG00010030103	C1QTNF1	2	0.190276994	0.128628929	1.479270607	0.139068005	0	0.037535488	0	1	CRA004775;PRJNA827465	Yang2025_Silkie_Wenchang	0.622860674
ENSGALG00010030104	P4HB	2	-0.046916997	0.216405487	-0.216801329	0.828363176	0.087932743	16.13469025	93.80217417	0.5	PRJNA827465;Yang2025_Silkie_Wenchang	CRA004775	0.956777964
ENSGALG00010030104	P4HB	2	-0.239936343	0.086184341	-2.783990012	0.005369469	0	0.79165635	0	1	CRA004775;Yang2025_Silkie_Wenchang	PRJNA827465	0.109629211
ENSGALG00010030104	P4HB	2	0.152657015	0.053963482	2.828894816	0.004670905	0	0.710684911	0	0.5	CRA004775;PRJNA827465	Yang2025_Silkie_Wenchang	0.100537004
ENSGALG00010030105	RAB40B	2	0.094041349	0.234768754	0.400570124	0.688736646	0.086935088	4.595264273	78.23846594	0.5	PRJNA827465;Yang2025_Silkie_Wenchang	CRA004775	0.919799842
ENSGALG00010030105	RAB40B	2	0.336295799	0.176345688	1.907025927	0.05651723	0	0.070024307	0	1	CRA004775;Yang2025_Silkie_Wenchang	PRJNA827465	0.411523553
ENSGALG00010030105	RAB40B	2	-0.108807657	0.11715025	-0.928787237	0.35299936	0	0.254275176	0	0.5	CRA004775;PRJNA827465	Yang2025_Silkie_Wenchang	0.811035834
ENSGALG00010030106	GPRC5C	2	0.245591358	0.118742998	2.06825971	0.038615608	0	0.450167875	0	1	PRJNA827465;Yang2025_Silkie_Wenchang	CRA004775	0.338965227
ENSGALG00010030106	GPRC5C	2	0.339071112	0.204934743	1.654532104	0.098019446	0	0.143241105	0	1	CRA004775;Yang2025_Silkie_Wenchang	PRJNA827465	0.534130658
ENSGALG00010030106	GPRC5C	2	0.206005612	0.126099623	1.633673493	0.102327427	0	0.004718316	0	1	CRA004775;PRJNA827465	Yang2025_Silkie_Wenchang	0.545029796
ENSGALG00010030107	RPTOR	2	-0.192257293	0.104340058	-1.842602905	0.06538701	0.014723173	3.053658913	67.2524002	1	PRJNA827465;Yang2025_Silkie_Wenchang	CRA004775	0.440704235
ENSGALG00010030107	RPTOR	2	-0.291634058	0.07250787	-4.022102102	5.7681E-05	0	0.020144134	0	1	CRA004775;Yang2025_Silkie_Wenchang	PRJNA827465	0.005336024
ENSGALG00010030107	RPTOR	2	-0.100985528	0.089753578	-1.125142091	0.260528827	0	0.611000264	0	1	CRA004775;PRJNA827465	Yang2025_Silkie_Wenchang	0.767694434
ENSGALG00010030108	LLGL2	2	0.127753575	0.077076506	1.657490483	0.097420351	0	0.549020608	0	1	PRJNA827465;Yang2025_Silkie_Wenchang	CRA004775	0.533126158
ENSGALG00010030108	LLGL2	2	0.077949439	0.149684148	0.520759475	0.602534337	0.021732998	1.895400634	47.24070563	0.5	CRA004775;Yang2025_Silkie_Wenchang	PRJNA827465	0.895518685
ENSGALG00010030108	LLGL2	2	0.044366829	0.084341569	0.526037511	0.598862144	0	0.826664974	0	0.5	CRA004775;PRJNA827465	Yang2025_Silkie_Wenchang	0.894792426
ENSGALG00010030109	FBF1	2	0.284759431	0.151015012	1.885636583	0.059343944	0.039339236	7.183253561	86.07873171	1	PRJNA827465;Yang2025_Silkie_Wenchang	CRA004775	0.422252444
ENSGALG00010030109	FBF1	2	0.236271181	0.248213061	0.951888592	0.341153486	0.095805899	4.026091086	75.16201251	0.5	CRA004775;Yang2025_Silkie_Wenchang	PRJNA827465	0.805646109
ENSGALG00010030109	FBF1	2	0.125515296	0.063351099	1.981264708	0.047561596	0	0.692567456	0	0.5	CRA004775;PRJNA827465	Yang2025_Silkie_Wenchang	0.376812254
