# Supplementary material for: Cross-cohort transcriptomic meta-analysis identifies conserved melanogenesis signatures and developmental background in black-boned chicken breast muscle
Source: Anim Biosci. 2026 Jun 24;39(10):260430. doi: 10.5713/ab.260430 (PMC13662945; doi:10.5713/ab.260430)

Supplement 7. Leave-one-cohort-out robustness of meta-analysis effects.

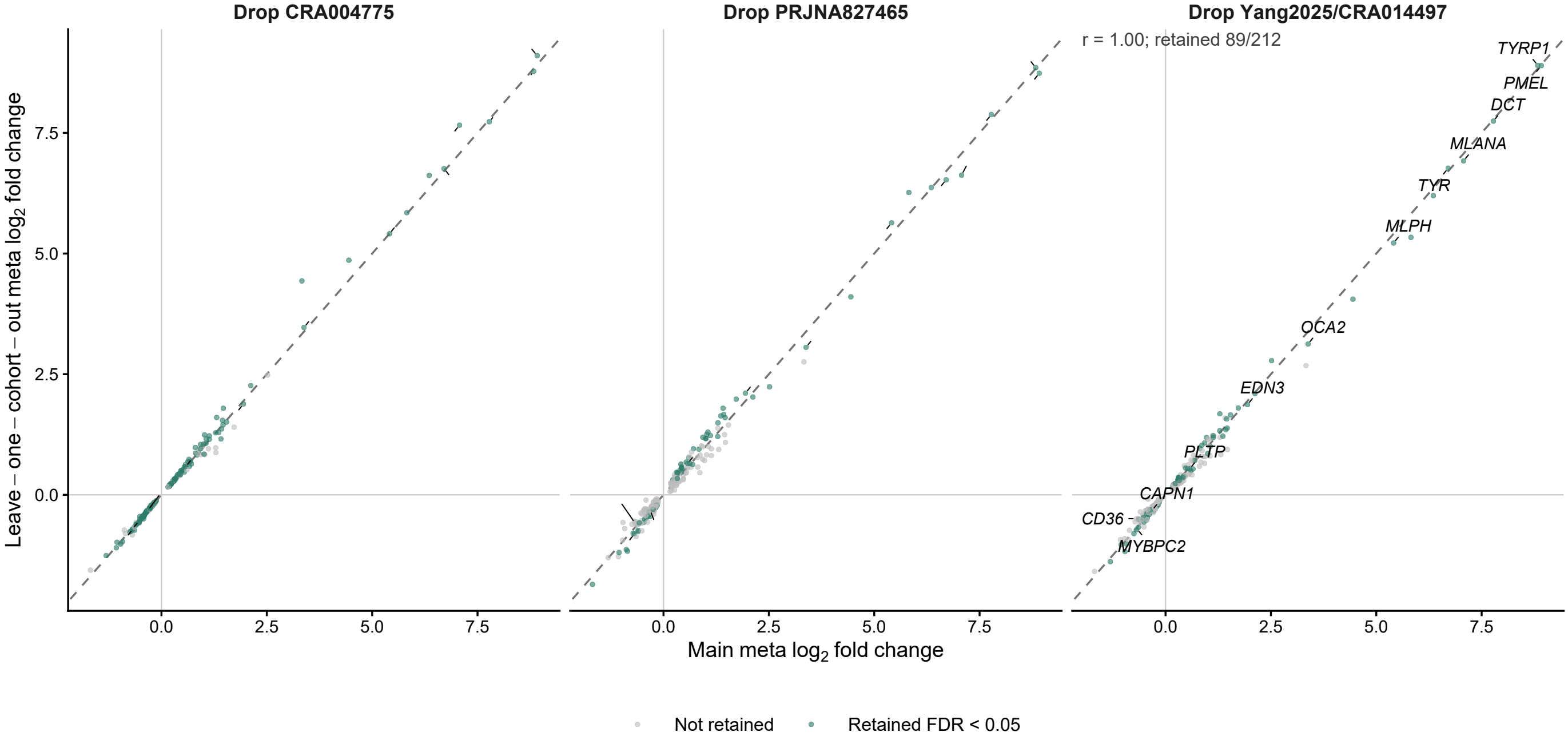

Supplement: Supplementary file 15 [file ab-260430-Supplementary-7.pdf]
