# Supplementary material for: Cross-cohort transcriptomic meta-analysis identifies conserved melanogenesis signatures and developmental background in black-boned chicken breast muscle
Source: Anim Biosci. 2026 Jun 24;39(10):260430. doi: 10.5713/ab.260430 (PMC13662945; doi:10.5713/ab.260430)
Supplement: Supplementary file 16 [file ab-260430-Supplementary-8.pdf]

Supplement 8. Gene-level summary of the strongest PRJNA729271 ordinary breast muscle background effect.

gene_id	symbol	cohort_id	log2FoldChange	padj	max_abs_log2FoldChange	strongest_background_contrast	strongest_background_type	significant_background_contrasts	n_significant_background_contrasts	background_development_gene
ENSGALG00010023167	GPD1	PRJNA729271	12.30674764	0	12.30674764	stage_E14_vs_E10	ordinary_muscle_development	D_vs_E10;stage_1W_vs_E10;stage_3W_vs_E10;stage	1	TRUE
ENSGALG00010000224	MYBPC2	PRJNA729271	11.51081654	0	11.51081654	stage_E14_vs_E10	ordinary_muscle_development	D_vs_E10;stage_1W_vs_E10;stage_3W_vs_E10;stage	1	TRUE
ENSGALG00010018839	RPL3L	PRJNA729271	11.40234531	0	11.40234531	stage_E14_vs_E10	ordinary_muscle_development	D_vs_E10;stage_1W_vs_E10;stage_3W_vs_E10;stage	1	TRUE
ENSGALG00010010323	FBP2	PRJNA729271	10.87699168	0	10.87699168	stage_E14_vs_E10	ordinary_muscle_development	D_vs_E10;stage_1W_vs_E10;stage_3W_vs_E10;stage	1	TRUE
ENSGALG00010025003	ENSGALG00010025003	PRJNA729271	10.81761032	0	10.81761032	stage_E14_vs_E10	ordinary_muscle_development	D_vs_E10;stage_1W_vs_E10;stage_3W_vs_E10;stage	1	TRUE
ENSGALG00010024547	PFKM	PRJNA729271	10.29363735	0	10.29363735	stage_E14_vs_E10	ordinary_muscle_development	D_vs_E10;stage_1W_vs_E10;stage_3W_vs_E10;stage	1	TRUE
ENSGALG00010004973	SLN	PRJNA729271	10.1500082	0	10.1500082	stage_E14_vs_E10	ordinary_muscle_development	D_vs_E10;stage_1W_vs_E10;stage_3W_vs_E10;stage	1	TRUE
ENSGALG00010010295	ENSGALG00010010295	PRJNA729271	8.978695392	0	8.978695392	stage_E14_vs_E10	ordinary_muscle_development	D_vs_E10;stage_1W_vs_E10;stage_3W_vs_E10;stage	1	TRUE
ENSGALG00010000476	ENSGALG00010000476	PRJNA729271	8.745614391	0	8.745614391	stage_E14_vs_E10	ordinary_muscle_development	D_vs_E10;stage_1W_vs_E10;stage_3W_vs_E10;stage	1	TRUE
ENSGALG00010027714	SYPL2	PRJNA729271	8.173892704	0	8.173892704	stage_E14_vs_E10	ordinary_muscle_development	D_vs_E10;stage_1W_vs_E10;stage_3W_vs_E10;stage	1	TRUE
ENSGALG00010024472	LDHA	PRJNA729271	8.151325055	0	8.151325055	stage_E14_vs_E10	ordinary_muscle_development	D_vs_E10;stage_1W_vs_E10;stage_3W_vs_E10;stage	1	TRUE
ENSGALG00010021224	MYOZ1	PRJNA729271	8.115042024	0	8.115042024	stage_E14_vs_E10	ordinary_muscle_development	D_vs_E10;stage_1W_vs_E10;stage_3W_vs_E10;stage	1	TRUE
ENSGALG00010017656	FSD2	PRJNA729271	7.997271248	0	7.997271248	stage_E14_vs_E10	ordinary_muscle_development	D_vs_E10;stage_1W_vs_E10;stage_3W_vs_E10;stage	1	TRUE
ENSGALG00010024350	PGM1	PRJNA729271	7.97523702	0	7.97523702	stage_E14_vs_E10	ordinary_muscle_development	D_vs_E10;stage_1W_vs_E10;stage_3W_vs_E10;stage	1	TRUE
ENSGALG00010029956	CCL5	PRJNA729271	7.264297588	0	7.264297588	stage_E14_vs_E10	ordinary_muscle_development	D_vs_E10;stage_1W_vs_E10;stage_3W_vs_E10;stage	1	TRUE
ENSGALG00010028082	AK1	PRJNA729271	7.263546077	0	7.263546077	stage_E14_vs_E10	ordinary_muscle_development	D_vs_E10;stage_1W_vs_E10;stage_3W_vs_E10;stage	1	TRUE
ENSGALG00010023180	EEF1A2	PRJNA729271	7.192914712	0	7.192914712	stage_E14_vs_E10	ordinary_muscle_development	D_vs_E10;stage_1W_vs_E10;stage_3W_vs_E10;stage	1	TRUE
ENSGALG00010005075	TRDN	PRJNA729271	7.120171621	0	7.120171621	stage_E14_vs_E10	ordinary_muscle_development	D_vs_E10;stage_1W_vs_E10;stage_3W_vs_E10;stage	1	TRUE
ENSGALG00010001526	PPP1R3A	PRJNA729271	6.839062246	0	6.839062246	stage_E14_vs_E10	ordinary_muscle_development	D_vs_E10;stage_1W_vs_E10;stage_3W_vs_E10;stage	1	TRUE
ENSGALG00010011505	MYL1	PRJNA729271	6.772346764	0	6.772346764	stage_E14_vs_E10	ordinary_muscle_development	D_vs_E10;stage_1W_vs_E10;stage_3W_vs_E10;stage	1	TRUE
ENSGALG00010029190	DHRS7C	PRJNA729271	6.733332342	0	6.733332342	stage_E14_vs_E10	ordinary_muscle_development	D_vs_E10;stage_1W_vs_E10;stage_3W_vs_E10;stage	1	TRUE
ENSGALG00010000285	MYLPF	PRJNA729271	6.729259844	0	6.729259844	stage_E14_vs_E10	ordinary_muscle_development	D_vs_E10;stage_1W_vs_E10;stage_3W_vs_E10;stage	1	TRUE
ENSGALG00010016292	PHKA1	PRJNA729271	6.66612829	0	6.66612829	stage_E14_vs_E10	ordinary_muscle_development	D_vs_E10;stage_1W_vs_E10;stage_3W_vs_E10;stage	1	TRUE
ENSGALG00010024570	TPM1	PRJNA729271	6.525205777	0	6.525205777	stage_E14_vs_E10	ordinary_muscle_development	D_vs_E10;stage_1W_vs_E10;stage_3W_vs_E10;stage	1	TRUE
ENSGALG00010026972	TNNT3	PRJNA729271	6.291084658	0	6.291084658	stage_E14_vs_E10	ordinary_muscle_development	D_vs_E10;stage_1W_vs_E10;stage_3W_vs_E10;stage	1	TRUE
ENSGALG00010029655	PHKG1	PRJNA729271	6.086570672	0	6.086570672	stage_E14_vs_E10	ordinary_muscle_development	D_vs_E10;stage_1W_vs_E10;stage_3W_vs_E10;stage	1	TRUE
ENSGALG00010029205	CACNG1	PRJNA729271	5.863519666	0	5.863519666	stage_E14_vs_E10	ordinary_muscle_development	D_vs_E10;stage_1W_vs_E10;stage_3W_vs_E10;stage	1	TRUE
ENSGALG00010020714	PGAM1	PRJNA729271	5.684299859	0	5.684299859	stage_E14_vs_E10	ordinary_muscle_development	D_vs_E10;stage_1W_vs_E10;stage_3W_vs_E10;stage	1	TRUE
ENSGALG00010017467	SFRP1	PRJNA729271	-5.562028494	0	5.562028494	stage_E14_vs_E10	ordinary_muscle_development	D_vs_E10;stage_1W_vs_E10;stage_3W_vs_E10;stage	1	TRUE
ENSGALG00010008168	CAVIN4	PRJNA729271	5.547254426	0	5.547254426	stage_E14_vs_E10	ordinary_muscle_development	D_vs_E10;stage_1W_vs_E10;stage_3W_vs_E10;stage	1	TRUE
ENSGALG00010008893	ENSGALG00010008893	PRJNA729271	5.490303573	0	5.490303573	stage_E14_vs_E10	ordinary_muscle_development	D_vs_E10;stage_1W_vs_E10;stage_3W_vs_E10;stage	1	TRUE
ENSGALG00010022038	GAPDH	PRJNA729271	5.458328455	0	5.458328455	stage_E14_vs_E10	ordinary_muscle_development	D_vs_E10;stage_1W_vs_E10;stage_3W_vs_E10;stage	1	TRUE
ENSGALG00010024024	AGL	PRJNA729271	5.430019196	0	5.430019196	stage_E14_vs_E10	ordinary_muscle_development	D_vs_E10;stage_1W_vs_E10;stage_3W_vs_E10;stage	1	TRUE
ENSGALG00010024263	PHKB	PRJNA729271	5.073041052	0	5.073041052	stage_E14_vs_E10	ordinary_muscle_development	D_vs_E10;stage_1W_vs_E10;stage_3W_vs_E10;stage	1	TRUE
ENSGALG00010015909	TNNC2	PRJNA729271	4.618831416	0	4.618831416	stage_E14_vs_E10	ordinary_muscle_development	D_vs_E10;stage_1W_vs_E10;stage_3W_vs_E10;stage	1	TRUE
ENSGALG00010021165	PTRF	PRJNA729271	4.091945257	0	4.091945257	stage_E14_vs_E10	ordinary_muscle_development	D_vs_E10;stage_1W_vs_E10;stage_3W_vs_E10;stage	1	TRUE
ENSGALG00010003543	SOX11	PRJNA729271	-3.733675533	0	3.733675533	stage_E14_vs_E10	ordinary_muscle_development	D_vs_E10;stage_1W_vs_E10;stage_3W_vs_E10;stage	1	TRUE
ENSGALG00010008756	ITSN2	PRJNA729271	3.011479785	0	3.011479785	stage_E14_vs_E10	ordinary_muscle_development	D_vs_E10;stage_1W_vs_E10;stage_3W_vs_E10;stage	1	TRUE
ENSGALG00010018756	EML4	PRJNA729271	-2.767475842	0	2.767475842	stage_E14_vs_E10	ordinary_muscle_development	D_vs_E10;stage_1W_vs_E10;stage_3W_vs_E10;stage	1	TRUE
ENSGALG00010012414	ENSGALG00010012414	PRJNA729271	7.802188474	1.5165E-319	7.802188474	stage_E14_vs_E10	ordinary_muscle_development	D_vs_E10;stage_1W_vs_E10;stage_3W_vs_E10;stage	1	TRUE
ENSGALG00010010869	ENSGALG00010010869	PRJNA729271	6.580318467	1.5211E-312	6.580318467	stage_E14_vs_E10	ordinary_muscle_development	D_vs_E10;stage_1W_vs_E10;stage_3W_vs_E10;stage	1	TRUE
ENSGALG00010013029	MYLK2	PRJNA729271	9.624272492	2.8526E-312	9.624272492	stage_E14_vs_E10	ordinary_muscle_development	D_vs_E10;stage_1W_vs_E10;stage_3W_vs_E10;stage	1	TRUE
ENSGALG00010010850	ENSGALG00010010850	PRJNA729271	3.989507827	3.0456E-309	3.989507827	stage_E14_vs_E10	ordinary_muscle_development	D_vs_E10;stage_1W_vs_E10;stage_3W_vs_E10;stage	1	TRUE
ENSGALG00010024534	VWF	PRJNA729271	4.657818093	2.3944E-299	4.657818093	stage_E14_vs_E10	ordinary_muscle_development	D_vs_E10;stage_1W_vs_E10;stage_3W_vs_E10;stage	1	TRUE
ENSGALG00010007143	CKMT2	PRJNA729271	11.7426673	3.0266E-298	11.7426673	stage_E14_vs_E10	ordinary_muscle_development	D_vs_E10;stage_1W_vs_E10;stage_3W_vs_E10;stage	1	TRUE
ENSGALG00010017318	ADSS1	PRJNA729271	4.559257504	3.9424E-295	4.559257504	stage_E14_vs_E10	ordinary_muscle_development	D_vs_E10;stage_1W_vs_E10;stage_3W_vs_E10;stage	1	TRUE
ENSGALG00010028598	TMOD4	PRJNA729271	9.199172342	4.8953E-293	9.199172342	stage_E14_vs_E10	ordinary_muscle_development	D_vs_E10;stage_1W_vs_E10;stage_3W_vs_E10;stage	1	TRUE
ENSGALG00010022819	PACSIN3	PRJNA729271	3.933400734	2.0918E-287	3.933400734	stage_E14_vs_E10	ordinary_muscle_development	D_vs_E10;stage_1W_vs_E10;stage_3W_vs_E10;stage	1	TRUE
ENSGALG00010027731	OLFML2A	PRJNA729271	3.681475189	7.2827E-287	3.681475189	stage_E14_vs_E10	ordinary_muscle_development	D_vs_E10;stage_1W_vs_E10;stage_3W_vs_E10;stage	1	TRUE
ENSGALG00010019065	CAND2	PRJNA729271	4.385499666	3.1811E-286	4.385499666	stage_E14_vs_E10	ordinary_muscle_development	D_vs_E10;stage_1W_vs_E10;stage_3W_vs_E10;stage	1	TRUE
ENSGALG00010012759	ATP1B4	PRJNA729271	7.172503105	7.2039E-286	7.172503105	stage_E14_vs_E10	ordinary_muscle_development	D_vs_E10;stage_1W_vs_E10;stage_3W_vs_E10;stage	1	TRUE
ENSGALG00010029264	COL26A1	PRJNA729271	-6.687798621	5.67E-284	6.687798621	stage_E14_vs_E10	ordinary_muscle_development	D_vs_E10;stage_1W_vs_E10;stage_3W_vs_E10;stage	1	TRUE
ENSGALG00010027059	ASB14	PRJNA729271	7.524276944	2.3787E-276	7.524276944	stage_E14_vs_E10	ordinary_muscle_development	D_vs_E10;stage_1W_vs_E10;stage_3W_vs_E10;stage	1	TRUE
ENSGALG00010017207	ENSGALG00010017207	PRJNA729271	9.955289318	1.1755E-273	9.955289318	stage_E14_vs_E10	ordinary_muscle_development	D_vs_E10;stage_1W_vs_E10;stage_3W_vs_E10;stage	1	TRUE
ENSGALG00010029215	ENSGALG00010029215	PRJNA729271	8.392843363	3.518E-273	8.392843363	stage_E14_vs_E10	ordinary_muscle_development	D_vs_E10;stage_1W_vs_E10;stage_3W_vs_E10;stage	1	TRUE
ENSGALG00010022890	MYPN	PRJNA729271	5.864557469	7.7167E-271	5.864557469	stage_E14_vs_E10	ordinary_muscle_development	D_vs_E10;stage_1W_vs_E10;stage_3W_vs_E10;stage	1	TRUE
ENSGALG00010025618	ADIPOQ	PRJNA729271	7.823163271	1.4989E-270	7.823163271	stage_E14_vs_E10	ordinary_muscle_development	D_vs_E10;stage_1W_vs_E10;stage_3W_vs_E10;stage	1	TRUE
ENSGALG00010021123	RYR3	PRJNA729271	7.623197242	4.4651E-270	7.623197242	stage_E14_vs_E10	ordinary_muscle_development	D_vs_E10;stage_1W_vs_E10;stage_3W_vs_E10;stage	1	TRUE
ENSGALG00010004312	PDLIM5	PRJNA729271	6.400664129	4.8802E-270	6.400664129	stage_E14_vs_E10	ordinary_muscle_development	D_vs_E10;stage_1W_vs_E10;stage_3W_vs_E10;stage	1	TRUE
ENSGALG00010024594	TNNI2	PRJNA729271	6.451275072	4.4029E-269	6.451275072	stage_E14_vs_E10	ordinary_muscle_development	D_vs_E10;stage_1W_vs_E10;stage_3W_vs_E10;stage	1	TRUE
ENSGALG00010021826	FBXO40	PRJNA729271	7.242218384	1.3611E-264	7.242218384	stage_E14_vs_E10	ordinary_muscle_development	D_vs_E10;stage_1W_vs_E10;stage_3W_vs_E10;stage	1	TRUE
ENSGALG00010005880	SLC25A4	PRJNA729271	6.256951224	3.7854E-263	6.256951224	stage_E14_vs_E10	ordinary_muscle_development	D_vs_E10;stage_1W_vs_E10;stage_3W_vs_E10;stage	1	TRUE
ENSGALG00010016543	TCEA3	PRJNA729271	6.035779471	6.0457E-262	6.035779471	stage_E14_vs_E10	ordinary_muscle_development	D_vs_E10;stage_1W_vs_E10;stage_3W_vs_E10;stage	1	TRUE
ENSGALG00010022228	FUBP1	PRJNA729271	-1.937309435	4.7446E-261	1.937309435	stage_E14_vs_E10	ordinary_muscle_development	D_vs_E10;stage_1W_vs_E10;stage_3W_vs_E10;stage	1	TRUE
ENSGALG00010012062	HMGA2	PRJNA729271	-6.694815462	5.0333E-259	6.694815462	stage_E14_vs_E10	ordinary_muscle_development	D_vs_E10;stage_1W_vs_E10;stage_3W_vs_E10;stage	1	TRUE
ENSGALG00010019040	HMGN5	PRJNA729271	-2.897100205	2.7149E-258	2.897100205	stage_E14_vs_E10	ordinary_muscle_development	D_vs_E10;stage_1W_vs_E10;stage_3W_vs_E10;stage	1	TRUE
ENSGALG00010023392	TPI1	PRJNA729271	5.131872254	8.1381E-256	5.131872254	stage_E14_vs_E10	ordinary_muscle_development	D_vs_E10;stage_1W_vs_E10;stage_3W_vs_E10;stage	1	TRUE
ENSGALG00010009112	ENSGALG00010009112	PRJNA729271	9.074365257	2.2578E-255	9.074365257	stage_E14_vs_E10	ordinary_muscle_development	D_vs_E10;stage_1W_vs_E10;stage_3W_vs_E10;stage	1	TRUE
ENSGALG00010025504	AMPD1	PRJNA729271	6.734639011	5.7206E-252	6.734639011	stage_E14_vs_E10	ordinary_muscle_development	D_vs_E10;stage_1W_vs_E10;stage_3W_vs_E10;stage	1	TRUE
ENSGALG00010019451	CAV3	PRJNA729271	4.847562588	4.7619E-250	4.847562588	stage_E14_vs_E10	ordinary_muscle_development	D_vs_E10;stage_1W_vs_E10;stage_3W_vs_E10;stage	1	TRUE
ENSGALG00010021110	PKLR	PRJNA729271	5.704630496	4.601E-248	5.704630496	stage_E14_vs_E10	ordinary_muscle_development	D_vs_E10;stage_1W_vs_E10;stage_3W_vs_E10;stage	1	TRUE
ENSGALG00010029553	MYH1C	PRJNA729271	6.568240029	8.4945E-247	6.568240029	stage_E14_vs_E10	ordinary_muscle_development	D_vs_E10;stage_1W_vs_E10;stage_3W_vs_E10;stage	1	TRUE
ENSGALG00010017431	FYCO1	PRJNA729271	3.949635112	8.922E-247	3.949635112	stage_E14_vs_E10	ordinary_muscle_development	D_vs_E10;stage_1W_vs_E10;stage_3W_vs_E10;stage	1	TRUE
ENSGALG00010007917	POGLUT1	PRJNA729271	-2.840920386	2.9598E-245	2.840920386	stage_E14_vs_E10	ordinary_muscle_development	D_vs_E10;stage_1W_vs_E10;stage_3W_vs_E10;stage	1	TRUE
ENSGALG00010004117	STBD1	PRJNA729271	6.331809846	3.9271E-243	6.331809846	stage_E14_vs_E10	ordinary_muscle_development	D_vs_E10;stage_1W_vs_E10;stage_3W_vs_E10;stage	1	TRUE
ENSGALG00010001699	ASB15	PRJNA729271	8.298588753	1.8674E-240	8.298588753	stage_E14_vs_E10	ordinary_muscle_development	D_vs_E10;stage_1W_vs_E10;stage_3W_vs_E10;stage	1	TRUE
ENSGALG00010029702	CORO6	PRJNA729271	5.638632725	3.2642E-240	5.638632725	stage_E14_vs_E10	ordinary_muscle_development	D_vs_E10;stage_1W_vs_E10;stage_3W_vs_E10;stage	1	TRUE
ENSGALG00010013767	PGK2	PRJNA729271	5.706937377	2.0894E-239	5.706937377	stage_E14_vs_E10	ordinary_muscle_development	D_vs_E10;stage_1W_vs_E10;stage_3W_vs_E10;stage	1	TRUE
ENSGALG00010020164	C10orf71	PRJNA729271	4.872609045	5.8279E-236	4.872609045	stage_E14_vs_E10	ordinary_muscle_development	D_vs_E10;stage_1W_vs_E10;stage_3W_vs_E10;stage	1	TRUE
ENSGALG00010013450	CD74	PRJNA729271	6.254919668	2.7356E-235	6.254919668	stage_E14_vs_E10	ordinary_muscle_development	D_vs_E10;stage_1W_vs_E10;stage_3W_vs_E10;stage	1	TRUE
ENSGALG00010018649	HAGH	PRJNA729271	3.43077863	4.9741E-233	3.43077863	stage_E14_vs_E10	ordinary_muscle_development	D_vs_E10;stage_1W_vs_E10;stage_3W_vs_E10;stage	1	TRUE
ENSGALG00010000402	PFKFB1	PRJNA729271	10.39834301	8.3109E-232	10.39834301	stage_E14_vs_E10	ordinary_muscle_development	D_vs_E10;stage_1W_vs_E10;stage_3W_vs_E10;stage	1	TRUE
ENSGALG00010012028	TMEM140	PRJNA729271	5.523803872	2.0961E-230	5.523803872	stage_E14_vs_E10	ordinary_muscle_development	D_vs_E10;stage_1W_vs_E10;stage_3W_vs_E10;stage	1	TRUE
ENSGALG00010016480	GPX3	PRJNA729271	4.325099986	8.6596E-229	4.325099986	stage_E14_vs_E10	ordinary_muscle_development	D_vs_E10;stage_1W_vs_E10;stage_3W_vs_E10;stage	1	TRUE
ENSGALG00010022649	PATZ1	PRJNA729271	-3.358631023	3.0108E-227	3.358631023	stage_E14_vs_E10	ordinary_muscle_development	D_vs_E10;stage_1W_vs_E10;stage_3W_vs_E10;stage	1	TRUE
ENSGALG00010025499	STAC3	PRJNA729271	5.122903926	6.0611E-227	5.122903926	stage_E14_vs_E10	ordinary_muscle_development	D_vs_E10;stage_1W_vs_E10;stage_3W_vs_E10;stage	1	TRUE
ENSGALG00010020854	ENSGALG00010020854	PRJNA729271	12.3025691	1.8141E-226	12.3025691	stage_E14_vs_E10	ordinary_muscle_development	D_vs_E10;stage_1W_vs_E10;stage_3W_vs_E10;stage	1	TRUE
ENSGALG00010023635	SCN4A	PRJNA729271	10.24942674	4.3774E-226	10.24942674	stage_E14_vs_E10	ordinary_muscle_development	D_vs_E10;stage_1W_vs_E10;stage_3W_vs_E10;stage	1	TRUE
ENSGALG00010022409	CARNS1	PRJNA729271	10.83492856	5.8483E-223	10.83492856	stage_E14_vs_E10	ordinary_muscle_development	D_vs_E10;stage_1W_vs_E10;stage_3W_vs_E10;stage	1	TRUE
ENSGALG00010019472	CAPN3	PRJNA729271	8.187296999	7.2125E-223	8.187296999	stage_E14_vs_E10	ordinary_muscle_development	D_vs_E10;stage_1W_vs_E10;stage_3W_vs_E10;stage	1	TRUE
ENSGALG00010005970	SHISA2	PRJNA729271	-3.994492899	9.0445E-223	3.994492899	stage_E14_vs_E10	ordinary_muscle_development	D_vs_E10;stage_1W_vs_E10;stage_3W_vs_E10;stage	1	TRUE
ENSGALG00010014192	KDM1A	PRJNA729271	-2.008679104	2.641E-222	2.008679104	stage_E14_vs_E10	ordinary_muscle_development	D_vs_E10;stage_1W_vs_E10;stage_3W_vs_E10;stage	1	TRUE
ENSGALG00010028601	ANO8	PRJNA729271	7.079602691	7.2707E-222	7.079602691	stage_E14_vs_E10	ordinary_muscle_development	D_vs_E10;stage_1W_vs_E10;stage_3W_vs_E10;stage	1	TRUE
ENSGALG00010022252	COQ9	PRJNA729271	4.189568238	1.1809E-220	4.189568238	stage_E14_vs_E10	ordinary_muscle_development	D_vs_E10;stage_1W_vs_E10;stage_3W_vs_E10;stage	1	TRUE
ENSGALG00010009118	ACTN2	PRJNA729271	4.581529698	3.4751E-220	4.581529698	stage_E14_vs_E10	ordinary_muscle_development	D_vs_E10;stage_1W_vs_E10;stage_3W_vs_E10;stage	1	TRUE
ENSGALG00010029146	NEK6	PRJNA729271	-1.877592434	3.6969E-217	1.877592434	stage_E14_vs_E10	ordinary_muscle_development	D_vs_E10;stage_1W_vs_E10;stage_3W_vs_E10;stage	1	TRUE
ENSGALG00010008330	EZH2	PRJNA729271	-2.655461697	6.5685E-217	2.655461697	stage_E14_vs_E10	ordinary_muscle_development	D_vs_E10;stage_1W_vs_E10;stage_3W_vs_E10;stage	1	TRUE
ENSGALG00010016373	SRL	PRJNA729271	4.15746093	9.2198E-217	4.15746093	stage_E14_vs_E10	ordinary_muscle_development	D_vs_E10;stage_1W_vs_E10;stage_3W_vs_E10;stage	1	TRUE
ENSGALG00010024773	APOA1	PRJNA729271	8.278481855	5.2461E-213	8.278481855	stage_E14_vs_E10	ordinary_muscle_development	D_vs_E10;stage_1W_vs_E10;stage_3W_vs_E10;stage	1	TRUE
ENSGALG00010028044	GAMT	PRJNA729271	4.796541973	8.9206E-213	4.796541973	stage_E14_vs_E10	ordinary_muscle_development	D_vs_E10;stage_1W_vs_E10;stage_3W_vs_E10;stage	1	TRUE
ENSGALG00010020978	BCL2L10	PRJNA729271	3.583447976	4.0738E-210	3.583447976	stage_E14_vs_E10	ordinary_muscle_development	D_vs_E10;stage_1W_vs_E10;stage_3W_vs_E10;stage	1	TRUE
ENSGALG00010028479	CASQ1	PRJNA729271	9.770596722	9.2045E-210	9.770596722	stage_E14_vs_E10	ordinary_muscle_development	D_vs_E10;stage_1W_vs_E10;stage_3W_vs_E10;stage	1	TRUE
ENSGALG00010011239	RALGPS2	PRJNA729271	-2.476822177	1.4452E-209	2.476822177	stage_E14_vs_E10	ordinary_muscle_development	D_vs_E10;stage_1W_vs_E10;stage_3W_vs_E10;stage	1	TRUE
ENSGALG00010015191	TOM1	PRJNA729271	5.788019486	6.6538E-209	5.788019486	stage_E14_vs_E10	ordinary_muscle_development	D_vs_E10;stage_1W_vs_E10;stage_3W_vs_E10;stage	1	TRUE
ENSGALG00010017747	DUSP26	PRJNA729271	3.640749596	1.0251E-208	3.640749596	stage_E14_vs_E10	ordinary_muscle_development	D_vs_E10;stage_1W_vs_E10;stage_3W_vs_E10;stage	1	TRUE
ENSGALG00010027322	CRABP2	PRJNA729271	-6.907586537	5.1553E-207	6.907586537	stage_E14_vs_E10	ordinary_muscle_development	D_vs_E10;stage_1W_vs_E10;stage_3W_vs_E10;stage	1	TRUE
ENSGALG00010000081	ENSGALG00010000081	PRJNA729271	9.211939997	1.4792E-206	9.211939997	stage_E14_vs_E10	ordinary_muscle_development	D_vs_E10;stage_1W_vs_E10;stage_3W_vs_E10;stage	1	TRUE
ENSGALG00010007365	MEMO1	PRJNA729271	2.646655199	1.0042E-204	2.646655199	stage_E14_vs_E10	ordinary_muscle_development	D_vs_E10;stage_1W_vs_E10;stage_3W_vs_E10;stage	1	TRUE
ENSGALG00010006517	ZBTB20	PRJNA729271	4.422670698	7.9699E-204	4.422670698	stage_E14_vs_E10	ordinary_muscle_development	D_vs_E10;stage_1W_vs_E10;stage_3W_vs_E10;stage	1	TRUE
ENSGALG00010017010	NONO	PRJNA729271	-1.700254599	1.7905E-203	1.700254599	stage_E14_vs_E10	ordinary_muscle_development	D_vs_E10;stage_1W_vs_E10;stage_3W_vs_E10;stage	1	TRUE
ENSGALG00010017826	PABPC4	PRJNA729271	4.274655073	4.1568E-203	4.274655073	stage_E14_vs_E10	ordinary_muscle_development	D_vs_E10;stage_1W_vs_E10;stage_3W_vs_E10;stage	1	TRUE
ENSGALG00010024856	SORT1	PRJNA729271	4.01483085	1.3305E-202	4.01483085	stage_E14_vs_E10	ordinary_muscle_development	D_vs_E10;stage_1W_vs_E10;stage_3W_vs_E10;stage	1	TRUE
ENSGALG00010027351	TMEM38A	PRJNA729271	4.497656746	3.8496E-201	4.497656746	stage_E14_vs_E10	ordinary_muscle_development	D_vs_E10;stage_1W_vs_E10;stage_3W_vs_E10;stage	1	TRUE
ENSGALG00010005119	MIB1	PRJNA729271	-1.769018257	7.3573E-201	1.769018257	stage_E14_vs_E10	ordinary_muscle_development	D_vs_E10;stage_1W_vs_E10;stage_3W_vs_E10;stage	1	TRUE
ENSGALG00010023600	CHST14	PRJNA729271	-2.623457482	2.4305E-200	2.623457482	stage_E14_vs_E10	ordinary_muscle_development	D_vs_E10;stage_1W_vs_E10;stage_3W_vs_E10;stage	1	TRUE
ENSGALG00010028514	FNBP1	PRJNA729271	1.768029971	1.0332E-199	1.768029971	stage_E14_vs_E10	ordinary_muscle_development	D_vs_E10;stage_1W_vs_E10;stage_3W_vs_E10;stage	1	TRUE
ENSGALG00010008355	MAFA	PRJNA729271	5.368567653	1.8389E-199	5.368567653	stage_E14_vs_E10	ordinary_muscle_development	D_vs_E10;stage_1W_vs_E10;stage_3W_vs_E10;stage	1	TRUE
ENSGALG00010010115	NEB	PRJNA729271	5.170327351	4.6087E-197	5.170327351	stage_E14_vs_E10	ordinary_muscle_development	D_vs_E10;stage_1W_vs_E10;stage_3W_vs_E10;stage	1	TRUE
ENSGALG00010000408	ENSGALG00010000408	PRJNA729271	10.53612493	4.7597E-197	10.53612493	stage_E14_vs_E10	ordinary_muscle_development	D_vs_E10;stage_1W_vs_E10;stage_3W_vs_E10;stage	1	TRUE
ENSGALG00010018306	DES	PRJNA729271	4.524000548	3.046E-195	4.524000548	stage_E14_vs_E10	ordinary_muscle_development	D_vs_E10;stage_1W_vs_E10;stage_3W_vs_E10;stage	1	TRUE
ENSGALG00010019278	JPT2	PRJNA729271	-2.223378453	6.3098E-195	2.223378453	stage_E14_vs_E10	ordinary_muscle_development	D_vs_E10;stage_1W_vs_E10;stage_3W_vs_E10;stage	1	TRUE
ENSGALG00010002024	KLHL31	PRJNA729271	4.961917312	1.0349E-194	4.961917312	stage_E14_vs_E10	ordinary_muscle_development	D_vs_E10;stage_1W_vs_E10;stage_3W_vs_E10;stage	1	TRUE
ENSGALG00010024744	KLF13	PRJNA729271	3.819541384	4.5774E-194	3.819541384	stage_E14_vs_E10	ordinary_muscle_development	D_vs_E10;stage_1W_vs_E10;stage_3W_vs_E10;stage	1	TRUE
ENSGALG00010028073	ST6GALNAC4	PRJNA729271	3.586596621	7.9217E-194	3.586596621	stage_E14_vs_E10	ordinary_muscle_development	D_vs_E10;stage_1W_vs_E10;stage_3W_vs_E10;stage	1	TRUE
ENSGALG00010001890	KLHL34	PRJNA729271	7.484842431	1.0464E-193	7.484842431	stage_E14_vs_E10	ordinary_muscle_development	D_vs_E10;stage_1W_vs_E10;stage_3W_vs_E10;stage	1	TRUE
ENSGALG00010022303	DUSP13A	PRJNA729271	6.049581821	3.8975E-193	6.049581821	stage_E14_vs_E10	ordinary_muscle_development	D_vs_E10;stage_1W_vs_E10;stage_3W_vs_E10;stage	1	TRUE
ENSGALG00010003903	COL28A1	PRJNA729271	8.255121785	6.7527E-193	8.255121785	stage_E14_vs_E10	ordinary_muscle_development	D_vs_E10;stage_1W_vs_E10;stage_3W_vs_E10;stage	1	TRUE
ENSGALG00010013828	PTN	PRJNA729271	-8.226725205	3.5861E-191	8.226725205	stage_E14_vs_E10	ordinary_muscle_development	D_vs_E10;stage_1W_vs_E10;stage_3W_vs_E10;stage	1	TRUE
ENSGALG00010018123	USP13	PRJNA729271	4.171470383	5.1414E-191	4.171470383	stage_E14_vs_E10	ordinary_muscle_development	D_vs_E10;stage_1W_vs_E10;stage_3W_vs_E10;stage	1	TRUE
ENSGALG00010017558	ENSGALG00010017558	PRJNA729271	-2.262712917	1.9451E-190	2.262712917	stage_E14_vs_E10	ordinary_muscle_development	D_vs_E10;stage_1W_vs_E10;stage_3W_vs_E10;stage	1	TRUE
ENSGALG00010001971	MLIP	PRJNA729271	6.032331786	3.0391E-190	6.032331786	stage_E14_vs_E10	ordinary_muscle_development	D_vs_E10;stage_1W_vs_E10;stage_3W_vs_E10;stage	1	TRUE
ENSGALG00010026192	SMIM29	PRJNA729271	3.490363814	5.1987E-190	3.490363814	stage_E14_vs_E10	ordinary_muscle_development	D_vs_E10;stage_1W_vs_E10;stage_3W_vs_E10;stage	1	TRUE
ENSGALG00010013354	WWP1	PRJNA729271	4.297196926	5.7848E-190	4.297196926	stage_E14_vs_E10	ordinary_muscle_development	D_vs_E10;stage_1W_vs_E10;stage_3W_vs_E10;stage	1	TRUE
ENSGALG00010029187	SRSF1	PRJNA729271	-1.566883044	7.4496E-190	1.566883044	stage_E14_vs_E10	ordinary_muscle_development	D_vs_E10;stage_1W_vs_E10;stage_3W_vs_E10;stage	1	TRUE
ENSGALG00010019391	GANC	PRJNA729271	3.81130479	8.9714E-185	3.81130479	stage_E14_vs_E10	ordinary_muscle_development	D_vs_E10;stage_1W_vs_E10;stage_3W_vs_E10;stage	1	TRUE
ENSGALG00010022769	FXYD6	PRJNA729271	2.820102723	2.0477E-183	2.820102723	stage_E14_vs_E10	ordinary_muscle_development	D_vs_E10;stage_1W_vs_E10;stage_3W_vs_E10;stage	1	TRUE
ENSGALG00010017415	HDAC1	PRJNA729271	-1.914661168	7.1818E-183	1.914661168	stage_E14_vs_E10	ordinary_muscle_development	D_vs_E10;stage_1W_vs_E10;stage_3W_vs_E10;stage	1	TRUE
ENSGALG00010027801	MBD3	PRJNA729271	-3.945709634	1.4642E-182	3.945709634	stage_E14_vs_E10	ordinary_muscle_development	D_vs_E10;stage_1W_vs_E10;stage_3W_vs_E10;stage	1	TRUE
ENSGALG00010029537	MYH1B	PRJNA729271	5.963971513	4.728E-182	5.963971513	stage_E14_vs_E10	ordinary_muscle_development	D_vs_E10;stage_1W_vs_E10;stage_3W_vs_E10;stage	1	TRUE
ENSGALG00010013428	BPGM	PRJNA729271	4.421312119	5.4146E-182	4.421312119	stage_E14_vs_E10	ordinary_muscle_development	D_vs_E10;stage_1W_vs_E10;stage_3W_vs_E10;stage	1	TRUE
ENSGALG00010002342	BAG2	PRJNA729271	3.563102004	1.2889E-181	3.563102004	stage_E14_vs_E10	ordinary_muscle_development	D_vs_E10;stage_1W_vs_E10;stage_3W_vs_E10;stage	1	TRUE
ENSGALG00010023643	SCN4B	PRJNA729271	4.239525955	1.1509E-180	4.239525955	stage_E14_vs_E10	ordinary_muscle_development	D_vs_E10;stage_1W_vs_E10;stage_3W_vs_E10;stage	1	TRUE
ENSGALG00010014145	NTMT2	PRJNA729271	7.2322403	5.437E-180	7.2322403	stage_E14_vs_E10	ordinary_muscle_development	D_vs_E10;stage_1W_vs_E10;stage_3W_vs_E10;stage	1	TRUE
ENSGALG00010024095	RBM12	PRJNA729271	-1.558254417	6.4193E-180	1.558254417	stage_E14_vs_E10	ordinary_muscle_development	D_vs_E10;stage_1W_vs_E10;stage_3W_vs_E10;stage	1	TRUE
ENSGALG00010020382	MFAP2	PRJNA729271	-4.856693305	1.6236E-179	4.856693305	stage_E14_vs_E10	ordinary_muscle_development	D_vs_E10;stage_1W_vs_E10;stage_3W_vs_E10;stage	1	TRUE
ENSGALG00010027086	CACNA1S	PRJNA729271	5.773446585	3.522E-179	5.773446585	stage_E14_vs_E10	ordinary_muscle_development	D_vs_E10;stage_1W_vs_E10;stage_3W_vs_E10;stage	1	TRUE
ENSGALG00010025247	SGCA	PRJNA729271	3.997393186	5.8849E-178	3.997393186	stage_E14_vs_E10	ordinary_muscle_development	D_vs_E10;stage_1W_vs_E10;stage_3W_vs_E10;stage	1	TRUE
ENSGALG00010015035	SLC35F1	PRJNA729271	-3.943142072	1.2075E-177	3.943142072	stage_E14_vs_E10	ordinary_muscle_development	D_vs_E10;stage_1W_vs_E10;stage_3W_vs_E10;stage	1	TRUE
ENSGALG00010006279	HACD1	PRJNA729271	4.038859984	1.3172E-177	4.038859984	stage_E14_vs_E10	ordinary_muscle_development	D_vs_E10;stage_1W_vs_E10;stage_3W_vs_E10;stage	1	TRUE
ENSGALG00010026740	DAB1	PRJNA729271	-5.692149831	1.7313E-176	5.692149831	stage_E14_vs_E10	ordinary_muscle_development	D_vs_E10;stage_1W_vs_E10;stage_3W_vs_E10;stage	1	TRUE
ENSGALG00010020833	RANBP1	PRJNA729271	-2.401982946	2.1289E-176	2.401982946	stage_E14_vs_E10	ordinary_muscle_development	D_vs_E10;stage_1W_vs_E10;stage_3W_vs_E10;stage	1	TRUE
ENSGALG00010013394	DNMT3B	PRJNA729271	-7.480440992	3.4377E-176	7.480440992	stage_E14_vs_E10	ordinary_muscle_development	D_vs_E10;stage_1W_vs_E10;stage_3W_vs_E10;stage	1	TRUE
ENSGALG00010027739	FGD3	PRJNA729271	-2.17494365	4.9825E-176	2.17494365	stage_E14_vs_E10	ordinary_muscle_development	D_vs_E10;stage_1W_vs_E10;stage_3W_vs_E10;stage	1	TRUE
ENSGALG00010029144	ENSGALG00010029144	PRJNA729271	6.206588419	7.1374E-176	6.206588419	stage_E14_vs_E10	ordinary_muscle_development	D_vs_E10;stage_1W_vs_E10;stage_3W_vs_E10;stage	1	TRUE
ENSGALG00010005941	TXLNB	PRJNA729271	5.130362318	5.0563E-174	5.130362318	stage_E14_vs_E10	ordinary_muscle_development	D_vs_E10;stage_1W_vs_E10;stage_3W_vs_E10;stage	1	TRUE
ENSGALG00010012829	RGS5	PRJNA729271	5.095991552	2.2028E-173	5.095991552	stage_E14_vs_E10	ordinary_muscle_development	D_vs_E10;stage_1W_vs_E10;stage_3W_vs_E10;stage	1	TRUE
ENSGALG00010007889	DCAF6	PRJNA729271	2.737462924	2.5233E-173	2.737462924	stage_E14_vs_E10	ordinary_muscle_development	D_vs_E10;stage_1W_vs_E10;stage_3W_vs_E10;stage	1	TRUE
ENSGALG00010017712	MSH2	PRJNA729271	-2.124907612	3.2244E-173	2.124907612	stage_E14_vs_E10	ordinary_muscle_development	D_vs_E10;stage_1W_vs_E10;stage_3W_vs_E10;stage	1	TRUE
ENSGALG00010018166	MSS51	PRJNA729271	6.656186968	5.0865E-173	6.656186968	stage_E14_vs_E10	ordinary_muscle_development	D_vs_E10;stage_1W_vs_E10;stage_3W_vs_E10;stage	1	TRUE
ENSGALG00010002705	JADE3	PRJNA729271	-1.591150649	1.853E-171	1.591150649	stage_E14_vs_E10	ordinary_muscle_development	D_vs_E10;stage_1W_vs_E10;stage_3W_vs_E10;stage	1	TRUE
ENSGALG00010016916	ACYP2	PRJNA729271	4.410404054	3.8569E-171	4.410404054	stage_E14_vs_E10	ordinary_muscle_development	D_vs_E10;stage_1W_vs_E10;stage_3W_vs_E10;stage	1	TRUE
ENSGALG00010011968	ENSGALG00010011968	PRJNA729271	6.268303775	3.9225E-171	6.268303775	stage_E14_vs_E10	ordinary_muscle_development	D_vs_E10;stage_1W_vs_E10;stage_3W_vs_E10;stage	1	TRUE
ENSGALG00010001982	DMD	PRJNA729271	3.405746138	4.1987E-170	3.405746138	stage_E14_vs_E10	ordinary_muscle_development	D_vs_E10;stage_1W_vs_E10;stage_3W_vs_E10;stage	1	TRUE
ENSGALG00010016258	CTNNB1	PRJNA729271	-2.161171217	1.0025E-169	2.161171217	stage_E14_vs_E10	ordinary_muscle_development	D_vs_E10;stage_1W_vs_E10;stage_3W_vs_E10;stage	1	TRUE
ENSGALG00010000574	ENSGALG00010000574	PRJNA729271	4.764966292	2.9694E-169	4.764966292	stage_E14_vs_E10	ordinary_muscle_development	D_vs_E10;stage_1W_vs_E10;stage_3W_vs_E10;stage	1	TRUE
ENSGALG00010004339	B4GALT6	PRJNA729271	-4.058057614	6.1393E-169	4.058057614	stage_E14_vs_E10	ordinary_muscle_development	D_vs_E10;stage_1W_vs_E10;stage_3W_vs_E10;stage	1	TRUE
ENSGALG00010024373	TCAP	PRJNA729271	13.31093467	2.1876E-168	13.31093467	stage_E14_vs_E10	ordinary_muscle_development	D_vs_E10;stage_1W_vs_E10;stage_3W_vs_E10;stage	1	TRUE
ENSGALG00010020756	MACROH2A2	PRJNA729271	-4.816699854	2.373E-168	4.816699854	stage_E14_vs_E10	ordinary_muscle_development	D_vs_E10;stage_1W_vs_E10;stage_3W_vs_E10;stage	1	TRUE
ENSGALG00010014726	PWP2	PRJNA729271	-1.802897438	3.0966E-168	1.802897438	stage_E14_vs_E10	ordinary_muscle_development	D_vs_E10;stage_1W_vs_E10;stage_3W_vs_E10;stage	1	TRUE
ENSGALG00010019726	PEBP4	PRJNA729271	6.159078182	5.1987E-167	6.159078182	stage_E14_vs_E10	ordinary_muscle_development	D_vs_E10;stage_1W_vs_E10;stage_3W_vs_E10;stage	1	TRUE
ENSGALG00010017937	PTPN21	PRJNA729271	3.288669095	2.0676E-166	3.288669095	stage_E14_vs_E10	ordinary_muscle_development	D_vs_E10;stage_1W_vs_E10;stage_3W_vs_E10;stage	1	TRUE
ENSGALG00010003205	ENSGALG00010003205	PRJNA729271	7.293176744	1.032E-165	7.293176744	stage_E14_vs_E10	ordinary_muscle_development	D_vs_E10;stage_1W_vs_E10;stage_3W_vs_E10;stage	1	TRUE
ENSGALG00010022662	GFRA1	PRJNA729271	-4.753539593	1.2109E-164	4.753539593	stage_E14_vs_E10	ordinary_muscle_development	D_vs_E10;stage_1W_vs_E10;stage_3W_vs_E10;stage	1	TRUE

ENSGALG00010012008	ZNF366	PRJNA729271	4.803797703	2.7971E-163	4.803797703	stage_E14_vs_E10	ordinary_muscle_development	D_vs_E10;stage_1W_vs_E10;stage_3W_vs_E10;stage	1	TRUE
ENSGALG00010016991	PDLIM3	PRJNA729271	5.453618948	4.6813E-163	5.453618948	stage_E14_vs_E10	ordinary_muscle_development	D_vs_E10;stage_1W_vs_E10;stage_3W_vs_E10;stage	1	TRUE
ENSGALG00010024280	ENSGALG00010024280	PRJNA729271	3.389325812	1.5732E-162	3.389325812	stage_E14_vs_E10	ordinary_muscle_development	D_vs_E10;stage_1W_vs_E10;stage_3W_vs_E10;stage	1	TRUE
ENSGALG00010022950	ITGA7	PRJNA729271	4.201970744	1.0035E-161	4.201970744	stage_E14_vs_E10	ordinary_muscle_development	D_vs_E10;stage_1W_vs_E10;stage_3W_vs_E10;stage	1	TRUE
ENSGALG00010028507	ANP32E	PRJNA729271	-1.56428816	1.4185E-161	1.56428816	stage_E14_vs_E10	ordinary_muscle_development	D_vs_E10;stage_1W_vs_E10;stage_3W_vs_E10;stage	1	TRUE
ENSGALG00010028148	ENSGALG00010028148	PRJNA729271	6.929762193	1.2144E-160	6.929762193	stage_E14_vs_E10	ordinary_muscle_development	D_vs_E10;stage_1W_vs_E10;stage_3W_vs_E10;stage	1	TRUE
ENSGALG00010014990	AGO1	PRJNA729271	-1.33016613	1.9534E-159	1.33016613	stage_E14_vs_E10	ordinary_muscle_development	D_vs_E10;stage_1W_vs_E10;stage_3W_vs_E10;stage	1	TRUE
ENSGALG00010016973	PLSCR1	PRJNA729271	3.250409248	3.786E-159	3.250409248	stage_E14_vs_E10	ordinary_muscle_development	D_vs_E10;stage_1W_vs_E10;stage_3W_vs_E10;stage	1	TRUE
ENSGALG00010023281	B2M	PRJNA729271	5.36243446	7.2145E-158	5.36243446	stage_E14_vs_E10	ordinary_muscle_development	D_vs_E10;stage_1W_vs_E10;stage_3W_vs_E10;stage	1	TRUE
ENSGALG00010005587	LEF1	PRJNA729271	-6.397862127	4.6332E-157	6.397862127	stage_E14_vs_E10	ordinary_muscle_development	D_vs_E10;stage_1W_vs_E10;stage_3W_vs_E10;stage	1	TRUE
ENSGALG00010022255	NOC4L	PRJNA729271	-2.539733937	7.5603E-157	2.539733937	stage_E14_vs_E10	ordinary_muscle_development	D_vs_E10;stage_1W_vs_E10;stage_3W_vs_E10;stage	1	TRUE
ENSGALG00010013846	YIPF7	PRJNA729271	8.549853195	3.1445E-156	8.549853195	stage_E14_vs_E10	ordinary_muscle_development	D_vs_E10;stage_1W_vs_E10;stage_3W_vs_E10;stage	1	TRUE
ENSGALG00010013451	PRKAB2	PRJNA729271	4.468790219	4.9513E-156	4.468790219	stage_E14_vs_E10	ordinary_muscle_development	D_vs_E10;stage_1W_vs_E10;stage_3W_vs_E10;stage	1	TRUE
ENSGALG00010005544	AQP4	PRJNA729271	8.245774597	6.1817E-155	8.245774597	stage_E14_vs_E10	ordinary_muscle_development	D_vs_E10;stage_1W_vs_E10;stage_3W_vs_E10;stage	1	TRUE
ENSGALG00010007781	MBP	PRJNA729271	6.27202769	6.1817E-155	6.27202769	stage_E14_vs_E10	ordinary_muscle_development	D_vs_E10;stage_1W_vs_E10;stage_3W_vs_E10;stage	1	TRUE
ENSGALG00010018577	NARFL	PRJNA729271	2.105738012	7.1024E-155	2.105738012	stage_E14_vs_E10	ordinary_muscle_development	D_vs_E10;stage_1W_vs_E10;stage_3W_vs_E10;stage	1	TRUE
ENSGALG00010022005	NLRC5	PRJNA729271	4.826048561	9.2579E-155	4.826048561	stage_E14_vs_E10	ordinary_muscle_development	D_vs_E10;stage_1W_vs_E10;stage_3W_vs_E10;stage	1	TRUE
ENSGALG00010014987	KLF9	PRJNA729271	7.720526335	3.2287E-154	7.720526335	stage_E14_vs_E10	ordinary_muscle_development	D_vs_E10;stage_1W_vs_E10;stage_3W_vs_E10;stage	1	TRUE
ENSGALG00010018048	GFRA4	PRJNA729271	-4.669671751	5.6002E-154	4.669671751	stage_E14_vs_E10	ordinary_muscle_development	D_vs_E10;stage_1W_vs_E10;stage_3W_vs_E10;stage	1	TRUE
ENSGALG00010029937	PMP22	PRJNA729271	3.287956864	1.0809E-153	3.287956864	stage_E14_vs_E10	ordinary_muscle_development	D_vs_E10;stage_1W_vs_E10;stage_3W_vs_E10;stage	1	TRUE
ENSGALG00010016370	TMEM255A	PRJNA729271	4.91686447	2.0945E-153	4.91686447	stage_E14_vs_E10	ordinary_muscle_development	D_vs_E10;stage_1W_vs_E10;stage_3W_vs_E10;stage	1	TRUE
ENSGALG00010008316	ADGRF5	PRJNA729271	3.54036565	6.3517E-153	3.54036565	stage_E14_vs_E10	ordinary_muscle_development	D_vs_E10;stage_1W_vs_E10;stage_3W_vs_E10;stage	1	TRUE
ENSGALG00010001850	MYOM2	PRJNA729271	4.979155005	6.9328E-153	4.979155005	stage_E14_vs_E10	ordinary_muscle_development	D_vs_E10;stage_1W_vs_E10;stage_3W_vs_E10;stage	1	TRUE
ENSGALG00010017642	MTARC1	PRJNA729271	3.223065194	8.6414E-153	3.223065194	stage_E14_vs_E10	ordinary_muscle_development	D_vs_E10;stage_1W_vs_E10;stage_3W_vs_E10;stage	1	TRUE
ENSGALG00010005743	ETNPPL	PRJNA729271	5.913619149	1.2516E-152	5.913619149	stage_E14_vs_E10	ordinary_muscle_development	D_vs_E10;stage_1W_vs_E10;stage_3W_vs_E10;stage	1	TRUE
ENSGALG00010001196	LSMEM1	PRJNA729271	5.431886247	2.6653E-152	5.431886247	stage_E14_vs_E10	ordinary_muscle_development	D_vs_E10;stage_1W_vs_E10;stage_3W_vs_E10;stage	1	TRUE
ENSGALG00010012436	ENSGALG00010012436	PRJNA729271	5.275352575	7.2666E-152	5.275352575	stage_E14_vs_E10	ordinary_muscle_development	D_vs_E10;stage_1W_vs_E10;stage_3W_vs_E10;stage	1	TRUE
ENSGALG00010019801	FIBIN	PRJNA729271	-4.99627616	1.2341E-151	4.99627616	stage_E14_vs_E10	ordinary_muscle_development	D_vs_E10;stage_1W_vs_E10;stage_3W_vs_E10;stage	1	TRUE
ENSGALG00010024428	SAAL1	PRJNA729271	-1.742822514	1.6463E-151	1.742822514	stage_E14_vs_E10	ordinary_muscle_development	D_vs_E10;stage_1W_vs_E10;stage_3W_vs_E10;stage	1	TRUE
ENSGALG00010016431	B3GNT5	PRJNA729271	-4.314799285	6.0784E-151	4.314799285	stage_E14_vs_E10	ordinary_muscle_development	D_vs_E10;stage_1W_vs_E10;stage_3W_vs_E10;stage	1	TRUE
ENSGALG00010009518	FGL2	PRJNA729271	7.587401526	8.6251E-151	7.587401526	stage_E14_vs_E10	ordinary_muscle_development	D_vs_E10;stage_1W_vs_E10;stage_3W_vs_E10;stage	1	TRUE
ENSGALG00010018747	FLRT3	PRJNA729271	-3.384442359	1.4037E-150	3.384442359	stage_E14_vs_E10	ordinary_muscle_development	D_vs_E10;stage_1W_vs_E10;stage_3W_vs_E10;stage	1	TRUE
ENSGALG00010008960	LRRC2	PRJNA729271	10.46217216	1.4559E-150	10.46217216	stage_E14_vs_E10	ordinary_muscle_development	D_vs_E10;stage_1W_vs_E10;stage_3W_vs_E10;stage	1	TRUE
ENSGALG00010027427	UBAC1	PRJNA729271	3.036785583	2.7134E-150	3.036785583	stage_E14_vs_E10	ordinary_muscle_development	D_vs_E10;stage_1W_vs_E10;stage_3W_vs_E10;stage	1	TRUE
ENSGALG00010021212	MYO18B	PRJNA729271	4.231830153	2.9777E-150	4.231830153	stage_E14_vs_E10	ordinary_muscle_development	D_vs_E10;stage_1W_vs_E10;stage_3W_vs_E10;stage	1	TRUE
ENSGALG00010029560	ENSGALG00010029560	PRJNA729271	4.385642563	4.2916E-150	4.385642563	stage_E14_vs_E10	ordinary_muscle_development	D_vs_E10;stage_1W_vs_E10;stage_3W_vs_E10;stage	1	TRUE
ENSGALG00010012709	PPP1R8	PRJNA729271	-1.573706746	4.8284E-150	1.573706746	stage_E14_vs_E10	ordinary_muscle_development	D_vs_E10;stage_1W_vs_E10;stage_3W_vs_E10;stage	1	TRUE
ENSGALG00010012019	ENSGALG00010012019	PRJNA729271	7.887222137	7.0693E-150	7.887222137	stage_E14_vs_E10	ordinary_muscle_development	D_vs_E10;stage_1W_vs_E10;stage_3W_vs_E10;stage	1	TRUE
ENSGALG00010003110	BF1	PRJNA729271	6.701907313	8.1157E-149	6.701907313	stage_E14_vs_E10	ordinary_muscle_development	D_vs_E10;stage_1W_vs_E10;stage_3W_vs_E10;stage	1	TRUE
ENSGALG00010017817	REEP1	PRJNA729271	3.044134261	1.0241E-148	3.044134261	stage_E14_vs_E10	ordinary_muscle_development	D_vs_E10;stage_1W_vs_E10;stage_3W_vs_E10;stage	1	TRUE
ENSGALG00010024107	NFE2L2	PRJNA729271	1.632948068	1.1238E-148	1.632948068	stage_E14_vs_E10	ordinary_muscle_development	D_vs_E10;stage_1W_vs_E10;stage_3W_vs_E10;stage	1	TRUE
ENSGALG00010028029	EFNA2	PRJNA729271	-7.193881808	2.6874E-148	7.193881808	stage_E14_vs_E10	ordinary_muscle_development	D_vs_E10;stage_1W_vs_E10;stage_3W_vs_E10;stage	1	TRUE
ENSGALG00010029554	ENSGALG00010029554	PRJNA729271	9.517476385	5.7975E-148	9.517476385	stage_E14_vs_E10	ordinary_muscle_development	D_vs_E10;stage_1W_vs_E10;stage_3W_vs_E10;stage	1	TRUE
ENSGALG00010016652	FXR1	PRJNA729271	2.576099923	7.4571E-148	2.576099923	stage_E14_vs_E10	ordinary_muscle_development	D_vs_E10;stage_1W_vs_E10;stage_3W_vs_E10;stage	1	TRUE
ENSGALG00010027932	POLE3	PRJNA729271	-2.026650914	8.8583E-148	2.026650914	stage_E14_vs_E10	ordinary_muscle_development	D_vs_E10;stage_1W_vs_E10;stage_3W_vs_E10;stage	1	TRUE
ENSGALG00010008217	ARHGEF37	PRJNA729271	4.202290601	9.1018E-148	4.202290601	stage_E14_vs_E10	ordinary_muscle_development	D_vs_E10;stage_1W_vs_E10;stage_3W_vs_E10;stage	1	TRUE
ENSGALG00010019120	DAGLB	PRJNA729271	2.012179109	1.094E-147	2.012179109	stage_E14_vs_E10	ordinary_muscle_development	D_vs_E10;stage_1W_vs_E10;stage_3W_vs_E10;stage	1	TRUE
ENSGALG00010021619	FHOD1	PRJNA729271	4.643681046	3.3359E-147	4.643681046	stage_E14_vs_E10	ordinary_muscle_development	D_vs_E10;stage_1W_vs_E10;stage_3W_vs_E10;stage	1	TRUE
ENSGALG00010003022	BLB2	PRJNA729271	6.90804765	3.9579E-147	6.90804765	stage_E14_vs_E10	ordinary_muscle_development	D_vs_E10;stage_1W_vs_E10;stage_3W_vs_E10;stage	1	TRUE
ENSGALG00010029349	MYH10	PRJNA729271	-2.799996582	3.97E-147	2.799996582	stage_E14_vs_E10	ordinary_muscle_development	D_vs_E10;stage_1W_vs_E10;stage_3W_vs_E10;stage	1	TRUE
ENSGALG00010021299	CYP27C1	PRJNA729271	-5.178703075	4.9346E-147	5.178703075	stage_E14_vs_E10	ordinary_muscle_development	D_vs_E10;stage_1W_vs_E10;stage_3W_vs_E10;stage	1	TRUE
ENSGALG00010005272	MBNL2	PRJNA729271	2.403081364	1.1234E-146	2.403081364	stage_E14_vs_E10	ordinary_muscle_development	D_vs_E10;stage_1W_vs_E10;stage_3W_vs_E10;stage	1	TRUE
ENSGALG00010012956	FABP3	PRJNA729271	4.367416758	1.5528E-146	4.367416758	stage_E14_vs_E10	ordinary_muscle_development	D_vs_E10;stage_1W_vs_E10;stage_3W_vs_E10;stage	1	TRUE
ENSGALG00010010572	AQP3	PRJNA729271	3.785988759	3.5618E-146	3.785988759	stage_E14_vs_E10	ordinary_muscle_development	D_vs_E10;stage_1W_vs_E10;stage_3W_vs_E10;stage	1	TRUE
ENSGALG00010028561	SET	PRJNA729271	-1.342626241	6.4981E-146	1.342626241	stage_E14_vs_E10	ordinary_muscle_development	D_vs_E10;stage_1W_vs_E10;stage_3W_vs_E10;stage	1	TRUE
ENSGALG00010014082	HNRNPR	PRJNA729271	-1.123760643	2.243E-145	1.123760643	stage_E14_vs_E10	ordinary_muscle_development	D_vs_E10;stage_1W_vs_E10;stage_3W_vs_E10;stage	1	TRUE
ENSGALG00010021684	BIN1	PRJNA729271	3.137899748	2.2989E-145	3.137899748	stage_E14_vs_E10	ordinary_muscle_development	D_vs_E10;stage_1W_vs_E10;stage_3W_vs_E10;stage	1	TRUE
ENSGALG00010017212	TINAGL1	PRJNA729271	3.481157877	3.8969E-145	3.481157877	stage_E14_vs_E10	ordinary_muscle_development	D_vs_E10;stage_1W_vs_E10;stage_3W_vs_E10;stage	1	TRUE
ENSGALG00010023135	GINS3	PRJNA729271	-1.910490073	6.3921E-145	1.910490073	stage_E14_vs_E10	ordinary_muscle_development	D_vs_E10;stage_1W_vs_E10;stage_3W_vs_E10;stage	1	TRUE
ENSGALG00010023411	ENSGALG00010023411	PRJNA729271	4.643492862	9.2711E-145	4.643492862	stage_E14_vs_E10	ordinary_muscle_development	D_vs_E10;stage_1W_vs_E10;stage_3W_vs_E10;stage	1	TRUE
ENSGALG00010000822	BASP1	PRJNA729271	-4.321491187	2.6643E-144	4.321491187	stage_E14_vs_E10	ordinary_muscle_development	D_vs_E10;stage_1W_vs_E10;stage_3W_vs_E10;stage	1	TRUE
ENSGALG00010016374	FRMD5	PRJNA729271	8.617149551	6.4104E-144	8.617149551	stage_E14_vs_E10	ordinary_muscle_development	D_vs_E10;stage_1W_vs_E10;stage_3W_vs_E10;stage	1	TRUE
ENSGALG00010005915	PAPSS1	PRJNA729271	-2.630649198	7.6179E-144	2.630649198	stage_E14_vs_E10	ordinary_muscle_development	D_vs_E10;stage_1W_vs_E10;stage_3W_vs_E10;stage	1	TRUE
ENSGALG00010003132	TPBG	PRJNA729271	-4.511232397	8.5301E-144	4.511232397	stage_E14_vs_E10	ordinary_muscle_development	D_vs_E10;stage_1W_vs_E10;stage_3W_vs_E10;stage	1	TRUE
ENSGALG00010001912	TMEM117	PRJNA729271	5.254341401	5.4384E-143	5.254341401	stage_E14_vs_E10	ordinary_muscle_development	D_vs_E10;stage_1W_vs_E10;stage_3W_vs_E10;stage	1	TRUE
ENSGALG00010028301	FBN3	PRJNA729271	-4.771397654	2.2281E-142	4.771397654	stage_E14_vs_E10	ordinary_muscle_development	D_vs_E10;stage_1W_vs_E10;stage_3W_vs_E10;stage	1	TRUE
ENSGALG00010009647	GALNT2	PRJNA729271	-2.03968568	5.4699E-142	2.03968568	stage_E14_vs_E10	ordinary_muscle_development	D_vs_E10;stage_1W_vs_E10;stage_3W_vs_E10;stage	1	TRUE
ENSGALG00010000821	ENSGALG00010000821	PRJNA729271	3.788912024	6.5381E-142	3.788912024	stage_E14_vs_E10	ordinary_muscle_development	D_vs_E10;stage_1W_vs_E10;stage_3W_vs_E10;stage	1	TRUE
ENSGALG00010028869	ADAMTS13	PRJNA729271	4.141936441	1.3003E-141	4.141936441	stage_E14_vs_E10	ordinary_muscle_development	D_vs_E10;stage_1W_vs_E10;stage_3W_vs_E10;stage	1	TRUE
ENSGALG00010023195	ENSGALG00010023195	PRJNA729271	-5.418282694	1.899E-141	5.418282694	stage_E14_vs_E10	ordinary_muscle_development	D_vs_E10;stage_1W_vs_E10;stage_3W_vs_E10;stage	1	TRUE
ENSGALG00010007936	ENSGALG00010007936	PRJNA729271	3.549267934	2.0229E-141	3.549267934	stage_E14_vs_E10	ordinary_muscle_development	D_vs_E10;stage_1W_vs_E10;stage_3W_vs_E10;stage	1	TRUE
ENSGALG00010011424	ACADL	PRJNA729271	2.470651734	2.4846E-141	2.470651734	stage_E14_vs_E10	ordinary_muscle_development	D_vs_E10;stage_1W_vs_E10;stage_3W_vs_E10;stage	1	TRUE
ENSGALG00010004233	NHSL1	PRJNA729271	-3.262435491	2.494E-141	3.262435491	stage_E14_vs_E10	ordinary_muscle_development	D_vs_E10;stage_1W_vs_E10;stage_3W_vs_E10;stage	1	TRUE
ENSGALG00010019645	ENSGALG00010019645	PRJNA729271	4.09252773	5.0406E-141	4.09252773	stage_E14_vs_E10	ordinary_muscle_development	D_vs_E10;stage_1W_vs_E10;stage_3W_vs_E10;stage	1	TRUE
ENSGALG00010014125	PLPP1	PRJNA729271	3.528089604	5.3698E-141	3.528089604	stage_E14_vs_E10	ordinary_muscle_development	D_vs_E10;stage_1W_vs_E10;stage_3W_vs_E10;stage	1	TRUE
ENSGALG00010016907	SQSTM1	PRJNA729271	4.04726701	1.5274E-140	4.04726701	stage_E14_vs_E10	ordinary_muscle_development	D_vs_E10;stage_1W_vs_E10;stage_3W_vs_E10;stage	1	TRUE
ENSGALG00010012093	ENSGALG00010012093	PRJNA729271	-1.76259038	3.2468E-140	1.76259038	stage_E14_vs_E10	ordinary_muscle_development	D_vs_E10;stage_1W_vs_E10;stage_3W_vs_E10;stage	1	TRUE
ENSGALG00010020770	B4GALNT4	PRJNA729271	-5.305702058	2.8959E-139	5.305702058	stage_E14_vs_E10	ordinary_muscle_development	D_vs_E10;stage_1W_vs_E10;stage_3W_vs_E10;stage	1	TRUE
ENSGALG00010024731	AOX1	PRJNA729271	4.98544945	6.8367E-139	4.98544945	stage_E14_vs_E10	ordinary_muscle_development	D_vs_E10;stage_1W_vs_E10;stage_3W_vs_E10;stage	1	TRUE
ENSGALG00010024025	CLCN1	PRJNA729271	8.417789319	8.3531E-139	8.417789319	stage_E14_vs_E10	ordinary_muscle_development	D_vs_E10;stage_1W_vs_E10;stage_3W_vs_E10;stage	1	TRUE
ENSGALG00010024552	CD81	PRJNA729271	1.67147236	1.9531E-138	1.67147236	stage_E14_vs_E10	ordinary_muscle_development	D_vs_E10;stage_1W_vs_E10;stage_3W_vs_E10;stage	1	TRUE
ENSGALG00010015896	LONRF2	PRJNA729271	1.952685473	5.4176E-138	1.952685473	stage_E14_vs_E10	ordinary_muscle_development	D_vs_E10;stage_1W_vs_E10;stage_3W_vs_E10;stage	1	TRUE
ENSGALG00010002878	P2RY2	PRJNA729271	6.280030343	1.7468E-137	6.280030343	stage_E14_vs_E10	ordinary_muscle_development	D_vs_E10;stage_1W_vs_E10;stage_3W_vs_E10;stage	1	TRUE
ENSGALG00010029214	ENSGALG00010029214	PRJNA729271	9.41353013	1.8473E-137	9.41353013	stage_E14_vs_E10	ordinary_muscle_development	D_vs_E10;stage_1W_vs_E10;stage_3W_vs_E10;stage	1	TRUE
ENSGALG00010025327	TMEM59	PRJNA729271	1.773841028	2.9587E-137	1.773841028	stage_E14_vs_E10	ordinary_muscle_development	D_vs_E10;stage_1W_vs_E10;stage_3W_vs_E10;stage	1	TRUE
ENSGALG00010014362	NAXD	PRJNA729271	2.661178444	4.0117E-137	2.661178444	stage_E14_vs_E10	ordinary_muscle_development	D_vs_E10;stage_1W_vs_E10;stage_3W_vs_E10;stage	1	TRUE
ENSGALG00010018053	DIAPH2	PRJNA729271	2.288192284	4.688E-137	2.288192284	stage_E14_vs_E10	ordinary_muscle_development	D_vs_E10;stage_1W_vs_E10;stage_3W_vs_E10;stage	1	TRUE
ENSGALG00010005383	OPTN	PRJNA729271	3.466140464	6.3839E-137	3.466140464	stage_E14_vs_E10	ordinary_muscle_development	D_vs_E10;stage_1W_vs_E10;stage_3W_vs_E10;stage	1	TRUE
ENSGALG00010002309	GADL1	PRJNA729271	8.152032438	1.4E-136	8.152032438	stage_E14_vs_E10	ordinary_muscle_development	D_vs_E10;stage_1W_vs_E10;stage_3W_vs_E10;stage	1	TRUE
ENSGALG00010021632	LIMD2	PRJNA729271	-4.272513944	3.3E-136	4.272513944	stage_E14_vs_E10	ordinary_muscle_development	D_vs_E10;stage_1W_vs_E10;stage_3W_vs_E10;stage	1	TRUE
ENSGALG00010009538	CRIM1	PRJNA729271	1.844772661	3.5143E-136	1.844772661	stage_E14_vs_E10	ordinary_muscle_development	D_vs_E10;stage_1W_vs_E10;stage_3W_vs_E10;stage	1	TRUE
ENSGALG00010013390	SLC16A2	PRJNA729271	-3.445374593	3.6148E-136	3.445374593	stage_E14_vs_E10	ordinary_muscle_development	D_vs_E10;stage_1W_vs_E10;stage_3W_vs_E10;stage	1	TRUE
ENSGALG00010022174	KCNMA1	PRJNA729271	3.082244705	3.6736E-136	3.082244705	stage_E14_vs_E10	ordinary_muscle_development	D_vs_E10;stage_1W_vs_E10;stage_3W_vs_E10;stage	1	TRUE
ENSGALG00010002998	LECT1	PRJNA729271	-7.563609488	3.7059E-136	7.563609488	stage_E14_vs_E10	ordinary_muscle_development	D_vs_E10;stage_1W_vs_E10;stage_3W_vs_E10;stage	1	TRUE
ENSGALG00010006767	ENSGALG00010006767	PRJNA729271	8.241389384	5.5836E-136	8.241389384	stage_E14_vs_E10	ordinary_muscle_development	D_vs_E10;stage_1W_vs_E10;stage_3W_vs_E10;stage	1	TRUE
ENSGALG00010028070	MEX3A	PRJNA729271	-4.325471865	5.5919E-136	4.325471865	stage_E14_vs_E10	ordinary_muscle_development	D_vs_E10;stage_1W_vs_E10;stage_3W_vs_E10;stage	1	TRUE
ENSGALG00010015731	PHF6	PRJNA729271	-1.790619171	8.9434E-136	1.790619171	stage_E14_vs_E10	ordinary_muscle_development	D_vs_E10;stage_1W_vs_E10;stage_3W_vs_E10;stage	1	TRUE
ENSGALG00010009297	CTNNA3	PRJNA729271	5.087470838	2.2204E-135	5.087470838	stage_E14_vs_E10	ordinary_muscle_development	D_vs_E10;stage_1W_vs_E10;stage_3W_vs_E10;stage	1	TRUE
ENSGALG00010005555	PRSS12	PRJNA729271	-4.861498748	2.2517E-135	4.861498748	stage_E14_vs_E10	ordinary_muscle_development	D_vs_E10;stage_1W_vs_E10;stage_3W_vs_E10;stage	1	TRUE
ENSGALG00010008036	SPOCK3	PRJNA729271	-4.947782309	6.55E-135	4.947782309	stage_E14_vs_E10	ordinary_muscle_development	D_vs_E10;stage_1W_vs_E10;stage_3W_vs_E10;stage	1	TRUE
ENSGALG00010029336	SYNGR2	PRJNA729271	2.737198167	7.2586E-135	2.737198167	stage_E14_vs_E10	ordinary_muscle_development	D_vs_E10;stage_1W_vs_E10;stage_3W_vs_E10;stage	1	TRUE
ENSGALG00010009653	ABLIM2	PRJNA729271	4.556458058	8.7208E-135	4.556458058	stage_E14_vs_E10	ordinary_muscle_development	D_vs_E10;stage_1W_vs_E10;stage_3W_vs_E10;stage	1	TRUE
ENSGALG00010017679	RASL12	PRJNA729271	4.363166829	3.4049E-134	4.363166829	stage_E14_vs_E10	ordinary_muscle_development	D_vs_E10;stage_1W_vs_E10;stage_3W_vs_E10;stage	1	TRUE
ENSGALG00010024751	MTMR10	PRJNA729271	1.383305855	2.8179E-133	1.383305855	stage_E14_vs_E10	ordinary_muscle_development	D_vs_E10;stage_1W_vs_E10;stage_3W_vs_E10;stage	1	TRUE
ENSGALG00010016807	VSIG4	PRJNA729271	5.678408951	3.0525E-133	5.678408951	stage_E14_vs_E10	ordinary_muscle_development	D_vs_E10;stage_1W_vs_E10;stage_3W_vs_E10;stage	1	TRUE
ENSGALG00010024665	MAP1LC3A	PRJNA729271	3.637804312	1.751E-132	3.637804312	stage_E14_vs_E10	ordinary_muscle_development	D_vs_E10;stage_1W_vs_E10;stage_3W_vs_E10;stage	1	TRUE
ENSGALG00010013095	ADSL	PRJNA729271	3.145460619	1.9496E-132	3.145460619	stage_E14_vs_E10	ordinary_muscle_development	D_vs_E10;stage_1W_vs_E10;stage_3W_vs_E10;stage	1	TRUE
ENSGALG00010008215	SELENOW	PRJNA729271	3.138581602	1.7596E-131	3.138581602	stage_E14_vs_E10	ordinary_muscle_development	D_vs_E10;stage_1W_vs_E10;stage_3W_vs_E10;stage	1	TRUE
ENSGALG00010014713	ST8SIA1	PRJNA729271	-3.597216636	1.842E-131	3.597216636	stage_E14_vs_E10	ordinary_muscle_development	D_vs_E10;stage_1W_vs_E10;stage_3W_vs_E10;stage	1	TRUE
ENSGALG00010027036	NUCKS1	PRJNA729271	-1.625547497	1.9897E-131	1.625547497	stage_E14_vs_E10	ordinary_muscle_development	D_vs_E10;stage_1W_vs_E10;stage_3W_vs_E10;stage	1	TRUE
ENSGALG00010016889	FUNDC2	PRJNA729271	2.132923009	2.097E-131	2.132923009	stage_E14_vs_E10	ordinary_muscle_development	D_vs_E10;stage_1W_vs_E10;stage_3W_vs_E10;stage	1	TRUE
ENSGALG00010026102	IPO9	PRJNA729271	-1.484267053	2.5545E-131	1.484267053	stage_E14_vs_E10	ordinary_muscle_development	D_vs_E10;stage_1W_vs_E10;stage_3W_vs_E10;stage	1	TRUE
ENSGALG00010025346	PXYLP1	PRJNA729271	-3.263211358	4.3147E-131	3.263211358	stage_E14_vs_E10	ordinary_muscle_development	D_vs_E10;stage_1W_vs_E10;stage_3W_vs_E10;stage	1	TRUE
ENSGALG00010028447	OLFM1	PRJNA729271	-4.068268719	7.1867E-131	4.068268719	stage_E14_vs_E10	ordinary_muscle_development	D_vs_E10;stage_1W_vs_E10;stage_3W_vs_E10;stage	1	TRUE
ENSGALG00010013259	PTPRB	PRJNA729271	3.402101641	1.4934E-130	3.402101641	stage_E14_vs_E10	ordinary_muscle_development	D_vs_E10;stage_1W_vs_E10;stage_3W_vs_E10;stage	1	TRUE
ENSGALG00010016940	FGFR3	PRJNA729271	-5.586470929	3.2212E-130	5.586470929	stage_E14_vs_E10	ordinary_muscle_development	D_vs_E10;stage_1W_vs_E10;stage_3W_vs_E10;stage	1	TRUE
ENSGALG00010009050	PIK3CG	PRJNA729271	2.2225952	1.4651E-129	2.2225952	stage_E14_vs_E10	ordinary_muscle_development	D_vs_E10;stage_1W_vs_E10;stage_3W_vs_E10;stage	1	TRUE
ENSGALG00010006948	ENSGALG00010006948	PRJNA729271	-10.84713713	1.8936E-129	10.84713713	stage_E14_vs_E10	ordinary_muscle_development	D_vs_E10;stage_1W_vs_E10;stage_3W_vs_E10;stage	1	TRUE
ENSGALG00010000247	ENSGALG00010000247	PRJNA729271	5.444414033	3.9378E-129	5.444414033	stage_E14_vs_E10	ordinary_muscle_development	D_vs_E10;stage_1W_vs_E10;stage_3W_vs_E10;stage	1	TRUE
ENSGALG00010016651	ENSGALG00010016651	PRJNA729271	4.331597951	6.1986E-129	4.331597951	stage_E14_vs_E10	ordinary_muscle_development	D_vs_E10;stage_1W_vs_E10;stage_3W_vs_E10;stage	1	TRUE
ENSGALG00010010300	DENND1B	PRJNA729271	2.201031351	7.7217E-129	2.201031351	stage_E14_vs_E10	ordinary_muscle_development	D_vs_E10;stage_1W_vs_E10;stage_3W_vs_E10;stage	1	TRUE
ENSGALG00010000146	ACADM	PRJNA729271	3.838119711	7.8348E-129	3.838119711	stage_E14_vs_E10	ordinary_muscle_development	D_vs_E10;stage_1W_vs_E10;stage_3W_vs_E10;stage	1	TRUE
ENSGALG00010027050	GSTT1	PRJNA729271	3.683432833	1.1402E-128	3.683432833	stage_E14_vs_E10	ordinary_muscle_development	D_vs_E10;stage_1W_vs_E10;stage_3W_vs_E10;stage	1	TRUE
ENSGALG00010020562	LRRN1	PRJNA729271	-6.25388992	3.5274E-128	6.25388992	stage_E14_vs_E10	ordinary_muscle_development	D_vs_E10;stage_1W_vs_E10;stage_3W_vs_E10;stage	1	TRUE
ENSGALG00010027419	PTBP1	PRJNA729271	-2.07289986	3.5736E-128	2.07289986	stage_E14_vs_E10	ordinary_muscle_development	D_vs_E10;stage_1W_vs_E10;stage_3W_vs_E10;stage	1	TRUE
ENSGALG00010017393	VDAC1	PRJNA729271	3.960268978	9.8961E-128	3.960268978	stage_E14_vs_E10	ordinary_muscle_development	D_vs_E10;stage_1W_vs_E10;stage_3W_vs_E10;stage	1	TRUE
ENSGALG00010017056	AGTR1	PRJNA729271	4.888478787	4.895E-127	4.888478787	stage_E14_vs_E10	ordinary_muscle_development	D_vs_E10;stage_1W_vs_E10;stage_3W_vs_E10;stage	1	TRUE
ENSGALG00010007929	LRRC14B	PRJNA729271	7.950484899	2.8461E-126	7.950484899	stage_E14_vs_E10	ordinary_muscle_development	D_vs_E10;stage_1W_vs_E10;stage_3W_vs_E10;stage	1	TRUE
ENSGALG00010007070	UBL3	PRJNA729271	1.251461623	2.9111E-126	1.251461623	stage_E14_vs_E10	ordinary_muscle_development	D_vs_E10;stage_1W_vs_E10;stage_3W_vs_E10;stage	1	TRUE
ENSGALG00010010868	SLC39A10	PRJNA729271	-2.727561992	8.6258E-126	2.727561992	stage_E14_vs_E10	ordinary_muscle_development	D_vs_E10;stage_1W_vs_E10;stage_3W_vs_E10;stage	1	TRUE
ENSGALG00010027706	GSN	PRJNA729271	4.130205047	9.5453E-126	4.130205047	stage_E14_vs_E10	ordinary_muscle_development	D_vs_E10;stage_1W_vs_E10;stage_3W_vs_E10;stage	1	TRUE
ENSGALG00010029588	RAB11FIP4	PRJNA729271	-3.963563354	2.1019E-125	3.963563354	stage_E14_vs_E10	ordinary_muscle_development	D_vs_E10;stage_1W_vs_E10;stage_3W_vs_E10;stage	1	TRUE
ENSGALG00010009147	HNMT	PRJNA729271	5.434323469	4.1113E-125	5.434323469	stage_E14_vs_E10	ordinary_muscle_development	D_vs_E10;stage_1W_vs_E10;stage_3W_vs_E10;stage	1	TRUE
ENSGALG00010012775	KCNE2	PRJNA729271	3.482940282	5.111E-125	3.482940282	stage_E14_vs_E10	ordinary_muscle_development	D_vs_E10;stage_1W_vs_E10;stage_3W_vs_E10;stage	1	TRUE
ENSGALG00010027265	IP6K3	PRJNA729271	4.974040871	5.1672E-125	4.974040871	stage_E14_vs_E10	ordinary_muscle_development	D_vs_E10;stage_1W_vs_E10;stage_3W_vs_E10;stage	1	TRUE
ENSGALG00010008058	USP25	PRJNA729271	3.304930379	6.5984E-125	3.304930379	stage_E14_vs_E10	ordinary_muscle_development	D_vs_E10;stage_1W_vs_E10;stage_3W_vs_E10;stage	1	TRUE
ENSGALG00010004921	RRAGD	PRJNA729271	3.167987233	1.8597E-124	3.167987233	stage_E14_vs_E10	ordinary_muscle_development	D_vs_E10;stage_1W_vs_E10;stage_3W_vs_E10;stage	1	TRUE
ENSGALG00010025421	NR4A1	PRJNA729271	9.475491548	2.0636E-124	9.475491548	stage_E14_vs_E10	ordinary_muscle_development	D_vs_E10;stage_1W_vs_E10;stage_3W_vs_E10;stage	1	TRUE
ENSGALG00010008182	CLIC5	PRJNA729271	6.788862675	5.3095E-124	6.788862675	stage_E14_vs_E10	ordinary_muscle_development	D_vs_E10;stage_1W_vs_E10;stage_3W_vs_E10;stage	1	TRUE
ENSGALG00010004798	PTPRK	PRJNA729271	-2.767051803	6.6156E-124	2.767051803	stage_E14_vs_E10	ordinary_muscle_development	D_vs_E10;stage_1W_vs_E10;stage_3W_vs_E10;stage	1	TRUE
ENSGALG00010013457	SH3BGR	PRJNA729271	3.517534633	7.2959E-124	3.517534633	stage_E14_vs_E10	ordinary_muscle_development	D_vs_E10;stage_1W_vs_E10;stage_3W_vs_E10;stage	1	TRUE
ENSGALG00010011118	GMPR2	PRJNA729271	3.229911463	9.6475E-124	3.229911463	stage_E14_vs_E10	ordinary_muscle_development	D_vs_E10;stage_1W_vs_E10;stage_3W_vs_E10;stage	1	TRUE
ENSGALG00010015174	ST6GAL2	PRJNA729271	-5.424062955	1.3388E-123	5.424062955	stage_E14_vs_E10	ordinary_muscle_development	D_vs_E10;stage_1W_vs_E10;stage_3W_vs_E10;stage	1	TRUE
ENSGALG00010023869	DOCK7	PRJNA729271	-1.140268845	1.3975E-123	1.140268845	stage_E14_vs_E10	ordinary_muscle_development	D_vs_E10;stage_1W_vs_E10;stage_3W_vs_E10;stage	1	TRUE
ENSGALG00010014056	FMO3	PRJNA729271	6.039071366	3.4464E-123	6.039071366	stage_E14_vs_E10	ordinary_muscle_development	D_vs_E10;stage_1W_vs_E10;stage_3W_vs_E10;stage	1	TRUE
ENSGALG00010025069	PODN	PRJNA729271	4.683279222	3.5942E-123	4.683279222	stage_E14_vs_E10	ordinary_muscle_development	D_vs_E10;stage_1W_vs_E10;stage_3W_vs_E10;stage	1	TRUE
ENSGALG00010009379	RYR1	PRJNA729271	5.21031428	8.182E-123	5.21031428	stage_E14_vs_E10	ordinary_muscle_development	D_vs_E10;stage_1W_vs_E10;stage_3W_vs_E10;stage	1	TRUE
ENSGALG00010019912	ARG2	PRJNA729271	-5.915684318	8.4758E-123	5.915684318	stage_E14_vs_E10	ordinary_muscle_development	D_vs_E10;stage_1W_vs_E10;stage_3W_vs_E10;stage	1	TRUE
ENSGALG00010016692	PRKCI	PRJNA729271	-1.790319513	9.0389E-123	1.790319513	stage_E14_vs_E10	ordinary_muscle_development	D_vs_E10;stage_1W_vs_E10;stage_3W_vs_E10;stage	1	TRUE
ENSGALG00010010438	LDB3	PRJNA729271	4.394766393	1.0754E-122	4.394766393	stage_E14_vs_E10	ordinary_muscle_development	D_vs_E10;stage_1W_vs_E10;stage_3W_vs_E10;stage	1	TRUE
ENSGALG00010022233	FAM46C	PRJNA729271	3.89459378	1.6081E-122	3.89459378	stage_E14_vs_E10	ordinary_muscle_development	D_vs_E10;stage_1W_vs_E10;stage_3W_vs_E10;stage	1	TRUE
ENSGALG00010021125	LY75	PRJNA729271	2.937825833	1.6538E-122	2.937825833	stage_E14_vs_E10	ordinary_muscle_development	D_vs_E10;stage_1W_vs_E10;stage_3W_vs_E10;stage	1	TRUE
ENSGALG00010022218	ENSGALG00010022218	PRJNA729271	5.842481986	1.9503E-122	5.842481986	stage_E14_vs_E10	ordinary_muscle_development	D_vs_E10;stage_1W_vs_E10;stage_3W_vs_E10;stage	1	TRUE
ENSGALG00010004852	ENSGALG00010004852	PRJNA729271	5.254159187	2.3376E-122	5.254159187	stage_E14_vs_E10	ordinary_muscle_development	D_vs_E10;stage_1W_vs_E10;stage_3W_vs_E10;stage	1	TRUE
ENSGALG00010014128	COL4A1	PRJNA729271	3.424010054	3.0021E-122	3.424010054	stage_E14_vs_E10	ordinary_muscle_development	D_vs_E10;stage_1W_vs_E10;stage_3W_vs_E10;stage	1	TRUE
ENSGALG00010019843	MCM6	PRJNA729271	-2.666958441	4.3039E-122	2.666958441	stage_E14_vs_E10	ordinary_muscle_development	D_vs_E10;stage_1W_vs_E10;stage_3W_vs_E10;stage	1	TRUE
ENSGALG00010007333	QTRT2	PRJNA729271	-1.864522525	9.2823E-122	1.864522525	stage_E14_vs_E10	ordinary_muscle_development	D_vs_E10;stage_1W_vs_E10;stage_3W_vs_E10;stage	1	TRUE
ENSGALG00010029409	ENSGALG00010029409	PRJNA729271	4.459273331	1.4458E-121	4.459273331	stage_E14_vs_E10	ordinary_muscle_development	D_vs_E10;stage_1W_vs_E10;stage_3W_vs_E10;stage	1	TRUE
ENSGALG00010013784	ANO6	PRJNA729271	2.227503624	1.7698E-121	2.227503624	stage_E14_vs_E10	ordinary_muscle_development	D_vs_E10;stage_1W_vs_E10;stage_3W_vs_E10;stage	1	TRUE
ENSGALG00010022332	KAT6B	PRJNA729271	-1.487981032	2.2785E-121	1.487981032	stage_E14_vs_E10	ordinary_muscle_development	D_vs_E10;stage_1W_vs_E10;stage_3W_vs_E10;stage	1	TRUE
ENSGALG00010014250	ENSGALG00010014250	PRJNA729271	5.975920378	2.2894E-121	5.975920378	stage_E14_vs_E10	ordinary_muscle_development	D_vs_E10;stage_1W_vs_E10;stage_3W_vs_E10;stage	1	TRUE
ENSGALG00010014324	COL4A2	PRJNA729271	3.354510012	2.9307E-121	3.354510012	stage_E14_vs_E10	ordinary_muscle_development	D_vs_E10;stage_1W_vs_E10;stage_3W_vs_E10;stage	1	TRUE
ENSGALG00010022144	SNTA1	PRJNA729271	4.399333648	2.9396E-121	4.399333648	stage_E14_vs_E10	ordinary_muscle_development	D_vs_E10;stage_1W_vs_E10;stage_3W_vs_E10;stage	1	TRUE
ENSGALG00010024482	USP3	PRJNA729271	-1.420769418	4.1487E-121	1.420769418	stage_E14_vs_E10	ordinary_muscle_development	D_vs_E10;stage_1W_vs_E10;stage_3W_vs_E10;stage	1	TRUE
ENSGALG00010007923	YAP1	PRJNA729271	-2.17094417	5.0294E-121	2.17094417	stage_E14_vs_E10	ordinary_muscle_development	D_vs_E10;stage_1W_vs_E10;stage_3W_vs_E10;stage	1	TRUE
ENSGALG00010002377	ART1A	PRJNA729271	5.703075842	9.7643E-121	5.703075842	stage_E14_vs_E10	ordinary_muscle_development	D_vs_E10;stage_1W_vs_E10;stage_3W_vs_E10;stage	1	TRUE
ENSGALG00010012405	MSRB3	PRJNA729271	2.949814098	1.4113E-120	2.949814098	stage_E14_vs_E10	ordinary_muscle_development	D_vs_E10;stage_1W_vs_E10;stage_3W_vs_E10;stage	1	TRUE
ENSGALG00010018420	RHOQ	PRJNA729271	3.089002399	2.5596E-120	3.089002399	stage_E14_vs_E10	ordinary_muscle_development	D_vs_E10;stage_1W_vs_E10;stage_3W_vs_E10;stage	1	TRUE
ENSGALG00010016955	ENSGALG00010016955	PRJNA729271	-7.277632149	2.5902E-120	7.277632149	stage_E14_vs_E10	ordinary_muscle_development	D_vs_E10;stage_1W_vs_E10;stage_3W_vs_E10;stage	1	TRUE
ENSGALG00010028747	ENSGALG00010028747	PRJNA729271	-4.533420463	2.7306E-120	4.533420463	stage_E14_vs_E10	ordinary_muscle_development	D_vs_E10;stage_1W_vs_E10;stage_3W_vs_E10;stage	1	TRUE
ENSGALG00010009200	ADGRA3	PRJNA729271	-2.247333174	4.9003E-120	2.247333174	stage_E14_vs_E10	ordinary_muscle_development	D_vs_E10;stage_1W_vs_E10;stage_3W_vs_E10;stage	1	TRUE
ENSGALG00010010274	MIS18A	PRJNA729271	-2.434474302	9.7356E-120	2.434474302	stage_E14_vs_E10	ordinary_muscle_development	D_vs_E10;stage_1W_vs_E10;stage_3W_vs_E10;stage	1	TRUE
ENSGALG00010003851	PHF14	PRJNA729271	-1.534253435	7.6611E-119	1.534253435	stage_E14_vs_E10	ordinary_muscle_development	D_vs_E10;stage_1W_vs_E10;stage_3W_vs_E10;stage	1	TRUE
ENSGALG00010028568	MLLT1	PRJNA729271	-1.593815665	8.142E-119	1.593815665	stage_E14_vs_E10	ordinary_muscle_development	D_vs_E10;stage_1W_vs_E10;stage_3W_vs_E10;stage	1	TRUE
ENSGALG00010021634	PLCD4	PRJNA729271	5.632314838	9.4601E-119	5.632314838	stage_E14_vs_E10	ordinary_muscle_development	D_vs_E10;stage_1W_vs_E10;stage_3W_vs_E10;stage	1	TRUE

ENSGALG00010024313	SERINC3	PRJNA729271	2.491752809	1.0452E-118	2.491752809	stage_E14_vs_E10	ordinary_muscle_development	D_vs_E10;stage_1W_vs_E10;stage_3W_vs_E10;stage	1	TRUE
ENSGALG00010016834	ENSGALG00010016834	PRJNA729271	1.727314913	1.0694E-118	1.727314913	stage_E14_vs_E10	ordinary_muscle_development	D_vs_E10;stage_1W_vs_E10;stage_3W_vs_E10;stage	1	TRUE
ENSGALG00010022392	EMILIN3	PRJNA729271	-4.566878337	2.7254E-118	4.566878337	stage_E14_vs_E10	ordinary_muscle_development	D_vs_E10;stage_1W_vs_E10;stage_3W_vs_E10;stage	1	TRUE
ENSGALG00010019441	LMCD1	PRJNA729271	3.180636458	7.006E-118	3.180636458	stage_E14_vs_E10	ordinary_muscle_development	D_vs_E10;stage_1W_vs_E10;stage_3W_vs_E10;stage	1	TRUE
ENSGALG00010012054	FAM60A	PRJNA729271	-2.166619925	1.0811E-117	2.166619925	stage_E14_vs_E10	ordinary_muscle_development	D_vs_E10;stage_1W_vs_E10;stage_3W_vs_E10;stage	1	TRUE
ENSGALG00010004060	ETV1	PRJNA729271	-2.229131471	1.1224E-117	2.229131471	stage_E14_vs_E10	ordinary_muscle_development	D_vs_E10;stage_1W_vs_E10;stage_3W_vs_E10;stage	1	TRUE
ENSGALG00010021196	ATP6V0A1	PRJNA729271	2.781476793	1.6776E-117	2.781476793	stage_E14_vs_E10	ordinary_muscle_development	D_vs_E10;stage_1W_vs_E10;stage_3W_vs_E10;stage	1	TRUE
ENSGALG00010027525	ENSGALG00010027525	PRJNA729271	6.120607994	2.225E-117	6.120607994	stage_E14_vs_E10	ordinary_muscle_development	D_vs_E10;stage_1W_vs_E10;stage_3W_vs_E10;stage	1	TRUE
ENSGALG00010009387	DEPTOR	PRJNA729271	3.199843345	6.6566E-117	3.199843345	stage_E14_vs_E10	ordinary_muscle_development	D_vs_E10;stage_1W_vs_E10;stage_3W_vs_E10;stage	1	TRUE
ENSGALG00010027704	MDFI	PRJNA729271	-5.064467289	6.7981E-117	5.064467289	stage_E14_vs_E10	ordinary_muscle_development	D_vs_E10;stage_1W_vs_E10;stage_3W_vs_E10;stage	1	TRUE
ENSGALG00010021022	ZFYVE1	PRJNA729271	1.79228051	8.452E-117	1.79228051	stage_E14_vs_E10	ordinary_muscle_development	D_vs_E10;stage_1W_vs_E10;stage_3W_vs_E10;stage	1	TRUE
ENSGALG00010001512	PLCL2	PRJNA729271	3.99535337	9.288E-117	3.99535337	stage_E14_vs_E10	ordinary_muscle_development	D_vs_E10;stage_1W_vs_E10;stage_3W_vs_E10;stage	1	TRUE
ENSGALG00010005984	IDH3B	PRJNA729271	2.713872141	1.0292E-116	2.713872141	stage_E14_vs_E10	ordinary_muscle_development	D_vs_E10;stage_1W_vs_E10;stage_3W_vs_E10;stage	1	TRUE
ENSGALG00010025418	CASQ2	PRJNA729271	4.84397206	1.1874E-116	4.84397206	stage_E14_vs_E10	ordinary_muscle_development	D_vs_E10;stage_1W_vs_E10;stage_3W_vs_E10;stage	1	TRUE
ENSGALG00010002478	PPME1	PRJNA729271	-1.349087183	1.1954E-116	1.349087183	stage_E14_vs_E10	ordinary_muscle_development	D_vs_E10;stage_1W_vs_E10;stage_3W_vs_E10;stage	1	TRUE
ENSGALG00010015921	SNX21	PRJNA729271	2.516150043	1.7267E-116	2.516150043	stage_E14_vs_E10	ordinary_muscle_development	D_vs_E10;stage_1W_vs_E10;stage_3W_vs_E10;stage	1	TRUE
ENSGALG00010002972	ME1	PRJNA729271	3.466412725	2.1134E-116	3.466412725	stage_E14_vs_E10	ordinary_muscle_development	D_vs_E10;stage_1W_vs_E10;stage_3W_vs_E10;stage	1	TRUE
ENSGALG00010013389	NAP1L1	PRJNA729271	-1.444114967	2.5477E-116	1.444114967	stage_E14_vs_E10	ordinary_muscle_development	D_vs_E10;stage_1W_vs_E10;stage_3W_vs_E10;stage	1	TRUE
ENSGALG00010005351	DTNA	PRJNA729271	3.765590481	4.0377E-116	3.765590481	stage_E14_vs_E10	ordinary_muscle_development	D_vs_E10;stage_1W_vs_E10;stage_3W_vs_E10;stage	1	TRUE
ENSGALG00010009679	ANK3	PRJNA729271	2.369466229	9.815E-116	2.369466229	stage_E14_vs_E10	ordinary_muscle_development	D_vs_E10;stage_1W_vs_E10;stage_3W_vs_E10;stage	1	TRUE
ENSGALG00010009385	GPI	PRJNA729271	4.095893304	1.568E-115	4.095893304	stage_E14_vs_E10	ordinary_muscle_development	D_vs_E10;stage_1W_vs_E10;stage_3W_vs_E10;stage	1	TRUE
ENSGALG00010020473	TMEM109	PRJNA729271	3.644826552	1.9736E-115	3.644826552	stage_E14_vs_E10	ordinary_muscle_development	D_vs_E10;stage_1W_vs_E10;stage_3W_vs_E10;stage	1	TRUE
ENSGALG00010023185	FBXW4	PRJNA729271	2.669898874	2.998E-115	2.669898874	stage_E14_vs_E10	ordinary_muscle_development	D_vs_E10;stage_1W_vs_E10;stage_3W_vs_E10;stage	1	TRUE
ENSGALG00010004127	EPHA7	PRJNA729271	-4.920774922	3.4077E-115	4.920774922	stage_E14_vs_E10	ordinary_muscle_development	D_vs_E10;stage_1W_vs_E10;stage_3W_vs_E10;stage	1	TRUE
ENSGALG00010024406	NUDT21	PRJNA729271	-1.555847229	4.5753E-115	1.555847229	stage_E14_vs_E10	ordinary_muscle_development	D_vs_E10;stage_1W_vs_E10;stage_3W_vs_E10;stage	1	TRUE
ENSGALG00010016547	SGCB	PRJNA729271	3.294385176	5.004E-115	3.294385176	stage_E14_vs_E10	ordinary_muscle_development	D_vs_E10;stage_1W_vs_E10;stage_3W_vs_E10;stage	1	TRUE
ENSGALG00010021433	ENSGALG00010021433	PRJNA729271	6.186527784	5.0208E-115	6.186527784	stage_E14_vs_E10	ordinary_muscle_development	D_vs_E10;stage_1W_vs_E10;stage_3W_vs_E10;stage	1	TRUE
ENSGALG00010006126	ENSGALG00010006126	PRJNA729271	6.456363792	5.0367E-115	6.456363792	stage_E14_vs_E10	ordinary_muscle_development	D_vs_E10;stage_1W_vs_E10;stage_3W_vs_E10;stage	1	TRUE
ENSGALG00010015776	SPOCK1	PRJNA729271	-6.316830842	5.9659E-115	6.316830842	stage_E14_vs_E10	ordinary_muscle_development	D_vs_E10;stage_1W_vs_E10;stage_3W_vs_E10;stage	1	TRUE
ENSGALG00010004705	ENSGALG00010004705	PRJNA729271	5.191577512	8.0957E-115	5.191577512	stage_E14_vs_E10	ordinary_muscle_development	D_vs_E10;stage_1W_vs_E10;stage_3W_vs_E10;stage	1	TRUE
ENSGALG00010008850	CYSTM1	PRJNA729271	2.824674579	8.9078E-115	2.824674579	stage_E14_vs_E10	ordinary_muscle_development	D_vs_E10;stage_1W_vs_E10;stage_3W_vs_E10;stage	1	TRUE
ENSGALG00010014537	GKAP1	PRJNA729271	2.747226921	1.0056E-114	2.747226921	stage_E14_vs_E10	ordinary_muscle_development	D_vs_E10;stage_1W_vs_E10;stage_3W_vs_E10;stage	1	TRUE
ENSGALG00010024697	IGF2BP1	PRJNA729271	-2.949110879	1.0119E-114	2.949110879	stage_E14_vs_E10	ordinary_muscle_development	D_vs_E10;stage_1W_vs_E10;stage_3W_vs_E10;stage	1	TRUE
ENSGALG00010022760	ELAVL4	PRJNA729271	-5.087620619	1.1734E-114	5.087620619	stage_E14_vs_E10	ordinary_muscle_development	D_vs_E10;stage_1W_vs_E10;stage_3W_vs_E10;stage	1	TRUE
ENSGALG00010023187	ENSGALG00010023187	PRJNA729271	5.267953076	1.8431E-114	5.267953076	stage_E14_vs_E10	ordinary_muscle_development	D_vs_E10;stage_1W_vs_E10;stage_3W_vs_E10;stage	1	TRUE
ENSGALG00010014238	PHLDA1	PRJNA729271	-2.808787821	2.5213E-114	2.808787821	stage_E14_vs_E10	ordinary_muscle_development	D_vs_E10;stage_1W_vs_E10;stage_3W_vs_E10;stage	1	TRUE
ENSGALG00010012915	ENSGALG00010012915	PRJNA729271	6.360278301	2.8387E-114	6.360278301	stage_E14_vs_E10	ordinary_muscle_development	D_vs_E10;stage_1W_vs_E10;stage_3W_vs_E10;stage	1	TRUE
ENSGALG00010023057	PAFAH1B2	PRJNA729271	-1.735257015	5.8057E-114	1.735257015	stage_E14_vs_E10	ordinary_muscle_development	D_vs_E10;stage_1W_vs_E10;stage_3W_vs_E10;stage	1	TRUE
ENSGALG00010002087	POLA1	PRJNA729271	-1.732469335	8.3148E-114	1.732469335	stage_E14_vs_E10	ordinary_muscle_development	D_vs_E10;stage_1W_vs_E10;stage_3W_vs_E10;stage	1	TRUE
ENSGALG00010001892	RAB12	PRJNA729271	1.606621825	1.162E-113	1.606621825	stage_E14_vs_E10	ordinary_muscle_development	D_vs_E10;stage_1W_vs_E10;stage_3W_vs_E10;stage	1	TRUE
ENSGALG00010002543	UCP3	PRJNA729271	11.45760752	3.1907E-113	11.45760752	stage_E14_vs_E10	ordinary_muscle_development	D_vs_E10;stage_1W_vs_E10;stage_3W_vs_E10;stage	1	TRUE
ENSGALG00010020202	MAPKBP1	PRJNA729271	2.050294909	4.6759E-113	2.050294909	stage_E14_vs_E10	ordinary_muscle_development	D_vs_E10;stage_1W_vs_E10;stage_3W_vs_E10;stage	1	TRUE
ENSGALG00010006183	PRKN	PRJNA729271	3.065467177	5.8389E-113	3.065467177	stage_E14_vs_E10	ordinary_muscle_development	D_vs_E10;stage_1W_vs_E10;stage_3W_vs_E10;stage	1	TRUE
ENSGALG00010001380	SCRN1	PRJNA729271	-4.412659921	6.9167E-113	4.412659921	stage_E14_vs_E10	ordinary_muscle_development	D_vs_E10;stage_1W_vs_E10;stage_3W_vs_E10;stage	1	TRUE
ENSGALG00010020142	SLC35E2B	PRJNA729271	-1.627173606	9.1026E-113	1.627173606	stage_E14_vs_E10	ordinary_muscle_development	D_vs_E10;stage_1W_vs_E10;stage_3W_vs_E10;stage	1	TRUE
ENSGALG00010017434	FGF13	PRJNA729271	3.374998552	9.8948E-113	3.374998552	stage_E14_vs_E10	ordinary_muscle_development	D_vs_E10;stage_1W_vs_E10;stage_3W_vs_E10;stage	1	TRUE
ENSGALG00010010080	CDC42EP3	PRJNA729271	2.681200891	9.9821E-113	2.681200891	stage_E14_vs_E10	ordinary_muscle_development	D_vs_E10;stage_1W_vs_E10;stage_3W_vs_E10;stage	1	TRUE
ENSGALG00010028344	LSM7	PRJNA729271	-2.248118294	1.0307E-112	2.248118294	stage_E14_vs_E10	ordinary_muscle_development	D_vs_E10;stage_1W_vs_E10;stage_3W_vs_E10;stage	1	TRUE
ENSGALG00010023112	PCMT1L	PRJNA729271	2.951407779	1.6774E-112	2.951407779	stage_E14_vs_E10	ordinary_muscle_development	D_vs_E10;stage_1W_vs_E10;stage_3W_vs_E10;stage	1	TRUE
ENSGALG00010015635	ABTB3	PRJNA729271	-3.12313252	1.9454E-112	3.12313252	stage_E14_vs_E10	ordinary_muscle_development	D_vs_E10;stage_1W_vs_E10;stage_3W_vs_E10;stage	1	TRUE
ENSGALG00010009411	SMARCA5	PRJNA729271	-2.275734739	2.1349E-112	2.275734739	stage_E14_vs_E10	ordinary_muscle_development	D_vs_E10;stage_1W_vs_E10;stage_3W_vs_E10;stage	1	TRUE
ENSGALG00010017658	WHAMM	PRJNA729271	3.654649544	4.0079E-112	3.654649544	stage_E14_vs_E10	ordinary_muscle_development	D_vs_E10;stage_1W_vs_E10;stage_3W_vs_E10;stage	1	TRUE
ENSGALG00010020873	PINK1	PRJNA729271	3.078279666	1.7235E-111	3.078279666	stage_E14_vs_E10	ordinary_muscle_development	D_vs_E10;stage_1W_vs_E10;stage_3W_vs_E10;stage	1	TRUE
ENSGALG00010001923	RAPGEF5	PRJNA729271	2.275063967	3.2344E-111	2.275063967	stage_E14_vs_E10	ordinary_muscle_development	D_vs_E10;stage_1W_vs_E10;stage_3W_vs_E10;stage	1	TRUE
ENSGALG00010012809	RHOBTB3	PRJNA729271	5.370558723	5.4358E-111	5.370558723	stage_E14_vs_E10	ordinary_muscle_development	D_vs_E10;stage_1W_vs_E10;stage_3W_vs_E10;stage	1	TRUE
ENSGALG00010017604	GATM	PRJNA729271	5.444986665	1.113E-110	5.444986665	stage_E14_vs_E10	ordinary_muscle_development	D_vs_E10;stage_1W_vs_E10;stage_3W_vs_E10;stage	1	TRUE
ENSGALG00010014603	ENSGALG00010014603	PRJNA729271	4.986868832	1.3387E-110	4.986868832	stage_E14_vs_E10	ordinary_muscle_development	D_vs_E10;stage_1W_vs_E10;stage_3W_vs_E10;stage	1	TRUE
ENSGALG00010029318	C17orf58	PRJNA729271	4.563156478	1.3597E-110	4.563156478	stage_E14_vs_E10	ordinary_muscle_development	D_vs_E10;stage_1W_vs_E10;stage_3W_vs_E10;stage	1	TRUE
ENSGALG00010021682	MYL4	PRJNA729271	-7.186030537	2.3404E-110	7.186030537	stage_E14_vs_E10	ordinary_muscle_development	D_vs_E10;stage_1W_vs_E10;stage_3W_vs_E10;stage	1	TRUE
ENSGALG00010005621	CDH2	PRJNA729271	-3.300744929	2.4519E-110	3.300744929	stage_E14_vs_E10	ordinary_muscle_development	D_vs_E10;stage_1W_vs_E10;stage_3W_vs_E10;stage	1	TRUE
ENSGALG00010021490	ACACB	PRJNA729271	7.393561362	3.5472E-110	7.393561362	stage_E14_vs_E10	ordinary_muscle_development	D_vs_E10;stage_1W_vs_E10;stage_3W_vs_E10;stage	1	TRUE
ENSGALG00010003817	BF2	PRJNA729271	6.329387627	4.7479E-110	6.329387627	stage_E14_vs_E10	ordinary_muscle_development	D_vs_E10;stage_1W_vs_E10;stage_3W_vs_E10;stage	1	TRUE
ENSGALG00010002928	ASB4	PRJNA729271	12.25122086	4.9073E-110	12.25122086	stage_E14_vs_E10	ordinary_muscle_development	D_vs_E10;stage_1W_vs_E10;stage_3W_vs_E10;stage	1	TRUE
ENSGALG00010002612	JPH1	PRJNA729271	4.807690353	8.9479E-110	4.807690353	stage_E14_vs_E10	ordinary_muscle_development	D_vs_E10;stage_1W_vs_E10;stage_3W_vs_E10;stage	1	TRUE
ENSGALG00010018150	AAGAB	PRJNA729271	-1.834350304	1.138E-109	1.834350304	stage_E14_vs_E10	ordinary_muscle_development	D_vs_E10;stage_1W_vs_E10;stage_3W_vs_E10;stage	1	TRUE
ENSGALG00010017563	ALK	PRJNA729271	3.381874951	1.3627E-109	3.381874951	stage_E14_vs_E10	ordinary_muscle_development	D_vs_E10;stage_1W_vs_E10;stage_3W_vs_E10;stage	1	TRUE
ENSGALG00010013301	PARVB	PRJNA729271	-3.192612243	1.787E-109	3.192612243	stage_E14_vs_E10	ordinary_muscle_development	D_vs_E10;stage_1W_vs_E10;stage_3W_vs_E10;stage	1	TRUE
ENSGALG00010012124	ERAP1	PRJNA729271	3.274421599	2.2622E-109	3.274421599	stage_E14_vs_E10	ordinary_muscle_development	D_vs_E10;stage_1W_vs_E10;stage_3W_vs_E10;stage	1	TRUE
ENSGALG00010028450	PTGDS	PRJNA729271	9.679208911	3.5565E-109	9.679208911	stage_E14_vs_E10	ordinary_muscle_development	D_vs_E10;stage_1W_vs_E10;stage_3W_vs_E10;stage	1	TRUE
ENSGALG00010021750	HELLS	PRJNA729271	-3.4605676	3.7373E-109	3.4605676	stage_E14_vs_E10	ordinary_muscle_development	D_vs_E10;stage_1W_vs_E10;stage_3W_vs_E10;stage	1	TRUE
ENSGALG00010027529	ACY1	PRJNA729271	3.121224861	4.1353E-109	3.121224861	stage_E14_vs_E10	ordinary_muscle_development	D_vs_E10;stage_1W_vs_E10;stage_3W_vs_E10;stage	1	TRUE
ENSGALG00010001104	MBNL1	PRJNA729271	2.739230077	4.6851E-109	2.739230077	stage_E14_vs_E10	ordinary_muscle_development	D_vs_E10;stage_1W_vs_E10;stage_3W_vs_E10;stage	1	TRUE
ENSGALG00010018582	RCC2	PRJNA729271	-2.526590466	8.7066E-109	2.526590466	stage_E14_vs_E10	ordinary_muscle_development	D_vs_E10;stage_1W_vs_E10;stage_3W_vs_E10;stage	1	TRUE
ENSGALG00010026982	ABTB1	PRJNA729271	1.889844606	9.4116E-109	1.889844606	stage_E14_vs_E10	ordinary_muscle_development	D_vs_E10;stage_1W_vs_E10;stage_3W_vs_E10;stage	1	TRUE
ENSGALG00010001778	KAT2B	PRJNA729271	3.282632872	9.9074E-109	3.282632872	stage_E14_vs_E10	ordinary_muscle_development	D_vs_E10;stage_1W_vs_E10;stage_3W_vs_E10;stage	1	TRUE
ENSGALG00010008185	PDGFC	PRJNA729271	-2.077653717	1.0912E-108	2.077653717	stage_E14_vs_E10	ordinary_muscle_development	D_vs_E10;stage_1W_vs_E10;stage_3W_vs_E10;stage	1	TRUE
ENSGALG00010022971	CKAP5	PRJNA729271	-1.307589249	1.0993E-108	1.307589249	stage_E14_vs_E10	ordinary_muscle_development	D_vs_E10;stage_1W_vs_E10;stage_3W_vs_E10;stage	1	TRUE
ENSGALG00010025629	ST6GAL1	PRJNA729271	3.197592666	1.7917E-108	3.197592666	stage_E14_vs_E10	ordinary_muscle_development	D_vs_E10;stage_1W_vs_E10;stage_3W_vs_E10;stage	1	TRUE
ENSGALG00010000802	SEMA5A	PRJNA729271	-2.86097232	2.0391E-108	2.86097232	stage_E14_vs_E10	ordinary_muscle_development	D_vs_E10;stage_1W_vs_E10;stage_3W_vs_E10;stage	1	TRUE
ENSGALG00010012607	LMNB1	PRJNA729271	-3.703995368	2.9745E-108	3.703995368	stage_E14_vs_E10	ordinary_muscle_development	D_vs_E10;stage_1W_vs_E10;stage_3W_vs_E10;stage	1	TRUE
ENSGALG00010027075	SLC41A1	PRJNA729271	2.090966051	3.4597E-108	2.090966051	stage_E14_vs_E10	ordinary_muscle_development	D_vs_E10;stage_1W_vs_E10;stage_3W_vs_E10;stage	1	TRUE
ENSGALG00010018576	PTK7	PRJNA729271	-2.589651571	3.5451E-108	2.589651571	stage_E14_vs_E10	ordinary_muscle_development	D_vs_E10;stage_1W_vs_E10;stage_3W_vs_E10;stage	1	TRUE
ENSGALG00010016458	ZC3H12A	PRJNA729271	1.956934471	5.4787E-108	1.956934471	stage_E14_vs_E10	ordinary_muscle_development	D_vs_E10;stage_1W_vs_E10;stage_3W_vs_E10;stage	1	TRUE
ENSGALG00010008285	ADSS2	PRJNA729271	-2.049171919	6.1857E-108	2.049171919	stage_E14_vs_E10	ordinary_muscle_development	D_vs_E10;stage_1W_vs_E10;stage_3W_vs_E10;stage	1	TRUE
ENSGALG00010004039	ALPK1	PRJNA729271	4.863942915	7.3901E-108	4.863942915	stage_E14_vs_E10	ordinary_muscle_development	D_vs_E10;stage_1W_vs_E10;stage_3W_vs_E10;stage	1	TRUE
ENSGALG00010029429	UBE2G1	PRJNA729271	1.987175117	7.5098E-108	1.987175117	stage_E14_vs_E10	ordinary_muscle_development	D_vs_E10;stage_1W_vs_E10;stage_3W_vs_E10;stage	1	TRUE
ENSGALG00010015837	GDA	PRJNA729271	3.511454844	8.1824E-108	3.511454844	stage_E14_vs_E10	ordinary_muscle_development	D_vs_E10;stage_1W_vs_E10;stage_3W_vs_E10;stage	1	TRUE
ENSGALG00010024143	UBE2Q2	PRJNA729271	2.585219326	1.0989E-107	2.585219326	stage_E14_vs_E10	ordinary_muscle_development	D_vs_E10;stage_1W_vs_E10;stage_3W_vs_E10;stage	1	TRUE
ENSGALG00010004721	LAMA2	PRJNA729271	3.536468265	1.2097E-107	3.536468265	stage_E14_vs_E10	ordinary_muscle_development	D_vs_E10;stage_1W_vs_E10;stage_3W_vs_E10;stage	1	TRUE
ENSGALG00010028201	ENSGALG00010028201	PRJNA729271	3.55130606	1.292E-107	3.55130606	stage_E14_vs_E10	ordinary_muscle_development	D_vs_E10;stage_1W_vs_E10;stage_3W_vs_E10;stage	1	TRUE
ENSGALG00010028414	KBTBD12	PRJNA729271	7.777624108	1.5295E-107	7.777624108	stage_E14_vs_E10	ordinary_muscle_development	D_vs_E10;stage_1W_vs_E10;stage_3W_vs_E10;stage	1	TRUE
ENSGALG00010016804	CSF1R	PRJNA729271	3.013696476	1.7094E-107	3.013696476	stage_E14_vs_E10	ordinary_muscle_development	D_vs_E10;stage_1W_vs_E10;stage_3W_vs_E10;stage	1	TRUE
ENSGALG00010017654	KBTBD13	PRJNA729271	6.897693548	2.3059E-107	6.897693548	stage_E14_vs_E10	ordinary_muscle_development	D_vs_E10;stage_1W_vs_E10;stage_3W_vs_E10;stage	1	TRUE
ENSGALG00010027122	DNASE1L3	PRJNA729271	5.489935361	2.4094E-107	5.489935361	stage_E14_vs_E10	ordinary_muscle_development	D_vs_E10;stage_1W_vs_E10;stage_3W_vs_E10;stage	1	TRUE
ENSGALG00010027171	ATG9B	PRJNA729271	6.060066445	3.2657E-107	6.060066445	stage_E14_vs_E10	ordinary_muscle_development	D_vs_E10;stage_1W_vs_E10;stage_3W_vs_E10;stage	1	TRUE
ENSGALG00010023053	MAN1A2	PRJNA729271	-1.083435791	4.7546E-107	1.083435791	stage_E14_vs_E10	ordinary_muscle_development	D_vs_E10;stage_1W_vs_E10;stage_3W_vs_E10;stage	1	TRUE
ENSGALG00010011628	MATN2	PRJNA729271	4.251734632	4.8511E-107	4.251734632	stage_E14_vs_E10	ordinary_muscle_development	D_vs_E10;stage_1W_vs_E10;stage_3W_vs_E10;stage	1	TRUE
ENSGALG00010028179	MCM2	PRJNA729271	-3.23918088	4.9179E-107	3.23918088	stage_E14_vs_E10	ordinary_muscle_development	D_vs_E10;stage_1W_vs_E10;stage_3W_vs_E10;stage	1	TRUE
ENSGALG00010027202	CHAF1A	PRJNA729271	-1.980462997	5.0378E-107	1.980462997	stage_E14_vs_E10	ordinary_muscle_development	D_vs_E10;stage_1W_vs_E10;stage_3W_vs_E10;stage	1	TRUE
ENSGALG00010008227	DUSP1	PRJNA729271	3.913843577	5.1715E-107	3.913843577	stage_E14_vs_E10	ordinary_muscle_development	D_vs_E10;stage_1W_vs_E10;stage_3W_vs_E10;stage	1	TRUE
ENSGALG00010005508	SS18	PRJNA729271	-1.456543796	6.6344E-107	1.456543796	stage_E14_vs_E10	ordinary_muscle_development	D_vs_E10;stage_1W_vs_E10;stage_3W_vs_E10;stage	1	TRUE
ENSGALG00010017713	ALKBH5	PRJNA729271	3.298529148	7.2809E-107	3.298529148	stage_E14_vs_E10	ordinary_muscle_development	D_vs_E10;stage_1W_vs_E10;stage_3W_vs_E10;stage	1	TRUE
ENSGALG00010008361	MATR3	PRJNA729271	-1.26596441	8.0787E-107	1.26596441	stage_E14_vs_E10	ordinary_muscle_development	D_vs_E10;stage_1W_vs_E10;stage_3W_vs_E10;stage	1	TRUE
ENSGALG00010005123	MTIF3	PRJNA729271	2.275762033	8.6625E-107	2.275762033	stage_E14_vs_E10	ordinary_muscle_development	D_vs_E10;stage_1W_vs_E10;stage_3W_vs_E10;stage	1	TRUE
ENSGALG00010005436	AQR	PRJNA729271	-1.25141053	1.0786E-106	1.25141053	stage_E14_vs_E10	ordinary_muscle_development	D_vs_E10;stage_1W_vs_E10;stage_3W_vs_E10;stage	1	TRUE
ENSGALG00010018322	NT5C1A	PRJNA729271	5.305952439	1.1503E-106	5.305952439	stage_E14_vs_E10	ordinary_muscle_development	D_vs_E10;stage_1W_vs_E10;stage_3W_vs_E10;stage	1	TRUE
ENSGALG00010003665	RDH14	PRJNA729271	2.012819644	1.3959E-106	2.012819644	stage_E14_vs_E10	ordinary_muscle_development	D_vs_E10;stage_1W_vs_E10;stage_3W_vs_E10;stage	1	TRUE
ENSGALG00010019697	EIF4EBP1	PRJNA729271	3.207466883	1.4746E-106	3.207466883	stage_E14_vs_E10	ordinary_muscle_development	D_vs_E10;stage_1W_vs_E10;stage_3W_vs_E10;stage	1	TRUE
ENSGALG00010005241	C4orf54	PRJNA729271	4.137678823	2.2097E-106	4.137678823	stage_E14_vs_E10	ordinary_muscle_development	D_vs_E10;stage_1W_vs_E10;stage_3W_vs_E10;stage	1	TRUE
ENSGALG00010009727	CDH20	PRJNA729271	4.683933518	2.4631E-106	4.683933518	stage_E14_vs_E10	ordinary_muscle_development	D_vs_E10;stage_1W_vs_E10;stage_3W_vs_E10;stage	1	TRUE
ENSGALG00010000684	COBL	PRJNA729271	2.970540301	3.3333E-106	2.970540301	stage_E14_vs_E10	ordinary_muscle_development	D_vs_E10;stage_1W_vs_E10;stage_3W_vs_E10;stage	1	TRUE
ENSGALG00010019009	NRAP	PRJNA729271	4.819745507	4.535E-106	4.819745507	stage_E14_vs_E10	ordinary_muscle_development	D_vs_E10;stage_1W_vs_E10;stage_3W_vs_E10;stage	1	TRUE
ENSGALG00010008130	RCSD1	PRJNA729271	2.632124469	6.4554E-106	2.632124469	stage_E14_vs_E10	ordinary_muscle_development	D_vs_E10;stage_1W_vs_E10;stage_3W_vs_E10;stage	1	TRUE
ENSGALG00010025882	LDHD	PRJNA729271	3.764351935	6.4716E-106	3.764351935	stage_E14_vs_E10	ordinary_muscle_development	D_vs_E10;stage_1W_vs_E10;stage_3W_vs_E10;stage	1	TRUE
ENSGALG00010008474	ENSGALG00010008474	PRJNA729271	3.027522059	6.6725E-106	3.027522059	stage_E14_vs_E10	ordinary_muscle_development	D_vs_E10;stage_1W_vs_E10;stage_3W_vs_E10;stage	1	TRUE
ENSGALG00010016460	LBR	PRJNA729271	-1.536323708	9.0829E-106	1.536323708	stage_E14_vs_E10	ordinary_muscle_development	D_vs_E10;stage_1W_vs_E10;stage_3W_vs_E10;stage	1	TRUE
ENSGALG00010024473	RAVER2	PRJNA729271	-1.345361134	9.8641E-106	1.345361134	stage_E14_vs_E10	ordinary_muscle_development	D_vs_E10;stage_1W_vs_E10;stage_3W_vs_E10;stage	1	TRUE
ENSGALG00010021618	NEO1	PRJNA729271	-2.381040204	1.2088E-105	2.381040204	stage_E14_vs_E10	ordinary_muscle_development	D_vs_E10;stage_1W_vs_E10;stage_3W_vs_E10;stage	1	TRUE
ENSGALG00010001157	TIMM21	PRJNA729271	1.9934159	1.304E-105	1.9934159	stage_E14_vs_E10	ordinary_muscle_development	D_vs_E10;stage_1W_vs_E10;stage_3W_vs_E10;stage	1	TRUE
ENSGALG00010028876	ADAMTSL2	PRJNA729271	4.272244892	1.4584E-105	4.272244892	stage_E14_vs_E10	ordinary_muscle_development	D_vs_E10;stage_1W_vs_E10;stage_3W_vs_E10;stage	1	TRUE
ENSGALG00010002904	PON1	PRJNA729271	3.928125212	1.4871E-105	3.928125212	stage_E14_vs_E10	ordinary_muscle_development	D_vs_E10;stage_1W_vs_E10;stage_3W_vs_E10;stage	1	TRUE
ENSGALG00010023184	SMARCD1	PRJNA729271	-1.246242226	1.5051E-105	1.246242226	stage_E14_vs_E10	ordinary_muscle_development	D_vs_E10;stage_1W_vs_E10;stage_3W_vs_E10;stage	1	TRUE
ENSGALG00010024683	SHISA4	PRJNA729271	3.809438482	1.5404E-105	3.809438482	stage_E14_vs_E10	ordinary_muscle_development	D_vs_E10;stage_1W_vs_E10;stage_3W_vs_E10;stage	1	TRUE
ENSGALG00010010351	NAMPTP1	PRJNA729271	4.671057749	1.7946E-105	4.671057749	stage_E14_vs_E10	ordinary_muscle_development	D_vs_E10;stage_1W_vs_E10;stage_3W_vs_E10;stage	1	TRUE
ENSGALG00010029241	STXBP4	PRJNA729271	-2.241233339	1.949E-105	2.241233339	stage_E14_vs_E10	ordinary_muscle_development	D_vs_E10;stage_1W_vs_E10;stage_3W_vs_E10;stage	1	TRUE
ENSGALG00010005172	OSBPL1A	PRJNA729271	1.754671194	2.3323E-105	1.754671194	stage_E14_vs_E10	ordinary_muscle_development	D_vs_E10;stage_1W_vs_E10;stage_3W_vs_E10;stage	1	TRUE
ENSGALG00010025691	ENSGALG00010025691	PRJNA729271	-4.119605633	2.7484E-105	4.119605633	stage_E14_vs_E10	ordinary_muscle_development	D_vs_E10;stage_1W_vs_E10;stage_3W_vs_E10;stage	1	TRUE
ENSGALG00010001528	NR1D2	PRJNA729271	3.953644008	3.7211E-105	3.953644008	stage_E14_vs_E10	ordinary_muscle_development	D_vs_E10;stage_1W_vs_E10;stage_3W_vs_E10;stage	1	TRUE
ENSGALG00010018519	OGDH	PRJNA729271	2.985491	4.3357E-105	2.985491	stage_E14_vs_E10	ordinary_muscle_development	D_vs_E10;stage_1W_vs_E10;stage_3W_vs_E10;stage	1	TRUE
ENSGALG00010029233	CBX2	PRJNA729271	-4.303304552	5.5318E-105	4.303304552	stage_E14_vs_E10	ordinary_muscle_development	D_vs_E10;stage_1W_vs_E10;stage_3W_vs_E10;stage	1	TRUE
ENSGALG00010001947	TINAG	PRJNA729271	6.609023526	6.4427E-105	6.609023526	stage_E14_vs_E10	ordinary_muscle_development	D_vs_E10;stage_1W_vs_E10;stage_3W_vs_E10;stage	1	TRUE
ENSGALG00010014578	ENSGALG00010014578	PRJNA729271	6.594332667	7.9347E-105	6.594332667	stage_E14_vs_E10	ordinary_muscle_development	D_vs_E10;stage_1W_vs_E10;stage_3W_vs_E10;stage	1	TRUE
ENSGALG00010008768	PRXL2C	PRJNA729271	3.149289794	1.1121E-104	3.149289794	stage_E14_vs_E10	ordinary_muscle_development	D_vs_E10;stage_1W_vs_E10;stage_3W_vs_E10;stage	1	TRUE
ENSGALG00010016399	ACSM5	PRJNA729271	7.166770563	1.3172E-104	7.166770563	stage_E14_vs_E10	ordinary_muscle_development	D_vs_E10;stage_1W_vs_E10;stage_3W_vs_E10;stage	1	TRUE
ENSGALG00010020374	PCNA	PRJNA729271	-2.121407764	1.5969E-104	2.121407764	stage_E14_vs_E10	ordinary_muscle_development	D_vs_E10;stage_1W_vs_E10;stage_3W_vs_E10;stage	1	TRUE
ENSGALG00010023221	AMY1A	PRJNA729271	2.97130607	1.8109E-104	2.97130607	stage_E14_vs_E10	ordinary_muscle_development	D_vs_E10;stage_1W_vs_E10;stage_3W_vs_E10;stage	1	TRUE
ENSGALG00010013335	POPDC3	PRJNA729271	3.843234413	2.1131E-104	3.843234413	stage_E14_vs_E10	ordinary_muscle_development	D_vs_E10;stage_1W_vs_E10;stage_3W_vs_E10;stage	1	TRUE
ENSGALG00010008066	PCNX2	PRJNA729271	-4.914227389	2.339E-104	4.914227389	stage_E14_vs_E10	ordinary_muscle_development	D_vs_E10;stage_1W_vs_E10;stage_3W_vs_E10;stage	1	TRUE
ENSGALG00010003472	FAM110B	PRJNA729271	-2.90111318	3.0877E-104	2.90111318	stage_E14_vs_E10	ordinary_muscle_development	D_vs_E10;stage_1W_vs_E10;stage_3W_vs_E10;stage	1	TRUE
ENSGALG00010012819	RANGAP1	PRJNA729271	-1.586164527	3.5791E-104	1.586164527	stage_E14_vs_E10	ordinary_muscle_development	D_vs_E10;stage_1W_vs_E10;stage_3W_vs_E10;stage	1	TRUE
ENSGALG00010001082	P2RY1	PRJNA729271	3.934627639	4.2176E-104	3.934627639	stage_E14_vs_E10	ordinary_muscle_development	D_vs_E10;stage_1W_vs_E10;stage_3W_vs_E10;stage	1	TRUE
ENSGALG00010028146	MAPKAPK3	PRJNA729271	2.274516158	4.3986E-104	2.274516158	stage_E14_vs_E10	ordinary_muscle_development	D_vs_E10;stage_1W_vs_E10;stage_3W_vs_E10;stage	1	TRUE
ENSGALG00010003596	CRISPLD1	PRJNA729271	-3.413436283	4.652E-104	3.413436283	stage_E14_vs_E10	ordinary_muscle_development	D_vs_E10;stage_1W_vs_E10;stage_3W_vs_E10;stage	1	TRUE
ENSGALG00010029787	H3-3B	PRJNA729271	-1.406547514	5.815E-104	1.406547514	stage_E14_vs_E10	ordinary_muscle_development	D_vs_E10;stage_1W_vs_E10;stage_3W_vs_E10;stage	1	TRUE
ENSGALG00010018922	EEF2K	PRJNA729271	2.491352983	6.6854E-104	2.491352983	stage_E14_vs_E10	ordinary_muscle_development	D_vs_E10;stage_1W_vs_E10;stage_3W_vs_E10;stage	1	TRUE
ENSGALG00010000048	MAN2A2	PRJNA729271	3.200621778	7.2414E-104	3.200621778	stage_E14_vs_E10	ordinary_muscle_development	D_vs_E10;stage_1W_vs_E10;stage_3W_vs_E10;stage	1	TRUE
ENSGALG00010025147	COX17	PRJNA729271	2.471499512	7.5833E-104	2.471499512	stage_E14_vs_E10	ordinary_muscle_development	D_vs_E10;stage_1W_vs_E10;stage_3W_vs_E10;stage	1	TRUE
ENSGALG00010018883	TBL3	PRJNA729271	-1.707948788	7.788E-104	1.707948788	stage_E14_vs_E10	ordinary_muscle_development	D_vs_E10;stage_1W_vs_E10;stage_3W_vs_E10;stage	1	TRUE
ENSGALG00010012429	CXCR7	PRJNA729271	2.194222614	1.1024E-103	2.194222614	stage_E14_vs_E10	ordinary_muscle_development	D_vs_E10;stage_1W_vs_E10;stage_3W_vs_E10;stage	1	TRUE
ENSGALG00010016769	BNC1	PRJNA729271	-9.517189986	1.1573E-103	9.517189986	stage_E14_vs_E10	ordinary_muscle_development	D_vs_E10;stage_1W_vs_E10;stage_3W_vs_E10;stage	1	TRUE
ENSGALG00010025538	ANO1	PRJNA729271	4.543653636	1.6222E-103	4.543653636	stage_E14_vs_E10	ordinary_muscle_development	D_vs_E10;stage_1W_vs_E10;stage_3W_vs_E10;stage	1	TRUE
ENSGALG00010017596	TDP1	PRJNA729271	-2.243928086	2.0716E-103	2.243928086	stage_E14_vs_E10	ordinary_muscle_development	D_vs_E10;stage_1W_vs_E10;stage_3W_vs_E10;stage	1	TRUE
ENSGALG00010027821	NMRK2	PRJNA729271	4.923674858	3.1087E-103	4.923674858	stage_E14_vs_E10	ordinary_muscle_development	D_vs_E10;stage_1W_vs_E10;stage_3W_vs_E10;stage	1	TRUE
ENSGALG00010020599	PARN	PRJNA729271	-1.518096598	3.8846E-103	1.518096598	stage_E14_vs_E10	ordinary_muscle_development	D_vs_E10;stage_1W_vs_E10;stage_3W_vs_E10;stage	1	TRUE
ENSGALG00010023312	UBR3	PRJNA729271	2.479252493	4.5739E-103	2.479252493	stage_E14_vs_E10	ordinary_muscle_development	D_vs_E10;stage_1W_vs_E10;stage_3W_vs_E10;stage	1	TRUE
ENSGALG00010026090	USP2	PRJNA729271	5.075894549	6.3581E-103	5.075894549	stage_E14_vs_E10	ordinary_muscle_development	D_vs_E10;stage_1W_vs_E10;stage_3W_vs_E10;stage	1	TRUE
ENSGALG00010024814	APBA2	PRJNA729271	-3.857491315	8.3249E-103	3.857491315	stage_E14_vs_E10	ordinary_muscle_development	D_vs_E10;stage_1W_vs_E10;stage_3W_vs_E10;stage	1	TRUE
ENSGALG00010026111	ENSGALG00010026111	PRJNA729271	-3.622165322	1.2879E-102	3.622165322	stage_E14_vs_E10	ordinary_muscle_development	D_vs_E10;stage_1W_vs_E10;stage_3W_vs_E10;stage	1	TRUE
ENSGALG00010020088	LMOD3	PRJNA729271	4.206491785	1.3683E-102	4.206491785	stage_E14_vs_E10	ordinary_muscle_development	D_vs_E10;stage_1W_vs_E10;stage_3W_vs_E10;stage	1	TRUE
ENSGALG00010021935	CETP	PRJNA729271	6.416979707	1.4243E-102	6.416979707	stage_E14_vs_E10	ordinary_muscle_development	D_vs_E10;stage_1W_vs_E10;stage_3W_vs_E10;stage	1	TRUE
ENSGALG00010014361	SSPN	PRJNA729271	3.230140512	3.6317E-102	3.230140512	stage_E14_vs_E10	ordinary_muscle_development	D_vs_E10;stage_1W_vs_E10;stage_3W_vs_E10;stage	1	TRUE
ENSGALG00010003948	SCD5	PRJNA729271	-3.236219525	5.2185E-102	3.236219525	stage_E14_vs_E10	ordinary_muscle_development	D_vs_E10;stage_1W_vs_E10;stage_3W_vs_E10;stage	1	TRUE
ENSGALG00010001754	APCDD1	PRJNA729271	-4.507365516	5.4144E-102	4.507365516	stage_E14_vs_E10	ordinary_muscle_development	D_vs_E10;stage_1W_vs_E10;stage_3W_vs_E10;stage	1	TRUE
ENSGALG00010022751	SLC25A12	PRJNA729271	4.125390671	5.5837E-102	4.125390671	stage_E14_vs_E10	ordinary_muscle_development	D_vs_E10;stage_1W_vs_E10;stage_3W_vs_E10;stage	1	TRUE
ENSGALG00010024688	CADM1	PRJNA729271	-2.922005364	6.4149E-102	2.922005364	stage_E14_vs_E10	ordinary_muscle_development	D_vs_E10;stage_1W_vs_E10;stage_3W_vs_E10;stage	1	TRUE
ENSGALG00010003560	ADGRL3	PRJNA729271	-3.071894018	6.9264E-102	3.071894018	stage_E14_vs_E10	ordinary_muscle_development	D_vs_E10;stage_1W_vs_E10;stage_3W_vs_E10;stage	1	TRUE
ENSGALG00010013392	HDAC2	PRJNA729271	-1.509697469	8.9053E-102	1.509697469	stage_E14_vs_E10	ordinary_muscle_development	D_vs_E10;stage_1W_vs_E10;stage_3W_vs_E10;stage	1	TRUE
ENSGALG00010008008	BTC	PRJNA729271	4.256260597	1.282E-101	4.256260597	stage_E14_vs_E10	ordinary_muscle_development	D_vs_E10;stage_1W_vs_E10;stage_3W_vs_E10;stage	1	TRUE
ENSGALG00010008133	CREG1	PRJNA729271	3.601920254	1.6315E-101	3.601920254	stage_E14_vs_E10	ordinary_muscle_development	D_vs_E10;stage_1W_vs_E10;stage_3W_vs_E10;stage	1	TRUE
ENSGALG00010009246	RUSC2	PRJNA729271	3.046727491	1.8718E-101	3.046727491	stage_E14_vs_E10	ordinary_muscle_development	D_vs_E10;stage_1W_vs_E10;stage_3W_vs_E10;stage	1	TRUE
ENSGALG00010016604	TP53BP2	PRJNA729271	-1.374413387	2.3209E-101	1.374413387	stage_E14_vs_E10	ordinary_muscle_development	D_vs_E10;stage_1W_vs_E10;stage_3W_vs_E10;stage	1	TRUE
ENSGALG00010025364	EPHA4	PRJNA729271	-4.171922672	2.5793E-101	4.171922672	stage_E14_vs_E10	ordinary_muscle_development	D_vs_E10;stage_1W_vs_E10;stage_3W_vs_E10;stage	1	TRUE
ENSGALG00010008636	AGTPBP1	PRJNA729271	5.132681033	3.494E-101	5.132681033	stage_E14_vs_E10	ordinary_muscle_development	D_vs_E10;stage_1W_vs_E10;stage_3W_vs_E10;stage	1	TRUE
ENSGALG00010009479	MTBP	PRJNA729271	-3.267700711	3.7946E-101	3.267700711	stage_E14_vs_E10	ordinary_muscle_development	D_vs_E10;stage_1W_vs_E10;stage_3W_vs_E10;stage	1	TRUE
ENSGALG00010028036	NDUFS7	PRJNA729271	2.616663175	9.0878E-101	2.616663175	stage_E14_vs_E10	ordinary_muscle_development	D_vs_E10;stage_1W_vs_E10;stage_3W_vs_E10;stage	1	TRUE

ENSGALG00010020794	STEAP3	PRJNA729271	2.787391158	9.7814E-101	2.787391158	stage_E14_vs_E10	ordinary_muscle_development	D_vs_E10;stage_1W_vs_E10;stage_3W_vs_E10;stage	1	TRUE
ENSGALG00010005281	TMED8	PRJNA729271	-1.528021911	1.01139E-100	1.528021911	stage_E14_vs_E10	ordinary_muscle_development	D_vs_E10;stage_1W_vs_E10;stage_3W_vs_E10;stage	1	TRUE
ENSGALG00010020700	MTHFD2	PRJNA729271	2.811429689	1.02461E-100	2.811429689	stage_E14_vs_E10	ordinary_muscle_development	D_vs_E10;stage_1W_vs_E10;stage_3W_vs_E10;stage	1	TRUE
ENSGALG00010018685	ARL3	PRJNA729271	-2.289265571	1.24068E-100	2.289265571	stage_E14_vs_E10	ordinary_muscle_development	D_vs_E10;stage_1W_vs_E10;stage_3W_vs_E10;stage	1	TRUE
ENSGALG00010005394	PHYH	PRJNA729271	2.902842427	1.52436E-100	2.902842427	stage_E14_vs_E10	ordinary_muscle_development	D_vs_E10;stage_1W_vs_E10;stage_3W_vs_E10;stage	1	TRUE
ENSGALG00010017322	KHDRBS1	PRJNA729271	-1.527177595	1.74796E-100	1.527177595	stage_E14_vs_E10	ordinary_muscle_development	D_vs_E10;stage_1W_vs_E10;stage_3W_vs_E10;stage	1	TRUE
ENSGALG00010015111	ADAMTS2	PRJNA729271	3.041681971	1.87504E-100	3.041681971	stage_E14_vs_E10	ordinary_muscle_development	D_vs_E10;stage_1W_vs_E10;stage_3W_vs_E10;stage	1	TRUE
ENSGALG00010009257	ENSGALG00010009257	PRJNA729271	3.993245027	2.53199E-100	3.993245027	stage_E14_vs_E10	ordinary_muscle_development	D_vs_E10;stage_1W_vs_E10;stage_3W_vs_E10;stage	1	TRUE
ENSGALG00010011321	MAP2	PRJNA729271	-3.452390865	2.67904E-100	3.452390865	stage_E14_vs_E10	ordinary_muscle_development	D_vs_E10;stage_1W_vs_E10;stage_3W_vs_E10;stage	1	TRUE
ENSGALG00010028436	S100A1	PRJNA729271	3.945678873	4.94013E-100	3.945678873	stage_E14_vs_E10	ordinary_muscle_development	D_vs_E10;stage_1W_vs_E10;stage_3W_vs_E10;stage	1	TRUE
ENSGALG00010012415	BID	PRJNA729271	-2.514209048	6.46951E-100	2.514209048	stage_E14_vs_E10	ordinary_muscle_development	D_vs_E10;stage_1W_vs_E10;stage_3W_vs_E10;stage	1	TRUE
ENSGALG00010018732	ENSGALG00010018732	PRJNA729271	4.665039026	8.77501E-100	4.665039026	stage_E14_vs_E10	ordinary_muscle_development	D_vs_E10;stage_1W_vs_E10;stage_3W_vs_E10;stage	1	TRUE
ENSGALG00010002556	ENSGALG00010002556	PRJNA729271	4.917723568	1.06376E-99	4.917723568	stage_E14_vs_E10	ordinary_muscle_development	D_vs_E10;stage_1W_vs_E10;stage_3W_vs_E10;stage	1	TRUE
ENSGALG00010023957	YBX3	PRJNA729271	2.961521809	1.9704E-99	2.961521809	stage_E14_vs_E10	ordinary_muscle_development	D_vs_E10;stage_1W_vs_E10;stage_3W_vs_E10;stage	1	TRUE
ENSGALG00010011886	NTNG1	PRJNA729271	-4.769279504	2.17067E-99	4.769279504	stage_E14_vs_E10	ordinary_muscle_development	D_vs_E10;stage_1W_vs_E10;stage_3W_vs_E10;stage	1	TRUE
ENSGALG00010021121	PLA2R1	PRJNA729271	1.94645893	2.49499E-99	1.94645893	stage_E14_vs_E10	ordinary_muscle_development	D_vs_E10;stage_1W_vs_E10;stage_3W_vs_E10;stage	1	TRUE
ENSGALG00010018008	ABCF3	PRJNA729271	2.805349622	2.94726E-99	2.805349622	stage_E14_vs_E10	ordinary_muscle_development	D_vs_E10;stage_1W_vs_E10;stage_3W_vs_E10;stage	1	TRUE
ENSGALG00010003660	IGFBP7	PRJNA729271	3.513383118	2.95297E-99	3.513383118	stage_E14_vs_E10	ordinary_muscle_development	D_vs_E10;stage_1W_vs_E10;stage_3W_vs_E10;stage	1	TRUE
ENSGALG00010029210	RSKR	PRJNA729271	5.392253683	5.04268E-99	5.392253683	stage_E14_vs_E10	ordinary_muscle_development	D_vs_E10;stage_1W_vs_E10;stage_3W_vs_E10;stage	1	TRUE
ENSGALG00010018753	SYNM	PRJNA729271	3.267409879	6.47315E-99	3.267409879	stage_E14_vs_E10	ordinary_muscle_development	D_vs_E10;stage_1W_vs_E10;stage_3W_vs_E10;stage	1	TRUE
ENSGALG00010024431	FEZ1	PRJNA729271	-3.432549455	6.63689E-99	3.432549455	stage_E14_vs_E10	ordinary_muscle_development	D_vs_E10;stage_1W_vs_E10;stage_3W_vs_E10;stage	1	TRUE
ENSGALG00010013686	UQCRQ	PRJNA729271	2.932842235	7.37095E-99	2.932842235	stage_E14_vs_E10	ordinary_muscle_development	D_vs_E10;stage_1W_vs_E10;stage_3W_vs_E10;stage	1	TRUE
ENSGALG00010001752	ARRB1	PRJNA729271	2.228681251	9.43356E-99	2.228681251	stage_E14_vs_E10	ordinary_muscle_development	D_vs_E10;stage_1W_vs_E10;stage_3W_vs_E10;stage	1	TRUE
ENSGALG00010017304	ANKRD9	PRJNA729271	6.319917995	1.15791E-98	6.319917995	stage_E14_vs_E10	ordinary_muscle_development	D_vs_E10;stage_1W_vs_E10;stage_3W_vs_E10;stage	1	TRUE
ENSGALG00010001820	PGM2L1	PRJNA729271	3.251989964	2.27262E-98	3.251989964	stage_E14_vs_E10	ordinary_muscle_development	D_vs_E10;stage_1W_vs_E10;stage_3W_vs_E10;stage	1	TRUE
ENSGALG00010011420	HMGB3	PRJNA729271	-2.123834329	2.87761E-98	2.123834329	stage_E14_vs_E10	ordinary_muscle_development	D_vs_E10;stage_1W_vs_E10;stage_3W_vs_E10;stage	1	TRUE
ENSGALG00010021169	CAMK2G	PRJNA729271	2.96376242	4.96639E-98	2.96376242	stage_E14_vs_E10	ordinary_muscle_development	D_vs_E10;stage_1W_vs_E10;stage_3W_vs_E10;stage	1	TRUE
ENSGALG00010023883	TBX21	PRJNA729271	7.220192089	5.56283E-98	7.220192089	stage_E14_vs_E10	ordinary_muscle_development	D_vs_E10;stage_1W_vs_E10;stage_3W_vs_E10;stage	1	TRUE
ENSGALG00010016224	MOSPD1	PRJNA729271	2.436395186	6.07148E-98	2.436395186	stage_E14_vs_E10	ordinary_muscle_development	D_vs_E10;stage_1W_vs_E10;stage_3W_vs_E10;stage	1	TRUE
ENSGALG00010006958	KIF26B	PRJNA729271	-5.386829415	6.49916E-98	5.386829415	stage_E14_vs_E10	ordinary_muscle_development	D_vs_E10;stage_1W_vs_E10;stage_3W_vs_E10;stage	1	TRUE
ENSGALG00010003858	SUCLA2	PRJNA729271	2.627099827	6.67536E-98	2.627099827	stage_E14_vs_E10	ordinary_muscle_development	D_vs_E10;stage_1W_vs_E10;stage_3W_vs_E10;stage	1	TRUE
ENSGALG00010003239	RRM1	PRJNA729271	-2.54283009	7.23414E-98	2.54283009	stage_E14_vs_E10	ordinary_muscle_development	D_vs_E10;stage_1W_vs_E10;stage_3W_vs_E10;stage	1	TRUE
ENSGALG00010005359	MEIS2	PRJNA729271	-2.935870435	1.04018E-97	2.935870435	stage_E14_vs_E10	ordinary_muscle_development	D_vs_E10;stage_1W_vs_E10;stage_3W_vs_E10;stage	1	TRUE
ENSGALG00010002994	DUSP4	PRJNA729271	-4.854339027	1.05152E-97	4.854339027	stage_E14_vs_E10	ordinary_muscle_development	D_vs_E10;stage_1W_vs_E10;stage_3W_vs_E10;stage	1	TRUE
ENSGALG00010009035	ENSGALG00010009035	PRJNA729271	-1.783732025	1.64685E-97	1.783732025	stage_E14_vs_E10	ordinary_muscle_development	D_vs_E10;stage_1W_vs_E10;stage_3W_vs_E10;stage	1	TRUE
ENSGALG00010023190	SF3B3	PRJNA729271	-1.193405327	1.67783E-97	1.193405327	stage_E14_vs_E10	ordinary_muscle_development	D_vs_E10;stage_1W_vs_E10;stage_3W_vs_E10;stage	1	TRUE
ENSGALG00010027200	G0S2	PRJNA729271	6.701599782	2.33718E-97	6.701599782	stage_E14_vs_E10	ordinary_muscle_development	D_vs_E10;stage_1W_vs_E10;stage_3W_vs_E10;stage	1	TRUE
ENSGALG00010018313	MYEF2	PRJNA729271	-1.739000735	2.82666E-97	1.739000735	stage_E14_vs_E10	ordinary_muscle_development	D_vs_E10;stage_1W_vs_E10;stage_3W_vs_E10;stage	1	TRUE
ENSGALG00010021237	FSCN1	PRJNA729271	-4.754685514	3.39172E-97	4.754685514	stage_E14_vs_E10	ordinary_muscle_development	D_vs_E10;stage_1W_vs_E10;stage_3W_vs_E10;stage	1	TRUE
ENSGALG00010028024	RBM3	PRJNA729271	-2.387671901	3.53054E-97	2.387671901	stage_E14_vs_E10	ordinary_muscle_development	D_vs_E10;stage_1W_vs_E10;stage_3W_vs_E10;stage	1	TRUE
ENSGALG00010001967	LAMA1	PRJNA729271	-5.174588306	4.1858E-97	5.174588306	stage_E14_vs_E10	ordinary_muscle_development	D_vs_E10;stage_1W_vs_E10;stage_3W_vs_E10;stage	1	TRUE
ENSGALG00010019985	UMPS	PRJNA729271	-1.890081605	4.30949E-97	1.890081605	stage_E14_vs_E10	ordinary_muscle_development	D_vs_E10;stage_1W_vs_E10;stage_3W_vs_E10;stage	1	TRUE
ENSGALG00010009543	MAF1	PRJNA729271	2.725799373	6.20803E-97	2.725799373	stage_E14_vs_E10	ordinary_muscle_development	D_vs_E10;stage_1W_vs_E10;stage_3W_vs_E10;stage	1	TRUE
ENSGALG00010018936	SYT14	PRJNA729271	4.489719378	1.11228E-96	4.489719378	stage_E14_vs_E10	ordinary_muscle_development	D_vs_E10;stage_1W_vs_E10;stage_3W_vs_E10;stage	1	TRUE
ENSGALG00010015075	ENSGALG00010015075	PRJNA729271	3.509020473	1.46649E-96	3.509020473	stage_E14_vs_E10	ordinary_muscle_development	D_vs_E10;stage_1W_vs_E10;stage_3W_vs_E10;stage	1	TRUE
ENSGALG00010001666	PIEZO2	PRJNA729271	-3.885222269	1.54437E-96	3.885222269	stage_E14_vs_E10	ordinary_muscle_development	D_vs_E10;stage_1W_vs_E10;stage_3W_vs_E10;stage	1	TRUE
ENSGALG00010014687	CUL4A	PRJNA729271	2.395072179	4.00683E-96	2.395072179	stage_E14_vs_E10	ordinary_muscle_development	D_vs_E10;stage_1W_vs_E10;stage_3W_vs_E10;stage	1	TRUE
ENSGALG00010019873	LGR4	PRJNA729271	-2.093018766	4.43245E-96	2.093018766	stage_E14_vs_E10	ordinary_muscle_development	D_vs_E10;stage_1W_vs_E10;stage_3W_vs_E10;stage	1	TRUE
ENSGALG00010021841	CENPT	PRJNA729271	-2.010689122	6.75624E-96	2.010689122	stage_E14_vs_E10	ordinary_muscle_development	D_vs_E10;stage_1W_vs_E10;stage_3W_vs_E10;stage	1	TRUE
ENSGALG00010007920	FAT3	PRJNA729271	-3.880095809	7.52548E-96	3.880095809	stage_E14_vs_E10	ordinary_muscle_development	D_vs_E10;stage_1W_vs_E10;stage_3W_vs_E10;stage	1	TRUE
ENSGALG00010001153	TSHZ1	PRJNA729271	-2.50326787	9.31003E-96	2.50326787	stage_E14_vs_E10	ordinary_muscle_development	D_vs_E10;stage_1W_vs_E10;stage_3W_vs_E10;stage	1	TRUE
ENSGALG00010004200	ABCB1	PRJNA729271	3.789167495	1.04883E-95	3.789167495	stage_E14_vs_E10	ordinary_muscle_development	D_vs_E10;stage_1W_vs_E10;stage_3W_vs_E10;stage	1	TRUE
ENSGALG00010003299	RAD23A	PRJNA729271	2.515036969	1.07165E-95	2.515036969	stage_E14_vs_E10	ordinary_muscle_development	D_vs_E10;stage_1W_vs_E10;stage_3W_vs_E10;stage	1	TRUE
ENSGALG00010023170	TCF12	PRJNA729271	-1.358011567	1.63292E-95	1.358011567	stage_E14_vs_E10	ordinary_muscle_development	D_vs_E10;stage_1W_vs_E10;stage_3W_vs_E10;stage	1	TRUE
ENSGALG00010027414	PBX3	PRJNA729271	-1.571100644	1.77567E-95	1.571100644	stage_E14_vs_E10	ordinary_muscle_development	D_vs_E10;stage_1W_vs_E10;stage_3W_vs_E10;stage	1	TRUE
ENSGALG00010005399	NDNF	PRJNA729271	-5.948957023	2.20532E-95	5.948957023	stage_E14_vs_E10	ordinary_muscle_development	D_vs_E10;stage_1W_vs_E10;stage_3W_vs_E10;stage	1	TRUE
ENSGALG00010011546	MAOA	PRJNA729271	2.624859409	2.26146E-95	2.624859409	stage_E14_vs_E10	ordinary_muscle_development	D_vs_E10;stage_1W_vs_E10;stage_3W_vs_E10;stage	1	TRUE
ENSGALG00010029733	ENSGALG00010029733	PRJNA729271	3.421110488	4.13265E-95	3.421110488	stage_E14_vs_E10	ordinary_muscle_development	D_vs_E10;stage_1W_vs_E10;stage_3W_vs_E10;stage	1	TRUE
ENSGALG00010012406	ENSGALG00010012406	PRJNA729271	4.952437487	1.32367E-94	4.952437487	stage_E14_vs_E10	ordinary_muscle_development	D_vs_E10;stage_1W_vs_E10;stage_3W_vs_E10;stage	1	TRUE
ENSGALG00010028193	BRINP1	PRJNA729271	-6.655268103	1.76311E-94	6.655268103	stage_E14_vs_E10	ordinary_muscle_development	D_vs_E10;stage_1W_vs_E10;stage_3W_vs_E10;stage	1	TRUE
ENSGALG00010017873	HSPG2	PRJNA729271	2.281378904	2.08219E-94	2.281378904	stage_E14_vs_E10	ordinary_muscle_development	D_vs_E10;stage_1W_vs_E10;stage_3W_vs_E10;stage	1	TRUE
ENSGALG00010026287	NASP	PRJNA729271	-1.910975409	2.98553E-94	1.910975409	stage_E14_vs_E10	ordinary_muscle_development	D_vs_E10;stage_1W_vs_E10;stage_3W_vs_E10;stage	1	TRUE
ENSGALG00010015643	ENSGALG00010015643	PRJNA729271	-2.545581356	3.83002E-94	2.545581356	stage_E14_vs_E10	ordinary_muscle_development	D_vs_E10;stage_1W_vs_E10;stage_3W_vs_E10;stage	1	TRUE
ENSGALG00010022194	ENSGALG00010022194	PRJNA729271	-1.953951105	4.15295E-94	1.953951105	stage_E14_vs_E10	ordinary_muscle_development	D_vs_E10;stage_1W_vs_E10;stage_3W_vs_E10;stage	1	TRUE
ENSGALG00010024973	RTEL1	PRJNA729271	-1.905122325	4.40859E-94	1.905122325	stage_E14_vs_E10	ordinary_muscle_development	D_vs_E10;stage_1W_vs_E10;stage_3W_vs_E10;stage	1	TRUE
ENSGALG00010001825	LIPT2	PRJNA729271	2.940587731	5.83842E-94	2.940587731	stage_E14_vs_E10	ordinary_muscle_development	D_vs_E10;stage_1W_vs_E10;stage_3W_vs_E10;stage	1	TRUE
ENSGALG00010015894	CEP78	PRJNA729271	-2.691048981	5.95606E-94	2.691048981	stage_E14_vs_E10	ordinary_muscle_development	D_vs_E10;stage_1W_vs_E10;stage_3W_vs_E10;stage	1	TRUE
ENSGALG00010023285	SLC48A1	PRJNA729271	2.298643843	7.08451E-94	2.298643843	stage_E14_vs_E10	ordinary_muscle_development	D_vs_E10;stage_1W_vs_E10;stage_3W_vs_E10;stage	1	TRUE
ENSGALG00010013137	TMEM121	PRJNA729271	-6.748967207	8.07458E-94	6.748967207	stage_E14_vs_E10	ordinary_muscle_development	D_vs_E10;stage_1W_vs_E10;stage_3W_vs_E10;stage	1	TRUE
ENSGALG00010006107	PGF	PRJNA729271	3.902297177	9.21614E-94	3.902297177	stage_E14_vs_E10	ordinary_muscle_development	D_vs_E10;stage_1W_vs_E10;stage_3W_vs_E10;stage	1	TRUE
ENSGALG00010002438	RARB	PRJNA729271	-1.976066804	9.66204E-94	1.976066804	stage_E14_vs_E10	ordinary_muscle_development	D_vs_E10;stage_1W_vs_E10;stage_3W_vs_E10;stage	1	TRUE
ENSGALG00010018021	ATRN	PRJNA729271	1.463888027	1.14359E-93	1.463888027	stage_E14_vs_E10	ordinary_muscle_development	D_vs_E10;stage_1W_vs_E10;stage_3W_vs_E10;stage	1	TRUE
ENSGALG00010013501	AKR1B10	PRJNA729271	-2.967565783	1.34312E-93	2.967565783	stage_E14_vs_E10	ordinary_muscle_development	D_vs_E10;stage_1W_vs_E10;stage_3W_vs_E10;stage	1	TRUE
ENSGALG00010026898	PNPLA2	PRJNA729271	5.625001813	1.54953E-93	5.625001813	stage_E14_vs_E10	ordinary_muscle_development	D_vs_E10;stage_1W_vs_E10;stage_3W_vs_E10;stage	1	TRUE
ENSGALG00010021153	PTPRF	PRJNA729271	-3.367387724	1.65524E-93	3.367387724	stage_E14_vs_E10	ordinary_muscle_development	D_vs_E10;stage_1W_vs_E10;stage_3W_vs_E10;stage	1	TRUE
ENSGALG00010010271	HUNK	PRJNA729271	-2.389197954	2.32702E-93	2.389197954	stage_E14_vs_E10	ordinary_muscle_development	D_vs_E10;stage_1W_vs_E10;stage_3W_vs_E10;stage	1	TRUE
ENSGALG00010015804	CKB	PRJNA729271	-3.349559318	2.9989E-93	3.349559318	stage_E14_vs_E10	ordinary_muscle_development	D_vs_E10;stage_1W_vs_E10;stage_3W_vs_E10;stage	1	TRUE
ENSGALG00010027870	HAX1	PRJNA729271	2.477252325	3.5599E-93	2.477252325	stage_E14_vs_E10	ordinary_muscle_development	D_vs_E10;stage_1W_vs_E10;stage_3W_vs_E10;stage	1	TRUE
ENSGALG00010002991	BLB1	PRJNA729271	6.332713641	4.55916E-93	6.332713641	stage_E14_vs_E10	ordinary_muscle_development	D_vs_E10;stage_1W_vs_E10;stage_3W_vs_E10;stage	1	TRUE
ENSGALG00010002113	GDPD4	PRJNA729271	-8.24015764	7.91177E-93	8.24015764	stage_E14_vs_E10	ordinary_muscle_development	D_vs_E10;stage_1W_vs_E10;stage_3W_vs_E10;stage	1	TRUE
ENSGALG00010021629	DHRS7B	PRJNA729271	2.072365533	9.04182E-93	2.072365533	stage_E14_vs_E10	ordinary_muscle_development	D_vs_E10;stage_1W_vs_E10;stage_3W_vs_E10;stage	1	TRUE
ENSGALG00010014194	LRIG3	PRJNA729271	-1.951554558	9.50795E-93	1.951554558	stage_E14_vs_E10	ordinary_muscle_development	D_vs_E10;stage_1W_vs_E10;stage_3W_vs_E10;stage	1	TRUE
ENSGALG00010015778	PABIR3	PRJNA729271	-1.825908347	1.04025E-92	1.825908347	stage_E14_vs_E10	ordinary_muscle_development	D_vs_E10;stage_1W_vs_E10;stage_3W_vs_E10;stage	1	TRUE
ENSGALG00010015979	ENSGALG00010015979	PRJNA729271	4.165097521	1.04458E-92	4.165097521	stage_E14_vs_E10	ordinary_muscle_development	D_vs_E10;stage_1W_vs_E10;stage_3W_vs_E10;stage	1	TRUE
ENSGALG00010009712	UBXN2A	PRJNA729271	3.661431373	1.27084E-92	3.661431373	stage_E14_vs_E10	ordinary_muscle_development	D_vs_E10;stage_1W_vs_E10;stage_3W_vs_E10;stage	1	TRUE
ENSGALG00010028337	ENSGALG00010028337	PRJNA729271	4.051768941	1.41427E-92	4.051768941	stage_E14_vs_E10	ordinary_muscle_development	D_vs_E10;stage_1W_vs_E10;stage_3W_vs_E10;stage	1	TRUE
ENSGALG00010003808	TWIST1	PRJNA729271	-3.527644028	2.15715E-92	3.527644028	stage_E14_vs_E10	ordinary_muscle_development	D_vs_E10;stage_1W_vs_E10;stage_3W_vs_E10;stage	1	TRUE
ENSGALG00010013890	SFPQ	PRJNA729271	-1.495488036	2.35934E-92	1.495488036	stage_E14_vs_E10	ordinary_muscle_development	D_vs_E10;stage_1W_vs_E10;stage_3W_vs_E10;stage	1	TRUE
ENSGALG00010026973	MGLL	PRJNA729271	2.533412437	2.66803E-92	2.533412437	stage_E14_vs_E10	ordinary_muscle_development	D_vs_E10;stage_1W_vs_E10;stage_3W_vs_E10;stage	1	TRUE
ENSGALG00010009347	TESK1	PRJNA729271	2.900242321	2.86376E-92	2.900242321	stage_E14_vs_E10	ordinary_muscle_development	D_vs_E10;stage_1W_vs_E10;stage_3W_vs_E10;stage	1	TRUE
ENSGALG00010022076	YWHAB	PRJNA729271	-1.443925431	3.35436E-92	1.443925431	stage_E14_vs_E10	ordinary_muscle_development	D_vs_E10;stage_1W_vs_E10;stage_3W_vs_E10;stage	1	TRUE
ENSGALG00010026960	SEC61A1	PRJNA729271	-2.114445694	3.45759E-92	2.114445694	stage_E14_vs_E10	ordinary_muscle_development	D_vs_E10;stage_1W_vs_E10;stage_3W_vs_E10;stage	1	TRUE
ENSGALG00010023354	SEPTIN2	PRJNA729271	-3.557993037	3.46674E-92	3.557993037	stage_E14_vs_E10	ordinary_muscle_development	D_vs_E10;stage_1W_vs_E10;stage_3W_vs_E10;stage	1	TRUE
ENSGALG00010026094	C6orf106	PRJNA729271	1.594371352	4.07334E-92	1.594371352	stage_E14_vs_E10	ordinary_muscle_development	D_vs_E10;stage_1W_vs_E10;stage_3W_vs_E10;stage	1	TRUE
ENSGALG00010017595	CCNF	PRJNA729271	-2.666247937	4.12191E-92	2.666247937	stage_E14_vs_E10	ordinary_muscle_development	D_vs_E10;stage_1W_vs_E10;stage_3W_vs_E10;stage	1	TRUE
ENSGALG00010028348	NES	PRJNA729271	-5.752736497	5.3513E-92	5.752736497	stage_E14_vs_E10	ordinary_muscle_development	D_vs_E10;stage_1W_vs_E10;stage_3W_vs_E10;stage	1	TRUE
ENSGALG00010025849	OBSCN	PRJNA729271	4.698021219	5.78312E-92	4.698021219	stage_E14_vs_E10	ordinary_muscle_development	D_vs_E10;stage_1W_vs_E10;stage_3W_vs_E10;stage	1	TRUE
ENSGALG00010006118	NPNT	PRJNA729271	-4.393049484	7.73925E-92	4.393049484	stage_E14_vs_E10	ordinary_muscle_development	D_vs_E10;stage_1W_vs_E10;stage_3W_vs_E10;stage	1	TRUE
ENSGALG00010024769	RALY	PRJNA729271	4.176392538	8.55563E-92	4.176392538	stage_E14_vs_E10	ordinary_muscle_development	D_vs_E10;stage_1W_vs_E10;stage_3W_vs_E10;stage	1	TRUE
ENSGALG00010018092	ENSGALG00010018092	PRJNA729271	-4.504558315	1.60635E-91	4.504558315	stage_E14_vs_E10	ordinary_muscle_development	D_vs_E10;stage_1W_vs_E10;stage_3W_vs_E10;stage	1	TRUE
ENSGALG00010018727	PARK7	PRJNA729271	2.693578086	1.71851E-91	2.693578086	stage_E14_vs_E10	ordinary_muscle_development	D_vs_E10;stage_1W_vs_E10;stage_3W_vs_E10;stage	1	TRUE
ENSGALG00010016999	ITGB1BP2	PRJNA729271	3.524612819	1.91198E-91	3.524612819	stage_E14_vs_E10	ordinary_muscle_development	D_vs_E10;stage_1W_vs_E10;stage_3W_vs_E10;stage	1	TRUE
ENSGALG00010017773	DVL3	PRJNA729271	-1.750660144	1.97454E-91	1.750660144	stage_E14_vs_E10	ordinary_muscle_development	D_vs_E10;stage_1W_vs_E10;stage_3W_vs_E10;stage	1	TRUE
ENSGALG00010017152	CYP4V2	PRJNA729271	3.993350297	2.15135E-91	3.993350297	stage_E14_vs_E10	ordinary_muscle_development	D_vs_E10;stage_1W_vs_E10;stage_3W_vs_E10;stage	1	TRUE
ENSGALG00010025996	CUL3	PRJNA729271	1.971285867	2.15135E-91	1.971285867	stage_E14_vs_E10	ordinary_muscle_development	D_vs_E10;stage_1W_vs_E10;stage_3W_vs_E10;stage	1	TRUE
ENSGALG00010024709	TEAD4	PRJNA729271	1.971512561	2.47238E-91	1.971512561	stage_E14_vs_E10	ordinary_muscle_development	D_vs_E10;stage_1W_vs_E10;stage_3W_vs_E10;stage	1	TRUE
ENSGALG00010025245	ENSGALG00010025245	PRJNA729271	1.389134085	3.03452E-91	1.389134085	stage_E14_vs_E10	ordinary_muscle_development	D_vs_E10;stage_1W_vs_E10;stage_3W_vs_E10;stage	1	TRUE
ENSGALG00010028592	SEMA6C	PRJNA729271	3.23197984	3.1542E-91	3.23197984	stage_E14_vs_E10	ordinary_muscle_development	D_vs_E10;stage_1W_vs_E10;stage_3W_vs_E10;stage	1	TRUE
ENSGALG00010016706	RTN4	PRJNA729271	2.561930327	3.1542E-91	2.561930327	stage_E14_vs_E10	ordinary_muscle_development	D_vs_E10;stage_1W_vs_E10;stage_3W_vs_E10;stage	1	TRUE
ENSGALG00010000818	KLF12	PRJNA729271	-1.677508421	3.42655E-91	1.677508421	stage_E14_vs_E10	ordinary_muscle_development	D_vs_E10;stage_1W_vs_E10;stage_3W_vs_E10;stage	1	TRUE
ENSGALG00010029654	EME1	PRJNA729271	-2.840527188	3.91083E-91	2.840527188	stage_E14_vs_E10	ordinary_muscle_development	D_vs_E10;stage_1W_vs_E10;stage_3W_vs_E10;stage	1	TRUE
ENSGALG00010022310	DCLRE1A	PRJNA729271	-2.120882895	5.45216E-91	2.120882895	stage_E14_vs_E10	ordinary_muscle_development	D_vs_E10;stage_1W_vs_E10;stage_3W_vs_E10;stage	1	TRUE
ENSGALG00010020065	TTYH3	PRJNA729271	-2.313843959	5.52144E-91	2.313843959	stage_E14_vs_E10	ordinary_muscle_development	D_vs_E10;stage_1W_vs_E10;stage_3W_vs_E10;stage	1	TRUE
ENSGALG00010016340	RGS12	PRJNA729271	-2.08999879	6.26029E-91	2.08999879	stage_E14_vs_E10	ordinary_muscle_development	D_vs_E10;stage_1W_vs_E10;stage_3W_vs_E10;stage	1	TRUE
ENSGALG00010023147	RAD51	PRJNA729271	-4.167577723	8.42633E-91	4.167577723	stage_E14_vs_E10	ordinary_muscle_development	D_vs_E10;stage_1W_vs_E10;stage_3W_vs_E10;stage	1	TRUE
ENSGALG00010007787	C1orf198	PRJNA729271	-2.456685662	1.20356E-90	2.456685662	stage_E14_vs_E10	ordinary_muscle_development	D_vs_E10;stage_1W_vs_E10;stage_3W_vs_E10;stage	1	TRUE
ENSGALG00010018852	NDUFB10	PRJNA729271	2.354940058	1.27522E-90	2.354940058	stage_E14_vs_E10	ordinary_muscle_development	D_vs_E10;stage_1W_vs_E10;stage_3W_vs_E10;stage	1	TRUE
ENSGALG00010014096	JAKMIP2	PRJNA729271	-3.372730589	3.05202E-90	3.372730589	stage_E14_vs_E10	ordinary_muscle_development	D_vs_E10;stage_1W_vs_E10;stage_3W_vs_E10;stage	1	TRUE
ENSGALG00010022454	ABLIM1	PRJNA729271	2.85359404	3.33348E-90	2.85359404	stage_E14_vs_E10	ordinary_muscle_development	D_vs_E10;stage_1W_vs_E10;stage_3W_vs_E10;stage	1	TRUE
ENSGALG00010019319	ACTR3	PRJNA729271	-1.366521088	3.61877E-90	1.366521088	stage_E14_vs_E10	ordinary_muscle_development	D_vs_E10;stage_1W_vs_E10;stage_3W_vs_E10;stage	1	TRUE
ENSGALG00010010528	GPD2	PRJNA729271	2.527634346	4.06209E-90	2.527634346	stage_E14_vs_E10	ordinary_muscle_development	D_vs_E10;stage_1W_vs_E10;stage_3W_vs_E10;stage	1	TRUE
ENSGALG00010027256	PHTF1	PRJNA729271	-1.91647113	4.93198E-90	1.91647113	stage_E14_vs_E10	ordinary_muscle_development	D_vs_E10;stage_1W_vs_E10;stage_3W_vs_E10;stage	1	TRUE
ENSGALG00010007414	ROBO2	PRJNA729271	-4.931905374	5.02728E-90	4.931905374	stage_E14_vs_E10	ordinary_muscle_development	D_vs_E10;stage_1W_vs_E10;stage_3W_vs_E10;stage	1	TRUE
ENSGALG00010013275	MAPRE1	PRJNA729271	-1.552090056	6.94675E-90	1.552090056	stage_E14_vs_E10	ordinary_muscle_development	D_vs_E10;stage_1W_vs_E10;stage_3W_vs_E10;stage	1	TRUE
ENSGALG00010001843	PRICKLE1	PRJNA729271	-2.513289086	7.53718E-90	2.513289086	stage_E14_vs_E10	ordinary_muscle_development	D_vs_E10;stage_1W_vs_E10;stage_3W_vs_E10;stage	1	TRUE
ENSGALG00010008318	ADPRH	PRJNA729271	2.691522631	7.85129E-90	2.691522631	stage_E14_vs_E10	ordinary_muscle_development	D_vs_E10;stage_1W_vs_E10;stage_3W_vs_E10;stage	1	TRUE
ENSGALG00010009309	TRIM54	PRJNA729271	3.015808067	8.04389E-90	3.015808067	stage_E14_vs_E10	ordinary_muscle_development	D_vs_E10;stage_1W_vs_E10;stage_3W_vs_E10;stage	1	TRUE
ENSGALG00010025405	TAMALIN	PRJNA729271	3.278022565	8.22863E-90	3.278022565	stage_E14_vs_E10	ordinary_muscle_development	D_vs_E10;stage_1W_vs_E10;stage_3W_vs_E10;stage	1	TRUE
ENSGALG00010028025	TNNC1	PRJNA729271	4.80318514	9.04486E-90	4.80318514	stage_E14_vs_E10	ordinary_muscle_development	D_vs_E10;stage_1W_vs_E10;stage_3W_vs_E10;stage	1	TRUE
ENSGALG00010024587	ENSGALG00010024587	PRJNA729271	3.610364954	1.04675E-89	3.610364954	stage_E14_vs_E10	ordinary_muscle_development	D_vs_E10;stage_1W_vs_E10;stage_3W_vs_E10;stage	1	TRUE
ENSGALG00010011888	CAND1	PRJNA729271	-1.437632441	1.06196E-89	1.437632441	stage_E14_vs_E10	ordinary_muscle_development	D_vs_E10;stage_1W_vs_E10;stage_3W_vs_E10;stage	1	TRUE
ENSGALG00010024794	DDX6	PRJNA729271	-1.326068768	1.13952E-89	1.326068768	stage_E14_vs_E10	ordinary_muscle_development	D_vs_E10;stage_1W_vs_E10;stage_3W_vs_E10;stage	1	TRUE
ENSGALG00010024626	TSHZ3	PRJNA729271	-1.991745547	1.3583E-89	1.991745547	stage_E14_vs_E10	ordinary_muscle_development	D_vs_E10;stage_1W_vs_E10;stage_3W_vs_E10;stage	1	TRUE
ENSGALG00010022317	PSME3	PRJNA729271	-1.508733657	1.44958E-89	1.508733657	stage_E14_vs_E10	ordinary_muscle_development	D_vs_E10;stage_1W_vs_E10;stage_3W_vs_E10;stage	1	TRUE
ENSGALG00010007388	CYP1B1	PRJNA729271	-4.25417292	1.51256E-89	4.25417292	stage_E14_vs_E10	ordinary_muscle_development	D_vs_E10;stage_1W_vs_E10;stage_3W_vs_E10;stage	1	TRUE
ENSGALG00010012314	ADRB2	PRJNA729271	5.289405733	1.81907E-89	5.289405733	stage_E14_vs_E10	ordinary_muscle_development	D_vs_E10;stage_1W_vs_E10;stage_3W_vs_E10;stage	1	TRUE
ENSGALG00010021803	TLL2	PRJNA729271	-3.515838951	2.82653E-89	3.515838951	stage_E14_vs_E10	ordinary_muscle_development	D_vs_E10;stage_1W_vs_E10;stage_3W_vs_E10;stage	1	TRUE
ENSGALG00010024994	NDUFB4	PRJNA729271	2.092085161	2.99334E-89	2.092085161	stage_E14_vs_E10	ordinary_muscle_development	D_vs_E10;stage_1W_vs_E10;stage_3W_vs_E10;stage	1	TRUE
ENSGALG00010008028	KCNK1	PRJNA729271	-3.457449489	3.73767E-89	3.457449489	stage_E14_vs_E10	ordinary_muscle_development	D_vs_E10;stage_1W_vs_E10;stage_3W_vs_E10;stage	1	TRUE
ENSGALG00010022021	TECPR1	PRJNA729271	1.558976737	4.02118E-89	1.558976737	stage_E14_vs_E10	ordinary_muscle_development	D_vs_E10;stage_1W_vs_E10;stage_3W_vs_E10;stage	1	TRUE
ENSGALG00010008418	TMEM245	PRJNA729271	1.784723009	4.4915E-89	1.784723009	stage_E14_vs_E10	ordinary_muscle_development	D_vs_E10;stage_1W_vs_E10;stage_3W_vs_E10;stage	1	TRUE
ENSGALG00010021565	CBX1	PRJNA729271	-2.155679906	4.56005E-89	2.155679906	stage_E14_vs_E10	ordinary_muscle_development	D_vs_E10;stage_1W_vs_E10;stage_3W_vs_E10;stage	1	TRUE
ENSGALG00010014179	CTBPL	PRJNA729271	-0.872650495	4.97506E-89	0.872650495	stage_E14_vs_E10	ordinary_muscle_development		0	FALSE
ENSGALG00010020759	ARHGAP19	PRJNA729271	-3.656917553	6.79562E-89	3.656917553	stage_E14_vs_E10	ordinary_muscle_development	D_vs_E10;stage_1W_vs_E10;stage_3W_vs_E10;stage	1	TRUE
ENSGALG00010006076	PODXL	PRJNA729271	3.286791832	7.03754E-89	3.286791832	stage_E14_vs_E10	ordinary_muscle_development	D_vs_E10;stage_1W_vs_E10;stage_3W_vs_E10;stage	1	TRUE
ENSGALG00010014120	SOX5	PRJNA729271	-2.848905829	7.54151E-89	2.848905829	stage_E14_vs_E10	ordinary_muscle_development	D_vs_E10;stage_1W_vs_E10;stage_3W_vs_E10;stage	1	TRUE
ENSGALG00010020096	GPHN	PRJNA729271	2.323475963	7.88572E-89	2.323475963	stage_E14_vs_E10	ordinary_muscle_development	D_vs_E10;stage_1W_vs_E10;stage_3W_vs_E10;stage	1	TRUE
ENSGALG00010015486	MTFR1L	PRJNA729271	2.386479118	8.39017E-89	2.386479118	stage_E14_vs_E10	ordinary_muscle_development	D_vs_E10;stage_1W_vs_E10;stage_3W_vs_E10;stage	1	TRUE
ENSGALG00010007360	ROBO1	PRJNA729271	-3.791339137	9.93505E-89	3.791339137	stage_E14_vs_E10	ordinary_muscle_development	D_vs_E10;stage_1W_vs_E10;stage_3W_vs_E10;stage	1	TRUE
ENSGALG00010002870	FABP5	PRJNA729271	4.353769146	1.10414E-88	4.353769146	stage_E14_vs_E10	ordinary_muscle_development	D_vs_E10;stage_1W_vs_E10;stage_3W_vs_E10;stage	1	TRUE
ENSGALG00010018944	NEFL	PRJNA729271	-5.831896064	1.82058E-88	5.831896064	stage_E14_vs_E10	ordinary_muscle_development	D_vs_E10;stage_1W_vs_E10;stage_3W_vs_E10;stage	1	TRUE
ENSGALG00010019282	IGFBP2	PRJNA729271	-3.205485586	1.89397E-88	3.205485586	stage_E14_vs_E10	ordinary_muscle_development	D_vs_E10;stage_1W_vs_E10;stage_3W_vs_E10;stage	1	TRUE
ENSGALG00010018991	RYBP	PRJNA729271	-2.751771376	1.99025E-88	2.751771376	stage_E14_vs_E10	ordinary_muscle_development	D_vs_E10;stage_1W_vs_E10;stage_3W_vs_E10;stage	1	TRUE
ENSGALG00010005913	NFKBIA	PRJNA729271	2.869620067	1.99679E-88	2.869620067	stage_E14_vs_E10	ordinary_muscle_development	D_vs_E10;stage_1W_vs_E10;stage_3W_vs_E10;stage	1	TRUE
ENSGALG00010005237	RAP2A	PRJNA729271	-2.277834219	2.04941E-88	2.277834219	stage_E14_vs_E10	ordinary_muscle_development	D_vs_E10;stage_1W_vs_E10;stage_3W_vs_E10;stage	1	TRUE
ENSGALG00010011447	LAMC1	PRJNA729271	2.626085804	2.1771E-88	2.626085804	stage_E14_vs_E10	ordinary_muscle_development	D_vs_E10;stage_1W_vs_E10;stage_3W_vs_E10;stage	1	TRUE
ENSGALG00010007567	TRIP13	PRJNA729271	-2.947991955	2.91311E-88	2.947991955	stage_E14_vs_E10	ordinary_muscle_development	D_vs_E10;stage_1W_vs_E10;stage_3W_vs_E10;stage	1	TRUE
ENSGALG00010002763	SEPTIN11	PRJNA729271	-2.335518634	2.92288E-88	2.335518634	stage_E14_vs_E10	ordinary_muscle_development	D_vs_E10;stage_1W_vs_E10;stage_3W_vs_E10;stage	1	TRUE
ENSGALG00010018701	SFXN2	PRJNA729271	-2.862857946	3.01812E-88	2.862857946	stage_E14_vs_E10	ordinary_muscle_development	D_vs_E10;stage_1W_vs_E10;stage_3W_vs_E10;stage	1	TRUE
ENSGALG00010005704	AKAP12	PRJNA729271	-1.884478505	4.7921E-88	1.884478505	stage_E14_vs_E10	ordinary_muscle_development	D_vs_E10;stage_1W_vs_E10;stage_3W_vs_E10;stage	1	TRUE
ENSGALG00010023193	PPDPF	PRJNA729271	6.267408655	5.43389E-88	6.267408655	stage_E14_vs_E10	ordinary_muscle_development	D_vs_E10;stage_1W_vs_E10;stage_3W_vs_E10;stage	1	TRUE
ENSGALG00010019144	IFT122	PRJNA729271	-1.527177589	5.97593E-88	1.527177589	stage_E14_vs_E10	ordinary_muscle_development	D_vs_E10;stage_1W_vs_E10;stage_3W_vs_E10;stage	1	TRUE
ENSGALG00010020984	PSEN1	PRJNA729271	-1.223922545	7.07945E-88	1.223922545	stage_E14_vs_E10	ordinary_muscle_development	D_vs_E10;stage_1W_vs_E10;stage_3W_vs_E10;stage	1	TRUE
ENSGALG00010018458	SEMA5B	PRJNA729271	-2.190739873	8.46299E-88	2.190739873	stage_E14_vs_E10	ordinary_muscle_development	D_vs_E10;stage_1W_vs_E10;stage_3W_vs_E10;stage	1	TRUE
ENSGALG00010018172	ACTL6A	PRJNA729271	-1.915096226	9.98104E-88	1.915096226	stage_E14_vs_E10	ordinary_muscle_development	D_vs_E10;stage_1W_vs_E10;stage_3W_vs_E10;stage	1	TRUE
ENSGALG00010001880	MTCL1	PRJNA729271	2.874975773	1.77786E-87	2.874975773	stage_E14_vs_E10	ordinary_muscle_development	D_vs_E10;stage_1W_vs_E10;stage_3W_vs_E10;stage	1	TRUE
ENSGALG00010007964	RECQL4	PRJNA729271	-3.779810456	2.57388E-87	3.779810456	stage_E14_vs_E10	ordinary_muscle_development	D_vs_E10;stage_1W_vs_E10;stage_3W_vs_E10;stage	1	TRUE
ENSGALG00010021871	ENSGALG00010021871	PRJNA729271	4.994270414	2.88336E-87	4.994270414	stage_E14_vs_E10	ordinary_muscle_development	D_vs_E10;stage_1W_vs_E10;stage_3W_vs_E10;stage	1	TRUE
ENSGALG00010000469	ENSGALG00010000469	PRJNA729271	7.695539078	3.14365E-87	7.695539078	stage_E14_vs_E10	ordinary_muscle_development	D_vs_E10;stage_1W_vs_E10;stage_3W_vs_E10;stage	1	TRUE
ENSGALG00010010122	THBS4	PRJNA729271	-5.031031903	3.1822E-87	5.031031903	stage_E14_vs_E10	ordinary_muscle_development	D_vs_E10;stage_1W_vs_E10;stage_3W_vs_E10;stage	1	TRUE
ENSGALG00010027448	ADAMTSL5	PRJNA729271	6.21031933	3.81207E-87	6.21031933	stage_E14_vs_E10	ordinary_muscle_development	D_vs_E10;stage_1W_vs_E10;stage_3W_vs_E10;stage	1	TRUE
ENSGALG00010027710	ENSGALG00010027710	PRJNA729271	5.601125114	4.03768E-87	5.601125114	stage_E14_vs_E10	ordinary_muscle_development	D_vs_E10;stage_1W_vs_E10;stage_3W_vs_E10;stage	1	TRUE
ENSGALG00010008284	BCKDHA	PRJNA729271	2.549220559	4.38055E-87	2.549220559	stage_E14_vs_E10	ordinary_muscle_development	D_vs_E10;stage_1W_vs_E10;stage_3W_vs_E10;stage	1	TRUE
ENSGALG00010009279	ELMOD2	PRJNA729271	-2.284137509	5.26982E-87	2.284137509	stage_E14_vs_E10	ordinary_muscle_development	D_vs_E10;stage_1W_vs_E10;stage_3W_vs_E10;stage	1	TRUE
ENSGALG00010016289	IRF1	PRJNA729271	5.09850073	5.47943E-87	5.09850073	stage_E14_vs_E10	ordinary_muscle_development	D_vs_E10;stage_1W_vs_E10;stage_3W_vs_E10;stage	1	TRUE

ENSGALG00010023984	FMNL1	PRJNA729271	3.908271768	5.52796E-87	3.908271768	stage_E14_vs_E10	ordinary_muscle_development	D_vs_E10;stage_1W_vs_E10;stage_3W_vs_E10;stage	1	TRUE
ENSGALG00010000045	TP53BP1	PRJNA729271	-1.484796003	1.22343E-86	1.484796003	stage_E14_vs_E10	ordinary_muscle_development	D_vs_E10;stage_1W_vs_E10;stage_3W_vs_E10;stage	1	TRUE
ENSGALG00010005720	COL25A1	PRJNA729271	-4.78457427	1.49794E-86	4.78457427	stage_E14_vs_E10	ordinary_muscle_development	D_vs_E10;stage_1W_vs_E10;stage_3W_vs_E10;stage	1	TRUE
ENSGALG00010009437	DPYSL5	PRJNA729271	-5.522364248	1.71652E-86	5.522364248	stage_E14_vs_E10	ordinary_muscle_development	D_vs_E10;stage_1W_vs_E10;stage_3W_vs_E10;stage	1	TRUE
ENSGALG00010002385	PALS2	PRJNA729271	-2.543529997	2.32795E-86	2.543529997	stage_E14_vs_E10	ordinary_muscle_development	D_vs_E10;stage_1W_vs_E10;stage_3W_vs_E10;stage	1	TRUE
ENSGALG00010016969	CHMP3	PRJNA729271	1.633379752	3.0143E-86	1.633379752	stage_E14_vs_E10	ordinary_muscle_development	D_vs_E10;stage_1W_vs_E10;stage_3W_vs_E10;stage	1	TRUE
ENSGALG00010003994	SDR16C5	PRJNA729271	1.664206167	3.10289E-86	1.664206167	stage_E14_vs_E10	ordinary_muscle_development	D_vs_E10;stage_1W_vs_E10;stage_3W_vs_E10;stage	1	TRUE
ENSGALG00010006203	MMR1L4	PRJNA729271	3.456438726	3.35106E-86	3.456438726	stage_E14_vs_E10	ordinary_muscle_development	D_vs_E10;stage_1W_vs_E10;stage_3W_vs_E10;stage	1	TRUE
ENSGALG00010005551	METTL14	PRJNA729271	-1.698287578	4.48208E-86	1.698287578	stage_E14_vs_E10	ordinary_muscle_development	D_vs_E10;stage_1W_vs_E10;stage_3W_vs_E10;stage	1	TRUE
ENSGALG00010009389	CPEB4	PRJNA729271	2.475347279	4.94016E-86	2.475347279	stage_E14_vs_E10	ordinary_muscle_development	D_vs_E10;stage_1W_vs_E10;stage_3W_vs_E10;stage	1	TRUE
ENSGALG00010010780	PARG	PRJNA729271	-0.991095385	5.40661E-86	0.991095385	stage_E14_vs_E10	ordinary_muscle_development		0	FALSE
ENSGALG00010005482	XPO4	PRJNA729271	-1.517462858	6.13091E-86	1.517462858	stage_E14_vs_E10	ordinary_muscle_development	D_vs_E10;stage_1W_vs_E10;stage_3W_vs_E10;stage	1	TRUE
ENSGALG00010024214	CRABP1	PRJNA729271	-10.39832292	6.17066E-86	10.39832292	stage_E14_vs_E10	ordinary_muscle_development	D_vs_E10;stage_1W_vs_E10;stage_3W_vs_E10;stage	1	TRUE
ENSGALG00010024010	ENSGALG00010024010	PRJNA729271	1.749939849	7.41452E-86	1.749939849	stage_E14_vs_E10	ordinary_muscle_development	D_vs_E10;stage_1W_vs_E10;stage_3W_vs_E10;stage	1	TRUE
ENSGALG00010027965	ACAD9	PRJNA729271	2.165980665	7.4906E-86	2.165980665	stage_E14_vs_E10	ordinary_muscle_development	D_vs_E10;stage_1W_vs_E10;stage_3W_vs_E10;stage	1	TRUE
ENSGALG00010027043	IL17RD	PRJNA729271	-3.55131865	7.56124E-86	3.55131865	stage_E14_vs_E10	ordinary_muscle_development	D_vs_E10;stage_1W_vs_E10;stage_3W_vs_E10;stage	1	TRUE
ENSGALG00010023746	TTC28	PRJNA729271	-1.790595194	8.07384E-86	1.790595194	stage_E14_vs_E10	ordinary_muscle_development	D_vs_E10;stage_1W_vs_E10;stage_3W_vs_E10;stage	1	TRUE
ENSGALG00010021561	TGIF2	PRJNA729271	-2.002790324	8.67612E-86	2.002790324	stage_E14_vs_E10	ordinary_muscle_development	D_vs_E10;stage_1W_vs_E10;stage_3W_vs_E10;stage	1	TRUE
ENSGALG00010030004	FLOT2	PRJNA729271	1.386303914	8.78922E-86	1.386303914	stage_E14_vs_E10	ordinary_muscle_development	D_vs_E10;stage_1W_vs_E10;stage_3W_vs_E10;stage	1	TRUE
ENSGALG00010017605	GFPT1	PRJNA729271	-1.42478246	1.06288E-85	1.42478246	stage_E14_vs_E10	ordinary_muscle_development	D_vs_E10;stage_1W_vs_E10;stage_3W_vs_E10;stage	1	TRUE
ENSGALG00010009462	NRBF2	PRJNA729271	2.361350504	1.14836E-85	2.361350504	stage_E14_vs_E10	ordinary_muscle_development	D_vs_E10;stage_1W_vs_E10;stage_3W_vs_E10;stage	1	TRUE
ENSGALG00010005437	PDE7B	PRJNA729271	3.121702227	1.29074E-85	3.121702227	stage_E14_vs_E10	ordinary_muscle_development	D_vs_E10;stage_1W_vs_E10;stage_3W_vs_E10;stage	1	TRUE
ENSGALG00010021593	DCAF7	PRJNA729271	-1.134196287	1.43053E-85	1.134196287	stage_E14_vs_E10	ordinary_muscle_development	D_vs_E10;stage_1W_vs_E10;stage_3W_vs_E10;stage	1	TRUE
ENSGALG00010028597	NUP188	PRJNA729271	-1.137792704	1.64692E-85	1.137792704	stage_E14_vs_E10	ordinary_muscle_development	D_vs_E10;stage_1W_vs_E10;stage_3W_vs_E10;stage	1	TRUE
ENSGALG00010023762	BBS5	PRJNA729271	3.004577367	2.21169E-85	3.004577367	stage_E14_vs_E10	ordinary_muscle_development	D_vs_E10;stage_1W_vs_E10;stage_3W_vs_E10;stage	1	TRUE
ENSGALG00010022202	CX3CL1	PRJNA729271	3.124533154	2.34731E-85	3.124533154	stage_E14_vs_E10	ordinary_muscle_development	D_vs_E10;stage_1W_vs_E10;stage_3W_vs_E10;stage	1	TRUE
ENSGALG00010008453	AMOTL1	PRJNA729271	2.363043096	2.49724E-85	2.363043096	stage_E14_vs_E10	ordinary_muscle_development	D_vs_E10;stage_1W_vs_E10;stage_3W_vs_E10;stage	1	TRUE
ENSGALG00010021158	ACTG1L	PRJNA729271	-2.552572447	5.24793E-85	2.552572447	stage_E14_vs_E10	ordinary_muscle_development	D_vs_E10;stage_1W_vs_E10;stage_3W_vs_E10;stage	1	TRUE
ENSGALG00010028752	MTMR11	PRJNA729271	2.562417902	7.11725E-85	2.562417902	stage_E14_vs_E10	ordinary_muscle_development	D_vs_E10;stage_1W_vs_E10;stage_3W_vs_E10;stage	1	TRUE
ENSGALG00010013897	AMOT	PRJNA729271	-1.521690856	7.56564E-85	1.521690856	stage_E14_vs_E10	ordinary_muscle_development	D_vs_E10;stage_1W_vs_E10;stage_3W_vs_E10;stage	1	TRUE
ENSGALG00010006288	ARF5	PRJNA729271	-3.870160177	1.02429E-84	3.870160177	stage_E14_vs_E10	ordinary_muscle_development	D_vs_E10;stage_1W_vs_E10;stage_3W_vs_E10;stage	1	TRUE
ENSGALG00010021719	BHLHE40	PRJNA729271	4.204626048	1.18334E-84	4.204626048	stage_E14_vs_E10	ordinary_muscle_development	D_vs_E10;stage_1W_vs_E10;stage_3W_vs_E10;stage	1	TRUE
ENSGALG00010015832	RAB39B	PRJNA729271	-1.717340506	1.25684E-84	1.717340506	stage_E14_vs_E10	ordinary_muscle_development	D_vs_E10;stage_1W_vs_E10;stage_3W_vs_E10;stage	1	TRUE
ENSGALG00010005879	FAM107B	PRJNA729271	-2.466491986	1.87318E-84	2.466491986	stage_E14_vs_E10	ordinary_muscle_development	D_vs_E10;stage_1W_vs_E10;stage_3W_vs_E10;stage	1	TRUE
ENSGALG00010015686	CNOT8	PRJNA729271	-1.086062724	3.24263E-84	1.086062724	stage_E14_vs_E10	ordinary_muscle_development	D_vs_E10;stage_1W_vs_E10;stage_3W_vs_E10;stage	1	TRUE
ENSGALG00010027119	NUB1	PRJNA729271	1.910116477	3.70838E-84	1.910116477	stage_E14_vs_E10	ordinary_muscle_development	D_vs_E10;stage_1W_vs_E10;stage_3W_vs_E10;stage	1	TRUE
ENSGALG00010018315	CLASP2	PRJNA729271	2.300957813	4.18359E-84	2.300957813	stage_E14_vs_E10	ordinary_muscle_development	D_vs_E10;stage_1W_vs_E10;stage_3W_vs_E10;stage	1	TRUE
ENSGALG00010021253	TMEM169	PRJNA729271	-2.356341011	5.56023E-84	2.356341011	stage_E14_vs_E10	ordinary_muscle_development	D_vs_E10;stage_1W_vs_E10;stage_3W_vs_E10;stage	1	TRUE
ENSGALG00010014984	LPP	PRJNA729271	1.778193881	6.76418E-84	1.778193881	stage_E14_vs_E10	ordinary_muscle_development	D_vs_E10;stage_1W_vs_E10;stage_3W_vs_E10;stage	1	TRUE
ENSGALG00010016923	ENSGALG00010016923	PRJNA729271	-3.621400371	7.25398E-84	3.621400371	stage_E14_vs_E10	ordinary_muscle_development	D_vs_E10;stage_1W_vs_E10;stage_3W_vs_E10;stage	1	TRUE
ENSGALG00010026237	KLHL22	PRJNA729271	2.678563901	8.61838E-84	2.678563901	stage_E14_vs_E10	ordinary_muscle_development	D_vs_E10;stage_1W_vs_E10;stage_3W_vs_E10;stage	1	TRUE
ENSGALG00010002371	BAG3	PRJNA729271	3.022904512	1.06432E-83	3.022904512	stage_E14_vs_E10	ordinary_muscle_development	D_vs_E10;stage_1W_vs_E10;stage_3W_vs_E10;stage	1	TRUE
ENSGALG00010008152	ENSGALG00010008152	PRJNA729271	-3.299572514	1.15946E-83	3.299572514	stage_E14_vs_E10	ordinary_muscle_development	D_vs_E10;stage_1W_vs_E10;stage_3W_vs_E10;stage	1	TRUE
ENSGALG00010011752	RNF2	PRJNA729271	-2.545925032	1.17109E-83	2.545925032	stage_E14_vs_E10	ordinary_muscle_development	D_vs_E10;stage_1W_vs_E10;stage_3W_vs_E10;stage	1	TRUE
ENSGALG00010004249	USP45	PRJNA729271	1.97037627	1.20821E-83	1.97037627	stage_E14_vs_E10	ordinary_muscle_development	D_vs_E10;stage_1W_vs_E10;stage_3W_vs_E10;stage	1	TRUE
ENSGALG00010014515	ETNK1	PRJNA729271	-2.232790493	1.35772E-83	2.232790493	stage_E14_vs_E10	ordinary_muscle_development	D_vs_E10;stage_1W_vs_E10;stage_3W_vs_E10;stage	1	TRUE
ENSGALG00010021594	ADPGK	PRJNA729271	-1.807922365	1.46694E-83	1.807922365	stage_E14_vs_E10	ordinary_muscle_development	D_vs_E10;stage_1W_vs_E10;stage_3W_vs_E10;stage	1	TRUE
ENSGALG00010024005	STYK1	PRJNA729271	4.86466541	1.64709E-83	4.86466541	stage_E14_vs_E10	ordinary_muscle_development	D_vs_E10;stage_1W_vs_E10;stage_3W_vs_E10;stage	1	TRUE
ENSGALG00010017475	STUM	PRJNA729271	4.817082867	2.0467E-83	4.817082867	stage_E14_vs_E10	ordinary_muscle_development	D_vs_E10;stage_1W_vs_E10;stage_3W_vs_E10;stage	1	TRUE
ENSGALG00010015507	AMMECR1	PRJNA729271	-1.434038696	2.21098E-83	1.434038696	stage_E14_vs_E10	ordinary_muscle_development	D_vs_E10;stage_1W_vs_E10;stage_3W_vs_E10;stage	1	TRUE
ENSGALG00010026008	NRP2	PRJNA729271	1.959817296	2.22967E-83	1.959817296	stage_E14_vs_E10	ordinary_muscle_development	D_vs_E10;stage_1W_vs_E10;stage_3W_vs_E10;stage	1	TRUE
ENSGALG00010008303	TEX10	PRJNA729271	-1.423705679	2.41381E-83	1.423705679	stage_E14_vs_E10	ordinary_muscle_development	D_vs_E10;stage_1W_vs_E10;stage_3W_vs_E10;stage	1	TRUE
ENSGALG00010011351	KANK1	PRJNA729271	-2.180349721	2.46323E-83	2.180349721	stage_E14_vs_E10	ordinary_muscle_development	D_vs_E10;stage_1W_vs_E10;stage_3W_vs_E10;stage	1	TRUE
ENSGALG00010001427	ASB11	PRJNA729271	9.026136086	2.53179E-83	9.026136086	stage_E14_vs_E10	ordinary_muscle_development	D_vs_E10;stage_1W_vs_E10;stage_3W_vs_E10;stage	1	TRUE
ENSGALG00010009558	TMEM26	PRJNA729271	-3.986333287	2.56036E-83	3.986333287	stage_E14_vs_E10	ordinary_muscle_development	D_vs_E10;stage_1W_vs_E10;stage_3W_vs_E10;stage	1	TRUE
ENSGALG00010014482	THOC3	PRJNA729271	-1.769781635	2.58684E-83	1.769781635	stage_E14_vs_E10	ordinary_muscle_development	D_vs_E10;stage_1W_vs_E10;stage_3W_vs_E10;stage	1	TRUE
ENSGALG00010013158	CRIP1	PRJNA729271	3.598103647	2.64696E-83	3.598103647	stage_E14_vs_E10	ordinary_muscle_development	D_vs_E10;stage_1W_vs_E10;stage_3W_vs_E10;stage	1	TRUE
ENSGALG00010010178	LGI2	PRJNA729271	-4.40843147	2.71773E-83	4.40843147	stage_E14_vs_E10	ordinary_muscle_development	D_vs_E10;stage_1W_vs_E10;stage_3W_vs_E10;stage	1	TRUE
ENSGALG00010007166	RASGRF2	PRJNA729271	3.522652866	2.73043E-83	3.522652866	stage_E14_vs_E10	ordinary_muscle_development	D_vs_E10;stage_1W_vs_E10;stage_3W_vs_E10;stage	1	TRUE
ENSGALG00010017008	RTN1	PRJNA729271	-3.666991159	2.97831E-83	3.666991159	stage_E14_vs_E10	ordinary_muscle_development	D_vs_E10;stage_1W_vs_E10;stage_3W_vs_E10;stage	1	TRUE
ENSGALG00010022667	PTEN	PRJNA729271	0.812382263	3.29442E-83	0.812382263	stage_E14_vs_E10	ordinary_muscle_development		0	FALSE
ENSGALG00010027741	RAB11B	PRJNA729271	-1.219106343	3.59914E-83	1.219106343	stage_E14_vs_E10	ordinary_muscle_development	D_vs_E10;stage_1W_vs_E10;stage_3W_vs_E10;stage	1	TRUE
ENSGALG00010015126	MCM5	PRJNA729271	-3.600406676	3.93954E-83	3.600406676	stage_E14_vs_E10	ordinary_muscle_development	D_vs_E10;stage_1W_vs_E10;stage_3W_vs_E10;stage	1	TRUE
ENSGALG00010021706	ENSGALG00010021706	PRJNA729271	3.087876459	4.50858E-83	3.087876459	stage_E14_vs_E10	ordinary_muscle_development	D_vs_E10;stage_1W_vs_E10;stage_3W_vs_E10;stage	1	TRUE
ENSGALG00010005236	TMTC4	PRJNA729271	-1.812488486	5.19576E-83	1.812488486	stage_E14_vs_E10	ordinary_muscle_development	D_vs_E10;stage_1W_vs_E10;stage_3W_vs_E10;stage	1	TRUE
ENSGALG00010002345	SCML2	PRJNA729271	-2.239496149	5.32794E-83	2.239496149	stage_E14_vs_E10	ordinary_muscle_development	D_vs_E10;stage_1W_vs_E10;stage_3W_vs_E10;stage	1	TRUE
ENSGALG00010025130	ATP2A2	PRJNA729271	-2.457496799	6.07295E-83	2.457496799	stage_E14_vs_E10	ordinary_muscle_development	D_vs_E10;stage_1W_vs_E10;stage_3W_vs_E10;stage	1	TRUE
ENSGALG00010013082	CHAF1B	PRJNA729271	-2.27975211	1.1036E-82	2.27975211	stage_E14_vs_E10	ordinary_muscle_development	D_vs_E10;stage_1W_vs_E10;stage_3W_vs_E10;stage	1	TRUE
ENSGALG00010019813	ENSGALG00010019813	PRJNA729271	4.195116992	4.75093E-82	4.195116992	stage_E14_vs_E10	ordinary_muscle_development	D_vs_E10;stage_1W_vs_E10;stage_3W_vs_E10;stage	1	TRUE
ENSGALG00010002194	SHROOM2	PRJNA729271	-2.614166692	6.16357E-82	2.614166692	stage_E14_vs_E10	ordinary_muscle_development	D_vs_E10;stage_1W_vs_E10;stage_3W_vs_E10;stage	1	TRUE
ENSGALG00010006544	MOB3B	PRJNA729271	-3.228355278	9.66578E-82	3.228355278	stage_E14_vs_E10	ordinary_muscle_development	D_vs_E10;stage_1W_vs_E10;stage_3W_vs_E10;stage	1	TRUE
ENSGALG00010012939	GJA1	PRJNA729271	-4.020764016	9.81334E-82	4.020764016	stage_E14_vs_E10	ordinary_muscle_development	D_vs_E10;stage_1W_vs_E10;stage_3W_vs_E10;stage	1	TRUE
ENSGALG00010020101	KIF15	PRJNA729271	-4.096289428	1.26102E-81	4.096289428	stage_E14_vs_E10	ordinary_muscle_development	D_vs_E10;stage_1W_vs_E10;stage_3W_vs_E10;stage	1	TRUE
ENSGALG00010028849	ENSGALG00010028849	PRJNA729271	6.282382119	1.38021E-81	6.282382119	stage_E14_vs_E10	ordinary_muscle_development	D_vs_E10;stage_1W_vs_E10;stage_3W_vs_E10;stage	1	TRUE
ENSGALG00010007916	MCM3	PRJNA729271	-4.0443904	1.46632E-81	4.0443904	stage_E14_vs_E10	ordinary_muscle_development	D_vs_E10;stage_1W_vs_E10;stage_3W_vs_E10;stage	1	TRUE
ENSGALG00010009850	RIF1	PRJNA729271	-2.052436137	1.61239E-81	2.052436137	stage_E14_vs_E10	ordinary_muscle_development	D_vs_E10;stage_1W_vs_E10;stage_3W_vs_E10;stage	1	TRUE
ENSGALG00010015895	RBMXL1	PRJNA729271	-1.654444884	1.65742E-81	1.654444884	stage_E14_vs_E10	ordinary_muscle_development	D_vs_E10;stage_1W_vs_E10;stage_3W_vs_E10;stage	1	TRUE
ENSGALG00010004385	ADAMTS3	PRJNA729271	-4.055422104	1.86467E-81	4.055422104	stage_E14_vs_E10	ordinary_muscle_development	D_vs_E10;stage_1W_vs_E10;stage_3W_vs_E10;stage	1	TRUE
ENSGALG00010001234	AASS	PRJNA729271	1.897543925	1.88174E-81	1.897543925	stage_E14_vs_E10	ordinary_muscle_development	D_vs_E10;stage_1W_vs_E10;stage_3W_vs_E10;stage	1	TRUE
ENSGALG00010022034	ENSGALG00010022034	PRJNA729271	2.020841947	2.33324E-81	2.020841947	stage_E14_vs_E10	ordinary_muscle_development	D_vs_E10;stage_1W_vs_E10;stage_3W_vs_E10;stage	1	TRUE
ENSGALG00010000714	FBXL7	PRJNA729271	-1.92287442	2.40178E-81	1.92287442	stage_E14_vs_E10	ordinary_muscle_development	D_vs_E10;stage_1W_vs_E10;stage_3W_vs_E10;stage	1	TRUE
ENSGALG00010022232	GPR27	PRJNA729271	-4.06992205	3.03453E-81	4.06992205	stage_E14_vs_E10	ordinary_muscle_development	D_vs_E10;stage_1W_vs_E10;stage_3W_vs_E10;stage	1	TRUE
ENSGALG00010018448	KLHDC3	PRJNA729271	3.160688775	3.09348E-81	3.160688775	stage_E14_vs_E10	ordinary_muscle_development	D_vs_E10;stage_1W_vs_E10;stage_3W_vs_E10;stage	1	TRUE
ENSGALG00010003159	TAP2	PRJNA729271	5.073657406	3.61964E-81	5.073657406	stage_E14_vs_E10	ordinary_muscle_development	D_vs_E10;stage_1W_vs_E10;stage_3W_vs_E10;stage	1	TRUE
ENSGALG00010024038	NKD1	PRJNA729271	-3.294354645	3.63069E-81	3.294354645	stage_E14_vs_E10	ordinary_muscle_development	D_vs_E10;stage_1W_vs_E10;stage_3W_vs_E10;stage	1	TRUE
ENSGALG00010001252	SNCAIP	PRJNA729271	-2.405507391	4.04513E-81	2.405507391	stage_E14_vs_E10	ordinary_muscle_development	D_vs_E10;stage_1W_vs_E10;stage_3W_vs_E10;stage	1	TRUE
ENSGALG00010005990	SLC2A12	PRJNA729271	2.884622796	4.17442E-81	2.884622796	stage_E14_vs_E10	ordinary_muscle_development	D_vs_E10;stage_1W_vs_E10;stage_3W_vs_E10;stage	1	TRUE
ENSGALG00010020753	SYDE2	PRJNA729271	1.829696135	4.95895E-81	1.829696135	stage_E14_vs_E10	ordinary_muscle_development	D_vs_E10;stage_1W_vs_E10;stage_3W_vs_E10;stage	1	TRUE
ENSGALG00010021220	SYNPO2L	PRJNA729271	3.961775578	5.11567E-81	3.961775578	stage_E14_vs_E10	ordinary_muscle_development	D_vs_E10;stage_1W_vs_E10;stage_3W_vs_E10;stage	1	TRUE
ENSGALG00010025182	PLA1A	PRJNA729271	3.692477014	5.56885E-81	3.692477014	stage_E14_vs_E10	ordinary_muscle_development	D_vs_E10;stage_1W_vs_E10;stage_3W_vs_E10;stage	1	TRUE
ENSGALG00010001517	CAV2	PRJNA729271	3.150036335	7.72109E-81	3.150036335	stage_E14_vs_E10	ordinary_muscle_development	D_vs_E10;stage_1W_vs_E10;stage_3W_vs_E10;stage	1	TRUE
ENSGALG00010006176	MMR1L3	PRJNA729271	4.093369773	8.09662E-81	4.093369773	stage_E14_vs_E10	ordinary_muscle_development	D_vs_E10;stage_1W_vs_E10;stage_3W_vs_E10;stage	1	TRUE
ENSGALG00010024771	AOX2	PRJNA729271	5.061307574	8.24916E-81	5.061307574	stage_E14_vs_E10	ordinary_muscle_development	D_vs_E10;stage_1W_vs_E10;stage_3W_vs_E10;stage	1	TRUE
ENSGALG00010025390	BCL7A	PRJNA729271	-1.736112436	8.4156E-81	1.736112436	stage_E14_vs_E10	ordinary_muscle_development	D_vs_E10;stage_1W_vs_E10;stage_3W_vs_E10;stage	1	TRUE
ENSGALG00010015306	BCAT1	PRJNA729271	1.738378607	9.67226E-81	1.738378607	stage_E14_vs_E10	ordinary_muscle_development	D_vs_E10;stage_1W_vs_E10;stage_3W_vs_E10;stage	1	TRUE
ENSGALG00010023422	WLS	PRJNA729271	-3.015198322	1.01787E-80	3.015198322	stage_E14_vs_E10	ordinary_muscle_development	D_vs_E10;stage_1W_vs_E10;stage_3W_vs_E10;stage	1	TRUE
ENSGALG00010014614	GHR	PRJNA729271	1.734360948	1.089E-80	1.734360948	stage_E14_vs_E10	ordinary_muscle_development	D_vs_E10;stage_1W_vs_E10;stage_3W_vs_E10;stage	1	TRUE
ENSGALG00010024438	RBL1	PRJNA729271	-2.377257383	1.51136E-80	2.377257383	stage_E14_vs_E10	ordinary_muscle_development	D_vs_E10;stage_1W_vs_E10;stage_3W_vs_E10;stage	1	TRUE
ENSGALG00010022547	SMARCE1	PRJNA729271	-1.194947041	1.81558E-80	1.194947041	stage_E14_vs_E10	ordinary_muscle_development	D_vs_E10;stage_1W_vs_E10;stage_3W_vs_E10;stage	1	TRUE
ENSGALG00010015145	CLIC2	PRJNA729271	2.373274745	2.40844E-80	2.373274745	stage_E14_vs_E10	ordinary_muscle_development	D_vs_E10;stage_1W_vs_E10;stage_3W_vs_E10;stage	1	TRUE
ENSGALG00010006803	COX4I1	PRJNA729271	1.987524951	2.51997E-80	1.987524951	stage_E14_vs_E10	ordinary_muscle_development	D_vs_E10;stage_1W_vs_E10;stage_3W_vs_E10;stage	1	TRUE
ENSGALG00010012756	RPA2	PRJNA729271	-2.062624452	3.63303E-80	2.062624452	stage_E14_vs_E10	ordinary_muscle_development	D_vs_E10;stage_1W_vs_E10;stage_3W_vs_E10;stage	1	TRUE
ENSGALG00010024767	CTTN	PRJNA729271	-1.336977713	3.77274E-80	1.336977713	stage_E14_vs_E10	ordinary_muscle_development	D_vs_E10;stage_1W_vs_E10;stage_3W_vs_E10;stage	1	TRUE
ENSGALG00010019255	ENSGALG00010019255	PRJNA729271	3.789465284	3.82483E-80	3.789465284	stage_E14_vs_E10	ordinary_muscle_development	D_vs_E10;stage_1W_vs_E10;stage_3W_vs_E10;stage	1	TRUE
ENSGALG00010016987	ASB2	PRJNA729271	6.069754665	6.56032E-80	6.069754665	stage_E14_vs_E10	ordinary_muscle_development	D_vs_E10;stage_1W_vs_E10;stage_3W_vs_E10;stage	1	TRUE
ENSGALG00010007529	CDH13	PRJNA729271	2.56331823	7.0326E-80	2.56331823	stage_E14_vs_E10	ordinary_muscle_development	D_vs_E10;stage_1W_vs_E10;stage_3W_vs_E10;stage	1	TRUE
ENSGALG00010009677	SLC26A5	PRJNA729271	3.064777076	8.00223E-80	3.064777076	stage_E14_vs_E10	ordinary_muscle_development	D_vs_E10;stage_1W_vs_E10;stage_3W_vs_E10;stage	1	TRUE
ENSGALG00010020960	CLEC19A	PRJNA729271	6.987134402	8.6627E-80	6.987134402	stage_E14_vs_E10	ordinary_muscle_development	D_vs_E10;stage_1W_vs_E10;stage_3W_vs_E10;stage	1	TRUE
ENSGALG00010023268	KCNJ11	PRJNA729271	3.432953774	9.33994E-80	3.432953774	stage_E14_vs_E10	ordinary_muscle_development	D_vs_E10;stage_1W_vs_E10;stage_3W_vs_E10;stage	1	TRUE
ENSGALG00010011887	PGM5	PRJNA729271	2.521767953	1.0119E-79	2.521767953	stage_E14_vs_E10	ordinary_muscle_development	D_vs_E10;stage_1W_vs_E10;stage_3W_vs_E10;stage	1	TRUE
ENSGALG00010002285	CAV1	PRJNA729271	3.006039804	1.13596E-79	3.006039804	stage_E14_vs_E10	ordinary_muscle_development	D_vs_E10;stage_1W_vs_E10;stage_3W_vs_E10;stage	1	TRUE
ENSGALG00010016519	MEX3B	PRJNA729271	-3.46714312	1.20315E-79	3.46714312	stage_E14_vs_E10	ordinary_muscle_development	D_vs_E10;stage_1W_vs_E10;stage_3W_vs_E10;stage	1	TRUE
ENSGALG00010003884	TYMS	PRJNA729271	-3.790862026	1.67757E-79	3.790862026	stage_E14_vs_E10	ordinary_muscle_development	D_vs_E10;stage_1W_vs_E10;stage_3W_vs_E10;stage	1	TRUE
ENSGALG00010018081	DIO3	PRJNA729271	-5.515941961	2.12418E-79	5.515941961	stage_E14_vs_E10	ordinary_muscle_development	D_vs_E10;stage_1W_vs_E10;stage_3W_vs_E10;stage	1	TRUE
ENSGALG00010007935	LARP1B	PRJNA729271	2.322618066	2.39678E-79	2.322618066	stage_E14_vs_E10	ordinary_muscle_development	D_vs_E10;stage_1W_vs_E10;stage_3W_vs_E10;stage	1	TRUE
ENSGALG00010020771	LINGO1	PRJNA729271	3.801261641	3.05322E-79	3.801261641	stage_E14_vs_E10	ordinary_muscle_development	D_vs_E10;stage_1W_vs_E10;stage_3W_vs_E10;stage	1	TRUE
ENSGALG00010024333	ETV4	PRJNA729271	-4.765732915	3.28108E-79	4.765732915	stage_E14_vs_E10	ordinary_muscle_development	D_vs_E10;stage_1W_vs_E10;stage_3W_vs_E10;stage	1	TRUE
ENSGALG00010029263	TOB1	PRJNA729271	3.578817855	3.41079E-79	3.578817855	stage_E14_vs_E10	ordinary_muscle_development	D_vs_E10;stage_1W_vs_E10;stage_3W_vs_E10;stage	1	TRUE
ENSGALG00010005489	G2E3	PRJNA729271	-1.654397119	3.49222E-79	1.654397119	stage_E14_vs_E10	ordinary_muscle_development	D_vs_E10;stage_1W_vs_E10;stage_3W_vs_E10;stage	1	TRUE
ENSGALG00010002722	TRIM7.1	PRJNA729271	2.831079044	3.77875E-79	2.831079044	stage_E14_vs_E10	ordinary_muscle_development	D_vs_E10;stage_1W_vs_E10;stage_3W_vs_E10;stage	1	TRUE
ENSGALG00010012352	PTPRC	PRJNA729271	3.662782487	4.06943E-79	3.662782487	stage_E14_vs_E10	ordinary_muscle_development	D_vs_E10;stage_1W_vs_E10;stage_3W_vs_E10;stage	1	TRUE
ENSGALG00010011271	ANGPTL1	PRJNA729271	-3.098399278	4.47418E-79	3.098399278	stage_E14_vs_E10	ordinary_muscle_development	D_vs_E10;stage_1W_vs_E10;stage_3W_vs_E10;stage	1	TRUE
ENSGALG00010007302	CDH11	PRJNA729271	-3.333117877	5.1181E-79	3.333117877	stage_E14_vs_E10	ordinary_muscle_development	D_vs_E10;stage_1W_vs_E10;stage_3W_vs_E10;stage	1	TRUE
ENSGALG00010002881	ENSGALG00010002881	PRJNA729271	5.921759933	6.32759E-79	5.921759933	stage_E14_vs_E10	ordinary_muscle_development	D_vs_E10;stage_1W_vs_E10;stage_3W_vs_E10;stage	1	TRUE
ENSGALG00010005309	ASXL3	PRJNA729271	-2.788335758	6.76463E-79	2.788335758	stage_E14_vs_E10	ordinary_muscle_development	D_vs_E10;stage_1W_vs_E10;stage_3W_vs_E10;stage	1	TRUE
ENSGALG00010027616	SLC38A3	PRJNA729271	5.06826321	7.87292E-79	5.06826321	stage_E14_vs_E10	ordinary_muscle_development	D_vs_E10;stage_1W_vs_E10;stage_3W_vs_E10;stage	1	TRUE
ENSGALG00010018323	GOT1	PRJNA729271	2.698237908	7.95593E-79	2.698237908	stage_E14_vs_E10	ordinary_muscle_development	D_vs_E10;stage_1W_vs_E10;stage_3W_vs_E10;stage	1	TRUE
ENSGALG00010029542	AATK	PRJNA729271	2.620218601	1.0635E-78	2.620218601	stage_E14_vs_E10	ordinary_muscle_development	D_vs_E10;stage_1W_vs_E10;stage_3W_vs_E10;stage	1	TRUE
ENSGALG00010026403	GUCD1	PRJNA729271	2.193637697	1.06482E-78	2.193637697	stage_E14_vs_E10	ordinary_muscle_development	D_vs_E10;stage_1W_vs_E10;stage_3W_vs_E10;stage	1	TRUE
ENSGALG00010020181	LEUTX	PRJNA729271	3.966951366	1.08419E-78	3.966951366	stage_E14_vs_E10	ordinary_muscle_development	D_vs_E10;stage_1W_vs_E10;stage_3W_vs_E10;stage	1	TRUE
ENSGALG00010021564	PRKAG3	PRJNA729271	11.92707295	1.18531E-78	11.92707295	stage_E14_vs_E10	ordinary_muscle_development	D_vs_E10;stage_1W_vs_E10;stage_3W_vs_E10;stage	1	TRUE
ENSGALG00010007011	HMGB1	PRJNA729271	-1.931837842	1.26807E-78	1.931837842	stage_E14_vs_E10	ordinary_muscle_development	D_vs_E10;stage_1W_vs_E10;stage_3W_vs_E10;stage	1	TRUE
ENSGALG00010024068	CASP2	PRJNA729271	-1.673510619	1.34527E-78	1.673510619	stage_E14_vs_E10	ordinary_muscle_development	D_vs_E10;stage_1W_vs_E10;stage_3W_vs_E10;stage	1	TRUE
ENSGALG00010018887	ADAMTS17	PRJNA729271	-3.972236659	1.54853E-78	3.972236659	stage_E14_vs_E10	ordinary_muscle_development	D_vs_E10;stage_1W_vs_E10;stage_3W_vs_E10;stage	1	TRUE
ENSGALG00010019832	MAD2L1BP	PRJNA729271	-2.785781268	1.59765E-78	2.785781268	stage_E14_vs_E10	ordinary_muscle_development	D_vs_E10;stage_1W_vs_E10;stage_3W_vs_E10;stage	1	TRUE
ENSGALG00010027984	ARHGAP45	PRJNA729271	3.11814685	1.72098E-78	3.11814685	stage_E14_vs_E10	ordinary_muscle_development	D_vs_E10;stage_1W_vs_E10;stage_3W_vs_E10;stage	1	TRUE
ENSGALG00010004102	PCMTD1	PRJNA729271	3.547190147	1.74284E-78	3.547190147	stage_E14_vs_E10	ordinary_muscle_development	D_vs_E10;stage_1W_vs_E10;stage_3W_vs_E10;stage	1	TRUE
ENSGALG00010008255	MSANTD3	PRJNA729271	-2.60182691	1.87227E-78	2.60182691	stage_E14_vs_E10	ordinary_muscle_development	D_vs_E10;stage_1W_vs_E10;stage_3W_vs_E10;stage	1	TRUE
ENSGALG00010027627	OARD1	PRJNA729271	1.756310348	1.99817E-78	1.756310348	stage_E14_vs_E10	ordinary_muscle_development	D_vs_E10;stage_1W_vs_E10;stage_3W_vs_E10;stage	1	TRUE
ENSGALG00010019392	NIN	PRJNA729271	-2.123402313	2.07282E-78	2.123402313	stage_E14_vs_E10	ordinary_muscle_development	D_vs_E10;stage_1W_vs_E10;stage_3W_vs_E10;stage	1	TRUE
ENSGALG00010003252	PHEX	PRJNA729271	-3.806707817	2.44816E-78	3.806707817	stage_E14_vs_E10	ordinary_muscle_development	D_vs_E10;stage_1W_vs_E10;stage_3W_vs_E10;stage	1	TRUE
ENSGALG00010005145	AKAP7	PRJNA729271	3.302213401	3.25994E-78	3.302213401	stage_E14_vs_E10	ordinary_muscle_development	D_vs_E10;stage_1W_vs_E10;stage_3W_vs_E10;stage	1	TRUE
ENSGALG00010014340	EFNB1	PRJNA729271	-2.621130326	3.67928E-78	2.621130326	stage_E14_vs_E10	ordinary_muscle_development	D_vs_E10;stage_1W_vs_E10;stage_3W_vs_E10;stage	1	TRUE
ENSGALG00010020155	MFGE8	PRJNA729271	2.531314149	3.89606E-78	2.531314149	stage_E14_vs_E10	ordinary_muscle_development	D_vs_E10;stage_1W_vs_E10;stage_3W_vs_E10;stage	1	TRUE
ENSGALG00010011433	RNASE6	PRJNA729271	6.225039307	4.35053E-78	6.225039307	stage_E14_vs_E10	ordinary_muscle_development	D_vs_E10;stage_1W_vs_E10;stage_3W_vs_E10;stage	1	TRUE
ENSGALG00010021613	ADGRL4	PRJNA729271	2.819924717	4.46086E-78	2.819924717	stage_E14_vs_E10	ordinary_muscle_development	D_vs_E10;stage_1W_vs_E10;stage_3W_vs_E10;stage	1	TRUE
ENSGALG00010012601	TUBA8A	PRJNA729271	4.474261372	4.73244E-78	4.474261372	stage_E14_vs_E10	ordinary_muscle_development	D_vs_E10;stage_1W_vs_E10;stage_3W_vs_E10;stage	1	TRUE
ENSGALG00010021931	ITGA6	PRJNA729271	2.309712414	5.2361E-78	2.309712414	stage_E14_vs_E10	ordinary_muscle_development	D_vs_E10;stage_1W_vs_E10;stage_3W_vs_E10;stage	1	TRUE
ENSGALG00010018087	ENSGALG00010018087	PRJNA729271	-1.081610987	5.37767E-78	1.081610987	stage_E14_vs_E10	ordinary_muscle_development	D_vs_E10;stage_1W_vs_E10;stage_3W_vs_E10;stage	1	TRUE
ENSGALG00010009199	SEMA4D	PRJNA729271	-3.088473883	5.66536E-78	3.088473883	stage_E14_vs_E10	ordinary_muscle_development	D_vs_E10;stage_1W_vs_E10;stage_3W_vs_E10;stage	1	TRUE
ENSGALG00010020649	MYH11	PRJNA729271	4.001960765	6.37183E-78	4.001960765	stage_E14_vs_E10	ordinary_muscle_development	D_vs_E10;stage_1W_vs_E10;stage_3W_vs_E10;stage	1	TRUE
ENSGALG00010022098	CDC7	PRJNA729271	-3.434391698	6.45952E-78	3.434391698	stage_E14_vs_E10	ordinary_muscle_development	D_vs_E10;stage_1W_vs_E10;stage_3W_vs_E10;stage	1	TRUE
ENSGALG00010012083	MGP	PRJNA729271	5.243611387	6.95285E-78	5.243611387	stage_E14_vs_E10	ordinary_muscle_development	D_vs_E10;stage_1W_vs_E10;stage_3W_vs_E10;stage	1	TRUE
ENSGALG00010008146	SOX4	PRJNA729271	-2.263043637	7.09882E-78	2.263043637	stage_E14_vs_E10	ordinary_muscle_development	D_vs_E10;stage_1W_vs_E10;stage_3W_vs_E10;stage	1	TRUE
ENSGALG00010009130	HEATR1	PRJNA729271	-1.416162496	7.23179E-78	1.416162496	stage_E14_vs_E10	ordinary_muscle_development	D_vs_E10;stage_1W_vs_E10;stage_3W_vs_E10;stage	1	TRUE
ENSGALG00010010410	CACNA2D1	PRJNA729271	2.977111415	8.57859E-78	2.977111415	stage_E14_vs_E10	ordinary_muscle_development	D_vs_E10;stage_1W_vs_E10;stage_3W_vs_E10;stage	1	TRUE
ENSGALG00010021378	KIF11	PRJNA729271	-3.559647296	9.1321E-78	3.559647296	stage_E14_vs_E10	ordinary_muscle_development	D_vs_E10;stage_1W_vs_E10;stage_3W_vs_E10;stage	1	TRUE
ENSGALG00010004929	ENSGALG00010004929	PRJNA729271	2.985886438	9.38155E-78	2.985886438	stage_E14_vs_E10	ordinary_muscle_development	D_vs_E10;stage_1W_vs_E10;stage_3W_vs_E10;stage	1	TRUE
ENSGALG00010024641	TRA2B	PRJNA729271	-1.681058607	9.69844E-78	1.681058607	stage_E14_vs_E10	ordinary_muscle_development	D_vs_E10;stage_1W_vs_E10;stage_3W_vs_E10;stage	1	TRUE
ENSGALG00010022250	SLC25A28	PRJNA729271	2.38641862	9.84523E-78	2.38641862	stage_E14_vs_E10	ordinary_muscle_development	D_vs_E10;stage_1W_vs_E10;stage_3W_vs_E10;stage	1	TRUE
ENSGALG00010027025	ORAI1	PRJNA729271	2.390242506	1.00828E-77	2.390242506	stage_E14_vs_E10	ordinary_muscle_development	D_vs_E10;stage_1W_vs_E10;stage_3W_vs_E10;stage	1	TRUE
ENSGALG00010028052	ENSGALG00010028052	PRJNA729271	-4.230720924	1.03617E-77	4.230720924	stage_E14_vs_E10	ordinary_muscle_development	D_vs_E10;stage_1W_vs_E10;stage_3W_vs_E10;stage	1	TRUE
ENSGALG00010022532	CBX5	PRJNA729271	-3.446992889	1.10639E-77	3.446992889	stage_E14_vs_E10	ordinary_muscle_development	D_vs_E10;stage_1W_vs_E10;stage_3W_vs_E10;stage	1	TRUE
ENSGALG00010018556	MRPL2	PRJNA729271	2.365231259	1.12286E-77	2.365231259	stage_E14_vs_E10	ordinary_muscle_development	D_vs_E10;stage_1W_vs_E10;stage_3W_vs_E10;stage	1	TRUE
ENSGALG00010029657	CHCHD2	PRJNA729271	2.76070867	1.24913E-77	2.76070867	stage_E14_vs_E10	ordinary_muscle_development	D_vs_E10;stage_1W_vs_E10;stage_3W_vs_E10;stage	1	TRUE
ENSGALG00010011957	ENSGALG00010011957	PRJNA729271	3.948288239	1.31307E-77	3.948288239	stage_E14_vs_E10	ordinary_muscle_development	D_vs_E10;stage_1W_vs_E10;stage_3W_vs_E10;stage	1	TRUE
ENSGALG00010024595	MYBPHL	PRJNA729271	5.947753237	1.66906E-77	5.947753237	stage_E14_vs_E10	ordinary_muscle_development	D_vs_E10;stage_1W_vs_E10;stage_3W_vs_E10;stage	1	TRUE
ENSGALG00010021518	CPEB3	PRJNA729271	2.742647247	1.80035E-77	2.742647247	stage_E14_vs_E10	ordinary_muscle_development	D_vs_E10;stage_1W_vs_E10;stage_3W_vs_E10;stage	1	TRUE
ENSGALG00010004325	LTBP2	PRJNA729271	2.125141627	2.18975E-77	2.125141627	stage_E14_vs_E10	ordinary_muscle_development	D_vs_E10;stage_1W_vs_E10;stage_3W_vs_E10;stage	1	TRUE
ENSGALG00010021332	OIT3	PRJNA729271	2.326330077	2.38608E-77	2.326330077	stage_E14_vs_E10	ordinary_muscle_development	D_vs_E10;stage_1W_vs_E10;stage_3W_vs_E10;stage	1	TRUE
ENSGALG00010028543	ANP32B	PRJNA729271	-1.767332024	2.41237E-77	1.767332024	stage_E14_vs_E10	ordinary_muscle_development	D_vs_E10;stage_1W_vs_E10;stage_3W_vs_E10;stage	1	TRUE
ENSGALG00010018193	ANK1	PRJNA729271	4.642000521	2.7289E-77	4.642000521	stage_E14_vs_E10	ordinary_muscle_development	D_vs_E10;stage_1W_vs_E10;stage_3W_vs_E10;stage	1	TRUE
ENSGALG00010016631	FAM53A	PRJNA729271	1.71255325	3.55877E-77	1.71255325	stage_E14_vs_E10	ordinary_muscle_development	D_vs_E10;stage_1W_vs_E10;stage_3W_vs_E10;stage	1	TRUE
ENSGALG00010012456	ENSGALG00010012456	PRJNA729271	-1.762871395	3.71288E-77	1.762871395	stage_E14_vs_E10	ordinary_muscle_development	D_vs_E10;stage_1W_vs_E10;stage_3W_vs_E10;stage	1	TRUE
ENSGALG00010028618	DOLPP1	PRJNA729271	-2.163771383	3.81923E-77	2.163771383	stage_E14_vs_E10	ordinary_muscle_development	D_vs_E10;stage_1W_vs_E10;stage_3W_vs_E10;stage	1	TRUE

ENSGALG00010029297	SH2B2	PRJNA729271	4.708570356	5.11939E-77	4.708570356	stage_E14_vs_E10	ordinary_muscle_development	D_vs_E10;stage_1W_vs_E10;stage_3W_vs_E10;stage	1	TRUE
ENSGALG00010002623	MID1IP1	PRJNA729271	2.536278895	6.04009E-77	2.536278895	stage_E14_vs_E10	ordinary_muscle_development	D_vs_E10;stage_1W_vs_E10;stage_3W_vs_E10;stage	1	TRUE
ENSGALG00010018455	RBM20	PRJNA729271	4.612883125	6.68639E-77	4.612883125	stage_E14_vs_E10	ordinary_muscle_development	D_vs_E10;stage_1W_vs_E10;stage_3W_vs_E10;stage	1	TRUE
ENSGALG00010020843	HMG20A	PRJNA729271	-1.595154027	8.14605E-77	1.595154027	stage_E14_vs_E10	ordinary_muscle_development	D_vs_E10;stage_1W_vs_E10;stage_3W_vs_E10;stage	1	TRUE
ENSGALG00010010809	CENPH	PRJNA729271	-3.123823006	9.04236E-77	3.123823006	stage_E14_vs_E10	ordinary_muscle_development	D_vs_E10;stage_1W_vs_E10;stage_3W_vs_E10;stage	1	TRUE
ENSGALG00010009362	IL15	PRJNA729271	5.768493906	9.30166E-77	5.768493906	stage_E14_vs_E10	ordinary_muscle_development	D_vs_E10;stage_1W_vs_E10;stage_3W_vs_E10;stage	1	TRUE
ENSGALG00010021211	FEN1	PRJNA729271	-2.982568008	1.0281E-76	2.982568008	stage_E14_vs_E10	ordinary_muscle_development	D_vs_E10;stage_1W_vs_E10;stage_3W_vs_E10;stage	1	TRUE
ENSGALG00010001897	TLR7	PRJNA729271	3.271708298	1.20228E-76	3.271708298	stage_E14_vs_E10	ordinary_muscle_development	D_vs_E10;stage_1W_vs_E10;stage_3W_vs_E10;stage	1	TRUE
ENSGALG00010021095	NT5C2	PRJNA729271	1.966360326	1.2067E-76	1.966360326	stage_E14_vs_E10	ordinary_muscle_development	D_vs_E10;stage_1W_vs_E10;stage_3W_vs_E10;stage	1	TRUE
ENSGALG00010010195	ENSGALG00010010195	PRJNA729271	5.174142673	1.27568E-76	5.174142673	stage_E14_vs_E10	ordinary_muscle_development	D_vs_E10;stage_1W_vs_E10;stage_3W_vs_E10;stage	1	TRUE
ENSGALG00010029649	PSPH	PRJNA729271	4.807759435	1.45971E-76	4.807759435	stage_E14_vs_E10	ordinary_muscle_development	D_vs_E10;stage_1W_vs_E10;stage_3W_vs_E10;stage	1	TRUE
ENSGALG00010021762	LAMB2	PRJNA729271	3.712770447	1.53358E-76	3.712770447	stage_E14_vs_E10	ordinary_muscle_development	D_vs_E10;stage_1W_vs_E10;stage_3W_vs_E10;stage	1	TRUE
ENSGALG00010016699	SYNPO	PRJNA729271	2.70409426	1.76797E-76	2.70409426	stage_E14_vs_E10	ordinary_muscle_development	D_vs_E10;stage_1W_vs_E10;stage_3W_vs_E10;stage	1	TRUE
ENSGALG00010028126	ZMYND10	PRJNA729271	1.484197498	2.00732E-76	1.484197498	stage_E14_vs_E10	ordinary_muscle_development	D_vs_E10;stage_1W_vs_E10;stage_3W_vs_E10;stage	1	TRUE
ENSGALG00010009336	LAMB1	PRJNA729271	2.05694641	2.06694E-76	2.05694641	stage_E14_vs_E10	ordinary_muscle_development	D_vs_E10;stage_1W_vs_E10;stage_3W_vs_E10;stage	1	TRUE
ENSGALG00010020184	RSL1D1	PRJNA729271	-1.350988434	2.81135E-76	1.350988434	stage_E14_vs_E10	ordinary_muscle_development	D_vs_E10;stage_1W_vs_E10;stage_3W_vs_E10;stage	1	TRUE
ENSGALG00010012599	DMGDH	PRJNA729271	-3.762757012	2.98052E-76	3.762757012	stage_E14_vs_E10	ordinary_muscle_development	D_vs_E10;stage_1W_vs_E10;stage_3W_vs_E10;stage	1	TRUE
ENSGALG00010001347	MYOM1	PRJNA729271	3.07151648	3.45723E-76	3.07151648	stage_E14_vs_E10	ordinary_muscle_development	D_vs_E10;stage_1W_vs_E10;stage_3W_vs_E10;stage	1	TRUE
ENSGALG00010007884	GLI3	PRJNA729271	-2.184950125	3.80448E-76	2.184950125	stage_E14_vs_E10	ordinary_muscle_development	D_vs_E10;stage_1W_vs_E10;stage_3W_vs_E10;stage	1	TRUE
ENSGALG00010008542	MOCOS	PRJNA729271	2.867034295	3.8828E-76	2.867034295	stage_E14_vs_E10	ordinary_muscle_development	D_vs_E10;stage_1W_vs_E10;stage_3W_vs_E10;stage	1	TRUE
ENSGALG00010021783	GIT2	PRJNA729271	-0.910332407	4.87448E-76	0.910332407	stage_E14_vs_E10	ordinary_muscle_development		0	FALSE
ENSGALG00010006992	SLC35B4	PRJNA729271	-1.98667599	4.88648E-76	1.98667599	stage_E14_vs_E10	ordinary_muscle_development	D_vs_E10;stage_1W_vs_E10;stage_3W_vs_E10;stage	1	TRUE
ENSGALG00010007821	FAM89A	PRJNA729271	-2.37541335	4.9206E-76	2.37541335	stage_E14_vs_E10	ordinary_muscle_development	D_vs_E10;stage_1W_vs_E10;stage_3W_vs_E10;stage	1	TRUE
ENSGALG00010029169	SBDS	PRJNA729271	1.441299946	5.33721E-76	1.441299946	stage_E14_vs_E10	ordinary_muscle_development	D_vs_E10;stage_1W_vs_E10;stage_3W_vs_E10;stage	1	TRUE
ENSGALG00010029417	ENSGALG00010029417	PRJNA729271	3.895433232	5.54796E-76	3.895433232	stage_E14_vs_E10	ordinary_muscle_development	D_vs_E10;stage_1W_vs_E10;stage_3W_vs_E10;stage	1	TRUE
ENSGALG00010025891	NUP93	PRJNA729271	-2.065606931	6.07949E-76	2.065606931	stage_E14_vs_E10	ordinary_muscle_development	D_vs_E10;stage_1W_vs_E10;stage_3W_vs_E10;stage	1	TRUE
ENSGALG00010022323	DUSP29	PRJNA729271	7.094209043	6.20421E-76	7.094209043	stage_E14_vs_E10	ordinary_muscle_development	D_vs_E10;stage_1W_vs_E10;stage_3W_vs_E10;stage	1	TRUE
ENSGALG00010002336	PRIM2	PRJNA729271	-2.303194248	7.22557E-76	2.303194248	stage_E14_vs_E10	ordinary_muscle_development	D_vs_E10;stage_1W_vs_E10;stage_3W_vs_E10;stage	1	TRUE
ENSGALG00010013012	TMEM182	PRJNA729271	2.821538492	7.23628E-76	2.821538492	stage_E14_vs_E10	ordinary_muscle_development	D_vs_E10;stage_1W_vs_E10;stage_3W_vs_E10;stage	1	TRUE
ENSGALG00010023212	RIMBP2	PRJNA729271	3.97957796	8.31044E-76	3.97957796	stage_E14_vs_E10	ordinary_muscle_development	D_vs_E10;stage_1W_vs_E10;stage_3W_vs_E10;stage	1	TRUE
ENSGALG00010026377	ENSGALG00010026377	PRJNA729271	-5.036810723	1.05399E-75	5.036810723	stage_E14_vs_E10	ordinary_muscle_development	D_vs_E10;stage_1W_vs_E10;stage_3W_vs_E10;stage	1	TRUE
ENSGALG00010001796	SLCO2B1	PRJNA729271	2.878291805	1.11083E-75	2.878291805	stage_E14_vs_E10	ordinary_muscle_development	D_vs_E10;stage_1W_vs_E10;stage_3W_vs_E10;stage	1	TRUE
ENSGALG00010005377	EMCN	PRJNA729271	2.608338465	1.27419E-75	2.608338465	stage_E14_vs_E10	ordinary_muscle_development	D_vs_E10;stage_1W_vs_E10;stage_3W_vs_E10;stage	1	TRUE
ENSGALG00010002814	TERF1	PRJNA729271	-2.94098368	1.49846E-75	2.94098368	stage_E14_vs_E10	ordinary_muscle_development	D_vs_E10;stage_1W_vs_E10;stage_3W_vs_E10;stage	1	TRUE
ENSGALG00010009498	ORC4	PRJNA729271	-2.188143085	1.66832E-75	2.188143085	stage_E14_vs_E10	ordinary_muscle_development	D_vs_E10;stage_1W_vs_E10;stage_3W_vs_E10;stage	1	TRUE
ENSGALG00010023545	PTPRT	PRJNA729271	-2.716465406	1.71565E-75	2.716465406	stage_E14_vs_E10	ordinary_muscle_development	D_vs_E10;stage_1W_vs_E10;stage_3W_vs_E10;stage	1	TRUE
ENSGALG00010004362	FBXL4	PRJNA729271	2.232737368	1.84768E-75	2.232737368	stage_E14_vs_E10	ordinary_muscle_development	D_vs_E10;stage_1W_vs_E10;stage_3W_vs_E10;stage	1	TRUE
ENSGALG00010012004	LGR5	PRJNA729271	-3.936464858	1.86973E-75	3.936464858	stage_E14_vs_E10	ordinary_muscle_development	D_vs_E10;stage_1W_vs_E10;stage_3W_vs_E10;stage	1	TRUE
ENSGALG00010004146	NAAA	PRJNA729271	3.156235702	1.96803E-75	3.156235702	stage_E14_vs_E10	ordinary_muscle_development	D_vs_E10;stage_1W_vs_E10;stage_3W_vs_E10;stage	1	TRUE
ENSGALG00010013397	MATN1	PRJNA729271	-10.51704016	2.05921E-75	10.51704016	stage_E14_vs_E10	ordinary_muscle_development	D_vs_E10;stage_1W_vs_E10;stage_3W_vs_E10;stage	1	TRUE
ENSGALG00010019026	CHSY1	PRJNA729271	-2.357727394	2.05921E-75	2.357727394	stage_E14_vs_E10	ordinary_muscle_development	D_vs_E10;stage_1W_vs_E10;stage_3W_vs_E10;stage	1	TRUE
ENSGALG00010015131	DOK5	PRJNA729271	4.455802518	2.12094E-75	4.455802518	stage_E14_vs_E10	ordinary_muscle_development	D_vs_E10;stage_1W_vs_E10;stage_3W_vs_E10;stage	1	TRUE
ENSGALG00010005429	ZDHHC20	PRJNA729271	-2.239839004	2.20189E-75	2.239839004	stage_E14_vs_E10	ordinary_muscle_development	D_vs_E10;stage_1W_vs_E10;stage_3W_vs_E10;stage	1	TRUE
ENSGALG00010029317	KPNA2	PRJNA729271	-3.832793765	3.128E-75	3.832793765	stage_E14_vs_E10	ordinary_muscle_development	D_vs_E10;stage_1W_vs_E10;stage_3W_vs_E10;stage	1	TRUE
ENSGALG00010017559	DUSP10	PRJNA729271	2.766552929	4.32776E-75	2.766552929	stage_E14_vs_E10	ordinary_muscle_development	D_vs_E10;stage_1W_vs_E10;stage_3W_vs_E10;stage	1	TRUE
ENSGALG00010013298	EPYC	PRJNA729271	-7.887907304	4.96771E-75	7.887907304	stage_E14_vs_E10	ordinary_muscle_development	D_vs_E10;stage_1W_vs_E10;stage_3W_vs_E10;stage	1	TRUE
ENSGALG00010016903	PPM1A	PRJNA729271	2.724263747	5.51078E-75	2.724263747	stage_E14_vs_E10	ordinary_muscle_development	D_vs_E10;stage_1W_vs_E10;stage_3W_vs_E10;stage	1	TRUE
ENSGALG00010025590	HRAS	PRJNA729271	2.102079984	5.80553E-75	2.102079984	stage_E14_vs_E10	ordinary_muscle_development	D_vs_E10;stage_1W_vs_E10;stage_3W_vs_E10;stage	1	TRUE
ENSGALG00010029841	SMIM5	PRJNA729271	2.380179201	6.915E-75	2.380179201	stage_E14_vs_E10	ordinary_muscle_development	D_vs_E10;stage_1W_vs_E10;stage_3W_vs_E10;stage	1	TRUE
ENSGALG00010004765	ENSGALG00010004765	PRJNA729271	3.672568119	6.97758E-75	3.672568119	stage_E14_vs_E10	ordinary_muscle_development	D_vs_E10;stage_1W_vs_E10;stage_3W_vs_E10;stage	1	TRUE
ENSGALG00010015655	ENSGALG00010015655	PRJNA729271	6.651176137	8.90014E-75	6.651176137	stage_E14_vs_E10	ordinary_muscle_development	D_vs_E10;stage_1W_vs_E10;stage_3W_vs_E10;stage	1	TRUE
ENSGALG00010011923	BARD1	PRJNA729271	-3.310920719	9.34734E-75	3.310920719	stage_E14_vs_E10	ordinary_muscle_development	D_vs_E10;stage_1W_vs_E10;stage_3W_vs_E10;stage	1	TRUE
ENSGALG00010002939	HMGB2	PRJNA729271	-3.194853956	9.87347E-75	3.194853956	stage_E14_vs_E10	ordinary_muscle_development	D_vs_E10;stage_1W_vs_E10;stage_3W_vs_E10;stage	1	TRUE
ENSGALG00010023347	STAT5A	PRJNA729271	3.121150515	1.04546E-74	3.121150515	stage_E14_vs_E10	ordinary_muscle_development	D_vs_E10;stage_1W_vs_E10;stage_3W_vs_E10;stage	1	TRUE
ENSGALG00010005871	EGLN3	PRJNA729271	3.606020035	1.08642E-74	3.606020035	stage_E14_vs_E10	ordinary_muscle_development	D_vs_E10;stage_1W_vs_E10;stage_3W_vs_E10;stage	1	TRUE
ENSGALG00010016287	BCL11B	PRJNA729271	-7.536585339	1.10859E-74	7.536585339	stage_E14_vs_E10	ordinary_muscle_development	D_vs_E10;stage_1W_vs_E10;stage_3W_vs_E10;stage	1	TRUE
ENSGALG00010010406	PTCH1	PRJNA729271	-1.765702636	1.2097E-74	1.765702636	stage_E14_vs_E10	ordinary_muscle_development	D_vs_E10;stage_1W_vs_E10;stage_3W_vs_E10;stage	1	TRUE
ENSGALG00010017470	HSPA2	PRJNA729271	3.134538213	1.27664E-74	3.134538213	stage_E14_vs_E10	ordinary_muscle_development	D_vs_E10;stage_1W_vs_E10;stage_3W_vs_E10;stage	1	TRUE
ENSGALG00010025407	MLXIP	PRJNA729271	2.361402914	1.54513E-74	2.361402914	stage_E14_vs_E10	ordinary_muscle_development	D_vs_E10;stage_1W_vs_E10;stage_3W_vs_E10;stage	1	TRUE
ENSGALG00010001705	HTRA1	PRJNA729271	2.855190385	1.92225E-74	2.855190385	stage_E14_vs_E10	ordinary_muscle_development	D_vs_E10;stage_1W_vs_E10;stage_3W_vs_E10;stage	1	TRUE
ENSGALG00010024194	NKAIN4	PRJNA729271	1.561363084	2.06161E-74	1.561363084	stage_E14_vs_E10	ordinary_muscle_development	D_vs_E10;stage_1W_vs_E10;stage_3W_vs_E10;stage	1	TRUE
ENSGALG00010018443	PLCB1	PRJNA729271	2.697076264	2.13009E-74	2.697076264	stage_E14_vs_E10	ordinary_muscle_development	D_vs_E10;stage_1W_vs_E10;stage_3W_vs_E10;stage	1	TRUE
ENSGALG00010027886	IL6R	PRJNA729271	3.524501147	2.61331E-74	3.524501147	stage_E14_vs_E10	ordinary_muscle_development	D_vs_E10;stage_1W_vs_E10;stage_3W_vs_E10;stage	1	TRUE
ENSGALG00010005435	MAD2L1	PRJNA729271	-3.280440532	2.83397E-74	3.280440532	stage_E14_vs_E10	ordinary_muscle_development	D_vs_E10;stage_1W_vs_E10;stage_3W_vs_E10;stage	1	TRUE
ENSGALG00010027282	ENSGALG00010027282	PRJNA729271	2.361243813	2.89771E-74	2.361243813	stage_E14_vs_E10	ordinary_muscle_development	D_vs_E10;stage_1W_vs_E10;stage_3W_vs_E10;stage	1	TRUE
ENSGALG00010005934	SLITRK6	PRJNA729271	-6.625878969	2.98592E-74	6.625878969	stage_E14_vs_E10	ordinary_muscle_development	D_vs_E10;stage_1W_vs_E10;stage_3W_vs_E10;stage	1	TRUE
ENSGALG00010001418	RNMT	PRJNA729271	-1.264961556	3.1139E-74	1.264961556	stage_E14_vs_E10	ordinary_muscle_development	D_vs_E10;stage_1W_vs_E10;stage_3W_vs_E10;stage	1	TRUE
ENSGALG00010009335	PTP4A3	PRJNA729271	3.586534828	3.36054E-74	3.586534828	stage_E14_vs_E10	ordinary_muscle_development	D_vs_E10;stage_1W_vs_E10;stage_3W_vs_E10;stage	1	TRUE
ENSGALG00010007207	EPM2A	PRJNA729271	1.888061893	4.08176E-74	1.888061893	stage_E14_vs_E10	ordinary_muscle_development	D_vs_E10;stage_1W_vs_E10;stage_3W_vs_E10;stage	1	TRUE
ENSGALG00010014099	TPX2	PRJNA729271	-2.660704644	4.24808E-74	2.660704644	stage_E14_vs_E10	ordinary_muscle_development	D_vs_E10;stage_1W_vs_E10;stage_3W_vs_E10;stage	1	TRUE
ENSGALG00010020019	PEX10	PRJNA729271	1.777723976	4.74913E-74	1.777723976	stage_E14_vs_E10	ordinary_muscle_development	D_vs_E10;stage_1W_vs_E10;stage_3W_vs_E10;stage	1	TRUE
ENSGALG00010013556	RASL11B	PRJNA729271	-3.282935109	5.02205E-74	3.282935109	stage_E14_vs_E10	ordinary_muscle_development	D_vs_E10;stage_1W_vs_E10;stage_3W_vs_E10;stage	1	TRUE
ENSGALG00010006200	GSTCD	PRJNA729271	-2.015100678	5.31244E-74	2.015100678	stage_E14_vs_E10	ordinary_muscle_development	D_vs_E10;stage_1W_vs_E10;stage_3W_vs_E10;stage	1	TRUE
ENSGALG00010014976	ITGB2	PRJNA729271	3.841507764	5.38126E-74	3.841507764	stage_E14_vs_E10	ordinary_muscle_development	D_vs_E10;stage_1W_vs_E10;stage_3W_vs_E10;stage	1	TRUE
ENSGALG00010025954	TUBA1A	PRJNA729271	-2.918852522	5.74987E-74	2.918852522	stage_E14_vs_E10	ordinary_muscle_development	D_vs_E10;stage_1W_vs_E10;stage_3W_vs_E10;stage	1	TRUE
ENSGALG00010006291	ENSGALG00010006291	PRJNA729271	3.520169119	6.7757E-74	3.520169119	stage_E14_vs_E10	ordinary_muscle_development	D_vs_E10;stage_1W_vs_E10;stage_3W_vs_E10;stage	1	TRUE
ENSGALG00010018564	DLGAP5	PRJNA729271	-2.853371372	7.00124E-74	2.853371372	stage_E14_vs_E10	ordinary_muscle_development	D_vs_E10;stage_1W_vs_E10;stage_3W_vs_E10;stage	1	TRUE
ENSGALG00010011962	CPSF6	PRJNA729271	-1.110658514	8.04262E-74	1.110658514	stage_E14_vs_E10	ordinary_muscle_development	D_vs_E10;stage_1W_vs_E10;stage_3W_vs_E10;stage	1	TRUE
ENSGALG00010014494	KLHL9	PRJNA729271	-1.265547424	8.50052E-74	1.265547424	stage_E14_vs_E10	ordinary_muscle_development	D_vs_E10;stage_1W_vs_E10;stage_3W_vs_E10;stage	1	TRUE
ENSGALG00010001545	SELENOO	PRJNA729271	2.358857	9.40991E-74	2.358857	stage_E14_vs_E10	ordinary_muscle_development	D_vs_E10;stage_1W_vs_E10;stage_3W_vs_E10;stage	1	TRUE
ENSGALG00010017024	BUB1	PRJNA729271	-4.099001503	1.01078E-73	4.099001503	stage_E14_vs_E10	ordinary_muscle_development	D_vs_E10;stage_1W_vs_E10;stage_3W_vs_E10;stage	1	TRUE
ENSGALG00010013044	ENSGALG00010013044	PRJNA729271	2.278680812	1.33692E-73	2.278680812	stage_E14_vs_E10	ordinary_muscle_development	D_vs_E10;stage_1W_vs_E10;stage_3W_vs_E10;stage	1	TRUE
ENSGALG00010025442	ABCF2	PRJNA729271	2.688492496	1.48541E-73	2.688492496	stage_E14_vs_E10	ordinary_muscle_development	D_vs_E10;stage_1W_vs_E10;stage_3W_vs_E10;stage	1	TRUE
ENSGALG00010018085	PGP	PRJNA729271	2.913444921	1.61537E-73	2.913444921	stage_E14_vs_E10	ordinary_muscle_development	D_vs_E10;stage_1W_vs_E10;stage_3W_vs_E10;stage	1	TRUE
ENSGALG00010013048	WDR75	PRJNA729271	-1.653887527	1.62654E-73	1.653887527	stage_E14_vs_E10	ordinary_muscle_development	D_vs_E10;stage_1W_vs_E10;stage_3W_vs_E10;stage	1	TRUE
ENSGALG00010004297	SULF1	PRJNA729271	-3.420268034	1.65815E-73	3.420268034	stage_E14_vs_E10	ordinary_muscle_development	D_vs_E10;stage_1W_vs_E10;stage_3W_vs_E10;stage	1	TRUE
ENSGALG00010004522	TSPAN5	PRJNA729271	-1.178537714	1.82885E-73	1.178537714	stage_E14_vs_E10	ordinary_muscle_development	D_vs_E10;stage_1W_vs_E10;stage_3W_vs_E10;stage	1	TRUE
ENSGALG00010001142	PPM1L	PRJNA729271	2.668418406	1.99837E-73	2.668418406	stage_E14_vs_E10	ordinary_muscle_development	D_vs_E10;stage_1W_vs_E10;stage_3W_vs_E10;stage	1	TRUE
ENSGALG00010027316	TRAIP	PRJNA729271	-4.358296419	2.04479E-73	4.358296419	stage_E14_vs_E10	ordinary_muscle_development	D_vs_E10;stage_1W_vs_E10;stage_3W_vs_E10;stage	1	TRUE
ENSGALG00010020175	ACAN	PRJNA729271	-11.2841594	2.06738E-73	11.2841594	stage_E14_vs_E10	ordinary_muscle_development	D_vs_E10;stage_1W_vs_E10;stage_3W_vs_E10;stage	1	TRUE
ENSGALG00010026697	CHN1	PRJNA729271	-2.200284103	2.31971E-73	2.200284103	stage_E14_vs_E10	ordinary_muscle_development	D_vs_E10;stage_1W_vs_E10;stage_3W_vs_E10;stage	1	TRUE
ENSGALG00010028528	UHRF1	PRJNA729271	-3.412562918	2.49364E-73	3.412562918	stage_E14_vs_E10	ordinary_muscle_development	D_vs_E10;stage_1W_vs_E10;stage_3W_vs_E10;stage	1	TRUE
ENSGALG00010005512	SMARCAD1	PRJNA729271	-2.069083815	2.69261E-73	2.069083815	stage_E14_vs_E10	ordinary_muscle_development	D_vs_E10;stage_1W_vs_E10;stage_3W_vs_E10;stage	1	TRUE
ENSGALG00010021161	ENSGALG00010021161	PRJNA729271	-2.076507275	2.8872E-73	2.076507275	stage_E14_vs_E10	ordinary_muscle_development	D_vs_E10;stage_1W_vs_E10;stage_3W_vs_E10;stage	1	TRUE
ENSGALG00010017396	CCR8L	PRJNA729271	4.933337109	3.63073E-73	4.933337109	stage_E14_vs_E10	ordinary_muscle_development	D_vs_E10;stage_1W_vs_E10;stage_3W_vs_E10;stage	1	TRUE
ENSGALG00010012521	HOMER1	PRJNA729271	2.350908681	3.98183E-73	2.350908681	stage_E14_vs_E10	ordinary_muscle_development	D_vs_E10;stage_1W_vs_E10;stage_3W_vs_E10;stage	1	TRUE
ENSGALG00010008906	DBN1	PRJNA729271	-3.934359645	4.03925E-73	3.934359645	stage_E14_vs_E10	ordinary_muscle_development	D_vs_E10;stage_1W_vs_E10;stage_3W_vs_E10;stage	1	TRUE
ENSGALG00010014847	PAICS	PRJNA729271	-1.69362256	4.12986E-73	1.69362256	stage_E14_vs_E10	ordinary_muscle_development	D_vs_E10;stage_1W_vs_E10;stage_3W_vs_E10;stage	1	TRUE
ENSGALG00010029399	PRKAR1A	PRJNA729271	-0.986038979	4.60094E-73	0.986038979	stage_E14_vs_E10	ordinary_muscle_development		0	FALSE
ENSGALG00010029616	TMEM120A	PRJNA729271	2.422906444	4.63292E-73	2.422906444	stage_E14_vs_E10	ordinary_muscle_development	D_vs_E10;stage_1W_vs_E10;stage_3W_vs_E10;stage	1	TRUE
ENSGALG00010008422	EXO1	PRJNA729271	-4.492397716	6.52981E-73	4.492397716	stage_E14_vs_E10	ordinary_muscle_development	D_vs_E10;stage_1W_vs_E10;stage_3W_vs_E10;stage	1	TRUE
ENSGALG00010001969	PLEKHA8	PRJNA729271	-2.40959315	7.92477E-73	2.40959315	stage_E14_vs_E10	ordinary_muscle_development	D_vs_E10;stage_1W_vs_E10;stage_3W_vs_E10;stage	1	TRUE
ENSGALG00010015957	ENSGALG00010015957	PRJNA729271	3.889020172	8.81327E-73	3.889020172	stage_E14_vs_E10	ordinary_muscle_development	D_vs_E10;stage_1W_vs_E10;stage_3W_vs_E10;stage	1	TRUE
ENSGALG00010023863	EYA2	PRJNA729271	-4.503442023	9.2783E-73	4.503442023	stage_E14_vs_E10	ordinary_muscle_development	D_vs_E10;stage_1W_vs_E10;stage_3W_vs_E10;stage	1	TRUE
ENSGALG00010028901	PAPPA	PRJNA729271	-2.269711135	9.48667E-73	2.269711135	stage_E14_vs_E10	ordinary_muscle_development	D_vs_E10;stage_1W_vs_E10;stage_3W_vs_E10;stage	1	TRUE
ENSGALG00010015632	COL4A5	PRJNA729271	2.947693658	9.54998E-73	2.947693658	stage_E14_vs_E10	ordinary_muscle_development	D_vs_E10;stage_1W_vs_E10;stage_3W_vs_E10;stage	1	TRUE
ENSGALG00010022377	ZHX3	PRJNA729271	1.627360226	9.75411E-73	1.627360226	stage_E14_vs_E10	ordinary_muscle_development	D_vs_E10;stage_1W_vs_E10;stage_3W_vs_E10;stage	1	TRUE
ENSGALG00010020574	DTL	PRJNA729271	-3.53097005	1.16789E-72	3.53097005	stage_E14_vs_E10	ordinary_muscle_development	D_vs_E10;stage_1W_vs_E10;stage_3W_vs_E10;stage	1	TRUE
ENSGALG00010021450	OLFML1	PRJNA729271	4.202599954	1.2452E-72	4.202599954	stage_E14_vs_E10	ordinary_muscle_development	D_vs_E10;stage_1W_vs_E10;stage_3W_vs_E10;stage	1	TRUE
ENSGALG00010013755	ANGPT4	PRJNA729271	2.777801029	1.28528E-72	2.777801029	stage_E14_vs_E10	ordinary_muscle_development	D_vs_E10;stage_1W_vs_E10;stage_3W_vs_E10;stage	1	TRUE
ENSGALG00010016393	BLM	PRJNA729271	-2.33875623	1.49838E-72	2.33875623	stage_E14_vs_E10	ordinary_muscle_development	D_vs_E10;stage_1W_vs_E10;stage_3W_vs_E10;stage	1	TRUE
ENSGALG00010008498	ENSGALG00010008498	PRJNA729271	2.683814477	1.57594E-72	2.683814477	stage_E14_vs_E10	ordinary_muscle_development	D_vs_E10;stage_1W_vs_E10;stage_3W_vs_E10;stage	1	TRUE
ENSGALG00010026581	DNER	PRJNA729271	-6.642889318	1.76264E-72	6.642889318	stage_E14_vs_E10	ordinary_muscle_development	D_vs_E10;stage_1W_vs_E10;stage_3W_vs_E10;stage	1	TRUE
ENSGALG00010004735	ENSGALG00010004735	PRJNA729271	2.987515334	1.77004E-72	2.987515334	stage_E14_vs_E10	ordinary_muscle_development	D_vs_E10;stage_1W_vs_E10;stage_3W_vs_E10;stage	1	TRUE
ENSGALG00010020622	ABCG2	PRJNA729271	2.10966651	2.17489E-72	2.10966651	stage_E14_vs_E10	ordinary_muscle_development	D_vs_E10;stage_1W_vs_E10;stage_3W_vs_E10;stage	1	TRUE
ENSGALG00010015310	PGM2	PRJNA729271	-1.748077708	2.24297E-72	1.748077708	stage_E14_vs_E10	ordinary_muscle_development	D_vs_E10;stage_1W_vs_E10;stage_3W_vs_E10;stage	1	TRUE
ENSGALG00010008030	GJA5	PRJNA729271	-2.778764651	2.28084E-72	2.778764651	stage_E14_vs_E10	ordinary_muscle_development	D_vs_E10;stage_1W_vs_E10;stage_3W_vs_E10;stage	1	TRUE
ENSGALG00010028671	CADM3	PRJNA729271	3.855651766	2.28202E-72	3.855651766	stage_E14_vs_E10	ordinary_muscle_development	D_vs_E10;stage_1W_vs_E10;stage_3W_vs_E10;stage	1	TRUE
ENSGALG00010027908	PBXIP1	PRJNA729271	1.925209585	2.41063E-72	1.925209585	stage_E14_vs_E10	ordinary_muscle_development	D_vs_E10;stage_1W_vs_E10;stage_3W_vs_E10;stage	1	TRUE
ENSGALG00010024415	SIK2	PRJNA729271	1.080414525	2.43957E-72	1.080414525	stage_E14_vs_E10	ordinary_muscle_development	D_vs_E10;stage_1W_vs_E10;stage_3W_vs_E10;stage	1	TRUE
ENSGALG00010028918	AGPAT2	PRJNA729271	3.322110416	2.80358E-72	3.322110416	stage_E14_vs_E10	ordinary_muscle_development	D_vs_E10;stage_1W_vs_E10;stage_3W_vs_E10;stage	1	TRUE
ENSGALG00010019838	ALX4	PRJNA729271	-4.029076745	2.84702E-72	4.029076745	stage_E14_vs_E10	ordinary_muscle_development	D_vs_E10;stage_1W_vs_E10;stage_3W_vs_E10;stage	1	TRUE
ENSGALG00010008516	MRE11	PRJNA729271	-1.468068654	3.09494E-72	1.468068654	stage_E14_vs_E10	ordinary_muscle_development	D_vs_E10;stage_1W_vs_E10;stage_3W_vs_E10;stage	1	TRUE
ENSGALG00010017786	KAZALD1	PRJNA729271	-3.123697604	4.26879E-72	3.123697604	stage_E14_vs_E10	ordinary_muscle_development	D_vs_E10;stage_1W_vs_E10;stage_3W_vs_E10;stage	1	TRUE
ENSGALG00010024508	RPS27L	PRJNA729271	3.421222702	4.35013E-72	3.421222702	stage_E14_vs_E10	ordinary_muscle_development	D_vs_E10;stage_1W_vs_E10;stage_3W_vs_E10;stage	1	TRUE
ENSGALG00010015122	ENSGALG00010015122	PRJNA729271	-3.996067971	4.36121E-72	3.996067971	stage_E14_vs_E10	ordinary_muscle_development	D_vs_E10;stage_1W_vs_E10;stage_3W_vs_E10;stage	1	TRUE
ENSGALG00010019376	ENSGALG00010019376	PRJNA729271	5.688138502	4.38715E-72	5.688138502	stage_E14_vs_E10	ordinary_muscle_development	D_vs_E10;stage_1W_vs_E10;stage_3W_vs_E10;stage	1	TRUE
ENSGALG00010017715	CILP	PRJNA729271	3.059743986	4.91765E-72	3.059743986	stage_E14_vs_E10	ordinary_muscle_development	D_vs_E10;stage_1W_vs_E10;stage_3W_vs_E10;stage	1	TRUE
ENSGALG00010009214	PCDH10	PRJNA729271	-5.964196215	5.39482E-72	5.964196215	stage_E14_vs_E10	ordinary_muscle_development	D_vs_E10;stage_1W_vs_E10;stage_3W_vs_E10;stage	1	TRUE
ENSGALG00010003431	PM20D2	PRJNA729271	5.171525105	6.21867E-72	5.171525105	stage_E14_vs_E10	ordinary_muscle_development	D_vs_E10;stage_1W_vs_E10;stage_3W_vs_E10;stage	1	TRUE
ENSGALG00010024692	DRAM2	PRJNA729271	1.886591872	6.52989E-72	1.886591872	stage_E14_vs_E10	ordinary_muscle_development	D_vs_E10;stage_1W_vs_E10;stage_3W_vs_E10;stage	1	TRUE
ENSGALG00010015733	COL4A6	PRJNA729271	2.763445054	6.54377E-72	2.763445054	stage_E14_vs_E10	ordinary_muscle_development	D_vs_E10;stage_1W_vs_E10;stage_3W_vs_E10;stage	1	TRUE
ENSGALG00010029261	IFT22	PRJNA729271	-1.919929545	6.5901E-72	1.919929545	stage_E14_vs_E10	ordinary_muscle_development	D_vs_E10;stage_1W_vs_E10;stage_3W_vs_E10;stage	1	TRUE
ENSGALG00010021062	MYO9A	PRJNA729271	-1.857273554	7.13135E-72	1.857273554	stage_E14_vs_E10	ordinary_muscle_development	D_vs_E10;stage_1W_vs_E10;stage_3W_vs_E10;stage	1	TRUE
ENSGALG00010001642	ENSGALG00010001642	PRJNA729271	2.157279918	9.00228E-72	2.157279918	stage_E14_vs_E10	ordinary_muscle_development	D_vs_E10;stage_1W_vs_E10;stage_3W_vs_E10;stage	1	TRUE
ENSGALG00010024413	AMFR	PRJNA729271	1.661870764	1.08898E-71	1.661870764	stage_E14_vs_E10	ordinary_muscle_development	D_vs_E10;stage_1W_vs_E10;stage_3W_vs_E10;stage	1	TRUE
ENSGALG00010027699	VANGL2	PRJNA729271	-2.370747603	1.12485E-71	2.370747603	stage_E14_vs_E10	ordinary_muscle_development	D_vs_E10;stage_1W_vs_E10;stage_3W_vs_E10;stage	1	TRUE
ENSGALG00010015869	SLC7A2	PRJNA729271	3.494165625	1.24567E-71	3.494165625	stage_E14_vs_E10	ordinary_muscle_development	D_vs_E10;stage_1W_vs_E10;stage_3W_vs_E10;stage	1	TRUE
ENSGALG00010003364	ANKRD6	PRJNA729271	-3.065885278	1.38183E-71	3.065885278	stage_E14_vs_E10	ordinary_muscle_development	D_vs_E10;stage_1W_vs_E10;stage_3W_vs_E10;stage	1	TRUE
ENSGALG00010022253	ALPK3	PRJNA729271	3.922510066	1.64882E-71	3.922510066	stage_E14_vs_E10	ordinary_muscle_development	D_vs_E10;stage_1W_vs_E10;stage_3W_vs_E10;stage	1	TRUE
ENSGALG00010005025	THOC1	PRJNA729271	-1.086606254	1.70714E-71	1.086606254	stage_E14_vs_E10	ordinary_muscle_development	D_vs_E10;stage_1W_vs_E10;stage_3W_vs_E10;stage	1	TRUE
ENSGALG00010025270	NDC1	PRJNA729271	-2.232994397	2.0941E-71	2.232994397	stage_E14_vs_E10	ordinary_muscle_development	D_vs_E10;stage_1W_vs_E10;stage_3W_vs_E10;stage	1	TRUE
ENSGALG00010000286	TCN2	PRJNA729271	2.459849951	2.14186E-71	2.459849951	stage_E14_vs_E10	ordinary_muscle_development	D_vs_E10;stage_1W_vs_E10;stage_3W_vs_E10;stage	1	TRUE
ENSGALG00010022024	MICAL2	PRJNA729271	3.29850636	2.17176E-71	3.29850636	stage_E14_vs_E10	ordinary_muscle_development	D_vs_E10;stage_1W_vs_E10;stage_3W_vs_E10;stage	1	TRUE
ENSGALG00010022167	WDR3	PRJNA729271	-1.362955021	2.17862E-71	1.362955021	stage_E14_vs_E10	ordinary_muscle_development	D_vs_E10;stage_1W_vs_E10;stage_3W_vs_E10;stage	1	TRUE
ENSGALG00010000639	SCN1B	PRJNA729271	4.795694071	2.66671E-71	4.795694071	stage_E14_vs_E10	ordinary_muscle_development	D_vs_E10;stage_1W_vs_E10;stage_3W_vs_E10;stage	1	TRUE
ENSGALG00010002930	SERAF	PRJNA729271	-5.407737804	2.7493E-71	5.407737804	stage_E14_vs_E10	ordinary_muscle_development	D_vs_E10;stage_1W_vs_E10;stage_3W_vs_E10;stage	1	TRUE
ENSGALG00010021755	ENSGALG00010021755	PRJNA729271	2.765953471	3.31653E-71	2.765953471	stage_E14_vs_E10	ordinary_muscle_development	D_vs_E10;stage_1W_vs_E10;stage_3W_vs_E10;stage	1	TRUE
ENSGALG00010005454	GPR161	PRJNA729271	-1.813124554	3.87641E-71	1.813124554	stage_E14_vs_E10	ordinary_muscle_development	D_vs_E10;stage_1W_vs_E10;stage_3W_vs_E10;stage	1	TRUE
ENSGALG00010017105	PLK1	PRJNA729271	-4.318777362	4.07637E-71	4.318777362	stage_E14_vs_E10	ordinary_muscle_development	D_vs_E10;stage_1W_vs_E10;stage_3W_vs_E10;stage	1	TRUE
ENSGALG00010021120	PDCD11	PRJNA729271	-1.591492026	4.91788E-71	1.591492026	stage_E14_vs_E10	ordinary_muscle_development	D_vs_E10;stage_1W_vs_E10;stage_3W_vs_E10;stage	1	TRUE
ENSGALG00010005302	PSPC1	PRJNA729271	-1.458796677	5.14635E-71	1.458796677	stage_E14_vs_E10	ordinary_muscle_development	D_vs_E10;stage_1W_vs_E10;stage_3W_vs_E10;stage	1	TRUE
ENSGALG00010017767	KIF4B	PRJNA729271	-4.050703074	5.8589E-71	4.050703074	stage_E14_vs_E10	ordinary_muscle_development	D_vs_E10;stage_1W_vs_E10;stage_3W_vs_E10;stage	1	TRUE
ENSGALG00010016412	TMSB15B	PRJNA729271	-3.894475147	6.56229E-71	3.894475147	stage_E14_vs_E10	ordinary_muscle_development	D_vs_E10;stage_1W_vs_E10;stage_3W_vs_E10;stage	1	TRUE
ENSGALG00010013254	SAR1B	PRJNA729271	2.541167268	7.05219E-71	2.541167268	stage_E14_vs_E10	ordinary_muscle_development	D_vs_E10;stage_1W_vs_E10;stage_3W_vs_E10;stage	1	TRUE
ENSGALG00010010393	FANCC	PRJNA729271	-2.740834888	7.8204E-71	2.740834888	stage_E14_vs_E10	ordinary_muscle_development	D_vs_E10;stage_1W_vs_E10;stage_3W_vs_E10;stage	1	TRUE
ENSGALG00010004934	CIP2A	PRJNA729271	-3.021163427	8.04678E-71	3.021163427	stage_E14_vs_E10	ordinary_muscle_development	D_vs_E10;stage_1W_vs_E10;stage_3W_vs_E10;stage	1	TRUE
ENSGALG00010015809	PCOLCE2	PRJNA729271	4.166827326	8.13647E-71	4.166827326	stage_E14_vs_E10	ordinary_muscle_development	D_vs_E10;stage_1W_vs_E10;stage_3W_vs_E10;stage	1	TRUE
ENSGALG00010024928	THAP4	PRJNA729271	1.5091555	9.91192E-71	1.5091555	stage_E14_vs_E10	ordinary_muscle_development	D_vs_E10;stage_1W_vs_E10;stage_3W_vs_E10;stage	1	TRUE
ENSGALG00010020094	CLEC3B	PRJNA729271	4.086874589	1.00475E-70	4.086874589	stage_E14_vs_E10	ordinary_muscle_development	D_vs_E10;stage_1W_vs_E10;stage_3W_vs_E10;stage	1	TRUE
ENSGALG00010016779	CTNNA2	PRJNA729271	-2.783410134	1.09795E-70	2.783410134	stage_E14_vs_E10	ordinary_muscle_development	D_vs_E10;stage_1W_vs_E10;stage_3W_vs_E10;stage	1	TRUE
ENSGALG00010027700	TCF3	PRJNA729271	-1.730277502	1.52903E-70	1.730277502	stage_E14_vs_E10	ordinary_muscle_development	D_vs_E10;stage_1W_vs_E10;stage_3W_vs_E10;stage	1	TRUE
ENSGALG00010022568	SCRN3	PRJNA729271	2.988938505	1.55358E-70	2.988938505	stage_E14_vs_E10	ordinary_muscle_development	D_vs_E10;stage_1W_vs_E10;stage_3W_vs_E10;stage	1	TRUE
ENSGALG00010011484	CHPT1	PRJNA729271	1.393162467	1.55583E-70	1.393162467	stage_E14_vs_E10	ordinary_muscle_development	D_vs_E10;stage_1W_vs_E10;stage_3W_vs_E10;stage	1	TRUE
ENSGALG00010012293	SRGAP1	PRJNA729271	-1.930713025	1.60784E-70	1.930713025	stage_E14_vs_E10	ordinary_muscle_development	D_vs_E10;stage_1W_vs_E10;stage_3W_vs_E10;stage	1	TRUE
ENSGALG00010027524	ENSGALG00010027524	PRJNA729271	1.985496948	1.72493E-70	1.985496948	stage_E14_vs_E10	ordinary_muscle_development	D_vs_E10;stage_1W_vs_E10;stage_3W_vs_E10;stage	1	TRUE
ENSGALG00010021988	FAM180B	PRJNA729271	4.702126424	1.73621E-70	4.702126424	stage_E14_vs_E10	ordinary_muscle_development	D_vs_E10;stage_1W_vs_E10;stage_3W_vs_E10;stage	1	TRUE
ENSGALG00010013612	TWIST2	PRJNA729271	-5.317250746	1.77028E-70	5.317250746	stage_E14_vs_E10	ordinary_muscle_development	D_vs_E10;stage_1W_vs_E10;stage_3W_vs_E10;stage	1	TRUE
ENSGALG00010024328	YDJC	PRJNA729271	-2.848923425	1.86987E-70	2.848923425	stage_E14_vs_E10	ordinary_muscle_development	D_vs_E10;stage_1W_vs_E10;stage_3W_vs_E10;stage	1	TRUE
ENSGALG00010016962	SQOR	PRJNA729271	2.434018312	1.9065E-70	2.434018312	stage_E14_vs_E10	ordinary_muscle_development	D_vs_E10;stage_1W_vs_E10;stage_3W_vs_E10;stage	1	TRUE
ENSGALG00010007555	NDUFS6	PRJNA729271	2.314198772	1.97993E-70	2.314198772	stage_E14_vs_E10	ordinary_muscle_development	D_vs_E10;stage_1W_vs_E10;stage_3W_vs_E10;stage	1	TRUE
ENSGALG00010027516	PPM1J	PRJNA729271	3.813583495	2.01998E-70	3.813583495	stage_E14_vs_E10	ordinary_muscle_development	D_vs_E10;stage_1W_vs_E10;stage_3W_vs_E10;stage	1	TRUE
ENSGALG00010017849	FBXO48	PRJNA729271	3.10210026	2.239E-70	3.10210026	stage_E14_vs_E10	ordinary_muscle_development	D_vs_E10;stage_1W_vs_E10;stage_3W_vs_E10;stage	1	TRUE
ENSGALG00010003701	SARAF	PRJNA729271	1.458377945	2.51624E-70	1.458377945	stage_E14_vs_E10	ordinary_muscle_development	D_vs_E10;stage_1W_vs_E10;stage_3W_vs_E10;stage	1	TRUE
ENSGALG00010022365	PLCG1	PRJNA729271	-2.002994258	3.6613E-70	2.002994258	stage_E14_vs_E10	ordinary_muscle_development	D_vs_E10;stage_1W_vs_E10;stage_3W_vs_E10;stage	1	TRUE

ENSGALG00010024869	NDUFB3	PRJNA729271	2.169040995	4.74014E-70	2.169040995	stage_E14_vs_E10	ordinary_muscle_development	D_vs_E10;stage_1W_vs_E10;stage_3W_vs_E10;stage	1	TRUE
ENSGALG00010016847	EPHB2	PRJNA729271	-1.923489204	5.20691E-70	1.923489204	stage_E14_vs_E10	ordinary_muscle_development	D_vs_E10;stage_1W_vs_E10;stage_3W_vs_E10;stage	1	TRUE
ENSGALG00010026537	FJX1	PRJNA729271	-3.318402721	5.21834E-70	3.318402721	stage_E14_vs_E10	ordinary_muscle_development	D_vs_E10;stage_1W_vs_E10;stage_3W_vs_E10;stage	1	TRUE
ENSGALG00010024188	TSSC4	PRJNA729271	-1.532626461	5.25868E-70	1.532626461	stage_E14_vs_E10	ordinary_muscle_development	D_vs_E10;stage_1W_vs_E10;stage_3W_vs_E10;stage	1	TRUE
ENSGALG00010005772	SVEP1	PRJNA729271	2.512817339	5.44149E-70	2.512817339	stage_E14_vs_E10	ordinary_muscle_development	D_vs_E10;stage_1W_vs_E10;stage_3W_vs_E10;stage	1	TRUE
ENSGALG00010012242	CCDC77	PRJNA729271	-2.825100436	5.63241E-70	2.825100436	stage_E14_vs_E10	ordinary_muscle_development	D_vs_E10;stage_1W_vs_E10;stage_3W_vs_E10;stage	1	TRUE
ENSGALG00010015800	BVES	PRJNA729271	3.236392381	5.93415E-70	3.236392381	stage_E14_vs_E10	ordinary_muscle_development	D_vs_E10;stage_1W_vs_E10;stage_3W_vs_E10;stage	1	TRUE
ENSGALG00010017188	ENSGALG00010017188	PRJNA729271	2.752108106	6.76921E-70	2.752108106	stage_E14_vs_E10	ordinary_muscle_development	D_vs_E10;stage_1W_vs_E10;stage_3W_vs_E10;stage	1	TRUE
ENSGALG00010015840	MTUS1	PRJNA729271	2.597845659	7.82553E-70	2.597845659	stage_E14_vs_E10	ordinary_muscle_development	D_vs_E10;stage_1W_vs_E10;stage_3W_vs_E10;stage	1	TRUE
ENSGALG00010028147	SLC25A42	PRJNA729271	3.525370299	7.85748E-70	3.525370299	stage_E14_vs_E10	ordinary_muscle_development	D_vs_E10;stage_1W_vs_E10;stage_3W_vs_E10;stage	1	TRUE
ENSGALG00010024817	UCKL1	PRJNA729271	3.912089298	8.15779E-70	3.912089298	stage_E14_vs_E10	ordinary_muscle_development	D_vs_E10;stage_1W_vs_E10;stage_3W_vs_E10;stage	1	TRUE
ENSGALG00010004639	HBS1L	PRJNA729271	1.409220217	8.86817E-70	1.409220217	stage_E14_vs_E10	ordinary_muscle_development	D_vs_E10;stage_1W_vs_E10;stage_3W_vs_E10;stage	1	TRUE
ENSGALG00010019146	KLHDC2	PRJNA729271	1.517371185	1.1418E-69	1.517371185	stage_E14_vs_E10	ordinary_muscle_development	D_vs_E10;stage_1W_vs_E10;stage_3W_vs_E10;stage	1	TRUE
ENSGALG00010017887	PROKR1	PRJNA729271	6.898174693	1.15534E-69	6.898174693	stage_E14_vs_E10	ordinary_muscle_development	D_vs_E10;stage_1W_vs_E10;stage_3W_vs_E10;stage	1	TRUE
ENSGALG00010008252	ZFP92	PRJNA729271	-2.396785873	1.15855E-69	2.396785873	stage_E14_vs_E10	ordinary_muscle_development	D_vs_E10;stage_1W_vs_E10;stage_3W_vs_E10;stage	1	TRUE
ENSGALG00010026167	CABIN1	PRJNA729271	-1.07803971	1.21267E-69	1.07803971	stage_E14_vs_E10	ordinary_muscle_development	D_vs_E10;stage_1W_vs_E10;stage_3W_vs_E10;stage	1	TRUE
ENSGALG00010006538	TEK	PRJNA729271	1.862861732	1.36383E-69	1.862861732	stage_E14_vs_E10	ordinary_muscle_development	D_vs_E10;stage_1W_vs_E10;stage_3W_vs_E10;stage	1	TRUE
ENSGALG00010025644	ENSGALG00010025644	PRJNA729271	5.027461307	1.38024E-69	5.027461307	stage_E14_vs_E10	ordinary_muscle_development	D_vs_E10;stage_1W_vs_E10;stage_3W_vs_E10;stage	1	TRUE
ENSGALG00010022824	PUS1	PRJNA729271	-1.551791149	1.46154E-69	1.551791149	stage_E14_vs_E10	ordinary_muscle_development	D_vs_E10;stage_1W_vs_E10;stage_3W_vs_E10;stage	1	TRUE
ENSGALG00010012940	FBN2	PRJNA729271	-4.001274015	1.82293E-69	4.001274015	stage_E14_vs_E10	ordinary_muscle_development	D_vs_E10;stage_1W_vs_E10;stage_3W_vs_E10;stage	1	TRUE
ENSGALG00010015986	NEURL2	PRJNA729271	4.050224916	1.8409E-69	4.050224916	stage_E14_vs_E10	ordinary_muscle_development	D_vs_E10;stage_1W_vs_E10;stage_3W_vs_E10;stage	1	TRUE
ENSGALG00010022997	ARHGAP1	PRJNA729271	-1.730924872	2.53089E-69	1.730924872	stage_E14_vs_E10	ordinary_muscle_development	D_vs_E10;stage_1W_vs_E10;stage_3W_vs_E10;stage	1	TRUE
ENSGALG00010025139	COPB2	PRJNA729271	-0.834140187	2.68033E-69	0.834140187	stage_E14_vs_E10	ordinary_muscle_development		0	FALSE
ENSGALG00010007699	SLC39A6	PRJNA729271	-1.922988425	2.93023E-69	1.922988425	stage_E14_vs_E10	ordinary_muscle_development	D_vs_E10;stage_1W_vs_E10;stage_3W_vs_E10;stage	1	TRUE
ENSGALG00010011843	BCL2	PRJNA729271	1.967422464	3.61749E-69	1.967422464	stage_E14_vs_E10	ordinary_muscle_development	D_vs_E10;stage_1W_vs_E10;stage_3W_vs_E10;stage	1	TRUE
ENSGALG00010022373	AK5	PRJNA729271	-4.480997598	4.14092E-69	4.480997598	stage_E14_vs_E10	ordinary_muscle_development	D_vs_E10;stage_1W_vs_E10;stage_3W_vs_E10;stage	1	TRUE
ENSGALG00010029390	RPA1	PRJNA729271	-1.446826105	4.30971E-69	1.446826105	stage_E14_vs_E10	ordinary_muscle_development	D_vs_E10;stage_1W_vs_E10;stage_3W_vs_E10;stage	1	TRUE
ENSGALG00010013175	HIPK2	PRJNA729271	2.886661151	4.4296E-69	2.886661151	stage_E14_vs_E10	ordinary_muscle_development	D_vs_E10;stage_1W_vs_E10;stage_3W_vs_E10;stage	1	TRUE
ENSGALG00010003400	PKIA	PRJNA729271	2.031404665	4.48155E-69	2.031404665	stage_E14_vs_E10	ordinary_muscle_development	D_vs_E10;stage_1W_vs_E10;stage_3W_vs_E10;stage	1	TRUE
ENSGALG00010028903	ASTN2	PRJNA729271	-5.050190034	5.26675E-69	5.050190034	stage_E14_vs_E10	ordinary_muscle_development	D_vs_E10;stage_1W_vs_E10;stage_3W_vs_E10;stage	1	TRUE
ENSGALG00010029671	GRIN2C	PRJNA729271	3.508177011	5.88659E-69	3.508177011	stage_E14_vs_E10	ordinary_muscle_development	D_vs_E10;stage_1W_vs_E10;stage_3W_vs_E10;stage	1	TRUE
ENSGALG00010002017	ARHGAP28	PRJNA729271	-2.120148124	6.65781E-69	2.120148124	stage_E14_vs_E10	ordinary_muscle_development	D_vs_E10;stage_1W_vs_E10;stage_3W_vs_E10;stage	1	TRUE
ENSGALG00010011378	TUBB2B	PRJNA729271	-3.46239872	6.78412E-69	3.46239872	stage_E14_vs_E10	ordinary_muscle_development	D_vs_E10;stage_1W_vs_E10;stage_3W_vs_E10;stage	1	TRUE
ENSGALG00010027914	BTBD2	PRJNA729271	2.205288326	8.62145E-69	2.205288326	stage_E14_vs_E10	ordinary_muscle_development	D_vs_E10;stage_1W_vs_E10;stage_3W_vs_E10;stage	1	TRUE
ENSGALG00010028833	GBGT1	PRJNA729271	2.936524623	8.92328E-69	2.936524623	stage_E14_vs_E10	ordinary_muscle_development	D_vs_E10;stage_1W_vs_E10;stage_3W_vs_E10;stage	1	TRUE
ENSGALG00010011353	ENSGALG00010011353	PRJNA729271	-3.884573941	9.54032E-69	3.884573941	stage_E14_vs_E10	ordinary_muscle_development	D_vs_E10;stage_1W_vs_E10;stage_3W_vs_E10;stage	1	TRUE
ENSGALG00010024609	PRR33	PRJNA729271	14.7926692	1.01544E-68	14.7926692	stage_E14_vs_E10	ordinary_muscle_development	D_vs_E10;stage_1W_vs_E10;stage_3W_vs_E10;stage	1	TRUE
ENSGALG00010009002	ENSGALG00010009002	PRJNA729271	1.694885325	1.08215E-68	1.694885325	stage_E14_vs_E10	ordinary_muscle_development	D_vs_E10;stage_1W_vs_E10;stage_3W_vs_E10;stage	1	TRUE
ENSGALG00010027041	MAPK14	PRJNA729271	1.303912184	1.08849E-68	1.303912184	stage_E14_vs_E10	ordinary_muscle_development	D_vs_E10;stage_1W_vs_E10;stage_3W_vs_E10;stage	1	TRUE
ENSGALG00010018559	DLG3	PRJNA729271	-2.465321175	1.11831E-68	2.465321175	stage_E14_vs_E10	ordinary_muscle_development	D_vs_E10;stage_1W_vs_E10;stage_3W_vs_E10;stage	1	TRUE
ENSGALG00010000151	TBX5	PRJNA729271	-4.521286177	1.15376E-68	4.521286177	stage_E14_vs_E10	ordinary_muscle_development	D_vs_E10;stage_1W_vs_E10;stage_3W_vs_E10;stage	1	TRUE
ENSGALG00010022293	DNAJC10	PRJNA729271	-1.160606673	1.52922E-68	1.160606673	stage_E14_vs_E10	ordinary_muscle_development	D_vs_E10;stage_1W_vs_E10;stage_3W_vs_E10;stage	1	TRUE
ENSGALG00010021438	GOT2	PRJNA729271	1.941600343	1.62866E-68	1.941600343	stage_E14_vs_E10	ordinary_muscle_development	D_vs_E10;stage_1W_vs_E10;stage_3W_vs_E10;stage	1	TRUE
ENSGALG00010028076	ST6GALNAC6	PRJNA729271	2.687811371	1.98117E-68	2.687811371	stage_E14_vs_E10	ordinary_muscle_development	D_vs_E10;stage_1W_vs_E10;stage_3W_vs_E10;stage	1	TRUE
ENSGALG00010023243	HK1	PRJNA729271	2.013531918	2.05619E-68	2.013531918	stage_E14_vs_E10	ordinary_muscle_development	D_vs_E10;stage_1W_vs_E10;stage_3W_vs_E10;stage	1	TRUE
ENSGALG00010028463	RPS28	PRJNA729271	2.98513525	2.19841E-68	2.98513525	stage_E14_vs_E10	ordinary_muscle_development	D_vs_E10;stage_1W_vs_E10;stage_3W_vs_E10;stage	1	TRUE
ENSGALG00010001145	TRIM36	PRJNA729271	-4.244200097	2.22991E-68	4.244200097	stage_E14_vs_E10	ordinary_muscle_development	D_vs_E10;stage_1W_vs_E10;stage_3W_vs_E10;stage	1	TRUE
ENSGALG00010018503	BMP2	PRJNA729271	-3.635025749	2.64962E-68	3.635025749	stage_E14_vs_E10	ordinary_muscle_development	D_vs_E10;stage_1W_vs_E10;stage_3W_vs_E10;stage	1	TRUE
ENSGALG00010018416	DUT	PRJNA729271	-2.387927069	3.06915E-68	2.387927069	stage_E14_vs_E10	ordinary_muscle_development	D_vs_E10;stage_1W_vs_E10;stage_3W_vs_E10;stage	1	TRUE
ENSGALG00010017608	RBBP4	PRJNA729271	-1.432513312	3.06915E-68	1.432513312	stage_E14_vs_E10	ordinary_muscle_development	D_vs_E10;stage_1W_vs_E10;stage_3W_vs_E10;stage	1	TRUE
ENSGALG00010021670	BLCAP	PRJNA729271	1.912517292	3.11342E-68	1.912517292	stage_E14_vs_E10	ordinary_muscle_development	D_vs_E10;stage_1W_vs_E10;stage_3W_vs_E10;stage	1	TRUE
ENSGALG00010023204	SRGN	PRJNA729271	5.389011539	3.27548E-68	5.389011539	stage_E14_vs_E10	ordinary_muscle_development	D_vs_E10;stage_1W_vs_E10;stage_3W_vs_E10;stage	1	TRUE
ENSGALG00010000586	ENSGALG00010000586	PRJNA729271	4.431992126	3.49701E-68	4.431992126	stage_E14_vs_E10	ordinary_muscle_development	D_vs_E10;stage_1W_vs_E10;stage_3W_vs_E10;stage	1	TRUE
ENSGALG00010012662	SLC35E3	PRJNA729271	-1.9331645	3.49879E-68	1.9331645	stage_E14_vs_E10	ordinary_muscle_development	D_vs_E10;stage_1W_vs_E10;stage_3W_vs_E10;stage	1	TRUE
ENSGALG00010015709	HNRNPA0	PRJNA729271	-2.018179966	3.79383E-68	2.018179966	stage_E14_vs_E10	ordinary_muscle_development	D_vs_E10;stage_1W_vs_E10;stage_3W_vs_E10;stage	1	TRUE
ENSGALG00010022162	POLE	PRJNA729271	-2.895446801	4.6861E-68	2.895446801	stage_E14_vs_E10	ordinary_muscle_development	D_vs_E10;stage_1W_vs_E10;stage_3W_vs_E10;stage	1	TRUE
ENSGALG00010012367	RAD52	PRJNA729271	-2.936157114	5.2177E-68	2.936157114	stage_E14_vs_E10	ordinary_muscle_development	D_vs_E10;stage_1W_vs_E10;stage_3W_vs_E10;stage	1	TRUE
ENSGALG00010010796	CAVIN2	PRJNA729271	2.574760377	5.94377E-68	2.574760377	stage_E14_vs_E10	ordinary_muscle_development	D_vs_E10;stage_1W_vs_E10;stage_3W_vs_E10;stage	1	TRUE
ENSGALG00010028538	FEM1A	PRJNA729271	2.056365836	6.24685E-68	2.056365836	stage_E14_vs_E10	ordinary_muscle_development	D_vs_E10;stage_1W_vs_E10;stage_3W_vs_E10;stage	1	TRUE
ENSGALG00010028189	GPSM1	PRJNA729271	1.839260897	7.63982E-68	1.839260897	stage_E14_vs_E10	ordinary_muscle_development	D_vs_E10;stage_1W_vs_E10;stage_3W_vs_E10;stage	1	TRUE
ENSGALG00010013531	ARSI	PRJNA729271	-4.777938171	9.64189E-68	4.777938171	stage_E14_vs_E10	ordinary_muscle_development	D_vs_E10;stage_1W_vs_E10;stage_3W_vs_E10;stage	1	TRUE
ENSGALG00010004265	PIP4K2A	PRJNA729271	1.613282887	1.03674E-67	1.613282887	stage_E14_vs_E10	ordinary_muscle_development	D_vs_E10;stage_1W_vs_E10;stage_3W_vs_E10;stage	1	TRUE
ENSGALG00010025748	ETNK2	PRJNA729271	3.376136393	1.14089E-67	3.376136393	stage_E14_vs_E10	ordinary_muscle_development	D_vs_E10;stage_1W_vs_E10;stage_3W_vs_E10;stage	1	TRUE
ENSGALG00010016361	NELFCD	PRJNA729271	-1.485231399	1.40027E-67	1.485231399	stage_E14_vs_E10	ordinary_muscle_development	D_vs_E10;stage_1W_vs_E10;stage_3W_vs_E10;stage	1	TRUE
ENSGALG00010004951	AFDN	PRJNA729271	-1.118996788	1.44462E-67	1.118996788	stage_E14_vs_E10	ordinary_muscle_development	D_vs_E10;stage_1W_vs_E10;stage_3W_vs_E10;stage	1	TRUE
ENSGALG00010021242	BCAR3	PRJNA729271	3.927248313	1.60523E-67	3.927248313	stage_E14_vs_E10	ordinary_muscle_development	D_vs_E10;stage_1W_vs_E10;stage_3W_vs_E10;stage	1	TRUE
ENSGALG00010008358	HPF1	PRJNA729271	-2.080736671	1.6412E-67	2.080736671	stage_E14_vs_E10	ordinary_muscle_development	D_vs_E10;stage_1W_vs_E10;stage_3W_vs_E10;stage	1	TRUE
ENSGALG00010028196	ENSGALG00010028196	PRJNA729271	-1.744694592	1.68891E-67	1.744694592	stage_E14_vs_E10	ordinary_muscle_development	D_vs_E10;stage_1W_vs_E10;stage_3W_vs_E10;stage	1	TRUE
ENSGALG00010014777	APCDD1L	PRJNA729271	-4.359910448	2.1664E-67	4.359910448	stage_E14_vs_E10	ordinary_muscle_development	D_vs_E10;stage_1W_vs_E10;stage_3W_vs_E10;stage	1	TRUE
ENSGALG00010005763	AKAP6	PRJNA729271	2.49449098	2.20153E-67	2.49449098	stage_E14_vs_E10	ordinary_muscle_development	D_vs_E10;stage_1W_vs_E10;stage_3W_vs_E10;stage	1	TRUE
ENSGALG00010018388	SMC3	PRJNA729271	-1.165224122	2.225E-67	1.165224122	stage_E14_vs_E10	ordinary_muscle_development	D_vs_E10;stage_1W_vs_E10;stage_3W_vs_E10;stage	1	TRUE
ENSGALG00010015071	SNAI1	PRJNA729271	-2.282104359	2.62569E-67	2.282104359	stage_E14_vs_E10	ordinary_muscle_development	D_vs_E10;stage_1W_vs_E10;stage_3W_vs_E10;stage	1	TRUE
ENSGALG00010015589	FRMD3	PRJNA729271	3.136040065	2.79434E-67	3.136040065	stage_E14_vs_E10	ordinary_muscle_development	D_vs_E10;stage_1W_vs_E10;stage_3W_vs_E10;stage	1	TRUE
ENSGALG00010003117	ID2	PRJNA729271	-2.22472932	2.80476E-67	2.22472932	stage_E14_vs_E10	ordinary_muscle_development	D_vs_E10;stage_1W_vs_E10;stage_3W_vs_E10;stage	1	TRUE
ENSGALG00010020284	HEG1	PRJNA729271	2.033854624	3.28858E-67	2.033854624	stage_E14_vs_E10	ordinary_muscle_development	D_vs_E10;stage_1W_vs_E10;stage_3W_vs_E10;stage	1	TRUE
ENSGALG00010002280	ST7	PRJNA729271	2.444997849	3.51055E-67	2.444997849	stage_E14_vs_E10	ordinary_muscle_development	D_vs_E10;stage_1W_vs_E10;stage_3W_vs_E10;stage	1	TRUE
ENSGALG00010001883	KBTBD11	PRJNA729271	-1.582153253	3.51948E-67	1.582153253	stage_E14_vs_E10	ordinary_muscle_development	D_vs_E10;stage_1W_vs_E10;stage_3W_vs_E10;stage	1	TRUE
ENSGALG00010017708	MEIS1	PRJNA729271	-1.839759323	3.8313E-67	1.839759323	stage_E14_vs_E10	ordinary_muscle_development	D_vs_E10;stage_1W_vs_E10;stage_3W_vs_E10;stage	1	TRUE
ENSGALG00010024546	KY	PRJNA729271	7.016262315	3.87261E-67	7.016262315	stage_E14_vs_E10	ordinary_muscle_development	D_vs_E10;stage_1W_vs_E10;stage_3W_vs_E10;stage	1	TRUE
ENSGALG00010022919	ACLY	PRJNA729271	-2.203965989	3.984E-67	2.203965989	stage_E14_vs_E10	ordinary_muscle_development	D_vs_E10;stage_1W_vs_E10;stage_3W_vs_E10;stage	1	TRUE
ENSGALG00010017778	PPP3R1	PRJNA729271	1.37201307	4.43168E-67	1.37201307	stage_E14_vs_E10	ordinary_muscle_development	D_vs_E10;stage_1W_vs_E10;stage_3W_vs_E10;stage	1	TRUE
ENSGALG00010007902	C7orf25	PRJNA729271	2.475178131	4.93599E-67	2.475178131	stage_E14_vs_E10	ordinary_muscle_development	D_vs_E10;stage_1W_vs_E10;stage_3W_vs_E10;stage	1	TRUE
ENSGALG00010021222	ENSGALG00010021222	PRJNA729271	5.281770049	5.00625E-67	5.281770049	stage_E14_vs_E10	ordinary_muscle_development	D_vs_E10;stage_1W_vs_E10;stage_3W_vs_E10;stage	1	TRUE
ENSGALG00010021526	KCNJ12	PRJNA729271	3.730777938	5.15304E-67	3.730777938	stage_E14_vs_E10	ordinary_muscle_development	D_vs_E10;stage_1W_vs_E10;stage_3W_vs_E10;stage	1	TRUE
ENSGALG00010018672	PKDCC	PRJNA729271	-1.733285727	5.23697E-67	1.733285727	stage_E14_vs_E10	ordinary_muscle_development	D_vs_E10;stage_1W_vs_E10;stage_3W_vs_E10;stage	1	TRUE
ENSGALG00010007891	GDI1	PRJNA729271	-0.978043934	6.84003E-67	0.978043934	stage_E14_vs_E10	ordinary_muscle_development		0	FALSE
ENSGALG00010012052	KLF10	PRJNA729271	2.145097921	6.95665E-67	2.145097921	stage_E14_vs_E10	ordinary_muscle_development	D_vs_E10;stage_1W_vs_E10;stage_3W_vs_E10;stage	1	TRUE
ENSGALG00010004446	ACAT2	PRJNA729271	-2.262415753	7.33483E-67	2.262415753	stage_E14_vs_E10	ordinary_muscle_development	D_vs_E10;stage_1W_vs_E10;stage_3W_vs_E10;stage	1	TRUE
ENSGALG00010022520	TMOD2	PRJNA729271	-3.955505967	7.73518E-67	3.955505967	stage_E14_vs_E10	ordinary_muscle_development	D_vs_E10;stage_1W_vs_E10;stage_3W_vs_E10;stage	1	TRUE
ENSGALG00010005430	MCM4	PRJNA729271	-2.391882012	7.74646E-67	2.391882012	stage_E14_vs_E10	ordinary_muscle_development	D_vs_E10;stage_1W_vs_E10;stage_3W_vs_E10;stage	1	TRUE
ENSGALG00010018075	MLST8	PRJNA729271	1.781798007	8.4625E-67	1.781798007	stage_E14_vs_E10	ordinary_muscle_development	D_vs_E10;stage_1W_vs_E10;stage_3W_vs_E10;stage	1	TRUE
ENSGALG00010009608	AFAP1	PRJNA729271	-1.875853606	1.02821E-66	1.875853606	stage_E14_vs_E10	ordinary_muscle_development	D_vs_E10;stage_1W_vs_E10;stage_3W_vs_E10;stage	1	TRUE
ENSGALG00010010813	EHD2	PRJNA729271	2.578164921	1.02831E-66	2.578164921	stage_E14_vs_E10	ordinary_muscle_development	D_vs_E10;stage_1W_vs_E10;stage_3W_vs_E10;stage	1	TRUE
ENSGALG00010002012	MID1	PRJNA729271	-2.505005282	1.0537E-66	2.505005282	stage_E14_vs_E10	ordinary_muscle_development	D_vs_E10;stage_1W_vs_E10;stage_3W_vs_E10;stage	1	TRUE
ENSGALG00010024330	SDF2L1	PRJNA729271	-1.822462119	1.10081E-66	1.822462119	stage_E14_vs_E10	ordinary_muscle_development	D_vs_E10;stage_1W_vs_E10;stage_3W_vs_E10;stage	1	TRUE
ENSGALG00010019030	CDR2	PRJNA729271	-2.733938283	1.19311E-66	2.733938283	stage_E14_vs_E10	ordinary_muscle_development	D_vs_E10;stage_1W_vs_E10;stage_3W_vs_E10;stage	1	TRUE
ENSGALG00010002848	TRIB2	PRJNA729271	-1.885606982	1.2464E-66	1.885606982	stage_E14_vs_E10	ordinary_muscle_development	D_vs_E10;stage_1W_vs_E10;stage_3W_vs_E10;stage	1	TRUE
ENSGALG00010021748	CDCA7	PRJNA729271	-3.140675202	1.28797E-66	3.140675202	stage_E14_vs_E10	ordinary_muscle_development	D_vs_E10;stage_1W_vs_E10;stage_3W_vs_E10;stage	1	TRUE
ENSGALG00010029396	WIPI1	PRJNA729271	2.680105021	1.34949E-66	2.680105021	stage_E14_vs_E10	ordinary_muscle_development	D_vs_E10;stage_1W_vs_E10;stage_3W_vs_E10;stage	1	TRUE
ENSGALG00010027425	CFD	PRJNA729271	4.144155281	1.36817E-66	4.144155281	stage_E14_vs_E10	ordinary_muscle_development	D_vs_E10;stage_1W_vs_E10;stage_3W_vs_E10;stage	1	TRUE
ENSGALG00010016247	KIF26A	PRJNA729271	-2.928049148	1.37545E-66	2.928049148	stage_E14_vs_E10	ordinary_muscle_development	D_vs_E10;stage_1W_vs_E10;stage_3W_vs_E10;stage	1	TRUE
ENSGALG00010005494	PPDPFL	PRJNA729271	7.548498536	1.44522E-66	7.548498536	stage_E14_vs_E10	ordinary_muscle_development	D_vs_E10;stage_1W_vs_E10;stage_3W_vs_E10;stage	1	TRUE
ENSGALG00010015974	VPS37A	PRJNA729271	2.833056398	1.46077E-66	2.833056398	stage_E14_vs_E10	ordinary_muscle_development	D_vs_E10;stage_1W_vs_E10;stage_3W_vs_E10;stage	1	TRUE
ENSGALG00010029621	TAF15	PRJNA729271	-2.136631333	1.98677E-66	2.136631333	stage_E14_vs_E10	ordinary_muscle_development	D_vs_E10;stage_1W_vs_E10;stage_3W_vs_E10;stage	1	TRUE
ENSGALG00010011657	ALDH1L2	PRJNA729271	2.49295271	2.02787E-66	2.49295271	stage_E14_vs_E10	ordinary_muscle_development	D_vs_E10;stage_1W_vs_E10;stage_3W_vs_E10;stage	1	TRUE
ENSGALG00010022863	FAM81A	PRJNA729271	4.778033105	2.13695E-66	4.778033105	stage_E14_vs_E10	ordinary_muscle_development	D_vs_E10;stage_1W_vs_E10;stage_3W_vs_E10;stage	1	TRUE
ENSGALG00010013381	DENND2A	PRJNA729271	-1.561007098	2.15308E-66	1.561007098	stage_E14_vs_E10	ordinary_muscle_development	D_vs_E10;stage_1W_vs_E10;stage_3W_vs_E10;stage	1	TRUE
ENSGALG00010001870	PLP2	PRJNA729271	2.846993826	2.30498E-66	2.846993826	stage_E14_vs_E10	ordinary_muscle_development	D_vs_E10;stage_1W_vs_E10;stage_3W_vs_E10;stage	1	TRUE
ENSGALG00010003449	BORCS6	PRJNA729271	1.890191823	2.73004E-66	1.890191823	stage_E14_vs_E10	ordinary_muscle_development	D_vs_E10;stage_1W_vs_E10;stage_3W_vs_E10;stage	1	TRUE
ENSGALG00010015390	CCNB3	PRJNA729271	-3.727337829	2.99179E-66	3.727337829	stage_E14_vs_E10	ordinary_muscle_development	D_vs_E10;stage_1W_vs_E10;stage_3W_vs_E10;stage	1	TRUE
ENSGALG00010025660	KIF2C	PRJNA729271	-3.629645413	3.20117E-66	3.629645413	stage_E14_vs_E10	ordinary_muscle_development	D_vs_E10;stage_1W_vs_E10;stage_3W_vs_E10;stage	1	TRUE
ENSGALG00010001164	KPNA4	PRJNA729271	1.291808109	3.95136E-66	1.291808109	stage_E14_vs_E10	ordinary_muscle_development	D_vs_E10;stage_1W_vs_E10;stage_3W_vs_E10;stage	1	TRUE
ENSGALG00010010434	PROM1	PRJNA729271	-2.696397368	4.6309E-66	2.696397368	stage_E14_vs_E10	ordinary_muscle_development	D_vs_E10;stage_1W_vs_E10;stage_3W_vs_E10;stage	1	TRUE
ENSGALG00010018941	KLHL21	PRJNA729271	2.491329627	4.89803E-66	2.491329627	stage_E14_vs_E10	ordinary_muscle_development	D_vs_E10;stage_1W_vs_E10;stage_3W_vs_E10;stage	1	TRUE
ENSGALG00010013289	TM9SF4	PRJNA729271	-1.319350479	4.97395E-66	1.319350479	stage_E14_vs_E10	ordinary_muscle_development	D_vs_E10;stage_1W_vs_E10;stage_3W_vs_E10;stage	1	TRUE
ENSGALG00010024760	ENSGALG00010024760	PRJNA729271	3.175686135	4.99322E-66	3.175686135	stage_E14_vs_E10	ordinary_muscle_development	D_vs_E10;stage_1W_vs_E10;stage_3W_vs_E10;stage	1	TRUE
ENSGALG00010010517	STMN4	PRJNA729271	-7.643524631	5.17197E-66	7.643524631	stage_E14_vs_E10	ordinary_muscle_development	D_vs_E10;stage_1W_vs_E10;stage_3W_vs_E10;stage	1	TRUE
ENSGALG00010027644	NAA80	PRJNA729271	2.41393884	5.95418E-66	2.41393884	stage_E14_vs_E10	ordinary_muscle_development	D_vs_E10;stage_1W_vs_E10;stage_3W_vs_E10;stage	1	TRUE
ENSGALG00010024735	OTUD7A	PRJNA729271	3.083782667	7.59521E-66	3.083782667	stage_E14_vs_E10	ordinary_muscle_development	D_vs_E10;stage_1W_vs_E10;stage_3W_vs_E10;stage	1	TRUE
ENSGALG00010015364	CLOCK	PRJNA729271	3.496621296	7.8878E-66	3.496621296	stage_E14_vs_E10	ordinary_muscle_development	D_vs_E10;stage_1W_vs_E10;stage_3W_vs_E10;stage	1	TRUE
ENSGALG00010023343	TMEM233	PRJNA729271	5.757415984	8.0479E-66	5.757415984	stage_E14_vs_E10	ordinary_muscle_development	D_vs_E10;stage_1W_vs_E10;stage_3W_vs_E10;stage	1	TRUE
ENSGALG00010002079	ARX	PRJNA729271	3.2659798	8.68017E-66	3.2659798	stage_E14_vs_E10	ordinary_muscle_development	D_vs_E10;stage_1W_vs_E10;stage_3W_vs_E10;stage	1	TRUE
ENSGALG00010020608	CAPZB	PRJNA729271	1.716666634	8.79713E-66	1.716666634	stage_E14_vs_E10	ordinary_muscle_development	D_vs_E10;stage_1W_vs_E10;stage_3W_vs_E10;stage	1	TRUE
ENSGALG00010023641	LRRC40	PRJNA729271	-1.995463173	8.91421E-66	1.995463173	stage_E14_vs_E10	ordinary_muscle_development	D_vs_E10;stage_1W_vs_E10;stage_3W_vs_E10;stage	1	TRUE
ENSGALG00010001146	DIPK1C	PRJNA729271	4.148803682	9.20961E-66	4.148803682	stage_E14_vs_E10	ordinary_muscle_development	D_vs_E10;stage_1W_vs_E10;stage_3W_vs_E10;stage	1	TRUE
ENSGALG00010015043	COQ10B	PRJNA729271	3.832649931	9.51469E-66	3.832649931	stage_E14_vs_E10	ordinary_muscle_development	D_vs_E10;stage_1W_vs_E10;stage_3W_vs_E10;stage	1	TRUE
ENSGALG00010021704	COPS7A	PRJNA729271	1.761532241	1.01245E-65	1.761532241	stage_E14_vs_E10	ordinary_muscle_development	D_vs_E10;stage_1W_vs_E10;stage_3W_vs_E10;stage	1	TRUE
ENSGALG00010013207	C1QTNF3	PRJNA729271	-3.464058013	1.03664E-65	3.464058013	stage_E14_vs_E10	ordinary_muscle_development	D_vs_E10;stage_1W_vs_E10;stage_3W_vs_E10;stage	1	TRUE
ENSGALG00010009503	IL11RA	PRJNA729271	-2.382170706	1.0498E-65	2.382170706	stage_E14_vs_E10	ordinary_muscle_development	D_vs_E10;stage_1W_vs_E10;stage_3W_vs_E10;stage	1	TRUE
ENSGALG00010018780	WDHD1	PRJNA729271	-3.717653608	1.07188E-65	3.717653608	stage_E14_vs_E10	ordinary_muscle_development	D_vs_E10;stage_1W_vs_E10;stage_3W_vs_E10;stage	1	TRUE
ENSGALG00010023485	BARX2	PRJNA729271	-5.746217836	1.07466E-65	5.746217836	stage_E14_vs_E10	ordinary_muscle_development	D_vs_E10;stage_1W_vs_E10;stage_3W_vs_E10;stage	1	TRUE
ENSGALG00010027730	ENSGALG00010027730	PRJNA729271	-2.740748441	1.13294E-65	2.740748441	stage_E14_vs_E10	ordinary_muscle_development	D_vs_E10;stage_1W_vs_E10;stage_3W_vs_E10;stage	1	TRUE
ENSGALG00010014658	SELENOP1	PRJNA729271	2.995366649	1.42631E-65	2.995366649	stage_E14_vs_E10	ordinary_muscle_development	D_vs_E10;stage_1W_vs_E10;stage_3W_vs_E10;stage	1	TRUE
ENSGALG00010018177	KIF20B	PRJNA729271	-3.307016156	1.67412E-65	3.307016156	stage_E14_vs_E10	ordinary_muscle_development	D_vs_E10;stage_1W_vs_E10;stage_3W_vs_E10;stage	1	TRUE
ENSGALG00010023182	EPHB6	PRJNA729271	-2.552961919	1.73569E-65	2.552961919	stage_E14_vs_E10	ordinary_muscle_development	D_vs_E10;stage_1W_vs_E10;stage_3W_vs_E10;stage	1	TRUE
ENSGALG00010028128	DNM1	PRJNA729271	-2.498279661	1.75033E-65	2.498279661	stage_E14_vs_E10	ordinary_muscle_development	D_vs_E10;stage_1W_vs_E10;stage_3W_vs_E10;stage	1	TRUE
ENSGALG00010010756	CENPL	PRJNA729271	-3.821042211	1.82224E-65	3.821042211	stage_E14_vs_E10	ordinary_muscle_development	D_vs_E10;stage_1W_vs_E10;stage_3W_vs_E10;stage	1	TRUE
ENSGALG00010010993	ENSGALG00010010993	PRJNA729271	5.686117199	1.85059E-65	5.686117199	stage_E14_vs_E10	ordinary_muscle_development	D_vs_E10;stage_1W_vs_E10;stage_3W_vs_E10;stage	1	TRUE
ENSGALG00010027586	HJV	PRJNA729271	4.974812865	1.91956E-65	4.974812865	stage_E14_vs_E10	ordinary_muscle_development	D_vs_E10;stage_1W_vs_E10;stage_3W_vs_E10;stage	1	TRUE
ENSGALG00010009371	PHTF2	PRJNA729271	2.666951583	1.9843E-65	2.666951583	stage_E14_vs_E10	ordinary_muscle_development	D_vs_E10;stage_1W_vs_E10;stage_3W_vs_E10;stage	1	TRUE
ENSGALG00010022274	CCDC91	PRJNA729271	3.000443588	2.21332E-65	3.000443588	stage_E14_vs_E10	ordinary_muscle_development	D_vs_E10;stage_1W_vs_E10;stage_3W_vs_E10;stage	1	TRUE
ENSGALG00010014736	KIF2A	PRJNA729271	-1.568147162	2.30276E-65	1.568147162	stage_E14_vs_E10	ordinary_muscle_development	D_vs_E10;stage_1W_vs_E10;stage_3W_vs_E10;stage	1	TRUE
ENSGALG00010012705	CPM	PRJNA729271	3.171074532	2.36056E-65	3.171074532	stage_E14_vs_E10	ordinary_muscle_development	D_vs_E10;stage_1W_vs_E10;stage_3W_vs_E10;stage	1	TRUE
ENSGALG00010009570	SHARPIN	PRJNA729271	2.322409907	2.91475E-65	2.322409907	stage_E14_vs_E10	ordinary_muscle_development	D_vs_E10;stage_1W_vs_E10;stage_3W_vs_E10;stage	1	TRUE
ENSGALG00010025092	COL1A1	PRJNA729271	3.818068681	3.29151E-65	3.818068681	stage_E14_vs_E10	ordinary_muscle_development	D_vs_E10;stage_1W_vs_E10;stage_3W_vs_E10;stage	1	TRUE
ENSGALG00010013181	DYRK1A	PRJNA729271	1.357019619	3.35463E-65	1.357019619	stage_E14_vs_E10	ordinary_muscle_development	D_vs_E10;stage_1W_vs_E10;stage_3W_vs_E10;stage	1	TRUE
ENSGALG00010021080	TTLL7	PRJNA729271	2.979351709	3.64191E-65	2.979351709	stage_E14_vs_E10	ordinary_muscle_development	D_vs_E10;stage_1W_vs_E10;stage_3W_vs_E10;stage	1	TRUE
ENSGALG00010019101	POLE2	PRJNA729271	-2.133879526	3.73739E-65	2.133879526	stage_E14_vs_E10	ordinary_muscle_development	D_vs_E10;stage_1W_vs_E10;stage_3W_vs_E10;stage	1	TRUE
ENSGALG00010027709	PSMA5	PRJNA729271	1.831736662	4.11776E-65	1.831736662	stage_E14_vs_E10	ordinary_muscle_development	D_vs_E10;stage_1W_vs_E10;stage_3W_vs_E10;stage	1	TRUE
ENSGALG00010009948	WBP2NL	PRJNA729271	1.108340721	4.49908E-65	1.108340721	stage_E14_vs_E10	ordinary_muscle_development	D_vs_E10;stage_1W_vs_E10;stage_3W_vs_E10;stage	1	TRUE
ENSGALG00010026272	LRRC58	PRJNA729271	1.633965544	4.5091E-65	1.633965544	stage_E14_vs_E10	ordinary_muscle_development	D_vs_E10;stage_1W_vs_E10;stage_3W_vs_E10;stage	1	TRUE
ENSGALG00010016181	FANCI	PRJNA729271	-3.173256833	4.85988E-65	3.173256833	stage_E14_vs_E10	ordinary_muscle_development	D_vs_E10;stage_1W_vs_E10;stage_3W_vs_E10;stage	1	TRUE
ENSGALG00010014716	TMEM35A	PRJNA729271	-2.437746928	5.28457E-65	2.437746928	stage_E14_vs_E10	ordinary_muscle_development	D_vs_E10;stage_1W_vs_E10;stage_3W_vs_E10;stage	1	TRUE
ENSGALG00010008027	C6orf62	PRJNA729271	1.84282976	5.69094E-65	1.84282976	stage_E14_vs_E10	ordinary_muscle_development	D_vs_E10;stage_1W_vs_E10;stage_3W_vs_E10;stage	1	TRUE
ENSGALG00010017660	CLIP4	PRJNA729271	4.24017752	5.97226E-65	4.24017752	stage_E14_vs_E10	ordinary_muscle_development	D_vs_E10;stage_1W_vs_E10;stage_3W_vs_E10;stage	1	TRUE
ENSGALG00010029267	ENSGALG00010029267	PRJNA729271	4.688967993	6.64711E-65	4.688967993	stage_E14_vs_E10	ordinary_muscle_development	D_vs_E10;stage_1W_vs_E10;stage_3W_vs_E10;stage	1	TRUE
ENSGALG00010027863	CHDH	PRJNA729271	-2.708781724	6.71725E-65	2.708781724	stage_E14_vs_E10	ordinary_muscle_development	D_vs_E10;stage_1W_vs_E10;stage_3W_vs_E10;stage	1	TRUE
ENSGALG00010001861	CBX3	PRJNA729271	-1.285474533	6.91471E-65	1.285474533	stage_E14_vs_E10	ordinary_muscle_development	D_vs_E10;stage_1W_vs_E10;stage_3W_vs_E10;stage	1	TRUE
ENSGALG00010001901	PRPS2	PRJNA729271	3.123707608	7.27757E-65	3.123707608	stage_E14_vs_E10	ordinary_muscle_development	D_vs_E10;stage_1W_vs_E10;stage_3W_vs_E10;stage	1	TRUE
ENSGALG00010017147	SRSF7L	PRJNA729271	-1.232822048	8.29016E-65	1.232822048	stage_E14_vs_E10	ordinary_muscle_development	D_vs_E10;stage_1W_vs_E10;stage_3W_vs_E10;stage	1	TRUE
ENSGALG00010015027	CLSPN	PRJNA729271	-3.300725324	8.41608E-65	3.300725324	stage_E14_vs_E10	ordinary_muscle_development	D_vs_E10;stage_1W_vs_E10;stage_3W_vs_E10;stage	1	TRUE
ENSGALG00010023891	ENSGALG00010023891	PRJNA729271	4.518694555	8.67823E-65	4.518694555	stage_E14_vs_E10	ordinary_muscle_development	D_vs_E10;stage_1W_vs_E10;stage_3W_vs_E10;stage	1	TRUE
ENSGALG00010015080	ASF1A	PRJNA729271	-1.384235957	9.43153E-65	1.384235957	stage_E14_vs_E10	ordinary_muscle_development	D_vs_E10;stage_1W_vs_E10;stage_3W_vs_E10;stage	1	TRUE
ENSGALG00010024326	KLHL24	PRJNA729271	3.064915799	1.08731E-64	3.064915799	stage_E14_vs_E10	ordinary_muscle_development	D_vs_E10;stage_1W_vs_E10;stage_3W_vs_E10;stage	1	TRUE
ENSGALG00010023230	ENSGALG00010023230	PRJNA729271	1.424208399	1.18029E-64	1.424208399	stage_E14_vs_E10	ordinary_muscle_development	D_vs_E10;stage_1W_vs_E10;stage_3W_vs_E10;stage	1	TRUE
ENSGALG00010002281	TGFBR2	PRJNA729271	2.65528053	1.39223E-64	2.65528053	stage_E14_vs_E10	ordinary_muscle_development	D_vs_E10;stage_1W_vs_E10;stage_3W_vs_E10;stage	1	TRUE
ENSGALG00010028476	ENSGALG00010028476	PRJNA729271	2.645567915	1.93499E-64	2.645567915	stage_E14_vs_E10	ordinary_muscle_development	D_vs_E10;stage_1W_vs_E10;stage_3W_vs_E10;stage	1	TRUE
ENSGALG00010015101	CAP1	PRJNA729271	-1.717254585	2.19951E-64	1.717254585	stage_E14_vs_E10	ordinary_muscle_development	D_vs_E10;stage_1W_vs_E10;stage_3W_vs_E10;stage	1	TRUE
ENSGALG00010028419	ENSGALG00010028419	PRJNA729271	6.155264821	2.40565E-64	6.155264821	stage_E14_vs_E10	ordinary_muscle_development	D_vs_E10;stage_1W_vs_E10;stage_3W_vs_E10;stage	1	TRUE
ENSGALG00010024729	FOXM1	PRJNA729271	-2.960697972	3.63365E-64	2.960697972	stage_E14_vs_E10	ordinary_muscle_development	D_vs_E10;stage_1W_vs_E10;stage_3W_vs_E10;stage	1	TRUE
ENSGALG00010025272	NCAPD3	PRJNA729271	-3.303258169	4.79458E-64	3.303258169	stage_E14_vs_E10	ordinary_muscle_development	D_vs_E10;stage_1W_vs_E10;stage_3W_vs_E10;stage	1	TRUE
ENSGALG00010007761	ENSGALG00010007761	PRJNA729271	1.955441563	4.79882E-64	1.955441563	stage_E14_vs_E10	ordinary_muscle_development	D_vs_E10;stage_1W_vs_E10;stage_3W_vs_E10;stage	1	TRUE
ENSGALG00010013293	TUBGCP3	PRJNA729271	-1.014424847	5.99195E-64	1.014424847	stage_E14_vs_E10	ordinary_muscle_development	D_vs_E10;stage_1W_vs_E10;stage_3W_vs_E10;stage	1	TRUE
ENSGALG00010016746	HDGFL3	PRJNA729271	-2.14111201	6.30313E-64	2.14111201	stage_E14_vs_E10	ordinary_muscle_development	D_vs_E10;stage_1W_vs_E10;stage_3W_vs_E10;stage	1	TRUE
ENSGALG00010019310	CRELD1	PRJNA729271	1.642227325	6.55934E-64	1.642227325	stage_E14_vs_E10	ordinary_muscle_development	D_vs_E10;stage_1W_vs_E10;stage_3W_vs_E10;stage	1	TRUE

ENSGALG00010021686	CCPG1	PRJNA729271	2.808992311	6.98582E-64	2.808992311	stage_E14_vs_E10	ordinary_muscle_development	D_vs_E10;stage_1W_vs_E10;stage_3W_vs_E10;stage	1	TRUE
ENSGALG00010022526	PPP1CA	PRJNA729271	2.338803621	7.76318E-64	2.338803621	stage_E14_vs_E10	ordinary_muscle_development	D_vs_E10;stage_1W_vs_E10;stage_3W_vs_E10;stage	1	TRUE
ENSGALG00010013230	ENSGALG00010013230	PRJNA729271	5.79546851	8.20597E-64	5.79546851	stage_E14_vs_E10	ordinary_muscle_development	D_vs_E10;stage_1W_vs_E10;stage_3W_vs_E10;stage	1	TRUE
ENSGALG00010022796	RIMKLB	PRJNA729271	-2.385817906	9.13416E-64	2.385817906	stage_E14_vs_E10	ordinary_muscle_development	D_vs_E10;stage_1W_vs_E10;stage_3W_vs_E10;stage	1	TRUE
ENSGALG00010009216	NSG1	PRJNA729271	-4.226832403	9.13699E-64	4.226832403	stage_E14_vs_E10	ordinary_muscle_development	D_vs_E10;stage_1W_vs_E10;stage_3W_vs_E10;stage	1	TRUE
ENSGALG00010024311	KLHL6	PRJNA729271	2.099534488	9.37386E-64	2.099534488	stage_E14_vs_E10	ordinary_muscle_development	D_vs_E10;stage_1W_vs_E10;stage_3W_vs_E10;stage	1	TRUE
ENSGALG00010021071	HIF1AN	PRJNA729271	1.616896649	9.54448E-64	1.616896649	stage_E14_vs_E10	ordinary_muscle_development	D_vs_E10;stage_1W_vs_E10;stage_3W_vs_E10;stage	1	TRUE
ENSGALG00010015069	HSPD1	PRJNA729271	-1.753218436	1.04088E-63	1.753218436	stage_E14_vs_E10	ordinary_muscle_development	D_vs_E10;stage_1W_vs_E10;stage_3W_vs_E10;stage	1	TRUE
ENSGALG00010008308	WWP2	PRJNA729271	-2.151157446	1.21547E-63	2.151157446	stage_E14_vs_E10	ordinary_muscle_development	D_vs_E10;stage_1W_vs_E10;stage_3W_vs_E10;stage	1	TRUE
ENSGALG00010001454	VEGFD	PRJNA729271	2.788474323	1.57584E-63	2.788474323	stage_E14_vs_E10	ordinary_muscle_development	D_vs_E10;stage_1W_vs_E10;stage_3W_vs_E10;stage	1	TRUE
ENSGALG00010017613	HAUS3	PRJNA729271	-2.098109365	1.73524E-63	2.098109365	stage_E14_vs_E10	ordinary_muscle_development	D_vs_E10;stage_1W_vs_E10;stage_3W_vs_E10;stage	1	TRUE
ENSGALG00010021810	ANKRD13A	PRJNA729271	-1.667297794	1.73606E-63	1.667297794	stage_E14_vs_E10	ordinary_muscle_development	D_vs_E10;stage_1W_vs_E10;stage_3W_vs_E10;stage	1	TRUE
ENSGALG00010024462	ZBTB32	PRJNA729271	7.292814443	1.80434E-63	7.292814443	stage_E14_vs_E10	ordinary_muscle_development	D_vs_E10;stage_1W_vs_E10;stage_3W_vs_E10;stage	1	TRUE
ENSGALG00010009809	SMDT1	PRJNA729271	1.51825586	1.90065E-63	1.51825586	stage_E14_vs_E10	ordinary_muscle_development	D_vs_E10;stage_1W_vs_E10;stage_3W_vs_E10;stage	1	TRUE
ENSGALG00010013156	ABCC9	PRJNA729271	2.201689944	1.9711E-63	2.201689944	stage_E14_vs_E10	ordinary_muscle_development	D_vs_E10;stage_1W_vs_E10;stage_3W_vs_E10;stage	1	TRUE
ENSGALG00010029771	SOX9	PRJNA729271	-4.604163777	2.11065E-63	4.604163777	stage_E14_vs_E10	ordinary_muscle_development	D_vs_E10;stage_1W_vs_E10;stage_3W_vs_E10;stage	1	TRUE
ENSGALG00010019948	TICRR	PRJNA729271	-2.263712842	2.11091E-63	2.263712842	stage_E14_vs_E10	ordinary_muscle_development	D_vs_E10;stage_1W_vs_E10;stage_3W_vs_E10;stage	1	TRUE
ENSGALG00010017198	TOPBP1	PRJNA729271	-1.202378499	2.2089E-63	1.202378499	stage_E14_vs_E10	ordinary_muscle_development	D_vs_E10;stage_1W_vs_E10;stage_3W_vs_E10;stage	1	TRUE
ENSGALG00010002437	TTK	PRJNA729271	-3.390003346	2.23204E-63	3.390003346	stage_E14_vs_E10	ordinary_muscle_development	D_vs_E10;stage_1W_vs_E10;stage_3W_vs_E10;stage	1	TRUE
ENSGALG00010021650	CD276	PRJNA729271	-1.906575149	2.37558E-63	1.906575149	stage_E14_vs_E10	ordinary_muscle_development	D_vs_E10;stage_1W_vs_E10;stage_3W_vs_E10;stage	1	TRUE
ENSGALG00010017390	DDX24	PRJNA729271	-1.796020684	2.42852E-63	1.796020684	stage_E14_vs_E10	ordinary_muscle_development	D_vs_E10;stage_1W_vs_E10;stage_3W_vs_E10;stage	1	TRUE
ENSGALG00010019233	ARPC2	PRJNA729271	-0.91598599	2.45989E-63	0.91598599	stage_E14_vs_E10	ordinary_muscle_development		0	FALSE
ENSGALG00010008159	SUPT3H	PRJNA729271	-2.45114212	2.57509E-63	2.45114212	stage_E14_vs_E10	ordinary_muscle_development	D_vs_E10;stage_1W_vs_E10;stage_3W_vs_E10;stage	1	TRUE
ENSGALG00010023441	ENSGALG00010023441	PRJNA729271	2.461364486	2.80941E-63	2.461364486	stage_E14_vs_E10	ordinary_muscle_development	D_vs_E10;stage_1W_vs_E10;stage_3W_vs_E10;stage	1	TRUE
ENSGALG00010013592	H3C33	PRJNA729271	-3.133338431	3.02094E-63	3.133338431	stage_E14_vs_E10	ordinary_muscle_development	D_vs_E10;stage_1W_vs_E10;stage_3W_vs_E10;stage	1	TRUE
ENSGALG00010001214	TES	PRJNA729271	-2.955181895	3.51534E-63	2.955181895	stage_E14_vs_E10	ordinary_muscle_development	D_vs_E10;stage_1W_vs_E10;stage_3W_vs_E10;stage	1	TRUE
ENSGALG00010008262	CYB5B	PRJNA729271	-1.241698056	3.92819E-63	1.241698056	stage_E14_vs_E10	ordinary_muscle_development	D_vs_E10;stage_1W_vs_E10;stage_3W_vs_E10;stage	1	TRUE
ENSGALG00010011283	LONRF3	PRJNA729271	2.364716486	5.20498E-63	2.364716486	stage_E14_vs_E10	ordinary_muscle_development	D_vs_E10;stage_1W_vs_E10;stage_3W_vs_E10;stage	1	TRUE
ENSGALG00010010882	CEP350	PRJNA729271	1.675590304	5.32738E-63	1.675590304	stage_E14_vs_E10	ordinary_muscle_development	D_vs_E10;stage_1W_vs_E10;stage_3W_vs_E10;stage	1	TRUE
ENSGALG00010017958	CAPN10	PRJNA729271	-1.610545886	6.35762E-63	1.610545886	stage_E14_vs_E10	ordinary_muscle_development	D_vs_E10;stage_1W_vs_E10;stage_3W_vs_E10;stage	1	TRUE
ENSGALG00010006454	SOD2	PRJNA729271	1.779871647	6.44433E-63	1.779871647	stage_E14_vs_E10	ordinary_muscle_development	D_vs_E10;stage_1W_vs_E10;stage_3W_vs_E10;stage	1	TRUE
ENSGALG00010006739	VCAN	PRJNA729271	-4.296251106	6.60178E-63	4.296251106	stage_E14_vs_E10	ordinary_muscle_development	D_vs_E10;stage_1W_vs_E10;stage_3W_vs_E10;stage	1	TRUE
ENSGALG00010003835	ENSGALG00010003835	PRJNA729271	2.757126974	7.90794E-63	2.757126974	stage_E14_vs_E10	ordinary_muscle_development	D_vs_E10;stage_1W_vs_E10;stage_3W_vs_E10;stage	1	TRUE
ENSGALG00010000695	FIGNL1	PRJNA729271	-2.662096555	8.00551E-63	2.662096555	stage_E14_vs_E10	ordinary_muscle_development	D_vs_E10;stage_1W_vs_E10;stage_3W_vs_E10;stage	1	TRUE
ENSGALG00010002884	ADHFE1	PRJNA729271	4.246854834	8.27447E-63	4.246854834	stage_E14_vs_E10	ordinary_muscle_development	D_vs_E10;stage_1W_vs_E10;stage_3W_vs_E10;stage	1	TRUE
ENSGALG00010022105	DLG5	PRJNA729271	-1.441759656	8.60657E-63	1.441759656	stage_E14_vs_E10	ordinary_muscle_development	D_vs_E10;stage_1W_vs_E10;stage_3W_vs_E10;stage	1	TRUE
ENSGALG00010009497	HS3ST1	PRJNA729271	3.145660971	9.01468E-63	3.145660971	stage_E14_vs_E10	ordinary_muscle_development	D_vs_E10;stage_1W_vs_E10;stage_3W_vs_E10;stage	1	TRUE
ENSGALG00010023134	KNL1	PRJNA729271	-3.709964143	1.06189E-62	3.709964143	stage_E14_vs_E10	ordinary_muscle_development	D_vs_E10;stage_1W_vs_E10;stage_3W_vs_E10;stage	1	TRUE
ENSGALG00010010358	ASPM	PRJNA729271	-3.082152934	1.10914E-62	3.082152934	stage_E14_vs_E10	ordinary_muscle_development	D_vs_E10;stage_1W_vs_E10;stage_3W_vs_E10;stage	1	TRUE
ENSGALG00010020607	ENSGALG00010020607	PRJNA729271	4.023675334	1.16546E-62	4.023675334	stage_E14_vs_E10	ordinary_muscle_development	D_vs_E10;stage_1W_vs_E10;stage_3W_vs_E10;stage	1	TRUE
ENSGALG00010026105	CNTF	PRJNA729271	3.061197897	1.23047E-62	3.061197897	stage_E14_vs_E10	ordinary_muscle_development	D_vs_E10;stage_1W_vs_E10;stage_3W_vs_E10;stage	1	TRUE
ENSGALG00010024756	CDK2	PRJNA729271	-2.89801772	1.5767E-62	2.89801772	stage_E14_vs_E10	ordinary_muscle_development	D_vs_E10;stage_1W_vs_E10;stage_3W_vs_E10;stage	1	TRUE
ENSGALG00010012212	RAD54B	PRJNA729271	-3.443719475	1.94879E-62	3.443719475	stage_E14_vs_E10	ordinary_muscle_development	D_vs_E10;stage_1W_vs_E10;stage_3W_vs_E10;stage	1	TRUE
ENSGALG00010021753	MTF2	PRJNA729271	-1.662761212	2.13548E-62	1.662761212	stage_E14_vs_E10	ordinary_muscle_development	D_vs_E10;stage_1W_vs_E10;stage_3W_vs_E10;stage	1	TRUE
ENSGALG00010017012	SYNGR3	PRJNA729271	2.991581591	2.15588E-62	2.991581591	stage_E14_vs_E10	ordinary_muscle_development	D_vs_E10;stage_1W_vs_E10;stage_3W_vs_E10;stage	1	TRUE
ENSGALG00010005148	TTC39C	PRJNA729271	-1.555359396	2.17691E-62	1.555359396	stage_E14_vs_E10	ordinary_muscle_development	D_vs_E10;stage_1W_vs_E10;stage_3W_vs_E10;stage	1	TRUE
ENSGALG00010012244	ENSGALG00010012244	PRJNA729271	-1.444084701	2.17929E-62	1.444084701	stage_E14_vs_E10	ordinary_muscle_development	D_vs_E10;stage_1W_vs_E10;stage_3W_vs_E10;stage	1	TRUE
ENSGALG00010003246	VWA8	PRJNA729271	1.827828205	2.30326E-62	1.827828205	stage_E14_vs_E10	ordinary_muscle_development	D_vs_E10;stage_1W_vs_E10;stage_3W_vs_E10;stage	1	TRUE
ENSGALG00010026724	OLA1	PRJNA729271	1.641548667	2.3104E-62	1.641548667	stage_E14_vs_E10	ordinary_muscle_development	D_vs_E10;stage_1W_vs_E10;stage_3W_vs_E10;stage	1	TRUE
ENSGALG00010029682	CDR2L	PRJNA729271	-2.46953896	2.56506E-62	2.46953896	stage_E14_vs_E10	ordinary_muscle_development	D_vs_E10;stage_1W_vs_E10;stage_3W_vs_E10;stage	1	TRUE
ENSGALG00010011496	ACTR6	PRJNA729271	-1.50691974	2.6299E-62	1.50691974	stage_E14_vs_E10	ordinary_muscle_development	D_vs_E10;stage_1W_vs_E10;stage_3W_vs_E10;stage	1	TRUE
ENSGALG00010006835	FRY	PRJNA729271	2.562683061	2.63724E-62	2.562683061	stage_E14_vs_E10	ordinary_muscle_development	D_vs_E10;stage_1W_vs_E10;stage_3W_vs_E10;stage	1	TRUE
ENSGALG00010023809	CDON	PRJNA729271	-1.687490297	2.91867E-62	1.687490297	stage_E14_vs_E10	ordinary_muscle_development	D_vs_E10;stage_1W_vs_E10;stage_3W_vs_E10;stage	1	TRUE
ENSGALG00010024596	EPS15	PRJNA729271	1.12073104	3.05151E-62	1.12073104	stage_E14_vs_E10	ordinary_muscle_development	D_vs_E10;stage_1W_vs_E10;stage_3W_vs_E10;stage	1	TRUE
ENSGALG00010006147	ENSGALG00010006147	PRJNA729271	3.620253081	3.11734E-62	3.620253081	stage_E14_vs_E10	ordinary_muscle_development	D_vs_E10;stage_1W_vs_E10;stage_3W_vs_E10;stage	1	TRUE
ENSGALG00010012742	ENSGALG00010012742	PRJNA729271	-2.942966288	3.36624E-62	2.942966288	stage_E14_vs_E10	ordinary_muscle_development	D_vs_E10;stage_1W_vs_E10;stage_3W_vs_E10;stage	1	TRUE
ENSGALG00010022471	ST6GALNAC5	PRJNA729271	-3.156966518	3.75778E-62	3.156966518	stage_E14_vs_E10	ordinary_muscle_development	D_vs_E10;stage_1W_vs_E10;stage_3W_vs_E10;stage	1	TRUE
ENSGALG00010022541	ITPKA	PRJNA729271	-2.182506383	3.94219E-62	2.182506383	stage_E14_vs_E10	ordinary_muscle_development	D_vs_E10;stage_1W_vs_E10;stage_3W_vs_E10;stage	1	TRUE
ENSGALG00010013435	SDC3	PRJNA729271	-3.074109648	3.95852E-62	3.074109648	stage_E14_vs_E10	ordinary_muscle_development	D_vs_E10;stage_1W_vs_E10;stage_3W_vs_E10;stage	1	TRUE
ENSGALG00010013427	NEXMIF	PRJNA729271	-3.675025684	4.0912E-62	3.675025684	stage_E14_vs_E10	ordinary_muscle_development	D_vs_E10;stage_1W_vs_E10;stage_3W_vs_E10;stage	1	TRUE
ENSGALG00010023852	LRRC39	PRJNA729271	3.194626666	4.22408E-62	3.194626666	stage_E14_vs_E10	ordinary_muscle_development	D_vs_E10;stage_1W_vs_E10;stage_3W_vs_E10;stage	1	TRUE
ENSGALG00010014465	SIMC1	PRJNA729271	2.461404647	4.42366E-62	2.461404647	stage_E14_vs_E10	ordinary_muscle_development	D_vs_E10;stage_1W_vs_E10;stage_3W_vs_E10;stage	1	TRUE
ENSGALG00010008455	FKBP1B	PRJNA729271	2.517702296	4.59774E-62	2.517702296	stage_E14_vs_E10	ordinary_muscle_development	D_vs_E10;stage_1W_vs_E10;stage_3W_vs_E10;stage	1	TRUE
ENSGALG00010002500	OSR1	PRJNA729271	-3.481557453	5.40126E-62	3.481557453	stage_E14_vs_E10	ordinary_muscle_development	D_vs_E10;stage_1W_vs_E10;stage_3W_vs_E10;stage	1	TRUE
ENSGALG00010004368	GJD4	PRJNA729271	-7.641286741	5.55553E-62	7.641286741	stage_E14_vs_E10	ordinary_muscle_development	D_vs_E10;stage_1W_vs_E10;stage_3W_vs_E10;stage	1	TRUE
ENSGALG00010001192	ENSGALG00010001192	PRJNA729271	3.700861264	5.89399E-62	3.700861264	stage_E14_vs_E10	ordinary_muscle_development	D_vs_E10;stage_1W_vs_E10;stage_3W_vs_E10;stage	1	TRUE
ENSGALG00010019477	NCAPH	PRJNA729271	-3.065605892	6.9817E-62	3.065605892	stage_E14_vs_E10	ordinary_muscle_development	D_vs_E10;stage_1W_vs_E10;stage_3W_vs_E10;stage	1	TRUE
ENSGALG00010017859	EXOC6	PRJNA729271	2.239870417	7.61455E-62	2.239870417	stage_E14_vs_E10	ordinary_muscle_development	D_vs_E10;stage_1W_vs_E10;stage_3W_vs_E10;stage	1	TRUE
ENSGALG00010022422	RPS6KB2	PRJNA729271	2.557498409	7.66084E-62	2.557498409	stage_E14_vs_E10	ordinary_muscle_development	D_vs_E10;stage_1W_vs_E10;stage_3W_vs_E10;stage	1	TRUE
ENSGALG00010016851	TEC	PRJNA729271	2.295919415	7.88326E-62	2.295919415	stage_E14_vs_E10	ordinary_muscle_development	D_vs_E10;stage_1W_vs_E10;stage_3W_vs_E10;stage	1	TRUE
ENSGALG00010014504	SEC63	PRJNA729271	-0.773056863	8.26114E-62	0.773056863	stage_E14_vs_E10	ordinary_muscle_development		0	FALSE
ENSGALG00010028375	LMNB2	PRJNA729271	-2.562564694	9.10725E-62	2.562564694	stage_E14_vs_E10	ordinary_muscle_development	D_vs_E10;stage_1W_vs_E10;stage_3W_vs_E10;stage	1	TRUE
ENSGALG00010014810	ENTREP1	PRJNA729271	3.615596681	9.75182E-62	3.615596681	stage_E14_vs_E10	ordinary_muscle_development	D_vs_E10;stage_1W_vs_E10;stage_3W_vs_E10;stage	1	TRUE
ENSGALG00010027284	CTTNBP2NL	PRJNA729271	1.500267456	1.0124E-61	1.500267456	stage_E14_vs_E10	ordinary_muscle_development	D_vs_E10;stage_1W_vs_E10;stage_3W_vs_E10;stage	1	TRUE
ENSGALG00010001501	CAPZA2	PRJNA729271	1.779146943	1.03248E-61	1.779146943	stage_E14_vs_E10	ordinary_muscle_development	D_vs_E10;stage_1W_vs_E10;stage_3W_vs_E10;stage	1	TRUE
ENSGALG00010023017	BUB1B	PRJNA729271	-3.427434062	1.06189E-61	3.427434062	stage_E14_vs_E10	ordinary_muscle_development	D_vs_E10;stage_1W_vs_E10;stage_3W_vs_E10;stage	1	TRUE
ENSGALG00010015485	BCAS1	PRJNA729271	3.053990898	1.07405E-61	3.053990898	stage_E14_vs_E10	ordinary_muscle_development	D_vs_E10;stage_1W_vs_E10;stage_3W_vs_E10;stage	1	TRUE
ENSGALG00010022135	CHST1	PRJNA729271	-4.742095206	1.13876E-61	4.742095206	stage_E14_vs_E10	ordinary_muscle_development	D_vs_E10;stage_1W_vs_E10;stage_3W_vs_E10;stage	1	TRUE
ENSGALG00010009596	URB2	PRJNA729271	-1.755355733	1.35481E-61	1.755355733	stage_E14_vs_E10	ordinary_muscle_development	D_vs_E10;stage_1W_vs_E10;stage_3W_vs_E10;stage	1	TRUE
ENSGALG00010029651	ENSGALG00010029651	PRJNA729271	4.593198489	1.45893E-61	4.593198489	stage_E14_vs_E10	ordinary_muscle_development	D_vs_E10;stage_1W_vs_E10;stage_3W_vs_E10;stage	1	TRUE
ENSGALG00010009123	ENSGALG00010009123	PRJNA729271	-2.300326692	1.46575E-61	2.300326692	stage_E14_vs_E10	ordinary_muscle_development	D_vs_E10;stage_1W_vs_E10;stage_3W_vs_E10;stage	1	TRUE
ENSGALG00010028043	NT5DC2	PRJNA729271	1.931997952	1.63625E-61	1.931997952	stage_E14_vs_E10	ordinary_muscle_development	D_vs_E10;stage_1W_vs_E10;stage_3W_vs_E10;stage	1	TRUE
ENSGALG00010016335	ABHD4	PRJNA729271	3.057393029	1.67939E-61	3.057393029	stage_E14_vs_E10	ordinary_muscle_development	D_vs_E10;stage_1W_vs_E10;stage_3W_vs_E10;stage	1	TRUE
ENSGALG00010022320	COL9A3	PRJNA729271	-9.622822114	1.70002E-61	9.622822114	stage_E14_vs_E10	ordinary_muscle_development	D_vs_E10;stage_1W_vs_E10;stage_3W_vs_E10;stage	1	TRUE
ENSGALG00010029153	OGFOD3	PRJNA729271	-1.403930467	1.78905E-61	1.403930467	stage_E14_vs_E10	ordinary_muscle_development	D_vs_E10;stage_1W_vs_E10;stage_3W_vs_E10;stage	1	TRUE
ENSGALG00010022483	TOP2A	PRJNA729271	-4.012281687	1.89982E-61	4.012281687	stage_E14_vs_E10	ordinary_muscle_development	D_vs_E10;stage_1W_vs_E10;stage_3W_vs_E10;stage	1	TRUE
ENSGALG00010009051	TROJANZ	PRJNA729271	3.939811282	2.31653E-61	3.939811282	stage_E14_vs_E10	ordinary_muscle_development	D_vs_E10;stage_1W_vs_E10;stage_3W_vs_E10;stage	1	TRUE
ENSGALG00010021934	EFTUD2	PRJNA729271	-1.124485509	2.33984E-61	1.124485509	stage_E14_vs_E10	ordinary_muscle_development	D_vs_E10;stage_1W_vs_E10;stage_3W_vs_E10;stage	1	TRUE
ENSGALG00010015227	FYTTD1	PRJNA729271	-1.279910892	2.35284E-61	1.279910892	stage_E14_vs_E10	ordinary_muscle_development	D_vs_E10;stage_1W_vs_E10;stage_3W_vs_E10;stage	1	TRUE
ENSGALG00010024009	CSE1L	PRJNA729271	-1.034508377	3.23663E-61	1.034508377	stage_E14_vs_E10	ordinary_muscle_development	D_vs_E10;stage_1W_vs_E10;stage_3W_vs_E10;stage	1	TRUE
ENSGALG00010016882	ENSGALG00010016882	PRJNA729271	-0.837436514	3.38679E-61	0.837436514	stage_E14_vs_E10	ordinary_muscle_development		0	FALSE
ENSGALG00010025577	PTH2R	PRJNA729271	-3.417356769	4.05804E-61	3.417356769	stage_E14_vs_E10	ordinary_muscle_development	D_vs_E10;stage_1W_vs_E10;stage_3W_vs_E10;stage	1	TRUE
ENSGALG00010021342	SFR1	PRJNA729271	-3.252426163	4.19019E-61	3.252426163	stage_E14_vs_E10	ordinary_muscle_development	D_vs_E10;stage_1W_vs_E10;stage_3W_vs_E10;stage	1	TRUE
ENSGALG00010014897	COL8A2	PRJNA729271	-4.260902808	4.30506E-61	4.260902808	stage_E14_vs_E10	ordinary_muscle_development	D_vs_E10;stage_1W_vs_E10;stage_3W_vs_E10;stage	1	TRUE
ENSGALG00010015861	PPP2CA	PRJNA729271	-1.132714375	4.30881E-61	1.132714375	stage_E14_vs_E10	ordinary_muscle_development	D_vs_E10;stage_1W_vs_E10;stage_3W_vs_E10;stage	1	TRUE
ENSGALG00010023767	ENSGALG00010023767	PRJNA729271	3.476831952	4.36202E-61	3.476831952	stage_E14_vs_E10	ordinary_muscle_development	D_vs_E10;stage_1W_vs_E10;stage_3W_vs_E10;stage	1	TRUE
ENSGALG00010016823	HSF3	PRJNA729271	1.310124728	5.08576E-61	1.310124728	stage_E14_vs_E10	ordinary_muscle_development	D_vs_E10;stage_1W_vs_E10;stage_3W_vs_E10;stage	1	TRUE
ENSGALG00010018694	MGME1	PRJNA729271	-1.781388538	5.18136E-61	1.781388538	stage_E14_vs_E10	ordinary_muscle_development	D_vs_E10;stage_1W_vs_E10;stage_3W_vs_E10;stage	1	TRUE
ENSGALG00010012720	ACO2	PRJNA729271	1.996041078	7.41674E-61	1.996041078	stage_E14_vs_E10	ordinary_muscle_development	D_vs_E10;stage_1W_vs_E10;stage_3W_vs_E10;stage	1	TRUE
ENSGALG00010018829	PTPN4	PRJNA729271	2.293567418	8.01058E-61	2.293567418	stage_E14_vs_E10	ordinary_muscle_development	D_vs_E10;stage_1W_vs_E10;stage_3W_vs_E10;stage	1	TRUE
ENSGALG00010000046	TUBGCP4	PRJNA729271	-1.088953631	8.64158E-61	1.088953631	stage_E14_vs_E10	ordinary_muscle_development	D_vs_E10;stage_1W_vs_E10;stage_3W_vs_E10;stage	1	TRUE
ENSGALG00010029736	ENSGALG00010029736	PRJNA729271	2.210342547	8.68807E-61	2.210342547	stage_E14_vs_E10	ordinary_muscle_development	D_vs_E10;stage_1W_vs_E10;stage_3W_vs_E10;stage	1	TRUE
ENSGALG00010021192	ENSGALG00010021192	PRJNA729271	-3.049061117	9.41477E-61	3.049061117	stage_E14_vs_E10	ordinary_muscle_development	D_vs_E10;stage_1W_vs_E10;stage_3W_vs_E10;stage	1	TRUE
ENSGALG00010015848	UBE2B	PRJNA729271	2.672894263	1.11534E-60	2.672894263	stage_E14_vs_E10	ordinary_muscle_development	D_vs_E10;stage_1W_vs_E10;stage_3W_vs_E10;stage	1	TRUE
ENSGALG00010023223	CEP164	PRJNA729271	-1.721013803	1.15883E-60	1.721013803	stage_E14_vs_E10	ordinary_muscle_development	D_vs_E10;stage_1W_vs_E10;stage_3W_vs_E10;stage	1	TRUE
ENSGALG00010024673	REXO2	PRJNA729271	1.614487112	1.16346E-60	1.614487112	stage_E14_vs_E10	ordinary_muscle_development	D_vs_E10;stage_1W_vs_E10;stage_3W_vs_E10;stage	1	TRUE
ENSGALG00010023276	ABCC8	PRJNA729271	2.689882102	1.3581E-60	2.689882102	stage_E14_vs_E10	ordinary_muscle_development	D_vs_E10;stage_1W_vs_E10;stage_3W_vs_E10;stage	1	TRUE
ENSGALG00010027776	TKT	PRJNA729271	-2.165808162	1.38842E-60	2.165808162	stage_E14_vs_E10	ordinary_muscle_development	D_vs_E10;stage_1W_vs_E10;stage_3W_vs_E10;stage	1	TRUE
ENSGALG00010025613	CDKN1A	PRJNA729271	4.520551372	1.38887E-60	4.520551372	stage_E14_vs_E10	ordinary_muscle_development	D_vs_E10;stage_1W_vs_E10;stage_3W_vs_E10;stage	1	TRUE
ENSGALG00010021797	SLC25A34	PRJNA729271	2.664217074	1.39492E-60	2.664217074	stage_E14_vs_E10	ordinary_muscle_development	D_vs_E10;stage_1W_vs_E10;stage_3W_vs_E10;stage	1	TRUE
ENSGALG00010024334	USP28	PRJNA729271	2.038262286	1.5602E-60	2.038262286	stage_E14_vs_E10	ordinary_muscle_development	D_vs_E10;stage_1W_vs_E10;stage_3W_vs_E10;stage	1	TRUE
ENSGALG00010017721	VWA1	PRJNA729271	1.994268894	1.5602E-60	1.994268894	stage_E14_vs_E10	ordinary_muscle_development	D_vs_E10;stage_1W_vs_E10;stage_3W_vs_E10;stage	1	TRUE
ENSGALG00010011992	YEATS4	PRJNA729271	-1.463750413	2.05267E-60	1.463750413	stage_E14_vs_E10	ordinary_muscle_development	D_vs_E10;stage_1W_vs_E10;stage_3W_vs_E10;stage	1	TRUE
ENSGALG00010001570	SMPD1	PRJNA729271	2.680725063	2.06329E-60	2.680725063	stage_E14_vs_E10	ordinary_muscle_development	D_vs_E10;stage_1W_vs_E10;stage_3W_vs_E10;stage	1	TRUE
ENSGALG00010025233	WDR12	PRJNA729271	-1.295455088	2.33478E-60	1.295455088	stage_E14_vs_E10	ordinary_muscle_development	D_vs_E10;stage_1W_vs_E10;stage_3W_vs_E10;stage	1	TRUE
ENSGALG00010009327	NID1	PRJNA729271	2.703392142	2.79801E-60	2.703392142	stage_E14_vs_E10	ordinary_muscle_development	D_vs_E10;stage_1W_vs_E10;stage_3W_vs_E10;stage	1	TRUE
ENSGALG00010020100	MOCS1	PRJNA729271	2.595722868	3.22572E-60	2.595722868	stage_E14_vs_E10	ordinary_muscle_development	D_vs_E10;stage_1W_vs_E10;stage_3W_vs_E10;stage	1	TRUE
ENSGALG00010003454	BPNT2	PRJNA729271	-2.037468854	3.39168E-60	2.037468854	stage_E14_vs_E10	ordinary_muscle_development	D_vs_E10;stage_1W_vs_E10;stage_3W_vs_E10;stage	1	TRUE
ENSGALG00010027368	EMC3	PRJNA729271	1.450582268	3.63235E-60	1.450582268	stage_E14_vs_E10	ordinary_muscle_development	D_vs_E10;stage_1W_vs_E10;stage_3W_vs_E10;stage	1	TRUE
ENSGALG00010029743	MYOCD	PRJNA729271	4.187369064	4.70369E-60	4.187369064	stage_E14_vs_E10	ordinary_muscle_development	D_vs_E10;stage_1W_vs_E10;stage_3W_vs_E10;stage	1	TRUE
ENSGALG00010010505	CD38	PRJNA729271	3.182374581	6.00743E-60	3.182374581	stage_E14_vs_E10	ordinary_muscle_development	D_vs_E10;stage_1W_vs_E10;stage_3W_vs_E10;stage	1	TRUE
ENSGALG00010022433	LNPK	PRJNA729271	1.760481505	6.08565E-60	1.760481505	stage_E14_vs_E10	ordinary_muscle_development	D_vs_E10;stage_1W_vs_E10;stage_3W_vs_E10;stage	1	TRUE
ENSGALG00010028178	ENSGALG00010028178	PRJNA729271	-2.790472924	6.53302E-60	2.790472924	stage_E14_vs_E10	ordinary_muscle_development	D_vs_E10;stage_1W_vs_E10;stage_3W_vs_E10;stage	1	TRUE
ENSGALG00010020945	NUMB	PRJNA729271	1.401977218	6.61576E-60	1.401977218	stage_E14_vs_E10	ordinary_muscle_development	D_vs_E10;stage_1W_vs_E10;stage_3W_vs_E10;stage	1	TRUE
ENSGALG00010001827	PDZRN4	PRJNA729271	-3.473417715	6.91628E-60	3.473417715	stage_E14_vs_E10	ordinary_muscle_development	D_vs_E10;stage_1W_vs_E10;stage_3W_vs_E10;stage	1	TRUE
ENSGALG00010016270	RFC4	PRJNA729271	-1.874825236	7.17559E-60	1.874825236	stage_E14_vs_E10	ordinary_muscle_development	D_vs_E10;stage_1W_vs_E10;stage_3W_vs_E10;stage	1	TRUE
ENSGALG00010002356	WNT11	PRJNA729271	-5.601709345	7.66816E-60	5.601709345	stage_E14_vs_E10	ordinary_muscle_development	D_vs_E10;stage_1W_vs_E10;stage_3W_vs_E10;stage	1	TRUE
ENSGALG00010003090	DMA	PRJNA729271	3.040901974	7.77335E-60	3.040901974	stage_E14_vs_E10	ordinary_muscle_development	D_vs_E10;stage_1W_vs_E10;stage_3W_vs_E10;stage	1	TRUE
ENSGALG00010006128	ACYP1	PRJNA729271	1.936517483	8.36027E-60	1.936517483	stage_E14_vs_E10	ordinary_muscle_development	D_vs_E10;stage_1W_vs_E10;stage_3W_vs_E10;stage	1	TRUE
ENSGALG00010021485	SMOC1	PRJNA729271	-3.87139658	8.58105E-60	3.87139658	stage_E14_vs_E10	ordinary_muscle_development	D_vs_E10;stage_1W_vs_E10;stage_3W_vs_E10;stage	1	TRUE
ENSGALG00010004158	JCHAIN	PRJNA729271	7.726233516	8.68842E-60	7.726233516	stage_E14_vs_E10	ordinary_muscle_development	D_vs_E10;stage_1W_vs_E10;stage_3W_vs_E10;stage	1	TRUE
ENSGALG00010002269	MCPH1	PRJNA729271	-2.59452215	8.7277E-60	2.59452215	stage_E14_vs_E10	ordinary_muscle_development	D_vs_E10;stage_1W_vs_E10;stage_3W_vs_E10;stage	1	TRUE
ENSGALG00010025705	ACSL3	PRJNA729271	-1.41854095	8.86205E-60	1.41854095	stage_E14_vs_E10	ordinary_muscle_development	D_vs_E10;stage_1W_vs_E10;stage_3W_vs_E10;stage	1	TRUE
ENSGALG00010027321	IFI30	PRJNA729271	3.343472011	9.09432E-60	3.343472011	stage_E14_vs_E10	ordinary_muscle_development	D_vs_E10;stage_1W_vs_E10;stage_3W_vs_E10;stage	1	TRUE
ENSGALG00010002868	PPP1R9A	PRJNA729271	1.097866939	9.61817E-60	1.097866939	stage_E14_vs_E10	ordinary_muscle_development	D_vs_E10;stage_1W_vs_E10;stage_3W_vs_E10;stage	1	TRUE
ENSGALG00010023526	KNSTRN	PRJNA729271	-3.210117168	9.64715E-60	3.210117168	stage_E14_vs_E10	ordinary_muscle_development	D_vs_E10;stage_1W_vs_E10;stage_3W_vs_E10;stage	1	TRUE
ENSGALG00010013249	AURKA	PRJNA729271	-3.859539384	1.01676E-59	3.859539384	stage_E14_vs_E10	ordinary_muscle_development	D_vs_E10;stage_1W_vs_E10;stage_3W_vs_E10;stage	1	TRUE
ENSGALG00010012976	SLC9A2	PRJNA729271	3.737245985	1.02189E-59	3.737245985	stage_E14_vs_E10	ordinary_muscle_development	D_vs_E10;stage_1W_vs_E10;stage_3W_vs_E10;stage	1	TRUE
ENSGALG00010028194	ENSGALG00010028194	PRJNA729271	2.987937982	1.03411E-59	2.987937982	stage_E14_vs_E10	ordinary_muscle_development	D_vs_E10;stage_1W_vs_E10;stage_3W_vs_E10;stage	1	TRUE
ENSGALG00010018517	ECT2	PRJNA729271	-3.381847099	1.05565E-59	3.381847099	stage_E14_vs_E10	ordinary_muscle_development	D_vs_E10;stage_1W_vs_E10;stage_3W_vs_E10;stage	1	TRUE
ENSGALG00010017494	TBC1D24	PRJNA729271	-1.610676126	1.06978E-59	1.610676126	stage_E14_vs_E10	ordinary_muscle_development	D_vs_E10;stage_1W_vs_E10;stage_3W_vs_E10;stage	1	TRUE
ENSGALG00010017104	FANCL	PRJNA729271	-3.021033434	1.17262E-59	3.021033434	stage_E14_vs_E10	ordinary_muscle_development	D_vs_E10;stage_1W_vs_E10;stage_3W_vs_E10;stage	1	TRUE
ENSGALG00010000591	SBK2	PRJNA729271	4.993134637	1.23776E-59	4.993134637	stage_E14_vs_E10	ordinary_muscle_development	D_vs_E10;stage_1W_vs_E10;stage_3W_vs_E10;stage	1	TRUE
ENSGALG00010015521	DKC1	PRJNA729271	-1.362108066	1.33517E-59	1.362108066	stage_E14_vs_E10	ordinary_muscle_development	D_vs_E10;stage_1W_vs_E10;stage_3W_vs_E10;stage	1	TRUE
ENSGALG00010021742	MATN4	PRJNA729271	-8.816621946	1.45279E-59	8.816621946	stage_E14_vs_E10	ordinary_muscle_development	D_vs_E10;stage_1W_vs_E10;stage_3W_vs_E10;stage	1	TRUE
ENSGALG00010028039	STAB1	PRJNA729271	2.668243431	1.48593E-59	2.668243431	stage_E14_vs_E10	ordinary_muscle_development	D_vs_E10;stage_1W_vs_E10;stage_3W_vs_E10;stage	1	TRUE
ENSGALG00010017534	ATP11C	PRJNA729271	-1.403069338	1.53153E-59	1.403069338	stage_E14_vs_E10	ordinary_muscle_development	D_vs_E10;stage_1W_vs_E10;stage_3W_vs_E10;stage	1	TRUE
ENSGALG00010001604	ENSGALG00010001604	PRJNA729271	6.885470948	1.68761E-59	6.885470948	stage_E14_vs_E10	ordinary_muscle_development	D_vs_E10;stage_1W_vs_E10;stage_3W_vs_E10;stage	1	TRUE
ENSGALG00010003806	EGF	PRJNA729271	5.185498785	1.72446E-59	5.185498785	stage_E14_vs_E10	ordinary_muscle_development	D_vs_E10;stage_1W_vs_E10;stage_3W_vs_E10;stage	1	TRUE
ENSGALG00010021245	ZFAND3	PRJNA729271	1.153629433	1.83526E-59	1.153629433	stage_E14_vs_E10	ordinary_muscle_development	D_vs_E10;stage_1W_vs_E10;stage_3W_vs_E10;stage	1	TRUE
ENSGALG00010017476	ATP6V0C	PRJNA729271	1.836651569	1.85251E-59	1.836651569	stage_E14_vs_E10	ordinary_muscle_development	D_vs_E10;stage_1W_vs_E10;stage_3W_vs_E10;stage	1	TRUE
ENSGALG00010018983	MDGA2	PRJNA729271	-4.379158969	1.93756E-59	4.379158969	stage_E14_vs_E10	ordinary_muscle_development	D_vs_E10;stage_1W_vs_E10;stage_3W_vs_E10;stage	1	TRUE
ENSGALG00010018045	PSEN2	PRJNA729271	1.201636534	1.97279E-59	1.201636534	stage_E14_vs_E10	ordinary_muscle_development	D_vs_E10;stage_1W_vs_E10;stage_3W_vs_E10;stage	1	TRUE
ENSGALG00010006730	HAPLN1	PRJNA729271	-8.867575702	2.16724E-59	8.867575702	stage_E14_vs_E10	ordinary_muscle_development	D_vs_E10;stage_1W_vs_E10;stage_3W_vs_E10;stage	1	TRUE
ENSGALG00010026354	ENSGALG00010026354	PRJNA729271	3.973144514	2.26226E-59	3.973144514	stage_E14_vs_E10	ordinary_muscle_development	D_vs_E10;stage_1W_vs_E10;stage_3W_vs_E10;stage	1	TRUE
ENSGALG00010020364	MRPL16	PRJNA729271	1.800502144	2.92492E-59	1.800502144	stage_E14_vs_E10	ordinary_muscle_development	D_vs_E10;stage_1W_vs_E10;stage_3W_vs_E10;stage	1	TRUE
ENSGALG00010020546	LRRC20	PRJNA729271	3.866573131	3.1076E-59	3.866573131	stage_E14_vs_E10	ordinary_muscle_development	D_vs_E10;stage_1W_vs_E10;stage_3W_vs_E10;stage	1	TRUE
ENSGALG00010017060	CLSTN1	PRJNA729271	-1.682075744	3.27611E-59	1.682075744	stage_E14_vs_E10	ordinary_muscle_development	D_vs_E10;stage_1W_vs_E10;stage_3W_vs_E10;stage	1	TRUE
ENSGALG00010002832	LPIN1	PRJNA729271	3.292738569	3.34864E-59	3.292738569	stage_E14_vs_E10	ordinary_muscle_development	D_vs_E10;stage_1W_vs_E10;stage_3W_vs_E10;stage	1	TRUE
ENSGALG00010001482	TRA2A	PRJNA729271	-1.086170037	4.355E-59	1.086170037	stage_E14_vs_E10	ordinary_muscle_development	D_vs_E10;stage_1W_vs_E10;stage_3W_vs_E10;stage	1	TRUE
ENSGALG00010024786	PDCD5	PRJNA729271	1.58313695	4.48588E-59	1.58313695	stage_E14_vs_E10	ordinary_muscle_development	D_vs_E10;stage_1W_vs_E10;stage_3W_vs_E10;stage	1	TRUE
ENSGALG00010017115	LGALSL	PRJNA729271	3.135589882	4.62986E-59	3.135589882	stage_E14_vs_E10	ordinary_muscle_development	D_vs_E10;stage_1W_vs_E10;stage_3W_vs_E10;stage	1	TRUE
ENSGALG00010001838	PPHLN1	PRJNA729271	-1.110804367	4.98131E-59	1.110804367	stage_E14_vs_E10	ordinary_muscle_development	D_vs_E10;stage_1W_vs_E10;stage_3W_vs_E10;stage	1	TRUE
ENSGALG00010012077	IFNGR2	PRJNA729271	1.111944678	5.08705E-59	1.111944678	stage_E14_vs_E10	ordinary_muscle_development	D_vs_E10;stage_1W_vs_E10;stage_3W_vs_E10;stage	1	TRUE
ENSGALG00010028141	CISH	PRJNA729271	3.046288464	5.25301E-59	3.046288464	stage_E14_vs_E10	ordinary_muscle_development	D_vs_E10;stage_1W_vs_E10;stage_3W_vs_E10;stage	1	TRUE
ENSGALG00010021473	SLC8A3	PRJNA729271	2.147177672	5.3863E-59	2.147177672	stage_E14_vs_E10	ordinary_muscle_development	D_vs_E10;stage_1W_vs_E10;stage_3W_vs_E10;stage	1	TRUE
ENSGALG00010028473	CDC34	PRJNA729271	2.244114275	5.39007E-59	2.244114275	stage_E14_vs_E10	ordinary_muscle_development	D_vs_E10;stage_1W_vs_E10;stage_3W_vs_E10;stage	1	TRUE
ENSGALG00010001378	POLA2	PRJNA729271	-2.284577518	6.08728E-59	2.284577518	stage_E14_vs_E10	ordinary_muscle_development	D_vs_E10;stage_1W_vs_E10;stage_3W_vs_E10;stage	1	TRUE
ENSGALG00010005401	BCLAF1	PRJNA729271	-1.431621974	6.26858E-59	1.431621974	stage_E14_vs_E10	ordinary_muscle_development	D_vs_E10;stage_1W_vs_E10;stage_3W_vs_E10;stage	1	TRUE
ENSGALG00010017729	MIEF2	PRJNA729271	2.276676207	6.89187E-59	2.276676207	stage_E14_vs_E10	ordinary_muscle_development	D_vs_E10;stage_1W_vs_E10;stage_3W_vs_E10;stage	1	TRUE
ENSGALG00010015742	HPRT1	PRJNA729271	1.398581537	7.20333E-59	1.398581537	stage_E14_vs_E10	ordinary_muscle_development	D_vs_E10;stage_1W_vs_E10;stage_3W_vs_E10;stage	1	TRUE
ENSGALG00010029257	YPEL2	PRJNA729271	2.627890578	7.24231E-59	2.627890578	stage_E14_vs_E10	ordinary_muscle_development	D_vs_E10;stage_1W_vs_E10;stage_3W_vs_E10;stage	1	TRUE
ENSGALG00010025837	ENSGALG00010025837	PRJNA729271	2.866592025	7.3862E-59	2.866592025	stage_E14_vs_E10	ordinary_muscle_development	D_vs_E10;stage_1W_vs_E10;stage_3W_vs_E10;stage	1	TRUE
ENSGALG00010023750	ENSGALG00010023750	PRJNA729271	-2.794580451	8.79235E-59	2.794580451	stage_E14_vs_E10	ordinary_muscle_development	D_vs_E10;stage_1W_vs_E10;stage_3W_vs_E10;stage	1	TRUE
ENSGALG00010029618	TEX2	PRJNA729271	1.83546248	8.84877E-59	1.83546248	stage_E14_vs_E10	ordinary_muscle_development	D_vs_E10;stage_1W_vs_E10;stage_3W_vs_E10;stage	1	TRUE

ENSGALG00010029516	ENSGALG00010029516	PRJNA729271	3.699595981	1.00889E-58	3.699595981	stage_E14_vs_E10	ordinary_muscle_development	D_vs_E10;stage_1W_vs_E10;stage_3W_vs_E10;stage	1	TRUE
ENSGALG00010026402	TFEB	PRJNA729271	2.67964236	1.05209E-58	2.67964236	stage_E14_vs_E10	ordinary_muscle_development	D_vs_E10;stage_1W_vs_E10;stage_3W_vs_E10;stage	1	TRUE
ENSGALG00010010959	TMEM263	PRJNA729271	-1.47625241	1.14544E-58	1.47625241	stage_E14_vs_E10	ordinary_muscle_development	D_vs_E10;stage_1W_vs_E10;stage_3W_vs_E10;stage	1	TRUE
ENSGALG00010024737	STK38	PRJNA729271	-1.035729849	1.37455E-58	1.035729849	stage_E14_vs_E10	ordinary_muscle_development	D_vs_E10;stage_1W_vs_E10;stage_3W_vs_E10;stage	1	TRUE
ENSGALG00010024282	HIC2	PRJNA729271	-2.488897407	1.45769E-58	2.488897407	stage_E14_vs_E10	ordinary_muscle_development	D_vs_E10;stage_1W_vs_E10;stage_3W_vs_E10;stage	1	TRUE
ENSGALG00010029594	CRLF3	PRJNA729271	-1.129848492	1.67703E-58	1.129848492	stage_E14_vs_E10	ordinary_muscle_development	D_vs_E10;stage_1W_vs_E10;stage_3W_vs_E10;stage	1	TRUE
ENSGALG00010013861	TCF15	PRJNA729271	2.530381045	1.68342E-58	2.530381045	stage_E14_vs_E10	ordinary_muscle_development	D_vs_E10;stage_1W_vs_E10;stage_3W_vs_E10;stage	1	TRUE
ENSGALG00010009016	DNAJC9	PRJNA729271	-1.858528843	1.77957E-58	1.858528843	stage_E14_vs_E10	ordinary_muscle_development	D_vs_E10;stage_1W_vs_E10;stage_3W_vs_E10;stage	1	TRUE
ENSGALG00010000595	MYO10	PRJNA729271	-2.298023179	1.80467E-58	2.298023179	stage_E14_vs_E10	ordinary_muscle_development	D_vs_E10;stage_1W_vs_E10;stage_3W_vs_E10;stage	1	TRUE
ENSGALG00010006254	PROS1	PRJNA729271	2.239587783	1.89476E-58	2.239587783	stage_E14_vs_E10	ordinary_muscle_development	D_vs_E10;stage_1W_vs_E10;stage_3W_vs_E10;stage	1	TRUE
ENSGALG00010000301	UQCR10	PRJNA729271	2.516286587	1.98712E-58	2.516286587	stage_E14_vs_E10	ordinary_muscle_development	D_vs_E10;stage_1W_vs_E10;stage_3W_vs_E10;stage	1	TRUE
ENSGALG00010012210	PPWD1	PRJNA729271	-1.259577093	2.00598E-58	1.259577093	stage_E14_vs_E10	ordinary_muscle_development	D_vs_E10;stage_1W_vs_E10;stage_3W_vs_E10;stage	1	TRUE
ENSGALG00010001457	NDC80	PRJNA729271	-3.75906419	2.02198E-58	3.75906419	stage_E14_vs_E10	ordinary_muscle_development	D_vs_E10;stage_1W_vs_E10;stage_3W_vs_E10;stage	1	TRUE
ENSGALG00010014032	PSD3	PRJNA729271	-1.291554902	2.43595E-58	1.291554902	stage_E14_vs_E10	ordinary_muscle_development	D_vs_E10;stage_1W_vs_E10;stage_3W_vs_E10;stage	1	TRUE
ENSGALG00010020906	SIRT3	PRJNA729271	1.766476973	2.46753E-58	1.766476973	stage_E14_vs_E10	ordinary_muscle_development	D_vs_E10;stage_1W_vs_E10;stage_3W_vs_E10;stage	1	TRUE
ENSGALG00010011361	NUP37	PRJNA729271	-1.500917052	2.63474E-58	1.500917052	stage_E14_vs_E10	ordinary_muscle_development	D_vs_E10;stage_1W_vs_E10;stage_3W_vs_E10;stage	1	TRUE
ENSGALG00010017223	NOC2L	PRJNA729271	-1.27324239	2.74078E-58	1.27324239	stage_E14_vs_E10	ordinary_muscle_development	D_vs_E10;stage_1W_vs_E10;stage_3W_vs_E10;stage	1	TRUE
ENSGALG00010029166	ZZEF1	PRJNA729271	2.00504157	3.34648E-58	2.00504157	stage_E14_vs_E10	ordinary_muscle_development	D_vs_E10;stage_1W_vs_E10;stage_3W_vs_E10;stage	1	TRUE
ENSGALG00010011896	SIK1	PRJNA729271	4.843349849	3.39019E-58	4.843349849	stage_E14_vs_E10	ordinary_muscle_development	D_vs_E10;stage_1W_vs_E10;stage_3W_vs_E10;stage	1	TRUE
ENSGALG00010004056	SHROOM3	PRJNA729271	-3.07826329	3.83204E-58	3.07826329	stage_E14_vs_E10	ordinary_muscle_development	D_vs_E10;stage_1W_vs_E10;stage_3W_vs_E10;stage	1	TRUE
ENSGALG00010018487	ENSGALG00010018487	PRJNA729271	-2.224488537	4.35853E-58	2.224488537	stage_E14_vs_E10	ordinary_muscle_development	D_vs_E10;stage_1W_vs_E10;stage_3W_vs_E10;stage	1	TRUE
ENSGALG00010020733	TMEM229B	PRJNA729271	-1.854951776	4.48972E-58	1.854951776	stage_E14_vs_E10	ordinary_muscle_development	D_vs_E10;stage_1W_vs_E10;stage_3W_vs_E10;stage	1	TRUE
ENSGALG00010007766	ENSGALG00010007766	PRJNA729271	2.048976081	4.75837E-58	2.048976081	stage_E14_vs_E10	ordinary_muscle_development	D_vs_E10;stage_1W_vs_E10;stage_3W_vs_E10;stage	1	TRUE
ENSGALG00010013517	STMN1	PRJNA729271	-3.537678066	5.23086E-58	3.537678066	stage_E14_vs_E10	ordinary_muscle_development	D_vs_E10;stage_1W_vs_E10;stage_3W_vs_E10;stage	1	TRUE
ENSGALG00010009578	KIF5C	PRJNA729271	-2.635484336	5.46342E-58	2.635484336	stage_E14_vs_E10	ordinary_muscle_development	D_vs_E10;stage_1W_vs_E10;stage_3W_vs_E10;stage	1	TRUE
ENSGALG00010023263	SRP14	PRJNA729271	-1.185771797	5.88239E-58	1.185771797	stage_E14_vs_E10	ordinary_muscle_development	D_vs_E10;stage_1W_vs_E10;stage_3W_vs_E10;stage	1	TRUE
ENSGALG00010013442	ENSGALG00010013442	PRJNA729271	1.679278188	6.11451E-58	1.679278188	stage_E14_vs_E10	ordinary_muscle_development	D_vs_E10;stage_1W_vs_E10;stage_3W_vs_E10;stage	1	TRUE
ENSGALG00010010740	CCDC134	PRJNA729271	1.534442153	6.8234E-58	1.534442153	stage_E14_vs_E10	ordinary_muscle_development	D_vs_E10;stage_1W_vs_E10;stage_3W_vs_E10;stage	1	TRUE
ENSGALG00010020437	SNX4	PRJNA729271	-1.389143175	6.8234E-58	1.389143175	stage_E14_vs_E10	ordinary_muscle_development	D_vs_E10;stage_1W_vs_E10;stage_3W_vs_E10;stage	1	TRUE
ENSGALG00010009593	NCBP1	PRJNA729271	-1.033475469	6.88457E-58	1.033475469	stage_E14_vs_E10	ordinary_muscle_development	D_vs_E10;stage_1W_vs_E10;stage_3W_vs_E10;stage	1	TRUE
ENSGALG00010023439	ACSS2	PRJNA729271	2.138108923	7.07053E-58	2.138108923	stage_E14_vs_E10	ordinary_muscle_development	D_vs_E10;stage_1W_vs_E10;stage_3W_vs_E10;stage	1	TRUE
ENSGALG00010003072	ELF1	PRJNA729271	1.193343017	7.7472E-58	1.193343017	stage_E14_vs_E10	ordinary_muscle_development	D_vs_E10;stage_1W_vs_E10;stage_3W_vs_E10;stage	1	TRUE
ENSGALG00010027953	SLC31A1	PRJNA729271	-1.988378292	8.22086E-58	1.988378292	stage_E14_vs_E10	ordinary_muscle_development	D_vs_E10;stage_1W_vs_E10;stage_3W_vs_E10;stage	1	TRUE
ENSGALG00010011143	AK3	PRJNA729271	2.634690054	8.53548E-58	2.634690054	stage_E14_vs_E10	ordinary_muscle_development	D_vs_E10;stage_1W_vs_E10;stage_3W_vs_E10;stage	1	TRUE
ENSGALG00010029895	FASN	PRJNA729271	-2.738436926	8.72316E-58	2.738436926	stage_E14_vs_E10	ordinary_muscle_development	D_vs_E10;stage_1W_vs_E10;stage_3W_vs_E10;stage	1	TRUE
ENSGALG00010005935	ATP8A2	PRJNA729271	-3.899231764	9.07992E-58	3.899231764	stage_E14_vs_E10	ordinary_muscle_development	D_vs_E10;stage_1W_vs_E10;stage_3W_vs_E10;stage	1	TRUE
ENSGALG00010008117	ENSGALG00010008117	PRJNA729271	2.804265258	1.02567E-57	2.804265258	stage_E14_vs_E10	ordinary_muscle_development	D_vs_E10;stage_1W_vs_E10;stage_3W_vs_E10;stage	1	TRUE
ENSGALG00010003375	YWHAQ	PRJNA729271	-1.396379194	1.05446E-57	1.396379194	stage_E14_vs_E10	ordinary_muscle_development	D_vs_E10;stage_1W_vs_E10;stage_3W_vs_E10;stage	1	TRUE
ENSGALG00010023798	ARCN1	PRJNA729271	-0.927283307	1.06048E-57	0.927283307	stage_E14_vs_E10	ordinary_muscle_development		0	FALSE
ENSGALG00010011751	ZNF706	PRJNA729271	1.768020666	1.06379E-57	1.768020666	stage_E14_vs_E10	ordinary_muscle_development	D_vs_E10;stage_1W_vs_E10;stage_3W_vs_E10;stage	1	TRUE
ENSGALG00010016377	FRMPD3	PRJNA729271	4.495594291	1.13836E-57	4.495594291	stage_E14_vs_E10	ordinary_muscle_development	D_vs_E10;stage_1W_vs_E10;stage_3W_vs_E10;stage	1	TRUE
ENSGALG00010029711	CASKIN2	PRJNA729271	1.78712436	1.23915E-57	1.78712436	stage_E14_vs_E10	ordinary_muscle_development	D_vs_E10;stage_1W_vs_E10;stage_3W_vs_E10;stage	1	TRUE
ENSGALG00010029425	SPNS2	PRJNA729271	2.631763704	1.30191E-57	2.631763704	stage_E14_vs_E10	ordinary_muscle_development	D_vs_E10;stage_1W_vs_E10;stage_3W_vs_E10;stage	1	TRUE
ENSGALG00010027909	TIMP4	PRJNA729271	-4.448066476	1.34704E-57	4.448066476	stage_E14_vs_E10	ordinary_muscle_development	D_vs_E10;stage_1W_vs_E10;stage_3W_vs_E10;stage	1	TRUE
ENSGALG00010004760	THBS2	PRJNA729271	-3.906674501	1.51158E-57	3.906674501	stage_E14_vs_E10	ordinary_muscle_development	D_vs_E10;stage_1W_vs_E10;stage_3W_vs_E10;stage	1	TRUE
ENSGALG00010018848	MEF2A	PRJNA729271	1.856675191	1.56791E-57	1.856675191	stage_E14_vs_E10	ordinary_muscle_development	D_vs_E10;stage_1W_vs_E10;stage_3W_vs_E10;stage	1	TRUE
ENSGALG00010022275	VDAC2	PRJNA729271	2.002539107	1.71682E-57	2.002539107	stage_E14_vs_E10	ordinary_muscle_development	D_vs_E10;stage_1W_vs_E10;stage_3W_vs_E10;stage	1	TRUE
ENSGALG00010011386	CTSB	PRJNA729271	2.035438437	1.74535E-57	2.035438437	stage_E14_vs_E10	ordinary_muscle_development	D_vs_E10;stage_1W_vs_E10;stage_3W_vs_E10;stage	1	TRUE
ENSGALG00010023836	NDUFS3	PRJNA729271	2.377494498	1.817E-57	2.377494498	stage_E14_vs_E10	ordinary_muscle_development	D_vs_E10;stage_1W_vs_E10;stage_3W_vs_E10;stage	1	TRUE
ENSGALG00010014710	NPEPL1	PRJNA729271	1.603569345	1.8662E-57	1.603569345	stage_E14_vs_E10	ordinary_muscle_development	D_vs_E10;stage_1W_vs_E10;stage_3W_vs_E10;stage	1	TRUE
ENSGALG00010005524	STRIP2	PRJNA729271	4.411535066	1.88613E-57	4.411535066	stage_E14_vs_E10	ordinary_muscle_development	D_vs_E10;stage_1W_vs_E10;stage_3W_vs_E10;stage	1	TRUE
ENSGALG00010011013	ENSGALG00010011013	PRJNA729271	5.282969933	1.88935E-57	5.282969933	stage_E14_vs_E10	ordinary_muscle_development	D_vs_E10;stage_1W_vs_E10;stage_3W_vs_E10;stage	1	TRUE
ENSGALG00010018774	MSRB1	PRJNA729271	1.969052973	1.93228E-57	1.969052973	stage_E14_vs_E10	ordinary_muscle_development	D_vs_E10;stage_1W_vs_E10;stage_3W_vs_E10;stage	1	TRUE
ENSGALG00010004225	CETN4	PRJNA729271	-2.130461542	2.54098E-57	2.130461542	stage_E14_vs_E10	ordinary_muscle_development	D_vs_E10;stage_1W_vs_E10;stage_3W_vs_E10;stage	1	TRUE
ENSGALG00010017430	CACNA1H	PRJNA729271	-3.771406837	2.59775E-57	3.771406837	stage_E14_vs_E10	ordinary_muscle_development	D_vs_E10;stage_1W_vs_E10;stage_3W_vs_E10;stage	1	TRUE
ENSGALG00010013281	BIRC8	PRJNA729271	2.231759962	2.84102E-57	2.231759962	stage_E14_vs_E10	ordinary_muscle_development	D_vs_E10;stage_1W_vs_E10;stage_3W_vs_E10;stage	1	TRUE
ENSGALG00010015186	ENSGALG00010015186	PRJNA729271	4.519457405	2.84756E-57	4.519457405	stage_E14_vs_E10	ordinary_muscle_development	D_vs_E10;stage_1W_vs_E10;stage_3W_vs_E10;stage	1	TRUE
ENSGALG00010028626	CRAT	PRJNA729271	2.791467134	2.86797E-57	2.791467134	stage_E14_vs_E10	ordinary_muscle_development	D_vs_E10;stage_1W_vs_E10;stage_3W_vs_E10;stage	1	TRUE
ENSGALG00010010310	NT5DC3	PRJNA729271	1.897898458	2.86797E-57	1.897898458	stage_E14_vs_E10	ordinary_muscle_development	D_vs_E10;stage_1W_vs_E10;stage_3W_vs_E10;stage	1	TRUE
ENSGALG00010025289	KDM2B	PRJNA729271	-1.213893478	2.96419E-57	1.213893478	stage_E14_vs_E10	ordinary_muscle_development	D_vs_E10;stage_1W_vs_E10;stage_3W_vs_E10;stage	1	TRUE
ENSGALG00010001918	CNKSR2	PRJNA729271	2.287396243	3.47136E-57	2.287396243	stage_E14_vs_E10	ordinary_muscle_development	D_vs_E10;stage_1W_vs_E10;stage_3W_vs_E10;stage	1	TRUE
ENSGALG00010003558	TPT1	PRJNA729271	2.970536157	4.21461E-57	2.970536157	stage_E14_vs_E10	ordinary_muscle_development	D_vs_E10;stage_1W_vs_E10;stage_3W_vs_E10;stage	1	TRUE
ENSGALG00010008623	TRPS1	PRJNA729271	-2.14064191	4.33813E-57	2.14064191	stage_E14_vs_E10	ordinary_muscle_development	D_vs_E10;stage_1W_vs_E10;stage_3W_vs_E10;stage	1	TRUE
ENSGALG00010020017	ENSGALG00010020017	PRJNA729271	4.55848183	4.44417E-57	4.55848183	stage_E14_vs_E10	ordinary_muscle_development	D_vs_E10;stage_1W_vs_E10;stage_3W_vs_E10;stage	1	TRUE
ENSGALG00010004283	DCK	PRJNA729271	-2.13580245	4.44417E-57	2.13580245	stage_E14_vs_E10	ordinary_muscle_development	D_vs_E10;stage_1W_vs_E10;stage_3W_vs_E10;stage	1	TRUE
ENSGALG00010004837	CENPC	PRJNA729271	-2.299252855	4.47787E-57	2.299252855	stage_E14_vs_E10	ordinary_muscle_development	D_vs_E10;stage_1W_vs_E10;stage_3W_vs_E10;stage	1	TRUE
ENSGALG00010001152	FEM1C	PRJNA729271	3.178277565	5.46025E-57	3.178277565	stage_E14_vs_E10	ordinary_muscle_development	D_vs_E10;stage_1W_vs_E10;stage_3W_vs_E10;stage	1	TRUE
ENSGALG00010028099	PCASP2	PRJNA729271	3.878828075	5.58275E-57	3.878828075	stage_E14_vs_E10	ordinary_muscle_development	D_vs_E10;stage_1W_vs_E10;stage_3W_vs_E10;stage	1	TRUE
ENSGALG00010012285	FBLN1	PRJNA729271	-3.21609146	5.7503E-57	3.21609146	stage_E14_vs_E10	ordinary_muscle_development	D_vs_E10;stage_1W_vs_E10;stage_3W_vs_E10;stage	1	TRUE
ENSGALG00010023956	KREMEN1	PRJNA729271	-2.149290694	5.8315E-57	2.149290694	stage_E14_vs_E10	ordinary_muscle_development	D_vs_E10;stage_1W_vs_E10;stage_3W_vs_E10;stage	1	TRUE
ENSGALG00010000816	TBC1D4	PRJNA729271	1.666555002	6.10561E-57	1.666555002	stage_E14_vs_E10	ordinary_muscle_development	D_vs_E10;stage_1W_vs_E10;stage_3W_vs_E10;stage	1	TRUE
ENSGALG00010023052	RAD54L	PRJNA729271	-2.257639387	6.27674E-57	2.257639387	stage_E14_vs_E10	ordinary_muscle_development	D_vs_E10;stage_1W_vs_E10;stage_3W_vs_E10;stage	1	TRUE
ENSGALG00010000599	RETREG1	PRJNA729271	6.016432519	6.56404E-57	6.016432519	stage_E14_vs_E10	ordinary_muscle_development	D_vs_E10;stage_1W_vs_E10;stage_3W_vs_E10;stage	1	TRUE
ENSGALG00010019891	PIGH	PRJNA729271	-1.77945547	6.86052E-57	1.77945547	stage_E14_vs_E10	ordinary_muscle_development	D_vs_E10;stage_1W_vs_E10;stage_3W_vs_E10;stage	1	TRUE
ENSGALG00010027583	GLE1	PRJNA729271	-1.20044139	6.88626E-57	1.20044139	stage_E14_vs_E10	ordinary_muscle_development	D_vs_E10;stage_1W_vs_E10;stage_3W_vs_E10;stage	1	TRUE
ENSGALG00010000039	PDIA3	PRJNA729271	-1.060355051	7.24669E-57	1.060355051	stage_E14_vs_E10	ordinary_muscle_development	D_vs_E10;stage_1W_vs_E10;stage_3W_vs_E10;stage	1	TRUE
ENSGALG00010017499	SNRPD1	PRJNA729271	-1.598877495	8.32426E-57	1.598877495	stage_E14_vs_E10	ordinary_muscle_development	D_vs_E10;stage_1W_vs_E10;stage_3W_vs_E10;stage	1	TRUE
ENSGALG00010029247	CCDC92B	PRJNA729271	8.420950373	8.61626E-57	8.420950373	stage_E14_vs_E10	ordinary_muscle_development	D_vs_E10;stage_1W_vs_E10;stage_3W_vs_E10;stage	1	TRUE
ENSGALG00010022813	USP24	PRJNA729271	1.955696322	8.63754E-57	1.955696322	stage_E14_vs_E10	ordinary_muscle_development	D_vs_E10;stage_1W_vs_E10;stage_3W_vs_E10;stage	1	TRUE
ENSGALG00010000859	BORA	PRJNA729271	-3.221344036	9.95351E-57	3.221344036	stage_E14_vs_E10	ordinary_muscle_development	D_vs_E10;stage_1W_vs_E10;stage_3W_vs_E10;stage	1	TRUE
ENSGALG00010019641	CAPN11	PRJNA729271	1.309305106	1.00162E-56	1.309305106	stage_E14_vs_E10	ordinary_muscle_development	D_vs_E10;stage_1W_vs_E10;stage_3W_vs_E10;stage	1	TRUE
ENSGALG00010018035	ITM2A	PRJNA729271	1.38406913	1.0653E-56	1.38406913	stage_E14_vs_E10	ordinary_muscle_development	D_vs_E10;stage_1W_vs_E10;stage_3W_vs_E10;stage	1	TRUE
ENSGALG00010008504	RNF139	PRJNA729271	1.397028435	1.07882E-56	1.397028435	stage_E14_vs_E10	ordinary_muscle_development	D_vs_E10;stage_1W_vs_E10;stage_3W_vs_E10;stage	1	TRUE
ENSGALG00010010218	PBK	PRJNA729271	-3.726762874	1.09425E-56	3.726762874	stage_E14_vs_E10	ordinary_muscle_development	D_vs_E10;stage_1W_vs_E10;stage_3W_vs_E10;stage	1	TRUE
ENSGALG00010025389	SNED1	PRJNA729271	-2.49396387	1.10231E-56	2.49396387	stage_E14_vs_E10	ordinary_muscle_development	D_vs_E10;stage_1W_vs_E10;stage_3W_vs_E10;stage	1	TRUE
ENSGALG00010021881	PHF20	PRJNA729271	-0.802254178	1.25314E-56	0.802254178	stage_E14_vs_E10	ordinary_muscle_development		0	FALSE
ENSGALG00010013309	ENSGALG00010013309	PRJNA729271	4.586089998	1.29198E-56	4.586089998	stage_E14_vs_E10	ordinary_muscle_development	D_vs_E10;stage_1W_vs_E10;stage_3W_vs_E10;stage	1	TRUE
ENSGALG00010029625	PECAM1	PRJNA729271	2.791873377	1.29346E-56	2.791873377	stage_E14_vs_E10	ordinary_muscle_development	D_vs_E10;stage_1W_vs_E10;stage_3W_vs_E10;stage	1	TRUE
ENSGALG00010025870	ENSGALG00010025870	PRJNA729271	2.589589453	1.33963E-56	2.589589453	stage_E14_vs_E10	ordinary_muscle_development	D_vs_E10;stage_1W_vs_E10;stage_3W_vs_E10;stage	1	TRUE
ENSGALG00010008148	ABCD2	PRJNA729271	2.210896514	1.33985E-56	2.210896514	stage_E14_vs_E10	ordinary_muscle_development	D_vs_E10;stage_1W_vs_E10;stage_3W_vs_E10;stage	1	TRUE
ENSGALG00010023482	MTCL2	PRJNA729271	1.569596429	1.47153E-56	1.569596429	stage_E14_vs_E10	ordinary_muscle_development	D_vs_E10;stage_1W_vs_E10;stage_3W_vs_E10;stage	1	TRUE
ENSGALG00010024164	SNAP29	PRJNA729271	1.793982184	1.47586E-56	1.793982184	stage_E14_vs_E10	ordinary_muscle_development	D_vs_E10;stage_1W_vs_E10;stage_3W_vs_E10;stage	1	TRUE
ENSGALG00010017855	SDR42E2	PRJNA729271	3.728923806	1.68765E-56	3.728923806	stage_E14_vs_E10	ordinary_muscle_development	D_vs_E10;stage_1W_vs_E10;stage_3W_vs_E10;stage	1	TRUE
ENSGALG00010017018	HP1BP3	PRJNA729271	-1.344219163	1.8814E-56	1.344219163	stage_E14_vs_E10	ordinary_muscle_development	D_vs_E10;stage_1W_vs_E10;stage_3W_vs_E10;stage	1	TRUE
ENSGALG00010010851	PNOC	PRJNA729271	11.90459353	1.88573E-56	11.90459353	stage_E14_vs_E10	ordinary_muscle_development	D_vs_E10;stage_1W_vs_E10;stage_3W_vs_E10;stage	1	TRUE
ENSGALG00010010349	RREB1	PRJNA729271	1.314164091	1.92628E-56	1.314164091	stage_E14_vs_E10	ordinary_muscle_development	D_vs_E10;stage_1W_vs_E10;stage_3W_vs_E10;stage	1	TRUE
ENSGALG00010026827	FAM117A	PRJNA729271	2.046509	1.97722E-56	2.046509	stage_E14_vs_E10	ordinary_muscle_development	D_vs_E10;stage_1W_vs_E10;stage_3W_vs_E10;stage	1	TRUE
ENSGALG00010023613	COPB1	PRJNA729271	-0.827191287	2.11349E-56	0.827191287	stage_E14_vs_E10	ordinary_muscle_development		0	FALSE
ENSGALG00010001407	FOXP2	PRJNA729271	-2.496648913	2.161E-56	2.496648913	stage_E14_vs_E10	ordinary_muscle_development	D_vs_E10;stage_1W_vs_E10;stage_3W_vs_E10;stage	1	TRUE
ENSGALG00010016496	PLP1	PRJNA729271	5.189391889	2.4613E-56	5.189391889	stage_E14_vs_E10	ordinary_muscle_development	D_vs_E10;stage_1W_vs_E10;stage_3W_vs_E10;stage	1	TRUE
ENSGALG00010009322	ENSGALG00010009322	PRJNA729271	2.635714172	2.90283E-56	2.635714172	stage_E14_vs_E10	ordinary_muscle_development	D_vs_E10;stage_1W_vs_E10;stage_3W_vs_E10;stage	1	TRUE
ENSGALG00010023041	CD151	PRJNA729271	1.90571015	2.90816E-56	1.90571015	stage_E14_vs_E10	ordinary_muscle_development	D_vs_E10;stage_1W_vs_E10;stage_3W_vs_E10;stage	1	TRUE
ENSGALG00010018397	FAM173A	PRJNA729271	2.537661392	3.00552E-56	2.537661392	stage_E14_vs_E10	ordinary_muscle_development	D_vs_E10;stage_1W_vs_E10;stage_3W_vs_E10;stage	1	TRUE
ENSGALG00010028177	ENSGALG00010028177	PRJNA729271	6.200051222	3.00965E-56	6.200051222	stage_E14_vs_E10	ordinary_muscle_development	D_vs_E10;stage_1W_vs_E10;stage_3W_vs_E10;stage	1	TRUE
ENSGALG00010009451	ROR2	PRJNA729271	-2.270822515	3.22664E-56	2.270822515	stage_E14_vs_E10	ordinary_muscle_development	D_vs_E10;stage_1W_vs_E10;stage_3W_vs_E10;stage	1	TRUE
ENSGALG00010007068	NCAM2	PRJNA729271	-5.104358348	3.39214E-56	5.104358348	stage_E14_vs_E10	ordinary_muscle_development	D_vs_E10;stage_1W_vs_E10;stage_3W_vs_E10;stage	1	TRUE
ENSGALG00010014390	SPARC	PRJNA729271	2.546162053	3.91906E-56	2.546162053	stage_E14_vs_E10	ordinary_muscle_development	D_vs_E10;stage_1W_vs_E10;stage_3W_vs_E10;stage	1	TRUE
ENSGALG00010023571	APLP2	PRJNA729271	1.577823858	4.08685E-56	1.577823858	stage_E14_vs_E10	ordinary_muscle_development	D_vs_E10;stage_1W_vs_E10;stage_3W_vs_E10;stage	1	TRUE
ENSGALG00010024900	DTYMK	PRJNA729271	-1.699748844	4.19177E-56	1.699748844	stage_E14_vs_E10	ordinary_muscle_development	D_vs_E10;stage_1W_vs_E10;stage_3W_vs_E10;stage	1	TRUE
ENSGALG00010002536	RGL2	PRJNA729271	2.226503501	4.46543E-56	2.226503501	stage_E14_vs_E10	ordinary_muscle_development	D_vs_E10;stage_1W_vs_E10;stage_3W_vs_E10;stage	1	TRUE
ENSGALG00010021774	FBXL20	PRJNA729271	1.140331569	4.63841E-56	1.140331569	stage_E14_vs_E10	ordinary_muscle_development	D_vs_E10;stage_1W_vs_E10;stage_3W_vs_E10;stage	1	TRUE
ENSGALG00010007996	NUDCD1	PRJNA729271	-1.226857576	4.65147E-56	1.226857576	stage_E14_vs_E10	ordinary_muscle_development	D_vs_E10;stage_1W_vs_E10;stage_3W_vs_E10;stage	1	TRUE
ENSGALG00010006457	NCAPH2	PRJNA729271	-3.49345456	4.89025E-56	3.49345456	stage_E14_vs_E10	ordinary_muscle_development	D_vs_E10;stage_1W_vs_E10;stage_3W_vs_E10;stage	1	TRUE
ENSGALG00010029194	DHRS11	PRJNA729271	-2.283189362	5.22495E-56	2.283189362	stage_E14_vs_E10	ordinary_muscle_development	D_vs_E10;stage_1W_vs_E10;stage_3W_vs_E10;stage	1	TRUE
ENSGALG00010025924	TMEM116	PRJNA729271	2.692747761	5.22705E-56	2.692747761	stage_E14_vs_E10	ordinary_muscle_development	D_vs_E10;stage_1W_vs_E10;stage_3W_vs_E10;stage	1	TRUE
ENSGALG00010024886	ABTB2	PRJNA729271	-2.368459701	5.35577E-56	2.368459701	stage_E14_vs_E10	ordinary_muscle_development	D_vs_E10;stage_1W_vs_E10;stage_3W_vs_E10;stage	1	TRUE
ENSGALG00010018465	NAALADL2	PRJNA729271	-3.421693648	5.47239E-56	3.421693648	stage_E14_vs_E10	ordinary_muscle_development	D_vs_E10;stage_1W_vs_E10;stage_3W_vs_E10;stage	1	TRUE
ENSGALG00010013311	RSPO1	PRJNA729271	-4.60314731	5.6629E-56	4.60314731	stage_E14_vs_E10	ordinary_muscle_development	D_vs_E10;stage_1W_vs_E10;stage_3W_vs_E10;stage	1	TRUE
ENSGALG00010028322	STRBP	PRJNA729271	-1.869806623	6.15629E-56	1.869806623	stage_E14_vs_E10	ordinary_muscle_development	D_vs_E10;stage_1W_vs_E10;stage_3W_vs_E10;stage	1	TRUE
ENSGALG00010013323	NOTCH2	PRJNA729271	-1.737777431	6.19722E-56	1.737777431	stage_E14_vs_E10	ordinary_muscle_development	D_vs_E10;stage_1W_vs_E10;stage_3W_vs_E10;stage	1	TRUE
ENSGALG00010003506	DYNC1I1	PRJNA729271	-3.171561073	6.41001E-56	3.171561073	stage_E14_vs_E10	ordinary_muscle_development	D_vs_E10;stage_1W_vs_E10;stage_3W_vs_E10;stage	1	TRUE
ENSGALG00010013225	GNE	PRJNA729271	-1.577217661	6.85046E-56	1.577217661	stage_E14_vs_E10	ordinary_muscle_development	D_vs_E10;stage_1W_vs_E10;stage_3W_vs_E10;stage	1	TRUE
ENSGALG00010014198	KDM3B	PRJNA729271	-0.738076843	7.68948E-56	0.738076843	stage_E14_vs_E10	ordinary_muscle_development		0	FALSE
ENSGALG00010006347	PPP6R2	PRJNA729271	2.153275639	7.74174E-56	2.153275639	stage_E14_vs_E10	ordinary_muscle_development	D_vs_E10;stage_1W_vs_E10;stage_3W_vs_E10;stage	1	TRUE
ENSGALG00010029576	ATP2A3	PRJNA729271	2.068427248	7.82633E-56	2.068427248	stage_E14_vs_E10	ordinary_muscle_development	D_vs_E10;stage_1W_vs_E10;stage_3W_vs_E10;stage	1	TRUE
ENSGALG00010007879	PLK4	PRJNA729271	-3.235885724	7.83754E-56	3.235885724	stage_E14_vs_E10	ordinary_muscle_development	D_vs_E10;stage_1W_vs_E10;stage_3W_vs_E10;stage	1	TRUE
ENSGALG00010024877	PRPF38A	PRJNA729271	-1.328130196	8.12013E-56	1.328130196	stage_E14_vs_E10	ordinary_muscle_development	D_vs_E10;stage_1W_vs_E10;stage_3W_vs_E10;stage	1	TRUE
ENSGALG00010017631	NAT8L	PRJNA729271	-4.548973299	8.23604E-56	4.548973299	stage_E14_vs_E10	ordinary_muscle_development	D_vs_E10;stage_1W_vs_E10;stage_3W_vs_E10;stage	1	TRUE
ENSGALG00010016724	PGD	PRJNA729271	-1.774180785	8.28093E-56	1.774180785	stage_E14_vs_E10	ordinary_muscle_development	D_vs_E10;stage_1W_vs_E10;stage_3W_vs_E10;stage	1	TRUE
ENSGALG00010005244	FBXO5	PRJNA729271	-1.597391927	8.39094E-56	1.597391927	stage_E14_vs_E10	ordinary_muscle_development	D_vs_E10;stage_1W_vs_E10;stage_3W_vs_E10;stage	1	TRUE
ENSGALG00010021042	LRRC38	PRJNA729271	1.507968355	8.58677E-56	1.507968355	stage_E14_vs_E10	ordinary_muscle_development	D_vs_E10;stage_1W_vs_E10;stage_3W_vs_E10;stage	1	TRUE
ENSGALG00010016825	ENSGALG00010016825	PRJNA729271	4.615472225	8.72285E-56	4.615472225	stage_E14_vs_E10	ordinary_muscle_development	D_vs_E10;stage_1W_vs_E10;stage_3W_vs_E10;stage	1	TRUE
ENSGALG00010014517	DCUN1D2	PRJNA729271	2.01251986	8.76109E-56	2.01251986	stage_E14_vs_E10	ordinary_muscle_development	D_vs_E10;stage_1W_vs_E10;stage_3W_vs_E10;stage	1	TRUE
ENSGALG00010002741	PDK4	PRJNA729271	9.147131433	9.29819E-56	9.147131433	stage_E14_vs_E10	ordinary_muscle_development	D_vs_E10;stage_1W_vs_E10;stage_3W_vs_E10;stage	1	TRUE
ENSGALG00010015153	ERCC6L	PRJNA729271	-4.136003033	1.05204E-55	4.136003033	stage_E14_vs_E10	ordinary_muscle_development	D_vs_E10;stage_1W_vs_E10;stage_3W_vs_E10;stage	1	TRUE
ENSGALG00010029549	KSR1	PRJNA729271	1.967849485	1.06901E-55	1.967849485	stage_E14_vs_E10	ordinary_muscle_development	D_vs_E10;stage_1W_vs_E10;stage_3W_vs_E10;stage	1	TRUE
ENSGALG00010018415	PELI2	PRJNA729271	-2.119192985	1.0951E-55	2.119192985	stage_E14_vs_E10	ordinary_muscle_development	D_vs_E10;stage_1W_vs_E10;stage_3W_vs_E10;stage	1	TRUE
ENSGALG00010017496	PARP1	PRJNA729271	-1.650610056	1.11428E-55	1.650610056	stage_E14_vs_E10	ordinary_muscle_development	D_vs_E10;stage_1W_vs_E10;stage_3W_vs_E10;stage	1	TRUE
ENSGALG00010011699	TIAM1	PRJNA729271	-2.357223619	1.18851E-55	2.357223619	stage_E14_vs_E10	ordinary_muscle_development	D_vs_E10;stage_1W_vs_E10;stage_3W_vs_E10;stage	1	TRUE
ENSGALG00010022593	MANBAL	PRJNA729271	-2.356582935	1.19926E-55	2.356582935	stage_E14_vs_E10	ordinary_muscle_development	D_vs_E10;stage_1W_vs_E10;stage_3W_vs_E10;stage	1	TRUE
ENSGALG00010010734	AMN1	PRJNA729271	2.231189175	1.21501E-55	2.231189175	stage_E14_vs_E10	ordinary_muscle_development	D_vs_E10;stage_1W_vs_E10;stage_3W_vs_E10;stage	1	TRUE
ENSGALG00010024664	DNAH10	PRJNA729271	-4.349337119	1.24958E-55	4.349337119	stage_E14_vs_E10	ordinary_muscle_development	D_vs_E10;stage_1W_vs_E10;stage_3W_vs_E10;stage	1	TRUE
ENSGALG00010011485	MTM1	PRJNA729271	1.204517955	1.53169E-55	1.204517955	stage_E14_vs_E10	ordinary_muscle_development	D_vs_E10;stage_1W_vs_E10;stage_3W_vs_E10;stage	1	TRUE
ENSGALG00010023828	CHEK1	PRJNA729271	-3.200948021	1.94451E-55	3.200948021	stage_E14_vs_E10	ordinary_muscle_development	D_vs_E10;stage_1W_vs_E10;stage_3W_vs_E10;stage	1	TRUE
ENSGALG00010029473	COG1	PRJNA729271	1.579166138	2.15457E-55	1.579166138	stage_E14_vs_E10	ordinary_muscle_development	D_vs_E10;stage_1W_vs_E10;stage_3W_vs_E10;stage	1	TRUE
ENSGALG00010024426	PHAF1	PRJNA729271	1.652061768	2.22083E-55	1.652061768	stage_E14_vs_E10	ordinary_muscle_development	D_vs_E10;stage_1W_vs_E10;stage_3W_vs_E10;stage	1	TRUE
ENSGALG00010010463	ACAA2	PRJNA729271	2.030095139	2.23E-55	2.030095139	stage_E14_vs_E10	ordinary_muscle_development	D_vs_E10;stage_1W_vs_E10;stage_3W_vs_E10;stage	1	TRUE
ENSGALG00010012264	MYF5	PRJNA729271	2.449792982	2.44975E-55	2.449792982	stage_E14_vs_E10	ordinary_muscle_development	D_vs_E10;stage_1W_vs_E10;stage_3W_vs_E10;stage	1	TRUE
ENSGALG00010013579	H2AC32	PRJNA729271	-2.813447597	2.45603E-55	2.813447597	stage_E14_vs_E10	ordinary_muscle_development	D_vs_E10;stage_1W_vs_E10;stage_3W_vs_E10;stage	1	TRUE
ENSGALG00010021540	STK4	PRJNA729271	-1.252984879	2.51948E-55	1.252984879	stage_E14_vs_E10	ordinary_muscle_development	D_vs_E10;stage_1W_vs_E10;stage_3W_vs_E10;stage	1	TRUE
ENSGALG00010009774	PIAS2	PRJNA729271	-1.000039925	2.61729E-55	1.000039925	stage_E14_vs_E10	ordinary_muscle_development	D_vs_E10;stage_1W_vs_E10;stage_3W_vs_E10;stage	1	TRUE
ENSGALG00010013458	LAMA4	PRJNA729271	2.282137304	2.61793E-55	2.282137304	stage_E14_vs_E10	ordinary_muscle_development	D_vs_E10;stage_1W_vs_E10;stage_3W_vs_E10;stage	1	TRUE
ENSGALG00010005381	FGF9	PRJNA729271	-4.735184231	2.67752E-55	4.735184231	stage_E14_vs_E10	ordinary_muscle_development	D_vs_E10;stage_1W_vs_E10;stage_3W_vs_E10;stage	1	TRUE
ENSGALG00010025415	ENSGALG00010025415	PRJNA729271	4.368642634	2.7011E-55	4.368642634	stage_E14_vs_E10	ordinary_muscle_development	D_vs_E10;stage_1W_vs_E10;stage_3W_vs_E10;stage	1	TRUE
ENSGALG00010016341	SLX4	PRJNA729271	-1.044951929	2.81991E-55	1.044951929	stage_E14_vs_E10	ordinary_muscle_development	D_vs_E10;stage_1W_vs_E10;stage_3W_vs_E10;stage	1	TRUE
ENSGALG00010029363	PIK3R6	PRJNA729271	2.999876145	2.86147E-55	2.999876145	stage_E14_vs_E10	ordinary_muscle_development	D_vs_E10;stage_1W_vs_E10;stage_3W_vs_E10;stage	1	TRUE
ENSGALG00010016664	TM6SF1	PRJNA729271	3.933517696	3.15345E-55	3.933517696	stage_E14_vs_E10	ordinary_muscle_development	D_vs_E10;stage_1W_vs_E10;stage_3W_vs_E10;stage	1	TRUE
ENSGALG00010005217	CCNA2	PRJNA729271	-2.298537755	3.18288E-55	2.298537755	stage_E14_vs_E10	ordinary_muscle_development	D_vs_E10;stage_1W_vs_E10;stage_3W_vs_E10;stage	1	TRUE
ENSGALG00010010981	RIC8B	PRJNA729271	-1.028613558	3.56627E-55	1.028613558	stage_E14_vs_E10	ordinary_muscle_development	D_vs_E10;stage_1W_vs_E10;stage_3W_vs_E10;stage	1	TRUE
ENSGALG00010028761	CTSS	PRJNA729271	3.734752705	3.69628E-55	3.734752705	stage_E14_vs_E10	ordinary_muscle_development	D_vs_E10;stage_1W_vs_E10;stage_3W_vs_E10;stage	1	TRUE
ENSGALG00010003363	HBE1	PRJNA729271	-9.323517301	3.7138E-55	9.323517301	stage_E14_vs_E10	ordinary_muscle_development	D_vs_E10;stage_1W_vs_E10;stage_3W_vs_E10;stage	1	TRUE
ENSGALG00010018655	BIRC5	PRJNA729271	-3.404846974	3.77089E-55	3.404846974	stage_E14_vs_E10	ordinary_muscle_development	D_vs_E10;stage_1W_vs_E10;stage_3W_vs_E10;stage	1	TRUE
ENSGALG00010004757	TRMT10A	PRJNA729271	1.155113437	3.81296E-55	1.155113437	stage_E14_vs_E10	ordinary_muscle_development	D_vs_E10;stage_1W_vs_E10;stage_3W_vs_E10;stage	1	TRUE
ENSGALG00010005092	ESCO1	PRJNA729271	2.204886875	4.32064E-55	2.204886875	stage_E14_vs_E10	ordinary_muscle_development	D_vs_E10;stage_1W_vs_E10;stage_3W_vs_E10;stage	1	TRUE
ENSGALG00010010254	WDR41	PRJNA729271	1.750685569	4.45218E-55	1.750685569	stage_E14_vs_E10	ordinary_muscle_development	D_vs_E10;stage_1W_vs_E10;stage_3W_vs_E10;stage	1	TRUE
ENSGALG00010020941	ENSGALG00010020941	PRJNA729271	2.893892703	4.53318E-55	2.893892703	stage_E14_vs_E10	ordinary_muscle_development	D_vs_E10;stage_1W_vs_E10;stage_3W_vs_E10;stage	1	TRUE
ENSGALG00010017643	CALM1	PRJNA729271	0.96187314	4.9946E-55	0.96187314	stage_E14_vs_E10	ordinary_muscle_development		0	FALSE
ENSGALG00010024597	DNAJB11	PRJNA729271	-1.623606032	5.31529E-55	1.623606032	stage_E14_vs_E10	ordinary_muscle_development	D_vs_E10;stage_1W_vs_E10;stage_3W_vs_E10;stage	1	TRUE
ENSGALG00010002978	DYNLT3	PRJNA729271	1.595144464	5.33309E-55	1.595144464	stage_E14_vs_E10	ordinary_muscle_development	D_vs_E10;stage_1W_vs_E10;stage_3W_vs_E10;stage	1	TRUE
ENSGALG00010024169	FAM168B	PRJNA729271	-1.152928876	5.56574E-55	1.152928876	stage_E14_vs_E10	ordinary_muscle_development	D_vs_E10;stage_1W_vs_E10;stage_3W_vs_E10;stage	1	TRUE
ENSGALG00010008067	MPC2	PRJNA729271	2.283255221	6.66781E-55	2.283255221	stage_E14_vs_E10	ordinary_muscle_development	D_vs_E10;stage_1W_vs_E10;stage_3W_vs_E10;stage	1	TRUE
ENSGALG00010002453	TOP2B	PRJNA729271	-0.999609763	7.13991E-55	0.999609763	stage_E14_vs_E10	ordinary_muscle_development		0	FALSE
ENSGALG00010016535	BNC2	PRJNA729271	-2.131211929	7.18365E-55	2.131211929	stage_E14_vs_E10	ordinary_muscle_development	D_vs_E10;stage_1W_vs_E10;stage_3W_vs_E10;stage	1	TRUE
ENSGALG00010015580	GEMIN5	PRJNA729271	-1.16635709	8.01499E-55	1.16635709	stage_E14_vs_E10	ordinary_muscle_development	D_vs_E10;stage_1W_vs_E10;stage_3W_vs_E10;stage	1	TRUE
ENSGALG00010017042	HNRNPLL	PRJNA729271	-1.118755567	8.81153E-55	1.118755567	stage_E14_vs_E10	ordinary_muscle_development	D_vs_E10;stage_1W_vs_E10;stage_3W_vs_E10;stage	1	TRUE

ENSGALG00010005484	GINM1	PRJNA729271	1.940941937	8.90746E-55	1.940941937	stage_E14_vs_E10	ordinary_muscle_development	D_vs_E10;stage_1W_vs_E10;stage_3W_vs_E10;stage	1	TRUE
ENSGALG00010024401	KCNC1	PRJNA729271	3.244784768	9.02268E-55	3.244784768	stage_E14_vs_E10	ordinary_muscle_development	D_vs_E10;stage_1W_vs_E10;stage_3W_vs_E10;stage	1	TRUE
ENSGALG00010021792	KLHDC8B	PRJNA729271	4.056650239	9.46573E-55	4.056650239	stage_E14_vs_E10	ordinary_muscle_development	D_vs_E10;stage_1W_vs_E10;stage_3W_vs_E10;stage	1	TRUE
ENSGALG00010011003	FECH	PRJNA729271	-1.816196594	9.74219E-55	1.816196594	stage_E14_vs_E10	ordinary_muscle_development	D_vs_E10;stage_1W_vs_E10;stage_3W_vs_E10;stage	1	TRUE
ENSGALG00010020880	PXK	PRJNA729271	2.574156663	9.76861E-55	2.574156663	stage_E14_vs_E10	ordinary_muscle_development	D_vs_E10;stage_1W_vs_E10;stage_3W_vs_E10;stage	1	TRUE
ENSGALG00010022770	HAT1	PRJNA729271	-1.470536119	1.0411E-54	1.470536119	stage_E14_vs_E10	ordinary_muscle_development	D_vs_E10;stage_1W_vs_E10;stage_3W_vs_E10;stage	1	TRUE
ENSGALG00010016878	SUCLG1	PRJNA729271	1.97208353	1.06793E-54	1.97208353	stage_E14_vs_E10	ordinary_muscle_development	D_vs_E10;stage_1W_vs_E10;stage_3W_vs_E10;stage	1	TRUE
ENSGALG00010001729	ENSGALG00010001729	PRJNA729271	3.759087384	1.11009E-54	3.759087384	stage_E14_vs_E10	ordinary_muscle_development	D_vs_E10;stage_1W_vs_E10;stage_3W_vs_E10;stage	1	TRUE
ENSGALG00010023818	ALOX5	PRJNA729271	2.878615227	1.12842E-54	2.878615227	stage_E14_vs_E10	ordinary_muscle_development	D_vs_E10;stage_1W_vs_E10;stage_3W_vs_E10;stage	1	TRUE
ENSGALG00010007439	HSD17B2	PRJNA729271	-7.093091349	1.16579E-54	7.093091349	stage_E14_vs_E10	ordinary_muscle_development	D_vs_E10;stage_1W_vs_E10;stage_3W_vs_E10;stage	1	TRUE
ENSGALG00010019123	PLEKHO2	PRJNA729271	1.850246212	1.32127E-54	1.850246212	stage_E14_vs_E10	ordinary_muscle_development	D_vs_E10;stage_1W_vs_E10;stage_3W_vs_E10;stage	1	TRUE
ENSGALG00010027906	LHX6	PRJNA729271	2.693280947	1.77276E-54	2.693280947	stage_E14_vs_E10	ordinary_muscle_development	D_vs_E10;stage_1W_vs_E10;stage_3W_vs_E10;stage	1	TRUE
ENSGALG00010015865	VRK1	PRJNA729271	-2.304269908	1.80404E-54	2.304269908	stage_E14_vs_E10	ordinary_muscle_development	D_vs_E10;stage_1W_vs_E10;stage_3W_vs_E10;stage	1	TRUE
ENSGALG00010016011	ADD1	PRJNA729271	-1.277360974	1.81482E-54	1.277360974	stage_E14_vs_E10	ordinary_muscle_development	D_vs_E10;stage_1W_vs_E10;stage_3W_vs_E10;stage	1	TRUE
ENSGALG00010011723	SPRY3	PRJNA729271	2.442283541	2.36397E-54	2.442283541	stage_E14_vs_E10	ordinary_muscle_development	D_vs_E10;stage_1W_vs_E10;stage_3W_vs_E10;stage	1	TRUE
ENSGALG00010013872	GLS2	PRJNA729271	3.086556096	2.58531E-54	3.086556096	stage_E14_vs_E10	ordinary_muscle_development	D_vs_E10;stage_1W_vs_E10;stage_3W_vs_E10;stage	1	TRUE
ENSGALG00010001448	GPR37	PRJNA729271	-6.249737946	2.76291E-54	6.249737946	stage_E14_vs_E10	ordinary_muscle_development	D_vs_E10;stage_1W_vs_E10;stage_3W_vs_E10;stage	1	TRUE
ENSGALG00010025164	C11orf52	PRJNA729271	1.943109218	2.84728E-54	1.943109218	stage_E14_vs_E10	ordinary_muscle_development	D_vs_E10;stage_1W_vs_E10;stage_3W_vs_E10;stage	1	TRUE
ENSGALG00010001368	WIPF3	PRJNA729271	2.191695116	2.85201E-54	2.191695116	stage_E14_vs_E10	ordinary_muscle_development	D_vs_E10;stage_1W_vs_E10;stage_3W_vs_E10;stage	1	TRUE
ENSGALG00010025983	RNF26	PRJNA729271	-1.350363353	2.94915E-54	1.350363353	stage_E14_vs_E10	ordinary_muscle_development	D_vs_E10;stage_1W_vs_E10;stage_3W_vs_E10;stage	1	TRUE
ENSGALG00010005405	CEBPD	PRJNA729271	7.426978676	2.98938E-54	7.426978676	stage_E14_vs_E10	ordinary_muscle_development	D_vs_E10;stage_1W_vs_E10;stage_3W_vs_E10;stage	1	TRUE
ENSGALG00010021234	ISLR2	PRJNA729271	5.058376229	3.01065E-54	5.058376229	stage_E14_vs_E10	ordinary_muscle_development	D_vs_E10;stage_1W_vs_E10;stage_3W_vs_E10;stage	1	TRUE
ENSGALG00010011150	FGD4	PRJNA729271	2.545836687	3.06134E-54	2.545836687	stage_E14_vs_E10	ordinary_muscle_development	D_vs_E10;stage_1W_vs_E10;stage_3W_vs_E10;stage	1	TRUE
ENSGALG00010029361	ALYREF	PRJNA729271	-2.139482421	3.37373E-54	2.139482421	stage_E14_vs_E10	ordinary_muscle_development	D_vs_E10;stage_1W_vs_E10;stage_3W_vs_E10;stage	1	TRUE
ENSGALG00010024648	MMP24	PRJNA729271	-2.006645395	3.37373E-54	2.006645395	stage_E14_vs_E10	ordinary_muscle_development	D_vs_E10;stage_1W_vs_E10;stage_3W_vs_E10;stage	1	TRUE
ENSGALG00010029403	FAM20A	PRJNA729271	2.462083587	3.51158E-54	2.462083587	stage_E14_vs_E10	ordinary_muscle_development	D_vs_E10;stage_1W_vs_E10;stage_3W_vs_E10;stage	1	TRUE
ENSGALG00010020386	PPM1B	PRJNA729271	1.126314033	3.51426E-54	1.126314033	stage_E14_vs_E10	ordinary_muscle_development	D_vs_E10;stage_1W_vs_E10;stage_3W_vs_E10;stage	1	TRUE
ENSGALG00010023136	MDK	PRJNA729271	-6.679185239	3.87148E-54	6.679185239	stage_E14_vs_E10	ordinary_muscle_development	D_vs_E10;stage_1W_vs_E10;stage_3W_vs_E10;stage	1	TRUE
ENSGALG00010005367	H2AZ1	PRJNA729271	-1.511193351	4.19143E-54	1.511193351	stage_E14_vs_E10	ordinary_muscle_development	D_vs_E10;stage_1W_vs_E10;stage_3W_vs_E10;stage	1	TRUE
ENSGALG00010023290	SLC16A14	PRJNA729271	-4.604988309	4.50048E-54	4.604988309	stage_E14_vs_E10	ordinary_muscle_development	D_vs_E10;stage_1W_vs_E10;stage_3W_vs_E10;stage	1	TRUE
ENSGALG00010002400	RBBP7	PRJNA729271	-2.564182859	4.56734E-54	2.564182859	stage_E14_vs_E10	ordinary_muscle_development	D_vs_E10;stage_1W_vs_E10;stage_3W_vs_E10;stage	1	TRUE
ENSGALG00010018895	SEPTIN7	PRJNA729271	-1.409917681	4.71713E-54	1.409917681	stage_E14_vs_E10	ordinary_muscle_development	D_vs_E10;stage_1W_vs_E10;stage_3W_vs_E10;stage	1	TRUE
ENSGALG00010020765	GLT1D1	PRJNA729271	-4.175831237	4.75481E-54	4.175831237	stage_E14_vs_E10	ordinary_muscle_development	D_vs_E10;stage_1W_vs_E10;stage_3W_vs_E10;stage	1	TRUE
ENSGALG00010014948	RBM38	PRJNA729271	4.049806547	5.10155E-54	4.049806547	stage_E14_vs_E10	ordinary_muscle_development	D_vs_E10;stage_1W_vs_E10;stage_3W_vs_E10;stage	1	TRUE
ENSGALG00010002379	NHS	PRJNA729271	-2.170426238	5.23E-54	2.170426238	stage_E14_vs_E10	ordinary_muscle_development	D_vs_E10;stage_1W_vs_E10;stage_3W_vs_E10;stage	1	TRUE
ENSGALG00010004421	RAP1GDS1	PRJNA729271	-1.181636689	5.32011E-54	1.181636689	stage_E14_vs_E10	ordinary_muscle_development	D_vs_E10;stage_1W_vs_E10;stage_3W_vs_E10;stage	1	TRUE
ENSGALG00010023954	PLEKHF1	PRJNA729271	1.480349477	5.84548E-54	1.480349477	stage_E14_vs_E10	ordinary_muscle_development	D_vs_E10;stage_1W_vs_E10;stage_3W_vs_E10;stage	1	TRUE
ENSGALG00010011837	VLDLR	PRJNA729271	2.0998299	6.2671E-54	2.0998299	stage_E14_vs_E10	ordinary_muscle_development	D_vs_E10;stage_1W_vs_E10;stage_3W_vs_E10;stage	1	TRUE
ENSGALG00010009135	TENM4	PRJNA729271	-2.924023574	6.3519E-54	2.924023574	stage_E14_vs_E10	ordinary_muscle_development	D_vs_E10;stage_1W_vs_E10;stage_3W_vs_E10;stage	1	TRUE
ENSGALG00010003074	TTPA	PRJNA729271	5.397754152	6.41132E-54	5.397754152	stage_E14_vs_E10	ordinary_muscle_development	D_vs_E10;stage_1W_vs_E10;stage_3W_vs_E10;stage	1	TRUE
ENSGALG00010027959	TPM4	PRJNA729271	-2.712564924	6.57353E-54	2.712564924	stage_E14_vs_E10	ordinary_muscle_development	D_vs_E10;stage_1W_vs_E10;stage_3W_vs_E10;stage	1	TRUE
ENSGALG00010004987	MAP3K8	PRJNA729271	4.014143371	7.68829E-54	4.014143371	stage_E14_vs_E10	ordinary_muscle_development	D_vs_E10;stage_1W_vs_E10;stage_3W_vs_E10;stage	1	TRUE
ENSGALG00010014045	GOLT1B	PRJNA729271	-1.756127881	7.79953E-54	1.756127881	stage_E14_vs_E10	ordinary_muscle_development	D_vs_E10;stage_1W_vs_E10;stage_3W_vs_E10;stage	1	TRUE
ENSGALG00010011178	RSPO2	PRJNA729271	-5.249697294	8.279E-54	5.249697294	stage_E14_vs_E10	ordinary_muscle_development	D_vs_E10;stage_1W_vs_E10;stage_3W_vs_E10;stage	1	TRUE
ENSGALG00010028134	INPP5E	PRJNA729271	-1.15337895	8.67072E-54	1.15337895	stage_E14_vs_E10	ordinary_muscle_development	D_vs_E10;stage_1W_vs_E10;stage_3W_vs_E10;stage	1	TRUE
ENSGALG00010008212	GTSE1	PRJNA729271	-3.695031807	9.13916E-54	3.695031807	stage_E14_vs_E10	ordinary_muscle_development	D_vs_E10;stage_1W_vs_E10;stage_3W_vs_E10;stage	1	TRUE
ENSGALG00010002638	FABP9	PRJNA729271	7.744308332	9.34386E-54	7.744308332	stage_E14_vs_E10	ordinary_muscle_development	D_vs_E10;stage_1W_vs_E10;stage_3W_vs_E10;stage	1	TRUE
ENSGALG00010011912	TCP11X2	PRJNA729271	1.957348023	9.51146E-54	1.957348023	stage_E14_vs_E10	ordinary_muscle_development	D_vs_E10;stage_1W_vs_E10;stage_3W_vs_E10;stage	1	TRUE
ENSGALG00010004707	OTUD1	PRJNA729271	4.632242042	9.51497E-54	4.632242042	stage_E14_vs_E10	ordinary_muscle_development	D_vs_E10;stage_1W_vs_E10;stage_3W_vs_E10;stage	1	TRUE
ENSGALG00010010313	GRID1	PRJNA729271	-3.664463466	9.5917E-54	3.664463466	stage_E14_vs_E10	ordinary_muscle_development	D_vs_E10;stage_1W_vs_E10;stage_3W_vs_E10;stage	1	TRUE
ENSGALG00010029739	P2RX5	PRJNA729271	3.173244192	1.00525E-53	3.173244192	stage_E14_vs_E10	ordinary_muscle_development	D_vs_E10;stage_1W_vs_E10;stage_3W_vs_E10;stage	1	TRUE
ENSGALG00010027752	SCAI	PRJNA729271	-1.358135611	1.07654E-53	1.358135611	stage_E14_vs_E10	ordinary_muscle_development	D_vs_E10;stage_1W_vs_E10;stage_3W_vs_E10;stage	1	TRUE
ENSGALG00010002419	CTBP2	PRJNA729271	-1.81190212	1.14635E-53	1.81190212	stage_E14_vs_E10	ordinary_muscle_development	D_vs_E10;stage_1W_vs_E10;stage_3W_vs_E10;stage	1	TRUE
ENSGALG00010009514	PRELID1	PRJNA729271	-1.444975275	1.15201E-53	1.444975275	stage_E14_vs_E10	ordinary_muscle_development	D_vs_E10;stage_1W_vs_E10;stage_3W_vs_E10;stage	1	TRUE
ENSGALG00010024402	GPC1	PRJNA729271	-1.87317057	1.19389E-53	1.87317057	stage_E14_vs_E10	ordinary_muscle_development	D_vs_E10;stage_1W_vs_E10;stage_3W_vs_E10;stage	1	TRUE
ENSGALG00010006657	PDS5B	PRJNA729271	-1.384392254	1.25548E-53	1.384392254	stage_E14_vs_E10	ordinary_muscle_development	D_vs_E10;stage_1W_vs_E10;stage_3W_vs_E10;stage	1	TRUE
ENSGALG00010016463	FREM1	PRJNA729271	-2.254245016	1.29041E-53	2.254245016	stage_E14_vs_E10	ordinary_muscle_development	D_vs_E10;stage_1W_vs_E10;stage_3W_vs_E10;stage	1	TRUE
ENSGALG00010012101	ENSGALG00010012101	PRJNA729271	-2.156411174	1.32624E-53	2.156411174	stage_E14_vs_E10	ordinary_muscle_development	D_vs_E10;stage_1W_vs_E10;stage_3W_vs_E10;stage	1	TRUE
ENSGALG00010029634	DDX5	PRJNA729271	-1.430543018	1.33239E-53	1.430543018	stage_E14_vs_E10	ordinary_muscle_development	D_vs_E10;stage_1W_vs_E10;stage_3W_vs_E10;stage	1	TRUE
ENSGALG00010028046	SMIM4	PRJNA729271	3.160421012	1.39239E-53	3.160421012	stage_E14_vs_E10	ordinary_muscle_development	D_vs_E10;stage_1W_vs_E10;stage_3W_vs_E10;stage	1	TRUE
ENSGALG00010014388	MFNG	PRJNA729271	3.393138611	1.45019E-53	3.393138611	stage_E14_vs_E10	ordinary_muscle_development	D_vs_E10;stage_1W_vs_E10;stage_3W_vs_E10;stage	1	TRUE
ENSGALG00010018581	SUFU	PRJNA729271	-1.451159944	1.45435E-53	1.451159944	stage_E14_vs_E10	ordinary_muscle_development	D_vs_E10;stage_1W_vs_E10;stage_3W_vs_E10;stage	1	TRUE
ENSGALG00010018302	RAB11FIP5	PRJNA729271	1.55410626	1.56267E-53	1.55410626	stage_E14_vs_E10	ordinary_muscle_development	D_vs_E10;stage_1W_vs_E10;stage_3W_vs_E10;stage	1	TRUE
ENSGALG00010016498	KIF23	PRJNA729271	-3.270331646	1.58379E-53	3.270331646	stage_E14_vs_E10	ordinary_muscle_development	D_vs_E10;stage_1W_vs_E10;stage_3W_vs_E10;stage	1	TRUE
ENSGALG00010021381	DNAJB2	PRJNA729271	2.287974818	1.64154E-53	2.287974818	stage_E14_vs_E10	ordinary_muscle_development	D_vs_E10;stage_1W_vs_E10;stage_3W_vs_E10;stage	1	TRUE
ENSGALG00010021238	NDUFS8	PRJNA729271	2.282916102	1.67277E-53	2.282916102	stage_E14_vs_E10	ordinary_muscle_development	D_vs_E10;stage_1W_vs_E10;stage_3W_vs_E10;stage	1	TRUE
ENSGALG00010017890	ANAPC1	PRJNA729271	-1.049291068	1.73852E-53	1.049291068	stage_E14_vs_E10	ordinary_muscle_development	D_vs_E10;stage_1W_vs_E10;stage_3W_vs_E10;stage	1	TRUE
ENSGALG00010012191	BEST3	PRJNA729271	5.803941899	1.76505E-53	5.803941899	stage_E14_vs_E10	ordinary_muscle_development	D_vs_E10;stage_1W_vs_E10;stage_3W_vs_E10;stage	1	TRUE
ENSGALG00010007925	STK17A	PRJNA729271	3.656885893	1.8159E-53	3.656885893	stage_E14_vs_E10	ordinary_muscle_development	D_vs_E10;stage_1W_vs_E10;stage_3W_vs_E10;stage	1	TRUE
ENSGALG00010008658	TSTA3	PRJNA729271	-1.959248083	1.89967E-53	1.959248083	stage_E14_vs_E10	ordinary_muscle_development	D_vs_E10;stage_1W_vs_E10;stage_3W_vs_E10;stage	1	TRUE
ENSGALG00010008832	MTSS1	PRJNA729271	1.924853446	1.90467E-53	1.924853446	stage_E14_vs_E10	ordinary_muscle_development	D_vs_E10;stage_1W_vs_E10;stage_3W_vs_E10;stage	1	TRUE
ENSGALG00010001826	HOXA7	PRJNA729271	2.06361124	2.14163E-53	2.06361124	stage_E14_vs_E10	ordinary_muscle_development	D_vs_E10;stage_1W_vs_E10;stage_3W_vs_E10;stage	1	TRUE
ENSGALG00010016526	EIF1B	PRJNA729271	1.759411014	2.1971E-53	1.759411014	stage_E14_vs_E10	ordinary_muscle_development	D_vs_E10;stage_1W_vs_E10;stage_3W_vs_E10;stage	1	TRUE
ENSGALG00010013276	POC1B	PRJNA729271	-1.73700339	2.21186E-53	1.73700339	stage_E14_vs_E10	ordinary_muscle_development	D_vs_E10;stage_1W_vs_E10;stage_3W_vs_E10;stage	1	TRUE
ENSGALG00010021489	CNPPD1	PRJNA729271	1.319413317	2.27458E-53	1.319413317	stage_E14_vs_E10	ordinary_muscle_development	D_vs_E10;stage_1W_vs_E10;stage_3W_vs_E10;stage	1	TRUE
ENSGALG00010005554	CRYL1	PRJNA729271	2.512359915	2.48228E-53	2.512359915	stage_E14_vs_E10	ordinary_muscle_development	D_vs_E10;stage_1W_vs_E10;stage_3W_vs_E10;stage	1	TRUE
ENSGALG00010026355	PPP1R12B	PRJNA729271	2.135268768	2.54714E-53	2.135268768	stage_E14_vs_E10	ordinary_muscle_development	D_vs_E10;stage_1W_vs_E10;stage_3W_vs_E10;stage	1	TRUE
ENSGALG00010029596	ATAD5	PRJNA729271	-2.064915063	2.60953E-53	2.064915063	stage_E14_vs_E10	ordinary_muscle_development	D_vs_E10;stage_1W_vs_E10;stage_3W_vs_E10;stage	1	TRUE
ENSGALG00010028012	PRRX2	PRJNA729271	-4.016961108	2.89208E-53	4.016961108	stage_E14_vs_E10	ordinary_muscle_development	D_vs_E10;stage_1W_vs_E10;stage_3W_vs_E10;stage	1	TRUE
ENSGALG00010022334	PDE1A	PRJNA729271	-1.932935351	2.98296E-53	1.932935351	stage_E14_vs_E10	ordinary_muscle_development	D_vs_E10;stage_1W_vs_E10;stage_3W_vs_E10;stage	1	TRUE
ENSGALG00010012361	WIF1	PRJNA729271	-7.811471638	3.01522E-53	7.811471638	stage_E14_vs_E10	ordinary_muscle_development	D_vs_E10;stage_1W_vs_E10;stage_3W_vs_E10;stage	1	TRUE
ENSGALG00010014399	GAS6	PRJNA729271	2.947181396	3.16432E-53	2.947181396	stage_E14_vs_E10	ordinary_muscle_development	D_vs_E10;stage_1W_vs_E10;stage_3W_vs_E10;stage	1	TRUE
ENSGALG00010029943	HS3ST3A1	PRJNA729271	-3.847187274	3.16448E-53	3.847187274	stage_E14_vs_E10	ordinary_muscle_development	D_vs_E10;stage_1W_vs_E10;stage_3W_vs_E10;stage	1	TRUE
ENSGALG00010026508	CSRP1	PRJNA729271	2.299846957	3.20824E-53	2.299846957	stage_E14_vs_E10	ordinary_muscle_development	D_vs_E10;stage_1W_vs_E10;stage_3W_vs_E10;stage	1	TRUE
ENSGALG00010028418	ENSGALG00010028418	PRJNA729271	1.924814299	3.52153E-53	1.924814299	stage_E14_vs_E10	ordinary_muscle_development	D_vs_E10;stage_1W_vs_E10;stage_3W_vs_E10;stage	1	TRUE
ENSGALG00010015300	ATXN3	PRJNA729271	1.965562771	3.52799E-53	1.965562771	stage_E14_vs_E10	ordinary_muscle_development	D_vs_E10;stage_1W_vs_E10;stage_3W_vs_E10;stage	1	TRUE
ENSGALG00010018876	GMFB	PRJNA729271	-1.533772851	3.69635E-53	1.533772851	stage_E14_vs_E10	ordinary_muscle_development	D_vs_E10;stage_1W_vs_E10;stage_3W_vs_E10;stage	1	TRUE
ENSGALG00010002446	RIMS1	PRJNA729271	-5.602292125	3.77677E-53	5.602292125	stage_E14_vs_E10	ordinary_muscle_development	D_vs_E10;stage_1W_vs_E10;stage_3W_vs_E10;stage	1	TRUE
ENSGALG00010015975	BTK	PRJNA729271	2.642922295	3.8165E-53	2.642922295	stage_E14_vs_E10	ordinary_muscle_development	D_vs_E10;stage_1W_vs_E10;stage_3W_vs_E10;stage	1	TRUE
ENSGALG00010003133	SAT1	PRJNA729271	2.589572676	4.16532E-53	2.589572676	stage_E14_vs_E10	ordinary_muscle_development	D_vs_E10;stage_1W_vs_E10;stage_3W_vs_E10;stage	1	TRUE
ENSGALG00010009003	ALG12	PRJNA729271	-2.14218874	4.3524E-53	2.14218874	stage_E14_vs_E10	ordinary_muscle_development	D_vs_E10;stage_1W_vs_E10;stage_3W_vs_E10;stage	1	TRUE
ENSGALG00010029174	CALN1	PRJNA729271	-10.56803953	4.59219E-53	10.56803953	stage_E14_vs_E10	ordinary_muscle_development	D_vs_E10;stage_1W_vs_E10;stage_3W_vs_E10;stage	1	TRUE
ENSGALG00010017789	IMMT	PRJNA729271	2.014748108	4.66342E-53	2.014748108	stage_E14_vs_E10	ordinary_muscle_development	D_vs_E10;stage_1W_vs_E10;stage_3W_vs_E10;stage	1	TRUE
ENSGALG00010010321	NUP133	PRJNA729271	-1.140554732	4.79581E-53	1.140554732	stage_E14_vs_E10	ordinary_muscle_development	D_vs_E10;stage_1W_vs_E10;stage_3W_vs_E10;stage	1	TRUE
ENSGALG00010012096	ENSGALG00010012096	PRJNA729271	-2.62148718	4.8945E-53	2.62148718	stage_E14_vs_E10	ordinary_muscle_development	D_vs_E10;stage_1W_vs_E10;stage_3W_vs_E10;stage	1	TRUE
ENSGALG00010023328	TCEA2	PRJNA729271	-1.754397952	4.90228E-53	1.754397952	stage_E14_vs_E10	ordinary_muscle_development	D_vs_E10;stage_1W_vs_E10;stage_3W_vs_E10;stage	1	TRUE
ENSGALG00010005411	STXBP5	PRJNA729271	1.47018526	5.13649E-53	1.47018526	stage_E14_vs_E10	ordinary_muscle_development	D_vs_E10;stage_1W_vs_E10;stage_3W_vs_E10;stage	1	TRUE
ENSGALG00010025600	ENSGALG00010025600	PRJNA729271	-1.783979518	5.18502E-53	1.783979518	stage_E14_vs_E10	ordinary_muscle_development	D_vs_E10;stage_1W_vs_E10;stage_3W_vs_E10;stage	1	TRUE
ENSGALG00010003183	NAA16	PRJNA729271	1.353810834	5.82637E-53	1.353810834	stage_E14_vs_E10	ordinary_muscle_development	D_vs_E10;stage_1W_vs_E10;stage_3W_vs_E10;stage	1	TRUE
ENSGALG00010024830	ORC2	PRJNA729271	-1.860114818	5.96601E-53	1.860114818	stage_E14_vs_E10	ordinary_muscle_development	D_vs_E10;stage_1W_vs_E10;stage_3W_vs_E10;stage	1	TRUE
ENSGALG00010008514	PIEZO1	PRJNA729271	2.566656342	6.75164E-53	2.566656342	stage_E14_vs_E10	ordinary_muscle_development	D_vs_E10;stage_1W_vs_E10;stage_3W_vs_E10;stage	1	TRUE
ENSGALG00010027933	ENSGALG00010027933	PRJNA729271	3.348512398	7.02404E-53	3.348512398	stage_E14_vs_E10	ordinary_muscle_development	D_vs_E10;stage_1W_vs_E10;stage_3W_vs_E10;stage	1	TRUE
ENSGALG00010014295	CACNA1C	PRJNA729271	2.6871522	7.39674E-53	2.6871522	stage_E14_vs_E10	ordinary_muscle_development	D_vs_E10;stage_1W_vs_E10;stage_3W_vs_E10;stage	1	TRUE
ENSGALG00010010467	FGFBP2	PRJNA729271	-10.16624943	8.11696E-53	10.16624943	stage_E14_vs_E10	ordinary_muscle_development	D_vs_E10;stage_1W_vs_E10;stage_3W_vs_E10;stage	1	TRUE
ENSGALG00010015047	NPY2R	PRJNA729271	-3.494946665	8.30723E-53	3.494946665	stage_E14_vs_E10	ordinary_muscle_development	D_vs_E10;stage_1W_vs_E10;stage_3W_vs_E10;stage	1	TRUE
ENSGALG00010018466	DYSF	PRJNA729271	2.825558513	8.36243E-53	2.825558513	stage_E14_vs_E10	ordinary_muscle_development	D_vs_E10;stage_1W_vs_E10;stage_3W_vs_E10;stage	1	TRUE
ENSGALG00010027533	WDR18	PRJNA729271	-1.539712222	8.57363E-53	1.539712222	stage_E14_vs_E10	ordinary_muscle_development	D_vs_E10;stage_1W_vs_E10;stage_3W_vs_E10;stage	1	TRUE
ENSGALG00010017139	COL9A2	PRJNA729271	-9.454402681	8.6466E-53	9.454402681	stage_E14_vs_E10	ordinary_muscle_development	D_vs_E10;stage_1W_vs_E10;stage_3W_vs_E10;stage	1	TRUE
ENSGALG00010015932	ACOT8	PRJNA729271	2.186444949	8.87593E-53	2.186444949	stage_E14_vs_E10	ordinary_muscle_development	D_vs_E10;stage_1W_vs_E10;stage_3W_vs_E10;stage	1	TRUE
ENSGALG00010002920	VCPIP1	PRJNA729271	1.56408567	1.03564E-52	1.56408567	stage_E14_vs_E10	ordinary_muscle_development	D_vs_E10;stage_1W_vs_E10;stage_3W_vs_E10;stage	1	TRUE
ENSGALG00010003761	UGT8	PRJNA729271	5.013040036	1.19207E-52	5.013040036	stage_E14_vs_E10	ordinary_muscle_development	D_vs_E10;stage_1W_vs_E10;stage_3W_vs_E10;stage	1	TRUE
ENSGALG00010018551	FBXO34	PRJNA729271	1.714491006	1.42061E-52	1.714491006	stage_E14_vs_E10	ordinary_muscle_development	D_vs_E10;stage_1W_vs_E10;stage_3W_vs_E10;stage	1	TRUE
ENSGALG00010016751	DFFA	PRJNA729271	-1.459151675	1.42913E-52	1.459151675	stage_E14_vs_E10	ordinary_muscle_development	D_vs_E10;stage_1W_vs_E10;stage_3W_vs_E10;stage	1	TRUE
ENSGALG00010016930	C10H15ORF48	PRJNA729271	5.9034298	1.45409E-52	5.9034298	stage_E14_vs_E10	ordinary_muscle_development	D_vs_E10;stage_1W_vs_E10;stage_3W_vs_E10;stage	1	TRUE
ENSGALG00010001822	KCNE3	PRJNA729271	4.594608372	1.53888E-52	4.594608372	stage_E14_vs_E10	ordinary_muscle_development	D_vs_E10;stage_1W_vs_E10;stage_3W_vs_E10;stage	1	TRUE
ENSGALG00010014929	FHL1	PRJNA729271	4.408113487	1.54463E-52	4.408113487	stage_E14_vs_E10	ordinary_muscle_development	D_vs_E10;stage_1W_vs_E10;stage_3W_vs_E10;stage	1	TRUE
ENSGALG00010020629	TRIM66	PRJNA729271	-4.10223741	1.82797E-52	4.10223741	stage_E14_vs_E10	ordinary_muscle_development	D_vs_E10;stage_1W_vs_E10;stage_3W_vs_E10;stage	1	TRUE
ENSGALG00010019371	TMEM87A	PRJNA729271	1.17795641	1.96647E-52	1.17795641	stage_E14_vs_E10	ordinary_muscle_development	D_vs_E10;stage_1W_vs_E10;stage_3W_vs_E10;stage	1	TRUE
ENSGALG00010015900	ENSGALG00010015900	PRJNA729271	2.416444189	1.9831E-52	2.416444189	stage_E14_vs_E10	ordinary_muscle_development	D_vs_E10;stage_1W_vs_E10;stage_3W_vs_E10;stage	1	TRUE
ENSGALG00010027626	SEMA3B	PRJNA729271	3.219819106	1.99309E-52	3.219819106	stage_E14_vs_E10	ordinary_muscle_development	D_vs_E10;stage_1W_vs_E10;stage_3W_vs_E10;stage	1	TRUE
ENSGALG00010020775	UBR1	PRJNA729271	1.245727431	2.00605E-52	1.245727431	stage_E14_vs_E10	ordinary_muscle_development	D_vs_E10;stage_1W_vs_E10;stage_3W_vs_E10;stage	1	TRUE
ENSGALG00010011898	ENSGALG00010011898	PRJNA729271	1.688423174	2.03353E-52	1.688423174	stage_E14_vs_E10	ordinary_muscle_development	D_vs_E10;stage_1W_vs_E10;stage_3W_vs_E10;stage	1	TRUE
ENSGALG00010001411	FGFR2	PRJNA729271	-5.769147285	2.05106E-52	5.769147285	stage_E14_vs_E10	ordinary_muscle_development	D_vs_E10;stage_1W_vs_E10;stage_3W_vs_E10;stage	1	TRUE
ENSGALG00010027510	RHOC	PRJNA729271	2.796850288	2.09539E-52	2.796850288	stage_E14_vs_E10	ordinary_muscle_development	D_vs_E10;stage_1W_vs_E10;stage_3W_vs_E10;stage	1	TRUE
ENSGALG00010003034	ERI1	PRJNA729271	-1.008509216	2.20129E-52	1.008509216	stage_E14_vs_E10	ordinary_muscle_development	D_vs_E10;stage_1W_vs_E10;stage_3W_vs_E10;stage	1	TRUE
ENSGALG00010029309	ALKBH4	PRJNA729271	2.501843431	2.32612E-52	2.501843431	stage_E14_vs_E10	ordinary_muscle_development	D_vs_E10;stage_1W_vs_E10;stage_3W_vs_E10;stage	1	TRUE
ENSGALG00010002883	MAP3K15	PRJNA729271	6.356094314	2.37916E-52	6.356094314	stage_E14_vs_E10	ordinary_muscle_development	D_vs_E10;stage_1W_vs_E10;stage_3W_vs_E10;stage	1	TRUE
ENSGALG00010005497	MKX	PRJNA729271	-3.187869193	2.39026E-52	3.187869193	stage_E14_vs_E10	ordinary_muscle_development	D_vs_E10;stage_1W_vs_E10;stage_3W_vs_E10;stage	1	TRUE
ENSGALG00010015298	ENSGALG00010015298	PRJNA729271	3.545852703	2.41159E-52	3.545852703	stage_E14_vs_E10	ordinary_muscle_development	D_vs_E10;stage_1W_vs_E10;stage_3W_vs_E10;stage	1	TRUE
ENSGALG00010012266	H3C33	PRJNA729271	-3.13200601	2.47658E-52	3.13200601	stage_E14_vs_E10	ordinary_muscle_development	D_vs_E10;stage_1W_vs_E10;stage_3W_vs_E10;stage	1	TRUE
ENSGALG00010023342	FITM2	PRJNA729271	2.064308777	2.74315E-52	2.064308777	stage_E14_vs_E10	ordinary_muscle_development	D_vs_E10;stage_1W_vs_E10;stage_3W_vs_E10;stage	1	TRUE
ENSGALG00010001701	NDUFA5	PRJNA729271	2.594911056	2.81675E-52	2.594911056	stage_E14_vs_E10	ordinary_muscle_development	D_vs_E10;stage_1W_vs_E10;stage_3W_vs_E10;stage	1	TRUE
ENSGALG00010029705	GRB2	PRJNA729271	-1.057037895	2.85793E-52	1.057037895	stage_E14_vs_E10	ordinary_muscle_development	D_vs_E10;stage_1W_vs_E10;stage_3W_vs_E10;stage	1	TRUE
ENSGALG00010015040	ZC4H2	PRJNA729271	-1.389845823	3.09359E-52	1.389845823	stage_E14_vs_E10	ordinary_muscle_development	D_vs_E10;stage_1W_vs_E10;stage_3W_vs_E10;stage	1	TRUE
ENSGALG00010013246	LNPEP	PRJNA729271	2.685998066	3.13801E-52	2.685998066	stage_E14_vs_E10	ordinary_muscle_development	D_vs_E10;stage_1W_vs_E10;stage_3W_vs_E10;stage	1	TRUE
ENSGALG00010013316	KDM7A	PRJNA729271	1.990053417	3.36142E-52	1.990053417	stage_E14_vs_E10	ordinary_muscle_development	D_vs_E10;stage_1W_vs_E10;stage_3W_vs_E10;stage	1	TRUE
ENSGALG00010005963	UGCG	PRJNA729271	1.576384076	3.82166E-52	1.576384076	stage_E14_vs_E10	ordinary_muscle_development	D_vs_E10;stage_1W_vs_E10;stage_3W_vs_E10;stage	1	TRUE
ENSGALG00010013370	CNKSR1	PRJNA729271	3.588816612	3.82613E-52	3.588816612	stage_E14_vs_E10	ordinary_muscle_development	D_vs_E10;stage_1W_vs_E10;stage_3W_vs_E10;stage	1	TRUE
ENSGALG00010018838	WFIKKN1	PRJNA729271	-6.738473295	3.87409E-52	6.738473295	stage_E14_vs_E10	ordinary_muscle_development	D_vs_E10;stage_1W_vs_E10;stage_3W_vs_E10;stage	1	TRUE
ENSGALG00010006939	FBXO31	PRJNA729271	1.945825951	3.93158E-52	1.945825951	stage_E14_vs_E10	ordinary_muscle_development	D_vs_E10;stage_1W_vs_E10;stage_3W_vs_E10;stage	1	TRUE
ENSGALG00010001232	TSPAN12	PRJNA729271	1.558194799	3.94963E-52	1.558194799	stage_E14_vs_E10	ordinary_muscle_development	D_vs_E10;stage_1W_vs_E10;stage_3W_vs_E10;stage	1	TRUE
ENSGALG00010016952	GINS1	PRJNA729271	-3.807967844	4.11467E-52	3.807967844	stage_E14_vs_E10	ordinary_muscle_development	D_vs_E10;stage_1W_vs_E10;stage_3W_vs_E10;stage	1	TRUE
ENSGALG00010009508	TOMM20	PRJNA729271	1.379278421	4.59905E-52	1.379278421	stage_E14_vs_E10	ordinary_muscle_development	D_vs_E10;stage_1W_vs_E10;stage_3W_vs_E10;stage	1	TRUE
ENSGALG00010002358	FREM2	PRJNA729271	-3.606675377	4.70584E-52	3.606675377	stage_E14_vs_E10	ordinary_muscle_development	D_vs_E10;stage_1W_vs_E10;stage_3W_vs_E10;stage	1	TRUE
ENSGALG00010009126	JPH3	PRJNA729271	2.076721693	4.93218E-52	2.076721693	stage_E14_vs_E10	ordinary_muscle_development	D_vs_E10;stage_1W_vs_E10;stage_3W_vs_E10;stage	1	TRUE
ENSGALG00010003777	ENSGALG00010003777	PRJNA729271	12.25545899	5.25796E-52	12.25545899	stage_E14_vs_E10	ordinary_muscle_development	D_vs_E10;stage_1W_vs_E10;stage_3W_vs_E10;stage	1	TRUE
ENSGALG00010010188	DSP	PRJNA729271	-5.495497743	5.31021E-52	5.495497743	stage_E14_vs_E10	ordinary_muscle_development	D_vs_E10;stage_1W_vs_E10;stage_3W_vs_E10;stage	1	TRUE
ENSGALG00010009392	MAPRE3	PRJNA729271	1.612827281	5.47617E-52	1.612827281	stage_E14_vs_E10	ordinary_muscle_development	D_vs_E10;stage_1W_vs_E10;stage_3W_vs_E10;stage	1	TRUE
ENSGALG00010018099	SMAD3	PRJNA729271	-2.013681286	5.72444E-52	2.013681286	stage_E14_vs_E10	ordinary_muscle_development	D_vs_E10;stage_1W_vs_E10;stage_3W_vs_E10;stage	1	TRUE
ENSGALG00010029371	AUTS2	PRJNA729271	-2.818455061	5.79888E-52	2.818455061	stage_E14_vs_E10	ordinary_muscle_development	D_vs_E10;stage_1W_vs_E10;stage_3W_vs_E10;stage	1	TRUE
ENSGALG00010016475	MCCC1	PRJNA729271	1.274786846	5.91267E-52	1.274786846	stage_E14_vs_E10	ordinary_muscle_development	D_vs_E10;stage_1W_vs_E10;stage_3W_vs_E10;stage	1	TRUE
ENSGALG00010015054	ENSGALG00010015054	PRJNA729271	-2.749353771	6.12343E-52	2.749353771	stage_E14_vs_E10	ordinary_muscle_development	D_vs_E10;stage_1W_vs_E10;stage_3W_vs_E10;stage	1	TRUE
ENSGALG00010010366	CLU	PRJNA729271	3.603660406	6.39308E-52	3.603660406	stage_E14_vs_E10	ordinary_muscle_development	D_vs_E10;stage_1W_vs_E10;stage_3W_vs_E10;stage	1	TRUE
ENSGALG00010019127	TMBIM1	PRJNA729271	1.864636314	6.41812E-52	1.864636314	stage_E14_vs_E10	ordinary_muscle_development	D_vs_E10;stage_1W_vs_E10;stage_3W_vs_E10;stage	1	TRUE
ENSGALG00010008101	CENPQ	PRJNA729271	-3.304136632	6.72224E-52	3.304136632	stage_E14_vs_E10	ordinary_muscle_development	D_vs_E10;stage_1W_vs_E10;stage_3W_vs_E10;stage	1	TRUE
ENSGALG00010013355	FAM171B	PRJNA729271	-2.340136732	7.07634E-52	2.340136732	stage_E14_vs_E10	ordinary_muscle_development	D_vs_E10;stage_1W_vs_E10;stage_3W_vs_E10;stage	1	TRUE
ENSGALG00010022780	DYNC1I2	PRJNA729271	-0.893918994	7.4506E-52	0.893918994	stage_E14_vs_E10	ordinary_muscle_development		0	FALSE
ENSGALG00010024233	FAIM2	PRJNA729271	-5.206609371	8.56283E-52	5.206609371	stage_E14_vs_E10	ordinary_muscle_development	D_vs_E10;stage_1W_vs_E10;stage_3W_vs_E10;stage	1	TRUE
ENSGALG00010027956	RAB8A	PRJNA729271	-1.033683504	8.63564E-52	1.033683504	stage_E14_vs_E10	ordinary_muscle_development	D_vs_E10;stage_1W_vs_E10;stage_3W_vs_E10;stage	1	TRUE
ENSGALG00010017519	NTN3	PRJNA729271	-3.633034561	8.86052E-52	3.633034561	stage_E14_vs_E10	ordinary_muscle_development	D_vs_E10;stage_1W_vs_E10;stage_3W_vs_E10;stage	1	TRUE
ENSGALG00010024371	YPEL1	PRJNA729271	1.602756289	9.00729E-52	1.602756289	stage_E14_vs_E10	ordinary_muscle_development	D_vs_E10;stage_1W_vs_E10;stage_3W_vs_E10;stage	1	TRUE
ENSGALG00010001925	FRMPD4	PRJNA729271	-3.496733903	9.11204E-52	3.496733903	stage_E14_vs_E10	ordinary_muscle_development	D_vs_E10;stage_1W_vs_E10;stage_3W_vs_E10;stage	1	TRUE
ENSGALG00010015121	UNC79	PRJNA729271	-4.818474565	9.27117E-52	4.818474565	stage_E14_vs_E10	ordinary_muscle_development	D_vs_E10;stage_1W_vs_E10;stage_3W_vs_E10;stage	1	TRUE
ENSGALG00010005558	TAGAP	PRJNA729271	3.127140415	9.28792E-52	3.127140415	stage_E14_vs_E10	ordinary_muscle_development	D_vs_E10;stage_1W_vs_E10;stage_3W_vs_E10;stage	1	TRUE
ENSGALG00010013191	ABRA	PRJNA729271	6.406463758	9.64253E-52	6.406463758	stage_E14_vs_E10	ordinary_muscle_development	D_vs_E10;stage_1W_vs_E10;stage_3W_vs_E10;stage	1	TRUE
ENSGALG00010016817	GGCT	PRJNA729271	-1.818548183	1.05556E-51	1.818548183	stage_E14_vs_E10	ordinary_muscle_development	D_vs_E10;stage_1W_vs_E10;stage_3W_vs_E10;stage	1	TRUE
ENSGALG00010010913	IFT56	PRJNA729271	-2.340889485	1.06434E-51	2.340889485	stage_E14_vs_E10	ordinary_muscle_development	D_vs_E10;stage_1W_vs_E10;stage_3W_vs_E10;stage	1	TRUE
ENSGALG00010019018	SH3BGRL	PRJNA729271	-1.696113843	1.1399E-51	1.696113843	stage_E14_vs_E10	ordinary_muscle_development	D_vs_E10;stage_1W_vs_E10;stage_3W_vs_E10;stage	1	TRUE
ENSGALG00010027487	MPZ	PRJNA729271	8.127201471	1.1789E-51	8.127201471	stage_E14_vs_E10	ordinary_muscle_development	D_vs_E10;stage_1W_vs_E10;stage_3W_vs_E10;stage	1	TRUE
ENSGALG00010006542	RFC3	PRJNA729271	-2.181613018	1.18089E-51	2.181613018	stage_E14_vs_E10	ordinary_muscle_development	D_vs_E10;stage_1W_vs_E10;stage_3W_vs_E10;stage	1	TRUE
ENSGALG00010017724	FLII	PRJNA729271	1.951693266	1.22611E-51	1.951693266	stage_E14_vs_E10	ordinary_muscle_development	D_vs_E10;stage_1W_vs_E10;stage_3W_vs_E10;stage	1	TRUE
ENSGALG00010002981	KLHL35	PRJNA729271	-5.729560331	1.48936E-51	5.729560331	stage_E14_vs_E10	ordinary_muscle_development	D_vs_E10;stage_1W_vs_E10;stage_3W_vs_E10;stage	1	TRUE
ENSGALG00010027165	ENSGALG00010027165	PRJNA729271	-3.56460306	1.53937E-51	3.56460306	stage_E14_vs_E10	ordinary_muscle_development	D_vs_E10;stage_1W_vs_E10;stage_3W_vs_E10;stage	1	TRUE
ENSGALG00010018583	PPL	PRJNA729271	3.250929033	1.57655E-51	3.250929033	stage_E14_vs_E10	ordinary_muscle_development	D_vs_E10;stage_1W_vs_E10;stage_3W_vs_E10;stage	1	TRUE
ENSGALG00010022347	SEC11A	PRJNA729271	-1.625035594	1.66257E-51	1.625035594	stage_E14_vs_E10	ordinary_muscle_development	D_vs_E10;stage_1W_vs_E10;stage_3W_vs_E10;stage	1	TRUE

ENSGALG00010024197	KRT7	PRJNA729271	-4.766629332	1.73818E-51	4.766629332	stage_E14_vs_E10	ordinary_muscle_development	D_vs_E10;stage_1W_vs_E10;stage_3W_vs_E10;stage	1	TRUE
ENSGALG00010002462	RRM2	PRJNA729271	-4.245573437	1.8271E-51	4.245573437	stage_E14_vs_E10	ordinary_muscle_development	D_vs_E10;stage_1W_vs_E10;stage_3W_vs_E10;stage	1	TRUE
ENSGALG00010016970	SYNE3	PRJNA729271	1.504429356	1.86248E-51	1.504429356	stage_E14_vs_E10	ordinary_muscle_development	D_vs_E10;stage_1W_vs_E10;stage_3W_vs_E10;stage	1	TRUE
ENSGALG00010012428	CCNE2	PRJNA729271	-3.730251452	1.9762E-51	3.730251452	stage_E14_vs_E10	ordinary_muscle_development	D_vs_E10;stage_1W_vs_E10;stage_3W_vs_E10;stage	1	TRUE
ENSGALG00010019149	COX5B	PRJNA729271	2.738620474	1.98557E-51	2.738620474	stage_E14_vs_E10	ordinary_muscle_development	D_vs_E10;stage_1W_vs_E10;stage_3W_vs_E10;stage	1	TRUE
ENSGALG00010026062	ITGA4	PRJNA729271	-2.472091617	1.98741E-51	2.472091617	stage_E14_vs_E10	ordinary_muscle_development	D_vs_E10;stage_1W_vs_E10;stage_3W_vs_E10;stage	1	TRUE
ENSGALG00010013282	SEC22B	PRJNA729271	-1.389507509	2.00655E-51	1.389507509	stage_E14_vs_E10	ordinary_muscle_development	D_vs_E10;stage_1W_vs_E10;stage_3W_vs_E10;stage	1	TRUE
ENSGALG00010022695	PIK3IP1	PRJNA729271	3.086982254	2.01762E-51	3.086982254	stage_E14_vs_E10	ordinary_muscle_development	D_vs_E10;stage_1W_vs_E10;stage_3W_vs_E10;stage	1	TRUE
ENSGALG00010014284	TMEM131L	PRJNA729271	-1.324517864	2.02486E-51	1.324517864	stage_E14_vs_E10	ordinary_muscle_development	D_vs_E10;stage_1W_vs_E10;stage_3W_vs_E10;stage	1	TRUE
ENSGALG00010025429	SCN3B	PRJNA729271	-4.279175514	2.07463E-51	4.279175514	stage_E14_vs_E10	ordinary_muscle_development	D_vs_E10;stage_1W_vs_E10;stage_3W_vs_E10;stage	1	TRUE
ENSGALG00010025650	PPP1R1A	PRJNA729271	6.947241719	2.11241E-51	6.947241719	stage_E14_vs_E10	ordinary_muscle_development	D_vs_E10;stage_1W_vs_E10;stage_3W_vs_E10;stage	1	TRUE
ENSGALG00010023775	ENSGALG00010023775	PRJNA729271	2.024668194	2.11605E-51	2.024668194	stage_E14_vs_E10	ordinary_muscle_development	D_vs_E10;stage_1W_vs_E10;stage_3W_vs_E10;stage	1	TRUE
ENSGALG00010016568	PAM16	PRJNA729271	2.03395302	2.24242E-51	2.03395302	stage_E14_vs_E10	ordinary_muscle_development	D_vs_E10;stage_1W_vs_E10;stage_3W_vs_E10;stage	1	TRUE
ENSGALG00010022650	NR1H3	PRJNA729271	1.501747133	2.27498E-51	1.501747133	stage_E14_vs_E10	ordinary_muscle_development	D_vs_E10;stage_1W_vs_E10;stage_3W_vs_E10;stage	1	TRUE
ENSGALG00010007001	SNX30	PRJNA729271	-1.4359845	2.55825E-51	1.4359845	stage_E14_vs_E10	ordinary_muscle_development	D_vs_E10;stage_1W_vs_E10;stage_3W_vs_E10;stage	1	TRUE
ENSGALG00010001957	NDUFC2	PRJNA729271	1.696393832	2.6989E-51	1.696393832	stage_E14_vs_E10	ordinary_muscle_development	D_vs_E10;stage_1W_vs_E10;stage_3W_vs_E10;stage	1	TRUE
ENSGALG00010029636	SPATA20	PRJNA729271	1.136322159	2.72511E-51	1.136322159	stage_E14_vs_E10	ordinary_muscle_development	D_vs_E10;stage_1W_vs_E10;stage_3W_vs_E10;stage	1	TRUE
ENSGALG00010014677	ENSGALG00010014677	PRJNA729271	-1.372847103	2.85439E-51	1.372847103	stage_E14_vs_E10	ordinary_muscle_development	D_vs_E10;stage_1W_vs_E10;stage_3W_vs_E10;stage	1	TRUE
ENSGALG00010016875	SESN2	PRJNA729271	3.317946856	2.85724E-51	3.317946856	stage_E14_vs_E10	ordinary_muscle_development	D_vs_E10;stage_1W_vs_E10;stage_3W_vs_E10;stage	1	TRUE
ENSGALG00010022142	TMEM208	PRJNA729271	1.584342731	3.25552E-51	1.584342731	stage_E14_vs_E10	ordinary_muscle_development	D_vs_E10;stage_1W_vs_E10;stage_3W_vs_E10;stage	1	TRUE
ENSGALG00010014942	PMEPA1	PRJNA729271	-2.267516456	3.26325E-51	2.267516456	stage_E14_vs_E10	ordinary_muscle_development	D_vs_E10;stage_1W_vs_E10;stage_3W_vs_E10;stage	1	TRUE
ENSGALG00010019546	HSD17B12	PRJNA729271	-1.199300403	3.41583E-51	1.199300403	stage_E14_vs_E10	ordinary_muscle_development	D_vs_E10;stage_1W_vs_E10;stage_3W_vs_E10;stage	1	TRUE
ENSGALG00010023854	SHCBP1	PRJNA729271	-3.473390461	3.97076E-51	3.473390461	stage_E14_vs_E10	ordinary_muscle_development	D_vs_E10;stage_1W_vs_E10;stage_3W_vs_E10;stage	1	TRUE
ENSGALG00010020106	ENSGALG00010020106	PRJNA729271	2.459642606	3.98208E-51	2.459642606	stage_E14_vs_E10	ordinary_muscle_development	D_vs_E10;stage_1W_vs_E10;stage_3W_vs_E10;stage	1	TRUE
ENSGALG00010022333	ENSGALG00010022333	PRJNA729271	1.965266411	4.02006E-51	1.965266411	stage_E14_vs_E10	ordinary_muscle_development	D_vs_E10;stage_1W_vs_E10;stage_3W_vs_E10;stage	1	TRUE
ENSGALG00010025358	INCENP	PRJNA729271	-3.155765215	4.26642E-51	3.155765215	stage_E14_vs_E10	ordinary_muscle_development	D_vs_E10;stage_1W_vs_E10;stage_3W_vs_E10;stage	1	TRUE
ENSGALG00010025960	CMKLR2	PRJNA729271	2.400773902	4.30541E-51	2.400773902	stage_E14_vs_E10	ordinary_muscle_development	D_vs_E10;stage_1W_vs_E10;stage_3W_vs_E10;stage	1	TRUE
ENSGALG00010010214	FAF2	PRJNA729271	-1.212244109	4.35311E-51	1.212244109	stage_E14_vs_E10	ordinary_muscle_development	D_vs_E10;stage_1W_vs_E10;stage_3W_vs_E10;stage	1	TRUE
ENSGALG00010020569	DTX3L	PRJNA729271	3.802108759	4.49419E-51	3.802108759	stage_E14_vs_E10	ordinary_muscle_development	D_vs_E10;stage_1W_vs_E10;stage_3W_vs_E10;stage	1	TRUE
ENSGALG00010005705	ECHDC3	PRJNA729271	1.859745639	4.75482E-51	1.859745639	stage_E14_vs_E10	ordinary_muscle_development	D_vs_E10;stage_1W_vs_E10;stage_3W_vs_E10;stage	1	TRUE
ENSGALG00010029180	CA4	PRJNA729271	6.487535276	4.94883E-51	6.487535276	stage_E14_vs_E10	ordinary_muscle_development	D_vs_E10;stage_1W_vs_E10;stage_3W_vs_E10;stage	1	TRUE
ENSGALG00010016828	LACTBL1	PRJNA729271	5.691814118	5.40083E-51	5.691814118	stage_E14_vs_E10	ordinary_muscle_development	D_vs_E10;stage_1W_vs_E10;stage_3W_vs_E10;stage	1	TRUE
ENSGALG00010013808	PLAAT1	PRJNA729271	2.054464604	5.65856E-51	2.054464604	stage_E14_vs_E10	ordinary_muscle_development	D_vs_E10;stage_1W_vs_E10;stage_3W_vs_E10;stage	1	TRUE
ENSGALG00010030104	P4HB	PRJNA729271	-1.674031552	5.66454E-51	1.674031552	stage_E14_vs_E10	ordinary_muscle_development	D_vs_E10;stage_1W_vs_E10;stage_3W_vs_E10;stage	1	TRUE
ENSGALG00010019325	SLITRK4	PRJNA729271	-3.288689445	5.86171E-51	3.288689445	stage_E14_vs_E10	ordinary_muscle_development	D_vs_E10;stage_1W_vs_E10;stage_3W_vs_E10;stage	1	TRUE
ENSGALG00010012753	GTPBP1	PRJNA729271	-0.814640691	6.18491E-51	0.814640691	stage_E14_vs_E10	ordinary_muscle_development		0	FALSE
ENSGALG00010003768	ARSJ	PRJNA729271	-2.059271633	6.87808E-51	2.059271633	stage_E14_vs_E10	ordinary_muscle_development	D_vs_E10;stage_1W_vs_E10;stage_3W_vs_E10;stage	1	TRUE
ENSGALG00010014573	CEBPB	PRJNA729271	5.26407962	7.09205E-51	5.26407962	stage_E14_vs_E10	ordinary_muscle_development	D_vs_E10;stage_1W_vs_E10;stage_3W_vs_E10;stage	1	TRUE
ENSGALG00010027126	ENSGALG00010027126	PRJNA729271	8.117381056	7.95374E-51	8.117381056	stage_E14_vs_E10	ordinary_muscle_development	D_vs_E10;stage_1W_vs_E10;stage_3W_vs_E10;stage	1	TRUE
ENSGALG00010010888	GNPAT	PRJNA729271	1.791710896	8.30987E-51	1.791710896	stage_E14_vs_E10	ordinary_muscle_development	D_vs_E10;stage_1W_vs_E10;stage_3W_vs_E10;stage	1	TRUE
ENSGALG00010020557	ENSGALG00010020557	PRJNA729271	3.878953161	8.83283E-51	3.878953161	stage_E14_vs_E10	ordinary_muscle_development	D_vs_E10;stage_1W_vs_E10;stage_3W_vs_E10;stage	1	TRUE
ENSGALG00010017351	CCR2	PRJNA729271	3.110178292	8.9688E-51	3.110178292	stage_E14_vs_E10	ordinary_muscle_development	D_vs_E10;stage_1W_vs_E10;stage_3W_vs_E10;stage	1	TRUE
ENSGALG00010027096	YIPF1	PRJNA729271	1.65194964	9.1193E-51	1.65194964	stage_E14_vs_E10	ordinary_muscle_development	D_vs_E10;stage_1W_vs_E10;stage_3W_vs_E10;stage	1	TRUE
ENSGALG00010013697	HDAC4	PRJNA729271	1.002450762	9.44702E-51	1.002450762	stage_E14_vs_E10	ordinary_muscle_development	D_vs_E10;stage_1W_vs_E10;stage_3W_vs_E10;stage	1	TRUE
ENSGALG00010015927	GLA	PRJNA729271	2.518004049	9.76069E-51	2.518004049	stage_E14_vs_E10	ordinary_muscle_development	D_vs_E10;stage_1W_vs_E10;stage_3W_vs_E10;stage	1	TRUE
ENSGALG00010023889	SAMD8	PRJNA729271	2.872274676	1.02164E-50	2.872274676	stage_E14_vs_E10	ordinary_muscle_development	D_vs_E10;stage_1W_vs_E10;stage_3W_vs_E10;stage	1	TRUE
ENSGALG00010005126	MED23	PRJNA729271	-0.893098541	1.05616E-50	0.893098541	stage_E14_vs_E10	ordinary_muscle_development		0	FALSE
ENSGALG00010005714	ARHGAP5	PRJNA729271	-1.038433824	1.09363E-50	1.038433824	stage_E14_vs_E10	ordinary_muscle_development	D_vs_E10;stage_1W_vs_E10;stage_3W_vs_E10;stage	1	TRUE
ENSGALG00010029365	PIK3R5	PRJNA729271	2.763289748	1.21062E-50	2.763289748	stage_E14_vs_E10	ordinary_muscle_development	D_vs_E10;stage_1W_vs_E10;stage_3W_vs_E10;stage	1	TRUE
ENSGALG00010016375	CCNI2	PRJNA729271	-2.219297203	1.21064E-50	2.219297203	stage_E14_vs_E10	ordinary_muscle_development	D_vs_E10;stage_1W_vs_E10;stage_3W_vs_E10;stage	1	TRUE
ENSGALG00010018044	INTS11	PRJNA729271	-0.927963748	1.21915E-50	0.927963748	stage_E14_vs_E10	ordinary_muscle_development		0	FALSE
ENSGALG00010029343	SCARF1	PRJNA729271	1.966080555	1.24962E-50	1.966080555	stage_E14_vs_E10	ordinary_muscle_development	D_vs_E10;stage_1W_vs_E10;stage_3W_vs_E10;stage	1	TRUE
ENSGALG00010006673	BAZ1A	PRJNA729271	-1.258804097	1.44731E-50	1.258804097	stage_E14_vs_E10	ordinary_muscle_development	D_vs_E10;stage_1W_vs_E10;stage_3W_vs_E10;stage	1	TRUE
ENSGALG00010028171	RNF123	PRJNA729271	2.383951114	1.50619E-50	2.383951114	stage_E14_vs_E10	ordinary_muscle_development	D_vs_E10;stage_1W_vs_E10;stage_3W_vs_E10;stage	1	TRUE
ENSGALG00010008341	DHODH	PRJNA729271	-1.68243968	1.50956E-50	1.68243968	stage_E14_vs_E10	ordinary_muscle_development	D_vs_E10;stage_1W_vs_E10;stage_3W_vs_E10;stage	1	TRUE
ENSGALG00010027607	ABL1	PRJNA729271	-1.207456037	1.55393E-50	1.207456037	stage_E14_vs_E10	ordinary_muscle_development	D_vs_E10;stage_1W_vs_E10;stage_3W_vs_E10;stage	1	TRUE
ENSGALG00010017730	TARP	PRJNA729271	8.120685934	1.62646E-50	8.120685934	stage_E14_vs_E10	ordinary_muscle_development	D_vs_E10;stage_1W_vs_E10;stage_3W_vs_E10;stage	1	TRUE
ENSGALG00010029208	CACNG5	PRJNA729271	-5.03036083	1.62926E-50	5.03036083	stage_E14_vs_E10	ordinary_muscle_development	D_vs_E10;stage_1W_vs_E10;stage_3W_vs_E10;stage	1	TRUE
ENSGALG00010016425	TRIM63	PRJNA729271	6.252793522	1.6428E-50	6.252793522	stage_E14_vs_E10	ordinary_muscle_development	D_vs_E10;stage_1W_vs_E10;stage_3W_vs_E10;stage	1	TRUE
ENSGALG00010007259	ANKRD34B	PRJNA729271	-1.477733437	1.6493E-50	1.477733437	stage_E14_vs_E10	ordinary_muscle_development	D_vs_E10;stage_1W_vs_E10;stage_3W_vs_E10;stage	1	TRUE
ENSGALG00010017162	BCL11A	PRJNA729271	-3.743531891	1.82392E-50	3.743531891	stage_E14_vs_E10	ordinary_muscle_development	D_vs_E10;stage_1W_vs_E10;stage_3W_vs_E10;stage	1	TRUE
ENSGALG00010022207	ENSGALG00010022207	PRJNA729271	4.613461936	1.83177E-50	4.613461936	stage_E14_vs_E10	ordinary_muscle_development	D_vs_E10;stage_1W_vs_E10;stage_3W_vs_E10;stage	1	TRUE
ENSGALG00010023942	NEGR1	PRJNA729271	3.330053866	1.83658E-50	3.330053866	stage_E14_vs_E10	ordinary_muscle_development	D_vs_E10;stage_1W_vs_E10;stage_3W_vs_E10;stage	1	TRUE
ENSGALG00010017652	C1orf115	PRJNA729271	2.563541312	1.89091E-50	2.563541312	stage_E14_vs_E10	ordinary_muscle_development	D_vs_E10;stage_1W_vs_E10;stage_3W_vs_E10;stage	1	TRUE
ENSGALG00010009658	NAPEPLD	PRJNA729271	1.84671564	1.93624E-50	1.84671564	stage_E14_vs_E10	ordinary_muscle_development	D_vs_E10;stage_1W_vs_E10;stage_3W_vs_E10;stage	1	TRUE
ENSGALG00010018405	DCLK3	PRJNA729271	-3.031126364	1.97438E-50	3.031126364	stage_E14_vs_E10	ordinary_muscle_development	D_vs_E10;stage_1W_vs_E10;stage_3W_vs_E10;stage	1	TRUE
ENSGALG00010020486	MRTFB	PRJNA729271	1.685011343	2.05942E-50	1.685011343	stage_E14_vs_E10	ordinary_muscle_development	D_vs_E10;stage_1W_vs_E10;stage_3W_vs_E10;stage	1	TRUE
ENSGALG00010004425	CREM	PRJNA729271	3.631239172	2.06999E-50	3.631239172	stage_E14_vs_E10	ordinary_muscle_development	D_vs_E10;stage_1W_vs_E10;stage_3W_vs_E10;stage	1	TRUE
ENSGALG00010016972	ASB3	PRJNA729271	-1.819999577	2.26448E-50	1.819999577	stage_E14_vs_E10	ordinary_muscle_development	D_vs_E10;stage_1W_vs_E10;stage_3W_vs_E10;stage	1	TRUE
ENSGALG00010012997	UBE2A	PRJNA729271	0.8656537	2.28269E-50	0.8656537	stage_E14_vs_E10	ordinary_muscle_development		0	FALSE
ENSGALG00010002039	AAMDC	PRJNA729271	1.851864442	2.28307E-50	1.851864442	stage_E14_vs_E10	ordinary_muscle_development	D_vs_E10;stage_1W_vs_E10;stage_3W_vs_E10;stage	1	TRUE
ENSGALG00010025580	RASSF7	PRJNA729271	2.094033694	2.29675E-50	2.094033694	stage_E14_vs_E10	ordinary_muscle_development	D_vs_E10;stage_1W_vs_E10;stage_3W_vs_E10;stage	1	TRUE
ENSGALG00010006748	FAM177A1	PRJNA729271	1.159166751	2.3244E-50	1.159166751	stage_E14_vs_E10	ordinary_muscle_development	D_vs_E10;stage_1W_vs_E10;stage_3W_vs_E10;stage	1	TRUE
ENSGALG00010025048	ESYT2	PRJNA729271	1.239039938	2.35179E-50	1.239039938	stage_E14_vs_E10	ordinary_muscle_development	D_vs_E10;stage_1W_vs_E10;stage_3W_vs_E10;stage	1	TRUE
ENSGALG00010011028	HMBOX1	PRJNA729271	-1.339501031	2.44952E-50	1.339501031	stage_E14_vs_E10	ordinary_muscle_development	D_vs_E10;stage_1W_vs_E10;stage_3W_vs_E10;stage	1	TRUE
ENSGALG00010016789	DISP3	PRJNA729271	1.675083004	2.6568E-50	1.675083004	stage_E14_vs_E10	ordinary_muscle_development	D_vs_E10;stage_1W_vs_E10;stage_3W_vs_E10;stage	1	TRUE
ENSGALG00010010829	LAMB4	PRJNA729271	-4.363577926	2.67242E-50	4.363577926	stage_E14_vs_E10	ordinary_muscle_development	D_vs_E10;stage_1W_vs_E10;stage_3W_vs_E10;stage	1	TRUE
ENSGALG00010017577	CLPX	PRJNA729271	2.075921158	2.81419E-50	2.075921158	stage_E14_vs_E10	ordinary_muscle_development	D_vs_E10;stage_1W_vs_E10;stage_3W_vs_E10;stage	1	TRUE
ENSGALG00010020168	COX7A2L	PRJNA729271	1.724741628	2.88673E-50	1.724741628	stage_E14_vs_E10	ordinary_muscle_development	D_vs_E10;stage_1W_vs_E10;stage_3W_vs_E10;stage	1	TRUE
ENSGALG00010002707	RELT	PRJNA729271	-4.997422428	2.89486E-50	4.997422428	stage_E14_vs_E10	ordinary_muscle_development	D_vs_E10;stage_1W_vs_E10;stage_3W_vs_E10;stage	1	TRUE
ENSGALG00010011441	CD99L2	PRJNA729271	1.200314215	2.94101E-50	1.200314215	stage_E14_vs_E10	ordinary_muscle_development	D_vs_E10;stage_1W_vs_E10;stage_3W_vs_E10;stage	1	TRUE
ENSGALG00010021136	WEE1	PRJNA729271	-2.271314086	2.94653E-50	2.271314086	stage_E14_vs_E10	ordinary_muscle_development	D_vs_E10;stage_1W_vs_E10;stage_3W_vs_E10;stage	1	TRUE
ENSGALG00010021387	SLC2A14	PRJNA729271	1.402321412	3.04233E-50	1.402321412	stage_E14_vs_E10	ordinary_muscle_development	D_vs_E10;stage_1W_vs_E10;stage_3W_vs_E10;stage	1	TRUE
ENSGALG00010001135	GYG1	PRJNA729271	2.398095181	3.1652E-50	2.398095181	stage_E14_vs_E10	ordinary_muscle_development	D_vs_E10;stage_1W_vs_E10;stage_3W_vs_E10;stage	1	TRUE
ENSGALG00010027746	UQCR11	PRJNA729271	2.774848504	3.39835E-50	2.774848504	stage_E14_vs_E10	ordinary_muscle_development	D_vs_E10;stage_1W_vs_E10;stage_3W_vs_E10;stage	1	TRUE
ENSGALG00010006946	PLPPR1	PRJNA729271	-7.16027822	3.42176E-50	7.16027822	stage_E14_vs_E10	ordinary_muscle_development	D_vs_E10;stage_1W_vs_E10;stage_3W_vs_E10;stage	1	TRUE
ENSGALG00010021498	B3GALT6	PRJNA729271	-1.742500902	3.5296E-50	1.742500902	stage_E14_vs_E10	ordinary_muscle_development	D_vs_E10;stage_1W_vs_E10;stage_3W_vs_E10;stage	1	TRUE
ENSGALG00010005282	FLT1	PRJNA729271	1.887569966	3.54977E-50	1.887569966	stage_E14_vs_E10	ordinary_muscle_development	D_vs_E10;stage_1W_vs_E10;stage_3W_vs_E10;stage	1	TRUE
ENSGALG00010001165	ENSGALG00010001165	PRJNA729271	2.382596875	3.62798E-50	2.382596875	stage_E14_vs_E10	ordinary_muscle_development	D_vs_E10;stage_1W_vs_E10;stage_3W_vs_E10;stage	1	TRUE
ENSGALG00010020272	ENSGALG00010020272	PRJNA729271	-1.428472517	3.65763E-50	1.428472517	stage_E14_vs_E10	ordinary_muscle_development	D_vs_E10;stage_1W_vs_E10;stage_3W_vs_E10;stage	1	TRUE
ENSGALG00010001551	FHIP1B	PRJNA729271	2.404618489	3.65788E-50	2.404618489	stage_E14_vs_E10	ordinary_muscle_development	D_vs_E10;stage_1W_vs_E10;stage_3W_vs_E10;stage	1	TRUE
ENSGALG00010012973	GLDC	PRJNA729271	-5.42757944	3.84532E-50	5.42757944	stage_E14_vs_E10	ordinary_muscle_development	D_vs_E10;stage_1W_vs_E10;stage_3W_vs_E10;stage	1	TRUE
ENSGALG00010024002	DGCR2	PRJNA729271	-0.931994887	3.87005E-50	0.931994887	stage_E14_vs_E10	ordinary_muscle_development		0	FALSE
ENSGALG00010010336	FMNL2	PRJNA729271	-1.363247879	4.10105E-50	1.363247879	stage_E14_vs_E10	ordinary_muscle_development	D_vs_E10;stage_1W_vs_E10;stage_3W_vs_E10;stage	1	TRUE
ENSGALG00010029207	SPAG5	PRJNA729271	-3.489540535	4.45608E-50	3.489540535	stage_E14_vs_E10	ordinary_muscle_development	D_vs_E10;stage_1W_vs_E10;stage_3W_vs_E10;stage	1	TRUE
ENSGALG00010011617	MAMLD1	PRJNA729271	1.439944608	4.49498E-50	1.439944608	stage_E14_vs_E10	ordinary_muscle_development	D_vs_E10;stage_1W_vs_E10;stage_3W_vs_E10;stage	1	TRUE
ENSGALG00010029614	MDH2	PRJNA729271	1.931843922	4.61248E-50	1.931843922	stage_E14_vs_E10	ordinary_muscle_development	D_vs_E10;stage_1W_vs_E10;stage_3W_vs_E10;stage	1	TRUE
ENSGALG00010010915	APP	PRJNA729271	-1.660694704	4.86656E-50	1.660694704	stage_E14_vs_E10	ordinary_muscle_development	D_vs_E10;stage_1W_vs_E10;stage_3W_vs_E10;stage	1	TRUE
ENSGALG00010007130	IRF2	PRJNA729271	1.829501937	5.04447E-50	1.829501937	stage_E14_vs_E10	ordinary_muscle_development	D_vs_E10;stage_1W_vs_E10;stage_3W_vs_E10;stage	1	TRUE
ENSGALG00010010444	TXNDC5	PRJNA729271	-2.110875086	5.3132E-50	2.110875086	stage_E14_vs_E10	ordinary_muscle_development	D_vs_E10;stage_1W_vs_E10;stage_3W_vs_E10;stage	1	TRUE
ENSGALG00010006175	IL2RA	PRJNA729271	7.391314169	5.71745E-50	7.391314169	stage_E14_vs_E10	ordinary_muscle_development	D_vs_E10;stage_1W_vs_E10;stage_3W_vs_E10;stage	1	TRUE
ENSGALG00010014654	LAMP1	PRJNA729271	1.568673407	5.82969E-50	1.568673407	stage_E14_vs_E10	ordinary_muscle_development	D_vs_E10;stage_1W_vs_E10;stage_3W_vs_E10;stage	1	TRUE
ENSGALG00010027117	RASSF5	PRJNA729271	3.427583394	6.16063E-50	3.427583394	stage_E14_vs_E10	ordinary_muscle_development	D_vs_E10;stage_1W_vs_E10;stage_3W_vs_E10;stage	1	TRUE
ENSGALG00010007978	JADE1	PRJNA729271	1.732715521	6.29312E-50	1.732715521	stage_E14_vs_E10	ordinary_muscle_development	D_vs_E10;stage_1W_vs_E10;stage_3W_vs_E10;stage	1	TRUE
ENSGALG00010024184	ENSGALG00010024184	PRJNA729271	-3.39826794	6.29929E-50	3.39826794	stage_E14_vs_E10	ordinary_muscle_development	D_vs_E10;stage_1W_vs_E10;stage_3W_vs_E10;stage	1	TRUE
ENSGALG00010018560	ENSGALG00010018560	PRJNA729271	1.203123268	6.30534E-50	1.203123268	stage_E14_vs_E10	ordinary_muscle_development	D_vs_E10;stage_1W_vs_E10;stage_3W_vs_E10;stage	1	TRUE
ENSGALG00010025291	DUS2	PRJNA729271	-1.553856908	7.15383E-50	1.553856908	stage_E14_vs_E10	ordinary_muscle_development	D_vs_E10;stage_1W_vs_E10;stage_3W_vs_E10;stage	1	TRUE
ENSGALG00010010625	FAM169A	PRJNA729271	-7.711724686	7.48481E-50	7.711724686	stage_E14_vs_E10	ordinary_muscle_development	D_vs_E10;stage_1W_vs_E10;stage_3W_vs_E10;stage	1	TRUE
ENSGALG00010017796	MXI1	PRJNA729271	1.979193127	7.98748E-50	1.979193127	stage_E14_vs_E10	ordinary_muscle_development	D_vs_E10;stage_1W_vs_E10;stage_3W_vs_E10;stage	1	TRUE
ENSGALG00010013184	LDHB	PRJNA729271	-1.725116497	8.01004E-50	1.725116497	stage_E14_vs_E10	ordinary_muscle_development	D_vs_E10;stage_1W_vs_E10;stage_3W_vs_E10;stage	1	TRUE
ENSGALG00010019789	ARNT2	PRJNA729271	-2.679472676	8.56404E-50	2.679472676	stage_E14_vs_E10	ordinary_muscle_development	D_vs_E10;stage_1W_vs_E10;stage_3W_vs_E10;stage	1	TRUE
ENSGALG00010021848	LARGE2	PRJNA729271	2.04886968	8.90247E-50	2.04886968	stage_E14_vs_E10	ordinary_muscle_development	D_vs_E10;stage_1W_vs_E10;stage_3W_vs_E10;stage	1	TRUE
ENSGALG00010002481	CKAP2	PRJNA729271	-3.715562696	9.31824E-50	3.715562696	stage_E14_vs_E10	ordinary_muscle_development	D_vs_E10;stage_1W_vs_E10;stage_3W_vs_E10;stage	1	TRUE
ENSGALG00010024251	ENSGALG00010024251	PRJNA729271	3.089311553	9.43211E-50	3.089311553	stage_E14_vs_E10	ordinary_muscle_development	D_vs_E10;stage_1W_vs_E10;stage_3W_vs_E10;stage	1	TRUE
ENSGALG00010009037	ENSGALG00010009037	PRJNA729271	2.69570826	1.018E-49	2.69570826	stage_E14_vs_E10	ordinary_muscle_development	D_vs_E10;stage_1W_vs_E10;stage_3W_vs_E10;stage	1	TRUE
ENSGALG00010005467	ENSGALG00010005467	PRJNA729271	3.754200297	1.1704E-49	3.754200297	stage_E14_vs_E10	ordinary_muscle_development	D_vs_E10;stage_1W_vs_E10;stage_3W_vs_E10;stage	1	TRUE
ENSGALG00010029694	ARMC7	PRJNA729271	1.315746924	1.26099E-49	1.315746924	stage_E14_vs_E10	ordinary_muscle_development	D_vs_E10;stage_1W_vs_E10;stage_3W_vs_E10;stage	1	TRUE
ENSGALG00010024823	ASB10	PRJNA729271	12.95842035	1.29014E-49	12.95842035	stage_E14_vs_E10	ordinary_muscle_development	D_vs_E10;stage_1W_vs_E10;stage_3W_vs_E10;stage	1	TRUE
ENSGALG00010003152	ENSGALG00010003152	PRJNA729271	1.90986932	1.30383E-49	1.90986932	stage_E14_vs_E10	ordinary_muscle_development	D_vs_E10;stage_1W_vs_E10;stage_3W_vs_E10;stage	1	TRUE
ENSGALG00010027185	ATRIP	PRJNA729271	-1.277893216	1.31454E-49	1.277893216	stage_E14_vs_E10	ordinary_muscle_development	D_vs_E10;stage_1W_vs_E10;stage_3W_vs_E10;stage	1	TRUE
ENSGALG00010013609	PIGC	PRJNA729271	2.403719126	1.36478E-49	2.403719126	stage_E14_vs_E10	ordinary_muscle_development	D_vs_E10;stage_1W_vs_E10;stage_3W_vs_E10;stage	1	TRUE
ENSGALG00010026264	FZD7	PRJNA729271	-2.14131266	1.41704E-49	2.14131266	stage_E14_vs_E10	ordinary_muscle_development	D_vs_E10;stage_1W_vs_E10;stage_3W_vs_E10;stage	1	TRUE
ENSGALG00010021156	DDOST	PRJNA729271	-1.215872147	1.47173E-49	1.215872147	stage_E14_vs_E10	ordinary_muscle_development	D_vs_E10;stage_1W_vs_E10;stage_3W_vs_E10;stage	1	TRUE
ENSGALG00010002609	PDIA6	PRJNA729271	-1.882555486	1.63861E-49	1.882555486	stage_E14_vs_E10	ordinary_muscle_development	D_vs_E10;stage_1W_vs_E10;stage_3W_vs_E10;stage	1	TRUE
ENSGALG00010023293	COL11A1	PRJNA729271	-7.353208777	1.67734E-49	7.353208777	stage_E14_vs_E10	ordinary_muscle_development	D_vs_E10;stage_1W_vs_E10;stage_3W_vs_E10;stage	1	TRUE
ENSGALG00010002807	P2RY6	PRJNA729271	3.198338503	1.68029E-49	3.198338503	stage_E14_vs_E10	ordinary_muscle_development	D_vs_E10;stage_1W_vs_E10;stage_3W_vs_E10;stage	1	TRUE
ENSGALG00010020323	ARHGAP25	PRJNA729271	2.258671855	1.78465E-49	2.258671855	stage_E14_vs_E10	ordinary_muscle_development	D_vs_E10;stage_1W_vs_E10;stage_3W_vs_E10;stage	1	TRUE
ENSGALG00010020744	CHST3	PRJNA729271	-1.803104981	1.87283E-49	1.803104981	stage_E14_vs_E10	ordinary_muscle_development	D_vs_E10;stage_1W_vs_E10;stage_3W_vs_E10;stage	1	TRUE
ENSGALG00010011745	AFAP1L1	PRJNA729271	2.853228538	1.99736E-49	2.853228538	stage_E14_vs_E10	ordinary_muscle_development	D_vs_E10;stage_1W_vs_E10;stage_3W_vs_E10;stage	1	TRUE
ENSGALG00010021075	DHRS3	PRJNA729271	-2.022979889	2.07541E-49	2.022979889	stage_E14_vs_E10	ordinary_muscle_development	D_vs_E10;stage_1W_vs_E10;stage_3W_vs_E10;stage	1	TRUE
ENSGALG00010001174	ENSGALG00010001174	PRJNA729271	-2.17035722	2.28356E-49	2.17035722	stage_E14_vs_E10	ordinary_muscle_development	D_vs_E10;stage_1W_vs_E10;stage_3W_vs_E10;stage	1	TRUE
ENSGALG00010013635	BCL6	PRJNA729271	2.344975993	2.36073E-49	2.344975993	stage_E14_vs_E10	ordinary_muscle_development	D_vs_E10;stage_1W_vs_E10;stage_3W_vs_E10;stage	1	TRUE
ENSGALG00010002137	PDK3	PRJNA729271	1.965806621	2.38431E-49	1.965806621	stage_E14_vs_E10	ordinary_muscle_development	D_vs_E10;stage_1W_vs_E10;stage_3W_vs_E10;stage	1	TRUE
ENSGALG00010022166	SDHB	PRJNA729271	1.608366668	2.39568E-49	1.608366668	stage_E14_vs_E10	ordinary_muscle_development	D_vs_E10;stage_1W_vs_E10;stage_3W_vs_E10;stage	1	TRUE
ENSGALG00010006091	RTKN	PRJNA729271	-1.564389478	2.42682E-49	1.564389478	stage_E14_vs_E10	ordinary_muscle_development	D_vs_E10;stage_1W_vs_E10;stage_3W_vs_E10;stage	1	TRUE
ENSGALG00010011384	PUM3	PRJNA729271	-1.23475069	2.52538E-49	1.23475069	stage_E14_vs_E10	ordinary_muscle_development	D_vs_E10;stage_1W_vs_E10;stage_3W_vs_E10;stage	1	TRUE
ENSGALG00010020638	IMPDH2	PRJNA729271	1.66071644	2.58407E-49	1.66071644	stage_E14_vs_E10	ordinary_muscle_development	D_vs_E10;stage_1W_vs_E10;stage_3W_vs_E10;stage	1	TRUE
ENSGALG00010026498	ENSGALG00010026498	PRJNA729271	-1.327040585	2.58549E-49	1.327040585	stage_E14_vs_E10	ordinary_muscle_development	D_vs_E10;stage_1W_vs_E10;stage_3W_vs_E10;stage	1	TRUE
ENSGALG00010010931	CYYR1	PRJNA729271	-2.983410703	2.60779E-49	2.983410703	stage_E14_vs_E10	ordinary_muscle_development	D_vs_E10;stage_1W_vs_E10;stage_3W_vs_E10;stage	1	TRUE
ENSGALG00010024633	NRGN	PRJNA729271	4.154173669	2.67747E-49	4.154173669	stage_E14_vs_E10	ordinary_muscle_development	D_vs_E10;stage_1W_vs_E10;stage_3W_vs_E10;stage	1	TRUE
ENSGALG00010019925	RDH11	PRJNA729271	-2.263562835	2.71717E-49	2.263562835	stage_E14_vs_E10	ordinary_muscle_development	D_vs_E10;stage_1W_vs_E10;stage_3W_vs_E10;stage	1	TRUE
ENSGALG00010017272	NRXN1	PRJNA729271	2.000931265	2.82223E-49	2.000931265	stage_E14_vs_E10	ordinary_muscle_development	D_vs_E10;stage_1W_vs_E10;stage_3W_vs_E10;stage	1	TRUE
ENSGALG00010016890	UBR7	PRJNA729271	-1.144658884	2.83286E-49	1.144658884	stage_E14_vs_E10	ordinary_muscle_development	D_vs_E10;stage_1W_vs_E10;stage_3W_vs_E10;stage	1	TRUE
ENSGALG00010017583	MTHFD1	PRJNA729271	-1.600074778	2.97849E-49	1.600074778	stage_E14_vs_E10	ordinary_muscle_development	D_vs_E10;stage_1W_vs_E10;stage_3W_vs_E10;stage	1	TRUE
ENSGALG00010029192	DTX2	PRJNA729271	1.502236336	3.01934E-49	1.502236336	stage_E14_vs_E10	ordinary_muscle_development	D_vs_E10;stage_1W_vs_E10;stage_3W_vs_E10;stage	1	TRUE
ENSGALG00010016550	FBLIM1	PRJNA729271	-2.184247219	3.04497E-49	2.184247219	stage_E14_vs_E10	ordinary_muscle_development	D_vs_E10;stage_1W_vs_E10;stage_3W_vs_E10;stage	1	TRUE
ENSGALG00010024682	ENSGALG00010024682	PRJNA729271	-2.676345805	3.30196E-49	2.676345805	stage_E14_vs_E10	ordinary_muscle_development	D_vs_E10;stage_1W_vs_E10;stage_3W_vs_E10;stage	1	TRUE
ENSGALG00010001202	CFTR	PRJNA729271	3.669158038	3.31831E-49	3.669158038	stage_E14_vs_E10	ordinary_muscle_development	D_vs_E10;stage_1W_vs_E10;stage_3W_vs_E10;stage	1	TRUE
ENSGALG00010018616	ENSGALG00010018616	PRJNA729271	6.98482304	3.36629E-49	6.98482304	stage_E14_vs_E10	ordinary_muscle_development	D_vs_E10;stage_1W_vs_E10;stage_3W_vs_E10;stage	1	TRUE
ENSGALG00010007224	DHFR	PRJNA729271	-3.27510876	3.55736E-49	3.27510876	stage_E14_vs_E10	ordinary_muscle_development	D_vs_E10;stage_1W_vs_E10;stage_3W_vs_E10;stage	1	TRUE
ENSGALG00010021383	MSI1	PRJNA729271	-1.811519442	3.5899E-49	1.811519442	stage_E14_vs_E10	ordinary_muscle_development	D_vs_E10;stage_1W_vs_E10;stage_3W_vs_E10;stage	1	TRUE
ENSGALG00010021842	ENSGALG00010021842	PRJNA729271	-2.894941154	3.94396E-49	2.894941154	stage_E14_vs_E10	ordinary_muscle_development	D_vs_E10;stage_1W_vs_E10;stage_3W_vs_E10;stage	1	TRUE
ENSGALG00010020887	ARPP19	PRJNA729271	-1.063725391	4.08072E-49	1.063725391	stage_E14_vs_E10	ordinary_muscle_development	D_vs_E10;stage_1W_vs_E10;stage_3W_vs_E10;stage	1	TRUE
ENSGALG00010027364	FSTL3	PRJNA729271	3.424486132	4.20159E-49	3.424486132	stage_E14_vs_E10	ordinary_muscle_development	D_vs_E10;stage_1W_vs_E10;stage_3W_vs_E10;stage	1	TRUE
ENSGALG00010017810	HIF1A	PRJNA729271	-1.18753164	4.27475E-49	1.18753164	stage_E14_vs_E10	ordinary_muscle_development	D_vs_E10;stage_1W_vs_E10;stage_3W_vs_E10;stage	1	TRUE
ENSGALG00010013402	MARCKS	PRJNA729271	-2.311550326	4.32098E-49	2.311550326	stage_E14_vs_E10	ordinary_muscle_development	D_vs_E10;stage_1W_vs_E10;stage_3W_vs_E10;stage	1	TRUE
ENSGALG00010004952	PEX1	PRJNA729271	1.784329705	4.54505E-49	1.784329705	stage_E14_vs_E10	ordinary_muscle_development	D_vs_E10;stage_1W_vs_E10;stage_3W_vs_E10;stage	1	TRUE
ENSGALG00010006345	SMC2	PRJNA729271	-3.735025188	4.57242E-49	3.735025188	stage_E14_vs_E10	ordinary_muscle_development	D_vs_E10;stage_1W_vs_E10;stage_3W_vs_E10;stage	1	TRUE
ENSGALG00010009034	SNX17	PRJNA729271	1.768327941	4.65786E-49	1.768327941	stage_E14_vs_E10	ordinary_muscle_development	D_vs_E10;stage_1W_vs_E10;stage_3W_vs_E10;stage	1	TRUE
ENSGALG00010022506	EBNA1BP2	PRJNA729271	-1.297091392	4.84684E-49	1.297091392	stage_E14_vs_E10	ordinary_muscle_development	D_vs_E10;stage_1W_vs_E10;stage_3W_vs_E10;stage	1	TRUE
ENSGALG00010005507	USP53	PRJNA729271	1.77364542	5.09449E-49	1.77364542	stage_E14_vs_E10	ordinary_muscle_development	D_vs_E10;stage_1W_vs_E10;stage_3W_vs_E10;stage	1	TRUE
ENSGALG00010008017	GMNN	PRJNA729271	-2.062964188	5.44212E-49	2.062964188	stage_E14_vs_E10	ordinary_muscle_development	D_vs_E10;stage_1W_vs_E10;stage_3W_vs_E10;stage	1	TRUE
ENSGALG00010005445	SFT2D2	PRJNA729271	-1.596746543	5.87466E-49	1.596746543	stage_E14_vs_E10	ordinary_muscle_development	D_vs_E10;stage_1W_vs_E10;stage_3W_vs_E10;stage	1	TRUE
ENSGALG00010026083	MUTYH	PRJNA729271	-1.989863656	6.02427E-49	1.989863656	stage_E14_vs_E10	ordinary_muscle_development	D_vs_E10;stage_1W_vs_E10;stage_3W_vs_E10;stage	1	TRUE
ENSGALG00010014505	CYTH4	PRJNA729271	3.109265642	6.04198E-49	3.109265642	stage_E14_vs_E10	ordinary_muscle_development	D_vs_E10;stage_1W_vs_E10;stage_3W_vs_E10;stage	1	TRUE
ENSGALG00010020653	NDUFAF3	PRJNA729271	1.729881779	6.13336E-49	1.729881779	stage_E14_vs_E10	ordinary_muscle_development	D_vs_E10;stage_1W_vs_E10;stage_3W_vs_E10;stage	1	TRUE
ENSGALG00010023778	FANCF	PRJNA729271	-3.560824089	6.18858E-49	3.560824089	stage_E14_vs_E10	ordinary_muscle_development	D_vs_E10;stage_1W_vs_E10;stage_3W_vs_E10;stage	1	TRUE
ENSGALG00010012122	GRIP1	PRJNA729271	-4.270838645	6.2491E-49	4.270838645	stage_E14_vs_E10	ordinary_muscle_development	D_vs_E10;stage_1W_vs_E10;stage_3W_vs_E10;stage	1	TRUE
ENSGALG00010024653	RFLNA	PRJNA729271	-3.899637678	6.44606E-49	3.899637678	stage_E14_vs_E10	ordinary_muscle_development	D_vs_E10;stage_1W_vs_E10;stage_3W_vs_E10;stage	1	TRUE
ENSGALG00010013252	PPM1H	PRJNA729271	-3.162155508	6.4884E-49	3.162155508	stage_E14_vs_E10	ordinary_muscle_development	D_vs_E10;stage_1W_vs_E10;stage_3W_vs_E10;stage	1	TRUE
ENSGALG00010006926	ID4	PRJNA729271	-3.501098918	6.94888E-49	3.501098918	stage_E14_vs_E10	ordinary_muscle_development	D_vs_E10;stage_1W_vs_E10;stage_3W_vs_E10;stage	1	TRUE
ENSGALG00010028875	DUSP23	PRJNA729271	1.768577129	6.97357E-49	1.768577129	stage_E14_vs_E10	ordinary_muscle_development	D_vs_E10;stage_1W_vs_E10;stage_3W_vs_E10;stage	1	TRUE
ENSGALG00010019807	ZDHHC6	PRJNA729271	1.156338479	7.04214E-49	1.156338479	stage_E14_vs_E10	ordinary_muscle_development	D_vs_E10;stage_1W_vs_E10;stage_3W_vs_E10;stage	1	TRUE
ENSGALG00010027885	PLEKHJ1	PRJNA729271	-1.121163648	7.2342E-49	1.121163648	stage_E14_vs_E10	ordinary_muscle_development	D_vs_E10;stage_1W_vs_E10;stage_3W_vs_E10;stage	1	TRUE
ENSGALG00010018498	ENSGALG00010018498	PRJNA729271	5.647678748	7.50647E-49	5.647678748	stage_E14_vs_E10	ordinary_muscle_development	D_vs_E10;stage_1W_vs_E10;stage_3W_vs_E10;stage	1	TRUE

ENSGALG00010019767	SUCLG2	PRJNA729271	-1.176544951	8.35555E-49	1.176544951	stage_E14_vs_E10	ordinary_muscle_development	D_vs_E10;stage_1W_vs_E10;stage_3W_vs_E10;stage	1	TRUE
ENSGALG00010024681	ATP6V0A2	PRJNA729271	1.26055555	8.6008E-49	1.26055555	stage_E14_vs_E10	ordinary_muscle_development	D_vs_E10;stage_1W_vs_E10;stage_3W_vs_E10;stage	1	TRUE
ENSGALG00010024946	KIF1A	PRJNA729271	-5.113062109	8.6953E-49	5.113062109	stage_E14_vs_E10	ordinary_muscle_development	D_vs_E10;stage_1W_vs_E10;stage_3W_vs_E10;stage	1	TRUE
ENSGALG00010014221	SCYL3	PRJNA729271	1.939140175	8.90661E-49	1.939140175	stage_E14_vs_E10	ordinary_muscle_development	D_vs_E10;stage_1W_vs_E10;stage_3W_vs_E10;stage	1	TRUE
ENSGALG00010027137	FKBP8	PRJNA729271	1.320834283	8.92744E-49	1.320834283	stage_E14_vs_E10	ordinary_muscle_development	D_vs_E10;stage_1W_vs_E10;stage_3W_vs_E10;stage	1	TRUE
ENSGALG00010020773	ZCCHC24	PRJNA729271	1.329819866	9.22326E-49	1.329819866	stage_E14_vs_E10	ordinary_muscle_development	D_vs_E10;stage_1W_vs_E10;stage_3W_vs_E10;stage	1	TRUE
ENSGALG00010002592	RPS6KA3	PRJNA729271	1.497671757	9.81457E-49	1.497671757	stage_E14_vs_E10	ordinary_muscle_development	D_vs_E10;stage_1W_vs_E10;stage_3W_vs_E10;stage	1	TRUE
ENSGALG00010016304	EIF3J	PRJNA729271	1.476797484	9.81457E-49	1.476797484	stage_E14_vs_E10	ordinary_muscle_development	D_vs_E10;stage_1W_vs_E10;stage_3W_vs_E10;stage	1	TRUE
ENSGALG00010001559	HDAC10	PRJNA729271	-2.009794061	9.92414E-49	2.009794061	stage_E14_vs_E10	ordinary_muscle_development	D_vs_E10;stage_1W_vs_E10;stage_3W_vs_E10;stage	1	TRUE
ENSGALG00010012968	LSM11	PRJNA729271	-1.165917681	9.96759E-49	1.165917681	stage_E14_vs_E10	ordinary_muscle_development	D_vs_E10;stage_1W_vs_E10;stage_3W_vs_E10;stage	1	TRUE
ENSGALG00010005412	MTFR2	PRJNA729271	-2.546287174	1.00029E-48	2.546287174	stage_E14_vs_E10	ordinary_muscle_development	D_vs_E10;stage_1W_vs_E10;stage_3W_vs_E10;stage	1	TRUE
ENSGALG00010004366	UNC5C	PRJNA729271	-6.043132801	1.0515E-48	6.043132801	stage_E14_vs_E10	ordinary_muscle_development	D_vs_E10;stage_1W_vs_E10;stage_3W_vs_E10;stage	1	TRUE
ENSGALG00010024833	MAPT	PRJNA729271	2.972533805	1.23529E-48	2.972533805	stage_E14_vs_E10	ordinary_muscle_development	D_vs_E10;stage_1W_vs_E10;stage_3W_vs_E10;stage	1	TRUE
ENSGALG00010015416	CAPN6	PRJNA729271	2.415009934	1.34947E-48	2.415009934	stage_E14_vs_E10	ordinary_muscle_development	D_vs_E10;stage_1W_vs_E10;stage_3W_vs_E10;stage	1	TRUE
ENSGALG00010008069	DLG2	PRJNA729271	-3.410632944	1.36871E-48	3.410632944	stage_E14_vs_E10	ordinary_muscle_development	D_vs_E10;stage_1W_vs_E10;stage_3W_vs_E10;stage	1	TRUE
ENSGALG00010015670	ENSGALG00010015670	PRJNA729271	-1.957186975	1.49665E-48	1.957186975	stage_E14_vs_E10	ordinary_muscle_development	D_vs_E10;stage_1W_vs_E10;stage_3W_vs_E10;stage	1	TRUE
ENSGALG00010017775	PRPSAP2	PRJNA729271	-1.291459793	1.49849E-48	1.291459793	stage_E14_vs_E10	ordinary_muscle_development	D_vs_E10;stage_1W_vs_E10;stage_3W_vs_E10;stage	1	TRUE
ENSGALG00010000263	MTMR3	PRJNA729271	1.358271605	1.51452E-48	1.358271605	stage_E14_vs_E10	ordinary_muscle_development	D_vs_E10;stage_1W_vs_E10;stage_3W_vs_E10;stage	1	TRUE
ENSGALG00010018548	RGMA	PRJNA729271	-1.857925227	1.62894E-48	1.857925227	stage_E14_vs_E10	ordinary_muscle_development	D_vs_E10;stage_1W_vs_E10;stage_3W_vs_E10;stage	1	TRUE
ENSGALG00010013362	ENSGALG00010013362	PRJNA729271	5.289464823	1.70906E-48	5.289464823	stage_E14_vs_E10	ordinary_muscle_development	D_vs_E10;stage_1W_vs_E10;stage_3W_vs_E10;stage	1	TRUE
ENSGALG00010025527	KNTC1	PRJNA729271	-3.598251248	1.70918E-48	3.598251248	stage_E14_vs_E10	ordinary_muscle_development	D_vs_E10;stage_1W_vs_E10;stage_3W_vs_E10;stage	1	TRUE
ENSGALG00010018414	EPAS1	PRJNA729271	2.037023631	1.78809E-48	2.037023631	stage_E14_vs_E10	ordinary_muscle_development	D_vs_E10;stage_1W_vs_E10;stage_3W_vs_E10;stage	1	TRUE
ENSGALG00010008932	VASP	PRJNA729271	1.76594377	1.80919E-48	1.76594377	stage_E14_vs_E10	ordinary_muscle_development	D_vs_E10;stage_1W_vs_E10;stage_3W_vs_E10;stage	1	TRUE
ENSGALG00010012600	NUP107	PRJNA729271	-1.512104913	1.92316E-48	1.512104913	stage_E14_vs_E10	ordinary_muscle_development	D_vs_E10;stage_1W_vs_E10;stage_3W_vs_E10;stage	1	TRUE
ENSGALG00010027156	ISYNA1	PRJNA729271	-2.25987115	2.09799E-48	2.25987115	stage_E14_vs_E10	ordinary_muscle_development	D_vs_E10;stage_1W_vs_E10;stage_3W_vs_E10;stage	1	TRUE
ENSGALG00010018808	MATCAP2	PRJNA729271	-1.367247024	2.17722E-48	1.367247024	stage_E14_vs_E10	ordinary_muscle_development	D_vs_E10;stage_1W_vs_E10;stage_3W_vs_E10;stage	1	TRUE
ENSGALG00010007067	CASP3	PRJNA729271	-1.613828877	2.18856E-48	1.613828877	stage_E14_vs_E10	ordinary_muscle_development	D_vs_E10;stage_1W_vs_E10;stage_3W_vs_E10;stage	1	TRUE
ENSGALG00010022186	DNAJB4	PRJNA729271	1.661231698	2.39129E-48	1.661231698	stage_E14_vs_E10	ordinary_muscle_development	D_vs_E10;stage_1W_vs_E10;stage_3W_vs_E10;stage	1	TRUE
ENSGALG00010005228	ZNF521	PRJNA729271	-1.959252846	2.46144E-48	1.959252846	stage_E14_vs_E10	ordinary_muscle_development	D_vs_E10;stage_1W_vs_E10;stage_3W_vs_E10;stage	1	TRUE
ENSGALG00010021647	ARHGAP11B	PRJNA729271	-2.061934345	2.63269E-48	2.061934345	stage_E14_vs_E10	ordinary_muscle_development	D_vs_E10;stage_1W_vs_E10;stage_3W_vs_E10;stage	1	TRUE
ENSGALG00010004040	LGALS1B	PRJNA729271	-3.352914182	2.816E-48	3.352914182	stage_E14_vs_E10	ordinary_muscle_development	D_vs_E10;stage_1W_vs_E10;stage_3W_vs_E10;stage	1	TRUE
ENSGALG00010020797	DGCR8	PRJNA729271	-0.990667206	2.8254E-48	0.990667206	stage_E14_vs_E10	ordinary_muscle_development		0	FALSE
ENSGALG00010027907	MKNK2	PRJNA729271	2.778448199	2.98191E-48	2.778448199	stage_E14_vs_E10	ordinary_muscle_development	D_vs_E10;stage_1W_vs_E10;stage_3W_vs_E10;stage	1	TRUE
ENSGALG00010027471	ENSGALG00010027471	PRJNA729271	1.902521345	3.02722E-48	1.902521345	stage_E14_vs_E10	ordinary_muscle_development	D_vs_E10;stage_1W_vs_E10;stage_3W_vs_E10;stage	1	TRUE
ENSGALG00010028170	COPE	PRJNA729271	-1.407072211	3.10492E-48	1.407072211	stage_E14_vs_E10	ordinary_muscle_development	D_vs_E10;stage_1W_vs_E10;stage_3W_vs_E10;stage	1	TRUE
ENSGALG00010023065	DNA2	PRJNA729271	-3.477169268	3.31805E-48	3.477169268	stage_E14_vs_E10	ordinary_muscle_development	D_vs_E10;stage_1W_vs_E10;stage_3W_vs_E10;stage	1	TRUE
ENSGALG00010029305	SMTNL2	PRJNA729271	2.800526769	3.37847E-48	2.800526769	stage_E14_vs_E10	ordinary_muscle_development	D_vs_E10;stage_1W_vs_E10;stage_3W_vs_E10;stage	1	TRUE
ENSGALG00010029740	HASPIN	PRJNA729271	-3.754214508	3.58834E-48	3.754214508	stage_E14_vs_E10	ordinary_muscle_development	D_vs_E10;stage_1W_vs_E10;stage_3W_vs_E10;stage	1	TRUE
ENSGALG00010020545	STYX	PRJNA729271	2.078034653	3.91481E-48	2.078034653	stage_E14_vs_E10	ordinary_muscle_development	D_vs_E10;stage_1W_vs_E10;stage_3W_vs_E10;stage	1	TRUE
ENSGALG00010004055	FILIP1	PRJNA729271	2.587705218	3.96706E-48	2.587705218	stage_E14_vs_E10	ordinary_muscle_development	D_vs_E10;stage_1W_vs_E10;stage_3W_vs_E10;stage	1	TRUE
ENSGALG00010008209	PPARGC1B	PRJNA729271	3.422769402	4.21661E-48	3.422769402	stage_E14_vs_E10	ordinary_muscle_development	D_vs_E10;stage_1W_vs_E10;stage_3W_vs_E10;stage	1	TRUE
ENSGALG00010019006	ENSGALG00010019006	PRJNA729271	-1.199951217	4.21757E-48	1.199951217	stage_E14_vs_E10	ordinary_muscle_development	D_vs_E10;stage_1W_vs_E10;stage_3W_vs_E10;stage	1	TRUE
ENSGALG00010001385	MME	PRJNA729271	3.712398504	4.24165E-48	3.712398504	stage_E14_vs_E10	ordinary_muscle_development	D_vs_E10;stage_1W_vs_E10;stage_3W_vs_E10;stage	1	TRUE
ENSGALG00010028888	WDR5B	PRJNA729271	-1.221869908	4.55374E-48	1.221869908	stage_E14_vs_E10	ordinary_muscle_development	D_vs_E10;stage_1W_vs_E10;stage_3W_vs_E10;stage	1	TRUE
ENSGALG00010024314	ITFG1	PRJNA729271	2.141541818	4.62526E-48	2.141541818	stage_E14_vs_E10	ordinary_muscle_development	D_vs_E10;stage_1W_vs_E10;stage_3W_vs_E10;stage	1	TRUE
ENSGALG00010009551	HADHA	PRJNA729271	1.919567506	4.71202E-48	1.919567506	stage_E14_vs_E10	ordinary_muscle_development	D_vs_E10;stage_1W_vs_E10;stage_3W_vs_E10;stage	1	TRUE
ENSGALG00010025984	NDUFS1	PRJNA729271	1.720944182	4.7436E-48	1.720944182	stage_E14_vs_E10	ordinary_muscle_development	D_vs_E10;stage_1W_vs_E10;stage_3W_vs_E10;stage	1	TRUE
ENSGALG00010027295	ENSGALG00010027295	PRJNA729271	3.4115479	4.85363E-48	3.4115479	stage_E14_vs_E10	ordinary_muscle_development	D_vs_E10;stage_1W_vs_E10;stage_3W_vs_E10;stage	1	TRUE
ENSGALG00010016835	CCDC88A	PRJNA729271	-2.252417813	4.91035E-48	2.252417813	stage_E14_vs_E10	ordinary_muscle_development	D_vs_E10;stage_1W_vs_E10;stage_3W_vs_E10;stage	1	TRUE
ENSGALG00010029487	CDC42EP4	PRJNA729271	-2.177493235	5.01216E-48	2.177493235	stage_E14_vs_E10	ordinary_muscle_development	D_vs_E10;stage_1W_vs_E10;stage_3W_vs_E10;stage	1	TRUE
ENSGALG00010013327	SLF1	PRJNA729271	-1.473086202	5.01216E-48	1.473086202	stage_E14_vs_E10	ordinary_muscle_development	D_vs_E10;stage_1W_vs_E10;stage_3W_vs_E10;stage	1	TRUE
ENSGALG00010028190	ILF2	PRJNA729271	-1.199775545	5.29108E-48	1.199775545	stage_E14_vs_E10	ordinary_muscle_development	D_vs_E10;stage_1W_vs_E10;stage_3W_vs_E10;stage	1	TRUE
ENSGALG00010028496	ENSGALG00010028496	PRJNA729271	2.042227848	6.58178E-48	2.042227848	stage_E14_vs_E10	ordinary_muscle_development	D_vs_E10;stage_1W_vs_E10;stage_3W_vs_E10;stage	1	TRUE
ENSGALG00010001292	LOX	PRJNA729271	2.091069987	6.73362E-48	2.091069987	stage_E14_vs_E10	ordinary_muscle_development	D_vs_E10;stage_1W_vs_E10;stage_3W_vs_E10;stage	1	TRUE
ENSGALG00010029151	CYBC1	PRJNA729271	-1.728196608	7.01775E-48	1.728196608	stage_E14_vs_E10	ordinary_muscle_development	D_vs_E10;stage_1W_vs_E10;stage_3W_vs_E10;stage	1	TRUE
ENSGALG00010005326	MASTL	PRJNA729271	-2.88688394	7.28551E-48	2.88688394	stage_E14_vs_E10	ordinary_muscle_development	D_vs_E10;stage_1W_vs_E10;stage_3W_vs_E10;stage	1	TRUE
ENSGALG00010005324	CENPJ	PRJNA729271	-3.071035416	7.63155E-48	3.071035416	stage_E14_vs_E10	ordinary_muscle_development	D_vs_E10;stage_1W_vs_E10;stage_3W_vs_E10;stage	1	TRUE
ENSGALG00010029545	OMG	PRJNA729271	4.272143618	7.76136E-48	4.272143618	stage_E14_vs_E10	ordinary_muscle_development	D_vs_E10;stage_1W_vs_E10;stage_3W_vs_E10;stage	1	TRUE
ENSGALG00010004344	SOSTDC1	PRJNA729271	-6.301142122	7.83179E-48	6.301142122	stage_E14_vs_E10	ordinary_muscle_development	D_vs_E10;stage_1W_vs_E10;stage_3W_vs_E10;stage	1	TRUE
ENSGALG00010007887	CDT1	PRJNA729271	-1.903624403	8.07091E-48	1.903624403	stage_E14_vs_E10	ordinary_muscle_development	D_vs_E10;stage_1W_vs_E10;stage_3W_vs_E10;stage	1	TRUE
ENSGALG00010005203	ENSGALG00010005203	PRJNA729271	1.557862592	8.42633E-48	1.557862592	stage_E14_vs_E10	ordinary_muscle_development	D_vs_E10;stage_1W_vs_E10;stage_3W_vs_E10;stage	1	TRUE
ENSGALG00010009777	ST3GAL1	PRJNA729271	-2.831185043	8.54519E-48	2.831185043	stage_E14_vs_E10	ordinary_muscle_development	D_vs_E10;stage_1W_vs_E10;stage_3W_vs_E10;stage	1	TRUE
ENSGALG00010009728	CCDC149	PRJNA729271	-1.573211904	8.54519E-48	1.573211904	stage_E14_vs_E10	ordinary_muscle_development	D_vs_E10;stage_1W_vs_E10;stage_3W_vs_E10;stage	1	TRUE
ENSGALG00010003019	TAPBP	PRJNA729271	3.693921306	8.55792E-48	3.693921306	stage_E14_vs_E10	ordinary_muscle_development	D_vs_E10;stage_1W_vs_E10;stage_3W_vs_E10;stage	1	TRUE
ENSGALG00010027040	CLIP1	PRJNA729271	1.584298653	8.76204E-48	1.584298653	stage_E14_vs_E10	ordinary_muscle_development	D_vs_E10;stage_1W_vs_E10;stage_3W_vs_E10;stage	1	TRUE
ENSGALG00010029591	SUZ12	PRJNA729271	-1.197054827	8.83924E-48	1.197054827	stage_E14_vs_E10	ordinary_muscle_development	D_vs_E10;stage_1W_vs_E10;stage_3W_vs_E10;stage	1	TRUE
ENSGALG00010025703	UBASH3B	PRJNA729271	-2.11757762	8.84243E-48	2.11757762	stage_E14_vs_E10	ordinary_muscle_development	D_vs_E10;stage_1W_vs_E10;stage_3W_vs_E10;stage	1	TRUE
ENSGALG00010005470	SEMA3A	PRJNA729271	-2.084270898	9.94102E-48	2.084270898	stage_E14_vs_E10	ordinary_muscle_development	D_vs_E10;stage_1W_vs_E10;stage_3W_vs_E10;stage	1	TRUE
ENSGALG00010005349	ACBD5	PRJNA729271	1.515954544	1.01887E-47	1.515954544	stage_E14_vs_E10	ordinary_muscle_development	D_vs_E10;stage_1W_vs_E10;stage_3W_vs_E10;stage	1	TRUE
ENSGALG00010007658	BIRC2	PRJNA729271	1.741408776	1.02969E-47	1.741408776	stage_E14_vs_E10	ordinary_muscle_development	D_vs_E10;stage_1W_vs_E10;stage_3W_vs_E10;stage	1	TRUE
ENSGALG00010004300	VNN2	PRJNA729271	3.599522212	1.0427E-47	3.599522212	stage_E14_vs_E10	ordinary_muscle_development	D_vs_E10;stage_1W_vs_E10;stage_3W_vs_E10;stage	1	TRUE
ENSGALG00010003202	FAM46A	PRJNA729271	1.509019953	1.20434E-47	1.509019953	stage_E14_vs_E10	ordinary_muscle_development	D_vs_E10;stage_1W_vs_E10;stage_3W_vs_E10;stage	1	TRUE
ENSGALG00010018698	MRPS16	PRJNA729271	1.65588462	1.21297E-47	1.65588462	stage_E14_vs_E10	ordinary_muscle_development	D_vs_E10;stage_1W_vs_E10;stage_3W_vs_E10;stage	1	TRUE
ENSGALG00010010974	NCAPG	PRJNA729271	-2.851092414	1.24455E-47	2.851092414	stage_E14_vs_E10	ordinary_muscle_development	D_vs_E10;stage_1W_vs_E10;stage_3W_vs_E10;stage	1	TRUE
ENSGALG00010022974	INPP5D	PRJNA729271	2.259827678	1.31753E-47	2.259827678	stage_E14_vs_E10	ordinary_muscle_development	D_vs_E10;stage_1W_vs_E10;stage_3W_vs_E10;stage	1	TRUE
ENSGALG00010000564	NAA40	PRJNA729271	-1.36038601	1.32048E-47	1.36038601	stage_E14_vs_E10	ordinary_muscle_development	D_vs_E10;stage_1W_vs_E10;stage_3W_vs_E10;stage	1	TRUE
ENSGALG00010016647	EGR1	PRJNA729271	2.992855626	1.32765E-47	2.992855626	stage_E14_vs_E10	ordinary_muscle_development	D_vs_E10;stage_1W_vs_E10;stage_3W_vs_E10;stage	1	TRUE
ENSGALG00010011685	NDUFA2	PRJNA729271	1.627575686	1.44475E-47	1.627575686	stage_E14_vs_E10	ordinary_muscle_development	D_vs_E10;stage_1W_vs_E10;stage_3W_vs_E10;stage	1	TRUE
ENSGALG00010020056	UACA	PRJNA729271	-2.442841124	1.61136E-47	2.442841124	stage_E14_vs_E10	ordinary_muscle_development	D_vs_E10;stage_1W_vs_E10;stage_3W_vs_E10;stage	1	TRUE
ENSGALG00010025573	CLMP	PRJNA729271	-2.486084921	1.66258E-47	2.486084921	stage_E14_vs_E10	ordinary_muscle_development	D_vs_E10;stage_1W_vs_E10;stage_3W_vs_E10;stage	1	TRUE
ENSGALG00010002601	PLCXD1	PRJNA729271	2.740173661	1.69618E-47	2.740173661	stage_E14_vs_E10	ordinary_muscle_development	D_vs_E10;stage_1W_vs_E10;stage_3W_vs_E10;stage	1	TRUE
ENSGALG00010019335	HIPK3	PRJNA729271	1.688352779	1.72261E-47	1.688352779	stage_E14_vs_E10	ordinary_muscle_development	D_vs_E10;stage_1W_vs_E10;stage_3W_vs_E10;stage	1	TRUE
ENSGALG00010001749	VAPA	PRJNA729271	1.550634183	1.7358E-47	1.550634183	stage_E14_vs_E10	ordinary_muscle_development	D_vs_E10;stage_1W_vs_E10;stage_3W_vs_E10;stage	1	TRUE
ENSGALG00010012730	CAP2	PRJNA729271	2.454017681	1.7725E-47	2.454017681	stage_E14_vs_E10	ordinary_muscle_development	D_vs_E10;stage_1W_vs_E10;stage_3W_vs_E10;stage	1	TRUE
ENSGALG00010005656	CYP2U1	PRJNA729271	1.902919637	1.91487E-47	1.902919637	stage_E14_vs_E10	ordinary_muscle_development	D_vs_E10;stage_1W_vs_E10;stage_3W_vs_E10;stage	1	TRUE
ENSGALG00010002573	MVB12A	PRJNA729271	1.372879541	1.99607E-47	1.372879541	stage_E14_vs_E10	ordinary_muscle_development	D_vs_E10;stage_1W_vs_E10;stage_3W_vs_E10;stage	1	TRUE
ENSGALG00010000449	PLBD2	PRJNA729271	1.571332944	2.01226E-47	1.571332944	stage_E14_vs_E10	ordinary_muscle_development	D_vs_E10;stage_1W_vs_E10;stage_3W_vs_E10;stage	1	TRUE
ENSGALG00010023087	SH3GLB1	PRJNA729271	1.720633243	2.04762E-47	1.720633243	stage_E14_vs_E10	ordinary_muscle_development	D_vs_E10;stage_1W_vs_E10;stage_3W_vs_E10;stage	1	TRUE
ENSGALG00010023913	ZMYND8	PRJNA729271	-1.235950291	2.14799E-47	1.235950291	stage_E14_vs_E10	ordinary_muscle_development	D_vs_E10;stage_1W_vs_E10;stage_3W_vs_E10;stage	1	TRUE
ENSGALG00010003804	PSME1	PRJNA729271	3.684423905	2.16343E-47	3.684423905	stage_E14_vs_E10	ordinary_muscle_development	D_vs_E10;stage_1W_vs_E10;stage_3W_vs_E10;stage	1	TRUE
ENSGALG00010017299	USP31	PRJNA729271	1.601811193	2.31603E-47	1.601811193	stage_E14_vs_E10	ordinary_muscle_development	D_vs_E10;stage_1W_vs_E10;stage_3W_vs_E10;stage	1	TRUE
ENSGALG00010012169	H3C33	PRJNA729271	-3.348927906	2.37951E-47	3.348927906	stage_E14_vs_E10	ordinary_muscle_development	D_vs_E10;stage_1W_vs_E10;stage_3W_vs_E10;stage	1	TRUE
ENSGALG00010001453	TAX1BP1	PRJNA729271	1.073123353	2.42758E-47	1.073123353	stage_E14_vs_E10	ordinary_muscle_development	D_vs_E10;stage_1W_vs_E10;stage_3W_vs_E10;stage	1	TRUE
ENSGALG00010019830	JAG2	PRJNA729271	1.433344496	2.60152E-47	1.433344496	stage_E14_vs_E10	ordinary_muscle_development	D_vs_E10;stage_1W_vs_E10;stage_3W_vs_E10;stage	1	TRUE
ENSGALG00010021112	TAF5	PRJNA729271	-1.064477808	2.62529E-47	1.064477808	stage_E14_vs_E10	ordinary_muscle_development	D_vs_E10;stage_1W_vs_E10;stage_3W_vs_E10;stage	1	TRUE
ENSGALG00010012324	ENSGALG00010012324	PRJNA729271	-2.404852352	2.65591E-47	2.404852352	stage_E14_vs_E10	ordinary_muscle_development	D_vs_E10;stage_1W_vs_E10;stage_3W_vs_E10;stage	1	TRUE
ENSGALG00010028364	MHM2	PRJNA729271	9.799601882	2.73931E-47	9.799601882	stage_E14_vs_E10	ordinary_muscle_development	D_vs_E10;stage_1W_vs_E10;stage_3W_vs_E10;stage	1	TRUE
ENSGALG00010008348	ENSGALG00010008348	PRJNA729271	-1.548486629	2.75832E-47	1.548486629	stage_E14_vs_E10	ordinary_muscle_development	D_vs_E10;stage_1W_vs_E10;stage_3W_vs_E10;stage	1	TRUE
ENSGALG00010013446	ATP5I	PRJNA729271	2.119175846	3.0154E-47	2.119175846	stage_E14_vs_E10	ordinary_muscle_development	D_vs_E10;stage_1W_vs_E10;stage_3W_vs_E10;stage	1	TRUE
ENSGALG00010028165	NFIC	PRJNA729271	2.251367838	3.01546E-47	2.251367838	stage_E14_vs_E10	ordinary_muscle_development	D_vs_E10;stage_1W_vs_E10;stage_3W_vs_E10;stage	1	TRUE
ENSGALG00010010923	F13A1	PRJNA729271	3.368416731	3.12931E-47	3.368416731	stage_E14_vs_E10	ordinary_muscle_development	D_vs_E10;stage_1W_vs_E10;stage_3W_vs_E10;stage	1	TRUE
ENSGALG00010020215	PRMT3	PRJNA729271	-1.248411008	3.22395E-47	1.248411008	stage_E14_vs_E10	ordinary_muscle_development	D_vs_E10;stage_1W_vs_E10;stage_3W_vs_E10;stage	1	TRUE
ENSGALG00010028455	ENSA	PRJNA729271	1.014494514	3.35043E-47	1.014494514	stage_E14_vs_E10	ordinary_muscle_development	D_vs_E10;stage_1W_vs_E10;stage_3W_vs_E10;stage	1	TRUE
ENSGALG00010024403	HMBS	PRJNA729271	-1.133696814	3.46169E-47	1.133696814	stage_E14_vs_E10	ordinary_muscle_development	D_vs_E10;stage_1W_vs_E10;stage_3W_vs_E10;stage	1	TRUE
ENSGALG00010023743	CD3D	PRJNA729271	3.175759138	3.48291E-47	3.175759138	stage_E14_vs_E10	ordinary_muscle_development	D_vs_E10;stage_1W_vs_E10;stage_3W_vs_E10;stage	1	TRUE
ENSGALG00010027635	CD48	PRJNA729271	3.28581609	3.959E-47	3.28581609	stage_E14_vs_E10	ordinary_muscle_development	D_vs_E10;stage_1W_vs_E10;stage_3W_vs_E10;stage	1	TRUE
ENSGALG00010012186	KIF20A	PRJNA729271	-3.643363325	4.00705E-47	3.643363325	stage_E14_vs_E10	ordinary_muscle_development	D_vs_E10;stage_1W_vs_E10;stage_3W_vs_E10;stage	1	TRUE
ENSGALG00010001301	GMPS	PRJNA729271	-1.125960302	4.21451E-47	1.125960302	stage_E14_vs_E10	ordinary_muscle_development	D_vs_E10;stage_1W_vs_E10;stage_3W_vs_E10;stage	1	TRUE
ENSGALG00010006131	ENSGALG00010006131	PRJNA729271	1.817284588	4.40416E-47	1.817284588	stage_E14_vs_E10	ordinary_muscle_development	D_vs_E10;stage_1W_vs_E10;stage_3W_vs_E10;stage	1	TRUE
ENSGALG00010025242	CLSTN2	PRJNA729271	-4.566685093	4.9567E-47	4.566685093	stage_E14_vs_E10	ordinary_muscle_development	D_vs_E10;stage_1W_vs_E10;stage_3W_vs_E10;stage	1	TRUE
ENSGALG00010015152	BOLL	PRJNA729271	3.994360071	5.0725E-47	3.994360071	stage_E14_vs_E10	ordinary_muscle_development	D_vs_E10;stage_1W_vs_E10;stage_3W_vs_E10;stage	1	TRUE
ENSGALG00010022788	CYBRD1	PRJNA729271	2.527286481	5.12884E-47	2.527286481	stage_E14_vs_E10	ordinary_muscle_development	D_vs_E10;stage_1W_vs_E10;stage_3W_vs_E10;stage	1	TRUE
ENSGALG00010028488	PTGES	PRJNA729271	1.878847114	5.17462E-47	1.878847114	stage_E14_vs_E10	ordinary_muscle_development	D_vs_E10;stage_1W_vs_E10;stage_3W_vs_E10;stage	1	TRUE
ENSGALG00010027623	APOBEC2	PRJNA729271	2.659477683	5.19671E-47	2.659477683	stage_E14_vs_E10	ordinary_muscle_development	D_vs_E10;stage_1W_vs_E10;stage_3W_vs_E10;stage	1	TRUE
ENSGALG00010028890	ENSGALG00010028890	PRJNA729271	-7.440875602	5.67369E-47	7.440875602	stage_E14_vs_E10	ordinary_muscle_development	D_vs_E10;stage_1W_vs_E10;stage_3W_vs_E10;stage	1	TRUE
ENSGALG00010009028	PDLIM7	PRJNA729271	-1.698924937	5.7326E-47	1.698924937	stage_E14_vs_E10	ordinary_muscle_development	D_vs_E10;stage_1W_vs_E10;stage_3W_vs_E10;stage	1	TRUE
ENSGALG00010027192	SH3GL1	PRJNA729271	-1.714206337	5.91624E-47	1.714206337	stage_E14_vs_E10	ordinary_muscle_development	D_vs_E10;stage_1W_vs_E10;stage_3W_vs_E10;stage	1	TRUE
ENSGALG00010018999	THUMPD3	PRJNA729271	-1.078265693	5.97967E-47	1.078265693	stage_E14_vs_E10	ordinary_muscle_development	D_vs_E10;stage_1W_vs_E10;stage_3W_vs_E10;stage	1	TRUE
ENSGALG00010023220	ST3GAL2	PRJNA729271	-1.286833688	6.30478E-47	1.286833688	stage_E14_vs_E10	ordinary_muscle_development	D_vs_E10;stage_1W_vs_E10;stage_3W_vs_E10;stage	1	TRUE
ENSGALG00010021989	NCAPD2	PRJNA729271	-2.22567532	6.52821E-47	2.22567532	stage_E14_vs_E10	ordinary_muscle_development	D_vs_E10;stage_1W_vs_E10;stage_3W_vs_E10;stage	1	TRUE
ENSGALG00010027269	CDC25A	PRJNA729271	-1.522613337	6.99608E-47	1.522613337	stage_E14_vs_E10	ordinary_muscle_development	D_vs_E10;stage_1W_vs_E10;stage_3W_vs_E10;stage	1	TRUE
ENSGALG00010016197	SNRPN	PRJNA729271	-1.69928054	7.23615E-47	1.69928054	stage_E14_vs_E10	ordinary_muscle_development	D_vs_E10;stage_1W_vs_E10;stage_3W_vs_E10;stage	1	TRUE
ENSGALG00010016947	CANX	PRJNA729271	-1.045399646	8.00661E-47	1.045399646	stage_E14_vs_E10	ordinary_muscle_development	D_vs_E10;stage_1W_vs_E10;stage_3W_vs_E10;stage	1	TRUE
ENSGALG00010020103	PSAP	PRJNA729271	1.525820925	8.40072E-47	1.525820925	stage_E14_vs_E10	ordinary_muscle_development	D_vs_E10;stage_1W_vs_E10;stage_3W_vs_E10;stage	1	TRUE
ENSGALG00010027153	ENSGALG00010027153	PRJNA729271	3.312368891	8.6666E-47	3.312368891	stage_E14_vs_E10	ordinary_muscle_development	D_vs_E10;stage_1W_vs_E10;stage_3W_vs_E10;stage	1	TRUE
ENSGALG00010010085	TDG	PRJNA729271	-1.523208342	8.80928E-47	1.523208342	stage_E14_vs_E10	ordinary_muscle_development	D_vs_E10;stage_1W_vs_E10;stage_3W_vs_E10;stage	1	TRUE
ENSGALG00010009525	ENSGALG00010009525	PRJNA729271	2.252029328	8.82204E-47	2.252029328	stage_E14_vs_E10	ordinary_muscle_development	D_vs_E10;stage_1W_vs_E10;stage_3W_vs_E10;stage	1	TRUE
ENSGALG00010021287	COL17A1	PRJNA729271	-7.24443059	9.09723E-47	7.24443059	stage_E14_vs_E10	ordinary_muscle_development	D_vs_E10;stage_1W_vs_E10;stage_3W_vs_E10;stage	1	TRUE
ENSGALG00010015370	ENSGALG00010015370	PRJNA729271	2.069755121	9.32539E-47	2.069755121	stage_E14_vs_E10	ordinary_muscle_development	D_vs_E10;stage_1W_vs_E10;stage_3W_vs_E10;stage	1	TRUE
ENSGALG00010017109	ITGB1BP3	PRJNA729271	1.848803304	9.74357E-47	1.848803304	stage_E14_vs_E10	ordinary_muscle_development	D_vs_E10;stage_1W_vs_E10;stage_3W_vs_E10;stage	1	TRUE
ENSGALG00010008047	ASAP1	PRJNA729271	-1.930990075	1.00312E-46	1.930990075	stage_E14_vs_E10	ordinary_muscle_development	D_vs_E10;stage_1W_vs_E10;stage_3W_vs_E10;stage	1	TRUE
ENSGALG00010008997	CHST6	PRJNA729271	-1.970759481	1.15507E-46	1.970759481	stage_E14_vs_E10	ordinary_muscle_development	D_vs_E10;stage_1W_vs_E10;stage_3W_vs_E10;stage	1	TRUE
ENSGALG00010005307	TIAM2	PRJNA729271	-2.302576822	1.18333E-46	2.302576822	stage_E14_vs_E10	ordinary_muscle_development	D_vs_E10;stage_1W_vs_E10;stage_3W_vs_E10;stage	1	TRUE
ENSGALG00010028889	FUBP3	PRJNA729271	-1.213237543	1.22744E-46	1.213237543	stage_E14_vs_E10	ordinary_muscle_development	D_vs_E10;stage_1W_vs_E10;stage_3W_vs_E10;stage	1	TRUE
ENSGALG00010012345	H2BC32	PRJNA729271	-2.399387285	1.23555E-46	2.399387285	stage_E14_vs_E10	ordinary_muscle_development	D_vs_E10;stage_1W_vs_E10;stage_3W_vs_E10;stage	1	TRUE
ENSGALG00010002566	TMSB4X	PRJNA729271	-1.682930991	1.2774E-46	1.682930991	stage_E14_vs_E10	ordinary_muscle_development	D_vs_E10;stage_1W_vs_E10;stage_3W_vs_E10;stage	1	TRUE
ENSGALG00010020787	TMEM132C	PRJNA729271	-4.529305981	1.29464E-46	4.529305981	stage_E14_vs_E10	ordinary_muscle_development	D_vs_E10;stage_1W_vs_E10;stage_3W_vs_E10;stage	1	TRUE
ENSGALG00010025639	MYL2	PRJNA729271	-7.247547482	1.31104E-46	7.247547482	stage_E14_vs_E10	ordinary_muscle_development	D_vs_E10;stage_1W_vs_E10;stage_3W_vs_E10;stage	1	TRUE
ENSGALG00010020963	CCDC93	PRJNA729271	1.43067467	1.34946E-46	1.43067467	stage_E14_vs_E10	ordinary_muscle_development	D_vs_E10;stage_1W_vs_E10;stage_3W_vs_E10;stage	1	TRUE
ENSGALG00010012726	IL7R	PRJNA729271	6.709445012	1.4336E-46	6.709445012	stage_E14_vs_E10	ordinary_muscle_development	D_vs_E10;stage_1W_vs_E10;stage_3W_vs_E10;stage	1	TRUE
ENSGALG00010019716	VEGFA	PRJNA729271	1.879830962	1.47497E-46	1.879830962	stage_E14_vs_E10	ordinary_muscle_development	D_vs_E10;stage_1W_vs_E10;stage_3W_vs_E10;stage	1	TRUE
ENSGALG00010020015	ATP6V1D	PRJNA729271	1.130451885	1.56569E-46	1.130451885	stage_E14_vs_E10	ordinary_muscle_development	D_vs_E10;stage_1W_vs_E10;stage_3W_vs_E10;stage	1	TRUE
ENSGALG00010002434	GPR34	PRJNA729271	3.558226509	1.5709E-46	3.558226509	stage_E14_vs_E10	ordinary_muscle_development	D_vs_E10;stage_1W_vs_E10;stage_3W_vs_E10;stage	1	TRUE
ENSGALG00010016836	HEPH	PRJNA729271	2.15126883	1.63377E-46	2.15126883	stage_E14_vs_E10	ordinary_muscle_development	D_vs_E10;stage_1W_vs_E10;stage_3W_vs_E10;stage	1	TRUE
ENSGALG00010024135	FBXO22	PRJNA729271	1.260725157	1.63477E-46	1.260725157	stage_E14_vs_E10	ordinary_muscle_development	D_vs_E10;stage_1W_vs_E10;stage_3W_vs_E10;stage	1	TRUE
ENSGALG00010022644	ARMC8	PRJNA729271	1.224310101	1.6584E-46	1.224310101	stage_E14_vs_E10	ordinary_muscle_development	D_vs_E10;stage_1W_vs_E10;stage_3W_vs_E10;stage	1	TRUE
ENSGALG00010020248	NOLC1	PRJNA729271	-1.187884315	1.66665E-46	1.187884315	stage_E14_vs_E10	ordinary_muscle_development	D_vs_E10;stage_1W_vs_E10;stage_3W_vs_E10;stage	1	TRUE
ENSGALG00010009361	LRRK2	PRJNA729271	1.260331965	1.82406E-46	1.260331965	stage_E14_vs_E10	ordinary_muscle_development	D_vs_E10;stage_1W_vs_E10;stage_3W_vs_E10;stage	1	TRUE
ENSGALG00010016402	SEPTIN8	PRJNA729271	-1.140565493	1.88395E-46	1.140565493	stage_E14_vs_E10	ordinary_muscle_development	D_vs_E10;stage_1W_vs_E10;stage_3W_vs_E10;stage	1	TRUE
ENSGALG00010025417	B3GAT1	PRJNA729271	-3.40111477	1.89184E-46	3.40111477	stage_E14_vs_E10	ordinary_muscle_development	D_vs_E10;stage_1W_vs_E10;stage_3W_vs_E10;stage	1	TRUE
ENSGALG00010025274	PDK2	PRJNA729271	3.891813557	2.08522E-46	3.891813557	stage_E14_vs_E10	ordinary_muscle_development	D_vs_E10;stage_1W_vs_E10;stage_3W_vs_E10;stage	1	TRUE
ENSGALG00010003330	HEY1	PRJNA729271	1.732141054	2.19776E-46	1.732141054	stage_E14_vs_E10	ordinary_muscle_development	D_vs_E10;stage_1W_vs_E10;stage_3W_vs_E10;stage	1	TRUE
ENSGALG00010017477	MCF2	PRJNA729271	2.641194614	2.22664E-46	2.641194614	stage_E14_vs_E10	ordinary_muscle_development	D_vs_E10;stage_1W_vs_E10;stage_3W_vs_E10;stage	1	TRUE
ENSGALG00010011868	SNRPF	PRJNA729271	-1.826824458	2.26691E-46	1.826824458	stage_E14_vs_E10	ordinary_muscle_development	D_vs_E10;stage_1W_vs_E10;stage_3W_vs_E10;stage	1	TRUE
ENSGALG00010028262	C1orf43	PRJNA729271	2.048426298	2.39377E-46	2.048426298	stage_E14_vs_E10	ordinary_muscle_development	D_vs_E10;stage_1W_vs_E10;stage_3W_vs_E10;stage	1	TRUE
ENSGALG00010028155	CIZ1	PRJNA729271	-1.155807931	2.40619E-46	1.155807931	stage_E14_vs_E10	ordinary_muscle_development	D_vs_E10;stage_1W_vs_E10;stage_3W_vs_E10;stage	1	TRUE
ENSGALG00010024300	MEOX1	PRJNA729271	-4.159601714	2.41632E-46	4.159601714	stage_E14_vs_E10	ordinary_muscle_development	D_vs_E10;stage_1W_vs_E10;stage_3W_vs_E10;stage	1	TRUE
ENSGALG00010016988	ECI1	PRJNA729271	1.578248003	2.60339E-46	1.578248003	stage_E14_vs_E10	ordinary_muscle_development	D_vs_E10;stage_1W_vs_E10;stage_3W_vs_E10;stage	1	TRUE
ENSGALG00010008246	TERF2	PRJNA729271	-0.863123528	2.60531E-46	0.863123528	stage_E14_vs_E10	ordinary_muscle_development		0	FALSE
ENSGALG00010004142	RBBP8	PRJNA729271	-1.702468188	2.80576E-46	1.702468188	stage_E14_vs_E10	ordinary_muscle_development	D_vs_E10;stage_1W_vs_E10;stage_3W_vs_E10;stage	1	TRUE
ENSGALG00010016504	SNRK	PRJNA729271	1.217602998	2.80655E-46	1.217602998	stage_E14_vs_E10	ordinary_muscle_development	D_vs_E10;stage_1W_vs_E10;stage_3W_vs_E10;stage	1	TRUE
ENSGALG00010012138	DDX11	PRJNA729271	-2.628057041	2.90572E-46	2.628057041	stage_E14_vs_E10	ordinary_muscle_development	D_vs_E10;stage_1W_vs_E10;stage_3W_vs_E10;stage	1	TRUE
ENSGALG00010015892	UBE2C	PRJNA729271	-3.342270148	2.97741E-46	3.342270148	stage_E14_vs_E10	ordinary_muscle_development	D_vs_E10;stage_1W_vs_E10;stage_3W_vs_E10;stage	1	TRUE
ENSGALG00010012582	CD24	PRJNA729271	-3.283266048	3.06165E-46	3.283266048	stage_E14_vs_E10	ordinary_muscle_development	D_vs_E10;stage_1W_vs_E10;stage_3W_vs_E10;stage	1	TRUE
ENSGALG00010029522	AKAP1	PRJNA729271	1.615403598	3.08067E-46	1.615403598	stage_E14_vs_E10	ordinary_muscle_development	D_vs_E10;stage_1W_vs_E10;stage_3W_vs_E10;stage	1	TRUE
ENSGALG00010026061	MPEG1	PRJNA729271	4.933775409	3.18985E-46	4.933775409	stage_E14_vs_E10	ordinary_muscle_development	D_vs_E10;stage_1W_vs_E10;stage_3W_vs_E10;stage	1	TRUE
ENSGALG00010004901	CUL5	PRJNA729271	1.32813189	3.21733E-46	1.32813189	stage_E14_vs_E10	ordinary_muscle_development	D_vs_E10;stage_1W_vs_E10;stage_3W_vs_E10;stage	1	TRUE
ENSGALG00010025488	SLC4A8	PRJNA729271	-3.060508178	3.28205E-46	3.060508178	stage_E14_vs_E10	ordinary_muscle_development	D_vs_E10;stage_1W_vs_E10;stage_3W_vs_E10;stage	1	TRUE
ENSGALG00010012555	ENSGALG00010012555	PRJNA729271	3.10939902	3.30297E-46	3.10939902	stage_E14_vs_E10	ordinary_muscle_development	D_vs_E10;stage_1W_vs_E10;stage_3W_vs_E10;stage	1	TRUE
ENSGALG00010015260	RASGEF1C	PRJNA729271	1.999070086	3.31045E-46	1.999070086	stage_E14_vs_E10	ordinary_muscle_development	D_vs_E10;stage_1W_vs_E10;stage_3W_vs_E10;stage	1	TRUE
ENSGALG00010004104	SCARB2	PRJNA729271	1.357849299	3.40987E-46	1.357849299	stage_E14_vs_E10	ordinary_muscle_development	D_vs_E10;stage_1W_vs_E10;stage_3W_vs_E10;stage	1	TRUE
ENSGALG00010020285	ADAMTS14	PRJNA729271	-3.974109646	3.4418E-46	3.974109646	stage_E14_vs_E10	ordinary_muscle_development	D_vs_E10;stage_1W_vs_E10;stage_3W_vs_E10;stage	1	TRUE
ENSGALG00010011438	ENSGALG00010011438	PRJNA729271	6.354775251	3.58434E-46	6.354775251	stage_E14_vs_E10	ordinary_muscle_development	D_vs_E10;stage_1W_vs_E10;stage_3W_vs_E10;stage	1	TRUE
ENSGALG00010022990	SLC25A22	PRJNA729271	-1.482322422	3.92628E-46	1.482322422	stage_E14_vs_E10	ordinary_muscle_development	D_vs_E10;stage_1W_vs_E10;stage_3W_vs_E10;stage	1	TRUE
ENSGALG00010024144	HNRNPA3	PRJNA729271	-1.830965246	4.03323E-46	1.830965246	stage_E14_vs_E10	ordinary_muscle_development	D_vs_E10;stage_1W_vs_E10;stage_3W_vs_E10;stage	1	TRUE

ENSGALG00010027720	DAB2IP	PRJNA729271	1.25612391	4.08627E-46	1.25612391	stage_E14_vs_E10	ordinary_muscle_development	D_vs_E10;stage_1W_vs_E10;stage_3W_vs_E10;stage	1	TRUE
ENSGALG00010028551	NCS1	PRJNA729271	-3.688254617	4.23654E-46	3.688254617	stage_E14_vs_E10	ordinary_muscle_development	D_vs_E10;stage_1W_vs_E10;stage_3W_vs_E10;stage	1	TRUE
ENSGALG00010013407	ZC3H15	PRJNA729271	1.619592274	4.70162E-46	1.619592274	stage_E14_vs_E10	ordinary_muscle_development	D_vs_E10;stage_1W_vs_E10;stage_3W_vs_E10;stage	1	TRUE
ENSGALG00010003525	NOL10	PRJNA729271	-0.993082752	4.89582E-46	0.993082752	stage_E14_vs_E10	ordinary_muscle_development		0	FALSE
ENSGALG00010023703	PITPNB	PRJNA729271	-1.17835467	4.94873E-46	1.17835467	stage_E14_vs_E10	ordinary_muscle_development	D_vs_E10;stage_1W_vs_E10;stage_3W_vs_E10;stage	1	TRUE
ENSGALG00010024829	ERBB3	PRJNA729271	-3.3262967	5.0531E-46	3.3262967	stage_E14_vs_E10	ordinary_muscle_development	D_vs_E10;stage_1W_vs_E10;stage_3W_vs_E10;stage	1	TRUE
ENSGALG00010019534	PPP2R5A	PRJNA729271	1.500047717	5.75116E-46	1.500047717	stage_E14_vs_E10	ordinary_muscle_development	D_vs_E10;stage_1W_vs_E10;stage_3W_vs_E10;stage	1	TRUE
ENSGALG00010001817	SLC25A6	PRJNA729271	-1.628289617	5.88216E-46	1.628289617	stage_E14_vs_E10	ordinary_muscle_development	D_vs_E10;stage_1W_vs_E10;stage_3W_vs_E10;stage	1	TRUE
ENSGALG00010027311	NFASC	PRJNA729271	3.436615717	6.52665E-46	3.436615717	stage_E14_vs_E10	ordinary_muscle_development	D_vs_E10;stage_1W_vs_E10;stage_3W_vs_E10;stage	1	TRUE
ENSGALG00010001885	SMPX	PRJNA729271	5.350623512	6.5516E-46	5.350623512	stage_E14_vs_E10	ordinary_muscle_development	D_vs_E10;stage_1W_vs_E10;stage_3W_vs_E10;stage	1	TRUE
ENSGALG00010025298	ENSGALG00010025298	PRJNA729271	-5.294686495	6.63991E-46	5.294686495	stage_E14_vs_E10	ordinary_muscle_development	D_vs_E10;stage_1W_vs_E10;stage_3W_vs_E10;stage	1	TRUE
ENSGALG00010020063	ENSGALG00010020063	PRJNA729271	1.848040558	6.74704E-46	1.848040558	stage_E14_vs_E10	ordinary_muscle_development	D_vs_E10;stage_1W_vs_E10;stage_3W_vs_E10;stage	1	TRUE
ENSGALG00010005463	SKA3	PRJNA729271	-3.822547757	6.82003E-46	3.822547757	stage_E14_vs_E10	ordinary_muscle_development	D_vs_E10;stage_1W_vs_E10;stage_3W_vs_E10;stage	1	TRUE
ENSGALG00010025653	IGSF11	PRJNA729271	2.77809122	7.48976E-46	2.77809122	stage_E14_vs_E10	ordinary_muscle_development	D_vs_E10;stage_1W_vs_E10;stage_3W_vs_E10;stage	1	TRUE
ENSGALG00010027273	HAUS8	PRJNA729271	-1.975261533	7.6055E-46	1.975261533	stage_E14_vs_E10	ordinary_muscle_development	D_vs_E10;stage_1W_vs_E10;stage_3W_vs_E10;stage	1	TRUE
ENSGALG00010008272	DCP2	PRJNA729271	-1.247582778	7.66098E-46	1.247582778	stage_E14_vs_E10	ordinary_muscle_development	D_vs_E10;stage_1W_vs_E10;stage_3W_vs_E10;stage	1	TRUE
ENSGALG00010015498	MID2	PRJNA729271	1.908150458	7.88616E-46	1.908150458	stage_E14_vs_E10	ordinary_muscle_development	D_vs_E10;stage_1W_vs_E10;stage_3W_vs_E10;stage	1	TRUE
ENSGALG00010003229	STIM1	PRJNA729271	1.506236188	8.02302E-46	1.506236188	stage_E14_vs_E10	ordinary_muscle_development	D_vs_E10;stage_1W_vs_E10;stage_3W_vs_E10;stage	1	TRUE
ENSGALG00010027146	KXD1	PRJNA729271	1.730514794	8.22211E-46	1.730514794	stage_E14_vs_E10	ordinary_muscle_development	D_vs_E10;stage_1W_vs_E10;stage_3W_vs_E10;stage	1	TRUE
ENSGALG00010024163	HS6ST1	PRJNA729271	-1.404886221	9.16359E-46	1.404886221	stage_E14_vs_E10	ordinary_muscle_development	D_vs_E10;stage_1W_vs_E10;stage_3W_vs_E10;stage	1	TRUE
ENSGALG00010014744	IL2RB	PRJNA729271	6.081553165	9.4324E-46	6.081553165	stage_E14_vs_E10	ordinary_muscle_development	D_vs_E10;stage_1W_vs_E10;stage_3W_vs_E10;stage	1	TRUE
ENSGALG00010024871	POLQ	PRJNA729271	-2.069609862	9.51124E-46	2.069609862	stage_E14_vs_E10	ordinary_muscle_development	D_vs_E10;stage_1W_vs_E10;stage_3W_vs_E10;stage	1	TRUE
ENSGALG00010001121	WWTR1	PRJNA729271	1.323854009	9.83876E-46	1.323854009	stage_E14_vs_E10	ordinary_muscle_development	D_vs_E10;stage_1W_vs_E10;stage_3W_vs_E10;stage	1	TRUE
ENSGALG00010025154	TMEM183A	PRJNA729271	1.732895572	1.02356E-45	1.732895572	stage_E14_vs_E10	ordinary_muscle_development	D_vs_E10;stage_1W_vs_E10;stage_3W_vs_E10;stage	1	TRUE
ENSGALG00010027391	TPM3	PRJNA729271	-1.650284978	1.04049E-45	1.650284978	stage_E14_vs_E10	ordinary_muscle_development	D_vs_E10;stage_1W_vs_E10;stage_3W_vs_E10;stage	1	TRUE
ENSGALG00010008534	RAB33B	PRJNA729271	1.031692887	1.08089E-45	1.031692887	stage_E14_vs_E10	ordinary_muscle_development	D_vs_E10;stage_1W_vs_E10;stage_3W_vs_E10;stage	1	TRUE
ENSGALG00010017891	GOLGA7B	PRJNA729271	4.629700968	1.20171E-45	4.629700968	stage_E14_vs_E10	ordinary_muscle_development	D_vs_E10;stage_1W_vs_E10;stage_3W_vs_E10;stage	1	TRUE
ENSGALG00010008179	SEMA6A	PRJNA729271	-1.407458689	1.21934E-45	1.407458689	stage_E14_vs_E10	ordinary_muscle_development	D_vs_E10;stage_1W_vs_E10;stage_3W_vs_E10;stage	1	TRUE
ENSGALG00010009368	DSCC1	PRJNA729271	-2.895681891	1.3285E-45	2.895681891	stage_E14_vs_E10	ordinary_muscle_development	D_vs_E10;stage_1W_vs_E10;stage_3W_vs_E10;stage	1	TRUE
ENSGALG00010007788	PALM2AKAP2	PRJNA729271	2.369137729	1.42021E-45	2.369137729	stage_E14_vs_E10	ordinary_muscle_development	D_vs_E10;stage_1W_vs_E10;stage_3W_vs_E10;stage	1	TRUE
ENSGALG00010000242	ENSGALG00010000242	PRJNA729271	2.768435625	1.4366E-45	2.768435625	stage_E14_vs_E10	ordinary_muscle_development	D_vs_E10;stage_1W_vs_E10;stage_3W_vs_E10;stage	1	TRUE
ENSGALG00010012749	RGS4	PRJNA729271	2.531152244	1.56518E-45	2.531152244	stage_E14_vs_E10	ordinary_muscle_development	D_vs_E10;stage_1W_vs_E10;stage_3W_vs_E10;stage	1	TRUE
ENSGALG00010013356	AMER1	PRJNA729271	-0.944527395	1.58594E-45	0.944527395	stage_E14_vs_E10	ordinary_muscle_development		0	FALSE
ENSGALG00010006901	TUBAL3	PRJNA729271	-3.781214176	1.60669E-45	3.781214176	stage_E14_vs_E10	ordinary_muscle_development	D_vs_E10;stage_1W_vs_E10;stage_3W_vs_E10;stage	1	TRUE
ENSGALG00010018033	COQ8A	PRJNA729271	3.059547534	1.61601E-45	3.059547534	stage_E14_vs_E10	ordinary_muscle_development	D_vs_E10;stage_1W_vs_E10;stage_3W_vs_E10;stage	1	TRUE
ENSGALG00010028457	KANK3	PRJNA729271	1.502299375	1.6453E-45	1.502299375	stage_E14_vs_E10	ordinary_muscle_development	D_vs_E10;stage_1W_vs_E10;stage_3W_vs_E10;stage	1	TRUE
ENSGALG00010017520	ZBTB1	PRJNA729271	-1.173187777	1.68185E-45	1.173187777	stage_E14_vs_E10	ordinary_muscle_development	D_vs_E10;stage_1W_vs_E10;stage_3W_vs_E10;stage	1	TRUE
ENSGALG00010013466	BRAF	PRJNA729271	0.873379652	1.76877E-45	0.873379652	stage_E14_vs_E10	ordinary_muscle_development		0	FALSE
ENSGALG00010026731	ENSGALG00010026731	PRJNA729271	6.338527495	1.79215E-45	6.338527495	stage_E14_vs_E10	ordinary_muscle_development	D_vs_E10;stage_1W_vs_E10;stage_3W_vs_E10;stage	1	TRUE
ENSGALG00010025628	ENSGALG00010025628	PRJNA729271	-5.855029208	1.97247E-45	5.855029208	stage_E14_vs_E10	ordinary_muscle_development	D_vs_E10;stage_1W_vs_E10;stage_3W_vs_E10;stage	1	TRUE
ENSGALG00010024939	COQ10A	PRJNA729271	2.892771332	2.01347E-45	2.892771332	stage_E14_vs_E10	ordinary_muscle_development	D_vs_E10;stage_1W_vs_E10;stage_3W_vs_E10;stage	1	TRUE
ENSGALG00010022831	ATCAY	PRJNA729271	-3.488926659	2.04498E-45	3.488926659	stage_E14_vs_E10	ordinary_muscle_development	D_vs_E10;stage_1W_vs_E10;stage_3W_vs_E10;stage	1	TRUE
ENSGALG00010007867	ZNF516	PRJNA729271	-1.53960386	2.07716E-45	1.53960386	stage_E14_vs_E10	ordinary_muscle_development	D_vs_E10;stage_1W_vs_E10;stage_3W_vs_E10;stage	1	TRUE
ENSGALG00010016317	CTDSPL2	PRJNA729271	-0.825964032	2.10839E-45	0.825964032	stage_E14_vs_E10	ordinary_muscle_development		0	FALSE
ENSGALG00010014308	ENSGALG00010014308	PRJNA729271	2.834079787	2.29424E-45	2.834079787	stage_E14_vs_E10	ordinary_muscle_development	D_vs_E10;stage_1W_vs_E10;stage_3W_vs_E10;stage	1	TRUE
ENSGALG00010018534	NCEH1	PRJNA729271	2.783969427	2.31651E-45	2.783969427	stage_E14_vs_E10	ordinary_muscle_development	D_vs_E10;stage_1W_vs_E10;stage_3W_vs_E10;stage	1	TRUE
ENSGALG00010015676	TGM2	PRJNA729271	2.753078698	2.32462E-45	2.753078698	stage_E14_vs_E10	ordinary_muscle_development	D_vs_E10;stage_1W_vs_E10;stage_3W_vs_E10;stage	1	TRUE
ENSGALG00010022702	CDC20	PRJNA729271	-3.396158557	2.41282E-45	3.396158557	stage_E14_vs_E10	ordinary_muscle_development	D_vs_E10;stage_1W_vs_E10;stage_3W_vs_E10;stage	1	TRUE
ENSGALG00010028108	IQGAP3	PRJNA729271	-3.143565276	2.45572E-45	3.143565276	stage_E14_vs_E10	ordinary_muscle_development	D_vs_E10;stage_1W_vs_E10;stage_3W_vs_E10;stage	1	TRUE
ENSGALG00010009575	RHOBTB1	PRJNA729271	-3.711122901	2.47607E-45	3.711122901	stage_E14_vs_E10	ordinary_muscle_development	D_vs_E10;stage_1W_vs_E10;stage_3W_vs_E10;stage	1	TRUE
ENSGALG00010018340	DNPEP	PRJNA729271	1.384434067	2.52593E-45	1.384434067	stage_E14_vs_E10	ordinary_muscle_development	D_vs_E10;stage_1W_vs_E10;stage_3W_vs_E10;stage	1	TRUE
ENSGALG00010013261	JADE2	PRJNA729271	2.812755462	2.53201E-45	2.812755462	stage_E14_vs_E10	ordinary_muscle_development	D_vs_E10;stage_1W_vs_E10;stage_3W_vs_E10;stage	1	TRUE
ENSGALG00010024678	AMOTL2	PRJNA729271	1.289348177	2.81416E-45	1.289348177	stage_E14_vs_E10	ordinary_muscle_development	D_vs_E10;stage_1W_vs_E10;stage_3W_vs_E10;stage	1	TRUE
ENSGALG00010022911	SMPD3	PRJNA729271	-5.444710342	2.81663E-45	5.444710342	stage_E14_vs_E10	ordinary_muscle_development	D_vs_E10;stage_1W_vs_E10;stage_3W_vs_E10;stage	1	TRUE
ENSGALG00010005010	NKAIN2	PRJNA729271	-5.0660822	2.85678E-45	5.0660822	stage_E14_vs_E10	ordinary_muscle_development	D_vs_E10;stage_1W_vs_E10;stage_3W_vs_E10;stage	1	TRUE
ENSGALG00010005660	UBAC2	PRJNA729271	1.432970785	2.96596E-45	1.432970785	stage_E14_vs_E10	ordinary_muscle_development	D_vs_E10;stage_1W_vs_E10;stage_3W_vs_E10;stage	1	TRUE
ENSGALG00010005245	KIAA1217	PRJNA729271	1.925220542	3.0266E-45	1.925220542	stage_E14_vs_E10	ordinary_muscle_development	D_vs_E10;stage_1W_vs_E10;stage_3W_vs_E10;stage	1	TRUE
ENSGALG00010002504	ENSGALG00010002504	PRJNA729271	0.936568461	3.08972E-45	0.936568461	stage_E14_vs_E10	ordinary_muscle_development		0	FALSE
ENSGALG00010028599	VPS72	PRJNA729271	1.677945194	3.41397E-45	1.677945194	stage_E14_vs_E10	ordinary_muscle_development	D_vs_E10;stage_1W_vs_E10;stage_3W_vs_E10;stage	1	TRUE
ENSGALG00010029638	EPN3	PRJNA729271	2.780792091	3.42757E-45	2.780792091	stage_E14_vs_E10	ordinary_muscle_development	D_vs_E10;stage_1W_vs_E10;stage_3W_vs_E10;stage	1	TRUE
ENSGALG00010021863	CLDN19	PRJNA729271	4.039242879	3.46462E-45	4.039242879	stage_E14_vs_E10	ordinary_muscle_development	D_vs_E10;stage_1W_vs_E10;stage_3W_vs_E10;stage	1	TRUE
ENSGALG00010027702	FOXP4	PRJNA729271	-3.734453287	3.66154E-45	3.734453287	stage_E14_vs_E10	ordinary_muscle_development	D_vs_E10;stage_1W_vs_E10;stage_3W_vs_E10;stage	1	TRUE
ENSGALG00010023332	PPP1R7	PRJNA729271	1.059445821	4.00534E-45	1.059445821	stage_E14_vs_E10	ordinary_muscle_development	D_vs_E10;stage_1W_vs_E10;stage_3W_vs_E10;stage	1	TRUE
ENSGALG00010025381	MRPL37	PRJNA729271	1.725891401	4.00918E-45	1.725891401	stage_E14_vs_E10	ordinary_muscle_development	D_vs_E10;stage_1W_vs_E10;stage_3W_vs_E10;stage	1	TRUE
ENSGALG00010016640	OCIAD1	PRJNA729271	1.474589049	4.07171E-45	1.474589049	stage_E14_vs_E10	ordinary_muscle_development	D_vs_E10;stage_1W_vs_E10;stage_3W_vs_E10;stage	1	TRUE
ENSGALG00010018137	NDUFB5	PRJNA729271	1.8162859	4.07577E-45	1.8162859	stage_E14_vs_E10	ordinary_muscle_development	D_vs_E10;stage_1W_vs_E10;stage_3W_vs_E10;stage	1	TRUE
ENSGALG00010027230	MAP2K2	PRJNA729271	-1.213747135	4.1322E-45	1.213747135	stage_E14_vs_E10	ordinary_muscle_development	D_vs_E10;stage_1W_vs_E10;stage_3W_vs_E10;stage	1	TRUE
ENSGALG00010009597	CDK1	PRJNA729271	-3.726548567	4.14122E-45	3.726548567	stage_E14_vs_E10	ordinary_muscle_development	D_vs_E10;stage_1W_vs_E10;stage_3W_vs_E10;stage	1	TRUE
ENSGALG00010010167	PUS7	PRJNA729271	-1.061933275	4.42645E-45	1.061933275	stage_E14_vs_E10	ordinary_muscle_development	D_vs_E10;stage_1W_vs_E10;stage_3W_vs_E10;stage	1	TRUE
ENSGALG00010023764	COX5A	PRJNA729271	2.515995602	4.52282E-45	2.515995602	stage_E14_vs_E10	ordinary_muscle_development	D_vs_E10;stage_1W_vs_E10;stage_3W_vs_E10;stage	1	TRUE
ENSGALG00010008448	TNS3	PRJNA729271	-1.160708138	4.70241E-45	1.160708138	stage_E14_vs_E10	ordinary_muscle_development	D_vs_E10;stage_1W_vs_E10;stage_3W_vs_E10;stage	1	TRUE
ENSGALG00010009213	DUS4L	PRJNA729271	-1.483254656	4.71153E-45	1.483254656	stage_E14_vs_E10	ordinary_muscle_development	D_vs_E10;stage_1W_vs_E10;stage_3W_vs_E10;stage	1	TRUE
ENSGALG00010016339	SETD3	PRJNA729271	1.740607943	4.71611E-45	1.740607943	stage_E14_vs_E10	ordinary_muscle_development	D_vs_E10;stage_1W_vs_E10;stage_3W_vs_E10;stage	1	TRUE
ENSGALG00010024977	LRP3	PRJNA729271	1.486614755	4.72881E-45	1.486614755	stage_E14_vs_E10	ordinary_muscle_development	D_vs_E10;stage_1W_vs_E10;stage_3W_vs_E10;stage	1	TRUE
ENSGALG00010027306	ENSGALG00010027306	PRJNA729271	4.465306172	4.77348E-45	4.465306172	stage_E14_vs_E10	ordinary_muscle_development	D_vs_E10;stage_1W_vs_E10;stage_3W_vs_E10;stage	1	TRUE
ENSGALG00010012277	H3C33	PRJNA729271	-2.996877785	5.39321E-45	2.996877785	stage_E14_vs_E10	ordinary_muscle_development	D_vs_E10;stage_1W_vs_E10;stage_3W_vs_E10;stage	1	TRUE
ENSGALG00010024175	DHRS9	PRJNA729271	5.6227028	5.46117E-45	5.6227028	stage_E14_vs_E10	ordinary_muscle_development	D_vs_E10;stage_1W_vs_E10;stage_3W_vs_E10;stage	1	TRUE
ENSGALG00010019675	MAFK	PRJNA729271	1.951190274	5.61629E-45	1.951190274	stage_E14_vs_E10	ordinary_muscle_development	D_vs_E10;stage_1W_vs_E10;stage_3W_vs_E10;stage	1	TRUE
ENSGALG00010010896	LY86	PRJNA729271	4.158504206	6.12432E-45	4.158504206	stage_E14_vs_E10	ordinary_muscle_development	D_vs_E10;stage_1W_vs_E10;stage_3W_vs_E10;stage	1	TRUE
ENSGALG00010007849	CXADR	PRJNA729271	-4.640792289	6.39285E-45	4.640792289	stage_E14_vs_E10	ordinary_muscle_development	D_vs_E10;stage_1W_vs_E10;stage_3W_vs_E10;stage	1	TRUE
ENSGALG00010022764	MFAP5	PRJNA729271	3.184219344	6.42505E-45	3.184219344	stage_E14_vs_E10	ordinary_muscle_development	D_vs_E10;stage_1W_vs_E10;stage_3W_vs_E10;stage	1	TRUE
ENSGALG00010021085	AMPD3	PRJNA729271	2.628174939	6.42505E-45	2.628174939	stage_E14_vs_E10	ordinary_muscle_development	D_vs_E10;stage_1W_vs_E10;stage_3W_vs_E10;stage	1	TRUE
ENSGALG00010007885	USP38	PRJNA729271	1.467407981	6.60874E-45	1.467407981	stage_E14_vs_E10	ordinary_muscle_development	D_vs_E10;stage_1W_vs_E10;stage_3W_vs_E10;stage	1	TRUE
ENSGALG00010021425	PTPN9	PRJNA729271	-1.840856936	6.72636E-45	1.840856936	stage_E14_vs_E10	ordinary_muscle_development	D_vs_E10;stage_1W_vs_E10;stage_3W_vs_E10;stage	1	TRUE
ENSGALG00010019635	LRRC57	PRJNA729271	1.080177033	7.04506E-45	1.080177033	stage_E14_vs_E10	ordinary_muscle_development	D_vs_E10;stage_1W_vs_E10;stage_3W_vs_E10;stage	1	TRUE
ENSGALG00010015006	HSPB3	PRJNA729271	4.704318184	7.10447E-45	4.704318184	stage_E14_vs_E10	ordinary_muscle_development	D_vs_E10;stage_1W_vs_E10;stage_3W_vs_E10;stage	1	TRUE
ENSGALG00010001708	AADAC	PRJNA729271	3.56064327	7.29874E-45	3.56064327	stage_E14_vs_E10	ordinary_muscle_development	D_vs_E10;stage_1W_vs_E10;stage_3W_vs_E10;stage	1	TRUE
ENSGALG00010011943	TTLL12	PRJNA729271	-0.797151691	7.3285E-45	0.797151691	stage_E14_vs_E10	ordinary_muscle_development		0	FALSE
ENSGALG00010028486	ARHGAP30	PRJNA729271	3.114271904	7.68369E-45	3.114271904	stage_E14_vs_E10	ordinary_muscle_development	D_vs_E10;stage_1W_vs_E10;stage_3W_vs_E10;stage	1	TRUE
ENSGALG00010023264	RACGAP1	PRJNA729271	-3.341130704	7.89174E-45	3.341130704	stage_E14_vs_E10	ordinary_muscle_development	D_vs_E10;stage_1W_vs_E10;stage_3W_vs_E10;stage	1	TRUE
ENSGALG00010023962	FPGT	PRJNA729271	-1.696901696	8.17299E-45	1.696901696	stage_E14_vs_E10	ordinary_muscle_development	D_vs_E10;stage_1W_vs_E10;stage_3W_vs_E10;stage	1	TRUE
ENSGALG00010024554	ST6GALNAC3	PRJNA729271	-3.047181578	8.26067E-45	3.047181578	stage_E14_vs_E10	ordinary_muscle_development	D_vs_E10;stage_1W_vs_E10;stage_3W_vs_E10;stage	1	TRUE
ENSGALG00010019270	ENSGALG00010019270	PRJNA729271	-3.488345615	8.40762E-45	3.488345615	stage_E14_vs_E10	ordinary_muscle_development	D_vs_E10;stage_1W_vs_E10;stage_3W_vs_E10;stage	1	TRUE
ENSGALG00010012061	AZIN1	PRJNA729271	1.704365927	9.08866E-45	1.704365927	stage_E14_vs_E10	ordinary_muscle_development	D_vs_E10;stage_1W_vs_E10;stage_3W_vs_E10;stage	1	TRUE
ENSGALG00010019724	HES5A	PRJNA729271	-7.436060187	9.34336E-45	7.436060187	stage_E14_vs_E10	ordinary_muscle_development	D_vs_E10;stage_1W_vs_E10;stage_3W_vs_E10;stage	1	TRUE
ENSGALG00010007859	CENPN	PRJNA729271	-2.348456559	9.35833E-45	2.348456559	stage_E14_vs_E10	ordinary_muscle_development	D_vs_E10;stage_1W_vs_E10;stage_3W_vs_E10;stage	1	TRUE
ENSGALG00010022923	CD63	PRJNA729271	1.794165776	9.52187E-45	1.794165776	stage_E14_vs_E10	ordinary_muscle_development	D_vs_E10;stage_1W_vs_E10;stage_3W_vs_E10;stage	1	TRUE
ENSGALG00010007051	E2F3	PRJNA729271	-2.213799719	9.67961E-45	2.213799719	stage_E14_vs_E10	ordinary_muscle_development	D_vs_E10;stage_1W_vs_E10;stage_3W_vs_E10;stage	1	TRUE
ENSGALG00010017973	PKD1	PRJNA729271	1.831513311	1.00355E-44	1.831513311	stage_E14_vs_E10	ordinary_muscle_development	D_vs_E10;stage_1W_vs_E10;stage_3W_vs_E10;stage	1	TRUE
ENSGALG00010024564	PPP1R3D	PRJNA729271	2.803451649	1.10865E-44	2.803451649	stage_E14_vs_E10	ordinary_muscle_development	D_vs_E10;stage_1W_vs_E10;stage_3W_vs_E10;stage	1	TRUE
ENSGALG00010025685	NCKAP1L	PRJNA729271	2.702661499	1.10946E-44	2.702661499	stage_E14_vs_E10	ordinary_muscle_development	D_vs_E10;stage_1W_vs_E10;stage_3W_vs_E10;stage	1	TRUE
ENSGALG00010001168	DOK6	PRJNA729271	-2.604533364	1.11156E-44	2.604533364	stage_E14_vs_E10	ordinary_muscle_development	D_vs_E10;stage_1W_vs_E10;stage_3W_vs_E10;stage	1	TRUE
ENSGALG00010007960	TSNAX	PRJNA729271	-0.896267055	1.11473E-44	0.896267055	stage_E14_vs_E10	ordinary_muscle_development		0	FALSE
ENSGALG00010014267	SCFD2	PRJNA729271	-1.111429393	1.19001E-44	1.111429393	stage_E14_vs_E10	ordinary_muscle_development	D_vs_E10;stage_1W_vs_E10;stage_3W_vs_E10;stage	1	TRUE
ENSGALG00010017811	HACD3	PRJNA729271	-0.948619146	1.23416E-44	0.948619146	stage_E14_vs_E10	ordinary_muscle_development		0	FALSE
ENSGALG00010018623	ARRDC4	PRJNA729271	1.859406816	1.29192E-44	1.859406816	stage_E14_vs_E10	ordinary_muscle_development	D_vs_E10;stage_1W_vs_E10;stage_3W_vs_E10;stage	1	TRUE
ENSGALG00010007514	VIT	PRJNA729271	-6.215660919	1.32479E-44	6.215660919	stage_E14_vs_E10	ordinary_muscle_development	D_vs_E10;stage_1W_vs_E10;stage_3W_vs_E10;stage	1	TRUE
ENSGALG00010011450	PTDSS1	PRJNA729271	-0.993924956	1.36127E-44	0.993924956	stage_E14_vs_E10	ordinary_muscle_development		0	FALSE
ENSGALG00010003107	GNG11	PRJNA729271	2.584243103	1.4519E-44	2.584243103	stage_E14_vs_E10	ordinary_muscle_development	D_vs_E10;stage_1W_vs_E10;stage_3W_vs_E10;stage	1	TRUE
ENSGALG00010023574	DEPDC1	PRJNA729271	-3.063454588	1.45875E-44	3.063454588	stage_E14_vs_E10	ordinary_muscle_development	D_vs_E10;stage_1W_vs_E10;stage_3W_vs_E10;stage	1	TRUE
ENSGALG00010013300	GNPDA1	PRJNA729271	-1.409918341	1.49083E-44	1.409918341	stage_E14_vs_E10	ordinary_muscle_development	D_vs_E10;stage_1W_vs_E10;stage_3W_vs_E10;stage	1	TRUE
ENSGALG00010013414	LAPTM5	PRJNA729271	2.449965347	1.49168E-44	2.449965347	stage_E14_vs_E10	ordinary_muscle_development	D_vs_E10;stage_1W_vs_E10;stage_3W_vs_E10;stage	1	TRUE
ENSGALG00010008382	ENSGALG00010008382	PRJNA729271	1.817530535	1.51398E-44	1.817530535	stage_E14_vs_E10	ordinary_muscle_development	D_vs_E10;stage_1W_vs_E10;stage_3W_vs_E10;stage	1	TRUE
ENSGALG00010012382	SH3PXD2B	PRJNA729271	-1.917605244	1.55564E-44	1.917605244	stage_E14_vs_E10	ordinary_muscle_development	D_vs_E10;stage_1W_vs_E10;stage_3W_vs_E10;stage	1	TRUE
ENSGALG00010011064	MYLIP	PRJNA729271	1.361521774	1.573E-44	1.361521774	stage_E14_vs_E10	ordinary_muscle_development	D_vs_E10;stage_1W_vs_E10;stage_3W_vs_E10;stage	1	TRUE
ENSGALG00010022874	EFCAB14	PRJNA729271	1.078729374	1.58801E-44	1.078729374	stage_E14_vs_E10	ordinary_muscle_development	D_vs_E10;stage_1W_vs_E10;stage_3W_vs_E10;stage	1	TRUE
ENSGALG00010013265	PACS2	PRJNA729271	1.731480446	1.66593E-44	1.731480446	stage_E14_vs_E10	ordinary_muscle_development	D_vs_E10;stage_1W_vs_E10;stage_3W_vs_E10;stage	1	TRUE
ENSGALG00010016927	MGAT4B	PRJNA729271	1.624070819	1.68706E-44	1.624070819	stage_E14_vs_E10	ordinary_muscle_development	D_vs_E10;stage_1W_vs_E10;stage_3W_vs_E10;stage	1	TRUE
ENSGALG00010022184	PRKAR1B	PRJNA729271	-1.340376884	1.69834E-44	1.340376884	stage_E14_vs_E10	ordinary_muscle_development	D_vs_E10;stage_1W_vs_E10;stage_3W_vs_E10;stage	1	TRUE
ENSGALG00010023992	FAM131B	PRJNA729271	-2.113477373	1.85743E-44	2.113477373	stage_E14_vs_E10	ordinary_muscle_development	D_vs_E10;stage_1W_vs_E10;stage_3W_vs_E10;stage	1	TRUE
ENSGALG00010020966	PAPLN	PRJNA729271	3.487085107	1.89338E-44	3.487085107	stage_E14_vs_E10	ordinary_muscle_development	D_vs_E10;stage_1W_vs_E10;stage_3W_vs_E10;stage	1	TRUE
ENSGALG00010002954	CHST7	PRJNA729271	1.714266864	1.91288E-44	1.714266864	stage_E14_vs_E10	ordinary_muscle_development	D_vs_E10;stage_1W_vs_E10;stage_3W_vs_E10;stage	1	TRUE
ENSGALG00010024722	BRCA1	PRJNA729271	-3.748373736	1.96399E-44	3.748373736	stage_E14_vs_E10	ordinary_muscle_development	D_vs_E10;stage_1W_vs_E10;stage_3W_vs_E10;stage	1	TRUE
ENSGALG00010027253	VGLL4	PRJNA729271	-1.255667333	1.98502E-44	1.255667333	stage_E14_vs_E10	ordinary_muscle_development	D_vs_E10;stage_1W_vs_E10;stage_3W_vs_E10;stage	1	TRUE
ENSGALG00010009841	GABRA1	PRJNA729271	3.231752478	2.09697E-44	3.231752478	stage_E14_vs_E10	ordinary_muscle_development	D_vs_E10;stage_1W_vs_E10;stage_3W_vs_E10;stage	1	TRUE
ENSGALG00010008031	MAP3K21	PRJNA729271	-2.727995785	2.14212E-44	2.727995785	stage_E14_vs_E10	ordinary_muscle_development	D_vs_E10;stage_1W_vs_E10;stage_3W_vs_E10;stage	1	TRUE
ENSGALG00010017746	MSH6	PRJNA729271	-1.271704219	2.18727E-44	1.271704219	stage_E14_vs_E10	ordinary_muscle_development	D_vs_E10;stage_1W_vs_E10;stage_3W_vs_E10;stage	1	TRUE
ENSGALG00010001461	ASB9	PRJNA729271	-3.356628184	2.32299E-44	3.356628184	stage_E14_vs_E10	ordinary_muscle_development	D_vs_E10;stage_1W_vs_E10;stage_3W_vs_E10;stage	1	TRUE
ENSGALG00010013429	PDE4DIP	PRJNA729271	2.65631561	2.34866E-44	2.65631561	stage_E14_vs_E10	ordinary_muscle_development	D_vs_E10;stage_1W_vs_E10;stage_3W_vs_E10;stage	1	TRUE
ENSGALG00010026097	ARF1	PRJNA729271	-0.793722189	2.40862E-44	0.793722189	stage_E14_vs_E10	ordinary_muscle_development		0	FALSE
ENSGALG00010024655	IGF2BP2	PRJNA729271	-2.387271898	2.46338E-44	2.387271898	stage_E14_vs_E10	ordinary_muscle_development	D_vs_E10;stage_1W_vs_E10;stage_3W_vs_E10;stage	1	TRUE
ENSGALG00010009686	MAT2B	PRJNA729271	1.225398075	2.50345E-44	1.225398075	stage_E14_vs_E10	ordinary_muscle_development	D_vs_E10;stage_1W_vs_E10;stage_3W_vs_E10;stage	1	TRUE
ENSGALG00010002527	LRRC3B	PRJNA729271	-3.315648149	2.58047E-44	3.315648149	stage_E14_vs_E10	ordinary_muscle_development	D_vs_E10;stage_1W_vs_E10;stage_3W_vs_E10;stage	1	TRUE
ENSGALG00010013444	OSBPL8	PRJNA729271	-1.466417406	2.58111E-44	1.466417406	stage_E14_vs_E10	ordinary_muscle_development	D_vs_E10;stage_1W_vs_E10;stage_3W_vs_E10;stage	1	TRUE
ENSGALG00010010768	ENSGALG00010010768	PRJNA729271	1.287870116	2.59518E-44	1.287870116	stage_E14_vs_E10	ordinary_muscle_development	D_vs_E10;stage_1W_vs_E10;stage_3W_vs_E10;stage	1	TRUE
ENSGALG00010010471	KCNJ3	PRJNA729271	2.041074453	2.6092E-44	2.041074453	stage_E14_vs_E10	ordinary_muscle_development	D_vs_E10;stage_1W_vs_E10;stage_3W_vs_E10;stage	1	TRUE
ENSGALG00010021708	CNTNAP5	PRJNA729271	-6.643693878	2.62402E-44	6.643693878	stage_E14_vs_E10	ordinary_muscle_development	D_vs_E10;stage_1W_vs_E10;stage_3W_vs_E10;stage	1	TRUE
ENSGALG00010018425	CYP26B1	PRJNA729271	-3.411969206	2.62827E-44	3.411969206	stage_E14_vs_E10	ordinary_muscle_development	D_vs_E10;stage_1W_vs_E10;stage_3W_vs_E10;stage	1	TRUE
ENSGALG00010018283	POF1B	PRJNA729271	2.427311447	2.65007E-44	2.427311447	stage_E14_vs_E10	ordinary_muscle_development	D_vs_E10;stage_1W_vs_E10;stage_3W_vs_E10;stage	1	TRUE
ENSGALG00010016858	ENSGALG00010016858	PRJNA729271	-2.893940232	2.7117E-44	2.893940232	stage_E14_vs_E10	ordinary_muscle_development	D_vs_E10;stage_1W_vs_E10;stage_3W_vs_E10;stage	1	TRUE
ENSGALG00010017741	SMCR8	PRJNA729271	1.952673125	2.86148E-44	1.952673125	stage_E14_vs_E10	ordinary_muscle_development	D_vs_E10;stage_1W_vs_E10;stage_3W_vs_E10;stage	1	TRUE
ENSGALG00010016978	FAM181A	PRJNA729271	4.927375656	3.0079E-44	4.927375656	stage_E14_vs_E10	ordinary_muscle_development	D_vs_E10;stage_1W_vs_E10;stage_3W_vs_E10;stage	1	TRUE
ENSGALG00010023260	ENSGALG00010023260	PRJNA729271	-4.863917412	3.06367E-44	4.863917412	stage_E14_vs_E10	ordinary_muscle_development	D_vs_E10;stage_1W_vs_E10;stage_3W_vs_E10;stage	1	TRUE
ENSGALG00010020803	ABCC6	PRJNA729271	2.214957011	3.07494E-44	2.214957011	stage_E14_vs_E10	ordinary_muscle_development	D_vs_E10;stage_1W_vs_E10;stage_3W_vs_E10;stage	1	TRUE
ENSGALG00010025195	P2RX7	PRJNA729271	5.614785631	3.34884E-44	5.614785631	stage_E14_vs_E10	ordinary_muscle_development	D_vs_E10;stage_1W_vs_E10;stage_3W_vs_E10;stage	1	TRUE
ENSGALG00010022834	CHRNA7	PRJNA729271	-4.0137805	3.47286E-44	4.0137805	stage_E14_vs_E10	ordinary_muscle_development	D_vs_E10;stage_1W_vs_E10;stage_3W_vs_E10;stage	1	TRUE
ENSGALG00010004781	ENSGALG00010004781	PRJNA729271	-1.190936805	3.53243E-44	1.190936805	stage_E14_vs_E10	ordinary_muscle_development	D_vs_E10;stage_1W_vs_E10;stage_3W_vs_E10;stage	1	TRUE
ENSGALG00010002554	EGFL6	PRJNA729271	-4.143087039	3.56587E-44	4.143087039	stage_E14_vs_E10	ordinary_muscle_development	D_vs_E10;stage_1W_vs_E10;stage_3W_vs_E10;stage	1	TRUE
ENSGALG00010013582	MAP4K4	PRJNA729271	-1.52116115	3.5724E-44	1.52116115	stage_E14_vs_E10	ordinary_muscle_development	D_vs_E10;stage_1W_vs_E10;stage_3W_vs_E10;stage	1	TRUE
ENSGALG00010004762	IFT57	PRJNA729271	-1.649741189	4.13801E-44	1.649741189	stage_E14_vs_E10	ordinary_muscle_development	D_vs_E10;stage_1W_vs_E10;stage_3W_vs_E10;stage	1	TRUE
ENSGALG00010016017	RAPGEF6	PRJNA729271	1.005371368	4.25914E-44	1.005371368	stage_E14_vs_E10	ordinary_muscle_development	D_vs_E10;stage_1W_vs_E10;stage_3W_vs_E10;stage	1	TRUE
ENSGALG00010024322	IDH3A	PRJNA729271	2.236430036	4.39295E-44	2.236430036	stage_E14_vs_E10	ordinary_muscle_development	D_vs_E10;stage_1W_vs_E10;stage_3W_vs_E10;stage	1	TRUE
ENSGALG00010022981	HNRNPH3	PRJNA729271	-1.830448377	4.4835E-44	1.830448377	stage_E14_vs_E10	ordinary_muscle_development	D_vs_E10;stage_1W_vs_E10;stage_3W_vs_E10;stage	1	TRUE
ENSGALG00010021685	DBNDD2	PRJNA729271	1.952775855	4.49262E-44	1.952775855	stage_E14_vs_E10	ordinary_muscle_development	D_vs_E10;stage_1W_vs_E10;stage_3W_vs_E10;stage	1	TRUE
ENSGALG00010003977	MYO6	PRJNA729271	-1.708278079	4.65759E-44	1.708278079	stage_E14_vs_E10	ordinary_muscle_development	D_vs_E10;stage_1W_vs_E10;stage_3W_vs_E10;stage	1	TRUE
ENSGALG00010017211	UBFD1	PRJNA729271	1.79194183	4.84497E-44	1.79194183	stage_E14_vs_E10	ordinary_muscle_development	D_vs_E10;stage_1W_vs_E10;stage_3W_vs_E10;stage	1	TRUE
ENSGALG00010020111	ADRA2B	PRJNA729271	1.695865479	5.40268E-44	1.695865479	stage_E14_vs_E10	ordinary_muscle_development	D_vs_E10;stage_1W_vs_E10;stage_3W_vs_E10;stage	1	TRUE
ENSGALG00010015933	ENSGALG00010015933	PRJNA729271	9.153345624	5.57672E-44	9.153345624	stage_E14_vs_E10	ordinary_muscle_development	D_vs_E10;stage_1W_vs_E10;stage_3W_vs_E10;stage	1	TRUE
ENSGALG00010012696	DNAL4	PRJNA729271	-1.373749315	5.75583E-44	1.373749315	stage_E14_vs_E10	ordinary_muscle_development	D_vs_E10;stage_1W_vs_E10;stage_3W_vs_E10;stage	1	TRUE
ENSGALG00010016416	CYP46A1	PRJNA729271	1.556875946	6.33623E-44	1.556875946	stage_E14_vs_E10	ordinary_muscle_development	D_vs_E10;stage_1W_vs_E10;stage_3W_vs_E10;stage	1	TRUE
ENSGALG00010017546	NFU1	PRJNA729271	1.515992135	6.34494E-44	1.515992135	stage_E14_vs_E10	ordinary_muscle_development	D_vs_E10;stage_1W_vs_E10;stage_3W_vs_E10;stage	1	TRUE
ENSGALG00010026458	DLEC1	PRJNA729271	2.466831118	6.47535E-44	2.466831118	stage_E14_vs_E10	ordinary_muscle_development	D_vs_E10;stage_1W_vs_E10;stage_3W_vs_E10;stage	1	TRUE
ENSGALG00010010330	ALPK2	PRJNA729271	2.16485159	6.50235E-44	2.16485159	stage_E14_vs_E10	ordinary_muscle_development	D_vs_E10;stage_1W_vs_E10;stage_3W_vs_E10;stage	1	TRUE
ENSGALG00010011682	USP15	PRJNA729271	1.719002946	7.215E-44	1.719002946	stage_E14_vs_E10	ordinary_muscle_development	D_vs_E10;stage_1W_vs_E10;stage_3W_vs_E10;stage	1	TRUE
ENSGALG00010005267	CYP51A1	PRJNA729271	-2.678907663	7.81161E-44	2.678907663	stage_E14_vs_E10	ordinary_muscle_development	D_vs_E10;stage_1W_vs_E10;stage_3W_vs_E10;stage	1	TRUE
ENSGALG00010006244	EPHA3	PRJNA729271	-2.28507863	8.0393E-44	2.28507863	stage_E14_vs_E10	ordinary_muscle_development	D_vs_E10;stage_1W_vs_E10;stage_3W_vs_E10;stage	1	TRUE
ENSGALG00010018263	SLX4IP	PRJNA729271	-1.664360269	8.14977E-44	1.664360269	stage_E14_vs_E10	ordinary_muscle_development	D_vs_E10;stage_1W_vs_E10;stage_3W_vs_E10;stage	1	TRUE
ENSGALG00010022655	NRIP3	PRJNA729271	-8.922312793	8.38069E-44	8.922312793	stage_E14_vs_E10	ordinary_muscle_development	D_vs_E10;stage_1W_vs_E10;stage_3W_vs_E10;stage	1	TRUE
ENSGALG00010028589	PHYHD1	PRJNA729271	2.506226664	8.38729E-44	2.506226664	stage_E14_vs_E10	ordinary_muscle_development	D_vs_E10;stage_1W_vs_E10;stage_3W_vs_E10;stage	1	TRUE
ENSGALG00010012569	C6orf203	PRJNA729271	1.691607496	8.67016E-44	1.691607496	stage_E14_vs_E10	ordinary_muscle_development	D_vs_E10;stage_1W_vs_E10;stage_3W_vs_E10;stage	1	TRUE
ENSGALG00010027789	UAP1L1	PRJNA729271	-1.905284126	8.78726E-44	1.905284126	stage_E14_vs_E10	ordinary_muscle_development	D_vs_E10;stage_1W_vs_E10;stage_3W_vs_E10;stage	1	TRUE
ENSGALG00010017510	HDC	PRJNA729271	5.278206852	8.79109E-44	5.278206852	stage_E14_vs_E10	ordinary_muscle_development	D_vs_E10;stage_1W_vs_E10;stage_3W_vs_E10;stage	1	TRUE
ENSGALG00010022569	KIAA1549L	PRJNA729271	-3.933349896	8.82888E-44	3.933349896	stage_E14_vs_E10	ordinary_muscle_development	D_vs_E10;stage_1W_vs_E10;stage_3W_vs_E10;stage	1	TRUE
ENSGALG00010014676	UBE2V1	PRJNA729271	-0.825401941	9.52339E-44	0.825401941	stage_E14_vs_E10	ordinary_muscle_development		0	FALSE
ENSGALG00010001839	ENSGALG00010001839	PRJNA729271	8.392177132	9.73937E-44	8.392177132	stage_E14_vs_E10	ordinary_muscle_development	D_vs_E10;stage_1W_vs_E10;stage_3W_vs_E10;stage	1	TRUE

ENSGALG00010024361	H2AX	PRJNA729271	-3.110296225	9.95434E-44	3.110296225	stage_E14_vs_E10	ordinary_muscle_development	D_vs_E10;stage_1W_vs_E10;stage_3W_vs_E10;stage	1	TRUE
ENSGALG00010025303	TMCC2	PRJNA729271	2.637188148	9.97179E-44	2.637188148	stage_E14_vs_E10	ordinary_muscle_development	D_vs_E10;stage_1W_vs_E10;stage_3W_vs_E10;stage	1	TRUE
ENSGALG00010012338	H3C33	PRJNA729271	-3.174947081	1.01706E-43	3.174947081	stage_E14_vs_E10	ordinary_muscle_development	D_vs_E10;stage_1W_vs_E10;stage_3W_vs_E10;stage	1	TRUE
ENSGALG00010006289	SKIDA1	PRJNA729271	-2.286390916	1.08696E-43	2.286390916	stage_E14_vs_E10	ordinary_muscle_development	D_vs_E10;stage_1W_vs_E10;stage_3W_vs_E10;stage	1	TRUE
ENSGALG00010006063	POGLUT2	PRJNA729271	-1.443155415	1.09579E-43	1.443155415	stage_E14_vs_E10	ordinary_muscle_development	D_vs_E10;stage_1W_vs_E10;stage_3W_vs_E10;stage	1	TRUE
ENSGALG00010001727	COL9A1	PRJNA729271	-11.86563844	1.09935E-43	11.86563844	stage_E14_vs_E10	ordinary_muscle_development	D_vs_E10;stage_1W_vs_E10;stage_3W_vs_E10;stage	1	TRUE
ENSGALG00010002441	USO1	PRJNA729271	0.949680276	1.10511E-43	0.949680276	stage_E14_vs_E10	ordinary_muscle_development		0	FALSE
ENSGALG00010013921	PPP2R2B	PRJNA729271	1.477122563	1.10678E-43	1.477122563	stage_E14_vs_E10	ordinary_muscle_development	D_vs_E10;stage_1W_vs_E10;stage_3W_vs_E10;stage	1	TRUE
ENSGALG00010012769	NEMP2	PRJNA729271	-1.212750242	1.18277E-43	1.212750242	stage_E14_vs_E10	ordinary_muscle_development	D_vs_E10;stage_1W_vs_E10;stage_3W_vs_E10;stage	1	TRUE
ENSGALG00010013363	HS3ST5	PRJNA729271	3.160007686	1.35596E-43	3.160007686	stage_E14_vs_E10	ordinary_muscle_development	D_vs_E10;stage_1W_vs_E10;stage_3W_vs_E10;stage	1	TRUE
ENSGALG00010027981	CNN2	PRJNA729271	-2.334793368	1.38077E-43	2.334793368	stage_E14_vs_E10	ordinary_muscle_development	D_vs_E10;stage_1W_vs_E10;stage_3W_vs_E10;stage	1	TRUE
ENSGALG00010011859	NTN4	PRJNA729271	2.783655068	1.38559E-43	2.783655068	stage_E14_vs_E10	ordinary_muscle_development	D_vs_E10;stage_1W_vs_E10;stage_3W_vs_E10;stage	1	TRUE
ENSGALG00010005991	FARP1	PRJNA729271	-1.283800492	1.3856E-43	1.283800492	stage_E14_vs_E10	ordinary_muscle_development	D_vs_E10;stage_1W_vs_E10;stage_3W_vs_E10;stage	1	TRUE
ENSGALG00010010843	ZNF395	PRJNA729271	2.50311674	1.46081E-43	2.50311674	stage_E14_vs_E10	ordinary_muscle_development	D_vs_E10;stage_1W_vs_E10;stage_3W_vs_E10;stage	1	TRUE
ENSGALG00010016129	CENPI	PRJNA729271	-3.438167714	1.46876E-43	3.438167714	stage_E14_vs_E10	ordinary_muscle_development	D_vs_E10;stage_1W_vs_E10;stage_3W_vs_E10;stage	1	TRUE
ENSGALG00010003097	TFPI2	PRJNA729271	1.916085593	1.50589E-43	1.916085593	stage_E14_vs_E10	ordinary_muscle_development	D_vs_E10;stage_1W_vs_E10;stage_3W_vs_E10;stage	1	TRUE
ENSGALG00010026964	SRPK1	PRJNA729271	-0.856038248	1.53085E-43	0.856038248	stage_E14_vs_E10	ordinary_muscle_development		0	FALSE
ENSGALG00010027359	ENSGALG00010027359	PRJNA729271	1.628909754	1.56764E-43	1.628909754	stage_E14_vs_E10	ordinary_muscle_development	D_vs_E10;stage_1W_vs_E10;stage_3W_vs_E10;stage	1	TRUE
ENSGALG00010022841	ENSGALG00010022841	PRJNA729271	-8.572341361	1.59286E-43	8.572341361	stage_E14_vs_E10	ordinary_muscle_development	D_vs_E10;stage_1W_vs_E10;stage_3W_vs_E10;stage	1	TRUE
ENSGALG00010024535	ENSGALG00010024535	PRJNA729271	2.113589285	1.62715E-43	2.113589285	stage_E14_vs_E10	ordinary_muscle_development	D_vs_E10;stage_1W_vs_E10;stage_3W_vs_E10;stage	1	TRUE
ENSGALG00010027803	SAPCD2	PRJNA729271	-2.415187957	1.63332E-43	2.415187957	stage_E14_vs_E10	ordinary_muscle_development	D_vs_E10;stage_1W_vs_E10;stage_3W_vs_E10;stage	1	TRUE
ENSGALG00010007890	ENSGALG00010007890	PRJNA729271	-1.770916584	1.65988E-43	1.770916584	stage_E14_vs_E10	ordinary_muscle_development	D_vs_E10;stage_1W_vs_E10;stage_3W_vs_E10;stage	1	TRUE
ENSGALG00010024551	RNF11	PRJNA729271	1.769924933	1.71049E-43	1.769924933	stage_E14_vs_E10	ordinary_muscle_development	D_vs_E10;stage_1W_vs_E10;stage_3W_vs_E10;stage	1	TRUE
ENSGALG00010008208	ILDR2	PRJNA729271	2.115534337	1.71138E-43	2.115534337	stage_E14_vs_E10	ordinary_muscle_development	D_vs_E10;stage_1W_vs_E10;stage_3W_vs_E10;stage	1	TRUE
ENSGALG00010022616	GLDN	PRJNA729271	4.620773815	1.74062E-43	4.620773815	stage_E14_vs_E10	ordinary_muscle_development	D_vs_E10;stage_1W_vs_E10;stage_3W_vs_E10;stage	1	TRUE
ENSGALG00010023829	USP1	PRJNA729271	-1.419796375	1.76865E-43	1.419796375	stage_E14_vs_E10	ordinary_muscle_development	D_vs_E10;stage_1W_vs_E10;stage_3W_vs_E10;stage	1	TRUE
ENSGALG00010021405	IDE	PRJNA729271	1.592288122	1.84072E-43	1.592288122	stage_E14_vs_E10	ordinary_muscle_development	D_vs_E10;stage_1W_vs_E10;stage_3W_vs_E10;stage	1	TRUE
ENSGALG00010003024	SUGT1	PRJNA729271	0.997125123	2.06109E-43	0.997125123	stage_E14_vs_E10	ordinary_muscle_development		0	FALSE
ENSGALG00010002588	FAM175A	PRJNA729271	-1.295379814	2.172E-43	1.295379814	stage_E14_vs_E10	ordinary_muscle_development	D_vs_E10;stage_1W_vs_E10;stage_3W_vs_E10;stage	1	TRUE
ENSGALG00010000423	ENSGALG00010000423	PRJNA729271	2.789417086	2.24034E-43	2.789417086	stage_E14_vs_E10	ordinary_muscle_development	D_vs_E10;stage_1W_vs_E10;stage_3W_vs_E10;stage	1	TRUE
ENSGALG00010016616	SLBP	PRJNA729271	-3.467963228	2.37599E-43	3.467963228	stage_E14_vs_E10	ordinary_muscle_development	D_vs_E10;stage_1W_vs_E10;stage_3W_vs_E10;stage	1	TRUE
ENSGALG00010004533	PAG1	PRJNA729271	1.567171649	2.378E-43	1.567171649	stage_E14_vs_E10	ordinary_muscle_development	D_vs_E10;stage_1W_vs_E10;stage_3W_vs_E10;stage	1	TRUE
ENSGALG00010023206	HSPB9	PRJNA729271	4.379295838	2.38328E-43	4.379295838	stage_E14_vs_E10	ordinary_muscle_development	D_vs_E10;stage_1W_vs_E10;stage_3W_vs_E10;stage	1	TRUE
ENSGALG00010015192	ENSGALG00010015192	PRJNA729271	2.96893112	2.52185E-43	2.96893112	stage_E14_vs_E10	ordinary_muscle_development	D_vs_E10;stage_1W_vs_E10;stage_3W_vs_E10;stage	1	TRUE
ENSGALG00010022307	ENSGALG00010022307	PRJNA729271	8.852662734	2.56927E-43	8.852662734	stage_E14_vs_E10	ordinary_muscle_development	D_vs_E10;stage_1W_vs_E10;stage_3W_vs_E10;stage	1	TRUE
ENSGALG00010015954	CTSA	PRJNA729271	1.344936588	2.58398E-43	1.344936588	stage_E14_vs_E10	ordinary_muscle_development	D_vs_E10;stage_1W_vs_E10;stage_3W_vs_E10;stage	1	TRUE
ENSGALG00010001858	ADAMTS20	PRJNA729271	2.2414225	2.5894E-43	2.2414225	stage_E14_vs_E10	ordinary_muscle_development	D_vs_E10;stage_1W_vs_E10;stage_3W_vs_E10;stage	1	TRUE
ENSGALG00010017858	GPR65	PRJNA729271	3.915943367	2.60293E-43	3.915943367	stage_E14_vs_E10	ordinary_muscle_development	D_vs_E10;stage_1W_vs_E10;stage_3W_vs_E10;stage	1	TRUE
ENSGALG00010016236	GPATCH2	PRJNA729271	-0.944957534	2.71053E-43	0.944957534	stage_E14_vs_E10	ordinary_muscle_development		0	FALSE
ENSGALG00010015083	CEP135	PRJNA729271	-2.793574068	2.78671E-43	2.793574068	stage_E14_vs_E10	ordinary_muscle_development	D_vs_E10;stage_1W_vs_E10;stage_3W_vs_E10;stage	1	TRUE
ENSGALG00010001832	BUB3	PRJNA729271	-1.226077214	2.82822E-43	1.226077214	stage_E14_vs_E10	ordinary_muscle_development	D_vs_E10;stage_1W_vs_E10;stage_3W_vs_E10;stage	1	TRUE
ENSGALG00010015980	EXOSC7	PRJNA729271	-1.323963333	2.95214E-43	1.323963333	stage_E14_vs_E10	ordinary_muscle_development	D_vs_E10;stage_1W_vs_E10;stage_3W_vs_E10;stage	1	TRUE
ENSGALG00010029612	HSPB1	PRJNA729271	2.738584104	3.00421E-43	2.738584104	stage_E14_vs_E10	ordinary_muscle_development	D_vs_E10;stage_1W_vs_E10;stage_3W_vs_E10;stage	1	TRUE
ENSGALG00010024480	CDC45	PRJNA729271	-3.072724093	3.07666E-43	3.072724093	stage_E14_vs_E10	ordinary_muscle_development	D_vs_E10;stage_1W_vs_E10;stage_3W_vs_E10;stage	1	TRUE
ENSGALG00010024170	NUP160	PRJNA729271	-1.223471966	3.10943E-43	1.223471966	stage_E14_vs_E10	ordinary_muscle_development	D_vs_E10;stage_1W_vs_E10;stage_3W_vs_E10;stage	1	TRUE
ENSGALG00010029411	SARM1	PRJNA729271	-3.841633524	3.23149E-43	3.841633524	stage_E14_vs_E10	ordinary_muscle_development	D_vs_E10;stage_1W_vs_E10;stage_3W_vs_E10;stage	1	TRUE
ENSGALG00010006953	MAP1LC3B	PRJNA729271	1.78095806	3.24076E-43	1.78095806	stage_E14_vs_E10	ordinary_muscle_development	D_vs_E10;stage_1W_vs_E10;stage_3W_vs_E10;stage	1	TRUE
ENSGALG00010017541	SREBF1	PRJNA729271	3.158949221	3.26092E-43	3.158949221	stage_E14_vs_E10	ordinary_muscle_development	D_vs_E10;stage_1W_vs_E10;stage_3W_vs_E10;stage	1	TRUE
ENSGALG00010002483	COQ2	PRJNA729271	1.886615594	3.29767E-43	1.886615594	stage_E14_vs_E10	ordinary_muscle_development	D_vs_E10;stage_1W_vs_E10;stage_3W_vs_E10;stage	1	TRUE
ENSGALG00010029536	BAZ1B	PRJNA729271	-1.096597882	3.33828E-43	1.096597882	stage_E14_vs_E10	ordinary_muscle_development	D_vs_E10;stage_1W_vs_E10;stage_3W_vs_E10;stage	1	TRUE
ENSGALG00010021329	PSMC3IP	PRJNA729271	-2.375735453	3.37866E-43	2.375735453	stage_E14_vs_E10	ordinary_muscle_development	D_vs_E10;stage_1W_vs_E10;stage_3W_vs_E10;stage	1	TRUE
ENSGALG00010011841	KCNMB4	PRJNA729271	-3.879203099	3.39243E-43	3.879203099	stage_E14_vs_E10	ordinary_muscle_development	D_vs_E10;stage_1W_vs_E10;stage_3W_vs_E10;stage	1	TRUE
ENSGALG00010019268	NSL1	PRJNA729271	-1.334183379	3.42436E-43	1.334183379	stage_E14_vs_E10	ordinary_muscle_development	D_vs_E10;stage_1W_vs_E10;stage_3W_vs_E10;stage	1	TRUE
ENSGALG00010019232	SOS2	PRJNA729271	1.294778569	3.45946E-43	1.294778569	stage_E14_vs_E10	ordinary_muscle_development	D_vs_E10;stage_1W_vs_E10;stage_3W_vs_E10;stage	1	TRUE
ENSGALG00010029744	CTNS	PRJNA729271	1.225500549	3.69322E-43	1.225500549	stage_E14_vs_E10	ordinary_muscle_development	D_vs_E10;stage_1W_vs_E10;stage_3W_vs_E10;stage	1	TRUE
ENSGALG00010027274	COPG1	PRJNA729271	-0.850054505	3.8515E-43	0.850054505	stage_E14_vs_E10	ordinary_muscle_development		0	FALSE
ENSGALG00010016932	OGT	PRJNA729271	1.283778127	3.85557E-43	1.283778127	stage_E14_vs_E10	ordinary_muscle_development	D_vs_E10;stage_1W_vs_E10;stage_3W_vs_E10;stage	1	TRUE
ENSGALG00010017676	SPON2	PRJNA729271	3.670106409	4.20291E-43	3.670106409	stage_E14_vs_E10	ordinary_muscle_development	D_vs_E10;stage_1W_vs_E10;stage_3W_vs_E10;stage	1	TRUE
ENSGALG00010012786	TEF	PRJNA729271	1.221794997	4.42714E-43	1.221794997	stage_E14_vs_E10	ordinary_muscle_development	D_vs_E10;stage_1W_vs_E10;stage_3W_vs_E10;stage	1	TRUE
ENSGALG00010020507	ANKRD1	PRJNA729271	8.148613512	4.58416E-43	8.148613512	stage_E14_vs_E10	ordinary_muscle_development	D_vs_E10;stage_1W_vs_E10;stage_3W_vs_E10;stage	1	TRUE
ENSGALG00010006581	CEP41	PRJNA729271	-1.470569279	4.63675E-43	1.470569279	stage_E14_vs_E10	ordinary_muscle_development	D_vs_E10;stage_1W_vs_E10;stage_3W_vs_E10;stage	1	TRUE
ENSGALG00010010472	MMRN2	PRJNA729271	1.897463447	4.70242E-43	1.897463447	stage_E14_vs_E10	ordinary_muscle_development	D_vs_E10;stage_1W_vs_E10;stage_3W_vs_E10;stage	1	TRUE
ENSGALG00010029240	MIS12	PRJNA729271	-3.73694254	4.75445E-43	3.73694254	stage_E14_vs_E10	ordinary_muscle_development	D_vs_E10;stage_1W_vs_E10;stage_3W_vs_E10;stage	1	TRUE
ENSGALG00010019113	KLHDC1	PRJNA729271	1.846053984	4.92056E-43	1.846053984	stage_E14_vs_E10	ordinary_muscle_development	D_vs_E10;stage_1W_vs_E10;stage_3W_vs_E10;stage	1	TRUE
ENSGALG00010029345	RILP	PRJNA729271	3.36195929	4.99326E-43	3.36195929	stage_E14_vs_E10	ordinary_muscle_development	D_vs_E10;stage_1W_vs_E10;stage_3W_vs_E10;stage	1	TRUE
ENSGALG00010016880	AR	PRJNA729271	2.799339635	5.42486E-43	2.799339635	stage_E14_vs_E10	ordinary_muscle_development	D_vs_E10;stage_1W_vs_E10;stage_3W_vs_E10;stage	1	TRUE
ENSGALG00010001547	PWWP2B	PRJNA729271	1.514643374	5.42996E-43	1.514643374	stage_E14_vs_E10	ordinary_muscle_development	D_vs_E10;stage_1W_vs_E10;stage_3W_vs_E10;stage	1	TRUE
ENSGALG00010011316	PARPBP	PRJNA729271	-3.242865288	5.43542E-43	3.242865288	stage_E14_vs_E10	ordinary_muscle_development	D_vs_E10;stage_1W_vs_E10;stage_3W_vs_E10;stage	1	TRUE
ENSGALG00010016816	MAP3K6	PRJNA729271	3.605098963	5.48363E-43	3.605098963	stage_E14_vs_E10	ordinary_muscle_development	D_vs_E10;stage_1W_vs_E10;stage_3W_vs_E10;stage	1	TRUE
ENSGALG00010028346	DENND1A	PRJNA729271	1.018371745	5.61074E-43	1.018371745	stage_E14_vs_E10	ordinary_muscle_development	D_vs_E10;stage_1W_vs_E10;stage_3W_vs_E10;stage	1	TRUE
ENSGALG00010009040	CRELD2	PRJNA729271	-2.04523036	5.6223E-43	2.04523036	stage_E14_vs_E10	ordinary_muscle_development	D_vs_E10;stage_1W_vs_E10;stage_3W_vs_E10;stage	1	TRUE
ENSGALG00010028594	ENSGALG00010028594	PRJNA729271	1.960884844	5.80549E-43	1.960884844	stage_E14_vs_E10	ordinary_muscle_development	D_vs_E10;stage_1W_vs_E10;stage_3W_vs_E10;stage	1	TRUE
ENSGALG00010015618	NCL	PRJNA729271	-1.405765352	6.9334E-43	1.405765352	stage_E14_vs_E10	ordinary_muscle_development	D_vs_E10;stage_1W_vs_E10;stage_3W_vs_E10;stage	1	TRUE
ENSGALG00010021533	SSX2IP	PRJNA729271	-5.05091868	7.529E-43	5.05091868	stage_E14_vs_E10	ordinary_muscle_development	D_vs_E10;stage_1W_vs_E10;stage_3W_vs_E10;stage	1	TRUE
ENSGALG00010013403	CPNE3	PRJNA729271	1.432618884	8.24181E-43	1.432618884	stage_E14_vs_E10	ordinary_muscle_development	D_vs_E10;stage_1W_vs_E10;stage_3W_vs_E10;stage	1	TRUE
ENSGALG00010019283	NT5C3A	PRJNA729271	3.54180657	8.63134E-43	3.54180657	stage_E14_vs_E10	ordinary_muscle_development	D_vs_E10;stage_1W_vs_E10;stage_3W_vs_E10;stage	1	TRUE
ENSGALG00010021302	P4HA1	PRJNA729271	-0.838935605	8.79083E-43	0.838935605	stage_E14_vs_E10	ordinary_muscle_development		0	FALSE
ENSGALG00010016276	ACTR2	PRJNA729271	-1.503810338	8.92582E-43	1.503810338	stage_E14_vs_E10	ordinary_muscle_development	D_vs_E10;stage_1W_vs_E10;stage_3W_vs_E10;stage	1	TRUE
ENSGALG00010015253	ENSGALG00010015253	PRJNA729271	-1.046213992	9.51274E-43	1.046213992	stage_E14_vs_E10	ordinary_muscle_development	D_vs_E10;stage_1W_vs_E10;stage_3W_vs_E10;stage	1	TRUE
ENSGALG00010023076	DNAJC7	PRJNA729271	1.108621334	9.53626E-43	1.108621334	stage_E14_vs_E10	ordinary_muscle_development	D_vs_E10;stage_1W_vs_E10;stage_3W_vs_E10;stage	1	TRUE
ENSGALG00010009562	HADHB	PRJNA729271	2.62976292	9.69442E-43	2.62976292	stage_E14_vs_E10	ordinary_muscle_development	D_vs_E10;stage_1W_vs_E10;stage_3W_vs_E10;stage	1	TRUE
ENSGALG00010004373	MMS22L	PRJNA729271	-1.825865372	1.02943E-42	1.825865372	stage_E14_vs_E10	ordinary_muscle_development	D_vs_E10;stage_1W_vs_E10;stage_3W_vs_E10;stage	1	TRUE
ENSGALG00010024533	CDKN2C	PRJNA729271	3.069038881	1.04516E-42	3.069038881	stage_E14_vs_E10	ordinary_muscle_development	D_vs_E10;stage_1W_vs_E10;stage_3W_vs_E10;stage	1	TRUE
ENSGALG00010019043	METTL9	PRJNA729271	-1.455065212	1.04576E-42	1.455065212	stage_E14_vs_E10	ordinary_muscle_development	D_vs_E10;stage_1W_vs_E10;stage_3W_vs_E10;stage	1	TRUE
ENSGALG00010007861	INTU	PRJNA729271	-2.527568222	1.05071E-42	2.527568222	stage_E14_vs_E10	ordinary_muscle_development	D_vs_E10;stage_1W_vs_E10;stage_3W_vs_E10;stage	1	TRUE
ENSGALG00010012060	ENSGALG00010012060	PRJNA729271	1.71672956	1.14333E-42	1.71672956	stage_E14_vs_E10	ordinary_muscle_development	D_vs_E10;stage_1W_vs_E10;stage_3W_vs_E10;stage	1	TRUE
ENSGALG00010007819	NRIP1	PRJNA729271	2.304272152	1.18393E-42	2.304272152	stage_E14_vs_E10	ordinary_muscle_development	D_vs_E10;stage_1W_vs_E10;stage_3W_vs_E10;stage	1	TRUE
ENSGALG00010029244	HLF	PRJNA729271	2.527411253	1.18396E-42	2.527411253	stage_E14_vs_E10	ordinary_muscle_development	D_vs_E10;stage_1W_vs_E10;stage_3W_vs_E10;stage	1	TRUE
ENSGALG00010022044	PXMP4	PRJNA729271	0.811547673	1.1867E-42	0.811547673	stage_E14_vs_E10	ordinary_muscle_development		0	FALSE
ENSGALG00010014165	LUZP1	PRJNA729271	-1.677148192	1.23522E-42	1.677148192	stage_E14_vs_E10	ordinary_muscle_development	D_vs_E10;stage_1W_vs_E10;stage_3W_vs_E10;stage	1	TRUE
ENSGALG00010018426	ENSGALG00010018426	PRJNA729271	4.779192942	1.27222E-42	4.779192942	stage_E14_vs_E10	ordinary_muscle_development	D_vs_E10;stage_1W_vs_E10;stage_3W_vs_E10;stage	1	TRUE
ENSGALG00010010932	GHITM	PRJNA729271	1.219480406	1.35674E-42	1.219480406	stage_E14_vs_E10	ordinary_muscle_development	D_vs_E10;stage_1W_vs_E10;stage_3W_vs_E10;stage	1	TRUE
ENSGALG00010029595	CLUH	PRJNA729271	1.867256679	1.40169E-42	1.867256679	stage_E14_vs_E10	ordinary_muscle_development	D_vs_E10;stage_1W_vs_E10;stage_3W_vs_E10;stage	1	TRUE
ENSGALG00010015567	ENSGALG00010015567	PRJNA729271	5.842685009	1.44841E-42	5.842685009	stage_E14_vs_E10	ordinary_muscle_development	D_vs_E10;stage_1W_vs_E10;stage_3W_vs_E10;stage	1	TRUE
ENSGALG00010012196	CENPK	PRJNA729271	-3.708614562	1.5273E-42	3.708614562	stage_E14_vs_E10	ordinary_muscle_development	D_vs_E10;stage_1W_vs_E10;stage_3W_vs_E10;stage	1	TRUE
ENSGALG00010008561	EEF1D	PRJNA729271	1.592637393	1.73529E-42	1.592637393	stage_E14_vs_E10	ordinary_muscle_development	D_vs_E10;stage_1W_vs_E10;stage_3W_vs_E10;stage	1	TRUE
ENSGALG00010017323	ENSGALG00010017323	PRJNA729271	1.442039189	1.73563E-42	1.442039189	stage_E14_vs_E10	ordinary_muscle_development	D_vs_E10;stage_1W_vs_E10;stage_3W_vs_E10;stage	1	TRUE
ENSGALG00010010006	PPARGC1A	PRJNA729271	3.662027824	1.7949E-42	3.662027824	stage_E14_vs_E10	ordinary_muscle_development	D_vs_E10;stage_1W_vs_E10;stage_3W_vs_E10;stage	1	TRUE
ENSGALG00010001520	KCNAB1	PRJNA729271	-3.235979214	1.7971E-42	3.235979214	stage_E14_vs_E10	ordinary_muscle_development	D_vs_E10;stage_1W_vs_E10;stage_3W_vs_E10;stage	1	TRUE
ENSGALG00010021036	E2F8	PRJNA729271	-2.876710721	1.80992E-42	2.876710721	stage_E14_vs_E10	ordinary_muscle_development	D_vs_E10;stage_1W_vs_E10;stage_3W_vs_E10;stage	1	TRUE
ENSGALG00010017228	RAP2C	PRJNA729271	-1.001689444	1.81077E-42	1.001689444	stage_E14_vs_E10	ordinary_muscle_development	D_vs_E10;stage_1W_vs_E10;stage_3W_vs_E10;stage	1	TRUE
ENSGALG00010021484	MARCHF5	PRJNA729271	0.976759249	1.87876E-42	0.976759249	stage_E14_vs_E10	ordinary_muscle_development		0	FALSE
ENSGALG00010013777	CPLANE1	PRJNA729271	-1.333877073	1.8807E-42	1.333877073	stage_E14_vs_E10	ordinary_muscle_development	D_vs_E10;stage_1W_vs_E10;stage_3W_vs_E10;stage	1	TRUE
ENSGALG00010009654	NDUFA12	PRJNA729271	1.925815602	1.96789E-42	1.925815602	stage_E14_vs_E10	ordinary_muscle_development	D_vs_E10;stage_1W_vs_E10;stage_3W_vs_E10;stage	1	TRUE
ENSGALG00010021892	NDUFV1	PRJNA729271	1.683825637	1.98789E-42	1.683825637	stage_E14_vs_E10	ordinary_muscle_development	D_vs_E10;stage_1W_vs_E10;stage_3W_vs_E10;stage	1	TRUE
ENSGALG00010024835	ENTREP2	PRJNA729271	-2.229591384	2.08706E-42	2.229591384	stage_E14_vs_E10	ordinary_muscle_development	D_vs_E10;stage_1W_vs_E10;stage_3W_vs_E10;stage	1	TRUE
ENSGALG00010006715	IRF8	PRJNA729271	2.21478295	2.14615E-42	2.21478295	stage_E14_vs_E10	ordinary_muscle_development	D_vs_E10;stage_1W_vs_E10;stage_3W_vs_E10;stage	1	TRUE
ENSGALG00010022881	CCNB1	PRJNA729271	-2.938909038	2.14685E-42	2.938909038	stage_E14_vs_E10	ordinary_muscle_development	D_vs_E10;stage_1W_vs_E10;stage_3W_vs_E10;stage	1	TRUE
ENSGALG00010007542	MRPL36	PRJNA729271	1.511583904	2.25578E-42	1.511583904	stage_E14_vs_E10	ordinary_muscle_development	D_vs_E10;stage_1W_vs_E10;stage_3W_vs_E10;stage	1	TRUE
ENSGALG00010028066	ENSGALG00010028066	PRJNA729271	2.509201157	2.31247E-42	2.509201157	stage_E14_vs_E10	ordinary_muscle_development	D_vs_E10;stage_1W_vs_E10;stage_3W_vs_E10;stage	1	TRUE
ENSGALG00010021483	UNG	PRJNA729271	-2.20320217	2.35608E-42	2.20320217	stage_E14_vs_E10	ordinary_muscle_development	D_vs_E10;stage_1W_vs_E10;stage_3W_vs_E10;stage	1	TRUE
ENSGALG00010012026	SYT1	PRJNA729271	-4.175092528	2.42223E-42	4.175092528	stage_E14_vs_E10	ordinary_muscle_development	D_vs_E10;stage_1W_vs_E10;stage_3W_vs_E10;stage	1	TRUE
ENSGALG00010009484	RTKN2	PRJNA729271	-1.881382057	2.42761E-42	1.881382057	stage_E14_vs_E10	ordinary_muscle_development	D_vs_E10;stage_1W_vs_E10;stage_3W_vs_E10;stage	1	TRUE
ENSGALG00010008193	CNOT6L	PRJNA729271	1.78748575	2.45096E-42	1.78748575	stage_E14_vs_E10	ordinary_muscle_development	D_vs_E10;stage_1W_vs_E10;stage_3W_vs_E10;stage	1	TRUE
ENSGALG00010021487	KLHL17	PRJNA729271	-2.281672844	2.48963E-42	2.281672844	stage_E14_vs_E10	ordinary_muscle_development	D_vs_E10;stage_1W_vs_E10;stage_3W_vs_E10;stage	1	TRUE
ENSGALG00010023305	PLCB2	PRJNA729271	2.166078372	2.58142E-42	2.166078372	stage_E14_vs_E10	ordinary_muscle_development	D_vs_E10;stage_1W_vs_E10;stage_3W_vs_E10;stage	1	TRUE
ENSGALG00010008939	RANBP9	PRJNA729271	1.413236872	2.7134E-42	1.413236872	stage_E14_vs_E10	ordinary_muscle_development	D_vs_E10;stage_1W_vs_E10;stage_3W_vs_E10;stage	1	TRUE
ENSGALG00010011333	ENSGALG00010011333	PRJNA729271	-1.839828507	2.75792E-42	1.839828507	stage_E14_vs_E10	ordinary_muscle_development	D_vs_E10;stage_1W_vs_E10;stage_3W_vs_E10;stage	1	TRUE
ENSGALG00010013533	SKP2	PRJNA729271	-1.138637097	2.76195E-42	1.138637097	stage_E14_vs_E10	ordinary_muscle_development	D_vs_E10;stage_1W_vs_E10;stage_3W_vs_E10;stage	1	TRUE
ENSGALG00010011538	ENSGALG00010011538	PRJNA729271	3.484651328	2.80876E-42	3.484651328	stage_E14_vs_E10	ordinary_muscle_development	D_vs_E10;stage_1W_vs_E10;stage_3W_vs_E10;stage	1	TRUE
ENSGALG00010005828	LPAR1	PRJNA729271	-2.161881373	2.82322E-42	2.161881373	stage_E14_vs_E10	ordinary_muscle_development	D_vs_E10;stage_1W_vs_E10;stage_3W_vs_E10;stage	1	TRUE
ENSGALG00010023880	ENSGALG00010023880	PRJNA729271	-2.825426159	2.88412E-42	2.825426159	stage_E14_vs_E10	ordinary_muscle_development	D_vs_E10;stage_1W_vs_E10;stage_3W_vs_E10;stage	1	TRUE
ENSGALG00010022395	PTGES3L	PRJNA729271	1.836885456	2.8971E-42	1.836885456	stage_E14_vs_E10	ordinary_muscle_development	D_vs_E10;stage_1W_vs_E10;stage_3W_vs_E10;stage	1	TRUE
ENSGALG00010023572	MYBL2	PRJNA729271	-1.787213468	3.33835E-42	1.787213468	stage_E14_vs_E10	ordinary_muscle_development	D_vs_E10;stage_1W_vs_E10;stage_3W_vs_E10;stage	1	TRUE
ENSGALG00010022231	ZNF503	PRJNA729271	-2.945678312	3.40736E-42	2.945678312	stage_E14_vs_E10	ordinary_muscle_development	D_vs_E10;stage_1W_vs_E10;stage_3W_vs_E10;stage	1	TRUE
ENSGALG00010013266	KIAA1755	PRJNA729271	2.590345015	3.42155E-42	2.590345015	stage_E14_vs_E10	ordinary_muscle_development	D_vs_E10;stage_1W_vs_E10;stage_3W_vs_E10;stage	1	TRUE
ENSGALG00010026334	ENSGALG00010026334	PRJNA729271	1.742156576	3.43812E-42	1.742156576	stage_E14_vs_E10	ordinary_muscle_development	D_vs_E10;stage_1W_vs_E10;stage_3W_vs_E10;stage	1	TRUE
ENSGALG00010001940	PTPRM	PRJNA729271	1.520564824	3.66222E-42	1.520564824	stage_E14_vs_E10	ordinary_muscle_development	D_vs_E10;stage_1W_vs_E10;stage_3W_vs_E10;stage	1	TRUE
ENSGALG00010029293	DNAH17	PRJNA729271	-2.068838518	3.72112E-42	2.068838518	stage_E14_vs_E10	ordinary_muscle_development	D_vs_E10;stage_1W_vs_E10;stage_3W_vs_E10;stage	1	TRUE
ENSGALG00010027567	PARP3	PRJNA729271	1.762312968	3.84917E-42	1.762312968	stage_E14_vs_E10	ordinary_muscle_development	D_vs_E10;stage_1W_vs_E10;stage_3W_vs_E10;stage	1	TRUE
ENSGALG00010018383	METRN	PRJNA729271	-2.328494263	4.01131E-42	2.328494263	stage_E14_vs_E10	ordinary_muscle_development	D_vs_E10;stage_1W_vs_E10;stage_3W_vs_E10;stage	1	TRUE
ENSGALG00010002752	NDUFA4	PRJNA729271	2.120272617	4.05324E-42	2.120272617	stage_E14_vs_E10	ordinary_muscle_development	D_vs_E10;stage_1W_vs_E10;stage_3W_vs_E10;stage	1	TRUE
ENSGALG00010000843	DIS3	PRJNA729271	-1.179235855	4.08862E-42	1.179235855	stage_E14_vs_E10	ordinary_muscle_development	D_vs_E10;stage_1W_vs_E10;stage_3W_vs_E10;stage	1	TRUE
ENSGALG00010023605	INSYN1	PRJNA729271	4.169338693	4.10093E-42	4.169338693	stage_E14_vs_E10	ordinary_muscle_development	D_vs_E10;stage_1W_vs_E10;stage_3W_vs_E10;stage	1	TRUE
ENSGALG00010027195	CENPP	PRJNA729271	-3.087893981	4.27346E-42	3.087893981	stage_E14_vs_E10	ordinary_muscle_development	D_vs_E10;stage_1W_vs_E10;stage_3W_vs_E10;stage	1	TRUE
ENSGALG00010001783	SGO1	PRJNA729271	-3.5186148	4.40729E-42	3.5186148	stage_E14_vs_E10	ordinary_muscle_development	D_vs_E10;stage_1W_vs_E10;stage_3W_vs_E10;stage	1	TRUE
ENSGALG00010007201	ENSGALG00010007201	PRJNA729271	3.476047465	4.44334E-42	3.476047465	stage_E14_vs_E10	ordinary_muscle_development	D_vs_E10;stage_1W_vs_E10;stage_3W_vs_E10;stage	1	TRUE
ENSGALG00010008764	ETFDH	PRJNA729271	1.700977993	4.8676E-42	1.700977993	stage_E14_vs_E10	ordinary_muscle_development	D_vs_E10;stage_1W_vs_E10;stage_3W_vs_E10;stage	1	TRUE
ENSGALG00010001365	ENSGALG00010001365	PRJNA729271	3.479045802	4.91645E-42	3.479045802	stage_E14_vs_E10	ordinary_muscle_development	D_vs_E10;stage_1W_vs_E10;stage_3W_vs_E10;stage	1	TRUE
ENSGALG00010007915	HECW1	PRJNA729271	1.789606887	5.04623E-42	1.789606887	stage_E14_vs_E10	ordinary_muscle_development	D_vs_E10;stage_1W_vs_E10;stage_3W_vs_E10;stage	1	TRUE
ENSGALG00010018894	SERTAD4	PRJNA729271	-1.292491111	5.19182E-42	1.292491111	stage_E14_vs_E10	ordinary_muscle_development	D_vs_E10;stage_1W_vs_E10;stage_3W_vs_E10;stage	1	TRUE
ENSGALG00010012300	NINJ2	PRJNA729271	-3.118158117	5.33382E-42	3.118158117	stage_E14_vs_E10	ordinary_muscle_development	D_vs_E10;stage_1W_vs_E10;stage_3W_vs_E10;stage	1	TRUE
ENSGALG00010021093	GRAMD2	PRJNA729271	3.011608396	5.47024E-42	3.011608396	stage_E14_vs_E10	ordinary_muscle_development	D_vs_E10;stage_1W_vs_E10;stage_3W_vs_E10;stage	1	TRUE
ENSGALG00010001476	IGF2BP3	PRJNA729271	-2.681787371	5.54734E-42	2.681787371	stage_E14_vs_E10	ordinary_muscle_development	D_vs_E10;stage_1W_vs_E10;stage_3W_vs_E10;stage	1	TRUE
ENSGALG00010007204	LANCL2	PRJNA729271	-1.210676293	5.76993E-42	1.210676293	stage_E14_vs_E10	ordinary_muscle_development	D_vs_E10;stage_1W_vs_E10;stage_3W_vs_E10;stage	1	TRUE
ENSGALG00010021541	CYP27A1	PRJNA729271	3.778194155	5.78063E-42	3.778194155	stage_E14_vs_E10	ordinary_muscle_development	D_vs_E10;stage_1W_vs_E10;stage_3W_vs_E10;stage	1	TRUE
ENSGALG00010008999	TRAPPC9	PRJNA729271	-0.914683511	5.80433E-42	0.914683511	stage_E14_vs_E10	ordinary_muscle_development		0	FALSE
ENSGALG00010012508	MDM1	PRJNA729271	-0.971834603	5.82206E-42	0.971834603	stage_E14_vs_E10	ordinary_muscle_development		0	FALSE
ENSGALG00010015827	ENSGALG00010015827	PRJNA729271	2.016852118	5.98872E-42	2.016852118	stage_E14_vs_E10	ordinary_muscle_development	D_vs_E10;stage_1W_vs_E10;stage_3W_vs_E10;stage	1	TRUE
ENSGALG00010021604	SPTB	PRJNA729271	2.598551866	6.03787E-42	2.598551866	stage_E14_vs_E10	ordinary_muscle_development	D_vs_E10;stage_1W_vs_E10;stage_3W_vs_E10;stage	1	TRUE
ENSGALG00010028445	SYT11	PRJNA729271	-2.820555727	6.12278E-42	2.820555727	stage_E14_vs_E10	ordinary_muscle_development	D_vs_E10;stage_1W_vs_E10;stage_3W_vs_E10;stage	1	TRUE
ENSGALG00010020436	CNTN4	PRJNA729271	-3.751476904	6.96593E-42	3.751476904	stage_E14_vs_E10	ordinary_muscle_development	D_vs_E10;stage_1W_vs_E10;stage_3W_vs_E10;stage	1	TRUE
ENSGALG00010017380	MARCKSL1	PRJNA729271	-1.859733264	6.98266E-42	1.859733264	stage_E14_vs_E10	ordinary_muscle_development	D_vs_E10;stage_1W_vs_E10;stage_3W_vs_E10;stage	1	TRUE
ENSGALG00010023807	MRPL10	PRJNA729271	1.70166323	7.24997E-42	1.70166323	stage_E14_vs_E10	ordinary_muscle_development	D_vs_E10;stage_1W_vs_E10;stage_3W_vs_E10;stage	1	TRUE
ENSGALG00010008202	KCNG2	PRJNA729271	2.634642602	7.35357E-42	2.634642602	stage_E14_vs_E10	ordinary_muscle_development	D_vs_E10;stage_1W_vs_E10;stage_3W_vs_E10;stage	1	TRUE
ENSGALG00010029315	TBC1D16	PRJNA729271	-1.366802614	7.41592E-42	1.366802614	stage_E14_vs_E10	ordinary_muscle_development	D_vs_E10;stage_1W_vs_E10;stage_3W_vs_E10;stage	1	TRUE
ENSGALG00010022326	COX19	PRJNA729271	1.287840319	7.55788E-42	1.287840319	stage_E14_vs_E10	ordinary_muscle_development	D_vs_E10;stage_1W_vs_E10;stage_3W_vs_E10;stage	1	TRUE
ENSGALG00010012157	H2BC32	PRJNA729271	-2.730722097	7.981E-42	2.730722097	stage_E14_vs_E10	ordinary_muscle_development	D_vs_E10;stage_1W_vs_E10;stage_3W_vs_E10;stage	1	TRUE
ENSGALG00010019483	SCUBE2	PRJNA729271	-3.678379173	8.7384E-42	3.678379173	stage_E14_vs_E10	ordinary_muscle_development	D_vs_E10;stage_1W_vs_E10;stage_3W_vs_E10;stage	1	TRUE
ENSGALG00010024509	SEPTIN5	PRJNA729271	1.469375903	9.15744E-42	1.469375903	stage_E14_vs_E10	ordinary_muscle_development	D_vs_E10;stage_1W_vs_E10;stage_3W_vs_E10;stage	1	TRUE
ENSGALG00010013930	SLC22A16	PRJNA729271	4.169050992	9.60111E-42	4.169050992	stage_E14_vs_E10	ordinary_muscle_development	D_vs_E10;stage_1W_vs_E10;stage_3W_vs_E10;stage	1	TRUE
ENSGALG00010015477	ARMC9	PRJNA729271	-1.479388869	9.92673E-42	1.479388869	stage_E14_vs_E10	ordinary_muscle_development	D_vs_E10;stage_1W_vs_E10;stage_3W_vs_E10;stage	1	TRUE
ENSGALG00010005398	SEPHS1	PRJNA729271	-1.190777479	9.96268E-42	1.190777479	stage_E14_vs_E10	ordinary_muscle_development	D_vs_E10;stage_1W_vs_E10;stage_3W_vs_E10;stage	1	TRUE
ENSGALG00010029526	BCL7B	PRJNA729271	1.52340266	1.025E-41	1.52340266	stage_E14_vs_E10	ordinary_muscle_development	D_vs_E10;stage_1W_vs_E10;stage_3W_vs_E10;stage	1	TRUE
ENSGALG00010029948	MYADML2	PRJNA729271	2.995172069	1.05259E-41	2.995172069	stage_E14_vs_E10	ordinary_muscle_development	D_vs_E10;stage_1W_vs_E10;stage_3W_vs_E10;stage	1	TRUE
ENSGALG00010005758	HIVEP2	PRJNA729271	1.440720694	1.08599E-41	1.440720694	stage_E14_vs_E10	ordinary_muscle_development	D_vs_E10;stage_1W_vs_E10;stage_3W_vs_E10;stage	1	TRUE
ENSGALG00010002431	BCKDHB	PRJNA729271	2.017055357	1.17588E-41	2.017055357	stage_E14_vs_E10	ordinary_muscle_development	D_vs_E10;stage_1W_vs_E10;stage_3W_vs_E10;stage	1	TRUE
ENSGALG00010010766	CENPO	PRJNA729271	-3.752907629	1.21311E-41	3.752907629	stage_E14_vs_E10	ordinary_muscle_development	D_vs_E10;stage_1W_vs_E10;stage_3W_vs_E10;stage	1	TRUE
ENSGALG00010002619	RAB6A	PRJNA729271	-0.941236653	1.24073E-41	0.941236653	stage_E14_vs_E10	ordinary_muscle_development		0	FALSE
ENSGALG00010011342	RASAL2	PRJNA729271	0.895924982	1.3299E-41	0.895924982	stage_E14_vs_E10	ordinary_muscle_development		0	FALSE
ENSGALG00010021154	CHST13	PRJNA729271	-2.961282134	1.33292E-41	2.961282134	stage_E14_vs_E10	ordinary_muscle_development	D_vs_E10;stage_1W_vs_E10;stage_3W_vs_E10;stage	1	TRUE
ENSGALG00010000820	KLF5	PRJNA729271	2.624148447	1.33786E-41	2.624148447	stage_E14_vs_E10	ordinary_muscle_development	D_vs_E10;stage_1W_vs_E10;stage_3W_vs_E10;stage	1	TRUE
ENSGALG00010017963	LPAR4	PRJNA729271	-2.334057384	1.37103E-41	2.334057384	stage_E14_vs_E10	ordinary_muscle_development	D_vs_E10;stage_1W_vs_E10;stage_3W_vs_E10;stage	1	TRUE
ENSGALG00010026166	WNT9A	PRJNA729271	-3.48332496	1.6092E-41	3.48332496	stage_E14_vs_E10	ordinary_muscle_development	D_vs_E10;stage_1W_vs_E10;stage_3W_vs_E10;stage	1	TRUE
ENSGALG00010009306	TBC1D9	PRJNA729271	-1.48611414	1.61767E-41	1.48611414	stage_E14_vs_E10	ordinary_muscle_development	D_vs_E10;stage_1W_vs_E10;stage_3W_vs_E10;stage	1	TRUE

ENSGALG00010019731	PANK4	PRJNA729271	1.814035758	1.97441E-41	1.814035758	stage_E14_vs_E10	ordinary_muscle_development	D_vs_E10;stage_1W_vs_E10;stage_3W_vs_E10;stage	1	TRUE
ENSGALG00010021243	DYL1	PRJNA729271	-1.300887144	1.9885E-41	1.300887144	stage_E14_vs_E10	ordinary_muscle_development	D_vs_E10;stage_1W_vs_E10;stage_3W_vs_E10;stage	1	TRUE
ENSGALG00010022363	KIF18A	PRJNA729271	-2.831170112	2.14516E-41	2.831170112	stage_E14_vs_E10	ordinary_muscle_development	D_vs_E10;stage_1W_vs_E10;stage_3W_vs_E10;stage	1	TRUE
ENSGALG00010004345	FAXC	PRJNA729271	-2.654283872	2.20863E-41	2.654283872	stage_E14_vs_E10	ordinary_muscle_development	D_vs_E10;stage_1W_vs_E10;stage_3W_vs_E10;stage	1	TRUE
ENSGALG00010013399	FGF1	PRJNA729271	4.384167857	2.2356E-41	4.384167857	stage_E14_vs_E10	ordinary_muscle_development	D_vs_E10;stage_1W_vs_E10;stage_3W_vs_E10;stage	1	TRUE
ENSGALG00010016410	TRAK1	PRJNA729271	1.31766838	2.28379E-41	1.31766838	stage_E14_vs_E10	ordinary_muscle_development	D_vs_E10;stage_1W_vs_E10;stage_3W_vs_E10;stage	1	TRUE
ENSGALG00010029726	SEPTIN9	PRJNA729271	-1.715928116	2.29025E-41	1.715928116	stage_E14_vs_E10	ordinary_muscle_development	D_vs_E10;stage_1W_vs_E10;stage_3W_vs_E10;stage	1	TRUE
ENSGALG00010012092	OC3	PRJNA729271	9.511516893	2.34866E-41	9.511516893	stage_E14_vs_E10	ordinary_muscle_development	D_vs_E10;stage_1W_vs_E10;stage_3W_vs_E10;stage	1	TRUE
ENSGALG00010005252	KLHL14	PRJNA729271	-3.294088754	2.34967E-41	3.294088754	stage_E14_vs_E10	ordinary_muscle_development	D_vs_E10;stage_1W_vs_E10;stage_3W_vs_E10;stage	1	TRUE
ENSGALG00010012449	SUB1	PRJNA729271	-1.496121595	2.52767E-41	1.496121595	stage_E14_vs_E10	ordinary_muscle_development	D_vs_E10;stage_1W_vs_E10;stage_3W_vs_E10;stage	1	TRUE
ENSGALG00010013944	METTL24	PRJNA729271	-3.47326408	2.5458E-41	3.47326408	stage_E14_vs_E10	ordinary_muscle_development	D_vs_E10;stage_1W_vs_E10;stage_3W_vs_E10;stage	1	TRUE
ENSGALG00010004000	ENSGALG00010004000	PRJNA729271	2.353512186	2.67545E-41	2.353512186	stage_E14_vs_E10	ordinary_muscle_development	D_vs_E10;stage_1W_vs_E10;stage_3W_vs_E10;stage	1	TRUE
ENSGALG00010020277	IDH2	PRJNA729271	1.696963968	2.73247E-41	1.696963968	stage_E14_vs_E10	ordinary_muscle_development	D_vs_E10;stage_1W_vs_E10;stage_3W_vs_E10;stage	1	TRUE
ENSGALG00010029200	GAS7	PRJNA729271	1.695598172	2.74079E-41	1.695598172	stage_E14_vs_E10	ordinary_muscle_development	D_vs_E10;stage_1W_vs_E10;stage_3W_vs_E10;stage	1	TRUE
ENSGALG00010008392	CD36	PRJNA729271	5.03585111	3.04456E-41	5.03585111	stage_E14_vs_E10	ordinary_muscle_development	D_vs_E10;stage_1W_vs_E10;stage_3W_vs_E10;stage	1	TRUE
ENSGALG00010024218	NGFR	PRJNA729271	-3.224653662	3.04989E-41	3.224653662	stage_E14_vs_E10	ordinary_muscle_development	D_vs_E10;stage_1W_vs_E10;stage_3W_vs_E10;stage	1	TRUE
ENSGALG00010023274	ZBTB38	PRJNA729271	1.55837484	3.06568E-41	1.55837484	stage_E14_vs_E10	ordinary_muscle_development	D_vs_E10;stage_1W_vs_E10;stage_3W_vs_E10;stage	1	TRUE
ENSGALG00010003485	RNGTT	PRJNA729271	-1.430768449	3.16364E-41	1.430768449	stage_E14_vs_E10	ordinary_muscle_development	D_vs_E10;stage_1W_vs_E10;stage_3W_vs_E10;stage	1	TRUE
ENSGALG00010022515	CHRNA1	PRJNA729271	-2.355302057	3.2117E-41	2.355302057	stage_E14_vs_E10	ordinary_muscle_development	D_vs_E10;stage_1W_vs_E10;stage_3W_vs_E10;stage	1	TRUE
ENSGALG00010005465	ENSGALG00010005465	PRJNA729271	-1.287910286	3.27479E-41	1.287910286	stage_E14_vs_E10	ordinary_muscle_development	D_vs_E10;stage_1W_vs_E10;stage_3W_vs_E10;stage	1	TRUE
ENSGALG00010016780	PRORSD1P	PRJNA729271	-1.960668786	3.3522E-41	1.960668786	stage_E14_vs_E10	ordinary_muscle_development	D_vs_E10;stage_1W_vs_E10;stage_3W_vs_E10;stage	1	TRUE
ENSGALG00010010061	CYP2D6	PRJNA729271	2.765495293	3.38056E-41	2.765495293	stage_E14_vs_E10	ordinary_muscle_development	D_vs_E10;stage_1W_vs_E10;stage_3W_vs_E10;stage	1	TRUE
ENSGALG00010016920	C1QA	PRJNA729271	4.320863695	3.40955E-41	4.320863695	stage_E14_vs_E10	ordinary_muscle_development	D_vs_E10;stage_1W_vs_E10;stage_3W_vs_E10;stage	1	TRUE
ENSGALG00010007408	HCLS1	PRJNA729271	3.294010228	3.40957E-41	3.294010228	stage_E14_vs_E10	ordinary_muscle_development	D_vs_E10;stage_1W_vs_E10;stage_3W_vs_E10;stage	1	TRUE
ENSGALG00010023652	SLC30A7	PRJNA729271	-1.328440507	3.55788E-41	1.328440507	stage_E14_vs_E10	ordinary_muscle_development	D_vs_E10;stage_1W_vs_E10;stage_3W_vs_E10;stage	1	TRUE
ENSGALG00010008433	EBAG9	PRJNA729271	1.285089062	3.59454E-41	1.285089062	stage_E14_vs_E10	ordinary_muscle_development	D_vs_E10;stage_1W_vs_E10;stage_3W_vs_E10;stage	1	TRUE
ENSGALG00010009378	ENSGALG00010009378	PRJNA729271	-1.157035766	3.76491E-41	1.157035766	stage_E14_vs_E10	ordinary_muscle_development	D_vs_E10;stage_1W_vs_E10;stage_3W_vs_E10;stage	1	TRUE
ENSGALG00010012362	COL6A3	PRJNA729271	3.994367574	3.81765E-41	3.994367574	stage_E14_vs_E10	ordinary_muscle_development	D_vs_E10;stage_1W_vs_E10;stage_3W_vs_E10;stage	1	TRUE
ENSGALG00010012248	H2AC39	PRJNA729271	-2.855309771	3.83201E-41	2.855309771	stage_E14_vs_E10	ordinary_muscle_development	D_vs_E10;stage_1W_vs_E10;stage_3W_vs_E10;stage	1	TRUE
ENSGALG00010014162	COL18A1	PRJNA729271	2.568423128	4.02371E-41	2.568423128	stage_E14_vs_E10	ordinary_muscle_development	D_vs_E10;stage_1W_vs_E10;stage_3W_vs_E10;stage	1	TRUE
ENSGALG00010025936	GOSR2	PRJNA729271	-0.948439524	4.29581E-41	0.948439524	stage_E14_vs_E10	ordinary_muscle_development		0	FALSE
ENSGALG00010007593	SEC61B	PRJNA729271	-1.626138221	4.6159E-41	1.626138221	stage_E14_vs_E10	ordinary_muscle_development	D_vs_E10;stage_1W_vs_E10;stage_3W_vs_E10;stage	1	TRUE
ENSGALG00010005323	DNAJB14	PRJNA729271	0.860257574	4.64192E-41	0.860257574	stage_E14_vs_E10	ordinary_muscle_development		0	FALSE
ENSGALG00010003925	SEC31A	PRJNA729271	-1.169157264	5.18536E-41	1.169157264	stage_E14_vs_E10	ordinary_muscle_development	D_vs_E10;stage_1W_vs_E10;stage_3W_vs_E10;stage	1	TRUE
ENSGALG00010027654	AIF1L	PRJNA729271	1.236539479	5.23418E-41	1.236539479	stage_E14_vs_E10	ordinary_muscle_development	D_vs_E10;stage_1W_vs_E10;stage_3W_vs_E10;stage	1	TRUE
ENSGALG00010018786	DSTN	PRJNA729271	-1.560918853	5.3316E-41	1.560918853	stage_E14_vs_E10	ordinary_muscle_development	D_vs_E10;stage_1W_vs_E10;stage_3W_vs_E10;stage	1	TRUE
ENSGALG00010027930	RAF1	PRJNA729271	1.209725184	5.43952E-41	1.209725184	stage_E14_vs_E10	ordinary_muscle_development	D_vs_E10;stage_1W_vs_E10;stage_3W_vs_E10;stage	1	TRUE
ENSGALG00010001556	EPB41L3	PRJNA729271	-1.597898336	5.73006E-41	1.597898336	stage_E14_vs_E10	ordinary_muscle_development	D_vs_E10;stage_1W_vs_E10;stage_3W_vs_E10;stage	1	TRUE
ENSGALG00010010823	TMEFF2	PRJNA729271	-2.512197527	5.77844E-41	2.512197527	stage_E14_vs_E10	ordinary_muscle_development	D_vs_E10;stage_1W_vs_E10;stage_3W_vs_E10;stage	1	TRUE
ENSGALG00010008354	TMEM71	PRJNA729271	2.933309017	5.97055E-41	2.933309017	stage_E14_vs_E10	ordinary_muscle_development	D_vs_E10;stage_1W_vs_E10;stage_3W_vs_E10;stage	1	TRUE
ENSGALG00010020226	TCIM	PRJNA729271	2.680956356	6.07568E-41	2.680956356	stage_E14_vs_E10	ordinary_muscle_development	D_vs_E10;stage_1W_vs_E10;stage_3W_vs_E10;stage	1	TRUE
ENSGALG00010007945	ENSGALG00010007945	PRJNA729271	-1.693239076	6.11756E-41	1.693239076	stage_E14_vs_E10	ordinary_muscle_development	D_vs_E10;stage_1W_vs_E10;stage_3W_vs_E10;stage	1	TRUE
ENSGALG00010025377	MYO1G	PRJNA729271	3.782689736	6.17063E-41	3.782689736	stage_E14_vs_E10	ordinary_muscle_development	D_vs_E10;stage_1W_vs_E10;stage_3W_vs_E10;stage	1	TRUE
ENSGALG00010015881	GAB3	PRJNA729271	2.366125113	6.26241E-41	2.366125113	stage_E14_vs_E10	ordinary_muscle_development	D_vs_E10;stage_1W_vs_E10;stage_3W_vs_E10;stage	1	TRUE
ENSGALG00010021474	PPFIBP2	PRJNA729271	2.455633052	6.3518E-41	2.455633052	stage_E14_vs_E10	ordinary_muscle_development	D_vs_E10;stage_1W_vs_E10;stage_3W_vs_E10;stage	1	TRUE
ENSGALG00010024708	BNIP2	PRJNA729271	0.844816096	6.59142E-41	0.844816096	stage_E14_vs_E10	ordinary_muscle_development		0	FALSE
ENSGALG00010014035	PSIP1	PRJNA729271	-1.642008095	6.60106E-41	1.642008095	stage_E14_vs_E10	ordinary_muscle_development	D_vs_E10;stage_1W_vs_E10;stage_3W_vs_E10;stage	1	TRUE
ENSGALG00010015917	TMEM63A	PRJNA729271	1.428941297	6.78241E-41	1.428941297	stage_E14_vs_E10	ordinary_muscle_development	D_vs_E10;stage_1W_vs_E10;stage_3W_vs_E10;stage	1	TRUE
ENSGALG00010021052	CEP15	PRJNA729271	-2.122007406	7.12293E-41	2.122007406	stage_E14_vs_E10	ordinary_muscle_development	D_vs_E10;stage_1W_vs_E10;stage_3W_vs_E10;stage	1	TRUE
ENSGALG00010005029	FAT4	PRJNA729271	-2.682736572	7.42477E-41	2.682736572	stage_E14_vs_E10	ordinary_muscle_development	D_vs_E10;stage_1W_vs_E10;stage_3W_vs_E10;stage	1	TRUE
ENSGALG00010021219	STRA6	PRJNA729271	-8.394320711	8.06718E-41	8.394320711	stage_E14_vs_E10	ordinary_muscle_development	D_vs_E10;stage_1W_vs_E10;stage_3W_vs_E10;stage	1	TRUE
ENSGALG00010005409	PRKCQ	PRJNA729271	2.946679605	8.25323E-41	2.946679605	stage_E14_vs_E10	ordinary_muscle_development	D_vs_E10;stage_1W_vs_E10;stage_3W_vs_E10;stage	1	TRUE
ENSGALG00010005389	MCM10	PRJNA729271	-2.566609797	8.43863E-41	2.566609797	stage_E14_vs_E10	ordinary_muscle_development	D_vs_E10;stage_1W_vs_E10;stage_3W_vs_E10;stage	1	TRUE
ENSGALG00010006546	SMOC2	PRJNA729271	-2.872389127	8.49869E-41	2.872389127	stage_E14_vs_E10	ordinary_muscle_development	D_vs_E10;stage_1W_vs_E10;stage_3W_vs_E10;stage	1	TRUE
ENSGALG00010012086	CIBAR1	PRJNA729271	-1.829902921	8.50751E-41	1.829902921	stage_E14_vs_E10	ordinary_muscle_development	D_vs_E10;stage_1W_vs_E10;stage_3W_vs_E10;stage	1	TRUE
ENSGALG00010016644	NMRAL1	PRJNA729271	-2.877934827	8.52958E-41	2.877934827	stage_E14_vs_E10	ordinary_muscle_development	D_vs_E10;stage_1W_vs_E10;stage_3W_vs_E10;stage	1	TRUE
ENSGALG00010024220	SIAH1	PRJNA729271	2.712105359	8.72411E-41	2.712105359	stage_E14_vs_E10	ordinary_muscle_development	D_vs_E10;stage_1W_vs_E10;stage_3W_vs_E10;stage	1	TRUE
ENSGALG00010008163	CHTF8	PRJNA729271	-3.329584431	8.91324E-41	3.329584431	stage_E14_vs_E10	ordinary_muscle_development	D_vs_E10;stage_1W_vs_E10;stage_3W_vs_E10;stage	1	TRUE
ENSGALG00010016707	KIF1B	PRJNA729271	1.765057048	9.09393E-41	1.765057048	stage_E14_vs_E10	ordinary_muscle_development	D_vs_E10;stage_1W_vs_E10;stage_3W_vs_E10;stage	1	TRUE
ENSGALG00010013170	COL3A1	PRJNA729271	3.193026545	9.18388E-41	3.193026545	stage_E14_vs_E10	ordinary_muscle_development	D_vs_E10;stage_1W_vs_E10;stage_3W_vs_E10;stage	1	TRUE
ENSGALG00010022248	FIGNL2	PRJNA729271	-3.247659894	9.30641E-41	3.247659894	stage_E14_vs_E10	ordinary_muscle_development	D_vs_E10;stage_1W_vs_E10;stage_3W_vs_E10;stage	1	TRUE
ENSGALG00010016022	EPHB3	PRJNA729271	-2.304899824	9.31844E-41	2.304899824	stage_E14_vs_E10	ordinary_muscle_development	D_vs_E10;stage_1W_vs_E10;stage_3W_vs_E10;stage	1	TRUE
ENSGALG00010011770	CHST11	PRJNA729271	-4.215611223	9.54505E-41	4.215611223	stage_E14_vs_E10	ordinary_muscle_development	D_vs_E10;stage_1W_vs_E10;stage_3W_vs_E10;stage	1	TRUE
ENSGALG00010026291	SERPING1	PRJNA729271	2.278350385	9.9853E-41	2.278350385	stage_E14_vs_E10	ordinary_muscle_development	D_vs_E10;stage_1W_vs_E10;stage_3W_vs_E10;stage	1	TRUE
ENSGALG00010004803	RMC1	PRJNA729271	1.882218452	1.02871E-40	1.882218452	stage_E14_vs_E10	ordinary_muscle_development	D_vs_E10;stage_1W_vs_E10;stage_3W_vs_E10;stage	1	TRUE
ENSGALG00010027186	PLXNA2	PRJNA729271	1.672162044	1.04333E-40	1.672162044	stage_E14_vs_E10	ordinary_muscle_development	D_vs_E10;stage_1W_vs_E10;stage_3W_vs_E10;stage	1	TRUE
ENSGALG00010012744	ARL4C	PRJNA729271	-3.205284662	1.04968E-40	3.205284662	stage_E14_vs_E10	ordinary_muscle_development	D_vs_E10;stage_1W_vs_E10;stage_3W_vs_E10;stage	1	TRUE
ENSGALG00010021756	USP42	PRJNA729271	-1.777316281	1.10229E-40	1.777316281	stage_E14_vs_E10	ordinary_muscle_development	D_vs_E10;stage_1W_vs_E10;stage_3W_vs_E10;stage	1	TRUE
ENSGALG00010014831	VAPB	PRJNA729271	0.81100269	1.13131E-40	0.81100269	stage_E14_vs_E10	ordinary_muscle_development		0	FALSE
ENSGALG00010013486	PLPP6	PRJNA729271	2.157712711	1.138E-40	2.157712711	stage_E14_vs_E10	ordinary_muscle_development	D_vs_E10;stage_1W_vs_E10;stage_3W_vs_E10;stage	1	TRUE
ENSGALG00010017057	NDUFAB1	PRJNA729271	1.634134761	1.14085E-40	1.634134761	stage_E14_vs_E10	ordinary_muscle_development	D_vs_E10;stage_1W_vs_E10;stage_3W_vs_E10;stage	1	TRUE
ENSGALG00010018554	PPIA	PRJNA729271	-1.672907463	1.15703E-40	1.672907463	stage_E14_vs_E10	ordinary_muscle_development	D_vs_E10;stage_1W_vs_E10;stage_3W_vs_E10;stage	1	TRUE
ENSGALG00010000020	COX2	PRJNA729271	3.22753283	1.18049E-40	3.22753283	stage_E14_vs_E10	ordinary_muscle_development	D_vs_E10;stage_1W_vs_E10;stage_3W_vs_E10;stage	1	TRUE
ENSGALG00010029373	GATSL2	PRJNA729271	2.279139793	1.19726E-40	2.279139793	stage_E14_vs_E10	ordinary_muscle_development	D_vs_E10;stage_1W_vs_E10;stage_3W_vs_E10;stage	1	TRUE
ENSGALG00010017201	AP5M1	PRJNA729271	-2.144597949	1.19808E-40	2.144597949	stage_E14_vs_E10	ordinary_muscle_development	D_vs_E10;stage_1W_vs_E10;stage_3W_vs_E10;stage	1	TRUE
ENSGALG00010017127	SOX8	PRJNA729271	-1.85642362	1.20674E-40	1.85642362	stage_E14_vs_E10	ordinary_muscle_development	D_vs_E10;stage_1W_vs_E10;stage_3W_vs_E10;stage	1	TRUE
ENSGALG00010002159	GALNT15	PRJNA729271	2.552575923	1.23741E-40	2.552575923	stage_E14_vs_E10	ordinary_muscle_development	D_vs_E10;stage_1W_vs_E10;stage_3W_vs_E10;stage	1	TRUE
ENSGALG00010014812	RWDD1	PRJNA729271	-1.180558065	1.24655E-40	1.180558065	stage_E14_vs_E10	ordinary_muscle_development	D_vs_E10;stage_1W_vs_E10;stage_3W_vs_E10;stage	1	TRUE
ENSGALG00010027877	OAZ1	PRJNA729271	1.542915142	1.26061E-40	1.542915142	stage_E14_vs_E10	ordinary_muscle_development	D_vs_E10;stage_1W_vs_E10;stage_3W_vs_E10;stage	1	TRUE
ENSGALG00010003131	TAP1	PRJNA729271	4.660040065	1.30302E-40	4.660040065	stage_E14_vs_E10	ordinary_muscle_development	D_vs_E10;stage_1W_vs_E10;stage_3W_vs_E10;stage	1	TRUE
ENSGALG00010012870	KCNC2	PRJNA729271	-2.843321482	1.33947E-40	2.843321482	stage_E14_vs_E10	ordinary_muscle_development	D_vs_E10;stage_1W_vs_E10;stage_3W_vs_E10;stage	1	TRUE
ENSGALG00010025486	B3GALT1	PRJNA729271	-2.924032835	1.35116E-40	2.924032835	stage_E14_vs_E10	ordinary_muscle_development	D_vs_E10;stage_1W_vs_E10;stage_3W_vs_E10;stage	1	TRUE
ENSGALG00010024616	ARVCF	PRJNA729271	-1.343215439	1.36455E-40	1.343215439	stage_E14_vs_E10	ordinary_muscle_development	D_vs_E10;stage_1W_vs_E10;stage_3W_vs_E10;stage	1	TRUE
ENSGALG00010024754	EIF2S2	PRJNA729271	1.950144203	1.37777E-40	1.950144203	stage_E14_vs_E10	ordinary_muscle_development	D_vs_E10;stage_1W_vs_E10;stage_3W_vs_E10;stage	1	TRUE
ENSGALG00010006287	ECPAS	PRJNA729271	1.652023937	1.42196E-40	1.652023937	stage_E14_vs_E10	ordinary_muscle_development	D_vs_E10;stage_1W_vs_E10;stage_3W_vs_E10;stage	1	TRUE
ENSGALG00010029722	SPECC1	PRJNA729271	1.914197542	1.59961E-40	1.914197542	stage_E14_vs_E10	ordinary_muscle_development	D_vs_E10;stage_1W_vs_E10;stage_3W_vs_E10;stage	1	TRUE
ENSGALG00010018004	CDC42BPA	PRJNA729271	0.836941364	1.61819E-40	0.836941364	stage_E14_vs_E10	ordinary_muscle_development		0	FALSE
ENSGALG00010022453	ENSGALG00010022453	PRJNA729271	3.142538167	1.71979E-40	3.142538167	stage_E14_vs_E10	ordinary_muscle_development	D_vs_E10;stage_1W_vs_E10;stage_3W_vs_E10;stage	1	TRUE
ENSGALG00010002177	FBXO9	PRJNA729271	2.084509897	1.78176E-40	2.084509897	stage_E14_vs_E10	ordinary_muscle_development	D_vs_E10;stage_1W_vs_E10;stage_3W_vs_E10;stage	1	TRUE
ENSGALG00010016417	NOP14	PRJNA729271	-1.500052393	1.78375E-40	1.500052393	stage_E14_vs_E10	ordinary_muscle_development	D_vs_E10;stage_1W_vs_E10;stage_3W_vs_E10;stage	1	TRUE
ENSGALG00010001237	MELK	PRJNA729271	-3.81204928	1.79194E-40	3.81204928	stage_E14_vs_E10	ordinary_muscle_development	D_vs_E10;stage_1W_vs_E10;stage_3W_vs_E10;stage	1	TRUE
ENSGALG00010014928	SUCO	PRJNA729271	-1.105012308	1.7965E-40	1.105012308	stage_E14_vs_E10	ordinary_muscle_development	D_vs_E10;stage_1W_vs_E10;stage_3W_vs_E10;stage	1	TRUE
ENSGALG00010006838	ASB13	PRJNA729271	1.626940693	1.83978E-40	1.626940693	stage_E14_vs_E10	ordinary_muscle_development	D_vs_E10;stage_1W_vs_E10;stage_3W_vs_E10;stage	1	TRUE
ENSGALG00010016956	CENPE	PRJNA729271	-3.476187974	1.8798E-40	3.476187974	stage_E14_vs_E10	ordinary_muscle_development	D_vs_E10;stage_1W_vs_E10;stage_3W_vs_E10;stage	1	TRUE
ENSGALG00010025558	GALNT3	PRJNA729271	-3.320004189	2.03121E-40	3.320004189	stage_E14_vs_E10	ordinary_muscle_development	D_vs_E10;stage_1W_vs_E10;stage_3W_vs_E10;stage	1	TRUE
ENSGALG00010024883	ING5	PRJNA729271	1.652334667	2.04567E-40	1.652334667	stage_E14_vs_E10	ordinary_muscle_development	D_vs_E10;stage_1W_vs_E10;stage_3W_vs_E10;stage	1	TRUE
ENSGALG00010013908	TBC1D20	PRJNA729271	1.548448382	2.10812E-40	1.548448382	stage_E14_vs_E10	ordinary_muscle_development	D_vs_E10;stage_1W_vs_E10;stage_3W_vs_E10;stage	1	TRUE
ENSGALG00010001716	TRIL	PRJNA729271	-2.05848057	2.25597E-40	2.05848057	stage_E14_vs_E10	ordinary_muscle_development	D_vs_E10;stage_1W_vs_E10;stage_3W_vs_E10;stage	1	TRUE
ENSGALG00010027597	EXOSC2	PRJNA729271	-1.600831263	2.30756E-40	1.600831263	stage_E14_vs_E10	ordinary_muscle_development	D_vs_E10;stage_1W_vs_E10;stage_3W_vs_E10;stage	1	TRUE
ENSGALG00010013574	NR2F1	PRJNA729271	-2.215202294	2.4151E-40	2.215202294	stage_E14_vs_E10	ordinary_muscle_development	D_vs_E10;stage_1W_vs_E10;stage_3W_vs_E10;stage	1	TRUE
ENSGALG00010006266	CENPW	PRJNA729271	-3.093100521	2.51648E-40	3.093100521	stage_E14_vs_E10	ordinary_muscle_development	D_vs_E10;stage_1W_vs_E10;stage_3W_vs_E10;stage	1	TRUE
ENSGALG00010004584	EIF4E	PRJNA729271	-0.911350227	2.5541E-40	0.911350227	stage_E14_vs_E10	ordinary_muscle_development		0	FALSE
ENSGALG00010023811	MRPL44	PRJNA729271	1.12862673	2.62246E-40	1.12862673	stage_E14_vs_E10	ordinary_muscle_development	D_vs_E10;stage_1W_vs_E10;stage_3W_vs_E10;stage	1	TRUE
ENSGALG00010002027	ENSGALG00010002027	PRJNA729271	7.209117786	2.71779E-40	7.209117786	stage_E14_vs_E10	ordinary_muscle_development	D_vs_E10;stage_1W_vs_E10;stage_3W_vs_E10;stage	1	TRUE
ENSGALG00010004122	TRIM55	PRJNA729271	2.650834113	2.7587E-40	2.650834113	stage_E14_vs_E10	ordinary_muscle_development	D_vs_E10;stage_1W_vs_E10;stage_3W_vs_E10;stage	1	TRUE
ENSGALG00010003933	TECR	PRJNA729271	2.770719079	2.7925E-40	2.770719079	stage_E14_vs_E10	ordinary_muscle_development	D_vs_E10;stage_1W_vs_E10;stage_3W_vs_E10;stage	1	TRUE
ENSGALG00010018329	ENSGALG00010018329	PRJNA729271	2.955393598	2.82285E-40	2.955393598	stage_E14_vs_E10	ordinary_muscle_development	D_vs_E10;stage_1W_vs_E10;stage_3W_vs_E10;stage	1	TRUE
ENSGALG00010017959	PDZD7	PRJNA729271	3.640686284	2.88984E-40	3.640686284	stage_E14_vs_E10	ordinary_muscle_development	D_vs_E10;stage_1W_vs_E10;stage_3W_vs_E10;stage	1	TRUE
ENSGALG00010016447	ID3	PRJNA729271	-1.526313637	3.05023E-40	1.526313637	stage_E14_vs_E10	ordinary_muscle_development	D_vs_E10;stage_1W_vs_E10;stage_3W_vs_E10;stage	1	TRUE
ENSGALG00010021232	ACTB	PRJNA729271	-1.473009679	3.12872E-40	1.473009679	stage_E14_vs_E10	ordinary_muscle_development	D_vs_E10;stage_1W_vs_E10;stage_3W_vs_E10;stage	1	TRUE
ENSGALG00010028088	NAXE	PRJNA729271	1.534631087	3.15915E-40	1.534631087	stage_E14_vs_E10	ordinary_muscle_development	D_vs_E10;stage_1W_vs_E10;stage_3W_vs_E10;stage	1	TRUE
ENSGALG00010016103	BPNT1	PRJNA729271	-1.252688167	3.2333E-40	1.252688167	stage_E14_vs_E10	ordinary_muscle_development	D_vs_E10;stage_1W_vs_E10;stage_3W_vs_E10;stage	1	TRUE
ENSGALG00010013690	CREB3L2	PRJNA729271	-1.377432086	3.29495E-40	1.377432086	stage_E14_vs_E10	ordinary_muscle_development	D_vs_E10;stage_1W_vs_E10;stage_3W_vs_E10;stage	1	TRUE
ENSGALG00010023712	ARHGAP40	PRJNA729271	-2.233223295	3.39046E-40	2.233223295	stage_E14_vs_E10	ordinary_muscle_development	D_vs_E10;stage_1W_vs_E10;stage_3W_vs_E10;stage	1	TRUE
ENSGALG00010021235	XRCC5	PRJNA729271	-1.88944727	3.58382E-40	1.88944727	stage_E14_vs_E10	ordinary_muscle_development	D_vs_E10;stage_1W_vs_E10;stage_3W_vs_E10;stage	1	TRUE
ENSGALG00010025214	LRP8	PRJNA729271	-3.693111445	3.60442E-40	3.693111445	stage_E14_vs_E10	ordinary_muscle_development	D_vs_E10;stage_1W_vs_E10;stage_3W_vs_E10;stage	1	TRUE
ENSGALG00010009732	HMMR	PRJNA729271	-3.334727446	3.62188E-40	3.334727446	stage_E14_vs_E10	ordinary_muscle_development	D_vs_E10;stage_1W_vs_E10;stage_3W_vs_E10;stage	1	TRUE
ENSGALG00010000717	NDFIP2	PRJNA729271	0.897811384	3.63534E-40	0.897811384	stage_E14_vs_E10	ordinary_muscle_development		0	FALSE
ENSGALG00010020724	TACR1	PRJNA729271	-4.034491281	3.64581E-40	4.034491281	stage_E14_vs_E10	ordinary_muscle_development	D_vs_E10;stage_1W_vs_E10;stage_3W_vs_E10;stage	1	TRUE
ENSGALG00010014173	MND1	PRJNA729271	-2.762992986	3.77949E-40	2.762992986	stage_E14_vs_E10	ordinary_muscle_development	D_vs_E10;stage_1W_vs_E10;stage_3W_vs_E10;stage	1	TRUE
ENSGALG00010027973	GAPVD1	PRJNA729271	0.864570071	3.78254E-40	0.864570071	stage_E14_vs_E10	ordinary_muscle_development		0	FALSE
ENSGALG00010011703	YWHAZ	PRJNA729271	-1.268535637	3.80003E-40	1.268535637	stage_E14_vs_E10	ordinary_muscle_development	D_vs_E10;stage_1W_vs_E10;stage_3W_vs_E10;stage	1	TRUE
ENSGALG00010027152	C4BPS	PRJNA729271	5.751648124	3.86258E-40	5.751648124	stage_E14_vs_E10	ordinary_muscle_development	D_vs_E10;stage_1W_vs_E10;stage_3W_vs_E10;stage	1	TRUE
ENSGALG00010003042	TOX	PRJNA729271	-5.157444346	4.1467E-40	5.157444346	stage_E14_vs_E10	ordinary_muscle_development	D_vs_E10;stage_1W_vs_E10;stage_3W_vs_E10;stage	1	TRUE
ENSGALG00010022505	ZNF511	PRJNA729271	1.583307372	4.16397E-40	1.583307372	stage_E14_vs_E10	ordinary_muscle_development	D_vs_E10;stage_1W_vs_E10;stage_3W_vs_E10;stage	1	TRUE
ENSGALG00010012486	RADX	PRJNA729271	1.432082745	4.17373E-40	1.432082745	stage_E14_vs_E10	ordinary_muscle_development	D_vs_E10;stage_1W_vs_E10;stage_3W_vs_E10;stage	1	TRUE
ENSGALG00010019221	VASH2	PRJNA729271	-3.131233243	4.57535E-40	3.131233243	stage_E14_vs_E10	ordinary_muscle_development	D_vs_E10;stage_1W_vs_E10;stage_3W_vs_E10;stage	1	TRUE
ENSGALG00010008389	GALNT1	PRJNA729271	-1.193537411	4.60848E-40	1.193537411	stage_E14_vs_E10	ordinary_muscle_development	D_vs_E10;stage_1W_vs_E10;stage_3W_vs_E10;stage	1	TRUE
ENSGALG00010023601	TPST2	PRJNA729271	1.403290922	4.75575E-40	1.403290922	stage_E14_vs_E10	ordinary_muscle_development	D_vs_E10;stage_1W_vs_E10;stage_3W_vs_E10;stage	1	TRUE
ENSGALG00010013129	RASSF3	PRJNA729271	1.499513917	4.895E-40	1.499513917	stage_E14_vs_E10	ordinary_muscle_development	D_vs_E10;stage_1W_vs_E10;stage_3W_vs_E10;stage	1	TRUE
ENSGALG00010011273	RUNX1T1	PRJNA729271	-1.332802181	5.15081E-40	1.332802181	stage_E14_vs_E10	ordinary_muscle_development	D_vs_E10;stage_1W_vs_E10;stage_3W_vs_E10;stage	1	TRUE
ENSGALG00010010874	ENSGALG00010010874	PRJNA729271	3.475383722	5.23824E-40	3.475383722	stage_E14_vs_E10	ordinary_muscle_development	D_vs_E10;stage_1W_vs_E10;stage_3W_vs_E10;stage	1	TRUE
ENSGALG00010024787	SARS1	PRJNA729271	1.325868648	5.29929E-40	1.325868648	stage_E14_vs_E10	ordinary_muscle_development	D_vs_E10;stage_1W_vs_E10;stage_3W_vs_E10;stage	1	TRUE
ENSGALG00010003803	LACTB2	PRJNA729271	1.768986124	5.31526E-40	1.768986124	stage_E14_vs_E10	ordinary_muscle_development	D_vs_E10;stage_1W_vs_E10;stage_3W_vs_E10;stage	1	TRUE
ENSGALG00010026706	KRT24	PRJNA729271	-8.591642606	5.49346E-40	8.591642606	stage_E14_vs_E10	ordinary_muscle_development	D_vs_E10;stage_1W_vs_E10;stage_3W_vs_E10;stage	1	TRUE
ENSGALG00010028585	MINDY1	PRJNA729271	1.618012765	5.49856E-40	1.618012765	stage_E14_vs_E10	ordinary_muscle_development	D_vs_E10;stage_1W_vs_E10;stage_3W_vs_E10;stage	1	TRUE
ENSGALG00010026690	KRT14	PRJNA729271	-6.469178639	5.52659E-40	6.469178639	stage_E14_vs_E10	ordinary_muscle_development	D_vs_E10;stage_1W_vs_E10;stage_3W_vs_E10;stage	1	TRUE
ENSGALG00010003412	ASB5	PRJNA729271	1.961547239	5.59583E-40	1.961547239	stage_E14_vs_E10	ordinary_muscle_development	D_vs_E10;stage_1W_vs_E10;stage_3W_vs_E10;stage	1	TRUE
ENSGALG00010016781	CDIP1	PRJNA729271	1.917871974	5.77523E-40	1.917871974	stage_E14_vs_E10	ordinary_muscle_development	D_vs_E10;stage_1W_vs_E10;stage_3W_vs_E10;stage	1	TRUE
ENSGALG00010014246	KRAS	PRJNA729271	-1.546165671	6.24225E-40	1.546165671	stage_E14_vs_E10	ordinary_muscle_development	D_vs_E10;stage_1W_vs_E10;stage_3W_vs_E10;stage	1	TRUE
ENSGALG00010013563	FKBP1A	PRJNA729271	1.32501076	6.30127E-40	1.32501076	stage_E14_vs_E10	ordinary_muscle_development	D_vs_E10;stage_1W_vs_E10;stage_3W_vs_E10;stage	1	TRUE
ENSGALG00010022797	EFHD1	PRJNA729271	1.760843391	6.36761E-40	1.760843391	stage_E14_vs_E10	ordinary_muscle_development	D_vs_E10;stage_1W_vs_E10;stage_3W_vs_E10;stage	1	TRUE
ENSGALG00010009833	SOD3	PRJNA729271	4.161360516	6.42116E-40	4.161360516	stage_E14_vs_E10	ordinary_muscle_development	D_vs_E10;stage_1W_vs_E10;stage_3W_vs_E10;stage	1	TRUE
ENSGALG00010023830	AGFG1	PRJNA729271	1.498543519	6.87905E-40	1.498543519	stage_E14_vs_E10	ordinary_muscle_development	D_vs_E10;stage_1W_vs_E10;stage_3W_vs_E10;stage	1	TRUE
ENSGALG00010022210	EZH1	PRJNA729271	1.106962208	6.88972E-40	1.106962208	stage_E14_vs_E10	ordinary_muscle_development	D_vs_E10;stage_1W_vs_E10;stage_3W_vs_E10;stage	1	TRUE
ENSGALG00010028032	ENSGALG00010028032	PRJNA729271	-4.577258102	6.90252E-40	4.577258102	stage_E14_vs_E10	ordinary_muscle_development	D_vs_E10;stage_1W_vs_E10;stage_3W_vs_E10;stage	1	TRUE
ENSGALG00010022888	OSBPL2	PRJNA729271	1.210361529	7.63012E-40	1.210361529	stage_E14_vs_E10	ordinary_muscle_development	D_vs_E10;stage_1W_vs_E10;stage_3W_vs_E10;stage	1	TRUE
ENSGALG00010012873	PTAFR	PRJNA729271	2.949535418	8.10366E-40	2.949535418	stage_E14_vs_E10	ordinary_muscle_development	D_vs_E10;stage_1W_vs_E10;stage_3W_vs_E10;stage	1	TRUE
ENSGALG00010018527	CCT4	PRJNA729271	-1.099167661	8.17099E-40	1.099167661	stage_E14_vs_E10	ordinary_muscle_development	D_vs_E10;stage_1W_vs_E10;stage_3W_vs_E10;stage	1	TRUE
ENSGALG00010012303	CA9	PRJNA729271	2.8117453	8.19408E-40	2.8117453	stage_E14_vs_E10	ordinary_muscle_development	D_vs_E10;stage_1W_vs_E10;stage_3W_vs_E10;stage	1	TRUE
ENSGALG00010027271	INKA2	PRJNA729271	1.887503807	8.33436E-40	1.887503807	stage_E14_vs_E10	ordinary_muscle_development	D_vs_E10;stage_1W_vs_E10;stage_3W_vs_E10;stage	1	TRUE
ENSGALG00010012442	CPLX1	PRJNA729271	-6.803306553	8.49682E-40	6.803306553	stage_E14_vs_E10	ordinary_muscle_development	D_vs_E10;stage_1W_vs_E10;stage_3W_vs_E10;stage	1	TRUE
ENSGALG00010011040	CDH6	PRJNA729271	-3.027306023	8.58349E-40	3.027306023	stage_E14_vs_E10	ordinary_muscle_development	D_vs_E10;stage_1W_vs_E10;stage_3W_vs_E10;stage	1	TRUE
ENSGALG00010016736	CENPS	PRJNA729271	-3.690382375	8.7041E-40	3.690382375	stage_E14_vs_E10	ordinary_muscle_development	D_vs_E10;stage_1W_vs_E10;stage_3W_vs_E10;stage	1	TRUE
ENSGALG00010020010	SORBS3	PRJNA729271	2.739131205	9.4701E-40	2.739131205	stage_E14_vs_E10	ordinary_muscle_development	D_vs_E10;stage_1W_vs_E10;stage_3W_vs_E10;stage	1	TRUE
ENSGALG00010007719	LTBP1	PRJNA729271	-2.553823099	9.47375E-40	2.553823099	stage_E14_vs_E10	ordinary_muscle_development	D_vs_E10;stage_1W_vs_E10;stage_3W_vs_E10;stage	1	TRUE
ENSGALG00010029600	RHOT1	PRJNA729271	1.447997586	9.47375E-40	1.447997586	stage_E14_vs_E10	ordinary_muscle_development	D_vs_E10;stage_1W_vs_E10;stage_3W_vs_E10;stage	1	TRUE
ENSGALG00010024001	ENSGALG00010024001	PRJNA729271	5.989012008	9.62282E-40	5.989012008	stage_E14_vs_E10	ordinary_muscle_development	D_vs_E10;stage_1W_vs_E10;stage_3W_vs_E10;stage	1	TRUE
ENSGALG00010015845	SAP30L	PRJNA729271	1.671717552	9.77562E-40	1.671717552	stage_E14_vs_E10	ordinary_muscle_development	D_vs_E10;stage_1W_vs_E10;stage_3W_vs_E10;stage	1	TRUE
ENSGALG00010005966	PLXDC2	PRJNA729271	2.045904638	9.78141E-40	2.045904638	stage_E14_vs_E10	ordinary_muscle_development	D_vs_E10;stage_1W_vs_E10;stage_3W_vs_E10;stage	1	TRUE
ENSGALG00010007321	CD200	PRJNA729271	2.009193216	9.81935E-40	2.009193216	stage_E14_vs_E10	ordinary_muscle_development	D_vs_E10;stage_1W_vs_E10;stage_3W_vs_E10;stage	1	TRUE
ENSGALG00010013318	KERA	PRJNA729271	-6.595389642	9.8525E-40	6.595389642	stage_E14_vs_E10	ordinary_muscle_development	D_vs_E10;stage_1W_vs_E10;stage_3W_vs_E10;stage	1	TRUE
ENSGALG00010002547	PBX4	PRJNA729271	-1.500987885	1.02538E-39	1.500987885	stage_E14_vs_E10	ordinary_muscle_development	D_vs_E10;stage_1W_vs_E10;stage_3W_vs_E10;stage	1	TRUE
ENSGALG00010005258	ITIH5	PRJNA729271	3.73856756	1.03446E-39	3.73856756	stage_E14_vs_E10	ordinary_muscle_development	D_vs_E10;stage_1W_vs_E10;stage_3W_vs_E10;stage	1	TRUE
ENSGALG00010000790	MARCHF6	PRJNA729271	1.31893594	1.10704E-39	1.31893594	stage_E14_vs_E10	ordinary_muscle_development	D_vs_E10;stage_1W_vs_E10;stage_3W_vs_E10;stage	1	TRUE
ENSGALG00010022046	CRY2	PRJNA729271	1.376545813	1.11073E-39	1.376545813	stage_E14_vs_E10	ordinary_muscle_development	D_vs_E10;stage_1W_vs_E10;stage_3W_vs_E10;stage	1	TRUE
ENSGALG00010017765	SYT16	PRJNA729271	-4.339966847	1.12029E-39	4.339966847	stage_E14_vs_E10	ordinary_muscle_development	D_vs_E10;stage_1W_vs_E10;stage_3W_vs_E10;stage	1	TRUE
ENSGALG00010013848	ITPA	PRJNA729271	-1.411740273	1.21609E-39	1.411740273	stage_E14_vs_E10	ordinary_muscle_development	D_vs_E10;stage_1W_vs_E10;stage_3W_vs_E10;stage	1	TRUE
ENSGALG00010025727	GLI1	PRJNA729271	-3.359562254	1.25403E-39	3.359562254	stage_E14_vs_E10	ordinary_muscle_development	D_vs_E10;stage_1W_vs_E10;stage_3W_vs_E10;stage	1	TRUE
ENSGALG00010029768	KCNJ2	PRJNA729271	2.336446353	1.29742E-39	2.336446353	stage_E14_vs_E10	ordinary_muscle_development	D_vs_E10;stage_1W_vs_E10;stage_3W_vs_E10;stage	1	TRUE
ENSGALG00010021090	ADM	PRJNA729271	3.40841617	1.31515E-39	3.40841617	stage_E14_vs_E10	ordinary_muscle_development	D_vs_E10;stage_1W_vs_E10;stage_3W_vs_E10;stage	1	TRUE
ENSGALG00010008347	PUF60	PRJNA729271	-0.741633476	1.38706E-39	0.741633476	stage_E14_vs_E10	ordinary_muscle_development		0	FALSE
ENSGALG00010009299	MAP4K1	PRJNA729271	3.867003007	1.40831E-39	3.867003007	stage_E14_vs_E10	ordinary_muscle_development	D_vs_E10;stage_1W_vs_E10;stage_3W_vs_E10;stage	1	TRUE
ENSGALG00010029216	RAB34	PRJNA729271	-1.828038307	1.51755E-39	1.828038307	stage_E14_vs_E10	ordinary_muscle_development	D_vs_E10;stage_1W_vs_E10;stage_3W_vs_E10;stage	1	TRUE
ENSGALG00010008275	CELSR1	PRJNA729271	-2.347125248	1.52222E-39	2.347125248	stage_E14_vs_E10	ordinary_muscle_development	D_vs_E10;stage_1W_vs_E10;stage_3W_vs_E10;stage	1	TRUE

ENSGALG00010002196	ENSGALG00010002196	PRJNA729271	-1.36257587	1.53035E-39	1.36257587	stage_E14_vs_E10	ordinary_muscle_development	D_vs_E10;stage_1W_vs_E10;stage_3W_vs_E10;stage	1	TRUE
ENSGALG00010010988	ANKRD44	PRJNA729271	-1.163804823	1.57288E-39	1.163804823	stage_E14_vs_E10	ordinary_muscle_development	D_vs_E10;stage_1W_vs_E10;stage_3W_vs_E10;stage	1	TRUE
ENSGALG00010016866	ZNRF2	PRJNA729271	1.328590619	1.57808E-39	1.328590619	stage_E14_vs_E10	ordinary_muscle_development	D_vs_E10;stage_1W_vs_E10;stage_3W_vs_E10;stage	1	TRUE
ENSGALG00010009224	ENSGALG00010009224	PRJNA729271	5.911049575	1.60772E-39	5.911049575	stage_E14_vs_E10	ordinary_muscle_development	D_vs_E10;stage_1W_vs_E10;stage_3W_vs_E10;stage	1	TRUE
ENSGALG00010027685	ENSGALG00010027685	PRJNA729271	3.91714898	1.61645E-39	3.91714898	stage_E14_vs_E10	ordinary_muscle_development	D_vs_E10;stage_1W_vs_E10;stage_3W_vs_E10;stage	1	TRUE
ENSGALG00010027624	ENSGALG00010027624	PRJNA729271	5.222376372	1.65052E-39	5.222376372	stage_E14_vs_E10	ordinary_muscle_development	D_vs_E10;stage_1W_vs_E10;stage_3W_vs_E10;stage	1	TRUE
ENSGALG00010012043	NUP50	PRJNA729271	-1.295507551	1.70858E-39	1.295507551	stage_E14_vs_E10	ordinary_muscle_development	D_vs_E10;stage_1W_vs_E10;stage_3W_vs_E10;stage	1	TRUE
ENSGALG00010011198	PINX1	PRJNA729271	-0.913251615	1.72132E-39	0.913251615	stage_E14_vs_E10	ordinary_muscle_development		0	FALSE
ENSGALG00010017948	PLAT	PRJNA729271	2.078136039	1.72321E-39	2.078136039	stage_E14_vs_E10	ordinary_muscle_development	D_vs_E10;stage_1W_vs_E10;stage_3W_vs_E10;stage	1	TRUE
ENSGALG00010000281	OSBP2	PRJNA729271	2.70742791	1.72527E-39	2.70742791	stage_E14_vs_E10	ordinary_muscle_development	D_vs_E10;stage_1W_vs_E10;stage_3W_vs_E10;stage	1	TRUE
ENSGALG00010024739	ASIP	PRJNA729271	-8.566065	1.93139E-39	8.566065	stage_E14_vs_E10	ordinary_muscle_development	D_vs_E10;stage_1W_vs_E10;stage_3W_vs_E10;stage	1	TRUE
ENSGALG00010018948	POLR3E	PRJNA729271	1.425493404	1.93681E-39	1.425493404	stage_E14_vs_E10	ordinary_muscle_development	D_vs_E10;stage_1W_vs_E10;stage_3W_vs_E10;stage	1	TRUE
ENSGALG00010005369	DPT	PRJNA729271	4.179714958	1.94911E-39	4.179714958	stage_E14_vs_E10	ordinary_muscle_development	D_vs_E10;stage_1W_vs_E10;stage_3W_vs_E10;stage	1	TRUE
ENSGALG00010008171	KIF21A	PRJNA729271	-2.474219	1.96413E-39	2.474219	stage_E14_vs_E10	ordinary_muscle_development	D_vs_E10;stage_1W_vs_E10;stage_3W_vs_E10;stage	1	TRUE
ENSGALG00010000485	MED13L	PRJNA729271	1.674429267	1.99782E-39	1.674429267	stage_E14_vs_E10	ordinary_muscle_development	D_vs_E10;stage_1W_vs_E10;stage_3W_vs_E10;stage	1	TRUE
ENSGALG00010028116	HYAL1	PRJNA729271	1.952251035	2.00938E-39	1.952251035	stage_E14_vs_E10	ordinary_muscle_development	D_vs_E10;stage_1W_vs_E10;stage_3W_vs_E10;stage	1	TRUE
ENSGALG00010005310	LAMTOR3	PRJNA729271	1.006561163	2.09626E-39	1.006561163	stage_E14_vs_E10	ordinary_muscle_development	D_vs_E10;stage_1W_vs_E10;stage_3W_vs_E10;stage	1	TRUE
ENSGALG00010020952	GNG5	PRJNA729271	-1.333551306	2.1619E-39	1.333551306	stage_E14_vs_E10	ordinary_muscle_development	D_vs_E10;stage_1W_vs_E10;stage_3W_vs_E10;stage	1	TRUE
ENSGALG00010021623	FAR1	PRJNA729271	1.720047274	2.22289E-39	1.720047274	stage_E14_vs_E10	ordinary_muscle_development	D_vs_E10;stage_1W_vs_E10;stage_3W_vs_E10;stage	1	TRUE
ENSGALG00010029742	TAX1BP3	PRJNA729271	-1.590734156	2.23086E-39	1.590734156	stage_E14_vs_E10	ordinary_muscle_development	D_vs_E10;stage_1W_vs_E10;stage_3W_vs_E10;stage	1	TRUE
ENSGALG00010012350	H2AC32	PRJNA729271	-2.557100867	2.23814E-39	2.557100867	stage_E14_vs_E10	ordinary_muscle_development	D_vs_E10;stage_1W_vs_E10;stage_3W_vs_E10;stage	1	TRUE
ENSGALG00010024953	NCAPG2	PRJNA729271	-3.030674949	2.2478E-39	3.030674949	stage_E14_vs_E10	ordinary_muscle_development	D_vs_E10;stage_1W_vs_E10;stage_3W_vs_E10;stage	1	TRUE
ENSGALG00010002866	ENSGALG00010002866	PRJNA729271	3.349033138	2.26088E-39	3.349033138	stage_E14_vs_E10	ordinary_muscle_development	D_vs_E10;stage_1W_vs_E10;stage_3W_vs_E10;stage	1	TRUE
ENSGALG00010023244	PPIB	PRJNA729271	-1.58020901	2.32256E-39	1.58020901	stage_E14_vs_E10	ordinary_muscle_development	D_vs_E10;stage_1W_vs_E10;stage_3W_vs_E10;stage	1	TRUE
ENSGALG00010026329	ENSGALG00010026329	PRJNA729271	3.575164044	2.49414E-39	3.575164044	stage_E14_vs_E10	ordinary_muscle_development	D_vs_E10;stage_1W_vs_E10;stage_3W_vs_E10;stage	1	TRUE
ENSGALG00010000526	POLD1	PRJNA729271	-2.288517302	2.61313E-39	2.288517302	stage_E14_vs_E10	ordinary_muscle_development	D_vs_E10;stage_1W_vs_E10;stage_3W_vs_E10;stage	1	TRUE
ENSGALG00010009340	SYK	PRJNA729271	1.825369538	2.71684E-39	1.825369538	stage_E14_vs_E10	ordinary_muscle_development	D_vs_E10;stage_1W_vs_E10;stage_3W_vs_E10;stage	1	TRUE
ENSGALG00010014093	PLD1	PRJNA729271	1.233280498	2.72908E-39	1.233280498	stage_E14_vs_E10	ordinary_muscle_development	D_vs_E10;stage_1W_vs_E10;stage_3W_vs_E10;stage	1	TRUE
ENSGALG00010013187	KDF1	PRJNA729271	-3.681912723	2.75581E-39	3.681912723	stage_E14_vs_E10	ordinary_muscle_development	D_vs_E10;stage_1W_vs_E10;stage_3W_vs_E10;stage	1	TRUE
ENSGALG00010015013	SNX18	PRJNA729271	-1.514796865	2.75822E-39	1.514796865	stage_E14_vs_E10	ordinary_muscle_development	D_vs_E10;stage_1W_vs_E10;stage_3W_vs_E10;stage	1	TRUE
ENSGALG00010016696	DOK7	PRJNA729271	1.729798784	2.79044E-39	1.729798784	stage_E14_vs_E10	ordinary_muscle_development	D_vs_E10;stage_1W_vs_E10;stage_3W_vs_E10;stage	1	TRUE
ENSGALG00010004534	CUBN	PRJNA729271	2.820395697	2.93196E-39	2.820395697	stage_E14_vs_E10	ordinary_muscle_development	D_vs_E10;stage_1W_vs_E10;stage_3W_vs_E10;stage	1	TRUE
ENSGALG00010005150	ALCAM	PRJNA729271	-2.523674977	2.96346E-39	2.523674977	stage_E14_vs_E10	ordinary_muscle_development	D_vs_E10;stage_1W_vs_E10;stage_3W_vs_E10;stage	1	TRUE
ENSGALG00010024017	TYW3	PRJNA729271	-1.867563422	3.0224E-39	1.867563422	stage_E14_vs_E10	ordinary_muscle_development	D_vs_E10;stage_1W_vs_E10;stage_3W_vs_E10;stage	1	TRUE
ENSGALG00010012307	ENSGALG00010012307	PRJNA729271	-2.298593386	3.04824E-39	2.298593386	stage_E14_vs_E10	ordinary_muscle_development	D_vs_E10;stage_1W_vs_E10;stage_3W_vs_E10;stage	1	TRUE
ENSGALG00010012011	HOOK3	PRJNA729271	0.903639317	3.24828E-39	0.903639317	stage_E14_vs_E10	ordinary_muscle_development		0	FALSE
ENSGALG00010021732	CASR	PRJNA729271	3.334042365	3.25951E-39	3.334042365	stage_E14_vs_E10	ordinary_muscle_development	D_vs_E10;stage_1W_vs_E10;stage_3W_vs_E10;stage	1	TRUE
ENSGALG00010022181	ENSGALG00010022181	PRJNA729271	-3.254609529	3.45814E-39	3.254609529	stage_E14_vs_E10	ordinary_muscle_development	D_vs_E10;stage_1W_vs_E10;stage_3W_vs_E10;stage	1	TRUE
ENSGALG00010011246	DNAJB5	PRJNA729271	3.087476746	3.66897E-39	3.087476746	stage_E14_vs_E10	ordinary_muscle_development	D_vs_E10;stage_1W_vs_E10;stage_3W_vs_E10;stage	1	TRUE
ENSGALG00010021772	TSN	PRJNA729271	-1.123860217	3.7538E-39	1.123860217	stage_E14_vs_E10	ordinary_muscle_development	D_vs_E10;stage_1W_vs_E10;stage_3W_vs_E10;stage	1	TRUE
ENSGALG00010029690	ENSGALG00010029690	PRJNA729271	2.122012554	3.83631E-39	2.122012554	stage_E14_vs_E10	ordinary_muscle_development	D_vs_E10;stage_1W_vs_E10;stage_3W_vs_E10;stage	1	TRUE
ENSGALG00010022082	TGFBR3	PRJNA729271	1.916709047	3.97303E-39	1.916709047	stage_E14_vs_E10	ordinary_muscle_development	D_vs_E10;stage_1W_vs_E10;stage_3W_vs_E10;stage	1	TRUE
ENSGALG00010006840	ENSGALG00010006840	PRJNA729271	-4.067284495	4.02901E-39	4.067284495	stage_E14_vs_E10	ordinary_muscle_development	D_vs_E10;stage_1W_vs_E10;stage_3W_vs_E10;stage	1	TRUE
ENSGALG00010005472	PFKP	PRJNA729271	-1.531890797	4.28422E-39	1.531890797	stage_E14_vs_E10	ordinary_muscle_development	D_vs_E10;stage_1W_vs_E10;stage_3W_vs_E10;stage	1	TRUE
ENSGALG00010019139	DPCD	PRJNA729271	-1.818839429	4.85613E-39	1.818839429	stage_E14_vs_E10	ordinary_muscle_development	D_vs_E10;stage_1W_vs_E10;stage_3W_vs_E10;stage	1	TRUE
ENSGALG00010011941	ENSGALG00010011941	PRJNA729271	-3.266012608	4.901E-39	3.266012608	stage_E14_vs_E10	ordinary_muscle_development	D_vs_E10;stage_1W_vs_E10;stage_3W_vs_E10;stage	1	TRUE
ENSGALG00010021231	STN1	PRJNA729271	-1.621926712	5.11723E-39	1.621926712	stage_E14_vs_E10	ordinary_muscle_development	D_vs_E10;stage_1W_vs_E10;stage_3W_vs_E10;stage	1	TRUE
ENSGALG00010022480	PIF1	PRJNA729271	-3.77744206	5.41505E-39	3.77744206	stage_E14_vs_E10	ordinary_muscle_development	D_vs_E10;stage_1W_vs_E10;stage_3W_vs_E10;stage	1	TRUE
ENSGALG00010018533	PCYOX1	PRJNA729271	2.278316989	5.64483E-39	2.278316989	stage_E14_vs_E10	ordinary_muscle_development	D_vs_E10;stage_1W_vs_E10;stage_3W_vs_E10;stage	1	TRUE
ENSGALG00010016984	RNF103	PRJNA729271	1.508248068	5.69571E-39	1.508248068	stage_E14_vs_E10	ordinary_muscle_development	D_vs_E10;stage_1W_vs_E10;stage_3W_vs_E10;stage	1	TRUE
ENSGALG00010023694	PPCDC	PRJNA729271	1.98444575	5.81065E-39	1.98444575	stage_E14_vs_E10	ordinary_muscle_development	D_vs_E10;stage_1W_vs_E10;stage_3W_vs_E10;stage	1	TRUE
ENSGALG00010016159	ASPG	PRJNA729271	-2.606264898	5.82094E-39	2.606264898	stage_E14_vs_E10	ordinary_muscle_development	D_vs_E10;stage_1W_vs_E10;stage_3W_vs_E10;stage	1	TRUE
ENSGALG00010000356	ENSGALG00010000356	PRJNA729271	1.583160112	5.93511E-39	1.583160112	stage_E14_vs_E10	ordinary_muscle_development	D_vs_E10;stage_1W_vs_E10;stage_3W_vs_E10;stage	1	TRUE
ENSGALG00010022949	INSC	PRJNA729271	-3.703109979	6.28305E-39	3.703109979	stage_E14_vs_E10	ordinary_muscle_development	D_vs_E10;stage_1W_vs_E10;stage_3W_vs_E10;stage	1	TRUE
ENSGALG00010009492	CDC42SE2	PRJNA729271	-1.683742794	6.44116E-39	1.683742794	stage_E14_vs_E10	ordinary_muscle_development	D_vs_E10;stage_1W_vs_E10;stage_3W_vs_E10;stage	1	TRUE
ENSGALG00010003837	ALKAL2	PRJNA729271	3.080824007	6.57427E-39	3.080824007	stage_E14_vs_E10	ordinary_muscle_development	D_vs_E10;stage_1W_vs_E10;stage_3W_vs_E10;stage	1	TRUE
ENSGALG00010001200	CTTNBP2	PRJNA729271	1.68030964	6.57695E-39	1.68030964	stage_E14_vs_E10	ordinary_muscle_development	D_vs_E10;stage_1W_vs_E10;stage_3W_vs_E10;stage	1	TRUE
ENSGALG00010003406	C1GALT1	PRJNA729271	-1.417415021	6.83799E-39	1.417415021	stage_E14_vs_E10	ordinary_muscle_development	D_vs_E10;stage_1W_vs_E10;stage_3W_vs_E10;stage	1	TRUE
ENSGALG00010023826	DBT	PRJNA729271	1.675786675	6.88808E-39	1.675786675	stage_E14_vs_E10	ordinary_muscle_development	D_vs_E10;stage_1W_vs_E10;stage_3W_vs_E10;stage	1	TRUE
ENSGALG00010000255	ENSGALG00010000255	PRJNA729271	1.900143165	6.88877E-39	1.900143165	stage_E14_vs_E10	ordinary_muscle_development	D_vs_E10;stage_1W_vs_E10;stage_3W_vs_E10;stage	1	TRUE
ENSGALG00010025163	LDLRAD3	PRJNA729271	-1.838528452	6.88877E-39	1.838528452	stage_E14_vs_E10	ordinary_muscle_development	D_vs_E10;stage_1W_vs_E10;stage_3W_vs_E10;stage	1	TRUE
ENSGALG00010029689	ENSGALG00010029689	PRJNA729271	2.317468611	7.02108E-39	2.317468611	stage_E14_vs_E10	ordinary_muscle_development	D_vs_E10;stage_1W_vs_E10;stage_3W_vs_E10;stage	1	TRUE
ENSGALG00010017344	RERE	PRJNA729271	2.018794714	7.27363E-39	2.018794714	stage_E14_vs_E10	ordinary_muscle_development	D_vs_E10;stage_1W_vs_E10;stage_3W_vs_E10;stage	1	TRUE
ENSGALG00010016502	YIPF5	PRJNA729271	-1.770174404	7.68235E-39	1.770174404	stage_E14_vs_E10	ordinary_muscle_development	D_vs_E10;stage_1W_vs_E10;stage_3W_vs_E10;stage	1	TRUE
ENSGALG00010020712	ZC3H7A	PRJNA729271	-0.94734275	7.86937E-39	0.94734275	stage_E14_vs_E10	ordinary_muscle_development		0	FALSE
ENSGALG00010025357	OLFML3	PRJNA729271	-1.604411158	8.03035E-39	1.604411158	stage_E14_vs_E10	ordinary_muscle_development	D_vs_E10;stage_1W_vs_E10;stage_3W_vs_E10;stage	1	TRUE
ENSGALG00010029370	SRSF2	PRJNA729271	-1.686691835	8.5086E-39	1.686691835	stage_E14_vs_E10	ordinary_muscle_development	D_vs_E10;stage_1W_vs_E10;stage_3W_vs_E10;stage	1	TRUE
ENSGALG00010017419	B9D1	PRJNA729271	-1.363905694	8.56228E-39	1.363905694	stage_E14_vs_E10	ordinary_muscle_development	D_vs_E10;stage_1W_vs_E10;stage_3W_vs_E10;stage	1	TRUE
ENSGALG00010001210	PANX2	PRJNA729271	-5.782722709	8.58756E-39	5.782722709	stage_E14_vs_E10	ordinary_muscle_development	D_vs_E10;stage_1W_vs_E10;stage_3W_vs_E10;stage	1	TRUE
ENSGALG00010021429	MED8	PRJNA729271	1.485404213	8.70521E-39	1.485404213	stage_E14_vs_E10	ordinary_muscle_development	D_vs_E10;stage_1W_vs_E10;stage_3W_vs_E10;stage	1	TRUE
ENSGALG00010026360	CTDSPL	PRJNA729271	1.671384086	9.03961E-39	1.671384086	stage_E14_vs_E10	ordinary_muscle_development	D_vs_E10;stage_1W_vs_E10;stage_3W_vs_E10;stage	1	TRUE
ENSGALG00010016591	HOMER2	PRJNA729271	-2.834490196	9.43298E-39	2.834490196	stage_E14_vs_E10	ordinary_muscle_development	D_vs_E10;stage_1W_vs_E10;stage_3W_vs_E10;stage	1	TRUE
ENSGALG00010018704	PGPEP1L	PRJNA729271	5.492353697	1.00327E-38	5.492353697	stage_E14_vs_E10	ordinary_muscle_development	D_vs_E10;stage_1W_vs_E10;stage_3W_vs_E10;stage	1	TRUE
ENSGALG00010004242	ADAM22	PRJNA729271	-2.366358766	1.03111E-38	2.366358766	stage_E14_vs_E10	ordinary_muscle_development	D_vs_E10;stage_1W_vs_E10;stage_3W_vs_E10;stage	1	TRUE
ENSGALG00010029167	ENSGALG00010029167	PRJNA729271	1.663886694	1.03278E-38	1.663886694	stage_E14_vs_E10	ordinary_muscle_development	D_vs_E10;stage_1W_vs_E10;stage_3W_vs_E10;stage	1	TRUE
ENSGALG00010006575	SSBP2	PRJNA729271	-1.554571977	1.0339E-38	1.554571977	stage_E14_vs_E10	ordinary_muscle_development	D_vs_E10;stage_1W_vs_E10;stage_3W_vs_E10;stage	1	TRUE
ENSGALG00010025938	ERP29	PRJNA729271	-1.192592764	1.04437E-38	1.192592764	stage_E14_vs_E10	ordinary_muscle_development	D_vs_E10;stage_1W_vs_E10;stage_3W_vs_E10;stage	1	TRUE
ENSGALG00010010223	ENSGALG00010010223	PRJNA729271	2.563812358	1.11699E-38	2.563812358	stage_E14_vs_E10	ordinary_muscle_development	D_vs_E10;stage_1W_vs_E10;stage_3W_vs_E10;stage	1	TRUE
ENSGALG00010012082	HAUS1	PRJNA729271	-2.8968426	1.23668E-38	2.8968426	stage_E14_vs_E10	ordinary_muscle_development	D_vs_E10;stage_1W_vs_E10;stage_3W_vs_E10;stage	1	TRUE
ENSGALG00010018215	CFAP70	PRJNA729271	3.478006301	1.25316E-38	3.478006301	stage_E14_vs_E10	ordinary_muscle_development	D_vs_E10;stage_1W_vs_E10;stage_3W_vs_E10;stage	1	TRUE
ENSGALG00010020675	SMYD2	PRJNA729271	-2.449453455	1.31652E-38	2.449453455	stage_E14_vs_E10	ordinary_muscle_development	D_vs_E10;stage_1W_vs_E10;stage_3W_vs_E10;stage	1	TRUE
ENSGALG00010024634	ENSGALG00010024634	PRJNA729271	2.146218573	1.32858E-38	2.146218573	stage_E14_vs_E10	ordinary_muscle_development	D_vs_E10;stage_1W_vs_E10;stage_3W_vs_E10;stage	1	TRUE
ENSGALG00010021519	FNTB	PRJNA729271	1.299992137	1.33121E-38	1.299992137	stage_E14_vs_E10	ordinary_muscle_development	D_vs_E10;stage_1W_vs_E10;stage_3W_vs_E10;stage	1	TRUE
ENSGALG00010006681	CRISPLD2	PRJNA729271	-2.96981878	1.40297E-38	2.96981878	stage_E14_vs_E10	ordinary_muscle_development	D_vs_E10;stage_1W_vs_E10;stage_3W_vs_E10;stage	1	TRUE
ENSGALG00010016799	WDTC1	PRJNA729271	1.734608129	1.42465E-38	1.734608129	stage_E14_vs_E10	ordinary_muscle_development	D_vs_E10;stage_1W_vs_E10;stage_3W_vs_E10;stage	1	TRUE
ENSGALG00010007660	FAM98A	PRJNA729271	1.269188221	1.48412E-38	1.269188221	stage_E14_vs_E10	ordinary_muscle_development	D_vs_E10;stage_1W_vs_E10;stage_3W_vs_E10;stage	1	TRUE
ENSGALG00010007376	CDKN2AIP	PRJNA729271	-0.807409337	1.49658E-38	0.807409337	stage_E14_vs_E10	ordinary_muscle_development		0	FALSE
ENSGALG00010007433	RNF144B	PRJNA729271	3.508155512	1.51611E-38	3.508155512	stage_E14_vs_E10	ordinary_muscle_development	D_vs_E10;stage_1W_vs_E10;stage_3W_vs_E10;stage	1	TRUE
ENSGALG00010009031	SPIN1	PRJNA729271	-1.049602725	1.53893E-38	1.049602725	stage_E14_vs_E10	ordinary_muscle_development	D_vs_E10;stage_1W_vs_E10;stage_3W_vs_E10;stage	1	TRUE
ENSGALG00010017993	REXO5	PRJNA729271	-1.294495969	1.56693E-38	1.294495969	stage_E14_vs_E10	ordinary_muscle_development	D_vs_E10;stage_1W_vs_E10;stage_3W_vs_E10;stage	1	TRUE
ENSGALG00010019050	TNS1	PRJNA729271	1.755324576	1.58363E-38	1.755324576	stage_E14_vs_E10	ordinary_muscle_development	D_vs_E10;stage_1W_vs_E10;stage_3W_vs_E10;stage	1	TRUE
ENSGALG00010023698	ENSGALG00010023698	PRJNA729271	-1.975825046	1.60489E-38	1.975825046	stage_E14_vs_E10	ordinary_muscle_development	D_vs_E10;stage_1W_vs_E10;stage_3W_vs_E10;stage	1	TRUE
ENSGALG00010009620	TMOD1	PRJNA729271	3.059956192	1.61581E-38	3.059956192	stage_E14_vs_E10	ordinary_muscle_development	D_vs_E10;stage_1W_vs_E10;stage_3W_vs_E10;stage	1	TRUE
ENSGALG00010015180	PITHD1	PRJNA729271	1.623654438	1.66159E-38	1.623654438	stage_E14_vs_E10	ordinary_muscle_development	D_vs_E10;stage_1W_vs_E10;stage_3W_vs_E10;stage	1	TRUE
ENSGALG00010005503	DYNLT1	PRJNA729271	-2.086442371	1.69158E-38	2.086442371	stage_E14_vs_E10	ordinary_muscle_development	D_vs_E10;stage_1W_vs_E10;stage_3W_vs_E10;stage	1	TRUE
ENSGALG00010001704	ENSGALG00010001704	PRJNA729271	-1.937627946	1.69691E-38	1.937627946	stage_E14_vs_E10	ordinary_muscle_development	D_vs_E10;stage_1W_vs_E10;stage_3W_vs_E10;stage	1	TRUE
ENSGALG00010008888	FBXO46	PRJNA729271	1.857652768	1.70733E-38	1.857652768	stage_E14_vs_E10	ordinary_muscle_development	D_vs_E10;stage_1W_vs_E10;stage_3W_vs_E10;stage	1	TRUE
ENSGALG00010017621	SYNE2	PRJNA729271	2.052377608	1.76967E-38	2.052377608	stage_E14_vs_E10	ordinary_muscle_development	D_vs_E10;stage_1W_vs_E10;stage_3W_vs_E10;stage	1	TRUE
ENSGALG00010021385	GDF5	PRJNA729271	-8.028630492	1.78652E-38	8.028630492	stage_E14_vs_E10	ordinary_muscle_development	D_vs_E10;stage_1W_vs_E10;stage_3W_vs_E10;stage	1	TRUE
ENSGALG00010028884	VAV2	PRJNA729271	-1.724333188	1.7989E-38	1.724333188	stage_E14_vs_E10	ordinary_muscle_development	D_vs_E10;stage_1W_vs_E10;stage_3W_vs_E10;stage	1	TRUE
ENSGALG00010009494	UBA2	PRJNA729271	-0.998138583	1.87268E-38	0.998138583	stage_E14_vs_E10	ordinary_muscle_development		0	FALSE
ENSGALG00010002805	FAM171A1	PRJNA729271	-1.326032021	1.93187E-38	1.326032021	stage_E14_vs_E10	ordinary_muscle_development	D_vs_E10;stage_1W_vs_E10;stage_3W_vs_E10;stage	1	TRUE
ENSGALG00010028097	TTC24	PRJNA729271	2.82981892	1.95198E-38	2.82981892	stage_E14_vs_E10	ordinary_muscle_development	D_vs_E10;stage_1W_vs_E10;stage_3W_vs_E10;stage	1	TRUE
ENSGALG00010016345	CTSZ	PRJNA729271	1.444324433	1.95474E-38	1.444324433	stage_E14_vs_E10	ordinary_muscle_development	D_vs_E10;stage_1W_vs_E10;stage_3W_vs_E10;stage	1	TRUE
ENSGALG00010029286	MAFG	PRJNA729271	2.386425984	2.0153E-38	2.386425984	stage_E14_vs_E10	ordinary_muscle_development	D_vs_E10;stage_1W_vs_E10;stage_3W_vs_E10;stage	1	TRUE
ENSGALG00010029710	BLMH	PRJNA729271	-1.104712238	2.07612E-38	1.104712238	stage_E14_vs_E10	ordinary_muscle_development	D_vs_E10;stage_1W_vs_E10;stage_3W_vs_E10;stage	1	TRUE
ENSGALG00010022215	ENSGALG00010022215	PRJNA729271	1.688160446	2.07912E-38	1.688160446	stage_E14_vs_E10	ordinary_muscle_development	D_vs_E10;stage_1W_vs_E10;stage_3W_vs_E10;stage	1	TRUE
ENSGALG00010013269	INPP5B	PRJNA729271	1.313693443	2.12454E-38	1.313693443	stage_E14_vs_E10	ordinary_muscle_development	D_vs_E10;stage_1W_vs_E10;stage_3W_vs_E10;stage	1	TRUE
ENSGALG00010016915	PLOD2	PRJNA729271	-2.209537792	2.21061E-38	2.209537792	stage_E14_vs_E10	ordinary_muscle_development	D_vs_E10;stage_1W_vs_E10;stage_3W_vs_E10;stage	1	TRUE
ENSGALG00010008126	CDH3	PRJNA729271	-4.361673505	2.21603E-38	4.361673505	stage_E14_vs_E10	ordinary_muscle_development	D_vs_E10;stage_1W_vs_E10;stage_3W_vs_E10;stage	1	TRUE
ENSGALG00010025222	FAM117B	PRJNA729271	-2.144317165	2.23525E-38	2.144317165	stage_E14_vs_E10	ordinary_muscle_development	D_vs_E10;stage_1W_vs_E10;stage_3W_vs_E10;stage	1	TRUE
ENSGALG00010008376	MMRN1	PRJNA729271	-3.687843346	2.36268E-38	3.687843346	stage_E14_vs_E10	ordinary_muscle_development	D_vs_E10;stage_1W_vs_E10;stage_3W_vs_E10;stage	1	TRUE
ENSGALG00010011871	CCT2	PRJNA729271	-1.289241407	2.47476E-38	1.289241407	stage_E14_vs_E10	ordinary_muscle_development	D_vs_E10;stage_1W_vs_E10;stage_3W_vs_E10;stage	1	TRUE
ENSGALG00010002523	DNAJB13	PRJNA729271	5.432601533	2.49346E-38	5.432601533	stage_E14_vs_E10	ordinary_muscle_development	D_vs_E10;stage_1W_vs_E10;stage_3W_vs_E10;stage	1	TRUE
ENSGALG00010013177	OXR1	PRJNA729271	0.907691964	2.55163E-38	0.907691964	stage_E14_vs_E10	ordinary_muscle_development		0	FALSE
ENSGALG00010027743	C1orf74	PRJNA729271	2.377556648	2.58372E-38	2.377556648	stage_E14_vs_E10	ordinary_muscle_development	D_vs_E10;stage_1W_vs_E10;stage_3W_vs_E10;stage	1	TRUE
ENSGALG00010017920	AP3M2	PRJNA729271	-1.075360827	2.5863E-38	1.075360827	stage_E14_vs_E10	ordinary_muscle_development	D_vs_E10;stage_1W_vs_E10;stage_3W_vs_E10;stage	1	TRUE
ENSGALG00010021255	EIF3F	PRJNA729271	1.200508949	2.78218E-38	1.200508949	stage_E14_vs_E10	ordinary_muscle_development	D_vs_E10;stage_1W_vs_E10;stage_3W_vs_E10;stage	1	TRUE
ENSGALG00010023336	GPR162	PRJNA729271	-2.062473355	2.80005E-38	2.062473355	stage_E14_vs_E10	ordinary_muscle_development	D_vs_E10;stage_1W_vs_E10;stage_3W_vs_E10;stage	1	TRUE
ENSGALG00010011825	TAFA2	PRJNA729271	-6.101665888	2.87209E-38	6.101665888	stage_E14_vs_E10	ordinary_muscle_development	D_vs_E10;stage_1W_vs_E10;stage_3W_vs_E10;stage	1	TRUE
ENSGALG00010017312	PTP4A2	PRJNA729271	2.023152591	2.9168E-38	2.023152591	stage_E14_vs_E10	ordinary_muscle_development	D_vs_E10;stage_1W_vs_E10;stage_3W_vs_E10;stage	1	TRUE
ENSGALG00010029519	STX1A	PRJNA729271	1.391430805	3.03621E-38	1.391430805	stage_E14_vs_E10	ordinary_muscle_development	D_vs_E10;stage_1W_vs_E10;stage_3W_vs_E10;stage	1	TRUE
ENSGALG00010029319	BRIP1	PRJNA729271	-1.695322653	3.22781E-38	1.695322653	stage_E14_vs_E10	ordinary_muscle_development	D_vs_E10;stage_1W_vs_E10;stage_3W_vs_E10;stage	1	TRUE
ENSGALG00010010146	PHACTR1	PRJNA729271	-4.833041447	3.23469E-38	4.833041447	stage_E14_vs_E10	ordinary_muscle_development	D_vs_E10;stage_1W_vs_E10;stage_3W_vs_E10;stage	1	TRUE
ENSGALG00010004135	NUP54	PRJNA729271	-0.877227166	3.2715E-38	0.877227166	stage_E14_vs_E10	ordinary_muscle_development		0	FALSE
ENSGALG00010019289	FKBP9	PRJNA729271	-2.181376056	3.55885E-38	2.181376056	stage_E14_vs_E10	ordinary_muscle_development	D_vs_E10;stage_1W_vs_E10;stage_3W_vs_E10;stage	1	TRUE
ENSGALG00010009079	KCTD15	PRJNA729271	-1.293937663	3.58028E-38	1.293937663	stage_E14_vs_E10	ordinary_muscle_development	D_vs_E10;stage_1W_vs_E10;stage_3W_vs_E10;stage	1	TRUE
ENSGALG00010024505	DNASE2B	PRJNA729271	2.742638497	3.59406E-38	2.742638497	stage_E14_vs_E10	ordinary_muscle_development	D_vs_E10;stage_1W_vs_E10;stage_3W_vs_E10;stage	1	TRUE
ENSGALG00010008107	TUBB3	PRJNA729271	-2.752833877	3.73256E-38	2.752833877	stage_E14_vs_E10	ordinary_muscle_development	D_vs_E10;stage_1W_vs_E10;stage_3W_vs_E10;stage	1	TRUE
ENSGALG00010009059	MED29	PRJNA729271	1.639018374	4.03168E-38	1.639018374	stage_E14_vs_E10	ordinary_muscle_development	D_vs_E10;stage_1W_vs_E10;stage_3W_vs_E10;stage	1	TRUE
ENSGALG00010024091	ETFA	PRJNA729271	1.517423602	4.05497E-38	1.517423602	stage_E14_vs_E10	ordinary_muscle_development	D_vs_E10;stage_1W_vs_E10;stage_3W_vs_E10;stage	1	TRUE
ENSGALG00010015624	ITGA1	PRJNA729271	1.6859426	4.27608E-38	1.6859426	stage_E14_vs_E10	ordinary_muscle_development	D_vs_E10;stage_1W_vs_E10;stage_3W_vs_E10;stage	1	TRUE
ENSGALG00010003103	YTHDF3	PRJNA729271	1.125451876	4.29816E-38	1.125451876	stage_E14_vs_E10	ordinary_muscle_development	D_vs_E10;stage_1W_vs_E10;stage_3W_vs_E10;stage	1	TRUE
ENSGALG00010006913	HSPH1	PRJNA729271	-2.503066264	4.42574E-38	2.503066264	stage_E14_vs_E10	ordinary_muscle_development	D_vs_E10;stage_1W_vs_E10;stage_3W_vs_E10;stage	1	TRUE
ENSGALG00010023275	TWIST3	PRJNA729271	-9.448209819	4.53126E-38	9.448209819	stage_E14_vs_E10	ordinary_muscle_development	D_vs_E10;stage_1W_vs_E10;stage_3W_vs_E10;stage	1	TRUE
ENSGALG00010018140	CHPF	PRJNA729271	-1.400010186	4.53431E-38	1.400010186	stage_E14_vs_E10	ordinary_muscle_development	D_vs_E10;stage_1W_vs_E10;stage_3W_vs_E10;stage	1	TRUE
ENSGALG00010010206	SCARA5	PRJNA729271	2.066501475	4.56133E-38	2.066501475	stage_E14_vs_E10	ordinary_muscle_development	D_vs_E10;stage_1W_vs_E10;stage_3W_vs_E10;stage	1	TRUE
ENSGALG00010028021	ATP5F1D	PRJNA729271	1.893573833	4.76971E-38	1.893573833	stage_E14_vs_E10	ordinary_muscle_development	D_vs_E10;stage_1W_vs_E10;stage_3W_vs_E10;stage	1	TRUE
ENSGALG00010024792	BZW1	PRJNA729271	-0.905171162	4.79993E-38	0.905171162	stage_E14_vs_E10	ordinary_muscle_development		0	FALSE
ENSGALG00010004993	SULT1B	PRJNA729271	3.142091445	4.88206E-38	3.142091445	stage_E14_vs_E10	ordinary_muscle_development	D_vs_E10;stage_1W_vs_E10;stage_3W_vs_E10;stage	1	TRUE
ENSGALG00010022314	DUSP13B	PRJNA729271	7.20733999	4.92802E-38	7.20733999	stage_E14_vs_E10	ordinary_muscle_development	D_vs_E10;stage_1W_vs_E10;stage_3W_vs_E10;stage	1	TRUE
ENSGALG00010026300	HMGA1	PRJNA729271	-1.452001804	4.95339E-38	1.452001804	stage_E14_vs_E10	ordinary_muscle_development	D_vs_E10;stage_1W_vs_E10;stage_3W_vs_E10;stage	1	TRUE
ENSGALG00010026492	SPECC1L	PRJNA729271	-0.989872501	5.05116E-38	0.989872501	stage_E14_vs_E10	ordinary_muscle_development		0	FALSE
ENSGALG00010013516	CSRP2	PRJNA729271	-1.765063698	5.19416E-38	1.765063698	stage_E14_vs_E10	ordinary_muscle_development	D_vs_E10;stage_1W_vs_E10;stage_3W_vs_E10;stage	1	TRUE
ENSGALG00010027136	MAPKAPK2	PRJNA729271	1.876682235	5.2263E-38	1.876682235	stage_E14_vs_E10	ordinary_muscle_development	D_vs_E10;stage_1W_vs_E10;stage_3W_vs_E10;stage	1	TRUE
ENSGALG00010022850	ULK1	PRJNA729271	3.254403475	5.27762E-38	3.254403475	stage_E14_vs_E10	ordinary_muscle_development	D_vs_E10;stage_1W_vs_E10;stage_3W_vs_E10;stage	1	TRUE
ENSGALG00010014903	MCF2L	PRJNA729271	2.00232705	5.3351E-38	2.00232705	stage_E14_vs_E10	ordinary_muscle_development	D_vs_E10;stage_1W_vs_E10;stage_3W_vs_E10;stage	1	TRUE
ENSGALG00010029278	MED31	PRJNA729271	-0.942216449	5.33779E-38	0.942216449	stage_E14_vs_E10	ordinary_muscle_development		0	FALSE
ENSGALG00010002308	GXYLT1	PRJNA729271	-1.363071898	5.34834E-38	1.363071898	stage_E14_vs_E10	ordinary_muscle_development	D_vs_E10;stage_1W_vs_E10;stage_3W_vs_E10;stage	1	TRUE
ENSGALG00010012848	SERINC1	PRJNA729271	1.20533151	5.44976E-38	1.20533151	stage_E14_vs_E10	ordinary_muscle_development	D_vs_E10;stage_1W_vs_E10;stage_3W_vs_E10;stage	1	TRUE
ENSGALG00010005464	SLC39A8	PRJNA729271	-4.599165459	5.60703E-38	4.599165459	stage_E14_vs_E10	ordinary_muscle_development	D_vs_E10;stage_1W_vs_E10;stage_3W_vs_E10;stage	1	TRUE
ENSGALG00010015785	FGL1	PRJNA729271	2.485318566	5.61907E-38	2.485318566	stage_E14_vs_E10	ordinary_muscle_development	D_vs_E10;stage_1W_vs_E10;stage_3W_vs_E10;stage	1	TRUE
ENSGALG00010006252	COX7C	PRJNA729271	1.779705768	5.80994E-38	1.779705768	stage_E14_vs_E10	ordinary_muscle_development	D_vs_E10;stage_1W_vs_E10;stage_3W_vs_E10;stage	1	TRUE
ENSGALG00010014707	KPNA5	PRJNA729271	1.685246371	5.84331E-38	1.685246371	stage_E14_vs_E10	ordinary_muscle_development	D_vs_E10;stage_1W_vs_E10;stage_3W_vs_E10;stage	1	TRUE
ENSGALG00010009228	ATP6V0E1	PRJNA729271	1.288682784	6.11429E-38	1.288682784	stage_E14_vs_E10	ordinary_muscle_development	D_vs_E10;stage_1W_vs_E10;stage_3W_vs_E10;stage	1	TRUE
ENSGALG00010007748	CSGALNACT1	PRJNA729271	-3.609606753	6.12447E-38	3.609606753	stage_E14_vs_E10	ordinary_muscle_development	D_vs_E10;stage_1W_vs_E10;stage_3W_vs_E10;stage	1	TRUE
ENSGALG00010028008	NTMT1	PRJNA729271	1.459964371	6.4196E-38	1.459964371	stage_E14_vs_E10	ordinary_muscle_development	D_vs_E10;stage_1W_vs_E10;stage_3W_vs_E10;stage	1	TRUE
ENSGALG00010017805	DPP8	PRJNA729271	1.382305075	6.69795E-38	1.382305075	stage_E14_vs_E10	ordinary_muscle_development	D_vs_E10;stage_1W_vs_E10;stage_3W_vs_E10;stage	1	TRUE
ENSGALG00010010246	CLTB	PRJNA729271	0.865886261	6.7778E-38	0.865886261	stage_E14_vs_E10	ordinary_muscle_development		0	FALSE
ENSGALG00010011464	FANCG	PRJNA729271	-2.213040696	6.80529E-38	2.213040696	stage_E14_vs_E10	ordinary_muscle_development	D_vs_E10;stage_1W_vs_E10;stage_3W_vs_E10;stage	1	TRUE
ENSGALG00010014856	MMGT1	PRJNA729271	0.924897433	6.89383E-38	0.924897433	stage_E14_vs_E10	ordinary_muscle_development		0	FALSE
ENSGALG00010023124	NSUN4	PRJNA729271	1.730796274	7.31576E-38	1.730796274	stage_E14_vs_E10	ordinary_muscle_development	D_vs_E10;stage_1W_vs_E10;stage_3W_vs_E10;stage	1	TRUE
ENSGALG00010006085	DLST	PRJNA729271	1.168874538	7.34701E-38	1.168874538	stage_E14_vs_E10	ordinary_muscle_development	D_vs_E10;stage_1W_vs_E10;stage_3W_vs_E10;stage	1	TRUE
ENSGALG00010023183	ENSGALG00010023183	PRJNA729271	5.353905775	7.37954E-38	5.353905775	stage_E14_vs_E10	ordinary_muscle_development	D_vs_E10;stage_1W_vs_E10;stage_3W_vs_E10;stage	1	TRUE
ENSGALG00010006653	CFL2	PRJNA729271	1.377080125	7.65382E-38	1.377080125	stage_E14_vs_E10	ordinary_muscle_development	D_vs_E10;stage_1W_vs_E10;stage_3W_vs_E10;stage	1	TRUE
ENSGALG00010001382	BNIP3	PRJNA729271	2.510118038	7.83636E-38	2.510118038	stage_E14_vs_E10	ordinary_muscle_development	D_vs_E10;stage_1W_vs_E10;stage_3W_vs_E10;stage	1	TRUE
ENSGALG00010024582	TLN2	PRJNA729271	1.397040319	7.86571E-38	1.397040319	stage_E14_vs_E10	ordinary_muscle_development	D_vs_E10;stage_1W_vs_E10;stage_3W_vs_E10;stage	1	TRUE
ENSGALG00010020193	VSTM4	PRJNA729271	-2.378722669	8.05889E-38	2.378722669	stage_E14_vs_E10	ordinary_muscle_development	D_vs_E10;stage_1W_vs_E10;stage_3W_vs_E10;stage	1	TRUE
ENSGALG00010023169	RAN	PRJNA729271	-1.271729422	8.07307E-38	1.271729422	stage_E14_vs_E10	ordinary_muscle_development	D_vs_E10;stage_1W_vs_E10;stage_3W_vs_E10;stage	1	TRUE
ENSGALG00010003517	GTF2F2	PRJNA729271	-1.162965002	8.24895E-38	1.162965002	stage_E14_vs_E10	ordinary_muscle_development	D_vs_E10;stage_1W_vs_E10;stage_3W_vs_E10;stage	1	TRUE
ENSGALG00010007931	GBE1	PRJNA729271	2.455807974	8.45522E-38	2.455807974	stage_E14_vs_E10	ordinary_muscle_development	D_vs_E10;stage_1W_vs_E10;stage_3W_vs_E10;stage	1	TRUE
ENSGALG00010025496	ATG101	PRJNA729271	1.429861585	8.46939E-38	1.429861585	stage_E14_vs_E10	ordinary_muscle_development	D_vs_E10;stage_1W_vs_E10;stage_3W_vs_E10;stage	1	TRUE
ENSGALG00010002779	FBXO8	PRJNA729271	1.083505347	9.03496E-38	1.083505347	stage_E14_vs_E10	ordinary_muscle_development	D_vs_E10;stage_1W_vs_E10;stage_3W_vs_E10;stage	1	TRUE
ENSGALG00010024259	ENSGALG00010024259	PRJNA729271	1.751002192	9.46981E-38	1.751002192	stage_E14_vs_E10	ordinary_muscle_development	D_vs_E10;stage_1W_vs_E10;stage_3W_vs_E10;stage	1	TRUE
ENSGALG00010018633	ENSGALG00010018633	PRJNA729271	2.308796095	9.68358E-38	2.308796095	stage_E14_vs_E10	ordinary_muscle_development	D_vs_E10;stage_1W_vs_E10;stage_3W_vs_E10;stage	1	TRUE

ENSGALG00010019659	MRPL14	PRJNA729271	1.619216411	9.79529E-38	1.619216411	stage_E14_vs_E10	ordinary_muscle_development	D_vs_E10;stage_1W_vs_E10;stage_3W_vs_E10;stage	1	TRUE
ENSGALG00010003791	ITGB8	PRJNA729271	-2.340437856	1.01449E-37	2.340437856	stage_E14_vs_E10	ordinary_muscle_development	D_vs_E10;stage_1W_vs_E10;stage_3W_vs_E10;stage	1	TRUE
ENSGALG00010027926	CKS1B	PRJNA729271	-3.353381862	1.06859E-37	3.353381862	stage_E14_vs_E10	ordinary_muscle_development	D_vs_E10;stage_1W_vs_E10;stage_3W_vs_E10;stage	1	TRUE
ENSGALG00010026002	FTH1	PRJNA729271	1.842946672	1.09233E-37	1.842946672	stage_E14_vs_E10	ordinary_muscle_development	D_vs_E10;stage_1W_vs_E10;stage_3W_vs_E10;stage	1	TRUE
ENSGALG00010009594	HINT2	PRJNA729271	2.069842463	1.12768E-37	2.069842463	stage_E14_vs_E10	ordinary_muscle_development	D_vs_E10;stage_1W_vs_E10;stage_3W_vs_E10;stage	1	TRUE
ENSGALG00010021580	KCTD10	PRJNA729271	-1.444939252	1.14915E-37	1.444939252	stage_E14_vs_E10	ordinary_muscle_development	D_vs_E10;stage_1W_vs_E10;stage_3W_vs_E10;stage	1	TRUE
ENSGALG00010010201	RIOK1	PRJNA729271	-1.357826463	1.16852E-37	1.357826463	stage_E14_vs_E10	ordinary_muscle_development	D_vs_E10;stage_1W_vs_E10;stage_3W_vs_E10;stage	1	TRUE
ENSGALG00010004085	RB1CC1	PRJNA729271	1.823350764	1.18231E-37	1.823350764	stage_E14_vs_E10	ordinary_muscle_development	D_vs_E10;stage_1W_vs_E10;stage_3W_vs_E10;stage	1	TRUE
ENSGALG00010008088	SLC35A4	PRJNA729271	0.9021272	1.19974E-37	0.9021272	stage_E14_vs_E10	ordinary_muscle_development		0	FALSE
ENSGALG00010025156	DLAT	PRJNA729271	1.496970396	1.21306E-37	1.496970396	stage_E14_vs_E10	ordinary_muscle_development	D_vs_E10;stage_1W_vs_E10;stage_3W_vs_E10;stage	1	TRUE
ENSGALG00010018434	HSPBAP1	PRJNA729271	2.060691699	1.21668E-37	2.060691699	stage_E14_vs_E10	ordinary_muscle_development	D_vs_E10;stage_1W_vs_E10;stage_3W_vs_E10;stage	1	TRUE
ENSGALG00010018042	ZWILCH	PRJNA729271	-1.83032444	1.23168E-37	1.83032444	stage_E14_vs_E10	ordinary_muscle_development	D_vs_E10;stage_1W_vs_E10;stage_3W_vs_E10;stage	1	TRUE
ENSGALG00010009086	UBE2D1	PRJNA729271	2.211423568	1.25052E-37	2.211423568	stage_E14_vs_E10	ordinary_muscle_development	D_vs_E10;stage_1W_vs_E10;stage_3W_vs_E10;stage	1	TRUE
ENSGALG00010023127	CERS5	PRJNA729271	-1.283407744	1.25895E-37	1.283407744	stage_E14_vs_E10	ordinary_muscle_development	D_vs_E10;stage_1W_vs_E10;stage_3W_vs_E10;stage	1	TRUE
ENSGALG00010015834	STARD8	PRJNA729271	1.726334924	1.39861E-37	1.726334924	stage_E14_vs_E10	ordinary_muscle_development	D_vs_E10;stage_1W_vs_E10;stage_3W_vs_E10;stage	1	TRUE
ENSGALG00010014026	SH3BP1	PRJNA729271	4.074348472	1.41988E-37	4.074348472	stage_E14_vs_E10	ordinary_muscle_development	D_vs_E10;stage_1W_vs_E10;stage_3W_vs_E10;stage	1	TRUE
ENSGALG00010001712	ATP6AP1	PRJNA729271	2.057982931	1.48588E-37	2.057982931	stage_E14_vs_E10	ordinary_muscle_development	D_vs_E10;stage_1W_vs_E10;stage_3W_vs_E10;stage	1	TRUE
ENSGALG00010020732	ARIH2	PRJNA729271	1.850741632	1.50601E-37	1.850741632	stage_E14_vs_E10	ordinary_muscle_development	D_vs_E10;stage_1W_vs_E10;stage_3W_vs_E10;stage	1	TRUE
ENSGALG00010026409	VILL	PRJNA729271	-3.54756073	1.55926E-37	3.54756073	stage_E14_vs_E10	ordinary_muscle_development	D_vs_E10;stage_1W_vs_E10;stage_3W_vs_E10;stage	1	TRUE
ENSGALG00010002750	ENSGALG00010002750	PRJNA729271	-3.052701365	1.56492E-37	3.052701365	stage_E14_vs_E10	ordinary_muscle_development	D_vs_E10;stage_1W_vs_E10;stage_3W_vs_E10;stage	1	TRUE
ENSGALG00010024563	ESPL1	PRJNA729271	-2.21328302	1.57803E-37	2.21328302	stage_E14_vs_E10	ordinary_muscle_development	D_vs_E10;stage_1W_vs_E10;stage_3W_vs_E10;stage	1	TRUE
ENSGALG00010003025	MFHAS1	PRJNA729271	1.589548975	1.59397E-37	1.589548975	stage_E14_vs_E10	ordinary_muscle_development	D_vs_E10;stage_1W_vs_E10;stage_3W_vs_E10;stage	1	TRUE
ENSGALG00010019999	ERLIN1	PRJNA729271	-0.834302297	1.61072E-37	0.834302297	stage_E14_vs_E10	ordinary_muscle_development		0	FALSE
ENSGALG00010022148	TALDO1	PRJNA729271	-1.148146556	1.61629E-37	1.148146556	stage_E14_vs_E10	ordinary_muscle_development	D_vs_E10;stage_1W_vs_E10;stage_3W_vs_E10;stage	1	TRUE
ENSGALG00010010834	KGD4	PRJNA729271	1.610875169	1.69409E-37	1.610875169	stage_E14_vs_E10	ordinary_muscle_development	D_vs_E10;stage_1W_vs_E10;stage_3W_vs_E10;stage	1	TRUE
ENSGALG00010028605	GTPBP3	PRJNA729271	2.740026333	1.6955E-37	2.740026333	stage_E14_vs_E10	ordinary_muscle_development	D_vs_E10;stage_1W_vs_E10;stage_3W_vs_E10;stage	1	TRUE
ENSGALG00010024277	ITGB3BP	PRJNA729271	-2.560315018	1.75046E-37	2.560315018	stage_E14_vs_E10	ordinary_muscle_development	D_vs_E10;stage_1W_vs_E10;stage_3W_vs_E10;stage	1	TRUE
ENSGALG00010005476	NFKB1	PRJNA729271	1.481013238	1.87099E-37	1.481013238	stage_E14_vs_E10	ordinary_muscle_development	D_vs_E10;stage_1W_vs_E10;stage_3W_vs_E10;stage	1	TRUE
ENSGALG00010022681	PPP2R3A	PRJNA729271	1.20580143	1.93423E-37	1.20580143	stage_E14_vs_E10	ordinary_muscle_development	D_vs_E10;stage_1W_vs_E10;stage_3W_vs_E10;stage	1	TRUE
ENSGALG00010010949	FZD3	PRJNA729271	-3.100189822	1.95338E-37	3.100189822	stage_E14_vs_E10	ordinary_muscle_development	D_vs_E10;stage_1W_vs_E10;stage_3W_vs_E10;stage	1	TRUE
ENSGALG00010004210	SYNCRIP	PRJNA729271	-0.922891551	1.97595E-37	0.922891551	stage_E14_vs_E10	ordinary_muscle_development		0	FALSE
ENSGALG00010017266	PMM2	PRJNA729271	-1.436312707	2.07141E-37	1.436312707	stage_E14_vs_E10	ordinary_muscle_development	D_vs_E10;stage_1W_vs_E10;stage_3W_vs_E10;stage	1	TRUE
ENSGALG00010027621	TSPO2	PRJNA729271	-6.468454989	2.08709E-37	6.468454989	stage_E14_vs_E10	ordinary_muscle_development	D_vs_E10;stage_1W_vs_E10;stage_3W_vs_E10;stage	1	TRUE
ENSGALG00010018837	RCOR3	PRJNA729271	-1.235136291	2.10012E-37	1.235136291	stage_E14_vs_E10	ordinary_muscle_development	D_vs_E10;stage_1W_vs_E10;stage_3W_vs_E10;stage	1	TRUE
ENSGALG00010023043	CNP	PRJNA729271	1.871015778	2.15775E-37	1.871015778	stage_E14_vs_E10	ordinary_muscle_development	D_vs_E10;stage_1W_vs_E10;stage_3W_vs_E10;stage	1	TRUE
ENSGALG00010016305	DENND4C	PRJNA729271	1.24047006	2.16085E-37	1.24047006	stage_E14_vs_E10	ordinary_muscle_development	D_vs_E10;stage_1W_vs_E10;stage_3W_vs_E10;stage	1	TRUE
ENSGALG00010018904	UGP2	PRJNA729271	3.507160103	2.17729E-37	3.507160103	stage_E14_vs_E10	ordinary_muscle_development	D_vs_E10;stage_1W_vs_E10;stage_3W_vs_E10;stage	1	TRUE
ENSGALG00010010605	NUF2	PRJNA729271	-3.517422041	2.24755E-37	3.517422041	stage_E14_vs_E10	ordinary_muscle_development	D_vs_E10;stage_1W_vs_E10;stage_3W_vs_E10;stage	1	TRUE
ENSGALG00010000786	ANKRD33B	PRJNA729271	3.91378179	2.31793E-37	3.91378179	stage_E14_vs_E10	ordinary_muscle_development	D_vs_E10;stage_1W_vs_E10;stage_3W_vs_E10;stage	1	TRUE
ENSGALG00010020177	KCNG3	PRJNA729271	-3.895259682	2.31839E-37	3.895259682	stage_E14_vs_E10	ordinary_muscle_development	D_vs_E10;stage_1W_vs_E10;stage_3W_vs_E10;stage	1	TRUE
ENSGALG00010014813	SASH3	PRJNA729271	3.233231133	2.38306E-37	3.233231133	stage_E14_vs_E10	ordinary_muscle_development	D_vs_E10;stage_1W_vs_E10;stage_3W_vs_E10;stage	1	TRUE
ENSGALG00010023720	SCAMP5	PRJNA729271	-1.29597156	2.41124E-37	1.29597156	stage_E14_vs_E10	ordinary_muscle_development	D_vs_E10;stage_1W_vs_E10;stage_3W_vs_E10;stage	1	TRUE
ENSGALG00010020655	RIC3	PRJNA729271	-1.847510921	2.44218E-37	1.847510921	stage_E14_vs_E10	ordinary_muscle_development	D_vs_E10;stage_1W_vs_E10;stage_3W_vs_E10;stage	1	TRUE
ENSGALG00010023110	LIMA1	PRJNA729271	-2.009683142	2.45524E-37	2.009683142	stage_E14_vs_E10	ordinary_muscle_development	D_vs_E10;stage_1W_vs_E10;stage_3W_vs_E10;stage	1	TRUE
ENSGALG00010029859	CSNK1D	PRJNA729271	-0.693527167	2.64014E-37	0.693527167	stage_E14_vs_E10	ordinary_muscle_development		0	FALSE
ENSGALG00010028180	ODF2	PRJNA729271	-1.372784439	2.72053E-37	1.372784439	stage_E14_vs_E10	ordinary_muscle_development	D_vs_E10;stage_1W_vs_E10;stage_3W_vs_E10;stage	1	TRUE
ENSGALG00010010405	EVA1C	PRJNA729271	2.93781679	2.72562E-37	2.93781679	stage_E14_vs_E10	ordinary_muscle_development	D_vs_E10;stage_1W_vs_E10;stage_3W_vs_E10;stage	1	TRUE
ENSGALG00010015620	PDRG1	PRJNA729271	1.84518235	2.73E-37	1.84518235	stage_E14_vs_E10	ordinary_muscle_development	D_vs_E10;stage_1W_vs_E10;stage_3W_vs_E10;stage	1	TRUE
ENSGALG00010022041	MAPK8IP1	PRJNA729271	-2.090259955	2.73425E-37	2.090259955	stage_E14_vs_E10	ordinary_muscle_development	D_vs_E10;stage_1W_vs_E10;stage_3W_vs_E10;stage	1	TRUE
ENSGALG00010020092	SDHAF2	PRJNA729271	1.761297252	2.80621E-37	1.761297252	stage_E14_vs_E10	ordinary_muscle_development	D_vs_E10;stage_1W_vs_E10;stage_3W_vs_E10;stage	1	TRUE
ENSGALG00010014883	PPAT	PRJNA729271	-1.622797189	2.82951E-37	1.622797189	stage_E14_vs_E10	ordinary_muscle_development	D_vs_E10;stage_1W_vs_E10;stage_3W_vs_E10;stage	1	TRUE
ENSGALG00010029930	ARHGAP44	PRJNA729271	2.638606122	2.93812E-37	2.638606122	stage_E14_vs_E10	ordinary_muscle_development	D_vs_E10;stage_1W_vs_E10;stage_3W_vs_E10;stage	1	TRUE
ENSGALG00010015695	ASAH1	PRJNA729271	1.209208798	2.93895E-37	1.209208798	stage_E14_vs_E10	ordinary_muscle_development	D_vs_E10;stage_1W_vs_E10;stage_3W_vs_E10;stage	1	TRUE
ENSGALG00010002368	UVRAG	PRJNA729271	1.226737892	3.00122E-37	1.226737892	stage_E14_vs_E10	ordinary_muscle_development	D_vs_E10;stage_1W_vs_E10;stage_3W_vs_E10;stage	1	TRUE
ENSGALG00010027178	C4BPM	PRJNA729271	1.038993642	3.01327E-37	1.038993642	stage_E14_vs_E10	ordinary_muscle_development	D_vs_E10;stage_1W_vs_E10;stage_3W_vs_E10;stage	1	TRUE
ENSGALG00010021801	VAMP1	PRJNA729271	3.118910853	3.19504E-37	3.118910853	stage_E14_vs_E10	ordinary_muscle_development	D_vs_E10;stage_1W_vs_E10;stage_3W_vs_E10;stage	1	TRUE
ENSGALG00010023871	UQCRFS1	PRJNA729271	1.809109253	3.31418E-37	1.809109253	stage_E14_vs_E10	ordinary_muscle_development	D_vs_E10;stage_1W_vs_E10;stage_3W_vs_E10;stage	1	TRUE
ENSGALG00010011998	MPPED1	PRJNA729271	-0.98571887	3.54536E-37	0.98571887	stage_E14_vs_E10	ordinary_muscle_development		0	FALSE
ENSGALG00010004236	DBF4	PRJNA729271	-1.645327956	3.61913E-37	1.645327956	stage_E14_vs_E10	ordinary_muscle_development	D_vs_E10;stage_1W_vs_E10;stage_3W_vs_E10;stage	1	TRUE
ENSGALG00010000024	COX3	PRJNA729271	2.855447375	3.69304E-37	2.855447375	stage_E14_vs_E10	ordinary_muscle_development	D_vs_E10;stage_1W_vs_E10;stage_3W_vs_E10;stage	1	TRUE
ENSGALG00010013463	ENSGALG00010013463	PRJNA729271	2.683085949	3.81114E-37	2.683085949	stage_E14_vs_E10	ordinary_muscle_development	D_vs_E10;stage_1W_vs_E10;stage_3W_vs_E10;stage	1	TRUE
ENSGALG00010026436	PLCD1	PRJNA729271	-0.995751824	3.88244E-37	0.995751824	stage_E14_vs_E10	ordinary_muscle_development		0	FALSE
ENSGALG00010012297	ABLIM3	PRJNA729271	2.403953359	3.91291E-37	2.403953359	stage_E14_vs_E10	ordinary_muscle_development	D_vs_E10;stage_1W_vs_E10;stage_3W_vs_E10;stage	1	TRUE
ENSGALG00010021229	GLO1	PRJNA729271	2.296121457	4.1725E-37	2.296121457	stage_E14_vs_E10	ordinary_muscle_development	D_vs_E10;stage_1W_vs_E10;stage_3W_vs_E10;stage	1	TRUE
ENSGALG00010029163	ANKFY1	PRJNA729271	-1.224125279	4.33467E-37	1.224125279	stage_E14_vs_E10	ordinary_muscle_development	D_vs_E10;stage_1W_vs_E10;stage_3W_vs_E10;stage	1	TRUE
ENSGALG00010006716	ENSGALG00010006716	PRJNA729271	1.246728011	4.37863E-37	1.246728011	stage_E14_vs_E10	ordinary_muscle_development	D_vs_E10;stage_1W_vs_E10;stage_3W_vs_E10;stage	1	TRUE
ENSGALG00010028600	PSMD4	PRJNA729271	1.697070355	4.47297E-37	1.697070355	stage_E14_vs_E10	ordinary_muscle_development	D_vs_E10;stage_1W_vs_E10;stage_3W_vs_E10;stage	1	TRUE
ENSGALG00010016240	EIF4A2	PRJNA729271	1.291747235	4.49165E-37	1.291747235	stage_E14_vs_E10	ordinary_muscle_development	D_vs_E10;stage_1W_vs_E10;stage_3W_vs_E10;stage	1	TRUE
ENSGALG00010005176	BAMBI	PRJNA729271	-4.191649125	4.5257E-37	4.191649125	stage_E14_vs_E10	ordinary_muscle_development	D_vs_E10;stage_1W_vs_E10;stage_3W_vs_E10;stage	1	TRUE
ENSGALG00010027492	PCP4L1	PRJNA729271	6.221958146	4.55719E-37	6.221958146	stage_E14_vs_E10	ordinary_muscle_development	D_vs_E10;stage_1W_vs_E10;stage_3W_vs_E10;stage	1	TRUE
ENSGALG00010028923	ENSGALG00010028923	PRJNA729271	2.314544129	4.56765E-37	2.314544129	stage_E14_vs_E10	ordinary_muscle_development	D_vs_E10;stage_1W_vs_E10;stage_3W_vs_E10;stage	1	TRUE
ENSGALG00010021791	PLEKHM2	PRJNA729271	2.367792265	4.86817E-37	2.367792265	stage_E14_vs_E10	ordinary_muscle_development	D_vs_E10;stage_1W_vs_E10;stage_3W_vs_E10;stage	1	TRUE
ENSGALG00010010781	NAPA	PRJNA729271	1.247918194	4.91167E-37	1.247918194	stage_E14_vs_E10	ordinary_muscle_development	D_vs_E10;stage_1W_vs_E10;stage_3W_vs_E10;stage	1	TRUE
ENSGALG00010006212	FOS	PRJNA729271	4.718872026	5.43293E-37	4.718872026	stage_E14_vs_E10	ordinary_muscle_development	D_vs_E10;stage_1W_vs_E10;stage_3W_vs_E10;stage	1	TRUE
ENSGALG00010000724	OBI1	PRJNA729271	-1.920926931	5.43445E-37	1.920926931	stage_E14_vs_E10	ordinary_muscle_development	D_vs_E10;stage_1W_vs_E10;stage_3W_vs_E10;stage	1	TRUE
ENSGALG00010001115	PFN2	PRJNA729271	-1.234716623	5.52694E-37	1.234716623	stage_E14_vs_E10	ordinary_muscle_development	D_vs_E10;stage_1W_vs_E10;stage_3W_vs_E10;stage	1	TRUE
ENSGALG00010005540	PCMT1	PRJNA729271	1.761935647	5.66476E-37	1.761935647	stage_E14_vs_E10	ordinary_muscle_development	D_vs_E10;stage_1W_vs_E10;stage_3W_vs_E10;stage	1	TRUE
ENSGALG00010027993	CLIC3	PRJNA729271	2.039678266	5.70178E-37	2.039678266	stage_E14_vs_E10	ordinary_muscle_development	D_vs_E10;stage_1W_vs_E10;stage_3W_vs_E10;stage	1	TRUE
ENSGALG00010019256	BBS9	PRJNA729271	-1.40094782	5.70395E-37	1.40094782	stage_E14_vs_E10	ordinary_muscle_development	D_vs_E10;stage_1W_vs_E10;stage_3W_vs_E10;stage	1	TRUE
ENSGALG00010019954	CYB561A3	PRJNA729271	1.591589906	5.72863E-37	1.591589906	stage_E14_vs_E10	ordinary_muscle_development	D_vs_E10;stage_1W_vs_E10;stage_3W_vs_E10;stage	1	TRUE
ENSGALG00010012250	NANS	PRJNA729271	-1.461295513	5.77878E-37	1.461295513	stage_E14_vs_E10	ordinary_muscle_development	D_vs_E10;stage_1W_vs_E10;stage_3W_vs_E10;stage	1	TRUE
ENSGALG00010015855	NIPAL3	PRJNA729271	-1.055223577	6.15991E-37	1.055223577	stage_E14_vs_E10	ordinary_muscle_development	D_vs_E10;stage_1W_vs_E10;stage_3W_vs_E10;stage	1	TRUE
ENSGALG00010024152	ZNF423	PRJNA729271	-1.389658593	6.20569E-37	1.389658593	stage_E14_vs_E10	ordinary_muscle_development	D_vs_E10;stage_1W_vs_E10;stage_3W_vs_E10;stage	1	TRUE
ENSGALG00010005265	DYNC2H1	PRJNA729271	-1.23661696	6.33403E-37	1.23661696	stage_E14_vs_E10	ordinary_muscle_development	D_vs_E10;stage_1W_vs_E10;stage_3W_vs_E10;stage	1	TRUE
ENSGALG00010027725	BTN1A1	PRJNA729271	3.213626543	6.35175E-37	3.213626543	stage_E14_vs_E10	ordinary_muscle_development	D_vs_E10;stage_1W_vs_E10;stage_3W_vs_E10;stage	1	TRUE
ENSGALG00010002712	PTPRE	PRJNA729271	1.586475484	6.35175E-37	1.586475484	stage_E14_vs_E10	ordinary_muscle_development	D_vs_E10;stage_1W_vs_E10;stage_3W_vs_E10;stage	1	TRUE
ENSGALG00010017449	TRMT6	PRJNA729271	-1.135645609	6.52539E-37	1.135645609	stage_E14_vs_E10	ordinary_muscle_development	D_vs_E10;stage_1W_vs_E10;stage_3W_vs_E10;stage	1	TRUE
ENSGALG00010012133	RAB3IP	PRJNA729271	-3.149899687	7.0829E-37	3.149899687	stage_E14_vs_E10	ordinary_muscle_development	D_vs_E10;stage_1W_vs_E10;stage_3W_vs_E10;stage	1	TRUE
ENSGALG00010009884	GFOD1	PRJNA729271	1.440794687	7.33791E-37	1.440794687	stage_E14_vs_E10	ordinary_muscle_development	D_vs_E10;stage_1W_vs_E10;stage_3W_vs_E10;stage	1	TRUE
ENSGALG00010018758	ENSGALG00010018758	PRJNA729271	-1.813690818	7.66391E-37	1.813690818	stage_E14_vs_E10	ordinary_muscle_development	D_vs_E10;stage_1W_vs_E10;stage_3W_vs_E10;stage	1	TRUE
ENSGALG00010006816	ENSGALG00010006816	PRJNA729271	6.442252773	7.7006E-37	6.442252773	stage_E14_vs_E10	ordinary_muscle_development	D_vs_E10;stage_1W_vs_E10;stage_3W_vs_E10;stage	1	TRUE
ENSGALG00010003308	MDN1	PRJNA729271	-1.279014462	7.77889E-37	1.279014462	stage_E14_vs_E10	ordinary_muscle_development	D_vs_E10;stage_1W_vs_E10;stage_3W_vs_E10;stage	1	TRUE
ENSGALG00010020369	CNTN3	PRJNA729271	-4.878692252	7.89946E-37	4.878692252	stage_E14_vs_E10	ordinary_muscle_development	D_vs_E10;stage_1W_vs_E10;stage_3W_vs_E10;stage	1	TRUE
ENSGALG00010015520	UBE3A	PRJNA729271	1.003470458	8.18113E-37	1.003470458	stage_E14_vs_E10	ordinary_muscle_development	D_vs_E10;stage_1W_vs_E10;stage_3W_vs_E10;stage	1	TRUE
ENSGALG00010020090	MORF4L2	PRJNA729271	-0.688672454	8.61967E-37	0.688672454	stage_E14_vs_E10	ordinary_muscle_development		0	FALSE
ENSGALG00010002239	AGPAT5	PRJNA729271	1.631527221	8.83639E-37	1.631527221	stage_E14_vs_E10	ordinary_muscle_development	D_vs_E10;stage_1W_vs_E10;stage_3W_vs_E10;stage	1	TRUE
ENSGALG00010003795	LCP1	PRJNA729271	2.570438191	9.01778E-37	2.570438191	stage_E14_vs_E10	ordinary_muscle_development	D_vs_E10;stage_1W_vs_E10;stage_3W_vs_E10;stage	1	TRUE
ENSGALG00010025320	JAM3	PRJNA729271	-1.859116352	9.31057E-37	1.859116352	stage_E14_vs_E10	ordinary_muscle_development	D_vs_E10;stage_1W_vs_E10;stage_3W_vs_E10;stage	1	TRUE
ENSGALG00010023781	COL4A3	PRJNA729271	2.901956444	9.31624E-37	2.901956444	stage_E14_vs_E10	ordinary_muscle_development	D_vs_E10;stage_1W_vs_E10;stage_3W_vs_E10;stage	1	TRUE
ENSGALG00010020684	P4HTM	PRJNA729271	1.533763919	9.54305E-37	1.533763919	stage_E14_vs_E10	ordinary_muscle_development	D_vs_E10;stage_1W_vs_E10;stage_3W_vs_E10;stage	1	TRUE
ENSGALG00010018284	ENSGALG00010018284	PRJNA729271	2.140188518	9.84042E-37	2.140188518	stage_E14_vs_E10	ordinary_muscle_development	D_vs_E10;stage_1W_vs_E10;stage_3W_vs_E10;stage	1	TRUE
ENSGALG00010023707	MPZL2	PRJNA729271	-5.058600821	9.91607E-37	5.058600821	stage_E14_vs_E10	ordinary_muscle_development	D_vs_E10;stage_1W_vs_E10;stage_3W_vs_E10;stage	1	TRUE
ENSGALG00010018189	MIS18BP1	PRJNA729271	-1.561648073	1.12828E-36	1.561648073	stage_E14_vs_E10	ordinary_muscle_development	D_vs_E10;stage_1W_vs_E10;stage_3W_vs_E10;stage	1	TRUE
ENSGALG00010012194	LRRFIP1	PRJNA729271	2.049930006	1.14339E-36	2.049930006	stage_E14_vs_E10	ordinary_muscle_development	D_vs_E10;stage_1W_vs_E10;stage_3W_vs_E10;stage	1	TRUE
ENSGALG00010001678	SYAP1	PRJNA729271	0.746324678	1.15834E-36	0.746324678	stage_E14_vs_E10	ordinary_muscle_development		0	FALSE
ENSGALG00010005017	ENSGALG00010005017	PRJNA729271	2.182276701	1.18633E-36	2.182276701	stage_E14_vs_E10	ordinary_muscle_development	D_vs_E10;stage_1W_vs_E10;stage_3W_vs_E10;stage	1	TRUE
ENSGALG00010021723	COPS3	PRJNA729271	1.356906977	1.19513E-36	1.356906977	stage_E14_vs_E10	ordinary_muscle_development	D_vs_E10;stage_1W_vs_E10;stage_3W_vs_E10;stage	1	TRUE
ENSGALG00010012880	CLK4	PRJNA729271	1.775792359	1.20005E-36	1.775792359	stage_E14_vs_E10	ordinary_muscle_development	D_vs_E10;stage_1W_vs_E10;stage_3W_vs_E10;stage	1	TRUE
ENSGALG00010017983	SLC4A11	PRJNA729271	2.587253924	1.21491E-36	2.587253924	stage_E14_vs_E10	ordinary_muscle_development	D_vs_E10;stage_1W_vs_E10;stage_3W_vs_E10;stage	1	TRUE
ENSGALG00010009943	DHX15	PRJNA729271	-0.894701889	1.22854E-36	0.894701889	stage_E14_vs_E10	ordinary_muscle_development		0	FALSE
ENSGALG00010007146	ENSGALG00010007146	PRJNA729271	1.805870572	1.34385E-36	1.805870572	stage_E14_vs_E10	ordinary_muscle_development	D_vs_E10;stage_1W_vs_E10;stage_3W_vs_E10;stage	1	TRUE
ENSGALG00010006378	CAAP1	PRJNA729271	-1.366209076	1.42146E-36	1.366209076	stage_E14_vs_E10	ordinary_muscle_development	D_vs_E10;stage_1W_vs_E10;stage_3W_vs_E10;stage	1	TRUE
ENSGALG00010027365	PLEKHA6	PRJNA729271	2.12084751	1.42831E-36	2.12084751	stage_E14_vs_E10	ordinary_muscle_development	D_vs_E10;stage_1W_vs_E10;stage_3W_vs_E10;stage	1	TRUE
ENSGALG00010024824	DNAJC5	PRJNA729271	-1.472898289	1.4871E-36	1.472898289	stage_E14_vs_E10	ordinary_muscle_development	D_vs_E10;stage_1W_vs_E10;stage_3W_vs_E10;stage	1	TRUE
ENSGALG00010012129	CRYZL1	PRJNA729271	-1.283721023	1.52494E-36	1.283721023	stage_E14_vs_E10	ordinary_muscle_development	D_vs_E10;stage_1W_vs_E10;stage_3W_vs_E10;stage	1	TRUE
ENSGALG00010008860	SQLE	PRJNA729271	-3.28092612	1.57007E-36	3.28092612	stage_E14_vs_E10	ordinary_muscle_development	D_vs_E10;stage_1W_vs_E10;stage_3W_vs_E10;stage	1	TRUE
ENSGALG00010029252	RPAIN	PRJNA729271	1.743120828	1.71824E-36	1.743120828	stage_E14_vs_E10	ordinary_muscle_development	D_vs_E10;stage_1W_vs_E10;stage_3W_vs_E10;stage	1	TRUE
ENSGALG00010021100	EMC7	PRJNA729271	1.089259577	1.73455E-36	1.089259577	stage_E14_vs_E10	ordinary_muscle_development	D_vs_E10;stage_1W_vs_E10;stage_3W_vs_E10;stage	1	TRUE
ENSGALG00010027929	CDK5RAP2	PRJNA729271	-1.785773951	1.86505E-36	1.785773951	stage_E14_vs_E10	ordinary_muscle_development	D_vs_E10;stage_1W_vs_E10;stage_3W_vs_E10;stage	1	TRUE
ENSGALG00010019087	PEX13	PRJNA729271	1.099298992	1.86945E-36	1.099298992	stage_E14_vs_E10	ordinary_muscle_development	D_vs_E10;stage_1W_vs_E10;stage_3W_vs_E10;stage	1	TRUE
ENSGALG00010023125	COL20A1	PRJNA729271	3.65099081	1.8854E-36	3.65099081	stage_E14_vs_E10	ordinary_muscle_development	D_vs_E10;stage_1W_vs_E10;stage_3W_vs_E10;stage	1	TRUE
ENSGALG00010017761	PARP16	PRJNA729271	1.350855365	2.01705E-36	1.350855365	stage_E14_vs_E10	ordinary_muscle_development	D_vs_E10;stage_1W_vs_E10;stage_3W_vs_E10;stage	1	TRUE
ENSGALG00010018638	SEC14L5	PRJNA729271	2.012066255	2.05807E-36	2.012066255	stage_E14_vs_E10	ordinary_muscle_development	D_vs_E10;stage_1W_vs_E10;stage_3W_vs_E10;stage	1	TRUE
ENSGALG00010022421	CMKLR1	PRJNA729271	2.229987324	2.16752E-36	2.229987324	stage_E14_vs_E10	ordinary_muscle_development	D_vs_E10;stage_1W_vs_E10;stage_3W_vs_E10;stage	1	TRUE
ENSGALG00010016453	MYCL	PRJNA729271	-2.401642125	2.2807E-36	2.401642125	stage_E14_vs_E10	ordinary_muscle_development	D_vs_E10;stage_1W_vs_E10;stage_3W_vs_E10;stage	1	TRUE
ENSGALG00010021000	NFKB2	PRJNA729271	1.453847682	2.4511E-36	1.453847682	stage_E14_vs_E10	ordinary_muscle_development	D_vs_E10;stage_1W_vs_E10;stage_3W_vs_E10;stage	1	TRUE
ENSGALG00010023394	HOXB5	PRJNA729271	-2.352248978	2.61258E-36	2.352248978	stage_E14_vs_E10	ordinary_muscle_development	D_vs_E10;stage_1W_vs_E10;stage_3W_vs_E10;stage	1	TRUE
ENSGALG00010018843	MCRIP2	PRJNA729271	3.001187649	2.75886E-36	3.001187649	stage_E14_vs_E10	ordinary_muscle_development	D_vs_E10;stage_1W_vs_E10;stage_3W_vs_E10;stage	1	TRUE
ENSGALG00010027076	VDHAP	PRJNA729271	2.184117162	2.79084E-36	2.184117162	stage_E14_vs_E10	ordinary_muscle_development	D_vs_E10;stage_1W_vs_E10;stage_3W_vs_E10;stage	1	TRUE
ENSGALG00010029331	TK1	PRJNA729271	-3.581320864	2.80265E-36	3.581320864	stage_E14_vs_E10	ordinary_muscle_development	D_vs_E10;stage_1W_vs_E10;stage_3W_vs_E10;stage	1	TRUE
ENSGALG00010016472	TFDP1	PRJNA729271	-1.563232759	2.8379E-36	1.563232759	stage_E14_vs_E10	ordinary_muscle_development	D_vs_E10;stage_1W_vs_E10;stage_3W_vs_E10;stage	1	TRUE
ENSGALG00010029466	SLC39A11	PRJNA729271	-1.634175838	2.87158E-36	1.634175838	stage_E14_vs_E10	ordinary_muscle_development	D_vs_E10;stage_1W_vs_E10;stage_3W_vs_E10;stage	1	TRUE
ENSGALG00010003879	CASP6	PRJNA729271	-1.541250033	2.98505E-36	1.541250033	stage_E14_vs_E10	ordinary_muscle_development	D_vs_E10;stage_1W_vs_E10;stage_3W_vs_E10;stage	1	TRUE
ENSGALG00010021615	FLVCR1	PRJNA729271	-2.387063152	3.28385E-36	2.387063152	stage_E14_vs_E10	ordinary_muscle_development	D_vs_E10;stage_1W_vs_E10;stage_3W_vs_E10;stage	1	TRUE
ENSGALG00010020705	EXOSC1	PRJNA729271	2.194928201	3.32541E-36	2.194928201	stage_E14_vs_E10	ordinary_muscle_development	D_vs_E10;stage_1W_vs_E10;stage_3W_vs_E10;stage	1	TRUE
ENSGALG00010013343	SHISAL1	PRJNA729271	-7.575842684	3.50589E-36	7.575842684	stage_E14_vs_E10	ordinary_muscle_development	D_vs_E10;stage_1W_vs_E10;stage_3W_vs_E10;stage	1	TRUE
ENSGALG00010029749	ASPA	PRJNA729271	3.585075225	3.8119E-36	3.585075225	stage_E14_vs_E10	ordinary_muscle_development	D_vs_E10;stage_1W_vs_E10;stage_3W_vs_E10;stage	1	TRUE
ENSGALG00010022828	PRKG1	PRJNA729271	-2.198828311	3.81439E-36	2.198828311	stage_E14_vs_E10	ordinary_muscle_development	D_vs_E10;stage_1W_vs_E10;stage_3W_vs_E10;stage	1	TRUE
ENSGALG00010014076	PRRX1	PRJNA729271	-3.175144321	3.86895E-36	3.175144321	stage_E14_vs_E10	ordinary_muscle_development	D_vs_E10;stage_1W_vs_E10;stage_3W_vs_E10;stage	1	TRUE
ENSGALG00010003796	ENSGALG00010003796	PRJNA729271	3.817126738	3.91723E-36	3.817126738	stage_E14_vs_E10	ordinary_muscle_development	D_vs_E10;stage_1W_vs_E10;stage_3W_vs_E10;stage	1	TRUE
ENSGALG00010027123	WDR86	PRJNA729271	-2.945100909	3.94524E-36	2.945100909	stage_E14_vs_E10	ordinary_muscle_development	D_vs_E10;stage_1W_vs_E10;stage_3W_vs_E10;stage	1	TRUE
ENSGALG00010004352	CHMP2A	PRJNA729271	0.713636662	3.95317E-36	0.713636662	stage_E14_vs_E10	ordinary_muscle_development		0	FALSE
ENSGALG00010015171	NHSL2	PRJNA729271	2.522818889	4.09143E-36	2.522818889	stage_E14_vs_E10	ordinary_muscle_development	D_vs_E10;stage_1W_vs_E10;stage_3W_vs_E10;stage	1	TRUE
ENSGALG00010002758	GREB1	PRJNA729271	-5.796410664	4.1332E-36	5.796410664	stage_E14_vs_E10	ordinary_muscle_development	D_vs_E10;stage_1W_vs_E10;stage_3W_vs_E10;stage	1	TRUE
ENSGALG00010021509	DDIT4	PRJNA729271	3.546219124	4.13581E-36	3.546219124	stage_E14_vs_E10	ordinary_muscle_development	D_vs_E10;stage_1W_vs_E10;stage_3W_vs_E10;stage	1	TRUE
ENSGALG00010029704	SLC25A19	PRJNA729271	1.253118419	4.25155E-36	1.253118419	stage_E14_vs_E10	ordinary_muscle_development	D_vs_E10;stage_1W_vs_E10;stage_3W_vs_E10;stage	1	TRUE
ENSGALG00010015133	RFTN2	PRJNA729271	-1.636156075	4.35014E-36	1.636156075	stage_E14_vs_E10	ordinary_muscle_development	D_vs_E10;stage_1W_vs_E10;stage_3W_vs_E10;stage	1	TRUE
ENSGALG00010028311	ENSGALG00010028311	PRJNA729271	-3.979158528	4.43065E-36	3.979158528	stage_E14_vs_E10	ordinary_muscle_development	D_vs_E10;stage_1W_vs_E10;stage_3W_vs_E10;stage	1	TRUE
ENSGALG00010027288	DAG1	PRJNA729271	2.140022094	4.48293E-36	2.140022094	stage_E14_vs_E10	ordinary_muscle_development	D_vs_E10;stage_1W_vs_E10;stage_3W_vs_E10;stage	1	TRUE
ENSGALG00010017100	SUSD5	PRJNA729271	-7.955954287	4.6647E-36	7.955954287	stage_E14_vs_E10	ordinary_muscle_development	D_vs_E10;stage_1W_vs_E10;stage_3W_vs_E10;stage	1	TRUE
ENSGALG00010021576	ERH	PRJNA729271	-1.558355581	4.69481E-36	1.558355581	stage_E14_vs_E10	ordinary_muscle_development	D_vs_E10;stage_1W_vs_E10;stage_3W_vs_E10;stage	1	TRUE
ENSGALG00010029632	TMEM132E	PRJNA729271	2.850034239	4.72612E-36	2.850034239	stage_E14_vs_E10	ordinary_muscle_development	D_vs_E10;stage_1W_vs_E10;stage_3W_vs_E10;stage	1	TRUE
ENSGALG00010024840	BTF3L4	PRJNA729271	-1.295250778	5.35938E-36	1.295250778	stage_E14_vs_E10	ordinary_muscle_development	D_vs_E10;stage_1W_vs_E10;stage_3W_vs_E10;stage	1	TRUE
ENSGALG00010017601	ENSGALG00010017601	PRJNA729271	1.45807586	5.9178E-36	1.45807586	stage_E14_vs_E10	ordinary_muscle_development	D_vs_E10;stage_1W_vs_E10;stage_3W_vs_E10;stage	1	TRUE
ENSGALG00010018404	IL2RG	PRJNA729271	3.210688677	5.93801E-36	3.210688677	stage_E14_vs_E10	ordinary_muscle_development	D_vs_E10;stage_1W_vs_E10;stage_3W_vs_E10;stage	1	TRUE
ENSGALG00010006078	SPATA13	PRJNA729271	-2.142427527	5.98E-36	2.142427527	stage_E14_vs_E10	ordinary_muscle_development	D_vs_E10;stage_1W_vs_E10;stage_3W_vs_E10;stage	1	TRUE
ENSGALG00010019231	ENSGALG00010019231	PRJNA729271	3.942331078	6.09456E-36	3.942331078	stage_E14_vs_E10	ordinary_muscle_development	D_vs_E10;stage_1W_vs_E10;stage_3W_vs_E10;stage	1	TRUE
ENSGALG00010027383	ASS1	PRJNA729271	3.576881059	6.20818E-36	3.576881059	stage_E14_vs_E10	ordinary_muscle_development	D_vs_E10;stage_1W_vs_E10;stage_3W_vs_E10;stage	1	TRUE
ENSGALG00010029449	UBALD2	PRJNA729271	2.290019658	6.40119E-36	2.290019658	stage_E14_vs_E10	ordinary_muscle_development	D_vs_E10;stage_1W_vs_E10;stage_3W_vs_E10;stage	1	TRUE
ENSGALG00010020147	ABHD2	PRJNA729271	1.847461435	6.47351E-36	1.847461435	stage_E14_vs_E10	ordinary_muscle_development	D_vs_E10;stage_1W_vs_E10;stage_3W_vs_E10;stage	1	TRUE
ENSGALG00010022933	UNC119B	PRJNA729271	-0.980871175	6.50721E-36	0.980871175	stage_E14_vs_E10	ordinary_muscle_development		0	FALSE
ENSGALG00010016865	CFAP36	PRJNA729271	-1.77725567	6.52538E-36	1.77725567	stage_E14_vs_E10	ordinary_muscle_development	D_vs_E10;stage_1W_vs_E10;stage_3W_vs_E10;stage	1	TRUE
ENSGALG00010021553	SLC39A9	PRJNA729271	-1.112015296	6.67951E-36	1.112015296	stage_E14_vs_E10	ordinary_muscle_development	D_vs_E10;stage_1W_vs_E10;stage_3W_vs_E10;stage	1	TRUE
ENSGALG00010029227	UTP18	PRJNA729271	-1.046897666	6.82911E-36	1.046897666	stage_E14_vs_E10	ordinary_muscle_development	D_vs_E10;stage_1W_vs_E10;stage_3W_vs_E10;stage	1	TRUE
ENSGALG00010025886	ALDH2	PRJNA729271	1.388434598	7.51021E-36	1.388434598	stage_E14_vs_E10	ordinary_muscle_development	D_vs_E10;stage_1W_vs_E10;stage_3W_vs_E10;stage	1	TRUE
ENSGALG00010013307	POFUT1	PRJNA729271	-1.144951362	7.60357E-36	1.144951362	stage_E14_vs_E10	ordinary_muscle_development	D_vs_E10;stage_1W_vs_E10;stage_3W_vs_E10;stage	1	TRUE
ENSGALG00010014992	ARL15	PRJNA729271	-1.249157474	7.67842E-36	1.249157474	stage_E14_vs_E10	ordinary_muscle_development	D_vs_E10;stage_1W_vs_E10;stage_3W_vs_E10;stage	1	TRUE
ENSGALG00010009103	AP3S1	PRJNA729271	-1.57698154	7.8331E-36	1.57698154	stage_E14_vs_E10	ordinary_muscle_development	D_vs_E10;stage_1W_vs_E10;stage_3W_vs_E10;stage	1	TRUE
ENSGALG00010023620	IRS1	PRJNA729271	1.78749149	8.188E-36	1.78749149	stage_E14_vs_E10	ordinary_muscle_development	D_vs_E10;stage_1W_vs_E10;stage_3W_vs_E10;stage	1	TRUE
ENSGALG00010020254	ENSGALG00010020254	PRJNA729271	-1.718632076	8.7361E-36	1.718632076	stage_E14_vs_E10	ordinary_muscle_development	D_vs_E10;stage_1W_vs_E10;stage_3W_vs_E10;stage	1	TRUE
ENSGALG00010024021	IGLL1	PRJNA729271	15.27035805	8.74499E-36	15.27035805	stage_E14_vs_E10	ordinary_muscle_development	D_vs_E10;stage_1W_vs_E10;stage_3W_vs_E10;stage	1	TRUE
ENSGALG00010012427	C7	PRJNA729271	5.657440245	9.13147E-36	5.657440245	stage_E14_vs_E10	ordinary_muscle_development	D_vs_E10;stage_1W_vs_E10;stage_3W_vs_E10;stage	1	TRUE
ENSGALG00010018807	RD3	PRJNA729271	-3.445026047	9.16796E-36	3.445026047	stage_E14_vs_E10	ordinary_muscle_development	D_vs_E10;stage_1W_vs_E10;stage_3W_vs_E10;stage	1	TRUE
ENSGALG00010008234	COMMD10	PRJNA729271	-1.542404012	9.3646E-36	1.542404012	stage_E14_vs_E10	ordinary_muscle_development	D_vs_E10;stage_1W_vs_E10;stage_3W_vs_E10;stage	1	TRUE
ENSGALG00010018309	DPYSL2	PRJNA729271	-1.57340892	9.47191E-36	1.57340892	stage_E14_vs_E10	ordinary_muscle_development	D_vs_E10;stage_1W_vs_E10;stage_3W_vs_E10;stage	1	TRUE

ENSGALG00010017140	UQCRC2	PRJNA729271	1.456545664	9.47409E-36	1.456545664	stage_E14_vs_E10	ordinary_muscle_development	D_vs_E10;stage_1W_vs_E10;stage_3W_vs_E10;stage	1	TRUE
ENSGALG00010022545	CSTF3	PRJNA729271	-0.863155846	9.93095E-36	0.863155846	stage_E14_vs_E10	ordinary_muscle_development		0	FALSE
ENSGALG00010005985	ENSGALG00010005985	PRJNA729271	-4.104181923	1.05622E-35	4.104181923	stage_E14_vs_E10	ordinary_muscle_development	D_vs_E10;stage_1W_vs_E10;stage_3W_vs_E10;stage	1	TRUE
ENSGALG00010004329	COQ3	PRJNA729271	1.510213652	1.06044E-35	1.510213652	stage_E14_vs_E10	ordinary_muscle_development	D_vs_E10;stage_1W_vs_E10;stage_3W_vs_E10;stage	1	TRUE
ENSGALG00010003836	CD1F	PRJNA729271	4.151578983	1.06045E-35	4.151578983	stage_E14_vs_E10	ordinary_muscle_development	D_vs_E10;stage_1W_vs_E10;stage_3W_vs_E10;stage	1	TRUE
ENSGALG00010005174	TPP2	PRJNA729271	2.335270129	1.06611E-35	2.335270129	stage_E14_vs_E10	ordinary_muscle_development	D_vs_E10;stage_1W_vs_E10;stage_3W_vs_E10;stage	1	TRUE
ENSGALG00010021617	SORBS1	PRJNA729271	1.402632377	1.07819E-35	1.402632377	stage_E14_vs_E10	ordinary_muscle_development	D_vs_E10;stage_1W_vs_E10;stage_3W_vs_E10;stage	1	TRUE
ENSGALG00010005522	SYNPO2	PRJNA729271	2.249635794	1.08591E-35	2.249635794	stage_E14_vs_E10	ordinary_muscle_development	D_vs_E10;stage_1W_vs_E10;stage_3W_vs_E10;stage	1	TRUE
ENSGALG00010028700	ANGPTL2	PRJNA729271	2.492724363	1.11152E-35	2.492724363	stage_E14_vs_E10	ordinary_muscle_development	D_vs_E10;stage_1W_vs_E10;stage_3W_vs_E10;stage	1	TRUE
ENSGALG00010013253	SHB	PRJNA729271	1.241138493	1.15545E-35	1.241138493	stage_E14_vs_E10	ordinary_muscle_development	D_vs_E10;stage_1W_vs_E10;stage_3W_vs_E10;stage	1	TRUE
ENSGALG00010021092	VPS13D	PRJNA729271	1.197434214	1.17997E-35	1.197434214	stage_E14_vs_E10	ordinary_muscle_development	D_vs_E10;stage_1W_vs_E10;stage_3W_vs_E10;stage	1	TRUE
ENSGALG00010018484	NLGN1	PRJNA729271	-3.648371578	1.22979E-35	3.648371578	stage_E14_vs_E10	ordinary_muscle_development	D_vs_E10;stage_1W_vs_E10;stage_3W_vs_E10;stage	1	TRUE
ENSGALG00010009795	NDUFA6	PRJNA729271	1.44262339	1.31907E-35	1.44262339	stage_E14_vs_E10	ordinary_muscle_development	D_vs_E10;stage_1W_vs_E10;stage_3W_vs_E10;stage	1	TRUE
ENSGALG00010014512	HMGN4	PRJNA729271	-1.402401676	1.33154E-35	1.402401676	stage_E14_vs_E10	ordinary_muscle_development	D_vs_E10;stage_1W_vs_E10;stage_3W_vs_E10;stage	1	TRUE
ENSGALG00010018231	SMYD5	PRJNA729271	-1.037002391	1.41522E-35	1.037002391	stage_E14_vs_E10	ordinary_muscle_development	D_vs_E10;stage_1W_vs_E10;stage_3W_vs_E10;stage	1	TRUE
ENSGALG00010016615	BCORL1	PRJNA729271	1.543912221	1.42067E-35	1.543912221	stage_E14_vs_E10	ordinary_muscle_development	D_vs_E10;stage_1W_vs_E10;stage_3W_vs_E10;stage	1	TRUE
ENSGALG00010017878	CHRNG	PRJNA729271	3.124197541	1.43349E-35	3.124197541	stage_E14_vs_E10	ordinary_muscle_development	D_vs_E10;stage_1W_vs_E10;stage_3W_vs_E10;stage	1	TRUE
ENSGALG00010006155	TNFRSF19	PRJNA729271	-2.477227622	1.50165E-35	2.477227622	stage_E14_vs_E10	ordinary_muscle_development	D_vs_E10;stage_1W_vs_E10;stage_3W_vs_E10;stage	1	TRUE
ENSGALG00010013286	RNF14	PRJNA729271	1.497799533	1.57278E-35	1.497799533	stage_E14_vs_E10	ordinary_muscle_development	D_vs_E10;stage_1W_vs_E10;stage_3W_vs_E10;stage	1	TRUE
ENSGALG00010021854	TAPBPL	PRJNA729271	1.924025554	1.64899E-35	1.924025554	stage_E14_vs_E10	ordinary_muscle_development	D_vs_E10;stage_1W_vs_E10;stage_3W_vs_E10;stage	1	TRUE
ENSGALG00010012767	JOSD1	PRJNA729271	-0.909549982	1.7576E-35	0.909549982	stage_E14_vs_E10	ordinary_muscle_development		0	FALSE
ENSGALG00010020183	XPNPEP1	PRJNA729271	-0.871905681	1.86054E-35	0.871905681	stage_E14_vs_E10	ordinary_muscle_development		0	FALSE
ENSGALG00010021700	FLCN	PRJNA729271	0.975970471	1.86647E-35	0.975970471	stage_E14_vs_E10	ordinary_muscle_development		0	FALSE
ENSGALG00010024630	CFAP410	PRJNA729271	-1.271042169	1.88561E-35	1.271042169	stage_E14_vs_E10	ordinary_muscle_development	D_vs_E10;stage_1W_vs_E10;stage_3W_vs_E10;stage	1	TRUE
ENSGALG00010011345	CRMP1	PRJNA729271	-2.948329151	1.95774E-35	2.948329151	stage_E14_vs_E10	ordinary_muscle_development	D_vs_E10;stage_1W_vs_E10;stage_3W_vs_E10;stage	1	TRUE
ENSGALG00010016763	UBALD1	PRJNA729271	2.161306796	2.06276E-35	2.161306796	stage_E14_vs_E10	ordinary_muscle_development	D_vs_E10;stage_1W_vs_E10;stage_3W_vs_E10;stage	1	TRUE
ENSGALG00010001670	SHOX	PRJNA729271	-2.134926194	2.13478E-35	2.134926194	stage_E14_vs_E10	ordinary_muscle_development	D_vs_E10;stage_1W_vs_E10;stage_3W_vs_E10;stage	1	TRUE
ENSGALG00010020572	PPP1R3C	PRJNA729271	3.416291642	2.19799E-35	3.416291642	stage_E14_vs_E10	ordinary_muscle_development	D_vs_E10;stage_1W_vs_E10;stage_3W_vs_E10;stage	1	TRUE
ENSGALG00010026643	YWHAH	PRJNA729271	-1.416797906	2.24501E-35	1.416797906	stage_E14_vs_E10	ordinary_muscle_development	D_vs_E10;stage_1W_vs_E10;stage_3W_vs_E10;stage	1	TRUE
ENSGALG00010002459	G3BP2	PRJNA729271	-0.937732163	2.36018E-35	0.937732163	stage_E14_vs_E10	ordinary_muscle_development		0	FALSE
ENSGALG00010015049	DCK2	PRJNA729271	-3.171549739	2.4689E-35	3.171549739	stage_E14_vs_E10	ordinary_muscle_development	D_vs_E10;stage_1W_vs_E10;stage_3W_vs_E10;stage	1	TRUE
ENSGALG00010017471	CRLS1	PRJNA729271	1.111254628	2.54451E-35	1.111254628	stage_E14_vs_E10	ordinary_muscle_development	D_vs_E10;stage_1W_vs_E10;stage_3W_vs_E10;stage	1	TRUE
ENSGALG00010002693	MSC	PRJNA729271	-2.136209698	2.63834E-35	2.136209698	stage_E14_vs_E10	ordinary_muscle_development	D_vs_E10;stage_1W_vs_E10;stage_3W_vs_E10;stage	1	TRUE
ENSGALG00010015573	MAN1C1	PRJNA729271	1.537341938	2.73887E-35	1.537341938	stage_E14_vs_E10	ordinary_muscle_development	D_vs_E10;stage_1W_vs_E10;stage_3W_vs_E10;stage	1	TRUE
ENSGALG00010025168	POPDC2	PRJNA729271	2.662704774	2.86803E-35	2.662704774	stage_E14_vs_E10	ordinary_muscle_development	D_vs_E10;stage_1W_vs_E10;stage_3W_vs_E10;stage	1	TRUE
ENSGALG00010024425	MRPL40	PRJNA729271	1.106108088	2.90998E-35	1.106108088	stage_E14_vs_E10	ordinary_muscle_development	D_vs_E10;stage_1W_vs_E10;stage_3W_vs_E10;stage	1	TRUE
ENSGALG00010005634	HADH	PRJNA729271	1.088111494	2.99307E-35	1.088111494	stage_E14_vs_E10	ordinary_muscle_development	D_vs_E10;stage_1W_vs_E10;stage_3W_vs_E10;stage	1	TRUE
ENSGALG00010025659	COBLL1	PRJNA729271	1.633453012	2.99754E-35	1.633453012	stage_E14_vs_E10	ordinary_muscle_development	D_vs_E10;stage_1W_vs_E10;stage_3W_vs_E10;stage	1	TRUE
ENSGALG00010013290	AKR1D1	PRJNA729271	-4.269843694	3.06238E-35	4.269843694	stage_E14_vs_E10	ordinary_muscle_development	D_vs_E10;stage_1W_vs_E10;stage_3W_vs_E10;stage	1	TRUE
ENSGALG00010023299	PASK	PRJNA729271	-1.834663944	3.19183E-35	1.834663944	stage_E14_vs_E10	ordinary_muscle_development	D_vs_E10;stage_1W_vs_E10;stage_3W_vs_E10;stage	1	TRUE
ENSGALG00010020913	TMEM132B	PRJNA729271	-3.541908582	3.21933E-35	3.541908582	stage_E14_vs_E10	ordinary_muscle_development	D_vs_E10;stage_1W_vs_E10;stage_3W_vs_E10;stage	1	TRUE
ENSGALG00010007294	TRAPPC11	PRJNA729271	0.801050015	3.25291E-35	0.801050015	stage_E14_vs_E10	ordinary_muscle_development		0	FALSE
ENSGALG00010011096	LDB2	PRJNA729271	-1.466513991	3.27132E-35	1.466513991	stage_E14_vs_E10	ordinary_muscle_development	D_vs_E10;stage_1W_vs_E10;stage_3W_vs_E10;stage	1	TRUE
ENSGALG00010015570	LIMCH1	PRJNA729271	1.624076351	3.35622E-35	1.624076351	stage_E14_vs_E10	ordinary_muscle_development	D_vs_E10;stage_1W_vs_E10;stage_3W_vs_E10;stage	1	TRUE
ENSGALG00010021367	GRIP2	PRJNA729271	2.231327283	3.38544E-35	2.231327283	stage_E14_vs_E10	ordinary_muscle_development	D_vs_E10;stage_1W_vs_E10;stage_3W_vs_E10;stage	1	TRUE
ENSGALG00010027215	ENSGALG00010027215	PRJNA729271	-2.533797557	3.40181E-35	2.533797557	stage_E14_vs_E10	ordinary_muscle_development	D_vs_E10;stage_1W_vs_E10;stage_3W_vs_E10;stage	1	TRUE
ENSGALG00010027868	CACNA2D3	PRJNA729271	-4.18742326	3.42939E-35	4.18742326	stage_E14_vs_E10	ordinary_muscle_development	D_vs_E10;stage_1W_vs_E10;stage_3W_vs_E10;stage	1	TRUE
ENSGALG00010005240	VIP	PRJNA729271	-5.491418909	3.461E-35	5.491418909	stage_E14_vs_E10	ordinary_muscle_development	D_vs_E10;stage_1W_vs_E10;stage_3W_vs_E10;stage	1	TRUE
ENSGALG00010014821	C3AR1L	PRJNA729271	2.852439884	3.59139E-35	2.852439884	stage_E14_vs_E10	ordinary_muscle_development	D_vs_E10;stage_1W_vs_E10;stage_3W_vs_E10;stage	1	TRUE
ENSGALG00010010646	MCM3AP	PRJNA729271	-0.742890018	3.62531E-35	0.742890018	stage_E14_vs_E10	ordinary_muscle_development		0	FALSE
ENSGALG00010027879	ENSGALG00010027879	PRJNA729271	5.212968187	4.30209E-35	5.212968187	stage_E14_vs_E10	ordinary_muscle_development	D_vs_E10;stage_1W_vs_E10;stage_3W_vs_E10;stage	1	TRUE
ENSGALG00010024393	OGFOD1	PRJNA729271	-1.359252374	4.36482E-35	1.359252374	stage_E14_vs_E10	ordinary_muscle_development	D_vs_E10;stage_1W_vs_E10;stage_3W_vs_E10;stage	1	TRUE
ENSGALG00010027134	DYRK3	PRJNA729271	1.865626885	4.53549E-35	1.865626885	stage_E14_vs_E10	ordinary_muscle_development	D_vs_E10;stage_1W_vs_E10;stage_3W_vs_E10;stage	1	TRUE
ENSGALG00010020584	PARP9	PRJNA729271	3.598956683	4.54458E-35	3.598956683	stage_E14_vs_E10	ordinary_muscle_development	D_vs_E10;stage_1W_vs_E10;stage_3W_vs_E10;stage	1	TRUE
ENSGALG00010027264	ENSGALG00010027264	PRJNA729271	1.171465019	4.58944E-35	1.171465019	stage_E14_vs_E10	ordinary_muscle_development	D_vs_E10;stage_1W_vs_E10;stage_3W_vs_E10;stage	1	TRUE
ENSGALG00010010381	MAST4	PRJNA729271	1.644486065	4.65909E-35	1.644486065	stage_E14_vs_E10	ordinary_muscle_development	D_vs_E10;stage_1W_vs_E10;stage_3W_vs_E10;stage	1	TRUE
ENSGALG00010027147	KMT2C	PRJNA729271	1.2961418	4.72222E-35	1.2961418	stage_E14_vs_E10	ordinary_muscle_development	D_vs_E10;stage_1W_vs_E10;stage_3W_vs_E10;stage	1	TRUE
ENSGALG00010005114	PHLDB2	PRJNA729271	-1.258215989	4.73816E-35	1.258215989	stage_E14_vs_E10	ordinary_muscle_development	D_vs_E10;stage_1W_vs_E10;stage_3W_vs_E10;stage	1	TRUE
ENSGALG00010016862	TXK	PRJNA729271	6.337087664	4.90972E-35	6.337087664	stage_E14_vs_E10	ordinary_muscle_development	D_vs_E10;stage_1W_vs_E10;stage_3W_vs_E10;stage	1	TRUE
ENSGALG00010005967	GJD2	PRJNA729271	-3.657411396	4.91449E-35	3.657411396	stage_E14_vs_E10	ordinary_muscle_development	D_vs_E10;stage_1W_vs_E10;stage_3W_vs_E10;stage	1	TRUE
ENSGALG00010019584	RAD18	PRJNA729271	-1.492646227	4.96536E-35	1.492646227	stage_E14_vs_E10	ordinary_muscle_development	D_vs_E10;stage_1W_vs_E10;stage_3W_vs_E10;stage	1	TRUE
ENSGALG00010011275	WDR4	PRJNA729271	-1.324870627	4.99021E-35	1.324870627	stage_E14_vs_E10	ordinary_muscle_development	D_vs_E10;stage_1W_vs_E10;stage_3W_vs_E10;stage	1	TRUE
ENSGALG00010023590	S1PR1	PRJNA729271	1.435637585	5.05786E-35	1.435637585	stage_E14_vs_E10	ordinary_muscle_development	D_vs_E10;stage_1W_vs_E10;stage_3W_vs_E10;stage	1	TRUE
ENSGALG00010029310	LRWD1	PRJNA729271	-1.159960669	5.06954E-35	1.159960669	stage_E14_vs_E10	ordinary_muscle_development	D_vs_E10;stage_1W_vs_E10;stage_3W_vs_E10;stage	1	TRUE
ENSGALG00010011193	FAM20B	PRJNA729271	-1.025874698	5.16131E-35	1.025874698	stage_E14_vs_E10	ordinary_muscle_development	D_vs_E10;stage_1W_vs_E10;stage_3W_vs_E10;stage	1	TRUE
ENSGALG00010003838	GAR1	PRJNA729271	-2.384346919	5.26687E-35	2.384346919	stage_E14_vs_E10	ordinary_muscle_development	D_vs_E10;stage_1W_vs_E10;stage_3W_vs_E10;stage	1	TRUE
ENSGALG00010023070	SIDT2	PRJNA729271	2.495651893	5.38521E-35	2.495651893	stage_E14_vs_E10	ordinary_muscle_development	D_vs_E10;stage_1W_vs_E10;stage_3W_vs_E10;stage	1	TRUE
ENSGALG00010024190	WARS2	PRJNA729271	-1.431490765	5.4056E-35	1.431490765	stage_E14_vs_E10	ordinary_muscle_development	D_vs_E10;stage_1W_vs_E10;stage_3W_vs_E10;stage	1	TRUE
ENSGALG00010021138	DDX18	PRJNA729271	-1.279567611	5.62483E-35	1.279567611	stage_E14_vs_E10	ordinary_muscle_development	D_vs_E10;stage_1W_vs_E10;stage_3W_vs_E10;stage	1	TRUE
ENSGALG00010019062	PUS10	PRJNA729271	-1.287436728	5.93545E-35	1.287436728	stage_E14_vs_E10	ordinary_muscle_development	D_vs_E10;stage_1W_vs_E10;stage_3W_vs_E10;stage	1	TRUE
ENSGALG00010012105	DONSON	PRJNA729271	-2.734399616	5.94301E-35	2.734399616	stage_E14_vs_E10	ordinary_muscle_development	D_vs_E10;stage_1W_vs_E10;stage_3W_vs_E10;stage	1	TRUE
ENSGALG00010008257	DESI2	PRJNA729271	-0.752903622	6.13581E-35	0.752903622	stage_E14_vs_E10	ordinary_muscle_development		0	FALSE
ENSGALG00010003986	FNDC3A	PRJNA729271	0.7904745	6.29213E-35	0.7904745	stage_E14_vs_E10	ordinary_muscle_development		0	FALSE
ENSGALG00010013772	KIAA0319L	PRJNA729271	1.784461114	6.40605E-35	1.784461114	stage_E14_vs_E10	ordinary_muscle_development	D_vs_E10;stage_1W_vs_E10;stage_3W_vs_E10;stage	1	TRUE
ENSGALG00010018509	ENSGALG00010018509	PRJNA729271	-1.409614852	6.44355E-35	1.409614852	stage_E14_vs_E10	ordinary_muscle_development	D_vs_E10;stage_1W_vs_E10;stage_3W_vs_E10;stage	1	TRUE
ENSGALG00010020981	CIT	PRJNA729271	-3.000282287	6.60736E-35	3.000282287	stage_E14_vs_E10	ordinary_muscle_development	D_vs_E10;stage_1W_vs_E10;stage_3W_vs_E10;stage	1	TRUE
ENSGALG00010005321	HS6ST3	PRJNA729271	-4.375801938	6.82567E-35	4.375801938	stage_E14_vs_E10	ordinary_muscle_development	D_vs_E10;stage_1W_vs_E10;stage_3W_vs_E10;stage	1	TRUE
ENSGALG00010009081	HBP1	PRJNA729271	1.518571867	6.83663E-35	1.518571867	stage_E14_vs_E10	ordinary_muscle_development	D_vs_E10;stage_1W_vs_E10;stage_3W_vs_E10;stage	1	TRUE
ENSGALG00010011530	ARPC5	PRJNA729271	-1.50116285	6.99484E-35	1.50116285	stage_E14_vs_E10	ordinary_muscle_development	D_vs_E10;stage_1W_vs_E10;stage_3W_vs_E10;stage	1	TRUE
ENSGALG00010027314	ENSGALG00010027314	PRJNA729271	4.344162843	7.00052E-35	4.344162843	stage_E14_vs_E10	ordinary_muscle_development	D_vs_E10;stage_1W_vs_E10;stage_3W_vs_E10;stage	1	TRUE
ENSGALG00010008293	PLA2G7	PRJNA729271	1.403692517	7.30669E-35	1.403692517	stage_E14_vs_E10	ordinary_muscle_development	D_vs_E10;stage_1W_vs_E10;stage_3W_vs_E10;stage	1	TRUE
ENSGALG00010009256	CREBRF	PRJNA729271	2.14873829	7.40424E-35	2.14873829	stage_E14_vs_E10	ordinary_muscle_development	D_vs_E10;stage_1W_vs_E10;stage_3W_vs_E10;stage	1	TRUE
ENSGALG00010027160	FASTK	PRJNA729271	2.067440444	7.41269E-35	2.067440444	stage_E14_vs_E10	ordinary_muscle_development	D_vs_E10;stage_1W_vs_E10;stage_3W_vs_E10;stage	1	TRUE
ENSGALG00010015181	VPS13A	PRJNA729271	1.496141227	7.59105E-35	1.496141227	stage_E14_vs_E10	ordinary_muscle_development	D_vs_E10;stage_1W_vs_E10;stage_3W_vs_E10;stage	1	TRUE
ENSGALG00010019646	VPS37C	PRJNA729271	1.562499658	7.59503E-35	1.562499658	stage_E14_vs_E10	ordinary_muscle_development	D_vs_E10;stage_1W_vs_E10;stage_3W_vs_E10;stage	1	TRUE
ENSGALG00010007941	MRPL19	PRJNA729271	-0.932821886	7.85146E-35	0.932821886	stage_E14_vs_E10	ordinary_muscle_development		0	FALSE
ENSGALG00010012654	TENT5B	PRJNA729271	3.844008007	7.9566E-35	3.844008007	stage_E14_vs_E10	ordinary_muscle_development	D_vs_E10;stage_1W_vs_E10;stage_3W_vs_E10;stage	1	TRUE
ENSGALG00010010825	MAK	PRJNA729271	4.532053855	7.97689E-35	4.532053855	stage_E14_vs_E10	ordinary_muscle_development	D_vs_E10;stage_1W_vs_E10;stage_3W_vs_E10;stage	1	TRUE
ENSGALG00010016752	ENSGALG00010016752	PRJNA729271	2.133326285	8.45212E-35	2.133326285	stage_E14_vs_E10	ordinary_muscle_development	D_vs_E10;stage_1W_vs_E10;stage_3W_vs_E10;stage	1	TRUE
ENSGALG00010024810	ADIPOR1	PRJNA729271	1.401082408	8.69791E-35	1.401082408	stage_E14_vs_E10	ordinary_muscle_development	D_vs_E10;stage_1W_vs_E10;stage_3W_vs_E10;stage	1	TRUE
ENSGALG00010016767	SLAIN2	PRJNA729271	1.145069076	8.75916E-35	1.145069076	stage_E14_vs_E10	ordinary_muscle_development	D_vs_E10;stage_1W_vs_E10;stage_3W_vs_E10;stage	1	TRUE
ENSGALG00010009078	PTTG2	PRJNA729271	-2.162416964	8.86225E-35	2.162416964	stage_E14_vs_E10	ordinary_muscle_development	D_vs_E10;stage_1W_vs_E10;stage_3W_vs_E10;stage	1	TRUE
ENSGALG00010002225	PRDX3	PRJNA729271	1.311897183	8.86447E-35	1.311897183	stage_E14_vs_E10	ordinary_muscle_development	D_vs_E10;stage_1W_vs_E10;stage_3W_vs_E10;stage	1	TRUE
ENSGALG00010025585	SARNP	PRJNA729271	-1.215156217	9.0992E-35	1.215156217	stage_E14_vs_E10	ordinary_muscle_development	D_vs_E10;stage_1W_vs_E10;stage_3W_vs_E10;stage	1	TRUE
ENSGALG00010016482	EML1	PRJNA729271	1.745165706	9.12339E-35	1.745165706	stage_E14_vs_E10	ordinary_muscle_development	D_vs_E10;stage_1W_vs_E10;stage_3W_vs_E10;stage	1	TRUE
ENSGALG00010025862	PTCH2	PRJNA729271	-4.175478199	9.55837E-35	4.175478199	stage_E14_vs_E10	ordinary_muscle_development	D_vs_E10;stage_1W_vs_E10;stage_3W_vs_E10;stage	1	TRUE
ENSGALG00010019029	CENPF	PRJNA729271	-3.257711079	9.72839E-35	3.257711079	stage_E14_vs_E10	ordinary_muscle_development	D_vs_E10;stage_1W_vs_E10;stage_3W_vs_E10;stage	1	TRUE
ENSGALG00010025338	FADD	PRJNA729271	-1.236764467	9.87435E-35	1.236764467	stage_E14_vs_E10	ordinary_muscle_development	D_vs_E10;stage_1W_vs_E10;stage_3W_vs_E10;stage	1	TRUE
ENSGALG00010021574	TTLL4	PRJNA729271	2.791789565	9.98231E-35	2.791789565	stage_E14_vs_E10	ordinary_muscle_development	D_vs_E10;stage_1W_vs_E10;stage_3W_vs_E10;stage	1	TRUE
ENSGALG00010027405	DCLRE1B	PRJNA729271	-2.425932221	1.05277E-34	2.425932221	stage_E14_vs_E10	ordinary_muscle_development	D_vs_E10;stage_1W_vs_E10;stage_3W_vs_E10;stage	1	TRUE
ENSGALG00010026481	DIXDC1	PRJNA729271	-1.735944429	1.08097E-34	1.735944429	stage_E14_vs_E10	ordinary_muscle_development	D_vs_E10;stage_1W_vs_E10;stage_3W_vs_E10;stage	1	TRUE
ENSGALG00010024705	SLCO2A1	PRJNA729271	2.901862156	1.12643E-34	2.901862156	stage_E14_vs_E10	ordinary_muscle_development	D_vs_E10;stage_1W_vs_E10;stage_3W_vs_E10;stage	1	TRUE
ENSGALG00010008429	IGFBP3	PRJNA729271	1.474835937	1.13783E-34	1.474835937	stage_E14_vs_E10	ordinary_muscle_development	D_vs_E10;stage_1W_vs_E10;stage_3W_vs_E10;stage	1	TRUE
ENSGALG00010012163	PDP1	PRJNA729271	2.74416707	1.15086E-34	2.74416707	stage_E14_vs_E10	ordinary_muscle_development	D_vs_E10;stage_1W_vs_E10;stage_3W_vs_E10;stage	1	TRUE
ENSGALG00010020896	MARCO	PRJNA729271	4.856992589	1.15993E-34	4.856992589	stage_E14_vs_E10	ordinary_muscle_development	D_vs_E10;stage_1W_vs_E10;stage_3W_vs_E10;stage	1	TRUE
ENSGALG00010025477	ENSGALG00010025477	PRJNA729271	0.874273492	1.19364E-34	0.874273492	stage_E14_vs_E10	ordinary_muscle_development		0	FALSE
ENSGALG00010021102	GOLGA3	PRJNA729271	1.392993109	1.22058E-34	1.392993109	stage_E14_vs_E10	ordinary_muscle_development	D_vs_E10;stage_1W_vs_E10;stage_3W_vs_E10;stage	1	TRUE
ENSGALG00010023598	IL10RA	PRJNA729271	4.33717558	1.23849E-34	4.33717558	stage_E14_vs_E10	ordinary_muscle_development	D_vs_E10;stage_1W_vs_E10;stage_3W_vs_E10;stage	1	TRUE
ENSGALG00010022504	RAD9A	PRJNA729271	1.766266581	1.259E-34	1.766266581	stage_E14_vs_E10	ordinary_muscle_development	D_vs_E10;stage_1W_vs_E10;stage_3W_vs_E10;stage	1	TRUE
ENSGALG00010018986	CTDSP1	PRJNA729271	2.373211286	1.31002E-34	2.373211286	stage_E14_vs_E10	ordinary_muscle_development	D_vs_E10;stage_1W_vs_E10;stage_3W_vs_E10;stage	1	TRUE
ENSGALG00010028137	NUP214	PRJNA729271	-0.835894647	1.34313E-34	0.835894647	stage_E14_vs_E10	ordinary_muscle_development		0	FALSE
ENSGALG00010019679	RHOBTB2	PRJNA729271	-2.208018113	1.43253E-34	2.208018113	stage_E14_vs_E10	ordinary_muscle_development	D_vs_E10;stage_1W_vs_E10;stage_3W_vs_E10;stage	1	TRUE
ENSGALG00010008151	SIPA1L2	PRJNA729271	1.500502623	1.45748E-34	1.500502623	stage_E14_vs_E10	ordinary_muscle_development	D_vs_E10;stage_1W_vs_E10;stage_3W_vs_E10;stage	1	TRUE
ENSGALG00010011792	HDHD5	PRJNA729271	1.74713066	1.53094E-34	1.74713066	stage_E14_vs_E10	ordinary_muscle_development	D_vs_E10;stage_1W_vs_E10;stage_3W_vs_E10;stage	1	TRUE
ENSGALG00010027980	NUP210	PRJNA729271	1.753870267	1.58069E-34	1.753870267	stage_E14_vs_E10	ordinary_muscle_development	D_vs_E10;stage_1W_vs_E10;stage_3W_vs_E10;stage	1	TRUE
ENSGALG00010008588	PYCR3	PRJNA729271	-2.327516167	1.58621E-34	2.327516167	stage_E14_vs_E10	ordinary_muscle_development	D_vs_E10;stage_1W_vs_E10;stage_3W_vs_E10;stage	1	TRUE
ENSGALG00010028169	DNLZ	PRJNA729271	1.757322584	1.62758E-34	1.757322584	stage_E14_vs_E10	ordinary_muscle_development	D_vs_E10;stage_1W_vs_E10;stage_3W_vs_E10;stage	1	TRUE
ENSGALG00010007393	PLCG2	PRJNA729271	1.559756659	1.64252E-34	1.559756659	stage_E14_vs_E10	ordinary_muscle_development	D_vs_E10;stage_1W_vs_E10;stage_3W_vs_E10;stage	1	TRUE
ENSGALG00010010942	NRN1	PRJNA729271	4.175175471	1.67034E-34	4.175175471	stage_E14_vs_E10	ordinary_muscle_development	D_vs_E10;stage_1W_vs_E10;stage_3W_vs_E10;stage	1	TRUE
ENSGALG00010027268	UQCRC1	PRJNA729271	1.574313195	1.68368E-34	1.574313195	stage_E14_vs_E10	ordinary_muscle_development	D_vs_E10;stage_1W_vs_E10;stage_3W_vs_E10;stage	1	TRUE
ENSGALG00010005790	MUSK	PRJNA729271	1.8243234	1.69971E-34	1.8243234	stage_E14_vs_E10	ordinary_muscle_development	D_vs_E10;stage_1W_vs_E10;stage_3W_vs_E10;stage	1	TRUE
ENSGALG00010010985	CERT1	PRJNA729271	1.336196986	1.74058E-34	1.336196986	stage_E14_vs_E10	ordinary_muscle_development	D_vs_E10;stage_1W_vs_E10;stage_3W_vs_E10;stage	1	TRUE
ENSGALG00010023544	SSB	PRJNA729271	-1.29400705	1.74877E-34	1.29400705	stage_E14_vs_E10	ordinary_muscle_development	D_vs_E10;stage_1W_vs_E10;stage_3W_vs_E10;stage	1	TRUE
ENSGALG00010014703	PCID2	PRJNA729271	-1.006216244	1.76008E-34	1.006216244	stage_E14_vs_E10	ordinary_muscle_development	D_vs_E10;stage_1W_vs_E10;stage_3W_vs_E10;stage	1	TRUE
ENSGALG00010017098	DAAM1	PRJNA729271	-1.564359144	1.78864E-34	1.564359144	stage_E14_vs_E10	ordinary_muscle_development	D_vs_E10;stage_1W_vs_E10;stage_3W_vs_E10;stage	1	TRUE
ENSGALG00010026681	PLXDC1	PRJNA729271	3.192619019	1.86853E-34	3.192619019	stage_E14_vs_E10	ordinary_muscle_development	D_vs_E10;stage_1W_vs_E10;stage_3W_vs_E10;stage	1	TRUE
ENSGALG00010024116	MCAM	PRJNA729271	2.266069962	1.92141E-34	2.266069962	stage_E14_vs_E10	ordinary_muscle_development	D_vs_E10;stage_1W_vs_E10;stage_3W_vs_E10;stage	1	TRUE
ENSGALG00010000047	IQGAP1	PRJNA729271	-1.208651667	1.98934E-34	1.208651667	stage_E14_vs_E10	ordinary_muscle_development	D_vs_E10;stage_1W_vs_E10;stage_3W_vs_E10;stage	1	TRUE
ENSGALG00010011564	ARAP3	PRJNA729271	1.505863074	2.00028E-34	1.505863074	stage_E14_vs_E10	ordinary_muscle_development	D_vs_E10;stage_1W_vs_E10;stage_3W_vs_E10;stage	1	TRUE
ENSGALG00010019051	IGSF6	PRJNA729271	3.086047837	2.05509E-34	3.086047837	stage_E14_vs_E10	ordinary_muscle_development	D_vs_E10;stage_1W_vs_E10;stage_3W_vs_E10;stage	1	TRUE
ENSGALG00010025098	ATP5B	PRJNA729271	1.711986759	2.06041E-34	1.711986759	stage_E14_vs_E10	ordinary_muscle_development	D_vs_E10;stage_1W_vs_E10;stage_3W_vs_E10;stage	1	TRUE
ENSGALG00010019145	RAC1	PRJNA729271	-0.968458141	2.0976E-34	0.968458141	stage_E14_vs_E10	ordinary_muscle_development		0	FALSE
ENSGALG00010011556	LAPTM4B	PRJNA729271	1.78896248	2.18684E-34	1.78896248	stage_E14_vs_E10	ordinary_muscle_development	D_vs_E10;stage_1W_vs_E10;stage_3W_vs_E10;stage	1	TRUE
ENSGALG00010002946	GDPD5	PRJNA729271	1.434319498	2.30239E-34	1.434319498	stage_E14_vs_E10	ordinary_muscle_development	D_vs_E10;stage_1W_vs_E10;stage_3W_vs_E10;stage	1	TRUE
ENSGALG00010029232	CA10	PRJNA729271	-5.979797013	2.30421E-34	5.979797013	stage_E14_vs_E10	ordinary_muscle_development	D_vs_E10;stage_1W_vs_E10;stage_3W_vs_E10;stage	1	TRUE
ENSGALG00010002790	BCLAF3	PRJNA729271	1.247870194	2.37815E-34	1.247870194	stage_E14_vs_E10	ordinary_muscle_development	D_vs_E10;stage_1W_vs_E10;stage_3W_vs_E10;stage	1	TRUE
ENSGALG00010004395	BOC	PRJNA729271	-2.399513089	2.45462E-34	2.399513089	stage_E14_vs_E10	ordinary_muscle_development	D_vs_E10;stage_1W_vs_E10;stage_3W_vs_E10;stage	1	TRUE
ENSGALG00010015898	ENSGALG00010015898	PRJNA729271	2.679528493	2.61672E-34	2.679528493	stage_E14_vs_E10	ordinary_muscle_development	D_vs_E10;stage_1W_vs_E10;stage_3W_vs_E10;stage	1	TRUE
ENSGALG00010004988	HJURP	PRJNA729271	-2.752752491	2.68475E-34	2.752752491	stage_E14_vs_E10	ordinary_muscle_development	D_vs_E10;stage_1W_vs_E10;stage_3W_vs_E10;stage	1	TRUE
ENSGALG00010019825	BCL2A1	PRJNA729271	3.400032997	2.74677E-34	3.400032997	stage_E14_vs_E10	ordinary_muscle_development	D_vs_E10;stage_1W_vs_E10;stage_3W_vs_E10;stage	1	TRUE
ENSGALG00010023171	ENSGALG00010023171	PRJNA729271	-1.445221911	2.83235E-34	1.445221911	stage_E14_vs_E10	ordinary_muscle_development	D_vs_E10;stage_1W_vs_E10;stage_3W_vs_E10;stage	1	TRUE
ENSGALG00010015218	PLCL1	PRJNA729271	-2.015739312	2.89733E-34	2.015739312	stage_E14_vs_E10	ordinary_muscle_development	D_vs_E10;stage_1W_vs_E10;stage_3W_vs_E10;stage	1	TRUE
ENSGALG00010025985	CHCHD10	PRJNA729271	3.929848865	2.92053E-34	3.929848865	stage_E14_vs_E10	ordinary_muscle_development	D_vs_E10;stage_1W_vs_E10;stage_3W_vs_E10;stage	1	TRUE
ENSGALG00010024675	NR1D1	PRJNA729271	5.05049439	2.97339E-34	5.05049439	stage_E14_vs_E10	ordinary_muscle_development	D_vs_E10;stage_1W_vs_E10;stage_3W_vs_E10;stage	1	TRUE
ENSGALG00010016286	ULK4	PRJNA729271	-1.824977557	2.98515E-34	1.824977557	stage_E14_vs_E10	ordinary_muscle_development	D_vs_E10;stage_1W_vs_E10;stage_3W_vs_E10;stage	1	TRUE
ENSGALG00010017421	GPR157	PRJNA729271	3.231053421	2.99883E-34	3.231053421	stage_E14_vs_E10	ordinary_muscle_development	D_vs_E10;stage_1W_vs_E10;stage_3W_vs_E10;stage	1	TRUE
ENSGALG00010007392	NAALAD2	PRJNA729271	2.470986629	3.1295E-34	2.470986629	stage_E14_vs_E10	ordinary_muscle_development	D_vs_E10;stage_1W_vs_E10;stage_3W_vs_E10;stage	1	TRUE
ENSGALG00010013274	MTF1	PRJNA729271	1.471403755	3.13587E-34	1.471403755	stage_E14_vs_E10	ordinary_muscle_development	D_vs_E10;stage_1W_vs_E10;stage_3W_vs_E10;stage	1	TRUE
ENSGALG00010002661	ACBD7	PRJNA729271	-2.723877608	3.17366E-34	2.723877608	stage_E14_vs_E10	ordinary_muscle_development	D_vs_E10;stage_1W_vs_E10;stage_3W_vs_E10;stage	1	TRUE
ENSGALG00010007416	DCTD	PRJNA729271	-3.144662184	3.18365E-34	3.144662184	stage_E14_vs_E10	ordinary_muscle_development	D_vs_E10;stage_1W_vs_E10;stage_3W_vs_E10;stage	1	TRUE
ENSGALG00010013480	TBC1D8	PRJNA729271	1.87267574	3.21543E-34	1.87267574	stage_E14_vs_E10	ordinary_muscle_development	D_vs_E10;stage_1W_vs_E10;stage_3W_vs_E10;stage	1	TRUE
ENSGALG00010004927	KIF25	PRJNA729271	-3.464897047	3.25113E-34	3.464897047	stage_E14_vs_E10	ordinary_muscle_development	D_vs_E10;stage_1W_vs_E10;stage_3W_vs_E10;stage	1	TRUE
ENSGALG00010012905	NKAIN1	PRJNA729271	-4.63922099	3.27107E-34	4.63922099	stage_E14_vs_E10	ordinary_muscle_development	D_vs_E10;stage_1W_vs_E10;stage_3W_vs_E10;stage	1	TRUE
ENSGALG00010014655	FHL3	PRJNA729271	-1.665955217	3.45442E-34	1.665955217	stage_E14_vs_E10	ordinary_muscle_development	D_vs_E10;stage_1W_vs_E10;stage_3W_vs_E10;stage	1	TRUE
ENSGALG00010017290	DACT1	PRJNA729271	-1.946105181	3.54391E-34	1.946105181	stage_E14_vs_E10	ordinary_muscle_development	D_vs_E10;stage_1W_vs_E10;stage_3W_vs_E10;stage	1	TRUE
ENSGALG00010005456	RPL7L1	PRJNA729271	-0.877076159	3.60868E-34	0.877076159	stage_E14_vs_E10	ordinary_muscle_development		0	FALSE
ENSGALG00010015916	COA8	PRJNA729271	1.777702031	3.6279E-34	1.777702031	stage_E14_vs_E10	ordinary_muscle_development	D_vs_E10;stage_1W_vs_E10;stage_3W_vs_E10;stage	1	TRUE
ENSGALG00010010698	GPRIN2	PRJNA729271	-2.714998266	3.64639E-34	2.714998266	stage_E14_vs_E10	ordinary_muscle_development	D_vs_E10;stage_1W_vs_E10;stage_3W_vs_E10;stage	1	TRUE
ENSGALG00010001167	SMC4	PRJNA729271	-1.908360986	3.77905E-34	1.908360986	stage_E14_vs_E10	ordinary_muscle_development	D_vs_E10;stage_1W_vs_E10;stage_3W_vs_E10;stage	1	TRUE
ENSGALG00010005527	NUP43	PRJNA729271	-1.194415461	3.93306E-34	1.194415461	stage_E14_vs_E10	ordinary_muscle_development	D_vs_E10;stage_1W_vs_E10;stage_3W_vs_E10;stage	1	TRUE
ENSGALG00010014184	SLC44A1	PRJNA729271	1.633708256	4.01954E-34	1.633708256	stage_E14_vs_E10	ordinary_muscle_development	D_vs_E10;stage_1W_vs_E10;stage_3W_vs_E10;stage	1	TRUE
ENSGALG00010021173	KLF15	PRJNA729271	3.551603582	4.06899E-34	3.551603582	stage_E14_vs_E10	ordinary_muscle_development	D_vs_E10;stage_1W_vs_E10;stage_3W_vs_E10;stage	1	TRUE
ENSGALG00010027078	PDE12	PRJNA729271	-1.616446094	4.17442E-34	1.616446094	stage_E14_vs_E10	ordinary_muscle_development	D_vs_E10;stage_1W_vs_E10;stage_3W_vs_E10;stage	1	TRUE
ENSGALG00010010399	FGF18	PRJNA729271	2.157039902	4.19896E-34	2.157039902	stage_E14_vs_E10	ordinary_muscle_development	D_vs_E10;stage_1W_vs_E10;stage_3W_vs_E10;stage	1	TRUE
ENSGALG00010002126	ENSGALG00010002126	PRJNA729271	3.648400537	4.27254E-34	3.648400537	stage_E14_vs_E10	ordinary_muscle_development	D_vs_E10;stage_1W_vs_E10;stage_3W_vs_E10;stage	1	TRUE
ENSGALG00010011981	SCUBE1	PRJNA729271	-4.406506525	4.41195E-34	4.406506525	stage_E14_vs_E10	ordinary_muscle_development	D_vs_E10;stage_1W_vs_E10;stage_3W_vs_E10;stage	1	TRUE
ENSGALG00010004076	COX7A2	PRJNA729271	-1.703115674	4.82866E-34	1.703115674	stage_E14_vs_E10	ordinary_muscle_development	D_vs_E10;stage_1W_vs_E10;stage_3W_vs_E10;stage	1	TRUE
ENSGALG00010006353	ABCE1	PRJNA729271	-1.00660907	4.90262E-34	1.00660907	stage_E14_vs_E10	ordinary_muscle_development	D_vs_E10;stage_1W_vs_E10;stage_3W_vs_E10;stage	1	TRUE
ENSGALG00010010742	LCP2	PRJNA729271	3.180444856	5.24767E-34	3.180444856	stage_E14_vs_E10	ordinary_muscle_development	D_vs_E10;stage_1W_vs_E10;stage_3W_vs_E10;stage	1	TRUE
ENSGALG00010007869	GCSH	PRJNA729271	-1.563150773	5.28301E-34	1.563150773	stage_E14_vs_E10	ordinary_muscle_development	D_vs_E10;stage_1W_vs_E10;stage_3W_vs_E10;stage	1	TRUE
ENSGALG00010004394	CCNY	PRJNA729271	-1.114052051	5.37894E-34	1.114052051	stage_E14_vs_E10	ordinary_muscle_development	D_vs_E10;stage_1W_vs_E10;stage_3W_vs_E10;stage	1	TRUE
ENSGALG00010027569	ATP5PB	PRJNA729271	1.542148503	5.45924E-34	1.542148503	stage_E14_vs_E10	ordinary_muscle_development	D_vs_E10;stage_1W_vs_E10;stage_3W_vs_E10;stage	1	TRUE
ENSGALG00010000808	ADCY2	PRJNA729271	-2.327575639	5.4781E-34	2.327575639	stage_E14_vs_E10	ordinary_muscle_development	D_vs_E10;stage_1W_vs_E10;stage_3W_vs_E10;stage	1	TRUE
ENSGALG00010021659	SPOCK2	PRJNA729271	-1.876286242	5.5853E-34	1.876286242	stage_E14_vs_E10	ordinary_muscle_development	D_vs_E10;stage_1W_vs_E10;stage_3W_vs_E10;stage	1	TRUE
ENSGALG00010013040	SLC25A43	PRJNA729271	2.504003502	5.80176E-34	2.504003502	stage_E14_vs_E10	ordinary_muscle_development	D_vs_E10;stage_1W_vs_E10;stage_3W_vs_E10;stage	1	TRUE

ENSGALG00010000143	SCARF2	PRJNA729271	-1.988898488	5.80176E-34	1.988898488	stage_E14_vs_E10	ordinary_muscle_development	D_vs_E10;stage_1W_vs_E10;stage_3W_vs_E10;stage	1	TRUE
ENSGALG00010024868	FAAP24	PRJNA729271	-2.950383621	5.8401E-34	2.950383621	stage_E14_vs_E10	ordinary_muscle_development	D_vs_E10;stage_1W_vs_E10;stage_3W_vs_E10;stage	1	TRUE
ENSGALG00010016908	CMC4	PRJNA729271	1.945233315	6.21515E-34	1.945233315	stage_E14_vs_E10	ordinary_muscle_development	D_vs_E10;stage_1W_vs_E10;stage_3W_vs_E10;stage	1	TRUE
ENSGALG00010002374	DST	PRJNA729271	1.408742929	6.22196E-34	1.408742929	stage_E14_vs_E10	ordinary_muscle_development	D_vs_E10;stage_1W_vs_E10;stage_3W_vs_E10;stage	1	TRUE
ENSGALG00010002415	PCDH17	PRJNA729271	-6.0226235	6.24339E-34	6.0226235	stage_E14_vs_E10	ordinary_muscle_development	D_vs_E10;stage_1W_vs_E10;stage_3W_vs_E10;stage	1	TRUE
ENSGALG00010013468	DEPDC1B	PRJNA729271	-3.483990781	6.30191E-34	3.483990781	stage_E14_vs_E10	ordinary_muscle_development	D_vs_E10;stage_1W_vs_E10;stage_3W_vs_E10;stage	1	TRUE
ENSGALG00010018566	ENSGALG00010018566	PRJNA729271	1.7476772	6.66539E-34	1.7476772	stage_E14_vs_E10	ordinary_muscle_development	D_vs_E10;stage_1W_vs_E10;stage_3W_vs_E10;stage	1	TRUE
ENSGALG00010016623	DNAJA3	PRJNA729271	1.733252367	6.77096E-34	1.733252367	stage_E14_vs_E10	ordinary_muscle_development	D_vs_E10;stage_1W_vs_E10;stage_3W_vs_E10;stage	1	TRUE
ENSGALG00010018314	SLC24A3	PRJNA729271	-1.639339965	6.96679E-34	1.639339965	stage_E14_vs_E10	ordinary_muscle_development	D_vs_E10;stage_1W_vs_E10;stage_3W_vs_E10;stage	1	TRUE
ENSGALG00010004042	PLAG1	PRJNA729271	-1.553194808	7.23242E-34	1.553194808	stage_E14_vs_E10	ordinary_muscle_development	D_vs_E10;stage_1W_vs_E10;stage_3W_vs_E10;stage	1	TRUE
ENSGALG00010016935	PRIMA1	PRJNA729271	-3.344841282	7.34883E-34	3.344841282	stage_E14_vs_E10	ordinary_muscle_development	D_vs_E10;stage_1W_vs_E10;stage_3W_vs_E10;stage	1	TRUE
ENSGALG00010014829	ENSGALG00010014829	PRJNA729271	4.743114368	7.44891E-34	4.743114368	stage_E14_vs_E10	ordinary_muscle_development	D_vs_E10;stage_1W_vs_E10;stage_3W_vs_E10;stage	1	TRUE
ENSGALG00010011019	NECAB1	PRJNA729271	-2.938493192	7.59779E-34	2.938493192	stage_E14_vs_E10	ordinary_muscle_development	D_vs_E10;stage_1W_vs_E10;stage_3W_vs_E10;stage	1	TRUE
ENSGALG00010012780	FABP7	PRJNA729271	-3.170979873	7.62346E-34	3.170979873	stage_E14_vs_E10	ordinary_muscle_development	D_vs_E10;stage_1W_vs_E10;stage_3W_vs_E10;stage	1	TRUE
ENSGALG00010029301	RNF213	PRJNA729271	5.700104995	8.31057E-34	5.700104995	stage_E14_vs_E10	ordinary_muscle_development	D_vs_E10;stage_1W_vs_E10;stage_3W_vs_E10;stage	1	TRUE
ENSGALG00010002330	KHDRBS2	PRJNA729271	-3.994943264	8.33338E-34	3.994943264	stage_E14_vs_E10	ordinary_muscle_development	D_vs_E10;stage_1W_vs_E10;stage_3W_vs_E10;stage	1	TRUE
ENSGALG00010004641	METAP1	PRJNA729271	-1.027215267	8.43866E-34	1.027215267	stage_E14_vs_E10	ordinary_muscle_development	D_vs_E10;stage_1W_vs_E10;stage_3W_vs_E10;stage	1	TRUE
ENSGALG00010028500	VPS45	PRJNA729271	0.8493108	8.58715E-34	0.8493108	stage_E14_vs_E10	ordinary_muscle_development		0	FALSE
ENSGALG00010000817	ENSGALG00010000817	PRJNA729271	1.415011634	8.69117E-34	1.415011634	stage_E14_vs_E10	ordinary_muscle_development	D_vs_E10;stage_1W_vs_E10;stage_3W_vs_E10;stage	1	TRUE
ENSGALG00010014149	LGALS2	PRJNA729271	6.158378607	8.77616E-34	6.158378607	stage_E14_vs_E10	ordinary_muscle_development	D_vs_E10;stage_1W_vs_E10;stage_3W_vs_E10;stage	1	TRUE
ENSGALG00010029693	HN1	PRJNA729271	-1.564231442	8.79446E-34	1.564231442	stage_E14_vs_E10	ordinary_muscle_development	D_vs_E10;stage_1W_vs_E10;stage_3W_vs_E10;stage	1	TRUE
ENSGALG00010019027	GNLY	PRJNA729271	3.814833484	9.10823E-34	3.814833484	stage_E14_vs_E10	ordinary_muscle_development	D_vs_E10;stage_1W_vs_E10;stage_3W_vs_E10;stage	1	TRUE
ENSGALG00010024884	ZBTB46	PRJNA729271	1.631415559	9.23031E-34	1.631415559	stage_E14_vs_E10	ordinary_muscle_development	D_vs_E10;stage_1W_vs_E10;stage_3W_vs_E10;stage	1	TRUE
ENSGALG00010029557	ENSGALG00010029557	PRJNA729271	6.687268327	9.29929E-34	6.687268327	stage_E14_vs_E10	ordinary_muscle_development	D_vs_E10;stage_1W_vs_E10;stage_3W_vs_E10;stage	1	TRUE
ENSGALG00010018943	ACKR4	PRJNA729271	4.202953297	9.44701E-34	4.202953297	stage_E14_vs_E10	ordinary_muscle_development	D_vs_E10;stage_1W_vs_E10;stage_3W_vs_E10;stage	1	TRUE
ENSGALG00010015677	GRHL3	PRJNA729271	-3.524196318	9.52109E-34	3.524196318	stage_E14_vs_E10	ordinary_muscle_development	D_vs_E10;stage_1W_vs_E10;stage_3W_vs_E10;stage	1	TRUE
ENSGALG00010019926	KIF7	PRJNA729271	-1.264013876	9.63563E-34	1.264013876	stage_E14_vs_E10	ordinary_muscle_development	D_vs_E10;stage_1W_vs_E10;stage_3W_vs_E10;stage	1	TRUE
ENSGALG00010010678	KCNIP1	PRJNA729271	4.209226214	9.88326E-34	4.209226214	stage_E14_vs_E10	ordinary_muscle_development	D_vs_E10;stage_1W_vs_E10;stage_3W_vs_E10;stage	1	TRUE
ENSGALG00010024968	TMEM101	PRJNA729271	1.597008309	1.00995E-33	1.597008309	stage_E14_vs_E10	ordinary_muscle_development	D_vs_E10;stage_1W_vs_E10;stage_3W_vs_E10;stage	1	TRUE
ENSGALG00010021177	OBSL1	PRJNA729271	2.317342637	1.06586E-33	2.317342637	stage_E14_vs_E10	ordinary_muscle_development	D_vs_E10;stage_1W_vs_E10;stage_3W_vs_E10;stage	1	TRUE
ENSGALG00010005526	STRN3	PRJNA729271	0.852411853	1.07179E-33	0.852411853	stage_E14_vs_E10	ordinary_muscle_development		0	FALSE
ENSGALG00010012426	MIEF1	PRJNA729271	-1.024576159	1.0806E-33	1.024576159	stage_E14_vs_E10	ordinary_muscle_development	D_vs_E10;stage_1W_vs_E10;stage_3W_vs_E10;stage	1	TRUE
ENSGALG00010014310	COL6A1	PRJNA729271	2.928496434	1.09069E-33	2.928496434	stage_E14_vs_E10	ordinary_muscle_development	D_vs_E10;stage_1W_vs_E10;stage_3W_vs_E10;stage	1	TRUE
ENSGALG00010018542	ZMIZ2	PRJNA729271	3.226641095	1.12114E-33	3.226641095	stage_E14_vs_E10	ordinary_muscle_development	D_vs_E10;stage_1W_vs_E10;stage_3W_vs_E10;stage	1	TRUE
ENSGALG00010022297	VPS18	PRJNA729271	-0.740061329	1.12114E-33	0.740061329	stage_E14_vs_E10	ordinary_muscle_development		0	FALSE
ENSGALG00010012672	RO60	PRJNA729271	-1.142204192	1.13032E-33	1.142204192	stage_E14_vs_E10	ordinary_muscle_development	D_vs_E10;stage_1W_vs_E10;stage_3W_vs_E10;stage	1	TRUE
ENSGALG00010015879	TRMT61A	PRJNA729271	-1.520845379	1.14849E-33	1.520845379	stage_E14_vs_E10	ordinary_muscle_development	D_vs_E10;stage_1W_vs_E10;stage_3W_vs_E10;stage	1	TRUE
ENSGALG00010012683	MDM2	PRJNA729271	1.051833317	1.18476E-33	1.051833317	stage_E14_vs_E10	ordinary_muscle_development	D_vs_E10;stage_1W_vs_E10;stage_3W_vs_E10;stage	1	TRUE
ENSGALG00010009307	DIRAS2	PRJNA729271	3.80467906	1.191E-33	3.80467906	stage_E14_vs_E10	ordinary_muscle_development	D_vs_E10;stage_1W_vs_E10;stage_3W_vs_E10;stage	1	TRUE
ENSGALG00010010371	SOCS1L	PRJNA729271	-2.669838154	1.24367E-33	2.669838154	stage_E14_vs_E10	ordinary_muscle_development	D_vs_E10;stage_1W_vs_E10;stage_3W_vs_E10;stage	1	TRUE
ENSGALG00010026917	AMT	PRJNA729271	-1.828979759	1.25551E-33	1.828979759	stage_E14_vs_E10	ordinary_muscle_development	D_vs_E10;stage_1W_vs_E10;stage_3W_vs_E10;stage	1	TRUE
ENSGALG00010027290	CSPG5	PRJNA729271	-5.353034355	1.25936E-33	5.353034355	stage_E14_vs_E10	ordinary_muscle_development	D_vs_E10;stage_1W_vs_E10;stage_3W_vs_E10;stage	1	TRUE
ENSGALG00010029730	TTC19	PRJNA729271	1.609226207	1.26056E-33	1.609226207	stage_E14_vs_E10	ordinary_muscle_development	D_vs_E10;stage_1W_vs_E10;stage_3W_vs_E10;stage	1	TRUE
ENSGALG00010008592	KIAA0513	PRJNA729271	1.697345658	1.26849E-33	1.697345658	stage_E14_vs_E10	ordinary_muscle_development	D_vs_E10;stage_1W_vs_E10;stage_3W_vs_E10;stage	1	TRUE
ENSGALG00010007326	RWDD4	PRJNA729271	1.412832322	1.27752E-33	1.412832322	stage_E14_vs_E10	ordinary_muscle_development	D_vs_E10;stage_1W_vs_E10;stage_3W_vs_E10;stage	1	TRUE
ENSGALG00010003259	VEGFC	PRJNA729271	1.69792925	1.27946E-33	1.69792925	stage_E14_vs_E10	ordinary_muscle_development	D_vs_E10;stage_1W_vs_E10;stage_3W_vs_E10;stage	1	TRUE
ENSGALG00010013434	CORIN	PRJNA729271	-5.876400709	1.30732E-33	5.876400709	stage_E14_vs_E10	ordinary_muscle_development	D_vs_E10;stage_1W_vs_E10;stage_3W_vs_E10;stage	1	TRUE
ENSGALG00010007817	CDYL2	PRJNA729271	-1.747921411	1.32229E-33	1.747921411	stage_E14_vs_E10	ordinary_muscle_development	D_vs_E10;stage_1W_vs_E10;stage_3W_vs_E10;stage	1	TRUE
ENSGALG00010012747	TOB2	PRJNA729271	1.777027361	1.32974E-33	1.777027361	stage_E14_vs_E10	ordinary_muscle_development	D_vs_E10;stage_1W_vs_E10;stage_3W_vs_E10;stage	1	TRUE
ENSGALG00010005042	SLC46A3	PRJNA729271	2.424815839	1.36699E-33	2.424815839	stage_E14_vs_E10	ordinary_muscle_development	D_vs_E10;stage_1W_vs_E10;stage_3W_vs_E10;stage	1	TRUE
ENSGALG00010005546	NRF1	PRJNA729271	1.853473535	1.37142E-33	1.853473535	stage_E14_vs_E10	ordinary_muscle_development	D_vs_E10;stage_1W_vs_E10;stage_3W_vs_E10;stage	1	TRUE
ENSGALG00010012706	TBC1D8B	PRJNA729271	1.267050183	1.38455E-33	1.267050183	stage_E14_vs_E10	ordinary_muscle_development	D_vs_E10;stage_1W_vs_E10;stage_3W_vs_E10;stage	1	TRUE
ENSGALG00010009502	ARID5B	PRJNA729271	-1.195460136	1.38827E-33	1.195460136	stage_E14_vs_E10	ordinary_muscle_development	D_vs_E10;stage_1W_vs_E10;stage_3W_vs_E10;stage	1	TRUE
ENSGALG00010020590	INTS7	PRJNA729271	-0.983061488	1.38936E-33	0.983061488	stage_E14_vs_E10	ordinary_muscle_development		0	FALSE
ENSGALG00010026886	JUN	PRJNA729271	2.363186177	1.39563E-33	2.363186177	stage_E14_vs_E10	ordinary_muscle_development	D_vs_E10;stage_1W_vs_E10;stage_3W_vs_E10;stage	1	TRUE
ENSGALG00010025473	PARS2	PRJNA729271	0.958456613	1.41909E-33	0.958456613	stage_E14_vs_E10	ordinary_muscle_development		0	FALSE
ENSGALG00010002005	COLQ	PRJNA729271	2.572746501	1.4291E-33	2.572746501	stage_E14_vs_E10	ordinary_muscle_development	D_vs_E10;stage_1W_vs_E10;stage_3W_vs_E10;stage	1	TRUE
ENSGALG00010007041	NAE1	PRJNA729271	-1.014662282	1.44388E-33	1.014662282	stage_E14_vs_E10	ordinary_muscle_development	D_vs_E10;stage_1W_vs_E10;stage_3W_vs_E10;stage	1	TRUE
ENSGALG00010020379	COL13A1	PRJNA729271	-2.467091687	1.44507E-33	2.467091687	stage_E14_vs_E10	ordinary_muscle_development	D_vs_E10;stage_1W_vs_E10;stage_3W_vs_E10;stage	1	TRUE
ENSGALG00010008731	NDUFB9	PRJNA729271	1.069145361	1.50031E-33	1.069145361	stage_E14_vs_E10	ordinary_muscle_development	D_vs_E10;stage_1W_vs_E10;stage_3W_vs_E10;stage	1	TRUE
ENSGALG00010000055	PRC1	PRJNA729271	-2.656368888	1.5053E-33	2.656368888	stage_E14_vs_E10	ordinary_muscle_development	D_vs_E10;stage_1W_vs_E10;stage_3W_vs_E10;stage	1	TRUE
ENSGALG00010006446	LSAMP	PRJNA729271	-2.305749773	1.5059E-33	2.305749773	stage_E14_vs_E10	ordinary_muscle_development	D_vs_E10;stage_1W_vs_E10;stage_3W_vs_E10;stage	1	TRUE
ENSGALG00010022671	DBR1	PRJNA729271	-0.711453962	1.54707E-33	0.711453962	stage_E14_vs_E10	ordinary_muscle_development		0	FALSE
ENSGALG00010017169	CCDC198	PRJNA729271	2.619090098	1.55456E-33	2.619090098	stage_E14_vs_E10	ordinary_muscle_development	D_vs_E10;stage_1W_vs_E10;stage_3W_vs_E10;stage	1	TRUE
ENSGALG00010018865	ENSGALG00010018865	PRJNA729271	-2.877554742	1.55476E-33	2.877554742	stage_E14_vs_E10	ordinary_muscle_development	D_vs_E10;stage_1W_vs_E10;stage_3W_vs_E10;stage	1	TRUE
ENSGALG00010008298	ENSGALG00010008298	PRJNA729271	-1.624952077	1.60091E-33	1.624952077	stage_E14_vs_E10	ordinary_muscle_development	D_vs_E10;stage_1W_vs_E10;stage_3W_vs_E10;stage	1	TRUE
ENSGALG00010024019	MAP3K14	PRJNA729271	1.292472728	1.71384E-33	1.292472728	stage_E14_vs_E10	ordinary_muscle_development	D_vs_E10;stage_1W_vs_E10;stage_3W_vs_E10;stage	1	TRUE
ENSGALG00010015136	TCEB3	PRJNA729271	1.098859683	1.73628E-33	1.098859683	stage_E14_vs_E10	ordinary_muscle_development	D_vs_E10;stage_1W_vs_E10;stage_3W_vs_E10;stage	1	TRUE
ENSGALG00010008276	DNAJC18	PRJNA729271	1.130491129	1.75543E-33	1.130491129	stage_E14_vs_E10	ordinary_muscle_development	D_vs_E10;stage_1W_vs_E10;stage_3W_vs_E10;stage	1	TRUE
ENSGALG00010029160	DUS1L	PRJNA729271	1.113789753	1.75859E-33	1.113789753	stage_E14_vs_E10	ordinary_muscle_development	D_vs_E10;stage_1W_vs_E10;stage_3W_vs_E10;stage	1	TRUE
ENSGALG00010026553	PLPP3	PRJNA729271	1.565139481	1.77597E-33	1.565139481	stage_E14_vs_E10	ordinary_muscle_development	D_vs_E10;stage_1W_vs_E10;stage_3W_vs_E10;stage	1	TRUE
ENSGALG00010028232	C9orf69	PRJNA729271	1.316736632	1.82342E-33	1.316736632	stage_E14_vs_E10	ordinary_muscle_development	D_vs_E10;stage_1W_vs_E10;stage_3W_vs_E10;stage	1	TRUE
ENSGALG00010027254	INSR	PRJNA729271	1.622235714	1.85082E-33	1.622235714	stage_E14_vs_E10	ordinary_muscle_development	D_vs_E10;stage_1W_vs_E10;stage_3W_vs_E10;stage	1	TRUE
ENSGALG00010017814	ENSGALG00010017814	PRJNA729271	-3.399481376	1.86068E-33	3.399481376	stage_E14_vs_E10	ordinary_muscle_development	D_vs_E10;stage_1W_vs_E10;stage_3W_vs_E10;stage	1	TRUE
ENSGALG00010025917	SCUBE3	PRJNA729271	-5.698212814	1.86232E-33	5.698212814	stage_E14_vs_E10	ordinary_muscle_development	D_vs_E10;stage_1W_vs_E10;stage_3W_vs_E10;stage	1	TRUE
ENSGALG00010021887	NUTF2	PRJNA729271	-1.361615053	1.86417E-33	1.361615053	stage_E14_vs_E10	ordinary_muscle_development	D_vs_E10;stage_1W_vs_E10;stage_3W_vs_E10;stage	1	TRUE
ENSGALG00010029530	ENSGALG00010029530	PRJNA729271	4.343334103	1.87663E-33	4.343334103	stage_E14_vs_E10	ordinary_muscle_development	D_vs_E10;stage_1W_vs_E10;stage_3W_vs_E10;stage	1	TRUE
ENSGALG00010001465	TACC2	PRJNA729271	1.925113195	1.89535E-33	1.925113195	stage_E14_vs_E10	ordinary_muscle_development	D_vs_E10;stage_1W_vs_E10;stage_3W_vs_E10;stage	1	TRUE
ENSGALG00010014244	SESN1	PRJNA729271	4.51380015	1.90527E-33	4.51380015	stage_E14_vs_E10	ordinary_muscle_development	D_vs_E10;stage_1W_vs_E10;stage_3W_vs_E10;stage	1	TRUE
ENSGALG00010002102	PAK1	PRJNA729271	-3.712455346	1.92344E-33	3.712455346	stage_E14_vs_E10	ordinary_muscle_development	D_vs_E10;stage_1W_vs_E10;stage_3W_vs_E10;stage	1	TRUE
ENSGALG00010024527	ATP6V0B	PRJNA729271	1.205238338	1.95999E-33	1.205238338	stage_E14_vs_E10	ordinary_muscle_development	D_vs_E10;stage_1W_vs_E10;stage_3W_vs_E10;stage	1	TRUE
ENSGALG00010007940	GLIPR2	PRJNA729271	-3.348392348	2.01256E-33	3.348392348	stage_E14_vs_E10	ordinary_muscle_development	D_vs_E10;stage_1W_vs_E10;stage_3W_vs_E10;stage	1	TRUE
ENSGALG00010019125	ENSGALG00010019125	PRJNA729271	2.402346165	2.09507E-33	2.402346165	stage_E14_vs_E10	ordinary_muscle_development	D_vs_E10;stage_1W_vs_E10;stage_3W_vs_E10;stage	1	TRUE
ENSGALG00010022002	KIF18B	PRJNA729271	-2.957399517	2.09685E-33	2.957399517	stage_E14_vs_E10	ordinary_muscle_development	D_vs_E10;stage_1W_vs_E10;stage_3W_vs_E10;stage	1	TRUE
ENSGALG00010012738	RAB21	PRJNA729271	0.833928638	2.13227E-33	0.833928638	stage_E14_vs_E10	ordinary_muscle_development		0	FALSE
ENSGALG00010013215	SF3A3	PRJNA729271	-1.012663239	2.14786E-33	1.012663239	stage_E14_vs_E10	ordinary_muscle_development	D_vs_E10;stage_1W_vs_E10;stage_3W_vs_E10;stage	1	TRUE
ENSGALG00010009999	GABRB2	PRJNA729271	-3.029015453	2.20997E-33	3.029015453	stage_E14_vs_E10	ordinary_muscle_development	D_vs_E10;stage_1W_vs_E10;stage_3W_vs_E10;stage	1	TRUE
ENSGALG00010021877	PLEKHN1	PRJNA729271	1.775555169	2.28618E-33	1.775555169	stage_E14_vs_E10	ordinary_muscle_development	D_vs_E10;stage_1W_vs_E10;stage_3W_vs_E10;stage	1	TRUE
ENSGALG00010027712	STOM	PRJNA729271	1.916112459	2.28648E-33	1.916112459	stage_E14_vs_E10	ordinary_muscle_development	D_vs_E10;stage_1W_vs_E10;stage_3W_vs_E10;stage	1	TRUE
ENSGALG00010006421	LMF2	PRJNA729271	-1.390098639	2.32096E-33	1.390098639	stage_E14_vs_E10	ordinary_muscle_development	D_vs_E10;stage_1W_vs_E10;stage_3W_vs_E10;stage	1	TRUE
ENSGALG00010028512	NDUFA13	PRJNA729271	1.492251063	2.32949E-33	1.492251063	stage_E14_vs_E10	ordinary_muscle_development	D_vs_E10;stage_1W_vs_E10;stage_3W_vs_E10;stage	1	TRUE
ENSGALG00010015858	CHST2	PRJNA729271	-1.136649826	2.33927E-33	1.136649826	stage_E14_vs_E10	ordinary_muscle_development	D_vs_E10;stage_1W_vs_E10;stage_3W_vs_E10;stage	1	TRUE
ENSGALG00010011451	CRHBP	PRJNA729271	-10.60132491	2.34238E-33	10.60132491	stage_E14_vs_E10	ordinary_muscle_development	D_vs_E10;stage_1W_vs_E10;stage_3W_vs_E10;stage	1	TRUE
ENSGALG00010006966	PGBD5	PRJNA729271	-3.571530964	2.44294E-33	3.571530964	stage_E14_vs_E10	ordinary_muscle_development	D_vs_E10;stage_1W_vs_E10;stage_3W_vs_E10;stage	1	TRUE
ENSGALG00010027329	MINDY4	PRJNA729271	-1.507039271	2.44521E-33	1.507039271	stage_E14_vs_E10	ordinary_muscle_development	D_vs_E10;stage_1W_vs_E10;stage_3W_vs_E10;stage	1	TRUE
ENSGALG00010014328	RASSF8	PRJNA729271	-1.123020036	2.49422E-33	1.123020036	stage_E14_vs_E10	ordinary_muscle_development	D_vs_E10;stage_1W_vs_E10;stage_3W_vs_E10;stage	1	TRUE
ENSGALG00010002546	ATP6AP2	PRJNA729271	0.765136455	2.51008E-33	0.765136455	stage_E14_vs_E10	ordinary_muscle_development		0	FALSE
ENSGALG00010019621	ENSGALG00010019621	PRJNA729271	1.865798863	2.56772E-33	1.865798863	stage_E14_vs_E10	ordinary_muscle_development	D_vs_E10;stage_1W_vs_E10;stage_3W_vs_E10;stage	1	TRUE
ENSGALG00010016905	C1QC	PRJNA729271	4.512982385	2.65279E-33	4.512982385	stage_E14_vs_E10	ordinary_muscle_development	D_vs_E10;stage_1W_vs_E10;stage_3W_vs_E10;stage	1	TRUE
ENSGALG00010008245	FBXO32	PRJNA729271	5.988865474	2.68593E-33	5.988865474	stage_E14_vs_E10	ordinary_muscle_development	D_vs_E10;stage_1W_vs_E10;stage_3W_vs_E10;stage	1	TRUE
ENSGALG00010013740	GNPDA2	PRJNA729271	-1.474110611	2.75237E-33	1.474110611	stage_E14_vs_E10	ordinary_muscle_development	D_vs_E10;stage_1W_vs_E10;stage_3W_vs_E10;stage	1	TRUE
ENSGALG00010017706	FGFRL1	PRJNA729271	-1.775733281	2.79363E-33	1.775733281	stage_E14_vs_E10	ordinary_muscle_development	D_vs_E10;stage_1W_vs_E10;stage_3W_vs_E10;stage	1	TRUE
ENSGALG00010011253	KDM4C	PRJNA729271	1.320440662	2.80416E-33	1.320440662	stage_E14_vs_E10	ordinary_muscle_development	D_vs_E10;stage_1W_vs_E10;stage_3W_vs_E10;stage	1	TRUE
ENSGALG00010007184	VGLL3	PRJNA729271	1.129660319	3.04442E-33	1.129660319	stage_E14_vs_E10	ordinary_muscle_development	D_vs_E10;stage_1W_vs_E10;stage_3W_vs_E10;stage	1	TRUE
ENSGALG00010010252	WDR7	PRJNA729271	1.159904443	3.08589E-33	1.159904443	stage_E14_vs_E10	ordinary_muscle_development	D_vs_E10;stage_1W_vs_E10;stage_3W_vs_E10;stage	1	TRUE
ENSGALG00010020597	ARID5A	PRJNA729271	2.878767995	3.11798E-33	2.878767995	stage_E14_vs_E10	ordinary_muscle_development	D_vs_E10;stage_1W_vs_E10;stage_3W_vs_E10;stage	1	TRUE
ENSGALG00010025268	ZBTB39	PRJNA729271	-1.37424691	3.14302E-33	1.37424691	stage_E14_vs_E10	ordinary_muscle_development	D_vs_E10;stage_1W_vs_E10;stage_3W_vs_E10;stage	1	TRUE
ENSGALG00010010186	TNPO1	PRJNA729271	0.875579282	3.42111E-33	0.875579282	stage_E14_vs_E10	ordinary_muscle_development		0	FALSE
ENSGALG00010023262	POLD4	PRJNA729271	2.501079805	3.4778E-33	2.501079805	stage_E14_vs_E10	ordinary_muscle_development	D_vs_E10;stage_1W_vs_E10;stage_3W_vs_E10;stage	1	TRUE
ENSGALG00010000823	PIBF1	PRJNA729271	1.935160132	3.71011E-33	1.935160132	stage_E14_vs_E10	ordinary_muscle_development	D_vs_E10;stage_1W_vs_E10;stage_3W_vs_E10;stage	1	TRUE
ENSGALG00010022237	SUN1	PRJNA729271	1.10515728	3.72223E-33	1.10515728	stage_E14_vs_E10	ordinary_muscle_development	D_vs_E10;stage_1W_vs_E10;stage_3W_vs_E10;stage	1	TRUE
ENSGALG00010025192	CRYAB	PRJNA729271	2.208573471	3.86054E-33	2.208573471	stage_E14_vs_E10	ordinary_muscle_development	D_vs_E10;stage_1W_vs_E10;stage_3W_vs_E10;stage	1	TRUE
ENSGALG00010006077	TBCK	PRJNA729271	-1.253600691	3.96317E-33	1.253600691	stage_E14_vs_E10	ordinary_muscle_development	D_vs_E10;stage_1W_vs_E10;stage_3W_vs_E10;stage	1	TRUE
ENSGALG00010023800	PEX5	PRJNA729271	0.867521659	3.97648E-33	0.867521659	stage_E14_vs_E10	ordinary_muscle_development		0	FALSE
ENSGALG00010001422	AP1S1	PRJNA729271	-1.729158125	4.1878E-33	1.729158125	stage_E14_vs_E10	ordinary_muscle_development	D_vs_E10;stage_1W_vs_E10;stage_3W_vs_E10;stage	1	TRUE
ENSGALG00010021135	CLK3	PRJNA729271	1.271012516	4.23235E-33	1.271012516	stage_E14_vs_E10	ordinary_muscle_development	D_vs_E10;stage_1W_vs_E10;stage_3W_vs_E10;stage	1	TRUE
ENSGALG00010021106	ITGB6	PRJNA729271	-2.63248332	4.26438E-33	2.63248332	stage_E14_vs_E10	ordinary_muscle_development	D_vs_E10;stage_1W_vs_E10;stage_3W_vs_E10;stage	1	TRUE
ENSGALG00010022572	ENSGALG00010022572	PRJNA729271	-6.324228743	4.32893E-33	6.324228743	stage_E14_vs_E10	ordinary_muscle_development	D_vs_E10;stage_1W_vs_E10;stage_3W_vs_E10;stage	1	TRUE
ENSGALG00010018864	ABCA3	PRJNA729271	1.442517023	4.42126E-33	1.442517023	stage_E14_vs_E10	ordinary_muscle_development	D_vs_E10;stage_1W_vs_E10;stage_3W_vs_E10;stage	1	TRUE
ENSGALG00010001657	SMAP1	PRJNA729271	-1.202689056	4.77076E-33	1.202689056	stage_E14_vs_E10	ordinary_muscle_development	D_vs_E10;stage_1W_vs_E10;stage_3W_vs_E10;stage	1	TRUE
ENSGALG00010015348	CPSF2	PRJNA729271	-0.749465091	4.79436E-33	0.749465091	stage_E14_vs_E10	ordinary_muscle_development		0	FALSE
ENSGALG00010011413	ZBTB37	PRJNA729271	1.254473165	4.88119E-33	1.254473165	stage_E14_vs_E10	ordinary_muscle_development	D_vs_E10;stage_1W_vs_E10;stage_3W_vs_E10;stage	1	TRUE
ENSGALG00010012771	ITGA2	PRJNA729271	-2.438772017	5.09987E-33	2.438772017	stage_E14_vs_E10	ordinary_muscle_development	D_vs_E10;stage_1W_vs_E10;stage_3W_vs_E10;stage	1	TRUE
ENSGALG00010011170	RBM24	PRJNA729271	1.74800927	5.15583E-33	1.74800927	stage_E14_vs_E10	ordinary_muscle_development	D_vs_E10;stage_1W_vs_E10;stage_3W_vs_E10;stage	1	TRUE
ENSGALG00010024011	AGPS	PRJNA729271	0.845234964	5.18981E-33	0.845234964	stage_E14_vs_E10	ordinary_muscle_development		0	FALSE
ENSGALG00010001185	PTPN2	PRJNA729271	-0.833367989	5.19899E-33	0.833367989	stage_E14_vs_E10	ordinary_muscle_development		0	FALSE
ENSGALG00010001179	GRHPR	PRJNA729271	2.222698834	5.38327E-33	2.222698834	stage_E14_vs_E10	ordinary_muscle_development	D_vs_E10;stage_1W_vs_E10;stage_3W_vs_E10;stage	1	TRUE
ENSGALG00010019169	GPR153	PRJNA729271	-3.167675358	5.47793E-33	3.167675358	stage_E14_vs_E10	ordinary_muscle_development	D_vs_E10;stage_1W_vs_E10;stage_3W_vs_E10;stage	1	TRUE
ENSGALG00010024119	AMMECR1L	PRJNA729271	-0.927508822	5.50848E-33	0.927508822	stage_E14_vs_E10	ordinary_muscle_development		0	FALSE
ENSGALG00010008075	SDHA	PRJNA729271	1.653159562	5.51043E-33	1.653159562	stage_E14_vs_E10	ordinary_muscle_development	D_vs_E10;stage_1W_vs_E10;stage_3W_vs_E10;stage	1	TRUE
ENSGALG00010018154	SLC16A12	PRJNA729271	-2.723036585	5.5211E-33	2.723036585	stage_E14_vs_E10	ordinary_muscle_development	D_vs_E10;stage_1W_vs_E10;stage_3W_vs_E10;stage	1	TRUE
ENSGALG00010024783	UBE2T	PRJNA729271	-2.991219735	5.56253E-33	2.991219735	stage_E14_vs_E10	ordinary_muscle_development	D_vs_E10;stage_1W_vs_E10;stage_3W_vs_E10;stage	1	TRUE
ENSGALG00010006580	ALDH6A1	PRJNA729271	1.679295229	5.56791E-33	1.679295229	stage_E14_vs_E10	ordinary_muscle_development	D_vs_E10;stage_1W_vs_E10;stage_3W_vs_E10;stage	1	TRUE
ENSGALG00010027982	ENTPD2	PRJNA729271	3.031770892	5.6922E-33	3.031770892	stage_E14_vs_E10	ordinary_muscle_development	D_vs_E10;stage_1W_vs_E10;stage_3W_vs_E10;stage	1	TRUE
ENSGALG00010012803	NUP155	PRJNA729271	-1.099983737	5.72369E-33	1.099983737	stage_E14_vs_E10	ordinary_muscle_development	D_vs_E10;stage_1W_vs_E10;stage_3W_vs_E10;stage	1	TRUE
ENSGALG00010016211	TACC3	PRJNA729271	-1.963710889	6.00254E-33	1.963710889	stage_E14_vs_E10	ordinary_muscle_development	D_vs_E10;stage_1W_vs_E10;stage_3W_vs_E10;stage	1	TRUE
ENSGALG00010013299	PLAGL2	PRJNA729271	-1.385600862	6.00254E-33	1.385600862	stage_E14_vs_E10	ordinary_muscle_development	D_vs_E10;stage_1W_vs_E10;stage_3W_vs_E10;stage	1	TRUE
ENSGALG00010021821	PPP1R19L	PRJNA729271	-1.461510632	6.35034E-33	1.461510632	stage_E14_vs_E10	ordinary_muscle_development	D_vs_E10;stage_1W_vs_E10;stage_3W_vs_E10;stage	1	TRUE
ENSGALG00010000412	RFC5	PRJNA729271	-1.326186619	6.36017E-33	1.326186619	stage_E14_vs_E10	ordinary_muscle_development	D_vs_E10;stage_1W_vs_E10;stage_3W_vs_E10;stage	1	TRUE
ENSGALG00010023060	ALDH1A2	PRJNA729271	-2.037549718	6.54971E-33	2.037549718	stage_E14_vs_E10	ordinary_muscle_development	D_vs_E10;stage_1W_vs_E10;stage_3W_vs_E10;stage	1	TRUE
ENSGALG00010022101	ENSGALG00010022101	PRJNA729271	2.163781575	6.67159E-33	2.163781575	stage_E14_vs_E10	ordinary_muscle_development	D_vs_E10;stage_1W_vs_E10;stage_3W_vs_E10;stage	1	TRUE
ENSGALG00010016904	EAF1	PRJNA729271	0.845686286	6.74797E-33	0.845686286	stage_E14_vs_E10	ordinary_muscle_development		0	FALSE
ENSGALG00010023359	CDCA3	PRJNA729271	-3.409297056	7.25952E-33	3.409297056	stage_E14_vs_E10	ordinary_muscle_development	D_vs_E10;stage_1W_vs_E10;stage_3W_vs_E10;stage	1	TRUE
ENSGALG00010007965	EGLN1	PRJNA729271	1.903578303	7.56748E-33	1.903578303	stage_E14_vs_E10	ordinary_muscle_development	D_vs_E10;stage_1W_vs_E10;stage_3W_vs_E10;stage	1	TRUE
ENSGALG00010012327	UBTD2	PRJNA729271	-1.090572751	7.58841E-33	1.090572751	stage_E14_vs_E10	ordinary_muscle_development	D_vs_E10;stage_1W_vs_E10;stage_3W_vs_E10;stage	1	TRUE
ENSGALG00010028870	CACFD1	PRJNA729271	-2.844770764	7.82695E-33	2.844770764	stage_E14_vs_E10	ordinary_muscle_development	D_vs_E10;stage_1W_vs_E10;stage_3W_vs_E10;stage	1	TRUE
ENSGALG00010015584	TTLL9	PRJNA729271	2.744500276	8.01887E-33	2.744500276	stage_E14_vs_E10	ordinary_muscle_development	D_vs_E10;stage_1W_vs_E10;stage_3W_vs_E10;stage	1	TRUE
ENSGALG00010005423	ST3GAL6	PRJNA729271	1.808344396	8.12406E-33	1.808344396	stage_E14_vs_E10	ordinary_muscle_development	D_vs_E10;stage_1W_vs_E10;stage_3W_vs_E10;stage	1	TRUE
ENSGALG00010024045	PSTPIP1	PRJNA729271	2.941233379	8.16117E-33	2.941233379	stage_E14_vs_E10	ordinary_muscle_development	D_vs_E10;stage_1W_vs_E10;stage_3W_vs_E10;stage	1	TRUE
ENSGALG00010017882	LZTS3	PRJNA729271	2.422964632	8.2025E-33	2.422964632	stage_E14_vs_E10	ordinary_muscle_development	D_vs_E10;stage_1W_vs_E10;stage_3W_vs_E10;stage	1	TRUE
ENSGALG00010009640	NPR2	PRJNA729271	2.266984943	9.03426E-33	2.266984943	stage_E14_vs_E10	ordinary_muscle_development	D_vs_E10;stage_1W_vs_E10;stage_3W_vs_E10;stage	1	TRUE
ENSGALG00010010922	DYM	PRJNA729271	1.222143637	9.38454E-33	1.222143637	stage_E14_vs_E10	ordinary_muscle_development	D_vs_E10;stage_1W_vs_E10;stage_3W_vs_E10;stage	1	TRUE
ENSGALG00010016404	ESRRG	PRJNA729271	1.800193365	9.78459E-33	1.800193365	stage_E14_vs_E10	ordinary_muscle_development	D_vs_E10;stage_1W_vs_E10;stage_3W_vs_E10;stage	1	TRUE
ENSGALG00010022931	RDH5	PRJNA729271	1.456252731	9.85873E-33	1.456252731	stage_E14_vs_E10	ordinary_muscle_development	D_vs_E10;stage_1W_vs_E10;stage_3W_vs_E10;stage	1	TRUE
ENSGALG00010018815	ENSGALG00010018815	PRJNA729271	2.055605592	9.93592E-33	2.055605592	stage_E14_vs_E10	ordinary_muscle_development	D_vs_E10;stage_1W_vs_E10;stage_3W_vs_E10;stage	1	TRUE
ENSGALG00010013209	KIAA1549	PRJNA729271	-1.263253928	1.01452E-32	1.263253928	stage_E14_vs_E10	ordinary_muscle_development	D_vs_E10;stage_1W_vs_E10;stage_3W_vs_E10;stage	1	TRUE
ENSGALG00010013369	MTMR8	PRJNA729271	-0.77062838	1.03335E-32	0.77062838	stage_E14_vs_E10	ordinary_muscle_development		0	FALSE
ENSGALG00010029439	ENSGALG00010029439	PRJNA729271	-2.687822729	1.05717E-32	2.687822729	stage_E14_vs_E10	ordinary_muscle_development	D_vs_E10;stage_1W_vs_E10;stage_3W_vs_E10;stage	1	TRUE
ENSGALG00010011671	SLC41A2	PRJNA729271	-2.388692713	1.06995E-32	2.388692713	stage_E14_vs_E10	ordinary_muscle_development	D_vs_E10;stage_1W_vs_E10;stage_3W_vs_E10;stage	1	TRUE
ENSGALG00010028160	ENSGALG00010028160	PRJNA729271	6.609003831	1.09195E-32	6.609003831	stage_E14_vs_E10	ordinary_muscle_development	D_vs_E10;stage_1W_vs_E10;stage_3W_vs_E10;stage	1	TRUE
ENSGALG00010022734	ENSGALG00010022734	PRJNA729271	1.432109695	1.14524E-32	1.432109695	stage_E14_vs_E10	ordinary_muscle_development	D_vs_E10;stage_1W_vs_E10;stage_3W_vs_E10;stage	1	TRUE
ENSGALG00010007432	NAT	PRJNA729271	2.23501458	1.14706E-32	2.23501458	stage_E14_vs_E10	ordinary_muscle_development	D_vs_E10;stage_1W_vs_E10;stage_3W_vs_E10;stage	1	TRUE
ENSGALG00010020055	R3HCC1	PRJNA729271	-1.027233175	1.15192E-32	1.027233175	stage_E14_vs_E10	ordinary_muscle_development	D_vs_E10;stage_1W_vs_E10;stage_3W_vs_E10;stage	1	TRUE
ENSGALG00010023172	DHX58	PRJNA729271	3.883644464	1.19893E-32	3.883644464	stage_E14_vs_E10	ordinary_muscle_development	D_vs_E10;stage_1W_vs_E10;stage_3W_vs_E10;stage	1	TRUE
ENSGALG00010002570	ADAM12	PRJNA729271	-3.279260056	1.20043E-32	3.279260056	stage_E14_vs_E10	ordinary_muscle_development	D_vs_E10;stage_1W_vs_E10;stage_3W_vs_E10;stage	1	TRUE
ENSGALG00010004154	COPS5	PRJNA729271	1.554908056	1.21221E-32	1.554908056	stage_E14_vs_E10	ordinary_muscle_development	D_vs_E10;stage_1W_vs_E10;stage_3W_vs_E10;stage	1	TRUE
ENSGALG00010023188	KIF1BP	PRJNA729271	0.692929979	1.22262E-32	0.692929979	stage_E14_vs_E10	ordinary_muscle_development		0	FALSE
ENSGALG00010003958	CYSLTR2	PRJNA729271	4.342251232	1.22385E-32	4.342251232	stage_E14_vs_E10	ordinary_muscle_development	D_vs_E10;stage_1W_vs_E10;stage_3W_vs_E10;stage	1	TRUE
ENSGALG00010021318	TP73	PRJNA729271	-3.594127104	1.22526E-32	3.594127104	stage_E14_vs_E10	ordinary_muscle_development	D_vs_E10;stage_1W_vs_E10;stage_3W_vs_E10;stage	1	TRUE
ENSGALG00010028734	ENSGALG00010028734	PRJNA729271	4.275844888	1.23174E-32	4.275844888	stage_E14_vs_E10	ordinary_muscle_development	D_vs_E10;stage_1W_vs_E10;stage_3W_vs_E10;stage	1	TRUE
ENSGALG00010027208	UBXN6	PRJNA729271	1.124552117	1.23339E-32	1.124552117	stage_E14_vs_E10	ordinary_muscle_development	D_vs_E10;stage_1W_vs_E10;stage_3W_vs_E10;stage	1	TRUE
ENSGALG00010022090	TRRAP	PRJNA729271	-1.099231862	1.32595E-32	1.099231862	stage_E14_vs_E10	ordinary_muscle_development	D_vs_E10;stage_1W_vs_E10;stage_3W_vs_E10;stage	1	TRUE
ENSGALG00010026154	ZNF385B	PRJNA729271	-2.230572712	1.39289E-32	2.230572712	stage_E14_vs_E10	ordinary_muscle_development	D_vs_E10;stage_1W_vs_E10;stage_3W_vs_E10;stage	1	TRUE
ENSGALG00010007007	ACSL1	PRJNA729271	2.618125306	1.39995E-32	2.618125306	stage_E14_vs_E10	ordinary_muscle_development	D_vs_E10;stage_1W_vs_E10;stage_3W_vs_E10;stage	1	TRUE
ENSGALG00010016369	MSANTD1	PRJNA729271	-5.210202948	1.4518E-32	5.210202948	stage_E14_vs_E10	ordinary_muscle_development	D_vs_E10;stage_1W_vs_E10;stage_3W_vs_E10;stage	1	TRUE

ENSGALG00010001224	DNAJB9	PRJNA729271	2.265610223	1.57156E-32	2.265610223	stage_E14_vs_E10	ordinary_muscle_development	D_vs_E10;stage_1W_vs_E10;stage_3W_vs_E10;stage	1	TRUE
ENSGALG00010003920	LPAR6	PRJNA729271	1.822100446	1.70358E-32	1.822100446	stage_E14_vs_E10	ordinary_muscle_development	D_vs_E10;stage_1W_vs_E10;stage_3W_vs_E10;stage	1	TRUE
ENSGALG00010020588	PALD1	PRJNA729271	-1.328432075	1.7045E-32	1.328432075	stage_E14_vs_E10	ordinary_muscle_development	D_vs_E10;stage_1W_vs_E10;stage_3W_vs_E10;stage	1	TRUE
ENSGALG00010011977	GATD3A	PRJNA729271	1.42174857	1.78521E-32	1.42174857	stage_E14_vs_E10	ordinary_muscle_development	D_vs_E10;stage_1W_vs_E10;stage_3W_vs_E10;stage	1	TRUE
ENSGALG00010000084	ENSGALG00010000084	PRJNA729271	3.063385087	1.83312E-32	3.063385087	stage_E14_vs_E10	ordinary_muscle_development	D_vs_E10;stage_1W_vs_E10;stage_3W_vs_E10;stage	1	TRUE
ENSGALG00010006548	TMEM209	PRJNA729271	-0.98044718	1.93916E-32	0.98044718	stage_E14_vs_E10	ordinary_muscle_development		0	FALSE
ENSGALG00010011551	GMDS	PRJNA729271	-1.892342393	1.94995E-32	1.892342393	stage_E14_vs_E10	ordinary_muscle_development	D_vs_E10;stage_1W_vs_E10;stage_3W_vs_E10;stage	1	TRUE
ENSGALG00010006359	SGK1	PRJNA729271	1.966697907	1.95252E-32	1.966697907	stage_E14_vs_E10	ordinary_muscle_development	D_vs_E10;stage_1W_vs_E10;stage_3W_vs_E10;stage	1	TRUE
ENSGALG00010029932	RASA4B	PRJNA729271	3.189988035	1.98513E-32	3.189988035	stage_E14_vs_E10	ordinary_muscle_development	D_vs_E10;stage_1W_vs_E10;stage_3W_vs_E10;stage	1	TRUE
ENSGALG00010014690	CSF3R	PRJNA729271	4.390423984	2.06909E-32	4.390423984	stage_E14_vs_E10	ordinary_muscle_development	D_vs_E10;stage_1W_vs_E10;stage_3W_vs_E10;stage	1	TRUE
ENSGALG00010021945	C1QTNF4	PRJNA729271	-2.711710409	2.10609E-32	2.711710409	stage_E14_vs_E10	ordinary_muscle_development	D_vs_E10;stage_1W_vs_E10;stage_3W_vs_E10;stage	1	TRUE
ENSGALG00010001287	PLPP4	PRJNA729271	-5.353368501	2.11524E-32	5.353368501	stage_E14_vs_E10	ordinary_muscle_development	D_vs_E10;stage_1W_vs_E10;stage_3W_vs_E10;stage	1	TRUE
ENSGALG00010027151	MICOS13	PRJNA729271	1.717862739	2.17096E-32	1.717862739	stage_E14_vs_E10	ordinary_muscle_development	D_vs_E10;stage_1W_vs_E10;stage_3W_vs_E10;stage	1	TRUE
ENSGALG00010029762	SLC47A2	PRJNA729271	5.563026247	2.17903E-32	5.563026247	stage_E14_vs_E10	ordinary_muscle_development	D_vs_E10;stage_1W_vs_E10;stage_3W_vs_E10;stage	1	TRUE
ENSGALG00010025708	HOXC8	PRJNA729271	-3.194423004	2.23785E-32	3.194423004	stage_E14_vs_E10	ordinary_muscle_development	D_vs_E10;stage_1W_vs_E10;stage_3W_vs_E10;stage	1	TRUE
ENSGALG00010021558	CHURC1	PRJNA729271	2.153084396	2.24506E-32	2.153084396	stage_E14_vs_E10	ordinary_muscle_development	D_vs_E10;stage_1W_vs_E10;stage_3W_vs_E10;stage	1	TRUE
ENSGALG00010003814	TPP1	PRJNA729271	1.586113726	2.27353E-32	1.586113726	stage_E14_vs_E10	ordinary_muscle_development	D_vs_E10;stage_1W_vs_E10;stage_3W_vs_E10;stage	1	TRUE
ENSGALG00010018377	RAP1GAP	PRJNA729271	3.073002618	2.3139E-32	3.073002618	stage_E14_vs_E10	ordinary_muscle_development	D_vs_E10;stage_1W_vs_E10;stage_3W_vs_E10;stage	1	TRUE
ENSGALG00010027092	ENSGALG00010027092	PRJNA729271	-2.483832883	2.35628E-32	2.483832883	stage_E14_vs_E10	ordinary_muscle_development	D_vs_E10;stage_1W_vs_E10;stage_3W_vs_E10;stage	1	TRUE
ENSGALG00010017672	SPRED2	PRJNA729271	-0.948723003	2.37985E-32	0.948723003	stage_E14_vs_E10	ordinary_muscle_development		0	FALSE
ENSGALG00010026980	LIMK2	PRJNA729271	-1.443586898	2.40367E-32	1.443586898	stage_E14_vs_E10	ordinary_muscle_development	D_vs_E10;stage_1W_vs_E10;stage_3W_vs_E10;stage	1	TRUE
ENSGALG00010016097	FNIP1	PRJNA729271	1.319118243	2.51026E-32	1.319118243	stage_E14_vs_E10	ordinary_muscle_development	D_vs_E10;stage_1W_vs_E10;stage_3W_vs_E10;stage	1	TRUE
ENSGALG00010005190	ENSGALG00010005190	PRJNA729271	3.284635614	2.55122E-32	3.284635614	stage_E14_vs_E10	ordinary_muscle_development	D_vs_E10;stage_1W_vs_E10;stage_3W_vs_E10;stage	1	TRUE
ENSGALG00010017568	CDCP1	PRJNA729271	-3.118286196	2.55523E-32	3.118286196	stage_E14_vs_E10	ordinary_muscle_development	D_vs_E10;stage_1W_vs_E10;stage_3W_vs_E10;stage	1	TRUE
ENSGALG00010029246	DHX33	PRJNA729271	-1.657163038	2.63507E-32	1.657163038	stage_E14_vs_E10	ordinary_muscle_development	D_vs_E10;stage_1W_vs_E10;stage_3W_vs_E10;stage	1	TRUE
ENSGALG00010001706	IQUB	PRJNA729271	4.056090392	2.6441E-32	4.056090392	stage_E14_vs_E10	ordinary_muscle_development	D_vs_E10;stage_1W_vs_E10;stage_3W_vs_E10;stage	1	TRUE
ENSGALG00010018729	HS3ST6	PRJNA729271	-7.304247036	2.77179E-32	7.304247036	stage_E14_vs_E10	ordinary_muscle_development	D_vs_E10;stage_1W_vs_E10;stage_3W_vs_E10;stage	1	TRUE
ENSGALG00010009266	PCDH7	PRJNA729271	-3.080654289	2.77728E-32	3.080654289	stage_E14_vs_E10	ordinary_muscle_development	D_vs_E10;stage_1W_vs_E10;stage_3W_vs_E10;stage	1	TRUE
ENSGALG00010028506	SHD	PRJNA729271	-8.157283113	2.78407E-32	8.157283113	stage_E14_vs_E10	ordinary_muscle_development	D_vs_E10;stage_1W_vs_E10;stage_3W_vs_E10;stage	1	TRUE
ENSGALG00010003214	PRDX4	PRJNA729271	-1.14544	2.79171E-32	1.14544	stage_E14_vs_E10	ordinary_muscle_development	D_vs_E10;stage_1W_vs_E10;stage_3W_vs_E10;stage	1	TRUE
ENSGALG00010008443	RSFR	PRJNA729271	5.263503428	2.81006E-32	5.263503428	stage_E14_vs_E10	ordinary_muscle_development	D_vs_E10;stage_1W_vs_E10;stage_3W_vs_E10;stage	1	TRUE
ENSGALG00010024178	N4BP1	PRJNA729271	1.441017191	2.83513E-32	1.441017191	stage_E14_vs_E10	ordinary_muscle_development	D_vs_E10;stage_1W_vs_E10;stage_3W_vs_E10;stage	1	TRUE
ENSGALG00010015103	ITPK1	PRJNA729271	-1.123262724	2.86393E-32	1.123262724	stage_E14_vs_E10	ordinary_muscle_development	D_vs_E10;stage_1W_vs_E10;stage_3W_vs_E10;stage	1	TRUE
ENSGALG00010025549	DHCR24	PRJNA729271	-3.06837399	2.87177E-32	3.06837399	stage_E14_vs_E10	ordinary_muscle_development	D_vs_E10;stage_1W_vs_E10;stage_3W_vs_E10;stage	1	TRUE
ENSGALG00010022386	SPI1	PRJNA729271	2.918624415	2.91082E-32	2.918624415	stage_E14_vs_E10	ordinary_muscle_development	D_vs_E10;stage_1W_vs_E10;stage_3W_vs_E10;stage	1	TRUE
ENSGALG00010004181	BBS12	PRJNA729271	-1.620345376	2.94545E-32	1.620345376	stage_E14_vs_E10	ordinary_muscle_development	D_vs_E10;stage_1W_vs_E10;stage_3W_vs_E10;stage	1	TRUE
ENSGALG00010027263	TAMM41	PRJNA729271	-1.557149263	2.96808E-32	1.557149263	stage_E14_vs_E10	ordinary_muscle_development	D_vs_E10;stage_1W_vs_E10;stage_3W_vs_E10;stage	1	TRUE
ENSGALG00010014400	RIPOR3	PRJNA729271	-1.247824345	3.02514E-32	1.247824345	stage_E14_vs_E10	ordinary_muscle_development	D_vs_E10;stage_1W_vs_E10;stage_3W_vs_E10;stage	1	TRUE
ENSGALG00010017822	VWA3A	PRJNA729271	3.116260722	3.03177E-32	3.116260722	stage_E14_vs_E10	ordinary_muscle_development	D_vs_E10;stage_1W_vs_E10;stage_3W_vs_E10;stage	1	TRUE
ENSGALG00010007985	APELA	PRJNA729271	-7.200209513	3.03541E-32	7.200209513	stage_E14_vs_E10	ordinary_muscle_development	D_vs_E10;stage_1W_vs_E10;stage_3W_vs_E10;stage	1	TRUE
ENSGALG00010015160	SIM1	PRJNA729271	-7.133288604	3.08266E-32	7.133288604	stage_E14_vs_E10	ordinary_muscle_development	D_vs_E10;stage_1W_vs_E10;stage_3W_vs_E10;stage	1	TRUE
ENSGALG00010029387	SERPINF1	PRJNA729271	2.107394511	3.09541E-32	2.107394511	stage_E14_vs_E10	ordinary_muscle_development	D_vs_E10;stage_1W_vs_E10;stage_3W_vs_E10;stage	1	TRUE
ENSGALG00010011021	PPP1R3G	PRJNA729271	2.193169711	3.10808E-32	2.193169711	stage_E14_vs_E10	ordinary_muscle_development	D_vs_E10;stage_1W_vs_E10;stage_3W_vs_E10;stage	1	TRUE
ENSGALG00010010338	CNST	PRJNA729271	1.856449007	3.20441E-32	1.856449007	stage_E14_vs_E10	ordinary_muscle_development	D_vs_E10;stage_1W_vs_E10;stage_3W_vs_E10;stage	1	TRUE
ENSGALG00010012911	CSNK1E	PRJNA729271	-1.025905283	3.23306E-32	1.025905283	stage_E14_vs_E10	ordinary_muscle_development	D_vs_E10;stage_1W_vs_E10;stage_3W_vs_E10;stage	1	TRUE
ENSGALG00010029245	SKA2	PRJNA729271	-1.38905456	3.30295E-32	1.38905456	stage_E14_vs_E10	ordinary_muscle_development	D_vs_E10;stage_1W_vs_E10;stage_3W_vs_E10;stage	1	TRUE
ENSGALG00010016831	AIDA	PRJNA729271	-0.809134509	3.33111E-32	0.809134509	stage_E14_vs_E10	ordinary_muscle_development		0	FALSE
ENSGALG00010024903	CASP8	PRJNA729271	-1.437482935	3.51991E-32	1.437482935	stage_E14_vs_E10	ordinary_muscle_development	D_vs_E10;stage_1W_vs_E10;stage_3W_vs_E10;stage	1	TRUE
ENSGALG00010008032	PUM2	PRJNA729271	-0.652057643	3.61227E-32	0.652057643	stage_E14_vs_E10	ordinary_muscle_development		0	FALSE
ENSGALG00010005618	PHACTR2	PRJNA729271	-0.822697553	3.77143E-32	0.822697553	stage_E14_vs_E10	ordinary_muscle_development		0	FALSE
ENSGALG00010028087	STIMATE	PRJNA729271	-1.08555282	3.81737E-32	1.08555282	stage_E14_vs_E10	ordinary_muscle_development	D_vs_E10;stage_1W_vs_E10;stage_3W_vs_E10;stage	1	TRUE
ENSGALG00010021315	COX6A1	PRJNA729271	1.615056856	3.86104E-32	1.615056856	stage_E14_vs_E10	ordinary_muscle_development	D_vs_E10;stage_1W_vs_E10;stage_3W_vs_E10;stage	1	TRUE
ENSGALG00010017400	GPC4	PRJNA729271	-1.424072913	4.09341E-32	1.424072913	stage_E14_vs_E10	ordinary_muscle_development	D_vs_E10;stage_1W_vs_E10;stage_3W_vs_E10;stage	1	TRUE
ENSGALG00010016422	KCTD3	PRJNA729271	1.088486116	4.11552E-32	1.088486116	stage_E14_vs_E10	ordinary_muscle_development	D_vs_E10;stage_1W_vs_E10;stage_3W_vs_E10;stage	1	TRUE
ENSGALG00010000812	ENSGALG00010000812	PRJNA729271	-1.160160103	4.20196E-32	1.160160103	stage_E14_vs_E10	ordinary_muscle_development	D_vs_E10;stage_1W_vs_E10;stage_3W_vs_E10;stage	1	TRUE
ENSGALG00010025102	ARPC3	PRJNA729271	-1.0405651	4.20196E-32	1.0405651	stage_E14_vs_E10	ordinary_muscle_development	D_vs_E10;stage_1W_vs_E10;stage_3W_vs_E10;stage	1	TRUE
ENSGALG00010022235	RAMP2	PRJNA729271	1.348418761	4.20368E-32	1.348418761	stage_E14_vs_E10	ordinary_muscle_development	D_vs_E10;stage_1W_vs_E10;stage_3W_vs_E10;stage	1	TRUE
ENSGALG00010004201	CPA6	PRJNA729271	-9.901717676	4.36281E-32	9.901717676	stage_E14_vs_E10	ordinary_muscle_development	D_vs_E10;stage_1W_vs_E10;stage_3W_vs_E10;stage	1	TRUE
ENSGALG00010026457	SSRP1	PRJNA729271	-1.185592276	4.4061E-32	1.185592276	stage_E14_vs_E10	ordinary_muscle_development	D_vs_E10;stage_1W_vs_E10;stage_3W_vs_E10;stage	1	TRUE
ENSGALG00010017592	GID4	PRJNA729271	1.296044186	4.63095E-32	1.296044186	stage_E14_vs_E10	ordinary_muscle_development	D_vs_E10;stage_1W_vs_E10;stage_3W_vs_E10;stage	1	TRUE
ENSGALG00010022622	MPPED2	PRJNA729271	-1.650318811	4.70671E-32	1.650318811	stage_E14_vs_E10	ordinary_muscle_development	D_vs_E10;stage_1W_vs_E10;stage_3W_vs_E10;stage	1	TRUE
ENSGALG00010007871	HSPA4L	PRJNA729271	-1.062487605	4.85489E-32	1.062487605	stage_E14_vs_E10	ordinary_muscle_development	D_vs_E10;stage_1W_vs_E10;stage_3W_vs_E10;stage	1	TRUE
ENSGALG00010016961	EIF4A3	PRJNA729271	-0.897305313	4.92187E-32	0.897305313	stage_E14_vs_E10	ordinary_muscle_development		0	FALSE
ENSGALG00010017831	PLEK	PRJNA729271	2.152802358	5.07359E-32	2.152802358	stage_E14_vs_E10	ordinary_muscle_development	D_vs_E10;stage_1W_vs_E10;stage_3W_vs_E10;stage	1	TRUE
ENSGALG00010021227	CHCHD4	PRJNA729271	-1.193768246	5.28938E-32	1.193768246	stage_E14_vs_E10	ordinary_muscle_development	D_vs_E10;stage_1W_vs_E10;stage_3W_vs_E10;stage	1	TRUE
ENSGALG00010023996	MYO7B	PRJNA729271	5.498179664	5.32969E-32	5.498179664	stage_E14_vs_E10	ordinary_muscle_development	D_vs_E10;stage_1W_vs_E10;stage_3W_vs_E10;stage	1	TRUE
ENSGALG00010028031	NISCH	PRJNA729271	0.963092294	5.32969E-32	0.963092294	stage_E14_vs_E10	ordinary_muscle_development		0	FALSE
ENSGALG00010001220	PLXNB2	PRJNA729271	-0.99082564	5.34866E-32	0.99082564	stage_E14_vs_E10	ordinary_muscle_development		0	FALSE
ENSGALG00010001419	KCND2	PRJNA729271	-4.817307094	5.38117E-32	4.817307094	stage_E14_vs_E10	ordinary_muscle_development	D_vs_E10;stage_1W_vs_E10;stage_3W_vs_E10;stage	1	TRUE
ENSGALG00010020720	ENSGALG00010020720	PRJNA729271	-6.191448294	5.46494E-32	6.191448294	stage_E14_vs_E10	ordinary_muscle_development	D_vs_E10;stage_1W_vs_E10;stage_3W_vs_E10;stage	1	TRUE
ENSGALG00010026209	MED19	PRJNA729271	2.029141916	5.55551E-32	2.029141916	stage_E14_vs_E10	ordinary_muscle_development	D_vs_E10;stage_1W_vs_E10;stage_3W_vs_E10;stage	1	TRUE
ENSGALG00010023078	TAGLN	PRJNA729271	2.338262953	5.56215E-32	2.338262953	stage_E14_vs_E10	ordinary_muscle_development	D_vs_E10;stage_1W_vs_E10;stage_3W_vs_E10;stage	1	TRUE
ENSGALG00010026229	CRHR2	PRJNA729271	2.499134821	5.58751E-32	2.499134821	stage_E14_vs_E10	ordinary_muscle_development	D_vs_E10;stage_1W_vs_E10;stage_3W_vs_E10;stage	1	TRUE
ENSGALG00010015149	ENSGALG00010015149	PRJNA729271	1.653844144	5.70183E-32	1.653844144	stage_E14_vs_E10	ordinary_muscle_development	D_vs_E10;stage_1W_vs_E10;stage_3W_vs_E10;stage	1	TRUE
ENSGALG00010023456	MPI	PRJNA729271	1.710427233	6.00444E-32	1.710427233	stage_E14_vs_E10	ordinary_muscle_development	D_vs_E10;stage_1W_vs_E10;stage_3W_vs_E10;stage	1	TRUE
ENSGALG00010021744	LITAF	PRJNA729271	4.03508488	6.02066E-32	4.03508488	stage_E14_vs_E10	ordinary_muscle_development	D_vs_E10;stage_1W_vs_E10;stage_3W_vs_E10;stage	1	TRUE
ENSGALG00010004430	TCEA1	PRJNA729271	-1.139990115	6.09363E-32	1.139990115	stage_E14_vs_E10	ordinary_muscle_development	D_vs_E10;stage_1W_vs_E10;stage_3W_vs_E10;stage	1	TRUE
ENSGALG00010005107	TTLL5	PRJNA729271	-0.887746549	6.89569E-32	0.887746549	stage_E14_vs_E10	ordinary_muscle_development		0	FALSE
ENSGALG00010007852	SLC20A2	PRJNA729271	2.471685167	7.69332E-32	2.471685167	stage_E14_vs_E10	ordinary_muscle_development	D_vs_E10;stage_1W_vs_E10;stage_3W_vs_E10;stage	1	TRUE
ENSGALG00010020994	DHCR7	PRJNA729271	-1.745105718	7.77613E-32	1.745105718	stage_E14_vs_E10	ordinary_muscle_development	D_vs_E10;stage_1W_vs_E10;stage_3W_vs_E10;stage	1	TRUE
ENSGALG00010017955	IKBKB	PRJNA729271	0.749291722	7.79496E-32	0.749291722	stage_E14_vs_E10	ordinary_muscle_development		0	FALSE
ENSGALG00010005537	GRIN3A	PRJNA729271	-4.634742002	7.99421E-32	4.634742002	stage_E14_vs_E10	ordinary_muscle_development	D_vs_E10;stage_1W_vs_E10;stage_3W_vs_E10;stage	1	TRUE
ENSGALG00010003995	MLNR	PRJNA729271	-3.495394147	8.44242E-32	3.495394147	stage_E14_vs_E10	ordinary_muscle_development	D_vs_E10;stage_1W_vs_E10;stage_3W_vs_E10;stage	1	TRUE
ENSGALG00010005247	THAP7	PRJNA729271	1.387917989	8.63023E-32	1.387917989	stage_E14_vs_E10	ordinary_muscle_development	D_vs_E10;stage_1W_vs_E10;stage_3W_vs_E10;stage	1	TRUE
ENSGALG00010007590	FBXW7	PRJNA729271	1.641072168	8.74063E-32	1.641072168	stage_E14_vs_E10	ordinary_muscle_development	D_vs_E10;stage_1W_vs_E10;stage_3W_vs_E10;stage	1	TRUE
ENSGALG00010019598	KCNAB2	PRJNA729271	-1.659704616	9.32666E-32	1.659704616	stage_E14_vs_E10	ordinary_muscle_development	D_vs_E10;stage_1W_vs_E10;stage_3W_vs_E10;stage	1	TRUE
ENSGALG00010010290	ENSGALG00010010290	PRJNA729271	1.928469036	9.5112E-32	1.928469036	stage_E14_vs_E10	ordinary_muscle_development	D_vs_E10;stage_1W_vs_E10;stage_3W_vs_E10;stage	1	TRUE
ENSGALG00010025204	TRAF6	PRJNA729271	1.383034428	9.64914E-32	1.383034428	stage_E14_vs_E10	ordinary_muscle_development	D_vs_E10;stage_1W_vs_E10;stage_3W_vs_E10;stage	1	TRUE
ENSGALG00010024193	GSTK1	PRJNA729271	1.502490846	9.80255E-32	1.502490846	stage_E14_vs_E10	ordinary_muscle_development	D_vs_E10;stage_1W_vs_E10;stage_3W_vs_E10;stage	1	TRUE
ENSGALG00010018505	CHTF18	PRJNA729271	-1.737999626	9.86389E-32	1.737999626	stage_E14_vs_E10	ordinary_muscle_development	D_vs_E10;stage_1W_vs_E10;stage_3W_vs_E10;stage	1	TRUE
ENSGALG00010027563	WDR77	PRJNA729271	-1.483512797	1.02521E-31	1.483512797	stage_E14_vs_E10	ordinary_muscle_development	D_vs_E10;stage_1W_vs_E10;stage_3W_vs_E10;stage	1	TRUE
ENSGALG00010012682	ENSGALG00010012682	PRJNA729271	-1.337975604	1.03944E-31	1.337975604	stage_E14_vs_E10	ordinary_muscle_development	D_vs_E10;stage_1W_vs_E10;stage_3W_vs_E10;stage	1	TRUE
ENSGALG00010027093	SLMAP	PRJNA729271	0.908335428	1.04352E-31	0.908335428	stage_E14_vs_E10	ordinary_muscle_development		0	FALSE
ENSGALG00010003844	TMEM18	PRJNA729271	-1.429040476	1.04454E-31	1.429040476	stage_E14_vs_E10	ordinary_muscle_development	D_vs_E10;stage_1W_vs_E10;stage_3W_vs_E10;stage	1	TRUE
ENSGALG00010029642	NAT9	PRJNA729271	1.707914238	1.06074E-31	1.707914238	stage_E14_vs_E10	ordinary_muscle_development	D_vs_E10;stage_1W_vs_E10;stage_3W_vs_E10;stage	1	TRUE
ENSGALG00010001111	SUCNR1	PRJNA729271	4.948964139	1.06733E-31	4.948964139	stage_E14_vs_E10	ordinary_muscle_development	D_vs_E10;stage_1W_vs_E10;stage_3W_vs_E10;stage	1	TRUE
ENSGALG00010029570	TNFAIP1	PRJNA729271	-0.952847377	1.07643E-31	0.952847377	stage_E14_vs_E10	ordinary_muscle_development		0	FALSE
ENSGALG00010005118	PRTFDC1	PRJNA729271	-1.675074511	1.08384E-31	1.675074511	stage_E14_vs_E10	ordinary_muscle_development	D_vs_E10;stage_1W_vs_E10;stage_3W_vs_E10;stage	1	TRUE
ENSGALG00010028392	ASH1L	PRJNA729271	1.434929648	1.17736E-31	1.434929648	stage_E14_vs_E10	ordinary_muscle_development	D_vs_E10;stage_1W_vs_E10;stage_3W_vs_E10;stage	1	TRUE
ENSGALG00010006037	METTL21EP	PRJNA729271	8.006939076	1.18843E-31	8.006939076	stage_E14_vs_E10	ordinary_muscle_development	D_vs_E10;stage_1W_vs_E10;stage_3W_vs_E10;stage	1	TRUE
ENSGALG00010001745	CADPS2	PRJNA729271	-1.355720681	1.21863E-31	1.355720681	stage_E14_vs_E10	ordinary_muscle_development	D_vs_E10;stage_1W_vs_E10;stage_3W_vs_E10;stage	1	TRUE
ENSGALG00010019115	DBNL	PRJNA729271	-1.227150099	1.28731E-31	1.227150099	stage_E14_vs_E10	ordinary_muscle_development	D_vs_E10;stage_1W_vs_E10;stage_3W_vs_E10;stage	1	TRUE
ENSGALG00010021583	NFE2L1	PRJNA729271	2.616220769	1.29087E-31	2.616220769	stage_E14_vs_E10	ordinary_muscle_development	D_vs_E10;stage_1W_vs_E10;stage_3W_vs_E10;stage	1	TRUE
ENSGALG00010003988	ENOPH1	PRJNA729271	-1.07326385	1.29584E-31	1.07326385	stage_E14_vs_E10	ordinary_muscle_development	D_vs_E10;stage_1W_vs_E10;stage_3W_vs_E10;stage	1	TRUE
ENSGALG00010025161	ENSGALG00010025161	PRJNA729271	-1.256661788	1.30929E-31	1.256661788	stage_E14_vs_E10	ordinary_muscle_development	D_vs_E10;stage_1W_vs_E10;stage_3W_vs_E10;stage	1	TRUE
ENSGALG00010017759	ENSGALG00010017759	PRJNA729271	9.930869629	1.37107E-31	9.930869629	stage_E14_vs_E10	ordinary_muscle_development	D_vs_E10;stage_1W_vs_E10;stage_3W_vs_E10;stage	1	TRUE
ENSGALG00010017687	TTC7B	PRJNA729271	1.135429099	1.40404E-31	1.135429099	stage_E14_vs_E10	ordinary_muscle_development	D_vs_E10;stage_1W_vs_E10;stage_3W_vs_E10;stage	1	TRUE
ENSGALG00010015613	PWP1	PRJNA729271	-0.984497242	1.4347E-31	0.984497242	stage_E14_vs_E10	ordinary_muscle_development		0	FALSE
ENSGALG00010000307	ZMAT5	PRJNA729271	1.321426584	1.48253E-31	1.321426584	stage_E14_vs_E10	ordinary_muscle_development	D_vs_E10;stage_1W_vs_E10;stage_3W_vs_E10;stage	1	TRUE
ENSGALG00010023612	GPT2	PRJNA729271	2.199159414	1.49169E-31	2.199159414	stage_E14_vs_E10	ordinary_muscle_development	D_vs_E10;stage_1W_vs_E10;stage_3W_vs_E10;stage	1	TRUE
ENSGALG00010025323	KLHDC8A	PRJNA729271	-2.280275025	1.52516E-31	2.280275025	stage_E14_vs_E10	ordinary_muscle_development	D_vs_E10;stage_1W_vs_E10;stage_3W_vs_E10;stage	1	TRUE
ENSGALG00010007404	FOLH1	PRJNA729271	2.012805554	1.56486E-31	2.012805554	stage_E14_vs_E10	ordinary_muscle_development	D_vs_E10;stage_1W_vs_E10;stage_3W_vs_E10;stage	1	TRUE
ENSGALG00010024440	PKNOX2	PRJNA729271	2.445569762	1.59443E-31	2.445569762	stage_E14_vs_E10	ordinary_muscle_development	D_vs_E10;stage_1W_vs_E10;stage_3W_vs_E10;stage	1	TRUE
ENSGALG00010029527	NDUFAF8	PRJNA729271	1.799502305	1.60516E-31	1.799502305	stage_E14_vs_E10	ordinary_muscle_development	D_vs_E10;stage_1W_vs_E10;stage_3W_vs_E10;stage	1	TRUE
ENSGALG00010029294	PGS1	PRJNA729271	-0.917778044	1.64914E-31	0.917778044	stage_E14_vs_E10	ordinary_muscle_development		0	FALSE
ENSGALG00010007856	PDCD2	PRJNA729271	1.512186457	1.7137E-31	1.512186457	stage_E14_vs_E10	ordinary_muscle_development	D_vs_E10;stage_1W_vs_E10;stage_3W_vs_E10;stage	1	TRUE
ENSGALG00010014717	ELOVL7	PRJNA729271	2.148886326	1.7447E-31	2.148886326	stage_E14_vs_E10	ordinary_muscle_development	D_vs_E10;stage_1W_vs_E10;stage_3W_vs_E10;stage	1	TRUE
ENSGALG00010016544	ADCY9	PRJNA729271	1.603063156	1.78263E-31	1.603063156	stage_E14_vs_E10	ordinary_muscle_development	D_vs_E10;stage_1W_vs_E10;stage_3W_vs_E10;stage	1	TRUE
ENSGALG00010022223	DOK4	PRJNA729271	-2.151086295	1.79068E-31	2.151086295	stage_E14_vs_E10	ordinary_muscle_development	D_vs_E10;stage_1W_vs_E10;stage_3W_vs_E10;stage	1	TRUE
ENSGALG00010017808	SFRP5	PRJNA729271	3.713850561	1.8015E-31	3.713850561	stage_E14_vs_E10	ordinary_muscle_development	D_vs_E10;stage_1W_vs_E10;stage_3W_vs_E10;stage	1	TRUE
ENSGALG00010002277	RGS10	PRJNA729271	-2.133023424	1.87307E-31	2.133023424	stage_E14_vs_E10	ordinary_muscle_development	D_vs_E10;stage_1W_vs_E10;stage_3W_vs_E10;stage	1	TRUE
ENSGALG00010018604	FAM43A	PRJNA729271	1.465573239	1.93093E-31	1.465573239	stage_E14_vs_E10	ordinary_muscle_development	D_vs_E10;stage_1W_vs_E10;stage_3W_vs_E10;stage	1	TRUE
ENSGALG00010024610	PFKL	PRJNA729271	-1.681497078	2.00535E-31	1.681497078	stage_E14_vs_E10	ordinary_muscle_development	D_vs_E10;stage_1W_vs_E10;stage_3W_vs_E10;stage	1	TRUE
ENSGALG00010029724	CACNA1G	PRJNA729271	-2.605776223	2.02703E-31	2.605776223	stage_E14_vs_E10	ordinary_muscle_development	D_vs_E10;stage_1W_vs_E10;stage_3W_vs_E10;stage	1	TRUE
ENSGALG00010017119	BTBD6	PRJNA729271	-1.795344477	2.09215E-31	1.795344477	stage_E14_vs_E10	ordinary_muscle_development	D_vs_E10;stage_1W_vs_E10;stage_3W_vs_E10;stage	1	TRUE
ENSGALG00010004489	LIG1	PRJNA729271	-3.191856343	2.11654E-31	3.191856343	stage_E14_vs_E10	ordinary_muscle_development	D_vs_E10;stage_1W_vs_E10;stage_3W_vs_E10;stage	1	TRUE
ENSGALG00010012100	UBE2F	PRJNA729271	1.310853171	2.2098E-31	1.310853171	stage_E14_vs_E10	ordinary_muscle_development	D_vs_E10;stage_1W_vs_E10;stage_3W_vs_E10;stage	1	TRUE
ENSGALG00010009139	CKS2	PRJNA729271	-3.718251215	2.2244E-31	3.718251215	stage_E14_vs_E10	ordinary_muscle_development	D_vs_E10;stage_1W_vs_E10;stage_3W_vs_E10;stage	1	TRUE
ENSGALG00010021675	DDX42	PRJNA729271	-0.704652977	2.2296E-31	0.704652977	stage_E14_vs_E10	ordinary_muscle_development		0	FALSE
ENSGALG00010011139	UBASH3A	PRJNA729271	6.658222098	2.23737E-31	6.658222098	stage_E14_vs_E10	ordinary_muscle_development	D_vs_E10;stage_1W_vs_E10;stage_3W_vs_E10;stage	1	TRUE
ENSGALG00010008222	STYXL2	PRJNA729271	3.24535911	2.30108E-31	3.24535911	stage_E14_vs_E10	ordinary_muscle_development	D_vs_E10;stage_1W_vs_E10;stage_3W_vs_E10;stage	1	TRUE
ENSGALG00010028374	FDPS	PRJNA729271	1.528491659	2.30844E-31	1.528491659	stage_E14_vs_E10	ordinary_muscle_development	D_vs_E10;stage_1W_vs_E10;stage_3W_vs_E10;stage	1	TRUE
ENSGALG00010029577	NOG	PRJNA729271	-7.013475644	2.34168E-31	7.013475644	stage_E14_vs_E10	ordinary_muscle_development	D_vs_E10;stage_1W_vs_E10;stage_3W_vs_E10;stage	1	TRUE
ENSGALG00010021656	SETD6	PRJNA729271	-1.006619282	2.35426E-31	1.006619282	stage_E14_vs_E10	ordinary_muscle_development	D_vs_E10;stage_1W_vs_E10;stage_3W_vs_E10;stage	1	TRUE
ENSGALG00010010599	F2RL1	PRJNA729271	-2.454058543	2.39999E-31	2.454058543	stage_E14_vs_E10	ordinary_muscle_development	D_vs_E10;stage_1W_vs_E10;stage_3W_vs_E10;stage	1	TRUE
ENSGALG00010012112	MBD2	PRJNA729271	2.235234202	2.40389E-31	2.235234202	stage_E14_vs_E10	ordinary_muscle_development	D_vs_E10;stage_1W_vs_E10;stage_3W_vs_E10;stage	1	TRUE
ENSGALG00010025968	FADS2	PRJNA729271	-1.912583099	2.4487E-31	1.912583099	stage_E14_vs_E10	ordinary_muscle_development	D_vs_E10;stage_1W_vs_E10;stage_3W_vs_E10;stage	1	TRUE
ENSGALG00010009197	FH	PRJNA729271	1.207171716	2.54658E-31	1.207171716	stage_E14_vs_E10	ordinary_muscle_development	D_vs_E10;stage_1W_vs_E10;stage_3W_vs_E10;stage	1	TRUE
ENSGALG00010023251	ENSGALG00010023251	PRJNA729271	10.81444612	2.59019E-31	10.81444612	stage_E14_vs_E10	ordinary_muscle_development	D_vs_E10;stage_1W_vs_E10;stage_3W_vs_E10;stage	1	TRUE
ENSGALG00010021994	ENSGALG00010021994	PRJNA729271	-4.923758384	2.648E-31	4.923758384	stage_E14_vs_E10	ordinary_muscle_development	D_vs_E10;stage_1W_vs_E10;stage_3W_vs_E10;stage	1	TRUE
ENSGALG00010024588	TTC39A	PRJNA729271	-4.988015331	2.71204E-31	4.988015331	stage_E14_vs_E10	ordinary_muscle_development	D_vs_E10;stage_1W_vs_E10;stage_3W_vs_E10;stage	1	TRUE
ENSGALG00010002632	HPGD	PRJNA729271	3.216223363	2.84094E-31	3.216223363	stage_E14_vs_E10	ordinary_muscle_development	D_vs_E10;stage_1W_vs_E10;stage_3W_vs_E10;stage	1	TRUE
ENSGALG00010007957	CDH15	PRJNA729271	1.945286614	2.91841E-31	1.945286614	stage_E14_vs_E10	ordinary_muscle_development	D_vs_E10;stage_1W_vs_E10;stage_3W_vs_E10;stage	1	TRUE
ENSGALG00010023390	HSPB8	PRJNA729271	2.953346553	3.06574E-31	2.953346553	stage_E14_vs_E10	ordinary_muscle_development	D_vs_E10;stage_1W_vs_E10;stage_3W_vs_E10;stage	1	TRUE
ENSGALG00010028891	SLC31A2	PRJNA729271	2.576231587	3.06907E-31	2.576231587	stage_E14_vs_E10	ordinary_muscle_development	D_vs_E10;stage_1W_vs_E10;stage_3W_vs_E10;stage	1	TRUE
ENSGALG00010013412	COMMD7	PRJNA729271	-1.220079407	3.0719E-31	1.220079407	stage_E14_vs_E10	ordinary_muscle_development	D_vs_E10;stage_1W_vs_E10;stage_3W_vs_E10;stage	1	TRUE
ENSGALG00010022245	PIKFYVE	PRJNA729271	1.176022401	3.15198E-31	1.176022401	stage_E14_vs_E10	ordinary_muscle_development	D_vs_E10;stage_1W_vs_E10;stage_3W_vs_E10;stage	1	TRUE
ENSGALG00010006616	COPG2	PRJNA729271	-0.818660131	3.18923E-31	0.818660131	stage_E14_vs_E10	ordinary_muscle_development		0	FALSE
ENSGALG00010029344	ENSGALG00010029344	PRJNA729271	1.925366228	3.26969E-31	1.925366228	stage_E14_vs_E10	ordinary_muscle_development	D_vs_E10;stage_1W_vs_E10;stage_3W_vs_E10;stage	1	TRUE
ENSGALG00010013227	CITED4	PRJNA729271	1.897200716	3.31912E-31	1.897200716	stage_E14_vs_E10	ordinary_muscle_development	D_vs_E10;stage_1W_vs_E10;stage_3W_vs_E10;stage	1	TRUE
ENSGALG00010028124	SUGP2	PRJNA729271	-1.061190452	3.36407E-31	1.061190452	stage_E14_vs_E10	ordinary_muscle_development	D_vs_E10;stage_1W_vs_E10;stage_3W_vs_E10;stage	1	TRUE
ENSGALG00010004586	ENSGALG00010004586	PRJNA729271	-5.604405641	3.41412E-31	5.604405641	stage_E14_vs_E10	ordinary_muscle_development	D_vs_E10;stage_1W_vs_E10;stage_3W_vs_E10;stage	1	TRUE
ENSGALG00010023455	CFDP1	PRJNA729271	-1.152276522	3.52177E-31	1.152276522	stage_E14_vs_E10	ordinary_muscle_development	D_vs_E10;stage_1W_vs_E10;stage_3W_vs_E10;stage	1	TRUE
ENSGALG00010016938	PSME4	PRJNA729271	1.955121276	3.53444E-31	1.955121276	stage_E14_vs_E10	ordinary_muscle_development	D_vs_E10;stage_1W_vs_E10;stage_3W_vs_E10;stage	1	TRUE
ENSGALG00010023766	ENSGALG00010023766	PRJNA729271	-3.967860002	3.66808E-31	3.967860002	stage_E14_vs_E10	ordinary_muscle_development	D_vs_E10;stage_1W_vs_E10;stage_3W_vs_E10;stage	1	TRUE
ENSGALG00010011117	SLC6A15	PRJNA729271	-8.906197518	3.78847E-31	8.906197518	stage_E14_vs_E10	ordinary_muscle_development	D_vs_E10;stage_1W_vs_E10;stage_3W_vs_E10;stage	1	TRUE
ENSGALG00010013277	YRDC	PRJNA729271	-1.141163991	3.79974E-31	1.141163991	stage_E14_vs_E10	ordinary_muscle_development	D_vs_E10;stage_1W_vs_E10;stage_3W_vs_E10;stage	1	TRUE
ENSGALG00010000200	GAL3ST1	PRJNA729271	-4.078239708	3.83189E-31	4.078239708	stage_E14_vs_E10	ordinary_muscle_development	D_vs_E10;stage_1W_vs_E10;stage_3W_vs_E10;stage	1	TRUE
ENSGALG00010011525	ENSGALG00010011525	PRJNA729271	-3.054044292	3.86154E-31	3.054044292	stage_E14_vs_E10	ordinary_muscle_development	D_vs_E10;stage_1W_vs_E10;stage_3W_vs_E10;stage	1	TRUE
ENSGALG00010013186	DDX46	PRJNA729271	-0.951264957	3.87665E-31	0.951264957	stage_E14_vs_E10	ordinary_muscle_development		0	FALSE
ENSGALG00010009798	YIPF4	PRJNA729271	0.906770633	3.88553E-31	0.906770633	stage_E14_vs_E10	ordinary_muscle_development		0	FALSE
ENSGALG00010028904	TRIM32	PRJNA729271	-1.024210192	3.90195E-31	1.024210192	stage_E14_vs_E10	ordinary_muscle_development	D_vs_E10;stage_1W_vs_E10;stage_3W_vs_E10;stage	1	TRUE
ENSGALG00010025976	NAA25	PRJNA729271	-1.18946094	3.93101E-31	1.18946094	stage_E14_vs_E10	ordinary_muscle_development	D_vs_E10;stage_1W_vs_E10;stage_3W_vs_E10;stage	1	TRUE
ENSGALG00010020624	MINOS1	PRJNA729271	1.602143909	3.98993E-31	1.602143909	stage_E14_vs_E10	ordinary_muscle_development	D_vs_E10;stage_1W_vs_E10;stage_3W_vs_E10;stage	1	TRUE
ENSGALG00010001849	NDUFV2	PRJNA729271	1.110365402	4.02965E-31	1.110365402	stage_E14_vs_E10	ordinary_muscle_development	D_vs_E10;stage_1W_vs_E10;stage_3W_vs_E10;stage	1	TRUE
ENSGALG00010023250	FZD10	PRJNA729271	-5.556774422	4.18092E-31	5.556774422	stage_E14_vs_E10	ordinary_muscle_development	D_vs_E10;stage_1W_vs_E10;stage_3W_vs_E10;stage	1	TRUE
ENSGALG00010022489	NUSAP1	PRJNA729271	-2.839281563	4.22043E-31	2.839281563	stage_E14_vs_E10	ordinary_muscle_development	D_vs_E10;stage_1W_vs_E10;stage_3W_vs_E10;stage	1	TRUE
ENSGALG00010020476	TMEM132A	PRJNA729271	-1.340774081	4.23652E-31	1.340774081	stage_E14_vs_E10	ordinary_muscle_development	D_vs_E10;stage_1W_vs_E10;stage_3W_vs_E10;stage	1	TRUE
ENSGALG00010017039	MAP2K5	PRJNA729271	-0.93425274	4.29075E-31	0.93425274	stage_E14_vs_E10	ordinary_muscle_development		0	FALSE
ENSGALG00010020990	UBB	PRJNA729271	-1.257668042	4.3299E-31	1.257668042	stage_E14_vs_E10	ordinary_muscle_development	D_vs_E10;stage_1W_vs_E10;stage_3W_vs_E10;stage	1	TRUE
ENSGALG00010021029	PRDM2	PRJNA729271	-0.898694895	4.37238E-31	0.898694895	stage_E14_vs_E10	ordinary_muscle_development		0	FALSE
ENSGALG00010014257	NOP16	PRJNA729271	-1.550886899	4.49963E-31	1.550886899	stage_E14_vs_E10	ordinary_muscle_development	D_vs_E10;stage_1W_vs_E10;stage_3W_vs_E10;stage	1	TRUE
ENSGALG00010017576	ENSGALG00010017576	PRJNA729271	-3.660449698	4.57058E-31	3.660449698	stage_E14_vs_E10	ordinary_muscle_development	D_vs_E10;stage_1W_vs_E10;stage_3W_vs_E10;stage	1	TRUE
ENSGALG00010009444	NFIL3	PRJNA729271	2.598100866	4.58897E-31	2.598100866	stage_E14_vs_E10	ordinary_muscle_development	D_vs_E10;stage_1W_vs_E10;stage_3W_vs_E10;stage	1	TRUE
ENSGALG00010026920	GATA2	PRJNA729271	2.333581016	4.68515E-31	2.333581016	stage_E14_vs_E10	ordinary_muscle_development	D_vs_E10;stage_1W_vs_E10;stage_3W_vs_E10;stage	1	TRUE

ENSGALG00010013416	NDUFB2	PRJNA729271	1.294651145	4.72535E-31	1.294651145	stage_E14_vs_E10	ordinary_muscle_development	D_vs_E10;stage_1W_vs_E10;stage_3W_vs_E10;stage	1	TRUE
ENSGALG00010009717	EDN1	PRJNA729271	2.917134348	4.9711E-31	2.917134348	stage_E14_vs_E10	ordinary_muscle_development	D_vs_E10;stage_1W_vs_E10;stage_3W_vs_E10;stage	1	TRUE
ENSGALG00010026595	PRKAA2	PRJNA729271	3.07317959	5.10296E-31	3.07317959	stage_E14_vs_E10	ordinary_muscle_development	D_vs_E10;stage_1W_vs_E10;stage_3W_vs_E10;stage	1	TRUE
ENSGALG00010002206	DPH3P1	PRJNA729271	1.270134664	5.12631E-31	1.270134664	stage_E14_vs_E10	ordinary_muscle_development	D_vs_E10;stage_1W_vs_E10;stage_3W_vs_E10;stage	1	TRUE
ENSGALG00010005534	ENSGALG00010005534	PRJNA729271	-1.951729599	5.38505E-31	1.951729599	stage_E14_vs_E10	ordinary_muscle_development	D_vs_E10;stage_1W_vs_E10;stage_3W_vs_E10;stage	1	TRUE
ENSGALG00010007441	STRN	PRJNA729271	1.584636291	5.57453E-31	1.584636291	stage_E14_vs_E10	ordinary_muscle_development	D_vs_E10;stage_1W_vs_E10;stage_3W_vs_E10;stage	1	TRUE
ENSGALG00010027998	SNX27	PRJNA729271	1.073865708	5.76767E-31	1.073865708	stage_E14_vs_E10	ordinary_muscle_development	D_vs_E10;stage_1W_vs_E10;stage_3W_vs_E10;stage	1	TRUE
ENSGALG00010015647	PDZD2	PRJNA729271	-1.667010064	6.01227E-31	1.667010064	stage_E14_vs_E10	ordinary_muscle_development	D_vs_E10;stage_1W_vs_E10;stage_3W_vs_E10;stage	1	TRUE
ENSGALG00010024254	ENSGALG00010024254	PRJNA729271	3.499365459	6.04402E-31	3.499365459	stage_E14_vs_E10	ordinary_muscle_development	D_vs_E10;stage_1W_vs_E10;stage_3W_vs_E10;stage	1	TRUE
ENSGALG00010023058	PPARA	PRJNA729271	2.370995175	6.16765E-31	2.370995175	stage_E14_vs_E10	ordinary_muscle_development	D_vs_E10;stage_1W_vs_E10;stage_3W_vs_E10;stage	1	TRUE
ENSGALG00010021155	RADIL	PRJNA729271	-2.757417373	6.61277E-31	2.757417373	stage_E14_vs_E10	ordinary_muscle_development	D_vs_E10;stage_1W_vs_E10;stage_3W_vs_E10;stage	1	TRUE
ENSGALG00010019735	ENSGALG00010019735	PRJNA729271	2.014333988	7.13389E-31	2.014333988	stage_E14_vs_E10	ordinary_muscle_development	D_vs_E10;stage_1W_vs_E10;stage_3W_vs_E10;stage	1	TRUE
ENSGALG00010023160	ENSGALG00010023160	PRJNA729271	-1.311947007	7.19735E-31	1.311947007	stage_E14_vs_E10	ordinary_muscle_development	D_vs_E10;stage_1W_vs_E10;stage_3W_vs_E10;stage	1	TRUE
ENSGALG00010018064	TTC8	PRJNA729271	-1.92018094	7.43262E-31	1.92018094	stage_E14_vs_E10	ordinary_muscle_development	D_vs_E10;stage_1W_vs_E10;stage_3W_vs_E10;stage	1	TRUE
ENSGALG00010012650	DSE	PRJNA729271	-1.248577925	7.47544E-31	1.248577925	stage_E14_vs_E10	ordinary_muscle_development	D_vs_E10;stage_1W_vs_E10;stage_3W_vs_E10;stage	1	TRUE
ENSGALG00010021189	ENSGALG00010021189	PRJNA729271	4.209458003	7.48249E-31	4.209458003	stage_E14_vs_E10	ordinary_muscle_development	D_vs_E10;stage_1W_vs_E10;stage_3W_vs_E10;stage	1	TRUE
ENSGALG00010022476	OIP5	PRJNA729271	-1.359650967	7.92912E-31	1.359650967	stage_E14_vs_E10	ordinary_muscle_development	D_vs_E10;stage_1W_vs_E10;stage_3W_vs_E10;stage	1	TRUE
ENSGALG00010019967	CEP170B	PRJNA729271	-1.285001356	7.98147E-31	1.285001356	stage_E14_vs_E10	ordinary_muscle_development	D_vs_E10;stage_1W_vs_E10;stage_3W_vs_E10;stage	1	TRUE
ENSGALG00010022247	GALNT9	PRJNA729271	-2.704095633	8.09023E-31	2.704095633	stage_E14_vs_E10	ordinary_muscle_development	D_vs_E10;stage_1W_vs_E10;stage_3W_vs_E10;stage	1	TRUE
ENSGALG00010021157	ENSGALG00010021157	PRJNA729271	9.400200253	8.10408E-31	9.400200253	stage_E14_vs_E10	ordinary_muscle_development	D_vs_E10;stage_1W_vs_E10;stage_3W_vs_E10;stage	1	TRUE
ENSGALG00010003027	SCIN	PRJNA729271	-3.017093568	8.30435E-31	3.017093568	stage_E14_vs_E10	ordinary_muscle_development	D_vs_E10;stage_1W_vs_E10;stage_3W_vs_E10;stage	1	TRUE
ENSGALG00010008745	GRAMD4	PRJNA729271	1.77747843	8.38563E-31	1.77747843	stage_E14_vs_E10	ordinary_muscle_development	D_vs_E10;stage_1W_vs_E10;stage_3W_vs_E10;stage	1	TRUE
ENSGALG00010012130	TMEM67	PRJNA729271	-2.037897719	8.39144E-31	2.037897719	stage_E14_vs_E10	ordinary_muscle_development	D_vs_E10;stage_1W_vs_E10;stage_3W_vs_E10;stage	1	TRUE
ENSGALG00010013867	RECQL	PRJNA729271	-1.561686413	8.40282E-31	1.561686413	stage_E14_vs_E10	ordinary_muscle_development	D_vs_E10;stage_1W_vs_E10;stage_3W_vs_E10;stage	1	TRUE
ENSGALG00010009439	B3GALNT2	PRJNA729271	1.139662102	8.68436E-31	1.139662102	stage_E14_vs_E10	ordinary_muscle_development	D_vs_E10;stage_1W_vs_E10;stage_3W_vs_E10;stage	1	TRUE
ENSGALG00010029697	GIT1	PRJNA729271	1.92156981	8.88393E-31	1.92156981	stage_E14_vs_E10	ordinary_muscle_development	D_vs_E10;stage_1W_vs_E10;stage_3W_vs_E10;stage	1	TRUE
ENSGALG00010023947	ENSGALG00010023947	PRJNA729271	-9.46659817	8.91058E-31	9.46659817	stage_E14_vs_E10	ordinary_muscle_development	D_vs_E10;stage_1W_vs_E10;stage_3W_vs_E10;stage	1	TRUE
ENSGALG00010029607	ZNF207	PRJNA729271	-0.763748907	9.47605E-31	0.763748907	stage_E14_vs_E10	ordinary_muscle_development		0	FALSE
ENSGALG00010016602	HSPB7	PRJNA729271	6.011981139	9.55628E-31	6.011981139	stage_E14_vs_E10	ordinary_muscle_development	D_vs_E10;stage_1W_vs_E10;stage_3W_vs_E10;stage	1	TRUE
ENSGALG00010011673	POP1	PRJNA729271	-1.487872037	9.76169E-31	1.487872037	stage_E14_vs_E10	ordinary_muscle_development	D_vs_E10;stage_1W_vs_E10;stage_3W_vs_E10;stage	1	TRUE
ENSGALG00010014170	CEMIP2	PRJNA729271	-1.263361989	9.77087E-31	1.263361989	stage_E14_vs_E10	ordinary_muscle_development	D_vs_E10;stage_1W_vs_E10;stage_3W_vs_E10;stage	1	TRUE
ENSGALG00010002929	PDHA2	PRJNA729271	1.317128204	9.81844E-31	1.317128204	stage_E14_vs_E10	ordinary_muscle_development	D_vs_E10;stage_1W_vs_E10;stage_3W_vs_E10;stage	1	TRUE
ENSGALG00010008339	FAM76B	PRJNA729271	-1.35551682	1.00403E-30	1.35551682	stage_E14_vs_E10	ordinary_muscle_development	D_vs_E10;stage_1W_vs_E10;stage_3W_vs_E10;stage	1	TRUE
ENSGALG00010021116	FAM21C	PRJNA729271	0.745931997	1.01622E-30	0.745931997	stage_E14_vs_E10	ordinary_muscle_development		0	FALSE
ENSGALG00010018168	ENSGALG00010018168	PRJNA729271	-8.705368458	1.02881E-30	8.705368458	stage_E14_vs_E10	ordinary_muscle_development	D_vs_E10;stage_1W_vs_E10;stage_3W_vs_E10;stage	1	TRUE
ENSGALG00010020915	EN1	PRJNA729271	-5.129426646	1.0573E-30	5.129426646	stage_E14_vs_E10	ordinary_muscle_development	D_vs_E10;stage_1W_vs_E10;stage_3W_vs_E10;stage	1	TRUE
ENSGALG00010004247	MEOX2	PRJNA729271	-1.228430677	1.0803E-30	1.228430677	stage_E14_vs_E10	ordinary_muscle_development	D_vs_E10;stage_1W_vs_E10;stage_3W_vs_E10;stage	1	TRUE
ENSGALG00010015972	FSTL4	PRJNA729271	-3.720042712	1.09464E-30	3.720042712	stage_E14_vs_E10	ordinary_muscle_development	D_vs_E10;stage_1W_vs_E10;stage_3W_vs_E10;stage	1	TRUE
ENSGALG00010008395	ENDOD1	PRJNA729271	1.691520667	1.11293E-30	1.691520667	stage_E14_vs_E10	ordinary_muscle_development	D_vs_E10;stage_1W_vs_E10;stage_3W_vs_E10;stage	1	TRUE
ENSGALG00010008033	ACOT13	PRJNA729271	1.386733722	1.15287E-30	1.386733722	stage_E14_vs_E10	ordinary_muscle_development	D_vs_E10;stage_1W_vs_E10;stage_3W_vs_E10;stage	1	TRUE
ENSGALG00010009617	SLC25A46	PRJNA729271	1.130273369	1.16909E-30	1.130273369	stage_E14_vs_E10	ordinary_muscle_development	D_vs_E10;stage_1W_vs_E10;stage_3W_vs_E10;stage	1	TRUE
ENSGALG00010028314	MEF2D	PRJNA729271	2.491341363	1.21197E-30	2.491341363	stage_E14_vs_E10	ordinary_muscle_development	D_vs_E10;stage_1W_vs_E10;stage_3W_vs_E10;stage	1	TRUE
ENSGALG00010028459	MCL1	PRJNA729271	2.447729695	1.22618E-30	2.447729695	stage_E14_vs_E10	ordinary_muscle_development	D_vs_E10;stage_1W_vs_E10;stage_3W_vs_E10;stage	1	TRUE
ENSGALG00010015906	BAG5	PRJNA729271	1.169235062	1.24323E-30	1.169235062	stage_E14_vs_E10	ordinary_muscle_development	D_vs_E10;stage_1W_vs_E10;stage_3W_vs_E10;stage	1	TRUE
ENSGALG00010001412	ENSGALG00010001412	PRJNA729271	-3.503118138	1.27887E-30	3.503118138	stage_E14_vs_E10	ordinary_muscle_development	D_vs_E10;stage_1W_vs_E10;stage_3W_vs_E10;stage	1	TRUE
ENSGALG00010013183	CLTA	PRJNA729271	-1.614940619	1.28076E-30	1.614940619	stage_E14_vs_E10	ordinary_muscle_development	D_vs_E10;stage_1W_vs_E10;stage_3W_vs_E10;stage	1	TRUE
ENSGALG00010006108	MKLN1	PRJNA729271	0.711162687	1.29369E-30	0.711162687	stage_E14_vs_E10	ordinary_muscle_development		0	FALSE
ENSGALG00010009524	FAM185A	PRJNA729271	1.862904552	1.30582E-30	1.862904552	stage_E14_vs_E10	ordinary_muscle_development	D_vs_E10;stage_1W_vs_E10;stage_3W_vs_E10;stage	1	TRUE
ENSGALG00010027285	PTPRS	PRJNA729271	-1.309085775	1.30818E-30	1.309085775	stage_E14_vs_E10	ordinary_muscle_development	D_vs_E10;stage_1W_vs_E10;stage_3W_vs_E10;stage	1	TRUE
ENSGALG00010011572	PTCD2	PRJNA729271	1.178407109	1.35561E-30	1.178407109	stage_E14_vs_E10	ordinary_muscle_development	D_vs_E10;stage_1W_vs_E10;stage_3W_vs_E10;stage	1	TRUE
ENSGALG00010001628	MAPK11	PRJNA729271	-2.526701067	1.36458E-30	2.526701067	stage_E14_vs_E10	ordinary_muscle_development	D_vs_E10;stage_1W_vs_E10;stage_3W_vs_E10;stage	1	TRUE
ENSGALG00010009328	ENSGALG00010009328	PRJNA729271	-1.648532419	1.41753E-30	1.648532419	stage_E14_vs_E10	ordinary_muscle_development	D_vs_E10;stage_1W_vs_E10;stage_3W_vs_E10;stage	1	TRUE
ENSGALG00010006068	PACRG	PRJNA729271	2.993010698	1.43178E-30	2.993010698	stage_E14_vs_E10	ordinary_muscle_development	D_vs_E10;stage_1W_vs_E10;stage_3W_vs_E10;stage	1	TRUE
ENSGALG00010025426	ENSGALG00010025426	PRJNA729271	1.790068604	1.43599E-30	1.790068604	stage_E14_vs_E10	ordinary_muscle_development	D_vs_E10;stage_1W_vs_E10;stage_3W_vs_E10;stage	1	TRUE
ENSGALG00010007123	ENSGALG00010007123	PRJNA729271	2.829047098	1.51125E-30	2.829047098	stage_E14_vs_E10	ordinary_muscle_development	D_vs_E10;stage_1W_vs_E10;stage_3W_vs_E10;stage	1	TRUE
ENSGALG00010022698	SEMA6D	PRJNA729271	1.363349492	1.51503E-30	1.363349492	stage_E14_vs_E10	ordinary_muscle_development	D_vs_E10;stage_1W_vs_E10;stage_3W_vs_E10;stage	1	TRUE
ENSGALG00010006740	BRCA2	PRJNA729271	-1.800302575	1.52411E-30	1.800302575	stage_E14_vs_E10	ordinary_muscle_development	D_vs_E10;stage_1W_vs_E10;stage_3W_vs_E10;stage	1	TRUE
ENSGALG00010016676	NLRC3	PRJNA729271	4.718008838	1.53753E-30	4.718008838	stage_E14_vs_E10	ordinary_muscle_development	D_vs_E10;stage_1W_vs_E10;stage_3W_vs_E10;stage	1	TRUE
ENSGALG00010029706	SSH2	PRJNA729271	2.517937072	1.56107E-30	2.517937072	stage_E14_vs_E10	ordinary_muscle_development	D_vs_E10;stage_1W_vs_E10;stage_3W_vs_E10;stage	1	TRUE
ENSGALG00010029327	CRK	PRJNA729271	-0.641223439	1.56271E-30	0.641223439	stage_E14_vs_E10	ordinary_muscle_development		0	FALSE
ENSGALG00010008187	ENSGALG00010008187	PRJNA729271	4.354411852	1.6207E-30	4.354411852	stage_E14_vs_E10	ordinary_muscle_development	D_vs_E10;stage_1W_vs_E10;stage_3W_vs_E10;stage	1	TRUE
ENSGALG00010019588	CHMP7	PRJNA729271	1.077102767	1.6468E-30	1.077102767	stage_E14_vs_E10	ordinary_muscle_development	D_vs_E10;stage_1W_vs_E10;stage_3W_vs_E10;stage	1	TRUE
ENSGALG00010007994	FAM65B	PRJNA729271	1.956697837	1.65054E-30	1.956697837	stage_E14_vs_E10	ordinary_muscle_development	D_vs_E10;stage_1W_vs_E10;stage_3W_vs_E10;stage	1	TRUE
ENSGALG00010020623	NEK2	PRJNA729271	-2.69996877	1.66301E-30	2.69996877	stage_E14_vs_E10	ordinary_muscle_development	D_vs_E10;stage_1W_vs_E10;stage_3W_vs_E10;stage	1	TRUE
ENSGALG00010013132	SKIC3	PRJNA729271	-1.050949479	1.70569E-30	1.050949479	stage_E14_vs_E10	ordinary_muscle_development	D_vs_E10;stage_1W_vs_E10;stage_3W_vs_E10;stage	1	TRUE
ENSGALG00010010220	LHX9	PRJNA729271	-9.381923261	1.78864E-30	9.381923261	stage_E14_vs_E10	ordinary_muscle_development	D_vs_E10;stage_1W_vs_E10;stage_3W_vs_E10;stage	1	TRUE
ENSGALG00010021913	ENSGALG00010021913	PRJNA729271	2.040933612	1.79776E-30	2.040933612	stage_E14_vs_E10	ordinary_muscle_development	D_vs_E10;stage_1W_vs_E10;stage_3W_vs_E10;stage	1	TRUE
ENSGALG00010028818	TSC1	PRJNA729271	1.375385572	1.81323E-30	1.375385572	stage_E14_vs_E10	ordinary_muscle_development	D_vs_E10;stage_1W_vs_E10;stage_3W_vs_E10;stage	1	TRUE
ENSGALG00010024666	MED24	PRJNA729271	-1.354454403	1.84902E-30	1.354454403	stage_E14_vs_E10	ordinary_muscle_development	D_vs_E10;stage_1W_vs_E10;stage_3W_vs_E10;stage	1	TRUE
ENSGALG00010003125	APOO	PRJNA729271	1.229329745	1.92897E-30	1.229329745	stage_E14_vs_E10	ordinary_muscle_development	D_vs_E10;stage_1W_vs_E10;stage_3W_vs_E10;stage	1	TRUE
ENSGALG00010021264	CBFB	PRJNA729271	-1.985617425	1.94635E-30	1.985617425	stage_E14_vs_E10	ordinary_muscle_development	D_vs_E10;stage_1W_vs_E10;stage_3W_vs_E10;stage	1	TRUE
ENSGALG00010001801	HOXA3	PRJNA729271	-1.8441813	1.97188E-30	1.8441813	stage_E14_vs_E10	ordinary_muscle_development	D_vs_E10;stage_1W_vs_E10;stage_3W_vs_E10;stage	1	TRUE
ENSGALG00010013344	OSMR	PRJNA729271	1.431399537	2.01456E-30	1.431399537	stage_E14_vs_E10	ordinary_muscle_development	D_vs_E10;stage_1W_vs_E10;stage_3W_vs_E10;stage	1	TRUE
ENSGALG00010003972	PENK	PRJNA729271	-2.801891315	2.1519E-30	2.801891315	stage_E14_vs_E10	ordinary_muscle_development	D_vs_E10;stage_1W_vs_E10;stage_3W_vs_E10;stage	1	TRUE
ENSGALG00010021550	MAP2K3	PRJNA729271	1.984885511	2.21211E-30	1.984885511	stage_E14_vs_E10	ordinary_muscle_development	D_vs_E10;stage_1W_vs_E10;stage_3W_vs_E10;stage	1	TRUE
ENSGALG00010010270	SSR1	PRJNA729271	-0.905701041	2.22897E-30	0.905701041	stage_E14_vs_E10	ordinary_muscle_development		0	FALSE
ENSGALG00010005679	TMEM45A	PRJNA729271	-2.422168933	2.25011E-30	2.422168933	stage_E14_vs_E10	ordinary_muscle_development	D_vs_E10;stage_1W_vs_E10;stage_3W_vs_E10;stage	1	TRUE
ENSGALG00010008264	CNTNAP2	PRJNA729271	-4.011664991	2.25316E-30	4.011664991	stage_E14_vs_E10	ordinary_muscle_development	D_vs_E10;stage_1W_vs_E10;stage_3W_vs_E10;stage	1	TRUE
ENSGALG00010010816	AP2S1	PRJNA729271	-1.329631259	2.29129E-30	1.329631259	stage_E14_vs_E10	ordinary_muscle_development	D_vs_E10;stage_1W_vs_E10;stage_3W_vs_E10;stage	1	TRUE
ENSGALG00010011574	GAS2L3	PRJNA729271	-1.866176347	2.2966E-30	1.866176347	stage_E14_vs_E10	ordinary_muscle_development	D_vs_E10;stage_1W_vs_E10;stage_3W_vs_E10;stage	1	TRUE
ENSGALG00010024493	JAK1	PRJNA729271	0.697658344	2.33684E-30	0.697658344	stage_E14_vs_E10	ordinary_muscle_development		0	FALSE
ENSGALG00010018968	GLI2	PRJNA729271	-2.338184272	2.42475E-30	2.338184272	stage_E14_vs_E10	ordinary_muscle_development	D_vs_E10;stage_1W_vs_E10;stage_3W_vs_E10;stage	1	TRUE
ENSGALG00010011904	KIF24	PRJNA729271	-3.961187722	2.43294E-30	3.961187722	stage_E14_vs_E10	ordinary_muscle_development	D_vs_E10;stage_1W_vs_E10;stage_3W_vs_E10;stage	1	TRUE
ENSGALG00010021072	ENSGALG00010021072	PRJNA729271	1.043006826	2.44454E-30	1.043006826	stage_E14_vs_E10	ordinary_muscle_development	D_vs_E10;stage_1W_vs_E10;stage_3W_vs_E10;stage	1	TRUE
ENSGALG00010003242	ASAP2	PRJNA729271	-1.491027772	2.46026E-30	1.491027772	stage_E14_vs_E10	ordinary_muscle_development	D_vs_E10;stage_1W_vs_E10;stage_3W_vs_E10;stage	1	TRUE
ENSGALG00010007588	TTC27	PRJNA729271	-1.215216063	2.47344E-30	1.215216063	stage_E14_vs_E10	ordinary_muscle_development	D_vs_E10;stage_1W_vs_E10;stage_3W_vs_E10;stage	1	TRUE
ENSGALG00010015362	ATP9A	PRJNA729271	1.467229333	2.49037E-30	1.467229333	stage_E14_vs_E10	ordinary_muscle_development	D_vs_E10;stage_1W_vs_E10;stage_3W_vs_E10;stage	1	TRUE
ENSGALG00010028033	ENSGALG00010028033	PRJNA729271	3.374671972	2.49432E-30	3.374671972	stage_E14_vs_E10	ordinary_muscle_development	D_vs_E10;stage_1W_vs_E10;stage_3W_vs_E10;stage	1	TRUE
ENSGALG00010003869	LY96	PRJNA729271	3.132112914	2.49693E-30	3.132112914	stage_E14_vs_E10	ordinary_muscle_development	D_vs_E10;stage_1W_vs_E10;stage_3W_vs_E10;stage	1	TRUE
ENSGALG00010018444	NUBP2	PRJNA729271	-1.249623169	2.51182E-30	1.249623169	stage_E14_vs_E10	ordinary_muscle_development	D_vs_E10;stage_1W_vs_E10;stage_3W_vs_E10;stage	1	TRUE
ENSGALG00010001440	CDCA7L	PRJNA729271	-1.759188226	2.59002E-30	1.759188226	stage_E14_vs_E10	ordinary_muscle_development	D_vs_E10;stage_1W_vs_E10;stage_3W_vs_E10;stage	1	TRUE
ENSGALG00010016953	GJA4	PRJNA729271	1.869960509	2.60444E-30	1.869960509	stage_E14_vs_E10	ordinary_muscle_development	D_vs_E10;stage_1W_vs_E10;stage_3W_vs_E10;stage	1	TRUE
ENSGALG00010005239	ENSGALG00010005239	PRJNA729271	-2.483228754	2.71555E-30	2.483228754	stage_E14_vs_E10	ordinary_muscle_development	D_vs_E10;stage_1W_vs_E10;stage_3W_vs_E10;stage	1	TRUE
ENSGALG00010017131	TLR3	PRJNA729271	4.339572615	2.78721E-30	4.339572615	stage_E14_vs_E10	ordinary_muscle_development	D_vs_E10;stage_1W_vs_E10;stage_3W_vs_E10;stage	1	TRUE
ENSGALG00010028007	STK11	PRJNA729271	1.379321246	2.87313E-30	1.379321246	stage_E14_vs_E10	ordinary_muscle_development	D_vs_E10;stage_1W_vs_E10;stage_3W_vs_E10;stage	1	TRUE
ENSGALG00010007970	SPRTN	PRJNA729271	1.222388793	2.92183E-30	1.222388793	stage_E14_vs_E10	ordinary_muscle_development	D_vs_E10;stage_1W_vs_E10;stage_3W_vs_E10;stage	1	TRUE
ENSGALG00010010183	NKX3-2	PRJNA729271	-5.274526548	2.96985E-30	5.274526548	stage_E14_vs_E10	ordinary_muscle_development	D_vs_E10;stage_1W_vs_E10;stage_3W_vs_E10;stage	1	TRUE
ENSGALG00010012506	TP53INP1	PRJNA729271	-1.492314497	3.01931E-30	1.492314497	stage_E14_vs_E10	ordinary_muscle_development	D_vs_E10;stage_1W_vs_E10;stage_3W_vs_E10;stage	1	TRUE
ENSGALG00010001233	CNDP2	PRJNA729271	-1.127654726	3.09431E-30	1.127654726	stage_E14_vs_E10	ordinary_muscle_development	D_vs_E10;stage_1W_vs_E10;stage_3W_vs_E10;stage	1	TRUE
ENSGALG00010004976	ENSGALG00010004976	PRJNA729271	2.614292921	3.16055E-30	2.614292921	stage_E14_vs_E10	ordinary_muscle_development	D_vs_E10;stage_1W_vs_E10;stage_3W_vs_E10;stage	1	TRUE
ENSGALG00010021210	ENSGALG00010021210	PRJNA729271	-0.742886966	3.20899E-30	0.742886966	stage_E14_vs_E10	ordinary_muscle_development		0	FALSE
ENSGALG00010015734	TSHZ2	PRJNA729271	-1.962428299	3.23699E-30	1.962428299	stage_E14_vs_E10	ordinary_muscle_development	D_vs_E10;stage_1W_vs_E10;stage_3W_vs_E10;stage	1	TRUE
ENSGALG00010004434	TCP1	PRJNA729271	-1.011921347	3.28792E-30	1.011921347	stage_E14_vs_E10	ordinary_muscle_development	D_vs_E10;stage_1W_vs_E10;stage_3W_vs_E10;stage	1	TRUE
ENSGALG00010022429	ADK	PRJNA729271	-1.527554925	3.42398E-30	1.527554925	stage_E14_vs_E10	ordinary_muscle_development	D_vs_E10;stage_1W_vs_E10;stage_3W_vs_E10;stage	1	TRUE
ENSGALG00010020540	PCGF5	PRJNA729271	1.866964085	3.59134E-30	1.866964085	stage_E14_vs_E10	ordinary_muscle_development	D_vs_E10;stage_1W_vs_E10;stage_3W_vs_E10;stage	1	TRUE
ENSGALG00010023387	ENSGALG00010023387	PRJNA729271	2.118216358	3.65798E-30	2.118216358	stage_E14_vs_E10	ordinary_muscle_development	D_vs_E10;stage_1W_vs_E10;stage_3W_vs_E10;stage	1	TRUE
ENSGALG00010021793	ENSGALG00010021793	PRJNA729271	1.834401633	3.83222E-30	1.834401633	stage_E14_vs_E10	ordinary_muscle_development	D_vs_E10;stage_1W_vs_E10;stage_3W_vs_E10;stage	1	TRUE
ENSGALG00010021033	RNF122	PRJNA729271	-2.735965538	3.95476E-30	2.735965538	stage_E14_vs_E10	ordinary_muscle_development	D_vs_E10;stage_1W_vs_E10;stage_3W_vs_E10;stage	1	TRUE
ENSGALG00010006352	GEMIN2	PRJNA729271	1.115516925	4.00039E-30	1.115516925	stage_E14_vs_E10	ordinary_muscle_development	D_vs_E10;stage_1W_vs_E10;stage_3W_vs_E10;stage	1	TRUE
ENSGALG00010024839	PA2G4	PRJNA729271	-1.151014341	4.01119E-30	1.151014341	stage_E14_vs_E10	ordinary_muscle_development	D_vs_E10;stage_1W_vs_E10;stage_3W_vs_E10;stage	1	TRUE
ENSGALG00010018355	PRKCB	PRJNA729271	-1.880671414	4.09821E-30	1.880671414	stage_E14_vs_E10	ordinary_muscle_development	D_vs_E10;stage_1W_vs_E10;stage_3W_vs_E10;stage	1	TRUE
ENSGALG00010017550	TM2D2	PRJNA729271	0.866104356	4.10955E-30	0.866104356	stage_E14_vs_E10	ordinary_muscle_development		0	FALSE
ENSGALG00010027497	KCNT1	PRJNA729271	3.961800101	4.18727E-30	3.961800101	stage_E14_vs_E10	ordinary_muscle_development	D_vs_E10;stage_1W_vs_E10;stage_3W_vs_E10;stage	1	TRUE
ENSGALG00010027977	BOLA1	PRJNA729271	1.790853817	4.26873E-30	1.790853817	stage_E14_vs_E10	ordinary_muscle_development	D_vs_E10;stage_1W_vs_E10;stage_3W_vs_E10;stage	1	TRUE
ENSGALG00010025114	CEBPG	PRJNA729271	1.116740455	4.3492E-30	1.116740455	stage_E14_vs_E10	ordinary_muscle_development	D_vs_E10;stage_1W_vs_E10;stage_3W_vs_E10;stage	1	TRUE
ENSGALG00010002918	LRATD1	PRJNA729271	-4.817326007	4.40626E-30	4.817326007	stage_E14_vs_E10	ordinary_muscle_development	D_vs_E10;stage_1W_vs_E10;stage_3W_vs_E10;stage	1	TRUE
ENSGALG00010025368	LRP1	PRJNA729271	1.606647411	4.44058E-30	1.606647411	stage_E14_vs_E10	ordinary_muscle_development	D_vs_E10;stage_1W_vs_E10;stage_3W_vs_E10;stage	1	TRUE
ENSGALG00010014522	FAT2	PRJNA729271	-6.501644082	4.45982E-30	6.501644082	stage_E14_vs_E10	ordinary_muscle_development	D_vs_E10;stage_1W_vs_E10;stage_3W_vs_E10;stage	1	TRUE
ENSGALG00010016985	ENSGALG00010016985	PRJNA729271	2.113785052	4.66473E-30	2.113785052	stage_E14_vs_E10	ordinary_muscle_development	D_vs_E10;stage_1W_vs_E10;stage_3W_vs_E10;stage	1	TRUE
ENSGALG00010001423	FAM210A	PRJNA729271	1.575426807	4.84414E-30	1.575426807	stage_E14_vs_E10	ordinary_muscle_development	D_vs_E10;stage_1W_vs_E10;stage_3W_vs_E10;stage	1	TRUE
ENSGALG00010002406	DIAPH3	PRJNA729271	-2.928105854	5.17066E-30	2.928105854	stage_E14_vs_E10	ordinary_muscle_development	D_vs_E10;stage_1W_vs_E10;stage_3W_vs_E10;stage	1	TRUE
ENSGALG00010026213	AKR1A1	PRJNA729271	-1.436821539	5.20001E-30	1.436821539	stage_E14_vs_E10	ordinary_muscle_development	D_vs_E10;stage_1W_vs_E10;stage_3W_vs_E10;stage	1	TRUE
ENSGALG00010008811	TERF2IP	PRJNA729271	1.085547711	5.3162E-30	1.085547711	stage_E14_vs_E10	ordinary_muscle_development	D_vs_E10;stage_1W_vs_E10;stage_3W_vs_E10;stage	1	TRUE
ENSGALG00010015706	ENSGALG00010015706	PRJNA729271	1.846788744	5.3742E-30	1.846788744	stage_E14_vs_E10	ordinary_muscle_development	D_vs_E10;stage_1W_vs_E10;stage_3W_vs_E10;stage	1	TRUE
ENSGALG00010016566	CNTLN	PRJNA729271	-2.523041429	5.49206E-30	2.523041429	stage_E14_vs_E10	ordinary_muscle_development	D_vs_E10;stage_1W_vs_E10;stage_3W_vs_E10;stage	1	TRUE
ENSGALG00010022656	FKBP10	PRJNA729271	-2.449899032	5.58035E-30	2.449899032	stage_E14_vs_E10	ordinary_muscle_development	D_vs_E10;stage_1W_vs_E10;stage_3W_vs_E10;stage	1	TRUE
ENSGALG00010027003	SEPTIN2L	PRJNA729271	-0.952198111	6.15076E-30	0.952198111	stage_E14_vs_E10	ordinary_muscle_development		0	FALSE
ENSGALG00010026098	STAT3	PRJNA729271	1.245047345	6.22755E-30	1.245047345	stage_E14_vs_E10	ordinary_muscle_development	D_vs_E10;stage_1W_vs_E10;stage_3W_vs_E10;stage	1	TRUE
ENSGALG00010023388	FARP2	PRJNA729271	-0.985293794	6.41508E-30	0.985293794	stage_E14_vs_E10	ordinary_muscle_development		0	FALSE
ENSGALG00010024873	HYOU1	PRJNA729271	-1.256367327	6.92257E-30	1.256367327	stage_E14_vs_E10	ordinary_muscle_development	D_vs_E10;stage_1W_vs_E10;stage_3W_vs_E10;stage	1	TRUE
ENSGALG00010000353	ENSGALG00010000353	PRJNA729271	1.270350496	6.93805E-30	1.270350496	stage_E14_vs_E10	ordinary_muscle_development	D_vs_E10;stage_1W_vs_E10;stage_3W_vs_E10;stage	1	TRUE
ENSGALG00010029605	C17orf75	PRJNA729271	-1.097828254	7.1091E-30	1.097828254	stage_E14_vs_E10	ordinary_muscle_development	D_vs_E10;stage_1W_vs_E10;stage_3W_vs_E10;stage	1	TRUE
ENSGALG00010028139	FZR1	PRJNA729271	-1.021533972	7.18291E-30	1.021533972	stage_E14_vs_E10	ordinary_muscle_development	D_vs_E10;stage_1W_vs_E10;stage_3W_vs_E10;stage	1	TRUE
ENSGALG00010026321	GPBP1L1	PRJNA729271	0.543555626	7.21274E-30	0.543555626	stage_E14_vs_E10	ordinary_muscle_development		0	FALSE
ENSGALG00010017184	CARD11	PRJNA729271	2.417281928	7.22394E-30	2.417281928	stage_E14_vs_E10	ordinary_muscle_development	D_vs_E10;stage_1W_vs_E10;stage_3W_vs_E10;stage	1	TRUE
ENSGALG00010024323	TTPAL	PRJNA729271	-1.193416685	7.3208E-30	1.193416685	stage_E14_vs_E10	ordinary_muscle_development	D_vs_E10;stage_1W_vs_E10;stage_3W_vs_E10;stage	1	TRUE
ENSGALG00010007873	ENSGALG00010007873	PRJNA729271	4.949964034	7.42246E-30	4.949964034	stage_E14_vs_E10	ordinary_muscle_development	D_vs_E10;stage_1W_vs_E10;stage_3W_vs_E10;stage	1	TRUE
ENSGALG00010009381	INPP4B	PRJNA729271	-1.362471356	7.43593E-30	1.362471356	stage_E14_vs_E10	ordinary_muscle_development	D_vs_E10;stage_1W_vs_E10;stage_3W_vs_E10;stage	1	TRUE
ENSGALG00010007695	RASGRP3	PRJNA729271	1.645481631	7.55987E-30	1.645481631	stage_E14_vs_E10	ordinary_muscle_development	D_vs_E10;stage_1W_vs_E10;stage_3W_vs_E10;stage	1	TRUE
ENSGALG00010028422	RXRA	PRJNA729271	2.389126367	7.80872E-30	2.389126367	stage_E14_vs_E10	ordinary_muscle_development	D_vs_E10;stage_1W_vs_E10;stage_3W_vs_E10;stage	1	TRUE
ENSGALG00010028425	DAP3	PRJNA729271	1.481828751	7.93404E-30	1.481828751	stage_E14_vs_E10	ordinary_muscle_development	D_vs_E10;stage_1W_vs_E10;stage_3W_vs_E10;stage	1	TRUE
ENSGALG00010014470	RPS6KA1	PRJNA729271	-1.510468002	7.96787E-30	1.510468002	stage_E14_vs_E10	ordinary_muscle_development	D_vs_E10;stage_1W_vs_E10;stage_3W_vs_E10;stage	1	TRUE
ENSGALG00010021244	PPP3CB	PRJNA729271	0.999754129	8.00396E-30	0.999754129	stage_E14_vs_E10	ordinary_muscle_development		0	FALSE
ENSGALG00010017763	POLR1A	PRJNA729271	-1.024849834	8.11638E-30	1.024849834	stage_E14_vs_E10	ordinary_muscle_development	D_vs_E10;stage_1W_vs_E10;stage_3W_vs_E10;stage	1	TRUE
ENSGALG00010030103	C1QTNF1	PRJNA729271	2.401717749	8.12902E-30	2.401717749	stage_E14_vs_E10	ordinary_muscle_development	D_vs_E10;stage_1W_vs_E10;stage_3W_vs_E10;stage	1	TRUE
ENSGALG00010003843	LRCH1	PRJNA729271	1.239891331	8.19621E-30	1.239891331	stage_E14_vs_E10	ordinary_muscle_development	D_vs_E10;stage_1W_vs_E10;stage_3W_vs_E10;stage	1	TRUE
ENSGALG00010015596	UCHL1	PRJNA729271	-5.925258557	8.55291E-30	5.925258557	stage_E14_vs_E10	ordinary_muscle_development	D_vs_E10;stage_1W_vs_E10;stage_3W_vs_E10;stage	1	TRUE
ENSGALG00010021735	PIGBOS1	PRJNA729271	1.661528406	8.62026E-30	1.661528406	stage_E14_vs_E10	ordinary_muscle_development	D_vs_E10;stage_1W_vs_E10;stage_3W_vs_E10;stage	1	TRUE
ENSGALG00010027012	RILPL1	PRJNA729271	2.205662521	8.77881E-30	2.205662521	stage_E14_vs_E10	ordinary_muscle_development	D_vs_E10;stage_1W_vs_E10;stage_3W_vs_E10;stage	1	TRUE
ENSGALG00010017077	CTNNBIP1	PRJNA729271	-1.222666815	8.7864E-30	1.222666815	stage_E14_vs_E10	ordinary_muscle_development	D_vs_E10;stage_1W_vs_E10;stage_3W_vs_E10;stage	1	TRUE
ENSGALG00010021416	PLEKHH3	PRJNA729271	1.396432918	8.93349E-30	1.396432918	stage_E14_vs_E10	ordinary_muscle_development	D_vs_E10;stage_1W_vs_E10;stage_3W_vs_E10;stage	1	TRUE
ENSGALG00010006157	SNX9	PRJNA729271	0.841640962	8.98209E-30	0.841640962	stage_E14_vs_E10	ordinary_muscle_development		0	FALSE
ENSGALG00010019426	GRM7	PRJNA729271	-3.118548069	9.07139E-30	3.118548069	stage_E14_vs_E10	ordinary_muscle_development	D_vs_E10;stage_1W_vs_E10;stage_3W_vs_E10;stage	1	TRUE
ENSGALG00010026438	TOMM6	PRJNA729271	1.384822388	9.08363E-30	1.384822388	stage_E14_vs_E10	ordinary_muscle_development	D_vs_E10;stage_1W_vs_E10;stage_3W_vs_E10;stage	1	TRUE
ENSGALG00010022946	KRT17	PRJNA729271	-6.471106649	9.16844E-30	6.471106649	stage_E14_vs_E10	ordinary_muscle_development	D_vs_E10;stage_1W_vs_E10;stage_3W_vs_E10;stage	1	TRUE
ENSGALG00010016459	PAQR5	PRJNA729271	3.124746229	9.21808E-30	3.124746229	stage_E14_vs_E10	ordinary_muscle_development	D_vs_E10;stage_1W_vs_E10;stage_3W_vs_E10;stage	1	TRUE
ENSGALG00010019595	GRIN2A	PRJNA729271	-2.734767307	9.28138E-30	2.734767307	stage_E14_vs_E10	ordinary_muscle_development	D_vs_E10;stage_1W_vs_E10;stage_3W_vs_E10;stage	1	TRUE
ENSGALG00010000815	ENSGALG00010000815	PRJNA729271	-2.129604421	9.43323E-30	2.129604421	stage_E14_vs_E10	ordinary_muscle_development	D_vs_E10;stage_1W_vs_E10;stage_3W_vs_E10;stage	1	TRUE
ENSGALG00010003740	PITX2	PRJNA729271	-4.217437308	9.53307E-30	4.217437308	stage_E14_vs_E10	ordinary_muscle_development	D_vs_E10;stage_1W_vs_E10;stage_3W_vs_E10;stage	1	TRUE
ENSGALG00010001291	ENSGALG00010001291	PRJNA729271	1.635715543	9.75563E-30	1.635715543	stage_E14_vs_E10	ordinary_muscle_development	D_vs_E10;stage_1W_vs_E10;stage_3W_vs_E10;stage	1	TRUE
ENSGALG00010009092	EED	PRJNA729271	-1.43071388	1.01874E-29	1.43071388	stage_E14_vs_E10	ordinary_muscle_development	D_vs_E10;stage_1W_vs_E10;stage_3W_vs_E10;stage	1	TRUE
ENSGALG00010011625	ERBB4	PRJNA729271	2.478270311	1.02563E-29	2.478270311	stage_E14_vs_E10	ordinary_muscle_development	D_vs_E10;stage_1W_vs_E10;stage_3W_vs_E10;stage	1	TRUE
ENSGALG00010027324	PIK3R2	PRJNA729271	-1.125603693	1.04703E-29	1.125603693	stage_E14_vs_E10	ordinary_muscle_development	D_vs_E10;stage_1W_vs_E10;stage_3W_vs_E10;stage	1	TRUE
ENSGALG00010004365	TPD52	PRJNA729271	-1.804392972	1.0648E-29	1.804392972	stage_E14_vs_E10	ordinary_muscle_development	D_vs_E10;stage_1W_vs_E10;stage_3W_vs_E10;stage	1	TRUE
ENSGALG00010012228	NHP2	PRJNA729271	-1.378411279	1.08982E-29	1.378411279	stage_E14_vs_E10	ordinary_muscle_development	D_vs_E10;stage_1W_vs_E10;stage_3W_vs_E10;stage	1	TRUE
ENSGALG00010016788	ANLN	PRJNA729271	-3.32848449	1.09445E-29	3.32848449	stage_E14_vs_E10	ordinary_muscle_development	D_vs_E10;stage_1W_vs_E10;stage_3W_vs_E10;stage	1	TRUE
ENSGALG00010023384	AP3B2	PRJNA729271	-2.802175922	1.09638E-29	2.802175922	stage_E14_vs_E10	ordinary_muscle_development	D_vs_E10;stage_1W_vs_E10;stage_3W_vs_E10;stage	1	TRUE
ENSGALG00010027421	PLPPR3	PRJNA729271	-3.180370327	1.11214E-29	3.180370327	stage_E14_vs_E10	ordinary_muscle_development	D_vs_E10;stage_1W_vs_E10;stage_3W_vs_E10;stage	1	TRUE
ENSGALG00010022440	SORD	PRJNA729271	1.420734269	1.11249E-29	1.420734269	stage_E14_vs_E10	ordinary_muscle_development	D_vs_E10;stage_1W_vs_E10;stage_3W_vs_E10;stage	1	TRUE
ENSGALG00010013783	NDUFA10	PRJNA729271	1.096312478	1.1173E-29	1.096312478	stage_E14_vs_E10	ordinary_muscle_development	D_vs_E10;stage_1W_vs_E10;stage_3W_vs_E10;stage	1	TRUE
ENSGALG00010017921	SPATA7	PRJNA729271	2.255947482	1.19878E-29	2.255947482	stage_E14_vs_E10	ordinary_muscle_development	D_vs_E10;stage_1W_vs_E10;stage_3W_vs_E10;stage	1	TRUE
ENSGALG00010019648	PRDM16	PRJNA729271	-2.348611666	1.20407E-29	2.348611666	stage_E14_vs_E10	ordinary_muscle_development	D_vs_E10;stage_1W_vs_E10;stage_3W_vs_E10;stage	1	TRUE
ENSGALG00010029175	CENPX	PRJNA729271	-1.548209722	1.21821E-29	1.548209722	stage_E14_vs_E10	ordinary_muscle_development	D_vs_E10;stage_1W_vs_E10;stage_3W_vs_E10;stage	1	TRUE
ENSGALG00010014001	CDC16	PRJNA729271	-0.997603566	1.2288E-29	0.997603566	stage_E14_vs_E10	ordinary_muscle_development		0	FALSE
ENSGALG00010018858	EPB41L5	PRJNA729271	-1.357115969	1.23522E-29	1.357115969	stage_E14_vs_E10	ordinary_muscle_development	D_vs_E10;stage_1W_vs_E10;stage_3W_vs_E10;stage	1	TRUE

ENSGALG00010015811	CTNNBL1	PRJNA729271	-0.635379068	1.24168E-29	0.635379068	stage_E14_vs_E10	ordinary_muscle_development		0	FALSE
ENSGALG00010016758	NR3C1	PRJNA729271	0.785742693	1.24472E-29	0.785742693	stage_E14_vs_E10	ordinary_muscle_development		0	FALSE
ENSGALG00010017526	PLEKHA2	PRJNA729271	1.949390918	1.24624E-29	1.949390918	stage_E14_vs_E10	ordinary_muscle_development	D_vs_E10;stage_1W_vs_E10;stage_3W_vs_E10;stage	1	TRUE
ENSGALG00010012893	ENSGALG00010012893	PRJNA729271	2.142888766	1.25875E-29	2.142888766	stage_E14_vs_E10	ordinary_muscle_development	D_vs_E10;stage_1W_vs_E10;stage_3W_vs_E10;stage	1	TRUE
ENSGALG00010015919	ENSGALG00010015919	PRJNA729271	2.55694403	1.31971E-29	2.55694403	stage_E14_vs_E10	ordinary_muscle_development	D_vs_E10;stage_1W_vs_E10;stage_3W_vs_E10;stage	1	TRUE
ENSGALG00010001141	MTAP	PRJNA729271	-1.306168249	1.32658E-29	1.306168249	stage_E14_vs_E10	ordinary_muscle_development	D_vs_E10;stage_1W_vs_E10;stage_3W_vs_E10;stage	1	TRUE
ENSGALG00010001514	MET	PRJNA729271	1.720941904	1.35066E-29	1.720941904	stage_E14_vs_E10	ordinary_muscle_development	D_vs_E10;stage_1W_vs_E10;stage_3W_vs_E10;stage	1	TRUE
ENSGALG00010008397	ATXN1L	PRJNA729271	-0.846304086	1.36924E-29	0.846304086	stage_E14_vs_E10	ordinary_muscle_development		0	FALSE
ENSGALG00010010779	DOCK2	PRJNA729271	2.122056878	1.3979E-29	2.122056878	stage_E14_vs_E10	ordinary_muscle_development	D_vs_E10;stage_1W_vs_E10;stage_3W_vs_E10;stage	1	TRUE
ENSGALG00010008385	CTNNA1	PRJNA729271	-1.049567021	1.40418E-29	1.049567021	stage_E14_vs_E10	ordinary_muscle_development	D_vs_E10;stage_1W_vs_E10;stage_3W_vs_E10;stage	1	TRUE
ENSGALG00010018185	GNB4	PRJNA729271	-0.798925615	1.45495E-29	0.798925615	stage_E14_vs_E10	ordinary_muscle_development		0	FALSE
ENSGALG00010024115	POLR2D	PRJNA729271	-0.869567425	1.49613E-29	0.869567425	stage_E14_vs_E10	ordinary_muscle_development		0	FALSE
ENSGALG00010025216	DPP6	PRJNA729271	-3.294244377	1.53104E-29	3.294244377	stage_E14_vs_E10	ordinary_muscle_development	D_vs_E10;stage_1W_vs_E10;stage_3W_vs_E10;stage	1	TRUE
ENSGALG00010012913	EGFLAM	PRJNA729271	-1.786691634	1.54019E-29	1.786691634	stage_E14_vs_E10	ordinary_muscle_development	D_vs_E10;stage_1W_vs_E10;stage_3W_vs_E10;stage	1	TRUE
ENSGALG00010029529	TEPSIN	PRJNA729271	1.430763695	1.54967E-29	1.430763695	stage_E14_vs_E10	ordinary_muscle_development	D_vs_E10;stage_1W_vs_E10;stage_3W_vs_E10;stage	1	TRUE
ENSGALG00010004896	UBA6	PRJNA729271	-0.97797607	1.563E-29	0.97797607	stage_E14_vs_E10	ordinary_muscle_development		0	FALSE
ENSGALG00010009210	PCDH15	PRJNA729271	-4.356773711	1.5834E-29	4.356773711	stage_E14_vs_E10	ordinary_muscle_development	D_vs_E10;stage_1W_vs_E10;stage_3W_vs_E10;stage	1	TRUE
ENSGALG00010005363	RDX	PRJNA729271	0.976800819	1.60601E-29	0.976800819	stage_E14_vs_E10	ordinary_muscle_development		0	FALSE
ENSGALG00010020160	SYT7	PRJNA729271	-3.987337999	1.61889E-29	3.987337999	stage_E14_vs_E10	ordinary_muscle_development	D_vs_E10;stage_1W_vs_E10;stage_3W_vs_E10;stage	1	TRUE
ENSGALG00010014418	RAC2	PRJNA729271	3.326284517	1.63085E-29	3.326284517	stage_E14_vs_E10	ordinary_muscle_development	D_vs_E10;stage_1W_vs_E10;stage_3W_vs_E10;stage	1	TRUE
ENSGALG00010029154	UTS2R	PRJNA729271	-1.678230797	1.72276E-29	1.678230797	stage_E14_vs_E10	ordinary_muscle_development	D_vs_E10;stage_1W_vs_E10;stage_3W_vs_E10;stage	1	TRUE
ENSGALG00010024433	ENSGALG00010024433	PRJNA729271	-1.938365949	1.73436E-29	1.938365949	stage_E14_vs_E10	ordinary_muscle_development	D_vs_E10;stage_1W_vs_E10;stage_3W_vs_E10;stage	1	TRUE
ENSGALG00010022391	ENSGALG00010022391	PRJNA729271	2.013417521	1.74414E-29	2.013417521	stage_E14_vs_E10	ordinary_muscle_development	D_vs_E10;stage_1W_vs_E10;stage_3W_vs_E10;stage	1	TRUE
ENSGALG00010000443	TBX3	PRJNA729271	-1.818111894	1.76705E-29	1.818111894	stage_E14_vs_E10	ordinary_muscle_development	D_vs_E10;stage_1W_vs_E10;stage_3W_vs_E10;stage	1	TRUE
ENSGALG00010021552	ENSGALG00010021552	PRJNA729271	1.958677053	1.79147E-29	1.958677053	stage_E14_vs_E10	ordinary_muscle_development	D_vs_E10;stage_1W_vs_E10;stage_3W_vs_E10;stage	1	TRUE
ENSGALG00010026001	ANKS1A	PRJNA729271	1.049529376	2.04785E-29	1.049529376	stage_E14_vs_E10	ordinary_muscle_development	D_vs_E10;stage_1W_vs_E10;stage_3W_vs_E10;stage	1	TRUE
ENSGALG00010022571	RIPOR1	PRJNA729271	1.220967587	2.05654E-29	1.220967587	stage_E14_vs_E10	ordinary_muscle_development	D_vs_E10;stage_1W_vs_E10;stage_3W_vs_E10;stage	1	TRUE
ENSGALG00010004338	ENSGALG00010004338	PRJNA729271	1.873745769	2.07361E-29	1.873745769	stage_E14_vs_E10	ordinary_muscle_development	D_vs_E10;stage_1W_vs_E10;stage_3W_vs_E10;stage	1	TRUE
ENSGALG00010018613	PADI3	PRJNA729271	4.009076375	2.08147E-29	4.009076375	stage_E14_vs_E10	ordinary_muscle_development	D_vs_E10;stage_1W_vs_E10;stage_3W_vs_E10;stage	1	TRUE
ENSGALG00010025419	CCND1	PRJNA729271	-1.53864599	2.19718E-29	1.53864599	stage_E14_vs_E10	ordinary_muscle_development	D_vs_E10;stage_1W_vs_E10;stage_3W_vs_E10;stage	1	TRUE
ENSGALG00010025931	TMEM136	PRJNA729271	-2.477909953	2.23352E-29	2.477909953	stage_E14_vs_E10	ordinary_muscle_development	D_vs_E10;stage_1W_vs_E10;stage_3W_vs_E10;stage	1	TRUE
ENSGALG00010025974	EEF1B2	PRJNA729271	1.089052886	2.32384E-29	1.089052886	stage_E14_vs_E10	ordinary_muscle_development	D_vs_E10;stage_1W_vs_E10;stage_3W_vs_E10;stage	1	TRUE
ENSGALG00010012839	TGFBI	PRJNA729271	-2.086298608	2.34419E-29	2.086298608	stage_E14_vs_E10	ordinary_muscle_development	D_vs_E10;stage_1W_vs_E10;stage_3W_vs_E10;stage	1	TRUE
ENSGALG00010025940	HECTD3	PRJNA729271	0.995524304	2.37169E-29	0.995524304	stage_E14_vs_E10	ordinary_muscle_development		0	FALSE
ENSGALG00010006220	SGCG	PRJNA729271	1.953578241	2.45349E-29	1.953578241	stage_E14_vs_E10	ordinary_muscle_development	D_vs_E10;stage_1W_vs_E10;stage_3W_vs_E10;stage	1	TRUE
ENSGALG00010005511	AHCYL2	PRJNA729271	1.114487812	2.4644E-29	1.114487812	stage_E14_vs_E10	ordinary_muscle_development	D_vs_E10;stage_1W_vs_E10;stage_3W_vs_E10;stage	1	TRUE
ENSGALG00010015939	SUN2	PRJNA729271	0.905367286	2.46975E-29	0.905367286	stage_E14_vs_E10	ordinary_muscle_development		0	FALSE
ENSGALG00010001194	POT1	PRJNA729271	-1.287707307	2.50542E-29	1.287707307	stage_E14_vs_E10	ordinary_muscle_development	D_vs_E10;stage_1W_vs_E10;stage_3W_vs_E10;stage	1	TRUE
ENSGALG00010006564	SLC10A7	PRJNA729271	-1.655921845	2.52038E-29	1.655921845	stage_E14_vs_E10	ordinary_muscle_development	D_vs_E10;stage_1W_vs_E10;stage_3W_vs_E10;stage	1	TRUE
ENSGALG00010023624	PSMA1	PRJNA729271	0.802557562	2.58971E-29	0.802557562	stage_E14_vs_E10	ordinary_muscle_development		0	FALSE
ENSGALG00010024668	ENSGALG00010024668	PRJNA729271	2.107852775	2.60266E-29	2.107852775	stage_E14_vs_E10	ordinary_muscle_development	D_vs_E10;stage_1W_vs_E10;stage_3W_vs_E10;stage	1	TRUE
ENSGALG00010021019	VSIG10	PRJNA729271	-1.116010093	2.65152E-29	1.116010093	stage_E14_vs_E10	ordinary_muscle_development	D_vs_E10;stage_1W_vs_E10;stage_3W_vs_E10;stage	1	TRUE
ENSGALG00010007595	ATP2C2	PRJNA729271	1.758701508	2.70607E-29	1.758701508	stage_E14_vs_E10	ordinary_muscle_development	D_vs_E10;stage_1W_vs_E10;stage_3W_vs_E10;stage	1	TRUE
ENSGALG00010021057	ZDHHC8	PRJNA729271	0.882573084	2.85031E-29	0.882573084	stage_E14_vs_E10	ordinary_muscle_development		0	FALSE
ENSGALG00010012051	ARK2C	PRJNA729271	-2.415994573	2.8566E-29	2.415994573	stage_E14_vs_E10	ordinary_muscle_development	D_vs_E10;stage_1W_vs_E10;stage_3W_vs_E10;stage	1	TRUE
ENSGALG00010028129	CYB561D2	PRJNA729271	1.1838099	2.99068E-29	1.1838099	stage_E14_vs_E10	ordinary_muscle_development	D_vs_E10;stage_1W_vs_E10;stage_3W_vs_E10;stage	1	TRUE
ENSGALG00010023054	AQP9	PRJNA729271	2.651934076	2.99377E-29	2.651934076	stage_E14_vs_E10	ordinary_muscle_development	D_vs_E10;stage_1W_vs_E10;stage_3W_vs_E10;stage	1	TRUE
ENSGALG00010021633	CCNJ	PRJNA729271	-1.255267114	3.1205E-29	1.255267114	stage_E14_vs_E10	ordinary_muscle_development	D_vs_E10;stage_1W_vs_E10;stage_3W_vs_E10;stage	1	TRUE
ENSGALG00010022743	ZNF326	PRJNA729271	-1.747878195	3.33216E-29	1.747878195	stage_E14_vs_E10	ordinary_muscle_development	D_vs_E10;stage_1W_vs_E10;stage_3W_vs_E10;stage	1	TRUE
ENSGALG00010004187	ARFGEF1	PRJNA729271	1.067794509	3.38208E-29	1.067794509	stage_E14_vs_E10	ordinary_muscle_development	D_vs_E10;stage_1W_vs_E10;stage_3W_vs_E10;stage	1	TRUE
ENSGALG00010016810	SH3GL3	PRJNA729271	-2.337931735	3.41277E-29	2.337931735	stage_E14_vs_E10	ordinary_muscle_development	D_vs_E10;stage_1W_vs_E10;stage_3W_vs_E10;stage	1	TRUE
ENSGALG00010000576	NOS1	PRJNA729271	4.779725104	3.5205E-29	4.779725104	stage_E14_vs_E10	ordinary_muscle_development	D_vs_E10;stage_1W_vs_E10;stage_3W_vs_E10;stage	1	TRUE
ENSGALG00010017992	ENSGALG00010017992	PRJNA729271	6.362279171	3.52734E-29	6.362279171	stage_E14_vs_E10	ordinary_muscle_development	D_vs_E10;stage_1W_vs_E10;stage_3W_vs_E10;stage	1	TRUE
ENSGALG00010029341	SLC43A2	PRJNA729271	3.716571018	3.66487E-29	3.716571018	stage_E14_vs_E10	ordinary_muscle_development	D_vs_E10;stage_1W_vs_E10;stage_3W_vs_E10;stage	1	TRUE
ENSGALG00010020388	MYOF	PRJNA729271	1.885309308	3.66656E-29	1.885309308	stage_E14_vs_E10	ordinary_muscle_development	D_vs_E10;stage_1W_vs_E10;stage_3W_vs_E10;stage	1	TRUE
ENSGALG00010021258	LSM3	PRJNA729271	-1.314401285	3.68315E-29	1.314401285	stage_E14_vs_E10	ordinary_muscle_development	D_vs_E10;stage_1W_vs_E10;stage_3W_vs_E10;stage	1	TRUE
ENSGALG00010019334	ATL1	PRJNA729271	-1.501301654	3.77085E-29	1.501301654	stage_E14_vs_E10	ordinary_muscle_development	D_vs_E10;stage_1W_vs_E10;stage_3W_vs_E10;stage	1	TRUE
ENSGALG00010011285	ENSGALG00010011285	PRJNA729271	2.891862251	3.9911E-29	2.891862251	stage_E14_vs_E10	ordinary_muscle_development	D_vs_E10;stage_1W_vs_E10;stage_3W_vs_E10;stage	1	TRUE
ENSGALG00010011399	JAKMIP1	PRJNA729271	-3.156190953	4.08203E-29	3.156190953	stage_E14_vs_E10	ordinary_muscle_development	D_vs_E10;stage_1W_vs_E10;stage_3W_vs_E10;stage	1	TRUE
ENSGALG00010005255	BZW2	PRJNA729271	1.053963557	4.21005E-29	1.053963557	stage_E14_vs_E10	ordinary_muscle_development	D_vs_E10;stage_1W_vs_E10;stage_3W_vs_E10;stage	1	TRUE
ENSGALG00010005366	AUP1	PRJNA729271	1.286431563	4.23591E-29	1.286431563	stage_E14_vs_E10	ordinary_muscle_development	D_vs_E10;stage_1W_vs_E10;stage_3W_vs_E10;stage	1	TRUE
ENSGALG00010023064	ATG13	PRJNA729271	0.777997588	4.24346E-29	0.777997588	stage_E14_vs_E10	ordinary_muscle_development		0	FALSE
ENSGALG00010023902	PTGER3	PRJNA729271	2.653880254	4.43176E-29	2.653880254	stage_E14_vs_E10	ordinary_muscle_development	D_vs_E10;stage_1W_vs_E10;stage_3W_vs_E10;stage	1	TRUE
ENSGALG00010013139	TMEM185A	PRJNA729271	0.931258115	4.46785E-29	0.931258115	stage_E14_vs_E10	ordinary_muscle_development		0	FALSE
ENSGALG00010025574	ARHGAP31	PRJNA729271	-1.137947835	4.47666E-29	1.137947835	stage_E14_vs_E10	ordinary_muscle_development	D_vs_E10;stage_1W_vs_E10;stage_3W_vs_E10;stage	1	TRUE
ENSGALG00010002864	RNASEH2B	PRJNA729271	-1.08050772	4.49289E-29	1.08050772	stage_E14_vs_E10	ordinary_muscle_development	D_vs_E10;stage_1W_vs_E10;stage_3W_vs_E10;stage	1	TRUE
ENSGALG00010023732	TM2D1	PRJNA729271	0.870113917	4.62106E-29	0.870113917	stage_E14_vs_E10	ordinary_muscle_development		0	FALSE
ENSGALG00010010420	NPM1	PRJNA729271	-1.275288624	4.65664E-29	1.275288624	stage_E14_vs_E10	ordinary_muscle_development	D_vs_E10;stage_1W_vs_E10;stage_3W_vs_E10;stage	1	TRUE
ENSGALG00010009320	GPR20	PRJNA729271	-2.874970932	4.65996E-29	2.874970932	stage_E14_vs_E10	ordinary_muscle_development	D_vs_E10;stage_1W_vs_E10;stage_3W_vs_E10;stage	1	TRUE
ENSGALG00010017956	TIPIN	PRJNA729271	-0.93295617	4.70835E-29	0.93295617	stage_E14_vs_E10	ordinary_muscle_development		0	FALSE
ENSGALG00010018832	EEPD1	PRJNA729271	2.098068551	4.72262E-29	2.098068551	stage_E14_vs_E10	ordinary_muscle_development	D_vs_E10;stage_1W_vs_E10;stage_3W_vs_E10;stage	1	TRUE
ENSGALG00010007575	MMP27	PRJNA729271	-4.073535986	4.73674E-29	4.073535986	stage_E14_vs_E10	ordinary_muscle_development	D_vs_E10;stage_1W_vs_E10;stage_3W_vs_E10;stage	1	TRUE
ENSGALG00010021728	TMED5	PRJNA729271	-0.746750368	4.91767E-29	0.746750368	stage_E14_vs_E10	ordinary_muscle_development		0	FALSE
ENSGALG00010022282	TMT1A	PRJNA729271	2.53026487	4.96133E-29	2.53026487	stage_E14_vs_E10	ordinary_muscle_development	D_vs_E10;stage_1W_vs_E10;stage_3W_vs_E10;stage	1	TRUE
ENSGALG00010028569	ZER1	PRJNA729271	0.775900193	5.12372E-29	0.775900193	stage_E14_vs_E10	ordinary_muscle_development		0	FALSE
ENSGALG00010020920	ACOX2	PRJNA729271	2.326517164	5.18703E-29	2.326517164	stage_E14_vs_E10	ordinary_muscle_development	D_vs_E10;stage_1W_vs_E10;stage_3W_vs_E10;stage	1	TRUE
ENSGALG00010004577	ZPLD1	PRJNA729271	-5.580997312	5.20571E-29	5.580997312	stage_E14_vs_E10	ordinary_muscle_development	D_vs_E10;stage_1W_vs_E10;stage_3W_vs_E10;stage	1	TRUE
ENSGALG00010015374	LIMS1	PRJNA729271	-1.72073304	5.33219E-29	1.72073304	stage_E14_vs_E10	ordinary_muscle_development	D_vs_E10;stage_1W_vs_E10;stage_3W_vs_E10;stage	1	TRUE
ENSGALG00010003210	EYA1	PRJNA729271	2.275934164	5.54884E-29	2.275934164	stage_E14_vs_E10	ordinary_muscle_development	D_vs_E10;stage_1W_vs_E10;stage_3W_vs_E10;stage	1	TRUE
ENSGALG00010023663	PHOSPHO2	PRJNA729271	1.011113502	5.59529E-29	1.011113502	stage_E14_vs_E10	ordinary_muscle_development	D_vs_E10;stage_1W_vs_E10;stage_3W_vs_E10;stage	1	TRUE
ENSGALG00010014907	ENSGALG00010014907	PRJNA729271	-0.807628555	5.62582E-29	0.807628555	stage_E14_vs_E10	ordinary_muscle_development		0	FALSE
ENSGALG00010014204	KIFAP3	PRJNA729271	-0.961445269	5.63385E-29	0.961445269	stage_E14_vs_E10	ordinary_muscle_development		0	FALSE
ENSGALG00010010659	ADAMTS1	PRJNA729271	1.719055437	5.68035E-29	1.719055437	stage_E14_vs_E10	ordinary_muscle_development	D_vs_E10;stage_1W_vs_E10;stage_3W_vs_E10;stage	1	TRUE
ENSGALG00010014389	COL6A2	PRJNA729271	3.012295034	5.7032E-29	3.012295034	stage_E14_vs_E10	ordinary_muscle_development	D_vs_E10;stage_1W_vs_E10;stage_3W_vs_E10;stage	1	TRUE
ENSGALG00010016704	CSTF2	PRJNA729271	-0.846821413	5.94245E-29	0.846821413	stage_E14_vs_E10	ordinary_muscle_development		0	FALSE
ENSGALG00010026305	ATP5MC1	PRJNA729271	1.895604982	6.01324E-29	1.895604982	stage_E14_vs_E10	ordinary_muscle_development	D_vs_E10;stage_1W_vs_E10;stage_3W_vs_E10;stage	1	TRUE
ENSGALG00010009868	LTN1	PRJNA729271	1.070423339	6.01332E-29	1.070423339	stage_E14_vs_E10	ordinary_muscle_development	D_vs_E10;stage_1W_vs_E10;stage_3W_vs_E10;stage	1	TRUE
ENSGALG00010021746	MTOR	PRJNA729271	1.24894344	6.08265E-29	1.24894344	stage_E14_vs_E10	ordinary_muscle_development	D_vs_E10;stage_1W_vs_E10;stage_3W_vs_E10;stage	1	TRUE
ENSGALG00010017452	ARMH4	PRJNA729271	-5.708262752	6.08826E-29	5.708262752	stage_E14_vs_E10	ordinary_muscle_development	D_vs_E10;stage_1W_vs_E10;stage_3W_vs_E10;stage	1	TRUE
ENSGALG00010002464	NGLY1	PRJNA729271	0.937294657	6.12332E-29	0.937294657	stage_E14_vs_E10	ordinary_muscle_development		0	FALSE
ENSGALG00010013549	PCP4	PRJNA729271	-4.744084927	6.42083E-29	4.744084927	stage_E14_vs_E10	ordinary_muscle_development	D_vs_E10;stage_1W_vs_E10;stage_3W_vs_E10;stage	1	TRUE
ENSGALG00010016738	NAA60	PRJNA729271	1.683662711	6.48237E-29	1.683662711	stage_E14_vs_E10	ordinary_muscle_development	D_vs_E10;stage_1W_vs_E10;stage_3W_vs_E10;stage	1	TRUE
ENSGALG00010024841	ENSGALG00010024841	PRJNA729271	-2.552550756	6.56921E-29	2.552550756	stage_E14_vs_E10	ordinary_muscle_development	D_vs_E10;stage_1W_vs_E10;stage_3W_vs_E10;stage	1	TRUE
ENSGALG00010029628	UNC45B	PRJNA729271	1.815718682	6.65562E-29	1.815718682	stage_E14_vs_E10	ordinary_muscle_development	D_vs_E10;stage_1W_vs_E10;stage_3W_vs_E10;stage	1	TRUE
ENSGALG00010015134	PLN	PRJNA729271	5.899671252	6.65902E-29	5.899671252	stage_E14_vs_E10	ordinary_muscle_development	D_vs_E10;stage_1W_vs_E10;stage_3W_vs_E10;stage	1	TRUE
ENSGALG00010022932	OPCML	PRJNA729271	-3.170471776	6.78478E-29	3.170471776	stage_E14_vs_E10	ordinary_muscle_development	D_vs_E10;stage_1W_vs_E10;stage_3W_vs_E10;stage	1	TRUE
ENSGALG00010026893	FKBP5	PRJNA729271	4.378409657	6.86166E-29	4.378409657	stage_E14_vs_E10	ordinary_muscle_development	D_vs_E10;stage_1W_vs_E10;stage_3W_vs_E10;stage	1	TRUE
ENSGALG00010028580	PRUNE1	PRJNA729271	1.702514354	6.86749E-29	1.702514354	stage_E14_vs_E10	ordinary_muscle_development	D_vs_E10;stage_1W_vs_E10;stage_3W_vs_E10;stage	1	TRUE
ENSGALG00010004082	FAM241A	PRJNA729271	1.373558596	6.92239E-29	1.373558596	stage_E14_vs_E10	ordinary_muscle_development	D_vs_E10;stage_1W_vs_E10;stage_3W_vs_E10;stage	1	TRUE
ENSGALG00010011950	PDXK	PRJNA729271	1.875231222	6.97181E-29	1.875231222	stage_E14_vs_E10	ordinary_muscle_development	D_vs_E10;stage_1W_vs_E10;stage_3W_vs_E10;stage	1	TRUE
ENSGALG00010019032	GNMT	PRJNA729271	2.146532789	6.98755E-29	2.146532789	stage_E14_vs_E10	ordinary_muscle_development	D_vs_E10;stage_1W_vs_E10;stage_3W_vs_E10;stage	1	TRUE
ENSGALG00010028516	GATAD2A	PRJNA729271	-0.720276509	7.11531E-29	0.720276509	stage_E14_vs_E10	ordinary_muscle_development		0	FALSE
ENSGALG00010000287	IQCD	PRJNA729271	-2.913866794	7.14415E-29	2.913866794	stage_E14_vs_E10	ordinary_muscle_development	D_vs_E10;stage_1W_vs_E10;stage_3W_vs_E10;stage	1	TRUE
ENSGALG00010008921	OPA3	PRJNA729271	1.410920411	7.31879E-29	1.410920411	stage_E14_vs_E10	ordinary_muscle_development	D_vs_E10;stage_1W_vs_E10;stage_3W_vs_E10;stage	1	TRUE
ENSGALG00010021747	AKTIP	PRJNA729271	1.298005043	7.5148E-29	1.298005043	stage_E14_vs_E10	ordinary_muscle_development	D_vs_E10;stage_1W_vs_E10;stage_3W_vs_E10;stage	1	TRUE
ENSGALG00010000092	INPPL1	PRJNA729271	1.91904819	7.58016E-29	1.91904819	stage_E14_vs_E10	ordinary_muscle_development	D_vs_E10;stage_1W_vs_E10;stage_3W_vs_E10;stage	1	TRUE
ENSGALG00010016067	AHSG	PRJNA729271	9.601063541	7.92682E-29	9.601063541	stage_E14_vs_E10	ordinary_muscle_development	D_vs_E10;stage_1W_vs_E10;stage_3W_vs_E10;stage	1	TRUE
ENSGALG00010021874	TMCC1	PRJNA729271	0.894989387	7.99161E-29	0.894989387	stage_E14_vs_E10	ordinary_muscle_development		0	FALSE
ENSGALG00010003830	DMB2	PRJNA729271	2.579377452	8.18078E-29	2.579377452	stage_E14_vs_E10	ordinary_muscle_development	D_vs_E10;stage_1W_vs_E10;stage_3W_vs_E10;stage	1	TRUE
ENSGALG00010012849	TRHDE	PRJNA729271	-4.294427417	8.367E-29	4.294427417	stage_E14_vs_E10	ordinary_muscle_development	D_vs_E10;stage_1W_vs_E10;stage_3W_vs_E10;stage	1	TRUE
ENSGALG00010010267	CCSER2	PRJNA729271	1.206723483	8.51358E-29	1.206723483	stage_E14_vs_E10	ordinary_muscle_development	D_vs_E10;stage_1W_vs_E10;stage_3W_vs_E10;stage	1	TRUE
ENSGALG00010029652	LRRC59	PRJNA729271	-1.174369501	8.57517E-29	1.174369501	stage_E14_vs_E10	ordinary_muscle_development	D_vs_E10;stage_1W_vs_E10;stage_3W_vs_E10;stage	1	TRUE
ENSGALG00010017825	PLPP5	PRJNA729271	-1.7749644	8.93135E-29	1.7749644	stage_E14_vs_E10	ordinary_muscle_development	D_vs_E10;stage_1W_vs_E10;stage_3W_vs_E10;stage	1	TRUE
ENSGALG00010005500	KATNA1	PRJNA729271	1.46117726	9.08938E-29	1.46117726	stage_E14_vs_E10	ordinary_muscle_development	D_vs_E10;stage_1W_vs_E10;stage_3W_vs_E10;stage	1	TRUE
ENSGALG00010000235	ENSGALG00010000235	PRJNA729271	-2.945364754	9.09534E-29	2.945364754	stage_E14_vs_E10	ordinary_muscle_development	D_vs_E10;stage_1W_vs_E10;stage_3W_vs_E10;stage	1	TRUE
ENSGALG00010014393	ATP8A1	PRJNA729271	1.622316763	9.15925E-29	1.622316763	stage_E14_vs_E10	ordinary_muscle_development	D_vs_E10;stage_1W_vs_E10;stage_3W_vs_E10;stage	1	TRUE
ENSGALG00010018811	VAMP3	PRJNA729271	1.386982056	9.16261E-29	1.386982056	stage_E14_vs_E10	ordinary_muscle_development	D_vs_E10;stage_1W_vs_E10;stage_3W_vs_E10;stage	1	TRUE
ENSGALG00010017359	EHD3	PRJNA729271	1.074285278	9.40544E-29	1.074285278	stage_E14_vs_E10	ordinary_muscle_development	D_vs_E10;stage_1W_vs_E10;stage_3W_vs_E10;stage	1	TRUE
ENSGALG00010004902	ANKRD29	PRJNA729271	3.36467033	9.41432E-29	3.36467033	stage_E14_vs_E10	ordinary_muscle_development	D_vs_E10;stage_1W_vs_E10;stage_3W_vs_E10;stage	1	TRUE
ENSGALG00010027209	TRAF3IP3	PRJNA729271	2.632680164	9.57196E-29	2.632680164	stage_E14_vs_E10	ordinary_muscle_development	D_vs_E10;stage_1W_vs_E10;stage_3W_vs_E10;stage	1	TRUE
ENSGALG00010029421	TBCD	PRJNA729271	-1.076150823	9.6521E-29	1.076150823	stage_E14_vs_E10	ordinary_muscle_development	D_vs_E10;stage_1W_vs_E10;stage_3W_vs_E10;stage	1	TRUE
ENSGALG00010003863	HTR1B	PRJNA729271	-4.058262416	9.9584E-29	4.058262416	stage_E14_vs_E10	ordinary_muscle_development	D_vs_E10;stage_1W_vs_E10;stage_3W_vs_E10;stage	1	TRUE
ENSGALG00010021195	GCLM	PRJNA729271	-1.041239202	1.01188E-28	1.041239202	stage_E14_vs_E10	ordinary_muscle_development	D_vs_E10;stage_1W_vs_E10;stage_3W_vs_E10;stage	1	TRUE
ENSGALG00010005402	ABCC4	PRJNA729271	1.898311027	1.01517E-28	1.898311027	stage_E14_vs_E10	ordinary_muscle_development	D_vs_E10;stage_1W_vs_E10;stage_3W_vs_E10;stage	1	TRUE
ENSGALG00010017187	ZNF839	PRJNA729271	1.54610314	1.03113E-28	1.54610314	stage_E14_vs_E10	ordinary_muscle_development	D_vs_E10;stage_1W_vs_E10;stage_3W_vs_E10;stage	1	TRUE
ENSGALG00010024887	CASP18	PRJNA729271	2.799047079	1.06658E-28	2.799047079	stage_E14_vs_E10	ordinary_muscle_development	D_vs_E10;stage_1W_vs_E10;stage_3W_vs_E10;stage	1	TRUE
ENSGALG00010027638	MRPS2	PRJNA729271	1.574816722	1.06728E-28	1.574816722	stage_E14_vs_E10	ordinary_muscle_development	D_vs_E10;stage_1W_vs_E10;stage_3W_vs_E10;stage	1	TRUE
ENSGALG00010017867	APLF	PRJNA729271	1.22263641	1.08381E-28	1.22263641	stage_E14_vs_E10	ordinary_muscle_development	D_vs_E10;stage_1W_vs_E10;stage_3W_vs_E10;stage	1	TRUE
ENSGALG00010028020	ENSGALG00010028020	PRJNA729271	1.637987237	1.08906E-28	1.637987237	stage_E14_vs_E10	ordinary_muscle_development	D_vs_E10;stage_1W_vs_E10;stage_3W_vs_E10;stage	1	TRUE
ENSGALG00010018012	DVL1	PRJNA729271	1.554433974	1.10793E-28	1.554433974	stage_E14_vs_E10	ordinary_muscle_development	D_vs_E10;stage_1W_vs_E10;stage_3W_vs_E10;stage	1	TRUE
ENSGALG00010011029	HMGCR	PRJNA729271	-1.437287948	1.11416E-28	1.437287948	stage_E14_vs_E10	ordinary_muscle_development	D_vs_E10;stage_1W_vs_E10;stage_3W_vs_E10;stage	1	TRUE
ENSGALG00010020304	ZFP36L2	PRJNA729271	1.751230869	1.11505E-28	1.751230869	stage_E14_vs_E10	ordinary_muscle_development	D_vs_E10;stage_1W_vs_E10;stage_3W_vs_E10;stage	1	TRUE
ENSGALG00010012376	VGLL2	PRJNA729271	2.097356127	1.16186E-28	2.097356127	stage_E14_vs_E10	ordinary_muscle_development	D_vs_E10;stage_1W_vs_E10;stage_3W_vs_E10;stage	1	TRUE
ENSGALG00010013349	ETS2	PRJNA729271	1.127422004	1.19668E-28	1.127422004	stage_E14_vs_E10	ordinary_muscle_development	D_vs_E10;stage_1W_vs_E10;stage_3W_vs_E10;stage	1	TRUE
ENSGALG00010004002	CDADC1	PRJNA729271	1.366144584	1.24626E-28	1.366144584	stage_E14_vs_E10	ordinary_muscle_development	D_vs_E10;stage_1W_vs_E10;stage_3W_vs_E10;stage	1	TRUE
ENSGALG00010002706	ROCK2	PRJNA729271	1.621952552	1.26106E-28	1.621952552	stage_E14_vs_E10	ordinary_muscle_development	D_vs_E10;stage_1W_vs_E10;stage_3W_vs_E10;stage	1	TRUE
ENSGALG00010008250	KLHL38	PRJNA729271	6.666457328	1.26333E-28	6.666457328	stage_E14_vs_E10	ordinary_muscle_development	D_vs_E10;stage_1W_vs_E10;stage_3W_vs_E10;stage	1	TRUE
ENSGALG00010017349	ENSGALG00010017349	PRJNA729271	1.274280202	1.28951E-28	1.274280202	stage_E14_vs_E10	ordinary_muscle_development	D_vs_E10;stage_1W_vs_E10;stage_3W_vs_E10;stage	1	TRUE
ENSGALG00010025485	DENND2C	PRJNA729271	1.804575988	1.29614E-28	1.804575988	stage_E14_vs_E10	ordinary_muscle_development	D_vs_E10;stage_1W_vs_E10;stage_3W_vs_E10;stage	1	TRUE
ENSGALG00010016558	NR2F2	PRJNA729271	-1.241223791	1.30129E-28	1.241223791	stage_E14_vs_E10	ordinary_muscle_development	D_vs_E10;stage_1W_vs_E10;stage_3W_vs_E10;stage	1	TRUE
ENSGALG00010004777	CDK14	PRJNA729271	-1.408058883	1.34513E-28	1.408058883	stage_E14_vs_E10	ordinary_muscle_development	D_vs_E10;stage_1W_vs_E10;stage_3W_vs_E10;stage	1	TRUE
ENSGALG00010014837	APBA1	PRJNA729271	-1.323018678	1.3484E-28	1.323018678	stage_E14_vs_E10	ordinary_muscle_development	D_vs_E10;stage_1W_vs_E10;stage_3W_vs_E10;stage	1	TRUE
ENSGALG00010013192	B4GALT5	PRJNA729271	0.913785339	1.35525E-28	0.913785339	stage_E14_vs_E10	ordinary_muscle_development		0	FALSE
ENSGALG00010029602	MTMR4	PRJNA729271	-0.991516077	1.35703E-28	0.991516077	stage_E14_vs_E10	ordinary_muscle_development		0	FALSE
ENSGALG00010022062	MITF	PRJNA729271	1.967004731	1.36149E-28	1.967004731	stage_E14_vs_E10	ordinary_muscle_development	D_vs_E10;stage_1W_vs_E10;stage_3W_vs_E10;stage	1	TRUE
ENSGALG00010017502	NAT8B	PRJNA729271	1.314175446	1.37252E-28	1.314175446	stage_E14_vs_E10	ordinary_muscle_development	D_vs_E10;stage_1W_vs_E10;stage_3W_vs_E10;stage	1	TRUE
ENSGALG00010022111	PCGF2	PRJNA729271	-2.303838273	1.39932E-28	2.303838273	stage_E14_vs_E10	ordinary_muscle_development	D_vs_E10;stage_1W_vs_E10;stage_3W_vs_E10;stage	1	TRUE
ENSGALG00010008020	COA6	PRJNA729271	1.46270395	1.447E-28	1.46270395	stage_E14_vs_E10	ordinary_muscle_development	D_vs_E10;stage_1W_vs_E10;stage_3W_vs_E10;stage	1	TRUE
ENSGALG00010001495	CCNL1	PRJNA729271	1.212201625	1.47577E-28	1.212201625	stage_E14_vs_E10	ordinary_muscle_development	D_vs_E10;stage_1W_vs_E10;stage_3W_vs_E10;stage	1	TRUE
ENSGALG00010001691	CTPS2	PRJNA729271	1.503492389	1.49317E-28	1.503492389	stage_E14_vs_E10	ordinary_muscle_development	D_vs_E10;stage_1W_vs_E10;stage_3W_vs_E10;stage	1	TRUE
ENSGALG00010009530	KIF3C	PRJNA729271	-1.8413468	1.5045E-28	1.8413468	stage_E14_vs_E10	ordinary_muscle_development	D_vs_E10;stage_1W_vs_E10;stage_3W_vs_E10;stage	1	TRUE
ENSGALG00010006853	NET1	PRJNA729271	-0.94672576	1.50794E-28	0.94672576	stage_E14_vs_E10	ordinary_muscle_development		0	FALSE
ENSGALG00010019800	EXT2	PRJNA729271	-0.685648029	1.51351E-28	0.685648029	stage_E14_vs_E10	ordinary_muscle_development		0	FALSE
ENSGALG00010005346	FBXO30	PRJNA729271	2.471241227	1.51352E-28	2.471241227	stage_E14_vs_E10	ordinary_muscle_development	D_vs_E10;stage_1W_vs_E10;stage_3W_vs_E10;stage	1	TRUE
ENSGALG00010009396	AUH	PRJNA729271	1.257824583	1.51902E-28	1.257824583	stage_E14_vs_E10	ordinary_muscle_development	D_vs_E10;stage_1W_vs_E10;stage_3W_vs_E10;stage	1	TRUE
ENSGALG00010002551	WDR35	PRJNA729271	-1.271543934	1.51939E-28	1.271543934	stage_E14_vs_E10	ordinary_muscle_development	D_vs_E10;stage_1W_vs_E10;stage_3W_vs_E10;stage	1	TRUE
ENSGALG00010020912	PDE8A	PRJNA729271	1.619113099	1.66118E-28	1.619113099	stage_E14_vs_E10	ordinary_muscle_development	D_vs_E10;stage_1W_vs_E10;stage_3W_vs_E10;stage	1	TRUE
ENSGALG00010007038	CENPU	PRJNA729271	-2.324250201	1.67599E-28	2.324250201	stage_E14_vs_E10	ordinary_muscle_development	D_vs_E10;stage_1W_vs_E10;stage_3W_vs_E10;stage	1	TRUE
ENSGALG00010019135	KIAA1841	PRJNA729271	-2.374774038	1.68318E-28	2.374774038	stage_E14_vs_E10	ordinary_muscle_development	D_vs_E10;stage_1W_vs_E10;stage_3W_vs_E10;stage	1	TRUE
ENSGALG00010001700	ENSGALG00010001700	PRJNA729271	2.228648011	1.68375E-28	2.228648011	stage_E14_vs_E10	ordinary_muscle_development	D_vs_E10;stage_1W_vs_E10;stage_3W_vs_E10;stage	1	TRUE
ENSGALG00010027732	EEF2	PRJNA729271	1.313595658	1.69262E-28	1.313595658	stage_E14_vs_E10	ordinary_muscle_development	D_vs_E10;stage_1W_vs_E10;stage_3W_vs_E10;stage	1	TRUE
ENSGALG00010000465	ENSGALG00010000465	PRJNA729271	2.147325822	1.69585E-28	2.147325822	stage_E14_vs_E10	ordinary_muscle_development	D_vs_E10;stage_1W_vs_E10;stage_3W_vs_E10;stage	1	TRUE
ENSGALG00010024775	ITFG2	PRJNA729271	1.059717624	1.70481E-28	1.059717624	stage_E14_vs_E10	ordinary_muscle_development	D_vs_E10;stage_1W_vs_E10;stage_3W_vs_E10;stage	1	TRUE
ENSGALG00010013125	POU3F3	PRJNA729271	-7.859049167	1.74468E-28	7.859049167	stage_E14_vs_E10	ordinary_muscle_development	D_vs_E10;stage_1W_vs_E10;stage_3W_vs_E10;stage	1	TRUE
ENSGALG00010003914	CAMK2D	PRJNA729271	1.186383477	1.83339E-28	1.186383477	stage_E14_vs_E10	ordinary_muscle_development	D_vs_E10;stage_1W_vs_E10;stage_3W_vs_E10;stage	1	TRUE
ENSGALG00010029325	LRRC75A	PRJNA729271	-1.993780801	1.85634E-28	1.993780801	stage_E14_vs_E10	ordinary_muscle_development	D_vs_E10;stage_1W_vs_E10;stage_3W_vs_E10;stage	1	TRUE
ENSGALG00010029357	ARHGDIA	PRJNA729271	-1.095999413	1.86023E-28	1.095999413	stage_E14_vs_E10	ordinary_muscle_development	D_vs_E10;stage_1W_vs_E10;stage_3W_vs_E10;stage	1	TRUE
ENSGALG00010016917	METTL6	PRJNA729271	1.899907281	1.92413E-28	1.899907281	stage_E14_vs_E10	ordinary_muscle_development	D_vs_E10;stage_1W_vs_E10;stage_3W_vs_E10;stage	1	TRUE
ENSGALG00010027619	JAK3	PRJNA729271	3.749352168	2.02616E-28	3.749352168	stage_E14_vs_E10	ordinary_muscle_development	D_vs_E10;stage_1W_vs_E10;stage_3W_vs_E10;stage	1	TRUE
ENSGALG00010014416	H2BC32	PRJNA729271	-3.027968718	2.02641E-28	3.027968718	stage_E14_vs_E10	ordinary_muscle_development	D_vs_E10;stage_1W_vs_E10;stage_3W_vs_E10;stage	1	TRUE
ENSGALG00010002653	EIF1AX	PRJNA729271	-0.722730947	2.02819E-28	0.722730947	stage_E14_vs_E10	ordinary_muscle_development		0	FALSE
ENSGALG00010016845	MIA3	PRJNA729271	1.539865089	2.0348E-28	1.539865089	stage_E14_vs_E10	ordinary_muscle_development	D_vs_E10;stage_1W_vs_E10;stage_3W_vs_E10;stage	1	TRUE
ENSGALG00010015129	PRRG3	PRJNA729271	-1.587944424	2.04296E-28	1.587944424	stage_E14_vs_E10	ordinary_muscle_development	D_vs_E10;stage_1W_vs_E10;stage_3W_vs_E10;stage	1	TRUE
ENSGALG00010008023	ATAD2	PRJNA729271	-0.89262309	2.04747E-28	0.89262309	stage_E14_vs_E10	ordinary_muscle_development		0	FALSE
ENSGALG00010003742	ZFHX4	PRJNA729271	-1.872291439	2.05699E-28	1.872291439	stage_E14_vs_E10	ordinary_muscle_development	D_vs_E10;stage_1W_vs_E10;stage_3W_vs_E10;stage	1	TRUE

ENSGALG00010012198	TMEM5	PRJNA729271	1.104297681	2.06347E-28	1.104297681	stage_E14_vs_E10	ordinary_muscle_development	D_vs_E10;stage_1W_vs_E10;stage_3W_vs_E10;stage	1	TRUE
ENSGALG00010001948	EMX2	PRJNA729271	-3.229512751	2.07778E-28	3.229512751	stage_E14_vs_E10	ordinary_muscle_development	D_vs_E10;stage_1W_vs_E10;stage_3W_vs_E10;stage	1	TRUE
ENSGALG00010027163	ENSGALG00010027163	PRJNA729271	-0.896264283	2.07778E-28	0.896264283	stage_E14_vs_E10	ordinary_muscle_development		0	FALSE
ENSGALG00010017406	IFT140	PRJNA729271	-1.425350679	2.11306E-28	1.425350679	stage_E14_vs_E10	ordinary_muscle_development	D_vs_E10;stage_1W_vs_E10;stage_3W_vs_E10;stage	1	TRUE
ENSGALG00010011478	HDAC3	PRJNA729271	-0.875548031	2.16289E-28	0.875548031	stage_E14_vs_E10	ordinary_muscle_development		0	FALSE
ENSGALG00010022055	E2F1	PRJNA729271	-1.862037824	2.16411E-28	1.862037824	stage_E14_vs_E10	ordinary_muscle_development	D_vs_E10;stage_1W_vs_E10;stage_3W_vs_E10;stage	1	TRUE
ENSGALG00010011643	C1orf21	PRJNA729271	0.896632775	2.16826E-28	0.896632775	stage_E14_vs_E10	ordinary_muscle_development		0	FALSE
ENSGALG00010013953	DHX29	PRJNA729271	-0.929258434	2.19405E-28	0.929258434	stage_E14_vs_E10	ordinary_muscle_development		0	FALSE
ENSGALG00010023478	TAOK3	PRJNA729271	1.371310031	2.29024E-28	1.371310031	stage_E14_vs_E10	ordinary_muscle_development	D_vs_E10;stage_1W_vs_E10;stage_3W_vs_E10;stage	1	TRUE
ENSGALG00010003676	POLR2B	PRJNA729271	0.86790034	2.30209E-28	0.86790034	stage_E14_vs_E10	ordinary_muscle_development		0	FALSE
ENSGALG00010011724	ARHGEF28	PRJNA729271	-1.777153557	2.304E-28	1.777153557	stage_E14_vs_E10	ordinary_muscle_development	D_vs_E10;stage_1W_vs_E10;stage_3W_vs_E10;stage	1	TRUE
ENSGALG00010029360	ACACA	PRJNA729271	-0.966596065	2.30547E-28	0.966596065	stage_E14_vs_E10	ordinary_muscle_development		0	FALSE
ENSGALG00010005215	VASH1	PRJNA729271	-1.594880939	2.40885E-28	1.594880939	stage_E14_vs_E10	ordinary_muscle_development	D_vs_E10;stage_1W_vs_E10;stage_3W_vs_E10;stage	1	TRUE
ENSGALG00010014880	SFRP2	PRJNA729271	-3.292317926	2.46061E-28	3.292317926	stage_E14_vs_E10	ordinary_muscle_development	D_vs_E10;stage_1W_vs_E10;stage_3W_vs_E10;stage	1	TRUE
ENSGALG00010008412	NEK1	PRJNA729271	-1.181235194	2.50281E-28	1.181235194	stage_E14_vs_E10	ordinary_muscle_development	D_vs_E10;stage_1W_vs_E10;stage_3W_vs_E10;stage	1	TRUE
ENSGALG00010003292	GADD45GIP1	PRJNA729271	1.378600176	2.58672E-28	1.378600176	stage_E14_vs_E10	ordinary_muscle_development	D_vs_E10;stage_1W_vs_E10;stage_3W_vs_E10;stage	1	TRUE
ENSGALG00010007318	RMDN2	PRJNA729271	1.380058248	2.6741E-28	1.380058248	stage_E14_vs_E10	ordinary_muscle_development	D_vs_E10;stage_1W_vs_E10;stage_3W_vs_E10;stage	1	TRUE
ENSGALG00010017433	RNPS1	PRJNA729271	-0.894231115	2.68982E-28	0.894231115	stage_E14_vs_E10	ordinary_muscle_development		0	FALSE
ENSGALG00010015886	AFF3	PRJNA729271	-2.433132209	2.69308E-28	2.433132209	stage_E14_vs_E10	ordinary_muscle_development	D_vs_E10;stage_1W_vs_E10;stage_3W_vs_E10;stage	1	TRUE
ENSGALG00010023228	GORASP2	PRJNA729271	1.135140104	2.70903E-28	1.135140104	stage_E14_vs_E10	ordinary_muscle_development	D_vs_E10;stage_1W_vs_E10;stage_3W_vs_E10;stage	1	TRUE
ENSGALG00010021532	DPP10	PRJNA729271	-2.078545056	2.71692E-28	2.078545056	stage_E14_vs_E10	ordinary_muscle_development	D_vs_E10;stage_1W_vs_E10;stage_3W_vs_E10;stage	1	TRUE
ENSGALG00010016761	GPR160	PRJNA729271	3.819388397	2.72787E-28	3.819388397	stage_E14_vs_E10	ordinary_muscle_development	D_vs_E10;stage_1W_vs_E10;stage_3W_vs_E10;stage	1	TRUE
ENSGALG00010011428	UQCRB	PRJNA729271	1.820955309	2.77115E-28	1.820955309	stage_E14_vs_E10	ordinary_muscle_development	D_vs_E10;stage_1W_vs_E10;stage_3W_vs_E10;stage	1	TRUE
ENSGALG00010005317	CHCHD3	PRJNA729271	1.222788449	2.77918E-28	1.222788449	stage_E14_vs_E10	ordinary_muscle_development	D_vs_E10;stage_1W_vs_E10;stage_3W_vs_E10;stage	1	TRUE
ENSGALG00010027301	SCAP	PRJNA729271	-0.734338865	2.78246E-28	0.734338865	stage_E14_vs_E10	ordinary_muscle_development		0	FALSE
ENSGALG00010029648	NIPSNAP2	PRJNA729271	1.362089383	2.81477E-28	1.362089383	stage_E14_vs_E10	ordinary_muscle_development	D_vs_E10;stage_1W_vs_E10;stage_3W_vs_E10;stage	1	TRUE
ENSGALG00010001172	TMEM200C	PRJNA729271	-3.373366675	2.83275E-28	3.373366675	stage_E14_vs_E10	ordinary_muscle_development	D_vs_E10;stage_1W_vs_E10;stage_3W_vs_E10;stage	1	TRUE
ENSGALG00010017352	GPCPD1	PRJNA729271	2.662913253	2.85517E-28	2.662913253	stage_E14_vs_E10	ordinary_muscle_development	D_vs_E10;stage_1W_vs_E10;stage_3W_vs_E10;stage	1	TRUE
ENSGALG00010017949	SFXN3	PRJNA729271	1.538907491	2.8731E-28	1.538907491	stage_E14_vs_E10	ordinary_muscle_development	D_vs_E10;stage_1W_vs_E10;stage_3W_vs_E10;stage	1	TRUE
ENSGALG00010000650	BCKDK	PRJNA729271	1.875721069	2.89185E-28	1.875721069	stage_E14_vs_E10	ordinary_muscle_development	D_vs_E10;stage_1W_vs_E10;stage_3W_vs_E10;stage	1	TRUE
ENSGALG00010025787	KCNE4	PRJNA729271	3.245029176	2.96809E-28	3.245029176	stage_E14_vs_E10	ordinary_muscle_development	D_vs_E10;stage_1W_vs_E10;stage_3W_vs_E10;stage	1	TRUE
ENSGALG00010026128	PRDX1	PRJNA729271	-1.589808753	3.03565E-28	1.589808753	stage_E14_vs_E10	ordinary_muscle_development	D_vs_E10;stage_1W_vs_E10;stage_3W_vs_E10;stage	1	TRUE
ENSGALG00010002365	RAI2	PRJNA729271	1.542436392	3.17259E-28	1.542436392	stage_E14_vs_E10	ordinary_muscle_development	D_vs_E10;stage_1W_vs_E10;stage_3W_vs_E10;stage	1	TRUE
ENSGALG00010012407	ATF4	PRJNA729271	1.437404133	3.24345E-28	1.437404133	stage_E14_vs_E10	ordinary_muscle_development	D_vs_E10;stage_1W_vs_E10;stage_3W_vs_E10;stage	1	TRUE
ENSGALG00010027653	WNT7A	PRJNA729271	-8.594880843	3.29013E-28	8.594880843	stage_E14_vs_E10	ordinary_muscle_development	D_vs_E10;stage_1W_vs_E10;stage_3W_vs_E10;stage	1	TRUE
ENSGALG00010009857	ORC5	PRJNA729271	-1.59519786	3.58228E-28	1.59519786	stage_E14_vs_E10	ordinary_muscle_development	D_vs_E10;stage_1W_vs_E10;stage_3W_vs_E10;stage	1	TRUE
ENSGALG00010027864	ACTR8	PRJNA729271	-0.958044887	3.60078E-28	0.958044887	stage_E14_vs_E10	ordinary_muscle_development		0	FALSE
ENSGALG00010022412	CHAC1	PRJNA729271	7.254437342	3.75564E-28	7.254437342	stage_E14_vs_E10	ordinary_muscle_development	D_vs_E10;stage_1W_vs_E10;stage_3W_vs_E10;stage	1	TRUE
ENSGALG00010001710	LCMT2	PRJNA729271	-0.910899998	3.88281E-28	0.910899998	stage_E14_vs_E10	ordinary_muscle_development		0	FALSE
ENSGALG00010011077	RASGEF1A	PRJNA729271	1.609698619	3.99711E-28	1.609698619	stage_E14_vs_E10	ordinary_muscle_development	D_vs_E10;stage_1W_vs_E10;stage_3W_vs_E10;stage	1	TRUE
ENSGALG00010017451	GABPB1	PRJNA729271	-0.875371097	4.10689E-28	0.875371097	stage_E14_vs_E10	ordinary_muscle_development		0	FALSE
ENSGALG00010019158	ENSGALG00010019158	PRJNA729271	2.788374581	4.11886E-28	2.788374581	stage_E14_vs_E10	ordinary_muscle_development	D_vs_E10;stage_1W_vs_E10;stage_3W_vs_E10;stage	1	TRUE
ENSGALG00010024118	TMEM266	PRJNA729271	-1.631487867	4.14886E-28	1.631487867	stage_E14_vs_E10	ordinary_muscle_development	D_vs_E10;stage_1W_vs_E10;stage_3W_vs_E10;stage	1	TRUE
ENSGALG00010019082	POLR3K	PRJNA729271	1.948249658	4.18693E-28	1.948249658	stage_E14_vs_E10	ordinary_muscle_development	D_vs_E10;stage_1W_vs_E10;stage_3W_vs_E10;stage	1	TRUE
ENSGALG00010013494	ASB1	PRJNA729271	-1.631219271	4.30076E-28	1.631219271	stage_E14_vs_E10	ordinary_muscle_development	D_vs_E10;stage_1W_vs_E10;stage_3W_vs_E10;stage	1	TRUE
ENSGALG00010015203	HMGXB4	PRJNA729271	-0.748677361	4.35332E-28	0.748677361	stage_E14_vs_E10	ordinary_muscle_development		0	FALSE
ENSGALG00010010648	CENPM	PRJNA729271	-3.191533215	4.44808E-28	3.191533215	stage_E14_vs_E10	ordinary_muscle_development	D_vs_E10;stage_1W_vs_E10;stage_3W_vs_E10;stage	1	TRUE
ENSGALG00010014865	TLR2	PRJNA729271	4.14500629	4.47809E-28	4.14500629	stage_E14_vs_E10	ordinary_muscle_development	D_vs_E10;stage_1W_vs_E10;stage_3W_vs_E10;stage	1	TRUE
ENSGALG00010005502	SCFD1	PRJNA729271	-0.814590954	4.48333E-28	0.814590954	stage_E14_vs_E10	ordinary_muscle_development		0	FALSE
ENSGALG00010012205	SLC6A13	PRJNA729271	-5.835917354	4.52464E-28	5.835917354	stage_E14_vs_E10	ordinary_muscle_development	D_vs_E10;stage_1W_vs_E10;stage_3W_vs_E10;stage	1	TRUE
ENSGALG00010004222	SPAG6	PRJNA729271	-2.917419501	4.64923E-28	2.917419501	stage_E14_vs_E10	ordinary_muscle_development	D_vs_E10;stage_1W_vs_E10;stage_3W_vs_E10;stage	1	TRUE
ENSGALG00010008416	MSX2	PRJNA729271	-5.924890784	4.76602E-28	5.924890784	stage_E14_vs_E10	ordinary_muscle_development	D_vs_E10;stage_1W_vs_E10;stage_3W_vs_E10;stage	1	TRUE
ENSGALG00010019602	SLC29A1	PRJNA729271	2.152940221	5.00008E-28	2.152940221	stage_E14_vs_E10	ordinary_muscle_development	D_vs_E10;stage_1W_vs_E10;stage_3W_vs_E10;stage	1	TRUE
ENSGALG00010008056	GTF2E2	PRJNA729271	-0.80416034	5.04371E-28	0.80416034	stage_E14_vs_E10	ordinary_muscle_development		0	FALSE
ENSGALG00010006621	DACT2	PRJNA729271	-2.34804303	5.1497E-28	2.34804303	stage_E14_vs_E10	ordinary_muscle_development	D_vs_E10;stage_1W_vs_E10;stage_3W_vs_E10;stage	1	TRUE
ENSGALG00010024585	TBCCD1	PRJNA729271	-1.488335313	5.27304E-28	1.488335313	stage_E14_vs_E10	ordinary_muscle_development	D_vs_E10;stage_1W_vs_E10;stage_3W_vs_E10;stage	1	TRUE
ENSGALG00010012207	GRIA3	PRJNA729271	-3.07123084	5.4094E-28	3.07123084	stage_E14_vs_E10	ordinary_muscle_development	D_vs_E10;stage_1W_vs_E10;stage_3W_vs_E10;stage	1	TRUE
ENSGALG00010010305	URB1	PRJNA729271	-1.150854189	5.4978E-28	1.150854189	stage_E14_vs_E10	ordinary_muscle_development	D_vs_E10;stage_1W_vs_E10;stage_3W_vs_E10;stage	1	TRUE
ENSGALG00010003737	SRP72	PRJNA729271	-0.637104821	5.64673E-28	0.637104821	stage_E14_vs_E10	ordinary_muscle_development		0	FALSE
ENSGALG00010018951	YKT6	PRJNA729271	-1.025459725	5.67936E-28	1.025459725	stage_E14_vs_E10	ordinary_muscle_development	D_vs_E10;stage_1W_vs_E10;stage_3W_vs_E10;stage	1	TRUE
ENSGALG00010016390	ENSGALG00010016390	PRJNA729271	3.426046563	6.22452E-28	3.426046563	stage_E14_vs_E10	ordinary_muscle_development	D_vs_E10;stage_1W_vs_E10;stage_3W_vs_E10;stage	1	TRUE
ENSGALG00010027366	USP4	PRJNA729271	0.912354565	6.33769E-28	0.912354565	stage_E14_vs_E10	ordinary_muscle_development		0	FALSE
ENSGALG00010005572	ENSGALG00010005572	PRJNA729271	1.689973049	6.35412E-28	1.689973049	stage_E14_vs_E10	ordinary_muscle_development	D_vs_E10;stage_1W_vs_E10;stage_3W_vs_E10;stage	1	TRUE
ENSGALG00010020537	CCDC167	PRJNA729271	-1.126196516	6.35412E-28	1.126196516	stage_E14_vs_E10	ordinary_muscle_development	D_vs_E10;stage_1W_vs_E10;stage_3W_vs_E10;stage	1	TRUE
ENSGALG00010006010	IPO5	PRJNA729271	-0.906645473	6.52542E-28	0.906645473	stage_E14_vs_E10	ordinary_muscle_development		0	FALSE
ENSGALG00010018351	RIN2	PRJNA729271	0.962995675	6.58415E-28	0.962995675	stage_E14_vs_E10	ordinary_muscle_development		0	FALSE
ENSGALG00010008688	HBEGF	PRJNA729271	2.535294564	6.64989E-28	2.535294564	stage_E14_vs_E10	ordinary_muscle_development	D_vs_E10;stage_1W_vs_E10;stage_3W_vs_E10;stage	1	TRUE
ENSGALG00010017450	ENSGALG00010017450	PRJNA729271	2.327421797	6.72273E-28	2.327421797	stage_E14_vs_E10	ordinary_muscle_development	D_vs_E10;stage_1W_vs_E10;stage_3W_vs_E10;stage	1	TRUE
ENSGALG00010001888	SNX10	PRJNA729271	2.239658569	6.82015E-28	2.239658569	stage_E14_vs_E10	ordinary_muscle_development	D_vs_E10;stage_1W_vs_E10;stage_3W_vs_E10;stage	1	TRUE
ENSGALG00010021362	ARPC1B	PRJNA729271	1.331482045	6.8271E-28	1.331482045	stage_E14_vs_E10	ordinary_muscle_development	D_vs_E10;stage_1W_vs_E10;stage_3W_vs_E10;stage	1	TRUE
ENSGALG00010023580	RDM1	PRJNA729271	-1.884962945	6.83738E-28	1.884962945	stage_E14_vs_E10	ordinary_muscle_development	D_vs_E10;stage_1W_vs_E10;stage_3W_vs_E10;stage	1	TRUE
ENSGALG00010022597	SLCO4A1	PRJNA729271	2.412240175	6.89494E-28	2.412240175	stage_E14_vs_E10	ordinary_muscle_development	D_vs_E10;stage_1W_vs_E10;stage_3W_vs_E10;stage	1	TRUE
ENSGALG00010003541	CNR1	PRJNA729271	2.458664884	6.93427E-28	2.458664884	stage_E14_vs_E10	ordinary_muscle_development	D_vs_E10;stage_1W_vs_E10;stage_3W_vs_E10;stage	1	TRUE
ENSGALG00010008188	VPS4A	PRJNA729271	1.249725766	6.94798E-28	1.249725766	stage_E14_vs_E10	ordinary_muscle_development	D_vs_E10;stage_1W_vs_E10;stage_3W_vs_E10;stage	1	TRUE
ENSGALG00010011936	CKAP4	PRJNA729271	-1.949407128	7.30463E-28	1.949407128	stage_E14_vs_E10	ordinary_muscle_development	D_vs_E10;stage_1W_vs_E10;stage_3W_vs_E10;stage	1	TRUE
ENSGALG00010018601	CPNE4	PRJNA729271	-6.003278162	7.45908E-28	6.003278162	stage_E14_vs_E10	ordinary_muscle_development	D_vs_E10;stage_1W_vs_E10;stage_3W_vs_E10;stage	1	TRUE
ENSGALG00010025897	MAPKAPK5	PRJNA729271	1.328106601	7.75138E-28	1.328106601	stage_E14_vs_E10	ordinary_muscle_development	D_vs_E10;stage_1W_vs_E10;stage_3W_vs_E10;stage	1	TRUE
ENSGALG00010021669	ENSGALG00010021669	PRJNA729271	-0.825060759	7.91894E-28	0.825060759	stage_E14_vs_E10	ordinary_muscle_development		0	FALSE
ENSGALG00010019700	HES5B	PRJNA729271	-2.970723019	8.00446E-28	2.970723019	stage_E14_vs_E10	ordinary_muscle_development	D_vs_E10;stage_1W_vs_E10;stage_3W_vs_E10;stage	1	TRUE
ENSGALG00010008166	RUNX2	PRJNA729271	-4.689656936	8.06014E-28	4.689656936	stage_E14_vs_E10	ordinary_muscle_development	D_vs_E10;stage_1W_vs_E10;stage_3W_vs_E10;stage	1	TRUE
ENSGALG00010022548	ENSGALG00010022548	PRJNA729271	1.258204406	8.2649E-28	1.258204406	stage_E14_vs_E10	ordinary_muscle_development	D_vs_E10;stage_1W_vs_E10;stage_3W_vs_E10;stage	1	TRUE
ENSGALG00010029423	MYBBP1A	PRJNA729271	-1.356664782	8.27632E-28	1.356664782	stage_E14_vs_E10	ordinary_muscle_development	D_vs_E10;stage_1W_vs_E10;stage_3W_vs_E10;stage	1	TRUE
ENSGALG00010012366	INTS8	PRJNA729271	-0.764474401	8.51985E-28	0.764474401	stage_E14_vs_E10	ordinary_muscle_development		0	FALSE
ENSGALG00010018579	FNDC3B	PRJNA729271	1.126996599	8.58748E-28	1.126996599	stage_E14_vs_E10	ordinary_muscle_development	D_vs_E10;stage_1W_vs_E10;stage_3W_vs_E10;stage	1	TRUE
ENSGALG00010018835	CAMK2B	PRJNA729271	1.910040847	8.6325E-28	1.910040847	stage_E14_vs_E10	ordinary_muscle_development	D_vs_E10;stage_1W_vs_E10;stage_3W_vs_E10;stage	1	TRUE
ENSGALG00010007835	GUCY1A1	PRJNA729271	2.071334979	8.77864E-28	2.071334979	stage_E14_vs_E10	ordinary_muscle_development	D_vs_E10;stage_1W_vs_E10;stage_3W_vs_E10;stage	1	TRUE
ENSGALG00010028548	MYO1F	PRJNA729271	2.74146367	8.81842E-28	2.74146367	stage_E14_vs_E10	ordinary_muscle_development	D_vs_E10;stage_1W_vs_E10;stage_3W_vs_E10;stage	1	TRUE
ENSGALG00010009068	AGO2	PRJNA729271	0.875375744	9.03509E-28	0.875375744	stage_E14_vs_E10	ordinary_muscle_development		0	FALSE
ENSGALG00010029221	AXIN2	PRJNA729271	-1.407155254	9.05197E-28	1.407155254	stage_E14_vs_E10	ordinary_muscle_development	D_vs_E10;stage_1W_vs_E10;stage_3W_vs_E10;stage	1	TRUE
ENSGALG00010019059	BRWD3	PRJNA729271	0.853366108	9.18557E-28	0.853366108	stage_E14_vs_E10	ordinary_muscle_development		0	FALSE
ENSGALG00010020120	DAAM2	PRJNA729271	-1.151838139	9.34425E-28	1.151838139	stage_E14_vs_E10	ordinary_muscle_development	D_vs_E10;stage_1W_vs_E10;stage_3W_vs_E10;stage	1	TRUE
ENSGALG00010029337	MYO1C	PRJNA729271	1.479993564	9.42778E-28	1.479993564	stage_E14_vs_E10	ordinary_muscle_development	D_vs_E10;stage_1W_vs_E10;stage_3W_vs_E10;stage	1	TRUE
ENSGALG00010018955	ZBTB48	PRJNA729271	1.550970265	9.44015E-28	1.550970265	stage_E14_vs_E10	ordinary_muscle_development	D_vs_E10;stage_1W_vs_E10;stage_3W_vs_E10;stage	1	TRUE
ENSGALG00010012796	MOCS2	PRJNA729271	1.329985649	9.59116E-28	1.329985649	stage_E14_vs_E10	ordinary_muscle_development	D_vs_E10;stage_1W_vs_E10;stage_3W_vs_E10;stage	1	TRUE
ENSGALG00010000797	ENSGALG00010000797	PRJNA729271	4.520201386	9.97111E-28	4.520201386	stage_E14_vs_E10	ordinary_muscle_development	D_vs_E10;stage_1W_vs_E10;stage_3W_vs_E10;stage	1	TRUE
ENSGALG00010026030	IBA57	PRJNA729271	0.915099891	1.0663E-27	0.915099891	stage_E14_vs_E10	ordinary_muscle_development		0	FALSE
ENSGALG00010012247	TRIM23	PRJNA729271	1.38626047	1.07592E-27	1.38626047	stage_E14_vs_E10	ordinary_muscle_development	D_vs_E10;stage_1W_vs_E10;stage_3W_vs_E10;stage	1	TRUE
ENSGALG00010029494	MYH7	PRJNA729271	-6.742772058	1.1371E-27	6.742772058	stage_E14_vs_E10	ordinary_muscle_development	D_vs_E10;stage_1W_vs_E10;stage_3W_vs_E10;stage	1	TRUE
ENSGALG00010026335	IPP	PRJNA729271	-1.16891037	1.15208E-27	1.16891037	stage_E14_vs_E10	ordinary_muscle_development	D_vs_E10;stage_1W_vs_E10;stage_3W_vs_E10;stage	1	TRUE
ENSGALG00010022236	SHF	PRJNA729271	2.017265658	1.17335E-27	2.017265658	stage_E14_vs_E10	ordinary_muscle_development	D_vs_E10;stage_1W_vs_E10;stage_3W_vs_E10;stage	1	TRUE
ENSGALG00010007763	FZD4	PRJNA729271	1.106986127	1.17335E-27	1.106986127	stage_E14_vs_E10	ordinary_muscle_development	D_vs_E10;stage_1W_vs_E10;stage_3W_vs_E10;stage	1	TRUE
ENSGALG00010023255	SNX22	PRJNA729271	3.330021569	1.18712E-27	3.330021569	stage_E14_vs_E10	ordinary_muscle_development	D_vs_E10;stage_1W_vs_E10;stage_3W_vs_E10;stage	1	TRUE
ENSGALG00010001400	SNX24	PRJNA729271	1.754561044	1.25534E-27	1.754561044	stage_E14_vs_E10	ordinary_muscle_development	D_vs_E10;stage_1W_vs_E10;stage_3W_vs_E10;stage	1	TRUE
ENSGALG00010028454	C8G	PRJNA729271	2.432344097	1.26188E-27	2.432344097	stage_E14_vs_E10	ordinary_muscle_development	D_vs_E10;stage_1W_vs_E10;stage_3W_vs_E10;stage	1	TRUE
ENSGALG00010016994	ATL2	PRJNA729271	1.275517582	1.26568E-27	1.275517582	stage_E14_vs_E10	ordinary_muscle_development	D_vs_E10;stage_1W_vs_E10;stage_3W_vs_E10;stage	1	TRUE
ENSGALG00010013351	EDAR	PRJNA729271	-4.91703882	1.27174E-27	4.91703882	stage_E14_vs_E10	ordinary_muscle_development	D_vs_E10;stage_1W_vs_E10;stage_3W_vs_E10;stage	1	TRUE
ENSGALG00010006219	CLEC14A	PRJNA729271	2.021555032	1.27533E-27	2.021555032	stage_E14_vs_E10	ordinary_muscle_development	D_vs_E10;stage_1W_vs_E10;stage_3W_vs_E10;stage	1	TRUE
ENSGALG00010015992	ATP10A	PRJNA729271	-1.778765877	1.28238E-27	1.778765877	stage_E14_vs_E10	ordinary_muscle_development	D_vs_E10;stage_1W_vs_E10;stage_3W_vs_E10;stage	1	TRUE
ENSGALG00010016881	C1QB	PRJNA729271	3.202088944	1.28642E-27	3.202088944	stage_E14_vs_E10	ordinary_muscle_development	D_vs_E10;stage_1W_vs_E10;stage_3W_vs_E10;stage	1	TRUE
ENSGALG00010024686	CMPK1	PRJNA729271	-1.483535662	1.28717E-27	1.483535662	stage_E14_vs_E10	ordinary_muscle_development	D_vs_E10;stage_1W_vs_E10;stage_3W_vs_E10;stage	1	TRUE
ENSGALG00010025301	MORN3	PRJNA729271	2.533350886	1.30131E-27	2.533350886	stage_E14_vs_E10	ordinary_muscle_development	D_vs_E10;stage_1W_vs_E10;stage_3W_vs_E10;stage	1	TRUE
ENSGALG00010020066	ENSGALG00010020066	PRJNA729271	4.098095149	1.34178E-27	4.098095149	stage_E14_vs_E10	ordinary_muscle_development	D_vs_E10;stage_1W_vs_E10;stage_3W_vs_E10;stage	1	TRUE
ENSGALG00010011922	PIK3R1	PRJNA729271	1.913992388	1.39425E-27	1.913992388	stage_E14_vs_E10	ordinary_muscle_development	D_vs_E10;stage_1W_vs_E10;stage_3W_vs_E10;stage	1	TRUE
ENSGALG00010004917	ENSGALG00010004917	PRJNA729271	0.972601672	1.40599E-27	0.972601672	stage_E14_vs_E10	ordinary_muscle_development		0	FALSE
ENSGALG00010020405	UBE2I	PRJNA729271	-0.908353564	1.40812E-27	0.908353564	stage_E14_vs_E10	ordinary_muscle_development		0	FALSE
ENSGALG00010013213	SH2D1A	PRJNA729271	3.881813738	1.4151E-27	3.881813738	stage_E14_vs_E10	ordinary_muscle_development	D_vs_E10;stage_1W_vs_E10;stage_3W_vs_E10;stage	1	TRUE
ENSGALG00010016262	SPG11	PRJNA729271	0.666520425	1.42124E-27	0.666520425	stage_E14_vs_E10	ordinary_muscle_development		0	FALSE
ENSGALG00010024567	IKZF3	PRJNA729271	4.188426932	1.42796E-27	4.188426932	stage_E14_vs_E10	ordinary_muscle_development	D_vs_E10;stage_1W_vs_E10;stage_3W_vs_E10;stage	1	TRUE
ENSGALG00010023245	TANC2	PRJNA729271	-1.667636346	1.43994E-27	1.667636346	stage_E14_vs_E10	ordinary_muscle_development	D_vs_E10;stage_1W_vs_E10;stage_3W_vs_E10;stage	1	TRUE
ENSGALG00010014840	NCF4	PRJNA729271	2.296407763	1.48241E-27	2.296407763	stage_E14_vs_E10	ordinary_muscle_development	D_vs_E10;stage_1W_vs_E10;stage_3W_vs_E10;stage	1	TRUE
ENSGALG00010004977	RNF217	PRJNA729271	1.454024351	1.49505E-27	1.454024351	stage_E14_vs_E10	ordinary_muscle_development	D_vs_E10;stage_1W_vs_E10;stage_3W_vs_E10;stage	1	TRUE
ENSGALG00010013374	RMDN1	PRJNA729271	1.814875092	1.49939E-27	1.814875092	stage_E14_vs_E10	ordinary_muscle_development	D_vs_E10;stage_1W_vs_E10;stage_3W_vs_E10;stage	1	TRUE
ENSGALG00010028481	ENSGALG00010028481	PRJNA729271	1.572092059	1.49987E-27	1.572092059	stage_E14_vs_E10	ordinary_muscle_development	D_vs_E10;stage_1W_vs_E10;stage_3W_vs_E10;stage	1	TRUE
ENSGALG00010023680	MPZL3	PRJNA729271	-8.1438973	1.52745E-27	8.1438973	stage_E14_vs_E10	ordinary_muscle_development	D_vs_E10;stage_1W_vs_E10;stage_3W_vs_E10;stage	1	TRUE
ENSGALG00010028069	NEK4	PRJNA729271	-1.284418943	1.55314E-27	1.284418943	stage_E14_vs_E10	ordinary_muscle_development	D_vs_E10;stage_1W_vs_E10;stage_3W_vs_E10;stage	1	TRUE
ENSGALG00010015410	HTR2B	PRJNA729271	-2.713213278	1.57389E-27	2.713213278	stage_E14_vs_E10	ordinary_muscle_development	D_vs_E10;stage_1W_vs_E10;stage_3W_vs_E10;stage	1	TRUE
ENSGALG00010027143	GALNT11	PRJNA729271	-1.360001303	1.58117E-27	1.360001303	stage_E14_vs_E10	ordinary_muscle_development	D_vs_E10;stage_1W_vs_E10;stage_3W_vs_E10;stage	1	TRUE
ENSGALG00010029750	TEN1	PRJNA729271	-2.894634666	1.59103E-27	2.894634666	stage_E14_vs_E10	ordinary_muscle_development	D_vs_E10;stage_1W_vs_E10;stage_3W_vs_E10;stage	1	TRUE
ENSGALG00010011389	RC3H1	PRJNA729271	0.94318667	1.61974E-27	0.94318667	stage_E14_vs_E10	ordinary_muscle_development		0	FALSE
ENSGALG00010013045	FSD1L	PRJNA729271	1.586534605	1.62323E-27	1.586534605	stage_E14_vs_E10	ordinary_muscle_development	D_vs_E10;stage_1W_vs_E10;stage_3W_vs_E10;stage	1	TRUE
ENSGALG00010021674	ING4	PRJNA729271	-1.411474195	1.63391E-27	1.411474195	stage_E14_vs_E10	ordinary_muscle_development	D_vs_E10;stage_1W_vs_E10;stage_3W_vs_E10;stage	1	TRUE
ENSGALG00010005364	MAP7	PRJNA729271	-4.752620676	1.63898E-27	4.752620676	stage_E14_vs_E10	ordinary_muscle_development	D_vs_E10;stage_1W_vs_E10;stage_3W_vs_E10;stage	1	TRUE
ENSGALG00010018216	FANCM	PRJNA729271	0.926291452	1.67252E-27	0.926291452	stage_E14_vs_E10	ordinary_muscle_development		0	FALSE
ENSGALG00010021096	ZFAND4	PRJNA729271	-1.192983639	1.67691E-27	1.192983639	stage_E14_vs_E10	ordinary_muscle_development	D_vs_E10;stage_1W_vs_E10;stage_3W_vs_E10;stage	1	TRUE
ENSGALG00010019130	RPS6KC1	PRJNA729271	-0.932671265	1.73829E-27	0.932671265	stage_E14_vs_E10	ordinary_muscle_development		0	FALSE
ENSGALG00010006075	SOX21	PRJNA729271	-4.846086706	1.77478E-27	4.846086706	stage_E14_vs_E10	ordinary_muscle_development	D_vs_E10;stage_1W_vs_E10;stage_3W_vs_E10;stage	1	TRUE
ENSGALG00010027860	ENSGALG00010027860	PRJNA729271	2.804078335	1.77531E-27	2.804078335	stage_E14_vs_E10	ordinary_muscle_development	D_vs_E10;stage_1W_vs_E10;stage_3W_vs_E10;stage	1	TRUE
ENSGALG00010027538	ABHD14A	PRJNA729271	1.435906969	1.81122E-27	1.435906969	stage_E14_vs_E10	ordinary_muscle_development	D_vs_E10;stage_1W_vs_E10;stage_3W_vs_E10;stage	1	TRUE
ENSGALG00010012832	CHADL	PRJNA729271	-4.168938988	1.87303E-27	4.168938988	stage_E14_vs_E10	ordinary_muscle_development	D_vs_E10;stage_1W_vs_E10;stage_3W_vs_E10;stage	1	TRUE
ENSGALG00010004393	ZBTB45	PRJNA729271	2.274083228	1.88365E-27	2.274083228	stage_E14_vs_E10	ordinary_muscle_development	D_vs_E10;stage_1W_vs_E10;stage_3W_vs_E10;stage	1	TRUE
ENSGALG00010024935	KLHL30	PRJNA729271	4.279140059	1.88523E-27	4.279140059	stage_E14_vs_E10	ordinary_muscle_development	D_vs_E10;stage_1W_vs_E10;stage_3W_vs_E10;stage	1	TRUE
ENSGALG00010017655	ENSGALG00010017655	PRJNA729271	-1.230772389	1.90213E-27	1.230772389	stage_E14_vs_E10	ordinary_muscle_development	D_vs_E10;stage_1W_vs_E10;stage_3W_vs_E10;stage	1	TRUE
ENSGALG00010013278	SAMM50	PRJNA729271	1.130053284	1.91634E-27	1.130053284	stage_E14_vs_E10	ordinary_muscle_development	D_vs_E10;stage_1W_vs_E10;stage_3W_vs_E10;stage	1	TRUE
ENSGALG00010019246	IL17RC	PRJNA729271	1.481302815	1.92042E-27	1.481302815	stage_E14_vs_E10	ordinary_muscle_development	D_vs_E10;stage_1W_vs_E10;stage_3W_vs_E10;stage	1	TRUE
ENSGALG00010027118	ABHD6	PRJNA729271	1.73483011	1.93072E-27	1.73483011	stage_E14_vs_E10	ordinary_muscle_development	D_vs_E10;stage_1W_vs_E10;stage_3W_vs_E10;stage	1	TRUE
ENSGALG00010024933	CS	PRJNA729271	1.729139086	1.954E-27	1.729139086	stage_E14_vs_E10	ordinary_muscle_development	D_vs_E10;stage_1W_vs_E10;stage_3W_vs_E10;stage	1	TRUE
ENSGALG00010019097	STC1	PRJNA729271	-3.260959599	1.97853E-27	3.260959599	stage_E14_vs_E10	ordinary_muscle_development	D_vs_E10;stage_1W_vs_E10;stage_3W_vs_E10;stage	1	TRUE
ENSGALG00010009441	ZNF518B	PRJNA729271	-1.091461447	1.98493E-27	1.091461447	stage_E14_vs_E10	ordinary_muscle_development	D_vs_E10;stage_1W_vs_E10;stage_3W_vs_E10;stage	1	TRUE
ENSGALG00010018822	TRAF5	PRJNA729271	2.094212956	1.99959E-27	2.094212956	stage_E14_vs_E10	ordinary_muscle_development	D_vs_E10;stage_1W_vs_E10;stage_3W_vs_E10;stage	1	TRUE
ENSGALG00010023822	CHEK2	PRJNA729271	-1.440582088	2.01792E-27	1.440582088	stage_E14_vs_E10	ordinary_muscle_development	D_vs_E10;stage_1W_vs_E10;stage_3W_vs_E10;stage	1	TRUE
ENSGALG00010023273	HDAC7	PRJNA729271	1.242823736	2.02299E-27	1.242823736	stage_E14_vs_E10	ordinary_muscle_development	D_vs_E10;stage_1W_vs_E10;stage_3W_vs_E10;stage	1	TRUE
ENSGALG00010005338	PCGF1	PRJNA729271	1.259331365	2.08902E-27	1.259331365	stage_E14_vs_E10	ordinary_muscle_development	D_vs_E10;stage_1W_vs_E10;stage_3W_vs_E10;stage	1	TRUE
ENSGALG00010004337	BMPR1B	PRJNA729271	-2.762203286	2.09355E-27	2.762203286	stage_E14_vs_E10	ordinary_muscle_development	D_vs_E10;stage_1W_vs_E10;stage_3W_vs_E10;stage	1	TRUE
ENSGALG00010024441	SAA	PRJNA729271	8.661322651	2.11223E-27	8.661322651	stage_E14_vs_E10	ordinary_muscle_development	D_vs_E10;stage_1W_vs_E10;stage_3W_vs_E10;stage	1	TRUE
ENSGALG00010008368	SNCA	PRJNA729271	3.44731128	2.16762E-27	3.44731128	stage_E14_vs_E10	ordinary_muscle_development	D_vs_E10;stage_1W_vs_E10;stage_3W_vs_E10;stage	1	TRUE
ENSGALG00010025579	CSRNP3	PRJNA729271	-3.849476437	2.17928E-27	3.849476437	stage_E14_vs_E10	ordinary_muscle_development	D_vs_E10;stage_1W_vs_E10;stage_3W_vs_E10;stage	1	TRUE
ENSGALG00010021064	LYVE1	PRJNA729271	3.123293978	2.30644E-27	3.123293978	stage_E14_vs_E10	ordinary_muscle_development	D_vs_E10;stage_1W_vs_E10;stage_3W_vs_E10;stage	1	TRUE
ENSGALG00010020342	CPPED1	PRJNA729271	-1.191627618	2.35427E-27	1.191627618	stage_E14_vs_E10	ordinary_muscle_development	D_vs_E10;stage_1W_vs_E10;stage_3W_vs_E10;stage	1	TRUE
ENSGALG00010026410	ENSGALG00010026410	PRJNA729271	3.483838699	2.35758E-27	3.483838699	stage_E14_vs_E10	ordinary_muscle_development	D_vs_E10;stage_1W_vs_E10;stage_3W_vs_E10;stage	1	TRUE
ENSGALG00010002065	CLNS1A	PRJNA729271	-0.70618741	2.37846E-27	0.70618741	stage_E14_vs_E10	ordinary_muscle_development		0	FALSE
ENSGALG00010006496	UBE2H	PRJNA729271	1.248529849	2.40504E-27	1.248529849	stage_E14_vs_E10	ordinary_muscle_development	D_vs_E10;stage_1W_vs_E10;stage_3W_vs_E10;stage	1	TRUE
ENSGALG00010003910	THAP9	PRJNA729271	1.615809109	2.41443E-27	1.615809109	stage_E14_vs_E10	ordinary_muscle_development	D_vs_E10;stage_1W_vs_E10;stage_3W_vs_E10;stage	1	TRUE
ENSGALG00010000614	KAT8	PRJNA729271	1.998778611	2.50066E-27	1.998778611	stage_E14_vs_E10	ordinary_muscle_development	D_vs_E10;stage_1W_vs_E10;stage_3W_vs_E10;stage	1	TRUE
ENSGALG00010027887	ERC2	PRJNA729271	-3.435254382	2.52192E-27	3.435254382	stage_E14_vs_E10	ordinary_muscle_development	D_vs_E10;stage_1W_vs_E10;stage_3W_vs_E10;stage	1	TRUE
ENSGALG00010013365	CEP85	PRJNA729271	2.492941955	2.5289E-27	2.492941955	stage_E14_vs_E10	ordinary_muscle_development	D_vs_E10;stage_1W_vs_E10;stage_3W_vs_E10;stage	1	TRUE
ENSGALG00010021094	ENSGALG00010021094	PRJNA729271	3.444373562	2.56395E-27	3.444373562	stage_E14_vs_E10	ordinary_muscle_development	D_vs_E10;stage_1W_vs_E10;stage_3W_vs_E10;stage	1	TRUE
ENSGALG00010001131	HPS3	PRJNA729271	1.084332369	2.60169E-27	1.084332369	stage_E14_vs_E10	ordinary_muscle_development	D_vs_E10;stage_1W_vs_E10;stage_3W_vs_E10;stage	1	TRUE
ENSGALG00010027811	MEX3D	PRJNA729271	-1.128817597	2.62607E-27	1.128817597	stage_E14_vs_E10	ordinary_muscle_development	D_vs_E10;stage_1W_vs_E10;stage_3W_vs_E10;stage	1	TRUE
ENSGALG00010011401	ANKRD46	PRJNA729271	-0.762632023	2.64427E-27	0.762632023	stage_E14_vs_E10	ordinary_muscle_development		0	FALSE
ENSGALG00010008041	ENSGALG00010008041	PRJNA729271	-2.705948679	2.65757E-27	2.705948679	stage_E14_vs_E10	ordinary_muscle_development	D_vs_E10;stage_1W_vs_E10;stage_3W_vs_E10;stage	1	TRUE
ENSGALG00010003503	NUFIP1	PRJNA729271	-0.994363439	2.66739E-27	0.994363439	stage_E14_vs_E10	ordinary_muscle_development		0	FALSE
ENSGALG00010010904	TOR1AIP1	PRJNA729271	-0.912794144	2.68239E-27	0.912794144	stage_E14_vs_E10	ordinary_muscle_development		0	FALSE
ENSGALG00010001140	NMD3	PRJNA729271	1.315164032	2.70846E-27	1.315164032	stage_E14_vs_E10	ordinary_muscle_development	D_vs_E10;stage_1W_vs_E10;stage_3W_vs_E10;stage	1	TRUE
ENSGALG00010011634	AVD	PRJNA729271	9.683435701	2.79451E-27	9.683435701	stage_E14_vs_E10	ordinary_muscle_development	D_vs_E10;stage_1W_vs_E10;stage_3W_vs_E10;stage	1	TRUE
ENSGALG00010026036	TUB5A	PRJNA729271	-2.279199562	2.79687E-27	2.279199562	stage_E14_vs_E10	ordinary_muscle_development	D_vs_E10;stage_1W_vs_E10;stage_3W_vs_E10;stage	1	TRUE

ENSGALG00010022439	IFI35	PRJNA729271	2.230099644	2.84586E-27	2.230099644	stage_E14_vs_E10	ordinary_muscle_development	D_vs_E10;stage_1W_vs_E10;stage_3W_vs_E10;stage	1	TRUE
ENSGALG00010020236	SLC35G1	PRJNA729271	3.118404955	2.86245E-27	3.118404955	stage_E14_vs_E10	ordinary_muscle_development	D_vs_E10;stage_1W_vs_E10;stage_3W_vs_E10;stage	1	TRUE
ENSGALG00010029280	USP36	PRJNA729271	1.991428539	2.91902E-27	1.991428539	stage_E14_vs_E10	ordinary_muscle_development	D_vs_E10;stage_1W_vs_E10;stage_3W_vs_E10;stage	1	TRUE
ENSGALG00010022229	RAPSN	PRJNA729271	1.976099398	3.08747E-27	1.976099398	stage_E14_vs_E10	ordinary_muscle_development	D_vs_E10;stage_1W_vs_E10;stage_3W_vs_E10;stage	1	TRUE
ENSGALG00010019183	ANGEL2	PRJNA729271	0.850835776	3.09124E-27	0.850835776	stage_E14_vs_E10	ordinary_muscle_development		0	FALSE
ENSGALG00010004341	ENSGALG00010004341	PRJNA729271	-1.892616792	3.11972E-27	1.892616792	stage_E14_vs_E10	ordinary_muscle_development	D_vs_E10;stage_1W_vs_E10;stage_3W_vs_E10;stage	1	TRUE
ENSGALG00010019110	MBD4	PRJNA729271	1.167569803	3.1383E-27	1.167569803	stage_E14_vs_E10	ordinary_muscle_development	D_vs_E10;stage_1W_vs_E10;stage_3W_vs_E10;stage	1	TRUE
ENSGALG00010004887	MYH15	PRJNA729271	-8.217408469	3.16912E-27	8.217408469	stage_E14_vs_E10	ordinary_muscle_development	D_vs_E10;stage_1W_vs_E10;stage_3W_vs_E10;stage	1	TRUE
ENSGALG00010017310	SAMD11	PRJNA729271	-2.00680885	3.18491E-27	2.00680885	stage_E14_vs_E10	ordinary_muscle_development	D_vs_E10;stage_1W_vs_E10;stage_3W_vs_E10;stage	1	TRUE
ENSGALG00010022213	IDH1	PRJNA729271	-0.885249393	3.19726E-27	0.885249393	stage_E14_vs_E10	ordinary_muscle_development		0	FALSE
ENSGALG00010019218	RMND5A	PRJNA729271	1.503914863	3.26071E-27	1.503914863	stage_E14_vs_E10	ordinary_muscle_development	D_vs_E10;stage_1W_vs_E10;stage_3W_vs_E10;stage	1	TRUE
ENSGALG00010020715	ENSGALG00010020715	PRJNA729271	7.675580237	3.31274E-27	7.675580237	stage_E14_vs_E10	ordinary_muscle_development	D_vs_E10;stage_1W_vs_E10;stage_3W_vs_E10;stage	1	TRUE
ENSGALG00010008381	C18orf21	PRJNA729271	1.227128315	3.31282E-27	1.227128315	stage_E14_vs_E10	ordinary_muscle_development	D_vs_E10;stage_1W_vs_E10;stage_3W_vs_E10;stage	1	TRUE
ENSGALG00010011660	ENSGALG00010011660	PRJNA729271	-0.98968226	3.33402E-27	0.98968226	stage_E14_vs_E10	ordinary_muscle_development		0	FALSE
ENSGALG00010024593	SRSF6	PRJNA729271	-0.823115209	3.38181E-27	0.823115209	stage_E14_vs_E10	ordinary_muscle_development		0	FALSE
ENSGALG00010003273	NFIX	PRJNA729271	2.157220988	3.39433E-27	2.157220988	stage_E14_vs_E10	ordinary_muscle_development	D_vs_E10;stage_1W_vs_E10;stage_3W_vs_E10;stage	1	TRUE
ENSGALG00010012412	PURA	PRJNA729271	1.435530753	3.41838E-27	1.435530753	stage_E14_vs_E10	ordinary_muscle_development	D_vs_E10;stage_1W_vs_E10;stage_3W_vs_E10;stage	1	TRUE
ENSGALG00010009069	GRID2	PRJNA729271	-3.899936252	3.47368E-27	3.899936252	stage_E14_vs_E10	ordinary_muscle_development	D_vs_E10;stage_1W_vs_E10;stage_3W_vs_E10;stage	1	TRUE
ENSGALG00010001611	MAPK12	PRJNA729271	-1.825525998	3.58945E-27	1.825525998	stage_E14_vs_E10	ordinary_muscle_development	D_vs_E10;stage_1W_vs_E10;stage_3W_vs_E10;stage	1	TRUE
ENSGALG00010002282	TSKU	PRJNA729271	-1.823552046	3.59847E-27	1.823552046	stage_E14_vs_E10	ordinary_muscle_development	D_vs_E10;stage_1W_vs_E10;stage_3W_vs_E10;stage	1	TRUE
ENSGALG00010006277	GNG10	PRJNA729271	-1.799052349	3.76555E-27	1.799052349	stage_E14_vs_E10	ordinary_muscle_development	D_vs_E10;stage_1W_vs_E10;stage_3W_vs_E10;stage	1	TRUE
ENSGALG00010011456	KIAA1328	PRJNA729271	-1.102381494	3.89418E-27	1.102381494	stage_E14_vs_E10	ordinary_muscle_development	D_vs_E10;stage_1W_vs_E10;stage_3W_vs_E10;stage	1	TRUE
ENSGALG00010018767	NDUFB6	PRJNA729271	1.847844576	3.91007E-27	1.847844576	stage_E14_vs_E10	ordinary_muscle_development	D_vs_E10;stage_1W_vs_E10;stage_3W_vs_E10;stage	1	TRUE
ENSGALG00010017190	STK26	PRJNA729271	-0.756335074	3.93976E-27	0.756335074	stage_E14_vs_E10	ordinary_muscle_development		0	FALSE
ENSGALG00010005283	ATP5C1	PRJNA729271	1.44404726	3.98168E-27	1.44404726	stage_E14_vs_E10	ordinary_muscle_development	D_vs_E10;stage_1W_vs_E10;stage_3W_vs_E10;stage	1	TRUE
ENSGALG00010020659	RAD51B	PRJNA729271	-1.552056878	3.98338E-27	1.552056878	stage_E14_vs_E10	ordinary_muscle_development	D_vs_E10;stage_1W_vs_E10;stage_3W_vs_E10;stage	1	TRUE
ENSGALG00010020950	EFHD2	PRJNA729271	-4.21981452	4.09561E-27	4.21981452	stage_E14_vs_E10	ordinary_muscle_development	D_vs_E10;stage_1W_vs_E10;stage_3W_vs_E10;stage	1	TRUE
ENSGALG00010022552	GPR155	PRJNA729271	1.149487726	4.118E-27	1.149487726	stage_E14_vs_E10	ordinary_muscle_development	D_vs_E10;stage_1W_vs_E10;stage_3W_vs_E10;stage	1	TRUE
ENSGALG00010026954	MFSD4A	PRJNA729271	1.90241503	4.27576E-27	1.90241503	stage_E14_vs_E10	ordinary_muscle_development	D_vs_E10;stage_1W_vs_E10;stage_3W_vs_E10;stage	1	TRUE
ENSGALG00010004246	C8orf34	PRJNA729271	-4.328885178	4.35523E-27	4.328885178	stage_E14_vs_E10	ordinary_muscle_development	D_vs_E10;stage_1W_vs_E10;stage_3W_vs_E10;stage	1	TRUE
ENSGALG00010018540	TNFSF10	PRJNA729271	1.188690874	4.37297E-27	1.188690874	stage_E14_vs_E10	ordinary_muscle_development	D_vs_E10;stage_1W_vs_E10;stage_3W_vs_E10;stage	1	TRUE
ENSGALG00010027663	COPA	PRJNA729271	-0.773944397	4.39494E-27	0.773944397	stage_E14_vs_E10	ordinary_muscle_development		0	FALSE
ENSGALG00010005181	IMPACT	PRJNA729271	1.417151803	4.71045E-27	1.417151803	stage_E14_vs_E10	ordinary_muscle_development	D_vs_E10;stage_1W_vs_E10;stage_3W_vs_E10;stage	1	TRUE
ENSGALG00010020951	ENSGALG00010020951	PRJNA729271	-4.143545287	4.73487E-27	4.143545287	stage_E14_vs_E10	ordinary_muscle_development	D_vs_E10;stage_1W_vs_E10;stage_3W_vs_E10;stage	1	TRUE
ENSGALG00010024128	PHLDA2	PRJNA729271	-2.585390772	4.76828E-27	2.585390772	stage_E14_vs_E10	ordinary_muscle_development	D_vs_E10;stage_1W_vs_E10;stage_3W_vs_E10;stage	1	TRUE
ENSGALG00010005416	PPP3CA	PRJNA729271	1.770201928	4.8464E-27	1.770201928	stage_E14_vs_E10	ordinary_muscle_development	D_vs_E10;stage_1W_vs_E10;stage_3W_vs_E10;stage	1	TRUE
ENSGALG00010011589	RERG	PRJNA729271	-2.47611141	4.84913E-27	2.47611141	stage_E14_vs_E10	ordinary_muscle_development	D_vs_E10;stage_1W_vs_E10;stage_3W_vs_E10;stage	1	TRUE
ENSGALG00010018163	CCT7	PRJNA729271	-0.888576453	4.84984E-27	0.888576453	stage_E14_vs_E10	ordinary_muscle_development		0	FALSE
ENSGALG00010021714	TSPAN18	PRJNA729271	1.262066459	4.85553E-27	1.262066459	stage_E14_vs_E10	ordinary_muscle_development	D_vs_E10;stage_1W_vs_E10;stage_3W_vs_E10;stage	1	TRUE
ENSGALG00010014344	LSG1	PRJNA729271	-0.979748195	4.87153E-27	0.979748195	stage_E14_vs_E10	ordinary_muscle_development		0	FALSE
ENSGALG00010005460	TGDS	PRJNA729271	-1.130467487	4.87924E-27	1.130467487	stage_E14_vs_E10	ordinary_muscle_development	D_vs_E10;stage_1W_vs_E10;stage_3W_vs_E10;stage	1	TRUE
ENSGALG00010011001	ANGPT1	PRJNA729271	-2.836452588	4.93463E-27	2.836452588	stage_E14_vs_E10	ordinary_muscle_development	D_vs_E10;stage_1W_vs_E10;stage_3W_vs_E10;stage	1	TRUE
ENSGALG00010011293	NDUFV3	PRJNA729271	1.825733283	5.04079E-27	1.825733283	stage_E14_vs_E10	ordinary_muscle_development	D_vs_E10;stage_1W_vs_E10;stage_3W_vs_E10;stage	1	TRUE
ENSGALG00010005459	ZC3H12D	PRJNA729271	5.472663443	5.10462E-27	5.472663443	stage_E14_vs_E10	ordinary_muscle_development	D_vs_E10;stage_1W_vs_E10;stage_3W_vs_E10;stage	1	TRUE
ENSGALG00010016772	TSPAN6	PRJNA729271	-1.389377797	5.16014E-27	1.389377797	stage_E14_vs_E10	ordinary_muscle_development	D_vs_E10;stage_1W_vs_E10;stage_3W_vs_E10;stage	1	TRUE
ENSGALG00010022037	DRG1	PRJNA729271	1.015619314	5.18049E-27	1.015619314	stage_E14_vs_E10	ordinary_muscle_development	D_vs_E10;stage_1W_vs_E10;stage_3W_vs_E10;stage	1	TRUE
ENSGALG00010025548	ENSGALG00010025548	PRJNA729271	-2.268342658	5.35037E-27	2.268342658	stage_E14_vs_E10	ordinary_muscle_development	D_vs_E10;stage_1W_vs_E10;stage_3W_vs_E10;stage	1	TRUE
ENSGALG00010005368	CCDC3	PRJNA729271	-2.740773336	5.44162E-27	2.740773336	stage_E14_vs_E10	ordinary_muscle_development	D_vs_E10;stage_1W_vs_E10;stage_3W_vs_E10;stage	1	TRUE
ENSGALG00010011418	RIPK1	PRJNA729271	-0.969202989	5.70607E-27	0.969202989	stage_E14_vs_E10	ordinary_muscle_development		0	FALSE
ENSGALG00010008459	EPB41L4B	PRJNA729271	-2.598396373	5.75242E-27	2.598396373	stage_E14_vs_E10	ordinary_muscle_development	D_vs_E10;stage_1W_vs_E10;stage_3W_vs_E10;stage	1	TRUE
ENSGALG00010028101	MRPL54	PRJNA729271	1.379360926	5.83336E-27	1.379360926	stage_E14_vs_E10	ordinary_muscle_development	D_vs_E10;stage_1W_vs_E10;stage_3W_vs_E10;stage	1	TRUE
ENSGALG00010021607	SLC9A5	PRJNA729271	2.010030308	5.88989E-27	2.010030308	stage_E14_vs_E10	ordinary_muscle_development	D_vs_E10;stage_1W_vs_E10;stage_3W_vs_E10;stage	1	TRUE
ENSGALG00010021743	SYT13	PRJNA729271	-6.265208733	6.14201E-27	6.265208733	stage_E14_vs_E10	ordinary_muscle_development	D_vs_E10;stage_1W_vs_E10;stage_3W_vs_E10;stage	1	TRUE
ENSGALG00010004137	CROT	PRJNA729271	-0.925678991	6.22711E-27	0.925678991	stage_E14_vs_E10	ordinary_muscle_development		0	FALSE
ENSGALG00010024793	FKBP4	PRJNA729271	-1.278495334	6.27239E-27	1.278495334	stage_E14_vs_E10	ordinary_muscle_development	D_vs_E10;stage_1W_vs_E10;stage_3W_vs_E10;stage	1	TRUE
ENSGALG00010027173	ENSGALG00010027173	PRJNA729271	-1.06840995	6.6188E-27	1.06840995	stage_E14_vs_E10	ordinary_muscle_development	D_vs_E10;stage_1W_vs_E10;stage_3W_vs_E10;stage	1	TRUE
ENSGALG00010008340	PDIA4	PRJNA729271	-1.154094613	6.70766E-27	1.154094613	stage_E14_vs_E10	ordinary_muscle_development	D_vs_E10;stage_1W_vs_E10;stage_3W_vs_E10;stage	1	TRUE
ENSGALG00010025610	RNH1	PRJNA729271	-1.467747091	6.72553E-27	1.467747091	stage_E14_vs_E10	ordinary_muscle_development	D_vs_E10;stage_1W_vs_E10;stage_3W_vs_E10;stage	1	TRUE
ENSGALG00010017904	MEGF11	PRJNA729271	-5.123890711	6.75737E-27	5.123890711	stage_E14_vs_E10	ordinary_muscle_development	D_vs_E10;stage_1W_vs_E10;stage_3W_vs_E10;stage	1	TRUE
ENSGALG00010018932	CDKN3	PRJNA729271	-2.60186783	6.76061E-27	2.60186783	stage_E14_vs_E10	ordinary_muscle_development	D_vs_E10;stage_1W_vs_E10;stage_3W_vs_E10;stage	1	TRUE
ENSGALG00010000660	ENSGALG00010000660	PRJNA729271	1.899848214	6.79891E-27	1.899848214	stage_E14_vs_E10	ordinary_muscle_development	D_vs_E10;stage_1W_vs_E10;stage_3W_vs_E10;stage	1	TRUE
ENSGALG00010012824	HIBCH	PRJNA729271	1.275734217	6.82711E-27	1.275734217	stage_E14_vs_E10	ordinary_muscle_development	D_vs_E10;stage_1W_vs_E10;stage_3W_vs_E10;stage	1	TRUE
ENSGALG00010021666	CCDC47	PRJNA729271	1.002217266	6.86326E-27	1.002217266	stage_E14_vs_E10	ordinary_muscle_development	D_vs_E10;stage_1W_vs_E10;stage_3W_vs_E10;stage	1	TRUE
ENSGALG00010023819	BIN2	PRJNA729271	2.460953932	6.93423E-27	2.460953932	stage_E14_vs_E10	ordinary_muscle_development	D_vs_E10;stage_1W_vs_E10;stage_3W_vs_E10;stage	1	TRUE
ENSGALG00010006944	MAB21L2	PRJNA729271	-6.091277624	7.26511E-27	6.091277624	stage_E14_vs_E10	ordinary_muscle_development	D_vs_E10;stage_1W_vs_E10;stage_3W_vs_E10;stage	1	TRUE
ENSGALG00010013491	CNOT11	PRJNA729271	1.199087486	7.41831E-27	1.199087486	stage_E14_vs_E10	ordinary_muscle_development	D_vs_E10;stage_1W_vs_E10;stage_3W_vs_E10;stage	1	TRUE
ENSGALG00010021758	RASSF10	PRJNA729271	-5.581645029	7.66164E-27	5.581645029	stage_E14_vs_E10	ordinary_muscle_development	D_vs_E10;stage_1W_vs_E10;stage_3W_vs_E10;stage	1	TRUE
ENSGALG00010026780	MYSM1	PRJNA729271	0.844541956	7.79969E-27	0.844541956	stage_E14_vs_E10	ordinary_muscle_development		0	FALSE
ENSGALG00010015813	ENSGALG00010015813	PRJNA729271	1.505072673	7.84307E-27	1.505072673	stage_E14_vs_E10	ordinary_muscle_development	D_vs_E10;stage_1W_vs_E10;stage_3W_vs_E10;stage	1	TRUE
ENSGALG00010029496	TTYH2	PRJNA729271	-1.628997877	7.84488E-27	1.628997877	stage_E14_vs_E10	ordinary_muscle_development	D_vs_E10;stage_1W_vs_E10;stage_3W_vs_E10;stage	1	TRUE
ENSGALG00010004505	ENSGALG00010004505	PRJNA729271	1.418686339	7.91971E-27	1.418686339	stage_E14_vs_E10	ordinary_muscle_development	D_vs_E10;stage_1W_vs_E10;stage_3W_vs_E10;stage	1	TRUE
ENSGALG00010006039	PCNP	PRJNA729271	-1.381789177	8.01749E-27	1.381789177	stage_E14_vs_E10	ordinary_muscle_development	D_vs_E10;stage_1W_vs_E10;stage_3W_vs_E10;stage	1	TRUE
ENSGALG00010013027	UHMK1	PRJNA729271	1.36174991	8.12456E-27	1.36174991	stage_E14_vs_E10	ordinary_muscle_development	D_vs_E10;stage_1W_vs_E10;stage_3W_vs_E10;stage	1	TRUE
ENSGALG00010017705	MAK16	PRJNA729271	1.119011609	8.15795E-27	1.119011609	stage_E14_vs_E10	ordinary_muscle_development	D_vs_E10;stage_1W_vs_E10;stage_3W_vs_E10;stage	1	TRUE
ENSGALG00010021027	PLA2G4A	PRJNA729271	1.814686761	8.17685E-27	1.814686761	stage_E14_vs_E10	ordinary_muscle_development	D_vs_E10;stage_1W_vs_E10;stage_3W_vs_E10;stage	1	TRUE
ENSGALG00010025355	STAT6	PRJNA729271	1.522108226	8.20092E-27	1.522108226	stage_E14_vs_E10	ordinary_muscle_development	D_vs_E10;stage_1W_vs_E10;stage_3W_vs_E10;stage	1	TRUE
ENSGALG00010027593	ENSGALG00010027593	PRJNA729271	4.586864718	8.27123E-27	4.586864718	stage_E14_vs_E10	ordinary_muscle_development	D_vs_E10;stage_1W_vs_E10;stage_3W_vs_E10;stage	1	TRUE
ENSGALG00010029250	SPAG9	PRJNA729271	0.947203375	8.27622E-27	0.947203375	stage_E14_vs_E10	ordinary_muscle_development		0	FALSE
ENSGALG00010024285	CCND2	PRJNA729271	-1.064325409	8.2904E-27	1.064325409	stage_E14_vs_E10	ordinary_muscle_development	D_vs_E10;stage_1W_vs_E10;stage_3W_vs_E10;stage	1	TRUE
ENSGALG00010025424	IRX6	PRJNA729271	-4.648388116	8.40562E-27	4.648388116	stage_E14_vs_E10	ordinary_muscle_development	D_vs_E10;stage_1W_vs_E10;stage_3W_vs_E10;stage	1	TRUE
ENSGALG00010022108	USP47	PRJNA729271	0.95560909	8.41554E-27	0.95560909	stage_E14_vs_E10	ordinary_muscle_development		0	FALSE
ENSGALG00010008290	FAM13A	PRJNA729271	1.783234941	8.44523E-27	1.783234941	stage_E14_vs_E10	ordinary_muscle_development	D_vs_E10;stage_1W_vs_E10;stage_3W_vs_E10;stage	1	TRUE
ENSGALG00010017219	EARS2	PRJNA729271	-1.662699387	8.72239E-27	1.662699387	stage_E14_vs_E10	ordinary_muscle_development	D_vs_E10;stage_1W_vs_E10;stage_3W_vs_E10;stage	1	TRUE
ENSGALG00010023030	HS2ST1	PRJNA729271	-0.931335503	9.18379E-27	0.931335503	stage_E14_vs_E10	ordinary_muscle_development		0	FALSE
ENSGALG00010017915	BFSP1	PRJNA729271	4.54946353	9.37727E-27	4.54946353	stage_E14_vs_E10	ordinary_muscle_development	D_vs_E10;stage_1W_vs_E10;stage_3W_vs_E10;stage	1	TRUE
ENSGALG00010028595	SCNM1	PRJNA729271	1.208140194	9.42376E-27	1.208140194	stage_E14_vs_E10	ordinary_muscle_development	D_vs_E10;stage_1W_vs_E10;stage_3W_vs_E10;stage	1	TRUE
ENSGALG00010016941	SLC8A1	PRJNA729271	1.844285253	9.59084E-27	1.844285253	stage_E14_vs_E10	ordinary_muscle_development	D_vs_E10;stage_1W_vs_E10;stage_3W_vs_E10;stage	1	TRUE
ENSGALG00010009743	CCNG1	PRJNA729271	1.87554599	9.60637E-27	1.87554599	stage_E14_vs_E10	ordinary_muscle_development	D_vs_E10;stage_1W_vs_E10;stage_3W_vs_E10;stage	1	TRUE
ENSGALG00010001845	ANKRD12	PRJNA729271	1.451427517	9.80878E-27	1.451427517	stage_E14_vs_E10	ordinary_muscle_development	D_vs_E10;stage_1W_vs_E10;stage_3W_vs_E10;stage	1	TRUE
ENSGALG00010007872	KLHL2	PRJNA729271	-0.861195677	9.97115E-27	0.861195677	stage_E14_vs_E10	ordinary_muscle_development		0	FALSE
ENSGALG00010019247	HBZ	PRJNA729271	-8.202865306	9.99849E-27	8.202865306	stage_E14_vs_E10	ordinary_muscle_development	D_vs_E10;stage_1W_vs_E10;stage_3W_vs_E10;stage	1	TRUE
ENSGALG00010020194	PRICKLE2	PRJNA729271	-1.720113824	1.02362E-26	1.720113824	stage_E14_vs_E10	ordinary_muscle_development	D_vs_E10;stage_1W_vs_E10;stage_3W_vs_E10;stage	1	TRUE
ENSGALG00010021139	ARHGAP29	PRJNA729271	1.036949521	1.03194E-26	1.036949521	stage_E14_vs_E10	ordinary_muscle_development	D_vs_E10;stage_1W_vs_E10;stage_3W_vs_E10;stage	1	TRUE
ENSGALG00010000061	SERINC4	PRJNA729271	3.567403064	1.04736E-26	3.567403064	stage_E14_vs_E10	ordinary_muscle_development	D_vs_E10;stage_1W_vs_E10;stage_3W_vs_E10;stage	1	TRUE
ENSGALG00010005138	ENSGALG00010005138	PRJNA729271	5.548289585	1.0901E-26	5.548289585	stage_E14_vs_E10	ordinary_muscle_development	D_vs_E10;stage_1W_vs_E10;stage_3W_vs_E10;stage	1	TRUE
ENSGALG00010001373	IMPA2	PRJNA729271	1.514186274	1.09749E-26	1.514186274	stage_E14_vs_E10	ordinary_muscle_development	D_vs_E10;stage_1W_vs_E10;stage_3W_vs_E10;stage	1	TRUE
ENSGALG00010009114	SETD7	PRJNA729271	1.625938378	1.12966E-26	1.625938378	stage_E14_vs_E10	ordinary_muscle_development	D_vs_E10;stage_1W_vs_E10;stage_3W_vs_E10;stage	1	TRUE
ENSGALG00010023079	SLC25A16	PRJNA729271	1.025126873	1.1807E-26	1.025126873	stage_E14_vs_E10	ordinary_muscle_development	D_vs_E10;stage_1W_vs_E10;stage_3W_vs_E10;stage	1	TRUE
ENSGALG00010008142	CD247	PRJNA729271	3.470202206	1.20033E-26	3.470202206	stage_E14_vs_E10	ordinary_muscle_development	D_vs_E10;stage_1W_vs_E10;stage_3W_vs_E10;stage	1	TRUE
ENSGALG00010002275	IL1RAPL1	PRJNA729271	-4.40251139	1.21805E-26	4.40251139	stage_E14_vs_E10	ordinary_muscle_development	D_vs_E10;stage_1W_vs_E10;stage_3W_vs_E10;stage	1	TRUE
ENSGALG00010011961	AGPAT3	PRJNA729271	1.131439993	1.22157E-26	1.131439993	stage_E14_vs_E10	ordinary_muscle_development	D_vs_E10;stage_1W_vs_E10;stage_3W_vs_E10;stage	1	TRUE
ENSGALG00010024905	GPX7	PRJNA729271	-2.283329856	1.2225E-26	2.283329856	stage_E14_vs_E10	ordinary_muscle_development	D_vs_E10;stage_1W_vs_E10;stage_3W_vs_E10;stage	1	TRUE
ENSGALG00010014824	ANXA6	PRJNA729271	0.996254324	1.23702E-26	0.996254324	stage_E14_vs_E10	ordinary_muscle_development		0	FALSE
ENSGALG00010013360	PRR5	PRJNA729271	1.218678952	1.24059E-26	1.218678952	stage_E14_vs_E10	ordinary_muscle_development	D_vs_E10;stage_1W_vs_E10;stage_3W_vs_E10;stage	1	TRUE
ENSGALG00010020944	MYO5A	PRJNA729271	-1.723255229	1.24147E-26	1.723255229	stage_E14_vs_E10	ordinary_muscle_development	D_vs_E10;stage_1W_vs_E10;stage_3W_vs_E10;stage	1	TRUE
ENSGALG00010000788	GATD3AL1	PRJNA729271	1.130790622	1.25151E-26	1.130790622	stage_E14_vs_E10	ordinary_muscle_development	D_vs_E10;stage_1W_vs_E10;stage_3W_vs_E10;stage	1	TRUE
ENSGALG00010006101	ENSGALG00010006101	PRJNA729271	1.230077882	1.25434E-26	1.230077882	stage_E14_vs_E10	ordinary_muscle_development	D_vs_E10;stage_1W_vs_E10;stage_3W_vs_E10;stage	1	TRUE
ENSGALG00010020976	GDF10	PRJNA729271	-3.679930138	1.27059E-26	3.679930138	stage_E14_vs_E10	ordinary_muscle_development	D_vs_E10;stage_1W_vs_E10;stage_3W_vs_E10;stage	1	TRUE
ENSGALG00010025587	LRRC56	PRJNA729271	-4.218810511	1.28538E-26	4.218810511	stage_E14_vs_E10	ordinary_muscle_development	D_vs_E10;stage_1W_vs_E10;stage_3W_vs_E10;stage	1	TRUE
ENSGALG00010018860	AGTR2	PRJNA729271	2.331490288	1.28984E-26	2.331490288	stage_E14_vs_E10	ordinary_muscle_development	D_vs_E10;stage_1W_vs_E10;stage_3W_vs_E10;stage	1	TRUE
ENSGALG00010020965	DHX37	PRJNA729271	-1.050609449	1.29051E-26	1.050609449	stage_E14_vs_E10	ordinary_muscle_development	D_vs_E10;stage_1W_vs_E10;stage_3W_vs_E10;stage	1	TRUE
ENSGALG00010013340	LIN28B	PRJNA729271	-6.886302832	1.29307E-26	6.886302832	stage_E14_vs_E10	ordinary_muscle_development	D_vs_E10;stage_1W_vs_E10;stage_3W_vs_E10;stage	1	TRUE
ENSGALG00010001330	ENSGALG00010001330	PRJNA729271	1.525063409	1.31667E-26	1.525063409	stage_E14_vs_E10	ordinary_muscle_development	D_vs_E10;stage_1W_vs_E10;stage_3W_vs_E10;stage	1	TRUE
ENSGALG00010025606	CCDC12	PRJNA729271	-1.457871406	1.35418E-26	1.457871406	stage_E14_vs_E10	ordinary_muscle_development	D_vs_E10;stage_1W_vs_E10;stage_3W_vs_E10;stage	1	TRUE
ENSGALG00010002835	CYFIP1	PRJNA729271	-0.917592153	1.3719E-26	0.917592153	stage_E14_vs_E10	ordinary_muscle_development		0	FALSE
ENSGALG00010018365	HPSE2	PRJNA729271	-2.993646301	1.39599E-26	2.993646301	stage_E14_vs_E10	ordinary_muscle_development	D_vs_E10;stage_1W_vs_E10;stage_3W_vs_E10;stage	1	TRUE
ENSGALG00010003017	PGM3	PRJNA729271	-1.702337997	1.40663E-26	1.702337997	stage_E14_vs_E10	ordinary_muscle_development	D_vs_E10;stage_1W_vs_E10;stage_3W_vs_E10;stage	1	TRUE
ENSGALG00010000555	OTUB1	PRJNA729271	-1.118246661	1.41093E-26	1.118246661	stage_E14_vs_E10	ordinary_muscle_development	D_vs_E10;stage_1W_vs_E10;stage_3W_vs_E10;stage	1	TRUE
ENSGALG00010024514	FAF1	PRJNA729271	0.735894291	1.44488E-26	0.735894291	stage_E14_vs_E10	ordinary_muscle_development		0	FALSE
ENSGALG00010000049	FES	PRJNA729271	2.034791438	1.45691E-26	2.034791438	stage_E14_vs_E10	ordinary_muscle_development	D_vs_E10;stage_1W_vs_E10;stage_3W_vs_E10;stage	1	TRUE
ENSGALG00010028144	FAM78A	PRJNA729271	2.019985741	1.49622E-26	2.019985741	stage_E14_vs_E10	ordinary_muscle_development	D_vs_E10;stage_1W_vs_E10;stage_3W_vs_E10;stage	1	TRUE
ENSGALG00010011578	MYLK4	PRJNA729271	3.55129306	1.53201E-26	3.55129306	stage_E14_vs_E10	ordinary_muscle_development	D_vs_E10;stage_1W_vs_E10;stage_3W_vs_E10;stage	1	TRUE
ENSGALG00010017821	ENSGALG00010017821	PRJNA729271	-5.230254935	1.54395E-26	5.230254935	stage_E14_vs_E10	ordinary_muscle_development	D_vs_E10;stage_1W_vs_E10;stage_3W_vs_E10;stage	1	TRUE
ENSGALG00010004881	MIPOL1	PRJNA729271	-1.723126753	1.55571E-26	1.723126753	stage_E14_vs_E10	ordinary_muscle_development	D_vs_E10;stage_1W_vs_E10;stage_3W_vs_E10;stage	1	TRUE
ENSGALG00010001995	LRRC1	PRJNA729271	-1.557033961	1.56481E-26	1.557033961	stage_E14_vs_E10	ordinary_muscle_development	D_vs_E10;stage_1W_vs_E10;stage_3W_vs_E10;stage	1	TRUE
ENSGALG00010015146	ENSGALG00010015146	PRJNA729271	1.374323332	1.59856E-26	1.374323332	stage_E14_vs_E10	ordinary_muscle_development	D_vs_E10;stage_1W_vs_E10;stage_3W_vs_E10;stage	1	TRUE
ENSGALG00010008251	AHRR	PRJNA729271	-3.822346687	1.60236E-26	3.822346687	stage_E14_vs_E10	ordinary_muscle_development	D_vs_E10;stage_1W_vs_E10;stage_3W_vs_E10;stage	1	TRUE
ENSGALG00010029270	TIMP2	PRJNA729271	1.614267999	1.61971E-26	1.614267999	stage_E14_vs_E10	ordinary_muscle_development	D_vs_E10;stage_1W_vs_E10;stage_3W_vs_E10;stage	1	TRUE
ENSGALG00010017442	LCK	PRJNA729271	2.575684566	1.63687E-26	2.575684566	stage_E14_vs_E10	ordinary_muscle_development	D_vs_E10;stage_1W_vs_E10;stage_3W_vs_E10;stage	1	TRUE
ENSGALG00010017609	TRIM71	PRJNA729271	-2.347623889	1.71276E-26	2.347623889	stage_E14_vs_E10	ordinary_muscle_development	D_vs_E10;stage_1W_vs_E10;stage_3W_vs_E10;stage	1	TRUE
ENSGALG00010000575	ENSGALG00010000575	PRJNA729271	1.703925932	1.80943E-26	1.703925932	stage_E14_vs_E10	ordinary_muscle_development	D_vs_E10;stage_1W_vs_E10;stage_3W_vs_E10;stage	1	TRUE
ENSGALG00010012857	NSDHL	PRJNA729271	-2.478257733	1.84664E-26	2.478257733	stage_E14_vs_E10	ordinary_muscle_development	D_vs_E10;stage_1W_vs_E10;stage_3W_vs_E10;stage	1	TRUE
ENSGALG00010008176	SLA	PRJNA729271	3.152664455	1.89182E-26	3.152664455	stage_E14_vs_E10	ordinary_muscle_development	D_vs_E10;stage_1W_vs_E10;stage_3W_vs_E10;stage	1	TRUE
ENSGALG00010015193	LYPLA2	PRJNA729271	-1.164282187	1.89591E-26	1.164282187	stage_E14_vs_E10	ordinary_muscle_development	D_vs_E10;stage_1W_vs_E10;stage_3W_vs_E10;stage	1	TRUE
ENSGALG00010022945	KCNA4	PRJNA729271	-6.63942654	1.91783E-26	6.63942654	stage_E14_vs_E10	ordinary_muscle_development	D_vs_E10;stage_1W_vs_E10;stage_3W_vs_E10;stage	1	TRUE
ENSGALG00010012869	ENSGALG00010012869	PRJNA729271	2.294114113	1.92811E-26	2.294114113	stage_E14_vs_E10	ordinary_muscle_development	D_vs_E10;stage_1W_vs_E10;stage_3W_vs_E10;stage	1	TRUE
ENSGALG00010009506	HAS2	PRJNA729271	-2.825758095	1.92832E-26	2.825758095	stage_E14_vs_E10	ordinary_muscle_development	D_vs_E10;stage_1W_vs_E10;stage_3W_vs_E10;stage	1	TRUE
ENSGALG00010008128	RARS	PRJNA729271	-0.765312939	1.93111E-26	0.765312939	stage_E14_vs_E10	ordinary_muscle_development		0	FALSE
ENSGALG00010004047	PDE7A	PRJNA729271	-1.310644808	1.94382E-26	1.310644808	stage_E14_vs_E10	ordinary_muscle_development	D_vs_E10;stage_1W_vs_E10;stage_3W_vs_E10;stage	1	TRUE
ENSGALG00010029168	RFNG	PRJNA729271	0.861055716	1.97111E-26	0.861055716	stage_E14_vs_E10	ordinary_muscle_development		0	FALSE
ENSGALG00010009415	HMP19	PRJNA729271	-5.075901795	1.99197E-26	5.075901795	stage_E14_vs_E10	ordinary_muscle_development	D_vs_E10;stage_1W_vs_E10;stage_3W_vs_E10;stage	1	TRUE
ENSGALG00010021504	ODR4	PRJNA729271	-0.862648528	2.05104E-26	0.862648528	stage_E14_vs_E10	ordinary_muscle_development		0	FALSE
ENSGALG00010020840	LPAR3	PRJNA729271	-4.00261689	2.12723E-26	4.00261689	stage_E14_vs_E10	ordinary_muscle_development	D_vs_E10;stage_1W_vs_E10;stage_3W_vs_E10;stage	1	TRUE
ENSGALG00010027249	C2CD4C	PRJNA729271	10.11306046	2.1387E-26	10.11306046	stage_E14_vs_E10	ordinary_muscle_development	D_vs_E10;stage_1W_vs_E10;stage_3W_vs_E10;stage	1	TRUE
ENSGALG00010012768	EPS8	PRJNA729271	-1.266928939	2.17012E-26	1.266928939	stage_E14_vs_E10	ordinary_muscle_development	D_vs_E10;stage_1W_vs_E10;stage_3W_vs_E10;stage	1	TRUE
ENSGALG00010020999	IFIH1	PRJNA729271	3.607968208	2.18711E-26	3.607968208	stage_E14_vs_E10	ordinary_muscle_development	D_vs_E10;stage_1W_vs_E10;stage_3W_vs_E10;stage	1	TRUE
ENSGALG00010015108	ENSGALG00010015108	PRJNA729271	0.938617336	2.1973E-26	0.938617336	stage_E14_vs_E10	ordinary_muscle_development		0	FALSE
ENSGALG00010021078	ENSGALG00010021078	PRJNA729271	-3.190155441	2.20771E-26	3.190155441	stage_E14_vs_E10	ordinary_muscle_development	D_vs_E10;stage_1W_vs_E10;stage_3W_vs_E10;stage	1	TRUE
ENSGALG00010025028	HES6	PRJNA729271	-1.48305747	2.24593E-26	1.48305747	stage_E14_vs_E10	ordinary_muscle_development	D_vs_E10;stage_1W_vs_E10;stage_3W_vs_E10;stage	1	TRUE
ENSGALG00010029737	ENSGALG00010029737	PRJNA729271	-6.268033181	2.26501E-26	6.268033181	stage_E14_vs_E10	ordinary_muscle_development	D_vs_E10;stage_1W_vs_E10;stage_3W_vs_E10;stage	1	TRUE
ENSGALG00010008177	NREP	PRJNA729271	-1.996703293	2.27972E-26	1.996703293	stage_E14_vs_E10	ordinary_muscle_development	D_vs_E10;stage_1W_vs_E10;stage_3W_vs_E10;stage	1	TRUE
ENSGALG00010006845	CALML3	PRJNA729271	-9.955627603	2.30507E-26	9.955627603	stage_E14_vs_E10	ordinary_muscle_development	D_vs_E10;stage_1W_vs_E10;stage_3W_vs_E10;stage	1	TRUE
ENSGALG00010029741	EMC6	PRJNA729271	1.739513681	2.37694E-26	1.739513681	stage_E14_vs_E10	ordinary_muscle_development	D_vs_E10;stage_1W_vs_E10;stage_3W_vs_E10;stage	1	TRUE
ENSGALG00010002678	CCNI	PRJNA729271	1.872201247	2.38871E-26	1.872201247	stage_E14_vs_E10	ordinary_muscle_development	D_vs_E10;stage_1W_vs_E10;stage_3W_vs_E10;stage	1	TRUE
ENSGALG00010028163	GBP	PRJNA729271	3.704550088	2.38872E-26	3.704550088	stage_E14_vs_E10	ordinary_muscle_development	D_vs_E10;stage_1W_vs_E10;stage_3W_vs_E10;stage	1	TRUE
ENSGALG00010023140	CCAR1	PRJNA729271	-0.917624303	2.40421E-26	0.917624303	stage_E14_vs_E10	ordinary_muscle_development		0	FALSE
ENSGALG00010022820	SHTN1	PRJNA729271	-1.993533639	2.46761E-26	1.993533639	stage_E14_vs_E10	ordinary_muscle_development	D_vs_E10;stage_1W_vs_E10;stage_3W_vs_E10;stage	1	TRUE
ENSGALG00010029661	KRABZFP	PRJNA729271	6.124502975	2.53911E-26	6.124502975	stage_E14_vs_E10	ordinary_muscle_development	D_vs_E10;stage_1W_vs_E10;stage_3W_vs_E10;stage	1	TRUE
ENSGALG00010028154	ENSGALG00010028154	PRJNA729271	-1.498907414	2.54147E-26	1.498907414	stage_E14_vs_E10	ordinary_muscle_development	D_vs_E10;stage_1W_vs_E10;stage_3W_vs_E10;stage	1	TRUE
ENSGALG00010018016	IFIT5	PRJNA729271	9.229454462	2.54511E-26	9.229454462	stage_E14_vs_E10	ordinary_muscle_development	D_vs_E10;stage_1W_vs_E10;stage_3W_vs_E10;stage	1	TRUE
ENSGALG00010012343	BCL2L13	PRJNA729271	1.831031461	2.65078E-26	1.831031461	stage_E14_vs_E10	ordinary_muscle_development	D_vs_E10;stage_1W_vs_E10;stage_3W_vs_E10;stage	1	TRUE
ENSGALG00010009127	SCOC	PRJNA729271	-0.817940807	2.6673E-26	0.817940807	stage_E14_vs_E10	ordinary_muscle_development		0	FALSE
ENSGALG00010003372	ENOX1	PRJNA729271	-1.551055198	2.66898E-26	1.551055198	stage_E14_vs_E10	ordinary_muscle_development	D_vs_E10;stage_1W_vs_E10;stage_3W_vs_E10;stage	1	TRUE
ENSGALG00010024131	ENSGALG00010024131	PRJNA729271	10.95443516	2.67236E-26	10.95443516	stage_E14_vs_E10	ordinary_muscle_development	D_vs_E10;stage_1W_vs_E10;stage_3W_vs_E10;stage	1	TRUE
ENSGALG00010011055	TOR3A	PRJNA729271	1.402278876	2.67555E-26	1.402278876	stage_E14_vs_E10	ordinary_muscle_development	D_vs_E10;stage_1W_vs_E10;stage_3W_vs_E10;stage	1	TRUE
ENSGALG00010027721	SUSD3	PRJNA729271	-2.175388312	2.70704E-26	2.175388312	stage_E14_vs_E10	ordinary_muscle_development	D_vs_E10;stage_1W_vs_E10;stage_3W_vs_E10;stage	1	TRUE
ENSGALG00010024623	NRDC	PRJNA729271	1.176390931	2.74228E-26	1.176390931	stage_E14_vs_E10	ordinary_muscle_development	D_vs_E10;stage_1W_vs_E10;stage_3W_vs_E10;stage	1	TRUE
ENSGALG00010013632	SLC1A3	PRJNA729271	-9.662092634	2.74885E-26	9.662092634	stage_E14_vs_E10	ordinary_muscle_development	D_vs_E10;stage_1W_vs_E10;stage_3W_vs_E10;stage	1	TRUE
ENSGALG00010020467	ALDH4A1	PRJNA729271	1.401794875	2.75859E-26	1.401794875	stage_E14_vs_E10	ordinary_muscle_development	D_vs_E10;stage_1W_vs_E10;stage_3W_vs_E10;stage	1	TRUE
ENSGALG00010023472	PTPN6	PRJNA729271	2.091713207	2.79203E-26	2.091713207	stage_E14_vs_E10	ordinary_muscle_development	D_vs_E10;stage_1W_vs_E10;stage_3W_vs_E10;stage	1	TRUE
ENSGALG00010012934	NDUFA1	PRJNA729271	1.62901935	2.79561E-26	1.62901935	stage_E14_vs_E10	ordinary_muscle_development	D_vs_E10;stage_1W_vs_E10;stage_3W_vs_E10;stage	1	TRUE
ENSGALG00010001114	MED12L	PRJNA729271	1.098317766	2.84975E-26	1.098317766	stage_E14_vs_E10	ordinary_muscle_development	D_vs_E10;stage_1W_vs_E10;stage_3W_vs_E10;stage	1	TRUE
ENSGALG00010012179	ENSGALG00010012179	PRJNA729271	-1.940520335	2.85697E-26	1.940520335	stage_E14_vs_E10	ordinary_muscle_development	D_vs_E10;stage_1W_vs_E10;stage_3W_vs_E10;stage	1	TRUE
ENSGALG00010022366	AARSD1	PRJNA729271	1.205413239	2.95097E-26	1.205413239	stage_E14_vs_E10	ordinary_muscle_development	D_vs_E10;stage_1W_vs_E10;stage_3W_vs_E10;stage	1	TRUE
ENSGALG00010019046	SELENOS	PRJNA729271	0.867356838	2.9626E-26	0.867356838	stage_E14_vs_E10	ordinary_muscle_development		0	FALSE

ENSGALG00010008879	HABP4	PRJNA729271	-1.932707952	3.01434E-26	1.932707952	stage_E14_vs_E10	ordinary_muscle_development	D_vs_E10;stage_1W_vs_E10;stage_3W_vs_E10;stage	1	TRUE
ENSGALG00010015766	TLE1	PRJNA729271	-1.123890394	3.09618E-26	1.123890394	stage_E14_vs_E10	ordinary_muscle_development	D_vs_E10;stage_1W_vs_E10;stage_3W_vs_E10;stage	1	TRUE
ENSGALG00010005969	HECA	PRJNA729271	0.709222695	3.12142E-26	0.709222695	stage_E14_vs_E10	ordinary_muscle_development		0	FALSE
ENSGALG00010006682	ARHGAP10	PRJNA729271	-1.321805411	3.12464E-26	1.321805411	stage_E14_vs_E10	ordinary_muscle_development	D_vs_E10;stage_1W_vs_E10;stage_3W_vs_E10;stage	1	TRUE
ENSGALG00010000124	ENSGALG00010000124	PRJNA729271	1.950836195	3.1566E-26	1.950836195	stage_E14_vs_E10	ordinary_muscle_development	D_vs_E10;stage_1W_vs_E10;stage_3W_vs_E10;stage	1	TRUE
ENSGALG00010020402	SLC3A1	PRJNA729271	3.32569569	3.1862E-26	3.32569569	stage_E14_vs_E10	ordinary_muscle_development	D_vs_E10;stage_1W_vs_E10;stage_3W_vs_E10;stage	1	TRUE
ENSGALG00010023874	HEXIM1	PRJNA729271	2.403444391	3.30493E-26	2.403444391	stage_E14_vs_E10	ordinary_muscle_development	D_vs_E10;stage_1W_vs_E10;stage_3W_vs_E10;stage	1	TRUE
ENSGALG00010005012	ENSGALG00010005012	PRJNA729271	11.38520733	3.32164E-26	11.38520733	stage_E14_vs_E10	ordinary_muscle_development	D_vs_E10;stage_1W_vs_E10;stage_3W_vs_E10;stage	1	TRUE
ENSGALG00010010168	SNCB	PRJNA729271	-5.148678874	3.36343E-26	5.148678874	stage_E14_vs_E10	ordinary_muscle_development	D_vs_E10;stage_1W_vs_E10;stage_3W_vs_E10;stage	1	TRUE
ENSGALG00010012055	ENSGALG00010012055	PRJNA729271	1.683920777	3.38385E-26	1.683920777	stage_E14_vs_E10	ordinary_muscle_development	D_vs_E10;stage_1W_vs_E10;stage_3W_vs_E10;stage	1	TRUE
ENSGALG00010013037	MORC4	PRJNA729271	0.960497152	3.42976E-26	0.960497152	stage_E14_vs_E10	ordinary_muscle_development		0	FALSE
ENSGALG00010024498	CCDC102A	PRJNA729271	-1.307977522	3.44332E-26	1.307977522	stage_E14_vs_E10	ordinary_muscle_development	D_vs_E10;stage_1W_vs_E10;stage_3W_vs_E10;stage	1	TRUE
ENSGALG00010002888	ENSGALG00010002888	PRJNA729271	-4.52164866	3.44475E-26	4.52164866	stage_E14_vs_E10	ordinary_muscle_development	D_vs_E10;stage_1W_vs_E10;stage_3W_vs_E10;stage	1	TRUE
ENSGALG00010013172	TNFSF13B	PRJNA729271	4.377692946	3.59199E-26	4.377692946	stage_E14_vs_E10	ordinary_muscle_development	D_vs_E10;stage_1W_vs_E10;stage_3W_vs_E10;stage	1	TRUE
ENSGALG00010029430	FN3KRP	PRJNA729271	-1.06406984	3.66056E-26	1.06406984	stage_E14_vs_E10	ordinary_muscle_development	D_vs_E10;stage_1W_vs_E10;stage_3W_vs_E10;stage	1	TRUE
ENSGALG00010024148	UGGT1	PRJNA729271	-0.639303726	3.70254E-26	0.639303726	stage_E14_vs_E10	ordinary_muscle_development		0	FALSE
ENSGALG00010027775	MVB12B	PRJNA729271	0.943952661	3.7204E-26	0.943952661	stage_E14_vs_E10	ordinary_muscle_development		0	FALSE
ENSGALG00010029183	ENSGALG00010029183	PRJNA729271	1.488332289	3.85619E-26	1.488332289	stage_E14_vs_E10	ordinary_muscle_development	D_vs_E10;stage_1W_vs_E10;stage_3W_vs_E10;stage	1	TRUE
ENSGALG00010023048	ARFGAP1	PRJNA729271	-0.907902872	3.87278E-26	0.907902872	stage_E14_vs_E10	ordinary_muscle_development		0	FALSE
ENSGALG00010008083	FANCA	PRJNA729271	-2.95303572	3.93964E-26	2.95303572	stage_E14_vs_E10	ordinary_muscle_development	D_vs_E10;stage_1W_vs_E10;stage_3W_vs_E10;stage	1	TRUE
ENSGALG00010004095	ANKRD50	PRJNA729271	-1.221400663	3.93964E-26	1.221400663	stage_E14_vs_E10	ordinary_muscle_development	D_vs_E10;stage_1W_vs_E10;stage_3W_vs_E10;stage	1	TRUE
ENSGALG00010018573	SEC23B	PRJNA729271	-0.857269736	4.01003E-26	0.857269736	stage_E14_vs_E10	ordinary_muscle_development		0	FALSE
ENSGALG00010029925	GGNBP2	PRJNA729271	1.328792337	4.02606E-26	1.328792337	stage_E14_vs_E10	ordinary_muscle_development	D_vs_E10;stage_1W_vs_E10;stage_3W_vs_E10;stage	1	TRUE
ENSGALG00010025645	NBEAL2	PRJNA729271	1.985673071	4.1273E-26	1.985673071	stage_E14_vs_E10	ordinary_muscle_development	D_vs_E10;stage_1W_vs_E10;stage_3W_vs_E10;stage	1	TRUE
ENSGALG00010022176	ARL2BPL	PRJNA729271	-1.638185049	4.35665E-26	1.638185049	stage_E14_vs_E10	ordinary_muscle_development	D_vs_E10;stage_1W_vs_E10;stage_3W_vs_E10;stage	1	TRUE
ENSGALG00010005861	MPC1L	PRJNA729271	1.416091944	4.40469E-26	1.416091944	stage_E14_vs_E10	ordinary_muscle_development	D_vs_E10;stage_1W_vs_E10;stage_3W_vs_E10;stage	1	TRUE
ENSGALG00010024667	RBM7	PRJNA729271	1.44486793	4.42519E-26	1.44486793	stage_E14_vs_E10	ordinary_muscle_development	D_vs_E10;stage_1W_vs_E10;stage_3W_vs_E10;stage	1	TRUE
ENSGALG00010015098	HAVCR1	PRJNA729271	2.279107792	4.52064E-26	2.279107792	stage_E14_vs_E10	ordinary_muscle_development	D_vs_E10;stage_1W_vs_E10;stage_3W_vs_E10;stage	1	TRUE
ENSGALG00010001177	SEH1L	PRJNA729271	-0.780666986	4.89108E-26	0.780666986	stage_E14_vs_E10	ordinary_muscle_development		0	FALSE
ENSGALG00010003191	CC2D1A	PRJNA729271	1.70592569	4.9626E-26	1.70592569	stage_E14_vs_E10	ordinary_muscle_development	D_vs_E10;stage_1W_vs_E10;stage_3W_vs_E10;stage	1	TRUE
ENSGALG00010017579	P3H2	PRJNA729271	1.515115982	5.33867E-26	1.515115982	stage_E14_vs_E10	ordinary_muscle_development	D_vs_E10;stage_1W_vs_E10;stage_3W_vs_E10;stage	1	TRUE
ENSGALG00010008183	ALG10	PRJNA729271	-0.945032018	5.42774E-26	0.945032018	stage_E14_vs_E10	ordinary_muscle_development		0	FALSE
ENSGALG00010005303	ENSGALG00010005303	PRJNA729271	2.957160945	5.44315E-26	2.957160945	stage_E14_vs_E10	ordinary_muscle_development	D_vs_E10;stage_1W_vs_E10;stage_3W_vs_E10;stage	1	TRUE
ENSGALG00010022224	NHEJ1	PRJNA729271	1.497151977	5.75334E-26	1.497151977	stage_E14_vs_E10	ordinary_muscle_development	D_vs_E10;stage_1W_vs_E10;stage_3W_vs_E10;stage	1	TRUE
ENSGALG00010002809	SH3KBP1	PRJNA729271	-1.189817996	5.76218E-26	1.189817996	stage_E14_vs_E10	ordinary_muscle_development	D_vs_E10;stage_1W_vs_E10;stage_3W_vs_E10;stage	1	TRUE
ENSGALG00010004147	SPRYD7	PRJNA729271	0.969471064	5.83423E-26	0.969471064	stage_E14_vs_E10	ordinary_muscle_development		0	FALSE
ENSGALG00010012334	ARHGEF39	PRJNA729271	-2.62167171	5.92654E-26	2.62167171	stage_E14_vs_E10	ordinary_muscle_development	D_vs_E10;stage_1W_vs_E10;stage_3W_vs_E10;stage	1	TRUE
ENSGALG00010013396	FYB1	PRJNA729271	1.986263523	5.92929E-26	1.986263523	stage_E14_vs_E10	ordinary_muscle_development	D_vs_E10;stage_1W_vs_E10;stage_3W_vs_E10;stage	1	TRUE
ENSGALG00010028015	DUSP7	PRJNA729271	-1.093831885	5.99084E-26	1.093831885	stage_E14_vs_E10	ordinary_muscle_development	D_vs_E10;stage_1W_vs_E10;stage_3W_vs_E10;stage	1	TRUE
ENSGALG00010010865	ENSGALG00010010865	PRJNA729271	1.671404576	6.04484E-26	1.671404576	stage_E14_vs_E10	ordinary_muscle_development	D_vs_E10;stage_1W_vs_E10;stage_3W_vs_E10;stage	1	TRUE
ENSGALG00010002633	ENSGALG00010002633	PRJNA729271	-1.446781868	6.05724E-26	1.446781868	stage_E14_vs_E10	ordinary_muscle_development	D_vs_E10;stage_1W_vs_E10;stage_3W_vs_E10;stage	1	TRUE
ENSGALG00010021545	CYB5R2	PRJNA729271	-1.528363062	6.1314E-26	1.528363062	stage_E14_vs_E10	ordinary_muscle_development	D_vs_E10;stage_1W_vs_E10;stage_3W_vs_E10;stage	1	TRUE
ENSGALG00010024192	ALG6	PRJNA729271	-1.0452122	6.18104E-26	1.0452122	stage_E14_vs_E10	ordinary_muscle_development	D_vs_E10;stage_1W_vs_E10;stage_3W_vs_E10;stage	1	TRUE
ENSGALG00010027722	GNAI3	PRJNA729271	-0.913668338	6.30438E-26	0.913668338	stage_E14_vs_E10	ordinary_muscle_development		0	FALSE
ENSGALG00010002430	NUMA1	PRJNA729271	1.861083025	6.31328E-26	1.861083025	stage_E14_vs_E10	ordinary_muscle_development	D_vs_E10;stage_1W_vs_E10;stage_3W_vs_E10;stage	1	TRUE
ENSGALG00010000306	ENSGALG00010000306	PRJNA729271	2.185878217	6.43545E-26	2.185878217	stage_E14_vs_E10	ordinary_muscle_development	D_vs_E10;stage_1W_vs_E10;stage_3W_vs_E10;stage	1	TRUE
ENSGALG00010023514	ULK3	PRJNA729271	1.104483678	6.45378E-26	1.104483678	stage_E14_vs_E10	ordinary_muscle_development	D_vs_E10;stage_1W_vs_E10;stage_3W_vs_E10;stage	1	TRUE
ENSGALG00010022831	LRRC8C	PRJNA729271	0.947777179	6.59395E-26	0.947777179	stage_E14_vs_E10	ordinary_muscle_development		0	FALSE
ENSGALG00010002760	RAB2A	PRJNA729271	0.986680314	6.59783E-26	0.986680314	stage_E14_vs_E10	ordinary_muscle_development		0	FALSE
ENSGALG00010023002	ATG16L1	PRJNA729271	1.426375194	6.64456E-26	1.426375194	stage_E14_vs_E10	ordinary_muscle_development	D_vs_E10;stage_1W_vs_E10;stage_3W_vs_E10;stage	1	TRUE
ENSGALG00010000468	TESC	PRJNA729271	-3.854493448	6.85395E-26	3.854493448	stage_E14_vs_E10	ordinary_muscle_development	D_vs_E10;stage_1W_vs_E10;stage_3W_vs_E10;stage	1	TRUE
ENSGALG00010017248	TECPR2	PRJNA729271	0.892749086	7.11045E-26	0.892749086	stage_E14_vs_E10	ordinary_muscle_development		0	FALSE
ENSGALG00010028113	PIP5K1C	PRJNA729271	-1.05470716	7.12608E-26	1.05470716	stage_E14_vs_E10	ordinary_muscle_development	D_vs_E10;stage_1W_vs_E10;stage_3W_vs_E10;stage	1	TRUE
ENSGALG00010017987	ERI2	PRJNA729271	-2.728964754	7.12811E-26	2.728964754	stage_E14_vs_E10	ordinary_muscle_development	D_vs_E10;stage_1W_vs_E10;stage_3W_vs_E10;stage	1	TRUE
ENSGALG00010029928	USP43	PRJNA729271	1.407216424	7.19353E-26	1.407216424	stage_E14_vs_E10	ordinary_muscle_development	D_vs_E10;stage_1W_vs_E10;stage_3W_vs_E10;stage	1	TRUE
ENSGALG00010003714	ORC3	PRJNA729271	-1.203791633	7.27128E-26	1.203791633	stage_E14_vs_E10	ordinary_muscle_development	D_vs_E10;stage_1W_vs_E10;stage_3W_vs_E10;stage	1	TRUE
ENSGALG00010023071	POLR2M	PRJNA729271	0.865116593	7.57336E-26	0.865116593	stage_E14_vs_E10	ordinary_muscle_development		0	FALSE
ENSGALG00010028078	LAMTOR2	PRJNA729271	1.388648698	7.86868E-26	1.388648698	stage_E14_vs_E10	ordinary_muscle_development	D_vs_E10;stage_1W_vs_E10;stage_3W_vs_E10;stage	1	TRUE
ENSGALG00010008043	IL17REL	PRJNA729271	-4.344285106	7.95489E-26	4.344285106	stage_E14_vs_E10	ordinary_muscle_development	D_vs_E10;stage_1W_vs_E10;stage_3W_vs_E10;stage	1	TRUE
ENSGALG00010020544	BMERB1	PRJNA729271	-1.764046284	8.01027E-26	1.764046284	stage_E14_vs_E10	ordinary_muscle_development	D_vs_E10;stage_1W_vs_E10;stage_3W_vs_E10;stage	1	TRUE
ENSGALG00010013251	MTA1	PRJNA729271	-0.884404927	8.2067E-26	0.884404927	stage_E14_vs_E10	ordinary_muscle_development		0	FALSE
ENSGALG00010015100	MOB4	PRJNA729271	0.94477921	8.21044E-26	0.94477921	stage_E14_vs_E10	ordinary_muscle_development		0	FALSE
ENSGALG00010029785	ENSGALG00010029785	PRJNA729271	2.617799043	8.26834E-26	2.617799043	stage_E14_vs_E10	ordinary_muscle_development	D_vs_E10;stage_1W_vs_E10;stage_3W_vs_E10;stage	1	TRUE
ENSGALG00010021727	MVK	PRJNA729271	-1.117043924	8.49778E-26	1.117043924	stage_E14_vs_E10	ordinary_muscle_development	D_vs_E10;stage_1W_vs_E10;stage_3W_vs_E10;stage	1	TRUE
ENSGALG00010010445	LMAN1L	PRJNA729271	-0.888855494	8.60558E-26	0.888855494	stage_E14_vs_E10	ordinary_muscle_development		0	FALSE
ENSGALG00010002982	HAND2	PRJNA729271	-3.356932655	8.76915E-26	3.356932655	stage_E14_vs_E10	ordinary_muscle_development	D_vs_E10;stage_1W_vs_E10;stage_3W_vs_E10;stage	1	TRUE
ENSGALG00010018885	EFEMP1	PRJNA729271	2.219483506	9.14113E-26	2.219483506	stage_E14_vs_E10	ordinary_muscle_development	D_vs_E10;stage_1W_vs_E10;stage_3W_vs_E10;stage	1	TRUE
ENSGALG00010007271	CADM2	PRJNA729271	-3.422970217	9.65755E-26	3.422970217	stage_E14_vs_E10	ordinary_muscle_development	D_vs_E10;stage_1W_vs_E10;stage_3W_vs_E10;stage	1	TRUE
ENSGALG00010010870	NOL6	PRJNA729271	-1.329396432	9.68402E-26	1.329396432	stage_E14_vs_E10	ordinary_muscle_development	D_vs_E10;stage_1W_vs_E10;stage_3W_vs_E10;stage	1	TRUE
ENSGALG00010018525	ST8SIA2	PRJNA729271	-3.125611084	9.79366E-26	3.125611084	stage_E14_vs_E10	ordinary_muscle_development	D_vs_E10;stage_1W_vs_E10;stage_3W_vs_E10;stage	1	TRUE
ENSGALG00010028084	ENG	PRJNA729271	2.040733214	9.89717E-26	2.040733214	stage_E14_vs_E10	ordinary_muscle_development	D_vs_E10;stage_1W_vs_E10;stage_3W_vs_E10;stage	1	TRUE
ENSGALG00010011111	MRPS14	PRJNA729271	1.237577189	9.95123E-26	1.237577189	stage_E14_vs_E10	ordinary_muscle_development	D_vs_E10;stage_1W_vs_E10;stage_3W_vs_E10;stage	1	TRUE
ENSGALG00010013859	MYOC	PRJNA729271	2.593393616	9.97996E-26	2.593393616	stage_E14_vs_E10	ordinary_muscle_development	D_vs_E10;stage_1W_vs_E10;stage_3W_vs_E10;stage	1	TRUE
ENSGALG00010021734	PARD3B	PRJNA729271	0.679278909	1.03127E-25	0.679278909	stage_E14_vs_E10	ordinary_muscle_development		0	FALSE
ENSGALG00010020300	EHD4	PRJNA729271	1.370388202	1.04065E-25	1.370388202	stage_E14_vs_E10	ordinary_muscle_development	D_vs_E10;stage_1W_vs_E10;stage_3W_vs_E10;stage	1	TRUE
ENSGALG00010004094	DGKB	PRJNA729271	-2.196855846	1.04306E-25	2.196855846	stage_E14_vs_E10	ordinary_muscle_development	D_vs_E10;stage_1W_vs_E10;stage_3W_vs_E10;stage	1	TRUE
ENSGALG00010026588	CCND3	PRJNA729271	1.514484096	1.04748E-25	1.514484096	stage_E14_vs_E10	ordinary_muscle_development	D_vs_E10;stage_1W_vs_E10;stage_3W_vs_E10;stage	1	TRUE
ENSGALG00010003268	SMS	PRJNA729271	-0.685345428	1.05347E-25	0.685345428	stage_E14_vs_E10	ordinary_muscle_development		0	FALSE
ENSGALG00010024854	C12orf65	PRJNA729271	1.585584022	1.06251E-25	1.585584022	stage_E14_vs_E10	ordinary_muscle_development	D_vs_E10;stage_1W_vs_E10;stage_3W_vs_E10;stage	1	TRUE
ENSGALG00010025351	CREB1	PRJNA729271	-0.718755593	1.12559E-25	0.718755593	stage_E14_vs_E10	ordinary_muscle_development		0	FALSE
ENSGALG00010006084	TMEM68	PRJNA729271	-1.653525081	1.15211E-25	1.653525081	stage_E14_vs_E10	ordinary_muscle_development	D_vs_E10;stage_1W_vs_E10;stage_3W_vs_E10;stage	1	TRUE
ENSGALG00010019017	IRAK2	PRJNA729271	0.871357867	1.182E-25	0.871357867	stage_E14_vs_E10	ordinary_muscle_development		0	FALSE
ENSGALG00010002914	SLC27A1	PRJNA729271	3.241261457	1.185E-25	3.241261457	stage_E14_vs_E10	ordinary_muscle_development	D_vs_E10;stage_1W_vs_E10;stage_3W_vs_E10;stage	1	TRUE
ENSGALG00010005800	FRMD4A	PRJNA729271	-1.167520016	1.18662E-25	1.167520016	stage_E14_vs_E10	ordinary_muscle_development	D_vs_E10;stage_1W_vs_E10;stage_3W_vs_E10;stage	1	TRUE
ENSGALG00010017898	NHERF2	PRJNA729271	1.216087117	1.19186E-25	1.216087117	stage_E14_vs_E10	ordinary_muscle_development	D_vs_E10;stage_1W_vs_E10;stage_3W_vs_E10;stage	1	TRUE
ENSGALG00010009359	REEP3	PRJNA729271	-1.183979569	1.1977E-25	1.183979569	stage_E14_vs_E10	ordinary_muscle_development	D_vs_E10;stage_1W_vs_E10;stage_3W_vs_E10;stage	1	TRUE
ENSGALG00010002409	CYCS	PRJNA729271	1.769824073	1.21591E-25	1.769824073	stage_E14_vs_E10	ordinary_muscle_development	D_vs_E10;stage_1W_vs_E10;stage_3W_vs_E10;stage	1	TRUE
ENSGALG00010013598	PAQR7	PRJNA729271	2.383882382	1.22611E-25	2.383882382	stage_E14_vs_E10	ordinary_muscle_development	D_vs_E10;stage_1W_vs_E10;stage_3W_vs_E10;stage	1	TRUE
ENSGALG00010019253	HBAD	PRJNA729271	-4.54084542	1.23377E-25	4.54084542	stage_E14_vs_E10	ordinary_muscle_development	D_vs_E10;stage_1W_vs_E10;stage_3W_vs_E10;stage	1	TRUE
ENSGALG00010000058	FURIN	PRJNA729271	1.822382907	1.23433E-25	1.822382907	stage_E14_vs_E10	ordinary_muscle_development	D_vs_E10;stage_1W_vs_E10;stage_3W_vs_E10;stage	1	TRUE
ENSGALG00010012898	FAM114A2	PRJNA729271	1.212963309	1.23452E-25	1.212963309	stage_E14_vs_E10	ordinary_muscle_development	D_vs_E10;stage_1W_vs_E10;stage_3W_vs_E10;stage	1	TRUE
ENSGALG00010017529	MFN2	PRJNA729271	1.256408649	1.24367E-25	1.256408649	stage_E14_vs_E10	ordinary_muscle_development	D_vs_E10;stage_1W_vs_E10;stage_3W_vs_E10;stage	1	TRUE
ENSGALG00010020341	DYNC2LI1	PRJNA729271	-1.407607575	1.2456E-25	1.407607575	stage_E14_vs_E10	ordinary_muscle_development	D_vs_E10;stage_1W_vs_E10;stage_3W_vs_E10;stage	1	TRUE
ENSGALG00010004433	AHR1A	PRJNA729271	-1.69966649	1.26306E-25	1.69966649	stage_E14_vs_E10	ordinary_muscle_development	D_vs_E10;stage_1W_vs_E10;stage_3W_vs_E10;stage	1	TRUE
ENSGALG00010013144	TEDC1	PRJNA729271	-1.874639119	1.26326E-25	1.874639119	stage_E14_vs_E10	ordinary_muscle_development	D_vs_E10;stage_1W_vs_E10;stage_3W_vs_E10;stage	1	TRUE
ENSGALG00010020831	MUL1	PRJNA729271	1.512165206	1.29802E-25	1.512165206	stage_E14_vs_E10	ordinary_muscle_development	D_vs_E10;stage_1W_vs_E10;stage_3W_vs_E10;stage	1	TRUE
ENSGALG00010023715	CD3E	PRJNA729271	3.606165864	1.31446E-25	3.606165864	stage_E14_vs_E10	ordinary_muscle_development	D_vs_E10;stage_1W_vs_E10;stage_3W_vs_E10;stage	1	TRUE
ENSGALG00010000721	ENSGALG00010000721	PRJNA729271	-1.689378538	1.31805E-25	1.689378538	stage_E14_vs_E10	ordinary_muscle_development	D_vs_E10;stage_1W_vs_E10;stage_3W_vs_E10;stage	1	TRUE
ENSGALG00010024902	LARP6	PRJNA729271	1.224798604	1.32951E-25	1.224798604	stage_E14_vs_E10	ordinary_muscle_development	D_vs_E10;stage_1W_vs_E10;stage_3W_vs_E10;stage	1	TRUE
ENSGALG00010005296	PAN3	PRJNA729271	1.071366773	1.33317E-25	1.071366773	stage_E14_vs_E10	ordinary_muscle_development	D_vs_E10;stage_1W_vs_E10;stage_3W_vs_E10;stage	1	TRUE
ENSGALG00010020691	ENSGALG00010020691	PRJNA729271	1.721844563	1.3904E-25	1.721844563	stage_E14_vs_E10	ordinary_muscle_development	D_vs_E10;stage_1W_vs_E10;stage_3W_vs_E10;stage	1	TRUE
ENSGALG00010016411	CTSH	PRJNA729271	1.303533081	1.40225E-25	1.303533081	stage_E14_vs_E10	ordinary_muscle_development	D_vs_E10;stage_1W_vs_E10;stage_3W_vs_E10;stage	1	TRUE
ENSGALG00010008195	ENSGALG00010008195	PRJNA729271	4.324717207	1.42671E-25	4.324717207	stage_E14_vs_E10	ordinary_muscle_development	D_vs_E10;stage_1W_vs_E10;stage_3W_vs_E10;stage	1	TRUE
ENSGALG00010024548	CDC6	PRJNA729271	-3.513225953	1.44716E-25	3.513225953	stage_E14_vs_E10	ordinary_muscle_development	D_vs_E10;stage_1W_vs_E10;stage_3W_vs_E10;stage	1	TRUE
ENSGALG00010005082	ENSGALG00010005082	PRJNA729271	-0.963431122	1.4531E-25	0.963431122	stage_E14_vs_E10	ordinary_muscle_development		0	FALSE
ENSGALG00010024451	PHOSPHO1	PRJNA729271	2.836574036	1.4983E-25	2.836574036	stage_E14_vs_E10	ordinary_muscle_development	D_vs_E10;stage_1W_vs_E10;stage_3W_vs_E10;stage	1	TRUE
ENSGALG00010021250	ISLR	PRJNA729271	1.545018257	1.52719E-25	1.545018257	stage_E14_vs_E10	ordinary_muscle_development	D_vs_E10;stage_1W_vs_E10;stage_3W_vs_E10;stage	1	TRUE
ENSGALG00010022171	FOXP1	PRJNA729271	-1.015335482	1.52781E-25	1.015335482	stage_E14_vs_E10	ordinary_muscle_development	D_vs_E10;stage_1W_vs_E10;stage_3W_vs_E10;stage	1	TRUE
ENSGALG00010017539	KPNA6	PRJNA729271	1.463654806	1.56525E-25	1.463654806	stage_E14_vs_E10	ordinary_muscle_development	D_vs_E10;stage_1W_vs_E10;stage_3W_vs_E10;stage	1	TRUE
ENSGALG00010024882	MYL6	PRJNA729271	-1.74733754	1.57249E-25	1.74733754	stage_E14_vs_E10	ordinary_muscle_development	D_vs_E10;stage_1W_vs_E10;stage_3W_vs_E10;stage	1	TRUE
ENSGALG00010023114	UQCRHL	PRJNA729271	1.772694792	1.58092E-25	1.772694792	stage_E14_vs_E10	ordinary_muscle_development	D_vs_E10;stage_1W_vs_E10;stage_3W_vs_E10;stage	1	TRUE
ENSGALG00010007798	HGSNAT	PRJNA729271	1.024301599	1.61051E-25	1.024301599	stage_E14_vs_E10	ordinary_muscle_development	D_vs_E10;stage_1W_vs_E10;stage_3W_vs_E10;stage	1	TRUE
ENSGALG00010016470	TNIP1	PRJNA729271	1.675173576	1.61936E-25	1.675173576	stage_E14_vs_E10	ordinary_muscle_development	D_vs_E10;stage_1W_vs_E10;stage_3W_vs_E10;stage	1	TRUE
ENSGALG00010011304	COMMD5	PRJNA729271	-0.990153617	1.6724E-25	0.990153617	stage_E14_vs_E10	ordinary_muscle_development		0	FALSE
ENSGALG00010024837	TNFRSF1A	PRJNA729271	1.660186046	1.71252E-25	1.660186046	stage_E14_vs_E10	ordinary_muscle_development	D_vs_E10;stage_1W_vs_E10;stage_3W_vs_E10;stage	1	TRUE
ENSGALG00010029673	TMEM248	PRJNA729271	-0.965406049	1.72911E-25	0.965406049	stage_E14_vs_E10	ordinary_muscle_development		0	FALSE
ENSGALG00010029295	SOCS3	PRJNA729271	2.625684709	1.77712E-25	2.625684709	stage_E14_vs_E10	ordinary_muscle_development	D_vs_E10;stage_1W_vs_E10;stage_3W_vs_E10;stage	1	TRUE
ENSGALG00010021662	RASGRP1	PRJNA729271	1.684773892	1.82237E-25	1.684773892	stage_E14_vs_E10	ordinary_muscle_development	D_vs_E10;stage_1W_vs_E10;stage_3W_vs_E10;stage	1	TRUE
ENSGALG00010007938	BCL9	PRJNA729271	-1.130301169	1.8331E-25	1.130301169	stage_E14_vs_E10	ordinary_muscle_development	D_vs_E10;stage_1W_vs_E10;stage_3W_vs_E10;stage	1	TRUE
ENSGALG00010017600	MXD4	PRJNA729271	1.097223968	1.83913E-25	1.097223968	stage_E14_vs_E10	ordinary_muscle_development	D_vs_E10;stage_1W_vs_E10;stage_3W_vs_E10;stage	1	TRUE
ENSGALG00010020149	FAHD2A	PRJNA729271	1.817386136	1.87306E-25	1.817386136	stage_E14_vs_E10	ordinary_muscle_development	D_vs_E10;stage_1W_vs_E10;stage_3W_vs_E10;stage	1	TRUE
ENSGALG00010010997	LAP3	PRJNA729271	-1.597388086	1.88131E-25	1.597388086	stage_E14_vs_E10	ordinary_muscle_development	D_vs_E10;stage_1W_vs_E10;stage_3W_vs_E10;stage	1	TRUE
ENSGALG00010027299	PGPEP1	PRJNA729271	1.716053073	1.88971E-25	1.716053073	stage_E14_vs_E10	ordinary_muscle_development	D_vs_E10;stage_1W_vs_E10;stage_3W_vs_E10;stage	1	TRUE
ENSGALG00010001183	RARRES1	PRJNA729271	1.988055729	1.89608E-25	1.988055729	stage_E14_vs_E10	ordinary_muscle_development	D_vs_E10;stage_1W_vs_E10;stage_3W_vs_E10;stage	1	TRUE
ENSGALG00010005165	METTL21C	PRJNA729271	5.900941612	1.90259E-25	5.900941612	stage_E14_vs_E10	ordinary_muscle_development	D_vs_E10;stage_1W_vs_E10;stage_3W_vs_E10;stage	1	TRUE
ENSGALG00010015866	ARHGEF6	PRJNA729271	0.746083109	1.90835E-25	0.746083109	stage_E14_vs_E10	ordinary_muscle_development		0	FALSE
ENSGALG00010019622	C2orf42	PRJNA729271	1.481378278	1.97087E-25	1.481378278	stage_E14_vs_E10	ordinary_muscle_development	D_vs_E10;stage_1W_vs_E10;stage_3W_vs_E10;stage	1	TRUE
ENSGALG00010002046	FAM126A	PRJNA729271	-0.939833831	1.99827E-25	0.939833831	stage_E14_vs_E10	ordinary_muscle_development		0	FALSE
ENSGALG00010027148	ELL	PRJNA729271	1.744475923	2.00136E-25	1.744475923	stage_E14_vs_E10	ordinary_muscle_development	D_vs_E10;stage_1W_vs_E10;stage_3W_vs_E10;stage	1	TRUE
ENSGALG00010005792	ADGRG6	PRJNA729271	-1.704317175	2.01785E-25	1.704317175	stage_E14_vs_E10	ordinary_muscle_development	D_vs_E10;stage_1W_vs_E10;stage_3W_vs_E10;stage	1	TRUE
ENSGALG00010021655	USP22	PRJNA729271	-0.99815767	2.02498E-25	0.99815767	stage_E14_vs_E10	ordinary_muscle_development		0	FALSE
ENSGALG00010010205	ARL5A	PRJNA729271	0.870752697	2.08301E-25	0.870752697	stage_E14_vs_E10	ordinary_muscle_development		0	FALSE
ENSGALG00010024855	CAPRIN1	PRJNA729271	-0.766003514	2.08701E-25	0.766003514	stage_E14_vs_E10	ordinary_muscle_development		0	FALSE
ENSGALG00010001695	LMOD2	PRJNA729271	4.984877589	2.08854E-25	4.984877589	stage_E14_vs_E10	ordinary_muscle_development	D_vs_E10;stage_1W_vs_E10;stage_3W_vs_E10;stage	1	TRUE
ENSGALG00010012640	PRRC1	PRJNA729271	-0.790650739	2.10028E-25	0.790650739	stage_E14_vs_E10	ordinary_muscle_development		0	FALSE
ENSGALG00010028591	GABPB2	PRJNA729271	-1.364205836	2.11423E-25	1.364205836	stage_E14_vs_E10	ordinary_muscle_development	D_vs_E10;stage_1W_vs_E10;stage_3W_vs_E10;stage	1	TRUE
ENSGALG00010002279	ENSGALG00010002279	PRJNA729271	7.359841995	2.1202E-25	7.359841995	stage_E14_vs_E10	ordinary_muscle_development	D_vs_E10;stage_1W_vs_E10;stage_3W_vs_E10;stage	1	TRUE
ENSGALG00010003896	MCUB	PRJNA729271	-1.436388829	2.12613E-25	1.436388829	stage_E14_vs_E10	ordinary_muscle_development	D_vs_E10;stage_1W_vs_E10;stage_3W_vs_E10;stage	1	TRUE
ENSGALG00010019297	SLC25A26	PRJNA729271	1.535884997	2.14733E-25	1.535884997	stage_E14_vs_E10	ordinary_muscle_development	D_vs_E10;stage_1W_vs_E10;stage_3W_vs_E10;stage	1	TRUE
ENSGALG00010003437	PNRC1	PRJNA729271	1.90257055	2.16087E-25	1.90257055	stage_E14_vs_E10	ordinary_muscle_development	D_vs_E10;stage_1W_vs_E10;stage_3W_vs_E10;stage	1	TRUE
ENSGALG00010002413	TSSC1	PRJNA729271	-0.926379497	2.17117E-25	0.926379497	stage_E14_vs_E10	ordinary_muscle_development		0	FALSE
ENSGALG00010008096	CPSF1	PRJNA729271	-1.651226762	2.18523E-25	1.651226762	stage_E14_vs_E10	ordinary_muscle_development	D_vs_E10;stage_1W_vs_E10;stage_3W_vs_E10;stage	1	TRUE
ENSGALG00010000800	LMO7	PRJNA729271	1.604089888	2.25403E-25	1.604089888	stage_E14_vs_E10	ordinary_muscle_development	D_vs_E10;stage_1W_vs_E10;stage_3W_vs_E10;stage	1	TRUE
ENSGALG00010024153	KCNQ1	PRJNA729271	2.123404189	2.2892E-25	2.123404189	stage_E14_vs_E10	ordinary_muscle_development	D_vs_E10;stage_1W_vs_E10;stage_3W_vs_E10;stage	1	TRUE
ENSGALG00010008259	MCC	PRJNA729271	-1.164129966	2.3553E-25	1.164129966	stage_E14_vs_E10	ordinary_muscle_development	D_vs_E10;stage_1W_vs_E10;stage_3W_vs_E10;stage	1	TRUE
ENSGALG00010029182	HEATR6	PRJNA729271	-0.760223362	2.36526E-25	0.760223362	stage_E14_vs_E10	ordinary_muscle_development		0	FALSE
ENSGALG00010027875	WNT5A	PRJNA729271	-1.790976553	2.41206E-25	1.790976553	stage_E14_vs_E10	ordinary_muscle_development	D_vs_E10;stage_1W_vs_E10;stage_3W_vs_E10;stage	1	TRUE
ENSGALG00010024918	ATG4B	PRJNA729271	-0.807996512	2.44477E-25	0.807996512	stage_E14_vs_E10	ordinary_muscle_development		0	FALSE
ENSGALG00010000901	PCDH9	PRJNA729271	-2.6773938	2.4642E-25	2.6773938	stage_E14_vs_E10	ordinary_muscle_development	D_vs_E10;stage_1W_vs_E10;stage_3W_vs_E10;stage	1	TRUE
ENSGALG00010010461	SLC7A5	PRJNA729271	2.754301889	2.55749E-25	2.754301889	stage_E14_vs_E10	ordinary_muscle_development	D_vs_E10;stage_1W_vs_E10;stage_3W_vs_E10;stage	1	TRUE
ENSGALG00010004622	ENSGALG00010004622	PRJNA729271	2.351001929	2.65592E-25	2.351001929	stage_E14_vs_E10	ordinary_muscle_development	D_vs_E10;stage_1W_vs_E10;stage_3W_vs_E10;stage	1	TRUE
ENSGALG00010012360	ROS1	PRJNA729271	3.863618436	2.69798E-25	3.863618436	stage_E14_vs_E10	ordinary_muscle_development	D_vs_E10;stage_1W_vs_E10;stage_3W_vs_E10;stage	1	TRUE
ENSGALG00010020530	SRSF5A	PRJNA729271	-0.734575094	2.69856E-25	0.734575094	stage_E14_vs_E10	ordinary_muscle_development		0	FALSE
ENSGALG00010028914	TLR4	PRJNA729271	2.395234048	2.72202E-25	2.395234048	stage_E14_vs_E10	ordinary_muscle_development	D_vs_E10;stage_1W_vs_E10;stage_3W_vs_E10;stage	1	TRUE
ENSGALG00010001193	PRELID3A	PRJNA729271	-2.832534505	2.74154E-25	2.832534505	stage_E14_vs_E10	ordinary_muscle_development	D_vs_E10;stage_1W_vs_E10;stage_3W_vs_E10;stage	1	TRUE
ENSGALG00010007895	GOLGB1	PRJNA729271	-1.186666168	2.74507E-25	1.186666168	stage_E14_vs_E10	ordinary_muscle_development	D_vs_E10;stage_1W_vs_E10;stage_3W_vs_E10;stage	1	TRUE
ENSGALG00010028221	TNFAIP8L1	PRJNA729271	-2.041157749	2.74772E-25	2.041157749	stage_E14_vs_E10	ordinary_muscle_development	D_vs_E10;stage_1W_vs_E10;stage_3W_vs_E10;stage	1	TRUE
ENSGALG00010011443	ENSGALG00010011443	PRJNA729271	-1.877137571	2.77388E-25	1.877137571	stage_E14_vs_E10	ordinary_muscle_development	D_vs_E10;stage_1W_vs_E10;stage_3W_vs_E10;stage	1	TRUE
ENSGALG00010000017	COX1	PRJNA729271	2.869849391	2.81829E-25	2.869849391	stage_E14_vs_E10	ordinary_muscle_development	D_vs_E10;stage_1W_vs_E10;stage_3W_vs_E10;stage	1	TRUE
ENSGALG00010008213	ISCA1	PRJNA729271	1.230620107	2.84729E-25	1.230620107	stage_E14_vs_E10	ordinary_muscle_development	D_vs_E10;stage_1W_vs_E10;stage_3W_vs_E10;stage	1	TRUE
ENSGALG00010025999	DBF4B	PRJNA729271	-1.769356703	2.85713E-25	1.769356703	stage_E14_vs_E10	ordinary_muscle_development	D_vs_E10;stage_1W_vs_E10;stage_3W_vs_E10;stage	1	TRUE
ENSGALG00010016400	ENSGALG00010016400	PRJNA729271	0.912891892	2.87334E-25	0.912891892	stage_E14_vs_E10	ordinary_muscle_development		0	FALSE
ENSGALG00010004907	ENSGALG00010004907	PRJNA729271	-1.263843075	2.90149E-25	1.263843075	stage_E14_vs_E10	ordinary_muscle_development	D_vs_E10;stage_1W_vs_E10;stage_3W_vs_E10;stage	1	TRUE
ENSGALG00010028011	ENSGALG00010028011	PRJNA729271	-1.046721812	2.93292E-25	1.046721812	stage_E14_vs_E10	ordinary_muscle_development	D_vs_E10;stage_1W_vs_E10;stage_3W_vs_E10;stage	1	TRUE
ENSGALG00010015749	ATR	PRJNA729271	-1.427394402	2.94176E-25	1.427394402	stage_E14_vs_E10	ordinary_muscle_development	D_vs_E10;stage_1W_vs_E10;stage_3W_vs_E10;stage	1	TRUE
ENSGALG00010005182	NEPRO	PRJNA729271	-0.980751883	2.98854E-25	0.980751883	stage_E14_vs_E10	ordinary_muscle_development		0	FALSE
ENSGALG00010015483	SGCD	PRJNA729271	1.70847499	3.04145E-25	1.70847499	stage_E14_vs_E10	ordinary_muscle_development	D_vs_E10;stage_1W_vs_E10;stage_3W_vs_E10;stage	1	TRUE
ENSGALG00010028872	SLC2A6	PRJNA729271	3.448748388	3.05193E-25	3.448748388	stage_E14_vs_E10	ordinary_muscle_development	D_vs_E10;stage_1W_vs_E10;stage_3W_vs_E10;stage	1	TRUE
ENSGALG00010024908	ENSGALG00010024908	PRJNA729271	1.324570222	3.05782E-25	1.324570222	stage_E14_vs_E10	ordinary_muscle_development	D_vs_E10;stage_1W_vs_E10;stage_3W_vs_E10;stage	1	TRUE
ENSGALG00010009098	CAD	PRJNA729271	-2.090064938	3.05784E-25	2.090064938	stage_E14_vs_E10	ordinary_muscle_development	D_vs_E10;stage_1W_vs_E10;stage_3W_vs_E10;stage	1	TRUE
ENSGALG00010022608	P3H4	PRJNA729271	-2.198277924	3.16538E-25	2.198277924	stage_E14_vs_E10	ordinary_muscle_development	D_vs_E10;stage_1W_vs_E10;stage_3W_vs_E10;stage	1	TRUE
ENSGALG00010024510	CNN3	PRJNA729271	-1.065667145	3.21686E-25	1.065667145	stage_E14_vs_E10	ordinary_muscle_development	D_vs_E10;stage_1W_vs_E10;stage_3W_vs_E10;stage	1	TRUE
ENSGALG00010003848	ESD	PRJNA729271	1.307171903	3.26857E-25	1.307171903	stage_E14_vs_E10	ordinary_muscle_development	D_vs_E10;stage_1W_vs_E10;stage_3W_vs_E10;stage	1	TRUE
ENSGALG00010004935	SVIL	PRJNA729271	0.731477763	3.28214E-25	0.731477763	stage_E14_vs_E10	ordinary_muscle_development		0	FALSE
ENSGALG00010006521	ZNF410	PRJNA729271	-1.134972991	3.28495E-25	1.134972991	stage_E14_vs_E10	ordinary_muscle_development	D_vs_E10;stage_1W_vs_E10;stage_3W_vs_E10;stage	1	TRUE

ENSGALG00010023377	OSER1	PRJNA729271	1.569503885	3.41301E-25	1.569503885	stage_E14_vs_E10	ordinary_muscle_development	D_vs_E10;stage_1W_vs_E10;stage_3W_vs_E10;stage	1	TRUE
ENSGALG00010013150	GNS	PRJNA729271	1.084606427	3.41813E-25	1.084606427	stage_E14_vs_E10	ordinary_muscle_development	D_vs_E10;stage_1W_vs_E10;stage_3W_vs_E10;stage	1	TRUE
ENSGALG00010023428	HOXB3	PRJNA729271	-2.088151508	3.45678E-25	2.088151508	stage_E14_vs_E10	ordinary_muscle_development	D_vs_E10;stage_1W_vs_E10;stage_3W_vs_E10;stage	1	TRUE
ENSGALG00010007564	IRX4	PRJNA729271	-9.364572528	3.60092E-25	9.364572528	stage_E14_vs_E10	ordinary_muscle_development	D_vs_E10;stage_1W_vs_E10;stage_3W_vs_E10;stage	1	TRUE
ENSGALG00010024132	ENSGALG00010024132	PRJNA729271	-1.592215165	3.65967E-25	1.592215165	stage_E14_vs_E10	ordinary_muscle_development	D_vs_E10;stage_1W_vs_E10;stage_3W_vs_E10;stage	1	TRUE
ENSGALG00010013226	DUSP6	PRJNA729271	-1.20744786	3.76562E-25	1.20744786	stage_E14_vs_E10	ordinary_muscle_development	D_vs_E10;stage_1W_vs_E10;stage_3W_vs_E10;stage	1	TRUE
ENSGALG00010019961	HTATSF1	PRJNA729271	-0.742340444	3.82771E-25	0.742340444	stage_E14_vs_E10	ordinary_muscle_development		0	FALSE
ENSGALG00010013523	TMEM178B	PRJNA729271	-2.106796647	3.8444E-25	2.106796647	stage_E14_vs_E10	ordinary_muscle_development	D_vs_E10;stage_1W_vs_E10;stage_3W_vs_E10;stage	1	TRUE
ENSGALG00010028474	PEX19	PRJNA729271	1.246558244	3.87758E-25	1.246558244	stage_E14_vs_E10	ordinary_muscle_development	D_vs_E10;stage_1W_vs_E10;stage_3W_vs_E10;stage	1	TRUE
ENSGALG00010020918	ENSGALG00010020918	PRJNA729271	-3.939365689	3.898E-25	3.939365689	stage_E14_vs_E10	ordinary_muscle_development	D_vs_E10;stage_1W_vs_E10;stage_3W_vs_E10;stage	1	TRUE
ENSGALG00010020117	AKAP13	PRJNA729271	-1.082525787	3.91445E-25	1.082525787	stage_E14_vs_E10	ordinary_muscle_development	D_vs_E10;stage_1W_vs_E10;stage_3W_vs_E10;stage	1	TRUE
ENSGALG00010022518	ECHS1	PRJNA729271	1.175493047	3.9156E-25	1.175493047	stage_E14_vs_E10	ordinary_muscle_development	D_vs_E10;stage_1W_vs_E10;stage_3W_vs_E10;stage	1	TRUE
ENSGALG00010012674	ENSGALG00010012674	PRJNA729271	1.335236072	3.94387E-25	1.335236072	stage_E14_vs_E10	ordinary_muscle_development	D_vs_E10;stage_1W_vs_E10;stage_3W_vs_E10;stage	1	TRUE
ENSGALG00010010688	IQGAP2	PRJNA729271	-1.794141473	3.95306E-25	1.794141473	stage_E14_vs_E10	ordinary_muscle_development	D_vs_E10;stage_1W_vs_E10;stage_3W_vs_E10;stage	1	TRUE
ENSGALG00010019336	DEXI	PRJNA729271	2.355653693	3.9991E-25	2.355653693	stage_E14_vs_E10	ordinary_muscle_development	D_vs_E10;stage_1W_vs_E10;stage_3W_vs_E10;stage	1	TRUE
ENSGALG00010024267	MAP3K12	PRJNA729271	-2.276574532	4.0025E-25	2.276574532	stage_E14_vs_E10	ordinary_muscle_development	D_vs_E10;stage_1W_vs_E10;stage_3W_vs_E10;stage	1	TRUE
ENSGALG00010027133	CELF5	PRJNA729271	-3.966212577	4.01854E-25	3.966212577	stage_E14_vs_E10	ordinary_muscle_development	D_vs_E10;stage_1W_vs_E10;stage_3W_vs_E10;stage	1	TRUE
ENSGALG00010001122	P2RY14	PRJNA729271	4.459211939	4.02483E-25	4.459211939	stage_E14_vs_E10	ordinary_muscle_development	D_vs_E10;stage_1W_vs_E10;stage_3W_vs_E10;stage	1	TRUE
ENSGALG00010021766	AGRN	PRJNA729271	-1.583040891	4.0689E-25	1.583040891	stage_E14_vs_E10	ordinary_muscle_development	D_vs_E10;stage_1W_vs_E10;stage_3W_vs_E10;stage	1	TRUE
ENSGALG00010014411	ATP13A3	PRJNA729271	-0.866953688	4.10125E-25	0.866953688	stage_E14_vs_E10	ordinary_muscle_development		0	FALSE
ENSGALG00010000674	GRB10	PRJNA729271	1.780067861	4.10725E-25	1.780067861	stage_E14_vs_E10	ordinary_muscle_development	D_vs_E10;stage_1W_vs_E10;stage_3W_vs_E10;stage	1	TRUE
ENSGALG00010002646	FOXO1	PRJNA729271	1.775831587	4.11187E-25	1.775831587	stage_E14_vs_E10	ordinary_muscle_development	D_vs_E10;stage_1W_vs_E10;stage_3W_vs_E10;stage	1	TRUE
ENSGALG00010012160	SYT4	PRJNA729271	-7.14286062	4.13822E-25	7.14286062	stage_E14_vs_E10	ordinary_muscle_development	D_vs_E10;stage_1W_vs_E10;stage_3W_vs_E10;stage	1	TRUE
ENSGALG00010018183	CYP17A1	PRJNA729271	2.977605988	4.15812E-25	2.977605988	stage_E14_vs_E10	ordinary_muscle_development	D_vs_E10;stage_1W_vs_E10;stage_3W_vs_E10;stage	1	TRUE
ENSGALG00010024407	HIRA	PRJNA729271	-0.868684691	4.21703E-25	0.868684691	stage_E14_vs_E10	ordinary_muscle_development		0	FALSE
ENSGALG00010017120	ERN2	PRJNA729271	1.481398081	4.2941E-25	1.481398081	stage_E14_vs_E10	ordinary_muscle_development	D_vs_E10;stage_1W_vs_E10;stage_3W_vs_E10;stage	1	TRUE
ENSGALG00010028541	ENSGALG00010028541	PRJNA729271	2.859097306	4.32386E-25	2.859097306	stage_E14_vs_E10	ordinary_muscle_development	D_vs_E10;stage_1W_vs_E10;stage_3W_vs_E10;stage	1	TRUE
ENSGALG00010008988	PPM1G	PRJNA729271	-0.848030046	4.3318E-25	0.848030046	stage_E14_vs_E10	ordinary_muscle_development		0	FALSE
ENSGALG00010008162	HSBP1L1	PRJNA729271	2.005535335	4.42935E-25	2.005535335	stage_E14_vs_E10	ordinary_muscle_development	D_vs_E10;stage_1W_vs_E10;stage_3W_vs_E10;stage	1	TRUE
ENSGALG00010009972	PTK2B	PRJNA729271	2.70600768	4.45062E-25	2.70600768	stage_E14_vs_E10	ordinary_muscle_development	D_vs_E10;stage_1W_vs_E10;stage_3W_vs_E10;stage	1	TRUE
ENSGALG00010012320	DPY19L4	PRJNA729271	-0.647439257	4.4588E-25	0.647439257	stage_E14_vs_E10	ordinary_muscle_development		0	FALSE
ENSGALG00010020003	ENSGALG00010020003	PRJNA729271	1.974271611	4.47646E-25	1.974271611	stage_E14_vs_E10	ordinary_muscle_development	D_vs_E10;stage_1W_vs_E10;stage_3W_vs_E10;stage	1	TRUE
ENSGALG00010029525	SLC38A10	PRJNA729271	-1.457539217	4.50567E-25	1.457539217	stage_E14_vs_E10	ordinary_muscle_development	D_vs_E10;stage_1W_vs_E10;stage_3W_vs_E10;stage	1	TRUE
ENSGALG00010011458	TPGS2	PRJNA729271	-1.400805894	4.55253E-25	1.400805894	stage_E14_vs_E10	ordinary_muscle_development	D_vs_E10;stage_1W_vs_E10;stage_3W_vs_E10;stage	1	TRUE
ENSGALG00010020636	NBL1	PRJNA729271	1.880456044	4.60798E-25	1.880456044	stage_E14_vs_E10	ordinary_muscle_development	D_vs_E10;stage_1W_vs_E10;stage_3W_vs_E10;stage	1	TRUE
ENSGALG00010004308	DSG2	PRJNA729271	-5.640497205	4.80936E-25	5.640497205	stage_E14_vs_E10	ordinary_muscle_development	D_vs_E10;stage_1W_vs_E10;stage_3W_vs_E10;stage	1	TRUE
ENSGALG00010028894	AKNA	PRJNA729271	-1.712412417	4.95821E-25	1.712412417	stage_E14_vs_E10	ordinary_muscle_development	D_vs_E10;stage_1W_vs_E10;stage_3W_vs_E10;stage	1	TRUE
ENSGALG00010025380	SPC25	PRJNA729271	-2.372892712	5.00646E-25	2.372892712	stage_E14_vs_E10	ordinary_muscle_development	D_vs_E10;stage_1W_vs_E10;stage_3W_vs_E10;stage	1	TRUE
ENSGALG00010016114	HAUS6	PRJNA729271	-1.596265271	5.04121E-25	1.596265271	stage_E14_vs_E10	ordinary_muscle_development	D_vs_E10;stage_1W_vs_E10;stage_3W_vs_E10;stage	1	TRUE
ENSGALG00010008223	ENSGALG00010008223	PRJNA729271	-1.613108599	5.07248E-25	1.613108599	stage_E14_vs_E10	ordinary_muscle_development	D_vs_E10;stage_1W_vs_E10;stage_3W_vs_E10;stage	1	TRUE
ENSGALG00010024880	NAT10	PRJNA729271	-1.481702025	5.0999E-25	1.481702025	stage_E14_vs_E10	ordinary_muscle_development	D_vs_E10;stage_1W_vs_E10;stage_3W_vs_E10;stage	1	TRUE
ENSGALG00010010828	SPDL1	PRJNA729271	-1.32539469	5.10511E-25	1.32539469	stage_E14_vs_E10	ordinary_muscle_development	D_vs_E10;stage_1W_vs_E10;stage_3W_vs_E10;stage	1	TRUE
ENSGALG00010024745	PMEL	PRJNA729271	-7.805510465	5.15437E-25	7.805510465	stage_E14_vs_E10	ordinary_muscle_development	D_vs_E10;stage_1W_vs_E10;stage_3W_vs_E10;stage	1	TRUE
ENSGALG00010017080	PALB2	PRJNA729271	-1.087909584	5.17484E-25	1.087909584	stage_E14_vs_E10	ordinary_muscle_development	D_vs_E10;stage_1W_vs_E10;stage_3W_vs_E10;stage	1	TRUE
ENSGALG00010009458	SCX	PRJNA729271	-5.398246262	5.20257E-25	5.398246262	stage_E14_vs_E10	ordinary_muscle_development	D_vs_E10;stage_1W_vs_E10;stage_3W_vs_E10;stage	1	TRUE
ENSGALG00010011431	DRAM1	PRJNA729271	4.192714829	5.20584E-25	4.192714829	stage_E14_vs_E10	ordinary_muscle_development	D_vs_E10;stage_1W_vs_E10;stage_3W_vs_E10;stage	1	TRUE
ENSGALG00010013100	ZFPM2	PRJNA729271	-1.99755814	5.20641E-25	1.99755814	stage_E14_vs_E10	ordinary_muscle_development	D_vs_E10;stage_1W_vs_E10;stage_3W_vs_E10;stage	1	TRUE
ENSGALG00010027958	ENSGALG00010027958	PRJNA729271	1.460224774	5.33368E-25	1.460224774	stage_E14_vs_E10	ordinary_muscle_development	D_vs_E10;stage_1W_vs_E10;stage_3W_vs_E10;stage	1	TRUE
ENSGALG00010017782	IGDCC4	PRJNA729271	-1.241434588	5.3787E-25	1.241434588	stage_E14_vs_E10	ordinary_muscle_development	D_vs_E10;stage_1W_vs_E10;stage_3W_vs_E10;stage	1	TRUE
ENSGALG00010010588	SLC25A3	PRJNA729271	1.057684119	5.39383E-25	1.057684119	stage_E14_vs_E10	ordinary_muscle_development	D_vs_E10;stage_1W_vs_E10;stage_3W_vs_E10;stage	1	TRUE
ENSGALG00010013364	OTOGL	PRJNA729271	3.672466075	5.40478E-25	3.672466075	stage_E14_vs_E10	ordinary_muscle_development	D_vs_E10;stage_1W_vs_E10;stage_3W_vs_E10;stage	1	TRUE
ENSGALG00010024327	PPP1R1B	PRJNA729271	4.335715947	5.64115E-25	4.335715947	stage_E14_vs_E10	ordinary_muscle_development	D_vs_E10;stage_1W_vs_E10;stage_3W_vs_E10;stage	1	TRUE
ENSGALG00010019028	TNFRSF25	PRJNA729271	7.344596047	5.68572E-25	7.344596047	stage_E14_vs_E10	ordinary_muscle_development	D_vs_E10;stage_1W_vs_E10;stage_3W_vs_E10;stage	1	TRUE
ENSGALG00010005495	TXN	PRJNA729271	-1.53585818	5.74415E-25	1.53585818	stage_E14_vs_E10	ordinary_muscle_development	D_vs_E10;stage_1W_vs_E10;stage_3W_vs_E10;stage	1	TRUE
ENSGALG00010023931	CSRNP2	PRJNA729271	-1.599857557	5.76421E-25	1.599857557	stage_E14_vs_E10	ordinary_muscle_development	D_vs_E10;stage_1W_vs_E10;stage_3W_vs_E10;stage	1	TRUE
ENSGALG00010016398	SEPTIN6	PRJNA729271	-0.721434051	5.76516E-25	0.721434051	stage_E14_vs_E10	ordinary_muscle_development		0	FALSE
ENSGALG00010009474	ACVR2A	PRJNA729271	-1.00876708	5.88389E-25	1.00876708	stage_E14_vs_E10	ordinary_muscle_development	D_vs_E10;stage_1W_vs_E10;stage_3W_vs_E10;stage	1	TRUE
ENSGALG00010002765	MAP7D2	PRJNA729271	-4.817534161	5.97645E-25	4.817534161	stage_E14_vs_E10	ordinary_muscle_development	D_vs_E10;stage_1W_vs_E10;stage_3W_vs_E10;stage	1	TRUE
ENSGALG00010017695	FNDC5	PRJNA729271	-2.206665381	6.09851E-25	2.206665381	stage_E14_vs_E10	ordinary_muscle_development	D_vs_E10;stage_1W_vs_E10;stage_3W_vs_E10;stage	1	TRUE
ENSGALG00010027501	BAP1	PRJNA729271	-0.706989224	6.29249E-25	0.706989224	stage_E14_vs_E10	ordinary_muscle_development		0	FALSE
ENSGALG00010008426	AP1G1	PRJNA729271	-0.58641292	6.47253E-25	0.58641292	stage_E14_vs_E10	ordinary_muscle_development		0	FALSE
ENSGALG00010028083	ELAVL1	PRJNA729271	-0.524289035	6.53125E-25	0.524289035	stage_E14_vs_E10	ordinary_muscle_development		0	FALSE
ENSGALG00010003815	RASAL3	PRJNA729271	5.871370921	6.54603E-25	5.871370921	stage_E14_vs_E10	ordinary_muscle_development	D_vs_E10;stage_1W_vs_E10;stage_3W_vs_E10;stage	1	TRUE
ENSGALG00010025149	IFT81	PRJNA729271	-1.505435099	6.58045E-25	1.505435099	stage_E14_vs_E10	ordinary_muscle_development	D_vs_E10;stage_1W_vs_E10;stage_3W_vs_E10;stage	1	TRUE
ENSGALG00010007786	SAMSN1	PRJNA729271	2.341548786	6.62355E-25	2.341548786	stage_E14_vs_E10	ordinary_muscle_development	D_vs_E10;stage_1W_vs_E10;stage_3W_vs_E10;stage	1	TRUE
ENSGALG00010024874	JMJD4	PRJNA729271	-0.87160261	6.63033E-25	0.87160261	stage_E14_vs_E10	ordinary_muscle_development		0	FALSE
ENSGALG00010014375	H2BC35	PRJNA729271	-2.584025159	6.65935E-25	2.584025159	stage_E14_vs_E10	ordinary_muscle_development	D_vs_E10;stage_1W_vs_E10;stage_3W_vs_E10;stage	1	TRUE
ENSGALG00010027150	YOD1	PRJNA729271	-0.962263897	6.69007E-25	0.962263897	stage_E14_vs_E10	ordinary_muscle_development		0	FALSE
ENSGALG00010014767	LRRC15	PRJNA729271	2.578922972	6.69282E-25	2.578922972	stage_E14_vs_E10	ordinary_muscle_development	D_vs_E10;stage_1W_vs_E10;stage_3W_vs_E10;stage	1	TRUE
ENSGALG00010004583	XKR4	PRJNA729271	1.123697862	6.79985E-25	1.123697862	stage_E14_vs_E10	ordinary_muscle_development	D_vs_E10;stage_1W_vs_E10;stage_3W_vs_E10;stage	1	TRUE
ENSGALG00010021361	WRAP73	PRJNA729271	-0.995598869	6.83177E-25	0.995598869	stage_E14_vs_E10	ordinary_muscle_development		0	FALSE
ENSGALG00010027713	ENSGALG00010027713	PRJNA729271	1.455998993	6.84643E-25	1.455998993	stage_E14_vs_E10	ordinary_muscle_development	D_vs_E10;stage_1W_vs_E10;stage_3W_vs_E10;stage	1	TRUE
ENSGALG00010025958	HOXC9	PRJNA729271	-5.103378307	6.85029E-25	5.103378307	stage_E14_vs_E10	ordinary_muscle_development	D_vs_E10;stage_1W_vs_E10;stage_3W_vs_E10;stage	1	TRUE
ENSGALG00010012337	ACSS3	PRJNA729271	1.583845361	6.91005E-25	1.583845361	stage_E14_vs_E10	ordinary_muscle_development	D_vs_E10;stage_1W_vs_E10;stage_3W_vs_E10;stage	1	TRUE
ENSGALG00010013679	MFSD4B	PRJNA729271	2.228687393	6.96366E-25	2.228687393	stage_E14_vs_E10	ordinary_muscle_development	D_vs_E10;stage_1W_vs_E10;stage_3W_vs_E10;stage	1	TRUE
ENSGALG00010023918	MFSD14A	PRJNA729271	-0.849264153	6.99874E-25	0.849264153	stage_E14_vs_E10	ordinary_muscle_development		0	FALSE
ENSGALG00010001203	TMX3	PRJNA729271	-1.054370095	7.00145E-25	1.054370095	stage_E14_vs_E10	ordinary_muscle_development	D_vs_E10;stage_1W_vs_E10;stage_3W_vs_E10;stage	1	TRUE
ENSGALG00010004751	PTER	PRJNA729271	1.300137949	7.08488E-25	1.300137949	stage_E14_vs_E10	ordinary_muscle_development	D_vs_E10;stage_1W_vs_E10;stage_3W_vs_E10;stage	1	TRUE
ENSGALG00010024181	FOXD3	PRJNA729271	3.28482537	7.20503E-25	3.28482537	stage_E14_vs_E10	ordinary_muscle_development	D_vs_E10;stage_1W_vs_E10;stage_3W_vs_E10;stage	1	TRUE
ENSGALG00010010884	DNAH7	PRJNA729271	-3.404309638	7.28823E-25	3.404309638	stage_E14_vs_E10	ordinary_muscle_development	D_vs_E10;stage_1W_vs_E10;stage_3W_vs_E10;stage	1	TRUE
ENSGALG00010022886	PLA2G15	PRJNA729271	2.936214728	7.29743E-25	2.936214728	stage_E14_vs_E10	ordinary_muscle_development	D_vs_E10;stage_1W_vs_E10;stage_3W_vs_E10;stage	1	TRUE
ENSGALG00010015937	ENSGALG00010015937	PRJNA729271	1.980774217	7.41884E-25	1.980774217	stage_E14_vs_E10	ordinary_muscle_development	D_vs_E10;stage_1W_vs_E10;stage_3W_vs_E10;stage	1	TRUE
ENSGALG00010015829	SMAD5	PRJNA729271	-0.503027435	7.49069E-25	0.503027435	stage_E14_vs_E10	ordinary_muscle_development		0	FALSE
ENSGALG00010028582	KYAT1	PRJNA729271	2.151576676	7.57401E-25	2.151576676	stage_E14_vs_E10	ordinary_muscle_development	D_vs_E10;stage_1W_vs_E10;stage_3W_vs_E10;stage	1	TRUE
ENSGALG00010028080	MUSTN1	PRJNA729271	4.229014454	7.74163E-25	4.229014454	stage_E14_vs_E10	ordinary_muscle_development	D_vs_E10;stage_1W_vs_E10;stage_3W_vs_E10;stage	1	TRUE
ENSGALG00010010989	TRIQK	PRJNA729271	-1.904422992	7.83951E-25	1.904422992	stage_E14_vs_E10	ordinary_muscle_development	D_vs_E10;stage_1W_vs_E10;stage_3W_vs_E10;stage	1	TRUE
ENSGALG00010016299	SRSF4	PRJNA729271	-1.158732622	7.8731E-25	1.158732622	stage_E14_vs_E10	ordinary_muscle_development	D_vs_E10;stage_1W_vs_E10;stage_3W_vs_E10;stage	1	TRUE
ENSGALG00010020701	PTPN11	PRJNA729271	-0.89469527	7.88104E-25	0.89469527	stage_E14_vs_E10	ordinary_muscle_development		0	FALSE
ENSGALG00010020514	CAMKMT	PRJNA729271	-1.493864374	7.97081E-25	1.493864374	stage_E14_vs_E10	ordinary_muscle_development	D_vs_E10;stage_1W_vs_E10;stage_3W_vs_E10;stage	1	TRUE
ENSGALG00010021087	THBS1	PRJNA729271	-3.790194703	8.01357E-25	3.790194703	stage_E14_vs_E10	ordinary_muscle_development	D_vs_E10;stage_1W_vs_E10;stage_3W_vs_E10;stage	1	TRUE
ENSGALG00010009600	LRRC17	PRJNA729271	-2.273366897	8.10383E-25	2.273366897	stage_E14_vs_E10	ordinary_muscle_development	D_vs_E10;stage_1W_vs_E10;stage_3W_vs_E10;stage	1	TRUE
ENSGALG00010028091	FPGS	PRJNA729271	-1.064218305	8.26829E-25	1.064218305	stage_E14_vs_E10	ordinary_muscle_development	D_vs_E10;stage_1W_vs_E10;stage_3W_vs_E10;stage	1	TRUE
ENSGALG00010023522	EMG1	PRJNA729271	-1.720461847	8.27712E-25	1.720461847	stage_E14_vs_E10	ordinary_muscle_development	D_vs_E10;stage_1W_vs_E10;stage_3W_vs_E10;stage	1	TRUE
ENSGALG00010015889	SH3BP5	PRJNA729271	1.720730123	8.45927E-25	1.720730123	stage_E14_vs_E10	ordinary_muscle_development	D_vs_E10;stage_1W_vs_E10;stage_3W_vs_E10;stage	1	TRUE
ENSGALG00010017591	ESR2	PRJNA729271	-3.268546583	8.50815E-25	3.268546583	stage_E14_vs_E10	ordinary_muscle_development	D_vs_E10;stage_1W_vs_E10;stage_3W_vs_E10;stage	1	TRUE
ENSGALG00010006843	STON2	PRJNA729271	1.999735552	8.82009E-25	1.999735552	stage_E14_vs_E10	ordinary_muscle_development	D_vs_E10;stage_1W_vs_E10;stage_3W_vs_E10;stage	1	TRUE
ENSGALG00010021534	KIFC3	PRJNA729271	1.762524378	9.08121E-25	1.762524378	stage_E14_vs_E10	ordinary_muscle_development	D_vs_E10;stage_1W_vs_E10;stage_3W_vs_E10;stage	1	TRUE
ENSGALG00010011475	NDP	PRJNA729271	-3.708358466	9.19331E-25	3.708358466	stage_E14_vs_E10	ordinary_muscle_development	D_vs_E10;stage_1W_vs_E10;stage_3W_vs_E10;stage	1	TRUE
ENSGALG00010027384	HHATL	PRJNA729271	2.284386469	9.29384E-25	2.284386469	stage_E14_vs_E10	ordinary_muscle_development	D_vs_E10;stage_1W_vs_E10;stage_3W_vs_E10;stage	1	TRUE
ENSGALG00010018062	GOLGA7	PRJNA729271	-0.990708826	9.49818E-25	0.990708826	stage_E14_vs_E10	ordinary_muscle_development		0	FALSE
ENSGALG00010012818	RGS18	PRJNA729271	1.998689453	9.51512E-25	1.998689453	stage_E14_vs_E10	ordinary_muscle_development	D_vs_E10;stage_1W_vs_E10;stage_3W_vs_E10;stage	1	TRUE
ENSGALG00010022860	MOB3C	PRJNA729271	-1.395909706	9.57476E-25	1.395909706	stage_E14_vs_E10	ordinary_muscle_development	D_vs_E10;stage_1W_vs_E10;stage_3W_vs_E10;stage	1	TRUE
ENSGALG00010029321	TBX2	PRJNA729271	-1.8474441	9.87135E-25	1.8474441	stage_E14_vs_E10	ordinary_muscle_development	D_vs_E10;stage_1W_vs_E10;stage_3W_vs_E10;stage	1	TRUE
ENSGALG00010023380	USP5	PRJNA729271	-1.023795329	9.89464E-25	1.023795329	stage_E14_vs_E10	ordinary_muscle_development	D_vs_E10;stage_1W_vs_E10;stage_3W_vs_E10;stage	1	TRUE
ENSGALG00010013648	NAV3	PRJNA729271	-1.244600355	9.92287E-25	1.244600355	stage_E14_vs_E10	ordinary_muscle_development	D_vs_E10;stage_1W_vs_E10;stage_3W_vs_E10;stage	1	TRUE
ENSGALG00010016015	ZDHHC3	PRJNA729271	0.854035139	1.00526E-24	0.854035139	stage_E14_vs_E10	ordinary_muscle_development		0	FALSE
ENSGALG00010017435	ACTR10	PRJNA729271	-0.949648864	1.03919E-24	0.949648864	stage_E14_vs_E10	ordinary_muscle_development		0	FALSE
ENSGALG00010016729	ELMO1	PRJNA729271	0.981261711	1.04225E-24	0.981261711	stage_E14_vs_E10	ordinary_muscle_development		0	FALSE
ENSGALG00010014314	ENSGALG00010014314	PRJNA729271	1.637018965	1.0525E-24	1.637018965	stage_E14_vs_E10	ordinary_muscle_development	D_vs_E10;stage_1W_vs_E10;stage_3W_vs_E10;stage	1	TRUE
ENSGALG00010029685	ATP5PD	PRJNA729271	1.20006209	1.07913E-24	1.20006209	stage_E14_vs_E10	ordinary_muscle_development	D_vs_E10;stage_1W_vs_E10;stage_3W_vs_E10;stage	1	TRUE
ENSGALG00010029284	CYTH1	PRJNA729271	0.967574095	1.09512E-24	0.967574095	stage_E14_vs_E10	ordinary_muscle_development		0	FALSE
ENSGALG00010003222	ITGA8	PRJNA729271	-3.237850454	1.15592E-24	3.237850454	stage_E14_vs_E10	ordinary_muscle_development	D_vs_E10;stage_1W_vs_E10;stage_3W_vs_E10;stage	1	TRUE
ENSGALG00010007579	GATB	PRJNA729271	1.854904185	1.16648E-24	1.854904185	stage_E14_vs_E10	ordinary_muscle_development	D_vs_E10;stage_1W_vs_E10;stage_3W_vs_E10;stage	1	TRUE
ENSGALG00010015959	TRNAU1AP	PRJNA729271	1.344333383	1.17151E-24	1.344333383	stage_E14_vs_E10	ordinary_muscle_development	D_vs_E10;stage_1W_vs_E10;stage_3W_vs_E10;stage	1	TRUE
ENSGALG00010013928	CCNO	PRJNA729271	4.748870435	1.17935E-24	4.748870435	stage_E14_vs_E10	ordinary_muscle_development	D_vs_E10;stage_1W_vs_E10;stage_3W_vs_E10;stage	1	TRUE
ENSGALG00010022052	IFFO1	PRJNA729271	1.079061351	1.19808E-24	1.079061351	stage_E14_vs_E10	ordinary_muscle_development	D_vs_E10;stage_1W_vs_E10;stage_3W_vs_E10;stage	1	TRUE
ENSGALG00010002685	CA8	PRJNA729271	1.84580265	1.20701E-24	1.84580265	stage_E14_vs_E10	ordinary_muscle_development	D_vs_E10;stage_1W_vs_E10;stage_3W_vs_E10;stage	1	TRUE
ENSGALG00010003889	ITM2B	PRJNA729271	0.915627917	1.21439E-24	0.915627917	stage_E14_vs_E10	ordinary_muscle_development		0	FALSE
ENSGALG00010016928	RGS11	PRJNA729271	3.904782134	1.21982E-24	3.904782134	stage_E14_vs_E10	ordinary_muscle_development	D_vs_E10;stage_1W_vs_E10;stage_3W_vs_E10;stage	1	TRUE
ENSGALG00010023689	WT1	PRJNA729271	-2.719484472	1.23139E-24	2.719484472	stage_E14_vs_E10	ordinary_muscle_development	D_vs_E10;stage_1W_vs_E10;stage_3W_vs_E10;stage	1	TRUE
ENSGALG00010012859	FBXL21	PRJNA729271	1.152021094	1.28668E-24	1.152021094	stage_E14_vs_E10	ordinary_muscle_development	D_vs_E10;stage_1W_vs_E10;stage_3W_vs_E10;stage	1	TRUE
ENSGALG00010021444	ATG9A	PRJNA729271	2.552676208	1.29232E-24	2.552676208	stage_E14_vs_E10	ordinary_muscle_development	D_vs_E10;stage_1W_vs_E10;stage_3W_vs_E10;stage	1	TRUE
ENSGALG00010029234	TOM1L1	PRJNA729271	-2.519169821	1.34893E-24	2.519169821	stage_E14_vs_E10	ordinary_muscle_development	D_vs_E10;stage_1W_vs_E10;stage_3W_vs_E10;stage	1	TRUE
ENSGALG00010028384	ENSGALG00010028384	PRJNA729271	8.621059779	1.40524E-24	8.621059779	stage_E14_vs_E10	ordinary_muscle_development	D_vs_E10;stage_1W_vs_E10;stage_3W_vs_E10;stage	1	TRUE
ENSGALG00010003305	CALR	PRJNA729271	-0.812952362	1.43151E-24	0.812952362	stage_E14_vs_E10	ordinary_muscle_development		0	FALSE
ENSGALG00010011883	HCFC2	PRJNA729271	1.107988235	1.50527E-24	1.107988235	stage_E14_vs_E10	ordinary_muscle_development	D_vs_E10;stage_1W_vs_E10;stage_3W_vs_E10;stage	1	TRUE
ENSGALG00010012571	AGAP1	PRJNA729271	0.853991707	1.51393E-24	0.853991707	stage_E14_vs_E10	ordinary_muscle_development		0	FALSE
ENSGALG00010018502	ARHGEF10L	PRJNA729271	1.263502528	1.54979E-24	1.263502528	stage_E14_vs_E10	ordinary_muscle_development	D_vs_E10;stage_1W_vs_E10;stage_3W_vs_E10;stage	1	TRUE
ENSGALG00010021622	PTHLH	PRJNA729271	-2.79534043	1.56662E-24	2.79534043	stage_E14_vs_E10	ordinary_muscle_development	D_vs_E10;stage_1W_vs_E10;stage_3W_vs_E10;stage	1	TRUE
ENSGALG00010011033	DROSHA	PRJNA729271	-1.057093742	1.59423E-24	1.057093742	stage_E14_vs_E10	ordinary_muscle_development	D_vs_E10;stage_1W_vs_E10;stage_3W_vs_E10;stage	1	TRUE
ENSGALG00010012970	UNC5A	PRJNA729271	-2.162391641	1.60737E-24	2.162391641	stage_E14_vs_E10	ordinary_muscle_development	D_vs_E10;stage_1W_vs_E10;stage_3W_vs_E10;stage	1	TRUE
ENSGALG00010024367	ENSGALG00010024367	PRJNA729271	-2.468197128	1.62996E-24	2.468197128	stage_E14_vs_E10	ordinary_muscle_development	D_vs_E10;stage_1W_vs_E10;stage_3W_vs_E10;stage	1	TRUE
ENSGALG00010008324	SCRIB	PRJNA729271	1.090140419	1.64998E-24	1.090140419	stage_E14_vs_E10	ordinary_muscle_development	D_vs_E10;stage_1W_vs_E10;stage_3W_vs_E10;stage	1	TRUE
ENSGALG00010003870	MED4	PRJNA729271	-1.027365289	1.74649E-24	1.027365289	stage_E14_vs_E10	ordinary_muscle_development	D_vs_E10;stage_1W_vs_E10;stage_3W_vs_E10;stage	1	TRUE
ENSGALG00010015449	HDX	PRJNA729271	-0.975485692	1.78192E-24	0.975485692	stage_E14_vs_E10	ordinary_muscle_development		0	FALSE
ENSGALG00010010466	CRADD	PRJNA729271	1.272637946	1.79743E-24	1.272637946	stage_E14_vs_E10	ordinary_muscle_development	D_vs_E10;stage_1W_vs_E10;stage_3W_vs_E10;stage	1	TRUE
ENSGALG00010021187	SEMA7A	PRJNA729271	2.897036243	1.80054E-24	2.897036243	stage_E14_vs_E10	ordinary_muscle_development	D_vs_E10;stage_1W_vs_E10;stage_3W_vs_E10;stage	1	TRUE
ENSGALG00010013958	TUSC3	PRJNA729271	1.768166422	1.83155E-24	1.768166422	stage_E14_vs_E10	ordinary_muscle_development	D_vs_E10;stage_1W_vs_E10;stage_3W_vs_E10;stage	1	TRUE
ENSGALG00010015585	BDKRB1	PRJNA729271	3.034356758	1.89687E-24	3.034356758	stage_E14_vs_E10	ordinary_muscle_development	D_vs_E10;stage_1W_vs_E10;stage_3W_vs_E10;stage	1	TRUE
ENSGALG00010027252	MAGI3	PRJNA729271	-0.757347638	1.94488E-24	0.757347638	stage_E14_vs_E10	ordinary_muscle_development		0	FALSE
ENSGALG00010013459	ATP10D	PRJNA729271	-1.530222546	1.94671E-24	1.530222546	stage_E14_vs_E10	ordinary_muscle_development	D_vs_E10;stage_1W_vs_E10;stage_3W_vs_E10;stage	1	TRUE
ENSGALG00010017787	CNRIP1	PRJNA729271	2.427481689	1.97112E-24	2.427481689	stage_E14_vs_E10	ordinary_muscle_development	D_vs_E10;stage_1W_vs_E10;stage_3W_vs_E10;stage	1	TRUE
ENSGALG00010008015	ARHGAP39	PRJNA729271	-1.550690252	1.97676E-24	1.550690252	stage_E14_vs_E10	ordinary_muscle_development	D_vs_E10;stage_1W_vs_E10;stage_3W_vs_E10;stage	1	TRUE
ENSGALG00010001386	FKBP14	PRJNA729271	-1.924940226	1.97784E-24	1.924940226	stage_E14_vs_E10	ordinary_muscle_development	D_vs_E10;stage_1W_vs_E10;stage_3W_vs_E10;stage	1	TRUE
ENSGALG00010008910	TRIB1	PRJNA729271	2.697132711	2.08996E-24	2.697132711	stage_E14_vs_E10	ordinary_muscle_development	D_vs_E10;stage_1W_vs_E10;stage_3W_vs_E10;stage	1	TRUE
ENSGALG00010004855	ZEB1	PRJNA729271	0.95461229	2.12486E-24	0.95461229	stage_E14_vs_E10	ordinary_muscle_development		0	FALSE
ENSGALG00010018611	NUDCD3	PRJNA729271	1.158688643	2.13528E-24	1.158688643	stage_E14_vs_E10	ordinary_muscle_development	D_vs_E10;stage_1W_vs_E10;stage_3W_vs_E10;stage	1	TRUE
ENSGALG00010018555	UBN1	PRJNA729271	-0.805701245	2.16741E-24	0.805701245	stage_E14_vs_E10	ordinary_muscle_development		0	FALSE
ENSGALG00010017482	CORO2B	PRJNA729271	-1.538861488	2.21111E-24	1.538861488	stage_E14_vs_E10	ordinary_muscle_development	D_vs_E10;stage_1W_vs_E10;stage_3W_vs_E10;stage	1	TRUE
ENSGALG00010006022	AFG2A	PRJNA729271	-0.962752504	2.22354E-24	0.962752504	stage_E14_vs_E10	ordinary_muscle_development		0	FALSE
ENSGALG00010016902	MRNIP	PRJNA729271	-1.526473649	2.22699E-24	1.526473649	stage_E14_vs_E10	ordinary_muscle_development	D_vs_E10;stage_1W_vs_E10;stage_3W_vs_E10;stage	1	TRUE
ENSGALG00010012048	PDE6H	PRJNA729271	4.615423535	2.25775E-24	4.615423535	stage_E14_vs_E10	ordinary_muscle_development	D_vs_E10;stage_1W_vs_E10;stage_3W_vs_E10;stage	1	TRUE
ENSGALG00010013198	CAMLG	PRJNA729271	-0.918855727	2.25959E-24	0.918855727	stage_E14_vs_E10	ordinary_muscle_development		0	FALSE
ENSGALG00010004791	FZD1	PRJNA729271	-1.227800993	2.26257E-24	1.227800993	stage_E14_vs_E10	ordinary_muscle_development	D_vs_E10;stage_1W_vs_E10;stage_3W_vs_E10;stage	1	TRUE
ENSGALG00010028286	KCTD12A	PRJNA729271	12.45386878	2.37168E-24	12.45386878	stage_E14_vs_E10	ordinary_muscle_development	D_vs_E10;stage_1W_vs_E10;stage_3W_vs_E10;stage	1	TRUE
ENSGALG00010003192	RGCC	PRJNA729271	3.179108931	2.38681E-24	3.179108931	stage_E14_vs_E10	ordinary_muscle_development	D_vs_E10;stage_1W_vs_E10;stage_3W_vs_E10;stage	1	TRUE
ENSGALG00010024286	ADA	PRJNA729271	1.850712478	2.39771E-24	1.850712478	stage_E14_vs_E10	ordinary_muscle_development	D_vs_E10;stage_1W_vs_E10;stage_3W_vs_E10;stage	1	TRUE
ENSGALG00010001414	CCDC126	PRJNA729271	1.762682689	2.44441E-24	1.762682689	stage_E14_vs_E10	ordinary_muscle_development	D_vs_E10;stage_1W_vs_E10;stage_3W_vs_E10;stage	1	TRUE
ENSGALG00010005193	EPB41L2	PRJNA729271	1.015690489	2.44647E-24	1.015690489	stage_E14_vs_E10	ordinary_muscle_development	D_vs_E10;stage_1W_vs_E10;stage_3W_vs_E10;stage	1	TRUE
ENSGALG00010005037	THNSL1	PRJNA729271	1.463613447	2.50273E-24	1.463613447	stage_E14_vs_E10	ordinary_muscle_development	D_vs_E10;stage_1W_vs_E10;stage_3W_vs_E10;stage	1	TRUE
ENSGALG00010012509	MICAL3	PRJNA729271	0.965315867	2.51845E-24	0.965315867	stage_E14_vs_E10	ordinary_muscle_development		0	FALSE
ENSGALG00010022749	HSPA12A	PRJNA729271	-1.353739502	2.51941E-24	1.353739502	stage_E14_vs_E10	ordinary_muscle_development	D_vs_E10;stage_1W_vs_E10;stage_3W_vs_E10;stage	1	TRUE
ENSGALG00010013672	ENSGALG00010013672	PRJNA729271	-5.624459566	2.53392E-24	5.624459566	stage_E14_vs_E10	ordinary_muscle_development	D_vs_E10;stage_1W_vs_E10;stage_3W_vs_E10;stage	1	TRUE
ENSGALG00010016209	ADAMTS7	PRJNA729271	-1.730897883	2.53826E-24	1.730897883	stage_E14_vs_E10	ordinary_muscle_development	D_vs_E10;stage_1W_vs_E10;stage_3W_vs_E10;stage	1	TRUE
ENSGALG00010003878	ARHGAP24	PRJNA729271	0.921849648	2.55992E-24	0.921849648	stage_E14_vs_E10	ordinary_muscle_development		0	FALSE
ENSGALG00010002728	RP2	PRJNA729271	-1.169608088	2.56516E-24	1.169608088	stage_E14_vs_E10	ordinary_muscle_development	D_vs_E10;stage_1W_vs_E10;stage_3W_vs_E10;stage	1	TRUE
ENSGALG00010020206	MRPS11	PRJNA729271	0.832991575	2.59792E-24	0.832991575	stage_E14_vs_E10	ordinary_muscle_development		0	FALSE
ENSGALG00010017691	LZTS1	PRJNA729271	-2.96920682	2.63755E-24	2.96920682	stage_E14_vs_E10	ordinary_muscle_development	D_vs_E10;stage_1W_vs_E10;stage_3W_vs_E10;stage	1	TRUE
ENSGALG00010004859	NPC1	PRJNA729271	1.972345798	2.69341E-24	1.972345798	stage_E14_vs_E10	ordinary_muscle_development	D_vs_E10;stage_1W_vs_E10;stage_3W_vs_E10;stage	1	TRUE
ENSGALG00010015759	EIF5	PRJNA729271	0.94425667	2.69422E-24	0.94425667	stage_E14_vs_E10	ordinary_muscle_development		0	FALSE
ENSGALG00010012565	SYNGR1	PRJNA729271	1.259439801	2.71055E-24	1.259439801	stage_E14_vs_E10	ordinary_muscle_development	D_vs_E10;stage_1W_vs_E10;stage_3W_vs_E10;stage	1	TRUE
ENSGALG00010000452	SLC8B1	PRJNA729271	2.308920516	2.73077E-24	2.308920516	stage_E14_vs_E10	ordinary_muscle_development	D_vs_E10;stage_1W_vs_E10;stage_3W_vs_E10;stage	1	TRUE
ENSGALG00010018255	MFN1	PRJNA729271	1.162421853	2.78367E-24	1.162421853	stage_E14_vs_E10	ordinary_muscle_development	D_vs_E10;stage_1W_vs_E10;stage_3W_vs_E10;stage	1	TRUE
ENSGALG00010025378	PAX3	PRJNA729271	-2.918667689	2.78784E-24	2.918667689	stage_E14_vs_E10	ordinary_muscle_development	D_vs_E10;stage_1W_vs_E10;stage_3W_vs_E10;stage	1	TRUE
ENSGALG00010002254	ENSGALG00010002254	PRJNA729271	4.672633371	2.79893E-24	4.672633371	stage_E14_vs_E10	ordinary_muscle_development	D_vs_E10;stage_1W_vs_E10;stage_3W_vs_E10;stage	1	TRUE

ENSGALG00010005975	ABRACL	PRJNA729271	-1.125956896	2.82332E-24	1.125956896	stage_E14_vs_E10	ordinary_muscle_development	D_vs_E10;stage_1W_vs_E10;stage_3W_vs_E10;stage	1	TRUE
ENSGALG00010019509	ZNF106	PRJNA729271	2.306717368	2.84224E-24	2.306717368	stage_E14_vs_E10	ordinary_muscle_development	D_vs_E10;stage_1W_vs_E10;stage_3W_vs_E10;stage	1	TRUE
ENSGALG00010002347	ZNF451	PRJNA729271	-0.653763877	2.84229E-24	0.653763877	stage_E14_vs_E10	ordinary_muscle_development		0	FALSE
ENSGALG00010013005	SLC40A1	PRJNA729271	0.93582329	2.86138E-24	0.93582329	stage_E14_vs_E10	ordinary_muscle_development		0	FALSE
ENSGALG00010019067	GTF3C1	PRJNA729271	-0.606268193	2.92206E-24	0.606268193	stage_E14_vs_E10	ordinary_muscle_development		0	FALSE
ENSGALG00010020340	DCAF5	PRJNA729271	0.990155976	2.9779E-24	0.990155976	stage_E14_vs_E10	ordinary_muscle_development		0	FALSE
ENSGALG00010021014	FBXL15	PRJNA729271	0.924411542	3.02109E-24	0.924411542	stage_E14_vs_E10	ordinary_muscle_development		0	FALSE
ENSGALG00010023806	PATJ	PRJNA729271	-1.815981696	3.02286E-24	1.815981696	stage_E14_vs_E10	ordinary_muscle_development	D_vs_E10;stage_1W_vs_E10;stage_3W_vs_E10;stage	1	TRUE
ENSGALG00010003109	ART5	PRJNA729271	5.753305034	3.06739E-24	5.753305034	stage_E14_vs_E10	ordinary_muscle_development	D_vs_E10;stage_1W_vs_E10;stage_3W_vs_E10;stage	1	TRUE
ENSGALG00010012034	ARHGEF9	PRJNA729271	1.560619765	3.1859E-24	1.560619765	stage_E14_vs_E10	ordinary_muscle_development	D_vs_E10;stage_1W_vs_E10;stage_3W_vs_E10;stage	1	TRUE
ENSGALG00010022891	NTM	PRJNA729271	2.092264514	3.19912E-24	2.092264514	stage_E14_vs_E10	ordinary_muscle_development	D_vs_E10;stage_1W_vs_E10;stage_3W_vs_E10;stage	1	TRUE
ENSGALG00010013614	ATRX	PRJNA729271	-0.895029842	3.21519E-24	0.895029842	stage_E14_vs_E10	ordinary_muscle_development		0	FALSE
ENSGALG00010026095	MIF	PRJNA729271	-2.084054554	3.29205E-24	2.084054554	stage_E14_vs_E10	ordinary_muscle_development	D_vs_E10;stage_1W_vs_E10;stage_3W_vs_E10;stage	1	TRUE
ENSGALG00010029764	NXN	PRJNA729271	-1.30636443	3.29916E-24	1.30636443	stage_E14_vs_E10	ordinary_muscle_development	D_vs_E10;stage_1W_vs_E10;stage_3W_vs_E10;stage	1	TRUE
ENSGALG00010025832	ENKD1	PRJNA729271	1.491364998	3.30757E-24	1.491364998	stage_E14_vs_E10	ordinary_muscle_development	D_vs_E10;stage_1W_vs_E10;stage_3W_vs_E10;stage	1	TRUE
ENSGALG00010000783	DAP	PRJNA729271	-1.685646383	3.32062E-24	1.685646383	stage_E14_vs_E10	ordinary_muscle_development	D_vs_E10;stage_1W_vs_E10;stage_3W_vs_E10;stage	1	TRUE
ENSGALG00010005485	PITRM1	PRJNA729271	-1.020282894	3.3486E-24	1.020282894	stage_E14_vs_E10	ordinary_muscle_development	D_vs_E10;stage_1W_vs_E10;stage_3W_vs_E10;stage	1	TRUE
ENSGALG00010021183	CFAP100	PRJNA729271	4.643568406	3.38298E-24	4.643568406	stage_E14_vs_E10	ordinary_muscle_development	D_vs_E10;stage_1W_vs_E10;stage_3W_vs_E10;stage	1	TRUE
ENSGALG00010003900	RB1	PRJNA729271	-0.931386481	3.4165E-24	0.931386481	stage_E14_vs_E10	ordinary_muscle_development		0	FALSE
ENSGALG00010016225	P4HA2	PRJNA729271	1.611477478	3.44144E-24	1.611477478	stage_E14_vs_E10	ordinary_muscle_development	D_vs_E10;stage_1W_vs_E10;stage_3W_vs_E10;stage	1	TRUE
ENSGALG00010009452	TBCE	PRJNA729271	-0.95618682	3.44145E-24	0.95618682	stage_E14_vs_E10	ordinary_muscle_development		0	FALSE
ENSGALG00010017313	MAP4K3	PRJNA729271	0.847180315	3.499E-24	0.847180315	stage_E14_vs_E10	ordinary_muscle_development		0	FALSE
ENSGALG00010002983	NSMAF	PRJNA729271	1.143979788	3.6065E-24	1.143979788	stage_E14_vs_E10	ordinary_muscle_development	D_vs_E10;stage_1W_vs_E10;stage_3W_vs_E10;stage	1	TRUE
ENSGALG00010023266	RFWD3	PRJNA729271	-1.484530704	3.68479E-24	1.484530704	stage_E14_vs_E10	ordinary_muscle_development	D_vs_E10;stage_1W_vs_E10;stage_3W_vs_E10;stage	1	TRUE
ENSGALG00010024351	SP1	PRJNA729271	1.576660884	3.7266E-24	1.576660884	stage_E14_vs_E10	ordinary_muscle_development	D_vs_E10;stage_1W_vs_E10;stage_3W_vs_E10;stage	1	TRUE
ENSGALG00010002916	SDCBP	PRJNA729271	-1.269141499	3.76639E-24	1.269141499	stage_E14_vs_E10	ordinary_muscle_development	D_vs_E10;stage_1W_vs_E10;stage_3W_vs_E10;stage	1	TRUE
ENSGALG00010024921	STRADB	PRJNA729271	-1.175230122	3.78054E-24	1.175230122	stage_E14_vs_E10	ordinary_muscle_development	D_vs_E10;stage_1W_vs_E10;stage_3W_vs_E10;stage	1	TRUE
ENSGALG00010012680	TRPM8	PRJNA729271	2.660510461	3.83905E-24	2.660510461	stage_E14_vs_E10	ordinary_muscle_development	D_vs_E10;stage_1W_vs_E10;stage_3W_vs_E10;stage	1	TRUE
ENSGALG00010025523	B4GALT4	PRJNA729271	-1.378215157	3.91531E-24	1.378215157	stage_E14_vs_E10	ordinary_muscle_development	D_vs_E10;stage_1W_vs_E10;stage_3W_vs_E10;stage	1	TRUE
ENSGALG00010010042	METTL25	PRJNA729271	-1.252578799	3.93406E-24	1.252578799	stage_E14_vs_E10	ordinary_muscle_development	D_vs_E10;stage_1W_vs_E10;stage_3W_vs_E10;stage	1	TRUE
ENSGALG00010016678	NOX1	PRJNA729271	3.203073832	3.98407E-24	3.203073832	stage_E14_vs_E10	ordinary_muscle_development	D_vs_E10;stage_1W_vs_E10;stage_3W_vs_E10;stage	1	TRUE
ENSGALG00010010111	CNOT4	PRJNA729271	1.364332123	4.18692E-24	1.364332123	stage_E14_vs_E10	ordinary_muscle_development	D_vs_E10;stage_1W_vs_E10;stage_3W_vs_E10;stage	1	TRUE
ENSGALG00010010909	SLIT3	PRJNA729271	-3.395753785	4.21701E-24	3.395753785	stage_E14_vs_E10	ordinary_muscle_development	D_vs_E10;stage_1W_vs_E10;stage_3W_vs_E10;stage	1	TRUE
ENSGALG00010016552	ENSGALG00010016552	PRJNA729271	4.532224338	4.2243E-24	4.532224338	stage_E14_vs_E10	ordinary_muscle_development	D_vs_E10;stage_1W_vs_E10;stage_3W_vs_E10;stage	1	TRUE
ENSGALG00010016892	MAL	PRJNA729271	4.146052165	4.24347E-24	4.146052165	stage_E14_vs_E10	ordinary_muscle_development	D_vs_E10;stage_1W_vs_E10;stage_3W_vs_E10;stage	1	TRUE
ENSGALG00010005424	ENSGALG00010005424	PRJNA729271	1.364087216	4.30231E-24	1.364087216	stage_E14_vs_E10	ordinary_muscle_development	D_vs_E10;stage_1W_vs_E10;stage_3W_vs_E10;stage	1	TRUE
ENSGALG00010016957	WNT4	PRJNA729271	-2.713476344	4.36205E-24	2.713476344	stage_E14_vs_E10	ordinary_muscle_development	D_vs_E10;stage_1W_vs_E10;stage_3W_vs_E10;stage	1	TRUE
ENSGALG00010021411	MCU	PRJNA729271	1.328392263	4.38743E-24	1.328392263	stage_E14_vs_E10	ordinary_muscle_development	D_vs_E10;stage_1W_vs_E10;stage_3W_vs_E10;stage	1	TRUE
ENSGALG00010029203	ALDOC	PRJNA729271	-1.783240381	4.47666E-24	1.783240381	stage_E14_vs_E10	ordinary_muscle_development	D_vs_E10;stage_1W_vs_E10;stage_3W_vs_E10;stage	1	TRUE
ENSGALG00010024904	ALS2CL	PRJNA729271	1.634090019	4.47742E-24	1.634090019	stage_E14_vs_E10	ordinary_muscle_development	D_vs_E10;stage_1W_vs_E10;stage_3W_vs_E10;stage	1	TRUE
ENSGALG00010028590	ENSGALG00010028590	PRJNA729271	2.335248278	4.55965E-24	2.335248278	stage_E14_vs_E10	ordinary_muscle_development	D_vs_E10;stage_1W_vs_E10;stage_3W_vs_E10;stage	1	TRUE
ENSGALG00010025232	PRIM1	PRJNA729271	-1.585131786	4.58787E-24	1.585131786	stage_E14_vs_E10	ordinary_muscle_development	D_vs_E10;stage_1W_vs_E10;stage_3W_vs_E10;stage	1	TRUE
ENSGALG00010017402	SLC2A5	PRJNA729271	2.33134768	4.60885E-24	2.33134768	stage_E14_vs_E10	ordinary_muscle_development	D_vs_E10;stage_1W_vs_E10;stage_3W_vs_E10;stage	1	TRUE
ENSGALG00010022664	TRPC4AP	PRJNA729271	0.998883395	4.61844E-24	0.998883395	stage_E14_vs_E10	ordinary_muscle_development		0	FALSE
ENSGALG00010023733	ORC6	PRJNA729271	-1.995166095	4.63855E-24	1.995166095	stage_E14_vs_E10	ordinary_muscle_development	D_vs_E10;stage_1W_vs_E10;stage_3W_vs_E10;stage	1	TRUE
ENSGALG00010011947	THAP1	PRJNA729271	1.143989481	4.66145E-24	1.143989481	stage_E14_vs_E10	ordinary_muscle_development	D_vs_E10;stage_1W_vs_E10;stage_3W_vs_E10;stage	1	TRUE
ENSGALG00010018380	SLC20A1	PRJNA729271	-1.49397222	4.70556E-24	1.49397222	stage_E14_vs_E10	ordinary_muscle_development	D_vs_E10;stage_1W_vs_E10;stage_3W_vs_E10;stage	1	TRUE
ENSGALG00010011312	VPS13B	PRJNA729271	-0.548960616	4.76946E-24	0.548960616	stage_E14_vs_E10	ordinary_muscle_development		0	FALSE
ENSGALG00010008161	TMEFF1	PRJNA729271	-4.086768115	4.7742E-24	4.086768115	stage_E14_vs_E10	ordinary_muscle_development	D_vs_E10;stage_1W_vs_E10;stage_3W_vs_E10;stage	1	TRUE
ENSGALG00010021045	DPP4	PRJNA729271	2.659802436	4.88945E-24	2.659802436	stage_E14_vs_E10	ordinary_muscle_development	D_vs_E10;stage_1W_vs_E10;stage_3W_vs_E10;stage	1	TRUE
ENSGALG00010020677	CYR61	PRJNA729271	2.388638562	4.9131E-24	2.388638562	stage_E14_vs_E10	ordinary_muscle_development	D_vs_E10;stage_1W_vs_E10;stage_3W_vs_E10;stage	1	TRUE
ENSGALG00010009847	ENSGALG00010009847	PRJNA729271	-1.232132335	4.91714E-24	1.232132335	stage_E14_vs_E10	ordinary_muscle_development	D_vs_E10;stage_1W_vs_E10;stage_3W_vs_E10;stage	1	TRUE
ENSGALG00010007624	TPPP	PRJNA729271	2.541622387	4.92629E-24	2.541622387	stage_E14_vs_E10	ordinary_muscle_development	D_vs_E10;stage_1W_vs_E10;stage_3W_vs_E10;stage	1	TRUE
ENSGALG00010028492	ENSGALG00010028492	PRJNA729271	2.535591253	4.93006E-24	2.535591253	stage_E14_vs_E10	ordinary_muscle_development	D_vs_E10;stage_1W_vs_E10;stage_3W_vs_E10;stage	1	TRUE
ENSGALG00010006227	AGPAT4	PRJNA729271	-1.522408848	5.04166E-24	1.522408848	stage_E14_vs_E10	ordinary_muscle_development	D_vs_E10;stage_1W_vs_E10;stage_3W_vs_E10;stage	1	TRUE
ENSGALG00010029405	POLDIP2	PRJNA729271	0.814735237	5.06019E-24	0.814735237	stage_E14_vs_E10	ordinary_muscle_development		0	FALSE
ENSGALG00010021680	PIGT	PRJNA729271	1.198207774	5.19882E-24	1.198207774	stage_E14_vs_E10	ordinary_muscle_development	D_vs_E10;stage_1W_vs_E10;stage_3W_vs_E10;stage	1	TRUE
ENSGALG00010025562	GRAMD1B	PRJNA729271	1.865110809	5.35237E-24	1.865110809	stage_E14_vs_E10	ordinary_muscle_development	D_vs_E10;stage_1W_vs_E10;stage_3W_vs_E10;stage	1	TRUE
ENSGALG00010016199	RLBP1	PRJNA729271	-6.61068611	5.3539E-24	6.61068611	stage_E14_vs_E10	ordinary_muscle_development	D_vs_E10;stage_1W_vs_E10;stage_3W_vs_E10;stage	1	TRUE
ENSGALG00010020140	SECISBP2L	PRJNA729271	0.968207571	5.35642E-24	0.968207571	stage_E14_vs_E10	ordinary_muscle_development		0	FALSE
ENSGALG00010021265	SIPA1L1	PRJNA729271	-0.927935301	5.42255E-24	0.927935301	stage_E14_vs_E10	ordinary_muscle_development		0	FALSE
ENSGALG00010008282	FAM83H	PRJNA729271	-3.057239092	5.43747E-24	3.057239092	stage_E14_vs_E10	ordinary_muscle_development	D_vs_E10;stage_1W_vs_E10;stage_3W_vs_E10;stage	1	TRUE
ENSGALG00010028125	HMG20B	PRJNA729271	-1.267973826	5.54029E-24	1.267973826	stage_E14_vs_E10	ordinary_muscle_development	D_vs_E10;stage_1W_vs_E10;stage_3W_vs_E10;stage	1	TRUE
ENSGALG00010013737	AFF4	PRJNA729271	0.865703231	5.58523E-24	0.865703231	stage_E14_vs_E10	ordinary_muscle_development		0	FALSE
ENSGALG00010001151	CCDC112	PRJNA729271	2.197915894	5.63573E-24	2.197915894	stage_E14_vs_E10	ordinary_muscle_development	D_vs_E10;stage_1W_vs_E10;stage_3W_vs_E10;stage	1	TRUE
ENSGALG00010020962	MYO5C	PRJNA729271	-4.113701704	5.65133E-24	4.113701704	stage_E14_vs_E10	ordinary_muscle_development	D_vs_E10;stage_1W_vs_E10;stage_3W_vs_E10;stage	1	TRUE
ENSGALG00010017757	ENSGALG00010017757	PRJNA729271	1.419672541	5.84186E-24	1.419672541	stage_E14_vs_E10	ordinary_muscle_development	D_vs_E10;stage_1W_vs_E10;stage_3W_vs_E10;stage	1	TRUE
ENSGALG00010027183	CD34	PRJNA729271	1.609431276	5.90777E-24	1.609431276	stage_E14_vs_E10	ordinary_muscle_development	D_vs_E10;stage_1W_vs_E10;stage_3W_vs_E10;stage	1	TRUE
ENSGALG00010021420	PLXNB3	PRJNA729271	-1.971294195	5.98795E-24	1.971294195	stage_E14_vs_E10	ordinary_muscle_development	D_vs_E10;stage_1W_vs_E10;stage_3W_vs_E10;stage	1	TRUE
ENSGALG00010019237	SORCS3	PRJNA729271	-3.935296574	6.03202E-24	3.935296574	stage_E14_vs_E10	ordinary_muscle_development	D_vs_E10;stage_1W_vs_E10;stage_3W_vs_E10;stage	1	TRUE
ENSGALG00010015213	RUBCN	PRJNA729271	1.193842856	6.05586E-24	1.193842856	stage_E14_vs_E10	ordinary_muscle_development	D_vs_E10;stage_1W_vs_E10;stage_3W_vs_E10;stage	1	TRUE
ENSGALG00010011562	ENSGALG00010011562	PRJNA729271	-9.170355174	6.06591E-24	9.170355174	stage_E14_vs_E10	ordinary_muscle_development	D_vs_E10;stage_1W_vs_E10;stage_3W_vs_E10;stage	1	TRUE
ENSGALG00010016861	FGR	PRJNA729271	1.243893902	6.12829E-24	1.243893902	stage_E14_vs_E10	ordinary_muscle_development	D_vs_E10;stage_1W_vs_E10;stage_3W_vs_E10;stage	1	TRUE
ENSGALG00010002788	ENSGALG00010002788	PRJNA729271	6.448019956	6.14012E-24	6.448019956	stage_E14_vs_E10	ordinary_muscle_development	D_vs_E10;stage_1W_vs_E10;stage_3W_vs_E10;stage	1	TRUE
ENSGALG00010029686	KCTD2	PRJNA729271	0.615521702	6.20375E-24	0.615521702	stage_E14_vs_E10	ordinary_muscle_development		0	FALSE
ENSGALG00010025386	TPCN2	PRJNA729271	-1.035311395	6.22889E-24	1.035311395	stage_E14_vs_E10	ordinary_muscle_development	D_vs_E10;stage_1W_vs_E10;stage_3W_vs_E10;stage	1	TRUE
ENSGALG00010007679	TMEM135	PRJNA729271	1.445921826	6.25953E-24	1.445921826	stage_E14_vs_E10	ordinary_muscle_development	D_vs_E10;stage_1W_vs_E10;stage_3W_vs_E10;stage	1	TRUE
ENSGALG00010011211	SLC37A1	PRJNA729271	1.522758895	6.30046E-24	1.522758895	stage_E14_vs_E10	ordinary_muscle_development	D_vs_E10;stage_1W_vs_E10;stage_3W_vs_E10;stage	1	TRUE
ENSGALG00010020057	RBKS	PRJNA729271	0.913338814	6.33922E-24	0.913338814	stage_E14_vs_E10	ordinary_muscle_development		0	FALSE
ENSGALG00010025925	KLHL18	PRJNA729271	0.935709403	6.41492E-24	0.935709403	stage_E14_vs_E10	ordinary_muscle_development		0	FALSE
ENSGALG00010025720	PHETA1	PRJNA729271	-2.798061317	6.42117E-24	2.798061317	stage_E14_vs_E10	ordinary_muscle_development	D_vs_E10;stage_1W_vs_E10;stage_3W_vs_E10;stage	1	TRUE
ENSGALG00010025340	METTL21A	PRJNA729271	1.184648426	6.55573E-24	1.184648426	stage_E14_vs_E10	ordinary_muscle_development	D_vs_E10;stage_1W_vs_E10;stage_3W_vs_E10;stage	1	TRUE
ENSGALG00010003307	COL1A2	PRJNA729271	2.269887673	6.6481E-24	2.269887673	stage_E14_vs_E10	ordinary_muscle_development	D_vs_E10;stage_1W_vs_E10;stage_3W_vs_E10;stage	1	TRUE
ENSGALG00010024304	PKIG	PRJNA729271	1.358027531	6.70996E-24	1.358027531	stage_E14_vs_E10	ordinary_muscle_development	D_vs_E10;stage_1W_vs_E10;stage_3W_vs_E10;stage	1	TRUE
ENSGALG00010015063	EEF1AKMT4	PRJNA729271	-2.782107929	6.74037E-24	2.782107929	stage_E14_vs_E10	ordinary_muscle_development	D_vs_E10;stage_1W_vs_E10;stage_3W_vs_E10;stage	1	TRUE
ENSGALG00010005562	HECTD1	PRJNA729271	0.872996035	6.81714E-24	0.872996035	stage_E14_vs_E10	ordinary_muscle_development		0	FALSE
ENSGALG00010021067	SLC4A10	PRJNA729271	-3.221487466	6.92747E-24	3.221487466	stage_E14_vs_E10	ordinary_muscle_development	D_vs_E10;stage_1W_vs_E10;stage_3W_vs_E10;stage	1	TRUE
ENSGALG00010000811	ENSGALG00010000811	PRJNA729271	10.67438974	6.96106E-24	10.67438974	stage_E14_vs_E10	ordinary_muscle_development	D_vs_E10;stage_1W_vs_E10;stage_3W_vs_E10;stage	1	TRUE
ENSGALG00010007413	GPATCH11	PRJNA729271	-1.029115396	6.98562E-24	1.029115396	stage_E14_vs_E10	ordinary_muscle_development	D_vs_E10;stage_1W_vs_E10;stage_3W_vs_E10;stage	1	TRUE
ENSGALG00010023911	TFCP2	PRJNA729271	-1.267912861	7.10103E-24	1.267912861	stage_E14_vs_E10	ordinary_muscle_development	D_vs_E10;stage_1W_vs_E10;stage_3W_vs_E10;stage	1	TRUE
ENSGALG00010003178	CENPA	PRJNA729271	-3.030628391	7.18438E-24	3.030628391	stage_E14_vs_E10	ordinary_muscle_development	D_vs_E10;stage_1W_vs_E10;stage_3W_vs_E10;stage	1	TRUE
ENSGALG00010011982	STK3	PRJNA729271	-1.084445015	7.22948E-24	1.084445015	stage_E14_vs_E10	ordinary_muscle_development	D_vs_E10;stage_1W_vs_E10;stage_3W_vs_E10;stage	1	TRUE
ENSGALG00010017375	BDH1A	PRJNA729271	-1.673912936	7.32119E-24	1.673912936	stage_E14_vs_E10	ordinary_muscle_development	D_vs_E10;stage_1W_vs_E10;stage_3W_vs_E10;stage	1	TRUE
ENSGALG00010026951	RUVBL1	PRJNA729271	-1.091339265	7.45658E-24	1.091339265	stage_E14_vs_E10	ordinary_muscle_development	D_vs_E10;stage_1W_vs_E10;stage_3W_vs_E10;stage	1	TRUE
ENSGALG00010012399	ERC1	PRJNA729271	-0.882383735	7.51481E-24	0.882383735	stage_E14_vs_E10	ordinary_muscle_development		0	FALSE
ENSGALG00010013325	CA2	PRJNA729271	3.259867166	7.51914E-24	3.259867166	stage_E14_vs_E10	ordinary_muscle_development	D_vs_E10;stage_1W_vs_E10;stage_3W_vs_E10;stage	1	TRUE
ENSGALG00010022575	TMEM41B	PRJNA729271	-1.151389632	7.53197E-24	1.151389632	stage_E14_vs_E10	ordinary_muscle_development	D_vs_E10;stage_1W_vs_E10;stage_3W_vs_E10;stage	1	TRUE
ENSGALG00010001831	CSF2RA	PRJNA729271	2.551921868	7.56865E-24	2.551921868	stage_E14_vs_E10	ordinary_muscle_development	D_vs_E10;stage_1W_vs_E10;stage_3W_vs_E10;stage	1	TRUE
ENSGALG00010026560	TNNI1	PRJNA729271	-6.060405845	7.71846E-24	6.060405845	stage_E14_vs_E10	ordinary_muscle_development	D_vs_E10;stage_1W_vs_E10;stage_3W_vs_E10;stage	1	TRUE
ENSGALG00010020038	SKI	PRJNA729271	-0.937379035	7.99279E-24	0.937379035	stage_E14_vs_E10	ordinary_muscle_development		0	FALSE
ENSGALG00010011076	PKP2	PRJNA729271	-3.267804784	8.0063E-24	3.267804784	stage_E14_vs_E10	ordinary_muscle_development	D_vs_E10;stage_1W_vs_E10;stage_3W_vs_E10;stage	1	TRUE
ENSGALG00010018544	HACD2	PRJNA729271	-1.059017071	8.06259E-24	1.059017071	stage_E14_vs_E10	ordinary_muscle_development	D_vs_E10;stage_1W_vs_E10;stage_3W_vs_E10;stage	1	TRUE
ENSGALG00010024964	ITM2C	PRJNA729271	-1.780692602	8.11744E-24	1.780692602	stage_E14_vs_E10	ordinary_muscle_development	D_vs_E10;stage_1W_vs_E10;stage_3W_vs_E10;stage	1	TRUE
ENSGALG00010015684	PDE6D	PRJNA729271	-1.045316601	8.1567E-24	1.045316601	stage_E14_vs_E10	ordinary_muscle_development	D_vs_E10;stage_1W_vs_E10;stage_3W_vs_E10;stage	1	TRUE
ENSGALG00010022304	ADAP1	PRJNA729271	-1.991471037	8.17472E-24	1.991471037	stage_E14_vs_E10	ordinary_muscle_development	D_vs_E10;stage_1W_vs_E10;stage_3W_vs_E10;stage	1	TRUE
ENSGALG00010011664	SERPINB10B	PRJNA729271	5.462322496	8.46192E-24	5.462322496	stage_E14_vs_E10	ordinary_muscle_development	D_vs_E10;stage_1W_vs_E10;stage_3W_vs_E10;stage	1	TRUE
ENSGALG00010024650	PRKAG1	PRJNA729271	1.229243965	8.48877E-24	1.229243965	stage_E14_vs_E10	ordinary_muscle_development	D_vs_E10;stage_1W_vs_E10;stage_3W_vs_E10;stage	1	TRUE
ENSGALG00010029644	MYCBPAP	PRJNA729271	1.640293304	8.49702E-24	1.640293304	stage_E14_vs_E10	ordinary_muscle_development	D_vs_E10;stage_1W_vs_E10;stage_3W_vs_E10;stage	1	TRUE
ENSGALG00010020943	FAM107A	PRJNA729271	3.416638146	8.57514E-24	3.416638146	stage_E14_vs_E10	ordinary_muscle_development	D_vs_E10;stage_1W_vs_E10;stage_3W_vs_E10;stage	1	TRUE
ENSGALG00010027570	NDUFS2	PRJNA729271	1.405442114	8.60591E-24	1.405442114	stage_E14_vs_E10	ordinary_muscle_development	D_vs_E10;stage_1W_vs_E10;stage_3W_vs_E10;stage	1	TRUE
ENSGALG00010008197	STARD4	PRJNA729271	-1.671959497	8.81684E-24	1.671959497	stage_E14_vs_E10	ordinary_muscle_development	D_vs_E10;stage_1W_vs_E10;stage_3W_vs_E10;stage	1	TRUE
ENSGALG00010028874	CRPL1	PRJNA729271	3.076248463	9.09981E-24	3.076248463	stage_E14_vs_E10	ordinary_muscle_development	D_vs_E10;stage_1W_vs_E10;stage_3W_vs_E10;stage	1	TRUE
ENSGALG00010017106	CRYM	PRJNA729271	-1.238589188	9.17675E-24	1.238589188	stage_E14_vs_E10	ordinary_muscle_development	D_vs_E10;stage_1W_vs_E10;stage_3W_vs_E10;stage	1	TRUE
ENSGALG00010029290	ENSGALG00010029290	PRJNA729271	-2.051485477	9.33503E-24	2.051485477	stage_E14_vs_E10	ordinary_muscle_development	D_vs_E10;stage_1W_vs_E10;stage_3W_vs_E10;stage	1	TRUE
ENSGALG00010024950	NCAM1	PRJNA729271	1.603059215	9.36525E-24	1.603059215	stage_E14_vs_E10	ordinary_muscle_development	D_vs_E10;stage_1W_vs_E10;stage_3W_vs_E10;stage	1	TRUE
ENSGALG00010022826	SNORC	PRJNA729271	-6.745492227	9.64885E-24	6.745492227	stage_E14_vs_E10	ordinary_muscle_development	D_vs_E10;stage_1W_vs_E10;stage_3W_vs_E10;stage	1	TRUE
ENSGALG00010018950	TTL	PRJNA729271	-0.995787427	9.85052E-24	0.995787427	stage_E14_vs_E10	ordinary_muscle_development		0	FALSE
ENSGALG00010011059	ENSGALG00010011059	PRJNA729271	-2.138277179	9.85762E-24	2.138277179	stage_E14_vs_E10	ordinary_muscle_development	D_vs_E10;stage_1W_vs_E10;stage_3W_vs_E10;stage	1	TRUE
ENSGALG00010013964	SLC38A1	PRJNA729271	-1.649917472	9.9344E-24	1.649917472	stage_E14_vs_E10	ordinary_muscle_development	D_vs_E10;stage_1W_vs_E10;stage_3W_vs_E10;stage	1	TRUE
ENSGALG00010016182	ELMO2	PRJNA729271	-0.639794231	1.03092E-23	0.639794231	stage_E14_vs_E10	ordinary_muscle_development		0	FALSE
ENSGALG00010008113	DBNDD1	PRJNA729271	-1.988923002	1.03278E-23	1.988923002	stage_E14_vs_E10	ordinary_muscle_development	D_vs_E10;stage_1W_vs_E10;stage_3W_vs_E10;stage	1	TRUE
ENSGALG00010007580	BRD9	PRJNA729271	-1.05955085	1.03499E-23	1.05955085	stage_E14_vs_E10	ordinary_muscle_development	D_vs_E10;stage_1W_vs_E10;stage_3W_vs_E10;stage	1	TRUE
ENSGALG00010026692	NMNAT3	PRJNA729271	5.060354015	1.04547E-23	5.060354015	stage_E14_vs_E10	ordinary_muscle_development	D_vs_E10;stage_1W_vs_E10;stage_3W_vs_E10;stage	1	TRUE
ENSGALG00010002627	MRPS31	PRJNA729271	1.487770046	1.05697E-23	1.487770046	stage_E14_vs_E10	ordinary_muscle_development	D_vs_E10;stage_1W_vs_E10;stage_3W_vs_E10;stage	1	TRUE
ENSGALG00010028495	LINGO3	PRJNA729271	-5.487543169	1.05787E-23	5.487543169	stage_E14_vs_E10	ordinary_muscle_development	D_vs_E10;stage_1W_vs_E10;stage_3W_vs_E10;stage	1	TRUE
ENSGALG00010005211	ESRRB	PRJNA729271	3.388080702	1.07971E-23	3.388080702	stage_E14_vs_E10	ordinary_muscle_development	D_vs_E10;stage_1W_vs_E10;stage_3W_vs_E10;stage	1	TRUE
ENSGALG00010024492	NTF3	PRJNA729271	-2.619457385	1.08036E-23	2.619457385	stage_E14_vs_E10	ordinary_muscle_development	D_vs_E10;stage_1W_vs_E10;stage_3W_vs_E10;stage	1	TRUE
ENSGALG00010012450	PPFIA2	PRJNA729271	-2.959539531	1.08353E-23	2.959539531	stage_E14_vs_E10	ordinary_muscle_development	D_vs_E10;stage_1W_vs_E10;stage_3W_vs_E10;stage	1	TRUE
ENSGALG00010028283	ENSGALG00010028283	PRJNA729271	1.548687397	1.0845E-23	1.548687397	stage_E14_vs_E10	ordinary_muscle_development	D_vs_E10;stage_1W_vs_E10;stage_3W_vs_E10;stage	1	TRUE
ENSGALG00010026822	PRELP	PRJNA729271	2.043654478	1.08757E-23	2.043654478	stage_E14_vs_E10	ordinary_muscle_development	D_vs_E10;stage_1W_vs_E10;stage_3W_vs_E10;stage	1	TRUE
ENSGALG00010012830	ARRDC3	PRJNA729271	1.516663792	1.10509E-23	1.516663792	stage_E14_vs_E10	ordinary_muscle_development	D_vs_E10;stage_1W_vs_E10;stage_3W_vs_E10;stage	1	TRUE
ENSGALG00010021658	STRADA	PRJNA729271	-0.947357405	1.11311E-23	0.947357405	stage_E14_vs_E10	ordinary_muscle_development		0	FALSE
ENSGALG00010028056	SLC25A25	PRJNA729271	2.403727364	1.12971E-23	2.403727364	stage_E14_vs_E10	ordinary_muscle_development	D_vs_E10;stage_1W_vs_E10;stage_3W_vs_E10;stage	1	TRUE
ENSGALG00010004340	SLC4A4	PRJNA729271	1.733627785	1.1424E-23	1.733627785	stage_E14_vs_E10	ordinary_muscle_development	D_vs_E10;stage_1W_vs_E10;stage_3W_vs_E10;stage	1	TRUE
ENSGALG00010000625	ENSGALG00010000625	PRJNA729271	3.600217541	1.17723E-23	3.600217541	stage_E14_vs_E10	ordinary_muscle_development	D_vs_E10;stage_1W_vs_E10;stage_3W_vs_E10;stage	1	TRUE
ENSGALG00010006794	PERPA	PRJNA729271	-6.194271435	1.17887E-23	6.194271435	stage_E14_vs_E10	ordinary_muscle_development	D_vs_E10;stage_1W_vs_E10;stage_3W_vs_E10;stage	1	TRUE
ENSGALG00010000810	UCHL3	PRJNA729271	-1.413625496	1.2112E-23	1.413625496	stage_E14_vs_E10	ordinary_muscle_development	D_vs_E10;stage_1W_vs_E10;stage_3W_vs_E10;stage	1	TRUE
ENSGALG00010013915	ENSGALG00010013915	PRJNA729271	1.926322524	1.23244E-23	1.926322524	stage_E14_vs_E10	ordinary_muscle_development	D_vs_E10;stage_1W_vs_E10;stage_3W_vs_E10;stage	1	TRUE
ENSGALG00010029306	CEP95	PRJNA729271	-1.658110332	1.24644E-23	1.658110332	stage_E14_vs_E10	ordinary_muscle_development	D_vs_E10;stage_1W_vs_E10;stage_3W_vs_E10;stage	1	TRUE
ENSGALG00010015409	FAM13B	PRJNA729271	1.342274847	1.26271E-23	1.342274847	stage_E14_vs_E10	ordinary_muscle_development	D_vs_E10;stage_1W_vs_E10;stage_3W_vs_E10;stage	1	TRUE
ENSGALG00010012761	SMPDL3A	PRJNA729271	2.285377292	1.28921E-23	2.285377292	stage_E14_vs_E10	ordinary_muscle_development	D_vs_E10;stage_1W_vs_E10;stage_3W_vs_E10;stage	1	TRUE
ENSGALG00010018497	ASTE1	PRJNA729271	-1.193352905	1.32653E-23	1.193352905	stage_E14_vs_E10	ordinary_muscle_development	D_vs_E10;stage_1W_vs_E10;stage_3W_vs_E10;stage	1	TRUE
ENSGALG00010004307	NPC2	PRJNA729271	1.42895827	1.33464E-23	1.42895827	stage_E14_vs_E10	ordinary_muscle_development	D_vs_E10;stage_1W_vs_E10;stage_3W_vs_E10;stage	1	TRUE
ENSGALG00010028187	TPRA1	PRJNA729271	0.707522351	1.33464E-23	0.707522351	stage_E14_vs_E10	ordinary_muscle_development		0	FALSE
ENSGALG00010015084	FOXO6	PRJNA729271	2.06320653	1.34351E-23	2.06320653	stage_E14_vs_E10	ordinary_muscle_development	D_vs_E10;stage_1W_vs_E10;stage_3W_vs_E10;stage	1	TRUE
ENSGALG00010016403	GRK4	PRJNA729271	-1.388170521	1.39518E-23	1.388170521	stage_E14_vs_E10	ordinary_muscle_development	D_vs_E10;stage_1W_vs_E10;stage_3W_vs_E10;stage	1	TRUE
ENSGALG00010027114	FLNB	PRJNA729271	-1.175061539	1.40189E-23	1.175061539	stage_E14_vs_E10	ordinary_muscle_development	D_vs_E10;stage_1W_vs_E10;stage_3W_vs_E10;stage	1	TRUE
ENSGALG00010001283	ENSGALG00010001283	PRJNA729271	1.779548035	1.41002E-23	1.779548035	stage_E14_vs_E10	ordinary_muscle_development	D_vs_E10;stage_1W_vs_E10;stage_3W_vs_E10;stage	1	TRUE
ENSGALG00010008469	NOCT	PRJNA729271	2.798165005	1.46451E-23	2.798165005	stage_E14_vs_E10	ordinary_muscle_development	D_vs_E10;stage_1W_vs_E10;stage_3W_vs_E10;stage	1	TRUE
ENSGALG00010011421	ENSGALG00010011421	PRJNA729271	1.185776692	1.48462E-23	1.185776692	stage_E14_vs_E10	ordinary_muscle_development	D_vs_E10;stage_1W_vs_E10;stage_3W_vs_E10;stage	1	TRUE
ENSGALG00010005372	CDIN1	PRJNA729271	-1.132181912	1.51676E-23	1.132181912	stage_E14_vs_E10	ordinary_muscle_development	D_vs_E10;stage_1W_vs_E10;stage_3W_vs_E10;stage	1	TRUE
ENSGALG00010022079	ABI2	PRJNA729271	-0.784315343	1.53061E-23	0.784315343	stage_E14_vs_E10	ordinary_muscle_development		0	FALSE
ENSGALG00010001456	LSM8	PRJNA729271	-1.197196433	1.5534E-23	1.197196433	stage_E14_vs_E10	ordinary_muscle_development	D_vs_E10;stage_1W_vs_E10;stage_3W_vs_E10;stage	1	TRUE
ENSGALG00010014062	GGA1	PRJNA729271	1.049804353	1.56014E-23	1.049804353	stage_E14_vs_E10	ordinary_muscle_development	D_vs_E10;stage_1W_vs_E10;stage_3W_vs_E10;stage	1	TRUE
ENSGALG00010015404	BEND4	PRJNA729271	-4.156976139	1.56895E-23	4.156976139	stage_E14_vs_E10	ordinary_muscle_development	D_vs_E10;stage_1W_vs_E10;stage_3W_vs_E10;stage	1	TRUE
ENSGALG00010010964	INTS9	PRJNA729271	-0.960575428	1.58245E-23	0.960575428	stage_E14_vs_E10	ordinary_muscle_development		0	FALSE
ENSGALG00010025554	DIP2C	PRJNA729271	0.718346935	1.59415E-23	0.718346935	stage_E14_vs_E10	ordinary_muscle_development		0	FALSE
ENSGALG00010014166	FGF12	PRJNA729271	-2.163157814	1.60914E-23	2.163157814	stage_E14_vs_E10	ordinary_muscle_development	D_vs_E10;stage_1W_vs_E10;stage_3W_vs_E10;stage	1	TRUE
ENSGALG00010029268	PCTP	PRJNA729271	1.807763388	1.63682E-23	1.807763388	stage_E14_vs_E10	ordinary_muscle_development	D_vs_E10;stage_1W_vs_E10;stage_3W_vs_E10;stage	1	TRUE
ENSGALG00010021257	RGS6	PRJNA729271	2.922750234	1.64149E-23	2.922750234	stage_E14_vs_E10	ordinary_muscle_development	D_vs_E10;stage_1W_vs_E10;stage_3W_vs_E10;stage	1	TRUE
ENSGALG00010014737	RMI1	PRJNA729271	-1.22183284	1.64149E-23	1.22183284	stage_E14_vs_E10	ordinary_muscle_development	D_vs_E10;stage_1W_vs_E10;stage_3W_vs_E10;stage	1	TRUE
ENSGALG00010021902	LMTK2	PRJNA729271	1.20560762	1.64682E-23	1.20560762	stage_E14_vs_E10	ordinary_muscle_development	D_vs_E10;stage_1W_vs_E10;stage_3W_vs_E10;stage	1	TRUE
ENSGALG00010020992	KCNH7	PRJNA729271	4.332688571	1.66635E-23	4.332688571	stage_E14_vs_E10	ordinary_muscle_development	D_vs_E10;stage_1W_vs_E10;stage_3W_vs_E10;stage	1	TRUE
ENSGALG00010012922	SNRNP40	PRJNA729271	-1.321246692	1.68445E-23	1.321246692	stage_E14_vs_E10	ordinary_muscle_development	D_vs_E10;stage_1W_vs_E10;stage_3W_vs_E10;stage	1	TRUE
ENSGALG00010026017	PLEKHM1	PRJNA729271	1.446666965	1.68919E-23	1.446666965	stage_E14_vs_E10	ordinary_muscle_development	D_vs_E10;stage_1W_vs_E10;stage_3W_vs_E10;stage	1	TRUE
ENSGALG00010004565	ENSGALG00010004565	PRJNA729271	3.832903126	1.6958E-23	3.832903126	stage_E14_vs_E10	ordinary_muscle_development	D_vs_E10;stage_1W_vs_E10;stage_3W_vs_E10;stage	1	TRUE
ENSGALG00010021676	TP53INP2	PRJNA729271	2.795258423	1.70117E-23	2.795258423	stage_E14_vs_E10	ordinary_muscle_development	D_vs_E10;stage_1W_vs_E10;stage_3W_vs_E10;stage	1	TRUE
ENSGALG00010019761	MRPS18A	PRJNA729271	0.878067681	1.7088E-23	0.878067681	stage_E14_vs_E10	ordinary_muscle_development		0	FALSE
ENSGALG00010011929	ELK3	PRJNA729271	-1.381233335	1.75973E-23	1.381233335	stage_E14_vs_E10	ordinary_muscle_development	D_vs_E10;stage_1W_vs_E10;stage_3W_vs_E10;stage	1	TRUE
ENSGALG00010021375	TUBG1	PRJNA729271	-1.219600104	1.78438E-23	1.219600104	stage_E14_vs_E10	ordinary_muscle_development	D_vs_E10;stage_1W_vs_E10;stage_3W_vs_E10;stage	1	TRUE
ENSGALG00010019081	PCSK6	PRJNA729271	1.36905832	1.80501E-23	1.36905832	stage_E14_vs_E10	ordinary_muscle_development	D_vs_E10;stage_1W_vs_E10;stage_3W_vs_E10;stage	1	TRUE
ENSGALG00010019875	PLEKHH1	PRJNA729271	-3.53060318	1.81632E-23	3.53060318	stage_E14_vs_E10	ordinary_muscle_development	D_vs_E10;stage_1W_vs_E10;stage_3W_vs_E10;stage	1	TRUE
ENSGALG00010001991	TOMM7	PRJNA729271	1.316980419	1.82829E-23	1.316980419	stage_E14_vs_E10	ordinary_muscle_development	D_vs_E10;stage_1W_vs_E10;stage_3W_vs_E10;stage	1	TRUE
ENSGALG00010000044	MAP1A	PRJNA729271	1.363576271	1.87378E-23	1.363576271	stage_E14_vs_E10	ordinary_muscle_development	D_vs_E10;stage_1W_vs_E10;stage_3W_vs_E10;stage	1	TRUE
ENSGALG00010002458	ENSGALG00010002458	PRJNA729271	2.034035331	1.87462E-23	2.034035331	stage_E14_vs_E10	ordinary_muscle_development	D_vs_E10;stage_1W_vs_E10;stage_3W_vs_E10;stage	1	TRUE

ENSGALG00010007090	CEBPZ	PRJNA729271	-0.979936082	1.87903E-23	0.979936082	stage_E14_vs_E10	ordinary_muscle_development		0	FALSE
ENSGALG00010008232	PYURF	PRJNA729271	1.289158663	1.88669E-23	1.289158663	stage_E14_vs_E10	ordinary_muscle_development	D_vs_E10;stage_1W_vs_E10;stage_3W_vs_E10;stage	1	TRUE
ENSGALG00010024852	CEP89	PRJNA729271	-0.987770857	1.91722E-23	0.987770857	stage_E14_vs_E10	ordinary_muscle_development		0	FALSE
ENSGALG00010027283	RAB7A	PRJNA729271	0.634821313	1.9452E-23	0.634821313	stage_E14_vs_E10	ordinary_muscle_development		0	FALSE
ENSGALG00010022242	USP19	PRJNA729271	1.270736752	1.95193E-23	1.270736752	stage_E14_vs_E10	ordinary_muscle_development	D_vs_E10;stage_1W_vs_E10;stage_3W_vs_E10;stage	1	TRUE
ENSGALG00010024453	HTR3A	PRJNA729271	5.633540021	1.97064E-23	5.633540021	stage_E14_vs_E10	ordinary_muscle_development	D_vs_E10;stage_1W_vs_E10;stage_3W_vs_E10;stage	1	TRUE
ENSGALG00010005382	RAB32	PRJNA729271	-1.314126183	1.97965E-23	1.314126183	stage_E14_vs_E10	ordinary_muscle_development	D_vs_E10;stage_1W_vs_E10;stage_3W_vs_E10;stage	1	TRUE
ENSGALG00010006059	BIVM	PRJNA729271	-0.568213548	2.02055E-23	0.568213548	stage_E14_vs_E10	ordinary_muscle_development		0	FALSE
ENSGALG00010002444	KLF11	PRJNA729271	1.830073059	2.04513E-23	1.830073059	stage_E14_vs_E10	ordinary_muscle_development	D_vs_E10;stage_1W_vs_E10;stage_3W_vs_E10;stage	1	TRUE
ENSGALG00010023066	TLR21	PRJNA729271	1.875553869	2.05061E-23	1.875553869	stage_E14_vs_E10	ordinary_muscle_development	D_vs_E10;stage_1W_vs_E10;stage_3W_vs_E10;stage	1	TRUE
ENSGALG00010023081	PRLHR2	PRJNA729271	-8.126227727	2.06437E-23	8.126227727	stage_E14_vs_E10	ordinary_muscle_development	D_vs_E10;stage_1W_vs_E10;stage_3W_vs_E10;stage	1	TRUE
ENSGALG00010006156	NFKBIZ	PRJNA729271	1.974596494	2.0935E-23	1.974596494	stage_E14_vs_E10	ordinary_muscle_development	D_vs_E10;stage_1W_vs_E10;stage_3W_vs_E10;stage	1	TRUE
ENSGALG00010003272	CPSF3	PRJNA729271	-0.682374052	2.1015E-23	0.682374052	stage_E14_vs_E10	ordinary_muscle_development		0	FALSE
ENSGALG00010021104	INA	PRJNA729271	2.613548034	2.11332E-23	2.613548034	stage_E14_vs_E10	ordinary_muscle_development	D_vs_E10;stage_1W_vs_E10;stage_3W_vs_E10;stage	1	TRUE
ENSGALG00010006614	TMEM184C	PRJNA729271	-0.574551801	2.13329E-23	0.574551801	stage_E14_vs_E10	ordinary_muscle_development		0	FALSE
ENSGALG00010012879	RCAN1	PRJNA729271	1.782140547	2.13495E-23	1.782140547	stage_E14_vs_E10	ordinary_muscle_development	D_vs_E10;stage_1W_vs_E10;stage_3W_vs_E10;stage	1	TRUE
ENSGALG00010028051	GNL3	PRJNA729271	-0.955035231	2.14593E-23	0.955035231	stage_E14_vs_E10	ordinary_muscle_development		0	FALSE
ENSGALG00010009269	ENSGALG00010009269	PRJNA729271	-5.920511324	2.14901E-23	5.920511324	stage_E14_vs_E10	ordinary_muscle_development	D_vs_E10;stage_1W_vs_E10;stage_3W_vs_E10;stage	1	TRUE
ENSGALG00010017641	GPRC5B	PRJNA729271	1.620745232	2.1709E-23	1.620745232	stage_E14_vs_E10	ordinary_muscle_development	D_vs_E10;stage_1W_vs_E10;stage_3W_vs_E10;stage	1	TRUE
ENSGALG00010027784	PEAR1	PRJNA729271	1.921870725	2.17563E-23	1.921870725	stage_E14_vs_E10	ordinary_muscle_development	D_vs_E10;stage_1W_vs_E10;stage_3W_vs_E10;stage	1	TRUE
ENSGALG00010007566	FHIP1A	PRJNA729271	1.737711762	2.1972E-23	1.737711762	stage_E14_vs_E10	ordinary_muscle_development	D_vs_E10;stage_1W_vs_E10;stage_3W_vs_E10;stage	1	TRUE
ENSGALG00010001470	GPR85	PRJNA729271	-3.42433182	2.24521E-23	3.42433182	stage_E14_vs_E10	ordinary_muscle_development	D_vs_E10;stage_1W_vs_E10;stage_3W_vs_E10;stage	1	TRUE
ENSGALG00010021298	RPGRIP1L	PRJNA729271	-1.51836377	2.29987E-23	1.51836377	stage_E14_vs_E10	ordinary_muscle_development	D_vs_E10;stage_1W_vs_E10;stage_3W_vs_E10;stage	1	TRUE
ENSGALG00010012653	CBX7	PRJNA729271	2.522018525	2.32766E-23	2.522018525	stage_E14_vs_E10	ordinary_muscle_development	D_vs_E10;stage_1W_vs_E10;stage_3W_vs_E10;stage	1	TRUE
ENSGALG00010010280	HSP90B1	PRJNA729271	-1.370334006	2.37006E-23	1.370334006	stage_E14_vs_E10	ordinary_muscle_development	D_vs_E10;stage_1W_vs_E10;stage_3W_vs_E10;stage	1	TRUE
ENSGALG00010020046	BDNF	PRJNA729271	-2.55484648	2.37642E-23	2.55484648	stage_E14_vs_E10	ordinary_muscle_development	D_vs_E10;stage_1W_vs_E10;stage_3W_vs_E10;stage	1	TRUE
ENSGALG00010018552	DTD1	PRJNA729271	1.035062614	2.40424E-23	1.035062614	stage_E14_vs_E10	ordinary_muscle_development	D_vs_E10;stage_1W_vs_E10;stage_3W_vs_E10;stage	1	TRUE
ENSGALG00010011368	ENSGALG00010011368	PRJNA729271	-3.38104777	2.46661E-23	3.38104777	stage_E14_vs_E10	ordinary_muscle_development	D_vs_E10;stage_1W_vs_E10;stage_3W_vs_E10;stage	1	TRUE
ENSGALG00010002689	COG6	PRJNA729271	-0.633094008	2.47165E-23	0.633094008	stage_E14_vs_E10	ordinary_muscle_development		0	FALSE
ENSGALG00010016469	MAVS	PRJNA729271	1.026000234	2.4847E-23	1.026000234	stage_E14_vs_E10	ordinary_muscle_development	D_vs_E10;stage_1W_vs_E10;stage_3W_vs_E10;stage	1	TRUE
ENSGALG00010020925	RIC8A	PRJNA729271	1.057817481	2.5211E-23	1.057817481	stage_E14_vs_E10	ordinary_muscle_development	D_vs_E10;stage_1W_vs_E10;stage_3W_vs_E10;stage	1	TRUE
ENSGALG00010004472	NEIL3	PRJNA729271	-2.332623714	2.5392E-23	2.332623714	stage_E14_vs_E10	ordinary_muscle_development	D_vs_E10;stage_1W_vs_E10;stage_3W_vs_E10;stage	1	TRUE
ENSGALG00010012288	MCTS1	PRJNA729271	1.140705586	2.54331E-23	1.140705586	stage_E14_vs_E10	ordinary_muscle_development	D_vs_E10;stage_1W_vs_E10;stage_3W_vs_E10;stage	1	TRUE
ENSGALG00010021538	TNFRSF18	PRJNA729271	3.994743556	2.55161E-23	3.994743556	stage_E14_vs_E10	ordinary_muscle_development	D_vs_E10;stage_1W_vs_E10;stage_3W_vs_E10;stage	1	TRUE
ENSGALG00010002487	BCCIP	PRJNA729271	-1.209649551	2.57397E-23	1.209649551	stage_E14_vs_E10	ordinary_muscle_development	D_vs_E10;stage_1W_vs_E10;stage_3W_vs_E10;stage	1	TRUE
ENSGALG00010020616	PI4K2A	PRJNA729271	1.312385376	2.58212E-23	1.312385376	stage_E14_vs_E10	ordinary_muscle_development	D_vs_E10;stage_1W_vs_E10;stage_3W_vs_E10;stage	1	TRUE
ENSGALG00010025157	NOP58	PRJNA729271	-1.424123031	2.60524E-23	1.424123031	stage_E14_vs_E10	ordinary_muscle_development	D_vs_E10;stage_1W_vs_E10;stage_3W_vs_E10;stage	1	TRUE
ENSGALG00010022455	C7orf50	PRJNA729271	-1.607920439	2.73907E-23	1.607920439	stage_E14_vs_E10	ordinary_muscle_development	D_vs_E10;stage_1W_vs_E10;stage_3W_vs_E10;stage	1	TRUE
ENSGALG00010004946	SH2D1B	PRJNA729271	4.197581896	2.74205E-23	4.197581896	stage_E14_vs_E10	ordinary_muscle_development	D_vs_E10;stage_1W_vs_E10;stage_3W_vs_E10;stage	1	TRUE
ENSGALG00010008237	GRIA2	PRJNA729271	-5.115611084	2.75236E-23	5.115611084	stage_E14_vs_E10	ordinary_muscle_development	D_vs_E10;stage_1W_vs_E10;stage_3W_vs_E10;stage	1	TRUE
ENSGALG00010017772	ENSGALG00010017772	PRJNA729271	3.350566239	2.77165E-23	3.350566239	stage_E14_vs_E10	ordinary_muscle_development	D_vs_E10;stage_1W_vs_E10;stage_3W_vs_E10;stage	1	TRUE
ENSGALG00010005226	MYCT1	PRJNA729271	1.675949958	2.78778E-23	1.675949958	stage_E14_vs_E10	ordinary_muscle_development	D_vs_E10;stage_1W_vs_E10;stage_3W_vs_E10;stage	1	TRUE
ENSGALG00010025305	GPR182	PRJNA729271	3.87827117	2.79505E-23	3.87827117	stage_E14_vs_E10	ordinary_muscle_development	D_vs_E10;stage_1W_vs_E10;stage_3W_vs_E10;stage	1	TRUE
ENSGALG00010019657	PPP1R17	PRJNA729271	-2.805799463	2.86942E-23	2.805799463	stage_E14_vs_E10	ordinary_muscle_development	D_vs_E10;stage_1W_vs_E10;stage_3W_vs_E10;stage	1	TRUE
ENSGALG00010005248	TMEM200A	PRJNA729271	-2.448359565	2.89889E-23	2.448359565	stage_E14_vs_E10	ordinary_muscle_development	D_vs_E10;stage_1W_vs_E10;stage_3W_vs_E10;stage	1	TRUE
ENSGALG00010002774	TBX18	PRJNA729271	-1.208399012	2.92092E-23	1.208399012	stage_E14_vs_E10	ordinary_muscle_development	D_vs_E10;stage_1W_vs_E10;stage_3W_vs_E10;stage	1	TRUE
ENSGALG00010010857	SEL1L3	PRJNA729271	0.608157743	2.94585E-23	0.608157743	stage_E14_vs_E10	ordinary_muscle_development		0	FALSE
ENSGALG00010024669	STIL	PRJNA729271	-1.907166789	2.95412E-23	1.907166789	stage_E14_vs_E10	ordinary_muscle_development	D_vs_E10;stage_1W_vs_E10;stage_3W_vs_E10;stage	1	TRUE
ENSGALG00010021530	MLF2	PRJNA729271	-0.614959649	3.08282E-23	0.614959649	stage_E14_vs_E10	ordinary_muscle_development		0	FALSE
ENSGALG00010011243	ALX1	PRJNA729271	-8.240828794	3.09095E-23	8.240828794	stage_E14_vs_E10	ordinary_muscle_development	D_vs_E10;stage_1W_vs_E10;stage_3W_vs_E10;stage	1	TRUE
ENSGALG00010025229	ENSGALG00010025229	PRJNA729271	-1.6877325	3.09095E-23	1.6877325	stage_E14_vs_E10	ordinary_muscle_development	D_vs_E10;stage_1W_vs_E10;stage_3W_vs_E10;stage	1	TRUE
ENSGALG00010005866	DNAJC1	PRJNA729271	1.486322093	3.11336E-23	1.486322093	stage_E14_vs_E10	ordinary_muscle_development	D_vs_E10;stage_1W_vs_E10;stage_3W_vs_E10;stage	1	TRUE
ENSGALG00010014218	IL31RA	PRJNA729271	1.672260802	3.11346E-23	1.672260802	stage_E14_vs_E10	ordinary_muscle_development	D_vs_E10;stage_1W_vs_E10;stage_3W_vs_E10;stage	1	TRUE
ENSGALG00010029177	ASPSCR1	PRJNA729271	0.80613177	3.13465E-23	0.80613177	stage_E14_vs_E10	ordinary_muscle_development		0	FALSE
ENSGALG00010013111	RFESD	PRJNA729271	2.740035061	3.14915E-23	2.740035061	stage_E14_vs_E10	ordinary_muscle_development	D_vs_E10;stage_1W_vs_E10;stage_3W_vs_E10;stage	1	TRUE
ENSGALG00010009683	ADAMTS19	PRJNA729271	2.409120949	3.15136E-23	2.409120949	stage_E14_vs_E10	ordinary_muscle_development	D_vs_E10;stage_1W_vs_E10;stage_3W_vs_E10;stage	1	TRUE
ENSGALG00010005068	PLCXD2	PRJNA729271	2.599867458	3.1598E-23	2.599867458	stage_E14_vs_E10	ordinary_muscle_development	D_vs_E10;stage_1W_vs_E10;stage_3W_vs_E10;stage	1	TRUE
ENSGALG00010011212	NCKAP1	PRJNA729271	-0.637311426	3.21953E-23	0.637311426	stage_E14_vs_E10	ordinary_muscle_development		0	FALSE
ENSGALG00010030106	GPRC5C	PRJNA729271	-2.151811892	3.25515E-23	2.151811892	stage_E14_vs_E10	ordinary_muscle_development	D_vs_E10;stage_1W_vs_E10;stage_3W_vs_E10;stage	1	TRUE
ENSGALG00010012123	ENSGALG00010012123	PRJNA729271	-2.148454411	3.25943E-23	2.148454411	stage_E14_vs_E10	ordinary_muscle_development	D_vs_E10;stage_1W_vs_E10;stage_3W_vs_E10;stage	1	TRUE
ENSGALG00010016514	GASK1A	PRJNA729271	-2.578015286	3.27592E-23	2.578015286	stage_E14_vs_E10	ordinary_muscle_development	D_vs_E10;stage_1W_vs_E10;stage_3W_vs_E10;stage	1	TRUE
ENSGALG00010022630	ENSGALG00010022630	PRJNA729271	-3.347577443	3.30136E-23	3.347577443	stage_E14_vs_E10	ordinary_muscle_development	D_vs_E10;stage_1W_vs_E10;stage_3W_vs_E10;stage	1	TRUE
ENSGALG00010021678	DNAAF4	PRJNA729271	1.537775503	3.32989E-23	1.537775503	stage_E14_vs_E10	ordinary_muscle_development	D_vs_E10;stage_1W_vs_E10;stage_3W_vs_E10;stage	1	TRUE
ENSGALG00010028462	EDF1	PRJNA729271	1.06074235	3.33166E-23	1.06074235	stage_E14_vs_E10	ordinary_muscle_development	D_vs_E10;stage_1W_vs_E10;stage_3W_vs_E10;stage	1	TRUE
ENSGALG00010029508	ENSGALG00010029508	PRJNA729271	-1.578574486	3.39171E-23	1.578574486	stage_E14_vs_E10	ordinary_muscle_development	D_vs_E10;stage_1W_vs_E10;stage_3W_vs_E10;stage	1	TRUE
ENSGALG00010025978	TRIM29	PRJNA729271	-8.703809721	3.39313E-23	8.703809721	stage_E14_vs_E10	ordinary_muscle_development	D_vs_E10;stage_1W_vs_E10;stage_3W_vs_E10;stage	1	TRUE
ENSGALG00010023925	NCOA3	PRJNA729271	-0.786609165	3.49404E-23	0.786609165	stage_E14_vs_E10	ordinary_muscle_development		0	FALSE
ENSGALG00010012790	PITX1	PRJNA729271	-6.189093697	3.50787E-23	6.189093697	stage_E14_vs_E10	ordinary_muscle_development	D_vs_E10;stage_1W_vs_E10;stage_3W_vs_E10;stage	1	TRUE
ENSGALG00010009749	NDRG1	PRJNA729271	1.685144472	3.51516E-23	1.685144472	stage_E14_vs_E10	ordinary_muscle_development	D_vs_E10;stage_1W_vs_E10;stage_3W_vs_E10;stage	1	TRUE
ENSGALG00010023231	ASIC1	PRJNA729271	-3.363576838	3.52524E-23	3.363576838	stage_E14_vs_E10	ordinary_muscle_development	D_vs_E10;stage_1W_vs_E10;stage_3W_vs_E10;stage	1	TRUE
ENSGALG00010003746	HOPX	PRJNA729271	4.093781771	3.52943E-23	4.093781771	stage_E14_vs_E10	ordinary_muscle_development	D_vs_E10;stage_1W_vs_E10;stage_3W_vs_E10;stage	1	TRUE
ENSGALG00010005250	SLC35A5	PRJNA729271	-0.945215892	3.63255E-23	0.945215892	stage_E14_vs_E10	ordinary_muscle_development		0	FALSE
ENSGALG00010011980	AvBD9	PRJNA729271	23.02417435	3.66309E-23	23.02417435	stage_E14_vs_E10	ordinary_muscle_development	D_vs_E10;stage_1W_vs_E10;stage_3W_vs_E10;stage	1	TRUE
ENSGALG00010022789	ENSGALG00010022789	PRJNA729271	-4.405469384	3.66311E-23	4.405469384	stage_E14_vs_E10	ordinary_muscle_development	D_vs_E10;stage_1W_vs_E10;stage_3W_vs_E10;stage	1	TRUE
ENSGALG00010029352	AATF	PRJNA729271	-1.134164256	3.9266E-23	1.134164256	stage_E14_vs_E10	ordinary_muscle_development	D_vs_E10;stage_1W_vs_E10;stage_3W_vs_E10;stage	1	TRUE
ENSGALG00010005858	CFAP97	PRJNA729271	-1.155143934	3.93306E-23	1.155143934	stage_E14_vs_E10	ordinary_muscle_development	D_vs_E10;stage_1W_vs_E10;stage_3W_vs_E10;stage	1	TRUE
ENSGALG00010017112	LZIC	PRJNA729271	-0.63861082	3.98483E-23	0.63861082	stage_E14_vs_E10	ordinary_muscle_development		0	FALSE
ENSGALG00010019263	SNRPB2	PRJNA729271	-1.044246596	3.99781E-23	1.044246596	stage_E14_vs_E10	ordinary_muscle_development	D_vs_E10;stage_1W_vs_E10;stage_3W_vs_E10;stage	1	TRUE
ENSGALG00010012962	SLC25A17	PRJNA729271	-2.149298944	4.00366E-23	2.149298944	stage_E14_vs_E10	ordinary_muscle_development	D_vs_E10;stage_1W_vs_E10;stage_3W_vs_E10;stage	1	TRUE
ENSGALG00010020244	SLC25A37	PRJNA729271	-2.202117062	4.02248E-23	2.202117062	stage_E14_vs_E10	ordinary_muscle_development	D_vs_E10;stage_1W_vs_E10;stage_3W_vs_E10;stage	1	TRUE
ENSGALG00010018973	NEFM	PRJNA729271	-7.608825108	4.03821E-23	7.608825108	stage_E14_vs_E10	ordinary_muscle_development	D_vs_E10;stage_1W_vs_E10;stage_3W_vs_E10;stage	1	TRUE
ENSGALG00010020314	THADA	PRJNA729271	-0.825423006	4.21651E-23	0.825423006	stage_E14_vs_E10	ordinary_muscle_development		0	FALSE
ENSGALG00010009560	RAB24	PRJNA729271	1.420123799	4.23135E-23	1.420123799	stage_E14_vs_E10	ordinary_muscle_development	D_vs_E10;stage_1W_vs_E10;stage_3W_vs_E10;stage	1	TRUE
ENSGALG00010026743	TNNT2	PRJNA729271	-5.927866111	4.28787E-23	5.927866111	stage_E14_vs_E10	ordinary_muscle_development	D_vs_E10;stage_1W_vs_E10;stage_3W_vs_E10;stage	1	TRUE
ENSGALG00010009605	ENSGALG00010009605	PRJNA729271	1.572113268	4.31493E-23	1.572113268	stage_E14_vs_E10	ordinary_muscle_development	D_vs_E10;stage_1W_vs_E10;stage_3W_vs_E10;stage	1	TRUE
ENSGALG00010017594	CEP68	PRJNA729271	1.010483067	4.35591E-23	1.010483067	stage_E14_vs_E10	ordinary_muscle_development	D_vs_E10;stage_1W_vs_E10;stage_3W_vs_E10;stage	1	TRUE
ENSGALG00010012995	SERINC2	PRJNA729271	1.495267003	4.36953E-23	1.495267003	stage_E14_vs_E10	ordinary_muscle_development	D_vs_E10;stage_1W_vs_E10;stage_3W_vs_E10;stage	1	TRUE
ENSGALG00010017976	GPR137C	PRJNA729271	-1.102465354	4.37338E-23	1.102465354	stage_E14_vs_E10	ordinary_muscle_development	D_vs_E10;stage_1W_vs_E10;stage_3W_vs_E10;stage	1	TRUE
ENSGALG00010019470	TGFB2	PRJNA729271	-2.019144826	4.44781E-23	2.019144826	stage_E14_vs_E10	ordinary_muscle_development	D_vs_E10;stage_1W_vs_E10;stage_3W_vs_E10;stage	1	TRUE
ENSGALG00010015508	FUCA1	PRJNA729271	1.035382794	4.4817E-23	1.035382794	stage_E14_vs_E10	ordinary_muscle_development	D_vs_E10;stage_1W_vs_E10;stage_3W_vs_E10;stage	1	TRUE
ENSGALG00010008268	ENSGALG00010008268	PRJNA729271	2.321370621	4.54189E-23	2.321370621	stage_E14_vs_E10	ordinary_muscle_development	D_vs_E10;stage_1W_vs_E10;stage_3W_vs_E10;stage	1	TRUE
ENSGALG00010023802	ENSGALG00010023802	PRJNA729271	2.722242062	4.55477E-23	2.722242062	stage_E14_vs_E10	ordinary_muscle_development	D_vs_E10;stage_1W_vs_E10;stage_3W_vs_E10;stage	1	TRUE
ENSGALG00010012891	CAPS2	PRJNA729271	-3.778880476	4.57098E-23	3.778880476	stage_E14_vs_E10	ordinary_muscle_development	D_vs_E10;stage_1W_vs_E10;stage_3W_vs_E10;stage	1	TRUE
ENSGALG00010027103	ACVR2B	PRJNA729271	-1.929359355	4.5924E-23	1.929359355	stage_E14_vs_E10	ordinary_muscle_development	D_vs_E10;stage_1W_vs_E10;stage_3W_vs_E10;stage	1	TRUE
ENSGALG00010025913	EIF2B3	PRJNA729271	-0.808968437	4.59914E-23	0.808968437	stage_E14_vs_E10	ordinary_muscle_development		0	FALSE
ENSGALG00010007830	SMIM19	PRJNA729271	1.305474391	4.64341E-23	1.305474391	stage_E14_vs_E10	ordinary_muscle_development	D_vs_E10;stage_1W_vs_E10;stage_3W_vs_E10;stage	1	TRUE
ENSGALG00010007544	MLYCD	PRJNA729271	1.016701216	4.64717E-23	1.016701216	stage_E14_vs_E10	ordinary_muscle_development	D_vs_E10;stage_1W_vs_E10;stage_3W_vs_E10;stage	1	TRUE
ENSGALG00010020861	CDA	PRJNA729271	1.740384358	4.67937E-23	1.740384358	stage_E14_vs_E10	ordinary_muscle_development	D_vs_E10;stage_1W_vs_E10;stage_3W_vs_E10;stage	1	TRUE
ENSGALG00010013081	NCOA5	PRJNA729271	-1.026603873	4.89493E-23	1.026603873	stage_E14_vs_E10	ordinary_muscle_development	D_vs_E10;stage_1W_vs_E10;stage_3W_vs_E10;stage	1	TRUE
ENSGALG00010005356	DNAJC3	PRJNA729271	0.843157804	4.913E-23	0.843157804	stage_E14_vs_E10	ordinary_muscle_development		0	FALSE
ENSGALG00010025360	THYN1	PRJNA729271	1.12749069	4.96358E-23	1.12749069	stage_E14_vs_E10	ordinary_muscle_development	D_vs_E10;stage_1W_vs_E10;stage_3W_vs_E10;stage	1	TRUE
ENSGALG00010017001	TPCN3	PRJNA729271	-1.228322229	4.97189E-23	1.228322229	stage_E14_vs_E10	ordinary_muscle_development	D_vs_E10;stage_1W_vs_E10;stage_3W_vs_E10;stage	1	TRUE
ENSGALG00010006472	DCLK1	PRJNA729271	-3.30000353	5.13173E-23	3.30000353	stage_E14_vs_E10	ordinary_muscle_development	D_vs_E10;stage_1W_vs_E10;stage_3W_vs_E10;stage	1	TRUE
ENSGALG00010028919	EGFL7	PRJNA729271	1.701654484	5.20493E-23	1.701654484	stage_E14_vs_E10	ordinary_muscle_development	D_vs_E10;stage_1W_vs_E10;stage_3W_vs_E10;stage	1	TRUE
ENSGALG00010006381	SPART	PRJNA729271	1.569666362	5.27312E-23	1.569666362	stage_E14_vs_E10	ordinary_muscle_development	D_vs_E10;stage_1W_vs_E10;stage_3W_vs_E10;stage	1	TRUE
ENSGALG00010024592	COMT	PRJNA729271	1.994513482	5.27519E-23	1.994513482	stage_E14_vs_E10	ordinary_muscle_development	D_vs_E10;stage_1W_vs_E10;stage_3W_vs_E10;stage	1	TRUE
ENSGALG00010014806	MAP7D1	PRJNA729271	2.26423957	5.2868E-23	2.26423957	stage_E14_vs_E10	ordinary_muscle_development	D_vs_E10;stage_1W_vs_E10;stage_3W_vs_E10;stage	1	TRUE
ENSGALG00010006331	SMAD9	PRJNA729271	-1.592986796	5.35904E-23	1.592986796	stage_E14_vs_E10	ordinary_muscle_development	D_vs_E10;stage_1W_vs_E10;stage_3W_vs_E10;stage	1	TRUE
ENSGALG00010020228	TM9SF3	PRJNA729271	-0.834811277	5.42868E-23	0.834811277	stage_E14_vs_E10	ordinary_muscle_development		0	FALSE
ENSGALG00010025948	WNT9B	PRJNA729271	-8.72449705	5.56938E-23	8.72449705	stage_E14_vs_E10	ordinary_muscle_development	D_vs_E10;stage_1W_vs_E10;stage_3W_vs_E10;stage	1	TRUE
ENSGALG00010014570	KIF27	PRJNA729271	-1.693021983	5.61519E-23	1.693021983	stage_E14_vs_E10	ordinary_muscle_development	D_vs_E10;stage_1W_vs_E10;stage_3W_vs_E10;stage	1	TRUE
ENSGALG00010019634	GNG2	PRJNA729271	-3.10652409	5.68688E-23	3.10652409	stage_E14_vs_E10	ordinary_muscle_development	D_vs_E10;stage_1W_vs_E10;stage_3W_vs_E10;stage	1	TRUE
ENSGALG00010021549	ANAPC16	PRJNA729271	0.901787915	5.80098E-23	0.901787915	stage_E14_vs_E10	ordinary_muscle_development		0	FALSE
ENSGALG00010027578	ENSGALG00010027578	PRJNA729271	2.376968422	5.83306E-23	2.376968422	stage_E14_vs_E10	ordinary_muscle_development	D_vs_E10;stage_1W_vs_E10;stage_3W_vs_E10;stage	1	TRUE
ENSGALG00010008995	ECD	PRJNA729271	-1.022379196	5.8613E-23	1.022379196	stage_E14_vs_E10	ordinary_muscle_development	D_vs_E10;stage_1W_vs_E10;stage_3W_vs_E10;stage	1	TRUE
ENSGALG00010025256	ANAPC5	PRJNA729271	-1.26709049	6.0743E-23	1.26709049	stage_E14_vs_E10	ordinary_muscle_development	D_vs_E10;stage_1W_vs_E10;stage_3W_vs_E10;stage	1	TRUE
ENSGALG00010017574	DYNC1LI1	PRJNA729271	-0.566607778	6.07891E-23	0.566607778	stage_E14_vs_E10	ordinary_muscle_development		0	FALSE
ENSGALG00010024989	TIMELESS	PRJNA729271	-1.662100015	6.14156E-23	1.662100015	stage_E14_vs_E10	ordinary_muscle_development	D_vs_E10;stage_1W_vs_E10;stage_3W_vs_E10;stage	1	TRUE
ENSGALG00010023923	CSNK1G1	PRJNA729271	-0.98209523	6.1825E-23	0.98209523	stage_E14_vs_E10	ordinary_muscle_development		0	FALSE
ENSGALG00010025250	GLIS1	PRJNA729271	-5.76078047	6.68473E-23	5.76078047	stage_E14_vs_E10	ordinary_muscle_development	D_vs_E10;stage_1W_vs_E10;stage_3W_vs_E10;stage	1	TRUE
ENSGALG00010006883	LRBA	PRJNA729271	-0.72118511	6.77768E-23	0.72118511	stage_E14_vs_E10	ordinary_muscle_development		0	FALSE
ENSGALG00010009416	COL14A1	PRJNA729271	-4.740493185	6.88499E-23	4.740493185	stage_E14_vs_E10	ordinary_muscle_development	D_vs_E10;stage_1W_vs_E10;stage_3W_vs_E10;stage	1	TRUE
ENSGALG00010024497	ABI3	PRJNA729271	1.777598328	6.91494E-23	1.777598328	stage_E14_vs_E10	ordinary_muscle_development	D_vs_E10;stage_1W_vs_E10;stage_3W_vs_E10;stage	1	TRUE
ENSGALG00010005415	PRKDC	PRJNA729271	-0.88373742	6.9806E-23	0.88373742	stage_E14_vs_E10	ordinary_muscle_development		0	FALSE
ENSGALG00010017850	TRMT5	PRJNA729271	-0.987714439	7.00546E-23	0.987714439	stage_E14_vs_E10	ordinary_muscle_development		0	FALSE
ENSGALG00010023603	C1S	PRJNA729271	3.927681495	7.2666E-23	3.927681495	stage_E14_vs_E10	ordinary_muscle_development	D_vs_E10;stage_1W_vs_E10;stage_3W_vs_E10;stage	1	TRUE
ENSGALG00010012219	KDM5A	PRJNA729271	-0.779760951	7.36915E-23	0.779760951	stage_E14_vs_E10	ordinary_muscle_development		0	FALSE
ENSGALG00010010460	SEMA3C	PRJNA729271	1.428113084	7.37794E-23	1.428113084	stage_E14_vs_E10	ordinary_muscle_development	D_vs_E10;stage_1W_vs_E10;stage_3W_vs_E10;stage	1	TRUE
ENSGALG00010009313	UNC13B	PRJNA729271	1.706883119	7.40859E-23	1.706883119	stage_E14_vs_E10	ordinary_muscle_development	D_vs_E10;stage_1W_vs_E10;stage_3W_vs_E10;stage	1	TRUE
ENSGALG00010017766	DNAAF10	PRJNA729271	1.529414874	7.55993E-23	1.529414874	stage_E14_vs_E10	ordinary_muscle_development	D_vs_E10;stage_1W_vs_E10;stage_3W_vs_E10;stage	1	TRUE
ENSGALG00010006239	EYA4	PRJNA729271	1.621770179	7.57828E-23	1.621770179	stage_E14_vs_E10	ordinary_muscle_development	D_vs_E10;stage_1W_vs_E10;stage_3W_vs_E10;stage	1	TRUE
ENSGALG00010009102	HTRA3	PRJNA729271	2.166294484	7.82434E-23	2.166294484	stage_E14_vs_E10	ordinary_muscle_development	D_vs_E10;stage_1W_vs_E10;stage_3W_vs_E10;stage	1	TRUE
ENSGALG00010024674	DHH	PRJNA729271	3.143077319	7.85682E-23	3.143077319	stage_E14_vs_E10	ordinary_muscle_development	D_vs_E10;stage_1W_vs_E10;stage_3W_vs_E10;stage	1	TRUE
ENSGALG00010017903	ENSGALG00010017903	PRJNA729271	-5.103893819	8.1157E-23	5.103893819	stage_E14_vs_E10	ordinary_muscle_development	D_vs_E10;stage_1W_vs_E10;stage_3W_vs_E10;stage	1	TRUE
ENSGALG00010004967	SLC35F2	PRJNA729271	-3.435446759	8.22862E-23	3.435446759	stage_E14_vs_E10	ordinary_muscle_development	D_vs_E10;stage_1W_vs_E10;stage_3W_vs_E10;stage	1	TRUE
ENSGALG00010012868	CTHRC1	PRJNA729271	-6.093321299	8.26333E-23	6.093321299	stage_E14_vs_E10	ordinary_muscle_development	D_vs_E10;stage_1W_vs_E10;stage_3W_vs_E10;stage	1	TRUE
ENSGALG00010023162	CHRNA4	PRJNA729271	-2.979503984	8.27681E-23	2.979503984	stage_E14_vs_E10	ordinary_muscle_development	D_vs_E10;stage_1W_vs_E10;stage_3W_vs_E10;stage	1	TRUE
ENSGALG00010020396	CHL1	PRJNA729271	-2.039178672	8.35516E-23	2.039178672	stage_E14_vs_E10	ordinary_muscle_development	D_vs_E10;stage_1W_vs_E10;stage_3W_vs_E10;stage	1	TRUE
ENSGALG00010020602	DENND2B	PRJNA729271	-0.977465457	8.44719E-23	0.977465457	stage_E14_vs_E10	ordinary_muscle_development		0	FALSE
ENSGALG00010029206	CACNG4	PRJNA729271	2.951683594	8.5358E-23	2.951683594	stage_E14_vs_E10	ordinary_muscle_development	D_vs_E10;stage_1W_vs_E10;stage_3W_vs_E10;stage	1	TRUE
ENSGALG00010005539	IFT88	PRJNA729271	-1.344288149	8.62515E-23	1.344288149	stage_E14_vs_E10	ordinary_muscle_development	D_vs_E10;stage_1W_vs_E10;stage_3W_vs_E10;stage	1	TRUE
ENSGALG00010000323	AP1B1	PRJNA729271	1.43685341	9.09595E-23	1.43685341	stage_E14_vs_E10	ordinary_muscle_development	D_vs_E10;stage_1W_vs_E10;stage_3W_vs_E10;stage	1	TRUE
ENSGALG00010013382	ASB12	PRJNA729271	2.213963603	9.21517E-23	2.213963603	stage_E14_vs_E10	ordinary_muscle_development	D_vs_E10;stage_1W_vs_E10;stage_3W_vs_E10;stage	1	TRUE
ENSGALG00010021247	GNAO1	PRJNA729271	-2.285446096	9.24965E-23	2.285446096	stage_E14_vs_E10	ordinary_muscle_development	D_vs_E10;stage_1W_vs_E10;stage_3W_vs_E10;stage	1	TRUE
ENSGALG00010024495	CLDN5	PRJNA729271	1.659733819	9.31007E-23	1.659733819	stage_E14_vs_E10	ordinary_muscle_development	D_vs_E10;stage_1W_vs_E10;stage_3W_vs_E10;stage	1	TRUE
ENSGALG00010009220	GADD45G	PRJNA729271	1.897456119	9.38888E-23	1.897456119	stage_E14_vs_E10	ordinary_muscle_development	D_vs_E10;stage_1W_vs_E10;stage_3W_vs_E10;stage	1	TRUE
ENSGALG00010013110	ANKRD10	PRJNA729271	1.537344231	9.4334E-23	1.537344231	stage_E14_vs_E10	ordinary_muscle_development	D_vs_E10;stage_1W_vs_E10;stage_3W_vs_E10;stage	1	TRUE
ENSGALG00010006251	SEMA3E	PRJNA729271	-3.86997852	9.45201E-23	3.86997852	stage_E14_vs_E10	ordinary_muscle_development	D_vs_E10;stage_1W_vs_E10;stage_3W_vs_E10;stage	1	TRUE
ENSGALG00010007626	DLK2	PRJNA729271	-3.942560244	9.46205E-23	3.942560244	stage_E14_vs_E10	ordinary_muscle_development	D_vs_E10;stage_1W_vs_E10;stage_3W_vs_E10;stage	1	TRUE
ENSGALG00010001776	LMBRD1	PRJNA729271	1.305601233	9.48264E-23	1.305601233	stage_E14_vs_E10	ordinary_muscle_development	D_vs_E10;stage_1W_vs_E10;stage_3W_vs_E10;stage	1	TRUE
ENSGALG00010014190	HACD4	PRJNA729271	2.189795283	9.48431E-23	2.189795283	stage_E14_vs_E10	ordinary_muscle_development	D_vs_E10;stage_1W_vs_E10;stage_3W_vs_E10;stage	1	TRUE
ENSGALG00010024078	KIRREL3	PRJNA729271	-2.588443278	9.49765E-23	2.588443278	stage_E14_vs_E10	ordinary_muscle_development	D_vs_E10;stage_1W_vs_E10;stage_3W_vs_E10;stage	1	TRUE
ENSGALG00010004046	ENSGALG00010004046	PRJNA729271	1.208169744	9.61172E-23	1.208169744	stage_E14_vs_E10	ordinary_muscle_development	D_vs_E10;stage_1W_vs_E10;stage_3W_vs_E10;stage	1	TRUE
ENSGALG00010015041	TFAP2C	PRJNA729271	-7.21758998	9.63906E-23	7.21758998	stage_E14_vs_E10	ordinary_muscle_development	D_vs_E10;stage_1W_vs_E10;stage_3W_vs_E10;stage	1	TRUE
ENSGALG00010001693	NKIRAS1	PRJNA729271	1.919966783	9.76188E-23	1.919966783	stage_E14_vs_E10	ordinary_muscle_development	D_vs_E10;stage_1W_vs_E10;stage_3W_vs_E10;stage	1	TRUE
ENSGALG00010024945	APIP	PRJNA729271	0.798560732	1.00773E-22	0.798560732	stage_E14_vs_E10	ordinary_muscle_development		0	FALSE
ENSGALG00010022081	FAS	PRJNA729271	1.71399843	1.01393E-22	1.71399843	stage_E14_vs_E10	ordinary_muscle_development	D_vs_E10;stage_1W_vs_E10;stage_3W_vs_E10;stage	1	TRUE
ENSGALG00010020319	NTRK3	PRJNA729271	-1.662767184	1.01434E-22	1.662767184	stage_E14_vs_E10	ordinary_muscle_development	D_vs_E10;stage_1W_vs_E10;stage_3W_vs_E10;stage	1	TRUE
ENSGALG00010018148	XRN2	PRJNA729271	-0.550683358	1.02676E-22	0.550683358	stage_E14_vs_E10	ordinary_muscle_development		0	FALSE
ENSGALG00010011776	IL17RA	PRJNA729271	-1.389767541	1.02849E-22	1.389767541	stage_E14_vs_E10	ordinary_muscle_development	D_vs_E10;stage_1W_vs_E10;stage_3W_vs_E10;stage	1	TRUE
ENSGALG00010009652	CIAO2B	PRJNA729271	0.789009357	1.02946E-22	0.789009357	stage_E14_vs_E10	ordinary_muscle_development		0	FALSE
ENSGALG00010026999	MLEC	PRJNA729271	-0.827373123	1.03002E-22	0.827373123	stage_E14_vs_E10	ordinary_muscle_development		0	FALSE
ENSGALG00010019591	ENSGALG00010019591	PRJNA729271	-2.27204189	1.03346E-22	2.27204189	stage_E14_vs_E10	ordinary_muscle_development	D_vs_E10;stage_1W_vs_E10;stage_3W_vs_E10;stage	1	TRUE
ENSGALG00010023740	PPP1R16B	PRJNA729271	0.993225837	1.03653E-22	0.993225837	stage_E14_vs_E10	ordinary_muscle_development		0	FALSE
ENSGALG00010011116	TAPT1	PRJNA729271	1.267798298	1.04899E-22	1.267798298	stage_E14_vs_E10	ordinary_muscle_development	D_vs_E10;stage_1W_vs_E10;stage_3W_vs_E10;stage	1	TRUE
ENSGALG00010007399	WWC2	PRJNA729271	-1.109815108	1.07981E-22	1.109815108	stage_E14_vs_E10	ordinary_muscle_development	D_vs_E10;stage_1W_vs_E10;stage_3W_vs_E10;stage	1	TRUE
ENSGALG00010024174	PLEKHB2	PRJNA729271	1.008799573	1.0844E-22	1.008799573	stage_E14_vs_E10	ordinary_muscle_development	D_vs_E10;stage_1W_vs_E10;stage_3W_vs_E10;stage	1	TRUE
ENSGALG00010010334	ZBTB41	PRJNA729271	0.87111626	1.08972E-22	0.87111626	stage_E14_vs_E10	ordinary_muscle_development		0	FALSE
ENSGALG00010021417	STK16	PRJNA729271	-1.658083637	1.09708E-22	1.658083637	stage_E14_vs_E10	ordinary_muscle_development	D_vs_E10;stage_1W_vs_E10;stage_3W_vs_E10;stage	1	TRUE
ENSGALG00010006285	FZD8	PRJNA729271	-1.556078802	1.18904E-22	1.556078802	stage_E14_vs_E10	ordinary_muscle_development	D_vs_E10;stage_1W_vs_E10;stage_3W_vs_E10;stage	1	TRUE
ENSGALG00010003582	RDH10	PRJNA729271	-1.418195783	1.20991E-22	1.418195783	stage_E14_vs_E10	ordinary_muscle_development	D_vs_E10;stage_1W_vs_E10;stage_3W_vs_E10;stage	1	TRUE
ENSGALG00010011363	FDFT1	PRJNA729271	1.449755661	1.22538E-22	1.449755661	stage_E14_vs_E10	ordinary_muscle_development	D_vs_E10;stage_1W_vs_E10;stage_3W_vs_E10;stage	1	TRUE
ENSGALG00010001938	ALG8	PRJNA729271	-1.068574871	1.2527E-22	1.068574871	stage_E14_vs_E10	ordinary_muscle_development	D_vs_E10;stage_1W_vs_E10;stage_3W_vs_E10;stage	1	TRUE
ENSGALG00010017830	DENND4A	PRJNA729271	1.268942876	1.29846E-22	1.268942876	stage_E14_vs_E10	ordinary_muscle_development	D_vs_E10;stage_1W_vs_E10;stage_3W_vs_E10;stage	1	TRUE
ENSGALG00010002084	HACL1	PRJNA729271	-0.927012655	1.3161E-22	0.927012655	stage_E14_vs_E10	ordinary_muscle_development		0	FALSE

ENSGALG00010004406	ENSGALG00010004406	PRJNA729271	-1.555827116	1.31632E-22	1.555827116	stage_E14_vs_E10	ordinary_muscle_development	D_vs_E10;stage_1W_vs_E10;stage_3W_vs_E10;stage	1	TRUE
ENSGALG00010022460	VAT1	PRJNA729271	-1.18419983	1.34229E-22	1.18419983	stage_E14_vs_E10	ordinary_muscle_development	D_vs_E10;stage_1W_vs_E10;stage_3W_vs_E10;stage	1	TRUE
ENSGALG00010018391	STAC	PRJNA729271	-2.288717167	1.35346E-22	2.288717167	stage_E14_vs_E10	ordinary_muscle_development	D_vs_E10;stage_1W_vs_E10;stage_3W_vs_E10;stage	1	TRUE
ENSGALG00010021353	IPO13	PRJNA729271	1.77911634	1.4007E-22	1.77911634	stage_E14_vs_E10	ordinary_muscle_development	D_vs_E10;stage_1W_vs_E10;stage_3W_vs_E10;stage	1	TRUE
ENSGALG00010008289	MTMR2	PRJNA729271	-0.726861674	1.40584E-22	0.726861674	stage_E14_vs_E10	ordinary_muscle_development		0	FALSE
ENSGALG00010020674	TRPV4	PRJNA729271	-8.789368279	1.41671E-22	8.789368279	stage_E14_vs_E10	ordinary_muscle_development	D_vs_E10;stage_1W_vs_E10;stage_3W_vs_E10;stage	1	TRUE
ENSGALG00010017711	LRRFIP2	PRJNA729271	1.330167009	1.42449E-22	1.330167009	stage_E14_vs_E10	ordinary_muscle_development	D_vs_E10;stage_1W_vs_E10;stage_3W_vs_E10;stage	1	TRUE
ENSGALG00010004096	COL12A1	PRJNA729271	-4.114345903	1.45502E-22	4.114345903	stage_E14_vs_E10	ordinary_muscle_development	D_vs_E10;stage_1W_vs_E10;stage_3W_vs_E10;stage	1	TRUE
ENSGALG00010001758	COL19A1	PRJNA729271	2.791655324	1.4613E-22	2.791655324	stage_E14_vs_E10	ordinary_muscle_development	D_vs_E10;stage_1W_vs_E10;stage_3W_vs_E10;stage	1	TRUE
ENSGALG00010027668	RANBP3	PRJNA729271	-0.539499464	1.47687E-22	0.539499464	stage_E14_vs_E10	ordinary_muscle_development		0	FALSE
ENSGALG00010000730	INTS5	PRJNA729271	2.192414074	1.48534E-22	2.192414074	stage_E14_vs_E10	ordinary_muscle_development	D_vs_E10;stage_1W_vs_E10;stage_3W_vs_E10;stage	1	TRUE
ENSGALG00010009234	PCDH18	PRJNA729271	-1.279331203	1.51829E-22	1.279331203	stage_E14_vs_E10	ordinary_muscle_development	D_vs_E10;stage_1W_vs_E10;stage_3W_vs_E10;stage	1	TRUE
ENSGALG00010022623	TOP1	PRJNA729271	-1.256484439	1.537E-22	1.256484439	stage_E14_vs_E10	ordinary_muscle_development	D_vs_E10;stage_1W_vs_E10;stage_3W_vs_E10;stage	1	TRUE
ENSGALG00010009515	MBD5	PRJNA729271	0.947561641	1.59939E-22	0.947561641	stage_E14_vs_E10	ordinary_muscle_development		0	FALSE
ENSGALG00010014912	ENSGALG00010014912	PRJNA729271	3.101419767	1.64648E-22	3.101419767	stage_E14_vs_E10	ordinary_muscle_development	D_vs_E10;stage_1W_vs_E10;stage_3W_vs_E10;stage	1	TRUE
ENSGALG00010016266	PDLIM4	PRJNA729271	-1.20653072	1.65782E-22	1.20653072	stage_E14_vs_E10	ordinary_muscle_development	D_vs_E10;stage_1W_vs_E10;stage_3W_vs_E10;stage	1	TRUE
ENSGALG00010023453	ARHGAP32	PRJNA729271	-1.85712186	1.67272E-22	1.85712186	stage_E14_vs_E10	ordinary_muscle_development	D_vs_E10;stage_1W_vs_E10;stage_3W_vs_E10;stage	1	TRUE
ENSGALG00010029723	ADORA2B	PRJNA729271	1.766055001	1.69856E-22	1.766055001	stage_E14_vs_E10	ordinary_muscle_development	D_vs_E10;stage_1W_vs_E10;stage_3W_vs_E10;stage	1	TRUE
ENSGALG00010022565	ARF3	PRJNA729271	-1.749305323	1.69977E-22	1.749305323	stage_E14_vs_E10	ordinary_muscle_development	D_vs_E10;stage_1W_vs_E10;stage_3W_vs_E10;stage	1	TRUE
ENSGALG00010028060	ENSGALG00010028060	PRJNA729271	1.38734321	1.70129E-22	1.38734321	stage_E14_vs_E10	ordinary_muscle_development	D_vs_E10;stage_1W_vs_E10;stage_3W_vs_E10;stage	1	TRUE
ENSGALG00010014338	BHLHE41	PRJNA729271	2.180579226	1.71372E-22	2.180579226	stage_E14_vs_E10	ordinary_muscle_development	D_vs_E10;stage_1W_vs_E10;stage_3W_vs_E10;stage	1	TRUE
ENSGALG00010029492	SDK2	PRJNA729271	-2.330629784	1.75822E-22	2.330629784	stage_E14_vs_E10	ordinary_muscle_development	D_vs_E10;stage_1W_vs_E10;stage_3W_vs_E10;stage	1	TRUE
ENSGALG00010005404	BEND7	PRJNA729271	1.84368706	1.77479E-22	1.84368706	stage_E14_vs_E10	ordinary_muscle_development	D_vs_E10;stage_1W_vs_E10;stage_3W_vs_E10;stage	1	TRUE
ENSGALG00010018529	UBR2	PRJNA729271	0.916491707	1.77696E-22	0.916491707	stage_E14_vs_E10	ordinary_muscle_development		0	FALSE
ENSGALG00010017670	FAM179A	PRJNA729271	3.243914499	1.78116E-22	3.243914499	stage_E14_vs_E10	ordinary_muscle_development	D_vs_E10;stage_1W_vs_E10;stage_3W_vs_E10;stage	1	TRUE
ENSGALG00010022862	ACADS	PRJNA729271	1.216380133	1.80535E-22	1.216380133	stage_E14_vs_E10	ordinary_muscle_development	D_vs_E10;stage_1W_vs_E10;stage_3W_vs_E10;stage	1	TRUE
ENSGALG00010024600	CD9	PRJNA729271	3.299332156	1.84001E-22	3.299332156	stage_E14_vs_E10	ordinary_muscle_development	D_vs_E10;stage_1W_vs_E10;stage_3W_vs_E10;stage	1	TRUE
ENSGALG00010012883	DCAF13	PRJNA729271	-0.672029189	1.85192E-22	0.672029189	stage_E14_vs_E10	ordinary_muscle_development		0	FALSE
ENSGALG00010028881	SARDH	PRJNA729271	2.311961453	1.85259E-22	2.311961453	stage_E14_vs_E10	ordinary_muscle_development	D_vs_E10;stage_1W_vs_E10;stage_3W_vs_E10;stage	1	TRUE
ENSGALG00010005184	FGF14	PRJNA729271	-3.284714588	1.87644E-22	3.284714588	stage_E14_vs_E10	ordinary_muscle_development	D_vs_E10;stage_1W_vs_E10;stage_3W_vs_E10;stage	1	TRUE
ENSGALG00010012671	NBN	PRJNA729271	-1.886766591	1.8855E-22	1.886766591	stage_E14_vs_E10	ordinary_muscle_development	D_vs_E10;stage_1W_vs_E10;stage_3W_vs_E10;stage	1	TRUE
ENSGALG00010017738	NDUFS5	PRJNA729271	1.104663571	1.89928E-22	1.104663571	stage_E14_vs_E10	ordinary_muscle_development	D_vs_E10;stage_1W_vs_E10;stage_3W_vs_E10;stage	1	TRUE
ENSGALG00010002148	POLR2A	PRJNA729271	1.749385088	1.96898E-22	1.749385088	stage_E14_vs_E10	ordinary_muscle_development	D_vs_E10;stage_1W_vs_E10;stage_3W_vs_E10;stage	1	TRUE
ENSGALG00010023760	ZW10	PRJNA729271	-0.927957845	1.97925E-22	0.927957845	stage_E14_vs_E10	ordinary_muscle_development		0	FALSE
ENSGALG00010003681	NT5C1B	PRJNA729271	-1.327349955	1.98436E-22	1.327349955	stage_E14_vs_E10	ordinary_muscle_development	D_vs_E10;stage_1W_vs_E10;stage_3W_vs_E10;stage	1	TRUE
ENSGALG00010015094	CDK5RAP1	PRJNA729271	2.106134222	1.99605E-22	2.106134222	stage_E14_vs_E10	ordinary_muscle_development	D_vs_E10;stage_1W_vs_E10;stage_3W_vs_E10;stage	1	TRUE
ENSGALG00010011571	ATOSB	PRJNA729271	1.132880743	2.01656E-22	1.132880743	stage_E14_vs_E10	ordinary_muscle_development	D_vs_E10;stage_1W_vs_E10;stage_3W_vs_E10;stage	1	TRUE
ENSGALG00010024920	VIPR2	PRJNA729271	-3.19434609	2.03199E-22	3.19434609	stage_E14_vs_E10	ordinary_muscle_development	D_vs_E10;stage_1W_vs_E10;stage_3W_vs_E10;stage	1	TRUE
ENSGALG00010002418	TRAPPC2	PRJNA729271	-0.848117799	2.06619E-22	0.848117799	stage_E14_vs_E10	ordinary_muscle_development		0	FALSE
ENSGALG00010023257	PAK6	PRJNA729271	2.850659605	2.07404E-22	2.850659605	stage_E14_vs_E10	ordinary_muscle_development	D_vs_E10;stage_1W_vs_E10;stage_3W_vs_E10;stage	1	TRUE
ENSGALG00010009804	XPR1	PRJNA729271	1.155381121	2.11068E-22	1.155381121	stage_E14_vs_E10	ordinary_muscle_development	D_vs_E10;stage_1W_vs_E10;stage_3W_vs_E10;stage	1	TRUE
ENSGALG00010028058	GLT8D1	PRJNA729271	-1.144432258	2.11316E-22	1.144432258	stage_E14_vs_E10	ordinary_muscle_development	D_vs_E10;stage_1W_vs_E10;stage_3W_vs_E10;stage	1	TRUE
ENSGALG00010013165	NUDC	PRJNA729271	-0.815200952	2.13764E-22	0.815200952	stage_E14_vs_E10	ordinary_muscle_development		0	FALSE
ENSGALG00010007465	PNAT10	PRJNA729271	1.91255325	2.15981E-22	1.91255325	stage_E14_vs_E10	ordinary_muscle_development	D_vs_E10;stage_1W_vs_E10;stage_3W_vs_E10;stage	1	TRUE
ENSGALG00010005362	ATP1B1	PRJNA729271	1.725989028	2.29582E-22	1.725989028	stage_E14_vs_E10	ordinary_muscle_development	D_vs_E10;stage_1W_vs_E10;stage_3W_vs_E10;stage	1	TRUE
ENSGALG00010019008	HERPUD2	PRJNA729271	0.439515342	2.30586E-22	0.439515342	stage_E14_vs_E10	ordinary_muscle_development		0	FALSE
ENSGALG00010001154	PTGR3	PRJNA729271	-0.859685408	2.37255E-22	0.859685408	stage_E14_vs_E10	ordinary_muscle_development		0	FALSE
ENSGALG00010013419	ENSGALG00010013419	PRJNA729271	3.283316399	2.38442E-22	3.283316399	stage_E14_vs_E10	ordinary_muscle_development	D_vs_E10;stage_1W_vs_E10;stage_3W_vs_E10;stage	1	TRUE
ENSGALG00010016910	NSD2	PRJNA729271	-1.09823663	2.46949E-22	1.09823663	stage_E14_vs_E10	ordinary_muscle_development	D_vs_E10;stage_1W_vs_E10;stage_3W_vs_E10;stage	1	TRUE
ENSGALG00010012390	MMP16	PRJNA729271	-1.727487116	2.4734E-22	1.727487116	stage_E14_vs_E10	ordinary_muscle_development	D_vs_E10;stage_1W_vs_E10;stage_3W_vs_E10;stage	1	TRUE
ENSGALG00010001506	DPYSL4	PRJNA729271	-3.041767123	2.50149E-22	3.041767123	stage_E14_vs_E10	ordinary_muscle_development	D_vs_E10;stage_1W_vs_E10;stage_3W_vs_E10;stage	1	TRUE
ENSGALG00010012064	RGS14	PRJNA729271	-1.810353423	2.53197E-22	1.810353423	stage_E14_vs_E10	ordinary_muscle_development	D_vs_E10;stage_1W_vs_E10;stage_3W_vs_E10;stage	1	TRUE
ENSGALG00010017803	ENSGALG00010017803	PRJNA729271	1.167008355	2.54712E-22	1.167008355	stage_E14_vs_E10	ordinary_muscle_development	D_vs_E10;stage_1W_vs_E10;stage_3W_vs_E10;stage	1	TRUE
ENSGALG00010025942	ENSGALG00010025942	PRJNA729271	10.26864088	2.5642E-22	10.26864088	stage_E14_vs_E10	ordinary_muscle_development	D_vs_E10;stage_1W_vs_E10;stage_3W_vs_E10;stage	1	TRUE
ENSGALG00010012015	ENSGALG00010012015	PRJNA729271	2.120227384	2.57558E-22	2.120227384	stage_E14_vs_E10	ordinary_muscle_development	D_vs_E10;stage_1W_vs_E10;stage_3W_vs_E10;stage	1	TRUE
ENSGALG00010012087	TMEM171	PRJNA729271	-2.020145326	2.72191E-22	2.020145326	stage_E14_vs_E10	ordinary_muscle_development	D_vs_E10;stage_1W_vs_E10;stage_3W_vs_E10;stage	1	TRUE
ENSGALG00010002825	TRIM41	PRJNA729271	1.177340839	2.72859E-22	1.177340839	stage_E14_vs_E10	ordinary_muscle_development	D_vs_E10;stage_1W_vs_E10;stage_3W_vs_E10;stage	1	TRUE
ENSGALG00010020940	ENSGALG00010020940	PRJNA729271	-1.357873044	2.73767E-22	1.357873044	stage_E14_vs_E10	ordinary_muscle_development	D_vs_E10;stage_1W_vs_E10;stage_3W_vs_E10;stage	1	TRUE
ENSGALG00010017296	SCNN1G	PRJNA729271	2.209162481	2.74898E-22	2.209162481	stage_E14_vs_E10	ordinary_muscle_development	D_vs_E10;stage_1W_vs_E10;stage_3W_vs_E10;stage	1	TRUE
ENSGALG00010008666	SLC16A9	PRJNA729271	1.855277864	2.76611E-22	1.855277864	stage_E14_vs_E10	ordinary_muscle_development	D_vs_E10;stage_1W_vs_E10;stage_3W_vs_E10;stage	1	TRUE
ENSGALG00010010663	EFR3B	PRJNA729271	-1.759772625	2.85168E-22	1.759772625	stage_E14_vs_E10	ordinary_muscle_development	D_vs_E10;stage_1W_vs_E10;stage_3W_vs_E10;stage	1	TRUE
ENSGALG00010002817	SHFM1	PRJNA729271	0.899443688	2.87836E-22	0.899443688	stage_E14_vs_E10	ordinary_muscle_development		0	FALSE
ENSGALG00010012099	UPK3A	PRJNA729271	-6.278580679	2.90065E-22	6.278580679	stage_E14_vs_E10	ordinary_muscle_development	D_vs_E10;stage_1W_vs_E10;stage_3W_vs_E10;stage	1	TRUE
ENSGALG00010012010	HENMT1	PRJNA729271	-1.938866351	2.91561E-22	1.938866351	stage_E14_vs_E10	ordinary_muscle_development	D_vs_E10;stage_1W_vs_E10;stage_3W_vs_E10;stage	1	TRUE
ENSGALG00010015356	ENSGALG00010015356	PRJNA729271	-0.874126992	2.92471E-22	0.874126992	stage_E14_vs_E10	ordinary_muscle_development		0	FALSE
ENSGALG00010001499	TIPARP	PRJNA729271	1.848045976	2.95851E-22	1.848045976	stage_E14_vs_E10	ordinary_muscle_development	D_vs_E10;stage_1W_vs_E10;stage_3W_vs_E10;stage	1	TRUE
ENSGALG00010021502	DNAJB12	PRJNA729271	1.433957911	2.97899E-22	1.433957911	stage_E14_vs_E10	ordinary_muscle_development	D_vs_E10;stage_1W_vs_E10;stage_3W_vs_E10;stage	1	TRUE
ENSGALG00010029532	EVI2A	PRJNA729271	4.396010212	3.00903E-22	4.396010212	stage_E14_vs_E10	ordinary_muscle_development	D_vs_E10;stage_1W_vs_E10;stage_3W_vs_E10;stage	1	TRUE
ENSGALG00010029629	ENSGALG00010029629	PRJNA729271	2.516359083	3.00972E-22	2.516359083	stage_E14_vs_E10	ordinary_muscle_development	D_vs_E10;stage_1W_vs_E10;stage_3W_vs_E10;stage	1	TRUE
ENSGALG00010017820	FLRT2	PRJNA729271	-1.356216055	3.03695E-22	1.356216055	stage_E14_vs_E10	ordinary_muscle_development	D_vs_E10;stage_1W_vs_E10;stage_3W_vs_E10;stage	1	TRUE
ENSGALG00010022554	SCG3	PRJNA729271	-3.521390152	3.04E-22	3.521390152	stage_E14_vs_E10	ordinary_muscle_development	D_vs_E10;stage_1W_vs_E10;stage_3W_vs_E10;stage	1	TRUE
ENSGALG00010010838	JAM2	PRJNA729271	1.705627681	3.04697E-22	1.705627681	stage_E14_vs_E10	ordinary_muscle_development	D_vs_E10;stage_1W_vs_E10;stage_3W_vs_E10;stage	1	TRUE
ENSGALG00010023606	LRRC7	PRJNA729271	-3.650121106	3.06143E-22	3.650121106	stage_E14_vs_E10	ordinary_muscle_development	D_vs_E10;stage_1W_vs_E10;stage_3W_vs_E10;stage	1	TRUE
ENSGALG00010005890	SNX32	PRJNA729271	-0.883430053	3.08211E-22	0.883430053	stage_E14_vs_E10	ordinary_muscle_development		0	FALSE
ENSGALG00010025592	HSPA8	PRJNA729271	-2.065663649	3.09809E-22	2.065663649	stage_E14_vs_E10	ordinary_muscle_development	D_vs_E10;stage_1W_vs_E10;stage_3W_vs_E10;stage	1	TRUE
ENSGALG00010005559	FILIP1L	PRJNA729271	1.286903446	3.12863E-22	1.286903446	stage_E14_vs_E10	ordinary_muscle_development	D_vs_E10;stage_1W_vs_E10;stage_3W_vs_E10;stage	1	TRUE
ENSGALG00010016491	DNAJC8	PRJNA729271	-0.534361906	3.13422E-22	0.534361906	stage_E14_vs_E10	ordinary_muscle_development		0	FALSE
ENSGALG00010022733	PCCB	PRJNA729271	1.128574162	3.14782E-22	1.128574162	stage_E14_vs_E10	ordinary_muscle_development	D_vs_E10;stage_1W_vs_E10;stage_3W_vs_E10;stage	1	TRUE
ENSGALG00010015989	ENAH	PRJNA729271	-1.609246262	3.20125E-22	1.609246262	stage_E14_vs_E10	ordinary_muscle_development	D_vs_E10;stage_1W_vs_E10;stage_3W_vs_E10;stage	1	TRUE
ENSGALG00010014745	LSM10	PRJNA729271	1.780906329	3.22722E-22	1.780906329	stage_E14_vs_E10	ordinary_muscle_development	D_vs_E10;stage_1W_vs_E10;stage_3W_vs_E10;stage	1	TRUE
ENSGALG00010021348	SLC4A3	PRJNA729271	1.132835084	3.26387E-22	1.132835084	stage_E14_vs_E10	ordinary_muscle_development	D_vs_E10;stage_1W_vs_E10;stage_3W_vs_E10;stage	1	TRUE
ENSGALG00010028434	COL5A1	PRJNA729271	1.940435131	3.27151E-22	1.940435131	stage_E14_vs_E10	ordinary_muscle_development	D_vs_E10;stage_1W_vs_E10;stage_3W_vs_E10;stage	1	TRUE
ENSGALG00010028786	GTF3C4	PRJNA729271	-0.673889501	3.30097E-22	0.673889501	stage_E14_vs_E10	ordinary_muscle_development		0	FALSE
ENSGALG00010020118	GNB1	PRJNA729271	-0.919699868	3.33201E-22	0.919699868	stage_E14_vs_E10	ordinary_muscle_development		0	FALSE
ENSGALG00010022246	RHOV	PRJNA729271	-4.892570878	3.34296E-22	4.892570878	stage_E14_vs_E10	ordinary_muscle_development	D_vs_E10;stage_1W_vs_E10;stage_3W_vs_E10;stage	1	TRUE
ENSGALG00010021771	ANKRD61	PRJNA729271	1.408676737	3.36591E-22	1.408676737	stage_E14_vs_E10	ordinary_muscle_development	D_vs_E10;stage_1W_vs_E10;stage_3W_vs_E10;stage	1	TRUE
ENSGALG00010017132	TMEM108	PRJNA729271	-3.162285302	3.49106E-22	3.162285302	stage_E14_vs_E10	ordinary_muscle_development	D_vs_E10;stage_1W_vs_E10;stage_3W_vs_E10;stage	1	TRUE
ENSGALG00010003652	MYCN	PRJNA729271	-1.412153387	3.49405E-22	1.412153387	stage_E14_vs_E10	ordinary_muscle_development	D_vs_E10;stage_1W_vs_E10;stage_3W_vs_E10;stage	1	TRUE
ENSGALG00010015028	MCM9	PRJNA729271	-2.705259571	3.49723E-22	2.705259571	stage_E14_vs_E10	ordinary_muscle_development	D_vs_E10;stage_1W_vs_E10;stage_3W_vs_E10;stage	1	TRUE
ENSGALG00010021496	MTMR14	PRJNA729271	1.129544404	3.54291E-22	1.129544404	stage_E14_vs_E10	ordinary_muscle_development	D_vs_E10;stage_1W_vs_E10;stage_3W_vs_E10;stage	1	TRUE
ENSGALG00010029346	ARL16	PRJNA729271	-1.350682656	3.61325E-22	1.350682656	stage_E14_vs_E10	ordinary_muscle_development	D_vs_E10;stage_1W_vs_E10;stage_3W_vs_E10;stage	1	TRUE
ENSGALG00010002393	GPM6B	PRJNA729271	-1.054880003	3.61606E-22	1.054880003	stage_E14_vs_E10	ordinary_muscle_development	D_vs_E10;stage_1W_vs_E10;stage_3W_vs_E10;stage	1	TRUE
ENSGALG00010005466	LATS2	PRJNA729271	0.872530675	3.75957E-22	0.872530675	stage_E14_vs_E10	ordinary_muscle_development		0	FALSE
ENSGALG00010017453	PPP1R2	PRJNA729271	1.356291167	3.8627E-22	1.356291167	stage_E14_vs_E10	ordinary_muscle_development	D_vs_E10;stage_1W_vs_E10;stage_3W_vs_E10;stage	1	TRUE
ENSGALG00010003456	CDS1	PRJNA729271	-4.082224825	3.87056E-22	4.082224825	stage_E14_vs_E10	ordinary_muscle_development	D_vs_E10;stage_1W_vs_E10;stage_3W_vs_E10;stage	1	TRUE
ENSGALG00010003002	PHKA2	PRJNA729271	1.816914726	3.90291E-22	1.816914726	stage_E14_vs_E10	ordinary_muscle_development	D_vs_E10;stage_1W_vs_E10;stage_3W_vs_E10;stage	1	TRUE
ENSGALG00010013324	PARVG	PRJNA729271	1.457085722	3.92689E-22	1.457085722	stage_E14_vs_E10	ordinary_muscle_development	D_vs_E10;stage_1W_vs_E10;stage_3W_vs_E10;stage	1	TRUE
ENSGALG00010011989	H1-010	PRJNA729271	-1.348337063	3.94796E-22	1.348337063	stage_E14_vs_E10	ordinary_muscle_development	D_vs_E10;stage_1W_vs_E10;stage_3W_vs_E10;stage	1	TRUE
ENSGALG00010002392	DFNA5	PRJNA729271	-2.056247412	3.97561E-22	2.056247412	stage_E14_vs_E10	ordinary_muscle_development	D_vs_E10;stage_1W_vs_E10;stage_3W_vs_E10;stage	1	TRUE
ENSGALG00010007357	NOX4	PRJNA729271	1.163666546	3.99792E-22	1.163666546	stage_E14_vs_E10	ordinary_muscle_development	D_vs_E10;stage_1W_vs_E10;stage_3W_vs_E10;stage	1	TRUE
ENSGALG00010015614	ATG2B	PRJNA729271	1.049335503	4.04163E-22	1.049335503	stage_E14_vs_E10	ordinary_muscle_development	D_vs_E10;stage_1W_vs_E10;stage_3W_vs_E10;stage	1	TRUE
ENSGALG00010003695	NOA1	PRJNA729271	0.984150996	4.1E-22	0.984150996	stage_E14_vs_E10	ordinary_muscle_development		0	FALSE
ENSGALG00010015908	CHRD	PRJNA729271	1.336060395	4.13059E-22	1.336060395	stage_E14_vs_E10	ordinary_muscle_development	D_vs_E10;stage_1W_vs_E10;stage_3W_vs_E10;stage	1	TRUE
ENSGALG00010011205	CYTL1	PRJNA729271	-9.683122119	4.1487E-22	9.683122119	stage_E14_vs_E10	ordinary_muscle_development	D_vs_E10;stage_1W_vs_E10;stage_3W_vs_E10;stage	1	TRUE
ENSGALG00010016322	EBF4	PRJNA729271	-1.336482756	4.16756E-22	1.336482756	stage_E14_vs_E10	ordinary_muscle_development	D_vs_E10;stage_1W_vs_E10;stage_3W_vs_E10;stage	1	TRUE
ENSGALG00010016872	EDA2R	PRJNA729271	2.747582517	4.24653E-22	2.747582517	stage_E14_vs_E10	ordinary_muscle_development	D_vs_E10;stage_1W_vs_E10;stage_3W_vs_E10;stage	1	TRUE
ENSGALG00010004250	MOB1B	PRJNA729271	-0.875902195	4.25768E-22	0.875902195	stage_E14_vs_E10	ordinary_muscle_development		0	FALSE
ENSGALG00010009769	TMCC3	PRJNA729271	2.1986266	4.2873E-22	2.1986266	stage_E14_vs_E10	ordinary_muscle_development	D_vs_E10;stage_1W_vs_E10;stage_3W_vs_E10;stage	1	TRUE
ENSGALG00010019811	UBXN4	PRJNA729271	1.083196805	4.31165E-22	1.083196805	stage_E14_vs_E10	ordinary_muscle_development	D_vs_E10;stage_1W_vs_E10;stage_3W_vs_E10;stage	1	TRUE
ENSGALG00010007784	INTS10	PRJNA729271	-0.948327012	4.48051E-22	0.948327012	stage_E14_vs_E10	ordinary_muscle_development		0	FALSE
ENSGALG00010016461	HPCAL4	PRJNA729271	-4.103234202	4.48509E-22	4.103234202	stage_E14_vs_E10	ordinary_muscle_development	D_vs_E10;stage_1W_vs_E10;stage_3W_vs_E10;stage	1	TRUE
ENSGALG00010002355	UFM1	PRJNA729271	-0.993690802	4.53539E-22	0.993690802	stage_E14_vs_E10	ordinary_muscle_development		0	FALSE
ENSGALG00010015562	IRF9	PRJNA729271	2.621368082	4.57018E-22	2.621368082	stage_E14_vs_E10	ordinary_muscle_development	D_vs_E10;stage_1W_vs_E10;stage_3W_vs_E10;stage	1	TRUE
ENSGALG00010025334	RHOF	PRJNA729271	2.12901613	4.58188E-22	2.12901613	stage_E14_vs_E10	ordinary_muscle_development	D_vs_E10;stage_1W_vs_E10;stage_3W_vs_E10;stage	1	TRUE
ENSGALG00010005163	GPC6	PRJNA729271	-4.016755232	4.68099E-22	4.016755232	stage_E14_vs_E10	ordinary_muscle_development	D_vs_E10;stage_1W_vs_E10;stage_3W_vs_E10;stage	1	TRUE
ENSGALG00010005333	PLXNA4	PRJNA729271	1.499607948	4.89153E-22	1.499607948	stage_E14_vs_E10	ordinary_muscle_development	D_vs_E10;stage_1W_vs_E10;stage_3W_vs_E10;stage	1	TRUE
ENSGALG00010027927	ENSGALG00010027927	PRJNA729271	1.913620336	5.01423E-22	1.913620336	stage_E14_vs_E10	ordinary_muscle_development	D_vs_E10;stage_1W_vs_E10;stage_3W_vs_E10;stage	1	TRUE
ENSGALG00010029573	MAP2K4	PRJNA729271	-0.600975295	5.04007E-22	0.600975295	stage_E14_vs_E10	ordinary_muscle_development		0	FALSE
ENSGALG00010021413	SYNJ2BP	PRJNA729271	1.463152996	5.06729E-22	1.463152996	stage_E14_vs_E10	ordinary_muscle_development	D_vs_E10;stage_1W_vs_E10;stage_3W_vs_E10;stage	1	TRUE
ENSGALG00010025379	ACAD8	PRJNA729271	1.812963549	5.11981E-22	1.812963549	stage_E14_vs_E10	ordinary_muscle_development	D_vs_E10;stage_1W_vs_E10;stage_3W_vs_E10;stage	1	TRUE
ENSGALG00010001125	CLRN1	PRJNA729271	2.561007659	5.23775E-22	2.561007659	stage_E14_vs_E10	ordinary_muscle_development	D_vs_E10;stage_1W_vs_E10;stage_3W_vs_E10;stage	1	TRUE
ENSGALG00010004149	SDAD1	PRJNA729271	-1.230471861	5.34628E-22	1.230471861	stage_E14_vs_E10	ordinary_muscle_development	D_vs_E10;stage_1W_vs_E10;stage_3W_vs_E10;stage	1	TRUE
ENSGALG00010027912	RGS3	PRJNA729271	-1.29635666	5.35591E-22	1.29635666	stage_E14_vs_E10	ordinary_muscle_development	D_vs_E10;stage_1W_vs_E10;stage_3W_vs_E10;stage	1	TRUE
ENSGALG00010016260	INTS6L	PRJNA729271	1.408616082	5.36032E-22	1.408616082	stage_E14_vs_E10	ordinary_muscle_development	D_vs_E10;stage_1W_vs_E10;stage_3W_vs_E10;stage	1	TRUE
ENSGALG00010020261	SSU72	PRJNA729271	0.886478409	5.3678E-22	0.886478409	stage_E14_vs_E10	ordinary_muscle_development		0	FALSE
ENSGALG00010004007	ENSGALG00010004007	PRJNA729271	-1.609533112	5.44876E-22	1.609533112	stage_E14_vs_E10	ordinary_muscle_development	D_vs_E10;stage_1W_vs_E10;stage_3W_vs_E10;stage	1	TRUE
ENSGALG00010024571	KCNA3	PRJNA729271	-4.493968153	5.51148E-22	4.493968153	stage_E14_vs_E10	ordinary_muscle_development	D_vs_E10;stage_1W_vs_E10;stage_3W_vs_E10;stage	1	TRUE
ENSGALG00010008394	CD2AP	PRJNA729271	1.082885355	5.6523E-22	1.082885355	stage_E14_vs_E10	ordinary_muscle_development	D_vs_E10;stage_1W_vs_E10;stage_3W_vs_E10;stage	1	TRUE
ENSGALG00010015793	RSRP1	PRJNA729271	2.276674838	5.71434E-22	2.276674838	stage_E14_vs_E10	ordinary_muscle_development	D_vs_E10;stage_1W_vs_E10;stage_3W_vs_E10;stage	1	TRUE
ENSGALG00010018049	SUGCT	PRJNA729271	-1.921541113	5.75437E-22	1.921541113	stage_E14_vs_E10	ordinary_muscle_development	D_vs_E10;stage_1W_vs_E10;stage_3W_vs_E10;stage	1	TRUE
ENSGALG00010016338	PTPRU	PRJNA729271	-1.486177057	5.8718E-22	1.486177057	stage_E14_vs_E10	ordinary_muscle_development	D_vs_E10;stage_1W_vs_E10;stage_3W_vs_E10;stage	1	TRUE
ENSGALG00010003589	SLC25A30	PRJNA729271	3.790298675	5.90789E-22	3.790298675	stage_E14_vs_E10	ordinary_muscle_development	D_vs_E10;stage_1W_vs_E10;stage_3W_vs_E10;stage	1	TRUE
ENSGALG00010028050	PBRM1	PRJNA729271	-0.858612903	6.04569E-22	0.858612903	stage_E14_vs_E10	ordinary_muscle_development		0	FALSE
ENSGALG00010011187	STK32B	PRJNA729271	-8.64988828	6.08656E-22	8.64988828	stage_E14_vs_E10	ordinary_muscle_development	D_vs_E10;stage_1W_vs_E10;stage_3W_vs_E10;stage	1	TRUE
ENSGALG00010029691	NUFIP2	PRJNA729271	1.366232112	6.33508E-22	1.366232112	stage_E14_vs_E10	ordinary_muscle_development	D_vs_E10;stage_1W_vs_E10;stage_3W_vs_E10;stage	1	TRUE
ENSGALG00010028909	ENSGALG00010028909	PRJNA729271	-1.385434952	6.34005E-22	1.385434952	stage_E14_vs_E10	ordinary_muscle_development	D_vs_E10;stage_1W_vs_E10;stage_3W_vs_E10;stage	1	TRUE
ENSGALG00010009699	POLR1C	PRJNA729271	-1.100443801	6.4925E-22	1.100443801	stage_E14_vs_E10	ordinary_muscle_development	D_vs_E10;stage_1W_vs_E10;stage_3W_vs_E10;stage	1	TRUE
ENSGALG00010027985	PSMB4	PRJNA729271	0.923495856	6.76578E-22	0.923495856	stage_E14_vs_E10	ordinary_muscle_development		0	FALSE
ENSGALG00010021751	MTCH2	PRJNA729271	-0.920349259	6.79684E-22	0.920349259	stage_E14_vs_E10	ordinary_muscle_development		0	FALSE
ENSGALG00010001908	FAM83B	PRJNA729271	-4.289916733	7.01983E-22	4.289916733	stage_E14_vs_E10	ordinary_muscle_development	D_vs_E10;stage_1W_vs_E10;stage_3W_vs_E10;stage	1	TRUE
ENSGALG00010015029	TFRC	PRJNA729271	-1.785960742	7.08796E-22	1.785960742	stage_E14_vs_E10	ordinary_muscle_development	D_vs_E10;stage_1W_vs_E10;stage_3W_vs_E10;stage	1	TRUE
ENSGALG00010015299	GALE	PRJNA729271	-0.946741077	7.09676E-22	0.946741077	stage_E14_vs_E10	ordinary_muscle_development		0	FALSE
ENSGALG00010017346	GM2A	PRJNA729271	-1.71389922	7.24471E-22	1.71389922	stage_E14_vs_E10	ordinary_muscle_development	D_vs_E10;stage_1W_vs_E10;stage_3W_vs_E10;stage	1	TRUE
ENSGALG00010019307	ATF3	PRJNA729271	4.74030849	7.27861E-22	4.74030849	stage_E14_vs_E10	ordinary_muscle_development	D_vs_E10;stage_1W_vs_E10;stage_3W_vs_E10;stage	1	TRUE
ENSGALG00010015801	VSTM2L	PRJNA729271	-3.064151218	7.27889E-22	3.064151218	stage_E14_vs_E10	ordinary_muscle_development	D_vs_E10;stage_1W_vs_E10;stage_3W_vs_E10;stage	1	TRUE
ENSGALG00010007581	LPCAT1	PRJNA729271	-1.170850334	7.47973E-22	1.170850334	stage_E14_vs_E10	ordinary_muscle_development	D_vs_E10;stage_1W_vs_E10;stage_3W_vs_E10;stage	1	TRUE
ENSGALG00010029674	FDXR	PRJNA729271	-1.133268327	7.63152E-22	1.133268327	stage_E14_vs_E10	ordinary_muscle_development	D_vs_E10;stage_1W_vs_E10;stage_3W_vs_E10;stage	1	TRUE
ENSGALG00010020143	KCNK5	PRJNA729271	-1.848762527	7.67204E-22	1.848762527	stage_E14_vs_E10	ordinary_muscle_development	D_vs_E10;stage_1W_vs_E10;stage_3W_vs_E10;stage	1	TRUE
ENSGALG00010006355	PTGR1	PRJNA729271	-1.495098203	7.79918E-22	1.495098203	stage_E14_vs_E10	ordinary_muscle_development	D_vs_E10;stage_1W_vs_E10;stage_3W_vs_E10;stage	1	TRUE
ENSGALG00010023837	STT3A	PRJNA729271	-0.907907523	7.91472E-22	0.907907523	stage_E14_vs_E10	ordinary_muscle_development		0	FALSE
ENSGALG00010025060	GPR156	PRJNA729271	1.488922594	7.95738E-22	1.488922594	stage_E14_vs_E10	ordinary_muscle_development	D_vs_E10;stage_1W_vs_E10;stage_3W_vs_E10;stage	1	TRUE
ENSGALG00010015156	CNOT6	PRJNA729271	-0.725419933	8.00048E-22	0.725419933	stage_E14_vs_E10	ordinary_muscle_development		0	FALSE
ENSGALG00010014018	DPYSL3	PRJNA729271	-2.342505665	8.09191E-22	2.342505665	stage_E14_vs_E10	ordinary_muscle_development	D_vs_E10;stage_1W_vs_E10;stage_3W_vs_E10;stage	1	TRUE
ENSGALG00010010266	EFNA5	PRJNA729271	-2.154848809	8.34031E-22	2.154848809	stage_E14_vs_E10	ordinary_muscle_development	D_vs_E10;stage_1W_vs_E10;stage_3W_vs_E10;stage	1	TRUE
ENSGALG00010023429	BMF	PRJNA729271	1.428372366	8.54266E-22	1.428372366	stage_E14_vs_E10	ordinary_muscle_development	D_vs_E10;stage_1W_vs_E10;stage_3W_vs_E10;stage	1	TRUE
ENSGALG00010018326	LCMT1	PRJNA729271	-1.104357043	8.60756E-22	1.104357043	stage_E14_vs_E10	ordinary_muscle_development	D_vs_E10;stage_1W_vs_E10;stage_3W_vs_E10;stage	1	TRUE
ENSGALG00010023102	RNF10	PRJNA729271	0.987689892	8.69481E-22	0.987689892	stage_E14_vs_E10	ordinary_muscle_development		0	FALSE
ENSGALG00010027232	ARRDC2	PRJNA729271	3.182829114	8.69842E-22	3.182829114	stage_E14_vs_E10	ordinary_muscle_development	D_vs_E10;stage_1W_vs_E10;stage_3W_vs_E10;stage	1	TRUE
ENSGALG00010015971	ENSGALG00010015971	PRJNA729271	-1.580028285	8.7437E-22	1.580028285	stage_E14_vs_E10	ordinary_muscle_development	D_vs_E10;stage_1W_vs_E10;stage_3W_vs_E10;stage	1	TRUE
ENSGALG00010022406	ITPRID2	PRJNA729271	1.014300498	8.78741E-22	1.014300498	stage_E14_vs_E10	ordinary_muscle_development	D_vs_E10;stage_1W_vs_E10;stage_3W_vs_E10;stage	1	TRUE
ENSGALG00010020287	GALNT16	PRJNA729271	1.566660409	8.80538E-22	1.566660409	stage_E14_vs_E10	ordinary_muscle_development	D_vs_E10;stage_1W_vs_E10;stage_3W_vs_E10;stage	1	TRUE
ENSGALG00010004420	ENSGALG00010004420	PRJNA729271	2.691948086	8.83611E-22	2.691948086	stage_E14_vs_E10	ordinary_muscle_development	D_vs_E10;stage_1W_vs_E10;stage_3W_vs_E10;stage	1	TRUE
ENSGALG00010029404	SRR	PRJNA729271	1.717702155	8.8928E-22	1.717702155	stage_E14_vs_E10	ordinary_muscle_development	D_vs_E10;stage_1W_vs_E10;stage_3W_vs_E10;stage	1	TRUE
ENSGALG00010016426	HELZ2	PRJNA729271	3.573569008	9.01229E-22	3.573569008	stage_E14_vs_E10	ordinary_muscle_development	D_vs_E10;stage_1W_vs_E10;stage_3W_vs_E10;stage	1	TRUE
ENSGALG00010003189	ANTXR2	PRJNA729271	1.236268416	9.11944E-22	1.236268416	stage_E14_vs_E10	ordinary_muscle_development	D_vs_E10;stage_1W_vs_E10;stage_3W_vs_E10;stage	1	TRUE
ENSGALG00010024519	MIEN1	PRJNA729271	1.343033608	9.24805E-22	1.343033608	stage_E14_vs_E10	ordinary_muscle_development	D_vs_E10;stage_1W_vs_E10;stage_3W_vs_E10;stage	1	TRUE
ENSGALG00010015353	RELL1	PRJNA729271	0.885212189	9.2952E-22	0.885212189	stage_E14_vs_E10	ordinary_muscle_development		0	FALSE
ENSGALG00010010662	MGST3	PRJNA729271	-1.503331654	9.31151E-22	1.503331654	stage_E14_vs_E10	ordinary_muscle_development	D_vs_E10;stage_1W_vs_E10;stage_3W_vs_E10;stage	1	TRUE
ENSGALG00010020018	ENSGALG00010020018	PRJNA729271	1.999200622	9.42332E-22	1.999200622	stage_E14_vs_E10	ordinary_muscle_development	D_vs_E10;stage_1W_vs_E10;stage_3W_vs_E10;stage	1	TRUE
ENSGALG00010021030	CSRP3	PRJNA729271	5.354217682	9.53758E-22	5.354217682	stage_E14_vs_E10	ordinary_muscle_development	D_vs_E10;stage_1W_vs_E10;stage_3W_vs_E10;stage	1	TRUE
ENSGALG00010014355	RAB20	PRJNA729271	1.599713961	9.66857E-22	1.599713961	stage_E14_vs_E10	ordinary_muscle_development	D_vs_E10;stage_1W_vs_E10;stage_3W_vs_E10;stage	1	TRUE
ENSGALG00010003164	C3	PRJNA729271	4.464126252	9.70208E-22	4.464126252	stage_E14_vs_E10	ordinary_muscle_development	D_vs_E10;stage_1W_vs_E10;stage_3W_vs_E10;stage	1	TRUE
ENSGALG00010021259	CEP104	PRJNA729271	-1.32466713	9.72006E-22	1.32466713	stage_E14_vs_E10	ordinary_muscle_development	D_vs_E10;stage_1W_vs_E10;stage_3W_vs_E10;stage	1	TRUE
ENSGALG00010025988	NSF	PRJNA729271	-0.588569275	9.7511E-22	0.588569275	stage_E14_vs_E10	ordinary_muscle_development		0	FALSE
ENSGALG00010008299	ANKRD66	PRJNA729271	2.396312157	9.7909E-22	2.396312157	stage_E14_vs_E10	ordinary_muscle_development	D_vs_E10;stage_1W_vs_E10;stage_3W_vs_E10;stage	1	TRUE

ENSGALG00010012967	ECRG4	PRJNA729271	-2.04377534	9.85373E-22	2.04377534	stage_E14_vs_E10	ordinary_muscle_development	D_vs_E10;stage_1W_vs_E10;stage_3W_vs_E10;stage	1	TRUE
ENSGALG00010026149	LASP1	PRJNA729271	-1.259432985	1.02353E-21	1.259432985	stage_E14_vs_E10	ordinary_muscle_development	D_vs_E10;stage_1W_vs_E10;stage_3W_vs_E10;stage	1	TRUE
ENSGALG00010007102	CMTM3	PRJNA729271	-1.61285124	1.02706E-21	1.61285124	stage_E14_vs_E10	ordinary_muscle_development	D_vs_E10;stage_1W_vs_E10;stage_3W_vs_E10;stage	1	TRUE
ENSGALG00010009623	XPO5	PRJNA729271	-1.086825929	1.03557E-21	1.086825929	stage_E14_vs_E10	ordinary_muscle_development	D_vs_E10;stage_1W_vs_E10;stage_3W_vs_E10;stage	1	TRUE
ENSGALG00010010014	GLUL	PRJNA729271	3.812985271	1.03604E-21	3.812985271	stage_E14_vs_E10	ordinary_muscle_development	D_vs_E10;stage_1W_vs_E10;stage_3W_vs_E10;stage	1	TRUE
ENSGALG00010012841	L3MBTL2	PRJNA729271	-0.921354502	1.04428E-21	0.921354502	stage_E14_vs_E10	ordinary_muscle_development		0	FALSE
ENSGALG00010025420	STK39	PRJNA729271	-2.066291817	1.04975E-21	2.066291817	stage_E14_vs_E10	ordinary_muscle_development	D_vs_E10;stage_1W_vs_E10;stage_3W_vs_E10;stage	1	TRUE
ENSGALG00010014091	CHIC2	PRJNA729271	0.948997844	1.06195E-21	0.948997844	stage_E14_vs_E10	ordinary_muscle_development		0	FALSE
ENSGALG00010011881	RIOK2	PRJNA729271	-0.951767759	1.06202E-21	0.951767759	stage_E14_vs_E10	ordinary_muscle_development		0	FALSE
ENSGALG00010018020	SNAPC5	PRJNA729271	-1.021464525	1.06759E-21	1.021464525	stage_E14_vs_E10	ordinary_muscle_development	D_vs_E10;stage_1W_vs_E10;stage_3W_vs_E10;stage	1	TRUE
ENSGALG00010006701	LINGO2	PRJNA729271	-6.813277774	1.068E-21	6.813277774	stage_E14_vs_E10	ordinary_muscle_development	D_vs_E10;stage_1W_vs_E10;stage_3W_vs_E10;stage	1	TRUE
ENSGALG00010010982	ZBTB21	PRJNA729271	1.159301808	1.06839E-21	1.159301808	stage_E14_vs_E10	ordinary_muscle_development	D_vs_E10;stage_1W_vs_E10;stage_3W_vs_E10;stage	1	TRUE
ENSGALG00010009989	TBC1D7	PRJNA729271	-1.338866131	1.06848E-21	1.338866131	stage_E14_vs_E10	ordinary_muscle_development	D_vs_E10;stage_1W_vs_E10;stage_3W_vs_E10;stage	1	TRUE
ENSGALG00010027490	ATP2B4	PRJNA729271	0.657886732	1.07817E-21	0.657886732	stage_E14_vs_E10	ordinary_muscle_development		0	FALSE
ENSGALG00010000153	NF2	PRJNA729271	0.905284132	1.10354E-21	0.905284132	stage_E14_vs_E10	ordinary_muscle_development		0	FALSE
ENSGALG00010016629	LARS1	PRJNA729271	-0.698171986	1.12227E-21	0.698171986	stage_E14_vs_E10	ordinary_muscle_development		0	FALSE
ENSGALG00010002405	KCNQ5	PRJNA729271	1.913690935	1.13468E-21	1.913690935	stage_E14_vs_E10	ordinary_muscle_development	D_vs_E10;stage_1W_vs_E10;stage_3W_vs_E10;stage	1	TRUE
ENSGALG00010023331	CD4	PRJNA729271	-3.573969004	1.17901E-21	3.573969004	stage_E14_vs_E10	ordinary_muscle_development	D_vs_E10;stage_1W_vs_E10;stage_3W_vs_E10;stage	1	TRUE
ENSGALG00010017457	ZMAT4	PRJNA729271	-2.759878691	1.19974E-21	2.759878691	stage_E14_vs_E10	ordinary_muscle_development	D_vs_E10;stage_1W_vs_E10;stage_3W_vs_E10;stage	1	TRUE
ENSGALG00010013995	REM1	PRJNA729271	2.024300883	1.21292E-21	2.024300883	stage_E14_vs_E10	ordinary_muscle_development	D_vs_E10;stage_1W_vs_E10;stage_3W_vs_E10;stage	1	TRUE
ENSGALG00010014292	NIM1K	PRJNA729271	4.293804648	1.24757E-21	4.293804648	stage_E14_vs_E10	ordinary_muscle_development	D_vs_E10;stage_1W_vs_E10;stage_3W_vs_E10;stage	1	TRUE
ENSGALG00010027911	PTPN22	PRJNA729271	3.197974307	1.2717E-21	3.197974307	stage_E14_vs_E10	ordinary_muscle_development	D_vs_E10;stage_1W_vs_E10;stage_3W_vs_E10;stage	1	TRUE
ENSGALG00010003700	REST	PRJNA729271	-0.656135092	1.31851E-21	0.656135092	stage_E14_vs_E10	ordinary_muscle_development		0	FALSE
ENSGALG00010008375	ENSGALG00010008375	PRJNA729271	-0.959728333	1.33252E-21	0.959728333	stage_E14_vs_E10	ordinary_muscle_development		0	FALSE
ENSGALG00010019650	KBTBD8	PRJNA729271	0.839924472	1.34786E-21	0.839924472	stage_E14_vs_E10	ordinary_muscle_development		0	FALSE
ENSGALG00010026775	BTG2	PRJNA729271	3.560534134	1.35896E-21	3.560534134	stage_E14_vs_E10	ordinary_muscle_development	D_vs_E10;stage_1W_vs_E10;stage_3W_vs_E10;stage	1	TRUE
ENSGALG00010023443	ENSGALG00010023443	PRJNA729271	1.723441303	1.36405E-21	1.723441303	stage_E14_vs_E10	ordinary_muscle_development	D_vs_E10;stage_1W_vs_E10;stage_3W_vs_E10;stage	1	TRUE
ENSGALG00010021134	CHFR	PRJNA729271	-0.913221711	1.38391E-21	0.913221711	stage_E14_vs_E10	ordinary_muscle_development		0	FALSE
ENSGALG00010018441	CRIPT	PRJNA729271	0.90282617	1.39618E-21	0.90282617	stage_E14_vs_E10	ordinary_muscle_development		0	FALSE
ENSGALG00010012760	PLEKHF2	PRJNA729271	1.017890253	1.4187E-21	1.017890253	stage_E14_vs_E10	ordinary_muscle_development	D_vs_E10;stage_1W_vs_E10;stage_3W_vs_E10;stage	1	TRUE
ENSGALG00010020179	PSMD6	PRJNA729271	1.301000543	1.45848E-21	1.301000543	stage_E14_vs_E10	ordinary_muscle_development	D_vs_E10;stage_1W_vs_E10;stage_3W_vs_E10;stage	1	TRUE
ENSGALG00010007243	BEAN1	PRJNA729271	-1.848418907	1.48111E-21	1.848418907	stage_E14_vs_E10	ordinary_muscle_development	D_vs_E10;stage_1W_vs_E10;stage_3W_vs_E10;stage	1	TRUE
ENSGALG00010021409	MEGF6	PRJNA729271	-2.723290792	1.51621E-21	2.723290792	stage_E14_vs_E10	ordinary_muscle_development	D_vs_E10;stage_1W_vs_E10;stage_3W_vs_E10;stage	1	TRUE
ENSGALG00010024974	CD44	PRJNA729271	-1.636397762	1.54414E-21	1.636397762	stage_E14_vs_E10	ordinary_muscle_development	D_vs_E10;stage_1W_vs_E10;stage_3W_vs_E10;stage	1	TRUE
ENSGALG00010003154	MTFR1	PRJNA729271	-0.796049152	1.5635E-21	0.796049152	stage_E14_vs_E10	ordinary_muscle_development		0	FALSE
ENSGALG00010029162	ELN	PRJNA729271	3.019367292	1.57408E-21	3.019367292	stage_E14_vs_E10	ordinary_muscle_development	D_vs_E10;stage_1W_vs_E10;stage_3W_vs_E10;stage	1	TRUE
ENSGALG00010002544	THSD1	PRJNA729271	1.214117532	1.57473E-21	1.214117532	stage_E14_vs_E10	ordinary_muscle_development	D_vs_E10;stage_1W_vs_E10;stage_3W_vs_E10;stage	1	TRUE
ENSGALG00010027891	CCDC66	PRJNA729271	-1.430728515	1.58033E-21	1.430728515	stage_E14_vs_E10	ordinary_muscle_development	D_vs_E10;stage_1W_vs_E10;stage_3W_vs_E10;stage	1	TRUE
ENSGALG00010021465	ABCB6	PRJNA729271	2.045374799	1.58181E-21	2.045374799	stage_E14_vs_E10	ordinary_muscle_development	D_vs_E10;stage_1W_vs_E10;stage_3W_vs_E10;stage	1	TRUE
ENSGALG00010025569	PPTC7	PRJNA729271	1.871392832	1.60411E-21	1.871392832	stage_E14_vs_E10	ordinary_muscle_development	D_vs_E10;stage_1W_vs_E10;stage_3W_vs_E10;stage	1	TRUE
ENSGALG00010010418	BRINP2	PRJNA729271	-8.904285956	1.60508E-21	8.904285956	stage_E14_vs_E10	ordinary_muscle_development	D_vs_E10;stage_1W_vs_E10;stage_3W_vs_E10;stage	1	TRUE
ENSGALG00010015469	TMEM33	PRJNA729271	-0.733941591	1.60597E-21	0.733941591	stage_E14_vs_E10	ordinary_muscle_development		0	FALSE
ENSGALG00010012042	FNDC7	PRJNA729271	-4.265545755	1.61545E-21	4.265545755	stage_E14_vs_E10	ordinary_muscle_development	D_vs_E10;stage_1W_vs_E10;stage_3W_vs_E10;stage	1	TRUE
ENSGALG00010012792	CBY1	PRJNA729271	-1.455839228	1.63635E-21	1.455839228	stage_E14_vs_E10	ordinary_muscle_development	D_vs_E10;stage_1W_vs_E10;stage_3W_vs_E10;stage	1	TRUE
ENSGALG00010000757	SCEL	PRJNA729271	-5.350021925	1.65205E-21	5.350021925	stage_E14_vs_E10	ordinary_muscle_development	D_vs_E10;stage_1W_vs_E10;stage_3W_vs_E10;stage	1	TRUE
ENSGALG00010020121	ENSGALG00010020121	PRJNA729271	-2.735722095	1.73081E-21	2.735722095	stage_E14_vs_E10	ordinary_muscle_development	D_vs_E10;stage_1W_vs_E10;stage_3W_vs_E10;stage	1	TRUE
ENSGALG00010010059	ENSGALG00010010059	PRJNA729271	-1.295528748	1.73589E-21	1.295528748	stage_E14_vs_E10	ordinary_muscle_development	D_vs_E10;stage_1W_vs_E10;stage_3W_vs_E10;stage	1	TRUE
ENSGALG00010029197	LHX1	PRJNA729271	5.355034587	1.7453E-21	5.355034587	stage_E14_vs_E10	ordinary_muscle_development	D_vs_E10;stage_1W_vs_E10;stage_3W_vs_E10;stage	1	TRUE
ENSGALG00010018385	SPR	PRJNA729271	-1.392789161	1.76089E-21	1.392789161	stage_E14_vs_E10	ordinary_muscle_development	D_vs_E10;stage_1W_vs_E10;stage_3W_vs_E10;stage	1	TRUE
ENSGALG00010028545	ENSGALG00010028545	PRJNA729271	12.84576392	1.77398E-21	12.84576392	stage_E14_vs_E10	ordinary_muscle_development	D_vs_E10;stage_1W_vs_E10;stage_3W_vs_E10;stage	1	TRUE
ENSGALG00010027079	PM20D1	PRJNA729271	0.910749792	1.80345E-21	0.910749792	stage_E14_vs_E10	ordinary_muscle_development		0	FALSE
ENSGALG00010004473	JDP2	PRJNA729271	1.272956532	1.81994E-21	1.272956532	stage_E14_vs_E10	ordinary_muscle_development	D_vs_E10;stage_1W_vs_E10;stage_3W_vs_E10;stage	1	TRUE
ENSGALG00010025505	ENSGALG00010025505	PRJNA729271	-2.890045925	1.87288E-21	2.890045925	stage_E14_vs_E10	ordinary_muscle_development	D_vs_E10;stage_1W_vs_E10;stage_3W_vs_E10;stage	1	TRUE
ENSGALG00010029262	CLTC	PRJNA729271	-0.630527817	1.87575E-21	0.630527817	stage_E14_vs_E10	ordinary_muscle_development		0	FALSE
ENSGALG00010010253	IL10RB	PRJNA729271	1.174317133	1.89181E-21	1.174317133	stage_E14_vs_E10	ordinary_muscle_development	D_vs_E10;stage_1W_vs_E10;stage_3W_vs_E10;stage	1	TRUE
ENSGALG00010016200	MICU3	PRJNA729271	1.128347966	1.94384E-21	1.128347966	stage_E14_vs_E10	ordinary_muscle_development	D_vs_E10;stage_1W_vs_E10;stage_3W_vs_E10;stage	1	TRUE
ENSGALG00010012561	OSGIN2	PRJNA729271	-1.279249028	1.94796E-21	1.279249028	stage_E14_vs_E10	ordinary_muscle_development	D_vs_E10;stage_1W_vs_E10;stage_3W_vs_E10;stage	1	TRUE
ENSGALG00010022401	LCAT	PRJNA729271	4.22453889	1.96233E-21	4.22453889	stage_E14_vs_E10	ordinary_muscle_development	D_vs_E10;stage_1W_vs_E10;stage_3W_vs_E10;stage	1	TRUE
ENSGALG00010019284	CHD5	PRJNA729271	1.270544166	2.00143E-21	1.270544166	stage_E14_vs_E10	ordinary_muscle_development	D_vs_E10;stage_1W_vs_E10;stage_3W_vs_E10;stage	1	TRUE
ENSGALG00010025341	VPS26B	PRJNA729271	-1.28857352	2.04596E-21	1.28857352	stage_E14_vs_E10	ordinary_muscle_development	D_vs_E10;stage_1W_vs_E10;stage_3W_vs_E10;stage	1	TRUE
ENSGALG00010003902	ENSGALG00010003902	PRJNA729271	1.200163915	2.07978E-21	1.200163915	stage_E14_vs_E10	ordinary_muscle_development	D_vs_E10;stage_1W_vs_E10;stage_3W_vs_E10;stage	1	TRUE
ENSGALG00010027381	KLHL40	PRJNA729271	1.934267941	2.14118E-21	1.934267941	stage_E14_vs_E10	ordinary_muscle_development	D_vs_E10;stage_1W_vs_E10;stage_3W_vs_E10;stage	1	TRUE
ENSGALG00010000407	WSB2	PRJNA729271	0.820411052	2.19786E-21	0.820411052	stage_E14_vs_E10	ordinary_muscle_development		0	FALSE
ENSGALG00010001201	TUBB6	PRJNA729271	-1.601272926	2.28292E-21	1.601272926	stage_E14_vs_E10	ordinary_muscle_development	D_vs_E10;stage_1W_vs_E10;stage_3W_vs_E10;stage	1	TRUE
ENSGALG00010013273	STAG2	PRJNA729271	1.11976018	2.28553E-21	1.11976018	stage_E14_vs_E10	ordinary_muscle_development	D_vs_E10;stage_1W_vs_E10;stage_3W_vs_E10;stage	1	TRUE
ENSGALG00010017744	ETAA1	PRJNA729271	-0.964161332	2.28607E-21	0.964161332	stage_E14_vs_E10	ordinary_muscle_development		0	FALSE
ENSGALG00010027305	LSM4	PRJNA729271	-1.278243983	2.37228E-21	1.278243983	stage_E14_vs_E10	ordinary_muscle_development	D_vs_E10;stage_1W_vs_E10;stage_3W_vs_E10;stage	1	TRUE
ENSGALG00010028465	ENSGALG00010028465	PRJNA729271	1.271907783	2.41764E-21	1.271907783	stage_E14_vs_E10	ordinary_muscle_development	D_vs_E10;stage_1W_vs_E10;stage_3W_vs_E10;stage	1	TRUE
ENSGALG00010017174	DHX57	PRJNA729271	-0.950647603	2.46025E-21	0.950647603	stage_E14_vs_E10	ordinary_muscle_development		0	FALSE
ENSGALG00010008174	SLC26A2	PRJNA729271	-1.214618013	2.46995E-21	1.214618013	stage_E14_vs_E10	ordinary_muscle_development	D_vs_E10;stage_1W_vs_E10;stage_3W_vs_E10;stage	1	TRUE
ENSGALG00010007944	ACSF3	PRJNA729271	1.275498439	2.51995E-21	1.275498439	stage_E14_vs_E10	ordinary_muscle_development	D_vs_E10;stage_1W_vs_E10;stage_3W_vs_E10;stage	1	TRUE
ENSGALG00010025219	NACA	PRJNA729271	-1.21237637	2.53424E-21	1.21237637	stage_E14_vs_E10	ordinary_muscle_development	D_vs_E10;stage_1W_vs_E10;stage_3W_vs_E10;stage	1	TRUE
ENSGALG00010021233	COASY	PRJNA729271	0.59387425	2.53431E-21	0.59387425	stage_E14_vs_E10	ordinary_muscle_development		0	FALSE
ENSGALG00010021126	ECE1	PRJNA729271	1.000564217	2.55585E-21	1.000564217	stage_E14_vs_E10	ordinary_muscle_development	D_vs_E10;stage_1W_vs_E10;stage_3W_vs_E10;stage	1	TRUE
ENSGALG00010010918	TOR1AIP2	PRJNA729271	1.144537406	2.56599E-21	1.144537406	stage_E14_vs_E10	ordinary_muscle_development	D_vs_E10;stage_1W_vs_E10;stage_3W_vs_E10;stage	1	TRUE
ENSGALG00010024896	RNF41	PRJNA729271	0.96213326	2.56665E-21	0.96213326	stage_E14_vs_E10	ordinary_muscle_development		0	FALSE
ENSGALG00010002331	ENSGALG00010002331	PRJNA729271	-3.504118048	2.5808E-21	3.504118048	stage_E14_vs_E10	ordinary_muscle_development	D_vs_E10;stage_1W_vs_E10;stage_3W_vs_E10;stage	1	TRUE
ENSGALG00010009117	ERGIC1	PRJNA729271	-0.654583787	2.61768E-21	0.654583787	stage_E14_vs_E10	ordinary_muscle_development		0	FALSE
ENSGALG00010026011	GJC2	PRJNA729271	-1.596956061	2.64609E-21	1.596956061	stage_E14_vs_E10	ordinary_muscle_development	D_vs_E10;stage_1W_vs_E10;stage_3W_vs_E10;stage	1	TRUE
ENSGALG00010000715	PCDH20	PRJNA729271	-2.796674643	2.66806E-21	2.796674643	stage_E14_vs_E10	ordinary_muscle_development	D_vs_E10;stage_1W_vs_E10;stage_3W_vs_E10;stage	1	TRUE
ENSGALG00010018142	DDHD1	PRJNA729271	-1.404314684	2.81282E-21	1.404314684	stage_E14_vs_E10	ordinary_muscle_development	D_vs_E10;stage_1W_vs_E10;stage_3W_vs_E10;stage	1	TRUE
ENSGALG00010008383	PLD5	PRJNA729271	-4.380775212	2.83231E-21	4.380775212	stage_E14_vs_E10	ordinary_muscle_development	D_vs_E10;stage_1W_vs_E10;stage_3W_vs_E10;stage	1	TRUE
ENSGALG00010011659	CD14	PRJNA729271	1.463381029	2.86028E-21	1.463381029	stage_E14_vs_E10	ordinary_muscle_development	D_vs_E10;stage_1W_vs_E10;stage_3W_vs_E10;stage	1	TRUE
ENSGALG00010019243	SERTAD2	PRJNA729271	1.467772668	2.91426E-21	1.467772668	stage_E14_vs_E10	ordinary_muscle_development	D_vs_E10;stage_1W_vs_E10;stage_3W_vs_E10;stage	1	TRUE
ENSGALG00010024031	LIMS2	PRJNA729271	1.11433551	2.9178E-21	1.11433551	stage_E14_vs_E10	ordinary_muscle_development	D_vs_E10;stage_1W_vs_E10;stage_3W_vs_E10;stage	1	TRUE
ENSGALG00010021034	NPM3	PRJNA729271	-2.762083282	2.93496E-21	2.762083282	stage_E14_vs_E10	ordinary_muscle_development	D_vs_E10;stage_1W_vs_E10;stage_3W_vs_E10;stage	1	TRUE
ENSGALG00010024700	TOLLIP	PRJNA729271	0.807167705	2.95954E-21	0.807167705	stage_E14_vs_E10	ordinary_muscle_development		0	FALSE
ENSGALG00010013914	NRG1	PRJNA729271	2.183693481	3.02968E-21	2.183693481	stage_E14_vs_E10	ordinary_muscle_development	D_vs_E10;stage_1W_vs_E10;stage_3W_vs_E10;stage	1	TRUE
ENSGALG00010015248	ENSGALG00010015248	PRJNA729271	1.264693163	3.11231E-21	1.264693163	stage_E14_vs_E10	ordinary_muscle_development	D_vs_E10;stage_1W_vs_E10;stage_3W_vs_E10;stage	1	TRUE
ENSGALG00010008905	GABARAPL2	PRJNA729271	0.894983319	3.14496E-21	0.894983319	stage_E14_vs_E10	ordinary_muscle_development		0	FALSE
ENSGALG00010020657	DALRD3	PRJNA729271	0.882585164	3.22201E-21	0.882585164	stage_E14_vs_E10	ordinary_muscle_development		0	FALSE
ENSGALG00010003064	VAV1	PRJNA729271	2.392211139	3.25218E-21	2.392211139	stage_E14_vs_E10	ordinary_muscle_development	D_vs_E10;stage_1W_vs_E10;stage_3W_vs_E10;stage	1	TRUE
ENSGALG00010023196	DGKZ	PRJNA729271	1.220204291	3.27491E-21	1.220204291	stage_E14_vs_E10	ordinary_muscle_development	D_vs_E10;stage_1W_vs_E10;stage_3W_vs_E10;stage	1	TRUE
ENSGALG00010003923	ENSGALG00010003923	PRJNA729271	-1.168843828	3.2852E-21	1.168843828	stage_E14_vs_E10	ordinary_muscle_development	D_vs_E10;stage_1W_vs_E10;stage_3W_vs_E10;stage	1	TRUE
ENSGALG00010018834	SAMD4A	PRJNA729271	1.650249382	3.30003E-21	1.650249382	stage_E14_vs_E10	ordinary_muscle_development	D_vs_E10;stage_1W_vs_E10;stage_3W_vs_E10;stage	1	TRUE
ENSGALG00010029567	IFT20	PRJNA729271	-1.16988217	3.31517E-21	1.16988217	stage_E14_vs_E10	ordinary_muscle_development	D_vs_E10;stage_1W_vs_E10;stage_3W_vs_E10;stage	1	TRUE
ENSGALG00010001535	RAP2B	PRJNA729271	-1.025956888	3.35662E-21	1.025956888	stage_E14_vs_E10	ordinary_muscle_development	D_vs_E10;stage_1W_vs_E10;stage_3W_vs_E10;stage	1	TRUE
ENSGALG00010027975	IQSEC1	PRJNA729271	-1.148219605	3.39035E-21	1.148219605	stage_E14_vs_E10	ordinary_muscle_development	D_vs_E10;stage_1W_vs_E10;stage_3W_vs_E10;stage	1	TRUE
ENSGALG00010022637	DMXL2	PRJNA729271	-1.06742899	3.40603E-21	1.06742899	stage_E14_vs_E10	ordinary_muscle_development	D_vs_E10;stage_1W_vs_E10;stage_3W_vs_E10;stage	1	TRUE
ENSGALG00010003389	ENSGALG00010003389	PRJNA729271	-4.586306382	3.42074E-21	4.586306382	stage_E14_vs_E10	ordinary_muscle_development	D_vs_E10;stage_1W_vs_E10;stage_3W_vs_E10;stage	1	TRUE
ENSGALG00010008793	C11orf54	PRJNA729271	1.086785905	3.42891E-21	1.086785905	stage_E14_vs_E10	ordinary_muscle_development	D_vs_E10;stage_1W_vs_E10;stage_3W_vs_E10;stage	1	TRUE
ENSGALG00010027410	ALAS1	PRJNA729271	0.835444617	3.44275E-21	0.835444617	stage_E14_vs_E10	ordinary_muscle_development		0	FALSE
ENSGALG00010005433	GPC5	PRJNA729271	-1.603543114	3.47316E-21	1.603543114	stage_E14_vs_E10	ordinary_muscle_development	D_vs_E10;stage_1W_vs_E10;stage_3W_vs_E10;stage	1	TRUE
ENSGALG00010003383	UBE2J1	PRJNA729271	0.824286703	3.49657E-21	0.824286703	stage_E14_vs_E10	ordinary_muscle_development		0	FALSE
ENSGALG00010023205	DNAJC17	PRJNA729271	-1.119475253	3.5241E-21	1.119475253	stage_E14_vs_E10	ordinary_muscle_development	D_vs_E10;stage_1W_vs_E10;stage_3W_vs_E10;stage	1	TRUE
ENSGALG00010015983	CNOT7	PRJNA729271	-0.626477602	3.55194E-21	0.626477602	stage_E14_vs_E10	ordinary_muscle_development		0	FALSE
ENSGALG00010013604	FYN	PRJNA729271	-1.078054559	3.64384E-21	1.078054559	stage_E14_vs_E10	ordinary_muscle_development	D_vs_E10;stage_1W_vs_E10;stage_3W_vs_E10;stage	1	TRUE
ENSGALG00010023414	SUDS3	PRJNA729271	-0.737560784	3.65239E-21	0.737560784	stage_E14_vs_E10	ordinary_muscle_development		0	FALSE
ENSGALG00010001487	CDH19	PRJNA729271	2.15698864	3.74811E-21	2.15698864	stage_E14_vs_E10	ordinary_muscle_development	D_vs_E10;stage_1W_vs_E10;stage_3W_vs_E10;stage	1	TRUE
ENSGALG00010025557	VPS29	PRJNA729271	-0.751358818	3.78583E-21	0.751358818	stage_E14_vs_E10	ordinary_muscle_development		0	FALSE
ENSGALG00010017668	ENSGALG00010017668	PRJNA729271	5.621148438	3.78658E-21	5.621148438	stage_E14_vs_E10	ordinary_muscle_development	D_vs_E10;stage_1W_vs_E10;stage_3W_vs_E10;stage	1	TRUE
ENSGALG00010014020	PDXP	PRJNA729271	-1.046942391	3.7907E-21	1.046942391	stage_E14_vs_E10	ordinary_muscle_development	D_vs_E10;stage_1W_vs_E10;stage_3W_vs_E10;stage	1	TRUE
ENSGALG00010019785	ACSL5	PRJNA729271	2.038725526	3.81837E-21	2.038725526	stage_E14_vs_E10	ordinary_muscle_development	D_vs_E10;stage_1W_vs_E10;stage_3W_vs_E10;stage	1	TRUE
ENSGALG00010019580	CD5	PRJNA729271	4.180412328	3.8731E-21	4.180412328	stage_E14_vs_E10	ordinary_muscle_development	D_vs_E10;stage_1W_vs_E10;stage_3W_vs_E10;stage	1	TRUE
ENSGALG00010018543	MRPL3	PRJNA729271	-0.754266532	4.01674E-21	0.754266532	stage_E14_vs_E10	ordinary_muscle_development		0	FALSE
ENSGALG00010024644	EIF6	PRJNA729271	-0.733795376	4.06693E-21	0.733795376	stage_E14_vs_E10	ordinary_muscle_development		0	FALSE
ENSGALG00010013528	PRDX6	PRJNA729271	1.253491733	4.15025E-21	1.253491733	stage_E14_vs_E10	ordinary_muscle_development	D_vs_E10;stage_1W_vs_E10;stage_3W_vs_E10;stage	1	TRUE
ENSGALG00010012016	WDR91	PRJNA729271	-0.616658426	4.16056E-21	0.616658426	stage_E14_vs_E10	ordinary_muscle_development		0	FALSE
ENSGALG00010015874	CLIC4	PRJNA729271	-1.099607526	4.16979E-21	1.099607526	stage_E14_vs_E10	ordinary_muscle_development	D_vs_E10;stage_1W_vs_E10;stage_3W_vs_E10;stage	1	TRUE
ENSGALG00010020370	CEP55	PRJNA729271	-2.2037883	4.17364E-21	2.2037883	stage_E14_vs_E10	ordinary_muscle_development	D_vs_E10;stage_1W_vs_E10;stage_3W_vs_E10;stage	1	TRUE
ENSGALG00010025373	CYB5RL	PRJNA729271	-0.881428566	4.17751E-21	0.881428566	stage_E14_vs_E10	ordinary_muscle_development		0	FALSE
ENSGALG00010017141	AFTPH	PRJNA729271	0.720782538	4.19073E-21	0.720782538	stage_E14_vs_E10	ordinary_muscle_development		0	FALSE
ENSGALG00010024828	TIMM17B	PRJNA729271	1.272846159	4.28484E-21	1.272846159	stage_E14_vs_E10	ordinary_muscle_development	D_vs_E10;stage_1W_vs_E10;stage_3W_vs_E10;stage	1	TRUE
ENSGALG00010013021	SLC25A5	PRJNA729271	-1.575855195	4.35111E-21	1.575855195	stage_E14_vs_E10	ordinary_muscle_development	D_vs_E10;stage_1W_vs_E10;stage_3W_vs_E10;stage	1	TRUE
ENSGALG00010013268	PTPRR	PRJNA729271	2.447896341	4.39839E-21	2.447896341	stage_E14_vs_E10	ordinary_muscle_development	D_vs_E10;stage_1W_vs_E10;stage_3W_vs_E10;stage	1	TRUE
ENSGALG00010028751	EHMT1	PRJNA729271	-0.918127514	4.51921E-21	0.918127514	stage_E14_vs_E10	ordinary_muscle_development		0	FALSE
ENSGALG00010012373	WNT7B	PRJNA729271	-9.229689837	4.53695E-21	9.229689837	stage_E14_vs_E10	ordinary_muscle_development	D_vs_E10;stage_1W_vs_E10;stage_3W_vs_E10;stage	1	TRUE
ENSGALG00010024811	NIF3L1	PRJNA729271	-1.372442408	4.54384E-21	1.372442408	stage_E14_vs_E10	ordinary_muscle_development	D_vs_E10;stage_1W_vs_E10;stage_3W_vs_E10;stage	1	TRUE
ENSGALG00010021142	WNT6	PRJNA729271	-7.594162488	4.57944E-21	7.594162488	stage_E14_vs_E10	ordinary_muscle_development	D_vs_E10;stage_1W_vs_E10;stage_3W_vs_E10;stage	1	TRUE
ENSGALG00010027842	SLAMF8	PRJNA729271	-2.522390994	4.58293E-21	2.522390994	stage_E14_vs_E10	ordinary_muscle_development	D_vs_E10;stage_1W_vs_E10;stage_3W_vs_E10;stage	1	TRUE
ENSGALG00010023693	SRSF11	PRJNA729271	-0.662840558	4.58556E-21	0.662840558	stage_E14_vs_E10	ordinary_muscle_development		0	FALSE
ENSGALG00010005549	RNF20	PRJNA729271	-1.094635993	4.5942E-21	1.094635993	stage_E14_vs_E10	ordinary_muscle_development	D_vs_E10;stage_1W_vs_E10;stage_3W_vs_E10;stage	1	TRUE
ENSGALG00010029698	NUP85	PRJNA729271	-0.82811405	4.70786E-21	0.82811405	stage_E14_vs_E10	ordinary_muscle_development		0	FALSE
ENSGALG00010012958	OLFML2B	PRJNA729271	1.970701913	4.72034E-21	1.970701913	stage_E14_vs_E10	ordinary_muscle_development	D_vs_E10;stage_1W_vs_E10;stage_3W_vs_E10;stage	1	TRUE
ENSGALG00010006492	GAP43	PRJNA729271	-4.91780357	4.73083E-21	4.91780357	stage_E14_vs_E10	ordinary_muscle_development	D_vs_E10;stage_1W_vs_E10;stage_3W_vs_E10;stage	1	TRUE
ENSGALG00010010465	BMPR1A	PRJNA729271	-0.939457961	4.83594E-21	0.939457961	stage_E14_vs_E10	ordinary_muscle_development		0	FALSE
ENSGALG00010012131	SLC14A1	PRJNA729271	-2.713987863	4.86532E-21	2.713987863	stage_E14_vs_E10	ordinary_muscle_development	D_vs_E10;stage_1W_vs_E10;stage_3W_vs_E10;stage	1	TRUE
ENSGALG00010005536	TAF4B	PRJNA729271	-2.040706464	5.02229E-21	2.040706464	stage_E14_vs_E10	ordinary_muscle_development	D_vs_E10;stage_1W_vs_E10;stage_3W_vs_E10;stage	1	TRUE
ENSGALG00010018451	PIK3R4	PRJNA729271	-1.104399538	5.13946E-21	1.104399538	stage_E14_vs_E10	ordinary_muscle_development	D_vs_E10;stage_1W_vs_E10;stage_3W_vs_E10;stage	1	TRUE
ENSGALG00010019938	TAFA4	PRJNA729271	-4.804449945	5.29571E-21	4.804449945	stage_E14_vs_E10	ordinary_muscle_development	D_vs_E10;stage_1W_vs_E10;stage_3W_vs_E10;stage	1	TRUE
ENSGALG00010000654	ENSGALG00010000654	PRJNA729271	-1.735688499	5.35449E-21	1.735688499	stage_E14_vs_E10	ordinary_muscle_development	D_vs_E10;stage_1W_vs_E10;stage_3W_vs_E10;stage	1	TRUE
ENSGALG00010011913	CXXC5	PRJNA729271	1.066002526	5.66653E-21	1.066002526	stage_E14_vs_E10	ordinary_muscle_development	D_vs_E10;stage_1W_vs_E10;stage_3W_vs_E10;stage	1	TRUE
ENSGALG00010013292	PRDM1	PRJNA729271	-2.891029053	5.67201E-21	2.891029053	stage_E14_vs_E10	ordinary_muscle_development	D_vs_E10;stage_1W_vs_E10;stage_3W_vs_E10;stage	1	TRUE
ENSGALG00010021643	ENSGALG00010021643	PRJNA729271	-1.326690948	5.71047E-21	1.326690948	stage_E14_vs_E10	ordinary_muscle_development	D_vs_E10;stage_1W_vs_E10;stage_3W_vs_E10;stage	1	TRUE
ENSGALG00010020839	PGGHG	PRJNA729271	-1.439722965	5.85101E-21	1.439722965	stage_E14_vs_E10	ordinary_muscle_development	D_vs_E10;stage_1W_vs_E10;stage_3W_vs_E10;stage	1	TRUE
ENSGALG00010020924	DNAJC16	PRJNA729271	1.139025045	5.91972E-21	1.139025045	stage_E14_vs_E10	ordinary_muscle_development	D_vs_E10;stage_1W_vs_E10;stage_3W_vs_E10;stage	1	TRUE
ENSGALG00010005427	ENSGALG00010005427	PRJNA729271	1.309236106	5.96049E-21	1.309236106	stage_E14_vs_E10	ordinary_muscle_development	D_vs_E10;stage_1W_vs_E10;stage_3W_vs_E10;stage	1	TRUE
ENSGALG00010027149	C1orf116	PRJNA729271	2.127449406	6.36973E-21	2.127449406	stage_E14_vs_E10	ordinary_muscle_development	D_vs_E10;stage_1W_vs_E10;stage_3W_vs_E10;stage	1	TRUE
ENSGALG00010012441	FST	PRJNA729271	2.281006005	6.45188E-21	2.281006005	stage_E14_vs_E10	ordinary_muscle_development	D_vs_E10;stage_1W_vs_E10;stage_3W_vs_E10;stage	1	TRUE
ENSGALG00010018265	PDCD4	PRJNA729271	-0.638366303	6.51033E-21	0.638366303	stage_E14_vs_E10	ordinary_muscle_development		0	FALSE
ENSGALG00010023927	KPNB1	PRJNA729271	-1.259776317	6.69634E-21	1.259776317	stage_E14_vs_E10	ordinary_muscle_development	D_vs_E10;stage_1W_vs_E10;stage_3W_vs_E10;stage	1	TRUE
ENSGALG00010012529	BEND3	PRJNA729271	-1.203173598	6.74027E-21	1.203173598	stage_E14_vs_E10	ordinary_muscle_development	D_vs_E10;stage_1W_vs_E10;stage_3W_vs_E10;stage	1	TRUE
ENSGALG00010001803	PPP4R1	PRJNA729271	-0.929764753	6.81357E-21	0.929764753	stage_E14_vs_E10	ordinary_muscle_development		0	FALSE
ENSGALG00010024646	RORA	PRJNA729271	1.835596547	6.97971E-21	1.835596547	stage_E14_vs_E10	ordinary_muscle_development	D_vs_E10;stage_1W_vs_E10;stage_3W_vs_E10;stage	1	TRUE
ENSGALG00010025998	SMARCB1	PRJNA729271	-1.380383584	7.04162E-21	1.380383584	stage_E14_vs_E10	ordinary_muscle_development	D_vs_E10;stage_1W_vs_E10;stage_3W_vs_E10;stage	1	TRUE
ENSGALG00010005417	LOXL3	PRJNA729271	1.560214796	7.16576E-21	1.560214796	stage_E14_vs_E10	ordinary_muscle_development	D_vs_E10;stage_1W_vs_E10;stage_3W_vs_E10;stage	1	TRUE
ENSGALG00010024542	ZNFX1	PRJNA729271	4.044281867	7.35246E-21	4.044281867	stage_E14_vs_E10	ordinary_muscle_development	D_vs_E10;stage_1W_vs_E10;stage_3W_vs_E10;stage	1	TRUE
ENSGALG00010021776	PMS2	PRJNA729271	-0.906573599	7.3641E-21	0.906573599	stage_E14_vs_E10	ordinary_muscle_development		0	FALSE
ENSGALG00010029669	HID1	PRJNA729271	0.700985884	7.38374E-21	0.700985884	stage_E14_vs_E10	ordinary_muscle_development		0	FALSE
ENSGALG00010005486	IRF5	PRJNA729271	2.964484593	7.41316E-21	2.964484593	stage_E14_vs_E10	ordinary_muscle_development	D_vs_E10;stage_1W_vs_E10;stage_3W_vs_E10;stage	1	TRUE
ENSGALG00010016027	COPS2	PRJNA729271	1.42451156	7.45774E-21	1.42451156	stage_E14_vs_E10	ordinary_muscle_development	D_vs_E10;stage_1W_vs_E10;stage_3W_vs_E10;stage	1	TRUE
ENSGALG00010026448	ADORA2A	PRJNA729271	-1.117437235	7.48363E-21	1.117437235	stage_E14_vs_E10	ordinary_muscle_development	D_vs_E10;stage_1W_vs_E10;stage_3W_vs_E10;stage	1	TRUE
ENSGALG00010000649	PAPD7	PRJNA729271	0.783507489	7.51652E-21	0.783507489	stage_E14_vs_E10	ordinary_muscle_development		0	FALSE
ENSGALG00010020497	OSBPL11	PRJNA729271	-0.672520793	7.57305E-21	0.672520793	stage_E14_vs_E10	ordinary_muscle_development		0	FALSE
ENSGALG00010015544	RTCB	PRJNA729271	-0.712814648	7.76991E-21	0.712814648	stage_E14_vs_E10	ordinary_muscle_development		0	FALSE
ENSGALG00010025271	RNF34	PRJNA729271	1.11664994	7.77012E-21	1.11664994	stage_E14_vs_E10	ordinary_muscle_development	D_vs_E10;stage_1W_vs_E10;stage_3W_vs_E10;stage	1	TRUE
ENSGALG00010018054	SMAD6	PRJNA729271	-1.555648022	7.82311E-21	1.555648022	stage_E14_vs_E10	ordinary_muscle_development	D_vs_E10;stage_1W_vs_E10;stage_3W_vs_E10;stage	1	TRUE
ENSGALG00010025594	UPK1B	PRJNA729271	-8.05852909	7.83906E-21	8.05852909	stage_E14_vs_E10	ordinary_muscle_development	D_vs_E10;stage_1W_vs_E10;stage_3W_vs_E10;stage	1	TRUE
ENSGALG00010025464	VPS33A	PRJNA729271	-0.9071422	7.84843E-21	0.9071422	stage_E14_vs_E10	ordinary_muscle_development		0	FALSE
ENSGALG00010008837	ENSGALG00010008837	PRJNA729271	-0.949863531	7.9525E-21	0.949863531	stage_E14_vs_E10	ordinary_muscle_development		0	FALSE
ENSGALG00010024146	CDKN1C	PRJNA729271	-2.331816954	8.15254E-21	2.331816954	stage_E14_vs_E10	ordinary_muscle_development	D_vs_E10;stage_1W_vs_E10;stage_3W_vs_E10;stage	1	TRUE
ENSGALG00010000799	ENSGALG00010000799	PRJNA729271	-2.908270933	8.31749E-21	2.908270933	stage_E14_vs_E10	ordinary_muscle_development	D_vs_E10;stage_1W_vs_E10;stage_3W_vs_E10;stage	1	TRUE
ENSGALG00010008674	RXFP1	PRJNA729271	2.540065614	8.37903E-21	2.540065614	stage_E14_vs_E10	ordinary_muscle_development	D_vs_E10;stage_1W_vs_E10;stage_3W_vs_E10;stage	1	TRUE
ENSGALG00010001689	ILK	PRJNA729271	0.635415724	8.63998E-21	0.635415724	stage_E14_vs_E10	ordinary_muscle_development		0	FALSE
ENSGALG00010029412	SLC46A1	PRJNA729271	1.009600716	8.71307E-21	1.009600716	stage_E14_vs_E10	ordinary_muscle_development	D_vs_E10;stage_1W_vs_E10;stage_3W_vs_E10;stage	1	TRUE

ENSGALG00010024853	TJP1	PRJNA729271	0.640218548	8.73707E-21	0.640218548	stage_E14_vs_E10	ordinary_muscle_development		0	FALSE
ENSGALG00010014769	CCDC69	PRJNA729271	3.106064743	8.75951E-21	3.106064743	stage_E14_vs_E10	ordinary_muscle_development	D_vs_E10;stage_1W_vs_E10;stage_3W_vs_E10;stage	1	TRUE
ENSGALG00010000376	ASCC2	PRJNA729271	-0.840881488	8.77418E-21	0.840881488	stage_E14_vs_E10	ordinary_muscle_development		0	FALSE
ENSGALG00010023005	IRF7	PRJNA729271	3.287375717	9.05506E-21	3.287375717	stage_E14_vs_E10	ordinary_muscle_development	D_vs_E10;stage_1W_vs_E10;stage_3W_vs_E10;stage	1	TRUE
ENSGALG00010007802	ENSGALG00010007802	PRJNA729271	6.838403155	9.1045E-21	6.838403155	stage_E14_vs_E10	ordinary_muscle_development	D_vs_E10;stage_1W_vs_E10;stage_3W_vs_E10;stage	1	TRUE
ENSGALG00010008019	PPP2CB	PRJNA729271	-0.959645361	9.47967E-21	0.959645361	stage_E14_vs_E10	ordinary_muscle_development		0	FALSE
ENSGALG00010020097	RETSAT	PRJNA729271	2.170216952	9.57509E-21	2.170216952	stage_E14_vs_E10	ordinary_muscle_development	D_vs_E10;stage_1W_vs_E10;stage_3W_vs_E10;stage	1	TRUE
ENSGALG00010021717	SDC4	PRJNA729271	1.87294132	9.94106E-21	1.87294132	stage_E14_vs_E10	ordinary_muscle_development	D_vs_E10;stage_1W_vs_E10;stage_3W_vs_E10;stage	1	TRUE
ENSGALG00010013203	AMACR	PRJNA729271	1.126892721	1.00812E-20	1.126892721	stage_E14_vs_E10	ordinary_muscle_development	D_vs_E10;stage_1W_vs_E10;stage_3W_vs_E10;stage	1	TRUE
ENSGALG00010009522	TAF5L	PRJNA729271	0.763475149	1.03515E-20	0.763475149	stage_E14_vs_E10	ordinary_muscle_development		0	FALSE
ENSGALG00010017536	GSC	PRJNA729271	-4.944512523	1.05895E-20	4.944512523	stage_E14_vs_E10	ordinary_muscle_development	D_vs_E10;stage_1W_vs_E10;stage_3W_vs_E10;stage	1	TRUE
ENSGALG00010004256	ENSGALG00010004256	PRJNA729271	-6.200437027	1.05948E-20	6.200437027	stage_E14_vs_E10	ordinary_muscle_development	D_vs_E10;stage_1W_vs_E10;stage_3W_vs_E10;stage	1	TRUE
ENSGALG00010018115	KLHL4	PRJNA729271	-1.917231415	1.09004E-20	1.917231415	stage_E14_vs_E10	ordinary_muscle_development	D_vs_E10;stage_1W_vs_E10;stage_3W_vs_E10;stage	1	TRUE
ENSGALG00010027369	ENSGALG00010027369	PRJNA729271	0.960858069	1.09004E-20	0.960858069	stage_E14_vs_E10	ordinary_muscle_development		0	FALSE
ENSGALG00010018937	INHBB	PRJNA729271	-4.160967545	1.10641E-20	4.160967545	stage_E14_vs_E10	ordinary_muscle_development	D_vs_E10;stage_1W_vs_E10;stage_3W_vs_E10;stage	1	TRUE
ENSGALG00010003585	PNPLA6	PRJNA729271	1.344731174	1.10797E-20	1.344731174	stage_E14_vs_E10	ordinary_muscle_development	D_vs_E10;stage_1W_vs_E10;stage_3W_vs_E10;stage	1	TRUE
ENSGALG00010021372	CD86	PRJNA729271	5.253607875	1.10814E-20	5.253607875	stage_E14_vs_E10	ordinary_muscle_development	D_vs_E10;stage_1W_vs_E10;stage_3W_vs_E10;stage	1	TRUE
ENSGALG00010029521	ACTG1	PRJNA729271	-0.931938544	1.13264E-20	0.931938544	stage_E14_vs_E10	ordinary_muscle_development		0	FALSE
ENSGALG00010006241	MRC1	PRJNA729271	2.311177752	1.13413E-20	2.311177752	stage_E14_vs_E10	ordinary_muscle_development	D_vs_E10;stage_1W_vs_E10;stage_3W_vs_E10;stage	1	TRUE
ENSGALG00010015532	NXT2	PRJNA729271	-1.063155585	1.14278E-20	1.063155585	stage_E14_vs_E10	ordinary_muscle_development	D_vs_E10;stage_1W_vs_E10;stage_3W_vs_E10;stage	1	TRUE
ENSGALG00010007751	HSPA13	PRJNA729271	-0.952180125	1.14838E-20	0.952180125	stage_E14_vs_E10	ordinary_muscle_development		0	FALSE
ENSGALG00010023966	PEAK1	PRJNA729271	0.927659843	1.15467E-20	0.927659843	stage_E14_vs_E10	ordinary_muscle_development		0	FALSE
ENSGALG00010002571	BCOR	PRJNA729271	1.326976128	1.15799E-20	1.326976128	stage_E14_vs_E10	ordinary_muscle_development	D_vs_E10;stage_1W_vs_E10;stage_3W_vs_E10;stage	1	TRUE
ENSGALG00010012291	NR5A2	PRJNA729271	-1.947816503	1.16432E-20	1.947816503	stage_E14_vs_E10	ordinary_muscle_development	D_vs_E10;stage_1W_vs_E10;stage_3W_vs_E10;stage	1	TRUE
ENSGALG00010015109	PPT1	PRJNA729271	1.016539603	1.17313E-20	1.016539603	stage_E14_vs_E10	ordinary_muscle_development	D_vs_E10;stage_1W_vs_E10;stage_3W_vs_E10;stage	1	TRUE
ENSGALG00010026485	ACAA1	PRJNA729271	0.999190915	1.18093E-20	0.999190915	stage_E14_vs_E10	ordinary_muscle_development		0	FALSE
ENSGALG00010010657	TMEM170B	PRJNA729271	-1.727522117	1.18287E-20	1.727522117	stage_E14_vs_E10	ordinary_muscle_development	D_vs_E10;stage_1W_vs_E10;stage_3W_vs_E10;stage	1	TRUE
ENSGALG00010024659	BEND5	PRJNA729271	1.013062302	1.18857E-20	1.013062302	stage_E14_vs_E10	ordinary_muscle_development	D_vs_E10;stage_1W_vs_E10;stage_3W_vs_E10;stage	1	TRUE
ENSGALG00010011997	DENND5B	PRJNA729271	1.237248259	1.21554E-20	1.237248259	stage_E14_vs_E10	ordinary_muscle_development	D_vs_E10;stage_1W_vs_E10;stage_3W_vs_E10;stage	1	TRUE
ENSGALG00010012046	LOXHD1	PRJNA729271	2.559347551	1.22577E-20	2.559347551	stage_E14_vs_E10	ordinary_muscle_development	D_vs_E10;stage_1W_vs_E10;stage_3W_vs_E10;stage	1	TRUE
ENSGALG00010023671	ENSGALG00010023671	PRJNA729271	5.987544796	1.22864E-20	5.987544796	stage_E14_vs_E10	ordinary_muscle_development	D_vs_E10;stage_1W_vs_E10;stage_3W_vs_E10;stage	1	TRUE
ENSGALG00010028357	SPPL2C	PRJNA729271	-0.89679872	1.23095E-20	0.89679872	stage_E14_vs_E10	ordinary_muscle_development		0	FALSE
ENSGALG00010017472	LZTFL1	PRJNA729271	-1.632961223	1.23566E-20	1.632961223	stage_E14_vs_E10	ordinary_muscle_development	D_vs_E10;stage_1W_vs_E10;stage_3W_vs_E10;stage	1	TRUE
ENSGALG00010024439	UFD1	PRJNA729271	1.059299916	1.24014E-20	1.059299916	stage_E14_vs_E10	ordinary_muscle_development	D_vs_E10;stage_1W_vs_E10;stage_3W_vs_E10;stage	1	TRUE
ENSGALG00010005695	KIF5B	PRJNA729271	-0.801534152	1.25506E-20	0.801534152	stage_E14_vs_E10	ordinary_muscle_development		0	FALSE
ENSGALG00010014077	SLITRK2	PRJNA729271	0.942908762	1.26326E-20	0.942908762	stage_E14_vs_E10	ordinary_muscle_development		0	FALSE
ENSGALG00010022674	ENSGALG00010022674	PRJNA729271	3.067008413	1.26424E-20	3.067008413	stage_E14_vs_E10	ordinary_muscle_development	D_vs_E10;stage_1W_vs_E10;stage_3W_vs_E10;stage	1	TRUE
ENSGALG00010029249	PRR11	PRJNA729271	-3.001217888	1.27309E-20	3.001217888	stage_E14_vs_E10	ordinary_muscle_development	D_vs_E10;stage_1W_vs_E10;stage_3W_vs_E10;stage	1	TRUE
ENSGALG00010007118	ENSGALG00010007118	PRJNA729271	-1.303736077	1.28196E-20	1.303736077	stage_E14_vs_E10	ordinary_muscle_development	D_vs_E10;stage_1W_vs_E10;stage_3W_vs_E10;stage	1	TRUE
ENSGALG00010022967	ADAMTS15	PRJNA729271	-1.550885293	1.28317E-20	1.550885293	stage_E14_vs_E10	ordinary_muscle_development	D_vs_E10;stage_1W_vs_E10;stage_3W_vs_E10;stage	1	TRUE
ENSGALG00010017972	RNPEPL1	PRJNA729271	1.621151746	1.29513E-20	1.621151746	stage_E14_vs_E10	ordinary_muscle_development	D_vs_E10;stage_1W_vs_E10;stage_3W_vs_E10;stage	1	TRUE
ENSGALG00010014000	STAT4	PRJNA729271	8.721431167	1.30333E-20	8.721431167	stage_E14_vs_E10	ordinary_muscle_development	D_vs_E10;stage_1W_vs_E10;stage_3W_vs_E10;stage	1	TRUE
ENSGALG00010006351	IGF2R	PRJNA729271	1.643792114	1.30554E-20	1.643792114	stage_E14_vs_E10	ordinary_muscle_development	D_vs_E10;stage_1W_vs_E10;stage_3W_vs_E10;stage	1	TRUE
ENSGALG00010002716	NHLRC3	PRJNA729271	-1.06316501	1.31493E-20	1.06316501	stage_E14_vs_E10	ordinary_muscle_development	D_vs_E10;stage_1W_vs_E10;stage_3W_vs_E10;stage	1	TRUE
ENSGALG00010006961	ZCCHC14	PRJNA729271	1.108554699	1.32177E-20	1.108554699	stage_E14_vs_E10	ordinary_muscle_development	D_vs_E10;stage_1W_vs_E10;stage_3W_vs_E10;stage	1	TRUE
ENSGALG00010016681	SUSD4	PRJNA729271	2.629573225	1.3253E-20	2.629573225	stage_E14_vs_E10	ordinary_muscle_development	D_vs_E10;stage_1W_vs_E10;stage_3W_vs_E10;stage	1	TRUE
ENSGALG00010029734	SEC14L1	PRJNA729271	-0.80337849	1.3328E-20	0.80337849	stage_E14_vs_E10	ordinary_muscle_development		0	FALSE
ENSGALG00010003552	PI15	PRJNA729271	-3.683859199	1.34439E-20	3.683859199	stage_E14_vs_E10	ordinary_muscle_development	D_vs_E10;stage_1W_vs_E10;stage_3W_vs_E10;stage	1	TRUE
ENSGALG00010005180	WAC	PRJNA729271	1.08947179	1.36187E-20	1.08947179	stage_E14_vs_E10	ordinary_muscle_development	D_vs_E10;stage_1W_vs_E10;stage_3W_vs_E10;stage	1	TRUE
ENSGALG00010000052	TTLL13	PRJNA729271	1.252210961	1.36742E-20	1.252210961	stage_E14_vs_E10	ordinary_muscle_development	D_vs_E10;stage_1W_vs_E10;stage_3W_vs_E10;stage	1	TRUE
ENSGALG00010021073	NDUFB8	PRJNA729271	1.014173832	1.37715E-20	1.014173832	stage_E14_vs_E10	ordinary_muscle_development	D_vs_E10;stage_1W_vs_E10;stage_3W_vs_E10;stage	1	TRUE
ENSGALG00010008343	TNFRSF21	PRJNA729271	2.10656213	1.37963E-20	2.10656213	stage_E14_vs_E10	ordinary_muscle_development	D_vs_E10;stage_1W_vs_E10;stage_3W_vs_E10;stage	1	TRUE
ENSGALG00010028432	RFXANK	PRJNA729271	1.255977035	1.40023E-20	1.255977035	stage_E14_vs_E10	ordinary_muscle_development	D_vs_E10;stage_1W_vs_E10;stage_3W_vs_E10;stage	1	TRUE
ENSGALG00010000653	NSUN2	PRJNA729271	-0.886933371	1.40222E-20	0.886933371	stage_E14_vs_E10	ordinary_muscle_development		0	FALSE
ENSGALG00010017616	PDCD7	PRJNA729271	1.462238143	1.40553E-20	1.462238143	stage_E14_vs_E10	ordinary_muscle_development	D_vs_E10;stage_1W_vs_E10;stage_3W_vs_E10;stage	1	TRUE
ENSGALG00010019064	ENSGALG00010019064	PRJNA729271	-1.63108118	1.4078E-20	1.63108118	stage_E14_vs_E10	ordinary_muscle_development	D_vs_E10;stage_1W_vs_E10;stage_3W_vs_E10;stage	1	TRUE
ENSGALG00010011669	ENSGALG00010011669	PRJNA729271	-9.163400521	1.43472E-20	9.163400521	stage_E14_vs_E10	ordinary_muscle_development	D_vs_E10;stage_1W_vs_E10;stage_3W_vs_E10;stage	1	TRUE
ENSGALG00010020084	ENSGALG00010020084	PRJNA729271	-0.838744146	1.44406E-20	0.838744146	stage_E14_vs_E10	ordinary_muscle_development		0	FALSE
ENSGALG00010008301	FAM91A1	PRJNA729271	-1.00362333	1.57045E-20	1.00362333	stage_E14_vs_E10	ordinary_muscle_development	D_vs_E10;stage_1W_vs_E10;stage_3W_vs_E10;stage	1	TRUE
ENSGALG00010011593	WRNIP1	PRJNA729271	-0.968395102	1.57776E-20	0.968395102	stage_E14_vs_E10	ordinary_muscle_development		0	FALSE
ENSGALG00010017116	ENSGALG00010017116	PRJNA729271	-3.10339165	1.58622E-20	3.10339165	stage_E14_vs_E10	ordinary_muscle_development	D_vs_E10;stage_1W_vs_E10;stage_3W_vs_E10;stage	1	TRUE
ENSGALG00010018724	RRBP1	PRJNA729271	-1.343019974	1.58957E-20	1.343019974	stage_E14_vs_E10	ordinary_muscle_development	D_vs_E10;stage_1W_vs_E10;stage_3W_vs_E10;stage	1	TRUE
ENSGALG00010029451	ENSGALG00010029451	PRJNA729271	-2.108771109	1.61708E-20	2.108771109	stage_E14_vs_E10	ordinary_muscle_development	D_vs_E10;stage_1W_vs_E10;stage_3W_vs_E10;stage	1	TRUE
ENSGALG00010010593	EIF3L	PRJNA729271	0.984777965	1.65236E-20	0.984777965	stage_E14_vs_E10	ordinary_muscle_development		0	FALSE
ENSGALG00010021609	ENSGALG00010021609	PRJNA729271	1.924191192	1.6733E-20	1.924191192	stage_E14_vs_E10	ordinary_muscle_development	D_vs_E10;stage_1W_vs_E10;stage_3W_vs_E10;stage	1	TRUE
ENSGALG00010011747	UTP15	PRJNA729271	-1.125406328	1.67406E-20	1.125406328	stage_E14_vs_E10	ordinary_muscle_development	D_vs_E10;stage_1W_vs_E10;stage_3W_vs_E10;stage	1	TRUE
ENSGALG00010029511	CUEDC1	PRJNA729271	0.681664103	1.69541E-20	0.681664103	stage_E14_vs_E10	ordinary_muscle_development		0	FALSE
ENSGALG00010012667	SCAMP1	PRJNA729271	-0.848514663	1.69968E-20	0.848514663	stage_E14_vs_E10	ordinary_muscle_development		0	FALSE
ENSGALG00010019398	SLC35F5	PRJNA729271	1.131359951	1.71278E-20	1.131359951	stage_E14_vs_E10	ordinary_muscle_development	D_vs_E10;stage_1W_vs_E10;stage_3W_vs_E10;stage	1	TRUE
ENSGALG00010012322	ENSGALG00010012322	PRJNA729271	-2.670525642	1.71385E-20	2.670525642	stage_E14_vs_E10	ordinary_muscle_development	D_vs_E10;stage_1W_vs_E10;stage_3W_vs_E10;stage	1	TRUE
ENSGALG00010029231	CBX8	PRJNA729271	-1.092521901	1.72544E-20	1.092521901	stage_E14_vs_E10	ordinary_muscle_development	D_vs_E10;stage_1W_vs_E10;stage_3W_vs_E10;stage	1	TRUE
ENSGALG00010027095	SCN5A	PRJNA729271	-4.074851065	1.7329E-20	4.074851065	stage_E14_vs_E10	ordinary_muscle_development	D_vs_E10;stage_1W_vs_E10;stage_3W_vs_E10;stage	1	TRUE
ENSGALG00010027445	PPM1M	PRJNA729271	1.542510087	1.7345E-20	1.542510087	stage_E14_vs_E10	ordinary_muscle_development	D_vs_E10;stage_1W_vs_E10;stage_3W_vs_E10;stage	1	TRUE
ENSGALG00010025413	NECAB3	PRJNA729271	1.140496562	1.74523E-20	1.140496562	stage_E14_vs_E10	ordinary_muscle_development	D_vs_E10;stage_1W_vs_E10;stage_3W_vs_E10;stage	1	TRUE
ENSGALG00010026093	TOE1	PRJNA729271	-1.259784753	1.74907E-20	1.259784753	stage_E14_vs_E10	ordinary_muscle_development	D_vs_E10;stage_1W_vs_E10;stage_3W_vs_E10;stage	1	TRUE
ENSGALG00010020389	ACTN1	PRJNA729271	-1.487609184	1.75129E-20	1.487609184	stage_E14_vs_E10	ordinary_muscle_development	D_vs_E10;stage_1W_vs_E10;stage_3W_vs_E10;stage	1	TRUE
ENSGALG00010007911	IFI6	PRJNA729271	7.543381535	1.78719E-20	7.543381535	stage_E14_vs_E10	ordinary_muscle_development	D_vs_E10;stage_1W_vs_E10;stage_3W_vs_E10;stage	1	TRUE
ENSGALG00010008468	EPPK1	PRJNA729271	-2.934742746	1.7973E-20	2.934742746	stage_E14_vs_E10	ordinary_muscle_development	D_vs_E10;stage_1W_vs_E10;stage_3W_vs_E10;stage	1	TRUE
ENSGALG00010014114	ANXA1	PRJNA729271	3.126886261	1.83717E-20	3.126886261	stage_E14_vs_E10	ordinary_muscle_development	D_vs_E10;stage_1W_vs_E10;stage_3W_vs_E10;stage	1	TRUE
ENSGALG00010013310	MYO16	PRJNA729271	-2.865578998	1.85314E-20	2.865578998	stage_E14_vs_E10	ordinary_muscle_development	D_vs_E10;stage_1W_vs_E10;stage_3W_vs_E10;stage	1	TRUE
ENSGALG00010025200	PEPD	PRJNA729271	-0.765668354	1.85549E-20	0.765668354	stage_E14_vs_E10	ordinary_muscle_development		0	FALSE
ENSGALG00010012876	KDELR3	PRJNA729271	-1.545465363	1.85805E-20	1.545465363	stage_E14_vs_E10	ordinary_muscle_development	D_vs_E10;stage_1W_vs_E10;stage_3W_vs_E10;stage	1	TRUE
ENSGALG00010023666	ENSGALG00010023666	PRJNA729271	2.332444767	1.86648E-20	2.332444767	stage_E14_vs_E10	ordinary_muscle_development	D_vs_E10;stage_1W_vs_E10;stage_3W_vs_E10;stage	1	TRUE
ENSGALG00010000231	ENSGALG00010000231	PRJNA729271	4.127446774	1.87251E-20	4.127446774	stage_E14_vs_E10	ordinary_muscle_development	D_vs_E10;stage_1W_vs_E10;stage_3W_vs_E10;stage	1	TRUE
ENSGALG00010018654	ENSGALG00010018654	PRJNA729271	2.031662481	1.9015E-20	2.031662481	stage_E14_vs_E10	ordinary_muscle_development	D_vs_E10;stage_1W_vs_E10;stage_3W_vs_E10;stage	1	TRUE
ENSGALG00010015151	ENSGALG00010015151	PRJNA729271	-4.709551346	1.96943E-20	4.709551346	stage_E14_vs_E10	ordinary_muscle_development	D_vs_E10;stage_1W_vs_E10;stage_3W_vs_E10;stage	1	TRUE
ENSGALG00010029773	TRIM65	PRJNA729271	1.239301032	1.97949E-20	1.239301032	stage_E14_vs_E10	ordinary_muscle_development	D_vs_E10;stage_1W_vs_E10;stage_3W_vs_E10;stage	1	TRUE
ENSGALG00010021720	CACNB1	PRJNA729271	2.448479956	1.99062E-20	2.448479956	stage_E14_vs_E10	ordinary_muscle_development	D_vs_E10;stage_1W_vs_E10;stage_3W_vs_E10;stage	1	TRUE
ENSGALG00010024917	TRAK2	PRJNA729271	1.151392708	2.01084E-20	1.151392708	stage_E14_vs_E10	ordinary_muscle_development	D_vs_E10;stage_1W_vs_E10;stage_3W_vs_E10;stage	1	TRUE
ENSGALG00010001793	IKZF5	PRJNA729271	0.969298557	2.02309E-20	0.969298557	stage_E14_vs_E10	ordinary_muscle_development		0	FALSE
ENSGALG00010019416	GPR39	PRJNA729271	3.89246901	2.02717E-20	3.89246901	stage_E14_vs_E10	ordinary_muscle_development	D_vs_E10;stage_1W_vs_E10;stage_3W_vs_E10;stage	1	TRUE
ENSGALG00010019108	KDELR2	PRJNA729271	-0.939933721	2.02806E-20	0.939933721	stage_E14_vs_E10	ordinary_muscle_development		0	FALSE
ENSGALG00010014431	ENSGALG00010014431	PRJNA729271	-1.491510236	2.12632E-20	1.491510236	stage_E14_vs_E10	ordinary_muscle_development	D_vs_E10;stage_1W_vs_E10;stage_3W_vs_E10;stage	1	TRUE
ENSGALG00010021577	ARIH1	PRJNA729271	0.718720034	2.14899E-20	0.718720034	stage_E14_vs_E10	ordinary_muscle_development		0	FALSE
ENSGALG00010021847	P3H1	PRJNA729271	-1.428397184	2.18042E-20	1.428397184	stage_E14_vs_E10	ordinary_muscle_development	D_vs_E10;stage_1W_vs_E10;stage_3W_vs_E10;stage	1	TRUE
ENSGALG00010017283	INF2	PRJNA729271	1.28230484	2.33904E-20	1.28230484	stage_E14_vs_E10	ordinary_muscle_development	D_vs_E10;stage_1W_vs_E10;stage_3W_vs_E10;stage	1	TRUE
ENSGALG00010012948	ST13P5	PRJNA729271	-0.896364215	2.34968E-20	0.896364215	stage_E14_vs_E10	ordinary_muscle_development		0	FALSE
ENSGALG00010022724	LUZP2	PRJNA729271	-4.235061693	2.35013E-20	4.235061693	stage_E14_vs_E10	ordinary_muscle_development	D_vs_E10;stage_1W_vs_E10;stage_3W_vs_E10;stage	1	TRUE
ENSGALG00010023216	SLC35D1	PRJNA729271	-1.189740358	2.35593E-20	1.189740358	stage_E14_vs_E10	ordinary_muscle_development	D_vs_E10;stage_1W_vs_E10;stage_3W_vs_E10;stage	1	TRUE
ENSGALG00010017834	ENSGALG00010017834	PRJNA729271	2.846695418	2.36113E-20	2.846695418	stage_E14_vs_E10	ordinary_muscle_development	D_vs_E10;stage_1W_vs_E10;stage_3W_vs_E10;stage	1	TRUE
ENSGALG00010001492	ENSGALG00010001492	PRJNA729271	2.586484561	2.37008E-20	2.586484561	stage_E14_vs_E10	ordinary_muscle_development	D_vs_E10;stage_1W_vs_E10;stage_3W_vs_E10;stage	1	TRUE
ENSGALG00010024742	PAK2	PRJNA729271	-0.664911204	2.37931E-20	0.664911204	stage_E14_vs_E10	ordinary_muscle_development		0	FALSE
ENSGALG00010008229	PIGY	PRJNA729271	1.146939544	2.38672E-20	1.146939544	stage_E14_vs_E10	ordinary_muscle_development	D_vs_E10;stage_1W_vs_E10;stage_3W_vs_E10;stage	1	TRUE
ENSGALG00010005450	ENSGALG00010005450	PRJNA729271	3.875971999	2.40493E-20	3.875971999	stage_E14_vs_E10	ordinary_muscle_development	D_vs_E10;stage_1W_vs_E10;stage_3W_vs_E10;stage	1	TRUE
ENSGALG00010010257	ENSGALG00010010257	PRJNA729271	1.25758933	2.42324E-20	1.25758933	stage_E14_vs_E10	ordinary_muscle_development	D_vs_E10;stage_1W_vs_E10;stage_3W_vs_E10;stage	1	TRUE
ENSGALG00010008938	ZBED4	PRJNA729271	-0.867168168	2.46159E-20	0.867168168	stage_E14_vs_E10	ordinary_muscle_development		0	FALSE
ENSGALG00010023791	VTI1B	PRJNA729271	0.750420404	2.47534E-20	0.750420404	stage_E14_vs_E10	ordinary_muscle_development		0	FALSE
ENSGALG00010025370	HPD	PRJNA729271	4.61221958	2.48866E-20	4.61221958	stage_E14_vs_E10	ordinary_muscle_development	D_vs_E10;stage_1W_vs_E10;stage_3W_vs_E10;stage	1	TRUE
ENSGALG00010017793	FAM83G	PRJNA729271	-6.434826873	2.49592E-20	6.434826873	stage_E14_vs_E10	ordinary_muscle_development	D_vs_E10;stage_1W_vs_E10;stage_3W_vs_E10;stage	1	TRUE
ENSGALG00010024515	C3orf70	PRJNA729271	-1.493992091	2.51869E-20	1.493992091	stage_E14_vs_E10	ordinary_muscle_development	D_vs_E10;stage_1W_vs_E10;stage_3W_vs_E10;stage	1	TRUE
ENSGALG00010013760	RPF2	PRJNA729271	-1.096571124	2.5859E-20	1.096571124	stage_E14_vs_E10	ordinary_muscle_development	D_vs_E10;stage_1W_vs_E10;stage_3W_vs_E10;stage	1	TRUE
ENSGALG00010007640	TMEM123	PRJNA729271	-2.080257913	2.58598E-20	2.080257913	stage_E14_vs_E10	ordinary_muscle_development	D_vs_E10;stage_1W_vs_E10;stage_3W_vs_E10;stage	1	TRUE
ENSGALG00010021860	ENSGALG00010021860	PRJNA729271	2.359211275	2.61483E-20	2.359211275	stage_E14_vs_E10	ordinary_muscle_development	D_vs_E10;stage_1W_vs_E10;stage_3W_vs_E10;stage	1	TRUE
ENSGALG00010012986	TMEM184B	PRJNA729271	1.320750781	2.62366E-20	1.320750781	stage_E14_vs_E10	ordinary_muscle_development	D_vs_E10;stage_1W_vs_E10;stage_3W_vs_E10;stage	1	TRUE
ENSGALG00010029340	NDEL1	PRJNA729271	0.970502992	2.63211E-20	0.970502992	stage_E14_vs_E10	ordinary_muscle_development		0	FALSE
ENSGALG00010023369	ACE	PRJNA729271	4.482521128	2.7496E-20	4.482521128	stage_E14_vs_E10	ordinary_muscle_development	D_vs_E10;stage_1W_vs_E10;stage_3W_vs_E10;stage	1	TRUE
ENSGALG00010018471	HHEX	PRJNA729271	1.68447937	2.80516E-20	1.68447937	stage_E14_vs_E10	ordinary_muscle_development	D_vs_E10;stage_1W_vs_E10;stage_3W_vs_E10;stage	1	TRUE
ENSGALG00010019898	NUDT1	PRJNA729271	-1.786452661	2.836E-20	1.786452661	stage_E14_vs_E10	ordinary_muscle_development	D_vs_E10;stage_1W_vs_E10;stage_3W_vs_E10;stage	1	TRUE
ENSGALG00010011326	COX6C	PRJNA729271	1.269216199	2.8959E-20	1.269216199	stage_E14_vs_E10	ordinary_muscle_development	D_vs_E10;stage_1W_vs_E10;stage_3W_vs_E10;stage	1	TRUE
ENSGALG00010025073	IL18	PRJNA729271	1.867142994	2.99121E-20	1.867142994	stage_E14_vs_E10	ordinary_muscle_development	D_vs_E10;stage_1W_vs_E10;stage_3W_vs_E10;stage	1	TRUE
ENSGALG00010011817	KDSR	PRJNA729271	0.835720895	2.99126E-20	0.835720895	stage_E14_vs_E10	ordinary_muscle_development		0	FALSE
ENSGALG00010009131	CDKL2	PRJNA729271	-2.992646146	3.05436E-20	2.992646146	stage_E14_vs_E10	ordinary_muscle_development	D_vs_E10;stage_1W_vs_E10;stage_3W_vs_E10;stage	1	TRUE
ENSGALG00010014801	MPST	PRJNA729271	-0.83028763	3.10157E-20	0.83028763	stage_E14_vs_E10	ordinary_muscle_development		0	FALSE
ENSGALG00010020635	NDE1	PRJNA729271	-1.268907934	3.11481E-20	1.268907934	stage_E14_vs_E10	ordinary_muscle_development	D_vs_E10;stage_1W_vs_E10;stage_3W_vs_E10;stage	1	TRUE
ENSGALG00010010077	CCT8	PRJNA729271	-1.09028421	3.12083E-20	1.09028421	stage_E14_vs_E10	ordinary_muscle_development	D_vs_E10;stage_1W_vs_E10;stage_3W_vs_E10;stage	1	TRUE
ENSGALG00010026040	GUK1	PRJNA729271	-1.20967926	3.15151E-20	1.20967926	stage_E14_vs_E10	ordinary_muscle_development	D_vs_E10;stage_1W_vs_E10;stage_3W_vs_E10;stage	1	TRUE
ENSGALG00010009193	EDARADD	PRJNA729271	-2.147959013	3.24145E-20	2.147959013	stage_E14_vs_E10	ordinary_muscle_development	D_vs_E10;stage_1W_vs_E10;stage_3W_vs_E10;stage	1	TRUE
ENSGALG00010010017	CCDC59	PRJNA729271	-1.029302902	3.26688E-20	1.029302902	stage_E14_vs_E10	ordinary_muscle_development	D_vs_E10;stage_1W_vs_E10;stage_3W_vs_E10;stage	1	TRUE
ENSGALG00010028898	TNFSF15	PRJNA729271	-2.482631004	3.32605E-20	2.482631004	stage_E14_vs_E10	ordinary_muscle_development	D_vs_E10;stage_1W_vs_E10;stage_3W_vs_E10;stage	1	TRUE
ENSGALG00010018296	PIK3CA	PRJNA729271	0.901565996	3.34092E-20	0.901565996	stage_E14_vs_E10	ordinary_muscle_development		0	FALSE
ENSGALG00010022093	CHD4	PRJNA729271	-1.288604705	3.4111E-20	1.288604705	stage_E14_vs_E10	ordinary_muscle_development	D_vs_E10;stage_1W_vs_E10;stage_3W_vs_E10;stage	1	TRUE
ENSGALG00010005396	MICU2	PRJNA729271	1.151986002	3.56828E-20	1.151986002	stage_E14_vs_E10	ordinary_muscle_development	D_vs_E10;stage_1W_vs_E10;stage_3W_vs_E10;stage	1	TRUE
ENSGALG00010022438	RPN2	PRJNA729271	-0.947759916	3.64095E-20	0.947759916	stage_E14_vs_E10	ordinary_muscle_development		0	FALSE
ENSGALG00010010425	BLOC1S5	PRJNA729271	0.733248708	3.68013E-20	0.733248708	stage_E14_vs_E10	ordinary_muscle_development		0	FALSE
ENSGALG00010012145	ENSGALG00010012145	PRJNA729271	8.090259433	3.70901E-20	8.090259433	stage_E14_vs_E10	ordinary_muscle_development	D_vs_E10;stage_1W_vs_E10;stage_3W_vs_E10;stage	1	TRUE
ENSGALG00010029515	NPLOC4	PRJNA729271	1.421572267	3.73327E-20	1.421572267	stage_E14_vs_E10	ordinary_muscle_development	D_vs_E10;stage_1W_vs_E10;stage_3W_vs_E10;stage	1	TRUE
ENSGALG00010020928	AACS	PRJNA729271	-1.437717752	3.75706E-20	1.437717752	stage_E14_vs_E10	ordinary_muscle_development	D_vs_E10;stage_1W_vs_E10;stage_3W_vs_E10;stage	1	TRUE
ENSGALG00010008201	ENPP4	PRJNA729271	2.244558939	3.8093E-20	2.244558939	stage_E14_vs_E10	ordinary_muscle_development	D_vs_E10;stage_1W_vs_E10;stage_3W_vs_E10;stage	1	TRUE
ENSGALG00010007375	CHORDC1	PRJNA729271	-0.715521856	3.86765E-20	0.715521856	stage_E14_vs_E10	ordinary_muscle_development		0	FALSE
ENSGALG00010007906	NAF1	PRJNA729271	-1.074876462	3.90954E-20	1.074876462	stage_E14_vs_E10	ordinary_muscle_development	D_vs_E10;stage_1W_vs_E10;stage_3W_vs_E10;stage	1	TRUE
ENSGALG00010005884	MYB	PRJNA729271	-2.994842919	4.06099E-20	2.994842919	stage_E14_vs_E10	ordinary_muscle_development	D_vs_E10;stage_1W_vs_E10;stage_3W_vs_E10;stage	1	TRUE
ENSGALG00010021327	IGSF1	PRJNA729271	3.868606013	4.09507E-20	3.868606013	stage_E14_vs_E10	ordinary_muscle_development	D_vs_E10;stage_1W_vs_E10;stage_3W_vs_E10;stage	1	TRUE
ENSGALG00010011282	CRY1	PRJNA729271	1.183531603	4.09723E-20	1.183531603	stage_E14_vs_E10	ordinary_muscle_development	D_vs_E10;stage_1W_vs_E10;stage_3W_vs_E10;stage	1	TRUE
ENSGALG00010027896	TOR4A	PRJNA729271	1.450753422	4.1597E-20	1.450753422	stage_E14_vs_E10	ordinary_muscle_development	D_vs_E10;stage_1W_vs_E10;stage_3W_vs_E10;stage	1	TRUE
ENSGALG00010001230	PTX3	PRJNA729271	-5.6061389	4.30352E-20	5.6061389	stage_E14_vs_E10	ordinary_muscle_development	D_vs_E10;stage_1W_vs_E10;stage_3W_vs_E10;stage	1	TRUE
ENSGALG00010025080	BAZ2A	PRJNA729271	1.885436682	4.31036E-20	1.885436682	stage_E14_vs_E10	ordinary_muscle_development	D_vs_E10;stage_1W_vs_E10;stage_3W_vs_E10;stage	1	TRUE
ENSGALG00010008473	CSMD3	PRJNA729271	-5.944456415	4.33481E-20	5.944456415	stage_E14_vs_E10	ordinary_muscle_development	D_vs_E10;stage_1W_vs_E10;stage_3W_vs_E10;stage	1	TRUE
ENSGALG00010025957	ENSGALG00010025957	PRJNA729271	-1.1062381	4.34282E-20	1.1062381	stage_E14_vs_E10	ordinary_muscle_development	D_vs_E10;stage_1W_vs_E10;stage_3W_vs_E10;stage	1	TRUE
ENSGALG00010002445	EDRF1	PRJNA729271	1.034812416	4.37818E-20	1.034812416	stage_E14_vs_E10	ordinary_muscle_development	D_vs_E10;stage_1W_vs_E10;stage_3W_vs_E10;stage	1	TRUE
ENSGALG00010011091	YARS2	PRJNA729271	0.918942889	4.41644E-20	0.918942889	stage_E14_vs_E10	ordinary_muscle_development		0	FALSE
ENSGALG00010009540	RAB10	PRJNA729271	0.838116047	4.41767E-20	0.838116047	stage_E14_vs_E10	ordinary_muscle_development		0	FALSE
ENSGALG00010001780	ENSGALG00010001780	PRJNA729271	2.214824631	4.46397E-20	2.214824631	stage_E14_vs_E10	ordinary_muscle_development	D_vs_E10;stage_1W_vs_E10;stage_3W_vs_E10;stage	1	TRUE
ENSGALG00010000191	NEFH	PRJNA729271	3.619598119	4.57986E-20	3.619598119	stage_E14_vs_E10	ordinary_muscle_development	D_vs_E10;stage_1W_vs_E10;stage_3W_vs_E10;stage	1	TRUE
ENSGALG00010027969	HSPA5	PRJNA729271	-1.207906155	4.58366E-20	1.207906155	stage_E14_vs_E10	ordinary_muscle_development	D_vs_E10;stage_1W_vs_E10;stage_3W_vs_E10;stage	1	TRUE
ENSGALG00010014729	MRPS15	PRJNA729271	0.885032978	4.76158E-20	0.885032978	stage_E14_vs_E10	ordinary_muscle_development		0	FALSE
ENSGALG00010005263	ENSGALG00010005263	PRJNA729271	2.92928915	4.84134E-20	2.92928915	stage_E14_vs_E10	ordinary_muscle_development	D_vs_E10;stage_1W_vs_E10;stage_3W_vs_E10;stage	1	TRUE
ENSGALG00010024637	DUSP8	PRJNA729271	2.585021441	4.93088E-20	2.585021441	stage_E14_vs_E10	ordinary_muscle_development	D_vs_E10;stage_1W_vs_E10;stage_3W_vs_E10;stage	1	TRUE
ENSGALG00010008137	HHLA1	PRJNA729271	4.131252103	4.95512E-20	4.131252103	stage_E14_vs_E10	ordinary_muscle_development	D_vs_E10;stage_1W_vs_E10;stage_3W_vs_E10;stage	1	TRUE
ENSGALG00010017364	AKT1	PRJNA729271	-0.699822423	4.99277E-20	0.699822423	stage_E14_vs_E10	ordinary_muscle_development		0	FALSE
ENSGALG00010000174	ENSGALG00010000174	PRJNA729271	0.910995605	5.04513E-20	0.910995605	stage_E14_vs_E10	ordinary_muscle_development		0	FALSE
ENSGALG00010018607	HAGHL	PRJNA729271	1.081401531	5.08483E-20	1.081401531	stage_E14_vs_E10	ordinary_muscle_development	D_vs_E10;stage_1W_vs_E10;stage_3W_vs_E10;stage	1	TRUE
ENSGALG00010017645	SYNC	PRJNA729271	2.517787322	5.17029E-20	2.517787322	stage_E14_vs_E10	ordinary_muscle_development	D_vs_E10;stage_1W_vs_E10;stage_3W_vs_E10;stage	1	TRUE
ENSGALG00010008903	ENSGALG00010008903	PRJNA729271	3.709450409	5.18242E-20	3.709450409	stage_E14_vs_E10	ordinary_muscle_development	D_vs_E10;stage_1W_vs_E10;stage_3W_vs_E10;stage	1	TRUE
ENSGALG00010014634	CCDC152	PRJNA729271	4.111344171	5.19199E-20	4.111344171	stage_E14_vs_E10	ordinary_muscle_development	D_vs_E10;stage_1W_vs_E10;stage_3W_vs_E10;stage	1	TRUE
ENSGALG00010028562	ACSBG2	PRJNA729271	1.854373701	5.19499E-20	1.854373701	stage_E14_vs_E10	ordinary_muscle_development	D_vs_E10;stage_1W_vs_E10;stage_3W_vs_E10;stage	1	TRUE
ENSGALG00010003632	TECRL	PRJNA729271	2.853610854	5.20337E-20	2.853610854	stage_E14_vs_E10	ordinary_muscle_development	D_vs_E10;stage_1W_vs_E10;stage_3W_vs_E10;stage	1	TRUE
ENSGALG00010003171	PAQR3	PRJNA729271	-1.03504463	5.2064E-20	1.03504463	stage_E14_vs_E10	ordinary_muscle_development	D_vs_E10;stage_1W_vs_E10;stage_3W_vs_E10;stage	1	TRUE
ENSGALG00010028862	ENSGALG00010028862	PRJNA729271	10.76905867	5.24602E-20	10.76905867	stage_E14_vs_E10	ordinary_muscle_development	D_vs_E10;stage_1W_vs_E10;stage_3W_vs_E10;stage	1	TRUE
ENSGALG00010015105	ENSGALG00010015105	PRJNA729271	8.735286726	5.25323E-20	8.735286726	stage_E14_vs_E10	ordinary_muscle_development	D_vs_E10;stage_1W_vs_E10;stage_3W_vs_E10;stage	1	TRUE
ENSGALG00010018010	ENSGALG00010018010	PRJNA729271	-1.120784082	5.30138E-20	1.120784082	stage_E14_vs_E10	ordinary_muscle_development	D_vs_E10;stage_1W_vs_E10;stage_3W_vs_E10;stage	1	TRUE
ENSGALG00010010494	ENSGALG00010010494	PRJNA729271	2.447180345	5.30361E-20	2.447180345	stage_E14_vs_E10	ordinary_muscle_development	D_vs_E10;stage_1W_vs_E10;stage_3W_vs_E10;stage	1	TRUE
ENSGALG00010001258	ENSGALG00010001258	PRJNA729271	-1.214854271	5.30386E-20	1.214854271	stage_E14_vs_E10	ordinary_muscle_development	D_vs_E10;stage_1W_vs_E10;stage_3W_vs_E10;stage	1	TRUE
ENSGALG00010011317	BLK	PRJNA729271	3.272706488	5.4341E-20	3.272706488	stage_E14_vs_E10	ordinary_muscle_development	D_vs_E10;stage_1W_vs_E10;stage_3W_vs_E10;stage	1	TRUE
ENSGALG00010013205	KCNJ6	PRJNA729271	3.085247411	5.47818E-20	3.085247411	stage_E14_vs_E10	ordinary_muscle_development	D_vs_E10;stage_1W_vs_E10;stage_3W_vs_E10;stage	1	TRUE
ENSGALG00010008312	ZBTB18	PRJNA729271	2.017778494	5.51135E-20	2.017778494	stage_E14_vs_E10	ordinary_muscle_development	D_vs_E10;stage_1W_vs_E10;stage_3W_vs_E10;stage	1	TRUE

ENSGALG00010001334	EBF3	PRJNA729271	-1.486693282	5.5276E-20	1.486693282	stage_E14_vs_E10	ordinary_muscle_development	D_vs_E10;stage_1W_vs_E10;stage_3W_vs_E10;stage	1	TRUE
ENSGALG00010021046	ENSGALG00010021046	PRJNA729271	-2.369857285	5.59023E-20	2.369857285	stage_E14_vs_E10	ordinary_muscle_development	D_vs_E10;stage_1W_vs_E10;stage_3W_vs_E10;stage	1	TRUE
ENSGALG00010008198	GLRB	PRJNA729271	-3.790921884	5.65276E-20	3.790921884	stage_E14_vs_E10	ordinary_muscle_development	D_vs_E10;stage_1W_vs_E10;stage_3W_vs_E10;stage	1	TRUE
ENSGALG00010022157	EPS8L2	PRJNA729271	-1.341016037	5.6606E-20	1.341016037	stage_E14_vs_E10	ordinary_muscle_development	D_vs_E10;stage_1W_vs_E10;stage_3W_vs_E10;stage	1	TRUE
ENSGALG00010027590	SEMA3F	PRJNA729271	-2.248376519	5.69525E-20	2.248376519	stage_E14_vs_E10	ordinary_muscle_development	D_vs_E10;stage_1W_vs_E10;stage_3W_vs_E10;stage	1	TRUE
ENSGALG00010020989	MAPK4	PRJNA729271	1.420669033	5.6992E-20	1.420669033	stage_E14_vs_E10	ordinary_muscle_development	D_vs_E10;stage_1W_vs_E10;stage_3W_vs_E10;stage	1	TRUE
ENSGALG00010027734	CSF1	PRJNA729271	1.291517378	5.74042E-20	1.291517378	stage_E14_vs_E10	ordinary_muscle_development	D_vs_E10;stage_1W_vs_E10;stage_3W_vs_E10;stage	1	TRUE
ENSGALG00010000793	ENSGALG00010000793	PRJNA729271	10.85318641	5.74404E-20	10.85318641	stage_E14_vs_E10	ordinary_muscle_development	D_vs_E10;stage_1W_vs_E10;stage_3W_vs_E10;stage	1	TRUE
ENSGALG00010003001	MAP6	PRJNA729271	-3.241374998	5.79989E-20	3.241374998	stage_E14_vs_E10	ordinary_muscle_development	D_vs_E10;stage_1W_vs_E10;stage_3W_vs_E10;stage	1	TRUE
ENSGALG00010008866	WASHC5	PRJNA729271	-0.703416934	5.83071E-20	0.703416934	stage_E14_vs_E10	ordinary_muscle_development		0	FALSE
ENSGALG00010009583	LYPD6B	PRJNA729271	-2.969661969	5.8325E-20	2.969661969	stage_E14_vs_E10	ordinary_muscle_development	D_vs_E10;stage_1W_vs_E10;stage_3W_vs_E10;stage	1	TRUE
ENSGALG00010027286	RPN1	PRJNA729271	0.587087655	5.91031E-20	0.587087655	stage_E14_vs_E10	ordinary_muscle_development		0	FALSE
ENSGALG00010029468	ENSGALG00010029468	PRJNA729271	-2.254212069	5.91845E-20	2.254212069	stage_E14_vs_E10	ordinary_muscle_development	D_vs_E10;stage_1W_vs_E10;stage_3W_vs_E10;stage	1	TRUE
ENSGALG00010028048	S100A10	PRJNA729271	2.027989494	6.21187E-20	2.027989494	stage_E14_vs_E10	ordinary_muscle_development	D_vs_E10;stage_1W_vs_E10;stage_3W_vs_E10;stage	1	TRUE
ENSGALG00010027099	GNA11	PRJNA729271	-0.800042358	6.33296E-20	0.800042358	stage_E14_vs_E10	ordinary_muscle_development		0	FALSE
ENSGALG00010022550	TRIM45	PRJNA729271	-1.581367484	6.50659E-20	1.581367484	stage_E14_vs_E10	ordinary_muscle_development	D_vs_E10;stage_1W_vs_E10;stage_3W_vs_E10;stage	1	TRUE
ENSGALG00010011969	TMEM173	PRJNA729271	2.683296553	6.55205E-20	2.683296553	stage_E14_vs_E10	ordinary_muscle_development	D_vs_E10;stage_1W_vs_E10;stage_3W_vs_E10;stage	1	TRUE
ENSGALG00010021512	CPNE9	PRJNA729271	3.669840854	6.64217E-20	3.669840854	stage_E14_vs_E10	ordinary_muscle_development	D_vs_E10;stage_1W_vs_E10;stage_3W_vs_E10;stage	1	TRUE
ENSGALG00010010430	ASTN1	PRJNA729271	-5.982869115	6.67689E-20	5.982869115	stage_E14_vs_E10	ordinary_muscle_development	D_vs_E10;stage_1W_vs_E10;stage_3W_vs_E10;stage	1	TRUE
ENSGALG00010025116	ANAPC7	PRJNA729271	-0.662147018	6.90305E-20	0.662147018	stage_E14_vs_E10	ordinary_muscle_development		0	FALSE
ENSGALG00010001540	TRABD	PRJNA729271	-1.234398985	6.90446E-20	1.234398985	stage_E14_vs_E10	ordinary_muscle_development	D_vs_E10;stage_1W_vs_E10;stage_3W_vs_E10;stage	1	TRUE
ENSGALG00010027278	H1-10	PRJNA729271	-1.205064537	6.94404E-20	1.205064537	stage_E14_vs_E10	ordinary_muscle_development	D_vs_E10;stage_1W_vs_E10;stage_3W_vs_E10;stage	1	TRUE
ENSGALG00010027217	SEMA6B	PRJNA729271	2.079281202	6.98868E-20	2.079281202	stage_E14_vs_E10	ordinary_muscle_development	D_vs_E10;stage_1W_vs_E10;stage_3W_vs_E10;stage	1	TRUE
ENSGALG00010022588	TCP11L1	PRJNA729271	-0.73823123	7.05702E-20	0.73823123	stage_E14_vs_E10	ordinary_muscle_development		0	FALSE
ENSGALG00010006634	PRMT9	PRJNA729271	0.882035223	7.06675E-20	0.882035223	stage_E14_vs_E10	ordinary_muscle_development		0	FALSE
ENSGALG00010001159	RTTN	PRJNA729271	-1.062815382	7.08341E-20	1.062815382	stage_E14_vs_E10	ordinary_muscle_development	D_vs_E10;stage_1W_vs_E10;stage_3W_vs_E10;stage	1	TRUE
ENSGALG00010001508	CD109	PRJNA729271	-2.766687234	7.14771E-20	2.766687234	stage_E14_vs_E10	ordinary_muscle_development	D_vs_E10;stage_1W_vs_E10;stage_3W_vs_E10;stage	1	TRUE
ENSGALG00010027112	XYLB	PRJNA729271	-1.800996923	7.21337E-20	1.800996923	stage_E14_vs_E10	ordinary_muscle_development	D_vs_E10;stage_1W_vs_E10;stage_3W_vs_E10;stage	1	TRUE
ENSGALG00010017999	GPR174	PRJNA729271	5.027301301	7.26848E-20	5.027301301	stage_E14_vs_E10	ordinary_muscle_development	D_vs_E10;stage_1W_vs_E10;stage_3W_vs_E10;stage	1	TRUE
ENSGALG00010027246	ZNF767	PRJNA729271	-0.940586558	7.3098E-20	0.940586558	stage_E14_vs_E10	ordinary_muscle_development		0	FALSE
ENSGALG00010008111	SYTL2	PRJNA729271	-2.510182875	7.31553E-20	2.510182875	stage_E14_vs_E10	ordinary_muscle_development	D_vs_E10;stage_1W_vs_E10;stage_3W_vs_E10;stage	1	TRUE
ENSGALG00010020778	SLIT1	PRJNA729271	-2.168372663	7.42554E-20	2.168372663	stage_E14_vs_E10	ordinary_muscle_development	D_vs_E10;stage_1W_vs_E10;stage_3W_vs_E10;stage	1	TRUE
ENSGALG00010009638	RND3	PRJNA729271	-1.399049179	7.49406E-20	1.399049179	stage_E14_vs_E10	ordinary_muscle_development	D_vs_E10;stage_1W_vs_E10;stage_3W_vs_E10;stage	1	TRUE
ENSGALG00010024836	TXNDC12	PRJNA729271	-0.992200176	7.52431E-20	0.992200176	stage_E14_vs_E10	ordinary_muscle_development		0	FALSE
ENSGALG00010015875	DNTTIP1	PRJNA729271	1.286968209	7.91856E-20	1.286968209	stage_E14_vs_E10	ordinary_muscle_development	D_vs_E10;stage_1W_vs_E10;stage_3W_vs_E10;stage	1	TRUE
ENSGALG00010007187	ENPP6	PRJNA729271	2.576751907	7.92196E-20	2.576751907	stage_E14_vs_E10	ordinary_muscle_development	D_vs_E10;stage_1W_vs_E10;stage_3W_vs_E10;stage	1	TRUE
ENSGALG00010022937	BLOC1S1	PRJNA729271	1.485755663	7.95373E-20	1.485755663	stage_E14_vs_E10	ordinary_muscle_development	D_vs_E10;stage_1W_vs_E10;stage_3W_vs_E10;stage	1	TRUE
ENSGALG00010016053	DRP2	PRJNA729271	4.241889218	7.99886E-20	4.241889218	stage_E14_vs_E10	ordinary_muscle_development	D_vs_E10;stage_1W_vs_E10;stage_3W_vs_E10;stage	1	TRUE
ENSGALG00010020493	DEPDC7	PRJNA729271	-1.501177868	8.06541E-20	1.501177868	stage_E14_vs_E10	ordinary_muscle_development	D_vs_E10;stage_1W_vs_E10;stage_3W_vs_E10;stage	1	TRUE
ENSGALG00010024383	DUSP28	PRJNA729271	1.740981661	8.07558E-20	1.740981661	stage_E14_vs_E10	ordinary_muscle_development	D_vs_E10;stage_1W_vs_E10;stage_3W_vs_E10;stage	1	TRUE
ENSGALG00010024663	ICE2	PRJNA729271	-1.158488143	8.30739E-20	1.158488143	stage_E14_vs_E10	ordinary_muscle_development	D_vs_E10;stage_1W_vs_E10;stage_3W_vs_E10;stage	1	TRUE
ENSGALG00010016549	PTPRA	PRJNA729271	-0.961547148	8.37286E-20	0.961547148	stage_E14_vs_E10	ordinary_muscle_development		0	FALSE
ENSGALG00010002006	ENSGALG00010002006	PRJNA729271	3.702204032	8.40936E-20	3.702204032	stage_E14_vs_E10	ordinary_muscle_development	D_vs_E10;stage_1W_vs_E10;stage_3W_vs_E10;stage	1	TRUE
ENSGALG00010008580	PANX1	PRJNA729271	-2.431540859	8.43297E-20	2.431540859	stage_E14_vs_E10	ordinary_muscle_development	D_vs_E10;stage_1W_vs_E10;stage_3W_vs_E10;stage	1	TRUE
ENSGALG00010010119	SRPK2	PRJNA729271	-1.162828984	8.50999E-20	1.162828984	stage_E14_vs_E10	ordinary_muscle_development	D_vs_E10;stage_1W_vs_E10;stage_3W_vs_E10;stage	1	TRUE
ENSGALG00010017941	DIS3L	PRJNA729271	1.252880163	8.88689E-20	1.252880163	stage_E14_vs_E10	ordinary_muscle_development	D_vs_E10;stage_1W_vs_E10;stage_3W_vs_E10;stage	1	TRUE
ENSGALG00010002655	FABP4	PRJNA729271	8.784851267	8.95424E-20	8.784851267	stage_E14_vs_E10	ordinary_muscle_development	D_vs_E10;stage_1W_vs_E10;stage_3W_vs_E10;stage	1	TRUE
ENSGALG00010024660	KMT2D	PRJNA729271	1.454238248	8.95424E-20	1.454238248	stage_E14_vs_E10	ordinary_muscle_development	D_vs_E10;stage_1W_vs_E10;stage_3W_vs_E10;stage	1	TRUE
ENSGALG00010019337	XPO1	PRJNA729271	-0.77858856	9.00593E-20	0.77858856	stage_E14_vs_E10	ordinary_muscle_development		0	FALSE
ENSGALG00010009043	TFAM	PRJNA729271	-0.78054839	9.03443E-20	0.78054839	stage_E14_vs_E10	ordinary_muscle_development		0	FALSE
ENSGALG00010019804	ELFN1	PRJNA729271	-2.9559403	9.04428E-20	2.9559403	stage_E14_vs_E10	ordinary_muscle_development	D_vs_E10;stage_1W_vs_E10;stage_3W_vs_E10;stage	1	TRUE
ENSGALG00010011515	SERINC5	PRJNA729271	0.79351416	9.13317E-20	0.79351416	stage_E14_vs_E10	ordinary_muscle_development		0	FALSE
ENSGALG00010002589	SLC4A7	PRJNA729271	1.334342789	9.16394E-20	1.334342789	stage_E14_vs_E10	ordinary_muscle_development	D_vs_E10;stage_1W_vs_E10;stage_3W_vs_E10;stage	1	TRUE
ENSGALG00010014104	ALDH1A1	PRJNA729271	4.779220794	9.36001E-20	4.779220794	stage_E14_vs_E10	ordinary_muscle_development	D_vs_E10;stage_1W_vs_E10;stage_3W_vs_E10;stage	1	TRUE
ENSGALG00010019670	ENSGALG00010019670	PRJNA729271	-1.169283051	9.39824E-20	1.169283051	stage_E14_vs_E10	ordinary_muscle_development	D_vs_E10;stage_1W_vs_E10;stage_3W_vs_E10;stage	1	TRUE
ENSGALG00010015798	IL22RA1	PRJNA729271	-3.074380808	9.41406E-20	3.074380808	stage_E14_vs_E10	ordinary_muscle_development	D_vs_E10;stage_1W_vs_E10;stage_3W_vs_E10;stage	1	TRUE
ENSGALG00010008342	PLD3	PRJNA729271	1.36635188	9.47392E-20	1.36635188	stage_E14_vs_E10	ordinary_muscle_development	D_vs_E10;stage_1W_vs_E10;stage_3W_vs_E10;stage	1	TRUE
ENSGALG00010003352	FARSA	PRJNA729271	0.930590907	9.65854E-20	0.930590907	stage_E14_vs_E10	ordinary_muscle_development		0	FALSE
ENSGALG00010011953	CDK17	PRJNA729271	0.536752029	9.74118E-20	0.536752029	stage_E14_vs_E10	ordinary_muscle_development		0	FALSE
ENSGALG00010023046	SLC7A6OS	PRJNA729271	-0.70045385	9.81323E-20	0.70045385	stage_E14_vs_E10	ordinary_muscle_development		0	FALSE
ENSGALG00010012259	IL1RAPL2	PRJNA729271	-2.206198819	9.90043E-20	2.206198819	stage_E14_vs_E10	ordinary_muscle_development	D_vs_E10;stage_1W_vs_E10;stage_3W_vs_E10;stage	1	TRUE
ENSGALG00010018165	DACH2	PRJNA729271	1.649203678	9.90043E-20	1.649203678	stage_E14_vs_E10	ordinary_muscle_development	D_vs_E10;stage_1W_vs_E10;stage_3W_vs_E10;stage	1	TRUE
ENSGALG00010017035	PIK3CD	PRJNA729271	1.850880084	9.96977E-20	1.850880084	stage_E14_vs_E10	ordinary_muscle_development	D_vs_E10;stage_1W_vs_E10;stage_3W_vs_E10;stage	1	TRUE
ENSGALG00010029385	CLIP2	PRJNA729271	-1.138026144	1.0075E-19	1.138026144	stage_E14_vs_E10	ordinary_muscle_development	D_vs_E10;stage_1W_vs_E10;stage_3W_vs_E10;stage	1	TRUE
ENSGALG00010024258	DNAJA4	PRJNA729271	1.395385245	1.02851E-19	1.395385245	stage_E14_vs_E10	ordinary_muscle_development	D_vs_E10;stage_1W_vs_E10;stage_3W_vs_E10;stage	1	TRUE
ENSGALG00010009710	ENSGALG00010009710	PRJNA729271	-1.051177545	1.03175E-19	1.051177545	stage_E14_vs_E10	ordinary_muscle_development	D_vs_E10;stage_1W_vs_E10;stage_3W_vs_E10;stage	1	TRUE
ENSGALG00010003978	ANK2	PRJNA729271	1.126214969	1.0366E-19	1.126214969	stage_E14_vs_E10	ordinary_muscle_development	D_vs_E10;stage_1W_vs_E10;stage_3W_vs_E10;stage	1	TRUE
ENSGALG00010021099	HMCN1	PRJNA729271	-2.445493542	1.0577E-19	2.445493542	stage_E14_vs_E10	ordinary_muscle_development	D_vs_E10;stage_1W_vs_E10;stage_3W_vs_E10;stage	1	TRUE
ENSGALG00010014260	ADNP	PRJNA729271	-0.69868752	1.05826E-19	0.69868752	stage_E14_vs_E10	ordinary_muscle_development		0	FALSE
ENSGALG00010025215	MYOG	PRJNA729271	-1.995051303	1.06085E-19	1.995051303	stage_E14_vs_E10	ordinary_muscle_development	D_vs_E10;stage_1W_vs_E10;stage_3W_vs_E10;stage	1	TRUE
ENSGALG00010021182	ZSWIM8	PRJNA729271	0.638237165	1.06417E-19	0.638237165	stage_E14_vs_E10	ordinary_muscle_development		0	FALSE
ENSGALG00010014011	SFN	PRJNA729271	-6.577870475	1.09085E-19	6.577870475	stage_E14_vs_E10	ordinary_muscle_development	D_vs_E10;stage_1W_vs_E10;stage_3W_vs_E10;stage	1	TRUE
ENSGALG00010026163	CTNND1	PRJNA729271	-0.758977935	1.10482E-19	0.758977935	stage_E14_vs_E10	ordinary_muscle_development		0	FALSE
ENSGALG00010001216	SHOX2	PRJNA729271	-6.897214947	1.11089E-19	6.897214947	stage_E14_vs_E10	ordinary_muscle_development	D_vs_E10;stage_1W_vs_E10;stage_3W_vs_E10;stage	1	TRUE
ENSGALG00010004958	TPD52L1	PRJNA729271	-4.802461266	1.13551E-19	4.802461266	stage_E14_vs_E10	ordinary_muscle_development	D_vs_E10;stage_1W_vs_E10;stage_3W_vs_E10;stage	1	TRUE
ENSGALG00010000089	ENSGALG00010000089	PRJNA729271	2.583602747	1.14508E-19	2.583602747	stage_E14_vs_E10	ordinary_muscle_development	D_vs_E10;stage_1W_vs_E10;stage_3W_vs_E10;stage	1	TRUE
ENSGALG00010010940	NRG3	PRJNA729271	-3.809386221	1.17803E-19	3.809386221	stage_E14_vs_E10	ordinary_muscle_development	D_vs_E10;stage_1W_vs_E10;stage_3W_vs_E10;stage	1	TRUE
ENSGALG00010019617	ANO3	PRJNA729271	-1.337123569	1.18376E-19	1.337123569	stage_E14_vs_E10	ordinary_muscle_development	D_vs_E10;stage_1W_vs_E10;stage_3W_vs_E10;stage	1	TRUE
ENSGALG00010029375	JMJD6	PRJNA729271	1.473138371	1.22719E-19	1.473138371	stage_E14_vs_E10	ordinary_muscle_development	D_vs_E10;stage_1W_vs_E10;stage_3W_vs_E10;stage	1	TRUE
ENSGALG00010022642	SEC13	PRJNA729271	-0.634924838	1.24019E-19	0.634924838	stage_E14_vs_E10	ordinary_muscle_development		0	FALSE
ENSGALG00010014967	HNRNPH1	PRJNA729271	-0.992754664	1.24118E-19	0.992754664	stage_E14_vs_E10	ordinary_muscle_development		0	FALSE
ENSGALG00010027083	DENND6A	PRJNA729271	-0.816245044	1.24118E-19	0.816245044	stage_E14_vs_E10	ordinary_muscle_development		0	FALSE
ENSGALG00010013793	METTL13	PRJNA729271	-1.22281236	1.24302E-19	1.22281236	stage_E14_vs_E10	ordinary_muscle_development	D_vs_E10;stage_1W_vs_E10;stage_3W_vs_E10;stage	1	TRUE
ENSGALG00010025342	TCEANC2	PRJNA729271	-1.136447185	1.24521E-19	1.136447185	stage_E14_vs_E10	ordinary_muscle_development	D_vs_E10;stage_1W_vs_E10;stage_3W_vs_E10;stage	1	TRUE
ENSGALG00010022845	HYDIN	PRJNA729271	2.921290233	1.26227E-19	2.921290233	stage_E14_vs_E10	ordinary_muscle_development	D_vs_E10;stage_1W_vs_E10;stage_3W_vs_E10;stage	1	TRUE
ENSGALG00010006205	CGGBP1	PRJNA729271	-0.686955245	1.28133E-19	0.686955245	stage_E14_vs_E10	ordinary_muscle_development		0	FALSE
ENSGALG00010027928	FLAD1	PRJNA729271	1.454186798	1.28628E-19	1.454186798	stage_E14_vs_E10	ordinary_muscle_development	D_vs_E10;stage_1W_vs_E10;stage_3W_vs_E10;stage	1	TRUE
ENSGALG00010026715	WDR48	PRJNA729271	-0.636570651	1.28628E-19	0.636570651	stage_E14_vs_E10	ordinary_muscle_development		0	FALSE
ENSGALG00010017732	RHOJ	PRJNA729271	-0.741479853	1.29886E-19	0.741479853	stage_E14_vs_E10	ordinary_muscle_development		0	FALSE
ENSGALG00010023815	PLCD3	PRJNA729271	1.248700936	1.3283E-19	1.248700936	stage_E14_vs_E10	ordinary_muscle_development	D_vs_E10;stage_1W_vs_E10;stage_3W_vs_E10;stage	1	TRUE
ENSGALG00010029524	ENSGALG00010029524	PRJNA729271	1.370707765	1.33428E-19	1.370707765	stage_E14_vs_E10	ordinary_muscle_development	D_vs_E10;stage_1W_vs_E10;stage_3W_vs_E10;stage	1	TRUE
ENSGALG00010025927	ENSGALG00010025927	PRJNA729271	7.928750757	1.37033E-19	7.928750757	stage_E14_vs_E10	ordinary_muscle_development	D_vs_E10;stage_1W_vs_E10;stage_3W_vs_E10;stage	1	TRUE
ENSGALG00010021570	KATNB1	PRJNA729271	-0.983649578	1.37108E-19	0.983649578	stage_E14_vs_E10	ordinary_muscle_development		0	FALSE
ENSGALG00010020259	ENSGALG00010020259	PRJNA729271	-2.137566251	1.39426E-19	2.137566251	stage_E14_vs_E10	ordinary_muscle_development	D_vs_E10;stage_1W_vs_E10;stage_3W_vs_E10;stage	1	TRUE
ENSGALG00010010980	POLK	PRJNA729271	1.14762453	1.39521E-19	1.14762453	stage_E14_vs_E10	ordinary_muscle_development	D_vs_E10;stage_1W_vs_E10;stage_3W_vs_E10;stage	1	TRUE
ENSGALG00010003680	TRAPPC5	PRJNA729271	0.972376866	1.39542E-19	0.972376866	stage_E14_vs_E10	ordinary_muscle_development		0	FALSE
ENSGALG00010011121	NUP35	PRJNA729271	-1.041817908	1.41273E-19	1.041817908	stage_E14_vs_E10	ordinary_muscle_development	D_vs_E10;stage_1W_vs_E10;stage_3W_vs_E10;stage	1	TRUE
ENSGALG00010019021	LRR1	PRJNA729271	-1.415763244	1.41992E-19	1.415763244	stage_E14_vs_E10	ordinary_muscle_development	D_vs_E10;stage_1W_vs_E10;stage_3W_vs_E10;stage	1	TRUE
ENSGALG00010024046	ENSGALG00010024046	PRJNA729271	-9.072379232	1.46142E-19	9.072379232	stage_E14_vs_E10	ordinary_muscle_development	D_vs_E10;stage_1W_vs_E10;stage_3W_vs_E10;stage	1	TRUE
ENSGALG00010015913	CHST10	PRJNA729271	-0.973000375	1.4757E-19	0.973000375	stage_E14_vs_E10	ordinary_muscle_development		0	FALSE
ENSGALG00010006558	USP10	PRJNA729271	-0.820229429	1.48911E-19	0.820229429	stage_E14_vs_E10	ordinary_muscle_development		0	FALSE
ENSGALG00010011789	TXNRD1	PRJNA729271	-1.278630558	1.49969E-19	1.278630558	stage_E14_vs_E10	ordinary_muscle_development	D_vs_E10;stage_1W_vs_E10;stage_3W_vs_E10;stage	1	TRUE
ENSGALG00010021864	GFI1	PRJNA729271	4.095447758	1.50162E-19	4.095447758	stage_E14_vs_E10	ordinary_muscle_development	D_vs_E10;stage_1W_vs_E10;stage_3W_vs_E10;stage	1	TRUE
ENSGALG00010008321	ZFHX3	PRJNA729271	-0.966178826	1.51377E-19	0.966178826	stage_E14_vs_E10	ordinary_muscle_development		0	FALSE
ENSGALG00010001585	ENSGALG00010001585	PRJNA729271	-1.305335566	1.52349E-19	1.305335566	stage_E14_vs_E10	ordinary_muscle_development	D_vs_E10;stage_1W_vs_E10;stage_3W_vs_E10;stage	1	TRUE
ENSGALG00010023513	CDCA9	PRJNA729271	-2.040244575	1.54526E-19	2.040244575	stage_E14_vs_E10	ordinary_muscle_development	D_vs_E10;stage_1W_vs_E10;stage_3W_vs_E10;stage	1	TRUE
ENSGALG00010011577	ENSGALG00010011577	PRJNA729271	1.476493269	1.57649E-19	1.476493269	stage_E14_vs_E10	ordinary_muscle_development	D_vs_E10;stage_1W_vs_E10;stage_3W_vs_E10;stage	1	TRUE
ENSGALG00010013233	LRRCC1	PRJNA729271	-1.23807808	1.58286E-19	1.23807808	stage_E14_vs_E10	ordinary_muscle_development	D_vs_E10;stage_1W_vs_E10;stage_3W_vs_E10;stage	1	TRUE
ENSGALG00010007283	STOX2	PRJNA729271	-1.130656054	1.58948E-19	1.130656054	stage_E14_vs_E10	ordinary_muscle_development	D_vs_E10;stage_1W_vs_E10;stage_3W_vs_E10;stage	1	TRUE
ENSGALG00010027717	ATXN7L2	PRJNA729271	2.064117967	1.63963E-19	2.064117967	stage_E14_vs_E10	ordinary_muscle_development	D_vs_E10;stage_1W_vs_E10;stage_3W_vs_E10;stage	1	TRUE
ENSGALG00010015583	PRDM4	PRJNA729271	1.233521469	1.64976E-19	1.233521469	stage_E14_vs_E10	ordinary_muscle_development	D_vs_E10;stage_1W_vs_E10;stage_3W_vs_E10;stage	1	TRUE
ENSGALG00010009160	ENSGALG00010009160	PRJNA729271	-3.628177197	1.66098E-19	3.628177197	stage_E14_vs_E10	ordinary_muscle_development	D_vs_E10;stage_1W_vs_E10;stage_3W_vs_E10;stage	1	TRUE
ENSGALG00010028061	NAIF1	PRJNA729271	-0.880062903	1.66098E-19	0.880062903	stage_E14_vs_E10	ordinary_muscle_development		0	FALSE
ENSGALG00010008194	PDF	PRJNA729271	1.483208749	1.69154E-19	1.483208749	stage_E14_vs_E10	ordinary_muscle_development	D_vs_E10;stage_1W_vs_E10;stage_3W_vs_E10;stage	1	TRUE
ENSGALG00010023569	LPCAT3	PRJNA729271	-1.005307171	1.70011E-19	1.005307171	stage_E14_vs_E10	ordinary_muscle_development	D_vs_E10;stage_1W_vs_E10;stage_3W_vs_E10;stage	1	TRUE
ENSGALG00010021505	IHH	PRJNA729271	-5.39370862	1.73725E-19	5.39370862	stage_E14_vs_E10	ordinary_muscle_development	D_vs_E10;stage_1W_vs_E10;stage_3W_vs_E10;stage	1	TRUE
ENSGALG00010001286	SRFBP1	PRJNA729271	-1.050299654	1.75086E-19	1.050299654	stage_E14_vs_E10	ordinary_muscle_development	D_vs_E10;stage_1W_vs_E10;stage_3W_vs_E10;stage	1	TRUE
ENSGALG00010005087	ENPP1	PRJNA729271	1.979141877	1.75525E-19	1.979141877	stage_E14_vs_E10	ordinary_muscle_development	D_vs_E10;stage_1W_vs_E10;stage_3W_vs_E10;stage	1	TRUE
ENSGALG00010008424	PPIP5K2	PRJNA729271	-1.129173432	1.75525E-19	1.129173432	stage_E14_vs_E10	ordinary_muscle_development	D_vs_E10;stage_1W_vs_E10;stage_3W_vs_E10;stage	1	TRUE
ENSGALG00010017491	CCDC28B	PRJNA729271	1.747195617	1.78646E-19	1.747195617	stage_E14_vs_E10	ordinary_muscle_development	D_vs_E10;stage_1W_vs_E10;stage_3W_vs_E10;stage	1	TRUE
ENSGALG00010013880	ZMYM4	PRJNA729271	-0.830345165	1.8384E-19	0.830345165	stage_E14_vs_E10	ordinary_muscle_development		0	FALSE
ENSGALG00010002381	TDRD3	PRJNA729271	0.882214867	1.85635E-19	0.882214867	stage_E14_vs_E10	ordinary_muscle_development		0	FALSE
ENSGALG00010020626	MORN4	PRJNA729271	-1.108830259	1.8784E-19	1.108830259	stage_E14_vs_E10	ordinary_muscle_development	D_vs_E10;stage_1W_vs_E10;stage_3W_vs_E10;stage	1	TRUE
ENSGALG00010001819	HOXA6	PRJNA729271	1.29585806	1.89352E-19	1.29585806	stage_E14_vs_E10	ordinary_muscle_development	D_vs_E10;stage_1W_vs_E10;stage_3W_vs_E10;stage	1	TRUE
ENSGALG00010021995	CD28	PRJNA729271	4.287210522	1.89596E-19	4.287210522	stage_E14_vs_E10	ordinary_muscle_development	D_vs_E10;stage_1W_vs_E10;stage_3W_vs_E10;stage	1	TRUE
ENSGALG00010016921	EFL1	PRJNA729271	-0.706893195	1.9153E-19	0.706893195	stage_E14_vs_E10	ordinary_muscle_development		0	FALSE
ENSGALG00010001871	ENSGALG00010001871	PRJNA729271	-1.144942354	1.92007E-19	1.144942354	stage_E14_vs_E10	ordinary_muscle_development	D_vs_E10;stage_1W_vs_E10;stage_3W_vs_E10;stage	1	TRUE
ENSGALG00010017815	VWA9	PRJNA729271	-0.807438307	1.92462E-19	0.807438307	stage_E14_vs_E10	ordinary_muscle_development		0	FALSE
ENSGALG00010009603	HINT1	PRJNA729271	1.621427976	1.96808E-19	1.621427976	stage_E14_vs_E10	ordinary_muscle_development	D_vs_E10;stage_1W_vs_E10;stage_3W_vs_E10;stage	1	TRUE
ENSGALG00010027233	ENSGALG00010027233	PRJNA729271	2.329215931	1.97304E-19	2.329215931	stage_E14_vs_E10	ordinary_muscle_development	D_vs_E10;stage_1W_vs_E10;stage_3W_vs_E10;stage	1	TRUE
ENSGALG00010020737	POLR3A	PRJNA729271	-1.088135011	1.98476E-19	1.088135011	stage_E14_vs_E10	ordinary_muscle_development	D_vs_E10;stage_1W_vs_E10;stage_3W_vs_E10;stage	1	TRUE
ENSGALG00010010095	C12orf73	PRJNA729271	0.895648743	2.00193E-19	0.895648743	stage_E14_vs_E10	ordinary_muscle_development		0	FALSE
ENSGALG00010014265	ZNF462	PRJNA729271	-1.283159245	2.01211E-19	1.283159245	stage_E14_vs_E10	ordinary_muscle_development	D_vs_E10;stage_1W_vs_E10;stage_3W_vs_E10;stage	1	TRUE
ENSGALG00010012340	ENHO	PRJNA729271	-1.530367492	2.04843E-19	1.530367492	stage_E14_vs_E10	ordinary_muscle_development	D_vs_E10;stage_1W_vs_E10;stage_3W_vs_E10;stage	1	TRUE
ENSGALG00010023695	CLSTN3	PRJNA729271	-1.213729976	2.05551E-19	1.213729976	stage_E14_vs_E10	ordinary_muscle_development	D_vs_E10;stage_1W_vs_E10;stage_3W_vs_E10;stage	1	TRUE
ENSGALG00010024866	TRAPPC4	PRJNA729271	0.774736146	2.06681E-19	0.774736146	stage_E14_vs_E10	ordinary_muscle_development		0	FALSE
ENSGALG00010028555	ENSGALG00010028555	PRJNA729271	10.81564733	2.07939E-19	10.81564733	stage_E14_vs_E10	ordinary_muscle_development	D_vs_E10;stage_1W_vs_E10;stage_3W_vs_E10;stage	1	TRUE
ENSGALG00010016500	ENSGALG00010016500	PRJNA729271	0.976164155	2.22081E-19	0.976164155	stage_E14_vs_E10	ordinary_muscle_development		0	FALSE
ENSGALG00010003755	SLC35A1	PRJNA729271	-0.8639723	2.22669E-19	0.8639723	stage_E14_vs_E10	ordinary_muscle_development		0	FALSE
ENSGALG00010020009	MACROD2	PRJNA729271	-1.106004912	2.30286E-19	1.106004912	stage_E14_vs_E10	ordinary_muscle_development	D_vs_E10;stage_1W_vs_E10;stage_3W_vs_E10;stage	1	TRUE
ENSGALG00010014302	FOXO3	PRJNA729271	2.140451171	2.31025E-19	2.140451171	stage_E14_vs_E10	ordinary_muscle_development	D_vs_E10;stage_1W_vs_E10;stage_3W_vs_E10;stage	1	TRUE
ENSGALG00010018446	FBN1	PRJNA729271	2.137388018	2.31885E-19	2.137388018	stage_E14_vs_E10	ordinary_muscle_development	D_vs_E10;stage_1W_vs_E10;stage_3W_vs_E10;stage	1	TRUE
ENSGALG00010023447	C12orf57	PRJNA729271	1.034591922	2.33355E-19	1.034591922	stage_E14_vs_E10	ordinary_muscle_development	D_vs_E10;stage_1W_vs_E10;stage_3W_vs_E10;stage	1	TRUE
ENSGALG00010017598	ENSGALG00010017598	PRJNA729271	1.070119816	2.36346E-19	1.070119816	stage_E14_vs_E10	ordinary_muscle_development	D_vs_E10;stage_1W_vs_E10;stage_3W_vs_E10;stage	1	TRUE
ENSGALG00010000279	ENSGALG00010000279	PRJNA729271	-1.294205301	2.36878E-19	1.294205301	stage_E14_vs_E10	ordinary_muscle_development	D_vs_E10;stage_1W_vs_E10;stage_3W_vs_E10;stage	1	TRUE
ENSGALG00010006237	ENSGALG00010006237	PRJNA729271	2.677081159	2.4074E-19	2.677081159	stage_E14_vs_E10	ordinary_muscle_development	D_vs_E10;stage_1W_vs_E10;stage_3W_vs_E10;stage	1	TRUE
ENSGALG00010024806	ANKRD27	PRJNA729271	0.667026278	2.41165E-19	0.667026278	stage_E14_vs_E10	ordinary_muscle_development		0	FALSE
ENSGALG00010017362	ENO1	PRJNA729271	1.19774236	2.4349E-19	1.19774236	stage_E14_vs_E10	ordinary_muscle_development	D_vs_E10;stage_1W_vs_E10;stage_3W_vs_E10;stage	1	TRUE
ENSGALG00010025160	MRPS22	PRJNA729271	1.184608997	2.46921E-19	1.184608997	stage_E14_vs_E10	ordinary_muscle_development	D_vs_E10;stage_1W_vs_E10;stage_3W_vs_E10;stage	1	TRUE
ENSGALG00010021077	SEC31B	PRJNA729271	0.718154239	2.47784E-19	0.718154239	stage_E14_vs_E10	ordinary_muscle_development		0	FALSE
ENSGALG00010003509	GPALPP1	PRJNA729271	-0.690093043	2.5336E-19	0.690093043	stage_E14_vs_E10	ordinary_muscle_development		0	FALSE
ENSGALG00010001773	NRCAM	PRJNA729271	-2.352173414	2.5433E-19	2.352173414	stage_E14_vs_E10	ordinary_muscle_development	D_vs_E10;stage_1W_vs_E10;stage_3W_vs_E10;stage	1	TRUE
ENSGALG00010004669	ELMOD1	PRJNA729271	-3.643091485	2.54617E-19	3.643091485	stage_E14_vs_E10	ordinary_muscle_development	D_vs_E10;stage_1W_vs_E10;stage_3W_vs_E10;stage	1	TRUE
ENSGALG00010016577	ZBTB17	PRJNA729271	1.687131083	2.57089E-19	1.687131083	stage_E14_vs_E10	ordinary_muscle_development	D_vs_E10;stage_1W_vs_E10;stage_3W_vs_E10;stage	1	TRUE
ENSGALG00010014203	TMEM38B	PRJNA729271	1.543170049	2.57343E-19	1.543170049	stage_E14_vs_E10	ordinary_muscle_development	D_vs_E10;stage_1W_vs_E10;stage_3W_vs_E10;stage	1	TRUE
ENSGALG00010028207	ENSGALG00010028207	PRJNA729271	2.212232745	2.59393E-19	2.212232745	stage_E14_vs_E10	ordinary_muscle_development	D_vs_E10;stage_1W_vs_E10;stage_3W_vs_E10;stage	1	TRUE
ENSGALG00010023158	ENSGALG00010023158	PRJNA729271	4.058489371	2.64846E-19	4.058489371	stage_E14_vs_E10	ordinary_muscle_development	D_vs_E10;stage_1W_vs_E10;stage_3W_vs_E10;stage	1	TRUE
ENSGALG00010026013	RABIF	PRJNA729271	-1.052935376	2.6538E-19	1.052935376	stage_E14_vs_E10	ordinary_muscle_development	D_vs_E10;stage_1W_vs_E10;stage_3W_vs_E10;stage	1	TRUE
ENSGALG00010005516	LARP4B	PRJNA729271	1.210295835	2.67341E-19	1.210295835	stage_E14_vs_E10	ordinary_muscle_development	D_vs_E10;stage_1W_vs_E10;stage_3W_vs_E10;stage	1	TRUE
ENSGALG00010029339	PITPNA	PRJNA729271	1.156046422	2.68757E-19	1.156046422	stage_E14_vs_E10	ordinary_muscle_development	D_vs_E10;stage_1W_vs_E10;stage_3W_vs_E10;stage	1	TRUE
ENSGALG00010020309	GXYLT2	PRJNA729271	-1.460263056	2.69529E-19	1.460263056	stage_E14_vs_E10	ordinary_muscle_development	D_vs_E10;stage_1W_vs_E10;stage_3W_vs_E10;stage	1	TRUE
ENSGALG00010007536	GABBR2	PRJNA729271	1.768546892	2.70046E-19	1.768546892	stage_E14_vs_E10	ordinary_muscle_development	D_vs_E10;stage_1W_vs_E10;stage_3W_vs_E10;stage	1	TRUE
ENSGALG00010017425	H6PD	PRJNA729271	1.148072264	2.7008E-19	1.148072264	stage_E14_vs_E10	ordinary_muscle_development	D_vs_E10;stage_1W_vs_E10;stage_3W_vs_E10;stage	1	TRUE
ENSGALG00010017868	CHRND	PRJNA729271	2.235413119	2.74579E-19	2.235413119	stage_E14_vs_E10	ordinary_muscle_development	D_vs_E10;stage_1W_vs_E10;stage_3W_vs_E10;stage	1	TRUE
ENSGALG00010001896	ARHGEF10	PRJNA729271	1.225432214	2.8038E-19	1.225432214	stage_E14_vs_E10	ordinary_muscle_development	D_vs_E10;stage_1W_vs_E10;stage_3W_vs_E10;stage	1	TRUE
ENSGALG00010006804	PSMA6	PRJNA729271	0.928676487	2.81687E-19	0.928676487	stage_E14_vs_E10	ordinary_muscle_development		0	FALSE
ENSGALG00010003928	ENSGALG00010003928	PRJNA729271	1.41361204	2.82702E-19	1.41361204	stage_E14_vs_E10	ordinary_muscle_development	D_vs_E10;stage_1W_vs_E10;stage_3W_vs_E10;stage	1	TRUE
ENSGALG00010014367	OPHN1	PRJNA729271	0.99676157	2.88372E-19	0.99676157	stage_E14_vs_E10	ordinary_muscle_development		0	FALSE
ENSGALG00010000592	CCT5	PRJNA729271	-1.091134409	2.9074E-19	1.091134409	stage_E14_vs_E10	ordinary_muscle_development	D_vs_E10;stage_1W_vs_E10;stage_3W_vs_E10;stage	1	TRUE
ENSGALG00010022452	AP3M1	PRJNA729271	0.744528499	2.91964E-19	0.744528499	stage_E14_vs_E10	ordinary_muscle_development		0	FALSE
ENSGALG00010011738	FGD6	PRJNA729271	1.06654385	2.93774E-19	1.06654385	stage_E14_vs_E10	ordinary_muscle_development	D_vs_E10;stage_1W_vs_E10;stage_3W_vs_E10;stage	1	TRUE
ENSGALG00010018546	SNRPGP15	PRJNA729271	-1.24821233	2.95731E-19	1.24821233	stage_E14_vs_E10	ordinary_muscle_development	D_vs_E10;stage_1W_vs_E10;stage_3W_vs_E10;stage	1	TRUE
ENSGALG00010012702	PTPRO	PRJNA729271	-3.508022377	2.96612E-19	3.508022377	stage_E14_vs_E10	ordinary_muscle_development	D_vs_E10;stage_1W_vs_E10;stage_3W_vs_E10;stage	1	TRUE
ENSGALG00010029329	ENSGALG00010029329	PRJNA729271	1.625527538	2.96765E-19	1.625527538	stage_E14_vs_E10	ordinary_muscle_development	D_vs_E10;stage_1W_vs_E10;stage_3W_vs_E10;stage	1	TRUE
ENSGALG00010019977	ZMIZ1	PRJNA729271	0.790656728	3.0269E-19	0.790656728	stage_E14_vs_E10	ordinary_muscle_development		0	FALSE

ENSGALG00010012944	NCK1	PRJNA729271	-0.481257377	3.03035E-19	0.481257377	stage_E14_vs_E10	ordinary_muscle_development		0	FALSE
ENSGALG00010010712	ADAMTS5	PRJNA729271	1.32572423	3.03306E-19	1.32572423	stage_E14_vs_E10	ordinary_muscle_development	D_vs_E10;stage_1W_vs_E10;stage_3W_vs_E10;stage	1	TRUE
ENSGALG00010020571	MRTO4	PRJNA729271	-0.758446206	3.04953E-19	0.758446206	stage_E14_vs_E10	ordinary_muscle_development		0	FALSE
ENSGALG00010010924	ENSGALG00010010924	PRJNA729271	-2.071447574	3.06011E-19	2.071447574	stage_E14_vs_E10	ordinary_muscle_development	D_vs_E10;stage_1W_vs_E10;stage_3W_vs_E10;stage	1	TRUE
ENSGALG00010001267	BCHE	PRJNA729271	2.283281546	3.0715E-19	2.283281546	stage_E14_vs_E10	ordinary_muscle_development	D_vs_E10;stage_1W_vs_E10;stage_3W_vs_E10;stage	1	TRUE
ENSGALG00010018726	EBF2	PRJNA729271	-1.429495096	3.08549E-19	1.429495096	stage_E14_vs_E10	ordinary_muscle_development	D_vs_E10;stage_1W_vs_E10;stage_3W_vs_E10;stage	1	TRUE
ENSGALG00010010421	POLR2F	PRJNA729271	0.821873921	3.15491E-19	0.821873921	stage_E14_vs_E10	ordinary_muscle_development		0	FALSE
ENSGALG00010017950	FBLN7	PRJNA729271	-3.083437588	3.18583E-19	3.083437588	stage_E14_vs_E10	ordinary_muscle_development	D_vs_E10;stage_1W_vs_E10;stage_3W_vs_E10;stage	1	TRUE
ENSGALG00010016792	HMOX2	PRJNA729271	-1.130444879	3.22009E-19	1.130444879	stage_E14_vs_E10	ordinary_muscle_development	D_vs_E10;stage_1W_vs_E10;stage_3W_vs_E10;stage	1	TRUE
ENSGALG00010022302	ENSGALG00010022302	PRJNA729271	-1.439177686	3.30456E-19	1.439177686	stage_E14_vs_E10	ordinary_muscle_development	D_vs_E10;stage_1W_vs_E10;stage_3W_vs_E10;stage	1	TRUE
ENSGALG00010025571	SRSF3	PRJNA729271	-2.09983225	3.32631E-19	2.09983225	stage_E14_vs_E10	ordinary_muscle_development	D_vs_E10;stage_1W_vs_E10;stage_3W_vs_E10;stage	1	TRUE
ENSGALG00010008824	GREM2	PRJNA729271	-2.940257052	3.344E-19	2.940257052	stage_E14_vs_E10	ordinary_muscle_development	D_vs_E10;stage_1W_vs_E10;stage_3W_vs_E10;stage	1	TRUE
ENSGALG00010028106	ARPC5L	PRJNA729271	0.925920105	3.39104E-19	0.925920105	stage_E14_vs_E10	ordinary_muscle_development		0	FALSE
ENSGALG00010006957	PALM2AKAP2	PRJNA729271	0.832135505	3.39247E-19	0.832135505	stage_E14_vs_E10	ordinary_muscle_development		0	FALSE
ENSGALG00010019422	IMMP1L	PRJNA729271	1.256333959	3.48769E-19	1.256333959	stage_E14_vs_E10	ordinary_muscle_development	D_vs_E10;stage_1W_vs_E10;stage_3W_vs_E10;stage	1	TRUE
ENSGALG00010002376	GEN1	PRJNA729271	-1.764985889	3.51531E-19	1.764985889	stage_E14_vs_E10	ordinary_muscle_development	D_vs_E10;stage_1W_vs_E10;stage_3W_vs_E10;stage	1	TRUE
ENSGALG00010014934	MAMDC2	PRJNA729271	1.890305492	3.60073E-19	1.890305492	stage_E14_vs_E10	ordinary_muscle_development	D_vs_E10;stage_1W_vs_E10;stage_3W_vs_E10;stage	1	TRUE
ENSGALG00010029217	CEP112	PRJNA729271	-0.850912031	3.60766E-19	0.850912031	stage_E14_vs_E10	ordinary_muscle_development		0	FALSE
ENSGALG00010029255	RAP1GAP2	PRJNA729271	1.673107073	3.62574E-19	1.673107073	stage_E14_vs_E10	ordinary_muscle_development	D_vs_E10;stage_1W_vs_E10;stage_3W_vs_E10;stage	1	TRUE
ENSGALG00010000798	ENSGALG00010000798	PRJNA729271	-6.776874796	3.63306E-19	6.776874796	stage_E14_vs_E10	ordinary_muscle_development	D_vs_E10;stage_1W_vs_E10;stage_3W_vs_E10;stage	1	TRUE
ENSGALG00010001195	ZNF608	PRJNA729271	-1.3087423	3.68042E-19	1.3087423	stage_E14_vs_E10	ordinary_muscle_development	D_vs_E10;stage_1W_vs_E10;stage_3W_vs_E10;stage	1	TRUE
ENSGALG00010014758	FXN	PRJNA729271	-0.941347731	3.68865E-19	0.941347731	stage_E14_vs_E10	ordinary_muscle_development		0	FALSE
ENSGALG00010027272	DDX20	PRJNA729271	-0.971022318	3.71505E-19	0.971022318	stage_E14_vs_E10	ordinary_muscle_development		0	FALSE
ENSGALG00010022150	CPNE2	PRJNA729271	-1.21373656	3.73142E-19	1.21373656	stage_E14_vs_E10	ordinary_muscle_development	D_vs_E10;stage_1W_vs_E10;stage_3W_vs_E10;stage	1	TRUE
ENSGALG00010016066	C14orf2	PRJNA729271	1.358278511	3.77857E-19	1.358278511	stage_E14_vs_E10	ordinary_muscle_development	D_vs_E10;stage_1W_vs_E10;stage_3W_vs_E10;stage	1	TRUE
ENSGALG00010021391	ENSGALG00010021391	PRJNA729271	3.265584854	3.82843E-19	3.265584854	stage_E14_vs_E10	ordinary_muscle_development	D_vs_E10;stage_1W_vs_E10;stage_3W_vs_E10;stage	1	TRUE
ENSGALG00010001143	CDKN2A	PRJNA729271	3.73583718	3.88156E-19	3.73583718	stage_E14_vs_E10	ordinary_muscle_development	D_vs_E10;stage_1W_vs_E10;stage_3W_vs_E10;stage	1	TRUE
ENSGALG00010024400	KCNA1	PRJNA729271	-4.154120372	3.97866E-19	4.154120372	stage_E14_vs_E10	ordinary_muscle_development	D_vs_E10;stage_1W_vs_E10;stage_3W_vs_E10;stage	1	TRUE
ENSGALG00010025737	ENSGALG00010025737	PRJNA729271	1.364318634	4.10198E-19	1.364318634	stage_E14_vs_E10	ordinary_muscle_development	D_vs_E10;stage_1W_vs_E10;stage_3W_vs_E10;stage	1	TRUE
ENSGALG00010011267	EVC2	PRJNA729271	-1.520429863	4.10671E-19	1.520429863	stage_E14_vs_E10	ordinary_muscle_development	D_vs_E10;stage_1W_vs_E10;stage_3W_vs_E10;stage	1	TRUE
ENSGALG00010024386	TPPP3	PRJNA729271	2.427826311	4.20915E-19	2.427826311	stage_E14_vs_E10	ordinary_muscle_development	D_vs_E10;stage_1W_vs_E10;stage_3W_vs_E10;stage	1	TRUE
ENSGALG00010029271	MYL10	PRJNA729271	-7.311220848	4.211E-19	7.311220848	stage_E14_vs_E10	ordinary_muscle_development	D_vs_E10;stage_1W_vs_E10;stage_3W_vs_E10;stage	1	TRUE
ENSGALG00010023189	RMDN3	PRJNA729271	-0.787710316	4.22057E-19	0.787710316	stage_E14_vs_E10	ordinary_muscle_development		0	FALSE
ENSGALG00010024113	ENSGALG00010024113	PRJNA729271	1.474039905	4.25921E-19	1.474039905	stage_E14_vs_E10	ordinary_muscle_development	D_vs_E10;stage_1W_vs_E10;stage_3W_vs_E10;stage	1	TRUE
ENSGALG00010008729	TMEM254	PRJNA729271	-1.097087926	4.27732E-19	1.097087926	stage_E14_vs_E10	ordinary_muscle_development	D_vs_E10;stage_1W_vs_E10;stage_3W_vs_E10;stage	1	TRUE
ENSGALG00010027270	ENSGALG00010027270	PRJNA729271	-4.43783879	4.39182E-19	4.43783879	stage_E14_vs_E10	ordinary_muscle_development	D_vs_E10;stage_1W_vs_E10;stage_3W_vs_E10;stage	1	TRUE
ENSGALG00010017771	PNO1	PRJNA729271	1.420761471	4.39182E-19	1.420761471	stage_E14_vs_E10	ordinary_muscle_development	D_vs_E10;stage_1W_vs_E10;stage_3W_vs_E10;stage	1	TRUE
ENSGALG00010001836	TWSG1	PRJNA729271	-1.274390227	4.47275E-19	1.274390227	stage_E14_vs_E10	ordinary_muscle_development	D_vs_E10;stage_1W_vs_E10;stage_3W_vs_E10;stage	1	TRUE
ENSGALG00010020790	PRPH2L	PRJNA729271	2.744574889	4.59699E-19	2.744574889	stage_E14_vs_E10	ordinary_muscle_development	D_vs_E10;stage_1W_vs_E10;stage_3W_vs_E10;stage	1	TRUE
ENSGALG00010029506	OXLD1	PRJNA729271	-1.608042762	4.70443E-19	1.608042762	stage_E14_vs_E10	ordinary_muscle_development	D_vs_E10;stage_1W_vs_E10;stage_3W_vs_E10;stage	1	TRUE
ENSGALG00010017327	CLN6	PRJNA729271	1.189247112	4.88319E-19	1.189247112	stage_E14_vs_E10	ordinary_muscle_development	D_vs_E10;stage_1W_vs_E10;stage_3W_vs_E10;stage	1	TRUE
ENSGALG00010023168	SPHKAP	PRJNA729271	-3.945589199	5.01898E-19	3.945589199	stage_E14_vs_E10	ordinary_muscle_development	D_vs_E10;stage_1W_vs_E10;stage_3W_vs_E10;stage	1	TRUE
ENSGALG00010004350	MOXD1	PRJNA729271	2.164637426	5.05094E-19	2.164637426	stage_E14_vs_E10	ordinary_muscle_development	D_vs_E10;stage_1W_vs_E10;stage_3W_vs_E10;stage	1	TRUE
ENSGALG00010029296	FBXO39	PRJNA729271	-1.465345593	5.05726E-19	1.465345593	stage_E14_vs_E10	ordinary_muscle_development	D_vs_E10;stage_1W_vs_E10;stage_3W_vs_E10;stage	1	TRUE
ENSGALG00010019994	PLD4	PRJNA729271	5.140354468	5.09155E-19	5.140354468	stage_E14_vs_E10	ordinary_muscle_development	D_vs_E10;stage_1W_vs_E10;stage_3W_vs_E10;stage	1	TRUE
ENSGALG00010004729	ENSGALG00010004729	PRJNA729271	9.801756456	5.24646E-19	9.801756456	stage_E14_vs_E10	ordinary_muscle_development	D_vs_E10;stage_1W_vs_E10;stage_3W_vs_E10;stage	1	TRUE
ENSGALG00010007188	MAB21L1	PRJNA729271	-2.019347276	5.25858E-19	2.019347276	stage_E14_vs_E10	ordinary_muscle_development	D_vs_E10;stage_1W_vs_E10;stage_3W_vs_E10;stage	1	TRUE
ENSGALG00010021457	USB1	PRJNA729271	1.018750566	5.26459E-19	1.018750566	stage_E14_vs_E10	ordinary_muscle_development	D_vs_E10;stage_1W_vs_E10;stage_3W_vs_E10;stage	1	TRUE
ENSGALG00010004507	CUL2	PRJNA729271	0.813459481	5.26972E-19	0.813459481	stage_E14_vs_E10	ordinary_muscle_development		0	FALSE
ENSGALG00010013898	EMB	PRJNA729271	-2.71697396	5.37769E-19	2.71697396	stage_E14_vs_E10	ordinary_muscle_development	D_vs_E10;stage_1W_vs_E10;stage_3W_vs_E10;stage	1	TRUE
ENSGALG00010027634	OASL	PRJNA729271	6.442610635	5.39879E-19	6.442610635	stage_E14_vs_E10	ordinary_muscle_development	D_vs_E10;stage_1W_vs_E10;stage_3W_vs_E10;stage	1	TRUE
ENSGALG00010005277	ENSGALG00010005277	PRJNA729271	1.810807214	5.40526E-19	1.810807214	stage_E14_vs_E10	ordinary_muscle_development	D_vs_E10;stage_1W_vs_E10;stage_3W_vs_E10;stage	1	TRUE
ENSGALG00010017096	DCTN5	PRJNA729271	-0.836354644	5.55559E-19	0.836354644	stage_E14_vs_E10	ordinary_muscle_development		0	FALSE
ENSGALG00010006627	SF3B5	PRJNA729271	-1.013556714	5.64683E-19	1.013556714	stage_E14_vs_E10	ordinary_muscle_development	D_vs_E10;stage_1W_vs_E10;stage_3W_vs_E10;stage	1	TRUE
ENSGALG00010029350	MRPL12	PRJNA729271	1.097001785	5.75893E-19	1.097001785	stage_E14_vs_E10	ordinary_muscle_development	D_vs_E10;stage_1W_vs_E10;stage_3W_vs_E10;stage	1	TRUE
ENSGALG00010012354	DCBLD1	PRJNA729271	1.350309207	5.78264E-19	1.350309207	stage_E14_vs_E10	ordinary_muscle_development	D_vs_E10;stage_1W_vs_E10;stage_3W_vs_E10;stage	1	TRUE
ENSGALG00010022837	ITGB3	PRJNA729271	-1.375990164	5.81325E-19	1.375990164	stage_E14_vs_E10	ordinary_muscle_development	D_vs_E10;stage_1W_vs_E10;stage_3W_vs_E10;stage	1	TRUE
ENSGALG00010006821	XRCC4	PRJNA729271	-1.187030032	5.90257E-19	1.187030032	stage_E14_vs_E10	ordinary_muscle_development	D_vs_E10;stage_1W_vs_E10;stage_3W_vs_E10;stage	1	TRUE
ENSGALG00010029416	UNC119	PRJNA729271	-4.729684105	5.91147E-19	4.729684105	stage_E14_vs_E10	ordinary_muscle_development	D_vs_E10;stage_1W_vs_E10;stage_3W_vs_E10;stage	1	TRUE
ENSGALG00010005004	CDK6	PRJNA729271	-0.893351857	5.96491E-19	0.893351857	stage_E14_vs_E10	ordinary_muscle_development		0	FALSE
ENSGALG00010002704	KCNB2	PRJNA729271	2.281708396	6.04479E-19	2.281708396	stage_E14_vs_E10	ordinary_muscle_development	D_vs_E10;stage_1W_vs_E10;stage_3W_vs_E10;stage	1	TRUE
ENSGALG00010027026	SLC45A3	PRJNA729271	1.771272513	6.12371E-19	1.771272513	stage_E14_vs_E10	ordinary_muscle_development	D_vs_E10;stage_1W_vs_E10;stage_3W_vs_E10;stage	1	TRUE
ENSGALG00010003729	RARS2	PRJNA729271	-0.960327332	6.14768E-19	0.960327332	stage_E14_vs_E10	ordinary_muscle_development		0	FALSE
ENSGALG00010009065	EIF2B4	PRJNA729271	0.84944286	6.15508E-19	0.84944286	stage_E14_vs_E10	ordinary_muscle_development		0	FALSE
ENSGALG00010001686	WASL	PRJNA729271	0.769320093	6.29344E-19	0.769320093	stage_E14_vs_E10	ordinary_muscle_development		0	FALSE
ENSGALG00010009615	ENSGALG00010009615	PRJNA729271	3.567927087	6.3527E-19	3.567927087	stage_E14_vs_E10	ordinary_muscle_development	D_vs_E10;stage_1W_vs_E10;stage_3W_vs_E10;stage	1	TRUE
ENSGALG00010005699	PRPF18	PRJNA729271	0.83383648	6.36053E-19	0.83383648	stage_E14_vs_E10	ordinary_muscle_development		0	FALSE
ENSGALG00010002896	OCA2	PRJNA729271	-2.90323112	6.5482E-19	2.90323112	stage_E14_vs_E10	ordinary_muscle_development	D_vs_E10;stage_1W_vs_E10;stage_3W_vs_E10;stage	1	TRUE
ENSGALG00010020152	ATXN7	PRJNA729271	0.968517619	6.56158E-19	0.968517619	stage_E14_vs_E10	ordinary_muscle_development		0	FALSE
ENSGALG00010026261	IFT70A	PRJNA729271	-0.986965885	6.59563E-19	0.986965885	stage_E14_vs_E10	ordinary_muscle_development		0	FALSE
ENSGALG00010021041	GLTP	PRJNA729271	-1.98599488	6.62776E-19	1.98599488	stage_E14_vs_E10	ordinary_muscle_development	D_vs_E10;stage_1W_vs_E10;stage_3W_vs_E10;stage	1	TRUE
ENSGALG00010002293	GYG2	PRJNA729271	-3.503009151	6.64434E-19	3.503009151	stage_E14_vs_E10	ordinary_muscle_development	D_vs_E10;stage_1W_vs_E10;stage_3W_vs_E10;stage	1	TRUE
ENSGALG00010020290	NELL1	PRJNA729271	-6.25638635	6.67086E-19	6.25638635	stage_E14_vs_E10	ordinary_muscle_development	D_vs_E10;stage_1W_vs_E10;stage_3W_vs_E10;stage	1	TRUE
ENSGALG00010000028	ND4L	PRJNA729271	2.445911529	6.72021E-19	2.445911529	stage_E14_vs_E10	ordinary_muscle_development	D_vs_E10;stage_1W_vs_E10;stage_3W_vs_E10;stage	1	TRUE
ENSGALG00010023282	SERBP1	PRJNA729271	-0.78917941	6.77389E-19	0.78917941	stage_E14_vs_E10	ordinary_muscle_development		0	FALSE
ENSGALG00010025067	SLC1A2	PRJNA729271	-3.114317615	6.79351E-19	3.114317615	stage_E14_vs_E10	ordinary_muscle_development	D_vs_E10;stage_1W_vs_E10;stage_3W_vs_E10;stage	1	TRUE
ENSGALG00010022221	CUTC	PRJNA729271	0.734109466	6.81027E-19	0.734109466	stage_E14_vs_E10	ordinary_muscle_development		0	FALSE
ENSGALG00010016409	GMEB2	PRJNA729271	1.855726555	6.8309E-19	1.855726555	stage_E14_vs_E10	ordinary_muscle_development	D_vs_E10;stage_1W_vs_E10;stage_3W_vs_E10;stage	1	TRUE
ENSGALG00010011126	ATXN1	PRJNA729271	1.248326568	6.94543E-19	1.248326568	stage_E14_vs_E10	ordinary_muscle_development	D_vs_E10;stage_1W_vs_E10;stage_3W_vs_E10;stage	1	TRUE
ENSGALG00010015091	MSN	PRJNA729271	-1.095082274	6.9831E-19	1.095082274	stage_E14_vs_E10	ordinary_muscle_development	D_vs_E10;stage_1W_vs_E10;stage_3W_vs_E10;stage	1	TRUE
ENSGALG00010016677	MEAF6	PRJNA729271	0.792122929	6.9831E-19	0.792122929	stage_E14_vs_E10	ordinary_muscle_development		0	FALSE
ENSGALG00010015905	ANPEP	PRJNA729271	1.960877126	7.04582E-19	1.960877126	stage_E14_vs_E10	ordinary_muscle_development	D_vs_E10;stage_1W_vs_E10;stage_3W_vs_E10;stage	1	TRUE
ENSGALG00010027944	WDR31	PRJNA729271	-3.399307994	7.07563E-19	3.399307994	stage_E14_vs_E10	ordinary_muscle_development	D_vs_E10;stage_1W_vs_E10;stage_3W_vs_E10;stage	1	TRUE
ENSGALG00010002730	E2F6	PRJNA729271	0.902080678	7.12106E-19	0.902080678	stage_E14_vs_E10	ordinary_muscle_development		0	FALSE
ENSGALG00010011427	TMTC3	PRJNA729271	-0.797195661	7.37551E-19	0.797195661	stage_E14_vs_E10	ordinary_muscle_development		0	FALSE
ENSGALG00010017799	GRAPL	PRJNA729271	2.713705845	7.38255E-19	2.713705845	stage_E14_vs_E10	ordinary_muscle_development	D_vs_E10;stage_1W_vs_E10;stage_3W_vs_E10;stage	1	TRUE
ENSGALG00010028318	NACC2	PRJNA729271	1.698611015	7.38929E-19	1.698611015	stage_E14_vs_E10	ordinary_muscle_development	D_vs_E10;stage_1W_vs_E10;stage_3W_vs_E10;stage	1	TRUE
ENSGALG00010028610	ENSGALG00010028610	PRJNA729271	4.770459795	7.44379E-19	4.770459795	stage_E14_vs_E10	ordinary_muscle_development	D_vs_E10;stage_1W_vs_E10;stage_3W_vs_E10;stage	1	TRUE
ENSGALG00010012253	MYF6	PRJNA729271	2.603511818	7.64315E-19	2.603511818	stage_E14_vs_E10	ordinary_muscle_development	D_vs_E10;stage_1W_vs_E10;stage_3W_vs_E10;stage	1	TRUE
ENSGALG00010005414	ENSGALG00010005414	PRJNA729271	-2.229641587	7.65317E-19	2.229641587	stage_E14_vs_E10	ordinary_muscle_development	D_vs_E10;stage_1W_vs_E10;stage_3W_vs_E10;stage	1	TRUE
ENSGALG00010021535	SUSD6	PRJNA729271	0.771114036	7.7128E-19	0.771114036	stage_E14_vs_E10	ordinary_muscle_development		0	FALSE
ENSGALG00010028421	ZBTB7A	PRJNA729271	1.69572942	7.72287E-19	1.69572942	stage_E14_vs_E10	ordinary_muscle_development	D_vs_E10;stage_1W_vs_E10;stage_3W_vs_E10;stage	1	TRUE
ENSGALG00010007106	SLC7A1	PRJNA729271	-2.187461257	7.80554E-19	2.187461257	stage_E14_vs_E10	ordinary_muscle_development	D_vs_E10;stage_1W_vs_E10;stage_3W_vs_E10;stage	1	TRUE
ENSGALG00010029563	ADPRM	PRJNA729271	1.119918377	7.83056E-19	1.119918377	stage_E14_vs_E10	ordinary_muscle_development	D_vs_E10;stage_1W_vs_E10;stage_3W_vs_E10;stage	1	TRUE
ENSGALG00010009143	MCCC2	PRJNA729271	1.21638596	8.10388E-19	1.21638596	stage_E14_vs_E10	ordinary_muscle_development	D_vs_E10;stage_1W_vs_E10;stage_3W_vs_E10;stage	1	TRUE
ENSGALG00010023592	BAHD1	PRJNA729271	-0.956360961	8.15291E-19	0.956360961	stage_E14_vs_E10	ordinary_muscle_development		0	FALSE
ENSGALG00010010011	EPB41L4A	PRJNA729271	1.097519146	8.17807E-19	1.097519146	stage_E14_vs_E10	ordinary_muscle_development	D_vs_E10;stage_1W_vs_E10;stage_3W_vs_E10;stage	1	TRUE
ENSGALG00010005760	GPR18	PRJNA729271	-3.616479384	8.23122E-19	3.616479384	stage_E14_vs_E10	ordinary_muscle_development	D_vs_E10;stage_1W_vs_E10;stage_3W_vs_E10;stage	1	TRUE
ENSGALG00010001363	HIBADH	PRJNA729271	1.892858903	8.3605E-19	1.892858903	stage_E14_vs_E10	ordinary_muscle_development	D_vs_E10;stage_1W_vs_E10;stage_3W_vs_E10;stage	1	TRUE
ENSGALG00010001198	IFRD1	PRJNA729271	1.583957998	8.38114E-19	1.583957998	stage_E14_vs_E10	ordinary_muscle_development	D_vs_E10;stage_1W_vs_E10;stage_3W_vs_E10;stage	1	TRUE
ENSGALG00010015563	B3GNT7	PRJNA729271	-2.95877533	8.61617E-19	2.95877533	stage_E14_vs_E10	ordinary_muscle_development	D_vs_E10;stage_1W_vs_E10;stage_3W_vs_E10;stage	1	TRUE
ENSGALG00010024621	THRA	PRJNA729271	1.665684921	8.7995E-19	1.665684921	stage_E14_vs_E10	ordinary_muscle_development	D_vs_E10;stage_1W_vs_E10;stage_3W_vs_E10;stage	1	TRUE
ENSGALG00010027188	KCNH2	PRJNA729271	2.295607227	8.88029E-19	2.295607227	stage_E14_vs_E10	ordinary_muscle_development	D_vs_E10;stage_1W_vs_E10;stage_3W_vs_E10;stage	1	TRUE
ENSGALG00010010642	CC2D2A	PRJNA729271	-1.092148304	9.02168E-19	1.092148304	stage_E14_vs_E10	ordinary_muscle_development	D_vs_E10;stage_1W_vs_E10;stage_3W_vs_E10;stage	1	TRUE
ENSGALG00010000076	ENSGALG00010000076	PRJNA729271	1.678107519	9.06328E-19	1.678107519	stage_E14_vs_E10	ordinary_muscle_development	D_vs_E10;stage_1W_vs_E10;stage_3W_vs_E10;stage	1	TRUE
ENSGALG00010016489	CCK	PRJNA729271	-7.915498773	9.19109E-19	7.915498773	stage_E14_vs_E10	ordinary_muscle_development	D_vs_E10;stage_1W_vs_E10;stage_3W_vs_E10;stage	1	TRUE
ENSGALG00010024712	LRIF1	PRJNA729271	0.879066898	9.20353E-19	0.879066898	stage_E14_vs_E10	ordinary_muscle_development		0	FALSE
ENSGALG00010020742	THAP10	PRJNA729271	-1.962358241	9.25773E-19	1.962358241	stage_E14_vs_E10	ordinary_muscle_development	D_vs_E10;stage_1W_vs_E10;stage_3W_vs_E10;stage	1	TRUE
ENSGALG00010019906	ENSGALG00010019906	PRJNA729271	2.091330428	9.30882E-19	2.091330428	stage_E14_vs_E10	ordinary_muscle_development	D_vs_E10;stage_1W_vs_E10;stage_3W_vs_E10;stage	1	TRUE
ENSGALG00010010154	IQSEC3	PRJNA729271	-7.33261172	9.4713E-19	7.33261172	stage_E14_vs_E10	ordinary_muscle_development	D_vs_E10;stage_1W_vs_E10;stage_3W_vs_E10;stage	1	TRUE
ENSGALG00010028442	CHTOP	PRJNA729271	-0.818984702	9.48374E-19	0.818984702	stage_E14_vs_E10	ordinary_muscle_development		0	FALSE
ENSGALG00010006201	SSTR1	PRJNA729271	-3.479972052	9.55712E-19	3.479972052	stage_E14_vs_E10	ordinary_muscle_development	D_vs_E10;stage_1W_vs_E10;stage_3W_vs_E10;stage	1	TRUE
ENSGALG00010029947	CCL5	PRJNA729271	8.768864045	9.69754E-19	8.768864045	stage_E14_vs_E10	ordinary_muscle_development	D_vs_E10;stage_1W_vs_E10;stage_3W_vs_E10;stage	1	TRUE
ENSGALG00010023881	VSTM2B	PRJNA729271	-2.180312692	9.79563E-19	2.180312692	stage_E14_vs_E10	ordinary_muscle_development	D_vs_E10;stage_1W_vs_E10;stage_3W_vs_E10;stage	1	TRUE
ENSGALG00010007991	PCF11	PRJNA729271	0.984065675	9.80183E-19	0.984065675	stage_E14_vs_E10	ordinary_muscle_development		0	FALSE
ENSGALG00010018440	MEA1	PRJNA729271	0.905132587	9.84912E-19	0.905132587	stage_E14_vs_E10	ordinary_muscle_development		0	FALSE
ENSGALG00010012538	RAP1B	PRJNA729271	-0.793235736	9.85154E-19	0.793235736	stage_E14_vs_E10	ordinary_muscle_development		0	FALSE
ENSGALG00010021932	RPAP2	PRJNA729271	1.448884527	9.92057E-19	1.448884527	stage_E14_vs_E10	ordinary_muscle_development	D_vs_E10;stage_1W_vs_E10;stage_3W_vs_E10;stage	1	TRUE
ENSGALG00010029457	ENSGALG00010029457	PRJNA729271	-1.725824905	9.95334E-19	1.725824905	stage_E14_vs_E10	ordinary_muscle_development	D_vs_E10;stage_1W_vs_E10;stage_3W_vs_E10;stage	1	TRUE
ENSGALG00010027455	R3HDM4	PRJNA729271	1.318260375	1.0093E-18	1.318260375	stage_E14_vs_E10	ordinary_muscle_development	D_vs_E10;stage_1W_vs_E10;stage_3W_vs_E10;stage	1	TRUE
ENSGALG00010008313	PSMD7	PRJNA729271	1.595179796	1.01181E-18	1.595179796	stage_E14_vs_E10	ordinary_muscle_development	D_vs_E10;stage_1W_vs_E10;stage_3W_vs_E10;stage	1	TRUE
ENSGALG00010026851	ENSGALG00010026851	PRJNA729271	10.08368057	1.02121E-18	10.08368057	stage_E14_vs_E10	ordinary_muscle_development	D_vs_E10;stage_1W_vs_E10;stage_3W_vs_E10;stage	1	TRUE
ENSGALG00010029366	MGAT5B	PRJNA729271	3.03966337	1.03E-18	3.03966337	stage_E14_vs_E10	ordinary_muscle_development	D_vs_E10;stage_1W_vs_E10;stage_3W_vs_E10;stage	1	TRUE
ENSGALG00010015494	CHRDL1	PRJNA729271	-2.225284249	1.03162E-18	2.225284249	stage_E14_vs_E10	ordinary_muscle_development	D_vs_E10;stage_1W_vs_E10;stage_3W_vs_E10;stage	1	TRUE
ENSGALG00010008143	DCTN6	PRJNA729271	1.029811512	1.03534E-18	1.029811512	stage_E14_vs_E10	ordinary_muscle_development	D_vs_E10;stage_1W_vs_E10;stage_3W_vs_E10;stage	1	TRUE
ENSGALG00010016564	SPEN	PRJNA729271	1.493223082	1.05138E-18	1.493223082	stage_E14_vs_E10	ordinary_muscle_development	D_vs_E10;stage_1W_vs_E10;stage_3W_vs_E10;stage	1	TRUE
ENSGALG00010013538	E2F7	PRJNA729271	-2.453974442	1.05491E-18	2.453974442	stage_E14_vs_E10	ordinary_muscle_development	D_vs_E10;stage_1W_vs_E10;stage_3W_vs_E10;stage	1	TRUE
ENSGALG00010028549	ADAMTS10	PRJNA729271	-1.620334117	1.05958E-18	1.620334117	stage_E14_vs_E10	ordinary_muscle_development	D_vs_E10;stage_1W_vs_E10;stage_3W_vs_E10;stage	1	TRUE
ENSGALG00010002886	HERC2	PRJNA729271	0.952289463	1.06083E-18	0.952289463	stage_E14_vs_E10	ordinary_muscle_development		0	FALSE
ENSGALG00010016281	SLC22A4	PRJNA729271	-2.120606295	1.12689E-18	2.120606295	stage_E14_vs_E10	ordinary_muscle_development	D_vs_E10;stage_1W_vs_E10;stage_3W_vs_E10;stage	1	TRUE
ENSGALG00010024857	SLC37A4	PRJNA729271	1.269249182	1.13935E-18	1.269249182	stage_E14_vs_E10	ordinary_muscle_development	D_vs_E10;stage_1W_vs_E10;stage_3W_vs_E10;stage	1	TRUE
ENSGALG00010011166	SOX7	PRJNA729271	1.272934585	1.14186E-18	1.272934585	stage_E14_vs_E10	ordinary_muscle_development	D_vs_E10;stage_1W_vs_E10;stage_3W_vs_E10;stage	1	TRUE
ENSGALG00010001534	SLITRK3	PRJNA729271	-3.695373067	1.14573E-18	3.695373067	stage_E14_vs_E10	ordinary_muscle_development	D_vs_E10;stage_1W_vs_E10;stage_3W_vs_E10;stage	1	TRUE
ENSGALG00010000053	HDDC3	PRJNA729271	1.016766519	1.1625E-18	1.016766519	stage_E14_vs_E10	ordinary_muscle_development	D_vs_E10;stage_1W_vs_E10;stage_3W_vs_E10;stage	1	TRUE
ENSGALG00010011996	LRRC70	PRJNA729271	1.953675953	1.1705E-18	1.953675953	stage_E14_vs_E10	ordinary_muscle_development	D_vs_E10;stage_1W_vs_E10;stage_3W_vs_E10;stage	1	TRUE
ENSGALG00010024694	GTF2H3	PRJNA729271	-0.89021571	1.17935E-18	0.89021571	stage_E14_vs_E10	ordinary_muscle_development		0	FALSE
ENSGALG00010019743	KCNU1	PRJNA729271	3.860461273	1.18046E-18	3.860461273	stage_E14_vs_E10	ordinary_muscle_development	D_vs_E10;stage_1W_vs_E10;stage_3W_vs_E10;stage	1	TRUE
ENSGALG00010024446	GRM4	PRJNA729271	-5.548780033	1.19004E-18	5.548780033	stage_E14_vs_E10	ordinary_muscle_development	D_vs_E10;stage_1W_vs_E10;stage_3W_vs_E10;stage	1	TRUE
ENSGALG00010012469	CETN2	PRJNA729271	-1.184096259	1.19295E-18	1.184096259	stage_E14_vs_E10	ordinary_muscle_development	D_vs_E10;stage_1W_vs_E10;stage_3W_vs_E10;stage	1	TRUE
ENSGALG00010003810	HMGN2P46	PRJNA729271	0.820802983	1.19492E-18	0.820802983	stage_E14_vs_E10	ordinary_muscle_development		0	FALSE
ENSGALG00010024110	CPNE1	PRJNA729271	-1.137767931	1.2093E-18	1.137767931	stage_E14_vs_E10	ordinary_muscle_development	D_vs_E10;stage_1W_vs_E10;stage_3W_vs_E10;stage	1	TRUE
ENSGALG00010029597	ADAP2	PRJNA729271	1.820082719	1.21686E-18	1.820082719	stage_E14_vs_E10	ordinary_muscle_development	D_vs_E10;stage_1W_vs_E10;stage_3W_vs_E10;stage	1	TRUE
ENSGALG00010029289	PITPNM3	PRJNA729271	-2.080556464	1.21761E-18	2.080556464	stage_E14_vs_E10	ordinary_muscle_development	D_vs_E10;stage_1W_vs_E10;stage_3W_vs_E10;stage	1	TRUE
ENSGALG00010026942	CDK18	PRJNA729271	-1.704495729	1.21956E-18	1.704495729	stage_E14_vs_E10	ordinary_muscle_development	D_vs_E10;stage_1W_vs_E10;stage_3W_vs_E10;stage	1	TRUE
ENSGALG00010012232	PCSK1	PRJNA729271	3.721805676	1.25938E-18	3.721805676	stage_E14_vs_E10	ordinary_muscle_development	D_vs_E10;stage_1W_vs_E10;stage_3W_vs_E10;stage	1	TRUE
ENSGALG00010021879	CREBL2	PRJNA729271	1.378660163	1.28845E-18	1.378660163	stage_E14_vs_E10	ordinary_muscle_development	D_vs_E10;stage_1W_vs_E10;stage_3W_vs_E10;stage	1	TRUE
ENSGALG00010017653	ENSGALG00010017653	PRJNA729271	1.543632316	1.30272E-18	1.543632316	stage_E14_vs_E10	ordinary_muscle_development	D_vs_E10;stage_1W_vs_E10;stage_3W_vs_E10;stage	1	TRUE
ENSGALG00010010951	ENSGALG00010010951	PRJNA729271	-1.211114414	1.32208E-18	1.211114414	stage_E14_vs_E10	ordinary_muscle_development	D_vs_E10;stage_1W_vs_E10;stage_3W_vs_E10;stage	1	TRUE
ENSGALG00010024147	ENSGALG00010024147	PRJNA729271	2.179178051	1.33317E-18	2.179178051	stage_E14_vs_E10	ordinary_muscle_development	D_vs_E10;stage_1W_vs_E10;stage_3W_vs_E10;stage	1	TRUE
ENSGALG00010012295	CUL4B	PRJNA729271	-0.597051934	1.35747E-18	0.597051934	stage_E14_vs_E10	ordinary_muscle_development		0	FALSE
ENSGALG00010021903	MORC2	PRJNA729271	-0.717135764	1.38245E-18	0.717135764	stage_E14_vs_E10	ordinary_muscle_development		0	FALSE
ENSGALG00010003371	ENSGALG00010003371	PRJNA729271	-3.493156278	1.38359E-18	3.493156278	stage_E14_vs_E10	ordinary_muscle_development	D_vs_E10;stage_1W_vs_E10;stage_3W_vs_E10;stage	1	TRUE
ENSGALG00010012614	LHFPL2	PRJNA729271	-1.30273844	1.40433E-18	1.30273844	stage_E14_vs_E10	ordinary_muscle_development	D_vs_E10;stage_1W_vs_E10;stage_3W_vs_E10;stage	1	TRUE
ENSGALG00010011692	ARL1	PRJNA729271	-0.804022495	1.42828E-18	0.804022495	stage_E14_vs_E10	ordinary_muscle_development		0	FALSE
ENSGALG00010000635	ENSGALG00010000635	PRJNA729271	-0.841787677	1.50886E-18	0.841787677	stage_E14_vs_E10	ordinary_muscle_development		0	FALSE
ENSGALG00010007789	WWOX	PRJNA729271	-1.030724048	1.5293E-18	1.030724048	stage_E14_vs_E10	ordinary_muscle_development	D_vs_E10;stage_1W_vs_E10;stage_3W_vs_E10;stage	1	TRUE
ENSGALG00010011124	CACYBP	PRJNA729271	0.601548406	1.53732E-18	0.601548406	stage_E14_vs_E10	ordinary_muscle_development		0	FALSE
ENSGALG00010002565	MRPS18C	PRJNA729271	1.018241881	1.54558E-18	1.018241881	stage_E14_vs_E10	ordinary_muscle_development	D_vs_E10;stage_1W_vs_E10;stage_3W_vs_E10;stage	1	TRUE
ENSGALG00010002118	ENSGALG00010002118	PRJNA729271	2.348080432	1.57732E-18	2.348080432	stage_E14_vs_E10	ordinary_muscle_development	D_vs_E10;stage_1W_vs_E10;stage_3W_vs_E10;stage	1	TRUE
ENSGALG00010027972	TMEM259	PRJNA729271	0.938396932	1.59326E-18	0.938396932	stage_E14_vs_E10	ordinary_muscle_development		0	FALSE
ENSGALG00010010754	DNAJ1	PRJNA729271	-2.68344149	1.60212E-18	2.68344149	stage_E14_vs_E10	ordinary_muscle_development	D_vs_E10;stage_1W_vs_E10;stage_3W_vs_E10;stage	1	TRUE
ENSGALG00010022033	RAPH1	PRJNA729271	-1.066671073	1.63193E-18	1.066671073	stage_E14_vs_E10	ordinary_muscle_development	D_vs_E10;stage_1W_vs_E10;stage_3W_vs_E10;stage	1	TRUE
ENSGALG00010008374	ZNF821	PRJNA729271	-0.786614085	1.63674E-18	0.786614085	stage_E14_vs_E10	ordinary_muscle_development		0	FALSE
ENSGALG00010026516	ENSGALG00010026516	PRJNA729271	1.495910028	1.6408E-18	1.495910028	stage_E14_vs_E10	ordinary_muscle_development	D_vs_E10;stage_1W_vs_E10;stage_3W_vs_E10;stage	1	TRUE
ENSGALG00010007175	FOXC2	PRJNA729271	-4.460090364	1.6621E-18	4.460090364	stage_E14_vs_E10	ordinary_muscle_development	D_vs_E10;stage_1W_vs_E10;stage_3W_vs_E10;stage	1	TRUE
ENSGALG00010013498	KDR	PRJNA729271	1.116845562	1.68711E-18	1.116845562	stage_E14_vs_E10	ordinary_muscle_development	D_vs_E10;stage_1W_vs_E10;stage_3W_vs_E10;stage	1	TRUE
ENSGALG00010020509	CRBN	PRJNA729271	0.915944988	1.69224E-18	0.915944988	stage_E14_vs_E10	ordinary_muscle_development		0	FALSE
ENSGALG00010005380	XCL1	PRJNA729271	9.037524926	1.70942E-18	9.037524926	stage_E14_vs_E10	ordinary_muscle_development	D_vs_E10;stage_1W_vs_E10;stage_3W_vs_E10;stage	1	TRUE
ENSGALG00010024051	ENSGALG00010024051	PRJNA729271	-7.730412603	1.71844E-18	7.730412603	stage_E14_vs_E10	ordinary_muscle_development	D_vs_E10;stage_1W_vs_E10;stage_3W_vs_E10;stage	1	TRUE
ENSGALG00010025637	C6orf89	PRJNA729271	1.132078373	1.72189E-18	1.132078373	stage_E14_vs_E10	ordinary_muscle_development	D_vs_E10;stage_1W_vs_E10;stage_3W_vs_E10;stage	1	TRUE
ENSGALG00010019673	NID2	PRJNA729271	1.190718703	1.72831E-18	1.190718703	stage_E14_vs_E10	ordinary_muscle_development	D_vs_E10;stage_1W_vs_E10;stage_3W_vs_E10;stage	1	TRUE
ENSGALG00010018060	CASKIN1	PRJNA729271	-3.689043642	1.74685E-18	3.689043642	stage_E14_vs_E10	ordinary_muscle_development	D_vs_E10;stage_1W_vs_E10;stage_3W_vs_E10;stage	1	TRUE
ENSGALG00010024845	HYCC2	PRJNA729271	0.969370002	1.77425E-18	0.969370002	stage_E14_vs_E10	ordinary_muscle_development		0	FALSE
ENSGALG00010028131	TMEM161A	PRJNA729271	1.800827742	1.79468E-18	1.800827742	stage_E14_vs_E10	ordinary_muscle_development	D_vs_E10;stage_1W_vs_E10;stage_3W_vs_E10;stage	1	TRUE
ENSGALG00010024238	ATF7	PRJNA729271	1.794946641	1.7962E-18	1.794946641	stage_E14_vs_E10	ordinary_muscle_development	D_vs_E10;stage_1W_vs_E10;stage_3W_vs_E10;stage	1	TRUE

ENSGALG00010013910	ADIPOR2	PRJNA729271	1.852114335	1.81368E-18	1.852114335	stage_E14_vs_E10	ordinary_muscle_development	D_vs_E10;stage_1W_vs_E10;stage_3W_vs_E10;stage	1	TRUE
ENSGALG00010027243	HRH1	PRJNA729271	-2.015230381	1.85157E-18	2.015230381	stage_E14_vs_E10	ordinary_muscle_development	D_vs_E10;stage_1W_vs_E10;stage_3W_vs_E10;stage	1	TRUE
ENSGALG00010013994	CHRM2	PRJNA729271	-5.500315376	1.85524E-18	5.500315376	stage_E14_vs_E10	ordinary_muscle_development	D_vs_E10;stage_1W_vs_E10;stage_3W_vs_E10;stage	1	TRUE
ENSGALG00010027386	RHOA	PRJNA729271	-0.591072772	1.87287E-18	0.591072772	stage_E14_vs_E10	ordinary_muscle_development		0	FALSE
ENSGALG00010018192	IQCH	PRJNA729271	-2.04944898	1.93475E-18	2.04944898	stage_E14_vs_E10	ordinary_muscle_development	D_vs_E10;stage_1W_vs_E10;stage_3W_vs_E10;stage	1	TRUE
ENSGALG00010005285	MPP7	PRJNA729271	1.135387177	1.93659E-18	1.135387177	stage_E14_vs_E10	ordinary_muscle_development	D_vs_E10;stage_1W_vs_E10;stage_3W_vs_E10;stage	1	TRUE
ENSGALG00010001488	GRPR	PRJNA729271	-4.614259057	1.94339E-18	4.614259057	stage_E14_vs_E10	ordinary_muscle_development	D_vs_E10;stage_1W_vs_E10;stage_3W_vs_E10;stage	1	TRUE
ENSGALG00010001546	CGAS	PRJNA729271	-1.568553685	1.9596E-18	1.568553685	stage_E14_vs_E10	ordinary_muscle_development	D_vs_E10;stage_1W_vs_E10;stage_3W_vs_E10;stage	1	TRUE
ENSGALG00010019072	AEBP1	PRJNA729271	1.894437561	1.97365E-18	1.894437561	stage_E14_vs_E10	ordinary_muscle_development	D_vs_E10;stage_1W_vs_E10;stage_3W_vs_E10;stage	1	TRUE
ENSGALG00010028386	NCAN	PRJNA729271	-7.862099443	1.97793E-18	7.862099443	stage_E14_vs_E10	ordinary_muscle_development	D_vs_E10;stage_1W_vs_E10;stage_3W_vs_E10;stage	1	TRUE
ENSGALG00010026746	HOXD10	PRJNA729271	-4.815071902	1.98772E-18	4.815071902	stage_E14_vs_E10	ordinary_muscle_development	D_vs_E10;stage_1W_vs_E10;stage_3W_vs_E10;stage	1	TRUE
ENSGALG00010025230	CHST8	PRJNA729271	-3.746623829	1.99278E-18	3.746623829	stage_E14_vs_E10	ordinary_muscle_development	D_vs_E10;stage_1W_vs_E10;stage_3W_vs_E10;stage	1	TRUE
ENSGALG00010018051	GNPNAT1	PRJNA729271	-1.242281203	2.00231E-18	1.242281203	stage_E14_vs_E10	ordinary_muscle_development	D_vs_E10;stage_1W_vs_E10;stage_3W_vs_E10;stage	1	TRUE
ENSGALG00010029226	MBTD1	PRJNA729271	-0.679863497	2.03364E-18	0.679863497	stage_E14_vs_E10	ordinary_muscle_development		0	FALSE
ENSGALG00010006341	HHIP	PRJNA729271	-2.020457141	2.0507E-18	2.020457141	stage_E14_vs_E10	ordinary_muscle_development	D_vs_E10;stage_1W_vs_E10;stage_3W_vs_E10;stage	1	TRUE
ENSGALG00010018891	GNRH1	PRJNA729271	-4.06035845	2.09739E-18	4.06035845	stage_E14_vs_E10	ordinary_muscle_development	D_vs_E10;stage_1W_vs_E10;stage_3W_vs_E10;stage	1	TRUE
ENSGALG00010022540	GPR146	PRJNA729271	2.120242285	2.18083E-18	2.120242285	stage_E14_vs_E10	ordinary_muscle_development	D_vs_E10;stage_1W_vs_E10;stage_3W_vs_E10;stage	1	TRUE
ENSGALG00010012867	A4GALT	PRJNA729271	0.908039017	2.19533E-18	0.908039017	stage_E14_vs_E10	ordinary_muscle_development		0	FALSE
ENSGALG00010023967	PRPH	PRJNA729271	-3.690952614	2.21699E-18	3.690952614	stage_E14_vs_E10	ordinary_muscle_development	D_vs_E10;stage_1W_vs_E10;stage_3W_vs_E10;stage	1	TRUE
ENSGALG00010005447	MYO3A	PRJNA729271	-4.54279065	2.21734E-18	4.54279065	stage_E14_vs_E10	ordinary_muscle_development	D_vs_E10;stage_1W_vs_E10;stage_3W_vs_E10;stage	1	TRUE
ENSGALG00010006531	FAM161B	PRJNA729271	-1.299798485	2.22865E-18	1.299798485	stage_E14_vs_E10	ordinary_muscle_development	D_vs_E10;stage_1W_vs_E10;stage_3W_vs_E10;stage	1	TRUE
ENSGALG00010023213	FCSK	PRJNA729271	1.004637918	2.23909E-18	1.004637918	stage_E14_vs_E10	ordinary_muscle_development	D_vs_E10;stage_1W_vs_E10;stage_3W_vs_E10;stage	1	TRUE
ENSGALG00010008121	MPZL1	PRJNA729271	-1.208976196	2.26937E-18	1.208976196	stage_E14_vs_E10	ordinary_muscle_development	D_vs_E10;stage_1W_vs_E10;stage_3W_vs_E10;stage	1	TRUE
ENSGALG00010023374	ENSGALG00010023374	PRJNA729271	-1.950757794	2.27529E-18	1.950757794	stage_E14_vs_E10	ordinary_muscle_development	D_vs_E10;stage_1W_vs_E10;stage_3W_vs_E10;stage	1	TRUE
ENSGALG00010011681	MAP1B	PRJNA729271	-3.194115978	2.27907E-18	3.194115978	stage_E14_vs_E10	ordinary_muscle_development	D_vs_E10;stage_1W_vs_E10;stage_3W_vs_E10;stage	1	TRUE
ENSGALG00010020453	ENSGALG00010020453	PRJNA729271	-1.316100136	2.28505E-18	1.316100136	stage_E14_vs_E10	ordinary_muscle_development	D_vs_E10;stage_1W_vs_E10;stage_3W_vs_E10;stage	1	TRUE
ENSGALG00010010778	UPP2	PRJNA729271	3.241657913	2.29548E-18	3.241657913	stage_E14_vs_E10	ordinary_muscle_development	D_vs_E10;stage_1W_vs_E10;stage_3W_vs_E10;stage	1	TRUE
ENSGALG00010027159	TMUB1	PRJNA729271	1.449192147	2.29663E-18	1.449192147	stage_E14_vs_E10	ordinary_muscle_development	D_vs_E10;stage_1W_vs_E10;stage_3W_vs_E10;stage	1	TRUE
ENSGALG00010011329	PCDHGC5	PRJNA729271	-1.042111638	2.31298E-18	1.042111638	stage_E14_vs_E10	ordinary_muscle_development	D_vs_E10;stage_1W_vs_E10;stage_3W_vs_E10;stage	1	TRUE
ENSGALG00010028502	PLEKHO1	PRJNA729271	1.355272062	2.32464E-18	1.355272062	stage_E14_vs_E10	ordinary_muscle_development	D_vs_E10;stage_1W_vs_E10;stage_3W_vs_E10;stage	1	TRUE
ENSGALG00010011184	FAM8A1	PRJNA729271	1.201000736	2.37371E-18	1.201000736	stage_E14_vs_E10	ordinary_muscle_development	D_vs_E10;stage_1W_vs_E10;stage_3W_vs_E10;stage	1	TRUE
ENSGALG00010009019	ZNF513	PRJNA729271	1.181579325	2.37968E-18	1.181579325	stage_E14_vs_E10	ordinary_muscle_development	D_vs_E10;stage_1W_vs_E10;stage_3W_vs_E10;stage	1	TRUE
ENSGALG00010029608	ENSGALG00010029608	PRJNA729271	-3.351629139	2.3803E-18	3.351629139	stage_E14_vs_E10	ordinary_muscle_development	D_vs_E10;stage_1W_vs_E10;stage_3W_vs_E10;stage	1	TRUE
ENSGALG00010003868	HBBA	PRJNA729271	-3.546654442	2.3818E-18	3.546654442	stage_E14_vs_E10	ordinary_muscle_development	D_vs_E10;stage_1W_vs_E10;stage_3W_vs_E10;stage	1	TRUE
ENSGALG00010028428	RABGAP1	PRJNA729271	0.593935768	2.39703E-18	0.593935768	stage_E14_vs_E10	ordinary_muscle_development		0	FALSE
ENSGALG00010017305	HS3ST2	PRJNA729271	4.123929803	2.41863E-18	4.123929803	stage_E14_vs_E10	ordinary_muscle_development	D_vs_E10;stage_1W_vs_E10;stage_3W_vs_E10;stage	1	TRUE
ENSGALG00010024172	ABCG4	PRJNA729271	-4.955319295	2.42512E-18	4.955319295	stage_E14_vs_E10	ordinary_muscle_development	D_vs_E10;stage_1W_vs_E10;stage_3W_vs_E10;stage	1	TRUE
ENSGALG00010002587	DOCK1	PRJNA729271	-0.594677936	2.44597E-18	0.594677936	stage_E14_vs_E10	ordinary_muscle_development		0	FALSE
ENSGALG00010002913	NXNL1	PRJNA729271	4.999799908	2.49578E-18	4.999799908	stage_E14_vs_E10	ordinary_muscle_development	D_vs_E10;stage_1W_vs_E10;stage_3W_vs_E10;stage	1	TRUE
ENSGALG00010017838	B3GNT2	PRJNA729271	-1.132918244	2.52508E-18	1.132918244	stage_E14_vs_E10	ordinary_muscle_development	D_vs_E10;stage_1W_vs_E10;stage_3W_vs_E10;stage	1	TRUE
ENSGALG00010029665	ASL1	PRJNA729271	1.675322812	2.52687E-18	1.675322812	stage_E14_vs_E10	ordinary_muscle_development	D_vs_E10;stage_1W_vs_E10;stage_3W_vs_E10;stage	1	TRUE
ENSGALG00010001218	ENSGALG00010001218	PRJNA729271	-1.001799166	2.54061E-18	1.001799166	stage_E14_vs_E10	ordinary_muscle_development	D_vs_E10;stage_1W_vs_E10;stage_3W_vs_E10;stage	1	TRUE
ENSGALG00010020008	EIF2S1	PRJNA729271	-0.713240291	2.54128E-18	0.713240291	stage_E14_vs_E10	ordinary_muscle_development		0	FALSE
ENSGALG00010000367	HSD17B10	PRJNA729271	1.229766466	2.56373E-18	1.229766466	stage_E14_vs_E10	ordinary_muscle_development	D_vs_E10;stage_1W_vs_E10;stage_3W_vs_E10;stage	1	TRUE
ENSGALG00010020558	EIF4EBP2	PRJNA729271	0.885231706	2.61815E-18	0.885231706	stage_E14_vs_E10	ordinary_muscle_development		0	FALSE
ENSGALG00010011422	KATNAL2	PRJNA729271	-2.351275584	2.62676E-18	2.351275584	stage_E14_vs_E10	ordinary_muscle_development	D_vs_E10;stage_1W_vs_E10;stage_3W_vs_E10;stage	1	TRUE
ENSGALG00010027988	HDAC11	PRJNA729271	1.134422045	2.63442E-18	1.134422045	stage_E14_vs_E10	ordinary_muscle_development	D_vs_E10;stage_1W_vs_E10;stage_3W_vs_E10;stage	1	TRUE
ENSGALG00010008400	MAP1LC3C	PRJNA729271	-2.38092493	2.65075E-18	2.38092493	stage_E14_vs_E10	ordinary_muscle_development	D_vs_E10;stage_1W_vs_E10;stage_3W_vs_E10;stage	1	TRUE
ENSGALG00010009470	ZNF469	PRJNA729271	-1.893428638	2.68492E-18	1.893428638	stage_E14_vs_E10	ordinary_muscle_development	D_vs_E10;stage_1W_vs_E10;stage_3W_vs_E10;stage	1	TRUE
ENSGALG00010006998	POLH	PRJNA729271	1.066100582	2.68896E-18	1.066100582	stage_E14_vs_E10	ordinary_muscle_development	D_vs_E10;stage_1W_vs_E10;stage_3W_vs_E10;stage	1	TRUE
ENSGALG00010025055	ALS2	PRJNA729271	1.365179685	2.69948E-18	1.365179685	stage_E14_vs_E10	ordinary_muscle_development	D_vs_E10;stage_1W_vs_E10;stage_3W_vs_E10;stage	1	TRUE
ENSGALG00010006031	ERCC5	PRJNA729271	0.853451333	2.69948E-18	0.853451333	stage_E14_vs_E10	ordinary_muscle_development		0	FALSE
ENSGALG00010002134	ANKRD28	PRJNA729271	1.069327663	2.7169E-18	1.069327663	stage_E14_vs_E10	ordinary_muscle_development	D_vs_E10;stage_1W_vs_E10;stage_3W_vs_E10;stage	1	TRUE
ENSGALG00010006361	SERTM1	PRJNA729271	-2.383690676	2.72644E-18	2.383690676	stage_E14_vs_E10	ordinary_muscle_development	D_vs_E10;stage_1W_vs_E10;stage_3W_vs_E10;stage	1	TRUE
ENSGALG00010027994	TUFT1	PRJNA729271	-1.565730137	2.75593E-18	1.565730137	stage_E14_vs_E10	ordinary_muscle_development	D_vs_E10;stage_1W_vs_E10;stage_3W_vs_E10;stage	1	TRUE
ENSGALG00010027559	ENSGALG00010027559	PRJNA729271	2.377461132	2.78067E-18	2.377461132	stage_E14_vs_E10	ordinary_muscle_development	D_vs_E10;stage_1W_vs_E10;stage_3W_vs_E10;stage	1	TRUE
ENSGALG00010022214	ENSGALG00010022214	PRJNA729271	-2.546709294	2.79894E-18	2.546709294	stage_E14_vs_E10	ordinary_muscle_development	D_vs_E10;stage_1W_vs_E10;stage_3W_vs_E10;stage	1	TRUE
ENSGALG00010002375	NPY	PRJNA729271	-5.183293025	2.90473E-18	5.183293025	stage_E14_vs_E10	ordinary_muscle_development	D_vs_E10;stage_1W_vs_E10;stage_3W_vs_E10;stage	1	TRUE
ENSGALG00010017135	ENSGALG00010017135	PRJNA729271	1.166359357	2.92599E-18	1.166359357	stage_E14_vs_E10	ordinary_muscle_development	D_vs_E10;stage_1W_vs_E10;stage_3W_vs_E10;stage	1	TRUE
ENSGALG00010006184	LBX2	PRJNA729271	-2.751010215	2.96748E-18	2.751010215	stage_E14_vs_E10	ordinary_muscle_development	D_vs_E10;stage_1W_vs_E10;stage_3W_vs_E10;stage	1	TRUE
ENSGALG00010018361	ASIC4	PRJNA729271	-2.767803544	2.96854E-18	2.767803544	stage_E14_vs_E10	ordinary_muscle_development	D_vs_E10;stage_1W_vs_E10;stage_3W_vs_E10;stage	1	TRUE
ENSGALG00010022154	CPN1	PRJNA729271	1.717892823	2.97793E-18	1.717892823	stage_E14_vs_E10	ordinary_muscle_development	D_vs_E10;stage_1W_vs_E10;stage_3W_vs_E10;stage	1	TRUE
ENSGALG00010008971	ANXA11	PRJNA729271	0.916821949	2.99062E-18	0.916821949	stage_E14_vs_E10	ordinary_muscle_development		0	FALSE
ENSGALG00010028746	MYDGF	PRJNA729271	-1.123640753	2.99424E-18	1.123640753	stage_E14_vs_E10	ordinary_muscle_development	D_vs_E10;stage_1W_vs_E10;stage_3W_vs_E10;stage	1	TRUE
ENSGALG00010014288	ARMC2	PRJNA729271	-2.273945275	3.02399E-18	2.273945275	stage_E14_vs_E10	ordinary_muscle_development	D_vs_E10;stage_1W_vs_E10;stage_3W_vs_E10;stage	1	TRUE
ENSGALG00010024035	OSBPL5	PRJNA729271	-1.222026268	3.04245E-18	1.222026268	stage_E14_vs_E10	ordinary_muscle_development	D_vs_E10;stage_1W_vs_E10;stage_3W_vs_E10;stage	1	TRUE
ENSGALG00010027227	ENSGALG00010027227	PRJNA729271	1.61830351	3.06522E-18	1.61830351	stage_E14_vs_E10	ordinary_muscle_development	D_vs_E10;stage_1W_vs_E10;stage_3W_vs_E10;stage	1	TRUE
ENSGALG00010022116	CYP20A1	PRJNA729271	-0.929275005	3.07374E-18	0.929275005	stage_E14_vs_E10	ordinary_muscle_development		0	FALSE
ENSGALG00010012312	SH3TC2	PRJNA729271	1.56439655	3.11934E-18	1.56439655	stage_E14_vs_E10	ordinary_muscle_development	D_vs_E10;stage_1W_vs_E10;stage_3W_vs_E10;stage	1	TRUE
ENSGALG00010021768	DIPK1A	PRJNA729271	1.316542345	3.1635E-18	1.316542345	stage_E14_vs_E10	ordinary_muscle_development	D_vs_E10;stage_1W_vs_E10;stage_3W_vs_E10;stage	1	TRUE
ENSGALG00010008145	ENSGALG00010008145	PRJNA729271	1.828860389	3.17008E-18	1.828860389	stage_E14_vs_E10	ordinary_muscle_development	D_vs_E10;stage_1W_vs_E10;stage_3W_vs_E10;stage	1	TRUE
ENSGALG00010029355	MCRIP1	PRJNA729271	1.524369288	3.18167E-18	1.524369288	stage_E14_vs_E10	ordinary_muscle_development	D_vs_E10;stage_1W_vs_E10;stage_3W_vs_E10;stage	1	TRUE
ENSGALG00010028468	PHPT1	PRJNA729271	-0.924375738	3.18249E-18	0.924375738	stage_E14_vs_E10	ordinary_muscle_development		0	FALSE
ENSGALG00010001458	JAZF1	PRJNA729271	-1.440489803	3.27048E-18	1.440489803	stage_E14_vs_E10	ordinary_muscle_development	D_vs_E10;stage_1W_vs_E10;stage_3W_vs_E10;stage	1	TRUE
ENSGALG00010016008	TAF7	PRJNA729271	-0.860570782	3.28128E-18	0.860570782	stage_E14_vs_E10	ordinary_muscle_development		0	FALSE
ENSGALG00010020246	ADAMTS9	PRJNA729271	-1.151534593	3.34471E-18	1.151534593	stage_E14_vs_E10	ordinary_muscle_development	D_vs_E10;stage_1W_vs_E10;stage_3W_vs_E10;stage	1	TRUE
ENSGALG00010027540	PPOX	PRJNA729271	-1.061195755	3.37233E-18	1.061195755	stage_E14_vs_E10	ordinary_muscle_development	D_vs_E10;stage_1W_vs_E10;stage_3W_vs_E10;stage	1	TRUE
ENSGALG00010022659	TIE1	PRJNA729271	1.335530109	3.37574E-18	1.335530109	stage_E14_vs_E10	ordinary_muscle_development	D_vs_E10;stage_1W_vs_E10;stage_3W_vs_E10;stage	1	TRUE
ENSGALG00010023686	MYLK3	PRJNA729271	1.94289989	3.37611E-18	1.94289989	stage_E14_vs_E10	ordinary_muscle_development	D_vs_E10;stage_1W_vs_E10;stage_3W_vs_E10;stage	1	TRUE
ENSGALG00010021003	KAZN	PRJNA729271	-1.09998557	3.38207E-18	1.09998557	stage_E14_vs_E10	ordinary_muscle_development	D_vs_E10;stage_1W_vs_E10;stage_3W_vs_E10;stage	1	TRUE
ENSGALG00010006070	TMEM242	PRJNA729271	1.003174836	3.3973E-18	1.003174836	stage_E14_vs_E10	ordinary_muscle_development	D_vs_E10;stage_1W_vs_E10;stage_3W_vs_E10;stage	1	TRUE
ENSGALG00010019265	RUFY4	PRJNA729271	5.787415872	3.42951E-18	5.787415872	stage_E14_vs_E10	ordinary_muscle_development	D_vs_E10;stage_1W_vs_E10;stage_3W_vs_E10;stage	1	TRUE
ENSGALG00010027642	DIPK1B	PRJNA729271	-1.592281615	3.45626E-18	1.592281615	stage_E14_vs_E10	ordinary_muscle_development	D_vs_E10;stage_1W_vs_E10;stage_3W_vs_E10;stage	1	TRUE
ENSGALG00010020986	CFAP20DC	PRJNA729271	-3.336402304	3.47224E-18	3.336402304	stage_E14_vs_E10	ordinary_muscle_development	D_vs_E10;stage_1W_vs_E10;stage_3W_vs_E10;stage	1	TRUE
ENSGALG00010026422	P2RX3	PRJNA729271	-3.291813492	3.52478E-18	3.291813492	stage_E14_vs_E10	ordinary_muscle_development	D_vs_E10;stage_1W_vs_E10;stage_3W_vs_E10;stage	1	TRUE
ENSGALG00010021965	PEX16	PRJNA729271	0.888253774	3.55243E-18	0.888253774	stage_E14_vs_E10	ordinary_muscle_development		0	FALSE
ENSGALG00010015516	SELENON	PRJNA729271	-1.001785001	3.65461E-18	1.001785001	stage_E14_vs_E10	ordinary_muscle_development	D_vs_E10;stage_1W_vs_E10;stage_3W_vs_E10;stage	1	TRUE
ENSGALG00010015392	TMEM165	PRJNA729271	-0.726955101	3.69272E-18	0.726955101	stage_E14_vs_E10	ordinary_muscle_development		0	FALSE
ENSGALG00010008527	CHUK	PRJNA729271	0.484037344	3.72801E-18	0.484037344	stage_E14_vs_E10	ordinary_muscle_development		0	FALSE
ENSGALG00010003468	ILVBL	PRJNA729271	1.189482971	3.74448E-18	1.189482971	stage_E14_vs_E10	ordinary_muscle_development	D_vs_E10;stage_1W_vs_E10;stage_3W_vs_E10;stage	1	TRUE
ENSGALG00010024449	CACHD1	PRJNA729271	-0.748276635	3.75103E-18	0.748276635	stage_E14_vs_E10	ordinary_muscle_development		0	FALSE
ENSGALG00010014579	NNT	PRJNA729271	1.281514567	3.77756E-18	1.281514567	stage_E14_vs_E10	ordinary_muscle_development	D_vs_E10;stage_1W_vs_E10;stage_3W_vs_E10;stage	1	TRUE
ENSGALG00010028613	ENSGALG00010028613	PRJNA729271	3.195082336	3.78963E-18	3.195082336	stage_E14_vs_E10	ordinary_muscle_development	D_vs_E10;stage_1W_vs_E10;stage_3W_vs_E10;stage	1	TRUE
ENSGALG00010001527	STK32C	PRJNA729271	1.937053193	3.83601E-18	1.937053193	stage_E14_vs_E10	ordinary_muscle_development	D_vs_E10;stage_1W_vs_E10;stage_3W_vs_E10;stage	1	TRUE
ENSGALG00010004172	ST8SIA6	PRJNA729271	-1.147793081	3.91638E-18	1.147793081	stage_E14_vs_E10	ordinary_muscle_development	D_vs_E10;stage_1W_vs_E10;stage_3W_vs_E10;stage	1	TRUE
ENSGALG00010013709	PSMF1	PRJNA729271	0.883907281	3.91804E-18	0.883907281	stage_E14_vs_E10	ordinary_muscle_development		0	FALSE
ENSGALG00010019355	ENTPD4	PRJNA729271	0.854338888	3.9391E-18	0.854338888	stage_E14_vs_E10	ordinary_muscle_development		0	FALSE
ENSGALG00010028896	ATP6V1G1	PRJNA729271	0.871021979	3.9473E-18	0.871021979	stage_E14_vs_E10	ordinary_muscle_development		0	FALSE
ENSGALG00010022311	DLL4	PRJNA729271	1.245338356	3.95814E-18	1.245338356	stage_E14_vs_E10	ordinary_muscle_development	D_vs_E10;stage_1W_vs_E10;stage_3W_vs_E10;stage	1	TRUE
ENSGALG00010001869	CRLF2	PRJNA729271	8.107566833	4.05989E-18	8.107566833	stage_E14_vs_E10	ordinary_muscle_development	D_vs_E10;stage_1W_vs_E10;stage_3W_vs_E10;stage	1	TRUE
ENSGALG00010013372	SPRY4	PRJNA729271	-2.732144465	4.07254E-18	2.732144465	stage_E14_vs_E10	ordinary_muscle_development	D_vs_E10;stage_1W_vs_E10;stage_3W_vs_E10;stage	1	TRUE
ENSGALG00010016963	CD8A	PRJNA729271	5.742284985	4.08481E-18	5.742284985	stage_E14_vs_E10	ordinary_muscle_development	D_vs_E10;stage_1W_vs_E10;stage_3W_vs_E10;stage	1	TRUE
ENSGALG00010014249	ENSGALG00010014249	PRJNA729271	0.886631288	4.08506E-18	0.886631288	stage_E14_vs_E10	ordinary_muscle_development		0	FALSE
ENSGALG00010014617	GRTP1	PRJNA729271	1.799044093	4.09358E-18	1.799044093	stage_E14_vs_E10	ordinary_muscle_development	D_vs_E10;stage_1W_vs_E10;stage_3W_vs_E10;stage	1	TRUE
ENSGALG00010005565	PGAP4	PRJNA729271	-1.596843887	4.15153E-18	1.596843887	stage_E14_vs_E10	ordinary_muscle_development	D_vs_E10;stage_1W_vs_E10;stage_3W_vs_E10;stage	1	TRUE
ENSGALG00010014878	TMCO1	PRJNA729271	1.136333876	4.19394E-18	1.136333876	stage_E14_vs_E10	ordinary_muscle_development	D_vs_E10;stage_1W_vs_E10;stage_3W_vs_E10;stage	1	TRUE
ENSGALG00010007753	PRSS23	PRJNA729271	2.008179871	4.19584E-18	2.008179871	stage_E14_vs_E10	ordinary_muscle_development	D_vs_E10;stage_1W_vs_E10;stage_3W_vs_E10;stage	1	TRUE
ENSGALG00010002411	ENSGALG00010002411	PRJNA729271	-1.767096478	4.21304E-18	1.767096478	stage_E14_vs_E10	ordinary_muscle_development	D_vs_E10;stage_1W_vs_E10;stage_3W_vs_E10;stage	1	TRUE
ENSGALG00010023366	GDAP1L1	PRJNA729271	-4.743171739	4.24776E-18	4.743171739	stage_E14_vs_E10	ordinary_muscle_development	D_vs_E10;stage_1W_vs_E10;stage_3W_vs_E10;stage	1	TRUE
ENSGALG00010026023	THY1	PRJNA729271	-2.763728337	4.27307E-18	2.763728337	stage_E14_vs_E10	ordinary_muscle_development	D_vs_E10;stage_1W_vs_E10;stage_3W_vs_E10;stage	1	TRUE
ENSGALG00010021159	MMD2	PRJNA729271	2.097148508	4.29508E-18	2.097148508	stage_E14_vs_E10	ordinary_muscle_development	D_vs_E10;stage_1W_vs_E10;stage_3W_vs_E10;stage	1	TRUE
ENSGALG00010012045	ENSGALG00010012045	PRJNA729271	1.910489145	4.2974E-18	1.910489145	stage_E14_vs_E10	ordinary_muscle_development	D_vs_E10;stage_1W_vs_E10;stage_3W_vs_E10;stage	1	TRUE
ENSGALG00010001525	EEF1A1	PRJNA729271	-1.556747527	4.4103E-18	1.556747527	stage_E14_vs_E10	ordinary_muscle_development	D_vs_E10;stage_1W_vs_E10;stage_3W_vs_E10;stage	1	TRUE
ENSGALG00010003333	ENSGALG00010003333	PRJNA729271	-24.95271153	4.44863E-18	24.95271153	stage_E14_vs_E10	ordinary_muscle_development	D_vs_E10;stage_1W_vs_E10;stage_3W_vs_E10;stage	1	TRUE
ENSGALG00010017085	ENSGALG00010017085	PRJNA729271	-1.073871037	4.45128E-18	1.073871037	stage_E14_vs_E10	ordinary_muscle_development	D_vs_E10;stage_1W_vs_E10;stage_3W_vs_E10;stage	1	TRUE
ENSGALG00010027568	NELFB	PRJNA729271	-0.687938209	4.55123E-18	0.687938209	stage_E14_vs_E10	ordinary_muscle_development		0	FALSE
ENSGALG00010016977	CLCN7	PRJNA729271	1.033558768	4.57215E-18	1.033558768	stage_E14_vs_E10	ordinary_muscle_development	D_vs_E10;stage_1W_vs_E10;stage_3W_vs_E10;stage	1	TRUE
ENSGALG00010005535	AP4S1	PRJNA729271	1.330656717	4.63926E-18	1.330656717	stage_E14_vs_E10	ordinary_muscle_development	D_vs_E10;stage_1W_vs_E10;stage_3W_vs_E10;stage	1	TRUE
ENSGALG00010025666	CUX2	PRJNA729271	-4.486719984	4.64507E-18	4.486719984	stage_E14_vs_E10	ordinary_muscle_development	D_vs_E10;stage_1W_vs_E10;stage_3W_vs_E10;stage	1	TRUE
ENSGALG00010029645	ENSGALG00010029645	PRJNA729271	-3.7501573	4.66049E-18	3.7501573	stage_E14_vs_E10	ordinary_muscle_development	D_vs_E10;stage_1W_vs_E10;stage_3W_vs_E10;stage	1	TRUE
ENSGALG00010024795	NRROS	PRJNA729271	1.807650554	4.66555E-18	1.807650554	stage_E14_vs_E10	ordinary_muscle_development	D_vs_E10;stage_1W_vs_E10;stage_3W_vs_E10;stage	1	TRUE
ENSGALG00010029347	PRPF8	PRJNA729271	-0.622535439	4.68938E-18	0.622535439	stage_E14_vs_E10	ordinary_muscle_development		0	FALSE
ENSGALG00010017252	EXOC5	PRJNA729271	-0.786927749	4.79164E-18	0.786927749	stage_E14_vs_E10	ordinary_muscle_development		0	FALSE
ENSGALG00010002995	RWDD2A	PRJNA729271	-1.140736024	4.8493E-18	1.140736024	stage_E14_vs_E10	ordinary_muscle_development	D_vs_E10;stage_1W_vs_E10;stage_3W_vs_E10;stage	1	TRUE
ENSGALG00010005538	ENSGALG00010005538	PRJNA729271	2.776881868	4.86098E-18	2.776881868	stage_E14_vs_E10	ordinary_muscle_development	D_vs_E10;stage_1W_vs_E10;stage_3W_vs_E10;stage	1	TRUE
ENSGALG00010001837	TAB3	PRJNA729271	1.214036234	4.88566E-18	1.214036234	stage_E14_vs_E10	ordinary_muscle_development	D_vs_E10;stage_1W_vs_E10;stage_3W_vs_E10;stage	1	TRUE
ENSGALG00010021681	BTBD10	PRJNA729271	-0.731685444	4.92014E-18	0.731685444	stage_E14_vs_E10	ordinary_muscle_development		0	FALSE
ENSGALG00010006100	RPS6KL1	PRJNA729271	-1.841082076	4.96265E-18	1.841082076	stage_E14_vs_E10	ordinary_muscle_development	D_vs_E10;stage_1W_vs_E10;stage_3W_vs_E10;stage	1	TRUE
ENSGALG00010016979	PLSCR5	PRJNA729271	3.798812527	5.07461E-18	3.798812527	stage_E14_vs_E10	ordinary_muscle_development	D_vs_E10;stage_1W_vs_E10;stage_3W_vs_E10;stage	1	TRUE
ENSGALG00010009383	ASXL2	PRJNA729271	0.978633493	5.08151E-18	0.978633493	stage_E14_vs_E10	ordinary_muscle_development		0	FALSE
ENSGALG00010018670	MRAS	PRJNA729271	1.880559343	5.09961E-18	1.880559343	stage_E14_vs_E10	ordinary_muscle_development	D_vs_E10;stage_1W_vs_E10;stage_3W_vs_E10;stage	1	TRUE
ENSGALG00010025982	SERPINE2	PRJNA729271	-1.753183872	5.23626E-18	1.753183872	stage_E14_vs_E10	ordinary_muscle_development	D_vs_E10;stage_1W_vs_E10;stage_3W_vs_E10;stage	1	TRUE
ENSGALG00010028859	SURF1	PRJNA729271	0.954653207	5.24814E-18	0.954653207	stage_E14_vs_E10	ordinary_muscle_development		0	FALSE
ENSGALG00010014033	SLC38A4	PRJNA729271	4.177764056	5.32422E-18	4.177764056	stage_E14_vs_E10	ordinary_muscle_development	D_vs_E10;stage_1W_vs_E10;stage_3W_vs_E10;stage	1	TRUE
ENSGALG00010000687	IKZF1	PRJNA729271	2.466493882	5.34149E-18	2.466493882	stage_E14_vs_E10	ordinary_muscle_development	D_vs_E10;stage_1W_vs_E10;stage_3W_vs_E10;stage	1	TRUE
ENSGALG00010005388	ENSGALG00010005388	PRJNA729271	4.005213379	5.47712E-18	4.005213379	stage_E14_vs_E10	ordinary_muscle_development	D_vs_E10;stage_1W_vs_E10;stage_3W_vs_E10;stage	1	TRUE
ENSGALG00010007883	DHTKD1	PRJNA729271	1.063439118	5.71174E-18	1.063439118	stage_E14_vs_E10	ordinary_muscle_development	D_vs_E10;stage_1W_vs_E10;stage_3W_vs_E10;stage	1	TRUE
ENSGALG00010019013	LRRK1	PRJNA729271	-0.937677684	5.8389E-18	0.937677684	stage_E14_vs_E10	ordinary_muscle_development		0	FALSE
ENSGALG00010012899	ENSGALG00010012899	PRJNA729271	1.13718108	5.95029E-18	1.13718108	stage_E14_vs_E10	ordinary_muscle_development	D_vs_E10;stage_1W_vs_E10;stage_3W_vs_E10;stage	1	TRUE
ENSGALG00010018078	NKX6-3	PRJNA729271	6.072676349	6.00891E-18	6.072676349	stage_E14_vs_E10	ordinary_muscle_development	D_vs_E10;stage_1W_vs_E10;stage_3W_vs_E10;stage	1	TRUE
ENSGALG00010011018	QDPR	PRJNA729271	-0.925498755	6.02885E-18	0.925498755	stage_E14_vs_E10	ordinary_muscle_development		0	FALSE
ENSGALG00010018516	LRRN4	PRJNA729271	1.353881788	6.0669E-18	1.353881788	stage_E14_vs_E10	ordinary_muscle_development	D_vs_E10;stage_1W_vs_E10;stage_3W_vs_E10;stage	1	TRUE
ENSGALG00010025899	ENSGALG00010025899	PRJNA729271	1.425078184	6.07498E-18	1.425078184	stage_E14_vs_E10	ordinary_muscle_development	D_vs_E10;stage_1W_vs_E10;stage_3W_vs_E10;stage	1	TRUE
ENSGALG00010017659	TNRC6A	PRJNA729271	-0.695836585	6.08669E-18	0.695836585	stage_E14_vs_E10	ordinary_muscle_development		0	FALSE
ENSGALG00010014109	AMIGO2	PRJNA729271	-1.472588445	6.08759E-18	1.472588445	stage_E14_vs_E10	ordinary_muscle_development	D_vs_E10;stage_1W_vs_E10;stage_3W_vs_E10;stage	1	TRUE
ENSGALG00010029312	SMURF2	PRJNA729271	0.815861491	6.22335E-18	0.815861491	stage_E14_vs_E10	ordinary_muscle_development		0	FALSE
ENSGALG00010018289	HEYL	PRJNA729271	-1.923246012	6.23375E-18	1.923246012	stage_E14_vs_E10	ordinary_muscle_development	D_vs_E10;stage_1W_vs_E10;stage_3W_vs_E10;stage	1	TRUE
ENSGALG00010002649	UFL1	PRJNA729271	0.691886091	6.25556E-18	0.691886091	stage_E14_vs_E10	ordinary_muscle_development		0	FALSE
ENSGALG00010021043	PTPRG	PRJNA729271	-0.799253559	6.30434E-18	0.799253559	stage_E14_vs_E10	ordinary_muscle_development		0	FALSE
ENSGALG00010001475	BMT2	PRJNA729271	-1.217319697	6.52304E-18	1.217319697	stage_E14_vs_E10	ordinary_muscle_development	D_vs_E10;stage_1W_vs_E10;stage_3W_vs_E10;stage	1	TRUE
ENSGALG00010002957	ADGRG2	PRJNA729271	-2.602623274	6.56771E-18	2.602623274	stage_E14_vs_E10	ordinary_muscle_development	D_vs_E10;stage_1W_vs_E10;stage_3W_vs_E10;stage	1	TRUE
ENSGALG00010003913	PSMB5	PRJNA729271	1.022977084	6.57112E-18	1.022977084	stage_E14_vs_E10	ordinary_muscle_development	D_vs_E10;stage_1W_vs_E10;stage_3W_vs_E10;stage	1	TRUE
ENSGALG00010013146	TNRC6B	PRJNA729271	1.045970408	6.61444E-18	1.045970408	stage_E14_vs_E10	ordinary_muscle_development	D_vs_E10;stage_1W_vs_E10;stage_3W_vs_E10;stage	1	TRUE
ENSGALG00010014735	STX16	PRJNA729271	0.915071479	6.65861E-18	0.915071479	stage_E14_vs_E10	ordinary_muscle_development		0	FALSE
ENSGALG00010019786	R3HDM1	PRJNA729271	0.764727415	6.67704E-18	0.764727415	stage_E14_vs_E10	ordinary_muscle_development		0	FALSE
ENSGALG00010005779	ARMT1	PRJNA729271	0.970123353	6.75997E-18	0.970123353	stage_E14_vs_E10	ordinary_muscle_development		0	FALSE
ENSGALG00010022546	MTG1	PRJNA729271	1.374781212	6.82647E-18	1.374781212	stage_E14_vs_E10	ordinary_muscle_development	D_vs_E10;stage_1W_vs_E10;stage_3W_vs_E10;stage	1	TRUE
ENSGALG00010029323	NOL11	PRJNA729271	0.793482114	6.8768E-18	0.793482114	stage_E14_vs_E10	ordinary_muscle_development		0	FALSE
ENSGALG00010027407	ENSGALG00010027407	PRJNA729271	7.209161462	6.91029E-18	7.209161462	stage_E14_vs_E10	ordinary_muscle_development	D_vs_E10;stage_1W_vs_E10;stage_3W_vs_E10;stage	1	TRUE
ENSGALG00010010302	WDR36	PRJNA729271	-0.928368632	6.95011E-18	0.928368632	stage_E14_vs_E10	ordinary_muscle_development		0	FALSE
ENSGALG00010028156	DOHH	PRJNA729271	0.839897666	6.9801E-18	0.839897666	stage_E14_vs_E10	ordinary_muscle_development		0	FALSE
ENSGALG00010007937	ANGPTL5	PRJNA729271	3.515759413	7.03149E-18	3.515759413	stage_E14_vs_E10	ordinary_muscle_development	D_vs_E10;stage_1W_vs_E10;stage_3W_vs_E10;stage	1	TRUE
ENSGALG00010010475	CCDC71L	PRJNA729271	0.937322958	7.15265E-18	0.937322958	stage_E14_vs_E10	ordinary_muscle_development		0	FALSE
ENSGALG00010009634	TBC1D31	PRJNA729271	-0.940069369	7.53033E-18	0.940069369	stage_E14_vs_E10	ordinary_muscle_development		0	FALSE
ENSGALG00010020878	XYLT1	PRJNA729271	-1.106570877	7.59822E-18	1.106570877	stage_E14_vs_E10	ordinary_muscle_development	D_vs_E10;stage_1W_vs_E10;stage_3W_vs_E10;stage	1	TRUE
ENSGALG00010001199	AFG3L2	PRJNA729271	0.786687731	7.63713E-18	0.786687731	stage_E14_vs_E10	ordinary_muscle_development		0	FALSE
ENSGALG00010019848	MAD1L1	PRJNA729271	-1.127899789	7.64776E-18	1.127899789	stage_E14_vs_E10	ordinary_muscle_development	D_vs_E10;stage_1W_vs_E10;stage_3W_vs_E10;stage	1	TRUE
ENSGALG00010016510	THG1L	PRJNA729271	-1.011329211	7.69372E-18	1.011329211	stage_E14_vs_E10	ordinary_muscle_development	D_vs_E10;stage_1W_vs_E10;stage_3W_vs_E10;stage	1	TRUE
ENSGALG00010015674	RFKL	PRJNA729271	1.688541251	7.7198E-18	1.688541251	stage_E14_vs_E10	ordinary_muscle_development	D_vs_E10;stage_1W_vs_E10;stage_3W_vs_E10;stage	1	TRUE
ENSGALG00010025801	PTPN7	PRJNA729271	3.145862503	7.72003E-18	3.145862503	stage_E14_vs_E10	ordinary_muscle_development	D_vs_E10;stage_1W_vs_E10;stage_3W_vs_E10;stage	1	TRUE
ENSGALG00010007500	MPHOSPH6	PRJNA729271	1.016278218	7.81368E-18	1.016278218	stage_E14_vs_E10	ordinary_muscle_development	D_vs_E10;stage_1W_vs_E10;stage_3W_vs_E10;stage	1	TRUE
ENSGALG00010018684	PADI2	PRJNA729271	8.451820983	7.81667E-18	8.451820983	stage_E14_vs_E10	ordinary_muscle_development	D_vs_E10;stage_1W_vs_E10;stage_3W_vs_E10;stage	1	TRUE
ENSGALG00010022027	EIF4ENIF1	PRJNA729271	-0.596671203	7.83121E-18	0.596671203	stage_E14_vs_E10	ordinary_muscle_development		0	FALSE
ENSGALG00010010977	FARS2	PRJNA729271	0.711539254	7.85134E-18	0.711539254	stage_E14_vs_E10	ordinary_muscle_development		0	FALSE
ENSGALG00010014455	C4orf19	PRJNA729271	-2.311197419	8.03451E-18	2.311197419	stage_E14_vs_E10	ordinary_muscle_development	D_vs_E10;stage_1W_vs_E10;stage_3W_vs_E10;stage	1	TRUE

ENSGALG00010019277	IGFBP5	PRJNA729271	1.164286505	8.03618E-18	1.164286505	stage_E14_vs_E10	ordinary_muscle_development	D_vs_E10;stage_1W_vs_E10;stage_3W_vs_E10;stage	1	TRUE
ENSGALG00010010771	ENSGALG00010010771	PRJNA729271	-0.633218574	8.19095E-18	0.633218574	stage_E14_vs_E10	ordinary_muscle_development		0	FALSE
ENSGALG00010026569	GNAZ	PRJNA729271	-1.872471947	8.24188E-18	1.872471947	stage_E14_vs_E10	ordinary_muscle_development	D_vs_E10;stage_1W_vs_E10;stage_3W_vs_E10;stage	1	TRUE
ENSGALG00010021152	STK11IP	PRJNA729271	1.10400047	8.27268E-18	1.10400047	stage_E14_vs_E10	ordinary_muscle_development	D_vs_E10;stage_1W_vs_E10;stage_3W_vs_E10;stage	1	TRUE
ENSGALG00010024104	PLPPR5	PRJNA729271	1.397365957	8.33336E-18	1.397365957	stage_E14_vs_E10	ordinary_muscle_development	D_vs_E10;stage_1W_vs_E10;stage_3W_vs_E10;stage	1	TRUE
ENSGALG00010005575	ENSGALG00010005575	PRJNA729271	3.807861489	8.36103E-18	3.807861489	stage_E14_vs_E10	ordinary_muscle_development	D_vs_E10;stage_1W_vs_E10;stage_3W_vs_E10;stage	1	TRUE
ENSGALG00010003240	PLACL2	PRJNA729271	7.481199875	8.36235E-18	7.481199875	stage_E14_vs_E10	ordinary_muscle_development	D_vs_E10;stage_1W_vs_E10;stage_3W_vs_E10;stage	1	TRUE
ENSGALG00010016654	SMIM3	PRJNA729271	1.602197401	8.42644E-18	1.602197401	stage_E14_vs_E10	ordinary_muscle_development	D_vs_E10;stage_1W_vs_E10;stage_3W_vs_E10;stage	1	TRUE
ENSGALG00010007800	MAF	PRJNA729271	0.998037401	8.5647E-18	0.998037401	stage_E14_vs_E10	ordinary_muscle_development		0	FALSE
ENSGALG00010012209	KIF14	PRJNA729271	-2.02199068	8.59405E-18	2.02199068	stage_E14_vs_E10	ordinary_muscle_development	D_vs_E10;stage_1W_vs_E10;stage_3W_vs_E10;stage	1	TRUE
ENSGALG00010014176	ABHD17B	PRJNA729271	-0.832252005	8.64327E-18	0.832252005	stage_E14_vs_E10	ordinary_muscle_development		0	FALSE
ENSGALG00010009779	PPP1R3E	PRJNA729271	2.998986474	8.65308E-18	2.998986474	stage_E14_vs_E10	ordinary_muscle_development	D_vs_E10;stage_1W_vs_E10;stage_3W_vs_E10;stage	1	TRUE
ENSGALG00010021693	PPA1	PRJNA729271	-0.810161486	8.73498E-18	0.810161486	stage_E14_vs_E10	ordinary_muscle_development		0	FALSE
ENSGALG00010000366	EMID1	PRJNA729271	-2.106096629	8.82661E-18	2.106096629	stage_E14_vs_E10	ordinary_muscle_development	D_vs_E10;stage_1W_vs_E10;stage_3W_vs_E10;stage	1	TRUE
ENSGALG00010005256	OPRM1	PRJNA729271	-7.68294245	8.83511E-18	7.68294245	stage_E14_vs_E10	ordinary_muscle_development	D_vs_E10;stage_1W_vs_E10;stage_3W_vs_E10;stage	1	TRUE
ENSGALG00010003052	ENSGALG00010003052	PRJNA729271	0.813101463	8.93511E-18	0.813101463	stage_E14_vs_E10	ordinary_muscle_development		0	FALSE
ENSGALG00010007877	MSMO1	PRJNA729271	-1.67683132	8.99384E-18	1.67683132	stage_E14_vs_E10	ordinary_muscle_development	D_vs_E10;stage_1W_vs_E10;stage_3W_vs_E10;stage	1	TRUE
ENSGALG00010000029	ND4	PRJNA729271	2.225141447	9.11797E-18	2.225141447	stage_E14_vs_E10	ordinary_muscle_development	D_vs_E10;stage_1W_vs_E10;stage_3W_vs_E10;stage	1	TRUE
ENSGALG00010007880	BCO1	PRJNA729271	2.449757033	9.47101E-18	2.449757033	stage_E14_vs_E10	ordinary_muscle_development	D_vs_E10;stage_1W_vs_E10;stage_3W_vs_E10;stage	1	TRUE
ENSGALG00010000042	CKMT1B	PRJNA729271	3.909585677	9.51142E-18	3.909585677	stage_E14_vs_E10	ordinary_muscle_development	D_vs_E10;stage_1W_vs_E10;stage_3W_vs_E10;stage	1	TRUE
ENSGALG00010011146	BMS1	PRJNA729271	-1.140614029	9.54792E-18	1.140614029	stage_E14_vs_E10	ordinary_muscle_development	D_vs_E10;stage_1W_vs_E10;stage_3W_vs_E10;stage	1	TRUE
ENSGALG00010022241	POLR2C	PRJNA729271	0.819562885	9.58955E-18	0.819562885	stage_E14_vs_E10	ordinary_muscle_development		0	FALSE
ENSGALG00010024531	CEP63	PRJNA729271	-1.161969315	9.63697E-18	1.161969315	stage_E14_vs_E10	ordinary_muscle_development	D_vs_E10;stage_1W_vs_E10;stage_3W_vs_E10;stage	1	TRUE
ENSGALG00010004131	NUDT6	PRJNA729271	1.361945959	9.67679E-18	1.361945959	stage_E14_vs_E10	ordinary_muscle_development	D_vs_E10;stage_1W_vs_E10;stage_3W_vs_E10;stage	1	TRUE
ENSGALG00010015430	HMGCL	PRJNA729271	1.132523892	9.6946E-18	1.132523892	stage_E14_vs_E10	ordinary_muscle_development	D_vs_E10;stage_1W_vs_E10;stage_3W_vs_E10;stage	1	TRUE
ENSGALG00010020743	PURB	PRJNA729271	1.418653636	9.77923E-18	1.418653636	stage_E14_vs_E10	ordinary_muscle_development	D_vs_E10;stage_1W_vs_E10;stage_3W_vs_E10;stage	1	TRUE
ENSGALG00010015650	ENSGALG00010015650	PRJNA729271	-6.471284676	9.78878E-18	6.471284676	stage_E14_vs_E10	ordinary_muscle_development	D_vs_E10;stage_1W_vs_E10;stage_3W_vs_E10;stage	1	TRUE
ENSGALG00010007855	RNF182	PRJNA729271	-1.969355618	9.95302E-18	1.969355618	stage_E14_vs_E10	ordinary_muscle_development	D_vs_E10;stage_1W_vs_E10;stage_3W_vs_E10;stage	1	TRUE
ENSGALG00010015985	TIMM8A	PRJNA729271	-0.985090538	1.00366E-17	0.985090538	stage_E14_vs_E10	ordinary_muscle_development		0	FALSE
ENSGALG00010011874	KCNS2	PRJNA729271	-7.876218279	1.01978E-17	7.876218279	stage_E14_vs_E10	ordinary_muscle_development	D_vs_E10;stage_1W_vs_E10;stage_3W_vs_E10;stage	1	TRUE
ENSGALG00010009205	SIRT2	PRJNA729271	0.847952611	1.02508E-17	0.847952611	stage_E14_vs_E10	ordinary_muscle_development		0	FALSE
ENSGALG00010003255	CALCR	PRJNA729271	-3.141113991	1.04505E-17	3.141113991	stage_E14_vs_E10	ordinary_muscle_development	D_vs_E10;stage_1W_vs_E10;stage_3W_vs_E10;stage	1	TRUE
ENSGALG00010002492	RPL8	PRJNA729271	1.137295988	1.0696E-17	1.137295988	stage_E14_vs_E10	ordinary_muscle_development	D_vs_E10;stage_1W_vs_E10;stage_3W_vs_E10;stage	1	TRUE
ENSGALG00010007862	ATMIN	PRJNA729271	0.745150781	1.08136E-17	0.745150781	stage_E14_vs_E10	ordinary_muscle_development		0	FALSE
ENSGALG00010020489	IFFO2	PRJNA729271	-1.697301235	1.09168E-17	1.697301235	stage_E14_vs_E10	ordinary_muscle_development	D_vs_E10;stage_1W_vs_E10;stage_3W_vs_E10;stage	1	TRUE
ENSGALG00010008762	SAE1	PRJNA729271	-0.89786554	1.10023E-17	0.89786554	stage_E14_vs_E10	ordinary_muscle_development		0	FALSE
ENSGALG00010018121	FERMT2	PRJNA729271	1.400830207	1.10087E-17	1.400830207	stage_E14_vs_E10	ordinary_muscle_development	D_vs_E10;stage_1W_vs_E10;stage_3W_vs_E10;stage	1	TRUE
ENSGALG00010027234	SLC6A11	PRJNA729271	-5.316262703	1.10154E-17	5.316262703	stage_E14_vs_E10	ordinary_muscle_development	D_vs_E10;stage_1W_vs_E10;stage_3W_vs_E10;stage	1	TRUE
ENSGALG00010021568	TADA3	PRJNA729271	1.223893125	1.10929E-17	1.223893125	stage_E14_vs_E10	ordinary_muscle_development	D_vs_E10;stage_1W_vs_E10;stage_3W_vs_E10;stage	1	TRUE
ENSGALG00010017163	CDV3	PRJNA729271	-1.09889278	1.1125E-17	1.09889278	stage_E14_vs_E10	ordinary_muscle_development	D_vs_E10;stage_1W_vs_E10;stage_3W_vs_E10;stage	1	TRUE
ENSGALG00010024654	CCDC92	PRJNA729271	-2.490835952	1.11589E-17	2.490835952	stage_E14_vs_E10	ordinary_muscle_development	D_vs_E10;stage_1W_vs_E10;stage_3W_vs_E10;stage	1	TRUE
ENSGALG00010007135	TK2	PRJNA729271	-1.034524776	1.12001E-17	1.034524776	stage_E14_vs_E10	ordinary_muscle_development	D_vs_E10;stage_1W_vs_E10;stage_3W_vs_E10;stage	1	TRUE
ENSGALG00010023831	SATB2	PRJNA729271	-4.953208734	1.14506E-17	4.953208734	stage_E14_vs_E10	ordinary_muscle_development	D_vs_E10;stage_1W_vs_E10;stage_3W_vs_E10;stage	1	TRUE
ENSGALG00010001351	CAPN1	PRJNA729271	1.350684254	1.15245E-17	1.350684254	stage_E14_vs_E10	ordinary_muscle_development	D_vs_E10;stage_1W_vs_E10;stage_3W_vs_E10;stage	1	TRUE
ENSGALG00010021523	CELF6	PRJNA729271	-1.439060565	1.15387E-17	1.439060565	stage_E14_vs_E10	ordinary_muscle_development	D_vs_E10;stage_1W_vs_E10;stage_3W_vs_E10;stage	1	TRUE
ENSGALG00010015142	HNRNPAB	PRJNA729271	-1.120537682	1.1554E-17	1.120537682	stage_E14_vs_E10	ordinary_muscle_development	D_vs_E10;stage_1W_vs_E10;stage_3W_vs_E10;stage	1	TRUE
ENSGALG00010020127	FRMD4B	PRJNA729271	-1.2288909	1.16046E-17	1.2288909	stage_E14_vs_E10	ordinary_muscle_development	D_vs_E10;stage_1W_vs_E10;stage_3W_vs_E10;stage	1	TRUE
ENSGALG00010025188	PPFIA4	PRJNA729271	-4.094191693	1.16118E-17	4.094191693	stage_E14_vs_E10	ordinary_muscle_development	D_vs_E10;stage_1W_vs_E10;stage_3W_vs_E10;stage	1	TRUE
ENSGALG00010027903	NDUFA8	PRJNA729271	1.090806457	1.17183E-17	1.090806457	stage_E14_vs_E10	ordinary_muscle_development	D_vs_E10;stage_1W_vs_E10;stage_3W_vs_E10;stage	1	TRUE
ENSGALG00010021739	EXOSC10	PRJNA729271	0.9543578	1.17442E-17	0.9543578	stage_E14_vs_E10	ordinary_muscle_development		0	FALSE
ENSGALG00010022290	NEXN	PRJNA729271	1.929489238	1.17664E-17	1.929489238	stage_E14_vs_E10	ordinary_muscle_development	D_vs_E10;stage_1W_vs_E10;stage_3W_vs_E10;stage	1	TRUE
ENSGALG00010023157	STOX1	PRJNA729271	-3.935089284	1.20102E-17	3.935089284	stage_E14_vs_E10	ordinary_muscle_development	D_vs_E10;stage_1W_vs_E10;stage_3W_vs_E10;stage	1	TRUE
ENSGALG00010008699	NAA35	PRJNA729271	0.866884964	1.20291E-17	0.866884964	stage_E14_vs_E10	ordinary_muscle_development		0	FALSE
ENSGALG00010025654	CRTAM	PRJNA729271	5.907869366	1.21847E-17	5.907869366	stage_E14_vs_E10	ordinary_muscle_development	D_vs_E10;stage_1W_vs_E10;stage_3W_vs_E10;stage	1	TRUE
ENSGALG00010023642	PAX6	PRJNA729271	4.353045883	1.21989E-17	4.353045883	stage_E14_vs_E10	ordinary_muscle_development	D_vs_E10;stage_1W_vs_E10;stage_3W_vs_E10;stage	1	TRUE
ENSGALG00010013532	WDR19	PRJNA729271	-0.668635815	1.23837E-17	0.668635815	stage_E14_vs_E10	ordinary_muscle_development		0	FALSE
ENSGALG00010005553	PPP1R14C	PRJNA729271	1.422853993	1.24617E-17	1.422853993	stage_E14_vs_E10	ordinary_muscle_development	D_vs_E10;stage_1W_vs_E10;stage_3W_vs_E10;stage	1	TRUE
ENSGALG00010017669	ENSGALG00010017669	PRJNA729271	1.512414907	1.25219E-17	1.512414907	stage_E14_vs_E10	ordinary_muscle_development	D_vs_E10;stage_1W_vs_E10;stage_3W_vs_E10;stage	1	TRUE
ENSGALG00010016257	ENSGALG00010016257	PRJNA729271	-0.993345885	1.25694E-17	0.993345885	stage_E14_vs_E10	ordinary_muscle_development		0	FALSE
ENSGALG00010022240	ENSGALG00010022240	PRJNA729271	-1.179499499	1.26246E-17	1.179499499	stage_E14_vs_E10	ordinary_muscle_development	D_vs_E10;stage_1W_vs_E10;stage_3W_vs_E10;stage	1	TRUE
ENSGALG00010010841	PMM1	PRJNA729271	1.132094883	1.29728E-17	1.132094883	stage_E14_vs_E10	ordinary_muscle_development	D_vs_E10;stage_1W_vs_E10;stage_3W_vs_E10;stage	1	TRUE
ENSGALG00010018719	ERRFI1	PRJNA729271	-0.8070697	1.30382E-17	0.8070697	stage_E14_vs_E10	ordinary_muscle_development		0	FALSE
ENSGALG00010013657	MAGT1	PRJNA729271	-0.913875096	1.31604E-17	0.913875096	stage_E14_vs_E10	ordinary_muscle_development		0	FALSE
ENSGALG00010024023	LHX8	PRJNA729271	-5.928169466	1.32227E-17	5.928169466	stage_E14_vs_E10	ordinary_muscle_development	D_vs_E10;stage_1W_vs_E10;stage_3W_vs_E10;stage	1	TRUE
ENSGALG00010020070	ARL6IP5	PRJNA729271	0.993058528	1.35021E-17	0.993058528	stage_E14_vs_E10	ordinary_muscle_development		0	FALSE
ENSGALG00010011133	CDC37L1	PRJNA729271	1.027704139	1.37124E-17	1.027704139	stage_E14_vs_E10	ordinary_muscle_development	D_vs_E10;stage_1W_vs_E10;stage_3W_vs_E10;stage	1	TRUE
ENSGALG00010003432	LACC1	PRJNA729271	2.677142548	1.38824E-17	2.677142548	stage_E14_vs_E10	ordinary_muscle_development	D_vs_E10;stage_1W_vs_E10;stage_3W_vs_E10;stage	1	TRUE
ENSGALG00010016975	MALL	PRJNA729271	1.841398776	1.3885E-17	1.841398776	stage_E14_vs_E10	ordinary_muscle_development	D_vs_E10;stage_1W_vs_E10;stage_3W_vs_E10;stage	1	TRUE
ENSGALG00010010859	ENSGALG00010010859	PRJNA729271	-2.476426464	1.41051E-17	2.476426464	stage_E14_vs_E10	ordinary_muscle_development	D_vs_E10;stage_1W_vs_E10;stage_3W_vs_E10;stage	1	TRUE
ENSGALG00010011400	BPHL	PRJNA729271	1.710533881	1.41908E-17	1.710533881	stage_E14_vs_E10	ordinary_muscle_development	D_vs_E10;stage_1W_vs_E10;stage_3W_vs_E10;stage	1	TRUE
ENSGALG00010007252	NFATC1	PRJNA729271	1.327766331	1.42263E-17	1.327766331	stage_E14_vs_E10	ordinary_muscle_development	D_vs_E10;stage_1W_vs_E10;stage_3W_vs_E10;stage	1	TRUE
ENSGALG00010008059	ENSGALG00010008059	PRJNA729271	0.800060083	1.4305E-17	0.800060083	stage_E14_vs_E10	ordinary_muscle_development		0	FALSE
ENSGALG00010016643	BTBD1	PRJNA729271	1.502151646	1.4369E-17	1.502151646	stage_E14_vs_E10	ordinary_muscle_development	D_vs_E10;stage_1W_vs_E10;stage_3W_vs_E10;stage	1	TRUE
ENSGALG00010012714	RGS2	PRJNA729271	1.504564858	1.43818E-17	1.504564858	stage_E14_vs_E10	ordinary_muscle_development	D_vs_E10;stage_1W_vs_E10;stage_3W_vs_E10;stage	1	TRUE
ENSGALG00010028000	MRPL9	PRJNA729271	1.437563274	1.45849E-17	1.437563274	stage_E14_vs_E10	ordinary_muscle_development	D_vs_E10;stage_1W_vs_E10;stage_3W_vs_E10;stage	1	TRUE
ENSGALG00010027962	SCAMP3	PRJNA729271	1.089201189	1.47106E-17	1.089201189	stage_E14_vs_E10	ordinary_muscle_development	D_vs_E10;stage_1W_vs_E10;stage_3W_vs_E10;stage	1	TRUE
ENSGALG00010030105	RAB40B	PRJNA729271	1.656726907	1.49924E-17	1.656726907	stage_E14_vs_E10	ordinary_muscle_development	D_vs_E10;stage_1W_vs_E10;stage_3W_vs_E10;stage	1	TRUE
ENSGALG00010001349	ING3	PRJNA729271	0.904259298	1.50666E-17	0.904259298	stage_E14_vs_E10	ordinary_muscle_development		0	FALSE
ENSGALG00010025964	HOXC10	PRJNA729271	-9.529149975	1.50964E-17	9.529149975	stage_E14_vs_E10	ordinary_muscle_development	D_vs_E10;stage_1W_vs_E10;stage_3W_vs_E10;stage	1	TRUE
ENSGALG00010012903	RGMB	PRJNA729271	0.797304784	1.51001E-17	0.797304784	stage_E14_vs_E10	ordinary_muscle_development		0	FALSE
ENSGALG00010028067	FAM102A	PRJNA729271	-0.994778213	1.51644E-17	0.994778213	stage_E14_vs_E10	ordinary_muscle_development		0	FALSE
ENSGALG00010016560	DCUN1D4	PRJNA729271	1.46576609	1.53192E-17	1.46576609	stage_E14_vs_E10	ordinary_muscle_development	D_vs_E10;stage_1W_vs_E10;stage_3W_vs_E10;stage	1	TRUE
ENSGALG00010003993	ENSGALG00010003993	PRJNA729271	1.159034447	1.54137E-17	1.159034447	stage_E14_vs_E10	ordinary_muscle_development	D_vs_E10;stage_1W_vs_E10;stage_3W_vs_E10;stage	1	TRUE
ENSGALG00010004892	HEY2	PRJNA729271	-3.731566864	1.54664E-17	3.731566864	stage_E14_vs_E10	ordinary_muscle_development	D_vs_E10;stage_1W_vs_E10;stage_3W_vs_E10;stage	1	TRUE
ENSGALG00010005440	ENSGALG00010005440	PRJNA729271	4.072241879	1.55024E-17	4.072241879	stage_E14_vs_E10	ordinary_muscle_development	D_vs_E10;stage_1W_vs_E10;stage_3W_vs_E10;stage	1	TRUE
ENSGALG00010024157	CHRNB4	PRJNA729271	-3.689844461	1.55108E-17	3.689844461	stage_E14_vs_E10	ordinary_muscle_development	D_vs_E10;stage_1W_vs_E10;stage_3W_vs_E10;stage	1	TRUE
ENSGALG00010024358	MAPK3	PRJNA729271	0.53596753	1.55272E-17	0.53596753	stage_E14_vs_E10	ordinary_muscle_development		0	FALSE
ENSGALG00010021111	AVEN	PRJNA729271	0.680924952	1.55279E-17	0.680924952	stage_E14_vs_E10	ordinary_muscle_development		0	FALSE
ENSGALG00010004319	TAAR1	PRJNA729271	7.055631718	1.5598E-17	7.055631718	stage_E14_vs_E10	ordinary_muscle_development	D_vs_E10;stage_1W_vs_E10;stage_3W_vs_E10;stage	1	TRUE
ENSGALG00010005831	SEC61A2	PRJNA729271	0.907419417	1.56826E-17	0.907419417	stage_E14_vs_E10	ordinary_muscle_development		0	FALSE
ENSGALG00010022276	BECN1	PRJNA729271	0.763878018	1.57762E-17	0.763878018	stage_E14_vs_E10	ordinary_muscle_development		0	FALSE
ENSGALG00010012930	IL6ST	PRJNA729271	0.787832696	1.58034E-17	0.787832696	stage_E14_vs_E10	ordinary_muscle_development		0	FALSE
ENSGALG00010005487	ENSGALG00010005487	PRJNA729271	-1.847318595	1.62481E-17	1.847318595	stage_E14_vs_E10	ordinary_muscle_development	D_vs_E10;stage_1W_vs_E10;stage_3W_vs_E10;stage	1	TRUE
ENSGALG00010005696	NUBPL	PRJNA729271	-1.240159611	1.62602E-17	1.240159611	stage_E14_vs_E10	ordinary_muscle_development	D_vs_E10;stage_1W_vs_E10;stage_3W_vs_E10;stage	1	TRUE
ENSGALG00010001808	HOXA4	PRJNA729271	-1.307007156	1.62825E-17	1.307007156	stage_E14_vs_E10	ordinary_muscle_development	D_vs_E10;stage_1W_vs_E10;stage_3W_vs_E10;stage	1	TRUE
ENSGALG00010027402	AP4B1	PRJNA729271	0.747291909	1.65247E-17	0.747291909	stage_E14_vs_E10	ordinary_muscle_development		0	FALSE
ENSGALG00010018344	SFXN5	PRJNA729271	-1.843639505	1.67149E-17	1.843639505	stage_E14_vs_E10	ordinary_muscle_development	D_vs_E10;stage_1W_vs_E10;stage_3W_vs_E10;stage	1	TRUE
ENSGALG00010024523	TOX3	PRJNA729271	-2.916965999	1.6794E-17	2.916965999	stage_E14_vs_E10	ordinary_muscle_development	D_vs_E10;stage_1W_vs_E10;stage_3W_vs_E10;stage	1	TRUE
ENSGALG00010027045	MAPK13	PRJNA729271	1.561764066	1.6878E-17	1.561764066	stage_E14_vs_E10	ordinary_muscle_development	D_vs_E10;stage_1W_vs_E10;stage_3W_vs_E10;stage	1	TRUE
ENSGALG00010020586	C11orf16	PRJNA729271	0.840570333	1.68987E-17	0.840570333	stage_E14_vs_E10	ordinary_muscle_development		0	FALSE
ENSGALG00010027063	RAB7L1	PRJNA729271	0.757962502	1.69014E-17	0.757962502	stage_E14_vs_E10	ordinary_muscle_development		0	FALSE
ENSGALG00010003249	YJU2B	PRJNA729271	2.66699251	1.70806E-17	2.66699251	stage_E14_vs_E10	ordinary_muscle_development	D_vs_E10;stage_1W_vs_E10;stage_3W_vs_E10;stage	1	TRUE
ENSGALG00010022271	SLC18A2	PRJNA729271	-2.160425818	1.78961E-17	2.160425818	stage_E14_vs_E10	ordinary_muscle_development	D_vs_E10;stage_1W_vs_E10;stage_3W_vs_E10;stage	1	TRUE
ENSGALG00010024494	DDAH1	PRJNA729271	-1.95586821	1.81132E-17	1.95586821	stage_E14_vs_E10	ordinary_muscle_development	D_vs_E10;stage_1W_vs_E10;stage_3W_vs_E10;stage	1	TRUE
ENSGALG00010009995	SEPTIN3	PRJNA729271	2.830732662	1.83648E-17	2.830732662	stage_E14_vs_E10	ordinary_muscle_development	D_vs_E10;stage_1W_vs_E10;stage_3W_vs_E10;stage	1	TRUE
ENSGALG00010016041	ENSGALG00010016041	PRJNA729271	0.775889212	1.84059E-17	0.775889212	stage_E14_vs_E10	ordinary_muscle_development		0	FALSE
ENSGALG00010015123	CEP85L	PRJNA729271	2.165263292	1.86553E-17	2.165263292	stage_E14_vs_E10	ordinary_muscle_development	D_vs_E10;stage_1W_vs_E10;stage_3W_vs_E10;stage	1	TRUE
ENSGALG00010002414	ENSGALG00010002414	PRJNA729271	3.076642811	1.87461E-17	3.076642811	stage_E14_vs_E10	ordinary_muscle_development	D_vs_E10;stage_1W_vs_E10;stage_3W_vs_E10;stage	1	TRUE
ENSGALG00010012977	NKRF	PRJNA729271	-0.887210978	1.88658E-17	0.887210978	stage_E14_vs_E10	ordinary_muscle_development		0	FALSE
ENSGALG00010022539	LYSMD2	PRJNA729271	0.876454846	1.92689E-17	0.876454846	stage_E14_vs_E10	ordinary_muscle_development		0	FALSE
ENSGALG00010002324	SNTG2	PRJNA729271	-7.018417318	1.93928E-17	7.018417318	stage_E14_vs_E10	ordinary_muscle_development	D_vs_E10;stage_1W_vs_E10;stage_3W_vs_E10;stage	1	TRUE
ENSGALG00010012693	UCHL5	PRJNA729271	-0.675950309	1.94323E-17	0.675950309	stage_E14_vs_E10	ordinary_muscle_development		0	FALSE
ENSGALG00010024580	IGF2	PRJNA729271	-1.735996822	1.96547E-17	1.735996822	stage_E14_vs_E10	ordinary_muscle_development	D_vs_E10;stage_1W_vs_E10;stage_3W_vs_E10;stage	1	TRUE
ENSGALG00010018436	FOXO4	PRJNA729271	1.251490315	1.99477E-17	1.251490315	stage_E14_vs_E10	ordinary_muscle_development	D_vs_E10;stage_1W_vs_E10;stage_3W_vs_E10;stage	1	TRUE
ENSGALG00010017707	AK2	PRJNA729271	-0.830505863	2.01002E-17	0.830505863	stage_E14_vs_E10	ordinary_muscle_development		0	FALSE
ENSGALG00010007767	VAT1L	PRJNA729271	-2.85988973	2.01798E-17	2.85988973	stage_E14_vs_E10	ordinary_muscle_development	D_vs_E10;stage_1W_vs_E10;stage_3W_vs_E10;stage	1	TRUE
ENSGALG00010018438	PPP2R2A	PRJNA729271	0.847757889	2.07263E-17	0.847757889	stage_E14_vs_E10	ordinary_muscle_development		0	FALSE
ENSGALG00010012833	JAK2	PRJNA729271	0.908367277	2.0882E-17	0.908367277	stage_E14_vs_E10	ordinary_muscle_development		0	FALSE
ENSGALG00010004787	RIOK3	PRJNA729271	0.982254325	2.10639E-17	0.982254325	stage_E14_vs_E10	ordinary_muscle_development		0	FALSE
ENSGALG00010014291	OSTN	PRJNA729271	-5.014118895	2.11612E-17	5.014118895	stage_E14_vs_E10	ordinary_muscle_development	D_vs_E10;stage_1W_vs_E10;stage_3W_vs_E10;stage	1	TRUE
ENSGALG00010016877	UBE2D3	PRJNA729271	1.168432768	2.13915E-17	1.168432768	stage_E14_vs_E10	ordinary_muscle_development	D_vs_E10;stage_1W_vs_E10;stage_3W_vs_E10;stage	1	TRUE
ENSGALG00010023338	HDLBP	PRJNA729271	1.069838162	2.1494E-17	1.069838162	stage_E14_vs_E10	ordinary_muscle_development	D_vs_E10;stage_1W_vs_E10;stage_3W_vs_E10;stage	1	TRUE
ENSGALG00010029728	TNRC6C	PRJNA729271	0.891554379	2.15443E-17	0.891554379	stage_E14_vs_E10	ordinary_muscle_development		0	FALSE
ENSGALG00010016683	RBM22	PRJNA729271	-0.643038148	2.15887E-17	0.643038148	stage_E14_vs_E10	ordinary_muscle_development		0	FALSE
ENSGALG00010002424	CASK	PRJNA729271	-0.49291411	2.18102E-17	0.49291411	stage_E14_vs_E10	ordinary_muscle_development		0	FALSE
ENSGALG00010027513	SEMA3G	PRJNA729271	1.569007548	2.22049E-17	1.569007548	stage_E14_vs_E10	ordinary_muscle_development	D_vs_E10;stage_1W_vs_E10;stage_3W_vs_E10;stage	1	TRUE
ENSGALG00010028573	KCNN1	PRJNA729271	2.267140062	2.2385E-17	2.267140062	stage_E14_vs_E10	ordinary_muscle_development	D_vs_E10;stage_1W_vs_E10;stage_3W_vs_E10;stage	1	TRUE
ENSGALG00010022533	TMEM119	PRJNA729271	-1.159122052	2.24749E-17	1.159122052	stage_E14_vs_E10	ordinary_muscle_development	D_vs_E10;stage_1W_vs_E10;stage_3W_vs_E10;stage	1	TRUE
ENSGALG00010009554	ABCB10	PRJNA729271	0.872843044	2.25631E-17	0.872843044	stage_E14_vs_E10	ordinary_muscle_development		0	FALSE
ENSGALG00010003165	BMP2K	PRJNA729271	0.961324229	2.26571E-17	0.961324229	stage_E14_vs_E10	ordinary_muscle_development		0	FALSE
ENSGALG00010028461	ENSGALG00010028461	PRJNA729271	2.722512758	2.3041E-17	2.722512758	stage_E14_vs_E10	ordinary_muscle_development	D_vs_E10;stage_1W_vs_E10;stage_3W_vs_E10;stage	1	TRUE
ENSGALG00010001127	SIAH2	PRJNA729271	0.856068388	2.35471E-17	0.856068388	stage_E14_vs_E10	ordinary_muscle_development		0	FALSE
ENSGALG00010010585	FBXL5	PRJNA729271	1.405921046	2.38478E-17	1.405921046	stage_E14_vs_E10	ordinary_muscle_development	D_vs_E10;stage_1W_vs_E10;stage_3W_vs_E10;stage	1	TRUE
ENSGALG00010011662	RIDA	PRJNA729271	-1.490280129	2.39506E-17	1.490280129	stage_E14_vs_E10	ordinary_muscle_development	D_vs_E10;stage_1W_vs_E10;stage_3W_vs_E10;stage	1	TRUE
ENSGALG00010013666	COX7B	PRJNA729271	1.234942198	2.41446E-17	1.234942198	stage_E14_vs_E10	ordinary_muscle_development	D_vs_E10;stage_1W_vs_E10;stage_3W_vs_E10;stage	1	TRUE
ENSGALG00010021217	FBXO2	PRJNA729271	-1.882663789	2.44414E-17	1.882663789	stage_E14_vs_E10	ordinary_muscle_development	D_vs_E10;stage_1W_vs_E10;stage_3W_vs_E10;stage	1	TRUE
ENSGALG00010027499	MOV10	PRJNA729271	3.438141139	2.47378E-17	3.438141139	stage_E14_vs_E10	ordinary_muscle_development	D_vs_E10;stage_1W_vs_E10;stage_3W_vs_E10;stage	1	TRUE
ENSGALG00010027291	MST1	PRJNA729271	2.34716202	2.48191E-17	2.34716202	stage_E14_vs_E10	ordinary_muscle_development	D_vs_E10;stage_1W_vs_E10;stage_3W_vs_E10;stage	1	TRUE
ENSGALG00010021023	MGEA5	PRJNA729271	0.835982055	2.49619E-17	0.835982055	stage_E14_vs_E10	ordinary_muscle_development		0	FALSE
ENSGALG00010022969	TTN	PRJNA729271	4.954214752	2.49645E-17	4.954214752	stage_E14_vs_E10	ordinary_muscle_development	D_vs_E10;stage_1W_vs_E10;stage_3W_vs_E10;stage	1	TRUE
ENSGALG00010018628	XDH	PRJNA729271	4.863487749	2.52873E-17	4.863487749	stage_E14_vs_E10	ordinary_muscle_development	D_vs_E10;stage_1W_vs_E10;stage_3W_vs_E10;stage	1	TRUE
ENSGALG00010022682	EIF1	PRJNA729271	1.338429631	2.56099E-17	1.338429631	stage_E14_vs_E10	ordinary_muscle_development	D_vs_E10;stage_1W_vs_E10;stage_3W_vs_E10;stage	1	TRUE
ENSGALG00010011772	GRHL2	PRJNA729271	-4.301549253	2.57571E-17	4.301549253	stage_E14_vs_E10	ordinary_muscle_development	D_vs_E10;stage_1W_vs_E10;stage_3W_vs_E10;stage	1	TRUE
ENSGALG00010027468	BSG	PRJNA729271	0.990921086	2.57625E-17	0.990921086	stage_E14_vs_E10	ordinary_muscle_development		0	FALSE
ENSGALG00010013387	NDST1	PRJNA729271	-0.808131314	2.63407E-17	0.808131314	stage_E14_vs_E10	ordinary_muscle_development		0	FALSE
ENSGALG00010016912	CCDC88C	PRJNA729271	1.37417845	2.65221E-17	1.37417845	stage_E14_vs_E10	ordinary_muscle_development	D_vs_E10;stage_1W_vs_E10;stage_3W_vs_E10;stage	1	TRUE
ENSGALG00010026767	ENSGALG00010026767	PRJNA729271	21.54756802	2.70761E-17	21.54756802	stage_E14_vs_E10	ordinary_muscle_development	D_vs_E10;stage_1W_vs_E10;stage_3W_vs_E10;stage	1	TRUE
ENSGALG00010024604	MFSD5	PRJNA729271	1.446658936	2.7291E-17	1.446658936	stage_E14_vs_E10	ordinary_muscle_development	D_vs_E10;stage_1W_vs_E10;stage_3W_vs_E10;stage	1	TRUE
ENSGALG00010007467	CHODL	PRJNA729271	-2.864651815	2.73098E-17	2.864651815	stage_E14_vs_E10	ordinary_muscle_development	D_vs_E10;stage_1W_vs_E10;stage_3W_vs_E10;stage	1	TRUE
ENSGALG00010003176	PRAG1	PRJNA729271	-2.330008353	2.7361E-17	2.330008353	stage_E14_vs_E10	ordinary_muscle_development	D_vs_E10;stage_1W_vs_E10;stage_3W_vs_E10;stage	1	TRUE
ENSGALG00010007270	CDH5	PRJNA729271	0.939147273	2.75007E-17	0.939147273	stage_E14_vs_E10	ordinary_muscle_development		0	FALSE
ENSGALG00010029218	RPL23A	PRJNA729271	1.302026971	2.7635E-17	1.302026971	stage_E14_vs_E10	ordinary_muscle_development	D_vs_E10;stage_1W_vs_E10;stage_3W_vs_E10;stage	1	TRUE
ENSGALG00010008943	ENSGALG00010008943	PRJNA729271	3.855222425	2.77039E-17	3.855222425	stage_E14_vs_E10	ordinary_muscle_development	D_vs_E10;stage_1W_vs_E10;stage_3W_vs_E10;stage	1	TRUE
ENSGALG00010016274	ZNF831	PRJNA729271	4.011682513	2.79239E-17	4.011682513	stage_E14_vs_E10	ordinary_muscle_development	D_vs_E10;stage_1W_vs_E10;stage_3W_vs_E10;stage	1	TRUE
ENSGALG00010029377	NCF1C	PRJNA729271	2.439818746	2.80923E-17	2.439818746	stage_E14_vs_E10	ordinary_muscle_development	D_vs_E10;stage_1W_vs_E10;stage_3W_vs_E10;stage	1	TRUE
ENSGALG00010010094	SLC2A9	PRJNA729271	2.084055657	2.81805E-17	2.084055657	stage_E14_vs_E10	ordinary_muscle_development	D_vs_E10;stage_1W_vs_E10;stage_3W_vs_E10;stage	1	TRUE
ENSGALG00010002231	TMEM14A	PRJNA729271	-1.199756629	2.83081E-17	1.199756629	stage_E14_vs_E10	ordinary_muscle_development	D_vs_E10;stage_1W_vs_E10;stage_3W_vs_E10;stage	1	TRUE
ENSGALG00010017278	CCR5	PRJNA729271	1.953389027	2.85911E-17	1.953389027	stage_E14_vs_E10	ordinary_muscle_development	D_vs_E10;stage_1W_vs_E10;stage_3W_vs_E10;stage	1	TRUE
ENSGALG00010021133	ENSGALG00010021133	PRJNA729271	-0.892869107	2.87165E-17	0.892869107	stage_E14_vs_E10	ordinary_muscle_development		0	FALSE
ENSGALG00010022991	TAL1	PRJNA729271	1.50264079	2.93122E-17	1.50264079	stage_E14_vs_E10	ordinary_muscle_development	D_vs_E10;stage_1W_vs_E10;stage_3W_vs_E10;stage	1	TRUE
ENSGALG00010001471	BMP5	PRJNA729271	-3.211511318	2.93434E-17	3.211511318	stage_E14_vs_E10	ordinary_muscle_development	D_vs_E10;stage_1W_vs_E10;stage_3W_vs_E10;stage	1	TRUE
ENSGALG00010015393	DCX	PRJNA729271	-3.255428551	2.93582E-17	3.255428551	stage_E14_vs_E10	ordinary_muscle_development	D_vs_E10;stage_1W_vs_E10;stage_3W_vs_E10;stage	1	TRUE
ENSGALG00010013016	QRSL1	PRJNA729271	1.046123725	2.94697E-17	1.046123725	stage_E14_vs_E10	ordinary_muscle_development	D_vs_E10;stage_1W_vs_E10;stage_3W_vs_E10;stage	1	TRUE
ENSGALG00010026137	LPXN	PRJNA729271	2.76208833	2.99551E-17	2.76208833	stage_E14_vs_E10	ordinary_muscle_development	D_vs_E10;stage_1W_vs_E10;stage_3W_vs_E10;stage	1	TRUE
ENSGALG00010006025	TNFAIP3	PRJNA729271	1.277539404	3.0307E-17	1.277539404	stage_E14_vs_E10	ordinary_muscle_development	D_vs_E10;stage_1W_vs_E10;stage_3W_vs_E10;stage	1	TRUE
ENSGALG00010025647	JHY	PRJNA729271	-4.569957777	3.04879E-17	4.569957777	stage_E14_vs_E10	ordinary_muscle_development	D_vs_E10;stage_1W_vs_E10;stage_3W_vs_E10;stage	1	TRUE
ENSGALG00010024931	UBE3C	PRJNA729271	0.759587759	3.07329E-17	0.759587759	stage_E14_vs_E10	ordinary_muscle_development		0	FALSE
ENSGALG00010016614	DNAJC19	PRJNA729271	1.191942838	3.0788E-17	1.191942838	stage_E14_vs_E10	ordinary_muscle_development	D_vs_E10;stage_1W_vs_E10;stage_3W_vs_E10;stage	1	TRUE
ENSGALG00010027205	OGN	PRJNA729271	1.920019632	3.0821E-17	1.920019632	stage_E14_vs_E10	ordinary_muscle_development	D_vs_E10;stage_1W_vs_E10;stage_3W_vs_E10;stage	1	TRUE
ENSGALG00010016100	SFRP4	PRJNA729271	-3.941384054	3.11639E-17	3.941384054	stage_E14_vs_E10	ordinary_muscle_development	D_vs_E10;stage_1W_vs_E10;stage_3W_vs_E10;stage	1	TRUE
ENSGALG00010023910	RNF7	PRJNA729271	0.862820014	3.13978E-17	0.862820014	stage_E14_vs_E10	ordinary_muscle_development		0	FALSE
ENSGALG00010008157	CTSO	PRJNA729271	-1.356424176	3.211E-17	1.356424176	stage_E14_vs_E10	ordinary_muscle_development	D_vs_E10;stage_1W_vs_E10;stage_3W_vs_E10;stage	1	TRUE
ENSGALG00010013228	RTFDC1	PRJNA729271	0.725335925	3.2526E-17	0.725335925	stage_E14_vs_E10	ordinary_muscle_development		0	FALSE
ENSGALG00010013371	FAT1	PRJNA729271	-1.345267667	3.30223E-17	1.345267667	stage_E14_vs_E10	ordinary_muscle_development	D_vs_E10;stage_1W_vs_E10;stage_3W_vs_E10;stage	1	TRUE
ENSGALG00010000173	NIPSNAP1	PRJNA729271	-1.103515465	3.31565E-17	1.103515465	stage_E14_vs_E10	ordinary_muscle_development	D_vs_E10;stage_1W_vs_E10;stage_3W_vs_E10;stage	1	TRUE

ENSGALG00010008233	CYRIB	PRJNA729271	-0.617879142	3.32541E-17	0.617879142	stage_E14_vs_E10	ordinary_muscle_development		0	FALSE
ENSGALG00010011814	ENSGALG00010011814	PRJNA729271	4.491834613	3.35157E-17	4.491834613	stage_E14_vs_E10	ordinary_muscle_development	D_vs_E10;stage_1W_vs_E10;stage_3W_vs_E10;stage	1	TRUE
ENSGALG00010016168	PLIN2	PRJNA729271	1.965217301	3.43125E-17	1.965217301	stage_E14_vs_E10	ordinary_muscle_development	D_vs_E10;stage_1W_vs_E10;stage_3W_vs_E10;stage	1	TRUE
ENSGALG00010012431	LAMP2	PRJNA729271	0.681598112	3.45557E-17	0.681598112	stage_E14_vs_E10	ordinary_muscle_development		0	FALSE
ENSGALG00010012801	DMC1	PRJNA729271	3.5828115	3.48119E-17	3.5828115	stage_E14_vs_E10	ordinary_muscle_development	D_vs_E10;stage_1W_vs_E10;stage_3W_vs_E10;stage	1	TRUE
ENSGALG00010023215	TRIP12	PRJNA729271	0.48149046	3.49192E-17	0.48149046	stage_E14_vs_E10	ordinary_muscle_development		0	FALSE
ENSGALG00010004296	ENSGALG00010004296	PRJNA729271	-8.624599321	3.51098E-17	8.624599321	stage_E14_vs_E10	ordinary_muscle_development	D_vs_E10;stage_1W_vs_E10;stage_3W_vs_E10;stage	1	TRUE
ENSGALG00010005499	PDE5A	PRJNA729271	-0.970450672	3.51777E-17	0.970450672	stage_E14_vs_E10	ordinary_muscle_development		0	FALSE
ENSGALG00010016624	MDH1	PRJNA729271	1.331153724	3.52819E-17	1.331153724	stage_E14_vs_E10	ordinary_muscle_development	D_vs_E10;stage_1W_vs_E10;stage_3W_vs_E10;stage	1	TRUE
ENSGALG00010021384	ARTN	PRJNA729271	2.981497151	3.57471E-17	2.981497151	stage_E14_vs_E10	ordinary_muscle_development	D_vs_E10;stage_1W_vs_E10;stage_3W_vs_E10;stage	1	TRUE
ENSGALG00010024781	CEP19	PRJNA729271	-1.481873521	3.5844E-17	1.481873521	stage_E14_vs_E10	ordinary_muscle_development	D_vs_E10;stage_1W_vs_E10;stage_3W_vs_E10;stage	1	TRUE
ENSGALG00010027388	URM1	PRJNA729271	1.241432693	3.65018E-17	1.241432693	stage_E14_vs_E10	ordinary_muscle_development	D_vs_E10;stage_1W_vs_E10;stage_3W_vs_E10;stage	1	TRUE
ENSGALG00010005619	CELF2	PRJNA729271	0.658488757	3.65585E-17	0.658488757	stage_E14_vs_E10	ordinary_muscle_development		0	FALSE
ENSGALG00010029593	PAFAH1B1	PRJNA729271	0.67560894	3.657E-17	0.67560894	stage_E14_vs_E10	ordinary_muscle_development		0	FALSE
ENSGALG00010023309	PIK3R3	PRJNA729271	-1.736223623	3.66916E-17	1.736223623	stage_E14_vs_E10	ordinary_muscle_development	D_vs_E10;stage_1W_vs_E10;stage_3W_vs_E10;stage	1	TRUE
ENSGALG00010019001	ALDH1A3	PRJNA729271	1.379294076	3.67342E-17	1.379294076	stage_E14_vs_E10	ordinary_muscle_development	D_vs_E10;stage_1W_vs_E10;stage_3W_vs_E10;stage	1	TRUE
ENSGALG00010006608	KL	PRJNA729271	3.493641905	3.68346E-17	3.493641905	stage_E14_vs_E10	ordinary_muscle_development	D_vs_E10;stage_1W_vs_E10;stage_3W_vs_E10;stage	1	TRUE
ENSGALG00010027460	WDR82	PRJNA729271	-0.631424968	3.72136E-17	0.631424968	stage_E14_vs_E10	ordinary_muscle_development		0	FALSE
ENSGALG00010028328	HNRNPM	PRJNA729271	-1.434327419	3.7674E-17	1.434327419	stage_E14_vs_E10	ordinary_muscle_development	D_vs_E10;stage_1W_vs_E10;stage_3W_vs_E10;stage	1	TRUE
ENSGALG00010021427	RPLP0	PRJNA729271	1.158586399	3.82138E-17	1.158586399	stage_E14_vs_E10	ordinary_muscle_development	D_vs_E10;stage_1W_vs_E10;stage_3W_vs_E10;stage	1	TRUE
ENSGALG00010017764	IGDCC3	PRJNA729271	-2.150011062	3.84379E-17	2.150011062	stage_E14_vs_E10	ordinary_muscle_development	D_vs_E10;stage_1W_vs_E10;stage_3W_vs_E10;stage	1	TRUE
ENSGALG00010005515	PCCA	PRJNA729271	0.81904648	3.84451E-17	0.81904648	stage_E14_vs_E10	ordinary_muscle_development		0	FALSE
ENSGALG00010014131	CDC23	PRJNA729271	-1.021263736	3.87063E-17	1.021263736	stage_E14_vs_E10	ordinary_muscle_development	D_vs_E10;stage_1W_vs_E10;stage_3W_vs_E10;stage	1	TRUE
ENSGALG00010008072	NRSN1	PRJNA729271	1.690422627	4.00777E-17	1.690422627	stage_E14_vs_E10	ordinary_muscle_development	D_vs_E10;stage_1W_vs_E10;stage_3W_vs_E10;stage	1	TRUE
ENSGALG00010015557	IRS4	PRJNA729271	1.640984138	4.02363E-17	1.640984138	stage_E14_vs_E10	ordinary_muscle_development	D_vs_E10;stage_1W_vs_E10;stage_3W_vs_E10;stage	1	TRUE
ENSGALG00010022407	RUNDC1	PRJNA729271	0.813482868	4.03759E-17	0.813482868	stage_E14_vs_E10	ordinary_muscle_development		0	FALSE
ENSGALG00010022792	DDB2	PRJNA729271	-1.088895533	4.11376E-17	1.088895533	stage_E14_vs_E10	ordinary_muscle_development	D_vs_E10;stage_1W_vs_E10;stage_3W_vs_E10;stage	1	TRUE
ENSGALG00010029776	WBP2	PRJNA729271	1.165922413	4.13926E-17	1.165922413	stage_E14_vs_E10	ordinary_muscle_development	D_vs_E10;stage_1W_vs_E10;stage_3W_vs_E10;stage	1	TRUE
ENSGALG00010024496	UEVLD	PRJNA729271	1.777870103	4.15668E-17	1.777870103	stage_E14_vs_E10	ordinary_muscle_development	D_vs_E10;stage_1W_vs_E10;stage_3W_vs_E10;stage	1	TRUE
ENSGALG00010014793	TLR2A	PRJNA729271	1.495647558	4.23943E-17	1.495647558	stage_E14_vs_E10	ordinary_muscle_development	D_vs_E10;stage_1W_vs_E10;stage_3W_vs_E10;stage	1	TRUE
ENSGALG00010001441	HMGCLL1	PRJNA729271	-2.840250996	4.27015E-17	2.840250996	stage_E14_vs_E10	ordinary_muscle_development	D_vs_E10;stage_1W_vs_E10;stage_3W_vs_E10;stage	1	TRUE
ENSGALG00010012610	RPL3	PRJNA729271	-1.326474472	4.28343E-17	1.326474472	stage_E14_vs_E10	ordinary_muscle_development	D_vs_E10;stage_1W_vs_E10;stage_3W_vs_E10;stage	1	TRUE
ENSGALG00010012193	TTLL1	PRJNA729271	-2.191320708	4.30577E-17	2.191320708	stage_E14_vs_E10	ordinary_muscle_development	D_vs_E10;stage_1W_vs_E10;stage_3W_vs_E10;stage	1	TRUE
ENSGALG00010027423	PEX11B	PRJNA729271	1.721125241	4.30917E-17	1.721125241	stage_E14_vs_E10	ordinary_muscle_development	D_vs_E10;stage_1W_vs_E10;stage_3W_vs_E10;stage	1	TRUE
ENSGALG00010005158	IFT43	PRJNA729271	-1.729375145	4.36626E-17	1.729375145	stage_E14_vs_E10	ordinary_muscle_development	D_vs_E10;stage_1W_vs_E10;stage_3W_vs_E10;stage	1	TRUE
ENSGALG00010020973	GNB5	PRJNA729271	-1.42343224	4.3709E-17	1.42343224	stage_E14_vs_E10	ordinary_muscle_development	D_vs_E10;stage_1W_vs_E10;stage_3W_vs_E10;stage	1	TRUE
ENSGALG00010029201	PIGS	PRJNA729271	-0.96609987	4.42536E-17	0.96609987	stage_E14_vs_E10	ordinary_muscle_development		0	FALSE
ENSGALG00010013376	ARHGAP8	PRJNA729271	-4.556739377	4.45261E-17	4.556739377	stage_E14_vs_E10	ordinary_muscle_development	D_vs_E10;stage_1W_vs_E10;stage_3W_vs_E10;stage	1	TRUE
ENSGALG00010004224	NT5E	PRJNA729271	2.246538928	4.47425E-17	2.246538928	stage_E14_vs_E10	ordinary_muscle_development	D_vs_E10;stage_1W_vs_E10;stage_3W_vs_E10;stage	1	TRUE
ENSGALG00010022839	SYT12	PRJNA729271	-3.018692676	4.49634E-17	3.018692676	stage_E14_vs_E10	ordinary_muscle_development	D_vs_E10;stage_1W_vs_E10;stage_3W_vs_E10;stage	1	TRUE
ENSGALG00010023655	C1R	PRJNA729271	1.582392287	4.53015E-17	1.582392287	stage_E14_vs_E10	ordinary_muscle_development	D_vs_E10;stage_1W_vs_E10;stage_3W_vs_E10;stage	1	TRUE
ENSGALG00010000033	ND5	PRJNA729271	1.989126529	4.54754E-17	1.989126529	stage_E14_vs_E10	ordinary_muscle_development	D_vs_E10;stage_1W_vs_E10;stage_3W_vs_E10;stage	1	TRUE
ENSGALG00010028587	ANKLE1	PRJNA729271	-1.844505907	4.59894E-17	1.844505907	stage_E14_vs_E10	ordinary_muscle_development	D_vs_E10;stage_1W_vs_E10;stage_3W_vs_E10;stage	1	TRUE
ENSGALG00010012391	NEURL1B	PRJNA729271	-1.360464392	4.59901E-17	1.360464392	stage_E14_vs_E10	ordinary_muscle_development	D_vs_E10;stage_1W_vs_E10;stage_3W_vs_E10;stage	1	TRUE
ENSGALG00010017544	SDE2	PRJNA729271	0.582924212	4.61086E-17	0.582924212	stage_E14_vs_E10	ordinary_muscle_development		0	FALSE
ENSGALG00010020969	RBP3	PRJNA729271	-3.939002783	4.63042E-17	3.939002783	stage_E14_vs_E10	ordinary_muscle_development	D_vs_E10;stage_1W_vs_E10;stage_3W_vs_E10;stage	1	TRUE
ENSGALG00010021573	NATD1	PRJNA729271	1.66062126	4.71487E-17	1.66062126	stage_E14_vs_E10	ordinary_muscle_development	D_vs_E10;stage_1W_vs_E10;stage_3W_vs_E10;stage	1	TRUE
ENSGALG00010018588	POLR3F	PRJNA729271	0.869661484	4.73848E-17	0.869661484	stage_E14_vs_E10	ordinary_muscle_development		0	FALSE
ENSGALG00010017185	NAA30	PRJNA729271	0.545550251	4.80372E-17	0.545550251	stage_E14_vs_E10	ordinary_muscle_development		0	FALSE
ENSGALG00010004097	ENSGALG00010004097	PRJNA729271	-1.030338717	4.85236E-17	1.030338717	stage_E14_vs_E10	ordinary_muscle_development	D_vs_E10;stage_1W_vs_E10;stage_3W_vs_E10;stage	1	TRUE
ENSGALG00010009499	MXD3	PRJNA729271	-2.17217468	4.88258E-17	2.17217468	stage_E14_vs_E10	ordinary_muscle_development	D_vs_E10;stage_1W_vs_E10;stage_3W_vs_E10;stage	1	TRUE
ENSGALG00010005601	TMEM215	PRJNA729271	-4.672010043	4.99456E-17	4.672010043	stage_E14_vs_E10	ordinary_muscle_development	D_vs_E10;stage_1W_vs_E10;stage_3W_vs_E10;stage	1	TRUE
ENSGALG00010015634	ENSGALG00010015634	PRJNA729271	-5.640428114	5.01399E-17	5.640428114	stage_E14_vs_E10	ordinary_muscle_development	D_vs_E10;stage_1W_vs_E10;stage_3W_vs_E10;stage	1	TRUE
ENSGALG00010029209	PRKCA	PRJNA729271	0.823918875	5.0374E-17	0.823918875	stage_E14_vs_E10	ordinary_muscle_development		0	FALSE
ENSGALG00010004372	SYNJ2	PRJNA729271	1.575561208	5.05761E-17	1.575561208	stage_E14_vs_E10	ordinary_muscle_development	D_vs_E10;stage_1W_vs_E10;stage_3W_vs_E10;stage	1	TRUE
ENSGALG00010013977	SLC38A2	PRJNA729271	1.64920444	5.10652E-17	1.64920444	stage_E14_vs_E10	ordinary_muscle_development	D_vs_E10;stage_1W_vs_E10;stage_3W_vs_E10;stage	1	TRUE
ENSGALG00010015645	MACO1	PRJNA729271	0.799817268	5.17725E-17	0.799817268	stage_E14_vs_E10	ordinary_muscle_development		0	FALSE
ENSGALG00010010456	BMP6	PRJNA729271	-1.561453876	5.23592E-17	1.561453876	stage_E14_vs_E10	ordinary_muscle_development	D_vs_E10;stage_1W_vs_E10;stage_3W_vs_E10;stage	1	TRUE
ENSGALG00010016859	RPIA	PRJNA729271	1.133792768	5.24315E-17	1.133792768	stage_E14_vs_E10	ordinary_muscle_development	D_vs_E10;stage_1W_vs_E10;stage_3W_vs_E10;stage	1	TRUE
ENSGALG00010026885	TULP1	PRJNA729271	5.308536264	5.29648E-17	5.308536264	stage_E14_vs_E10	ordinary_muscle_development	D_vs_E10;stage_1W_vs_E10;stage_3W_vs_E10;stage	1	TRUE
ENSGALG00010004237	RUFY3	PRJNA729271	0.775836715	5.30932E-17	0.775836715	stage_E14_vs_E10	ordinary_muscle_development		0	FALSE
ENSGALG00010029332	YWHAE	PRJNA729271	-1.269424429	5.37449E-17	1.269424429	stage_E14_vs_E10	ordinary_muscle_development	D_vs_E10;stage_1W_vs_E10;stage_3W_vs_E10;stage	1	TRUE
ENSGALG00010002533	LPAR2	PRJNA729271	-1.265423964	5.5417E-17	1.265423964	stage_E14_vs_E10	ordinary_muscle_development	D_vs_E10;stage_1W_vs_E10;stage_3W_vs_E10;stage	1	TRUE
ENSGALG00010002700	LHFPL6	PRJNA729271	-1.458334433	5.60525E-17	1.458334433	stage_E14_vs_E10	ordinary_muscle_development	D_vs_E10;stage_1W_vs_E10;stage_3W_vs_E10;stage	1	TRUE
ENSGALG00010002520	TTC32	PRJNA729271	1.384844228	5.62913E-17	1.384844228	stage_E14_vs_E10	ordinary_muscle_development	D_vs_E10;stage_1W_vs_E10;stage_3W_vs_E10;stage	1	TRUE
ENSGALG00010001966	ARHGAP6	PRJNA729271	1.203063614	5.65466E-17	1.203063614	stage_E14_vs_E10	ordinary_muscle_development	D_vs_E10;stage_1W_vs_E10;stage_3W_vs_E10;stage	1	TRUE
ENSGALG00010014372	CCDC171	PRJNA729271	-1.093825477	5.78598E-17	1.093825477	stage_E14_vs_E10	ordinary_muscle_development	D_vs_E10;stage_1W_vs_E10;stage_3W_vs_E10;stage	1	TRUE
ENSGALG00010007803	ME3	PRJNA729271	1.507733231	5.82352E-17	1.507733231	stage_E14_vs_E10	ordinary_muscle_development	D_vs_E10;stage_1W_vs_E10;stage_3W_vs_E10;stage	1	TRUE
ENSGALG00010018823	RAB40C	PRJNA729271	-0.657261288	5.92355E-17	0.657261288	stage_E14_vs_E10	ordinary_muscle_development		0	FALSE
ENSGALG00010024894	TUT4	PRJNA729271	1.038018492	5.95756E-17	1.038018492	stage_E14_vs_E10	ordinary_muscle_development	D_vs_E10;stage_1W_vs_E10;stage_3W_vs_E10;stage	1	TRUE
ENSGALG00010014945	TXN2	PRJNA729271	0.887611716	5.97994E-17	0.887611716	stage_E14_vs_E10	ordinary_muscle_development		0	FALSE
ENSGALG00010015716	MGAT4A	PRJNA729271	-1.156482515	5.98825E-17	1.156482515	stage_E14_vs_E10	ordinary_muscle_development	D_vs_E10;stage_1W_vs_E10;stage_3W_vs_E10;stage	1	TRUE
ENSGALG00010014030	WASF1	PRJNA729271	-3.541623068	5.99311E-17	3.541623068	stage_E14_vs_E10	ordinary_muscle_development	D_vs_E10;stage_1W_vs_E10;stage_3W_vs_E10;stage	1	TRUE
ENSGALG00010023179	KAT2A	PRJNA729271	-1.176567335	6.01167E-17	1.176567335	stage_E14_vs_E10	ordinary_muscle_development	D_vs_E10;stage_1W_vs_E10;stage_3W_vs_E10;stage	1	TRUE
ENSGALG00010021947	CDK2AP2	PRJNA729271	2.081044236	6.04356E-17	2.081044236	stage_E14_vs_E10	ordinary_muscle_development	D_vs_E10;stage_1W_vs_E10;stage_3W_vs_E10;stage	1	TRUE
ENSGALG00010011465	PPP2R2C	PRJNA729271	-3.096764634	6.1393E-17	3.096764634	stage_E14_vs_E10	ordinary_muscle_development	D_vs_E10;stage_1W_vs_E10;stage_3W_vs_E10;stage	1	TRUE
ENSGALG00010014449	ENSGALG00010014449	PRJNA729271	-1.318671344	6.17289E-17	1.318671344	stage_E14_vs_E10	ordinary_muscle_development	D_vs_E10;stage_1W_vs_E10;stage_3W_vs_E10;stage	1	TRUE
ENSGALG00010015039	FAM114A1	PRJNA729271	-1.093171217	6.21184E-17	1.093171217	stage_E14_vs_E10	ordinary_muscle_development	D_vs_E10;stage_1W_vs_E10;stage_3W_vs_E10;stage	1	TRUE
ENSGALG00010027107	KIF21B	PRJNA729271	-1.782457101	6.26989E-17	1.782457101	stage_E14_vs_E10	ordinary_muscle_development	D_vs_E10;stage_1W_vs_E10;stage_3W_vs_E10;stage	1	TRUE
ENSGALG00010022170	RSPRY1	PRJNA729271	-1.24502189	6.27123E-17	1.24502189	stage_E14_vs_E10	ordinary_muscle_development	D_vs_E10;stage_1W_vs_E10;stage_3W_vs_E10;stage	1	TRUE
ENSGALG00010025544	CSDE1	PRJNA729271	0.730801025	6.38004E-17	0.730801025	stage_E14_vs_E10	ordinary_muscle_development		0	FALSE
ENSGALG00010010956	AXDND1	PRJNA729271	4.648198409	6.47443E-17	4.648198409	stage_E14_vs_E10	ordinary_muscle_development	D_vs_E10;stage_1W_vs_E10;stage_3W_vs_E10;stage	1	TRUE
ENSGALG00010010945	PI4K2B	PRJNA729271	-0.860221214	6.4921E-17	0.860221214	stage_E14_vs_E10	ordinary_muscle_development		0	FALSE
ENSGALG00010028001	SBNO2	PRJNA729271	0.986902067	6.52135E-17	0.986902067	stage_E14_vs_E10	ordinary_muscle_development		0	FALSE
ENSGALG00010012511	OXCT1	PRJNA729271	-1.048237495	6.54167E-17	1.048237495	stage_E14_vs_E10	ordinary_muscle_development	D_vs_E10;stage_1W_vs_E10;stage_3W_vs_E10;stage	1	TRUE
ENSGALG00010008871	PRR7	PRJNA729271	-1.680947604	6.65687E-17	1.680947604	stage_E14_vs_E10	ordinary_muscle_development	D_vs_E10;stage_1W_vs_E10;stage_3W_vs_E10;stage	1	TRUE
ENSGALG00010020696	MOB1A	PRJNA729271	-0.866886775	6.65972E-17	0.866886775	stage_E14_vs_E10	ordinary_muscle_development		0	FALSE
ENSGALG00010026696	CSRNP1	PRJNA729271	-1.287305399	6.73535E-17	1.287305399	stage_E14_vs_E10	ordinary_muscle_development	D_vs_E10;stage_1W_vs_E10;stage_3W_vs_E10;stage	1	TRUE
ENSGALG00010005490	DCBLD2	PRJNA729271	-0.981369618	6.75722E-17	0.981369618	stage_E14_vs_E10	ordinary_muscle_development		0	FALSE
ENSGALG00010006004	DCLRE1C	PRJNA729271	-1.334115717	6.77357E-17	1.334115717	stage_E14_vs_E10	ordinary_muscle_development	D_vs_E10;stage_1W_vs_E10;stage_3W_vs_E10;stage	1	TRUE
ENSGALG00010024332	EFCAB7	PRJNA729271	-1.445373471	6.90404E-17	1.445373471	stage_E14_vs_E10	ordinary_muscle_development	D_vs_E10;stage_1W_vs_E10;stage_3W_vs_E10;stage	1	TRUE
ENSGALG00010019262	TMC3	PRJNA729271	-5.235237461	6.91791E-17	5.235237461	stage_E14_vs_E10	ordinary_muscle_development	D_vs_E10;stage_1W_vs_E10;stage_3W_vs_E10;stage	1	TRUE
ENSGALG00010006735	NR3C2	PRJNA729271	-0.987074826	6.98028E-17	0.987074826	stage_E14_vs_E10	ordinary_muscle_development		0	FALSE
ENSGALG00010008752	GOLM1	PRJNA729271	-1.473901471	6.98258E-17	1.473901471	stage_E14_vs_E10	ordinary_muscle_development	D_vs_E10;stage_1W_vs_E10;stage_3W_vs_E10;stage	1	TRUE
ENSGALG00010022379	METTL15	PRJNA729271	-1.053228801	6.98657E-17	1.053228801	stage_E14_vs_E10	ordinary_muscle_development	D_vs_E10;stage_1W_vs_E10;stage_3W_vs_E10;stage	1	TRUE
ENSGALG00010008123	ENSGALG00010008123	PRJNA729271	-1.424031496	6.98882E-17	1.424031496	stage_E14_vs_E10	ordinary_muscle_development	D_vs_E10;stage_1W_vs_E10;stage_3W_vs_E10;stage	1	TRUE
ENSGALG00010021736	EDEM1	PRJNA729271	0.701059407	7.00416E-17	0.701059407	stage_E14_vs_E10	ordinary_muscle_development		0	FALSE
ENSGALG00010025661	SNRPE	PRJNA729271	-1.113399142	7.02701E-17	1.113399142	stage_E14_vs_E10	ordinary_muscle_development	D_vs_E10;stage_1W_vs_E10;stage_3W_vs_E10;stage	1	TRUE
ENSGALG00010016131	SMYD1	PRJNA729271	1.887391338	7.09029E-17	1.887391338	stage_E14_vs_E10	ordinary_muscle_development	D_vs_E10;stage_1W_vs_E10;stage_3W_vs_E10;stage	1	TRUE
ENSGALG00010027356	ENSGALG00010027356	PRJNA729271	1.834937978	7.17218E-17	1.834937978	stage_E14_vs_E10	ordinary_muscle_development	D_vs_E10;stage_1W_vs_E10;stage_3W_vs_E10;stage	1	TRUE
ENSGALG00010026141	WNT3A	PRJNA729271	-8.199010373	7.2191E-17	8.199010373	stage_E14_vs_E10	ordinary_muscle_development	D_vs_E10;stage_1W_vs_E10;stage_3W_vs_E10;stage	1	TRUE
ENSGALG00010026112	ZFP91	PRJNA729271	1.023527978	7.27307E-17	1.023527978	stage_E14_vs_E10	ordinary_muscle_development	D_vs_E10;stage_1W_vs_E10;stage_3W_vs_E10;stage	1	TRUE
ENSGALG00010027077	ARF4	PRJNA729271	-0.889896974	7.30622E-17	0.889896974	stage_E14_vs_E10	ordinary_muscle_development		0	FALSE
ENSGALG00010005738	ELAVL2	PRJNA729271	-4.654131623	7.40738E-17	4.654131623	stage_E14_vs_E10	ordinary_muscle_development	D_vs_E10;stage_1W_vs_E10;stage_3W_vs_E10;stage	1	TRUE
ENSGALG00010000796	ENSGALG00010000796	PRJNA729271	-1.823710428	7.48429E-17	1.823710428	stage_E14_vs_E10	ordinary_muscle_development	D_vs_E10;stage_1W_vs_E10;stage_3W_vs_E10;stage	1	TRUE
ENSGALG00010029248	C1QBP	PRJNA729271	-0.695588378	7.55245E-17	0.695588378	stage_E14_vs_E10	ordinary_muscle_development		0	FALSE
ENSGALG00010013258	RBIS	PRJNA729271	1.223529941	7.61816E-17	1.223529941	stage_E14_vs_E10	ordinary_muscle_development	D_vs_E10;stage_1W_vs_E10;stage_3W_vs_E10;stage	1	TRUE
ENSGALG00010005875	CDK8	PRJNA729271	-1.332980457	7.63677E-17	1.332980457	stage_E14_vs_E10	ordinary_muscle_development	D_vs_E10;stage_1W_vs_E10;stage_3W_vs_E10;stage	1	TRUE
ENSGALG00010027964	RABEPK	PRJNA729271	-1.185816582	7.64542E-17	1.185816582	stage_E14_vs_E10	ordinary_muscle_development	D_vs_E10;stage_1W_vs_E10;stage_3W_vs_E10;stage	1	TRUE
ENSGALG00010014900	UBLCP1	PRJNA729271	-0.736202803	7.651E-17	0.736202803	stage_E14_vs_E10	ordinary_muscle_development		0	FALSE
ENSGALG00010022193	GDAP2	PRJNA729271	-0.702006788	7.66529E-17	0.702006788	stage_E14_vs_E10	ordinary_muscle_development		0	FALSE
ENSGALG00010022340	GFOD2	PRJNA729271	-0.810713119	7.66584E-17	0.810713119	stage_E14_vs_E10	ordinary_muscle_development		0	FALSE
ENSGALG00010013934	STAT1	PRJNA729271	2.396713341	7.68241E-17	2.396713341	stage_E14_vs_E10	ordinary_muscle_development	D_vs_E10;stage_1W_vs_E10;stage_3W_vs_E10;stage	1	TRUE
ENSGALG00010028071	DPM2	PRJNA729271	-0.991917996	7.75099E-17	0.991917996	stage_E14_vs_E10	ordinary_muscle_development		0	FALSE
ENSGALG00010023335	WDR59	PRJNA729271	0.579884379	7.77344E-17	0.579884379	stage_E14_vs_E10	ordinary_muscle_development		0	FALSE
ENSGALG00010007423	HEATR5B	PRJNA729271	-0.573467511	7.77932E-17	0.573467511	stage_E14_vs_E10	ordinary_muscle_development		0	FALSE
ENSGALG00010024753	HOXD8	PRJNA729271	-1.541617594	7.8771E-17	1.541617594	stage_E14_vs_E10	ordinary_muscle_development	D_vs_E10;stage_1W_vs_E10;stage_3W_vs_E10;stage	1	TRUE
ENSGALG00010021572	MYL9	PRJNA729271	0.967714402	7.88193E-17	0.967714402	stage_E14_vs_E10	ordinary_muscle_development		0	FALSE
ENSGALG00010011231	PLBD1	PRJNA729271	1.736052062	7.96797E-17	1.736052062	stage_E14_vs_E10	ordinary_muscle_development	D_vs_E10;stage_1W_vs_E10;stage_3W_vs_E10;stage	1	TRUE
ENSGALG00010014019	IRAG2	PRJNA729271	1.86414878	8.01945E-17	1.86414878	stage_E14_vs_E10	ordinary_muscle_development	D_vs_E10;stage_1W_vs_E10;stage_3W_vs_E10;stage	1	TRUE
ENSGALG00010010837	RBPJ	PRJNA729271	-1.050942037	8.04457E-17	1.050942037	stage_E14_vs_E10	ordinary_muscle_development	D_vs_E10;stage_1W_vs_E10;stage_3W_vs_E10;stage	1	TRUE
ENSGALG00010001307	CALU	PRJNA729271	-1.332354982	8.0559E-17	1.332354982	stage_E14_vs_E10	ordinary_muscle_development	D_vs_E10;stage_1W_vs_E10;stage_3W_vs_E10;stage	1	TRUE
ENSGALG00010011570	RGL1	PRJNA729271	-1.161936977	8.07112E-17	1.161936977	stage_E14_vs_E10	ordinary_muscle_development	D_vs_E10;stage_1W_vs_E10;stage_3W_vs_E10;stage	1	TRUE
ENSGALG00010020954	PLIN1	PRJNA729271	11.96120795	8.10804E-17	11.96120795	stage_E14_vs_E10	ordinary_muscle_development	D_vs_E10;stage_1W_vs_E10;stage_3W_vs_E10;stage	1	TRUE
ENSGALG00010010521	SNCG	PRJNA729271	3.234231974	8.11688E-17	3.234231974	stage_E14_vs_E10	ordinary_muscle_development	D_vs_E10;stage_1W_vs_E10;stage_3W_vs_E10;stage	1	TRUE
ENSGALG00010023879	EI24	PRJNA729271	0.988064454	8.25813E-17	0.988064454	stage_E14_vs_E10	ordinary_muscle_development		0	FALSE
ENSGALG00010007485	CTSC	PRJNA729271	1.316020712	8.27732E-17	1.316020712	stage_E14_vs_E10	ordinary_muscle_development	D_vs_E10;stage_1W_vs_E10;stage_3W_vs_E10;stage	1	TRUE
ENSGALG00010001599	TBLX1	PRJNA729271	-0.638498185	8.28473E-17	0.638498185	stage_E14_vs_E10	ordinary_muscle_development		0	FALSE
ENSGALG00010017264	PIAS1	PRJNA729271	0.725710579	8.38057E-17	0.725710579	stage_E14_vs_E10	ordinary_muscle_development		0	FALSE
ENSGALG00010010697	NEDD9	PRJNA729271	-1.206616918	8.46434E-17	1.206616918	stage_E14_vs_E10	ordinary_muscle_development	D_vs_E10;stage_1W_vs_E10;stage_3W_vs_E10;stage	1	TRUE
ENSGALG00010013497	FAM172A	PRJNA729271	-0.922390989	8.52219E-17	0.922390989	stage_E14_vs_E10	ordinary_muscle_development		0	FALSE
ENSGALG00010021431	CPSF4	PRJNA729271	-0.923579613	8.52584E-17	0.923579613	stage_E14_vs_E10	ordinary_muscle_development		0	FALSE
ENSGALG00010004968	TRAT1	PRJNA729271	7.85070333	8.62158E-17	7.85070333	stage_E14_vs_E10	ordinary_muscle_development	D_vs_E10;stage_1W_vs_E10;stage_3W_vs_E10;stage	1	TRUE
ENSGALG00010027744	ENSGALG00010027744	PRJNA729271	9.028682619	8.81889E-17	9.028682619	stage_E14_vs_E10	ordinary_muscle_development	D_vs_E10;stage_1W_vs_E10;stage_3W_vs_E10;stage	1	TRUE
ENSGALG00010019810	ZFAND6	PRJNA729271	1.054502581	8.87499E-17	1.054502581	stage_E14_vs_E10	ordinary_muscle_development	D_vs_E10;stage_1W_vs_E10;stage_3W_vs_E10;stage	1	TRUE
ENSGALG00010018470	IQCB1	PRJNA729271	1.33208129	8.87981E-17	1.33208129	stage_E14_vs_E10	ordinary_muscle_development	D_vs_E10;stage_1W_vs_E10;stage_3W_vs_E10;stage	1	TRUE
ENSGALG00010010153	ENSGALG00010010153	PRJNA729271	3.669648288	8.89992E-17	3.669648288	stage_E14_vs_E10	ordinary_muscle_development	D_vs_E10;stage_1W_vs_E10;stage_3W_vs_E10;stage	1	TRUE
ENSGALG00010023948	SULF2	PRJNA729271	-1.91185099	8.90786E-17	1.91185099	stage_E14_vs_E10	ordinary_muscle_development	D_vs_E10;stage_1W_vs_E10;stage_3W_vs_E10;stage	1	TRUE
ENSGALG00010001285	ENSGALG00010001285	PRJNA729271	1.802508332	8.98275E-17	1.802508332	stage_E14_vs_E10	ordinary_muscle_development	D_vs_E10;stage_1W_vs_E10;stage_3W_vs_E10;stage	1	TRUE
ENSGALG00010016505	KCTD16	PRJNA729271	-4.012152016	8.9922E-17	4.012152016	stage_E14_vs_E10	ordinary_muscle_development	D_vs_E10;stage_1W_vs_E10;stage_3W_vs_E10;stage	1	TRUE
ENSGALG00010014278	G3BP1	PRJNA729271	-1.191436574	9.05932E-17	1.191436574	stage_E14_vs_E10	ordinary_muscle_development	D_vs_E10;stage_1W_vs_E10;stage_3W_vs_E10;stage	1	TRUE
ENSGALG00010000098	ENSGALG00010000098	PRJNA729271	1.610761305	9.27481E-17	1.610761305	stage_E14_vs_E10	ordinary_muscle_development	D_vs_E10;stage_1W_vs_E10;stage_3W_vs_E10;stage	1	TRUE
ENSGALG00010025125	PIK3CB	PRJNA729271	1.109069141	9.32436E-17	1.109069141	stage_E14_vs_E10	ordinary_muscle_development	D_vs_E10;stage_1W_vs_E10;stage_3W_vs_E10;stage	1	TRUE
ENSGALG00010001187	GFM1	PRJNA729271	1.534843225	9.34882E-17	1.534843225	stage_E14_vs_E10	ordinary_muscle_development	D_vs_E10;stage_1W_vs_E10;stage_3W_vs_E10;stage	1	TRUE
ENSGALG00010016462	RNF24	PRJNA729271	-1.352916957	9.35145E-17	1.352916957	stage_E14_vs_E10	ordinary_muscle_development	D_vs_E10;stage_1W_vs_E10;stage_3W_vs_E10;stage	1	TRUE
ENSGALG00010017664	ENSGALG00010017664	PRJNA729271	0.627519093	9.37743E-17	0.627519093	stage_E14_vs_E10	ordinary_muscle_development		0	FALSE
ENSGALG00010024434	B3GNT9	PRJNA729271	-1.352351218	9.44889E-17	1.352351218	stage_E14_vs_E10	ordinary_muscle_development	D_vs_E10;stage_1W_vs_E10;stage_3W_vs_E10;stage	1	TRUE
ENSGALG00010023593	CSK	PRJNA729271	-0.71187063	9.61056E-17	0.71187063	stage_E14_vs_E10	ordinary_muscle_development		0	FALSE
ENSGALG00010005446	ENSGALG00010005446	PRJNA729271	3.124813713	9.69505E-17	3.124813713	stage_E14_vs_E10	ordinary_muscle_development	D_vs_E10;stage_1W_vs_E10;stage_3W_vs_E10;stage	1	TRUE
ENSGALG00010016382	UGDH	PRJNA729271	-1.574466124	9.80021E-17	1.574466124	stage_E14_vs_E10	ordinary_muscle_development	D_vs_E10;stage_1W_vs_E10;stage_3W_vs_E10;stage	1	TRUE
ENSGALG00010022956	PBLD	PRJNA729271	1.022665296	9.95031E-17	1.022665296	stage_E14_vs_E10	ordinary_muscle_development	D_vs_E10;stage_1W_vs_E10;stage_3W_vs_E10;stage	1	TRUE
ENSGALG00010023265	RAB5C	PRJNA729271	-0.605931681	1.00545E-16	0.605931681	stage_E14_vs_E10	ordinary_muscle_development		0	FALSE
ENSGALG00010006516	NBEA	PRJNA729271	-1.075943327	1.01671E-16	1.075943327	stage_E14_vs_E10	ordinary_muscle_development	D_vs_E10;stage_1W_vs_E10;stage_3W_vs_E10;stage	1	TRUE
ENSGALG00010004065	TIFA	PRJNA729271	2.101660925	1.01679E-16	2.101660925	stage_E14_vs_E10	ordinary_muscle_development	D_vs_E10;stage_1W_vs_E10;stage_3W_vs_E10;stage	1	TRUE
ENSGALG00010027547	B4GALT3	PRJNA729271	1.867559105	1.02668E-16	1.867559105	stage_E14_vs_E10	ordinary_muscle_development	D_vs_E10;stage_1W_vs_E10;stage_3W_vs_E10;stage	1	TRUE
ENSGALG00010012688	NDUFAF2	PRJNA729271	0.946180306	1.03195E-16	0.946180306	stage_E14_vs_E10	ordinary_muscle_development		0	FALSE
ENSGALG00010012113	WDR47	PRJNA729271	-0.815776456	1.04341E-16	0.815776456	stage_E14_vs_E10	ordinary_muscle_development		0	FALSE
ENSGALG00010013488	UPRT	PRJNA729271	-1.040348257	1.04519E-16	1.040348257	stage_E14_vs_E10	ordinary_muscle_development	D_vs_E10;stage_1W_vs_E10;stage_3W_vs_E10;stage	1	TRUE
ENSGALG00010017372	ENSGALG00010017372	PRJNA729271	0.928420402	1.06709E-16	0.928420402	stage_E14_vs_E10	ordinary_muscle_development		0	FALSE
ENSGALG00010013047	CTPS1	PRJNA729271	-0.872998077	1.07917E-16	0.872998077	stage_E14_vs_E10	ordinary_muscle_development		0	FALSE
ENSGALG00010000491	ENSGALG00010000491	PRJNA729271	1.30798563	1.08583E-16	1.30798563	stage_E14_vs_E10	ordinary_muscle_development	D_vs_E10;stage_1W_vs_E10;stage_3W_vs_E10;stage	1	TRUE
ENSGALG00010017743	KCNH5	PRJNA729271	-4.633945033	1.09138E-16	4.633945033	stage_E14_vs_E10	ordinary_muscle_development	D_vs_E10;stage_1W_vs_E10;stage_3W_vs_E10;stage	1	TRUE
ENSGALG00010008169	HTR4	PRJNA729271	2.558909131	1.09155E-16	2.558909131	stage_E14_vs_E10	ordinary_muscle_development	D_vs_E10;stage_1W_vs_E10;stage_3W_vs_E10;stage	1	TRUE
ENSGALG00010019399	CD93	PRJNA729271	1.285656405	1.09269E-16	1.285656405	stage_E14_vs_E10	ordinary_muscle_development	D_vs_E10;stage_1W_vs_E10;stage_3W_vs_E10;stage	1	TRUE
ENSGALG00010000603	ZNF622	PRJNA729271	-0.947250978	1.09853E-16	0.947250978	stage_E14_vs_E10	ordinary_muscle_development		0	FALSE
ENSGALG00010008346	NR4A3	PRJNA729271	4.40646965	1.09931E-16	4.40646965	stage_E14_vs_E10	ordinary_muscle_development	D_vs_E10;stage_1W_vs_E10;stage_3W_vs_E10;stage	1	TRUE
ENSGALG00010017518	PEMT	PRJNA729271	1.241394639	1.12796E-16	1.241394639	stage_E14_vs_E10	ordinary_muscle_development	D_vs_E10;stage_1W_vs_E10;stage_3W_vs_E10;stage	1	TRUE
ENSGALG00010024555	EPHB1	PRJNA729271	1.378270591	1.12945E-16	1.378270591	stage_E14_vs_E10	ordinary_muscle_development	D_vs_E10;stage_1W_vs_E10;stage_3W_vs_E10;stage	1	TRUE
ENSGALG00010001738	PUDP	PRJNA729271	1.004607962	1.13065E-16	1.004607962	stage_E14_vs_E10	ordinary_muscle_development	D_vs_E10;stage_1W_vs_E10;stage_3W_vs_E10;stage	1	TRUE
ENSGALG00010023850	DAZAP2	PRJNA729271	1.69560815	1.14137E-16	1.69560815	stage_E14_vs_E10	ordinary_muscle_development	D_vs_E10;stage_1W_vs_E10;stage_3W_vs_E10;stage	1	TRUE
ENSGALG00010022312	ZNF592	PRJNA729271	0.45164762	1.1437E-16	0.45164762	stage_E14_vs_E10	ordinary_muscle_development		0	FALSE
ENSGALG00010027816	ZMYND19	PRJNA729271	-1.046936429	1.15653E-16	1.046936429	stage_E14_vs_E10	ordinary_muscle_development	D_vs_E10;stage_1W_vs_E10;stage_3W_vs_E10;stage	1	TRUE
ENSGALG00010020779	SLC15A4	PRJNA729271	-0.565517742	1.16034E-16	0.565517742	stage_E14_vs_E10	ordinary_muscle_development		0	FALSE
ENSGALG00010018619	MAPK1IP1L	PRJNA729271	-0.657199328	1.16234E-16	0.657199328	stage_E14_vs_E10	ordinary_muscle_development		0	FALSE
ENSGALG00010003797	ENSGALG00010003797	PRJNA729271	1.437323815	1.16758E-16	1.437323815	stage_E14_vs_E10	ordinary_muscle_development	D_vs_E10;stage_1W_vs_E10;stage_3W_vs_E10;stage	1	TRUE

ENSGALG00010027224	RARRES2	PRJNA729271	1.018462262	1.17155E-16	1.018462262	stage_E14_vs_E10	ordinary_muscle_development	D_vs_E10;stage_1W_vs_E10;stage_3W_vs_E10;stage	1	TRUE
ENSGALG00010029941	COX10	PRJNA729271	1.450026314	1.18061E-16	1.450026314	stage_E14_vs_E10	ordinary_muscle_development	D_vs_E10;stage_1W_vs_E10;stage_3W_vs_E10;stage	1	TRUE
ENSGALG00010023317	RAPGEF3	PRJNA729271	1.256945184	1.18318E-16	1.256945184	stage_E14_vs_E10	ordinary_muscle_development	D_vs_E10;stage_1W_vs_E10;stage_3W_vs_E10;stage	1	TRUE
ENSGALG00010024223	ENSGALG00010024223	PRJNA729271	-3.450891857	1.18512E-16	3.450891857	stage_E14_vs_E10	ordinary_muscle_development	D_vs_E10;stage_1W_vs_E10;stage_3W_vs_E10;stage	1	TRUE
ENSGALG00010017554	FAM131A	PRJNA729271	-3.28136233	1.18652E-16	3.28136233	stage_E14_vs_E10	ordinary_muscle_development	D_vs_E10;stage_1W_vs_E10;stage_3W_vs_E10;stage	1	TRUE
ENSGALG00010013063	PARP12	PRJNA729271	1.828600155	1.19246E-16	1.828600155	stage_E14_vs_E10	ordinary_muscle_development	D_vs_E10;stage_1W_vs_E10;stage_3W_vs_E10;stage	1	TRUE
ENSGALG00010022396	PPP1R1C	PRJNA729271	2.759769865	1.20242E-16	2.759769865	stage_E14_vs_E10	ordinary_muscle_development	D_vs_E10;stage_1W_vs_E10;stage_3W_vs_E10;stage	1	TRUE
ENSGALG00010010431	SOCS2	PRJNA729271	-2.25954403	1.22714E-16	2.25954403	stage_E14_vs_E10	ordinary_muscle_development	D_vs_E10;stage_1W_vs_E10;stage_3W_vs_E10;stage	1	TRUE
ENSGALG00010010337	SCARA3	PRJNA729271	-1.325865705	1.23549E-16	1.325865705	stage_E14_vs_E10	ordinary_muscle_development	D_vs_E10;stage_1W_vs_E10;stage_3W_vs_E10;stage	1	TRUE
ENSGALG00010002369	GEMIN8	PRJNA729271	-1.027174626	1.24341E-16	1.027174626	stage_E14_vs_E10	ordinary_muscle_development	D_vs_E10;stage_1W_vs_E10;stage_3W_vs_E10;stage	1	TRUE
ENSGALG00010013989	ENSGALG00010013989	PRJNA729271	1.70502744	1.2566E-16	1.70502744	stage_E14_vs_E10	ordinary_muscle_development	D_vs_E10;stage_1W_vs_E10;stage_3W_vs_E10;stage	1	TRUE
ENSGALG00010005909	BRMS1L	PRJNA729271	-0.869984837	1.27658E-16	0.869984837	stage_E14_vs_E10	ordinary_muscle_development		0	FALSE
ENSGALG00010017714	ATAD3A	PRJNA729271	-1.321316584	1.29116E-16	1.321316584	stage_E14_vs_E10	ordinary_muscle_development	D_vs_E10;stage_1W_vs_E10;stage_3W_vs_E10;stage	1	TRUE
ENSGALG00010029145	RC3H2	PRJNA729271	1.121987459	1.30263E-16	1.121987459	stage_E14_vs_E10	ordinary_muscle_development	D_vs_E10;stage_1W_vs_E10;stage_3W_vs_E10;stage	1	TRUE
ENSGALG00010023208	BACE1	PRJNA729271	-0.667402541	1.30379E-16	0.667402541	stage_E14_vs_E10	ordinary_muscle_development		0	FALSE
ENSGALG00010028380	RUSC1	PRJNA729271	1.185810171	1.32423E-16	1.185810171	stage_E14_vs_E10	ordinary_muscle_development	D_vs_E10;stage_1W_vs_E10;stage_3W_vs_E10;stage	1	TRUE
ENSGALG00010007214	MTHFSD	PRJNA729271	1.166699079	1.32496E-16	1.166699079	stage_E14_vs_E10	ordinary_muscle_development	D_vs_E10;stage_1W_vs_E10;stage_3W_vs_E10;stage	1	TRUE
ENSGALG00010019771	VWCE	PRJNA729271	-3.869838098	1.32938E-16	3.869838098	stage_E14_vs_E10	ordinary_muscle_development	D_vs_E10;stage_1W_vs_E10;stage_3W_vs_E10;stage	1	TRUE
ENSGALG00010007826	ENSGALG00010007826	PRJNA729271	11.54056804	1.33723E-16	11.54056804	stage_E14_vs_E10	ordinary_muscle_development	D_vs_E10;stage_1W_vs_E10;stage_3W_vs_E10;stage	1	TRUE
ENSGALG00010019461	MGAT5	PRJNA729271	0.588074098	1.34953E-16	0.588074098	stage_E14_vs_E10	ordinary_muscle_development		0	FALSE
ENSGALG00010013573	ENSGALG00010013573	PRJNA729271	3.933105312	1.3505E-16	3.933105312	stage_E14_vs_E10	ordinary_muscle_development	D_vs_E10;stage_1W_vs_E10;stage_3W_vs_E10;stage	1	TRUE
ENSGALG00010024960	ARFRP1	PRJNA729271	-0.874746954	1.39973E-16	0.874746954	stage_E14_vs_E10	ordinary_muscle_development		0	FALSE
ENSGALG00010019855	TTC7A	PRJNA729271	1.045983193	1.40018E-16	1.045983193	stage_E14_vs_E10	ordinary_muscle_development	D_vs_E10;stage_1W_vs_E10;stage_3W_vs_E10;stage	1	TRUE
ENSGALG00010027145	CELSR3	PRJNA729271	-1.466652975	1.41389E-16	1.466652975	stage_E14_vs_E10	ordinary_muscle_development	D_vs_E10;stage_1W_vs_E10;stage_3W_vs_E10;stage	1	TRUE
ENSGALG00010013837	IFI27L2	PRJNA729271	2.810779829	1.41717E-16	2.810779829	stage_E14_vs_E10	ordinary_muscle_development	D_vs_E10;stage_1W_vs_E10;stage_3W_vs_E10;stage	1	TRUE
ENSGALG00010002146	CACUL1	PRJNA729271	0.736827055	1.42483E-16	0.736827055	stage_E14_vs_E10	ordinary_muscle_development		0	FALSE
ENSGALG00010020393	SHISA9	PRJNA729271	-2.756812587	1.4304E-16	2.756812587	stage_E14_vs_E10	ordinary_muscle_development	D_vs_E10;stage_1W_vs_E10;stage_3W_vs_E10;stage	1	TRUE
ENSGALG00010015137	AMN	PRJNA729271	3.344531505	1.44771E-16	3.344531505	stage_E14_vs_E10	ordinary_muscle_development	D_vs_E10;stage_1W_vs_E10;stage_3W_vs_E10;stage	1	TRUE
ENSGALG00010004933	SPTLC2	PRJNA729271	-0.773770559	1.45985E-16	0.773770559	stage_E14_vs_E10	ordinary_muscle_development		0	FALSE
ENSGALG00010002149	ENSGALG00010002149	PRJNA729271	-3.331051067	1.4718E-16	3.331051067	stage_E14_vs_E10	ordinary_muscle_development	D_vs_E10;stage_1W_vs_E10;stage_3W_vs_E10;stage	1	TRUE
ENSGALG00010017368	ERLIN2	PRJNA729271	-0.429920463	1.4744E-16	0.429920463	stage_E14_vs_E10	ordinary_muscle_development		0	FALSE
ENSGALG00010015984	PDCL3	PRJNA729271	0.922039458	1.47452E-16	0.922039458	stage_E14_vs_E10	ordinary_muscle_development		0	FALSE
ENSGALG00010028111	TRUB2	PRJNA729271	-0.781640155	1.47761E-16	0.781640155	stage_E14_vs_E10	ordinary_muscle_development		0	FALSE
ENSGALG00010013160	CRYBG1	PRJNA729271	-5.200844828	1.49238E-16	5.200844828	stage_E14_vs_E10	ordinary_muscle_development	D_vs_E10;stage_1W_vs_E10;stage_3W_vs_E10;stage	1	TRUE
ENSGALG00010010439	HGF	PRJNA729271	2.006158922	1.50777E-16	2.006158922	stage_E14_vs_E10	ordinary_muscle_development	D_vs_E10;stage_1W_vs_E10;stage_3W_vs_E10;stage	1	TRUE
ENSGALG00010018901	TCF7L2	PRJNA729271	-1.256570705	1.53391E-16	1.256570705	stage_E14_vs_E10	ordinary_muscle_development	D_vs_E10;stage_1W_vs_E10;stage_3W_vs_E10;stage	1	TRUE
ENSGALG00010005521	SYTL3	PRJNA729271	3.203245366	1.54816E-16	3.203245366	stage_E14_vs_E10	ordinary_muscle_development	D_vs_E10;stage_1W_vs_E10;stage_3W_vs_E10;stage	1	TRUE
ENSGALG00010028911	LMX1B	PRJNA729271	-7.681343126	1.56349E-16	7.681343126	stage_E14_vs_E10	ordinary_muscle_development	D_vs_E10;stage_1W_vs_E10;stage_3W_vs_E10;stage	1	TRUE
ENSGALG00010002467	SART3	PRJNA729271	-0.985190653	1.59315E-16	0.985190653	stage_E14_vs_E10	ordinary_muscle_development		0	FALSE
ENSGALG00010016141	RRP15	PRJNA729271	-1.509465081	1.59378E-16	1.509465081	stage_E14_vs_E10	ordinary_muscle_development	D_vs_E10;stage_1W_vs_E10;stage_3W_vs_E10;stage	1	TRUE
ENSGALG00010000034	CYTB	PRJNA729271	1.844039228	1.61245E-16	1.844039228	stage_E14_vs_E10	ordinary_muscle_development	D_vs_E10;stage_1W_vs_E10;stage_3W_vs_E10;stage	1	TRUE
ENSGALG00010020886	GBF1	PRJNA729271	-0.734877408	1.63146E-16	0.734877408	stage_E14_vs_E10	ordinary_muscle_development		0	FALSE
ENSGALG00010001248	PTPRZ1	PRJNA729271	-2.187705369	1.64821E-16	2.187705369	stage_E14_vs_E10	ordinary_muscle_development	D_vs_E10;stage_1W_vs_E10;stage_3W_vs_E10;stage	1	TRUE
ENSGALG00010022590	ENSGALG00010022590	PRJNA729271	-1.393526464	1.66281E-16	1.393526464	stage_E14_vs_E10	ordinary_muscle_development	D_vs_E10;stage_1W_vs_E10;stage_3W_vs_E10;stage	1	TRUE
ENSGALG00010025333	FZD5	PRJNA729271	1.618109114	1.66502E-16	1.618109114	stage_E14_vs_E10	ordinary_muscle_development	D_vs_E10;stage_1W_vs_E10;stage_3W_vs_E10;stage	1	TRUE
ENSGALG00010003787	SMIM8	PRJNA729271	1.571572187	1.66804E-16	1.571572187	stage_E14_vs_E10	ordinary_muscle_development	D_vs_E10;stage_1W_vs_E10;stage_3W_vs_E10;stage	1	TRUE
ENSGALG00010027925	MKRN2	PRJNA729271	-0.594847432	1.6866E-16	0.594847432	stage_E14_vs_E10	ordinary_muscle_development		0	FALSE
ENSGALG00010016379	TFAP4	PRJNA729271	-1.32039669	1.72603E-16	1.32039669	stage_E14_vs_E10	ordinary_muscle_development	D_vs_E10;stage_1W_vs_E10;stage_3W_vs_E10;stage	1	TRUE
ENSGALG00010017003	CPZ	PRJNA729271	3.352983198	1.72846E-16	3.352983198	stage_E14_vs_E10	ordinary_muscle_development	D_vs_E10;stage_1W_vs_E10;stage_3W_vs_E10;stage	1	TRUE
ENSGALG00010009232	ECH1	PRJNA729271	1.374705245	1.7306E-16	1.374705245	stage_E14_vs_E10	ordinary_muscle_development	D_vs_E10;stage_1W_vs_E10;stage_3W_vs_E10;stage	1	TRUE
ENSGALG00010013119	RNF114	PRJNA729271	0.659393564	1.74533E-16	0.659393564	stage_E14_vs_E10	ordinary_muscle_development		0	FALSE
ENSGALG00010029313	CCDC40	PRJNA729271	-2.377039424	1.7466E-16	2.377039424	stage_E14_vs_E10	ordinary_muscle_development	D_vs_E10;stage_1W_vs_E10;stage_3W_vs_E10;stage	1	TRUE
ENSGALG00010014243	ENSGALG00010014243	PRJNA729271	1.202938178	1.77851E-16	1.202938178	stage_E14_vs_E10	ordinary_muscle_development	D_vs_E10;stage_1W_vs_E10;stage_3W_vs_E10;stage	1	TRUE
ENSGALG00010016040	RPSA2	PRJNA729271	0.886868886	1.77885E-16	0.886868886	stage_E14_vs_E10	ordinary_muscle_development		0	FALSE
ENSGALG00010022731	LRRC8B	PRJNA729271	-1.543400127	1.79098E-16	1.543400127	stage_E14_vs_E10	ordinary_muscle_development	D_vs_E10;stage_1W_vs_E10;stage_3W_vs_E10;stage	1	TRUE
ENSGALG00010022190	ACVR1B	PRJNA729271	1.368102362	1.8194E-16	1.368102362	stage_E14_vs_E10	ordinary_muscle_development	D_vs_E10;stage_1W_vs_E10;stage_3W_vs_E10;stage	1	TRUE
ENSGALG00010008464	SF3B6	PRJNA729271	-0.940220679	1.89813E-16	0.940220679	stage_E14_vs_E10	ordinary_muscle_development		0	FALSE
ENSGALG00010018030	POLB	PRJNA729271	0.840318361	1.90983E-16	0.840318361	stage_E14_vs_E10	ordinary_muscle_development		0	FALSE
ENSGALG00010015124	UXS1	PRJNA729271	0.802640761	1.9156E-16	0.802640761	stage_E14_vs_E10	ordinary_muscle_development		0	FALSE
ENSGALG00010029324	BCAS3	PRJNA729271	1.055895734	1.91971E-16	1.055895734	stage_E14_vs_E10	ordinary_muscle_development	D_vs_E10;stage_1W_vs_E10;stage_3W_vs_E10;stage	1	TRUE
ENSGALG00010007896	TIMMDC1	PRJNA729271	0.877252973	1.94646E-16	0.877252973	stage_E14_vs_E10	ordinary_muscle_development		0	FALSE
ENSGALG00010025404	CERS6	PRJNA729271	-2.443797609	1.97052E-16	2.443797609	stage_E14_vs_E10	ordinary_muscle_development	D_vs_E10;stage_1W_vs_E10;stage_3W_vs_E10;stage	1	TRUE
ENSGALG00010013493	ENSGALG00010013493	PRJNA729271	1.072128703	1.98468E-16	1.072128703	stage_E14_vs_E10	ordinary_muscle_development	D_vs_E10;stage_1W_vs_E10;stage_3W_vs_E10;stage	1	TRUE
ENSGALG00010016295	UBE2K	PRJNA729271	-0.779843397	1.99154E-16	0.779843397	stage_E14_vs_E10	ordinary_muscle_development		0	FALSE
ENSGALG00010016324	TUBB1	PRJNA729271	-1.801289811	1.99517E-16	1.801289811	stage_E14_vs_E10	ordinary_muscle_development	D_vs_E10;stage_1W_vs_E10;stage_3W_vs_E10;stage	1	TRUE
ENSGALG00010006120	MOGS	PRJNA729271	1.115357028	2.00659E-16	1.115357028	stage_E14_vs_E10	ordinary_muscle_development	D_vs_E10;stage_1W_vs_E10;stage_3W_vs_E10;stage	1	TRUE
ENSGALG00010019390	TVP23A	PRJNA729271	-2.279242357	2.03688E-16	2.279242357	stage_E14_vs_E10	ordinary_muscle_development	D_vs_E10;stage_1W_vs_E10;stage_3W_vs_E10;stage	1	TRUE
ENSGALG00010004966	LAMA3	PRJNA729271	1.764888806	2.04928E-16	1.764888806	stage_E14_vs_E10	ordinary_muscle_development	D_vs_E10;stage_1W_vs_E10;stage_3W_vs_E10;stage	1	TRUE
ENSGALG00010019267	STARD5	PRJNA729271	-1.759266128	2.0508E-16	1.759266128	stage_E14_vs_E10	ordinary_muscle_development	D_vs_E10;stage_1W_vs_E10;stage_3W_vs_E10;stage	1	TRUE
ENSGALG00010027100	MIER2	PRJNA729271	-0.789359701	2.07806E-16	0.789359701	stage_E14_vs_E10	ordinary_muscle_development		0	FALSE
ENSGALG00010005421	ENSGALG00010005421	PRJNA729271	2.688484409	2.10494E-16	2.688484409	stage_E14_vs_E10	ordinary_muscle_development	D_vs_E10;stage_1W_vs_E10;stage_3W_vs_E10;stage	1	TRUE
ENSGALG00010017677	WDR43	PRJNA729271	-0.925703243	2.13166E-16	0.925703243	stage_E14_vs_E10	ordinary_muscle_development		0	FALSE
ENSGALG00010017625	YBX1	PRJNA729271	-0.76600314	2.18969E-16	0.76600314	stage_E14_vs_E10	ordinary_muscle_development		0	FALSE
ENSGALG00010019990	MARCHF8	PRJNA729271	0.897078549	2.2046E-16	0.897078549	stage_E14_vs_E10	ordinary_muscle_development		0	FALSE
ENSGALG00010008105	MMUT	PRJNA729271	0.804259871	2.20753E-16	0.804259871	stage_E14_vs_E10	ordinary_muscle_development		0	FALSE
ENSGALG00010015112	CBLN4	PRJNA729271	-3.582162889	2.21304E-16	3.582162889	stage_E14_vs_E10	ordinary_muscle_development	D_vs_E10;stage_1W_vs_E10;stage_3W_vs_E10;stage	1	TRUE
ENSGALG00010001268	CBLN2	PRJNA729271	-3.613338266	2.22947E-16	3.613338266	stage_E14_vs_E10	ordinary_muscle_development	D_vs_E10;stage_1W_vs_E10;stage_3W_vs_E10;stage	1	TRUE
ENSGALG00010017387	HS6ST2	PRJNA729271	-0.960253079	2.24343E-16	0.960253079	stage_E14_vs_E10	ordinary_muscle_development		0	FALSE
ENSGALG00010013677	GABRG1	PRJNA729271	-4.50324915	2.25024E-16	4.50324915	stage_E14_vs_E10	ordinary_muscle_development	D_vs_E10;stage_1W_vs_E10;stage_3W_vs_E10;stage	1	TRUE
ENSGALG00010001133	EIF2A	PRJNA729271	-0.63808134	2.25616E-16	0.63808134	stage_E14_vs_E10	ordinary_muscle_development		0	FALSE
ENSGALG00010027289	SMARCC1	PRJNA729271	-1.166225816	2.25821E-16	1.166225816	stage_E14_vs_E10	ordinary_muscle_development	D_vs_E10;stage_1W_vs_E10;stage_3W_vs_E10;stage	1	TRUE
ENSGALG00010024865	SNAP47	PRJNA729271	1.185180144	2.27697E-16	1.185180144	stage_E14_vs_E10	ordinary_muscle_development	D_vs_E10;stage_1W_vs_E10;stage_3W_vs_E10;stage	1	TRUE
ENSGALG00010002467	NEK3	PRJNA729271	-1.904296312	2.31517E-16	1.904296312	stage_E14_vs_E10	ordinary_muscle_development	D_vs_E10;stage_1W_vs_E10;stage_3W_vs_E10;stage	1	TRUE
ENSGALG00010002561	PAAF1	PRJNA729271	-0.703764152	2.33135E-16	0.703764152	stage_E14_vs_E10	ordinary_muscle_development		0	FALSE
ENSGALG00010023003	ENSGALG00010023003	PRJNA729271	-6.770275593	2.33784E-16	6.770275593	stage_E14_vs_E10	ordinary_muscle_development	D_vs_E10;stage_1W_vs_E10;stage_3W_vs_E10;stage	1	TRUE
ENSGALG00010008091	ENSGALG00010008091	PRJNA729271	2.26084976	2.34368E-16	2.26084976	stage_E14_vs_E10	ordinary_muscle_development	D_vs_E10;stage_1W_vs_E10;stage_3W_vs_E10;stage	1	TRUE
ENSGALG00010029760	SHISA6	PRJNA729271	-7.210194331	2.35717E-16	7.210194331	stage_E14_vs_E10	ordinary_muscle_development	D_vs_E10;stage_1W_vs_E10;stage_3W_vs_E10;stage	1	TRUE
ENSGALG00010027648	NFYA	PRJNA729271	-0.622728706	2.35767E-16	0.622728706	stage_E14_vs_E10	ordinary_muscle_development		0	FALSE
ENSGALG00010022234	COX15	PRJNA729271	0.775954537	2.36561E-16	0.775954537	stage_E14_vs_E10	ordinary_muscle_development		0	FALSE
ENSGALG00010008274	NFAT5	PRJNA729271	-0.925421155	2.36627E-16	0.925421155	stage_E14_vs_E10	ordinary_muscle_development		0	FALSE
ENSGALG00010005649	TOMM70	PRJNA729271	-0.661575956	2.38283E-16	0.661575956	stage_E14_vs_E10	ordinary_muscle_development		0	FALSE
ENSGALG00010017439	PPP4R4	PRJNA729271	-2.350743166	2.38522E-16	2.350743166	stage_E14_vs_E10	ordinary_muscle_development	D_vs_E10;stage_1W_vs_E10;stage_3W_vs_E10;stage	1	TRUE
ENSGALG00010024475	ADGRG1	PRJNA729271	1.310044897	2.41946E-16	1.310044897	stage_E14_vs_E10	ordinary_muscle_development	D_vs_E10;stage_1W_vs_E10;stage_3W_vs_E10;stage	1	TRUE
ENSGALG00010021602	PLEKHG4	PRJNA729271	0.815469351	2.42907E-16	0.815469351	stage_E14_vs_E10	ordinary_muscle_development		0	FALSE
ENSGALG00010001136	SERP1	PRJNA729271	-1.061578454	2.43773E-16	1.061578454	stage_E14_vs_E10	ordinary_muscle_development	D_vs_E10;stage_1W_vs_E10;stage_3W_vs_E10;stage	1	TRUE
ENSGALG00010009866	NAGA	PRJNA729271	0.954080958	2.44852E-16	0.954080958	stage_E14_vs_E10	ordinary_muscle_development		0	FALSE
ENSGALG00010002334	OSBPL10	PRJNA729271	-1.424851521	2.4501E-16	1.424851521	stage_E14_vs_E10	ordinary_muscle_development	D_vs_E10;stage_1W_vs_E10;stage_3W_vs_E10;stage	1	TRUE
ENSGALG00010027931	ABHD17A	PRJNA729271	1.429619718	2.45713E-16	1.429619718	stage_E14_vs_E10	ordinary_muscle_development	D_vs_E10;stage_1W_vs_E10;stage_3W_vs_E10;stage	1	TRUE
ENSGALG00010029291	RNFT1	PRJNA729271	-0.971712436	2.46292E-16	0.971712436	stage_E14_vs_E10	ordinary_muscle_development		0	FALSE
ENSGALG00010026584	ENSGALG00010026584	PRJNA729271	-0.941173339	2.46594E-16	0.941173339	stage_E14_vs_E10	ordinary_muscle_development		0	FALSE
ENSGALG00010009352	HSF1	PRJNA729271	1.48712728	2.49612E-16	1.48712728	stage_E14_vs_E10	ordinary_muscle_development	D_vs_E10;stage_1W_vs_E10;stage_3W_vs_E10;stage	1	TRUE
ENSGALG00010027520	FANCE	PRJNA729271	-1.477836308	2.49997E-16	1.477836308	stage_E14_vs_E10	ordinary_muscle_development	D_vs_E10;stage_1W_vs_E10;stage_3W_vs_E10;stage	1	TRUE
ENSGALG00010021701	DR1	PRJNA729271	-0.653563047	2.5148E-16	0.653563047	stage_E14_vs_E10	ordinary_muscle_development		0	FALSE
ENSGALG00010015738	CRACDL	PRJNA729271	-3.742995757	2.52768E-16	3.742995757	stage_E14_vs_E10	ordinary_muscle_development	D_vs_E10;stage_1W_vs_E10;stage_3W_vs_E10;stage	1	TRUE
ENSGALG00010018337	TMEM151B	PRJNA729271	-2.927613693	2.52937E-16	2.927613693	stage_E14_vs_E10	ordinary_muscle_development	D_vs_E10;stage_1W_vs_E10;stage_3W_vs_E10;stage	1	TRUE
ENSGALG00010002855	RACK1	PRJNA729271	1.052783034	2.53884E-16	1.052783034	stage_E14_vs_E10	ordinary_muscle_development	D_vs_E10;stage_1W_vs_E10;stage_3W_vs_E10;stage	1	TRUE
ENSGALG00010008386	SESN3	PRJNA729271	-1.904835397	2.55743E-16	1.904835397	stage_E14_vs_E10	ordinary_muscle_development	D_vs_E10;stage_1W_vs_E10;stage_3W_vs_E10;stage	1	TRUE
ENSGALG00010000054	VPS33B	PRJNA729271	0.825708426	2.56722E-16	0.825708426	stage_E14_vs_E10	ordinary_muscle_development		0	FALSE
ENSGALG00010006602	EDNRA	PRJNA729271	-0.989297297	2.60566E-16	0.989297297	stage_E14_vs_E10	ordinary_muscle_development		0	FALSE
ENSGALG00010022842	PHC1	PRJNA729271	-1.195990184	2.64198E-16	1.195990184	stage_E14_vs_E10	ordinary_muscle_development	D_vs_E10;stage_1W_vs_E10;stage_3W_vs_E10;stage	1	TRUE
ENSGALG00010014728	INTS13	PRJNA729271	0.931182462	2.64298E-16	0.931182462	stage_E14_vs_E10	ordinary_muscle_development		0	FALSE
ENSGALG00010007942	PGRMC2	PRJNA729271	-0.724276245	2.67734E-16	0.724276245	stage_E14_vs_E10	ordinary_muscle_development		0	FALSE
ENSGALG00010010682	SMIM13	PRJNA729271	-0.883481468	2.67799E-16	0.883481468	stage_E14_vs_E10	ordinary_muscle_development		0	FALSE
ENSGALG00010011403	SV2C	PRJNA729271	-6.658192188	2.70048E-16	6.658192188	stage_E14_vs_E10	ordinary_muscle_development	D_vs_E10;stage_1W_vs_E10;stage_3W_vs_E10;stage	1	TRUE
ENSGALG00010028893	STXBP1	PRJNA729271	1.637516351	2.73651E-16	1.637516351	stage_E14_vs_E10	ordinary_muscle_development	D_vs_E10;stage_1W_vs_E10;stage_3W_vs_E10;stage	1	TRUE
ENSGALG00010016328	USH2A	PRJNA729271	5.077782994	2.74646E-16	5.077782994	stage_E14_vs_E10	ordinary_muscle_development	D_vs_E10;stage_1W_vs_E10;stage_3W_vs_E10;stage	1	TRUE
ENSGALG00010015931	RBM47	PRJNA729271	2.272063227	2.76017E-16	2.272063227	stage_E14_vs_E10	ordinary_muscle_development	D_vs_E10;stage_1W_vs_E10;stage_3W_vs_E10;stage	1	TRUE
ENSGALG00010029578	C17orf67	PRJNA729271	-8.098101127	2.76276E-16	8.098101127	stage_E14_vs_E10	ordinary_muscle_development	D_vs_E10;stage_1W_vs_E10;stage_3W_vs_E10;stage	1	TRUE
ENSGALG00010003650	SPACA1	PRJNA729271	4.02534568	2.76441E-16	4.02534568	stage_E14_vs_E10	ordinary_muscle_development	D_vs_E10;stage_1W_vs_E10;stage_3W_vs_E10;stage	1	TRUE
ENSGALG00010029509	DNAJC30	PRJNA729271	1.396076263	2.78363E-16	1.396076263	stage_E14_vs_E10	ordinary_muscle_development	D_vs_E10;stage_1W_vs_E10;stage_3W_vs_E10;stage	1	TRUE
ENSGALG00010013379	ZNF593	PRJNA729271	1.104778775	2.79181E-16	1.104778775	stage_E14_vs_E10	ordinary_muscle_development	D_vs_E10;stage_1W_vs_E10;stage_3W_vs_E10;stage	1	TRUE
ENSGALG00010027943	RAB43	PRJNA729271	-0.795162311	2.80421E-16	0.795162311	stage_E14_vs_E10	ordinary_muscle_development		0	FALSE
ENSGALG00010002360	DHRSX	PRJNA729271	1.368098253	2.86031E-16	1.368098253	stage_E14_vs_E10	ordinary_muscle_development	D_vs_E10;stage_1W_vs_E10;stage_3W_vs_E10;stage	1	TRUE
ENSGALG00010021239	USP54	PRJNA729271	0.583934952	2.88546E-16	0.583934952	stage_E14_vs_E10	ordinary_muscle_development		0	FALSE
ENSGALG00010017300	TRPM7	PRJNA729271	-0.887090956	2.89919E-16	0.887090956	stage_E14_vs_E10	ordinary_muscle_development		0	FALSE
ENSGALG00010007436	PNAT3	PRJNA729271	2.166208345	2.90702E-16	2.166208345	stage_E14_vs_E10	ordinary_muscle_development	D_vs_E10;stage_1W_vs_E10;stage_3W_vs_E10;stage	1	TRUE
ENSGALG00010002762	PPAN	PRJNA729271	-1.255008981	2.91164E-16	1.255008981	stage_E14_vs_E10	ordinary_muscle_development	D_vs_E10;stage_1W_vs_E10;stage_3W_vs_E10;stage	1	TRUE
ENSGALG00010019655	RTRAF	PRJNA729271	0.817207141	2.91883E-16	0.817207141	stage_E14_vs_E10	ordinary_muscle_development		0	FALSE
ENSGALG00010017485	LBH	PRJNA729271	-0.901908567	2.98898E-16	0.901908567	stage_E14_vs_E10	ordinary_muscle_development		0	FALSE
ENSGALG00010022875	SPON1	PRJNA729271	-2.08522574	3.05816E-16	2.08522574	stage_E14_vs_E10	ordinary_muscle_development	D_vs_E10;stage_1W_vs_E10;stage_3W_vs_E10;stage	1	TRUE
ENSGALG00010010963	TSPAN14	PRJNA729271	-0.622499313	3.06665E-16	0.622499313	stage_E14_vs_E10	ordinary_muscle_development		0	FALSE
ENSGALG00010022425	VWA2	PRJNA729271	-4.755772007	3.09196E-16	4.755772007	stage_E14_vs_E10	ordinary_muscle_development	D_vs_E10;stage_1W_vs_E10;stage_3W_vs_E10;stage	1	TRUE
ENSGALG00010020764	PRKAR2A	PRJNA729271	-0.844174623	3.11246E-16	0.844174623	stage_E14_vs_E10	ordinary_muscle_development		0	FALSE
ENSGALG00010002335	TMEM47	PRJNA729271	1.117104766	3.1159E-16	1.117104766	stage_E14_vs_E10	ordinary_muscle_development	D_vs_E10;stage_1W_vs_E10;stage_3W_vs_E10;stage	1	TRUE
ENSGALG00010013042	SLC25A48	PRJNA729271	-2.95034599	3.12406E-16	2.95034599	stage_E14_vs_E10	ordinary_muscle_development	D_vs_E10;stage_1W_vs_E10;stage_3W_vs_E10;stage	1	TRUE
ENSGALG00010009465	GGPS1	PRJNA729271	0.817512252	3.12972E-16	0.817512252	stage_E14_vs_E10	ordinary_muscle_development		0	FALSE
ENSGALG00010024162	SRCIN1	PRJNA729271	-3.653782931	3.13225E-16	3.653782931	stage_E14_vs_E10	ordinary_muscle_development	D_vs_E10;stage_1W_vs_E10;stage_3W_vs_E10;stage	1	TRUE
ENSGALG00010003960	ASPH	PRJNA729271	1.628410287	3.16604E-16	1.628410287	stage_E14_vs_E10	ordinary_muscle_development	D_vs_E10;stage_1W_vs_E10;stage_3W_vs_E10;stage	1	TRUE
ENSGALG00010025422	SSBP3	PRJNA729271	-1.31597507	3.1853E-16	1.31597507	stage_E14_vs_E10	ordinary_muscle_development	D_vs_E10;stage_1W_vs_E10;stage_3W_vs_E10;stage	1	TRUE
ENSGALG00010021388	DPH2	PRJNA729271	-1.324078403	3.23183E-16	1.324078403	stage_E14_vs_E10	ordinary_muscle_development	D_vs_E10;stage_1W_vs_E10;stage_3W_vs_E10;stage	1	TRUE
ENSGALG00010017424	APOD	PRJNA729271	3.983814679	3.29921E-16	3.983814679	stage_E14_vs_E10	ordinary_muscle_development	D_vs_E10;stage_1W_vs_E10;stage_3W_vs_E10;stage	1	TRUE
ENSGALG00010020970	ARMH3	PRJNA729271	0.651174777	3.31634E-16	0.651174777	stage_E14_vs_E10	ordinary_muscle_development		0	FALSE
ENSGALG00010017985	ABCC5	PRJNA729271	2.229668689	3.35275E-16	2.229668689	stage_E14_vs_E10	ordinary_muscle_development	D_vs_E10;stage_1W_vs_E10;stage_3W_vs_E10;stage	1	TRUE
ENSGALG00010010288	ENSGALG00010010288	PRJNA729271	1.354366373	3.46971E-16	1.354366373	stage_E14_vs_E10	ordinary_muscle_development	D_vs_E10;stage_1W_vs_E10;stage_3W_vs_E10;stage	1	TRUE
ENSGALG00010029378	ST6GALNAC1	PRJNA729271	-3.120382439	3.48493E-16	3.120382439	stage_E14_vs_E10	ordinary_muscle_development	D_vs_E10;stage_1W_vs_E10;stage_3W_vs_E10;stage	1	TRUE
ENSGALG00010014197	SH3GL2	PRJNA729271	-3.107338616	3.50643E-16	3.107338616	stage_E14_vs_E10	ordinary_muscle_development	D_vs_E10;stage_1W_vs_E10;stage_3W_vs_E10;stage	1	TRUE
ENSGALG00010002283	ENSGALG00010002283	PRJNA729271	5.440373851	3.54065E-16	5.440373851	stage_E14_vs_E10	ordinary_muscle_development	D_vs_E10;stage_1W_vs_E10;stage_3W_vs_E10;stage	1	TRUE
ENSGALG00010003201	MBOAT2	PRJNA729271	-1.076749643	3.63854E-16	1.076749643	stage_E14_vs_E10	ordinary_muscle_development	D_vs_E10;stage_1W_vs_E10;stage_3W_vs_E10;stage	1	TRUE
ENSGALG00010007894	CD88	PRJNA729271	2.151866225	3.65614E-16	2.151866225	stage_E14_vs_E10	ordinary_muscle_development	D_vs_E10;stage_1W_vs_E10;stage_3W_vs_E10;stage	1	TRUE
ENSGALG00010023702	COL4A4	PRJNA729271	2.059421559	3.67472E-16	2.059421559	stage_E14_vs_E10	ordinary_muscle_development	D_vs_E10;stage_1W_vs_E10;stage_3W_vs_E10;stage	1	TRUE
ENSGALG00010013866	KCTD8	PRJNA729271	-4.110492277	3.68869E-16	4.110492277	stage_E14_vs_E10	ordinary_muscle_development	D_vs_E10;stage_1W_vs_E10;stage_3W_vs_E10;stage	1	TRUE
ENSGALG00010003298	IL7	PRJNA729271	8.044858178	3.72267E-16	8.044858178	stage_E14_vs_E10	ordinary_muscle_development	D_vs_E10;stage_1W_vs_E10;stage_3W_vs_E10;stage	1	TRUE
ENSGALG00010010279	CCSAP	PRJNA729271	1.983632823	3.79321E-16	1.983632823	stage_E14_vs_E10	ordinary_muscle_development	D_vs_E10;stage_1W_vs_E10;stage_3W_vs_E10;stage	1	TRUE
ENSGALG00010029269	WSCD1	PRJNA729271	1.288933503	3.87988E-16	1.288933503	stage_E14_vs_E10	ordinary_muscle_development	D_vs_E10;stage_1W_vs_E10;stage_3W_vs_E10;stage	1	TRUE
ENSGALG00010027693	RAB14	PRJNA729271	-0.498814045	3.91264E-16	0.498814045	stage_E14_vs_E10	ordinary_muscle_development		0	FALSE
ENSGALG00010019206	RNF207	PRJNA729271	2.330062279	3.95173E-16	2.330062279	stage_E14_vs_E10	ordinary_muscle_development	D_vs_E10;stage_1W_vs_E10;stage_3W_vs_E10;stage	1	TRUE
ENSGALG00010018486	ENSGALG00010018486	PRJNA729271	0.829567084	3.97147E-16	0.829567084	stage_E14_vs_E10	ordinary_muscle_development		0	FALSE
ENSGALG00010005568	PLEKHG1	PRJNA729271	1.099284921	4.00545E-16	1.099284921	stage_E14_vs_E10	ordinary_muscle_development	D_vs_E10;stage_1W_vs_E10;stage_3W_vs_E10;stage	1	TRUE
ENSGALG00010005420	DHRS4	PRJNA729271	0.98629337	4.0592E-16	0.98629337	stage_E14_vs_E10	ordinary_muscle_development		0	FALSE
ENSGALG00010026104	DDX51	PRJNA729271	-0.899233569	4.12324E-16	0.899233569	stage_E14_vs_E10	ordinary_muscle_development		0	FALSE
ENSGALG00010007133	BLVRA	PRJNA729271	1.559771094	4.13946E-16	1.559771094	stage_E14_vs_E10	ordinary_muscle_development	D_vs_E10;stage_1W_vs_E10;stage_3W_vs_E10;stage	1	TRUE
ENSGALG00010025809	ENSGALG00010025809	PRJNA729271	1.58695745	4.17952E-16	1.58695745	stage_E14_vs_E10	ordinary_muscle_development	D_vs_E10;stage_1W_vs_E10;stage_3W_vs_E10;stage	1	TRUE
ENSGALG00010024476	KCNB1	PRJNA729271	-1.593542786	4.23131E-16	1.593542786	stage_E14_vs_E10	ordinary_muscle_development	D_vs_E10;stage_1W_vs_E10;stage_3W_vs_E10;stage	1	TRUE
ENSGALG00010027194	ENSGALG00010027194	PRJNA729271	-2.217025142	4.23222E-16	2.217025142	stage_E14_vs_E10	ordinary_muscle_development	D_vs_E10;stage_1W_vs_E10;stage_3W_vs_E10;stage	1	TRUE
ENSGALG00010016918	RCOR1	PRJNA729271	-0.630386272	4.23311E-16	0.630386272	stage_E14_vs_E10	ordinary_muscle_development		0	FALSE
ENSGALG00010022538	WIPF1	PRJNA729271	1.324461652	4.25488E-16	1.324461652	stage_E14_vs_E10	ordinary_muscle_development	D_vs_E10;stage_1W_vs_E10;stage_3W_vs_E10;stage	1	TRUE
ENSGALG00010029326	NCBP3	PRJNA729271	-0.680442395	4.25953E-16	0.680442395	stage_E14_vs_E10	ordinary_muscle_development		0	FALSE
ENSGALG00010003818	GAB2	PRJNA729271	1.299655868	4.35017E-16	1.299655868	stage_E14_vs_E10	ordinary_muscle_development	D_vs_E10;stage_1W_vs_E10;stage_3W_vs_E10;stage	1	TRUE
ENSGALG00010010499	NR4A2	PRJNA729271	3.738365298	4.37943E-16	3.738365298	stage_E14_vs_E10	ordinary_muscle_development	D_vs_E10;stage_1W_vs_E10;stage_3W_vs_E10;stage	1	TRUE
ENSGALG00010007642	ZNF318	PRJNA729271	0.882505214	4.40331E-16	0.882505214	stage_E14_vs_E10	ordinary_muscle_development		0	FALSE
ENSGALG00010011094	CSGALNACT2	PRJNA729271	-0.760853402	4.53787E-16	0.760853402	stage_E14_vs_E10	ordinary_muscle_development		0	FALSE
ENSGALG00010007907	IRX2	PRJNA729271	-8.518415702	4.54316E-16	8.518415702	stage_E14_vs_E10	ordinary_muscle_development	D_vs_E10;stage_1W_vs_E10;stage_3W_vs_E10;stage	1	TRUE
ENSGALG00010027212	IRF6	PRJNA729271	-4.976405066	4.55653E-16	4.976405066	stage_E14_vs_E10	ordinary_muscle_development	D_vs_E10;stage_1W_vs_E10;stage_3W_vs_E10;stage	1	TRUE
ENSGALG00010028544	ZNF414	PRJNA729271	1.29069251	4.57863E-16	1.29069251	stage_E14_vs_E10	ordinary_muscle_development	D_vs_E10;stage_1W_vs_E10;stage_3W_vs_E10;stage	1	TRUE
ENSGALG00010009157	ABHD1	PRJNA729271	1.129452343	4.67486E-16	1.129452343	stage_E14_vs_E10	ordinary_muscle_development	D_vs_E10;stage_1W_vs_E10;stage_3W_vs_E10;stage	1	TRUE

ENSGALG00010005821	DOCK9	PRJNA729271	1.272366184	4.73757E-16	1.272366184	stage_E14_vs_E10	ordinary_muscle_development	D_vs_E10;stage_1W_vs_E10;stage_3W_vs_E10;stage	1	TRUE
ENSGALG00010016215	SPATA17	PRJNA729271	-3.279884451	4.7411E-16	3.279884451	stage_E14_vs_E10	ordinary_muscle_development	D_vs_E10;stage_1W_vs_E10;stage_3W_vs_E10;stage	1	TRUE
ENSGALG00010000069	ENSGALG00010000069	PRJNA729271	1.935856919	4.78192E-16	1.935856919	stage_E14_vs_E10	ordinary_muscle_development	D_vs_E10;stage_1W_vs_E10;stage_3W_vs_E10;stage	1	TRUE
ENSGALG00010018969	DIEXF	PRJNA729271	-0.754594146	4.83816E-16	0.754594146	stage_E14_vs_E10	ordinary_muscle_development		0	FALSE
ENSGALG00010001264	FAM3C	PRJNA729271	0.931785699	4.93846E-16	0.931785699	stage_E14_vs_E10	ordinary_muscle_development		0	FALSE
ENSGALG00010014317	RAD23B	PRJNA729271	1.206794806	4.93989E-16	1.206794806	stage_E14_vs_E10	ordinary_muscle_development	D_vs_E10;stage_1W_vs_E10;stage_3W_vs_E10;stage	1	TRUE
ENSGALG00010003821	RUBCNL	PRJNA729271	1.285524435	4.98208E-16	1.285524435	stage_E14_vs_E10	ordinary_muscle_development	D_vs_E10;stage_1W_vs_E10;stage_3W_vs_E10;stage	1	TRUE
ENSGALG00010002261	NR0B1	PRJNA729271	-3.966361292	4.98407E-16	3.966361292	stage_E14_vs_E10	ordinary_muscle_development	D_vs_E10;stage_1W_vs_E10;stage_3W_vs_E10;stage	1	TRUE
ENSGALG00010007899	APRT	PRJNA729271	-1.292918654	4.99347E-16	1.292918654	stage_E14_vs_E10	ordinary_muscle_development	D_vs_E10;stage_1W_vs_E10;stage_3W_vs_E10;stage	1	TRUE
ENSGALG00010029422	EIF4H	PRJNA729271	-0.721450663	5.01967E-16	0.721450663	stage_E14_vs_E10	ordinary_muscle_development		0	FALSE
ENSGALG00010003035	DGAT2	PRJNA729271	3.764641909	5.03057E-16	3.764641909	stage_E14_vs_E10	ordinary_muscle_development	D_vs_E10;stage_1W_vs_E10;stage_3W_vs_E10;stage	1	TRUE
ENSGALG00010027711	WNK2	PRJNA729271	1.540653001	5.03655E-16	1.540653001	stage_E14_vs_E10	ordinary_muscle_development	D_vs_E10;stage_1W_vs_E10;stage_3W_vs_E10;stage	1	TRUE
ENSGALG00010011192	RPL17	PRJNA729271	1.027302312	5.0666E-16	1.027302312	stage_E14_vs_E10	ordinary_muscle_development	D_vs_E10;stage_1W_vs_E10;stage_3W_vs_E10;stage	1	TRUE
ENSGALG00010009333	ADRA1B	PRJNA729271	2.570132411	5.22281E-16	2.570132411	stage_E14_vs_E10	ordinary_muscle_development	D_vs_E10;stage_1W_vs_E10;stage_3W_vs_E10;stage	1	TRUE
ENSGALG00010025365	SIKE1	PRJNA729271	-0.768754023	5.3293E-16	0.768754023	stage_E14_vs_E10	ordinary_muscle_development		0	FALSE
ENSGALG00010029606	ABHD11	PRJNA729271	1.64389276	5.33297E-16	1.64389276	stage_E14_vs_E10	ordinary_muscle_development	D_vs_E10;stage_1W_vs_E10;stage_3W_vs_E10;stage	1	TRUE
ENSGALG00010021477	ZFAND2B	PRJNA729271	0.961724339	5.34826E-16	0.961724339	stage_E14_vs_E10	ordinary_muscle_development		0	FALSE
ENSGALG00010010534	SYNJ1	PRJNA729271	0.98845146	5.38888E-16	0.98845146	stage_E14_vs_E10	ordinary_muscle_development		0	FALSE
ENSGALG00010004281	VIM	PRJNA729271	-1.421485707	5.4029E-16	1.421485707	stage_E14_vs_E10	ordinary_muscle_development	D_vs_E10;stage_1W_vs_E10;stage_3W_vs_E10;stage	1	TRUE
ENSGALG00010016465	STK35	PRJNA729271	-1.891820535	5.4859E-16	1.891820535	stage_E14_vs_E10	ordinary_muscle_development	D_vs_E10;stage_1W_vs_E10;stage_3W_vs_E10;stage	1	TRUE
ENSGALG00010001805	ENSGALG00010001805	PRJNA729271	2.106834947	5.5278E-16	2.106834947	stage_E14_vs_E10	ordinary_muscle_development	D_vs_E10;stage_1W_vs_E10;stage_3W_vs_E10;stage	1	TRUE
ENSGALG00010001639	ENSGALG00010001639	PRJNA729271	-1.358365337	5.55736E-16	1.358365337	stage_E14_vs_E10	ordinary_muscle_development	D_vs_E10;stage_1W_vs_E10;stage_3W_vs_E10;stage	1	TRUE
ENSGALG00010028867	REXO4	PRJNA729271	-1.011482479	5.57317E-16	1.011482479	stage_E14_vs_E10	ordinary_muscle_development	D_vs_E10;stage_1W_vs_E10;stage_3W_vs_E10;stage	1	TRUE
ENSGALG00010007868	INHBA	PRJNA729271	-2.524830618	5.57949E-16	2.524830618	stage_E14_vs_E10	ordinary_muscle_development	D_vs_E10;stage_1W_vs_E10;stage_3W_vs_E10;stage	1	TRUE
ENSGALG00010023890	SASS6	PRJNA729271	-0.991179741	5.59999E-16	0.991179741	stage_E14_vs_E10	ordinary_muscle_development		0	FALSE
ENSGALG00010029480	ENSGALG00010029480	PRJNA729271	0.944104737	5.61632E-16	0.944104737	stage_E14_vs_E10	ordinary_muscle_development		0	FALSE
ENSGALG00010015076	ITK	PRJNA729271	4.625587535	5.75838E-16	4.625587535	stage_E14_vs_E10	ordinary_muscle_development	D_vs_E10;stage_1W_vs_E10;stage_3W_vs_E10;stage	1	TRUE
ENSGALG00010014997	RAE1	PRJNA729271	-0.752803877	5.85106E-16	0.752803877	stage_E14_vs_E10	ordinary_muscle_development		0	FALSE
ENSGALG00010009668	TFAP2A	PRJNA729271	-7.951648299	5.91984E-16	7.951648299	stage_E14_vs_E10	ordinary_muscle_development	D_vs_E10;stage_1W_vs_E10;stage_3W_vs_E10;stage	1	TRUE
ENSGALG00010024133	PSMD11	PRJNA729271	1.1563915	5.95468E-16	1.1563915	stage_E14_vs_E10	ordinary_muscle_development	D_vs_E10;stage_1W_vs_E10;stage_3W_vs_E10;stage	1	TRUE
ENSGALG00010024719	PYM1	PRJNA729271	-1.152485038	5.99531E-16	1.152485038	stage_E14_vs_E10	ordinary_muscle_development	D_vs_E10;stage_1W_vs_E10;stage_3W_vs_E10;stage	1	TRUE
ENSGALG00010021529	BTAF1	PRJNA729271	0.620727365	5.99785E-16	0.620727365	stage_E14_vs_E10	ordinary_muscle_development		0	FALSE
ENSGALG00010022647	SVIP	PRJNA729271	-1.115684088	6.04316E-16	1.115684088	stage_E14_vs_E10	ordinary_muscle_development	D_vs_E10;stage_1W_vs_E10;stage_3W_vs_E10;stage	1	TRUE
ENSGALG00010012063	PTPRD	PRJNA729271	-1.629034379	6.13407E-16	1.629034379	stage_E14_vs_E10	ordinary_muscle_development	D_vs_E10;stage_1W_vs_E10;stage_3W_vs_E10;stage	1	TRUE
ENSGALG00010028377	PDCL	PRJNA729271	0.474864665	6.18664E-16	0.474864665	stage_E14_vs_E10	ordinary_muscle_development		0	FALSE
ENSGALG00010018342	BNIP3L	PRJNA729271	-0.876524392	6.18913E-16	0.876524392	stage_E14_vs_E10	ordinary_muscle_development		0	FALSE
ENSGALG00010001856	CHST15	PRJNA729271	-0.989846617	6.26364E-16	0.989846617	stage_E14_vs_E10	ordinary_muscle_development		0	FALSE
ENSGALG00010026113	MFRP	PRJNA729271	3.856872042	6.27018E-16	3.856872042	stage_E14_vs_E10	ordinary_muscle_development	D_vs_E10;stage_1W_vs_E10;stage_3W_vs_E10;stage	1	TRUE
ENSGALG00010007780	SIRT5	PRJNA729271	1.167344015	6.32783E-16	1.167344015	stage_E14_vs_E10	ordinary_muscle_development	D_vs_E10;stage_1W_vs_E10;stage_3W_vs_E10;stage	1	TRUE
ENSGALG00010022707	SALL1	PRJNA729271	-4.528328719	6.43788E-16	4.528328719	stage_E14_vs_E10	ordinary_muscle_development	D_vs_E10;stage_1W_vs_E10;stage_3W_vs_E10;stage	1	TRUE
ENSGALG00010023368	ENSGALG00010023368	PRJNA729271	-1.940074749	6.45079E-16	1.940074749	stage_E14_vs_E10	ordinary_muscle_development	D_vs_E10;stage_1W_vs_E10;stage_3W_vs_E10;stage	1	TRUE
ENSGALG00010008488	CCSER1	PRJNA729271	-1.303162508	6.53851E-16	1.303162508	stage_E14_vs_E10	ordinary_muscle_development	D_vs_E10;stage_1W_vs_E10;stage_3W_vs_E10;stage	1	TRUE
ENSGALG00010006537	DRD3	PRJNA729271	-5.643636229	6.57692E-16	5.643636229	stage_E14_vs_E10	ordinary_muscle_development	D_vs_E10;stage_1W_vs_E10;stage_3W_vs_E10;stage	1	TRUE
ENSGALG00010008192	HNRNPU	PRJNA729271	-0.681928496	6.60177E-16	0.681928496	stage_E14_vs_E10	ordinary_muscle_development		0	FALSE
ENSGALG00010012808	EYA3	PRJNA729271	-0.779776557	6.61078E-16	0.779776557	stage_E14_vs_E10	ordinary_muscle_development		0	FALSE
ENSGALG00010022205	GALNT18	PRJNA729271	-0.899020038	6.83735E-16	0.899020038	stage_E14_vs_E10	ordinary_muscle_development		0	FALSE
ENSGALG00010018267	ADPRHL2	PRJNA729271	-0.635467356	6.89111E-16	0.635467356	stage_E14_vs_E10	ordinary_muscle_development		0	FALSE
ENSGALG00010024463	FBXL22	PRJNA729271	1.888840196	6.91786E-16	1.888840196	stage_E14_vs_E10	ordinary_muscle_development	D_vs_E10;stage_1W_vs_E10;stage_3W_vs_E10;stage	1	TRUE
ENSGALG00010029418	ABCA5	PRJNA729271	1.081066366	6.92436E-16	1.081066366	stage_E14_vs_E10	ordinary_muscle_development	D_vs_E10;stage_1W_vs_E10;stage_3W_vs_E10;stage	1	TRUE
ENSGALG00010014329	IER5	PRJNA729271	1.265067507	6.92833E-16	1.265067507	stage_E14_vs_E10	ordinary_muscle_development	D_vs_E10;stage_1W_vs_E10;stage_3W_vs_E10;stage	1	TRUE
ENSGALG00010021074	TTC17	PRJNA729271	0.568871023	7.19713E-16	0.568871023	stage_E14_vs_E10	ordinary_muscle_development		0	FALSE
ENSGALG00010018325	ENSGALG00010018325	PRJNA729271	1.570823979	7.29299E-16	1.570823979	stage_E14_vs_E10	ordinary_muscle_development	D_vs_E10;stage_1W_vs_E10;stage_3W_vs_E10;stage	1	TRUE
ENSGALG00010009125	CD72L1	PRJNA729271	7.619652321	7.46393E-16	7.619652321	stage_E14_vs_E10	ordinary_muscle_development	D_vs_E10;stage_1W_vs_E10;stage_3W_vs_E10;stage	1	TRUE
ENSGALG00010010792	XRCC6	PRJNA729271	-1.225839919	7.50906E-16	1.225839919	stage_E14_vs_E10	ordinary_muscle_development	D_vs_E10;stage_1W_vs_E10;stage_3W_vs_E10;stage	1	TRUE
ENSGALG00010003583	RSAD2	PRJNA729271	6.556662736	7.6237E-16	6.556662736	stage_E14_vs_E10	ordinary_muscle_development	D_vs_E10;stage_1W_vs_E10;stage_3W_vs_E10;stage	1	TRUE
ENSGALG00010000651	ENSGALG00010000651	PRJNA729271	-5.373771715	7.76877E-16	5.373771715	stage_E14_vs_E10	ordinary_muscle_development	D_vs_E10;stage_1W_vs_E10;stage_3W_vs_E10;stage	1	TRUE
ENSGALG00010002934	VWDE	PRJNA729271	-8.094942861	7.84862E-16	8.094942861	stage_E14_vs_E10	ordinary_muscle_development	D_vs_E10;stage_1W_vs_E10;stage_3W_vs_E10;stage	1	TRUE
ENSGALG00010027258	ENSGALG00010027258	PRJNA729271	-1.460490738	7.9398E-16	1.460490738	stage_E14_vs_E10	ordinary_muscle_development	D_vs_E10;stage_1W_vs_E10;stage_3W_vs_E10;stage	1	TRUE
ENSGALG00010014917	EIF3D	PRJNA729271	0.8673117	8.06696E-16	0.8673117	stage_E14_vs_E10	ordinary_muscle_development		0	FALSE
ENSGALG00010027692	PHF2	PRJNA729271	0.916262318	8.09652E-16	0.916262318	stage_E14_vs_E10	ordinary_muscle_development		0	FALSE
ENSGALG00010008094	PIM3	PRJNA729271	1.157801824	8.10609E-16	1.157801824	stage_E14_vs_E10	ordinary_muscle_development	D_vs_E10;stage_1W_vs_E10;stage_3W_vs_E10;stage	1	TRUE
ENSGALG00010027751	ANGPTL4	PRJNA729271	-1.744017632	8.14265E-16	1.744017632	stage_E14_vs_E10	ordinary_muscle_development	D_vs_E10;stage_1W_vs_E10;stage_3W_vs_E10;stage	1	TRUE
ENSGALG00010017208	CINP	PRJNA729271	-1.451989105	8.14517E-16	1.451989105	stage_E14_vs_E10	ordinary_muscle_development	D_vs_E10;stage_1W_vs_E10;stage_3W_vs_E10;stage	1	TRUE
ENSGALG00010017646	MAEA	PRJNA729271	-0.716398549	8.16265E-16	0.716398549	stage_E14_vs_E10	ordinary_muscle_development		0	FALSE
ENSGALG00010012245	ENSGALG00010012245	PRJNA729271	-0.652316875	8.30632E-16	0.652316875	stage_E14_vs_E10	ordinary_muscle_development		0	FALSE
ENSGALG00010024672	DYNLRB1	PRJNA729271	-0.870774743	8.44365E-16	0.870774743	stage_E14_vs_E10	ordinary_muscle_development		0	FALSE
ENSGALG00010008024	MRS2	PRJNA729271	0.77566257	8.46084E-16	0.77566257	stage_E14_vs_E10	ordinary_muscle_development		0	FALSE
ENSGALG00010015876	SKP1	PRJNA729271	-0.635502526	8.46415E-16	0.635502526	stage_E14_vs_E10	ordinary_muscle_development		0	FALSE
ENSGALG00010028192	ENSGALG00010028192	PRJNA729271	2.540421912	8.51634E-16	2.540421912	stage_E14_vs_E10	ordinary_muscle_development	D_vs_E10;stage_1W_vs_E10;stage_3W_vs_E10;stage	1	TRUE
ENSGALG00010005899	ENSGALG00010005899	PRJNA729271	-0.727875859	8.55884E-16	0.727875859	stage_E14_vs_E10	ordinary_muscle_development		0	FALSE
ENSGALG00010007850	ENSGALG00010007850	PRJNA729271	-2.481933233	8.79147E-16	2.481933233	stage_E14_vs_E10	ordinary_muscle_development	D_vs_E10;stage_1W_vs_E10;stage_3W_vs_E10;stage	1	TRUE
ENSGALG00010011926	DYRK2	PRJNA729271	1.60742419	8.81706E-16	1.60742419	stage_E14_vs_E10	ordinary_muscle_development	D_vs_E10;stage_1W_vs_E10;stage_3W_vs_E10;stage	1	TRUE
ENSGALG00010027902	UBE2Q1	PRJNA729271	0.571057256	8.86699E-16	0.571057256	stage_E14_vs_E10	ordinary_muscle_development		0	FALSE
ENSGALG00010018752	PLS3	PRJNA729271	-1.154695317	8.87485E-16	1.154695317	stage_E14_vs_E10	ordinary_muscle_development	D_vs_E10;stage_1W_vs_E10;stage_3W_vs_E10;stage	1	TRUE
ENSGALG00010025800	ARL2BP	PRJNA729271	-1.146016155	8.94123E-16	1.146016155	stage_E14_vs_E10	ordinary_muscle_development	D_vs_E10;stage_1W_vs_E10;stage_3W_vs_E10;stage	1	TRUE
ENSGALG00010015320	TRPM6	PRJNA729271	-3.869455402	8.96232E-16	3.869455402	stage_E14_vs_E10	ordinary_muscle_development	D_vs_E10;stage_1W_vs_E10;stage_3W_vs_E10;stage	1	TRUE
ENSGALG00010021462	SETD5	PRJNA729271	-0.781130488	9.06302E-16	0.781130488	stage_E14_vs_E10	ordinary_muscle_development		0	FALSE
ENSGALG00010019956	SLC29A3	PRJNA729271	-1.534852092	9.11516E-16	1.534852092	stage_E14_vs_E10	ordinary_muscle_development	D_vs_E10;stage_1W_vs_E10;stage_3W_vs_E10;stage	1	TRUE
ENSGALG00010008653	ENSGALG00010008653	PRJNA729271	-1.32579895	9.18155E-16	1.32579895	stage_E14_vs_E10	ordinary_muscle_development	D_vs_E10;stage_1W_vs_E10;stage_3W_vs_E10;stage	1	TRUE
ENSGALG00010003937	ENSGALG00010003937	PRJNA729271	1.696608267	9.43743E-16	1.696608267	stage_E14_vs_E10	ordinary_muscle_development	D_vs_E10;stage_1W_vs_E10;stage_3W_vs_E10;stage	1	TRUE
ENSGALG00010011311	DOCK8	PRJNA729271	1.548646813	9.53276E-16	1.548646813	stage_E14_vs_E10	ordinary_muscle_development	D_vs_E10;stage_1W_vs_E10;stage_3W_vs_E10;stage	1	TRUE
ENSGALG00010013753	PLEKHA5	PRJNA729271	1.013294295	9.60012E-16	1.013294295	stage_E14_vs_E10	ordinary_muscle_development	D_vs_E10;stage_1W_vs_E10;stage_3W_vs_E10;stage	1	TRUE
ENSGALG00010012772	TOMM22	PRJNA729271	-0.611986025	9.60603E-16	0.611986025	stage_E14_vs_E10	ordinary_muscle_development		0	FALSE
ENSGALG00010010998	SOAT1	PRJNA729271	1.366956221	9.64021E-16	1.366956221	stage_E14_vs_E10	ordinary_muscle_development	D_vs_E10;stage_1W_vs_E10;stage_3W_vs_E10;stage	1	TRUE
ENSGALG00010011987	H2BC32	PRJNA729271	-3.07349242	9.70516E-16	3.07349242	stage_E14_vs_E10	ordinary_muscle_development	D_vs_E10;stage_1W_vs_E10;stage_3W_vs_E10;stage	1	TRUE
ENSGALG00010027168	SSBP4	PRJNA729271	-1.518748731	9.7652E-16	1.518748731	stage_E14_vs_E10	ordinary_muscle_development	D_vs_E10;stage_1W_vs_E10;stage_3W_vs_E10;stage	1	TRUE
ENSGALG00010007651	CTNNAL1	PRJNA729271	-1.14397358	9.77187E-16	1.14397358	stage_E14_vs_E10	ordinary_muscle_development	D_vs_E10;stage_1W_vs_E10;stage_3W_vs_E10;stage	1	TRUE
ENSGALG00010027574	GNG7	PRJNA729271	-0.788113318	9.80806E-16	0.788113318	stage_E14_vs_E10	ordinary_muscle_development		0	FALSE
ENSGALG00010021390	GSTO2	PRJNA729271	-1.805520071	9.88779E-16	1.805520071	stage_E14_vs_E10	ordinary_muscle_development	D_vs_E10;stage_1W_vs_E10;stage_3W_vs_E10;stage	1	TRUE
ENSGALG00010005529	ENSGALG00010005529	PRJNA729271	2.951981951	9.98749E-16	2.951981951	stage_E14_vs_E10	ordinary_muscle_development	D_vs_E10;stage_1W_vs_E10;stage_3W_vs_E10;stage	1	TRUE
ENSGALG00010022864	CSTPP1	PRJNA729271	-1.798700702	1.0192E-15	1.798700702	stage_E14_vs_E10	ordinary_muscle_development	D_vs_E10;stage_1W_vs_E10;stage_3W_vs_E10;stage	1	TRUE
ENSGALG00010009038	RAD21	PRJNA729271	-1.016198759	1.02395E-15	1.016198759	stage_E14_vs_E10	ordinary_muscle_development	D_vs_E10;stage_1W_vs_E10;stage_3W_vs_E10;stage	1	TRUE
ENSGALG00010010171	ENSGALG00010010171	PRJNA729271	3.084763591	1.02615E-15	3.084763591	stage_E14_vs_E10	ordinary_muscle_development	D_vs_E10;stage_1W_vs_E10;stage_3W_vs_E10;stage	1	TRUE
ENSGALG00010027206	ENSGALG00010027206	PRJNA729271	7.9508303	1.02845E-15	7.9508303	stage_E14_vs_E10	ordinary_muscle_development	D_vs_E10;stage_1W_vs_E10;stage_3W_vs_E10;stage	1	TRUE
ENSGALG00010024716	GTF2A2	PRJNA729271	-0.642694744	1.03841E-15	0.642694744	stage_E14_vs_E10	ordinary_muscle_development		0	FALSE
ENSGALG00010011991	CSNK1A1	PRJNA729271	0.468531772	1.043E-15	0.468531772	stage_E14_vs_E10	ordinary_muscle_development		0	FALSE
ENSGALG00010023730	SP2	PRJNA729271	1.453061995	1.04904E-15	1.453061995	stage_E14_vs_E10	ordinary_muscle_development	D_vs_E10;stage_1W_vs_E10;stage_3W_vs_E10;stage	1	TRUE
ENSGALG00010007924	ABHD18	PRJNA729271	0.947125877	1.05092E-15	0.947125877	stage_E14_vs_E10	ordinary_muscle_development		0	FALSE
ENSGALG00010010216	ATXN7L1	PRJNA729271	1.04318203	1.05491E-15	1.04318203	stage_E14_vs_E10	ordinary_muscle_development	D_vs_E10;stage_1W_vs_E10;stage_3W_vs_E10;stage	1	TRUE
ENSGALG00010001557	THRB	PRJNA729271	-2.948030292	1.06085E-15	2.948030292	stage_E14_vs_E10	ordinary_muscle_development	D_vs_E10;stage_1W_vs_E10;stage_3W_vs_E10;stage	1	TRUE
ENSGALG00010019423	LYPD1	PRJNA729271	-1.492767107	1.06194E-15	1.492767107	stage_E14_vs_E10	ordinary_muscle_development	D_vs_E10;stage_1W_vs_E10;stage_3W_vs_E10;stage	1	TRUE
ENSGALG00010018428	PPP2R5D	PRJNA729271	-0.529107304	1.07482E-15	0.529107304	stage_E14_vs_E10	ordinary_muscle_development		0	FALSE
ENSGALG00010009119	CISD1	PRJNA729271	1.249197406	1.08444E-15	1.249197406	stage_E14_vs_E10	ordinary_muscle_development	D_vs_E10;stage_1W_vs_E10;stage_3W_vs_E10;stage	1	TRUE
ENSGALG00010010100	IRF2BP2	PRJNA729271	1.334525136	1.0861E-15	1.334525136	stage_E14_vs_E10	ordinary_muscle_development	D_vs_E10;stage_1W_vs_E10;stage_3W_vs_E10;stage	1	TRUE
ENSGALG00010016936	RCC1	PRJNA729271	-1.352777534	1.09446E-15	1.352777534	stage_E14_vs_E10	ordinary_muscle_development	D_vs_E10;stage_1W_vs_E10;stage_3W_vs_E10;stage	1	TRUE
ENSGALG00010029767	GLOD4	PRJNA729271	-0.719731965	1.1063E-15	0.719731965	stage_E14_vs_E10	ordinary_muscle_development		0	FALSE
ENSGALG00010021668	PYGO1	PRJNA729271	-1.080617359	1.10974E-15	1.080617359	stage_E14_vs_E10	ordinary_muscle_development	D_vs_E10;stage_1W_vs_E10;stage_3W_vs_E10;stage	1	TRUE
ENSGALG00010025674	GRB14	PRJNA729271	3.463479524	1.11345E-15	3.463479524	stage_E14_vs_E10	ordinary_muscle_development	D_vs_E10;stage_1W_vs_E10;stage_3W_vs_E10;stage	1	TRUE
ENSGALG00010016613	SNIP1	PRJNA729271	-0.642817458	1.12229E-15	0.642817458	stage_E14_vs_E10	ordinary_muscle_development		0	FALSE
ENSGALG00010019931	ZFYVE26	PRJNA729271	1.095930894	1.12668E-15	1.095930894	stage_E14_vs_E10	ordinary_muscle_development	D_vs_E10;stage_1W_vs_E10;stage_3W_vs_E10;stage	1	TRUE
ENSGALG00010028583	USHBP1	PRJNA729271	1.619158202	1.12692E-15	1.619158202	stage_E14_vs_E10	ordinary_muscle_development	D_vs_E10;stage_1W_vs_E10;stage_3W_vs_E10;stage	1	TRUE
ENSGALG00010025743	SH2B3	PRJNA729271	0.879843515	1.14102E-15	0.879843515	stage_E14_vs_E10	ordinary_muscle_development		0	FALSE
ENSGALG00010027231	ENSGALG00010027231	PRJNA729271	-1.299992913	1.15153E-15	1.299992913	stage_E14_vs_E10	ordinary_muscle_development	D_vs_E10;stage_1W_vs_E10;stage_3W_vs_E10;stage	1	TRUE
ENSGALG00010011979	NEDD1	PRJNA729271	-0.648993777	1.15814E-15	0.648993777	stage_E14_vs_E10	ordinary_muscle_development		0	FALSE
ENSGALG00010011463	IMP3	PRJNA729271	-1.123169717	1.16216E-15	1.123169717	stage_E14_vs_E10	ordinary_muscle_development	D_vs_E10;stage_1W_vs_E10;stage_3W_vs_E10;stage	1	TRUE
ENSGALG00010002781	EIF3G	PRJNA729271	1.130723927	1.16655E-15	1.130723927	stage_E14_vs_E10	ordinary_muscle_development	D_vs_E10;stage_1W_vs_E10;stage_3W_vs_E10;stage	1	TRUE
ENSGALG00010010908	STK17B	PRJNA729271	1.000971125	1.16823E-15	1.000971125	stage_E14_vs_E10	ordinary_muscle_development	D_vs_E10;stage_1W_vs_E10;stage_3W_vs_E10;stage	1	TRUE
ENSGALG00010016235	DDX27	PRJNA729271	-1.028111651	1.17845E-15	1.028111651	stage_E14_vs_E10	ordinary_muscle_development	D_vs_E10;stage_1W_vs_E10;stage_3W_vs_E10;stage	1	TRUE
ENSGALG00010021725	SH2D6	PRJNA729271	1.708764463	1.17893E-15	1.708764463	stage_E14_vs_E10	ordinary_muscle_development	D_vs_E10;stage_1W_vs_E10;stage_3W_vs_E10;stage	1	TRUE
ENSGALG00010006024	AMER2	PRJNA729271	-3.734501544	1.19562E-15	3.734501544	stage_E14_vs_E10	ordinary_muscle_development	D_vs_E10;stage_1W_vs_E10;stage_3W_vs_E10;stage	1	TRUE
ENSGALG00010008100	ENSGALG00010008100	PRJNA729271	1.909378701	1.1987E-15	1.909378701	stage_E14_vs_E10	ordinary_muscle_development	D_vs_E10;stage_1W_vs_E10;stage_3W_vs_E10;stage	1	TRUE
ENSGALG00010023527	TMEM98	PRJNA729271	-1.943410395	1.22105E-15	1.943410395	stage_E14_vs_E10	ordinary_muscle_development	D_vs_E10;stage_1W_vs_E10;stage_3W_vs_E10;stage	1	TRUE
ENSGALG00010023648	SCN2B	PRJNA729271	2.928905368	1.22256E-15	2.928905368	stage_E14_vs_E10	ordinary_muscle_development	D_vs_E10;stage_1W_vs_E10;stage_3W_vs_E10;stage	1	TRUE
ENSGALG00010005434	PRDM5	PRJNA729271	-1.828891501	1.2279E-15	1.828891501	stage_E14_vs_E10	ordinary_muscle_development	D_vs_E10;stage_1W_vs_E10;stage_3W_vs_E10;stage	1	TRUE
ENSGALG00010016315	RPS4Y1	PRJNA729271	1.119995932	1.23114E-15	1.119995932	stage_E14_vs_E10	ordinary_muscle_development	D_vs_E10;stage_1W_vs_E10;stage_3W_vs_E10;stage	1	TRUE
ENSGALG00010001818	ADGRB3	PRJNA729271	-2.228608016	1.2514E-15	2.228608016	stage_E14_vs_E10	ordinary_muscle_development	D_vs_E10;stage_1W_vs_E10;stage_3W_vs_E10;stage	1	TRUE
ENSGALG00010029946	METRNL	PRJNA729271	-1.118443505	1.26434E-15	1.118443505	stage_E14_vs_E10	ordinary_muscle_development	D_vs_E10;stage_1W_vs_E10;stage_3W_vs_E10;stage	1	TRUE
ENSGALG00010002357	RAB31	PRJNA729271	-1.174201643	1.28842E-15	1.174201643	stage_E14_vs_E10	ordinary_muscle_development	D_vs_E10;stage_1W_vs_E10;stage_3W_vs_E10;stage	1	TRUE
ENSGALG00010003898	NDST4	PRJNA729271	-5.567475186	1.29269E-15	5.567475186	stage_E14_vs_E10	ordinary_muscle_development	D_vs_E10;stage_1W_vs_E10;stage_3W_vs_E10;stage	1	TRUE
ENSGALG00010025946	KRT5	PRJNA729271	-10.35503788	1.2989E-15	10.35503788	stage_E14_vs_E10	ordinary_muscle_development	D_vs_E10;stage_1W_vs_E10;stage_3W_vs_E10;stage	1	TRUE
ENSGALG00010016894	BTBD7	PRJNA729271	0.744899446	1.30547E-15	0.744899446	stage_E14_vs_E10	ordinary_muscle_development		0	FALSE
ENSGALG00010010243	ESCO2	PRJNA729271	-0.93093379	1.31136E-15	0.93093379	stage_E14_vs_E10	ordinary_muscle_development		0	FALSE
ENSGALG00010012587	MBLAC2	PRJNA729271	1.179143955	1.31211E-15	1.179143955	stage_E14_vs_E10	ordinary_muscle_development	D_vs_E10;stage_1W_vs_E10;stage_3W_vs_E10;stage	1	TRUE
ENSGALG00010007577	WFDC1	PRJNA729271	0.975448824	1.37442E-15	0.975448824	stage_E14_vs_E10	ordinary_muscle_development		0	FALSE
ENSGALG00010007480	TENM3	PRJNA729271	-0.881602794	1.38641E-15	0.881602794	stage_E14_vs_E10	ordinary_muscle_development		0	FALSE
ENSGALG00010013452	LSS	PRJNA729271	-1.889176832	1.38921E-15	1.889176832	stage_E14_vs_E10	ordinary_muscle_development	D_vs_E10;stage_1W_vs_E10;stage_3W_vs_E10;stage	1	TRUE
ENSGALG00010017050	L3HYPDH	PRJNA729271	-1.287460991	1.40205E-15	1.287460991	stage_E14_vs_E10	ordinary_muscle_development	D_vs_E10;stage_1W_vs_E10;stage_3W_vs_E10;stage	1	TRUE
ENSGALG00010004379	ZBTB10	PRJNA729271	-0.495580263	1.40803E-15	0.495580263	stage_E14_vs_E10	ordinary_muscle_development		0	FALSE
ENSGALG00010026242	CCDC141	PRJNA729271	2.068046345	1.43861E-15	2.068046345	stage_E14_vs_E10	ordinary_muscle_development	D_vs_E10;stage_1W_vs_E10;stage_3W_vs_E10;stage	1	TRUE
ENSGALG00010025491	ZMYND11	PRJNA729271	-0.523981725	1.43909E-15	0.523981725	stage_E14_vs_E10	ordinary_muscle_development		0	FALSE
ENSGALG00010028763	CTSK	PRJNA729271	1.779503067	1.45783E-15	1.779503067	stage_E14_vs_E10	ordinary_muscle_development	D_vs_E10;stage_1W_vs_E10;stage_3W_vs_E10;stage	1	TRUE
ENSGALG00010017454	ENSGALG00010017454	PRJNA729271	1.282675809	1.46137E-15	1.282675809	stage_E14_vs_E10	ordinary_muscle_development	D_vs_E10;stage_1W_vs_E10;stage_3W_vs_E10;stage	1	TRUE
ENSGALG00010012116	RBM12B	PRJNA729271	-0.637708438	1.47531E-15	0.637708438	stage_E14_vs_E10	ordinary_muscle_development		0	FALSE
ENSGALG00010017347	TMEM39B	PRJNA729271	-0.996550365	1.49362E-15	0.996550365	stage_E14_vs_E10	ordinary_muscle_development		0	FALSE
ENSGALG00010013511	FAM78B	PRJNA729271	1.831485184	1.49606E-15	1.831485184	stage_E14_vs_E10	ordinary_muscle_development	D_vs_E10;stage_1W_vs_E10;stage_3W_vs_E10;stage	1	TRUE
ENSGALG00010005724	PROSER2	PRJNA729271	0.955330899	1.51246E-15	0.955330899	stage_E14_vs_E10	ordinary_muscle_development		0	FALSE
ENSGALG00010024970	CAB39	PRJNA729271	0.629990771	1.5204E-15	0.629990771	stage_E14_vs_E10	ordinary_muscle_development		0	FALSE
ENSGALG00010001298	DOCK4	PRJNA729271	1.065734719	1.52637E-15	1.065734719	stage_E14_vs_E10	ordinary_muscle_development	D_vs_E10;stage_1W_vs_E10;stage_3W_vs_E10;stage	1	TRUE
ENSGALG00010022666	ENSGALG00010022666	PRJNA729271	-0.862933872	1.54751E-15	0.862933872	stage_E14_vs_E10	ordinary_muscle_development		0	FALSE
ENSGALG00010008120	CDC5L	PRJNA729271	-0.532561278	1.55292E-15	0.532561278	stage_E14_vs_E10	ordinary_muscle_development		0	FALSE
ENSGALG00010002621	SLC25A15	PRJNA729271	-1.342572007	1.55721E-15	1.342572007	stage_E14_vs_E10	ordinary_muscle_development	D_vs_E10;stage_1W_vs_E10;stage_3W_vs_E10;stage	1	TRUE
ENSGALG00010005519	PTBP3	PRJNA729271	-2.142311567	1.56133E-15	2.142311567	stage_E14_vs_E10	ordinary_muscle_development	D_vs_E10;stage_1W_vs_E10;stage_3W_vs_E10;stage	1	TRUE
ENSGALG00010028053	ORM1	PRJNA729271	-19.03364215	1.56868E-15	19.03364215	stage_E14_vs_E10	ordinary_muscle_development	D_vs_E10;stage_1W_vs_E10;stage_3W_vs_E10;stage	1	TRUE
ENSGALG00010027304	PTPN23	PRJNA729271	1.110679296	1.58024E-15	1.110679296	stage_E14_vs_E10	ordinary_muscle_development	D_vs_E10;stage_1W_vs_E10;stage_3W_vs_E10;stage	1	TRUE
ENSGALG00010008002	GJA8	PRJNA729271	-1.324454944	1.59564E-15	1.324454944	stage_E14_vs_E10	ordinary_muscle_development	D_vs_E10;stage_1W_vs_E10;stage_3W_vs_E10;stage	1	TRUE
ENSGALG00010023104	ENSGALG00010023104	PRJNA729271	1.275684344	1.60171E-15	1.275684344	stage_E14_vs_E10	ordinary_muscle_development	D_vs_E10;stage_1W_vs_E10;stage_3W_vs_E10;stage	1	TRUE
ENSGALG00010022737	CYLD	PRJNA729271	0.641889459	1.60251E-15	0.641889459	stage_E14_vs_E10	ordinary_muscle_development		0	FALSE
ENSGALG00010027830	CACNA1D	PRJNA729271	-3.500557437	1.62212E-15	3.500557437	stage_E14_vs_E10	ordinary_muscle_development	D_vs_E10;stage_1W_vs_E10;stage_3W_vs_E10;stage	1	TRUE
ENSGALG00010010284	CCDC25	PRJNA729271	0.764972074	1.65087E-15	0.764972074	stage_E14_vs_E10	ordinary_muscle_development		0	FALSE
ENSGALG00010022069	NOP2	PRJNA729271	-1.102777145	1.6516E-15	1.102777145	stage_E14_vs_E10	ordinary_muscle_development	D_vs_E10;stage_1W_vs_E10;stage_3W_vs_E10;stage	1	TRUE
ENSGALG00010003929	RCBTB2	PRJNA729271	0.786028276	1.65602E-15	0.786028276	stage_E14_vs_E10	ordinary_muscle_development		0	FALSE
ENSGALG00010011630	DELE1	PRJNA729271	0.816131547	1.66303E-15	0.816131547	stage_E14_vs_E10	ordinary_muscle_development		0	FALSE
ENSGALG00010029653	CCT6A	PRJNA729271	-0.735503047	1.6654E-15	0.735503047	stage_E14_vs_E10	ordinary_muscle_development		0	FALSE
ENSGALG00010010706	SREBF2	PRJNA729271	1.121520951	1.68041E-15	1.121520951	stage_E14_vs_E10	ordinary_muscle_development	D_vs_E10;stage_1W_vs_E10;stage_3W_vs_E10;stage	1	TRUE
ENSGALG00010025451	LRRC43	PRJNA729271	2.550720807	1.70616E-15	2.550720807	stage_E14_vs_E10	ordinary_muscle_development	D_vs_E10;stage_1W_vs_E10;stage_3W_vs_E10;stage	1	TRUE
ENSGALG00010004034	NEDD8	PRJNA729271	0.901481917	1.71685E-15	0.901481917	stage_E14_vs_E10	ordinary_muscle_development		0	FALSE
ENSGALG00010029660	CD300LG	PRJNA729271	2.439953954	1.80941E-15	2.439953954	stage_E14_vs_E10	ordinary_muscle_development	D_vs_E10;stage_1W_vs_E10;stage_3W_vs_E10;stage	1	TRUE
ENSGALG00010012341	GOPC	PRJNA729271	-0.658673719	1.81349E-15	0.658673719	stage_E14_vs_E10	ordinary_muscle_development		0	FALSE
ENSGALG00010005799	UFSP2	PRJNA729271	-0.802309778	1.8275E-15	0.802309778	stage_E14_vs_E10	ordinary_muscle_development		0	FALSE
ENSGALG00010013569	ENSGALG00010013569	PRJNA729271	-4.791799359	1.83187E-15	4.791799359	stage_E14_vs_E10	ordinary_muscle_development	D_vs_E10;stage_1W_vs_E10;stage_3W_vs_E10;stage	1	TRUE
ENSGALG00010006001	NOP56	PRJNA729271	-1.142015088	1.83449E-15	1.142015088	stage_E14_vs_E10	ordinary_muscle_development	D_vs_E10;stage_1W_vs_E10;stage_3W_vs_E10;stage	1	TRUE
ENSGALG00010009007	TMEM65	PRJNA729271	-0.814990465	1.83576E-15	0.814990465	stage_E14_vs_E10	ordinary_muscle_development		0	FALSE
ENSGALG00010005386	CPOX	PRJNA729271	-1.155486113	1.86104E-15	1.155486113	stage_E14_vs_E10	ordinary_muscle_development	D_vs_E10;stage_1W_vs_E10;stage_3W_vs_E10;stage	1	TRUE
ENSGALG00010023546	SLC11A2	PRJNA729271	1.247028171	1.86442E-15	1.247028171	stage_E14_vs_E10	ordinary_muscle_development	D_vs_E10;stage_1W_vs_E10;stage_3W_vs_E10;stage	1	TRUE
ENSGALG00010012413	NEK7	PRJNA729271	0.585688778	1.86778E-15	0.585688778	stage_E14_vs_E10	ordinary_muscle_development		0	FALSE

ENSGALG00010012110	ENSGALG00010012110	PRJNA729271	0.911637352	1.86926E-15	0.911637352	stage_E14_vs_E10	ordinary_muscle_development		0	FALSE
ENSGALG00010010920	UBN2	PRJNA729271	0.918401302	1.872E-15	0.918401302	stage_E14_vs_E10	ordinary_muscle_development		0	FALSE
ENSGALG00010017280	SOS1	PRJNA729271	1.253655209	1.87482E-15	1.253655209	stage_E14_vs_E10	ordinary_muscle_development	D_vs_E10;stage_1W_vs_E10;stage_3W_vs_E10;stage	1	TRUE
ENSGALG00010024291	ENSGALG00010024291	PRJNA729271	4.026974982	1.88016E-15	4.026974982	stage_E14_vs_E10	ordinary_muscle_development	D_vs_E10;stage_1W_vs_E10;stage_3W_vs_E10;stage	1	TRUE
ENSGALG00010007403	SDR42E1	PRJNA729271	-2.161339054	1.90522E-15	2.161339054	stage_E14_vs_E10	ordinary_muscle_development	D_vs_E10;stage_1W_vs_E10;stage_3W_vs_E10;stage	1	TRUE
ENSGALG00010004851	MTCL3	PRJNA729271	-5.070439066	1.93706E-15	5.070439066	stage_E14_vs_E10	ordinary_muscle_development	D_vs_E10;stage_1W_vs_E10;stage_3W_vs_E10;stage	1	TRUE
ENSGALG00010025980	HECTD4	PRJNA729271	-0.524606326	1.97713E-15	0.524606326	stage_E14_vs_E10	ordinary_muscle_development		0	FALSE
ENSGALG00010026315	SYT2	PRJNA729271	-4.373672204	1.98373E-15	4.373672204	stage_E14_vs_E10	ordinary_muscle_development	D_vs_E10;stage_1W_vs_E10;stage_3W_vs_E10;stage	1	TRUE
ENSGALG00010024816	NFIA	PRJNA729271	-0.994992986	1.98544E-15	0.994992986	stage_E14_vs_E10	ordinary_muscle_development		0	FALSE
ENSGALG00010021630	VTG1	PRJNA729271	-2.768717362	1.98773E-15	2.768717362	stage_E14_vs_E10	ordinary_muscle_development	D_vs_E10;stage_1W_vs_E10;stage_3W_vs_E10;stage	1	TRUE
ENSGALG00010013794	GTF3C6	PRJNA729271	-1.474121681	1.99735E-15	1.474121681	stage_E14_vs_E10	ordinary_muscle_development	D_vs_E10;stage_1W_vs_E10;stage_3W_vs_E10;stage	1	TRUE
ENSGALG00010018558	ENSGALG00010018558	PRJNA729271	1.813338251	2.00122E-15	1.813338251	stage_E14_vs_E10	ordinary_muscle_development	D_vs_E10;stage_1W_vs_E10;stage_3W_vs_E10;stage	1	TRUE
ENSGALG00010002894	FCHSD2	PRJNA729271	-0.59084888	2.00447E-15	0.59084888	stage_E14_vs_E10	ordinary_muscle_development		0	FALSE
ENSGALG00010016452	GLCE	PRJNA729271	-0.933496574	2.02774E-15	0.933496574	stage_E14_vs_E10	ordinary_muscle_development		0	FALSE
ENSGALG00010028608	MIGA2	PRJNA729271	1.271724968	2.03596E-15	1.271724968	stage_E14_vs_E10	ordinary_muscle_development	D_vs_E10;stage_1W_vs_E10;stage_3W_vs_E10;stage	1	TRUE
ENSGALG00010023668	RCN1	PRJNA729271	-1.304739921	2.04186E-15	1.304739921	stage_E14_vs_E10	ordinary_muscle_development	D_vs_E10;stage_1W_vs_E10;stage_3W_vs_E10;stage	1	TRUE
ENSGALG00010019005	ENSGALG00010019005	PRJNA729271	-1.788549689	2.05999E-15	1.788549689	stage_E14_vs_E10	ordinary_muscle_development	D_vs_E10;stage_1W_vs_E10;stage_3W_vs_E10;stage	1	TRUE
ENSGALG00010017089	CRTAP	PRJNA729271	1.275586254	2.0801E-15	1.275586254	stage_E14_vs_E10	ordinary_muscle_development	D_vs_E10;stage_1W_vs_E10;stage_3W_vs_E10;stage	1	TRUE
ENSGALG00010007549	SH3D19	PRJNA729271	1.283522559	2.09625E-15	1.283522559	stage_E14_vs_E10	ordinary_muscle_development	D_vs_E10;stage_1W_vs_E10;stage_3W_vs_E10;stage	1	TRUE
ENSGALG00010008258	CCDC82	PRJNA729271	1.054532921	2.10363E-15	1.054532921	stage_E14_vs_E10	ordinary_muscle_development	D_vs_E10;stage_1W_vs_E10;stage_3W_vs_E10;stage	1	TRUE
ENSGALG00010024937	ILKAP	PRJNA729271	-1.027813523	2.11096E-15	1.027813523	stage_E14_vs_E10	ordinary_muscle_development	D_vs_E10;stage_1W_vs_E10;stage_3W_vs_E10;stage	1	TRUE
ENSGALG00010001119	COMMD2	PRJNA729271	-0.80532008	2.14498E-15	0.80532008	stage_E14_vs_E10	ordinary_muscle_development		0	FALSE
ENSGALG00010016205	CD40LG	PRJNA729271	8.098713154	2.153E-15	8.098713154	stage_E14_vs_E10	ordinary_muscle_development	D_vs_E10;stage_1W_vs_E10;stage_3W_vs_E10;stage	1	TRUE
ENSGALG00010029624	RASL10B	PRJNA729271	-2.841382902	2.16362E-15	2.841382902	stage_E14_vs_E10	ordinary_muscle_development	D_vs_E10;stage_1W_vs_E10;stage_3W_vs_E10;stage	1	TRUE
ENSGALG00010005596	PARD3	PRJNA729271	-0.441280334	2.2141E-15	0.441280334	stage_E14_vs_E10	ordinary_muscle_development		0	FALSE
ENSGALG00010029275	PCYT2	PRJNA729271	0.571847825	2.22168E-15	0.571847825	stage_E14_vs_E10	ordinary_muscle_development		0	FALSE
ENSGALG00010013775	DBX2	PRJNA729271	-3.660222963	2.23124E-15	3.660222963	stage_E14_vs_E10	ordinary_muscle_development	D_vs_E10;stage_1W_vs_E10;stage_3W_vs_E10;stage	1	TRUE
ENSGALG00010028552	ENSGALG00010028552	PRJNA729271	5.25078839	2.23748E-15	5.25078839	stage_E14_vs_E10	ordinary_muscle_development	D_vs_E10;stage_1W_vs_E10;stage_3W_vs_E10;stage	1	TRUE
ENSGALG00010013206	AFF2	PRJNA729271	-0.918414372	2.23752E-15	0.918414372	stage_E14_vs_E10	ordinary_muscle_development		0	FALSE
ENSGALG00010013194	GRAP2	PRJNA729271	-7.385064325	2.24852E-15	7.385064325	stage_E14_vs_E10	ordinary_muscle_development	D_vs_E10;stage_1W_vs_E10;stage_3W_vs_E10;stage	1	TRUE
ENSGALG00010024142	DNAJC6	PRJNA729271	-2.267419587	2.24865E-15	2.267419587	stage_E14_vs_E10	ordinary_muscle_development	D_vs_E10;stage_1W_vs_E10;stage_3W_vs_E10;stage	1	TRUE
ENSGALG00010021738	PRDM11	PRJNA729271	0.577480166	2.28568E-15	0.577480166	stage_E14_vs_E10	ordinary_muscle_development		0	FALSE
ENSGALG00010008780	KARS1	PRJNA729271	0.461172447	2.37069E-15	0.461172447	stage_E14_vs_E10	ordinary_muscle_development		0	FALSE
ENSGALG00010003447	ZC2HC1A	PRJNA729271	1.01325339	2.39621E-15	1.01325339	stage_E14_vs_E10	ordinary_muscle_development	D_vs_E10;stage_1W_vs_E10;stage_3W_vs_E10;stage	1	TRUE
ENSGALG00010018631	KAT14	PRJNA729271	-0.436263695	2.40127E-15	0.436263695	stage_E14_vs_E10	ordinary_muscle_development		0	FALSE
ENSGALG00010025565	ENSGALG00010025565	PRJNA729271	1.957590381	2.4177E-15	1.957590381	stage_E14_vs_E10	ordinary_muscle_development	D_vs_E10;stage_1W_vs_E10;stage_3W_vs_E10;stage	1	TRUE
ENSGALG00010029611	YWHAG	PRJNA729271	1.051923299	2.41805E-15	1.051923299	stage_E14_vs_E10	ordinary_muscle_development	D_vs_E10;stage_1W_vs_E10;stage_3W_vs_E10;stage	1	TRUE
ENSGALG00010013257	STX12	PRJNA729271	-0.731097751	2.41805E-15	0.731097751	stage_E14_vs_E10	ordinary_muscle_development		0	FALSE
ENSGALG00010024583	ITGB7	PRJNA729271	3.837745663	2.42587E-15	3.837745663	stage_E14_vs_E10	ordinary_muscle_development	D_vs_E10;stage_1W_vs_E10;stage_3W_vs_E10;stage	1	TRUE
ENSGALG00010029907	NTN1	PRJNA729271	-2.443052647	2.43581E-15	2.443052647	stage_E14_vs_E10	ordinary_muscle_development	D_vs_E10;stage_1W_vs_E10;stage_3W_vs_E10;stage	1	TRUE
ENSGALG00010021163	INHA	PRJNA729271	-3.565132498	2.46375E-15	3.565132498	stage_E14_vs_E10	ordinary_muscle_development	D_vs_E10;stage_1W_vs_E10;stage_3W_vs_E10;stage	1	TRUE
ENSGALG00010020517	UBR4	PRJNA729271	0.758578174	2.47245E-15	0.758578174	stage_E14_vs_E10	ordinary_muscle_development		0	FALSE
ENSGALG00010029672	KCTD7	PRJNA729271	-1.939444173	2.48584E-15	1.939444173	stage_E14_vs_E10	ordinary_muscle_development	D_vs_E10;stage_1W_vs_E10;stage_3W_vs_E10;stage	1	TRUE
ENSGALG00010019045	LBX1	PRJNA729271	2.132672622	2.55645E-15	2.132672622	stage_E14_vs_E10	ordinary_muscle_development	D_vs_E10;stage_1W_vs_E10;stage_3W_vs_E10;stage	1	TRUE
ENSGALG00010010007	CTIF	PRJNA729271	0.761535212	2.55685E-15	0.761535212	stage_E14_vs_E10	ordinary_muscle_development		0	FALSE
ENSGALG00010001279	LPIN2	PRJNA729271	-1.122206925	2.55697E-15	1.122206925	stage_E14_vs_E10	ordinary_muscle_development	D_vs_E10;stage_1W_vs_E10;stage_3W_vs_E10;stage	1	TRUE
ENSGALG00010018381	HAPSTR1	PRJNA729271	0.543265805	2.56796E-15	0.543265805	stage_E14_vs_E10	ordinary_muscle_development		0	FALSE
ENSGALG00010012483	ENSGALG00010012483	PRJNA729271	2.027407596	2.58947E-15	2.027407596	stage_E14_vs_E10	ordinary_muscle_development	D_vs_E10;stage_1W_vs_E10;stage_3W_vs_E10;stage	1	TRUE
ENSGALG00010003254	DGKH	PRJNA729271	1.34964894	2.63368E-15	1.34964894	stage_E14_vs_E10	ordinary_muscle_development	D_vs_E10;stage_1W_vs_E10;stage_3W_vs_E10;stage	1	TRUE
ENSGALG00010002812	TRIM13	PRJNA729271	-0.82955671	2.65776E-15	0.82955671	stage_E14_vs_E10	ordinary_muscle_development		0	FALSE
ENSGALG00010006528	ZC3HC1	PRJNA729271	-0.694628593	2.70604E-15	0.694628593	stage_E14_vs_E10	ordinary_muscle_development		0	FALSE
ENSGALG00010002956	SCRG1	PRJNA729271	2.666946573	2.71717E-15	2.666946573	stage_E14_vs_E10	ordinary_muscle_development	D_vs_E10;stage_1W_vs_E10;stage_3W_vs_E10;stage	1	TRUE
ENSGALG00010026638	LAD1	PRJNA729271	-4.799364732	2.72321E-15	4.799364732	stage_E14_vs_E10	ordinary_muscle_development	D_vs_E10;stage_1W_vs_E10;stage_3W_vs_E10;stage	1	TRUE
ENSGALG00010004001	LARP7	PRJNA729271	-0.921427689	2.7374E-15	0.921427689	stage_E14_vs_E10	ordinary_muscle_development		0	FALSE
ENSGALG00010022806	SPPL3	PRJNA729271	-0.512551853	2.75743E-15	0.512551853	stage_E14_vs_E10	ordinary_muscle_development		0	FALSE
ENSGALG00010026550	MYD88	PRJNA729271	0.845296208	2.76438E-15	0.845296208	stage_E14_vs_E10	ordinary_muscle_development		0	FALSE
ENSGALG00010028878	IGSF9	PRJNA729271	1.249393399	2.82894E-15	1.249393399	stage_E14_vs_E10	ordinary_muscle_development	D_vs_E10;stage_1W_vs_E10;stage_3W_vs_E10;stage	1	TRUE
ENSGALG00010012311	ENSGALG00010012311	PRJNA729271	-4.454150537	2.84148E-15	4.454150537	stage_E14_vs_E10	ordinary_muscle_development	D_vs_E10;stage_1W_vs_E10;stage_3W_vs_E10;stage	1	TRUE
ENSGALG00010023214	SUPV3L1	PRJNA729271	-0.746935578	2.86292E-15	0.746935578	stage_E14_vs_E10	ordinary_muscle_development		0	FALSE
ENSGALG00010005617	DTD2	PRJNA729271	1.686472707	2.86706E-15	1.686472707	stage_E14_vs_E10	ordinary_muscle_development	D_vs_E10;stage_1W_vs_E10;stage_3W_vs_E10;stage	1	TRUE
ENSGALG00010009720	RELN	PRJNA729271	-1.390836939	2.89045E-15	1.390836939	stage_E14_vs_E10	ordinary_muscle_development	D_vs_E10;stage_1W_vs_E10;stage_3W_vs_E10;stage	1	TRUE
ENSGALG00010012812	DECR1	PRJNA729271	1.933162891	2.93614E-15	1.933162891	stage_E14_vs_E10	ordinary_muscle_development	D_vs_E10;stage_1W_vs_E10;stage_3W_vs_E10;stage	1	TRUE
ENSGALG00010021401	PDAP1	PRJNA729271	-0.765630783	2.93635E-15	0.765630783	stage_E14_vs_E10	ordinary_muscle_development		0	FALSE
ENSGALG00010011810	FGFR4	PRJNA729271	1.030078049	3.01347E-15	1.030078049	stage_E14_vs_E10	ordinary_muscle_development	D_vs_E10;stage_1W_vs_E10;stage_3W_vs_E10;stage	1	TRUE
ENSGALG00010007758	DEK	PRJNA729271	-0.62316418	3.01969E-15	0.62316418	stage_E14_vs_E10	ordinary_muscle_development		0	FALSE
ENSGALG00010024479	ERBB2	PRJNA729271	1.02380454	3.02364E-15	1.02380454	stage_E14_vs_E10	ordinary_muscle_development	D_vs_E10;stage_1W_vs_E10;stage_3W_vs_E10;stage	1	TRUE
ENSGALG00010025325	DPEP2	PRJNA729271	1.92916928	3.09234E-15	1.92916928	stage_E14_vs_E10	ordinary_muscle_development	D_vs_E10;stage_1W_vs_E10;stage_3W_vs_E10;stage	1	TRUE
ENSGALG00010002927	GABRA5	PRJNA729271	-7.412694799	3.10016E-15	7.412694799	stage_E14_vs_E10	ordinary_muscle_development	D_vs_E10;stage_1W_vs_E10;stage_3W_vs_E10;stage	1	TRUE
ENSGALG00010005350	ARHGAP20	PRJNA729271	-2.343977745	3.12157E-15	2.343977745	stage_E14_vs_E10	ordinary_muscle_development	D_vs_E10;stage_1W_vs_E10;stage_3W_vs_E10;stage	1	TRUE
ENSGALG00010022879	ENSGALG00010022879	PRJNA729271	2.224027266	3.13367E-15	2.224027266	stage_E14_vs_E10	ordinary_muscle_development	D_vs_E10;stage_1W_vs_E10;stage_3W_vs_E10;stage	1	TRUE
ENSGALG00010001518	SLC17A5	PRJNA729271	-1.346009582	3.15172E-15	1.346009582	stage_E14_vs_E10	ordinary_muscle_development	D_vs_E10;stage_1W_vs_E10;stage_3W_vs_E10;stage	1	TRUE
ENSGALG00010023334	RPS17	PRJNA729271	1.226959946	3.17605E-15	1.226959946	stage_E14_vs_E10	ordinary_muscle_development	D_vs_E10;stage_1W_vs_E10;stage_3W_vs_E10;stage	1	TRUE
ENSGALG00010010984	MAT1A	PRJNA729271	3.205709616	3.18717E-15	3.205709616	stage_E14_vs_E10	ordinary_muscle_development	D_vs_E10;stage_1W_vs_E10;stage_3W_vs_E10;stage	1	TRUE
ENSGALG00010023946	ZNRF3	PRJNA729271	-1.0360388	3.23465E-15	1.0360388	stage_E14_vs_E10	ordinary_muscle_development	D_vs_E10;stage_1W_vs_E10;stage_3W_vs_E10;stage	1	TRUE
ENSGALG00010017848	CYP3A4	PRJNA729271	1.583197286	3.2361E-15	1.583197286	stage_E14_vs_E10	ordinary_muscle_development	D_vs_E10;stage_1W_vs_E10;stage_3W_vs_E10;stage	1	TRUE
ENSGALG00010007765	NUDT7	PRJNA729271	0.762808329	3.29869E-15	0.762808329	stage_E14_vs_E10	ordinary_muscle_development		0	FALSE
ENSGALG00010022180	PLLP	PRJNA729271	2.958910875	3.30657E-15	2.958910875	stage_E14_vs_E10	ordinary_muscle_development	D_vs_E10;stage_1W_vs_E10;stage_3W_vs_E10;stage	1	TRUE
ENSGALG00010021697	PIANP	PRJNA729271	-2.060823746	3.31486E-15	2.060823746	stage_E14_vs_E10	ordinary_muscle_development	D_vs_E10;stage_1W_vs_E10;stage_3W_vs_E10;stage	1	TRUE
ENSGALG00010018489	SLC7A3	PRJNA729271	-1.675262236	3.37511E-15	1.675262236	stage_E14_vs_E10	ordinary_muscle_development	D_vs_E10;stage_1W_vs_E10;stage_3W_vs_E10;stage	1	TRUE
ENSGALG00010015904	MTMR7	PRJNA729271	2.077753037	3.3785E-15	2.077753037	stage_E14_vs_E10	ordinary_muscle_development	D_vs_E10;stage_1W_vs_E10;stage_3W_vs_E10;stage	1	TRUE
ENSGALG00010024925	GTF2E1	PRJNA729271	-0.679504597	3.4043E-15	0.679504597	stage_E14_vs_E10	ordinary_muscle_development		0	FALSE
ENSGALG00010012532	FAM174A	PRJNA729271	1.205003431	3.41945E-15	1.205003431	stage_E14_vs_E10	ordinary_muscle_development	D_vs_E10;stage_1W_vs_E10;stage_3W_vs_E10;stage	1	TRUE
ENSGALG00010029599	RNF43	PRJNA729271	-3.91659536	3.4603E-15	3.91659536	stage_E14_vs_E10	ordinary_muscle_development	D_vs_E10;stage_1W_vs_E10;stage_3W_vs_E10;stage	1	TRUE
ENSGALG00010025658	PDE1B	PRJNA729271	-3.67093705	3.46661E-15	3.67093705	stage_E14_vs_E10	ordinary_muscle_development	D_vs_E10;stage_1W_vs_E10;stage_3W_vs_E10;stage	1	TRUE
ENSGALG00010003331	EPSTI1	PRJNA729271	4.899719146	3.54368E-15	4.899719146	stage_E14_vs_E10	ordinary_muscle_development	D_vs_E10;stage_1W_vs_E10;stage_3W_vs_E10;stage	1	TRUE
ENSGALG00010013749	NELL2	PRJNA729271	-3.748019194	3.56878E-15	3.748019194	stage_E14_vs_E10	ordinary_muscle_development	D_vs_E10;stage_1W_vs_E10;stage_3W_vs_E10;stage	1	TRUE
ENSGALG00010015751	MYOM3	PRJNA729271	-4.367035355	3.57321E-15	4.367035355	stage_E14_vs_E10	ordinary_muscle_development	D_vs_E10;stage_1W_vs_E10;stage_3W_vs_E10;stage	1	TRUE
ENSGALG00010028110	COQ4	PRJNA729271	0.809313184	3.61721E-15	0.809313184	stage_E14_vs_E10	ordinary_muscle_development		0	FALSE
ENSGALG00010005223	GRIA4	PRJNA729271	-4.023427539	3.65471E-15	4.023427539	stage_E14_vs_E10	ordinary_muscle_development	D_vs_E10;stage_1W_vs_E10;stage_3W_vs_E10;stage	1	TRUE
ENSGALG00010028556	SPTAN1	PRJNA729271	0.765185651	3.66037E-15	0.765185651	stage_E14_vs_E10	ordinary_muscle_development		0	FALSE
ENSGALG00010023340	P3H3	PRJNA729271	-1.287639815	3.68015E-15	1.287639815	stage_E14_vs_E10	ordinary_muscle_development	D_vs_E10;stage_1W_vs_E10;stage_3W_vs_E10;stage	1	TRUE
ENSGALG00010014750	LONRF1	PRJNA729271	1.443335852	3.71035E-15	1.443335852	stage_E14_vs_E10	ordinary_muscle_development	D_vs_E10;stage_1W_vs_E10;stage_3W_vs_E10;stage	1	TRUE
ENSGALG00010021514	TPR	PRJNA729271	-0.636289965	3.78019E-15	0.636289965	stage_E14_vs_E10	ordinary_muscle_development		0	FALSE
ENSGALG00010028900	TNC	PRJNA729271	-4.40837494	3.78518E-15	4.40837494	stage_E14_vs_E10	ordinary_muscle_development	D_vs_E10;stage_1W_vs_E10;stage_3W_vs_E10;stage	1	TRUE
ENSGALG00010018831	WBP1L	PRJNA729271	1.937436947	3.8072E-15	1.937436947	stage_E14_vs_E10	ordinary_muscle_development	D_vs_E10;stage_1W_vs_E10;stage_3W_vs_E10;stage	1	TRUE
ENSGALG00010002919	ENSGALG00010002919	PRJNA729271	1.627989584	3.80793E-15	1.627989584	stage_E14_vs_E10	ordinary_muscle_development	D_vs_E10;stage_1W_vs_E10;stage_3W_vs_E10;stage	1	TRUE
ENSGALG00010012074	KIAA0930	PRJNA729271	-0.715834316	3.82932E-15	0.715834316	stage_E14_vs_E10	ordinary_muscle_development		0	FALSE
ENSGALG00010025044	TMEM9	PRJNA729271	1.702781171	3.84709E-15	1.702781171	stage_E14_vs_E10	ordinary_muscle_development	D_vs_E10;stage_1W_vs_E10;stage_3W_vs_E10;stage	1	TRUE
ENSGALG00010011915	RRM2B	PRJNA729271	0.966866234	3.85234E-15	0.966866234	stage_E14_vs_E10	ordinary_muscle_development		0	FALSE
ENSGALG00010027813	LRRC71	PRJNA729271	-2.934856898	3.86089E-15	2.934856898	stage_E14_vs_E10	ordinary_muscle_development	D_vs_E10;stage_1W_vs_E10;stage_3W_vs_E10;stage	1	TRUE
ENSGALG00010004991	FAM133B	PRJNA729271	-0.508651254	3.86222E-15	0.508651254	stage_E14_vs_E10	ordinary_muscle_development		0	FALSE
ENSGALG00010014722	KCTD17	PRJNA729271	-3.728201664	3.86442E-15	3.728201664	stage_E14_vs_E10	ordinary_muscle_development	D_vs_E10;stage_1W_vs_E10;stage_3W_vs_E10;stage	1	TRUE
ENSGALG00010007033	CEBPZOS	PRJNA729271	1.496162712	3.87939E-15	1.496162712	stage_E14_vs_E10	ordinary_muscle_development	D_vs_E10;stage_1W_vs_E10;stage_3W_vs_E10;stage	1	TRUE
ENSGALG00010018350	CNNM1	PRJNA729271	-2.380729431	3.91921E-15	2.380729431	stage_E14_vs_E10	ordinary_muscle_development	D_vs_E10;stage_1W_vs_E10;stage_3W_vs_E10;stage	1	TRUE
ENSGALG00010024910	UBTF	PRJNA729271	-0.990081277	3.94641E-15	0.990081277	stage_E14_vs_E10	ordinary_muscle_development		0	FALSE
ENSGALG00010009080	TLL1	PRJNA729271	-1.386460101	3.96031E-15	1.386460101	stage_E14_vs_E10	ordinary_muscle_development	D_vs_E10;stage_1W_vs_E10;stage_3W_vs_E10;stage	1	TRUE
ENSGALG00010014220	C9orf85	PRJNA729271	-1.076639346	3.9703E-15	1.076639346	stage_E14_vs_E10	ordinary_muscle_development	D_vs_E10;stage_1W_vs_E10;stage_3W_vs_E10;stage	1	TRUE
ENSGALG00010023131	SOX6	PRJNA729271	1.094861103	3.97994E-15	1.094861103	stage_E14_vs_E10	ordinary_muscle_development	D_vs_E10;stage_1W_vs_E10;stage_3W_vs_E10;stage	1	TRUE
ENSGALG00010013431	TMEM175	PRJNA729271	-0.705099944	4.03208E-15	0.705099944	stage_E14_vs_E10	ordinary_muscle_development		0	FALSE
ENSGALG00010023847	PIK3AP1	PRJNA729271	1.456783928	4.03289E-15	1.456783928	stage_E14_vs_E10	ordinary_muscle_development	D_vs_E10;stage_1W_vs_E10;stage_3W_vs_E10;stage	1	TRUE
ENSGALG00010028501	USP20	PRJNA729271	0.699341385	4.07599E-15	0.699341385	stage_E14_vs_E10	ordinary_muscle_development		0	FALSE
ENSGALG00010029287	ANKRD40	PRJNA729271	0.982330576	4.13871E-15	0.982330576	stage_E14_vs_E10	ordinary_muscle_development		0	FALSE
ENSGALG00010019038	PLEKHS1	PRJNA729271	-4.420916172	4.15276E-15	4.420916172	stage_E14_vs_E10	ordinary_muscle_development	D_vs_E10;stage_1W_vs_E10;stage_3W_vs_E10;stage	1	TRUE
ENSGALG00010021638	SRC	PRJNA729271	-1.436346924	4.1777E-15	1.436346924	stage_E14_vs_E10	ordinary_muscle_development	D_vs_E10;stage_1W_vs_E10;stage_3W_vs_E10;stage	1	TRUE
ENSGALG00010017754	PDZD11	PRJNA729271	-1.29556926	4.22098E-15	1.29556926	stage_E14_vs_E10	ordinary_muscle_development	D_vs_E10;stage_1W_vs_E10;stage_3W_vs_E10;stage	1	TRUE
ENSGALG00010012755	CLVS2	PRJNA729271	-3.35372833	4.25543E-15	3.35372833	stage_E14_vs_E10	ordinary_muscle_development	D_vs_E10;stage_1W_vs_E10;stage_3W_vs_E10;stage	1	TRUE
ENSGALG00010005014	SULT1B1	PRJNA729271	1.210407457	4.28066E-15	1.210407457	stage_E14_vs_E10	ordinary_muscle_development	D_vs_E10;stage_1W_vs_E10;stage_3W_vs_E10;stage	1	TRUE
ENSGALG00010008311	SRP19	PRJNA729271	-0.926884966	4.29691E-15	0.926884966	stage_E14_vs_E10	ordinary_muscle_development		0	FALSE
ENSGALG00010009355	GARRE1	PRJNA729271	0.574755467	4.29756E-15	0.574755467	stage_E14_vs_E10	ordinary_muscle_development		0	FALSE
ENSGALG00010003666	SLC25A13	PRJNA729271	-0.624776491	4.31322E-15	0.624776491	stage_E14_vs_E10	ordinary_muscle_development		0	FALSE
ENSGALG00010027889	RBM8A	PRJNA729271	-1.336086505	4.49458E-15	1.336086505	stage_E14_vs_E10	ordinary_muscle_development	D_vs_E10;stage_1W_vs_E10;stage_3W_vs_E10;stage	1	TRUE
ENSGALG00010023362	GADD45A	PRJNA729271	1.196373268	4.5093E-15	1.196373268	stage_E14_vs_E10	ordinary_muscle_development	D_vs_E10;stage_1W_vs_E10;stage_3W_vs_E10;stage	1	TRUE
ENSGALG00010027320	MON1A	PRJNA729271	1.278898908	4.63157E-15	1.278898908	stage_E14_vs_E10	ordinary_muscle_development	D_vs_E10;stage_1W_vs_E10;stage_3W_vs_E10;stage	1	TRUE
ENSGALG00010012685	NPTXR	PRJNA729271	1.900471043	4.63205E-15	1.900471043	stage_E14_vs_E10	ordinary_muscle_development	D_vs_E10;stage_1W_vs_E10;stage_3W_vs_E10;stage	1	TRUE
ENSGALG00010021358	MMP15	PRJNA729271	1.029560976	4.71142E-15	1.029560976	stage_E14_vs_E10	ordinary_muscle_development	D_vs_E10;stage_1W_vs_E10;stage_3W_vs_E10;stage	1	TRUE
ENSGALG00010028783	DDX31	PRJNA729271	-0.613467186	4.74149E-15	0.613467186	stage_E14_vs_E10	ordinary_muscle_development		0	FALSE
ENSGALG00010012606	ATP5PO	PRJNA729271	0.909774924	4.75166E-15	0.909774924	stage_E14_vs_E10	ordinary_muscle_development		0	FALSE
ENSGALG00010024122	SOCS7	PRJNA729271	0.831156723	4.79586E-15	0.831156723	stage_E14_vs_E10	ordinary_muscle_development		0	FALSE
ENSGALG00010024619	ENSGALG00010024619	PRJNA729271	-1.879835445	4.81001E-15	1.879835445	stage_E14_vs_E10	ordinary_muscle_development	D_vs_E10;stage_1W_vs_E10;stage_3W_vs_E10;stage	1	TRUE
ENSGALG00010003754	FAM110C	PRJNA729271	1.958804196	4.8161E-15	1.958804196	stage_E14_vs_E10	ordinary_muscle_development	D_vs_E10;stage_1W_vs_E10;stage_3W_vs_E10;stage	1	TRUE
ENSGALG00010025297	SPSB4	PRJNA729271	-1.1749253	4.8448E-15	1.1749253	stage_E14_vs_E10	ordinary_muscle_development	D_vs_E10;stage_1W_vs_E10;stage_3W_vs_E10;stage	1	TRUE
ENSGALG00010028105	PRKCD	PRJNA729271	-1.148601948	4.85149E-15	1.148601948	stage_E14_vs_E10	ordinary_muscle_development	D_vs_E10;stage_1W_vs_E10;stage_3W_vs_E10;stage	1	TRUE
ENSGALG00010005755	ZMYM2	PRJNA729271	0.773299519	4.9025E-15	0.773299519	stage_E14_vs_E10	ordinary_muscle_development		0	FALSE
ENSGALG00010010897	CEP170	PRJNA729271	-0.734636497	4.91285E-15	0.734636497	stage_E14_vs_E10	ordinary_muscle_development		0	FALSE
ENSGALG00010009635	TDRD7	PRJNA729271	1.101649895	4.99717E-15	1.101649895	stage_E14_vs_E10	ordinary_muscle_development	D_vs_E10;stage_1W_vs_E10;stage_3W_vs_E10;stage	1	TRUE
ENSGALG00010020211	ADD3	PRJNA729271	-0.664917582	5.00721E-15	0.664917582	stage_E14_vs_E10	ordinary_muscle_development		0	FALSE
ENSGALG00010000271	RTN4R	PRJNA729271	-3.905728929	5.04259E-15	3.905728929	stage_E14_vs_E10	ordinary_muscle_development	D_vs_E10;stage_1W_vs_E10;stage_3W_vs_E10;stage	1	TRUE
ENSGALG00010023556	IVD	PRJNA729271	0.966552824	5.06295E-15	0.966552824	stage_E14_vs_E10	ordinary_muscle_development		0	FALSE
ENSGALG00010022579	TNFAIP8L3	PRJNA729271	-1.187269434	5.11224E-15	1.187269434	stage_E14_vs_E10	ordinary_muscle_development	D_vs_E10;stage_1W_vs_E10;stage_3W_vs_E10;stage	1	TRUE
ENSGALG00010012925	SETD4	PRJNA729271	-1.550681538	5.11458E-15	1.550681538	stage_E14_vs_E10	ordinary_muscle_development	D_vs_E10;stage_1W_vs_E10;stage_3W_vs_E10;stage	1	TRUE
ENSGALG00010027866	KCNA2	PRJNA729271	-1.115783183	5.21583E-15	1.115783183	stage_E14_vs_E10	ordinary_muscle_development	D_vs_E10;stage_1W_vs_E10;stage_3W_vs_E10;stage	1	TRUE
ENSGALG00010020136	KCNK17	PRJNA729271	2.18148354	5.23049E-15	2.18148354	stage_E14_vs_E10	ordinary_muscle_development	D_vs_E10;stage_1W_vs_E10;stage_3W_vs_E10;stage	1	TRUE
ENSGALG00010000289	QPRT	PRJNA729271	-3.936565904	5.23077E-15	3.936565904	stage_E14_vs_E10	ordinary_muscle_development	D_vs_E10;stage_1W_vs_E10;stage_3W_vs_E10;stage	1	TRUE
ENSGALG00010028188	ENSGALG00010028188	PRJNA729271	0.860406905	5.23525E-15	0.860406905	stage_E14_vs_E10	ordinary_muscle_development		0	FALSE
ENSGALG00010013576	CAMK2A	PRJNA729271	1.512412564	5.25686E-15	1.512412564	stage_E14_vs_E10	ordinary_muscle_development	D_vs_E10;stage_1W_vs_E10;stage_3W_vs_E10;stage	1	TRUE
ENSGALG00010008270	ENSGALG00010008270	PRJNA729271	1.292331929	5.33115E-15	1.292331929	stage_E14_vs_E10	ordinary_muscle_development	D_vs_E10;stage_1W_vs_E10;stage_3W_vs_E10;stage	1	TRUE
ENSGALG00010024544	ZNF536	PRJNA729271	-2.001007733	5.34392E-15	2.001007733	stage_E14_vs_E10	ordinary_muscle_development	D_vs_E10;stage_1W_vs_E10;stage_3W_vs_E10;stage	1	TRUE
ENSGALG00010021452	SLC25A1	PRJNA729271	-0.955694864	5.36252E-15	0.955694864	stage_E14_vs_E10	ordinary_muscle_development		0	FALSE
ENSGALG00010019041	KATNIP	PRJNA729271	-0.737501831	5.44568E-15	0.737501831	stage_E14_vs_E10	ordinary_muscle_development		0	FALSE
ENSGALG00010004758	FLT3	PRJNA729271	6.088949787	5.53597E-15	6.088949787	stage_E14_vs_E10	ordinary_muscle_development	D_vs_E10;stage_1W_vs_E10;stage_3W_vs_E10;stage	1	TRUE
ENSGALG00010012971	ENSGALG00010012971	PRJNA729271	3.132303011	5.61278E-15	3.132303011	stage_E14_vs_E10	ordinary_muscle_development	D_vs_E10;stage_1W_vs_E10;stage_3W_vs_E10;stage	1	TRUE
ENSGALG00010022528	SIDT1	PRJNA729271	-4.354122484	5.62736E-15	4.354122484	stage_E14_vs_E10	ordinary_muscle_development	D_vs_E10;stage_1W_vs_E10;stage_3W_vs_E10;stage	1	TRUE
ENSGALG00010000507	TRPT1	PRJNA729271	2.247856902	5.67387E-15	2.247856902	stage_E14_vs_E10	ordinary_muscle_development	D_vs_E10;stage_1W_vs_E10;stage_3W_vs_E10;stage	1	TRUE
ENSGALG00010006083	CACNB2	PRJNA729271	-1.433397768	5.72051E-15	1.433397768	stage_E14_vs_E10	ordinary_muscle_development	D_vs_E10;stage_1W_vs_E10;stage_3W_vs_E10;stage	1	TRUE
ENSGALG00010020991	ALG14	PRJNA729271	1.08234089	5.79884E-15	1.08234089	stage_E14_vs_E10	ordinary_muscle_development	D_vs_E10;stage_1W_vs_E10;stage_3W_vs_E10;stage	1	TRUE
ENSGALG00010010161	SEPSECS	PRJNA729271	0.505830885	5.8045E-15	0.505830885	stage_E14_vs_E10	ordinary_muscle_development		0	FALSE
ENSGALG00010008057	BAG1	PRJNA729271	-1.075425745	5.86114E-15	1.075425745	stage_E14_vs_E10	ordinary_muscle_development	D_vs_E10;stage_1W_vs_E10;stage_3W_vs_E10;stage	1	TRUE
ENSGALG00010001483	ENSGALG00010001483	PRJNA729271	-3.450050928	5.86657E-15	3.450050928	stage_E14_vs_E10	ordinary_muscle_development	D_vs_E10;stage_1W_vs_E10;stage_3W_vs_E10;stage	1	TRUE
ENSGALG00010004965	JCAD	PRJNA729271	0.806736836	5.90177E-15	0.806736836	stage_E14_vs_E10	ordinary_muscle_development		0	FALSE
ENSGALG00010008884	FAM135B	PRJNA729271	3.542695415	5.94991E-15	3.542695415	stage_E14_vs_E10	ordinary_muscle_development	D_vs_E10;stage_1W_vs_E10;stage_3W_vs_E10;stage	1	TRUE
ENSGALG00010012059	ARHGDIB	PRJNA729271	-1.166570958	6.02896E-15	1.166570958	stage_E14_vs_E10	ordinary_muscle_development	D_vs_E10;stage_1W_vs_E10;stage_3W_vs_E10;stage	1	TRUE
ENSGALG00010005567	NDST3	PRJNA729271	-1.968193032	6.03463E-15	1.968193032	stage_E14_vs_E10	ordinary_muscle_development	D_vs_E10;stage_1W_vs_E10;stage_3W_vs_E10;stage	1	TRUE
ENSGALG00010006193	RBM17	PRJNA729271	-0.797023747	6.0484E-15	0.797023747	stage_E14_vs_E10	ordinary_muscle_development		0	FALSE
ENSGALG00010009761	PDP2	PRJNA729271	0.920072819	6.0996E-15	0.920072819	stage_E14_vs_E10	ordinary_muscle_development		0	FALSE
ENSGALG00010027129	NCLN	PRJNA729271	1.020078234	6.12188E-15	1.020078234	stage_E14_vs_E10	ordinary_muscle_development	D_vs_E10;stage_1W_vs_E10;stage_3W_vs_E10;stage	1	TRUE
ENSGALG00010015165	KLF3	PRJNA729271	0.923997335	6.21983E-15	0.923997335	stage_E14_vs_E10	ordinary_muscle_development		0	FALSE
ENSGALG00010012618	B3GALT2	PRJNA729271	2.598057466	6.28918E-15	2.598057466	stage_E14_vs_E10	ordinary_muscle_development	D_vs_E10;stage_1W_vs_E10;stage_3W_vs_E10;stage	1	TRUE
ENSGALG00010013398	CHAMP1	PRJNA729271	1.234829547	6.37826E-15	1.234829547	stage_E14_vs_E10	ordinary_muscle_development	D_vs_E10;stage_1W_vs_E10;stage_3W_vs_E10;stage	1	TRUE
ENSGALG00010010586	CYTIP	PRJNA729271	-2.703417084	6.39551E-15	2.703417084	stage_E14_vs_E10	ordinary_muscle_development	D_vs_E10;stage_1W_vs_E10;stage_3W_vs_E10;stage	1	TRUE
ENSGALG00010025994	INO80D	PRJNA729271	0.855463686	6.40661E-15	0.855463686	stage_E14_vs_E10	ordinary_muscle_development		0	FALSE
ENSGALG00010007337	PRORP	PRJNA729271	-0.798816055	6.42044E-15	0.798816055	stage_E14_vs_E10	ordinary_muscle_development		0	FALSE
ENSGALG00010010947	LUC7L2	PRJNA729271	-0.567668925	6.54421E-15	0.567668925	stage_E14_vs_E10	ordinary_muscle_development		0	FALSE
ENSGALG00010002958	ENSGALG00010002958	PRJNA729271	1.364982579	6.64894E-15	1.364982579	stage_E14_vs_E10	ordinary_muscle_development	D_vs_E10;stage_1W_vs_E10;stage_3W_vs_E10;stage	1	TRUE
ENSGALG00010005246	ENSGALG00010005246	PRJNA729271	-1.581785816	6.67417E-15	1.581785816	stage_E14_vs_E10	ordinary_muscle_development	D_vs_E10;stage_1W_vs_E10;stage_3W_vs_E10;stage	1	TRUE
ENSGALG00010010960	POLDIP3	PRJNA729271	-0.560165491	6.67584E-15	0.560165491	stage_E14_vs_E10	ordinary_muscle_development		0	FALSE

ENSGALG00010026081	KPNA1	PRJNA729271	-0.882682138	6.6843E-15	0.882682138	stage_E14_vs_E10	ordinary_muscle_development		0	FALSE
ENSGALG00010010155	BRINP3	PRJNA729271	-4.797036165	6.68832E-15	4.797036165	stage_E14_vs_E10	ordinary_muscle_development	D_vs_E10;stage_1W_vs_E10;stage_3W_vs_E10;stage	1	TRUE
ENSGALG00010016626	CAPN2	PRJNA729271	-1.894633622	6.69988E-15	1.894633622	stage_E14_vs_E10	ordinary_muscle_development	D_vs_E10;stage_1W_vs_E10;stage_3W_vs_E10;stage	1	TRUE
ENSGALG00010017377	USP8	PRJNA729271	0.596659214	6.72168E-15	0.596659214	stage_E14_vs_E10	ordinary_muscle_development		0	FALSE
ENSGALG00010021775	CCDC36	PRJNA729271	2.47462415	6.73724E-15	2.47462415	stage_E14_vs_E10	ordinary_muscle_development	D_vs_E10;stage_1W_vs_E10;stage_3W_vs_E10;stage	1	TRUE
ENSGALG00010012908	STRAP	PRJNA729271	-0.669319725	6.75662E-15	0.669319725	stage_E14_vs_E10	ordinary_muscle_development		0	FALSE
ENSGALG00010029864	SLC16A3	PRJNA729271	2.678221749	6.81427E-15	2.678221749	stage_E14_vs_E10	ordinary_muscle_development	D_vs_E10;stage_1W_vs_E10;stage_3W_vs_E10;stage	1	TRUE
ENSGALG00010012339	WNK1	PRJNA729271	1.22752345	6.81763E-15	1.22752345	stage_E14_vs_E10	ordinary_muscle_development	D_vs_E10;stage_1W_vs_E10;stage_3W_vs_E10;stage	1	TRUE
ENSGALG00010018036	RPL4	PRJNA729271	0.855804175	6.84531E-15	0.855804175	stage_E14_vs_E10	ordinary_muscle_development		0	FALSE
ENSGALG00010009391	RSBN1L	PRJNA729271	0.839349786	6.99761E-15	0.839349786	stage_E14_vs_E10	ordinary_muscle_development		0	FALSE
ENSGALG00010015388	PSMD1	PRJNA729271	-0.982644635	7.02718E-15	0.982644635	stage_E14_vs_E10	ordinary_muscle_development		0	FALSE
ENSGALG00010019732	HBA1	PRJNA729271	-3.20208133	7.0338E-15	3.20208133	stage_E14_vs_E10	ordinary_muscle_development	D_vs_E10;stage_1W_vs_E10;stage_3W_vs_E10;stage	1	TRUE
ENSGALG00010022759	THSD4	PRJNA729271	-0.963537434	7.08649E-15	0.963537434	stage_E14_vs_E10	ordinary_muscle_development		0	FALSE
ENSGALG00010012952	BRD10	PRJNA729271	1.476824018	7.19047E-15	1.476824018	stage_E14_vs_E10	ordinary_muscle_development	D_vs_E10;stage_1W_vs_E10;stage_3W_vs_E10;stage	1	TRUE
ENSGALG00010008134	SLC2A13	PRJNA729271	-0.632718603	7.35482E-15	0.632718603	stage_E14_vs_E10	ordinary_muscle_development		0	FALSE
ENSGALG00010003875	AKAP8	PRJNA729271	-0.885152969	7.41849E-15	0.885152969	stage_E14_vs_E10	ordinary_muscle_development		0	FALSE
ENSGALG00010012103	ENSGALG00010012103	PRJNA729271	1.639997254	7.42518E-15	1.639997254	stage_E14_vs_E10	ordinary_muscle_development	D_vs_E10;stage_1W_vs_E10;stage_3W_vs_E10;stage	1	TRUE
ENSGALG00010003478	COLEC11	PRJNA729271	1.409692612	7.51953E-15	1.409692612	stage_E14_vs_E10	ordinary_muscle_development	D_vs_E10;stage_1W_vs_E10;stage_3W_vs_E10;stage	1	TRUE
ENSGALG00010003057	DDX1	PRJNA729271	-0.714996292	7.5286E-15	0.714996292	stage_E14_vs_E10	ordinary_muscle_development		0	FALSE
ENSGALG00010024112	NAP1L4	PRJNA729271	0.716095333	7.58266E-15	0.716095333	stage_E14_vs_E10	ordinary_muscle_development		0	FALSE
ENSGALG00010000225	LIF	PRJNA729271	3.608177291	7.58498E-15	3.608177291	stage_E14_vs_E10	ordinary_muscle_development	D_vs_E10;stage_1W_vs_E10;stage_3W_vs_E10;stage	1	TRUE
ENSGALG00010000293	ENSGALG00010000293	PRJNA729271	2.773085352	7.61424E-15	2.773085352	stage_E14_vs_E10	ordinary_muscle_development	D_vs_E10;stage_1W_vs_E10;stage_3W_vs_E10;stage	1	TRUE
ENSGALG00010024799	ENSGALG00010024799	PRJNA729271	1.467016794	7.62643E-15	1.467016794	stage_E14_vs_E10	ordinary_muscle_development	D_vs_E10;stage_1W_vs_E10;stage_3W_vs_E10;stage	1	TRUE
ENSGALG00010001442	LDLRAD4	PRJNA729271	0.908375458	7.68973E-15	0.908375458	stage_E14_vs_E10	ordinary_muscle_development		0	FALSE
ENSGALG00010000634	ENSGALG00010000634	PRJNA729271	1.456236496	7.70312E-15	1.456236496	stage_E14_vs_E10	ordinary_muscle_development	D_vs_E10;stage_1W_vs_E10;stage_3W_vs_E10;stage	1	TRUE
ENSGALG00010021501	SLC23A3	PRJNA729271	4.375063753	7.70581E-15	4.375063753	stage_E14_vs_E10	ordinary_muscle_development	D_vs_E10;stage_1W_vs_E10;stage_3W_vs_E10;stage	1	TRUE
ENSGALG00010023646	DPH5	PRJNA729271	1.12262121	7.76236E-15	1.12262121	stage_E14_vs_E10	ordinary_muscle_development	D_vs_E10;stage_1W_vs_E10;stage_3W_vs_E10;stage	1	TRUE
ENSGALG00010017571	ADAM9	PRJNA729271	1.188931914	7.89949E-15	1.188931914	stage_E14_vs_E10	ordinary_muscle_development	D_vs_E10;stage_1W_vs_E10;stage_3W_vs_E10;stage	1	TRUE
ENSGALG00010012049	IFNAR1	PRJNA729271	1.142185855	7.91315E-15	1.142185855	stage_E14_vs_E10	ordinary_muscle_development	D_vs_E10;stage_1W_vs_E10;stage_3W_vs_E10;stage	1	TRUE
ENSGALG00010013465	CALD1	PRJNA729271	-1.274195712	8.13699E-15	1.274195712	stage_E14_vs_E10	ordinary_muscle_development	D_vs_E10;stage_1W_vs_E10;stage_3W_vs_E10;stage	1	TRUE
ENSGALG00010009793	TNFAIP6	PRJNA729271	-2.345137392	8.24458E-15	2.345137392	stage_E14_vs_E10	ordinary_muscle_development	D_vs_E10;stage_1W_vs_E10;stage_3W_vs_E10;stage	1	TRUE
ENSGALG00010009212	NXPH2	PRJNA729271	-2.376422643	8.29277E-15	2.376422643	stage_E14_vs_E10	ordinary_muscle_development	D_vs_E10;stage_1W_vs_E10;stage_3W_vs_E10;stage	1	TRUE
ENSGALG00010015540	ACSL4	PRJNA729271	1.197584038	8.29555E-15	1.197584038	stage_E14_vs_E10	ordinary_muscle_development	D_vs_E10;stage_1W_vs_E10;stage_3W_vs_E10;stage	1	TRUE
ENSGALG00010019312	KBTBD2	PRJNA729271	0.567449218	8.29555E-15	0.567449218	stage_E14_vs_E10	ordinary_muscle_development		0	FALSE
ENSGALG00010024657	DPY19L3	PRJNA729271	-1.021372606	8.32301E-15	1.021372606	stage_E14_vs_E10	ordinary_muscle_development	D_vs_E10;stage_1W_vs_E10;stage_3W_vs_E10;stage	1	TRUE
ENSGALG00010026203	TMX2	PRJNA729271	0.812748973	8.33035E-15	0.812748973	stage_E14_vs_E10	ordinary_muscle_development		0	FALSE
ENSGALG00010016606	VASN	PRJNA729271	-1.441351862	8.42976E-15	1.441351862	stage_E14_vs_E10	ordinary_muscle_development	D_vs_E10;stage_1W_vs_E10;stage_3W_vs_E10;stage	1	TRUE
ENSGALG00010016733	PHC3	PRJNA729271	1.202166698	8.60897E-15	1.202166698	stage_E14_vs_E10	ordinary_muscle_development	D_vs_E10;stage_1W_vs_E10;stage_3W_vs_E10;stage	1	TRUE
ENSGALG00010018379	SRBD1	PRJNA729271	0.994598766	8.61239E-15	0.994598766	stage_E14_vs_E10	ordinary_muscle_development		0	FALSE
ENSGALG00010007707	ENSGALG00010007707	PRJNA729271	5.582016927	8.67605E-15	5.582016927	stage_E14_vs_E10	ordinary_muscle_development	D_vs_E10;stage_1W_vs_E10;stage_3W_vs_E10;stage	1	TRUE
ENSGALG00010022872	KRT13	PRJNA729271	-4.030075415	8.6778E-15	4.030075415	stage_E14_vs_E10	ordinary_muscle_development	D_vs_E10;stage_1W_vs_E10;stage_3W_vs_E10;stage	1	TRUE
ENSGALG00010011605	APPL2	PRJNA729271	-1.130056753	8.81321E-15	1.130056753	stage_E14_vs_E10	ordinary_muscle_development	D_vs_E10;stage_1W_vs_E10;stage_3W_vs_E10;stage	1	TRUE
ENSGALG00010022259	GET4	PRJNA729271	0.670620047	8.86379E-15	0.670620047	stage_E14_vs_E10	ordinary_muscle_development		0	FALSE
ENSGALG00010003466	TSC22D1	PRJNA729271	-0.801812838	8.92746E-15	0.801812838	stage_E14_vs_E10	ordinary_muscle_development		0	FALSE
ENSGALG00010027257	TAFA3	PRJNA729271	2.081604876	8.9763E-15	2.081604876	stage_E14_vs_E10	ordinary_muscle_development	D_vs_E10;stage_1W_vs_E10;stage_3W_vs_E10;stage	1	TRUE
ENSGALG00010007796	MCUR1	PRJNA729271	-0.83407309	8.99586E-15	0.83407309	stage_E14_vs_E10	ordinary_muscle_development		0	FALSE
ENSGALG00010020582	KANSL3	PRJNA729271	1.165990591	9.127E-15	1.165990591	stage_E14_vs_E10	ordinary_muscle_development	D_vs_E10;stage_1W_vs_E10;stage_3W_vs_E10;stage	1	TRUE
ENSGALG00010013242	SULT4A1	PRJNA729271	-1.291055418	9.13875E-15	1.291055418	stage_E14_vs_E10	ordinary_muscle_development	D_vs_E10;stage_1W_vs_E10;stage_3W_vs_E10;stage	1	TRUE
ENSGALG00010029230	PPM1E	PRJNA729271	1.296321273	9.13876E-15	1.296321273	stage_E14_vs_E10	ordinary_muscle_development	D_vs_E10;stage_1W_vs_E10;stage_3W_vs_E10;stage	1	TRUE
ENSGALG00010024008	CCNE1	PRJNA729271	-0.882966255	9.25005E-15	0.882966255	stage_E14_vs_E10	ordinary_muscle_development		0	FALSE
ENSGALG00010019225	PROK2	PRJNA729271	-7.165032572	9.30514E-15	7.165032572	stage_E14_vs_E10	ordinary_muscle_development	D_vs_E10;stage_1W_vs_E10;stage_3W_vs_E10;stage	1	TRUE
ENSGALG00010016565	NVL	PRJNA729271	-0.790319082	9.35918E-15	0.790319082	stage_E14_vs_E10	ordinary_muscle_development		0	FALSE
ENSGALG00010000475	RNFT2	PRJNA729271	-0.877639138	9.43036E-15	0.877639138	stage_E14_vs_E10	ordinary_muscle_development		0	FALSE
ENSGALG00010028554	ENSGALG00010028554	PRJNA729271	6.04925229	9.44047E-15	6.04925229	stage_E14_vs_E10	ordinary_muscle_development	D_vs_E10;stage_1W_vs_E10;stage_3W_vs_E10;stage	1	TRUE
ENSGALG00010020866	LCOR	PRJNA729271	-0.788845567	9.45745E-15	0.788845567	stage_E14_vs_E10	ordinary_muscle_development		0	FALSE
ENSGALG00010018179	PRADC1	PRJNA729271	0.913897753	9.50864E-15	0.913897753	stage_E14_vs_E10	ordinary_muscle_development		0	FALSE
ENSGALG00010024670	SENP2	PRJNA729271	0.740178761	9.55242E-15	0.740178761	stage_E14_vs_E10	ordinary_muscle_development		0	FALSE
ENSGALG00010004778	ENSGALG00010004778	PRJNA729271	-4.273156473	9.61209E-15	4.273156473	stage_E14_vs_E10	ordinary_muscle_development	D_vs_E10;stage_1W_vs_E10;stage_3W_vs_E10;stage	1	TRUE
ENSGALG00010019411	LSM5	PRJNA729271	-1.257170256	9.67307E-15	1.257170256	stage_E14_vs_E10	ordinary_muscle_development	D_vs_E10;stage_1W_vs_E10;stage_3W_vs_E10;stage	1	TRUE
ENSGALG00010021419	MANSC1	PRJNA729271	-1.595577286	9.69076E-15	1.595577286	stage_E14_vs_E10	ordinary_muscle_development	D_vs_E10;stage_1W_vs_E10;stage_3W_vs_E10;stage	1	TRUE
ENSGALG00010016812	LRRC31	PRJNA729271	-1.040277027	9.72588E-15	1.040277027	stage_E14_vs_E10	ordinary_muscle_development	D_vs_E10;stage_1W_vs_E10;stage_3W_vs_E10;stage	1	TRUE
ENSGALG00010005132	LNX2	PRJNA729271	-0.766915008	1.00272E-14	0.766915008	stage_E14_vs_E10	ordinary_muscle_development		0	FALSE
ENSGALG00010029514	MRPS23	PRJNA729271	0.762800902	1.02635E-14	0.762800902	stage_E14_vs_E10	ordinary_muscle_development		0	FALSE
ENSGALG00010004501	TAGLN3	PRJNA729271	-2.828545832	1.03135E-14	2.828545832	stage_E14_vs_E10	ordinary_muscle_development	D_vs_E10;stage_1W_vs_E10;stage_3W_vs_E10;stage	1	TRUE
ENSGALG00010024321	UBE2L3	PRJNA729271	-0.656735114	1.03347E-14	0.656735114	stage_E14_vs_E10	ordinary_muscle_development		0	FALSE
ENSGALG00010008596	ENSGALG00010008596	PRJNA729271	-1.292302706	1.04336E-14	1.292302706	stage_E14_vs_E10	ordinary_muscle_development	D_vs_E10;stage_1W_vs_E10;stage_3W_vs_E10;stage	1	TRUE
ENSGALG00010001767	ENSGALG00010001767	PRJNA729271	1.333988897	1.04615E-14	1.333988897	stage_E14_vs_E10	ordinary_muscle_development	D_vs_E10;stage_1W_vs_E10;stage_3W_vs_E10;stage	1	TRUE
ENSGALG00010023108	TET1	PRJNA729271	0.486340233	1.05006E-14	0.486340233	stage_E14_vs_E10	ordinary_muscle_development		0	FALSE
ENSGALG00010023864	EPHA1	PRJNA729271	-7.61434653	1.06912E-14	7.61434653	stage_E14_vs_E10	ordinary_muscle_development	D_vs_E10;stage_1W_vs_E10;stage_3W_vs_E10;stage	1	TRUE
ENSGALG00010003719	RPGR	PRJNA729271	-0.913349715	1.11654E-14	0.913349715	stage_E14_vs_E10	ordinary_muscle_development		0	FALSE
ENSGALG00010011702	NUP205	PRJNA729271	-0.660869862	1.13256E-14	0.660869862	stage_E14_vs_E10	ordinary_muscle_development		0	FALSE
ENSGALG00010004650	GTPBP10	PRJNA729271	-1.021845925	1.16051E-14	1.021845925	stage_E14_vs_E10	ordinary_muscle_development	D_vs_E10;stage_1W_vs_E10;stage_3W_vs_E10;stage	1	TRUE
ENSGALG00010027394	SLC27A4	PRJNA729271	-0.690780905	1.16982E-14	0.690780905	stage_E14_vs_E10	ordinary_muscle_development		0	FALSE
ENSGALG00010017565	ENSGALG00010017565	PRJNA729271	-1.519515879	1.18084E-14	1.519515879	stage_E14_vs_E10	ordinary_muscle_development	D_vs_E10;stage_1W_vs_E10;stage_3W_vs_E10;stage	1	TRUE
ENSGALG00010001472	ENSGALG00010001472	PRJNA729271	1.386868188	1.18133E-14	1.386868188	stage_E14_vs_E10	ordinary_muscle_development	D_vs_E10;stage_1W_vs_E10;stage_3W_vs_E10;stage	1	TRUE
ENSGALG00010024718	ENSGALG00010024718	PRJNA729271	1.523724449	1.18624E-14	1.523724449	stage_E14_vs_E10	ordinary_muscle_development	D_vs_E10;stage_1W_vs_E10;stage_3W_vs_E10;stage	1	TRUE
ENSGALG00010003428	SNX16	PRJNA729271	1.204105606	1.19974E-14	1.204105606	stage_E14_vs_E10	ordinary_muscle_development	D_vs_E10;stage_1W_vs_E10;stage_3W_vs_E10;stage	1	TRUE
ENSGALG00010021053	ENSGALG00010021053	PRJNA729271	-5.494061369	1.21407E-14	5.494061369	stage_E14_vs_E10	ordinary_muscle_development	D_vs_E10;stage_1W_vs_E10;stage_3W_vs_E10;stage	1	TRUE
ENSGALG00010012965	ENSGALG00010012965	PRJNA729271	-0.676114825	1.21783E-14	0.676114825	stage_E14_vs_E10	ordinary_muscle_development		0	FALSE
ENSGALG00010017285	IL21R	PRJNA729271	6.349581546	1.22147E-14	6.349581546	stage_E14_vs_E10	ordinary_muscle_development	D_vs_E10;stage_1W_vs_E10;stage_3W_vs_E10;stage	1	TRUE
ENSGALG00010019227	CLCN5	PRJNA729271	0.566467479	1.2435E-14	0.566467479	stage_E14_vs_E10	ordinary_muscle_development		0	FALSE
ENSGALG00010008716	TATDN1	PRJNA729271	-0.894607342	1.25098E-14	0.894607342	stage_E14_vs_E10	ordinary_muscle_development		0	FALSE
ENSGALG00010005740	ENSGALG00010005740	PRJNA729271	2.507991351	1.25963E-14	2.507991351	stage_E14_vs_E10	ordinary_muscle_development	D_vs_E10;stage_1W_vs_E10;stage_3W_vs_E10;stage	1	TRUE
ENSGALG00010003271	AKAP11	PRJNA729271	0.656766839	1.28865E-14	0.656766839	stage_E14_vs_E10	ordinary_muscle_development		0	FALSE
ENSGALG00010008852	KHDRBS3	PRJNA729271	0.654388462	1.30399E-14	0.654388462	stage_E14_vs_E10	ordinary_muscle_development		0	FALSE
ENSGALG00010008004	ENY2	PRJNA729271	-1.350298653	1.31803E-14	1.350298653	stage_E14_vs_E10	ordinary_muscle_development	D_vs_E10;stage_1W_vs_E10;stage_3W_vs_E10;stage	1	TRUE
ENSGALG00010024305	ENSGALG00010024305	PRJNA729271	5.667334191	1.31952E-14	5.667334191	stage_E14_vs_E10	ordinary_muscle_development	D_vs_E10;stage_1W_vs_E10;stage_3W_vs_E10;stage	1	TRUE
ENSGALG00010026507	APLNR	PRJNA729271	-2.358903908	1.31977E-14	2.358903908	stage_E14_vs_E10	ordinary_muscle_development	D_vs_E10;stage_1W_vs_E10;stage_3W_vs_E10;stage	1	TRUE
ENSGALG00010019706	NPRL3	PRJNA729271	0.767248494	1.32782E-14	0.767248494	stage_E14_vs_E10	ordinary_muscle_development		0	FALSE
ENSGALG00010024872	RUNDC3A	PRJNA729271	-1.636526797	1.33691E-14	1.636526797	stage_E14_vs_E10	ordinary_muscle_development	D_vs_E10;stage_1W_vs_E10;stage_3W_vs_E10;stage	1	TRUE
ENSGALG00010004424	MRPL18	PRJNA729271	1.135635695	1.34576E-14	1.135635695	stage_E14_vs_E10	ordinary_muscle_development	D_vs_E10;stage_1W_vs_E10;stage_3W_vs_E10;stage	1	TRUE
ENSGALG00010019322	EGR3	PRJNA729271	2.358795853	1.35681E-14	2.358795853	stage_E14_vs_E10	ordinary_muscle_development	D_vs_E10;stage_1W_vs_E10;stage_3W_vs_E10;stage	1	TRUE
ENSGALG00010012798	MFSD6	PRJNA729271	0.746809073	1.35783E-14	0.746809073	stage_E14_vs_E10	ordinary_muscle_development		0	FALSE
ENSGALG00010005561	CLYBL	PRJNA729271	1.502647386	1.36353E-14	1.502647386	stage_E14_vs_E10	ordinary_muscle_development	D_vs_E10;stage_1W_vs_E10;stage_3W_vs_E10;stage	1	TRUE
ENSGALG00010024643	ETV7	PRJNA729271	2.933377435	1.38853E-14	2.933377435	stage_E14_vs_E10	ordinary_muscle_development	D_vs_E10;stage_1W_vs_E10;stage_3W_vs_E10;stage	1	TRUE
ENSGALG00010019124	BMPER	PRJNA729271	-1.976474177	1.40799E-14	1.976474177	stage_E14_vs_E10	ordinary_muscle_development	D_vs_E10;stage_1W_vs_E10;stage_3W_vs_E10;stage	1	TRUE
ENSGALG00010014102	TRIM2	PRJNA729271	1.074608737	1.42565E-14	1.074608737	stage_E14_vs_E10	ordinary_muscle_development	D_vs_E10;stage_1W_vs_E10;stage_3W_vs_E10;stage	1	TRUE
ENSGALG00010010435	GAS1	PRJNA729271	-1.301738275	1.43436E-14	1.301738275	stage_E14_vs_E10	ordinary_muscle_development	D_vs_E10;stage_1W_vs_E10;stage_3W_vs_E10;stage	1	TRUE
ENSGALG00010012172	CNOT2	PRJNA729271	-0.706126033	1.47417E-14	0.706126033	stage_E14_vs_E10	ordinary_muscle_development		0	FALSE
ENSGALG00010011866	KLF8	PRJNA729271	1.449277349	1.48569E-14	1.449277349	stage_E14_vs_E10	ordinary_muscle_development	D_vs_E10;stage_1W_vs_E10;stage_3W_vs_E10;stage	1	TRUE
ENSGALG00010009235	KLHDC4	PRJNA729271	1.018203367	1.49791E-14	1.018203367	stage_E14_vs_E10	ordinary_muscle_development	D_vs_E10;stage_1W_vs_E10;stage_3W_vs_E10;stage	1	TRUE
ENSGALG00010027350	INSRR	PRJNA729271	2.576124062	1.51469E-14	2.576124062	stage_E14_vs_E10	ordinary_muscle_development	D_vs_E10;stage_1W_vs_E10;stage_3W_vs_E10;stage	1	TRUE
ENSGALG00010016383	FAM110D	PRJNA729271	1.364977094	1.51558E-14	1.364977094	stage_E14_vs_E10	ordinary_muscle_development	D_vs_E10;stage_1W_vs_E10;stage_3W_vs_E10;stage	1	TRUE
ENSGALG00010008327	TGFB1	PRJNA729271	0.995742391	1.52495E-14	0.995742391	stage_E14_vs_E10	ordinary_muscle_development		0	FALSE
ENSGALG00010022627	CTCF	PRJNA729271	-0.773055645	1.53106E-14	0.773055645	stage_E14_vs_E10	ordinary_muscle_development		0	FALSE
ENSGALG00010017503	MED9	PRJNA729271	0.98063139	1.54454E-14	0.98063139	stage_E14_vs_E10	ordinary_muscle_development		0	FALSE
ENSGALG00010021718	SST7	PRJNA729271	-4.086675559	1.55375E-14	4.086675559	stage_E14_vs_E10	ordinary_muscle_development	D_vs_E10;stage_1W_vs_E10;stage_3W_vs_E10;stage	1	TRUE
ENSGALG00010013262	CA13	PRJNA729271	-2.074305256	1.57677E-14	2.074305256	stage_E14_vs_E10	ordinary_muscle_development	D_vs_E10;stage_1W_vs_E10;stage_3W_vs_E10;stage	1	TRUE
ENSGALG00010009595	ENSGALG00010009595	PRJNA729271	1.45000467	1.59884E-14	1.45000467	stage_E14_vs_E10	ordinary_muscle_development	D_vs_E10;stage_1W_vs_E10;stage_3W_vs_E10;stage	1	TRUE
ENSGALG00010027893	DOT1L	PRJNA729271	1.24208029	1.60764E-14	1.24208029	stage_E14_vs_E10	ordinary_muscle_development	D_vs_E10;stage_1W_vs_E10;stage_3W_vs_E10;stage	1	TRUE
ENSGALG00010026121	ENSGALG00010026121	PRJNA729271	2.014710116	1.61287E-14	2.014710116	stage_E14_vs_E10	ordinary_muscle_development	D_vs_E10;stage_1W_vs_E10;stage_3W_vs_E10;stage	1	TRUE
ENSGALG00010021488	HYI	PRJNA729271	-1.141067362	1.62043E-14	1.141067362	stage_E14_vs_E10	ordinary_muscle_development	D_vs_E10;stage_1W_vs_E10;stage_3W_vs_E10;stage	1	TRUE
ENSGALG00010007854	FAM181B	PRJNA729271	0.917419738	1.63716E-14	0.917419738	stage_E14_vs_E10	ordinary_muscle_development		0	FALSE
ENSGALG00010006615	GRAMD1C	PRJNA729271	-0.817257305	1.67334E-14	0.817257305	stage_E14_vs_E10	ordinary_muscle_development		0	FALSE
ENSGALG00010001161	B3GALNT1	PRJNA729271	0.75444863	1.6788E-14	0.75444863	stage_E14_vs_E10	ordinary_muscle_development		0	FALSE
ENSGALG00010024825	UBXN7	PRJNA729271	-0.596564664	1.68372E-14	0.596564664	stage_E14_vs_E10	ordinary_muscle_development		0	FALSE
ENSGALG00010013288	CA3A	PRJNA729271	6.063816526	1.68586E-14	6.063816526	stage_E14_vs_E10	ordinary_muscle_development	D_vs_E10;stage_1W_vs_E10;stage_3W_vs_E10;stage	1	TRUE
ENSGALG00010009721	ZHX1	PRJNA729271	1.219104065	1.68586E-14	1.219104065	stage_E14_vs_E10	ordinary_muscle_development	D_vs_E10;stage_1W_vs_E10;stage_3W_vs_E10;stage	1	TRUE
ENSGALG00010002959	DLX5	PRJNA729271	-6.615550583	1.68791E-14	6.615550583	stage_E14_vs_E10	ordinary_muscle_development	D_vs_E10;stage_1W_vs_E10;stage_3W_vs_E10;stage	1	TRUE
ENSGALG00010006081	ZDHHC14	PRJNA729271	0.611069816	1.69007E-14	0.611069816	stage_E14_vs_E10	ordinary_muscle_development		0	FALSE
ENSGALG00010013051	XPOT	PRJNA729271	0.612927628	1.71292E-14	0.612927628	stage_E14_vs_E10	ordinary_muscle_development		0	FALSE
ENSGALG00010012588	PEX26	PRJNA729271	0.814187428	1.71995E-14	0.814187428	stage_E14_vs_E10	ordinary_muscle_development		0	FALSE
ENSGALG00010027155	ENSGALG00010027155	PRJNA729271	2.26028332	1.72162E-14	2.26028332	stage_E14_vs_E10	ordinary_muscle_development	D_vs_E10;stage_1W_vs_E10;stage_3W_vs_E10;stage	1	TRUE
ENSGALG00010024822	RPS26	PRJNA729271	1.070335306	1.73917E-14	1.070335306	stage_E14_vs_E10	ordinary_muscle_development	D_vs_E10;stage_1W_vs_E10;stage_3W_vs_E10;stage	1	TRUE
ENSGALG00010024677	LMBR1L	PRJNA729271	-3.84111083	1.74907E-14	3.84111083	stage_E14_vs_E10	ordinary_muscle_development	D_vs_E10;stage_1W_vs_E10;stage_3W_vs_E10;stage	1	TRUE
ENSGALG00010024343	POLR2L	PRJNA729271	0.984267996	1.76429E-14	0.984267996	stage_E14_vs_E10	ordinary_muscle_development		0	FALSE
ENSGALG00010010722	TBC1D19	PRJNA729271	-1.326658367	1.78552E-14	1.326658367	stage_E14_vs_E10	ordinary_muscle_development	D_vs_E10;stage_1W_vs_E10;stage_3W_vs_E10;stage	1	TRUE
ENSGALG00010024064	GPR17	PRJNA729271	-1.740732847	1.80406E-14	1.740732847	stage_E14_vs_E10	ordinary_muscle_development	D_vs_E10;stage_1W_vs_E10;stage_3W_vs_E10;stage	1	TRUE
ENSGALG00010009141	MAML3	PRJNA729271	0.905907229	1.81002E-14	0.905907229	stage_E14_vs_E10	ordinary_muscle_development		0	FALSE
ENSGALG00010015918	BICD1	PRJNA729271	-1.536808688	1.81012E-14	1.536808688	stage_E14_vs_E10	ordinary_muscle_development	D_vs_E10;stage_1W_vs_E10;stage_3W_vs_E10;stage	1	TRUE
ENSGALG00010028899	TNFSF8	PRJNA729271	4.036916301	1.81912E-14	4.036916301	stage_E14_vs_E10	ordinary_muscle_development	D_vs_E10;stage_1W_vs_E10;stage_3W_vs_E10;stage	1	TRUE
ENSGALG00010024878	RHPN2	PRJNA729271	-2.290956857	1.83138E-14	2.290956857	stage_E14_vs_E10	ordinary_muscle_development	D_vs_E10;stage_1W_vs_E10;stage_3W_vs_E10;stage	1	TRUE
ENSGALG00010007908	IRX1	PRJNA729271	-7.738648867	1.83296E-14	7.738648867	stage_E14_vs_E10	ordinary_muscle_development	D_vs_E10;stage_1W_vs_E10;stage_3W_vs_E10;stage	1	TRUE
ENSGALG00010028114	PFKFB4	PRJNA729271	0.707084843	1.86372E-14	0.707084843	stage_E14_vs_E10	ordinary_muscle_development		0	FALSE
ENSGALG00010022172	GIPC2	PRJNA729271	1.414569892	1.88325E-14	1.414569892	stage_E14_vs_E10	ordinary_muscle_development	D_vs_E10;stage_1W_vs_E10;stage_3W_vs_E10;stage	1	TRUE
ENSGALG00010021269	NUDT13	PRJNA729271	1.269186671	1.88715E-14	1.269186671	stage_E14_vs_E10	ordinary_muscle_development	D_vs_E10;stage_1W_vs_E10;stage_3W_vs_E10;stage	1	TRUE
ENSGALG00010007999	SDC1	PRJNA729271	-1.625805906	1.89027E-14	1.625805906	stage_E14_vs_E10	ordinary_muscle_development	D_vs_E10;stage_1W_vs_E10;stage_3W_vs_E10;stage	1	TRUE
ENSGALG00010027534	KCNN3	PRJNA729271	1.465525917	1.90949E-14	1.465525917	stage_E14_vs_E10	ordinary_muscle_development	D_vs_E10;stage_1W_vs_E10;stage_3W_vs_E10;stage	1	TRUE
ENSGALG00010005395	ENSGALG00010005395	PRJNA729271	-3.171282002	1.92696E-14	3.171282002	stage_E14_vs_E10	ordinary_muscle_development	D_vs_E10;stage_1W_vs_E10;stage_3W_vs_E10;stage	1	TRUE
ENSGALG00010018913	ENSGALG00010018913	PRJNA729271	1.692666128	1.95149E-14	1.692666128	stage_E14_vs_E10	ordinary_muscle_development	D_vs_E10;stage_1W_vs_E10;stage_3W_vs_E10;stage	1	TRUE
ENSGALG00010022838	ARFGAP2	PRJNA729271	0.616943429	1.95351E-14	0.616943429	stage_E14_vs_E10	ordinary_muscle_development		0	FALSE
ENSGALG00010018157	FBXL2	PRJNA729271	-1.21162712	1.95951E-14	1.21162712	stage_E14_vs_E10	ordinary_muscle_development	D_vs_E10;stage_1W_vs_E10;stage_3W_vs_E10;stage	1	TRUE
ENSGALG00010014555	VGLL1	PRJNA729271	-7.936133482	1.96106E-14	7.936133482	stage_E14_vs_E10	ordinary_muscle_development	D_vs_E10;stage_1W_vs_E10;stage_3W_vs_E10;stage	1	TRUE
ENSGALG00010003471	ADI1	PRJNA729271	1.037728726	1.96121E-14	1.037728726	stage_E14_vs_E10	ordinary_muscle_development	D_vs_E10;stage_1W_vs_E10;stage_3W_vs_E10;stage	1	TRUE
ENSGALG00010022508	USF3	PRJNA729271	0.939289526	1.99536E-14	0.939289526	stage_E14_vs_E10	ordinary_muscle_development		0	FALSE
ENSGALG00010009695	ATAD2B	PRJNA729271	0.589433866	1.99957E-14	0.589433866	stage_E14_vs_E10	ordinary_muscle_development		0	FALSE
ENSGALG00010009323	RNF150	PRJNA729271	0.833706058	2.0046E-14	0.833706058	stage_E14_vs_E10	ordinary_muscle_development		0	FALSE
ENSGALG00010027175	NOS3	PRJNA729271	1.272971456	2.02359E-14	1.272971456	stage_E14_vs_E10	ordinary_muscle_development	D_vs_E10;stage_1W_vs_E10;stage_3W_vs_E10;stage	1	TRUE
ENSGALG00010003874	YES1	PRJNA729271	-0.764758082	2.03208E-14	0.764758082	stage_E14_vs_E10	ordinary_muscle_development		0	FALSE
ENSGALG00010017644	RBBP6	PRJNA729271	0.754402847	2.03402E-14	0.754402847	stage_E14_vs_E10	ordinary_muscle_development		0	FALSE
ENSGALG00010023164	NKIRAS2	PRJNA729271	1.03246314	2.08754E-14	1.03246314	stage_E14_vs_E10	ordinary_muscle_development	D_vs_E10;stage_1W_vs_E10;stage_3W_vs_E10;stage	1	TRUE
ENSGALG00010005964	HSPA14	PRJNA729271	-0.913145252	2.09263E-14	0.913145252	stage_E14_vs_E10	ordinary_muscle_development		0	FALSE
ENSGALG00010008334	STX17	PRJNA729271	1.034984781	2.09768E-14	1.034984781	stage_E14_vs_E10	ordinary_muscle_development	D_vs_E10;stage_1W_vs_E10;stage_3W_vs_E10;stage	1	TRUE
ENSGALG00010000593	ATPSCKMT	PRJNA729271	-1.192801267	2.11532E-14	1.192801267	stage_E14_vs_E10	ordinary_muscle_development	D_vs_E10;stage_1W_vs_E10;stage_3W_vs_E10;stage	1	TRUE
ENSGALG00010010933	ANAPC4	PRJNA729271	-0.879262091	2.14602E-14	0.879262091	stage_E14_vs_E10	ordinary_muscle_development		0	FALSE
ENSGALG00010028195	ENSGALG00010028195	PRJNA729271	2.316392808	2.14927E-14	2.316392808	stage_E14_vs_E10	ordinary_muscle_development	D_vs_E10;stage_1W_vs_E10;stage_3W_vs_E10;stage	1	TRUE
ENSGALG00010027537	SGTA	PRJNA729271	-0.530994115	2.1774E-14	0.530994115	stage_E14_vs_E10	ordinary_muscle_development		0	FALSE
ENSGALG00010017101	CSMD2	PRJNA729271	-1.955605418	2.18019E-14	1.955605418	stage_E14_vs_E10	ordinary_muscle_development	D_vs_E10;stage_1W_vs_E10;stage_3W_vs_E10;stage	1	TRUE
ENSGALG00010020303	EXD2	PRJNA729271	-0.580795234	2.18239E-14	0.580795234	stage_E14_vs_E10	ordinary_muscle_development		0	FALSE
ENSGALG00010028072	ENSGALG00010028072	PRJNA729271	-7.746205239	2.1948E-14	7.746205239	stage_E14_vs_E10	ordinary_muscle_development	D_vs_E10;stage_1W_vs_E10;stage_3W_vs_E10;stage	1	TRUE
ENSGALG00010000155	MAZ	PRJNA729271	-1.32259928	2.21895E-14	1.32259928	stage_E14_vs_E10	ordinary_muscle_development	D_vs_E10;stage_1W_vs_E10;stage_3W_vs_E10;stage	1	TRUE
ENSGALG00010023744	MT4	PRJNA729271	2.515127402	2.22744E-14	2.515127402	stage_E14_vs_E10	ordinary_muscle_development	D_vs_E10;stage_1W_vs_E10;stage_3W_vs_E10;stage	1	TRUE
ENSGALG00010017516	AAK1	PRJNA729271	-0.632409296	2.2369E-14	0.632409296	stage_E14_vs_E10	ordinary_muscle_development		0	FALSE
ENSGALG00010009991	LHFPL3	PRJNA729271	-5.245875041	2.24913E-14	5.245875041	stage_E14_vs_E10	ordinary_muscle_development	D_vs_E10;stage_1W_vs_E10;stage_3W_vs_E10;stage	1	TRUE
ENSGALG00010022322	ENSGALG00010022322	PRJNA729271	2.884710837	2.2556E-14	2.884710837	stage_E14_vs_E10	ordinary_muscle_development	D_vs_E10;stage_1W_vs_E10;stage_3W_vs_E10;stage	1	TRUE
ENSGALG00010028034	MUM1L1	PRJNA729271	-0.618576857	2.27855E-14	0.618576857	stage_E14_vs_E10	ordinary_muscle_development		0	FALSE
ENSGALG00010009344	ENPP2	PRJNA729271	-2.067768981	2.29136E-14	2.067768981	stage_E14_vs_E10	ordinary_muscle_development	D_vs_E10;stage_1W_vs_E10;stage_3W_vs_E10;stage	1	TRUE
ENSGALG00010016665	ENSGALG00010016665	PRJNA729271	7.675966294	2.29367E-14	7.675966294	stage_E14_vs_E10	ordinary_muscle_development	D_vs_E10;stage_1W_vs_E10;stage_3W_vs_E10;stage	1	TRUE
ENSGALG00010018229	AS3MT	PRJNA729271	1.471898522	2.30052E-14	1.471898522	stage_E14_vs_E10	ordinary_muscle_development	D_vs_E10;stage_1W_vs_E10;stage_3W_vs_E10;stage	1	TRUE
ENSGALG00010015104	CNGA2	PRJNA729271	2.069386238	2.32395E-14	2.069386238	stage_E14_vs_E10	ordinary_muscle_development	D_vs_E10;stage_1W_vs_E10;stage_3W_vs_E10;stage	1	TRUE
ENSGALG00010011449	DUSP22	PRJNA729271	-1.130983923	2.33722E-14	1.130983923	stage_E14_vs_E10	ordinary_muscle_development	D_vs_E10;stage_1W_vs_E10;stage_3W_vs_E10;stage	1	TRUE
ENSGALG00010018387	NT5DC4	PRJNA729271	-2.894664531	2.34095E-14	2.894664531	stage_E14_vs_E10	ordinary_muscle_development	D_vs_E10;stage_1W_vs_E10;stage_3W_vs_E10;stage	1	TRUE
ENSGALG00010008686	ENSGALG00010008686	PRJNA729271	-3.672106778	2.34132E-14	3.672106778	stage_E14_vs_E10	ordinary_muscle_development	D_vs_E10;stage_1W_vs_E10;stage_3W_vs_E10;stage	1	TRUE
ENSGALG00010014083	ENSGALG00010014083	PRJNA729271	-1.144767425	2.3427E-14	1.144767425	stage_E14_vs_E10	ordinary_muscle_development	D_vs_E10;stage_1W_vs_E10;stage_3W_vs_E10;stage	1	TRUE
ENSGALG00010004398	COX18	PRJNA729271	-0.989415245	2.34407E-14	0.989415245	stage_E14_vs_E10	ordinary_muscle_development		0	FALSE

ENSGALG00010016457	SMOX	PRJNA729271	1.37727224	2.34418E-14	1.37727224	stage_E14_vs_E10	ordinary_muscle_development	D_vs_E10;stage_1W_vs_E10;stage_3W_vs_E10;stage	1	TRUE
ENSGALG00010004736	CD47	PRJNA729271	0.803326108	2.35997E-14	0.803326108	stage_E14_vs_E10	ordinary_muscle_development		0	FALSE
ENSGALG00010000223	CASTOR1	PRJNA729271	1.190440698	2.3769E-14	1.190440698	stage_E14_vs_E10	ordinary_muscle_development	D_vs_E10;stage_1W_vs_E10;stage_3W_vs_E10;stage	1	TRUE
ENSGALG00010015610	ENSGALG00010015610	PRJNA729271	2.235409692	2.38916E-14	2.235409692	stage_E14_vs_E10	ordinary_muscle_development	D_vs_E10;stage_1W_vs_E10;stage_3W_vs_E10;stage	1	TRUE
ENSGALG00010001313	EMILIN2	PRJNA729271	1.369764923	2.39335E-14	1.369764923	stage_E14_vs_E10	ordinary_muscle_development	D_vs_E10;stage_1W_vs_E10;stage_3W_vs_E10;stage	1	TRUE
ENSGALG00010024986	PTS	PRJNA729271	-1.341314389	2.42454E-14	1.341314389	stage_E14_vs_E10	ordinary_muscle_development	D_vs_E10;stage_1W_vs_E10;stage_3W_vs_E10;stage	1	TRUE
ENSGALG00010024502	VPS8	PRJNA729271	0.567127638	2.52923E-14	0.567127638	stage_E14_vs_E10	ordinary_muscle_development		0	FALSE
ENSGALG00010018799	ENSGALG00010018799	PRJNA729271	-3.278680081	2.53257E-14	3.278680081	stage_E14_vs_E10	ordinary_muscle_development	D_vs_E10;stage_1W_vs_E10;stage_3W_vs_E10;stage	1	TRUE
ENSGALG00010016735	ENSGALG00010016735	PRJNA729271	2.522937687	2.54689E-14	2.522937687	stage_E14_vs_E10	ordinary_muscle_development	D_vs_E10;stage_1W_vs_E10;stage_3W_vs_E10;stage	1	TRUE
ENSGALG00010028517	RNF208	PRJNA729271	-2.660893969	2.5474E-14	2.660893969	stage_E14_vs_E10	ordinary_muscle_development	D_vs_E10;stage_1W_vs_E10;stage_3W_vs_E10;stage	1	TRUE
ENSGALG00010007805	ENSGALG00010007805	PRJNA729271	11.75883499	2.56367E-14	11.75883499	stage_E14_vs_E10	ordinary_muscle_development	D_vs_E10;stage_1W_vs_E10;stage_3W_vs_E10;stage	1	TRUE
ENSGALG00010010855	ATP5PF	PRJNA729271	1.151705392	2.56816E-14	1.151705392	stage_E14_vs_E10	ordinary_muscle_development	D_vs_E10;stage_1W_vs_E10;stage_3W_vs_E10;stage	1	TRUE
ENSGALG00010018531	ADCY5	PRJNA729271	1.049654367	2.61421E-14	1.049654367	stage_E14_vs_E10	ordinary_muscle_development	D_vs_E10;stage_1W_vs_E10;stage_3W_vs_E10;stage	1	TRUE
ENSGALG00010017004	CX3CR1	PRJNA729271	-5.926213631	2.61805E-14	5.926213631	stage_E14_vs_E10	ordinary_muscle_development	D_vs_E10;stage_1W_vs_E10;stage_3W_vs_E10;stage	1	TRUE
ENSGALG00010019036	REL	PRJNA729271	0.847758664	2.64228E-14	0.847758664	stage_E14_vs_E10	ordinary_muscle_development		0	FALSE
ENSGALG00010027309	ENSGALG00010027309	PRJNA729271	5.047828394	2.65484E-14	5.047828394	stage_E14_vs_E10	ordinary_muscle_development	D_vs_E10;stage_1W_vs_E10;stage_3W_vs_E10;stage	1	TRUE
ENSGALG00010026115	MMACHC	PRJNA729271	1.341759575	2.68671E-14	1.341759575	stage_E14_vs_E10	ordinary_muscle_development	D_vs_E10;stage_1W_vs_E10;stage_3W_vs_E10;stage	1	TRUE
ENSGALG00010018348	TOGARAM1	PRJNA729271	0.82955006	2.69011E-14	0.82955006	stage_E14_vs_E10	ordinary_muscle_development		0	FALSE
ENSGALG00010014186	MOCS3	PRJNA729271	-0.872734229	2.75744E-14	0.872734229	stage_E14_vs_E10	ordinary_muscle_development		0	FALSE
ENSGALG00010024770	CDC27	PRJNA729271	0.557733803	2.76761E-14	0.557733803	stage_E14_vs_E10	ordinary_muscle_development		0	FALSE
ENSGALG00010024566	MTSS2	PRJNA729271	1.703028352	2.79732E-14	1.703028352	stage_E14_vs_E10	ordinary_muscle_development	D_vs_E10;stage_1W_vs_E10;stage_3W_vs_E10;stage	1	TRUE
ENSGALG00010006903	B3GLCT	PRJNA729271	-1.134474171	2.79962E-14	1.134474171	stage_E14_vs_E10	ordinary_muscle_development	D_vs_E10;stage_1W_vs_E10;stage_3W_vs_E10;stage	1	TRUE
ENSGALG00010028202	QSOX2	PRJNA729271	-0.524276454	2.79966E-14	0.524276454	stage_E14_vs_E10	ordinary_muscle_development		0	FALSE
ENSGALG00010024058	SLC44A5	PRJNA729271	0.813972742	2.82067E-14	0.813972742	stage_E14_vs_E10	ordinary_muscle_development		0	FALSE
ENSGALG00010024488	ADGRG5	PRJNA729271	3.196254516	2.82981E-14	3.196254516	stage_E14_vs_E10	ordinary_muscle_development	D_vs_E10;stage_1W_vs_E10;stage_3W_vs_E10;stage	1	TRUE
ENSGALG00010004003	HNRNPDL	PRJNA729271	-0.535139778	2.83248E-14	0.535139778	stage_E14_vs_E10	ordinary_muscle_development		0	FALSE
ENSGALG00010009129	ENSGALG00010009129	PRJNA729271	2.778676796	2.86239E-14	2.778676796	stage_E14_vs_E10	ordinary_muscle_development	D_vs_E10;stage_1W_vs_E10;stage_3W_vs_E10;stage	1	TRUE
ENSGALG00010029609	METTL27	PRJNA729271	-1.224262886	2.86838E-14	1.224262886	stage_E14_vs_E10	ordinary_muscle_development	D_vs_E10;stage_1W_vs_E10;stage_3W_vs_E10;stage	1	TRUE
ENSGALG00010028339	BCAN	PRJNA729271	-4.036517315	2.89312E-14	4.036517315	stage_E14_vs_E10	ordinary_muscle_development	D_vs_E10;stage_1W_vs_E10;stage_3W_vs_E10;stage	1	TRUE
ENSGALG00010020550	HECTD2	PRJNA729271	1.643296347	2.92346E-14	1.643296347	stage_E14_vs_E10	ordinary_muscle_development	D_vs_E10;stage_1W_vs_E10;stage_3W_vs_E10;stage	1	TRUE
ENSGALG00010021978	NR2C2	PRJNA729271	0.570517983	2.92917E-14	0.570517983	stage_E14_vs_E10	ordinary_muscle_development		0	FALSE
ENSGALG00010016820	EIF2AK3	PRJNA729271	-0.851495843	2.93719E-14	0.851495843	stage_E14_vs_E10	ordinary_muscle_development		0	FALSE
ENSGALG00010029288	PYCR1	PRJNA729271	-2.2830792	2.94211E-14	2.2830792	stage_E14_vs_E10	ordinary_muscle_development	D_vs_E10;stage_1W_vs_E10;stage_3W_vs_E10;stage	1	TRUE
ENSGALG00010025348	HIPK1	PRJNA729271	0.718039245	2.96485E-14	0.718039245	stage_E14_vs_E10	ordinary_muscle_development		0	FALSE
ENSGALG00010019257	SLC1A4	PRJNA729271	-1.234870095	2.98463E-14	1.234870095	stage_E14_vs_E10	ordinary_muscle_development	D_vs_E10;stage_1W_vs_E10;stage_3W_vs_E10;stage	1	TRUE
ENSGALG00010000134	ENSGALG00010000134	PRJNA729271	0.979458123	3.01083E-14	0.979458123	stage_E14_vs_E10	ordinary_muscle_development		0	FALSE
ENSGALG00010020346	PCBD1	PRJNA729271	-2.291466275	3.03083E-14	2.291466275	stage_E14_vs_E10	ordinary_muscle_development	D_vs_E10;stage_1W_vs_E10;stage_3W_vs_E10;stage	1	TRUE
ENSGALG00010024199	SLC35B1	PRJNA729271	-0.806649136	3.04862E-14	0.806649136	stage_E14_vs_E10	ordinary_muscle_development		0	FALSE
ENSGALG00010021639	SCG5	PRJNA729271	-2.928773325	3.08521E-14	2.928773325	stage_E14_vs_E10	ordinary_muscle_development	D_vs_E10;stage_1W_vs_E10;stage_3W_vs_E10;stage	1	TRUE
ENSGALG00010026782	HOXB9	PRJNA729271	-5.805459469	3.08832E-14	5.805459469	stage_E14_vs_E10	ordinary_muscle_development	D_vs_E10;stage_1W_vs_E10;stage_3W_vs_E10;stage	1	TRUE
ENSGALG00010019287	AHSA2	PRJNA729271	-0.767641935	3.09799E-14	0.767641935	stage_E14_vs_E10	ordinary_muscle_development		0	FALSE
ENSGALG00010020481	RPP30	PRJNA729271	-0.966130899	3.10281E-14	0.966130899	stage_E14_vs_E10	ordinary_muscle_development		0	FALSE
ENSGALG00010017593	MTHFR	PRJNA729271	1.018911291	3.11787E-14	1.018911291	stage_E14_vs_E10	ordinary_muscle_development	D_vs_E10;stage_1W_vs_E10;stage_3W_vs_E10;stage	1	TRUE
ENSGALG00010023063	ENSGALG00010023063	PRJNA729271	-0.811894848	3.12336E-14	0.811894848	stage_E14_vs_E10	ordinary_muscle_development		0	FALSE
ENSGALG00010001865	CYBB	PRJNA729271	2.434110218	3.13031E-14	2.434110218	stage_E14_vs_E10	ordinary_muscle_development	D_vs_E10;stage_1W_vs_E10;stage_3W_vs_E10;stage	1	TRUE
ENSGALG00010027946	AP1M1	PRJNA729271	-0.727895993	3.13253E-14	0.727895993	stage_E14_vs_E10	ordinary_muscle_development		0	FALSE
ENSGALG00010018278	PUSL1	PRJNA729271	1.123159745	3.1943E-14	1.123159745	stage_E14_vs_E10	ordinary_muscle_development	D_vs_E10;stage_1W_vs_E10;stage_3W_vs_E10;stage	1	TRUE
ENSGALG00010016567	E2F2	PRJNA729271	-2.172984158	3.21405E-14	2.172984158	stage_E14_vs_E10	ordinary_muscle_development	D_vs_E10;stage_1W_vs_E10;stage_3W_vs_E10;stage	1	TRUE
ENSGALG00010021168	IVNS1ABP	PRJNA729271	0.816046677	3.22368E-14	0.816046677	stage_E14_vs_E10	ordinary_muscle_development		0	FALSE
ENSGALG00010024834	INSIG1	PRJNA729271	1.996849627	3.24131E-14	1.996849627	stage_E14_vs_E10	ordinary_muscle_development	D_vs_E10;stage_1W_vs_E10;stage_3W_vs_E10;stage	1	TRUE
ENSGALG00010025427	SLC35G2	PRJNA729271	-1.037157743	3.25579E-14	1.037157743	stage_E14_vs_E10	ordinary_muscle_development	D_vs_E10;stage_1W_vs_E10;stage_3W_vs_E10;stage	1	TRUE
ENSGALG00010006245	PFKFB3	PRJNA729271	3.929065213	3.25618E-14	3.929065213	stage_E14_vs_E10	ordinary_muscle_development	D_vs_E10;stage_1W_vs_E10;stage_3W_vs_E10;stage	1	TRUE
ENSGALG00010014886	RABGAP1L	PRJNA729271	0.539866181	3.28654E-14	0.539866181	stage_E14_vs_E10	ordinary_muscle_development		0	FALSE
ENSGALG00010008277	MZB1	PRJNA729271	7.899502299	3.3022E-14	7.899502299	stage_E14_vs_E10	ordinary_muscle_development	D_vs_E10;stage_1W_vs_E10;stage_3W_vs_E10;stage	1	TRUE
ENSGALG00010021525	ENSGALG00010021525	PRJNA729271	5.58024715	3.3156E-14	5.58024715	stage_E14_vs_E10	ordinary_muscle_development	D_vs_E10;stage_1W_vs_E10;stage_3W_vs_E10;stage	1	TRUE
ENSGALG00010020153	AXL	PRJNA729271	-0.945083201	3.33876E-14	0.945083201	stage_E14_vs_E10	ordinary_muscle_development		0	FALSE
ENSGALG00010017340	SIVA1	PRJNA729271	-0.831641471	3.34402E-14	0.831641471	stage_E14_vs_E10	ordinary_muscle_development		0	FALSE
ENSGALG00010020257	ZNF710	PRJNA729271	1.239960255	3.35649E-14	1.239960255	stage_E14_vs_E10	ordinary_muscle_development	D_vs_E10;stage_1W_vs_E10;stage_3W_vs_E10;stage	1	TRUE
ENSGALG00010025243	PAXIP1	PRJNA729271	-0.696163262	3.37757E-14	0.696163262	stage_E14_vs_E10	ordinary_muscle_development		0	FALSE
ENSGALG00010021081	KCNH6	PRJNA729271	-1.873776575	3.38121E-14	1.873776575	stage_E14_vs_E10	ordinary_muscle_development	D_vs_E10;stage_1W_vs_E10;stage_3W_vs_E10;stage	1	TRUE
ENSGALG00010015157	CDC42BPB	PRJNA729271	-0.541902552	3.39347E-14	0.541902552	stage_E14_vs_E10	ordinary_muscle_development		0	FALSE
ENSGALG00010013089	TBK1	PRJNA729271	0.588531611	3.39471E-14	0.588531611	stage_E14_vs_E10	ordinary_muscle_development		0	FALSE
ENSGALG00010015066	RUFY1	PRJNA729271	-0.556246301	3.40377E-14	0.556246301	stage_E14_vs_E10	ordinary_muscle_development		0	FALSE
ENSGALG00010008165	MRPL1	PRJNA729271	0.652197202	3.44578E-14	0.652197202	stage_E14_vs_E10	ordinary_muscle_development		0	FALSE
ENSGALG00010006197	ENSGALG00010006197	PRJNA729271	2.836020091	3.44837E-14	2.836020091	stage_E14_vs_E10	ordinary_muscle_development	D_vs_E10;stage_1W_vs_E10;stage_3W_vs_E10;stage	1	TRUE
ENSGALG00010024387	PPIL2	PRJNA729271	0.502073592	3.45498E-14	0.502073592	stage_E14_vs_E10	ordinary_muscle_development		0	FALSE
ENSGALG00010005903	PRR18	PRJNA729271	-2.344127682	3.45662E-14	2.344127682	stage_E14_vs_E10	ordinary_muscle_development	D_vs_E10;stage_1W_vs_E10;stage_3W_vs_E10;stage	1	TRUE
ENSGALG00010004468	LYPLA1	PRJNA729271	0.942684975	3.51518E-14	0.942684975	stage_E14_vs_E10	ordinary_muscle_development		0	FALSE
ENSGALG00010021627	NCOA6	PRJNA729271	0.798800511	3.52127E-14	0.798800511	stage_E14_vs_E10	ordinary_muscle_development		0	FALSE
ENSGALG00010004219	FGF2	PRJNA729271	1.411208905	3.5249E-14	1.411208905	stage_E14_vs_E10	ordinary_muscle_development	D_vs_E10;stage_1W_vs_E10;stage_3W_vs_E10;stage	1	TRUE
ENSGALG00010029659	VKORC1L1	PRJNA729271	-1.134172234	3.53419E-14	1.134172234	stage_E14_vs_E10	ordinary_muscle_development	D_vs_E10;stage_1W_vs_E10;stage_3W_vs_E10;stage	1	TRUE
ENSGALG00010021722	AARS1	PRJNA729271	1.224389741	3.56033E-14	1.224389741	stage_E14_vs_E10	ordinary_muscle_development	D_vs_E10;stage_1W_vs_E10;stage_3W_vs_E10;stage	1	TRUE
ENSGALG00010028467	ENSGALG00010028467	PRJNA729271	1.799501283	3.57794E-14	1.799501283	stage_E14_vs_E10	ordinary_muscle_development	D_vs_E10;stage_1W_vs_E10;stage_3W_vs_E10;stage	1	TRUE
ENSGALG00010009365	CA5B	PRJNA729271	1.234227319	3.5974E-14	1.234227319	stage_E14_vs_E10	ordinary_muscle_development	D_vs_E10;stage_1W_vs_E10;stage_3W_vs_E10;stage	1	TRUE
ENSGALG00010029709	SLC6A4	PRJNA729271	1.501866003	3.62869E-14	1.501866003	stage_E14_vs_E10	ordinary_muscle_development	D_vs_E10;stage_1W_vs_E10;stage_3W_vs_E10;stage	1	TRUE
ENSGALG00010024586	DLGAP4	PRJNA729271	0.746899649	3.64235E-14	0.746899649	stage_E14_vs_E10	ordinary_muscle_development		0	FALSE
ENSGALG00010015814	PAQR9	PRJNA729271	3.112881281	3.67839E-14	3.112881281	stage_E14_vs_E10	ordinary_muscle_development	D_vs_E10;stage_1W_vs_E10;stage_3W_vs_E10;stage	1	TRUE
ENSGALG00010023835	INAFM2	PRJNA729271	0.928587843	3.69522E-14	0.928587843	stage_E14_vs_E10	ordinary_muscle_development		0	FALSE
ENSGALG00010008320	GPRIN3	PRJNA729271	0.883015034	3.7496E-14	0.883015034	stage_E14_vs_E10	ordinary_muscle_development		0	FALSE
ENSGALG00010007777	KIF13A	PRJNA729271	0.962292465	3.75975E-14	0.962292465	stage_E14_vs_E10	ordinary_muscle_development		0	FALSE
ENSGALG00010016169	RHOH	PRJNA729271	0.91543123	3.76332E-14	0.91543123	stage_E14_vs_E10	ordinary_muscle_development		0	FALSE
ENSGALG00010013357	RICTOR	PRJNA729271	0.846564471	3.78072E-14	0.846564471	stage_E14_vs_E10	ordinary_muscle_development		0	FALSE
ENSGALG00010019586	GLYR1	PRJNA729271	-0.700355089	3.7999E-14	0.700355089	stage_E14_vs_E10	ordinary_muscle_development		0	FALSE
ENSGALG00010023350	ZNRF1	PRJNA729271	1.776527731	3.82318E-14	1.776527731	stage_E14_vs_E10	ordinary_muscle_development	D_vs_E10;stage_1W_vs_E10;stage_3W_vs_E10;stage	1	TRUE
ENSGALG00010029477	FAM104A	PRJNA729271	-0.455394806	3.85602E-14	0.455394806	stage_E14_vs_E10	ordinary_muscle_development		0	FALSE
ENSGALG00010025113	SDHD	PRJNA729271	0.925686726	3.8596E-14	0.925686726	stage_E14_vs_E10	ordinary_muscle_development		0	FALSE
ENSGALG00010025568	MYL3	PRJNA729271	-7.809367952	3.88052E-14	7.809367952	stage_E14_vs_E10	ordinary_muscle_development	D_vs_E10;stage_1W_vs_E10;stage_3W_vs_E10;stage	1	TRUE
ENSGALG00010005311	YME1L1	PRJNA729271	0.601613766	3.89985E-14	0.601613766	stage_E14_vs_E10	ordinary_muscle_development		0	FALSE
ENSGALG00010008862	ZNF367	PRJNA729271	-1.149249688	3.90916E-14	1.149249688	stage_E14_vs_E10	ordinary_muscle_development	D_vs_E10;stage_1W_vs_E10;stage_3W_vs_E10;stage	1	TRUE
ENSGALG00010023631	ENSGALG00010023631	PRJNA729271	3.404206158	3.96337E-14	3.404206158	stage_E14_vs_E10	ordinary_muscle_development	D_vs_E10;stage_1W_vs_E10;stage_3W_vs_E10;stage	1	TRUE
ENSGALG00010028086	MATK	PRJNA729271	1.922390958	3.97145E-14	1.922390958	stage_E14_vs_E10	ordinary_muscle_development	D_vs_E10;stage_1W_vs_E10;stage_3W_vs_E10;stage	1	TRUE
ENSGALG00010000215	SF3A1	PRJNA729271	-0.698084219	4.03021E-14	0.698084219	stage_E14_vs_E10	ordinary_muscle_development		0	FALSE
ENSGALG00010024250	AP2A2	PRJNA729271	-0.414675295	4.06043E-14	0.414675295	stage_E14_vs_E10	ordinary_muscle_development		0	FALSE
ENSGALG00010029729	RASL10A	PRJNA729271	5.874015765	4.08513E-14	5.874015765	stage_E14_vs_E10	ordinary_muscle_development	D_vs_E10;stage_1W_vs_E10;stage_3W_vs_E10;stage	1	TRUE
ENSGALG00010013084	SGSM3	PRJNA729271	0.88735508	4.12166E-14	0.88735508	stage_E14_vs_E10	ordinary_muscle_development		0	FALSE
ENSGALG00010024605	LSP1P1	PRJNA729271	-1.510260131	4.12445E-14	1.510260131	stage_E14_vs_E10	ordinary_muscle_development	D_vs_E10;stage_1W_vs_E10;stage_3W_vs_E10;stage	1	TRUE
ENSGALG00010016976	ENSGALG00010016976	PRJNA729271	6.930894935	4.14232E-14	6.930894935	stage_E14_vs_E10	ordinary_muscle_development	D_vs_E10;stage_1W_vs_E10;stage_3W_vs_E10;stage	1	TRUE
ENSGALG00010001295	ENSGALG00010001295	PRJNA729271	0.980844785	4.17559E-14	0.980844785	stage_E14_vs_E10	ordinary_muscle_development		0	FALSE
ENSGALG00010026068	BLTP3A	PRJNA729271	0.855731385	4.17647E-14	0.855731385	stage_E14_vs_E10	ordinary_muscle_development		0	FALSE
ENSGALG00010029945	CCL4	PRJNA729271	5.244150685	4.18533E-14	5.244150685	stage_E14_vs_E10	ordinary_muscle_development	D_vs_E10;stage_1W_vs_E10;stage_3W_vs_E10;stage	1	TRUE
ENSGALG00010013256	BRIX1	PRJNA729271	-0.913030762	4.238E-14	0.913030762	stage_E14_vs_E10	ordinary_muscle_development		0	FALSE
ENSGALG00010023119	NDRG4	PRJNA729271	-1.355684836	4.24641E-14	1.355684836	stage_E14_vs_E10	ordinary_muscle_development	D_vs_E10;stage_1W_vs_E10;stage_3W_vs_E10;stage	1	TRUE
ENSGALG00010018707	SLC45A1	PRJNA729271	0.768941455	4.2474E-14	0.768941455	stage_E14_vs_E10	ordinary_muscle_development		0	FALSE
ENSGALG00010022755	MINPP1	PRJNA729271	-0.898229908	4.25408E-14	0.898229908	stage_E14_vs_E10	ordinary_muscle_development		0	FALSE
ENSGALG00010026010	ZSWIM5	PRJNA729271	-1.003298076	4.2678E-14	1.003298076	stage_E14_vs_E10	ordinary_muscle_development	D_vs_E10;stage_1W_vs_E10;stage_3W_vs_E10;stage	1	TRUE
ENSGALG00010021454	ATP5J2	PRJNA729271	1.345781592	4.26821E-14	1.345781592	stage_E14_vs_E10	ordinary_muscle_development	D_vs_E10;stage_1W_vs_E10;stage_3W_vs_E10;stage	1	TRUE
ENSGALG00010018308	ACAP3	PRJNA729271	-1.383576562	4.31834E-14	1.383576562	stage_E14_vs_E10	ordinary_muscle_development	D_vs_E10;stage_1W_vs_E10;stage_3W_vs_E10;stage	1	TRUE
ENSGALG00010020728	TMEM37	PRJNA729271	1.598923438	4.33275E-14	1.598923438	stage_E14_vs_E10	ordinary_muscle_development	D_vs_E10;stage_1W_vs_E10;stage_3W_vs_E10;stage	1	TRUE
ENSGALG00010026630	XIRP1	PRJNA729271	1.890972899	4.34966E-14	1.890972899	stage_E14_vs_E10	ordinary_muscle_development	D_vs_E10;stage_1W_vs_E10;stage_3W_vs_E10;stage	1	TRUE
ENSGALG00010010022	KMT2E	PRJNA729271	0.779403749	4.36017E-14	0.779403749	stage_E14_vs_E10	ordinary_muscle_development		0	FALSE
ENSGALG00010024625	SPRYD3	PRJNA729271	0.606258821	4.3725E-14	0.606258821	stage_E14_vs_E10	ordinary_muscle_development		0	FALSE
ENSGALG00010013638	COMMD8	PRJNA729271	-0.923069435	4.40402E-14	0.923069435	stage_E14_vs_E10	ordinary_muscle_development		0	FALSE
ENSGALG00010004238	CCNC	PRJNA729271	-0.9635131	4.41097E-14	0.9635131	stage_E14_vs_E10	ordinary_muscle_development		0	FALSE
ENSGALG00010015167	RAB3C	PRJNA729271	-3.843602818	4.4632E-14	3.843602818	stage_E14_vs_E10	ordinary_muscle_development	D_vs_E10;stage_1W_vs_E10;stage_3W_vs_E10;stage	1	TRUE
ENSGALG00010004495	MRPL15	PRJNA729271	0.869457376	4.51198E-14	0.869457376	stage_E14_vs_E10	ordinary_muscle_development		0	FALSE
ENSGALG00010016786	DISP1	PRJNA729271	-0.864341249	4.54128E-14	0.864341249	stage_E14_vs_E10	ordinary_muscle_development		0	FALSE
ENSGALG00010013328	SLC37A3	PRJNA729271	-0.612486722	4.60554E-14	0.612486722	stage_E14_vs_E10	ordinary_muscle_development		0	FALSE
ENSGALG00010003726	ENPEP	PRJNA729271	-4.580076042	4.61276E-14	4.580076042	stage_E14_vs_E10	ordinary_muscle_development	D_vs_E10;stage_1W_vs_E10;stage_3W_vs_E10;stage	1	TRUE
ENSGALG00010014213	ETFRF1	PRJNA729271	1.170374871	4.65143E-14	1.170374871	stage_E14_vs_E10	ordinary_muscle_development	D_vs_E10;stage_1W_vs_E10;stage_3W_vs_E10;stage	1	TRUE
ENSGALG00010023363	CPEB1	PRJNA729271	-1.859028911	4.68263E-14	1.859028911	stage_E14_vs_E10	ordinary_muscle_development	D_vs_E10;stage_1W_vs_E10;stage_3W_vs_E10;stage	1	TRUE
ENSGALG00010008186	NOP53	PRJNA729271	0.929840844	4.76347E-14	0.929840844	stage_E14_vs_E10	ordinary_muscle_development		0	FALSE
ENSGALG00010018376	PYGB	PRJNA729271	-1.127803179	4.80903E-14	1.127803179	stage_E14_vs_E10	ordinary_muscle_development	D_vs_E10;stage_1W_vs_E10;stage_3W_vs_E10;stage	1	TRUE
ENSGALG00010009985	USP16	PRJNA729271	-0.908707363	4.82513E-14	0.908707363	stage_E14_vs_E10	ordinary_muscle_development		0	FALSE
ENSGALG00010024888	PITPNM2	PRJNA729271	0.975984971	4.89091E-14	0.975984971	stage_E14_vs_E10	ordinary_muscle_development		0	FALSE
ENSGALG00010017762	FBXO11	PRJNA729271	-0.431336596	4.9248E-14	0.431336596	stage_E14_vs_E10	ordinary_muscle_development		0	FALSE
ENSGALG00010014632	NWD2	PRJNA729271	-1.332384961	4.9815E-14	1.332384961	stage_E14_vs_E10	ordinary_muscle_development	D_vs_E10;stage_1W_vs_E10;stage_3W_vs_E10;stage	1	TRUE
ENSGALG00010011909	C8orf48	PRJNA729271	-1.678491837	4.99091E-14	1.678491837	stage_E14_vs_E10	ordinary_muscle_development	D_vs_E10;stage_1W_vs_E10;stage_3W_vs_E10;stage	1	TRUE
ENSGALG00010016479	ENSGALG00010016479	PRJNA729271	-4.068900213	5.09055E-14	4.068900213	stage_E14_vs_E10	ordinary_muscle_development	D_vs_E10;stage_1W_vs_E10;stage_3W_vs_E10;stage	1	TRUE
ENSGALG00010020601	AKIP1	PRJNA729271	-1.390739387	5.11141E-14	1.390739387	stage_E14_vs_E10	ordinary_muscle_development	D_vs_E10;stage_1W_vs_E10;stage_3W_vs_E10;stage	1	TRUE
ENSGALG00010011949	ABCA12	PRJNA729271	-3.701931215	5.14534E-14	3.701931215	stage_E14_vs_E10	ordinary_muscle_development	D_vs_E10;stage_1W_vs_E10;stage_3W_vs_E10;stage	1	TRUE
ENSGALG00010022725	KRT222	PRJNA729271	-6.993384513	5.16603E-14	6.993384513	stage_E14_vs_E10	ordinary_muscle_development	D_vs_E10;stage_1W_vs_E10;stage_3W_vs_E10;stage	1	TRUE
ENSGALG00010008108	ENSGALG00010008108	PRJNA729271	2.364640721	5.25799E-14	2.364640721	stage_E14_vs_E10	ordinary_muscle_development	D_vs_E10;stage_1W_vs_E10;stage_3W_vs_E10;stage	1	TRUE
ENSGALG00010011734	ENSGALG00010011734	PRJNA729271	-2.371027305	5.28259E-14	2.371027305	stage_E14_vs_E10	ordinary_muscle_development	D_vs_E10;stage_1W_vs_E10;stage_3W_vs_E10;stage	1	TRUE
ENSGALG00010022244	ENTPD7	PRJNA729271	-1.372228612	5.32634E-14	1.372228612	stage_E14_vs_E10	ordinary_muscle_development	D_vs_E10;stage_1W_vs_E10;stage_3W_vs_E10;stage	1	TRUE
ENSGALG00010023639	KLHL23	PRJNA729271	1.651264617	5.36203E-14	1.651264617	stage_E14_vs_E10	ordinary_muscle_development	D_vs_E10;stage_1W_vs_E10;stage_3W_vs_E10;stage	1	TRUE
ENSGALG00010018947	ENSGALG00010018947	PRJNA729271	1.590973283	5.38057E-14	1.590973283	stage_E14_vs_E10	ordinary_muscle_development	D_vs_E10;stage_1W_vs_E10;stage_3W_vs_E10;stage	1	TRUE
ENSGALG00010029781	UNC13D	PRJNA729271	1.868647354	5.38556E-14	1.868647354	stage_E14_vs_E10	ordinary_muscle_development	D_vs_E10;stage_1W_vs_E10;stage_3W_vs_E10;stage	1	TRUE
ENSGALG00010013757	DGKI	PRJNA729271	-2.523048855	5.43569E-14	2.523048855	stage_E14_vs_E10	ordinary_muscle_development	D_vs_E10;stage_1W_vs_E10;stage_3W_vs_E10;stage	1	TRUE
ENSGALG00010015852	ZFAND5	PRJNA729271	2.187872113	5.48935E-14	2.187872113	stage_E14_vs_E10	ordinary_muscle_development	D_vs_E10;stage_1W_vs_E10;stage_3W_vs_E10;stage	1	TRUE
ENSGALG00010017639	ENSGALG00010017639	PRJNA729271	-1.081542565	5.49432E-14	1.081542565	stage_E14_vs_E10	ordinary_muscle_development	D_vs_E10;stage_1W_vs_E10;stage_3W_vs_E10;stage	1	TRUE
ENSGALG00010010547	BST1	PRJNA729271	1.258910843	5.49544E-14	1.258910843	stage_E14_vs_E10	ordinary_muscle_development	D_vs_E10;stage_1W_vs_E10;stage_3W_vs_E10;stage	1	TRUE
ENSGALG00010016968	C15orf61	PRJNA729271	1.121928833	5.49781E-14	1.121928833	stage_E14_vs_E10	ordinary_muscle_development	D_vs_E10;stage_1W_vs_E10;stage_3W_vs_E10;stage	1	TRUE
ENSGALG00010013476	ZDHHC17	PRJNA729271	-1.00413102	5.55753E-14	1.00413102	stage_E14_vs_E10	ordinary_muscle_development	D_vs_E10;stage_1W_vs_E10;stage_3W_vs_E10;stage	1	TRUE
ENSGALG00010021851	ENSGALG00010021851	PRJNA729271	1.628510707	5.56882E-14	1.628510707	stage_E14_vs_E10	ordinary_muscle_development	D_vs_E10;stage_1W_vs_E10;stage_3W_vs_E10;stage	1	TRUE
ENSGALG00010022084	SELENOM	PRJNA729271	-1.5564149	5.59577E-14	1.5564149	stage_E14_vs_E10	ordinary_muscle_development	D_vs_E10;stage_1W_vs_E10;stage_3W_vs_E10;stage	1	TRUE
ENSGALG00010011050	SYT10	PRJNA729271	3.107265621	5.66266E-14	3.107265621	stage_E14_vs_E10	ordinary_muscle_development	D_vs_E10;stage_1W_vs_E10;stage_3W_vs_E10;stage	1	TRUE
ENSGALG00010029170	DCXR	PRJNA729271	-1.104877986	5.67537E-14	1.104877986	stage_E14_vs_E10	ordinary_muscle_development	D_vs_E10;stage_1W_vs_E10;stage_3W_vs_E10;stage	1	TRUE
ENSGALG00010024540	PTPN5	PRJNA729271	-2.323019894	5.6767E-14	2.323019894	stage_E14_vs_E10	ordinary_muscle_development	D_vs_E10;stage_1W_vs_E10;stage_3W_vs_E10;stage	1	TRUE
ENSGALG00010029147	RABL6	PRJNA729271	-0.798592195	5.67824E-14	0.798592195	stage_E14_vs_E10	ordinary_muscle_development		0	FALSE
ENSGALG00010009619	ENSGALG00010009619	PRJNA729271	1.439588298	5.72765E-14	1.439588298	stage_E14_vs_E10	ordinary_muscle_development	D_vs_E10;stage_1W_vs_E10;stage_3W_vs_E10;stage	1	TRUE
ENSGALG00010014205	DPM1	PRJNA729271	-0.670552626	5.72765E-14	0.670552626	stage_E14_vs_E10	ordinary_muscle_development		0	FALSE
ENSGALG00010016744	CRYBG2	PRJNA729271	-7.965418212	5.73889E-14	7.965418212	stage_E14_vs_E10	ordinary_muscle_development	D_vs_E10;stage_1W_vs_E10;stage_3W_vs_E10;stage	1	TRUE
ENSGALG00010029204	HELZ	PRJNA729271	0.746792372	5.7945E-14	0.746792372	stage_E14_vs_E10	ordinary_muscle_development		0	FALSE
ENSGALG00010012058	ENSGALG00010012058	PRJNA729271	-2.54937316	5.80148E-14	2.54937316	stage_E14_vs_E10	ordinary_muscle_development	D_vs_E10;stage_1W_vs_E10;stage_3W_vs_E10;stage	1	TRUE
ENSGALG00010010150	NTRK2	PRJNA729271	2.288375486	5.86276E-14	2.288375486	stage_E14_vs_E10	ordinary_muscle_development	D_vs_E10;stage_1W_vs_E10;stage_3W_vs_E10;stage	1	TRUE
ENSGALG00010018288	CRNKL1	PRJNA729271	-0.484222886	5.86276E-14	0.484222886	stage_E14_vs_E10	ordinary_muscle_development		0	FALSE
ENSGALG00010012889	RIC1	PRJNA729271	0.800827751	5.91769E-14	0.800827751	stage_E14_vs_E10	ordinary_muscle_development		0	FALSE
ENSGALG00010022427	RPL27	PRJNA729271	1.069606745	5.96431E-14	1.069606745	stage_E14_vs_E10	ordinary_muscle_development	D_vs_E10;stage_1W_vs_E10;stage_3W_vs_E10;stage	1	TRUE
ENSGALG00010011154	SLC26A7	PRJNA729271	-7.216803467	6.05509E-14	7.216803467	stage_E14_vs_E10	ordinary_muscle_development	D_vs_E10;stage_1W_vs_E10;stage_3W_vs_E10;stage	1	TRUE
ENSGALG00010008492	ANKRD49	PRJNA729271	0.93570891	6.05808E-14	0.93570891	stage_E14_vs_E10	ordinary_muscle_development		0	FALSE
ENSGALG00010024798	FBXO45	PRJNA729271	-0.654492216	6.18958E-14	0.654492216	stage_E14_vs_E10	ordinary_muscle_development		0	FALSE
ENSGALG00010028572	TBC1D13	PRJNA729271	-0.530926323	6.22718E-14	0.530926323	stage_E14_vs_E10	ordinary_muscle_development		0	FALSE
ENSGALG00010007853	C1orf131	PRJNA729271	0.835724429	6.23961E-14	0.835724429	stage_E14_vs_E10	ordinary_muscle_development		0	FALSE
ENSGALG00010010285	ENSGALG00010010285	PRJNA729271	4.339967744	6.25798E-14	4.339967744	stage_E14_vs_E10	ordinary_muscle_development	D_vs_E10;stage_1W_vs_E10;stage_3W_vs_E10;stage	1	TRUE
ENSGALG00010020125	NAV2	PRJNA729271	0.999482416	6.38547E-14	0.999482416	stage_E14_vs_E10	ordinary_muscle_development		0	FALSE
ENSGALG00010008292	REEP5	PRJNA729271	0.865887131	6.4107E-14	0.865887131	stage_E14_vs_E10	ordinary_muscle_development		0	FALSE
ENSGALG00010014427	TMEM267	PRJNA729271	-1.06012358	6.43895E-14	1.06012358	stage_E14_vs_E10	ordinary_muscle_development	D_vs_E10;stage_1W_vs_E10;stage_3W_vs_E10;stage	1	TRUE
ENSGALG00010002522	HPSE	PRJNA729271	2.168899272	6.44313E-14	2.168899272	stage_E14_vs_E10	ordinary_muscle_development	D_vs_E10;stage_1W_vs_E10;stage_3W_vs_E10;stage	1	TRUE
ENSGALG00010025353	SLC12A4	PRJNA729271	-0.603021835	6.48649E-14	0.603021835	stage_E14_vs_E10	ordinary_muscle_development		0	FALSE
ENSGALG00010008153	SMU1	PRJNA729271	-0.473514432	6.49752E-14	0.473514432	stage_E14_vs_E10	ordinary_muscle_development		0	FALSE
ENSGALG00010001741	SATB1	PRJNA729271	-1.586679555	6.57943E-14	1.586679555	stage_E14_vs_E10	ordinary_muscle_development	D_vs_E10;stage_1W_vs_E10;stage_3W_vs_E10;stage	1	TRUE
ENSGALG00010015196	PDE4D	PRJNA729271	1.661822262	6.61496E-14	1.661822262	stage_E14_vs_E10	ordinary_muscle_development	D_vs_E10;stage_1W_vs_E10;stage_3W_vs_E10;stage	1	TRUE
ENSGALG00010023857	ACBD4	PRJNA729271	1.149338041	6.63627E-14	1.149338041	stage_E14_vs_E10	ordinary_muscle_development	D_vs_E10;stage_1W_vs_E10;stage_3W_vs_E10;stage	1	TRUE
ENSGALG00010010573	CEP83	PRJNA729271	-1.258280165	6.65677E-14	1.258280165	stage_E14_vs_E10	ordinary_muscle_development	D_vs_E10;stage_1W_vs_E10;stage_3W_vs_E10;stage	1	TRUE
ENSGALG00010017537	MANBA	PRJNA729271	1.440175015	6.74644E-14	1.440175015	stage_E14_vs_E10	ordinary_muscle_development	D_vs_E10;stage_1W_vs_E10;stage_3W_vs_E10;stage	1	TRUE

ENSGALG00010013461	S100B	PRJNA729271	4.333292437	6.74833E-14	4.333292437	stage_E14_vs_E10	ordinary_muscle_development	D_vs_E10;stage_1W_vs_E10;stage_3W_vs_E10;stage	1	TRUE
ENSGALG00010019834	PLCH2	PRJNA729271	-1.894098255	6.78929E-14	1.894098255	stage_E14_vs_E10	ordinary_muscle_development	D_vs_E10;stage_1W_vs_E10;stage_3W_vs_E10;stage	1	TRUE
ENSGALG00010005609	GJB6	PRJNA729271	-4.615544957	6.81828E-14	4.615544957	stage_E14_vs_E10	ordinary_muscle_development	D_vs_E10;stage_1W_vs_E10;stage_3W_vs_E10;stage	1	TRUE
ENSGALG00010008074	RHAG	PRJNA729271	-3.430966438	6.85817E-14	3.430966438	stage_E14_vs_E10	ordinary_muscle_development	D_vs_E10;stage_1W_vs_E10;stage_3W_vs_E10;stage	1	TRUE
ENSGALG00010007604	ENSGALG00010007604	PRJNA729271	-5.035504666	6.86634E-14	5.035504666	stage_E14_vs_E10	ordinary_muscle_development	D_vs_E10;stage_1W_vs_E10;stage_3W_vs_E10;stage	1	TRUE
ENSGALG00010018514	EAF2	PRJNA729271	-1.916693576	6.89492E-14	1.916693576	stage_E14_vs_E10	ordinary_muscle_development	D_vs_E10;stage_1W_vs_E10;stage_3W_vs_E10;stage	1	TRUE
ENSGALG00010017337	ABAT	PRJNA729271	-1.600870929	6.90283E-14	1.600870929	stage_E14_vs_E10	ordinary_muscle_development	D_vs_E10;stage_1W_vs_E10;stage_3W_vs_E10;stage	1	TRUE
ENSGALG00010027759	ENSGALG00010027759	PRJNA729271	5.425539093	6.9363E-14	5.425539093	stage_E14_vs_E10	ordinary_muscle_development	D_vs_E10;stage_1W_vs_E10;stage_3W_vs_E10;stage	1	TRUE
ENSGALG00010024052	ENSGALG00010024052	PRJNA729271	-1.385574944	6.98114E-14	1.385574944	stage_E14_vs_E10	ordinary_muscle_development	D_vs_E10;stage_1W_vs_E10;stage_3W_vs_E10;stage	1	TRUE
ENSGALG00010020619	QARS	PRJNA729271	-0.778561614	7.03343E-14	0.778561614	stage_E14_vs_E10	ordinary_muscle_development		0	FALSE
ENSGALG00010010333	WAPL	PRJNA729271	0.480986208	7.06463E-14	0.480986208	stage_E14_vs_E10	ordinary_muscle_development		0	FALSE
ENSGALG00010015935	EPHX1	PRJNA729271	0.844320997	7.11643E-14	0.844320997	stage_E14_vs_E10	ordinary_muscle_development		0	FALSE
ENSGALG00010021262	MDGA1	PRJNA729271	-3.130970151	7.13236E-14	3.130970151	stage_E14_vs_E10	ordinary_muscle_development	D_vs_E10;stage_1W_vs_E10;stage_3W_vs_E10;stage	1	TRUE
ENSGALG00010021516	SRSF5	PRJNA729271	-1.169953553	7.37902E-14	1.169953553	stage_E14_vs_E10	ordinary_muscle_development	D_vs_E10;stage_1W_vs_E10;stage_3W_vs_E10;stage	1	TRUE
ENSGALG00010029626	AP2B1	PRJNA729271	-0.835451179	7.47177E-14	0.835451179	stage_E14_vs_E10	ordinary_muscle_development		0	FALSE
ENSGALG00010029394	CYGB	PRJNA729271	-1.091039902	7.61497E-14	1.091039902	stage_E14_vs_E10	ordinary_muscle_development	D_vs_E10;stage_1W_vs_E10;stage_3W_vs_E10;stage	1	TRUE
ENSGALG00010007921	FAM120B	PRJNA729271	-0.784962164	7.6425E-14	0.784962164	stage_E14_vs_E10	ordinary_muscle_development		0	FALSE
ENSGALG00010029333	ENSGALG00010029333	PRJNA729271	-1.340475518	7.65888E-14	1.340475518	stage_E14_vs_E10	ordinary_muscle_development	D_vs_E10;stage_1W_vs_E10;stage_3W_vs_E10;stage	1	TRUE
ENSGALG00010009571	DNAJC2	PRJNA729271	-1.446280877	7.67846E-14	1.446280877	stage_E14_vs_E10	ordinary_muscle_development	D_vs_E10;stage_1W_vs_E10;stage_3W_vs_E10;stage	1	TRUE
ENSGALG00010018401	ENSGALG00010018401	PRJNA729271	-0.785023498	7.68982E-14	0.785023498	stage_E14_vs_E10	ordinary_muscle_development		0	FALSE
ENSGALG00010021178	NDST2	PRJNA729271	-0.750164818	7.75295E-14	0.750164818	stage_E14_vs_E10	ordinary_muscle_development		0	FALSE
ENSGALG00010007251	SPAST	PRJNA729271	-0.487980706	7.82984E-14	0.487980706	stage_E14_vs_E10	ordinary_muscle_development		0	FALSE
ENSGALG00010003405	GABRR2	PRJNA729271	-2.233767716	7.93772E-14	2.233767716	stage_E14_vs_E10	ordinary_muscle_development	D_vs_E10;stage_1W_vs_E10;stage_3W_vs_E10;stage	1	TRUE
ENSGALG00010008444	SLC7A11	PRJNA729271	-3.986926984	8.01647E-14	3.986926984	stage_E14_vs_E10	ordinary_muscle_development	D_vs_E10;stage_1W_vs_E10;stage_3W_vs_E10;stage	1	TRUE
ENSGALG00010004494	WTAP	PRJNA729271	-0.440827064	8.02296E-14	0.440827064	stage_E14_vs_E10	ordinary_muscle_development		0	FALSE
ENSGALG00010025403	ENSGALG00010025403	PRJNA729271	0.971010251	8.04444E-14	0.971010251	stage_E14_vs_E10	ordinary_muscle_development		0	FALSE
ENSGALG00010000814	CDH18	PRJNA729271	-2.282864559	8.24282E-14	2.282864559	stage_E14_vs_E10	ordinary_muscle_development	D_vs_E10;stage_1W_vs_E10;stage_3W_vs_E10;stage	1	TRUE
ENSGALG00010006132	MIPEP	PRJNA729271	-1.030249025	8.32295E-14	1.030249025	stage_E14_vs_E10	ordinary_muscle_development	D_vs_E10;stage_1W_vs_E10;stage_3W_vs_E10;stage	1	TRUE
ENSGALG00010008351	IST1	PRJNA729271	-0.524272373	8.32573E-14	0.524272373	stage_E14_vs_E10	ordinary_muscle_development		0	FALSE
ENSGALG00010004217	CCDC28A	PRJNA729271	0.860508095	8.38497E-14	0.860508095	stage_E14_vs_E10	ordinary_muscle_development		0	FALSE
ENSGALG00010026666	ENSGALG00010026666	PRJNA729271	3.207233143	8.40089E-14	3.207233143	stage_E14_vs_E10	ordinary_muscle_development	D_vs_E10;stage_1W_vs_E10;stage_3W_vs_E10;stage	1	TRUE
ENSGALG00010011903	LTA4H	PRJNA729271	-0.888121942	8.41819E-14	0.888121942	stage_E14_vs_E10	ordinary_muscle_development		0	FALSE
ENSGALG00010025623	ENSGALG00010025623	PRJNA729271	2.937589532	8.50589E-14	2.937589532	stage_E14_vs_E10	ordinary_muscle_development	D_vs_E10;stage_1W_vs_E10;stage_3W_vs_E10;stage	1	TRUE
ENSGALG00010012200	GEM	PRJNA729271	1.82947146	8.52697E-14	1.82947146	stage_E14_vs_E10	ordinary_muscle_development	D_vs_E10;stage_1W_vs_E10;stage_3W_vs_E10;stage	1	TRUE
ENSGALG00010025635	CHKA	PRJNA729271	-1.252954921	8.55938E-14	1.252954921	stage_E14_vs_E10	ordinary_muscle_development	D_vs_E10;stage_1W_vs_E10;stage_3W_vs_E10;stage	1	TRUE
ENSGALG00010025648	ENSGALG00010025648	PRJNA729271	3.687546177	8.64691E-14	3.687546177	stage_E14_vs_E10	ordinary_muscle_development	D_vs_E10;stage_1W_vs_E10;stage_3W_vs_E10;stage	1	TRUE
ENSGALG00010009582	TIGD5	PRJNA729271	-1.060190382	8.66306E-14	1.060190382	stage_E14_vs_E10	ordinary_muscle_development	D_vs_E10;stage_1W_vs_E10;stage_3W_vs_E10;stage	1	TRUE
ENSGALG00010021759	NIFK	PRJNA729271	-0.725320998	8.67242E-14	0.725320998	stage_E14_vs_E10	ordinary_muscle_development		0	FALSE
ENSGALG00010020450	ANKRD39	PRJNA729271	1.363233056	8.70072E-14	1.363233056	stage_E14_vs_E10	ordinary_muscle_development	D_vs_E10;stage_1W_vs_E10;stage_3W_vs_E10;stage	1	TRUE
ENSGALG00010001132	SELENOT	PRJNA729271	0.717963163	8.75366E-14	0.717963163	stage_E14_vs_E10	ordinary_muscle_development		0	FALSE
ENSGALG00010029580	P2RX1	PRJNA729271	2.4552747	8.85895E-14	2.4552747	stage_E14_vs_E10	ordinary_muscle_development	D_vs_E10;stage_1W_vs_E10;stage_3W_vs_E10;stage	1	TRUE
ENSGALG00010025400	SLC22A15	PRJNA729271	-1.244549065	8.9571E-14	1.244549065	stage_E14_vs_E10	ordinary_muscle_development	D_vs_E10;stage_1W_vs_E10;stage_3W_vs_E10;stage	1	TRUE
ENSGALG00010027641	HYAL3	PRJNA729271	1.240023677	8.9684E-14	1.240023677	stage_E14_vs_E10	ordinary_muscle_development	D_vs_E10;stage_1W_vs_E10;stage_3W_vs_E10;stage	1	TRUE
ENSGALG00010015831	LYG2	PRJNA729271	4.759454234	9.07427E-14	4.759454234	stage_E14_vs_E10	ordinary_muscle_development	D_vs_E10;stage_1W_vs_E10;stage_3W_vs_E10;stage	1	TRUE
ENSGALG00010018915	DOCK5	PRJNA729271	-1.474556633	9.12727E-14	1.474556633	stage_E14_vs_E10	ordinary_muscle_development	D_vs_E10;stage_1W_vs_E10;stage_3W_vs_E10;stage	1	TRUE
ENSGALG00010027360	SMIM7	PRJNA729271	0.860761479	9.19687E-14	0.860761479	stage_E14_vs_E10	ordinary_muscle_development		0	FALSE
ENSGALG00010023209	PIK3C2A	PRJNA729271	-0.820045642	9.27131E-14	0.820045642	stage_E14_vs_E10	ordinary_muscle_development		0	FALSE
ENSGALG00010027211	HDGFRP2	PRJNA729271	-0.775997793	9.34976E-14	0.775997793	stage_E14_vs_E10	ordinary_muscle_development		0	FALSE
ENSGALG00010016455	RBM27	PRJNA729271	-0.700529615	9.35086E-14	0.700529615	stage_E14_vs_E10	ordinary_muscle_development		0	FALSE
ENSGALG00010022609	RBBP8NL	PRJNA729271	-8.052839093	9.35354E-14	8.052839093	stage_E14_vs_E10	ordinary_muscle_development	D_vs_E10;stage_1W_vs_E10;stage_3W_vs_E10;stage	1	TRUE
ENSGALG00010017933	SIX1	PRJNA729271	1.483595081	9.47742E-14	1.483595081	stage_E14_vs_E10	ordinary_muscle_development	D_vs_E10;stage_1W_vs_E10;stage_3W_vs_E10;stage	1	TRUE
ENSGALG00010015698	TNFAIP2	PRJNA729271	-1.560774294	9.62043E-14	1.560774294	stage_E14_vs_E10	ordinary_muscle_development	D_vs_E10;stage_1W_vs_E10;stage_3W_vs_E10;stage	1	TRUE
ENSGALG00010021805	FUT8	PRJNA729271	-0.84277026	9.74973E-14	0.84277026	stage_E14_vs_E10	ordinary_muscle_development		0	FALSE
ENSGALG00010007108	MBOAT1	PRJNA729271	-1.502823512	9.77855E-14	1.502823512	stage_E14_vs_E10	ordinary_muscle_development	D_vs_E10;stage_1W_vs_E10;stage_3W_vs_E10;stage	1	TRUE
ENSGALG00010016791	LRRTM1	PRJNA729271	-6.039259592	9.79767E-14	6.039259592	stage_E14_vs_E10	ordinary_muscle_development	D_vs_E10;stage_1W_vs_E10;stage_3W_vs_E10;stage	1	TRUE
ENSGALG00010022776	CDH4	PRJNA729271	-1.376823669	9.80496E-14	1.376823669	stage_E14_vs_E10	ordinary_muscle_development	D_vs_E10;stage_1W_vs_E10;stage_3W_vs_E10;stage	1	TRUE
ENSGALG00010007898	GAB1	PRJNA729271	0.681382722	9.81573E-14	0.681382722	stage_E14_vs_E10	ordinary_muscle_development		0	FALSE
ENSGALG00010007092	DYNC1LI2	PRJNA729271	0.464089401	9.85084E-14	0.464089401	stage_E14_vs_E10	ordinary_muscle_development		0	FALSE
ENSGALG00010023884	C2orf69	PRJNA729271	-0.980379745	9.85869E-14	0.980379745	stage_E14_vs_E10	ordinary_muscle_development		0	FALSE
ENSGALG00010021228	ESRP2	PRJNA729271	-8.970939261	1.0019E-13	8.970939261	stage_E14_vs_E10	ordinary_muscle_development	D_vs_E10;stage_1W_vs_E10;stage_3W_vs_E10;stage	1	TRUE
ENSGALG00010008323	AKT3	PRJNA729271	0.785444881	1.00863E-13	0.785444881	stage_E14_vs_E10	ordinary_muscle_development		0	FALSE
ENSGALG00010016433	TMED3	PRJNA729271	-1.127048215	1.01139E-13	1.127048215	stage_E14_vs_E10	ordinary_muscle_development	D_vs_E10;stage_1W_vs_E10;stage_3W_vs_E10;stage	1	TRUE
ENSGALG00010000341	EWSR1	PRJNA729271	-1.123315926	1.0156E-13	1.123315926	stage_E14_vs_E10	ordinary_muscle_development	D_vs_E10;stage_1W_vs_E10;stage_3W_vs_E10;stage	1	TRUE
ENSGALG00010003511	UBXN2B	PRJNA729271	-1.101473842	1.03282E-13	1.101473842	stage_E14_vs_E10	ordinary_muscle_development	D_vs_E10;stage_1W_vs_E10;stage_3W_vs_E10;stage	1	TRUE
ENSGALG00010020265	ENSGALG00010020265	PRJNA729271	-1.779423415	1.05587E-13	1.779423415	stage_E14_vs_E10	ordinary_muscle_development	D_vs_E10;stage_1W_vs_E10;stage_3W_vs_E10;stage	1	TRUE
ENSGALG00010019104	POLL	PRJNA729271	0.915337778	1.05587E-13	0.915337778	stage_E14_vs_E10	ordinary_muscle_development		0	FALSE
ENSGALG00010001502	LGSN	PRJNA729271	-4.746531463	1.06802E-13	4.746531463	stage_E14_vs_E10	ordinary_muscle_development	D_vs_E10;stage_1W_vs_E10;stage_3W_vs_E10;stage	1	TRUE
ENSGALG00010027645	SLAMF1	PRJNA729271	5.905216407	1.07872E-13	5.905216407	stage_E14_vs_E10	ordinary_muscle_development	D_vs_E10;stage_1W_vs_E10;stage_3W_vs_E10;stage	1	TRUE
ENSGALG00010027373	MLN	PRJNA729271	7.946935002	1.08576E-13	7.946935002	stage_E14_vs_E10	ordinary_muscle_development	D_vs_E10;stage_1W_vs_E10;stage_3W_vs_E10;stage	1	TRUE
ENSGALG00010003604	ODC	PRJNA729271	-1.63993701	1.12869E-13	1.63993701	stage_E14_vs_E10	ordinary_muscle_development	D_vs_E10;stage_1W_vs_E10;stage_3W_vs_E10;stage	1	TRUE
ENSGALG00010027614	LONP1	PRJNA729271	-0.61570678	1.14176E-13	0.61570678	stage_E14_vs_E10	ordinary_muscle_development		0	FALSE
ENSGALG00010022825	SS18L1	PRJNA729271	1.955448061	1.14963E-13	1.955448061	stage_E14_vs_E10	ordinary_muscle_development	D_vs_E10;stage_1W_vs_E10;stage_3W_vs_E10;stage	1	TRUE
ENSGALG00010011726	WDR55	PRJNA729271	-0.671143806	1.16688E-13	0.671143806	stage_E14_vs_E10	ordinary_muscle_development		0	FALSE
ENSGALG00010020417	ENSGALG00010020417	PRJNA729271	5.633140652	1.16797E-13	5.633140652	stage_E14_vs_E10	ordinary_muscle_development	D_vs_E10;stage_1W_vs_E10;stage_3W_vs_E10;stage	1	TRUE
ENSGALG00010015911	TM9SF2L	PRJNA729271	0.589636116	1.16872E-13	0.589636116	stage_E14_vs_E10	ordinary_muscle_development		0	FALSE
ENSGALG00010023091	ENSGALG00010023091	PRJNA729271	0.991004905	1.17223E-13	0.991004905	stage_E14_vs_E10	ordinary_muscle_development		0	FALSE
ENSGALG00010021626	ZNF142	PRJNA729271	1.601924471	1.17475E-13	1.601924471	stage_E14_vs_E10	ordinary_muscle_development	D_vs_E10;stage_1W_vs_E10;stage_3W_vs_E10;stage	1	TRUE
ENSGALG00010006260	MAP3K4	PRJNA729271	1.202342533	1.18024E-13	1.202342533	stage_E14_vs_E10	ordinary_muscle_development	D_vs_E10;stage_1W_vs_E10;stage_3W_vs_E10;stage	1	TRUE
ENSGALG00010002626	CHD7	PRJNA729271	1.025352524	1.18524E-13	1.025352524	stage_E14_vs_E10	ordinary_muscle_development	D_vs_E10;stage_1W_vs_E10;stage_3W_vs_E10;stage	1	TRUE
ENSGALG00010024573	ENSGALG00010024573	PRJNA729271	2.006110973	1.18879E-13	2.006110973	stage_E14_vs_E10	ordinary_muscle_development	D_vs_E10;stage_1W_vs_E10;stage_3W_vs_E10;stage	1	TRUE
ENSGALG00010011487	MAN2B2	PRJNA729271	-1.209413535	1.19161E-13	1.209413535	stage_E14_vs_E10	ordinary_muscle_development	D_vs_E10;stage_1W_vs_E10;stage_3W_vs_E10;stage	1	TRUE
ENSGALG00010008509	MGARP	PRJNA729271	2.662511637	1.19205E-13	2.662511637	stage_E14_vs_E10	ordinary_muscle_development	D_vs_E10;stage_1W_vs_E10;stage_3W_vs_E10;stage	1	TRUE
ENSGALG00010027267	MYO9B	PRJNA729271	-0.727335801	1.19296E-13	0.727335801	stage_E14_vs_E10	ordinary_muscle_development		0	FALSE
ENSGALG00010017685	PHC2	PRJNA729271	1.382760643	1.21409E-13	1.382760643	stage_E14_vs_E10	ordinary_muscle_development	D_vs_E10;stage_1W_vs_E10;stage_3W_vs_E10;stage	1	TRUE
ENSGALG00010010311	CTSV	PRJNA729271	1.430513281	1.21468E-13	1.430513281	stage_E14_vs_E10	ordinary_muscle_development	D_vs_E10;stage_1W_vs_E10;stage_3W_vs_E10;stage	1	TRUE
ENSGALG00010005692	SEC24B	PRJNA729271	1.084863693	1.22166E-13	1.084863693	stage_E14_vs_E10	ordinary_muscle_development	D_vs_E10;stage_1W_vs_E10;stage_3W_vs_E10;stage	1	TRUE
ENSGALG00010011802	VPS4B	PRJNA729271	-0.717395203	1.22961E-13	0.717395203	stage_E14_vs_E10	ordinary_muscle_development		0	FALSE
ENSGALG00010017513	TNIP2	PRJNA729271	1.086589494	1.2308E-13	1.086589494	stage_E14_vs_E10	ordinary_muscle_development	D_vs_E10;stage_1W_vs_E10;stage_3W_vs_E10;stage	1	TRUE
ENSGALG00010000432	ENSGALG00010000432	PRJNA729271	1.372475132	1.2408E-13	1.372475132	stage_E14_vs_E10	ordinary_muscle_development	D_vs_E10;stage_1W_vs_E10;stage_3W_vs_E10;stage	1	TRUE
ENSGALG00010013583	FASLG	PRJNA729271	2.321388814	1.24797E-13	2.321388814	stage_E14_vs_E10	ordinary_muscle_development	D_vs_E10;stage_1W_vs_E10;stage_3W_vs_E10;stage	1	TRUE
ENSGALG00010008401	PSMD8	PRJNA729271	1.175033825	1.25539E-13	1.175033825	stage_E14_vs_E10	ordinary_muscle_development	D_vs_E10;stage_1W_vs_E10;stage_3W_vs_E10;stage	1	TRUE
ENSGALG00010029541	ENSGALG00010029541	PRJNA729271	1.660626897	1.25572E-13	1.660626897	stage_E14_vs_E10	ordinary_muscle_development	D_vs_E10;stage_1W_vs_E10;stage_3W_vs_E10;stage	1	TRUE
ENSGALG00010022717	ELOVL1	PRJNA729271	1.294672339	1.26026E-13	1.294672339	stage_E14_vs_E10	ordinary_muscle_development	D_vs_E10;stage_1W_vs_E10;stage_3W_vs_E10;stage	1	TRUE
ENSGALG00010029202	PSMD12	PRJNA729271	1.074747125	1.29429E-13	1.074747125	stage_E14_vs_E10	ordinary_muscle_development	D_vs_E10;stage_1W_vs_E10;stage_3W_vs_E10;stage	1	TRUE
ENSGALG00010024614	ENSGALG00010024614	PRJNA729271	-7.197030819	1.29708E-13	7.197030819	stage_E14_vs_E10	ordinary_muscle_development	D_vs_E10;stage_1W_vs_E10;stage_3W_vs_E10;stage	1	TRUE
ENSGALG00010029569	TMEM220	PRJNA729271	1.523134704	1.30353E-13	1.523134704	stage_E14_vs_E10	ordinary_muscle_development	D_vs_E10;stage_1W_vs_E10;stage_3W_vs_E10;stage	1	TRUE
ENSGALG00010005895	MLLT10	PRJNA729271	0.746905926	1.30742E-13	0.746905926	stage_E14_vs_E10	ordinary_muscle_development		0	FALSE
ENSGALG00010003186	CYP21A1	PRJNA729271	-2.127817982	1.31257E-13	2.127817982	stage_E14_vs_E10	ordinary_muscle_development	D_vs_E10;stage_1W_vs_E10;stage_3W_vs_E10;stage	1	TRUE
ENSGALG00010021546	OGG1	PRJNA729271	-1.400649353	1.31476E-13	1.400649353	stage_E14_vs_E10	ordinary_muscle_development	D_vs_E10;stage_1W_vs_E10;stage_3W_vs_E10;stage	1	TRUE
ENSGALG00010019166	PNPT1	PRJNA729271	-0.847151679	1.32731E-13	0.847151679	stage_E14_vs_E10	ordinary_muscle_development		0	FALSE
ENSGALG00010024907	PHLDB1	PRJNA729271	1.004931356	1.33293E-13	1.004931356	stage_E14_vs_E10	ordinary_muscle_development	D_vs_E10;stage_1W_vs_E10;stage_3W_vs_E10;stage	1	TRUE
ENSGALG00010001178	ZBTB5	PRJNA729271	-0.494115108	1.33985E-13	0.494115108	stage_E14_vs_E10	ordinary_muscle_development		0	FALSE
ENSGALG00010016657	SLC25A29	PRJNA729271	2.10722536	1.34131E-13	2.10722536	stage_E14_vs_E10	ordinary_muscle_development	D_vs_E10;stage_1W_vs_E10;stage_3W_vs_E10;stage	1	TRUE
ENSGALG00010013264	DNAJC21	PRJNA729271	-0.955311178	1.34755E-13	0.955311178	stage_E14_vs_E10	ordinary_muscle_development		0	FALSE
ENSGALG00010023793	RBM45	PRJNA729271	-0.503869722	1.35293E-13	0.503869722	stage_E14_vs_E10	ordinary_muscle_development		0	FALSE
ENSGALG00010014816	TST	PRJNA729271	0.815710657	1.35621E-13	0.815710657	stage_E14_vs_E10	ordinary_muscle_development		0	FALSE
ENSGALG00010025433	CHPF2	PRJNA729271	0.916605856	1.3643E-13	0.916605856	stage_E14_vs_E10	ordinary_muscle_development		0	FALSE
ENSGALG00010001595	AKAIN1	PRJNA729271	1.905649562	1.36925E-13	1.905649562	stage_E14_vs_E10	ordinary_muscle_development	D_vs_E10;stage_1W_vs_E10;stage_3W_vs_E10;stage	1	TRUE
ENSGALG00010024102	SGF29	PRJNA729271	0.677370454	1.38038E-13	0.677370454	stage_E14_vs_E10	ordinary_muscle_development		0	FALSE
ENSGALG00010005232	GATA3	PRJNA729271	-3.977796949	1.3823E-13	3.977796949	stage_E14_vs_E10	ordinary_muscle_development	D_vs_E10;stage_1W_vs_E10;stage_3W_vs_E10;stage	1	TRUE
ENSGALG00010016648	ADRA2C	PRJNA729271	-2.192074752	1.41364E-13	2.192074752	stage_E14_vs_E10	ordinary_muscle_development	D_vs_E10;stage_1W_vs_E10;stage_3W_vs_E10;stage	1	TRUE
ENSGALG00010012837	PKIB	PRJNA729271	1.968594374	1.41949E-13	1.968594374	stage_E14_vs_E10	ordinary_muscle_development	D_vs_E10;stage_1W_vs_E10;stage_3W_vs_E10;stage	1	TRUE
ENSGALG00010028136	C3orf18	PRJNA729271	-1.231842429	1.43354E-13	1.231842429	stage_E14_vs_E10	ordinary_muscle_development	D_vs_E10;stage_1W_vs_E10;stage_3W_vs_E10;stage	1	TRUE
ENSGALG00010015032	C1orf216	PRJNA729271	-2.041249598	1.43875E-13	2.041249598	stage_E14_vs_E10	ordinary_muscle_development	D_vs_E10;stage_1W_vs_E10;stage_3W_vs_E10;stage	1	TRUE
ENSGALG00010011365	PSMG4	PRJNA729271	1.347299868	1.46575E-13	1.347299868	stage_E14_vs_E10	ordinary_muscle_development	D_vs_E10;stage_1W_vs_E10;stage_3W_vs_E10;stage	1	TRUE
ENSGALG00010023174	KCNQ2	PRJNA729271	-2.807543511	1.46686E-13	2.807543511	stage_E14_vs_E10	ordinary_muscle_development	D_vs_E10;stage_1W_vs_E10;stage_3W_vs_E10;stage	1	TRUE
ENSGALG00010011049	ABL2	PRJNA729271	0.982921195	1.47811E-13	0.982921195	stage_E14_vs_E10	ordinary_muscle_development		0	FALSE
ENSGALG00010027941	KLF2	PRJNA729271	1.137223873	1.49288E-13	1.137223873	stage_E14_vs_E10	ordinary_muscle_development	D_vs_E10;stage_1W_vs_E10;stage_3W_vs_E10;stage	1	TRUE
ENSGALG00010009702	ENSGALG00010009702	PRJNA729271	-0.940237237	1.51074E-13	0.940237237	stage_E14_vs_E10	ordinary_muscle_development		0	FALSE
ENSGALG00010009744	DAPK1	PRJNA729271	-0.798692985	1.54195E-13	0.798692985	stage_E14_vs_E10	ordinary_muscle_development		0	FALSE
ENSGALG00010022258	ENSGALG00010022258	PRJNA729271	5.070874757	1.54453E-13	5.070874757	stage_E14_vs_E10	ordinary_muscle_development	D_vs_E10;stage_1W_vs_E10;stage_3W_vs_E10;stage	1	TRUE
ENSGALG00010010941	RIPK4	PRJNA729271	-6.441592733	1.54504E-13	6.441592733	stage_E14_vs_E10	ordinary_muscle_development	D_vs_E10;stage_1W_vs_E10;stage_3W_vs_E10;stage	1	TRUE
ENSGALG00010021782	ADAM11	PRJNA729271	2.124485467	1.55484E-13	2.124485467	stage_E14_vs_E10	ordinary_muscle_development	D_vs_E10;stage_1W_vs_E10;stage_3W_vs_E10;stage	1	TRUE
ENSGALG00010018562	ENSGALG00010018562	PRJNA729271	-8.453419202	1.57685E-13	8.453419202	stage_E14_vs_E10	ordinary_muscle_development	D_vs_E10;stage_1W_vs_E10;stage_3W_vs_E10;stage	1	TRUE
ENSGALG00010005378	ENSGALG00010005378	PRJNA729271	-7.936186353	1.58285E-13	7.936186353	stage_E14_vs_E10	ordinary_muscle_development	D_vs_E10;stage_1W_vs_E10;stage_3W_vs_E10;stage	1	TRUE
ENSGALG00010002501	TCEANC	PRJNA729271	0.695572432	1.58709E-13	0.695572432	stage_E14_vs_E10	ordinary_muscle_development		0	FALSE
ENSGALG00010015413	CARNMT1	PRJNA729271	0.797550604	1.60747E-13	0.797550604	stage_E14_vs_E10	ordinary_muscle_development		0	FALSE
ENSGALG00010005523	ENSGALG00010005523	PRJNA729271	4.476202435	1.61107E-13	4.476202435	stage_E14_vs_E10	ordinary_muscle_development	D_vs_E10;stage_1W_vs_E10;stage_3W_vs_E10;stage	1	TRUE
ENSGALG00010010486	ENSGALG00010010486	PRJNA729271	-3.639635351	1.62141E-13	3.639635351	stage_E14_vs_E10	ordinary_muscle_development	D_vs_E10;stage_1W_vs_E10;stage_3W_vs_E10;stage	1	TRUE
ENSGALG00010007596	ENSGALG00010007596	PRJNA729271	-4.034342269	1.6301E-13	4.034342269	stage_E14_vs_E10	ordinary_muscle_development	D_vs_E10;stage_1W_vs_E10;stage_3W_vs_E10;stage	1	TRUE
ENSGALG00010027355	ADCYAP1R1	PRJNA729271	-2.022440996	1.63575E-13	2.022440996	stage_E14_vs_E10	ordinary_muscle_development	D_vs_E10;stage_1W_vs_E10;stage_3W_vs_E10;stage	1	TRUE
ENSGALG00010013297	FCHO2	PRJNA729271	0.929557317	1.64596E-13	0.929557317	stage_E14_vs_E10	ordinary_muscle_development		0	FALSE
ENSGALG00010000511	FERMT3	PRJNA729271	1.171171169	1.6737E-13	1.171171169	stage_E14_vs_E10	ordinary_muscle_development	D_vs_E10;stage_1W_vs_E10;stage_3W_vs_E10;stage	1	TRUE
ENSGALG00010014908	ENSGALG00010014908	PRJNA729271	-0.77112056	1.68405E-13	0.77112056	stage_E14_vs_E10	ordinary_muscle_development		0	FALSE
ENSGALG00010020343	PATL1	PRJNA729271	1.307807196	1.6956E-13	1.307807196	stage_E14_vs_E10	ordinary_muscle_development	D_vs_E10;stage_1W_vs_E10;stage_3W_vs_E10;stage	1	TRUE
ENSGALG00010016279	PRELID3B	PRJNA729271	-0.802828239	1.69702E-13	0.802828239	stage_E14_vs_E10	ordinary_muscle_development		0	FALSE
ENSGALG00010024821	NECAP1	PRJNA729271	-0.801277023	1.70973E-13	0.801277023	stage_E14_vs_E10	ordinary_muscle_development		0	FALSE
ENSGALG00010007901	FSTL5	PRJNA729271	-2.511887401	1.72233E-13	2.511887401	stage_E14_vs_E10	ordinary_muscle_development	D_vs_E10;stage_1W_vs_E10;stage_3W_vs_E10;stage	1	TRUE
ENSGALG00010006480	IFT74	PRJNA729271	-0.903670253	1.74822E-13	0.903670253	stage_E14_vs_E10	ordinary_muscle_development		0	FALSE
ENSGALG00010013664	REV3L	PRJNA729271	0.722325	1.77467E-13	0.722325	stage_E14_vs_E10	ordinary_muscle_development		0	FALSE
ENSGALG00010027082	SLC26A9	PRJNA729271	-5.638515162	1.78172E-13	5.638515162	stage_E14_vs_E10	ordinary_muscle_development	D_vs_E10;stage_1W_vs_E10;stage_3W_vs_E10;stage	1	TRUE
ENSGALG00010009788	PAK1IP1	PRJNA729271	0.686414987	1.78648E-13	0.686414987	stage_E14_vs_E10	ordinary_muscle_development		0	FALSE
ENSGALG00010026116	DLX3	PRJNA729271	-7.385278918	1.8141E-13	7.385278918	stage_E14_vs_E10	ordinary_muscle_development	D_vs_E10;stage_1W_vs_E10;stage_3W_vs_E10;stage	1	TRUE
ENSGALG00010029253	NUP88	PRJNA729271	-0.657826973	1.81871E-13	0.657826973	stage_E14_vs_E10	ordinary_muscle_development		0	FALSE
ENSGALG00010012719	COL10A1	PRJNA729271	-6.348843721	1.83016E-13	6.348843721	stage_E14_vs_E10	ordinary_muscle_development	D_vs_E10;stage_1W_vs_E10;stage_3W_vs_E10;stage	1	TRUE
ENSGALG00010027575	FCER1G	PRJNA729271	2.479109614	1.84071E-13	2.479109614	stage_E14_vs_E10	ordinary_muscle_development	D_vs_E10;stage_1W_vs_E10;stage_3W_vs_E10;stage	1	TRUE
ENSGALG00010016415	RASGRF1	PRJNA729271	3.816134017	1.86012E-13	3.816134017	stage_E14_vs_E10	ordinary_muscle_development	D_vs_E10;stage_1W_vs_E10;stage_3W_vs_E10;stage	1	TRUE
ENSGALG00010027450	CAMSAP1	PRJNA729271	1.068301518	1.87157E-13	1.068301518	stage_E14_vs_E10	ordinary_muscle_development	D_vs_E10;stage_1W_vs_E10;stage_3W_vs_E10;stage	1	TRUE
ENSGALG00010002460	LAMTOR1	PRJNA729271	1.350661579	1.87751E-13	1.350661579	stage_E14_vs_E10	ordinary_muscle_development	D_vs_E10;stage_1W_vs_E10;stage_3W_vs_E10;stage	1	TRUE
ENSGALG00010003528	GDAP1	PRJNA729271	-1.553056821	1.87986E-13	1.553056821	stage_E14_vs_E10	ordinary_muscle_development	D_vs_E10;stage_1W_vs_E10;stage_3W_vs_E10;stage	1	TRUE
ENSGALG00010016378	WDR76	PRJNA729271	-1.32135803	1.89225E-13	1.32135803	stage_E14_vs_E10	ordinary_muscle_development	D_vs_E10;stage_1W_vs_E10;stage_3W_vs_E10;stage	1	TRUE
ENSGALG00010006263	EXOSC8	PRJNA729271	-1.133102943	1.90414E-13	1.133102943	stage_E14_vs_E10	ordinary_muscle_development	D_vs_E10;stage_1W_vs_E10;stage_3W_vs_E10;stage	1	TRUE
ENSGALG00010024338	NDUFA9	PRJNA729271	0.854339483	1.90748E-13	0.854339483	stage_E14_vs_E10	ordinary_muscle_development		0	FALSE
ENSGALG00010020975	HCCS	PRJNA729271	0.975311319	1.91913E-13	0.975311319	stage_E14_vs_E10	ordinary_muscle_development		0	FALSE
ENSGALG00010023142	TLK2	PRJNA729271	1.259145812	1.96202E-13	1.259145812	stage_E14_vs_E10	ordinary_muscle_development	D_vs_E10;stage_1W_vs_E10;stage_3W_vs_E10;stage	1	TRUE
ENSGALG00010013506	RNF149	PRJNA729271	-0.806403736	1.96884E-13	0.806403736	stage_E14_vs_E10	ordinary_muscle_development		0	FALSE
ENSGALG00010018199	BORCS7	PRJNA729271	-0.91084697	1.97438E-13	0.91084697	stage_E14_vs_E10	ordinary_muscle_development		0	FALSE
ENSGALG00010009740	LRRC73	PRJNA729271	-2.802754578	1.97551E-13	2.802754578	stage_E14_vs_E10	ordinary_muscle_development	D_vs_E10;stage_1W_vs_E10;stage_3W_vs_E10;stage	1	TRUE
ENSGALG00010008052	RBFA	PRJNA729271	0.907100736	1.98292E-13	0.907100736	stage_E14_vs_E10	ordinary_muscle_development		0	FALSE
ENSGALG00010001209	CDH7	PRJNA729271	-1.862680262	1.99318E-13	1.862680262	stage_E14_vs_E10	ordinary_muscle_development	D_vs_E10;stage_1W_vs_E10;stage_3W_vs_E10;stage	1	TRUE
ENSGALG00010006568	ENTPD5	PRJNA729271	-1.520243152	2.00132E-13	1.520243152	stage_E14_vs_E10	ordinary_muscle_development	D_vs_E10;stage_1W_vs_E10;stage_3W_vs_E10;stage	1	TRUE
ENSGALG00010028420	ENSGALG00010028420	PRJNA729271	6.180970104	2.00559E-13	6.180970104	stage_E14_vs_E10	ordinary_muscle_development	D_vs_E10;stage_1W_vs_E10;stage_3W_vs_E10;stage	1	TRUE
ENSGALG00010013330	ERG	PRJNA729271	-0.994336702	2.01433E-13	0.994336702	stage_E14_vs_E10	ordinary_muscle_development		0	FALSE
ENSGALG00010003965	SNTG1	PRJNA729271	-4.450516716	2.0365E-13	4.450516716	stage_E14_vs_E10	ordinary_muscle_development	D_vs_E10;stage_1W_vs_E10;stage_3W_vs_E10;stage	1	TRUE
ENSGALG00010023024	PRMT7	PRJNA729271	-0.881614772	2.03827E-13	0.881614772	stage_E14_vs_E10	ordinary_muscle_development		0	FALSE
ENSGALG00010027869	NSMF	PRJNA729271	-1.247457678	2.06633E-13	1.247457678	stage_E14_vs_E10	ordinary_muscle_development	D_vs_E10;stage_1W_vs_E10;stage_3W_vs_E10;stage	1	TRUE
ENSGALG00010018561	ACTR1A	PRJNA729271	0.452213552	2.0739E-13	0.452213552	stage_E14_vs_E10	ordinary_muscle_development		0	FALSE
ENSGALG00010009239	STX18	PRJNA729271	0.609260049	2.09071E-13	0.609260049	stage_E14_vs_E10	ordinary_muscle_development		0	FALSE
ENSGALG00010014042	SGCZ	PRJNA729271	-3.074211748	2.09648E-13	3.074211748	stage_E14_vs_E10	ordinary_muscle_development	D_vs_E10;stage_1W_vs_E10;stage_3W_vs_E10;stage	1	TRUE
ENSGALG00010024524	TMEM41A	PRJNA729271	0.991757336	2.10346E-13	0.991757336	stage_E14_vs_E10	ordinary_muscle_development		0	FALSE
ENSGALG00010008010	CERK	PRJNA729271	-0.696512526	2.12065E-13	0.696512526	stage_E14_vs_E10	ordinary_muscle_development		0	FALSE
ENSGALG00010021320	SLC6A6	PRJNA729271	1.472345802	2.13884E-13	1.472345802	stage_E14_vs_E10	ordinary_muscle_development	D_vs_E10;stage_1W_vs_E10;stage_3W_vs_E10;stage	1	TRUE
ENSGALG00010004903	ENSGALG00010004903	PRJNA729271	3.453721792	2.14068E-13	3.453721792	stage_E14_vs_E10	ordinary_muscle_development	D_vs_E10;stage_1W_vs_E10;stage_3W_vs_E10;stage	1	TRUE
ENSGALG00010024061	ENSGALG00010024061	PRJNA729271	9.150561386	2.14983E-13	9.150561386	stage_E14_vs_E10	ordinary_muscle_development	D_vs_E10;stage_1W_vs_E10;stage_3W_vs_E10;stage	1	TRUE
ENSGALG00010028362	TMPRSS9	PRJNA729271	-3.832607586	2.16055E-13	3.832607586	stage_E14_vs_E10	ordinary_muscle_development	D_vs_E10;stage_1W_vs_E10;stage_3W_vs_E10;stage	1	TRUE

ENSGALG00010028854	RPL7A	PRJNA729271	1.058389563	2.17191E-13	1.058389563	stage_E14_vs_E10	ordinary_muscle_development	D_vs_E10;stage_1W_vs_E10;stage_3W_vs_E10;stage	1	TRUE
ENSGALG00010013326	FGF20	PRJNA729271	-7.774293193	2.1724E-13	7.774293193	stage_E14_vs_E10	ordinary_muscle_development	D_vs_E10;stage_1W_vs_E10;stage_3W_vs_E10;stage	1	TRUE
ENSGALG00010024424	SAMHD1	PRJNA729271	1.340519903	2.19034E-13	1.340519903	stage_E14_vs_E10	ordinary_muscle_development	D_vs_E10;stage_1W_vs_E10;stage_3W_vs_E10;stage	1	TRUE
ENSGALG00010019816	ENSGALG00010019816	PRJNA729271	5.127051756	2.20747E-13	5.127051756	stage_E14_vs_E10	ordinary_muscle_development	D_vs_E10;stage_1W_vs_E10;stage_3W_vs_E10;stage	1	TRUE
ENSGALG00010010053	CHRNA2	PRJNA729271	-7.041201141	2.21073E-13	7.041201141	stage_E14_vs_E10	ordinary_muscle_development	D_vs_E10;stage_1W_vs_E10;stage_3W_vs_E10;stage	1	TRUE
ENSGALG00010022953	ENSGALG00010022953	PRJNA729271	6.291963547	2.22265E-13	6.291963547	stage_E14_vs_E10	ordinary_muscle_development	D_vs_E10;stage_1W_vs_E10;stage_3W_vs_E10;stage	1	TRUE
ENSGALG00010010713	NSA2	PRJNA729271	0.676543527	2.25861E-13	0.676543527	stage_E14_vs_E10	ordinary_muscle_development		0	FALSE
ENSGALG00010013936	DLGAP3	PRJNA729271	-3.943539871	2.2769E-13	3.943539871	stage_E14_vs_E10	ordinary_muscle_development	D_vs_E10;stage_1W_vs_E10;stage_3W_vs_E10;stage	1	TRUE
ENSGALG00010022718	C12orf43	PRJNA729271	-1.09304479	2.28845E-13	1.09304479	stage_E14_vs_E10	ordinary_muscle_development	D_vs_E10;stage_1W_vs_E10;stage_3W_vs_E10;stage	1	TRUE
ENSGALG00010011471	NMNAT2	PRJNA729271	-3.36100006	2.2893E-13	3.36100006	stage_E14_vs_E10	ordinary_muscle_development	D_vs_E10;stage_1W_vs_E10;stage_3W_vs_E10;stage	1	TRUE
ENSGALG00010016787	PPP2R5C	PRJNA729271	-0.566304813	2.29537E-13	0.566304813	stage_E14_vs_E10	ordinary_muscle_development		0	FALSE
ENSGALG00010024789	SUOX	PRJNA729271	1.280817813	2.30195E-13	1.280817813	stage_E14_vs_E10	ordinary_muscle_development	D_vs_E10;stage_1W_vs_E10;stage_3W_vs_E10;stage	1	TRUE
ENSGALG00010010382	SEC11C	PRJNA729271	1.122273384	2.30458E-13	1.122273384	stage_E14_vs_E10	ordinary_muscle_development	D_vs_E10;stage_1W_vs_E10;stage_3W_vs_E10;stage	1	TRUE
ENSGALG00010027317	MPV17L2	PRJNA729271	1.086009285	2.30838E-13	1.086009285	stage_E14_vs_E10	ordinary_muscle_development	D_vs_E10;stage_1W_vs_E10;stage_3W_vs_E10;stage	1	TRUE
ENSGALG00010012712	EEF1E1	PRJNA729271	0.990720118	2.32394E-13	0.990720118	stage_E14_vs_E10	ordinary_muscle_development		0	FALSE
ENSGALG00010021721	TP53I11	PRJNA729271	-1.073282269	2.33486E-13	1.073282269	stage_E14_vs_E10	ordinary_muscle_development	D_vs_E10;stage_1W_vs_E10;stage_3W_vs_E10;stage	1	TRUE
ENSGALG00010005902	EAPP	PRJNA729271	-0.774747597	2.34689E-13	0.774747597	stage_E14_vs_E10	ordinary_muscle_development		0	FALSE
ENSGALG00010011128	GCNT4	PRJNA729271	1.764080597	2.34872E-13	1.764080597	stage_E14_vs_E10	ordinary_muscle_development	D_vs_E10;stage_1W_vs_E10;stage_3W_vs_E10;stage	1	TRUE
ENSGALG00010001976	THRSP	PRJNA729271	10.67160302	2.36602E-13	10.67160302	stage_E14_vs_E10	ordinary_muscle_development	D_vs_E10;stage_1W_vs_E10;stage_3W_vs_E10;stage	1	TRUE
ENSGALG00010015923	RCAN3	PRJNA729271	-0.912660965	2.36784E-13	0.912660965	stage_E14_vs_E10	ordinary_muscle_development		0	FALSE
ENSGALG00010021657	JAGN1	PRJNA729271	1.055512133	2.38384E-13	1.055512133	stage_E14_vs_E10	ordinary_muscle_development	D_vs_E10;stage_1W_vs_E10;stage_3W_vs_E10;stage	1	TRUE
ENSGALG00010029237	COX11	PRJNA729271	-0.826911658	2.41328E-13	0.826911658	stage_E14_vs_E10	ordinary_muscle_development		0	FALSE
ENSGALG00010022921	ENSGALG00010022921	PRJNA729271	1.456800029	2.44426E-13	1.456800029	stage_E14_vs_E10	ordinary_muscle_development	D_vs_E10;stage_1W_vs_E10;stage_3W_vs_E10;stage	1	TRUE
ENSGALG00010000760	CDH10	PRJNA729271	-2.821175016	2.46692E-13	2.821175016	stage_E14_vs_E10	ordinary_muscle_development	D_vs_E10;stage_1W_vs_E10;stage_3W_vs_E10;stage	1	TRUE
ENSGALG00010024167	CHRNA3	PRJNA729271	2.885550007	2.47577E-13	2.885550007	stage_E14_vs_E10	ordinary_muscle_development	D_vs_E10;stage_1W_vs_E10;stage_3W_vs_E10;stage	1	TRUE
ENSGALG00010024155	SNX7	PRJNA729271	-1.245235467	2.48724E-13	1.245235467	stage_E14_vs_E10	ordinary_muscle_development	D_vs_E10;stage_1W_vs_E10;stage_3W_vs_E10;stage	1	TRUE
ENSGALG00010019298	PGAP6	PRJNA729271	0.699165998	2.49948E-13	0.699165998	stage_E14_vs_E10	ordinary_muscle_development		0	FALSE
ENSGALG00010000479	C12orf49	PRJNA729271	-0.87350265	2.51388E-13	0.87350265	stage_E14_vs_E10	ordinary_muscle_development		0	FALSE
ENSGALG00010026907	ENSGALG00010026907	PRJNA729271	-1.013517388	2.53103E-13	1.013517388	stage_E14_vs_E10	ordinary_muscle_development	D_vs_E10;stage_1W_vs_E10;stage_3W_vs_E10;stage	1	TRUE
ENSGALG00010027312	ENSGALG00010027312	PRJNA729271	4.533078387	2.53995E-13	4.533078387	stage_E14_vs_E10	ordinary_muscle_development	D_vs_E10;stage_1W_vs_E10;stage_3W_vs_E10;stage	1	TRUE
ENSGALG00010012586	CDC73	PRJNA729271	0.995252536	2.55719E-13	0.995252536	stage_E14_vs_E10	ordinary_muscle_development		0	FALSE
ENSGALG00010005376	HTRA2	PRJNA729271	0.704553774	2.558E-13	0.704553774	stage_E14_vs_E10	ordinary_muscle_development		0	FALSE
ENSGALG00010021190	ENSGALG00010021190	PRJNA729271	0.759600257	2.57693E-13	0.759600257	stage_E14_vs_E10	ordinary_muscle_development		0	FALSE
ENSGALG00010019738	ADRA2A	PRJNA729271	-2.714461684	2.58995E-13	2.714461684	stage_E14_vs_E10	ordinary_muscle_development	D_vs_E10;stage_1W_vs_E10;stage_3W_vs_E10;stage	1	TRUE
ENSGALG00010018488	ENSGALG00010018488	PRJNA729271	1.146627575	2.61156E-13	1.146627575	stage_E14_vs_E10	ordinary_muscle_development	D_vs_E10;stage_1W_vs_E10;stage_3W_vs_E10;stage	1	TRUE
ENSGALG00010020108	RPAP1	PRJNA729271	-0.878113235	2.61652E-13	0.878113235	stage_E14_vs_E10	ordinary_muscle_development		0	FALSE
ENSGALG00010001197	CEP76	PRJNA729271	-1.123322184	2.61701E-13	1.123322184	stage_E14_vs_E10	ordinary_muscle_development	D_vs_E10;stage_1W_vs_E10;stage_3W_vs_E10;stage	1	TRUE
ENSGALG00010005221	ENSGALG00010005221	PRJNA729271	2.242591763	2.61889E-13	2.242591763	stage_E14_vs_E10	ordinary_muscle_development	D_vs_E10;stage_1W_vs_E10;stage_3W_vs_E10;stage	1	TRUE
ENSGALG00010019016	PLEKHG5	PRJNA729271	1.064785776	2.62312E-13	1.064785776	stage_E14_vs_E10	ordinary_muscle_development	D_vs_E10;stage_1W_vs_E10;stage_3W_vs_E10;stage	1	TRUE
ENSGALG00010015097	MFSD2A	PRJNA729271	-1.253128424	2.62541E-13	1.253128424	stage_E14_vs_E10	ordinary_muscle_development	D_vs_E10;stage_1W_vs_E10;stage_3W_vs_E10;stage	1	TRUE
ENSGALG00010003081	DOP1A	PRJNA729271	0.769615087	2.62966E-13	0.769615087	stage_E14_vs_E10	ordinary_muscle_development		0	FALSE
ENSGALG00010021127	SWAP70	PRJNA729271	-0.591796266	2.63532E-13	0.591796266	stage_E14_vs_E10	ordinary_muscle_development		0	FALSE
ENSGALG00010004226	RUNDC3B	PRJNA729271	-1.547681426	2.64457E-13	1.547681426	stage_E14_vs_E10	ordinary_muscle_development	D_vs_E10;stage_1W_vs_E10;stage_3W_vs_E10;stage	1	TRUE
ENSGALG00010027198	ECM2	PRJNA729271	1.519041062	2.65022E-13	1.519041062	stage_E14_vs_E10	ordinary_muscle_development	D_vs_E10;stage_1W_vs_E10;stage_3W_vs_E10;stage	1	TRUE
ENSGALG00010005462	AHI1	PRJNA729271	1.112716117	2.6687E-13	1.112716117	stage_E14_vs_E10	ordinary_muscle_development	D_vs_E10;stage_1W_vs_E10;stage_3W_vs_E10;stage	1	TRUE
ENSGALG00010013820	MASP1	PRJNA729271	-3.763813773	2.69764E-13	3.763813773	stage_E14_vs_E10	ordinary_muscle_development	D_vs_E10;stage_1W_vs_E10;stage_3W_vs_E10;stage	1	TRUE
ENSGALG00010025145	CPT2	PRJNA729271	-0.715984712	2.71548E-13	0.715984712	stage_E14_vs_E10	ordinary_muscle_development		0	FALSE
ENSGALG00010010784	CCDC148	PRJNA729271	2.148942712	2.73552E-13	2.148942712	stage_E14_vs_E10	ordinary_muscle_development	D_vs_E10;stage_1W_vs_E10;stage_3W_vs_E10;stage	1	TRUE
ENSGALG00010011531	MTDH	PRJNA729271	0.534575889	2.74253E-13	0.534575889	stage_E14_vs_E10	ordinary_muscle_development		0	FALSE
ENSGALG00010029770	TLCD3A	PRJNA729271	-1.758774216	2.7468E-13	1.758774216	stage_E14_vs_E10	ordinary_muscle_development	D_vs_E10;stage_1W_vs_E10;stage_3W_vs_E10;stage	1	TRUE
ENSGALG00010006603	MEST	PRJNA729271	-1.795971453	2.78352E-13	1.795971453	stage_E14_vs_E10	ordinary_muscle_development	D_vs_E10;stage_1W_vs_E10;stage_3W_vs_E10;stage	1	TRUE
ENSGALG00010021691	PIGB	PRJNA729271	-1.146101586	2.78478E-13	1.146101586	stage_E14_vs_E10	ordinary_muscle_development	D_vs_E10;stage_1W_vs_E10;stage_3W_vs_E10;stage	1	TRUE
ENSGALG00010019989	ENSGALG00010019989	PRJNA729271	2.030288093	2.84413E-13	2.030288093	stage_E14_vs_E10	ordinary_muscle_development	D_vs_E10;stage_1W_vs_E10;stage_3W_vs_E10;stage	1	TRUE
ENSGALG00010000601	ENSGALG00010000601	PRJNA729271	-2.813403041	2.84436E-13	2.813403041	stage_E14_vs_E10	ordinary_muscle_development	D_vs_E10;stage_1W_vs_E10;stage_3W_vs_E10;stage	1	TRUE
ENSGALG00010023028	TMEM61	PRJNA729271	-2.493802015	2.87022E-13	2.493802015	stage_E14_vs_E10	ordinary_muscle_development	D_vs_E10;stage_1W_vs_E10;stage_3W_vs_E10;stage	1	TRUE
ENSGALG00010007951	TGFBR1	PRJNA729271	-0.851960967	2.87042E-13	0.851960967	stage_E14_vs_E10	ordinary_muscle_development		0	FALSE
ENSGALG00010016948	CCR8	PRJNA729271	-3.710518763	2.9293E-13	3.710518763	stage_E14_vs_E10	ordinary_muscle_development	D_vs_E10;stage_1W_vs_E10;stage_3W_vs_E10;stage	1	TRUE
ENSGALG00010007098	NDUFAF7	PRJNA729271	0.94241306	2.9377E-13	0.94241306	stage_E14_vs_E10	ordinary_muscle_development		0	FALSE
ENSGALG00010019188	ENSGALG00010019188	PRJNA729271	2.793883413	2.9466E-13	2.793883413	stage_E14_vs_E10	ordinary_muscle_development	D_vs_E10;stage_1W_vs_E10;stage_3W_vs_E10;stage	1	TRUE
ENSGALG00010000441	SLC7A4	PRJNA729271	-1.632835876	2.94981E-13	1.632835876	stage_E14_vs_E10	ordinary_muscle_development	D_vs_E10;stage_1W_vs_E10;stage_3W_vs_E10;stage	1	TRUE
ENSGALG00010018568	FAM136A	PRJNA729271	-1.467785656	2.96052E-13	1.467785656	stage_E14_vs_E10	ordinary_muscle_development	D_vs_E10;stage_1W_vs_E10;stage_3W_vs_E10;stage	1	TRUE
ENSGALG00010012410	CACNA1I	PRJNA729271	-3.337714946	2.97479E-13	3.337714946	stage_E14_vs_E10	ordinary_muscle_development	D_vs_E10;stage_1W_vs_E10;stage_3W_vs_E10;stage	1	TRUE
ENSGALG00010009173	LYAR	PRJNA729271	-1.08582808	2.9877E-13	1.08582808	stage_E14_vs_E10	ordinary_muscle_development	D_vs_E10;stage_1W_vs_E10;stage_3W_vs_E10;stage	1	TRUE
ENSGALG00010014347	KIAA0040	PRJNA729271	2.46424716	2.99406E-13	2.46424716	stage_E14_vs_E10	ordinary_muscle_development	D_vs_E10;stage_1W_vs_E10;stage_3W_vs_E10;stage	1	TRUE
ENSGALG00010014147	ENSGALG00010014147	PRJNA729271	-3.111774164	2.99848E-13	3.111774164	stage_E14_vs_E10	ordinary_muscle_development	D_vs_E10;stage_1W_vs_E10;stage_3W_vs_E10;stage	1	TRUE
ENSGALG00010005680	ENSGALG00010005680	PRJNA729271	3.260714655	3.00744E-13	3.260714655	stage_E14_vs_E10	ordinary_muscle_development	D_vs_E10;stage_1W_vs_E10;stage_3W_vs_E10;stage	1	TRUE
ENSGALG00010002599	KIFC1	PRJNA729271	-1.635223826	3.05464E-13	1.635223826	stage_E14_vs_E10	ordinary_muscle_development	D_vs_E10;stage_1W_vs_E10;stage_3W_vs_E10;stage	1	TRUE
ENSGALG00010022929	LRP4	PRJNA729271	0.846945998	3.06354E-13	0.846945998	stage_E14_vs_E10	ordinary_muscle_development		0	FALSE
ENSGALG00010016387	CCDC85C	PRJNA729271	-1.218848256	3.06438E-13	1.218848256	stage_E14_vs_E10	ordinary_muscle_development	D_vs_E10;stage_1W_vs_E10;stage_3W_vs_E10;stage	1	TRUE
ENSGALG00010013893	CSNK2A1	PRJNA729271	-0.596637892	3.13419E-13	0.596637892	stage_E14_vs_E10	ordinary_muscle_development		0	FALSE
ENSGALG00010026003	NECTIN1	PRJNA729271	-1.164170895	3.18507E-13	1.164170895	stage_E14_vs_E10	ordinary_muscle_development	D_vs_E10;stage_1W_vs_E10;stage_3W_vs_E10;stage	1	TRUE
ENSGALG00010023432	TMOD3	PRJNA729271	-1.300493948	3.19573E-13	1.300493948	stage_E14_vs_E10	ordinary_muscle_development	D_vs_E10;stage_1W_vs_E10;stage_3W_vs_E10;stage	1	TRUE
ENSGALG00010018430	LOXL4	PRJNA729271	-2.119281578	3.20954E-13	2.119281578	stage_E14_vs_E10	ordinary_muscle_development	D_vs_E10;stage_1W_vs_E10;stage_3W_vs_E10;stage	1	TRUE
ENSGALG00010026148	RPS10	PRJNA729271	1.003934655	3.24114E-13	1.003934655	stage_E14_vs_E10	ordinary_muscle_development	D_vs_E10;stage_1W_vs_E10;stage_3W_vs_E10;stage	1	TRUE
ENSGALG00010006283	DNAJC25	PRJNA729271	0.788801723	3.26096E-13	0.788801723	stage_E14_vs_E10	ordinary_muscle_development		0	FALSE
ENSGALG00010016856	NOD1	PRJNA729271	-1.06437367	3.26225E-13	1.06437367	stage_E14_vs_E10	ordinary_muscle_development	D_vs_E10;stage_1W_vs_E10;stage_3W_vs_E10;stage	1	TRUE
ENSGALG00010027169	ABCB8	PRJNA729271	0.824041593	3.26225E-13	0.824041593	stage_E14_vs_E10	ordinary_muscle_development		0	FALSE
ENSGALG00010025212	P2RX4	PRJNA729271	1.318848849	3.28453E-13	1.318848849	stage_E14_vs_E10	ordinary_muscle_development	D_vs_E10;stage_1W_vs_E10;stage_3W_vs_E10;stage	1	TRUE
ENSGALG00010008291	PTGFRN	PRJNA729271	1.224684949	3.3103E-13	1.224684949	stage_E14_vs_E10	ordinary_muscle_development	D_vs_E10;stage_1W_vs_E10;stage_3W_vs_E10;stage	1	TRUE
ENSGALG00010011619	PCDH1	PRJNA729271	1.187214651	3.33616E-13	1.187214651	stage_E14_vs_E10	ordinary_muscle_development	D_vs_E10;stage_1W_vs_E10;stage_3W_vs_E10;stage	1	TRUE
ENSGALG00010025874	MDH1B	PRJNA729271	-2.445033219	3.41618E-13	2.445033219	stage_E14_vs_E10	ordinary_muscle_development	D_vs_E10;stage_1W_vs_E10;stage_3W_vs_E10;stage	1	TRUE
ENSGALG00010024629	ENSGALG00010024629	PRJNA729271	1.286609938	3.47363E-13	1.286609938	stage_E14_vs_E10	ordinary_muscle_development	D_vs_E10;stage_1W_vs_E10;stage_3W_vs_E10;stage	1	TRUE
ENSGALG00010005413	GAD2	PRJNA729271	-6.234502963	3.49114E-13	6.234502963	stage_E14_vs_E10	ordinary_muscle_development	D_vs_E10;stage_1W_vs_E10;stage_3W_vs_E10;stage	1	TRUE
ENSGALG00010023759	CALCA	PRJNA729271	-2.393977472	3.49752E-13	2.393977472	stage_E14_vs_E10	ordinary_muscle_development	D_vs_E10;stage_1W_vs_E10;stage_3W_vs_E10;stage	1	TRUE
ENSGALG00010023383	HOXB6	PRJNA729271	-1.33081363	3.50285E-13	1.33081363	stage_E14_vs_E10	ordinary_muscle_development	D_vs_E10;stage_1W_vs_E10;stage_3W_vs_E10;stage	1	TRUE
ENSGALG00010001477	NLGN4	PRJNA729271	-1.716735374	3.54185E-13	1.716735374	stage_E14_vs_E10	ordinary_muscle_development	D_vs_E10;stage_1W_vs_E10;stage_3W_vs_E10;stage	1	TRUE
ENSGALG00010005227	EXOSC9	PRJNA729271	-0.789787217	3.57583E-13	0.789787217	stage_E14_vs_E10	ordinary_muscle_development		0	FALSE
ENSGALG00010029777	ENSGALG00010029777	PRJNA729271	-1.071759861	3.58374E-13	1.071759861	stage_E14_vs_E10	ordinary_muscle_development	D_vs_E10;stage_1W_vs_E10;stage_3W_vs_E10;stage	1	TRUE
ENSGALG00010018338	PDCD6IP	PRJNA729271	0.632816706	3.58999E-13	0.632816706	stage_E14_vs_E10	ordinary_muscle_development		0	FALSE
ENSGALG00010008399	INO80C	PRJNA729271	-0.843795551	3.60034E-13	0.843795551	stage_E14_vs_E10	ordinary_muscle_development		0	FALSE
ENSGALG00010024166	ENSGALG00010024166	PRJNA729271	6.538284498	3.63E-13	6.538284498	stage_E14_vs_E10	ordinary_muscle_development	D_vs_E10;stage_1W_vs_E10;stage_3W_vs_E10;stage	1	TRUE
ENSGALG00010029251	ENGASE	PRJNA729271	-1.649277427	3.65171E-13	1.649277427	stage_E14_vs_E10	ordinary_muscle_development	D_vs_E10;stage_1W_vs_E10;stage_3W_vs_E10;stage	1	TRUE
ENSGALG00010027727	GSTM2	PRJNA729271	1.265662928	3.67094E-13	1.265662928	stage_E14_vs_E10	ordinary_muscle_development	D_vs_E10;stage_1W_vs_E10;stage_3W_vs_E10;stage	1	TRUE
ENSGALG00010004630	ENSGALG00010004630	PRJNA729271	2.26933342	3.68073E-13	2.26933342	stage_E14_vs_E10	ordinary_muscle_development	D_vs_E10;stage_1W_vs_E10;stage_3W_vs_E10;stage	1	TRUE
ENSGALG00010027695	TREMB2	PRJNA729271	2.182715812	3.69555E-13	2.182715812	stage_E14_vs_E10	ordinary_muscle_development	D_vs_E10;stage_1W_vs_E10;stage_3W_vs_E10;stage	1	TRUE
ENSGALG00010018241	RALGAPA2	PRJNA729271	0.674029974	3.72681E-13	0.674029974	stage_E14_vs_E10	ordinary_muscle_development		0	FALSE
ENSGALG00010001402	NUP42	PRJNA729271	-1.076419483	3.74067E-13	1.076419483	stage_E14_vs_E10	ordinary_muscle_development	D_vs_E10;stage_1W_vs_E10;stage_3W_vs_E10;stage	1	TRUE
ENSGALG00010018452	SPSB3	PRJNA729271	0.877348759	3.74403E-13	0.877348759	stage_E14_vs_E10	ordinary_muscle_development		0	FALSE
ENSGALG00010025910	HEATR3	PRJNA729271	-0.855854359	3.80906E-13	0.855854359	stage_E14_vs_E10	ordinary_muscle_development		0	FALSE
ENSGALG00010022164	PDGFA	PRJNA729271	-0.808047825	3.82081E-13	0.808047825	stage_E14_vs_E10	ordinary_muscle_development		0	FALSE
ENSGALG00010014241	ENSGALG00010014241	PRJNA729271	-8.863969723	3.85617E-13	8.863969723	stage_E14_vs_E10	ordinary_muscle_development	D_vs_E10;stage_1W_vs_E10;stage_3W_vs_E10;stage	1	TRUE
ENSGALG00010012326	SGTB	PRJNA729271	-0.899587696	3.86137E-13	0.899587696	stage_E14_vs_E10	ordinary_muscle_development		0	FALSE
ENSGALG00010024528	TBX1	PRJNA729271	-1.654454298	3.8848E-13	1.654454298	stage_E14_vs_E10	ordinary_muscle_development	D_vs_E10;stage_1W_vs_E10;stage_3W_vs_E10;stage	1	TRUE
ENSGALG00010023075	PKDREJ	PRJNA729271	-2.386142199	3.89483E-13	2.386142199	stage_E14_vs_E10	ordinary_muscle_development	D_vs_E10;stage_1W_vs_E10;stage_3W_vs_E10;stage	1	TRUE
ENSGALG00010011621	WASHC4	PRJNA729271	0.648643573	3.90456E-13	0.648643573	stage_E14_vs_E10	ordinary_muscle_development		0	FALSE
ENSGALG00010002380	CMPK2	PRJNA729271	5.486267222	3.99252E-13	5.486267222	stage_E14_vs_E10	ordinary_muscle_development	D_vs_E10;stage_1W_vs_E10;stage_3W_vs_E10;stage	1	TRUE
ENSGALG00010021026	ADGRA2	PRJNA729271	-1.00296216	4.01248E-13	1.00296216	stage_E14_vs_E10	ordinary_muscle_development	D_vs_E10;stage_1W_vs_E10;stage_3W_vs_E10;stage	1	TRUE
ENSGALG00010010407	SOX10	PRJNA729271	-2.295290761	4.01835E-13	2.295290761	stage_E14_vs_E10	ordinary_muscle_development	D_vs_E10;stage_1W_vs_E10;stage_3W_vs_E10;stage	1	TRUE
ENSGALG00010009367	DMXL1	PRJNA729271	0.884275816	4.02767E-13	0.884275816	stage_E14_vs_E10	ordinary_muscle_development		0	FALSE
ENSGALG00010021118	USMG5	PRJNA729271	1.328581533	4.04002E-13	1.328581533	stage_E14_vs_E10	ordinary_muscle_development	D_vs_E10;stage_1W_vs_E10;stage_3W_vs_E10;stage	1	TRUE
ENSGALG00010003279	ENSGALG00010003279	PRJNA729271	2.631067228	4.07492E-13	2.631067228	stage_E14_vs_E10	ordinary_muscle_development	D_vs_E10;stage_1W_vs_E10;stage_3W_vs_E10;stage	1	TRUE
ENSGALG00010028068	POLE4	PRJNA729271	-0.841365011	4.11088E-13	0.841365011	stage_E14_vs_E10	ordinary_muscle_development		0	FALSE
ENSGALG00010020087	ENSGALG00010020087	PRJNA729271	3.498779049	4.1223E-13	3.498779049	stage_E14_vs_E10	ordinary_muscle_development	D_vs_E10;stage_1W_vs_E10;stage_3W_vs_E10;stage	1	TRUE
ENSGALG00010005978	VPS16	PRJNA729271	0.842342156	4.14341E-13	0.842342156	stage_E14_vs_E10	ordinary_muscle_development		0	FALSE
ENSGALG00010017122	NCBP2	PRJNA729271	-1.443179804	4.14414E-13	1.443179804	stage_E14_vs_E10	ordinary_muscle_development	D_vs_E10;stage_1W_vs_E10;stage_3W_vs_E10;stage	1	TRUE
ENSGALG00010026801	FMOD	PRJNA729271	3.63762676	4.14787E-13	3.63762676	stage_E14_vs_E10	ordinary_muscle_development	D_vs_E10;stage_1W_vs_E10;stage_3W_vs_E10;stage	1	TRUE
ENSGALG00010005509	COCH	PRJNA729271	3.292826748	4.15622E-13	3.292826748	stage_E14_vs_E10	ordinary_muscle_development	D_vs_E10;stage_1W_vs_E10;stage_3W_vs_E10;stage	1	TRUE
ENSGALG00010008035	TDP2	PRJNA729271	-0.79170343	4.15787E-13	0.79170343	stage_E14_vs_E10	ordinary_muscle_development		0	FALSE
ENSGALG00010013204	ENSGALG00010013204	PRJNA729271	2.366844216	4.18654E-13	2.366844216	stage_E14_vs_E10	ordinary_muscle_development	D_vs_E10;stage_1W_vs_E10;stage_3W_vs_E10;stage	1	TRUE
ENSGALG00010025006	ENSGALG00010025006	PRJNA729271	1.921050249	4.23381E-13	1.921050249	stage_E14_vs_E10	ordinary_muscle_development	D_vs_E10;stage_1W_vs_E10;stage_3W_vs_E10;stage	1	TRUE
ENSGALG00010008158	CLPTM1L	PRJNA729271	-0.567570529	4.23381E-13	0.567570529	stage_E14_vs_E10	ordinary_muscle_development		0	FALSE
ENSGALG00010011306	ECI2	PRJNA729271	1.017499859	4.25202E-13	1.017499859	stage_E14_vs_E10	ordinary_muscle_development	D_vs_E10;stage_1W_vs_E10;stage_3W_vs_E10;stage	1	TRUE
ENSGALG00010023178	CFAP20	PRJNA729271	-0.737204885	4.26013E-13	0.737204885	stage_E14_vs_E10	ordinary_muscle_development		0	FALSE
ENSGALG00010016900	CISD2	PRJNA729271	-0.680681277	4.28553E-13	0.680681277	stage_E14_vs_E10	ordinary_muscle_development		0	FALSE
ENSGALG00010002398	ENSGALG00010002398	PRJNA729271	-4.046736862	4.29999E-13	4.046736862	stage_E14_vs_E10	ordinary_muscle_development	D_vs_E10;stage_1W_vs_E10;stage_3W_vs_E10;stage	1	TRUE
ENSGALG00010016876	MTURN	PRJNA729271	-1.452585193	4.30147E-13	1.452585193	stage_E14_vs_E10	ordinary_muscle_development	D_vs_E10;stage_1W_vs_E10;stage_3W_vs_E10;stage	1	TRUE
ENSGALG00010027600	KIRREL1	PRJNA729271	-1.04819883	4.35642E-13	1.04819883	stage_E14_vs_E10	ordinary_muscle_development	D_vs_E10;stage_1W_vs_E10;stage_3W_vs_E10;stage	1	TRUE
ENSGALG00010023930	SRPRA	PRJNA729271	0.966589772	4.4322E-13	0.966589772	stage_E14_vs_E10	ordinary_muscle_development		0	FALSE
ENSGALG00010021795	NDRG3	PRJNA729271	-0.805121185	4.434E-13	0.805121185	stage_E14_vs_E10	ordinary_muscle_development		0	FALSE
ENSGALG00010006172	ENSGALG00010006172	PRJNA729271	1.406745427	4.45376E-13	1.406745427	stage_E14_vs_E10	ordinary_muscle_development	D_vs_E10;stage_1W_vs_E10;stage_3W_vs_E10;stage	1	TRUE
ENSGALG00010005355	FDX1	PRJNA729271	0.756895039	4.48209E-13	0.756895039	stage_E14_vs_E10	ordinary_muscle_development		0	FALSE
ENSGALG00010002423	ENSGALG00010002423	PRJNA729271	4.168052164	4.49111E-13	4.168052164	stage_E14_vs_E10	ordinary_muscle_development	D_vs_E10;stage_1W_vs_E10;stage_3W_vs_E10;stage	1	TRUE
ENSGALG00010003358	DNAJC15	PRJNA729271	0.980319158	4.51015E-13	0.980319158	stage_E14_vs_E10	ordinary_muscle_development		0	FALSE
ENSGALG00010024207	KCTD18	PRJNA729271	-0.935264146	4.52246E-13	0.935264146	stage_E14_vs_E10	ordinary_muscle_development		0	FALSE
ENSGALG00010024049	RCN2	PRJNA729271	-1.065720729	4.53973E-13	1.065720729	stage_E14_vs_E10	ordinary_muscle_development	D_vs_E10;stage_1W_vs_E10;stage_3W_vs_E10;stage	1	TRUE
ENSGALG00010017016	ZIC1	PRJNA729271	-6.776282246	4.54443E-13	6.776282246	stage_E14_vs_E10	ordinary_muscle_development	D_vs_E10;stage_1W_vs_E10;stage_3W_vs_E10;stage	1	TRUE
ENSGALG00010027260	LEMD2	PRJNA729271	0.742415548	4.57575E-13	0.742415548	stage_E14_vs_E10	ordinary_muscle_development		0	FALSE
ENSGALG00010007966	TMEM74	PRJNA729271	-1.671426909	4.57732E-13	1.671426909	stage_E14_vs_E10	ordinary_muscle_development		0	FALSE
ENSGALG00010016756	NUDT16L1	PRJNA729271	-0.800027243	4.59161E-13	0.800027243	stage_E14_vs_E10	ordinary_muscle_development		0	FALSE
ENSGALG00010013502	PAFAH2	PRJNA729271	1.525225518	4.61473E-13	1.525225518	stage_E14_vs_E10	ordinary_muscle_development	D_vs_E10;stage_1W_vs_E10;stage_3W_vs_E10;stage	1	TRUE
ENSGALG00010027612	BAK1	PRJNA729271	-1.217901089	4.62698E-13	1.217901089	stage_E14_vs_E10	ordinary_muscle_development	D_vs_E10;stage_1W_vs_E10;stage_3W_vs_E10;stage	1	TRUE
ENSGALG00010028059	GZMM	PRJNA729271	4.053326687	4.70656E-13	4.053326687	stage_E14_vs_E10	ordinary_muscle_development	D_vs_E10;stage_1W_vs_E10;stage_3W_vs_E10;stage	1	TRUE
ENSGALG00010012854	LECT2	PRJNA729271	5.515915134	4.71733E-13	5.515915134	stage_E14_vs_E10	ordinary_muscle_development	D_vs_E10;stage_1W_vs_E10;stage_3W_vs_E10;stage	1	TRUE
ENSGALG00010023823	MUC2	PRJNA729271	3.799775407	4.727E-13	3.799775407	stage_E14_vs_E10	ordinary_muscle_development	D_vs_E10;stage_1W_vs_E10;stage_3W_vs_E10;stage	1	TRUE
ENSGALG00010020030	MRPL33	PRJNA729271	0.990036527	4.73299E-13	0.990036527	stage_E14_vs_E10	ordinary_muscle_development		0	FALSE
ENSGALG00010006850	IRF2BPL	PRJNA729271	-1.184300481	4.79836E-13	1.184300481	stage_E14_vs_E10	ordinary_muscle_development	D_vs_E10;stage_1W_vs_E10;stage_3W_vs_E10;stage	1	TRUE
ENSGALG00010011875	PSD2	PRJNA729271	-2.196468047	4.83438E-13	2.196468047	stage_E14_vs_E10	ordinary_muscle_development	D_vs_E10;stage_1W_vs_E10;stage_3W_vs_E10;stage	1	TRUE
ENSGALG00010027339	LAMTOR5	PRJNA729271	0.788937174	4.86881E-13	0.788937174	stage_E14_vs_E10	ordinary_muscle_development		0	FALSE
ENSGALG00010028433	MSTO1	PRJNA729271	-0.704788689	4.9171E-13	0.704788689	stage_E14_vs_E10	ordinary_muscle_development		0	FALSE
ENSGALG00010017930	TSC2	PRJNA729271	-0.579831248	4.93376E-13	0.579831248	stage_E14_vs_E10	ordinary_muscle_development		0	FALSE
ENSGALG00010023412	GSS	PRJNA729271	-0.801270159	4.95958E-13	0.801270159	stage_E14_vs_E10	ordinary_muscle_development		0	FALSE
ENSGALG00010006641	ENSGALG00010006641	PRJNA729271	-5.366561824	4.97918E-13	5.366561824	stage_E14_vs_E10	ordinary_muscle_development	D_vs_E10;stage_1W_vs_E10;stage_3W_vs_E10;stage	1	TRUE
ENSGALG00010011300	EVC	PRJNA729271	-1.200408549	5.03121E-13	1.200408549	stage_E14_vs_E10	ordinary_muscle_development	D_vs_E10;stage_1W_vs_E10;stage_3W_vs_E10;stage	1	TRUE
ENSGALG00010013017	SCMH1	PRJNA729271	1.160053609	5.04265E-13	1.160053609	stage_E14_vs_E10	ordinary_muscle_development	D_vs_E10;stage_1W_vs_E10;stage_3W_vs_E10;stage	1	TRUE
ENSGALG00010009149	FUT4	PRJNA729271	-1.567861196	5.06192E-13	1.567861196	stage_E14_vs_E10	ordinary_muscle_development	D_vs_E10;stage_1W_vs_E10;stage_3W_vs_E10;stage	1	TRUE
ENSGALG00010010578	GLUD2	PRJNA729271	0.819346994	5.09692E-13	0.819346994	stage_E14_vs_E10	ordinary_muscle_development		0	FALSE
ENSGALG00010029311	ENSGALG00010029311	PRJNA729271	1.206689104	5.12871E-13	1.206689104	stage_E14_vs_E10	ordinary_muscle_development	D_vs_E10;stage_1W_vs_E10;stage_3W_vs_E10;stage	1	TRUE
ENSGALG00010028200	COG8	PRJNA729271	0.920761328	5.15952E-13	0.920761328	stage_E14_vs_E10	ordinary_muscle_development		0	FALSE
ENSGALG00010001066	SLC33A1	PRJNA729271	-0.656668139	5.17396E-13	0.656668139	stage_E14_vs_E10	ordinary_muscle_development		0	FALSE
ENSGALG00010017927	PCSK2	PRJNA729271	-0.982348405	5.17949E-13	0.982348405	stage_E14_vs_E10	ordinary_muscle_development		0	FALSE
ENSGALG00010023968	TYW5	PRJNA729271	0.827236273	5.25226E-13	0.827236273	stage_E14_vs_E10	ordinary_muscle_development		0	FALSE
ENSGALG00010023588	FARSB	PRJNA729271	-1.009105194	5.30573E-13	1.009105194	stage_E14_vs_E10	ordinary_muscle_development	D_vs_E10;stage_1W_vs_E10;stage_3W_vs_E10;stage	1	TRUE
ENSGALG00010022745	PAPSS2	PRJNA729271	-1.80630738	5.33399E-13	1.80630738	stage_E14_vs_E10	ordinary_muscle_development	D_vs_E10;stage_1W_vs_E10;stage_3W_vs_E10;stage	1	TRUE
ENSGALG00010016312	MECR	PRJNA729271	-1.178292642	5.34034E-13	1.178292642	stage_E14_vs_E10	ordinary_muscle_development	D_vs_E10;stage_1W_vs_E10;stage_3W_vs_E10;stage	1	TRUE
ENSGALG00010002526	ZBTB22	PRJNA729271	0.773034121	5.34454E-13	0.773034121	stage_E14_vs_E10	ordinary_muscle_development		0	FALSE
ENSGALG00010012260	ENSGALG00010012260	PRJNA729271	2.222872883	5.36885E-13	2.222872883	stage_E14_vs_E10	ordinary_muscle_development	D_vs_E10;stage_1W_vs_E10;stage_3W_vs_E10;stage	1	TRUE
ENSGALG00010001790	HOXA2	PRJNA729271	-1.957729091	5.37505E-13	1.957729091	stage_E14_vs_E10	ordinary_muscle_development	D_vs_E10;stage_1W_vs_E10;stage_3W_vs_E10;stage	1	TRUE
ENSGALG00010011213	XKR6	PRJNA729271	1.255236205	5.40021E-13	1.255236205	stage_E14_vs_E10	ordinary_muscle_development	D_vs_E10;stage_1W_vs_E10;stage_3W_vs_E10;stage	1	TRUE
ENSGALG00010001542	MTO1	PRJNA729271	0.939117814	5.49723E-13	0.939117814	stage_E14_vs_E10	ordinary_muscle_development		0	FALSE
ENSGALG00010010635	ALDH9A1	PRJNA729271	-0.567400016	5.59137E-13	0.567400016	stage_E14_vs_E10	ordinary_muscle_development		0	FALSE
ENSGALG00010005015	NRXN3	PRJNA729271	-3.225107597	5.59564E-13	3.225107597	stage_E14_vs_E10	ordinary_muscle_development	D_vs_E10;stage_1W_vs_E10;stage_3W_vs_E10;stage	1	TRUE
ENSGALG00010029260	DHX40	PRJNA729271	-0.778915163	5.60903E-13	0.778915163	stage_E14_vs_E10	ordinary_muscle_development		0	FALSE
ENSGALG00010029658	XYLT2	PRJNA729271	0.910040579	5.61738E-13	0.910040579	stage_E14_vs_E10	ordinary_muscle_development		0	FALSE
ENSGALG00010027341	ENSGALG00010027341	PRJNA729271	3.99939186	5.63661E-13	3.99939186	stage_E14_vs_E10	ordinary_muscle_development	D_vs_E10;stage_1W_vs_E10;stage_3W_vs_E10;stage	1	TRUE
ENSGALG00010023974	PREX1	PRJNA729271	-0.773129447	5.72554E-13	0.773129447	stage_E14_vs_E10	ordinary_muscle_development		0	FALSE

ENSGALG00010028865	STKLD1	PRJNA729271	1.221126896	5.73857E-13	1.221126896	stage_E14_vs_E10	ordinary_muscle_development	D_vs_E10;stage_1W_vs_E10;stage_3W_vs_E10;stage	1	TRUE
ENSGALG00010006015	CDC123	PRJNA729271	-0.828771647	5.74957E-13	0.828771647	stage_E14_vs_E10	ordinary_muscle_development		0	FALSE
ENSGALG00010007979	RAB30	PRJNA729271	-0.664878217	5.77335E-13	0.664878217	stage_E14_vs_E10	ordinary_muscle_development		0	FALSE
ENSGALG00010013115	ENSGALG00010013115	PRJNA729271	-2.830442485	5.7734E-13	2.830442485	stage_E14_vs_E10	ordinary_muscle_development	D_vs_E10;stage_1W_vs_E10;stage_3W_vs_E10;stage	1	TRUE
ENSGALG00010016700	CLUAP1	PRJNA729271	-0.93295475	5.81705E-13	0.93295475	stage_E14_vs_E10	ordinary_muscle_development		0	FALSE
ENSGALG00010008500	ENSGALG00010008500	PRJNA729271	1.111387903	5.84717E-13	1.111387903	stage_E14_vs_E10	ordinary_muscle_development	D_vs_E10;stage_1W_vs_E10;stage_3W_vs_E10;stage	1	TRUE
ENSGALG00010029622	MMP28	PRJNA729271	1.17181353	5.85081E-13	1.17181353	stage_E14_vs_E10	ordinary_muscle_development	D_vs_E10;stage_1W_vs_E10;stage_3W_vs_E10;stage	1	TRUE
ENSGALG00010020377	TMEM230	PRJNA729271	-0.744264035	5.85795E-13	0.744264035	stage_E14_vs_E10	ordinary_muscle_development		0	FALSE
ENSGALG00010002367	STK31	PRJNA729271	-7.514564191	5.85849E-13	7.514564191	stage_E14_vs_E10	ordinary_muscle_development	D_vs_E10;stage_1W_vs_E10;stage_3W_vs_E10;stage	1	TRUE
ENSGALG00010014010	EIF5A2	PRJNA729271	-0.760667851	5.88723E-13	0.760667851	stage_E14_vs_E10	ordinary_muscle_development		0	FALSE
ENSGALG00010017768	FOXN2	PRJNA729271	-0.670083427	5.88723E-13	0.670083427	stage_E14_vs_E10	ordinary_muscle_development		0	FALSE
ENSGALG00010012454	IQCA1	PRJNA729271	2.954712208	5.90337E-13	2.954712208	stage_E14_vs_E10	ordinary_muscle_development	D_vs_E10;stage_1W_vs_E10;stage_3W_vs_E10;stage	1	TRUE
ENSGALG00010019079	CAPG	PRJNA729271	1.768181188	5.95574E-13	1.768181188	stage_E14_vs_E10	ordinary_muscle_development	D_vs_E10;stage_1W_vs_E10;stage_3W_vs_E10;stage	1	TRUE
ENSGALG00010011095	TNN	PRJNA729271	-3.696912117	5.99738E-13	3.696912117	stage_E14_vs_E10	ordinary_muscle_development	D_vs_E10;stage_1W_vs_E10;stage_3W_vs_E10;stage	1	TRUE
ENSGALG00010023704	ANKRD13C	PRJNA729271	-0.51396389	6.01486E-13	0.51396389	stage_E14_vs_E10	ordinary_muscle_development		0	FALSE
ENSGALG00010002819	BMP3	PRJNA729271	-2.590057202	6.01491E-13	2.590057202	stage_E14_vs_E10	ordinary_muscle_development	D_vs_E10;stage_1W_vs_E10;stage_3W_vs_E10;stage	1	TRUE
ENSGALG00010024627	CEPT1	PRJNA729271	0.573942039	6.01491E-13	0.573942039	stage_E14_vs_E10	ordinary_muscle_development		0	FALSE
ENSGALG00010021098	SDK1	PRJNA729271	-1.0474626	6.01849E-13	1.0474626	stage_E14_vs_E10	ordinary_muscle_development	D_vs_E10;stage_1W_vs_E10;stage_3W_vs_E10;stage	1	TRUE
ENSGALG00010012084	TMEM50B	PRJNA729271	0.774741139	6.05516E-13	0.774741139	stage_E14_vs_E10	ordinary_muscle_development		0	FALSE
ENSGALG00010024757	PPFIA1	PRJNA729271	-0.557831955	6.14627E-13	0.557831955	stage_E14_vs_E10	ordinary_muscle_development		0	FALSE
ENSGALG00010022169	TOX2	PRJNA729271	-2.373367715	6.15794E-13	2.373367715	stage_E14_vs_E10	ordinary_muscle_development	D_vs_E10;stage_1W_vs_E10;stage_3W_vs_E10;stage	1	TRUE
ENSGALG00010015002	ADAM19	PRJNA729271	-1.478790407	6.17297E-13	1.478790407	stage_E14_vs_E10	ordinary_muscle_development	D_vs_E10;stage_1W_vs_E10;stage_3W_vs_E10;stage	1	TRUE
ENSGALG00010020576	AKR7L	PRJNA729271	0.763578895	6.18645E-13	0.763578895	stage_E14_vs_E10	ordinary_muscle_development		0	FALSE
ENSGALG00010015140	ASCC3	PRJNA729271	-0.554293818	6.22613E-13	0.554293818	stage_E14_vs_E10	ordinary_muscle_development		0	FALSE
ENSGALG00010017957	THUMPD1	PRJNA729271	-0.733845153	6.26256E-13	0.733845153	stage_E14_vs_E10	ordinary_muscle_development		0	FALSE
ENSGALG00010020682	ZDHHC16	PRJNA729271	1.219154277	6.27086E-13	1.219154277	stage_E14_vs_E10	ordinary_muscle_development	D_vs_E10;stage_1W_vs_E10;stage_3W_vs_E10;stage	1	TRUE
ENSGALG00010004087	NCOA7	PRJNA729271	-0.817862798	6.27387E-13	0.817862798	stage_E14_vs_E10	ordinary_muscle_development		0	FALSE
ENSGALG00010027319	MST1R	PRJNA729271	2.333462329	6.28091E-13	2.333462329	stage_E14_vs_E10	ordinary_muscle_development	D_vs_E10;stage_1W_vs_E10;stage_3W_vs_E10;stage	1	TRUE
ENSGALG00010015087	GABRA3	PRJNA729271	-6.679052927	6.29115E-13	6.679052927	stage_E14_vs_E10	ordinary_muscle_development	D_vs_E10;stage_1W_vs_E10;stage_3W_vs_E10;stage	1	TRUE
ENSGALG00010017054	GALM	PRJNA729271	-1.127059989	6.32028E-13	1.127059989	stage_E14_vs_E10	ordinary_muscle_development	D_vs_E10;stage_1W_vs_E10;stage_3W_vs_E10;stage	1	TRUE
ENSGALG00010028607	ENSGALG00010028607	PRJNA729271	1.75610992	6.36547E-13	1.75610992	stage_E14_vs_E10	ordinary_muscle_development	D_vs_E10;stage_1W_vs_E10;stage_3W_vs_E10;stage	1	TRUE
ENSGALG00010015057	LAS1L	PRJNA729271	-0.833459475	6.43355E-13	0.833459475	stage_E14_vs_E10	ordinary_muscle_development		0	FALSE
ENSGALG00010027287	DHX30	PRJNA729271	-0.658488939	6.44036E-13	0.658488939	stage_E14_vs_E10	ordinary_muscle_development		0	FALSE
ENSGALG00010007845	TFAP2B	PRJNA729271	-8.058541692	6.47069E-13	8.058541692	stage_E14_vs_E10	ordinary_muscle_development	D_vs_E10;stage_1W_vs_E10;stage_3W_vs_E10;stage	1	TRUE
ENSGALG00010004568	RSU1	PRJNA729271	0.490838615	6.51889E-13	0.490838615	stage_E14_vs_E10	ordinary_muscle_development		0	FALSE
ENSGALG00010004445	EPHA6	PRJNA729271	-4.696886523	6.54291E-13	4.696886523	stage_E14_vs_E10	ordinary_muscle_development	D_vs_E10;stage_1W_vs_E10;stage_3W_vs_E10;stage	1	TRUE
ENSGALG00010013553	TADA1	PRJNA729271	0.805304382	6.54603E-13	0.805304382	stage_E14_vs_E10	ordinary_muscle_development		0	FALSE
ENSGALG00010018536	ENSGALG00010018536	PRJNA729271	1.735715494	6.63826E-13	1.735715494	stage_E14_vs_E10	ordinary_muscle_development	D_vs_E10;stage_1W_vs_E10;stage_3W_vs_E10;stage	1	TRUE
ENSGALG00010005187	KBTBD3	PRJNA729271	1.820777078	6.67943E-13	1.820777078	stage_E14_vs_E10	ordinary_muscle_development	D_vs_E10;stage_1W_vs_E10;stage_3W_vs_E10;stage	1	TRUE
ENSGALG00010014217	RASA3	PRJNA729271	0.809157903	6.72301E-13	0.809157903	stage_E14_vs_E10	ordinary_muscle_development		0	FALSE
ENSGALG00010004367	TSPAN13	PRJNA729271	-1.355373051	6.8903E-13	1.355373051	stage_E14_vs_E10	ordinary_muscle_development	D_vs_E10;stage_1W_vs_E10;stage_3W_vs_E10;stage	1	TRUE
ENSGALG00010002402	TXLNG	PRJNA729271	0.927215378	6.95205E-13	0.927215378	stage_E14_vs_E10	ordinary_muscle_development		0	FALSE
ENSGALG00010024195	PSMA4	PRJNA729271	0.881674579	6.958E-13	0.881674579	stage_E14_vs_E10	ordinary_muscle_development		0	FALSE
ENSGALG00010011536	FCHSD1	PRJNA729271	-1.352981375	7.11486E-13	1.352981375	stage_E14_vs_E10	ordinary_muscle_development	D_vs_E10;stage_1W_vs_E10;stage_3W_vs_E10;stage	1	TRUE
ENSGALG00010012089	ENSGALG00010012089	PRJNA729271	-2.110167123	7.11506E-13	2.110167123	stage_E14_vs_E10	ordinary_muscle_development	D_vs_E10;stage_1W_vs_E10;stage_3W_vs_E10;stage	1	TRUE
ENSGALG00010028158	DDX49	PRJNA729271	-0.743373657	7.11593E-13	0.743373657	stage_E14_vs_E10	ordinary_muscle_development		0	FALSE
ENSGALG00010021432	SYT9	PRJNA729271	-2.372008877	7.19393E-13	2.372008877	stage_E14_vs_E10	ordinary_muscle_development	D_vs_E10;stage_1W_vs_E10;stage_3W_vs_E10;stage	1	TRUE
ENSGALG00010003887	GIPC1	PRJNA729271	-1.064735119	7.21975E-13	1.064735119	stage_E14_vs_E10	ordinary_muscle_development	D_vs_E10;stage_1W_vs_E10;stage_3W_vs_E10;stage	1	TRUE
ENSGALG00010011235	ENSGALG00010011235	PRJNA729271	-0.679145114	7.28577E-13	0.679145114	stage_E14_vs_E10	ordinary_muscle_development		0	FALSE
ENSGALG00010004866	SLC25A21	PRJNA729271	-2.400677846	7.32736E-13	2.400677846	stage_E14_vs_E10	ordinary_muscle_development	D_vs_E10;stage_1W_vs_E10;stage_3W_vs_E10;stage	1	TRUE
ENSGALG00010016942	ZBTB40	PRJNA729271	1.100735736	7.3513E-13	1.100735736	stage_E14_vs_E10	ordinary_muscle_development	D_vs_E10;stage_1W_vs_E10;stage_3W_vs_E10;stage	1	TRUE
ENSGALG00010001647	ARL8BL	PRJNA729271	1.110545207	7.35656E-13	1.110545207	stage_E14_vs_E10	ordinary_muscle_development	D_vs_E10;stage_1W_vs_E10;stage_3W_vs_E10;stage	1	TRUE
ENSGALG00010024691	TCTN2	PRJNA729271	-1.120041301	7.3578E-13	1.120041301	stage_E14_vs_E10	ordinary_muscle_development	D_vs_E10;stage_1W_vs_E10;stage_3W_vs_E10;stage	1	TRUE
ENSGALG00010017774	ASH2L	PRJNA729271	1.188387106	7.41461E-13	1.188387106	stage_E14_vs_E10	ordinary_muscle_development	D_vs_E10;stage_1W_vs_E10;stage_3W_vs_E10;stage	1	TRUE
ENSGALG00010008207	CAMK4	PRJNA729271	-1.193256509	7.44086E-13	1.193256509	stage_E14_vs_E10	ordinary_muscle_development	D_vs_E10;stage_1W_vs_E10;stage_3W_vs_E10;stage	1	TRUE
ENSGALG00010000373	ENSGALG00010000373	PRJNA729271	3.351836073	7.44596E-13	3.351836073	stage_E14_vs_E10	ordinary_muscle_development	D_vs_E10;stage_1W_vs_E10;stage_3W_vs_E10;stage	1	TRUE
ENSGALG00010029392	RTN4RL1	PRJNA729271	3.630087775	7.47195E-13	3.630087775	stage_E14_vs_E10	ordinary_muscle_development	D_vs_E10;stage_1W_vs_E10;stage_3W_vs_E10;stage	1	TRUE
ENSGALG00010010298	KCNIP4	PRJNA729271	-3.421758937	7.4859E-13	3.421758937	stage_E14_vs_E10	ordinary_muscle_development	D_vs_E10;stage_1W_vs_E10;stage_3W_vs_E10;stage	1	TRUE
ENSGALG00010017626	COPS7B	PRJNA729271	-1.04562699	7.53598E-13	1.04562699	stage_E14_vs_E10	ordinary_muscle_development	D_vs_E10;stage_1W_vs_E10;stage_3W_vs_E10;stage	1	TRUE
ENSGALG00010012304	ESRP1	PRJNA729271	-6.993984402	7.57947E-13	6.993984402	stage_E14_vs_E10	ordinary_muscle_development	D_vs_E10;stage_1W_vs_E10;stage_3W_vs_E10;stage	1	TRUE
ENSGALG00010007958	ENSGALG00010007958	PRJNA729271	-4.601495894	7.6886E-13	4.601495894	stage_E14_vs_E10	ordinary_muscle_development	D_vs_E10;stage_1W_vs_E10;stage_3W_vs_E10;stage	1	TRUE
ENSGALG00010005910	ENSGALG00010005910	PRJNA729271	3.056222261	7.73127E-13	3.056222261	stage_E14_vs_E10	ordinary_muscle_development	D_vs_E10;stage_1W_vs_E10;stage_3W_vs_E10;stage	1	TRUE
ENSGALG00010026755	ENSGALG00010026755	PRJNA729271	-1.683143386	7.75184E-13	1.683143386	stage_E14_vs_E10	ordinary_muscle_development	D_vs_E10;stage_1W_vs_E10;stage_3W_vs_E10;stage	1	TRUE
ENSGALG00010001853	ENSGALG00010001853	PRJNA729271	1.353689783	7.77827E-13	1.353689783	stage_E14_vs_E10	ordinary_muscle_development	D_vs_E10;stage_1W_vs_E10;stage_3W_vs_E10;stage	1	TRUE
ENSGALG00010007419	RABL2A	PRJNA729271	-1.218124582	7.84359E-13	1.218124582	stage_E14_vs_E10	ordinary_muscle_development	D_vs_E10;stage_1W_vs_E10;stage_3W_vs_E10;stage	1	TRUE
ENSGALG00010017547	RNF4	PRJNA729271	-0.494014475	7.85649E-13	0.494014475	stage_E14_vs_E10	ordinary_muscle_development		0	FALSE
ENSGALG00010017634	PPIH	PRJNA729271	-0.721560178	7.89135E-13	0.721560178	stage_E14_vs_E10	ordinary_muscle_development		0	FALSE
ENSGALG00010017398	SLC22A5	PRJNA729271	-1.374377372	7.91245E-13	1.374377372	stage_E14_vs_E10	ordinary_muscle_development	D_vs_E10;stage_1W_vs_E10;stage_3W_vs_E10;stage	1	TRUE
ENSGALG00010024536	URI1	PRJNA729271	-0.455580822	7.95522E-13	0.455580822	stage_E14_vs_E10	ordinary_muscle_development		0	FALSE
ENSGALG00010021578	ENSGALG00010021578	PRJNA729271	6.797444126	7.9728E-13	6.797444126	stage_E14_vs_E10	ordinary_muscle_development	D_vs_E10;stage_1W_vs_E10;stage_3W_vs_E10;stage	1	TRUE
ENSGALG00010022599	RNLS	PRJNA729271	-2.14893923	8.00372E-13	2.14893923	stage_E14_vs_E10	ordinary_muscle_development	D_vs_E10;stage_1W_vs_E10;stage_3W_vs_E10;stage	1	TRUE
ENSGALG00010005304	ENSGALG00010005304	PRJNA729271	1.509585931	8.00584E-13	1.509585931	stage_E14_vs_E10	ordinary_muscle_development	D_vs_E10;stage_1W_vs_E10;stage_3W_vs_E10;stage	1	TRUE
ENSGALG00010009831	N6AMT1	PRJNA729271	-0.893074122	8.10008E-13	0.893074122	stage_E14_vs_E10	ordinary_muscle_development		0	FALSE
ENSGALG00010023259	TSPAN15	PRJNA729271	2.098931028	8.17662E-13	2.098931028	stage_E14_vs_E10	ordinary_muscle_development	D_vs_E10;stage_1W_vs_E10;stage_3W_vs_E10;stage	1	TRUE
ENSGALG00010017649	ENSGALG00010017649	PRJNA729271	-0.836582233	8.18268E-13	0.836582233	stage_E14_vs_E10	ordinary_muscle_development		0	FALSE
ENSGALG00010027664	TREMB1	PRJNA729271	2.206254967	8.24098E-13	2.206254967	stage_E14_vs_E10	ordinary_muscle_development	D_vs_E10;stage_1W_vs_E10;stage_3W_vs_E10;stage	1	TRUE
ENSGALG00010028558	ENSGALG00010028558	PRJNA729271	10.1510308	8.36495E-13	10.1510308	stage_E14_vs_E10	ordinary_muscle_development	D_vs_E10;stage_1W_vs_E10;stage_3W_vs_E10;stage	1	TRUE
ENSGALG00010028151	DOCK3	PRJNA729271	-2.028351091	8.40224E-13	2.028351091	stage_E14_vs_E10	ordinary_muscle_development	D_vs_E10;stage_1W_vs_E10;stage_3W_vs_E10;stage	1	TRUE
ENSGALG00010028107	NOTCH1	PRJNA729271	-0.911066167	8.40224E-13	0.911066167	stage_E14_vs_E10	ordinary_muscle_development		0	FALSE
ENSGALG00010029547	HIP1	PRJNA729271	-0.737474926	8.43604E-13	0.737474926	stage_E14_vs_E10	ordinary_muscle_development		0	FALSE
ENSGALG00010013334	SH3BGRL3	PRJNA729271	0.889501339	8.4444E-13	0.889501339	stage_E14_vs_E10	ordinary_muscle_development		0	FALSE
ENSGALG00010029517	MSI2	PRJNA729271	0.81081206	8.45618E-13	0.81081206	stage_E14_vs_E10	ordinary_muscle_development		0	FALSE
ENSGALG00010017489	TNFRSF1B	PRJNA729271	1.294976653	8.51235E-13	1.294976653	stage_E14_vs_E10	ordinary_muscle_development	D_vs_E10;stage_1W_vs_E10;stage_3W_vs_E10;stage	1	TRUE
ENSGALG00010013285	ATP2B1	PRJNA729271	-0.798923701	8.53793E-13	0.798923701	stage_E14_vs_E10	ordinary_muscle_development		0	FALSE
ENSGALG00010025331	ENSGALG00010025331	PRJNA729271	2.455874577	8.7024E-13	2.455874577	stage_E14_vs_E10	ordinary_muscle_development	D_vs_E10;stage_1W_vs_E10;stage_3W_vs_E10;stage	1	TRUE
ENSGALG00010001504	RBMS3	PRJNA729271	-0.72862709	8.8859E-13	0.72862709	stage_E14_vs_E10	ordinary_muscle_development		0	FALSE
ENSGALG00010024732	AHCY	PRJNA729271	1.683750283	8.93468E-13	1.683750283	stage_E14_vs_E10	ordinary_muscle_development	D_vs_E10;stage_1W_vs_E10;stage_3W_vs_E10;stage	1	TRUE
ENSGALG00010015055	ZNF335	PRJNA729271	1.323704624	8.98001E-13	1.323704624	stage_E14_vs_E10	ordinary_muscle_development	D_vs_E10;stage_1W_vs_E10;stage_3W_vs_E10;stage	1	TRUE
ENSGALG00010002041	GCLC	PRJNA729271	1.016624924	9.00562E-13	1.016624924	stage_E14_vs_E10	ordinary_muscle_development	D_vs_E10;stage_1W_vs_E10;stage_3W_vs_E10;stage	1	TRUE
ENSGALG00010013393	ADCK2	PRJNA729271	-1.221071302	9.11683E-13	1.221071302	stage_E14_vs_E10	ordinary_muscle_development	D_vs_E10;stage_1W_vs_E10;stage_3W_vs_E10;stage	1	TRUE
ENSGALG00010001238	GOLIM4	PRJNA729271	-0.892320475	9.16905E-13	0.892320475	stage_E14_vs_E10	ordinary_muscle_development		0	FALSE
ENSGALG00010000149	ENSGALG00010000149	PRJNA729271	-1.036251911	9.18678E-13	1.036251911	stage_E14_vs_E10	ordinary_muscle_development	D_vs_E10;stage_1W_vs_E10;stage_3W_vs_E10;stage	1	TRUE
ENSGALG00010003595	SNX13	PRJNA729271	0.687568744	9.23567E-13	0.687568744	stage_E14_vs_E10	ordinary_muscle_development		0	FALSE
ENSGALG00010002634	RGN	PRJNA729271	1.330897813	9.27364E-13	1.330897813	stage_E14_vs_E10	ordinary_muscle_development	D_vs_E10;stage_1W_vs_E10;stage_3W_vs_E10;stage	1	TRUE
ENSGALG00010023677	MN1	PRJNA729271	1.176481669	9.33987E-13	1.176481669	stage_E14_vs_E10	ordinary_muscle_development	D_vs_E10;stage_1W_vs_E10;stage_3W_vs_E10;stage	1	TRUE
ENSGALG00010021197	CHCHD1	PRJNA729271	0.765157323	9.3959E-13	0.765157323	stage_E14_vs_E10	ordinary_muscle_development		0	FALSE
ENSGALG00010010856	CSDC2	PRJNA729271	-6.644028243	9.41109E-13	6.644028243	stage_E14_vs_E10	ordinary_muscle_development	D_vs_E10;stage_1W_vs_E10;stage_3W_vs_E10;stage	1	TRUE
ENSGALG00010006052	RALGAPA1	PRJNA729271	-0.596128757	9.41739E-13	0.596128757	stage_E14_vs_E10	ordinary_muscle_development		0	FALSE
ENSGALG00010010127	FAM13C	PRJNA729271	2.387484439	9.46033E-13	2.387484439	stage_E14_vs_E10	ordinary_muscle_development	D_vs_E10;stage_1W_vs_E10;stage_3W_vs_E10;stage	1	TRUE
ENSGALG00010025846	GRIK4	PRJNA729271	-1.912901627	9.47948E-13	1.912901627	stage_E14_vs_E10	ordinary_muscle_development	D_vs_E10;stage_1W_vs_E10;stage_3W_vs_E10;stage	1	TRUE
ENSGALG00010020385	SLC12A8	PRJNA729271	-4.22124464	9.52691E-13	4.22124464	stage_E14_vs_E10	ordinary_muscle_development	D_vs_E10;stage_1W_vs_E10;stage_3W_vs_E10;stage	1	TRUE
ENSGALG00010016896	SPTBN1	PRJNA729271	-0.718787555	9.656E-13	0.718787555	stage_E14_vs_E10	ordinary_muscle_development		0	FALSE
ENSGALG00010013250	E2F5	PRJNA729271	-0.715002085	9.7561E-13	0.715002085	stage_E14_vs_E10	ordinary_muscle_development		0	FALSE
ENSGALG00010009116	S1PR3	PRJNA729271	-1.347132172	9.82734E-13	1.347132172	stage_E14_vs_E10	ordinary_muscle_development	D_vs_E10;stage_1W_vs_E10;stage_3W_vs_E10;stage	1	TRUE
ENSGALG00010002840	SRPX	PRJNA729271	1.235210427	9.97555E-13	1.235210427	stage_E14_vs_E10	ordinary_muscle_development	D_vs_E10;stage_1W_vs_E10;stage_3W_vs_E10;stage	1	TRUE
ENSGALG00010021648	BRE	PRJNA729271	-0.500164995	1.00256E-12	0.500164995	stage_E14_vs_E10	ordinary_muscle_development		0	FALSE
ENSGALG00010029348	HGS	PRJNA729271	0.894430465	1.00496E-12	0.894430465	stage_E14_vs_E10	ordinary_muscle_development		0	FALSE
ENSGALG00010021867	CXCL12	PRJNA729271	1.335000946	1.01177E-12	1.335000946	stage_E14_vs_E10	ordinary_muscle_development	D_vs_E10;stage_1W_vs_E10;stage_3W_vs_E10;stage	1	TRUE
ENSGALG00010011885	NCALD	PRJNA729271	-1.563791763	1.0138E-12	1.563791763	stage_E14_vs_E10	ordinary_muscle_development	D_vs_E10;stage_1W_vs_E10;stage_3W_vs_E10;stage	1	TRUE
ENSGALG00010001761	ELOB	PRJNA729271	1.079472213	1.0162E-12	1.079472213	stage_E14_vs_E10	ordinary_muscle_development	D_vs_E10;stage_1W_vs_E10;stage_3W_vs_E10;stage	1	TRUE
ENSGALG00010008167	UTP4	PRJNA729271	1.253637078	1.02065E-12	1.253637078	stage_E14_vs_E10	ordinary_muscle_development	D_vs_E10;stage_1W_vs_E10;stage_3W_vs_E10;stage	1	TRUE
ENSGALG00010002647	FAM168A	PRJNA729271	-0.719590435	1.02636E-12	0.719590435	stage_E14_vs_E10	ordinary_muscle_development		0	FALSE
ENSGALG00010013949	PRRC2C	PRJNA729271	-0.927310116	1.02662E-12	0.927310116	stage_E14_vs_E10	ordinary_muscle_development		0	FALSE
ENSGALG00010025172	RBP1	PRJNA729271	6.529473025	1.03288E-12	6.529473025	stage_E14_vs_E10	ordinary_muscle_development	D_vs_E10;stage_1W_vs_E10;stage_3W_vs_E10;stage	1	TRUE
ENSGALG00010022087	SLC35C1	PRJNA729271	-0.549130834	1.0332E-12	0.549130834	stage_E14_vs_E10	ordinary_muscle_development		0	FALSE
ENSGALG00010016766	AOAH	PRJNA729271	2.349356958	1.03326E-12	2.349356958	stage_E14_vs_E10	ordinary_muscle_development	D_vs_E10;stage_1W_vs_E10;stage_3W_vs_E10;stage	1	TRUE
ENSGALG00010028168	PTGES2	PRJNA729271	0.99873412	1.05762E-12	0.99873412	stage_E14_vs_E10	ordinary_muscle_development		0	FALSE
ENSGALG00010012974	SLC49A3	PRJNA729271	-1.624220976	1.05824E-12	1.624220976	stage_E14_vs_E10	ordinary_muscle_development	D_vs_E10;stage_1W_vs_E10;stage_3W_vs_E10;stage	1	TRUE
ENSGALG00010010379	SLC35F6	PRJNA729271	1.356131443	1.07247E-12	1.356131443	stage_E14_vs_E10	ordinary_muscle_development	D_vs_E10;stage_1W_vs_E10;stage_3W_vs_E10;stage	1	TRUE
ENSGALG00010011663	UTP20	PRJNA729271	-1.010555527	1.07921E-12	1.010555527	stage_E14_vs_E10	ordinary_muscle_development	D_vs_E10;stage_1W_vs_E10;stage_3W_vs_E10;stage	1	TRUE
ENSGALG00010008309	ENSGALG00010008309	PRJNA729271	1.983511308	1.09005E-12	1.983511308	stage_E14_vs_E10	ordinary_muscle_development	D_vs_E10;stage_1W_vs_E10;stage_3W_vs_E10;stage	1	TRUE
ENSGALG00010005681	EPC1	PRJNA729271	0.477876208	1.09246E-12	0.477876208	stage_E14_vs_E10	ordinary_muscle_development		0	FALSE
ENSGALG00010017661	NHSL3	PRJNA729271	1.203884749	1.09255E-12	1.203884749	stage_E14_vs_E10	ordinary_muscle_development	D_vs_E10;stage_1W_vs_E10;stage_3W_vs_E10;stage	1	TRUE
ENSGALG00010013339	ATP6V0D2	PRJNA729271	3.890547313	1.0939E-12	3.890547313	stage_E14_vs_E10	ordinary_muscle_development	D_vs_E10;stage_1W_vs_E10;stage_3W_vs_E10;stage	1	TRUE
ENSGALG00010013650	BACE2	PRJNA729271	-1.179550864	1.09492E-12	1.179550864	stage_E14_vs_E10	ordinary_muscle_development	D_vs_E10;stage_1W_vs_E10;stage_3W_vs_E10;stage	1	TRUE
ENSGALG00010010655	C1QTNF7	PRJNA729271	-1.966678693	1.10004E-12	1.966678693	stage_E14_vs_E10	ordinary_muscle_development	D_vs_E10;stage_1W_vs_E10;stage_3W_vs_E10;stage	1	TRUE
ENSGALG00010016562	GLIS2	PRJNA729271	1.595097291	1.11265E-12	1.595097291	stage_E14_vs_E10	ordinary_muscle_development	D_vs_E10;stage_1W_vs_E10;stage_3W_vs_E10;stage	1	TRUE
ENSGALG00010011549	KIAA0232	PRJNA729271	1.047024714	1.12291E-12	1.047024714	stage_E14_vs_E10	ordinary_muscle_development	D_vs_E10;stage_1W_vs_E10;stage_3W_vs_E10;stage	1	TRUE
ENSGALG00010003860	TMEM70	PRJNA729271	0.82343093	1.12612E-12	0.82343093	stage_E14_vs_E10	ordinary_muscle_development		0	FALSE
ENSGALG00010016849	WASF2	PRJNA729271	1.106392456	1.13845E-12	1.106392456	stage_E14_vs_E10	ordinary_muscle_development	D_vs_E10;stage_1W_vs_E10;stage_3W_vs_E10;stage	1	TRUE
ENSGALG00010024545	PHACTR3	PRJNA729271	-2.224723084	1.15385E-12	2.224723084	stage_E14_vs_E10	ordinary_muscle_development	D_vs_E10;stage_1W_vs_E10;stage_3W_vs_E10;stage	1	TRUE
ENSGALG00010021325	ARPC1A	PRJNA729271	-0.479925186	1.18111E-12	0.479925186	stage_E14_vs_E10	ordinary_muscle_development		0	FALSE
ENSGALG00010005057	FLVCR2	PRJNA729271	-2.185122393	1.18692E-12	2.185122393	stage_E14_vs_E10	ordinary_muscle_development	D_vs_E10;stage_1W_vs_E10;stage_3W_vs_E10;stage	1	TRUE
ENSGALG00010021919	PARVA	PRJNA729271	0.903898308	1.18726E-12	0.903898308	stage_E14_vs_E10	ordinary_muscle_development		0	FALSE
ENSGALG00010022120	FOXL3	PRJNA729271	-6.771829066	1.19494E-12	6.771829066	stage_E14_vs_E10	ordinary_muscle_development	D_vs_E10;stage_1W_vs_E10;stage_3W_vs_E10;stage	1	TRUE
ENSGALG00010024243	MPP3	PRJNA729271	1.444865208	1.20256E-12	1.444865208	stage_E14_vs_E10	ordinary_muscle_development	D_vs_E10;stage_1W_vs_E10;stage_3W_vs_E10;stage	1	TRUE
ENSGALG00010006819	EMC8	PRJNA729271	0.950586228	1.20699E-12	0.950586228	stage_E14_vs_E10	ordinary_muscle_development		0	FALSE
ENSGALG00010008013	ENSGALG00010008013	PRJNA729271	1.651860421	1.20781E-12	1.651860421	stage_E14_vs_E10	ordinary_muscle_development	D_vs_E10;stage_1W_vs_E10;stage_3W_vs_E10;stage	1	TRUE
ENSGALG00010009136	KHK	PRJNA729271	2.488095821	1.21691E-12	2.488095821	stage_E14_vs_E10	ordinary_muscle_development	D_vs_E10;stage_1W_vs_E10;stage_3W_vs_E10;stage	1	TRUE
ENSGALG00010023788	RTCA	PRJNA729271	-0.788140672	1.22481E-12	0.788140672	stage_E14_vs_E10	ordinary_muscle_development		0	FALSE
ENSGALG00010025992	MMP11	PRJNA729271	-3.077923441	1.24354E-12	3.077923441	stage_E14_vs_E10	ordinary_muscle_development	D_vs_E10;stage_1W_vs_E10;stage_3W_vs_E10;stage	1	TRUE
ENSGALG00010022073	PTPMT1	PRJNA729271	0.957784133	1.24381E-12	0.957784133	stage_E14_vs_E10	ordinary_muscle_development		0	FALSE
ENSGALG00010011908	ENSGALG00010011908	PRJNA729271	-1.630064441	1.2515E-12	1.630064441	stage_E14_vs_E10	ordinary_muscle_development	D_vs_E10;stage_1W_vs_E10;stage_3W_vs_E10;stage	1	TRUE
ENSGALG00010029774	RFLNB	PRJNA729271	-0.913194487	1.27498E-12	0.913194487	stage_E14_vs_E10	ordinary_muscle_development		0	FALSE
ENSGALG00010017795	MACF1	PRJNA729271	-0.827314636	1.27525E-12	0.827314636	stage_E14_vs_E10	ordinary_muscle_development		0	FALSE
ENSGALG00010004623	MBIP	PRJNA729271	-0.939496672	1.28734E-12	0.939496672	stage_E14_vs_E10	ordinary_muscle_development		0	FALSE
ENSGALG00010024234	ENSGALG00010024234	PRJNA729271	1.970943087	1.28976E-12	1.970943087	stage_E14_vs_E10	ordinary_muscle_development	D_vs_E10;stage_1W_vs_E10;stage_3W_vs_E10;stage	1	TRUE
ENSGALG00010009181	ARID3C	PRJNA729271	-6.748982984	1.30152E-12	6.748982984	stage_E14_vs_E10	ordinary_muscle_development	D_vs_E10;stage_1W_vs_E10;stage_3W_vs_E10;stage	1	TRUE
ENSGALG00010013625	SSBP1	PRJNA729271	-0.821562751	1.304E-12	0.821562751	stage_E14_vs_E10	ordinary_muscle_development		0	FALSE
ENSGALG00010028372	PTGS1	PRJNA729271	1.18137431	1.31545E-12	1.18137431	stage_E14_vs_E10	ordinary_muscle_development	D_vs_E10;stage_1W_vs_E10;stage_3W_vs_E10;stage	1	TRUE
ENSGALG00010000127	ENSGALG00010000127	PRJNA729271	1.476549413	1.32435E-12	1.476549413	stage_E14_vs_E10	ordinary_muscle_development	D_vs_E10;stage_1W_vs_E10;stage_3W_vs_E10;stage	1	TRUE
ENSGALG00010027332	HDGF	PRJNA729271	1.275289862	1.33079E-12	1.275289862	stage_E14_vs_E10	ordinary_muscle_development	D_vs_E10;stage_1W_vs_E10;stage_3W_vs_E10;stage	1	TRUE
ENSGALG00010001877	OAT	PRJNA729271	0.987459822	1.33549E-12	0.987459822	stage_E14_vs_E10	ordinary_muscle_development		0	FALSE
ENSGALG00010020745	ABCC1	PRJNA729271	1.202865273	1.34032E-12	1.202865273	stage_E14_vs_E10	ordinary_muscle_development	D_vs_E10;stage_1W_vs_E10;stage_3W_vs_E10;stage	1	TRUE
ENSGALG00010017405	XCR1	PRJNA729271	6.518137171	1.34216E-12	6.518137171	stage_E14_vs_E10	ordinary_muscle_development	D_vs_E10;stage_1W_vs_E10;stage_3W_vs_E10;stage	1	TRUE
ENSGALG00010018312	PRPF39	PRJNA729271	-0.575594005	1.34332E-12	0.575594005	stage_E14_vs_E10	ordinary_muscle_development		0	FALSE
ENSGALG00010003438	KCNS3	PRJNA729271	-1.278707936	1.34397E-12	1.278707936	stage_E14_vs_E10	ordinary_muscle_development	D_vs_E10;stage_1W_vs_E10;stage_3W_vs_E10;stage	1	TRUE
ENSGALG00010009722	GBA2	PRJNA729271	0.690211214	1.35375E-12	0.690211214	stage_E14_vs_E10	ordinary_muscle_development		0	FALSE
ENSGALG00010022137	PDDC1	PRJNA729271	-0.938031717	1.3596E-12	0.938031717	stage_E14_vs_E10	ordinary_muscle_development		0	FALSE
ENSGALG00010029273	SLC13A5	PRJNA729271	-3.607603327	1.35972E-12	3.607603327	stage_E14_vs_E10	ordinary_muscle_development	D_vs_E10;stage_1W_vs_E10;stage_3W_vs_E10;stage	1	TRUE
ENSGALG00010002348	THAP12	PRJNA729271	-1.168534477	1.36122E-12	1.168534477	stage_E14_vs_E10	ordinary_muscle_development	D_vs_E10;stage_1W_vs_E10;stage_3W_vs_E10;stage	1	TRUE
ENSGALG00010020916	ARL6IP1	PRJNA729271	0.719162375	1.36326E-12	0.719162375	stage_E14_vs_E10	ordinary_muscle_development		0	FALSE
ENSGALG00010002433	GRHL1	PRJNA729271	-2.426259059	1.3729E-12	2.426259059	stage_E14_vs_E10	ordinary_muscle_development	D_vs_E10;stage_1W_vs_E10;stage_3W_vs_E10;stage	1	TRUE
ENSGALG00010025330	USH1C	PRJNA729271	-3.065305365	1.40586E-12	3.065305365	stage_E14_vs_E10	ordinary_muscle_development	D_vs_E10;stage_1W_vs_E10;stage_3W_vs_E10;stage	1	TRUE
ENSGALG00010024844	TM4SF19	PRJNA729271	2.694778696	1.41283E-12	2.694778696	stage_E14_vs_E10	ordinary_muscle_development	D_vs_E10;stage_1W_vs_E10;stage_3W_vs_E10;stage	1	TRUE
ENSGALG00010011893	LIX1	PRJNA729271	-6.243928718	1.42496E-12	6.243928718	stage_E14_vs_E10	ordinary_muscle_development	D_vs_E10;stage_1W_vs_E10;stage_3W_vs_E10;stage	1	TRUE
ENSGALG00010001138	SPTSSB	PRJNA729271	-7.217341426	1.42738E-12	7.217341426	stage_E14_vs_E10	ordinary_muscle_development	D_vs_E10;stage_1W_vs_E10;stage_3W_vs_E10;stage	1	TRUE
ENSGALG00010022747	NT5C3B	PRJNA729271	-1.014754522	1.4346E-12	1.014754522	stage_E14_vs_E10	ordinary_muscle_development	D_vs_E10;stage_1W_vs_E10;stage_3W_vs_E10;stage	1	TRUE
ENSGALG00010006833	LTV1	PRJNA729271	-0.634089606	1.44694E-12	0.634089606	stage_E14_vs_E10	ordinary_muscle_development		0	FALSE
ENSGALG00010016731	C4orf48	PRJNA729271	-2.018485521	1.45577E-12	2.018485521	stage_E14_vs_E10	ordinary_muscle_development	D_vs_E10;stage_1W_vs_E10;stage_3W_vs_E10;stage	1	TRUE
ENSGALG00010021731	SRM	PRJNA729271	-0.883964629	1.45723E-12	0.883964629	stage_E14_vs_E10	ordinary_muscle_development		0	FALSE
ENSGALG00010019823	LCT	PRJNA729271	-1.638829528	1.45853E-12	1.638829528	stage_E14_vs_E10	ordinary_muscle_development	D_vs_E10;stage_1W_vs_E10;stage_3W_vs_E10;stage	1	TRUE
ENSGALG00010008928	CFC1	PRJNA729271	-6.612298555	1.45974E-12	6.612298555	stage_E14_vs_E10	ordinary_muscle_development	D_vs_E10;stage_1W_vs_E10;stage_3W_vs_E10;stage	1	TRUE
ENSGALG00010022803	ENSGALG00010022803	PRJNA729271	1.110825999	1.46091E-12	1.110825999	stage_E14_vs_E10	ordinary_muscle_development	D_vs_E10;stage_1W_vs_E10;stage_3W_vs_E10;stage	1	TRUE
ENSGALG00010017671	ANTXR1	PRJNA729271	-1.342429992	1.46548E-12	1.342429992	stage_E14_vs_E10	ordinary_muscle_development	D_vs_E10;stage_1W_vs_E10;stage_3W_vs_E10;stage	1	TRUE

ENSGALG00010029367	DUSP14	PRJNA729271	-0.854915528	1.49283E-12	0.854915528	stage_E14_vs_E10	ordinary_muscle_development		0	FALSE
ENSGALG00010024611	ENSGALG00010024611	PRJNA729271	-1.991020735	1.50192E-12	1.991020735	stage_E14_vs_E10	ordinary_muscle_development	D_vs_E10;stage_1W_vs_E10;stage_3W_vs_E10;stage	1	TRUE
ENSGALG00010029419	RFC2	PRJNA729271	-0.965536116	1.50364E-12	0.965536116	stage_E14_vs_E10	ordinary_muscle_development		0	FALSE
ENSGALG00010000267	ENSGALG00010000267	PRJNA729271	0.866976791	1.5144E-12	0.866976791	stage_E14_vs_E10	ordinary_muscle_development		0	FALSE
ENSGALG00010029696	SUMO2	PRJNA729271	-0.793100719	1.51649E-12	0.793100719	stage_E14_vs_E10	ordinary_muscle_development		0	FALSE
ENSGALG00010029259	PTRH2	PRJNA729271	-0.791028372	1.57809E-12	0.791028372	stage_E14_vs_E10	ordinary_muscle_development		0	FALSE
ENSGALG00010007956	HS1BP3	PRJNA729271	0.956857009	1.60235E-12	0.956857009	stage_E14_vs_E10	ordinary_muscle_development		0	FALSE
ENSGALG00010020875	ENSGALG00010020875	PRJNA729271	1.244033389	1.60754E-12	1.244033389	stage_E14_vs_E10	ordinary_muscle_development	D_vs_E10;stage_1W_vs_E10;stage_3W_vs_E10;stage	1	TRUE
ENSGALG00010009388	TMED7	PRJNA729271	-0.537878799	1.61205E-12	0.537878799	stage_E14_vs_E10	ordinary_muscle_development		0	FALSE
ENSGALG00010016793	SLC9A1	PRJNA729271	1.112454357	1.6144E-12	1.112454357	stage_E14_vs_E10	ordinary_muscle_development	D_vs_E10;stage_1W_vs_E10;stage_3W_vs_E10;stage	1	TRUE
ENSGALG00010017922	MERTK	PRJNA729271	0.93822586	1.62863E-12	0.93822586	stage_E14_vs_E10	ordinary_muscle_development		0	FALSE
ENSGALG00010019997	FSHR	PRJNA729271	2.918111335	1.63401E-12	2.918111335	stage_E14_vs_E10	ordinary_muscle_development	D_vs_E10;stage_1W_vs_E10;stage_3W_vs_E10;stage	1	TRUE
ENSGALG00010015867	PICALML	PRJNA729271	0.407386258	1.64861E-12	0.407386258	stage_E14_vs_E10	ordinary_muscle_development		0	FALSE
ENSGALG00010002622	AZI2	PRJNA729271	0.573048148	1.66378E-12	0.573048148	stage_E14_vs_E10	ordinary_muscle_development		0	FALSE
ENSGALG00010014742	PROZ	PRJNA729271	20.49415553	1.68523E-12	20.49415553	stage_E14_vs_E10	ordinary_muscle_development	D_vs_E10;stage_1W_vs_E10;stage_3W_vs_E10;stage	1	TRUE
ENSGALG00010000501	ENSGALG00010000501	PRJNA729271	1.159857684	1.68624E-12	1.159857684	stage_E14_vs_E10	ordinary_muscle_development	D_vs_E10;stage_1W_vs_E10;stage_3W_vs_E10;stage	1	TRUE
ENSGALG00010004358	GC	PRJNA729271	-16.35242069	1.69065E-12	16.35242069	stage_E14_vs_E10	ordinary_muscle_development	D_vs_E10;stage_1W_vs_E10;stage_3W_vs_E10;stage	1	TRUE
ENSGALG00010018882	HHAT	PRJNA729271	-1.211783946	1.69205E-12	1.211783946	stage_E14_vs_E10	ordinary_muscle_development	D_vs_E10;stage_1W_vs_E10;stage_3W_vs_E10;stage	1	TRUE
ENSGALG00010011819	USP44	PRJNA729271	0.995347028	1.69459E-12	0.995347028	stage_E14_vs_E10	ordinary_muscle_development		0	FALSE
ENSGALG00010021788	RPL5	PRJNA729271	0.899326226	1.72686E-12	0.899326226	stage_E14_vs_E10	ordinary_muscle_development		0	FALSE
ENSGALG00010012088	ATP5F1AZ	PRJNA729271	1.227628941	1.734E-12	1.227628941	stage_E14_vs_E10	ordinary_muscle_development	D_vs_E10;stage_1W_vs_E10;stage_3W_vs_E10;stage	1	TRUE
ENSGALG00010014209	CCDC50	PRJNA729271	-0.774347595	1.73582E-12	0.774347595	stage_E14_vs_E10	ordinary_muscle_development		0	FALSE
ENSGALG00010020692	TUB	PRJNA729271	1.124988053	1.74668E-12	1.124988053	stage_E14_vs_E10	ordinary_muscle_development	D_vs_E10;stage_1W_vs_E10;stage_3W_vs_E10;stage	1	TRUE
ENSGALG00010018061	NDUFAF5	PRJNA729271	0.970762904	1.78999E-12	0.970762904	stage_E14_vs_E10	ordinary_muscle_development		0	FALSE
ENSGALG00010029469	ENSGALG00010029469	PRJNA729271	-3.008435859	1.80727E-12	3.008435859	stage_E14_vs_E10	ordinary_muscle_development	D_vs_E10;stage_1W_vs_E10;stage_3W_vs_E10;stage	1	TRUE
ENSGALG00010011971	RNF170	PRJNA729271	-0.655283866	1.80902E-12	0.655283866	stage_E14_vs_E10	ordinary_muscle_development		0	FALSE
ENSGALG00010029664	RAB37	PRJNA729271	2.663695146	1.82173E-12	2.663695146	stage_E14_vs_E10	ordinary_muscle_development	D_vs_E10;stage_1W_vs_E10;stage_3W_vs_E10;stage	1	TRUE
ENSGALG00010020170	SORCS1	PRJNA729271	-2.247134122	1.8259E-12	2.247134122	stage_E14_vs_E10	ordinary_muscle_development	D_vs_E10;stage_1W_vs_E10;stage_3W_vs_E10;stage	1	TRUE
ENSGALG00010009541	PMPCB	PRJNA729271	0.658193138	1.83372E-12	0.658193138	stage_E14_vs_E10	ordinary_muscle_development		0	FALSE
ENSGALG00010019998	PLEK2	PRJNA729271	-6.862772039	1.83442E-12	6.862772039	stage_E14_vs_E10	ordinary_muscle_development	D_vs_E10;stage_1W_vs_E10;stage_3W_vs_E10;stage	1	TRUE
ENSGALG00010009806	WDCP	PRJNA729271	-1.269098743	1.83746E-12	1.269098743	stage_E14_vs_E10	ordinary_muscle_development	D_vs_E10;stage_1W_vs_E10;stage_3W_vs_E10;stage	1	TRUE
ENSGALG00010002122	ENSGALG00010002122	PRJNA729271	3.636229002	1.8401E-12	3.636229002	stage_E14_vs_E10	ordinary_muscle_development	D_vs_E10;stage_1W_vs_E10;stage_3W_vs_E10;stage	1	TRUE
ENSGALG00010010408	GAS8	PRJNA729271	-0.864759594	1.84017E-12	0.864759594	stage_E14_vs_E10	ordinary_muscle_development		0	FALSE
ENSGALG00010009097	C1QTNF2	PRJNA729271	1.313317449	1.84337E-12	1.313317449	stage_E14_vs_E10	ordinary_muscle_development	D_vs_E10;stage_1W_vs_E10;stage_3W_vs_E10;stage	1	TRUE
ENSGALG00010009490	SNTB1	PRJNA729271	1.150802765	1.84653E-12	1.150802765	stage_E14_vs_E10	ordinary_muscle_development	D_vs_E10;stage_1W_vs_E10;stage_3W_vs_E10;stage	1	TRUE
ENSGALG00010006270	STAM	PRJNA729271	0.662853356	1.85359E-12	0.662853356	stage_E14_vs_E10	ordinary_muscle_development		0	FALSE
ENSGALG00010029281	KIAA0753	PRJNA729271	-0.973842488	1.86051E-12	0.973842488	stage_E14_vs_E10	ordinary_muscle_development		0	FALSE
ENSGALG00010024208	SERPIND1	PRJNA729271	4.175448387	1.86399E-12	4.175448387	stage_E14_vs_E10	ordinary_muscle_development	D_vs_E10;stage_1W_vs_E10;stage_3W_vs_E10;stage	1	TRUE
ENSGALG00010016372	DNAAF6	PRJNA729271	-1.497578465	1.87216E-12	1.497578465	stage_E14_vs_E10	ordinary_muscle_development	D_vs_E10;stage_1W_vs_E10;stage_3W_vs_E10;stage	1	TRUE
ENSGALG00010022657	MRPS35	PRJNA729271	0.797825992	1.87286E-12	0.797825992	stage_E14_vs_E10	ordinary_muscle_development		0	FALSE
ENSGALG00010021862	PDK1	PRJNA729271	-0.769969955	1.88383E-12	0.769969955	stage_E14_vs_E10	ordinary_muscle_development		0	FALSE
ENSGALG00010014303	GABRA2	PRJNA729271	-3.922811669	1.89667E-12	3.922811669	stage_E14_vs_E10	ordinary_muscle_development	D_vs_E10;stage_1W_vs_E10;stage_3W_vs_E10;stage	1	TRUE
ENSGALG00010005997	ENSGALG00010005997	PRJNA729271	1.658450554	1.92001E-12	1.658450554	stage_E14_vs_E10	ordinary_muscle_development	D_vs_E10;stage_1W_vs_E10;stage_3W_vs_E10;stage	1	TRUE
ENSGALG00010021757	ZP4	PRJNA729271	2.157769691	1.9465E-12	2.157769691	stage_E14_vs_E10	ordinary_muscle_development	D_vs_E10;stage_1W_vs_E10;stage_3W_vs_E10;stage	1	TRUE
ENSGALG00010016518	TMEM42	PRJNA729271	-1.119788485	1.98772E-12	1.119788485	stage_E14_vs_E10	ordinary_muscle_development	D_vs_E10;stage_1W_vs_E10;stage_3W_vs_E10;stage	1	TRUE
ENSGALG00010023621	CD79B	PRJNA729271	6.213888451	2.02753E-12	6.213888451	stage_E14_vs_E10	ordinary_muscle_development	D_vs_E10;stage_1W_vs_E10;stage_3W_vs_E10;stage	1	TRUE
ENSGALG00010005035	NECTIN3	PRJNA729271	0.703027454	2.05658E-12	0.703027454	stage_E14_vs_E10	ordinary_muscle_development		0	FALSE
ENSGALG00010027733	RPL35	PRJNA729271	1.114130279	2.05788E-12	1.114130279	stage_E14_vs_E10	ordinary_muscle_development	D_vs_E10;stage_1W_vs_E10;stage_3W_vs_E10;stage	1	TRUE
ENSGALG00010026707	GORASP1	PRJNA729271	-0.60017494	2.0802E-12	0.60017494	stage_E14_vs_E10	ordinary_muscle_development		0	FALSE
ENSGALG00010020595	ZFYVE27	PRJNA729271	0.93611346	2.08065E-12	0.93611346	stage_E14_vs_E10	ordinary_muscle_development		0	FALSE
ENSGALG00010022583	FKBP11	PRJNA729271	0.839097709	2.08787E-12	0.839097709	stage_E14_vs_E10	ordinary_muscle_development		0	FALSE
ENSGALG00010022868	VAC14	PRJNA729271	-0.73582598	2.11747E-12	0.73582598	stage_E14_vs_E10	ordinary_muscle_development		0	FALSE
ENSGALG00010011388	GPR50	PRJNA729271	1.162813443	2.13723E-12	1.162813443	stage_E14_vs_E10	ordinary_muscle_development	D_vs_E10;stage_1W_vs_E10;stage_3W_vs_E10;stage	1	TRUE
ENSGALG00010026427	PGC	PRJNA729271	7.535809921	2.13879E-12	7.535809921	stage_E14_vs_E10	ordinary_muscle_development	D_vs_E10;stage_1W_vs_E10;stage_3W_vs_E10;stage	1	TRUE
ENSGALG00010017970	AMPH	PRJNA729271	4.730790082	2.15311E-12	4.730790082	stage_E14_vs_E10	ordinary_muscle_development	D_vs_E10;stage_1W_vs_E10;stage_3W_vs_E10;stage	1	TRUE
ENSGALG00010024349	R3HDML	PRJNA729271	-5.277186799	2.16522E-12	5.277186799	stage_E14_vs_E10	ordinary_muscle_development	D_vs_E10;stage_1W_vs_E10;stage_3W_vs_E10;stage	1	TRUE
ENSGALG00010006259	PPA2	PRJNA729271	0.622671029	2.19437E-12	0.622671029	stage_E14_vs_E10	ordinary_muscle_development		0	FALSE
ENSGALG00010021396	MED6	PRJNA729271	-0.584170664	2.20702E-12	0.584170664	stage_E14_vs_E10	ordinary_muscle_development		0	FALSE
ENSGALG00010023320	ENSGALG00010023320	PRJNA729271	-1.005946422	2.22909E-12	1.005946422	stage_E14_vs_E10	ordinary_muscle_development	D_vs_E10;stage_1W_vs_E10;stage_3W_vs_E10;stage	1	TRUE
ENSGALG00010011480	BLTP3B	PRJNA729271	-0.471176107	2.23358E-12	0.471176107	stage_E14_vs_E10	ordinary_muscle_development		0	FALSE
ENSGALG00010029460	ENSGALG00010029460	PRJNA729271	-2.062379821	2.2418E-12	2.062379821	stage_E14_vs_E10	ordinary_muscle_development	D_vs_E10;stage_1W_vs_E10;stage_3W_vs_E10;stage	1	TRUE
ENSGALG00010010356	STAB2	PRJNA729271	-2.246645207	2.24928E-12	2.246645207	stage_E14_vs_E10	ordinary_muscle_development	D_vs_E10;stage_1W_vs_E10;stage_3W_vs_E10;stage	1	TRUE
ENSGALG00010000284	ENSGALG00010000284	PRJNA729271	-1.125821889	2.26261E-12	1.125821889	stage_E14_vs_E10	ordinary_muscle_development	D_vs_E10;stage_1W_vs_E10;stage_3W_vs_E10;stage	1	TRUE
ENSGALG00010027530	PHF19	PRJNA729271	0.969764177	2.2628E-12	0.969764177	stage_E14_vs_E10	ordinary_muscle_development		0	FALSE
ENSGALG00010017894	KAT6A	PRJNA729271	0.580437802	2.32103E-12	0.580437802	stage_E14_vs_E10	ordinary_muscle_development		0	FALSE
ENSGALG00010020133	ENSGALG00010020133	PRJNA729271	2.642179452	2.34988E-12	2.642179452	stage_E14_vs_E10	ordinary_muscle_development	D_vs_E10;stage_1W_vs_E10;stage_3W_vs_E10;stage	1	TRUE
ENSGALG00010016559	ROGDI	PRJNA729271	-0.84314307	2.36011E-12	0.84314307	stage_E14_vs_E10	ordinary_muscle_development		0	FALSE
ENSGALG00010016423	TRMT44	PRJNA729271	-0.807110004	2.37409E-12	0.807110004	stage_E14_vs_E10	ordinary_muscle_development		0	FALSE
ENSGALG00010003597	RNF144A	PRJNA729271	-1.053082152	2.3992E-12	1.053082152	stage_E14_vs_E10	ordinary_muscle_development	D_vs_E10;stage_1W_vs_E10;stage_3W_vs_E10;stage	1	TRUE
ENSGALG00010028743	ARRDC1	PRJNA729271	1.334881804	2.41027E-12	1.334881804	stage_E14_vs_E10	ordinary_muscle_development	D_vs_E10;stage_1W_vs_E10;stage_3W_vs_E10;stage	1	TRUE
ENSGALG00010001895	P4HA3	PRJNA729271	-1.719647174	2.41063E-12	1.719647174	stage_E14_vs_E10	ordinary_muscle_development	D_vs_E10;stage_1W_vs_E10;stage_3W_vs_E10;stage	1	TRUE
ENSGALG00010003166	PGAP2	PRJNA729271	0.781646769	2.42344E-12	0.781646769	stage_E14_vs_E10	ordinary_muscle_development		0	FALSE
ENSGALG00010011575	ENSGALG00010011575	PRJNA729271	-4.483419597	2.43003E-12	4.483419597	stage_E14_vs_E10	ordinary_muscle_development	D_vs_E10;stage_1W_vs_E10;stage_3W_vs_E10;stage	1	TRUE
ENSGALG00010023533	FKBP7	PRJNA729271	-1.167766655	2.44988E-12	1.167766655	stage_E14_vs_E10	ordinary_muscle_development	D_vs_E10;stage_1W_vs_E10;stage_3W_vs_E10;stage	1	TRUE
ENSGALG00010013352	ASXL1	PRJNA729271	-0.637037736	2.45965E-12	0.637037736	stage_E14_vs_E10	ordinary_muscle_development		0	FALSE
ENSGALG00010017866	RALA	PRJNA729271	-0.685818553	2.46204E-12	0.685818553	stage_E14_vs_E10	ordinary_muscle_development		0	FALSE
ENSGALG00010007948	ENSGALG00010007948	PRJNA729271	-3.893134876	2.50341E-12	3.893134876	stage_E14_vs_E10	ordinary_muscle_development	D_vs_E10;stage_1W_vs_E10;stage_3W_vs_E10;stage	1	TRUE
ENSGALG00010023261	SNX1	PRJNA729271	-0.688559312	2.5043E-12	0.688559312	stage_E14_vs_E10	ordinary_muscle_development		0	FALSE
ENSGALG00010022397	ATF1	PRJNA729271	-0.345492413	2.51189E-12	0.345492413	stage_E14_vs_E10	ordinary_muscle_development		0	FALSE
ENSGALG00010028861	SURF4	PRJNA729271	-0.535021509	2.52018E-12	0.535021509	stage_E14_vs_E10	ordinary_muscle_development		0	FALSE
ENSGALG00010019264	HK2	PRJNA729271	1.529433623	2.53681E-12	1.529433623	stage_E14_vs_E10	ordinary_muscle_development	D_vs_E10;stage_1W_vs_E10;stage_3W_vs_E10;stage	1	TRUE
ENSGALG00010023896	POP4	PRJNA729271	-0.932390801	2.55421E-12	0.932390801	stage_E14_vs_E10	ordinary_muscle_development		0	FALSE
ENSGALG00010005224	ENSGALG00010005224	PRJNA729271	2.707705127	2.58623E-12	2.707705127	stage_E14_vs_E10	ordinary_muscle_development	D_vs_E10;stage_1W_vs_E10;stage_3W_vs_E10;stage	1	TRUE
ENSGALG00010021330	COMMD4	PRJNA729271	-0.946848266	2.59325E-12	0.946848266	stage_E14_vs_E10	ordinary_muscle_development		0	FALSE
ENSGALG00010019600	MAP3K19	PRJNA729271	-1.920060692	2.59487E-12	1.920060692	stage_E14_vs_E10	ordinary_muscle_development	D_vs_E10;stage_1W_vs_E10;stage_3W_vs_E10;stage	1	TRUE
ENSGALG00010001277	ENSGALG00010001277	PRJNA729271	1.151029576	2.59887E-12	1.151029576	stage_E14_vs_E10	ordinary_muscle_development	D_vs_E10;stage_1W_vs_E10;stage_3W_vs_E10;stage	1	TRUE
ENSGALG00010001987	KCTD14	PRJNA729271	-4.383762881	2.60263E-12	4.383762881	stage_E14_vs_E10	ordinary_muscle_development	D_vs_E10;stage_1W_vs_E10;stage_3W_vs_E10;stage	1	TRUE
ENSGALG00010028438	MED27	PRJNA729271	-0.527214284	2.61444E-12	0.527214284	stage_E14_vs_E10	ordinary_muscle_development		0	FALSE
ENSGALG00010006328	SYNDIG1L	PRJNA729271	-3.635252058	2.62358E-12	3.635252058	stage_E14_vs_E10	ordinary_muscle_development	D_vs_E10;stage_1W_vs_E10;stage_3W_vs_E10;stage	1	TRUE
ENSGALG00010009700	SH3TC1	PRJNA729271	1.478229828	2.63757E-12	1.478229828	stage_E14_vs_E10	ordinary_muscle_development	D_vs_E10;stage_1W_vs_E10;stage_3W_vs_E10;stage	1	TRUE
ENSGALG00010012315	ATP6V1E1	PRJNA729271	0.71792376	2.63866E-12	0.71792376	stage_E14_vs_E10	ordinary_muscle_development		0	FALSE
ENSGALG00010029804	ITGB4	PRJNA729271	1.6118842	2.64553E-12	1.6118842	stage_E14_vs_E10	ordinary_muscle_development	D_vs_E10;stage_1W_vs_E10;stage_3W_vs_E10;stage	1	TRUE
ENSGALG00010001113	IGSF10	PRJNA729271	-2.116979236	2.6599E-12	2.116979236	stage_E14_vs_E10	ordinary_muscle_development	D_vs_E10;stage_1W_vs_E10;stage_3W_vs_E10;stage	1	TRUE
ENSGALG00010000298	SLC35E4	PRJNA729271	1.518640872	2.66728E-12	1.518640872	stage_E14_vs_E10	ordinary_muscle_development	D_vs_E10;stage_1W_vs_E10;stage_3W_vs_E10;stage	1	TRUE
ENSGALG00010015199	ENSGALG00010015199	PRJNA729271	-1.288289183	2.68504E-12	1.288289183	stage_E14_vs_E10	ordinary_muscle_development	D_vs_E10;stage_1W_vs_E10;stage_3W_vs_E10;stage	1	TRUE
ENSGALG00010028493	TOR1A	PRJNA729271	-0.634985414	2.72365E-12	0.634985414	stage_E14_vs_E10	ordinary_muscle_development		0	FALSE
ENSGALG00010008936	IFT172	PRJNA729271	-0.684493658	2.73004E-12	0.684493658	stage_E14_vs_E10	ordinary_muscle_development		0	FALSE
ENSGALG00010014396	ING1	PRJNA729271	0.831672234	2.75957E-12	0.831672234	stage_E14_vs_E10	ordinary_muscle_development		0	FALSE
ENSGALG00010010383	SLIT2	PRJNA729271	-1.14656893	2.76013E-12	1.14656893	stage_E14_vs_E10	ordinary_muscle_development	D_vs_E10;stage_1W_vs_E10;stage_3W_vs_E10;stage	1	TRUE
ENSGALG00010004928	HDDC2	PRJNA729271	-0.88245002	2.76858E-12	0.88245002	stage_E14_vs_E10	ordinary_muscle_development		0	FALSE
ENSGALG00010020646	CEP70	PRJNA729271	-0.839414809	2.7772E-12	0.839414809	stage_E14_vs_E10	ordinary_muscle_development		0	FALSE
ENSGALG00010013698	TFAP2E	PRJNA729271	-6.871927816	2.8059E-12	6.871927816	stage_E14_vs_E10	ordinary_muscle_development	D_vs_E10;stage_1W_vs_E10;stage_3W_vs_E10;stage	1	TRUE
ENSGALG00010005352	ENSGALG00010005352	PRJNA729271	4.169586962	2.81385E-12	4.169586962	stage_E14_vs_E10	ordinary_muscle_development	D_vs_E10;stage_1W_vs_E10;stage_3W_vs_E10;stage	1	TRUE
ENSGALG00010029402	SMG6	PRJNA729271	0.644366921	2.84117E-12	0.644366921	stage_E14_vs_E10	ordinary_muscle_development		0	FALSE
ENSGALG00010024724	HOXD3	PRJNA729271	-1.87138459	2.88592E-12	1.87138459	stage_E14_vs_E10	ordinary_muscle_development	D_vs_E10;stage_1W_vs_E10;stage_3W_vs_E10;stage	1	TRUE
ENSGALG00010029738	TRPV2	PRJNA729271	1.187217798	2.89099E-12	1.187217798	stage_E14_vs_E10	ordinary_muscle_development	D_vs_E10;stage_1W_vs_E10;stage_3W_vs_E10;stage	1	TRUE
ENSGALG00010017667	SLC2A1	PRJNA729271	-0.797693458	2.89099E-12	0.797693458	stage_E14_vs_E10	ordinary_muscle_development		0	FALSE
ENSGALG00010029300	TEKT1	PRJNA729271	2.43942685	2.90144E-12	2.43942685	stage_E14_vs_E10	ordinary_muscle_development	D_vs_E10;stage_1W_vs_E10;stage_3W_vs_E10;stage	1	TRUE
ENSGALG00010005492	TNPO3	PRJNA729271	0.686538805	2.91268E-12	0.686538805	stage_E14_vs_E10	ordinary_muscle_development		0	FALSE
ENSGALG00010016173	ENSGALG00010016173	PRJNA729271	-3.836826878	2.91988E-12	3.836826878	stage_E14_vs_E10	ordinary_muscle_development	D_vs_E10;stage_1W_vs_E10;stage_3W_vs_E10;stage	1	TRUE
ENSGALG00010027918	CSNK1G2	PRJNA729271	0.714923533	2.9257E-12	0.714923533	stage_E14_vs_E10	ordinary_muscle_development		0	FALSE
ENSGALG00010029555	LYRM9	PRJNA729271	1.095270361	2.93928E-12	1.095270361	stage_E14_vs_E10	ordinary_muscle_development	D_vs_E10;stage_1W_vs_E10;stage_3W_vs_E10;stage	1	TRUE
ENSGALG00010029635	ENSGALG00010029635	PRJNA729271	4.088245382	2.96625E-12	4.088245382	stage_E14_vs_E10	ordinary_muscle_development	D_vs_E10;stage_1W_vs_E10;stage_3W_vs_E10;stage	1	TRUE
ENSGALG00010017792	LSM1	PRJNA729271	-0.950441947	2.96705E-12	0.950441947	stage_E14_vs_E10	ordinary_muscle_development		0	FALSE
ENSGALG00010003009	TNKS	PRJNA729271	-0.414864072	2.97243E-12	0.414864072	stage_E14_vs_E10	ordinary_muscle_development		0	FALSE
ENSGALG00010009852	ENSGALG00010009852	PRJNA729271	-1.986126814	3.01473E-12	1.986126814	stage_E14_vs_E10	ordinary_muscle_development	D_vs_E10;stage_1W_vs_E10;stage_3W_vs_E10;stage	1	TRUE
ENSGALG00010001533	ENSGALG00010001533	PRJNA729271	1.047059105	3.02118E-12	1.047059105	stage_E14_vs_E10	ordinary_muscle_development	D_vs_E10;stage_1W_vs_E10;stage_3W_vs_E10;stage	1	TRUE
ENSGALG00010020905	KCTD6	PRJNA729271	-0.643696564	3.03261E-12	0.643696564	stage_E14_vs_E10	ordinary_muscle_development		0	FALSE
ENSGALG00010009192	LSM14A	PRJNA729271	-0.425222607	3.03817E-12	0.425222607	stage_E14_vs_E10	ordinary_muscle_development		0	FALSE
ENSGALG00010009876	SELENOI	PRJNA729271	-0.789537616	3.04909E-12	0.789537616	stage_E14_vs_E10	ordinary_muscle_development		0	FALSE
ENSGALG00010022559	SSH1	PRJNA729271	0.60753407	3.05753E-12	0.60753407	stage_E14_vs_E10	ordinary_muscle_development		0	FALSE
ENSGALG00010025487	FGF3	PRJNA729271	-6.668104926	3.0677E-12	6.668104926	stage_E14_vs_E10	ordinary_muscle_development	D_vs_E10;stage_1W_vs_E10;stage_3W_vs_E10;stage	1	TRUE
ENSGALG00010007539	RPS3A	PRJNA729271	1.258383718	3.08455E-12	1.258383718	stage_E14_vs_E10	ordinary_muscle_development	D_vs_E10;stage_1W_vs_E10;stage_3W_vs_E10;stage	1	TRUE
ENSGALG00010028566	ZDHHC12	PRJNA729271	0.875292051	3.13291E-12	0.875292051	stage_E14_vs_E10	ordinary_muscle_development		0	FALSE
ENSGALG00010017464	SH3BP2	PRJNA729271	0.915624729	3.14396E-12	0.915624729	stage_E14_vs_E10	ordinary_muscle_development		0	FALSE
ENSGALG00010000349	CHIR-IG1-5	PRJNA729271	3.087900933	3.15627E-12	3.087900933	stage_E14_vs_E10	ordinary_muscle_development	D_vs_E10;stage_1W_vs_E10;stage_3W_vs_E10;stage	1	TRUE
ENSGALG00010018462	KTN1	PRJNA729271	0.826681447	3.158E-12	0.826681447	stage_E14_vs_E10	ordinary_muscle_development		0	FALSE
ENSGALG00010009394	BANP	PRJNA729271	0.656862001	3.16388E-12	0.656862001	stage_E14_vs_E10	ordinary_muscle_development		0	FALSE
ENSGALG00010009189	ENSGALG00010009189	PRJNA729271	-1.841567599	3.20763E-12	1.841567599	stage_E14_vs_E10	ordinary_muscle_development	D_vs_E10;stage_1W_vs_E10;stage_3W_vs_E10;stage	1	TRUE
ENSGALG00010021086	PSMD14	PRJNA729271	-0.791382004	3.20842E-12	0.791382004	stage_E14_vs_E10	ordinary_muscle_development		0	FALSE
ENSGALG00010003895	THSD7A	PRJNA729271	1.118706452	3.21197E-12	1.118706452	stage_E14_vs_E10	ordinary_muscle_development	D_vs_E10;stage_1W_vs_E10;stage_3W_vs_E10;stage	1	TRUE
ENSGALG00010015200	TBC1D1	PRJNA729271	-0.613834469	3.21816E-12	0.613834469	stage_E14_vs_E10	ordinary_muscle_development		0	FALSE
ENSGALG00010004880	STAP1	PRJNA729271	4.034292455	3.23615E-12	4.034292455	stage_E14_vs_E10	ordinary_muscle_development	D_vs_E10;stage_1W_vs_E10;stage_3W_vs_E10;stage	1	TRUE
ENSGALG00010024864	MPHOSPH9	PRJNA729271	-0.936213275	3.23985E-12	0.936213275	stage_E14_vs_E10	ordinary_muscle_development		0	FALSE
ENSGALG00010002942	GABRB3	PRJNA729271	-4.672621163	3.25888E-12	4.672621163	stage_E14_vs_E10	ordinary_muscle_development	D_vs_E10;stage_1W_vs_E10;stage_3W_vs_E10;stage	1	TRUE
ENSGALG00010020959	ENSGALG00010020959	PRJNA729271	-2.744635178	3.27097E-12	2.744635178	stage_E14_vs_E10	ordinary_muscle_development	D_vs_E10;stage_1W_vs_E10;stage_3W_vs_E10;stage	1	TRUE
ENSGALG00010022691	ASIC2	PRJNA729271	-4.805094074	3.2872E-12	4.805094074	stage_E14_vs_E10	ordinary_muscle_development	D_vs_E10;stage_1W_vs_E10;stage_3W_vs_E10;stage	1	TRUE
ENSGALG00010006983	GTPBP2	PRJNA729271	0.890672591	3.29527E-12	0.890672591	stage_E14_vs_E10	ordinary_muscle_development		0	FALSE
ENSGALG00010012732	MGST1	PRJNA729271	-1.251289794	3.31188E-12	1.251289794	stage_E14_vs_E10	ordinary_muscle_development	D_vs_E10;stage_1W_vs_E10;stage_3W_vs_E10;stage	1	TRUE
ENSGALG00010007698	ADAMTS18	PRJNA729271	-2.891994113	3.31195E-12	2.891994113	stage_E14_vs_E10	ordinary_muscle_development	D_vs_E10;stage_1W_vs_E10;stage_3W_vs_E10;stage	1	TRUE
ENSGALG00010014923	CIMIP1	PRJNA729271	-4.287798258	3.33321E-12	4.287798258	stage_E14_vs_E10	ordinary_muscle_development	D_vs_E10;stage_1W_vs_E10;stage_3W_vs_E10;stage	1	TRUE
ENSGALG00010023237	GAD1	PRJNA729271	-4.978975785	3.34075E-12	4.978975785	stage_E14_vs_E10	ordinary_muscle_development	D_vs_E10;stage_1W_vs_E10;stage_3W_vs_E10;stage	1	TRUE
ENSGALG00010016944	OPRD1	PRJNA729271	-6.412705824	3.35973E-12	6.412705824	stage_E14_vs_E10	ordinary_muscle_development	D_vs_E10;stage_1W_vs_E10;stage_3W_vs_E10;stage	1	TRUE
ENSGALG00010001753	PNPLA8	PRJNA729271	1.44931152	3.37283E-12	1.44931152	stage_E14_vs_E10	ordinary_muscle_development	D_vs_E10;stage_1W_vs_E10;stage_3W_vs_E10;stage	1	TRUE
ENSGALG00010006557	COQ6	PRJNA729271	0.767118884	3.39771E-12	0.767118884	stage_E14_vs_E10	ordinary_muscle_development		0	FALSE
ENSGALG00010018002	ZC3H8	PRJNA729271	0.56219644	3.45425E-12	0.56219644	stage_E14_vs_E10	ordinary_muscle_development		0	FALSE
ENSGALG00010018641	ENSGALG00010018641	PRJNA729271	-7.494075238	3.47031E-12	7.494075238	stage_E14_vs_E10	ordinary_muscle_development	D_vs_E10;stage_1W_vs_E10;stage_3W_vs_E10;stage	1	TRUE
ENSGALG00010005853	COMMD3	PRJNA729271	-1.013950427	3.51478E-12	1.013950427	stage_E14_vs_E10	ordinary_muscle_development	D_vs_E10;stage_1W_vs_E10;stage_3W_vs_E10;stage	1	TRUE
ENSGALG00010018000	MAP2K1	PRJNA729271	0.517843678	3.53173E-12	0.517843678	stage_E14_vs_E10	ordinary_muscle_development		0	FALSE
ENSGALG00010016750	SYTL4	PRJNA729271	0.88679878	3.54685E-12	0.88679878	stage_E14_vs_E10	ordinary_muscle_development		0	FALSE
ENSGALG00010025597	SCN2A	PRJNA729271	-6.709252173	3.56685E-12	6.709252173	stage_E14_vs_E10	ordinary_muscle_development	D_vs_E10;stage_1W_vs_E10;stage_3W_vs_E10;stage	1	TRUE
ENSGALG00010015086	FOXN3	PRJNA729271	0.681559403	3.5754E-12	0.681559403	stage_E14_vs_E10	ordinary_muscle_development		0	FALSE
ENSGALG00010029328	ENSGALG00010029328	PRJNA729271	0.575606642	3.5754E-12	0.575606642	stage_E14_vs_E10	ordinary_muscle_development		0	FALSE
ENSGALG00010021025	CUEDC2	PRJNA729271	0.816210372	3.57607E-12	0.816210372	stage_E14_vs_E10	ordinary_muscle_development		0	FALSE
ENSGALG00010018959	UBA5	PRJNA729271	-0.845845933	3.60858E-12	0.845845933	stage_E14_vs_E10	ordinary_muscle_development		0	FALSE
ENSGALG00010028658	PTPA	PRJNA729271	-0.584602752	3.63264E-12	0.584602752	stage_E14_vs_E10	ordinary_muscle_development		0	FALSE
ENSGALG00010019443	NCKAP5	PRJNA729271	-0.964492845	3.64544E-12	0.964492845	stage_E14_vs_E10	ordinary_muscle_development		0	FALSE
ENSGALG00010011452	KITLG	PRJNA729271	1.336845834	3.72615E-12	1.336845834	stage_E14_vs_E10	ordinary_muscle_development	D_vs_E10;stage_1W_vs_E10;stage_3W_vs_E10;stage	1	TRUE
ENSGALG00010024559	ENSGALG00010024559	PRJNA729271	-2.525622719	3.74693E-12	2.525622719	stage_E14_vs_E10	ordinary_muscle_development	D_vs_E10;stage_1W_vs_E10;stage_3W_vs_E10;stage	1	TRUE
ENSGALG00010011972	IPO11	PRJNA729271	-0.582843788	3.80966E-12	0.582843788	stage_E14_vs_E10	ordinary_muscle_development		0	FALSE
ENSGALG00010011658	TENM1	PRJNA729271	-2.281400626	3.84648E-12	2.281400626	stage_E14_vs_E10	ordinary_muscle_development	D_vs_E10;stage_1W_vs_E10;stage_3W_vs_E10;stage	1	TRUE
ENSGALG00010019373	DENND5A	PRJNA729271	0.432680726	3.8651E-12	0.432680726	stage_E14_vs_E10	ordinary_muscle_development		0	FALSE
ENSGALG00010000589	ENSGALG00010000589	PRJNA729271	2.152017984	3.86673E-12	2.152017984	stage_E14_vs_E10	ordinary_muscle_development	D_vs_E10;stage_1W_vs_E10;stage_3W_vs_E10;stage	1	TRUE
ENSGALG00010010976	DYDC2	PRJNA729271	4.284439559	3.87184E-12	4.284439559	stage_E14_vs_E10	ordinary_muscle_development	D_vs_E10;stage_1W_vs_E10;stage_3W_vs_E10;stage	1	TRUE
ENSGALG00010011426	NQO2	PRJNA729271	0.896471961	3.87656E-12	0.896471961	stage_E14_vs_E10	ordinary_muscle_development		0	FALSE
ENSGALG00010002582	ENSGALG00010002582	PRJNA729271	-4.257532778	3.89009E-12	4.257532778	stage_E14_vs_E10	ordinary_muscle_development	D_vs_E10;stage_1W_vs_E10;stage_3W_vs_E10;stage	1	TRUE
ENSGALG00010026106	TESK2	PRJNA729271	0.840275097	3.91214E-12	0.840275097	stage_E14_vs_E10	ordinary_muscle_development		0	FALSE
ENSGALG00010011419	RNF180	PRJNA729271	1.04840258	3.92218E-12	1.04840258	stage_E14_vs_E10	ordinary_muscle_development	D_vs_E10;stage_1W_vs_E10;stage_3W_vs_E10;stage	1	TRUE
ENSGALG00010022088	DKK3	PRJNA729271	-1.918279153	3.98802E-12	1.918279153	stage_E14_vs_E10	ordinary_muscle_development	D_vs_E10;stage_1W_vs_E10;stage_3W_vs_E10;stage	1	TRUE
ENSGALG00010019024	CNPY3	PRJNA729271	-0.740550859	3.99823E-12	0.740550859	stage_E14_vs_E10	ordinary_muscle_development		0	FALSE
ENSGALG00010012308	TRABD2A	PRJNA729271	2.39636848	4.02176E-12	2.39636848	stage_E14_vs_E10	ordinary_muscle_development	D_vs_E10;stage_1W_vs_E10;stage_3W_vs_E10;stage	1	TRUE
ENSGALG00010006146	CCDC142	PRJNA729271	1.266434571	4.03527E-12	1.266434571	stage_E14_vs_E10	ordinary_muscle_development	D_vs_E10;stage_1W_vs_E10;stage_3W_vs_E10;stage	1	TRUE
ENSGALG00010014611	TMEM189	PRJNA729271	0.731460897	4.03719E-12	0.731460897	stage_E14_vs_E10	ordinary_muscle_development		0	FALSE
ENSGALG00010028130	TMEM115	PRJNA729271	0.894732853	4.06998E-12	0.894732853	stage_E14_vs_E10	ordinary_muscle_development		0	FALSE
ENSGALG00010015542	CNGA3	PRJNA729271	-2.413317761	4.08272E-12	2.413317761	stage_E14_vs_E10	ordinary_muscle_development	D_vs_E10;stage_1W_vs_E10;stage_3W_vs_E10;stage	1	TRUE

ENSGALG00010015987	POLR2H	PRJNA729271	-0.695323452	4.09187E-12	0.695323452	stage_E14_vs_E10	ordinary_muscle_development		0	FALSE
ENSGALG00010011461	IRF4	PRJNA729271	2.42501587	4.0971E-12	2.42501587	stage_E14_vs_E10	ordinary_muscle_development	D_vs_E10;stage_1W_vs_E10;stage_3W_vs_E10;stage	1	TRUE
ENSGALG00010007989	SPG7	PRJNA729271	0.68710864	4.12564E-12	0.68710864	stage_E14_vs_E10	ordinary_muscle_development		0	FALSE
ENSGALG00010022771	SGMS1	PRJNA729271	1.556844319	4.14361E-12	1.556844319	stage_E14_vs_E10	ordinary_muscle_development	D_vs_E10;stage_1W_vs_E10;stage_3W_vs_E10;stage	1	TRUE
ENSGALG00010005841	ENSGALG00010005841	PRJNA729271	2.162074696	4.16911E-12	2.162074696	stage_E14_vs_E10	ordinary_muscle_development	D_vs_E10;stage_1W_vs_E10;stage_3W_vs_E10;stage	1	TRUE
ENSGALG00010020109	WDFY4	PRJNA729271	2.538537494	4.18343E-12	2.538537494	stage_E14_vs_E10	ordinary_muscle_development	D_vs_E10;stage_1W_vs_E10;stage_3W_vs_E10;stage	1	TRUE
ENSGALG00010009765	STX10	PRJNA729271	-0.703218589	4.18421E-12	0.703218589	stage_E14_vs_E10	ordinary_muscle_development		0	FALSE
ENSGALG00010028326	CRB2	PRJNA729271	-1.887496162	4.23569E-12	1.887496162	stage_E14_vs_E10	ordinary_muscle_development	D_vs_E10;stage_1W_vs_E10;stage_3W_vs_E10;stage	1	TRUE
ENSGALG00010027473	DNAH1	PRJNA729271	-3.141802027	4.23956E-12	3.141802027	stage_E14_vs_E10	ordinary_muscle_development	D_vs_E10;stage_1W_vs_E10;stage_3W_vs_E10;stage	1	TRUE
ENSGALG00010002340	RAB23	PRJNA729271	-0.981995706	4.2523E-12	0.981995706	stage_E14_vs_E10	ordinary_muscle_development		0	FALSE
ENSGALG00010009317	LRRTM3	PRJNA729271	-4.797449499	4.25328E-12	4.797449499	stage_E14_vs_E10	ordinary_muscle_development	D_vs_E10;stage_1W_vs_E10;stage_3W_vs_E10;stage	1	TRUE
ENSGALG00010000232	ENSGALG00010000232	PRJNA729271	1.138592205	4.26684E-12	1.138592205	stage_E14_vs_E10	ordinary_muscle_development	D_vs_E10;stage_1W_vs_E10;stage_3W_vs_E10;stage	1	TRUE
ENSGALG00010022385	ENSGALG00010022385	PRJNA729271	4.311142228	4.3196E-12	4.311142228	stage_E14_vs_E10	ordinary_muscle_development	D_vs_E10;stage_1W_vs_E10;stage_3W_vs_E10;stage	1	TRUE
ENSGALG00010021928	ENSGALG00010021928	PRJNA729271	6.135502343	4.32737E-12	6.135502343	stage_E14_vs_E10	ordinary_muscle_development	D_vs_E10;stage_1W_vs_E10;stage_3W_vs_E10;stage	1	TRUE
ENSGALG00010029164	CYB5D2	PRJNA729271	0.578586217	4.33876E-12	0.578586217	stage_E14_vs_E10	ordinary_muscle_development		0	FALSE
ENSGALG00010027457	CAPZA1	PRJNA729271	-0.910645526	4.36021E-12	0.910645526	stage_E14_vs_E10	ordinary_muscle_development		0	FALSE
ENSGALG00010000303	ENSGALG00010000303	PRJNA729271	-0.805095029	4.36177E-12	0.805095029	stage_E14_vs_E10	ordinary_muscle_development		0	FALSE
ENSGALG00010017356	NSMCE1	PRJNA729271	-0.858631431	4.4418E-12	0.858631431	stage_E14_vs_E10	ordinary_muscle_development		0	FALSE
ENSGALG00010010930	FAM163A	PRJNA729271	-5.464003088	4.47459E-12	5.464003088	stage_E14_vs_E10	ordinary_muscle_development	D_vs_E10;stage_1W_vs_E10;stage_3W_vs_E10;stage	1	TRUE
ENSGALG00010018857	ENSGALG00010018857	PRJNA729271	0.578245478	4.62376E-12	0.578245478	stage_E14_vs_E10	ordinary_muscle_development		0	FALSE
ENSGALG00010024149	TCIRG1	PRJNA729271	1.072941726	4.63337E-12	1.072941726	stage_E14_vs_E10	ordinary_muscle_development	D_vs_E10;stage_1W_vs_E10;stage_3W_vs_E10;stage	1	TRUE
ENSGALG00010001353	GLRX3	PRJNA729271	-0.561289585	4.6852E-12	0.561289585	stage_E14_vs_E10	ordinary_muscle_development		0	FALSE
ENSGALG00010024077	SFT2D3	PRJNA729271	0.807537097	4.68938E-12	0.807537097	stage_E14_vs_E10	ordinary_muscle_development		0	FALSE
ENSGALG00010002656	GLRA3	PRJNA729271	3.773040441	4.70048E-12	3.773040441	stage_E14_vs_E10	ordinary_muscle_development	D_vs_E10;stage_1W_vs_E10;stage_3W_vs_E10;stage	1	TRUE
ENSGALG00010000026	ND3	PRJNA729271	2.239514231	4.73197E-12	2.239514231	stage_E14_vs_E10	ordinary_muscle_development	D_vs_E10;stage_1W_vs_E10;stage_3W_vs_E10;stage	1	TRUE
ENSGALG00010029316	INTS2	PRJNA729271	-0.671915958	4.75938E-12	0.671915958	stage_E14_vs_E10	ordinary_muscle_development		0	FALSE
ENSGALG00010018730	IGF1R	PRJNA729271	-0.621742474	4.76287E-12	0.621742474	stage_E14_vs_E10	ordinary_muscle_development		0	FALSE
ENSGALG00010000405	ENSGALG00010000405	PRJNA729271	2.721854631	4.83148E-12	2.721854631	stage_E14_vs_E10	ordinary_muscle_development	D_vs_E10;stage_1W_vs_E10;stage_3W_vs_E10;stage	1	TRUE
ENSGALG00010005177	AASDHPPT	PRJNA729271	0.894592302	4.84765E-12	0.894592302	stage_E14_vs_E10	ordinary_muscle_development		0	FALSE
ENSGALG00010010234	ENSGALG00010010234	PRJNA729271	1.89168592	4.88383E-12	1.89168592	stage_E14_vs_E10	ordinary_muscle_development	D_vs_E10;stage_1W_vs_E10;stage_3W_vs_E10;stage	1	TRUE
ENSGALG00010017517	LIN9	PRJNA729271	-0.725049767	4.9208E-12	0.725049767	stage_E14_vs_E10	ordinary_muscle_development		0	FALSE
ENSGALG00010021105	ENSGALG00010021105	PRJNA729271	2.873457679	4.92822E-12	2.873457679	stage_E14_vs_E10	ordinary_muscle_development	D_vs_E10;stage_1W_vs_E10;stage_3W_vs_E10;stage	1	TRUE
ENSGALG00010023442	STK25	PRJNA729271	-0.804706305	4.92902E-12	0.804706305	stage_E14_vs_E10	ordinary_muscle_development		0	FALSE
ENSGALG00010028871	PIP5K1A	PRJNA729271	-0.771886636	4.96309E-12	0.771886636	stage_E14_vs_E10	ordinary_muscle_development		0	FALSE
ENSGALG00010028553	NFILZ	PRJNA729271	6.440972283	4.9833E-12	6.440972283	stage_E14_vs_E10	ordinary_muscle_development	D_vs_E10;stage_1W_vs_E10;stage_3W_vs_E10;stage	1	TRUE
ENSGALG00010003108	PPP1R3B	PRJNA729271	1.304984625	5.01321E-12	1.304984625	stage_E14_vs_E10	ordinary_muscle_development	D_vs_E10;stage_1W_vs_E10;stage_3W_vs_E10;stage	1	TRUE
ENSGALG00010029583	LIMK1	PRJNA729271	-1.521920208	5.0189E-12	1.521920208	stage_E14_vs_E10	ordinary_muscle_development	D_vs_E10;stage_1W_vs_E10;stage_3W_vs_E10;stage	1	TRUE
ENSGALG00010021150	ENSGALG00010021150	PRJNA729271	1.761641286	5.1307E-12	1.761641286	stage_E14_vs_E10	ordinary_muscle_development	D_vs_E10;stage_1W_vs_E10;stage_3W_vs_E10;stage	1	TRUE
ENSGALG00010011850	SPAG16	PRJNA729271	-2.810949118	5.15157E-12	2.810949118	stage_E14_vs_E10	ordinary_muscle_development	D_vs_E10;stage_1W_vs_E10;stage_3W_vs_E10;stage	1	TRUE
ENSGALG00010013940	UPF3A	PRJNA729271	-1.884495499	5.16174E-12	1.884495499	stage_E14_vs_E10	ordinary_muscle_development	D_vs_E10;stage_1W_vs_E10;stage_3W_vs_E10;stage	1	TRUE
ENSGALG00010015183	SLC5A7	PRJNA729271	-7.060021788	5.16873E-12	7.060021788	stage_E14_vs_E10	ordinary_muscle_development	D_vs_E10;stage_1W_vs_E10;stage_3W_vs_E10;stage	1	TRUE
ENSGALG00010015190	FBLN5	PRJNA729271	-1.396943855	5.20473E-12	1.396943855	stage_E14_vs_E10	ordinary_muscle_development	D_vs_E10;stage_1W_vs_E10;stage_3W_vs_E10;stage	1	TRUE
ENSGALG00010021004	PSD	PRJNA729271	1.183215974	5.20473E-12	1.183215974	stage_E14_vs_E10	ordinary_muscle_development	D_vs_E10;stage_1W_vs_E10;stage_3W_vs_E10;stage	1	TRUE
ENSGALG00010017215	FRMD7	PRJNA729271	4.102370991	5.22625E-12	4.102370991	stage_E14_vs_E10	ordinary_muscle_development	D_vs_E10;stage_1W_vs_E10;stage_3W_vs_E10;stage	1	TRUE
ENSGALG00010011696	ZFC3H1	PRJNA729271	0.768511473	5.27565E-12	0.768511473	stage_E14_vs_E10	ordinary_muscle_development		0	FALSE
ENSGALG00010015914	SRRM1	PRJNA729271	-0.83028304	5.32141E-12	0.83028304	stage_E14_vs_E10	ordinary_muscle_development		0	FALSE
ENSGALG00010005219	NALCN	PRJNA729271	-1.335403024	5.34963E-12	1.335403024	stage_E14_vs_E10	ordinary_muscle_development	D_vs_E10;stage_1W_vs_E10;stage_3W_vs_E10;stage	1	TRUE
ENSGALG00010005547	LRP11	PRJNA729271	1.514185319	5.36594E-12	1.514185319	stage_E14_vs_E10	ordinary_muscle_development	D_vs_E10;stage_1W_vs_E10;stage_3W_vs_E10;stage	1	TRUE
ENSGALG00010021131	EDC3	PRJNA729271	-0.822251668	5.38601E-12	0.822251668	stage_E14_vs_E10	ordinary_muscle_development		0	FALSE
ENSGALG00010021020	ZDHHC13	PRJNA729271	0.829991657	5.43849E-12	0.829991657	stage_E14_vs_E10	ordinary_muscle_development		0	FALSE
ENSGALG00010018532	MFSD13A	PRJNA729271	-0.93488967	5.44878E-12	0.93488967	stage_E14_vs_E10	ordinary_muscle_development		0	FALSE
ENSGALG00010001621	DLGAP2	PRJNA729271	-1.943985159	5.45071E-12	1.943985159	stage_E14_vs_E10	ordinary_muscle_development	D_vs_E10;stage_1W_vs_E10;stage_3W_vs_E10;stage	1	TRUE
ENSGALG00010024911	CAT	PRJNA729271	1.08232526	5.45434E-12	1.08232526	stage_E14_vs_E10	ordinary_muscle_development	D_vs_E10;stage_1W_vs_E10;stage_3W_vs_E10;stage	1	TRUE
ENSGALG00010028444	MEF2B	PRJNA729271	2.176444745	5.50755E-12	2.176444745	stage_E14_vs_E10	ordinary_muscle_development	D_vs_E10;stage_1W_vs_E10;stage_3W_vs_E10;stage	1	TRUE
ENSGALG00010019132	ENSGALG00010019132	PRJNA729271	-1.082279122	5.51016E-12	1.082279122	stage_E14_vs_E10	ordinary_muscle_development	D_vs_E10;stage_1W_vs_E10;stage_3W_vs_E10;stage	1	TRUE
ENSGALG00010001854	CHRDL2	PRJNA729271	2.002500241	5.56201E-12	2.002500241	stage_E14_vs_E10	ordinary_muscle_development	D_vs_E10;stage_1W_vs_E10;stage_3W_vs_E10;stage	1	TRUE
ENSGALG00010009011	ACP6	PRJNA729271	-0.936489524	5.56303E-12	0.936489524	stage_E14_vs_E10	ordinary_muscle_development		0	FALSE
ENSGALG00010012877	SLC25A32	PRJNA729271	-0.68564771	5.57463E-12	0.68564771	stage_E14_vs_E10	ordinary_muscle_development		0	FALSE
ENSGALG00010021040	FGF8	PRJNA729271	-6.340399689	5.57701E-12	6.340399689	stage_E14_vs_E10	ordinary_muscle_development	D_vs_E10;stage_1W_vs_E10;stage_3W_vs_E10;stage	1	TRUE
ENSGALG00010020315	ANO5	PRJNA729271	-0.785326995	5.60699E-12	0.785326995	stage_E14_vs_E10	ordinary_muscle_development		0	FALSE
ENSGALG00010027861	PNPLA7	PRJNA729271	-0.571315941	5.63595E-12	0.571315941	stage_E14_vs_E10	ordinary_muscle_development		0	FALSE
ENSGALG00010014734	ZUP1	PRJNA729271	-0.706322371	5.65105E-12	0.706322371	stage_E14_vs_E10	ordinary_muscle_development		0	FALSE
ENSGALG00010007455	TRPC6	PRJNA729271	1.46786923	5.71712E-12	1.46786923	stage_E14_vs_E10	ordinary_muscle_development	D_vs_E10;stage_1W_vs_E10;stage_3W_vs_E10;stage	1	TRUE
ENSGALG00010001496	PTP4A1	PRJNA729271	1.335088944	5.72461E-12	1.335088944	stage_E14_vs_E10	ordinary_muscle_development	D_vs_E10;stage_1W_vs_E10;stage_3W_vs_E10;stage	1	TRUE
ENSGALG00010013086	CXCL14	PRJNA729271	-3.639631857	5.76775E-12	3.639631857	stage_E14_vs_E10	ordinary_muscle_development	D_vs_E10;stage_1W_vs_E10;stage_3W_vs_E10;stage	1	TRUE
ENSGALG00010000467	RNASEK	PRJNA729271	1.396121968	5.76991E-12	1.396121968	stage_E14_vs_E10	ordinary_muscle_development	D_vs_E10;stage_1W_vs_E10;stage_3W_vs_E10;stage	1	TRUE
ENSGALG00010011839	CBS	PRJNA729271	-3.249042323	5.78194E-12	3.249042323	stage_E14_vs_E10	ordinary_muscle_development	D_vs_E10;stage_1W_vs_E10;stage_3W_vs_E10;stage	1	TRUE
ENSGALG00010014477	ITPR2	PRJNA729271	0.68815406	5.79283E-12	0.68815406	stage_E14_vs_E10	ordinary_muscle_development		0	FALSE
ENSGALG00010024652	PUS3	PRJNA729271	-0.929792513	5.84009E-12	0.929792513	stage_E14_vs_E10	ordinary_muscle_development		0	FALSE
ENSGALG00010001244	ENSGALG00010001244	PRJNA729271	1.065329015	5.84608E-12	1.065329015	stage_E14_vs_E10	ordinary_muscle_development	D_vs_E10;stage_1W_vs_E10;stage_3W_vs_E10;stage	1	TRUE
ENSGALG00010009243	BCAP29	PRJNA729271	-0.707893162	5.86685E-12	0.707893162	stage_E14_vs_E10	ordinary_muscle_development		0	FALSE
ENSGALG00010027102	DUS3L	PRJNA729271	-1.031138484	5.89715E-12	1.031138484	stage_E14_vs_E10	ordinary_muscle_development	D_vs_E10;stage_1W_vs_E10;stage_3W_vs_E10;stage	1	TRUE
ENSGALG00010005477	TMEM161B	PRJNA729271	1.134133758	5.93245E-12	1.134133758	stage_E14_vs_E10	ordinary_muscle_development	D_vs_E10;stage_1W_vs_E10;stage_3W_vs_E10;stage	1	TRUE
ENSGALG00010013878	ARID2	PRJNA729271	-0.578835967	5.95372E-12	0.578835967	stage_E14_vs_E10	ordinary_muscle_development		0	FALSE
ENSGALG00010015141	PFDN4	PRJNA729271	0.951045641	5.96712E-12	0.951045641	stage_E14_vs_E10	ordinary_muscle_development		0	FALSE
ENSGALG00010023050	RUFY2	PRJNA729271	-0.675738425	5.97646E-12	0.675738425	stage_E14_vs_E10	ordinary_muscle_development		0	FALSE
ENSGALG00010019323	LRIG1	PRJNA729271	0.838705308	5.97899E-12	0.838705308	stage_E14_vs_E10	ordinary_muscle_development		0	FALSE
ENSGALG00010021151	WNT10A	PRJNA729271	-6.759566263	6.10264E-12	6.759566263	stage_E14_vs_E10	ordinary_muscle_development	D_vs_E10;stage_1W_vs_E10;stage_3W_vs_E10;stage	1	TRUE
ENSGALG00010011545	USP12-like	PRJNA729271	-0.540540143	6.11428E-12	0.540540143	stage_E14_vs_E10	ordinary_muscle_development		0	FALSE
ENSGALG00010002138	MYO7A	PRJNA729271	-1.225407909	6.1644E-12	1.225407909	stage_E14_vs_E10	ordinary_muscle_development	D_vs_E10;stage_1W_vs_E10;stage_3W_vs_E10;stage	1	TRUE
ENSGALG00010002455	NYX	PRJNA729271	2.474404765	6.19367E-12	2.474404765	stage_E14_vs_E10	ordinary_muscle_development	D_vs_E10;stage_1W_vs_E10;stage_3W_vs_E10;stage	1	TRUE
ENSGALG00010024965	STAT2	PRJNA729271	1.525311538	6.20758E-12	1.525311538	stage_E14_vs_E10	ordinary_muscle_development	D_vs_E10;stage_1W_vs_E10;stage_3W_vs_E10;stage	1	TRUE
ENSGALG00010021338	PDIA5	PRJNA729271	-1.114303251	6.26152E-12	1.114303251	stage_E14_vs_E10	ordinary_muscle_development	D_vs_E10;stage_1W_vs_E10;stage_3W_vs_E10;stage	1	TRUE
ENSGALG00010002917	PGLS	PRJNA729271	0.677068646	6.31482E-12	0.677068646	stage_E14_vs_E10	ordinary_muscle_development		0	FALSE
ENSGALG00010010952	ENSGALG00010010952	PRJNA729271	3.276030066	6.33373E-12	3.276030066	stage_E14_vs_E10	ordinary_muscle_development	D_vs_E10;stage_1W_vs_E10;stage_3W_vs_E10;stage	1	TRUE
ENSGALG00010017703	RPS6KA5	PRJNA729271	0.979759079	6.40167E-12	0.979759079	stage_E14_vs_E10	ordinary_muscle_development		0	FALSE
ENSGALG00010009106	THSD7B	PRJNA729271	-1.674381622	6.41623E-12	1.674381622	stage_E14_vs_E10	ordinary_muscle_development	D_vs_E10;stage_1W_vs_E10;stage_3W_vs_E10;stage	1	TRUE
ENSGALG00010023059	SELENOF	PRJNA729271	-0.656364481	6.41703E-12	0.656364481	stage_E14_vs_E10	ordinary_muscle_development		0	FALSE
ENSGALG00010025790	LRP5	PRJNA729271	0.544943684	6.50246E-12	0.544943684	stage_E14_vs_E10	ordinary_muscle_development		0	FALSE
ENSGALG00010015090	GZMA	PRJNA729271	3.48778613	6.5544E-12	3.48778613	stage_E14_vs_E10	ordinary_muscle_development	D_vs_E10;stage_1W_vs_E10;stage_3W_vs_E10;stage	1	TRUE
ENSGALG00010023240	PIWIL1	PRJNA729271	-3.675251196	6.58591E-12	3.675251196	stage_E14_vs_E10	ordinary_muscle_development	D_vs_E10;stage_1W_vs_E10;stage_3W_vs_E10;stage	1	TRUE
ENSGALG00010029562	ENSGALG00010029562	PRJNA729271	4.732739335	6.60214E-12	4.732739335	stage_E14_vs_E10	ordinary_muscle_development	D_vs_E10;stage_1W_vs_E10;stage_3W_vs_E10;stage	1	TRUE
ENSGALG00010011262	ENSGALG00010011262	PRJNA729271	-1.459864552	6.61348E-12	1.459864552	stage_E14_vs_E10	ordinary_muscle_development	D_vs_E10;stage_1W_vs_E10;stage_3W_vs_E10;stage	1	TRUE
ENSGALG00010018877	CST7	PRJNA729271	5.533126262	6.62297E-12	5.533126262	stage_E14_vs_E10	ordinary_muscle_development	D_vs_E10;stage_1W_vs_E10;stage_3W_vs_E10;stage	1	TRUE
ENSGALG00010029534	NF1	PRJNA729271	-0.638258841	6.63138E-12	0.638258841	stage_E14_vs_E10	ordinary_muscle_development		0	FALSE
ENSGALG00010009332	ENSGALG00010009332	PRJNA729271	1.135806234	6.63575E-12	1.135806234	stage_E14_vs_E10	ordinary_muscle_development	D_vs_E10;stage_1W_vs_E10;stage_3W_vs_E10;stage	1	TRUE
ENSGALG00010018333	ENSGALG00010018333	PRJNA729271	1.453725195	6.63778E-12	1.453725195	stage_E14_vs_E10	ordinary_muscle_development	D_vs_E10;stage_1W_vs_E10;stage_3W_vs_E10;stage	1	TRUE
ENSGALG00010027241	LRRN2	PRJNA729271	-1.906359391	6.63985E-12	1.906359391	stage_E14_vs_E10	ordinary_muscle_development	D_vs_E10;stage_1W_vs_E10;stage_3W_vs_E10;stage	1	TRUE
ENSGALG00010004336	ENSGALG00010004336	PRJNA729271	-4.648086273	6.65907E-12	4.648086273	stage_E14_vs_E10	ordinary_muscle_development	D_vs_E10;stage_1W_vs_E10;stage_3W_vs_E10;stage	1	TRUE
ENSGALG00010027222	GUCA1B	PRJNA729271	5.813749981	6.69958E-12	5.813749981	stage_E14_vs_E10	ordinary_muscle_development	D_vs_E10;stage_1W_vs_E10;stage_3W_vs_E10;stage	1	TRUE
ENSGALG00010024927	NOM1	PRJNA729271	-0.830305162	6.72611E-12	0.830305162	stage_E14_vs_E10	ordinary_muscle_development		0	FALSE
ENSGALG00010005848	WASF3	PRJNA729271	1.307876958	6.79512E-12	1.307876958	stage_E14_vs_E10	ordinary_muscle_development	D_vs_E10;stage_1W_vs_E10;stage_3W_vs_E10;stage	1	TRUE
ENSGALG00010013778	ENSGALG00010013778	PRJNA729271	-5.205581258	6.82403E-12	5.205581258	stage_E14_vs_E10	ordinary_muscle_development	D_vs_E10;stage_1W_vs_E10;stage_3W_vs_E10;stage	1	TRUE
ENSGALG00010016561	DEGS2	PRJNA729271	3.825917304	6.84862E-12	3.825917304	stage_E14_vs_E10	ordinary_muscle_development	D_vs_E10;stage_1W_vs_E10;stage_3W_vs_E10;stage	1	TRUE
ENSGALG00010011638	GRPEL1	PRJNA729271	0.56864901	6.90628E-12	0.56864901	stage_E14_vs_E10	ordinary_muscle_development		0	FALSE
ENSGALG00010015068	ESM1	PRJNA729271	-2.325300581	7.01212E-12	2.325300581	stage_E14_vs_E10	ordinary_muscle_development	D_vs_E10;stage_1W_vs_E10;stage_3W_vs_E10;stage	1	TRUE
ENSGALG00010022251	WNK4	PRJNA729271	-1.099622571	7.03752E-12	1.099622571	stage_E14_vs_E10	ordinary_muscle_development	D_vs_E10;stage_1W_vs_E10;stage_3W_vs_E10;stage	1	TRUE
ENSGALG00010010752	ENSGALG00010010752	PRJNA729271	-1.917545179	7.04671E-12	1.917545179	stage_E14_vs_E10	ordinary_muscle_development	D_vs_E10;stage_1W_vs_E10;stage_3W_vs_E10;stage	1	TRUE
ENSGALG00010001420	FAM221A	PRJNA729271	-5.307281443	7.05068E-12	5.307281443	stage_E14_vs_E10	ordinary_muscle_development	D_vs_E10;stage_1W_vs_E10;stage_3W_vs_E10;stage	1	TRUE
ENSGALG00010010776	GCAT	PRJNA729271	1.35383945	7.1115E-12	1.35383945	stage_E14_vs_E10	ordinary_muscle_development	D_vs_E10;stage_1W_vs_E10;stage_3W_vs_E10;stage	1	TRUE
ENSGALG00010008178	PGR	PRJNA729271	-2.488178995	7.11355E-12	2.488178995	stage_E14_vs_E10	ordinary_muscle_development	D_vs_E10;stage_1W_vs_E10;stage_3W_vs_E10;stage	1	TRUE
ENSGALG00010012419	HACE1	PRJNA729271	0.672180573	7.14035E-12	0.672180573	stage_E14_vs_E10	ordinary_muscle_development		0	FALSE
ENSGALG00010011902	TP53I3	PRJNA729271	-1.867780494	7.24312E-12	1.867780494	stage_E14_vs_E10	ordinary_muscle_development	D_vs_E10;stage_1W_vs_E10;stage_3W_vs_E10;stage	1	TRUE
ENSGALG00010000277	ENSGALG00010000277	PRJNA729271	3.968208291	7.25331E-12	3.968208291	stage_E14_vs_E10	ordinary_muscle_development	D_vs_E10;stage_1W_vs_E10;stage_3W_vs_E10;stage	1	TRUE
ENSGALG00010011141	MSX1	PRJNA729271	-7.823453567	7.27866E-12	7.823453567	stage_E14_vs_E10	ordinary_muscle_development	D_vs_E10;stage_1W_vs_E10;stage_3W_vs_E10;stage	1	TRUE
ENSGALG00010004240	ENSGALG00010004240	PRJNA729271	1.213269865	7.33434E-12	1.213269865	stage_E14_vs_E10	ordinary_muscle_development	D_vs_E10;stage_1W_vs_E10;stage_3W_vs_E10;stage	1	TRUE
ENSGALG00010012865	EP300	PRJNA729271	0.861300377	7.41076E-12	0.861300377	stage_E14_vs_E10	ordinary_muscle_development		0	FALSE
ENSGALG00010004891	ANKIB1	PRJNA729271	-0.544101447	7.41325E-12	0.544101447	stage_E14_vs_E10	ordinary_muscle_development		0	FALSE
ENSGALG00010022165	FAM192A	PRJNA729271	-0.629913397	7.4889E-12	0.629913397	stage_E14_vs_E10	ordinary_muscle_development		0	FALSE
ENSGALG00010024309	ABCB11	PRJNA729271	2.724561084	7.53642E-12	2.724561084	stage_E14_vs_E10	ordinary_muscle_development	D_vs_E10;stage_1W_vs_E10;stage_3W_vs_E10;stage	1	TRUE
ENSGALG00010029372	METTL23	PRJNA729271	-1.180174874	7.54157E-12	1.180174874	stage_E14_vs_E10	ordinary_muscle_development	D_vs_E10;stage_1W_vs_E10;stage_3W_vs_E10;stage	1	TRUE
ENSGALG00010007548	HSBP1	PRJNA729271	-0.612339334	7.5711E-12	0.612339334	stage_E14_vs_E10	ordinary_muscle_development		0	FALSE
ENSGALG00010008515	TAFA5	PRJNA729271	-1.563469727	7.62782E-12	1.563469727	stage_E14_vs_E10	ordinary_muscle_development	D_vs_E10;stage_1W_vs_E10;stage_3W_vs_E10;stage	1	TRUE
ENSGALG00010028412	ENSGALG00010028412	PRJNA729271	2.196343064	7.67884E-12	2.196343064	stage_E14_vs_E10	ordinary_muscle_development	D_vs_E10;stage_1W_vs_E10;stage_3W_vs_E10;stage	1	TRUE
ENSGALG00010022216	ROMO1	PRJNA729271	1.004862221	7.77297E-12	1.004862221	stage_E14_vs_E10	ordinary_muscle_development	D_vs_E10;stage_1W_vs_E10;stage_3W_vs_E10;stage	1	TRUE
ENSGALG00010018510	ENSGALG00010018510	PRJNA729271	0.876451247	7.77432E-12	0.876451247	stage_E14_vs_E10	ordinary_muscle_development		0	FALSE
ENSGALG00010005425	ELAPOR2	PRJNA729271	-1.839547675	7.78174E-12	1.839547675	stage_E14_vs_E10	ordinary_muscle_development	D_vs_E10;stage_1W_vs_E10;stage_3W_vs_E10;stage	1	TRUE
ENSGALG00010006526	LSM6	PRJNA729271	-1.129296456	7.81507E-12	1.129296456	stage_E14_vs_E10	ordinary_muscle_development	D_vs_E10;stage_1W_vs_E10;stage_3W_vs_E10;stage	1	TRUE
ENSGALG00010017418	CHGB	PRJNA729271	-2.035175243	7.836E-12	2.035175243	stage_E14_vs_E10	ordinary_muscle_development	D_vs_E10;stage_1W_vs_E10;stage_3W_vs_E10;stage	1	TRUE
ENSGALG00010007190	ENSGALG00010007190	PRJNA729271	-1.076529759	7.85684E-12	1.076529759	stage_E14_vs_E10	ordinary_muscle_development	D_vs_E10;stage_1W_vs_E10;stage_3W_vs_E10;stage	1	TRUE
ENSGALG00010010361	PIGM	PRJNA729271	-1.216669068	7.8915E-12	1.216669068	stage_E14_vs_E10	ordinary_muscle_development	D_vs_E10;stage_1W_vs_E10;stage_3W_vs_E10;stage	1	TRUE
ENSGALG00010015753	RPRD1B	PRJNA729271	-0.550349309	7.92715E-12	0.550349309	stage_E14_vs_E10	ordinary_muscle_development		0	FALSE
ENSGALG00010012734	TMEM19	PRJNA729271	-1.02324445	8.03349E-12	1.02324445	stage_E14_vs_E10	ordinary_muscle_development	D_vs_E10;stage_1W_vs_E10;stage_3W_vs_E10;stage	1	TRUE
ENSGALG00010022619	MADD	PRJNA729271	-0.936066358	8.24309E-12	0.936066358	stage_E14_vs_E10	ordinary_muscle_development		0	FALSE
ENSGALG00010009609	MMADHC	PRJNA729271	-0.506716506	8.30706E-12	0.506716506	stage_E14_vs_E10	ordinary_muscle_development		0	FALSE
ENSGALG00010000288	VARS2	PRJNA729271	1.05672861	8.31078E-12	1.05672861	stage_E14_vs_E10	ordinary_muscle_development	D_vs_E10;stage_1W_vs_E10;stage_3W_vs_E10;stage	1	TRUE
ENSGALG00010006478	ENSGALG00010006478	PRJNA729271	0.916093534	8.31273E-12	0.916093534	stage_E14_vs_E10	ordinary_muscle_development		0	FALSE
ENSGALG00010011374	ENSGALG00010011374	PRJNA729271	-0.65128516	8.38487E-12	0.65128516	stage_E14_vs_E10	ordinary_muscle_development		0	FALSE
ENSGALG00010013472	ENSGALG00010013472	PRJNA729271	1.589942932	8.40859E-12	1.589942932	stage_E14_vs_E10	ordinary_muscle_development	D_vs_E10;stage_1W_vs_E10;stage_3W_vs_E10;stage	1	TRUE
ENSGALG00010027989	ENSGALG00010027989	PRJNA729271	0.959553487	8.43666E-12	0.959553487	stage_E14_vs_E10	ordinary_muscle_development		0	FALSE
ENSGALG00010010397	PICK1	PRJNA729271	-0.647631676	8.48486E-12	0.647631676	stage_E14_vs_E10	ordinary_muscle_development		0	FALSE
ENSGALG00010003198	PRKG2	PRJNA729271	-2.463944936	8.48858E-12	2.463944936	stage_E14_vs_E10	ordinary_muscle_development	D_vs_E10;stage_1W_vs_E10;stage_3W_vs_E10;stage	1	TRUE
ENSGALG00010001911	USP35	PRJNA729271	-3.727293593	8.52863E-12	3.727293593	stage_E14_vs_E10	ordinary_muscle_development	D_vs_E10;stage_1W_vs_E10;stage_3W_vs_E10;stage	1	TRUE
ENSGALG00010027124	IP6K2	PRJNA729271	1.211914233	8.54881E-12	1.211914233	stage_E14_vs_E10	ordinary_muscle_development	D_vs_E10;stage_1W_vs_E10;stage_3W_vs_E10;stage	1	TRUE
ENSGALG00010005431	GRM3	PRJNA729271	-7.209699724	8.55266E-12	7.209699724	stage_E14_vs_E10	ordinary_muscle_development	D_vs_E10;stage_1W_vs_E10;stage_3W_vs_E10;stage	1	TRUE
ENSGALG00010007017	SRD5A2	PRJNA729271	-2.656117052	8.61538E-12	2.656117052	stage_E14_vs_E10	ordinary_muscle_development	D_vs_E10;stage_1W_vs_E10;stage_3W_vs_E10;stage	1	TRUE
ENSGALG00010018261	PRNP	PRJNA729271	1.351142938	8.70086E-12	1.351142938	stage_E14_vs_E10	ordinary_muscle_development	D_vs_E10;stage_1W_vs_E10;stage_3W_vs_E10;stage	1	TRUE
ENSGALG00010024711	DDX55	PRJNA729271	-0.652229635	8.801E-12	0.652229635	stage_E14_vs_E10	ordinary_muscle_development		0	FALSE
ENSGALG00010026625	ENSGALG00010026625	PRJNA729271	4.386657217	8.86105E-12	4.386657217	stage_E14_vs_E10	ordinary_muscle_development	D_vs_E10;stage_1W_vs_E10;stage_3W_vs_E10;stage	1	TRUE
ENSGALG00010008079	CCDC127	PRJNA729271	0.751080821	8.97766E-12	0.751080821	stage_E14_vs_E10	ordinary_muscle_development		0	FALSE
ENSGALG00010014492	CPLX2	PRJNA729271	-2.717433762	9.02185E-12	2.717433762	stage_E14_vs_E10	ordinary_muscle_development	D_vs_E10;stage_1W_vs_E10;stage_3W_vs_E10;stage	1	TRUE
ENSGALG00010009643	PSMC2	PRJNA729271	0.755018951	9.04476E-12	0.755018951	stage_E14_vs_E10	ordinary_muscle_development		0	FALSE
ENSGALG00010029149	ENSGALG00010029149	PRJNA729271	-1.684171786	9.10922E-12	1.684171786	stage_E14_vs_E10	ordinary_muscle_development	D_vs_E10;stage_1W_vs_E10;stage_3W_vs_E10;stage	1	TRUE
ENSGALG00010010710	UCK2	PRJNA729271	-0.706768742	9.12013E-12	0.706768742	stage_E14_vs_E10	ordinary_muscle_development		0	FALSE
ENSGALG00010021191	MYRF	PRJNA729271	-1.510037744	9.16421E-12	1.510037744	stage_E14_vs_E10	ordinary_muscle_development	D_vs_E10;stage_1W_vs_E10;stage_3W_vs_E10;stage	1	TRUE
ENSGALG00010003442	SPCS3	PRJNA729271	0.620126189	9.16928E-12	0.620126189	stage_E14_vs_E10	ordinary_muscle_development		0	FALSE
ENSGALG00010029211	SDF2	PRJNA729271	-1.178402281	9.21067E-12	1.178402281	stage_E14_vs_E10	ordinary_muscle_development	D_vs_E10;stage_1W_vs_E10;stage_3W_vs_E10;stage	1	TRUE
ENSGALG00010012119	EPG5	PRJNA729271	0.852283333	9.21591E-12	0.852283333	stage_E14_vs_E10	ordinary_muscle_development		0	FALSE
ENSGALG00010004081	RPS20	PRJNA729271	1.178644565	9.29028E-12	1.178644565	stage_E14_vs_E10	ordinary_muscle_development		1	TRUE
ENSGALG00010024690	TUBA1C	PRJNA729271	-4.534984308	9.29037E-12	4.534984308	stage_E14_vs_E10	ordinary_muscle_development		1	TRUE
ENSGALG00010000688	ENSGALG00010000688	PRJNA729271	1.928832174	9.2967E-12	1.928832174	stage_E14_vs_E10	ordinary_muscle_development	D_vs_E10;stage_1W_vs_E10;stage_3W_vs_E10;stage	1	TRUE
ENSGALG00010026277	SMARCD2	PRJNA729271	0.862393926	9.33585E-12	0.862393926	stage_E14_vs_E10	ordinary_muscle_development		0	FALSE
ENSGALG00010021843	ENSGALG00010021843	PRJNA729271	2.6191337	9.3496E-12	2.6191337	stage_E14_vs_E10	ordinary_muscle_development	D_vs_E10;stage_1W_vs_E10;stage_3W_vs_E10;stage	1	TRUE
ENSGALG00010017648	ENSGALG00010017648	PRJNA729271	-1.110165674	9.46618E-12	1.110165674	stage_E14_vs_E10	ordinary_muscle_development	D_vs_E10;stage_1W_vs_E10;stage_3W_vs_E10;stage	1	TRUE
ENSGALG00010023752	C1QL1	PRJNA729271	-2.440888663	9.50868E-12	2.440888663	stage_E14_vs_E10	ordinary_muscle_development	D_vs_E10;stage_1W_vs_E10;stage_3W_vs_E10;stage	1	TRUE
ENSGALG00010011090	GRIN2B	PRJNA729271	-3.406106295	9.64969E-12	3.406106295	stage_E14_vs_E10	ordinary_muscle_development	D_vs_E10;stage_1W_vs_E10;stage_3W_vs_E10;stage	1	TRUE
ENSGALG00010016087	IARS2	PRJNA729271	0.624453115	9.66169E-12	0.624453115	stage_E14_vs_E10	ordinary_muscle_development		0	FALSE
ENSGALG00010011775	VEZT	PRJNA729271	0.553378575	9.67518E-12	0.553378575	stage_E14_vs_E10	ordinary_muscle_development		0	FALSE
ENSGALG00010024240	WDR61	PRJNA729271	-0.884604584	9.78578E-12	0.884604584	stage_E14_vs_E10	ordinary_muscle_development		0	FALSE
ENSGALG00010020422	ERCC4	PRJNA729271	-0.642895096	9.9406E-12	0.642895096	stage_E14_vs_E10	ordinary_muscle_development		0	FALSE
ENSGALG00010004890	ACAT1	PRJNA729271	-0.686864517	9.9818E-12	0.686864517	stage_E14_vs_E10	ordinary_muscle_development		0	FALSE
ENSGALG00010017284	ENSGALG00010017284	PRJNA729271	1.3565271	1.00097E-11	1.3565271	stage_E14_vs_E10	ordinary_muscle_development	D_vs_E10;stage_1W_vs_E10;stage_3W_vs_E10;stage	1	TRUE
ENSGALG00010027367	SEC22C	PRJNA729271	-0.664517139	1.00368E-11	0.664517139	stage_E14_vs_E10	ordinary_muscle_development		0	FALSE
ENSGALG00010015856	TXNDC9	PRJNA729271	0.923123738	1.00672E-11	0.923123738	stage_E14_vs_E10	ordinary_muscle_development		0	FALSE
ENSGALG00010023888	CTH	PRJNA729271	-1.811857218	1.0079E-11	1.811857218	stage_E14_vs_E10	ordinary_muscle_development	D_vs_E10;stage_1W_vs_E10;stage_3W_vs_E10;stage	1	TRUE

ENSGALG00010029369	MFSD11	PRJNA729271	-0.595360557	1.01142E-11	0.595360557	stage_E14_vs_E10	ordinary_muscle_development		0	FALSE
ENSGALG00010021461	SNX33	PRJNA729271	0.75787148	1.01636E-11	0.75787148	stage_E14_vs_E10	ordinary_muscle_development		0	FALSE
ENSGALG00010014795	THRAP3	PRJNA729271	-0.663859313	1.03098E-11	0.663859313	stage_E14_vs_E10	ordinary_muscle_development		0	FALSE
ENSGALG00010008294	NOB1	PRJNA729271	1.064940099	1.03331E-11	1.064940099	stage_E14_vs_E10	ordinary_muscle_development	D_vs_E10;stage_1W_vs_E10;stage_3W_vs_E10;stage	1	TRUE
ENSGALG00010015161	GFPT2	PRJNA729271	-1.398398347	1.04864E-11	1.398398347	stage_E14_vs_E10	ordinary_muscle_development	D_vs_E10;stage_1W_vs_E10;stage_3W_vs_E10;stage	1	TRUE
ENSGALG00010016661	UBE4B	PRJNA729271	-0.905356232	1.05146E-11	0.905356232	stage_E14_vs_E10	ordinary_muscle_development		0	FALSE
ENSGALG00010008457	MARVELD3	PRJNA729271	0.822255	1.05612E-11	0.822255	stage_E14_vs_E10	ordinary_muscle_development		0	FALSE
ENSGALG00010011203	ENSGALG00010011203	PRJNA729271	-0.729486625	1.05692E-11	0.729486625	stage_E14_vs_E10	ordinary_muscle_development		0	FALSE
ENSGALG00010029784	CLDN3	PRJNA729271	-6.742973414	1.06059E-11	6.742973414	stage_E14_vs_E10	ordinary_muscle_development	D_vs_E10;stage_1W_vs_E10;stage_3W_vs_E10;stage	1	TRUE
ENSGALG00010012725	ENSGALG00010012725	PRJNA729271	-1.897516316	1.09002E-11	1.897516316	stage_E14_vs_E10	ordinary_muscle_development	D_vs_E10;stage_1W_vs_E10;stage_3W_vs_E10;stage	1	TRUE
ENSGALG00010005475	TMEM181	PRJNA729271	-0.642646394	1.10829E-11	0.642646394	stage_E14_vs_E10	ordinary_muscle_development		0	FALSE
ENSGALG00010009067	ENSGALG00010009067	PRJNA729271	6.694761497	1.10839E-11	6.694761497	stage_E14_vs_E10	ordinary_muscle_development	D_vs_E10;stage_1W_vs_E10;stage_3W_vs_E10;stage	1	TRUE
ENSGALG00010002949	ENSGALG00010002949	PRJNA729271	4.877867169	1.10839E-11	4.877867169	stage_E14_vs_E10	ordinary_muscle_development	D_vs_E10;stage_1W_vs_E10;stage_3W_vs_E10;stage	1	TRUE
ENSGALG00010020282	MAGI1	PRJNA729271	-0.956494842	1.1126E-11	0.956494842	stage_E14_vs_E10	ordinary_muscle_development		0	FALSE
ENSGALG00010008104	TCF25	PRJNA729271	0.652174397	1.11273E-11	0.652174397	stage_E14_vs_E10	ordinary_muscle_development		0	FALSE
ENSGALG00010001798	PSTK	PRJNA729271	0.881188884	1.11333E-11	0.881188884	stage_E14_vs_E10	ordinary_muscle_development		0	FALSE
ENSGALG00010029752	ENSGALG00010029752	PRJNA729271	1.182881368	1.11529E-11	1.182881368	stage_E14_vs_E10	ordinary_muscle_development	D_vs_E10;stage_1W_vs_E10;stage_3W_vs_E10;stage	1	TRUE
ENSGALG00010008522	NUDT12	PRJNA729271	1.043230189	1.12712E-11	1.043230189	stage_E14_vs_E10	ordinary_muscle_development	D_vs_E10;stage_1W_vs_E10;stage_3W_vs_E10;stage	1	TRUE
ENSGALG00010029934	TEKT3	PRJNA729271	3.117332073	1.13223E-11	3.117332073	stage_E14_vs_E10	ordinary_muscle_development	D_vs_E10;stage_1W_vs_E10;stage_3W_vs_E10;stage	1	TRUE
ENSGALG00010019614	MICALL2	PRJNA729271	1.078307832	1.13661E-11	1.078307832	stage_E14_vs_E10	ordinary_muscle_development	D_vs_E10;stage_1W_vs_E10;stage_3W_vs_E10;stage	1	TRUE
ENSGALG00010023375	ST14	PRJNA729271	1.026559463	1.14713E-11	1.026559463	stage_E14_vs_E10	ordinary_muscle_development	D_vs_E10;stage_1W_vs_E10;stage_3W_vs_E10;stage	1	TRUE
ENSGALG00010012890	MFAP3	PRJNA729271	-0.487140961	1.15703E-11	0.487140961	stage_E14_vs_E10	ordinary_muscle_development		0	FALSE
ENSGALG00010005885	SLITRK5	PRJNA729271	2.314730603	1.15935E-11	2.314730603	stage_E14_vs_E10	ordinary_muscle_development	D_vs_E10;stage_1W_vs_E10;stage_3W_vs_E10;stage	1	TRUE
ENSGALG00010025753	SORL1	PRJNA729271	-1.206144417	1.16639E-11	1.206144417	stage_E14_vs_E10	ordinary_muscle_development	D_vs_E10;stage_1W_vs_E10;stage_3W_vs_E10;stage	1	TRUE
ENSGALG00010021999	ANKRD22	PRJNA729271	-4.538060622	1.1725E-11	4.538060622	stage_E14_vs_E10	ordinary_muscle_development	D_vs_E10;stage_1W_vs_E10;stage_3W_vs_E10;stage	1	TRUE
ENSGALG00010001844	HOXA10	PRJNA729271	-1.18433442	1.17379E-11	1.18433442	stage_E14_vs_E10	ordinary_muscle_development	D_vs_E10;stage_1W_vs_E10;stage_3W_vs_E10;stage	1	TRUE
ENSGALG00010015241	ENSGALG00010015241	PRJNA729271	1.048477953	1.18658E-11	1.048477953	stage_E14_vs_E10	ordinary_muscle_development	D_vs_E10;stage_1W_vs_E10;stage_3W_vs_E10;stage	1	TRUE
ENSGALG00010016895	ENSGALG00010016895	PRJNA729271	1.0777957	1.20418E-11	1.0777957	stage_E14_vs_E10	ordinary_muscle_development	D_vs_E10;stage_1W_vs_E10;stage_3W_vs_E10;stage	1	TRUE
ENSGALG00010011445	MTERF3	PRJNA729271	0.549789398	1.21795E-11	0.549789398	stage_E14_vs_E10	ordinary_muscle_development		0	FALSE
ENSGALG00010003982	GLCCI1	PRJNA729271	-0.846011417	1.22539E-11	0.846011417	stage_E14_vs_E10	ordinary_muscle_development		0	FALSE
ENSGALG00010024201	IREB2	PRJNA729271	-0.489689934	1.2302E-11	0.489689934	stage_E14_vs_E10	ordinary_muscle_development		0	FALSE
ENSGALG00010015555	PNRC2	PRJNA729271	-0.709570408	1.24433E-11	0.709570408	stage_E14_vs_E10	ordinary_muscle_development		0	FALSE
ENSGALG00010013271	GULP1	PRJNA729271	0.963554456	1.25107E-11	0.963554456	stage_E14_vs_E10	ordinary_muscle_development		0	FALSE
ENSGALG00010025640	SLC38A11	PRJNA729271	-6.872897442	1.25122E-11	6.872897442	stage_E14_vs_E10	ordinary_muscle_development	D_vs_E10;stage_1W_vs_E10;stage_3W_vs_E10;stage	1	TRUE
ENSGALG00010020725	FOPNL	PRJNA729271	-0.785299251	1.25875E-11	0.785299251	stage_E14_vs_E10	ordinary_muscle_development		0	FALSE
ENSGALG00010005893	SFT2D1	PRJNA729271	0.459226851	1.2703E-11	0.459226851	stage_E14_vs_E10	ordinary_muscle_development		0	FALSE
ENSGALG00010024419	PGAP3	PRJNA729271	0.854817458	1.28521E-11	0.854817458	stage_E14_vs_E10	ordinary_muscle_development		0	FALSE
ENSGALG00010024490	TGM3	PRJNA729271	5.589284875	1.2862E-11	5.589284875	stage_E14_vs_E10	ordinary_muscle_development	D_vs_E10;stage_1W_vs_E10;stage_3W_vs_E10;stage	1	TRUE
ENSGALG00010028472	RPRD2	PRJNA729271	0.957055488	1.29007E-11	0.957055488	stage_E14_vs_E10	ordinary_muscle_development		0	FALSE
ENSGALG00010004780	ENSGALG00010004780	PRJNA729271	1.799068002	1.29279E-11	1.799068002	stage_E14_vs_E10	ordinary_muscle_development	D_vs_E10;stage_1W_vs_E10;stage_3W_vs_E10;stage	1	TRUE
ENSGALG00010013562	RERGL	PRJNA729271	1.08995276	1.29653E-11	1.08995276	stage_E14_vs_E10	ordinary_muscle_development	D_vs_E10;stage_1W_vs_E10;stage_3W_vs_E10;stage	1	TRUE
ENSGALG00010008391	ENSGALG00010008391	PRJNA729271	-0.522025765	1.32191E-11	0.522025765	stage_E14_vs_E10	ordinary_muscle_development		0	FALSE
ENSGALG00010018328	ENSGALG00010018328	PRJNA729271	3.723365214	1.32397E-11	3.723365214	stage_E14_vs_E10	ordinary_muscle_development	D_vs_E10;stage_1W_vs_E10;stage_3W_vs_E10;stage	1	TRUE
ENSGALG00010011412	KANSL1L	PRJNA729271	0.831835015	1.32425E-11	0.831835015	stage_E14_vs_E10	ordinary_muscle_development		0	FALSE
ENSGALG00010015605	LDLRAP1	PRJNA729271	-0.404686244	1.3262E-11	0.404686244	stage_E14_vs_E10	ordinary_muscle_development		0	FALSE
ENSGALG00010014274	FIRRM	PRJNA729271	-1.066375866	1.33705E-11	1.066375866	stage_E14_vs_E10	ordinary_muscle_development	D_vs_E10;stage_1W_vs_E10;stage_3W_vs_E10;stage	1	TRUE
ENSGALG00010005442	SASH1	PRJNA729271	0.67429	1.342E-11	0.67429	stage_E14_vs_E10	ordinary_muscle_development		0	FALSE
ENSGALG00010025472	ZCCHC8	PRJNA729271	0.665750951	1.34203E-11	0.665750951	stage_E14_vs_E10	ordinary_muscle_development		0	FALSE
ENSGALG00010017459	FAM167B	PRJNA729271	1.954211726	1.34341E-11	1.954211726	stage_E14_vs_E10	ordinary_muscle_development	D_vs_E10;stage_1W_vs_E10;stage_3W_vs_E10;stage	1	TRUE
ENSGALG00010029188	ENSGALG00010029188	PRJNA729271	1.659434719	1.34796E-11	1.659434719	stage_E14_vs_E10	ordinary_muscle_development	D_vs_E10;stage_1W_vs_E10;stage_3W_vs_E10;stage	1	TRUE
ENSGALG00010029438	ENSGALG00010029438	PRJNA729271	-1.880671811	1.34826E-11	1.880671811	stage_E14_vs_E10	ordinary_muscle_development	D_vs_E10;stage_1W_vs_E10;stage_3W_vs_E10;stage	1	TRUE
ENSGALG00010018849	KCNH1	PRJNA729271	-3.919838072	1.35483E-11	3.919838072	stage_E14_vs_E10	ordinary_muscle_development	D_vs_E10;stage_1W_vs_E10;stage_3W_vs_E10;stage	1	TRUE
ENSGALG00010026234	ZDHHC5	PRJNA729271	1.102547713	1.38892E-11	1.102547713	stage_E14_vs_E10	ordinary_muscle_development	D_vs_E10;stage_1W_vs_E10;stage_3W_vs_E10;stage	1	TRUE
ENSGALG00010022138	SPAG17	PRJNA729271	1.439517094	1.38995E-11	1.439517094	stage_E14_vs_E10	ordinary_muscle_development	D_vs_E10;stage_1W_vs_E10;stage_3W_vs_E10;stage	1	TRUE
ENSGALG00010018046	CAPN15	PRJNA729271	0.718038033	1.40609E-11	0.718038033	stage_E14_vs_E10	ordinary_muscle_development		0	FALSE
ENSGALG00010014193	CARD10	PRJNA729271	-2.728925129	1.42242E-11	2.728925129	stage_E14_vs_E10	ordinary_muscle_development	D_vs_E10;stage_1W_vs_E10;stage_3W_vs_E10;stage	1	TRUE
ENSGALG00010002590	ATP6V1C2	PRJNA729271	-1.8075954	1.43187E-11	1.8075954	stage_E14_vs_E10	ordinary_muscle_development	D_vs_E10;stage_1W_vs_E10;stage_3W_vs_E10;stage	1	TRUE
ENSGALG00010014550	SCML4	PRJNA729271	-6.787126018	1.43422E-11	6.787126018	stage_E14_vs_E10	ordinary_muscle_development	D_vs_E10;stage_1W_vs_E10;stage_3W_vs_E10;stage	1	TRUE
ENSGALG00010011025	POLR3B	PRJNA729271	-0.645185433	1.46358E-11	0.645185433	stage_E14_vs_E10	ordinary_muscle_development		0	FALSE
ENSGALG00010029564	NLK	PRJNA729271	0.597869387	1.4689E-11	0.597869387	stage_E14_vs_E10	ordinary_muscle_development		0	FALSE
ENSGALG00010013079	ENSGALG00010013079	PRJNA729271	-1.42827472	1.48385E-11	1.42827472	stage_E14_vs_E10	ordinary_muscle_development	D_vs_E10;stage_1W_vs_E10;stage_3W_vs_E10;stage	1	TRUE
ENSGALG00010023319	EIF2AK4	PRJNA729271	-0.432524973	1.48417E-11	0.432524973	stage_E14_vs_E10	ordinary_muscle_development		0	FALSE
ENSGALG00010021400	B4GALT2	PRJNA729271	0.638367066	1.48522E-11	0.638367066	stage_E14_vs_E10	ordinary_muscle_development		0	FALSE
ENSGALG00010001633	B3GAT2	PRJNA729271	-2.817888492	1.49315E-11	2.817888492	stage_E14_vs_E10	ordinary_muscle_development	D_vs_E10;stage_1W_vs_E10;stage_3W_vs_E10;stage	1	TRUE
ENSGALG00010013842	CDK19	PRJNA729271	0.763240651	1.49364E-11	0.763240651	stage_E14_vs_E10	ordinary_muscle_development		0	FALSE
ENSGALG00010021749	CLASP1	PRJNA729271	0.642453627	1.50104E-11	0.642453627	stage_E14_vs_E10	ordinary_muscle_development		0	FALSE
ENSGALG00010004251	CCN2	PRJNA729271	-1.894824134	1.50279E-11	1.894824134	stage_E14_vs_E10	ordinary_muscle_development	D_vs_E10;stage_1W_vs_E10;stage_3W_vs_E10;stage	1	TRUE
ENSGALG00010002597	ENSGALG00010002597	PRJNA729271	-0.840427302	1.50351E-11	0.840427302	stage_E14_vs_E10	ordinary_muscle_development		0	FALSE
ENSGALG00010021971	ICOS	PRJNA729271	-3.42953541	1.51417E-11	3.42953541	stage_E14_vs_E10	ordinary_muscle_development	D_vs_E10;stage_1W_vs_E10;stage_3W_vs_E10;stage	1	TRUE
ENSGALG00010027097	ENSGALG00010027097	PRJNA729271	-1.034593744	1.52828E-11	1.034593744	stage_E14_vs_E10	ordinary_muscle_development	D_vs_E10;stage_1W_vs_E10;stage_3W_vs_E10;stage	1	TRUE
ENSGALG00010001567	INPP5A	PRJNA729271	-0.575907273	1.56313E-11	0.575907273	stage_E14_vs_E10	ordinary_muscle_development		0	FALSE
ENSGALG00010017524	ACBD3	PRJNA729271	-0.413671154	1.57169E-11	0.413671154	stage_E14_vs_E10	ordinary_muscle_development		0	FALSE
ENSGALG00010015009	AGO4	PRJNA729271	0.718054179	1.57578E-11	0.718054179	stage_E14_vs_E10	ordinary_muscle_development		0	FALSE
ENSGALG00010028027	ENSGALG00010028027	PRJNA729271	1.580676722	1.57663E-11	1.580676722	stage_E14_vs_E10	ordinary_muscle_development	D_vs_E10;stage_1W_vs_E10;stage_3W_vs_E10;stage	1	TRUE
ENSGALG00010015132	GCNT1	PRJNA729271	-2.077653352	1.59538E-11	2.077653352	stage_E14_vs_E10	ordinary_muscle_development	D_vs_E10;stage_1W_vs_E10;stage_3W_vs_E10;stage	1	TRUE
ENSGALG00010023724	CYP2R1	PRJNA729271	-0.878702571	1.59538E-11	0.878702571	stage_E14_vs_E10	ordinary_muscle_development		0	FALSE
ENSGALG00010016004	XRCC3	PRJNA729271	-1.187117417	1.60501E-11	1.187117417	stage_E14_vs_E10	ordinary_muscle_development	D_vs_E10;stage_1W_vs_E10;stage_3W_vs_E10;stage	1	TRUE
ENSGALG00010008336	PAIP2B	PRJNA729271	0.749164288	1.63149E-11	0.749164288	stage_E14_vs_E10	ordinary_muscle_development		0	FALSE
ENSGALG00010023638	DCTN2	PRJNA729271	0.897500246	1.6565E-11	0.897500246	stage_E14_vs_E10	ordinary_muscle_development		0	FALSE
ENSGALG00010024466	ADGRG3	PRJNA729271	4.678603449	1.65753E-11	4.678603449	stage_E14_vs_E10	ordinary_muscle_development	D_vs_E10;stage_1W_vs_E10;stage_3W_vs_E10;stage	1	TRUE
ENSGALG00010001731	ENSGALG00010001731	PRJNA729271	1.308248752	1.69184E-11	1.308248752	stage_E14_vs_E10	ordinary_muscle_development	D_vs_E10;stage_1W_vs_E10;stage_3W_vs_E10;stage	1	TRUE
ENSGALG00010026536	BCR	PRJNA729271	-0.673493746	1.70945E-11	0.673493746	stage_E14_vs_E10	ordinary_muscle_development		0	FALSE
ENSGALG00010016471	POMGNT2	PRJNA729271	0.931662005	1.73572E-11	0.931662005	stage_E14_vs_E10	ordinary_muscle_development		0	FALSE
ENSGALG00010020240	SV2B	PRJNA729271	-3.308959291	1.73596E-11	3.308959291	stage_E14_vs_E10	ordinary_muscle_development	D_vs_E10;stage_1W_vs_E10;stage_3W_vs_E10;stage	1	TRUE
ENSGALG00010001785	CNTN1	PRJNA729271	1.464169416	1.76438E-11	1.464169416	stage_E14_vs_E10	ordinary_muscle_development	D_vs_E10;stage_1W_vs_E10;stage_3W_vs_E10;stage	1	TRUE
ENSGALG00010017388	PLPBP	PRJNA729271	0.566168656	1.76438E-11	0.566168656	stage_E14_vs_E10	ordinary_muscle_development		0	FALSE
ENSGALG00010027881	ENSGALG00010027881	PRJNA729271	-2.279447178	1.78204E-11	2.279447178	stage_E14_vs_E10	ordinary_muscle_development	D_vs_E10;stage_1W_vs_E10;stage_3W_vs_E10;stage	1	TRUE
ENSGALG00010003793	TRAM1L1	PRJNA729271	-0.585486095	1.79096E-11	0.585486095	stage_E14_vs_E10	ordinary_muscle_development		0	FALSE
ENSGALG00010016473	RAB9B	PRJNA729271	-1.126516432	1.80118E-11	1.126516432	stage_E14_vs_E10	ordinary_muscle_development	D_vs_E10;stage_1W_vs_E10;stage_3W_vs_E10;stage	1	TRUE
ENSGALG00010010906	ATP6V1C1	PRJNA729271	0.951295249	1.81138E-11	0.951295249	stage_E14_vs_E10	ordinary_muscle_development		0	FALSE
ENSGALG00010011544	STOML2	PRJNA729271	-0.646214784	1.83569E-11	0.646214784	stage_E14_vs_E10	ordinary_muscle_development		0	FALSE
ENSGALG00010015616	NADK2	PRJNA729271	-0.817972987	1.83643E-11	0.817972987	stage_E14_vs_E10	ordinary_muscle_development		0	FALSE
ENSGALG00010003753	SLCO5A1	PRJNA729271	-1.149279683	1.83662E-11	1.149279683	stage_E14_vs_E10	ordinary_muscle_development	D_vs_E10;stage_1W_vs_E10;stage_3W_vs_E10;stage	1	TRUE
ENSGALG00010028355	RBM18	PRJNA729271	-0.514025073	1.84145E-11	0.514025073	stage_E14_vs_E10	ordinary_muscle_development		0	FALSE
ENSGALG00010011210	NUP153	PRJNA729271	-0.685499096	1.84223E-11	0.685499096	stage_E14_vs_E10	ordinary_muscle_development		0	FALSE
ENSGALG00010029330	AFMID	PRJNA729271	0.863760982	1.8428E-11	0.863760982	stage_E14_vs_E10	ordinary_muscle_development		0	FALSE
ENSGALG00010023337	FLI1	PRJNA729271	-0.828397448	1.84557E-11	0.828397448	stage_E14_vs_E10	ordinary_muscle_development		0	FALSE
ENSGALG00010012704	SMIM15	PRJNA729271	-0.470230497	1.86468E-11	0.470230497	stage_E14_vs_E10	ordinary_muscle_development		0	FALSE
ENSGALG00010020568	PLA2G4F	PRJNA729271	2.605265322	1.88617E-11	2.605265322	stage_E14_vs_E10	ordinary_muscle_development	D_vs_E10;stage_1W_vs_E10;stage_3W_vs_E10;stage	1	TRUE
ENSGALG00010014122	ABCA1	PRJNA729271	0.963506325	1.88832E-11	0.963506325	stage_E14_vs_E10	ordinary_muscle_development		0	FALSE
ENSGALG00010025932	AP1S3	PRJNA729271	-2.374564255	1.89018E-11	2.374564255	stage_E14_vs_E10	ordinary_muscle_development	D_vs_E10;stage_1W_vs_E10;stage_3W_vs_E10;stage	1	TRUE
ENSGALG00010017540	EIF4G1	PRJNA729271	1.126297396	1.89355E-11	1.126297396	stage_E14_vs_E10	ordinary_muscle_development	D_vs_E10;stage_1W_vs_E10;stage_3W_vs_E10;stage	1	TRUE
ENSGALG00010018083	PAX1	PRJNA729271	-7.545223273	1.90014E-11	7.545223273	stage_E14_vs_E10	ordinary_muscle_development	D_vs_E10;stage_1W_vs_E10;stage_3W_vs_E10;stage	1	TRUE
ENSGALG00010013669	NPR3	PRJNA729271	-1.840409775	1.90285E-11	1.840409775	stage_E14_vs_E10	ordinary_muscle_development	D_vs_E10;stage_1W_vs_E10;stage_3W_vs_E10;stage	1	TRUE
ENSGALG00010013766	DNM3	PRJNA729271	-0.771727465	1.91043E-11	0.771727465	stage_E14_vs_E10	ordinary_muscle_development		0	FALSE
ENSGALG00010016436	SLC30A2	PRJNA729271	3.558073783	1.9147E-11	3.558073783	stage_E14_vs_E10	ordinary_muscle_development	D_vs_E10;stage_1W_vs_E10;stage_3W_vs_E10;stage	1	TRUE
ENSGALG00010025881	GAL	PRJNA729271	3.655442153	1.9188E-11	3.655442153	stage_E14_vs_E10	ordinary_muscle_development	D_vs_E10;stage_1W_vs_E10;stage_3W_vs_E10;stage	1	TRUE
ENSGALG00010018089	TASP1	PRJNA729271	0.621249697	1.92053E-11	0.621249697	stage_E14_vs_E10	ordinary_muscle_development		0	FALSE
ENSGALG00010008356	ENSGALG00010008356	PRJNA729271	-0.929653921	1.92843E-11	0.929653921	stage_E14_vs_E10	ordinary_muscle_development		0	FALSE
ENSGALG00010026346	FZD2	PRJNA729271	1.114894442	1.93273E-11	1.114894442	stage_E14_vs_E10	ordinary_muscle_development	D_vs_E10;stage_1W_vs_E10;stage_3W_vs_E10;stage	1	TRUE
ENSGALG00010016569	YY1	PRJNA729271	0.593099394	1.93831E-11	0.593099394	stage_E14_vs_E10	ordinary_muscle_development		0	FALSE
ENSGALG00010027229	MDM4	PRJNA729271	0.550184592	1.95405E-11	0.550184592	stage_E14_vs_E10	ordinary_muscle_development		0	FALSE
ENSGALG00010023748	ENSGALG00010023748	PRJNA729271	-1.208866667	1.9682E-11	1.208866667	stage_E14_vs_E10	ordinary_muscle_development	D_vs_E10;stage_1W_vs_E10;stage_3W_vs_E10;stage	1	TRUE
ENSGALG00010019854	CCDC14	PRJNA729271	-1.030530107	1.97768E-11	1.030530107	stage_E14_vs_E10	ordinary_muscle_development	D_vs_E10;stage_1W_vs_E10;stage_3W_vs_E10;stage	1	TRUE
ENSGALG00010028370	S100A12	PRJNA729271	4.516818983	1.97925E-11	4.516818983	stage_E14_vs_E10	ordinary_muscle_development	D_vs_E10;stage_1W_vs_E10;stage_3W_vs_E10;stage	1	TRUE
ENSGALG00010028559	DYNC2I2	PRJNA729271	-0.877298083	1.98792E-11	0.877298083	stage_E14_vs_E10	ordinary_muscle_development		0	FALSE
ENSGALG00010012066	STXBP3	PRJNA729271	-0.849582376	1.98799E-11	0.849582376	stage_E14_vs_E10	ordinary_muscle_development		0	FALSE
ENSGALG00010015490	RANBP2	PRJNA729271	-0.575650492	1.99486E-11	0.575650492	stage_E14_vs_E10	ordinary_muscle_development		0	FALSE
ENSGALG00010007353	ING2	PRJNA729271	-0.90028112	1.99975E-11	0.90028112	stage_E14_vs_E10	ordinary_muscle_development		0	FALSE
ENSGALG00010011952	UBE2D2	PRJNA729271	1.187829291	2.00958E-11	1.187829291	stage_E14_vs_E10	ordinary_muscle_development	D_vs_E10;stage_1W_vs_E10;stage_3W_vs_E10;stage	1	TRUE
ENSGALG00010007671	FAM206A	PRJNA729271	0.683025137	2.02873E-11	0.683025137	stage_E14_vs_E10	ordinary_muscle_development		0	FALSE
ENSGALG00010016760	DLK1	PRJNA729271	1.792448026	2.03095E-11	1.792448026	stage_E14_vs_E10	ordinary_muscle_development	D_vs_E10;stage_1W_vs_E10;stage_3W_vs_E10;stage	1	TRUE
ENSGALG00010010322	ENSGALG00010010322	PRJNA729271	0.45845548	2.04002E-11	0.45845548	stage_E14_vs_E10	ordinary_muscle_development		0	FALSE
ENSGALG00010000093	ENSGALG00010000093	PRJNA729271	2.252158649	2.05652E-11	2.252158649	stage_E14_vs_E10	ordinary_muscle_development	D_vs_E10;stage_1W_vs_E10;stage_3W_vs_E10;stage	1	TRUE
ENSGALG00010012102	SON	PRJNA729271	0.716499194	2.06246E-11	0.716499194	stage_E14_vs_E10	ordinary_muscle_development		0	FALSE
ENSGALG00010024699	FBXL8	PRJNA729271	-0.927285398	2.08979E-11	0.927285398	stage_E14_vs_E10	ordinary_muscle_development		0	FALSE
ENSGALG00010029584	TRIM25	PRJNA729271	1.592211636	2.10288E-11	1.592211636	stage_E14_vs_E10	ordinary_muscle_development	D_vs_E10;stage_1W_vs_E10;stage_3W_vs_E10;stage	1	TRUE
ENSGALG00010001486	WNT2	PRJNA729271	1.811991506	2.11161E-11	1.811991506	stage_E14_vs_E10	ordinary_muscle_development	D_vs_E10;stage_1W_vs_E10;stage_3W_vs_E10;stage	1	TRUE
ENSGALG00010021794	ENSGALG00010021794	PRJNA729271	-1.407653358	2.13251E-11	1.407653358	stage_E14_vs_E10	ordinary_muscle_development	D_vs_E10;stage_1W_vs_E10;stage_3W_vs_E10;stage	1	TRUE
ENSGALG00010020484	TRNT1	PRJNA729271	0.843859302	2.13579E-11	0.843859302	stage_E14_vs_E10	ordinary_muscle_development		0	FALSE
ENSGALG00010008099	PANK3	PRJNA729271	-0.534061798	2.14794E-11	0.534061798	stage_E14_vs_E10	ordinary_muscle_development		0	FALSE
ENSGALG00010013436	NPAS2	PRJNA729271	-1.610082273	2.16233E-11	1.610082273	stage_E14_vs_E10	ordinary_muscle_development	D_vs_E10;stage_1W_vs_E10;stage_3W_vs_E10;stage	1	TRUE
ENSGALG00010020620	MARF1	PRJNA729271	0.704444977	2.1879E-11	0.704444977	stage_E14_vs_E10	ordinary_muscle_development		0	FALSE
ENSGALG00010020321	SZRD1	PRJNA729271	1.156366492	2.22336E-11	1.156366492	stage_E14_vs_E10	ordinary_muscle_development	D_vs_E10;stage_1W_vs_E10;stage_3W_vs_E10;stage	1	TRUE
ENSGALG00010006041	QKI	PRJNA729271	0.588205885	2.23876E-11	0.588205885	stage_E14_vs_E10	ordinary_muscle_development		0	FALSE
ENSGALG00010018644	ESF1	PRJNA729271	-0.680337896	2.24805E-11	0.680337896	stage_E14_vs_E10	ordinary_muscle_development		0	FALSE
ENSGALG00010008425	MAGI2	PRJNA729271	-0.753256121	2.25095E-11	0.753256121	stage_E14_vs_E10	ordinary_muscle_development		0	FALSE
ENSGALG00010002404	OSBPL3	PRJNA729271	-1.787138553	2.31444E-11	1.787138553	stage_E14_vs_E10	ordinary_muscle_development	D_vs_E10;stage_1W_vs_E10;stage_3W_vs_E10;stage	1	TRUE
ENSGALG00010013383	SEPTIN10	PRJNA729271	0.942871986	2.32973E-11	0.942871986	stage_E14_vs_E10	ordinary_muscle_development		0	FALSE
ENSGALG00010010840	RGS16	PRJNA729271	3.666235052	2.36338E-11	3.666235052	stage_E14_vs_E10	ordinary_muscle_development	D_vs_E10;stage_1W_vs_E10;stage_3W_vs_E10;stage	1	TRUE
ENSGALG00010027511	POLRMT	PRJNA729271	0.552768113	2.38214E-11	0.552768113	stage_E14_vs_E10	ordinary_muscle_development		0	FALSE
ENSGALG00010011423	GDF6	PRJNA729271	-3.454159164	2.38423E-11	3.454159164	stage_E14_vs_E10	ordinary_muscle_development	D_vs_E10;stage_1W_vs_E10;stage_3W_vs_E10;stage	1	TRUE
ENSGALG00010023469	ENSGALG00010023469	PRJNA729271	1.435986626	2.38769E-11	1.435986626	stage_E14_vs_E10	ordinary_muscle_development	D_vs_E10;stage_1W_vs_E10;stage_3W_vs_E10;stage	1	TRUE
ENSGALG00010011006	TMEM64	PRJNA729271	-0.691839657	2.40564E-11	0.691839657	stage_E14_vs_E10	ordinary_muscle_development		0	FALSE
ENSGALG00010024063	BRD7	PRJNA729271	-0.722671	2.41319E-11	0.722671	stage_E14_vs_E10	ordinary_muscle_development		0	FALSE
ENSGALG00010024631	EPB41L1	PRJNA729271	-1.027653269	2.4202E-11	1.027653269	stage_E14_vs_E10	ordinary_muscle_development	D_vs_E10;stage_1W_vs_E10;stage_3W_vs_E10;stage	1	TRUE
ENSGALG00010000607	ENSGALG00010000607	PRJNA729271	-2.034747521	2.43604E-11	2.034747521	stage_E14_vs_E10	ordinary_muscle_development	D_vs_E10;stage_1W_vs_E10;stage_3W_vs_E10;stage	1	TRUE
ENSGALG00010001620	MOSPD2	PRJNA729271	0.984816532	2.44353E-11	0.984816532	stage_E14_vs_E10	ordinary_muscle_development		0	FALSE
ENSGALG00010008180	POU2F1	PRJNA729271	1.064745391	2.44408E-11	1.064745391	stage_E14_vs_E10	ordinary_muscle_development	D_vs_E10;stage_1W_vs_E10;stage_3W_vs_E10;stage	1	TRUE
ENSGALG00010020786	DDX19	PRJNA729271	-0.908629535	2.45014E-11	0.908629535	stage_E14_vs_E10	ordinary_muscle_development		0	FALSE
ENSGALG00010009030	ZDHHC7	PRJNA729271	-0.629548502	2.45372E-11	0.629548502	stage_E14_vs_E10	ordinary_muscle_development		0	FALSE
ENSGALG00010024345	STARD3	PRJNA729271	0.62183845	2.45669E-11	0.62183845	stage_E14_vs_E10	ordinary_muscle_development		0	FALSE
ENSGALG00010019034	CASP7	PRJNA729271	1.133943077	2.48213E-11	1.133943077	stage_E14_vs_E10	ordinary_muscle_development	D_vs_E10;stage_1W_vs_E10;stage_3W_vs_E10;stage	1	TRUE
ENSGALG00010024628	TANGO2	PRJNA729271	0.760721422	2.49625E-11	0.760721422	stage_E14_vs_E10	ordinary_muscle_development		0	FALSE
ENSGALG00010013135	TTC3	PRJNA729271	-0.583386448	2.50318E-11	0.583386448	stage_E14_vs_E10	ordinary_muscle_development		0	FALSE
ENSGALG00010028132	CACNA2D2	PRJNA729271	0.87242025	2.53404E-11	0.87242025	stage_E14_vs_E10	ordinary_muscle_development		0	FALSE
ENSGALG00010012831	ENSGALG00010012831	PRJNA729271	-1.668523305	2.54018E-11	1.668523305	stage_E14_vs_E10	ordinary_muscle_development	D_vs_E10;stage_1W_vs_E10;stage_3W_vs_E10;stage	1	TRUE
ENSGALG00010021199	TMEM258	PRJNA729271	-0.938789993	2.55012E-11	0.938789993	stage_E14_vs_E10	ordinary_muscle_development		0	FALSE
ENSGALG00010028448	RIT1	PRJNA729271	-0.394943653	2.5564E-11	0.394943653	stage_E14_vs_E10	ordinary_muscle_development		0	FALSE
ENSGALG00010019585	PDE1C	PRJNA729271	-1.491944958	2.55738E-11	1.491944958	stage_E14_vs_E10	ordinary_muscle_development	D_vs_E10;stage_1W_vs_E10;stage_3W_vs_E10;stage	1	TRUE
ENSGALG00010014918	PLRG1	PRJNA729271	-0.581102976	2.55911E-11	0.581102976	stage_E14_vs_E10	ordinary_muscle_development		0	FALSE
ENSGALG00010004044	RNF138	PRJNA729271	-0.925660179	2.56971E-11	0.925660179	stage_E14_vs_E10	ordinary_muscle_development		0	FALSE
ENSGALG00010027723	ENSGALG00010027723	PRJNA729271	6.531582137	2.58247E-11	6.531582137	stage_E14_vs_E10	ordinary_muscle_development	D_vs_E10;stage_1W_vs_E10;stage_3W_vs_E10;stage	1	TRUE
ENSGALG00010015839	U2SURP	PRJNA729271	-0.487217859	2.58358E-11	0.487217859	stage_E14_vs_E10	ordinary_muscle_development		0	FALSE
ENSGALG00010019292	ENSGALG00010019292	PRJNA729271	-0.665084349	2.58854E-11	0.665084349	stage_E14_vs_E10	ordinary_muscle_development		0	FALSE
ENSGALG00010015899	SMARCA1	PRJNA729271	-0.790138136	2.60481E-11	0.790138136	stage_E14_vs_E10	ordinary_muscle_development		0	FALSE
ENSGALG00010024412	PIGU	PRJNA729271	-0.711401005	2.63761E-11	0.711401005	stage_E14_vs_E10	ordinary_muscle_development		0	FALSE
ENSGALG00010009842	PHETA2	PRJNA729271	0.617675116	2.65664E-11	0.617675116	stage_E14_vs_E10	ordinary_muscle_development		0	FALSE
ENSGALG00010029158	CD7	PRJNA729271	2.722034982	2.65941E-11	2.722034982	stage_E14_vs_E10	ordinary_muscle_development	D_vs_E10;stage_1W_vs_E10;stage_3W_vs_E10;stage	1	TRUE
ENSGALG00010021800	TMEM82	PRJNA729271	2.253672921	2.66008E-11	2.253672921	stage_E14_vs_E10	ordinary_muscle_development	D_vs_E10;stage_1W_vs_E10;stage_3W_vs_E10;stage	1	TRUE
ENSGALG00010001632	FANCB	PRJNA729271	1.851499122	2.66008E-11	1.851499122	stage_E14_vs_E10	ordinary_muscle_development	D_vs_E10;stage_1W_vs_E10;stage_3W_vs_E10;stage	1	TRUE
ENSGALG00010027672	CNTRL	PRJNA729271	-1.20456779	2.66738E-11	1.20456779	stage_E14_vs_E10	ordinary_muscle_development	D_vs_E10;stage_1W_vs_E10;stage_3W_vs_E10;stage	1	TRUE
ENSGALG00010012875	HSF2	PRJNA729271	-0.924014212	2.66949E-11	0.924014212	stage_E14_vs_E10	ordinary_muscle_development		0	FALSE
ENSGALG00010028860	SURF2	PRJNA729271	1.161424923	2.70047E-11	1.161424923	stage_E14_vs_E10	ordinary_muscle_development	D_vs_E10;stage_1W_vs_E10;stage_3W_vs_E10;stage	1	TRUE
ENSGALG00010011343	PXDC1	PRJNA729271	-0.96558723	2.70058E-11	0.96558723	stage_E14_vs_E10	ordinary_muscle_development		0	FALSE

ENSGALG00010005033	USP14	PRJNA729271	-0.939206056	2.70757E-11	0.939206056	stage_E14_vs_E10	ordinary_muscle_development		0	FALSE
ENSGALG00010007584	ELP2	PRJNA729271	-0.81312399	2.75684E-11	0.81312399	stage_E14_vs_E10	ordinary_muscle_development		0	FALSE
ENSGALG00010001242	ENSGALG00010001242	PRJNA729271	-2.719897371	2.76734E-11	2.719897371	stage_E14_vs_E10	ordinary_muscle_development	D_vs_E10;stage_1W_vs_E10;stage_3W_vs_E10;stage	1	TRUE
ENSGALG00010029285	CUX1	PRJNA729271	0.477029603	2.78118E-11	0.477029603	stage_E14_vs_E10	ordinary_muscle_development		0	FALSE
ENSGALG00010009446	ENSGALG00010009446	PRJNA729271	1.695458649	2.81923E-11	1.695458649	stage_E14_vs_E10	ordinary_muscle_development	D_vs_E10;stage_1W_vs_E10;stage_3W_vs_E10;stage	1	TRUE
ENSGALG00010011780	SERPINB5	PRJNA729271	-7.052003776	2.82777E-11	7.052003776	stage_E14_vs_E10	ordinary_muscle_development	D_vs_E10;stage_1W_vs_E10;stage_3W_vs_E10;stage	1	TRUE
ENSGALG00010022900	MMP17	PRJNA729271	1.35916004	2.84247E-11	1.35916004	stage_E14_vs_E10	ordinary_muscle_development	D_vs_E10;stage_1W_vs_E10;stage_3W_vs_E10;stage	1	TRUE
ENSGALG00010007066	INIP	PRJNA729271	-1.059714714	2.85077E-11	1.059714714	stage_E14_vs_E10	ordinary_muscle_development	D_vs_E10;stage_1W_vs_E10;stage_3W_vs_E10;stage	1	TRUE
ENSGALG00010004116	SPRY1	PRJNA729271	0.648141111	2.88062E-11	0.648141111	stage_E14_vs_E10	ordinary_muscle_development		0	FALSE
ENSGALG00010019717	ZRANB3	PRJNA729271	-0.969063362	2.88159E-11	0.969063362	stage_E14_vs_E10	ordinary_muscle_development		0	FALSE
ENSGALG00010017895	MNAT1	PRJNA729271	0.546650409	2.88583E-11	0.546650409	stage_E14_vs_E10	ordinary_muscle_development		0	FALSE
ENSGALG00010001116	RNF13	PRJNA729271	0.475603579	2.88597E-11	0.475603579	stage_E14_vs_E10	ordinary_muscle_development		0	FALSE
ENSGALG00010017727	AZIN2	PRJNA729271	1.027124916	2.90055E-11	1.027124916	stage_E14_vs_E10	ordinary_muscle_development	D_vs_E10;stage_1W_vs_E10;stage_3W_vs_E10;stage	1	TRUE
ENSGALG00010016805	ENSGALG00010016805	PRJNA729271	-7.261667245	2.90675E-11	7.261667245	stage_E14_vs_E10	ordinary_muscle_development	D_vs_E10;stage_1W_vs_E10;stage_3W_vs_E10;stage	1	TRUE
ENSGALG00010022472	QSER1	PRJNA729271	0.594093027	2.91884E-11	0.594093027	stage_E14_vs_E10	ordinary_muscle_development		0	FALSE
ENSGALG00010029320	TBX4	PRJNA729271	-6.809992744	2.95203E-11	6.809992744	stage_E14_vs_E10	ordinary_muscle_development	D_vs_E10;stage_1W_vs_E10;stage_3W_vs_E10;stage	1	TRUE
ENSGALG00010011371	ENSGALG00010011371	PRJNA729271	1.300065413	2.9822E-11	1.300065413	stage_E14_vs_E10	ordinary_muscle_development	D_vs_E10;stage_1W_vs_E10;stage_3W_vs_E10;stage	1	TRUE
ENSGALG00010021740	PDLIM1	PRJNA729271	1.073619596	3.00708E-11	1.073619596	stage_E14_vs_E10	ordinary_muscle_development	D_vs_E10;stage_1W_vs_E10;stage_3W_vs_E10;stage	1	TRUE
ENSGALG00010009180	NFKBIB	PRJNA729271	0.992612194	3.00987E-11	0.992612194	stage_E14_vs_E10	ordinary_muscle_development		0	FALSE
ENSGALG00010027259	ENSGALG00010027259	PRJNA729271	-0.582971544	3.03019E-11	0.582971544	stage_E14_vs_E10	ordinary_muscle_development		0	FALSE
ENSGALG00010027280	HMCES	PRJNA729271	-0.874286599	3.04563E-11	0.874286599	stage_E14_vs_E10	ordinary_muscle_development		0	FALSE
ENSGALG00010027990	ENSGALG00010027990	PRJNA729271	1.293515713	3.05084E-11	1.293515713	stage_E14_vs_E10	ordinary_muscle_development	D_vs_E10;stage_1W_vs_E10;stage_3W_vs_E10;stage	1	TRUE
ENSGALG00010013056	ENSGALG00010013056	PRJNA729271	3.662829805	3.07283E-11	3.662829805	stage_E14_vs_E10	ordinary_muscle_development	D_vs_E10;stage_1W_vs_E10;stage_3W_vs_E10;stage	1	TRUE
ENSGALG00010024204	PTBP2	PRJNA729271	0.728873725	3.08831E-11	0.728873725	stage_E14_vs_E10	ordinary_muscle_development		0	FALSE
ENSGALG00010015443	PAK3	PRJNA729271	-0.704249684	3.11593E-11	0.704249684	stage_E14_vs_E10	ordinary_muscle_development		0	FALSE
ENSGALG00010010416	SLC35B3	PRJNA729271	-0.875717694	3.13158E-11	0.875717694	stage_E14_vs_E10	ordinary_muscle_development		0	FALSE
ENSGALG00010015946	TLE4	PRJNA729271	0.666207283	3.13869E-11	0.666207283	stage_E14_vs_E10	ordinary_muscle_development		0	FALSE
ENSGALG00010018508	ENSGALG00010018508	PRJNA729271	-0.706342379	3.14415E-11	0.706342379	stage_E14_vs_E10	ordinary_muscle_development		0	FALSE
ENSGALG00010020131	SNRNP200	PRJNA729271	-0.846571526	3.15414E-11	0.846571526	stage_E14_vs_E10	ordinary_muscle_development		0	FALSE
ENSGALG00010011349	ENSGALG00010011349	PRJNA729271	-0.621449274	3.15721E-11	0.621449274	stage_E14_vs_E10	ordinary_muscle_development		0	FALSE
ENSGALG00010024180	HINFP	PRJNA729271	0.935707176	3.16276E-11	0.935707176	stage_E14_vs_E10	ordinary_muscle_development		0	FALSE
ENSGALG00010007792	ENSGALG00010007792	PRJNA729271	1.507703197	3.16828E-11	1.507703197	stage_E14_vs_E10	ordinary_muscle_development	D_vs_E10;stage_1W_vs_E10;stage_3W_vs_E10;stage	1	TRUE
ENSGALG00010027157	ENSGALG00010027157	PRJNA729271	1.689771172	3.17117E-11	1.689771172	stage_E14_vs_E10	ordinary_muscle_development	D_vs_E10;stage_1W_vs_E10;stage_3W_vs_E10;stage	1	TRUE
ENSGALG00010005360	CAMK1D	PRJNA729271	-1.035166281	3.17778E-11	1.035166281	stage_E14_vs_E10	ordinary_muscle_development	D_vs_E10;stage_1W_vs_E10;stage_3W_vs_E10;stage	1	TRUE
ENSGALG00010012136	ENSGALG00010012136	PRJNA729271	1.164342776	3.19091E-11	1.164342776	stage_E14_vs_E10	ordinary_muscle_development	D_vs_E10;stage_1W_vs_E10;stage_3W_vs_E10;stage	1	TRUE
ENSGALG00010014171	DLC1	PRJNA729271	0.902685636	3.192E-11	0.902685636	stage_E14_vs_E10	ordinary_muscle_development		0	FALSE
ENSGALG00010003269	ENSGALG00010003269	PRJNA729271	2.765828398	3.22535E-11	2.765828398	stage_E14_vs_E10	ordinary_muscle_development	D_vs_E10;stage_1W_vs_E10;stage_3W_vs_E10;stage	1	TRUE
ENSGALG00010018615	HTR2C	PRJNA729271	1.594326685	3.23E-11	1.594326685	stage_E14_vs_E10	ordinary_muscle_development	D_vs_E10;stage_1W_vs_E10;stage_3W_vs_E10;stage	1	TRUE
ENSGALG00010009164	TMEM128	PRJNA729271	0.677748451	3.23646E-11	0.677748451	stage_E14_vs_E10	ordinary_muscle_development		0	FALSE
ENSGALG00010025933	SKIC2	PRJNA729271	0.687998297	3.23868E-11	0.687998297	stage_E14_vs_E10	ordinary_muscle_development		0	FALSE
ENSGALG00010017034	ZMYM3	PRJNA729271	0.602036941	3.25742E-11	0.602036941	stage_E14_vs_E10	ordinary_muscle_development		0	FALSE
ENSGALG00010011482	DMRT2	PRJNA729271	-1.106581379	3.26494E-11	1.106581379	stage_E14_vs_E10	ordinary_muscle_development	D_vs_E10;stage_1W_vs_E10;stage_3W_vs_E10;stage	1	TRUE
ENSGALG00010024717	TF	PRJNA729271	4.456229353	3.30248E-11	4.456229353	stage_E14_vs_E10	ordinary_muscle_development	D_vs_E10;stage_1W_vs_E10;stage_3W_vs_E10;stage	1	TRUE
ENSGALG00010004370	ATP6V1H	PRJNA729271	0.499794517	3.30334E-11	0.499794517	stage_E14_vs_E10	ordinary_muscle_development		0	FALSE
ENSGALG00010020949	HPS6	PRJNA729271	0.743513695	3.30802E-11	0.743513695	stage_E14_vs_E10	ordinary_muscle_development		0	FALSE
ENSGALG00010005742	GPR183	PRJNA729271	-1.439201196	3.31016E-11	1.439201196	stage_E14_vs_E10	ordinary_muscle_development	D_vs_E10;stage_1W_vs_E10;stage_3W_vs_E10;stage	1	TRUE
ENSGALG00010003841	UBE2W	PRJNA729271	0.98265397	3.33298E-11	0.98265397	stage_E14_vs_E10	ordinary_muscle_development		0	FALSE
ENSGALG00010026124	PACSIN1	PRJNA729271	-4.085505198	3.37281E-11	4.085505198	stage_E14_vs_E10	ordinary_muscle_development	D_vs_E10;stage_1W_vs_E10;stage_3W_vs_E10;stage	1	TRUE
ENSGALG00010015431	RIN3	PRJNA729271	-1.093207327	3.37564E-11	1.093207327	stage_E14_vs_E10	ordinary_muscle_development	D_vs_E10;stage_1W_vs_E10;stage_3W_vs_E10;stage	1	TRUE
ENSGALG00010023972	ENSGALG00010023972	PRJNA729271	3.417761578	3.3924E-11	3.417761578	stage_E14_vs_E10	ordinary_muscle_development	D_vs_E10;stage_1W_vs_E10;stage_3W_vs_E10;stage	1	TRUE
ENSGALG00010014180	SLC19A1	PRJNA729271	-0.991425612	3.40939E-11	0.991425612	stage_E14_vs_E10	ordinary_muscle_development		0	FALSE
ENSGALG00010015825	RUNX3	PRJNA729271	-6.298719794	3.4105E-11	6.298719794	stage_E14_vs_E10	ordinary_muscle_development	D_vs_E10;stage_1W_vs_E10;stage_3W_vs_E10;stage	1	TRUE
ENSGALG00010021335	NFATC3	PRJNA729271	0.687964156	3.45408E-11	0.687964156	stage_E14_vs_E10	ordinary_muscle_development		0	FALSE
ENSGALG00010024018	KRT18	PRJNA729271	-8.442204263	3.46382E-11	8.442204263	stage_E14_vs_E10	ordinary_muscle_development	D_vs_E10;stage_1W_vs_E10;stage_3W_vs_E10;stage	1	TRUE
ENSGALG00010028692	RALGPS1	PRJNA729271	0.753176548	3.46503E-11	0.753176548	stage_E14_vs_E10	ordinary_muscle_development		0	FALSE
ENSGALG00010005253	POMT2	PRJNA729271	-0.660688336	3.47905E-11	0.660688336	stage_E14_vs_E10	ordinary_muscle_development		0	FALSE
ENSGALG00010026566	ENSGALG00010026566	PRJNA729271	-0.854397322	3.51474E-11	0.854397322	stage_E14_vs_E10	ordinary_muscle_development		0	FALSE
ENSGALG00010001677	SDHAF4	PRJNA729271	0.717433911	3.5304E-11	0.717433911	stage_E14_vs_E10	ordinary_muscle_development		0	FALSE
ENSGALG00010029766	ENSGALG00010029766	PRJNA729271	0.9776704	3.5878E-11	0.9776704	stage_E14_vs_E10	ordinary_muscle_development		0	FALSE
ENSGALG00010007998	RPL13	PRJNA729271	0.987296121	3.62068E-11	0.987296121	stage_E14_vs_E10	ordinary_muscle_development		0	FALSE
ENSGALG00010000292	ABHD16A	PRJNA729271	0.869756489	3.6267E-11	0.869756489	stage_E14_vs_E10	ordinary_muscle_development		0	FALSE
ENSGALG00010021798	RANBP10	PRJNA729271	1.216820385	3.65124E-11	1.216820385	stage_E14_vs_E10	ordinary_muscle_development	D_vs_E10;stage_1W_vs_E10;stage_3W_vs_E10;stage	1	TRUE
ENSGALG00010010293	ENSGALG00010010293	PRJNA729271	3.215288293	3.66132E-11	3.215288293	stage_E14_vs_E10	ordinary_muscle_development	D_vs_E10;stage_1W_vs_E10;stage_3W_vs_E10;stage	1	TRUE
ENSGALG00010016888	ENSGALG00010016888	PRJNA729271	-0.605212147	3.66887E-11	0.605212147	stage_E14_vs_E10	ordinary_muscle_development		0	FALSE
ENSGALG00010007673	FHDC1	PRJNA729271	-2.328518594	3.67973E-11	2.328518594	stage_E14_vs_E10	ordinary_muscle_development	D_vs_E10;stage_1W_vs_E10;stage_3W_vs_E10;stage	1	TRUE
ENSGALG00010014226	SLC16A7	PRJNA729271	1.750348184	3.70383E-11	1.750348184	stage_E14_vs_E10	ordinary_muscle_development	D_vs_E10;stage_1W_vs_E10;stage_3W_vs_E10;stage	1	TRUE
ENSGALG00010028047	ENSGALG00010028047	PRJNA729271	1.936931027	3.72373E-11	1.936931027	stage_E14_vs_E10	ordinary_muscle_development	D_vs_E10;stage_1W_vs_E10;stage_3W_vs_E10;stage	1	TRUE
ENSGALG00010029718	ABR	PRJNA729271	0.742027621	3.72501E-11	0.742027621	stage_E14_vs_E10	ordinary_muscle_development		0	FALSE
ENSGALG00010017755	C1D	PRJNA729271	-0.953286863	3.73346E-11	0.953286863	stage_E14_vs_E10	ordinary_muscle_development		0	FALSE
ENSGALG00010018602	MCTP2	PRJNA729271	-1.537997557	3.76128E-11	1.537997557	stage_E14_vs_E10	ordinary_muscle_development	D_vs_E10;stage_1W_vs_E10;stage_3W_vs_E10;stage	1	TRUE
ENSGALG00010019876	MRM2	PRJNA729271	1.500700295	3.8227E-11	1.500700295	stage_E14_vs_E10	ordinary_muscle_development	D_vs_E10;stage_1W_vs_E10;stage_3W_vs_E10;stage	1	TRUE
ENSGALG00010001834	ZCRB1	PRJNA729271	0.561793353	3.82323E-11	0.561793353	stage_E14_vs_E10	ordinary_muscle_development		0	FALSE
ENSGALG00010027210	ENSGALG00010027210	PRJNA729271	-1.898194448	3.83793E-11	1.898194448	stage_E14_vs_E10	ordinary_muscle_development	D_vs_E10;stage_1W_vs_E10;stage_3W_vs_E10;stage	1	TRUE
ENSGALG00010022567	PAOX	PRJNA729271	0.836620189	3.84529E-11	0.836620189	stage_E14_vs_E10	ordinary_muscle_development		0	FALSE
ENSGALG00010004970	RBM48	PRJNA729271	1.110772311	3.8824E-11	1.110772311	stage_E14_vs_E10	ordinary_muscle_development	D_vs_E10;stage_1W_vs_E10;stage_3W_vs_E10;stage	1	TRUE
ENSGALG00010014753	ENSGALG00010014753	PRJNA729271	-4.350340445	3.90512E-11	4.350340445	stage_E14_vs_E10	ordinary_muscle_development	D_vs_E10;stage_1W_vs_E10;stage_3W_vs_E10;stage	1	TRUE
ENSGALG00010024624	CTSD	PRJNA729271	0.867524151	3.91205E-11	0.867524151	stage_E14_vs_E10	ordinary_muscle_development		0	FALSE
ENSGALG00010025598	ENSGALG00010025598	PRJNA729271	2.661137082	3.91617E-11	2.661137082	stage_E14_vs_E10	ordinary_muscle_development	D_vs_E10;stage_1W_vs_E10;stage_3W_vs_E10;stage	1	TRUE
ENSGALG00010024410	MYG1	PRJNA729271	0.711121648	3.94035E-11	0.711121648	stage_E14_vs_E10	ordinary_muscle_development		0	FALSE
ENSGALG00010012861	WDR70	PRJNA729271	-0.571124953	3.95071E-11	0.571124953	stage_E14_vs_E10	ordinary_muscle_development		0	FALSE
ENSGALG00010014224	NMUR2	PRJNA729271	-6.519787199	3.97644E-11	6.519787199	stage_E14_vs_E10	ordinary_muscle_development	D_vs_E10;stage_1W_vs_E10;stage_3W_vs_E10;stage	1	TRUE
ENSGALG00010011774	ANKRA2	PRJNA729271	-0.633849288	4.06367E-11	0.633849288	stage_E14_vs_E10	ordinary_muscle_development		0	FALSE
ENSGALG00010014334	HMGCS1	PRJNA729271	-1.08433781	4.08998E-11	1.08433781	stage_E14_vs_E10	ordinary_muscle_development	D_vs_E10;stage_1W_vs_E10;stage_3W_vs_E10;stage	1	TRUE
ENSGALG00010022449	ENSGALG00010022449	PRJNA729271	-0.69388392	4.11592E-11	0.69388392	stage_E14_vs_E10	ordinary_muscle_development		0	FALSE
ENSGALG00010017684	TRMT61B	PRJNA729271	0.636913574	4.13346E-11	0.636913574	stage_E14_vs_E10	ordinary_muscle_development		0	FALSE
ENSGALG00010029579	GALNT17	PRJNA729271	1.24668568	4.17129E-11	1.24668568	stage_E14_vs_E10	ordinary_muscle_development	D_vs_E10;stage_1W_vs_E10;stage_3W_vs_E10;stage	1	TRUE
ENSGALG00010018911	CNIH1	PRJNA729271	0.628845245	4.18077E-11	0.628845245	stage_E14_vs_E10	ordinary_muscle_development		0	FALSE
ENSGALG00010022867	ATPAF1	PRJNA729271	-0.929913103	4.18669E-11	0.929913103	stage_E14_vs_E10	ordinary_muscle_development		0	FALSE
ENSGALG00010028490	NECTIN4	PRJNA729271	-2.481335643	4.24023E-11	2.481335643	stage_E14_vs_E10	ordinary_muscle_development	D_vs_E10;stage_1W_vs_E10;stage_3W_vs_E10;stage	1	TRUE
ENSGALG00010024651	PSMD3	PRJNA729271	-0.872393991	4.24287E-11	0.872393991	stage_E14_vs_E10	ordinary_muscle_development		0	FALSE
ENSGALG00010023981	ENSGALG00010023981	PRJNA729271	2.43235899	4.28646E-11	2.43235899	stage_E14_vs_E10	ordinary_muscle_development	D_vs_E10;stage_1W_vs_E10;stage_3W_vs_E10;stage	1	TRUE
ENSGALG00010015774	PLS1	PRJNA729271	-2.777527022	4.3352E-11	2.777527022	stage_E14_vs_E10	ordinary_muscle_development	D_vs_E10;stage_1W_vs_E10;stage_3W_vs_E10;stage	1	TRUE
ENSGALG00010016508	CREBBP	PRJNA729271	0.807658901	4.38004E-11	0.807658901	stage_E14_vs_E10	ordinary_muscle_development		0	FALSE
ENSGALG00010024791	SHANK2	PRJNA729271	-3.122845267	4.42381E-11	3.122845267	stage_E14_vs_E10	ordinary_muscle_development	D_vs_E10;stage_1W_vs_E10;stage_3W_vs_E10;stage	1	TRUE
ENSGALG00010017962	TXNDC16	PRJNA729271	-1.021670959	4.44237E-11	1.021670959	stage_E14_vs_E10	ordinary_muscle_development	D_vs_E10;stage_1W_vs_E10;stage_3W_vs_E10;stage	1	TRUE
ENSGALG00010017020	GJB5	PRJNA729271	-3.622173878	4.44829E-11	3.622173878	stage_E14_vs_E10	ordinary_muscle_development	D_vs_E10;stage_1W_vs_E10;stage_3W_vs_E10;stage	1	TRUE
ENSGALG00010023437	KCNJ5	PRJNA729271	1.694614293	4.46662E-11	1.694614293	stage_E14_vs_E10	ordinary_muscle_development	D_vs_E10;stage_1W_vs_E10;stage_3W_vs_E10;stage	1	TRUE
ENSGALG00010020617	DGUOK	PRJNA729271	-0.762331866	4.53235E-11	0.762331866	stage_E14_vs_E10	ordinary_muscle_development		0	FALSE
ENSGALG00010025329	MYO1A	PRJNA729271	-1.423003591	4.55167E-11	1.423003591	stage_E14_vs_E10	ordinary_muscle_development	D_vs_E10;stage_1W_vs_E10;stage_3W_vs_E10;stage	1	TRUE
ENSGALG00010005299	ODAD2	PRJNA729271	-6.286672903	4.6192E-11	6.286672903	stage_E14_vs_E10	ordinary_muscle_development	D_vs_E10;stage_1W_vs_E10;stage_3W_vs_E10;stage	1	TRUE
ENSGALG00010025210	HSPB2	PRJNA729271	1.985460814	4.65649E-11	1.985460814	stage_E14_vs_E10	ordinary_muscle_development	D_vs_E10;stage_1W_vs_E10;stage_3W_vs_E10;stage	1	TRUE
ENSGALG00010009360	TNFAIP8	PRJNA729271	-0.89631014	4.65906E-11	0.89631014	stage_E14_vs_E10	ordinary_muscle_development		0	FALSE
ENSGALG00010016588	ATP11B	PRJNA729271	-0.676486124	4.71554E-11	0.676486124	stage_E14_vs_E10	ordinary_muscle_development		0	FALSE
ENSGALG00010016385	HTT	PRJNA729271	0.716946621	4.7237E-11	0.716946621	stage_E14_vs_E10	ordinary_muscle_development		0	FALSE
ENSGALG00010021493	UNC93B1	PRJNA729271	0.84425158	4.73368E-11	0.84425158	stage_E14_vs_E10	ordinary_muscle_development		0	FALSE
ENSGALG00010007663	ARFIP1	PRJNA729271	-0.745049348	4.74505E-11	0.745049348	stage_E14_vs_E10	ordinary_muscle_development		0	FALSE
ENSGALG00010025907	ARHGEF12	PRJNA729271	0.481299858	4.7457E-11	0.481299858	stage_E14_vs_E10	ordinary_muscle_development		0	FALSE
ENSGALG00010009219	TNFRSF11B	PRJNA729271	1.213286272	4.77317E-11	1.213286272	stage_E14_vs_E10	ordinary_muscle_development	D_vs_E10;stage_1W_vs_E10;stage_3W_vs_E10;stage	1	TRUE
ENSGALG00010003531	PPM1K	PRJNA729271	-1.017916973	4.79982E-11	1.017916973	stage_E14_vs_E10	ordinary_muscle_development	D_vs_E10;stage_1W_vs_E10;stage_3W_vs_E10;stage	1	TRUE
ENSGALG00010023555	HPS4	PRJNA729271	-1.153056386	4.90075E-11	1.153056386	stage_E14_vs_E10	ordinary_muscle_development	D_vs_E10;stage_1W_vs_E10;stage_3W_vs_E10;stage	1	TRUE
ENSGALG00010017531	SACM1L	PRJNA729271	-0.767416425	4.90755E-11	0.767416425	stage_E14_vs_E10	ordinary_muscle_development		0	FALSE
ENSGALG00010023103	DGKD	PRJNA729271	0.841820199	4.94695E-11	0.841820199	stage_E14_vs_E10	ordinary_muscle_development		0	FALSE
ENSGALG00010021779	UNC13C	PRJNA729271	-1.720378201	4.97747E-11	1.720378201	stage_E14_vs_E10	ordinary_muscle_development	D_vs_E10;stage_1W_vs_E10;stage_3W_vs_E10;stage	1	TRUE
ENSGALG00010029647	RSAD1	PRJNA729271	1.000938064	5.02052E-11	1.000938064	stage_E14_vs_E10	ordinary_muscle_development	D_vs_E10;stage_1W_vs_E10;stage_3W_vs_E10;stage	1	TRUE
ENSGALG00010002428	IBSP	PRJNA729271	-5.381649956	5.02734E-11	5.381649956	stage_E14_vs_E10	ordinary_muscle_development	D_vs_E10;stage_1W_vs_E10;stage_3W_vs_E10;stage	1	TRUE
ENSGALG00010020640	PTPN14	PRJNA729271	-0.622911959	5.08786E-11	0.622911959	stage_E14_vs_E10	ordinary_muscle_development		0	FALSE
ENSGALG00010016993	ENSGALG00010016993	PRJNA729271	1.211640519	5.08918E-11	1.211640519	stage_E14_vs_E10	ordinary_muscle_development	D_vs_E10;stage_1W_vs_E10;stage_3W_vs_E10;stage	1	TRUE
ENSGALG00010013967	MLLT3	PRJNA729271	-1.069131643	5.13889E-11	1.069131643	stage_E14_vs_E10	ordinary_muscle_development	D_vs_E10;stage_1W_vs_E10;stage_3W_vs_E10;stage	1	TRUE
ENSGALG00010024776	MPHOSPH10	PRJNA729271	-0.733875411	5.15441E-11	0.733875411	stage_E14_vs_E10	ordinary_muscle_development		0	FALSE
ENSGALG00010015318	SALL4	PRJNA729271	1.108481547	5.16561E-11	1.108481547	stage_E14_vs_E10	ordinary_muscle_development	D_vs_E10;stage_1W_vs_E10;stage_3W_vs_E10;stage	1	TRUE
ENSGALG00010024074	ADCY7	PRJNA729271	-1.557344622	5.17719E-11	1.557344622	stage_E14_vs_E10	ordinary_muscle_development	D_vs_E10;stage_1W_vs_E10;stage_3W_vs_E10;stage	1	TRUE
ENSGALG00010025459	DIABLO	PRJNA729271	-0.535077214	5.17754E-11	0.535077214	stage_E14_vs_E10	ordinary_muscle_development		0	FALSE
ENSGALG00010010314	CRB1	PRJNA729271	1.904624662	5.21783E-11	1.904624662	stage_E14_vs_E10	ordinary_muscle_development	D_vs_E10;stage_1W_vs_E10;stage_3W_vs_E10;stage	1	TRUE
ENSGALG00010029761	MRPL38	PRJNA729271	0.787773025	5.22721E-11	0.787773025	stage_E14_vs_E10	ordinary_muscle_development		0	FALSE
ENSGALG00010023118	PIDD1	PRJNA729271	0.908888737	5.28308E-11	0.908888737	stage_E14_vs_E10	ordinary_muscle_development		0	FALSE
ENSGALG00010015025	TRAF3IP1	PRJNA729271	-1.321153605	5.32344E-11	1.321153605	stage_E14_vs_E10	ordinary_muscle_development	D_vs_E10;stage_1W_vs_E10;stage_3W_vs_E10;stage	1	TRUE
ENSGALG00010019592	ALKBH3	PRJNA729271	0.869006388	5.33929E-11	0.869006388	stage_E14_vs_E10	ordinary_muscle_development		0	FALSE
ENSGALG00010013809	MX1	PRJNA729271	8.122167451	5.35616E-11	8.122167451	stage_E14_vs_E10	ordinary_muscle_development	D_vs_E10;stage_1W_vs_E10;stage_3W_vs_E10;stage	1	TRUE
ENSGALG00010021827	CCZ1	PRJNA729271	-0.648124144	5.38985E-11	0.648124144	stage_E14_vs_E10	ordinary_muscle_development		0	FALSE
ENSGALG00010015397	SRD5A3	PRJNA729271	-0.599726731	5.40949E-11	0.599726731	stage_E14_vs_E10	ordinary_muscle_development		0	FALSE
ENSGALG00010020900	PDHB	PRJNA729271	0.779904124	5.45795E-11	0.779904124	stage_E14_vs_E10	ordinary_muscle_development		0	FALSE
ENSGALG00010022448	FERRY3	PRJNA729271	-0.819258316	5.53148E-11	0.819258316	stage_E14_vs_E10	ordinary_muscle_development		0	FALSE
ENSGALG00010011129	ENSGALG00010011129	PRJNA729271	0.773967504	5.56019E-11	0.773967504	stage_E14_vs_E10	ordinary_muscle_development		0	FALSE
ENSGALG00010010805	PTRHD1	PRJNA729271	-1.156420662	5.60133E-11	1.156420662	stage_E14_vs_E10	ordinary_muscle_development	D_vs_E10;stage_1W_vs_E10;stage_3W_vs_E10;stage	1	TRUE
ENSGALG00010021531	RAB15	PRJNA729271	-2.01651155	5.62193E-11	2.01651155	stage_E14_vs_E10	ordinary_muscle_development	D_vs_E10;stage_1W_vs_E10;stage_3W_vs_E10;stage	1	TRUE
ENSGALG00010018709	SNX5	PRJNA729271	-0.778409741	5.62997E-11	0.778409741	stage_E14_vs_E10	ordinary_muscle_development		0	FALSE
ENSGALG00010009715	NUDCD2	PRJNA729271	-0.631408033	5.71155E-11	0.631408033	stage_E14_vs_E10	ordinary_muscle_development		0	FALSE
ENSGALG00010015621	IDNK	PRJNA729271	1.001248773	5.72064E-11	1.001248773	stage_E14_vs_E10	ordinary_muscle_development	D_vs_E10;stage_1W_vs_E10;stage_3W_vs_E10;stage	1	TRUE
ENSGALG00010017338	MBNL3	PRJNA729271	0.499129256	5.75567E-11	0.499129256	stage_E14_vs_E10	ordinary_muscle_development		0	FALSE
ENSGALG00010000741	FAM105A	PRJNA729271	-1.334931915	5.78461E-11	1.334931915	stage_E14_vs_E10	ordinary_muscle_development	D_vs_E10;stage_1W_vs_E10;stage_3W_vs_E10;stage	1	TRUE
ENSGALG00010013589	DSCAM	PRJNA729271	-6.213123529	5.78852E-11	6.213123529	stage_E14_vs_E10	ordinary_muscle_development	D_vs_E10;stage_1W_vs_E10;stage_3W_vs_E10;stage	1	TRUE
ENSGALG00010024618	VPS13C	PRJNA729271	-0.847317038	5.80803E-11	0.847317038	stage_E14_vs_E10	ordinary_muscle_development		0	FALSE
ENSGALG00010005636	ENSGALG00010005636	PRJNA729271	3.847557279	5.83305E-11	3.847557279	stage_E14_vs_E10	ordinary_muscle_development	D_vs_E10;stage_1W_vs_E10;stage_3W_vs_E10;stage	1	TRUE
ENSGALG00010002926	GALNTL6	PRJNA729271	-2.480918999	5.84845E-11	2.480918999	stage_E14_vs_E10	ordinary_muscle_development	D_vs_E10;stage_1W_vs_E10;stage_3W_vs_E10;stage	1	TRUE
ENSGALG00010012114	TAL2	PRJNA729271	2.029759336	5.85435E-11	2.029759336	stage_E14_vs_E10	ordinary_muscle_development	D_vs_E10;stage_1W_vs_E10;stage_3W_vs_E10;stage	1	TRUE
ENSGALG00010008396	LRRTM2	PRJNA729271	-3.166515325	5.90236E-11	3.166515325	stage_E14_vs_E10	ordinary_muscle_development	D_vs_E10;stage_1W_vs_E10;stage_3W_vs_E10;stage	1	TRUE
ENSGALG00010017341	TA3	PRJNA729271	-0.541437399	5.93045E-11	0.541437399	stage_E14_vs_E10	ordinary_muscle_development		0	FALSE
ENSGALG00010013263	ATP11A	PRJNA729271	-0.759330104	5.97131E-11	0.759330104	stage_E14_vs_E10	ordinary_muscle_development		0	FALSE
ENSGALG00010021649	PXN	PRJNA729271	0.642572655	5.98978E-11	0.642572655	stage_E14_vs_E10	ordinary_muscle_development		0	FALSE
ENSGALG00010002243	ARSH	PRJNA729271	-1.233912805	6.00599E-11	1.233912805	stage_E14_vs_E10	ordinary_muscle_development	D_vs_E10;stage_1W_vs_E10;stage_3W_vs_E10;stage	1	TRUE
ENSGALG00010009348	ERGIC2	PRJNA729271	-0.562176266	6.02633E-11	0.562176266	stage_E14_vs_E10	ordinary_muscle_development		0	FALSE
ENSGALG00010019208	PLXND1	PRJNA729271	1.065036368	6.04996E-11	1.065036368	stage_E14_vs_E10	ordinary_muscle_development	D_vs_E10;stage_1W_vs_E10;stage_3W_vs_E10;stage	1	TRUE
ENSGALG00010003962	UMAD1	PRJNA729271	1.357277574	6.09197E-11	1.357277574	stage_E14_vs_E10	ordinary_muscle_development	D_vs_E10;stage_1W_vs_E10;stage_3W_vs_E10;stage	1	TRUE
ENSGALG00010006465	PLAA	PRJNA729271	-0.795092926	6.10472E-11	0.795092926	stage_E14_vs_E10	ordinary_muscle_development		0	FALSE
ENSGALG00010016428	ADRA1D	PRJNA729271	1.323780842	6.15033E-11	1.323780842	stage_E14_vs_E10	ordinary_muscle_development	D_vs_E10;stage_1W_vs_E10;stage_3W_vs_E10;stage	1	TRUE
ENSGALG00010003261	PEX2	PRJNA729271	-1.15999578	6.18491E-11	1.15999578	stage_E14_vs_E10	ordinary_muscle_development	D_vs_E10;stage_1W_vs_E10;stage_3W_vs_E10;stage	1	TRUE
ENSGALG00010010651	NUAK1	PRJNA729271	0.895941234	6.20629E-11	0.895941234	stage_E14_vs_E10	ordinary_muscle_development		0	FALSE
ENSGALG00010004006	NKAIN3	PRJNA729271	-2.31546576	6.21004E-11	2.31546576	stage_E14_vs_E10	ordinary_muscle_development	D_vs_E10;stage_1W_vs_E10;stage_3W_vs_E10;stage	1	TRUE
ENSGALG00010004451	CLDND1	PRJNA729271	-0.572274842	6.2261E-11	0.572274842	stage_E14_vs_E10	ordinary_muscle_development		0	FALSE
ENSGALG00010009637	CYBA	PRJNA729271	0.91302403	6.27365E-11	0.91302403	stage_E14_vs_E10	ordinary_muscle_development		0	FALSE
ENSGALG00010029279	ENSGALG00010029279	PRJNA729271	1.05662886	6.30414E-11	1.05662886	stage_E14_vs_E10	ordinary_muscle_development	D_vs_E10;stage_1W_vs_E10;stage_3W_vs_E10;stage	1	TRUE
ENSGALG00010017633	HLX	PRJNA729271	0.988800522	6.35349E-11	0.988800522	stage_E14_vs_E10	ordinary_muscle_development		0	FALSE
ENSGALG00010010116	DRD5	PRJNA729271	-6.047361318	6.38253E-11	6.047361318	stage_E14_vs_E10	ordinary_muscle_development	D_vs_E10;stage_1W_vs_E10;stage_3W_vs_E10;stage	1	TRUE
ENSGALG00010014906	IFT27	PRJNA729271	-0.743541432	6.38986E-11	0.743541432	stage_E14_vs_E10	ordinary_muscle_development		0	FALSE
ENSGALG00010024820	NUDT19	PRJNA729271	0.924670117	6.42158E-11	0.924670117	stage_E14_vs_E10	ordinary_muscle_development		0	FALSE
ENSGALG00010019300	MESDC2	PRJNA729271	-0.443663495	6.44812E-11	0.443663495	stage_E14_vs_E10	ordinary_muscle_development		0	FALSE
ENSGALG00010011287	ENSGALG00010011287	PRJNA729271	-0.810391988	6.46149E-11	0.810391988	stage_E14_vs_E10	ordinary_muscle_development		0	FALSE
ENSGALG00010022357	ENSGALG00010022357	PRJNA729271	-6.163753904	6.47669E-11	6.163753904	stage_E14_vs_E10	ordinary_muscle_development	D_vs_E10;stage_1W_vs_E10;stage_3W_vs_E10;stage	1	TRUE
ENSGALG00010029938	ENSGALG00010029938	PRJNA729271	-2.141996597	6.49309E-11	2.141996597	stage_E14_vs_E10	ordinary_muscle_development	D_vs_E10;stage_1W_vs_E10;stage_3W_vs_E10;stage	1	TRUE
ENSGALG00010027162	SLC4A2	PRJNA729271	0.863495657	6.5101E-11	0.863495657	stage_E14_vs_E10	ordinary_muscle_development		0	FALSE
ENSGALG00010008034	DPEP1	PRJNA729271	-3.020888085	6.60431E-11	3.020888085	stage_E14_vs_E10	ordinary_muscle_development	D_vs_E10;stage_1W_vs_E10;stage_3W_vs_E10;stage	1	TRUE
ENSGALG00010007730	COG2	PRJNA729271	-0.44340618	6.63787E-11	0.44340618	stage_E14_vs_E10	ordinary_muscle_development		0	FALSE
ENSGALG00010017553	EDA	PRJNA729271	0.733360674	6.64248E-11	0.733360674	stage_E14_vs_E10	ordinary_muscle_development		0	FALSE
ENSGALG00010018149	ENSGALG00010018149	PRJNA729271	1.941954987	6.6658E-11	1.941954987	stage_E14_vs_E10	ordinary_muscle_development	D_vs_E10;stage_1W_vs_E10;stage_3W_vs_E10;stage	1	TRUE
ENSGALG00010009170	RPL26L1	PRJNA729271	1.286448102	6.72087E-11	1.286448102	stage_E14_vs_E10	ordinary_muscle_development	D_vs_E10;stage_1W_vs_E10;stage_3W_vs_E10;stage	1	TRUE

ENSGALG00010000643	VKORC1	PRJNA729271	1.33834422	6.75185E-11	1.33834422	stage_E14_vs_E10	ordinary_muscle_development	D_vs_E10;stage_1W_vs_E10;stage_3W_vs_E10;stage	1	TRUE
ENSGALG00010020642	ANKRD2	PRJNA729271	4.191808283	6.75519E-11	4.191808283	stage_E14_vs_E10	ordinary_muscle_development	D_vs_E10;stage_1W_vs_E10;stage_3W_vs_E10;stage	1	TRUE
ENSGALG00010023234	ENSGALG00010023234	PRJNA729271	-0.64109482	6.77203E-11	0.64109482	stage_E14_vs_E10	ordinary_muscle_development		0	FALSE
ENSGALG00010003985	PRMT5	PRJNA729271	0.887862781	6.77436E-11	0.887862781	stage_E14_vs_E10	ordinary_muscle_development		0	FALSE
ENSGALG00010028464	ENSGALG00010028464	PRJNA729271	0.746304085	6.82239E-11	0.746304085	stage_E14_vs_E10	ordinary_muscle_development		0	FALSE
ENSGALG00010019042	PEX6	PRJNA729271	0.703198717	6.87306E-11	0.703198717	stage_E14_vs_E10	ordinary_muscle_development		0	FALSE
ENSGALG00010007430	MEF2C	PRJNA729271	1.008126302	6.8741E-11	1.008126302	stage_E14_vs_E10	ordinary_muscle_development	D_vs_E10;stage_1W_vs_E10;stage_3W_vs_E10;stage	1	TRUE
ENSGALG00010028546	ENSGALG00010028546	PRJNA729271	10.41617262	6.91987E-11	10.41617262	stage_E14_vs_E10	ordinary_muscle_development	D_vs_E10;stage_1W_vs_E10;stage_3W_vs_E10;stage	1	TRUE
ENSGALG00010021101	TMEM240	PRJNA729271	-4.372442171	6.96574E-11	4.372442171	stage_E14_vs_E10	ordinary_muscle_development	D_vs_E10;stage_1W_vs_E10;stage_3W_vs_E10;stage	1	TRUE
ENSGALG00010017723	KCNK12	PRJNA729271	-3.046951273	6.97569E-11	3.046951273	stage_E14_vs_E10	ordinary_muscle_development	D_vs_E10;stage_1W_vs_E10;stage_3W_vs_E10;stage	1	TRUE
ENSGALG00010018410	EXOC6B	PRJNA729271	0.649755395	6.98673E-11	0.649755395	stage_E14_vs_E10	ordinary_muscle_development		0	FALSE
ENSGALG00010010544	ENSGALG00010010544	PRJNA729271	-1.151674894	7.01083E-11	1.151674894	stage_E14_vs_E10	ordinary_muscle_development	D_vs_E10;stage_1W_vs_E10;stage_3W_vs_E10;stage	1	TRUE
ENSGALG00010018693	ENSGALG00010018693	PRJNA729271	1.08656961	7.05172E-11	1.08656961	stage_E14_vs_E10	ordinary_muscle_development	D_vs_E10;stage_1W_vs_E10;stage_3W_vs_E10;stage	1	TRUE
ENSGALG00010002539	SNAP91	PRJNA729271	-1.409962426	7.09985E-11	1.409962426	stage_E14_vs_E10	ordinary_muscle_development	D_vs_E10;stage_1W_vs_E10;stage_3W_vs_E10;stage	1	TRUE
ENSGALG00010011764	PCYOX1L	PRJNA729271	-0.905042042	7.10184E-11	0.905042042	stage_E14_vs_E10	ordinary_muscle_development		0	FALSE
ENSGALG00010022343	GATA5	PRJNA729271	-4.539785707	7.19786E-11	4.539785707	stage_E14_vs_E10	ordinary_muscle_development	D_vs_E10;stage_1W_vs_E10;stage_3W_vs_E10;stage	1	TRUE
ENSGALG00010003839	ENSGALG00010003839	PRJNA729271	4.766927931	7.19858E-11	4.766927931	stage_E14_vs_E10	ordinary_muscle_development	D_vs_E10;stage_1W_vs_E10;stage_3W_vs_E10;stage	1	TRUE
ENSGALG00010029314	MED13	PRJNA729271	0.837866455	7.21021E-11	0.837866455	stage_E14_vs_E10	ordinary_muscle_development		0	FALSE
ENSGALG00010000140	SEPHS3	PRJNA729271	-0.852785657	7.22236E-11	0.852785657	stage_E14_vs_E10	ordinary_muscle_development		0	FALSE
ENSGALG00010016946	TAF1	PRJNA729271	0.707688737	7.31482E-11	0.707688737	stage_E14_vs_E10	ordinary_muscle_development		0	FALSE
ENSGALG00010024846	LMO2	PRJNA729271	0.947469035	7.31712E-11	0.947469035	stage_E14_vs_E10	ordinary_muscle_development		0	FALSE
ENSGALG00010010071	GLT8D2	PRJNA729271	-1.79669874	7.33095E-11	1.79669874	stage_E14_vs_E10	ordinary_muscle_development	D_vs_E10;stage_1W_vs_E10;stage_3W_vs_E10;stage	1	TRUE
ENSGALG00010018506	ENSGALG00010018506	PRJNA729271	0.944467751	7.34027E-11	0.944467751	stage_E14_vs_E10	ordinary_muscle_development		0	FALSE
ENSGALG00010008693	C4orf46	PRJNA729271	-0.703320947	7.35822E-11	0.703320947	stage_E14_vs_E10	ordinary_muscle_development		0	FALSE
ENSGALG00010002399	CSMD1	PRJNA729271	-2.693782654	7.37351E-11	2.693782654	stage_E14_vs_E10	ordinary_muscle_development	D_vs_E10;stage_1W_vs_E10;stage_3W_vs_E10;stage	1	TRUE
ENSGALG00010009152	RPP25L	PRJNA729271	0.814495606	7.39765E-11	0.814495606	stage_E14_vs_E10	ordinary_muscle_development		0	FALSE
ENSGALG00010028074	ENSGALG00010028074	PRJNA729271	2.977745003	7.40277E-11	2.977745003	stage_E14_vs_E10	ordinary_muscle_development	D_vs_E10;stage_1W_vs_E10;stage_3W_vs_E10;stage	1	TRUE
ENSGALG00010028925	ENSGALG00010028925	PRJNA729271	2.132491065	7.40963E-11	2.132491065	stage_E14_vs_E10	ordinary_muscle_development	D_vs_E10;stage_1W_vs_E10;stage_3W_vs_E10;stage	1	TRUE
ENSGALG00010027440	TWF2	PRJNA729271	1.26369138	7.43397E-11	1.26369138	stage_E14_vs_E10	ordinary_muscle_development	D_vs_E10;stage_1W_vs_E10;stage_3W_vs_E10;stage	1	TRUE
ENSGALG00010013654	SLC6A7	PRJNA729271	-6.086802561	7.45935E-11	6.086802561	stage_E14_vs_E10	ordinary_muscle_development	D_vs_E10;stage_1W_vs_E10;stage_3W_vs_E10;stage	1	TRUE
ENSGALG00010026260	ENSGALG00010026260	PRJNA729271	20.97472475	7.48425E-11	20.97472475	stage_E14_vs_E10	ordinary_muscle_development	D_vs_E10;stage_1W_vs_E10;stage_3W_vs_E10;stage	1	TRUE
ENSGALG00010008497	PAM	PRJNA729271	-0.865273323	7.49217E-11	0.865273323	stage_E14_vs_E10	ordinary_muscle_development		0	FALSE
ENSGALG00010005291	IFNGR1	PRJNA729271	0.644087495	7.51356E-11	0.644087495	stage_E14_vs_E10	ordinary_muscle_development		0	FALSE
ENSGALG00010025745	COX14	PRJNA729271	1.50098602	7.52957E-11	1.50098602	stage_E14_vs_E10	ordinary_muscle_development	D_vs_E10;stage_1W_vs_E10;stage_3W_vs_E10;stage	1	TRUE
ENSGALG00010005238	TMEM63C	PRJNA729271	-1.137435995	7.61994E-11	1.137435995	stage_E14_vs_E10	ordinary_muscle_development	D_vs_E10;stage_1W_vs_E10;stage_3W_vs_E10;stage	1	TRUE
ENSGALG00010018796	PER3	PRJNA729271	2.051291838	7.62502E-11	2.051291838	stage_E14_vs_E10	ordinary_muscle_development	D_vs_E10;stage_1W_vs_E10;stage_3W_vs_E10;stage	1	TRUE
ENSGALG00010012072	CAPRIN2	PRJNA729271	-1.027515892	7.66799E-11	1.027515892	stage_E14_vs_E10	ordinary_muscle_development	D_vs_E10;stage_1W_vs_E10;stage_3W_vs_E10;stage	1	TRUE
ENSGALG00010016873	ENTPD6	PRJNA729271	0.923289731	7.75786E-11	0.923289731	stage_E14_vs_E10	ordinary_muscle_development		0	FALSE
ENSGALG00010003101	KLHL15	PRJNA729271	-0.491151927	7.83426E-11	0.491151927	stage_E14_vs_E10	ordinary_muscle_development		0	FALSE
ENSGALG00010021010	LEO1	PRJNA729271	-0.761213329	7.89884E-11	0.761213329	stage_E14_vs_E10	ordinary_muscle_development		0	FALSE
ENSGALG00010001406	EML3	PRJNA729271	1.057419597	7.91289E-11	1.057419597	stage_E14_vs_E10	ordinary_muscle_development	D_vs_E10;stage_1W_vs_E10;stage_3W_vs_E10;stage	1	TRUE
ENSGALG00010007783	NOL7	PRJNA729271	-0.737980979	7.9268E-11	0.737980979	stage_E14_vs_E10	ordinary_muscle_development		0	FALSE
ENSGALG00010011468	EFHC2	PRJNA729271	-2.141897489	7.98078E-11	2.141897489	stage_E14_vs_E10	ordinary_muscle_development	D_vs_E10;stage_1W_vs_E10;stage_3W_vs_E10;stage	1	TRUE
ENSGALG00010019921	CALM2	PRJNA729271	0.74764724	8.17526E-11	0.74764724	stage_E14_vs_E10	ordinary_muscle_development		0	FALSE
ENSGALG00010000795	ENSGALG00010000795	PRJNA729271	-1.70984174	8.28181E-11	1.70984174	stage_E14_vs_E10	ordinary_muscle_development	D_vs_E10;stage_1W_vs_E10;stage_3W_vs_E10;stage	1	TRUE
ENSGALG00010014739	TRMT2B	PRJNA729271	1.15836656	8.31135E-11	1.15836656	stage_E14_vs_E10	ordinary_muscle_development	D_vs_E10;stage_1W_vs_E10;stage_3W_vs_E10;stage	1	TRUE
ENSGALG00010027935	ISY1	PRJNA729271	-0.593313711	8.41776E-11	0.593313711	stage_E14_vs_E10	ordinary_muscle_development		0	FALSE
ENSGALG00010022585	CIR1	PRJNA729271	-0.643731079	8.56616E-11	0.643731079	stage_E14_vs_E10	ordinary_muscle_development		0	FALSE
ENSGALG00010005387	CLDN10	PRJNA729271	-5.589777954	8.6356E-11	5.589777954	stage_E14_vs_E10	ordinary_muscle_development	D_vs_E10;stage_1W_vs_E10;stage_3W_vs_E10;stage	1	TRUE
ENSGALG00010010064	ENSGALG00010010064	PRJNA729271	-0.604120353	8.72105E-11	0.604120353	stage_E14_vs_E10	ordinary_muscle_development		0	FALSE
ENSGALG00010021188	TNRC18	PRJNA729271	-0.885521972	8.73515E-11	0.885521972	stage_E14_vs_E10	ordinary_muscle_development		0	FALSE
ENSGALG00010012358	STK10	PRJNA729271	0.984029213	8.79366E-11	0.984029213	stage_E14_vs_E10	ordinary_muscle_development		0	FALSE
ENSGALG00010006134	K123	PRJNA729271	4.675016642	8.88987E-11	4.675016642	stage_E14_vs_E10	ordinary_muscle_development	D_vs_E10;stage_1W_vs_E10;stage_3W_vs_E10;stage	1	TRUE
ENSGALG00010009941	CACNA1E	PRJNA729271	-3.304324575	8.8954E-11	3.304324575	stage_E14_vs_E10	ordinary_muscle_development	D_vs_E10;stage_1W_vs_E10;stage_3W_vs_E10;stage	1	TRUE
ENSGALG00010023786	ENSGALG00010023786	PRJNA729271	-3.532609502	8.92475E-11	3.532609502	stage_E14_vs_E10	ordinary_muscle_development	D_vs_E10;stage_1W_vs_E10;stage_3W_vs_E10;stage	1	TRUE
ENSGALG00010013348	MKRN4P	PRJNA729271	0.462925106	9.17873E-11	0.462925106	stage_E14_vs_E10	ordinary_muscle_development		0	FALSE
ENSGALG00010015052	ENSGALG00010015052	PRJNA729271	-0.722964214	9.27512E-11	0.722964214	stage_E14_vs_E10	ordinary_muscle_development		0	FALSE
ENSGALG00010019213	RHBDF1	PRJNA729271	0.731226811	9.28257E-11	0.731226811	stage_E14_vs_E10	ordinary_muscle_development		0	FALSE
ENSGALG00010020938	SMG1	PRJNA729271	0.528572082	9.2971E-11	0.528572082	stage_E14_vs_E10	ordinary_muscle_development		0	FALSE
ENSGALG00010022353	SLC39A13	PRJNA729271	0.628833362	9.31222E-11	0.628833362	stage_E14_vs_E10	ordinary_muscle_development		0	FALSE
ENSGALG00010018022	XPO6	PRJNA729271	-0.79244165	9.34149E-11	0.79244165	stage_E14_vs_E10	ordinary_muscle_development		0	FALSE
ENSGALG00010010891	POLR3H	PRJNA729271	-0.713676994	9.38618E-11	0.713676994	stage_E14_vs_E10	ordinary_muscle_development		0	FALSE
ENSGALG00010022544	SPICE1	PRJNA729271	0.458146349	9.39283E-11	0.458146349	stage_E14_vs_E10	ordinary_muscle_development		0	FALSE
ENSGALG00010011655	SORCS2	PRJNA729271	-1.227105719	9.41771E-11	1.227105719	stage_E14_vs_E10	ordinary_muscle_development	D_vs_E10;stage_1W_vs_E10;stage_3W_vs_E10;stage	1	TRUE
ENSGALG00010021606	RNF8	PRJNA729271	-0.642900804	9.43133E-11	0.642900804	stage_E14_vs_E10	ordinary_muscle_development		0	FALSE
ENSGALG00010012834	TPH2	PRJNA729271	-6.434833247	9.48864E-11	6.434833247	stage_E14_vs_E10	ordinary_muscle_development	D_vs_E10;stage_1W_vs_E10;stage_3W_vs_E10;stage	1	TRUE
ENSGALG00010013317	PREP	PRJNA729271	-0.73393569	9.55072E-11	0.73393569	stage_E14_vs_E10	ordinary_muscle_development		0	FALSE
ENSGALG00010000525	ENSGALG00010000525	PRJNA729271	-4.093231398	9.57821E-11	4.093231398	stage_E14_vs_E10	ordinary_muscle_development	D_vs_E10;stage_1W_vs_E10;stage_3W_vs_E10;stage	1	TRUE
ENSGALG00010017521	MIIP	PRJNA729271	0.753410002	9.59573E-11	0.753410002	stage_E14_vs_E10	ordinary_muscle_development		0	FALSE
ENSGALG00010021172	SLC29A4	PRJNA729271	-2.018593784	9.61658E-11	2.018593784	stage_E14_vs_E10	ordinary_muscle_development	D_vs_E10;stage_1W_vs_E10;stage_3W_vs_E10;stage	1	TRUE
ENSGALG00010020373	MS4A4A	PRJNA729271	-1.44677215	9.61973E-11	1.44677215	stage_E14_vs_E10	ordinary_muscle_development	D_vs_E10;stage_1W_vs_E10;stage_3W_vs_E10;stage	1	TRUE
ENSGALG00010023914	CCDC117	PRJNA729271	-0.439258025	9.72323E-11	0.439258025	stage_E14_vs_E10	ordinary_muscle_development		0	FALSE
ENSGALG00010028019	RPL29	PRJNA729271	1.514660719	9.78243E-11	1.514660719	stage_E14_vs_E10	ordinary_muscle_development	D_vs_E10;stage_1W_vs_E10;stage_3W_vs_E10;stage	1	TRUE
ENSGALG00010019345	ENSGALG00010019345	PRJNA729271	1.212823975	9.81603E-11	1.212823975	stage_E14_vs_E10	ordinary_muscle_development	D_vs_E10;stage_1W_vs_E10;stage_3W_vs_E10;stage	1	TRUE
ENSGALG00010026367	RBP	PRJNA729271	3.460417937	9.84033E-11	3.460417937	stage_E14_vs_E10	ordinary_muscle_development	D_vs_E10;stage_1W_vs_E10;stage_3W_vs_E10;stage	1	TRUE
ENSGALG00010011679	OVAL	PRJNA729271	-8.019757279	9.94463E-11	8.019757279	stage_E14_vs_E10	ordinary_muscle_development	D_vs_E10;stage_1W_vs_E10;stage_3W_vs_E10;stage	1	TRUE
ENSGALG00010001205	CEP192	PRJNA729271	-0.707890544	9.98125E-11	0.707890544	stage_E14_vs_E10	ordinary_muscle_development		0	FALSE
ENSGALG00010027938	ENSGALG00010027938	PRJNA729271	-0.805728224	9.98702E-11	0.805728224	stage_E14_vs_E10	ordinary_muscle_development		0	FALSE
ENSGALG00010013087	GPN2	PRJNA729271	-0.625568971	1.00155E-10	0.625568971	stage_E14_vs_E10	ordinary_muscle_development		0	FALSE
ENSGALG00010002669	IL4I1	PRJNA729271	7.221166013	1.00847E-10	7.221166013	stage_E14_vs_E10	ordinary_muscle_development	D_vs_E10;stage_1W_vs_E10;stage_3W_vs_E10;stage	1	TRUE
ENSGALG00010014346	AFG1L	PRJNA729271	1.046563709	1.00978E-10	1.046563709	stage_E14_vs_E10	ordinary_muscle_development	D_vs_E10;stage_1W_vs_E10;stage_3W_vs_E10;stage	1	TRUE
ENSGALG00010023246	SP5	PRJNA729271	-6.430563656	1.01022E-10	6.430563656	stage_E14_vs_E10	ordinary_muscle_development	D_vs_E10;stage_1W_vs_E10;stage_3W_vs_E10;stage	1	TRUE
ENSGALG00010024121	NRG4	PRJNA729271	1.108023611	1.01115E-10	1.108023611	stage_E14_vs_E10	ordinary_muscle_development	D_vs_E10;stage_1W_vs_E10;stage_3W_vs_E10;stage	1	TRUE
ENSGALG00010018892	APMAP	PRJNA729271	-0.822154922	1.0216E-10	0.822154922	stage_E14_vs_E10	ordinary_muscle_development		0	FALSE
ENSGALG00010022022	STAMBPL1	PRJNA729271	-1.782646947	1.02305E-10	1.782646947	stage_E14_vs_E10	ordinary_muscle_development	D_vs_E10;stage_1W_vs_E10;stage_3W_vs_E10;stage	1	TRUE
ENSGALG00010014861	MED21	PRJNA729271	-0.659775142	1.02481E-10	0.659775142	stage_E14_vs_E10	ordinary_muscle_development		0	FALSE
ENSGALG00010021186	SWT1	PRJNA729271	0.592680829	1.02648E-10	0.592680829	stage_E14_vs_E10	ordinary_muscle_development		0	FALSE
ENSGALG00010002197	GSTA2	PRJNA729271	-1.255857613	1.03234E-10	1.255857613	stage_E14_vs_E10	ordinary_muscle_development	D_vs_E10;stage_1W_vs_E10;stage_3W_vs_E10;stage	1	TRUE
ENSGALG00010016814	HMGXB3	PRJNA729271	0.656737325	1.03533E-10	0.656737325	stage_E14_vs_E10	ordinary_muscle_development		0	FALSE
ENSGALG00010021778	RBL2	PRJNA729271	0.684204267	1.04759E-10	0.684204267	stage_E14_vs_E10	ordinary_muscle_development		0	FALSE
ENSGALG00010005335	AHSA1	PRJNA729271	-0.90110383	1.05282E-10	0.90110383	stage_E14_vs_E10	ordinary_muscle_development		0	FALSE
ENSGALG00010007900	FHOD3	PRJNA729271	1.33017852	1.05377E-10	1.33017852	stage_E14_vs_E10	ordinary_muscle_development	D_vs_E10;stage_1W_vs_E10;stage_3W_vs_E10;stage	1	TRUE
ENSGALG00010011894	VWC2L	PRJNA729271	-2.584218664	1.05542E-10	2.584218664	stage_E14_vs_E10	ordinary_muscle_development	D_vs_E10;stage_1W_vs_E10;stage_3W_vs_E10;stage	1	TRUE
ENSGALG00010008116	URAH	PRJNA729271	2.468276696	1.06079E-10	2.468276696	stage_E14_vs_E10	ordinary_muscle_development	D_vs_E10;stage_1W_vs_E10;stage_3W_vs_E10;stage	1	TRUE
ENSGALG00010009830	RNASEL	PRJNA729271	-1.317035246	1.06559E-10	1.317035246	stage_E14_vs_E10	ordinary_muscle_development	D_vs_E10;stage_1W_vs_E10;stage_3W_vs_E10;stage	1	TRUE
ENSGALG00010001222	ZNF800	PRJNA729271	-0.678950776	1.06589E-10	0.678950776	stage_E14_vs_E10	ordinary_muscle_development		0	FALSE
ENSGALG00010003507	WDFY3	PRJNA729271	0.642888998	1.07171E-10	0.642888998	stage_E14_vs_E10	ordinary_muscle_development		0	FALSE
ENSGALG00010017886	DDRGK1	PRJNA729271	0.668947978	1.08862E-10	0.668947978	stage_E14_vs_E10	ordinary_muscle_development		0	FALSE
ENSGALG00010011497	CPQ	PRJNA729271	1.649213335	1.10369E-10	1.649213335	stage_E14_vs_E10	ordinary_muscle_development	D_vs_E10;stage_1W_vs_E10;stage_3W_vs_E10;stage	1	TRUE
ENSGALG00010001462	DHX36	PRJNA729271	-0.728834842	1.10437E-10	0.728834842	stage_E14_vs_E10	ordinary_muscle_development		0	FALSE
ENSGALG00010024537	EPB42	PRJNA729271	-1.216869045	1.10485E-10	1.216869045	stage_E14_vs_E10	ordinary_muscle_development	D_vs_E10;stage_1W_vs_E10;stage_3W_vs_E10;stage	1	TRUE
ENSGALG00010013302	ENSGALG00010013302	PRJNA729271	3.672056225	1.10494E-10	3.672056225	stage_E14_vs_E10	ordinary_muscle_development	D_vs_E10;stage_1W_vs_E10;stage_3W_vs_E10;stage	1	TRUE
ENSGALG00010020614	FAIM	PRJNA729271	-0.734827671	1.10752E-10	0.734827671	stage_E14_vs_E10	ordinary_muscle_development		0	FALSE
ENSGALG00010002349	ZBED1	PRJNA729271	0.85323726	1.10771E-10	0.85323726	stage_E14_vs_E10	ordinary_muscle_development		0	FALSE
ENSGALG00010024467	ENSGALG00010024467	PRJNA729271	1.827518501	1.10783E-10	1.827518501	stage_E14_vs_E10	ordinary_muscle_development	D_vs_E10;stage_1W_vs_E10;stage_3W_vs_E10;stage	1	TRUE
ENSGALG00010019640	MGRN1	PRJNA729271	-0.778995566	1.11337E-10	0.778995566	stage_E14_vs_E10	ordinary_muscle_development		0	FALSE
ENSGALG00010017479	SSTR5	PRJNA729271	3.091346245	1.12049E-10	3.091346245	stage_E14_vs_E10	ordinary_muscle_development	D_vs_E10;stage_1W_vs_E10;stage_3W_vs_E10;stage	1	TRUE
ENSGALG00010018186	SPTLC3	PRJNA729271	-4.547717709	1.1338E-10	4.547717709	stage_E14_vs_E10	ordinary_muscle_development	D_vs_E10;stage_1W_vs_E10;stage_3W_vs_E10;stage	1	TRUE
ENSGALG00010016474	MAPK8IP3	PRJNA729271	-0.735969503	1.13929E-10	0.735969503	stage_E14_vs_E10	ordinary_muscle_development		0	FALSE
ENSGALG00010024183	MTX2	PRJNA729271	0.699163654	1.1411E-10	0.699163654	stage_E14_vs_E10	ordinary_muscle_development		0	FALSE
ENSGALG00010008065	CHMP5	PRJNA729271	-0.585595232	1.14325E-10	0.585595232	stage_E14_vs_E10	ordinary_muscle_development		0	FALSE
ENSGALG00010011244	PDE9A	PRJNA729271	-1.141325905	1.15309E-10	1.141325905	stage_E14_vs_E10	ordinary_muscle_development	D_vs_E10;stage_1W_vs_E10;stage_3W_vs_E10;stage	1	TRUE
ENSGALG00010021588	RNF25	PRJNA729271	0.908015705	1.15927E-10	0.908015705	stage_E14_vs_E10	ordinary_muscle_development		0	FALSE
ENSGALG00010014953	ENSGALG00010014953	PRJNA729271	2.144079264	1.16415E-10	2.144079264	stage_E14_vs_E10	ordinary_muscle_development	D_vs_E10;stage_1W_vs_E10;stage_3W_vs_E10;stage	1	TRUE
ENSGALG00010027996	GPX4	PRJNA729271	0.800861645	1.16889E-10	0.800861645	stage_E14_vs_E10	ordinary_muscle_development		0	FALSE
ENSGALG00010007863	AvBD1	PRJNA729271	5.421555168	1.18033E-10	5.421555168	stage_E14_vs_E10	ordinary_muscle_development	D_vs_E10;stage_1W_vs_E10;stage_3W_vs_E10;stage	1	TRUE
ENSGALG00010004212	RSPO3	PRJNA729271	-2.560105606	1.1824E-10	2.560105606	stage_E14_vs_E10	ordinary_muscle_development	D_vs_E10;stage_1W_vs_E10;stage_3W_vs_E10;stage	1	TRUE
ENSGALG00010028518	MAU2	PRJNA729271	0.754824239	1.18809E-10	0.754824239	stage_E14_vs_E10	ordinary_muscle_development		0	FALSE
ENSGALG00010016599	ENSGALG00010016599	PRJNA729271	1.778599016	1.19512E-10	1.778599016	stage_E14_vs_E10	ordinary_muscle_development	D_vs_E10;stage_1W_vs_E10;stage_3W_vs_E10;stage	1	TRUE
ENSGALG00010024471	CCDC61	PRJNA729271	-0.77676693	1.2003E-10	0.77676693	stage_E14_vs_E10	ordinary_muscle_development		0	FALSE
ENSGALG00010026759	OMA1	PRJNA729271	-0.744118183	1.20151E-10	0.744118183	stage_E14_vs_E10	ordinary_muscle_development		0	FALSE
ENSGALG00010023751	PRRG4	PRJNA729271	-2.079972977	1.20839E-10	2.079972977	stage_E14_vs_E10	ordinary_muscle_development	D_vs_E10;stage_1W_vs_E10;stage_3W_vs_E10;stage	1	TRUE
ENSGALG00010018624	ENSGALG00010018624	PRJNA729271	2.50727323	1.21253E-10	2.50727323	stage_E14_vs_E10	ordinary_muscle_development	D_vs_E10;stage_1W_vs_E10;stage_3W_vs_E10;stage	1	TRUE
ENSGALG00010010690	MTPN	PRJNA729271	-0.890399676	1.21543E-10	0.890399676	stage_E14_vs_E10	ordinary_muscle_development		0	FALSE
ENSGALG00010018080	BRICD5	PRJNA729271	2.097500316	1.21813E-10	2.097500316	stage_E14_vs_E10	ordinary_muscle_development	D_vs_E10;stage_1W_vs_E10;stage_3W_vs_E10;stage	1	TRUE
ENSGALG00010014380	SNX3	PRJNA729271	0.771956326	1.21827E-10	0.771956326	stage_E14_vs_E10	ordinary_muscle_development		0	FALSE
ENSGALG00010010966	ENSGALG00010010966	PRJNA729271	3.337383609	1.21876E-10	3.337383609	stage_E14_vs_E10	ordinary_muscle_development	D_vs_E10;stage_1W_vs_E10;stage_3W_vs_E10;stage	1	TRUE
ENSGALG00010015037	CYFIP2	PRJNA729271	1.257459104	1.24179E-10	1.257459104	stage_E14_vs_E10	ordinary_muscle_development	D_vs_E10;stage_1W_vs_E10;stage_3W_vs_E10;stage	1	TRUE
ENSGALG00010028873	TMEM8C	PRJNA729271	1.848684239	1.25187E-10	1.848684239	stage_E14_vs_E10	ordinary_muscle_development	D_vs_E10;stage_1W_vs_E10;stage_3W_vs_E10;stage	1	TRUE
ENSGALG00010027942	KHDC4	PRJNA729271	0.807549043	1.26126E-10	0.807549043	stage_E14_vs_E10	ordinary_muscle_development		0	FALSE
ENSGALG00010011586	ENSGALG00010011586	PRJNA729271	1.707273209	1.27062E-10	1.707273209	stage_E14_vs_E10	ordinary_muscle_development	D_vs_E10;stage_1W_vs_E10;stage_3W_vs_E10;stage	1	TRUE
ENSGALG00010022485	ATF2	PRJNA729271	-0.38214613	1.27567E-10	0.38214613	stage_E14_vs_E10	ordinary_muscle_development		0	FALSE
ENSGALG00010012969	SLC12A2	PRJNA729271	-0.966556808	1.28216E-10	0.966556808	stage_E14_vs_E10	ordinary_muscle_development		0	FALSE
ENSGALG00010002953	WDFY2	PRJNA729271	-0.890402122	1.28339E-10	0.890402122	stage_E14_vs_E10	ordinary_muscle_development		0	FALSE
ENSGALG00010011729	FAM129A	PRJNA729271	-1.527463509	1.28754E-10	1.527463509	stage_E14_vs_E10	ordinary_muscle_development	D_vs_E10;stage_1W_vs_E10;stage_3W_vs_E10;stage	1	TRUE
ENSGALG00010000633	VWC2	PRJNA729271	-5.832674152	1.28835E-10	5.832674152	stage_E14_vs_E10	ordinary_muscle_development	D_vs_E10;stage_1W_vs_E10;stage_3W_vs_E10;stage	1	TRUE
ENSGALG00010008407	GNAI1	PRJNA729271	-1.278155686	1.29222E-10	1.278155686	stage_E14_vs_E10	ordinary_muscle_development	D_vs_E10;stage_1W_vs_E10;stage_3W_vs_E10;stage	1	TRUE
ENSGALG00010020399	ATP13A2	PRJNA729271	-0.900353432	1.29267E-10	0.900353432	stage_E14_vs_E10	ordinary_muscle_development		0	FALSE
ENSGALG00010020782	PPIF	PRJNA729271	1.030609113	1.29438E-10	1.030609113	stage_E14_vs_E10	ordinary_muscle_development	D_vs_E10;stage_1W_vs_E10;stage_3W_vs_E10;stage	1	TRUE
ENSGALG00010004871	C11orf87	PRJNA729271	-2.192454805	1.30361E-10	2.192454805	stage_E14_vs_E10	ordinary_muscle_development	D_vs_E10;stage_1W_vs_E10;stage_3W_vs_E10;stage	1	TRUE
ENSGALG00010015033	BMP7	PRJNA729271	-2.168954804	1.30909E-10	2.168954804	stage_E14_vs_E10	ordinary_muscle_development	D_vs_E10;stage_1W_vs_E10;stage_3W_vs_E10;stage	1	TRUE
ENSGALG00010009258	DNAJC12	PRJNA729271	-1.142859876	1.31174E-10	1.142859876	stage_E14_vs_E10	ordinary_muscle_development	D_vs_E10;stage_1W_vs_E10;stage_3W_vs_E10;stage	1	TRUE
ENSGALG00010013945	SCAF11	PRJNA729271	-0.507593791	1.31256E-10	0.507593791	stage_E14_vs_E10	ordinary_muscle_development		0	FALSE
ENSGALG00010005361	ENSGALG00010005361	PRJNA729271	2.722457957	1.31283E-10	2.722457957	stage_E14_vs_E10	ordinary_muscle_development	D_vs_E10;stage_1W_vs_E10;stage_3W_vs_E10;stage	1	TRUE
ENSGALG00010022219	EIF4E3	PRJNA729271	0.895250099	1.34266E-10	0.895250099	stage_E14_vs_E10	ordinary_muscle_development		0	FALSE
ENSGALG00010005273	ENSGALG00010005273	PRJNA729271	4.093963409	1.34557E-10	4.093963409	stage_E14_vs_E10	ordinary_muscle_development	D_vs_E10;stage_1W_vs_E10;stage_3W_vs_E10;stage	1	TRUE
ENSGALG00010013053	MRTFA	PRJNA729271	1.142143733	1.35323E-10	1.142143733	stage_E14_vs_E10	ordinary_muscle_development	D_vs_E10;stage_1W_vs_E10;stage_3W_vs_E10;stage	1	TRUE
ENSGALG00010008218	ARHGAP42	PRJNA729271	-0.679070894	1.36457E-10	0.679070894	stage_E14_vs_E10	ordinary_muscle_development		0	FALSE
ENSGALG00010021082	SENP8	PRJNA729271	-1.097935601	1.36535E-10	1.097935601	stage_E14_vs_E10	ordinary_muscle_development	D_vs_E10;stage_1W_vs_E10;stage_3W_vs_E10;stage	1	TRUE
ENSGALG00010024671	KCTD20	PRJNA729271	1.835352238	1.37309E-10	1.835352238	stage_E14_vs_E10	ordinary_muscle_development	D_vs_E10;stage_1W_vs_E10;stage_3W_vs_E10;stage	1	TRUE
ENSGALG00010024518	ENSGALG00010024518	PRJNA729271	7.119770652	1.37335E-10	7.119770652	stage_E14_vs_E10	ordinary_muscle_development	D_vs_E10;stage_1W_vs_E10;stage_3W_vs_E10;stage	1	TRUE
ENSGALG00010003403	GCDH	PRJNA729271	0.96609396	1.37515E-10	0.96609396	stage_E14_vs_E10	ordinary_muscle_development		0	FALSE
ENSGALG00010001181	MFSD1	PRJNA729271	0.685500944	1.38228E-10	0.685500944	stage_E14_vs_E10	ordinary_muscle_development		0	FALSE
ENSGALG00010019344	DNAJC24	PRJNA729271	-0.577281486	1.38339E-10	0.577281486	stage_E14_vs_E10	ordinary_muscle_development		0	FALSE
ENSGALG00010012125	RNF152	PRJNA729271	0.974166656	1.39023E-10	0.974166656	stage_E14_vs_E10	ordinary_muscle_development		0	FALSE
ENSGALG00010027882	CHIA	PRJNA729271	9.07587484	1.39415E-10	9.07587484	stage_E14_vs_E10	ordinary_muscle_development	D_vs_E10;stage_1W_vs_E10;stage_3W_vs_E10;stage	1	TRUE
ENSGALG00010016310	HDAC8	PRJNA729271	-0.611635524	1.39764E-10	0.611635524	stage_E14_vs_E10	ordinary_muscle_development		0	FALSE
ENSGALG00010010390	KCNK3	PRJNA729271	2.723478703	1.40123E-10	2.723478703	stage_E14_vs_E10	ordinary_muscle_development	D_vs_E10;stage_1W_vs_E10;stage_3W_vs_E10;stage	1	TRUE
ENSGALG00010028411	LHX2	PRJNA729271	-6.43554749	1.40162E-10	6.43554749	stage_E14_vs_E10	ordinary_muscle_development	D_vs_E10;stage_1W_vs_E10;stage_3W_vs_E10;stage	1	TRUE
ENSGALG00010009676	ACO1	PRJNA729271	-0.631593034	1.42574E-10	0.631593034	stage_E14_vs_E10	ordinary_muscle_development		0	FALSE
ENSGALG00010010263	RAB4A	PRJNA729271	-0.905139056	1.4261E-10	0.905139056	stage_E14_vs_E10	ordinary_muscle_development		0	FALSE
ENSGALG00010007521	SLC12A7	PRJNA729271	-0.906313808	1.42903E-10	0.906313808	stage_E14_vs_E10	ordinary_muscle_development		0	FALSE
ENSGALG00010000346	ENSGALG00010000346	PRJNA729271	-0.756572359	1.43094E-10	0.756572359	stage_E14_vs_E10	ordinary_muscle_development		0	FALSE
ENSGALG00010022059	NPTX2	PRJNA729271	-1.649840284	1.4373E-10	1.649840284	stage_E14_vs_E10	ordinary_muscle_development	D_vs_E10;stage_1W_vs_E10;stage_3W_vs_E10;stage	1	TRUE
ENSGALG00010010052	ZNF346	PRJNA729271	-0.755721283	1.45043E-10	0.755721283	stage_E14_vs_E10	ordinary_muscle_development		0	FALSE
ENSGALG00010022239	DEAF1	PRJNA729271	0.731648163	1.45122E-10	0.731648163	stage_E14_vs_E10	ordinary_muscle_development		0	FALSE
ENSGALG00010005127	GATA6	PRJNA729271	-2.161759107	1.4523E-10	2.161759107	stage_E14_vs_E10	ordinary_muscle_development	D_vs_E10;stage_1W_vs_E10;stage_3W_vs_E10;stage	1	TRUE
ENSGALG00010019833	MTHFS	PRJNA729271	-0.871656147	1.45653E-10	0.871656147	stage_E14_vs_E10	ordinary_muscle_development		0	FALSE
ENSGALG00010024723	ITCH	PRJNA729271	0.657674501	1.46905E-10	0.657674501	stage_E14_vs_E10	ordinary_muscle_development		0	FALSE
ENSGALG00010004356	TRAPPC8	PRJNA729271	0.505165088	1.47179E-10	0.505165088	stage_E14_vs_E10	ordinary_muscle_development		0	FALSE
ENSGALG00010017692	EPCAM	PRJNA729271	-6.848265551	1.47313E-10	6.848265551	stage_E14_vs_E10	ordinary_muscle_development	D_vs_E10;stage_1W_vs_E10;stage_3W_vs_E10;stage	1	TRUE
ENSGALG00010028360	ENSGALG00010028360	PRJNA729271	-6.471284703	1.47685E-10	6.471284703	stage_E14_vs_E10	ordinary_muscle_development	D_vs_E10;stage_1W_vs_E10;stage_3W_vs_E10;stage	1	TRUE
ENSGALG00010011993	RELCH	PRJNA729271	-0.346572692	1.47722E-10	0.346572692	stage_E14_vs_E10	ordinary_muscle_development		0	FALSE
ENSGALG00010028924	TMEM203	PRJNA729271	0.780100535	1.48138E-10	0.780100535	stage_E14_vs_E10	ordinary_muscle_development		0	FALSE
ENSGALG00010029631	LIG3	PRJNA729271	-0.575828097	1.49832E-10	0.575828097	stage_E14_vs_E10	ordinary_muscle_development		0	FALSE

ENSGALG00010021539	HEXA	PRJNA729271	-0.793969957	1.50625E-10	0.793969957	stage_E14_vs_E10	ordinary_muscle_development		0	FALSE
ENSGALG00010019660	ENSGALG00010019660	PRJNA729271	2.945875509	1.50704E-10	2.945875509	stage_E14_vs_E10	ordinary_muscle_development	D_vs_E10;stage_1W_vs_E10;stage_3W_vs_E10;stage	1	TRUE
ENSGALG00010005216	ENSGALG00010005216	PRJNA729271	-1.124286896	1.50772E-10	1.124286896	stage_E14_vs_E10	ordinary_muscle_development	D_vs_E10;stage_1W_vs_E10;stage_3W_vs_E10;stage	1	TRUE
ENSGALG00010028191	KLHL26	PRJNA729271	0.64814914	1.51657E-10	0.64814914	stage_E14_vs_E10	ordinary_muscle_development		0	FALSE
ENSGALG00010022426	EXD1	PRJNA729271	-1.597050787	1.52021E-10	1.597050787	stage_E14_vs_E10	ordinary_muscle_development	D_vs_E10;stage_1W_vs_E10;stage_3W_vs_E10;stage	1	TRUE
ENSGALG00010027277	KCND3	PRJNA729271	2.49854758	1.53722E-10	2.49854758	stage_E14_vs_E10	ordinary_muscle_development	D_vs_E10;stage_1W_vs_E10;stage_3W_vs_E10;stage	1	TRUE
ENSGALG00010005336	PEX7	PRJNA729271	-0.845632055	1.54337E-10	0.845632055	stage_E14_vs_E10	ordinary_muscle_development		0	FALSE
ENSGALG00010020760	TXNDC11	PRJNA729271	-0.634721732	1.56136E-10	0.634721732	stage_E14_vs_E10	ordinary_muscle_development		0	FALSE
ENSGALG00010024680	BRSK1	PRJNA729271	-1.859902693	1.57254E-10	1.859902693	stage_E14_vs_E10	ordinary_muscle_development	D_vs_E10;stage_1W_vs_E10;stage_3W_vs_E10;stage	1	TRUE
ENSGALG00010014407	YIPF6	PRJNA729271	-0.598545163	1.59103E-10	0.598545163	stage_E14_vs_E10	ordinary_muscle_development		0	FALSE
ENSGALG00010023401	LRRC23	PRJNA729271	2.344616941	1.5955E-10	2.344616941	stage_E14_vs_E10	ordinary_muscle_development	D_vs_E10;stage_1W_vs_E10;stage_3W_vs_E10;stage	1	TRUE
ENSGALG00010022100	ENTPD1	PRJNA729271	-1.094878544	1.6055E-10	1.094878544	stage_E14_vs_E10	ordinary_muscle_development	D_vs_E10;stage_1W_vs_E10;stage_3W_vs_E10;stage	1	TRUE
ENSGALG00010027223	SIRT6	PRJNA729271	-0.553080371	1.62399E-10	0.553080371	stage_E14_vs_E10	ordinary_muscle_development		0	FALSE
ENSGALG00010027106	CYP2J23	PRJNA729271	0.924992735	1.6273E-10	0.924992735	stage_E14_vs_E10	ordinary_muscle_development		0	FALSE
ENSGALG00010025005	HIP1R	PRJNA729271	-0.817919793	1.64266E-10	0.817919793	stage_E14_vs_E10	ordinary_muscle_development		0	FALSE
ENSGALG00010013537	LMO3	PRJNA729271	-3.075663374	1.65016E-10	3.075663374	stage_E14_vs_E10	ordinary_muscle_development	D_vs_E10;stage_1W_vs_E10;stage_3W_vs_E10;stage	1	TRUE
ENSGALG00010024565	FAM217B	PRJNA729271	0.902087783	1.66455E-10	0.902087783	stage_E14_vs_E10	ordinary_muscle_development		0	FALSE
ENSGALG00010002384	ENSGALG00010002384	PRJNA729271	-2.098753323	1.6675E-10	2.098753323	stage_E14_vs_E10	ordinary_muscle_development	D_vs_E10;stage_1W_vs_E10;stage_3W_vs_E10;stage	1	TRUE
ENSGALG00010024741	RILPL2	PRJNA729271	-1.034127913	1.67029E-10	1.034127913	stage_E14_vs_E10	ordinary_muscle_development	D_vs_E10;stage_1W_vs_E10;stage_3W_vs_E10;stage	1	TRUE
ENSGALG00010026950	LHFPL5	PRJNA729271	1.728412534	1.67284E-10	1.728412534	stage_E14_vs_E10	ordinary_muscle_development	D_vs_E10;stage_1W_vs_E10;stage_3W_vs_E10;stage	1	TRUE
ENSGALG00010003764	HDAC9	PRJNA729271	1.271106571	1.67383E-10	1.271106571	stage_E14_vs_E10	ordinary_muscle_development	D_vs_E10;stage_1W_vs_E10;stage_3W_vs_E10;stage	1	TRUE
ENSGALG00010023926	ZYX	PRJNA729271	-0.921945825	1.68439E-10	0.921945825	stage_E14_vs_E10	ordinary_muscle_development		0	FALSE
ENSGALG00010008221	ENSGALG00010008221	PRJNA729271	3.387518433	1.68477E-10	3.387518433	stage_E14_vs_E10	ordinary_muscle_development	D_vs_E10;stage_1W_vs_E10;stage_3W_vs_E10;stage	1	TRUE
ENSGALG00010007932	NPY5R	PRJNA729271	1.152436105	1.6852E-10	1.152436105	stage_E14_vs_E10	ordinary_muscle_development	D_vs_E10;stage_1W_vs_E10;stage_3W_vs_E10;stage	1	TRUE
ENSGALG00010024083	PALMD	PRJNA729271	1.274387204	1.7138E-10	1.274387204	stage_E14_vs_E10	ordinary_muscle_development	D_vs_E10;stage_1W_vs_E10;stage_3W_vs_E10;stage	1	TRUE
ENSGALG00010024620	MSANTD2	PRJNA729271	-0.861768047	1.71505E-10	0.861768047	stage_E14_vs_E10	ordinary_muscle_development		0	FALSE
ENSGALG00010019172	NEMF	PRJNA729271	0.562997416	1.72059E-10	0.562997416	stage_E14_vs_E10	ordinary_muscle_development		0	FALSE
ENSGALG00010008026	PURG	PRJNA729271	0.79877096	1.74005E-10	0.79877096	stage_E14_vs_E10	ordinary_muscle_development		0	FALSE
ENSGALG00010016964	MAML1	PRJNA729271	0.55112875	1.74715E-10	0.55112875	stage_E14_vs_E10	ordinary_muscle_development		0	FALSE
ENSGALG00010009334	SS18L2	PRJNA729271	1.060973259	1.75198E-10	1.060973259	stage_E14_vs_E10	ordinary_muscle_development	D_vs_E10;stage_1W_vs_E10;stage_3W_vs_E10;stage	1	TRUE
ENSGALG00010008486	CTU2	PRJNA729271	-0.916764243	1.76572E-10	0.916764243	stage_E14_vs_E10	ordinary_muscle_development		0	FALSE
ENSGALG00010009466	FAM193B	PRJNA729271	-0.731239191	1.76925E-10	0.731239191	stage_E14_vs_E10	ordinary_muscle_development		0	FALSE
ENSGALG00010007643	COTL1	PRJNA729271	-1.069229936	1.77248E-10	1.069229936	stage_E14_vs_E10	ordinary_muscle_development	D_vs_E10;stage_1W_vs_E10;stage_3W_vs_E10;stage	1	TRUE
ENSGALG00010024054	ENSGALG00010024054	PRJNA729271	-6.726345172	1.77397E-10	6.726345172	stage_E14_vs_E10	ordinary_muscle_development	D_vs_E10;stage_1W_vs_E10;stage_3W_vs_E10;stage	1	TRUE
ENSGALG00010024457	CHD6	PRJNA729271	-0.659436706	1.77397E-10	0.659436706	stage_E14_vs_E10	ordinary_muscle_development		0	FALSE
ENSGALG00010005403	ENSGALG00010005403	PRJNA729271	-0.967684342	1.78565E-10	0.967684342	stage_E14_vs_E10	ordinary_muscle_development		0	FALSE
ENSGALG00010028002	ABCA2	PRJNA729271	0.781932932	1.79709E-10	0.781932932	stage_E14_vs_E10	ordinary_muscle_development		0	FALSE
ENSGALG00010013015	PLA2G6	PRJNA729271	0.968496849	1.8087E-10	0.968496849	stage_E14_vs_E10	ordinary_muscle_development		0	FALSE
ENSGALG00010000453	ACHE	PRJNA729271	1.590870724	1.81498E-10	1.590870724	stage_E14_vs_E10	ordinary_muscle_development	D_vs_E10;stage_1W_vs_E10;stage_3W_vs_E10;stage	1	TRUE
ENSGALG00010017221	SPPL2A	PRJNA729271	0.507163074	1.8308E-10	0.507163074	stage_E14_vs_E10	ordinary_muscle_development		0	FALSE
ENSGALG00010009424	ENSGALG00010009424	PRJNA729271	6.569046691	1.8382E-10	6.569046691	stage_E14_vs_E10	ordinary_muscle_development	D_vs_E10;stage_1W_vs_E10;stage_3W_vs_E10;stage	1	TRUE
ENSGALG00010005670	SGMS2	PRJNA729271	-1.395567675	1.8414E-10	1.395567675	stage_E14_vs_E10	ordinary_muscle_development	D_vs_E10;stage_1W_vs_E10;stage_3W_vs_E10;stage	1	TRUE
ENSGALG00010023163	CRTAC1	PRJNA729271	-1.899599661	1.84163E-10	1.899599661	stage_E14_vs_E10	ordinary_muscle_development	D_vs_E10;stage_1W_vs_E10;stage_3W_vs_E10;stage	1	TRUE
ENSGALG00010025126	ENSGALG00010025126	PRJNA729271	-0.917015527	1.85208E-10	0.917015527	stage_E14_vs_E10	ordinary_muscle_development		0	FALSE
ENSGALG00010012148	SETBP1	PRJNA729271	-0.716209826	1.85952E-10	0.716209826	stage_E14_vs_E10	ordinary_muscle_development		0	FALSE
ENSGALG00010008877	FNDC4	PRJNA729271	1.584450503	1.86289E-10	1.584450503	stage_E14_vs_E10	ordinary_muscle_development	D_vs_E10;stage_1W_vs_E10;stage_3W_vs_E10;stage	1	TRUE
ENSGALG00010027397	RFX5	PRJNA729271	0.812932972	1.86529E-10	0.812932972	stage_E14_vs_E10	ordinary_muscle_development		0	FALSE
ENSGALG00010023207	MNS1	PRJNA729271	-1.097519697	1.86901E-10	1.097519697	stage_E14_vs_E10	ordinary_muscle_development	D_vs_E10;stage_1W_vs_E10;stage_3W_vs_E10;stage	1	TRUE
ENSGALG00010026612	MFF	PRJNA729271	-0.562323305	1.87816E-10	0.562323305	stage_E14_vs_E10	ordinary_muscle_development		0	FALSE
ENSGALG00010004990	ENSGALG00010004990	PRJNA729271	-16.87739022	1.88054E-10	16.87739022	stage_E14_vs_E10	ordinary_muscle_development	D_vs_E10;stage_1W_vs_E10;stage_3W_vs_E10;stage	1	TRUE
ENSGALG00010008149	DNAJA1	PRJNA729271	1.832347487	1.88506E-10	1.832347487	stage_E14_vs_E10	ordinary_muscle_development	D_vs_E10;stage_1W_vs_E10;stage_3W_vs_E10;stage	1	TRUE
ENSGALG00010027444	SELENBP1	PRJNA729271	0.7915477	1.89446E-10	0.7915477	stage_E14_vs_E10	ordinary_muscle_development		0	FALSE
ENSGALG00010005545	RPL34	PRJNA729271	1.085125863	1.89475E-10	1.085125863	stage_E14_vs_E10	ordinary_muscle_development	D_vs_E10;stage_1W_vs_E10;stage_3W_vs_E10;stage	1	TRUE
ENSGALG00010018011	LIPA	PRJNA729271	1.206026529	1.90235E-10	1.206026529	stage_E14_vs_E10	ordinary_muscle_development	D_vs_E10;stage_1W_vs_E10;stage_3W_vs_E10;stage	1	TRUE
ENSGALG00010023324	LAG3	PRJNA729271	6.138680856	1.90863E-10	6.138680856	stage_E14_vs_E10	ordinary_muscle_development	D_vs_E10;stage_1W_vs_E10;stage_3W_vs_E10;stage	1	TRUE
ENSGALG00010028550	ZAP70	PRJNA729271	-4.302010309	1.92902E-10	4.302010309	stage_E14_vs_E10	ordinary_muscle_development	D_vs_E10;stage_1W_vs_E10;stage_3W_vs_E10;stage	1	TRUE
ENSGALG00010018574	ENSGALG00010018574	PRJNA729271	-1.701838848	1.94719E-10	1.701838848	stage_E14_vs_E10	ordinary_muscle_development	D_vs_E10;stage_1W_vs_E10;stage_3W_vs_E10;stage	1	TRUE
ENSGALG00010017413	ZIC3	PRJNA729271	-6.478339303	1.94811E-10	6.478339303	stage_E14_vs_E10	ordinary_muscle_development	D_vs_E10;stage_1W_vs_E10;stage_3W_vs_E10;stage	1	TRUE
ENSGALG00010024362	DHX8	PRJNA729271	-0.388964841	1.94897E-10	0.388964841	stage_E14_vs_E10	ordinary_muscle_development		0	FALSE
ENSGALG00010021752	CSTB	PRJNA729271	-0.72583137	1.959E-10	0.72583137	stage_E14_vs_E10	ordinary_muscle_development		0	FALSE
ENSGALG00010022209	ZFYVE19	PRJNA729271	-0.792292458	1.96105E-10	0.792292458	stage_E14_vs_E10	ordinary_muscle_development		0	FALSE
ENSGALG00010012917	RUNX1	PRJNA729271	-1.746786495	1.9673E-10	1.746786495	stage_E14_vs_E10	ordinary_muscle_development	D_vs_E10;stage_1W_vs_E10;stage_3W_vs_E10;stage	1	TRUE
ENSGALG00010009195	ENSGALG00010009195	PRJNA729271	3.967676073	1.99207E-10	3.967676073	stage_E14_vs_E10	ordinary_muscle_development	D_vs_E10;stage_1W_vs_E10;stage_3W_vs_E10;stage	1	TRUE
ENSGALG00010009858	ST8SIA5	PRJNA729271	-1.639929438	1.9969E-10	1.639929438	stage_E14_vs_E10	ordinary_muscle_development	D_vs_E10;stage_1W_vs_E10;stage_3W_vs_E10;stage	1	TRUE
ENSGALG00010011086	OTUD6A	PRJNA729271	0.734467441	2.01301E-10	0.734467441	stage_E14_vs_E10	ordinary_muscle_development		0	FALSE
ENSGALG00010016541	UBE2J2	PRJNA729271	-0.523943124	2.0235E-10	0.523943124	stage_E14_vs_E10	ordinary_muscle_development		0	FALSE
ENSGALG00010017522	XXYLT1	PRJNA729271	-0.701666928	2.03457E-10	0.701666928	stage_E14_vs_E10	ordinary_muscle_development		0	FALSE
ENSGALG00010014014	PARP8	PRJNA729271	1.272934979	2.04433E-10	1.272934979	stage_E14_vs_E10	ordinary_muscle_development	D_vs_E10;stage_1W_vs_E10;stage_3W_vs_E10;stage	1	TRUE
ENSGALG00010025262	CARF	PRJNA729271	-0.744759344	2.04988E-10	0.744759344	stage_E14_vs_E10	ordinary_muscle_development		0	FALSE
ENSGALG00010028542	ENSGALG00010028542	PRJNA729271	12.15576689	2.07892E-10	12.15576689	stage_E14_vs_E10	ordinary_muscle_development	D_vs_E10;stage_1W_vs_E10;stage_3W_vs_E10;stage	1	TRUE
ENSGALG00010028096	TOR2A	PRJNA729271	-0.998178521	2.07908E-10	0.998178521	stage_E14_vs_E10	ordinary_muscle_development		0	FALSE
ENSGALG00010005557	CMSS1	PRJNA729271	-1.126625297	2.0828E-10	1.126625297	stage_E14_vs_E10	ordinary_muscle_development	D_vs_E10;stage_1W_vs_E10;stage_3W_vs_E10;stage	1	TRUE
ENSGALG00010004143	TCF24	PRJNA729271	-3.242699065	2.09002E-10	3.242699065	stage_E14_vs_E10	ordinary_muscle_development	D_vs_E10;stage_1W_vs_E10;stage_3W_vs_E10;stage	1	TRUE
ENSGALG00010001235	CCDC102B	PRJNA729271	-2.090274153	2.0913E-10	2.090274153	stage_E14_vs_E10	ordinary_muscle_development	D_vs_E10;stage_1W_vs_E10;stage_3W_vs_E10;stage	1	TRUE
ENSGALG00010006851	TMEM167A	PRJNA729271	-0.682956709	2.09881E-10	0.682956709	stage_E14_vs_E10	ordinary_muscle_development		0	FALSE
ENSGALG00010017742	DGLUCY	PRJNA729271	1.559060507	2.11267E-10	1.559060507	stage_E14_vs_E10	ordinary_muscle_development	D_vs_E10;stage_1W_vs_E10;stage_3W_vs_E10;stage	1	TRUE
ENSGALG00010000784	FBXL3	PRJNA729271	0.618339806	2.13185E-10	0.618339806	stage_E14_vs_E10	ordinary_muscle_development		0	FALSE
ENSGALG00010024726	TMED2	PRJNA729271	-0.381590044	2.15777E-10	0.381590044	stage_E14_vs_E10	ordinary_muscle_development		0	FALSE
ENSGALG00010024569	ASCL2	PRJNA729271	-2.072642202	2.16509E-10	2.072642202	stage_E14_vs_E10	ordinary_muscle_development	D_vs_E10;stage_1W_vs_E10;stage_3W_vs_E10;stage	1	TRUE
ENSGALG00010027141	PIGR	PRJNA729271	8.991340885	2.16782E-10	8.991340885	stage_E14_vs_E10	ordinary_muscle_development	D_vs_E10;stage_1W_vs_E10;stage_3W_vs_E10;stage	1	TRUE
ENSGALG00010016251	MPDZ	PRJNA729271	-0.475154387	2.19337E-10	0.475154387	stage_E14_vs_E10	ordinary_muscle_development		0	FALSE
ENSGALG00010027890	ENSGALG00010027890	PRJNA729271	5.337915607	2.19824E-10	5.337915607	stage_E14_vs_E10	ordinary_muscle_development	D_vs_E10;stage_1W_vs_E10;stage_3W_vs_E10;stage	1	TRUE
ENSGALG00010009088	ENSGALG00010009088	PRJNA729271	-0.960025683	2.20464E-10	0.960025683	stage_E14_vs_E10	ordinary_muscle_development		0	FALSE
ENSGALG00010012215	RAB17	PRJNA729271	4.978202214	2.22602E-10	4.978202214	stage_E14_vs_E10	ordinary_muscle_development	D_vs_E10;stage_1W_vs_E10;stage_3W_vs_E10;stage	1	TRUE
ENSGALG00010013482	ENSGALG00010013482	PRJNA729271	-1.552362235	2.23578E-10	1.552362235	stage_E14_vs_E10	ordinary_muscle_development	D_vs_E10;stage_1W_vs_E10;stage_3W_vs_E10;stage	1	TRUE
ENSGALG00010027418	ENSGALG00010027418	PRJNA729271	5.46201603	2.23892E-10	5.46201603	stage_E14_vs_E10	ordinary_muscle_development	D_vs_E10;stage_1W_vs_E10;stage_3W_vs_E10;stage	1	TRUE
ENSGALG00010008505	PTPN3	PRJNA729271	-1.795224191	2.25482E-10	1.795224191	stage_E14_vs_E10	ordinary_muscle_development	D_vs_E10;stage_1W_vs_E10;stage_3W_vs_E10;stage	1	TRUE
ENSGALG00010025432	IRX3	PRJNA729271	-5.198324153	2.27222E-10	5.198324153	stage_E14_vs_E10	ordinary_muscle_development	D_vs_E10;stage_1W_vs_E10;stage_3W_vs_E10;stage	1	TRUE
ENSGALG00010003861	NUDT15	PRJNA729271	-0.682714521	2.29048E-10	0.682714521	stage_E14_vs_E10	ordinary_muscle_development		0	FALSE
ENSGALG00010014403	H2BC37	PRJNA729271	-1.221579031	2.29232E-10	1.221579031	stage_E14_vs_E10	ordinary_muscle_development	D_vs_E10;stage_1W_vs_E10;stage_3W_vs_E10;stage	1	TRUE
ENSGALG00010007922	IL17A	PRJNA729271	2.470664276	2.30696E-10	2.470664276	stage_E14_vs_E10	ordinary_muscle_development	D_vs_E10;stage_1W_vs_E10;stage_3W_vs_E10;stage	1	TRUE
ENSGALG00010001124	GPR171	PRJNA729271	3.649164052	2.3077E-10	3.649164052	stage_E14_vs_E10	ordinary_muscle_development	D_vs_E10;stage_1W_vs_E10;stage_3W_vs_E10;stage	1	TRUE
ENSGALG00010006503	LRRC19	PRJNA729271	6.403066142	2.31133E-10	6.403066142	stage_E14_vs_E10	ordinary_muscle_development	D_vs_E10;stage_1W_vs_E10;stage_3W_vs_E10;stage	1	TRUE
ENSGALG00010021448	SZT2	PRJNA729271	0.648489497	2.31379E-10	0.648489497	stage_E14_vs_E10	ordinary_muscle_development		0	FALSE
ENSGALG00010014178	LRTM2	PRJNA729271	-2.560526933	2.33947E-10	2.560526933	stage_E14_vs_E10	ordinary_muscle_development	D_vs_E10;stage_1W_vs_E10;stage_3W_vs_E10;stage	1	TRUE
ENSGALG00010028895	WHRN	PRJNA729271	-6.057448549	2.34444E-10	6.057448549	stage_E14_vs_E10	ordinary_muscle_development	D_vs_E10;stage_1W_vs_E10;stage_3W_vs_E10;stage	1	TRUE
ENSGALG00010026244	CLP1	PRJNA729271	-0.853894228	2.35729E-10	0.853894228	stage_E14_vs_E10	ordinary_muscle_development		0	FALSE
ENSGALG00010018317	ZMAT3	PRJNA729271	-0.937599034	2.36896E-10	0.937599034	stage_E14_vs_E10	ordinary_muscle_development		0	FALSE
ENSGALG00010001254	NETO1	PRJNA729271	-2.892155617	2.38059E-10	2.892155617	stage_E14_vs_E10	ordinary_muscle_development	D_vs_E10;stage_1W_vs_E10;stage_3W_vs_E10;stage	1	TRUE
ENSGALG00010015782	ATG4A	PRJNA729271	0.528506946	2.38745E-10	0.528506946	stage_E14_vs_E10	ordinary_muscle_development		0	FALSE
ENSGALG00010021055	IRAG1	PRJNA729271	-1.499262649	2.39771E-10	1.499262649	stage_E14_vs_E10	ordinary_muscle_development	D_vs_E10;stage_1W_vs_E10;stage_3W_vs_E10;stage	1	TRUE
ENSGALG00010016395	ENSGALG00010016395	PRJNA729271	-1.058022938	2.40179E-10	1.058022938	stage_E14_vs_E10	ordinary_muscle_development	D_vs_E10;stage_1W_vs_E10;stage_3W_vs_E10;stage	1	TRUE
ENSGALG00010000442	DGCR6	PRJNA729271	-0.848231993	2.40806E-10	0.848231993	stage_E14_vs_E10	ordinary_muscle_development		0	FALSE
ENSGALG00010008199	TPRKB	PRJNA729271	0.722918783	2.42808E-10	0.722918783	stage_E14_vs_E10	ordinary_muscle_development		0	FALSE
ENSGALG00010001245	SERPINI1	PRJNA729271	-1.470654183	2.4296E-10	1.470654183	stage_E14_vs_E10	ordinary_muscle_development	D_vs_E10;stage_1W_vs_E10;stage_3W_vs_E10;stage	1	TRUE
ENSGALG00010019979	ENSGALG00010019979	PRJNA729271	-3.651247108	2.4358E-10	3.651247108	stage_E14_vs_E10	ordinary_muscle_development	D_vs_E10;stage_1W_vs_E10;stage_3W_vs_E10;stage	1	TRUE
ENSGALG00010004477	TULP4	PRJNA729271	-0.820677805	2.51838E-10	0.820677805	stage_E14_vs_E10	ordinary_muscle_development		0	FALSE
ENSGALG00010016943	PCNX4	PRJNA729271	-0.718876748	2.53881E-10	0.718876748	stage_E14_vs_E10	ordinary_muscle_development		0	FALSE
ENSGALG00010029683	MRPL58	PRJNA729271	0.703814699	2.56886E-10	0.703814699	stage_E14_vs_E10	ordinary_muscle_development		0	FALSE
ENSGALG00010007973	CARMIL1	PRJNA729271	-0.877750707	2.56965E-10	0.877750707	stage_E14_vs_E10	ordinary_muscle_development		0	FALSE
ENSGALG00010001170	DCAF10	PRJNA729271	-0.468623823	2.57394E-10	0.468623823	stage_E14_vs_E10	ordinary_muscle_development		0	FALSE
ENSGALG00010009294	ACOX3	PRJNA729271	0.898021705	2.57726E-10	0.898021705	stage_E14_vs_E10	ordinary_muscle_development		0	FALSE
ENSGALG00010013791	AEBP2	PRJNA729271	0.407222196	2.57932E-10	0.407222196	stage_E14_vs_E10	ordinary_muscle_development		0	FALSE
ENSGALG00010008037	ENSGALG00010008037	PRJNA729271	5.836365513	2.59651E-10	5.836365513	stage_E14_vs_E10	ordinary_muscle_development	D_vs_E10;stage_1W_vs_E10;stage_3W_vs_E10;stage	1	TRUE
ENSGALG00010028009	RRP9	PRJNA729271	-1.165286009	2.60219E-10	1.165286009	stage_E14_vs_E10	ordinary_muscle_development	D_vs_E10;stage_1W_vs_E10;stage_3W_vs_E10;stage	1	TRUE
ENSGALG00010011647	FBXO38	PRJNA729271	0.53316919	2.60513E-10	0.53316919	stage_E14_vs_E10	ordinary_muscle_development		0	FALSE
ENSGALG00010023518	METTL5	PRJNA729271	-0.787524601	2.62018E-10	0.787524601	stage_E14_vs_E10	ordinary_muscle_development		0	FALSE
ENSGALG00010021562	LRP6	PRJNA729271	0.529939556	2.62215E-10	0.529939556	stage_E14_vs_E10	ordinary_muscle_development		0	FALSE
ENSGALG00010016464	PANK2	PRJNA729271	-0.593015619	2.63669E-10	0.593015619	stage_E14_vs_E10	ordinary_muscle_development		0	FALSE
ENSGALG00010007085	PRIMPOL	PRJNA729271	1.029227454	2.6556E-10	1.029227454	stage_E14_vs_E10	ordinary_muscle_development	D_vs_E10;stage_1W_vs_E10;stage_3W_vs_E10;stage	1	TRUE
ENSGALG00010013001	ADGRV1	PRJNA729271	-2.240472315	2.67547E-10	2.240472315	stage_E14_vs_E10	ordinary_muscle_development	D_vs_E10;stage_1W_vs_E10;stage_3W_vs_E10;stage	1	TRUE
ENSGALG00010021960	CDKN1B	PRJNA729271	0.93563055	2.68527E-10	0.93563055	stage_E14_vs_E10	ordinary_muscle_development		0	FALSE
ENSGALG00010025791	FIGN	PRJNA729271	1.524633601	2.69653E-10	1.524633601	stage_E14_vs_E10	ordinary_muscle_development	D_vs_E10;stage_1W_vs_E10;stage_3W_vs_E10;stage	1	TRUE
ENSGALG00010011667	ENSGALG00010011667	PRJNA729271	1.615711891	2.70333E-10	1.615711891	stage_E14_vs_E10	ordinary_muscle_development	D_vs_E10;stage_1W_vs_E10;stage_3W_vs_E10;stage	1	TRUE
ENSGALG00010021230	C1orf174	PRJNA729271	-0.398579674	2.712E-10	0.398579674	stage_E14_vs_E10	ordinary_muscle_development		0	FALSE
ENSGALG00010023200	VPS26A	PRJNA729271	0.53129967	2.71214E-10	0.53129967	stage_E14_vs_E10	ordinary_muscle_development		0	FALSE
ENSGALG00010017542	PLOD1	PRJNA729271	-0.808761524	2.72158E-10	0.808761524	stage_E14_vs_E10	ordinary_muscle_development		0	FALSE
ENSGALG00010028010	ASB6	PRJNA729271	-0.471311537	2.72337E-10	0.471311537	stage_E14_vs_E10	ordinary_muscle_development		0	FALSE
ENSGALG00010001698	RPL15	PRJNA729271	1.14882181	2.73271E-10	1.14882181	stage_E14_vs_E10	ordinary_muscle_development	D_vs_E10;stage_1W_vs_E10;stage_3W_vs_E10;stage	1	TRUE
ENSGALG00010022954	ADRM1	PRJNA729271	0.923216877	2.75229E-10	0.923216877	stage_E14_vs_E10	ordinary_muscle_development		0	FALSE
ENSGALG00010024685	ENSGALG00010024685	PRJNA729271	-1.441370292	2.81811E-10	1.441370292	stage_E14_vs_E10	ordinary_muscle_development	D_vs_E10;stage_1W_vs_E10;stage_3W_vs_E10;stage	1	TRUE
ENSGALG00010011034	FAM180A	PRJNA729271	2.481304964	2.82271E-10	2.481304964	stage_E14_vs_E10	ordinary_muscle_development	D_vs_E10;stage_1W_vs_E10;stage_3W_vs_E10;stage	1	TRUE
ENSGALG00010010546	MICALL1	PRJNA729271	1.107194469	2.83308E-10	1.107194469	stage_E14_vs_E10	ordinary_muscle_development	D_vs_E10;stage_1W_vs_E10;stage_3W_vs_E10;stage	1	TRUE
ENSGALG00010002235	MXRA5	PRJNA729271	-1.29663727	2.83399E-10	1.29663727	stage_E14_vs_E10	ordinary_muscle_development	D_vs_E10;stage_1W_vs_E10;stage_3W_vs_E10;stage	1	TRUE
ENSGALG00010004108	PERPB	PRJNA729271	1.550840297	2.85257E-10	1.550840297	stage_E14_vs_E10	ordinary_muscle_development	D_vs_E10;stage_1W_vs_E10;stage_3W_vs_E10;stage	1	TRUE
ENSGALG00010007955	TMA16	PRJNA729271	-0.796230561	2.85494E-10	0.796230561	stage_E14_vs_E10	ordinary_muscle_development		0	FALSE
ENSGALG00010027245	LRIG2	PRJNA729271	0.421848468	2.85823E-10	0.421848468	stage_E14_vs_E10	ordinary_muscle_development		0	FALSE
ENSGALG00010005077	ERG28	PRJNA729271	-0.690155971	2.86297E-10	0.690155971	stage_E14_vs_E10	ordinary_muscle_development		0	FALSE
ENSGALG00010024513	SPTY2D1	PRJNA729271	0.892682077	2.86422E-10	0.892682077	stage_E14_vs_E10	ordinary_muscle_development		0	FALSE
ENSGALG00010026737	SCN11A	PRJNA729271	-7.41334819	2.90243E-10	7.41334819	stage_E14_vs_E10	ordinary_muscle_development	D_vs_E10;stage_1W_vs_E10;stage_3W_vs_E10;stage	1	TRUE
ENSGALG00010023710	CDC14A	PRJNA729271	0.771027508	2.91359E-10	0.771027508	stage_E14_vs_E10	ordinary_muscle_development		0	FALSE
ENSGALG00010003832	ART1B	PRJNA729271	6.018195761	2.92113E-10	6.018195761	stage_E14_vs_E10	ordinary_muscle_development	D_vs_E10;stage_1W_vs_E10;stage_3W_vs_E10;stage	1	TRUE
ENSGALG00010024635	ENSGALG00010024635	PRJNA729271	-1.312870343	2.96103E-10	1.312870343	stage_E14_vs_E10	ordinary_muscle_development	D_vs_E10;stage_1W_vs_E10;stage_3W_vs_E10;stage	1	TRUE
ENSGALG00010016542	ENOX2	PRJNA729271	-0.510155007	2.97201E-10	0.510155007	stage_E14_vs_E10	ordinary_muscle_development		0	FALSE
ENSGALG00010027393	TEX264	PRJNA729271	1.214515325	2.9776E-10	1.214515325	stage_E14_vs_E10	ordinary_muscle_development	D_vs_E10;stage_1W_vs_E10;stage_3W_vs_E10;stage	1	TRUE
ENSGALG00010030107	RPTOR	PRJNA729271	0.888133221	3.00827E-10	0.888133221	stage_E14_vs_E10	ordinary_muscle_development		0	FALSE
ENSGALG00010012071	ENSGALG00010012071	PRJNA729271	-1.002193281	3.01082E-10	1.002193281	stage_E14_vs_E10	ordinary_muscle_development	D_vs_E10;stage_1W_vs_E10;stage_3W_vs_E10;stage	1	TRUE
ENSGALG00010011313	RASSF9	PRJNA729271	-1.684497637	3.07885E-10	1.684497637	stage_E14_vs_E10	ordinary_muscle_development	D_vs_E10;stage_1W_vs_E10;stage_3W_vs_E10;stage	1	TRUE
ENSGALG00010019962	SNX8	PRJNA729271	0.910636661	3.08802E-10	0.910636661	stage_E14_vs_E10	ordinary_muscle_development		0	FALSE
ENSGALG00010001426	ATE1	PRJNA729271	0.58481899	3.08804E-10	0.58481899	stage_E14_vs_E10	ordinary_muscle_development		0	FALSE
ENSGALG00010027671	BARX1	PRJNA729271	-5.871137444	3.11161E-10	5.871137444	stage_E14_vs_E10	ordinary_muscle_development	D_vs_E10;stage_1W_vs_E10;stage_3W_vs_E10;stage	1	TRUE
ENSGALG00010013617	PIK3C2G	PRJNA729271	1.018413867	3.11551E-10	1.018413867	stage_E14_vs_E10	ordinary_muscle_development	D_vs_E10;stage_1W_vs_E10;stage_3W_vs_E10;stage	1	TRUE
ENSGALG00010012966	GLIPR1L	PRJNA729271	1.699944791	3.12976E-10	1.699944791	stage_E14_vs_E10	ordinary_muscle_development	D_vs_E10;stage_1W_vs_E10;stage_3W_vs_E10;stage	1	TRUE
ENSGALG00010021293	PCNX1	PRJNA729271	0.443054702	3.13784E-10	0.443054702	stage_E14_vs_E10	ordinary_muscle_development		0	FALSE
ENSGALG00010028584	LRRC8A	PRJNA729271	0.485296826	3.15643E-10	0.485296826	stage_E14_vs_E10	ordinary_muscle_development		0	FALSE
ENSGALG00010008333	APC	PRJNA729271	-0.593253461	3.18049E-10	0.593253461	stage_E14_vs_E10	ordinary_muscle_development		0	FALSE
ENSGALG00010002466	UROS	PRJNA729271	1.131189641	3.18193E-10	1.131189641	stage_E14_vs_E10	ordinary_muscle_development	D_vs_E10;stage_1W_vs_E10;stage_3W_vs_E10;stage	1	TRUE
ENSGALG00010017841	GALC	PRJNA729271	-1.751480581	3.18493E-10	1.751480581	stage_E14_vs_E10	ordinary_muscle_development	D_vs_E10;stage_1W_vs_E10;stage_3W_vs_E10;stage	1	TRUE
ENSGALG00010021215	SH3PXD2A	PRJNA729271	-0.718305228	3.18827E-10	0.718305228	stage_E14_vs_E10	ordinary_muscle_development		0	FALSE
ENSGALG00010013901	NFXL1	PRJNA729271	-0.808804657	3.19241E-10	0.808804657	stage_E14_vs_E10	ordinary_muscle_development		0	FALSE
ENSGALG00010002097	PCYT1B	PRJNA729271	-3.34185239	3.21955E-10	3.34185239	stage_E14_vs_E10	ordinary_muscle_development	D_vs_E10;stage_1W_vs_E10;stage_3W_vs_E10;stage	1	TRUE
ENSGALG00010002625	FHL5	PRJNA729271	1.402891408	3.23943E-10	1.402891408	stage_E14_vs_E10	ordinary_muscle_development	D_vs_E10;stage_1W_vs_E10;stage_3W_vs_E10;stage	1	TRUE
ENSGALG00010020123	ENSGALG00010020123	PRJNA729271	-1.077883675	3.24409E-10	1.077883675	stage_E14_vs_E10	ordinary_muscle_development	D_vs_E10;stage_1W_vs_E10;stage_3W_vs_E10;stage	1	TRUE
ENSGALG00010015553	ASCL4	PRJNA729271	-7.278012023	3.27517E-10	7.278012023	stage_E14_vs_E10	ordinary_muscle_development	D_vs_E10;stage_1W_vs_E10;stage_3W_vs_E10;stage	1	TRUE
ENSGALG00010006111	WBP1	PRJNA729271	-0.550850577	3.30199E-10	0.550850577	stage_E14_vs_E10	ordinary_muscle_development		0	FALSE
ENSGALG00010003816	KLHL8	PRJNA729271	-1.17427957	3.33964E-10	1.17427957	stage_E14_vs_E10	ordinary_muscle_development	D_vs_E10;stage_1W_vs_E10;stage_3W_vs_E10;stage	1	TRUE
ENSGALG00010014719	ENSGALG00010014719	PRJNA729271	5.486413122	3.38258E-10	5.486413122	stage_E14_vs_E10	ordinary_muscle_development	D_vs_E10;stage_1W_vs_E10;stage_3W_vs_E10;stage	1	TRUE
ENSGALG00010008007	CBR4	PRJNA729271	0.776995449	3.41716E-10	0.776995449	stage_E14_vs_E10	ordinary_muscle_development		0	FALSE
ENSGALG00010022473	ZNF385A	PRJNA729271	2.519730239	3.41789E-10	2.519730239	stage_E14_vs_E10	ordinary_muscle_development	D_vs_E10;stage_1W_vs_E10;stage_3W_vs_E10;stage	1	TRUE
ENSGALG00010004588	MINDY3	PRJNA729271	-0.99224365	3.42351E-10	0.99224365	stage_E14_vs_E10	ordinary_muscle_development		0	FALSE
ENSGALG00010015046	PSMB2	PRJNA729271	-0.607647089	3.42645E-10	0.607647089	stage_E14_vs_E10	ordinary_muscle_development		0	FALSE
ENSGALG00010021065	CNNM2	PRJNA729271	-0.737519007	3.44405E-10	0.737519007	stage_E14_vs_E10	ordinary_muscle_development		0	FALSE
ENSGALG00010026388	SNRPD3	PRJNA729271	-0.514759255	3.47404E-10	0.514759255	stage_E14_vs_E10	ordinary_muscle_development		0	FALSE
ENSGALG00010021011	SCARB1	PRJNA729271	0.683078727	3.50402E-10	0.683078727	stage_E14_vs_E10	ordinary_muscle_development		0	FALSE
ENSGALG00010004063	ENSGALG00010004063	PRJNA729271	2.58610224	3.52709E-10	2.58610224	stage_E14_vs_E10	ordinary_muscle_development	D_vs_E10;stage_1W_vs_E10;stage_3W_vs_E10;stage	1	TRUE

ENSGALG00010016622	KIAA1143	PRJNA729271	-0.867113747	3.5457E-10	0.867113747	stage_E14_vs_E10	ordinary_muscle_development		0	FALSE
ENSGALG00010021240	SLK	PRJNA729271	-0.512896187	3.59302E-10	0.512896187	stage_E14_vs_E10	ordinary_muscle_development		0	FALSE
ENSGALG00010002215	YLEC18	PRJNA729271	2.294389873	3.61406E-10	2.294389873	stage_E14_vs_E10	ordinary_muscle_development	D_vs_E10;stage_1W_vs_E10;stage_3W_vs_E10;stage	1	TRUE
ENSGALG00010024941	PDHX	PRJNA729271	0.756044256	3.6259E-10	0.756044256	stage_E14_vs_E10	ordinary_muscle_development		0	FALSE
ENSGALG00010004440	GTF2H5	PRJNA729271	-0.674953394	3.62636E-10	0.674953394	stage_E14_vs_E10	ordinary_muscle_development		0	FALSE
ENSGALG00010011144	ATF7IP	PRJNA729271	0.397869857	3.62821E-10	0.397869857	stage_E14_vs_E10	ordinary_muscle_development		0	FALSE
ENSGALG00010002768	TRIM39.2	PRJNA729271	5.823858754	3.63446E-10	5.823858754	stage_E14_vs_E10	ordinary_muscle_development	D_vs_E10;stage_1W_vs_E10;stage_3W_vs_E10;stage	1	TRUE
ENSGALG00010008380	SIL1	PRJNA729271	1.104891083	3.65897E-10	1.104891083	stage_E14_vs_E10	ordinary_muscle_development	D_vs_E10;stage_1W_vs_E10;stage_3W_vs_E10;stage	1	TRUE
ENSGALG00010009094	DARS	PRJNA729271	-0.615366606	3.66886E-10	0.615366606	stage_E14_vs_E10	ordinary_muscle_development		0	FALSE
ENSGALG00010010995	HEBP1	PRJNA729271	-1.181404577	3.67803E-10	1.181404577	stage_E14_vs_E10	ordinary_muscle_development	D_vs_E10;stage_1W_vs_E10;stage_3W_vs_E10;stage	1	TRUE
ENSGALG00010029401	HIC1	PRJNA729271	-1.295989317	3.70366E-10	1.295989317	stage_E14_vs_E10	ordinary_muscle_development	D_vs_E10;stage_1W_vs_E10;stage_3W_vs_E10;stage	1	TRUE
ENSGALG00010021635	FOXJ2	PRJNA729271	0.728743479	3.71128E-10	0.728743479	stage_E14_vs_E10	ordinary_muscle_development		0	FALSE
ENSGALG00010021044	EIF4G2	PRJNA729271	-0.440140926	3.72886E-10	0.440140926	stage_E14_vs_E10	ordinary_muscle_development		0	FALSE
ENSGALG00010002341	FAM49A	PRJNA729271	-0.714306708	3.74988E-10	0.714306708	stage_E14_vs_E10	ordinary_muscle_development		0	FALSE
ENSGALG00010013981	RLF	PRJNA729271	0.683550451	3.7512E-10	0.683550451	stage_E14_vs_E10	ordinary_muscle_development		0	FALSE
ENSGALG00010013477	RFC1	PRJNA729271	-0.585877581	3.76639E-10	0.585877581	stage_E14_vs_E10	ordinary_muscle_development		0	FALSE
ENSGALG00010015797	PIP5K1B	PRJNA729271	-1.068240684	3.76986E-10	1.068240684	stage_E14_vs_E10	ordinary_muscle_development	D_vs_E10;stage_1W_vs_E10;stage_3W_vs_E10;stage	1	TRUE
ENSGALG00010023249	IL12RB2	PRJNA729271	2.663583123	3.7826E-10	2.663583123	stage_E14_vs_E10	ordinary_muscle_development	D_vs_E10;stage_1W_vs_E10;stage_3W_vs_E10;stage	1	TRUE
ENSGALG00010025031	ECHDC2	PRJNA729271	0.774540494	3.80546E-10	0.774540494	stage_E14_vs_E10	ordinary_muscle_development		0	FALSE
ENSGALG00010028104	GOLGA1	PRJNA729271	0.732290358	3.84961E-10	0.732290358	stage_E14_vs_E10	ordinary_muscle_development		0	FALSE
ENSGALG00010024858	SLC25A39	PRJNA729271	-0.803355672	3.91037E-10	0.803355672	stage_E14_vs_E10	ordinary_muscle_development		0	FALSE
ENSGALG00010008410	NAPRT	PRJNA729271	-0.857514334	3.91475E-10	0.857514334	stage_E14_vs_E10	ordinary_muscle_development		0	FALSE
ENSGALG00010011434	NPL	PRJNA729271	1.147673803	3.93818E-10	1.147673803	stage_E14_vs_E10	ordinary_muscle_development	D_vs_E10;stage_1W_vs_E10;stage_3W_vs_E10;stage	1	TRUE
ENSGALG00010004974	MTPAP	PRJNA729271	-0.396464252	3.96822E-10	0.396464252	stage_E14_vs_E10	ordinary_muscle_development		0	FALSE
ENSGALG00010010911	ENSGALG00010010911	PRJNA729271	-2.536094298	3.99196E-10	2.536094298	stage_E14_vs_E10	ordinary_muscle_development	D_vs_E10;stage_1W_vs_E10;stage_3W_vs_E10;stage	1	TRUE
ENSGALG00010025259	SHH	PRJNA729271	-6.727375874	4.01402E-10	6.727375874	stage_E14_vs_E10	ordinary_muscle_development	D_vs_E10;stage_1W_vs_E10;stage_3W_vs_E10;stage	1	TRUE
ENSGALG00010019991	EIF3B	PRJNA729271	0.479747636	4.06175E-10	0.479747636	stage_E14_vs_E10	ordinary_muscle_development		0	FALSE
ENSGALG00010025465	ENSGALG00010025465	PRJNA729271	-0.541879625	4.08198E-10	0.541879625	stage_E14_vs_E10	ordinary_muscle_development		0	FALSE
ENSGALG00010008434	PTCHD4	PRJNA729271	-5.938532863	4.08895E-10	5.938532863	stage_E14_vs_E10	ordinary_muscle_development	D_vs_E10;stage_1W_vs_E10;stage_3W_vs_E10;stage	1	TRUE
ENSGALG00010000043	PPIP5K1	PRJNA729271	0.790006491	4.10305E-10	0.790006491	stage_E14_vs_E10	ordinary_muscle_development		0	FALSE
ENSGALG00010024347	OTOG	PRJNA729271	-1.901369053	4.11601E-10	1.901369053	stage_E14_vs_E10	ordinary_muscle_development	D_vs_E10;stage_1W_vs_E10;stage_3W_vs_E10;stage	1	TRUE
ENSGALG00010013212	ENSGALG00010013212	PRJNA729271	0.973349103	4.12332E-10	0.973349103	stage_E14_vs_E10	ordinary_muscle_development		0	FALSE
ENSGALG00010006249	C9orf72	PRJNA729271	0.665603479	4.17146E-10	0.665603479	stage_E14_vs_E10	ordinary_muscle_development		0	FALSE
ENSGALG00010024363	EDEM2	PRJNA729271	-0.755593672	4.18893E-10	0.755593672	stage_E14_vs_E10	ordinary_muscle_development		0	FALSE
ENSGALG00010019316	CLEC16A	PRJNA729271	0.559484257	4.22987E-10	0.559484257	stage_E14_vs_E10	ordinary_muscle_development		0	FALSE
ENSGALG00010024360	FTL	PRJNA729271	-1.573120591	4.25463E-10	1.573120591	stage_E14_vs_E10	ordinary_muscle_development	D_vs_E10;stage_1W_vs_E10;stage_3W_vs_E10;stage	1	TRUE
ENSGALG00010022359	ZZZ3	PRJNA729271	-0.508723247	4.28027E-10	0.508723247	stage_E14_vs_E10	ordinary_muscle_development		0	FALSE
ENSGALG00010006053	TMEM243	PRJNA729271	-1.664682657	4.34391E-10	1.664682657	stage_E14_vs_E10	ordinary_muscle_development	D_vs_E10;stage_1W_vs_E10;stage_3W_vs_E10;stage	1	TRUE
ENSGALG00010017589	ATPAF2	PRJNA729271	0.922343586	4.34569E-10	0.922343586	stage_E14_vs_E10	ordinary_muscle_development		0	FALSE
ENSGALG00010002289	ENSGALG00010002289	PRJNA729271	5.974939555	4.38613E-10	5.974939555	stage_E14_vs_E10	ordinary_muscle_development	D_vs_E10;stage_1W_vs_E10;stage_3W_vs_E10;stage	1	TRUE
ENSGALG00010023202	PID1	PRJNA729271	1.003800391	4.39222E-10	1.003800391	stage_E14_vs_E10	ordinary_muscle_development	D_vs_E10;stage_1W_vs_E10;stage_3W_vs_E10;stage	1	TRUE
ENSGALG00010029758	DNAH9	PRJNA729271	1.923629477	4.43346E-10	1.923629477	stage_E14_vs_E10	ordinary_muscle_development	D_vs_E10;stage_1W_vs_E10;stage_3W_vs_E10;stage	1	TRUE
ENSGALG00010026704	WDFY1	PRJNA729271	-0.449328507	4.44285E-10	0.449328507	stage_E14_vs_E10	ordinary_muscle_development		0	FALSE
ENSGALG00010023953	SLC35A3	PRJNA729271	0.980549783	4.45948E-10	0.980549783	stage_E14_vs_E10	ordinary_muscle_development		0	FALSE
ENSGALG00010004374	ILDR1	PRJNA729271	-6.318522027	4.46363E-10	6.318522027	stage_E14_vs_E10	ordinary_muscle_development	D_vs_E10;stage_1W_vs_E10;stage_3W_vs_E10;stage	1	TRUE
ENSGALG00010010671	SIGMAR1	PRJNA729271	-0.916317762	4.4887E-10	0.916317762	stage_E14_vs_E10	ordinary_muscle_development		0	FALSE
ENSGALG00010022742	IFT46	PRJNA729271	-0.809235652	4.48973E-10	0.809235652	stage_E14_vs_E10	ordinary_muscle_development		0	FALSE
ENSGALG00010027399	MAPKAP1	PRJNA729271	0.702425642	4.49982E-10	0.702425642	stage_E14_vs_E10	ordinary_muscle_development		0	FALSE
ENSGALG00010020827	ENSGALG00010020827	PRJNA729271	-0.588774118	4.54515E-10	0.588774118	stage_E14_vs_E10	ordinary_muscle_development		0	FALSE
ENSGALG00010007141	PRKD3	PRJNA729271	-0.542668762	4.57835E-10	0.542668762	stage_E14_vs_E10	ordinary_muscle_development		0	FALSE
ENSGALG00010001491	PHF3	PRJNA729271	0.56381576	4.59078E-10	0.56381576	stage_E14_vs_E10	ordinary_muscle_development		0	FALSE
ENSGALG00010024956	VPS37B	PRJNA729271	-0.885538028	4.59743E-10	0.885538028	stage_E14_vs_E10	ordinary_muscle_development		0	FALSE
ENSGALG00010025621	PPP1CC	PRJNA729271	-0.750739162	4.61223E-10	0.750739162	stage_E14_vs_E10	ordinary_muscle_development		0	FALSE
ENSGALG00010024369	MYOD1	PRJNA729271	1.752449918	4.61747E-10	1.752449918	stage_E14_vs_E10	ordinary_muscle_development	D_vs_E10;stage_1W_vs_E10;stage_3W_vs_E10;stage	1	TRUE
ENSGALG00010005318	SLC35D3	PRJNA729271	-3.976863794	4.62025E-10	3.976863794	stage_E14_vs_E10	ordinary_muscle_development	D_vs_E10;stage_1W_vs_E10;stage_3W_vs_E10;stage	1	TRUE
ENSGALG00010026977	ELK4	PRJNA729271	0.615092362	4.64056E-10	0.615092362	stage_E14_vs_E10	ordinary_muscle_development		0	FALSE
ENSGALG00010006151	ENSGALG00010006151	PRJNA729271	-1.07216353	4.65974E-10	1.07216353	stage_E14_vs_E10	ordinary_muscle_development	D_vs_E10;stage_1W_vs_E10;stage_3W_vs_E10;stage	1	TRUE
ENSGALG00010014240	PIGV	PRJNA729271	0.80804198	4.66851E-10	0.80804198	stage_E14_vs_E10	ordinary_muscle_development		0	FALSE
ENSGALG00010010893	CCL21	PRJNA729271	-3.439717852	4.66947E-10	3.439717852	stage_E14_vs_E10	ordinary_muscle_development	D_vs_E10;stage_1W_vs_E10;stage_3W_vs_E10;stage	1	TRUE
ENSGALG00010021595	UBE3B	PRJNA729271	0.535082498	4.67677E-10	0.535082498	stage_E14_vs_E10	ordinary_muscle_development		0	FALSE
ENSGALG00010012788	RGS1	PRJNA729271	4.00420679	4.68443E-10	4.00420679	stage_E14_vs_E10	ordinary_muscle_development	D_vs_E10;stage_1W_vs_E10;stage_3W_vs_E10;stage	1	TRUE
ENSGALG00010017816	PRKCH	PRJNA729271	-2.092557506	4.68779E-10	2.092557506	stage_E14_vs_E10	ordinary_muscle_development	D_vs_E10;stage_1W_vs_E10;stage_3W_vs_E10;stage	1	TRUE
ENSGALG00010019230	SPG21	PRJNA729271	-0.627786774	4.71648E-10	0.627786774	stage_E14_vs_E10	ordinary_muscle_development		0	FALSE
ENSGALG00010013331	KIF3B	PRJNA729271	-0.962321115	4.72152E-10	0.962321115	stage_E14_vs_E10	ordinary_muscle_development		0	FALSE
ENSGALG00010009142	LGALS8	PRJNA729271	-0.874934517	4.74152E-10	0.874934517	stage_E14_vs_E10	ordinary_muscle_development		0	FALSE
ENSGALG00010005769	TFG	PRJNA729271	-0.677495216	4.76602E-10	0.677495216	stage_E14_vs_E10	ordinary_muscle_development		0	FALSE
ENSGALG00010027015	ENSGALG00010027015	PRJNA729271	3.261285208	4.76702E-10	3.261285208	stage_E14_vs_E10	ordinary_muscle_development	D_vs_E10;stage_1W_vs_E10;stage_3W_vs_E10;stage	1	TRUE
ENSGALG00010028121	TUSC2	PRJNA729271	0.608061065	4.77662E-10	0.608061065	stage_E14_vs_E10	ordinary_muscle_development		0	FALSE
ENSGALG00010014889	PVALB	PRJNA729271	2.616288633	4.79225E-10	2.616288633	stage_E14_vs_E10	ordinary_muscle_development	D_vs_E10;stage_1W_vs_E10;stage_3W_vs_E10;stage	1	TRUE
ENSGALG00010014754	DIMT1	PRJNA729271	-0.934880582	4.82607E-10	0.934880582	stage_E14_vs_E10	ordinary_muscle_development		0	FALSE
ENSGALG00010028853	ENSGALG00010028853	PRJNA729271	7.741328934	4.85384E-10	7.741328934	stage_E14_vs_E10	ordinary_muscle_development	D_vs_E10;stage_1W_vs_E10;stage_3W_vs_E10;stage	1	TRUE
ENSGALG00010021175	IPO7	PRJNA729271	0.619978642	4.8963E-10	0.619978642	stage_E14_vs_E10	ordinary_muscle_development		0	FALSE
ENSGALG00010024622	PRMT8	PRJNA729271	-1.368631651	4.89704E-10	1.368631651	stage_E14_vs_E10	ordinary_muscle_development	D_vs_E10;stage_1W_vs_E10;stage_3W_vs_E10;stage	1	TRUE
ENSGALG00010012190	CAST	PRJNA729271	0.845609726	4.90091E-10	0.845609726	stage_E14_vs_E10	ordinary_muscle_development		0	FALSE
ENSGALG00010008267	MAML2	PRJNA729271	-0.763083412	4.91297E-10	0.763083412	stage_E14_vs_E10	ordinary_muscle_development		0	FALSE
ENSGALG00010002507	C2CD3	PRJNA729271	-0.716716184	4.94987E-10	0.716716184	stage_E14_vs_E10	ordinary_muscle_development		0	FALSE
ENSGALG00010022173	RPL23	PRJNA729271	0.667700617	4.96613E-10	0.667700617	stage_E14_vs_E10	ordinary_muscle_development		0	FALSE
ENSGALG00010013260	FAM210B	PRJNA729271	-0.534689995	4.98643E-10	0.534689995	stage_E14_vs_E10	ordinary_muscle_development		0	FALSE
ENSGALG00010013942	CACNA2D4	PRJNA729271	2.854932529	5.00337E-10	2.854932529	stage_E14_vs_E10	ordinary_muscle_development	D_vs_E10;stage_1W_vs_E10;stage_3W_vs_E10;stage	1	TRUE
ENSGALG00010008278	CYP39A1	PRJNA729271	1.017067397	5.00802E-10	1.017067397	stage_E14_vs_E10	ordinary_muscle_development	D_vs_E10;stage_1W_vs_E10;stage_3W_vs_E10;stage	1	TRUE
ENSGALG00010017562	SLC30A10	PRJNA729271	-3.965157751	5.02371E-10	3.965157751	stage_E14_vs_E10	ordinary_muscle_development	D_vs_E10;stage_1W_vs_E10;stage_3W_vs_E10;stage	1	TRUE
ENSGALG00010014796	PRLR	PRJNA729271	-1.608674458	5.09333E-10	1.608674458	stage_E14_vs_E10	ordinary_muscle_development	D_vs_E10;stage_1W_vs_E10;stage_3W_vs_E10;stage	1	TRUE
ENSGALG00010021252	XPC	PRJNA729271	0.83302107	5.09981E-10	0.83302107	stage_E14_vs_E10	ordinary_muscle_development		0	FALSE
ENSGALG00010008046	RHOB	PRJNA729271	-1.074385106	5.19901E-10	1.074385106	stage_E14_vs_E10	ordinary_muscle_development	D_vs_E10;stage_1W_vs_E10;stage_3W_vs_E10;stage	1	TRUE
ENSGALG00010016094	FGF7	PRJNA729271	1.177108826	5.20029E-10	1.177108826	stage_E14_vs_E10	ordinary_muscle_development	D_vs_E10;stage_1W_vs_E10;stage_3W_vs_E10;stage	1	TRUE
ENSGALG00010007772	CLEC3A	PRJNA729271	4.655960947	5.22373E-10	4.655960947	stage_E14_vs_E10	ordinary_muscle_development	D_vs_E10;stage_1W_vs_E10;stage_3W_vs_E10;stage	1	TRUE
ENSGALG00010000444	RBM19	PRJNA729271	-0.768335048	5.2914E-10	0.768335048	stage_E14_vs_E10	ordinary_muscle_development		0	FALSE
ENSGALG00010026686	ENSGALG00010026686	PRJNA729271	1.601546963	5.28586E-10	1.601546963	stage_E14_vs_E10	ordinary_muscle_development	D_vs_E10;stage_1W_vs_E10;stage_3W_vs_E10;stage	1	TRUE
ENSGALG00010028270	RAPGEF1	PRJNA729271	1.14925569	5.31142E-10	1.14925569	stage_E14_vs_E10	ordinary_muscle_development	D_vs_E10;stage_1W_vs_E10;stage_3W_vs_E10;stage	1	TRUE
ENSGALG00010025599	COPS9	PRJNA729271	0.90421008	5.34136E-10	0.90421008	stage_E14_vs_E10	ordinary_muscle_development		0	FALSE
ENSGALG00010016785	PDGFRB	PRJNA729271	1.232193848	5.34215E-10	1.232193848	stage_E14_vs_E10	ordinary_muscle_development	D_vs_E10;stage_1W_vs_E10;stage_3W_vs_E10;stage	1	TRUE
ENSGALG00010012147	ARFGAP3	PRJNA729271	-0.784909853	5.34679E-10	0.784909853	stage_E14_vs_E10	ordinary_muscle_development		0	FALSE
ENSGALG00010011469	CPS1	PRJNA729271	2.114317704	5.36604E-10	2.114317704	stage_E14_vs_E10	ordinary_muscle_development	D_vs_E10;stage_1W_vs_E10;stage_3W_vs_E10;stage	1	TRUE
ENSGALG00010005358	SHPRH	PRJNA729271	0.564409009	5.37803E-10	0.564409009	stage_E14_vs_E10	ordinary_muscle_development		0	FALSE
ENSGALG00010027718	ENSGALG00010027718	PRJNA729271	2.907490572	5.38057E-10	2.907490572	stage_E14_vs_E10	ordinary_muscle_development	D_vs_E10;stage_1W_vs_E10;stage_3W_vs_E10;stage	1	TRUE
ENSGALG00010014319	ENSGALG00010014319	PRJNA729271	0.73713552	5.38411E-10	0.73713552	stage_E14_vs_E10	ordinary_muscle_development		0	FALSE
ENSGALG00010008708	ZC3H4	PRJNA729271	1.544540497	5.39716E-10	1.544540497	stage_E14_vs_E10	ordinary_muscle_development	D_vs_E10;stage_1W_vs_E10;stage_3W_vs_E10;stage	1	TRUE
ENSGALG00010021408	TTC9	PRJNA729271	1.295384725	5.41604E-10	1.295384725	stage_E14_vs_E10	ordinary_muscle_development	D_vs_E10;stage_1W_vs_E10;stage_3W_vs_E10;stage	1	TRUE
ENSGALG00010029440	FOXK2	PRJNA729271	1.149133742	5.4374E-10	1.149133742	stage_E14_vs_E10	ordinary_muscle_development	D_vs_E10;stage_1W_vs_E10;stage_3W_vs_E10;stage	1	TRUE
ENSGALG00010014705	ARAP2	PRJNA729271	-1.4440394	5.44362E-10	1.4440394	stage_E14_vs_E10	ordinary_muscle_development	D_vs_E10;stage_1W_vs_E10;stage_3W_vs_E10;stage	1	TRUE
ENSGALG00010008373	MACIR	PRJNA729271	-1.06568651	5.48005E-10	1.06568651	stage_E14_vs_E10	ordinary_muscle_development	D_vs_E10;stage_1W_vs_E10;stage_3W_vs_E10;stage	1	TRUE
ENSGALG00010008081	TOP1MT	PRJNA729271	-0.903859054	5.4976E-10	0.903859054	stage_E14_vs_E10	ordinary_muscle_development		0	FALSE
ENSGALG00010008283	NQO1	PRJNA729271	-1.275278473	5.54793E-10	1.275278473	stage_E14_vs_E10	ordinary_muscle_development	D_vs_E10;stage_1W_vs_E10;stage_3W_vs_E10;stage	1	TRUE
ENSGALG00010013550	KLHL5	PRJNA729271	-0.795843764	5.58974E-10	0.795843764	stage_E14_vs_E10	ordinary_muscle_development		0	FALSE
ENSGALG00010026325	TMEM69	PRJNA729271	0.868455657	5.60664E-10	0.868455657	stage_E14_vs_E10	ordinary_muscle_development		0	FALSE
ENSGALG00010026073	HPDL	PRJNA729271	-1.783778606	5.61663E-10	1.783778606	stage_E14_vs_E10	ordinary_muscle_development	D_vs_E10;stage_1W_vs_E10;stage_3W_vs_E10;stage	1	TRUE
ENSGALG00010020739	PKP3	PRJNA729271	-4.91995717	5.62586E-10	4.91995717	stage_E14_vs_E10	ordinary_muscle_development	D_vs_E10;stage_1W_vs_E10;stage_3W_vs_E10;stage	1	TRUE
ENSGALG00010006281	TRMT11	PRJNA729271	0.511695606	5.63553E-10	0.511695606	stage_E14_vs_E10	ordinary_muscle_development		0	FALSE
ENSGALG00010021711	ENSGALG00010021711	PRJNA729271	1.37766658	5.65777E-10	1.37766658	stage_E14_vs_E10	ordinary_muscle_development	D_vs_E10;stage_1W_vs_E10;stage_3W_vs_E10;stage	1	TRUE
ENSGALG00010005530	IL17D	PRJNA729271	1.378937864	5.70364E-10	1.378937864	stage_E14_vs_E10	ordinary_muscle_development	D_vs_E10;stage_1W_vs_E10;stage_3W_vs_E10;stage	1	TRUE
ENSGALG00010024942	DNAJB6	PRJNA729271	0.418543716	5.75925E-10	0.418543716	stage_E14_vs_E10	ordinary_muscle_development		0	FALSE
ENSGALG00010004962	ENSGALG00010004962	PRJNA729271	-0.600846049	5.81063E-10	0.600846049	stage_E14_vs_E10	ordinary_muscle_development		0	FALSE
ENSGALG00010017525	ENSGALG00010017525	PRJNA729271	-6.189992561	5.85198E-10	6.189992561	stage_E14_vs_E10	ordinary_muscle_development	D_vs_E10;stage_1W_vs_E10;stage_3W_vs_E10;stage	1	TRUE
ENSGALG00010024676	ENSGALG00010024676	PRJNA729271	1.033629721	5.88596E-10	1.033629721	stage_E14_vs_E10	ordinary_muscle_development	D_vs_E10;stage_1W_vs_E10;stage_3W_vs_E10;stage	1	TRUE
ENSGALG00010013464	ABCB7	PRJNA729271	-0.485126337	5.92052E-10	0.485126337	stage_E14_vs_E10	ordinary_muscle_development		0	FALSE
ENSGALG00010014653	FAM162B	PRJNA729271	3.030075044	5.92543E-10	3.030075044	stage_E14_vs_E10	ordinary_muscle_development	D_vs_E10;stage_1W_vs_E10;stage_3W_vs_E10;stage	1	TRUE
ENSGALG00010029544	ENSGALG00010029544	PRJNA729271	-1.13433395	5.94477E-10	1.13433395	stage_E14_vs_E10	ordinary_muscle_development	D_vs_E10;stage_1W_vs_E10;stage_3W_vs_E10;stage	1	TRUE
ENSGALG00010013768	FAM110A	PRJNA729271	-1.539562972	5.94738E-10	1.539562972	stage_E14_vs_E10	ordinary_muscle_development	D_vs_E10;stage_1W_vs_E10;stage_3W_vs_E10;stage	1	TRUE
ENSGALG00010014797	ZDHHC9	PRJNA729271	0.587405722	5.96279E-10	0.587405722	stage_E14_vs_E10	ordinary_muscle_development		0	FALSE
ENSGALG00010011932	VAV3	PRJNA729271	-1.097109095	5.96766E-10	1.097109095	stage_E14_vs_E10	ordinary_muscle_development	D_vs_E10;stage_1W_vs_E10;stage_3W_vs_E10;stage	1	TRUE
ENSGALG00010018367	PAK5	PRJNA729271	-1.450525069	5.9745E-10	1.450525069	stage_E14_vs_E10	ordinary_muscle_development	D_vs_E10;stage_1W_vs_E10;stage_3W_vs_E10;stage	1	TRUE
ENSGALG00010024696	TRADD	PRJNA729271	-0.750353296	6.05979E-10	0.750353296	stage_E14_vs_E10	ordinary_muscle_development		0	FALSE
ENSGALG00010025483	FGF4	PRJNA729271	-2.337800935	6.0772E-10	2.337800935	stage_E14_vs_E10	ordinary_muscle_development	D_vs_E10;stage_1W_vs_E10;stage_3W_vs_E10;stage	1	TRUE
ENSGALG00010017423	TMEM204	PRJNA729271	0.845422284	6.11596E-10	0.845422284	stage_E14_vs_E10	ordinary_muscle_development		0	FALSE
ENSGALG00010007162	ENSGALG00010007162	PRJNA729271	2.595727213	6.15071E-10	2.595727213	stage_E14_vs_E10	ordinary_muscle_development	D_vs_E10;stage_1W_vs_E10;stage_3W_vs_E10;stage	1	TRUE
ENSGALG00010021494	SLC2A10	PRJNA729271	-1.57542088	6.15821E-10	1.57542088	stage_E14_vs_E10	ordinary_muscle_development	D_vs_E10;stage_1W_vs_E10;stage_3W_vs_E10;stage	1	TRUE
ENSGALG00010020316	PDPK1	PRJNA729271	0.708569794	6.21526E-10	0.708569794	stage_E14_vs_E10	ordinary_muscle_development		0	FALSE
ENSGALG00010017538	H3-3A	PRJNA729271	-0.481536481	6.24247E-10	0.481536481	stage_E14_vs_E10	ordinary_muscle_development		0	FALSE
ENSGALG00010010948	PRDM15	PRJNA729271	-0.555184161	6.27408E-10	0.555184161	stage_E14_vs_E10	ordinary_muscle_development		0	FALSE
ENSGALG00010005316	PARP4	PRJNA729271	0.679567646	6.3166E-10	0.679567646	stage_E14_vs_E10	ordinary_muscle_development		0	FALSE
ENSGALG00010002310	PRRG1	PRJNA729271	-0.675526152	6.31913E-10	0.675526152	stage_E14_vs_E10	ordinary_muscle_development		0	FALSE
ENSGALG00010010800	BICRA	PRJNA729271	1.185301059	6.36317E-10	1.185301059	stage_E14_vs_E10	ordinary_muscle_development	D_vs_E10;stage_1W_vs_E10;stage_3W_vs_E10;stage	1	TRUE
ENSGALG00010022709	RNF5	PRJNA729271	-0.538051215	6.44525E-10	0.538051215	stage_E14_vs_E10	ordinary_muscle_development		0	FALSE
ENSGALG00010018406	TMEM260	PRJNA729271	-0.835262739	6.4535E-10	0.835262739	stage_E14_vs_E10	ordinary_muscle_development		0	FALSE
ENSGALG00010028174	GMPPB	PRJNA729271	-0.986694809	6.50606E-10	0.986694809	stage_E14_vs_E10	ordinary_muscle_development		0	FALSE
ENSGALG00010022400	NHLRC2	PRJNA729271	0.460665947	6.51395E-10	0.460665947	stage_E14_vs_E10	ordinary_muscle_development		0	FALSE
ENSGALG00010003347	GPM6A	PRJNA729271	-2.422212905	6.51889E-10	2.422212905	stage_E14_vs_E10	ordinary_muscle_development	D_vs_E10;stage_1W_vs_E10;stage_3W_vs_E10;stage	1	TRUE
ENSGALG00010006694	ENSGALG00010006694	PRJNA729271	-6.188058927	6.5233E-10	6.188058927	stage_E14_vs_E10	ordinary_muscle_development	D_vs_E10;stage_1W_vs_E10;stage_3W_vs_E10;stage	1	TRUE
ENSGALG00010011746	SOD1	PRJNA729271	-0.907665322	6.54459E-10	0.907665322	stage_E14_vs_E10	ordinary_muscle_development		0	FALSE
ENSGALG00010005159	ARHGAP21	PRJNA729271	0.626107348	6.60868E-10	0.626107348	stage_E14_vs_E10	ordinary_muscle_development		0	FALSE
ENSGALG00010027738	ENSGALG00010027738	PRJNA729271	1.535715304	6.65292E-10	1.535715304	stage_E14_vs_E10	ordinary_muscle_development	D_vs_E10;stage_1W_vs_E10;stage_3W_vs_E10;stage	1	TRUE
ENSGALG00010001221	GRAMD2B	PRJNA729271	-0.993889532	6.67204E-10	0.993889532	stage_E14_vs_E10	ordinary_muscle_development		0	FALSE
ENSGALG00010012404	VAMP7	PRJNA729271	0.327397385	6.67822E-10	0.327397385	stage_E14_vs_E10	ordinary_muscle_development		0	FALSE
ENSGALG00010015127	RPL11	PRJNA729271	0.965601173	6.6784E-10	0.965601173	stage_E14_vs_E10	ordinary_muscle_development		0	FALSE
ENSGALG00010008431	SH3RF1	PRJNA729271	-0.804274951	6.69595E-10	0.804274951	stage_E14_vs_E10	ordinary_muscle_development		0	FALSE
ENSGALG00010019141	ACOT7	PRJNA729271	-1.004193893	6.71691E-10	1.004193893	stage_E14_vs_E10	ordinary_muscle_development	D_vs_E10;stage_1W_vs_E10;stage_3W_vs_E10;stage	1	TRUE
ENSGALG00010018608	TRIM8	PRJNA729271	0.81409969	6.75057E-10	0.81409969	stage_E14_vs_E10	ordinary_muscle_development		0	FALSE
ENSGALG00010024004	EIF4B	PRJNA729271	0.796449064	6.81227E-10	0.796449064	stage_E14_vs_E10	ordinary_muscle_development		0	FALSE
ENSGALG00010029398	RHBDF2	PRJNA729271	0.773207341	6.8171E-10	0.773207341	stage_E14_vs_E10	ordinary_muscle_development		0	FALSE
ENSGALG00010006598	ENSGALG00010006598	PRJNA729271	0.954639164	6.88994E-10	0.954639164	stage_E14_vs_E10	ordinary_muscle_development		0	FALSE
ENSGALG00010012751	AP3B1	PRJNA729271	-0.481104299	6.90091E-10	0.481104299	stage_E14_vs_E10	ordinary_muscle_development		0	FALSE
ENSGALG00010000578	ENSGALG00010000578	PRJNA729271	2.272766487	6.9737E-10	2.272766487	stage_E14_vs_E10	ordinary_muscle_development	D_vs_E10;stage_1W_vs_E10;stage_3W_vs_E10;stage	1	TRUE
ENSGALG00010023349	ENSGALG00010023349	PRJNA729271	3.554968812	7.02628E-10	3.554968812	stage_E14_vs_E10	ordinary_muscle_development	D_vs_E10;stage_1W_vs_E10;stage_3W_vs_E10;stage	1	TRUE
ENSGALG00010016630	ENSGALG00010016630	PRJNA729271	2.082768299	7.04448E-10	2.082768299	stage_E14_vs_E10	ordinary_muscle_development	D_vs_E10;stage_1W_vs_E10;stage_3W_vs_E10;stage	1	TRUE
ENSGALG00010015059	ENSGALG00010015059	PRJNA729271	1.014584339	7.07023E-10	1.014584339	stage_E14_vs_E10	ordinary_muscle_development	D_vs_E10;stage_1W_vs_E10;stage_3W_vs_E10;stage	1	TRUE
ENSGALG00010019294	SMARCAL1	PRJNA729271	-0.626437748	7.15103E-10	0.626437748	stage_E14_vs_E10	ordinary_muscle_development		0	FALSE
ENSGALG00010013580	RANBP3L	PRJNA729271	1.914201928	7.15914E-10	1.914201928	stage_E14_vs_E10	ordinary_muscle_development	D_vs_E10;stage_1W_vs_E10;stage_3W_vs_E10;stage	1	TRUE
ENSGALG00010000610	ENSGALG00010000610	PRJNA729271	-0.505156388	7.17819E-10	0.505156388	stage_E14_vs_E10	ordinary_muscle_development		0	FALSE
ENSGALG00010022348	G6PC1	PRJNA729271	3.849586114	7.21408E-10	3.849586114	stage_E14_vs_E10	ordinary_muscle_development	D_vs_E10;stage_1W_vs_E10;stage_3W_vs_E10;stage	1	TRUE
ENSGALG00010018618	FAHD1	PRJNA729271	1.079540716	7.22075E-10	1.079540716	stage_E14_vs_E10	ordinary_muscle_development	D_vs_E10;stage_1W_vs_E10;stage_3W_vs_E10;stage	1	TRUE
ENSGALG00010024339	TOP3B	PRJNA729271	-0.568558662	7.23941E-10	0.568558662	stage_E14_vs_E10	ordinary_muscle_development		0	FALSE
ENSGALG00010024861	ORC1	PRJNA729271	-0.413237711	7.30797E-10	0.413237711	stage_E14_vs_E10	ordinary_muscle_development		0	FALSE
ENSGALG00010023074	CCNT1	PRJNA729271	1.032239229	7.31365E-10	1.032239229	stage_E14_vs_E10	ordinary_muscle_development	D_vs_E10;stage_1W_vs_E10;stage_3W_vs_E10;stage	1	TRUE
ENSGALG00010012188	ADAMTS6	PRJNA729271	-1.172669547	7.34964E-10	1.172669547	stage_E14_vs_E10	ordinary_muscle_development	D_vs_E10;stage_1W_vs_E10;stage_3W_vs_E10;stage	1	TRUE
ENSGALG00010007183	VOPP1	PRJNA729271	1.976441566	7.35465E-10	1.976441566	stage_E14_vs_E10	ordinary_muscle_development	D_vs_E10;stage_1W_vs_E10;stage_3W_vs_E10;stage	1	TRUE
ENSGALG00010023235	ENSGALG00010023235	PRJNA729271	-2.479354549	7.38886E-10	2.479354549	stage_E14_vs_E10	ordinary_muscle_development	D_vs_E10;stage_1W_vs_E10;stage_3W_vs_E10;stage	1	TRUE
ENSGALG00010009657	MSMP	PRJNA729271	4.047772064	7.47215E-10	4.047772064	stage_E14_vs_E10	ordinary_muscle_development	D_vs_E10;stage_1W_vs_E10;stage_3W_vs_E10;stage	1	TRUE
ENSGALG00010010623	SMAD2	PRJNA729271	-0.48890993	7.4724E-10	0.48890993	stage_E14_vs_E10	ordinary_muscle_development		0	FALSE
ENSGALG00010029142	ENSGALG00010029142	PRJNA729271	1.151378577	7.49518E-10	1.151378577	stage_E14_vs_E10	ordinary_muscle_development	D_vs_E10;stage_1W_vs_E10;stage_3W_vs_E10;stage	1	TRUE
ENSGALG00010000436	ENSGALG00010000436	PRJNA729271	2.280799803	7.49584E-10	2.280799803	stage_E14_vs_E10	ordinary_muscle_development	D_vs_E10;stage_1W_vs_E10;stage_3W_vs_E10;stage	1	TRUE
ENSGALG00010010303	ETF1	PRJNA729271	1.160174825	7.51076E-10	1.160174825	stage_E14_vs_E10	ordinary_muscle_development	D_vs_E10;stage_1W_vs_E10;stage_3W_vs_E10;stage	1	TRUE
ENSGALG00010025318	NUAK2	PRJNA729271	-3.062289449	7.53457E-10	3.062289449	stage_E14_vs_E10	ordinary_muscle_development	D_vs_E10;stage_1W_vs_E10;stage_3W_vs_E10;stage	1	TRUE
ENSGALG00010016242	PDS5A	PRJNA729271	0.579086822	7.5412E-10	0.579086822	stage_E14_vs_E10	ordinary_muscle_development		0	FALSE
ENSGALG00010019011	VIL1	PRJNA729271	2.443673503	7.57137E-10	2.443673503	stage_E14_vs_E10	ordinary_muscle_development	D_vs_E10;stage_1W_vs_E10;stage_3W_vs_E10;stage	1	TRUE
ENSGALG00010017870	RAB11A	PRJNA729271	0.462451462	7.7041E-10	0.462451462	stage_E14_vs_E10	ordinary_muscle_development		0	FALSE

ENSGALG00010011360	TCF4	PRJNA729271	1.069091863	7.7272E-10	1.069091863	stage_E14_vs_E10	ordinary_muscle_development	D_vs_E10;stage_1W_vs_E10;stage_3W_vs_E10;stage	1	TRUE
ENSGALG00010000768	ENSGALG00010000768	PRJNA729271	-2.56796348	7.76272E-10	2.56796348	stage_E14_vs_E10	ordinary_muscle_development	D_vs_E10;stage_1W_vs_E10;stage_3W_vs_E10;stage	1	TRUE
ENSGALG00010024687	RYK	PRJNA729271	-0.678902671	7.80044E-10	0.678902671	stage_E14_vs_E10	ordinary_muscle_development		0	FALSE
ENSGALG00010016796	BROX	PRJNA729271	0.528282003	7.80507E-10	0.528282003	stage_E14_vs_E10	ordinary_muscle_development		0	FALSE
ENSGALG00010027605	FCHO1	PRJNA729271	1.911428771	7.99374E-10	1.911428771	stage_E14_vs_E10	ordinary_muscle_development	D_vs_E10;stage_1W_vs_E10;stage_3W_vs_E10;stage	1	TRUE
ENSGALG00010024215	ENSGALG00010024215	PRJNA729271	0.977567935	8.00562E-10	0.977567935	stage_E14_vs_E10	ordinary_muscle_development		0	FALSE
ENSGALG00010010821	RGS8	PRJNA729271	-2.904221336	8.02189E-10	2.904221336	stage_E14_vs_E10	ordinary_muscle_development	D_vs_E10;stage_1W_vs_E10;stage_3W_vs_E10;stage	1	TRUE
ENSGALG00010021933	PRR14L	PRJNA729271	-0.565342376	8.08161E-10	0.565342376	stage_E14_vs_E10	ordinary_muscle_development		0	FALSE
ENSGALG00010009024	PMAIP1	PRJNA729271	1.583431029	8.09533E-10	1.583431029	stage_E14_vs_E10	ordinary_muscle_development	D_vs_E10;stage_1W_vs_E10;stage_3W_vs_E10;stage	1	TRUE
ENSGALG00010029266	CANT1	PRJNA729271	0.691278392	8.10558E-10	0.691278392	stage_E14_vs_E10	ordinary_muscle_development		0	FALSE
ENSGALG00010011683	NIPAL2	PRJNA729271	-1.832552901	8.11033E-10	1.832552901	stage_E14_vs_E10	ordinary_muscle_development	D_vs_E10;stage_1W_vs_E10;stage_3W_vs_E10;stage	1	TRUE
ENSGALG00010009718	ENSGALG00010009718	PRJNA729271	-1.17841826	8.11104E-10	1.17841826	stage_E14_vs_E10	ordinary_muscle_development	D_vs_E10;stage_1W_vs_E10;stage_3W_vs_E10;stage	1	TRUE
ENSGALG00010023113	MYZAP	PRJNA729271	-0.864422051	8.22496E-10	0.864422051	stage_E14_vs_E10	ordinary_muscle_development		0	FALSE
ENSGALG00010028460	CRLF1	PRJNA729271	-1.57646401	8.38768E-10	1.57646401	stage_E14_vs_E10	ordinary_muscle_development	D_vs_E10;stage_1W_vs_E10;stage_3W_vs_E10;stage	1	TRUE
ENSGALG00010027707	HIGD1C	PRJNA729271	1.325659885	8.40056E-10	1.325659885	stage_E14_vs_E10	ordinary_muscle_development	D_vs_E10;stage_1W_vs_E10;stage_3W_vs_E10;stage	1	TRUE
ENSGALG00010021575	ADGRL2	PRJNA729271	-0.520947269	8.47342E-10	0.520947269	stage_E14_vs_E10	ordinary_muscle_development		0	FALSE
ENSGALG00010012079	ENSGALG00010012079	PRJNA729271	-1.658835809	8.51767E-10	1.658835809	stage_E14_vs_E10	ordinary_muscle_development	D_vs_E10;stage_1W_vs_E10;stage_3W_vs_E10;stage	1	TRUE
ENSGALG00010026874	TEAD3	PRJNA729271	1.036013314	8.54201E-10	1.036013314	stage_E14_vs_E10	ordinary_muscle_development	D_vs_E10;stage_1W_vs_E10;stage_3W_vs_E10;stage	1	TRUE
ENSGALG00010003990	ICA1	PRJNA729271	-1.09902152	8.56254E-10	1.09902152	stage_E14_vs_E10	ordinary_muscle_development	D_vs_E10;stage_1W_vs_E10;stage_3W_vs_E10;stage	1	TRUE
ENSGALG00010009678	JARID2	PRJNA729271	-0.636639639	8.74456E-10	0.636639639	stage_E14_vs_E10	ordinary_muscle_development		0	FALSE
ENSGALG00010004808	THEMIS	PRJNA729271	4.204836028	8.74576E-10	4.204836028	stage_E14_vs_E10	ordinary_muscle_development	D_vs_E10;stage_1W_vs_E10;stage_3W_vs_E10;stage	1	TRUE
ENSGALG00010010215	ENSGALG00010010215	PRJNA729271	0.775296128	8.78756E-10	0.775296128	stage_E14_vs_E10	ordinary_muscle_development		0	FALSE
ENSGALG00010020679	OTUD3	PRJNA729271	0.671013284	8.81336E-10	0.671013284	stage_E14_vs_E10	ordinary_muscle_development		0	FALSE
ENSGALG00010014323	KLF4	PRJNA729271	1.109945938	8.84468E-10	1.109945938	stage_E14_vs_E10	ordinary_muscle_development	D_vs_E10;stage_1W_vs_E10;stage_3W_vs_E10;stage	1	TRUE
ENSGALG00010023623	OSBPL6	PRJNA729271	-1.43957336	8.85713E-10	1.43957336	stage_E14_vs_E10	ordinary_muscle_development	D_vs_E10;stage_1W_vs_E10;stage_3W_vs_E10;stage	1	TRUE
ENSGALG00010012852	MSTN	PRJNA729271	2.647882491	9.00624E-10	2.647882491	stage_E14_vs_E10	ordinary_muscle_development	D_vs_E10;stage_1W_vs_E10;stage_3W_vs_E10;stage	1	TRUE
ENSGALG00010029650	ACSF2	PRJNA729271	-0.858173237	9.05519E-10	0.858173237	stage_E14_vs_E10	ordinary_muscle_development		0	FALSE
ENSGALG00010028649	ENSGALG00010028649	PRJNA729271	1.067935019	9.09613E-10	1.067935019	stage_E14_vs_E10	ordinary_muscle_development	D_vs_E10;stage_1W_vs_E10;stage_3W_vs_E10;stage	1	TRUE
ENSGALG00010022866	KLHL11	PRJNA729271	-0.624622388	9.2403E-10	0.624622388	stage_E14_vs_E10	ordinary_muscle_development		0	FALSE
ENSGALG00010016121	SLC35C2	PRJNA729271	-0.639142434	9.25694E-10	0.639142434	stage_E14_vs_E10	ordinary_muscle_development		0	FALSE
ENSGALG00010009796	ENSGALG00010009796	PRJNA729271	-1.25065793	9.26922E-10	1.25065793	stage_E14_vs_E10	ordinary_muscle_development	D_vs_E10;stage_1W_vs_E10;stage_3W_vs_E10;stage	1	TRUE
ENSGALG00010005385	PDSS1	PRJNA729271	0.617446119	9.29957E-10	0.617446119	stage_E14_vs_E10	ordinary_muscle_development		0	FALSE
ENSGALG00010011367	IER3IP1	PRJNA729271	0.701858318	9.33732E-10	0.701858318	stage_E14_vs_E10	ordinary_muscle_development		0	FALSE
ENSGALG00010004241	PREX2	PRJNA729271	-0.746554362	9.38447E-10	0.746554362	stage_E14_vs_E10	ordinary_muscle_development		0	FALSE
ENSGALG00010003727	FBXO25	PRJNA729271	0.953541952	9.4701E-10	0.953541952	stage_E14_vs_E10	ordinary_muscle_development		0	FALSE
ENSGALG00010025228	FDXACB1	PRJNA729271	0.667805397	9.49215E-10	0.667805397	stage_E14_vs_E10	ordinary_muscle_development		0	FALSE
ENSGALG00010020685	RPL6	PRJNA729271	0.732609197	9.56192E-10	0.732609197	stage_E14_vs_E10	ordinary_muscle_development		0	FALSE
ENSGALG00010021983	CTLA4	PRJNA729271	-6.02995033	9.58121E-10	6.02995033	stage_E14_vs_E10	ordinary_muscle_development	D_vs_E10;stage_1W_vs_E10;stage_3W_vs_E10;stage	1	TRUE
ENSGALG00010006532	KLHDC10	PRJNA729271	0.642186241	9.60214E-10	0.642186241	stage_E14_vs_E10	ordinary_muscle_development		0	FALSE
ENSGALG00010003260	ACP5	PRJNA729271	1.651843773	9.6238E-10	1.651843773	stage_E14_vs_E10	ordinary_muscle_development	D_vs_E10;stage_1W_vs_E10;stage_3W_vs_E10;stage	1	TRUE
ENSGALG00010024013	PTPRJ	PRJNA729271	0.747888042	9.64706E-10	0.747888042	stage_E14_vs_E10	ordinary_muscle_development		0	FALSE
ENSGALG00010012842	DDX17	PRJNA729271	0.31444038	9.70157E-10	0.31444038	stage_E14_vs_E10	ordinary_muscle_development		0	FALSE
ENSGALG00010022419	DUOX2	PRJNA729271	4.184669704	9.73932E-10	4.184669704	stage_E14_vs_E10	ordinary_muscle_development	D_vs_E10;stage_1W_vs_E10;stage_3W_vs_E10;stage	1	TRUE
ENSGALG00010004255	RPS12	PRJNA729271	0.953437513	9.74954E-10	0.953437513	stage_E14_vs_E10	ordinary_muscle_development		0	FALSE
ENSGALG00010002259	TASL	PRJNA729271	3.011288017	9.81049E-10	3.011288017	stage_E14_vs_E10	ordinary_muscle_development	D_vs_E10;stage_1W_vs_E10;stage_3W_vs_E10;stage	1	TRUE
ENSGALG00010008219	NIP7	PRJNA729271	-0.755453443	9.81433E-10	0.755453443	stage_E14_vs_E10	ordinary_muscle_development		0	FALSE
ENSGALG00010009780	MFSD2B	PRJNA729271	-1.658529965	9.84842E-10	1.658529965	stage_E14_vs_E10	ordinary_muscle_development	D_vs_E10;stage_1W_vs_E10;stage_3W_vs_E10;stage	1	TRUE
ENSGALG00010012085	IPO8	PRJNA729271	-0.613223514	9.8833E-10	0.613223514	stage_E14_vs_E10	ordinary_muscle_development		0	FALSE
ENSGALG00010012860	ENSGALG00010012860	PRJNA729271	1.58915089	9.90129E-10	1.58915089	stage_E14_vs_E10	ordinary_muscle_development	D_vs_E10;stage_1W_vs_E10;stage_3W_vs_E10;stage	1	TRUE
ENSGALG00010015472	LGMN	PRJNA729271	0.836532063	1.00152E-09	0.836532063	stage_E14_vs_E10	ordinary_muscle_development		0	FALSE
ENSGALG00010012001	EEIG2	PRJNA729271	-0.957328554	1.00692E-09	0.957328554	stage_E14_vs_E10	ordinary_muscle_development		0	FALSE
ENSGALG00010007860	DEFB4A	PRJNA729271	5.556980515	1.00797E-09	5.556980515	stage_E14_vs_E10	ordinary_muscle_development	D_vs_E10;stage_1W_vs_E10;stage_3W_vs_E10;stage	1	TRUE
ENSGALG00010009811	ENSGALG00010009811	PRJNA729271	0.542038414	1.00991E-09	0.542038414	stage_E14_vs_E10	ordinary_muscle_development		0	FALSE
ENSGALG00010013836	IL18R1	PRJNA729271	1.568269986	1.0129E-09	1.568269986	stage_E14_vs_E10	ordinary_muscle_development	D_vs_E10;stage_1W_vs_E10;stage_3W_vs_E10;stage	1	TRUE
ENSGALG00010017832	POU3F1	PRJNA729271	-3.836296042	1.0208E-09	3.836296042	stage_E14_vs_E10	ordinary_muscle_development	D_vs_E10;stage_1W_vs_E10;stage_3W_vs_E10;stage	1	TRUE
ENSGALG00010023979	FAM118B	PRJNA729271	-0.733340393	1.02168E-09	0.733340393	stage_E14_vs_E10	ordinary_muscle_development		0	FALSE
ENSGALG00010028206	RBM15B	PRJNA729271	-0.598163967	1.03069E-09	0.598163967	stage_E14_vs_E10	ordinary_muscle_development		0	FALSE
ENSGALG00010018068	ADAM33	PRJNA729271	0.867907969	1.05564E-09	0.867907969	stage_E14_vs_E10	ordinary_muscle_development		0	FALSE
ENSGALG00010010191	RINT1	PRJNA729271	-0.588307628	1.06017E-09	0.588307628	stage_E14_vs_E10	ordinary_muscle_development		0	FALSE
ENSGALG00010018492	ENSGALG00010018492	PRJNA729271	1.066777016	1.0819E-09	1.066777016	stage_E14_vs_E10	ordinary_muscle_development	D_vs_E10;stage_1W_vs_E10;stage_3W_vs_E10;stage	1	TRUE
ENSGALG00010012384	NRK	PRJNA729271	0.759042091	1.08508E-09	0.759042091	stage_E14_vs_E10	ordinary_muscle_development		0	FALSE
ENSGALG00010029592	MNT	PRJNA729271	1.028066632	1.08779E-09	1.028066632	stage_E14_vs_E10	ordinary_muscle_development	D_vs_E10;stage_1W_vs_E10;stage_3W_vs_E10;stage	1	TRUE
ENSGALG00010024026	TIRAP	PRJNA729271	0.646312139	1.09181E-09	0.646312139	stage_E14_vs_E10	ordinary_muscle_development		0	FALSE
ENSGALG00010015820	ENSGALG00010015820	PRJNA729271	3.187806126	1.09199E-09	3.187806126	stage_E14_vs_E10	ordinary_muscle_development	D_vs_E10;stage_1W_vs_E10;stage_3W_vs_E10;stage	1	TRUE
ENSGALG00010020693	PTGFR	PRJNA729271	-1.216142482	1.09725E-09	1.216142482	stage_E14_vs_E10	ordinary_muscle_development	D_vs_E10;stage_1W_vs_E10;stage_3W_vs_E10;stage	1	TRUE
ENSGALG00010021784	ANGPTL7	PRJNA729271	5.275581793	1.105E-09	5.275581793	stage_E14_vs_E10	ordinary_muscle_development	D_vs_E10;stage_1W_vs_E10;stage_3W_vs_E10;stage	1	TRUE
ENSGALG00010001612	HPX	PRJNA729271	5.76256011	1.10766E-09	5.76256011	stage_E14_vs_E10	ordinary_muscle_development	D_vs_E10;stage_1W_vs_E10;stage_3W_vs_E10;stage	1	TRUE
ENSGALG00010008750	PFDN1	PRJNA729271	1.043186621	1.11922E-09	1.043186621	stage_E14_vs_E10	ordinary_muscle_development	D_vs_E10;stage_1W_vs_E10;stage_3W_vs_E10;stage	1	TRUE
ENSGALG00010002247	RFTN1	PRJNA729271	-1.164282467	1.12049E-09	1.164282467	stage_E14_vs_E10	ordinary_muscle_development	D_vs_E10;stage_1W_vs_E10;stage_3W_vs_E10;stage	1	TRUE
ENSGALG00010020894	PSMD13	PRJNA729271	0.751204329	1.13116E-09	0.751204329	stage_E14_vs_E10	ordinary_muscle_development		0	FALSE
ENSGALG00010001532	ENSGALG00010001532	PRJNA729271	-1.726465199	1.13334E-09	1.726465199	stage_E14_vs_E10	ordinary_muscle_development	D_vs_E10;stage_1W_vs_E10;stage_3W_vs_E10;stage	1	TRUE
ENSGALG00010000391	ENSGALG00010000391	PRJNA729271	3.898972782	1.15095E-09	3.898972782	stage_E14_vs_E10	ordinary_muscle_development	D_vs_E10;stage_1W_vs_E10;stage_3W_vs_E10;stage	1	TRUE
ENSGALG00010013033	MAP3K1	PRJNA729271	-0.784905593	1.15459E-09	0.784905593	stage_E14_vs_E10	ordinary_muscle_development		0	FALSE
ENSGALG00010005887	CCNH	PRJNA729271	-0.545975644	1.15536E-09	0.545975644	stage_E14_vs_E10	ordinary_muscle_development		0	FALSE
ENSGALG00010027131	UBA52	PRJNA729271	0.956252102	1.1636E-09	0.956252102	stage_E14_vs_E10	ordinary_muscle_development		0	FALSE
ENSGALG00010018347	CTXN2	PRJNA729271	-5.993365689	1.16419E-09	5.993365689	stage_E14_vs_E10	ordinary_muscle_development	D_vs_E10;stage_1W_vs_E10;stage_3W_vs_E10;stage	1	TRUE
ENSGALG00010005716	ZBTB2	PRJNA729271	-0.71139878	1.17224E-09	0.71139878	stage_E14_vs_E10	ordinary_muscle_development		0	FALSE
ENSGALG00010010638	ACVR1C	PRJNA729271	2.697635075	1.17854E-09	2.697635075	stage_E14_vs_E10	ordinary_muscle_development	D_vs_E10;stage_1W_vs_E10;stage_3W_vs_E10;stage	1	TRUE
ENSGALG00010007304	F5	PRJNA729271	2.204962645	1.18782E-09	2.204962645	stage_E14_vs_E10	ordinary_muscle_development	D_vs_E10;stage_1W_vs_E10;stage_3W_vs_E10;stage	1	TRUE
ENSGALG00010005392	ENSGALG00010005392	PRJNA729271	0.661333236	1.19253E-09	0.661333236	stage_E14_vs_E10	ordinary_muscle_development		0	FALSE
ENSGALG00010017514	TXLNA	PRJNA729271	-0.606275896	1.19682E-09	0.606275896	stage_E14_vs_E10	ordinary_muscle_development		0	FALSE
ENSGALG00010024430	ZNF652	PRJNA729271	-0.405248975	1.20342E-09	0.405248975	stage_E14_vs_E10	ordinary_muscle_development		0	FALSE
ENSGALG00010025664	GPR55	PRJNA729271	3.701989345	1.20653E-09	3.701989345	stage_E14_vs_E10	ordinary_muscle_development	D_vs_E10;stage_1W_vs_E10;stage_3W_vs_E10;stage	1	TRUE
ENSGALG00010017556	TOM1L2	PRJNA729271	0.718182269	1.21694E-09	0.718182269	stage_E14_vs_E10	ordinary_muscle_development		0	FALSE
ENSGALG00010009477	ZNF365	PRJNA729271	1.507642958	1.22407E-09	1.507642958	stage_E14_vs_E10	ordinary_muscle_development	D_vs_E10;stage_1W_vs_E10;stage_3W_vs_E10;stage	1	TRUE
ENSGALG00010013199	RALYL	PRJNA729271	-5.348360924	1.23482E-09	5.348360924	stage_E14_vs_E10	ordinary_muscle_development	D_vs_E10;stage_1W_vs_E10;stage_3W_vs_E10;stage	1	TRUE
ENSGALG00010028756	ENSGALG00010028756	PRJNA729271	-6.215593168	1.23744E-09	6.215593168	stage_E14_vs_E10	ordinary_muscle_development	D_vs_E10;stage_1W_vs_E10;stage_3W_vs_E10;stage	1	TRUE
ENSGALG00010023921	GRK7	PRJNA729271	1.111422244	1.24189E-09	1.111422244	stage_E14_vs_E10	ordinary_muscle_development	D_vs_E10;stage_1W_vs_E10;stage_3W_vs_E10;stage	1	TRUE
ENSGALG00010028748	PIAS3	PRJNA729271	1.224385415	1.24594E-09	1.224385415	stage_E14_vs_E10	ordinary_muscle_development	D_vs_E10;stage_1W_vs_E10;stage_3W_vs_E10;stage	1	TRUE
ENSGALG00010014199	LNX1	PRJNA729271	-1.614330426	1.25705E-09	1.614330426	stage_E14_vs_E10	ordinary_muscle_development	D_vs_E10;stage_1W_vs_E10;stage_3W_vs_E10;stage	1	TRUE
ENSGALG00010020383	BAAT	PRJNA729271	-0.660263642	1.25988E-09	0.660263642	stage_E14_vs_E10	ordinary_muscle_development		0	FALSE
ENSGALG00010009346	STC2	PRJNA729271	1.913090785	1.27503E-09	1.913090785	stage_E14_vs_E10	ordinary_muscle_development	D_vs_E10;stage_1W_vs_E10;stage_3W_vs_E10;stage	1	TRUE
ENSGALG00010012871	ENSGALG00010012871	PRJNA729271	2.158483423	1.28641E-09	2.158483423	stage_E14_vs_E10	ordinary_muscle_development	D_vs_E10;stage_1W_vs_E10;stage_3W_vs_E10;stage	1	TRUE
ENSGALG00010022238	COMTD1	PRJNA729271	-1.094496691	1.29244E-09	1.094496691	stage_E14_vs_E10	ordinary_muscle_development	D_vs_E10;stage_1W_vs_E10;stage_3W_vs_E10;stage	1	TRUE
ENSGALG00010014740	TRMT9B	PRJNA729271	-3.697774059	1.30402E-09	3.697774059	stage_E14_vs_E10	ordinary_muscle_development	D_vs_E10;stage_1W_vs_E10;stage_3W_vs_E10;stage	1	TRUE
ENSGALG00010017246	GGA2	PRJNA729271	0.804338652	1.31338E-09	0.804338652	stage_E14_vs_E10	ordinary_muscle_development		0	FALSE
ENSGALG00010014762	STK40	PRJNA729271	-0.668557925	1.32392E-09	0.668557925	stage_E14_vs_E10	ordinary_muscle_development		0	FALSE
ENSGALG00010024158	ENSGALG00010024158	PRJNA729271	2.021132268	1.32457E-09	2.021132268	stage_E14_vs_E10	ordinary_muscle_development	D_vs_E10;stage_1W_vs_E10;stage_3W_vs_E10;stage	1	TRUE
ENSGALG00010028026	ANKMY1	PRJNA729271	1.018682788	1.3272E-09	1.018682788	stage_E14_vs_E10	ordinary_muscle_development	D_vs_E10;stage_1W_vs_E10;stage_3W_vs_E10;stage	1	TRUE
ENSGALG00010027239	ENSGALG00010027239	PRJNA729271	-0.5256271	1.32981E-09	0.5256271	stage_E14_vs_E10	ordinary_muscle_development		0	FALSE
ENSGALG00010005860	ABI3BP	PRJNA729271	1.322166238	1.34346E-09	1.322166238	stage_E14_vs_E10	ordinary_muscle_development	D_vs_E10;stage_1W_vs_E10;stage_3W_vs_E10;stage	1	TRUE
ENSGALG00010001759	C10orf88	PRJNA729271	-0.581074872	1.3449E-09	0.581074872	stage_E14_vs_E10	ordinary_muscle_development		0	FALSE
ENSGALG00010013131	SPATA2	PRJNA729271	0.718565191	1.34831E-09	0.718565191	stage_E14_vs_E10	ordinary_muscle_development		0	FALSE
ENSGALG00010021781	RAPGEF4	PRJNA729271	-1.746205662	1.35099E-09	1.746205662	stage_E14_vs_E10	ordinary_muscle_development	D_vs_E10;stage_1W_vs_E10;stage_3W_vs_E10;stage	1	TRUE
ENSGALG00010026568	BYSL	PRJNA729271	0.682557048	1.3699E-09	0.682557048	stage_E14_vs_E10	ordinary_muscle_development		0	FALSE
ENSGALG00010012168	ENSGALG00010012168	PRJNA729271	-3.814620547	1.37573E-09	3.814620547	stage_E14_vs_E10	ordinary_muscle_development	D_vs_E10;stage_1W_vs_E10;stage_3W_vs_E10;stage	1	TRUE
ENSGALG00010027901	MOB3A	PRJNA729271	-0.617494142	1.37712E-09	0.617494142	stage_E14_vs_E10	ordinary_muscle_development		0	FALSE
ENSGALG00010019625	INTS1	PRJNA729271	-0.475044149	1.38303E-09	0.475044149	stage_E14_vs_E10	ordinary_muscle_development		0	FALSE
ENSGALG00010017533	FAM193A	PRJNA729271	0.475293414	1.38639E-09	0.475293414	stage_E14_vs_E10	ordinary_muscle_development		0	FALSE
ENSGALG00010025679	ENSGALG00010025679	PRJNA729271	6.959705842	1.39329E-09	6.959705842	stage_E14_vs_E10	ordinary_muscle_development	D_vs_E10;stage_1W_vs_E10;stage_3W_vs_E10;stage	1	TRUE
ENSGALG00010028456	FBXW5	PRJNA729271	0.575260888	1.40172E-09	0.575260888	stage_E14_vs_E10	ordinary_muscle_development		0	FALSE
ENSGALG00010011635	PCDH12	PRJNA729271	-1.239278868	1.41913E-09	1.239278868	stage_E14_vs_E10	ordinary_muscle_development	D_vs_E10;stage_1W_vs_E10;stage_3W_vs_E10;stage	1	TRUE
ENSGALG00010011251	FRZB	PRJNA729271	1.08392438	1.42158E-09	1.08392438	stage_E14_vs_E10	ordinary_muscle_development	D_vs_E10;stage_1W_vs_E10;stage_3W_vs_E10;stage	1	TRUE
ENSGALG00010008864	ADAT1	PRJNA729271	0.851784688	1.4324E-09	0.851784688	stage_E14_vs_E10	ordinary_muscle_development		0	FALSE
ENSGALG00010018526	ENSGALG00010018526	PRJNA729271	0.62403189	1.4435E-09	0.62403189	stage_E14_vs_E10	ordinary_muscle_development		0	FALSE
ENSGALG00010014839	TM7SF3	PRJNA729271	0.658716506	1.46598E-09	0.658716506	stage_E14_vs_E10	ordinary_muscle_development		0	FALSE
ENSGALG00010002192	ENSGALG00010002192	PRJNA729271	1.207513044	1.47598E-09	1.207513044	stage_E14_vs_E10	ordinary_muscle_development	D_vs_E10;stage_1W_vs_E10;stage_3W_vs_E10;stage	1	TRUE
ENSGALG00010015533	PSAT1	PRJNA729271	-1.725622384	1.48472E-09	1.725622384	stage_E14_vs_E10	ordinary_muscle_development	D_vs_E10;stage_1W_vs_E10;stage_3W_vs_E10;stage	1	TRUE
ENSGALG00010021141	SGSM1	PRJNA729271	-1.000054291	1.49775E-09	1.000054291	stage_E14_vs_E10	ordinary_muscle_development	D_vs_E10;stage_1W_vs_E10;stage_3W_vs_E10;stage	1	TRUE
ENSGALG00010018587	SRF	PRJNA729271	1.070201896	1.50388E-09	1.070201896	stage_E14_vs_E10	ordinary_muscle_development	D_vs_E10;stage_1W_vs_E10;stage_3W_vs_E10;stage	1	TRUE
ENSGALG00010000338	ENSGALG00010000338	PRJNA729271	-1.221382561	1.50788E-09	1.221382561	stage_E14_vs_E10	ordinary_muscle_development	D_vs_E10;stage_1W_vs_E10;stage_3W_vs_E10;stage	1	TRUE
ENSGALG00010012979	HTR1A	PRJNA729271	3.214070019	1.50986E-09	3.214070019	stage_E14_vs_E10	ordinary_muscle_development	D_vs_E10;stage_1W_vs_E10;stage_3W_vs_E10;stage	1	TRUE
ENSGALG00010021481	MICU1	PRJNA729271	1.087981844	1.5116E-09	1.087981844	stage_E14_vs_E10	ordinary_muscle_development	D_vs_E10;stage_1W_vs_E10;stage_3W_vs_E10;stage	1	TRUE
ENSGALG00010018998	RPS29	PRJNA729271	2.745781662	1.51401E-09	2.745781662	stage_E14_vs_E10	ordinary_muscle_development	D_vs_E10;stage_1W_vs_E10;stage_3W_vs_E10;stage	1	TRUE
ENSGALG00010024662	MOB2	PRJNA729271	0.440020789	1.51533E-09	0.440020789	stage_E14_vs_E10	ordinary_muscle_development		0	FALSE
ENSGALG00010015031	MYH9	PRJNA729271	0.676809872	1.51839E-09	0.676809872	stage_E14_vs_E10	ordinary_muscle_development		0	FALSE
ENSGALG00010017458	ENSGALG00010017458	PRJNA729271	1.06033767	1.52382E-09	1.06033767	stage_E14_vs_E10	ordinary_muscle_development	D_vs_E10;stage_1W_vs_E10;stage_3W_vs_E10;stage	1	TRUE
ENSGALG00010027920	SCAMP4	PRJNA729271	-0.706607427	1.52454E-09	0.706607427	stage_E14_vs_E10	ordinary_muscle_development		0	FALSE
ENSGALG00010018258	OSCP1	PRJNA729271	-1.456567727	1.52491E-09	1.456567727	stage_E14_vs_E10	ordinary_muscle_development	D_vs_E10;stage_1W_vs_E10;stage_3W_vs_E10;stage	1	TRUE
ENSGALG00010008859	ENSGALG00010008859	PRJNA729271	-1.419010992	1.52702E-09	1.419010992	stage_E14_vs_E10	ordinary_muscle_development	D_vs_E10;stage_1W_vs_E10;stage_3W_vs_E10;stage	1	TRUE
ENSGALG00010009467	EGR2	PRJNA729271	1.852747621	1.53554E-09	1.852747621	stage_E14_vs_E10	ordinary_muscle_development	D_vs_E10;stage_1W_vs_E10;stage_3W_vs_E10;stage	1	TRUE
ENSGALG00010013413	WRB	PRJNA729271	-0.526562145	1.53704E-09	0.526562145	stage_E14_vs_E10	ordinary_muscle_development		0	FALSE
ENSGALG00010012773	XKR8	PRJNA729271	-0.800595083	1.54976E-09	0.800595083	stage_E14_vs_E10	ordinary_muscle_development		0	FALSE
ENSGALG00010029426	ZNF750	PRJNA729271	-6.943845257	1.55461E-09	6.943845257	stage_E14_vs_E10	ordinary_muscle_development	D_vs_E10;stage_1W_vs_E10;stage_3W_vs_E10;stage	1	TRUE
ENSGALG00010011823	ENSGALG00010011823	PRJNA729271	-4.779093409	1.55818E-09	4.779093409	stage_E14_vs_E10	ordinary_muscle_development	D_vs_E10;stage_1W_vs_E10;stage_3W_vs_E10;stage	1	TRUE
ENSGALG00010024501	ARL8A	PRJNA729271	0.552311063	1.56663E-09	0.552311063	stage_E14_vs_E10	ordinary_muscle_development		0	FALSE
ENSGALG00010008071	SMIM18	PRJNA729271	-2.392979921	1.57198E-09	2.392979921	stage_E14_vs_E10	ordinary_muscle_development	D_vs_E10;stage_1W_vs_E10;stage_3W_vs_E10;stage	1	TRUE
ENSGALG00010018627	MYLK	PRJNA729271	-1.112366249	1.57208E-09	1.112366249	stage_E14_vs_E10	ordinary_muscle_development	D_vs_E10;stage_1W_vs_E10;stage_3W_vs_E10;stage	1	TRUE
ENSGALG00010006635	ENSGALG00010006635	PRJNA729271	-3.014174849	1.58313E-09	3.014174849	stage_E14_vs_E10	ordinary_muscle_development	D_vs_E10;stage_1W_vs_E10;stage_3W_vs_E10;stage	1	TRUE
ENSGALG00010016086	TDRD9	PRJNA729271	-2.28528732	1.59458E-09	2.28528732	stage_E14_vs_E10	ordinary_muscle_development	D_vs_E10;stage_1W_vs_E10;stage_3W_vs_E10;stage	1	TRUE
ENSGALG00010015628	UBQLN1	PRJNA729271	0.729516537	1.59619E-09	0.729516537	stage_E14_vs_E10	ordinary_muscle_development		0	FALSE
ENSGALG00010018299	SHOC2	PRJNA729271	0.509741908	1.60575E-09	0.509741908	stage_E14_vs_E10	ordinary_muscle_development		0	FALSE
ENSGALG00010017694	ATP6V1B2	PRJNA729271	-0.447729809	1.62034E-09	0.447729809	stage_E14_vs_E10	ordinary_muscle_development		0	FALSE
ENSGALG00010015386	SLC24A4	PRJNA729271	1.497606601	1.62358E-09	1.497606601	stage_E14_vs_E10	ordinary_muscle_development	D_vs_E10;stage_1W_vs_E10;stage_3W_vs_E10;stage	1	TRUE
ENSGALG00010024924	MNX1	PRJNA729271	-5.877789214	1.62396E-09	5.877789214	stage_E14_vs_E10	ordinary_muscle_development	D_vs_E10;stage_1W_vs_E10;stage_3W_vs_E10;stage	1	TRUE
ENSGALG00010025292	ATP1A1	PRJNA729271	0.786567468	1.62475E-09	0.786567468	stage_E14_vs_E10	ordinary_muscle_development		0	FALSE
ENSGALG00010029235	TRIM37	PRJNA729271	-0.475411	1.6326E-09	0.475411	stage_E14_vs_E10	ordinary_muscle_development		0	FALSE
ENSGALG00010017720	ENSGALG00010017720	PRJNA729271	-2.574988283	1.64031E-09	2.574988283	stage_E14_vs_E10	ordinary_muscle_development	D_vs_E10;stage_1W_vs_E10;stage_3W_vs_E10;stage	1	TRUE
ENSGALG00010008409	CWC15	PRJNA729271	0.521002296	1.65413E-09	0.521002296	stage_E14_vs_E10	ordinary_muscle_development		0	FALSE
ENSGALG00010026088	SNRPC	PRJNA729271	-0.50767943	1.65504E-09	0.50767943	stage_E14_vs_E10	ordinary_muscle_development		0	FALSE
ENSGALG00010016253	EDN3	PRJNA729271	-1.326133363	1.66143E-09	1.326133363	stage_E14_vs_E10	ordinary_muscle_development	D_vs_E10;stage_1W_vs_E10;stage_3W_vs_E10;stage	1	TRUE
ENSGALG00010021147	ARID3B	PRJNA729271	-0.529348542	1.66381E-09	0.529348542	stage_E14_vs_E10	ordinary_muscle_development		0	FALSE
ENSGALG00010011796	METAP2	PRJNA729271	0.549305063	1.66756E-09	0.549305063	stage_E14_vs_E10	ordinary_muscle_development		0	FALSE
ENSGALG00010019498	ENSGALG00010019498	PRJNA729271	1.605197845	1.6737E-09	1.605197845	stage_E14_vs_E10	ordinary_muscle_development	D_vs_E10;stage_1W_vs_E10;stage_3W_vs_E10;stage	1	TRUE
ENSGALG00010028216	NKX2-3	PRJNA729271	8.581779666	1.67923E-09	8.581779666	stage_E14_vs_E10	ordinary_muscle_development	D_vs_E10;stage_1W_vs_E10;stage_3W_vs_E10;stage	1	TRUE
ENSGALG00010027080	TBC1D22B	PRJNA729271	0.956710438	1.69132E-09	0.956710438	stage_E14_vs_E10	ordinary_muscle_development		0	FALSE
ENSGALG00010007851	AvBD7	PRJNA729271	5.532518394	1.698E-09	5.532518394	stage_E14_vs_E10	ordinary_muscle_development	D_vs_E10;stage_1W_vs_E10;stage_3W_vs_E10;stage	1	TRUE
ENSGALG00010022481	CORO1B	PRJNA729271	-0.931814906	1.69836E-09	0.931814906	stage_E14_vs_E10	ordinary_muscle_development		0	FALSE
ENSGALG00010004141	KPNA3	PRJNA729271	0.867628867	1.712E-09	0.867628867	stage_E14_vs_E10	ordinary_muscle_development		0	FALSE
ENSGALG00010017033	LDAF1	PRJNA729271	0.6823667	1.71409E-09	0.6823667	stage_E14_vs_E10	ordinary_muscle_development		0	FALSE
ENSGALG00010004610	TGS1	PRJNA729271	-0.765606384	1.71702E-09	0.765606384	stage_E14_vs_E10	ordinary_muscle_development		0	FALSE
ENSGALG00010029359	ENSGALG00010029359	PRJNA729271	-3.285644954	1.75166E-09	3.285644954	stage_E14_vs_E10	ordinary_muscle_development	D_vs_E10;stage_1W_vs_E10;stage_3W_vs_E10;stage	1	TRUE
ENSGALG00010028800	ENSGALG00010028800	PRJNA729271	2.471069074	1.75646E-09	2.471069074	stage_E14_vs_E10	ordinary_muscle_development	D_vs_E10;stage_1W_vs_E10;stage_3W_vs_E10;stage	1	TRUE
ENSGALG00010010810	GALT	PRJNA729271	0.694999326	1.75925E-09	0.694999326	stage_E14_vs_E10	ordinary_muscle_development		0	FALSE
ENSGALG00010004924	ENSGALG00010004924	PRJNA729271	2.081549755	1.76007E-09	2.081549755	stage_E14_vs_E10	ordinary_muscle_development	D_vs_E10;stage_1W_vs_E10;stage_3W_vs_E10;stage	1	TRUE
ENSGALG00010007972	EXOC8	PRJNA729271	-0.710969061	1.76288E-09	0.710969061	stage_E14_vs_E10	ordinary_muscle_development		0	FALSE
ENSGALG00010003187	RHOG	PRJNA729271	1.473814265	1.77094E-09	1.473814265	stage_E14_vs_E10	ordinary_muscle_development	D_vs_E10;stage_1W_vs_E10;stage_3W_vs_E10;stage	1	TRUE
ENSGALG00010027262	USE1	PRJNA729271	-0.846205123	1.79489E-09	0.846205123	stage_E14_vs_E10	ordinary_muscle_development		0	FALSE
ENSGALG00010024698	ENSGALG00010024698	PRJNA729271	-2.515195776	1.79756E-09	2.515195776	stage_E14_vs_E10	ordinary_muscle_development	D_vs_E10;stage_1W_vs_E10;stage_3W_vs_E10;stage	1	TRUE
ENSGALG00010004481	ZNF704	PRJNA729271	0.489465075	1.80277E-09	0.489465075	stage_E14_vs_E10	ordinary_muscle_development		0	FALSE
ENSGALG00010013596	SDCBP2	PRJNA729271	0.745851629	1.81692E-09	0.745851629	stage_E14_vs_E10	ordinary_muscle_development		0	FALSE
ENSGALG00010020816	TRMT2A	PRJNA729271	0.548244468	1.82237E-09	0.548244468	stage_E14_vs_E10	ordinary_muscle_development		0	FALSE
ENSGALG00010020433	GNPTG	PRJNA729271	-0.450098926	1.82559E-09	0.450098926	stage_E14_vs_E10	ordinary_muscle_development		0	FALSE
ENSGALG00010004231	ARMC3	PRJNA729271	2.159970004	1.83357E-09	2.159970004	stage_E14_vs_E10	ordinary_muscle_development	D_vs_E10;stage_1W_vs_E10;stage_3W_vs_E10;stage	1	TRUE
ENSGALG00010029388	ST6GALNAC2	PRJNA729271	1.928683621	1.83745E-09	1.928683621	stage_E14_vs_E10	ordinary_muscle_development	D_vs_E10;stage_1W_vs_E10;stage_3W_vs_E10;stage	1	TRUE

ENSGALG00010025257	LPCAT2	PRJNA729271	-1.055914253	1.84618E-09	1.055914253	stage_E14_vs_E10	ordinary_muscle_development	D_vs_E10;stage_1W_vs_E10;stage_3W_vs_E10;stage	1	TRUE
ENSGALG00010027767	ENSGALG00010027767	PRJNA729271	5.3287134	1.8613E-09	5.3287134	stage_E14_vs_E10	ordinary_muscle_development	D_vs_E10;stage_1W_vs_E10;stage_3W_vs_E10;stage	1	TRUE
ENSGALG00010001958	RAB11FIP2	PRJNA729271	-0.691623423	1.8629E-09	0.691623423	stage_E14_vs_E10	ordinary_muscle_development		0	FALSE
ENSGALG00010021021	GCG	PRJNA729271	2.113551739	1.87039E-09	2.113551739	stage_E14_vs_E10	ordinary_muscle_development	D_vs_E10;stage_1W_vs_E10;stage_3W_vs_E10;stage	1	TRUE
ENSGALG00010029756	ELAC2	PRJNA729271	-0.533256013	1.87255E-09	0.533256013	stage_E14_vs_E10	ordinary_muscle_development		0	FALSE
ENSGALG00010024636	PANX3	PRJNA729271	-1.912387227	1.87289E-09	1.912387227	stage_E14_vs_E10	ordinary_muscle_development	D_vs_E10;stage_1W_vs_E10;stage_3W_vs_E10;stage	1	TRUE
ENSGALG00010029754	NLE1	PRJNA729271	-1.116897758	1.87409E-09	1.116897758	stage_E14_vs_E10	ordinary_muscle_development	D_vs_E10;stage_1W_vs_E10;stage_3W_vs_E10;stage	1	TRUE
ENSGALG00010009168	MRPS12	PRJNA729271	1.07167673	1.88381E-09	1.07167673	stage_E14_vs_E10	ordinary_muscle_development	D_vs_E10;stage_1W_vs_E10;stage_3W_vs_E10;stage	1	TRUE
ENSGALG00010024802	CD59	PRJNA729271	1.006452474	1.89384E-09	1.006452474	stage_E14_vs_E10	ordinary_muscle_development	D_vs_E10;stage_1W_vs_E10;stage_3W_vs_E10;stage	1	TRUE
ENSGALG00010023490	PHB2	PRJNA729271	0.630907027	1.90504E-09	0.630907027	stage_E14_vs_E10	ordinary_muscle_development		0	FALSE
ENSGALG00010003220	SPP1	PRJNA729271	-6.17346867	1.90987E-09	6.17346867	stage_E14_vs_E10	ordinary_muscle_development	D_vs_E10;stage_1W_vs_E10;stage_3W_vs_E10;stage	1	TRUE
ENSGALG00010022054	ENSGALG00010022054	PRJNA729271	2.522116287	1.91094E-09	2.522116287	stage_E14_vs_E10	ordinary_muscle_development	D_vs_E10;stage_1W_vs_E10;stage_3W_vs_E10;stage	1	TRUE
ENSGALG00010027892	SHE	PRJNA729271	1.035220516	1.92424E-09	1.035220516	stage_E14_vs_E10	ordinary_muscle_development	D_vs_E10;stage_1W_vs_E10;stage_3W_vs_E10;stage	1	TRUE
ENSGALG00010028391	MED26	PRJNA729271	0.691226629	1.92669E-09	0.691226629	stage_E14_vs_E10	ordinary_muscle_development		0	FALSE
ENSGALG00010021599	ARPC4	PRJNA729271	-0.640328429	1.93263E-09	0.640328429	stage_E14_vs_E10	ordinary_muscle_development		0	FALSE
ENSGALG00010006173	TBPL1	PRJNA729271	-0.528322472	1.93406E-09	0.528322472	stage_E14_vs_E10	ordinary_muscle_development		0	FALSE
ENSGALG00010004216	SNX14	PRJNA729271	0.4511456	1.93757E-09	0.4511456	stage_E14_vs_E10	ordinary_muscle_development		0	FALSE
ENSGALG00010018593	LGALS3	PRJNA729271	-2.234328088	1.95594E-09	2.234328088	stage_E14_vs_E10	ordinary_muscle_development	D_vs_E10;stage_1W_vs_E10;stage_3W_vs_E10;stage	1	TRUE
ENSGALG00010015633	CCM2L	PRJNA729271	1.132116875	1.96114E-09	1.132116875	stage_E14_vs_E10	ordinary_muscle_development	D_vs_E10;stage_1W_vs_E10;stage_3W_vs_E10;stage	1	TRUE
ENSGALG00010009138	ENSGALG00010009138	PRJNA729271	0.649574594	1.96164E-09	0.649574594	stage_E14_vs_E10	ordinary_muscle_development		0	FALSE
ENSGALG00010029362	TADA2A	PRJNA729271	-0.702675854	1.97755E-09	0.702675854	stage_E14_vs_E10	ordinary_muscle_development		0	FALSE
ENSGALG00010025480	FGF19	PRJNA729271	-5.486727328	1.9959E-09	5.486727328	stage_E14_vs_E10	ordinary_muscle_development	D_vs_E10;stage_1W_vs_E10;stage_3W_vs_E10;stage	1	TRUE
ENSGALG00010018102	ZDHHC4	PRJNA729271	1.015638603	1.99719E-09	1.015638603	stage_E14_vs_E10	ordinary_muscle_development	D_vs_E10;stage_1W_vs_E10;stage_3W_vs_E10;stage	1	TRUE
ENSGALG00010011626	SERPINB1	PRJNA729271	3.395332477	1.99782E-09	3.395332477	stage_E14_vs_E10	ordinary_muscle_development	D_vs_E10;stage_1W_vs_E10;stage_3W_vs_E10;stage	1	TRUE
ENSGALG00010003862	PCK2	PRJNA729271	-0.895263786	2.006E-09	0.895263786	stage_E14_vs_E10	ordinary_muscle_development		0	FALSE
ENSGALG00010026633	SLC5A1	PRJNA729271	3.086309375	2.01046E-09	3.086309375	stage_E14_vs_E10	ordinary_muscle_development	D_vs_E10;stage_1W_vs_E10;stage_3W_vs_E10;stage	1	TRUE
ENSGALG00010000186	THOC5	PRJNA729271	-0.49205266	2.0125E-09	0.49205266	stage_E14_vs_E10	ordinary_muscle_development		0	FALSE
ENSGALG00010016960	GJB3	PRJNA729271	-6.541446616	2.01289E-09	6.541446616	stage_E14_vs_E10	ordinary_muscle_development	D_vs_E10;stage_1W_vs_E10;stage_3W_vs_E10;stage	1	TRUE
ENSGALG00010015910	C5orf15	PRJNA729271	0.569047516	2.0169E-09	0.569047516	stage_E14_vs_E10	ordinary_muscle_development		0	FALSE
ENSGALG00010020252	DMTN	PRJNA729271	1.167450476	2.02785E-09	1.167450476	stage_E14_vs_E10	ordinary_muscle_development	D_vs_E10;stage_1W_vs_E10;stage_3W_vs_E10;stage	1	TRUE
ENSGALG00010014312	KIAA1191	PRJNA729271	-0.523118433	2.0525E-09	0.523118433	stage_E14_vs_E10	ordinary_muscle_development		0	FALSE
ENSGALG00010005560	RSPH3	PRJNA729271	-2.648617797	2.06469E-09	2.648617797	stage_E14_vs_E10	ordinary_muscle_development	D_vs_E10;stage_1W_vs_E10;stage_3W_vs_E10;stage	1	TRUE
ENSGALG00010012804	ENSGALG00010012804	PRJNA729271	0.973844778	2.06477E-09	0.973844778	stage_E14_vs_E10	ordinary_muscle_development		0	FALSE
ENSGALG00010020449	PRPF19	PRJNA729271	-0.598432226	2.07046E-09	0.598432226	stage_E14_vs_E10	ordinary_muscle_development		0	FALSE
ENSGALG00010029757	ULK2	PRJNA729271	1.280350211	2.07509E-09	1.280350211	stage_E14_vs_E10	ordinary_muscle_development	D_vs_E10;stage_1W_vs_E10;stage_3W_vs_E10;stage	1	TRUE
ENSGALG00010006139	GAREM1	PRJNA729271	-0.838080027	2.09439E-09	0.838080027	stage_E14_vs_E10	ordinary_muscle_development		0	FALSE
ENSGALG00010018345	WDR24	PRJNA729271	1.042761376	2.10159E-09	1.042761376	stage_E14_vs_E10	ordinary_muscle_development	D_vs_E10;stage_1W_vs_E10;stage_3W_vs_E10;stage	1	TRUE
ENSGALG00010014397	PRKAA1	PRJNA729271	-0.471579411	2.10761E-09	0.471579411	stage_E14_vs_E10	ordinary_muscle_development		0	FALSE
ENSGALG00010029456	RNF157	PRJNA729271	-3.36831697	2.11036E-09	3.36831697	stage_E14_vs_E10	ordinary_muscle_development	D_vs_E10;stage_1W_vs_E10;stage_3W_vs_E10;stage	1	TRUE
ENSGALG00010002766	NANOS1	PRJNA729271	-2.103107592	2.11036E-09	2.103107592	stage_E14_vs_E10	ordinary_muscle_development	D_vs_E10;stage_1W_vs_E10;stage_3W_vs_E10;stage	1	TRUE
ENSGALG00010012458	MRPS30	PRJNA729271	0.658852909	2.11111E-09	0.658852909	stage_E14_vs_E10	ordinary_muscle_development		0	FALSE
ENSGALG00010011973	TRAPPC10	PRJNA729271	0.303623877	2.12521E-09	0.303623877	stage_E14_vs_E10	ordinary_muscle_development		0	FALSE
ENSGALG00010021290	ERCC3	PRJNA729271	-0.431118951	2.12776E-09	0.431118951	stage_E14_vs_E10	ordinary_muscle_development		0	FALSE
ENSGALG00010012578	NDUFS4	PRJNA729271	0.93468644	2.15804E-09	0.93468644	stage_E14_vs_E10	ordinary_muscle_development		0	FALSE
ENSGALG00010012673	ENSGALG00010012673	PRJNA729271	-2.082445113	2.16463E-09	2.082445113	stage_E14_vs_E10	ordinary_muscle_development	D_vs_E10;stage_1W_vs_E10;stage_3W_vs_E10;stage	1	TRUE
ENSGALG00010002824	NXPH1	PRJNA729271	-3.887363801	2.17216E-09	3.887363801	stage_E14_vs_E10	ordinary_muscle_development	D_vs_E10;stage_1W_vs_E10;stage_3W_vs_E10;stage	1	TRUE
ENSGALG00010026262	NAV1	PRJNA729271	0.985831515	2.17717E-09	0.985831515	stage_E14_vs_E10	ordinary_muscle_development		0	FALSE
ENSGALG00010013551	UBE2N	PRJNA729271	-0.68749209	2.20347E-09	0.68749209	stage_E14_vs_E10	ordinary_muscle_development		0	FALSE
ENSGALG00010024863	LMOD1	PRJNA729271	1.766905548	2.2228E-09	1.766905548	stage_E14_vs_E10	ordinary_muscle_development	D_vs_E10;stage_1W_vs_E10;stage_3W_vs_E10;stage	1	TRUE
ENSGALG00010025336	ADAM10	PRJNA729271	-0.393638836	2.22756E-09	0.393638836	stage_E14_vs_E10	ordinary_muscle_development		0	FALSE
ENSGALG00010002605	EOMES	PRJNA729271	4.141566095	2.23471E-09	4.141566095	stage_E14_vs_E10	ordinary_muscle_development	D_vs_E10;stage_1W_vs_E10;stage_3W_vs_E10;stage	1	TRUE
ENSGALG00010014980	ENSGALG00010014980	PRJNA729271	2.687483325	2.23954E-09	2.687483325	stage_E14_vs_E10	ordinary_muscle_development	D_vs_E10;stage_1W_vs_E10;stage_3W_vs_E10;stage	1	TRUE
ENSGALG00010013076	RTN4IP1	PRJNA729271	-0.523924186	2.24408E-09	0.523924186	stage_E14_vs_E10	ordinary_muscle_development		0	FALSE
ENSGALG00010013378	SH3RF3	PRJNA729271	-0.893100009	2.25325E-09	0.893100009	stage_E14_vs_E10	ordinary_muscle_development		0	FALSE
ENSGALG00010025876	ENSGALG00010025876	PRJNA729271	9.985348849	2.25631E-09	9.985348849	stage_E14_vs_E10	ordinary_muscle_development	D_vs_E10;stage_1W_vs_E10;stage_3W_vs_E10;stage	1	TRUE
ENSGALG00010011347	RGS22	PRJNA729271	-2.757904068	2.25631E-09	2.757904068	stage_E14_vs_E10	ordinary_muscle_development	D_vs_E10;stage_1W_vs_E10;stage_3W_vs_E10;stage	1	TRUE
ENSGALG00010019871	TAFA1	PRJNA729271	-4.891462939	2.27156E-09	4.891462939	stage_E14_vs_E10	ordinary_muscle_development	D_vs_E10;stage_1W_vs_E10;stage_3W_vs_E10;stage	1	TRUE
ENSGALG00010015072	MAP9	PRJNA729271	-0.874682689	2.28746E-09	0.874682689	stage_E14_vs_E10	ordinary_muscle_development		0	FALSE
ENSGALG00010001697	TAF10	PRJNA729271	-0.736094162	2.33071E-09	0.736094162	stage_E14_vs_E10	ordinary_muscle_development		0	FALSE
ENSGALG00010029619	ENSGALG00010029619	PRJNA729271	0.623949755	2.33672E-09	0.623949755	stage_E14_vs_E10	ordinary_muscle_development		0	FALSE
ENSGALG00010007275	DPY30	PRJNA729271	-0.618405606	2.34303E-09	0.618405606	stage_E14_vs_E10	ordinary_muscle_development		0	FALSE
ENSGALG00010002136	ACAP1	PRJNA729271	-1.661967004	2.35124E-09	1.661967004	stage_E14_vs_E10	ordinary_muscle_development	D_vs_E10;stage_1W_vs_E10;stage_3W_vs_E10;stage	1	TRUE
ENSGALG00010020365	UNC5B	PRJNA729271	-0.862916873	2.36168E-09	0.862916873	stage_E14_vs_E10	ordinary_muscle_development		0	FALSE
ENSGALG00010001381	MDFIC	PRJNA729271	-0.798813872	2.37604E-09	0.798813872	stage_E14_vs_E10	ordinary_muscle_development		0	FALSE
ENSGALG00010000483	ENSGALG00010000483	PRJNA729271	1.467463583	2.38019E-09	1.467463583	stage_E14_vs_E10	ordinary_muscle_development	D_vs_E10;stage_1W_vs_E10;stage_3W_vs_E10;stage	1	TRUE
ENSGALG00010017569	CLMN	PRJNA729271	-1.50640969	2.38195E-09	1.50640969	stage_E14_vs_E10	ordinary_muscle_development	D_vs_E10;stage_1W_vs_E10;stage_3W_vs_E10;stage	1	TRUE
ENSGALG00010028513	ENSGALG00010028513	PRJNA729271	6.44314893	2.41573E-09	6.44314893	stage_E14_vs_E10	ordinary_muscle_development	D_vs_E10;stage_1W_vs_E10;stage_3W_vs_E10;stage	1	TRUE
ENSGALG00010019982	PLCE1	PRJNA729271	0.692323825	2.41656E-09	0.692323825	stage_E14_vs_E10	ordinary_muscle_development		0	FALSE
ENSGALG00010029566	TMEM97	PRJNA729271	-0.899565471	2.42241E-09	0.899565471	stage_E14_vs_E10	ordinary_muscle_development		0	FALSE
ENSGALG00010007982	RECK	PRJNA729271	-0.896434403	2.42802E-09	0.896434403	stage_E14_vs_E10	ordinary_muscle_development		0	FALSE
ENSGALG00010005543	SEC24D	PRJNA729271	-1.235612371	2.43195E-09	1.235612371	stage_E14_vs_E10	ordinary_muscle_development	D_vs_E10;stage_1W_vs_E10;stage_3W_vs_E10;stage	1	TRUE
ENSGALG00010021002	SLC44A3	PRJNA729271	-1.00152845	2.43213E-09	1.00152845	stage_E14_vs_E10	ordinary_muscle_development	D_vs_E10;stage_1W_vs_E10;stage_3W_vs_E10;stage	1	TRUE
ENSGALG00010010979	ENSGALG00010010979	PRJNA729271	-2.827988188	2.4386E-09	2.827988188	stage_E14_vs_E10	ordinary_muscle_development	D_vs_E10;stage_1W_vs_E10;stage_3W_vs_E10;stage	1	TRUE
ENSGALG00010027650	CCDC124	PRJNA729271	1.162244482	2.46091E-09	1.162244482	stage_E14_vs_E10	ordinary_muscle_development	D_vs_E10;stage_1W_vs_E10;stage_3W_vs_E10;stage	1	TRUE
ENSGALG00010001188	PSMG2	PRJNA729271	-0.72499212	2.47743E-09	0.72499212	stage_E14_vs_E10	ordinary_muscle_development		0	FALSE
ENSGALG00010024862	METTL16	PRJNA729271	0.729222028	2.49761E-09	0.729222028	stage_E14_vs_E10	ordinary_muscle_development		0	FALSE
ENSGALG00010025738	SOX13	PRJNA729271	0.933307674	2.50349E-09	0.933307674	stage_E14_vs_E10	ordinary_muscle_development		0	FALSE
ENSGALG00010028004	ENSGALG00010028004	PRJNA729271	-2.146425656	2.50937E-09	2.146425656	stage_E14_vs_E10	ordinary_muscle_development	D_vs_E10;stage_1W_vs_E10;stage_3W_vs_E10;stage	1	TRUE
ENSGALG00010013214	RAI14	PRJNA729271	-1.044390779	2.54514E-09	1.044390779	stage_E14_vs_E10	ordinary_muscle_development	D_vs_E10;stage_1W_vs_E10;stage_3W_vs_E10;stage	1	TRUE
ENSGALG00010008086	RHPN1	PRJNA729271	1.26990443	2.56245E-09	1.26990443	stage_E14_vs_E10	ordinary_muscle_development	D_vs_E10;stage_1W_vs_E10;stage_3W_vs_E10;stage	1	TRUE
ENSGALG00010013814	AMD1	PRJNA729271	-0.676107866	2.58289E-09	0.676107866	stage_E14_vs_E10	ordinary_muscle_development		0	FALSE
ENSGALG00010024227	MUC6	PRJNA729271	2.555712899	2.58697E-09	2.555712899	stage_E14_vs_E10	ordinary_muscle_development	D_vs_E10;stage_1W_vs_E10;stage_3W_vs_E10;stage	1	TRUE
ENSGALG00010014200	DCP1B	PRJNA729271	0.546217842	2.62992E-09	0.546217842	stage_E14_vs_E10	ordinary_muscle_development		0	FALSE
ENSGALG00010002416	SH3BGRL2	PRJNA729271	-1.753642227	2.6305E-09	1.753642227	stage_E14_vs_E10	ordinary_muscle_development	D_vs_E10;stage_1W_vs_E10;stage_3W_vs_E10;stage	1	TRUE
ENSGALG00010027847	NRTN	PRJNA729271	-2.731910238	2.63546E-09	2.731910238	stage_E14_vs_E10	ordinary_muscle_development	D_vs_E10;stage_1W_vs_E10;stage_3W_vs_E10;stage	1	TRUE
ENSGALG00010014645	SLC36A1	PRJNA729271	-0.815226427	2.65304E-09	0.815226427	stage_E14_vs_E10	ordinary_muscle_development		0	FALSE
ENSGALG00010005191	MSANTD4	PRJNA729271	-0.645602257	2.65652E-09	0.645602257	stage_E14_vs_E10	ordinary_muscle_development		0	FALSE
ENSGALG00010021556	CAMK1	PRJNA729271	-0.890150282	2.68721E-09	0.890150282	stage_E14_vs_E10	ordinary_muscle_development		0	FALSE
ENSGALG00010016553	SLC25A14	PRJNA729271	-0.590885373	2.70069E-09	0.590885373	stage_E14_vs_E10	ordinary_muscle_development		0	FALSE
ENSGALG00010011503	NCF2	PRJNA729271	2.210124682	2.72726E-09	2.210124682	stage_E14_vs_E10	ordinary_muscle_development	D_vs_E10;stage_1W_vs_E10;stage_3W_vs_E10;stage	1	TRUE
ENSGALG00010010991	C2CD2	PRJNA729271	0.990999376	2.75198E-09	0.990999376	stage_E14_vs_E10	ordinary_muscle_development		0	FALSE
ENSGALG00010021969	CCDC103	PRJNA729271	-1.749803261	2.7586E-09	1.749803261	stage_E14_vs_E10	ordinary_muscle_development	D_vs_E10;stage_1W_vs_E10;stage_3W_vs_E10;stage	1	TRUE
ENSGALG00010013032	BAIAP2L2	PRJNA729271	2.302179158	2.7669E-09	2.302179158	stage_E14_vs_E10	ordinary_muscle_development	D_vs_E10;stage_1W_vs_E10;stage_3W_vs_E10;stage	1	TRUE
ENSGALG00010022685	ATAD1	PRJNA729271	0.655343616	2.79194E-09	0.655343616	stage_E14_vs_E10	ordinary_muscle_development		0	FALSE
ENSGALG00010024173	CBLN1	PRJNA729271	-2.551654769	2.79598E-09	2.551654769	stage_E14_vs_E10	ordinary_muscle_development	D_vs_E10;stage_1W_vs_E10;stage_3W_vs_E10;stage	1	TRUE
ENSGALG00010006363	TRAPPC6A	PRJNA729271	-0.632465258	2.80929E-09	0.632465258	stage_E14_vs_E10	ordinary_muscle_development		0	FALSE
ENSGALG00010020025	LOXL2	PRJNA729271	-0.863607247	2.81835E-09	0.863607247	stage_E14_vs_E10	ordinary_muscle_development		0	FALSE
ENSGALG00010024459	AHCYL1	PRJNA729271	-0.57272238	2.83521E-09	0.57272238	stage_E14_vs_E10	ordinary_muscle_development		0	FALSE
ENSGALG00010029513	WBSCR22	PRJNA729271	-0.531667202	2.84335E-09	0.531667202	stage_E14_vs_E10	ordinary_muscle_development		0	FALSE
ENSGALG00010029353	GCGR	PRJNA729271	5.399403328	2.84381E-09	5.399403328	stage_E14_vs_E10	ordinary_muscle_development	D_vs_E10;stage_1W_vs_E10;stage_3W_vs_E10;stage	1	TRUE
ENSGALG00010026120	C1QTNF5	PRJNA729271	1.175306416	2.84735E-09	1.175306416	stage_E14_vs_E10	ordinary_muscle_development	D_vs_E10;stage_1W_vs_E10;stage_3W_vs_E10;stage	1	TRUE
ENSGALG00010017263	COG7	PRJNA729271	-0.443430459	2.84735E-09	0.443430459	stage_E14_vs_E10	ordinary_muscle_development		0	FALSE
ENSGALG00010000704	ENSGALG00010000704	PRJNA729271	-0.592088796	2.86869E-09	0.592088796	stage_E14_vs_E10	ordinary_muscle_development		0	FALSE
ENSGALG00010000546	FLRT1	PRJNA729271	-4.88882832	2.89824E-09	4.88882832	stage_E14_vs_E10	ordinary_muscle_development	D_vs_E10;stage_1W_vs_E10;stage_3W_vs_E10;stage	1	TRUE
ENSGALG00010008054	CRISP3	PRJNA729271	-5.916906185	2.90974E-09	5.916906185	stage_E14_vs_E10	ordinary_muscle_development	D_vs_E10;stage_1W_vs_E10;stage_3W_vs_E10;stage	1	TRUE
ENSGALG00010018501	ATG14	PRJNA729271	0.815380261	2.91476E-09	0.815380261	stage_E14_vs_E10	ordinary_muscle_development		0	FALSE
ENSGALG00010027158	XRCC2	PRJNA729271	-0.832727365	2.92187E-09	0.832727365	stage_E14_vs_E10	ordinary_muscle_development		0	FALSE
ENSGALG00010011509	EXOC2	PRJNA729271	0.406018628	2.94044E-09	0.406018628	stage_E14_vs_E10	ordinary_muscle_development		0	FALSE
ENSGALG00010022522	TNS4	PRJNA729271	-2.419508491	2.9518E-09	2.419508491	stage_E14_vs_E10	ordinary_muscle_development	D_vs_E10;stage_1W_vs_E10;stage_3W_vs_E10;stage	1	TRUE
ENSGALG00010016874	ADAMTSL3	PRJNA729271	0.888702189	2.95248E-09	0.888702189	stage_E14_vs_E10	ordinary_muscle_development		0	FALSE
ENSGALG00010014225	ZMPSTE24	PRJNA729271	-0.471731365	2.97502E-09	0.471731365	stage_E14_vs_E10	ordinary_muscle_development		0	FALSE
ENSGALG00010021713	RAB27A	PRJNA729271	0.484423924	3.00976E-09	0.484423924	stage_E14_vs_E10	ordinary_muscle_development		0	FALSE
ENSGALG00010010511	GABRP	PRJNA729271	-4.55054266	3.022E-09	4.55054266	stage_E14_vs_E10	ordinary_muscle_development	D_vs_E10;stage_1W_vs_E10;stage_3W_vs_E10;stage	1	TRUE
ENSGALG00010014792	ENSGALG00010014792	PRJNA729271	1.601547902	3.03618E-09	1.601547902	stage_E14_vs_E10	ordinary_muscle_development	D_vs_E10;stage_1W_vs_E10;stage_3W_vs_E10;stage	1	TRUE
ENSGALG00010004983	CLXN	PRJNA729271	-1.687227396	3.044E-09	1.687227396	stage_E14_vs_E10	ordinary_muscle_development	D_vs_E10;stage_1W_vs_E10;stage_3W_vs_E10;stage	1	TRUE
ENSGALG00010003379	ENSGALG00010003379	PRJNA729271	-3.221954278	3.04891E-09	3.221954278	stage_E14_vs_E10	ordinary_muscle_development	D_vs_E10;stage_1W_vs_E10;stage_3W_vs_E10;stage	1	TRUE
ENSGALG00010022028	FGD5	PRJNA729271	-0.726070076	3.05345E-09	0.726070076	stage_E14_vs_E10	ordinary_muscle_development		0	FALSE
ENSGALG00010011104	ABCG1	PRJNA729271	1.49703886	3.07657E-09	1.49703886	stage_E14_vs_E10	ordinary_muscle_development	D_vs_E10;stage_1W_vs_E10;stage_3W_vs_E10;stage	1	TRUE
ENSGALG00010022729	ENSGALG00010022729	PRJNA729271	-0.830462752	3.07991E-09	0.830462752	stage_E14_vs_E10	ordinary_muscle_development		0	FALSE
ENSGALG00010017798	BAG4	PRJNA729271	0.949111366	3.08055E-09	0.949111366	stage_E14_vs_E10	ordinary_muscle_development		0	FALSE
ENSGALG00010028877	FAM163B	PRJNA729271	-5.741714827	3.0922E-09	5.741714827	stage_E14_vs_E10	ordinary_muscle_development	D_vs_E10;stage_1W_vs_E10;stage_3W_vs_E10;stage	1	TRUE
ENSGALG00010021974	GFAP	PRJNA729271	4.796999732	3.09277E-09	4.796999732	stage_E14_vs_E10	ordinary_muscle_development	D_vs_E10;stage_1W_vs_E10;stage_3W_vs_E10;stage	1	TRUE
ENSGALG00010010970	TPM2	PRJNA729271	2.158198688	3.0972E-09	2.158198688	stage_E14_vs_E10	ordinary_muscle_development	D_vs_E10;stage_1W_vs_E10;stage_3W_vs_E10;stage	1	TRUE
ENSGALG00010024780	HOXD11	PRJNA729271	-6.771275515	3.11439E-09	6.771275515	stage_E14_vs_E10	ordinary_muscle_development	D_vs_E10;stage_1W_vs_E10;stage_3W_vs_E10;stage	1	TRUE
ENSGALG00010020903	AGMAT	PRJNA729271	2.280512496	3.12101E-09	2.280512496	stage_E14_vs_E10	ordinary_muscle_development	D_vs_E10;stage_1W_vs_E10;stage_3W_vs_E10;stage	1	TRUE
ENSGALG00010027665	TRAF1	PRJNA729271	-0.914758966	3.12896E-09	0.914758966	stage_E14_vs_E10	ordinary_muscle_development		0	FALSE
ENSGALG00010024607	RARG	PRJNA729271	-0.863188986	3.14813E-09	0.863188986	stage_E14_vs_E10	ordinary_muscle_development		0	FALSE
ENSGALG00010010992	PCDHA6	PRJNA729271	1.764274185	3.18927E-09	1.764274185	stage_E14_vs_E10	ordinary_muscle_development	D_vs_E10;stage_1W_vs_E10;stage_3W_vs_E10;stage	1	TRUE
ENSGALG00010024191	DPYD	PRJNA729271	-0.675949072	3.19633E-09	0.675949072	stage_E14_vs_E10	ordinary_muscle_development		0	FALSE
ENSGALG00010023239	RBPMS2	PRJNA729271	1.1897991	3.21817E-09	1.1897991	stage_E14_vs_E10	ordinary_muscle_development	D_vs_E10;stage_1W_vs_E10;stage_3W_vs_E10;stage	1	TRUE
ENSGALG00010016773	RPS27A	PRJNA729271	0.986441266	3.21853E-09	0.986441266	stage_E14_vs_E10	ordinary_muscle_development		0	FALSE
ENSGALG00010002801	FGF5	PRJNA729271	-3.408860631	3.234E-09	3.408860631	stage_E14_vs_E10	ordinary_muscle_development	D_vs_E10;stage_1W_vs_E10;stage_3W_vs_E10;stage	1	TRUE
ENSGALG00010019649	TMEM63B	PRJNA729271	-0.479036869	3.23408E-09	0.479036869	stage_E14_vs_E10	ordinary_muscle_development		0	FALSE
ENSGALG00010027947	GP9	PRJNA729271	1.95061515	3.27032E-09	1.95061515	stage_E14_vs_E10	ordinary_muscle_development	D_vs_E10;stage_1W_vs_E10;stage_3W_vs_E10;stage	1	TRUE
ENSGALG00010029552	NOS2	PRJNA729271	0.759643993	3.27928E-09	0.759643993	stage_E14_vs_E10	ordinary_muscle_development		0	FALSE
ENSGALG00010017414	ENSGALG00010017414	PRJNA729271	1.202417363	3.30334E-09	1.202417363	stage_E14_vs_E10	ordinary_muscle_development	D_vs_E10;stage_1W_vs_E10;stage_3W_vs_E10;stage	1	TRUE
ENSGALG00010029539	FKBP6	PRJNA729271	-2.638384431	3.30563E-09	2.638384431	stage_E14_vs_E10	ordinary_muscle_development	D_vs_E10;stage_1W_vs_E10;stage_3W_vs_E10;stage	1	TRUE
ENSGALG00010013358	RPS16	PRJNA729271	1.064166158	3.33682E-09	1.064166158	stage_E14_vs_E10	ordinary_muscle_development	D_vs_E10;stage_1W_vs_E10;stage_3W_vs_E10;stage	1	TRUE
ENSGALG00010023475	ENSGALG00010023475	PRJNA729271	-4.132580786	3.34049E-09	4.132580786	stage_E14_vs_E10	ordinary_muscle_development	D_vs_E10;stage_1W_vs_E10;stage_3W_vs_E10;stage	1	TRUE
ENSGALG00010018276	ARHGAP17	PRJNA729271	-0.441969149	3.38387E-09	0.441969149	stage_E14_vs_E10	ordinary_muscle_development		0	FALSE
ENSGALG00010010512	PLXNC1	PRJNA729271	-1.218650548	3.39326E-09	1.218650548	stage_E14_vs_E10	ordinary_muscle_development	D_vs_E10;stage_1W_vs_E10;stage_3W_vs_E10;stage	1	TRUE
ENSGALG00010016691	ENSGALG00010016691	PRJNA729271	1.71553499	3.41675E-09	1.71553499	stage_E14_vs_E10	ordinary_muscle_development	D_vs_E10;stage_1W_vs_E10;stage_3W_vs_E10;stage	1	TRUE
ENSGALG00010020946	CTBS	PRJNA729271	1.313453608	3.42644E-09	1.313453608	stage_E14_vs_E10	ordinary_muscle_development	D_vs_E10;stage_1W_vs_E10;stage_3W_vs_E10;stage	1	TRUE
ENSGALG00010013190	SLC45A2	PRJNA729271	-4.483941927	3.4676E-09	4.483941927	stage_E14_vs_E10	ordinary_muscle_development	D_vs_E10;stage_1W_vs_E10;stage_3W_vs_E10;stage	1	TRUE
ENSGALG00010002161	CDKL5	PRJNA729271	1.07699165	3.47485E-09	1.07699165	stage_E14_vs_E10	ordinary_muscle_development	D_vs_E10;stage_1W_vs_E10;stage_3W_vs_E10;stage	1	TRUE
ENSGALG00010010449	PAPPA2	PRJNA729271	-5.270902396	3.47495E-09	5.270902396	stage_E14_vs_E10	ordinary_muscle_development	D_vs_E10;stage_1W_vs_E10;stage_3W_vs_E10;stage	1	TRUE
ENSGALG00010005275	L3MBTL3	PRJNA729271	1.038708287	3.49814E-09	1.038708287	stage_E14_vs_E10	ordinary_muscle_development	D_vs_E10;stage_1W_vs_E10;stage_3W_vs_E10;stage	1	TRUE
ENSGALG00010007926	EIF3E	PRJNA729271	0.792569873	3.58906E-09	0.792569873	stage_E14_vs_E10	ordinary_muscle_development		0	FALSE
ENSGALG00010022199	RBM39	PRJNA729271	-0.373735028	3.59965E-09	0.373735028	stage_E14_vs_E10	ordinary_muscle_development		0	FALSE
ENSGALG00010017662	GOLGA4	PRJNA729271	1.002603108	3.60413E-09	1.002603108	stage_E14_vs_E10	ordinary_muscle_development	D_vs_E10;stage_1W_vs_E10;stage_3W_vs_E10;stage	1	TRUE
ENSGALG00010021703	CD82	PRJNA729271	-1.054227214	3.63103E-09	1.054227214	stage_E14_vs_E10	ordinary_muscle_development	D_vs_E10;stage_1W_vs_E10;stage_3W_vs_E10;stage	1	TRUE
ENSGALG00010027113	PIAS4	PRJNA729271	-0.852780763	3.63122E-09	0.852780763	stage_E14_vs_E10	ordinary_muscle_development		0	FALSE
ENSGALG00010021352	CFAP43	PRJNA729271	-1.43305961	3.64513E-09	1.43305961	stage_E14_vs_E10	ordinary_muscle_development	D_vs_E10;stage_1W_vs_E10;stage_3W_vs_E10;stage	1	TRUE
ENSGALG00010010733	GFM2	PRJNA729271	-0.78587824	3.66409E-09	0.78587824	stage_E14_vs_E10	ordinary_muscle_development		0	FALSE
ENSGALG00010021660	RNF223	PRJNA729271	2.702865292	3.6658E-09	2.702865292	stage_E14_vs_E10	ordinary_muscle_development	D_vs_E10;stage_1W_vs_E10;stage_3W_vs_E10;stage	1	TRUE
ENSGALG00010012517	MGAT3	PRJNA729271	1.057435998	3.70977E-09	1.057435998	stage_E14_vs_E10	ordinary_muscle_development	D_vs_E10;stage_1W_vs_E10;stage_3W_vs_E10;stage	1	TRUE
ENSGALG00010004443	ANKRD17	PRJNA729271	-0.566617079	3.76696E-09	0.566617079	stage_E14_vs_E10	ordinary_muscle_development		0	FALSE
ENSGALG00010026295	SMPD4	PRJNA729271	-0.830100692	3.79182E-09	0.830100692	stage_E14_vs_E10	ordinary_muscle_development		0	FALSE
ENSGALG00010023238	NUCB2	PRJNA729271	-0.472268209	3.80936E-09	0.472268209	stage_E14_vs_E10	ordinary_muscle_development		0	FALSE
ENSGALG00010011394	SERPINC1	PRJNA729271	5.485159018	3.81159E-09	5.485159018	stage_E14_vs_E10	ordinary_muscle_development	D_vs_E10;stage_1W_vs_E10;stage_3W_vs_E10;stage	1	TRUE
ENSGALG00010017587	MB21D2	PRJNA729271	-0.896437511	3.82422E-09	0.896437511	stage_E14_vs_E10	ordinary_muscle_development		0	FALSE
ENSGALG00010012013	FOXD1	PRJNA729271	-6.350316235	3.85236E-09	6.350316235	stage_E14_vs_E10	ordinary_muscle_development	D_vs_E10;stage_1W_vs_E10;stage_3W_vs_E10;stage	1	TRUE
ENSGALG00010024761	NECAB2	PRJNA729271	1.40178664	3.86727E-09	1.40178664	stage_E14_vs_E10	ordinary_muscle_development	D_vs_E10;stage_1W_vs_E10;stage_3W_vs_E10;stage	1	TRUE
ENSGALG00010007429	C21orf91	PRJNA729271	1.25982508	3.88561E-09	1.25982508	stage_E14_vs_E10	ordinary_muscle_development	D_vs_E10;stage_1W_vs_E10;stage_3W_vs_E10;stage	1	TRUE
ENSGALG00010017063	TMPPE	PRJNA729271	0.633355692	3.91866E-09	0.633355692	stage_E14_vs_E10	ordinary_muscle_development		0	FALSE
ENSGALG00010028915	ADGRD2	PRJNA729271	2.768058224	3.92084E-09	2.768058224	stage_E14_vs_E10	ordinary_muscle_development	D_vs_E10;stage_1W_vs_E10;stage_3W_vs_E10;stage	1	TRUE
ENSGALG00010028451	TMEM59L	PRJNA729271	-2.087539023	3.93654E-09	2.087539023	stage_E14_vs_E10	ordinary_muscle_development	D_vs_E10;stage_1W_vs_E10;stage_3W_vs_E10;stage	1	TRUE
ENSGALG00010015668	ZNF217	PRJNA729271	0.810544538	3.93803E-09	0.810544538	stage_E14_vs_E10	ordinary_muscle_development		0	FALSE
ENSGALG00010021267	LRRC47	PRJNA729271	0.471331467	3.93906E-09	0.471331467	stage_E14_vs_E10	ordinary_muscle_development		0	FALSE
ENSGALG00010020810	IFITM5	PRJNA729271	1.462706856	3.94287E-09	1.462706856	stage_E14_vs_E10	ordinary_muscle_development	D_vs_E10;stage_1W_vs_E10;stage_3W_vs_E10;stage	1	TRUE
ENSGALG00010013437	ENSGALG00010013437	PRJNA729271	0.745642659	3.95224E-09	0.745642659	stage_E14_vs_E10	ordinary_muscle_development		0	FALSE
ENSGALG00010016049	ZFYVE21	PRJNA729271	0.742687943	3.95907E-09	0.742687943	stage_E14_vs_E10	ordinary_muscle_development		0	FALSE
ENSGALG00010020036	TMF1	PRJNA729271	0.502387226	3.96833E-09	0.502387226	stage_E14_vs_E10	ordinary_muscle_development		0	FALSE
ENSGALG00010002987	PCDH8	PRJNA729271	-2.276968249	3.98537E-09	2.276968249	stage_E14_vs_E10	ordinary_muscle_development	D_vs_E10;stage_1W_vs_E10;stage_3W_vs_E10;stage	1	TRUE
ENSGALG00010023821	KANK4	PRJNA729271	-0.771721124	3.98791E-09	0.771721124	stage_E14_vs_E10	ordinary_muscle_development		0	FALSE

ENSGALG00010020818	TMEM62	PRJNA729271	-1.234794475	3.99965E-09	1.234794475	stage_E14_vs_E10	ordinary_muscle_development	D_vs_E10;stage_1W_vs_E10;stage_3W_vs_E10;stage	1	TRUE
ENSGALG00010011379	CELF4	PRJNA729271	-5.517156316	4.01168E-09	5.517156316	stage_E14_vs_E10	ordinary_muscle_development	D_vs_E10;stage_1W_vs_E10;stage_3W_vs_E10;stage	1	TRUE
ENSGALG00010024391	TBC1D2B	PRJNA729271	-0.66950394	4.01833E-09	0.66950394	stage_E14_vs_E10	ordinary_muscle_development		0	FALSE
ENSGALG00010012918	GLRX	PRJNA729271	1.66557831	4.02565E-09	1.66557831	stage_E14_vs_E10	ordinary_muscle_development	D_vs_E10;stage_1W_vs_E10;stage_3W_vs_E10;stage	1	TRUE
ENSGALG00010028489	ENSGALG00010028489	PRJNA729271	4.648283657	4.0329E-09	4.648283657	stage_E14_vs_E10	ordinary_muscle_development	D_vs_E10;stage_1W_vs_E10;stage_3W_vs_E10;stage	1	TRUE
ENSGALG00010028184	TMEM79	PRJNA729271	1.487584807	4.06595E-09	1.487584807	stage_E14_vs_E10	ordinary_muscle_development	D_vs_E10;stage_1W_vs_E10;stage_3W_vs_E10;stage	1	TRUE
ENSGALG00010015128	OCRL	PRJNA729271	0.639869937	4.07122E-09	0.639869937	stage_E14_vs_E10	ordinary_muscle_development		0	FALSE
ENSGALG00010008955	KRTCAP3	PRJNA729271	-2.59246458	4.07672E-09	2.59246458	stage_E14_vs_E10	ordinary_muscle_development	D_vs_E10;stage_1W_vs_E10;stage_3W_vs_E10;stage	1	TRUE
ENSGALG00010029531	CEP131	PRJNA729271	-1.036267905	4.0957E-09	1.036267905	stage_E14_vs_E10	ordinary_muscle_development	D_vs_E10;stage_1W_vs_E10;stage_3W_vs_E10;stage	1	TRUE
ENSGALG00010016394	PDIK1L	PRJNA729271	0.58120523	4.10444E-09	0.58120523	stage_E14_vs_E10	ordinary_muscle_development		0	FALSE
ENSGALG00010010760	FOXI1	PRJNA729271	3.830240651	4.13334E-09	3.830240651	stage_E14_vs_E10	ordinary_muscle_development	D_vs_E10;stage_1W_vs_E10;stage_3W_vs_E10;stage	1	TRUE
ENSGALG00010022930	GTF2B	PRJNA729271	-0.729471765	4.14357E-09	0.729471765	stage_E14_vs_E10	ordinary_muscle_development		0	FALSE
ENSGALG00010021070	RNF141	PRJNA729271	0.514368015	4.16115E-09	0.514368015	stage_E14_vs_E10	ordinary_muscle_development		0	FALSE
ENSGALG00010017314	CALML4	PRJNA729271	-1.362653255	4.17873E-09	1.362653255	stage_E14_vs_E10	ordinary_muscle_development	D_vs_E10;stage_1W_vs_E10;stage_3W_vs_E10;stage	1	TRUE
ENSGALG00010009225	HNRNPL	PRJNA729271	-0.754571503	4.18082E-09	0.754571503	stage_E14_vs_E10	ordinary_muscle_development		0	FALSE
ENSGALG00010019083	OTOA	PRJNA729271	-1.466913853	4.1854E-09	1.466913853	stage_E14_vs_E10	ordinary_muscle_development	D_vs_E10;stage_1W_vs_E10;stage_3W_vs_E10;stage	1	TRUE
ENSGALG00010009000	FAM149B1	PRJNA729271	-0.633549789	4.19965E-09	0.633549789	stage_E14_vs_E10	ordinary_muscle_development		0	FALSE
ENSGALG00010018874	ENSGALG00010018874	PRJNA729271	-0.70301087	4.25398E-09	0.70301087	stage_E14_vs_E10	ordinary_muscle_development		0	FALSE
ENSGALG00010027161	C4BPG	PRJNA729271	1.217839511	4.27855E-09	1.217839511	stage_E14_vs_E10	ordinary_muscle_development	D_vs_E10;stage_1W_vs_E10;stage_3W_vs_E10;stage	1	TRUE
ENSGALG00010028162	CARD9	PRJNA729271	1.051724033	4.3509E-09	1.051724033	stage_E14_vs_E10	ordinary_muscle_development	D_vs_E10;stage_1W_vs_E10;stage_3W_vs_E10;stage	1	TRUE
ENSGALG00010017905	EIF4E2	PRJNA729271	-0.530331705	4.35517E-09	0.530331705	stage_E14_vs_E10	ordinary_muscle_development		0	FALSE
ENSGALG00010003722	MHCY11	PRJNA729271	3.914425387	4.3674E-09	3.914425387	stage_E14_vs_E10	ordinary_muscle_development	D_vs_E10;stage_1W_vs_E10;stage_3W_vs_E10;stage	1	TRUE
ENSGALG00010024926	ELF5	PRJNA729271	-5.996997085	4.39233E-09	5.996997085	stage_E14_vs_E10	ordinary_muscle_development	D_vs_E10;stage_1W_vs_E10;stage_3W_vs_E10;stage	1	TRUE
ENSGALG00010000604	ENSGALG00010000604	PRJNA729271	-0.823194996	4.40561E-09	0.823194996	stage_E14_vs_E10	ordinary_muscle_development		0	FALSE
ENSGALG00010020336	OSBP	PRJNA729271	0.805290348	4.42224E-09	0.805290348	stage_E14_vs_E10	ordinary_muscle_development		0	FALSE
ENSGALG00010006106	SHOC1	PRJNA729271	2.22556291	4.43244E-09	2.22556291	stage_E14_vs_E10	ordinary_muscle_development	D_vs_E10;stage_1W_vs_E10;stage_3W_vs_E10;stage	1	TRUE
ENSGALG00010025959	UROD	PRJNA729271	-0.637777954	4.45552E-09	0.637777954	stage_E14_vs_E10	ordinary_muscle_development		0	FALSE
ENSGALG00010012445	SOBP	PRJNA729271	-1.011025692	4.47305E-09	1.011025692	stage_E14_vs_E10	ordinary_muscle_development	D_vs_E10;stage_1W_vs_E10;stage_3W_vs_E10;stage	1	TRUE
ENSGALG00010017133	WDR20	PRJNA729271	0.673238905	4.52323E-09	0.673238905	stage_E14_vs_E10	ordinary_muscle_development		0	FALSE
ENSGALG00010023089	ENSGALG00010023089	PRJNA729271	4.097652598	4.57551E-09	4.097652598	stage_E14_vs_E10	ordinary_muscle_development	D_vs_E10;stage_1W_vs_E10;stage_3W_vs_E10;stage	1	TRUE
ENSGALG00010023479	MCCC2L	PRJNA729271	0.93590201	4.6575E-09	0.93590201	stage_E14_vs_E10	ordinary_muscle_development		0	FALSE
ENSGALG00010022801	LSM14B	PRJNA729271	0.619091699	4.6816E-09	0.619091699	stage_E14_vs_E10	ordinary_muscle_development		0	FALSE
ENSGALG00010015511	BPIFB4	PRJNA729271	-3.544732697	4.69682E-09	3.544732697	stage_E14_vs_E10	ordinary_muscle_development	D_vs_E10;stage_1W_vs_E10;stage_3W_vs_E10;stage	1	TRUE
ENSGALG00010006342	RFXAP	PRJNA729271	-0.514380369	4.71293E-09	0.514380369	stage_E14_vs_E10	ordinary_muscle_development		0	FALSE
ENSGALG00010022067	TMEM130	PRJNA729271	-1.527320349	4.72671E-09	1.527320349	stage_E14_vs_E10	ordinary_muscle_development	D_vs_E10;stage_1W_vs_E10;stage_3W_vs_E10;stage	1	TRUE
ENSGALG00010027303	APEH	PRJNA729271	0.828806307	4.77842E-09	0.828806307	stage_E14_vs_E10	ordinary_muscle_development		0	FALSE
ENSGALG00010002907	GABRG3	PRJNA729271	-2.296136694	4.8055E-09	2.296136694	stage_E14_vs_E10	ordinary_muscle_development	D_vs_E10;stage_1W_vs_E10;stage_3W_vs_E10;stage	1	TRUE
ENSGALG00010003295	IAH1	PRJNA729271	-0.815822892	4.82946E-09	0.815822892	stage_E14_vs_E10	ordinary_muscle_development		0	FALSE
ENSGALG00010009649	TENM2	PRJNA729271	-1.890594273	4.88128E-09	1.890594273	stage_E14_vs_E10	ordinary_muscle_development	D_vs_E10;stage_1W_vs_E10;stage_3W_vs_E10;stage	1	TRUE
ENSGALG00010018985	NOL9	PRJNA729271	-0.536470879	4.89194E-09	0.536470879	stage_E14_vs_E10	ordinary_muscle_development		0	FALSE
ENSGALG00010015118	GPX8	PRJNA729271	-1.120369958	4.91392E-09	1.120369958	stage_E14_vs_E10	ordinary_muscle_development	D_vs_E10;stage_1W_vs_E10;stage_3W_vs_E10;stage	1	TRUE
ENSGALG00010007614	BIRC6	PRJNA729271	0.528812977	4.92287E-09	0.528812977	stage_E14_vs_E10	ordinary_muscle_development		0	FALSE
ENSGALG00010028858	ENSGALG00010028858	PRJNA729271	-1.664115279	4.92795E-09	1.664115279	stage_E14_vs_E10	ordinary_muscle_development	D_vs_E10;stage_1W_vs_E10;stage_3W_vs_E10;stage	1	TRUE
ENSGALG00010027166	CDK5	PRJNA729271	0.586994838	4.95491E-09	0.586994838	stage_E14_vs_E10	ordinary_muscle_development		0	FALSE
ENSGALG00010009591	LYPD6	PRJNA729271	-3.227396958	4.96023E-09	3.227396958	stage_E14_vs_E10	ordinary_muscle_development	D_vs_E10;stage_1W_vs_E10;stage_3W_vs_E10;stage	1	TRUE
ENSGALG00010000585	SPTBN2	PRJNA729271	-4.743899754	4.99108E-09	4.743899754	stage_E14_vs_E10	ordinary_muscle_development	D_vs_E10;stage_1W_vs_E10;stage_3W_vs_E10;stage	1	TRUE
ENSGALG00010029364	ANAPC11	PRJNA729271	-0.795959441	5.02187E-09	0.795959441	stage_E14_vs_E10	ordinary_muscle_development		0	FALSE
ENSGALG00010020611	LPGAT1	PRJNA729271	-0.607818735	5.07361E-09	0.607818735	stage_E14_vs_E10	ordinary_muscle_development		0	FALSE
ENSGALG00010027128	EIF2D	PRJNA729271	0.53749288	5.07927E-09	0.53749288	stage_E14_vs_E10	ordinary_muscle_development		0	FALSE
ENSGALG00010009659	APOLD1	PRJNA729271	1.514789701	5.09162E-09	1.514789701	stage_E14_vs_E10	ordinary_muscle_development	D_vs_E10;stage_1W_vs_E10;stage_3W_vs_E10;stage	1	TRUE
ENSGALG00010011601	ANO4	PRJNA729271	-5.548626982	5.11018E-09	5.548626982	stage_E14_vs_E10	ordinary_muscle_development	D_vs_E10;stage_1W_vs_E10;stage_3W_vs_E10;stage	1	TRUE
ENSGALG00010012070	DNAJC28	PRJNA729271	0.952199658	5.12477E-09	0.952199658	stage_E14_vs_E10	ordinary_muscle_development		0	FALSE
ENSGALG00010005688	ENSGALG00010005688	PRJNA729271	2.903937594	5.14118E-09	2.903937594	stage_E14_vs_E10	ordinary_muscle_development	D_vs_E10;stage_1W_vs_E10;stage_3W_vs_E10;stage	1	TRUE
ENSGALG00010020307	SHQ1	PRJNA729271	-1.373675197	5.16088E-09	1.373675197	stage_E14_vs_E10	ordinary_muscle_development	D_vs_E10;stage_1W_vs_E10;stage_3W_vs_E10;stage	1	TRUE
ENSGALG00010026671	TAF8	PRJNA729271	0.93455564	5.16779E-09	0.93455564	stage_E14_vs_E10	ordinary_muscle_development		0	FALSE
ENSGALG00010017581	EFCAB11	PRJNA729271	-2.80149813	5.17634E-09	2.80149813	stage_E14_vs_E10	ordinary_muscle_development	D_vs_E10;stage_1W_vs_E10;stage_3W_vs_E10;stage	1	TRUE
ENSGALG00010028772	TTF1	PRJNA729271	-0.456659297	5.18757E-09	0.456659297	stage_E14_vs_E10	ordinary_muscle_development		0	FALSE
ENSGALG00010023840	SMAGP	PRJNA729271	-4.851496483	5.22396E-09	4.851496483	stage_E14_vs_E10	ordinary_muscle_development	D_vs_E10;stage_1W_vs_E10;stage_3W_vs_E10;stage	1	TRUE
ENSGALG00010027201	ASPN	PRJNA729271	-1.966362974	5.27415E-09	1.966362974	stage_E14_vs_E10	ordinary_muscle_development	D_vs_E10;stage_1W_vs_E10;stage_3W_vs_E10;stage	1	TRUE
ENSGALG00010014305	ARL10	PRJNA729271	-1.987465484	5.28071E-09	1.987465484	stage_E14_vs_E10	ordinary_muscle_development	D_vs_E10;stage_1W_vs_E10;stage_3W_vs_E10;stage	1	TRUE
ENSGALG00010006942	ALOX5AP	PRJNA729271	-1.20297402	5.28974E-09	1.20297402	stage_E14_vs_E10	ordinary_muscle_development	D_vs_E10;stage_1W_vs_E10;stage_3W_vs_E10;stage	1	TRUE
ENSGALG00010027899	PMVK	PRJNA729271	-1.091686742	5.3166E-09	1.091686742	stage_E14_vs_E10	ordinary_muscle_development	D_vs_E10;stage_1W_vs_E10;stage_3W_vs_E10;stage	1	TRUE
ENSGALG00010001156	ALDH7A1	PRJNA729271	-0.654326254	5.33347E-09	0.654326254	stage_E14_vs_E10	ordinary_muscle_development		0	FALSE
ENSGALG00010020596	SUMF1	PRJNA729271	0.716388453	5.36511E-09	0.716388453	stage_E14_vs_E10	ordinary_muscle_development		0	FALSE
ENSGALG00010001813	HOXA5	PRJNA729271	-0.635414568	5.37787E-09	0.635414568	stage_E14_vs_E10	ordinary_muscle_development		0	FALSE
ENSGALG00010021382	TPRG1L	PRJNA729271	0.535028199	5.419E-09	0.535028199	stage_E14_vs_E10	ordinary_muscle_development		0	FALSE
ENSGALG00010022097	INPP5J	PRJNA729271	-1.650896721	5.42769E-09	1.650896721	stage_E14_vs_E10	ordinary_muscle_development	D_vs_E10;stage_1W_vs_E10;stage_3W_vs_E10;stage	1	TRUE
ENSGALG00010021716	TFCP2L1	PRJNA729271	2.316010409	5.43059E-09	2.316010409	stage_E14_vs_E10	ordinary_muscle_development	D_vs_E10;stage_1W_vs_E10;stage_3W_vs_E10;stage	1	TRUE
ENSGALG00010020632	BLOC1S2	PRJNA729271	-0.68706338	5.47725E-09	0.68706338	stage_E14_vs_E10	ordinary_muscle_development		0	FALSE
ENSGALG00010012398	ENSGALG00010012398	PRJNA729271	-5.533617668	5.48129E-09	5.533617668	stage_E14_vs_E10	ordinary_muscle_development	D_vs_E10;stage_1W_vs_E10;stage_3W_vs_E10;stage	1	TRUE
ENSGALG00010016671	CASS4	PRJNA729271	1.500748548	5.5079E-09	1.500748548	stage_E14_vs_E10	ordinary_muscle_development	D_vs_E10;stage_1W_vs_E10;stage_3W_vs_E10;stage	1	TRUE
ENSGALG00010021103	RBMS1	PRJNA729271	-0.445055148	5.51898E-09	0.445055148	stage_E14_vs_E10	ordinary_muscle_development		0	FALSE
ENSGALG00010006877	GSE1	PRJNA729271	1.440019387	5.55163E-09	1.440019387	stage_E14_vs_E10	ordinary_muscle_development	D_vs_E10;stage_1W_vs_E10;stage_3W_vs_E10;stage	1	TRUE
ENSGALG00010028103	APBA3	PRJNA729271	0.771370085	5.59645E-09	0.771370085	stage_E14_vs_E10	ordinary_muscle_development		0	FALSE
ENSGALG00010013164	FAM83F	PRJNA729271	-3.120756032	5.62728E-09	3.120756032	stage_E14_vs_E10	ordinary_muscle_development	D_vs_E10;stage_1W_vs_E10;stage_3W_vs_E10;stage	1	TRUE
ENSGALG00010021866	SCNN1A	PRJNA729271	2.150973012	5.75207E-09	2.150973012	stage_E14_vs_E10	ordinary_muscle_development	D_vs_E10;stage_1W_vs_E10;stage_3W_vs_E10;stage	1	TRUE
ENSGALG00010014738	BRWD1	PRJNA729271	0.514868809	5.77885E-09	0.514868809	stage_E14_vs_E10	ordinary_muscle_development		0	FALSE
ENSGALG00010026433	VPS11	PRJNA729271	-0.442311174	5.87543E-09	0.442311174	stage_E14_vs_E10	ordinary_muscle_development		0	FALSE
ENSGALG00010028606	PRPF3	PRJNA729271	0.805649018	5.92827E-09	0.805649018	stage_E14_vs_E10	ordinary_muscle_development		0	FALSE
ENSGALG00010011793	TLN1	PRJNA729271	0.678707757	5.9553E-09	0.678707757	stage_E14_vs_E10	ordinary_muscle_development		0	FALSE
ENSGALG00010015110	ENSGALG00010015110	PRJNA729271	4.065977521	6.02572E-09	4.065977521	stage_E14_vs_E10	ordinary_muscle_development	D_vs_E10;stage_1W_vs_E10;stage_3W_vs_E10;stage	1	TRUE
ENSGALG00010005315	ENSGALG00010005315	PRJNA729271	2.943776975	6.02572E-09	2.943776975	stage_E14_vs_E10	ordinary_muscle_development	D_vs_E10;stage_1W_vs_E10;stage_3W_vs_E10;stage	1	TRUE
ENSGALG00010007028	KATNAL1	PRJNA729271	-0.872033419	6.05811E-09	0.872033419	stage_E14_vs_E10	ordinary_muscle_development		0	FALSE
ENSGALG00010010291	TCF20	PRJNA729271	-0.701037405	6.06565E-09	0.701037405	stage_E14_vs_E10	ordinary_muscle_development		0	FALSE
ENSGALG00010027110	IKBKE	PRJNA729271	1.198978115	6.09849E-09	1.198978115	stage_E14_vs_E10	ordinary_muscle_development	D_vs_E10;stage_1W_vs_E10;stage_3W_vs_E10;stage	1	TRUE
ENSGALG00010029720	TIMM22	PRJNA729271	0.629997392	6.09894E-09	0.629997392	stage_E14_vs_E10	ordinary_muscle_development		0	FALSE
ENSGALG00010026266	ENSGALG00010026266	PRJNA729271	-2.386678599	6.09898E-09	2.386678599	stage_E14_vs_E10	ordinary_muscle_development	D_vs_E10;stage_1W_vs_E10;stage_3W_vs_E10;stage	1	TRUE
ENSGALG00010000156	ENSGALG00010000156	PRJNA729271	2.849293403	6.10104E-09	2.849293403	stage_E14_vs_E10	ordinary_muscle_development	D_vs_E10;stage_1W_vs_E10;stage_3W_vs_E10;stage	1	TRUE
ENSGALG00010029601	HSF5	PRJNA729271	-3.402686103	6.1288E-09	3.402686103	stage_E14_vs_E10	ordinary_muscle_development	D_vs_E10;stage_1W_vs_E10;stage_3W_vs_E10;stage	1	TRUE
ENSGALG00010020023	FOSL2	PRJNA729271	-1.296906892	6.14529E-09	1.296906892	stage_E14_vs_E10	ordinary_muscle_development	D_vs_E10;stage_1W_vs_E10;stage_3W_vs_E10;stage	1	TRUE
ENSGALG00010008184	SLC66A2	PRJNA729271	-0.517693122	6.1838E-09	0.517693122	stage_E14_vs_E10	ordinary_muscle_development		0	FALSE
ENSGALG00010010185	PHYHIPL	PRJNA729271	-2.124357212	6.18965E-09	2.124357212	stage_E14_vs_E10	ordinary_muscle_development	D_vs_E10;stage_1W_vs_E10;stage_3W_vs_E10;stage	1	TRUE
ENSGALG00010001915	EEF1AKMT2	PRJNA729271	-0.918365131	6.20025E-09	0.918365131	stage_E14_vs_E10	ordinary_muscle_development		0	FALSE
ENSGALG00010011457	ENSGALG00010011457	PRJNA729271	1.529302697	6.21406E-09	1.529302697	stage_E14_vs_E10	ordinary_muscle_development	D_vs_E10;stage_1W_vs_E10;stage_3W_vs_E10;stage	1	TRUE
ENSGALG00010022516	TRUB1	PRJNA729271	-0.90732171	6.23849E-09	0.90732171	stage_E14_vs_E10	ordinary_muscle_development		0	FALSE
ENSGALG00010020603	VPS39	PRJNA729271	0.551875817	6.24775E-09	0.551875817	stage_E14_vs_E10	ordinary_muscle_development		0	FALSE
ENSGALG00010029617	POR	PRJNA729271	-0.568220821	6.29385E-09	0.568220821	stage_E14_vs_E10	ordinary_muscle_development		0	FALSE
ENSGALG00010024465	SENP1	PRJNA729271	-0.979194118	6.3352E-09	0.979194118	stage_E14_vs_E10	ordinary_muscle_development		0	FALSE
ENSGALG00010017383	ENSGALG00010017383	PRJNA729271	-0.922059857	6.39685E-09	0.922059857	stage_E14_vs_E10	ordinary_muscle_development		0	FALSE
ENSGALG00010017438	ADRB3	PRJNA729271	4.593477462	6.4144E-09	4.593477462	stage_E14_vs_E10	ordinary_muscle_development	D_vs_E10;stage_1W_vs_E10;stage_3W_vs_E10;stage	1	TRUE
ENSGALG00010003366	CASD1	PRJNA729271	0.642587888	6.43269E-09	0.642587888	stage_E14_vs_E10	ordinary_muscle_development		0	FALSE
ENSGALG00010020870	RIOX1	PRJNA729271	0.868049148	6.44283E-09	0.868049148	stage_E14_vs_E10	ordinary_muscle_development		0	FALSE
ENSGALG00010004377	AGR2	PRJNA729271	-5.849682416	6.45343E-09	5.849682416	stage_E14_vs_E10	ordinary_muscle_development	D_vs_E10;stage_1W_vs_E10;stage_3W_vs_E10;stage	1	TRUE
ENSGALG00010016931	SLC30A4	PRJNA729271	0.487957804	6.4698E-09	0.487957804	stage_E14_vs_E10	ordinary_muscle_development		0	FALSE
ENSGALG00010025056	RBMS2	PRJNA729271	0.976464756	6.47326E-09	0.976464756	stage_E14_vs_E10	ordinary_muscle_development		0	FALSE
ENSGALG00010013802	PYROXD1	PRJNA729271	0.830297557	6.47691E-09	0.830297557	stage_E14_vs_E10	ordinary_muscle_development		0	FALSE
ENSGALG00010012982	DDR2	PRJNA729271	-1.455966922	6.49174E-09	1.455966922	stage_E14_vs_E10	ordinary_muscle_development	D_vs_E10;stage_1W_vs_E10;stage_3W_vs_E10;stage	1	TRUE
ENSGALG00010015792	MARK3	PRJNA729271	0.482809133	6.52481E-09	0.482809133	stage_E14_vs_E10	ordinary_muscle_development		0	FALSE
ENSGALG00010015092	HSPE1	PRJNA729271	-0.729168443	6.56261E-09	0.729168443	stage_E14_vs_E10	ordinary_muscle_development		0	FALSE
ENSGALG00010029745	SHPK	PRJNA729271	1.008845576	6.56465E-09	1.008845576	stage_E14_vs_E10	ordinary_muscle_development	D_vs_E10;stage_1W_vs_E10;stage_3W_vs_E10;stage	1	TRUE
ENSGALG00010012795	TBCA	PRJNA729271	-0.814308817	6.56802E-09	0.814308817	stage_E14_vs_E10	ordinary_muscle_development		0	FALSE
ENSGALG00010027880	AMH	PRJNA729271	2.037667465	6.56844E-09	2.037667465	stage_E14_vs_E10	ordinary_muscle_development	D_vs_E10;stage_1W_vs_E10;stage_3W_vs_E10;stage	1	TRUE
ENSGALG00010019471	NUBP1	PRJNA729271	0.547692738	6.61082E-09	0.547692738	stage_E14_vs_E10	ordinary_muscle_development		0	FALSE
ENSGALG00010024859	TARSL2	PRJNA729271	0.610022518	6.63427E-09	0.610022518	stage_E14_vs_E10	ordinary_muscle_development		0	FALSE
ENSGALG00010025042	BCO2	PRJNA729271	-5.783931579	6.64508E-09	5.783931579	stage_E14_vs_E10	ordinary_muscle_development	D_vs_E10;stage_1W_vs_E10;stage_3W_vs_E10;stage	1	TRUE
ENSGALG00010016777	TNMD	PRJNA729271	-2.905800725	6.67491E-09	2.905800725	stage_E14_vs_E10	ordinary_muscle_development	D_vs_E10;stage_1W_vs_E10;stage_3W_vs_E10;stage	1	TRUE
ENSGALG00010022808	PSMA7	PRJNA729271	-0.824099785	6.6771E-09	0.824099785	stage_E14_vs_E10	ordinary_muscle_development		0	FALSE
ENSGALG00010008247	GASK1B	PRJNA729271	0.964204856	6.70715E-09	0.964204856	stage_E14_vs_E10	ordinary_muscle_development		0	FALSE
ENSGALG00010006061	RPL24	PRJNA729271	0.952890613	6.76795E-09	0.952890613	stage_E14_vs_E10	ordinary_muscle_development		0	FALSE
ENSGALG00010005712	ENSGALG00010005712	PRJNA729271	0.642396663	6.78153E-09	0.642396663	stage_E14_vs_E10	ordinary_muscle_development		0	FALSE
ENSGALG00010011007	ENSGALG00010011007	PRJNA729271	-1.08966157	6.80586E-09	1.08966157	stage_E14_vs_E10	ordinary_muscle_development	D_vs_E10;stage_1W_vs_E10;stage_3W_vs_E10;stage	1	TRUE
ENSGALG00010003790	ELOVL6	PRJNA729271	-1.081199208	6.83539E-09	1.081199208	stage_E14_vs_E10	ordinary_muscle_development	D_vs_E10;stage_1W_vs_E10;stage_3W_vs_E10;stage	1	TRUE
ENSGALG00010010946	SH2D4B	PRJNA729271	1.79732753	6.87591E-09	1.79732753	stage_E14_vs_E10	ordinary_muscle_development	D_vs_E10;stage_1W_vs_E10;stage_3W_vs_E10;stage	1	TRUE
ENSGALG00010023706	CDK5RAP3	PRJNA729271	-0.770167987	6.92579E-09	0.770167987	stage_E14_vs_E10	ordinary_muscle_development		0	FALSE
ENSGALG00010006549	ZDHHC23	PRJNA729271	-1.135750953	6.93587E-09	1.135750953	stage_E14_vs_E10	ordinary_muscle_development	D_vs_E10;stage_1W_vs_E10;stage_3W_vs_E10;stage	1	TRUE
ENSGALG00010022058	NLRP3	PRJNA729271	-0.767575748	6.95814E-09	0.767575748	stage_E14_vs_E10	ordinary_muscle_development		0	FALSE
ENSGALG00010002449	ALG11	PRJNA729271	-0.85121825	6.97526E-09	0.85121825	stage_E14_vs_E10	ordinary_muscle_development		0	FALSE
ENSGALG00010017535	LIMD1	PRJNA729271	-0.539265365	7.07819E-09	0.539265365	stage_E14_vs_E10	ordinary_muscle_development		0	FALSE
ENSGALG00010024784	ENSGALG00010024784	PRJNA729271	0.827026289	7.08867E-09	0.827026289	stage_E14_vs_E10	ordinary_muscle_development		0	FALSE
ENSGALG00010005407	DPH6	PRJNA729271	-0.725328175	7.15019E-09	0.725328175	stage_E14_vs_E10	ordinary_muscle_development		0	FALSE
ENSGALG00010020898	ENSGALG00010020898	PRJNA729271	0.729878787	7.17385E-09	0.729878787	stage_E14_vs_E10	ordinary_muscle_development		0	FALSE
ENSGALG00010012474	ASB18	PRJNA729271	5.538487244	7.17439E-09	5.538487244	stage_E14_vs_E10	ordinary_muscle_development	D_vs_E10;stage_1W_vs_E10;stage_3W_vs_E10;stage	1	TRUE
ENSGALG00010013805	HSPA4	PRJNA729271	0.858673567	7.21745E-09	0.858673567	stage_E14_vs_E10	ordinary_muscle_development		0	FALSE
ENSGALG00010024230	LONP2	PRJNA729271	0.88862211	7.27264E-09	0.88862211	stage_E14_vs_E10	ordinary_muscle_development		0	FALSE
ENSGALG00010016619	SLC25A47	PRJNA729271	1.936326065	7.27764E-09	1.936326065	stage_E14_vs_E10	ordinary_muscle_development	D_vs_E10;stage_1W_vs_E10;stage_3W_vs_E10;stage	1	TRUE
ENSGALG00010007893	MFSD8	PRJNA729271	0.872111272	7.33589E-09	0.872111272	stage_E14_vs_E10	ordinary_muscle_development		0	FALSE
ENSGALG00010007320	ENSGALG00010007320	PRJNA729271	1.790256639	7.34323E-09	1.790256639	stage_E14_vs_E10	ordinary_muscle_development	D_vs_E10;stage_1W_vs_E10;stage_3W_vs_E10;stage	1	TRUE
ENSGALG00010020578	TNKS2	PRJNA729271	0.372658021	7.42673E-09	0.372658021	stage_E14_vs_E10	ordinary_muscle_development		0	FALSE
ENSGALG00010021646	RPUSD3	PRJNA729271	0.971937347	7.45887E-09	0.971937347	stage_E14_vs_E10	ordinary_muscle_development		0	FALSE
ENSGALG00010018897	CCDC85A	PRJNA729271	-1.023838362	7.52822E-09	1.023838362	stage_E14_vs_E10	ordinary_muscle_development	D_vs_E10;stage_1W_vs_E10;stage_3W_vs_E10;stage	1	TRUE
ENSGALG00010023415	HOXB4	PRJNA729271	-1.026694076	7.57585E-09	1.026694076	stage_E14_vs_E10	ordinary_muscle_development	D_vs_E10;stage_1W_vs_E10;stage_3W_vs_E10;stage	1	TRUE
ENSGALG00010003571	RPL7	PRJNA729271	0.940301042	7.62566E-09	0.940301042	stage_E14_vs_E10	ordinary_muscle_development		0	FALSE
ENSGALG00010012346	NLN	PRJNA729271	-0.838669627	7.6386E-09	0.838669627	stage_E14_vs_E10	ordinary_muscle_development		0	FALSE
ENSGALG00010017530	TLR1B	PRJNA729271	1.247800875	7.65687E-09	1.247800875	stage_E14_vs_E10	ordinary_muscle_development	D_vs_E10;stage_1W_vs_E10;stage_3W_vs_E10;stage	1	TRUE
ENSGALG00010009386	TMEM60	PRJNA729271	0.655269306	7.69948E-09	0.655269306	stage_E14_vs_E10	ordinary_muscle_development		0	FALSE
ENSGALG00010021341	L3MBTL1	PRJNA729271	-0.948932876	7.72206E-09	0.948932876	stage_E14_vs_E10	ordinary_muscle_development		0	FALSE
ENSGALG00010008344	MFAP3L	PRJNA729271	1.657844365	7.74058E-09	1.657844365	stage_E14_vs_E10	ordinary_muscle_development	D_vs_E10;stage_1W_vs_E10;stage_3W_vs_E10;stage	1	TRUE
ENSGALG00010003006	CYP7B1	PRJNA729271	-1.192351048	7.76317E-09	1.192351048	stage_E14_vs_E10	ordinary_muscle_development	D_vs_E10;stage_1W_vs_E10;stage_3W_vs_E10;stage	1	TRUE
ENSGALG00010016672	DCTN4	PRJNA729271	0.474349241	7.76354E-09	0.474349241	stage_E14_vs_E10	ordinary_muscle_development		0	FALSE
ENSGALG00010014469	TMCO3	PRJNA729271	-0.760546828	7.78666E-09	0.760546828	stage_E14_vs_E10	ordinary_muscle_development		0	FALSE
ENSGALG00010001760	RAB5A	PRJNA729271	0.564039833	7.95126E-09	0.564039833	stage_E14_vs_E10	ordinary_muscle_development		0	FALSE
ENSGALG00010012763	SMPDL3B	PRJNA729271	-0.820019068	7.95422E-09	0.820019068	stage_E14_vs_E10	ordinary_muscle_development		0	FALSE
ENSGALG00010022669	MANSC4	PRJNA729271	-6.762522966	7.97464E-09	6.762522966	stage_E14_vs_E10	ordinary_muscle_development	D_vs_E10;stage_1W_vs_E10;stage_3W_vs_E10;stage	1	TRUE
ENSGALG00010027417	WNT2B	PRJNA729271	-2.17703401	8.044E-09	2.17703401	stage_E14_vs_E10	ordinary_muscle_development	D_vs_E10;stage_1W_vs_E10;stage_3W_vs_E10;stage	1	TRUE
ENSGALG00010020138	ENSGALG00010020138	PRJNA729271	0.501098799	8.06206E-09	0.501098799	stage_E14_vs_E10	ordinary_muscle_development		0	FALSE
ENSGALG00010021031	DDX54	PRJNA729271	-0.66594966	8.10724E-09	0.66594966	stage_E14_vs_E10	ordinary_muscle_development		0	FALSE
ENSGALG00010029505	VEZF1	PRJNA729271	0.646735026	8.13176E-09	0.646735026	stage_E14_vs_E10	ordinary_muscle_development		0	FALSE
ENSGALG00010011106	USP40	PRJNA729271	0.680549713	8.13716E-09	0.680549713	stage_E14_vs_E10	ordinary_muscle_development		0	FALSE
ENSGALG00010001219	TFEC	PRJNA729271	2.290991966	8.14197E-09	2.290991966	stage_E14_vs_E10	ordinary_muscle_development	D_vs_E10;stage_1W_vs_E10;stage_3W_vs_E10;stage	1	TRUE
ENSGALG00010025935	CATH3	PRJNA729271	2.221020048	8.14266E-09	2.221020048	stage_E14_vs_E10	ordinary_muscle_development	D_vs_E10;stage_1W_vs_E10;stage_3W_vs_E10;stage	1	TRUE
ENSGALG00010024812	SBNO1	PRJNA729271	0.659601918	8.16611E-09	0.659601918	stage_E14_vs_E10	ordinary_muscle_development		0	FALSE
ENSGALG00010023203	COG4	PRJNA729271	-0.382821816	8.23594E-09	0.382821816	stage_E14_vs_E10	ordinary_muscle_development		0	FALSE
ENSGALG00010006344	HINT3	PRJNA729271	0.620578767	8.30514E-09	0.620578767	stage_E14_vs_E10	ordinary_muscle_development		0	FALSE
ENSGALG00010016047	SRP9	PRJNA729271	-0.536269163	8.33894E-09	0.536269163	stage_E14_vs_E10	ordinary_muscle_development		0	FALSE
ENSGALG00010005757	ARHGAP12	PRJNA729271	0.794088802	8.40609E-09	0.794088802	stage_E14_vs_E10	ordinary_muscle_development		0	FALSE
ENSGALG00010006592	STARD13	PRJNA729271	0.696634992	8.43463E-09	0.696634992	stage_E14_vs_E10	ordinary_muscle_development		0	FALSE
ENSGALG00010021137	PLAU	PRJNA729271	-1.207313647	8.548E-09	1.207313647	stage_E14_vs_E10	ordinary_muscle_development	D_vs_E10;stage_1W_vs_E10;stage_3W_vs_E10;stage	1	TRUE
ENSGALG00010025032	FSTL1	PRJNA729271	-1.067244406	8.55192E-09	1.067244406	stage_E14_vs_E10	ordinary_muscle_development	D_vs_E10;stage_1W_vs_E10;stage_3W_vs_E10;stage	1	TRUE
ENSGALG00010009435	GTDC1	PRJNA729271	-0.535291657	8.57123E-09	0.535291657	stage_E14_vs_E10	ordinary_muscle_development		0	FALSE
ENSGALG00010003323	STMN2	PRJNA729271	-3.093448572	8.57716E-09	3.093448572	stage_E14_vs_E10	ordinary_muscle_development	D_vs_E10;stage_1W_vs_E10;stage_3W_vs_E10;stage	1	TRUE
ENSGALG00010021129	TXNRD3	PRJNA729271	-0.509919483	8.58037E-09	0.509919483	stage_E14_vs_E10	ordinary_muscle_development		0	FALSE
ENSGALG00010009373	ARHGAP15	PRJNA729271	-1.342086768	8.5966E-09	1.342086768	stage_E14_vs_E10	ordinary_muscle_development	D_vs_E10;stage_1W_vs_E10;stage_3W_vs_E10;stage	1	TRUE
ENSGALG00010020390	KLHL25	PRJNA729271	1.320489599	8.66171E-09	1.320489599	stage_E14_vs_E10	ordinary_muscle_development	D_vs_E10;stage_1W_vs_E10;stage_3W_vs_E10;stage	1	TRUE
ENSGALG00010001223	GRM8	PRJNA729271	-4.104416982	8.67046E-09	4.104416982	stage_E14_vs_E10	ordinary_muscle_development	D_vs_E10;stage_1W_vs_E10;stage_3W_vs_E10;stage	1	TRUE
ENSGALG00010024511	ENSGALG00010024511	PRJNA729271	-1.011230643	8.6966E-09	1.011230643	stage_E14_vs_E10	ordinary_muscle_development	D_vs_E10;stage_1W_vs_E10;stage_3W_vs_E10;stage	1	TRUE

ENSGALG00010024930	TMEM237	PRJNA729271	0.35932266	8.77445E-09	0.35932266	stage_E14_vs_E10	ordinary_muscle_development		0	FALSE
ENSGALG00010007724	RBM11	PRJNA729271	1.929743599	8.81031E-09	1.929743599	stage_E14_vs_E10	ordinary_muscle_development	D_vs_E10;stage_1W_vs_E10;stage_3W_vs_E10;stage	1	TRUE
ENSGALG00010027649	FBLN2	PRJNA729271	-1.127399536	8.84815E-09	1.127399536	stage_E14_vs_E10	ordinary_muscle_development	D_vs_E10;stage_1W_vs_E10;stage_3W_vs_E10;stage	1	TRUE
ENSGALG00010009208	SLC5A6	PRJNA729271	-1.20087814	8.87412E-09	1.20087814	stage_E14_vs_E10	ordinary_muscle_development	D_vs_E10;stage_1W_vs_E10;stage_3W_vs_E10;stage	1	TRUE
ENSGALG00010007995	KIAA0319	PRJNA729271	-6.031398743	8.90408E-09	6.031398743	stage_E14_vs_E10	ordinary_muscle_development	D_vs_E10;stage_1W_vs_E10;stage_3W_vs_E10;stage	1	TRUE
ENSGALG00010029725	TSEN54	PRJNA729271	-0.410587165	8.93667E-09	0.410587165	stage_E14_vs_E10	ordinary_muscle_development		0	FALSE
ENSGALG00010013337	RAB19	PRJNA729271	1.484148305	8.96567E-09	1.484148305	stage_E14_vs_E10	ordinary_muscle_development	D_vs_E10;stage_1W_vs_E10;stage_3W_vs_E10;stage	1	TRUE
ENSGALG00010020967	VPS51	PRJNA729271	0.438392739	9.02637E-09	0.438392739	stage_E14_vs_E10	ordinary_muscle_development		0	FALSE
ENSGALG00010027331	SLC16A4	PRJNA729271	0.955360908	9.03017E-09	0.955360908	stage_E14_vs_E10	ordinary_muscle_development		0	FALSE
ENSGALG00010010620	ANKRD54	PRJNA729271	0.64946727	9.03949E-09	0.64946727	stage_E14_vs_E10	ordinary_muscle_development		0	FALSE
ENSGALG00010005474	COL8A1	PRJNA729271	-2.12475531	9.06766E-09	2.12475531	stage_E14_vs_E10	ordinary_muscle_development	D_vs_E10;stage_1W_vs_E10;stage_3W_vs_E10;stage	1	TRUE
ENSGALG00010013179	TBC1D30	PRJNA729271	-1.166122372	9.15018E-09	1.166122372	stage_E14_vs_E10	ordinary_muscle_development	D_vs_E10;stage_1W_vs_E10;stage_3W_vs_E10;stage	1	TRUE
ENSGALG00010029699	ANKRD13B	PRJNA729271	-1.041990919	9.1567E-09	1.041990919	stage_E14_vs_E10	ordinary_muscle_development	D_vs_E10;stage_1W_vs_E10;stage_3W_vs_E10;stage	1	TRUE
ENSGALG00010008637	RGS7	PRJNA729271	-6.041158497	9.17181E-09	6.041158497	stage_E14_vs_E10	ordinary_muscle_development	D_vs_E10;stage_1W_vs_E10;stage_3W_vs_E10;stage	1	TRUE
ENSGALG00010023716	FASTKD1	PRJNA729271	-0.625998128	9.19512E-09	0.625998128	stage_E14_vs_E10	ordinary_muscle_development		0	FALSE
ENSGALG00010021966	ADGRA1	PRJNA729271	0.962627683	9.22463E-09	0.962627683	stage_E14_vs_E10	ordinary_muscle_development		0	FALSE
ENSGALG00010019520	CD6	PRJNA729271	5.684604279	9.28983E-09	5.684604279	stage_E14_vs_E10	ordinary_muscle_development	D_vs_E10;stage_1W_vs_E10;stage_3W_vs_E10;stage	1	TRUE
ENSGALG00010013527	ADARB1	PRJNA729271	0.5871797	9.35105E-09	0.5871797	stage_E14_vs_E10	ordinary_muscle_development		0	FALSE
ENSGALG00010004072	SGK3	PRJNA729271	-0.824559774	9.35645E-09	0.824559774	stage_E14_vs_E10	ordinary_muscle_development		0	FALSE
ENSGALG00010026009	ENSGALG00010026009	PRJNA729271	-1.826539088	9.36054E-09	1.826539088	stage_E14_vs_E10	ordinary_muscle_development	D_vs_E10;stage_1W_vs_E10;stage_3W_vs_E10;stage	1	TRUE
ENSGALG00010002186	CILK1	PRJNA729271	-0.444284793	9.36547E-09	0.444284793	stage_E14_vs_E10	ordinary_muscle_development		0	FALSE
ENSGALG00010004899	GATAD1	PRJNA729271	-0.653315419	9.38656E-09	0.653315419	stage_E14_vs_E10	ordinary_muscle_development		0	FALSE
ENSGALG00010016939	FAM234A	PRJNA729271	0.477728767	9.39868E-09	0.477728767	stage_E14_vs_E10	ordinary_muscle_development		0	FALSE
ENSGALG00010001617	TRIM3	PRJNA729271	-0.553997711	9.5187E-09	0.553997711	stage_E14_vs_E10	ordinary_muscle_development		0	FALSE
ENSGALG00010002443	TYK2	PRJNA729271	0.798659496	9.62632E-09	0.798659496	stage_E14_vs_E10	ordinary_muscle_development		0	FALSE
ENSGALG00010017909	NTHL1	PRJNA729271	-0.838499035	9.63811E-09	0.838499035	stage_E14_vs_E10	ordinary_muscle_development		0	FALSE
ENSGALG00010013391	HMGN1	PRJNA729271	-0.519127195	9.65407E-09	0.519127195	stage_E14_vs_E10	ordinary_muscle_development		0	FALSE
ENSGALG00010024423	SERGEF	PRJNA729271	0.688069923	9.67956E-09	0.688069923	stage_E14_vs_E10	ordinary_muscle_development		0	FALSE
ENSGALG00010015746	PAPOLB	PRJNA729271	-0.429864993	9.68128E-09	0.429864993	stage_E14_vs_E10	ordinary_muscle_development		0	FALSE
ENSGALG00010025972	WNT3	PRJNA729271	-7.781135955	9.69537E-09	7.781135955	stage_E14_vs_E10	ordinary_muscle_development	D_vs_E10;stage_1W_vs_E10;stage_3W_vs_E10;stage	1	TRUE
ENSGALG00010025186	TMEM120B	PRJNA729271	-1.224424849	9.74524E-09	1.224424849	stage_E14_vs_E10	ordinary_muscle_development	D_vs_E10;stage_1W_vs_E10;stage_3W_vs_E10;stage	1	TRUE
ENSGALG00010002383	INPP5F	PRJNA729271	0.727380011	9.81509E-09	0.727380011	stage_E14_vs_E10	ordinary_muscle_development		0	FALSE
ENSGALG00010016868	NIPAL1	PRJNA729271	2.730773058	9.81823E-09	2.730773058	stage_E14_vs_E10	ordinary_muscle_development	D_vs_E10;stage_1W_vs_E10;stage_3W_vs_E10;stage	1	TRUE
ENSGALG00010018147	ISM1	PRJNA729271	1.681283871	9.95454E-09	1.681283871	stage_E14_vs_E10	ordinary_muscle_development	D_vs_E10;stage_1W_vs_E10;stage_3W_vs_E10;stage	1	TRUE
ENSGALG00010017991	RAB26	PRJNA729271	1.456285909	9.95844E-09	1.456285909	stage_E14_vs_E10	ordinary_muscle_development	D_vs_E10;stage_1W_vs_E10;stage_3W_vs_E10;stage	1	TRUE
ENSGALG00010027438	ST7L	PRJNA729271	0.486594105	9.97144E-09	0.486594105	stage_E14_vs_E10	ordinary_muscle_development		0	FALSE
ENSGALG00010003296	ENSGALG00010003296	PRJNA729271	3.154178679	9.99062E-09	3.154178679	stage_E14_vs_E10	ordinary_muscle_development	D_vs_E10;stage_1W_vs_E10;stage_3W_vs_E10;stage	1	TRUE
ENSGALG00010006720	SRP54	PRJNA729271	-0.545700169	1.003E-08	0.545700169	stage_E14_vs_E10	ordinary_muscle_development		0	FALSE
ENSGALG00010025445	B3GNT4	PRJNA729271	1.767628735	1.00337E-08	1.767628735	stage_E14_vs_E10	ordinary_muscle_development	D_vs_E10;stage_1W_vs_E10;stage_3W_vs_E10;stage	1	TRUE
ENSGALG00010001786	P2RY8	PRJNA729271	1.204227153	1.00381E-08	1.204227153	stage_E14_vs_E10	ordinary_muscle_development	D_vs_E10;stage_1W_vs_E10;stage_3W_vs_E10;stage	1	TRUE
ENSGALG00010025430	IRX5	PRJNA729271	-5.8582024	1.00876E-08	5.8582024	stage_E14_vs_E10	ordinary_muscle_development	D_vs_E10;stage_1W_vs_E10;stage_3W_vs_E10;stage	1	TRUE
ENSGALG00010005629	NRP1	PRJNA729271	1.020711279	1.01059E-08	1.020711279	stage_E14_vs_E10	ordinary_muscle_development	D_vs_E10;stage_1W_vs_E10;stage_3W_vs_E10;stage	1	TRUE
ENSGALG00010013099	SIM2	PRJNA729271	1.188063962	1.01174E-08	1.188063962	stage_E14_vs_E10	ordinary_muscle_development	D_vs_E10;stage_1W_vs_E10;stage_3W_vs_E10;stage	1	TRUE
ENSGALG00010022203	DNAAF5	PRJNA729271	-0.810386045	1.02679E-08	0.810386045	stage_E14_vs_E10	ordinary_muscle_development		0	FALSE
ENSGALG00010020754	SLC25A20	PRJNA729271	-0.675494336	1.03578E-08	0.675494336	stage_E14_vs_E10	ordinary_muscle_development		0	FALSE
ENSGALG00010026184	NUDT3	PRJNA729271	0.629743365	1.0551E-08	0.629743365	stage_E14_vs_E10	ordinary_muscle_development		0	FALSE
ENSGALG00010022415	DIP2B	PRJNA729271	-0.642983334	1.06627E-08	0.642983334	stage_E14_vs_E10	ordinary_muscle_development		0	FALSE
ENSGALG00010005756	TET2	PRJNA729271	0.961900931	1.06877E-08	0.961900931	stage_E14_vs_E10	ordinary_muscle_development		0	FALSE
ENSGALG00010027214	PLIN4	PRJNA729271	1.574211365	1.072E-08	1.574211365	stage_E14_vs_E10	ordinary_muscle_development	D_vs_E10;stage_1W_vs_E10;stage_3W_vs_E10;stage	1	TRUE
ENSGALG00010025315	LRRC42	PRJNA729271	-0.719229055	1.07872E-08	0.719229055	stage_E14_vs_E10	ordinary_muscle_development		0	FALSE
ENSGALG00010020113	CIAO1	PRJNA729271	-0.622188663	1.07952E-08	0.622188663	stage_E14_vs_E10	ordinary_muscle_development		0	FALSE
ENSGALG00010025986	RAB3IL1	PRJNA729271	-0.635218493	1.08154E-08	0.635218493	stage_E14_vs_E10	ordinary_muscle_development		0	FALSE
ENSGALG00010023015	METTL2B	PRJNA729271	-0.605968028	1.08261E-08	0.605968028	stage_E14_vs_E10	ordinary_muscle_development		0	FALSE
ENSGALG00010020275	ELOVL3	PRJNA729271	-5.36753054	1.0865E-08	5.36753054	stage_E14_vs_E10	ordinary_muscle_development	D_vs_E10;stage_1W_vs_E10;stage_3W_vs_E10;stage	1	TRUE
ENSGALG00010003126	ENSGALG00010003126	PRJNA729271	-3.208602056	1.08703E-08	3.208602056	stage_E14_vs_E10	ordinary_muscle_development	D_vs_E10;stage_1W_vs_E10;stage_3W_vs_E10;stage	1	TRUE
ENSGALG00010029283	RPS6KB1	PRJNA729271	-0.462628237	1.08728E-08	0.462628237	stage_E14_vs_E10	ordinary_muscle_development		0	FALSE
ENSGALG00010027073	DNAH12	PRJNA729271	-1.918272161	1.08918E-08	1.918272161	stage_E14_vs_E10	ordinary_muscle_development	D_vs_E10;stage_1W_vs_E10;stage_3W_vs_E10;stage	1	TRUE
ENSGALG00010018370	ZBTB8A	PRJNA729271	-0.674782035	1.08986E-08	0.674782035	stage_E14_vs_E10	ordinary_muscle_development		0	FALSE
ENSGALG00010018598	ENSGALG00010018598	PRJNA729271	0.457786461	1.09018E-08	0.457786461	stage_E14_vs_E10	ordinary_muscle_development		0	FALSE
ENSGALG00010002100	BTD	PRJNA729271	0.681415445	1.1082E-08	0.681415445	stage_E14_vs_E10	ordinary_muscle_development		0	FALSE
ENSGALG00010012106	TMTC1	PRJNA729271	-0.817687066	1.11011E-08	0.817687066	stage_E14_vs_E10	ordinary_muscle_development		0	FALSE
ENSGALG00010010386	PRPF40A	PRJNA729271	0.854130505	1.11392E-08	0.854130505	stage_E14_vs_E10	ordinary_muscle_development		0	FALSE
ENSGALG00010022787	ENO4	PRJNA729271	-2.205025613	1.12981E-08	2.205025613	stage_E14_vs_E10	ordinary_muscle_development	D_vs_E10;stage_1W_vs_E10;stage_3W_vs_E10;stage	1	TRUE
ENSGALG00010026320	ENSGALG00010026320	PRJNA729271	1.020025107	1.13737E-08	1.020025107	stage_E14_vs_E10	ordinary_muscle_development	D_vs_E10;stage_1W_vs_E10;stage_3W_vs_E10;stage	1	TRUE
ENSGALG00010002271	ENSGALG00010002271	PRJNA729271	3.903792853	1.13836E-08	3.903792853	stage_E14_vs_E10	ordinary_muscle_development	D_vs_E10;stage_1W_vs_E10;stage_3W_vs_E10;stage	1	TRUE
ENSGALG00010001460	MALSU1	PRJNA729271	-0.639154515	1.13949E-08	0.639154515	stage_E14_vs_E10	ordinary_muscle_development		0	FALSE
ENSGALG00010026114	CWC22	PRJNA729271	-0.489735162	1.14261E-08	0.489735162	stage_E14_vs_E10	ordinary_muscle_development		0	FALSE
ENSGALG00010017918	SIX4	PRJNA729271	1.170365159	1.14514E-08	1.170365159	stage_E14_vs_E10	ordinary_muscle_development	D_vs_E10;stage_1W_vs_E10;stage_3W_vs_E10;stage	1	TRUE
ENSGALG00010019093	RPL32	PRJNA729271	0.885748176	1.14514E-08	0.885748176	stage_E14_vs_E10	ordinary_muscle_development		0	FALSE
ENSGALG00010022684	ENSGALG00010022684	PRJNA729271	-3.153571483	1.14526E-08	3.153571483	stage_E14_vs_E10	ordinary_muscle_development	D_vs_E10;stage_1W_vs_E10;stage_3W_vs_E10;stage	1	TRUE
ENSGALG00010027898	TASOR	PRJNA729271	-0.541894472	1.14821E-08	0.541894472	stage_E14_vs_E10	ordinary_muscle_development		0	FALSE
ENSGALG00010000050	CIB1	PRJNA729271	0.758320177	1.15111E-08	0.758320177	stage_E14_vs_E10	ordinary_muscle_development		0	FALSE
ENSGALG00010027081	PDE4B	PRJNA729271	1.696353346	1.16262E-08	1.696353346	stage_E14_vs_E10	ordinary_muscle_development	D_vs_E10;stage_1W_vs_E10;stage_3W_vs_E10;stage	1	TRUE
ENSGALG00010010535	LMX1A	PRJNA729271	-2.698480892	1.16461E-08	2.698480892	stage_E14_vs_E10	ordinary_muscle_development	D_vs_E10;stage_1W_vs_E10;stage_3W_vs_E10;stage	1	TRUE
ENSGALG00010004515	ENSGALG00010004515	PRJNA729271	-1.565812818	1.16964E-08	1.565812818	stage_E14_vs_E10	ordinary_muscle_development	D_vs_E10;stage_1W_vs_E10;stage_3W_vs_E10;stage	1	TRUE
ENSGALG00010028353	ENSGALG00010028353	PRJNA729271	8.424113945	1.1742E-08	8.424113945	stage_E14_vs_E10	ordinary_muscle_development	D_vs_E10;stage_1W_vs_E10;stage_3W_vs_E10;stage	1	TRUE
ENSGALG00010029386	SLC16A6	PRJNA729271	-1.402942329	1.18901E-08	1.402942329	stage_E14_vs_E10	ordinary_muscle_development	D_vs_E10;stage_1W_vs_E10;stage_3W_vs_E10;stage	1	TRUE
ENSGALG00010022536	SMUG1	PRJNA729271	0.588772446	1.19618E-08	0.588772446	stage_E14_vs_E10	ordinary_muscle_development		0	FALSE
ENSGALG00010005594	MRPL50	PRJNA729271	0.874976349	1.20592E-08	0.874976349	stage_E14_vs_E10	ordinary_muscle_development		0	FALSE
ENSGALG00010021464	ENSGALG00010021464	PRJNA729271	2.349853601	1.21445E-08	2.349853601	stage_E14_vs_E10	ordinary_muscle_development	D_vs_E10;stage_1W_vs_E10;stage_3W_vs_E10;stage	1	TRUE
ENSGALG00010026839	FGGY	PRJNA729271	0.68634165	1.215E-08	0.68634165	stage_E14_vs_E10	ordinary_muscle_development		0	FALSE
ENSGALG00010021769	ENSGALG00010021769	PRJNA729271	-1.079748883	1.22323E-08	1.079748883	stage_E14_vs_E10	ordinary_muscle_development	D_vs_E10;stage_1W_vs_E10;stage_3W_vs_E10;stage	1	TRUE
ENSGALG00010013645	RRAGC	PRJNA729271	-0.731658098	1.22432E-08	0.731658098	stage_E14_vs_E10	ordinary_muscle_development		0	FALSE
ENSGALG00010012574	PHAX	PRJNA729271	-0.616456724	1.22485E-08	0.616456724	stage_E14_vs_E10	ordinary_muscle_development		0	FALSE
ENSGALG00010019695	PGA5	PRJNA729271	-15.21873674	1.25447E-08	15.21873674	stage_E14_vs_E10	ordinary_muscle_development	D_vs_E10;stage_1W_vs_E10;stage_3W_vs_E10;stage	1	TRUE
ENSGALG00010025836	ENSGALG00010025836	PRJNA729271	0.663702065	1.26031E-08	0.663702065	stage_E14_vs_E10	ordinary_muscle_development		0	FALSE
ENSGALG00010011061	KIF13B	PRJNA729271	0.904365799	1.26334E-08	0.904365799	stage_E14_vs_E10	ordinary_muscle_development		0	FALSE
ENSGALG00010026064	ENSGALG00010026064	PRJNA729271	0.861623898	1.27538E-08	0.861623898	stage_E14_vs_E10	ordinary_muscle_development		0	FALSE
ENSGALG00010022328	ENSGALG00010022328	PRJNA729271	-1.70525453	1.28253E-08	1.70525453	stage_E14_vs_E10	ordinary_muscle_development	D_vs_E10;stage_1W_vs_E10;stage_3W_vs_E10;stage	1	TRUE
ENSGALG00010024899	OGFOD2	PRJNA729271	-0.775961574	1.29376E-08	0.775961574	stage_E14_vs_E10	ordinary_muscle_development		0	FALSE
ENSGALG00010027052	APPL1	PRJNA729271	0.491371478	1.29573E-08	0.491371478	stage_E14_vs_E10	ordinary_muscle_development		0	FALSE
ENSGALG00010015680	ENSGALG00010015680	PRJNA729271	-0.611451082	1.29801E-08	0.611451082	stage_E14_vs_E10	ordinary_muscle_development		0	FALSE
ENSGALG00010020292	NPM2	PRJNA729271	-1.497134775	1.30139E-08	1.497134775	stage_E14_vs_E10	ordinary_muscle_development	D_vs_E10;stage_1W_vs_E10;stage_3W_vs_E10;stage	1	TRUE
ENSGALG00010018259	WDR90	PRJNA729271	-0.764409891	1.30252E-08	0.764409891	stage_E14_vs_E10	ordinary_muscle_development		0	FALSE
ENSGALG00010016421	ENSGALG00010016421	PRJNA729271	-5.154886466	1.30547E-08	5.154886466	stage_E14_vs_E10	ordinary_muscle_development	D_vs_E10;stage_1W_vs_E10;stage_3W_vs_E10;stage	1	TRUE
ENSGALG00010021435	GCN1	PRJNA729271	0.518144491	1.30711E-08	0.518144491	stage_E14_vs_E10	ordinary_muscle_development		0	FALSE
ENSGALG00010019613	FRMD6	PRJNA729271	-0.728777932	1.31732E-08	0.728777932	stage_E14_vs_E10	ordinary_muscle_development		0	FALSE
ENSGALG00010016366	KIF3A	PRJNA729271	-0.576175002	1.32027E-08	0.576175002	stage_E14_vs_E10	ordinary_muscle_development		0	FALSE
ENSGALG00010023448	FAM219B	PRJNA729271	0.681049782	1.32446E-08	0.681049782	stage_E14_vs_E10	ordinary_muscle_development		0	FALSE
ENSGALG00010024165	LEPR	PRJNA729271	-1.66367417	1.32662E-08	1.66367417	stage_E14_vs_E10	ordinary_muscle_development	D_vs_E10;stage_1W_vs_E10;stage_3W_vs_E10;stage	1	TRUE
ENSGALG00010019947	ENSGALG00010019947	PRJNA729271	-1.806070921	1.32881E-08	1.806070921	stage_E14_vs_E10	ordinary_muscle_development	D_vs_E10;stage_1W_vs_E10;stage_3W_vs_E10;stage	1	TRUE
ENSGALG00010015177	TC2N	PRJNA729271	0.921469961	1.32897E-08	0.921469961	stage_E14_vs_E10	ordinary_muscle_development		0	FALSE
ENSGALG00010006963	ENSGALG00010006963	PRJNA729271	2.406512378	1.33928E-08	2.406512378	stage_E14_vs_E10	ordinary_muscle_development	D_vs_E10;stage_1W_vs_E10;stage_3W_vs_E10;stage	1	TRUE
ENSGALG00010027237	CTC-575C13.4	PRJNA729271	0.878868024	1.34284E-08	0.878868024	stage_E14_vs_E10	ordinary_muscle_development		0	FALSE
ENSGALG00010021051	GDE1	PRJNA729271	0.783917617	1.34478E-08	0.783917617	stage_E14_vs_E10	ordinary_muscle_development		0	FALSE
ENSGALG00010018847	CGRRF1	PRJNA729271	-0.982763087	1.34765E-08	0.982763087	stage_E14_vs_E10	ordinary_muscle_development		0	FALSE
ENSGALG00010027346	SIN3B	PRJNA729271	0.514951915	1.34885E-08	0.514951915	stage_E14_vs_E10	ordinary_muscle_development		0	FALSE
ENSGALG00010024800	ENSGALG00010024800	PRJNA729271	0.756640211	1.35909E-08	0.756640211	stage_E14_vs_E10	ordinary_muscle_development		0	FALSE
ENSGALG00010027594	ITPR3	PRJNA729271	0.805412422	1.3698E-08	0.805412422	stage_E14_vs_E10	ordinary_muscle_development		0	FALSE
ENSGALG00010029481	ENSGALG00010029481	PRJNA729271	-1.634198997	1.38169E-08	1.634198997	stage_E14_vs_E10	ordinary_muscle_development	D_vs_E10;stage_1W_vs_E10;stage_3W_vs_E10;stage	1	TRUE
ENSGALG00010000569	SIPA1	PRJNA729271	1.303552932	1.38373E-08	1.303552932	stage_E14_vs_E10	ordinary_muscle_development	D_vs_E10;stage_1W_vs_E10;stage_3W_vs_E10;stage	1	TRUE
ENSGALG00010009784	ENSGALG00010009784	PRJNA729271	-1.046371815	1.40239E-08	1.046371815	stage_E14_vs_E10	ordinary_muscle_development	D_vs_E10;stage_1W_vs_E10;stage_3W_vs_E10;stage	1	TRUE
ENSGALG00010016581	H2AFYMACROH2A1	PRJNA729271	-0.459269151	1.40924E-08	0.459269151	stage_E14_vs_E10	ordinary_muscle_development		0	FALSE
ENSGALG00010021114	SBF2	PRJNA729271	-0.574378557	1.41255E-08	0.574378557	stage_E14_vs_E10	ordinary_muscle_development		0	FALSE
ENSGALG00010022382	CALB2	PRJNA729271	-6.372348789	1.42594E-08	6.372348789	stage_E14_vs_E10	ordinary_muscle_development	D_vs_E10;stage_1W_vs_E10;stage_3W_vs_E10;stage	1	TRUE
ENSGALG00010005768	CXXC4	PRJNA729271	-5.52899519	1.42714E-08	5.52899519	stage_E14_vs_E10	ordinary_muscle_development	D_vs_E10;stage_1W_vs_E10;stage_3W_vs_E10;stage	1	TRUE
ENSGALG00010021579	C20H20ORF24	PRJNA729271	-0.662576808	1.42897E-08	0.662576808	stage_E14_vs_E10	ordinary_muscle_development		0	FALSE
ENSGALG00010009501	HGH1	PRJNA729271	0.642647613	1.43139E-08	0.642647613	stage_E14_vs_E10	ordinary_muscle_development		0	FALSE
ENSGALG00010020098	GABRD	PRJNA729271	1.916727304	1.43503E-08	1.916727304	stage_E14_vs_E10	ordinary_muscle_development	D_vs_E10;stage_1W_vs_E10;stage_3W_vs_E10;stage	1	TRUE
ENSGALG00010027199	LAMB3	PRJNA729271	-2.185576829	1.43728E-08	2.185576829	stage_E14_vs_E10	ordinary_muscle_development	D_vs_E10;stage_1W_vs_E10;stage_3W_vs_E10;stage	1	TRUE
ENSGALG00010021949	ENSGALG00010021949	PRJNA729271	-1.448636984	1.44345E-08	1.448636984	stage_E14_vs_E10	ordinary_muscle_development	D_vs_E10;stage_1W_vs_E10;stage_3W_vs_E10;stage	1	TRUE
ENSGALG00010001469	ARHGEF26	PRJNA729271	-0.717757993	1.446E-08	0.717757993	stage_E14_vs_E10	ordinary_muscle_development		0	FALSE
ENSGALG00010027189	ENSGALG00010027189	PRJNA729271	-1.910950736	1.45802E-08	1.910950736	stage_E14_vs_E10	ordinary_muscle_development	D_vs_E10;stage_1W_vs_E10;stage_3W_vs_E10;stage	1	TRUE
ENSGALG00010001262	ENSGALG00010001262	PRJNA729271	0.836631327	1.45884E-08	0.836631327	stage_E14_vs_E10	ordinary_muscle_development		0	FALSE
ENSGALG00010024710	HSDL1	PRJNA729271	-0.753238861	1.46646E-08	0.753238861	stage_E14_vs_E10	ordinary_muscle_development		0	FALSE
ENSGALG00010028065	REEP6	PRJNA729271	4.405716644	1.46911E-08	4.405716644	stage_E14_vs_E10	ordinary_muscle_development	D_vs_E10;stage_1W_vs_E10;stage_3W_vs_E10;stage	1	TRUE
ENSGALG00010011597	TBC1D14	PRJNA729271	-0.758940767	1.47252E-08	0.758940767	stage_E14_vs_E10	ordinary_muscle_development		0	FALSE
ENSGALG00010006619	LIN52	PRJNA729271	-0.60270142	1.48866E-08	0.60270142	stage_E14_vs_E10	ordinary_muscle_development		0	FALSE
ENSGALG00010016869	HSP90AA1	PRJNA729271	-0.916313458	1.49657E-08	0.916313458	stage_E14_vs_E10	ordinary_muscle_development		0	FALSE
ENSGALG00010019266	ATP5S	PRJNA729271	-0.953486489	1.49664E-08	0.953486489	stage_E14_vs_E10	ordinary_muscle_development		0	FALSE
ENSGALG00010020137	NTAN1	PRJNA729271	-0.493886671	1.49725E-08	0.493886671	stage_E14_vs_E10	ordinary_muscle_development		0	FALSE
ENSGALG00010027576	GADD45B	PRJNA729271	2.413897442	1.4981E-08	2.413897442	stage_E14_vs_E10	ordinary_muscle_development	D_vs_E10;stage_1W_vs_E10;stage_3W_vs_E10;stage	1	TRUE
ENSGALG00010024389	AAAS	PRJNA729271	0.616982777	1.51371E-08	0.616982777	stage_E14_vs_E10	ordinary_muscle_development		0	FALSE
ENSGALG00010018174	BMP4	PRJNA729271	1.177007159	1.51612E-08	1.177007159	stage_E14_vs_E10	ordinary_muscle_development	D_vs_E10;stage_1W_vs_E10;stage_3W_vs_E10;stage	1	TRUE
ENSGALG00010024525	GP1BB	PRJNA729271	1.088624022	1.51633E-08	1.088624022	stage_E14_vs_E10	ordinary_muscle_development	D_vs_E10;stage_1W_vs_E10;stage_3W_vs_E10;stage	1	TRUE
ENSGALG00010021980	GLMN	PRJNA729271	-0.806106834	1.52104E-08	0.806106834	stage_E14_vs_E10	ordinary_muscle_development		0	FALSE
ENSGALG00010027876	ANAPC2	PRJNA729271	0.647610494	1.53237E-08	0.647610494	stage_E14_vs_E10	ordinary_muscle_development		0	FALSE
ENSGALG00010024168	BIRC7	PRJNA729271	-2.901877945	1.54227E-08	2.901877945	stage_E14_vs_E10	ordinary_muscle_development	D_vs_E10;stage_1W_vs_E10;stage_3W_vs_E10;stage	1	TRUE
ENSGALG00010018294	FKBP3	PRJNA729271	1.065718203	1.54587E-08	1.065718203	stage_E14_vs_E10	ordinary_muscle_development	D_vs_E10;stage_1W_vs_E10;stage_3W_vs_E10;stage	1	TRUE
ENSGALG00010001358	TCERG1L	PRJNA729271	-3.194102751	1.54859E-08	3.194102751	stage_E14_vs_E10	ordinary_muscle_development	D_vs_E10;stage_1W_vs_E10;stage_3W_vs_E10;stage	1	TRUE
ENSGALG00010015877	REV1	PRJNA729271	0.597636329	1.55971E-08	0.597636329	stage_E14_vs_E10	ordinary_muscle_development		0	FALSE
ENSGALG00010010786	NABP1	PRJNA729271	0.890179038	1.56113E-08	0.890179038	stage_E14_vs_E10	ordinary_muscle_development		0	FALSE
ENSGALG00010029548	BAIAP2	PRJNA729271	1.27194797	1.58053E-08	1.27194797	stage_E14_vs_E10	ordinary_muscle_development	D_vs_E10;stage_1W_vs_E10;stage_3W_vs_E10;stage	1	TRUE
ENSGALG00010015631	GSKIP	PRJNA729271	-0.626116111	1.58111E-08	0.626116111	stage_E14_vs_E10	ordinary_muscle_development		0	FALSE
ENSGALG00010021415	SIRT4	PRJNA729271	0.863106929	1.5829E-08	0.863106929	stage_E14_vs_E10	ordinary_muscle_development		0	FALSE
ENSGALG00010013732	FNTA	PRJNA729271	0.434802778	1.58477E-08	0.434802778	stage_E14_vs_E10	ordinary_muscle_development		0	FALSE
ENSGALG00010029156	ENSGALG00010029156	PRJNA729271	-7.422589715	1.6E-08	7.422589715	stage_E14_vs_E10	ordinary_muscle_development	D_vs_E10;stage_1W_vs_E10;stage_3W_vs_E10;stage	1	TRUE
ENSGALG00010011307	ENSGALG00010011307	PRJNA729271	-2.779610565	1.60266E-08	2.779610565	stage_E14_vs_E10	ordinary_muscle_development	D_vs_E10;stage_1W_vs_E10;stage_3W_vs_E10;stage	1	TRUE
ENSGALG00010021113	SYP	PRJNA729271	-1.822031363	1.60296E-08	1.822031363	stage_E14_vs_E10	ordinary_muscle_development	D_vs_E10;stage_1W_vs_E10;stage_3W_vs_E10;stage	1	TRUE
ENSGALG00010008739	VSTM5	PRJNA729271	-1.656485395	1.61025E-08	1.656485395	stage_E14_vs_E10	ordinary_muscle_development	D_vs_E10;stage_1W_vs_E10;stage_3W_vs_E10;stage	1	TRUE
ENSGALG00010002742	PROSER1	PRJNA729271	0.681638903	1.62202E-08	0.681638903	stage_E14_vs_E10	ordinary_muscle_development		0	FALSE
ENSGALG00010024138	CBL	PRJNA729271	0.859074624	1.62707E-08	0.859074624	stage_E14_vs_E10	ordinary_muscle_development		0	FALSE
ENSGALG00010025869	FASTKD2	PRJNA729271	-0.557545412	1.63779E-08	0.557545412	stage_E14_vs_E10	ordinary_muscle_development		0	FALSE
ENSGALG00010025586	TCTN1	PRJNA729271	0.720512638	1.64529E-08	0.720512638	stage_E14_vs_E10	ordinary_muscle_development		0	FALSE
ENSGALG00010013410	KIAA0825	PRJNA729271	-0.600450999	1.64546E-08	0.600450999	stage_E14_vs_E10	ordinary_muscle_development		0	FALSE
ENSGALG00010003785	ENSGALG00010003785	PRJNA729271	6.022652807	1.64904E-08	6.022652807	stage_E14_vs_E10	ordinary_muscle_development	D_vs_E10;stage_1W_vs_E10;stage_3W_vs_E10;stage	1	TRUE
ENSGALG00010021414	BUD31	PRJNA729271	0.641810004	1.6531E-08	0.641810004	stage_E14_vs_E10	ordinary_muscle_development		0	FALSE
ENSGALG00010028102	CERCAM	PRJNA729271	1.045806016	1.65574E-08	1.045806016	stage_E14_vs_E10	ordinary_muscle_development	D_vs_E10;stage_1W_vs_E10;stage_3W_vs_E10;stage	1	TRUE
ENSGALG00010012593	TUBA8B	PRJNA729271	5.5163596	1.6596E-08	5.5163596	stage_E14_vs_E10	ordinary_muscle_development	D_vs_E10;stage_1W_vs_E10;stage_3W_vs_E10;stage	1	TRUE
ENSGALG00010021624	HSD11B2	PRJNA729271	2.048366175	1.66504E-08	2.048366175	stage_E14_vs_E10	ordinary_muscle_development	D_vs_E10;stage_1W_vs_E10;stage_3W_vs_E10;stage	1	TRUE
ENSGALG00010011107	ENSGALG00010011107	PRJNA729271	1.079877321	1.66757E-08	1.079877321	stage_E14_vs_E10	ordinary_muscle_development	D_vs_E10;stage_1W_vs_E10;stage_3W_vs_E10;stage	1	TRUE
ENSGALG00010012371	ATP6V1G3	PRJNA729271	-4.310553962	1.67576E-08	4.310553962	stage_E14_vs_E10	ordinary_muscle_development	D_vs_E10;stage_1W_vs_E10;stage_3W_vs_E10;stage	1	TRUE
ENSGALG00010022470	ENSGALG00010022470	PRJNA729271	-1.043737844	1.67837E-08	1.043737844	stage_E14_vs_E10	ordinary_muscle_development	D_vs_E10;stage_1W_vs_E10;stage_3W_vs_E10;stage	1	TRUE
ENSGALG00010014567	UTP11	PRJNA729271	-0.691115671	1.67858E-08	0.691115671	stage_E14_vs_E10	ordinary_muscle_development		0	FALSE
ENSGALG00010006261	PCLO	PRJNA729271	-3.564431889	1.6792E-08	3.564431889	stage_E14_vs_E10	ordinary_muscle_development	D_vs_E10;stage_1W_vs_E10;stage_3W_vs_E10;stage	1	TRUE
ENSGALG00010028183	CRTC1	PRJNA729271	0.55395237	1.69989E-08	0.55395237	stage_E14_vs_E10	ordinary_muscle_development		0	FALSE
ENSGALG00010009202	FABP6	PRJNA729271	1.644226747	1.70264E-08	1.644226747	stage_E14_vs_E10	ordinary_muscle_development	D_vs_E10;stage_1W_vs_E10;stage_3W_vs_E10;stage	1	TRUE
ENSGALG00010021164	SLC41A3	PRJNA729271	1.223003378	1.72184E-08	1.223003378	stage_E14_vs_E10	ordinary_muscle_development	D_vs_E10;stage_1W_vs_E10;stage_3W_vs_E10;stage	1	TRUE
ENSGALG00010009326	DLD	PRJNA729271	0.75987509	1.72806E-08	0.75987509	stage_E14_vs_E10	ordinary_muscle_development		0	FALSE
ENSGALG00010005070	ALB	PRJNA729271	8.18471507	1.73908E-08	8.18471507	stage_E14_vs_E10	ordinary_muscle_development	D_vs_E10;stage_1W_vs_E10;stage_3W_vs_E10;stage	1	TRUE
ENSGALG00010018219	SLC24A5	PRJNA729271	-4.480577751	1.73995E-08	4.480577751	stage_E14_vs_E10	ordinary_muscle_development	D_vs_E10;stage_1W_vs_E10;stage_3W_vs_E10;stage	1	TRUE
ENSGALG00010028771	SETX	PRJNA729271	-0.430150825	1.74917E-08	0.430150825	stage_E14_vs_E10	ordinary_muscle_development		0	FALSE
ENSGALG00010011045	ENSGALG00010011045	PRJNA729271	-2.494680272	1.75864E-08	2.494680272	stage_E14_vs_E10	ordinary_muscle_development	D_vs_E10;stage_1W_vs_E10;stage_3W_vs_E10;stage	1	TRUE
ENSGALG00010021510	NTSR1	PRJNA729271	2.23872871	1.7618E-08	2.23872871	stage_E14_vs_E10	ordinary_muscle_development	D_vs_E10;stage_1W_vs_E10;stage_3W_vs_E10;stage	1	TRUE
ENSGALG00010010374	CMTM4	PRJNA729271	-0.669396277	1.76371E-08	0.669396277	stage_E14_vs_E10	ordinary_muscle_development		0	FALSE

ENSGALG00010028426	GPR21	PRJNA729271	-0.921234541	1.78726E-08	0.921234541	stage_E14_vs_E10	ordinary_muscle_development		0	FALSE
ENSGALG00010000085	ENSGALG00010000085	PRJNA729271	-0.895934996	1.7957E-08	0.895934996	stage_E14_vs_E10	ordinary_muscle_development		0	FALSE
ENSGALG00010005668	LNP1	PRJNA729271	-3.187272032	1.80545E-08	3.187272032	stage_E14_vs_E10	ordinary_muscle_development	D_vs_E10;stage_1W_vs_E10;stage_3W_vs_E10;stage	1	TRUE
ENSGALG00010001823	RALBP1	PRJNA729271	-0.445169218	1.8063E-08	0.445169218	stage_E14_vs_E10	ordinary_muscle_development		0	FALSE
ENSGALG00010025057	SCP2	PRJNA729271	-0.393504961	1.80895E-08	0.393504961	stage_E14_vs_E10	ordinary_muscle_development		0	FALSE
ENSGALG00010025312	PLEKHM3	PRJNA729271	0.651816033	1.81383E-08	0.651816033	stage_E14_vs_E10	ordinary_muscle_development		0	FALSE
ENSGALG00010023117	RPUSD2	PRJNA729271	-0.622003289	1.81769E-08	0.622003289	stage_E14_vs_E10	ordinary_muscle_development		0	FALSE
ENSGALG00010004969	YTHDC1	PRJNA729271	0.446483575	1.82997E-08	0.446483575	stage_E14_vs_E10	ordinary_muscle_development		0	FALSE
ENSGALG00010014442	SHISA3	PRJNA729271	-5.746118505	1.83887E-08	5.746118505	stage_E14_vs_E10	ordinary_muscle_development	D_vs_E10;stage_1W_vs_E10;stage_3W_vs_E10;stage	1	TRUE
ENSGALG00010008063	KCNV1	PRJNA729271	-1.90675736	1.84838E-08	1.90675736	stage_E14_vs_E10	ordinary_muscle_development	D_vs_E10;stage_1W_vs_E10;stage_3W_vs_E10;stage	1	TRUE
ENSGALG00010020180	TCF7L1	PRJNA729271	-1.265515593	1.84935E-08	1.265515593	stage_E14_vs_E10	ordinary_muscle_development	D_vs_E10;stage_1W_vs_E10;stage_3W_vs_E10;stage	1	TRUE
ENSGALG00010010853	SMIM20	PRJNA729271	0.838003883	1.87192E-08	0.838003883	stage_E14_vs_E10	ordinary_muscle_development		0	FALSE
ENSGALG00010028609	MRPS21	PRJNA729271	0.83325861	1.87276E-08	0.83325861	stage_E14_vs_E10	ordinary_muscle_development		0	FALSE
ENSGALG00010013758	FAM3B	PRJNA729271	-5.126513535	1.87713E-08	5.126513535	stage_E14_vs_E10	ordinary_muscle_development	D_vs_E10;stage_1W_vs_E10;stage_3W_vs_E10;stage	1	TRUE
ENSGALG00010021204	NAGLU	PRJNA729271	0.54547498	1.88452E-08	0.54547498	stage_E14_vs_E10	ordinary_muscle_development		0	FALSE
ENSGALG00010024560	A4GNT	PRJNA729271	2.385016371	1.89262E-08	2.385016371	stage_E14_vs_E10	ordinary_muscle_development	D_vs_E10;stage_1W_vs_E10;stage_3W_vs_E10;stage	1	TRUE
ENSGALG00010013305	THOC2	PRJNA729271	-0.525770236	1.89939E-08	0.525770236	stage_E14_vs_E10	ordinary_muscle_development		0	FALSE
ENSGALG00010028669	ZBTB34	PRJNA729271	0.639043889	1.90398E-08	0.639043889	stage_E14_vs_E10	ordinary_muscle_development		0	FALSE
ENSGALG00010016319	RBM41	PRJNA729271	1.058488806	1.91905E-08	1.058488806	stage_E14_vs_E10	ordinary_muscle_development	D_vs_E10;stage_1W_vs_E10;stage_3W_vs_E10;stage	1	TRUE
ENSGALG00010024150	NHERF4	PRJNA729271	1.967124115	1.92681E-08	1.967124115	stage_E14_vs_E10	ordinary_muscle_development	D_vs_E10;stage_1W_vs_E10;stage_3W_vs_E10;stage	1	TRUE
ENSGALG00010027872	KDM5B	PRJNA729271	-0.528457634	1.94949E-08	0.528457634	stage_E14_vs_E10	ordinary_muscle_development		0	FALSE
ENSGALG00010023670	EXTL2	PRJNA729271	0.727907431	1.95514E-08	0.727907431	stage_E14_vs_E10	ordinary_muscle_development		0	FALSE
ENSGALG00010021024	ENSGALG00010021024	PRJNA729271	1.063631022	1.95752E-08	1.063631022	stage_E14_vs_E10	ordinary_muscle_development	D_vs_E10;stage_1W_vs_E10;stage_3W_vs_E10;stage	1	TRUE
ENSGALG00010007927	DLL1	PRJNA729271	-1.030406235	1.96296E-08	1.030406235	stage_E14_vs_E10	ordinary_muscle_development	D_vs_E10;stage_1W_vs_E10;stage_3W_vs_E10;stage	1	TRUE
ENSGALG00010018668	OVOL2	PRJNA729271	-6.274099399	1.98345E-08	6.274099399	stage_E14_vs_E10	ordinary_muscle_development	D_vs_E10;stage_1W_vs_E10;stage_3W_vs_E10;stage	1	TRUE
ENSGALG00010005231	ENSGALG00010005231	PRJNA729271	2.412382518	1.98398E-08	2.412382518	stage_E14_vs_E10	ordinary_muscle_development	D_vs_E10;stage_1W_vs_E10;stage_3W_vs_E10;stage	1	TRUE
ENSGALG00010021584	OVCH2	PRJNA729271	2.035117427	1.98635E-08	2.035117427	stage_E14_vs_E10	ordinary_muscle_development	D_vs_E10;stage_1W_vs_E10;stage_3W_vs_E10;stage	1	TRUE
ENSGALG00010000598	ENSGALG00010000598	PRJNA729271	-3.513447403	2.01307E-08	3.513447403	stage_E14_vs_E10	ordinary_muscle_development	D_vs_E10;stage_1W_vs_E10;stage_3W_vs_E10;stage	1	TRUE
ENSGALG00010025345	CCM2	PRJNA729271	-0.519301376	2.05597E-08	0.519301376	stage_E14_vs_E10	ordinary_muscle_development		0	FALSE
ENSGALG00010018717	MEIOB	PRJNA729271	-5.659349565	2.06461E-08	5.659349565	stage_E14_vs_E10	ordinary_muscle_development	D_vs_E10;stage_1W_vs_E10;stage_3W_vs_E10;stage	1	TRUE
ENSGALG00010014592	QNG1	PRJNA729271	-0.541308266	2.07651E-08	0.541308266	stage_E14_vs_E10	ordinary_muscle_development		0	FALSE
ENSGALG00010004872	KIAA0408	PRJNA729271	-1.479217622	2.07928E-08	1.479217622	stage_E14_vs_E10	ordinary_muscle_development	D_vs_E10;stage_1W_vs_E10;stage_3W_vs_E10;stage	1	TRUE
ENSGALG00010009644	SLC27A6	PRJNA729271	-1.418663336	2.08279E-08	1.418663336	stage_E14_vs_E10	ordinary_muscle_development	D_vs_E10;stage_1W_vs_E10;stage_3W_vs_E10;stage	1	TRUE
ENSGALG00010028494	ENSGALG00010028494	PRJNA729271	-1.180448039	2.08879E-08	1.180448039	stage_E14_vs_E10	ordinary_muscle_development	D_vs_E10;stage_1W_vs_E10;stage_3W_vs_E10;stage	1	TRUE
ENSGALG00010021729	RBPJL	PRJNA729271	-4.019782008	2.1003E-08	4.019782008	stage_E14_vs_E10	ordinary_muscle_development	D_vs_E10;stage_1W_vs_E10;stage_3W_vs_E10;stage	1	TRUE
ENSGALG00010000803	MTRR	PRJNA729271	-0.589998278	2.10115E-08	0.589998278	stage_E14_vs_E10	ordinary_muscle_development		0	FALSE
ENSGALG00010008362	RAMP3	PRJNA729271	1.508274548	2.10713E-08	1.508274548	stage_E14_vs_E10	ordinary_muscle_development	D_vs_E10;stage_1W_vs_E10;stage_3W_vs_E10;stage	1	TRUE
ENSGALG00010010084	HK3	PRJNA729271	1.214189437	2.10744E-08	1.214189437	stage_E14_vs_E10	ordinary_muscle_development	D_vs_E10;stage_1W_vs_E10;stage_3W_vs_E10;stage	1	TRUE
ENSGALG00010021492	PARP6	PRJNA729271	0.885512436	2.10964E-08	0.885512436	stage_E14_vs_E10	ordinary_muscle_development		0	FALSE
ENSGALG00010027219	ENSGALG00010027219	PRJNA729271	0.787572718	2.11233E-08	0.787572718	stage_E14_vs_E10	ordinary_muscle_development		0	FALSE
ENSGALG00010005141	CCDC170	PRJNA729271	-1.872475435	2.11977E-08	1.872475435	stage_E14_vs_E10	ordinary_muscle_development	D_vs_E10;stage_1W_vs_E10;stage_3W_vs_E10;stage	1	TRUE
ENSGALG00010001879	IRAK4	PRJNA729271	0.936966206	2.12132E-08	0.936966206	stage_E14_vs_E10	ordinary_muscle_development		0	FALSE
ENSGALG00010005581	GR42L5	PRJNA729271	4.142960594	2.12321E-08	4.142960594	stage_E14_vs_E10	ordinary_muscle_development	D_vs_E10;stage_1W_vs_E10;stage_3W_vs_E10;stage	1	TRUE
ENSGALG00010002935	SYTL5	PRJNA729271	-5.168761551	2.12392E-08	5.168761551	stage_E14_vs_E10	ordinary_muscle_development	D_vs_E10;stage_1W_vs_E10;stage_3W_vs_E10;stage	1	TRUE
ENSGALG00010005468	PPIL4	PRJNA729271	-0.60675174	2.12597E-08	0.60675174	stage_E14_vs_E10	ordinary_muscle_development		0	FALSE
ENSGALG00010028753	SV2A	PRJNA729271	3.512194068	2.12776E-08	3.512194068	stage_E14_vs_E10	ordinary_muscle_development	D_vs_E10;stage_1W_vs_E10;stage_3W_vs_E10;stage	1	TRUE
ENSGALG00010017055	GPR135	PRJNA729271	-2.045162642	2.14108E-08	2.045162642	stage_E14_vs_E10	ordinary_muscle_development	D_vs_E10;stage_1W_vs_E10;stage_3W_vs_E10;stage	1	TRUE
ENSGALG00010012439	TENT2	PRJNA729271	0.57984757	2.14152E-08	0.57984757	stage_E14_vs_E10	ordinary_muscle_development		0	FALSE
ENSGALG00010025904	RPL10A	PRJNA729271	0.804129419	2.16415E-08	0.804129419	stage_E14_vs_E10	ordinary_muscle_development		0	FALSE
ENSGALG00010027193	CAMK1G	PRJNA729271	-4.848676471	2.16476E-08	4.848676471	stage_E14_vs_E10	ordinary_muscle_development	D_vs_E10;stage_1W_vs_E10;stage_3W_vs_E10;stage	1	TRUE
ENSGALG00010012932	ATF6	PRJNA729271	0.677350055	2.17309E-08	0.677350055	stage_E14_vs_E10	ordinary_muscle_development		0	FALSE
ENSGALG00010007980	MARCHF1	PRJNA729271	-1.332373349	2.1819E-08	1.332373349	stage_E14_vs_E10	ordinary_muscle_development	D_vs_E10;stage_1W_vs_E10;stage_3W_vs_E10;stage	1	TRUE
ENSGALG00010002614	GPAT3	PRJNA729271	0.836648226	2.18245E-08	0.836648226	stage_E14_vs_E10	ordinary_muscle_development		0	FALSE
ENSGALG00010029575	ANKFN1	PRJNA729271	-5.231730041	2.22154E-08	5.231730041	stage_E14_vs_E10	ordinary_muscle_development	D_vs_E10;stage_1W_vs_E10;stage_3W_vs_E10;stage	1	TRUE
ENSGALG00010027362	ENSGALG00010027362	PRJNA729271	0.83549853	2.22707E-08	0.83549853	stage_E14_vs_E10	ordinary_muscle_development		0	FALSE
ENSGALG00010022256	PSMC3	PRJNA729271	-0.531799999	2.22815E-08	0.531799999	stage_E14_vs_E10	ordinary_muscle_development		0	FALSE
ENSGALG00010008974	SMCO4	PRJNA729271	-0.605591082	2.23524E-08	0.605591082	stage_E14_vs_E10	ordinary_muscle_development		0	FALSE
ENSGALG00010005170	GPATCH2L	PRJNA729271	0.338865897	2.23581E-08	0.338865897	stage_E14_vs_E10	ordinary_muscle_development		0	FALSE
ENSGALG00010029615	ENSGALG00010029615	PRJNA729271	-2.069612717	2.25166E-08	2.069612717	stage_E14_vs_E10	ordinary_muscle_development	D_vs_E10;stage_1W_vs_E10;stage_3W_vs_E10;stage	1	TRUE
ENSGALG00010011414	PABPC1	PRJNA729271	0.656324178	2.26955E-08	0.656324178	stage_E14_vs_E10	ordinary_muscle_development		0	FALSE
ENSGALG00010014222	TBX22	PRJNA729271	-2.568741489	2.29232E-08	2.568741489	stage_E14_vs_E10	ordinary_muscle_development	D_vs_E10;stage_1W_vs_E10;stage_3W_vs_E10;stage	1	TRUE
ENSGALG00010022529	CCR7	PRJNA729271	-2.336087275	2.29404E-08	2.336087275	stage_E14_vs_E10	ordinary_muscle_development	D_vs_E10;stage_1W_vs_E10;stage_3W_vs_E10;stage	1	TRUE
ENSGALG00010018273	PROM2	PRJNA729271	-6.200539356	2.29444E-08	6.200539356	stage_E14_vs_E10	ordinary_muscle_development	D_vs_E10;stage_1W_vs_E10;stage_3W_vs_E10;stage	1	TRUE
ENSGALG00010020462	CNNM4	PRJNA729271	1.030052406	2.29654E-08	1.030052406	stage_E14_vs_E10	ordinary_muscle_development	D_vs_E10;stage_1W_vs_E10;stage_3W_vs_E10;stage	1	TRUE
ENSGALG00010015902	ENSGALG00010015902	PRJNA729271	2.547270702	2.3017E-08	2.547270702	stage_E14_vs_E10	ordinary_muscle_development	D_vs_E10;stage_1W_vs_E10;stage_3W_vs_E10;stage	1	TRUE
ENSGALG00010029150	NARF	PRJNA729271	-0.71132918	2.30847E-08	0.71132918	stage_E14_vs_E10	ordinary_muscle_development		0	FALSE
ENSGALG00010018521	ENSGALG00010018521	PRJNA729271	0.907165964	2.31465E-08	0.907165964	stage_E14_vs_E10	ordinary_muscle_development		0	FALSE
ENSGALG00010004980	FNDC1	PRJNA729271	2.181429798	2.32316E-08	2.181429798	stage_E14_vs_E10	ordinary_muscle_development	D_vs_E10;stage_1W_vs_E10;stage_3W_vs_E10;stage	1	TRUE
ENSGALG00010006273	ARL13B	PRJNA729271	-0.570205198	2.3252E-08	0.570205198	stage_E14_vs_E10	ordinary_muscle_development		0	FALSE
ENSGALG00010005204	ITGBL1	PRJNA729271	-1.890323937	2.33246E-08	1.890323937	stage_E14_vs_E10	ordinary_muscle_development	D_vs_E10;stage_1W_vs_E10;stage_3W_vs_E10;stage	1	TRUE
ENSGALG00010001416	AP4M1	PRJNA729271	0.526894736	2.34998E-08	0.526894736	stage_E14_vs_E10	ordinary_muscle_development		0	FALSE
ENSGALG00010002274	ENSGALG00010002274	PRJNA729271	4.442758439	2.354E-08	4.442758439	stage_E14_vs_E10	ordinary_muscle_development	D_vs_E10;stage_1W_vs_E10;stage_3W_vs_E10;stage	1	TRUE
ENSGALG00010008136	ELP1	PRJNA729271	-0.658938672	2.36276E-08	0.658938672	stage_E14_vs_E10	ordinary_muscle_development		0	FALSE
ENSGALG00010029763	GEMIN4	PRJNA729271	-0.561357121	2.38034E-08	0.561357121	stage_E14_vs_E10	ordinary_muscle_development		0	FALSE
ENSGALG00010028140	HEMK1	PRJNA729271	-0.635207617	2.38356E-08	0.635207617	stage_E14_vs_E10	ordinary_muscle_development		0	FALSE
ENSGALG00010011846	UBAP1	PRJNA729271	0.598265799	2.39308E-08	0.598265799	stage_E14_vs_E10	ordinary_muscle_development		0	FALSE
ENSGALG00010017673	WDR89	PRJNA729271	-0.524455207	2.40744E-08	0.524455207	stage_E14_vs_E10	ordinary_muscle_development		0	FALSE
ENSGALG00010029929	GDPD1	PRJNA729271	0.536215655	2.40765E-08	0.536215655	stage_E14_vs_E10	ordinary_muscle_development		0	FALSE
ENSGALG00010027936	ENSGALG00010027936	PRJNA729271	-0.502895578	2.42445E-08	0.502895578	stage_E14_vs_E10	ordinary_muscle_development		0	FALSE
ENSGALG00010017651	PPCS	PRJNA729271	-0.7308257	2.44249E-08	0.7308257	stage_E14_vs_E10	ordinary_muscle_development		0	FALSE
ENSGALG00010022524	SOX18	PRJNA729271	1.29998116	2.45256E-08	1.29998116	stage_E14_vs_E10	ordinary_muscle_development	D_vs_E10;stage_1W_vs_E10;stage_3W_vs_E10;stage	1	TRUE
ENSGALG00010025962	ENSGALG00010025962	PRJNA729271	1.055298318	2.4724E-08	1.055298318	stage_E14_vs_E10	ordinary_muscle_development	D_vs_E10;stage_1W_vs_E10;stage_3W_vs_E10;stage	1	TRUE
ENSGALG00010005748	AIG1	PRJNA729271	1.051732333	2.47981E-08	1.051732333	stage_E14_vs_E10	ordinary_muscle_development	D_vs_E10;stage_1W_vs_E10;stage_3W_vs_E10;stage	1	TRUE
ENSGALG00010018145	ENSGALG00010018145	PRJNA729271	-2.15147187	2.48375E-08	2.15147187	stage_E14_vs_E10	ordinary_muscle_development	D_vs_E10;stage_1W_vs_E10;stage_3W_vs_E10;stage	1	TRUE
ENSGALG00010002782	GSTA3	PRJNA729271	-0.850610144	2.48772E-08	0.850610144	stage_E14_vs_E10	ordinary_muscle_development		0	FALSE
ENSGALG00010015466	NMRK1	PRJNA729271	0.708659666	2.49237E-08	0.708659666	stage_E14_vs_E10	ordinary_muscle_development		0	FALSE
ENSGALG00010003317	LYRM2	PRJNA729271	1.020183933	2.5073E-08	1.020183933	stage_E14_vs_E10	ordinary_muscle_development	D_vs_E10;stage_1W_vs_E10;stage_3W_vs_E10;stage	1	TRUE
ENSGALG00010021363	ENSGALG00010021363	PRJNA729271	-0.43603228	2.50865E-08	0.43603228	stage_E14_vs_E10	ordinary_muscle_development		0	FALSE
ENSGALG00010014894	RAB22A	PRJNA729271	-0.466752501	2.51514E-08	0.466752501	stage_E14_vs_E10	ordinary_muscle_development		0	FALSE
ENSGALG00010003439	GMIP	PRJNA729271	-1.39387168	2.51745E-08	1.39387168	stage_E14_vs_E10	ordinary_muscle_development	D_vs_E10;stage_1W_vs_E10;stage_3W_vs_E10;stage	1	TRUE
ENSGALG00010010156	ENSGALG00010010156	PRJNA729271	-0.561570594	2.51783E-08	0.561570594	stage_E14_vs_E10	ordinary_muscle_development		0	FALSE
ENSGALG00010001227	ENSGALG00010001227	PRJNA729271	-4.611055409	2.53637E-08	4.611055409	stage_E14_vs_E10	ordinary_muscle_development	D_vs_E10;stage_1W_vs_E10;stage_3W_vs_E10;stage	1	TRUE
ENSGALG00010007300	CCDC125	PRJNA729271	-1.382380518	2.53851E-08	1.382380518	stage_E14_vs_E10	ordinary_muscle_development	D_vs_E10;stage_1W_vs_E10;stage_3W_vs_E10;stage	1	TRUE
ENSGALG00010002389	COL21A1	PRJNA729271	-1.855556987	2.54136E-08	1.855556987	stage_E14_vs_E10	ordinary_muscle_development	D_vs_E10;stage_1W_vs_E10;stage_3W_vs_E10;stage	1	TRUE
ENSGALG00010012095	RNF38	PRJNA729271	-0.515551479	2.54476E-08	0.515551479	stage_E14_vs_E10	ordinary_muscle_development		0	FALSE
ENSGALG00010015872	TMC1	PRJNA729271	-2.442964446	2.55981E-08	2.442964446	stage_E14_vs_E10	ordinary_muscle_development	D_vs_E10;stage_1W_vs_E10;stage_3W_vs_E10;stage	1	TRUE
ENSGALG00010011935	ST8SIA4	PRJNA729271	-0.726901778	2.56474E-08	0.726901778	stage_E14_vs_E10	ordinary_muscle_development		0	FALSE
ENSGALG00010022708	SPRED1	PRJNA729271	-0.747757216	2.56745E-08	0.747757216	stage_E14_vs_E10	ordinary_muscle_development		0	FALSE
ENSGALG00010019846	ENSGALG00010019846	PRJNA729271	0.407170737	2.57912E-08	0.407170737	stage_E14_vs_E10	ordinary_muscle_development		0	FALSE
ENSGALG00010024151	LEPROT	PRJNA729271	-0.845636718	2.58027E-08	0.845636718	stage_E14_vs_E10	ordinary_muscle_development		0	FALSE
ENSGALG00010018987	MRPS10	PRJNA729271	0.607230167	2.58772E-08	0.607230167	stage_E14_vs_E10	ordinary_muscle_development		0	FALSE
ENSGALG00010018198	BTBD3	PRJNA729271	-0.58744558	2.61156E-08	0.58744558	stage_E14_vs_E10	ordinary_muscle_development		0	FALSE
ENSGALG00010023381	HOXB7	PRJNA729271	-0.971315045	2.62416E-08	0.971315045	stage_E14_vs_E10	ordinary_muscle_development		0	FALSE
ENSGALG00010010457	AGGF1	PRJNA729271	-0.523147185	2.66034E-08	0.523147185	stage_E14_vs_E10	ordinary_muscle_development		0	FALSE
ENSGALG00010024029	SCAPER	PRJNA729271	0.843192164	2.66356E-08	0.843192164	stage_E14_vs_E10	ordinary_muscle_development		0	FALSE
ENSGALG00010004101	ARID1B	PRJNA729271	0.945450847	2.66524E-08	0.945450847	stage_E14_vs_E10	ordinary_muscle_development		0	FALSE
ENSGALG00010018359	CACNG3	PRJNA729271	-4.680711572	2.668E-08	4.680711572	stage_E14_vs_E10	ordinary_muscle_development	D_vs_E10;stage_1W_vs_E10;stage_3W_vs_E10;stage	1	TRUE
ENSGALG00010016057	SOHO1	PRJNA729271	-4.531914145	2.68662E-08	4.531914145	stage_E14_vs_E10	ordinary_muscle_development	D_vs_E10;stage_1W_vs_E10;stage_3W_vs_E10;stage	1	TRUE
ENSGALG00010027561	ADAMTS4	PRJNA729271	1.895316759	2.6928E-08	1.895316759	stage_E14_vs_E10	ordinary_muscle_development	D_vs_E10;stage_1W_vs_E10;stage_3W_vs_E10;stage	1	TRUE
ENSGALG00010003634	CYP4F11	PRJNA729271	1.703212774	2.69802E-08	1.703212774	stage_E14_vs_E10	ordinary_muscle_development	D_vs_E10;stage_1W_vs_E10;stage_3W_vs_E10;stage	1	TRUE
ENSGALG00010017307	TMEM186	PRJNA729271	0.891380051	2.6983E-08	0.891380051	stage_E14_vs_E10	ordinary_muscle_development		0	FALSE
ENSGALG00010007532	DCUN1D5	PRJNA729271	1.512281935	2.70904E-08	1.512281935	stage_E14_vs_E10	ordinary_muscle_development	D_vs_E10;stage_1W_vs_E10;stage_3W_vs_E10;stage	1	TRUE
ENSGALG00010008127	TBC1D2	PRJNA729271	-0.786905081	2.72069E-08	0.786905081	stage_E14_vs_E10	ordinary_muscle_development		0	FALSE
ENSGALG00010021241	TMEM43	PRJNA729271	0.984755653	2.72252E-08	0.984755653	stage_E14_vs_E10	ordinary_muscle_development		0	FALSE
ENSGALG00010015887	SLC9A9	PRJNA729271	1.036501656	2.72626E-08	1.036501656	stage_E14_vs_E10	ordinary_muscle_development	D_vs_E10;stage_1W_vs_E10;stage_3W_vs_E10;stage	1	TRUE
ENSGALG00010008922	ENSGALG00010008922	PRJNA729271	6.517123489	2.73844E-08	6.517123489	stage_E14_vs_E10	ordinary_muscle_development	D_vs_E10;stage_1W_vs_E10;stage_3W_vs_E10;stage	1	TRUE
ENSGALG00010010577	PBX1	PRJNA729271	-0.860236291	2.75526E-08	0.860236291	stage_E14_vs_E10	ordinary_muscle_development		0	FALSE
ENSGALG00010010765	ENSGALG00010010765	PRJNA729271	4.381103589	2.76806E-08	4.381103589	stage_E14_vs_E10	ordinary_muscle_development	D_vs_E10;stage_1W_vs_E10;stage_3W_vs_E10;stage	1	TRUE
ENSGALG00010013678	ATP7A	PRJNA729271	0.578496759	2.77264E-08	0.578496759	stage_E14_vs_E10	ordinary_muscle_development		0	FALSE
ENSGALG00010020041	CDH23	PRJNA729271	0.894824973	2.77863E-08	0.894824973	stage_E14_vs_E10	ordinary_muscle_development		0	FALSE
ENSGALG00010026218	FTSJ3	PRJNA729271	-0.969837955	2.781E-08	0.969837955	stage_E14_vs_E10	ordinary_muscle_development		0	FALSE
ENSGALG00010001696	FAM135A	PRJNA729271	0.514155586	2.78331E-08	0.514155586	stage_E14_vs_E10	ordinary_muscle_development		0	FALSE
ENSGALG00010026268	GARS1	PRJNA729271	-0.870547955	2.78458E-08	0.870547955	stage_E14_vs_E10	ordinary_muscle_development		0	FALSE
ENSGALG00010018160	ENSGALG00010018160	PRJNA729271	0.936765708	2.8417E-08	0.936765708	stage_E14_vs_E10	ordinary_muscle_development		0	FALSE
ENSGALG00010027109	C19orf44	PRJNA729271	0.693861905	2.86209E-08	0.693861905	stage_E14_vs_E10	ordinary_muscle_development		0	FALSE
ENSGALG00010007382	EIF2AK2	PRJNA729271	1.456373453	2.87789E-08	1.456373453	stage_E14_vs_E10	ordinary_muscle_development	D_vs_E10;stage_1W_vs_E10;stage_3W_vs_E10;stage	1	TRUE
ENSGALG00010001468	ANOS1	PRJNA729271	-0.898692573	2.87805E-08	0.898692573	stage_E14_vs_E10	ordinary_muscle_development		0	FALSE
ENSGALG00010024656	HYLS1	PRJNA729271	-1.069550613	2.88101E-08	1.069550613	stage_E14_vs_E10	ordinary_muscle_development	D_vs_E10;stage_1W_vs_E10;stage_3W_vs_E10;stage	1	TRUE
ENSGALG00010013284	MANEAL	PRJNA729271	-1.93275966	2.88403E-08	1.93275966	stage_E14_vs_E10	ordinary_muscle_development	D_vs_E10;stage_1W_vs_E10;stage_3W_vs_E10;stage	1	TRUE
ENSGALG00010021115	ALPL	PRJNA729271	-1.41551211	2.89617E-08	1.41551211	stage_E14_vs_E10	ordinary_muscle_development	D_vs_E10;stage_1W_vs_E10;stage_3W_vs_E10;stage	1	TRUE
ENSGALG00010001372	ENSGALG00010001372	PRJNA729271	1.589156077	2.89873E-08	1.589156077	stage_E14_vs_E10	ordinary_muscle_development	D_vs_E10;stage_1W_vs_E10;stage_3W_vs_E10;stage	1	TRUE
ENSGALG00010009150	ENSGALG00010009150	PRJNA729271	2.198581137	2.93832E-08	2.198581137	stage_E14_vs_E10	ordinary_muscle_development	D_vs_E10;stage_1W_vs_E10;stage_3W_vs_E10;stage	1	TRUE
ENSGALG00010010106	C7orf73	PRJNA729271	-1.177200879	2.94718E-08	1.177200879	stage_E14_vs_E10	ordinary_muscle_development	D_vs_E10;stage_1W_vs_E10;stage_3W_vs_E10;stage	1	TRUE
ENSGALG00010028912	EXFABP	PRJNA729271	2.818606115	2.9487E-08	2.818606115	stage_E14_vs_E10	ordinary_muscle_development	D_vs_E10;stage_1W_vs_E10;stage_3W_vs_E10;stage	1	TRUE
ENSGALG00010028239	EDMPN2	PRJNA729271	8.375952362	2.96819E-08	8.375952362	stage_E14_vs_E10	ordinary_muscle_development	D_vs_E10;stage_1W_vs_E10;stage_3W_vs_E10;stage	1	TRUE
ENSGALG00010013283	PAWR	PRJNA729271	-1.635981001	2.97104E-08	1.635981001	stage_E14_vs_E10	ordinary_muscle_development	D_vs_E10;stage_1W_vs_E10;stage_3W_vs_E10;stage	1	TRUE
ENSGALG00010002370	ENSGALG00010002370	PRJNA729271	0.851683962	2.98611E-08	0.851683962	stage_E14_vs_E10	ordinary_muscle_development		0	FALSE
ENSGALG00010016025	PCIF1	PRJNA729271	0.610729398	3.00379E-08	0.610729398	stage_E14_vs_E10	ordinary_muscle_development		0	FALSE
ENSGALG00010005060	POMP	PRJNA729271	0.783109471	3.0531E-08	0.783109471	stage_E14_vs_E10	ordinary_muscle_development		0	FALSE
ENSGALG00010000340	NAA10	PRJNA729271	-0.778396041	3.05694E-08	0.778396041	stage_E14_vs_E10	ordinary_muscle_development		0	FALSE
ENSGALG00010010799	DARS2	PRJNA729271	-0.610401779	3.08521E-08	0.610401779	stage_E14_vs_E10	ordinary_muscle_development		0	FALSE
ENSGALG00010009284	CCN3	PRJNA729271	-3.866723714	3.10214E-08	3.866723714	stage_E14_vs_E10	ordinary_muscle_development	D_vs_E10;stage_1W_vs_E10;stage_3W_vs_E10;stage	1	TRUE
ENSGALG00010007811	PRCP	PRJNA729271	1.044806506	3.1367E-08	1.044806506	stage_E14_vs_E10	ordinary_muscle_development	D_vs_E10;stage_1W_vs_E10;stage_3W_vs_E10;stage	1	TRUE
ENSGALG00010009786	DIPK2B	PRJNA729271	0.901242381	3.1394E-08	0.901242381	stage_E14_vs_E10	ordinary_muscle_development		0	FALSE
ENSGALG00010004245	ARFGEF3	PRJNA729271	-1.29739663	3.17487E-08	1.29739663	stage_E14_vs_E10	ordinary_muscle_development	D_vs_E10;stage_1W_vs_E10;stage_3W_vs_E10;stage	1	TRUE
ENSGALG00010024421	DAPK2	PRJNA729271	1.529359418	3.20751E-08	1.529359418	stage_E14_vs_E10	ordinary_muscle_development	D_vs_E10;stage_1W_vs_E10;stage_3W_vs_E10;stage	1	TRUE
ENSGALG00010013619	IL1R2	PRJNA729271	1.108666375	3.22328E-08	1.108666375	stage_E14_vs_E10	ordinary_muscle_development	D_vs_E10;stage_1W_vs_E10;stage_3W_vs_E10;stage	1	TRUE
ENSGALG00010018025	CPTP	PRJNA729271	-0.779883692	3.22803E-08	0.779883692	stage_E14_vs_E10	ordinary_muscle_development		0	FALSE
ENSGALG00010023218	SSH3	PRJNA729271	0.74128304	3.23478E-08	0.74128304	stage_E14_vs_E10	ordinary_muscle_development		0	FALSE
ENSGALG00010014818	ENSGALG00010014818	PRJNA729271	2.195151013	3.25568E-08	2.195151013	stage_E14_vs_E10	ordinary_muscle_development	D_vs_E10;stage_1W_vs_E10;stage_3W_vs_E10;stage	1	TRUE
ENSGALG00010008141	APTX	PRJNA729271	-0.912635261	3.25874E-08	0.912635261	stage_E14_vs_E10	ordinary_muscle_development		0	FALSE
ENSGALG00010013484	TCOF1	PRJNA729271	-0.578482833	3.26374E-08	0.578482833	stage_E14_vs_E10	ordinary_muscle_development		0	FALSE
ENSGALG00010016397	LIAS	PRJNA729271	0.585662159	3.28177E-08	0.585662159	stage_E14_vs_E10	ordinary_muscle_development		0	FALSE
ENSGALG00010004211	RNF146	PRJNA729271	0.422916847	3.29855E-08	0.422916847	stage_E14_vs_E10	ordinary_muscle_development		0	FALSE
ENSGALG00010005379	DZIP1	PRJNA729271	-0.958895958	3.33511E-08	0.958895958	stage_E14_vs_E10	ordinary_muscle_development		0	FALSE
ENSGALG00010024762	FAN1	PRJNA729271	-0.525557824	3.37023E-08	0.525557824	stage_E14_vs_E10	ordinary_muscle_development		0	FALSE
ENSGALG00010012900	RBX1	PRJNA729271	0.970546894	3.37615E-08	0.970546894	stage_E14_vs_E10	ordinary_muscle_development		0	FALSE
ENSGALG00010016922	ENSGALG00010016922	PRJNA729271	1.079896197	3.39338E-08	1.079896197	stage_E14_vs_E10	ordinary_muscle_development	D_vs_E10;stage_1W_vs_E10;stage_3W_vs_E10;stage	1	TRUE
ENSGALG00010010124	ENSGALG00010010124	PRJNA729271	0.827416415	3.42632E-08	0.827416415	stage_E14_vs_E10	ordinary_muscle_development		0	FALSE
ENSGALG00010027745	BICD2	PRJNA729271	0.811272447	3.4308E-08	0.811272447	stage_E14_vs_E10	ordinary_muscle_development		0	FALSE
ENSGALG00010027074	BRPF3	PRJNA729271	0.449406515	3.43593E-08	0.449406515	stage_E14_vs_E10	ordinary_muscle_development		0	FALSE
ENSGALG00010027895	LIX1L	PRJNA729271	1.004346399	3.44893E-08	1.004346399	stage_E14_vs_E10	ordinary_muscle_development	D_vs_E10;stage_1W_vs_E10;stage_3W_vs_E10;stage	1	TRUE
ENSGALG00010002923	SERPINH1	PRJNA729271	-0.895694466	3.45318E-08	0.895694466	stage_E14_vs_E10	ordinary_muscle_development		0	FALSE
ENSGALG00010013345	DCN	PRJNA729271	1.220734381	3.45894E-08	1.220734381	stage_E14_vs_E10	ordinary_muscle_development	D_vs_E10;stage_1W_vs_E10;stage_3W_vs_E10;stage	1	TRUE
ENSGALG00010002512	DAXX	PRJNA729271	1.288362244	3.46699E-08	1.288362244	stage_E14_vs_E10	ordinary_muscle_development	D_vs_E10;stage_1W_vs_E10;stage_3W_vs_E10;stage	1	TRUE
ENSGALG00010022196	PLXNA1	PRJNA729271	-0.615604925	3.47667E-08	0.615604925	stage_E14_vs_E10	ordinary_muscle_development		0	FALSE
ENSGALG00010015107	MB	PRJNA729271	-3.507689051	3.47753E-08	3.507689051	stage_E14_vs_E10	ordinary_muscle_development	D_vs_E10;stage_1W_vs_E10;stage_3W_vs_E10;stage	1	TRUE
ENSGALG00010024541	WIPF2	PRJNA729271	1.135592703	3.48455E-08	1.135592703	stage_E14_vs_E10	ordinary_muscle_development	D_vs_E10;stage_1W_vs_E10;stage_3W_vs_E10;stage	1	TRUE
ENSGALG00010023405	DNAJA2	PRJNA729271	-0.442201524	3.49024E-08	0.442201524	stage_E14_vs_E10	ordinary_muscle_development		0	FALSE
ENSGALG00010016885	CDCA4	PRJNA729271	-0.86834691	3.4977E-08	0.86834691	stage_E14_vs_E10	ordinary_muscle_development		0	FALSE
ENSGALG00010011937	SLC1A1	PRJNA729271	-1.897847498	3.50229E-08	1.897847498	stage_E14_vs_E10	ordinary_muscle_development	D_vs_E10;stage_1W_vs_E10;stage_3W_vs_E10;stage	1	TRUE
ENSGALG00010028042	DPP7	PRJNA729271	1.078945667	3.50248E-08	1.078945667	stage_E14_vs_E10	ordinary_muscle_development	D_vs_E10;stage_1W_vs_E10;stage_3W_vs_E10;stage	1	TRUE
ENSGALG00010028321	RHBG	PRJNA729271	1.047718729	3.51013E-08	1.047718729	stage_E14_vs_E10	ordinary_muscle_development	D_vs_E10;stage_1W_vs_E10;stage_3W_vs_E10;stage	1	TRUE
ENSGALG00010018764	TMEM177	PRJNA729271	-0.532540074	3.54288E-08	0.532540074	stage_E14_vs_E10	ordinary_muscle_development		0	FALSE
ENSGALG00010011044	MBL2	PRJNA729271	3.43582	3.55558E-08	3.43582	stage_E14_vs_E10	ordinary_muscle_development	D_vs_E10;stage_1W_vs_E10;stage_3W_vs_E10;stage	1	TRUE

ENSGALG00010001265	OPN1LW	PRJNA729271	3.133762592	3.56E-08	3.133762592	stage_E14_vs_E10	ordinary_muscle_development	D_vs_E10;stage_1W_vs_E10;stage_3W_vs_E10;stage	1	TRUE
ENSGALG00010001914	ENSGALG00010001914	PRJNA729271	-0.8900132	3.61941E-08	0.8900132	stage_E14_vs_E10	ordinary_muscle_development		0	FALSE
ENSGALG00010004285	SLC18B1	PRJNA729271	-0.705935255	3.66021E-08	0.705935255	stage_E14_vs_E10	ordinary_muscle_development		0	FALSE
ENSGALG00010027276	ENSGALG00010027276	PRJNA729271	0.779520124	3.6859E-08	0.779520124	stage_E14_vs_E10	ordinary_muscle_development		0	FALSE
ENSGALG00010012975	KRR1	PRJNA729271	-0.529920299	3.68613E-08	0.529920299	stage_E14_vs_E10	ordinary_muscle_development		0	FALSE
ENSGALG00010025236	ENSGALG00010025236	PRJNA729271	1.392715518	3.69174E-08	1.392715518	stage_E14_vs_E10	ordinary_muscle_development	D_vs_E10;stage_1W_vs_E10;stage_3W_vs_E10;stage	1	TRUE
ENSGALG00010022257	KCNK18	PRJNA729271	4.770213161	3.70742E-08	4.770213161	stage_E14_vs_E10	ordinary_muscle_development	D_vs_E10;stage_1W_vs_E10;stage_3W_vs_E10;stage	1	TRUE
ENSGALG00010021760	CSTA	PRJNA729271	2.211741791	3.71199E-08	2.211741791	stage_E14_vs_E10	ordinary_muscle_development	D_vs_E10;stage_1W_vs_E10;stage_3W_vs_E10;stage	1	TRUE
ENSGALG00010013954	LPL	PRJNA729271	1.883342273	3.72821E-08	1.883342273	stage_E14_vs_E10	ordinary_muscle_development	D_vs_E10;stage_1W_vs_E10;stage_3W_vs_E10;stage	1	TRUE
ENSGALG00010015669	INPP4A	PRJNA729271	0.733973237	3.7436E-08	0.733973237	stage_E14_vs_E10	ordinary_muscle_development		0	FALSE
ENSGALG00010013899	WNT5B	PRJNA729271	-0.831208007	3.77807E-08	0.831208007	stage_E14_vs_E10	ordinary_muscle_development		0	FALSE
ENSGALG00010027204	OMD	PRJNA729271	-5.512762756	3.84241E-08	5.512762756	stage_E14_vs_E10	ordinary_muscle_development	D_vs_E10;stage_1W_vs_E10;stage_3W_vs_E10;stage	1	TRUE
ENSGALG00010027736	ENSGALG00010027736	PRJNA729271	8.398668113	3.85501E-08	8.398668113	stage_E14_vs_E10	ordinary_muscle_development	D_vs_E10;stage_1W_vs_E10;stage_3W_vs_E10;stage	1	TRUE
ENSGALG00010019285	BATF3	PRJNA729271	-1.660303865	3.85939E-08	1.660303865	stage_E14_vs_E10	ordinary_muscle_development	D_vs_E10;stage_1W_vs_E10;stage_3W_vs_E10;stage	1	TRUE
ENSGALG00010021601	LPAR5	PRJNA729271	1.965496568	3.87191E-08	1.965496568	stage_E14_vs_E10	ordinary_muscle_development	D_vs_E10;stage_1W_vs_E10;stage_3W_vs_E10;stage	1	TRUE
ENSGALG00010016926	ENSGALG00010016926	PRJNA729271	1.026173492	3.8776E-08	1.026173492	stage_E14_vs_E10	ordinary_muscle_development	D_vs_E10;stage_1W_vs_E10;stage_3W_vs_E10;stage	1	TRUE
ENSGALG00010010929	FMC1	PRJNA729271	0.840475303	3.90408E-08	0.840475303	stage_E14_vs_E10	ordinary_muscle_development		0	FALSE
ENSGALG00010008360	ZC3H3	PRJNA729271	0.347339104	3.90966E-08	0.347339104	stage_E14_vs_E10	ordinary_muscle_development		0	FALSE
ENSGALG00010013405	RPS14	PRJNA729271	0.997385604	3.91288E-08	0.997385604	stage_E14_vs_E10	ordinary_muscle_development		0	FALSE
ENSGALG00010024336	TSPAN4	PRJNA729271	0.679463927	3.94024E-08	0.679463927	stage_E14_vs_E10	ordinary_muscle_development		0	FALSE
ENSGALG00010024838	EN2	PRJNA729271	-5.482919877	3.94846E-08	5.482919877	stage_E14_vs_E10	ordinary_muscle_development	D_vs_E10;stage_1W_vs_E10;stage_3W_vs_E10;stage	1	TRUE
ENSGALG00010009705	DTNBP1	PRJNA729271	-0.405979271	3.95031E-08	0.405979271	stage_E14_vs_E10	ordinary_muscle_development		0	FALSE
ENSGALG00010002436	ENSGALG00010002436	PRJNA729271	4.912865272	3.95983E-08	4.912865272	stage_E14_vs_E10	ordinary_muscle_development	D_vs_E10;stage_1W_vs_E10;stage_3W_vs_E10;stage	1	TRUE
ENSGALG00010006229	INTS12	PRJNA729271	-0.782212929	3.99428E-08	0.782212929	stage_E14_vs_E10	ordinary_muscle_development		0	FALSE
ENSGALG00010010774	KPTN	PRJNA729271	-0.780120989	4.03117E-08	0.780120989	stage_E14_vs_E10	ordinary_muscle_development		0	FALSE
ENSGALG00010021345	NEIL1	PRJNA729271	0.845440612	4.0504E-08	0.845440612	stage_E14_vs_E10	ordinary_muscle_development		0	FALSE
ENSGALG00010006236	C3orf38	PRJNA729271	0.471722901	4.06128E-08	0.471722901	stage_E14_vs_E10	ordinary_muscle_development		0	FALSE
ENSGALG00010006038	ENSGALG00010006038	PRJNA729271	1.07824689	4.06451E-08	1.07824689	stage_E14_vs_E10	ordinary_muscle_development	D_vs_E10;stage_1W_vs_E10;stage_3W_vs_E10;stage	1	TRUE
ENSGALG00010027986	CGN	PRJNA729271	-1.757084567	4.0684E-08	1.757084567	stage_E14_vs_E10	ordinary_muscle_development	D_vs_E10;stage_1W_vs_E10;stage_3W_vs_E10;stage	1	TRUE
ENSGALG00010011318	PRP4K	PRJNA729271	-0.552040585	4.0721E-08	0.552040585	stage_E14_vs_E10	ordinary_muscle_development		0	FALSE
ENSGALG00010025434	SYT6	PRJNA729271	-1.386953845	4.07568E-08	1.386953845	stage_E14_vs_E10	ordinary_muscle_development	D_vs_E10;stage_1W_vs_E10;stage_3W_vs_E10;stage	1	TRUE
ENSGALG00010009791	GABRG2	PRJNA729271	-2.095537023	4.07702E-08	2.095537023	stage_E14_vs_E10	ordinary_muscle_development	D_vs_E10;stage_1W_vs_E10;stage_3W_vs_E10;stage	1	TRUE
ENSGALG00010022325	MIGA1	PRJNA729271	-0.651099001	4.09236E-08	0.651099001	stage_E14_vs_E10	ordinary_muscle_development		0	FALSE
ENSGALG00010012690	NT5DC1	PRJNA729271	1.881707558	4.10465E-08	1.881707558	stage_E14_vs_E10	ordinary_muscle_development	D_vs_E10;stage_1W_vs_E10;stage_3W_vs_E10;stage	1	TRUE
ENSGALG00010006411	RGS17	PRJNA729271	-2.676270825	4.16266E-08	2.676270825	stage_E14_vs_E10	ordinary_muscle_development	D_vs_E10;stage_1W_vs_E10;stage_3W_vs_E10;stage	1	TRUE
ENSGALG00010012263	SHLD3	PRJNA729271	0.708007018	4.16351E-08	0.708007018	stage_E14_vs_E10	ordinary_muscle_development		0	FALSE
ENSGALG00010013071	ENSGALG00010013071	PRJNA729271	-1.268574229	4.1637E-08	1.268574229	stage_E14_vs_E10	ordinary_muscle_development	D_vs_E10;stage_1W_vs_E10;stage_3W_vs_E10;stage	1	TRUE
ENSGALG00010010422	CDH1	PRJNA729271	-5.304479345	4.17902E-08	5.304479345	stage_E14_vs_E10	ordinary_muscle_development	D_vs_E10;stage_1W_vs_E10;stage_3W_vs_E10;stage	1	TRUE
ENSGALG00010002388	ENSGALG00010002388	PRJNA729271	4.087543303	4.18807E-08	4.087543303	stage_E14_vs_E10	ordinary_muscle_development	D_vs_E10;stage_1W_vs_E10;stage_3W_vs_E10;stage	1	TRUE
ENSGALG00010003077	EIF2S3	PRJNA729271	0.458803495	4.1904E-08	0.458803495	stage_E14_vs_E10	ordinary_muscle_development		0	FALSE
ENSGALG00010008456	NHLRC1	PRJNA729271	0.714430872	4.19291E-08	0.714430872	stage_E14_vs_E10	ordinary_muscle_development		0	FALSE
ENSGALG00010001432	NSMCE4A	PRJNA729271	0.632563269	4.19918E-08	0.632563269	stage_E14_vs_E10	ordinary_muscle_development		0	FALSE
ENSGALG00010024185	CHRNA5	PRJNA729271	0.862989564	4.20606E-08	0.862989564	stage_E14_vs_E10	ordinary_muscle_development		0	FALSE
ENSGALG00010013078	COL5A2	PRJNA729271	-1.358003976	4.20846E-08	1.358003976	stage_E14_vs_E10	ordinary_muscle_development	D_vs_E10;stage_1W_vs_E10;stage_3W_vs_E10;stage	1	TRUE
ENSGALG00010012470	CHD1	PRJNA729271	-0.604435614	4.22458E-08	0.604435614	stage_E14_vs_E10	ordinary_muscle_development		0	FALSE
ENSGALG00010016355	MCF2L2	PRJNA729271	1.279591888	4.24172E-08	1.279591888	stage_E14_vs_E10	ordinary_muscle_development	D_vs_E10;stage_1W_vs_E10;stage_3W_vs_E10;stage	1	TRUE
ENSGALG00010013514	LMBRD2	PRJNA729271	0.759668541	4.27301E-08	0.759668541	stage_E14_vs_E10	ordinary_muscle_development		0	FALSE
ENSGALG00010018349	KCNMB2	PRJNA729271	1.075275096	4.28298E-08	1.075275096	stage_E14_vs_E10	ordinary_muscle_development	D_vs_E10;stage_1W_vs_E10;stage_3W_vs_E10;stage	1	TRUE
ENSGALG00010000126	ENSGALG00010000126	PRJNA729271	1.120152892	4.28704E-08	1.120152892	stage_E14_vs_E10	ordinary_muscle_development	D_vs_E10;stage_1W_vs_E10;stage_3W_vs_E10;stage	1	TRUE
ENSGALG00010009089	AGBL5	PRJNA729271	0.851794636	4.31011E-08	0.851794636	stage_E14_vs_E10	ordinary_muscle_development		0	FALSE
ENSGALG00010005157	IMPA1	PRJNA729271	-0.619254956	4.31042E-08	0.619254956	stage_E14_vs_E10	ordinary_muscle_development		0	FALSE
ENSGALG00010029389	SMYD4	PRJNA729271	0.53104486	4.31276E-08	0.53104486	stage_E14_vs_E10	ordinary_muscle_development		0	FALSE
ENSGALG00010028247	MVP	PRJNA729271	0.535606994	4.31789E-08	0.535606994	stage_E14_vs_E10	ordinary_muscle_development		0	FALSE
ENSGALG00010004079	AP1AR	PRJNA729271	-0.851137615	4.32448E-08	0.851137615	stage_E14_vs_E10	ordinary_muscle_development		0	FALSE
ENSGALG00010017801	SMNDC1	PRJNA729271	-0.404948666	4.32613E-08	0.404948666	stage_E14_vs_E10	ordinary_muscle_development		0	FALSE
ENSGALG00010027939	BSPRY	PRJNA729271	-3.561898626	4.33705E-08	3.561898626	stage_E14_vs_E10	ordinary_muscle_development	D_vs_E10;stage_1W_vs_E10;stage_3W_vs_E10;stage	1	TRUE
ENSGALG00010008016	GSR	PRJNA729271	-0.809740381	4.33839E-08	0.809740381	stage_E14_vs_E10	ordinary_muscle_development		0	FALSE
ENSGALG00010027937	HDHD3	PRJNA729271	0.537110275	4.34187E-08	0.537110275	stage_E14_vs_E10	ordinary_muscle_development		0	FALSE
ENSGALG00010002912	RPS3	PRJNA729271	1.089867761	4.39157E-08	1.089867761	stage_E14_vs_E10	ordinary_muscle_development	D_vs_E10;stage_1W_vs_E10;stage_3W_vs_E10;stage	1	TRUE
ENSGALG00010012926	OSGEPL1	PRJNA729271	0.918069056	4.40211E-08	0.918069056	stage_E14_vs_E10	ordinary_muscle_development		0	FALSE
ENSGALG00010001800	ENSGALG00010001800	PRJNA729271	1.010887577	4.4063E-08	1.010887577	stage_E14_vs_E10	ordinary_muscle_development	D_vs_E10;stage_1W_vs_E10;stage_3W_vs_E10;stage	1	TRUE
ENSGALG00010013667	SOWAHA	PRJNA729271	-5.201676618	4.42648E-08	5.201676618	stage_E14_vs_E10	ordinary_muscle_development	D_vs_E10;stage_1W_vs_E10;stage_3W_vs_E10;stage	1	TRUE
ENSGALG00010022986	ALG9	PRJNA729271	-0.604994461	4.43514E-08	0.604994461	stage_E14_vs_E10	ordinary_muscle_development		0	FALSE
ENSGALG00010027376	DSTYK	PRJNA729271	0.445152358	4.45609E-08	0.445152358	stage_E14_vs_E10	ordinary_muscle_development		0	FALSE
ENSGALG00010011907	BIK	PRJNA729271	-1.515208043	4.51216E-08	1.515208043	stage_E14_vs_E10	ordinary_muscle_development	D_vs_E10;stage_1W_vs_E10;stage_3W_vs_E10;stage	1	TRUE
ENSGALG00010027164	ENSGALG00010027164	PRJNA729271	2.5841707	4.5495E-08	2.5841707	stage_E14_vs_E10	ordinary_muscle_development	D_vs_E10;stage_1W_vs_E10;stage_3W_vs_E10;stage	1	TRUE
ENSGALG00010015175	MAPK9	PRJNA729271	0.389432279	4.55814E-08	0.389432279	stage_E14_vs_E10	ordinary_muscle_development		0	FALSE
ENSGALG00010023851	HSCB	PRJNA729271	0.830698375	4.5743E-08	0.830698375	stage_E14_vs_E10	ordinary_muscle_development		0	FALSE
ENSGALG00010021610	TMEM11	PRJNA729271	0.511180783	4.59186E-08	0.511180783	stage_E14_vs_E10	ordinary_muscle_development		0	FALSE
ENSGALG00010022072	SMTN	PRJNA729271	1.759074751	4.59974E-08	1.759074751	stage_E14_vs_E10	ordinary_muscle_development	D_vs_E10;stage_1W_vs_E10;stage_3W_vs_E10;stage	1	TRUE
ENSGALG00010008204	ENSGALG00010008204	PRJNA729271	0.854083538	4.67141E-08	0.854083538	stage_E14_vs_E10	ordinary_muscle_development		0	FALSE
ENSGALG00010024140	KRT6A	PRJNA729271	-5.893883264	4.67861E-08	5.893883264	stage_E14_vs_E10	ordinary_muscle_development	D_vs_E10;stage_1W_vs_E10;stage_3W_vs_E10;stage	1	TRUE
ENSGALG00010018549	GHSR	PRJNA729271	-5.050209876	4.69871E-08	5.050209876	stage_E14_vs_E10	ordinary_muscle_development	D_vs_E10;stage_1W_vs_E10;stage_3W_vs_E10;stage	1	TRUE
ENSGALG00010023097	LURAP1	PRJNA729271	1.196668149	4.71168E-08	1.196668149	stage_E14_vs_E10	ordinary_muscle_development	D_vs_E10;stage_1W_vs_E10;stage_3W_vs_E10;stage	1	TRUE
ENSGALG00010003397	SPATA4	PRJNA729271	2.197329704	4.71512E-08	2.197329704	stage_E14_vs_E10	ordinary_muscle_development	D_vs_E10;stage_1W_vs_E10;stage_3W_vs_E10;stage	1	TRUE
ENSGALG00010015024	CRACD	PRJNA729271	-2.194283234	4.72936E-08	2.194283234	stage_E14_vs_E10	ordinary_muscle_development	D_vs_E10;stage_1W_vs_E10;stage_3W_vs_E10;stage	1	TRUE
ENSGALG00010024785	MCEE	PRJNA729271	1.117104494	4.76964E-08	1.117104494	stage_E14_vs_E10	ordinary_muscle_development	D_vs_E10;stage_1W_vs_E10;stage_3W_vs_E10;stage	1	TRUE
ENSGALG00010000141	ENSGALG00010000141	PRJNA729271	-0.782727614	4.78196E-08	0.782727614	stage_E14_vs_E10	ordinary_muscle_development		0	FALSE
ENSGALG00010020053	LFNG	PRJNA729271	0.761537774	4.80271E-08	0.761537774	stage_E14_vs_E10	ordinary_muscle_development		0	FALSE
ENSGALG00010020186	MGA	PRJNA729271	0.531448045	4.82713E-08	0.531448045	stage_E14_vs_E10	ordinary_muscle_development		0	FALSE
ENSGALG00010005308	ENSGALG00010005308	PRJNA729271	2.988420794	4.83618E-08	2.988420794	stage_E14_vs_E10	ordinary_muscle_development	D_vs_E10;stage_1W_vs_E10;stage_3W_vs_E10;stage	1	TRUE
ENSGALG00010025865	LGR6	PRJNA729271	-3.794062326	4.84199E-08	3.794062326	stage_E14_vs_E10	ordinary_muscle_development	D_vs_E10;stage_1W_vs_E10;stage_3W_vs_E10;stage	1	TRUE
ENSGALG00010010732	CCKAR	PRJNA729271	-6.583188259	4.85163E-08	6.583188259	stage_E14_vs_E10	ordinary_muscle_development	D_vs_E10;stage_1W_vs_E10;stage_3W_vs_E10;stage	1	TRUE
ENSGALG00010008087	CPNE8	PRJNA729271	-1.066177383	4.87173E-08	1.066177383	stage_E14_vs_E10	ordinary_muscle_development	D_vs_E10;stage_1W_vs_E10;stage_3W_vs_E10;stage	1	TRUE
ENSGALG00010005301	ENSGALG00010005301	PRJNA729271	1.950538263	4.89352E-08	1.950538263	stage_E14_vs_E10	ordinary_muscle_development	D_vs_E10;stage_1W_vs_E10;stage_3W_vs_E10;stage	1	TRUE
ENSGALG00010002837	DNMT1	PRJNA729271	-0.729929185	4.90039E-08	0.729929185	stage_E14_vs_E10	ordinary_muscle_development		0	FALSE
ENSGALG00010028535	TICAM1	PRJNA729271	0.590164566	4.90397E-08	0.590164566	stage_E14_vs_E10	ordinary_muscle_development		0	FALSE
ENSGALG00010024919	ANKRD52	PRJNA729271	-0.703667726	4.90416E-08	0.703667726	stage_E14_vs_E10	ordinary_muscle_development		0	FALSE
ENSGALG00010005911	TBXT	PRJNA729271	-3.143587073	4.94206E-08	3.143587073	stage_E14_vs_E10	ordinary_muscle_development	D_vs_E10;stage_1W_vs_E10;stage_3W_vs_E10;stage	1	TRUE
ENSGALG00010029687	SLC16A5	PRJNA729271	-3.63013292	4.99172E-08	3.63013292	stage_E14_vs_E10	ordinary_muscle_development	D_vs_E10;stage_1W_vs_E10;stage_3W_vs_E10;stage	1	TRUE
ENSGALG00010000787	SNRNP48	PRJNA729271	0.585689572	5.01567E-08	0.585689572	stage_E14_vs_E10	ordinary_muscle_development		0	FALSE
ENSGALG00010010849	ACBD6	PRJNA729271	-0.616104206	5.01599E-08	0.616104206	stage_E14_vs_E10	ordinary_muscle_development		0	FALSE
ENSGALG00010012078	TMBIM4	PRJNA729271	-0.46698661	5.03425E-08	0.46698661	stage_E14_vs_E10	ordinary_muscle_development		0	FALSE
ENSGALG00010020392	SLC23A2	PRJNA729271	-0.46684198	5.07058E-08	0.46684198	stage_E14_vs_E10	ordinary_muscle_development		0	FALSE
ENSGALG00010020676	CYP2C23a	PRJNA729271	7.66270945	5.0721E-08	7.66270945	stage_E14_vs_E10	ordinary_muscle_development	D_vs_E10;stage_1W_vs_E10;stage_3W_vs_E10;stage	1	TRUE
ENSGALG00010024797	IKZF4	PRJNA729271	1.291577313	5.09688E-08	1.291577313	stage_E14_vs_E10	ordinary_muscle_development	D_vs_E10;stage_1W_vs_E10;stage_3W_vs_E10;stage	1	TRUE
ENSGALG00010014682	IL13RA1	PRJNA729271	0.700943419	5.11305E-08	0.700943419	stage_E14_vs_E10	ordinary_muscle_development		0	FALSE
ENSGALG00010013359	BTG1	PRJNA729271	0.743185042	5.13336E-08	0.743185042	stage_E14_vs_E10	ordinary_muscle_development		0	FALSE
ENSGALG00010025448	CHD1L	PRJNA729271	-0.610941015	5.14164E-08	0.610941015	stage_E14_vs_E10	ordinary_muscle_development		0	FALSE
ENSGALG00010026825	ADAM23	PRJNA729271	-0.760211303	5.19165E-08	0.760211303	stage_E14_vs_E10	ordinary_muscle_development		0	FALSE
ENSGALG00010020681	BCL10	PRJNA729271	-0.507537048	5.19398E-08	0.507537048	stage_E14_vs_E10	ordinary_muscle_development		0	FALSE
ENSGALG00010024947	MPP4	PRJNA729271	1.750422158	5.21852E-08	1.750422158	stage_E14_vs_E10	ordinary_muscle_development	D_vs_E10;stage_1W_vs_E10;stage_3W_vs_E10;stage	1	TRUE
ENSGALG00010023107	ZC3HAV1	PRJNA729271	1.624427426	5.26499E-08	1.624427426	stage_E14_vs_E10	ordinary_muscle_development	D_vs_E10;stage_1W_vs_E10;stage_3W_vs_E10;stage	1	TRUE
ENSGALG00010012144	H1-01	PRJNA729271	-0.879800686	5.27112E-08	0.879800686	stage_E14_vs_E10	ordinary_muscle_development		0	FALSE
ENSGALG00010014089	FIG4	PRJNA729271	-0.670771194	5.29297E-08	0.670771194	stage_E14_vs_E10	ordinary_muscle_development		0	FALSE
ENSGALG00010005441	UBE2V2	PRJNA729271	-0.545266569	5.30948E-08	0.545266569	stage_E14_vs_E10	ordinary_muscle_development		0	FALSE
ENSGALG00010021254	FNBP1L	PRJNA729271	-0.604709428	5.31168E-08	0.604709428	stage_E14_vs_E10	ordinary_muscle_development		0	FALSE
ENSGALG00010022356	TCFL5	PRJNA729271	-1.38985264	5.37246E-08	1.38985264	stage_E14_vs_E10	ordinary_muscle_development	D_vs_E10;stage_1W_vs_E10;stage_3W_vs_E10;stage	1	TRUE
ENSGALG00010003470	METTL3	PRJNA729271	1.038197229	5.37683E-08	1.038197229	stage_E14_vs_E10	ordinary_muscle_development	D_vs_E10;stage_1W_vs_E10;stage_3W_vs_E10;stage	1	TRUE
ENSGALG00010002456	ELOVL4	PRJNA729271	-3.684872971	5.41148E-08	3.684872971	stage_E14_vs_E10	ordinary_muscle_development	D_vs_E10;stage_1W_vs_E10;stage_3W_vs_E10;stage	1	TRUE
ENSGALG00010010411	CD180	PRJNA729271	2.339720721	5.41414E-08	2.339720721	stage_E14_vs_E10	ordinary_muscle_development	D_vs_E10;stage_1W_vs_E10;stage_3W_vs_E10;stage	1	TRUE
ENSGALG00010017509	RASD1	PRJNA729271	1.660362694	5.42337E-08	1.660362694	stage_E14_vs_E10	ordinary_muscle_development	D_vs_E10;stage_1W_vs_E10;stage_3W_vs_E10;stage	1	TRUE
ENSGALG00010023805	GALNT6	PRJNA729271	-2.160061808	5.43143E-08	2.160061808	stage_E14_vs_E10	ordinary_muscle_development	D_vs_E10;stage_1W_vs_E10;stage_3W_vs_E10;stage	1	TRUE
ENSGALG00010016809	SYTL1	PRJNA729271	-3.422472833	5.47564E-08	3.422472833	stage_E14_vs_E10	ordinary_muscle_development	D_vs_E10;stage_1W_vs_E10;stage_3W_vs_E10;stage	1	TRUE
ENSGALG00010028739	GARNL3	PRJNA729271	-0.434931414	5.47728E-08	0.434931414	stage_E14_vs_E10	ordinary_muscle_development		0	FALSE
ENSGALG00010020091	SUPT7L	PRJNA729271	-0.474065715	5.48056E-08	0.474065715	stage_E14_vs_E10	ordinary_muscle_development		0	FALSE
ENSGALG00010008012	CCDC90B	PRJNA729271	-0.451610958	5.49913E-08	0.451610958	stage_E14_vs_E10	ordinary_muscle_development		0	FALSE
ENSGALG00010022727	ACP2	PRJNA729271	0.462893708	5.50418E-08	0.462893708	stage_E14_vs_E10	ordinary_muscle_development		0	FALSE
ENSGALG00010011960	CFAP54	PRJNA729271	-1.735150559	5.52104E-08	1.735150559	stage_E14_vs_E10	ordinary_muscle_development	D_vs_E10;stage_1W_vs_E10;stage_3W_vs_E10;stage	1	TRUE
ENSGALG00010026836	ENSGALG00010026836	PRJNA729271	3.279663181	5.55133E-08	3.279663181	stage_E14_vs_E10	ordinary_muscle_development	D_vs_E10;stage_1W_vs_E10;stage_3W_vs_E10;stage	1	TRUE
ENSGALG00010020766	C2orf76	PRJNA729271	0.517867665	5.58013E-08	0.517867665	stage_E14_vs_E10	ordinary_muscle_development		0	FALSE
ENSGALG00010017745	SHMT1	PRJNA729271	-1.056816838	5.61831E-08	1.056816838	stage_E14_vs_E10	ordinary_muscle_development	D_vs_E10;stage_1W_vs_E10;stage_3W_vs_E10;stage	1	TRUE
ENSGALG00010003981	TMEM150C	PRJNA729271	-0.950327461	5.6368E-08	0.950327461	stage_E14_vs_E10	ordinary_muscle_development		0	FALSE
ENSGALG00010024089	ENSGALG00010024089	PRJNA729271	9.739629966	5.64558E-08	9.739629966	stage_E14_vs_E10	ordinary_muscle_development	D_vs_E10;stage_1W_vs_E10;stage_3W_vs_E10;stage	1	TRUE
ENSGALG00010023304	ENSGALG00010023304	PRJNA729271	-0.983283351	5.65572E-08	0.983283351	stage_E14_vs_E10	ordinary_muscle_development		0	FALSE
ENSGALG00010016980	NPHP1	PRJNA729271	-0.570473412	5.73019E-08	0.570473412	stage_E14_vs_E10	ordinary_muscle_development		0	FALSE
ENSGALG00010000734	SLAIN1	PRJNA729271	-2.158356675	5.73684E-08	2.158356675	stage_E14_vs_E10	ordinary_muscle_development	D_vs_E10;stage_1W_vs_E10;stage_3W_vs_E10;stage	1	TRUE
ENSGALG00010026565	RSPH14	PRJNA729271	-3.306741607	5.75937E-08	3.306741607	stage_E14_vs_E10	ordinary_muscle_development	D_vs_E10;stage_1W_vs_E10;stage_3W_vs_E10;stage	1	TRUE
ENSGALG00010015207	AKR1E2	PRJNA729271	-1.003808852	5.77798E-08	1.003808852	stage_E14_vs_E10	ordinary_muscle_development	D_vs_E10;stage_1W_vs_E10;stage_3W_vs_E10;stage	1	TRUE
ENSGALG00010016043	ADAMTSL1	PRJNA729271	1.077647151	5.81935E-08	1.077647151	stage_E14_vs_E10	ordinary_muscle_development	D_vs_E10;stage_1W_vs_E10;stage_3W_vs_E10;stage	1	TRUE
ENSGALG00010012583	NDUFAF6	PRJNA729271	0.562246199	5.82086E-08	0.562246199	stage_E14_vs_E10	ordinary_muscle_development		0	FALSE
ENSGALG00010017954	PTGER2	PRJNA729271	1.427615407	5.83868E-08	1.427615407	stage_E14_vs_E10	ordinary_muscle_development	D_vs_E10;stage_1W_vs_E10;stage_3W_vs_E10;stage	1	TRUE
ENSGALG00010029408	KIF12	PRJNA729271	2.581558327	5.84903E-08	2.581558327	stage_E14_vs_E10	ordinary_muscle_development	D_vs_E10;stage_1W_vs_E10;stage_3W_vs_E10;stage	1	TRUE
ENSGALG00010014487	OSTM1	PRJNA729271	0.463237626	5.88803E-08	0.463237626	stage_E14_vs_E10	ordinary_muscle_development		0	FALSE
ENSGALG00010020651	LMO1	PRJNA729271	-1.069056381	5.938E-08	1.069056381	stage_E14_vs_E10	ordinary_muscle_development	D_vs_E10;stage_1W_vs_E10;stage_3W_vs_E10;stage	1	TRUE
ENSGALG00010008656	CCDC6	PRJNA729271	0.396715625	5.96303E-08	0.396715625	stage_E14_vs_E10	ordinary_muscle_development		0	FALSE
ENSGALG00010024788	SIK3	PRJNA729271	0.919447732	5.97311E-08	0.919447732	stage_E14_vs_E10	ordinary_muscle_development		0	FALSE
ENSGALG00010005989	FCF1	PRJNA729271	-0.562847043	5.98324E-08	0.562847043	stage_E14_vs_E10	ordinary_muscle_development		0	FALSE
ENSGALG00010014159	CDC42EP1	PRJNA729271	-0.834493049	6.00976E-08	0.834493049	stage_E14_vs_E10	ordinary_muscle_development		0	FALSE
ENSGALG00010001661	TCTN3	PRJNA729271	-0.587982572	6.01909E-08	0.587982572	stage_E14_vs_E10	ordinary_muscle_development		0	FALSE
ENSGALG00010014922	MAN1A1	PRJNA729271	-0.952712194	6.10103E-08	0.952712194	stage_E14_vs_E10	ordinary_muscle_development		0	FALSE
ENSGALG00010024843	BCL9L	PRJNA729271	0.740426754	6.10244E-08	0.740426754	stage_E14_vs_E10	ordinary_muscle_development		0	FALSE
ENSGALG00010003775	NCOA2	PRJNA729271	0.633370021	6.10696E-08	0.633370021	stage_E14_vs_E10	ordinary_muscle_development		0	FALSE
ENSGALG00010016576	MRPS26	PRJNA729271	0.58911326	6.12871E-08	0.58911326	stage_E14_vs_E10	ordinary_muscle_development		0	FALSE
ENSGALG00010002439	GPR82	PRJNA729271	4.702028193	6.14641E-08	4.702028193	stage_E14_vs_E10	ordinary_muscle_development	D_vs_E10;stage_1W_vs_E10;stage_3W_vs_E10;stage	1	TRUE
ENSGALG00010027323	ISG20L2	PRJNA729271	-0.738667568	6.16797E-08	0.738667568	stage_E14_vs_E10	ordinary_muscle_development		0	FALSE
ENSGALG00010021180	TMEM198	PRJNA729271	-0.812589288	6.17014E-08	0.812589288	stage_E14_vs_E10	ordinary_muscle_development		0	FALSE
ENSGALG00010006922	SMYD3	PRJNA729271	-0.909043471	6.17412E-08	0.909043471	stage_E14_vs_E10	ordinary_muscle_development		0	FALSE
ENSGALG00010009104	MTR	PRJNA729271	0.678475588	6.19455E-08	0.678475588	stage_E14_vs_E10	ordinary_muscle_development		0	FALSE
ENSGALG00010025012	ENSGALG00010025012	PRJNA729271	4.143499138	6.20872E-08	4.143499138	stage_E14_vs_E10	ordinary_muscle_development	D_vs_E10;stage_1W_vs_E10;stage_3W_vs_E10;stage	1	TRUE
ENSGALG00010006202	SACS	PRJNA729271	-1.111538849	6.22635E-08	1.111538849	stage_E14_vs_E10	ordinary_muscle_development	D_vs_E10;stage_1W_vs_E10;stage_3W_vs_E10;stage	1	TRUE
ENSGALG00010021741	RPL19	PRJNA729271	0.818442618	6.25156E-08	0.818442618	stage_E14_vs_E10	ordinary_muscle_development		0	FALSE
ENSGALG00010025512	NRAS	PRJNA729271	-0.43396335	6.27003E-08	0.43396335	stage_E14_vs_E10	ordinary_muscle_development		0	FALSE
ENSGALG00010007930	CBFA2T3	PRJNA729271	1.12554951	6.32028E-08	1.12554951	stage_E14_vs_E10	ordinary_muscle_development	D_vs_E10;stage_1W_vs_E10;stage_3W_vs_E10;stage	1	TRUE
ENSGALG00010008170	PICALM	PRJNA729271	-0.394164383	6.3527E-08	0.394164383	stage_E14_vs_E10	ordinary_muscle_development		0	FALSE
ENSGALG00010023937	XBP1	PRJNA729271	-0.613776439	6.37125E-08	0.613776439	stage_E14_vs_E10	ordinary_muscle_development		0	FALSE
ENSGALG00010013871	ENSGALG00010013871	PRJNA729271	1.245640238	6.44014E-08	1.245640238	stage_E14_vs_E10	ordinary_muscle_development	D_vs_E10;stage_1W_vs_E10;stage_3W_vs_E10;stage	1	TRUE
ENSGALG00010024399	ROR1	PRJNA729271	-0.966351617	6.45907E-08	0.966351617	stage_E14_vs_E10	ordinary_muscle_development		0	FALSE
ENSGALG00010002050	ENSGALG00010002050	PRJNA729271	6.092282231	6.46605E-08	6.092282231	stage_E14_vs_E10	ordinary_muscle_development	D_vs_E10;stage_1W_vs_E10;stage_3W_vs_E10;stage	1	TRUE
ENSGALG00010011038	GSG1	PRJNA729271	-2.95669166	6.47978E-08	2.95669166	stage_E14_vs_E10	ordinary_muscle_development	D_vs_E10;stage_1W_vs_E10;stage_3W_vs_E10;stage	1	TRUE
ENSGALG00010019799	DOK2	PRJNA729271	1.459769895	6.48076E-08	1.459769895	stage_E14_vs_E10	ordinary_muscle_development	D_vs_E10;stage_1W_vs_E10;stage_3W_vs_E10;stage	1	TRUE
ENSGALG00010016607	FAM103A1	PRJNA729271	-0.647151046	6.49031E-08	0.647151046	stage_E14_vs_E10	ordinary_muscle_development		0	FALSE
ENSGALG00010008265	ENSGALG00010008265	PRJNA729271	-5.829624051	6.50926E-08	5.829624051	stage_E14_vs_E10	ordinary_muscle_development	D_vs_E10;stage_1W_vs_E10;stage_3W_vs_E10;stage	1	TRUE
ENSGALG00010001409	GPNMB	PRJNA729271	-3.937692635	6.52063E-08	3.937692635	stage_E14_vs_E10	ordinary_muscle_development	D_vs_E10;stage_1W_vs_E10;stage_3W_vs_E10;stage	1	TRUE
ENSGALG00010018958	PELI3	PRJNA729271	-0.656513749	6.52496E-08	0.656513749	stage_E14_vs_E10	ordinary_muscle_development		0	FALSE
ENSGALG00010001654	RPS11	PRJNA729271	0.948091908	6.53193E-08	0.948091908	stage_E14_vs_E10	ordinary_muscle_development		0	FALSE
ENSGALG00010002857	DCAF15	PRJNA729271	-0.829361604	6.54894E-08	0.829361604	stage_E14_vs_E10	ordinary_muscle_development		0	FALSE
ENSGALG00010016556	ASAP3	PRJNA729271	-1.091497992	6.55147E-08	1.091497992	stage_E14_vs_E10	ordinary_muscle_development	D_vs_E10;stage_1W_vs_E10;stage_3W_vs_E10;stage	1	TRUE
ENSGALG00010006515	FBXO33	PRJNA729271	-0.59292096	6.56958E-08	0.59292096	stage_E14_vs_E10	ordinary_muscle_development		0	FALSE
ENSGALG00010022976	MINDY2	PRJNA729271	-0.368278448	6.57142E-08	0.368278448	stage_E14_vs_E10	ordinary_muscle_development		0	FALSE
ENSGALG00010011976	TNFRSF11A	PRJNA729271	0.495186976	6.57344E-08	0.495186976	stage_E14_vs_E10	ordinary_muscle_development		0	FALSE
ENSGALG00010021970	ERGIC3	PRJNA729271	-0.506269623	6.62801E-08	0.506269623	stage_E14_vs_E10	ordinary_muscle_development		0	FALSE
ENSGALG00010008839	ZNF512	PRJNA729271	-0.412034858	6.633E-08	0.412034858	stage_E14_vs_E10	ordinary_muscle_development		0	FALSE
ENSGALG00010005261	ATG3	PRJNA729271	-0.615266303	6.65764E-08	0.615266303	stage_E14_vs_E10	ordinary_muscle_development		0	FALSE
ENSGALG00010012437	ENSGALG00010012437	PRJNA729271	-21.55372565	6.66447E-08	21.55372565	stage_E14_vs_E10	ordinary_muscle_development	D_vs_E10;stage_1W_vs_E10;stage_3W_vs_E10;stage	1	TRUE

ENSGALG00010016165	KK34	PRJNA729271	4.959024413	6.68271E-08	4.959024413	stage_E14_vs_E10	ordinary_muscle_development	D_vs_E10;stage_1W_vs_E10;stage_3W_vs_E10;stage	1	TRUE
ENSGALG00010021521	ENSGALG00010021521	PRJNA729271	1.438522232	6.7912E-08	1.438522232	stage_E14_vs_E10	ordinary_muscle_development	D_vs_E10;stage_1W_vs_E10;stage_3W_vs_E10;stage	1	TRUE
ENSGALG00010002106	ENSGALG00010002106	PRJNA729271	-2.935006787	6.81289E-08	2.935006787	stage_E14_vs_E10	ordinary_muscle_development	D_vs_E10;stage_1W_vs_E10;stage_3W_vs_E10;stage	1	TRUE
ENSGALG00010028435	ATP8B3	PRJNA729271	1.808334546	6.83918E-08	1.808334546	stage_E14_vs_E10	ordinary_muscle_development	D_vs_E10;stage_1W_vs_E10;stage_3W_vs_E10;stage	1	TRUE
ENSGALG00010005550	ENSGALG00010005550	PRJNA729271	2.978738965	6.84274E-08	2.978738965	stage_E14_vs_E10	ordinary_muscle_development	D_vs_E10;stage_1W_vs_E10;stage_3W_vs_E10;stage	1	TRUE
ENSGALG00010010975	ATP8B1	PRJNA729271	-6.582092744	6.84314E-08	6.582092744	stage_E14_vs_E10	ordinary_muscle_development	D_vs_E10;stage_1W_vs_E10;stage_3W_vs_E10;stage	1	TRUE
ENSGALG00010017865	UBOX5	PRJNA729271	0.732572585	6.94989E-08	0.732572585	stage_E14_vs_E10	ordinary_muscle_development		0	FALSE
ENSGALG00010012964	CBR3	PRJNA729271	-0.401922155	6.98492E-08	0.401922155	stage_E14_vs_E10	ordinary_muscle_development		0	FALSE
ENSGALG00010028596	DDA1	PRJNA729271	-0.493580349	7.05487E-08	0.493580349	stage_E14_vs_E10	ordinary_muscle_development		0	FALSE
ENSGALG00010020416	ENSGALG00010020416	PRJNA729271	-6.094424469	7.10073E-08	6.094424469	stage_E14_vs_E10	ordinary_muscle_development	D_vs_E10;stage_1W_vs_E10;stage_3W_vs_E10;stage	1	TRUE
ENSGALG00010015155	HMOX1	PRJNA729271	-0.996202726	7.14005E-08	0.996202726	stage_E14_vs_E10	ordinary_muscle_development		0	FALSE
ENSGALG00010007981	TDRD15	PRJNA729271	-5.296976752	7.23423E-08	5.296976752	stage_E14_vs_E10	ordinary_muscle_development	D_vs_E10;stage_1W_vs_E10;stage_3W_vs_E10;stage	1	TRUE
ENSGALG00010024504	CASC3	PRJNA729271	1.026982609	7.26925E-08	1.026982609	stage_E14_vs_E10	ordinary_muscle_development	D_vs_E10;stage_1W_vs_E10;stage_3W_vs_E10;stage	1	TRUE
ENSGALG00010027697	CATH2	PRJNA729271	4.867094235	7.27009E-08	4.867094235	stage_E14_vs_E10	ordinary_muscle_development	D_vs_E10;stage_1W_vs_E10;stage_3W_vs_E10;stage	1	TRUE
ENSGALG00010000697	ENSGALG00010000697	PRJNA729271	1.343939807	7.31297E-08	1.343939807	stage_E14_vs_E10	ordinary_muscle_development	D_vs_E10;stage_1W_vs_E10;stage_3W_vs_E10;stage	1	TRUE
ENSGALG00010001305	WNT16	PRJNA729271	-2.19474298	7.31672E-08	2.19474298	stage_E14_vs_E10	ordinary_muscle_development	D_vs_E10;stage_1W_vs_E10;stage_3W_vs_E10;stage	1	TRUE
ENSGALG00010001270	DRAP1	PRJNA729271	-0.615186258	7.33123E-08	0.615186258	stage_E14_vs_E10	ordinary_muscle_development		0	FALSE
ENSGALG00010005408	APBB1IP	PRJNA729271	1.114686517	7.38427E-08	1.114686517	stage_E14_vs_E10	ordinary_muscle_development	D_vs_E10;stage_1W_vs_E10;stage_3W_vs_E10;stage	1	TRUE
ENSGALG00010009453	GSAP	PRJNA729271	0.632402939	7.4163E-08	0.632402939	stage_E14_vs_E10	ordinary_muscle_development		0	FALSE
ENSGALG00010013375	PSMG1	PRJNA729271	-0.498066804	7.41776E-08	0.498066804	stage_E14_vs_E10	ordinary_muscle_development		0	FALSE
ENSGALG00010015863	ENSGALG00010015863	PRJNA729271	-3.284848742	7.46025E-08	3.284848742	stage_E14_vs_E10	ordinary_muscle_development	D_vs_E10;stage_1W_vs_E10;stage_3W_vs_E10;stage	1	TRUE
ENSGALG00010009612	DERL1	PRJNA729271	-0.3519661	7.50103E-08	0.3519661	stage_E14_vs_E10	ordinary_muscle_development		0	FALSE
ENSGALG00010023626	LOXL1	PRJNA729271	1.073767073	7.53024E-08	1.073767073	stage_E14_vs_E10	ordinary_muscle_development	D_vs_E10;stage_1W_vs_E10;stage_3W_vs_E10;stage	1	TRUE
ENSGALG00010021597	GGT7	PRJNA729271	-1.043914052	7.55879E-08	1.043914052	stage_E14_vs_E10	ordinary_muscle_development	D_vs_E10;stage_1W_vs_E10;stage_3W_vs_E10;stage	1	TRUE
ENSGALG00010015182	PHYKPL	PRJNA729271	-1.860856277	7.68752E-08	1.860856277	stage_E14_vs_E10	ordinary_muscle_development	D_vs_E10;stage_1W_vs_E10;stage_3W_vs_E10;stage	1	TRUE
ENSGALG00010001480	JAKMIP3	PRJNA729271	-1.807239801	7.69791E-08	1.807239801	stage_E14_vs_E10	ordinary_muscle_development	D_vs_E10;stage_1W_vs_E10;stage_3W_vs_E10;stage	1	TRUE
ENSGALG00010003334	TAC1	PRJNA729271	-5.984716865	7.72181E-08	5.984716865	stage_E14_vs_E10	ordinary_muscle_development	D_vs_E10;stage_1W_vs_E10;stage_3W_vs_E10;stage	1	TRUE
ENSGALG00010011529	SCYL2	PRJNA729271	0.450633559	7.75352E-08	0.450633559	stage_E14_vs_E10	ordinary_muscle_development		0	FALSE
ENSGALG00010004730	CLDN12	PRJNA729271	-0.353779803	7.7602E-08	0.353779803	stage_E14_vs_E10	ordinary_muscle_development		0	FALSE
ENSGALG00010022134	CH25H	PRJNA729271	-1.405080376	7.77921E-08	1.405080376	stage_E14_vs_E10	ordinary_muscle_development	D_vs_E10;stage_1W_vs_E10;stage_3W_vs_E10;stage	1	TRUE
ENSGALG00010007909	GALNS	PRJNA729271	-0.560529091	7.86851E-08	0.560529091	stage_E14_vs_E10	ordinary_muscle_development		0	FALSE
ENSGALG00010023783	VPS35	PRJNA729271	0.34917122	7.95781E-08	0.34917122	stage_E14_vs_E10	ordinary_muscle_development		0	FALSE
ENSGALG00010001388	MPPE1	PRJNA729271	0.916255817	7.96804E-08	0.916255817	stage_E14_vs_E10	ordinary_muscle_development		0	FALSE
ENSGALG00010024040	ENSGALG00010024040	PRJNA729271	-5.168644385	8.07348E-08	5.168644385	stage_E14_vs_E10	ordinary_muscle_development	D_vs_E10;stage_1W_vs_E10;stage_3W_vs_E10;stage	1	TRUE
ENSGALG00010012195	ITSN1	PRJNA729271	-0.411212596	8.2329E-08	0.411212596	stage_E14_vs_E10	ordinary_muscle_development		0	FALSE
ENSGALG00010010666	C12orf75	PRJNA729271	2.091248019	8.25745E-08	2.091248019	stage_E14_vs_E10	ordinary_muscle_development	D_vs_E10;stage_1W_vs_E10;stage_3W_vs_E10;stage	1	TRUE
ENSGALG00010013696	CETN3	PRJNA729271	-0.617954945	8.27793E-08	0.617954945	stage_E14_vs_E10	ordinary_muscle_development		0	FALSE
ENSGALG00010012128	ENSGALG00010012128	PRJNA729271	4.069769695	8.36925E-08	4.069769695	stage_E14_vs_E10	ordinary_muscle_development	D_vs_E10;stage_1W_vs_E10;stage_3W_vs_E10;stage	1	TRUE
ENSGALG00010022983	PKN2	PRJNA729271	0.335633947	8.40342E-08	0.335633947	stage_E14_vs_E10	ordinary_muscle_development		0	FALSE
ENSGALG00010017407	EPN2	PRJNA729271	0.46696128	8.4222E-08	0.46696128	stage_E14_vs_E10	ordinary_muscle_development		0	FALSE
ENSGALG00010028133	ENSGALG00010028133	PRJNA729271	1.607951785	8.44544E-08	1.607951785	stage_E14_vs_E10	ordinary_muscle_development	D_vs_E10;stage_1W_vs_E10;stage_3W_vs_E10;stage	1	TRUE
ENSGALG00010006096	EDIL3	PRJNA729271	1.308372007	8.46814E-08	1.308372007	stage_E14_vs_E10	ordinary_muscle_development	D_vs_E10;stage_1W_vs_E10;stage_3W_vs_E10;stage	1	TRUE
ENSGALG00010023421	OLFM3	PRJNA729271	-1.396632709	8.5308E-08	1.396632709	stage_E14_vs_E10	ordinary_muscle_development	D_vs_E10;stage_1W_vs_E10;stage_3W_vs_E10;stage	1	TRUE
ENSGALG00010001910	FAM53B	PRJNA729271	0.534589587	8.53734E-08	0.534589587	stage_E14_vs_E10	ordinary_muscle_development		0	FALSE
ENSGALG00010011860	CECR1	PRJNA729271	0.852162687	8.56074E-08	0.852162687	stage_E14_vs_E10	ordinary_muscle_development		0	FALSE
ENSGALG00010005836	NEBL	PRJNA729271	-1.416068245	8.63442E-08	1.416068245	stage_E14_vs_E10	ordinary_muscle_development	D_vs_E10;stage_1W_vs_E10;stage_3W_vs_E10;stage	1	TRUE
ENSGALG00010009724	ISOC1	PRJNA729271	0.781396235	8.64279E-08	0.781396235	stage_E14_vs_E10	ordinary_muscle_development		0	FALSE
ENSGALG00010006073	CEP97	PRJNA729271	-0.608950945	8.66478E-08	0.608950945	stage_E14_vs_E10	ordinary_muscle_development		0	FALSE
ENSGALG00010015541	RASEF	PRJNA729271	-1.993186165	8.69501E-08	1.993186165	stage_E14_vs_E10	ordinary_muscle_development	D_vs_E10;stage_1W_vs_E10;stage_3W_vs_E10;stage	1	TRUE
ENSGALG00010012329	ATXN10	PRJNA729271	-0.607348359	8.76433E-08	0.607348359	stage_E14_vs_E10	ordinary_muscle_development		0	FALSE
ENSGALG00010019610	CORO7	PRJNA729271	0.564335218	8.83637E-08	0.564335218	stage_E14_vs_E10	ordinary_muscle_development		0	FALSE
ENSGALG00010001206	RSRC1	PRJNA729271	-0.427661465	8.87093E-08	0.427661465	stage_E14_vs_E10	ordinary_muscle_development		0	FALSE
ENSGALG00010001807	ASMTL	PRJNA729271	0.563463096	8.88949E-08	0.563463096	stage_E14_vs_E10	ordinary_muscle_development		0	FALSE
ENSGALG00010028245	JAC1	PRJNA729271	7.01709518	8.90183E-08	7.01709518	stage_E14_vs_E10	ordinary_muscle_development	D_vs_E10;stage_1W_vs_E10;stage_3W_vs_E10;stage	1	TRUE
ENSGALG00010024850	CDK2AP1	PRJNA729271	-0.645049376	8.92891E-08	0.645049376	stage_E14_vs_E10	ordinary_muscle_development		0	FALSE
ENSGALG00010017180	ENSGALG00010017180	PRJNA729271	-4.159825816	8.99333E-08	4.159825816	stage_E14_vs_E10	ordinary_muscle_development	D_vs_E10;stage_1W_vs_E10;stage_3W_vs_E10;stage	1	TRUE
ENSGALG00010005208	ENSGALG00010005208	PRJNA729271	-0.620953579	9.03766E-08	0.620953579	stage_E14_vs_E10	ordinary_muscle_development		0	FALSE
ENSGALG00010018467	TLX1	PRJNA729271	-6.387222332	9.04962E-08	6.387222332	stage_E14_vs_E10	ordinary_muscle_development	D_vs_E10;stage_1W_vs_E10;stage_3W_vs_E10;stage	1	TRUE
ENSGALG00010011372	VMA21	PRJNA729271	-0.508645085	9.32654E-08	0.508645085	stage_E14_vs_E10	ordinary_muscle_development		0	FALSE
ENSGALG00010014453	NR2E1	PRJNA729271	1.830864045	9.37049E-08	1.830864045	stage_E14_vs_E10	ordinary_muscle_development	D_vs_E10;stage_1W_vs_E10;stage_3W_vs_E10;stage	1	TRUE
ENSGALG00010018974	TAS1R1	PRJNA729271	1.497567223	9.37384E-08	1.497567223	stage_E14_vs_E10	ordinary_muscle_development	D_vs_E10;stage_1W_vs_E10;stage_3W_vs_E10;stage	1	TRUE
ENSGALG00010012325	MLPH	PRJNA729271	-3.534547562	9.41315E-08	3.534547562	stage_E14_vs_E10	ordinary_muscle_development	D_vs_E10;stage_1W_vs_E10;stage_3W_vs_E10;stage	1	TRUE
ENSGALG00010018422	ENSGALG00010018422	PRJNA729271	1.177597223	9.43977E-08	1.177597223	stage_E14_vs_E10	ordinary_muscle_development	D_vs_E10;stage_1W_vs_E10;stage_3W_vs_E10;stage	1	TRUE
ENSGALG00010007847	CMC2	PRJNA729271	0.626120284	9.445E-08	0.626120284	stage_E14_vs_E10	ordinary_muscle_development		0	FALSE
ENSGALG00010024733	MBTPS1	PRJNA729271	-0.483415754	9.5024E-08	0.483415754	stage_E14_vs_E10	ordinary_muscle_development		0	FALSE
ENSGALG00010024557	TSPAN32	PRJNA729271	1.555807334	9.66976E-08	1.555807334	stage_E14_vs_E10	ordinary_muscle_development	D_vs_E10;stage_1W_vs_E10;stage_3W_vs_E10;stage	1	TRUE
ENSGALG00010022795	TAF4	PRJNA729271	0.601923651	9.68503E-08	0.601923651	stage_E14_vs_E10	ordinary_muscle_development		0	FALSE
ENSGALG00010012024	UBR5	PRJNA729271	0.415629676	9.71761E-08	0.415629676	stage_E14_vs_E10	ordinary_muscle_development		0	FALSE
ENSGALG00010027127	S1PR4	PRJNA729271	3.251251887	9.9099E-08	3.251251887	stage_E14_vs_E10	ordinary_muscle_development	D_vs_E10;stage_1W_vs_E10;stage_3W_vs_E10;stage	1	TRUE
ENSGALG00010025736	KIF9	PRJNA729271	1.770064327	9.93118E-08	1.770064327	stage_E14_vs_E10	ordinary_muscle_development	D_vs_E10;stage_1W_vs_E10;stage_3W_vs_E10;stage	1	TRUE
ENSGALG00010009137	IPMK	PRJNA729271	0.506704556	9.93118E-08	0.506704556	stage_E14_vs_E10	ordinary_muscle_development		0	FALSE
ENSGALG00010013771	PELO	PRJNA729271	-0.58194345	9.9568E-08	0.58194345	stage_E14_vs_E10	ordinary_muscle_development		0	FALSE
ENSGALG00010006728	ENSGALG00010006728	PRJNA729271	0.750395095	9.9634E-08	0.750395095	stage_E14_vs_E10	ordinary_muscle_development		0	FALSE
ENSGALG00010005637	TOPORS	PRJNA729271	-0.398036686	9.9674E-08	0.398036686	stage_E14_vs_E10	ordinary_muscle_development		0	FALSE
ENSGALG00010023086	PCSK7	PRJNA729271	-0.527964059	9.99124E-08	0.527964059	stage_E14_vs_E10	ordinary_muscle_development		0	FALSE
ENSGALG00010021236	BTBD9	PRJNA729271	0.402777829	1.00675E-07	0.402777829	stage_E14_vs_E10	ordinary_muscle_development		0	FALSE
ENSGALG00010010496	MRPL17	PRJNA729271	0.775660047	1.00832E-07	0.775660047	stage_E14_vs_E10	ordinary_muscle_development		0	FALSE
ENSGALG00010025522	SUMO3	PRJNA729271	-0.580667789	1.01002E-07	0.580667789	stage_E14_vs_E10	ordinary_muscle_development		0	FALSE
ENSGALG00010025007	ZYG11B	PRJNA729271	0.553270502	1.0149E-07	0.553270502	stage_E14_vs_E10	ordinary_muscle_development		0	FALSE
ENSGALG00010009093	SLU7	PRJNA729271	0.716902907	1.02023E-07	0.716902907	stage_E14_vs_E10	ordinary_muscle_development		0	FALSE
ENSGALG00010025943	CATH1	PRJNA729271	3.935943555	1.03632E-07	3.935943555	stage_E14_vs_E10	ordinary_muscle_development	D_vs_E10;stage_1W_vs_E10;stage_3W_vs_E10;stage	1	TRUE
ENSGALG00010023217	CREB3L1	PRJNA729271	-1.13164134	1.0411E-07	1.13164134	stage_E14_vs_E10	ordinary_muscle_development	D_vs_E10;stage_1W_vs_E10;stage_3W_vs_E10;stage	1	TRUE
ENSGALG00010017881	KCNK10	PRJNA729271	-3.965890063	1.04247E-07	3.965890063	stage_E14_vs_E10	ordinary_muscle_development	D_vs_E10;stage_1W_vs_E10;stage_3W_vs_E10;stage	1	TRUE
ENSGALG00010001898	LHPP	PRJNA729271	0.715790433	1.05759E-07	0.715790433	stage_E14_vs_E10	ordinary_muscle_development		0	FALSE
ENSGALG00010028826	CEL	PRJNA729271	-16.11845486	1.05886E-07	16.11845486	stage_E14_vs_E10	ordinary_muscle_development	D_vs_E10;stage_1W_vs_E10;stage_3W_vs_E10;stage	1	TRUE
ENSGALG00010017888	MPLKIP	PRJNA729271	-0.623948321	1.05971E-07	0.623948321	stage_E14_vs_E10	ordinary_muscle_development		0	FALSE
ENSGALG00010015636	APBB2	PRJNA729271	0.676625559	1.06323E-07	0.676625559	stage_E14_vs_E10	ordinary_muscle_development		0	FALSE
ENSGALG00010011067	TMTC2	PRJNA729271	-0.560741022	1.06903E-07	0.560741022	stage_E14_vs_E10	ordinary_muscle_development		0	FALSE
ENSGALG00010013524	ZDHHC15	PRJNA729271	0.661252539	1.0724E-07	0.661252539	stage_E14_vs_E10	ordinary_muscle_development		0	FALSE
ENSGALG00010002034	WWC3	PRJNA729271	-0.544103036	1.07393E-07	0.544103036	stage_E14_vs_E10	ordinary_muscle_development		0	FALSE
ENSGALG00010024598	LRRC3C	PRJNA729271	1.289428205	1.07649E-07	1.289428205	stage_E14_vs_E10	ordinary_muscle_development	D_vs_E10;stage_1W_vs_E10;stage_3W_vs_E10;stage	1	TRUE
ENSGALG00010023225	PDPR	PRJNA729271	0.653086524	1.08359E-07	0.653086524	stage_E14_vs_E10	ordinary_muscle_development		0	FALSE
ENSGALG00010029504	GPR142	PRJNA729271	-4.820816001	1.08571E-07	4.820816001	stage_E14_vs_E10	ordinary_muscle_development	D_vs_E10;stage_1W_vs_E10;stage_3W_vs_E10;stage	1	TRUE
ENSGALG00010017946	PTGDR	PRJNA729271	1.350704998	1.09647E-07	1.350704998	stage_E14_vs_E10	ordinary_muscle_development	D_vs_E10;stage_1W_vs_E10;stage_3W_vs_E10;stage	1	TRUE
ENSGALG00010029351	ENSGALG00010029351	PRJNA729271	-1.093589251	1.10969E-07	1.093589251	stage_E14_vs_E10	ordinary_muscle_development	D_vs_E10;stage_1W_vs_E10;stage_3W_vs_E10;stage	1	TRUE
ENSGALG00010018067	ENSGALG00010018067	PRJNA729271	1.004117891	1.11826E-07	1.004117891	stage_E14_vs_E10	ordinary_muscle_development	D_vs_E10;stage_1W_vs_E10;stage_3W_vs_E10;stage	1	TRUE
ENSGALG00010023862	TRMT13	PRJNA729271	-0.736370385	1.12351E-07	0.736370385	stage_E14_vs_E10	ordinary_muscle_development		0	FALSE
ENSGALG00010024206	KRT80	PRJNA729271	-5.015624644	1.12704E-07	5.015624644	stage_E14_vs_E10	ordinary_muscle_development	D_vs_E10;stage_1W_vs_E10;stage_3W_vs_E10;stage	1	TRUE
ENSGALG00010003865	AKAP8L	PRJNA729271	-1.037634946	1.12889E-07	1.037634946	stage_E14_vs_E10	ordinary_muscle_development	D_vs_E10;stage_1W_vs_E10;stage_3W_vs_E10;stage	1	TRUE
ENSGALG00010024581	RARA	PRJNA729271	0.933462463	1.14036E-07	0.933462463	stage_E14_vs_E10	ordinary_muscle_development		0	FALSE
ENSGALG00010029184	DYNLL2	PRJNA729271	0.519073105	1.15306E-07	0.519073105	stage_E14_vs_E10	ordinary_muscle_development		0	FALSE
ENSGALG00010017455	CCR9	PRJNA729271	3.700946769	1.15404E-07	3.700946769	stage_E14_vs_E10	ordinary_muscle_development	D_vs_E10;stage_1W_vs_E10;stage_3W_vs_E10;stage	1	TRUE
ENSGALG00010003169	MTRF1	PRJNA729271	0.586966821	1.15748E-07	0.586966821	stage_E14_vs_E10	ordinary_muscle_development		0	FALSE
ENSGALG00010024522	DNTTIP2	PRJNA729271	0.40634879	1.16049E-07	0.40634879	stage_E14_vs_E10	ordinary_muscle_development		0	FALSE
ENSGALG00010003772	ENSGALG00010003772	PRJNA729271	2.391174497	1.16384E-07	2.391174497	stage_E14_vs_E10	ordinary_muscle_development	D_vs_E10;stage_1W_vs_E10;stage_3W_vs_E10;stage	1	TRUE
ENSGALG00010027187	ENSGALG00010027187	PRJNA729271	-0.766368531	1.16401E-07	0.766368531	stage_E14_vs_E10	ordinary_muscle_development		0	FALSE
ENSGALG00010026943	EEFSEC	PRJNA729271	-0.388131379	1.17141E-07	0.388131379	stage_E14_vs_E10	ordinary_muscle_development		0	FALSE
ENSGALG00010000870	DACH1	PRJNA729271	0.770701725	1.17906E-07	0.770701725	stage_E14_vs_E10	ordinary_muscle_development		0	FALSE
ENSGALG00010012007	MREG	PRJNA729271	-1.252939968	1.18135E-07	1.252939968	stage_E14_vs_E10	ordinary_muscle_development	D_vs_E10;stage_1W_vs_E10;stage_3W_vs_E10;stage	1	TRUE
ENSGALG00010016190	THNSL2	PRJNA729271	0.826274772	1.18323E-07	0.826274772	stage_E14_vs_E10	ordinary_muscle_development		0	FALSE
ENSGALG00010015481	GOLGA5	PRJNA729271	0.389589546	1.18428E-07	0.389589546	stage_E14_vs_E10	ordinary_muscle_development		0	FALSE
ENSGALG00010005473	ENSGALG00010005473	PRJNA729271	3.478951631	1.19111E-07	3.478951631	stage_E14_vs_E10	ordinary_muscle_development	D_vs_E10;stage_1W_vs_E10;stage_3W_vs_E10;stage	1	TRUE
ENSGALG00010001692	ALDH18A1	PRJNA729271	-1.38827506	1.19216E-07	1.38827506	stage_E14_vs_E10	ordinary_muscle_development	D_vs_E10;stage_1W_vs_E10;stage_3W_vs_E10;stage	1	TRUE
ENSGALG00010005510	MYOZ2	PRJNA729271	1.790039879	1.19241E-07	1.790039879	stage_E14_vs_E10	ordinary_muscle_development	D_vs_E10;stage_1W_vs_E10;stage_3W_vs_E10;stage	1	TRUE
ENSGALG00010028744	HOMER3	PRJNA729271	-1.14045559	1.20007E-07	1.14045559	stage_E14_vs_E10	ordinary_muscle_development	D_vs_E10;stage_1W_vs_E10;stage_3W_vs_E10;stage	1	TRUE
ENSGALG00010020375	LRPPRC	PRJNA729271	0.550930835	1.20833E-07	0.550930835	stage_E14_vs_E10	ordinary_muscle_development		0	FALSE
ENSGALG00010013353	NDFIP1	PRJNA729271	-0.461024876	1.21664E-07	0.461024876	stage_E14_vs_E10	ordinary_muscle_development		0	FALSE
ENSGALG00010007975	ANKRD11	PRJNA729271	0.958316439	1.22477E-07	0.958316439	stage_E14_vs_E10	ordinary_muscle_development		0	FALSE
ENSGALG00010016958	TLR15	PRJNA729271	2.671207059	1.22959E-07	2.671207059	stage_E14_vs_E10	ordinary_muscle_development	D_vs_E10;stage_1W_vs_E10;stage_3W_vs_E10;stage	1	TRUE
ENSGALG00010006207	ENSGALG00010006207	PRJNA729271	-1.176252154	1.22966E-07	1.176252154	stage_E14_vs_E10	ordinary_muscle_development	D_vs_E10;stage_1W_vs_E10;stage_3W_vs_E10;stage	1	TRUE
ENSGALG00010028482	CD320	PRJNA729271	0.948926307	1.23019E-07	0.948926307	stage_E14_vs_E10	ordinary_muscle_development		0	FALSE
ENSGALG00010010136	EPHX2	PRJNA729271	0.634261297	1.23735E-07	0.634261297	stage_E14_vs_E10	ordinary_muscle_development		0	FALSE
ENSGALG00010019572	NFKBIE	PRJNA729271	-1.072055155	1.24459E-07	1.072055155	stage_E14_vs_E10	ordinary_muscle_development	D_vs_E10;stage_1W_vs_E10;stage_3W_vs_E10;stage	1	TRUE
ENSGALG00010008996	CHRM3	PRJNA729271	-1.315107182	1.24516E-07	1.315107182	stage_E14_vs_E10	ordinary_muscle_development	D_vs_E10;stage_1W_vs_E10;stage_3W_vs_E10;stage	1	TRUE
ENSGALG00010027826	ARHGEF11	PRJNA729271	0.889795168	1.24665E-07	0.889795168	stage_E14_vs_E10	ordinary_muscle_development		0	FALSE
ENSGALG00010008228	TMED6	PRJNA729271	3.551131631	1.24831E-07	3.551131631	stage_E14_vs_E10	ordinary_muscle_development	D_vs_E10;stage_1W_vs_E10;stage_3W_vs_E10;stage	1	TRUE
ENSGALG00010006570	ENSGALG00010006570	PRJNA729271	2.625500167	1.25018E-07	2.625500167	stage_E14_vs_E10	ordinary_muscle_development	D_vs_E10;stage_1W_vs_E10;stage_3W_vs_E10;stage	1	TRUE
ENSGALG00010024469	GTF2H1	PRJNA729271	0.690687101	1.25541E-07	0.690687101	stage_E14_vs_E10	ordinary_muscle_development		0	FALSE
ENSGALG00010018419	USP7	PRJNA729271	0.723877488	1.25742E-07	0.723877488	stage_E14_vs_E10	ordinary_muscle_development		0	FALSE
ENSGALG00010014805	SPEF2	PRJNA729271	-2.166824626	1.266E-07	2.166824626	stage_E14_vs_E10	ordinary_muscle_development	D_vs_E10;stage_1W_vs_E10;stage_3W_vs_E10;stage	1	TRUE
ENSGALG00010012896	STK38L	PRJNA729271	0.536639514	1.28161E-07	0.536639514	stage_E14_vs_E10	ordinary_muscle_development		0	FALSE
ENSGALG00010010972	EXOSC3	PRJNA729271	-0.917870137	1.28436E-07	0.917870137	stage_E14_vs_E10	ordinary_muscle_development		0	FALSE
ENSGALG00010024576	CSAD	PRJNA729271	-0.530760909	1.28639E-07	0.530760909	stage_E14_vs_E10	ordinary_muscle_development		0	FALSE
ENSGALG00010006016	MTMR6	PRJNA729271	0.412856799	1.28736E-07	0.412856799	stage_E14_vs_E10	ordinary_muscle_development		0	FALSE
ENSGALG00010007986	MYC	PRJNA729271	-1.065211972	1.28839E-07	1.065211972	stage_E14_vs_E10	ordinary_muscle_development	D_vs_E10;stage_1W_vs_E10;stage_3W_vs_E10;stage	1	TRUE
ENSGALG00010018721	LRCH2	PRJNA729271	1.030645478	1.29145E-07	1.030645478	stage_E14_vs_E10	ordinary_muscle_development	D_vs_E10;stage_1W_vs_E10;stage_3W_vs_E10;stage	1	TRUE
ENSGALG00010008129	TANGO6	PRJNA729271	0.632545232	1.29587E-07	0.632545232	stage_E14_vs_E10	ordinary_muscle_development		0	FALSE
ENSGALG00010024590	MRPL23	PRJNA729271	-0.578727684	1.30081E-07	0.578727684	stage_E14_vs_E10	ordinary_muscle_development		0	FALSE
ENSGALG00010018490	NME3	PRJNA729271	-0.659669025	1.30395E-07	0.659669025	stage_E14_vs_E10	ordinary_muscle_development		0	FALSE
ENSGALG00010024983	APOF	PRJNA729271	2.622618093	1.30502E-07	2.622618093	stage_E14_vs_E10	ordinary_muscle_development	D_vs_E10;stage_1W_vs_E10;stage_3W_vs_E10;stage	1	TRUE
ENSGALG00010003577	ENSGALG00010003577	PRJNA729271	8.383989156	1.30837E-07	8.383989156	stage_E14_vs_E10	ordinary_muscle_development	D_vs_E10;stage_1W_vs_E10;stage_3W_vs_E10;stage	1	TRUE
ENSGALG00010002880	RASGEF1B	PRJNA729271	-1.137842927	1.31718E-07	1.137842927	stage_E14_vs_E10	ordinary_muscle_development	D_vs_E10;stage_1W_vs_E10;stage_3W_vs_E10;stage	1	TRUE
ENSGALG00010021261	JUP	PRJNA729271	0.701354267	1.31873E-07	0.701354267	stage_E14_vs_E10	ordinary_muscle_development		0	FALSE
ENSGALG00010017428	PSMA3	PRJNA729271	-0.59487381	1.32462E-07	0.59487381	stage_E14_vs_E10	ordinary_muscle_development		0	FALSE
ENSGALG00010012268	ENSGALG00010012268	PRJNA729271	-0.915177183	1.32729E-07	0.915177183	stage_E14_vs_E10	ordinary_muscle_development		0	FALSE
ENSGALG00010017700	VPS35L	PRJNA729271	-0.42991655	1.32865E-07	0.42991655	stage_E14_vs_E10	ordinary_muscle_development		0	FALSE
ENSGALG00010006633	FBXO18	PRJNA729271	0.510116724	1.33148E-07	0.510116724	stage_E14_vs_E10	ordinary_muscle_development		0	FALSE
ENSGALG00010000735	ENSGALG00010000735	PRJNA729271	2.84661763	1.33748E-07	2.84661763	stage_E14_vs_E10	ordinary_muscle_development	D_vs_E10;stage_1W_vs_E10;stage_3W_vs_E10;stage	1	TRUE
ENSGALG00010028578	SPOUT1	PRJNA729271	-0.680103634	1.34005E-07	0.680103634	stage_E14_vs_E10	ordinary_muscle_development		0	FALSE
ENSGALG00010017610	PSMC1	PRJNA729271	0.642524516	1.34089E-07	0.642524516	stage_E14_vs_E10	ordinary_muscle_development		0	FALSE
ENSGALG00010008364	ENSGALG00010008364	PRJNA729271	-3.527232846	1.34166E-07	3.527232846	stage_E14_vs_E10	ordinary_muscle_development	D_vs_E10;stage_1W_vs_E10;stage_3W_vs_E10;stage	1	TRUE
ENSGALG00010016529	DCUN1D1	PRJNA729271	-0.539257552	1.34167E-07	0.539257552	stage_E14_vs_E10	ordinary_muscle_development		0	FALSE
ENSGALG00010027729	EPS8L3	PRJNA729271	1.31769128	1.35564E-07	1.31769128	stage_E14_vs_E10	ordinary_muscle_development	D_vs_E10;stage_1W_vs_E10;stage_3W_vs_E10;stage	1	TRUE
ENSGALG00010022208	CCL17	PRJNA729271	3.659763016	1.3642E-07	3.659763016	stage_E14_vs_E10	ordinary_muscle_development	D_vs_E10;stage_1W_vs_E10;stage_3W_vs_E10;stage	1	TRUE
ENSGALG00010014143	NFATC2	PRJNA729271	-0.854465516	1.36511E-07	0.854465516	stage_E14_vs_E10	ordinary_muscle_development		0	FALSE
ENSGALG00010023434	ATN1	PRJNA729271	-1.046542085	1.36562E-07	1.046542085	stage_E14_vs_E10	ordinary_muscle_development	D_vs_E10;stage_1W_vs_E10;stage_3W_vs_E10;stage	1	TRUE
ENSGALG00010014626	C2CD5	PRJNA729271	-0.49708696	1.36591E-07	0.49708696	stage_E14_vs_E10	ordinary_muscle_development		0	FALSE
ENSGALG00010020075	CPSF7	PRJNA729271	-0.804635057	1.38045E-07	0.804635057	stage_E14_vs_E10	ordinary_muscle_development		0	FALSE
ENSGALG00010015204	ENSGALG00010015204	PRJNA729271	0.753766737	1.3833E-07	0.753766737	stage_E14_vs_E10	ordinary_muscle_development		0	FALSE
ENSGALG00010027910	CHRNB2	PRJNA729271	-1.138470123	1.38548E-07	1.138470123	stage_E14_vs_E10	ordinary_muscle_development	D_vs_E10;stage_1W_vs_E10;stage_3W_vs_E10;stage	1	TRUE
ENSGALG00010007633	ENSGALG00010007633	PRJNA729271	0.554093615	1.39074E-07	0.554093615	stage_E14_vs_E10	ordinary_muscle_development		0	FALSE
ENSGALG00010021048	API5	PRJNA729271	-0.409681578	1.39114E-07	0.409681578	stage_E14_vs_E10	ordinary_muscle_development		0	FALSE
ENSGALG00010023068	CDPF1	PRJNA729271	-0.690124084	1.39115E-07	0.690124084	stage_E14_vs_E10	ordinary_muscle_development		0	FALSE
ENSGALG00010020187	MMP23B	PRJNA729271	-1.128188203	1.39182E-07	1.128188203	stage_E14_vs_E10	ordinary_muscle_development	D_vs_E10;stage_1W_vs_E10;stage_3W_vs_E10;stage	1	TRUE
ENSGALG00010025138	CFAP91	PRJNA729271	-1.597264435	1.40527E-07	1.597264435	stage_E14_vs_E10	ordinary_muscle_development	D_vs_E10;stage_1W_vs_E10;stage_3W_vs_E10;stage	1	TRUE
ENSGALG00010002616	ZNFB3	PRJNA729271	-0.926367144	1.40683E-07	0.926367144	stage_E14_vs_E10	ordinary_muscle_development		0	FALSE
ENSGALG00010017996	ZC3H14	PRJNA729271	-0.51139993	1.40705E-07	0.51139993	stage_E14_vs_E10	ordinary_muscle_development		0	FALSE
ENSGALG00010001288	TGIF1	PRJNA729271	-0.846147494	1.41932E-07	0.846147494	stage_E14_vs_E10	ordinary_muscle_development		0	FALSE
ENSGALG00010005789	RNASET2	PRJNA729271	0.83357809	1.42735E-07	0.83357809	stage_E14_vs_E10	ordinary_muscle_development		0	FALSE
ENSGALG00010028905	COLGALT1	PRJNA729271	0.468654853	1.4361E-07	0.468654853	stage_E14_vs_E10	ordinary_muscle_development		0	FALSE
ENSGALG00010012234	C1GALT1C1	PRJNA729271	-0.339589117	1.43951E-07	0.339589117	stage_E14_vs_E10	ordinary_muscle_development		0	FALSE
ENSGALG00010001899	TWF1	PRJNA729271	-0.609342292	1.46432E-07	0.609342292	stage_E14_vs_E10	ordinary_muscle_development		0	FALSE
ENSGALG00010007374	CMIP	PRJNA729271	-0.733035707	1.46498E-07	0.733035707	stage_E14_vs_E10	ordinary_muscle_development		0	FALSE
ENSGALG00010027207	NOL8	PRJNA729271	-0.68193449	1.48273E-07	0.68193449	stage_E14_vs_E10	ordinary_muscle_development		0	FALSE
ENSGALG00010025692	ENSGALG00010025692	PRJNA729271	5.140167917	1.49401E-07	5.140167917	stage_E14_vs_E10	ordinary_muscle_development	D_vs_E10;stage_1W_vs_E10;stage_3W_vs_E10;stage	1	TRUE

ENSGALG00010018491	TIA1	PRJNA729271	0.646074454	1.49417E-07	0.646074454	stage_E14_vs_E10	ordinary_muscle_development		0	FALSE
ENSGALG00010012988	RPL18A	PRJNA729271	0.734494352	1.49617E-07	0.734494352	stage_E14_vs_E10	ordinary_muscle_development		0	FALSE
ENSGALG00010005493	KLF6	PRJNA729271	0.9750396	1.50334E-07	0.9750396	stage_E14_vs_E10	ordinary_muscle_development		0	FALSE
ENSGALG00010017578	BSDC1	PRJNA729271	0.538267652	1.50948E-07	0.538267652	stage_E14_vs_E10	ordinary_muscle_development		0	FALSE
ENSGALG00010021354	ENSGALG00010021354	PRJNA729271	0.622426738	1.51019E-07	0.622426738	stage_E14_vs_E10	ordinary_muscle_development		0	FALSE
ENSGALG00010001302	ENSGALG00010001302	PRJNA729271	-2.750367443	1.52308E-07	2.750367443	stage_E14_vs_E10	ordinary_muscle_development	D_vs_E10;stage_1W_vs_E10;stage_3W_vs_E10;stage	1	TRUE
ENSGALG00010029778	DOC2B	PRJNA729271	1.862496501	1.53116E-07	1.862496501	stage_E14_vs_E10	ordinary_muscle_development	D_vs_E10;stage_1W_vs_E10;stage_3W_vs_E10;stage	1	TRUE
ENSGALG00010013013	DCSTAMP	PRJNA729271	5.71561615	1.53775E-07	5.71561615	stage_E14_vs_E10	ordinary_muscle_development	D_vs_E10;stage_1W_vs_E10;stage_3W_vs_E10;stage	1	TRUE
ENSGALG00010010451	RANBP17	PRJNA729271	-3.085243007	1.53963E-07	3.085243007	stage_E14_vs_E10	ordinary_muscle_development	D_vs_E10;stage_1W_vs_E10;stage_3W_vs_E10;stage	1	TRUE
ENSGALG00010002410	RNF121	PRJNA729271	0.560390266	1.54096E-07	0.560390266	stage_E14_vs_E10	ordinary_muscle_development		0	FALSE
ENSGALG00010028458	TRAF2	PRJNA729271	0.602979408	1.54122E-07	0.602979408	stage_E14_vs_E10	ordinary_muscle_development		0	FALSE
ENSGALG00010020927	ENSGALG00010020927	PRJNA729271	-0.639714917	1.55258E-07	0.639714917	stage_E14_vs_E10	ordinary_muscle_development		0	FALSE
ENSGALG00010017545	DICER1	PRJNA729271	-0.674857616	1.55365E-07	0.674857616	stage_E14_vs_E10	ordinary_muscle_development		0	FALSE
ENSGALG00010014898	ENSGALG00010014898	PRJNA729271	3.442623087	1.55636E-07	3.442623087	stage_E14_vs_E10	ordinary_muscle_development	D_vs_E10;stage_1W_vs_E10;stage_3W_vs_E10;stage	1	TRUE
ENSGALG00010001180	SPIRE1	PRJNA729271	-0.972050426	1.55733E-07	0.972050426	stage_E14_vs_E10	ordinary_muscle_development		0	FALSE
ENSGALG00010012156	ENSGALG00010012156	PRJNA729271	-4.402286481	1.56456E-07	4.402286481	stage_E14_vs_E10	ordinary_muscle_development	D_vs_E10;stage_1W_vs_E10;stage_3W_vs_E10;stage	1	TRUE
ENSGALG00010021434	ANKZF1	PRJNA729271	0.947668534	1.57016E-07	0.947668534	stage_E14_vs_E10	ordinary_muscle_development		0	FALSE
ENSGALG00010009736	WISP1	PRJNA729271	-1.792346666	1.58143E-07	1.792346666	stage_E14_vs_E10	ordinary_muscle_development	D_vs_E10;stage_1W_vs_E10;stage_3W_vs_E10;stage	1	TRUE
ENSGALG00010008408	SMARCA4	PRJNA729271	-0.828020736	1.58551E-07	0.828020736	stage_E14_vs_E10	ordinary_muscle_development		0	FALSE
ENSGALG00010020116	GNA12	PRJNA729271	-0.390898366	1.58794E-07	0.390898366	stage_E14_vs_E10	ordinary_muscle_development		0	FALSE
ENSGALG00010029376	ENSGALG00010029376	PRJNA729271	-1.126857045	1.59669E-07	1.126857045	stage_E14_vs_E10	ordinary_muscle_development	D_vs_E10;stage_1W_vs_E10;stage_3W_vs_E10;stage	1	TRUE
ENSGALG00010016332	ENSGALG00010016332	PRJNA729271	-1.373774045	1.59843E-07	1.373774045	stage_E14_vs_E10	ordinary_muscle_development	D_vs_E10;stage_1W_vs_E10;stage_3W_vs_E10;stage	1	TRUE
ENSGALG00010002940	BG1	PRJNA729271	2.171870431	1.60175E-07	2.171870431	stage_E14_vs_E10	ordinary_muscle_development	D_vs_E10;stage_1W_vs_E10;stage_3W_vs_E10;stage	1	TRUE
ENSGALG00010009555	EPC2	PRJNA729271	0.407818436	1.60673E-07	0.407818436	stage_E14_vs_E10	ordinary_muscle_development		0	FALSE
ENSGALG00010009172	SPOPL	PRJNA729271	0.710033639	1.61205E-07	0.710033639	stage_E14_vs_E10	ordinary_muscle_development		0	FALSE
ENSGALG00010001928	KCTD21	PRJNA729271	-0.962160501	1.61306E-07	0.962160501	stage_E14_vs_E10	ordinary_muscle_development		0	FALSE
ENSGALG00010004083	PHF11	PRJNA729271	-1.029947821	1.62146E-07	1.029947821	stage_E14_vs_E10	ordinary_muscle_development	D_vs_E10;stage_1W_vs_E10;stage_3W_vs_E10;stage	1	TRUE
ENSGALG00010028054	RPS15	PRJNA729271	0.764128013	1.63773E-07	0.764128013	stage_E14_vs_E10	ordinary_muscle_development		0	FALSE
ENSGALG00010017473	EIF3I	PRJNA729271	0.56027214	1.63887E-07	0.56027214	stage_E14_vs_E10	ordinary_muscle_development		0	FALSE
ENSGALG00010004689	BBX	PRJNA729271	0.449910518	1.64368E-07	0.449910518	stage_E14_vs_E10	ordinary_muscle_development		0	FALSE
ENSGALG00010006912	ENSGALG00010006912	PRJNA729271	-0.994913837	1.64506E-07	0.994913837	stage_E14_vs_E10	ordinary_muscle_development		0	FALSE
ENSGALG00010008310	EXOSC5	PRJNA729271	0.953895514	1.64758E-07	0.953895514	stage_E14_vs_E10	ordinary_muscle_development		0	FALSE
ENSGALG00010000458	ENSGALG00010000458	PRJNA729271	1.06792705	1.65369E-07	1.06792705	stage_E14_vs_E10	ordinary_muscle_development	D_vs_E10;stage_1W_vs_E10;stage_3W_vs_E10;stage	1	TRUE
ENSGALG00010024377	CIB2	PRJNA729271	0.935174973	1.66699E-07	0.935174973	stage_E14_vs_E10	ordinary_muscle_development		0	FALSE
ENSGALG00010025642	RPS8	PRJNA729271	0.629794522	1.68821E-07	0.629794522	stage_E14_vs_E10	ordinary_muscle_development		0	FALSE
ENSGALG00010001609	ENSGALG00010001609	PRJNA729271	2.42404358	1.70342E-07	2.42404358	stage_E14_vs_E10	ordinary_muscle_development	D_vs_E10;stage_1W_vs_E10;stage_3W_vs_E10;stage	1	TRUE
ENSGALG00010004996	GPR158	PRJNA729271	1.151925807	1.70724E-07	1.151925807	stage_E14_vs_E10	ordinary_muscle_development	D_vs_E10;stage_1W_vs_E10;stage_3W_vs_E10;stage	1	TRUE
ENSGALG00010027027	ARHGEF3	PRJNA729271	0.545743929	1.72312E-07	0.545743929	stage_E14_vs_E10	ordinary_muscle_development		0	FALSE
ENSGALG00010012041	ICOSLG	PRJNA729271	-0.830500515	1.72631E-07	0.830500515	stage_E14_vs_E10	ordinary_muscle_development		0	FALSE
ENSGALG00010007160	ENSGALG00010007160	PRJNA729271	1.573145124	1.73434E-07	1.573145124	stage_E14_vs_E10	ordinary_muscle_development	D_vs_E10;stage_1W_vs_E10;stage_3W_vs_E10;stage	1	TRUE
ENSGALG00010005564	TM9SF2	PRJNA729271	0.310323493	1.75553E-07	0.310323493	stage_E14_vs_E10	ordinary_muscle_development		0	FALSE
ENSGALG00010015478	FBXO7	PRJNA729271	0.337476972	1.75847E-07	0.337476972	stage_E14_vs_E10	ordinary_muscle_development		0	FALSE
ENSGALG00010020666	TMCO4	PRJNA729271	1.100266541	1.77039E-07	1.100266541	stage_E14_vs_E10	ordinary_muscle_development	D_vs_E10;stage_1W_vs_E10;stage_3W_vs_E10;stage	1	TRUE
ENSGALG00010015148	ENSGALG00010015148	PRJNA729271	-0.411608273	1.77128E-07	0.411608273	stage_E14_vs_E10	ordinary_muscle_development		0	FALSE
ENSGALG00010022525	ISCU	PRJNA729271	1.038256434	1.77172E-07	1.038256434	stage_E14_vs_E10	ordinary_muscle_development	D_vs_E10;stage_1W_vs_E10;stage_3W_vs_E10;stage	1	TRUE
ENSGALG00010002382	ART1D	PRJNA729271	2.807356691	1.7749E-07	2.807356691	stage_E14_vs_E10	ordinary_muscle_development	D_vs_E10;stage_1W_vs_E10;stage_3W_vs_E10;stage	1	TRUE
ENSGALG00010028199	DPP9	PRJNA729271	-0.58592886	1.80001E-07	0.58592886	stage_E14_vs_E10	ordinary_muscle_development		0	FALSE
ENSGALG00010023042	ENSGALG00010023042	PRJNA729271	-5.592332169	1.82273E-07	5.592332169	stage_E14_vs_E10	ordinary_muscle_development	D_vs_E10;stage_1W_vs_E10;stage_3W_vs_E10;stage	1	TRUE
ENSGALG00010000023	ATP6	PRJNA729271	1.068529358	1.831E-07	1.068529358	stage_E14_vs_E10	ordinary_muscle_development	D_vs_E10;stage_1W_vs_E10;stage_3W_vs_E10;stage	1	TRUE
ENSGALG00010011407	CEP290	PRJNA729271	-0.970747884	1.83219E-07	0.970747884	stage_E14_vs_E10	ordinary_muscle_development		0	FALSE
ENSGALG00010017748	ENSGALG00010017748	PRJNA729271	-4.673492452	1.837E-07	4.673492452	stage_E14_vs_E10	ordinary_muscle_development	D_vs_E10;stage_1W_vs_E10;stage_3W_vs_E10;stage	1	TRUE
ENSGALG00010025343	NAB2	PRJNA729271	1.541332587	1.83723E-07	1.541332587	stage_E14_vs_E10	ordinary_muscle_development	D_vs_E10;stage_1W_vs_E10;stage_3W_vs_E10;stage	1	TRUE
ENSGALG00010012240	TRIM14	PRJNA729271	-1.418386561	1.83892E-07	1.418386561	stage_E14_vs_E10	ordinary_muscle_development	D_vs_E10;stage_1W_vs_E10;stage_3W_vs_E10;stage	1	TRUE
ENSGALG00010015085	PSMD2	PRJNA729271	-0.888793877	1.84966E-07	0.888793877	stage_E14_vs_E10	ordinary_muscle_development		0	FALSE
ENSGALG00010001707	ENSGALG00010001707	PRJNA729271	5.559805023	1.85052E-07	5.559805023	stage_E14_vs_E10	ordinary_muscle_development	D_vs_E10;stage_1W_vs_E10;stage_3W_vs_E10;stage	1	TRUE
ENSGALG00010000435	P2RX6	PRJNA729271	-1.915181205	1.85519E-07	1.915181205	stage_E14_vs_E10	ordinary_muscle_development	D_vs_E10;stage_1W_vs_E10;stage_3W_vs_E10;stage	1	TRUE
ENSGALG00010005457	SEMA3D	PRJNA729271	-0.85791791	1.86643E-07	0.85791791	stage_E14_vs_E10	ordinary_muscle_development		0	FALSE
ENSGALG00010017681	ENSGALG00010017681	PRJNA729271	6.574351537	1.87904E-07	6.574351537	stage_E14_vs_E10	ordinary_muscle_development	D_vs_E10;stage_1W_vs_E10;stage_3W_vs_E10;stage	1	TRUE
ENSGALG00010025595	ERI3	PRJNA729271	-0.50581249	1.90151E-07	0.50581249	stage_E14_vs_E10	ordinary_muscle_development		0	FALSE
ENSGALG00010022858	HERC4	PRJNA729271	0.678429314	1.9035E-07	0.678429314	stage_E14_vs_E10	ordinary_muscle_development		0	FALSE
ENSGALG00010003980	CHCHD7	PRJNA729271	0.632825126	1.90416E-07	0.632825126	stage_E14_vs_E10	ordinary_muscle_development		0	FALSE
ENSGALG00010012904	ERMP1	PRJNA729271	-0.750142103	1.91316E-07	0.750142103	stage_E14_vs_E10	ordinary_muscle_development		0	FALSE
ENSGALG00010009096	ENSGALG00010009096	PRJNA729271	5.993297604	1.91906E-07	5.993297604	stage_E14_vs_E10	ordinary_muscle_development	D_vs_E10;stage_1W_vs_E10;stage_3W_vs_E10;stage	1	TRUE
ENSGALG00010018609	ENSGALG00010018609	PRJNA729271	0.66306457	1.92029E-07	0.66306457	stage_E14_vs_E10	ordinary_muscle_development		0	FALSE
ENSGALG00010007137	ENSGALG00010007137	PRJNA729271	1.915234004	1.92579E-07	1.915234004	stage_E14_vs_E10	ordinary_muscle_development	D_vs_E10;stage_1W_vs_E10;stage_3W_vs_E10;stage	1	TRUE
ENSGALG00010023794	MT3	PRJNA729271	2.44808676	1.93802E-07	2.44808676	stage_E14_vs_E10	ordinary_muscle_development	D_vs_E10;stage_1W_vs_E10;stage_3W_vs_E10;stage	1	TRUE
ENSGALG00010023777	OAZ2	PRJNA729271	0.70511772	1.93993E-07	0.70511772	stage_E14_vs_E10	ordinary_muscle_development		0	FALSE
ENSGALG00010024867	TM2D3	PRJNA729271	0.561913715	1.94083E-07	0.561913715	stage_E14_vs_E10	ordinary_muscle_development		0	FALSE
ENSGALG00010002890	VXN	PRJNA729271	0.874383966	1.9424E-07	0.874383966	stage_E14_vs_E10	ordinary_muscle_development		0	FALSE
ENSGALG00010022200	DNMBP	PRJNA729271	0.589282614	1.94916E-07	0.589282614	stage_E14_vs_E10	ordinary_muscle_development		0	FALSE
ENSGALG00010005143	GTF3A	PRJNA729271	-0.747931035	1.96343E-07	0.747931035	stage_E14_vs_E10	ordinary_muscle_development		0	FALSE
ENSGALG00010002471	OGFRL1	PRJNA729271	0.831526158	1.96529E-07	0.831526158	stage_E14_vs_E10	ordinary_muscle_development		0	FALSE
ENSGALG00010029461	ENSGALG00010029461	PRJNA729271	-1.089432554	1.97675E-07	1.089432554	stage_E14_vs_E10	ordinary_muscle_development	D_vs_E10;stage_1W_vs_E10;stage_3W_vs_E10;stage	1	TRUE
ENSGALG00010008214	CCNG2	PRJNA729271	-0.725835012	1.9927E-07	0.725835012	stage_E14_vs_E10	ordinary_muscle_development		0	FALSE
ENSGALG00010011861	ENSGALG00010011861	PRJNA729271	-3.886309607	2.02352E-07	3.886309607	stage_E14_vs_E10	ordinary_muscle_development	D_vs_E10;stage_1W_vs_E10;stage_3W_vs_E10;stage	1	TRUE
ENSGALG00010008807	BRD1	PRJNA729271	0.32937935	2.02362E-07	0.32937935	stage_E14_vs_E10	ordinary_muscle_development		0	FALSE
ENSGALG00010016808	ENTPD3	PRJNA729271	-4.183719517	2.02521E-07	4.183719517	stage_E14_vs_E10	ordinary_muscle_development	D_vs_E10;stage_1W_vs_E10;stage_3W_vs_E10;stage	1	TRUE
ENSGALG00010006187	TCF21	PRJNA729271	-3.640330567	2.03303E-07	3.640330567	stage_E14_vs_E10	ordinary_muscle_development	D_vs_E10;stage_1W_vs_E10;stage_3W_vs_E10;stage	1	TRUE
ENSGALG00010001886	NEU3	PRJNA729271	-0.453050897	2.03496E-07	0.453050897	stage_E14_vs_E10	ordinary_muscle_development		0	FALSE
ENSGALG00010014971	SMC5	PRJNA729271	-0.670673864	2.03614E-07	0.670673864	stage_E14_vs_E10	ordinary_muscle_development		0	FALSE
ENSGALG00010004624	PHF10	PRJNA729271	0.403794209	2.04929E-07	0.403794209	stage_E14_vs_E10	ordinary_muscle_development		0	FALSE
ENSGALG00010016580	MARS2	PRJNA729271	-0.601681161	2.05272E-07	0.601681161	stage_E14_vs_E10	ordinary_muscle_development		0	FALSE
ENSGALG00010006103	NXPE3	PRJNA729271	-0.653711603	2.05502E-07	0.653711603	stage_E14_vs_E10	ordinary_muscle_development		0	FALSE
ENSGALG00010026559	RAB36	PRJNA729271	-3.570569638	2.06201E-07	3.570569638	stage_E14_vs_E10	ordinary_muscle_development	D_vs_E10;stage_1W_vs_E10;stage_3W_vs_E10;stage	1	TRUE
ENSGALG00010001333	PLCH1	PRJNA729271	-1.65632341	2.06201E-07	1.65632341	stage_E14_vs_E10	ordinary_muscle_development	D_vs_E10;stage_1W_vs_E10;stage_3W_vs_E10;stage	1	TRUE
ENSGALG00010010845	FKRP	PRJNA729271	1.333414028	2.07137E-07	1.333414028	stage_E14_vs_E10	ordinary_muscle_development	D_vs_E10;stage_1W_vs_E10;stage_3W_vs_E10;stage	1	TRUE
ENSGALG00010002160	CAPN5	PRJNA729271	0.89071666	2.0739E-07	0.89071666	stage_E14_vs_E10	ordinary_muscle_development		0	FALSE
ENSGALG00010016083	CDH22	PRJNA729271	-3.311157763	2.07964E-07	3.311157763	stage_E14_vs_E10	ordinary_muscle_development	D_vs_E10;stage_1W_vs_E10;stage_3W_vs_E10;stage	1	TRUE
ENSGALG00010018539	CHD2	PRJNA729271	-0.473130165	2.08833E-07	0.473130165	stage_E14_vs_E10	ordinary_muscle_development		0	FALSE
ENSGALG00010019836	MINAR1	PRJNA729271	2.555931172	2.09824E-07	2.555931172	stage_E14_vs_E10	ordinary_muscle_development	D_vs_E10;stage_1W_vs_E10;stage_3W_vs_E10;stage	1	TRUE
ENSGALG00010003294	ENSGALG00010003294	PRJNA729271	-23.93606849	2.10324E-07	23.93606849	stage_E14_vs_E10	ordinary_muscle_development	D_vs_E10;stage_1W_vs_E10;stage_3W_vs_E10;stage	1	TRUE
ENSGALG00010029835	RECQL5	PRJNA729271	-0.600130708	2.10622E-07	0.600130708	stage_E14_vs_E10	ordinary_muscle_development		0	FALSE
ENSGALG00010022992	SLC7A6	PRJNA729271	-0.582036545	2.10622E-07	0.582036545	stage_E14_vs_E10	ordinary_muscle_development		0	FALSE
ENSGALG00010013241	SEC24A	PRJNA729271	0.720258254	2.11156E-07	0.720258254	stage_E14_vs_E10	ordinary_muscle_development		0	FALSE
ENSGALG00010029397	OVCA2	PRJNA729271	-0.844423333	2.1155E-07	0.844423333	stage_E14_vs_E10	ordinary_muscle_development		0	FALSE
ENSGALG00010006033	NSUN6	PRJNA729271	-0.965245463	2.1335E-07	0.965245463	stage_E14_vs_E10	ordinary_muscle_development		0	FALSE
ENSGALG00010000428	GNL3L	PRJNA729271	-0.79446261	2.1497E-07	0.79446261	stage_E14_vs_E10	ordinary_muscle_development		0	FALSE
ENSGALG00010022085	MLLT6	PRJNA729271	0.952673727	2.15266E-07	0.952673727	stage_E14_vs_E10	ordinary_muscle_development		0	FALSE
ENSGALG00010005288	ENSGALG00010005288	PRJNA729271	-0.507738727	2.15339E-07	0.507738727	stage_E14_vs_E10	ordinary_muscle_development		0	FALSE
ENSGALG00010005268	MTTP	PRJNA729271	4.68729694	2.17042E-07	4.68729694	stage_E14_vs_E10	ordinary_muscle_development	D_vs_E10;stage_1W_vs_E10;stage_3W_vs_E10;stage	1	TRUE
ENSGALG00010012017	N4BP3	PRJNA729271	-0.702711503	2.17369E-07	0.702711503	stage_E14_vs_E10	ordinary_muscle_development		0	FALSE
ENSGALG00010020625	SLC30A1	PRJNA729271	-0.408446405	2.17467E-07	0.408446405	stage_E14_vs_E10	ordinary_muscle_development		0	FALSE
ENSGALG00010029379	GTF2I	PRJNA729271	0.595381348	2.17824E-07	0.595381348	stage_E14_vs_E10	ordinary_muscle_development		0	FALSE
ENSGALG00010002457	ENSGALG00010002457	PRJNA729271	2.345124405	2.1825E-07	2.345124405	stage_E14_vs_E10	ordinary_muscle_development	D_vs_E10;stage_1W_vs_E10;stage_3W_vs_E10;stage	1	TRUE
ENSGALG00010013322	IRS2	PRJNA729271	2.712170396	2.18353E-07	2.712170396	stage_E14_vs_E10	ordinary_muscle_development	D_vs_E10;stage_1W_vs_E10;stage_3W_vs_E10;stage	1	TRUE
ENSGALG00010005552	WDR37	PRJNA729271	-0.463266758	2.18458E-07	0.463266758	stage_E14_vs_E10	ordinary_muscle_development		0	FALSE
ENSGALG00010029556	NPTX1	PRJNA729271	-3.04800165	2.19428E-07	3.04800165	stage_E14_vs_E10	ordinary_muscle_development	D_vs_E10;stage_1W_vs_E10;stage_3W_vs_E10;stage	1	TRUE
ENSGALG00010021421	GLB1L	PRJNA729271	0.87720968	2.20155E-07	0.87720968	stage_E14_vs_E10	ordinary_muscle_development		0	FALSE
ENSGALG00010007968	SLC22A31	PRJNA729271	1.071609173	2.22883E-07	1.071609173	stage_E14_vs_E10	ordinary_muscle_development	D_vs_E10;stage_1W_vs_E10;stage_3W_vs_E10;stage	1	TRUE
ENSGALG00010010618	APAF1	PRJNA729271	-0.731705526	2.25568E-07	0.731705526	stage_E14_vs_E10	ordinary_muscle_development		0	FALSE
ENSGALG00010024826	RBM33	PRJNA729271	0.591673457	2.27407E-07	0.591673457	stage_E14_vs_E10	ordinary_muscle_development		0	FALSE
ENSGALG00010013976	CDC40	PRJNA729271	-0.410791465	2.289E-07	0.410791465	stage_E14_vs_E10	ordinary_muscle_development		0	FALSE
ENSGALG00010016732	ARHGAP26	PRJNA729271	0.409684137	2.29094E-07	0.409684137	stage_E14_vs_E10	ordinary_muscle_development		0	FALSE
ENSGALG00010028537	ENSGALG00010028537	PRJNA729271	10.78187594	2.31925E-07	10.78187594	stage_E14_vs_E10	ordinary_muscle_development	D_vs_E10;stage_1W_vs_E10;stage_3W_vs_E10;stage	1	TRUE
ENSGALG00010005353	ENSGALG00010005353	PRJNA729271	0.554543998	2.3451E-07	0.554543998	stage_E14_vs_E10	ordinary_muscle_development		0	FALSE
ENSGALG00010015493	OSTF1	PRJNA729271	-0.757092408	2.34645E-07	0.757092408	stage_E14_vs_E10	ordinary_muscle_development		0	FALSE
ENSGALG00010019693	HAUS2	PRJNA729271	-0.638220523	2.35005E-07	0.638220523	stage_E14_vs_E10	ordinary_muscle_development		0	FALSE
ENSGALG00010024578	ENSGALG00010024578	PRJNA729271	3.038056378	2.36967E-07	3.038056378	stage_E14_vs_E10	ordinary_muscle_development	D_vs_E10;stage_1W_vs_E10;stage_3W_vs_E10;stage	1	TRUE
ENSGALG00010025905	CPT1A	PRJNA729271	0.925954334	2.37536E-07	0.925954334	stage_E14_vs_E10	ordinary_muscle_development		0	FALSE
ENSGALG00010019713	NUDT14	PRJNA729271	0.610989343	2.37646E-07	0.610989343	stage_E14_vs_E10	ordinary_muscle_development		0	FALSE
ENSGALG00010001935	ABRAXAS2	PRJNA729271	0.683012122	2.3814E-07	0.683012122	stage_E14_vs_E10	ordinary_muscle_development		0	FALSE
ENSGALG00010003087	TWISTNB	PRJNA729271	0.938585898	2.38346E-07	0.938585898	stage_E14_vs_E10	ordinary_muscle_development		0	FALSE
ENSGALG00010021699	SYS1	PRJNA729271	-0.459475482	2.38935E-07	0.459475482	stage_E14_vs_E10	ordinary_muscle_development		0	FALSE
ENSGALG00010028857	MED22	PRJNA729271	0.422212473	2.39092E-07	0.422212473	stage_E14_vs_E10	ordinary_muscle_development		0	FALSE
ENSGALG00010012127	ENSGALG00010012127	PRJNA729271	-0.620687788	2.39527E-07	0.620687788	stage_E14_vs_E10	ordinary_muscle_development		0	FALSE
ENSGALG00010020440	PAX7	PRJNA729271	1.021177928	2.40276E-07	1.021177928	stage_E14_vs_E10	ordinary_muscle_development	D_vs_E10;stage_1W_vs_E10;stage_3W_vs_E10;stage	1	TRUE
ENSGALG00010008466	C11orf97	PRJNA729271	-2.438474355	2.40421E-07	2.438474355	stage_E14_vs_E10	ordinary_muscle_development	D_vs_E10;stage_1W_vs_E10;stage_3W_vs_E10;stage	1	TRUE
ENSGALG00010005448	ENSGALG00010005448	PRJNA729271	0.780110122	2.40542E-07	0.780110122	stage_E14_vs_E10	ordinary_muscle_development		0	FALSE
ENSGALG00010028014	ENSGALG00010028014	PRJNA729271	1.540085425	2.42174E-07	1.540085425	stage_E14_vs_E10	ordinary_muscle_development	D_vs_E10;stage_1W_vs_E10;stage_3W_vs_E10;stage	1	TRUE
ENSGALG00010017017	ENSGALG00010017017	PRJNA729271	1.380829664	2.43129E-07	1.380829664	stage_E14_vs_E10	ordinary_muscle_development	D_vs_E10;stage_1W_vs_E10;stage_3W_vs_E10;stage	1	TRUE
ENSGALG00010026232	PSMC5	PRJNA729271	0.692270688	2.44052E-07	0.692270688	stage_E14_vs_E10	ordinary_muscle_development		0	FALSE
ENSGALG00010005347	DOK1	PRJNA729271	-0.828288956	2.4442E-07	0.828288956	stage_E14_vs_E10	ordinary_muscle_development		0	FALSE
ENSGALG00010005131	IL8L2	PRJNA729271	3.436488904	2.4482E-07	3.436488904	stage_E14_vs_E10	ordinary_muscle_development	D_vs_E10;stage_1W_vs_E10;stage_3W_vs_E10;stage	1	TRUE
ENSGALG00010027940	ZBTB7B	PRJNA729271	2.084092633	2.44975E-07	2.084092633	stage_E14_vs_E10	ordinary_muscle_development	D_vs_E10;stage_1W_vs_E10;stage_3W_vs_E10;stage	1	TRUE
ENSGALG00010003850	PLA2G12A	PRJNA729271	-0.530621493	2.47045E-07	0.530621493	stage_E14_vs_E10	ordinary_muscle_development		0	FALSE
ENSGALG00010010917	TANC1	PRJNA729271	0.362748374	2.47801E-07	0.362748374	stage_E14_vs_E10	ordinary_muscle_development		0	FALSE
ENSGALG00010013423	ZNF804A	PRJNA729271	-2.499050847	2.49099E-07	2.499050847	stage_E14_vs_E10	ordinary_muscle_development	D_vs_E10;stage_1W_vs_E10;stage_3W_vs_E10;stage	1	TRUE
ENSGALG00010028764	ARNT	PRJNA729271	0.662514772	2.5E-07	0.662514772	stage_E14_vs_E10	ordinary_muscle_development		0	FALSE
ENSGALG00010027632	LAMC3	PRJNA729271	-2.343856637	2.51479E-07	2.343856637	stage_E14_vs_E10	ordinary_muscle_development	D_vs_E10;stage_1W_vs_E10;stage_3W_vs_E10;stage	1	TRUE
ENSGALG00010023637	RHBDD1	PRJNA729271	0.55323349	2.52865E-07	0.55323349	stage_E14_vs_E10	ordinary_muscle_development		0	FALSE
ENSGALG00010019690	ENSGALG00010019690	PRJNA729271	-0.4864266	2.53371E-07	0.4864266	stage_E14_vs_E10	ordinary_muscle_development		0	FALSE
ENSGALG00010027326	MRPL24	PRJNA729271	0.621132594	2.54011E-07	0.621132594	stage_E14_vs_E10	ordinary_muscle_development		0	FALSE
ENSGALG00010018371	ENSGALG00010018371	PRJNA729271	0.719997873	2.54697E-07	0.719997873	stage_E14_vs_E10	ordinary_muscle_development		0	FALSE
ENSGALG00010022463	DUSP12	PRJNA729271	0.610857992	2.54961E-07	0.610857992	stage_E14_vs_E10	ordinary_muscle_development		0	FALSE
ENSGALG00010012923	MLANA	PRJNA729271	-5.322206599	2.55083E-07	5.322206599	stage_E14_vs_E10	ordinary_muscle_development	D_vs_E10;stage_1W_vs_E10;stage_3W_vs_E10;stage	1	TRUE
ENSGALG00010007381	EGFR	PRJNA729271	-0.519524299	2.56605E-07	0.519524299	stage_E14_vs_E10	ordinary_muscle_development		0	FALSE
ENSGALG00010000377	ENSGALG00010000377	PRJNA729271	1.211232438	2.56785E-07	1.211232438	stage_E14_vs_E10	ordinary_muscle_development	D_vs_E10;stage_1W_vs_E10;stage_3W_vs_E10;stage	1	TRUE
ENSGALG00010028695	VSIG8	PRJNA729271	4.368959224	2.58756E-07	4.368959224	stage_E14_vs_E10	ordinary_muscle_development	D_vs_E10;stage_1W_vs_E10;stage_3W_vs_E10;stage	1	TRUE
ENSGALG00010024198	PI4KA	PRJNA729271	0.339667478	2.59714E-07	0.339667478	stage_E14_vs_E10	ordinary_muscle_development		0	FALSE
ENSGALG00010012044	ENSGALG00010012044	PRJNA729271	3.187756203	2.59718E-07	3.187756203	stage_E14_vs_E10	ordinary_muscle_development	D_vs_E10;stage_1W_vs_E10;stage_3W_vs_E10;stage	1	TRUE
ENSGALG00010003669	ENSGALG00010003669	PRJNA729271	7.721836318	2.60232E-07	7.721836318	stage_E14_vs_E10	ordinary_muscle_development	D_vs_E10;stage_1W_vs_E10;stage_3W_vs_E10;stage	1	TRUE
ENSGALG00010027976	LRSAM1	PRJNA729271	-0.32057016	2.65463E-07	0.32057016	stage_E14_vs_E10	ordinary_muscle_development		0	FALSE
ENSGALG00010008266	ECSCR	PRJNA729271	0.987994047	2.65698E-07	0.987994047	stage_E14_vs_E10	ordinary_muscle_development		0	FALSE
ENSGALG00010010745	ATVR1	PRJNA729271	-0.337280443	2.67803E-07	0.337280443	stage_E14_vs_E10	ordinary_muscle_development		0	FALSE
ENSGALG00010012985	ARNTL2	PRJNA729271	-0.938710616	2.68917E-07	0.938710616	stage_E14_vs_E10	ordinary_muscle_development		0	FALSE
ENSGALG00010002796	PRDM8	PRJNA729271	3.693404864	2.69495E-07	3.693404864	stage_E14_vs_E10	ordinary_muscle_development	D_vs_E10;stage_1W_vs_E10;stage_3W_vs_E10;stage	1	TRUE
ENSGALG00010025953	POU2F3	PRJNA729271	3.819705744	2.69963E-07	3.819705744	stage_E14_vs_E10	ordinary_muscle_development	D_vs_E10;stage_1W_vs_E10;stage_3W_vs_E10;stage	1	TRUE
ENSGALG00010008240	RCAN2	PRJNA729271	-0.75742224	2.71902E-07	0.75742224	stage_E14_vs_E10	ordinary_muscle_development		0	FALSE
ENSGALG00010016883	TMEM251	PRJNA729271	0.404709817	2.74349E-07	0.404709817	stage_E14_vs_E10	ordinary_muscle_development		0	FALSE
ENSGALG00010021466	HES4	PRJNA729271	-0.961809783	2.75512E-07	0.961809783	stage_E14_vs_E10	ordinary_muscle_development		0	FALSE
ENSGALG00010004755	PAX9	PRJNA729271	-5.156389069	2.76111E-07	5.156389069	stage_E14_vs_E10	ordinary_muscle_development	D_vs_E10;stage_1W_vs_E10;stage_3W_vs_E10;stage	1	TRUE
ENSGALG00010012943	ASNSD1	PRJNA729271	-0.665331247	2.76183E-07	0.665331247	stage_E14_vs_E10	ordinary_muscle_development		0	FALSE
ENSGALG00010011906	ENSGALG00010011906	PRJNA729271	-5.343610919	2.77501E-07	5.343610919	stage_E14_vs_E10	ordinary_muscle_development	D_vs_E10;stage_1W_vs_E10;stage_3W_vs_E10;stage	1	TRUE
ENSGALG00010008018	ENSGALG00010008018	PRJNA729271	1.090764613	2.77539E-07	1.090764613	stage_E14_vs_E10	ordinary_muscle_development	D_vs_E10;stage_1W_vs_E10;stage_3W_vs_E10;stage	1	TRUE
ENSGALG00010023917	ZRANB2	PRJNA729271	-0.423072798	2.78394E-07	0.423072798	stage_E14_vs_E10	ordinary_muscle_development		0	FALSE
ENSGALG00010023860	TRIP4	PRJNA729271	0.488231331	2.79637E-07	0.488231331	stage_E14_vs_E10	ordinary_muscle_development		0	FALSE
ENSGALG00010011921	MEGF10	PRJNA729271	0.916092177	2.81294E-07	0.916092177	stage_E14_vs_E10	ordinary_muscle_development		0	FALSE
ENSGALG00010023093	POP5	PRJNA729271	-0.700135778	2.81548E-07	0.700135778	stage_E14_vs_E10	ordinary_muscle_development		0	FALSE
ENSGALG00010029775	ENSGALG00010029775	PRJNA729271	-0.78083245	2.81824E-07	0.78083245	stage_E14_vs_E10	ordinary_muscle_development		0	FALSE
ENSGALG00010012318	POLR1D	PRJNA729271	-0.761050678	2.82424E-07	0.761050678	stage_E14_vs_E10	ordinary_muscle_development		0	FALSE
ENSGALG00010001711	CREB5	PRJNA729271	-0.963876775	2.82627E-07	0.963876775	stage_E14_vs_E10	ordinary_muscle_development		0	FALSE
ENSGALG00010029735	SRP68	PRJNA729271	-0.604451125	2.82821E-07	0.604451125	stage_E14_vs_E10	ordinary_muscle_development		0	FALSE
ENSGALG00010012624	PDGFB	PRJNA729271	0.963552417	2.82983E-07	0.963552417	stage_E14_vs_E10	ordinary_muscle_development		0	FALSE
ENSGALG00010020855	DNAL1	PRJNA729271	-0.553065162	2.84053E-07	0.553065162	stage_E14_vs_E10	ordinary_muscle_development		0	FALSE
ENSGALG00010022280	PDZD8	PRJNA729271	-0.371421345	2.84053E-07	0.371421345	stage_E14_vs_E10	ordinary_muscle_development		0	FALSE

ENSGALG00010016597	FRYL	PRJNA729271	0.663368133	2.85896E-07	0.663368133	stage_E14_vs_E10	ordinary_muscle_development		0	FALSE
ENSGALG00010021069	PGAM5	PRJNA729271	0.373768747	2.87126E-07	0.373768747	stage_E14_vs_E10	ordinary_muscle_development		0	FALSE
ENSGALG00010018854	LYSMD4	PRJNA729271	0.89997525	2.87255E-07	0.89997525	stage_E14_vs_E10	ordinary_muscle_development		0	FALSE
ENSGALG00010010619	LRRC52	PRJNA729271	-1.513295138	2.88573E-07	1.513295138	stage_E14_vs_E10	ordinary_muscle_development	D_vs_E10;stage_1W_vs_E10;stage_3W_vs_E10;stage	1	TRUE
ENSGALG00010024529	GRB7	PRJNA729271	-6.380948798	2.88693E-07	6.380948798	stage_E14_vs_E10	ordinary_muscle_development	D_vs_E10;stage_1W_vs_E10;stage_3W_vs_E10;stage	1	TRUE
ENSGALG00010018926	SLC22A7	PRJNA729271	4.544078474	2.88872E-07	4.544078474	stage_E14_vs_E10	ordinary_muscle_development	D_vs_E10;stage_1W_vs_E10;stage_3W_vs_E10;stage	1	TRUE
ENSGALG00010020856	VTG2	PRJNA729271	-5.186489558	2.91892E-07	5.186489558	stage_E14_vs_E10	ordinary_muscle_development	D_vs_E10;stage_1W_vs_E10;stage_3W_vs_E10;stage	1	TRUE
ENSGALG00010008946	COL22A1	PRJNA729271	-3.295950902	2.92325E-07	3.295950902	stage_E14_vs_E10	ordinary_muscle_development	D_vs_E10;stage_1W_vs_E10;stage_3W_vs_E10;stage	1	TRUE
ENSGALG00010018806	GCH1	PRJNA729271	1.624104506	2.95884E-07	1.624104506	stage_E14_vs_E10	ordinary_muscle_development	D_vs_E10;stage_1W_vs_E10;stage_3W_vs_E10;stage	1	TRUE
ENSGALG00010021812	TEAD1	PRJNA729271	0.440361767	2.95989E-07	0.440361767	stage_E14_vs_E10	ordinary_muscle_development		0	FALSE
ENSGALG00010029561	SCO1	PRJNA729271	-0.474944662	3.00197E-07	0.474944662	stage_E14_vs_E10	ordinary_muscle_development		0	FALSE
ENSGALG00010016592	TMEM129	PRJNA729271	0.539421854	3.00532E-07	0.539421854	stage_E14_vs_E10	ordinary_muscle_development		0	FALSE
ENSGALG00010019288	RPL37A	PRJNA729271	0.83297016	3.00841E-07	0.83297016	stage_E14_vs_E10	ordinary_muscle_development		0	FALSE
ENSGALG00010011335	VCP	PRJNA729271	0.912550235	3.01182E-07	0.912550235	stage_E14_vs_E10	ordinary_muscle_development		0	FALSE
ENSGALG00010003356	ENSGALG00010003356	PRJNA729271	6.059253238	3.02642E-07	6.059253238	stage_E14_vs_E10	ordinary_muscle_development	D_vs_E10;stage_1W_vs_E10;stage_3W_vs_E10;stage	1	TRUE
ENSGALG00010007387	ENSGALG00010007387	PRJNA729271	-3.073256552	3.03138E-07	3.073256552	stage_E14_vs_E10	ordinary_muscle_development	D_vs_E10;stage_1W_vs_E10;stage_3W_vs_E10;stage	1	TRUE
ENSGALG00010005901	ENSGALG00010005901	PRJNA729271	1.264466589	3.03294E-07	1.264466589	stage_E14_vs_E10	ordinary_muscle_development	D_vs_E10;stage_1W_vs_E10;stage_3W_vs_E10;stage	1	TRUE
ENSGALG00010002992	ENSGALG00010002992	PRJNA729271	-1.382253371	3.04044E-07	1.382253371	stage_E14_vs_E10	ordinary_muscle_development	D_vs_E10;stage_1W_vs_E10;stage_3W_vs_E10;stage	1	TRUE
ENSGALG00010013006	GRIK2	PRJNA729271	-6.164462944	3.04248E-07	6.164462944	stage_E14_vs_E10	ordinary_muscle_development	D_vs_E10;stage_1W_vs_E10;stage_3W_vs_E10;stage	1	TRUE
ENSGALG00010029732	PIGL	PRJNA729271	0.931784533	3.0434E-07	0.931784533	stage_E14_vs_E10	ordinary_muscle_development		0	FALSE
ENSGALG00010023322	PRKAB1	PRJNA729271	-0.487355952	3.08303E-07	0.487355952	stage_E14_vs_E10	ordinary_muscle_development		0	FALSE
ENSGALG00010010711	ENSGALG00010010711	PRJNA729271	-2.338254861	3.08437E-07	2.338254861	stage_E14_vs_E10	ordinary_muscle_development	D_vs_E10;stage_1W_vs_E10;stage_3W_vs_E10;stage	1	TRUE
ENSGALG00010011965	ATIC	PRJNA729271	0.774668003	3.08541E-07	0.774668003	stage_E14_vs_E10	ordinary_muscle_development		0	FALSE
ENSGALG00010021469	CSPG4	PRJNA729271	-0.852062075	3.09519E-07	0.852062075	stage_E14_vs_E10	ordinary_muscle_development		0	FALSE
ENSGALG00010000702	DDC	PRJNA729271	-2.932208326	3.10422E-07	2.932208326	stage_E14_vs_E10	ordinary_muscle_development	D_vs_E10;stage_1W_vs_E10;stage_3W_vs_E10;stage	1	TRUE
ENSGALG00010024851	CPNE5	PRJNA729271	-5.184117619	3.11085E-07	5.184117619	stage_E14_vs_E10	ordinary_muscle_development	D_vs_E10;stage_1W_vs_E10;stage_3W_vs_E10;stage	1	TRUE
ENSGALG00010001398	WDR11	PRJNA729271	-0.517187717	3.1183E-07	0.517187717	stage_E14_vs_E10	ordinary_muscle_development		0	FALSE
ENSGALG00010018264	CFAP61	PRJNA729271	-2.05732636	3.12772E-07	2.05732636	stage_E14_vs_E10	ordinary_muscle_development	D_vs_E10;stage_1W_vs_E10;stage_3W_vs_E10;stage	1	TRUE
ENSGALG00010012098	ART4	PRJNA729271	2.708547969	3.13319E-07	2.708547969	stage_E14_vs_E10	ordinary_muscle_development	D_vs_E10;stage_1W_vs_E10;stage_3W_vs_E10;stage	1	TRUE
ENSGALG00010020351	CROCC	PRJNA729271	-0.8123891	3.1495E-07	0.8123891	stage_E14_vs_E10	ordinary_muscle_development		0	FALSE
ENSGALG00010026016	SLC2A11	PRJNA729271	-2.618857752	3.17062E-07	2.618857752	stage_E14_vs_E10	ordinary_muscle_development	D_vs_E10;stage_1W_vs_E10;stage_3W_vs_E10;stage	1	TRUE
ENSGALG00010007418	NME6	PRJNA729271	0.764538561	3.18445E-07	0.764538561	stage_E14_vs_E10	ordinary_muscle_development		0	FALSE
ENSGALG00010024179	ATG4C	PRJNA729271	-0.620248219	3.18636E-07	0.620248219	stage_E14_vs_E10	ordinary_muscle_development		0	FALSE
ENSGALG00010015212	GNAQ	PRJNA729271	0.510467923	3.1866E-07	0.510467923	stage_E14_vs_E10	ordinary_muscle_development		0	FALSE
ENSGALG00010005229	CIPC	PRJNA729271	-0.398868852	3.18694E-07	0.398868852	stage_E14_vs_E10	ordinary_muscle_development		0	FALSE
ENSGALG00010000440	ENSGALG00010000440	PRJNA729271	-2.594201129	3.19619E-07	2.594201129	stage_E14_vs_E10	ordinary_muscle_development	D_vs_E10;stage_1W_vs_E10;stage_3W_vs_E10;stage	1	TRUE
ENSGALG00010020594	PQLC2	PRJNA729271	-0.844794133	3.20451E-07	0.844794133	stage_E14_vs_E10	ordinary_muscle_development		0	FALSE
ENSGALG00010022117	FAM20C	PRJNA729271	0.639964551	3.21334E-07	0.639964551	stage_E14_vs_E10	ordinary_muscle_development		0	FALSE
ENSGALG00010022482	PRTG	PRJNA729271	-1.743971795	3.21349E-07	1.743971795	stage_E14_vs_E10	ordinary_muscle_development	D_vs_E10;stage_1W_vs_E10;stage_3W_vs_E10;stage	1	TRUE
ENSGALG00010013546	IDUA	PRJNA729271	-0.597704294	3.22972E-07	0.597704294	stage_E14_vs_E10	ordinary_muscle_development		0	FALSE
ENSGALG00010011377	SEC16B	PRJNA729271	1.567761421	3.25055E-07	1.567761421	stage_E14_vs_E10	ordinary_muscle_development	D_vs_E10;stage_1W_vs_E10;stage_3W_vs_E10;stage	1	TRUE
ENSGALG00010011247	ENSGALG00010011247	PRJNA729271	-0.510709553	3.25793E-07	0.510709553	stage_E14_vs_E10	ordinary_muscle_development		0	FALSE
ENSGALG00010002810	TRIM27.1	PRJNA729271	1.612539763	3.28794E-07	1.612539763	stage_E14_vs_E10	ordinary_muscle_development	D_vs_E10;stage_1W_vs_E10;stage_3W_vs_E10;stage	1	TRUE
ENSGALG00010020750	AIFM2	PRJNA729271	-1.494775637	3.30058E-07	1.494775637	stage_E14_vs_E10	ordinary_muscle_development	D_vs_E10;stage_1W_vs_E10;stage_3W_vs_E10;stage	1	TRUE
ENSGALG00010028185	GLMP	PRJNA729271	0.455894143	3.33487E-07	0.455894143	stage_E14_vs_E10	ordinary_muscle_development		0	FALSE
ENSGALG00010023149	ADGRD1	PRJNA729271	-0.970234915	3.35769E-07	0.970234915	stage_E14_vs_E10	ordinary_muscle_development		0	FALSE
ENSGALG00010010905	ENC1	PRJNA729271	-0.477004374	3.35808E-07	0.477004374	stage_E14_vs_E10	ordinary_muscle_development		0	FALSE
ENSGALG00010017110	BRF1	PRJNA729271	-0.382263737	3.36394E-07	0.382263737	stage_E14_vs_E10	ordinary_muscle_development		0	FALSE
ENSGALG00010001182	ZCCHC7	PRJNA729271	-0.447661377	3.37928E-07	0.447661377	stage_E14_vs_E10	ordinary_muscle_development		0	FALSE
ENSGALG00010029410	VTN	PRJNA729271	4.65560924	3.38973E-07	4.65560924	stage_E14_vs_E10	ordinary_muscle_development	D_vs_E10;stage_1W_vs_E10;stage_3W_vs_E10;stage	1	TRUE
ENSGALG00010000711	SPRY2	PRJNA729271	0.581427716	3.41569E-07	0.581427716	stage_E14_vs_E10	ordinary_muscle_development		0	FALSE
ENSGALG00010010934	ENSGALG00010010934	PRJNA729271	0.580628601	3.41611E-07	0.580628601	stage_E14_vs_E10	ordinary_muscle_development		0	FALSE
ENSGALG00010026901	FAM212A	PRJNA729271	-0.922808984	3.44389E-07	0.922808984	stage_E14_vs_E10	ordinary_muscle_development		0	FALSE
ENSGALG00010027609	RPL36	PRJNA729271	0.987876259	3.44721E-07	0.987876259	stage_E14_vs_E10	ordinary_muscle_development		0	FALSE
ENSGALG00010006885	RHOU	PRJNA729271	-0.802205728	3.45993E-07	0.802205728	stage_E14_vs_E10	ordinary_muscle_development		0	FALSE
ENSGALG00010017678	YARS	PRJNA729271	-0.734144809	3.47879E-07	0.734144809	stage_E14_vs_E10	ordinary_muscle_development		0	FALSE
ENSGALG00010022928	RNF111	PRJNA729271	0.433336932	3.4871E-07	0.433336932	stage_E14_vs_E10	ordinary_muscle_development		0	FALSE
ENSGALG00010009731	NMI	PRJNA729271	1.263208021	3.50135E-07	1.263208021	stage_E14_vs_E10	ordinary_muscle_development	D_vs_E10;stage_1W_vs_E10;stage_3W_vs_E10;stage	1	TRUE
ENSGALG00010029400	AANAT	PRJNA729271	3.21200581	3.51118E-07	3.21200581	stage_E14_vs_E10	ordinary_muscle_development	D_vs_E10;stage_1W_vs_E10;stage_3W_vs_E10;stage	1	TRUE
ENSGALG00010011004	UMODL1	PRJNA729271	-2.334100185	3.5185E-07	2.334100185	stage_E14_vs_E10	ordinary_muscle_development	D_vs_E10;stage_1W_vs_E10;stage_3W_vs_E10;stage	1	TRUE
ENSGALG00010016073	SAXO1	PRJNA729271	5.085868498	3.55829E-07	5.085868498	stage_E14_vs_E10	ordinary_muscle_development	D_vs_E10;stage_1W_vs_E10;stage_3W_vs_E10;stage	1	TRUE
ENSGALG00010024126	CDK5R1	PRJNA729271	-1.577938568	3.5615E-07	1.577938568	stage_E14_vs_E10	ordinary_muscle_development	D_vs_E10;stage_1W_vs_E10;stage_3W_vs_E10;stage	1	TRUE
ENSGALG00010017585	ENSGALG00010017585	PRJNA729271	-0.647618017	3.56502E-07	0.647618017	stage_E14_vs_E10	ordinary_muscle_development		0	FALSE
ENSGALG00010019557	TRIM9	PRJNA729271	-1.928725679	3.56553E-07	1.928725679	stage_E14_vs_E10	ordinary_muscle_development	D_vs_E10;stage_1W_vs_E10;stage_3W_vs_E10;stage	1	TRUE
ENSGALG00010026916	ENSGALG00010026916	PRJNA729271	1.107457032	3.57098E-07	1.107457032	stage_E14_vs_E10	ordinary_muscle_development	D_vs_E10;stage_1W_vs_E10;stage_3W_vs_E10;stage	1	TRUE
ENSGALG00010006387	OTUD4	PRJNA729271	-0.723797321	3.57567E-07	0.723797321	stage_E14_vs_E10	ordinary_muscle_development		0	FALSE
ENSGALG00010020130	PDXDC1	PRJNA729271	-0.395482645	3.60482E-07	0.395482645	stage_E14_vs_E10	ordinary_muscle_development		0	FALSE
ENSGALG00010002295	YAF2	PRJNA729271	0.591296792	3.62752E-07	0.591296792	stage_E14_vs_E10	ordinary_muscle_development		0	FALSE
ENSGALG00010015044	ZC3H12B	PRJNA729271	-0.696313941	3.63019E-07	0.696313941	stage_E14_vs_E10	ordinary_muscle_development		0	FALSE
ENSGALG00010022573	AP4E1	PRJNA729271	-0.433342714	3.64452E-07	0.433342714	stage_E14_vs_E10	ordinary_muscle_development		0	FALSE
ENSGALG00010017437	DRG2	PRJNA729271	0.725444766	3.68265E-07	0.725444766	stage_E14_vs_E10	ordinary_muscle_development		0	FALSE
ENSGALG00010013470	RPL31	PRJNA729271	0.771990413	3.69243E-07	0.771990413	stage_E14_vs_E10	ordinary_muscle_development		0	FALSE
ENSGALG00010000154	ENSGALG00010000154	PRJNA729271	-21.09418731	3.7118E-07	21.09418731	stage_E14_vs_E10	ordinary_muscle_development	D_vs_E10;stage_1W_vs_E10;stage_3W_vs_E10;stage	1	TRUE
ENSGALG00010021554	SNX11	PRJNA729271	0.767324032	3.72536E-07	0.767324032	stage_E14_vs_E10	ordinary_muscle_development		0	FALSE
ENSGALG00010029765	KCNJ16	PRJNA729271	-1.621757407	3.74401E-07	1.621757407	stage_E14_vs_E10	ordinary_muscle_development	D_vs_E10;stage_1W_vs_E10;stage_3W_vs_E10;stage	1	TRUE
ENSGALG00010029753	AKAP10	PRJNA729271	-0.404847326	3.75902E-07	0.404847326	stage_E14_vs_E10	ordinary_muscle_development		0	FALSE
ENSGALG00010015119	TLR1A	PRJNA729271	1.107861264	3.76248E-07	1.107861264	stage_E14_vs_E10	ordinary_muscle_development	D_vs_E10;stage_1W_vs_E10;stage_3W_vs_E10;stage	1	TRUE
ENSGALG00010007992	LAPTM4A	PRJNA729271	0.515348732	3.77467E-07	0.515348732	stage_E14_vs_E10	ordinary_muscle_development		0	FALSE
ENSGALG00010028762	CACNA1B	PRJNA729271	-5.148104722	3.78905E-07	5.148104722	stage_E14_vs_E10	ordinary_muscle_development	D_vs_E10;stage_1W_vs_E10;stage_3W_vs_E10;stage	1	TRUE
ENSGALG00010027728	CRNN	PRJNA729271	7.920763099	3.80354E-07	7.920763099	stage_E14_vs_E10	ordinary_muscle_development	D_vs_E10;stage_1W_vs_E10;stage_3W_vs_E10;stage	1	TRUE
ENSGALG00010011425	WASHC3	PRJNA729271	-0.420155849	3.83252E-07	0.420155849	stage_E14_vs_E10	ordinary_muscle_development		0	FALSE
ENSGALG00010004275	ADARB2	PRJNA729271	-5.07812021	3.86149E-07	5.07812021	stage_E14_vs_E10	ordinary_muscle_development	D_vs_E10;stage_1W_vs_E10;stage_3W_vs_E10;stage	1	TRUE
ENSGALG00010009337	ZNF330	PRJNA729271	0.329792752	3.87046E-07	0.329792752	stage_E14_vs_E10	ordinary_muscle_development		0	FALSE
ENSGALG00010026786	ENSGALG00010026786	PRJNA729271	2.973321011	3.87473E-07	2.973321011	stage_E14_vs_E10	ordinary_muscle_development	D_vs_E10;stage_1W_vs_E10;stage_3W_vs_E10;stage	1	TRUE
ENSGALG00010016867	FLT4	PRJNA729271	0.621673879	3.87681E-07	0.621673879	stage_E14_vs_E10	ordinary_muscle_development		0	FALSE
ENSGALG00010026298	SNF8	PRJNA729271	-0.612847041	3.88338E-07	0.612847041	stage_E14_vs_E10	ordinary_muscle_development		0	FALSE
ENSGALG00010003276	TNFSF11	PRJNA729271	-2.601568912	3.90859E-07	2.601568912	stage_E14_vs_E10	ordinary_muscle_development	D_vs_E10;stage_1W_vs_E10;stage_3W_vs_E10;stage	1	TRUE
ENSGALG00010016407	RPL9	PRJNA729271	0.951412196	3.9155E-07	0.951412196	stage_E14_vs_E10	ordinary_muscle_development		0	FALSE
ENSGALG00010027674	TREM2	PRJNA729271	1.460599159	3.92421E-07	1.460599159	stage_E14_vs_E10	ordinary_muscle_development	D_vs_E10;stage_1W_vs_E10;stage_3W_vs_E10;stage	1	TRUE
ENSGALG00010020083	CFAP74	PRJNA729271	-2.514856774	3.94055E-07	2.514856774	stage_E14_vs_E10	ordinary_muscle_development	D_vs_E10;stage_1W_vs_E10;stage_3W_vs_E10;stage	1	TRUE
ENSGALG00010012980	KICS2	PRJNA729271	-0.407220601	3.95427E-07	0.407220601	stage_E14_vs_E10	ordinary_muscle_development		0	FALSE
ENSGALG00010009447	DDX41	PRJNA729271	-0.564921136	3.95763E-07	0.564921136	stage_E14_vs_E10	ordinary_muscle_development		0	FALSE
ENSGALG00010013329	LIFR	PRJNA729271	-0.638663365	3.96198E-07	0.638663365	stage_E14_vs_E10	ordinary_muscle_development		0	FALSE
ENSGALG00010005597	ENSGALG00010005597	PRJNA729271	2.428840042	3.96999E-07	2.428840042	stage_E14_vs_E10	ordinary_muscle_development	D_vs_E10;stage_1W_vs_E10;stage_3W_vs_E10;stage	1	TRUE
ENSGALG00010001806	ENSGALG00010001806	PRJNA729271	4.683364731	3.97829E-07	4.683364731	stage_E14_vs_E10	ordinary_muscle_development	D_vs_E10;stage_1W_vs_E10;stage_3W_vs_E10;stage	1	TRUE
ENSGALG00010012676	ENSGALG00010012676	PRJNA729271	1.094175288	3.98465E-07	1.094175288	stage_E14_vs_E10	ordinary_muscle_development	D_vs_E10;stage_1W_vs_E10;stage_3W_vs_E10;stage	1	TRUE
ENSGALG00010009303	TTC1	PRJNA729271	0.498877921	3.99746E-07	0.498877921	stage_E14_vs_E10	ordinary_muscle_development		0	FALSE
ENSGALG00010027579	C26H6ORF125	PRJNA729271	-0.833848939	3.99849E-07	0.833848939	stage_E14_vs_E10	ordinary_muscle_development		0	FALSE
ENSGALG00010024860	NEU4	PRJNA729271	-4.678017181	4.00056E-07	4.678017181	stage_E14_vs_E10	ordinary_muscle_development	D_vs_E10;stage_1W_vs_E10;stage_3W_vs_E10;stage	1	TRUE
ENSGALG00010013779	SLC52A3	PRJNA729271	-1.509828896	4.01778E-07	1.509828896	stage_E14_vs_E10	ordinary_muscle_development	D_vs_E10;stage_1W_vs_E10;stage_3W_vs_E10;stage	1	TRUE
ENSGALG00010003115	UBE3D	PRJNA729271	-0.817289896	4.02305E-07	0.817289896	stage_E14_vs_E10	ordinary_muscle_development		0	FALSE
ENSGALG00010009511	ZFPM1	PRJNA729271	-1.376209903	4.03088E-07	1.376209903	stage_E14_vs_E10	ordinary_muscle_development	D_vs_E10;stage_1W_vs_E10;stage_3W_vs_E10;stage	1	TRUE
ENSGALG00010000584	RBM14	PRJNA729271	1.088600939	4.04608E-07	1.088600939	stage_E14_vs_E10	ordinary_muscle_development	D_vs_E10;stage_1W_vs_E10;stage_3W_vs_E10;stage	1	TRUE
ENSGALG00010012535	JMY	PRJNA729271	-0.545218608	4.05417E-07	0.545218608	stage_E14_vs_E10	ordinary_muscle_development		0	FALSE
ENSGALG00010004436	NOVA1	PRJNA729271	0.782973398	4.05728E-07	0.782973398	stage_E14_vs_E10	ordinary_muscle_development		0	FALSE
ENSGALG00010009410	JMJD1C	PRJNA729271	0.567806424	4.06958E-07	0.567806424	stage_E14_vs_E10	ordinary_muscle_development		0	FALSE
ENSGALG00010023664	SLC6A9	PRJNA729271	-1.713132613	4.08293E-07	1.713132613	stage_E14_vs_E10	ordinary_muscle_development	D_vs_E10;stage_1W_vs_E10;stage_3W_vs_E10;stage	1	TRUE
ENSGALG00010019916	NOC3L	PRJNA729271	-0.604125746	4.09311E-07	0.604125746	stage_E14_vs_E10	ordinary_muscle_development		0	FALSE
ENSGALG00010007513	GALNT12	PRJNA729271	-0.680896387	4.11487E-07	0.680896387	stage_E14_vs_E10	ordinary_muscle_development		0	FALSE
ENSGALG00010012551	PCGF3	PRJNA729271	-0.813325351	4.13768E-07	0.813325351	stage_E14_vs_E10	ordinary_muscle_development		0	FALSE
ENSGALG00010003193	ACOT9	PRJNA729271	0.709726763	4.14239E-07	0.709726763	stage_E14_vs_E10	ordinary_muscle_development		0	FALSE
ENSGALG00010002792	ENSGALG00010002792	PRJNA729271	-1.212194698	4.17419E-07	1.212194698	stage_E14_vs_E10	ordinary_muscle_development	D_vs_E10;stage_1W_vs_E10;stage_3W_vs_E10;stage	1	TRUE
ENSGALG00010001521	TBC1D5	PRJNA729271	-0.394702336	4.18795E-07	0.394702336	stage_E14_vs_E10	ordinary_muscle_development		0	FALSE
ENSGALG00010012778	TBC1D15	PRJNA729271	0.784975181	4.1969E-07	0.784975181	stage_E14_vs_E10	ordinary_muscle_development		0	FALSE
ENSGALG00010027335	MAST3	PRJNA729271	-0.948545495	4.20993E-07	0.948545495	stage_E14_vs_E10	ordinary_muscle_development		0	FALSE
ENSGALG00010000729	EDNRB	PRJNA729271	-0.821083132	4.2513E-07	0.821083132	stage_E14_vs_E10	ordinary_muscle_development		0	FALSE
ENSGALG00010028567	ENSGALG00010028567	PRJNA729271	4.687899667	4.28391E-07	4.687899667	stage_E14_vs_E10	ordinary_muscle_development	D_vs_E10;stage_1W_vs_E10;stage_3W_vs_E10;stage	1	TRUE
ENSGALG00010016118	EPRS	PRJNA729271	0.453375066	4.29731E-07	0.453375066	stage_E14_vs_E10	ordinary_muscle_development		0	FALSE
ENSGALG00010017615	AGTRAP	PRJNA729271	0.706953962	4.34447E-07	0.706953962	stage_E14_vs_E10	ordinary_muscle_development		0	FALSE
ENSGALG00010001249	PDCD10	PRJNA729271	0.718660843	4.35769E-07	0.718660843	stage_E14_vs_E10	ordinary_muscle_development		0	FALSE
ENSGALG00010028727	ENSGALG00010028727	PRJNA729271	-0.945027546	4.37755E-07	0.945027546	stage_E14_vs_E10	ordinary_muscle_development		0	FALSE
ENSGALG00010009057	NXNL2	PRJNA729271	1.863595825	4.37987E-07	1.863595825	stage_E14_vs_E10	ordinary_muscle_development	D_vs_E10;stage_1W_vs_E10;stage_3W_vs_E10;stage	1	TRUE
ENSGALG00010000294	DUSP21	PRJNA729271	-1.624761375	4.38624E-07	1.624761375	stage_E14_vs_E10	ordinary_muscle_development	D_vs_E10;stage_1W_vs_E10;stage_3W_vs_E10;stage	1	TRUE
ENSGALG00010022264	SCN8A	PRJNA729271	-1.358779096	4.38803E-07	1.358779096	stage_E14_vs_E10	ordinary_muscle_development	D_vs_E10;stage_1W_vs_E10;stage_3W_vs_E10;stage	1	TRUE
ENSGALG00010020580	ENSGALG00010020580	PRJNA729271	-0.60407235	4.39448E-07	0.60407235	stage_E14_vs_E10	ordinary_muscle_development		0	FALSE
ENSGALG00010018482	TWNK	PRJNA729271	-0.677644665	4.40368E-07	0.677644665	stage_E14_vs_E10	ordinary_muscle_development		0	FALSE
ENSGALG00010013116	TARS	PRJNA729271	-0.872700673	4.42723E-07	0.872700673	stage_E14_vs_E10	ordinary_muscle_development		0	FALSE
ENSGALG00010000670	ENSGALG00010000670	PRJNA729271	-3.947399981	4.44332E-07	3.947399981	stage_E14_vs_E10	ordinary_muscle_development	D_vs_E10;stage_1W_vs_E10;stage_3W_vs_E10;stage	1	TRUE
ENSGALG00010012372	LEMD3	PRJNA729271	0.382684642	4.44424E-07	0.382684642	stage_E14_vs_E10	ordinary_muscle_development		0	FALSE
ENSGALG00010000269	ENSGALG00010000269	PRJNA729271	-3.618883868	4.45414E-07	3.618883868	stage_E14_vs_E10	ordinary_muscle_development	D_vs_E10;stage_1W_vs_E10;stage_3W_vs_E10;stage	1	TRUE
ENSGALG00010008150	TERT	PRJNA729271	-3.810346772	4.47278E-07	3.810346772	stage_E14_vs_E10	ordinary_muscle_development	D_vs_E10;stage_1W_vs_E10;stage_3W_vs_E10;stage	1	TRUE
ENSGALG00010024141	YTHDF1	PRJNA729271	-0.498519405	4.50893E-07	0.498519405	stage_E14_vs_E10	ordinary_muscle_development		0	FALSE
ENSGALG00010027307	JUND	PRJNA729271	0.910899793	4.51794E-07	0.910899793	stage_E14_vs_E10	ordinary_muscle_development		0	FALSE
ENSGALG00010007952	EMC2	PRJNA729271	-0.570760783	4.53198E-07	0.570760783	stage_E14_vs_E10	ordinary_muscle_development		0	FALSE
ENSGALG00010018585	GDPD2	PRJNA729271	-4.732589524	4.53565E-07	4.732589524	stage_E14_vs_E10	ordinary_muscle_development	D_vs_E10;stage_1W_vs_E10;stage_3W_vs_E10;stage	1	TRUE
ENSGALG00010025578	ORMDL2	PRJNA729271	0.747631826	4.58097E-07	0.747631826	stage_E14_vs_E10	ordinary_muscle_development		0	FALSE
ENSGALG00010016147	FABP1	PRJNA729271	4.087658197	4.59323E-07	4.087658197	stage_E14_vs_E10	ordinary_muscle_development	D_vs_E10;stage_1W_vs_E10;stage_3W_vs_E10;stage	1	TRUE
ENSGALG00010001505	SSR3	PRJNA729271	-0.503118858	4.60617E-07	0.503118858	stage_E14_vs_E10	ordinary_muscle_development		0	FALSE
ENSGALG00010018413	SLC15A2	PRJNA729271	-1.866981109	4.60921E-07	1.866981109	stage_E14_vs_E10	ordinary_muscle_development	D_vs_E10;stage_1W_vs_E10;stage_3W_vs_E10;stage	1	TRUE
ENSGALG00010015843	ENSGALG00010015843	PRJNA729271	-2.945647381	4.62141E-07	2.945647381	stage_E14_vs_E10	ordinary_muscle_development	D_vs_E10;stage_1W_vs_E10;stage_3W_vs_E10;stage	1	TRUE
ENSGALG00010005752	RMND1	PRJNA729271	0.506284722	4.62675E-07	0.506284722	stage_E14_vs_E10	ordinary_muscle_development		0	FALSE
ENSGALG00010019643	RAB3GAP1	PRJNA729271	-0.367755552	4.6531E-07	0.367755552	stage_E14_vs_E10	ordinary_muscle_development		0	FALSE
ENSGALG00010025362	G6PC2	PRJNA729271	-1.331872158	4.66449E-07	1.331872158	stage_E14_vs_E10	ordinary_muscle_development	D_vs_E10;stage_1W_vs_E10;stage_3W_vs_E10;stage	1	TRUE
ENSGALG00010011297	OSR2	PRJNA729271	-0.963891021	4.67164E-07	0.963891021	stage_E14_vs_E10	ordinary_muscle_development		0	FALSE
ENSGALG00010012206	RIBC2	PRJNA729271	-4.686473011	4.67237E-07	4.686473011	stage_E14_vs_E10	ordinary_muscle_development	D_vs_E10;stage_1W_vs_E10;stage_3W_vs_E10;stage	1	TRUE
ENSGALG00010007953	SCGN	PRJNA729271	-4.155429608	4.6752E-07	4.155429608	stage_E14_vs_E10	ordinary_muscle_development	D_vs_E10;stage_1W_vs_E10;stage_3W_vs_E10;stage	1	TRUE
ENSGALG00010007265	CD200R1A	PRJNA729271	-1.633350907	4.68938E-07	1.633350907	stage_E14_vs_E10	ordinary_muscle_development	D_vs_E10;stage_1W_vs_E10;stage_3W_vs_E10;stage	1	TRUE
ENSGALG00010027116	CCDC94	PRJNA729271	0.524887089	4.70125E-07	0.524887089	stage_E14_vs_E10	ordinary_muscle_development		0	FALSE
ENSGALG00010023552	SGPP2	PRJNA729271	1.660278229	4.71008E-07	1.660278229	stage_E14_vs_E10	ordinary_muscle_development	D_vs_E10;stage_1W_vs_E10;stage_3W_vs_E10;stage	1	TRUE
ENSGALG00010024640	GSDMA	PRJNA729271	-1.609596656	4.75286E-07	1.609596656	stage_E14_vs_E10	ordinary_muscle_development	D_vs_E10;stage_1W_vs_E10;stage_3W_vs_E10;stage	1	TRUE
ENSGALG00010028485	USF1	PRJNA729271	0.927282505	4.75417E-07	0.927282505	stage_E14_vs_E10	ordinary_muscle_development		0	FALSE
ENSGALG00010027244	ARHGEF18	PRJNA729271	0.465440371	4.76117E-07	0.465440371	stage_E14_vs_E10	ordinary_muscle_development		0	FALSE
ENSGALG00010005243	NGB	PRJNA729271	-2.839518807	4.76575E-07	2.839518807	stage_E14_vs_E10	ordinary_muscle_development	D_vs_E10;stage_1W_vs_E10;stage_3W_vs_E10;stage	1	TRUE
ENSGALG00010023269	OPRL1	PRJNA729271	-3.592599678	4.79824E-07	3.592599678	stage_E14_vs_E10	ordinary_muscle_development	D_vs_E10;stage_1W_vs_E10;stage_3W_vs_E10;stage	1	TRUE
ENSGALG00010004995	PIT54	PRJNA729271	11.32812394	4.83964E-07	11.32812394	stage_E14_vs_E10	ordinary_muscle_development	D_vs_E10;stage_1W_vs_E10;stage_3W_vs_E10;stage	1	TRUE
ENSGALG00010002312	ENSGALG00010002312	PRJNA729271	-1.017348002	4.84429E-07	1.017348002	stage_E14_vs_E10	ordinary_muscle_development	D_vs_E10;stage_1W_vs_E10;stage_3W_vs_E10;stage	1	TRUE
ENSGALG00010014350	PTGER4	PRJNA729271	-0.927144178	4.8542E-07	0.927144178	stage_E14_vs_E10	ordinary_muscle_development		0	FALSE
ENSGALG00010002454	NEK5	PRJNA729271	-4.451178354	4.8811E-07	4.451178354	stage_E14_vs_E10	ordinary_muscle_development	D_vs_E10;stage_1W_vs_E10;stage_3W_vs_E10;stage	1	TRUE
ENSGALG00010004235	ADAD1	PRJNA729271	-2.460243138	4.90909E-07	2.460243138	stage_E14_vs_E10	ordinary_muscle_development	D_vs_E10;stage_1W_vs_E10;stage_3W_vs_E10;stage	1	TRUE
ENSGALG00010005212	TAF13	PRJNA729271	0.520456018	4.92291E-07	0.520456018	stage_E14_vs_E10	ordinary_muscle_development		0	FALSE
ENSGALG00010029436	WDR45B	PRJNA729271	-0.413747141	4.97222E-07	0.413747141	stage_E14_vs_E10	ordinary_muscle_development		0	FALSE
ENSGALG00010025892	ZC3H11A	PRJNA729271	0.443306108	4.97623E-07	0.443306108	stage_E14_vs_E10	ordinary_muscle_development		0	FALSE
ENSGALG00010007182	ENSGALG00010007182	PRJNA729271	1.515215863	4.97649E-07	1.515215863	stage_E14_vs_E10	ordinary_muscle_development	D_vs_E10;stage_1W_vs_E10;stage_3W_vs_E10;stage	1	TRUE
ENSGALG00010015719	XRN1	PRJNA729271	0.514281768	4.99603E-07	0.514281768	stage_E14_vs_E10	ordinary_muscle_development		0	FALSE
ENSGALG00010000659	ENSGALG00010000659	PRJNA729271	2.810243074	4.99676E-07	2.810243074	stage_E14_vs_E10	ordinary_muscle_development	D_vs_E10;stage_1W_vs_E10;stage_3W_vs_E10;stage	1	TRUE
ENSGALG00010018356	ADRA1A	PRJNA729271	-2.467904748	5.02558E-07	2.467904748	stage_E14_vs_E10	ordinary_muscle_development	D_vs_E10;stage_1W_vs_E10;stage_3W_vs_E10;stage	1	TRUE
ENSGALG00010013367	NOL4L	PRJNA729271	0.86866923	5.03507E-07	0.86866923	stage_E14_vs_E10	ordinary_muscle_development		0	FALSE
ENSGALG00010009250	ERO1B	PRJNA729271	-0.563569768	5.04758E-07	0.563569768	stage_E14_vs_E10	ordinary_muscle_development		0	FALSE
ENSGALG00010019752	GPAM	PRJNA729271	0.862467744	5.058E-07	0.862467744	stage_E14_vs_E10	ordinary_muscle_development		0	FALSE
ENSGALG00010006651	ENSGALG00010006651	PRJNA729271	0.579684854	5.11302E-07	0.579684854	stage_E14_vs_E10	ordinary_muscle_development		0	FALSE
ENSGALG00010013303	TFPI	PRJNA729271	-1.264914742	5.11425E-07	1.264914742	stage_E14_vs_E10	ordinary_muscle_development	D_vs_E10;stage_1W_vs_E10;stage_3W_vs_E10;stage	1	TRUE
ENSGALG00010017226	PEF1	PRJNA729271	0.387703681	5.12645E-07	0.387703681	stage_E14_vs_E10	ordinary_muscle_development		0	FALSE
ENSGALG00010009054	RYR2	PRJNA729271	-2.717989598	5.13797E-07	2.717989598	stage_E14_vs_E10	ordinary_muscle_development	D_vs_E10;stage_1W_vs_E10;stage_3W_vs_E10;stage	1	TRUE
ENSGALG00010029322	BPTF	PRJNA729271	-0.507685619	5.14926E-07	0.507685619	stage_E14_vs_E10	ordinary_muscle_development		0	FALSE
ENSGALG00010022513	NDUFAF1	PRJNA729271	0.436385363	5.16171E-07	0.436385363	stage_E14_vs_E10	ordinary_muscle_development		0	FALSE

ENSGALG00010020935	BRI3BP	PRJNA729271	-0.344295768	5.18591E-07	0.344295768	stage_E14_vs_E10	ordinary_muscle_development		0	FALSE
ENSGALG00010001606	PIGA	PRJNA729271	-0.570410079	5.20646E-07	0.570410079	stage_E14_vs_E10	ordinary_muscle_development		0	FALSE
ENSGALG00010029276	TUBD1	PRJNA729271	0.894198313	5.20781E-07	0.894198313	stage_E14_vs_E10	ordinary_muscle_development		0	FALSE
ENSGALG00010016600	GNL2	PRJNA729271	-0.576627174	5.21536E-07	0.576627174	stage_E14_vs_E10	ordinary_muscle_development		0	FALSE
ENSGALG00010018523	TEX11	PRJNA729271	-0.943460316	5.21987E-07	0.943460316	stage_E14_vs_E10	ordinary_muscle_development		0	FALSE
ENSGALG00010001166	TRIM59	PRJNA729271	-0.506216767	5.23139E-07	0.506216767	stage_E14_vs_E10	ordinary_muscle_development		0	FALSE
ENSGALG00010022309	WSCD2	PRJNA729271	-2.079925071	5.23878E-07	2.079925071	stage_E14_vs_E10	ordinary_muscle_development	D_vs_E10;stage_1W_vs_E10;stage_3W_vs_E10;stage	1	TRUE
ENSGALG00010017806	ENSGALG00010017806	PRJNA729271	3.85021108	5.24494E-07	3.85021108	stage_E14_vs_E10	ordinary_muscle_development	D_vs_E10;stage_1W_vs_E10;stage_3W_vs_E10;stage	1	TRUE
ENSGALG00010000309	ENSGALG00010000309	PRJNA729271	-22.56039938	5.2733E-07	22.56039938	stage_E14_vs_E10	ordinary_muscle_development	D_vs_E10;stage_1W_vs_E10;stage_3W_vs_E10;stage	1	TRUE
ENSGALG00010024847	PPIL1	PRJNA729271	-0.794500487	5.31693E-07	0.794500487	stage_E14_vs_E10	ordinary_muscle_development		0	FALSE
ENSGALG00010021412	ZNF319	PRJNA729271	0.63447989	5.34215E-07	0.63447989	stage_E14_vs_E10	ordinary_muscle_development		0	FALSE
ENSGALG00010016618	TRPM3	PRJNA729271	-2.828380354	5.38983E-07	2.828380354	stage_E14_vs_E10	ordinary_muscle_development	D_vs_E10;stage_1W_vs_E10;stage_3W_vs_E10;stage	1	TRUE
ENSGALG00010010423	C21orf59	PRJNA729271	0.49747652	5.38996E-07	0.49747652	stage_E14_vs_E10	ordinary_muscle_development		0	FALSE
ENSGALG00010030108	LLGL2	PRJNA729271	0.60957102	5.43493E-07	0.60957102	stage_E14_vs_E10	ordinary_muscle_development		0	FALSE
ENSGALG00010006301	ENSGALG00010006301	PRJNA729271	0.751226491	5.46072E-07	0.751226491	stage_E14_vs_E10	ordinary_muscle_development		0	FALSE
ENSGALG00010013483	ENSGALG00010013483	PRJNA729271	-1.077764555	5.46582E-07	1.077764555	stage_E14_vs_E10	ordinary_muscle_development	D_vs_E10;stage_1W_vs_E10;stage_3W_vs_E10;stage	1	TRUE
ENSGALG00010017580	LYPLAL1	PRJNA729271	-0.80807863	5.54537E-07	0.80807863	stage_E14_vs_E10	ordinary_muscle_development		0	FALSE
ENSGALG00010002056	PRLHR	PRJNA729271	-2.81467348	5.55242E-07	2.81467348	stage_E14_vs_E10	ordinary_muscle_development	D_vs_E10;stage_1W_vs_E10;stage_3W_vs_E10;stage	1	TRUE
ENSGALG00010017378	ENSGALG00010017378	PRJNA729271	-3.420582121	5.55746E-07	3.420582121	stage_E14_vs_E10	ordinary_muscle_development	D_vs_E10;stage_1W_vs_E10;stage_3W_vs_E10;stage	1	TRUE
ENSGALG00010014142	PPIL6	PRJNA729271	2.489969893	5.57656E-07	2.489969893	stage_E14_vs_E10	ordinary_muscle_development	D_vs_E10;stage_1W_vs_E10;stage_3W_vs_E10;stage	1	TRUE
ENSGALG00010000312	CABP7	PRJNA729271	-3.245705281	5.60573E-07	3.245705281	stage_E14_vs_E10	ordinary_muscle_development	D_vs_E10;stage_1W_vs_E10;stage_3W_vs_E10;stage	1	TRUE
ENSGALG00010027240	SLC6A1	PRJNA729271	-1.855665868	5.61983E-07	1.855665868	stage_E14_vs_E10	ordinary_muscle_development	D_vs_E10;stage_1W_vs_E10;stage_3W_vs_E10;stage	1	TRUE
ENSGALG00010018374	HPS1	PRJNA729271	-0.71080933	5.62019E-07	0.71080933	stage_E14_vs_E10	ordinary_muscle_development		0	FALSE
ENSGALG00010010125	AHCTF1	PRJNA729271	-0.349210375	5.62479E-07	0.349210375	stage_E14_vs_E10	ordinary_muscle_development		0	FALSE
ENSGALG00010023496	PLEKHA3	PRJNA729271	0.531217116	5.65376E-07	0.531217116	stage_E14_vs_E10	ordinary_muscle_development		0	FALSE
ENSGALG00010027375	FUT7	PRJNA729271	2.107890179	5.71462E-07	2.107890179	stage_E14_vs_E10	ordinary_muscle_development	D_vs_E10;stage_1W_vs_E10;stage_3W_vs_E10;stage	1	TRUE
ENSGALG00010023233	SEZ6L	PRJNA729271	3.850363293	5.72572E-07	3.850363293	stage_E14_vs_E10	ordinary_muscle_development	D_vs_E10;stage_1W_vs_E10;stage_3W_vs_E10;stage	1	TRUE
ENSGALG00010013008	STEEP1	PRJNA729271	-0.579054761	5.72966E-07	0.579054761	stage_E14_vs_E10	ordinary_muscle_development		0	FALSE
ENSGALG00010004243	BLTP1	PRJNA729271	0.578481698	5.74458E-07	0.578481698	stage_E14_vs_E10	ordinary_muscle_development		0	FALSE
ENSGALG00010012274	B4GALNT3	PRJNA729271	1.42580139	5.78908E-07	1.42580139	stage_E14_vs_E10	ordinary_muscle_development	D_vs_E10;stage_1W_vs_E10;stage_3W_vs_E10;stage	1	TRUE
ENSGALG00010020069	TMEM216	PRJNA729271	-1.051094193	5.79261E-07	1.051094193	stage_E14_vs_E10	ordinary_muscle_development	D_vs_E10;stage_1W_vs_E10;stage_3W_vs_E10;stage	1	TRUE
ENSGALG00010029427	SPNS3	PRJNA729271	-1.737667703	5.80954E-07	1.737667703	stage_E14_vs_E10	ordinary_muscle_development	D_vs_E10;stage_1W_vs_E10;stage_3W_vs_E10;stage	1	TRUE
ENSGALG00010027144	ENSGALG00010027144	PRJNA729271	-5.02138573	5.87174E-07	5.02138573	stage_E14_vs_E10	ordinary_muscle_development	D_vs_E10;stage_1W_vs_E10;stage_3W_vs_E10;stage	1	TRUE
ENSGALG00010009704	RGP1	PRJNA729271	0.664341621	5.87712E-07	0.664341621	stage_E14_vs_E10	ordinary_muscle_development		0	FALSE
ENSGALG00010027475	ARID3A	PRJNA729271	-0.871420193	5.89251E-07	0.871420193	stage_E14_vs_E10	ordinary_muscle_development		0	FALSE
ENSGALG00010004334	ENSGALG00010004334	PRJNA729271	1.172844931	5.89259E-07	1.172844931	stage_E14_vs_E10	ordinary_muscle_development	D_vs_E10;stage_1W_vs_E10;stage_3W_vs_E10;stage	1	TRUE
ENSGALG00010010862	STRN4	PRJNA729271	0.763529796	5.89594E-07	0.763529796	stage_E14_vs_E10	ordinary_muscle_development		0	FALSE
ENSGALG00010029695	TAOK1	PRJNA729271	0.444133151	5.90191E-07	0.444133151	stage_E14_vs_E10	ordinary_muscle_development		0	FALSE
ENSGALG00010000722	ANKH	PRJNA729271	0.873079288	5.97397E-07	0.873079288	stage_E14_vs_E10	ordinary_muscle_development		0	FALSE
ENSGALG00010010596	SHLD2	PRJNA729271	0.680750189	5.98874E-07	0.680750189	stage_E14_vs_E10	ordinary_muscle_development		0	FALSE
ENSGALG00010024370	HERPUD1	PRJNA729271	0.469832142	6.00687E-07	0.469832142	stage_E14_vs_E10	ordinary_muscle_development		0	FALSE
ENSGALG00010005610	MTHFD1L	PRJNA729271	-0.98993903	6.02193E-07	0.98993903	stage_E14_vs_E10	ordinary_muscle_development		0	FALSE
ENSGALG00010005334	NME7	PRJNA729271	-0.692069324	6.0315E-07	0.692069324	stage_E14_vs_E10	ordinary_muscle_development		0	FALSE
ENSGALG00010022625	ENSGALG00010022625	PRJNA729271	0.645051908	6.05789E-07	0.645051908	stage_E14_vs_E10	ordinary_muscle_development		0	FALSE
ENSGALG00010002291	RNASEH1	PRJNA729271	0.592603869	6.15692E-07	0.592603869	stage_E14_vs_E10	ordinary_muscle_development		0	FALSE
ENSGALG00010029140	NPDC1	PRJNA729271	0.972715121	6.32809E-07	0.972715121	stage_E14_vs_E10	ordinary_muscle_development		0	FALSE
ENSGALG00010009663	FER	PRJNA729271	-0.629229184	6.32934E-07	0.629229184	stage_E14_vs_E10	ordinary_muscle_development		0	FALSE
ENSGALG00010025131	SUMO1	PRJNA729271	-0.415755442	6.36903E-07	0.415755442	stage_E14_vs_E10	ordinary_muscle_development		0	FALSE
ENSGALG00010012485	C6	PRJNA729271	6.215179379	6.37863E-07	6.215179379	stage_E14_vs_E10	ordinary_muscle_development	D_vs_E10;stage_1W_vs_E10;stage_3W_vs_E10;stage	1	TRUE
ENSGALG00010016573	DEGS1	PRJNA729271	-0.661339553	6.38252E-07	0.661339553	stage_E14_vs_E10	ordinary_muscle_development		0	FALSE
ENSGALG00010008248	TPK1	PRJNA729271	-0.840933656	6.39075E-07	0.840933656	stage_E14_vs_E10	ordinary_muscle_development		0	FALSE
ENSGALG00010021698	GYPC	PRJNA729271	0.696220538	6.40108E-07	0.696220538	stage_E14_vs_E10	ordinary_muscle_development		0	FALSE
ENSGALG00010018990	GCK	PRJNA729271	-2.19992566	6.42167E-07	2.19992566	stage_E14_vs_E10	ordinary_muscle_development	D_vs_E10;stage_1W_vs_E10;stage_3W_vs_E10;stage	1	TRUE
ENSGALG00010024575	TXNRD2	PRJNA729271	0.631288001	6.43063E-07	0.631288001	stage_E14_vs_E10	ordinary_muscle_development		0	FALSE
ENSGALG00010021637	ATP6V0D1	PRJNA729271	-0.546661858	6.44558E-07	0.546661858	stage_E14_vs_E10	ordinary_muscle_development		0	FALSE
ENSGALG00010021407	SIN3A	PRJNA729271	-0.573322474	6.46252E-07	0.573322474	stage_E14_vs_E10	ordinary_muscle_development		0	FALSE
ENSGALG00010010749	NCOA4	PRJNA729271	-0.505599352	6.47637E-07	0.505599352	stage_E14_vs_E10	ordinary_muscle_development		0	FALSE
ENSGALG00010019935	ENSGALG00010019935	PRJNA729271	6.142677851	6.477E-07	6.142677851	stage_E14_vs_E10	ordinary_muscle_development	D_vs_E10;stage_1W_vs_E10;stage_3W_vs_E10;stage	1	TRUE
ENSGALG00010010699	DNAJC27	PRJNA729271	-0.783547892	6.51552E-07	0.783547892	stage_E14_vs_E10	ordinary_muscle_development		0	FALSE
ENSGALG00010027105	CHERP	PRJNA729271	-0.658944228	6.53479E-07	0.658944228	stage_E14_vs_E10	ordinary_muscle_development		0	FALSE
ENSGALG00010020798	C1QL2	PRJNA729271	-3.449378835	6.53836E-07	3.449378835	stage_E14_vs_E10	ordinary_muscle_development	D_vs_E10;stage_1W_vs_E10;stage_3W_vs_E10;stage	1	TRUE
ENSGALG00010018459	SNX12	PRJNA729271	0.522830212	6.54542E-07	0.522830212	stage_E14_vs_E10	ordinary_muscle_development		0	FALSE
ENSGALG00010012550	TAB1	PRJNA729271	0.489263145	6.5602E-07	0.489263145	stage_E14_vs_E10	ordinary_muscle_development		0	FALSE
ENSGALG00010027961	TINCR	PRJNA729271	-5.382015546	6.62389E-07	5.382015546	stage_E14_vs_E10	ordinary_muscle_development	D_vs_E10;stage_1W_vs_E10;stage_3W_vs_E10;stage	1	TRUE
ENSGALG00010023714	EIF3M	PRJNA729271	-0.564538401	6.65269E-07	0.564538401	stage_E14_vs_E10	ordinary_muscle_development		0	FALSE
ENSGALG00010018378	ARPP21	PRJNA729271	0.748396403	6.70026E-07	0.748396403	stage_E14_vs_E10	ordinary_muscle_development		0	FALSE
ENSGALG00010009091	EXT1	PRJNA729271	-0.573023696	6.75584E-07	0.573023696	stage_E14_vs_E10	ordinary_muscle_development		0	FALSE
ENSGALG00010025344	ENSGALG00010025344	PRJNA729271	2.706301316	6.7781E-07	2.706301316	stage_E14_vs_E10	ordinary_muscle_development	D_vs_E10;stage_1W_vs_E10;stage_3W_vs_E10;stage	1	TRUE
ENSGALG00010006029	HSDL2	PRJNA729271	0.68892535	6.79866E-07	0.68892535	stage_E14_vs_E10	ordinary_muscle_development		0	FALSE
ENSGALG00010006339	ENSGALG00010006339	PRJNA729271	3.638823356	6.83856E-07	3.638823356	stage_E14_vs_E10	ordinary_muscle_development	D_vs_E10;stage_1W_vs_E10;stage_3W_vs_E10;stage	1	TRUE
ENSGALG00010015942	AP3S2	PRJNA729271	-0.677176927	6.8473E-07	0.677176927	stage_E14_vs_E10	ordinary_muscle_development		0	FALSE
ENSGALG00010000731	ENSGALG00010000731	PRJNA729271	-2.27557509	6.88659E-07	2.27557509	stage_E14_vs_E10	ordinary_muscle_development	D_vs_E10;stage_1W_vs_E10;stage_3W_vs_E10;stage	1	TRUE
ENSGALG00010024520	MAP3K13	PRJNA729271	-0.724436567	6.95453E-07	0.724436567	stage_E14_vs_E10	ordinary_muscle_development		0	FALSE
ENSGALG00010027354	ENSGALG00010027354	PRJNA729271	4.154770149	6.96936E-07	4.154770149	stage_E14_vs_E10	ordinary_muscle_development	D_vs_E10;stage_1W_vs_E10;stage_3W_vs_E10;stage	1	TRUE
ENSGALG00010024568	DZIP1L	PRJNA729271	1.3323417	7.00223E-07	1.3323417	stage_E14_vs_E10	ordinary_muscle_development	D_vs_E10;stage_1W_vs_E10;stage_3W_vs_E10;stage	1	TRUE
ENSGALG00010024758	SPATA6	PRJNA729271	-0.549108569	7.02462E-07	0.549108569	stage_E14_vs_E10	ordinary_muscle_development		0	FALSE
ENSGALG00010005357	TNIP3	PRJNA729271	-4.632563766	7.04908E-07	4.632563766	stage_E14_vs_E10	ordinary_muscle_development	D_vs_E10;stage_1W_vs_E10;stage_3W_vs_E10;stage	1	TRUE
ENSGALG00010007809	ARV1	PRJNA729271	-0.623989563	7.05842E-07	0.623989563	stage_E14_vs_E10	ordinary_muscle_development		0	FALSE
ENSGALG00010021938	CABP2	PRJNA729271	-0.996075042	7.07588E-07	0.996075042	stage_E14_vs_E10	ordinary_muscle_development		0	FALSE
ENSGALG00010014189	DDX4	PRJNA729271	-4.244809868	7.08994E-07	4.244809868	stage_E14_vs_E10	ordinary_muscle_development	D_vs_E10;stage_1W_vs_E10;stage_3W_vs_E10;stage	1	TRUE
ENSGALG00010015095	RBFOX2	PRJNA729271	-0.677465724	7.17317E-07	0.677465724	stage_E14_vs_E10	ordinary_muscle_development		0	FALSE
ENSGALG00010020922	INSIG2	PRJNA729271	0.409010262	7.19745E-07	0.409010262	stage_E14_vs_E10	ordinary_muscle_development		0	FALSE
ENSGALG00010023438	HOXB2	PRJNA729271	-1.324529069	7.2167E-07	1.324529069	stage_E14_vs_E10	ordinary_muscle_development	D_vs_E10;stage_1W_vs_E10;stage_3W_vs_E10;stage	1	TRUE
ENSGALG00010022978	ARL14EP	PRJNA729271	-0.44416964	7.2465E-07	0.44416964	stage_E14_vs_E10	ordinary_muscle_development		0	FALSE
ENSGALG00010013661	GABRA4	PRJNA729271	-1.996887087	7.2881E-07	1.996887087	stage_E14_vs_E10	ordinary_muscle_development	D_vs_E10;stage_1W_vs_E10;stage_3W_vs_E10;stage	1	TRUE
ENSGALG00010007650	KLHL36	PRJNA729271	-0.454129058	7.28812E-07	0.454129058	stage_E14_vs_E10	ordinary_muscle_development		0	FALSE
ENSGALG00010010867	QSOX1	PRJNA729271	-0.833807092	7.29038E-07	0.833807092	stage_E14_vs_E10	ordinary_muscle_development		0	FALSE
ENSGALG00010024695	TRABD2B	PRJNA729271	-1.034834136	7.36071E-07	1.034834136	stage_E14_vs_E10	ordinary_muscle_development	D_vs_E10;stage_1W_vs_E10;stage_3W_vs_E10;stage	1	TRUE
ENSGALG00010024506	RAB8B	PRJNA729271	0.411958492	7.38256E-07	0.411958492	stage_E14_vs_E10	ordinary_muscle_development		0	FALSE
ENSGALG00010021835	NUDT8	PRJNA729271	0.876352084	7.4097E-07	0.876352084	stage_E14_vs_E10	ordinary_muscle_development		0	FALSE
ENSGALG00010016720	INTS15	PRJNA729271	-0.534000084	7.41383E-07	0.534000084	stage_E14_vs_E10	ordinary_muscle_development		0	FALSE
ENSGALG00010015588	BDKRB2	PRJNA729271	-2.739355869	7.43491E-07	2.739355869	stage_E14_vs_E10	ordinary_muscle_development	D_vs_E10;stage_1W_vs_E10;stage_3W_vs_E10;stage	1	TRUE
ENSGALG00010024486	GPR37L1	PRJNA729271	-1.805580587	7.46633E-07	1.805580587	stage_E14_vs_E10	ordinary_muscle_development	D_vs_E10;stage_1W_vs_E10;stage_3W_vs_E10;stage	1	TRUE
ENSGALG00010017599	KCNK13	PRJNA729271	1.137334733	7.49561E-07	1.137334733	stage_E14_vs_E10	ordinary_muscle_development	D_vs_E10;stage_1W_vs_E10;stage_3W_vs_E10;stage	1	TRUE
ENSGALG00010021916	MRPS25	PRJNA729271	0.75213766	7.49604E-07	0.75213766	stage_E14_vs_E10	ordinary_muscle_development		0	FALSE
ENSGALG00010000753	TRIO	PRJNA729271	-0.347625304	7.54489E-07	0.347625304	stage_E14_vs_E10	ordinary_muscle_development		0	FALSE
ENSGALG00010028062	ENSGALG00010028062	PRJNA729271	-1.534287139	7.5895E-07	1.534287139	stage_E14_vs_E10	ordinary_muscle_development	D_vs_E10;stage_1W_vs_E10;stage_3W_vs_E10;stage	1	TRUE
ENSGALG00010018319	STUB1	PRJNA729271	1.021894752	7.61075E-07	1.021894752	stage_E14_vs_E10	ordinary_muscle_development	D_vs_E10;stage_1W_vs_E10;stage_3W_vs_E10;stage	1	TRUE
ENSGALG00010010983	MED28	PRJNA729271	0.447933051	7.61598E-07	0.447933051	stage_E14_vs_E10	ordinary_muscle_development		0	FALSE
ENSGALG00010001605	APBB1	PRJNA729271	0.589965659	7.67547E-07	0.589965659	stage_E14_vs_E10	ordinary_muscle_development		0	FALSE
ENSGALG00010010973	PGAP1	PRJNA729271	-0.576057041	7.67816E-07	0.576057041	stage_E14_vs_E10	ordinary_muscle_development		0	FALSE
ENSGALG00010029446	PRPSAP1	PRJNA729271	0.415397432	7.71243E-07	0.415397432	stage_E14_vs_E10	ordinary_muscle_development		0	FALSE
ENSGALG00010007479	NFX1	PRJNA729271	-0.495448165	7.78392E-07	0.495448165	stage_E14_vs_E10	ordinary_muscle_development		0	FALSE
ENSGALG00010000597	CDH9	PRJNA729271	-4.938067469	7.78764E-07	4.938067469	stage_E14_vs_E10	ordinary_muscle_development	D_vs_E10;stage_1W_vs_E10;stage_3W_vs_E10;stage	1	TRUE
ENSGALG00010006013	TRMT10C	PRJNA729271	-0.55315901	7.80567E-07	0.55315901	stage_E14_vs_E10	ordinary_muscle_development		0	FALSE
ENSGALG00010017807	MRPL35	PRJNA729271	0.742718708	7.82362E-07	0.742718708	stage_E14_vs_E10	ordinary_muscle_development		0	FALSE
ENSGALG00010005839	RPS6KA2	PRJNA729271	-0.961816625	7.88853E-07	0.961816625	stage_E14_vs_E10	ordinary_muscle_development		0	FALSE
ENSGALG00010014156	CD164	PRJNA729271	0.467474336	7.89198E-07	0.467474336	stage_E14_vs_E10	ordinary_muscle_development		0	FALSE
ENSGALG00010000606	UBE2QL1	PRJNA729271	0.780919131	7.90033E-07	0.780919131	stage_E14_vs_E10	ordinary_muscle_development		0	FALSE
ENSGALG00010022375	NMB	PRJNA729271	-2.233715956	7.96084E-07	2.233715956	stage_E14_vs_E10	ordinary_muscle_development	D_vs_E10;stage_1W_vs_E10;stage_3W_vs_E10;stage	1	TRUE
ENSGALG00010009689	ENSGALG00010009689	PRJNA729271	-3.454786592	7.984E-07	3.454786592	stage_E14_vs_E10	ordinary_muscle_development	D_vs_E10;stage_1W_vs_E10;stage_3W_vs_E10;stage	1	TRUE
ENSGALG00010017167	SMAP2	PRJNA729271	-0.49525985	8.0157E-07	0.49525985	stage_E14_vs_E10	ordinary_muscle_development		0	FALSE
ENSGALG00010017797	PPP1R21	PRJNA729271	-0.471683775	8.0157E-07	0.471683775	stage_E14_vs_E10	ordinary_muscle_development		0	FALSE
ENSGALG00010006178	RPS23	PRJNA729271	0.721983543	8.06555E-07	0.721983543	stage_E14_vs_E10	ordinary_muscle_development		0	FALSE
ENSGALG00010026484	TNKS1BP1	PRJNA729271	-0.57079502	8.06701E-07	0.57079502	stage_E14_vs_E10	ordinary_muscle_development		0	FALSE
ENSGALG00010001860	RNF169	PRJNA729271	0.646476031	8.08417E-07	0.646476031	stage_E14_vs_E10	ordinary_muscle_development		0	FALSE
ENSGALG00010000205	RNF215	PRJNA729271	0.565104212	8.09644E-07	0.565104212	stage_E14_vs_E10	ordinary_muscle_development		0	FALSE
ENSGALG00010027979	RPL12	PRJNA729271	0.604681236	8.10501E-07	0.604681236	stage_E14_vs_E10	ordinary_muscle_development		0	FALSE
ENSGALG00010005280	TMEM244	PRJNA729271	-4.787433904	8.14109E-07	4.787433904	stage_E14_vs_E10	ordinary_muscle_development	D_vs_E10;stage_1W_vs_E10;stage_3W_vs_E10;stage	1	TRUE
ENSGALG00010009167	DCTN3	PRJNA729271	-0.565576989	8.19825E-07	0.565576989	stage_E14_vs_E10	ordinary_muscle_development		0	FALSE
ENSGALG00010024639	AAR2	PRJNA729271	-0.387973538	8.20317E-07	0.387973538	stage_E14_vs_E10	ordinary_muscle_development		0	FALSE
ENSGALG00010010943	WDSUB1	PRJNA729271	0.384159807	8.25091E-07	0.384159807	stage_E14_vs_E10	ordinary_muscle_development		0	FALSE
ENSGALG00010015117	RASD2	PRJNA729271	1.0372381	8.2885E-07	1.0372381	stage_E14_vs_E10	ordinary_muscle_development	D_vs_E10;stage_1W_vs_E10;stage_3W_vs_E10;stage	1	TRUE
ENSGALG00010010346	TFB2M	PRJNA729271	0.70340764	8.29476E-07	0.70340764	stage_E14_vs_E10	ordinary_muscle_development		0	FALSE
ENSGALG00010000806	CFAP90	PRJNA729271	1.514013	8.33434E-07	1.514013	stage_E14_vs_E10	ordinary_muscle_development	D_vs_E10;stage_1W_vs_E10;stage_3W_vs_E10;stage	1	TRUE
ENSGALG00010004666	C6orf120	PRJNA729271	-0.601138161	8.35578E-07	0.601138161	stage_E14_vs_E10	ordinary_muscle_development		0	FALSE
ENSGALG00010007943	BLOC1S4	PRJNA729271	-0.515588486	8.42105E-07	0.515588486	stage_E14_vs_E10	ordinary_muscle_development		0	FALSE
ENSGALG00010010062	RGS7BP	PRJNA729271	-0.895428934	8.48042E-07	0.895428934	stage_E14_vs_E10	ordinary_muscle_development		0	FALSE
ENSGALG00010010889	CCL19	PRJNA729271	3.229581171	8.48379E-07	3.229581171	stage_E14_vs_E10	ordinary_muscle_development	D_vs_E10;stage_1W_vs_E10;stage_3W_vs_E10;stage	1	TRUE
ENSGALG00010000007	ND1	PRJNA729271	1.234489947	8.49944E-07	1.234489947	stage_E14_vs_E10	ordinary_muscle_development	D_vs_E10;stage_1W_vs_E10;stage_3W_vs_E10;stage	1	TRUE
ENSGALG00010020460	MAPK8	PRJNA729271	0.71328727	8.54294E-07	0.71328727	stage_E14_vs_E10	ordinary_muscle_development		0	FALSE
ENSGALG00010016229	ENSGALG00010016229	PRJNA729271	1.124953421	8.56644E-07	1.124953421	stage_E14_vs_E10	ordinary_muscle_development	D_vs_E10;stage_1W_vs_E10;stage_3W_vs_E10;stage	1	TRUE
ENSGALG00010002425	FDX2	PRJNA729271	0.673747111	8.63965E-07	0.673747111	stage_E14_vs_E10	ordinary_muscle_development		0	FALSE
ENSGALG00010024245	TARBP2	PRJNA729271	0.826942932	8.67909E-07	0.826942932	stage_E14_vs_E10	ordinary_muscle_development		0	FALSE
ENSGALG00010022230	NFS1	PRJNA729271	-0.440969114	8.80644E-07	0.440969114	stage_E14_vs_E10	ordinary_muscle_development		0	FALSE
ENSGALG00010024649	NCOR2	PRJNA729271	0.656384064	8.84124E-07	0.656384064	stage_E14_vs_E10	ordinary_muscle_development		0	FALSE
ENSGALG00010008880	FMN2	PRJNA729271	-1.494244791	8.844E-07	1.494244791	stage_E14_vs_E10	ordinary_muscle_development	D_vs_E10;stage_1W_vs_E10;stage_3W_vs_E10;stage	1	TRUE
ENSGALG00010013333	PPP1R12A	PRJNA729271	0.496150088	8.8516E-07	0.496150088	stage_E14_vs_E10	ordinary_muscle_development		0	FALSE
ENSGALG00010008025	TBC1D22A	PRJNA729271	-0.493692971	8.86838E-07	0.493692971	stage_E14_vs_E10	ordinary_muscle_development		0	FALSE
ENSGALG00010013651	ENSGALG00010013651	PRJNA729271	-0.854813191	8.8759E-07	0.854813191	stage_E14_vs_E10	ordinary_muscle_development		0	FALSE
ENSGALG00010021726	ARNTL	PRJNA729271	-0.751351115	8.88449E-07	0.751351115	stage_E14_vs_E10	ordinary_muscle_development		0	FALSE
ENSGALG00010011308	SPIC	PRJNA729271	6.902599217	8.91268E-07	6.902599217	stage_E14_vs_E10	ordinary_muscle_development	D_vs_E10;stage_1W_vs_E10;stage_3W_vs_E10;stage	1	TRUE
ENSGALG00010021663	CNOT9	PRJNA729271	0.703750105	8.93072E-07	0.703750105	stage_E14_vs_E10	ordinary_muscle_development		0	FALSE
ENSGALG00010011398	ENSGALG00010011398	PRJNA729271	0.914958098	9.02482E-07	0.914958098	stage_E14_vs_E10	ordinary_muscle_development		0	FALSE
ENSGALG00010017250	COL16A1	PRJNA729271	0.859903565	9.02482E-07	0.859903565	stage_E14_vs_E10	ordinary_muscle_development		0	FALSE
ENSGALG00010016913	BRCC3	PRJNA729271	-0.566915438	9.04777E-07	0.566915438	stage_E14_vs_E10	ordinary_muscle_development		0	FALSE
ENSGALG00010027719	GPR61	PRJNA729271	-5.632286433	9.06354E-07	5.632286433	stage_E14_vs_E10	ordinary_muscle_development	D_vs_E10;stage_1W_vs_E10;stage_3W_vs_E10;stage	1	TRUE
ENSGALG00010018143	MRPL47	PRJNA729271	0.62068465	9.08487E-07	0.62068465	stage_E14_vs_E10	ordinary_muscle_development		0	FALSE
ENSGALG00010003822	ENSGALG00010003822	PRJNA729271	-0.845856392	9.10031E-07	0.845856392	stage_E14_vs_E10	ordinary_muscle_development		0	FALSE
ENSGALG00010025438	EXOSC6	PRJNA729271	0.599103177	9.13608E-07	0.599103177	stage_E14_vs_E10	ordinary_muscle_development		0	FALSE
ENSGALG00010027715	NINJ1	PRJNA729271	-0.629944009	9.14881E-07	0.629944009	stage_E14_vs_E10	ordinary_muscle_development		0	FALSE
ENSGALG00010007339	ENSGALG00010007339	PRJNA729271	1.125410399	9.16386E-07	1.125410399	stage_E14_vs_E10	ordinary_muscle_development	D_vs_E10;stage_1W_vs_E10;stage_3W_vs_E10;stage	1	TRUE
ENSGALG00010016863	MYNN	PRJNA729271	-0.42826067	9.2166E-07	0.42826067	stage_E14_vs_E10	ordinary_muscle_development		0	FALSE
ENSGALG00010008001	ALDH5A1	PRJNA729271	-0.702199628	9.23445E-07	0.702199628	stage_E14_vs_E10	ordinary_muscle_development		0	FALSE
ENSGALG00010021528	BRPF1	PRJNA729271	0.835734138	9.23912E-07	0.835734138	stage_E14_vs_E10	ordinary_muscle_development		0	FALSE
ENSGALG00010001702	PLEKHA1	PRJNA729271	0.418569467	9.25531E-07	0.418569467	stage_E14_vs_E10	ordinary_muscle_development		0	FALSE
ENSGALG00010000611	C7orf57	PRJNA729271	1.611180367	9.2559E-07	1.611180367	stage_E14_vs_E10	ordinary_muscle_development	D_vs_E10;stage_1W_vs_E10;stage_3W_vs_E10;stage	1	TRUE
ENSGALG00010024517	LACTB	PRJNA729271	-0.521592724	9.2606E-07	0.521592724	stage_E14_vs_E10	ordinary_muscle_development		0	FALSE
ENSGALG00010011493	ENSGALG00010011493	PRJNA729271	1.316582781	9.26403E-07	1.316582781	stage_E14_vs_E10	ordinary_muscle_development	D_vs_E10;stage_1W_vs_E10;stage_3W_vs_E10;stage	1	TRUE
ENSGALG00010027522	PSMD5	PRJNA729271	0.525285827	9.27676E-07	0.525285827	stage_E14_vs_E10	ordinary_muscle_development		0	FALSE
ENSGALG00010017038	ZNF598	PRJNA729271	-0.674979251	9.27924E-07	0.674979251	stage_E14_vs_E10	ordinary_muscle_development		0	FALSE
ENSGALG00010005481	ENSGALG00010005481	PRJNA729271	-2.91061913	9.44288E-07	2.91061913	stage_E14_vs_E10	ordinary_muscle_development	D_vs_E10;stage_1W_vs_E10;stage_3W_vs_E10;stage	1	TRUE
ENSGALG00010029656	MRPL27	PRJNA729271	0.568484728	9.44459E-07	0.568484728	stage_E14_vs_E10	ordinary_muscle_development		0	FALSE
ENSGALG00010021167	KIAA1671	PRJNA729271	0.640558551	9.45075E-07	0.640558551	stage_E14_vs_E10	ordinary_muscle_development		0	FALSE
ENSGALG00010028079	ITIH3	PRJNA729271	7.687499712	9.45262E-07	7.687499712	stage_E14_vs_E10	ordinary_muscle_development	D_vs_E10;stage_1W_vs_E10;stage_3W_vs_E10;stage	1	TRUE
ENSGALG00010009275	CBLL1	PRJNA729271	0.697465813	9.49715E-07	0.697465813	stage_E14_vs_E10	ordinary_muscle_development		0	FALSE
ENSGALG00010010347	FBXW11	PRJNA729271	-0.43107586	9.50795E-07	0.43107586	stage_E14_vs_E10	ordinary_muscle_development		0	FALSE
ENSGALG00010013539	NSFL1C	PRJNA729271	-0.468107291	9.52138E-07	0.468107291	stage_E14_vs_E10	ordinary_muscle_development		0	FALSE
ENSGALG00010005542	KCTD1	PRJNA729271	-0.701427595	9.52671E-07	0.701427595	stage_E14_vs_E10	ordinary_muscle_development		0	FALSE
ENSGALG00010028745	ENSGALG00010028745	PRJNA729271	0.712721201	9.55767E-07	0.712721201	stage_E14_vs_E10	ordinary_muscle_development		0	FALSE
ENSGALG00010024876	CFLAR	PRJNA729271	0.477197335	9.56681E-07	0.477197335	stage_E14_vs_E10	ordinary_muscle_development		0	FALSE
ENSGALG00010002838	ENSGALG00010002838	PRJNA729271	1.293547522	9.61603E-07	1.293547522	stage_E14_vs_E10	ordinary_muscle_development	D_vs_E10;stage_1W_vs_E10;stage_3W_vs_E10;stage	1	TRUE
ENSGALG00010004939	DDX10	PRJNA729271	-0.560207917	9.62929E-07	0.560207917	stage_E14_vs_E10	ordinary_muscle_development		0	FALSE
ENSGALG00010019968	RHCG	PRJNA729271	4.012800436	9.64875E-07	4.012800436	stage_E14_vs_E10	ordinary_muscle_development	D_vs_E10;stage_1W_vs_E10;stage_3W_vs_E10;stage	1	TRUE
ENSGALG00010021346	RPS21	PRJNA729271	1.41730551	9.64875E-07	1.41730551	stage_E14_vs_E10	ordinary_muscle_development	D_vs_E10;stage_1W_vs_E10;stage_3W_vs_E10;stage	1	TRUE

ENSGALG00010028738	MIDN	PRJNA729271	-0.936136094	9.65035E-07	0.936136094	stage_E14_vs_E10	ordinary_muscle_development		0	FALSE
ENSGALG00010002576	HELQ	PRJNA729271	-0.55064982	9.66076E-07	0.55064982	stage_E14_vs_E10	ordinary_muscle_development		0	FALSE
ENSGALG00010016884	SBK1	PRJNA729271	-1.450151473	9.66807E-07	1.450151473	stage_E14_vs_E10	ordinary_muscle_development	D_vs_E10;stage_1W_vs_E10;stage_3W_vs_E10;stage	1	TRUE
ENSGALG00010001112	TSC22D2	PRJNA729271	-0.457049013	9.67086E-07	0.457049013	stage_E14_vs_E10	ordinary_muscle_development		0	FALSE
ENSGALG00010008302	ENSGALG00010008302	PRJNA729271	0.728221169	9.70489E-07	0.728221169	stage_E14_vs_E10	ordinary_muscle_development		0	FALSE
ENSGALG00010009166	CLGN	PRJNA729271	-1.22612514	9.72626E-07	1.22612514	stage_E14_vs_E10	ordinary_muscle_development	D_vs_E10;stage_1W_vs_E10;stage_3W_vs_E10;stage	1	TRUE
ENSGALG00010029713	CPD	PRJNA729271	0.584804728	9.76511E-07	0.584804728	stage_E14_vs_E10	ordinary_muscle_development		0	FALSE
ENSGALG00010016832	DYNC1H1	PRJNA729271	-0.479421112	9.80521E-07	0.479421112	stage_E14_vs_E10	ordinary_muscle_development		0	FALSE
ENSGALG00010011573	CMAS	PRJNA729271	-0.910028416	9.80837E-07	0.910028416	stage_E14_vs_E10	ordinary_muscle_development		0	FALSE
ENSGALG00010016548	EVL	PRJNA729271	0.5659136	9.81293E-07	0.5659136	stage_E14_vs_E10	ordinary_muscle_development		0	FALSE
ENSGALG00010012336	ENSGALG00010012336	PRJNA729271	-21.11578489	9.85652E-07	21.11578489	stage_E14_vs_E10	ordinary_muscle_development	D_vs_E10;stage_1W_vs_E10;stage_3W_vs_E10;stage	1	TRUE
ENSGALG00010017979	ENSGALG00010017979	PRJNA729271	-0.86461645	9.87516E-07	0.86461645	stage_E14_vs_E10	ordinary_muscle_development		0	FALSE
ENSGALG00010004609	CFAP69	PRJNA729271	-3.4148535	9.88491E-07	3.4148535	stage_E14_vs_E10	ordinary_muscle_development	D_vs_E10;stage_1W_vs_E10;stage_3W_vs_E10;stage	1	TRUE
ENSGALG00010018290	RHOT2	PRJNA729271	-0.70627502	9.92654E-07	0.70627502	stage_E14_vs_E10	ordinary_muscle_development		0	FALSE
ENSGALG00010029791	GALK1	PRJNA729271	-0.576794049	1.00286E-06	0.576794049	stage_E14_vs_E10	ordinary_muscle_development		0	FALSE
ENSGALG00010016778	MTIF2	PRJNA729271	-0.56070341	1.00761E-06	0.56070341	stage_E14_vs_E10	ordinary_muscle_development		0	FALSE
ENSGALG00010020104	BRAT1	PRJNA729271	0.561533608	1.01985E-06	0.561533608	stage_E14_vs_E10	ordinary_muscle_development		0	FALSE
ENSGALG00010002476	DDX3X	PRJNA729271	0.726626242	1.02145E-06	0.726626242	stage_E14_vs_E10	ordinary_muscle_development		0	FALSE
ENSGALG00010016655	TRANK1	PRJNA729271	1.623311428	1.02774E-06	1.623311428	stage_E14_vs_E10	ordinary_muscle_development	D_vs_E10;stage_1W_vs_E10;stage_3W_vs_E10;stage	1	TRUE
ENSGALG00010004380	ISCA2	PRJNA729271	0.422920076	1.03489E-06	0.422920076	stage_E14_vs_E10	ordinary_muscle_development		0	FALSE
ENSGALG00010017726	TTI2	PRJNA729271	0.602838923	1.03508E-06	0.602838923	stage_E14_vs_E10	ordinary_muscle_development		0	FALSE
ENSGALG00010018217	ENSGALG00010018217	PRJNA729271	-3.449888714	1.0395E-06	3.449888714	stage_E14_vs_E10	ordinary_muscle_development	D_vs_E10;stage_1W_vs_E10;stage_3W_vs_E10;stage	1	TRUE
ENSGALG00010002226	OXNAD1	PRJNA729271	-0.702665417	1.0403E-06	0.702665417	stage_E14_vs_E10	ordinary_muscle_development		0	FALSE
ENSGALG00010020005	EOGT	PRJNA729271	0.819236134	1.04067E-06	0.819236134	stage_E14_vs_E10	ordinary_muscle_development		0	FALSE
ENSGALG00010029159	SSC4D	PRJNA729271	-5.348505414	1.04183E-06	5.348505414	stage_E14_vs_E10	ordinary_muscle_development	D_vs_E10;stage_1W_vs_E10;stage_3W_vs_E10;stage	1	TRUE
ENSGALG00010010197	SCCPDH	PRJNA729271	-0.610080293	1.04845E-06	0.610080293	stage_E14_vs_E10	ordinary_muscle_development		0	FALSE
ENSGALG00010007878	PSMB1	PRJNA729271	-0.622774423	1.06514E-06	0.622774423	stage_E14_vs_E10	ordinary_muscle_development		0	FALSE
ENSGALG00010026344	TIMM10	PRJNA729271	0.857423825	1.06518E-06	0.857423825	stage_E14_vs_E10	ordinary_muscle_development		0	FALSE
ENSGALG00010002591	ENSGALG00010002591	PRJNA729271	1.073635418	1.07248E-06	1.073635418	stage_E14_vs_E10	ordinary_muscle_development	D_vs_E10;stage_1W_vs_E10;stage_3W_vs_E10;stage	1	TRUE
ENSGALG00010010836	DIP2A	PRJNA729271	-0.532471649	1.07492E-06	0.532471649	stage_E14_vs_E10	ordinary_muscle_development		0	FALSE
ENSGALG00010027995	POLR2E	PRJNA729271	-0.421926369	1.07649E-06	0.421926369	stage_E14_vs_E10	ordinary_muscle_development		0	FALSE
ENSGALG00010021754	SLC17A9	PRJNA729271	-2.641040588	1.08017E-06	2.641040588	stage_E14_vs_E10	ordinary_muscle_development	D_vs_E10;stage_1W_vs_E10;stage_3W_vs_E10;stage	1	TRUE
ENSGALG00010000166	PFDN6	PRJNA729271	0.646259357	1.10049E-06	0.646259357	stage_E14_vs_E10	ordinary_muscle_development		0	FALSE
ENSGALG00010016924	LETM1	PRJNA729271	-0.596665053	1.10115E-06	0.596665053	stage_E14_vs_E10	ordinary_muscle_development		0	FALSE
ENSGALG00010002943	SAP30	PRJNA729271	-0.771239044	1.10404E-06	0.771239044	stage_E14_vs_E10	ordinary_muscle_development		0	FALSE
ENSGALG00010019020	KCNK2	PRJNA729271	2.226558208	1.11654E-06	2.226558208	stage_E14_vs_E10	ordinary_muscle_development	D_vs_E10;stage_1W_vs_E10;stage_3W_vs_E10;stage	1	TRUE
ENSGALG00010009144	CREBZF	PRJNA729271	0.777251126	1.12197E-06	0.777251126	stage_E14_vs_E10	ordinary_muscle_development		0	FALSE
ENSGALG00010025591	OTOS	PRJNA729271	1.71007208	1.12819E-06	1.71007208	stage_E14_vs_E10	ordinary_muscle_development	D_vs_E10;stage_1W_vs_E10;stage_3W_vs_E10;stage	1	TRUE
ENSGALG00010004113	ECHDC1	PRJNA729271	0.930812319	1.12994E-06	0.930812319	stage_E14_vs_E10	ordinary_muscle_development		0	FALSE
ENSGALG00010029368	DDX52	PRJNA729271	-0.476409139	1.13239E-06	0.476409139	stage_E14_vs_E10	ordinary_muscle_development		0	FALSE
ENSGALG00010018233	RASSF2	PRJNA729271	-0.95189982	1.13779E-06	0.95189982	stage_E14_vs_E10	ordinary_muscle_development		0	FALSE
ENSGALG00010019388	GPR75	PRJNA729271	-0.690262763	1.14063E-06	0.690262763	stage_E14_vs_E10	ordinary_muscle_development		0	FALSE
ENSGALG00010007741	ENSGALG00010007741	PRJNA729271	-1.081991674	1.14086E-06	1.081991674	stage_E14_vs_E10	ordinary_muscle_development	D_vs_E10;stage_1W_vs_E10;stage_3W_vs_E10;stage	1	TRUE
ENSGALG00010020612	RPL27A	PRJNA729271	0.974728769	1.14448E-06	0.974728769	stage_E14_vs_E10	ordinary_muscle_development		0	FALSE
ENSGALG00010014893	SLC9A6	PRJNA729271	-0.449112217	1.14848E-06	0.449112217	stage_E14_vs_E10	ordinary_muscle_development		0	FALSE
ENSGALG00010029482	DDX25	PRJNA729271	-0.954515737	1.15405E-06	0.954515737	stage_E14_vs_E10	ordinary_muscle_development		0	FALSE
ENSGALG00010010689	POFUT2	PRJNA729271	-0.749669314	1.15603E-06	0.749669314	stage_E14_vs_E10	ordinary_muscle_development		0	FALSE
ENSGALG00010000063	SERF2	PRJNA729271	0.635564657	1.15889E-06	0.635564657	stage_E14_vs_E10	ordinary_muscle_development		0	FALSE
ENSGALG00010022816	METTL8	PRJNA729271	-0.786260227	1.15964E-06	0.786260227	stage_E14_vs_E10	ordinary_muscle_development		0	FALSE
ENSGALG00010017376	GALNT14	PRJNA729271	-1.514752869	1.16704E-06	1.514752869	stage_E14_vs_E10	ordinary_muscle_development	D_vs_E10;stage_1W_vs_E10;stage_3W_vs_E10;stage	1	TRUE
ENSGALG00010007962	ADCY8	PRJNA729271	-2.534352684	1.17464E-06	2.534352684	stage_E14_vs_E10	ordinary_muscle_development	D_vs_E10;stage_1W_vs_E10;stage_3W_vs_E10;stage	1	TRUE
ENSGALG00010029443	ENSGALG00010029443	PRJNA729271	-1.229311255	1.18071E-06	1.229311255	stage_E14_vs_E10	ordinary_muscle_development	D_vs_E10;stage_1W_vs_E10;stage_3W_vs_E10;stage	1	TRUE
ENSGALG00010011808	SMARCA2	PRJNA729271	0.615107554	1.1934E-06	0.615107554	stage_E14_vs_E10	ordinary_muscle_development		0	FALSE
ENSGALG00010010887	GABPA	PRJNA729271	-0.336671131	1.19342E-06	0.336671131	stage_E14_vs_E10	ordinary_muscle_development		0	FALSE
ENSGALG00010008226	COX20	PRJNA729271	-0.842408386	1.19519E-06	0.842408386	stage_E14_vs_E10	ordinary_muscle_development		0	FALSE
ENSGALG00010023007	LMO4	PRJNA729271	-0.721840284	1.20388E-06	0.721840284	stage_E14_vs_E10	ordinary_muscle_development		0	FALSE
ENSGALG00010029380	RGS9	PRJNA729271	0.742009709	1.20402E-06	0.742009709	stage_E14_vs_E10	ordinary_muscle_development		0	FALSE
ENSGALG00010028142	PMPCA	PRJNA729271	0.610241612	1.20558E-06	0.610241612	stage_E14_vs_E10	ordinary_muscle_development		0	FALSE
ENSGALG00010014994	ENSGALG00010014994	PRJNA729271	0.600205372	1.2081E-06	0.600205372	stage_E14_vs_E10	ordinary_muscle_development		0	FALSE
ENSGALG00010005222	ABCB1	PRJNA729271	4.849832691	1.20999E-06	4.849832691	stage_E14_vs_E10	ordinary_muscle_development	D_vs_E10;stage_1W_vs_E10;stage_3W_vs_E10;stage	1	TRUE
ENSGALG00010006177	NEK9	PRJNA729271	-0.575565447	1.21517E-06	0.575565447	stage_E14_vs_E10	ordinary_muscle_development		0	FALSE
ENSGALG00010018399	ABHD12	PRJNA729271	0.820162585	1.22047E-06	0.820162585	stage_E14_vs_E10	ordinary_muscle_development		0	FALSE
ENSGALG00010014148	KCNG1	PRJNA729271	-1.848192054	1.23122E-06	1.848192054	stage_E14_vs_E10	ordinary_muscle_development	D_vs_E10;stage_1W_vs_E10;stage_3W_vs_E10;stage	1	TRUE
ENSGALG00010021166	NEURL1	PRJNA729271	1.455758528	1.23912E-06	1.455758528	stage_E14_vs_E10	ordinary_muscle_development	D_vs_E10;stage_1W_vs_E10;stage_3W_vs_E10;stage	1	TRUE
ENSGALG00010016520	CHMP1B	PRJNA729271	0.411715559	1.24041E-06	0.411715559	stage_E14_vs_E10	ordinary_muscle_development		0	FALSE
ENSGALG00010008160	TXNL4A	PRJNA729271	-0.463129088	1.2447E-06	0.463129088	stage_E14_vs_E10	ordinary_muscle_development		0	FALSE
ENSGALG00010021015	RBM25	PRJNA729271	-0.685680496	1.24943E-06	0.685680496	stage_E14_vs_E10	ordinary_muscle_development		0	FALSE
ENSGALG00010024881	CASP10	PRJNA729271	-1.621534492	1.25144E-06	1.621534492	stage_E14_vs_E10	ordinary_muscle_development	D_vs_E10;stage_1W_vs_E10;stage_3W_vs_E10;stage	1	TRUE
ENSGALG00010021671	TNFRSF13B	PRJNA729271	3.589674081	1.25988E-06	3.589674081	stage_E14_vs_E10	ordinary_muscle_development	D_vs_E10;stage_1W_vs_E10;stage_3W_vs_E10;stage	1	TRUE
ENSGALG00010019524	HHIPL2	PRJNA729271	1.764706171	1.26766E-06	1.764706171	stage_E14_vs_E10	ordinary_muscle_development	D_vs_E10;stage_1W_vs_E10;stage_3W_vs_E10;stage	1	TRUE
ENSGALG00010018427	PIGF	PRJNA729271	-0.753416712	1.2682E-06	0.753416712	stage_E14_vs_E10	ordinary_muscle_development		0	FALSE
ENSGALG00010009277	ACTN4	PRJNA729271	-0.639578753	1.27992E-06	0.639578753	stage_E14_vs_E10	ordinary_muscle_development		0	FALSE
ENSGALG00010025155	CZIB	PRJNA729271	0.454919789	1.28885E-06	0.454919789	stage_E14_vs_E10	ordinary_muscle_development		0	FALSE
ENSGALG00010019148	ENSGALG00010019148	PRJNA729271	1.057751172	1.30046E-06	1.057751172	stage_E14_vs_E10	ordinary_muscle_development	D_vs_E10;stage_1W_vs_E10;stage_3W_vs_E10;stage	1	TRUE
ENSGALG00010016006	PLTP	PRJNA729271	-1.611623359	1.31314E-06	1.611623359	stage_E14_vs_E10	ordinary_muscle_development	D_vs_E10;stage_1W_vs_E10;stage_3W_vs_E10;stage	1	TRUE
ENSGALG00010005295	ENSGALG00010005295	PRJNA729271	2.720666225	1.31444E-06	2.720666225	stage_E14_vs_E10	ordinary_muscle_development	D_vs_E10;stage_1W_vs_E10;stage_3W_vs_E10;stage	1	TRUE
ENSGALG00010025047	PER2	PRJNA729271	1.454956191	1.31602E-06	1.454956191	stage_E14_vs_E10	ordinary_muscle_development	D_vs_E10;stage_1W_vs_E10;stage_3W_vs_E10;stage	1	TRUE
ENSGALG00010009186	PREB	PRJNA729271	0.490639164	1.3294E-06	0.490639164	stage_E14_vs_E10	ordinary_muscle_development		0	FALSE
ENSGALG00010021611	GREM1	PRJNA729271	-3.029917032	1.32948E-06	3.029917032	stage_E14_vs_E10	ordinary_muscle_development	D_vs_E10;stage_1W_vs_E10;stage_3W_vs_E10;stage	1	TRUE
ENSGALG00010025428	VANGL1	PRJNA729271	-0.557675664	1.3334E-06	0.557675664	stage_E14_vs_E10	ordinary_muscle_development		0	FALSE
ENSGALG00010028686	TAGLN2	PRJNA729271	0.592506838	1.3394E-06	0.592506838	stage_E14_vs_E10	ordinary_muscle_development		0	FALSE
ENSGALG00010025248	C11orf74	PRJNA729271	-1.100471742	1.34116E-06	1.100471742	stage_E14_vs_E10	ordinary_muscle_development	D_vs_E10;stage_1W_vs_E10;stage_3W_vs_E10;stage	1	TRUE
ENSGALG00010028316	TIMM44	PRJNA729271	-0.401846456	1.34563E-06	0.401846456	stage_E14_vs_E10	ordinary_muscle_development		0	FALSE
ENSGALG00010001320	CPED1	PRJNA729271	-1.025517799	1.35466E-06	1.025517799	stage_E14_vs_E10	ordinary_muscle_development	D_vs_E10;stage_1W_vs_E10;stage_3W_vs_E10;stage	1	TRUE
ENSGALG00010015061	MMP9	PRJNA729271	-2.729665398	1.36304E-06	2.729665398	stage_E14_vs_E10	ordinary_muscle_development	D_vs_E10;stage_1W_vs_E10;stage_3W_vs_E10;stage	1	TRUE
ENSGALG00010011352	NTS	PRJNA729271	-3.376490994	1.36523E-06	3.376490994	stage_E14_vs_E10	ordinary_muscle_development	D_vs_E10;stage_1W_vs_E10;stage_3W_vs_E10;stage	1	TRUE
ENSGALG00010027543	TUBB4B	PRJNA729271	-0.754555239	1.36557E-06	0.754555239	stage_E14_vs_E10	ordinary_muscle_development		0	FALSE
ENSGALG00010018057	SSTR4	PRJNA729271	0.892245885	1.37274E-06	0.892245885	stage_E14_vs_E10	ordinary_muscle_development		0	FALSE
ENSGALG00010018662	PADI1	PRJNA729271	3.878995977	1.37567E-06	3.878995977	stage_E14_vs_E10	ordinary_muscle_development	D_vs_E10;stage_1W_vs_E10;stage_3W_vs_E10;stage	1	TRUE
ENSGALG00010003556	ENSGALG00010003556	PRJNA729271	9.066896434	1.37862E-06	9.066896434	stage_E14_vs_E10	ordinary_muscle_development	D_vs_E10;stage_1W_vs_E10;stage_3W_vs_E10;stage	1	TRUE
ENSGALG00010021260	KDM4A	PRJNA729271	0.334475789	1.3816E-06	0.334475789	stage_E14_vs_E10	ordinary_muscle_development		0	FALSE
ENSGALG00010029759	ALDH3A2	PRJNA729271	0.533131839	1.38306E-06	0.533131839	stage_E14_vs_E10	ordinary_muscle_development		0	FALSE
ENSGALG00010027190	IPPK	PRJNA729271	-0.590724309	1.38731E-06	0.590724309	stage_E14_vs_E10	ordinary_muscle_development		0	FALSE
ENSGALG00010017094	SORBS2	PRJNA729271	0.734108554	1.38883E-06	0.734108554	stage_E14_vs_E10	ordinary_muscle_development		0	FALSE
ENSGALG00010013624	SST1	PRJNA729271	-8.67699827	1.39442E-06	8.67699827	stage_E14_vs_E10	ordinary_muscle_development	D_vs_E10;stage_1W_vs_E10;stage_3W_vs_E10;stage	1	TRUE
ENSGALG00010020957	RPF1	PRJNA729271	-0.511909443	1.39919E-06	0.511909443	stage_E14_vs_E10	ordinary_muscle_development		0	FALSE
ENSGALG00010022652	CARMIL2	PRJNA729271	-1.423480522	1.41241E-06	1.423480522	stage_E14_vs_E10	ordinary_muscle_development	D_vs_E10;stage_1W_vs_E10;stage_3W_vs_E10;stage	1	TRUE
ENSGALG00010018916	CERS3	PRJNA729271	-1.724525713	1.41779E-06	1.724525713	stage_E14_vs_E10	ordinary_muscle_development	D_vs_E10;stage_1W_vs_E10;stage_3W_vs_E10;stage	1	TRUE
ENSGALG00010003731	ENSGALG00010003731	PRJNA729271	2.839107245	1.42161E-06	2.839107245	stage_E14_vs_E10	ordinary_muscle_development	D_vs_E10;stage_1W_vs_E10;stage_3W_vs_E10;stage	1	TRUE
ENSGALG00010010861	ENSGALG00010010861	PRJNA729271	1.169554415	1.43381E-06	1.169554415	stage_E14_vs_E10	ordinary_muscle_development	D_vs_E10;stage_1W_vs_E10;stage_3W_vs_E10;stage	1	TRUE
ENSGALG00010016384	GNAS	PRJNA729271	0.367626357	1.43644E-06	0.367626357	stage_E14_vs_E10	ordinary_muscle_development		0	FALSE
ENSGALG00010028093	SFMBT1	PRJNA729271	-0.730389728	1.44507E-06	0.730389728	stage_E14_vs_E10	ordinary_muscle_development		0	FALSE
ENSGALG00010001260	ENSGALG00010001260	PRJNA729271	-5.471372211	1.44871E-06	5.471372211	stage_E14_vs_E10	ordinary_muscle_development	D_vs_E10;stage_1W_vs_E10;stage_3W_vs_E10;stage	1	TRUE
ENSGALG00010000705	ENSGALG00010000705	PRJNA729271	5.048488085	1.47236E-06	5.048488085	stage_E14_vs_E10	ordinary_muscle_development	D_vs_E10;stage_1W_vs_E10;stage_3W_vs_E10;stage	1	TRUE
ENSGALG00010005214	BBS7	PRJNA729271	-0.599210201	1.48198E-06	0.599210201	stage_E14_vs_E10	ordinary_muscle_development		0	FALSE
ENSGALG00010021837	ENSGALG00010021837	PRJNA729271	-1.000353412	1.48442E-06	1.000353412	stage_E14_vs_E10	ordinary_muscle_development	D_vs_E10;stage_1W_vs_E10;stage_3W_vs_E10;stage	1	TRUE
ENSGALG00010014877	ENSGALG00010014877	PRJNA729271	-0.46860012	1.48979E-06	0.46860012	stage_E14_vs_E10	ordinary_muscle_development		0	FALSE
ENSGALG00010017781	CYP26C1	PRJNA729271	1.482240749	1.49231E-06	1.482240749	stage_E14_vs_E10	ordinary_muscle_development	D_vs_E10;stage_1W_vs_E10;stage_3W_vs_E10;stage	1	TRUE
ENSGALG00010021088	ANKLE2	PRJNA729271	-0.439929283	1.50419E-06	0.439929283	stage_E14_vs_E10	ordinary_muscle_development		0	FALSE
ENSGALG00010017409	GPC3	PRJNA729271	-1.699628104	1.50799E-06	1.699628104	stage_E14_vs_E10	ordinary_muscle_development	D_vs_E10;stage_1W_vs_E10;stage_3W_vs_E10;stage	1	TRUE
ENSGALG00010025871	ENSGALG00010025871	PRJNA729271	7.042876029	1.51111E-06	7.042876029	stage_E14_vs_E10	ordinary_muscle_development	D_vs_E10;stage_1W_vs_E10;stage_3W_vs_E10;stage	1	TRUE
ENSGALG00010004408	SERAC1	PRJNA729271	-0.56837108	1.51344E-06	0.56837108	stage_E14_vs_E10	ordinary_muscle_development		0	FALSE
ENSGALG00010013917	TRPC5	PRJNA729271	-4.620926711	1.51783E-06	4.620926711	stage_E14_vs_E10	ordinary_muscle_development	D_vs_E10;stage_1W_vs_E10;stage_3W_vs_E10;stage	1	TRUE
ENSGALG00010001852	CPXM2	PRJNA729271	-1.925780456	1.5255E-06	1.925780456	stage_E14_vs_E10	ordinary_muscle_development	D_vs_E10;stage_1W_vs_E10;stage_3W_vs_E10;stage	1	TRUE
ENSGALG00010005826	LRP2BP	PRJNA729271	1.007283892	1.5266E-06	1.007283892	stage_E14_vs_E10	ordinary_muscle_development	D_vs_E10;stage_1W_vs_E10;stage_3W_vs_E10;stage	1	TRUE
ENSGALG00010021148	GPATCH8	PRJNA729271	0.51852265	1.52754E-06	0.51852265	stage_E14_vs_E10	ordinary_muscle_development		0	FALSE
ENSGALG00010007984	EIF3H	PRJNA729271	0.609797923	1.54293E-06	0.609797923	stage_E14_vs_E10	ordinary_muscle_development		0	FALSE
ENSGALG00010012748	ENSGALG00010012748	PRJNA729271	1.015227941	1.54307E-06	1.015227941	stage_E14_vs_E10	ordinary_muscle_development	D_vs_E10;stage_1W_vs_E10;stage_3W_vs_E10;stage	1	TRUE
ENSGALG00010007818	ENSGALG00010007818	PRJNA729271	1.076268423	1.55105E-06	1.076268423	stage_E14_vs_E10	ordinary_muscle_development	D_vs_E10;stage_1W_vs_E10;stage_3W_vs_E10;stage	1	TRUE
ENSGALG00010025097	LMBR1	PRJNA729271	0.604310972	1.55218E-06	0.604310972	stage_E14_vs_E10	ordinary_muscle_development		0	FALSE
ENSGALG00010027340	ENSGALG00010027340	PRJNA729271	3.599538326	1.5533E-06	3.599538326	stage_E14_vs_E10	ordinary_muscle_development	D_vs_E10;stage_1W_vs_E10;stage_3W_vs_E10;stage	1	TRUE
ENSGALG00010002202	SFXN4	PRJNA729271	0.496537807	1.56133E-06	0.496537807	stage_E14_vs_E10	ordinary_muscle_development		0	FALSE
ENSGALG00010023115	RPLP2	PRJNA729271	0.680831907	1.56587E-06	0.680831907	stage_E14_vs_E10	ordinary_muscle_development		0	FALSE
ENSGALG00010024123	PLPPR4	PRJNA729271	-2.213154291	1.56973E-06	2.213154291	stage_E14_vs_E10	ordinary_muscle_development	D_vs_E10;stage_1W_vs_E10;stage_3W_vs_E10;stage	1	TRUE
ENSGALG00010016680	BPIFB3	PRJNA729271	3.744742444	1.58483E-06	3.744742444	stage_E14_vs_E10	ordinary_muscle_development	D_vs_E10;stage_1W_vs_E10;stage_3W_vs_E10;stage	1	TRUE
ENSGALG00010017289	ADGRB2	PRJNA729271	-1.579096133	1.58516E-06	1.579096133	stage_E14_vs_E10	ordinary_muscle_development	D_vs_E10;stage_1W_vs_E10;stage_3W_vs_E10;stage	1	TRUE
ENSGALG00010016059	EPDR1	PRJNA729271	0.989119413	1.58863E-06	0.989119413	stage_E14_vs_E10	ordinary_muscle_development		0	FALSE
ENSGALG00010003061	ENSGALG00010003061	PRJNA729271	-1.760733169	1.59306E-06	1.760733169	stage_E14_vs_E10	ordinary_muscle_development	D_vs_E10;stage_1W_vs_E10;stage_3W_vs_E10;stage	1	TRUE
ENSGALG00010020295	LGI1	PRJNA729271	-0.719795772	1.5962E-06	0.719795772	stage_E14_vs_E10	ordinary_muscle_development		0	FALSE
ENSGALG00010026091	DTX4	PRJNA729271	0.799866817	1.59651E-06	0.799866817	stage_E14_vs_E10	ordinary_muscle_development		0	FALSE
ENSGALG00010024714	SRPRB	PRJNA729271	-0.86305636	1.59651E-06	0.86305636	stage_E14_vs_E10	ordinary_muscle_development		0	FALSE
ENSGALG00010011708	IK	PRJNA729271	0.450828924	1.60182E-06	0.450828924	stage_E14_vs_E10	ordinary_muscle_development		0	FALSE
ENSGALG00010017022	CCR4	PRJNA729271	2.591806493	1.60454E-06	2.591806493	stage_E14_vs_E10	ordinary_muscle_development	D_vs_E10;stage_1W_vs_E10;stage_3W_vs_E10;stage	1	TRUE
ENSGALG00010018594	BICRAL	PRJNA729271	0.514942298	1.61195E-06	0.514942298	stage_E14_vs_E10	ordinary_muscle_development		0	FALSE
ENSGALG00010026894	IP6K1	PRJNA729271	-0.331292824	1.61479E-06	0.331292824	stage_E14_vs_E10	ordinary_muscle_development		0	FALSE
ENSGALG00010020311	SGPL1	PRJNA729271	-0.664326361	1.61818E-06	0.664326361	stage_E14_vs_E10	ordinary_muscle_development		0	FALSE
ENSGALG00010024060	FRRS1	PRJNA729271	0.793498246	1.62824E-06	0.793498246	stage_E14_vs_E10	ordinary_muscle_development		0	FALSE
ENSGALG00010024901	ZGPAT	PRJNA729271	-0.650654	1.63112E-06	0.650654	stage_E14_vs_E10	ordinary_muscle_development		0	FALSE
ENSGALG00010024416	VDR	PRJNA729271	-1.638736757	1.63406E-06	1.638736757	stage_E14_vs_E10	ordinary_muscle_development	D_vs_E10;stage_1W_vs_E10;stage_3W_vs_E10;stage	1	TRUE
ENSGALG00010022521	RTF1	PRJNA729271	-0.455761856	1.63551E-06	0.455761856	stage_E14_vs_E10	ordinary_muscle_development		0	FALSE
ENSGALG00010026846	MICAL1	PRJNA729271	1.100090061	1.65098E-06	1.100090061	stage_E14_vs_E10	ordinary_muscle_development	D_vs_E10;stage_1W_vs_E10;stage_3W_vs_E10;stage	1	TRUE
ENSGALG00010024499	CA12	PRJNA729271	-3.719305443	1.65178E-06	3.719305443	stage_E14_vs_E10	ordinary_muscle_development	D_vs_E10;stage_1W_vs_E10;stage_3W_vs_E10;stage	1	TRUE
ENSGALG00010006223	ATG10	PRJNA729271	-0.565825444	1.65357E-06	0.565825444	stage_E14_vs_E10	ordinary_muscle_development		0	FALSE
ENSGALG00010010478	ENSGALG00010010478	PRJNA729271	1.041006778	1.65992E-06	1.041006778	stage_E14_vs_E10	ordinary_muscle_development	D_vs_E10;stage_1W_vs_E10;stage_3W_vs_E10;stage	1	TRUE
ENSGALG00010012547	ENSGALG00010012547	PRJNA729271	-2.402556053	1.66006E-06	2.402556053	stage_E14_vs_E10	ordinary_muscle_development	D_vs_E10;stage_1W_vs_E10;stage_3W_vs_E10;stage	1	TRUE
ENSGALG00010017045	GLB1	PRJNA729271	0.347620234	1.66458E-06	0.347620234	stage_E14_vs_E10	ordinary_muscle_development		0	FALSE
ENSGALG00010005905	CITED2	PRJNA729271	0.985760358	1.68926E-06	0.985760358	stage_E14_vs_E10	ordinary_muscle_development		0	FALSE
ENSGALG00010024200	GCHFR	PRJNA729271	-3.799417607	1.69468E-06	3.799417607	stage_E14_vs_E10	ordinary_muscle_development	D_vs_E10;stage_1W_vs_E10;stage_3W_vs_E10;stage	1	TRUE
ENSGALG00010009286	ENSGALG00010009286	PRJNA729271	0.892276448	1.705E-06	0.892276448	stage_E14_vs_E10	ordinary_muscle_development		0	FALSE
ENSGALG00010014272	PARD6B	PRJNA729271	-1.258867667	1.70895E-06	1.258867667	stage_E14_vs_E10	ordinary_muscle_development	D_vs_E10;stage_1W_vs_E10;stage_3W_vs_E10;stage	1	TRUE
ENSGALG00010010447	BTF3	PRJNA729271	0.472288886	1.71238E-06	0.472288886	stage_E14_vs_E10	ordinary_muscle_development		0	FALSE
ENSGALG00010003683	ENSGALG00010003683	PRJNA729271	7.61895774	1.71657E-06	7.61895774	stage_E14_vs_E10	ordinary_muscle_development	D_vs_E10;stage_1W_vs_E10;stage_3W_vs_E10;stage	1	TRUE
ENSGALG00010025990	FAM124B	PRJNA729271	1.176655482	1.71657E-06	1.176655482	stage_E14_vs_E10	ordinary_muscle_development	D_vs_E10;stage_1W_vs_E10;stage_3W_vs_E10;stage	1	TRUE
ENSGALG00010021091	TANK	PRJNA729271	0.400388422	1.72185E-06	0.400388422	stage_E14_vs_E10	ordinary_muscle_development		0	FALSE
ENSGALG00010015391	SYN3	PRJNA729271	-2.930175822	1.72519E-06	2.930175822	stage_E14_vs_E10	ordinary_muscle_development	D_vs_E10;stage_1W_vs_E10;stage_3W_vs_E10;stage	1	TRUE
ENSGALG00010013558	SNAPC3	PRJNA729271	-0.42810323	1.72782E-06	0.42810323	stage_E14_vs_E10	ordinary_muscle_development		0	FALSE
ENSGALG00010013959	STK32A	PRJNA729271	-2.512854528	1.74251E-06	2.512854528	stage_E14_vs_E10	ordinary_muscle_development	D_vs_E10;stage_1W_vs_E10;stage_3W_vs_E10;stage	1	TRUE
ENSGALG00010005872	STK24	PRJNA729271	0.661841151	1.7428E-06	0.661841151	stage_E14_vs_E10	ordinary_muscle_development		0	FALSE
ENSGALG00010027385	CNTN2	PRJNA729271	2.830495378	1.74368E-06	2.830495378	stage_E14_vs_E10	ordinary_muscle_development	D_vs_E10;stage_1W_vs_E10;stage_3W_vs_E10;stage	1	TRUE
ENSGALG00010024963	TNFRSF6B	PRJNA729271	2.827226434	1.74609E-06	2.827226434	stage_E14_vs_E10	ordinary_muscle_development	D_vs_E10;stage_1W_vs_E10;stage_3W_vs_E10;stage	1	TRUE
ENSGALG00010022815	LRRC8D	PRJNA729271	-1.207101539	1.74626E-06	1.207101539	stage_E14_vs_E10	ordinary_muscle_development	D_vs_E10;stage_1W_vs_E10;stage_3W_vs_E10;stage	1	TRUE
ENSGALG00010024809	KANSL1	PRJNA729271	0.835778274	1.74961E-06	0.835778274	stage_E14_vs_E10	ordinary_muscle_development		0	FALSE
ENSGALG00010000720	RBM26	PRJNA729271	-0.253295306	1.77521E-06	0.253295306	stage_E14_vs_E10	ordinary_muscle_development		0	FALSE
ENSGALG00010028064	SPCS1	PRJNA729271	-0.433251341	1.784E-06	0.433251341	stage_E14_vs_E10	ordinary_muscle_development		0	FALSE
ENSGALG00010008352	ENSGALG00010008352	PRJNA729271	0.745802184	1.7846E-06	0.745802184	stage_E14_vs_E10	ordinary_muscle_development		0	FALSE
ENSGALG00010001228	THAP5	PRJNA729271	-0.550963775	1.79199E-06	0.550963775	stage_E14_vs_E10	ordinary_muscle_development		0	FALSE
ENSGALG00010017753	GNRHR	PRJNA729271	1.581712115	1.79881E-06	1.581712115	stage_E14_vs_E10	ordinary_muscle_development	D_vs_E10;stage_1W_vs_E10;stage_3W_vs_E10;stage	1	TRUE
ENSGALG00010028119	HYAL2	PRJNA729271	0.623507932	1.81131E-06	0.623507932	stage_E14_vs_E10	ordinary_muscle_development		0	FALSE
ENSGALG00010028885	BRD3	PRJNA729271	-0.697088816	1.81403E-06	0.697088816	stage_E14_vs_E10	ordinary_muscle_development		0	FALSE
ENSGALG00010024034	LRP2	PRJNA729271	-1.224793787	1.81931E-06	1.224793787	stage_E14_vs_E10	ordinary_muscle_development	D_vs_E10;stage_1W_vs_E10;stage_3W_vs_E10;stage	1	TRUE
ENSGALG00010017876	COMMD1	PRJNA729271	-0.679214357	1.82184E-06	0.679214357	stage_E14_vs_E10	ordinary_muscle_development		0	FALSE
ENSGALG00010006060	ENSGALG00010006060	PRJNA729271	1.659910662	1.83597E-06	1.659910662	stage_E14_vs_E10	ordinary_muscle_development	D_vs_E10;stage_1W_vs_E10;stage_3W_vs_E10;stage	1	TRUE
ENSGALG00010012464	ENSGALG00010012464	PRJNA729271	1.154730598	1.84022E-06	1.154730598	stage_E14_vs_E10	ordinary_muscle_development	D_vs_E10;stage_1W_vs_E10;stage_3W_vs_E10;stage	1	TRUE
ENSGALG00010001271	ENSGALG00010001271	PRJNA729271	0.915171765	1.84263E-06	0.915171765	stage_E14_vs_E10	ordinary_muscle_development		0	FALSE
ENSGALG00010023859	ENSGALG00010023859	PRJNA729271	3.650226163	1.84285E-06	3.650226163	stage_E14_vs_E10	ordinary_muscle_development	D_vs_E10;stage_1W_vs_E10;stage_3W_vs_E10;stage	1	TRUE

ENSGALG00010018398	LRFN5	PRJNA729271	0.900975705	1.85019E-06	0.900975705	stage_E14_vs_E10	ordinary_muscle_development		0	FALSE
ENSGALG00010000789	ROPN1L	PRJNA729271	1.115933192	1.8569E-06	1.115933192	stage_E14_vs_E10	ordinary_muscle_development	D_vs_E10;stage_1W_vs_E10;stage_3W_vs_E10;stage	1	TRUE
ENSGALG00010000636	ENSGALG00010000636	PRJNA729271	0.688836671	1.86691E-06	0.688836671	stage_E14_vs_E10	ordinary_muscle_development		0	FALSE
ENSGALG00010012949	ENSGALG00010012949	PRJNA729271	-0.896369178	1.87346E-06	0.896369178	stage_E14_vs_E10	ordinary_muscle_development		0	FALSE
ENSGALG00010009083	MED30	PRJNA729271	0.620292934	1.87438E-06	0.620292934	stage_E14_vs_E10	ordinary_muscle_development		0	FALSE
ENSGALG00010000532	ENSGALG00010000532	PRJNA729271	2.301869549	1.87796E-06	2.301869549	stage_E14_vs_E10	ordinary_muscle_development	D_vs_E10;stage_1W_vs_E10;stage_3W_vs_E10;stage	1	TRUE
ENSGALG00010017590	ENSGALG00010017590	PRJNA729271	-0.955425732	1.87851E-06	0.955425732	stage_E14_vs_E10	ordinary_muscle_development		0	FALSE
ENSGALG00010015893	TCF7	PRJNA729271	-2.28304232	1.88602E-06	2.28304232	stage_E14_vs_E10	ordinary_muscle_development	D_vs_E10;stage_1W_vs_E10;stage_3W_vs_E10;stage	1	TRUE
ENSGALG00010007000	LRGUK	PRJNA729271	-3.538086461	1.88723E-06	3.538086461	stage_E14_vs_E10	ordinary_muscle_development	D_vs_E10;stage_1W_vs_E10;stage_3W_vs_E10;stage	1	TRUE
ENSGALG00010008064	DDX60	PRJNA729271	3.070697448	1.89328E-06	3.070697448	stage_E14_vs_E10	ordinary_muscle_development	D_vs_E10;stage_1W_vs_E10;stage_3W_vs_E10;stage	1	TRUE
ENSGALG00010022830	MTG2	PRJNA729271	-0.644812836	1.89555E-06	0.644812836	stage_E14_vs_E10	ordinary_muscle_development		0	FALSE
ENSGALG00010021424	PTCD1	PRJNA729271	0.522686533	1.89611E-06	0.522686533	stage_E14_vs_E10	ordinary_muscle_development		0	FALSE
ENSGALG00010009237	DENND3	PRJNA729271	-0.875435147	1.91992E-06	0.875435147	stage_E14_vs_E10	ordinary_muscle_development		0	FALSE
ENSGALG00010027363	VIPR1	PRJNA729271	-1.323971897	1.93036E-06	1.323971897	stage_E14_vs_E10	ordinary_muscle_development	D_vs_E10;stage_1W_vs_E10;stage_3W_vs_E10;stage	1	TRUE
ENSGALG00010018393	PRKCE	PRJNA729271	-0.387434426	1.93438E-06	0.387434426	stage_E14_vs_E10	ordinary_muscle_development		0	FALSE
ENSGALG00010013018	ID1	PRJNA729271	-0.986676111	1.94158E-06	0.986676111	stage_E14_vs_E10	ordinary_muscle_development		0	FALSE
ENSGALG00010028022	ENSGALG00010028022	PRJNA729271	-0.705216264	1.9419E-06	0.705216264	stage_E14_vs_E10	ordinary_muscle_development		0	FALSE
ENSGALG00010004454	SRI	PRJNA729271	-0.808365359	1.94795E-06	0.808365359	stage_E14_vs_E10	ordinary_muscle_development		0	FALSE
ENSGALG00010011713	AVPR1A	PRJNA729271	-4.84650991	1.94892E-06	4.84650991	stage_E14_vs_E10	ordinary_muscle_development	D_vs_E10;stage_1W_vs_E10;stage_3W_vs_E10;stage	1	TRUE
ENSGALG00010012075	PIGN	PRJNA729271	-0.48148916	1.95018E-06	0.48148916	stage_E14_vs_E10	ordinary_muscle_development		0	FALSE
ENSGALG00010016934	SLC25A38	PRJNA729271	-0.695182022	1.97041E-06	0.695182022	stage_E14_vs_E10	ordinary_muscle_development		0	FALSE
ENSGALG00010014830	TRAPPC3L	PRJNA729271	-2.899188092	1.9969E-06	2.899188092	stage_E14_vs_E10	ordinary_muscle_development	D_vs_E10;stage_1W_vs_E10;stage_3W_vs_E10;stage	1	TRUE
ENSGALG00010011222	RCL1	PRJNA729271	-0.441559023	1.99935E-06	0.441559023	stage_E14_vs_E10	ordinary_muscle_development		0	FALSE
ENSGALG00010024099	ENSGALG00010024099	PRJNA729271	9.321971377	2.0007E-06	9.321971377	stage_E14_vs_E10	ordinary_muscle_development	D_vs_E10;stage_1W_vs_E10;stage_3W_vs_E10;stage	1	TRUE
ENSGALG00010006163	ENSGALG00010006163	PRJNA729271	2.351419918	2.00287E-06	2.351419918	stage_E14_vs_E10	ordinary_muscle_development	D_vs_E10;stage_1W_vs_E10;stage_3W_vs_E10;stage	1	TRUE
ENSGALG00010027315	RAB3A	PRJNA729271	1.086588254	2.00508E-06	1.086588254	stage_E14_vs_E10	ordinary_muscle_development	D_vs_E10;stage_1W_vs_E10;stage_3W_vs_E10;stage	1	TRUE
ENSGALG00010004016	ENSGALG00010004016	PRJNA729271	-4.257036405	2.02302E-06	4.257036405	stage_E14_vs_E10	ordinary_muscle_development	D_vs_E10;stage_1W_vs_E10;stage_3W_vs_E10;stage	1	TRUE
ENSGALG00010010890	NOL12	PRJNA729271	-0.676344996	2.02897E-06	0.676344996	stage_E14_vs_E10	ordinary_muscle_development		0	FALSE
ENSGALG00010023116	PROC	PRJNA729271	4.958835161	2.0299E-06	4.958835161	stage_E14_vs_E10	ordinary_muscle_development	D_vs_E10;stage_1W_vs_E10;stage_3W_vs_E10;stage	1	TRUE
ENSGALG00010016612	GRIA1	PRJNA729271	-1.681896044	2.0362E-06	1.681896044	stage_E14_vs_E10	ordinary_muscle_development	D_vs_E10;stage_1W_vs_E10;stage_3W_vs_E10;stage	1	TRUE
ENSGALG00010022042	AIP	PRJNA729271	0.413664001	2.04039E-06	0.413664001	stage_E14_vs_E10	ordinary_muscle_development		0	FALSE
ENSGALG00010010645	FAM219A	PRJNA729271	0.416515397	2.04737E-06	0.416515397	stage_E14_vs_E10	ordinary_muscle_development		0	FALSE
ENSGALG00010008045	DCDC2	PRJNA729271	-4.476899508	2.05179E-06	4.476899508	stage_E14_vs_E10	ordinary_muscle_development	D_vs_E10;stage_1W_vs_E10;stage_3W_vs_E10;stage	1	TRUE
ENSGALG00010001259	ENSGALG00010001259	PRJNA729271	0.80725687	2.06801E-06	0.80725687	stage_E14_vs_E10	ordinary_muscle_development		0	FALSE
ENSGALG00010007713	SCLT1	PRJNA729271	-1.052445477	2.07853E-06	1.052445477	stage_E14_vs_E10	ordinary_muscle_development	D_vs_E10;stage_1W_vs_E10;stage_3W_vs_E10;stage	1	TRUE
ENSGALG00010011375	C12orf50	PRJNA729271	0.665406064	2.09038E-06	0.665406064	stage_E14_vs_E10	ordinary_muscle_development		0	FALSE
ENSGALG00010020237	MIB2	PRJNA729271	0.367234776	2.09211E-06	0.367234776	stage_E14_vs_E10	ordinary_muscle_development		0	FALSE
ENSGALG00010022784	DMRTA2	PRJNA729271	-4.837791259	2.0933E-06	4.837791259	stage_E14_vs_E10	ordinary_muscle_development	D_vs_E10;stage_1W_vs_E10;stage_3W_vs_E10;stage	1	TRUE
ENSGALG00010002366	ENSGALG00010002366	PRJNA729271	3.684079869	2.09439E-06	3.684079869	stage_E14_vs_E10	ordinary_muscle_development	D_vs_E10;stage_1W_vs_E10;stage_3W_vs_E10;stage	1	TRUE
ENSGALG00010003275	MBTPS2	PRJNA729271	0.620422669	2.09877E-06	0.620422669	stage_E14_vs_E10	ordinary_muscle_development		0	FALSE
ENSGALG00010018960	RBFOX1	PRJNA729271	-1.120599375	2.10751E-06	1.120599375	stage_E14_vs_E10	ordinary_muscle_development	D_vs_E10;stage_1W_vs_E10;stage_3W_vs_E10;stage	1	TRUE
ENSGALG00010014995	LRAT	PRJNA729271	-1.219456848	2.11044E-06	1.219456848	stage_E14_vs_E10	ordinary_muscle_development	D_vs_E10;stage_1W_vs_E10;stage_3W_vs_E10;stage	1	TRUE
ENSGALG00010024943	ABCB9	PRJNA729271	-0.974676381	2.11084E-06	0.974676381	stage_E14_vs_E10	ordinary_muscle_development		0	FALSE
ENSGALG00010016127	ENSGALG00010016127	PRJNA729271	0.816571403	2.13241E-06	0.816571403	stage_E14_vs_E10	ordinary_muscle_development		0	FALSE
ENSGALG00010012743	ENSGALG00010012743	PRJNA729271	2.41309442	2.13437E-06	2.41309442	stage_E14_vs_E10	ordinary_muscle_development	D_vs_E10;stage_1W_vs_E10;stage_3W_vs_E10;stage	1	TRUE
ENSGALG00010017411	RAB11FIP1	PRJNA729271	-0.683183521	2.14033E-06	0.683183521	stage_E14_vs_E10	ordinary_muscle_development		0	FALSE
ENSGALG00010016702	WDR25	PRJNA729271	0.46169361	2.15306E-06	0.46169361	stage_E14_vs_E10	ordinary_muscle_development		0	FALSE
ENSGALG00010006072	ENSGALG00010006072	PRJNA729271	1.13191137	2.17202E-06	1.13191137	stage_E14_vs_E10	ordinary_muscle_development	D_vs_E10;stage_1W_vs_E10;stage_3W_vs_E10;stage	1	TRUE
ENSGALG00010023080	MCRS1	PRJNA729271	0.542936459	2.19077E-06	0.542936459	stage_E14_vs_E10	ordinary_muscle_development		0	FALSE
ENSGALG00010024474	STRIP1	PRJNA729271	-0.503766303	2.20215E-06	0.503766303	stage_E14_vs_E10	ordinary_muscle_development		0	FALSE
ENSGALG00010008050	CDK10	PRJNA729271	-0.766562198	2.21007E-06	0.766562198	stage_E14_vs_E10	ordinary_muscle_development		0	FALSE
ENSGALG00010023123	RNF214	PRJNA729271	0.6926758	2.23945E-06	0.6926758	stage_E14_vs_E10	ordinary_muscle_development		0	FALSE
ENSGALG00010011720	IKZF2	PRJNA729271	-0.88104621	2.25101E-06	0.88104621	stage_E14_vs_E10	ordinary_muscle_development		0	FALSE
ENSGALG00010027330	ENSGALG00010027330	PRJNA729271	0.958602523	2.25398E-06	0.958602523	stage_E14_vs_E10	ordinary_muscle_development		0	FALSE
ENSGALG00010029550	CHMP6	PRJNA729271	1.161965662	2.26884E-06	1.161965662	stage_E14_vs_E10	ordinary_muscle_development	D_vs_E10;stage_1W_vs_E10;stage_3W_vs_E10;stage	1	TRUE
ENSGALG00010018188	UBP1	PRJNA729271	-0.254838298	2.28407E-06	0.254838298	stage_E14_vs_E10	ordinary_muscle_development		0	FALSE
ENSGALG00010002343	GPD1L	PRJNA729271	0.402410545	2.28607E-06	0.402410545	stage_E14_vs_E10	ordinary_muscle_development		0	FALSE
ENSGALG00010018917	DNAJC11	PRJNA729271	-0.524368455	2.28614E-06	0.524368455	stage_E14_vs_E10	ordinary_muscle_development		0	FALSE
ENSGALG00010029721	EXOC7	PRJNA729271	0.509030282	2.29326E-06	0.509030282	stage_E14_vs_E10	ordinary_muscle_development		0	FALSE
ENSGALG00010008892	ENSGALG00010008892	PRJNA729271	-0.638393181	2.3004E-06	0.638393181	stage_E14_vs_E10	ordinary_muscle_development		0	FALSE
ENSGALG00010003756	MHCY7	PRJNA729271	1.803186966	2.31329E-06	1.803186966	stage_E14_vs_E10	ordinary_muscle_development	D_vs_E10;stage_1W_vs_E10;stage_3W_vs_E10;stage	1	TRUE
ENSGALG00010003489	SGCE	PRJNA729271	-0.581688844	2.31399E-06	0.581688844	stage_E14_vs_E10	ordinary_muscle_development		0	FALSE
ENSGALG00010012699	RNF128	PRJNA729271	0.716858345	2.32475E-06	0.716858345	stage_E14_vs_E10	ordinary_muscle_development		0	FALSE
ENSGALG00010021765	EIF2AK1	PRJNA729271	0.504937407	2.32868E-06	0.504937407	stage_E14_vs_E10	ordinary_muscle_development		0	FALSE
ENSGALG00010003627	MCOLN1	PRJNA729271	0.580431844	2.34264E-06	0.580431844	stage_E14_vs_E10	ordinary_muscle_development		0	FALSE
ENSGALG00010026719	ENSGALG00010026719	PRJNA729271	-5.238630434	2.34605E-06	5.238630434	stage_E14_vs_E10	ordinary_muscle_development	D_vs_E10;stage_1W_vs_E10;stage_3W_vs_E10;stage	1	TRUE
ENSGALG00010022006	SFI1	PRJNA729271	0.523903162	2.34845E-06	0.523903162	stage_E14_vs_E10	ordinary_muscle_development		0	FALSE
ENSGALG00010010483	FGFBP1	PRJNA729271	-5.285811429	2.35411E-06	5.285811429	stage_E14_vs_E10	ordinary_muscle_development	D_vs_E10;stage_1W_vs_E10;stage_3W_vs_E10;stage	1	TRUE
ENSGALG00010018027	EML5	PRJNA729271	-1.516538377	2.3546E-06	1.516538377	stage_E14_vs_E10	ordinary_muscle_development	D_vs_E10;stage_1W_vs_E10;stage_3W_vs_E10;stage	1	TRUE
ENSGALG00010029950	RFFL	PRJNA729271	-0.529892688	2.3546E-06	0.529892688	stage_E14_vs_E10	ordinary_muscle_development		0	FALSE
ENSGALG00010005784	RASL11A	PRJNA729271	-2.52228463	2.35734E-06	2.52228463	stage_E14_vs_E10	ordinary_muscle_development	D_vs_E10;stage_1W_vs_E10;stage_3W_vs_E10;stage	1	TRUE
ENSGALG00010000791	ACOD1	PRJNA729271	2.394772423	2.37038E-06	2.394772423	stage_E14_vs_E10	ordinary_muscle_development	D_vs_E10;stage_1W_vs_E10;stage_3W_vs_E10;stage	1	TRUE
ENSGALG00010022017	CNP1	PRJNA729271	2.827579845	2.38249E-06	2.827579845	stage_E14_vs_E10	ordinary_muscle_development	D_vs_E10;stage_1W_vs_E10;stage_3W_vs_E10;stage	1	TRUE
ENSGALG00010006049	AIMP1	PRJNA729271	-0.612695003	2.40695E-06	0.612695003	stage_E14_vs_E10	ordinary_muscle_development		0	FALSE
ENSGALG00010015929	SLC6A12	PRJNA729271	-5.464865929	2.41286E-06	5.464865929	stage_E14_vs_E10	ordinary_muscle_development	D_vs_E10;stage_1W_vs_E10;stage_3W_vs_E10;stage	1	TRUE
ENSGALG00010020613	ACTG2	PRJNA729271	1.165764952	2.41427E-06	1.165764952	stage_E14_vs_E10	ordinary_muscle_development	D_vs_E10;stage_1W_vs_E10;stage_3W_vs_E10;stage	1	TRUE
ENSGALG00010020890	RPS15A	PRJNA729271	0.923949503	2.41541E-06	0.923949503	stage_E14_vs_E10	ordinary_muscle_development		0	FALSE
ENSGALG00010022578	ZNF512B	PRJNA729271	-0.657575998	2.41755E-06	0.657575998	stage_E14_vs_E10	ordinary_muscle_development		0	FALSE
ENSGALG00010010935	LGALS1A	PRJNA729271	1.116264914	2.42339E-06	1.116264914	stage_E14_vs_E10	ordinary_muscle_development	D_vs_E10;stage_1W_vs_E10;stage_3W_vs_E10;stage	1	TRUE
ENSGALG00010021124	CALHM2	PRJNA729271	0.642544274	2.44428E-06	0.642544274	stage_E14_vs_E10	ordinary_muscle_development		0	FALSE
ENSGALG00010015936	RPL36AL	PRJNA729271	0.683963575	2.45038E-06	0.683963575	stage_E14_vs_E10	ordinary_muscle_development		0	FALSE
ENSGALG00010004384	ENSGALG00010004384	PRJNA729271	2.47321058	2.45392E-06	2.47321058	stage_E14_vs_E10	ordinary_muscle_development	D_vs_E10;stage_1W_vs_E10;stage_3W_vs_E10;stage	1	TRUE
ENSGALG00010007892	ENSGALG00010007892	PRJNA729271	1.431186262	2.45791E-06	1.431186262	stage_E14_vs_E10	ordinary_muscle_development	D_vs_E10;stage_1W_vs_E10;stage_3W_vs_E10;stage	1	TRUE
ENSGALG00010018226	JAG1	PRJNA729271	-0.515394911	2.47232E-06	0.515394911	stage_E14_vs_E10	ordinary_muscle_development		0	FALSE
ENSGALG00010001857	HNRNPA2B1	PRJNA729271	-0.920762875	2.47355E-06	0.920762875	stage_E14_vs_E10	ordinary_muscle_development		0	FALSE
ENSGALG00010007771	CAPN9	PRJNA729271	-2.928687792	2.47548E-06	2.928687792	stage_E14_vs_E10	ordinary_muscle_development	D_vs_E10;stage_1W_vs_E10;stage_3W_vs_E10;stage	1	TRUE
ENSGALG00010016368	CCNK	PRJNA729271	-0.745466827	2.47548E-06	0.745466827	stage_E14_vs_E10	ordinary_muscle_development		0	FALSE
ENSGALG00010002140	ENSGALG00010002140	PRJNA729271	2.001150356	2.4784E-06	2.001150356	stage_E14_vs_E10	ordinary_muscle_development	D_vs_E10;stage_1W_vs_E10;stage_3W_vs_E10;stage	1	TRUE
ENSGALG00010003206	ENSGALG00010003206	PRJNA729271	-4.680561902	2.49598E-06	4.680561902	stage_E14_vs_E10	ordinary_muscle_development	D_vs_E10;stage_1W_vs_E10;stage_3W_vs_E10;stage	1	TRUE
ENSGALG00010028175	POMT1	PRJNA729271	0.444817583	2.51631E-06	0.444817583	stage_E14_vs_E10	ordinary_muscle_development		0	FALSE
ENSGALG00010011595	ENSGALG00010011595	PRJNA729271	0.785109053	2.51802E-06	0.785109053	stage_E14_vs_E10	ordinary_muscle_development		0	FALSE
ENSGALG00010007021	SULT6B1	PRJNA729271	-1.358907643	2.5269E-06	1.358907643	stage_E14_vs_E10	ordinary_muscle_development	D_vs_E10;stage_1W_vs_E10;stage_3W_vs_E10;stage	1	TRUE
ENSGALG00010022308	OGFR	PRJNA729271	-0.908996515	2.53026E-06	0.908996515	stage_E14_vs_E10	ordinary_muscle_development		0	FALSE
ENSGALG00010019193	ENSGALG00010019193	PRJNA729271	4.883633272	2.53515E-06	4.883633272	stage_E14_vs_E10	ordinary_muscle_development	D_vs_E10;stage_1W_vs_E10;stage_3W_vs_E10;stage	1	TRUE
ENSGALG00010000406	NSMCE3	PRJNA729271	-0.480589939	2.53588E-06	0.480589939	stage_E14_vs_E10	ordinary_muscle_development		0	FALSE
ENSGALG00010021799	ENSGALG00010021799	PRJNA729271	-1.17478632	2.55376E-06	1.17478632	stage_E14_vs_E10	ordinary_muscle_development	D_vs_E10;stage_1W_vs_E10;stage_3W_vs_E10;stage	1	TRUE
ENSGALG00010015764	TTI1	PRJNA729271	0.460988365	2.59885E-06	0.460988365	stage_E14_vs_E10	ordinary_muscle_development		0	FALSE
ENSGALG00010018902	NOXO1	PRJNA729271	-3.391918267	2.60603E-06	3.391918267	stage_E14_vs_E10	ordinary_muscle_development	D_vs_E10;stage_1W_vs_E10;stage_3W_vs_E10;stage	1	TRUE
ENSGALG00010021730	ARL8B	PRJNA729271	-0.355372188	2.60985E-06	0.355372188	stage_E14_vs_E10	ordinary_muscle_development		0	FALSE
ENSGALG00010003703	TDRP	PRJNA729271	-1.120800574	2.613E-06	1.120800574	stage_E14_vs_E10	ordinary_muscle_development	D_vs_E10;stage_1W_vs_E10;stage_3W_vs_E10;stage	1	TRUE
ENSGALG00010017335	THUMPD2	PRJNA729271	0.655684871	2.61378E-06	0.655684871	stage_E14_vs_E10	ordinary_muscle_development		0	FALSE
ENSGALG00010001840	HOXA9	PRJNA729271	0.696270387	2.62251E-06	0.696270387	stage_E14_vs_E10	ordinary_muscle_development		0	FALSE
ENSGALG00010002530	STAG3	PRJNA729271	-0.594336088	2.6283E-06	0.594336088	stage_E14_vs_E10	ordinary_muscle_development		0	FALSE
ENSGALG00010017852	LETM2	PRJNA729271	0.548116293	2.64904E-06	0.548116293	stage_E14_vs_E10	ordinary_muscle_development		0	FALSE
ENSGALG00010002386	MHCY2B2	PRJNA729271	-2.125604598	2.65172E-06	2.125604598	stage_E14_vs_E10	ordinary_muscle_development	D_vs_E10;stage_1W_vs_E10;stage_3W_vs_E10;stage	1	TRUE
ENSGALG00010004746	LYN	PRJNA729271	0.681148036	2.65848E-06	0.681148036	stage_E14_vs_E10	ordinary_muscle_development		0	FALSE
ENSGALG00010013572	DENND11	PRJNA729271	0.497153977	2.67465E-06	0.497153977	stage_E14_vs_E10	ordinary_muscle_development		0	FALSE
ENSGALG00010025688	BEST4	PRJNA729271	2.359776696	2.68215E-06	2.359776696	stage_E14_vs_E10	ordinary_muscle_development	D_vs_E10;stage_1W_vs_E10;stage_3W_vs_E10;stage	1	TRUE
ENSGALG00010030102	GPS1	PRJNA729271	0.399999504	2.6858E-06	0.399999504	stage_E14_vs_E10	ordinary_muscle_development		0	FALSE
ENSGALG00010019015	GSG1L	PRJNA729271	-5.17550442	2.70318E-06	5.17550442	stage_E14_vs_E10	ordinary_muscle_development	D_vs_E10;stage_1W_vs_E10;stage_3W_vs_E10;stage	1	TRUE
ENSGALG00010013171	SLC9A8	PRJNA729271	0.654083145	2.73963E-06	0.654083145	stage_E14_vs_E10	ordinary_muscle_development		0	FALSE
ENSGALG00010028098	RFT1	PRJNA729271	-0.582856401	2.77301E-06	0.582856401	stage_E14_vs_E10	ordinary_muscle_development		0	FALSE
ENSGALG00010017505	TNFRSF8	PRJNA729271	1.43294621	2.77512E-06	1.43294621	stage_E14_vs_E10	ordinary_muscle_development	D_vs_E10;stage_1W_vs_E10;stage_3W_vs_E10;stage	1	TRUE
ENSGALG00010005814	UPF2	PRJNA729271	-0.60003377	2.78042E-06	0.60003377	stage_E14_vs_E10	ordinary_muscle_development		0	FALSE
ENSGALG00010007574	ENSGALG00010007574	PRJNA729271	0.885045795	2.78263E-06	0.885045795	stage_E14_vs_E10	ordinary_muscle_development		0	FALSE
ENSGALG00010010844	CDC14B	PRJNA729271	0.83968969	2.78577E-06	0.83968969	stage_E14_vs_E10	ordinary_muscle_development		0	FALSE
ENSGALG00010009029	IGSF3	PRJNA729271	-0.737909397	2.78703E-06	0.737909397	stage_E14_vs_E10	ordinary_muscle_development		0	FALSE
ENSGALG00010021710	ENSGALG00010021710	PRJNA729271	-1.590036086	2.79441E-06	1.590036086	stage_E14_vs_E10	ordinary_muscle_development	D_vs_E10;stage_1W_vs_E10;stage_3W_vs_E10;stage	1	TRUE
ENSGALG00010003829	ENSGALG00010003829	PRJNA729271	-0.666806802	2.80825E-06	0.666806802	stage_E14_vs_E10	ordinary_muscle_development		0	FALSE
ENSGALG00010012121	SREK1	PRJNA729271	-0.484380138	2.81177E-06	0.484380138	stage_E14_vs_E10	ordinary_muscle_development		0	FALSE
ENSGALG00010028483	EXD3	PRJNA729271	0.441779038	2.81267E-06	0.441779038	stage_E14_vs_E10	ordinary_muscle_development		0	FALSE
ENSGALG00010024003	ERICH3	PRJNA729271	-1.509772003	2.81331E-06	1.509772003	stage_E14_vs_E10	ordinary_muscle_development	D_vs_E10;stage_1W_vs_E10;stage_3W_vs_E10;stage	1	TRUE
ENSGALG00010013787	ZCCHC10	PRJNA729271	-0.506504701	2.81335E-06	0.506504701	stage_E14_vs_E10	ordinary_muscle_development		0	FALSE
ENSGALG00010014488	ENSGALG00010014488	PRJNA729271	-1.9744019	2.82152E-06	1.9744019	stage_E14_vs_E10	ordinary_muscle_development	D_vs_E10;stage_1W_vs_E10;stage_3W_vs_E10;stage	1	TRUE
ENSGALG00010019271	CDKL1	PRJNA729271	-0.967407692	2.8254E-06	0.967407692	stage_E14_vs_E10	ordinary_muscle_development		0	FALSE
ENSGALG00010001274	DLGAP1	PRJNA729271	-2.354425298	2.82975E-06	2.354425298	stage_E14_vs_E10	ordinary_muscle_development	D_vs_E10;stage_1W_vs_E10;stage_3W_vs_E10;stage	1	TRUE
ENSGALG00010028153	MANF	PRJNA729271	-0.712433547	2.83286E-06	0.712433547	stage_E14_vs_E10	ordinary_muscle_development		0	FALSE
ENSGALG00010024346	PPM1F	PRJNA729271	0.436563647	2.84516E-06	0.436563647	stage_E14_vs_E10	ordinary_muscle_development		0	FALSE
ENSGALG00010009527	ENSGALG00010009527	PRJNA729271	-2.879205707	2.84655E-06	2.879205707	stage_E14_vs_E10	ordinary_muscle_development	D_vs_E10;stage_1W_vs_E10;stage_3W_vs_E10;stage	1	TRUE
ENSGALG00010017422	ENSGALG00010017422	PRJNA729271	1.072522255	2.8529E-06	1.072522255	stage_E14_vs_E10	ordinary_muscle_development	D_vs_E10;stage_1W_vs_E10;stage_3W_vs_E10;stage	1	TRUE
ENSGALG00010021652	BORCS5	PRJNA729271	-0.476102967	2.87207E-06	0.476102967	stage_E14_vs_E10	ordinary_muscle_development		0	FALSE
ENSGALG00010019305	SNRNP27	PRJNA729271	0.568238531	2.89427E-06	0.568238531	stage_E14_vs_E10	ordinary_muscle_development		0	FALSE
ENSGALG00010006927	UCN3	PRJNA729271	-5.031616045	2.90294E-06	5.031616045	stage_E14_vs_E10	ordinary_muscle_development	D_vs_E10;stage_1W_vs_E10;stage_3W_vs_E10;stage	1	TRUE
ENSGALG00010000627	ENSGALG00010000627	PRJNA729271	1.090717508	2.91034E-06	1.090717508	stage_E14_vs_E10	ordinary_muscle_development	D_vs_E10;stage_1W_vs_E10;stage_3W_vs_E10;stage	1	TRUE
ENSGALG00010026221	CRKL	PRJNA729271	0.70107299	2.91177E-06	0.70107299	stage_E14_vs_E10	ordinary_muscle_development		0	FALSE
ENSGALG00010016986	GFER	PRJNA729271	0.411392829	2.91831E-06	0.411392829	stage_E14_vs_E10	ordinary_muscle_development		0	FALSE
ENSGALG00010015250	RORB	PRJNA729271	-2.742795889	2.93344E-06	2.742795889	stage_E14_vs_E10	ordinary_muscle_development	D_vs_E10;stage_1W_vs_E10;stage_3W_vs_E10;stage	1	TRUE
ENSGALG00010026984	PGR2/3	PRJNA729271	4.290970681	2.94622E-06	4.290970681	stage_E14_vs_E10	ordinary_muscle_development	D_vs_E10;stage_1W_vs_E10;stage_3W_vs_E10;stage	1	TRUE
ENSGALG00010008241	HERC3	PRJNA729271	0.40008141	2.95247E-06	0.40008141	stage_E14_vs_E10	ordinary_muscle_development		0	FALSE
ENSGALG00010029384	WDR81	PRJNA729271	-0.362733338	2.98632E-06	0.362733338	stage_E14_vs_E10	ordinary_muscle_development		0	FALSE
ENSGALG00010016919	ARHGDIG	PRJNA729271	0.953697506	2.99512E-06	0.953697506	stage_E14_vs_E10	ordinary_muscle_development		0	FALSE
ENSGALG00010015711	LBFABP	PRJNA729271	3.329701969	3.00027E-06	3.329701969	stage_E14_vs_E10	ordinary_muscle_development	D_vs_E10;stage_1W_vs_E10;stage_3W_vs_E10;stage	1	TRUE
ENSGALG00010013973	ENSGALG00010013973	PRJNA729271	1.138721131	3.00627E-06	1.138721131	stage_E14_vs_E10	ordinary_muscle_development	D_vs_E10;stage_1W_vs_E10;stage_3W_vs_E10;stage	1	TRUE
ENSGALG00010001123	CP	PRJNA729271	-0.727048229	3.01691E-06	0.727048229	stage_E14_vs_E10	ordinary_muscle_development		0	FALSE
ENSGALG00010021223	DFFB	PRJNA729271	0.53642466	3.02273E-06	0.53642466	stage_E14_vs_E10	ordinary_muscle_development		0	FALSE
ENSGALG00010023267	DSCAML1	PRJNA729271	-4.357820609	3.0325E-06	4.357820609	stage_E14_vs_E10	ordinary_muscle_development	D_vs_E10;stage_1W_vs_E10;stage_3W_vs_E10;stage	1	TRUE
ENSGALG00010019295	MAP4K5	PRJNA729271	0.620806554	3.0332E-06	0.620806554	stage_E14_vs_E10	ordinary_muscle_development		0	FALSE
ENSGALG00010024213	ENSGALG00010024213	PRJNA729271	-0.952321028	3.05917E-06	0.952321028	stage_E14_vs_E10	ordinary_muscle_development		0	FALSE
ENSGALG00010027292	KDM4B	PRJNA729271	0.296113931	3.06023E-06	0.296113931	stage_E14_vs_E10	ordinary_muscle_development		0	FALSE
ENSGALG00010016307	RAD50	PRJNA729271	-0.618772429	3.06777E-06	0.618772429	stage_E14_vs_E10	ordinary_muscle_development		0	FALSE
ENSGALG00010021213	FBXO44	PRJNA729271	0.928100483	3.0709E-06	0.928100483	stage_E14_vs_E10	ordinary_muscle_development		0	FALSE
ENSGALG00010009914	RWDD2B	PRJNA729271	-0.601900327	3.1146E-06	0.601900327	stage_E14_vs_E10	ordinary_muscle_development		0	FALSE
ENSGALG00010006327	DENND6B	PRJNA729271	0.544167593	3.11764E-06	0.544167593	stage_E14_vs_E10	ordinary_muscle_development		0	FALSE
ENSGALG00010008332	DHX38	PRJNA729271	-0.456754928	3.13627E-06	0.456754928	stage_E14_vs_E10	ordinary_muscle_development		0	FALSE
ENSGALG00010016451	USP46	PRJNA729271	-0.722528426	3.15939E-06	0.722528426	stage_E14_vs_E10	ordinary_muscle_development		0	FALSE
ENSGALG00010027085	PIM1	PRJNA729271	0.714375867	3.16174E-06	0.714375867	stage_E14_vs_E10	ordinary_muscle_development		0	FALSE
ENSGALG00010021143	ABCA4	PRJNA729271	-1.953888554	3.16307E-06	1.953888554	stage_E14_vs_E10	ordinary_muscle_development	D_vs_E10;stage_1W_vs_E10;stage_3W_vs_E10;stage	1	TRUE
ENSGALG00010023199	RPS13	PRJNA729271	0.953746751	3.17558E-06	0.953746751	stage_E14_vs_E10	ordinary_muscle_development		0	FALSE
ENSGALG00010015705	NPY6R	PRJNA729271	-5.634164471	3.18728E-06	5.634164471	stage_E14_vs_E10	ordinary_muscle_development	D_vs_E10;stage_1W_vs_E10;stage_3W_vs_E10;stage	1	TRUE
ENSGALG00010006585	TTC29	PRJNA729271	-0.926268702	3.1979E-06	0.926268702	stage_E14_vs_E10	ordinary_muscle_development		0	FALSE
ENSGALG00010003698	MACC1	PRJNA729271	-6.410640174	3.19982E-06	6.410640174	stage_E14_vs_E10	ordinary_muscle_development	D_vs_E10;stage_1W_vs_E10;stage_3W_vs_E10;stage	1	TRUE
ENSGALG00010013438	RPL37	PRJNA729271	0.908564987	3.2253E-06	0.908564987	stage_E14_vs_E10	ordinary_muscle_development		0	FALSE
ENSGALG00010016981	ENSGALG00010016981	PRJNA729271	2.110075505	3.22579E-06	2.110075505	stage_E14_vs_E10	ordinary_muscle_development	D_vs_E10;stage_1W_vs_E10;stage_3W_vs_E10;stage	1	TRUE
ENSGALG00010008295	DMAC2	PRJNA729271	0.627656188	3.24652E-06	0.627656188	stage_E14_vs_E10	ordinary_muscle_development		0	FALSE
ENSGALG00010002473	ENSGALG00010002473	PRJNA729271	3.000869864	3.25917E-06	3.000869864	stage_E14_vs_E10	ordinary_muscle_development	D_vs_E10;stage_1W_vs_E10;stage_3W_vs_E10;stage	1	TRUE
ENSGALG00010022313	ENSGALG00010022313	PRJNA729271	-2.191314959	3.26367E-06	2.191314959	stage_E14_vs_E10	ordinary_muscle_development	D_vs_E10;stage_1W_vs_E10;stage_3W_vs_E10;stage	1	TRUE
ENSGALG00010000148	ENSGALG00010000148	PRJNA729271	1.327961505	3.27848E-06	1.327961505	stage_E14_vs_E10	ordinary_muscle_development	D_vs_E10;stage_1W_vs_E10;stage_3W_vs_E10;stage	1	TRUE
ENSGALG00010016303	ENSGALG00010016303	PRJNA729271	0.978562133	3.28136E-06	0.978562133	stage_E14_vs_E10	ordinary_muscle_development		0	FALSE
ENSGALG00010026078	UBE2E3	PRJNA729271	0.510749471	3.28184E-06	0.510749471	stage_E14_vs_E10	ordinary_muscle_development		0	FALSE
ENSGALG00010000770	ENSGALG00010000770	PRJNA729271	-4.60527918	3.2831E-06	4.60527918	stage_E14_vs_E10	ordinary_muscle_development	D_vs_E10;stage_1W_vs_E10;stage_3W_vs_E10;stage	1	TRUE
ENSGALG00010015163	PLK2	PRJNA729271	-1.003212953	3.29532E-06	1.003212953	stage_E14_vs_E10	ordinary_muscle_development	D_vs_E10;stage_1W_vs_E10;stage_3W_vs_E10;stage	1	TRUE
ENSGALG00010022451	CHP1	PRJNA729271	-0.590095909	3.31603E-06	0.590095909	stage_E14_vs_E10	ordinary_muscle_development		0	FALSE
ENSGALG00010025941	ENSGALG00010025941	PRJNA729271	-4.061789976	3.32834E-06	4.061789976	stage_E14_vs_E10	ordinary_muscle_development	D_vs_E10;stage_1W_vs_E10;stage_3W_vs_E10;stage	1	TRUE
ENSGALG00010010954	MTERF2	PRJNA729271	1.074757056	3.34253E-06	1.074757056	stage_E14_vs_E10	ordinary_muscle_development	D_vs_E10;stage_1W_vs_E10;stage_3W_vs_E10;stage	1	TRUE
ENSGALG00010006855	DCLK2	PRJNA729271	-0.611389973	3.3623E-06	0.611389973	stage_E14_vs_E10	ordinary_muscle_development		0	FALSE

ENSGALG00010007457	SHANK3	PRJNA729271	0.846205793	3.36504E-06	0.846205793	stage_E14_vs_E10	ordinary_muscle_development		0	FALSE
ENSGALG00010023120	CHRM4	PRJNA729271	-1.21126273	3.37522E-06	1.21126273	stage_E14_vs_E10	ordinary_muscle_development	D_vs_E10;stage_1W_vs_E10;stage_3W_vs_E10;stage	1	TRUE
ENSGALG00010028368	TIMM13	PRJNA729271	0.584243278	3.37547E-06	0.584243278	stage_E14_vs_E10	ordinary_muscle_development		0	FALSE
ENSGALG00010029428	FN3K	PRJNA729271	1.213459643	3.37914E-06	1.213459643	stage_E14_vs_E10	ordinary_muscle_development	D_vs_E10;stage_1W_vs_E10;stage_3W_vs_E10;stage	1	TRUE
ENSGALG00010025167	PTGES3	PRJNA729271	-0.914622296	3.39148E-06	0.914622296	stage_E14_vs_E10	ordinary_muscle_development		0	FALSE
ENSGALG00010010473	TRIM35	PRJNA729271	0.84510116	3.40594E-06	0.84510116	stage_E14_vs_E10	ordinary_muscle_development		0	FALSE
ENSGALG00010015158	PIN4	PRJNA729271	0.781578344	3.41272E-06	0.781578344	stage_E14_vs_E10	ordinary_muscle_development		0	FALSE
ENSGALG00010027167	ENSGALG00010027167	PRJNA729271	-2.369418987	3.41801E-06	2.369418987	stage_E14_vs_E10	ordinary_muscle_development	D_vs_E10;stage_1W_vs_E10;stage_3W_vs_E10;stage	1	TRUE
ENSGALG00010023597	MOGAT1	PRJNA729271	-1.491857745	3.42E-06	1.491857745	stage_E14_vs_E10	ordinary_muscle_development	D_vs_E10;stage_1W_vs_E10;stage_3W_vs_E10;stage	1	TRUE
ENSGALG00010005443	BANK1	PRJNA729271	2.006733754	3.42865E-06	2.006733754	stage_E14_vs_E10	ordinary_muscle_development	D_vs_E10;stage_1W_vs_E10;stage_3W_vs_E10;stage	1	TRUE
ENSGALG00010001688	ENSGALG00010001688	PRJNA729271	-0.795300201	3.4444E-06	0.795300201	stage_E14_vs_E10	ordinary_muscle_development		0	FALSE
ENSGALG00010000196	KSR2	PRJNA729271	-4.846682945	3.44932E-06	4.846682945	stage_E14_vs_E10	ordinary_muscle_development	D_vs_E10;stage_1W_vs_E10;stage_3W_vs_E10;stage	1	TRUE
ENSGALG00010022436	AFAP1L2	PRJNA729271	0.748300595	3.45915E-06	0.748300595	stage_E14_vs_E10	ordinary_muscle_development		0	FALSE
ENSGALG00010021394	MAN2C1	PRJNA729271	-0.469648737	3.50995E-06	0.469648737	stage_E14_vs_E10	ordinary_muscle_development		0	FALSE
ENSGALG00010022817	KRT23	PRJNA729271	-5.026069894	3.51752E-06	5.026069894	stage_E14_vs_E10	ordinary_muscle_development	D_vs_E10;stage_1W_vs_E10;stage_3W_vs_E10;stage	1	TRUE
ENSGALG00010012132	MC4R	PRJNA729271	-5.499988269	3.51879E-06	5.499988269	stage_E14_vs_E10	ordinary_muscle_development	D_vs_E10;stage_1W_vs_E10;stage_3W_vs_E10;stage	1	TRUE
ENSGALG00010013492	B3GALT5	PRJNA729271	-3.268422076	3.52333E-06	3.268422076	stage_E14_vs_E10	ordinary_muscle_development	D_vs_E10;stage_1W_vs_E10;stage_3W_vs_E10;stage	1	TRUE
ENSGALG00010020324	PLEKHH2	PRJNA729271	-0.915381479	3.54649E-06	0.915381479	stage_E14_vs_E10	ordinary_muscle_development		0	FALSE
ENSGALG00010004944	ALKBH1	PRJNA729271	0.457878749	3.54725E-06	0.457878749	stage_E14_vs_E10	ordinary_muscle_development		0	FALSE
ENSGALG00010011142	DUSP19	PRJNA729271	0.450282534	3.57163E-06	0.450282534	stage_E14_vs_E10	ordinary_muscle_development		0	FALSE
ENSGALG00010025898	ENSGALG00010025898	PRJNA729271	9.079434752	3.57406E-06	9.079434752	stage_E14_vs_E10	ordinary_muscle_development	D_vs_E10;stage_1W_vs_E10;stage_3W_vs_E10;stage	1	TRUE
ENSGALG00010002577	MRPL48	PRJNA729271	0.488787119	3.58279E-06	0.488787119	stage_E14_vs_E10	ordinary_muscle_development		0	FALSE
ENSGALG00010023585	TFIP11	PRJNA729271	-0.471446437	3.58314E-06	0.471446437	stage_E14_vs_E10	ordinary_muscle_development		0	FALSE
ENSGALG00010007866	TBP	PRJNA729271	-0.412607054	3.59405E-06	0.412607054	stage_E14_vs_E10	ordinary_muscle_development		0	FALSE
ENSGALG00010021184	DRAXIN	PRJNA729271	-2.664120917	3.5992E-06	2.664120917	stage_E14_vs_E10	ordinary_muscle_development	D_vs_E10;stage_1W_vs_E10;stage_3W_vs_E10;stage	1	TRUE
ENSGALG00010024879	D2HGDH	PRJNA729271	0.710318608	3.6017E-06	0.710318608	stage_E14_vs_E10	ordinary_muscle_development		0	FALSE
ENSGALG00010019275	CTAGE1	PRJNA729271	2.749166793	3.63072E-06	2.749166793	stage_E14_vs_E10	ordinary_muscle_development	D_vs_E10;stage_1W_vs_E10;stage_3W_vs_E10;stage	1	TRUE
ENSGALG00010010919	CDHR1	PRJNA729271	-3.119780729	3.63479E-06	3.119780729	stage_E14_vs_E10	ordinary_muscle_development	D_vs_E10;stage_1W_vs_E10;stage_3W_vs_E10;stage	1	TRUE
ENSGALG00010029420	LAT2	PRJNA729271	1.434635193	3.63844E-06	1.434635193	stage_E14_vs_E10	ordinary_muscle_development	D_vs_E10;stage_1W_vs_E10;stage_3W_vs_E10;stage	1	TRUE
ENSGALG00010002911	MYBL1	PRJNA729271	-1.135381998	3.64554E-06	1.135381998	stage_E14_vs_E10	ordinary_muscle_development	D_vs_E10;stage_1W_vs_E10;stage_3W_vs_E10;stage	1	TRUE
ENSGALG00010013123	PPFIBP1	PRJNA729271	0.514741452	3.64632E-06	0.514741452	stage_E14_vs_E10	ordinary_muscle_development		0	FALSE
ENSGALG00010023151	ENSGALG00010023151	PRJNA729271	-2.642024792	3.6652E-06	2.642024792	stage_E14_vs_E10	ordinary_muscle_development	D_vs_E10;stage_1W_vs_E10;stage_3W_vs_E10;stage	1	TRUE
ENSGALG00010012317	ENSGALG00010012317	PRJNA729271	-5.496096188	3.68817E-06	5.496096188	stage_E14_vs_E10	ordinary_muscle_development	D_vs_E10;stage_1W_vs_E10;stage_3W_vs_E10;stage	1	TRUE
ENSGALG00010006293	SLC22A3	PRJNA729271	-2.474567236	3.69119E-06	2.474567236	stage_E14_vs_E10	ordinary_muscle_development	D_vs_E10;stage_1W_vs_E10;stage_3W_vs_E10;stage	1	TRUE
ENSGALG00010000470	FBXW8	PRJNA729271	0.511955748	3.6973E-06	0.511955748	stage_E14_vs_E10	ordinary_muscle_development		0	FALSE
ENSGALG00010005988	NUP58	PRJNA729271	-0.428360047	3.71355E-06	0.428360047	stage_E14_vs_E10	ordinary_muscle_development		0	FALSE
ENSGALG00010016983	ZFAND2A	PRJNA729271	1.343601271	3.72004E-06	1.343601271	stage_E14_vs_E10	ordinary_muscle_development	D_vs_E10;stage_1W_vs_E10;stage_3W_vs_E10;stage	1	TRUE
ENSGALG00010012229	FSBP	PRJNA729271	0.998123329	3.76062E-06	0.998123329	stage_E14_vs_E10	ordinary_muscle_development		0	FALSE
ENSGALG00010002608	RPP38	PRJNA729271	-0.628707691	3.78245E-06	0.628707691	stage_E14_vs_E10	ordinary_muscle_development		0	FALSE
ENSGALG00010025280	HSPB11	PRJNA729271	-0.747882358	3.78746E-06	0.747882358	stage_E14_vs_E10	ordinary_muscle_development		0	FALSE
ENSGALG00010007401	SEC61G	PRJNA729271	-1.054521256	3.79962E-06	1.054521256	stage_E14_vs_E10	ordinary_muscle_development	D_vs_E10;stage_1W_vs_E10;stage_3W_vs_E10;stage	1	TRUE
ENSGALG00010014802	ENSGALG00010014802	PRJNA729271	-2.210676054	3.8048E-06	2.210676054	stage_E14_vs_E10	ordinary_muscle_development	D_vs_E10;stage_1W_vs_E10;stage_3W_vs_E10;stage	1	TRUE
ENSGALG00010010822	OGDHL	PRJNA729271	-1.993326133	3.814E-06	1.993326133	stage_E14_vs_E10	ordinary_muscle_development	D_vs_E10;stage_1W_vs_E10;stage_3W_vs_E10;stage	1	TRUE
ENSGALG00010020338	NECAP2	PRJNA729271	-0.802595471	3.82834E-06	0.802595471	stage_E14_vs_E10	ordinary_muscle_development		0	FALSE
ENSGALG00010013104	HLCS	PRJNA729271	-0.521927445	3.84656E-06	0.521927445	stage_E14_vs_E10	ordinary_muscle_development		0	FALSE
ENSGALG00010021360	PTPRN	PRJNA729271	-1.41938393	3.85745E-06	1.41938393	stage_E14_vs_E10	ordinary_muscle_development	D_vs_E10;stage_1W_vs_E10;stage_3W_vs_E10;stage	1	TRUE
ENSGALG00010024042	DCPS	PRJNA729271	-0.625757306	3.86975E-06	0.625757306	stage_E14_vs_E10	ordinary_muscle_development		0	FALSE
ENSGALG00010011633	TADA2B	PRJNA729271	-0.417068203	3.88162E-06	0.417068203	stage_E14_vs_E10	ordinary_muscle_development		0	FALSE
ENSGALG00010020847	MIDEAS	PRJNA729271	0.426594886	3.89622E-06	0.426594886	stage_E14_vs_E10	ordinary_muscle_development		0	FALSE
ENSGALG00010029374	RCC1L	PRJNA729271	-0.564093283	3.90666E-06	0.564093283	stage_E14_vs_E10	ordinary_muscle_development		0	FALSE
ENSGALG00010000645	ENSGALG00010000645	PRJNA729271	-2.920184501	3.91194E-06	2.920184501	stage_E14_vs_E10	ordinary_muscle_development	D_vs_E10;stage_1W_vs_E10;stage_3W_vs_E10;stage	1	TRUE
ENSGALG00010023808	DHX35	PRJNA729271	-0.575041793	3.91414E-06	0.575041793	stage_E14_vs_E10	ordinary_muscle_development		0	FALSE
ENSGALG00010005266	SLC19A2	PRJNA729271	1.253642666	3.92318E-06	1.253642666	stage_E14_vs_E10	ordinary_muscle_development	D_vs_E10;stage_1W_vs_E10;stage_3W_vs_E10;stage	1	TRUE
ENSGALG00010014414	TMEM255B	PRJNA729271	2.304919728	3.9379E-06	2.304919728	stage_E14_vs_E10	ordinary_muscle_development	D_vs_E10;stage_1W_vs_E10;stage_3W_vs_E10;stage	1	TRUE
ENSGALG00010009351	GTF3C2	PRJNA729271	0.576561665	3.94354E-06	0.576561665	stage_E14_vs_E10	ordinary_muscle_development		0	FALSE
ENSGALG00010025026	DENR	PRJNA729271	-0.556600506	3.95792E-06	0.556600506	stage_E14_vs_E10	ordinary_muscle_development		0	FALSE
ENSGALG00010023397	ENSGALG00010023397	PRJNA729271	-1.278715154	3.95879E-06	1.278715154	stage_E14_vs_E10	ordinary_muscle_development	D_vs_E10;stage_1W_vs_E10;stage_3W_vs_E10;stage	1	TRUE
ENSGALG00010004483	STEAP4	PRJNA729271	2.767339199	3.9634E-06	2.767339199	stage_E14_vs_E10	ordinary_muscle_development	D_vs_E10;stage_1W_vs_E10;stage_3W_vs_E10;stage	1	TRUE
ENSGALG00010024073	RABGGTB	PRJNA729271	0.591459243	3.97699E-06	0.591459243	stage_E14_vs_E10	ordinary_muscle_development		0	FALSE
ENSGALG00010020971	TMEM51	PRJNA729271	-1.426640823	3.97784E-06	1.426640823	stage_E14_vs_E10	ordinary_muscle_development	D_vs_E10;stage_1W_vs_E10;stage_3W_vs_E10;stage	1	TRUE
ENSGALG00010012955	RPL39L	PRJNA729271	0.74470206	4.02008E-06	0.74470206	stage_E14_vs_E10	ordinary_muscle_development		0	FALSE
ENSGALG00010009542	WDR1	PRJNA729271	-0.687800599	4.0571E-06	0.687800599	stage_E14_vs_E10	ordinary_muscle_development		0	FALSE
ENSGALG00010000387	DTX1	PRJNA729271	-2.689344527	4.08195E-06	2.689344527	stage_E14_vs_E10	ordinary_muscle_development	D_vs_E10;stage_1W_vs_E10;stage_3W_vs_E10;stage	1	TRUE
ENSGALG00010023254	MYO3B	PRJNA729271	-1.513086545	4.0855E-06	1.513086545	stage_E14_vs_E10	ordinary_muscle_development	D_vs_E10;stage_1W_vs_E10;stage_3W_vs_E10;stage	1	TRUE
ENSGALG00010014539	PAIP1	PRJNA729271	-0.404842496	4.08936E-06	0.404842496	stage_E14_vs_E10	ordinary_muscle_development		0	FALSE
ENSGALG00010001144	CDKN2B	PRJNA729271	6.456598183	4.09011E-06	6.456598183	stage_E14_vs_E10	ordinary_muscle_development	D_vs_E10;stage_1W_vs_E10;stage_3W_vs_E10;stage	1	TRUE
ENSGALG00010005630	NIT2	PRJNA729271	0.784433946	4.10089E-06	0.784433946	stage_E14_vs_E10	ordinary_muscle_development		0	FALSE
ENSGALG00010009236	DTWD2	PRJNA729271	-0.562026748	4.1047E-06	0.562026748	stage_E14_vs_E10	ordinary_muscle_development		0	FALSE
ENSGALG00010008690	YIF1B	PRJNA729271	-0.589707039	4.11606E-06	0.589707039	stage_E14_vs_E10	ordinary_muscle_development		0	FALSE
ENSGALG00010011677	EDEM3	PRJNA729271	-0.387213583	4.14396E-06	0.387213583	stage_E14_vs_E10	ordinary_muscle_development		0	FALSE
ENSGALG00010004487	ABHD10	PRJNA729271	-0.478804635	4.15186E-06	0.478804635	stage_E14_vs_E10	ordinary_muscle_development		0	FALSE
ENSGALG00010029455	ENSGALG00010029455	PRJNA729271	-1.228218445	4.15672E-06	1.228218445	stage_E14_vs_E10	ordinary_muscle_development	D_vs_E10;stage_1W_vs_E10;stage_3W_vs_E10;stage	1	TRUE
ENSGALG00010029783	RAD51C	PRJNA729271	-0.582768737	4.20946E-06	0.582768737	stage_E14_vs_E10	ordinary_muscle_development		0	FALSE
ENSGALG00010020707	SLC4A5	PRJNA729271	-2.745864895	4.21803E-06	2.745864895	stage_E14_vs_E10	ordinary_muscle_development	D_vs_E10;stage_1W_vs_E10;stage_3W_vs_E10;stage	1	TRUE
ENSGALG00010027618	PPP1R26	PRJNA729271	0.372000775	4.21924E-06	0.372000775	stage_E14_vs_E10	ordinary_muscle_development		0	FALSE
ENSGALG00010000381	ENSGALG00010000381	PRJNA729271	-1.491227534	4.21974E-06	1.491227534	stage_E14_vs_E10	ordinary_muscle_development	D_vs_E10;stage_1W_vs_E10;stage_3W_vs_E10;stage	1	TRUE
ENSGALG00010024615	SYT8	PRJNA729271	-3.827682323	4.23676E-06	3.827682323	stage_E14_vs_E10	ordinary_muscle_development	D_vs_E10;stage_1W_vs_E10;stage_3W_vs_E10;stage	1	TRUE
ENSGALG00010028016	PRF1	PRJNA729271	5.805400182	4.23797E-06	5.805400182	stage_E14_vs_E10	ordinary_muscle_development	D_vs_E10;stage_1W_vs_E10;stage_3W_vs_E10;stage	1	TRUE
ENSGALG00010024521	HEPACAM	PRJNA729271	-2.757382222	4.24108E-06	2.757382222	stage_E14_vs_E10	ordinary_muscle_development	D_vs_E10;stage_1W_vs_E10;stage_3W_vs_E10;stage	1	TRUE
ENSGALG00010012069	ERP27	PRJNA729271	1.966074691	4.28818E-06	1.966074691	stage_E14_vs_E10	ordinary_muscle_development	D_vs_E10;stage_1W_vs_E10;stage_3W_vs_E10;stage	1	TRUE
ENSGALG00010001160	TRMT10B	PRJNA729271	-0.613421117	4.30085E-06	0.613421117	stage_E14_vs_E10	ordinary_muscle_development		0	FALSE
ENSGALG00010018001	TRAF7	PRJNA729271	0.413451017	4.32119E-06	0.413451017	stage_E14_vs_E10	ordinary_muscle_development		0	FALSE
ENSGALG00010027888	PFKFB2	PRJNA729271	-0.708872314	4.35854E-06	0.708872314	stage_E14_vs_E10	ordinary_muscle_development		0	FALSE
ENSGALG00010028470	AJM1	PRJNA729271	2.043597185	4.38679E-06	2.043597185	stage_E14_vs_E10	ordinary_muscle_development	D_vs_E10;stage_1W_vs_E10;stage_3W_vs_E10;stage	1	TRUE
ENSGALG00010014214	REEP2	PRJNA729271	-0.665134749	4.40191E-06	0.665134749	stage_E14_vs_E10	ordinary_muscle_development		0	FALSE
ENSGALG00010011453	GNPTAB	PRJNA729271	-0.614398965	4.44414E-06	0.614398965	stage_E14_vs_E10	ordinary_muscle_development		0	FALSE
ENSGALG00010001694	ENSGALG00010001694	PRJNA729271	-1.051987878	4.50575E-06	1.051987878	stage_E14_vs_E10	ordinary_muscle_development	D_vs_E10;stage_1W_vs_E10;stage_3W_vs_E10;stage	1	TRUE
ENSGALG00010027170	TLE2	PRJNA729271	-0.846992302	4.54282E-06	0.846992302	stage_E14_vs_E10	ordinary_muscle_development		0	FALSE
ENSGALG00010017462	MCM8	PRJNA729271	-0.568848097	4.57371E-06	0.568848097	stage_E14_vs_E10	ordinary_muscle_development		0	FALSE
ENSGALG00010029633	POLG2	PRJNA729271	0.832499357	4.57469E-06	0.832499357	stage_E14_vs_E10	ordinary_muscle_development		0	FALSE
ENSGALG00010014134	RPAP3	PRJNA729271	-0.549411024	4.62888E-06	0.549411024	stage_E14_vs_E10	ordinary_muscle_development		0	FALSE
ENSGALG00010027539	DDR2L	PRJNA729271	0.509149505	4.6363E-06	0.509149505	stage_E14_vs_E10	ordinary_muscle_development		0	FALSE
ENSGALG00010001828	ENSGALG00010001828	PRJNA729271	3.591356965	4.64009E-06	3.591356965	stage_E14_vs_E10	ordinary_muscle_development	D_vs_E10;stage_1W_vs_E10;stage_3W_vs_E10;stage	1	TRUE
ENSGALG00010017399	CPXM1	PRJNA729271	1.382671264	4.66276E-06	1.382671264	stage_E14_vs_E10	ordinary_muscle_development	D_vs_E10;stage_1W_vs_E10;stage_3W_vs_E10;stage	1	TRUE
ENSGALG00010023604	ENSGALG00010023604	PRJNA729271	-1.221605615	4.66532E-06	1.221605615	stage_E14_vs_E10	ordinary_muscle_development	D_vs_E10;stage_1W_vs_E10;stage_3W_vs_E10;stage	1	TRUE
ENSGALG00010024779	ENSGALG00010024779	PRJNA729271	-5.664399847	4.68017E-06	5.664399847	stage_E14_vs_E10	ordinary_muscle_development	D_vs_E10;stage_1W_vs_E10;stage_3W_vs_E10;stage	1	TRUE
ENSGALG00010024427	C22orf39	PRJNA729271	0.657616979	4.69406E-06	0.657616979	stage_E14_vs_E10	ordinary_muscle_development		0	FALSE
ENSGALG00010017445	F9	PRJNA729271	5.522625697	4.7125E-06	5.522625697	stage_E14_vs_E10	ordinary_muscle_development	D_vs_E10;stage_1W_vs_E10;stage_3W_vs_E10;stage	1	TRUE
ENSGALG00010001289	ENSGALG00010001289	PRJNA729271	1.621455972	4.71823E-06	1.621455972	stage_E14_vs_E10	ordinary_muscle_development	D_vs_E10;stage_1W_vs_E10;stage_3W_vs_E10;stage	1	TRUE
ENSGALG00010020095	LRFN2	PRJNA729271	-1.618828312	4.73192E-06	1.618828312	stage_E14_vs_E10	ordinary_muscle_development	D_vs_E10;stage_1W_vs_E10;stage_3W_vs_E10;stage	1	TRUE
ENSGALG00010017665	CTBP1	PRJNA729271	-0.298258415	4.74253E-06	0.298258415	stage_E14_vs_E10	ordinary_muscle_development		0	FALSE
ENSGALG00010015653	HCK	PRJNA729271	2.422901629	4.77132E-06	2.422901629	stage_E14_vs_E10	ordinary_muscle_development	D_vs_E10;stage_1W_vs_E10;stage_3W_vs_E10;stage	1	TRUE
ENSGALG00010019878	MCFD2	PRJNA729271	0.713905309	4.79724E-06	0.713905309	stage_E14_vs_E10	ordinary_muscle_development		0	FALSE
ENSGALG00010003212	LTB4R	PRJNA729271	3.319774201	4.80102E-06	3.319774201	stage_E14_vs_E10	ordinary_muscle_development	D_vs_E10;stage_1W_vs_E10;stage_3W_vs_E10;stage	1	TRUE
ENSGALG00010005269	KIN	PRJNA729271	0.494322254	4.81855E-06	0.494322254	stage_E14_vs_E10	ordinary_muscle_development		0	FALSE
ENSGALG00010020478	ENSGALG00010020478	PRJNA729271	3.111504455	4.83518E-06	3.111504455	stage_E14_vs_E10	ordinary_muscle_development	D_vs_E10;stage_1W_vs_E10;stage_3W_vs_E10;stage	1	TRUE
ENSGALG00010029495	RPL38	PRJNA729271	0.897696793	4.8396E-06	0.897696793	stage_E14_vs_E10	ordinary_muscle_development		0	FALSE
ENSGALG00010002303	ENSGALG00010002303	PRJNA729271	3.357554186	4.84079E-06	3.357554186	stage_E14_vs_E10	ordinary_muscle_development	D_vs_E10;stage_1W_vs_E10;stage_3W_vs_E10;stage	1	TRUE
ENSGALG00010025929	NELFE	PRJNA729271	0.431618757	4.86348E-06	0.431618757	stage_E14_vs_E10	ordinary_muscle_development		0	FALSE
ENSGALG00010027724	TTLL11	PRJNA729271	-0.53377817	4.8883E-06	0.53377817	stage_E14_vs_E10	ordinary_muscle_development		0	FALSE
ENSGALG00010012387	ERBIN	PRJNA729271	-0.419066437	4.92104E-06	0.419066437	stage_E14_vs_E10	ordinary_muscle_development		0	FALSE
ENSGALG00010027327	RBM6	PRJNA729271	-0.419088358	4.94637E-06	0.419088358	stage_E14_vs_E10	ordinary_muscle_development		0	FALSE
ENSGALG00010024891	PKP1	PRJNA729271	-2.303062335	4.95436E-06	2.303062335	stage_E14_vs_E10	ordinary_muscle_development	D_vs_E10;stage_1W_vs_E10;stage_3W_vs_E10;stage	1	TRUE
ENSGALG00010013417	AKR1B1	PRJNA729271	-0.415071721	4.96103E-06	0.415071721	stage_E14_vs_E10	ordinary_muscle_development		0	FALSE
ENSGALG00010009672	DDX47	PRJNA729271	-0.565897792	4.96182E-06	0.565897792	stage_E14_vs_E10	ordinary_muscle_development		0	FALSE
ENSGALG00010022783	STAG1	PRJNA729271	0.265249519	5.01192E-06	0.265249519	stage_E14_vs_E10	ordinary_muscle_development		0	FALSE
ENSGALG00010016483	WDR26	PRJNA729271	0.481447026	5.02547E-06	0.481447026	stage_E14_vs_E10	ordinary_muscle_development		0	FALSE
ENSGALG00010014379	CARS2	PRJNA729271	-0.42725407	5.03303E-06	0.42725407	stage_E14_vs_E10	ordinary_muscle_development		0	FALSE
ENSGALG00010027213	KCNC4	PRJNA729271	1.560917282	5.03532E-06	1.560917282	stage_E14_vs_E10	ordinary_muscle_development	D_vs_E10;stage_1W_vs_E10;stage_3W_vs_E10;stage	1	TRUE
ENSGALG00010015064	RFK	PRJNA729271	-0.729093572	5.04154E-06	0.729093572	stage_E14_vs_E10	ordinary_muscle_development		0	FALSE
ENSGALG00010014371	TTC33	PRJNA729271	-0.4931083	5.06221E-06	0.4931083	stage_E14_vs_E10	ordinary_muscle_development		0	FALSE
ENSGALG00010007203	SLC30A6	PRJNA729271	0.489640816	5.08956E-06	0.489640816	stage_E14_vs_E10	ordinary_muscle_development		0	FALSE
ENSGALG00010022587	ENSGALG00010022587	PRJNA729271	-5.415711078	5.11677E-06	5.415711078	stage_E14_vs_E10	ordinary_muscle_development	D_vs_E10;stage_1W_vs_E10;stage_3W_vs_E10;stage	1	TRUE
ENSGALG00010025559	SLC6A17	PRJNA729271	-5.617759333	5.11858E-06	5.617759333	stage_E14_vs_E10	ordinary_muscle_development	D_vs_E10;stage_1W_vs_E10;stage_3W_vs_E10;stage	1	TRUE
ENSGALG00010023278	ENSGALG00010023278	PRJNA729271	-1.442407532	5.12441E-06	1.442407532	stage_E14_vs_E10	ordinary_muscle_development	D_vs_E10;stage_1W_vs_E10;stage_3W_vs_E10;stage	1	TRUE
ENSGALG00010003674	AKIRIN2	PRJNA729271	0.617284237	5.14036E-06	0.617284237	stage_E14_vs_E10	ordinary_muscle_development		0	FALSE
ENSGALG00010011381	RLIG1	PRJNA729271	-0.59061125	5.16507E-06	0.59061125	stage_E14_vs_E10	ordinary_muscle_development		0	FALSE
ENSGALG00010009697	ENSGALG00010009697	PRJNA729271	-1.403947122	5.19304E-06	1.403947122	stage_E14_vs_E10	ordinary_muscle_development	D_vs_E10;stage_1W_vs_E10;stage_3W_vs_E10;stage	1	TRUE
ENSGALG00010006382	PNN	PRJNA729271	-0.93738899	5.19366E-06	0.93738899	stage_E14_vs_E10	ordinary_muscle_development		0	FALSE
ENSGALG00010016802	TMEM222	PRJNA729271	-0.399336103	5.19834E-06	0.399336103	stage_E14_vs_E10	ordinary_muscle_development		0	FALSE
ENSGALG00010019350	SAV1	PRJNA729271	-0.418384998	5.2457E-06	0.418384998	stage_E14_vs_E10	ordinary_muscle_development		0	FALSE
ENSGALG00010013449	LCA5L	PRJNA729271	-1.115106766	5.27327E-06	1.115106766	stage_E14_vs_E10	ordinary_muscle_development	D_vs_E10;stage_1W_vs_E10;stage_3W_vs_E10;stage	1	TRUE
ENSGALG00010023726	PNPO	PRJNA729271	0.844266735	5.28917E-06	0.844266735	stage_E14_vs_E10	ordinary_muscle_development		0	FALSE
ENSGALG00010027966	CLK2	PRJNA729271	0.459283965	5.30906E-06	0.459283965	stage_E14_vs_E10	ordinary_muscle_development		0	FALSE
ENSGALG00010018394	KLHL28	PRJNA729271	-0.470271805	5.35724E-06	0.470271805	stage_E14_vs_E10	ordinary_muscle_development		0	FALSE
ENSGALG00010015487	RPS6KA6	PRJNA729271	-0.570107351	5.39441E-06	0.570107351	stage_E14_vs_E10	ordinary_muscle_development		0	FALSE
ENSGALG00010022243	ENSGALG00010022243	PRJNA729271	2.600344425	5.44375E-06	2.600344425	stage_E14_vs_E10	ordinary_muscle_development	D_vs_E10;stage_1W_vs_E10;stage_3W_vs_E10;stage	1	TRUE
ENSGALG00010021028	ENSGALG00010021028	PRJNA729271	1.45843624	5.47509E-06	1.45843624	stage_E14_vs_E10	ordinary_muscle_development	D_vs_E10;stage_1W_vs_E10;stage_3W_vs_E10;stage	1	TRUE
ENSGALG00010020809	ENSGALG00010020809	PRJNA729271	1.079669363	5.48684E-06	1.079669363	stage_E14_vs_E10	ordinary_muscle_development	D_vs_E10;stage_1W_vs_E10;stage_3W_vs_E10;stage	1	TRUE
ENSGALG00010000272	ENSGALG00010000272	PRJNA729271	1.56164919	5.5029E-06	1.56164919	stage_E14_vs_E10	ordinary_muscle_development	D_vs_E10;stage_1W_vs_E10;stage_3W_vs_E10;stage	1	TRUE
ENSGALG00010016418	NFIB	PRJNA729271	0.459866175	5.53856E-06	0.459866175	stage_E14_vs_E10	ordinary_muscle_development		0	FALSE
ENSGALG00010004038	SENP6	PRJNA729271	0.408347816	5.54362E-06	0.408347816	stage_E14_vs_E10	ordinary_muscle_development		0	FALSE
ENSGALG00010004133	CRH	PRJNA729271	4.461399448	5.6217E-06	4.461399448	stage_E14_vs_E10	ordinary_muscle_development	D_vs_E10;stage_1W_vs_E10;stage_3W_vs_E10;stage	1	TRUE
ENSGALG00010008450	GIN1	PRJNA729271	-0.842156458	5.63218E-06	0.842156458	stage_E14_vs_E10	ordinary_muscle_development		0	FALSE
ENSGALG00010023978	TFDP2	PRJNA729271	-0.450847272	5.65955E-06	0.450847272	stage_E14_vs_E10	ordinary_muscle_development		0	FALSE
ENSGALG00010024242	KMT2A	PRJNA729271	0.585089668	5.66803E-06	0.585089668	stage_E14_vs_E10	ordinary_muscle_development		0	FALSE
ENSGALG00010027337	AQP1	PRJNA729271	-1.24201462	5.66841E-06	1.24201462	stage_E14_vs_E10	ordinary_muscle_development	D_vs_E10;stage_1W_vs_E10;stage_3W_vs_E10;stage	1	TRUE
ENSGALG00010020708	FAM214A	PRJNA729271	-0.63898003	5.68352E-06	0.63898003	stage_E14_vs_E10	ordinary_muscle_development		0	FALSE
ENSGALG00010008501	ENSGALG00010008501	PRJNA729271	-2.066447396	5.71488E-06	2.066447396	stage_E14_vs_E10	ordinary_muscle_development	D_vs_E10;stage_1W_vs_E10;stage_3W_vs_E10;stage	1	TRUE
ENSGALG00010025064	GPN3	PRJNA729271	0.383331415	5.74591E-06	0.383331415	stage_E14_vs_E10	ordinary_muscle_development		0	FALSE
ENSGALG00010002210	GPR143	PRJNA729271	-5.740149746	5.7541E-06	5.740149746	stage_E14_vs_E10	ordinary_muscle_development	D_vs_E10;stage_1W_vs_E10;stage_3W_vs_E10;stage	1	TRUE
ENSGALG00010006066	ARHGAP18	PRJNA729271	-0.565458668	5.76988E-06	0.565458668	stage_E14_vs_E10	ordinary_muscle_development		0	FALSE
ENSGALG00010017739	MRPL20	PRJNA729271	-0.473765907	5.81788E-06	0.473765907	stage_E14_vs_E10	ordinary_muscle_development		0	FALSE
ENSGALG00010011674	ENSGALG00010011674	PRJNA729271	-2.514383086	5.83041E-06	2.514383086	stage_E14_vs_E10	ordinary_muscle_development	D_vs_E10;stage_1W_vs_E10;stage_3W_vs_E10;stage	1	TRUE
ENSGALG00010019481	PYGL	PRJNA729271	0.463392165	5.84763E-06	0.463392165	stage_E14_vs_E10	ordinary_muscle_development		0	FALSE
ENSGALG00010001215	PRDM6	PRJNA729271	-3.416569757	5.86734E-06	3.416569757	stage_E14_vs_E10	ordinary_muscle_development	D_vs_E10;stage_1W_vs_E10;stage_3W_vs_E10;stage	1	TRUE
ENSGALG00010020535	LMLN	PRJNA729271	-0.72089743	5.86889E-06	0.72089743	stage_E14_vs_E10	ordinary_muscle_development		0	FALSE
ENSGALG00010010769	HEXB	PRJNA729271	-0.572232632	5.87365E-06	0.572232632	stage_E14_vs_E10	ordinary_muscle_development		0	FALSE
ENSGALG00010017480	DCDC2B	PRJNA729271	0.898530002	5.90419E-06	0.898530002	stage_E14_vs_E10	ordinary_muscle_development		0	FALSE
ENSGALG00010023945	LETMD1	PRJNA729271	0.965699033	5.96363E-06	0.965699033	stage_E14_vs_E10	ordinary_muscle_development		0	FALSE
ENSGALG00010002361	ENSGALG00010002361	PRJNA729271	0.460722979	5.96453E-06	0.460722979	stage_E14_vs_E10	ordinary_muscle_development		0	FALSE
ENSGALG00010014936	FOXRED2	PRJNA729271	-0.802894047	5.99435E-06	0.802894047	stage_E14_vs_E10	ordinary_muscle_development		0	FALSE
ENSGALG00010017301	LHCGR	PRJNA729271	-2.942790362	5.99493E-06	2.942790362	stage_E14_vs_E10	ordinary_muscle_development	D_vs_E10;stage_1W_vs_E10;stage_3W_vs_E10;stage	1	TRUE
ENSGALG00010007328	NOVA1	PRJNA729271	0.620256693	6.01736E-06	0.620256693	stage_E14_vs_E10	ordinary_muscle_development		0	FALSE
ENSGALG00010015870	EIF5B	PRJNA729271	-0.73942768	6.03564E-06	0.73942768	stage_E14_vs_E10	ordinary_muscle_development		0	FALSE
ENSGALG00010019934	TKFC	PRJNA729271	-0.740346493	6.07467E-06	0.740346493	stage_E14_vs_E10	ordinary_muscle_development		0	FALSE
ENSGALG00010012097	GART	PRJNA729271	-0.909415715	6.12494E-06	0.909415715	stage_E14_vs_E10	ordinary_muscle_development		0	FALSE
ENSGALG00010003899	LIN54	PRJNA729271	0.765796237	6.16815E-06	0.765796237	stage_E14_vs_E10	ordinary_muscle_development		0	FALSE
ENSGALG00010005345	NOX3	PRJNA729271	2.284924489	6.19119E-06	2.284924489	stage_E14_vs_E10	ordinary_muscle_development	D_vs_E10;stage_1W_vs_E10;stage_3W_vs_E10;stage	1	TRUE
ENSGALG00010012466	PDSS2	PRJNA729271	0.56184478	6.21629E-06	0.56184478	stage_E14_vs_E10	ordinary_muscle_development		0	FALSE
ENSGALG00010017065	VRK2	PRJNA729271	-0.635016369	6.24324E-06	0.635016369	stage_E14_vs_E10	ordinary_muscle_development		0	FALSE
ENSGALG00010000254	ENSGALG00010000254	PRJNA729271	-3.360887701	6.24713E-06	3.360887701	stage_E14_vs_E10	ordinary_muscle_development	D_vs_E10;stage_1W_vs_E10;stage_3W_vs_E10;stage	1	TRUE
ENSGALG00010024642	RAB3B	PRJNA729271	1.029915989	6.25853E-06	1.029915989	stage_E14_vs_E10	ordinary_muscle_development	D_vs_E10;stage_1W_vs_E10;stage_3W_vs_E10;stage	1	TRUE
ENSGALG00010013180	C5orf24	PRJNA729271	-0.276654643	6.2642E-06	0.276654643	stage_E14_vs_E10	ordinary_muscle_development		0	FALSE
ENSGALG00010019094	SNRNP25	PRJNA729271	0.787527997	6.28681E-06	0.787527997	stage_E14_vs_E10	ordinary_muscle_development		0	FALSE

ENSGALG00010005999	PDE10A	PRJNA729271	1.358446588	6.29497E-06	1.358446588	stage_E14_vs_E10	ordinary_muscle_development	D_vs_E10;stage_1W_vs_E10;stage_3W_vs_E10;stage	1	TRUE
ENSGALG00010009330	EIF3K	PRJNA729271	0.665408804	6.3273E-06	0.665408804	stage_E14_vs_E10	ordinary_muscle_development		0	FALSE
ENSGALG00010029755	ACOX1	PRJNA729271	0.553621426	6.36899E-06	0.553621426	stage_E14_vs_E10	ordinary_muscle_development		0	FALSE
ENSGALG00010014318	ENSGALG00010014318	PRJNA729271	2.153961782	6.3798E-06	2.153961782	stage_E14_vs_E10	ordinary_muscle_development	D_vs_E10;stage_1W_vs_E10;stage_3W_vs_E10;stage	1	TRUE
ENSGALG00010015934	TMC2	PRJNA729271	4.675405268	6.39067E-06	4.675405268	stage_E14_vs_E10	ordinary_muscle_development	D_vs_E10;stage_1W_vs_E10;stage_3W_vs_E10;stage	1	TRUE
ENSGALG00010024813	RNF168	PRJNA729271	-0.448444018	6.39842E-06	0.448444018	stage_E14_vs_E10	ordinary_muscle_development		0	FALSE
ENSGALG00010028563	ENSGALG00010028563	PRJNA729271	10.42809496	6.40986E-06	10.42809496	stage_E14_vs_E10	ordinary_muscle_development	D_vs_E10;stage_1W_vs_E10;stage_3W_vs_E10;stage	1	TRUE
ENSGALG00010001250	ENSGALG00010001250	PRJNA729271	-3.552646421	6.42718E-06	3.552646421	stage_E14_vs_E10	ordinary_muscle_development	D_vs_E10;stage_1W_vs_E10;stage_3W_vs_E10;stage	1	TRUE
ENSGALG00010025652	KMT5B	PRJNA729271	-0.358089692	6.43117E-06	0.358089692	stage_E14_vs_E10	ordinary_muscle_development		0	FALSE
ENSGALG00010013792	FGF10	PRJNA729271	-1.148460176	6.439E-06	1.148460176	stage_E14_vs_E10	ordinary_muscle_development	D_vs_E10;stage_1W_vs_E10;stage_3W_vs_E10;stage	1	TRUE
ENSGALG00010023749	RPP25	PRJNA729271	1.142471588	6.4646E-06	1.142471588	stage_E14_vs_E10	ordinary_muscle_development	D_vs_E10;stage_1W_vs_E10;stage_3W_vs_E10;stage	1	TRUE
ENSGALG00010024129	GID8	PRJNA729271	0.470039891	6.46831E-06	0.470039891	stage_E14_vs_E10	ordinary_muscle_development		0	FALSE
ENSGALG00010002298	ENSGALG00010002298	PRJNA729271	-2.491212029	6.46936E-06	2.491212029	stage_E14_vs_E10	ordinary_muscle_development	D_vs_E10;stage_1W_vs_E10;stage_3W_vs_E10;stage	1	TRUE
ENSGALG00010016658	ZAR1	PRJNA729271	-3.222856432	6.48065E-06	3.222856432	stage_E14_vs_E10	ordinary_muscle_development	D_vs_E10;stage_1W_vs_E10;stage_3W_vs_E10;stage	1	TRUE
ENSGALG00010007762	SH2D4A	PRJNA729271	-1.664194459	6.49216E-06	1.664194459	stage_E14_vs_E10	ordinary_muscle_development	D_vs_E10;stage_1W_vs_E10;stage_3W_vs_E10;stage	1	TRUE
ENSGALG00010023489	ETV5	PRJNA729271	-1.013242587	6.49517E-06	1.013242587	stage_E14_vs_E10	ordinary_muscle_development	D_vs_E10;stage_1W_vs_E10;stage_3W_vs_E10;stage	1	TRUE
ENSGALG00010013041	LRP12	PRJNA729271	-0.356142481	6.49582E-06	0.356142481	stage_E14_vs_E10	ordinary_muscle_development		0	FALSE
ENSGALG00010000318	ENSGALG00010000318	PRJNA729271	-22.39108995	6.49674E-06	22.39108995	stage_E14_vs_E10	ordinary_muscle_development	D_vs_E10;stage_1W_vs_E10;stage_3W_vs_E10;stage	1	TRUE
ENSGALG00010002174	ENSGALG00010002174	PRJNA729271	2.16836384	6.50765E-06	2.16836384	stage_E14_vs_E10	ordinary_muscle_development	D_vs_E10;stage_1W_vs_E10;stage_3W_vs_E10;stage	1	TRUE
ENSGALG00010027554	ENSGALG00010027554	PRJNA729271	0.673166677	6.52042E-06	0.673166677	stage_E14_vs_E10	ordinary_muscle_development		0	FALSE
ENSGALG00010022479	ENSGALG00010022479	PRJNA729271	2.982316762	6.54306E-06	2.982316762	stage_E14_vs_E10	ordinary_muscle_development	D_vs_E10;stage_1W_vs_E10;stage_3W_vs_E10;stage	1	TRUE
ENSGALG00010008790	GPN1	PRJNA729271	0.438777435	6.551E-06	0.438777435	stage_E14_vs_E10	ordinary_muscle_development		0	FALSE
ENSGALG00010027313	BSN	PRJNA729271	-1.155562861	6.58199E-06	1.155562861	stage_E14_vs_E10	ordinary_muscle_development	D_vs_E10;stage_1W_vs_E10;stage_3W_vs_E10;stage	1	TRUE
ENSGALG00010026132	DDTL	PRJNA729271	-0.862373431	6.59025E-06	0.862373431	stage_E14_vs_E10	ordinary_muscle_development		0	FALSE
ENSGALG00010017606	ENSGALG00010017606	PRJNA729271	1.132928317	6.60174E-06	1.132928317	stage_E14_vs_E10	ordinary_muscle_development	D_vs_E10;stage_1W_vs_E10;stage_3W_vs_E10;stage	1	TRUE
ENSGALG00010020011	TLE3	PRJNA729271	-0.946798569	6.60185E-06	0.946798569	stage_E14_vs_E10	ordinary_muscle_development		0	FALSE
ENSGALG00010024325	PCBP2	PRJNA729271	0.787529122	6.60731E-06	0.787529122	stage_E14_vs_E10	ordinary_muscle_development		0	FALSE
ENSGALG00010027221	LRRC61	PRJNA729271	-1.302482589	6.62314E-06	1.302482589	stage_E14_vs_E10	ordinary_muscle_development	D_vs_E10;stage_1W_vs_E10;stage_3W_vs_E10;stage	1	TRUE
ENSGALG00010002211	ACER3	PRJNA729271	-0.809495298	6.63172E-06	0.809495298	stage_E14_vs_E10	ordinary_muscle_development		0	FALSE
ENSGALG00010016466	TMEM164	PRJNA729271	-1.103598487	6.67149E-06	1.103598487	stage_E14_vs_E10	ordinary_muscle_development	D_vs_E10;stage_1W_vs_E10;stage_3W_vs_E10;stage	1	TRUE
ENSGALG00010029701	MRPS7	PRJNA729271	0.305344712	6.68431E-06	0.305344712	stage_E14_vs_E10	ordinary_muscle_development		0	FALSE
ENSGALG00010002062	GSTA4	PRJNA729271	-1.73845007	6.70854E-06	1.73845007	stage_E14_vs_E10	ordinary_muscle_development	D_vs_E10;stage_1W_vs_E10;stage_3W_vs_E10;stage	1	TRUE
ENSGALG00010011062	TMEM55A	PRJNA729271	-0.568897267	6.71431E-06	0.568897267	stage_E14_vs_E10	ordinary_muscle_development		0	FALSE
ENSGALG00010019905	ENSGALG00010019905	PRJNA729271	-0.577912677	6.72261E-06	0.577912677	stage_E14_vs_E10	ordinary_muscle_development		0	FALSE
ENSGALG00010028523	SUGP1	PRJNA729271	-0.536521383	6.83576E-06	0.536521383	stage_E14_vs_E10	ordinary_muscle_development		0	FALSE
ENSGALG00010022459	FGF6	PRJNA729271	3.429669517	6.85137E-06	3.429669517	stage_E14_vs_E10	ordinary_muscle_development	D_vs_E10;stage_1W_vs_E10;stage_3W_vs_E10;stage	1	TRUE
ENSGALG00010020163	CDC2L1	PRJNA729271	-0.446082266	6.8654E-06	0.446082266	stage_E14_vs_E10	ordinary_muscle_development		0	FALSE
ENSGALG00010021272	ENSGALG00010021272	PRJNA729271	1.643464328	6.87097E-06	1.643464328	stage_E14_vs_E10	ordinary_muscle_development	D_vs_E10;stage_1W_vs_E10;stage_3W_vs_E10;stage	1	TRUE
ENSGALG00010019515	ENSGALG00010019515	PRJNA729271	-0.509045312	6.88029E-06	0.509045312	stage_E14_vs_E10	ordinary_muscle_development		0	FALSE
ENSGALG00010028118	ENSGALG00010028118	PRJNA729271	0.357684271	6.88923E-06	0.357684271	stage_E14_vs_E10	ordinary_muscle_development		0	FALSE
ENSGALG00010013280	FRS2	PRJNA729271	-0.523331913	6.88943E-06	0.523331913	stage_E14_vs_E10	ordinary_muscle_development		0	FALSE
ENSGALG00010006552	CPA2	PRJNA729271	7.571042827	6.89665E-06	7.571042827	stage_E14_vs_E10	ordinary_muscle_development	D_vs_E10;stage_1W_vs_E10;stage_3W_vs_E10;stage	1	TRUE
ENSGALG00010021908	EDC4	PRJNA729271	-0.512748782	6.9029E-06	0.512748782	stage_E14_vs_E10	ordinary_muscle_development		0	FALSE
ENSGALG00010022299	MRGBP	PRJNA729271	-0.373981365	6.92607E-06	0.373981365	stage_E14_vs_E10	ordinary_muscle_development		0	FALSE
ENSGALG00010005322	TFB1M	PRJNA729271	0.563692184	6.92864E-06	0.563692184	stage_E14_vs_E10	ordinary_muscle_development		0	FALSE
ENSGALG00010024549	GNB1L	PRJNA729271	-0.64280698	6.93902E-06	0.64280698	stage_E14_vs_E10	ordinary_muscle_development		0	FALSE
ENSGALG00010009363	TAF2	PRJNA729271	-0.358799315	6.97684E-06	0.358799315	stage_E14_vs_E10	ordinary_muscle_development		0	FALSE
ENSGALG00010027921	MKRN2OS	PRJNA729271	1.06872644	6.97992E-06	1.06872644	stage_E14_vs_E10	ordinary_muscle_development	D_vs_E10;stage_1W_vs_E10;stage_3W_vs_E10;stage	1	TRUE
ENSGALG00010014974	LIG4	PRJNA729271	0.513908028	7.02964E-06	0.513908028	stage_E14_vs_E10	ordinary_muscle_development		0	FALSE
ENSGALG00010012159	WASHC1	PRJNA729271	0.471958172	7.05311E-06	0.471958172	stage_E14_vs_E10	ordinary_muscle_development		0	FALSE
ENSGALG00010003734	ZC3H13	PRJNA729271	-0.665131736	7.11749E-06	0.665131736	stage_E14_vs_E10	ordinary_muscle_development		0	FALSE
ENSGALG00010017395	BRF2	PRJNA729271	-0.614489078	7.11899E-06	0.614489078	stage_E14_vs_E10	ordinary_muscle_development		0	FALSE
ENSGALG00010001902	WDR45	PRJNA729271	0.653101018	7.13306E-06	0.653101018	stage_E14_vs_E10	ordinary_muscle_development		0	FALSE
ENSGALG00010004333	DIO2	PRJNA729271	-1.555389112	7.16123E-06	1.555389112	stage_E14_vs_E10	ordinary_muscle_development	D_vs_E10;stage_1W_vs_E10;stage_3W_vs_E10;stage	1	TRUE
ENSGALG00010022805	ENSGALG00010022805	PRJNA729271	-1.312803395	7.20264E-06	1.312803395	stage_E14_vs_E10	ordinary_muscle_development	D_vs_E10;stage_1W_vs_E10;stage_3W_vs_E10;stage	1	TRUE
ENSGALG00010012401	RIPK2	PRJNA729271	0.603365927	7.20925E-06	0.603365927	stage_E14_vs_E10	ordinary_muscle_development		0	FALSE
ENSGALG00010023175	GRK2	PRJNA729271	0.631987775	7.21948E-06	0.631987775	stage_E14_vs_E10	ordinary_muscle_development		0	FALSE
ENSGALG00010022614	DAO	PRJNA729271	-0.95373483	7.27146E-06	0.95373483	stage_E14_vs_E10	ordinary_muscle_development		0	FALSE
ENSGALG00010008973	KCNK9	PRJNA729271	-4.298996491	7.28488E-06	4.298996491	stage_E14_vs_E10	ordinary_muscle_development	D_vs_E10;stage_1W_vs_E10;stage_3W_vs_E10;stage	1	TRUE
ENSGALG00010028829	GTF3C5	PRJNA729271	-0.52956718	7.28798E-06	0.52956718	stage_E14_vs_E10	ordinary_muscle_development		0	FALSE
ENSGALG00010005284	ENSGALG00010005284	PRJNA729271	0.968527837	7.32759E-06	0.968527837	stage_E14_vs_E10	ordinary_muscle_development		0	FALSE
ENSGALG00010024145	RAP1GAP3	PRJNA729271	0.851594461	7.33047E-06	0.851594461	stage_E14_vs_E10	ordinary_muscle_development		0	FALSE
ENSGALG00010022979	LAMA5	PRJNA729271	0.69481266	7.39309E-06	0.69481266	stage_E14_vs_E10	ordinary_muscle_development		0	FALSE
ENSGALG00010003539	ENSGALG00010003539	PRJNA729271	0.951538039	7.43713E-06	0.951538039	stage_E14_vs_E10	ordinary_muscle_development		0	FALSE
ENSGALG00010024205	ANKRD13D	PRJNA729271	0.773180459	7.47809E-06	0.773180459	stage_E14_vs_E10	ordinary_muscle_development		0	FALSE
ENSGALG00010017068	ZP2	PRJNA729271	-5.554404044	7.48861E-06	5.554404044	stage_E14_vs_E10	ordinary_muscle_development	D_vs_E10;stage_1W_vs_E10;stage_3W_vs_E10;stage	1	TRUE
ENSGALG00010000733	ENSGALG00010000733	PRJNA729271	-2.432036138	7.50752E-06	2.432036138	stage_E14_vs_E10	ordinary_muscle_development	D_vs_E10;stage_1W_vs_E10;stage_3W_vs_E10;stage	1	TRUE
ENSGALG00010014935	PCYT1A	PRJNA729271	0.613745957	7.52045E-06	0.613745957	stage_E14_vs_E10	ordinary_muscle_development		0	FALSE
ENSGALG00010013112	CD40	PRJNA729271	0.741017425	7.5255E-06	0.741017425	stage_E14_vs_E10	ordinary_muscle_development		0	FALSE
ENSGALG00010029186	ZNF302	PRJNA729271	0.642313701	7.52905E-06	0.642313701	stage_E14_vs_E10	ordinary_muscle_development		0	FALSE
ENSGALG00010004043	CAB39L	PRJNA729271	0.930339901	7.53368E-06	0.930339901	stage_E14_vs_E10	ordinary_muscle_development		0	FALSE
ENSGALG00010029256	RABEP1	PRJNA729271	0.444366937	7.53414E-06	0.444366937	stage_E14_vs_E10	ordinary_muscle_development		0	FALSE
ENSGALG00010008317	CEP57	PRJNA729271	-0.536369579	7.54634E-06	0.536369579	stage_E14_vs_E10	ordinary_muscle_development		0	FALSE
ENSGALG00010009557	CIDEC	PRJNA729271	4.877831506	7.57964E-06	4.877831506	stage_E14_vs_E10	ordinary_muscle_development	D_vs_E10;stage_1W_vs_E10;stage_3W_vs_E10;stage	1	TRUE
ENSGALG00010016901	MECOM	PRJNA729271	-0.926340708	7.62215E-06	0.926340708	stage_E14_vs_E10	ordinary_muscle_development		0	FALSE
ENSGALG00010029277	WFIKKN2	PRJNA729271	3.394610739	7.62365E-06	3.394610739	stage_E14_vs_E10	ordinary_muscle_development	D_vs_E10;stage_1W_vs_E10;stage_3W_vs_E10;stage	1	TRUE
ENSGALG00010024684	ENSGALG00010024684	PRJNA729271	-1.820119121	7.63669E-06	1.820119121	stage_E14_vs_E10	ordinary_muscle_development	D_vs_E10;stage_1W_vs_E10;stage_3W_vs_E10;stage	1	TRUE
ENSGALG00010012758	NAB1	PRJNA729271	-0.59401816	7.65161E-06	0.59401816	stage_E14_vs_E10	ordinary_muscle_development		0	FALSE
ENSGALG00010009646	MVD	PRJNA729271	0.768510213	7.68939E-06	0.768510213	stage_E14_vs_E10	ordinary_muscle_development		0	FALSE
ENSGALG00010023077	KANSL2	PRJNA729271	0.693705933	7.71942E-06	0.693705933	stage_E14_vs_E10	ordinary_muscle_development		0	FALSE
ENSGALG00010013640	SNPH	PRJNA729271	-3.996656009	7.72963E-06	3.996656009	stage_E14_vs_E10	ordinary_muscle_development	D_vs_E10;stage_1W_vs_E10;stage_3W_vs_E10;stage	1	TRUE
ENSGALG00010029239	RBFOX3	PRJNA729271	-3.322790641	7.73767E-06	3.322790641	stage_E14_vs_E10	ordinary_muscle_development	D_vs_E10;stage_1W_vs_E10;stage_3W_vs_E10;stage	1	TRUE
ENSGALG00010008039	CHMP1A	PRJNA729271	-0.423852997	7.73852E-06	0.423852997	stage_E14_vs_E10	ordinary_muscle_development		0	FALSE
ENSGALG00010022158	CWC25	PRJNA729271	-0.379185753	7.76017E-06	0.379185753	stage_E14_vs_E10	ordinary_muscle_development		0	FALSE
ENSGALG00010027412	ENSGALG00010027412	PRJNA729271	1.261192978	7.77257E-06	1.261192978	stage_E14_vs_E10	ordinary_muscle_development	D_vs_E10;stage_1W_vs_E10;stage_3W_vs_E10;stage	1	TRUE
ENSGALG00010024961	LSM12	PRJNA729271	0.930902125	7.78912E-06	0.930902125	stage_E14_vs_E10	ordinary_muscle_development		0	FALSE
ENSGALG00010022143	MATCAP1	PRJNA729271	-0.933865591	7.80354E-06	0.933865591	stage_E14_vs_E10	ordinary_muscle_development		0	FALSE
ENSGALG00010021246	PECR	PRJNA729271	0.526039642	7.81351E-06	0.526039642	stage_E14_vs_E10	ordinary_muscle_development		0	FALSE
ENSGALG00010008405	LRATD2	PRJNA729271	-0.687261767	7.82091E-06	0.687261767	stage_E14_vs_E10	ordinary_muscle_development		0	FALSE
ENSGALG00010021479	SKAP1	PRJNA729271	-2.154212442	7.859E-06	2.154212442	stage_E14_vs_E10	ordinary_muscle_development	D_vs_E10;stage_1W_vs_E10;stage_3W_vs_E10;stage	1	TRUE
ENSGALG00010011348	SLC22A23	PRJNA729271	0.74674626	7.86807E-06	0.74674626	stage_E14_vs_E10	ordinary_muscle_development		0	FALSE
ENSGALG00010022494	FHIP2A	PRJNA729271	-0.314847403	7.87078E-06	0.314847403	stage_E14_vs_E10	ordinary_muscle_development		0	FALSE
ENSGALG00010025541	TTC21B	PRJNA729271	-0.592497835	7.8849E-06	0.592497835	stage_E14_vs_E10	ordinary_muscle_development		0	FALSE
ENSGALG00010008618	EIF4EBP3	PRJNA729271	0.535211779	7.91281E-06	0.535211779	stage_E14_vs_E10	ordinary_muscle_development		0	FALSE
ENSGALG00010023736	KIF5A	PRJNA729271	-2.970271691	8.00202E-06	2.970271691	stage_E14_vs_E10	ordinary_muscle_development	D_vs_E10;stage_1W_vs_E10;stage_3W_vs_E10;stage	1	TRUE
ENSGALG00010008189	EXOC3	PRJNA729271	0.522853409	8.01698E-06	0.522853409	stage_E14_vs_E10	ordinary_muscle_development		0	FALSE
ENSGALG00010022071	ACTA2	PRJNA729271	-0.910089112	8.02888E-06	0.910089112	stage_E14_vs_E10	ordinary_muscle_development		0	FALSE
ENSGALG00010023364	CTRB1	PRJNA729271	10.14735755	8.0307E-06	10.14735755	stage_E14_vs_E10	ordinary_muscle_development	D_vs_E10;stage_1W_vs_E10;stage_3W_vs_E10;stage	1	TRUE
ENSGALG00010007506	RAB38	PRJNA729271	-3.027674408	8.05639E-06	3.027674408	stage_E14_vs_E10	ordinary_muscle_development	D_vs_E10;stage_1W_vs_E10;stage_3W_vs_E10;stage	1	TRUE
ENSGALG00010021924	ENSGALG00010021924	PRJNA729271	-2.575227304	8.07457E-06	2.575227304	stage_E14_vs_E10	ordinary_muscle_development	D_vs_E10;stage_1W_vs_E10;stage_3W_vs_E10;stage	1	TRUE
ENSGALG00010003921	COLEC12	PRJNA729271	-0.772993697	8.07791E-06	0.772993697	stage_E14_vs_E10	ordinary_muscle_development		0	FALSE
ENSGALG00010015198	NME5	PRJNA729271	0.99784345	8.08266E-06	0.99784345	stage_E14_vs_E10	ordinary_muscle_development		0	FALSE
ENSGALG00010021307	MAP3K9	PRJNA729271	-0.642257016	8.08552E-06	0.642257016	stage_E14_vs_E10	ordinary_muscle_development		0	FALSE
ENSGALG00010002353	CMTM8	PRJNA729271	0.630310201	8.17879E-06	0.630310201	stage_E14_vs_E10	ordinary_muscle_development		0	FALSE
ENSGALG00010015333	MED7	PRJNA729271	-0.317923273	8.18199E-06	0.317923273	stage_E14_vs_E10	ordinary_muscle_development		0	FALSE
ENSGALG00010030014	ENSGALG00010030014	PRJNA729271	-1.43799462	8.20353E-06	1.43799462	stage_E14_vs_E10	ordinary_muscle_development	D_vs_E10;stage_1W_vs_E10;stage_3W_vs_E10;stage	1	TRUE
ENSGALG00010015928	CAPN7	PRJNA729271	0.55773407	8.20655E-06	0.55773407	stage_E14_vs_E10	ordinary_muscle_development		0	FALSE
ENSGALG00010001672	ENSGALG00010001672	PRJNA729271	2.482870874	8.23084E-06	2.482870874	stage_E14_vs_E10	ordinary_muscle_development	D_vs_E10;stage_1W_vs_E10;stage_3W_vs_E10;stage	1	TRUE
ENSGALG00010028100	ENSGALG00010028100	PRJNA729271	2.02962321	8.24146E-06	2.02962321	stage_E14_vs_E10	ordinary_muscle_development	D_vs_E10;stage_1W_vs_E10;stage_3W_vs_E10;stage	1	TRUE
ENSGALG00010027174	SHISA5	PRJNA729271	-0.610246167	8.24469E-06	0.610246167	stage_E14_vs_E10	ordinary_muscle_development		0	FALSE
ENSGALG00010019859	BIN3	PRJNA729271	-0.630314415	8.27785E-06	0.630314415	stage_E14_vs_E10	ordinary_muscle_development		0	FALSE
ENSGALG00010009070	ENSGALG00010009070	PRJNA729271	-5.919026022	8.29481E-06	5.919026022	stage_E14_vs_E10	ordinary_muscle_development	D_vs_E10;stage_1W_vs_E10;stage_3W_vs_E10;stage	1	TRUE
ENSGALG00010009133	BOD1L1	PRJNA729271	0.595140764	8.34265E-06	0.595140764	stage_E14_vs_E10	ordinary_muscle_development		0	FALSE
ENSGALG00010005270	GSTZ1	PRJNA729271	-1.008580479	8.37711E-06	1.008580479	stage_E14_vs_E10	ordinary_muscle_development	D_vs_E10;stage_1W_vs_E10;stage_3W_vs_E10;stage	1	TRUE
ENSGALG00010026005	DERL3	PRJNA729271	0.521039074	8.42882E-06	0.521039074	stage_E14_vs_E10	ordinary_muscle_development		0	FALSE
ENSGALG00010015065	F11	PRJNA729271	5.523002118	8.43161E-06	5.523002118	stage_E14_vs_E10	ordinary_muscle_development	D_vs_E10;stage_1W_vs_E10;stage_3W_vs_E10;stage	1	TRUE
ENSGALG00010024713	RND2	PRJNA729271	-2.263021553	8.43298E-06	2.263021553	stage_E14_vs_E10	ordinary_muscle_development	D_vs_E10;stage_1W_vs_E10;stage_3W_vs_E10;stage	1	TRUE
ENSGALG00010028642	APH1A	PRJNA729271	0.633706857	8.47524E-06	0.633706857	stage_E14_vs_E10	ordinary_muscle_development		0	FALSE
ENSGALG00010015571	DUSP15	PRJNA729271	1.359761885	8.48018E-06	1.359761885	stage_E14_vs_E10	ordinary_muscle_development	D_vs_E10;stage_1W_vs_E10;stage_3W_vs_E10;stage	1	TRUE
ENSGALG00010023920	ENSGALG00010023920	PRJNA729271	-2.112540946	8.48582E-06	2.112540946	stage_E14_vs_E10	ordinary_muscle_development	D_vs_E10;stage_1W_vs_E10;stage_3W_vs_E10;stage	1	TRUE
ENSGALG00010024556	ENSGALG00010024556	PRJNA729271	-0.982019846	8.51002E-06	0.982019846	stage_E14_vs_E10	ordinary_muscle_development		0	FALSE
ENSGALG00010002315	ENSGALG00010002315	PRJNA729271	-0.82985838	8.5316E-06	0.82985838	stage_E14_vs_E10	ordinary_muscle_development		0	FALSE
ENSGALG00010009565	TMEM8B	PRJNA729271	1.005466026	8.54436E-06	1.005466026	stage_E14_vs_E10	ordinary_muscle_development	D_vs_E10;stage_1W_vs_E10;stage_3W_vs_E10;stage	1	TRUE
ENSGALG00010024602	PROCR	PRJNA729271	1.102972351	8.59412E-06	1.102972351	stage_E14_vs_E10	ordinary_muscle_development	D_vs_E10;stage_1W_vs_E10;stage_3W_vs_E10;stage	1	TRUE
ENSGALG00010020204	GSPT2	PRJNA729271	-0.403771899	8.66564E-06	0.403771899	stage_E14_vs_E10	ordinary_muscle_development		0	FALSE
ENSGALG00010017856	FASTKD5	PRJNA729271	0.528213376	8.67865E-06	0.528213376	stage_E14_vs_E10	ordinary_muscle_development		0	FALSE
ENSGALG00010004950	SLIRP	PRJNA729271	0.501879069	8.68668E-06	0.501879069	stage_E14_vs_E10	ordinary_muscle_development		0	FALSE
ENSGALG00010018530	FAM161A	PRJNA729271	-1.061170495	8.76241E-06	1.061170495	stage_E14_vs_E10	ordinary_muscle_development	D_vs_E10;stage_1W_vs_E10;stage_3W_vs_E10;stage	1	TRUE
ENSGALG00010000177	ENSGALG00010000177	PRJNA729271	0.953200033	8.76605E-06	0.953200033	stage_E14_vs_E10	ordinary_muscle_development		0	FALSE
ENSGALG00010025286	ITGA3	PRJNA729271	-0.815417014	8.76661E-06	0.815417014	stage_E14_vs_E10	ordinary_muscle_development		0	FALSE
ENSGALG00010011622	BACH1	PRJNA729271	0.736006226	8.768E-06	0.736006226	stage_E14_vs_E10	ordinary_muscle_development		0	FALSE
ENSGALG00010029582	SCPEP1	PRJNA729271	-0.693461918	8.77269E-06	0.693461918	stage_E14_vs_E10	ordinary_muscle_development		0	FALSE
ENSGALG00010016797	SEC62	PRJNA729271	-0.501009536	8.78769E-06	0.501009536	stage_E14_vs_E10	ordinary_muscle_development		0	FALSE
ENSGALG00010008000	C8orf82	PRJNA729271	-0.81058755	8.80171E-06	0.81058755	stage_E14_vs_E10	ordinary_muscle_development		0	FALSE
ENSGALG00010016813	ENSGALG00010016813	PRJNA729271	-2.795318734	8.81557E-06	2.795318734	stage_E14_vs_E10	ordinary_muscle_development	D_vs_E10;stage_1W_vs_E10;stage_3W_vs_E10;stage	1	TRUE
ENSGALG00010011641	ENSGALG00010011641	PRJNA729271	-3.937422055	8.89902E-06	3.937422055	stage_E14_vs_E10	ordinary_muscle_development	D_vs_E10;stage_1W_vs_E10;stage_3W_vs_E10;stage	1	TRUE
ENSGALG00010011954	ENSGALG00010011954	PRJNA729271	1.987449467	8.91823E-06	1.987449467	stage_E14_vs_E10	ordinary_muscle_development	D_vs_E10;stage_1W_vs_E10;stage_3W_vs_E10;stage	1	TRUE
ENSGALG00010021058	CCP110	PRJNA729271	-0.435029648	8.92731E-06	0.435029648	stage_E14_vs_E10	ordinary_muscle_development		0	FALSE
ENSGALG00010009457	BDP1	PRJNA729271	-0.604169193	8.93786E-06	0.604169193	stage_E14_vs_E10	ordinary_muscle_development		0	FALSE
ENSGALG00010012963	UIMC1	PRJNA729271	-0.427149555	8.95655E-06	0.427149555	stage_E14_vs_E10	ordinary_muscle_development		0	FALSE
ENSGALG00010005186	ESR1	PRJNA729271	0.87039001	8.97658E-06	0.87039001	stage_E14_vs_E10	ordinary_muscle_development		0	FALSE
ENSGALG00010018251	USP48	PRJNA729271	0.757990099	8.99389E-06	0.757990099	stage_E14_vs_E10	ordinary_muscle_development		0	FALSE
ENSGALG00010002332	ENSGALG00010002332	PRJNA729271	-2.924818079	9.00796E-06	2.924818079	stage_E14_vs_E10	ordinary_muscle_development	D_vs_E10;stage_1W_vs_E10;stage_3W_vs_E10;stage	1	TRUE
ENSGALG00010020485	LMAN2L	PRJNA729271	-0.613246942	9.00974E-06	0.613246942	stage_E14_vs_E10	ordinary_muscle_development		0	FALSE
ENSGALG00010007277	FAM151B	PRJNA729271	-0.880197806	9.0132E-06	0.880197806	stage_E14_vs_E10	ordinary_muscle_development		0	FALSE
ENSGALG00010002701	TMEM196	PRJNA729271	-3.495039308	9.02072E-06	3.495039308	stage_E14_vs_E10	ordinary_muscle_development	D_vs_E10;stage_1W_vs_E10;stage_3W_vs_E10;stage	1	TRUE
ENSGALG00010013411	ENSGALG00010013411	PRJNA729271	2.008443772	9.03831E-06	2.008443772	stage_E14_vs_E10	ordinary_muscle_development	D_vs_E10;stage_1W_vs_E10;stage_3W_vs_E10;stage	1	TRUE
ENSGALG00010022032	KBTBD4	PRJNA729271	0.373278866	9.05813E-06	0.373278866	stage_E14_vs_E10	ordinary_muscle_development		0	FALSE
ENSGALG00010008476	RNF166	PRJNA729271	0.614597208	9.07552E-06	0.614597208	stage_E14_vs_E10	ordinary_muscle_development		0	FALSE
ENSGALG00010028740	DAZAP1	PRJNA729271	-0.487287518	9.14644E-06	0.487287518	stage_E14_vs_E10	ordinary_muscle_development		0	FALSE
ENSGALG00010020835	PTGR2	PRJNA729271	0.544528938	9.2186E-06	0.544528938	stage_E14_vs_E10	ordinary_muscle_development		0	FALSE
ENSGALG00010005548	EZR	PRJNA729271	-0.822541625	9.22567E-06	0.822541625	stage_E14_vs_E10	ordinary_muscle_development		0	FALSE
ENSGALG00010028006	ENSGALG00010028006	PRJNA729271	3.650866413	9.33812E-06	3.650866413	stage_E14_vs_E10	ordinary_muscle_development	D_vs_E10;stage_1W_vs_E10;stage_3W_vs_E10;stage	1	TRUE
ENSGALG00010011911	NUDT2	PRJNA729271	0.63574294	9.34751E-06	0.63574294	stage_E14_vs_E10	ordinary_muscle_development		0	FALSE
ENSGALG00010000150	B3GAT3	PRJNA729271	0.581549247	9.35749E-06	0.581549247	stage_E14_vs_E10	ordinary_muscle_development		0	FALSE
ENSGALG00010027557	C6orf132	PRJNA729271	-4.983600697	9.39684E-06	4.983600697	stage_E14_vs_E10	ordinary_muscle_development	D_vs_E10;stage_1W_vs_E10;stage_3W_vs_E10;stage	1	TRUE
ENSGALG00010003909	DNAJB1	PRJNA729271	-0.733122114	9.42715E-06	0.733122114	stage_E14_vs_E10	ordinary_muscle_development		0	FALSE
ENSGALG00010024693	ANXA2	PRJNA729271	0.930044913	9.43129E-06	0.930044913	stage_E14_vs_E10	ordinary_muscle_development		0	FALSE
ENSGALG00010022316	INO80	PRJNA729271	0.315868934	9.45563E-06	0.315868934	stage_E14_vs_E10	ordinary_muscle_development		0	FALSE
ENSGALG00010005843	GPR12	PRJNA729271	-2.138631304	9.46337E-06	2.138631304	stage_E14_vs_E10	ordinary_muscle_development	D_vs_E10;stage_1W_vs_E10;stage_3W_vs_E10;stage	1	TRUE
ENSGALG00010001771	ENSGALG00010001771	PRJNA729271	0.615283836	9.47497E-06	0.615283836	stage_E14_vs_E10	ordinary_muscle_development		0	FALSE
ENSGALG00010022456	ATP5G3	PRJNA729271	0.719672978	9.48808E-06	0.719672978	stage_E14_vs_E10	ordinary_muscle_development		0	FALSE
ENSGALG00010004188	BMI1	PRJNA729271	-0.531080117	9.53439E-06	0.531080117	stage_E14_vs_E10	ordinary_muscle_development		0	FALSE
ENSGALG00010020648	TET3	PRJNA729271	-0.687131763	9.53911E-06	0.687131763	stage_E14_vs_E10	ordinary_muscle_development		0	FALSE
ENSGALG00010018783	ENSGALG00010018783	PRJNA729271	-0.820865312	9.5958E-06	0.820865312	stage_E14_vs_E10	ordinary_muscle_development		0	FALSE
ENSGALG00010013296	AGXT2	PRJNA729271	-2.219771289	9.67206E-06	2.219771289	stage_E14_vs_E10	ordinary_muscle_development	D_vs_E10;stage_1W_vs_E10;stage_3W_vs_E10;stage	1	TRUE
ENSGALG00010012633	USP18	PRJNA729271	2.464280253	9.68405E-06	2.464280253	stage_E14_vs_E10	ordinary_muscle_development	D_vs_E10;stage_1W_vs_E10;stage_3W_vs_E10;stage	1	TRUE
ENSGALG00010015172	PPP4R3A	PRJNA729271	0.348942462	9.71274E-06	0.348942462	stage_E14_vs_E10	ordinary_muscle_development		0	FALSE
ENSGALG00010016429	ENSGALG00010016429	PRJNA729271	-2.308640549	9.7647E-06	2.308640549	stage_E14_vs_E10	ordinary_muscle_development	D_vs_E10;stage_1W_vs_E10;stage_3W_vs_E10;stage	1	TRUE
ENSGALG00010020533	ENSGALG00010020533	PRJNA729271	-0.45538818	9.77541E-06	0.45538818	stage_E14_vs_E10	ordinary_muscle_development		0	FALSE
ENSGALG00010028581	NR2F6	PRJNA729271	0.588634247	9.9249E-06	0.588634247	stage_E14_vs_E10	ordinary_muscle_development		0	FALSE
ENSGALG00010014135	ZBTB24	PRJNA729271	0.694510443	9.9375E-06	0.694510443	stage_E14_vs_E10	ordinary_muscle_development		0	FALSE
ENSGALG00010020059	PRKCZ	PRJNA729271	-0.669280188	9.99579E-06	0.669280188	stage_E14_vs_E10	ordinary_muscle_development		0	FALSE
ENSGALG00010028588	ABHD8	PRJNA729271	-3.980224986	1.00065E-05	3.980224986	stage_E14_vs_E10	ordinary_muscle_development	D_vs_E10;stage_1W_vs_E10;stage_3W_vs_E10;stage	1	TRUE
ENSGALG00010005504	LATS1	PRJNA729271	-0.36427559	1.00319E-05	0.36427559	stage_E14_vs_E10	ordinary_muscle_development		0	FALSE

ENSGALG00010001171	POLR1E	PRJNA729271	-0.590343051	1.00444E-05	0.590343051	stage_E14_vs_E10	ordinary_muscle_development		0	FALSE
ENSGALG00010019540	UNC5D	PRJNA729271	-1.8040944	1.00589E-05	1.8040944	stage_E14_vs_E10	ordinary_muscle_development	D_vs_E10;stage_1W_vs_E10;stage_3W_vs_E10;stage	1	TRUE
ENSGALG00010027247	ATG7	PRJNA729271	-0.378796073	1.00603E-05	0.378796073	stage_E14_vs_E10	ordinary_muscle_development		0	FALSE
ENSGALG00010021480	ENSGALG00010021480	PRJNA729271	-0.350647734	1.00799E-05	0.350647734	stage_E14_vs_E10	ordinary_muscle_development		0	FALSE
ENSGALG00010025799	SC5D	PRJNA729271	1.205021111	1.02519E-05	1.205021111	stage_E14_vs_E10	ordinary_muscle_development	D_vs_E10;stage_1W_vs_E10;stage_3W_vs_E10;stage	1	TRUE
ENSGALG00010021439	ITPRIP	PRJNA729271	-0.888387784	1.02592E-05	0.888387784	stage_E14_vs_E10	ordinary_muscle_development		0	FALSE
ENSGALG00010006346	NSUN3	PRJNA729271	0.587500646	1.03111E-05	0.587500646	stage_E14_vs_E10	ordinary_muscle_development		0	FALSE
ENSGALG00010009738	ENSGALG00010009738	PRJNA729271	-1.187173362	1.03729E-05	1.187173362	stage_E14_vs_E10	ordinary_muscle_development	D_vs_E10;stage_1W_vs_E10;stage_3W_vs_E10;stage	1	TRUE
ENSGALG00010004369	UBE2M	PRJNA729271	1.057367105	1.04505E-05	1.057367105	stage_E14_vs_E10	ordinary_muscle_development	D_vs_E10;stage_1W_vs_E10;stage_3W_vs_E10;stage	1	TRUE
ENSGALG00010018128	SEPTIN12	PRJNA729271	-0.904272977	1.04505E-05	0.904272977	stage_E14_vs_E10	ordinary_muscle_development		0	FALSE
ENSGALG00010010575	PAXBP1	PRJNA729271	0.556139109	1.04677E-05	0.556139109	stage_E14_vs_E10	ordinary_muscle_development		0	FALSE
ENSGALG00010012447	COPS8	PRJNA729271	-0.54118932	1.04861E-05	0.54118932	stage_E14_vs_E10	ordinary_muscle_development		0	FALSE
ENSGALG00010028579	OCLN	PRJNA729271	1.226136397	1.05246E-05	1.226136397	stage_E14_vs_E10	ordinary_muscle_development	D_vs_E10;stage_1W_vs_E10;stage_3W_vs_E10;stage	1	TRUE
ENSGALG00010011119	ENSGALG00010011119	PRJNA729271	-1.349901351	1.05574E-05	1.349901351	stage_E14_vs_E10	ordinary_muscle_development	D_vs_E10;stage_1W_vs_E10;stage_3W_vs_E10;stage	1	TRUE
ENSGALG00010023311	ETS1	PRJNA729271	-0.607043768	1.05794E-05	0.607043768	stage_E14_vs_E10	ordinary_muscle_development		0	FALSE
ENSGALG00010019865	ENSGALG00010019865	PRJNA729271	0.610167384	1.06033E-05	0.610167384	stage_E14_vs_E10	ordinary_muscle_development		0	FALSE
ENSGALG00010003852	TCEB1	PRJNA729271	-0.490633758	1.06333E-05	0.490633758	stage_E14_vs_E10	ordinary_muscle_development		0	FALSE
ENSGALG00010028198	UCK1	PRJNA729271	-0.433910557	1.0658E-05	0.433910557	stage_E14_vs_E10	ordinary_muscle_development		0	FALSE
ENSGALG00010013093	GPATCH3	PRJNA729271	0.706985836	1.06784E-05	0.706985836	stage_E14_vs_E10	ordinary_muscle_development		0	FALSE
ENSGALG00010001952	ENSGALG00010001952	PRJNA729271	0.32872624	1.06825E-05	0.32872624	stage_E14_vs_E10	ordinary_muscle_development		0	FALSE
ENSGALG00010014916	DYNLT2B	PRJNA729271	-0.718751933	1.06923E-05	0.718751933	stage_E14_vs_E10	ordinary_muscle_development		0	FALSE
ENSGALG00010021605	MAP3K3	PRJNA729271	-0.533533284	1.07071E-05	0.533533284	stage_E14_vs_E10	ordinary_muscle_development		0	FALSE
ENSGALG00010000123	ENSGALG00010000123	PRJNA729271	1.059572436	1.07195E-05	1.059572436	stage_E14_vs_E10	ordinary_muscle_development	D_vs_E10;stage_1W_vs_E10;stage_3W_vs_E10;stage	1	TRUE
ENSGALG00010007997	TMEM192	PRJNA729271	-0.634235214	1.07516E-05	0.634235214	stage_E14_vs_E10	ordinary_muscle_development		0	FALSE
ENSGALG00010010275	PDE8B	PRJNA729271	-4.962009749	1.07611E-05	4.962009749	stage_E14_vs_E10	ordinary_muscle_development	D_vs_E10;stage_1W_vs_E10;stage_3W_vs_E10;stage	1	TRUE
ENSGALG00010020362	CDS2	PRJNA729271	-0.577027089	1.07768E-05	0.577027089	stage_E14_vs_E10	ordinary_muscle_development		0	FALSE
ENSGALG00010015050	HIVEP3	PRJNA729271	-0.817794833	1.0839E-05	0.817794833	stage_E14_vs_E10	ordinary_muscle_development		0	FALSE
ENSGALG00010011759	CREB3	PRJNA729271	0.588673566	1.08461E-05	0.588673566	stage_E14_vs_E10	ordinary_muscle_development		0	FALSE
ENSGALG00010012933	ZCCHC17	PRJNA729271	-0.673877221	1.08513E-05	0.673877221	stage_E14_vs_E10	ordinary_muscle_development		0	FALSE
ENSGALG00010026834	ENSGALG00010026834	PRJNA729271	9.144563585	1.0969E-05	9.144563585	stage_E14_vs_E10	ordinary_muscle_development	D_vs_E10;stage_1W_vs_E10;stage_3W_vs_E10;stage	1	TRUE
ENSGALG00010004029	ARL4A	PRJNA729271	0.921214421	1.10377E-05	0.921214421	stage_E14_vs_E10	ordinary_muscle_development		0	FALSE
ENSGALG00010005817	VTA1	PRJNA729271	-0.319320121	1.10668E-05	0.319320121	stage_E14_vs_E10	ordinary_muscle_development		0	FALSE
ENSGALG00010013121	PCBD2	PRJNA729271	-0.761049946	1.10787E-05	0.761049946	stage_E14_vs_E10	ordinary_muscle_development		0	FALSE
ENSGALG00010023909	FOXRED1	PRJNA729271	0.688366096	1.11034E-05	0.688366096	stage_E14_vs_E10	ordinary_muscle_development		0	FALSE
ENSGALG00010012851	ENSGALG00010012851	PRJNA729271	-1.223303109	1.11508E-05	1.223303109	stage_E14_vs_E10	ordinary_muscle_development	D_vs_E10;stage_1W_vs_E10;stage_3W_vs_E10;stage	1	TRUE
ENSGALG00010014914	PTAR1	PRJNA729271	0.811613441	1.1159E-05	0.811613441	stage_E14_vs_E10	ordinary_muscle_development		0	FALSE
ENSGALG00010018082	RAB11FIP3	PRJNA729271	-0.838523318	1.13073E-05	0.838523318	stage_E14_vs_E10	ordinary_muscle_development		0	FALSE
ENSGALG00010009163	SAMD12	PRJNA729271	-0.811242079	1.14109E-05	0.811242079	stage_E14_vs_E10	ordinary_muscle_development		0	FALSE
ENSGALG00010004503	ENSGALG00010004503	PRJNA729271	0.84643514	1.14167E-05	0.84643514	stage_E14_vs_E10	ordinary_muscle_development		0	FALSE
ENSGALG00010024944	ENSGALG00010024944	PRJNA729271	0.504680515	1.1417E-05	0.504680515	stage_E14_vs_E10	ordinary_muscle_development		0	FALSE
ENSGALG00010008048	ENSGALG00010008048	PRJNA729271	3.355964401	1.14208E-05	3.355964401	stage_E14_vs_E10	ordinary_muscle_development	D_vs_E10;stage_1W_vs_E10;stage_3W_vs_E10;stage	1	TRUE
ENSGALG00010028511	YJEFN3	PRJNA729271	1.767790249	1.14369E-05	1.767790249	stage_E14_vs_E10	ordinary_muscle_development	D_vs_E10;stage_1W_vs_E10;stage_3W_vs_E10;stage	1	TRUE
ENSGALG00010002598	NMT2	PRJNA729271	0.460064525	1.14415E-05	0.460064525	stage_E14_vs_E10	ordinary_muscle_development		0	FALSE
ENSGALG00010017528	RAI1	PRJNA729271	0.807270016	1.14456E-05	0.807270016	stage_E14_vs_E10	ordinary_muscle_development		0	FALSE
ENSGALG00010024579	ZNF740	PRJNA729271	-0.481213806	1.15004E-05	0.481213806	stage_E14_vs_E10	ordinary_muscle_development		0	FALSE
ENSGALG00010001554	ENSGALG00010001554	PRJNA729271	0.834465391	1.1515E-05	0.834465391	stage_E14_vs_E10	ordinary_muscle_development		0	FALSE
ENSGALG00010001191	TOMM5	PRJNA729271	0.69089232	1.15486E-05	0.69089232	stage_E14_vs_E10	ordinary_muscle_development		0	FALSE
ENSGALG00010025506	R3HDM2	PRJNA729271	0.797109134	1.16063E-05	0.797109134	stage_E14_vs_E10	ordinary_muscle_development		0	FALSE
ENSGALG00010009822	ENSGALG00010009822	PRJNA729271	-0.923928151	1.16808E-05	0.923928151	stage_E14_vs_E10	ordinary_muscle_development		0	FALSE
ENSGALG00010012921	EBF1	PRJNA729271	-0.635845869	1.17262E-05	0.635845869	stage_E14_vs_E10	ordinary_muscle_development		0	FALSE
ENSGALG00010010037	NAA15	PRJNA729271	-0.633520738	1.17476E-05	0.633520738	stage_E14_vs_E10	ordinary_muscle_development		0	FALSE
ENSGALG00010012978	HSD17B7	PRJNA729271	-0.967574148	1.178E-05	0.967574148	stage_E14_vs_E10	ordinary_muscle_development		0	FALSE
ENSGALG00010022957	SLTM	PRJNA729271	-0.657215669	1.17853E-05	0.657215669	stage_E14_vs_E10	ordinary_muscle_development		0	FALSE
ENSGALG00010007993	LRRC14	PRJNA729271	0.771752493	1.17883E-05	0.771752493	stage_E14_vs_E10	ordinary_muscle_development		0	FALSE
ENSGALG00010009393	MARVELD2	PRJNA729271	-0.608232567	1.17894E-05	0.608232567	stage_E14_vs_E10	ordinary_muscle_development		0	FALSE
ENSGALG00010000161	CDC42EP2	PRJNA729271	1.413730651	1.1827E-05	1.413730651	stage_E14_vs_E10	ordinary_muscle_development	D_vs_E10;stage_1W_vs_E10;stage_3W_vs_E10;stage	1	TRUE
ENSGALG00010011650	RPL30	PRJNA729271	0.709435504	1.18435E-05	0.709435504	stage_E14_vs_E10	ordinary_muscle_development		0	FALSE
ENSGALG00010014105	AHR1B	PRJNA729271	-3.667081172	1.18563E-05	3.667081172	stage_E14_vs_E10	ordinary_muscle_development	D_vs_E10;stage_1W_vs_E10;stage_3W_vs_E10;stage	1	TRUE
ENSGALG00010027348	ENSGALG00010027348	PRJNA729271	0.925883703	1.18568E-05	0.925883703	stage_E14_vs_E10	ordinary_muscle_development		0	FALSE
ENSGALG00010016122	AFG2B	PRJNA729271	0.587256466	1.19051E-05	0.587256466	stage_E14_vs_E10	ordinary_muscle_development		0	FALSE
ENSGALG00010013526	SIRPA	PRJNA729271	1.02558432	1.19337E-05	1.02558432	stage_E14_vs_E10	ordinary_muscle_development	D_vs_E10;stage_1W_vs_E10;stage_3W_vs_E10;stage	1	TRUE
ENSGALG00010001788	HOXA1	PRJNA729271	-2.019894221	1.19734E-05	2.019894221	stage_E14_vs_E10	ordinary_muscle_development	D_vs_E10;stage_1W_vs_E10;stage_3W_vs_E10;stage	1	TRUE
ENSGALG00010024763	HOXD9	PRJNA729271	-1.444678897	1.19828E-05	1.444678897	stage_E14_vs_E10	ordinary_muscle_development	D_vs_E10;stage_1W_vs_E10;stage_3W_vs_E10;stage	1	TRUE
ENSGALG00010016346	TRAP1	PRJNA729271	-0.475185196	1.1985E-05	0.475185196	stage_E14_vs_E10	ordinary_muscle_development		0	FALSE
ENSGALG00010012180	DDX59	PRJNA729271	-0.485059794	1.19918E-05	0.485059794	stage_E14_vs_E10	ordinary_muscle_development		0	FALSE
ENSGALG00010015138	ENSGALG00010015138	PRJNA729271	-2.291671259	1.20069E-05	2.291671259	stage_E14_vs_E10	ordinary_muscle_development	D_vs_E10;stage_1W_vs_E10;stage_3W_vs_E10;stage	1	TRUE
ENSGALG00010008335	HNRNPUL1	PRJNA729271	-0.862170419	1.20539E-05	0.862170419	stage_E14_vs_E10	ordinary_muscle_development		0	FALSE
ENSGALG00010019023	AP2M1	PRJNA729271	-0.322361901	1.21083E-05	0.322361901	stage_E14_vs_E10	ordinary_muscle_development		0	FALSE
ENSGALG00010029395	DPH1	PRJNA729271	-0.549786888	1.21822E-05	0.549786888	stage_E14_vs_E10	ordinary_muscle_development		0	FALSE
ENSGALG00010020074	ENSGALG00010020074	PRJNA729271	-0.944484065	1.22118E-05	0.944484065	stage_E14_vs_E10	ordinary_muscle_development		0	FALSE
ENSGALG00010022066	DAD1	PRJNA729271	-0.630494473	1.22437E-05	0.630494473	stage_E14_vs_E10	ordinary_muscle_development		0	FALSE
ENSGALG00010022462	ITGA5	PRJNA729271	0.588709906	1.22907E-05	0.588709906	stage_E14_vs_E10	ordinary_muscle_development		0	FALSE
ENSGALG00010001169	ENSGALG00010001169	PRJNA729271	-0.468355642	1.23705E-05	0.468355642	stage_E14_vs_E10	ordinary_muscle_development		0	FALSE
ENSGALG00010015208	GNA14	PRJNA729271	2.576796094	1.24204E-05	2.576796094	stage_E14_vs_E10	ordinary_muscle_development	D_vs_E10;stage_1W_vs_E10;stage_3W_vs_E10;stage	1	TRUE
ENSGALG00010003388	PXDN	PRJNA729271	-0.80492711	1.25048E-05	0.80492711	stage_E14_vs_E10	ordinary_muscle_development		0	FALSE
ENSGALG00010013152	TBXAS1	PRJNA729271	-1.052855687	1.25134E-05	1.052855687	stage_E14_vs_E10	ordinary_muscle_development	D_vs_E10;stage_1W_vs_E10;stage_3W_vs_E10;stage	1	TRUE
ENSGALG00010003762	EVI5L	PRJNA729271	0.46343352	1.25183E-05	0.46343352	stage_E14_vs_E10	ordinary_muscle_development		0	FALSE
ENSGALG00010008403	GNAT3	PRJNA729271	-1.526742436	1.25396E-05	1.526742436	stage_E14_vs_E10	ordinary_muscle_development	D_vs_E10;stage_1W_vs_E10;stage_3W_vs_E10;stage	1	TRUE
ENSGALG00010026522	MED20	PRJNA729271	0.404752736	1.25396E-05	0.404752736	stage_E14_vs_E10	ordinary_muscle_development		0	FALSE
ENSGALG00010005319	NOL4	PRJNA729271	-4.561646291	1.25558E-05	4.561646291	stage_E14_vs_E10	ordinary_muscle_development	D_vs_E10;stage_1W_vs_E10;stage_3W_vs_E10;stage	1	TRUE
ENSGALG00010008273	TMEM144	PRJNA729271	0.545735524	1.26034E-05	0.545735524	stage_E14_vs_E10	ordinary_muscle_development		0	FALSE
ENSGALG00010016456	LAMP3	PRJNA729271	-1.749609056	1.26105E-05	1.749609056	stage_E14_vs_E10	ordinary_muscle_development	D_vs_E10;stage_1W_vs_E10;stage_3W_vs_E10;stage	1	TRUE
ENSGALG00010025723	SETD2	PRJNA729271	0.551538299	1.26201E-05	0.551538299	stage_E14_vs_E10	ordinary_muscle_development		0	FALSE
ENSGALG00010000411	ENSGALG00010000411	PRJNA729271	4.000236194	1.27043E-05	4.000236194	stage_E14_vs_E10	ordinary_muscle_development	D_vs_E10;stage_1W_vs_E10;stage_3W_vs_E10;stage	1	TRUE
ENSGALG00010002338	CD99	PRJNA729271	0.431362135	1.27126E-05	0.431362135	stage_E14_vs_E10	ordinary_muscle_development		0	FALSE
ENSGALG00010020520	ARHGAP22	PRJNA729271	-0.9154154	1.27538E-05	0.9154154	stage_E14_vs_E10	ordinary_muscle_development		0	FALSE
ENSGALG00010010761	MEI1	PRJNA729271	1.170381054	1.27801E-05	1.170381054	stage_E14_vs_E10	ordinary_muscle_development	D_vs_E10;stage_1W_vs_E10;stage_3W_vs_E10;stage	1	TRUE
ENSGALG00010016949	LUC7L	PRJNA729271	0.421504628	1.29406E-05	0.421504628	stage_E14_vs_E10	ordinary_muscle_development		0	FALSE
ENSGALG00010013815	SRXN1	PRJNA729271	1.117111449	1.29457E-05	1.117111449	stage_E14_vs_E10	ordinary_muscle_development	D_vs_E10;stage_1W_vs_E10;stage_3W_vs_E10;stage	1	TRUE
ENSGALG00010003520	ENSGALG00010003520	PRJNA729271	7.242863496	1.29577E-05	7.242863496	stage_E14_vs_E10	ordinary_muscle_development	D_vs_E10;stage_1W_vs_E10;stage_3W_vs_E10;stage	1	TRUE
ENSGALG00010001929	POLR2I	PRJNA729271	-0.571331276	1.30129E-05	0.571331276	stage_E14_vs_E10	ordinary_muscle_development		0	FALSE
ENSGALG00010023887	RASA2	PRJNA729271	-0.602642316	1.30805E-05	0.602642316	stage_E14_vs_E10	ordinary_muscle_development		0	FALSE
ENSGALG00010007882	CPE	PRJNA729271	-1.019493044	1.31334E-05	1.019493044	stage_E14_vs_E10	ordinary_muscle_development	D_vs_E10;stage_1W_vs_E10;stage_3W_vs_E10;stage	1	TRUE
ENSGALG00010001848	HOXA11	PRJNA729271	-1.680589437	1.31387E-05	1.680589437	stage_E14_vs_E10	ordinary_muscle_development	D_vs_E10;stage_1W_vs_E10;stage_3W_vs_E10;stage	1	TRUE
ENSGALG00010004148	ENSGALG00010004148	PRJNA729271	0.688569794	1.31387E-05	0.688569794	stage_E14_vs_E10	ordinary_muscle_development		0	FALSE
ENSGALG00010004058	TMEM30A	PRJNA729271	-0.278526011	1.32608E-05	0.278526011	stage_E14_vs_E10	ordinary_muscle_development		0	FALSE
ENSGALG00010021905	RBSN	PRJNA729271	-0.342099028	1.33129E-05	0.342099028	stage_E14_vs_E10	ordinary_muscle_development		0	FALSE
ENSGALG00010004999	ADCK1	PRJNA729271	-0.908371084	1.33411E-05	0.908371084	stage_E14_vs_E10	ordinary_muscle_development		0	FALSE
ENSGALG00010021214	FBXL18	PRJNA729271	-0.533007253	1.34946E-05	0.533007253	stage_E14_vs_E10	ordinary_muscle_development		0	FALSE
ENSGALG00010005249	PDGFD	PRJNA729271	-1.024396902	1.35476E-05	1.024396902	stage_E14_vs_E10	ordinary_muscle_development	D_vs_E10;stage_1W_vs_E10;stage_3W_vs_E10;stage	1	TRUE
ENSGALG00010024801	CXCR5	PRJNA729271	4.586720453	1.36392E-05	4.586720453	stage_E14_vs_E10	ordinary_muscle_development	D_vs_E10;stage_1W_vs_E10;stage_3W_vs_E10;stage	1	TRUE
ENSGALG00010014599	SSTR3	PRJNA729271	-2.00893241	1.36665E-05	2.00893241	stage_E14_vs_E10	ordinary_muscle_development	D_vs_E10;stage_1W_vs_E10;stage_3W_vs_E10;stage	1	TRUE
ENSGALG00010005829	NPAS3	PRJNA729271	-0.623187846	1.36908E-05	0.623187846	stage_E14_vs_E10	ordinary_muscle_development		0	FALSE
ENSGALG00010010987	MARCHF7	PRJNA729271	-0.363333763	1.36908E-05	0.363333763	stage_E14_vs_E10	ordinary_muscle_development		0	FALSE
ENSGALG00010029342	TMC6	PRJNA729271	-0.589387878	1.37056E-05	0.589387878	stage_E14_vs_E10	ordinary_muscle_development		0	FALSE
ENSGALG00010019682	TMEM158	PRJNA729271	-1.665542365	1.37229E-05	1.665542365	stage_E14_vs_E10	ordinary_muscle_development	D_vs_E10;stage_1W_vs_E10;stage_3W_vs_E10;stage	1	TRUE
ENSGALG00010018076	FOXA2	PRJNA729271	-7.047778292	1.37612E-05	7.047778292	stage_E14_vs_E10	ordinary_muscle_development	D_vs_E10;stage_1W_vs_E10;stage_3W_vs_E10;stage	1	TRUE
ENSGALG00010023130	DYNLT5	PRJNA729271	-4.240505195	1.37698E-05	4.240505195	stage_E14_vs_E10	ordinary_muscle_development	D_vs_E10;stage_1W_vs_E10;stage_3W_vs_E10;stage	1	TRUE
ENSGALG00010022152	ZNF644	PRJNA729271	-0.451148803	1.38099E-05	0.451148803	stage_E14_vs_E10	ordinary_muscle_development		0	FALSE
ENSGALG00010005532	GTPBP4	PRJNA729271	0.434313115	1.39321E-05	0.434313115	stage_E14_vs_E10	ordinary_muscle_development		0	FALSE
ENSGALG00010025939	TMEM136-1	PRJNA729271	-3.981735909	1.39422E-05	3.981735909	stage_E14_vs_E10	ordinary_muscle_development	D_vs_E10;stage_1W_vs_E10;stage_3W_vs_E10;stage	1	TRUE
ENSGALG00010002511	VPS36	PRJNA729271	-0.348000234	1.3957E-05	0.348000234	stage_E14_vs_E10	ordinary_muscle_development		0	FALSE
ENSGALG00010021144	FOXK1	PRJNA729271	0.484491468	1.40418E-05	0.484491468	stage_E14_vs_E10	ordinary_muscle_development		0	FALSE
ENSGALG00010028602	ZNF687	PRJNA729271	0.753829807	1.40653E-05	0.753829807	stage_E14_vs_E10	ordinary_muscle_development		0	FALSE
ENSGALG00010029717	TRARG1	PRJNA729271	-2.914264126	1.40678E-05	2.914264126	stage_E14_vs_E10	ordinary_muscle_development	D_vs_E10;stage_1W_vs_E10;stage_3W_vs_E10;stage	1	TRUE
ENSGALG00010026143	RNPEP	PRJNA729271	0.63347105	1.40952E-05	0.63347105	stage_E14_vs_E10	ordinary_muscle_development		0	FALSE
ENSGALG00010022517	HNRNPA1	PRJNA729271	-0.811097594	1.4104E-05	0.811097594	stage_E14_vs_E10	ordinary_muscle_development		0	FALSE
ENSGALG00010018971	POLR1B	PRJNA729271	-0.447538323	1.43023E-05	0.447538323	stage_E14_vs_E10	ordinary_muscle_development		0	FALSE
ENSGALG00010003068	BRD2	PRJNA729271	0.563374356	1.43307E-05	0.563374356	stage_E14_vs_E10	ordinary_muscle_development		0	FALSE
ENSGALG00010021017	COQ7	PRJNA729271	0.710355018	1.437E-05	0.710355018	stage_E14_vs_E10	ordinary_muscle_development		0	FALSE
ENSGALG00010008825	PPID	PRJNA729271	-0.490974594	1.44135E-05	0.490974594	stage_E14_vs_E10	ordinary_muscle_development		0	FALSE
ENSGALG00010008181	DISC1	PRJNA729271	-0.610169977	1.44561E-05	0.610169977	stage_E14_vs_E10	ordinary_muscle_development		0	FALSE
ENSGALG00010029590	UTP6	PRJNA729271	-0.448781551	1.44586E-05	0.448781551	stage_E14_vs_E10	ordinary_muscle_development		0	FALSE
ENSGALG00010021202	UROC1	PRJNA729271	-3.078727912	1.44758E-05	3.078727912	stage_E14_vs_E10	ordinary_muscle_development	D_vs_E10;stage_1W_vs_E10;stage_3W_vs_E10;stage	1	TRUE
ENSGALG00010013797	IL1RL1	PRJNA729271	1.407996022	1.44935E-05	1.407996022	stage_E14_vs_E10	ordinary_muscle_development	D_vs_E10;stage_1W_vs_E10;stage_3W_vs_E10;stage	1	TRUE
ENSGALG00010000864	MZT1	PRJNA729271	-0.581607354	1.45585E-05	0.581607354	stage_E14_vs_E10	ordinary_muscle_development		0	FALSE
ENSGALG00010005342	MAP3K5	PRJNA729271	0.647128919	1.45638E-05	0.647128919	stage_E14_vs_E10	ordinary_muscle_development		0	FALSE
ENSGALG00010024354	C2CD2L	PRJNA729271	-0.363905085	1.45983E-05	0.363905085	stage_E14_vs_E10	ordinary_muscle_development		0	FALSE
ENSGALG00010013719	NCDN	PRJNA729271	-0.643055228	1.46066E-05	0.643055228	stage_E14_vs_E10	ordinary_muscle_development		0	FALSE
ENSGALG00010005590	TBC1D23	PRJNA729271	-0.255209021	1.46319E-05	0.255209021	stage_E14_vs_E10	ordinary_muscle_development		0	FALSE
ENSGALG00010003819	PHIP	PRJNA729271	-0.427848007	1.46325E-05	0.427848007	stage_E14_vs_E10	ordinary_muscle_development		0	FALSE
ENSGALG00010015154	CYP24A1	PRJNA729271	-4.00925646	1.46824E-05	4.00925646	stage_E14_vs_E10	ordinary_muscle_development	D_vs_E10;stage_1W_vs_E10;stage_3W_vs_E10;stage	1	TRUE
ENSGALG00010008279	MAPK15	PRJNA729271	-0.777135262	1.47108E-05	0.777135262	stage_E14_vs_E10	ordinary_muscle_development		0	FALSE
ENSGALG00010002395	CLN8	PRJNA729271	0.409444736	1.49179E-05	0.409444736	stage_E14_vs_E10	ordinary_muscle_development		0	FALSE
ENSGALG00010027401	ENSGALG00010027401	PRJNA729271	1.393181095	1.49284E-05	1.393181095	stage_E14_vs_E10	ordinary_muscle_development	D_vs_E10;stage_1W_vs_E10;stage_3W_vs_E10;stage	1	TRUE
ENSGALG00010011510	ENSGALG00010011510	PRJNA729271	1.158187998	1.49462E-05	1.158187998	stage_E14_vs_E10	ordinary_muscle_development	D_vs_E10;stage_1W_vs_E10;stage_3W_vs_E10;stage	1	TRUE
ENSGALG00010009223	ENSGALG00010009223	PRJNA729271	0.490390216	1.49578E-05	0.490390216	stage_E14_vs_E10	ordinary_muscle_development		0	FALSE
ENSGALG00010015617	SRSF10	PRJNA729271	-0.250740255	1.50231E-05	0.250740255	stage_E14_vs_E10	ordinary_muscle_development		0	FALSE
ENSGALG00010022582	ENSGALG00010022582	PRJNA729271	0.955727259	1.50568E-05	0.955727259	stage_E14_vs_E10	ordinary_muscle_development		0	FALSE
ENSGALG00010015963	DIPK2A	PRJNA729271	0.489232682	1.50911E-05	0.489232682	stage_E14_vs_E10	ordinary_muscle_development		0	FALSE
ENSGALG00010011983	LYZ	PRJNA729271	2.345810007	1.521E-05	2.345810007	stage_E14_vs_E10	ordinary_muscle_development	D_vs_E10;stage_1W_vs_E10;stage_3W_vs_E10;stage	1	TRUE
ENSGALG00010011984	FN1	PRJNA729271	-1.068523175	1.52168E-05	1.068523175	stage_E14_vs_E10	ordinary_muscle_development	D_vs_E10;stage_1W_vs_E10;stage_3W_vs_E10;stage	1	TRUE
ENSGALG00010029646	MRPS17	PRJNA729271	-0.488499986	1.5302E-05	0.488499986	stage_E14_vs_E10	ordinary_muscle_development		0	FALSE
ENSGALG00010009526	ENSGALG00010009526	PRJNA729271	-1.145770569	1.53183E-05	1.145770569	stage_E14_vs_E10	ordinary_muscle_development	D_vs_E10;stage_1W_vs_E10;stage_3W_vs_E10;stage	1	TRUE
ENSGALG00010013306	ARSB	PRJNA729271	-0.673585016	1.54083E-05	0.673585016	stage_E14_vs_E10	ordinary_muscle_development		0	FALSE
ENSGALG00010005438	GPR180	PRJNA729271	-0.625693708	1.54399E-05	0.625693708	stage_E14_vs_E10	ordinary_muscle_development		0	FALSE
ENSGALG00010029452	ENSGALG00010029452	PRJNA729271	-2.767711588	1.54416E-05	2.767711588	stage_E14_vs_E10	ordinary_muscle_development	D_vs_E10;stage_1W_vs_E10;stage_3W_vs_E10;stage	1	TRUE
ENSGALG00010029641	ENSGALG00010029641	PRJNA729271	-3.391243189	1.54607E-05	3.391243189	stage_E14_vs_E10	ordinary_muscle_development	D_vs_E10;stage_1W_vs_E10;stage_3W_vs_E10;stage	1	TRUE
ENSGALG00010015802	TRPC7	PRJNA729271	-5.039848032	1.55324E-05	5.039848032	stage_E14_vs_E10	ordinary_muscle_development	D_vs_E10;stage_1W_vs_E10;stage_3W_vs_E10;stage	1	TRUE
ENSGALG00010024934	G6PC3	PRJNA729271	0.983746266	1.55327E-05	0.983746266	stage_E14_vs_E10	ordinary_muscle_development		0	FALSE
ENSGALG00010019114	TENT5D	PRJNA729271	-1.688401468	1.55474E-05	1.688401468	stage_E14_vs_E10	ordinary_muscle_development	D_vs_E10;stage_1W_vs_E10;stage_3W_vs_E10;stage	1	TRUE
ENSGALG00010024807	PPIL3	PRJNA729271	-0.878131121	1.55555E-05	0.878131121	stage_E14_vs_E10	ordinary_muscle_development		0	FALSE
ENSGALG00010013663	OPA1	PRJNA729271	0.471978042	1.55835E-05	0.471978042	stage_E14_vs_E10	ordinary_muscle_development		0	FALSE
ENSGALG00010016150	POLG	PRJNA729271	0.419264503	1.5696E-05	0.419264503	stage_E14_vs_E10	ordinary_muscle_development		0	FALSE
ENSGALG00010027135	PRKAG2	PRJNA729271	-0.617297377	1.57398E-05	0.617297377	stage_E14_vs_E10	ordinary_muscle_development		0	FALSE
ENSGALG00010009101	HIKESHI	PRJNA729271	0.434056968	1.58807E-05	0.434056968	stage_E14_vs_E10	ordinary_muscle_development		0	FALSE
ENSGALG00010010871	GCNT2	PRJNA729271	-5.308702849	1.59124E-05	5.308702849	stage_E14_vs_E10	ordinary_muscle_development	D_vs_E10;stage_1W_vs_E10;stage_3W_vs_E10;stage	1	TRUE
ENSGALG00010000147	ENSGALG00010000147	PRJNA729271	0.493662416	1.59229E-05	0.493662416	stage_E14_vs_E10	ordinary_muscle_development		0	FALSE
ENSGALG00010005824	NMBR	PRJNA729271	-5.915919677	1.59865E-05	5.915919677	stage_E14_vs_E10	ordinary_muscle_development	D_vs_E10;stage_1W_vs_E10;stage_3W_vs_E10;stage	1	TRUE
ENSGALG00010004036	ADCYAP1	PRJNA729271	-4.144351927	1.60936E-05	4.144351927	stage_E14_vs_E10	ordinary_muscle_development	D_vs_E10;stage_1W_vs_E10;stage_3W_vs_E10;stage	1	TRUE
ENSGALG00010007903	PSMA2	PRJNA729271	-0.464182746	1.6111E-05	0.464182746	stage_E14_vs_E10	ordinary_muscle_development		0	FALSE
ENSGALG00010020129	MGAT2	PRJNA729271	-0.520301669	1.62745E-05	0.520301669	stage_E14_vs_E10	ordinary_muscle_development		0	FALSE
ENSGALG00010009111	LRFN1	PRJNA729271	1.074249372	1.62839E-05	1.074249372	stage_E14_vs_E10	ordinary_muscle_development	D_vs_E10;stage_1W_vs_E10;stage_3W_vs_E10;stage	1	TRUE
ENSGALG00010000466	FBXO21	PRJNA729271	0.349900586	1.63137E-05	0.349900586	stage_E14_vs_E10	ordinary_muscle_development		0	FALSE
ENSGALG00010014873	CALHM6	PRJNA729271	2.988990837	1.63227E-05	2.988990837	stage_E14_vs_E10	ordinary_muscle_development	D_vs_E10;stage_1W_vs_E10;stage_3W_vs_E10;stage	1	TRUE
ENSGALG00010000246	ENSGALG00010000246	PRJNA729271	-19.27784589	1.63543E-05	19.27784589	stage_E14_vs_E10	ordinary_muscle_development	D_vs_E10;stage_1W_vs_E10;stage_3W_vs_E10;stage	1	TRUE
ENSGALG00010020662	BFAR	PRJNA729271	-0.392754385	1.64014E-05	0.392754385	stage_E14_vs_E10	ordinary_muscle_development		0	FALSE
ENSGALG00010012498	KCNT2	PRJNA729271	0.947188578	1.64118E-05	0.947188578	stage_E14_vs_E10	ordinary_muscle_development		0	FALSE
ENSGALG00010026628	FYB2	PRJNA729271	-4.882023937	1.64143E-05	4.882023937	stage_E14_vs_E10	ordinary_muscle_development	D_vs_E10;stage_1W_vs_E10;stage_3W_vs_E10;stage	1	TRUE
ENSGALG00010007169	QPCT	PRJNA729271	-1.019282313	1.6417E-05	1.019282313	stage_E14_vs_E10	ordinary_muscle_development	D_vs_E10;stage_1W_vs_E10;stage_3W_vs_E10;stage	1	TRUE
ENSGALG00010010308	PACRGL	PRJNA729271	-1.002166709	1.64213E-05	1.002166709	stage_E14_vs_E10	ordinary_muscle_development	D_vs_E10;stage_1W_vs_E10;stage_3W_vs_E10;stage	1	TRUE
ENSGALG00010001997	INTS4	PRJNA729271	-0.347086952	1.64979E-05	0.347086952	stage_E14_vs_E10	ordinary_muscle_development		0	FALSE
ENSGALG00010016546	C1QTNF12	PRJNA729271	-1.961549831	1.66556E-05	1.961549831	stage_E14_vs_E10	ordinary_muscle_development	D_vs_E10;stage_1W_vs_E10;stage_3W_vs_E10;stage	1	TRUE
ENSGALG00010016768	GALNT10	PRJNA729271	0.447506861	1.6719E-05	0.447506861	stage_E14_vs_E10	ordinary_muscle_development		0	FALSE
ENSGALG00010018354	AARS2	PRJNA729271	0.53243384	1.67392E-05	0.53243384	stage_E14_vs_E10	ordinary_muscle_development		0	FALSE

ENSGALG00010017780	TAF9	PRJNA729271	0.60385524	1.68231E-05	0.60385524	stage_E14_vs_E10	ordinary_muscle_development		0	FALSE
ENSGALG00010007322	AK6	PRJNA729271	-0.493346948	1.69185E-05	0.493346948	stage_E14_vs_E10	ordinary_muscle_development		0	FALSE
ENSGALG00010029382	GTF2IRD1	PRJNA729271	0.739411874	1.69498E-05	0.739411874	stage_E14_vs_E10	ordinary_muscle_development		0	FALSE
ENSGALG00010025252	PPP1R9B	PRJNA729271	-0.648995609	1.69838E-05	0.648995609	stage_E14_vs_E10	ordinary_muscle_development		0	FALSE
ENSGALG00010003671	ENSGALG00010003671	PRJNA729271	1.164927103	1.69905E-05	1.164927103	stage_E14_vs_E10	ordinary_muscle_development	D_vs_E10;stage_1W_vs_E10;stage_3W_vs_E10;stage	1	TRUE
ENSGALG00010018867	RPS2	PRJNA729271	0.699748282	1.69943E-05	0.699748282	stage_E14_vs_E10	ordinary_muscle_development		0	FALSE
ENSGALG00010013746	ENSGALG00010013746	PRJNA729271	0.386030835	1.70407E-05	0.386030835	stage_E14_vs_E10	ordinary_muscle_development		0	FALSE
ENSGALG00010017701	PPP2R5E	PRJNA729271	0.438847491	1.7097E-05	0.438847491	stage_E14_vs_E10	ordinary_muscle_development		0	FALSE
ENSGALG00010008350	LRRC6	PRJNA729271	-0.819383137	1.7123E-05	0.819383137	stage_E14_vs_E10	ordinary_muscle_development		0	FALSE
ENSGALG00010007774	ENSGALG00010007774	PRJNA729271	1.182463673	1.71711E-05	1.182463673	stage_E14_vs_E10	ordinary_muscle_development	D_vs_E10;stage_1W_vs_E10;stage_3W_vs_E10;stage	1	TRUE
ENSGALG00010027197	ENSGALG00010027197	PRJNA729271	-2.690143253	1.72341E-05	2.690143253	stage_E14_vs_E10	ordinary_muscle_development	D_vs_E10;stage_1W_vs_E10;stage_3W_vs_E10;stage	1	TRUE
ENSGALG00010010724	ADCY3	PRJNA729271	-0.682023275	1.72513E-05	0.682023275	stage_E14_vs_E10	ordinary_muscle_development		0	FALSE
ENSGALG00010019019	TBX20	PRJNA729271	1.131052369	1.73146E-05	1.131052369	stage_E14_vs_E10	ordinary_muscle_development	D_vs_E10;stage_1W_vs_E10;stage_3W_vs_E10;stage	1	TRUE
ENSGALG00010007547	ENSGALG00010007547	PRJNA729271	2.399963704	1.74368E-05	2.399963704	stage_E14_vs_E10	ordinary_muscle_development	D_vs_E10;stage_1W_vs_E10;stage_3W_vs_E10;stage	1	TRUE
ENSGALG00010005900	SPTSSA	PRJNA729271	-0.387648711	1.75608E-05	0.387648711	stage_E14_vs_E10	ordinary_muscle_development		0	FALSE
ENSGALG00010023627	IFT52	PRJNA729271	-0.453766627	1.75667E-05	0.453766627	stage_E14_vs_E10	ordinary_muscle_development		0	FALSE
ENSGALG00010004346	CEP128	PRJNA729271	-0.475873092	1.75735E-05	0.475873092	stage_E14_vs_E10	ordinary_muscle_development		0	FALSE
ENSGALG00010014748	FGFR1OP2	PRJNA729271	-0.496681695	1.77344E-05	0.496681695	stage_E14_vs_E10	ordinary_muscle_development		0	FALSE
ENSGALG00010024808	CELSR2	PRJNA729271	-1.14557038	1.77648E-05	1.14557038	stage_E14_vs_E10	ordinary_muscle_development	D_vs_E10;stage_1W_vs_E10;stage_3W_vs_E10;stage	1	TRUE
ENSGALG00010002860	NIPA2	PRJNA729271	-0.48565983	1.77737E-05	0.48565983	stage_E14_vs_E10	ordinary_muscle_development		0	FALSE
ENSGALG00010020141	THOC7	PRJNA729271	-0.569030359	1.79392E-05	0.569030359	stage_E14_vs_E10	ordinary_muscle_development		0	FALSE
ENSGALG00010029528	TBL2	PRJNA729271	-0.431885071	1.79865E-05	0.431885071	stage_E14_vs_E10	ordinary_muscle_development		0	FALSE
ENSGALG00010023056	SAG	PRJNA729271	1.906926369	1.81649E-05	1.906926369	stage_E14_vs_E10	ordinary_muscle_development	D_vs_E10;stage_1W_vs_E10;stage_3W_vs_E10;stage	1	TRUE
ENSGALG00010005220	ANGEL1	PRJNA729271	0.394938059	1.82653E-05	0.394938059	stage_E14_vs_E10	ordinary_muscle_development		0	FALSE
ENSGALG00010005518	HPGDS	PRJNA729271	-3.048087689	1.83073E-05	3.048087689	stage_E14_vs_E10	ordinary_muscle_development	D_vs_E10;stage_1W_vs_E10;stage_3W_vs_E10;stage	1	TRUE
ENSGALG00010020166	HAPLN3	PRJNA729271	-0.788875753	1.83243E-05	0.788875753	stage_E14_vs_E10	ordinary_muscle_development		0	FALSE
ENSGALG00010011466	MTMR1	PRJNA729271	-0.301533938	1.84949E-05	0.301533938	stage_E14_vs_E10	ordinary_muscle_development		0	FALSE
ENSGALG00010011607	TSEN15	PRJNA729271	-0.803873523	1.85297E-05	0.803873523	stage_E14_vs_E10	ordinary_muscle_development		0	FALSE
ENSGALG00010025316	SLC25A36	PRJNA729271	-0.494170192	1.85567E-05	0.494170192	stage_E14_vs_E10	ordinary_muscle_development		0	FALSE
ENSGALG00010020806	HTD2	PRJNA729271	0.759840286	1.86395E-05	0.759840286	stage_E14_vs_E10	ordinary_muscle_development		0	FALSE
ENSGALG00010027108	ENSGALG00010027108	PRJNA729271	-0.662951837	1.86656E-05	0.662951837	stage_E14_vs_E10	ordinary_muscle_development		0	FALSE
ENSGALG00010001721	TM4SF1	PRJNA729271	0.626540301	1.86971E-05	0.626540301	stage_E14_vs_E10	ordinary_muscle_development		0	FALSE
ENSGALG00010006123	MLH3	PRJNA729271	-0.467727896	1.87466E-05	0.467727896	stage_E14_vs_E10	ordinary_muscle_development		0	FALSE
ENSGALG00010022431	LARP4	PRJNA729271	-0.595181234	1.87737E-05	0.595181234	stage_E14_vs_E10	ordinary_muscle_development		0	FALSE
ENSGALG00010014691	PABIR2	PRJNA729271	0.241732578	1.87803E-05	0.241732578	stage_E14_vs_E10	ordinary_muscle_development		0	FALSE
ENSGALG00010022159	KIAA2013	PRJNA729271	0.394443624	1.88089E-05	0.394443624	stage_E14_vs_E10	ordinary_muscle_development		0	FALSE
ENSGALG00010013857	IL18RAP	PRJNA729271	2.529492855	1.88249E-05	2.529492855	stage_E14_vs_E10	ordinary_muscle_development	D_vs_E10;stage_1W_vs_E10;stage_3W_vs_E10;stage	1	TRUE
ENSGALG00010021503	KCNS1	PRJNA729271	-3.149055846	1.88286E-05	3.149055846	stage_E14_vs_E10	ordinary_muscle_development	D_vs_E10;stage_1W_vs_E10;stage_3W_vs_E10;stage	1	TRUE
ENSGALG00010023287	RGS19	PRJNA729271	-0.91471774	1.88534E-05	0.91471774	stage_E14_vs_E10	ordinary_muscle_development		0	FALSE
ENSGALG00010003519	KCTD4	PRJNA729271	-3.499608606	1.88703E-05	3.499608606	stage_E14_vs_E10	ordinary_muscle_development	D_vs_E10;stage_1W_vs_E10;stage_3W_vs_E10;stage	1	TRUE
ENSGALG00010022531	ENSGALG00010022531	PRJNA729271	-2.048900948	1.90119E-05	2.048900948	stage_E14_vs_E10	ordinary_muscle_development	D_vs_E10;stage_1W_vs_E10;stage_3W_vs_E10;stage	1	TRUE
ENSGALG00010027370	NUP210L	PRJNA729271	-1.615830886	1.90992E-05	1.615830886	stage_E14_vs_E10	ordinary_muscle_development	D_vs_E10;stage_1W_vs_E10;stage_3W_vs_E10;stage	1	TRUE
ENSGALG00010024081	CARS	PRJNA729271	-0.444108289	1.91377E-05	0.444108289	stage_E14_vs_E10	ordinary_muscle_development		0	FALSE
ENSGALG00010015758	PCDH19	PRJNA729271	0.75653352	1.92484E-05	0.75653352	stage_E14_vs_E10	ordinary_muscle_development		0	FALSE
ENSGALG00010017880	CDK13	PRJNA729271	0.372202739	1.92664E-05	0.372202739	stage_E14_vs_E10	ordinary_muscle_development		0	FALSE
ENSGALG00010011190	ENSGALG00010011190	PRJNA729271	-0.955617613	1.92711E-05	0.955617613	stage_E14_vs_E10	ordinary_muscle_development		0	FALSE
ENSGALG00010008172	SNTB2	PRJNA729271	-0.58912516	1.92786E-05	0.58912516	stage_E14_vs_E10	ordinary_muscle_development		0	FALSE
ENSGALG00010001162	ENSGALG00010001162	PRJNA729271	1.74263733	1.93998E-05	1.74263733	stage_E14_vs_E10	ordinary_muscle_development	D_vs_E10;stage_1W_vs_E10;stage_3W_vs_E10;stage	1	TRUE
ENSGALG00010016308	RPS6	PRJNA729271	-0.618961134	1.94424E-05	0.618961134	stage_E14_vs_E10	ordinary_muscle_development		0	FALSE
ENSGALG00010020013	ADAM8	PRJNA729271	-3.306148441	1.95244E-05	3.306148441	stage_E14_vs_E10	ordinary_muscle_development	D_vs_E10;stage_1W_vs_E10;stage_3W_vs_E10;stage	1	TRUE
ENSGALG00010027571	RBM10	PRJNA729271	0.452882635	1.95943E-05	0.452882635	stage_E14_vs_E10	ordinary_muscle_development		0	FALSE
ENSGALG00010028536	GPR107	PRJNA729271	-0.282916759	1.95973E-05	0.282916759	stage_E14_vs_E10	ordinary_muscle_development		0	FALSE
ENSGALG00010022823	IGHMBP2	PRJNA729271	0.500381661	1.96692E-05	0.500381661	stage_E14_vs_E10	ordinary_muscle_development		0	FALSE
ENSGALG00010020939	CTRC	PRJNA729271	9.273531567	1.97091E-05	9.273531567	stage_E14_vs_E10	ordinary_muscle_development	D_vs_E10;stage_1W_vs_E10;stage_3W_vs_E10;stage	1	TRUE
ENSGALG00010017386	ENSGALG00010017386	PRJNA729271	-4.1089786	1.99171E-05	4.1089786	stage_E14_vs_E10	ordinary_muscle_development	D_vs_E10;stage_1W_vs_E10;stage_3W_vs_E10;stage	1	TRUE
ENSGALG00010002673	SLC66A3	PRJNA729271	-0.67123075	1.9997E-05	0.67123075	stage_E14_vs_E10	ordinary_muscle_development		0	FALSE
ENSGALG00010009408	ENSGALG00010009408	PRJNA729271	-0.410640054	2.00413E-05	0.410640054	stage_E14_vs_E10	ordinary_muscle_development		0	FALSE
ENSGALG00010016974	CFAP96	PRJNA729271	-4.302873401	2.01438E-05	4.302873401	stage_E14_vs_E10	ordinary_muscle_development	D_vs_E10;stage_1W_vs_E10;stage_3W_vs_E10;stage	1	TRUE
ENSGALG00010005384	MAPRE2	PRJNA729271	0.758016046	2.01533E-05	0.758016046	stage_E14_vs_E10	ordinary_muscle_development		0	FALSE
ENSGALG00010018515	SEMA4G	PRJNA729271	0.774490663	2.01788E-05	0.774490663	stage_E14_vs_E10	ordinary_muscle_development		0	FALSE
ENSGALG00010002463	ASRGL1	PRJNA729271	-0.462917912	2.02157E-05	0.462917912	stage_E14_vs_E10	ordinary_muscle_development		0	FALSE
ENSGALG00010006113	EIF2B2	PRJNA729271	0.735611939	2.02868E-05	0.735611939	stage_E14_vs_E10	ordinary_muscle_development		0	FALSE
ENSGALG00010001764	ENSGALG00010001764	PRJNA729271	1.674837429	2.02978E-05	1.674837429	stage_E14_vs_E10	ordinary_muscle_development	D_vs_E10;stage_1W_vs_E10;stage_3W_vs_E10;stage	1	TRUE
ENSGALG00010007928	C11orf70	PRJNA729271	-3.589033526	2.03512E-05	3.589033526	stage_E14_vs_E10	ordinary_muscle_development	D_vs_E10;stage_1W_vs_E10;stage_3W_vs_E10;stage	1	TRUE
ENSGALG00010005426	ENSGALG00010005426	PRJNA729271	3.830246987	2.03751E-05	3.830246987	stage_E14_vs_E10	ordinary_muscle_development	D_vs_E10;stage_1W_vs_E10;stage_3W_vs_E10;stage	1	TRUE
ENSGALG00010027251	ENSGALG00010027251	PRJNA729271	0.861332356	2.0416E-05	0.861332356	stage_E14_vs_E10	ordinary_muscle_development		0	FALSE
ENSGALG00010015551	VWA3B	PRJNA729271	-2.390151099	2.05554E-05	2.390151099	stage_E14_vs_E10	ordinary_muscle_development	D_vs_E10;stage_1W_vs_E10;stage_3W_vs_E10;stage	1	TRUE
ENSGALG00010019803	FAH	PRJNA729271	-2.24116684	2.0598E-05	2.24116684	stage_E14_vs_E10	ordinary_muscle_development	D_vs_E10;stage_1W_vs_E10;stage_3W_vs_E10;stage	1	TRUE
ENSGALG00010018119	GRID2IP	PRJNA729271	-4.124354473	2.06229E-05	4.124354473	stage_E14_vs_E10	ordinary_muscle_development	D_vs_E10;stage_1W_vs_E10;stage_3W_vs_E10;stage	1	TRUE
ENSGALG00010026066	SELENOP2	PRJNA729271	2.516292139	2.063E-05	2.516292139	stage_E14_vs_E10	ordinary_muscle_development	D_vs_E10;stage_1W_vs_E10;stage_3W_vs_E10;stage	1	TRUE
ENSGALG00010025951	TBRG4	PRJNA729271	0.497464557	2.07279E-05	0.497464557	stage_E14_vs_E10	ordinary_muscle_development		0	FALSE
ENSGALG00010007731	ENSGALG00010007731	PRJNA729271	-1.210078767	2.07829E-05	1.210078767	stage_E14_vs_E10	ordinary_muscle_development	D_vs_E10;stage_1W_vs_E10;stage_3W_vs_E10;stage	1	TRUE
ENSGALG00010004404	RGS20	PRJNA729271	-2.135543429	2.07946E-05	2.135543429	stage_E14_vs_E10	ordinary_muscle_development	D_vs_E10;stage_1W_vs_E10;stage_3W_vs_E10;stage	1	TRUE
ENSGALG00010016091	HMX1	PRJNA729271	-4.356786264	2.08413E-05	4.356786264	stage_E14_vs_E10	ordinary_muscle_development	D_vs_E10;stage_1W_vs_E10;stage_3W_vs_E10;stage	1	TRUE
ENSGALG00010022124	ENSGALG00010022124	PRJNA729271	0.497555799	2.0848E-05	0.497555799	stage_E14_vs_E10	ordinary_muscle_development		0	FALSE
ENSGALG00010014764	ENSGALG00010014764	PRJNA729271	10.01677958	2.08487E-05	10.01677958	stage_E14_vs_E10	ordinary_muscle_development	D_vs_E10;stage_1W_vs_E10;stage_3W_vs_E10;stage	1	TRUE
ENSGALG00010017791	CCNL2	PRJNA729271	0.490662278	2.09095E-05	0.490662278	stage_E14_vs_E10	ordinary_muscle_development		0	FALSE
ENSGALG00010022999	ENSGALG00010022999	PRJNA729271	2.085952632	2.10698E-05	2.085952632	stage_E14_vs_E10	ordinary_muscle_development	D_vs_E10;stage_1W_vs_E10;stage_3W_vs_E10;stage	1	TRUE
ENSGALG00010008367	ENSGALG00010008367	PRJNA729271	-2.922266324	2.13426E-05	2.922266324	stage_E14_vs_E10	ordinary_muscle_development	D_vs_E10;stage_1W_vs_E10;stage_3W_vs_E10;stage	1	TRUE
ENSGALG00010012120	ENSGALG00010012120	PRJNA729271	-0.838932142	2.15076E-05	0.838932142	stage_E14_vs_E10	ordinary_muscle_development		0	FALSE
ENSGALG00010013174	ADAMTS12	PRJNA729271	0.732763769	2.16018E-05	0.732763769	stage_E14_vs_E10	ordinary_muscle_development		0	FALSE
ENSGALG00010012140	GLOD5	PRJNA729271	2.519733858	2.16976E-05	2.519733858	stage_E14_vs_E10	ordinary_muscle_development	D_vs_E10;stage_1W_vs_E10;stage_3W_vs_E10;stage	1	TRUE
ENSGALG00010024748	TRPM1	PRJNA729271	1.705868613	2.1713E-05	1.705868613	stage_E14_vs_E10	ordinary_muscle_development	D_vs_E10;stage_1W_vs_E10;stage_3W_vs_E10;stage	1	TRUE
ENSGALG00010016245	FAM96A	PRJNA729271	-0.466258207	2.17366E-05	0.466258207	stage_E14_vs_E10	ordinary_muscle_development		0	FALSE
ENSGALG00010017441	SPSB1	PRJNA729271	1.119930445	2.17872E-05	1.119930445	stage_E14_vs_E10	ordinary_muscle_development	D_vs_E10;stage_1W_vs_E10;stage_3W_vs_E10;stage	1	TRUE
ENSGALG00010015339	ENSGALG00010015339	PRJNA729271	3.258963073	2.18414E-05	3.258963073	stage_E14_vs_E10	ordinary_muscle_development	D_vs_E10;stage_1W_vs_E10;stage_3W_vs_E10;stage	1	TRUE
ENSGALG00010027281	ENSGALG00010027281	PRJNA729271	-1.214893017	2.19443E-05	1.214893017	stage_E14_vs_E10	ordinary_muscle_development	D_vs_E10;stage_1W_vs_E10;stage_3W_vs_E10;stage	1	TRUE
ENSGALG00010018209	GMPPA	PRJNA729271	0.57086122	2.21589E-05	0.57086122	stage_E14_vs_E10	ordinary_muscle_development		0	FALSE
ENSGALG00010015042	EFNB2	PRJNA729271	-0.583896297	2.21697E-05	0.583896297	stage_E14_vs_E10	ordinary_muscle_development		0	FALSE
ENSGALG00010028902	ENSGALG00010028902	PRJNA729271	-2.791950685	2.22827E-05	2.791950685	stage_E14_vs_E10	ordinary_muscle_development	D_vs_E10;stage_1W_vs_E10;stage_3W_vs_E10;stage	1	TRUE
ENSGALG00010004191	CABLES1	PRJNA729271	0.551330237	2.24353E-05	0.551330237	stage_E14_vs_E10	ordinary_muscle_development		0	FALSE
ENSGALG00010025239	RAG2	PRJNA729271	-3.500027658	2.25588E-05	3.500027658	stage_E14_vs_E10	ordinary_muscle_development	D_vs_E10;stage_1W_vs_E10;stage_3W_vs_E10;stage	1	TRUE
ENSGALG00010028366	MRRF	PRJNA729271	0.375997551	2.25588E-05	0.375997551	stage_E14_vs_E10	ordinary_muscle_development		0	FALSE
ENSGALG00010011653	NR2C1	PRJNA729271	-0.397142814	2.25604E-05	0.397142814	stage_E14_vs_E10	ordinary_muscle_development		0	FALSE
ENSGALG00010017041	JKAMP	PRJNA729271	0.38885325	2.26113E-05	0.38885325	stage_E14_vs_E10	ordinary_muscle_development		0	FALSE
ENSGALG00010025190	PRR5L	PRJNA729271	1.041449727	2.26171E-05	1.041449727	stage_E14_vs_E10	ordinary_muscle_development	D_vs_E10;stage_1W_vs_E10;stage_3W_vs_E10;stage	1	TRUE
ENSGALG00010024069	ENSGALG00010024069	PRJNA729271	9.662818723	2.26557E-05	9.662818723	stage_E14_vs_E10	ordinary_muscle_development	D_vs_E10;stage_1W_vs_E10;stage_3W_vs_E10;stage	1	TRUE
ENSGALG00010028519	ENSGALG00010028519	PRJNA729271	8.844369398	2.27889E-05	8.844369398	stage_E14_vs_E10	ordinary_muscle_development	D_vs_E10;stage_1W_vs_E10;stage_3W_vs_E10;stage	1	TRUE
ENSGALG00010027382	PALM	PRJNA729271	0.879309735	2.29059E-05	0.879309735	stage_E14_vs_E10	ordinary_muscle_development		0	FALSE
ENSGALG00010006069	TTR	PRJNA729271	6.140383764	2.29196E-05	6.140383764	stage_E14_vs_E10	ordinary_muscle_development	D_vs_E10;stage_1W_vs_E10;stage_3W_vs_E10;stage	1	TRUE
ENSGALG00010028161	ENSGALG00010028161	PRJNA729271	2.570658992	2.29552E-05	2.570658992	stage_E14_vs_E10	ordinary_muscle_development	D_vs_E10;stage_1W_vs_E10;stage_3W_vs_E10;stage	1	TRUE
ENSGALG00010005785	RPL21	PRJNA729271	0.617879863	2.30415E-05	0.617879863	stage_E14_vs_E10	ordinary_muscle_development		0	FALSE
ENSGALG00010022562	GHRH	PRJNA729271	-5.29548675	2.30608E-05	5.29548675	stage_E14_vs_E10	ordinary_muscle_development	D_vs_E10;stage_1W_vs_E10;stage_3W_vs_E10;stage	1	TRUE
ENSGALG00010014931	RNF145	PRJNA729271	-0.4748836	2.31873E-05	0.4748836	stage_E14_vs_E10	ordinary_muscle_development		0	FALSE
ENSGALG00010018043	PSMC6	PRJNA729271	0.415645367	2.32468E-05	0.415645367	stage_E14_vs_E10	ordinary_muscle_development		0	FALSE
ENSGALG00010002470	OXSM	PRJNA729271	0.695604915	2.33043E-05	0.695604915	stage_E14_vs_E10	ordinary_muscle_development		0	FALSE
ENSGALG00010029510	PDE6G	PRJNA729271	-1.489435815	2.3317E-05	1.489435815	stage_E14_vs_E10	ordinary_muscle_development	D_vs_E10;stage_1W_vs_E10;stage_3W_vs_E10;stage	1	TRUE
ENSGALG00010017751	ST3GAL5	PRJNA729271	-0.570751412	2.33389E-05	0.570751412	stage_E14_vs_E10	ordinary_muscle_development		0	FALSE
ENSGALG00010009461	ZEB2	PRJNA729271	0.564066973	2.3392E-05	0.564066973	stage_E14_vs_E10	ordinary_muscle_development		0	FALSE
ENSGALG00010023289	NFRKB	PRJNA729271	-0.380235252	2.34011E-05	0.380235252	stage_E14_vs_E10	ordinary_muscle_development		0	FALSE
ENSGALG00010022558	NYAP2	PRJNA729271	-3.218715873	2.38728E-05	3.218715873	stage_E14_vs_E10	ordinary_muscle_development	D_vs_E10;stage_1W_vs_E10;stage_3W_vs_E10;stage	1	TRUE
ENSGALG00010028211	GATAD2B	PRJNA729271	-0.675074929	2.38728E-05	0.675074929	stage_E14_vs_E10	ordinary_muscle_development		0	FALSE
ENSGALG00010002145	ENSGALG00010002145	PRJNA729271	2.035728926	2.39318E-05	2.035728926	stage_E14_vs_E10	ordinary_muscle_development	D_vs_E10;stage_1W_vs_E10;stage_3W_vs_E10;stage	1	TRUE
ENSGALG00010022497	CORO1C	PRJNA729271	-0.654729992	2.42224E-05	0.654729992	stage_E14_vs_E10	ordinary_muscle_development		0	FALSE
ENSGALG00010024755	PIGX	PRJNA729271	0.692378381	2.43211E-05	0.692378381	stage_E14_vs_E10	ordinary_muscle_development		0	FALSE
ENSGALG00010019239	CYTH3	PRJNA729271	0.352625001	2.43409E-05	0.352625001	stage_E14_vs_E10	ordinary_muscle_development		0	FALSE
ENSGALG00010025008	ENSGALG00010025008	PRJNA729271	-2.689467858	2.44142E-05	2.689467858	stage_E14_vs_E10	ordinary_muscle_development	D_vs_E10;stage_1W_vs_E10;stage_3W_vs_E10;stage	1	TRUE
ENSGALG00010015296	RPL35A	PRJNA729271	0.860430416	2.46768E-05	0.860430416	stage_E14_vs_E10	ordinary_muscle_development		0	FALSE
ENSGALG00010025889	TESMIN	PRJNA729271	2.337312184	2.47024E-05	2.337312184	stage_E14_vs_E10	ordinary_muscle_development	D_vs_E10;stage_1W_vs_E10;stage_3W_vs_E10;stage	1	TRUE
ENSGALG00010008155	MAP10	PRJNA729271	0.958076137	2.47181E-05	0.958076137	stage_E14_vs_E10	ordinary_muscle_development		0	FALSE
ENSGALG00010005341	UGGT2	PRJNA729271	-0.480928415	2.47705E-05	0.480928415	stage_E14_vs_E10	ordinary_muscle_development		0	FALSE
ENSGALG00010028115	CACTIN	PRJNA729271	0.759017368	2.48738E-05	0.759017368	stage_E14_vs_E10	ordinary_muscle_development		0	FALSE
ENSGALG00010012992	HARS1	PRJNA729271	-0.554470228	2.48855E-05	0.554470228	stage_E14_vs_E10	ordinary_muscle_development		0	FALSE
ENSGALG00010022809	MYO1E	PRJNA729271	-0.679766566	2.49389E-05	0.679766566	stage_E14_vs_E10	ordinary_muscle_development		0	FALSE
ENSGALG00010014668	GP5	PRJNA729271	-0.705560876	2.49725E-05	0.705560876	stage_E14_vs_E10	ordinary_muscle_development		0	FALSE
ENSGALG00010016353	C1orf232	PRJNA729271	0.623765781	2.49858E-05	0.623765781	stage_E14_vs_E10	ordinary_muscle_development		0	FALSE
ENSGALG00010001204	SNX2	PRJNA729271	0.353086613	2.50122E-05	0.353086613	stage_E14_vs_E10	ordinary_muscle_development		0	FALSE
ENSGALG00010008211	ADGRB1	PRJNA729271	-1.041569203	2.50472E-05	1.041569203	stage_E14_vs_E10	ordinary_muscle_development	D_vs_E10;stage_1W_vs_E10;stage_3W_vs_E10;stage	1	TRUE
ENSGALG00010009113	RPLP1	PRJNA729271	0.775875521	2.51733E-05	0.775875521	stage_E14_vs_E10	ordinary_muscle_development		0	FALSE
ENSGALG00010016840	LRRC34	PRJNA729271	-0.961273265	2.51808E-05	0.961273265	stage_E14_vs_E10	ordinary_muscle_development		0	FALSE
ENSGALG00010002288	ANGPT2	PRJNA729271	-0.720433818	2.52528E-05	0.720433818	stage_E14_vs_E10	ordinary_muscle_development		0	FALSE
ENSGALG00010009039	SLC36A4	PRJNA729271	-0.756089314	2.53879E-05	0.756089314	stage_E14_vs_E10	ordinary_muscle_development		0	FALSE
ENSGALG00010013675	CHRNA6	PRJNA729271	1.618783428	2.54341E-05	1.618783428	stage_E14_vs_E10	ordinary_muscle_development	D_vs_E10;stage_1W_vs_E10;stage_3W_vs_E10;stage	1	TRUE
ENSGALG00010024283	UBE4A	PRJNA729271	0.477667234	2.54517E-05	0.477667234	stage_E14_vs_E10	ordinary_muscle_development		0	FALSE
ENSGALG00010019692	MMEL1	PRJNA729271	-1.384755407	2.55938E-05	1.384755407	stage_E14_vs_E10	ordinary_muscle_development	D_vs_E10;stage_1W_vs_E10;stage_3W_vs_E10;stage	1	TRUE
ENSGALG00010002391	REPS2	PRJNA729271	-0.701897107	2.56957E-05	0.701897107	stage_E14_vs_E10	ordinary_muscle_development		0	FALSE
ENSGALG00010025608	ENSGALG00010025608	PRJNA729271	-2.362958601	2.57128E-05	2.362958601	stage_E14_vs_E10	ordinary_muscle_development	D_vs_E10;stage_1W_vs_E10;stage_3W_vs_E10;stage	1	TRUE
ENSGALG00010016434	GPR139	PRJNA729271	-3.255497532	2.57497E-05	3.255497532	stage_E14_vs_E10	ordinary_muscle_development	D_vs_E10;stage_1W_vs_E10;stage_3W_vs_E10;stage	1	TRUE
ENSGALG00010019820	BBOX1	PRJNA729271	-0.683725343	2.5795E-05	0.683725343	stage_E14_vs_E10	ordinary_muscle_development		0	FALSE
ENSGALG00010006623	ENSGALG00010006623	PRJNA729271	0.800023364	2.58454E-05	0.800023364	stage_E14_vs_E10	ordinary_muscle_development		0	FALSE
ENSGALG00010017828	ENSGALG00010017828	PRJNA729271	1.021732069	2.58922E-05	1.021732069	stage_E14_vs_E10	ordinary_muscle_development	D_vs_E10;stage_1W_vs_E10;stage_3W_vs_E10;stage	1	TRUE
ENSGALG00010007585	KCNG4	PRJNA729271	-4.029127056	2.59134E-05	4.029127056	stage_E14_vs_E10	ordinary_muscle_development	D_vs_E10;stage_1W_vs_E10;stage_3W_vs_E10;stage	1	TRUE
ENSGALG00010001149	CIDEA	PRJNA729271	2.683557466	2.59704E-05	2.683557466	stage_E14_vs_E10	ordinary_muscle_development	D_vs_E10;stage_1W_vs_E10;stage_3W_vs_E10;stage	1	TRUE
ENSGALG00010000282	ENSGALG00010000282	PRJNA729271	-21.77393171	2.59904E-05	21.77393171	stage_E14_vs_E10	ordinary_muscle_development	D_vs_E10;stage_1W_vs_E10;stage_3W_vs_E10;stage	1	TRUE
ENSGALG00010020556	EMC1	PRJNA729271	-0.37946652	2.61129E-05	0.37946652	stage_E14_vs_E10	ordinary_muscle_development		0	FALSE
ENSGALG00010005262	ENSGALG00010005262	PRJNA729271	-0.541952827	2.62329E-05	0.541952827	stage_E14_vs_E10	ordinary_muscle_development		0	FALSE
ENSGALG00010014258	ENSGALG00010014258	PRJNA729271	-2.805025254	2.62635E-05	2.805025254	stage_E14_vs_E10	ordinary_muscle_development	D_vs_E10;stage_1W_vs_E10;stage_3W_vs_E10;stage	1	TRUE
ENSGALG00010008011	ADNP2	PRJNA729271	-0.436971567	2.63231E-05	0.436971567	stage_E14_vs_E10	ordinary_muscle_development		0	FALSE
ENSGALG00010009289	GPR137B	PRJNA729271	0.624774067	2.63966E-05	0.624774067	stage_E14_vs_E10	ordinary_muscle_development		0	FALSE
ENSGALG00010024679	MSL1	PRJNA729271	0.479700888	2.64138E-05	0.479700888	stage_E14_vs_E10	ordinary_muscle_development		0	FALSE
ENSGALG00010003813	LRIT3	PRJNA729271	-3.781930025	2.64553E-05	3.781930025	stage_E14_vs_E10	ordinary_muscle_development	D_vs_E10;stage_1W_vs_E10;stage_3W_vs_E10;stage	1	TRUE
ENSGALG00010023083	HARBI1	PRJNA729271	0.666796649	2.64656E-05	0.666796649	stage_E14_vs_E10	ordinary_muscle_development		0	FALSE
ENSGALG00010008331	CCDC97	PRJNA729271	1.133756543	2.6467E-05	1.133756543	stage_E14_vs_E10	ordinary_muscle_development	D_vs_E10;stage_1W_vs_E10;stage_3W_vs_E10;stage	1	TRUE
ENSGALG00010015143	EXOC1	PRJNA729271	-0.357640032	2.64963E-05	0.357640032	stage_E14_vs_E10	ordinary_muscle_development		0	FALSE
ENSGALG00010029677	PHF12	PRJNA729271	0.439700509	2.65722E-05	0.439700509	stage_E14_vs_E10	ordinary_muscle_development		0	FALSE
ENSGALG00010021642	NPTN	PRJNA729271	-0.392584101	2.67083E-05	0.392584101	stage_E14_vs_E10	ordinary_muscle_development		0	FALSE
ENSGALG00010008236	ENSGALG00010008236	PRJNA729271	2.7039141	2.69127E-05	2.7039141	stage_E14_vs_E10	ordinary_muscle_development	D_vs_E10;stage_1W_vs_E10;stage_3W_vs_E10;stage	1	TRUE
ENSGALG00010025887	ZNF76	PRJNA729271	-0.503255979	2.69172E-05	0.503255979	stage_E14_vs_E10	ordinary_muscle_development		0	FALSE
ENSGALG00010005293	BLZF1	PRJNA729271	-0.428907198	2.70141E-05	0.428907198	stage_E14_vs_E10	ordinary_muscle_development		0	FALSE
ENSGALG00010022122	PSMB3	PRJNA729271	-0.689033346	2.7054E-05	0.689033346	stage_E14_vs_E10	ordinary_muscle_development		0	FALSE
ENSGALG00010014576	HRH2	PRJNA729271	1.136714557	2.71503E-05	1.136714557	stage_E14_vs_E10	ordinary_muscle_development	D_vs_E10;stage_1W_vs_E10;stage_3W_vs_E10;stage	1	TRUE
ENSGALG00010019403	PACC1	PRJNA729271	-0.465720173	2.72106E-05	0.465720173	stage_E14_vs_E10	ordinary_muscle_development		0	FALSE
ENSGALG00010007344	RAD17	PRJNA729271	0.463689056	2.73697E-05	0.463689056	stage_E14_vs_E10	ordinary_muscle_development		0	FALSE
ENSGALG00010006055	ENSGALG00010006055	PRJNA729271	2.449696752	2.74949E-05	2.449696752	stage_E14_vs_E10	ordinary_muscle_development	D_vs_E10;stage_1W_vs_E10;stage_3W_vs_E10;stage	1	TRUE
ENSGALG00010017487	C1QTNF8	PRJNA729271	-2.2586945	2.76556E-05	2.2586945	stage_E14_vs_E10	ordinary_muscle_development	D_vs_E10;stage_1W_vs_E10;stage_3W_vs_E10;stage	1	TRUE
ENSGALG00010013312	ENSGALG00010013312	PRJNA729271	-3.850294907	2.77159E-05	3.850294907	stage_E14_vs_E10	ordinary_muscle_development	D_vs_E10;stage_1W_vs_E10;stage_3W_vs_E10;stage	1	TRUE
ENSGALG00010008387	PHF20L1	PRJNA729271	0.483872767	2.77235E-05	0.483872767	stage_E14_vs_E10	ordinary_muscle_development		0	FALSE
ENSGALG00010021695	FNBP4	PRJNA729271	0.30492226	2.78116E-05	0.30492226	stage_E14_vs_E10	ordinary_muscle_development		0	FALSE
ENSGALG00010010916	ENSGALG00010010916	PRJNA729271	-3.829678907	2.78254E-05	3.829678907	stage_E14_vs_E10	ordinary_muscle_development	D_vs_E10;stage_1W_vs_E10;stage_3W_vs_E10;stage	1	TRUE
ENSGALG00010027527	ENSGALG00010027527	PRJNA729271	-2.866322546	2.80362E-05	2.866322546	stage_E14_vs_E10	ordinary_muscle_development	D_vs_E10;stage_1W_vs_E10;stage_3W_vs_E10;stage	1	TRUE
ENSGALG00010010633	EPCIP	PRJNA729271	1.391891252	2.80495E-05	1.391891252	stage_E14_vs_E10	ordinary_muscle_development	D_vs_E10;stage_1W_vs_E10;stage_3W_vs_E10;stage	1	TRUE
ENSGALG00010022227	TMEM80	PRJNA729271	0.678189233	2.82161E-05	0.678189233	stage_E14_vs_E10	ordinary_muscle_development		0	FALSE
ENSGALG00010017843	NSD3	PRJNA729271	-0.3090562	2.82362E-05	0.3090562	stage_E14_vs_E10	ordinary_muscle_development		0	FALSE
ENSGALG00010002853	CEP162	PRJNA729271	-0.586639319	2.84815E-05	0.586639319	stage_E14_vs_E10	ordinary_muscle_development		0	FALSE
ENSGALG00010011417	WFS1	PRJNA729271	0.626664214	2.86564E-05	0.626664214	stage_E14_vs_E10	ordinary_muscle_development		0	FALSE

ENSGALG00010029454	ENSGALG00010029454	PRJNA729271	-1.259239175	2.86896E-05	1.259239175	stage_E14_vs_E10	ordinary_muscle_development	D_vs_E10;stage_1W_vs_E10;stage_3W_vs_E10;stage	1	TRUE
ENSGALG00010017575	CLCN2	PRJNA729271	3.027122873	2.86939E-05	3.027122873	stage_E14_vs_E10	ordinary_muscle_development	D_vs_E10;stage_1W_vs_E10;stage_3W_vs_E10;stage	1	TRUE
ENSGALG00010008230	TRMU	PRJNA729271	0.489321055	2.87331E-05	0.489321055	stage_E14_vs_E10	ordinary_muscle_development		0	FALSE
ENSGALG00010018750	SOCS4	PRJNA729271	0.548429656	2.88267E-05	0.548429656	stage_E14_vs_E10	ordinary_muscle_development		0	FALSE
ENSGALG00010016583	VPS41	PRJNA729271	-0.361727701	2.90097E-05	0.361727701	stage_E14_vs_E10	ordinary_muscle_development		0	FALSE
ENSGALG00010023499	PEBP1	PRJNA729271	-0.606577039	2.92354E-05	0.606577039	stage_E14_vs_E10	ordinary_muscle_development		0	FALSE
ENSGALG00010015328	ENSGALG00010015328	PRJNA729271	1.641755796	2.92994E-05	1.641755796	stage_E14_vs_E10	ordinary_muscle_development	D_vs_E10;stage_1W_vs_E10;stage_3W_vs_E10;stage	1	TRUE
ENSGALG00010018920	ACAD11	PRJNA729271	-0.504953227	2.93138E-05	0.504953227	stage_E14_vs_E10	ordinary_muscle_development		0	FALSE
ENSGALG00010003475	ENSGALG00010003475	PRJNA729271	8.922784145	2.94809E-05	8.922784145	stage_E14_vs_E10	ordinary_muscle_development	D_vs_E10;stage_1W_vs_E10;stage_3W_vs_E10;stage	1	TRUE
ENSGALG00010000805	FASTKD3	PRJNA729271	0.557670355	2.96602E-05	0.557670355	stage_E14_vs_E10	ordinary_muscle_development		0	FALSE
ENSGALG00010024885	SLC4A1	PRJNA729271	-1.02251955	2.97058E-05	1.02251955	stage_E14_vs_E10	ordinary_muscle_development	D_vs_E10;stage_1W_vs_E10;stage_3W_vs_E10;stage	1	TRUE
ENSGALG00010006760	TASOR2	PRJNA729271	0.4421869	2.9745E-05	0.4421869	stage_E14_vs_E10	ordinary_muscle_development		0	FALSE
ENSGALG00010021170	UBL7	PRJNA729271	-0.614040682	2.98918E-05	0.614040682	stage_E14_vs_E10	ordinary_muscle_development		0	FALSE
ENSGALG00010005998	YLPM1	PRJNA729271	0.573599748	3.00453E-05	0.573599748	stage_E14_vs_E10	ordinary_muscle_development		0	FALSE
ENSGALG00010008164	TG	PRJNA729271	1.271262419	3.01905E-05	1.271262419	stage_E14_vs_E10	ordinary_muscle_development	D_vs_E10;stage_1W_vs_E10;stage_3W_vs_E10;stage	1	TRUE
ENSGALG00010009574	TAF1C	PRJNA729271	-0.656929752	3.0327E-05	0.656929752	stage_E14_vs_E10	ordinary_muscle_development		0	FALSE
ENSGALG00010029484	ENSGALG00010029484	PRJNA729271	-2.975787578	3.03343E-05	2.975787578	stage_E14_vs_E10	ordinary_muscle_development	D_vs_E10;stage_1W_vs_E10;stage_3W_vs_E10;stage	1	TRUE
ENSGALG00010022942	NEU2	PRJNA729271	-2.249295545	3.04275E-05	2.249295545	stage_E14_vs_E10	ordinary_muscle_development	D_vs_E10;stage_1W_vs_E10;stage_3W_vs_E10;stage	1	TRUE
ENSGALG00010024020	MAIP1	PRJNA729271	0.523078165	3.05754E-05	0.523078165	stage_E14_vs_E10	ordinary_muscle_development		0	FALSE
ENSGALG00010008156	HAS3	PRJNA729271	-4.956499205	3.06629E-05	4.956499205	stage_E14_vs_E10	ordinary_muscle_development	D_vs_E10;stage_1W_vs_E10;stage_3W_vs_E10;stage	1	TRUE
ENSGALG00010001155	CYB5A	PRJNA729271	0.416681904	3.08579E-05	0.416681904	stage_E14_vs_E10	ordinary_muscle_development		0	FALSE
ENSGALG00010008190	TSNARE1	PRJNA729271	-0.738203423	3.09238E-05	0.738203423	stage_E14_vs_E10	ordinary_muscle_development		0	FALSE
ENSGALG00010020554	ZFP36L1	PRJNA729271	0.904247728	3.09846E-05	0.904247728	stage_E14_vs_E10	ordinary_muscle_development		0	FALSE
ENSGALG00010005111	RASSF6	PRJNA729271	-1.846310795	3.11504E-05	1.846310795	stage_E14_vs_E10	ordinary_muscle_development	D_vs_E10;stage_1W_vs_E10;stage_3W_vs_E10;stage	1	TRUE
ENSGALG00010027919	TSEN2	PRJNA729271	-0.531166415	3.12809E-05	0.531166415	stage_E14_vs_E10	ordinary_muscle_development		0	FALSE
ENSGALG00010016507	SPATA18	PRJNA729271	0.610790985	3.13183E-05	0.610790985	stage_E14_vs_E10	ordinary_muscle_development		0	FALSE
ENSGALG00010028279	EDBETA	PRJNA729271	6.355276545	3.13902E-05	6.355276545	stage_E14_vs_E10	ordinary_muscle_development	D_vs_E10;stage_1W_vs_E10;stage_3W_vs_E10;stage	1	TRUE
ENSGALG00010013295	CA3B	PRJNA729271	-1.790356449	3.16136E-05	1.790356449	stage_E14_vs_E10	ordinary_muscle_development	D_vs_E10;stage_1W_vs_E10;stage_3W_vs_E10;stage	1	TRUE
ENSGALG00010004257	ENSGALG00010004257	PRJNA729271	-2.836244684	3.16331E-05	2.836244684	stage_E14_vs_E10	ordinary_muscle_development	D_vs_E10;stage_1W_vs_E10;stage_3W_vs_E10;stage	1	TRUE
ENSGALG00010025829	ENSGALG00010025829	PRJNA729271	-1.112474696	3.16413E-05	1.112474696	stage_E14_vs_E10	ordinary_muscle_development	D_vs_E10;stage_1W_vs_E10;stage_3W_vs_E10;stage	1	TRUE
ENSGALG00010011130	DNM1L	PRJNA729271	-0.50288876	3.16415E-05	0.50288876	stage_E14_vs_E10	ordinary_muscle_development		0	FALSE
ENSGALG00010019932	KALRN	PRJNA729271	-0.525978494	3.19792E-05	0.525978494	stage_E14_vs_E10	ordinary_muscle_development		0	FALSE
ENSGALG00010013036	ENSGALG00010013036	PRJNA729271	1.720918293	3.20802E-05	1.720918293	stage_E14_vs_E10	ordinary_muscle_development	D_vs_E10;stage_1W_vs_E10;stage_3W_vs_E10;stage	1	TRUE
ENSGALG00010013154	ENSGALG00010013154	PRJNA729271	-2.113627276	3.21059E-05	2.113627276	stage_E14_vs_E10	ordinary_muscle_development	D_vs_E10;stage_1W_vs_E10;stage_3W_vs_E10;stage	1	TRUE
ENSGALG00010000813	ENSGALG00010000813	PRJNA729271	-2.030162631	3.22079E-05	2.030162631	stage_E14_vs_E10	ordinary_muscle_development	D_vs_E10;stage_1W_vs_E10;stage_3W_vs_E10;stage	1	TRUE
ENSGALG00010003092	ART1F	PRJNA729271	5.765101359	3.2375E-05	5.765101359	stage_E14_vs_E10	ordinary_muscle_development	D_vs_E10;stage_1W_vs_E10;stage_3W_vs_E10;stage	1	TRUE
ENSGALG00010017111	GJB1	PRJNA729271	-2.138254155	3.26381E-05	2.138254155	stage_E14_vs_E10	ordinary_muscle_development	D_vs_E10;stage_1W_vs_E10;stage_3W_vs_E10;stage	1	TRUE
ENSGALG00010025311	ENSGALG00010025311	PRJNA729271	2.443134273	3.27807E-05	2.443134273	stage_E14_vs_E10	ordinary_muscle_development	D_vs_E10;stage_1W_vs_E10;stage_3W_vs_E10;stage	1	TRUE
ENSGALG00010021644	TARDBP	PRJNA729271	-0.659369313	3.2822E-05	0.659369313	stage_E14_vs_E10	ordinary_muscle_development		0	FALSE
ENSGALG00010014161	GFRA3	PRJNA729271	1.067734467	3.28532E-05	1.067734467	stage_E14_vs_E10	ordinary_muscle_development	D_vs_E10;stage_1W_vs_E10;stage_3W_vs_E10;stage	1	TRUE
ENSGALG00010012199	ENSGALG00010012199	PRJNA729271	1.216835492	3.28562E-05	1.216835492	stage_E14_vs_E10	ordinary_muscle_development	D_vs_E10;stage_1W_vs_E10;stage_3W_vs_E10;stage	1	TRUE
ENSGALG00010019212	RPL22	PRJNA729271	0.493819815	3.29261E-05	0.493819815	stage_E14_vs_E10	ordinary_muscle_development		0	FALSE
ENSGALG00010024075	SPATS2L	PRJNA729271	-0.586697914	3.29514E-05	0.586697914	stage_E14_vs_E10	ordinary_muscle_development		0	FALSE
ENSGALG00010029223	NME2	PRJNA729271	0.62265504	3.29613E-05	0.62265504	stage_E14_vs_E10	ordinary_muscle_development		0	FALSE
ENSGALG00010007115	ENSGALG00010007115	PRJNA729271	1.2055925	3.30789E-05	1.2055925	stage_E14_vs_E10	ordinary_muscle_development	D_vs_E10;stage_1W_vs_E10;stage_3W_vs_E10;stage	1	TRUE
ENSGALG00010025435	SCLY	PRJNA729271	-0.433267155	3.31464E-05	0.433267155	stage_E14_vs_E10	ordinary_muscle_development		0	FALSE
ENSGALG00010015096	GABRE	PRJNA729271	-5.344025292	3.31683E-05	5.344025292	stage_E14_vs_E10	ordinary_muscle_development	D_vs_E10;stage_1W_vs_E10;stage_3W_vs_E10;stage	1	TRUE
ENSGALG00010003218	MAP3K7	PRJNA729271	0.266551427	3.3184E-05	0.266551427	stage_E14_vs_E10	ordinary_muscle_development		0	FALSE
ENSGALG00010008991	ENSGALG00010008991	PRJNA729271	0.548484414	3.32323E-05	0.548484414	stage_E14_vs_E10	ordinary_muscle_development		0	FALSE
ENSGALG00010008966	NRBP1	PRJNA729271	0.515461778	3.33339E-05	0.515461778	stage_E14_vs_E10	ordinary_muscle_development		0	FALSE
ENSGALG00010009263	COLEC10	PRJNA729271	-0.708259669	3.33526E-05	0.708259669	stage_E14_vs_E10	ordinary_muscle_development		0	FALSE
ENSGALG00010008040	ENSGALG00010008040	PRJNA729271	1.112291668	3.35476E-05	1.112291668	stage_E14_vs_E10	ordinary_muscle_development	D_vs_E10;stage_1W_vs_E10;stage_3W_vs_E10;stage	1	TRUE
ENSGALG00010011784	GRPEL2	PRJNA729271	0.575164881	3.37536E-05	0.575164881	stage_E14_vs_E10	ordinary_muscle_development		0	FALSE
ENSGALG00010002072	NEURL4	PRJNA729271	0.682224337	3.39063E-05	0.682224337	stage_E14_vs_E10	ordinary_muscle_development		0	FALSE
ENSGALG00010000725	ENSGALG00010000725	PRJNA729271	-4.110560803	3.39358E-05	4.110560803	stage_E14_vs_E10	ordinary_muscle_development	D_vs_E10;stage_1W_vs_E10;stage_3W_vs_E10;stage	1	TRUE
ENSGALG00010011406	UBE2R2	PRJNA729271	-0.498381807	3.40315E-05	0.498381807	stage_E14_vs_E10	ordinary_muscle_development		0	FALSE
ENSGALG00010022279	EP400	PRJNA729271	0.639570137	3.40463E-05	0.639570137	stage_E14_vs_E10	ordinary_muscle_development		0	FALSE
ENSGALG00010006028	ARL5B	PRJNA729271	-0.548944478	3.43679E-05	0.548944478	stage_E14_vs_E10	ordinary_muscle_development		0	FALSE
ENSGALG00010025392	SLC6A2	PRJNA729271	-1.061383156	3.43903E-05	1.061383156	stage_E14_vs_E10	ordinary_muscle_development	D_vs_E10;stage_1W_vs_E10;stage_3W_vs_E10;stage	1	TRUE
ENSGALG00010027502	THOP1	PRJNA729271	-0.594332206	3.44613E-05	0.594332206	stage_E14_vs_E10	ordinary_muscle_development		0	FALSE
ENSGALG00010002600	TRPA1	PRJNA729271	-2.648415069	3.47679E-05	2.648415069	stage_E14_vs_E10	ordinary_muscle_development	D_vs_E10;stage_1W_vs_E10;stage_3W_vs_E10;stage	1	TRUE
ENSGALG00010009253	SLC45A4	PRJNA729271	-0.614411225	3.49221E-05	0.614411225	stage_E14_vs_E10	ordinary_muscle_development		0	FALSE
ENSGALG00010012489	GBX2	PRJNA729271	-4.58778123	3.49553E-05	4.58778123	stage_E14_vs_E10	ordinary_muscle_development	D_vs_E10;stage_1W_vs_E10;stage_3W_vs_E10;stage	1	TRUE
ENSGALG00010022917	CYP4B7	PRJNA729271	-2.148939917	3.49659E-05	2.148939917	stage_E14_vs_E10	ordinary_muscle_development	D_vs_E10;stage_1W_vs_E10;stage_3W_vs_E10;stage	1	TRUE
ENSGALG00010017429	TELO2	PRJNA729271	-0.448573957	3.49973E-05	0.448573957	stage_E14_vs_E10	ordinary_muscle_development		0	FALSE
ENSGALG00010003251	ELOF1	PRJNA729271	-0.668554687	3.51635E-05	0.668554687	stage_E14_vs_E10	ordinary_muscle_development		0	FALSE
ENSGALG00010016656	CAPN8	PRJNA729271	-5.099978395	3.5292E-05	5.099978395	stage_E14_vs_E10	ordinary_muscle_development	D_vs_E10;stage_1W_vs_E10;stage_3W_vs_E10;stage	1	TRUE
ENSGALG00010016893	TBC1D9B	PRJNA729271	-0.222251467	3.53349E-05	0.222251467	stage_E14_vs_E10	ordinary_muscle_development		0	FALSE
ENSGALG00010009370	ENSGALG00010009370	PRJNA729271	1.930776357	3.56642E-05	1.930776357	stage_E14_vs_E10	ordinary_muscle_development	D_vs_E10;stage_1W_vs_E10;stage_3W_vs_E10;stage	1	TRUE
ENSGALG00010021972	PISD	PRJNA729271	-0.802094313	3.56765E-05	0.802094313	stage_E14_vs_E10	ordinary_muscle_development		0	FALSE
ENSGALG00010024340	NETO2	PRJNA729271	-1.62853806	3.57554E-05	1.62853806	stage_E14_vs_E10	ordinary_muscle_development	D_vs_E10;stage_1W_vs_E10;stage_3W_vs_E10;stage	1	TRUE
ENSGALG00010021207	MAD2L2	PRJNA729271	-0.527095574	3.57974E-05	0.527095574	stage_E14_vs_E10	ordinary_muscle_development		0	FALSE
ENSGALG00010004306	PNISR	PRJNA729271	0.615243224	3.58645E-05	0.615243224	stage_E14_vs_E10	ordinary_muscle_development		0	FALSE
ENSGALG00010006486	CEP43	PRJNA729271	-0.493819916	3.59038E-05	0.493819916	stage_E14_vs_E10	ordinary_muscle_development		0	FALSE
ENSGALG00010021203	FUT11	PRJNA729271	0.351091781	3.59147E-05	0.351091781	stage_E14_vs_E10	ordinary_muscle_development		0	FALSE
ENSGALG00010017718	ATOH8	PRJNA729271	1.270609956	3.59651E-05	1.270609956	stage_E14_vs_E10	ordinary_muscle_development	D_vs_E10;stage_1W_vs_E10;stage_3W_vs_E10;stage	1	TRUE
ENSGALG00010028806	SPACA9	PRJNA729271	1.630223435	3.59669E-05	1.630223435	stage_E14_vs_E10	ordinary_muscle_development	D_vs_E10;stage_1W_vs_E10;stage_3W_vs_E10;stage	1	TRUE
ENSGALG00010019049	POLD2	PRJNA729271	-0.696975606	3.62183E-05	0.696975606	stage_E14_vs_E10	ordinary_muscle_development		0	FALSE
ENSGALG00010004728	ENSGALG00010004728	PRJNA729271	-0.928180855	3.6301E-05	0.928180855	stage_E14_vs_E10	ordinary_muscle_development		0	FALSE
ENSGALG00010029518	FAAP100	PRJNA729271	-0.801558709	3.63308E-05	0.801558709	stage_E14_vs_E10	ordinary_muscle_development		0	FALSE
ENSGALG00010009109	ENSGALG00010009109	PRJNA729271	-0.998808433	3.63516E-05	0.998808433	stage_E14_vs_E10	ordinary_muscle_development		0	FALSE
ENSGALG00010009190	ZBTB49	PRJNA729271	0.58554801	3.6727E-05	0.58554801	stage_E14_vs_E10	ordinary_muscle_development		0	FALSE
ENSGALG00010014909	FBXL14	PRJNA729271	-0.429551324	3.6887E-05	0.429551324	stage_E14_vs_E10	ordinary_muscle_development		0	FALSE
ENSGALG00010023069	ENSGALG00010023069	PRJNA729271	1.166717801	3.6942E-05	1.166717801	stage_E14_vs_E10	ordinary_muscle_development	D_vs_E10;stage_1W_vs_E10;stage_3W_vs_E10;stage	1	TRUE
ENSGALG00010004576	STEAP2	PRJNA729271	0.892494518	3.69421E-05	0.892494518	stage_E14_vs_E10	ordinary_muscle_development		0	FALSE
ENSGALG00010004602	DYNLT2	PRJNA729271	-1.69469716	3.72484E-05	1.69469716	stage_E14_vs_E10	ordinary_muscle_development	D_vs_E10;stage_1W_vs_E10;stage_3W_vs_E10;stage	1	TRUE
ENSGALG00010013720	SLC16A10	PRJNA729271	0.806137923	3.75112E-05	0.806137923	stage_E14_vs_E10	ordinary_muscle_development		0	FALSE
ENSGALG00010001811	ACADSB	PRJNA729271	0.636063848	3.76918E-05	0.636063848	stage_E14_vs_E10	ordinary_muscle_development		0	FALSE
ENSGALG00010017818	SLC38A6	PRJNA729271	-0.587464086	3.79419E-05	0.587464086	stage_E14_vs_E10	ordinary_muscle_development		0	FALSE
ENSGALG00010008814	ENSGALG00010008814	PRJNA729271	0.97546466	3.81676E-05	0.97546466	stage_E14_vs_E10	ordinary_muscle_development		0	FALSE
ENSGALG00010014867	TP63	PRJNA729271	-1.167258937	3.8585E-05	1.167258937	stage_E14_vs_E10	ordinary_muscle_development	D_vs_E10;stage_1W_vs_E10;stage_3W_vs_E10;stage	1	TRUE
ENSGALG00010018522	NEK11	PRJNA729271	-1.213768046	3.86585E-05	1.213768046	stage_E14_vs_E10	ordinary_muscle_development	D_vs_E10;stage_1W_vs_E10;stage_3W_vs_E10;stage	1	TRUE
ENSGALG00010003113	WBP4	PRJNA729271	-0.501840022	3.86585E-05	0.501840022	stage_E14_vs_E10	ordinary_muscle_development		0	FALSE
ENSGALG00010024096	MYO1D	PRJNA729271	-0.98651592	3.86639E-05	0.98651592	stage_E14_vs_E10	ordinary_muscle_development		0	FALSE
ENSGALG00010011295	FAM167A	PRJNA729271	-1.714254513	3.88299E-05	1.714254513	stage_E14_vs_E10	ordinary_muscle_development	D_vs_E10;stage_1W_vs_E10;stage_3W_vs_E10;stage	1	TRUE
ENSGALG00010003248	MRI1	PRJNA729271	0.405962868	3.89652E-05	0.405962868	stage_E14_vs_E10	ordinary_muscle_development		0	FALSE
ENSGALG00010006506	ZNF827	PRJNA729271	-0.45053524	3.90719E-05	0.45053524	stage_E14_vs_E10	ordinary_muscle_development		0	FALSE
ENSGALG00010014946	PCK1	PRJNA729271	4.399196474	3.91463E-05	4.399196474	stage_E14_vs_E10	ordinary_muscle_development	D_vs_E10;stage_1W_vs_E10;stage_3W_vs_E10;stage	1	TRUE
ENSGALG00010022873	NGEF	PRJNA729271	-1.726867119	3.91668E-05	1.726867119	stage_E14_vs_E10	ordinary_muscle_development	D_vs_E10;stage_1W_vs_E10;stage_3W_vs_E10;stage	1	TRUE
ENSGALG00010006310	ALG5	PRJNA729271	-0.473327128	3.92221E-05	0.473327128	stage_E14_vs_E10	ordinary_muscle_development		0	FALSE
ENSGALG00010024030	STAU1	PRJNA729271	0.397556516	3.92955E-05	0.397556516	stage_E14_vs_E10	ordinary_muscle_development		0	FALSE
ENSGALG00010015233	ENSGALG00010015233	PRJNA729271	2.634011275	3.93682E-05	2.634011275	stage_E14_vs_E10	ordinary_muscle_development	D_vs_E10;stage_1W_vs_E10;stage_3W_vs_E10;stage	1	TRUE
ENSGALG00010008377	AKT2	PRJNA729271	0.493257002	3.93883E-05	0.493257002	stage_E14_vs_E10	ordinary_muscle_development		0	FALSE
ENSGALG00010016572	RAB33A	PRJNA729271	0.481788054	3.94659E-05	0.481788054	stage_E14_vs_E10	ordinary_muscle_development		0	FALSE
ENSGALG00010008139	ENSGALG00010008139	PRJNA729271	1.685063251	3.95051E-05	1.685063251	stage_E14_vs_E10	ordinary_muscle_development	D_vs_E10;stage_1W_vs_E10;stage_3W_vs_E10;stage	1	TRUE
ENSGALG00010008799	ENSGALG00010008799	PRJNA729271	0.741329026	3.95291E-05	0.741329026	stage_E14_vs_E10	ordinary_muscle_development		0	FALSE
ENSGALG00010010824	PKP4	PRJNA729271	-0.474489337	3.95519E-05	0.474489337	stage_E14_vs_E10	ordinary_muscle_development		0	FALSE
ENSGALG00010028917	NR6A1	PRJNA729271	-0.555144806	3.95998E-05	0.555144806	stage_E14_vs_E10	ordinary_muscle_development		0	FALSE
ENSGALG00010020293	SEMA4B	PRJNA729271	-0.480947804	3.96014E-05	0.480947804	stage_E14_vs_E10	ordinary_muscle_development		0	FALSE
ENSGALG00010014780	EVA1B	PRJNA729271	0.529507004	3.96856E-05	0.529507004	stage_E14_vs_E10	ordinary_muscle_development		0	FALSE
ENSGALG00010014065	CHUNK-1	PRJNA729271	-4.188549853	3.9741E-05	4.188549853	stage_E14_vs_E10	ordinary_muscle_development	D_vs_E10;stage_1W_vs_E10;stage_3W_vs_E10;stage	1	TRUE
ENSGALG00010002432	RAVER1	PRJNA729271	-0.783261889	3.99297E-05	0.783261889	stage_E14_vs_E10	ordinary_muscle_development		0	FALSE
ENSGALG00010007230	ENSGALG00010007230	PRJNA729271	-1.500955703	3.9978E-05	1.500955703	stage_E14_vs_E10	ordinary_muscle_development	D_vs_E10;stage_1W_vs_E10;stage_3W_vs_E10;stage	1	TRUE
ENSGALG00010027905	SYN2	PRJNA729271	1.12011703	4.02011E-05	1.12011703	stage_E14_vs_E10	ordinary_muscle_development	D_vs_E10;stage_1W_vs_E10;stage_3W_vs_E10;stage	1	TRUE
ENSGALG00010014066	BCL2L1	PRJNA729271	-0.500820673	4.02288E-05	0.500820673	stage_E14_vs_E10	ordinary_muscle_development		0	FALSE
ENSGALG00010007114	CKLF	PRJNA729271	-0.650302108	4.02834E-05	0.650302108	stage_E14_vs_E10	ordinary_muscle_development		0	FALSE
ENSGALG00010013106	SETD9	PRJNA729271	-0.796047079	4.0341E-05	0.796047079	stage_E14_vs_E10	ordinary_muscle_development		0	FALSE
ENSGALG00010001231	CNDP1	PRJNA729271	2.197486362	4.06095E-05	2.197486362	stage_E14_vs_E10	ordinary_muscle_development	D_vs_E10;stage_1W_vs_E10;stage_3W_vs_E10;stage	1	TRUE
ENSGALG00010006752	ZAR1L	PRJNA729271	-1.858123916	4.07808E-05	1.858123916	stage_E14_vs_E10	ordinary_muscle_development	D_vs_E10;stage_1W_vs_E10;stage_3W_vs_E10;stage	1	TRUE
ENSGALG00010004330	STX7	PRJNA729271	0.435336328	4.07841E-05	0.435336328	stage_E14_vs_E10	ordinary_muscle_development		0	FALSE
ENSGALG00010021831	ENSGALG00010021831	PRJNA729271	1.533109093	4.09215E-05	1.533109093	stage_E14_vs_E10	ordinary_muscle_development	D_vs_E10;stage_1W_vs_E10;stage_3W_vs_E10;stage	1	TRUE
ENSGALG00010007897	EFHC1	PRJNA729271	-1.966538254	4.117E-05	1.966538254	stage_E14_vs_E10	ordinary_muscle_development	D_vs_E10;stage_1W_vs_E10;stage_3W_vs_E10;stage	1	TRUE
ENSGALG00010010614	IKBIP	PRJNA729271	-0.612804248	4.14591E-05	0.612804248	stage_E14_vs_E10	ordinary_muscle_development		0	FALSE
ENSGALG00010004949	RAB39A	PRJNA729271	-1.455648654	4.15835E-05	1.455648654	stage_E14_vs_E10	ordinary_muscle_development	D_vs_E10;stage_1W_vs_E10;stage_3W_vs_E10;stage	1	TRUE
ENSGALG00010010990	MED18	PRJNA729271	0.50345984	4.17738E-05	0.50345984	stage_E14_vs_E10	ordinary_muscle_development		0	FALSE
ENSGALG00010019068	SNRPA1	PRJNA729271	0.68802628	4.18041E-05	0.68802628	stage_E14_vs_E10	ordinary_muscle_development		0	FALSE
ENSGALG00010011857	ENSGALG00010011857	PRJNA729271	1.033598772	4.22002E-05	1.033598772	stage_E14_vs_E10	ordinary_muscle_development	D_vs_E10;stage_1W_vs_E10;stage_3W_vs_E10;stage	1	TRUE
ENSGALG00010007912	TRAPPC2L	PRJNA729271	-0.488785613	4.24315E-05	0.488785613	stage_E14_vs_E10	ordinary_muscle_development		0	FALSE
ENSGALG00010001549	ENSGALG00010001549	PRJNA729271	-2.403368075	4.24354E-05	2.403368075	stage_E14_vs_E10	ordinary_muscle_development	D_vs_E10;stage_1W_vs_E10;stage_3W_vs_E10;stage	1	TRUE
ENSGALG00010029308	GAA	PRJNA729271	0.586105495	4.24488E-05	0.586105495	stage_E14_vs_E10	ordinary_muscle_development		0	FALSE
ENSGALG00010015960	CHRNA9	PRJNA729271	3.560912324	4.25101E-05	3.560912324	stage_E14_vs_E10	ordinary_muscle_development	D_vs_E10;stage_1W_vs_E10;stage_3W_vs_E10;stage	1	TRUE
ENSGALG00010023987	TNNI3K	PRJNA729271	-2.019249438	4.25202E-05	2.019249438	stage_E14_vs_E10	ordinary_muscle_development	D_vs_E10;stage_1W_vs_E10;stage_3W_vs_E10;stage	1	TRUE
ENSGALG00010010937	CDK7	PRJNA729271	0.483972349	4.25267E-05	0.483972349	stage_E14_vs_E10	ordinary_muscle_development		0	FALSE
ENSGALG00010018524	SEC22A	PRJNA729271	-0.49686427	4.26505E-05	0.49686427	stage_E14_vs_E10	ordinary_muscle_development		0	FALSE
ENSGALG00010027184	MPND	PRJNA729271	-1.667569587	4.27124E-05	1.667569587	stage_E14_vs_E10	ordinary_muscle_development	D_vs_E10;stage_1W_vs_E10;stage_3W_vs_E10;stage	1	TRUE
ENSGALG00010003474	RPS7	PRJNA729271	0.710416696	4.27521E-05	0.710416696	stage_E14_vs_E10	ordinary_muscle_development		0	FALSE
ENSGALG00010003786	ENSGALG00010003786	PRJNA729271	1.956558455	4.28102E-05	1.956558455	stage_E14_vs_E10	ordinary_muscle_development	D_vs_E10;stage_1W_vs_E10;stage_3W_vs_E10;stage	1	TRUE
ENSGALG00010005675	GJA3	PRJNA729271	-1.300040065	4.28337E-05	1.300040065	stage_E14_vs_E10	ordinary_muscle_development	D_vs_E10;stage_1W_vs_E10;stage_3W_vs_E10;stage	1	TRUE
ENSGALG00010008931	FNIP2	PRJNA729271	0.563135108	4.28671E-05	0.563135108	stage_E14_vs_E10	ordinary_muscle_development		0	FALSE
ENSGALG00010010035	ATP10B	PRJNA729271	-1.43315708	4.30625E-05	1.43315708	stage_E14_vs_E10	ordinary_muscle_development	D_vs_E10;stage_1W_vs_E10;stage_3W_vs_E10;stage	1	TRUE
ENSGALG00010027960	PPP6C	PRJNA729271	0.503463005	4.32821E-05	0.503463005	stage_E14_vs_E10	ordinary_muscle_development		0	FALSE
ENSGALG00010027203	ENSGALG00010027203	PRJNA729271	-1.308344575	4.35057E-05	1.308344575	stage_E14_vs_E10	ordinary_muscle_development	D_vs_E10;stage_1W_vs_E10;stage_3W_vs_E10;stage	1	TRUE
ENSGALG00010002604	BLEC3	PRJNA729271	1.042626171	4.37689E-05	1.042626171	stage_E14_vs_E10	ordinary_muscle_development	D_vs_E10;stage_1W_vs_E10;stage_3W_vs_E10;stage	1	TRUE
ENSGALG00010011022	C5orf22	PRJNA729271	-0.514918933	4.37689E-05	0.514918933	stage_E14_vs_E10	ordinary_muscle_development		0	FALSE
ENSGALG00010009491	RBM34	PRJNA729271	0.271130898	4.37689E-05	0.271130898	stage_E14_vs_E10	ordinary_muscle_development		0	FALSE
ENSGALG00010006372	SBF1	PRJNA729271	0.477203512	4.39122E-05	0.477203512	stage_E14_vs_E10	ordinary_muscle_development		0	FALSE
ENSGALG00010000509	ENSGALG00010000509	PRJNA729271	-4.910366333	4.41794E-05	4.910366333	stage_E14_vs_E10	ordinary_muscle_development	D_vs_E10;stage_1W_vs_E10;stage_3W_vs_E10;stage	1	TRUE
ENSGALG00010009546	GAREM2	PRJNA729271	-3.536144489	4.41888E-05	3.536144489	stage_E14_vs_E10	ordinary_muscle_development	D_vs_E10;stage_1W_vs_E10;stage_3W_vs_E10;stage	1	TRUE
ENSGALG00010019184	PNKD	PRJNA729271	-0.813421994	4.41888E-05	0.813421994	stage_E14_vs_E10	ordinary_muscle_development		0	FALSE
ENSGALG00010022811	GIGYF2	PRJNA729271	0.413688082	4.42456E-05	0.413688082	stage_E14_vs_E10	ordinary_muscle_development		0	FALSE
ENSGALG00010029406	TMEM199	PRJNA729271	0.428034193	4.43785E-05	0.428034193	stage_E14_vs_E10	ordinary_muscle_development		0	FALSE
ENSGALG00010001829	POLD3	PRJNA729271	-0.513060161	4.46248E-05	0.513060161	stage_E14_vs_E10	ordinary_muscle_development		0	FALSE
ENSGALG00010021251	RNF216	PRJNA729271	-0.361183647	4.47291E-05	0.361183647	stage_E14_vs_E10	ordinary_muscle_development		0	FALSE
ENSGALG00010021707	DLX2	PRJNA729271	-4.768620609	4.47362E-05	4.768620609	stage_E14_vs_E10	ordinary_muscle_development	D_vs_E10;stage_1W_vs_E10;stage_3W_vs_E10;stage	1	TRUE
ENSGALG00010008372	CLCN3	PRJNA729271	-0.299095963	4.49487E-05	0.299095963	stage_E14_vs_E10	ordinary_muscle_development		0	FALSE
ENSGALG00010025553	UBE2G2	PRJNA729271	-0.408136429	4.57602E-05	0.408136429	stage_E14_vs_E10	ordinary_muscle_development		0	FALSE
ENSGALG00010028926	ENSGALG00010028926	PRJNA729271	-2.79727628	4.59835E-05	2.79727628	stage_E14_vs_E10	ordinary_muscle_development	D_vs_E10;stage_1W_vs_E10;stage_3W_vs_E10;stage	1	TRUE
ENSGALG00010010693	KCNMB1	PRJNA729271	1.384719123	4.5997E-05	1.384719123	stage_E14_vs_E10	ordinary_muscle_development	D_vs_E10;stage_1W_vs_E10;stage_3W_vs_E10;stage	1	TRUE
ENSGALG00010017326	STON1	PRJNA729271	0.506351317	4.60056E-05	0.506351317	stage_E14_vs_E10	ordinary_muscle_development		0	FALSE
ENSGALG00010018069	ENSGALG00010018069	PRJNA729271	-1.245261094	4.64192E-05	1.245261094	stage_E14_vs_E10	ordinary_muscle_development	D_vs_E10;stage_1W_vs_E10;stage_3W_vs_E10;stage	1	TRUE
ENSGALG00010014294	TMPO	PRJNA729271	-0.428137402	4.69145E-05	0.428137402	stage_E14_vs_E10	ordinary_muscle_development		0	FALSE
ENSGALG00010002362	TRPC4	PRJNA729271	-1.815468409	4.69564E-05	1.815468409	stage_E14_vs_E10	ordinary_muscle_development	D_vs_E10;stage_1W_vs_E10;stage_3W_vs_E10;stage	1	TRUE
ENSGALG00010008415	ENSGALG00010008415	PRJNA729271	1.493177914	4.69564E-05	1.493177914	stage_E14_vs_E10	ordinary_muscle_development	D_vs_E10;stage_1W_vs_E10;stage_3W_vs_E10;stage	1	TRUE
ENSGALG00010002787	CEP44	PRJNA729271	-0.565195555	4.70284E-05	0.565195555	stage_E14_vs_E10	ordinary_muscle_development		0	FALSE
ENSGALG00010022502	ENSGALG00010022502	PRJNA729271	-2.64273463	4.72527E-05	2.64273463	stage_E14_vs_E10	ordinary_muscle_development	D_vs_E10;stage_1W_vs_E10;stage_3W_vs_E10;stage	1	TRUE
ENSGALG00010025847	DEF6	PRJNA729271	-0.539049563	4.75256E-05	0.539049563	stage_E14_vs_E10	ordinary_muscle_development		0	FALSE
ENSGALG00010015967	ZSWIM1	PRJNA729271	-0.944574016	4.76037E-05	0.944574016	stage_E14_vs_E10	ordinary_muscle_development		0	FALSE
ENSGALG00010001548	AKAP17A	PRJNA729271	-0.41423481	4.77092E-05	0.41423481	stage_E14_vs_E10	ordinary_muscle_development		0	FALSE
ENSGALG00010027328	RBM15	PRJNA729271	-0.56293837	4.80819E-05	0.56293837	stage_E14_vs_E10	ordinary_muscle_development		0	FALSE
ENSGALG00010008775	MED17	PRJNA729271	-0.386412951	4.80852E-05	0.386412951	stage_E14_vs_E10	ordinary_muscle_development		0	FALSE
ENSGALG00010014956	FGG	PRJNA729271	6.472816511	4.80951E-05	6.472816511	stage_E14_vs_E10	ordinary_muscle_development	D_vs_E10;stage_1W_vs_E10;stage_3W_vs_E10;stage	1	TRUE
ENSGALG00010021016	FHIT	PRJNA729271	-0.934065125	4.8658E-05	0.934065125	stage_E14_vs_E10	ordinary_muscle_development		0	FALSE
ENSGALG00010018485	ATP2C1	PRJNA729271	-0.249302002	4.91071E-05	0.249302002	stage_E14_vs_E10	ordinary_muscle_development		0	FALSE
ENSGALG00010003320	OR51E2	PRJNA729271	1.905739477	4.92686E-05	1.905739477	stage_E14_vs_E10	ordinary_muscle_development	D_vs_E10;stage_1W_vs_E10;stage_3W_vs_E10;stage	1	TRUE
ENSGALG00010008131	OC90	PRJNA729271	-4.03109918	4.9338E-05	4.03109918	stage_E14_vs_E10	ordinary_muscle_development	D_vs_E10;stage_1W_vs_E10;stage_3W_vs_E10;stage	1	TRUE

ENSGALG00010006865	EFCAB2	PRJNA729271	-1.549754137	4.96116E-05	1.549754137	stage_E14_vs_E10	ordinary_muscle_development	D_vs_E10;stage_1W_vs_E10;stage_3W_vs_E10;stage	1	TRUE
ENSGALG00010013162	IDS	PRJNA729271	0.468776331	4.96542E-05	0.468776331	stage_E14_vs_E10	ordinary_muscle_development		0	FALSE
ENSGALG00010028446	ENSGALG00010028446	PRJNA729271	-0.931458755	4.97702E-05	0.931458755	stage_E14_vs_E10	ordinary_muscle_development		0	FALSE
ENSGALG00010003262	ENSGALG00010003262	PRJNA729271	-2.577701544	4.98343E-05	2.577701544	stage_E14_vs_E10	ordinary_muscle_development	D_vs_E10;stage_1W_vs_E10;stage_3W_vs_E10;stage	1	TRUE
ENSGALG00010011437	DMRT1	PRJNA729271	-2.500963032	5.00674E-05	2.500963032	stage_E14_vs_E10	ordinary_muscle_development	D_vs_E10;stage_1W_vs_E10;stage_3W_vs_E10;stage	1	TRUE
ENSGALG00010028013	CBARP	PRJNA729271	0.773892792	5.03087E-05	0.773892792	stage_E14_vs_E10	ordinary_muscle_development		0	FALSE
ENSGALG00010029354	TLCD2	PRJNA729271	-1.252793365	5.047E-05	1.252793365	stage_E14_vs_E10	ordinary_muscle_development	D_vs_E10;stage_1W_vs_E10;stage_3W_vs_E10;stage	1	TRUE
ENSGALG00010013487	ENSGALG00010013487	PRJNA729271	-0.398610672	5.05192E-05	0.398610672	stage_E14_vs_E10	ordinary_muscle_development		0	FALSE
ENSGALG00010017586	ZFYVE28	PRJNA729271	0.686867454	5.06133E-05	0.686867454	stage_E14_vs_E10	ordinary_muscle_development		0	FALSE
ENSGALG00010018855	SYNDIG1	PRJNA729271	-3.510258627	5.06313E-05	3.510258627	stage_E14_vs_E10	ordinary_muscle_development	D_vs_E10;stage_1W_vs_E10;stage_3W_vs_E10;stage	1	TRUE
ENSGALG00010004591	RRS1	PRJNA729271	0.673323331	5.10247E-05	0.673323331	stage_E14_vs_E10	ordinary_muscle_development		0	FALSE
ENSGALG00010018819	LRRC28	PRJNA729271	0.327446897	5.10247E-05	0.327446897	stage_E14_vs_E10	ordinary_muscle_development		0	FALSE
ENSGALG00010017403	ITGA11	PRJNA729271	-1.227188247	5.10663E-05	1.227188247	stage_E14_vs_E10	ordinary_muscle_development	D_vs_E10;stage_1W_vs_E10;stage_3W_vs_E10;stage	1	TRUE
ENSGALG00010012108	PSTPIP2	PRJNA729271	-1.530320499	5.11128E-05	1.530320499	stage_E14_vs_E10	ordinary_muscle_development	D_vs_E10;stage_1W_vs_E10;stage_3W_vs_E10;stage	1	TRUE
ENSGALG00010023155	CFAP263	PRJNA729271	-1.609891373	5.14662E-05	1.609891373	stage_E14_vs_E10	ordinary_muscle_development	D_vs_E10;stage_1W_vs_E10;stage_3W_vs_E10;stage	1	TRUE
ENSGALG00010018063	DECR2	PRJNA729271	0.4280738	5.15058E-05	0.4280738	stage_E14_vs_E10	ordinary_muscle_development		0	FALSE
ENSGALG00010016350	CASC4	PRJNA729271	0.26174743	5.20273E-05	0.26174743	stage_E14_vs_E10	ordinary_muscle_development		0	FALSE
ENSGALG00010017144	CPB1	PRJNA729271	3.014805843	5.20714E-05	3.014805843	stage_E14_vs_E10	ordinary_muscle_development	D_vs_E10;stage_1W_vs_E10;stage_3W_vs_E10;stage	1	TRUE
ENSGALG00010025416	ENSGALG00010025416	PRJNA729271	2.499657958	5.28266E-05	2.499657958	stage_E14_vs_E10	ordinary_muscle_development	D_vs_E10;stage_1W_vs_E10;stage_3W_vs_E10;stage	1	TRUE
ENSGALG00010027125	CRYGN	PRJNA729271	1.668432831	5.28819E-05	1.668432831	stage_E14_vs_E10	ordinary_muscle_development	D_vs_E10;stage_1W_vs_E10;stage_3W_vs_E10;stage	1	TRUE
ENSGALG00010009073	PRKAR2B	PRJNA729271	1.702885866	5.31071E-05	1.702885866	stage_E14_vs_E10	ordinary_muscle_development	D_vs_E10;stage_1W_vs_E10;stage_3W_vs_E10;stage	1	TRUE
ENSGALG00010027867	UBAP2L	PRJNA729271	-0.704709025	5.32978E-05	0.704709025	stage_E14_vs_E10	ordinary_muscle_development		0	FALSE
ENSGALG00010016575	CWH43	PRJNA729271	1.569976278	5.382E-05	1.569976278	stage_E14_vs_E10	ordinary_muscle_development	D_vs_E10;stage_1W_vs_E10;stage_3W_vs_E10;stage	1	TRUE
ENSGALG00010005314	IL20RA	PRJNA729271	-2.768633536	5.42968E-05	2.768633536	stage_E14_vs_E10	ordinary_muscle_development	D_vs_E10;stage_1W_vs_E10;stage_3W_vs_E10;stage	1	TRUE
ENSGALG00010014672	ISL1	PRJNA729271	-5.119373635	5.45815E-05	5.119373635	stage_E14_vs_E10	ordinary_muscle_development	D_vs_E10;stage_1W_vs_E10;stage_3W_vs_E10;stage	1	TRUE
ENSGALG00010004673	RAB18	PRJNA729271	0.352688777	5.47961E-05	0.352688777	stage_E14_vs_E10	ordinary_muscle_development		0	FALSE
ENSGALG00010024940	RABL3	PRJNA729271	-0.383954474	5.51241E-05	0.383954474	stage_E14_vs_E10	ordinary_muscle_development		0	FALSE
ENSGALG00010023636	PDE3B	PRJNA729271	0.337471114	5.51907E-05	0.337471114	stage_E14_vs_E10	ordinary_muscle_development		0	FALSE
ENSGALG00010000022	ATP8	PRJNA729271	1.128341656	5.53619E-05	1.128341656	stage_E14_vs_E10	ordinary_muscle_development	D_vs_E10;stage_1W_vs_E10;stage_3W_vs_E10;stage	1	TRUE
ENSGALG00010001251	ZNF277	PRJNA729271	-0.516771783	5.56233E-05	0.516771783	stage_E14_vs_E10	ordinary_muscle_development		0	FALSE
ENSGALG00010003419	ENSGALG00010003419	PRJNA729271	-4.972756275	5.58205E-05	4.972756275	stage_E14_vs_E10	ordinary_muscle_development	D_vs_E10;stage_1W_vs_E10;stage_3W_vs_E10;stage	1	TRUE
ENSGALG00010018026	CCDC39	PRJNA729271	-4.225357054	5.59211E-05	4.225357054	stage_E14_vs_E10	ordinary_muscle_development	D_vs_E10;stage_1W_vs_E10;stage_3W_vs_E10;stage	1	TRUE
ENSGALG00010021870	ENSGALG00010021870	PRJNA729271	-0.706730561	5.60505E-05	0.706730561	stage_E14_vs_E10	ordinary_muscle_development		0	FALSE
ENSGALG00010010826	NCOA1	PRJNA729271	0.68163317	5.6118E-05	0.68163317	stage_E14_vs_E10	ordinary_muscle_development		0	FALSE
ENSGALG00010017334	KDM8	PRJNA729271	-0.654352185	5.61331E-05	0.654352185	stage_E14_vs_E10	ordinary_muscle_development		0	FALSE
ENSGALG00010009482	ARID4B	PRJNA729271	0.449765767	5.61414E-05	0.449765767	stage_E14_vs_E10	ordinary_muscle_development		0	FALSE
ENSGALG00010004429	ENSGALG00010004429	PRJNA729271	1.167194182	5.63783E-05	1.167194182	stage_E14_vs_E10	ordinary_muscle_development	D_vs_E10;stage_1W_vs_E10;stage_3W_vs_E10;stage	1	TRUE
ENSGALG00010028750	OTUD7B	PRJNA729271	0.499459144	5.64266E-05	0.499459144	stage_E14_vs_E10	ordinary_muscle_development		0	FALSE
ENSGALG00010021689	MPRIP	PRJNA729271	0.476585816	5.6531E-05	0.476585816	stage_E14_vs_E10	ordinary_muscle_development		0	FALSE
ENSGALG00010029196	MRM1	PRJNA729271	-0.428826276	5.66639E-05	0.428826276	stage_E14_vs_E10	ordinary_muscle_development		0	FALSE
ENSGALG00010022049	EPHX4	PRJNA729271	-2.814588574	5.6755E-05	2.814588574	stage_E14_vs_E10	ordinary_muscle_development	D_vs_E10;stage_1W_vs_E10;stage_3W_vs_E10;stage	1	TRUE
ENSGALG00010014298	METTL18	PRJNA729271	-0.945888705	5.69834E-05	0.945888705	stage_E14_vs_E10	ordinary_muscle_development		0	FALSE
ENSGALG00010009671	MINAR2	PRJNA729271	3.342814696	5.70999E-05	3.342814696	stage_E14_vs_E10	ordinary_muscle_development	D_vs_E10;stage_1W_vs_E10;stage_3W_vs_E10;stage	1	TRUE
ENSGALG00010018494	HAO1	PRJNA729271	1.169498086	5.76092E-05	1.169498086	stage_E14_vs_E10	ordinary_muscle_development	D_vs_E10;stage_1W_vs_E10;stage_3W_vs_E10;stage	1	TRUE
ENSGALG00010029407	UBE2O	PRJNA729271	0.780179637	5.77222E-05	0.780179637	stage_E14_vs_E10	ordinary_muscle_development		0	FALSE
ENSGALG00010011027	FAM234B	PRJNA729271	-0.425870135	5.78089E-05	0.425870135	stage_E14_vs_E10	ordinary_muscle_development		0	FALSE
ENSGALG00010003274	BACH2	PRJNA729271	0.614301215	5.79169E-05	0.614301215	stage_E14_vs_E10	ordinary_muscle_development		0	FALSE
ENSGALG00010022511	IGFBP4	PRJNA729271	-1.12563318	5.82965E-05	1.12563318	stage_E14_vs_E10	ordinary_muscle_development	D_vs_E10;stage_1W_vs_E10;stage_3W_vs_E10;stage	1	TRUE
ENSGALG00010016864	ENSGALG00010016864	PRJNA729271	1.09709454	5.85151E-05	1.09709454	stage_E14_vs_E10	ordinary_muscle_development	D_vs_E10;stage_1W_vs_E10;stage_3W_vs_E10;stage	1	TRUE
ENSGALG00010010885	SNRPD2	PRJNA729271	-1.164552522	5.86806E-05	1.164552522	stage_E14_vs_E10	ordinary_muscle_development	D_vs_E10;stage_1W_vs_E10;stage_3W_vs_E10;stage	1	TRUE
ENSGALG00010017200	KCNQ4	PRJNA729271	1.270897474	5.86906E-05	1.270897474	stage_E14_vs_E10	ordinary_muscle_development	D_vs_E10;stage_1W_vs_E10;stage_3W_vs_E10;stage	1	TRUE
ENSGALG00010019418	ZNF703	PRJNA729271	0.955016535	5.87676E-05	0.955016535	stage_E14_vs_E10	ordinary_muscle_development		0	FALSE
ENSGALG00010013831	TCERG1	PRJNA729271	-0.477461133	5.88231E-05	0.477461133	stage_E14_vs_E10	ordinary_muscle_development		0	FALSE
ENSGALG00010001190	LXN	PRJNA729271	-2.021191484	5.8967E-05	2.021191484	stage_E14_vs_E10	ordinary_muscle_development	D_vs_E10;stage_1W_vs_E10;stage_3W_vs_E10;stage	1	TRUE
ENSGALG00010009775	KIAA1614	PRJNA729271	-0.600654698	5.9523E-05	0.600654698	stage_E14_vs_E10	ordinary_muscle_development		0	FALSE
ENSGALG00010018923	VPS54	PRJNA729271	-0.384984535	5.95513E-05	0.384984535	stage_E14_vs_E10	ordinary_muscle_development		0	FALSE
ENSGALG00010009726	PRR16	PRJNA729271	0.583047714	5.9902E-05	0.583047714	stage_E14_vs_E10	ordinary_muscle_development		0	FALSE
ENSGALG00010029442	ENSGALG00010029442	PRJNA729271	-1.179122544	6.0021E-05	1.179122544	stage_E14_vs_E10	ordinary_muscle_development	D_vs_E10;stage_1W_vs_E10;stage_3W_vs_E10;stage	1	TRUE
ENSGALG00010014155	ENSGALG00010014155	PRJNA729271	-6.243034003	6.00402E-05	6.243034003	stage_E14_vs_E10	ordinary_muscle_development	D_vs_E10;stage_1W_vs_E10;stage_3W_vs_E10;stage	1	TRUE
ENSGALG00010007603	ALG2	PRJNA729271	-0.439012864	6.05334E-05	0.439012864	stage_E14_vs_E10	ordinary_muscle_development		0	FALSE
ENSGALG00010023617	ENSGALG00010023617	PRJNA729271	1.212790165	6.05729E-05	1.212790165	stage_E14_vs_E10	ordinary_muscle_development	D_vs_E10;stage_1W_vs_E10;stage_3W_vs_E10;stage	1	TRUE
ENSGALG00010009188	ENSGALG00010009188	PRJNA729271	-2.23603347	6.06422E-05	2.23603347	stage_E14_vs_E10	ordinary_muscle_development	D_vs_E10;stage_1W_vs_E10;stage_3W_vs_E10;stage	1	TRUE
ENSGALG00010022498	NAA50	PRJNA729271	0.469927514	6.09146E-05	0.469927514	stage_E14_vs_E10	ordinary_muscle_development		0	FALSE
ENSGALG00010028415	FSD1	PRJNA729271	-0.701522004	6.11683E-05	0.701522004	stage_E14_vs_E10	ordinary_muscle_development		0	FALSE
ENSGALG00010023396	ENSGALG00010023396	PRJNA729271	0.590074076	6.20306E-05	0.590074076	stage_E14_vs_E10	ordinary_muscle_development		0	FALSE
ENSGALG00010018052	PPP1RL	PRJNA729271	-0.543815505	6.21272E-05	0.543815505	stage_E14_vs_E10	ordinary_muscle_development		0	FALSE
ENSGALG00010028150	ARMC6	PRJNA729271	-0.346816602	6.2314E-05	0.346816602	stage_E14_vs_E10	ordinary_muscle_development		0	FALSE
ENSGALG00010021677	ENSGALG00010021677	PRJNA729271	-0.641096028	6.2581E-05	0.641096028	stage_E14_vs_E10	ordinary_muscle_development		0	FALSE
ENSGALG00010003968	DSN1	PRJNA729271	0.708684222	6.2594E-05	0.708684222	stage_E14_vs_E10	ordinary_muscle_development		0	FALSE
ENSGALG00010021171	ENSGALG00010021171	PRJNA729271	-0.886626922	6.26738E-05	0.886626922	stage_E14_vs_E10	ordinary_muscle_development		0	FALSE
ENSGALG00010003733	STAU2	PRJNA729271	0.303220868	6.27143E-05	0.303220868	stage_E14_vs_E10	ordinary_muscle_development		0	FALSE
ENSGALG00010005167	GUCY1A2	PRJNA729271	0.732935005	6.27234E-05	0.732935005	stage_E14_vs_E10	ordinary_muscle_development		0	FALSE
ENSGALG00010021122	CYP1A1	PRJNA729271	3.780128956	6.27622E-05	3.780128956	stage_E14_vs_E10	ordinary_muscle_development	D_vs_E10;stage_1W_vs_E10;stage_3W_vs_E10;stage	1	TRUE
ENSGALG00010014408	ENSGALG00010014408	PRJNA729271	-1.562065885	6.28517E-05	1.562065885	stage_E14_vs_E10	ordinary_muscle_development	D_vs_E10;stage_1W_vs_E10;stage_3W_vs_E10;stage	1	TRUE
ENSGALG00010005943	PROX2	PRJNA729271	-4.379014482	6.29894E-05	4.379014482	stage_E14_vs_E10	ordinary_muscle_development	D_vs_E10;stage_1W_vs_E10;stage_3W_vs_E10;stage	1	TRUE
ENSGALG00010025121	DYNC2I1	PRJNA729271	-0.648168843	6.30163E-05	0.648168843	stage_E14_vs_E10	ordinary_muscle_development		0	FALSE
ENSGALG00010002435	ATP7B	PRJNA729271	-3.104083913	6.31118E-05	3.104083913	stage_E14_vs_E10	ordinary_muscle_development	D_vs_E10;stage_1W_vs_E10;stage_3W_vs_E10;stage	1	TRUE
ENSGALG00010028149	SNAPC4	PRJNA729271	0.361247882	6.31638E-05	0.361247882	stage_E14_vs_E10	ordinary_muscle_development		0	FALSE
ENSGALG00010000011	ND2	PRJNA729271	1.04719932	6.36023E-05	1.04719932	stage_E14_vs_E10	ordinary_muscle_development	D_vs_E10;stage_1W_vs_E10;stage_3W_vs_E10;stage	1	TRUE
ENSGALG00010029747	TRPV3	PRJNA729271	1.392891972	6.37021E-05	1.392891972	stage_E14_vs_E10	ordinary_muscle_development	D_vs_E10;stage_1W_vs_E10;stage_3W_vs_E10;stage	1	TRUE
ENSGALG00010017717	LLGL1	PRJNA729271	-0.623348521	6.42207E-05	0.623348521	stage_E14_vs_E10	ordinary_muscle_development		0	FALSE
ENSGALG00010015816	MITD1	PRJNA729271	-0.919026353	6.42273E-05	0.919026353	stage_E14_vs_E10	ordinary_muscle_development		0	FALSE
ENSGALG00010025359	CDCP2	PRJNA729271	1.556424704	6.4246E-05	1.556424704	stage_E14_vs_E10	ordinary_muscle_development	D_vs_E10;stage_1W_vs_E10;stage_3W_vs_E10;stage	1	TRUE
ENSGALG00010028429	REXO1	PRJNA729271	-0.374236864	6.43782E-05	0.374236864	stage_E14_vs_E10	ordinary_muscle_development		0	FALSE
ENSGALG00010017901	HTR7L	PRJNA729271	-3.231374467	6.4902E-05	3.231374467	stage_E14_vs_E10	ordinary_muscle_development	D_vs_E10;stage_1W_vs_E10;stage_3W_vs_E10;stage	1	TRUE
ENSGALG00010010567	ERMN	PRJNA729271	-3.370431777	6.50751E-05	3.370431777	stage_E14_vs_E10	ordinary_muscle_development	D_vs_E10;stage_1W_vs_E10;stage_3W_vs_E10;stage	1	TRUE
ENSGALG00010012040	B4GALT7	PRJNA729271	0.614825039	6.51283E-05	0.614825039	stage_E14_vs_E10	ordinary_muscle_development		0	FALSE
ENSGALG00010029538	FZD9	PRJNA729271	0.710614184	6.51639E-05	0.710614184	stage_E14_vs_E10	ordinary_muscle_development		0	FALSE
ENSGALG00010025963	OAF	PRJNA729271	-1.01474219	6.53778E-05	1.01474219	stage_E14_vs_E10	ordinary_muscle_development	D_vs_E10;stage_1W_vs_E10;stage_3W_vs_E10;stage	1	TRUE
ENSGALG00010022500	ENSGALG00010022500	PRJNA729271	1.688278137	6.54878E-05	1.688278137	stage_E14_vs_E10	ordinary_muscle_development	D_vs_E10;stage_1W_vs_E10;stage_3W_vs_E10;stage	1	TRUE
ENSGALG00010014167	SLC38A9	PRJNA729271	0.444981876	6.56815E-05	0.444981876	stage_E14_vs_E10	ordinary_muscle_development		0	FALSE
ENSGALG00010027772	ENSGALG00010027772	PRJNA729271	3.599673815	6.57573E-05	3.599673815	stage_E14_vs_E10	ordinary_muscle_development	D_vs_E10;stage_1W_vs_E10;stage_3W_vs_E10;stage	1	TRUE
ENSGALG00010000777	MYCBP2	PRJNA729271	0.339541012	6.57583E-05	0.339541012	stage_E14_vs_E10	ordinary_muscle_development		0	FALSE
ENSGALG00010000137	ENSGALG00010000137	PRJNA729271	-2.190295928	6.5825E-05	2.190295928	stage_E14_vs_E10	ordinary_muscle_development	D_vs_E10;stage_1W_vs_E10;stage_3W_vs_E10;stage	1	TRUE
ENSGALG00010017698	ENSGALG00010017698	PRJNA729271	2.900664948	6.66128E-05	2.900664948	stage_E14_vs_E10	ordinary_muscle_development	D_vs_E10;stage_1W_vs_E10;stage_3W_vs_E10;stage	1	TRUE
ENSGALG00010021478	SLC13A3	PRJNA729271	-0.816006958	6.66128E-05	0.816006958	stage_E14_vs_E10	ordinary_muscle_development		0	FALSE
ENSGALG00010019624	SRGAP3	PRJNA729271	0.5409033	6.66155E-05	0.5409033	stage_E14_vs_E10	ordinary_muscle_development		0	FALSE
ENSGALG00010001835	MECP2	PRJNA729271	0.781083355	6.67041E-05	0.781083355	stage_E14_vs_E10	ordinary_muscle_development		0	FALSE
ENSGALG00010012347	ENSGALG00010012347	PRJNA729271	-2.066271871	6.70511E-05	2.066271871	stage_E14_vs_E10	ordinary_muscle_development	D_vs_E10;stage_1W_vs_E10;stage_3W_vs_E10;stage	1	TRUE
ENSGALG00010000605	MARCHF11	PRJNA729271	-4.08525474	6.70708E-05	4.08525474	stage_E14_vs_E10	ordinary_muscle_development	D_vs_E10;stage_1W_vs_E10;stage_3W_vs_E10;stage	1	TRUE
ENSGALG00010028604	PI4KB	PRJNA729271	0.362419056	6.74408E-05	0.362419056	stage_E14_vs_E10	ordinary_muscle_development		0	FALSE
ENSGALG00010020006	PLB1	PRJNA729271	-1.600802207	6.74758E-05	1.600802207	stage_E14_vs_E10	ordinary_muscle_development	D_vs_E10;stage_1W_vs_E10;stage_3W_vs_E10;stage	1	TRUE
ENSGALG00010004018	HNRNPD	PRJNA729271	-0.676083852	6.75729E-05	0.676083852	stage_E14_vs_E10	ordinary_muscle_development		0	FALSE
ENSGALG00010003243	VPS50	PRJNA729271	-0.38983086	6.76252E-05	0.38983086	stage_E14_vs_E10	ordinary_muscle_development		0	FALSE
ENSGALG00010010448	GALNT13	PRJNA729271	-1.568643776	6.78414E-05	1.568643776	stage_E14_vs_E10	ordinary_muscle_development	D_vs_E10;stage_1W_vs_E10;stage_3W_vs_E10;stage	1	TRUE
ENSGALG00010025470	ORAOV1	PRJNA729271	0.506650697	6.78572E-05	0.506650697	stage_E14_vs_E10	ordinary_muscle_development		0	FALSE
ENSGALG00010008110	DEF8	PRJNA729271	0.625741342	6.79233E-05	0.625741342	stage_E14_vs_E10	ordinary_muscle_development		0	FALSE
ENSGALG00010021130	ENSGALG00010021130	PRJNA729271	4.526319895	6.79495E-05	4.526319895	stage_E14_vs_E10	ordinary_muscle_development	D_vs_E10;stage_1W_vs_E10;stage_3W_vs_E10;stage	1	TRUE
ENSGALG00010016757	SRPX2	PRJNA729271	-1.155828825	6.80384E-05	1.155828825	stage_E14_vs_E10	ordinary_muscle_development	D_vs_E10;stage_1W_vs_E10;stage_3W_vs_E10;stage	1	TRUE
ENSGALG00010024632	IFITM10	PRJNA729271	-2.245789674	6.83408E-05	2.245789674	stage_E14_vs_E10	ordinary_muscle_development	D_vs_E10;stage_1W_vs_E10;stage_3W_vs_E10;stage	1	TRUE
ENSGALG00010010009	ELF2	PRJNA729271	-0.370079053	6.88845E-05	0.370079053	stage_E14_vs_E10	ordinary_muscle_development		0	FALSE
ENSGALG00010018418	PLCB4	PRJNA729271	-0.499995624	6.89449E-05	0.499995624	stage_E14_vs_E10	ordinary_muscle_development		0	FALSE
ENSGALG00010009531	FBXL13	PRJNA729271	-2.305200694	6.89619E-05	2.305200694	stage_E14_vs_E10	ordinary_muscle_development	D_vs_E10;stage_1W_vs_E10;stage_3W_vs_E10;stage	1	TRUE
ENSGALG00010028095	CDK9	PRJNA729271	-0.389433054	6.89765E-05	0.389433054	stage_E14_vs_E10	ordinary_muscle_development		0	FALSE
ENSGALG00010022661	ENSGALG00010022661	PRJNA729271	-1.708854194	6.90538E-05	1.708854194	stage_E14_vs_E10	ordinary_muscle_development	D_vs_E10;stage_1W_vs_E10;stage_3W_vs_E10;stage	1	TRUE
ENSGALG00010012797	ZC3H7B	PRJNA729271	0.288427309	6.91502E-05	0.288427309	stage_E14_vs_E10	ordinary_muscle_development		0	FALSE
ENSGALG00010023229	HKDC1	PRJNA729271	-2.002621298	6.92244E-05	2.002621298	stage_E14_vs_E10	ordinary_muscle_development	D_vs_E10;stage_1W_vs_E10;stage_3W_vs_E10;stage	1	TRUE
ENSGALG00010029572	TSPOAP1	PRJNA729271	0.419459998	6.93613E-05	0.419459998	stage_E14_vs_E10	ordinary_muscle_development		0	FALSE
ENSGALG00010004963	ENSGALG00010004963	PRJNA729271	0.957154057	6.9385E-05	0.957154057	stage_E14_vs_E10	ordinary_muscle_development		0	FALSE
ENSGALG00010004248	GRSF1	PRJNA729271	0.56287293	6.94143E-05	0.56287293	stage_E14_vs_E10	ordinary_muscle_development		0	FALSE
ENSGALG00010005332	ENSGALG00010005332	PRJNA729271	2.78728926	6.94499E-05	2.78728926	stage_E14_vs_E10	ordinary_muscle_development	D_vs_E10;stage_1W_vs_E10;stage_3W_vs_E10;stage	1	TRUE
ENSGALG00010009021	HNF4beta	PRJNA729271	-2.22331611	6.95793E-05	2.22331611	stage_E14_vs_E10	ordinary_muscle_development	D_vs_E10;stage_1W_vs_E10;stage_3W_vs_E10;stage	1	TRUE
ENSGALG00010022885	M6PR	PRJNA729271	0.407017064	7.00934E-05	0.407017064	stage_E14_vs_E10	ordinary_muscle_development		0	FALSE
ENSGALG00010000384	ENSGALG00010000384	PRJNA729271	0.589633496	7.01537E-05	0.589633496	stage_E14_vs_E10	ordinary_muscle_development		0	FALSE
ENSGALG00010023227	RNPC3	PRJNA729271	0.503535175	7.01537E-05	0.503535175	stage_E14_vs_E10	ordinary_muscle_development		0	FALSE
ENSGALG00010022804	SNX19	PRJNA729271	0.457562086	7.03415E-05	0.457562086	stage_E14_vs_E10	ordinary_muscle_development		0	FALSE
ENSGALG00010024608	DENND2D	PRJNA729271	1.362878189	7.03525E-05	1.362878189	stage_E14_vs_E10	ordinary_muscle_development	D_vs_E10;stage_1W_vs_E10;stage_3W_vs_E10;stage	1	TRUE
ENSGALG00010017804	DUSP5	PRJNA729271	-1.046695461	7.05387E-05	1.046695461	stage_E14_vs_E10	ordinary_muscle_development	D_vs_E10;stage_1W_vs_E10;stage_3W_vs_E10;stage	1	TRUE
ENSGALG00010012954	RIMS2	PRJNA729271	-1.502668704	7.07192E-05	1.502668704	stage_E14_vs_E10	ordinary_muscle_development	D_vs_E10;stage_1W_vs_E10;stage_3W_vs_E10;stage	1	TRUE
ENSGALG00010006576	CCDC191	PRJNA729271	-0.482704348	7.12121E-05	0.482704348	stage_E14_vs_E10	ordinary_muscle_development		0	FALSE
ENSGALG00010009666	RRAD	PRJNA729271	1.4923398	7.14949E-05	1.4923398	stage_E14_vs_E10	ordinary_muscle_development	D_vs_E10;stage_1W_vs_E10;stage_3W_vs_E10;stage	1	TRUE
ENSGALG00010008203	CXCL13	PRJNA729271	5.165334468	7.16595E-05	5.165334468	stage_E14_vs_E10	ordinary_muscle_development	D_vs_E10;stage_1W_vs_E10;stage_3W_vs_E10;stage	1	TRUE
ENSGALG00010027951	FAM32A	PRJNA729271	0.426956553	7.19585E-05	0.426956553	stage_E14_vs_E10	ordinary_muscle_development		0	FALSE
ENSGALG00010024645	PARP11	PRJNA729271	0.482457006	7.20181E-05	0.482457006	stage_E14_vs_E10	ordinary_muscle_development		0	FALSE
ENSGALG00010016527	MRPS34	PRJNA729271	-0.406336632	7.24346E-05	0.406336632	stage_E14_vs_E10	ordinary_muscle_development		0	FALSE
ENSGALG00010027954	CFAP92	PRJNA729271	-0.987715911	7.25377E-05	0.987715911	stage_E14_vs_E10	ordinary_muscle_development		0	FALSE
ENSGALG00010028565	PKN1	PRJNA729271	0.489657625	7.26453E-05	0.489657625	stage_E14_vs_E10	ordinary_muscle_development		0	FALSE
ENSGALG00010021266	COQ5	PRJNA729271	0.406064791	7.28E-05	0.406064791	stage_E14_vs_E10	ordinary_muscle_development		0	FALSE
ENSGALG00010006330	STX19	PRJNA729271	1.274612152	7.2887E-05	1.274612152	stage_E14_vs_E10	ordinary_muscle_development	D_vs_E10;stage_1W_vs_E10;stage_3W_vs_E10;stage	1	TRUE
ENSGALG00010000626	ABCA13	PRJNA729271	1.822975031	7.32232E-05	1.822975031	stage_E14_vs_E10	ordinary_muscle_development	D_vs_E10;stage_1W_vs_E10;stage_3W_vs_E10;stage	1	TRUE
ENSGALG00010010196	EFCAB10	PRJNA729271	1.34967449	7.32312E-05	1.34967449	stage_E14_vs_E10	ordinary_muscle_development	D_vs_E10;stage_1W_vs_E10;stage_3W_vs_E10;stage	1	TRUE
ENSGALG00010025734	PLK3	PRJNA729271	0.727696028	7.33759E-05	0.727696028	stage_E14_vs_E10	ordinary_muscle_development		0	FALSE
ENSGALG00010023578	SRRD	PRJNA729271	-0.42133048	7.4218E-05	0.42133048	stage_E14_vs_E10	ordinary_muscle_development		0	FALSE
ENSGALG00010019683	TMEM184A	PRJNA729271	1.147079626	7.43992E-05	1.147079626	stage_E14_vs_E10	ordinary_muscle_development	D_vs_E10;stage_1W_vs_E10;stage_3W_vs_E10;stage	1	TRUE
ENSGALG00010018665	IL13RA2	PRJNA729271	-1.373802217	7.44961E-05	1.373802217	stage_E14_vs_E10	ordinary_muscle_development	D_vs_E10;stage_1W_vs_E10;stage_3W_vs_E10;stage	1	TRUE
ENSGALG00010010107	MGST2	PRJNA729271	-1.462725308	7.45375E-05	1.462725308	stage_E14_vs_E10	ordinary_muscle_development	D_vs_E10;stage_1W_vs_E10;stage_3W_vs_E10;stage	1	TRUE
ENSGALG00010010526	ENSGALG00010010526	PRJNA729271	0.996512266	7.47181E-05	0.996512266	stage_E14_vs_E10	ordinary_muscle_development		0	FALSE
ENSGALG00010022204	ENSGALG00010022204	PRJNA729271	0.93416712	7.5319E-05	0.93416712	stage_E14_vs_E10	ordinary_muscle_development		0	FALSE
ENSGALG00010025604	HVCN1	PRJNA729271	-0.565703354	7.54191E-05	0.565703354	stage_E14_vs_E10	ordinary_muscle_development		0	FALSE
ENSGALG00010014641	DOCK11	PRJNA729271	-0.396211158	7.54311E-05	0.396211158	stage_E14_vs_E10	ordinary_muscle_development		0	FALSE
ENSGALG00010002359	CMTM6	PRJNA729271	0.471174074	7.55477E-05	0.471174074	stage_E14_vs_E10	ordinary_muscle_development		0	FALSE
ENSGALG00010017240	MELTF	PRJNA729271	0.968525681	7.57223E-05	0.968525681	stage_E14_vs_E10	ordinary_muscle_development		0	FALSE
ENSGALG00010008300	CUL1	PRJNA729271	-0.355240616	7.5801E-05	0.355240616	stage_E14_vs_E10	ordinary_muscle_development		0	FALSE
ENSGALG00010020932	CASP9	PRJNA729271	-0.514025241	7.58278E-05	0.514025241	stage_E14_vs_E10	ordinary_muscle_development		0	FALSE
ENSGALG00010024778	RAB5B	PRJNA729271	0.570213006	7.61772E-05	0.570213006	stage_E14_vs_E10	ordinary_muscle_development		0	FALSE
ENSGALG00010023861	ENSGALG00010023861	PRJNA729271	-0.631303071	7.62106E-05	0.631303071	stage_E14_vs_E10	ordinary_muscle_development		0	FALSE
ENSGALG00010007875	AvBD4	PRJNA729271	5.666119901	7.6995E-05	5.666119901	stage_E14_vs_E10	ordinary_muscle_development	D_vs_E10;stage_1W_vs_E10;stage_3W_vs_E10;stage	1	TRUE
ENSGALG00010026239	GIP	PRJNA729271	-4.748817408	7.71096E-05	4.748817408	stage_E14_vs_E10	ordinary_muscle_development	D_vs_E10;stage_1W_vs_E10;stage_3W_vs_E10;stage	1	TRUE
ENSGALG00010002401	ZRANB1	PRJNA729271	0.662249187	7.72262E-05	0.662249187	stage_E14_vs_E10	ordinary_muscle_development		0	FALSE
ENSGALG00010025520	ACOT11	PRJNA729271	1.204162571	7.73789E-05	1.204162571	stage_E14_vs_E10	ordinary_muscle_development	D_vs_E10;stage_1W_vs_E10;stage_3W_vs_E10;stage	1	TRUE
ENSGALG00010001713	PNPLA4	PRJNA729271	0.893182081	7.7539E-05	0.893182081	stage_E14_vs_E10	ordinary_muscle_development		0	FALSE
ENSGALG00010024420	TMEM106C	PRJNA729271	-0.693230694	7.76022E-05	0.693230694	stage_E14_vs_E10	ordinary_muscle_development		0	FALSE
ENSGALG00010027101	RNF220	PRJNA729271	-0.325899709	7.79216E-05	0.325899709	stage_E14_vs_E10	ordinary_muscle_development		0	FALSE
ENSGALG00010028678	ENSGALG00010028678	PRJNA729271	1.466892666	7.80499E-05	1.466892666	stage_E14_vs_E10	ordinary_muscle_development	D_vs_E10;stage_1W_vs_E10;stage_3W_vs_E10;stage	1	TRUE
ENSGALG00010027999	ENSGALG00010027999	PRJNA729271	-2.333074153	7.87991E-05	2.333074153	stage_E14_vs_E10	ordinary_muscle_development	D_vs_E10;stage_1W_vs_E10;stage_3W_vs_E10;stage	1	TRUE
ENSGALG00010010783	F2RL2	PRJNA729271	-0.604931312	7.92549E-05	0.604931312	stage_E14_vs_E10	ordinary_muscle_development		0	FALSE
ENSGALG00010012236	PRLH	PRJNA729271	-3.159566205	7.92658E-05	3.159566205	stage_E14_vs_E10	ordinary_muscle_development	D_vs_E10;stage_1W_vs_E10;stage_3W_vs_E10;stage	1	TRUE
ENSGALG00010000422	ITIH6	PRJNA729271	-2.426548795	7.93552E-05	2.426548795	stage_E14_vs_E10	ordinary_muscle_development	D_vs_E10;stage_1W_vs_E10;stage_3W_vs_E10;stage	1	TRUE
ENSGALG00010001466	PRKX	PRJNA729271	-0.534666746	8.02312E-05	0.534666746	stage_E14_vs_E10	ordinary_muscle_development		0	FALSE
ENSGALG00010013320	CALCRL	PRJNA729271	0.506756618	8.03951E-05	0.506756618	stage_E14_vs_E10	ordinary_muscle_development		0	FALSE
ENSGALG00010018310	APOOL	PRJNA729271	0.457872884	8.13309E-05	0.457872884	stage_E14_vs_E10	ordinary_muscle_development		0	FALSE
ENSGALG00010006685	ENSGALG00010006685	PRJNA729271	-0.498814058	8.14694E-05	0.498814058	stage_E14_vs_E10	ordinary_muscle_development		0	FALSE
ENSGALG00010021275	MAP3K2	PRJNA729271	0.370829092	8.16333E-05	0.370829092	stage_E14_vs_E10	ordinary_muscle_development		0	FALSE
ENSGALG00010017734	TOP3A	PRJNA729271	-0.4858653	8.21791E-05	0.4858653	stage_E14_vs_E10	ordinary_muscle_development		0	FALSE
ENSGALG00010027279	F2RL3	PRJNA729271	1.414744074	8.27709E-05	1.414744074	stage_E14_vs_E10	ordinary_muscle_development	D_vs_E10;stage_1W_vs_E10;stage_3W_vs_E10;stage	1	TRUE

ENSGALG00010002586	GPR63	PRJNA729271	-0.601181608	8.30535E-05	0.601181608	stage_E14_vs_E10	ordinary_muscle_development		0	FALSE
ENSGALG00010022493	ENSGALG00010022493	PRJNA729271	-0.69015078	8.31286E-05	0.69015078	stage_E14_vs_E10	ordinary_muscle_development		0	FALSE
ENSGALG00010016336	SLC24A2	PRJNA729271	-0.96160996	8.34637E-05	0.96160996	stage_E14_vs_E10	ordinary_muscle_development		0	FALSE
ENSGALG00010018384	LAMP5	PRJNA729271	1.001171536	8.36277E-05	1.001171536	stage_E14_vs_E10	ordinary_muscle_development	D_vs_E10;stage_1W_vs_E10;stage_3W_vs_E10;stage	1	TRUE
ENSGALG00010015564	PKD2L2	PRJNA729271	-1.315354082	8.41692E-05	1.315354082	stage_E14_vs_E10	ordinary_muscle_development	D_vs_E10;stage_1W_vs_E10;stage_3W_vs_E10;stage	1	TRUE
ENSGALG00010024189	KAT7	PRJNA729271	-0.581275193	8.42526E-05	0.581275193	stage_E14_vs_E10	ordinary_muscle_development		0	FALSE
ENSGALG00010010965	BAZ2B	PRJNA729271	0.538021195	8.43597E-05	0.538021195	stage_E14_vs_E10	ordinary_muscle_development		0	FALSE
ENSGALG00010006021	ENSGALG00010006021	PRJNA729271	0.508856541	8.53035E-05	0.508856541	stage_E14_vs_E10	ordinary_muscle_development		0	FALSE
ENSGALG00010001794	ENSGALG00010001794	PRJNA729271	-2.927731398	8.53792E-05	2.927731398	stage_E14_vs_E10	ordinary_muscle_development	D_vs_E10;stage_1W_vs_E10;stage_3W_vs_E10;stage	1	TRUE
ENSGALG00010010320	SYPL1	PRJNA729271	2.305734098	8.54402E-05	2.305734098	stage_E14_vs_E10	ordinary_muscle_development	D_vs_E10;stage_1W_vs_E10;stage_3W_vs_E10;stage	1	TRUE
ENSGALG00010005081	GREB1L	PRJNA729271	0.61141917	8.5784E-05	0.61141917	stage_E14_vs_E10	ordinary_muscle_development		0	FALSE
ENSGALG00010024720	BUD13	PRJNA729271	-0.438760332	8.57997E-05	0.438760332	stage_E14_vs_E10	ordinary_muscle_development		0	FALSE
ENSGALG00010008103	RBPMS	PRJNA729271	0.434423221	8.59803E-05	0.434423221	stage_E14_vs_E10	ordinary_muscle_development		0	FALSE
ENSGALG00010021350	CABLES2	PRJNA729271	-0.552469729	8.63148E-05	0.552469729	stage_E14_vs_E10	ordinary_muscle_development		0	FALSE
ENSGALG00010022104	EXOC3L1	PRJNA729271	0.581214117	8.69518E-05	0.581214117	stage_E14_vs_E10	ordinary_muscle_development		0	FALSE
ENSGALG00010017647	ITGA9	PRJNA729271	-0.452047187	8.70292E-05	0.452047187	stage_E14_vs_E10	ordinary_muscle_development		0	FALSE
ENSGALG00010020548	CMTR1	PRJNA729271	-0.763612835	8.72108E-05	0.763612835	stage_E14_vs_E10	ordinary_muscle_development		0	FALSE
ENSGALG00010027904	MORN5	PRJNA729271	-2.858857702	8.74491E-05	2.858857702	stage_E14_vs_E10	ordinary_muscle_development	D_vs_E10;stage_1W_vs_E10;stage_3W_vs_E10;stage	1	TRUE
ENSGALG00010002681	PLEKHB1	PRJNA729271	-0.669045266	8.77954E-05	0.669045266	stage_E14_vs_E10	ordinary_muscle_development		0	FALSE
ENSGALG00010013149	MCTP1	PRJNA729271	0.796362478	8.81379E-05	0.796362478	stage_E14_vs_E10	ordinary_muscle_development		0	FALSE
ENSGALG00010020064	TMEM138	PRJNA729271	-0.548901516	8.84003E-05	0.548901516	stage_E14_vs_E10	ordinary_muscle_development		0	FALSE
ENSGALG00010000037	ND6	PRJNA729271	0.776718149	8.95001E-05	0.776718149	stage_E14_vs_E10	ordinary_muscle_development		0	FALSE
ENSGALG00010027531	UFC1	PRJNA729271	0.67384621	9.04895E-05	0.67384621	stage_E14_vs_E10	ordinary_muscle_development		0	FALSE
ENSGALG00010006188	TMED10	PRJNA729271	0.317503696	9.14602E-05	0.317503696	stage_E14_vs_E10	ordinary_muscle_development		0	FALSE
ENSGALG00010020686	C1orf52	PRJNA729271	0.490288147	9.20889E-05	0.490288147	stage_E14_vs_E10	ordinary_muscle_development		0	FALSE
ENSGALG00010016069	GALK2	PRJNA729271	0.587960544	9.28547E-05	0.587960544	stage_E14_vs_E10	ordinary_muscle_development		0	FALSE
ENSGALG00010014092	TYRP1	PRJNA729271	-3.520624506	9.28852E-05	3.520624506	stage_E14_vs_E10	ordinary_muscle_development	D_vs_E10;stage_1W_vs_E10;stage_3W_vs_E10;stage	1	TRUE
ENSGALG00010007914	PAQR8	PRJNA729271	0.497102082	9.3552E-05	0.497102082	stage_E14_vs_E10	ordinary_muscle_development		0	FALSE
ENSGALG00010022416	CCDC186	PRJNA729271	0.352775481	9.36244E-05	0.352775481	stage_E14_vs_E10	ordinary_muscle_development		0	FALSE
ENSGALG00010002584	ENSGALG00010002584	PRJNA729271	1.755507724	9.40252E-05	1.755507724	stage_E14_vs_E10	ordinary_muscle_development	D_vs_E10;stage_1W_vs_E10;stage_3W_vs_E10;stage	1	TRUE
ENSGALG00010026393	SLC43A3	PRJNA729271	0.483242726	9.58323E-05	0.483242726	stage_E14_vs_E10	ordinary_muscle_development		0	FALSE
ENSGALG00010027218	IARS1	PRJNA729271	-0.672429567	9.63642E-05	0.672429567	stage_E14_vs_E10	ordinary_muscle_development		0	FALSE
ENSGALG00010018472	LMF1	PRJNA729271	0.589570554	9.63855E-05	0.589570554	stage_E14_vs_E10	ordinary_muscle_development		0	FALSE
ENSGALG00010010433	ENSGALG00010010433	PRJNA729271	4.209148206	9.71205E-05	4.209148206	stage_E14_vs_E10	ordinary_muscle_development	D_vs_E10;stage_1W_vs_E10;stage_3W_vs_E10;stage	1	TRUE
ENSGALG00010029181	MYO19	PRJNA729271	0.412595801	9.71375E-05	0.412595801	stage_E14_vs_E10	ordinary_muscle_development		0	FALSE
ENSGALG00010005146	TGFB3	PRJNA729271	-0.829770425	9.7289E-05	0.829770425	stage_E14_vs_E10	ordinary_muscle_development		0	FALSE
ENSGALG00010000621	UPP1	PRJNA729271	-0.97388673	9.73827E-05	0.97388673	stage_E14_vs_E10	ordinary_muscle_development		0	FALSE
ENSGALG00010028030	PFDN2	PRJNA729271	0.498611045	9.74871E-05	0.498611045	stage_E14_vs_E10	ordinary_muscle_development		0	FALSE
ENSGALG00010015166	YTHDF2	PRJNA729271	0.725033822	9.75769E-05	0.725033822	stage_E14_vs_E10	ordinary_muscle_development		0	FALSE
ENSGALG00010024603	ENSGALG00010024603	PRJNA729271	3.98547691	9.79177E-05	3.98547691	stage_E14_vs_E10	ordinary_muscle_development	D_vs_E10;stage_1W_vs_E10;stage_3W_vs_E10;stage	1	TRUE
ENSGALG00010010953	EXTL3	PRJNA729271	0.464130718	9.80639E-05	0.464130718	stage_E14_vs_E10	ordinary_muscle_development		0	FALSE
ENSGALG00010020716	RRAS	PRJNA729271	0.720082827	9.84831E-05	0.720082827	stage_E14_vs_E10	ordinary_muscle_development		0	FALSE
ENSGALG00010018909	TTBK1	PRJNA729271	0.339026385	9.8713E-05	0.339026385	stage_E14_vs_E10	ordinary_muscle_development		0	FALSE
ENSGALG00010009372	RAB28	PRJNA729271	-0.397964075	9.93371E-05	0.397964075	stage_E14_vs_E10	ordinary_muscle_development		0	FALSE
ENSGALG00010012226	SLCO4C1	PRJNA729271	1.975632538	0.000100114	1.975632538	stage_E14_vs_E10	ordinary_muscle_development	D_vs_E10;stage_1W_vs_E10;stage_3W_vs_E10;stage	1	TRUE
ENSGALG00010002251	GRK5	PRJNA729271	-0.632721701	0.000100502	0.632721701	stage_E14_vs_E10	ordinary_muscle_development		0	FALSE
ENSGALG00010010854	LRIT1	PRJNA729271	2.214295011	0.000100576	2.214295011	stage_E14_vs_E10	ordinary_muscle_development	D_vs_E10;stage_1W_vs_E10;stage_3W_vs_E10;stage	1	TRUE
ENSGALG00010004139	PPEF2	PRJNA729271	2.37603173	0.0001009	2.37603173	stage_E14_vs_E10	ordinary_muscle_development	D_vs_E10;stage_1W_vs_E10;stage_3W_vs_E10;stage	1	TRUE
ENSGALG00010025582	DMAP1	PRJNA729271	-0.46232236	0.000101231	0.46232236	stage_E14_vs_E10	ordinary_muscle_development		0	FALSE
ENSGALG00010025458	BCAS2	PRJNA729271	0.484615793	0.000101478	0.484615793	stage_E14_vs_E10	ordinary_muscle_development		0	FALSE
ENSGALG00010010685	STIM2	PRJNA729271	-0.457858334	0.000102183	0.457858334	stage_E14_vs_E10	ordinary_muscle_development		0	FALSE
ENSGALG00010024210	NXPH3	PRJNA729271	-4.997449528	0.000102293	4.997449528	stage_E14_vs_E10	ordinary_muscle_development	D_vs_E10;stage_1W_vs_E10;stage_3W_vs_E10;stage	1	TRUE
ENSGALG00010020199	DET1	PRJNA729271	-0.341887254	0.000102331	0.341887254	stage_E14_vs_E10	ordinary_muscle_development		0	FALSE
ENSGALG00010006080	ENSGALG00010006080	PRJNA729271	-0.834388514	0.000102717	0.834388514	stage_E14_vs_E10	ordinary_muscle_development		0	FALSE
ENSGALG00010021458	USP30	PRJNA729271	0.32077485	0.000102817	0.32077485	stage_E14_vs_E10	ordinary_muscle_development		0	FALSE
ENSGALG00010027098	EXOG	PRJNA729271	0.582122917	0.000102899	0.582122917	stage_E14_vs_E10	ordinary_muscle_development		0	FALSE
ENSGALG00010007806	DYNLRB2	PRJNA729271	-1.761330975	0.000103109	1.761330975	stage_E14_vs_E10	ordinary_muscle_development	D_vs_E10;stage_1W_vs_E10;stage_3W_vs_E10;stage	1	TRUE
ENSGALG00010012838	C2orf88	PRJNA729271	-0.594453916	0.000103191	0.594453916	stage_E14_vs_E10	ordinary_muscle_development		0	FALSE
ENSGALG00010018496	GNG13	PRJNA729271	-2.29289537	0.000103387	2.29289537	stage_E14_vs_E10	ordinary_muscle_development	D_vs_E10;stage_1W_vs_E10;stage_3W_vs_E10;stage	1	TRUE
ENSGALG00010000197	ENSGALG00010000197	PRJNA729271	2.017552703	0.000103411	2.017552703	stage_E14_vs_E10	ordinary_muscle_development	D_vs_E10;stage_1W_vs_E10;stage_3W_vs_E10;stage	1	TRUE
ENSGALG00010017710	RAB41	PRJNA729271	-0.473083939	0.0001038	0.473083939	stage_E14_vs_E10	ordinary_muscle_development		0	FALSE
ENSGALG00010024211	TRPM5	PRJNA729271	2.689898567	0.000103857	2.689898567	stage_E14_vs_E10	ordinary_muscle_development	D_vs_E10;stage_1W_vs_E10;stage_3W_vs_E10;stage	1	TRUE
ENSGALG00010024750	APOA4	PRJNA729271	6.199940524	0.000104414	6.199940524	stage_E14_vs_E10	ordinary_muscle_development	D_vs_E10;stage_1W_vs_E10;stage_3W_vs_E10;stage	1	TRUE
ENSGALG00010007284	ENSGALG00010007284	PRJNA729271	-0.345715783	0.000104588	0.345715783	stage_E14_vs_E10	ordinary_muscle_development		0	FALSE
ENSGALG00010003823	PTPN13	PRJNA729271	-0.608398922	0.000104719	0.608398922	stage_E14_vs_E10	ordinary_muscle_development		0	FALSE
ENSGALG00010000590	DNAH5	PRJNA729271	1.706083602	0.000105372	1.706083602	stage_E14_vs_E10	ordinary_muscle_development	D_vs_E10;stage_1W_vs_E10;stage_3W_vs_E10;stage	1	TRUE
ENSGALG00010020037	MPP5	PRJNA729271	-0.361261673	0.00010543	0.361261673	stage_E14_vs_E10	ordinary_muscle_development		0	FALSE
ENSGALG00010019652	TNFRSF10B	PRJNA729271	1.264678356	0.000105804	1.264678356	stage_E14_vs_E10	ordinary_muscle_development	D_vs_E10;stage_1W_vs_E10;stage_3W_vs_E10;stage	1	TRUE
ENSGALG00010024589	ADORA1	PRJNA729271	1.913282098	0.000106381	1.913282098	stage_E14_vs_E10	ordinary_muscle_development	D_vs_E10;stage_1W_vs_E10;stage_3W_vs_E10;stage	1	TRUE
ENSGALG00010015558	MPP1	PRJNA729271	1.035939678	0.000106386	1.035939678	stage_E14_vs_E10	ordinary_muscle_development	D_vs_E10;stage_1W_vs_E10;stage_3W_vs_E10;stage	1	TRUE
ENSGALG00010021773	ENSGALG00010021773	PRJNA729271	-4.134127038	0.00010663	4.134127038	stage_E14_vs_E10	ordinary_muscle_development	D_vs_E10;stage_1W_vs_E10;stage_3W_vs_E10;stage	1	TRUE
ENSGALG00010012477	ENSGALG00010012477	PRJNA729271	-1.057268453	0.000106663	1.057268453	stage_E14_vs_E10	ordinary_muscle_development	D_vs_E10;stage_1W_vs_E10;stage_3W_vs_E10;stage	1	TRUE
ENSGALG00010006198	ENSGALG00010006198	PRJNA729271	2.680140326	0.000106739	2.680140326	stage_E14_vs_E10	ordinary_muscle_development	D_vs_E10;stage_1W_vs_E10;stage_3W_vs_E10;stage	1	TRUE
ENSGALG00010003918	HTR1E	PRJNA729271	-3.050571743	0.00010779	3.050571743	stage_E14_vs_E10	ordinary_muscle_development	D_vs_E10;stage_1W_vs_E10;stage_3W_vs_E10;stage	1	TRUE
ENSGALG00010013173	KCNJ8	PRJNA729271	0.623999212	0.000108041	0.623999212	stage_E14_vs_E10	ordinary_muscle_development		0	FALSE
ENSGALG00010017463	PPP1R36	PRJNA729271	1.32324512	0.000108557	1.32324512	stage_E14_vs_E10	ordinary_muscle_development	D_vs_E10;stage_1W_vs_E10;stage_3W_vs_E10;stage	1	TRUE
ENSGALG00010003382	ASNS	PRJNA729271	2.392115091	0.000108807	2.392115091	stage_E14_vs_E10	ordinary_muscle_development	D_vs_E10;stage_1W_vs_E10;stage_3W_vs_E10;stage	1	TRUE
ENSGALG00010007934	PKHD1	PRJNA729271	-1.459067225	0.000108952	1.459067225	stage_E14_vs_E10	ordinary_muscle_development	D_vs_E10;stage_1W_vs_E10;stage_3W_vs_E10;stage	1	TRUE
ENSGALG00010009047	SUPT5H	PRJNA729271	0.438281246	0.000109433	0.438281246	stage_E14_vs_E10	ordinary_muscle_development		0	FALSE
ENSGALG00010010164	ENSGALG00010010164	PRJNA729271	1.562547739	0.000109954	1.562547739	stage_E14_vs_E10	ordinary_muscle_development	D_vs_E10;stage_1W_vs_E10;stage_3W_vs_E10;stage	1	TRUE
ENSGALG00010024746	HOXD4	PRJNA729271	-1.32435733	0.000110209	1.32435733	stage_E14_vs_E10	ordinary_muscle_development	D_vs_E10;stage_1W_vs_E10;stage_3W_vs_E10;stage	1	TRUE
ENSGALG00010009159	PTK2	PRJNA729271	0.357936625	0.000110349	0.357936625	stage_E14_vs_E10	ordinary_muscle_development		0	FALSE
ENSGALG00010000450	ENSGALG00010000450	PRJNA729271	0.752847586	0.000110405	0.752847586	stage_E14_vs_E10	ordinary_muscle_development		0	FALSE
ENSGALG00010013313	GAK	PRJNA729271	-0.37883913	0.000110704	0.37883913	stage_E14_vs_E10	ordinary_muscle_development		0	FALSE
ENSGALG00010022182	CNTNAP1	PRJNA729271	-1.215205162	0.000110854	1.215205162	stage_E14_vs_E10	ordinary_muscle_development	D_vs_E10;stage_1W_vs_E10;stage_3W_vs_E10;stage	1	TRUE
ENSGALG00010020415	TSR3	PRJNA729271	0.364668089	0.000110947	0.364668089	stage_E14_vs_E10	ordinary_muscle_development		0	FALSE
ENSGALG00010024818	FBXO3	PRJNA729271	-0.312835209	0.000111633	0.312835209	stage_E14_vs_E10	ordinary_muscle_development		0	FALSE
ENSGALG00010005491	GGACT	PRJNA729271	0.676273026	0.000112322	0.676273026	stage_E14_vs_E10	ordinary_muscle_development		0	FALSE
ENSGALG00010004769	HTR2A	PRJNA729271	-1.529418576	0.000112422	1.529418576	stage_E14_vs_E10	ordinary_muscle_development	D_vs_E10;stage_1W_vs_E10;stage_3W_vs_E10;stage	1	TRUE
ENSGALG00010020132	SHC4	PRJNA729271	-0.934430618	0.000112744	0.934430618	stage_E14_vs_E10	ordinary_muscle_development		0	FALSE
ENSGALG00010006019	SLC10A2	PRJNA729271	-4.720506613	0.000113638	4.720506613	stage_E14_vs_E10	ordinary_muscle_development	D_vs_E10;stage_1W_vs_E10;stage_3W_vs_E10;stage	1	TRUE
ENSGALG00010005458	PRKD1	PRJNA729271	0.694409171	0.000113873	0.694409171	stage_E14_vs_E10	ordinary_muscle_development		0	FALSE
ENSGALG00010017142	RBP7	PRJNA729271	2.785734268	0.000114342	2.785734268	stage_E14_vs_E10	ordinary_muscle_development	D_vs_E10;stage_1W_vs_E10;stage_3W_vs_E10;stage	1	TRUE
ENSGALG00010025508	ENSGALG00010025508	PRJNA729271	0.528826821	0.000114921	0.528826821	stage_E14_vs_E10	ordinary_muscle_development		0	FALSE
ENSGALG00010006761	PPP2R3C	PRJNA729271	-0.382424769	0.000115354	0.382424769	stage_E14_vs_E10	ordinary_muscle_development		0	FALSE
ENSGALG00010008449	SRA1	PRJNA729271	0.44949771	0.00011544	0.44949771	stage_E14_vs_E10	ordinary_muscle_development		0	FALSE
ENSGALG00010000091	ENSGALG00010000091	PRJNA729271	3.533054517	0.00011592	3.533054517	stage_E14_vs_E10	ordinary_muscle_development	D_vs_E10;stage_1W_vs_E10;stage_3W_vs_E10;stage	1	TRUE
ENSGALG00010005573	CHST9	PRJNA729271	-1.198367287	0.000116475	1.198367287	stage_E14_vs_E10	ordinary_muscle_development	D_vs_E10;stage_1W_vs_E10;stage_3W_vs_E10;stage	1	TRUE
ENSGALG00010005632	ITGB1	PRJNA729271	0.364105185	0.000116712	0.364105185	stage_E14_vs_E10	ordinary_muscle_development		0	FALSE
ENSGALG00010016950	ENSGALG00010016950	PRJNA729271	3.903180381	0.000117061	3.903180381	stage_E14_vs_E10	ordinary_muscle_development	D_vs_E10;stage_1W_vs_E10;stage_3W_vs_E10;stage	1	TRUE
ENSGALG00010003713	SPERT	PRJNA729271	1.014744636	0.000117298	1.014744636	stage_E14_vs_E10	ordinary_muscle_development	D_vs_E10;stage_1W_vs_E10;stage_3W_vs_E10;stage	1	TRUE
ENSGALG00010027361	FANCD2	PRJNA729271	0.762613634	0.000118064	0.762613634	stage_E14_vs_E10	ordinary_muscle_development		0	FALSE
ENSGALG00010029558	ENSGALG00010029558	PRJNA729271	-2.986426413	0.000118504	2.986426413	stage_E14_vs_E10	ordinary_muscle_development	D_vs_E10;stage_1W_vs_E10;stage_3W_vs_E10;stage	1	TRUE
ENSGALG00010029307	SGSH	PRJNA729271	0.65276908	0.000119097	0.65276908	stage_E14_vs_E10	ordinary_muscle_development		0	FALSE
ENSGALG00010001507	METTL4	PRJNA729271	-0.405495678	0.00012025	0.405495678	stage_E14_vs_E10	ordinary_muscle_development		0	FALSE
ENSGALG00010025481	TMEM39A	PRJNA729271	-0.36156493	0.000120399	0.36156493	stage_E14_vs_E10	ordinary_muscle_development		0	FALSE
ENSGALG00010027983	POGZ	PRJNA729271	-0.582990032	0.000120606	0.582990032	stage_E14_vs_E10	ordinary_muscle_development		0	FALSE
ENSGALG00010020736	LRRTM4	PRJNA729271	-0.926666687	0.000120826	0.926666687	stage_E14_vs_E10	ordinary_muscle_development		0	FALSE
ENSGALG00010029229	CBX4	PRJNA729271	0.873130124	0.000122144	0.873130124	stage_E14_vs_E10	ordinary_muscle_development		0	FALSE
ENSGALG00010022354	FAR2	PRJNA729271	-0.54577728	0.000123457	0.54577728	stage_E14_vs_E10	ordinary_muscle_development		0	FALSE
ENSGALG00010006499	CCR6	PRJNA729271	2.656484859	0.000123467	2.656484859	stage_E14_vs_E10	ordinary_muscle_development	D_vs_E10;stage_1W_vs_E10;stage_3W_vs_E10;stage	1	TRUE
ENSGALG00010000646	ENSGALG00010000646	PRJNA729271	1.318742835	0.000123509	1.318742835	stage_E14_vs_E10	ordinary_muscle_development	D_vs_E10;stage_1W_vs_E10;stage_3W_vs_E10;stage	1	TRUE
ENSGALG00010021084	ENSGALG00010021084	PRJNA729271	-3.643698138	0.000123534	3.643698138	stage_E14_vs_E10	ordinary_muscle_development	D_vs_E10;stage_1W_vs_E10;stage_3W_vs_E10;stage	1	TRUE
ENSGALG00010020102	TMEM127	PRJNA729271	0.793405352	0.000124024	0.793405352	stage_E14_vs_E10	ordinary_muscle_development		0	FALSE
ENSGALG00010025902	ENSGALG00010025902	PRJNA729271	7.046045334	0.000124189	7.046045334	stage_E14_vs_E10	ordinary_muscle_development	D_vs_E10;stage_1W_vs_E10;stage_3W_vs_E10;stage	1	TRUE
ENSGALG00010008119	CCDC83	PRJNA729271	-2.364436496	0.000124569	2.364436496	stage_E14_vs_E10	ordinary_muscle_development	D_vs_E10;stage_1W_vs_E10;stage_3W_vs_E10;stage	1	TRUE
ENSGALG00010006269	ENSGALG00010006269	PRJNA729271	4.879482678	0.000124589	4.879482678	stage_E14_vs_E10	ordinary_muscle_development	D_vs_E10;stage_1W_vs_E10;stage_3W_vs_E10;stage	1	TRUE
ENSGALG00010020664	ESYT3	PRJNA729271	1.710751242	0.000125075	1.710751242	stage_E14_vs_E10	ordinary_muscle_development	D_vs_E10;stage_1W_vs_E10;stage_3W_vs_E10;stage	1	TRUE
ENSGALG00010024516	EHHADH	PRJNA729271	-0.976490469	0.000125912	0.976490469	stage_E14_vs_E10	ordinary_muscle_development		0	FALSE
ENSGALG00010013400	BBS10	PRJNA729271	0.656617162	0.000126059	0.656617162	stage_E14_vs_E10	ordinary_muscle_development		0	FALSE
ENSGALG00010000457	TPCN1	PRJNA729271	-0.324358773	0.000126059	0.324358773	stage_E14_vs_E10	ordinary_muscle_development		0	FALSE
ENSGALG00010027948	PRPF4	PRJNA729271	-0.453531689	0.00012624	0.453531689	stage_E14_vs_E10	ordinary_muscle_development		0	FALSE
ENSGALG00010007990	TRHR	PRJNA729271	-4.569890442	0.00012632	4.569890442	stage_E14_vs_E10	ordinary_muscle_development	D_vs_E10;stage_1W_vs_E10;stage_3W_vs_E10;stage	1	TRUE
ENSGALG00010015018	MAP7D3	PRJNA729271	-0.274028449	0.00012632	0.274028449	stage_E14_vs_E10	ordinary_muscle_development		0	FALSE
ENSGALG00010026924	TPH1	PRJNA729271	-1.52769929	0.000126489	1.52769929	stage_E14_vs_E10	ordinary_muscle_development	D_vs_E10;stage_1W_vs_E10;stage_3W_vs_E10;stage	1	TRUE
ENSGALG00010021958	BHLHA15	PRJNA729271	1.478399194	0.000126537	1.478399194	stage_E14_vs_E10	ordinary_muscle_development	D_vs_E10;stage_1W_vs_E10;stage_3W_vs_E10;stage	1	TRUE
ENSGALG00010007610	TLDC1	PRJNA729271	-0.634828049	0.000126718	0.634828049	stage_E14_vs_E10	ordinary_muscle_development		0	FALSE
ENSGALG00010017573	ENSGALG00010017573	PRJNA729271	0.873891894	0.00012694	0.873891894	stage_E14_vs_E10	ordinary_muscle_development		0	FALSE
ENSGALG00010017551	NALF2	PRJNA729271	-1.14285461	0.000127043	1.14285461	stage_E14_vs_E10	ordinary_muscle_development	D_vs_E10;stage_1W_vs_E10;stage_3W_vs_E10;stage	1	TRUE
ENSGALG00010010751	SLC30A5	PRJNA729271	-0.323523301	0.000127682	0.323523301	stage_E14_vs_E10	ordinary_muscle_development		0	FALSE
ENSGALG00010029543	NSUN5	PRJNA729271	0.52502984	0.000128167	0.52502984	stage_E14_vs_E10	ordinary_muscle_development		0	FALSE
ENSGALG00010028437	ENSGALG00010028437	PRJNA729271	0.265160199	0.000129082	0.265160199	stage_E14_vs_E10	ordinary_muscle_development		0	FALSE
ENSGALG00010023991	TSPAN3	PRJNA729271	0.419361611	0.000129273	0.419361611	stage_E14_vs_E10	ordinary_muscle_development		0	FALSE
ENSGALG00010022754	SNX20	PRJNA729271	-0.777809065	0.000129343	0.777809065	stage_E14_vs_E10	ordinary_muscle_development		0	FALSE
ENSGALG00010001229	ZNF407	PRJNA729271	0.407545575	0.000130784	0.407545575	stage_E14_vs_E10	ordinary_muscle_development		0	FALSE
ENSGALG00010024715	DNAAF1	PRJNA729271	0.677277222	0.000131276	0.677277222	stage_E14_vs_E10	ordinary_muscle_development		0	FALSE
ENSGALG00010000792	ENSGALG00010000792	PRJNA729271	6.811397771	0.000131512	6.811397771	stage_E14_vs_E10	ordinary_muscle_development	D_vs_E10;stage_1W_vs_E10;stage_3W_vs_E10;stage	1	TRUE
ENSGALG00010001450	GPR149	PRJNA729271	1.505820128	0.000131514	1.505820128	stage_E14_vs_E10	ordinary_muscle_development	D_vs_E10;stage_1W_vs_E10;stage_3W_vs_E10;stage	1	TRUE
ENSGALG00010005109	ABHD3	PRJNA729271	1.430140163	0.000132149	1.430140163	stage_E14_vs_E10	ordinary_muscle_development	D_vs_E10;stage_1W_vs_E10;stage_3W_vs_E10;stage	1	TRUE
ENSGALG00010003871	ENSGALG00010003871	PRJNA729271	-0.810148209	0.000132678	0.810148209	stage_E14_vs_E10	ordinary_muscle_development		0	FALSE
ENSGALG00010023510	ASPHD2	PRJNA729271	-1.018334594	0.000133494	1.018334594	stage_E14_vs_E10	ordinary_muscle_development	D_vs_E10;stage_1W_vs_E10;stage_3W_vs_E10;stage	1	TRUE
ENSGALG00010001306	CEP120	PRJNA729271	-0.347173823	0.000134506	0.347173823	stage_E14_vs_E10	ordinary_muscle_development		0	FALSE
ENSGALG00010029520	MKS1	PRJNA729271	-0.43950494	0.000134586	0.43950494	stage_E14_vs_E10	ordinary_muscle_development		0	FALSE
ENSGALG00010017657	DIS3L2	PRJNA729271	0.386813717	0.000134593	0.386813717	stage_E14_vs_E10	ordinary_muscle_development		0	FALSE
ENSGALG00010022051	BAIAP2L1	PRJNA729271	0.444347595	0.000134659	0.444347595	stage_E14_vs_E10	ordinary_muscle_development		0	FALSE
ENSGALG00010015523	MRPL22	PRJNA729271	-0.58681826	0.00013498	0.58681826	stage_E14_vs_E10	ordinary_muscle_development		0	FALSE
ENSGALG00010022660	METAP1D	PRJNA729271	0.716892178	0.000134983	0.716892178	stage_E14_vs_E10	ordinary_muscle_development		0	FALSE
ENSGALG00010027987	TMEM141	PRJNA729271	0.54791768	0.000135116	0.54791768	stage_E14_vs_E10	ordinary_muscle_development		0	FALSE
ENSGALG00010009345	ZFAT	PRJNA729271	0.490879447	0.000135463	0.490879447	stage_E14_vs_E10	ordinary_muscle_development		0	FALSE
ENSGALG00010029604	ENSGALG00010029604	PRJNA729271	-0.487816331	0.000135558	0.487816331	stage_E14_vs_E10	ordinary_muscle_development		0	FALSE
ENSGALG00010020268	CPLANE2	PRJNA729271	-0.396110896	0.000136407	0.396110896	stage_E14_vs_E10	ordinary_muscle_development		0	FALSE
ENSGALG00010005949	SLITRK1	PRJNA729271	1.368533886	0.000137115	1.368533886	stage_E14_vs_E10	ordinary_muscle_development	D_vs_E10;stage_1W_vs_E10;stage_3W_vs_E10;stage	1	TRUE
ENSGALG00010000561	ENSGALG00010000561	PRJNA729271	-1.10067074	0.000137543	1.10067074	stage_E14_vs_E10	ordinary_muscle_development	D_vs_E10;stage_1W_vs_E10;stage_3W_vs_E10;stage	1	TRUE
ENSGALG00010001226	VEPH1	PRJNA729271	-2.59031604	0.0001384	2.59031604	stage_E14_vs_E10	ordinary_muscle_development	D_vs_E10;stage_1W_vs_E10;stage_3W_vs_E10;stage	1	TRUE
ENSGALG00010004244	TMEM241	PRJNA729271	-0.324941338	0.000138619	0.324941338	stage_E14_vs_E10	ordinary_muscle_development		0	FALSE
ENSGALG00010002703	PIN1	PRJNA729271	0.377865103	0.000138751	0.377865103	stage_E14_vs_E10	ordinary_muscle_development		0	FALSE
ENSGALG00010013510	MRPS33	PRJNA729271	-0.34935511	0.000139401	0.34935511	stage_E14_vs_E10	ordinary_muscle_development		0	FALSE
ENSGALG00010016117	ENSGALG00010016117	PRJNA729271	0.93163863	0.000139911	0.93163863	stage_E14_vs_E10	ordinary_muscle_development		0	FALSE
ENSGALG00010027196	ENSGALG00010027196	PRJNA729271	1.284315777	0.000140204	1.284315777	stage_E14_vs_E10	ordinary_muscle_development	D_vs_E10;stage_1W_vs_E10;stage_3W_vs_E10;stage	1	TRUE
ENSGALG00010020747	DBI	PRJNA729271	-0.477129947	0.000140692	0.477129947	stage_E14_vs_E10	ordinary_muscle_development		0	FALSE
ENSGALG00010014982	ENSGALG00010014982	PRJNA729271	1.275128872	0.000141096	1.275128872	stage_E14_vs_E10	ordinary_muscle_development	D_vs_E10;stage_1W_vs_E10;stage_3W_vs_E10;stage	1	TRUE
ENSGALG00010006135	ZC2HC1C	PRJNA729271	-0.601278406	0.000141321	0.601278406	stage_E14_vs_E10	ordinary_muscle_development		0	FALSE
ENSGALG00010004942	GABRR1	PRJNA729271	-3.195455678	0.000141407	3.195455678	stage_E14_vs_E10	ordinary_muscle_development	D_vs_E10;stage_1W_vs_E10;stage_3W_vs_E10;stage	1	TRUE
ENSGALG00010008451	OPN3	PRJNA729271	1.200516415	0.00014148	1.200516415	stage_E14_vs_E10	ordinary_muscle_development	D_vs_E10;stage_1W_vs_E10;stage_3W_vs_E10;stage	1	TRUE
ENSGALG00010007181	GJB2	PRJNA729271	2.264344952	0.00014157	2.264344952	stage_E14_vs_E10	ordinary_muscle_development	D_vs_E10;stage_1W_vs_E10;stage_3W_vs_E10;stage	1	TRUE
ENSGALG00010001868	ENSGALG00010001868	PRJNA729271	0.478418162	0.000141964	0.478418162	stage_E14_vs_E10	ordinary_muscle_development		0	FALSE
ENSGALG00010008144	NTPCR	PRJNA729271	0.519723418	0.000142387	0.519723418	stage_E14_vs_E10	ordinary_muscle_development		0	FALSE
ENSGALG00010008700	ENSGALG00010008700	PRJNA729271	-1.099234605	0.000143093	1.099234605	stage_E14_vs_E10	ordinary_muscle_development	D_vs_E10;stage_1W_vs_E10;stage_3W_vs_E10;stage	1	TRUE
ENSGALG00010023181	PLEKHA7	PRJNA729271	0.647053447	0.000144186	0.647053447	stage_E14_vs_E10	ordinary_muscle_development		0	FALSE
ENSGALG00010016911	AXIN1	PRJNA729271	-0.391803463	0.000144791	0.391803463	stage_E14_vs_E10	ordinary_muscle_development		0	FALSE
ENSGALG00010003407	CHMP4C	PRJNA729271	0.822572007	0.000144902	0.822572007	stage_E14_vs_E10	ordinary_muscle_development		0	FALSE
ENSGALG00010004386	CFAP44	PRJNA729271	-1.750184299	0.000145444	1.750184299	stage_E14_vs_E10	ordinary_muscle_development	D_vs_E10;stage_1W_vs_E10;stage_3W_vs_E10;stage	1	TRUE
ENSGALG00010001126	MINDY4B	PRJNA729271	4.641714021	0.00014593	4.641714021	stage_E14_vs_E10	ordinary_muscle_development	D_vs_E10;stage_1W_vs_E10;stage_3W_vs_E10;stage	1	TRUE
ENSGALG00010001273	ENSGALG00010001273	PRJNA729271	-2.433067169	0.000147301	2.433067169	stage_E14_vs_E10	ordinary_muscle_development	D_vs_E10;stage_1W_vs_E10;stage_3W_vs_E10;stage	1	TRUE
ENSGALG00010019259	L2HGDH	PRJNA729271	0.426049242	0.000147472	0.426049242	stage_E14_vs_E10	ordinary_muscle_development		0	FALSE

ENSGALG00010000302	ENSGALG00010000302	PRJNA729271	0.905582476	0.000147544	0.905582476	stage_E14_vs_E10	ordinary_muscle_development		0	FALSE
ENSGALG00010003250	CD1G	PRJNA729271	2.678658709	0.00014771	2.678658709	stage_E14_vs_E10	ordinary_muscle_development	D_vs_E10;stage_1W_vs_E10;stage_3W_vs_E10;stage	1	TRUE
ENSGALG00010021200	FTO	PRJNA729271	-0.654890922	0.00014888	0.654890922	stage_E14_vs_E10	ordinary_muscle_development		0	FALSE
ENSGALG00010003522	ENSGALG00010003522	PRJNA729271	2.614123147	0.000149233	2.614123147	stage_E14_vs_E10	ordinary_muscle_development	D_vs_E10;stage_1W_vs_E10;stage_3W_vs_E10;stage	1	TRUE
ENSGALG00010008092	SPIRE2	PRJNA729271	2.432859751	0.000149359	2.432859751	stage_E14_vs_E10	ordinary_muscle_development	D_vs_E10;stage_1W_vs_E10;stage_3W_vs_E10;stage	1	TRUE
ENSGALG00010029383	GNA13	PRJNA729271	0.308452835	0.000149722	0.308452835	stage_E14_vs_E10	ordinary_muscle_development		0	FALSE
ENSGALG00010023454	HOXB1	PRJNA729271	1.519019946	0.000149913	1.519019946	stage_E14_vs_E10	ordinary_muscle_development	D_vs_E10;stage_1W_vs_E10;stage_3W_vs_E10;stage	1	TRUE
ENSGALG00010029727	ZSWIM7	PRJNA729271	0.530179865	0.000149943	0.530179865	stage_E14_vs_E10	ordinary_muscle_development		0	FALSE
ENSGALG00010014648	C1QTNF6	PRJNA729271	-1.139179674	0.000149971	1.139179674	stage_E14_vs_E10	ordinary_muscle_development	D_vs_E10;stage_1W_vs_E10;stage_3W_vs_E10;stage	1	TRUE
ENSGALG00010025127	PAMR1	PRJNA729271	-0.634257466	0.000150022	0.634257466	stage_E14_vs_E10	ordinary_muscle_development		0	FALSE
ENSGALG00010027334	PRCC	PRJNA729271	-0.449854838	0.000151258	0.449854838	stage_E14_vs_E10	ordinary_muscle_development		0	FALSE
ENSGALG00010019260	TRAIL-LIKE	PRJNA729271	-0.691510004	0.000152661	0.691510004	stage_E14_vs_E10	ordinary_muscle_development		0	FALSE
ENSGALG00010004504	ZNF804B	PRJNA729271	-3.012910091	0.000152923	3.012910091	stage_E14_vs_E10	ordinary_muscle_development	D_vs_E10;stage_1W_vs_E10;stage_3W_vs_E10;stage	1	TRUE
ENSGALG00010019972	PPP1CB	PRJNA729271	0.360033312	0.00015294	0.360033312	stage_E14_vs_E10	ordinary_muscle_development		0	FALSE
ENSGALG00010018395	MED12	PRJNA729271	0.308017808	0.000153304	0.308017808	stage_E14_vs_E10	ordinary_muscle_development		0	FALSE
ENSGALG00010029966	RABGEF1	PRJNA729271	0.315011569	0.00015447	0.315011569	stage_E14_vs_E10	ordinary_muscle_development		0	FALSE
ENSGALG00010015020	NIPAL4	PRJNA729271	2.865748889	0.000154864	2.865748889	stage_E14_vs_E10	ordinary_muscle_development	D_vs_E10;stage_1W_vs_E10;stage_3W_vs_E10;stage	1	TRUE
ENSGALG00010024066	ENSGALG00010024066	PRJNA729271	8.246281372	0.000155026	8.246281372	stage_E14_vs_E10	ordinary_muscle_development	D_vs_E10;stage_1W_vs_E10;stage_3W_vs_E10;stage	1	TRUE
ENSGALG00010021373	CEP250	PRJNA729271	-0.601594637	0.000155317	0.601594637	stage_E14_vs_E10	ordinary_muscle_development		0	FALSE
ENSGALG00010015862	PDGFRL	PRJNA729271	0.828824497	0.000155529	0.828824497	stage_E14_vs_E10	ordinary_muscle_development		0	FALSE
ENSGALG00010024890	SMARCC2	PRJNA729271	0.570520287	0.000155539	0.570520287	stage_E14_vs_E10	ordinary_muscle_development		0	FALSE
ENSGALG00010002373	POSTN	PRJNA729271	1.054757121	0.000156564	1.054757121	stage_E14_vs_E10	ordinary_muscle_development	D_vs_E10;stage_1W_vs_E10;stage_3W_vs_E10;stage	1	TRUE
ENSGALG00010014783	ENSGALG00010014783	PRJNA729271	0.462268031	0.000159749	0.462268031	stage_E14_vs_E10	ordinary_muscle_development		0	FALSE
ENSGALG00010025971	TRAFD1	PRJNA729271	0.772170143	0.000159772	0.772170143	stage_E14_vs_E10	ordinary_muscle_development		0	FALSE
ENSGALG00010026316	ENSGALG00010026316	PRJNA729271	1.351981253	0.000160659	1.351981253	stage_E14_vs_E10	ordinary_muscle_development	D_vs_E10;stage_1W_vs_E10;stage_3W_vs_E10;stage	1	TRUE
ENSGALG00010009076	TMEM214	PRJNA729271	-0.471384945	0.000161371	0.471384945	stage_E14_vs_E10	ordinary_muscle_development		0	FALSE
ENSGALG00010013080	ARHGEF7	PRJNA729271	0.392197295	0.000161396	0.392197295	stage_E14_vs_E10	ordinary_muscle_development		0	FALSE
ENSGALG00010028547	ZNF618	PRJNA729271	-0.410962593	0.000162301	0.410962593	stage_E14_vs_E10	ordinary_muscle_development		0	FALSE
ENSGALG00010016848	ENSGALG00010016848	PRJNA729271	0.668041183	0.000163677	0.668041183	stage_E14_vs_E10	ordinary_muscle_development		0	FALSE
ENSGALG00010000078	ENSGALG00010000078	PRJNA729271	1.812258469	0.000164022	1.812258469	stage_E14_vs_E10	ordinary_muscle_development	D_vs_E10;stage_1W_vs_E10;stage_3W_vs_E10;stage	1	TRUE
ENSGALG00010017588	NOX5	PRJNA729271	-2.114642879	0.000165085	2.114642879	stage_E14_vs_E10	ordinary_muscle_development	D_vs_E10;stage_1W_vs_E10;stage_3W_vs_E10;stage	1	TRUE
ENSGALG00010019618	NPHP4	PRJNA729271	-0.410554189	0.000165163	0.410554189	stage_E14_vs_E10	ordinary_muscle_development		0	FALSE
ENSGALG00010029523	FSCN2	PRJNA729271	2.593954262	0.000165348	2.593954262	stage_E14_vs_E10	ordinary_muscle_development	D_vs_E10;stage_1W_vs_E10;stage_3W_vs_E10;stage	1	TRUE
ENSGALG00010023094	CCDC32	PRJNA729271	-0.398585943	0.000165547	0.398585943	stage_E14_vs_E10	ordinary_muscle_development		0	FALSE
ENSGALG00010003204	PKD2	PRJNA729271	0.555664017	0.000165766	0.555664017	stage_E14_vs_E10	ordinary_muscle_development		0	FALSE
ENSGALG00010028112	SWI5	PRJNA729271	-0.329312616	0.000166393	0.329312616	stage_E14_vs_E10	ordinary_muscle_development		0	FALSE
ENSGALG00010018244	CHM	PRJNA729271	0.317478843	0.000166442	0.317478843	stage_E14_vs_E10	ordinary_muscle_development		0	FALSE
ENSGALG00010005213	ENSGALG00010005213	PRJNA729271	-0.619908457	0.000167252	0.619908457	stage_E14_vs_E10	ordinary_muscle_development		0	FALSE
ENSGALG00010017776	GPR68	PRJNA729271	1.401853976	0.000168354	1.401853976	stage_E14_vs_E10	ordinary_muscle_development	D_vs_E10;stage_1W_vs_E10;stage_3W_vs_E10;stage	1	TRUE
ENSGALG00010024171	ENSGALG00010024171	PRJNA729271	-2.592127731	0.000169678	2.592127731	stage_E14_vs_E10	ordinary_muscle_development	D_vs_E10;stage_1W_vs_E10;stage_3W_vs_E10;stage	1	TRUE
ENSGALG00010005455	ENSGALG00010005455	PRJNA729271	2.6328006	0.00017168	2.6328006	stage_E14_vs_E10	ordinary_muscle_development	D_vs_E10;stage_1W_vs_E10;stage_3W_vs_E10;stage	1	TRUE
ENSGALG00010003184	BET1	PRJNA729271	0.322850191	0.000172087	0.322850191	stage_E14_vs_E10	ordinary_muscle_development		0	FALSE
ENSGALG00010021543	PLEKHD1	PRJNA729271	0.917490535	0.000172525	0.917490535	stage_E14_vs_E10	ordinary_muscle_development		0	FALSE
ENSGALG00010005286	ENSGALG00010005286	PRJNA729271	2.284790476	0.000175699	2.284790476	stage_E14_vs_E10	ordinary_muscle_development	D_vs_E10;stage_1W_vs_E10;stage_3W_vs_E10;stage	1	TRUE
ENSGALG00010019274	ENSGALG00010019274	PRJNA729271	-3.847948491	0.000175944	3.847948491	stage_E14_vs_E10	ordinary_muscle_development	D_vs_E10;stage_1W_vs_E10;stage_3W_vs_E10;stage	1	TRUE
ENSGALG00010010928	HECW2	PRJNA729271	-0.455096565	0.000175944	0.455096565	stage_E14_vs_E10	ordinary_muscle_development		0	FALSE
ENSGALG00010012331	ENSGALG00010012331	PRJNA729271	-1.030018968	0.000175982	1.030018968	stage_E14_vs_E10	ordinary_muscle_development	D_vs_E10;stage_1W_vs_E10;stage_3W_vs_E10;stage	1	TRUE
ENSGALG00010017629	ENSGALG00010017629	PRJNA729271	-0.560270021	0.000176822	0.560270021	stage_E14_vs_E10	ordinary_muscle_development		0	FALSE
ENSGALG00010016712	ENSGALG00010016712	PRJNA729271	0.607524526	0.000179061	0.607524526	stage_E14_vs_E10	ordinary_muscle_development		0	FALSE
ENSGALG00010010403	ENSGALG00010010403	PRJNA729271	-1.902958805	0.000179421	1.902958805	stage_E14_vs_E10	ordinary_muscle_development	D_vs_E10;stage_1W_vs_E10;stage_3W_vs_E10;stage	1	TRUE
ENSGALG00010011895	PHF21B	PRJNA729271	-3.613676205	0.000179433	3.613676205	stage_E14_vs_E10	ordinary_muscle_development	D_vs_E10;stage_1W_vs_E10;stage_3W_vs_E10;stage	1	TRUE
ENSGALG00010028452	GOLPH3L	PRJNA729271	-3.44084941	0.000179589	3.44084941	stage_E14_vs_E10	ordinary_muscle_development	D_vs_E10;stage_1W_vs_E10;stage_3W_vs_E10;stage	1	TRUE
ENSGALG00010002733	RDH8	PRJNA729271	1.395268789	0.000179783	1.395268789	stage_E14_vs_E10	ordinary_muscle_development	D_vs_E10;stage_1W_vs_E10;stage_3W_vs_E10;stage	1	TRUE
ENSGALG00010010262	HSPA9	PRJNA729271	0.567574624	0.00018003	0.567574624	stage_E14_vs_E10	ordinary_muscle_development		0	FALSE
ENSGALG00010012039	ODF1	PRJNA729271	-3.785492182	0.000180243	3.785492182	stage_E14_vs_E10	ordinary_muscle_development	D_vs_E10;stage_1W_vs_E10;stage_3W_vs_E10;stage	1	TRUE
ENSGALG00010017790	R3HCC1L	PRJNA729271	0.700014912	0.000180348	0.700014912	stage_E14_vs_E10	ordinary_muscle_development		0	FALSE
ENSGALG00010027154	PLPP2	PRJNA729271	0.537705219	0.000181302	0.537705219	stage_E14_vs_E10	ordinary_muscle_development		0	FALSE
ENSGALG00010024612	ENSGALG00010024612	PRJNA729271	-2.852576996	0.000182508	2.852576996	stage_E14_vs_E10	ordinary_muscle_development	D_vs_E10;stage_1W_vs_E10;stage_3W_vs_E10;stage	1	TRUE
ENSGALG00010013923	FMR1NB	PRJNA729271	1.928141842	0.000183105	1.928141842	stage_E14_vs_E10	ordinary_muscle_development	D_vs_E10;stage_1W_vs_E10;stage_3W_vs_E10;stage	1	TRUE
ENSGALG00010025398	TSPAN2	PRJNA729271	-0.565464462	0.000183162	0.565464462	stage_E14_vs_E10	ordinary_muscle_development		0	FALSE
ENSGALG00010027974	ENSGALG00010027974	PRJNA729271	-1.133951531	0.000183486	1.133951531	stage_E14_vs_E10	ordinary_muscle_development	D_vs_E10;stage_1W_vs_E10;stage_3W_vs_E10;stage	1	TRUE
ENSGALG00010012271	ENSGALG00010012271	PRJNA729271	1.013897889	0.000183685	1.013897889	stage_E14_vs_E10	ordinary_muscle_development	D_vs_E10;stage_1W_vs_E10;stage_3W_vs_E10;stage	1	TRUE
ENSGALG00010024288	CHID1	PRJNA729271	0.525430145	0.000183733	0.525430145	stage_E14_vs_E10	ordinary_muscle_development		0	FALSE
ENSGALG00010001497	LEKR1	PRJNA729271	-1.124789231	0.000184084	1.124789231	stage_E14_vs_E10	ordinary_muscle_development	D_vs_E10;stage_1W_vs_E10;stage_3W_vs_E10;stage	1	TRUE
ENSGALG00010008314	INVS	PRJNA729271	0.414414025	0.000184804	0.414414025	stage_E14_vs_E10	ordinary_muscle_development		0	FALSE
ENSGALG00010015810	LIPT1	PRJNA729271	-0.412766122	0.000184909	0.412766122	stage_E14_vs_E10	ordinary_muscle_development		0	FALSE
ENSGALG00010024689	FOXD2	PRJNA729271	1.162305445	0.000185279	1.162305445	stage_E14_vs_E10	ordinary_muscle_development	D_vs_E10;stage_1W_vs_E10;stage_3W_vs_E10;stage	1	TRUE
ENSGALG00010029222	TRAF4	PRJNA729271	-0.931017791	0.000185849	0.931017791	stage_E14_vs_E10	ordinary_muscle_development		0	FALSE
ENSGALG00010005533	OPN4-1	PRJNA729271	-3.999750909	0.000185979	3.999750909	stage_E14_vs_E10	ordinary_muscle_development	D_vs_E10;stage_1W_vs_E10;stage_3W_vs_E10;stage	1	TRUE
ENSGALG00010014151	ATP23	PRJNA729271	0.345703611	0.00018743	0.345703611	stage_E14_vs_E10	ordinary_muscle_development		0	FALSE
ENSGALG00010019296	ENSGALG00010019296	PRJNA729271	0.390127766	0.000187592	0.390127766	stage_E14_vs_E10	ordinary_muscle_development		0	FALSE
ENSGALG00010006323	ANAPC10	PRJNA729271	0.304400968	0.000187783	0.304400968	stage_E14_vs_E10	ordinary_muscle_development		0	FALSE
ENSGALG00010012722	ENSGALG00010012722	PRJNA729271	0.37800332	0.000189384	0.37800332	stage_E14_vs_E10	ordinary_muscle_development		0	FALSE
ENSGALG00010008175	ENSGALG00010008175	PRJNA729271	-1.779853388	0.000189765	1.779853388	stage_E14_vs_E10	ordinary_muscle_development	D_vs_E10;stage_1W_vs_E10;stage_3W_vs_E10;stage	1	TRUE
ENSGALG00010008296	PALLD	PRJNA729271	-0.589905185	0.000190614	0.589905185	stage_E14_vs_E10	ordinary_muscle_development		0	FALSE
ENSGALG00010012171	CDH17	PRJNA729271	1.505847822	0.000191712	1.505847822	stage_E14_vs_E10	ordinary_muscle_development	D_vs_E10;stage_1W_vs_E10;stage_3W_vs_E10;stage	1	TRUE
ENSGALG00010026434	ENSGALG00010026434	PRJNA729271	0.547475715	0.000192039	0.547475715	stage_E14_vs_E10	ordinary_muscle_development		0	FALSE
ENSGALG00010019667	ITPRID1	PRJNA729271	-1.485519879	0.000192265	1.485519879	stage_E14_vs_E10	ordinary_muscle_development	D_vs_E10;stage_1W_vs_E10;stage_3W_vs_E10;stage	1	TRUE
ENSGALG00010012850	CALB1	PRJNA729271	4.178927484	0.000192836	4.178927484	stage_E14_vs_E10	ordinary_muscle_development	D_vs_E10;stage_1W_vs_E10;stage_3W_vs_E10;stage	1	TRUE
ENSGALG00010016603	ENSGALG00010016603	PRJNA729271	0.483611703	0.000193182	0.483611703	stage_E14_vs_E10	ordinary_muscle_development		0	FALSE
ENSGALG00010028726	SLC2A8	PRJNA729271	-0.275390035	0.000193317	0.275390035	stage_E14_vs_E10	ordinary_muscle_development		0	FALSE
ENSGALG00010027403	ACKR2	PRJNA729271	1.413910461	0.000193368	1.413910461	stage_E14_vs_E10	ordinary_muscle_development	D_vs_E10;stage_1W_vs_E10;stage_3W_vs_E10;stage	1	TRUE
ENSGALG00010011966	SLC25A24	PRJNA729271	-0.532401279	0.000194889	0.532401279	stage_E14_vs_E10	ordinary_muscle_development		0	FALSE
ENSGALG00010016673	LRPAP1	PRJNA729271	0.305099109	0.000195224	0.305099109	stage_E14_vs_E10	ordinary_muscle_development		0	FALSE
ENSGALG00010002420	TMEM272	PRJNA729271	-4.047197869	0.000195498	4.047197869	stage_E14_vs_E10	ordinary_muscle_development	D_vs_E10;stage_1W_vs_E10;stage_3W_vs_E10;stage	1	TRUE
ENSGALG00010012993	DOP1B	PRJNA729271	-0.391233007	0.000196539	0.391233007	stage_E14_vs_E10	ordinary_muscle_development		0	FALSE
ENSGALG00010001120	GPR87	PRJNA729271	-4.407826803	0.000197361	4.407826803	stage_E14_vs_E10	ordinary_muscle_development	D_vs_E10;stage_1W_vs_E10;stage_3W_vs_E10;stage	1	TRUE
ENSGALG00010028197	INTS3	PRJNA729271	0.598537954	0.000197681	0.598537954	stage_E14_vs_E10	ordinary_muscle_development		0	FALSE
ENSGALG00010023126	CGNL1	PRJNA729271	0.530934249	0.00019834	0.530934249	stage_E14_vs_E10	ordinary_muscle_development		0	FALSE
ENSGALG00010005498	SMO	PRJNA729271	0.52484757	0.000199616	0.52484757	stage_E14_vs_E10	ordinary_muscle_development		0	FALSE
ENSGALG00010022468	MYBPC3	PRJNA729271	1.522786835	0.000200913	1.522786835	stage_E14_vs_E10	ordinary_muscle_development	D_vs_E10;stage_1W_vs_E10;stage_3W_vs_E10;stage	1	TRUE
ENSGALG00010023665	C16orf87	PRJNA729271	-0.536758638	0.000201579	0.536758638	stage_E14_vs_E10	ordinary_muscle_development		0	FALSE
ENSGALG00010005593	ENSGALG00010005593	PRJNA729271	4.037301217	0.00020216	4.037301217	stage_E14_vs_E10	ordinary_muscle_development	D_vs_E10;stage_1W_vs_E10;stage_3W_vs_E10;stage	1	TRUE
ENSGALG00010002447	YLEC12	PRJNA729271	2.208895525	0.000202417	2.208895525	stage_E14_vs_E10	ordinary_muscle_development	D_vs_E10;stage_1W_vs_E10;stage_3W_vs_E10;stage	1	TRUE
ENSGALG00010026909	ENSGALG00010026909	PRJNA729271	0.593981486	0.000203319	0.593981486	stage_E14_vs_E10	ordinary_muscle_development		0	FALSE
ENSGALG00010022151	PIP4K2B	PRJNA729271	0.623080542	0.000203335	0.623080542	stage_E14_vs_E10	ordinary_muscle_development		0	FALSE
ENSGALG00010011152	SLC18A3	PRJNA729271	1.782698001	0.000203556	1.782698001	stage_E14_vs_E10	ordinary_muscle_development	D_vs_E10;stage_1W_vs_E10;stage_3W_vs_E10;stage	1	TRUE
ENSGALG00010002581	ATP13A1	PRJNA729271	-0.305570592	0.00020441	0.305570592	stage_E14_vs_E10	ordinary_muscle_development		0	FALSE
ENSGALG00010013520	CREG2	PRJNA729271	-0.736523002	0.000205037	0.736523002	stage_E14_vs_E10	ordinary_muscle_development		0	FALSE
ENSGALG00010013971	ENSGALG00010013971	PRJNA729271	-1.55794469	0.000205278	1.55794469	stage_E14_vs_E10	ordinary_muscle_development	D_vs_E10;stage_1W_vs_E10;stage_3W_vs_E10;stage	1	TRUE
ENSGALG00010002329	STT3B	PRJNA729271	-0.283385947	0.000205484	0.283385947	stage_E14_vs_E10	ordinary_muscle_development		0	FALSE
ENSGALG00010003257	ENSGALG00010003257	PRJNA729271	3.035535853	0.000206394	3.035535853	stage_E14_vs_E10	ordinary_muscle_development	D_vs_E10;stage_1W_vs_E10;stage_3W_vs_E10;stage	1	TRUE
ENSGALG00010021467	LHFPL4	PRJNA729271	0.929275176	0.000208618	0.929275176	stage_E14_vs_E10	ordinary_muscle_development		0	FALSE
ENSGALG00010028675	ZBTB43	PRJNA729271	0.269585399	0.000208738	0.269585399	stage_E14_vs_E10	ordinary_muscle_development		0	FALSE
ENSGALG00010025533	INHBC	PRJNA729271	-2.589747629	0.000209142	2.589747629	stage_E14_vs_E10	ordinary_muscle_development	D_vs_E10;stage_1W_vs_E10;stage_3W_vs_E10;stage	1	TRUE
ENSGALG00010027395	BCL2L15	PRJNA729271	0.967527859	0.000209161	0.967527859	stage_E14_vs_E10	ordinary_muscle_development		0	FALSE
ENSGALG00010011648	ENSGALG00010011648	PRJNA729271	0.781243005	0.000209181	0.781243005	stage_E14_vs_E10	ordinary_muscle_development		0	FALSE
ENSGALG00010012187	ENSGALG00010012187	PRJNA729271	-3.371388323	0.000209282	3.371388323	stage_E14_vs_E10	ordinary_muscle_development	D_vs_E10;stage_1W_vs_E10;stage_3W_vs_E10;stage	1	TRUE
ENSGALG00010018039	TTC14	PRJNA729271	0.502161675	0.000209606	0.502161675	stage_E14_vs_E10	ordinary_muscle_development		0	FALSE
ENSGALG00010016637	POU6F2	PRJNA729271	-3.303247063	0.000213809	3.303247063	stage_E14_vs_E10	ordinary_muscle_development	D_vs_E10;stage_1W_vs_E10;stage_3W_vs_E10;stage	1	TRUE
ENSGALG00010026205	ENSGALG00010026205	PRJNA729271	4.981110426	0.000214179	4.981110426	stage_E14_vs_E10	ordinary_muscle_development	D_vs_E10;stage_1W_vs_E10;stage_3W_vs_E10;stage	1	TRUE
ENSGALG00010026854	SMPD2	PRJNA729271	0.571603094	0.000214678	0.571603094	stage_E14_vs_E10	ordinary_muscle_development		0	FALSE
ENSGALG00010011870	AvBD6	PRJNA729271	5.156564225	0.000216109	5.156564225	stage_E14_vs_E10	ordinary_muscle_development	D_vs_E10;stage_1W_vs_E10;stage_3W_vs_E10;stage	1	TRUE
ENSGALG00010004471	C1H3ORF52	PRJNA729271	0.968414799	0.000216142	0.968414799	stage_E14_vs_E10	ordinary_muscle_development		0	FALSE
ENSGALG00010023177	LZTS2	PRJNA729271	0.651816549	0.000217467	0.651816549	stage_E14_vs_E10	ordinary_muscle_development		0	FALSE
ENSGALG00010007870	AvBD3	PRJNA729271	-3.601946648	0.000218291	3.601946648	stage_E14_vs_E10	ordinary_muscle_development	D_vs_E10;stage_1W_vs_E10;stage_3W_vs_E10;stage	1	TRUE
ENSGALG00010002350	BEND6	PRJNA729271	0.941230701	0.000218509	0.941230701	stage_E14_vs_E10	ordinary_muscle_development		0	FALSE
ENSGALG00010010515	RXRG	PRJNA729271	-1.496404052	0.000218513	1.496404052	stage_E14_vs_E10	ordinary_muscle_development	D_vs_E10;stage_1W_vs_E10;stage_3W_vs_E10;stage	1	TRUE
ENSGALG00010021664	TYSND1	PRJNA729271	-0.640895104	0.00021876	0.640895104	stage_E14_vs_E10	ordinary_muscle_development		0	FALSE
ENSGALG00010023799	NMT1	PRJNA729271	0.482579185	0.000219444	0.482579185	stage_E14_vs_E10	ordinary_muscle_development		0	FALSE
ENSGALG00010001725	MTA2	PRJNA729271	-0.729388419	0.0002209	0.729388419	stage_E14_vs_E10	ordinary_muscle_development		0	FALSE
ENSGALG00010027971	ENSGALG00010027971	PRJNA729271	1.457629002	0.000221561	1.457629002	stage_E14_vs_E10	ordinary_muscle_development	D_vs_E10;stage_1W_vs_E10;stage_3W_vs_E10;stage	1	TRUE
ENSGALG00010016445	ZDHHC21	PRJNA729271	-0.541479027	0.000221735	0.541479027	stage_E14_vs_E10	ordinary_muscle_development		0	FALSE
ENSGALG00010005859	ENSGALG00010005859	PRJNA729271	1.972426338	0.00022234	1.972426338	stage_E14_vs_E10	ordinary_muscle_development	D_vs_E10;stage_1W_vs_E10;stage_3W_vs_E10;stage	1	TRUE
ENSGALG00010014154	DNAI7	PRJNA729271	-1.850915978	0.000222402	1.850915978	stage_E14_vs_E10	ordinary_muscle_development	D_vs_E10;stage_1W_vs_E10;stage_3W_vs_E10;stage	1	TRUE
ENSGALG00010005578	HEATR5A	PRJNA729271	0.461834161	0.000222781	0.461834161	stage_E14_vs_E10	ordinary_muscle_development		0	FALSE
ENSGALG00010011584	ENSGALG00010011584	PRJNA729271	-4.351597912	0.000222988	4.351597912	stage_E14_vs_E10	ordinary_muscle_development	D_vs_E10;stage_1W_vs_E10;stage_3W_vs_E10;stage	1	TRUE
ENSGALG00010029716	GOSR1	PRJNA729271	0.244835637	0.000223177	0.244835637	stage_E14_vs_E10	ordinary_muscle_development		0	FALSE
ENSGALG00010007864	TYR	PRJNA729271	-4.247909911	0.000223267	4.247909911	stage_E14_vs_E10	ordinary_muscle_development	D_vs_E10;stage_1W_vs_E10;stage_3W_vs_E10;stage	1	TRUE
ENSGALG00010012230	PTPRQ	PRJNA729271	-1.617060081	0.00022339	1.617060081	stage_E14_vs_E10	ordinary_muscle_development	D_vs_E10;stage_1W_vs_E10;stage_3W_vs_E10;stage	1	TRUE
ENSGALG00010025144	PIH1D2	PRJNA729271	0.594983899	0.000224664	0.594983899	stage_E14_vs_E10	ordinary_muscle_development		0	FALSE
ENSGALG00010009606	CHSY3	PRJNA729271	-0.824650933	0.00022485	0.824650933	stage_E14_vs_E10	ordinary_muscle_development		0	FALSE
ENSGALG00010003853	ENSGALG00010003853	PRJNA729271	0.861682508	0.000224858	0.861682508	stage_E14_vs_E10	ordinary_muscle_development		0	FALSE
ENSGALG00010014111	GORAB	PRJNA729271	-0.315514313	0.000225043	0.315514313	stage_E14_vs_E10	ordinary_muscle_development		0	FALSE
ENSGALG00010011963	ZCCHC2	PRJNA729271	0.383453516	0.000226462	0.383453516	stage_E14_vs_E10	ordinary_muscle_development		0	FALSE
ENSGALG00010025449	ENSGALG00010025449	PRJNA729271	0.451415113	0.000227258	0.451415113	stage_E14_vs_E10	ordinary_muscle_development		0	FALSE
ENSGALG00010024460	IMP4	PRJNA729271	-0.608368513	0.000228498	0.608368513	stage_E14_vs_E10	ordinary_muscle_development		0	FALSE
ENSGALG00010005513	TMEM30CP	PRJNA729271	0.976255458	0.000229352	0.976255458	stage_E14_vs_E10	ordinary_muscle_development		0	FALSE
ENSGALG00010016800	RPL14	PRJNA729271	0.653048298	0.000231846	0.653048298	stage_E14_vs_E10	ordinary_muscle_development		0	FALSE
ENSGALG00010029926	BLTP2	PRJNA729271	0.502056633	0.000232347	0.502056633	stage_E14_vs_E10	ordinary_muscle_development		0	FALSE
ENSGALG00010015964	KLC1	PRJNA729271	0.347470374	0.000232512	0.347470374	stage_E14_vs_E10	ordinary_muscle_development		0	FALSE
ENSGALG00010002528	MED14	PRJNA729271	-0.456442172	0.000232889	0.456442172	stage_E14_vs_E10	ordinary_muscle_development		0	FALSE
ENSGALG00010011916	MCAT	PRJNA729271	0.369393309	0.000233057	0.369393309	stage_E14_vs_E10	ordinary_muscle_development		0	FALSE
ENSGALG00010002352	ENSGALG00010002352	PRJNA729271	0.405508939	0.000233347	0.405508939	stage_E14_vs_E10	ordinary_muscle_development		0	FALSE
ENSGALG00010014474	PTPN1	PRJNA729271	-0.296032909	0.000233848	0.296032909	stage_E14_vs_E10	ordinary_muscle_development		0	FALSE
ENSGALG00010014263	ZNF131	PRJNA729271	-0.467572296	0.000233881	0.467572296	stage_E14_vs_E10	ordinary_muscle_development		0	FALSE
ENSGALG00010009099	CXCR4	PRJNA729271	-0.740576637	0.000233984	0.740576637	stage_E14_vs_E10	ordinary_muscle_development		0	FALSE
ENSGALG00010019331	CEMIP	PRJNA729271	1.164270089	0.000234408	1.164270089	stage_E14_vs_E10	ordinary_muscle_development	D_vs_E10;stage_1W_vs_E10;stage_3W_vs_E10;stage	1	TRUE
ENSGALG00010027873	RFX2	PRJNA729271	-0.788800988	0.000234991	0.788800988	stage_E14_vs_E10	ordinary_muscle_development		0	FALSE
ENSGALG00010016682	GPR141	PRJNA729271	1.629638157	0.000235545	1.629638157	stage_E14_vs_E10	ordinary_muscle_development	D_vs_E10;stage_1W_vs_E10;stage_3W_vs_E10;stage	1	TRUE
ENSGALG00010027894	RSBN1	PRJNA729271	0.252856296	0.000236655	0.252856296	stage_E14_vs_E10	ordinary_muscle_development		0	FALSE
ENSGALG00010021715	MMAB	PRJNA729271	0.503155177	0.000236905	0.503155177	stage_E14_vs_E10	ordinary_muscle_development		0	FALSE
ENSGALG00010019919	LIN7C	PRJNA729271	-0.417039571	0.000237973	0.417039571	stage_E14_vs_E10	ordinary_muscle_development		0	FALSE
ENSGALG00010019035	ENSGALG00010019035	PRJNA729271	-2.190429186	0.000238159	2.190429186	stage_E14_vs_E10	ordinary_muscle_development	D_vs_E10;stage_1W_vs_E10;stage_3W_vs_E10;stage	1	TRUE
ENSGALG00010027139	HSD11B1L	PRJNA729271	0.533619168	0.00023871	0.533619168	stage_E14_vs_E10	ordinary_muscle_development		0	FALSE
ENSGALG00010003311	ENSGALG00010003311	PRJNA729271	4.397696281	0.000239132	4.397696281	stage_E14_vs_E10	ordinary_muscle_development	D_vs_E10;stage_1W_vs_E10;stage_3W_vs_E10;stage	1	TRUE
ENSGALG00010023997	ARFGEF2	PRJNA729271	-0.240112293	0.000239381	0.240112293	stage_E14_vs_E10	ordinary_muscle_development		0	FALSE
ENSGALG00010005444	ACTC1	PRJNA729271	1.714661635	0.00023948	1.714661635	stage_E14_vs_E10	ordinary_muscle_development	D_vs_E10;stage_1W_vs_E10;stage_3W_vs_E10;stage	1	TRUE
ENSGALG00010008366	RPRD1A	PRJNA729271	-0.41265113	0.00023981	0.41265113	stage_E14_vs_E10	ordinary_muscle_development		0	FALSE
ENSGALG00010029585	ENSGALG00010029585	PRJNA729271	1.316647198	0.0002409	1.316647198	stage_E14_vs_E10	ordinary_muscle_development	D_vs_E10;stage_1W_vs_E10;stage_3W_vs_E10;stage	1	TRUE
ENSGALG00010029148	RALGDS	PRJNA729271	0.457275707	0.00024172	0.457275707	stage_E14_vs_E10	ordinary_muscle_development		0	FALSE
ENSGALG00010004457	AGA	PRJNA729271	0.486977805	0.000242108	0.486977805	stage_E14_vs_E10	ordinary_muscle_development		0	FALSE
ENSGALG00010001558	CCKBR	PRJNA729271	-3.299652296	0.000242217	3.299652296	stage_E14_vs_E10	ordinary_muscle_development	D_vs_E10;stage_1W_vs_E10;stage_3W_vs_E10;stage	1	TRUE
ENSGALG00010001728	STS	PRJNA729271	1.228063912	0.000242812	1.228063912	stage_E14_vs_E10	ordinary_muscle_development	D_vs_E10;stage_1W_vs_E10;stage_3W_vs_E10;stage	1	TRUE
ENSGALG00010006828	GTF2A1	PRJNA729271	-0.362070578	0.000243051	0.362070578	stage_E14_vs_E10	ordinary_muscle_development		0	FALSE
ENSGALG00010019204	ARF6	PRJNA729271	-0.888803576	0.000243428	0.888803576	stage_E14_vs_E10	ordinary_muscle_development		0	FALSE
ENSGALG00010012882	ENSGALG00010012882	PRJNA729271	2.102505087	0.000244187	2.102505087	stage_E14_vs_E10	ordinary_muscle_development	D_vs_E10;stage_1W_vs_E10;stage_3W_vs_E10;stage	1	TRUE
ENSGALG00010029586	COIL	PRJNA729271	-0.735823042	0.000244736	0.735823042	stage_E14_vs_E10	ordinary_muscle_development		0	FALSE
ENSGALG00010018899	ACSS1	PRJNA729271	-0.362549454	0.000244838	0.362549454	stage_E14_vs_E10	ordinary_muscle_development		0	FALSE
ENSGALG00010028242	ENSGALG00010028242	PRJNA729271	1.072759956	0.000245371	1.072759956	stage_E14_vs_E10	ordinary_muscle_development	D_vs_E10;stage_1W_vs_E10;stage_3W_vs_E10;stage	1	TRUE
ENSGALG00010023658	ENSGALG00010023658	PRJNA729271	-1.316750003	0.000245954	1.316750003	stage_E14_vs_E10	ordinary_muscle_development	D_vs_E10;stage_1W_vs_E10;stage_3W_vs_E10;stage	1	TRUE
ENSGALG00010027884	SF3A2	PRJNA729271	-0.725428484	0.000246037	0.725428484	stage_E14_vs_E10	ordinary_muscle_development		0	FALSE
ENSGALG00010010719	SH3BP4	PRJNA729271	0.631104017	0.000246504	0.631104017	stage_E14_vs_E10	ordinary_muscle_development		0	FALSE
ENSGALG00010010912	BAALC	PRJNA729271	-1.366286887	0.000246528	1.366286887	stage_E14_vs_E10	ordinary_muscle_development	D_vs_E10;stage_1W_vs_E10;stage_3W_vs_E10;stage	1	TRUE
ENSGALG00010006678	ENSGALG00010006678	PRJNA729271	-5.3482642	0.000247448	5.3482642	stage_E14_vs_E10	ordinary_muscle_development	D_vs_E10;stage_1W_vs_E10;stage_3W_vs_E10;stage	1	TRUE

ENSGALG00010011250	ENSGALG00010011250	PRJNA729271	-0.397670823	0.000247631	0.397670823	stage_E14_vs_E10	ordinary_muscle_development		0	FALSE
ENSGALG00010024356	PARL	PRJNA729271	0.444601821	0.00024782	0.444601821	stage_E14_vs_E10	ordinary_muscle_development		0	FALSE
ENSGALG00010000732	ENSGALG00010000732	PRJNA729271	-0.559616938	0.000249197	0.559616938	stage_E14_vs_E10	ordinary_muscle_development		0	FALSE
ENSGALG00010000728	OTULIN	PRJNA729271	-1.195237235	0.000250696	1.195237235	stage_E14_vs_E10	ordinary_muscle_development	D_vs_E10;stage_1W_vs_E10;stage_3W_vs_E10;stage	1	TRUE
ENSGALG00010024749	APOA5	PRJNA729271	2.525218701	0.000250699	2.525218701	stage_E14_vs_E10	ordinary_muscle_development	D_vs_E10;stage_1W_vs_E10;stage_3W_vs_E10;stage	1	TRUE
ENSGALG00010022711	LRRC49	PRJNA729271	-0.739393595	0.000251652	0.739393595	stage_E14_vs_E10	ordinary_muscle_development		0	FALSE
ENSGALG00010029479	ENSGALG00010029479	PRJNA729271	-2.113065006	0.000252205	2.113065006	stage_E14_vs_E10	ordinary_muscle_development	D_vs_E10;stage_1W_vs_E10;stage_3W_vs_E10;stage	1	TRUE
ENSGALG00010021181	DAGLA	PRJNA729271	0.48218379	0.000255382	0.48218379	stage_E14_vs_E10	ordinary_muscle_development		0	FALSE
ENSGALG00010004397	ENSGALG00010004397	PRJNA729271	0.629809655	0.000256852	0.629809655	stage_E14_vs_E10	ordinary_muscle_development		0	FALSE
ENSGALG00010022581	TMEM125	PRJNA729271	-2.830706354	0.000260458	2.830706354	stage_E14_vs_E10	ordinary_muscle_development	D_vs_E10;stage_1W_vs_E10;stage_3W_vs_E10;stage	1	TRUE
ENSGALG00010026796	DCAKD	PRJNA729271	0.804646856	0.000260972	0.804646856	stage_E14_vs_E10	ordinary_muscle_development		0	FALSE
ENSGALG00010008147	CDKAL1	PRJNA729271	0.424382019	0.000261925	0.424382019	stage_E14_vs_E10	ordinary_muscle_development		0	FALSE
ENSGALG00010027358	RNF126	PRJNA729271	0.375392532	0.000262985	0.375392532	stage_E14_vs_E10	ordinary_muscle_development		0	FALSE
ENSGALG00010002725	TSPAN7	PRJNA729271	0.672320217	0.000263719	0.672320217	stage_E14_vs_E10	ordinary_muscle_development		0	FALSE
ENSGALG00010028085	ENSGALG00010028085	PRJNA729271	-1.096217269	0.000263754	1.096217269	stage_E14_vs_E10	ordinary_muscle_development	D_vs_E10;stage_1W_vs_E10;stage_3W_vs_E10;stage	1	TRUE
ENSGALG00010024392	CHMP4B	PRJNA729271	-0.327740322	0.000264112	0.327740322	stage_E14_vs_E10	ordinary_muscle_development		0	FALSE
ENSGALG00010029663	GUSB	PRJNA729271	-0.367157611	0.000266654	0.367157611	stage_E14_vs_E10	ordinary_muscle_development		0	FALSE
ENSGALG00010020048	ENSGALG00010020048	PRJNA729271	-1.009880557	0.000268773	1.009880557	stage_E14_vs_E10	ordinary_muscle_development	D_vs_E10;stage_1W_vs_E10;stage_3W_vs_E10;stage	1	TRUE
ENSGALG00010018311	NKX2-3	PRJNA729271	2.731656027	0.000270586	2.731656027	stage_E14_vs_E10	ordinary_muscle_development	D_vs_E10;stage_1W_vs_E10;stage_3W_vs_E10;stage	1	TRUE
ENSGALG00010025804	ENSGALG00010025804	PRJNA729271	8.37372853	0.000272023	8.37372853	stage_E14_vs_E10	ordinary_muscle_development	D_vs_E10;stage_1W_vs_E10;stage_3W_vs_E10;stage	1	TRUE
ENSGALG00010029220	NEK8	PRJNA729271	0.481694867	0.000272076	0.481694867	stage_E14_vs_E10	ordinary_muscle_development		0	FALSE
ENSGALG00010007319	ATP9B	PRJNA729271	0.382064749	0.000274205	0.382064749	stage_E14_vs_E10	ordinary_muscle_development		0	FALSE
ENSGALG00010009178	ENSGALG00010009178	PRJNA729271	0.960530797	0.000274815	0.960530797	stage_E14_vs_E10	ordinary_muscle_development		0	FALSE
ENSGALG00010015606	TMEM131	PRJNA729271	0.410681319	0.000275504	0.410681319	stage_E14_vs_E10	ordinary_muscle_development		0	FALSE
ENSGALG00010017699	RNF19B	PRJNA729271	0.903073819	0.000276117	0.903073819	stage_E14_vs_E10	ordinary_muscle_development		0	FALSE
ENSGALG00010017555	CELA2A	PRJNA729271	4.506392869	0.000277458	4.506392869	stage_E14_vs_E10	ordinary_muscle_development	D_vs_E10;stage_1W_vs_E10;stage_3W_vs_E10;stage	1	TRUE
ENSGALG00010018275	ZNF639	PRJNA729271	-0.317086481	0.000278916	0.317086481	stage_E14_vs_E10	ordinary_muscle_development		0	FALSE
ENSGALG00010021047	TMC5	PRJNA729271	-2.176941484	0.000279249	2.176941484	stage_E14_vs_E10	ordinary_muscle_development	D_vs_E10;stage_1W_vs_E10;stage_3W_vs_E10;stage	1	TRUE
ENSGALG00010002960	BLEC2	PRJNA729271	2.56181678	0.000279249	2.56181678	stage_E14_vs_E10	ordinary_muscle_development	D_vs_E10;stage_1W_vs_E10;stage_3W_vs_E10;stage	1	TRUE
ENSGALG00010017570	DRC3	PRJNA729271	-1.692210502	0.000279664	1.692210502	stage_E14_vs_E10	ordinary_muscle_development	D_vs_E10;stage_1W_vs_E10;stage_3W_vs_E10;stage	1	TRUE
ENSGALG00010021256	ENSGALG00010021256	PRJNA729271	1.085116152	0.000281655	1.085116152	stage_E14_vs_E10	ordinary_muscle_development	D_vs_E10;stage_1W_vs_E10;stage_3W_vs_E10;stage	1	TRUE
ENSGALG00010020171	RRN3	PRJNA729271	-0.293486938	0.000282359	0.293486938	stage_E14_vs_E10	ordinary_muscle_development		0	FALSE
ENSGALG00010023122	ENSGALG00010023122	PRJNA729271	2.523541895	0.000283071	2.523541895	stage_E14_vs_E10	ordinary_muscle_development	D_vs_E10;stage_1W_vs_E10;stage_3W_vs_E10;stage	1	TRUE
ENSGALG00010002429	ENSGALG00010002429	PRJNA729271	2.133953532	0.000284243	2.133953532	stage_E14_vs_E10	ordinary_muscle_development	D_vs_E10;stage_1W_vs_E10;stage_3W_vs_E10;stage	1	TRUE
ENSGALG00010026051	TAF11	PRJNA729271	-0.487580417	0.000284372	0.487580417	stage_E14_vs_E10	ordinary_muscle_development		0	FALSE
ENSGALG00010000641	ENSGALG00010000641	PRJNA729271	-1.889277995	0.000285683	1.889277995	stage_E14_vs_E10	ordinary_muscle_development	D_vs_E10;stage_1W_vs_E10;stage_3W_vs_E10;stage	1	TRUE
ENSGALG00010016609	SOX2	PRJNA729271	-2.533012187	0.000286228	2.533012187	stage_E14_vs_E10	ordinary_muscle_development	D_vs_E10;stage_1W_vs_E10;stage_3W_vs_E10;stage	1	TRUE
ENSGALG00010017622	RAB1B	PRJNA729271	-0.428991337	0.000288901	0.428991337	stage_E14_vs_E10	ordinary_muscle_development		0	FALSE
ENSGALG00010026919	NICN1	PRJNA729271	-0.64436115	0.000291202	0.64436115	stage_E14_vs_E10	ordinary_muscle_development		0	FALSE
ENSGALG00010000192	ENSGALG00010000192	PRJNA729271	2.620812965	0.000293166	2.620812965	stage_E14_vs_E10	ordinary_muscle_development	D_vs_E10;stage_1W_vs_E10;stage_3W_vs_E10;stage	1	TRUE
ENSGALG00010009357	LYST	PRJNA729271	-0.462855224	0.000293389	0.462855224	stage_E14_vs_E10	ordinary_muscle_development		0	FALSE
ENSGALG00010020329	PPP4R2	PRJNA729271	-0.405514856	0.000293819	0.405514856	stage_E14_vs_E10	ordinary_muscle_development		0	FALSE
ENSGALG00010024080	TENT4B	PRJNA729271	0.326851201	0.000294216	0.326851201	stage_E14_vs_E10	ordinary_muscle_development		0	FALSE
ENSGALG00010003894	COPS4	PRJNA729271	0.464867958	0.000294714	0.464867958	stage_E14_vs_E10	ordinary_muscle_development		0	FALSE
ENSGALG00010021790	PHF21A	PRJNA729271	0.478519659	0.000294805	0.478519659	stage_E14_vs_E10	ordinary_muscle_development		0	FALSE
ENSGALG00010001516	CLTRN	PRJNA729271	-2.42796332	0.000295032	2.42796332	stage_E14_vs_E10	ordinary_muscle_development	D_vs_E10;stage_1W_vs_E10;stage_3W_vs_E10;stage	1	TRUE
ENSGALG00010012897	ENSGALG00010012897	PRJNA729271	1.080057673	0.000295625	1.080057673	stage_E14_vs_E10	ordinary_muscle_development	D_vs_E10;stage_1W_vs_E10;stage_3W_vs_E10;stage	1	TRUE
ENSGALG00010016879	RNF130	PRJNA729271	-0.380325844	0.000295633	0.380325844	stage_E14_vs_E10	ordinary_muscle_development		0	FALSE
ENSGALG00010012275	VIRMA	PRJNA729271	-0.282010507	0.000296197	0.282010507	stage_E14_vs_E10	ordinary_muscle_development		0	FALSE
ENSGALG00010005218	GTPBP8	PRJNA729271	0.438204705	0.000296236	0.438204705	stage_E14_vs_E10	ordinary_muscle_development		0	FALSE
ENSGALG00010016193	TCAIM	PRJNA729271	0.505429734	0.000296531	0.505429734	stage_E14_vs_E10	ordinary_muscle_development		0	FALSE
ENSGALG00010026199	TBX6	PRJNA729271	-1.804163149	0.000296567	1.804163149	stage_E14_vs_E10	ordinary_muscle_development	D_vs_E10;stage_1W_vs_E10;stage_3W_vs_E10;stage	1	TRUE
ENSGALG00010001586	ZBTB14	PRJNA729271	0.289167079	0.000297441	0.289167079	stage_E14_vs_E10	ordinary_muscle_development		0	FALSE
ENSGALG00010009259	MGAT4D	PRJNA729271	2.897098569	0.000297475	2.897098569	stage_E14_vs_E10	ordinary_muscle_development	D_vs_E10;stage_1W_vs_E10;stage_3W_vs_E10;stage	1	TRUE
ENSGALG00010021313	KPNA7	PRJNA729271	4.530353088	0.000298017	4.530353088	stage_E14_vs_E10	ordinary_muscle_development	D_vs_E10;stage_1W_vs_E10;stage_3W_vs_E10;stage	1	TRUE
ENSGALG00010016206	KNG1	PRJNA729271	4.356957055	0.000300686	4.356957055	stage_E14_vs_E10	ordinary_muscle_development	D_vs_E10;stage_1W_vs_E10;stage_3W_vs_E10;stage	1	TRUE
ENSGALG00010001795	ENSGALG00010001795	PRJNA729271	1.804729341	0.000300926	1.804729341	stage_E14_vs_E10	ordinary_muscle_development	D_vs_E10;stage_1W_vs_E10;stage_3W_vs_E10;stage	1	TRUE
ENSGALG00010023084	ENSGALG00010023084	PRJNA729271	0.900062625	0.00030217	0.900062625	stage_E14_vs_E10	ordinary_muscle_development		0	FALSE
ENSGALG00010003300	CASP8AP2	PRJNA729271	-0.438899707	0.000303631	0.438899707	stage_E14_vs_E10	ordinary_muscle_development		0	FALSE
ENSGALG00010028498	C9orf78	PRJNA729271	0.386096152	0.000303759	0.386096152	stage_E14_vs_E10	ordinary_muscle_development		0	FALSE
ENSGALG00010024702	INAVA	PRJNA729271	-3.571504479	0.000305372	3.571504479	stage_E14_vs_E10	ordinary_muscle_development	D_vs_E10;stage_1W_vs_E10;stage_3W_vs_E10;stage	1	TRUE
ENSGALG00010023389	GNG12	PRJNA729271	-0.411252145	0.000308771	0.411252145	stage_E14_vs_E10	ordinary_muscle_development		0	FALSE
ENSGALG00010021651	RNF113B	PRJNA729271	0.568929201	0.000309351	0.568929201	stage_E14_vs_E10	ordinary_muscle_development		0	FALSE
ENSGALG00010022226	FBRSL1	PRJNA729271	0.491614239	0.000309694	0.491614239	stage_E14_vs_E10	ordinary_muscle_development		0	FALSE
ENSGALG00010023541	PRSS2	PRJNA729271	9.238243836	0.000310384	9.238243836	stage_E14_vs_E10	ordinary_muscle_development	D_vs_E10;stage_1W_vs_E10;stage_3W_vs_E10;stage	1	TRUE
ENSGALG00010005140	DCT	PRJNA729271	-4.15001051	0.000311439	4.15001051	stage_E14_vs_E10	ordinary_muscle_development	D_vs_E10;stage_1W_vs_E10;stage_3W_vs_E10;stage	1	TRUE
ENSGALG00010027374	NKTR	PRJNA729271	0.440038947	0.000314109	0.440038947	stage_E14_vs_E10	ordinary_muscle_development		0	FALSE
ENSGALG00010010876	ENSGALG00010010876	PRJNA729271	0.587822121	0.000314653	0.587822121	stage_E14_vs_E10	ordinary_muscle_development		0	FALSE
ENSGALG00010029437	ENSGALG00010029437	PRJNA729271	-7.306890685	0.000314784	7.306890685	stage_E14_vs_E10	ordinary_muscle_development	D_vs_E10;stage_1W_vs_E10;stage_3W_vs_E10;stage	1	TRUE
ENSGALG00010024139	SLC22A18	PRJNA729271	-3.571815583	0.000316378	3.571815583	stage_E14_vs_E10	ordinary_muscle_development	D_vs_E10;stage_1W_vs_E10;stage_3W_vs_E10;stage	1	TRUE
ENSGALG00010012283	CUPIN1A	PRJNA729271	1.853827307	0.000318007	1.853827307	stage_E14_vs_E10	ordinary_muscle_development	D_vs_E10;stage_1W_vs_E10;stage_3W_vs_E10;stage	1	TRUE
ENSGALG00010015502	BPIFB6	PRJNA729271	4.794363656	0.00031841	4.794363656	stage_E14_vs_E10	ordinary_muscle_development	D_vs_E10;stage_1W_vs_E10;stage_3W_vs_E10;stage	1	TRUE
ENSGALG00010007812	POMK	PRJNA729271	0.448116843	0.000320978	0.448116843	stage_E14_vs_E10	ordinary_muscle_development		0	FALSE
ENSGALG00010022782	TMPRSS13	PRJNA729271	0.845326505	0.000321041	0.845326505	stage_E14_vs_E10	ordinary_muscle_development		0	FALSE
ENSGALG00010019630	AJAP1	PRJNA729271	-1.448847758	0.000321932	1.448847758	stage_E14_vs_E10	ordinary_muscle_development	D_vs_E10;stage_1W_vs_E10;stage_3W_vs_E10;stage	1	TRUE
ENSGALG00010005048	ROCK1	PRJNA729271	-0.426842289	0.000323259	0.426842289	stage_E14_vs_E10	ordinary_muscle_development		0	FALSE
ENSGALG00010009910	ENSGALG00010009910	PRJNA729271	0.651553472	0.000323948	0.651553472	stage_E14_vs_E10	ordinary_muscle_development		0	FALSE
ENSGALG00010021786	FAM162A	PRJNA729271	0.641253726	0.000324504	0.641253726	stage_E14_vs_E10	ordinary_muscle_development		0	FALSE
ENSGALG00010007597	SMKR1	PRJNA729271	-3.752249677	0.000325166	3.752249677	stage_E14_vs_E10	ordinary_muscle_development	D_vs_E10;stage_1W_vs_E10;stage_3W_vs_E10;stage	1	TRUE
ENSGALG00010002364	VSNL1	PRJNA729271	-1.181392306	0.00032665	1.181392306	stage_E14_vs_E10	ordinary_muscle_development	D_vs_E10;stage_1W_vs_E10;stage_3W_vs_E10;stage	1	TRUE
ENSGALG00010021733	ENSGALG00010021733	PRJNA729271	2.174323777	0.000329118	2.174323777	stage_E14_vs_E10	ordinary_muscle_development	D_vs_E10;stage_1W_vs_E10;stage_3W_vs_E10;stage	1	TRUE
ENSGALG00010029574	SGSM2	PRJNA729271	-0.51414033	0.000330697	0.51414033	stage_E14_vs_E10	ordinary_muscle_development		0	FALSE
ENSGALG00010007226	ENSGALG00010007226	PRJNA729271	5.584385647	0.000332142	5.584385647	stage_E14_vs_E10	ordinary_muscle_development	D_vs_E10;stage_1W_vs_E10;stage_3W_vs_E10;stage	1	TRUE
ENSGALG00010010345	ENSGALG00010010345	PRJNA729271	-0.522981019	0.000332189	0.522981019	stage_E14_vs_E10	ordinary_muscle_development		0	FALSE
ENSGALG00010019076	ESPN	PRJNA729271	-1.691931819	0.000333351	1.691931819	stage_E14_vs_E10	ordinary_muscle_development	D_vs_E10;stage_1W_vs_E10;stage_3W_vs_E10;stage	1	TRUE
ENSGALG00010012631	GLRX2	PRJNA729271	0.573898623	0.000333937	0.573898623	stage_E14_vs_E10	ordinary_muscle_development		0	FALSE
ENSGALG00010003641	ENSGALG00010003641	PRJNA729271	9.04687018	0.000337305	9.04687018	stage_E14_vs_E10	ordinary_muscle_development	D_vs_E10;stage_1W_vs_E10;stage_3W_vs_E10;stage	1	TRUE
ENSGALG00010021443	SNUPN	PRJNA729271	-0.511613148	0.000337595	0.511613148	stage_E14_vs_E10	ordinary_muscle_development		0	FALSE
ENSGALG00010002739	HEP21	PRJNA729271	-2.905279374	0.000337879	2.905279374	stage_E14_vs_E10	ordinary_muscle_development	D_vs_E10;stage_1W_vs_E10;stage_3W_vs_E10;stage	1	TRUE
ENSGALG00010009534	XPA	PRJNA729271	0.44145349	0.000340355	0.44145349	stage_E14_vs_E10	ordinary_muscle_development		0	FALSE
ENSGALG00010000431	AIFM3	PRJNA729271	-4.395844561	0.000341439	4.395844561	stage_E14_vs_E10	ordinary_muscle_development	D_vs_E10;stage_1W_vs_E10;stage_3W_vs_E10;stage	1	TRUE
ENSGALG00010023211	TLK1	PRJNA729271	-0.284782565	0.000341444	0.284782565	stage_E14_vs_E10	ordinary_muscle_development		0	FALSE
ENSGALG00010023676	ENSGALG00010023676	PRJNA729271	-1.128666921	0.000342462	1.128666921	stage_E14_vs_E10	ordinary_muscle_development	D_vs_E10;stage_1W_vs_E10;stage_3W_vs_E10;stage	1	TRUE
ENSGALG00010029165	RAC3	PRJNA729271	-0.446995878	0.000342857	0.446995878	stage_E14_vs_E10	ordinary_muscle_development		0	FALSE
ENSGALG00010000462	ENSGALG00010000462	PRJNA729271	-0.924001214	0.000343538	0.924001214	stage_E14_vs_E10	ordinary_muscle_development		0	FALSE
ENSGALG00010019236	MPG	PRJNA729271	-0.789582446	0.000346051	0.789582446	stage_E14_vs_E10	ordinary_muscle_development		0	FALSE
ENSGALG00010000655	ENSGALG00010000655	PRJNA729271	-3.935634581	0.000347375	3.935634581	stage_E14_vs_E10	ordinary_muscle_development	D_vs_E10;stage_1W_vs_E10;stage_3W_vs_E10;stage	1	TRUE
ENSGALG00010025572	ENSGALG00010025572	PRJNA729271	-0.41569737	0.000348948	0.41569737	stage_E14_vs_E10	ordinary_muscle_development		0	FALSE
ENSGALG00010014602	FRK	PRJNA729271	-5.352296095	0.000349305	5.352296095	stage_E14_vs_E10	ordinary_muscle_development	D_vs_E10;stage_1W_vs_E10;stage_3W_vs_E10;stage	1	TRUE
ENSGALG00010009883	ENSGALG00010009883	PRJNA729271	-0.75400924	0.000350006	0.75400924	stage_E14_vs_E10	ordinary_muscle_development		0	FALSE
ENSGALG00010020982	TLCD4	PRJNA729271	0.561339032	0.000350032	0.561339032	stage_E14_vs_E10	ordinary_muscle_development		0	FALSE
ENSGALG00010029939	ENSGALG00010029939	PRJNA729271	4.666537766	0.000352879	4.666537766	stage_E14_vs_E10	ordinary_muscle_development	D_vs_E10;stage_1W_vs_E10;stage_3W_vs_E10;stage	1	TRUE
ENSGALG00010007954	PSCA	PRJNA729271	-2.657787616	0.000352879	2.657787616	stage_E14_vs_E10	ordinary_muscle_development	D_vs_E10;stage_1W_vs_E10;stage_3W_vs_E10;stage	1	TRUE
ENSGALG00010018553	ENSGALG00010018553	PRJNA729271	-2.526614095	0.000353722	2.526614095	stage_E14_vs_E10	ordinary_muscle_development	D_vs_E10;stage_1W_vs_E10;stage_3W_vs_E10;stage	1	TRUE
ENSGALG00010029498	ENSGALG00010029498	PRJNA729271	0.578551324	0.000356835	0.578551324	stage_E14_vs_E10	ordinary_muscle_development		0	FALSE
ENSGALG00010002135	ELOVL5	PRJNA729271	-0.626937242	0.000357829	0.626937242	stage_E14_vs_E10	ordinary_muscle_development		0	FALSE
ENSGALG00010004879	ENSGALG00010004879	PRJNA729271	-2.815788378	0.000358816	2.815788378	stage_E14_vs_E10	ordinary_muscle_development	D_vs_E10;stage_1W_vs_E10;stage_3W_vs_E10;stage	1	TRUE
ENSGALG00010001903	NARS2	PRJNA729271	-0.43710137	0.000358947	0.43710137	stage_E14_vs_E10	ordinary_muscle_development		0	FALSE
ENSGALG00010016982	GLRX5	PRJNA729271	-0.52236243	0.000360096	0.52236243	stage_E14_vs_E10	ordinary_muscle_development		0	FALSE
ENSGALG00010010368	ENSGALG00010010368	PRJNA729271	-0.295468493	0.000360109	0.295468493	stage_E14_vs_E10	ordinary_muscle_development		0	FALSE
ENSGALG00010004516	ENSGALG00010004516	PRJNA729271	1.092798177	0.000362395	1.092798177	stage_E14_vs_E10	ordinary_muscle_development	D_vs_E10;stage_1W_vs_E10;stage_3W_vs_E10;stage	1	TRUE
ENSGALG00010025588	ENSGALG00010025588	PRJNA729271	-1.209795997	0.000362735	1.209795997	stage_E14_vs_E10	ordinary_muscle_development	D_vs_E10;stage_1W_vs_E10;stage_3W_vs_E10;stage	1	TRUE
ENSGALG00010005777	ENSGALG00010005777	PRJNA729271	2.341032429	0.000362804	2.341032429	stage_E14_vs_E10	ordinary_muscle_development	D_vs_E10;stage_1W_vs_E10;stage_3W_vs_E10;stage	1	TRUE
ENSGALG00010024736	DGKA	PRJNA729271	-0.562108711	0.00036494	0.562108711	stage_E14_vs_E10	ordinary_muscle_development		0	FALSE
ENSGALG00010015283	ENSGALG00010015283	PRJNA729271	-3.476949827	0.0003654	3.476949827	stage_E14_vs_E10	ordinary_muscle_development	D_vs_E10;stage_1W_vs_E10;stage_3W_vs_E10;stage	1	TRUE
ENSGALG00010020721	PTDSS2	PRJNA729271	0.625133985	0.000365539	0.625133985	stage_E14_vs_E10	ordinary_muscle_development		0	FALSE
ENSGALG00010020979	ENSGALG00010020979	PRJNA729271	0.897394227	0.000366749	0.897394227	stage_E14_vs_E10	ordinary_muscle_development		0	FALSE
ENSGALG00010028157	PRRC2B	PRJNA729271	-0.534459346	0.000367339	0.534459346	stage_E14_vs_E10	ordinary_muscle_development		0	FALSE
ENSGALG00010002937	GALNT7	PRJNA729271	-0.408444489	0.000367506	0.408444489	stage_E14_vs_E10	ordinary_muscle_development		0	FALSE
ENSGALG00010003223	AREG	PRJNA729271	-3.70303808	0.000368771	3.70303808	stage_E14_vs_E10	ordinary_muscle_development	D_vs_E10;stage_1W_vs_E10;stage_3W_vs_E10;stage	1	TRUE
ENSGALG00010018715	DNAJC13	PRJNA729271	-0.38744505	0.000369606	0.38744505	stage_E14_vs_E10	ordinary_muscle_development		0	FALSE
ENSGALG00010021097	KATNBL1	PRJNA729271	-0.478411817	0.000370506	0.478411817	stage_E14_vs_E10	ordinary_muscle_development		0	FALSE
ENSGALG00010001213	PPIC	PRJNA729271	-0.811791303	0.000370653	0.811791303	stage_E14_vs_E10	ordinary_muscle_development		0	FALSE
ENSGALG00010009376	CDO1	PRJNA729271	-0.97810441	0.000371225	0.97810441	stage_E14_vs_E10	ordinary_muscle_development		0	FALSE
ENSGALG00010028117	ENSGALG00010028117	PRJNA729271	0.840236422	0.000372212	0.840236422	stage_E14_vs_E10	ordinary_muscle_development		0	FALSE
ENSGALG00010012290	ENSGALG00010012290	PRJNA729271	0.34919839	0.000372614	0.34919839	stage_E14_vs_E10	ordinary_muscle_development		0	FALSE
ENSGALG00010023408	ZBTB44	PRJNA729271	0.469110541	0.000372971	0.469110541	stage_E14_vs_E10	ordinary_muscle_development		0	FALSE
ENSGALG00010008238	NTAQ1	PRJNA729271	-0.630868396	0.000374301	0.630868396	stage_E14_vs_E10	ordinary_muscle_development		0	FALSE
ENSGALG00010020269	ENSGALG00010020269	PRJNA729271	-3.522342465	0.000374322	3.522342465	stage_E14_vs_E10	ordinary_muscle_development	D_vs_E10;stage_1W_vs_E10;stage_3W_vs_E10;stage	1	TRUE
ENSGALG00010005104	ENPP3	PRJNA729271	-0.744139785	0.000375266	0.744139785	stage_E14_vs_E10	ordinary_muscle_development		0	FALSE
ENSGALG00010004309	MRPS28	PRJNA729271	-0.507655838	0.000377051	0.507655838	stage_E14_vs_E10	ordinary_muscle_development		0	FALSE
ENSGALG00010010789	ENSGALG00010010789	PRJNA729271	-1.095286146	0.000377296	1.095286146	stage_E14_vs_E10	ordinary_muscle_development	D_vs_E10;stage_1W_vs_E10;stage_3W_vs_E10;stage	1	TRUE
ENSGALG00010006246	SUPT20H	PRJNA729271	-0.370036315	0.000377911	0.370036315	stage_E14_vs_E10	ordinary_muscle_development		0	FALSE
ENSGALG00010010301	ENSGALG00010010301	PRJNA729271	-2.056984885	0.000378927	2.056984885	stage_E14_vs_E10	ordinary_muscle_development	D_vs_E10;stage_1W_vs_E10;stage_3W_vs_E10;stage	1	TRUE
ENSGALG00010008269	ENSGALG00010008269	PRJNA729271	1.096976762	0.000380002	1.096976762	stage_E14_vs_E10	ordinary_muscle_development	D_vs_E10;stage_1W_vs_E10;stage_3W_vs_E10;stage	1	TRUE
ENSGALG00010020071	ENSGALG00010020071	PRJNA729271	0.527574708	0.000380309	0.527574708	stage_E14_vs_E10	ordinary_muscle_development		0	FALSE
ENSGALG00010003820	SH3YL1	PRJNA729271	-1.11940627	0.000380501	1.11940627	stage_E14_vs_E10	ordinary_muscle_development	D_vs_E10;stage_1W_vs_E10;stage_3W_vs_E10;stage	1	TRUE
ENSGALG00010013232	FHL2	PRJNA729271	1.571101445	0.000381291	1.571101445	stage_E14_vs_E10	ordinary_muscle_development	D_vs_E10;stage_1W_vs_E10;stage_3W_vs_E10;stage	1	TRUE
ENSGALG00010022786	KRT40	PRJNA729271	1.942382864	0.000381528	1.942382864	stage_E14_vs_E10	ordinary_muscle_development	D_vs_E10;stage_1W_vs_E10;stage_3W_vs_E10;stage	1	TRUE
ENSGALG00010013742	RSPO4	PRJNA729271	-5.043599538	0.000382534	5.043599538	stage_E14_vs_E10	ordinary_muscle_development	D_vs_E10;stage_1W_vs_E10;stage_3W_vs_E10;stage	1	TRUE
ENSGALG00010011533	ENSGALG00010011533	PRJNA729271	-1.066093626	0.000383066	1.066093626	stage_E14_vs_E10	ordinary_muscle_development	D_vs_E10;stage_1W_vs_E10;stage_3W_vs_E10;stage	1	TRUE
ENSGALG00010024226	POU2AF1	PRJNA729271	4.574081303	0.000383765	4.574081303	stage_E14_vs_E10	ordinary_muscle_development	D_vs_E10;stage_1W_vs_E10;stage_3W_vs_E10;stage	1	TRUE
ENSGALG00010023165	CSNK2A2	PRJNA729271	0.476681459	0.000383919	0.476681459	stage_E14_vs_E10	ordinary_muscle_development		0	FALSE
ENSGALG00010007881	AvBD14	PRJNA729271	-3.505079117	0.000384461	3.505079117	stage_E14_vs_E10	ordinary_muscle_development	D_vs_E10;stage_1W_vs_E10;stage_3W_vs_E10;stage	1	TRUE
ENSGALG00010012149	SFXN1	PRJNA729271	-0.642527105	0.000384626	0.642527105	stage_E14_vs_E10	ordinary_muscle_development		0	FALSE
ENSGALG00010023559	PJVK	PRJNA729271	2.502703755	0.000386727	2.502703755	stage_E14_vs_E10	ordinary_muscle_development	D_vs_E10;stage_1W_vs_E10;stage_3W_vs_E10;stage	1	TRUE
ENSGALG00010024849	CENATAC	PRJNA729271	0.695708671	0.000386902	0.695708671	stage_E14_vs_E10	ordinary_muscle_development		0	FALSE
ENSGALG00010015691	EXOC3L4	PRJNA729271	-3.837185411	0.000387036	3.837185411	stage_E14_vs_E10	ordinary_muscle_development	D_vs_E10;stage_1W_vs_E10;stage_3W_vs_E10;stage	1	TRUE
ENSGALG00010014300	ELFN2	PRJNA729271	-2.183591926	0.000388368	2.183591926	stage_E14_vs_E10	ordinary_muscle_development	D_vs_E10;stage_1W_vs_E10;stage_3W_vs_E10;stage	1	TRUE
ENSGALG00010027378	ZBTB47	PRJNA729271	0.612698867	0.000389071	0.612698867	stage_E14_vs_E10	ordinary_muscle_development		0	FALSE
ENSGALG00010004364	TSHR	PRJNA729271	1.677095799	0.000389189	1.677095799	stage_E14_vs_E10	ordinary_muscle_development	D_vs_E10;stage_1W_vs_E10;stage_3W_vs_E10;stage	1	TRUE
ENSGALG00010024374	ENSGALG00010024374	PRJNA729271	2.158343139	0.00038992	2.158343139	stage_E14_vs_E10	ordinary_muscle_development	D_vs_E10;stage_1W_vs_E10;stage_3W_vs_E10;stage	1	TRUE
ENSGALG00010027608	GNAI2	PRJNA729271	-0.529720737	0.000389958	0.529720737	stage_E14_vs_E10	ordinary_muscle_development		0	FALSE
ENSGALG00010004800	ZC3H12C	PRJNA729271	-1.049997682	0.000390416	1.049997682	stage_E14_vs_E10	ordinary_muscle_development	D_vs_E10;stage_1W_vs_E10;stage_3W_vs_E10;stage	1	TRUE
ENSGALG00010017070	BLOC1S6	PRJNA729271	0.384592382	0.000393504	0.384592382	stage_E14_vs_E10	ordinary_muscle_development		0	FALSE
ENSGALG00010018520	ENSGALG00010018520	PRJNA729271	0.455547291	0.000394658	0.455547291	stage_E14_vs_E10	ordinary_muscle_development		0	FALSE
ENSGALG00010005428	ZNF770	PRJNA729271	0.373520061	0.000397462	0.373520061	stage_E14_vs_E10	ordinary_muscle_development		0	FALSE
ENSGALG00010015407	TIMP3	PRJNA729271	0.652873341	0.000400114	0.652873341	stage_E14_vs_E10	ordinary_muscle_development		0	FALSE
ENSGALG00010021397	ENSGALG00010021397	PRJNA729271	6.17191421	0.00040141	6.17191421	stage_E14_vs_E10	ordinary_muscle_development	D_vs_E10;stage_1W_vs_E10;stage_3W_vs_E10;stage	1	TRUE
ENSGALG00010024707	ENSGALG00010024707	PRJNA729271	-1.376643107	0.000403625	1.376643107	stage_E14_vs_E10	ordinary_muscle_development	D_vs_E10;stage_1W_vs_E10;stage_3W_vs_E10;stage	1	TRUE
ENSGALG00010017584	WNT11B	PRJNA729271	-3.449154862	0.000406403	3.449154862	stage_E14_vs_E10	ordinary_muscle_development	D_vs_E10;stage_1W_vs_E10;stage_3W_vs_E10;stage	1	TRUE
ENSGALG00010021586	BBS4	PRJNA729271	-0.452795044	0.000407564	0.452795044	stage_E14_vs_E10	ordinary_muscle_development		0	FALSE
ENSGALG00010027172	ENSGALG00010027172	PRJNA729271	-2.014184006	0.000408854	2.014184006	stage_E14_vs_E10	ordinary_muscle_development	D_vs_E10;stage_1W_vs_E10;stage_3W_vs_E10;stage	1	TRUE
ENSGALG00010001649	UBE2E2	PRJNA729271	-0.85861438	0.000410313	0.85861438	stage_E14_vs_E10	ordinary_muscle_development		0	FALSE
ENSGALG00010009567	NSD1	PRJNA729271	0.391155571	0.000411336	0.391155571	stage_E14_vs_E10	ordinary_muscle_development		0	FALSE
ENSGALG00010011649	MRPS27	PRJNA729271	0.498980455	0.000411752	0.498980455	stage_E14_vs_E10	ordinary_muscle_development		0	FALSE
ENSGALG00010027536	USP21	PRJNA729271	1.105989726	0.000413191	1.105989726	stage_E14_vs_E10	ordinary_muscle_development	D_vs_E10;stage_1W_vs_E10;stage_3W_vs_E10;stage	1	TRUE
ENSGALG00010018103	BTRC	PRJNA729271	-0.394878189	0.000413896	0.394878189	stage_E14_vs_E10	ordinary_muscle_development		0	FALSE
ENSGALG00010012175	PACSIN2	PRJNA729271	0.563566059	0.000415996	0.563566059	stage_E14_vs_E10	ordinary_muscle_development		0	FALSE
ENSGALG00010027636	SLC1A6	PRJNA729271	2.16242287	0.000416186	2.16242287	stage_E14_vs_E10	ordinary_muscle_development	D_vs_E10;stage_1W_vs_E10;stage_3W_vs_E10;stage	1	TRUE
ENSGALG00010022435	PIGK	PRJNA729271	0.59676401	0.000416963	0.59676401	stage_E14_vs_E10	ordinary_muscle_development		0	FALSE
ENSGALG00010013117	ARSK	PRJNA729271	-0.510294337	0.000419007	0.510294337	stage_E14_vs_E10	ordinary_muscle_development		0	FALSE
ENSGALG00010009751	ENSGALG00010009751	PRJNA729271	0.745414522	0.000419425	0.745414522	stage_E14_vs_E10	ordinary_muscle_development		0	FALSE
ENSGALG00010002468	ENSGALG00010002468	PRJNA729271	2.06815583	0.000420008	2.06815583	stage_E14_vs_E10	ordinary_muscle_development	D_vs_E10;stage_1W_vs_E10;stage_3W_vs_E10;stage	1	TRUE
ENSGALG00010015574	C14orf132	PRJNA729271	-0.775619281	0.000422014	0.775619281	stage_E14_vs_E10	ordinary_muscle_development		0	FALSE

ENSGALG00010016605	WDPCP	PRJNA729271	-0.616963195	0.00042268	0.616963195	stage_E14_vs_E10	ordinary_muscle_development		0	FALSE
ENSGALG00010018925	LINS1	PRJNA729271	-0.487220968	0.000422961	0.487220968	stage_E14_vs_E10	ordinary_muscle_development		0	FALSE
ENSGALG00010029298	ENDOV	PRJNA729271	0.529546669	0.000423982	0.529546669	stage_E14_vs_E10	ordinary_muscle_development		0	FALSE
ENSGALG00010012741	ENSGALG00010012741	PRJNA729271	0.550284713	0.000424436	0.550284713	stage_E14_vs_E10	ordinary_muscle_development		0	FALSE
ENSGALG00010020298	ENSGALG00010020298	PRJNA729271	0.643131215	0.000424508	0.643131215	stage_E14_vs_E10	ordinary_muscle_development		0	FALSE
ENSGALG00010013408	RLIM	PRJNA729271	-0.242726541	0.000424639	0.242726541	stage_E14_vs_E10	ordinary_muscle_development		0	FALSE
ENSGALG00010000221	TBC1D10A	PRJNA729271	-0.324276313	0.000425082	0.324276313	stage_E14_vs_E10	ordinary_muscle_development		0	FALSE
ENSGALG00010019794	RSPH9	PRJNA729271	-1.943317209	0.000430265	1.943317209	stage_E14_vs_E10	ordinary_muscle_development	D_vs_E10;stage_1W_vs_E10;stage_3W_vs_E10;stage	1	TRUE
ENSGALG00010017391	CRAMP1	PRJNA729271	0.424730709	0.00043108	0.424730709	stage_E14_vs_E10	ordinary_muscle_development		0	FALSE
ENSGALG00010011639	ENSGALG00010011639	PRJNA729271	0.548648172	0.000436258	0.548648172	stage_E14_vs_E10	ordinary_muscle_development		0	FALSE
ENSGALG00010020457	IL5RA	PRJNA729271	3.380863788	0.000436405	3.380863788	stage_E14_vs_E10	ordinary_muscle_development	D_vs_E10;stage_1W_vs_E10;stage_3W_vs_E10;stage	1	TRUE
ENSGALG00010011339	ENSGALG00010011339	PRJNA729271	-1.156746981	0.000441975	1.156746981	stage_E14_vs_E10	ordinary_muscle_development	D_vs_E10;stage_1W_vs_E10;stage_3W_vs_E10;stage	1	TRUE
ENSGALG00010007168	MINA	PRJNA729271	-0.393593488	0.000445713	0.393593488	stage_E14_vs_E10	ordinary_muscle_development		0	FALSE
ENSGALG00010007946	ENSGALG00010007946	PRJNA729271	-0.606899622	0.000446041	0.606899622	stage_E14_vs_E10	ordinary_muscle_development		0	FALSE
ENSGALG00010004460	ENSGALG00010004460	PRJNA729271	-3.059885007	0.00044934	3.059885007	stage_E14_vs_E10	ordinary_muscle_development	D_vs_E10;stage_1W_vs_E10;stage_3W_vs_E10;stage	1	TRUE
ENSGALG00010028055	ENSGALG00010028055	PRJNA729271	-0.43151311	0.000449481	0.43151311	stage_E14_vs_E10	ordinary_muscle_development		0	FALSE
ENSGALG00010011136	ENSGALG00010011136	PRJNA729271	-1.487819307	0.000453159	1.487819307	stage_E14_vs_E10	ordinary_muscle_development	D_vs_E10;stage_1W_vs_E10;stage_3W_vs_E10;stage	1	TRUE
ENSGALG00010023841	OSBPL7	PRJNA729271	0.535206433	0.000453391	0.535206433	stage_E14_vs_E10	ordinary_muscle_development		0	FALSE
ENSGALG00010002658	FBXL12	PRJNA729271	-0.551430582	0.000453635	0.551430582	stage_E14_vs_E10	ordinary_muscle_development		0	FALSE
ENSGALG00010023198	PTK6	PRJNA729271	-2.159168294	0.000454061	2.159168294	stage_E14_vs_E10	ordinary_muscle_development	D_vs_E10;stage_1W_vs_E10;stage_3W_vs_E10;stage	1	TRUE
ENSGALG00010016852	AHDC1	PRJNA729271	0.616433004	0.000454249	0.616433004	stage_E14_vs_E10	ordinary_muscle_development		0	FALSE
ENSGALG00010017823	PPIE	PRJNA729271	-0.329774882	0.000456276	0.329774882	stage_E14_vs_E10	ordinary_muscle_development		0	FALSE
ENSGALG00010027176	ENSGALG00010027176	PRJNA729271	0.617511057	0.000456376	0.617511057	stage_E14_vs_E10	ordinary_muscle_development		0	FALSE
ENSGALG00010027255	ENSGALG00010027255	PRJNA729271	-0.670267012	0.000457559	0.670267012	stage_E14_vs_E10	ordinary_muscle_development		0	FALSE
ENSGALG00010019173	RHO	PRJNA729271	-3.776782649	0.000463651	3.776782649	stage_E14_vs_E10	ordinary_muscle_development	D_vs_E10;stage_1W_vs_E10;stage_3W_vs_E10;stage	1	TRUE
ENSGALG00010018528	ENSGALG00010018528	PRJNA729271	2.24366763	0.000465133	2.24366763	stage_E14_vs_E10	ordinary_muscle_development	D_vs_E10;stage_1W_vs_E10;stage_3W_vs_E10;stage	1	TRUE
ENSGALG00010021248	ANXA7	PRJNA729271	0.612404617	0.000469439	0.612404617	stage_E14_vs_E10	ordinary_muscle_development		0	FALSE
ENSGALG00010008206	CXCL13L2	PRJNA729271	-3.774295939	0.000469772	3.774295939	stage_E14_vs_E10	ordinary_muscle_development	D_vs_E10;stage_1W_vs_E10;stage_3W_vs_E10;stage	1	TRUE
ENSGALG00010013544	ENSGALG00010013544	PRJNA729271	-1.056286212	0.000470849	1.056286212	stage_E14_vs_E10	ordinary_muscle_development	D_vs_E10;stage_1W_vs_E10;stage_3W_vs_E10;stage	1	TRUE
ENSGALG00010003749	MHCY15	PRJNA729271	3.473768164	0.000473324	3.473768164	stage_E14_vs_E10	ordinary_muscle_development	D_vs_E10;stage_1W_vs_E10;stage_3W_vs_E10;stage	1	TRUE
ENSGALG00010023379	CCDC60	PRJNA729271	1.726469846	0.000474148	1.726469846	stage_E14_vs_E10	ordinary_muscle_development	D_vs_E10;stage_1W_vs_E10;stage_3W_vs_E10;stage	1	TRUE
ENSGALG00010029670	TPST1	PRJNA729271	0.616992084	0.000474418	0.616992084	stage_E14_vs_E10	ordinary_muscle_development		0	FALSE
ENSGALG00010018372	NLGN3	PRJNA729271	-1.738621108	0.000474438	1.738621108	stage_E14_vs_E10	ordinary_muscle_development	D_vs_E10;stage_1W_vs_E10;stage_3W_vs_E10;stage	1	TRUE
ENSGALG00010021194	CYP11A1	PRJNA729271	-1.487645421	0.000475351	1.487645421	stage_E14_vs_E10	ordinary_muscle_development	D_vs_E10;stage_1W_vs_E10;stage_3W_vs_E10;stage	1	TRUE
ENSGALG00010000596	ENSGALG00010000596	PRJNA729271	-2.932490268	0.000475953	2.932490268	stage_E14_vs_E10	ordinary_muscle_development	D_vs_E10;stage_1W_vs_E10;stage_3W_vs_E10;stage	1	TRUE
ENSGALG00010028005	IER5L	PRJNA729271	0.525588038	0.000477553	0.525588038	stage_E14_vs_E10	ordinary_muscle_development		0	FALSE
ENSGALG00010001510	AP1S2	PRJNA729271	0.354010581	0.000480836	0.354010581	stage_E14_vs_E10	ordinary_muscle_development		0	FALSE
ENSGALG00010028028	FAM174C	PRJNA729271	-0.624568941	0.000481009	0.624568941	stage_E14_vs_E10	ordinary_muscle_development		0	FALSE
ENSGALG00010022673	KLHL42	PRJNA729271	0.54583473	0.000481009	0.54583473	stage_E14_vs_E10	ordinary_muscle_development		0	FALSE
ENSGALG00010017532	MIXL1	PRJNA729271	1.144227344	0.000483964	1.144227344	stage_E14_vs_E10	ordinary_muscle_development	D_vs_E10;stage_1W_vs_E10;stage_3W_vs_E10;stage	1	TRUE
ENSGALG00010003063	GGH	PRJNA729271	0.324166605	0.000486962	0.324166605	stage_E14_vs_E10	ordinary_muscle_development		0	FALSE
ENSGALG00010017861	PRSS56	PRJNA729271	1.767205847	0.000487662	1.767205847	stage_E14_vs_E10	ordinary_muscle_development	D_vs_E10;stage_1W_vs_E10;stage_3W_vs_E10;stage	1	TRUE
ENSGALG00010022922	ATOH7	PRJNA729271	1.227901094	0.000491692	1.227901094	stage_E14_vs_E10	ordinary_muscle_development	D_vs_E10;stage_1W_vs_E10;stage_3W_vs_E10;stage	1	TRUE
ENSGALG00010018480	EME2	PRJNA729271	0.871079938	0.000492585	0.871079938	stage_E14_vs_E10	ordinary_muscle_development		0	FALSE
ENSGALG00010028707	ENSGALG00010028707	PRJNA729271	-4.481834392	0.000493827	4.481834392	stage_E14_vs_E10	ordinary_muscle_development	D_vs_E10;stage_1W_vs_E10;stage_3W_vs_E10;stage	1	TRUE
ENSGALG00010026127	ENSGALG00010026127	PRJNA729271	2.152983585	0.000497339	2.152983585	stage_E14_vs_E10	ordinary_muscle_development	D_vs_E10;stage_1W_vs_E10;stage_3W_vs_E10;stage	1	TRUE
ENSGALG00010011175	GPR52	PRJNA729271	-0.73249126	0.000498158	0.73249126	stage_E14_vs_E10	ordinary_muscle_development		0	FALSE
ENSGALG00010000145	ENSGALG00010000145	PRJNA729271	1.065477967	0.000499259	1.065477967	stage_E14_vs_E10	ordinary_muscle_development	D_vs_E10;stage_1W_vs_E10;stage_3W_vs_E10;stage	1	TRUE
ENSGALG00010001208	MOV10L1	PRJNA729271	-2.970428611	0.000499936	2.970428611	stage_E14_vs_E10	ordinary_muscle_development	D_vs_E10;stage_1W_vs_E10;stage_3W_vs_E10;stage	1	TRUE
ENSGALG00010012910	XPNPEP3	PRJNA729271	0.35636488	0.000502828	0.35636488	stage_E14_vs_E10	ordinary_muscle_development		0	FALSE
ENSGALG00010015234	TRIP11	PRJNA729271	0.520410874	0.000502975	0.520410874	stage_E14_vs_E10	ordinary_muscle_development		0	FALSE
ENSGALG00010024382	ENSGALG00010024382	PRJNA729271	1.510797495	0.000503663	1.510797495	stage_E14_vs_E10	ordinary_muscle_development	D_vs_E10;stage_1W_vs_E10;stage_3W_vs_E10;stage	1	TRUE
ENSGALG00010018933	PHF13	PRJNA729271	0.314425368	0.000505265	0.314425368	stage_E14_vs_E10	ordinary_muscle_development		0	FALSE
ENSGALG00010015660	KLHL3	PRJNA729271	0.451923195	0.000506427	0.451923195	stage_E14_vs_E10	ordinary_muscle_development		0	FALSE
ENSGALG00010019291	ADD2	PRJNA729271	-0.996059073	0.000507233	0.996059073	stage_E14_vs_E10	ordinary_muscle_development		0	FALSE
ENSGALG00010021311	TRIAP1	PRJNA729271	0.432442227	0.000507413	0.432442227	stage_E14_vs_E10	ordinary_muscle_development		0	FALSE
ENSGALG00010022478	ATP6V1A	PRJNA729271	-0.348571523	0.000512103	0.348571523	stage_E14_vs_E10	ordinary_muscle_development		0	FALSE
ENSGALG00010006574	CPA1	PRJNA729271	9.164181656	0.000513853	9.164181656	stage_E14_vs_E10	ordinary_muscle_development	D_vs_E10;stage_1W_vs_E10;stage_3W_vs_E10;stage	1	TRUE
ENSGALG00010002448	RAB9A	PRJNA729271	0.487501796	0.000513875	0.487501796	stage_E14_vs_E10	ordinary_muscle_development		0	FALSE
ENSGALG00010008263	C1orf101	PRJNA729271	-2.070889131	0.000514573	2.070889131	stage_E14_vs_E10	ordinary_muscle_development	D_vs_E10;stage_1W_vs_E10;stage_3W_vs_E10;stage	1	TRUE
ENSGALG00010020230	ENSGALG00010020230	PRJNA729271	0.776259948	0.000516454	0.776259948	stage_E14_vs_E10	ordinary_muscle_development		0	FALSE
ENSGALG00010003473	ENSGALG00010003473	PRJNA729271	-0.959253765	0.000517919	0.959253765	stage_E14_vs_E10	ordinary_muscle_development		0	FALSE
ENSGALG00010016745	BEGAIN	PRJNA729271	1.32057925	0.00051989	1.32057925	stage_E14_vs_E10	ordinary_muscle_development	D_vs_E10;stage_1W_vs_E10;stage_3W_vs_E10;stage	1	TRUE
ENSGALG00010014866	FAM26E	PRJNA729271	0.558510659	0.000520217	0.558510659	stage_E14_vs_E10	ordinary_muscle_development		0	FALSE
ENSGALG00010004070	ARL11	PRJNA729271	-1.488756495	0.000520703	1.488756495	stage_E14_vs_E10	ordinary_muscle_development	D_vs_E10;stage_1W_vs_E10;stage_3W_vs_E10;stage	1	TRUE
ENSGALG00010020713	SCTR	PRJNA729271	-4.772716313	0.000521151	4.772716313	stage_E14_vs_E10	ordinary_muscle_development	D_vs_E10;stage_1W_vs_E10;stage_3W_vs_E10;stage	1	TRUE
ENSGALG00010029700	GGA3	PRJNA729271	0.286483121	0.000521991	0.286483121	stage_E14_vs_E10	ordinary_muscle_development		0	FALSE
ENSGALG00010012135	CAMSAP2	PRJNA729271	-0.33182422	0.000523457	0.33182422	stage_E14_vs_E10	ordinary_muscle_development		0	FALSE
ENSGALG00010022994	LIPC	PRJNA729271	-3.245039694	0.000524164	3.245039694	stage_E14_vs_E10	ordinary_muscle_development	D_vs_E10;stage_1W_vs_E10;stage_3W_vs_E10;stage	1	TRUE
ENSGALG00010005694	ENSGALG00010005694	PRJNA729271	-0.274990542	0.000526286	0.274990542	stage_E14_vs_E10	ordinary_muscle_development		0	FALSE
ENSGALG00010010958	LCORL	PRJNA729271	0.578331259	0.000526353	0.578331259	stage_E14_vs_E10	ordinary_muscle_development		0	FALSE
ENSGALG00010002372	SMC6	PRJNA729271	-0.457016729	0.000528753	0.457016729	stage_E14_vs_E10	ordinary_muscle_development		0	FALSE
ENSGALG00010017997	ENSGALG00010017997	PRJNA729271	-1.620068039	0.000531308	1.620068039	stage_E14_vs_E10	ordinary_muscle_development	D_vs_E10;stage_1W_vs_E10;stage_3W_vs_E10;stage	1	TRUE
ENSGALG00010023473	BOK	PRJNA729271	-0.722865673	0.000531388	0.722865673	stage_E14_vs_E10	ordinary_muscle_development		0	FALSE
ENSGALG00010019992	CHAC2	PRJNA729271	-0.545716536	0.000534462	0.545716536	stage_E14_vs_E10	ordinary_muscle_development		0	FALSE
ENSGALG00010010142	ENSGALG00010010142	PRJNA729271	1.033906476	0.000537469	1.033906476	stage_E14_vs_E10	ordinary_muscle_development	D_vs_E10;stage_1W_vs_E10;stage_3W_vs_E10;stage	1	TRUE
ENSGALG00010021263	STOML1	PRJNA729271	-0.914017538	0.000539402	0.914017538	stage_E14_vs_E10	ordinary_muscle_development		0	FALSE
ENSGALG00010016598	AIFM1	PRJNA729271	0.316155822	0.000540151	0.316155822	stage_E14_vs_E10	ordinary_muscle_development		0	FALSE
ENSGALG00010024177	ENSGALG00010024177	PRJNA729271	1.435064221	0.000540447	1.435064221	stage_E14_vs_E10	ordinary_muscle_development	D_vs_E10;stage_1W_vs_E10;stage_3W_vs_E10;stage	1	TRUE
ENSGALG00010028477	NRARP	PRJNA729271	-0.781665775	0.000540518	0.781665775	stage_E14_vs_E10	ordinary_muscle_development		0	FALSE
ENSGALG00010029668	CRCP	PRJNA729271	-0.652875056	0.000542134	0.652875056	stage_E14_vs_E10	ordinary_muscle_development		0	FALSE
ENSGALG00010016741	NELFA	PRJNA729271	-0.32118862	0.000542166	0.32118862	stage_E14_vs_E10	ordinary_muscle_development		0	FALSE
ENSGALG00010025926	ENSGALG00010025926	PRJNA729271	0.986922478	0.000545729	0.986922478	stage_E14_vs_E10	ordinary_muscle_development		0	FALSE
ENSGALG00010012111	DRD1	PRJNA729271	-1.306571444	0.000546832	1.306571444	stage_E14_vs_E10	ordinary_muscle_development	D_vs_E10;stage_1W_vs_E10;stage_3W_vs_E10;stage	1	TRUE
ENSGALG00010005194	ENSGALG00010005194	PRJNA729271	4.179567512	0.000547974	4.179567512	stage_E14_vs_E10	ordinary_muscle_development	D_vs_E10;stage_1W_vs_E10;stage_3W_vs_E10;stage	1	TRUE
ENSGALG00010027622	FIBCD1	PRJNA729271	-4.442564502	0.000550372	4.442564502	stage_E14_vs_E10	ordinary_muscle_development	D_vs_E10;stage_1W_vs_E10;stage_3W_vs_E10;stage	1	TRUE
ENSGALG00010010327	FBP1	PRJNA729271	-2.105771966	0.00055309	2.105771966	stage_E14_vs_E10	ordinary_muscle_development	D_vs_E10;stage_1W_vs_E10;stage_3W_vs_E10;stage	1	TRUE
ENSGALG00010010728	ELOVL2	PRJNA729271	3.018335491	0.000557163	3.018335491	stage_E14_vs_E10	ordinary_muscle_development	D_vs_E10;stage_1W_vs_E10;stage_3W_vs_E10;stage	1	TRUE
ENSGALG00010009227	ENSGALG00010009227	PRJNA729271	0.628258249	0.000561002	0.628258249	stage_E14_vs_E10	ordinary_muscle_development		0	FALSE
ENSGALG00010002834	MRPL53	PRJNA729271	0.400093435	0.000561268	0.400093435	stage_E14_vs_E10	ordinary_muscle_development		0	FALSE
ENSGALG00010013304	KCNJ15	PRJNA729271	0.878134669	0.000561435	0.878134669	stage_E14_vs_E10	ordinary_muscle_development		0	FALSE
ENSGALG00010027970	GRIN3B	PRJNA729271	0.634119806	0.000563861	0.634119806	stage_E14_vs_E10	ordinary_muscle_development		0	FALSE
ENSGALG00010010231	C1orf53	PRJNA729271	0.82913559	0.000565269	0.82913559	stage_E14_vs_E10	ordinary_muscle_development		0	FALSE
ENSGALG00010011340	PKNOX1	PRJNA729271	-0.388718732	0.000565302	0.388718732	stage_E14_vs_E10	ordinary_muscle_development		0	FALSE
ENSGALG00010010468	ENSGALG00010010468	PRJNA729271	4.70100679	0.000565819	4.70100679	stage_E14_vs_E10	ordinary_muscle_development	D_vs_E10;stage_1W_vs_E10;stage_3W_vs_E10;stage	1	TRUE
ENSGALG00010024378	ZDHHC1	PRJNA729271	-0.640557314	0.000565819	0.640557314	stage_E14_vs_E10	ordinary_muscle_development		0	FALSE
ENSGALG00010006065	WDR54	PRJNA729271	0.585744056	0.000570042	0.585744056	stage_E14_vs_E10	ordinary_muscle_development		0	FALSE
ENSGALG00010028886	ENSGALG00010028886	PRJNA729271	6.324473736	0.000570536	6.324473736	stage_E14_vs_E10	ordinary_muscle_development	D_vs_E10;stage_1W_vs_E10;stage_3W_vs_E10;stage	1	TRUE
ENSGALG00010016139	LURAP1L	PRJNA729271	-0.729191259	0.000572173	0.729191259	stage_E14_vs_E10	ordinary_muscle_development		0	FALSE
ENSGALG00010019209	VCPKMT	PRJNA729271	0.309826214	0.000573002	0.309826214	stage_E14_vs_E10	ordinary_muscle_development		0	FALSE
ENSGALG00010000256	ENSGALG00010000256	PRJNA729271	-1.592227027	0.000574115	1.592227027	stage_E14_vs_E10	ordinary_muscle_development	D_vs_E10;stage_1W_vs_E10;stage_3W_vs_E10;stage	1	TRUE
ENSGALG00010025919	RGS9BP	PRJNA729271	-1.243787239	0.000575282	1.243787239	stage_E14_vs_E10	ordinary_muscle_development	D_vs_E10;stage_1W_vs_E10;stage_3W_vs_E10;stage	1	TRUE
ENSGALG00010024895	ENSGALG00010024895	PRJNA729271	1.450998462	0.000577318	1.450998462	stage_E14_vs_E10	ordinary_muscle_development	D_vs_E10;stage_1W_vs_E10;stage_3W_vs_E10;stage	1	TRUE
ENSGALG00010020889	PPRC1	PRJNA729271	-1.035220617	0.000578144	1.035220617	stage_E14_vs_E10	ordinary_muscle_development	D_vs_E10;stage_1W_vs_E10;stage_3W_vs_E10;stage	1	TRUE
ENSGALG00010008846	SLC35D2	PRJNA729271	-1.017094658	0.000578312	1.017094658	stage_E14_vs_E10	ordinary_muscle_development	D_vs_E10;stage_1W_vs_E10;stage_3W_vs_E10;stage	1	TRUE
ENSGALG00010022411	ADRB1	PRJNA729271	1.033114235	0.000578925	1.033114235	stage_E14_vs_E10	ordinary_muscle_development	D_vs_E10;stage_1W_vs_E10;stage_3W_vs_E10;stage	1	TRUE
ENSGALG00010027874	FRS3	PRJNA729271	-0.482317886	0.000579243	0.482317886	stage_E14_vs_E10	ordinary_muscle_development		0	FALSE
ENSGALG00010000608	ENSGALG00010000608	PRJNA729271	-4.265159917	0.000579682	4.265159917	stage_E14_vs_E10	ordinary_muscle_development	D_vs_E10;stage_1W_vs_E10;stage_3W_vs_E10;stage	1	TRUE
ENSGALG00010027822	DPH7	PRJNA729271	0.320570051	0.000579725	0.320570051	stage_E14_vs_E10	ordinary_muscle_development		0	FALSE
ENSGALG00010024530	ENSGALG00010024530	PRJNA729271	1.832027249	0.000580446	1.832027249	stage_E14_vs_E10	ordinary_muscle_development	D_vs_E10;stage_1W_vs_E10;stage_3W_vs_E10;stage	1	TRUE
ENSGALG00010010831	DESI1	PRJNA729271	-0.331141324	0.000584822	0.331141324	stage_E14_vs_E10	ordinary_muscle_development		0	FALSE
ENSGALG00010002967	OLFM4	PRJNA729271	-3.433999739	0.000586326	3.433999739	stage_E14_vs_E10	ordinary_muscle_development	D_vs_E10;stage_1W_vs_E10;stage_3W_vs_E10;stage	1	TRUE
ENSGALG00010009198	KCNN2	PRJNA729271	0.629982397	0.000587128	0.629982397	stage_E14_vs_E10	ordinary_muscle_development		0	FALSE
ENSGALG00010013346	HMGCS2	PRJNA729271	2.474957101	0.000587876	2.474957101	stage_E14_vs_E10	ordinary_muscle_development	D_vs_E10;stage_1W_vs_E10;stage_3W_vs_E10;stage	1	TRUE
ENSGALG00010028329	HAPLN2	PRJNA729271	2.039299616	0.000589224	2.039299616	stage_E14_vs_E10	ordinary_muscle_development	D_vs_E10;stage_1W_vs_E10;stage_3W_vs_E10;stage	1	TRUE
ENSGALG00010020921	LDB1	PRJNA729271	-0.543260702	0.000589562	0.543260702	stage_E14_vs_E10	ordinary_muscle_development		0	FALSE
ENSGALG00010013980	FMO4	PRJNA729271	1.3209441	0.000590167	1.3209441	stage_E14_vs_E10	ordinary_muscle_development	D_vs_E10;stage_1W_vs_E10;stage_3W_vs_E10;stage	1	TRUE
ENSGALG00010004125	MANEA	PRJNA729271	0.329028952	0.000590233	0.329028952	stage_E14_vs_E10	ordinary_muscle_development		0	FALSE
ENSGALG00010014359	TMEM271	PRJNA729271	1.266295606	0.000590615	1.266295606	stage_E14_vs_E10	ordinary_muscle_development	D_vs_E10;stage_1W_vs_E10;stage_3W_vs_E10;stage	1	TRUE
ENSGALG00010023161	ODF2L	PRJNA729271	-1.623986361	0.000595205	1.623986361	stage_E14_vs_E10	ordinary_muscle_development	D_vs_E10;stage_1W_vs_E10;stage_3W_vs_E10;stage	1	TRUE
ENSGALG00010002469	MEPE	PRJNA729271	-5.232595875	0.000595216	5.232595875	stage_E14_vs_E10	ordinary_muscle_development	D_vs_E10;stage_1W_vs_E10;stage_3W_vs_E10;stage	1	TRUE
ENSGALG00010010629	ENSGALG00010010629	PRJNA729271	-1.288029919	0.00059739	1.288029919	stage_E14_vs_E10	ordinary_muscle_development	D_vs_E10;stage_1W_vs_E10;stage_3W_vs_E10;stage	1	TRUE
ENSGALG00010018961	ASB7	PRJNA729271	-0.357391223	0.000598261	0.357391223	stage_E14_vs_E10	ordinary_muscle_development		0	FALSE
ENSGALG00010006336	ALDH8A1	PRJNA729271	3.026694431	0.000600794	3.026694431	stage_E14_vs_E10	ordinary_muscle_development	D_vs_E10;stage_1W_vs_E10;stage_3W_vs_E10;stage	1	TRUE
ENSGALG00010019240	ENSGALG00010019240	PRJNA729271	0.715847213	0.000601283	0.715847213	stage_E14_vs_E10	ordinary_muscle_development		0	FALSE
ENSGALG00010021513	SDF4	PRJNA729271	0.35037514	0.000602182	0.35037514	stage_E14_vs_E10	ordinary_muscle_development		0	FALSE
ENSGALG00010002644	KCNF1	PRJNA729271	-1.807425693	0.000603213	1.807425693	stage_E14_vs_E10	ordinary_muscle_development	D_vs_E10;stage_1W_vs_E10;stage_3W_vs_E10;stage	1	TRUE
ENSGALG00010025822	KLF7	PRJNA729271	-0.354900622	0.000603359	0.354900622	stage_E14_vs_E10	ordinary_muscle_development		0	FALSE
ENSGALG00010016016	ZDHHC2	PRJNA729271	0.975935491	0.000604059	0.975935491	stage_E14_vs_E10	ordinary_muscle_development		0	FALSE
ENSGALG00010022574	ITPRIPL2	PRJNA729271	0.387991578	0.000605903	0.387991578	stage_E14_vs_E10	ordinary_muscle_development		0	FALSE
ENSGALG00010016392	STMN3	PRJNA729271	1.118886316	0.000609676	1.118886316	stage_E14_vs_E10	ordinary_muscle_development	D_vs_E10;stage_1W_vs_E10;stage_3W_vs_E10;stage	1	TRUE
ENSGALG00010011790	AMY2A	PRJNA729271	3.924442278	0.000612582	3.924442278	stage_E14_vs_E10	ordinary_muscle_development	D_vs_E10;stage_1W_vs_E10;stage_3W_vs_E10;stage	1	TRUE
ENSGALG00010018504	SLCO3A1	PRJNA729271	-0.647278314	0.000612984	0.647278314	stage_E14_vs_E10	ordinary_muscle_development		0	FALSE
ENSGALG00010007770	ENSGALG00010007770	PRJNA729271	-1.637564995	0.000613596	1.637564995	stage_E14_vs_E10	ordinary_muscle_development	D_vs_E10;stage_1W_vs_E10;stage_3W_vs_E10;stage	1	TRUE
ENSGALG00010007977	APOB	PRJNA729271	4.961049055	0.000613727	4.961049055	stage_E14_vs_E10	ordinary_muscle_development	D_vs_E10;stage_1W_vs_E10;stage_3W_vs_E10;stage	1	TRUE
ENSGALG00010018095	E4F1	PRJNA729271	-0.25011386	0.000617065	0.25011386	stage_E14_vs_E10	ordinary_muscle_development		0	FALSE
ENSGALG00010027387	ENSGALG00010027387	PRJNA729271	-0.9685297	0.000618647	0.9685297	stage_E14_vs_E10	ordinary_muscle_development		0	FALSE
ENSGALG00010027950	CDC26	PRJNA729271	0.45943459	0.000618841	0.45943459	stage_E14_vs_E10	ordinary_muscle_development		0	FALSE
ENSGALG00010003833	ACP1	PRJNA729271	0.561996673	0.000619571	0.561996673	stage_E14_vs_E10	ordinary_muscle_development		0	FALSE
ENSGALG00010028921	MRPL41	PRJNA729271	0.425129201	0.000620169	0.425129201	stage_E14_vs_E10	ordinary_muscle_development		0	FALSE
ENSGALG00010008205	ENSGALG00010008205	PRJNA729271	-0.647381865	0.000627424	0.647381865	stage_E14_vs_E10	ordinary_muscle_development		0	FALSE
ENSGALG00010027698	FAM120A	PRJNA729271	0.234825534	0.000627816	0.234825534	stage_E14_vs_E10	ordinary_muscle_development		0	FALSE
ENSGALG00010017363	TIMM9	PRJNA729271	0.498022937	0.00062873	0.498022937	stage_E14_vs_E10	ordinary_muscle_development		0	FALSE
ENSGALG00010021829	ERCC6	PRJNA729271	-0.362557369	0.000630728	0.362557369	stage_E14_vs_E10	ordinary_muscle_development		0	FALSE
ENSGALG00010001864	ENSGALG00010001864	PRJNA729271	0.458759626	0.00063206	0.458759626	stage_E14_vs_E10	ordinary_muscle_development		0	FALSE
ENSGALG00010025238	IGSF9B	PRJNA729271	1.002563451	0.000634262	1.002563451	stage_E14_vs_E10	ordinary_muscle_development	D_vs_E10;stage_1W_vs_E10;stage_3W_vs_E10;stage	1	TRUE
ENSGALG00010011157	ENSGALG00010011157	PRJNA729271	-5.619228264	0.000636607	5.619228264	stage_E14_vs_E10	ordinary_muscle_development	D_vs_E10;stage_1W_vs_E10;stage_3W_vs_E10;stage	1	TRUE
ENSGALG00010027913	PYGO2	PRJNA729271	-0.830819977	0.000637246	0.830819977	stage_E14_vs_E10	ordinary_muscle_development		0	FALSE
ENSGALG00010010255	CACNB4	PRJNA729271	0.618232356	0.000638698	0.618232356	stage_E14_vs_E10	ordinary_muscle_development		0	FALSE
ENSGALG00010005453	UST	PRJNA729271	-0.429589025	0.000638909	0.429589025	stage_E14_vs_E10	ordinary_muscle_development		0	FALSE
ENSGALG00010000615	ENSGALG00010000615	PRJNA729271	-2.343457594	0.00064074	2.343457594	stage_E14_vs_E10	ordinary_muscle_development	D_vs_E10;stage_1W_vs_E10;stage_3W_vs_E10;stage	1	TRUE
ENSGALG00010004835	HHLA2	PRJNA729271	-1.457668693	0.000645686	1.457668693	stage_E14_vs_E10	ordinary_muscle_development	D_vs_E10;stage_1W_vs_E10;stage_3W_vs_E10;stage	1	TRUE
ENSGALG00010028510	CILP2	PRJNA729271	2.073495612	0.000647858	2.073495612	stage_E14_vs_E10	ordinary_muscle_development	D_vs_E10;stage_1W_vs_E10;stage_3W_vs_E10;stage	1	TRUE
ENSGALG00010025225	DMRTB1	PRJNA729271	-3.315930322	0.000648288	3.315930322	stage_E14_vs_E10	ordinary_muscle_development	D_vs_E10;stage_1W_vs_E10;stage_3W_vs_E10;stage	1	TRUE
ENSGALG00010009209	SLC6A19	PRJNA729271	5.965628562	0.000649387	5.965628562	stage_E14_vs_E10	ordinary_muscle_development	D_vs_E10;stage_1W_vs_E10;stage_3W_vs_E10;stage	1	TRUE
ENSGALG00010011009	SFTPA1	PRJNA729271	4.022416667	0.000649949	4.022416667	stage_E14_vs_E10	ordinary_muscle_development	D_vs_E10;stage_1W_vs_E10;stage_3W_vs_E10;stage	1	TRUE
ENSGALG00010023090	LRRC41	PRJNA729271	-0.33336115	0.000652139	0.33336115	stage_E14_vs_E10	ordinary_muscle_development		0	FALSE
ENSGALG00010020429	ZNF148	PRJNA729271	0.358116428	0.000652576	0.358116428	stage_E14_vs_E10	ordinary_muscle_development		0	FALSE
ENSGALG00010014766	F10	PRJNA729271	0.76337258	0.000656925	0.76337258	stage_E14_vs_E10	ordinary_muscle_development		0	FALSE
ENSGALG00010001364	SMCHD1	PRJNA729271	-0.662849081	0.000656925	0.662849081	stage_E14_vs_E10	ordinary_muscle_development		0	FALSE
ENSGALG00010009252	SIRT1	PRJNA729271	-0.347489429	0.000661192	0.347489429	stage_E14_vs_E10	ordinary_muscle_development		0	FALSE
ENSGALG00010007905	MRPL32	PRJNA729271	0.546299646	0.000662516	0.546299646	stage_E14_vs_E10	ordinary_muscle_development		0	FALSE
ENSGALG00010028586	BABAM1	PRJNA729271	-0.43226142	0.000665023	0.43226142	stage_E14_vs_E10	ordinary_muscle_development		0	FALSE
ENSGALG00010013361	ENSGALG00010013361	PRJNA729271	-0.874352381	0.000665385	0.874352381	stage_E14_vs_E10	ordinary_muscle_development		0	FALSE
ENSGALG00010017737	ENSGALG00010017737	PRJNA729271	-0.715366899	0.000665873	0.715366899	stage_E14_vs_E10	ordinary_muscle_development		0	FALSE
ENSGALG00010009012	DEUP1	PRJNA729271	-3.767670018	0.000667632	3.767670018	stage_E14_vs_E10	ordinary_muscle_development	D_vs_E10;stage_1W_vs_E10;stage_3W_vs_E10;stage	1	TRUE
ENSGALG00010025414	MMP2	PRJNA729271	-0.778964456	0.00066768	0.778964456	stage_E14_vs_E10	ordinary_muscle_development		0	FALSE
ENSGALG00010027916	ADAR	PRJNA729271	0.451962832	0.000669887	0.451962832	stage_E14_vs_E10	ordinary_muscle_development		0	FALSE
ENSGALG00010016966	KDM3A	PRJNA729271	0.556209108	0.000670244	0.556209108	stage_E14_vs_E10	ordinary_muscle_development		0	FALSE
ENSGALG00010005122	IL8L1	PRJNA729271	3.157502586	0.000672636	3.157502586	stage_E14_vs_E10	ordinary_muscle_development	D_vs_E10;stage_1W_vs_E10;stage_3W_vs_E10;stage	1	TRUE
ENSGALG00010010226	BICC1	PRJNA729271	-0.606903937	0.000672736	0.606903937	stage_E14_vs_E10	ordinary_muscle_development		0	FALSE
ENSGALG00010002559	NEK10	PRJNA729271	-2.155107797	0.000672787	2.155107797	stage_E14_vs_E10	ordinary_muscle_development	D_vs_E10;stage_1W_vs_E10;stage_3W_vs_E10;stage	1	TRUE
ENSGALG00010022719	MSL2	PRJNA729271	0.342043522	0.000674683	0.342043522	stage_E14_vs_E10	ordinary_muscle_development		0	FALSE
ENSGALG00010005260	ANXA5	PRJNA729271	-0.496291489	0.000679078	0.496291489	stage_E14_vs_E10	ordinary_muscle_development		0	FALSE
ENSGALG00010014859	TRAPPC3	PRJNA729271	-0.461717733	0.00068052	0.461717733	stage_E14_vs_E10	ordinary_muscle_development		0	FALSE

ENSGALG00010011370	MGAT4C	PRJNA729271	-5.535214555	0.000681351	5.535214555	stage_E14_vs_E10	ordinary_muscle_development	D_vs_E10;stage_1W_vs_E10;stage_3W_vs_E10;stage	1	TRUE
ENSGALG00010005065	ENKUR	PRJNA729271	-1.639792446	0.000681687	1.639792446	stage_E14_vs_E10	ordinary_muscle_development	D_vs_E10;stage_1W_vs_E10;stage_3W_vs_E10;stage	1	TRUE
ENSGALG00010015201	GCC2	PRJNA729271	0.381130167	0.000682213	0.381130167	stage_E14_vs_E10	ordinary_muscle_development		0	FALSE
ENSGALG00010013085	LYSMD3	PRJNA729271	0.317108962	0.000683234	0.317108962	stage_E14_vs_E10	ordinary_muscle_development		0	FALSE
ENSGALG00010024212	RIMS4	PRJNA729271	-0.580238924	0.000685297	0.580238924	stage_E14_vs_E10	ordinary_muscle_development		0	FALSE
ENSGALG00010003720	ENSGALG00010003720	PRJNA729271	9.146953666	0.000688408	9.146953666	stage_E14_vs_E10	ordinary_muscle_development	D_vs_E10;stage_1W_vs_E10;stage_3W_vs_E10;stage	1	TRUE
ENSGALG00010027915	GSTM3	PRJNA729271	0.298732534	0.000691604	0.298732534	stage_E14_vs_E10	ordinary_muscle_development		0	FALSE
ENSGALG00010015657	NMUR1	PRJNA729271	-4.197038096	0.000693685	4.197038096	stage_E14_vs_E10	ordinary_muscle_development	D_vs_E10;stage_1W_vs_E10;stage_3W_vs_E10;stage	1	TRUE
ENSGALG00010005297	IL22RA2	PRJNA729271	-2.926471453	0.000694333	2.926471453	stage_E14_vs_E10	ordinary_muscle_development	D_vs_E10;stage_1W_vs_E10;stage_3W_vs_E10;stage	1	TRUE
ENSGALG00010012938	UPF3B	PRJNA729271	0.527348846	0.00069655	0.527348846	stage_E14_vs_E10	ordinary_muscle_development		0	FALSE
ENSGALG00010021218	DPF3	PRJNA729271	-0.655960845	0.000701526	0.655960845	stage_E14_vs_E10	ordinary_muscle_development		0	FALSE
ENSGALG00010013196	MRPS9	PRJNA729271	0.398219645	0.000703182	0.398219645	stage_E14_vs_E10	ordinary_muscle_development		0	FALSE
ENSGALG00010025382	MAB21L3	PRJNA729271	-1.153112255	0.000704173	1.153112255	stage_E14_vs_E10	ordinary_muscle_development	D_vs_E10;stage_1W_vs_E10;stage_3W_vs_E10;stage	1	TRUE
ENSGALG00010011460	LAMC2	PRJNA729271	-2.37394913	0.000706728	2.37394913	stage_E14_vs_E10	ordinary_muscle_development	D_vs_E10;stage_1W_vs_E10;stage_3W_vs_E10;stage	1	TRUE
ENSGALG00010011322	GATA4	PRJNA729271	-5.005663881	0.000707153	5.005663881	stage_E14_vs_E10	ordinary_muscle_development	D_vs_E10;stage_1W_vs_E10;stage_3W_vs_E10;stage	1	TRUE
ENSGALG00010019033	CYP2AB4	PRJNA729271	-4.098360366	0.000707182	4.098360366	stage_E14_vs_E10	ordinary_muscle_development	D_vs_E10;stage_1W_vs_E10;stage_3W_vs_E10;stage	1	TRUE
ENSGALG00010029189	ENSGALG00010029189	PRJNA729271	1.115632485	0.000710373	1.115632485	stage_E14_vs_E10	ordinary_muscle_development	D_vs_E10;stage_1W_vs_E10;stage_3W_vs_E10;stage	1	TRUE
ENSGALG00010028742	ENSGALG00010028742	PRJNA729271	0.495143169	0.000711405	0.495143169	stage_E14_vs_E10	ordinary_muscle_development		0	FALSE
ENSGALG00010007987	ENSGALG00010007987	PRJNA729271	1.044816701	0.000713007	1.044816701	stage_E14_vs_E10	ordinary_muscle_development	D_vs_E10;stage_1W_vs_E10;stage_3W_vs_E10;stage	1	TRUE
ENSGALG00010017153	SLC35F4	PRJNA729271	-2.18063618	0.000715318	2.18063618	stage_E14_vs_E10	ordinary_muscle_development	D_vs_E10;stage_1W_vs_E10;stage_3W_vs_E10;stage	1	TRUE
ENSGALG00010002682	UBL5	PRJNA729271	0.579367877	0.000718982	0.579367877	stage_E14_vs_E10	ordinary_muscle_development		0	FALSE
ENSGALG00010019458	ELP4	PRJNA729271	-0.370958318	0.000719524	0.370958318	stage_E14_vs_E10	ordinary_muscle_development		0	FALSE
ENSGALG00010011603	ENSGALG00010011603	PRJNA729271	0.956130546	0.000721001	0.956130546	stage_E14_vs_E10	ordinary_muscle_development		0	FALSE
ENSGALG00010021176	ENSGALG00010021176	PRJNA729271	-0.421766628	0.000721367	0.421766628	stage_E14_vs_E10	ordinary_muscle_development		0	FALSE
ENSGALG00010007886	GRM5	PRJNA729271	-3.385353775	0.000721745	3.385353775	stage_E14_vs_E10	ordinary_muscle_development	D_vs_E10;stage_1W_vs_E10;stage_3W_vs_E10;stage	1	TRUE
ENSGALG00010012733	ENSGALG00010012733	PRJNA729271	-4.429067129	0.000722173	4.429067129	stage_E14_vs_E10	ordinary_muscle_development	D_vs_E10;stage_1W_vs_E10;stage_3W_vs_E10;stage	1	TRUE
ENSGALG00010009431	SMN	PRJNA729271	-0.538892095	0.000725983	0.538892095	stage_E14_vs_E10	ordinary_muscle_development		0	FALSE
ENSGALG00010024222	ENSGALG00010024222	PRJNA729271	1.033520457	0.000728211	1.033520457	stage_E14_vs_E10	ordinary_muscle_development	D_vs_E10;stage_1W_vs_E10;stage_3W_vs_E10;stage	1	TRUE
ENSGALG00010010626	ADTRP	PRJNA729271	2.008333309	0.000728596	2.008333309	stage_E14_vs_E10	ordinary_muscle_development	D_vs_E10;stage_1W_vs_E10;stage_3W_vs_E10;stage	1	TRUE
ENSGALG00010010759	ENSGALG00010010759	PRJNA729271	-1.157660731	0.000729245	1.157660731	stage_E14_vs_E10	ordinary_muscle_development	D_vs_E10;stage_1W_vs_E10;stage_3W_vs_E10;stage	1	TRUE
ENSGALG00010021393	CCDC174	PRJNA729271	-0.412928096	0.000732358	0.412928096	stage_E14_vs_E10	ordinary_muscle_development		0	FALSE
ENSGALG00010005278	DAPP1	PRJNA729271	-0.464341577	0.000733136	0.464341577	stage_E14_vs_E10	ordinary_muscle_development		0	FALSE
ENSGALG00010017143	FAM149A	PRJNA729271	-1.028495932	0.000733245	1.028495932	stage_E14_vs_E10	ordinary_muscle_development	D_vs_E10;stage_1W_vs_E10;stage_3W_vs_E10;stage	1	TRUE
ENSGALG00010021683	HFM1	PRJNA729271	-1.550055766	0.000735046	1.550055766	stage_E14_vs_E10	ordinary_muscle_development	D_vs_E10;stage_1W_vs_E10;stage_3W_vs_E10;stage	1	TRUE
ENSGALG00010020126	NADK	PRJNA729271	-0.367522245	0.000737492	0.367522245	stage_E14_vs_E10	ordinary_muscle_development		0	FALSE
ENSGALG00010022768	ENSGALG00010022768	PRJNA729271	-3.334503646	0.000739921	3.334503646	stage_E14_vs_E10	ordinary_muscle_development	D_vs_E10;stage_1W_vs_E10;stage_3W_vs_E10;stage	1	TRUE
ENSGALG00010025115	MGAT4F	PRJNA729271	-0.927535745	0.000740715	0.927535745	stage_E14_vs_E10	ordinary_muscle_development		0	FALSE
ENSGALG00010023159	DAW1	PRJNA729271	-2.156964446	0.000741763	2.156964446	stage_E14_vs_E10	ordinary_muscle_development	D_vs_E10;stage_1W_vs_E10;stage_3W_vs_E10;stage	1	TRUE
ENSGALG00010008132	TDO2	PRJNA729271	2.252580125	0.000741786	2.252580125	stage_E14_vs_E10	ordinary_muscle_development	D_vs_E10;stage_1W_vs_E10;stage_3W_vs_E10;stage	1	TRUE
ENSGALG00010013621	PDGFRA	PRJNA729271	0.579474764	0.000746019	0.579474764	stage_E14_vs_E10	ordinary_muscle_development		0	FALSE
ENSGALG00010002553	ENSGALG00010002553	PRJNA729271	0.76127264	0.000747224	0.76127264	stage_E14_vs_E10	ordinary_muscle_development		0	FALSE
ENSGALG00010018469	KLHDC7A	PRJNA729271	-0.730240493	0.000747281	0.730240493	stage_E14_vs_E10	ordinary_muscle_development		0	FALSE
ENSGALG00010009085	PAF1	PRJNA729271	0.325747664	0.000747786	0.325747664	stage_E14_vs_E10	ordinary_muscle_development		0	FALSE
ENSGALG00010017343	DLG1	PRJNA729271	0.333102482	0.000749191	0.333102482	stage_E14_vs_E10	ordinary_muscle_development		0	FALSE
ENSGALG00010003842	ENSGALG00010003842	PRJNA729271	-0.506492646	0.000749974	0.506492646	stage_E14_vs_E10	ordinary_muscle_development		0	FALSE
ENSGALG00010013627	MYCBP	PRJNA729271	0.521932035	0.000753575	0.521932035	stage_E14_vs_E10	ordinary_muscle_development		0	FALSE
ENSGALG00010013332	LUM	PRJNA729271	-1.117525804	0.00075367	1.117525804	stage_E14_vs_E10	ordinary_muscle_development	D_vs_E10;stage_1W_vs_E10;stage_3W_vs_E10;stage	1	TRUE
ENSGALG00010024532	TMEM86A	PRJNA729271	0.747137546	0.000754331	0.747137546	stage_E14_vs_E10	ordinary_muscle_development		0	FALSE
ENSGALG00010027275	CPAMD8	PRJNA729271	0.805274004	0.000755166	0.805274004	stage_E14_vs_E10	ordinary_muscle_development		0	FALSE
ENSGALG00010027001	SLC26A8	PRJNA729271	0.877632415	0.000755676	0.877632415	stage_E14_vs_E10	ordinary_muscle_development		0	FALSE
ENSGALG00010022551	GPER1	PRJNA729271	-2.431660604	0.000756863	2.431660604	stage_E14_vs_E10	ordinary_muscle_development	D_vs_E10;stage_1W_vs_E10;stage_3W_vs_E10;stage	1	TRUE
ENSGALG00010023696	LRRC4C	PRJNA729271	-1.979646053	0.000756863	1.979646053	stage_E14_vs_E10	ordinary_muscle_development	D_vs_E10;stage_1W_vs_E10;stage_3W_vs_E10;stage	1	TRUE
ENSGALG00010028430	NR2C2AP	PRJNA729271	0.421738596	0.000756863	0.421738596	stage_E14_vs_E10	ordinary_muscle_development		0	FALSE
ENSGALG00010001253	ENSGALG00010001253	PRJNA729271	-3.854472798	0.000758387	3.854472798	stage_E14_vs_E10	ordinary_muscle_development	D_vs_E10;stage_1W_vs_E10;stage_3W_vs_E10;stage	1	TRUE
ENSGALG00010022496	TUBGCP2	PRJNA729271	0.264166844	0.000760707	0.264166844	stage_E14_vs_E10	ordinary_muscle_development		0	FALSE
ENSGALG00010017779	SNAPC1	PRJNA729271	0.430414821	0.000762252	0.430414821	stage_E14_vs_E10	ordinary_muscle_development		0	FALSE
ENSGALG00010024734	ZPR1	PRJNA729271	0.396613618	0.000763725	0.396613618	stage_E14_vs_E10	ordinary_muscle_development		0	FALSE
ENSGALG00010025956	ENSGALG00010025956	PRJNA729271	1.713315046	0.000774704	1.713315046	stage_E14_vs_E10	ordinary_muscle_development	D_vs_E10;stage_1W_vs_E10;stage_3W_vs_E10;stage	1	TRUE
ENSGALG00010022266	COA3	PRJNA729271	0.53296872	0.000774871	0.53296872	stage_E14_vs_E10	ordinary_muscle_development		0	FALSE
ENSGALG00010026254	ENSGALG00010026254	PRJNA729271	-2.538698357	0.000775964	2.538698357	stage_E14_vs_E10	ordinary_muscle_development	D_vs_E10;stage_1W_vs_E10;stage_3W_vs_E10;stage	1	TRUE
ENSGALG00010003247	ENSGALG00010003247	PRJNA729271	0.732583613	0.000778835	0.732583613	stage_E14_vs_E10	ordinary_muscle_development		0	FALSE
ENSGALG00010020224	MRPL46	PRJNA729271	0.301295292	0.000779331	0.301295292	stage_E14_vs_E10	ordinary_muscle_development		0	FALSE
ENSGALG00010020865	BICDL1	PRJNA729271	-3.64338526	0.000779456	3.64338526	stage_E14_vs_E10	ordinary_muscle_development	D_vs_E10;stage_1W_vs_E10;stage_3W_vs_E10;stage	1	TRUE
ENSGALG00010000807	ENSGALG00010000807	PRJNA729271	2.683500951	0.000779684	2.683500951	stage_E14_vs_E10	ordinary_muscle_development	D_vs_E10;stage_1W_vs_E10;stage_3W_vs_E10;stage	1	TRUE
ENSGALG00010018336	JMJD8	PRJNA729271	-0.539273173	0.000782067	0.539273173	stage_E14_vs_E10	ordinary_muscle_development		0	FALSE
ENSGALG00010017860	CYSLTR1	PRJNA729271	-0.766898724	0.000786753	0.766898724	stage_E14_vs_E10	ordinary_muscle_development		0	FALSE
ENSGALG00010007983	RCHY1	PRJNA729271	-0.307288182	0.000792297	0.307288182	stage_E14_vs_E10	ordinary_muscle_development		0	FALSE
ENSGALG00010021146	AP5Z1	PRJNA729271	-0.310609101	0.00079319	0.310609101	stage_E14_vs_E10	ordinary_muscle_development		0	FALSE
ENSGALG00010002185	ENSGALG00010002185	PRJNA729271	0.750518089	0.000794091	0.750518089	stage_E14_vs_E10	ordinary_muscle_development		0	FALSE
ENSGALG00010028181	BGLAP	PRJNA729271	-0.986084121	0.000796576	0.986084121	stage_E14_vs_E10	ordinary_muscle_development		0	FALSE
ENSGALG00010014476	C5orf34	PRJNA729271	0.603009703	0.000800479	0.603009703	stage_E14_vs_E10	ordinary_muscle_development		0	FALSE
ENSGALG00010029707	NSRP1	PRJNA729271	0.772980891	0.000801792	0.772980891	stage_E14_vs_E10	ordinary_muscle_development		0	FALSE
ENSGALG00010013509	ENSGALG00010013509	PRJNA729271	-0.684755778	0.000812542	0.684755778	stage_E14_vs_E10	ordinary_muscle_development		0	FALSE
ENSGALG00010024638	ZNF507	PRJNA729271	0.447521119	0.000821471	0.447521119	stage_E14_vs_E10	ordinary_muscle_development		0	FALSE
ENSGALG00010016533	CNIH4	PRJNA729271	0.379728866	0.000825655	0.379728866	stage_E14_vs_E10	ordinary_muscle_development		0	FALSE
ENSGALG00010029172	LRRC45	PRJNA729271	-0.260554122	0.000829519	0.260554122	stage_E14_vs_E10	ordinary_muscle_development		0	FALSE
ENSGALG00010029571	EPX	PRJNA729271	-3.728664469	0.000832904	3.728664469	stage_E14_vs_E10	ordinary_muscle_development	D_vs_E10;stage_1W_vs_E10;stage_3W_vs_E10;stage	1	TRUE
ENSGALG00010001297	MYL12A	PRJNA729271	-0.583439457	0.000834433	0.583439457	stage_E14_vs_E10	ordinary_muscle_development		0	FALSE
ENSGALG00010021145	EIF4G3	PRJNA729271	-0.558288578	0.000834866	0.558288578	stage_E14_vs_E10	ordinary_muscle_development		0	FALSE
ENSGALG00010015017	FAM184A	PRJNA729271	-0.70496397	0.000839625	0.70496397	stage_E14_vs_E10	ordinary_muscle_development		0	FALSE
ENSGALG00010022466	NEDD4	PRJNA729271	-0.506568345	0.000848419	0.506568345	stage_E14_vs_E10	ordinary_muscle_development		0	FALSE
ENSGALG00010021160	ZNF143	PRJNA729271	0.566338747	0.000851112	0.566338747	stage_E14_vs_E10	ordinary_muscle_development		0	FALSE
ENSGALG00010000121	ENSGALG00010000121	PRJNA729271	0.463475144	0.000853281	0.463475144	stage_E14_vs_E10	ordinary_muscle_development		0	FALSE
ENSGALG00010024647	RPUSD4	PRJNA729271	-0.468665084	0.000855691	0.468665084	stage_E14_vs_E10	ordinary_muscle_development		0	FALSE
ENSGALG00010022050	ENSGALG00010022050	PRJNA729271	0.697673821	0.000856613	0.697673821	stage_E14_vs_E10	ordinary_muscle_development		0	FALSE
ENSGALG00010026897	ENSGALG00010026897	PRJNA729271	-0.802857033	0.000859538	0.802857033	stage_E14_vs_E10	ordinary_muscle_development		0	FALSE
ENSGALG00010012378	RPS19BP1	PRJNA729271	-0.319306935	0.000859864	0.319306935	stage_E14_vs_E10	ordinary_muscle_development		0	FALSE
ENSGALG00010003180	IBTK	PRJNA729271	0.437818147	0.000860536	0.437818147	stage_E14_vs_E10	ordinary_muscle_development		0	FALSE
ENSGALG00010017332	FGFR1	PRJNA729271	-0.548466278	0.000862269	0.548466278	stage_E14_vs_E10	ordinary_muscle_development		0	FALSE
ENSGALG00010015642	ENSGALG00010015642	PRJNA729271	-3.278669067	0.000862851	3.278669067	stage_E14_vs_E10	ordinary_muscle_development	D_vs_E10;stage_1W_vs_E10;stage_3W_vs_E10;stage	1	TRUE
ENSGALG00010024848	ESYT1	PRJNA729271	0.311644671	0.000865299	0.311644671	stage_E14_vs_E10	ordinary_muscle_development		0	FALSE
ENSGALG00010018590	TGFA	PRJNA729271	-2.284455215	0.000865733	2.284455215	stage_E14_vs_E10	ordinary_muscle_development	D_vs_E10;stage_1W_vs_E10;stage_3W_vs_E10;stage	1	TRUE
ENSGALG00010029708	TMEM94	PRJNA729271	0.309030506	0.00086902	0.309030506	stage_E14_vs_E10	ordinary_muscle_development		0	FALSE
ENSGALG00010028491	TOR1B	PRJNA729271	-0.341933547	0.000869391	0.341933547	stage_E14_vs_E10	ordinary_muscle_development		0	FALSE
ENSGALG00010018464	ENSGALG00010018464	PRJNA729271	-4.101739199	0.000869648	4.101739199	stage_E14_vs_E10	ordinary_muscle_development	D_vs_E10;stage_1W_vs_E10;stage_3W_vs_E10;stage	1	TRUE
ENSGALG00010009015	TMEM231	PRJNA729271	-0.332540793	0.000870796	0.332540793	stage_E14_vs_E10	ordinary_muscle_development		0	FALSE
ENSGALG00010017500	ACAP2	PRJNA729271	0.346071098	0.000874668	0.346071098	stage_E14_vs_E10	ordinary_muscle_development		0	FALSE
ENSGALG00010022030	ZNF341	PRJNA729271	0.527497602	0.00087673	0.527497602	stage_E14_vs_E10	ordinary_muscle_development		0	FALSE
ENSGALG00010021012	KCNIP2	PRJNA729271	-3.437482047	0.000877258	3.437482047	stage_E14_vs_E10	ordinary_muscle_development	D_vs_E10;stage_1W_vs_E10;stage_3W_vs_E10;stage	1	TRUE
ENSGALG00010005178	EPHA5	PRJNA729271	-0.690140048	0.000882587	0.690140048	stage_E14_vs_E10	ordinary_muscle_development		0	FALSE
ENSGALG00010001369	PPP2R2D	PRJNA729271	-0.436969673	0.000884892	0.436969673	stage_E14_vs_E10	ordinary_muscle_development		0	FALSE
ENSGALG00010007025	ENSGALG00010007025	PRJNA729271	2.471736545	0.000892912	2.471736545	stage_E14_vs_E10	ordinary_muscle_development	D_vs_E10;stage_1W_vs_E10;stage_3W_vs_E10;stage	1	TRUE
ENSGALG00010021249	ENSGALG00010021249	PRJNA729271	-1.473988019	0.000894171	1.473988019	stage_E14_vs_E10	ordinary_muscle_development	D_vs_E10;stage_1W_vs_E10;stage_3W_vs_E10;stage	1	TRUE
ENSGALG00010009690	RBM43	PRJNA729271	-0.536378638	0.000894317	0.536378638	stage_E14_vs_E10	ordinary_muscle_development		0	FALSE
ENSGALG00010011477	SDC2	PRJNA729271	0.584933674	0.000897267	0.584933674	stage_E14_vs_E10	ordinary_muscle_development		0	FALSE
ENSGALG00010007950	ARC	PRJNA729271	-2.146442297	0.000898155	2.146442297	stage_E14_vs_E10	ordinary_muscle_development	D_vs_E10;stage_1W_vs_E10;stage_3W_vs_E10;stage	1	TRUE
ENSGALG00010014320	UTS2B	PRJNA729271	-3.842133578	0.000906651	3.842133578	stage_E14_vs_E10	ordinary_muscle_development	D_vs_E10;stage_1W_vs_E10;stage_3W_vs_E10;stage	1	TRUE
ENSGALG00010013217	TGFBRAP1	PRJNA729271	0.286243334	0.000915658	0.286243334	stage_E14_vs_E10	ordinary_muscle_development		0	FALSE
ENSGALG00010023186	MIER1	PRJNA729271	-0.342379702	0.000916489	0.342379702	stage_E14_vs_E10	ordinary_muscle_development		0	FALSE
ENSGALG00010021193	USP37	PRJNA729271	-0.553431813	0.000917767	0.553431813	stage_E14_vs_E10	ordinary_muscle_development		0	FALSE
ENSGALG00010015795	PSMD10	PRJNA729271	-0.417273759	0.000918354	0.417273759	stage_E14_vs_E10	ordinary_muscle_development		0	FALSE
ENSGALG00010027628	PIERCE1	PRJNA729271	-0.870042677	0.000919198	0.870042677	stage_E14_vs_E10	ordinary_muscle_development		0	FALSE
ENSGALG00010023356	ENSGALG00010023356	PRJNA729271	-0.711536547	0.000920164	0.711536547	stage_E14_vs_E10	ordinary_muscle_development		0	FALSE
ENSGALG00010020704	PROX1	PRJNA729271	0.799385757	0.000922045	0.799385757	stage_E14_vs_E10	ordinary_muscle_development		0	FALSE
ENSGALG00010011559	MTX3	PRJNA729271	0.432171518	0.000924436	0.432171518	stage_E14_vs_E10	ordinary_muscle_development		0	FALSE
ENSGALG00010024558	PFDN5	PRJNA729271	0.370501014	0.000929325	0.370501014	stage_E14_vs_E10	ordinary_muscle_development		0	FALSE
ENSGALG00010027555	ENSGALG00010027555	PRJNA729271	1.179069466	0.000930434	1.179069466	stage_E14_vs_E10	ordinary_muscle_development	D_vs_E10;stage_1W_vs_E10;stage_3W_vs_E10;stage	1	TRUE
ENSGALG00010001281	LRRN3	PRJNA729271	-0.533055744	0.000932416	0.533055744	stage_E14_vs_E10	ordinary_muscle_development		0	FALSE
ENSGALG00010020521	ENSGALG00010020521	PRJNA729271	2.174589614	0.000937474	2.174589614	stage_E14_vs_E10	ordinary_muscle_development	D_vs_E10;stage_1W_vs_E10;stage_3W_vs_E10;stage	1	TRUE
ENSGALG00010013425	ENSGALG00010013425	PRJNA729271	0.758310922	0.000940998	0.758310922	stage_E14_vs_E10	ordinary_muscle_development		0	FALSE
ENSGALG00010015697	FAXDC2	PRJNA729271	1.129612753	0.000941312	1.129612753	stage_E14_vs_E10	ordinary_muscle_development	D_vs_E10;stage_1W_vs_E10;stage_3W_vs_E10;stage	1	TRUE
ENSGALG00010022486	FAM183A	PRJNA729271	-2.581141549	0.000941727	2.581141549	stage_E14_vs_E10	ordinary_muscle_development	D_vs_E10;stage_1W_vs_E10;stage_3W_vs_E10;stage	1	TRUE
ENSGALG00010028760	SSR2	PRJNA729271	0.338782587	0.000942818	0.338782587	stage_E14_vs_E10	ordinary_muscle_development		0	FALSE
ENSGALG00010023770	SCRN2	PRJNA729271	-0.490095181	0.000950656	0.490095181	stage_E14_vs_E10	ordinary_muscle_development		0	FALSE
ENSGALG00010029258	TMEM100	PRJNA729271	0.800860974	0.000950686	0.800860974	stage_E14_vs_E10	ordinary_muscle_development		0	FALSE
ENSGALG00010009472	ADO	PRJNA729271	-0.299910556	0.000954497	0.299910556	stage_E14_vs_E10	ordinary_muscle_development		0	FALSE
ENSGALG00010000056	UNC45A	PRJNA729271	0.279902754	0.000957924	0.279902754	stage_E14_vs_E10	ordinary_muscle_development		0	FALSE
ENSGALG00010000853	ENSGALG00010000853	PRJNA729271	1.046854541	0.000961413	1.046854541	stage_E14_vs_E10	ordinary_muscle_development	D_vs_E10;stage_1W_vs_E10;stage_3W_vs_E10;stage	1	TRUE
ENSGALG00010016909	MRPS5	PRJNA729271	-0.371614791	0.000962511	0.371614791	stage_E14_vs_E10	ordinary_muscle_development		0	FALSE
ENSGALG00010016218	N4BP2	PRJNA729271	0.263097959	0.000965607	0.263097959	stage_E14_vs_E10	ordinary_muscle_development		0	FALSE
ENSGALG00010001974	ENSGALG00010001974	PRJNA729271	1.372906076	0.000966747	1.372906076	stage_E14_vs_E10	ordinary_muscle_development	D_vs_E10;stage_1W_vs_E10;stage_3W_vs_E10;stage	1	TRUE
ENSGALG00010007437	TUSC1	PRJNA729271	0.481234318	0.000968188	0.481234318	stage_E14_vs_E10	ordinary_muscle_development		0	FALSE
ENSGALG00010015703	GK5	PRJNA729271	0.638344737	0.000969534	0.638344737	stage_E14_vs_E10	ordinary_muscle_development		0	FALSE
ENSGALG00010028440	PSMB7	PRJNA729271	0.267280775	0.00097476	0.267280775	stage_E14_vs_E10	ordinary_muscle_development		0	FALSE
ENSGALG00010027897	AP3D1	PRJNA729271	-0.342950468	0.000977513	0.342950468	stage_E14_vs_E10	ordinary_muscle_development		0	FALSE
ENSGALG00010002603	INSYN2A	PRJNA729271	-0.934484737	0.000985339	0.934484737	stage_E14_vs_E10	ordinary_muscle_development		0	FALSE
ENSGALG00010019248	RMI2	PRJNA729271	-0.58324093	0.000999307	0.58324093	stage_E14_vs_E10	ordinary_muscle_development		0	FALSE
ENSGALG00010018014	LYRM1	PRJNA729271	0.326119448	0.001002594	0.326119448	stage_E14_vs_E10	ordinary_muscle_development		0	FALSE
ENSGALG00010016449	FMR1	PRJNA729271	0.363266733	0.001006296	0.363266733	stage_E14_vs_E10	ordinary_muscle_development		0	FALSE
ENSGALG00010021426	SLC38A7	PRJNA729271	-0.500757609	0.00101263	0.500757609	stage_E14_vs_E10	ordinary_muscle_development		0	FALSE
ENSGALG00010025920	GTSF1	PRJNA729271	0.884184489	0.001019391	0.884184489	stage_E14_vs_E10	ordinary_muscle_development		0	FALSE
ENSGALG00010029630	RAD51D	PRJNA729271	0.413206872	0.001020145	0.413206872	stage_E14_vs_E10	ordinary_muscle_development		0	FALSE
ENSGALG00010029675	DHRS13	PRJNA729271	-0.840487819	0.001023105	0.840487819	stage_E14_vs_E10	ordinary_muscle_development		0	FALSE
ENSGALG00010017468	AMDHD2	PRJNA729271	-0.443056011	0.00102324	0.443056011	stage_E14_vs_E10	ordinary_muscle_development		0	FALSE
ENSGALG00010000710	ENSGALG00010000710	PRJNA729271	1.65974124	0.001024055	1.65974124	stage_E14_vs_E10	ordinary_muscle_development	D_vs_E10;stage_1W_vs_E10;stage_3W_vs_E10;stage	1	TRUE
ENSGALG00010024062	ST3GAL4	PRJNA729271	-0.457882544	0.001027736	0.457882544	stage_E14_vs_E10	ordinary_muscle_development		0	FALSE
ENSGALG00010014972	FGA	PRJNA729271	5.726756945	0.001032622	5.726756945	stage_E14_vs_E10	ordinary_muscle_development	D_vs_E10;stage_1W_vs_E10;stage_3W_vs_E10;stage	1	TRUE
ENSGALG00010018512	FERMT1	PRJNA729271	-0.617642432	0.001038505	0.617642432	stage_E14_vs_E10	ordinary_muscle_development		0	FALSE
ENSGALG00010016937	DHRS7	PRJNA729271	0.584944896	0.001039056	0.584944896	stage_E14_vs_E10	ordinary_muscle_development		0	FALSE
ENSGALG00010015021	RBM46	PRJNA729271	-3.698180391	0.00104544	3.698180391	stage_E14_vs_E10	ordinary_muscle_development	D_vs_E10;stage_1W_vs_E10;stage_3W_vs_E10;stage	1	TRUE
ENSGALG00010017733	TRIM62	PRJNA729271	-0.452604826	0.001045645	0.452604826	stage_E14_vs_E10	ordinary_muscle_development		0	FALSE
ENSGALG00010024526	TGM6	PRJNA729271	4.936790436	0.001049176	4.936790436	stage_E14_vs_E10	ordinary_muscle_development	D_vs_E10;stage_1W_vs_E10;stage_3W_vs_E10;stage	1	TRUE
ENSGALG00010016898	ENSGALG00010016898	PRJNA729271	-2.900845872	0.001063484	2.900845872	stage_E14_vs_E10	ordinary_muscle_development	D_vs_E10;stage_1W_vs_E10;stage_3W_vs_E10;stage	1	TRUE
ENSGALG00010013096	CLDN14	PRJNA729271	-1.549976095	0.001067885	1.549976095	stage_E14_vs_E10	ordinary_muscle_development	D_vs_E10;stage_1W_vs_E10;stage_3W_vs_E10;stage	1	TRUE
ENSGALG00010021645	ASCC1	PRJNA729271	-0.475603327	0.00107354	0.475603327	stage_E14_vs_E10	ordinary_muscle_development		0	FALSE
ENSGALG00010011015	LYRM4	PRJNA729271	0.654437025	0.001076432	0.654437025	stage_E14_vs_E10	ordinary_muscle_development		0	FALSE
ENSGALG00010001150	ENSGALG00010001150	PRJNA729271	2.033430948	0.001077529	2.033430948	stage_E14_vs_E10	ordinary_muscle_development	D_vs_E10;stage_1W_vs_E10;stage_3W_vs_E10;stage	1	TRUE
ENSGALG00010001117	P2RY13	PRJNA729271	1.066329554	0.001078664	1.066329554	stage_E14_vs_E10	ordinary_muscle_development	D_vs_E10;stage_1W_vs_E10;stage_3W_vs_E10;stage	1	TRUE
ENSGALG00010021694	ENSGALG00010021694	PRJNA729271	3.728429831	0.001080367	3.728429831	stage_E14_vs_E10	ordinary_muscle_development	D_vs_E10;stage_1W_vs_E10;stage_3W_vs_E10;stage	1	TRUE
ENSGALG00010029213	APOH	PRJNA729271	5.218210295	0.001082444	5.218210295	stage_E14_vs_E10	ordinary_muscle_development	D_vs_E10;stage_1W_vs_E10;stage_3W_vs_E10;stage	1	TRUE
ENSGALG00010015756	PCM1	PRJNA729271	-0.367625286	0.001082515	0.367625286	stage_E14_vs_E10	ordinary_muscle_development		0	FALSE
ENSGALG00010019996	ITGB5	PRJNA729271	0.421515523	0.001083708	0.421515523	stage_E14_vs_E10	ordinary_muscle_development		0	FALSE
ENSGALG00010013114	PIGP	PRJNA729271	0.274447971	0.001085676	0.274447971	stage_E14_vs_E10	ordinary_muscle_development		0	FALSE
ENSGALG00010027261	ENSGALG00010027261	PRJNA729271	-0.383991556	0.001091802	0.383991556	stage_E14_vs_E10	ordinary_muscle_development		0	FALSE
ENSGALG00010011988	HINTW	PRJNA729271	-11.06706335	0.001094011	11.06706335	stage_E14_vs_E10	ordinary_muscle_development	D_vs_E10;stage_1W_vs_E10;stage_3W_vs_E10;stage	1	TRUE
ENSGALG00010027392	RBBP5	PRJNA729271	-0.439199523	0.001099146	0.439199523	stage_E14_vs_E10	ordinary_muscle_development		0	FALSE
ENSGALG00010014210	ENSGALG00010014210	PRJNA729271	0.835679599	0.00110107	0.835679599	stage_E14_vs_E10	ordinary_muscle_development		0	FALSE
ENSGALG00010003287	ENSGALG00010003287	PRJNA729271	-6.659005011	0.001103289	6.659005011	stage_E14_vs_E10	ordinary_muscle_development	D_vs_E10;stage_1W_vs_E10;stage_3W_vs_E10;stage	1	TRUE
ENSGALG00010007155	FOXL1	PRJNA729271	-2.570440321	0.001104115	2.570440321	stage_E14_vs_E10	ordinary_muscle_development	D_vs_E10;stage_1W_vs_E10;stage_3W_vs_E10;stage	1	TRUE
ENSGALG00010023993	C19orf12	PRJNA729271	-0.598939163	0.001105607	0.598939163	stage_E14_vs_E10	ordinary_muscle_development		0	FALSE
ENSGALG00010023773	OVOA	PRJNA729271	1.800859812	0.001108096	1.800859812	stage_E14_vs_E10	ordinary_muscle_development	D_vs_E10;stage_1W_vs_E10;stage_3W_vs_E10;stage	1	TRUE
ENSGALG00010027115	C9ORF152	PRJNA729271	-1.614018596	0.001108331	1.614018596	stage_E14_vs_E10	ordinary_muscle_development	D_vs_E10;stage_1W_vs_E10;stage_3W_vs_E10;stage	1	TRUE
ENSGALG00010025702	GTSF2	PRJNA729271	0.594691156	0.001118328	0.594691156	stage_E14_vs_E10	ordinary_muscle_development		0	FALSE
ENSGALG00010024827	ENSGALG00010024827	PRJNA729271	0.855803676	0.001118837	0.855803676	stage_E14_vs_E10	ordinary_muscle_development		0	FALSE
ENSGALG00010016589	FBXO28	PRJNA729271	0.26082816	0.001121628	0.26082816	stage_E14_vs_E10	ordinary_muscle_development		0	FALSE

ENSGALG00010028897	TMEM268	PRJNA729271	0.93947009	0.001122616	0.93947009	stage_E14_vs_E10	ordinary_muscle_development		0	FALSE
ENSGALG00010002948	DLX6	PRJNA729271	-5.405856545	0.001129529	5.405856545	stage_E14_vs_E10	ordinary_muscle_development	D_vs_E10;stage_1W_vs_E10;stage_3W_vs_E10;stage	1	TRUE
ENSGALG00010002422	TRAPPC12	PRJNA729271	0.347763367	0.001131225	0.347763367	stage_E14_vs_E10	ordinary_muscle_development		0	FALSE
ENSGALG00010022512	PRAP1	PRJNA729271	2.800899052	0.001131244	2.800899052	stage_E14_vs_E10	ordinary_muscle_development	D_vs_E10;stage_1W_vs_E10;stage_3W_vs_E10;stage	1	TRUE
ENSGALG00010027923	AMPD2	PRJNA729271	-0.554024839	0.001137814	0.554024839	stage_E14_vs_E10	ordinary_muscle_development		0	FALSE
ENSGALG00010015291	ZFP64	PRJNA729271	0.310017223	0.001153601	0.310017223	stage_E14_vs_E10	ordinary_muscle_development		0	FALSE
ENSGALG00010013235	GCNT7	PRJNA729271	0.833500455	0.00115523	0.833500455	stage_E14_vs_E10	ordinary_muscle_development		0	FALSE
ENSGALG00010016762	EML6	PRJNA729271	0.419330892	0.001162967	0.419330892	stage_E14_vs_E10	ordinary_muscle_development		0	FALSE
ENSGALG00010015693	COA5	PRJNA729271	-0.373819771	0.001162967	0.373819771	stage_E14_vs_E10	ordinary_muscle_development		0	FALSE
ENSGALG00010029335	RNF222	PRJNA729271	4.555232344	0.001163693	4.555232344	stage_E14_vs_E10	ordinary_muscle_development	D_vs_E10;stage_1W_vs_E10;stage_3W_vs_E10;stage	1	TRUE
ENSGALG00010027545	PPARD	PRJNA729271	0.65031731	0.001167156	0.65031731	stage_E14_vs_E10	ordinary_muscle_development		0	FALSE
ENSGALG00010011280	FAM199X	PRJNA729271	-0.278446758	0.001169697	0.278446758	stage_E14_vs_E10	ordinary_muscle_development		0	FALSE
ENSGALG00010019302	USP34	PRJNA729271	-0.334779101	0.001174292	0.334779101	stage_E14_vs_E10	ordinary_muscle_development		0	FALSE
ENSGALG00010024209	RWDD3	PRJNA729271	-0.485925526	0.00117626	0.485925526	stage_E14_vs_E10	ordinary_muscle_development		0	FALSE
ENSGALG00010025830	TBCEL	PRJNA729271	0.653973993	0.001177426	0.653973993	stage_E14_vs_E10	ordinary_muscle_development		0	FALSE
ENSGALG00010000737	ENSGALG00010000737	PRJNA729271	-3.616225169	0.001177511	3.616225169	stage_E14_vs_E10	ordinary_muscle_development	D_vs_E10;stage_1W_vs_E10;stage_3W_vs_E10;stage	1	TRUE
ENSGALG00010013922	ENSGALG00010013922	PRJNA729271	-3.70956704	0.001187417	3.70956704	stage_E14_vs_E10	ordinary_muscle_development	D_vs_E10;stage_1W_vs_E10;stage_3W_vs_E10;stage	1	TRUE
ENSGALG00010028049	MAN1B1	PRJNA729271	-0.204648785	0.001190156	0.204648785	stage_E14_vs_E10	ordinary_muscle_development		0	FALSE
ENSGALG00010013380	PHGDH	PRJNA729271	-2.056181439	0.001190401	2.056181439	stage_E14_vs_E10	ordinary_muscle_development	D_vs_E10;stage_1W_vs_E10;stage_3W_vs_E10;stage	1	TRUE
ENSGALG00010004542	ERMARD	PRJNA729271	-0.445751533	0.001190577	0.445751533	stage_E14_vs_E10	ordinary_muscle_development		0	FALSE
ENSGALG00010029703	MIF4GD	PRJNA729271	-0.388864348	0.001191127	0.388864348	stage_E14_vs_E10	ordinary_muscle_development		0	FALSE
ENSGALG00010010962	ANKDD1B	PRJNA729271	0.891953451	0.001197387	0.891953451	stage_E14_vs_E10	ordinary_muscle_development		0	FALSE
ENSGALG00010016667	WARS1	PRJNA729271	-0.492253735	0.001197931	0.492253735	stage_E14_vs_E10	ordinary_muscle_development		0	FALSE
ENSGALG00010017367	TIMD4	PRJNA729271	-1.100467767	0.001200188	1.100467767	stage_E14_vs_E10	ordinary_muscle_development	D_vs_E10;stage_1W_vs_E10;stage_3W_vs_E10;stage	1	TRUE
ENSGALG00010027667	PTPDC1	PRJNA729271	0.504036917	0.001203955	0.504036917	stage_E14_vs_E10	ordinary_muscle_development		0	FALSE
ENSGALG00010001304	SYVN1	PRJNA729271	0.591818096	0.001208106	0.591818096	stage_E14_vs_E10	ordinary_muscle_development		0	FALSE
ENSGALG00010029927	ENSGALG00010029927	PRJNA729271	-2.821296212	0.001211886	2.821296212	stage_E14_vs_E10	ordinary_muscle_development	D_vs_E10;stage_1W_vs_E10;stage_3W_vs_E10;stage	1	TRUE
ENSGALG00010000226	ENSGALG00010000226	PRJNA729271	1.377928338	0.001212996	1.377928338	stage_E14_vs_E10	ordinary_muscle_development	D_vs_E10;stage_1W_vs_E10;stage_3W_vs_E10;stage	1	TRUE
ENSGALG00010002333	ENSGALG00010002333	PRJNA729271	2.341059624	0.001214616	2.341059624	stage_E14_vs_E10	ordinary_muscle_development	D_vs_E10;stage_1W_vs_E10;stage_3W_vs_E10;stage	1	TRUE
ENSGALG00010006434	MMAA	PRJNA729271	0.396894576	0.001215077	0.396894576	stage_E14_vs_E10	ordinary_muscle_development		0	FALSE
ENSGALG00010021201	TRMT1L	PRJNA729271	-0.298871411	0.001218921	0.298871411	stage_E14_vs_E10	ordinary_muscle_development		0	FALSE
ENSGALG00010003479	SYDE1	PRJNA729271	0.675795526	0.001219741	0.675795526	stage_E14_vs_E10	ordinary_muscle_development		0	FALSE
ENSGALG00010007801	TTC13	PRJNA729271	0.535300882	0.001221476	0.535300882	stage_E14_vs_E10	ordinary_muscle_development		0	FALSE
ENSGALG00010017725	ENSGALG00010017725	PRJNA729271	1.325893941	0.001222597	1.325893941	stage_E14_vs_E10	ordinary_muscle_development	D_vs_E10;stage_1W_vs_E10;stage_3W_vs_E10;stage	1	TRUE
ENSGALG00010029921	STX8	PRJNA729271	0.348499131	0.00122264	0.348499131	stage_E14_vs_E10	ordinary_muscle_development		0	FALSE
ENSGALG00010016221	TMEM200B	PRJNA729271	0.716298326	0.001222853	0.716298326	stage_E14_vs_E10	ordinary_muscle_development		0	FALSE
ENSGALG00010016347	ANO10	PRJNA729271	0.301937713	0.001226438	0.301937713	stage_E14_vs_E10	ordinary_muscle_development		0	FALSE
ENSGALG00010017373	RP1-27O5.3	PRJNA729271	-0.361486477	0.001236362	0.361486477	stage_E14_vs_E10	ordinary_muscle_development		0	FALSE
ENSGALG00010001616	OPNVA	PRJNA729271	-2.583755757	0.001241676	2.583755757	stage_E14_vs_E10	ordinary_muscle_development	D_vs_E10;stage_1W_vs_E10;stage_3W_vs_E10;stage	1	TRUE
ENSGALG00010027814	ENSGALG00010027814	PRJNA729271	0.447863554	0.001242559	0.447863554	stage_E14_vs_E10	ordinary_muscle_development		0	FALSE
ENSGALG00010022807	ADCY6	PRJNA729271	-0.498580943	0.001244406	0.498580943	stage_E14_vs_E10	ordinary_muscle_development		0	FALSE
ENSGALG00010014882	XPNPEP2	PRJNA729271	1.986136396	0.001248926	1.986136396	stage_E14_vs_E10	ordinary_muscle_development	D_vs_E10;stage_1W_vs_E10;stage_3W_vs_E10;stage	1	TRUE
ENSGALG00010004957	ENSGALG00010004957	PRJNA729271	0.47684773	0.001250968	0.47684773	stage_E14_vs_E10	ordinary_muscle_development		0	FALSE
ENSGALG00010001443	ANKRD26	PRJNA729271	-0.518384139	0.001254141	0.518384139	stage_E14_vs_E10	ordinary_muscle_development		0	FALSE
ENSGALG00010005045	UTRN	PRJNA729271	-0.344297658	0.001256075	0.344297658	stage_E14_vs_E10	ordinary_muscle_development		0	FALSE
ENSGALG00010005313	MPHOSPH8	PRJNA729271	-0.502658422	0.001258402	0.502658422	stage_E14_vs_E10	ordinary_muscle_development		0	FALSE
ENSGALG00010016959	PERCC1	PRJNA729271	3.64582571	0.001270411	3.64582571	stage_E14_vs_E10	ordinary_muscle_development	D_vs_E10;stage_1W_vs_E10;stage_3W_vs_E10;stage	1	TRUE
ENSGALG00010022840	MARVELD1	PRJNA729271	0.6311335	0.001277924	0.6311335	stage_E14_vs_E10	ordinary_muscle_development		0	FALSE
ENSGALG00010001635	TIMM10B	PRJNA729271	0.49484343	0.001278227	0.49484343	stage_E14_vs_E10	ordinary_muscle_development		0	FALSE
ENSGALG00010025337	NEMP1	PRJNA729271	-0.524330153	0.001278513	0.524330153	stage_E14_vs_E10	ordinary_muscle_development		0	FALSE
ENSGALG00010000581	ENSGALG00010000581	PRJNA729271	-0.438141153	0.001282404	0.438141153	stage_E14_vs_E10	ordinary_muscle_development		0	FALSE
ENSGALG00010022125	CBFA2T2	PRJNA729271	0.626222963	0.001288324	0.626222963	stage_E14_vs_E10	ordinary_muscle_development		0	FALSE
ENSGALG00010000059	MFAP1	PRJNA729271	-0.372535394	0.001292202	0.372535394	stage_E14_vs_E10	ordinary_muscle_development		0	FALSE
ENSGALG00010014572	IL1RAP	PRJNA729271	-0.426867229	0.001297141	0.426867229	stage_E14_vs_E10	ordinary_muscle_development		0	FALSE
ENSGALG00010008918	PLAC9	PRJNA729271	0.873816042	0.001298409	0.873816042	stage_E14_vs_E10	ordinary_muscle_development		0	FALSE
ENSGALG00010003348	ADAM17	PRJNA729271	-0.284608704	0.001300341	0.284608704	stage_E14_vs_E10	ordinary_muscle_development		0	FALSE
ENSGALG00010002952	NBAS	PRJNA729271	0.21883121	0.001300454	0.21883121	stage_E14_vs_E10	ordinary_muscle_development		0	FALSE
ENSGALG00010021761	MAFB	PRJNA729271	-0.59312243	0.001307748	0.59312243	stage_E14_vs_E10	ordinary_muscle_development		0	FALSE
ENSGALG00010003882	ENSGALG00010003882	PRJNA729271	-0.406010386	0.001313625	0.406010386	stage_E14_vs_E10	ordinary_muscle_development		0	FALSE
ENSGALG00010029501	ENSGALG00010029501	PRJNA729271	-1.315324226	0.001315657	1.315324226	stage_E14_vs_E10	ordinary_muscle_development	D_vs_E10;stage_1W_vs_E10;stage_3W_vs_E10;stage	1	TRUE
ENSGALG00010023252	GLG1	PRJNA729271	-0.266009159	0.001320526	0.266009159	stage_E14_vs_E10	ordinary_muscle_development		0	FALSE
ENSGALG00010000686	ENSGALG00010000686	PRJNA729271	-0.36030328	0.001339161	0.36030328	stage_E14_vs_E10	ordinary_muscle_development		0	FALSE
ENSGALG00010011069	ENSGALG00010011069	PRJNA729271	-1.838857583	0.001341225	1.838857583	stage_E14_vs_E10	ordinary_muscle_development	D_vs_E10;stage_1W_vs_E10;stage_3W_vs_E10;stage	1	TRUE
ENSGALG00010016774	PEX14	PRJNA729271	-0.417806621	0.001342444	0.417806621	stage_E14_vs_E10	ordinary_muscle_development		0	FALSE
ENSGALG00010008243	YTHDC2	PRJNA729271	-0.353192488	0.001344319	0.353192488	stage_E14_vs_E10	ordinary_muscle_development		0	FALSE
ENSGALG00010029176	ENSGALG00010029176	PRJNA729271	-1.750454196	0.001346252	1.750454196	stage_E14_vs_E10	ordinary_muscle_development	D_vs_E10;stage_1W_vs_E10;stage_3W_vs_E10;stage	1	TRUE
ENSGALG00010012021	ENSGALG00010012021	PRJNA729271	-0.441959411	0.001347435	0.441959411	stage_E14_vs_E10	ordinary_muscle_development		0	FALSE
ENSGALG00010014949	AGO3	PRJNA729271	-0.385290392	0.001347953	0.385290392	stage_E14_vs_E10	ordinary_muscle_development		0	FALSE
ENSGALG00010029731	NCOR1	PRJNA729271	0.612549877	0.001357981	0.612549877	stage_E14_vs_E10	ordinary_muscle_development		0	FALSE
ENSGALG00010027766	ENSGALG00010027766	PRJNA729271	-3.117614234	0.001365434	3.117614234	stage_E14_vs_E10	ordinary_muscle_development	D_vs_E10;stage_1W_vs_E10;stage_3W_vs_E10;stage	1	TRUE
ENSGALG00010027111	CALR3	PRJNA729271	-2.901405926	0.0013712	2.901405926	stage_E14_vs_E10	ordinary_muscle_development	D_vs_E10;stage_1W_vs_E10;stage_3W_vs_E10;stage	1	TRUE
ENSGALG00010021687	ZNF384	PRJNA729271	-0.355269734	0.001372028	0.355269734	stage_E14_vs_E10	ordinary_muscle_development		0	FALSE
ENSGALG00010029453	ENSGALG00010029453	PRJNA729271	-5.683854841	0.001375518	5.683854841	stage_E14_vs_E10	ordinary_muscle_development	D_vs_E10;stage_1W_vs_E10;stage_3W_vs_E10;stage	1	TRUE
ENSGALG00010027553	FGF22	PRJNA729271	0.971393287	0.001376993	0.971393287	stage_E14_vs_E10	ordinary_muscle_development		0	FALSE
ENSGALG00010009464	SPTLC1	PRJNA729271	0.493212495	0.001382565	0.493212495	stage_E14_vs_E10	ordinary_muscle_development		0	FALSE
ENSGALG00010005166	CBLB	PRJNA729271	-0.437204445	0.001385836	0.437204445	stage_E14_vs_E10	ordinary_muscle_development		0	FALSE
ENSGALG00010022092	CTRL	PRJNA729271	5.886791064	0.001388844	5.886791064	stage_E14_vs_E10	ordinary_muscle_development	D_vs_E10;stage_1W_vs_E10;stage_3W_vs_E10;stage	1	TRUE
ENSGALG00010000661	ENSGALG00010000661	PRJNA729271	-2.73954051	0.001391361	2.73954051	stage_E14_vs_E10	ordinary_muscle_development	D_vs_E10;stage_1W_vs_E10;stage_3W_vs_E10;stage	1	TRUE
ENSGALG00010014301	ARID1A	PRJNA729271	0.397275582	0.001394038	0.397275582	stage_E14_vs_E10	ordinary_muscle_development		0	FALSE
ENSGALG00010024915	STXBP5L	PRJNA729271	-0.583375954	0.001398154	0.583375954	stage_E14_vs_E10	ordinary_muscle_development		0	FALSE
ENSGALG00010020498	FRMPD2	PRJNA729271	-2.080045867	0.001410644	2.080045867	stage_E14_vs_E10	ordinary_muscle_development	D_vs_E10;stage_1W_vs_E10;stage_3W_vs_E10;stage	1	TRUE
ENSGALG00010011278	ENSGALG00010011278	PRJNA729271	-1.767043989	0.001412075	1.767043989	stage_E14_vs_E10	ordinary_muscle_development	D_vs_E10;stage_1W_vs_E10;stage_3W_vs_E10;stage	1	TRUE
ENSGALG00010009115	COG5	PRJNA729271	-0.389310013	0.001416756	0.389310013	stage_E14_vs_E10	ordinary_muscle_development		0	FALSE
ENSGALG00010009013	LIPG	PRJNA729271	-0.744617772	0.001421795	0.744617772	stage_E14_vs_E10	ordinary_muscle_development		0	FALSE
ENSGALG00010029812	SAP30BP	PRJNA729271	-0.384795144	0.001428816	0.384795144	stage_E14_vs_E10	ordinary_muscle_development		0	FALSE
ENSGALG00010019314	GKN2	PRJNA729271	-4.317679086	0.001432642	4.317679086	stage_E14_vs_E10	ordinary_muscle_development	D_vs_E10;stage_1W_vs_E10;stage_3W_vs_E10;stage	1	TRUE
ENSGALG00010019281	ENSGALG00010019281	PRJNA729271	-4.863275125	0.001441422	4.863275125	stage_E14_vs_E10	ordinary_muscle_development	D_vs_E10;stage_1W_vs_E10;stage_3W_vs_E10;stage	1	TRUE
ENSGALG00010009273	MAL2	PRJNA729271	-1.76492933	0.001445738	1.76492933	stage_E14_vs_E10	ordinary_muscle_development	D_vs_E10;stage_1W_vs_E10;stage_3W_vs_E10;stage	1	TRUE
ENSGALG00010002294	DAZL	PRJNA729271	2.663278474	0.001446982	2.663278474	stage_E14_vs_E10	ordinary_muscle_development	D_vs_E10;stage_1W_vs_E10;stage_3W_vs_E10;stage	1	TRUE
ENSGALG00010009268	PWWP2A	PRJNA729271	0.436153073	0.001449868	0.436153073	stage_E14_vs_E10	ordinary_muscle_development		0	FALSE
ENSGALG00010024805	ENSGALG00010024805	PRJNA729271	1.215641171	0.001453556	1.215641171	stage_E14_vs_E10	ordinary_muscle_development	D_vs_E10;stage_1W_vs_E10;stage_3W_vs_E10;stage	1	TRUE
ENSGALG00010019012	ADAM28	PRJNA729271	-1.247430398	0.001456393	1.247430398	stage_E14_vs_E10	ordinary_muscle_development	D_vs_E10;stage_1W_vs_E10;stage_3W_vs_E10;stage	1	TRUE
ENSGALG00010020654	SAYSD1	PRJNA729271	-0.503151148	0.001457923	0.503151148	stage_E14_vs_E10	ordinary_muscle_development		0	FALSE
ENSGALG00010008073	ZNF276	PRJNA729271	0.203828154	0.001476877	0.203828154	stage_E14_vs_E10	ordinary_muscle_development		0	FALSE
ENSGALG00010018511	SOCS5	PRJNA729271	-0.333048326	0.001487311	0.333048326	stage_E14_vs_E10	ordinary_muscle_development		0	FALSE
ENSGALG00010002999	CMTM7	PRJNA729271	-0.707434413	0.00149251	0.707434413	stage_E14_vs_E10	ordinary_muscle_development		0	FALSE
ENSGALG00010024057	HSD3B1	PRJNA729271	-2.864444976	0.001493245	2.864444976	stage_E14_vs_E10	ordinary_muscle_development	D_vs_E10;stage_1W_vs_E10;stage_3W_vs_E10;stage	1	TRUE
ENSGALG00010027922	SHC1	PRJNA729271	0.371884087	0.00149468	0.371884087	stage_E14_vs_E10	ordinary_muscle_development		0	FALSE
ENSGALG00010013004	UAP1	PRJNA729271	-0.508644283	0.001497977	0.508644283	stage_E14_vs_E10	ordinary_muscle_development		0	FALSE
ENSGALG00010011927	ENSGALG00010011927	PRJNA729271	-1.715131718	0.001498267	1.715131718	stage_E14_vs_E10	ordinary_muscle_development	D_vs_E10;stage_1W_vs_E10;stage_3W_vs_E10;stage	1	TRUE
ENSGALG00010020080	SLC4A1AP	PRJNA729271	-0.35120935	0.001500573	0.35120935	stage_E14_vs_E10	ordinary_muscle_development		0	FALSE
ENSGALG00010015070	EDN2	PRJNA729271	1.402895015	0.001513914	1.402895015	stage_E14_vs_E10	ordinary_muscle_development	D_vs_E10;stage_1W_vs_E10;stage_3W_vs_E10;stage	1	TRUE
ENSGALG00010022019	BTBD8	PRJNA729271	-0.527004948	0.001517661	0.527004948	stage_E14_vs_E10	ordinary_muscle_development		0	FALSE
ENSGALG00010029623	ENSGALG00010029623	PRJNA729271	1.31993565	0.001523119	1.31993565	stage_E14_vs_E10	ordinary_muscle_development	D_vs_E10;stage_1W_vs_E10;stage_3W_vs_E10;stage	1	TRUE
ENSGALG00010008106	ENSGALG00010008106	PRJNA729271	0.851609729	0.001523119	0.851609729	stage_E14_vs_E10	ordinary_muscle_development		0	FALSE
ENSGALG00010025914	ENSGALG00010025914	PRJNA729271	-5.788040555	0.001531097	5.788040555	stage_E14_vs_E10	ordinary_muscle_development	D_vs_E10;stage_1W_vs_E10;stage_3W_vs_E10;stage	1	TRUE
ENSGALG00010022474	VCL	PRJNA729271	-0.393349309	0.001534613	0.393349309	stage_E14_vs_E10	ordinary_muscle_development		0	FALSE
ENSGALG00010002022	ERNI	PRJNA729271	-2.538144816	0.001538027	2.538144816	stage_E14_vs_E10	ordinary_muscle_development	D_vs_E10;stage_1W_vs_E10;stage_3W_vs_E10;stage	1	TRUE
ENSGALG00010021705	CCDC18	PRJNA729271	-0.632154278	0.001539817	0.632154278	stage_E14_vs_E10	ordinary_muscle_development		0	FALSE
ENSGALG00010025319	LDLRAD1	PRJNA729271	1.127908508	0.001542777	1.127908508	stage_E14_vs_E10	ordinary_muscle_development	D_vs_E10;stage_1W_vs_E10;stage_3W_vs_E10;stage	1	TRUE
ENSGALG00010001225	SOCS6	PRJNA729271	-0.327947782	0.001546996	0.327947782	stage_E14_vs_E10	ordinary_muscle_development		0	FALSE
ENSGALG00010011688	PDE3A	PRJNA729271	0.486312627	0.001547734	0.486312627	stage_E14_vs_E10	ordinary_muscle_development		0	FALSE
ENSGALG00010022827	CMTR2	PRJNA729271	-0.31094246	0.001552854	0.31094246	stage_E14_vs_E10	ordinary_muscle_development		0	FALSE
ENSGALG00010019867	PEX11A	PRJNA729271	-1.256870236	0.001553125	1.256870236	stage_E14_vs_E10	ordinary_muscle_development	D_vs_E10;stage_1W_vs_E10;stage_3W_vs_E10;stage	1	TRUE
ENSGALG00010019529	SNAP23	PRJNA729271	-0.226886008	0.001562602	0.226886008	stage_E14_vs_E10	ordinary_muscle_development		0	FALSE
ENSGALG00010021140	SLC11A1	PRJNA729271	-1.385406879	0.001587486	1.385406879	stage_E14_vs_E10	ordinary_muscle_development	D_vs_E10;stage_1W_vs_E10;stage_3W_vs_E10;stage	1	TRUE
ENSGALG00010022115	E2F4	PRJNA729271	-0.625253118	0.001597969	0.625253118	stage_E14_vs_E10	ordinary_muscle_development		0	FALSE
ENSGALG00010012708	SPP2	PRJNA729271	3.641522514	0.001605693	3.641522514	stage_E14_vs_E10	ordinary_muscle_development	D_vs_E10;stage_1W_vs_E10;stage_3W_vs_E10;stage	1	TRUE
ENSGALG00010020762	TTBK2	PRJNA729271	-0.494845054	0.00161282	0.494845054	stage_E14_vs_E10	ordinary_muscle_development		0	FALSE
ENSGALG00010010921	ENSGALG00010010921	PRJNA729271	-0.431904798	0.001613479	0.431904798	stage_E14_vs_E10	ordinary_muscle_development		0	FALSE
ENSGALG00010001463	DSEL	PRJNA729271	-0.520264577	0.001614142	0.520264577	stage_E14_vs_E10	ordinary_muscle_development		0	FALSE
ENSGALG00010021108	PCGF6	PRJNA729271	0.344388559	0.001614783	0.344388559	stage_E14_vs_E10	ordinary_muscle_development		0	FALSE
ENSGALG00010018673	NAGPA	PRJNA729271	-0.301887536	0.001629566	0.301887536	stage_E14_vs_E10	ordinary_muscle_development		0	FALSE
ENSGALG00010008388	ADCY1	PRJNA729271	-1.416559002	0.001632552	1.416559002	stage_E14_vs_E10	ordinary_muscle_development	D_vs_E10;stage_1W_vs_E10;stage_3W_vs_E10;stage	1	TRUE
ENSGALG00010028394	ZBTB26	PRJNA729271	-0.346674711	0.001638851	0.346674711	stage_E14_vs_E10	ordinary_muscle_development		0	FALSE
ENSGALG00010011002	RPRM	PRJNA729271	-2.676327751	0.001641553	2.676327751	stage_E14_vs_E10	ordinary_muscle_development	D_vs_E10;stage_1W_vs_E10;stage_3W_vs_E10;stage	1	TRUE
ENSGALG00010025425	ENSGALG00010025425	PRJNA729271	0.462425269	0.00164171	0.462425269	stage_E14_vs_E10	ordinary_muscle_development		0	FALSE
ENSGALG00010028109	TJP3	PRJNA729271	-0.734359102	0.00164683	0.734359102	stage_E14_vs_E10	ordinary_muscle_development		0	FALSE
ENSGALG00010020630	QRICH1	PRJNA729271	0.410065855	0.00165207	0.410065855	stage_E14_vs_E10	ordinary_muscle_development		0	FALSE
ENSGALG00010028057	APC2	PRJNA729271	-1.270852223	0.001654163	1.270852223	stage_E14_vs_E10	ordinary_muscle_development	D_vs_E10;stage_1W_vs_E10;stage_3W_vs_E10;stage	1	TRUE
ENSGALG00010008261	CRX	PRJNA729271	-1.390680187	0.001662404	1.390680187	stage_E14_vs_E10	ordinary_muscle_development	D_vs_E10;stage_1W_vs_E10;stage_3W_vs_E10;stage	1	TRUE
ENSGALG00010018713	ENSGALG00010018713	PRJNA729271	-0.451006888	0.001662443	0.451006888	stage_E14_vs_E10	ordinary_muscle_development		0	FALSE
ENSGALG00010011758	SERPINB11	PRJNA729271	1.504502541	0.001667641	1.504502541	stage_E14_vs_E10	ordinary_muscle_development	D_vs_E10;stage_1W_vs_E10;stage_3W_vs_E10;stage	1	TRUE
ENSGALG00010024728	SLC5A9	PRJNA729271	-3.365418602	0.001677607	3.365418602	stage_E14_vs_E10	ordinary_muscle_development	D_vs_E10;stage_1W_vs_E10;stage_3W_vs_E10;stage	1	TRUE
ENSGALG00010008231	ZCCHC6	PRJNA729271	0.360791085	0.00167851	0.360791085	stage_E14_vs_E10	ordinary_muscle_development		0	FALSE
ENSGALG00010020413	CYP26A1	PRJNA729271	1.023471889	0.001686046	1.023471889	stage_E14_vs_E10	ordinary_muscle_development	D_vs_E10;stage_1W_vs_E10;stage_3W_vs_E10;stage	1	TRUE
ENSGALG00010022178	ENSGALG00010022178	PRJNA729271	1.073463795	0.001686329	1.073463795	stage_E14_vs_E10	ordinary_muscle_development	D_vs_E10;stage_1W_vs_E10;stage_3W_vs_E10;stage	1	TRUE
ENSGALG00010018977	NPHP3	PRJNA729271	-0.366868538	0.001689648	0.366868538	stage_E14_vs_E10	ordinary_muscle_development		0	FALSE
ENSGALG00010003170	C4	PRJNA729271	1.323706822	0.001689846	1.323706822	stage_E14_vs_E10	ordinary_muscle_development	D_vs_E10;stage_1W_vs_E10;stage_3W_vs_E10;stage	1	TRUE
ENSGALG00010023699	PPIG	PRJNA729271	-0.675907987	0.001700078	0.675907987	stage_E14_vs_E10	ordinary_muscle_development		0	FALSE
ENSGALG00010014031	RPL22L1	PRJNA729271	-0.614585551	0.00170159	0.614585551	stage_E14_vs_E10	ordinary_muscle_development		0	FALSE
ENSGALG00010016675	SKIL	PRJNA729271	-0.48438072	0.001701852	0.48438072	stage_E14_vs_E10	ordinary_muscle_development		0	FALSE
ENSGALG00010008830	DHX34	PRJNA729271	0.632066224	0.001706471	0.632066224	stage_E14_vs_E10	ordinary_muscle_development		0	FALSE
ENSGALG00010023820	CYP1C1	PRJNA729271	2.541025034	0.001719052	2.541025034	stage_E14_vs_E10	ordinary_muscle_development	D_vs_E10;stage_1W_vs_E10;stage_3W_vs_E10;stage	1	TRUE
ENSGALG00010019000	TRERF1	PRJNA729271	0.387267839	0.00171967	0.387267839	stage_E14_vs_E10	ordinary_muscle_development		0	FALSE
ENSGALG00010029666	SLC9A3R1	PRJNA729271	-0.659337724	0.001724479	0.659337724	stage_E14_vs_E10	ordinary_muscle_development		0	FALSE
ENSGALG00010005926	NUDT5	PRJNA729271	0.60211788	0.001726386	0.60211788	stage_E14_vs_E10	ordinary_muscle_development		0	FALSE
ENSGALG00010012885	PMS1	PRJNA729271	0.419656649	0.001732339	0.419656649	stage_E14_vs_E10	ordinary_muscle_development		0	FALSE
ENSGALG00010021679	MASP2	PRJNA729271	-4.096124845	0.001733768	4.096124845	stage_E14_vs_E10	ordinary_muscle_development	D_vs_E10;stage_1W_vs_E10;stage_3W_vs_E10;stage	1	TRUE
ENSGALG00010005449	TIPRL	PRJNA729271	0.278509005	0.001738307	0.278509005	stage_E14_vs_E10	ordinary_muscle_development		0	FALSE
ENSGALG00010003724	ENSGALG00010003724	PRJNA729271	0.725800656	0.0017451	0.725800656	stage_E14_vs_E10	ordinary_muscle_development		0	FALSE
ENSGALG00010002387	MCMBP	PRJNA729271	-0.361454078	0.001745411	0.361454078	stage_E14_vs_E10	ordinary_muscle_development		0	FALSE
ENSGALG00010003408	ENSGALG00010003408	PRJNA729271	12.71626137	0.001749886	12.71626137	stage_E14_vs_E10	ordinary_muscle_development	D_vs_E10;stage_1W_vs_E10;stage_3W_vs_E10;stage	1	TRUE
ENSGALG00010027957	ENTREP3	PRJNA729271	-1.701519004	0.001750254	1.701519004	stage_E14_vs_E10	ordinary_muscle_development	D_vs_E10;stage_1W_vs_E10;stage_3W_vs_E10;stage	1	TRUE
ENSGALG00010000583	CCS	PRJNA729271	0.350028652	0.001752072	0.350028652	stage_E14_vs_E10	ordinary_muscle_development		0	FALSE
ENSGALG00010029935	SYNRG	PRJNA729271	-0.24014388	0.001757597	0.24014388	stage_E14_vs_E10	ordinary_muscle_development		0	FALSE
ENSGALG00010002569	GTPBP6	PRJNA729271	0.376465502	0.001757637	0.376465502	stage_E14_vs_E10	ordinary_muscle_development		0	FALSE
ENSGALG00010011357	UNC80	PRJNA729271	-1.425710849	0.001760583	1.425710849	stage_E14_vs_E10	ordinary_muscle_development	D_vs_E10;stage_1W_vs_E10;stage_3W_vs_E10;stage	1	TRUE
ENSGALG00010025900	FAM237A	PRJNA729271	-1.82536797	0.001764613	1.82536797	stage_E14_vs_E10	ordinary_muscle_development	D_vs_E10;stage_1W_vs_E10;stage_3W_vs_E10;stage	1	TRUE
ENSGALG00010013103	AIRIM	PRJNA729271	0.463216628	0.001772951	0.463216628	stage_E14_vs_E10	ordinary_muscle_development		0	FALSE
ENSGALG00010029143	SH2D3C	PRJNA729271	-0.358730319	0.00178057	0.358730319	stage_E14_vs_E10	ordinary_muscle_development		0	FALSE
ENSGALG00010023279	FA2H	PRJNA729271	1.408881661	0.001781289	1.408881661	stage_E14_vs_E10	ordinary_muscle_development	D_vs_E10;stage_1W_vs_E10;stage_3W_vs_E10;stage	1	TRUE
ENSGALG00010016783	CLBA1	PRJNA729271	-1.040383541	0.001781299	1.040383541	stage_E14_vs_E10	ordinary_muscle_development	D_vs_E10;stage_1W_vs_E10;stage_3W_vs_E10;stage	1	TRUE
ENSGALG00010011429	UBAP2	PRJNA729271	0.349903292	0.00178725	0.349903292	stage_E14_vs_E10	ordinary_muscle_development		0	FALSE
ENSGALG00010015440	ENSGALG00010015440	PRJNA729271	1.17679667	0.001790157	1.17679667	stage_E14_vs_E10	ordinary_muscle_development	D_vs_E10;stage_1W_vs_E10;stage_3W_vs_E10;stage	1	TRUE
ENSGALG00010009463	MRPL13	PRJNA729271	-0.355310087	0.001801962	0.355310087	stage_E14_vs_E10	ordinary_muscle_development		0	FALSE
ENSGALG00010018606	DZANK1	PRJNA729271	-0.808509751	0.001806434	0.808509751	stage_E14_vs_E10	ordinary_muscle_development		0	FALSE
ENSGALG00010011085	UGT1A1	PRJNA729271	4.254041785	0.001807017	4.254041785	stage_E14_vs_E10	ordinary_muscle_development	D_vs_E10;stage_1W_vs_E10;stage_3W_vs_E10;stage	1	TRUE
ENSGALG00010011148	ENSGALG00010011148	PRJNA729271	-0.607427175	0.001809335	0.607427175	stage_E14_vs_E10	ordinary_muscle_development		0	FALSE
ENSGALG00010013386	ITGAV	PRJNA729271	-0.356844507	0.001811761	0.356844507	stage_E14_vs_E10	ordinary_muscle_development		0	FALSE
ENSGALG00010025710	ENSGALG00010025710	PRJNA729271	6.538828822	0.001814278	6.538828822	stage_E14_vs_E10	ordinary_muscle_development	D_vs_E10;stage_1W_vs_E10;stage_3W_vs_E10;stage	1	TRUE
ENSGALG00010028768	NTNG2	PRJNA729271	-1.213368994	0.001818443	1.213368994	stage_E14_vs_E10	ordinary_muscle_development	D_vs_E10;stage_1W_vs_E10;stage_3W_vs_E10;stage	1	TRUE
ENSGALG00010021309	ST3GAL3	PRJNA729271	0.4363968	0.001824607	0.4363968	stage_E14_vs_E10	ordinary_muscle_development		0	FALSE
ENSGALG00010016030	TGM4	PRJNA729271	2.276602582	0.001827758	2.276602582	stage_E14_vs_E10	ordinary_muscle_development	D_vs_E10;stage_1W_vs_E10;stage_3W_vs_E10;stage	1	TRUE
ENSGALG00010020061	UBA3	PRJNA729271	0.233371287	0.00183283	0.233371287	stage_E14_vs_E10	ordinary_muscle_development		0	FALSE

ENSGALG00010015803	SYF2	PRJNA729271	0.516907943	0.001833038	0.516907943	stage_E14_vs_E10	ordinary_muscle_development		0	FALSE
ENSGALG00010016860	VSX1	PRJNA729271	-1.661510332	0.001840232	1.661510332	stage_E14_vs_E10	ordinary_muscle_development	D_vs_E10;stage_1W_vs_E10;stage_3W_vs_E10;stage	1	TRUE
ENSGALG00010010894	SPAG1	PRJNA729271	0.451007901	0.001847305	0.451007901	stage_E14_vs_E10	ordinary_muscle_development		0	FALSE
ENSGALG00010028484	NOXA1	PRJNA729271	-2.053056392	0.001863935	2.053056392	stage_E14_vs_E10	ordinary_muscle_development	D_vs_E10;stage_1W_vs_E10;stage_3W_vs_E10;stage	1	TRUE
ENSGALG00010000133	USP39	PRJNA729271	0.450926812	0.001866134	0.450926812	stage_E14_vs_E10	ordinary_muscle_development		0	FALSE
ENSGALG00010020027	RER1	PRJNA729271	-0.230024569	0.001867547	0.230024569	stage_E14_vs_E10	ordinary_muscle_development		0	FALSE
ENSGALG00010012216	BRD8	PRJNA729271	-0.186803035	0.001875317	0.186803035	stage_E14_vs_E10	ordinary_muscle_development		0	FALSE
ENSGALG00010028164	BBLN	PRJNA729271	0.453549764	0.001876892	0.453549764	stage_E14_vs_E10	ordinary_muscle_development		0	FALSE
ENSGALG00010010938	FZD6	PRJNA729271	-0.400299318	0.001886578	0.400299318	stage_E14_vs_E10	ordinary_muscle_development		0	FALSE
ENSGALG00010010971	LCORL	PRJNA729271	0.409370264	0.001890223	0.409370264	stage_E14_vs_E10	ordinary_muscle_development		0	FALSE
ENSGALG00010021665	CNOT1	PRJNA729271	0.294305974	0.00189182	0.294305974	stage_E14_vs_E10	ordinary_muscle_development		0	FALSE
ENSGALG00010012151	MYRFL	PRJNA729271	-1.930703487	0.001894684	1.930703487	stage_E14_vs_E10	ordinary_muscle_development	D_vs_E10;stage_1W_vs_E10;stage_3W_vs_E10;stage	1	TRUE
ENSGALG00010002925	INTS6	PRJNA729271	-0.41997577	0.001895967	0.41997577	stage_E14_vs_E10	ordinary_muscle_development		0	FALSE
ENSGALG00010007680	CHMP2B	PRJNA729271	-0.409838116	0.001896042	0.409838116	stage_E14_vs_E10	ordinary_muscle_development		0	FALSE
ENSGALG00010013715	IL1R1	PRJNA729271	0.751349504	0.001912028	0.751349504	stage_E14_vs_E10	ordinary_muscle_development		0	FALSE
ENSGALG00010010394	MRPL42	PRJNA729271	0.39677336	0.001916831	0.39677336	stage_E14_vs_E10	ordinary_muscle_development		0	FALSE
ENSGALG00010012843	ENSGALG00010012843	PRJNA729271	0.504447877	0.001918319	0.504447877	stage_E14_vs_E10	ordinary_muscle_development		0	FALSE
ENSGALG00010018298	ENSGALG00010018298	PRJNA729271	0.635679992	0.001926967	0.635679992	stage_E14_vs_E10	ordinary_muscle_development		0	FALSE
ENSGALG00010011609	ENSGALG00010011609	PRJNA729271	2.623141503	0.001938437	2.623141503	stage_E14_vs_E10	ordinary_muscle_development	D_vs_E10;stage_1W_vs_E10;stage_3W_vs_E10;stage	1	TRUE
ENSGALG00010014940	VAMP4	PRJNA729271	-0.287260671	0.001946233	0.287260671	stage_E14_vs_E10	ordinary_muscle_development		0	FALSE
ENSGALG00010017770	PTCD3	PRJNA729271	-0.227227943	0.001969744	0.227227943	stage_E14_vs_E10	ordinary_muscle_development		0	FALSE
ENSGALG00010024752	MPP2	PRJNA729271	0.681807998	0.001971617	0.681807998	stage_E14_vs_E10	ordinary_muscle_development		0	FALSE
ENSGALG00010016441	ENSGALG00010016441	PRJNA729271	-2.385861248	0.001973453	2.385861248	stage_E14_vs_E10	ordinary_muscle_development	D_vs_E10;stage_1W_vs_E10;stage_3W_vs_E10;stage	1	TRUE
ENSGALG00010029274	VMP1	PRJNA729271	-0.484525978	0.00198048	0.484525978	stage_E14_vs_E10	ordinary_muscle_development		0	FALSE
ENSGALG00010009569	ZC3H18	PRJNA729271	-0.473817657	0.001981626	0.473817657	stage_E14_vs_E10	ordinary_muscle_development		0	FALSE
ENSGALG00010011899	PHLPP1	PRJNA729271	0.271749544	0.001984227	0.271749544	stage_E14_vs_E10	ordinary_muscle_development		0	FALSE
ENSGALG00010005651	USP6NL	PRJNA729271	0.390150224	0.001984478	0.390150224	stage_E14_vs_E10	ordinary_muscle_development		0	FALSE
ENSGALG00010021566	ENSGALG00010021566	PRJNA729271	0.306840951	0.001988434	0.306840951	stage_E14_vs_E10	ordinary_muscle_development		0	FALSE
ENSGALG00010019680	CCDC34	PRJNA729271	-0.653240578	0.001989164	0.653240578	stage_E14_vs_E10	ordinary_muscle_development		0	FALSE
ENSGALG00010026006	ITGA2B	PRJNA729271	-0.874181523	0.001995675	0.874181523	stage_E14_vs_E10	ordinary_muscle_development		0	FALSE
ENSGALG00010018032	ERO1A	PRJNA729271	0.385602949	0.001997813	0.385602949	stage_E14_vs_E10	ordinary_muscle_development		0	FALSE
ENSGALG00010004049	SETDB2	PRJNA729271	-0.456280043	0.002007618	0.456280043	stage_E14_vs_E10	ordinary_muscle_development		0	FALSE
ENSGALG00010010806	INSYN2B	PRJNA729271	-2.571194079	0.002011534	2.571194079	stage_E14_vs_E10	ordinary_muscle_development	D_vs_E10;stage_1W_vs_E10;stage_3W_vs_E10;stage	1	TRUE
ENSGALG00010016362	NUP62	PRJNA729271	-0.545456436	0.002025033	0.545456436	stage_E14_vs_E10	ordinary_muscle_development		0	FALSE
ENSGALG00010000202	ENSGALG00010000202	PRJNA729271	-0.750711616	0.002025788	0.750711616	stage_E14_vs_E10	ordinary_muscle_development		0	FALSE
ENSGALG00010018058	ITPKB	PRJNA729271	-0.296041314	0.00204208	0.296041314	stage_E14_vs_E10	ordinary_muscle_development		0	FALSE
ENSGALG00010024721	ENSGALG00010024721	PRJNA729271	2.36122117	0.002043042	2.36122117	stage_E14_vs_E10	ordinary_muscle_development	D_vs_E10;stage_1W_vs_E10;stage_3W_vs_E10;stage	1	TRUE
ENSGALG00010001866	PUS7L	PRJNA729271	-0.389543784	0.002047073	0.389543784	stage_E14_vs_E10	ordinary_muscle_development		0	FALSE
ENSGALG00010029684	ENSGALG00010029684	PRJNA729271	1.597611691	0.002064822	1.597611691	stage_E14_vs_E10	ordinary_muscle_development	D_vs_E10;stage_1W_vs_E10;stage_3W_vs_E10;stage	1	TRUE
ENSGALG00010012141	ENSGALG00010012141	PRJNA729271	0.299956038	0.002067571	0.299956038	stage_E14_vs_E10	ordinary_muscle_development		0	FALSE
ENSGALG00010011199	ENSGALG00010011199	PRJNA729271	-5.544047396	0.002076201	5.544047396	stage_E14_vs_E10	ordinary_muscle_development	D_vs_E10;stage_1W_vs_E10;stage_3W_vs_E10;stage	1	TRUE
ENSGALG00010009741	C2H6orf52	PRJNA729271	-0.23497918	0.002087485	0.23497918	stage_E14_vs_E10	ordinary_muscle_development		0	FALSE
ENSGALG00010006047	ZBTB11	PRJNA729271	-0.44384137	0.002091909	0.44384137	stage_E14_vs_E10	ordinary_muscle_development		0	FALSE
ENSGALG00010012316	ENSGALG00010012316	PRJNA729271	-3.514486283	0.002096109	3.514486283	stage_E14_vs_E10	ordinary_muscle_development	D_vs_E10;stage_1W_vs_E10;stage_3W_vs_E10;stage	1	TRUE
ENSGALG00010012094	ENSGALG00010012094	PRJNA729271	-1.180325857	0.002098673	1.180325857	stage_E14_vs_E10	ordinary_muscle_development	D_vs_E10;stage_1W_vs_E10;stage_3W_vs_E10;stage	1	TRUE
ENSGALG00010009730	C8orf76	PRJNA729271	-0.377800084	0.002103052	0.377800084	stage_E14_vs_E10	ordinary_muscle_development		0	FALSE
ENSGALG00010011967	MTMR9	PRJNA729271	0.437535644	0.002105781	0.437535644	stage_E14_vs_E10	ordinary_muscle_development		0	FALSE
ENSGALG00010017315	HS3ST4	PRJNA729271	-3.363842112	0.002118859	3.363842112	stage_E14_vs_E10	ordinary_muscle_development	D_vs_E10;stage_1W_vs_E10;stage_3W_vs_E10;stage	1	TRUE
ENSGALG00010008325	AADAT	PRJNA729271	0.427908896	0.002122282	0.427908896	stage_E14_vs_E10	ordinary_muscle_development		0	FALSE
ENSGALG00010028405	S100A6	PRJNA729271	0.891403766	0.002139138	0.891403766	stage_E14_vs_E10	ordinary_muscle_development		0	FALSE
ENSGALG00010000227	ENSGALG00010000227	PRJNA729271	0.888022331	0.002140479	0.888022331	stage_E14_vs_E10	ordinary_muscle_development		0	FALSE
ENSGALG00010000656	ENSGALG00010000656	PRJNA729271	1.30282524	0.002146421	1.30282524	stage_E14_vs_E10	ordinary_muscle_development	D_vs_E10;stage_1W_vs_E10;stage_3W_vs_E10;stage	1	TRUE
ENSGALG00010021447	OCSTAMP	PRJNA729271	1.985780496	0.002147168	1.985780496	stage_E14_vs_E10	ordinary_muscle_development	D_vs_E10;stage_1W_vs_E10;stage_3W_vs_E10;stage	1	TRUE
ENSGALG00010029242	DERL2	PRJNA729271	-0.317138227	0.00215509	0.317138227	stage_E14_vs_E10	ordinary_muscle_development		0	FALSE
ENSGALG00010024701	EIF2B1	PRJNA729271	-0.325807901	0.002169412	0.325807901	stage_E14_vs_E10	ordinary_muscle_development		0	FALSE
ENSGALG00010024124	DIDO1	PRJNA729271	0.213438694	0.002180118	0.213438694	stage_E14_vs_E10	ordinary_muscle_development		0	FALSE
ENSGALG00010022408	RAD51AP1	PRJNA729271	-0.438560724	0.002186846	0.438560724	stage_E14_vs_E10	ordinary_muscle_development		0	FALSE
ENSGALG00010020719	RPH3A	PRJNA729271	-2.129399472	0.002189411	2.129399472	stage_E14_vs_E10	ordinary_muscle_development	D_vs_E10;stage_1W_vs_E10;stage_3W_vs_E10;stage	1	TRUE
ENSGALG00010022848	SGIP1	PRJNA729271	-3.197379893	0.002195476	3.197379893	stage_E14_vs_E10	ordinary_muscle_development	D_vs_E10;stage_1W_vs_E10;stage_3W_vs_E10;stage	1	TRUE
ENSGALG00010009616	ARMC10	PRJNA729271	-0.593196943	0.002209086	0.593196943	stage_E14_vs_E10	ordinary_muscle_development		0	FALSE
ENSGALG00010029627	PEX12	PRJNA729271	-0.473436529	0.002210827	0.473436529	stage_E14_vs_E10	ordinary_muscle_development		0	FALSE
ENSGALG00010008093	EFR3A	PRJNA729271	-0.313854226	0.002213735	0.313854226	stage_E14_vs_E10	ordinary_muscle_development		0	FALSE
ENSGALG00010018513	RPUSD1	PRJNA729271	-0.508570228	0.002214453	0.508570228	stage_E14_vs_E10	ordinary_muscle_development		0	FALSE
ENSGALG00010020987	NADSYN1	PRJNA729271	-0.298034095	0.002226189	0.298034095	stage_E14_vs_E10	ordinary_muscle_development		0	FALSE
ENSGALG00010016674	SLC10A4	PRJNA729271	-1.253498396	0.002241607	1.253498396	stage_E14_vs_E10	ordinary_muscle_development	D_vs_E10;stage_1W_vs_E10;stage_3W_vs_E10;stage	1	TRUE
ENSGALG00010005514	EEF1AKMT1	PRJNA729271	0.455241601	0.002242769	0.455241601	stage_E14_vs_E10	ordinary_muscle_development		0	FALSE
ENSGALG00010024747	TULP3	PRJNA729271	-0.27691037	0.002262896	0.27691037	stage_E14_vs_E10	ordinary_muscle_development		0	FALSE
ENSGALG00010018111	NKX2-2	PRJNA729271	-2.62111562	0.00226478	2.62111562	stage_E14_vs_E10	ordinary_muscle_development	D_vs_E10;stage_1W_vs_E10;stage_3W_vs_E10;stage	1	TRUE
ENSGALG00010024039	MRPL45	PRJNA729271	-0.348049446	0.00226478	0.348049446	stage_E14_vs_E10	ordinary_muscle_development		0	FALSE
ENSGALG00010001972	FAM204A	PRJNA729271	0.483401576	0.00226808	0.483401576	stage_E14_vs_E10	ordinary_muscle_development		0	FALSE
ENSGALG00010006566	CPA5	PRJNA729271	8.86741647	0.00226907	8.86741647	stage_E14_vs_E10	ordinary_muscle_development	D_vs_E10;stage_1W_vs_E10;stage_3W_vs_E10;stage	1	TRUE
ENSGALG00010000233	HORMAD2	PRJNA729271	-1.763277534	0.002280644	1.763277534	stage_E14_vs_E10	ordinary_muscle_development	D_vs_E10;stage_1W_vs_E10;stage_3W_vs_E10;stage	1	TRUE
ENSGALG00010018007	DNAAF9	PRJNA729271	-0.549656777	0.0022902	0.549656777	stage_E14_vs_E10	ordinary_muscle_development		0	FALSE
ENSGALG00010019581	SLC35B2	PRJNA729271	-0.434333713	0.002300001	0.434333713	stage_E14_vs_E10	ordinary_muscle_development		0	FALSE
ENSGALG00010024274	ACSBG1	PRJNA729271	1.003143105	0.002307951	1.003143105	stage_E14_vs_E10	ordinary_muscle_development	D_vs_E10;stage_1W_vs_E10;stage_3W_vs_E10;stage	1	TRUE
ENSGALG00010001395	GNAL	PRJNA729271	0.335108924	0.002310129	0.335108924	stage_E14_vs_E10	ordinary_muscle_development		0	FALSE
ENSGALG00010008210	CTDP1	PRJNA729271	0.269609197	0.002327152	0.269609197	stage_E14_vs_E10	ordinary_muscle_development		0	FALSE
ENSGALG00010023543	ENSGALG00010023543	PRJNA729271	-2.886138394	0.002333967	2.886138394	stage_E14_vs_E10	ordinary_muscle_development	D_vs_E10;stage_1W_vs_E10;stage_3W_vs_E10;stage	1	TRUE
ENSGALG00010024949	PAN2	PRJNA729271	-0.365274238	0.002336551	0.365274238	stage_E14_vs_E10	ordinary_muscle_development		0	FALSE
ENSGALG00010024765	SNRNP35	PRJNA729271	-0.427650775	0.002337956	0.427650775	stage_E14_vs_E10	ordinary_muscle_development		0	FALSE
ENSGALG00010012912	NKAPL	PRJNA729271	0.313333678	0.00234467	0.313333678	stage_E14_vs_E10	ordinary_muscle_development		0	FALSE
ENSGALG00010020350	PDZRN3	PRJNA729271	0.340942648	0.002345968	0.340942648	stage_E14_vs_E10	ordinary_muscle_development		0	FALSE
ENSGALG00010017619	POLN	PRJNA729271	0.682318286	0.002360492	0.682318286	stage_E14_vs_E10	ordinary_muscle_development		0	FALSE
ENSGALG00010008437	SHKBP1	PRJNA729271	0.52079591	0.002377674	0.52079591	stage_E14_vs_E10	ordinary_muscle_development		0	FALSE
ENSGALG00010006242	ENSGALG00010006242	PRJNA729271	1.664198733	0.002380543	1.664198733	stage_E14_vs_E10	ordinary_muscle_development	D_vs_E10;stage_1W_vs_E10;stage_3W_vs_E10;stage	1	TRUE
ENSGALG00010014153	FOCAD	PRJNA729271	0.417292729	0.002397797	0.417292729	stage_E14_vs_E10	ordinary_muscle_development		0	FALSE
ENSGALG00010012309	TRAPPC13	PRJNA729271	-0.397857869	0.002398342	0.397857869	stage_E14_vs_E10	ordinary_muscle_development		0	FALSE
ENSGALG00010026601	ENSGALG00010026601	PRJNA729271	1.420648822	0.002399569	1.420648822	stage_E14_vs_E10	ordinary_muscle_development	D_vs_E10;stage_1W_vs_E10;stage_3W_vs_E10;stage	1	TRUE
ENSGALG00010025263	SAMD14	PRJNA729271	-2.803113809	0.002401563	2.803113809	stage_E14_vs_E10	ordinary_muscle_development	D_vs_E10;stage_1W_vs_E10;stage_3W_vs_E10;stage	1	TRUE
ENSGALG00010016477	CNIH3	PRJNA729271	0.600990079	0.002404497	0.600990079	stage_E14_vs_E10	ordinary_muscle_development		0	FALSE
ENSGALG00010016899	SAXO2	PRJNA729271	-2.001256143	0.002424355	2.001256143	stage_E14_vs_E10	ordinary_muscle_development	D_vs_E10;stage_1W_vs_E10;stage_3W_vs_E10;stage	1	TRUE
ENSGALG00010029292	ENSGALG00010029292	PRJNA729271	-0.798689015	0.002428457	0.798689015	stage_E14_vs_E10	ordinary_muscle_development		0	FALSE
ENSGALG00010010404	COP1	PRJNA729271	0.287196899	0.00242909	0.287196899	stage_E14_vs_E10	ordinary_muscle_development		0	FALSE
ENSGALG00010001397	KLHL7	PRJNA729271	-0.202839819	0.002444174	0.202839819	stage_E14_vs_E10	ordinary_muscle_development		0	FALSE
ENSGALG00010022560	SAMD10	PRJNA729271	-4.554805507	0.002450356	4.554805507	stage_E14_vs_E10	ordinary_muscle_development	D_vs_E10;stage_1W_vs_E10;stage_3W_vs_E10;stage	1	TRUE
ENSGALG00010016967	ERLEC1	PRJNA729271	0.235013768	0.002450356	0.235013768	stage_E14_vs_E10	ordinary_muscle_development		0	FALSE
ENSGALG00010021840	ENSGALG00010021840	PRJNA729271	0.697082422	0.002451033	0.697082422	stage_E14_vs_E10	ordinary_muscle_development		0	FALSE
ENSGALG00010020357	RBP4	PRJNA729271	2.320635179	0.002460572	2.320635179	stage_E14_vs_E10	ordinary_muscle_development	D_vs_E10;stage_1W_vs_E10;stage_3W_vs_E10;stage	1	TRUE
ENSGALG00010028427	ENSGALG00010028427	PRJNA729271	0.645452921	0.002467546	0.645452921	stage_E14_vs_E10	ordinary_muscle_development		0	FALSE
ENSGALG00010017628	NRDE2	PRJNA729271	-0.338843835	0.002486737	0.338843835	stage_E14_vs_E10	ordinary_muscle_development		0	FALSE
ENSGALG00010005371	GRM1	PRJNA729271	-4.201527145	0.00249175	4.201527145	stage_E14_vs_E10	ordinary_muscle_development	D_vs_E10;stage_1W_vs_E10;stage_3W_vs_E10;stage	1	TRUE
ENSGALG00010028122	SEC16A	PRJNA729271	0.279978674	0.002495134	0.279978674	stage_E14_vs_E10	ordinary_muscle_development		0	FALSE
ENSGALG00010002557	ENSGALG00010002557	PRJNA729271	1.277250203	0.002495438	1.277250203	stage_E14_vs_E10	ordinary_muscle_development	D_vs_E10;stage_1W_vs_E10;stage_3W_vs_E10;stage	1	TRUE
ENSGALG00010015788	TRPC1	PRJNA729271	-0.430298205	0.002504346	0.430298205	stage_E14_vs_E10	ordinary_muscle_development		0	FALSE
ENSGALG00010004230	TAF1B	PRJNA729271	0.361869326	0.002504391	0.361869326	stage_E14_vs_E10	ordinary_muscle_development		0	FALSE
ENSGALG00010017154	PIGZ	PRJNA729271	0.584353753	0.002505767	0.584353753	stage_E14_vs_E10	ordinary_muscle_development		0	FALSE
ENSGALG00010004123	NDUFAF4	PRJNA729271	-0.322926088	0.002525807	0.322926088	stage_E14_vs_E10	ordinary_muscle_development		0	FALSE
ENSGALG00010015384	TAF12	PRJNA729271	0.386691881	0.002528408	0.386691881	stage_E14_vs_E10	ordinary_muscle_development		0	FALSE
ENSGALG00010007586	MMP7	PRJNA729271	4.654016909	0.002569174	4.654016909	stage_E14_vs_E10	ordinary_muscle_development	D_vs_E10;stage_1W_vs_E10;stage_3W_vs_E10;stage	1	TRUE
ENSGALG00010020455	ENSGALG00010020455	PRJNA729271	-2.905747087	0.002574835	2.905747087	stage_E14_vs_E10	ordinary_muscle_development	D_vs_E10;stage_1W_vs_E10;stage_3W_vs_E10;stage	1	TRUE
ENSGALG00010028123	RASSF1	PRJNA729271	0.290909755	0.002575962	0.290909755	stage_E14_vs_E10	ordinary_muscle_development		0	FALSE
ENSGALG00010027216	ENSGALG00010027216	PRJNA729271	1.774903972	0.002600333	1.774903972	stage_E14_vs_E10	ordinary_muscle_development	D_vs_E10;stage_1W_vs_E10;stage_3W_vs_E10;stage	1	TRUE
ENSGALG00010004302	CRPPA	PRJNA729271	0.398695722	0.002608216	0.398695722	stage_E14_vs_E10	ordinary_muscle_development		0	FALSE
ENSGALG00010002142	CLDN34	PRJNA729271	0.763618598	0.00261302	0.763618598	stage_E14_vs_E10	ordinary_muscle_development		0	FALSE
ENSGALG00010028159	DCAF1	PRJNA729271	0.283049358	0.002615631	0.283049358	stage_E14_vs_E10	ordinary_muscle_development		0	FALSE
ENSGALG00010021953	PITPNM1	PRJNA729271	-0.599832809	0.002621304	0.599832809	stage_E14_vs_E10	ordinary_muscle_development		0	FALSE
ENSGALG00010024196	MRPL21	PRJNA729271	0.339685295	0.002623113	0.339685295	stage_E14_vs_E10	ordinary_muscle_development		0	FALSE
ENSGALG00010000324	ENSGALG00010000324	PRJNA729271	-0.477823274	0.002624191	0.477823274	stage_E14_vs_E10	ordinary_muscle_development		0	FALSE
ENSGALG00010000579	ENSGALG00010000579	PRJNA729271	-3.111430038	0.00262888	3.111430038	stage_E14_vs_E10	ordinary_muscle_development	D_vs_E10;stage_1W_vs_E10;stage_3W_vs_E10;stage	1	TRUE
ENSGALG00010022603	ATRNL1	PRJNA729271	-0.530024829	0.002645423	0.530024829	stage_E14_vs_E10	ordinary_muscle_development		0	FALSE
ENSGALG00010023487	GRN	PRJNA729271	-0.463437058	0.002647002	0.463437058	stage_E14_vs_E10	ordinary_muscle_development		0	FALSE
ENSGALG00010023536	ENSGALG00010023536	PRJNA729271	-0.710639469	0.002652574	0.710639469	stage_E14_vs_E10	ordinary_muscle_development		0	FALSE
ENSGALG00010022713	PNLIPRP1	PRJNA729271	8.687787021	0.002670818	8.687787021	stage_E14_vs_E10	ordinary_muscle_development	D_vs_E10;stage_1W_vs_E10;stage_3W_vs_E10;stage	1	TRUE
ENSGALG00010005344	ENSGALG00010005344	PRJNA729271	2.13953996	0.002673147	2.13953996	stage_E14_vs_E10	ordinary_muscle_development	D_vs_E10;stage_1W_vs_E10;stage_3W_vs_E10;stage	1	TRUE
ENSGALG00010004378	TRIM28	PRJNA729271	0.479447635	0.002678866	0.479447635	stage_E14_vs_E10	ordinary_muscle_development		0	FALSE
ENSGALG00010011080	EMP1	PRJNA729271	-0.802789492	0.002680773	0.802789492	stage_E14_vs_E10	ordinary_muscle_development		0	FALSE
ENSGALG00010029772	VPS53	PRJNA729271	0.219002555	0.002683235	0.219002555	stage_E14_vs_E10	ordinary_muscle_development		0	FALSE
ENSGALG00010020444	ENSGALG00010020444	PRJNA729271	-3.428253958	0.002699754	3.428253958	stage_E14_vs_E10	ordinary_muscle_development	D_vs_E10;stage_1W_vs_E10;stage_3W_vs_E10;stage	1	TRUE
ENSGALG00010016314	ACER2	PRJNA729271	0.487467688	0.002719651	0.487467688	stage_E14_vs_E10	ordinary_muscle_development		0	FALSE
ENSGALG00010003339	ENSGALG00010003339	PRJNA729271	-1.230531713	0.002719695	1.230531713	stage_E14_vs_E10	ordinary_muscle_development	D_vs_E10;stage_1W_vs_E10;stage_3W_vs_E10;stage	1	TRUE
ENSGALG00010000331	ENSGALG00010000331	PRJNA729271	-0.576355402	0.002724896	0.576355402	stage_E14_vs_E10	ordinary_muscle_development		0	FALSE
ENSGALG00010017693	MTFMT	PRJNA729271	0.370631814	0.002737462	0.370631814	stage_E14_vs_E10	ordinary_muscle_development		0	FALSE
ENSGALG00010013058	ENSGALG00010013058	PRJNA729271	0.853005554	0.002737918	0.853005554	stage_E14_vs_E10	ordinary_muscle_development		0	FALSE
ENSGALG00010005329	VIPAS39	PRJNA729271	0.364434231	0.002743103	0.364434231	stage_E14_vs_E10	ordinary_muscle_development		0	FALSE
ENSGALG00010026899	HOOK1	PRJNA729271	-0.554874993	0.002745916	0.554874993	stage_E14_vs_E10	ordinary_muscle_development		0	FALSE
ENSGALG00010021459	ARHGEF16	PRJNA729271	0.395482053	0.002750573	0.395482053	stage_E14_vs_E10	ordinary_muscle_development		0	FALSE
ENSGALG00010019189	ICMT	PRJNA729271	-0.303630999	0.002750573	0.303630999	stage_E14_vs_E10	ordinary_muscle_development		0	FALSE
ENSGALG00010028735	ENSGALG00010028735	PRJNA729271	-1.12846777	0.002753812	1.12846777	stage_E14_vs_E10	ordinary_muscle_development	D_vs_E10;stage_1W_vs_E10;stage_3W_vs_E10;stage	1	TRUE
ENSGALG00010025349	SETD1A	PRJNA729271	0.395223478	0.002764649	0.395223478	stage_E14_vs_E10	ordinary_muscle_development		0	FALSE
ENSGALG00010018037	GZF1	PRJNA729271	0.26074265	0.002772175	0.26074265	stage_E14_vs_E10	ordinary_muscle_development		0	FALSE
ENSGALG00010014887	MTMR12	PRJNA729271	-0.411283155	0.002787565	0.411283155	stage_E14_vs_E10	ordinary_muscle_development		0	FALSE
ENSGALG00010007110	ARL6	PRJNA729271	-0.319548272	0.002794645	0.319548272	stage_E14_vs_E10	ordinary_muscle_development		0	FALSE
ENSGALG00010009404	GNG4	PRJNA729271	-1.264042008	0.002800507	1.264042008	stage_E14_vs_E10	ordinary_muscle_development	D_vs_E10;stage_1W_vs_E10;stage_3W_vs_E10;stage	1	TRUE
ENSGALG00010000210	CCDC157	PRJNA729271	-1.801181175	0.002801627	1.801181175	stage_E14_vs_E10	ordinary_muscle_development	D_vs_E10;stage_1W_vs_E10;stage_3W_vs_E10;stage	1	TRUE
ENSGALG00010008062	VPS9D1	PRJNA729271	0.42542107	0.002803761	0.42542107	stage_E14_vs_E10	ordinary_muscle_development		0	FALSE
ENSGALG00010001717	STARD10	PRJNA729271	-2.298634173	0.002804713	2.298634173	stage_E14_vs_E10	ordinary_muscle_development	D_vs_E10;stage_1W_vs_E10;stage_3W_vs_E10;stage	1	TRUE
ENSGALG00010005016	STX11	PRJNA729271	-0.543912787	0.002806432	0.543912787	stage_E14_vs_E10	ordinary_muscle_development		0	FALSE
ENSGALG00010001524	ACE2	PRJNA729271	4.500280554	0.002806704	4.500280554	stage_E14_vs_E10	ordinary_muscle_development	D_vs_E10;stage_1W_vs_E10;stage_3W_vs_E10;stage	1	TRUE
ENSGALG00010005259	SLC27A5	PRJNA729271	2.073850354	0.002814841	2.073850354	stage_E14_vs_E10	ordinary_muscle_development	D_vs_E10;stage_1W_vs_E10;stage_3W_vs_E10;stage	1	TRUE
ENSGALG00010010880	ELP3	PRJNA729271	0.328061351	0.002848338	0.328061351	stage_E14_vs_E10	ordinary_muscle_development		0	FALSE
ENSGALG00010024344	YEATS2	PRJNA729271	0.410933536	0.002861915	0.410933536	stage_E14_vs_E10	ordinary_muscle_development		0	FALSE
ENSGALG00010029178	NOTUM	PRJNA729271	0.635468579	0.002873002	0.635468579	stage_E14_vs_E10	ordinary_muscle_development		0	FALSE
ENSGALG00010016795	GUCA2B	PRJNA729271	3.778716409	0.002878368	3.778716409	stage_E14_vs_E10	ordinary_muscle_development	D_vs_E10;stage_1W_vs_E10;stage_3W_vs_E10;stage	1	TRUE
ENSGALG00010013238	RAD1	PRJNA729271	-0.434705729	0.002913894	0.434705729	stage_E14_vs_E10	ordinary_muscle_development		0	FALSE
ENSGALG00010007601	TMEM154	PRJNA729271	-0.981416646	0.002917559	0.981416646	stage_E14_vs_E10	ordinary_muscle_development		0	FALSE
ENSGALG00010029152	HEXD	PRJNA729271	-0.40363087	0.002933514	0.40363087	stage_E14_vs_E10	ordinary_muscle_development		0	FALSE
ENSGALG00010028782	MLLT11	PRJNA729271	0.776622925	0.00294311	0.776622925	stage_E14_vs_E10	ordinary_muscle_development		0	FALSE
ENSGALG00010002408	OFD1	PRJNA729271	-0.361169552	0.002967644	0.361169552	stage_E14_vs_E10	ordinary_muscle_development		0	FALSE
ENSGALG00010001674	ENSGALG00010001674	PRJNA729271	0.323011015	0.002972188	0.323011015	stage_E14_vs_E10	ordinary_muscle_development		0	FALSE
ENSGALG00010019684	PIGQ	PRJNA729271	-0.406726678	0.00298045	0.406726678	stage_E14_vs_E10	ordinary_muscle_development		0	FALSE
ENSGALG00010000189	ENSGALG00010000189	PRJNA729271	1.758609308	0.002990155	1.758609308	stage_E14_vs_E10	ordinary_muscle_development	D_vs_E10;stage_1W_vs_E10;stage_3W_vs_E10;stage	1	TRUE
ENSGALG00010029692	CRYBA1	PRJNA729271	-2.13144151	0.002990215	2.13144151	stage_E14_vs_E10	ordinary_muscle_development	D_vs_E10;stage_1W_vs_E10;stage_3W_vs_E10;stage	1	TRUE
ENSGALG00010015796	LARP1	PRJNA729271	0.438510848	0.002991635	0.438510848	stage_E14_vs_E10	ordinary_muscle_development		0	FALSE
ENSGALG00010004883	KRIT1	PRJNA729271	-0.242269532	0.003056917	0.242269532	stage_E14_vs_E10	ordinary_muscle_development		0	FALSE
ENSGALG00010021625	HCN4	PRJNA729271	-3.161964025	0.003061083	3.161964025	stage_E14_vs_E10	ordinary_muscle_development	D_vs_E10;stage_1W_vs_E10;stage_3W_vs_E10;stage	1	TRUE
ENSGALG00010015701	UNC50	PRJNA729271	-0.2713993	0.0030666	0.2713993	stage_E14_vs_E10	ordinary_muscle_development		0	FALSE
ENSGALG00010010259	IGF1	PRJNA729271	0.873817397	0.003068338	0.873817397	stage_E14_vs_E10	ordinary_muscle_development		0	FALSE
ENSGALG00010020609	AVPI1	PRJNA729271	-0.732766006	0.003077779	0.732766006	stage_E14_vs_E10	ordinary_muscle_development		0	FALSE
ENSGALG00010009493	ENSGALG00010009493	PRJNA729271	1.186852041	0.003081911	1.186852041	stage_E14_vs_E10	ordinary_muscle_development	D_vs_E10;stage_1W_vs_E10;stage_3W_vs_E10;stage	1	TRUE
ENSGALG00010009407	PTPN12	PRJNA729271	0.272538629	0.00308925	0.272538629	stage_E14_vs_E10	ordinary_muscle_development		0	FALSE
ENSGALG00010027467	GLYCTK	PRJNA729271	-0.423072434	0.003112598	0.423072434	stage_E14_vs_E10	ordinary_muscle_development		0	FALSE
ENSGALG00010022628	SVOP	PRJNA729271	1.680647899	0.003114871	1.680647899	stage_E14_vs_E10	ordinary_muscle_development	D_vs_E10;stage_1W_vs_E10;stage_3W_vs_E10;stage	1	TRUE
ENSGALG00010024764	ENSGALG00010024764	PRJNA729271	0.788512384	0.003140741	0.788512384	stage_E14_vs_E10	ordinary_muscle_development		0	FALSE
ENSGALG00010028292	ENSGALG00010028292	PRJNA729271	-1.731225979	0.003142503	1.731225979	stage_E14_vs_E10	ordinary_muscle_development	D_vs_E10;stage_1W_vs_E10;stage_3W_vs_E10;stage	1	TRUE
ENSGALG00010013062	ENSGALG00010013062	PRJNA729271	-0.335220871	0.003143087	0.335220871	stage_E14_vs_E10	ordinary_muscle_development		0	FALSE

ENSGALG00010006311	ABCD4	PRJNA729271	-0.359351132	0.003147275	0.359351132	stage_E14_vs_E10	ordinary_muscle_development		0	FALSE
ENSGALG00010020936	BET1L	PRJNA729271	-0.387256184	0.003195405	0.387256184	stage_E14_vs_E10	ordinary_muscle_development		0	FALSE
ENSGALG00010010762	MRPL39	PRJNA729271	0.218424691	0.003224169	0.218424691	stage_E14_vs_E10	ordinary_muscle_development		0	FALSE
ENSGALG00010026673	DEPDC5	PRJNA729271	0.373069763	0.003225964	0.373069763	stage_E14_vs_E10	ordinary_muscle_development		0	FALSE
ENSGALG00010001147	PGGT1B	PRJNA729271	-0.357092338	0.003227528	0.357092338	stage_E14_vs_E10	ordinary_muscle_development		0	FALSE
ENSGALG00010014236	WDR44	PRJNA729271	-0.252648074	0.003235026	0.252648074	stage_E14_vs_E10	ordinary_muscle_development		0	FALSE
ENSGALG00010018396	TBL1XR1	PRJNA729271	-0.23436132	0.003242801	0.23436132	stage_E14_vs_E10	ordinary_muscle_development		0	FALSE
ENSGALG00010008135	WWC1	PRJNA729271	-0.410313709	0.003259933	0.410313709	stage_E14_vs_E10	ordinary_muscle_development		0	FALSE
ENSGALG00010002396	SEC23IP	PRJNA729271	-0.278140633	0.003260313	0.278140633	stage_E14_vs_E10	ordinary_muscle_development		0	FALSE
ENSGALG00010007910	ANKS6	PRJNA729271	0.458004055	0.003262732	0.458004055	stage_E14_vs_E10	ordinary_muscle_development		0	FALSE
ENSGALG00010028399	SLC35E1	PRJNA729271	0.241731739	0.003271143	0.241731739	stage_E14_vs_E10	ordinary_muscle_development		0	FALSE
ENSGALG00010025858	ENSGALG00010025858	PRJNA729271	9.367981901	0.003291488	9.367981901	stage_E14_vs_E10	ordinary_muscle_development	D_vs_E10;stage_1W_vs_E10;stage_3W_vs_E10;stage	1	TRUE
ENSGALG00010024613	ENSGALG00010024613	PRJNA729271	-0.29394994	0.003291881	0.29394994	stage_E14_vs_E10	ordinary_muscle_development		0	FALSE
ENSGALG00010010453	DRC1	PRJNA729271	-1.443976436	0.003309059	1.443976436	stage_E14_vs_E10	ordinary_muscle_development	D_vs_E10;stage_1W_vs_E10;stage_3W_vs_E10;stage	1	TRUE
ENSGALG00010022712	DDX23	PRJNA729271	0.392321617	0.003318805	0.392321617	stage_E14_vs_E10	ordinary_muscle_development		0	FALSE
ENSGALG00010001763	ENSGALG00010001763	PRJNA729271	0.620939069	0.003345766	0.620939069	stage_E14_vs_E10	ordinary_muscle_development		0	FALSE
ENSGALG00010002440	LRRC51	PRJNA729271	0.825977421	0.003360122	0.825977421	stage_E14_vs_E10	ordinary_muscle_development		0	FALSE
ENSGALG00010021640	PLEKHG3	PRJNA729271	0.331143653	0.003360122	0.331143653	stage_E14_vs_E10	ordinary_muscle_development		0	FALSE
ENSGALG00010014080	MYO1B	PRJNA729271	-0.448129601	0.003360578	0.448129601	stage_E14_vs_E10	ordinary_muscle_development		0	FALSE
ENSGALG00010022161	CELF1	PRJNA729271	0.307063469	0.00338715	0.307063469	stage_E14_vs_E10	ordinary_muscle_development		0	FALSE
ENSGALG00010006343	SEC23A	PRJNA729271	-0.291519441	0.003392215	0.291519441	stage_E14_vs_E10	ordinary_muscle_development		0	FALSE
ENSGALG00010015422	ENSGALG00010015422	PRJNA729271	-1.667860984	0.003394707	1.667860984	stage_E14_vs_E10	ordinary_muscle_development	D_vs_E10;stage_1W_vs_E10;stage_3W_vs_E10;stage	1	TRUE
ENSGALG00010028176	UPF1	PRJNA729271	0.290135936	0.003407093	0.290135936	stage_E14_vs_E10	ordinary_muscle_development		0	FALSE
ENSGALG00010009778	ENSGALG00010009778	PRJNA729271	0.339110865	0.003412391	0.339110865	stage_E14_vs_E10	ordinary_muscle_development		0	FALSE
ENSGALG00010028855	SURF6	PRJNA729271	-0.517068921	0.003416883	0.517068921	stage_E14_vs_E10	ordinary_muscle_development		0	FALSE
ENSGALG00010011237	ENSGALG00010011237	PRJNA729271	-0.806976774	0.003435606	0.806976774	stage_E14_vs_E10	ordinary_muscle_development		0	FALSE
ENSGALG00010009762	MAN2A1	PRJNA729271	0.313910959	0.003496395	0.313910959	stage_E14_vs_E10	ordinary_muscle_development		0	FALSE
ENSGALG00010020828	CAMK2N1	PRJNA729271	-0.693011171	0.003502174	0.693011171	stage_E14_vs_E10	ordinary_muscle_development		0	FALSE
ENSGALG00010001681	NAPG	PRJNA729271	-0.285592945	0.003510892	0.285592945	stage_E14_vs_E10	ordinary_muscle_development		0	FALSE
ENSGALG00010015194	TMLHE	PRJNA729271	0.325741703	0.003514075	0.325741703	stage_E14_vs_E10	ordinary_muscle_development		0	FALSE
ENSGALG00010000785	CLN5	PRJNA729271	0.283066394	0.003525351	0.283066394	stage_E14_vs_E10	ordinary_muscle_development		0	FALSE
ENSGALG00010010994	NAA20	PRJNA729271	-0.28952079	0.003532834	0.28952079	stage_E14_vs_E10	ordinary_muscle_development		0	FALSE
ENSGALG00010023867	ENSGALG00010023867	PRJNA729271	1.473246058	0.003545838	1.473246058	stage_E14_vs_E10	ordinary_muscle_development	D_vs_E10;stage_1W_vs_E10;stage_3W_vs_E10;stage	1	TRUE
ENSGALG00010025207	BMPR2	PRJNA729271	-0.312302526	0.003546694	0.312302526	stage_E14_vs_E10	ordinary_muscle_development		0	FALSE
ENSGALG00010009629	ENSGALG00010009629	PRJNA729271	1.619241616	0.003578858	1.619241616	stage_E14_vs_E10	ordinary_muscle_development	D_vs_E10;stage_1W_vs_E10;stage_3W_vs_E10;stage	1	TRUE
ENSGALG00010011917	HSF2BP	PRJNA729271	-0.544249257	0.003585397	0.544249257	stage_E14_vs_E10	ordinary_muscle_development		0	FALSE
ENSGALG00010021162	WIPI2	PRJNA729271	-0.220105449	0.003590476	0.220105449	stage_E14_vs_E10	ordinary_muscle_development		0	FALSE
ENSGALG00010001756	ENSGALG00010001756	PRJNA729271	0.478453248	0.003596097	0.478453248	stage_E14_vs_E10	ordinary_muscle_development		0	FALSE
ENSGALG00010015912	ENSGALG00010015912	PRJNA729271	0.783021191	0.003605037	0.783021191	stage_E14_vs_E10	ordinary_muscle_development		0	FALSE
ENSGALG00010003256	ENSGALG00010003256	PRJNA729271	4.249995849	0.003612577	4.249995849	stage_E14_vs_E10	ordinary_muscle_development	D_vs_E10;stage_1W_vs_E10;stage_3W_vs_E10;stage	1	TRUE
ENSGALG00010005154	CWF19L2	PRJNA729271	-0.452303044	0.003619164	0.452303044	stage_E14_vs_E10	ordinary_muscle_development		0	FALSE
ENSGALG00010026443	UPB1	PRJNA729271	1.113930601	0.00361978	1.113930601	stage_E14_vs_E10	ordinary_muscle_development	D_vs_E10;stage_1W_vs_E10;stage_3W_vs_E10;stage	1	TRUE
ENSGALG00010003280	ENSGALG00010003280	PRJNA729271	-0.363829635	0.003631269	0.363829635	stage_E14_vs_E10	ordinary_muscle_development		0	FALSE
ENSGALG00010008038	WRN	PRJNA729271	-0.239983839	0.003641373	0.239983839	stage_E14_vs_E10	ordinary_muscle_development		0	FALSE
ENSGALG00010017632	AWAT1	PRJNA729271	1.793914206	0.00371229	1.793914206	stage_E14_vs_E10	ordinary_muscle_development	D_vs_E10;stage_1W_vs_E10;stage_3W_vs_E10;stage	1	TRUE
ENSGALG00010021038	PTGS2	PRJNA729271	-0.916747042	0.003720188	0.916747042	stage_E14_vs_E10	ordinary_muscle_development		0	FALSE
ENSGALG00010015505	CHGA	PRJNA729271	-1.549387681	0.003726832	1.549387681	stage_E14_vs_E10	ordinary_muscle_development	D_vs_E10;stage_1W_vs_E10;stage_3W_vs_E10;stage	1	TRUE
ENSGALG00010025169	RBP2	PRJNA729271	6.312003888	0.003735707	6.312003888	stage_E14_vs_E10	ordinary_muscle_development	D_vs_E10;stage_1W_vs_E10;stage_3W_vs_E10;stage	1	TRUE
ENSGALG00010006404	SMAD1	PRJNA729271	0.335390463	0.003738307	0.335390463	stage_E14_vs_E10	ordinary_muscle_development		0	FALSE
ENSGALG00010013406	ENSGALG00010013406	PRJNA729271	0.833905051	0.003740167	0.833905051	stage_E14_vs_E10	ordinary_muscle_development		0	FALSE
ENSGALG00010024772	ELAPOR1	PRJNA729271	-0.713716888	0.003740189	0.713716888	stage_E14_vs_E10	ordinary_muscle_development		0	FALSE
ENSGALG00010024738	NBR1	PRJNA729271	0.237785982	0.003740189	0.237785982	stage_E14_vs_E10	ordinary_muscle_development		0	FALSE
ENSGALG00010026770	ENSGALG00010026770	PRJNA729271	-1.93863406	0.003741973	1.93863406	stage_E14_vs_E10	ordinary_muscle_development	D_vs_E10;stage_1W_vs_E10;stage_3W_vs_E10;stage	1	TRUE
ENSGALG00010021018	F3	PRJNA729271	0.665211239	0.003776903	0.665211239	stage_E14_vs_E10	ordinary_muscle_development		0	FALSE
ENSGALG00010004355	ANKMY2	PRJNA729271	0.373234888	0.003799588	0.373234888	stage_E14_vs_E10	ordinary_muscle_development		0	FALSE
ENSGALG00010008029	LY6E	PRJNA729271	0.946200591	0.003803833	0.946200591	stage_E14_vs_E10	ordinary_muscle_development		0	FALSE
ENSGALG00010019495	EMP2	PRJNA729271	0.602961859	0.003823686	0.602961859	stage_E14_vs_E10	ordinary_muscle_development		0	FALSE
ENSGALG00010029489	ENSGALG00010029489	PRJNA729271	-3.51099191	0.003835297	3.51099191	stage_E14_vs_E10	ordinary_muscle_development	D_vs_E10;stage_1W_vs_E10;stage_3W_vs_E10;stage	1	TRUE
ENSGALG00010024159	NLRX1	PRJNA729271	0.498915496	0.003838453	0.498915496	stage_E14_vs_E10	ordinary_muscle_development		0	FALSE
ENSGALG00010003395	ENSGALG00010003395	PRJNA729271	2.981445479	0.003847294	2.981445479	stage_E14_vs_E10	ordinary_muscle_development	D_vs_E10;stage_1W_vs_E10;stage_3W_vs_E10;stage	1	TRUE
ENSGALG00010000652	SRD5A1	PRJNA729271	0.691114268	0.003848735	0.691114268	stage_E14_vs_E10	ordinary_muscle_development		0	FALSE
ENSGALG00010000762	ENSGALG00010000762	PRJNA729271	0.892472439	0.003852418	0.892472439	stage_E14_vs_E10	ordinary_muscle_development		0	FALSE
ENSGALG00010021789	CHD9	PRJNA729271	0.275020722	0.003852418	0.275020722	stage_E14_vs_E10	ordinary_muscle_development		0	FALSE
ENSGALG00010016998	DNASE1L2	PRJNA729271	1.045910535	0.003855718	1.045910535	stage_E14_vs_E10	ordinary_muscle_development	D_vs_E10;stage_1W_vs_E10;stage_3W_vs_E10;stage	1	TRUE
ENSGALG00010020250	ENSGALG00010020250	PRJNA729271	0.661881972	0.003877635	0.661881972	stage_E14_vs_E10	ordinary_muscle_development		0	FALSE
ENSGALG00010001118	P2RY12	PRJNA729271	-1.049882922	0.003881519	1.049882922	stage_E14_vs_E10	ordinary_muscle_development	D_vs_E10;stage_1W_vs_E10;stage_3W_vs_E10;stage	1	TRUE
ENSGALG00010029141	C5	PRJNA729271	-1.748879678	0.003885346	1.748879678	stage_E14_vs_E10	ordinary_muscle_development	D_vs_E10;stage_1W_vs_E10;stage_3W_vs_E10;stage	1	TRUE
ENSGALG00010007126	ENSGALG00010007126	PRJNA729271	3.244166439	0.003886689	3.244166439	stage_E14_vs_E10	ordinary_muscle_development	D_vs_E10;stage_1W_vs_E10;stage_3W_vs_E10;stage	1	TRUE
ENSGALG00010026220	ENSGALG00010026220	PRJNA729271	0.186836146	0.003887885	0.186836146	stage_E14_vs_E10	ordinary_muscle_development		0	FALSE
ENSGALG00010029304	ORAI3	PRJNA729271	-0.364273894	0.003907597	0.364273894	stage_E14_vs_E10	ordinary_muscle_development		0	FALSE
ENSGALG00010022129	ELMO3	PRJNA729271	0.71618448	0.003923072	0.71618448	stage_E14_vs_E10	ordinary_muscle_development		0	FALSE
ENSGALG00010020438	HTR7	PRJNA729271	-0.923125728	0.003940278	0.923125728	stage_E14_vs_E10	ordinary_muscle_development		0	FALSE
ENSGALG00010028593	DOLK	PRJNA729271	-0.376852673	0.00395462	0.376852673	stage_E14_vs_E10	ordinary_muscle_development		0	FALSE
ENSGALG00010023833	PIP4K2C	PRJNA729271	0.338752087	0.003962767	0.338752087	stage_E14_vs_E10	ordinary_muscle_development		0	FALSE
ENSGALG00010024255	CFAP97D1	PRJNA729271	2.008913611	0.003963661	2.008913611	stage_E14_vs_E10	ordinary_muscle_development	D_vs_E10;stage_1W_vs_E10;stage_3W_vs_E10;stage	1	TRUE
ENSGALG00010002664	ZNFB1	PRJNA729271	0.433643848	0.003963756	0.433643848	stage_E14_vs_E10	ordinary_muscle_development		0	FALSE
ENSGALG00010021807	CDC42	PRJNA729271	-0.250826136	0.003967879	0.250826136	stage_E14_vs_E10	ordinary_muscle_development		0	FALSE
ENSGALG00010028892	NIBAN2	PRJNA729271	0.421445559	0.003973569	0.421445559	stage_E14_vs_E10	ordinary_muscle_development		0	FALSE
ENSGALG00010009840	ENSGALG00010009840	PRJNA729271	0.537172867	0.003983753	0.537172867	stage_E14_vs_E10	ordinary_muscle_development		0	FALSE
ENSGALG00010023210	ZNHIT6	PRJNA729271	-0.430215682	0.00398394	0.430215682	stage_E14_vs_E10	ordinary_muscle_development		0	FALSE
ENSGALG00010008124	ENSGALG00010008124	PRJNA729271	-0.695777371	0.003997508	0.695777371	stage_E14_vs_E10	ordinary_muscle_development		0	FALSE
ENSGALG00010028186	CCT3	PRJNA729271	-0.403899858	0.003997772	0.403899858	stage_E14_vs_E10	ordinary_muscle_development		0	FALSE
ENSGALG00010010961	C2orf66	PRJNA729271	1.086028966	0.00401338	1.086028966	stage_E14_vs_E10	ordinary_muscle_development	D_vs_E10;stage_1W_vs_E10;stage_3W_vs_E10;stage	1	TRUE
ENSGALG00010026076	C1orf35	PRJNA729271	0.413714716	0.004015302	0.413714716	stage_E14_vs_E10	ordinary_muscle_development		0	FALSE
ENSGALG00010000718	EEF1G	PRJNA729271	0.455150982	0.004054037	0.455150982	stage_E14_vs_E10	ordinary_muscle_development		0	FALSE
ENSGALG00010028182	SMG5	PRJNA729271	0.437088075	0.004075258	0.437088075	stage_E14_vs_E10	ordinary_muscle_development		0	FALSE
ENSGALG00010003778	LCA5	PRJNA729271	0.365707181	0.004075748	0.365707181	stage_E14_vs_E10	ordinary_muscle_development		0	FALSE
ENSGALG00010025970	KLHL12	PRJNA729271	-0.33239925	0.004079335	0.33239925	stage_E14_vs_E10	ordinary_muscle_development		0	FALSE
ENSGALG00010018287	MKKS	PRJNA729271	0.305428208	0.004134157	0.305428208	stage_E14_vs_E10	ordinary_muscle_development		0	FALSE
ENSGALG00010023878	ENSGALG00010023878	PRJNA729271	4.258111569	0.004134697	4.258111569	stage_E14_vs_E10	ordinary_muscle_development	D_vs_E10;stage_1W_vs_E10;stage_3W_vs_E10;stage	1	TRUE
ENSGALG00010008118	ENSGALG00010008118	PRJNA729271	1.585468345	0.004137424	1.585468345	stage_E14_vs_E10	ordinary_muscle_development	D_vs_E10;stage_1W_vs_E10;stage_3W_vs_E10;stage	1	TRUE
ENSGALG00010013185	ENSGALG00010013185	PRJNA729271	-0.972951361	0.004144021	0.972951361	stage_E14_vs_E10	ordinary_muscle_development		0	FALSE
ENSGALG00010002054	RSF1	PRJNA729271	-0.318078963	0.004166844	0.318078963	stage_E14_vs_E10	ordinary_muscle_development		0	FALSE
ENSGALG00010025513	RSRC2	PRJNA729271	-0.247295381	0.004183788	0.247295381	stage_E14_vs_E10	ordinary_muscle_development		0	FALSE
ENSGALG00010029512	TSPAN10	PRJNA729271	-2.269098162	0.004204522	2.269098162	stage_E14_vs_E10	ordinary_muscle_development	D_vs_E10;stage_1W_vs_E10;stage_3W_vs_E10;stage	1	TRUE
ENSGALG00010024897	ADAL	PRJNA729271	0.57071123	0.004211106	0.57071123	stage_E14_vs_E10	ordinary_muscle_development		0	FALSE
ENSGALG00010021661	DUSP16	PRJNA729271	-0.386797357	0.004216311	0.386797357	stage_E14_vs_E10	ordinary_muscle_development		0	FALSE
ENSGALG00010005956	TXNL4B	PRJNA729271	0.330855933	0.004222385	0.330855933	stage_E14_vs_E10	ordinary_muscle_development		0	FALSE
ENSGALG00010027248	ENSGALG00010027248	PRJNA729271	-2.790786888	0.004260836	2.790786888	stage_E14_vs_E10	ordinary_muscle_development	D_vs_E10;stage_1W_vs_E10;stage_3W_vs_E10;stage	1	TRUE
ENSGALG00010013947	ENSGALG00010013947	PRJNA729271	-0.561373835	0.004283876	0.561373835	stage_E14_vs_E10	ordinary_muscle_development		0	FALSE
ENSGALG00010005643	ENSGALG00010005643	PRJNA729271	-3.999030862	0.004300453	3.999030862	stage_E14_vs_E10	ordinary_muscle_development	D_vs_E10;stage_1W_vs_E10;stage_3W_vs_E10;stage	1	TRUE
ENSGALG00010029568	ENSGALG00010029568	PRJNA729271	-2.856740778	0.004303019	2.856740778	stage_E14_vs_E10	ordinary_muscle_development	D_vs_E10;stage_1W_vs_E10;stage_3W_vs_E10;stage	1	TRUE
ENSGALG00010017756	EIF2B5	PRJNA729271	-0.35412076	0.004303705	0.35412076	stage_E14_vs_E10	ordinary_muscle_development		0	FALSE
ENSGALG00010006664	ENSGALG00010006664	PRJNA729271	3.946636016	0.004329901	3.946636016	stage_E14_vs_E10	ordinary_muscle_development	D_vs_E10;stage_1W_vs_E10;stage_3W_vs_E10;stage	1	TRUE
ENSGALG00010024365	ENSGALG00010024365	PRJNA729271	-0.319383241	0.004335692	0.319383241	stage_E14_vs_E10	ordinary_muscle_development		0	FALSE
ENSGALG00010015327	LARGE1	PRJNA729271	0.295122841	0.004337735	0.295122841	stage_E14_vs_E10	ordinary_muscle_development		0	FALSE
ENSGALG00010011797	SCAF4	PRJNA729271	0.465067501	0.004355778	0.465067501	stage_E14_vs_E10	ordinary_muscle_development		0	FALSE
ENSGALG00010015729	RHCE	PRJNA729271	0.566146808	0.004444622	0.566146808	stage_E14_vs_E10	ordinary_muscle_development		0	FALSE
ENSGALG00010002421	ENSGALG00010002421	PRJNA729271	-0.389081066	0.004460976	0.389081066	stage_E14_vs_E10	ordinary_muscle_development		0	FALSE
ENSGALG00010013552	FGF16	PRJNA729271	-0.842167167	0.004499142	0.842167167	stage_E14_vs_E10	ordinary_muscle_development		0	FALSE
ENSGALG00010024454	HPS5	PRJNA729271	0.355637843	0.004503718	0.355637843	stage_E14_vs_E10	ordinary_muscle_development		0	FALSE
ENSGALG00010021296	GATC	PRJNA729271	-0.366851971	0.004510243	0.366851971	stage_E14_vs_E10	ordinary_muscle_development		0	FALSE
ENSGALG00010025072	ENSGALG00010025072	PRJNA729271	-3.041767974	0.004516929	3.041767974	stage_E14_vs_E10	ordinary_muscle_development	D_vs_E10;stage_1W_vs_E10;stage_3W_vs_E10;stage	1	TRUE
ENSGALG00010000727	ENSGALG00010000727	PRJNA729271	-0.444543985	0.004524362	0.444543985	stage_E14_vs_E10	ordinary_muscle_development		0	FALSE
ENSGALG00010026183	SESTD1	PRJNA729271	0.369295002	0.004534789	0.369295002	stage_E14_vs_E10	ordinary_muscle_development		0	FALSE
ENSGALG00010006009	MALRD1	PRJNA729271	1.784327585	0.00453669	1.784327585	stage_E14_vs_E10	ordinary_muscle_development	D_vs_E10;stage_1W_vs_E10;stage_3W_vs_E10;stage	1	TRUE
ENSGALG00010009548	PIK3C3	PRJNA729271	-0.242775015	0.004540175	0.242775015	stage_E14_vs_E10	ordinary_muscle_development		0	FALSE
ENSGALG00010013336	MTNR1A	PRJNA729271	2.024462842	0.004548148	2.024462842	stage_E14_vs_E10	ordinary_muscle_development	D_vs_E10;stage_1W_vs_E10;stage_3W_vs_E10;stage	1	TRUE
ENSGALG00010011440	FUNDC1	PRJNA729271	-0.333291237	0.004571605	0.333291237	stage_E14_vs_E10	ordinary_muscle_development		0	FALSE
ENSGALG00010005556	IYD	PRJNA729271	-2.900214679	0.004614469	2.900214679	stage_E14_vs_E10	ordinary_muscle_development	D_vs_E10;stage_1W_vs_E10;stage_3W_vs_E10;stage	1	TRUE
ENSGALG00010024452	SLC16A1	PRJNA729271	-0.800025773	0.004623156	0.800025773	stage_E14_vs_E10	ordinary_muscle_development		0	FALSE
ENSGALG00010002922	TMEM106B	PRJNA729271	0.26997021	0.00462424	0.26997021	stage_E14_vs_E10	ordinary_muscle_development		0	FALSE
ENSGALG00010016887	TJP2	PRJNA729271	0.412574119	0.004630169	0.412574119	stage_E14_vs_E10	ordinary_muscle_development		0	FALSE
ENSGALG00010005331	ENSGALG00010005331	PRJNA729271	2.312921987	0.004631845	2.312921987	stage_E14_vs_E10	ordinary_muscle_development	D_vs_E10;stage_1W_vs_E10;stage_3W_vs_E10;stage	1	TRUE
ENSGALG00010025304	DDX28	PRJNA729271	-0.284743373	0.004632408	0.284743373	stage_E14_vs_E10	ordinary_muscle_development		0	FALSE
ENSGALG00010012031	GRK6	PRJNA729271	-0.322928615	0.004644304	0.322928615	stage_E14_vs_E10	ordinary_muscle_development		0	FALSE
ENSGALG00010009100	ENSGALG00010009100	PRJNA729271	-0.457208072	0.004668871	0.457208072	stage_E14_vs_E10	ordinary_muscle_development		0	FALSE
ENSGALG00010003831	IRAK1BP1	PRJNA729271	-0.503495163	0.004669587	0.503495163	stage_E14_vs_E10	ordinary_muscle_development		0	FALSE
ENSGALG00010028574	ENDOG	PRJNA729271	-0.359020521	0.004694303	0.359020521	stage_E14_vs_E10	ordinary_muscle_development		0	FALSE
ENSGALG00010019269	SOCS1	PRJNA729271	0.742956053	0.004697555	0.742956053	stage_E14_vs_E10	ordinary_muscle_development		0	FALSE
ENSGALG00010025624	ARMH1	PRJNA729271	1.029044735	0.004724223	1.029044735	stage_E14_vs_E10	ordinary_muscle_development	D_vs_E10;stage_1W_vs_E10;stage_3W_vs_E10;stage	1	TRUE
ENSGALG00010004715	WDR27	PRJNA729271	-1.062674034	0.004731209	1.062674034	stage_E14_vs_E10	ordinary_muscle_development	D_vs_E10;stage_1W_vs_E10;stage_3W_vs_E10;stage	1	TRUE
ENSGALG00010028431	ENSGALG00010028431	PRJNA729271	-0.810853685	0.004751176	0.810853685	stage_E14_vs_E10	ordinary_muscle_development		0	FALSE
ENSGALG00010020781	SNN	PRJNA729271	-0.409786236	0.004756683	0.409786236	stage_E14_vs_E10	ordinary_muscle_development		0	FALSE
ENSGALG00010009121	SYCN	PRJNA729271	2.153618844	0.004758041	2.153618844	stage_E14_vs_E10	ordinary_muscle_development	D_vs_E10;stage_1W_vs_E10;stage_3W_vs_E10;stage	1	TRUE
ENSGALG00010026252	ENO2	PRJNA729271	0.94115161	0.004759593	0.94115161	stage_E14_vs_E10	ordinary_muscle_development		0	FALSE
ENSGALG00010010287	STAM2	PRJNA729271	-0.292466199	0.004790978	0.292466199	stage_E14_vs_E10	ordinary_muscle_development		0	FALSE
ENSGALG00010017812	ENSGALG00010017812	PRJNA729271	5.058421733	0.004803734	5.058421733	stage_E14_vs_E10	ordinary_muscle_development	D_vs_E10;stage_1W_vs_E10;stage_3W_vs_E10;stage	1	TRUE
ENSGALG00010020605	STAMBP	PRJNA729271	-0.261080514	0.004813459	0.261080514	stage_E14_vs_E10	ordinary_muscle_development		0	FALSE
ENSGALG00010024842	ZFYVE9	PRJNA729271	0.143436012	0.00481568	0.143436012	stage_E14_vs_E10	ordinary_muscle_development		0	FALSE
ENSGALG00010021582	STK36	PRJNA729271	0.423833274	0.004817503	0.423833274	stage_E14_vs_E10	ordinary_muscle_development		0	FALSE
ENSGALG00010027883	ATP8B2	PRJNA729271	0.350496136	0.004817656	0.350496136	stage_E14_vs_E10	ordinary_muscle_development		0	FALSE
ENSGALG00010009254	SLC26A4	PRJNA729271	-2.569402558	0.004843032	2.569402558	stage_E14_vs_E10	ordinary_muscle_development	D_vs_E10;stage_1W_vs_E10;stage_3W_vs_E10;stage	1	TRUE
ENSGALG00010024016	CRYZ	PRJNA729271	0.31988275	0.004861714	0.31988275	stage_E14_vs_E10	ordinary_muscle_development		0	FALSE
ENSGALG00010001627	ARFIP2	PRJNA729271	-0.27819998	0.004873213	0.27819998	stage_E14_vs_E10	ordinary_muscle_development		0	FALSE
ENSGALG00010012107	CLCC1	PRJNA729271	-0.227197648	0.004873213	0.227197648	stage_E14_vs_E10	ordinary_muscle_development		0	FALSE
ENSGALG00010015170	EXOC1L	PRJNA729271	-2.896910181	0.004873956	2.896910181	stage_E14_vs_E10	ordinary_muscle_development	D_vs_E10;stage_1W_vs_E10;stage_3W_vs_E10;stage	1	TRUE
ENSGALG00010019896	DDB1	PRJNA729271	-0.272264251	0.004877798	0.272264251	stage_E14_vs_E10	ordinary_muscle_development		0	FALSE
ENSGALG00010029507	VPS37D	PRJNA729271	-0.516924015	0.004879108	0.516924015	stage_E14_vs_E10	ordinary_muscle_development		0	FALSE
ENSGALG00010002575	BGV	PRJNA729271	1.161952194	0.004920587	1.161952194	stage_E14_vs_E10	ordinary_muscle_development	D_vs_E10;stage_1W_vs_E10;stage_3W_vs_E10;stage	1	TRUE
ENSGALG00010029155	ENSGALG00010029155	PRJNA729271	-1.713802141	0.004932084	1.713802141	stage_E14_vs_E10	ordinary_muscle_development	D_vs_E10;stage_1W_vs_E10;stage_3W_vs_E10;stage	1	TRUE
ENSGALG00010023817	BBS2	PRJNA729271	-0.357762477	0.00495312	0.357762477	stage_E14_vs_E10	ordinary_muscle_development		0	FALSE
ENSGALG00010015016	ARGLU1	PRJNA729271	0.233429662	0.00495765	0.233429662	stage_E14_vs_E10	ordinary_muscle_development		0	FALSE
ENSGALG00010023501	ENSGALG00010023501	PRJNA729271	8.654818782	0.004977512	8.654818782	stage_E14_vs_E10	ordinary_muscle_development	D_vs_E10;stage_1W_vs_E10;stage_3W_vs_E10;stage	1	TRUE
ENSGALG00010010604	HIVEP1	PRJNA729271	0.292034879	0.00498192	0.292034879	stage_E14_vs_E10	ordinary_muscle_development		0	FALSE
ENSGALG00010026416	ENSGALG00010026416	PRJNA729271	4.123397816	0.005005742	4.123397816	stage_E14_vs_E10	ordinary_muscle_development	D_vs_E10;stage_1W_vs_E10;stage_3W_vs_E10;stage	1	TRUE
ENSGALG00010000580	ENSGALG00010000580	PRJNA729271	-1.925731849	0.005007187	1.925731849	stage_E14_vs_E10	ordinary_muscle_development	D_vs_E10;stage_1W_vs_E10;stage_3W_vs_E10;stage	1	TRUE
ENSGALG00010023295	ENSGALG00010023295	PRJNA729271	1.457242854	0.005016662	1.457242854	stage_E14_vs_E10	ordinary_muscle_development	D_vs_E10;stage_1W_vs_E10;stage_3W_vs_E10;stage	1	TRUE
ENSGALG00010027955	ENSGALG00010027955	PRJNA729271	0.341496072	0.005024592	0.341496072	stage_E14_vs_E10	ordinary_muscle_development		0	FALSE
ENSGALG00010027293	ELP6	PRJNA729271	-0.446963995	0.005047411	0.446963995	stage_E14_vs_E10	ordinary_muscle_development		0	FALSE
ENSGALG00010011099	CCBE1	PRJNA729271	-0.583566766	0.005054078	0.583566766	stage_E14_vs_E10	ordinary_muscle_development		0	FALSE
ENSGALG00010006814	ENSGALG00010006814	PRJNA729271	-1.240592193	0.005057053	1.240592193	stage_E14_vs_E10	ordinary_muscle_development	D_vs_E10;stage_1W_vs_E10;stage_3W_vs_E10;stage	1	TRUE
ENSGALG00010022959	ADAMTS8	PRJNA729271	0.82720211	0.005066172	0.82720211	stage_E14_vs_E10	ordinary_muscle_development		0	FALSE
ENSGALG00010027336	RAD54L2	PRJNA729271	-0.414714653	0.005083105	0.414714653	stage_E14_vs_E10	ordinary_muscle_development		0	FALSE
ENSGALG00010008078	GUCY1B1	PRJNA729271	0.411829697	0.005090469	0.411829697	stage_E14_vs_E10	ordinary_muscle_development		0	FALSE
ENSGALG00010021301	SMURF1	PRJNA729271	-0.237465037	0.005115353	0.237465037	stage_E14_vs_E10	ordinary_muscle_development		0	FALSE
ENSGALG00010005198	TRPC3	PRJNA729271	-1.58614558	0.005123481	1.58614558	stage_E14_vs_E10	ordinary_muscle_development	D_vs_E10;stage_1W_vs_E10;stage_3W_vs_E10;stage	1	TRUE
ENSGALG00010023506	RPE65	PRJNA729271	-2.116813079	0.005141491	2.116813079	stage_E14_vs_E10	ordinary_muscle_development	D_vs_E10;stage_1W_vs_E10;stage_3W_vs_E10;stage	1	TRUE
ENSGALG00010025696	MTCH1	PRJNA729271	0.300913793	0.005158254	0.300913793	stage_E14_vs_E10	ordinary_muscle_development		0	FALSE
ENSGALG00010013440	EEA1	PRJNA729271	-0.3073663	0.005165957	0.3073663	stage_E14_vs_E10	ordinary_muscle_development		0	FALSE
ENSGALG00010004342	ZNF438	PRJNA729271	0.386359345	0.00516633	0.386359345	stage_E14_vs_E10	ordinary_muscle_development		0	FALSE
ENSGALG00010019194	ENSGALG00010019194	PRJNA729271	0.568614962	0.005186303	0.568614962	stage_E14_vs_E10	ordinary_muscle_development		0	FALSE
ENSGALG00010027748	ENSGALG00010027748	PRJNA729271	8.651700895	0.005250332	8.651700895	stage_E14_vs_E10	ordinary_muscle_development	D_vs_E10;stage_1W_vs_E10;stage_3W_vs_E10;stage	1	TRUE
ENSGALG00010000612	ENSGALG00010000612	PRJNA729271	-1.349772641	0.005253199	1.349772641	stage_E14_vs_E10	ordinary_muscle_development	D_vs_E10;stage_1W_vs_E10;stage_3W_vs_E10;stage	1	TRUE
ENSGALG00010004399	POU3F2	PRJNA729271	-2.012041558	0.005266489	2.012041558	stage_E14_vs_E10	ordinary_muscle_development	D_vs_E10;stage_1W_vs_E10;stage_3W_vs_E10;stage	1	TRUE

ENSGALG00010011539	LANCL1	PRJNA729271	-0.317177835	0.005277602	0.317177835	stage_E14_vs_E10	ordinary_muscle_development		0	FALSE
ENSGALG00010013314	KIT	PRJNA729271	-0.645104563	0.005283267	0.645104563	stage_E14_vs_E10	ordinary_muscle_development		0	FALSE
ENSGALG00010021149	ENSGALG00010021149	PRJNA729271	-3.9432211	0.005305559	3.9432211	stage_E14_vs_E10	ordinary_muscle_development	D_vs_E10;stage_1W_vs_E10;stage_3W_vs_E10;stage	1	TRUE
ENSGALG00010012884	ENSGALG00010012884	PRJNA729271	0.412646876	0.005315229	0.412646876	stage_E14_vs_E10	ordinary_muscle_development		0	FALSE
ENSGALG00010011880	U2AF1	PRJNA729271	-0.457940292	0.005315382	0.457940292	stage_E14_vs_E10	ordinary_muscle_development		0	FALSE
ENSGALG00010027991	ENSGALG00010027991	PRJNA729271	0.432462225	0.005321205	0.432462225	stage_E14_vs_E10	ordinary_muscle_development		0	FALSE
ENSGALG00010002346	FBXO15	PRJNA729271	-1.09827961	0.005323299	1.09827961	stage_E14_vs_E10	ordinary_muscle_development	D_vs_E10;stage_1W_vs_E10;stage_3W_vs_E10;stage	1	TRUE
ENSGALG00010023013	ENSGALG00010023013	PRJNA729271	1.164345805	0.00532599	1.164345805	stage_E14_vs_E10	ordinary_muscle_development	D_vs_E10;stage_1W_vs_E10;stage_3W_vs_E10;stage	1	TRUE
ENSGALG00010027865	SELENOK	PRJNA729271	-0.359927407	0.005353806	0.359927407	stage_E14_vs_E10	ordinary_muscle_development		0	FALSE
ENSGALG00010027562	SLC39A3	PRJNA729271	0.422792474	0.005363327	0.422792474	stage_E14_vs_E10	ordinary_muscle_development		0	FALSE
ENSGALG00010011274	ENSGALG00010011274	PRJNA729271	-1.344141933	0.005385581	1.344141933	stage_E14_vs_E10	ordinary_muscle_development	D_vs_E10;stage_1W_vs_E10;stage_3W_vs_E10;stage	1	TRUE
ENSGALG00010012648	MRPS6	PRJNA729271	-0.385521199	0.00539194	0.385521199	stage_E14_vs_E10	ordinary_muscle_development		0	FALSE
ENSGALG00010024266	ENSGALG00010024266	PRJNA729271	0.699143749	0.005396283	0.699143749	stage_E14_vs_E10	ordinary_muscle_development		0	FALSE
ENSGALG00010006588	ACOT12	PRJNA729271	-2.223195471	0.005432726	2.223195471	stage_E14_vs_E10	ordinary_muscle_development	D_vs_E10;stage_1W_vs_E10;stage_3W_vs_E10;stage	1	TRUE
ENSGALG00010028127	MFSD12	PRJNA729271	-0.529252972	0.005432973	0.529252972	stage_E14_vs_E10	ordinary_muscle_development		0	FALSE
ENSGALG00010019488	TEKT5	PRJNA729271	-3.884629163	0.005439092	3.884629163	stage_E14_vs_E10	ordinary_muscle_development	D_vs_E10;stage_1W_vs_E10;stage_3W_vs_E10;stage	1	TRUE
ENSGALG00010007287	ENSGALG00010007287	PRJNA729271	1.828950846	0.005445966	1.828950846	stage_E14_vs_E10	ordinary_muscle_development	D_vs_E10;stage_1W_vs_E10;stage_3W_vs_E10;stage	1	TRUE
ENSGALG00010012745	MARCHF3	PRJNA729271	-0.935104507	0.005462154	0.935104507	stage_E14_vs_E10	ordinary_muscle_development		0	FALSE
ENSGALG00010025894	DYTN	PRJNA729271	-1.783950984	0.005469434	1.783950984	stage_E14_vs_E10	ordinary_muscle_development	D_vs_E10;stage_1W_vs_E10;stage_3W_vs_E10;stage	1	TRUE
ENSGALG00010011411	ENSGALG00010011411	PRJNA729271	-0.651665975	0.005474845	0.651665975	stage_E14_vs_E10	ordinary_muscle_development		0	FALSE
ENSGALG00010024437	HERC1	PRJNA729271	-0.240898393	0.005474936	0.240898393	stage_E14_vs_E10	ordinary_muscle_development		0	FALSE
ENSGALG00010019313	MRPL28	PRJNA729271	0.286358242	0.005486209	0.286358242	stage_E14_vs_E10	ordinary_muscle_development		0	FALSE
ENSGALG00010002427	CYB5R4	PRJNA729271	-0.338259653	0.005511599	0.338259653	stage_E14_vs_E10	ordinary_muscle_development		0	FALSE
ENSGALG00010003512	ENSGALG00010003512	PRJNA729271	-1.460517977	0.005532474	1.460517977	stage_E14_vs_E10	ordinary_muscle_development	D_vs_E10;stage_1W_vs_E10;stage_3W_vs_E10;stage	1	TRUE
ENSGALG00010025997	CCDC43	PRJNA729271	0.276383588	0.00553523	0.276383588	stage_E14_vs_E10	ordinary_muscle_development		0	FALSE
ENSGALG00010027333	PROK1	PRJNA729271	-0.911898037	0.005549681	0.911898037	stage_E14_vs_E10	ordinary_muscle_development		0	FALSE
ENSGALG00010020345	AGBL1	PRJNA729271	-1.31809379	0.005553503	1.31809379	stage_E14_vs_E10	ordinary_muscle_development	D_vs_E10;stage_1W_vs_E10;stage_3W_vs_E10;stage	1	TRUE
ENSGALG00010027325	METTL25B	PRJNA729271	0.82851778	0.005562306	0.82851778	stage_E14_vs_E10	ordinary_muscle_development		0	FALSE
ENSGALG00010020673	MMS19	PRJNA729271	-0.381193528	0.005564689	0.381193528	stage_E14_vs_E10	ordinary_muscle_development		0	FALSE
ENSGALG00010017561	CFAP99	PRJNA729271	-2.428508479	0.005572969	2.428508479	stage_E14_vs_E10	ordinary_muscle_development	D_vs_E10;stage_1W_vs_E10;stage_3W_vs_E10;stage	1	TRUE
ENSGALG00010020776	MCOLN3	PRJNA729271	-1.379256517	0.005593652	1.379256517	stage_E14_vs_E10	ordinary_muscle_development	D_vs_E10;stage_1W_vs_E10;stage_3W_vs_E10;stage	1	TRUE
ENSGALG00010029391	ARSG	PRJNA729271	0.424847269	0.005632257	0.424847269	stage_E14_vs_E10	ordinary_muscle_development		0	FALSE
ENSGALG00010010986	NARS	PRJNA729271	-0.382933795	0.00563961	0.382933795	stage_E14_vs_E10	ordinary_muscle_development		0	FALSE
ENSGALG00010009432	BOP1	PRJNA729271	-0.556635937	0.005670826	0.556635937	stage_E14_vs_E10	ordinary_muscle_development		0	FALSE
ENSGALG00010006062	ENSGALG00010006062	PRJNA729271	1.857666469	0.00569086	1.857666469	stage_E14_vs_E10	ordinary_muscle_development	D_vs_E10;stage_1W_vs_E10;stage_3W_vs_E10;stage	1	TRUE
ENSGALG00010029502	BTBD17	PRJNA729271	-1.690348651	0.005704489	1.690348651	stage_E14_vs_E10	ordinary_muscle_development	D_vs_E10;stage_1W_vs_E10;stage_3W_vs_E10;stage	1	TRUE
ENSGALG00010024106	WDR33	PRJNA729271	-0.184177449	0.005708809	0.184177449	stage_E14_vs_E10	ordinary_muscle_development		0	FALSE
ENSGALG00010005531	SUSD1	PRJNA729271	0.729276217	0.005752132	0.729276217	stage_E14_vs_E10	ordinary_muscle_development		0	FALSE
ENSGALG00010025406	ENSGALG00010025406	PRJNA729271	0.424042647	0.005796084	0.424042647	stage_E14_vs_E10	ordinary_muscle_development		0	FALSE
ENSGALG00010013385	PGRMC1	PRJNA729271	-0.456366384	0.005796963	0.456366384	stage_E14_vs_E10	ordinary_muscle_development		0	FALSE
ENSGALG00010007846	ENSGALG00010007846	PRJNA729271	0.43658763	0.005805263	0.43658763	stage_E14_vs_E10	ordinary_muscle_development		0	FALSE
ENSGALG00010003691	ERICH1	PRJNA729271	0.413486206	0.005807647	0.413486206	stage_E14_vs_E10	ordinary_muscle_development		0	FALSE
ENSGALG00010013833	TMPRSS2	PRJNA729271	-4.109341879	0.005807858	4.109341879	stage_E14_vs_E10	ordinary_muscle_development	D_vs_E10;stage_1W_vs_E10;stage_3W_vs_E10;stage	1	TRUE
ENSGALG00010001668	UBE2E1	PRJNA729271	-0.2480607	0.005866424	0.2480607	stage_E14_vs_E10	ordinary_muscle_development		0	FALSE
ENSGALG00010028449	EFCAB12	PRJNA729271	-0.967536391	0.005875004	0.967536391	stage_E14_vs_E10	ordinary_muscle_development		0	FALSE
ENSGALG00010027084	OSBPL9	PRJNA729271	0.271374376	0.005933793	0.271374376	stage_E14_vs_E10	ordinary_muscle_development		0	FALSE
ENSGALG00010002713	OLFM2	PRJNA729271	0.501584276	0.005937978	0.501584276	stage_E14_vs_E10	ordinary_muscle_development		0	FALSE
ENSGALG00010018903	CAMTA1	PRJNA729271	-0.278845132	0.005967013	0.278845132	stage_E14_vs_E10	ordinary_muscle_development		0	FALSE
ENSGALG00010011281	ENSGALG00010011281	PRJNA729271	-1.947071937	0.005969871	1.947071937	stage_E14_vs_E10	ordinary_muscle_development	D_vs_E10;stage_1W_vs_E10;stage_3W_vs_E10;stage	1	TRUE
ENSGALG00010025387	PSMD9	PRJNA729271	-0.198962076	0.005973932	0.198962076	stage_E14_vs_E10	ordinary_muscle_development		0	FALSE
ENSGALG00010012669	ERCC8	PRJNA729271	-0.429666326	0.005976211	0.429666326	stage_E14_vs_E10	ordinary_muscle_development		0	FALSE
ENSGALG00010010791	H-10	PRJNA729271	0.854622452	0.005999267	0.854622452	stage_E14_vs_E10	ordinary_muscle_development		0	FALSE
ENSGALG00010024893	ARL6IP4	PRJNA729271	-0.405166571	0.006028001	0.405166571	stage_E14_vs_E10	ordinary_muscle_development		0	FALSE
ENSGALG00010008154	KCNQ3	PRJNA729271	-0.695160697	0.006049321	0.695160697	stage_E14_vs_E10	ordinary_muscle_development		0	FALSE
ENSGALG00010024803	CLK1	PRJNA729271	-0.423681371	0.006062399	0.423681371	stage_E14_vs_E10	ordinary_muscle_development		0	FALSE
ENSGALG00010021446	HOXB8	PRJNA729271	-1.822137535	0.006087373	1.822137535	stage_E14_vs_E10	ordinary_muscle_development	D_vs_E10;stage_1W_vs_E10;stage_3W_vs_E10;stage	1	TRUE
ENSGALG00010028205	PRRT1B	PRJNA729271	-1.442712138	0.006142552	1.442712138	stage_E14_vs_E10	ordinary_muscle_development	D_vs_E10;stage_1W_vs_E10;stage_3W_vs_E10;stage	1	TRUE
ENSGALG00010011499	SMG7	PRJNA729271	0.421144129	0.006166332	0.421144129	stage_E14_vs_E10	ordinary_muscle_development		0	FALSE
ENSGALG00010014943	APLN	PRJNA729271	0.876431291	0.006179489	0.876431291	stage_E14_vs_E10	ordinary_muscle_development		0	FALSE
ENSGALG00010008098	ENSGALG00010008098	PRJNA729271	0.475539464	0.006183588	0.475539464	stage_E14_vs_E10	ordinary_muscle_development		0	FALSE
ENSGALG00010027297	ENSGALG00010027297	PRJNA729271	-3.815518789	0.006193109	3.815518789	stage_E14_vs_E10	ordinary_muscle_development	D_vs_E10;stage_1W_vs_E10;stage_3W_vs_E10;stage	1	TRUE
ENSGALG00010004416	STXBP6	PRJNA729271	-0.653210273	0.006193732	0.653210273	stage_E14_vs_E10	ordinary_muscle_development		0	FALSE
ENSGALG00010013046	SLC12A5	PRJNA729271	0.826964446	0.006211118	0.826964446	stage_E14_vs_E10	ordinary_muscle_development		0	FALSE
ENSGALG00010015822	WFDC2	PRJNA729271	-1.735979006	0.006223637	1.735979006	stage_E14_vs_E10	ordinary_muscle_development	D_vs_E10;stage_1W_vs_E10;stage_3W_vs_E10;stage	1	TRUE
ENSGALG00010012957	DERA	PRJNA729271	-0.658087282	0.006238404	0.658087282	stage_E14_vs_E10	ordinary_muscle_development		0	FALSE
ENSGALG00010010996	RFX4	PRJNA729271	-1.896543976	0.00629993	1.896543976	stage_E14_vs_E10	ordinary_muscle_development	D_vs_E10;stage_1W_vs_E10;stage_3W_vs_E10;stage	1	TRUE
ENSGALG00010017813	DDHD2	PRJNA729271	-0.478574264	0.006300986	0.478574264	stage_E14_vs_E10	ordinary_muscle_development		0	FALSE
ENSGALG00010024512	CCNDBP1	PRJNA729271	0.327655434	0.006312804	0.327655434	stage_E14_vs_E10	ordinary_muscle_development		0	FALSE
ENSGALG00010020291	SNX29	PRJNA729271	0.411967711	0.006325149	0.411967711	stage_E14_vs_E10	ordinary_muscle_development		0	FALSE
ENSGALG00010013418	HCN1	PRJNA729271	-2.528426908	0.0063283	2.528426908	stage_E14_vs_E10	ordinary_muscle_development	D_vs_E10;stage_1W_vs_E10;stage_3W_vs_E10;stage	1	TRUE
ENSGALG00010022643	SP3	PRJNA729271	-0.219505304	0.006328644	0.219505304	stage_E14_vs_E10	ordinary_muscle_development		0	FALSE
ENSGALG00010001664	SPAM1	PRJNA729271	5.817647998	0.00633946	5.817647998	stage_E14_vs_E10	ordinary_muscle_development	D_vs_E10;stage_1W_vs_E10;stage_3W_vs_E10;stage	1	TRUE
ENSGALG00010023551	ENSGALG00010023551	PRJNA729271	0.508648461	0.006370021	0.508648461	stage_E14_vs_E10	ordinary_muscle_development		0	FALSE
ENSGALG00010024553	ASB8	PRJNA729271	0.338604003	0.006385899	0.338604003	stage_E14_vs_E10	ordinary_muscle_development		0	FALSE
ENSGALG00010011651	SERPINB6	PRJNA729271	-0.352341883	0.006392481	0.352341883	stage_E14_vs_E10	ordinary_muscle_development		0	FALSE
ENSGALG00010000623	ENSGALG00010000623	PRJNA729271	0.674822282	0.006435643	0.674822282	stage_E14_vs_E10	ordinary_muscle_development		0	FALSE
ENSGALG00010020998	SYT17	PRJNA729271	0.790457193	0.006451398	0.790457193	stage_E14_vs_E10	ordinary_muscle_development		0	FALSE
ENSGALG00010010939	ZCCHC4	PRJNA729271	-0.403120788	0.006453623	0.403120788	stage_E14_vs_E10	ordinary_muscle_development		0	FALSE
ENSGALG00010009400	CNTFR	PRJNA729271	0.805459566	0.006459728	0.805459566	stage_E14_vs_E10	ordinary_muscle_development		0	FALSE
ENSGALG00010004545	TMPRSS7	PRJNA729271	1.591778332	0.006500384	1.591778332	stage_E14_vs_E10	ordinary_muscle_development	D_vs_E10;stage_1W_vs_E10;stage_3W_vs_E10;stage	1	TRUE
ENSGALG00010004832	C6orf58	PRJNA729271	5.220055583	0.006537429	5.220055583	stage_E14_vs_E10	ordinary_muscle_development	D_vs_E10;stage_1W_vs_E10;stage_3W_vs_E10;stage	1	TRUE
ENSGALG00010008980	ENSGALG00010008980	PRJNA729271	-0.826019057	0.006541244	0.826019057	stage_E14_vs_E10	ordinary_muscle_development		0	FALSE
ENSGALG00010011612	ENSGALG00010011612	PRJNA729271	-1.441427944	0.006638934	1.441427944	stage_E14_vs_E10	ordinary_muscle_development	D_vs_E10;stage_1W_vs_E10;stage_3W_vs_E10;stage	1	TRUE
ENSGALG00010024966	AGAP3	PRJNA729271	-0.328256978	0.00666877	0.328256978	stage_E14_vs_E10	ordinary_muscle_development		0	FALSE
ENSGALG00010029243	NME1	PRJNA729271	-1.083606866	0.006673254	1.083606866	stage_E14_vs_E10	ordinary_muscle_development	D_vs_E10;stage_1W_vs_E10;stage_3W_vs_E10;stage	1	TRUE
ENSGALG00010029491	ENSGALG00010029491	PRJNA729271	-0.569245367	0.006710648	0.569245367	stage_E14_vs_E10	ordinary_muscle_development		0	FALSE
ENSGALG00010002628	TRIM7.2	PRJNA729271	0.581376752	0.006723481	0.581376752	stage_E14_vs_E10	ordinary_muscle_development		0	FALSE
ENSGALG00010006079	ENSGALG00010006079	PRJNA729271	-2.83438122	0.006730862	2.83438122	stage_E14_vs_E10	ordinary_muscle_development	D_vs_E10;stage_1W_vs_E10;stage_3W_vs_E10;stage	1	TRUE
ENSGALG00010002965	PRSS35	PRJNA729271	0.634582908	0.006743395	0.634582908	stage_E14_vs_E10	ordinary_muscle_development		0	FALSE
ENSGALG00010021688	ITPR1	PRJNA729271	-0.331266069	0.006746762	0.331266069	stage_E14_vs_E10	ordinary_muscle_development		0	FALSE
ENSGALG00010008782	CCDC9	PRJNA729271	0.770249446	0.006760596	0.770249446	stage_E14_vs_E10	ordinary_muscle_development		0	FALSE
ENSGALG00010011654	CLDN8	PRJNA729271	-3.88865136	0.006773817	3.88865136	stage_E14_vs_E10	ordinary_muscle_development	D_vs_E10;stage_1W_vs_E10;stage_3W_vs_E10;stage	1	TRUE
ENSGALG00010013188	ENSGALG00010013188	PRJNA729271	1.021180719	0.006777294	1.021180719	stage_E14_vs_E10	ordinary_muscle_development	D_vs_E10;stage_1W_vs_E10;stage_3W_vs_E10;stage	1	TRUE
ENSGALG00010006463	SCO2	PRJNA729271	0.386513436	0.006789894	0.386513436	stage_E14_vs_E10	ordinary_muscle_development		0	FALSE
ENSGALG00010003678	ZNFY1	PRJNA729271	0.804875755	0.006800554	0.804875755	stage_E14_vs_E10	ordinary_muscle_development		0	FALSE
ENSGALG00010016107	UVSSA	PRJNA729271	0.25414443	0.006839588	0.25414443	stage_E14_vs_E10	ordinary_muscle_development		0	FALSE
ENSGALG00010029173	ZNHIT3	PRJNA729271	0.378675662	0.006903933	0.378675662	stage_E14_vs_E10	ordinary_muscle_development		0	FALSE
ENSGALG00010004570	ENSGALG00010004570	PRJNA729271	-2.011238348	0.006921864	2.011238348	stage_E14_vs_E10	ordinary_muscle_development	D_vs_E10;stage_1W_vs_E10;stage_3W_vs_E10;stage	1	TRUE
ENSGALG00010025575	TRPM2	PRJNA729271	-1.443728364	0.006927364	1.443728364	stage_E14_vs_E10	ordinary_muscle_development	D_vs_E10;stage_1W_vs_E10;stage_3W_vs_E10;stage	1	TRUE
ENSGALG00010021404	UQCC1	PRJNA729271	0.269652302	0.006941916	0.269652302	stage_E14_vs_E10	ordinary_muscle_development		0	FALSE
ENSGALG00010023731	ACTR5	PRJNA729271	0.273298687	0.006951025	0.273298687	stage_E14_vs_E10	ordinary_muscle_development		0	FALSE
ENSGALG00010005439	TBX19	PRJNA729271	1.397489825	0.007044708	1.397489825	stage_E14_vs_E10	ordinary_muscle_development	D_vs_E10;stage_1W_vs_E10;stage_3W_vs_E10;stage	1	TRUE
ENSGALG00010019474	TMEM163	PRJNA729271	-1.711253327	0.007044772	1.711253327	stage_E14_vs_E10	ordinary_muscle_development	D_vs_E10;stage_1W_vs_E10;stage_3W_vs_E10;stage	1	TRUE
ENSGALG00010002747	FUT9	PRJNA729271	-1.585029131	0.00706591	1.585029131	stage_E14_vs_E10	ordinary_muscle_development	D_vs_E10;stage_1W_vs_E10;stage_3W_vs_E10;stage	1	TRUE
ENSGALG00010002378	ENSGALG00010002378	PRJNA729271	1.25600518	0.007068132	1.25600518	stage_E14_vs_E10	ordinary_muscle_development	D_vs_E10;stage_1W_vs_E10;stage_3W_vs_E10;stage	1	TRUE
ENSGALG00010014881	ENSGALG00010014881	PRJNA729271	1.164690461	0.007076809	1.164690461	stage_E14_vs_E10	ordinary_muscle_development	D_vs_E10;stage_1W_vs_E10;stage_3W_vs_E10;stage	1	TRUE
ENSGALG00010018453	TMX4	PRJNA729271	-0.215382422	0.007089281	0.215382422	stage_E14_vs_E10	ordinary_muscle_development		0	FALSE
ENSGALG00010019430	ENSGALG00010019430	PRJNA729271	1.132890076	0.007141632	1.132890076	stage_E14_vs_E10	ordinary_muscle_development	D_vs_E10;stage_1W_vs_E10;stage_3W_vs_E10;stage	1	TRUE
ENSGALG00010015285	ALG13	PRJNA729271	1.152130869	0.007149637	1.152130869	stage_E14_vs_E10	ordinary_muscle_development	D_vs_E10;stage_1W_vs_E10;stage_3W_vs_E10;stage	1	TRUE
ENSGALG00010018881	ENSGALG00010018881	PRJNA729271	1.687155311	0.00722278	1.687155311	stage_E14_vs_E10	ordinary_muscle_development	D_vs_E10;stage_1W_vs_E10;stage_3W_vs_E10;stage	1	TRUE
ENSGALG00010013247	ATG5	PRJNA729271	-0.26259915	0.007247866	0.26259915	stage_E14_vs_E10	ordinary_muscle_development		0	FALSE
ENSGALG00010023476	SCAMP2	PRJNA729271	0.421242672	0.007250022	0.421242672	stage_E14_vs_E10	ordinary_muscle_development		0	FALSE
ENSGALG00010008956	LVRN	PRJNA729271	-1.46331626	0.007274119	1.46331626	stage_E14_vs_E10	ordinary_muscle_development	D_vs_E10;stage_1W_vs_E10;stage_3W_vs_E10;stage	1	TRUE
ENSGALG00010007678	CDH8	PRJNA729271	0.789359262	0.007324446	0.789359262	stage_E14_vs_E10	ordinary_muscle_development		0	FALSE
ENSGALG00010018495	ENSGALG00010018495	PRJNA729271	1.790212467	0.007333158	1.790212467	stage_E14_vs_E10	ordinary_muscle_development	D_vs_E10;stage_1W_vs_E10;stage_3W_vs_E10;stage	1	TRUE
ENSGALG00010007551	FEZ2	PRJNA729271	0.333542969	0.007337061	0.333542969	stage_E14_vs_E10	ordinary_muscle_development		0	FALSE
ENSGALG00010029254	MMD	PRJNA729271	-0.339575931	0.007376419	0.339575931	stage_E14_vs_E10	ordinary_muscle_development		0	FALSE
ENSGALG00010019025	DNAAF2	PRJNA729271	-0.691832324	0.007419639	0.691832324	stage_E14_vs_E10	ordinary_muscle_development		0	FALSE
ENSGALG00010024053	ISL2	PRJNA729271	-1.701699823	0.007446289	1.701699823	stage_E14_vs_E10	ordinary_muscle_development	D_vs_E10;stage_1W_vs_E10;stage_3W_vs_E10;stage	1	TRUE
ENSGALG00010017183	NFYC	PRJNA729271	0.271497707	0.00750562	0.271497707	stage_E14_vs_E10	ordinary_muscle_development		0	FALSE
ENSGALG00010027046	HESX1	PRJNA729271	-0.92126782	0.00755938	0.92126782	stage_E14_vs_E10	ordinary_muscle_development		0	FALSE
ENSGALG00010017543	ZBTB25	PRJNA729271	0.521785072	0.007565106	0.521785072	stage_E14_vs_E10	ordinary_muscle_development		0	FALSE
ENSGALG00010017809	MOSMO	PRJNA729271	0.287135056	0.007588285	0.287135056	stage_E14_vs_E10	ordinary_muscle_development		0	FALSE
ENSGALG00010006035	ADH1C	PRJNA729271	2.066625983	0.007598105	2.066625983	stage_E14_vs_E10	ordinary_muscle_development	D_vs_E10;stage_1W_vs_E10;stage_3W_vs_E10;stage	1	TRUE
ENSGALG00010007918	NPY1R	PRJNA729271	-2.098679949	0.00760176	2.098679949	stage_E14_vs_E10	ordinary_muscle_development	D_vs_E10;stage_1W_vs_E10;stage_3W_vs_E10;stage	1	TRUE
ENSGALG00010010957	ZMAT2	PRJNA729271	0.20923635	0.007619797	0.20923635	stage_E14_vs_E10	ordinary_muscle_development		0	FALSE
ENSGALG00010016115	RD3L	PRJNA729271	-1.345274468	0.007650704	1.345274468	stage_E14_vs_E10	ordinary_muscle_development	D_vs_E10;stage_1W_vs_E10;stage_3W_vs_E10;stage	1	TRUE
ENSGALG00010010967	PRXL2A	PRJNA729271	1.092574567	0.007717466	1.092574567	stage_E14_vs_E10	ordinary_muscle_development	D_vs_E10;stage_1W_vs_E10;stage_3W_vs_E10;stage	1	TRUE
ENSGALG00010014234	GLRA1	PRJNA729271	-2.556752006	0.007723677	2.556752006	stage_E14_vs_E10	ordinary_muscle_development	D_vs_E10;stage_1W_vs_E10;stage_3W_vs_E10;stage	1	TRUE
ENSGALG00010023989	ENSGALG00010023989	PRJNA729271	-0.288903568	0.007763885	0.288903568	stage_E14_vs_E10	ordinary_muscle_development		0	FALSE
ENSGALG00010024154	VPS29L	PRJNA729271	1.286883591	0.007825502	1.286883591	stage_E14_vs_E10	ordinary_muscle_development	D_vs_E10;stage_1W_vs_E10;stage_3W_vs_E10;stage	1	TRUE
ENSGALG00010001479	TMEM168	PRJNA729271	-0.440996881	0.007845747	0.440996881	stage_E14_vs_E10	ordinary_muscle_development		0	FALSE
ENSGALG00010021745	RSL24D1	PRJNA729271	-0.35769796	0.007846419	0.35769796	stage_E14_vs_E10	ordinary_muscle_development		0	FALSE
ENSGALG00010013373	ENSGALG00010013373	PRJNA729271	1.016099176	0.007896071	1.016099176	stage_E14_vs_E10	ordinary_muscle_development	D_vs_E10;stage_1W_vs_E10;stage_3W_vs_E10;stage	1	TRUE
ENSGALG00010019587	CCNT2	PRJNA729271	0.354884921	0.007907602	0.354884921	stage_E14_vs_E10	ordinary_muscle_development		0	FALSE
ENSGALG00010005343	RNF17	PRJNA729271	-4.939285036	0.007908052	4.939285036	stage_E14_vs_E10	ordinary_muscle_development	D_vs_E10;stage_1W_vs_E10;stage_3W_vs_E10;stage	1	TRUE
ENSGALG00010025423	NGF	PRJNA729271	-0.979537886	0.0079355	0.979537886	stage_E14_vs_E10	ordinary_muscle_development		0	FALSE
ENSGALG00010009280	HSD17B4	PRJNA729271	-0.247703902	0.007950207	0.247703902	stage_E14_vs_E10	ordinary_muscle_development		0	FALSE
ENSGALG00010001300	MRPL49	PRJNA729271	0.499291248	0.007967022	0.499291248	stage_E14_vs_E10	ordinary_muscle_development		0	FALSE
ENSGALG00010008115	ENSGALG00010008115	PRJNA729271	-0.484404515	0.007983799	0.484404515	stage_E14_vs_E10	ordinary_muscle_development		0	FALSE
ENSGALG00010004846	TTLL2	PRJNA729271	0.819309743	0.00799064	0.819309743	stage_E14_vs_E10	ordinary_muscle_development		0	FALSE
ENSGALG00010003824	SP4	PRJNA729271	0.438145693	0.008027276	0.438145693	stage_E14_vs_E10	ordinary_muscle_development		0	FALSE
ENSGALG00010006300	GCC1	PRJNA729271	0.350112872	0.008092367	0.350112872	stage_E14_vs_E10	ordinary_muscle_development		0	FALSE
ENSGALG00010014807	HES1	PRJNA729271	-0.630414859	0.008100388	0.630414859	stage_E14_vs_E10	ordinary_muscle_development		0	FALSE
ENSGALG00010005254	ENSGALG00010005254	PRJNA729271	-2.158826706	0.008158555	2.158826706	stage_E14_vs_E10	ordinary_muscle_development	D_vs_E10;stage_1W_vs_E10;stage_3W_vs_E10;stage	1	TRUE
ENSGALG00010025831	BRAP	PRJNA729271	0.192688831	0.008166141	0.192688831	stage_E14_vs_E10	ordinary_muscle_development		0	FALSE
ENSGALG00010003030	PPEF1	PRJNA729271	-2.298788228	0.00818832	2.298788228	stage_E14_vs_E10	ordinary_muscle_development	D_vs_E10;stage_1W_vs_E10;stage_3W_vs_E10;stage	1	TRUE
ENSGALG00010029228	ERAL1	PRJNA729271	0.326007698	0.00818935	0.326007698	stage_E14_vs_E10	ordinary_muscle_development		0	FALSE
ENSGALG00010023333	ENDOU	PRJNA729271	-1.627454536	0.008232032	1.627454536	stage_E14_vs_E10	ordinary_muscle_development	D_vs_E10;stage_1W_vs_E10;stage_3W_vs_E10;stage	1	TRUE
ENSGALG00010010446	C22orf23	PRJNA729271	-1.735548767	0.008263403	1.735548767	stage_E14_vs_E10	ordinary_muscle_development	D_vs_E10;stage_1W_vs_E10;stage_3W_vs_E10;stage	1	TRUE
ENSGALG00010005505	FABP2	PRJNA729271	-3.51588386	0.008291203	3.51588386	stage_E14_vs_E10	ordinary_muscle_development	D_vs_E10;stage_1W_vs_E10;stage_3W_vs_E10;stage	1	TRUE
ENSGALG00010029942	ENSGALG00010029942	PRJNA729271	-4.683582537	0.008309644	4.683582537	stage_E14_vs_E10	ordinary_muscle_development	D_vs_E10;stage_1W_vs_E10;stage_3W_vs_E10;stage	1	TRUE
ENSGALG00010012670	ENSGALG00010012670	PRJNA729271	-0.62044071	0.008344664	0.62044071	stage_E14_vs_E10	ordinary_muscle_development		0	FALSE
ENSGALG00010017582	CLCN6	PRJNA729271	0.282242844	0.008347674	0.282242844	stage_E14_vs_E10	ordinary_muscle_development		0	FALSE
ENSGALG00010009469	DNMT3A	PRJNA729271	0.578569046	0.008354264	0.578569046	stage_E14_vs_E10	ordinary_muscle_development		0	FALSE
ENSGALG00010028120	TBXA2R	PRJNA729271	0.625104964	0.008365918	0.625104964	stage_E14_vs_E10	ordinary_muscle_development		0	FALSE
ENSGALG00010021770	RALGAPB	PRJNA729271	0.268419186	0.008394097	0.268419186	stage_E14_vs_E10	ordinary_muscle_development		0	FALSE
ENSGALG00010002474	HPCAL1	PRJNA729271	-0.391129699	0.008396396	0.391129699	stage_E14_vs_E10	ordinary_muscle_development		0	FALSE
ENSGALG00010023286	HCRT	PRJNA729271	-1.904274262	0.008420808	1.904274262	stage_E14_vs_E10	ordinary_muscle_development	D_vs_E10;stage_1W_vs_E10;stage_3W_vs_E10;stage	1	TRUE
ENSGALG00010020670	ENSGALG00010020670	PRJNA729271	-0.370599289	0.008440788	0.370599289	stage_E14_vs_E10	ordinary_muscle_development		0	FALSE
ENSGALG00010012553	ENSGALG00010012553	PRJNA729271	0.613671888	0.008446874	0.613671888	stage_E14_vs_E10	ordinary_muscle_development		0	FALSE
ENSGALG00010010848	RGR	PRJNA729271	1.644666238	0.008471542	1.644666238	stage_E14_vs_E10	ordinary_muscle_development	D_vs_E10;stage_1W_vs_E10;stage_3W_vs_E10;stage	1	TRUE
ENSGALG00010020767	UBXN10	PRJNA729271	-0.488855253	0.008471741	0.488855253	stage_E14_vs_E10	ordinary_muscle_development		0	FALSE
ENSGALG00010008224	ENSGALG00010008224	PRJNA729271	0.580125608	0.008475242	0.580125608	stage_E14_vs_E10	ordinary_muscle_development		0	FALSE
ENSGALG00010010190	RNF44	PRJNA729271	0.490017783	0.008489084	0.490017783	stage_E14_vs_E10	ordinary_muscle_development		0	FALSE
ENSGALG00010024938	ENSGALG00010024938	PRJNA729271	0.479606605	0.008624845	0.479606605	stage_E14_vs_E10	ordinary_muscle_development		0	FALSE
ENSGALG00010003344	ZFAND1	PRJNA729271	0.519990402	0.008672592	0.519990402	stage_E14_vs_E10	ordinary_muscle_development		0	FALSE
ENSGALG00010005881	CORO2A	PRJNA729271	-0.822852318	0.008696535	0.822852318	stage_E14_vs_E10	ordinary_muscle_development		0	FALSE
ENSGALG00010026635	C8A	PRJNA729271	7.033095696	0.008754478	7.033095696	stage_E14_vs_E10	ordinary_muscle_development	D_vs_E10;stage_1W_vs_E10;stage_3W_vs_E10;stage	1	TRUE
ENSGALG00010021817	EVI5	PRJNA729271	0.274494483	0.00876012	0.274494483	stage_E14_vs_E10	ordinary_muscle_development		0	FALSE
ENSGALG00010028443	ENSGALG00010028443	PRJNA729271	0.887507601	0.008764009	0.887507601	stage_E14_vs_E10	ordinary_muscle_development		0	FALSE
ENSGALG00010005003	EXPH5	PRJNA729271	-1.074803761	0.008798813	1.074803761	stage_E14_vs_E10	ordinary_muscle_development	D_vs_E10;stage_1W_vs_E10;stage_3W_vs_E10;stage	1	TRUE
ENSGALG00010013561	AGK	PRJNA729271	0.294866361	0.008883909	0.294866361	stage_E14_vs_E10	ordinary_muscle_development		0	FALSE
ENSGALG00010015994	ENSGALG00010015994	PRJNA729271	-0.302486941	0.008893767	0.302486941	stage_E14_vs_E10	ordinary_muscle_development		0	FALSE
ENSGALG00010013938	ENSGALG00010013938	PRJNA729271	1.119668701	0.008895779	1.119668701	stage_E14_vs_E10	ordinary_muscle_development	D_vs_E10;stage_1W_vs_E10;stage_3W_vs_E10;stage	1	TRUE

ENSGALG00010028539	ENSGALG00010028539	PRJNA729271	9.545073275	0.008906521	9.545073275	stage_E14_vs_E10	ordinary_muscle_development	D_vs_E10;stage_1W_vs_E10;stage_3W_vs_E10;stage	1	TRUE
ENSGALG00010024706	ENSGALG00010024706	PRJNA729271	2.533584904	0.008929348	2.533584904	stage_E14_vs_E10	ordinary_muscle_development	D_vs_E10;stage_1W_vs_E10;stage_3W_vs_E10;stage	1	TRUE
ENSGALG00010017006	DNAH3	PRJNA729271	0.680699219	0.008969197	0.680699219	stage_E14_vs_E10	ordinary_muscle_development		0	FALSE
ENSGALG00010007434	ENSGALG00010007434	PRJNA729271	0.653332126	0.008991697	0.653332126	stage_E14_vs_E10	ordinary_muscle_development		0	FALSE
ENSGALG00010023219	TEX9	PRJNA729271	0.414302127	0.009026483	0.414302127	stage_E14_vs_E10	ordinary_muscle_development		0	FALSE
ENSGALG00010008945	FER1L6	PRJNA729271	1.462784553	0.009040954	1.462784553	stage_E14_vs_E10	ordinary_muscle_development	D_vs_E10;stage_1W_vs_E10;stage_3W_vs_E10;stage	1	TRUE
ENSGALG00010024997	HES6	PRJNA729271	0.684321537	0.009079269	0.684321537	stage_E14_vs_E10	ordinary_muscle_development		0	FALSE
ENSGALG00010026958	CLTCL1	PRJNA729271	-0.231564578	0.00910624	0.231564578	stage_E14_vs_E10	ordinary_muscle_development		0	FALSE
ENSGALG00010002475	ENSGALG00010002475	PRJNA729271	-0.551422246	0.009116281	0.551422246	stage_E14_vs_E10	ordinary_muscle_development		0	FALSE
ENSGALG00010008441	PHLPP2	PRJNA729271	-0.245490046	0.009128949	0.245490046	stage_E14_vs_E10	ordinary_muscle_development		0	FALSE
ENSGALG00010019365	ENSGALG00010019365	PRJNA729271	0.482303743	0.009163172	0.482303743	stage_E14_vs_E10	ordinary_muscle_development		0	FALSE
ENSGALG00010026362	ENSGALG00010026362	PRJNA729271	-1.151648639	0.009252422	1.151648639	stage_E14_vs_E10	ordinary_muscle_development	D_vs_E10;stage_1W_vs_E10;stage_3W_vs_E10;stage	1	TRUE
ENSGALG00010005300	QRFPR	PRJNA729271	-2.365894882	0.009254084	2.365894882	stage_E14_vs_E10	ordinary_muscle_development	D_vs_E10;stage_1W_vs_E10;stage_3W_vs_E10;stage	1	TRUE
ENSGALG00010016454	ANKS3	PRJNA729271	0.255611219	0.009254678	0.255611219	stage_E14_vs_E10	ordinary_muscle_development		0	FALSE
ENSGALG00010016076	RAB3GAP2	PRJNA729271	0.245470547	0.009283133	0.245470547	stage_E14_vs_E10	ordinary_muscle_development		0	FALSE
ENSGALG00010028145	ENSGALG00010028145	PRJNA729271	0.379028089	0.009313758	0.379028089	stage_E14_vs_E10	ordinary_muscle_development		0	FALSE
ENSGALG00010019223	AAMP	PRJNA729271	0.363137834	0.009348844	0.363137834	stage_E14_vs_E10	ordinary_muscle_development		0	FALSE
ENSGALG00010025701	ENSGALG00010025701	PRJNA729271	9.208680089	0.009353107	9.208680089	stage_E14_vs_E10	ordinary_muscle_development	D_vs_E10;stage_1W_vs_E10;stage_3W_vs_E10;stage	1	TRUE
ENSGALG00010010743	KLHL20	PRJNA729271	-0.22870353	0.009360683	0.22870353	stage_E14_vs_E10	ordinary_muscle_development		0	FALSE
ENSGALG00010009151	CCNJL	PRJNA729271	0.972269402	0.009374251	0.972269402	stage_E14_vs_E10	ordinary_muscle_development		0	FALSE
ENSGALG00010018162	ENSGALG00010018162	PRJNA729271	-1.095495069	0.009398319	1.095495069	stage_E14_vs_E10	ordinary_muscle_development	D_vs_E10;stage_1W_vs_E10;stage_3W_vs_E10;stage	1	TRUE
ENSGALG00010002753	SLC9A7	PRJNA729271	-0.421463919	0.0094285	0.421463919	stage_E14_vs_E10	ordinary_muscle_development		0	FALSE
ENSGALG00010029424	MAP2K6	PRJNA729271	-0.591904505	0.009435792	0.591904505	stage_E14_vs_E10	ordinary_muscle_development		0	FALSE
ENSGALG00010023073	POMGNT1	PRJNA729271	-0.233947468	0.009438734	0.233947468	stage_E14_vs_E10	ordinary_muscle_development		0	FALSE
ENSGALG00010016754	ENSGALG00010016754	PRJNA729271	0.501462123	0.009484484	0.501462123	stage_E14_vs_E10	ordinary_muscle_development		0	FALSE
ENSGALG00010004841	AKAP9	PRJNA729271	0.414705828	0.009494975	0.414705828	stage_E14_vs_E10	ordinary_muscle_development		0	FALSE
ENSGALG00010008006	CPNE7	PRJNA729271	-2.259284886	0.009504274	2.259284886	stage_E14_vs_E10	ordinary_muscle_development	D_vs_E10;stage_1W_vs_E10;stage_3W_vs_E10;stage	1	TRUE
ENSGALG00010029933	TVP23B	PRJNA729271	0.165750471	0.009532625	0.165750471	stage_E14_vs_E10	ordinary_muscle_development		0	FALSE
ENSGALG00010010537	ENSGALG00010010537	PRJNA729271	0.559740509	0.009567687	0.559740509	stage_E14_vs_E10	ordinary_muscle_development		0	FALSE
ENSGALG00010024366	PARD6A	PRJNA729271	1.13223743	0.009581705	1.13223743	stage_E14_vs_E10	ordinary_muscle_development	D_vs_E10;stage_1W_vs_E10;stage_3W_vs_E10;stage	1	TRUE
ENSGALG00010028856	ETV3	PRJNA729271	-0.297404002	0.009583504	0.297404002	stage_E14_vs_E10	ordinary_muscle_development		0	FALSE
ENSGALG00010021506	MAX	PRJNA729271	0.679249795	0.009596168	0.679249795	stage_E14_vs_E10	ordinary_muscle_development		0	FALSE
ENSGALG00010013220	CSTF1	PRJNA729271	0.181209026	0.009605573	0.181209026	stage_E14_vs_E10	ordinary_muscle_development		0	FALSE
ENSGALG00010015188	SULT1C3	PRJNA729271	-3.581689268	0.009617805	3.581689268	stage_E14_vs_E10	ordinary_muscle_development	D_vs_E10;stage_1W_vs_E10;stage_3W_vs_E10;stage	1	TRUE
ENSGALG00010012137	ENSGALG00010012137	PRJNA729271	-3.378011596	0.009648876	3.378011596	stage_E14_vs_E10	ordinary_muscle_development	D_vs_E10;stage_1W_vs_E10;stage_3W_vs_E10;stage	1	TRUE
ENSGALG00010024270	RAB18L	PRJNA729271	2.111815917	0.009649616	2.111815917	stage_E14_vs_E10	ordinary_muscle_development	D_vs_E10;stage_1W_vs_E10;stage_3W_vs_E10;stage	1	TRUE
ENSGALG00010024936	EHF	PRJNA729271	1.474459542	0.009701994	1.474459542	stage_E14_vs_E10	ordinary_muscle_development	D_vs_E10;stage_1W_vs_E10;stage_3W_vs_E10;stage	1	TRUE
ENSGALG00010019252	TATDN3	PRJNA729271	-0.204095268	0.009743353	0.204095268	stage_E14_vs_E10	ordinary_muscle_development		0	FALSE
ENSGALG00010021032	TMC7	PRJNA729271	-0.326525822	0.009758782	0.326525822	stage_E14_vs_E10	ordinary_muscle_development		0	FALSE
ENSGALG00010003834	MAPK10	PRJNA729271	-1.078230054	0.009782731	1.078230054	stage_E14_vs_E10	ordinary_muscle_development	D_vs_E10;stage_1W_vs_E10;stage_3W_vs_E10;stage	1	TRUE
ENSGALG00010024870	TPD52L2	PRJNA729271	0.229764759	0.009829956	0.229764759	stage_E14_vs_E10	ordinary_muscle_development		0	FALSE
ENSGALG00010014870	ENSGALG00010014870	PRJNA729271	5.114301581	0.009909736	5.114301581	stage_E14_vs_E10	ordinary_muscle_development	D_vs_E10;stage_1W_vs_E10;stage_3W_vs_E10;stage	1	TRUE
ENSGALG00010012519	ENSGALG00010012519	PRJNA729271	-4.991471432	0.009948466	4.991471432	stage_E14_vs_E10	ordinary_muscle_development	D_vs_E10;stage_1W_vs_E10;stage_3W_vs_E10;stage	1	TRUE
ENSGALG00010003264	ENSGALG00010003264	PRJNA729271	-1.065256393	0.009983619	1.065256393	stage_E14_vs_E10	ordinary_muscle_development	D_vs_E10;stage_1W_vs_E10;stage_3W_vs_E10;stage	1	TRUE
ENSGALG00010022507	ENSGALG00010022507	PRJNA729271	-0.588337375	0.010009152	0.588337375	stage_E14_vs_E10	ordinary_muscle_development		0	FALSE
ENSGALG00010017636	ZBTB8OS	PRJNA729271	-0.430741631	0.01003711	0.430741631	stage_E14_vs_E10	ordinary_muscle_development		0	FALSE
ENSGALG00010022222	ENSGALG00010022222	PRJNA729271	0.291501036	0.010040776	0.291501036	stage_E14_vs_E10	ordinary_muscle_development		0	FALSE
ENSGALG00010018945	HABP2	PRJNA729271	-1.906947578	0.010047055	1.906947578	stage_E14_vs_E10	ordinary_muscle_development	D_vs_E10;stage_1W_vs_E10;stage_3W_vs_E10;stage	1	TRUE
ENSGALG00010027443	MED16	PRJNA729271	-0.420668807	0.010071463	0.420668807	stage_E14_vs_E10	ordinary_muscle_development		0	FALSE
ENSGALG00010027548	PCBP4	PRJNA729271	0.401511053	0.010081125	0.401511053	stage_E14_vs_E10	ordinary_muscle_development		0	FALSE
ENSGALG00010001751	AVPR2C	PRJNA729271	-1.265193672	0.01010508	1.265193672	stage_E14_vs_E10	ordinary_muscle_development	D_vs_E10;stage_1W_vs_E10;stage_3W_vs_E10;stage	1	TRUE
ENSGALG00010011020	SFTPA2	PRJNA729271	2.307529416	0.010127708	2.307529416	stage_E14_vs_E10	ordinary_muscle_development	D_vs_E10;stage_1W_vs_E10;stage_3W_vs_E10;stage	1	TRUE
ENSGALG00010008447	FRRS1L	PRJNA729271	-0.272346409	0.010166537	0.272346409	stage_E14_vs_E10	ordinary_muscle_development		0	FALSE
ENSGALG00010009486	B4GALT1	PRJNA729271	0.368761365	0.010178075	0.368761365	stage_E14_vs_E10	ordinary_muscle_development		0	FALSE
ENSGALG00010013911	TNIK	PRJNA729271	0.427675719	0.010188403	0.427675719	stage_E14_vs_E10	ordinary_muscle_development		0	FALSE
ENSGALG00010029212	SUPT6H	PRJNA729271	0.304493088	0.010195801	0.304493088	stage_E14_vs_E10	ordinary_muscle_development		0	FALSE
ENSGALG00010005391	ADGB	PRJNA729271	1.023796466	0.010240195	1.023796466	stage_E14_vs_E10	ordinary_muscle_development	D_vs_E10;stage_1W_vs_E10;stage_3W_vs_E10;stage	1	TRUE
ENSGALG00010003160	KIDINS220	PRJNA729271	-0.370714909	0.010313831	0.370714909	stage_E14_vs_E10	ordinary_muscle_development		0	FALSE
ENSGALG00010005588	PLAGL1	PRJNA729271	0.345593037	0.010547677	0.345593037	stage_E14_vs_E10	ordinary_muscle_development		0	FALSE
ENSGALG00010015765	ENSGALG00010015765	PRJNA729271	0.88272348	0.010581763	0.88272348	stage_E14_vs_E10	ordinary_muscle_development		0	FALSE
ENSGALG00010011856	NFYB	PRJNA729271	-0.330267583	0.010596237	0.330267583	stage_E14_vs_E10	ordinary_muscle_development		0	FALSE
ENSGALG00010021702	PLD6	PRJNA729271	-0.940398204	0.01063874	0.940398204	stage_E14_vs_E10	ordinary_muscle_development		0	FALSE
ENSGALG00010029879	ENSGALG00010029879	PRJNA729271	-0.920221436	0.010696011	0.920221436	stage_E14_vs_E10	ordinary_muscle_development		0	FALSE
ENSGALG00010023738	ENSGALG00010023738	PRJNA729271	1.025693848	0.010715508	1.025693848	stage_E14_vs_E10	ordinary_muscle_development	D_vs_E10;stage_1W_vs_E10;stage_3W_vs_E10;stage	1	TRUE
ENSGALG00010012286	ENSGALG00010012286	PRJNA729271	-7.406616901	0.01081171	7.406616901	stage_E14_vs_E10	ordinary_muscle_development	D_vs_E10;stage_1W_vs_E10;stage_3W_vs_E10;stage	1	TRUE
ENSGALG00010018919	RALB	PRJNA729271	-0.289370447	0.010816512	0.289370447	stage_E14_vs_E10	ordinary_muscle_development		0	FALSE
ENSGALG00010021767	CCDC58	PRJNA729271	0.56389281	0.010825143	0.56389281	stage_E14_vs_E10	ordinary_muscle_development		0	FALSE
ENSGALG00010027372	PIK3C2B	PRJNA729271	-0.460532572	0.010870719	0.460532572	stage_E14_vs_E10	ordinary_muscle_development		0	FALSE
ENSGALG00010008452	ENSGALG00010008452	PRJNA729271	1.054064755	0.010893627	1.054064755	stage_E14_vs_E10	ordinary_muscle_development	D_vs_E10;stage_1W_vs_E10;stage_3W_vs_E10;stage	1	TRUE
ENSGALG00010023144	C11orf58	PRJNA729271	-0.260408182	0.01089932	0.260408182	stage_E14_vs_E10	ordinary_muscle_development		0	FALSE
ENSGALG00010003716	YLEC8	PRJNA729271	1.948449655	0.010902906	1.948449655	stage_E14_vs_E10	ordinary_muscle_development	D_vs_E10;stage_1W_vs_E10;stage_3W_vs_E10;stage	1	TRUE
ENSGALG00010026117	LZTR1	PRJNA729271	0.193721928	0.010907294	0.193721928	stage_E14_vs_E10	ordinary_muscle_development		0	FALSE
ENSGALG00010009338	KYNU	PRJNA729271	1.506098487	0.010908176	1.506098487	stage_E14_vs_E10	ordinary_muscle_development	D_vs_E10;stage_1W_vs_E10;stage_3W_vs_E10;stage	1	TRUE
ENSGALG00010004389	ENSGALG00010004389	PRJNA729271	0.358343144	0.010936411	0.358343144	stage_E14_vs_E10	ordinary_muscle_development		0	FALSE
ENSGALG00010029282	LUC7L3	PRJNA729271	-0.375279811	0.010975015	0.375279811	stage_E14_vs_E10	ordinary_muscle_development		0	FALSE
ENSGALG00010009120	ATG12	PRJNA729271	-0.246948461	0.010977028	0.246948461	stage_E14_vs_E10	ordinary_muscle_development		0	FALSE
ENSGALG00010008077	ENSGALG00010008077	PRJNA729271	2.550506247	0.01098717	2.550506247	stage_E14_vs_E10	ordinary_muscle_development	D_vs_E10;stage_1W_vs_E10;stage_3W_vs_E10;stage	1	TRUE
ENSGALG00010021823	ENSGALG00010021823	PRJNA729271	0.658034879	0.011024805	0.658034879	stage_E14_vs_E10	ordinary_muscle_development		0	FALSE
ENSGALG00010011637	CECR2	PRJNA729271	0.434491436	0.011036233	0.434491436	stage_E14_vs_E10	ordinary_muscle_development		0	FALSE
ENSGALG00010028439	BORCS8	PRJNA729271	-0.323909936	0.01107763	0.323909936	stage_E14_vs_E10	ordinary_muscle_development		0	FALSE
ENSGALG00010022225	ENSGALG00010022225	PRJNA729271	-1.815599581	0.011112123	1.815599581	stage_E14_vs_E10	ordinary_muscle_development	D_vs_E10;stage_1W_vs_E10;stage_3W_vs_E10;stage	1	TRUE
ENSGALG00010009801	ENSGALG00010009801	PRJNA729271	-1.728215451	0.011125099	1.728215451	stage_E14_vs_E10	ordinary_muscle_development	D_vs_E10;stage_1W_vs_E10;stage_3W_vs_E10;stage	1	TRUE
ENSGALG00010020305	XPO7	PRJNA729271	-0.335618721	0.011153729	0.335618721	stage_E14_vs_E10	ordinary_muscle_development		0	FALSE
ENSGALG00010011448	AvBD10	PRJNA729271	7.038620465	0.011173843	7.038620465	stage_E14_vs_E10	ordinary_muscle_development	D_vs_E10;stage_1W_vs_E10;stage_3W_vs_E10;stage	1	TRUE
ENSGALG00010019772	ENSGALG00010019772	PRJNA729271	-0.412946799	0.011184439	0.412946799	stage_E14_vs_E10	ordinary_muscle_development		0	FALSE
ENSGALG00010024601	ENSGALG00010024601	PRJNA729271	-1.387993258	0.011210732	1.387993258	stage_E14_vs_E10	ordinary_muscle_development	D_vs_E10;stage_1W_vs_E10;stage_3W_vs_E10;stage	1	TRUE
ENSGALG00010009500	CCDC146	PRJNA729271	1.488722934	0.011212545	1.488722934	stage_E14_vs_E10	ordinary_muscle_development	D_vs_E10;stage_1W_vs_E10;stage_3W_vs_E10;stage	1	TRUE
ENSGALG00010012821	ZBTB33	PRJNA729271	0.146292126	0.011222461	0.146292126	stage_E14_vs_E10	ordinary_muscle_development		0	FALSE
ENSGALG00010004177	CSPP1	PRJNA729271	0.273922098	0.01123093	0.273922098	stage_E14_vs_E10	ordinary_muscle_development		0	FALSE
ENSGALG00010026659	LIPH	PRJNA729271	-0.713839159	0.011231091	0.713839159	stage_E14_vs_E10	ordinary_muscle_development		0	FALSE
ENSGALG00010008042	ENSGALG00010008042	PRJNA729271	2.07868763	0.011233296	2.07868763	stage_E14_vs_E10	ordinary_muscle_development	D_vs_E10;stage_1W_vs_E10;stage_3W_vs_E10;stage	1	TRUE
ENSGALG00010023096	SPATS2	PRJNA729271	0.451266881	0.011239224	0.451266881	stage_E14_vs_E10	ordinary_muscle_development		0	FALSE
ENSGALG00010004553	BATF	PRJNA729271	0.896858902	0.011285255	0.896858902	stage_E14_vs_E10	ordinary_muscle_development		0	FALSE
ENSGALG00010025612	TMEM53	PRJNA729271	-0.422361543	0.011383076	0.422361543	stage_E14_vs_E10	ordinary_muscle_development		0	FALSE
ENSGALG00010005292	TAF3	PRJNA729271	-0.220376131	0.011478474	0.220376131	stage_E14_vs_E10	ordinary_muscle_development		0	FALSE
ENSGALG00010018557	TBCC	PRJNA729271	0.28488651	0.011485754	0.28488651	stage_E14_vs_E10	ordinary_muscle_development		0	FALSE
ENSGALG00010004716	ADH6	PRJNA729271	2.702472304	0.011486472	2.702472304	stage_E14_vs_E10	ordinary_muscle_development	D_vs_E10;stage_1W_vs_E10;stage_3W_vs_E10;stage	1	TRUE
ENSGALG00010009398	WTIP	PRJNA729271	-0.393619089	0.011689473	0.393619089	stage_E14_vs_E10	ordinary_muscle_development		0	FALSE
ENSGALG00010011056	ENSGALG00010011056	PRJNA729271	-1.428453057	0.011699842	1.428453057	stage_E14_vs_E10	ordinary_muscle_development	D_vs_E10;stage_1W_vs_E10;stage_3W_vs_E10;stage	1	TRUE
ENSGALG00010018476	ENSGALG00010018476	PRJNA729271	-0.63291321	0.011711294	0.63291321	stage_E14_vs_E10	ordinary_muscle_development		0	FALSE
ENSGALG00010013208	ENSGALG00010013208	PRJNA729271	0.782424671	0.011720504	0.782424671	stage_E14_vs_E10	ordinary_muscle_development		0	FALSE
ENSGALG00010024996	COA7	PRJNA729271	0.326552451	0.011773449	0.326552451	stage_E14_vs_E10	ordinary_muscle_development		0	FALSE
ENSGALG00010023660	CRYBB1	PRJNA729271	-1.999093289	0.011788644	1.999093289	stage_E14_vs_E10	ordinary_muscle_development	D_vs_E10;stage_1W_vs_E10;stage_3W_vs_E10;stage	1	TRUE
ENSGALG00010017974	MXRA8	PRJNA729271	-0.522982545	0.011849335	0.522982545	stage_E14_vs_E10	ordinary_muscle_development		0	FALSE
ENSGALG00010025719	HOXC11	PRJNA729271	-7.116498271	0.01187057	7.116498271	stage_E14_vs_E10	ordinary_muscle_development	D_vs_E10;stage_1W_vs_E10;stage_3W_vs_E10;stage	1	TRUE
ENSGALG00010012998	MAFF	PRJNA729271	0.56523543	0.011870756	0.56523543	stage_E14_vs_E10	ordinary_muscle_development		0	FALSE
ENSGALG00010012863	KCNE1	PRJNA729271	1.071746887	0.012043942	1.071746887	stage_E14_vs_E10	ordinary_muscle_development	D_vs_E10;stage_1W_vs_E10;stage_3W_vs_E10;stage	1	TRUE
ENSGALG00010007904	ENSGALG00010007904	PRJNA729271	-0.337036472	0.012128294	0.337036472	stage_E14_vs_E10	ordinary_muscle_development		0	FALSE
ENSGALG00010016584	EHBP1	PRJNA729271	0.420269729	0.012135519	0.420269729	stage_E14_vs_E10	ordinary_muscle_development		0	FALSE
ENSGALG00010017371	TMEM114	PRJNA729271	-3.521867824	0.012147739	3.521867824	stage_E14_vs_E10	ordinary_muscle_development	D_vs_E10;stage_1W_vs_E10;stage_3W_vs_E10;stage	1	TRUE
ENSGALG00010025805	ATXN2	PRJNA729271	0.247856245	0.012164423	0.247856245	stage_E14_vs_E10	ordinary_muscle_development		0	FALSE
ENSGALG00010003717	LEPROTL1	PRJNA729271	0.25341396	0.012190751	0.25341396	stage_E14_vs_E10	ordinary_muscle_development		0	FALSE
ENSGALG00010013639	ENSGALG00010013639	PRJNA729271	1.177596852	0.01221628	1.177596852	stage_E14_vs_E10	ordinary_muscle_development	D_vs_E10;stage_1W_vs_E10;stage_3W_vs_E10;stage	1	TRUE
ENSGALG00010023351	GNB3	PRJNA729271	0.975097537	0.012253623	0.975097537	stage_E14_vs_E10	ordinary_muscle_development		0	FALSE
ENSGALG00010028172	CERS1	PRJNA729271	0.54949803	0.012279135	0.54949803	stage_E14_vs_E10	ordinary_muscle_development		0	FALSE
ENSGALG00010004332	ENSGALG00010004332	PRJNA729271	-2.424221921	0.012279541	2.424221921	stage_E14_vs_E10	ordinary_muscle_development	D_vs_E10;stage_1W_vs_E10;stage_3W_vs_E10;stage	1	TRUE
ENSGALG00010024398	PHB	PRJNA729271	-0.231535922	0.012286659	0.231535922	stage_E14_vs_E10	ordinary_muscle_development		0	FALSE
ENSGALG00010012243	ENSGALG00010012243	PRJNA729271	-4.439042636	0.01235903	4.439042636	stage_E14_vs_E10	ordinary_muscle_development	D_vs_E10;stage_1W_vs_E10;stage_3W_vs_E10;stage	1	TRUE
ENSGALG00010000107	ENSGALG00010000107	PRJNA729271	-1.506020573	0.012379403	1.506020573	stage_E14_vs_E10	ordinary_muscle_development	D_vs_E10;stage_1W_vs_E10;stage_3W_vs_E10;stage	1	TRUE
ENSGALG00010001163	ARL14	PRJNA729271	-1.518169846	0.012470892	1.518169846	stage_E14_vs_E10	ordinary_muscle_development	D_vs_E10;stage_1W_vs_E10;stage_3W_vs_E10;stage	1	TRUE
ENSGALG00010023832	ENSGALG00010023832	PRJNA729271	-1.675784582	0.012519447	1.675784582	stage_E14_vs_E10	ordinary_muscle_development	D_vs_E10;stage_1W_vs_E10;stage_3W_vs_E10;stage	1	TRUE
ENSGALG00010020176	ENSGALG00010020176	PRJNA729271	0.632108204	0.012572071	0.632108204	stage_E14_vs_E10	ordinary_muscle_development		0	FALSE
ENSGALG00010010222	PAH	PRJNA729271	2.488774026	0.012647745	2.488774026	stage_E14_vs_E10	ordinary_muscle_development	D_vs_E10;stage_1W_vs_E10;stage_3W_vs_E10;stage	1	TRUE
ENSGALG00010022689	MPL	PRJNA729271	1.061420625	0.012670335	1.061420625	stage_E14_vs_E10	ordinary_muscle_development	D_vs_E10;stage_1W_vs_E10;stage_3W_vs_E10;stage	1	TRUE
ENSGALG00010006087	ENSGALG00010006087	PRJNA729271	1.467730438	0.01282808	1.467730438	stage_E14_vs_E10	ordinary_muscle_development	D_vs_E10;stage_1W_vs_E10;stage_3W_vs_E10;stage	1	TRUE
ENSGALG00010016742	TRMT12	PRJNA729271	-0.372834812	0.012831029	0.372834812	stage_E14_vs_E10	ordinary_muscle_development		0	FALSE
ENSGALG00010020352	ABCG5	PRJNA729271	-2.001666792	0.012910385	2.001666792	stage_E14_vs_E10	ordinary_muscle_development	D_vs_E10;stage_1W_vs_E10;stage_3W_vs_E10;stage	1	TRUE
ENSGALG00010023792	ZNF609	PRJNA729271	-0.459235635	0.012957109	0.459235635	stage_E14_vs_E10	ordinary_muscle_development		0	FALSE
ENSGALG00010020934	ABCD3	PRJNA729271	-0.408322975	0.012968981	0.408322975	stage_E14_vs_E10	ordinary_muscle_development		0	FALSE
ENSGALG00010027088	AGBL4	PRJNA729271	0.697044113	0.01303775	0.697044113	stage_E14_vs_E10	ordinary_muscle_development		0	FALSE
ENSGALG00010000171	DPF2	PRJNA729271	0.518953792	0.013086986	0.518953792	stage_E14_vs_E10	ordinary_muscle_development		0	FALSE
ENSGALG00010018339	FAM76A	PRJNA729271	0.29929974	0.01314824	0.29929974	stage_E14_vs_E10	ordinary_muscle_development		0	FALSE
ENSGALG00010010049	ZBTB7C	PRJNA729271	-0.797731109	0.013194241	0.797731109	stage_E14_vs_E10	ordinary_muscle_development		0	FALSE
ENSGALG00010011670	TMCO6	PRJNA729271	0.44037841	0.013230271	0.44037841	stage_E14_vs_E10	ordinary_muscle_development		0	FALSE
ENSGALG00010020699	PLA2G10	PRJNA729271	-0.702426021	0.01327863	0.702426021	stage_E14_vs_E10	ordinary_muscle_development		0	FALSE
ENSGALG00010003812	BG8	PRJNA729271	1.031380289	0.01340756	1.031380289	stage_E14_vs_E10	ordinary_muscle_development	D_vs_E10;stage_1W_vs_E10;stage_3W_vs_E10;stage	1	TRUE
ENSGALG00010024186	HAO2	PRJNA729271	-1.993499072	0.013432915	1.993499072	stage_E14_vs_E10	ordinary_muscle_development	D_vs_E10;stage_1W_vs_E10;stage_3W_vs_E10;stage	1	TRUE
ENSGALG00010027934	CNBP	PRJNA729271	0.219423221	0.013442419	0.219423221	stage_E14_vs_E10	ordinary_muscle_development		0	FALSE
ENSGALG00010019356	AVL9	PRJNA729271	-0.248282167	0.013445292	0.248282167	stage_E14_vs_E10	ordinary_muscle_development		0	FALSE
ENSGALG00010025852	BTBD19	PRJNA729271	-0.675692184	0.013659927	0.675692184	stage_E14_vs_E10	ordinary_muscle_development		0	FALSE
ENSGALG00010018301	ENSGALG00010018301	PRJNA729271	-0.851737894	0.013677937	0.851737894	stage_E14_vs_E10	ordinary_muscle_development		0	FALSE
ENSGALG00010012035	ENSGALG00010012035	PRJNA729271	1.177891557	0.013681287	1.177891557	stage_E14_vs_E10	ordinary_muscle_development	D_vs_E10;stage_1W_vs_E10;stage_3W_vs_E10;stage	1	TRUE
ENSGALG00010014876	PCNT	PRJNA729271	-0.266077473	0.01371445	0.266077473	stage_E14_vs_E10	ordinary_muscle_development		0	FALSE
ENSGALG00010025313	DIO1	PRJNA729271	-2.804242971	0.013736823	2.804242971	stage_E14_vs_E10	ordinary_muscle_development	D_vs_E10;stage_1W_vs_E10;stage_3W_vs_E10;stage	1	TRUE
ENSGALG00010002613	ENSGALG00010002613	PRJNA729271	0.265979653	0.013740529	0.265979653	stage_E14_vs_E10	ordinary_muscle_development		0	FALSE
ENSGALG00010011200	PCDHB4	PRJNA729271	-1.596939184	0.01375631	1.596939184	stage_E14_vs_E10	ordinary_muscle_development	D_vs_E10;stage_1W_vs_E10;stage_3W_vs_E10;stage	1	TRUE
ENSGALG00010009440	DOK3	PRJNA729271	-0.593184341	0.013819047	0.593184341	stage_E14_vs_E10	ordinary_muscle_development		0	FALSE
ENSGALG00010027130	NCKIPSD	PRJNA729271	0.386332537	0.013824764	0.386332537	stage_E14_vs_E10	ordinary_muscle_development		0	FALSE
ENSGALG00010014702	ENSGALG00010014702	PRJNA729271	0.707066214	0.013839517	0.707066214	stage_E14_vs_E10	ordinary_muscle_development		0	FALSE
ENSGALG00010021402	ENSGALG00010021402	PRJNA729271	0.22548816	0.013949986	0.22548816	stage_E14_vs_E10	ordinary_muscle_development		0	FALSE
ENSGALG00010002947	ENSGALG00010002947	PRJNA729271	-1.827489974	0.013951884	1.827489974	stage_E14_vs_E10	ordinary_muscle_development	D_vs_E10;stage_1W_vs_E10;stage_3W_vs_E10;stage	1	TRUE
ENSGALG00010009601	ZHX2	PRJNA729271	0.349250954	0.014003601	0.349250954	stage_E14_vs_E10	ordinary_muscle_development		0	FALSE
ENSGALG00010020443	PREPL	PRJNA729271	0.346908404	0.014045067	0.346908404	stage_E14_vs_E10	ordinary_muscle_development		0	FALSE
ENSGALG00010019778	ABHD17C	PRJNA729271	0.336282778	0.014228297	0.336282778	stage_E14_vs_E10	ordinary_muscle_development		0	FALSE
ENSGALG00010014862	ENSGALG00010014862	PRJNA729271	-0.891564133	0.014273369	0.891564133	stage_E14_vs_E10	ordinary_muscle_development		0	FALSE
ENSGALG00010012847	RLN3	PRJNA729271	-2.504041108	0.014373966	2.504041108	stage_E14_vs_E10	ordinary_muscle_development	D_vs_E10;stage_1W_vs_E10;stage_3W_vs_E10;stage	1	TRUE
ENSGALG00010021869	GJC3	PRJNA729271	0.312702127	0.014399096	0.312702127	stage_E14_vs_E10	ordinary_muscle_development		0	FALSE
ENSGALG00010028757	MAT2A	PRJNA729271	-3.513594953	0.01445915	3.513594953	stage_E14_vs_E10	ordinary_muscle_development	D_vs_E10;stage_1W_vs_E10;stage_3W_vs_E10;stage	1	TRUE
ENSGALG00010025784	ENSGALG00010025784	PRJNA729271	6.416790336	0.0144846	6.416790336	stage_E14_vs_E10	ordinary_muscle_development	D_vs_E10;stage_1W_vs_E10;stage_3W_vs_E10;stage	1	TRUE
ENSGALG00010022645	ENSGALG00010022645	PRJNA729271	-1.979429294	0.01465818	1.979429294	stage_E14_vs_E10	ordinary_muscle_development	D_vs_E10;stage_1W_vs_E10;stage_3W_vs_E10;stage	1	TRUE
ENSGALG00010007223	FOXF1	PRJNA729271	-1.377945438	0.014705439	1.377945438	stage_E14_vs_E10	ordinary_muscle_development	D_vs_E10;stage_1W_vs_E10;stage_3W_vs_E10;stage	1	TRUE
ENSGALG00010013504	IGSF5	PRJNA729271	-2.201178589	0.014796681	2.201178589	stage_E14_vs_E10	ordinary_muscle_development	D_vs_E10;stage_1W_vs_E10;stage_3W_vs_E10;stage	1	TRUE
ENSGALG00010011712	DCAF12L2	PRJNA729271	0.207322834	0.014858745	0.207322834	stage_E14_vs_E10	ordinary_muscle_development		0	FALSE
ENSGALG00010012827	ENSGALG00010012827	PRJNA729271	0.476856232	0.014913914	0.476856232	stage_E14_vs_E10	ordinary_muscle_development		0	FALSE
ENSGALG00010002403	ZNFY3	PRJNA729271	-0.701810363	0.014958593	0.701810363	stage_E14_vs_E10	ordinary_muscle_development		0	FALSE
ENSGALG00010000388	ENSGALG00010000388	PRJNA729271	-0.48445596	0.015061271	0.48445596	stage_E14_vs_E10	ordinary_muscle_development		0	FALSE
ENSGALG00010021050	CTR9	PRJNA729271	-0.246493421	0.015113092	0.246493421	stage_E14_vs_E10	ordinary_muscle_development		0	FALSE
ENSGALG00010022149	NBEAL1	PRJNA729271	0.272347439	0.015126617	0.272347439	stage_E14_vs_E10	ordinary_muscle_development		0	FALSE
ENSGALG00010007411	ENSGALG00010007411	PRJNA729271	1.469082721	0.015270037	1.469082721	stage_E14_vs_E10	ordinary_muscle_development	D_vs_E10;stage_1W_vs_E10;stage_3W_vs_E10;stage	1	TRUE
ENSGALG00010022877	KYAT3	PRJNA729271	-0.246077719	0.015409523	0.246077719	stage_E14_vs_E10	ordinary_muscle_development		0	FALSE
ENSGALG00010012081	CHIC1	PRJNA729271	-0.341122952	0.015440944	0.341122952	stage_E14_vs_E10	ordinary_muscle_development		0	FALSE
ENSGALG00010022767	CACNB3	PRJNA729271	-1.249328375	0.015445144	1.249328375	stage_E14_vs_E10	ordinary_muscle_development	D_vs_E10;stage_1W_vs_E10;stage_3W_vs_E10;stage	1	TRUE
ENSGALG00010025071	CDK15	PRJNA729271	0.828055538	0.015456621	0.828055538	stage_E14_vs_E10	ordinary_muscle_development		0	FALSE
ENSGALG00010006847	SEL1L	PRJNA729271	0.154368042	0.015540694	0.154368042	stage_E14_vs_E10	ordinary_muscle_development		0	FALSE
ENSGALG00010025755	ENSGALG00010025755	PRJNA729271	-0.715737736	0.015605502	0.715737736	stage_E14_vs_E10	ordinary_muscle_development		0	FALSE

ENSGALG00010025949	GPATCH1	PRJNA729271	-0.206785903	0.015625098	0.206785903	stage_E14_vs_E10	ordinary_muscle_development		0	FALSE
ENSGALG00010003692	ZNFY2	PRJNA729271	-0.680378327	0.015641848	0.680378327	stage_E14_vs_E10	ordinary_muscle_development		0	FALSE
ENSGALG00010003315	EPGN	PRJNA729271	-0.933979781	0.015653516	0.933979781	stage_E14_vs_E10	ordinary_muscle_development		0	FALSE
ENSGALG00010005479	ENSGALG00010005479	PRJNA729271	1.558064594	0.015681691	1.558064594	stage_E14_vs_E10	ordinary_muscle_development	D_vs_E10;stage_1W_vs_E10;stage_3W_vs_E10;stage	1	TRUE
ENSGALG00010011446	ENSGALG00010011446	PRJNA729271	-0.53996862	0.015683754	0.53996862	stage_E14_vs_E10	ordinary_muscle_development		0	FALSE
ENSGALG00010008076	ENSGALG00010008076	PRJNA729271	4.430615073	0.015711172	4.430615073	stage_E14_vs_E10	ordinary_muscle_development	D_vs_E10;stage_1W_vs_E10;stage_3W_vs_E10;stage	1	TRUE
ENSGALG00010011359	RNF19A	PRJNA729271	0.223028833	0.01589958	0.223028833	stage_E14_vs_E10	ordinary_muscle_development		0	FALSE
ENSGALG00010019594	TMX1	PRJNA729271	0.805870571	0.015906696	0.805870571	stage_E14_vs_E10	ordinary_muscle_development		0	FALSE
ENSGALG00010001513	ZRSR2	PRJNA729271	0.151765508	0.01591139	0.151765508	stage_E14_vs_E10	ordinary_muscle_development		0	FALSE
ENSGALG00010028868	ENSGALG00010028868	PRJNA729271	11.35363887	0.016006422	11.35363887	stage_E14_vs_E10	ordinary_muscle_development	D_vs_E10;stage_1W_vs_E10;stage_3W_vs_E10;stage	1	TRUE
ENSGALG00010024225	BCDIN3D	PRJNA729271	0.443869224	0.01604746	0.443869224	stage_E14_vs_E10	ordinary_muscle_development		0	FALSE
ENSGALG00010028173	GDF3	PRJNA729271	0.571839194	0.016096795	0.571839194	stage_E14_vs_E10	ordinary_muscle_development		0	FALSE
ENSGALG00010023026	PHRF1	PRJNA729271	0.21690044	0.016113307	0.21690044	stage_E14_vs_E10	ordinary_muscle_development		0	FALSE
ENSGALG00010000350	ENSGALG00010000350	PRJNA729271	0.41584526	0.016115118	0.41584526	stage_E14_vs_E10	ordinary_muscle_development		0	FALSE
ENSGALG00010028037	ENSGALG00010028037	PRJNA729271	0.46214698	0.016117144	0.46214698	stage_E14_vs_E10	ordinary_muscle_development		0	FALSE
ENSGALG00010026856	UBE2Z	PRJNA729271	0.205313199	0.016120448	0.205313199	stage_E14_vs_E10	ordinary_muscle_development		0	FALSE
ENSGALG00010008319	ERP44	PRJNA729271	-0.164088162	0.016185072	0.164088162	stage_E14_vs_E10	ordinary_muscle_development		0	FALSE
ENSGALG00010013019	DPYS	PRJNA729271	-2.633453602	0.016189705	2.633453602	stage_E14_vs_E10	ordinary_muscle_development	D_vs_E10;stage_1W_vs_E10;stage_3W_vs_E10;stage	1	TRUE
ENSGALG00010013267	PNPLA3	PRJNA729271	-0.458233773	0.016287206	0.458233773	stage_E14_vs_E10	ordinary_muscle_development		0	FALSE
ENSGALG00010014306	ENSGALG00010014306	PRJNA729271	-0.862001614	0.016490119	0.862001614	stage_E14_vs_E10	ordinary_muscle_development		0	FALSE
ENSGALG00010023137	ETV6	PRJNA729271	-0.287888681	0.016490152	0.287888681	stage_E14_vs_E10	ordinary_muscle_development		0	FALSE
ENSGALG00010007391	NANP	PRJNA729271	0.348134398	0.016522675	0.348134398	stage_E14_vs_E10	ordinary_muscle_development		0	FALSE
ENSGALG00010024804	WDR53	PRJNA729271	0.263017415	0.016664847	0.263017415	stage_E14_vs_E10	ordinary_muscle_development		0	FALSE
ENSGALG00010013744	ENSGALG00010013744	PRJNA729271	-0.803030053	0.016673688	0.803030053	stage_E14_vs_E10	ordinary_muscle_development		0	FALSE
ENSGALG00010008022	SLC35F3	PRJNA729271	-0.994124364	0.016685266	0.994124364	stage_E14_vs_E10	ordinary_muscle_development		0	FALSE
ENSGALG00010029639	ENSGALG00010029639	PRJNA729271	-1.985673235	0.016701615	1.985673235	stage_E14_vs_E10	ordinary_muscle_development	D_vs_E10;stage_1W_vs_E10;stage_3W_vs_E10;stage	1	TRUE
ENSGALG00010019280	TLNRD1	PRJNA729271	-0.292205346	0.016733603	0.292205346	stage_E14_vs_E10	ordinary_muscle_development		0	FALSE
ENSGALG00010025354	ENSGALG00010025354	PRJNA729271	-4.259685645	0.016746298	4.259685645	stage_E14_vs_E10	ordinary_muscle_development	D_vs_E10;stage_1W_vs_E10;stage_3W_vs_E10;stage	1	TRUE
ENSGALG00010002518	MAST1	PRJNA729271	0.879356475	0.016809195	0.879356475	stage_E14_vs_E10	ordinary_muscle_development		0	FALSE
ENSGALG00010016109	MLH1	PRJNA729271	-0.263646414	0.01685844	0.263646414	stage_E14_vs_E10	ordinary_muscle_development		0	FALSE
ENSGALG00010028612	DIRAS1	PRJNA729271	-1.979209384	0.01686674	1.979209384	stage_E14_vs_E10	ordinary_muscle_development	D_vs_E10;stage_1W_vs_E10;stage_3W_vs_E10;stage	1	TRUE
ENSGALG00010007097	ZCCHC9	PRJNA729271	0.276582954	0.016869442	0.276582954	stage_E14_vs_E10	ordinary_muscle_development		0	FALSE
ENSGALG00010028075	RAB25	PRJNA729271	-1.465877555	0.016937678	1.465877555	stage_E14_vs_E10	ordinary_muscle_development	D_vs_E10;stage_1W_vs_E10;stage_3W_vs_E10;stage	1	TRUE
ENSGALG00010024136	SAP130	PRJNA729271	-0.439090188	0.016991438	0.439090188	stage_E14_vs_E10	ordinary_muscle_development		0	FALSE
ENSGALG00010029472	ENSGALG00010029472	PRJNA729271	-2.14360565	0.017040923	2.14360565	stage_E14_vs_E10	ordinary_muscle_development	D_vs_E10;stage_1W_vs_E10;stage_3W_vs_E10;stage	1	TRUE
ENSGALG00010019250	CXCR1	PRJNA729271	0.607395405	0.017120942	0.607395405	stage_E14_vs_E10	ordinary_muscle_development		0	FALSE
ENSGALG00010022294	ENSGALG00010022294	PRJNA729271	1.587666677	0.017188638	1.587666677	stage_E14_vs_E10	ordinary_muscle_development	D_vs_E10;stage_1W_vs_E10;stage_3W_vs_E10;stage	1	TRUE
ENSGALG00010013145	ENSGALG00010013145	PRJNA729271	-0.777128747	0.017195601	0.777128747	stage_E14_vs_E10	ordinary_muscle_development		0	FALSE
ENSGALG00010013221	C2orf49	PRJNA729271	0.294140198	0.017345125	0.294140198	stage_E14_vs_E10	ordinary_muscle_development		0	FALSE
ENSGALG00010019975	ENSGALG00010019975	PRJNA729271	-0.533281326	0.017411085	0.533281326	stage_E14_vs_E10	ordinary_muscle_development		0	FALSE
ENSGALG00010026162	MED1	PRJNA729271	-0.377801281	0.017420431	0.377801281	stage_E14_vs_E10	ordinary_muscle_development		0	FALSE
ENSGALG00010003788	NUDT9	PRJNA729271	0.216056369	0.017438457	0.216056369	stage_E14_vs_E10	ordinary_muscle_development		0	FALSE
ENSGALG00010022958	SFSWAP	PRJNA729271	0.247816622	0.017482382	0.247816622	stage_E14_vs_E10	ordinary_muscle_development		0	FALSE
ENSGALG00010024203	SPOP	PRJNA729271	0.179080037	0.017489389	0.179080037	stage_E14_vs_E10	ordinary_muscle_development		0	FALSE
ENSGALG00010009489	MROH1	PRJNA729271	-0.344351982	0.017508046	0.344351982	stage_E14_vs_E10	ordinary_muscle_development		0	FALSE
ENSGALG00010007330	MMP13	PRJNA729271	-1.775660335	0.017526728	1.775660335	stage_E14_vs_E10	ordinary_muscle_development	D_vs_E10;stage_1W_vs_E10;stage_3W_vs_E10;stage	1	TRUE
ENSGALG00010004981	ENSGALG00010004981	PRJNA729271	0.49449219	0.017528531	0.49449219	stage_E14_vs_E10	ordinary_muscle_development		0	FALSE
ENSGALG00010015056	ENSGALG00010015056	PRJNA729271	-1.259953719	0.017641176	1.259953719	stage_E14_vs_E10	ordinary_muscle_development	D_vs_E10;stage_1W_vs_E10;stage_3W_vs_E10;stage	1	TRUE
ENSGALG00010001343	ENSGALG00010001343	PRJNA729271	-0.818139237	0.017732742	0.818139237	stage_E14_vs_E10	ordinary_muscle_development		0	FALSE
ENSGALG00010029581	DGKE	PRJNA729271	-0.269904832	0.017830156	0.269904832	stage_E14_vs_E10	ordinary_muscle_development		0	FALSE
ENSGALG00010002270	IMMP2L	PRJNA729271	0.59693592	0.017852243	0.59693592	stage_E14_vs_E10	ordinary_muscle_development		0	FALSE
ENSGALG00010020811	MCOLN2	PRJNA729271	-1.974758793	0.017937278	1.974758793	stage_E14_vs_E10	ordinary_muscle_development	D_vs_E10;stage_1W_vs_E10;stage_3W_vs_E10;stage	1	TRUE
ENSGALG00010003617	COG3	PRJNA729271	-0.219410001	0.018024036	0.219410001	stage_E14_vs_E10	ordinary_muscle_development		0	FALSE
ENSGALG00010000495	ENSGALG00010000495	PRJNA729271	1.204521659	0.018026467	1.204521659	stage_E14_vs_E10	ordinary_muscle_development	D_vs_E10;stage_1W_vs_E10;stage_3W_vs_E10;stage	1	TRUE
ENSGALG00010005305	SCAF8	PRJNA729271	0.137491937	0.01808327	0.137491937	stage_E14_vs_E10	ordinary_muscle_development		0	FALSE
ENSGALG00010012887	KCNJ4	PRJNA729271	0.958348399	0.018124978	0.958348399	stage_E14_vs_E10	ordinary_muscle_development		0	FALSE
ENSGALG00010003736	ENSGALG00010003736	PRJNA729271	7.422541788	0.018269415	7.422541788	stage_E14_vs_E10	ordinary_muscle_development	D_vs_E10;stage_1W_vs_E10;stage_3W_vs_E10;stage	1	TRUE
ENSGALG00010005891	RNF6	PRJNA729271	-0.300016312	0.018329136	0.300016312	stage_E14_vs_E10	ordinary_muscle_development		0	FALSE
ENSGALG00010012856	AVR2	PRJNA729271	2.589237911	0.018376327	2.589237911	stage_E14_vs_E10	ordinary_muscle_development	D_vs_E10;stage_1W_vs_E10;stage_3W_vs_E10;stage	1	TRUE
ENSGALG00010011288	ENSGALG00010011288	PRJNA729271	-0.745076973	0.018427747	0.745076973	stage_E14_vs_E10	ordinary_muscle_development		0	FALSE
ENSGALG00010023940	ATP1B3	PRJNA729271	0.176289528	0.018428381	0.176289528	stage_E14_vs_E10	ordinary_muscle_development		0	FALSE
ENSGALG00010006988	USPL1	PRJNA729271	-0.263329567	0.018462313	0.263329567	stage_E14_vs_E10	ordinary_muscle_development		0	FALSE
ENSGALG00010002800	TUBGCP5	PRJNA729271	-0.166582292	0.018537701	0.166582292	stage_E14_vs_E10	ordinary_muscle_development		0	FALSE
ENSGALG00010005979	SUV39H2	PRJNA729271	-0.235031749	0.018662076	0.235031749	stage_E14_vs_E10	ordinary_muscle_development		0	FALSE
ENSGALG00010023330	TSPAN1	PRJNA729271	3.276780283	0.01869852	3.276780283	stage_E14_vs_E10	ordinary_muscle_development	D_vs_E10;stage_1W_vs_E10;stage_3W_vs_E10;stage	1	TRUE
ENSGALG00010018535	FAM174B	PRJNA729271	-0.258114537	0.018787934	0.258114537	stage_E14_vs_E10	ordinary_muscle_development		0	FALSE
ENSGALG00010002090	AQP11	PRJNA729271	-0.769912697	0.018833639	0.769912697	stage_E14_vs_E10	ordinary_muscle_development		0	FALSE
ENSGALG00010004390	PNLDC1	PRJNA729271	-1.60203721	0.018872954	1.60203721	stage_E14_vs_E10	ordinary_muscle_development	D_vs_E10;stage_1W_vs_E10;stage_3W_vs_E10;stage	1	TRUE
ENSGALG00010008009	GPLD1	PRJNA729271	-3.678971263	0.018895486	3.678971263	stage_E14_vs_E10	ordinary_muscle_development	D_vs_E10;stage_1W_vs_E10;stage_3W_vs_E10;stage	1	TRUE
ENSGALG00010009650	KLHL29	PRJNA729271	0.504776706	0.019089564	0.504776706	stage_E14_vs_E10	ordinary_muscle_development		0	FALSE
ENSGALG00010011131	RET	PRJNA729271	-1.320677665	0.019095909	1.320677665	stage_E14_vs_E10	ordinary_muscle_development	D_vs_E10;stage_1W_vs_E10;stage_3W_vs_E10;stage	1	TRUE
ENSGALG00010017620	UBAP1L	PRJNA729271	0.784197335	0.019140735	0.784197335	stage_E14_vs_E10	ordinary_muscle_development		0	FALSE
ENSGALG00010029338	INPP5K	PRJNA729271	-0.319218866	0.019193405	0.319218866	stage_E14_vs_E10	ordinary_muscle_development		0	FALSE
ENSGALG00010024932	ANKK1	PRJNA729271	4.140157824	0.019208181	4.140157824	stage_E14_vs_E10	ordinary_muscle_development	D_vs_E10;stage_1W_vs_E10;stage_3W_vs_E10;stage	1	TRUE
ENSGALG00010016137	STARD3NL	PRJNA729271	-0.348881365	0.019239119	0.348881365	stage_E14_vs_E10	ordinary_muscle_development		0	FALSE
ENSGALG00010000313	ENSGALG00010000313	PRJNA729271	-6.9980282	0.019273539	6.9980282	stage_E14_vs_E10	ordinary_muscle_development	D_vs_E10;stage_1W_vs_E10;stage_3W_vs_E10;stage	1	TRUE
ENSGALG00010017603	CYP3A5	PRJNA729271	2.296211298	0.019323579	2.296211298	stage_E14_vs_E10	ordinary_muscle_development	D_vs_E10;stage_1W_vs_E10;stage_3W_vs_E10;stage	1	TRUE
ENSGALG00010004824	ATM	PRJNA729271	-0.273202913	0.019372935	0.273202913	stage_E14_vs_E10	ordinary_muscle_development		0	FALSE
ENSGALG00010006923	ENSGALG00010006923	PRJNA729271	0.541148166	0.019381646	0.541148166	stage_E14_vs_E10	ordinary_muscle_development		0	FALSE
ENSGALG00010021839	C1orf50	PRJNA729271	0.293713308	0.01938188	0.293713308	stage_E14_vs_E10	ordinary_muscle_development		0	FALSE
ENSGALG00010003769	C6orf163	PRJNA729271	1.178449663	0.019391308	1.178449663	stage_E14_vs_E10	ordinary_muscle_development	D_vs_E10;stage_1W_vs_E10;stage_3W_vs_E10;stage	1	TRUE
ENSGALG00010021785	CCDC71	PRJNA729271	-0.220291723	0.019432409	0.220291723	stage_E14_vs_E10	ordinary_muscle_development		0	FALSE
ENSGALG00010014547	DHDDS	PRJNA729271	0.262325176	0.019461668	0.262325176	stage_E14_vs_E10	ordinary_muscle_development		0	FALSE
ENSGALG00010027220	ANKRD24	PRJNA729271	-1.553755161	0.019502604	1.553755161	stage_E14_vs_E10	ordinary_muscle_development	D_vs_E10;stage_1W_vs_E10;stage_3W_vs_E10;stage	1	TRUE
ENSGALG00010011323	GUCY2C	PRJNA729271	-2.506295778	0.019583585	2.506295778	stage_E14_vs_E10	ordinary_muscle_development	D_vs_E10;stage_1W_vs_E10;stage_3W_vs_E10;stage	1	TRUE
ENSGALG00010029470	SSTR2	PRJNA729271	2.270183657	0.019619666	2.270183657	stage_E14_vs_E10	ordinary_muscle_development	D_vs_E10;stage_1W_vs_E10;stage_3W_vs_E10;stage	1	TRUE
ENSGALG00010001415	MC5R	PRJNA729271	-0.57459301	0.019707353	0.57459301	stage_E14_vs_E10	ordinary_muscle_development		0	FALSE
ENSGALG00010002611	NPS	PRJNA729271	-1.075390583	0.019753533	1.075390583	stage_E14_vs_E10	ordinary_muscle_development	D_vs_E10;stage_1W_vs_E10;stage_3W_vs_E10;stage	1	TRUE
ENSGALG00010001588	ENSGALG00010001588	PRJNA729271	1.415300221	0.019821778	1.415300221	stage_E14_vs_E10	ordinary_muscle_development	D_vs_E10;stage_1W_vs_E10;stage_3W_vs_E10;stage	1	TRUE
ENSGALG00010003253	ITGB1BP1	PRJNA729271	-0.281122473	0.019847867	0.281122473	stage_E14_vs_E10	ordinary_muscle_development		0	FALSE
ENSGALG00010028480	F11R	PRJNA729271	0.536917311	0.019912247	0.536917311	stage_E14_vs_E10	ordinary_muscle_development		0	FALSE
ENSGALG00010012845	HDHD2	PRJNA729271	0.24817943	0.019921333	0.24817943	stage_E14_vs_E10	ordinary_muscle_development		0	FALSE
ENSGALG00010021764	FAM222A	PRJNA729271	-2.222723922	0.019924095	2.222723922	stage_E14_vs_E10	ordinary_muscle_development	D_vs_E10;stage_1W_vs_E10;stage_3W_vs_E10;stage	1	TRUE
ENSGALG00010017049	TRAF3	PRJNA729271	0.21643188	0.019949067	0.21643188	stage_E14_vs_E10	ordinary_muscle_development		0	FALSE
ENSGALG00010021559	MYO1H	PRJNA729271	-1.053932971	0.01997169	1.053932971	stage_E14_vs_E10	ordinary_muscle_development	D_vs_E10;stage_1W_vs_E10;stage_3W_vs_E10;stage	1	TRUE
ENSGALG00010017597	CNOT10	PRJNA729271	-0.341339342	0.020091741	0.341339342	stage_E14_vs_E10	ordinary_muscle_development		0	FALSE
ENSGALG00010027787	DCP1A	PRJNA729271	-0.290143016	0.020118093	0.290143016	stage_E14_vs_E10	ordinary_muscle_development		0	FALSE
ENSGALG00010025039	CEBPA	PRJNA729271	-0.752491263	0.020153225	0.752491263	stage_E14_vs_E10	ordinary_muscle_development		0	FALSE
ENSGALG00010007569	GPR89A	PRJNA729271	-0.199920539	0.020153561	0.199920539	stage_E14_vs_E10	ordinary_muscle_development		0	FALSE
ENSGALG00010001308	MGMT	PRJNA729271	-0.340686164	0.02030338	0.340686164	stage_E14_vs_E10	ordinary_muscle_development		0	FALSE
ENSGALG00010024396	ENSGALG00010024396	PRJNA729271	0.790466004	0.020337398	0.790466004	stage_E14_vs_E10	ordinary_muscle_development		0	FALSE
ENSGALG00010002227	ENSGALG00010002227	PRJNA729271	2.923631596	0.020490743	2.923631596	stage_E14_vs_E10	ordinary_muscle_development	D_vs_E10;stage_1W_vs_E10;stage_3W_vs_E10;stage	1	TRUE
ENSGALG00010020667	UBTD1	PRJNA729271	-0.405631512	0.020502689	0.405631512	stage_E14_vs_E10	ordinary_muscle_development		0	FALSE
ENSGALG00010017354	FEM1B	PRJNA729271	-0.223852147	0.020539488	0.223852147	stage_E14_vs_E10	ordinary_muscle_development		0	FALSE
ENSGALG00010000105	CACNA1F	PRJNA729271	0.6931612	0.020751118	0.6931612	stage_E14_vs_E10	ordinary_muscle_development		0	FALSE
ENSGALG00010014985	ENSGALG00010014985	PRJNA729271	0.260724221	0.020850489	0.260724221	stage_E14_vs_E10	ordinary_muscle_development		0	FALSE
ENSGALG00010003826	ENSGALG00010003826	PRJNA729271	-0.258491936	0.020856398	0.258491936	stage_E14_vs_E10	ordinary_muscle_development		0	FALSE
ENSGALG00010007865	GABRR3	PRJNA729271	1.048237013	0.020864079	1.048237013	stage_E14_vs_E10	ordinary_muscle_development	D_vs_E10;stage_1W_vs_E10;stage_3W_vs_E10;stage	1	TRUE
ENSGALG00010002869	ENSGALG00010002869	PRJNA729271	0.744402961	0.020875318	0.744402961	stage_E14_vs_E10	ordinary_muscle_development		0	FALSE
ENSGALG00010017704	ZNF362	PRJNA729271	-0.516461153	0.020992585	0.516461153	stage_E14_vs_E10	ordinary_muscle_development		0	FALSE
ENSGALG00010029782	UNK	PRJNA729271	0.293111109	0.02101232	0.293111109	stage_E14_vs_E10	ordinary_muscle_development		0	FALSE
ENSGALG00010022029	ENSGALG00010022029	PRJNA729271	1.515680397	0.02103167	1.515680397	stage_E14_vs_E10	ordinary_muscle_development	D_vs_E10;stage_1W_vs_E10;stage_3W_vs_E10;stage	1	TRUE
ENSGALG00010000616	PSENEN	PRJNA729271	1.198038193	0.021091399	1.198038193	stage_E14_vs_E10	ordinary_muscle_development	D_vs_E10;stage_1W_vs_E10;stage_3W_vs_E10;stage	1	TRUE
ENSGALG00010025835	PPP6R3	PRJNA729271	0.287010537	0.021193014	0.287010537	stage_E14_vs_E10	ordinary_muscle_development		0	FALSE
ENSGALG00010000386	ENSGALG00010000386	PRJNA729271	0.856512484	0.021226552	0.856512484	stage_E14_vs_E10	ordinary_muscle_development		0	FALSE
ENSGALG00010016965	SLC25A33	PRJNA729271	-0.841187988	0.021266073	0.841187988	stage_E14_vs_E10	ordinary_muscle_development		0	FALSE
ENSGALG00010021470	ENSGALG00010021470	PRJNA729271	-1.55592385	0.021316557	1.55592385	stage_E14_vs_E10	ordinary_muscle_development	D_vs_E10;stage_1W_vs_E10;stage_3W_vs_E10;stage	1	TRUE
ENSGALG00010027592	MAP1S	PRJNA729271	-0.407848771	0.02135607	0.407848771	stage_E14_vs_E10	ordinary_muscle_development		0	FALSE
ENSGALG00010023128	IWS1	PRJNA729271	0.284370434	0.021365365	0.284370434	stage_E14_vs_E10	ordinary_muscle_development		0	FALSE
ENSGALG00010012924	GLIPR1	PRJNA729271	-0.529676772	0.021419725	0.529676772	stage_E14_vs_E10	ordinary_muscle_development		0	FALSE
ENSGALG00010004876	ENSGALG00010004876	PRJNA729271	-1.851307281	0.021599841	1.851307281	stage_E14_vs_E10	ordinary_muscle_development	D_vs_E10;stage_1W_vs_E10;stage_3W_vs_E10;stage	1	TRUE
ENSGALG00010017663	ZMYND12	PRJNA729271	-0.538396447	0.021615336	0.538396447	stage_E14_vs_E10	ordinary_muscle_development		0	FALSE
ENSGALG00010002253	ENSGALG00010002253	PRJNA729271	0.777228444	0.021888275	0.777228444	stage_E14_vs_E10	ordinary_muscle_development		0	FALSE
ENSGALG00010005251	MTRF1L	PRJNA729271	0.246481874	0.021911303	0.246481874	stage_E14_vs_E10	ordinary_muscle_development		0	FALSE
ENSGALG00010029199	APPBP2	PRJNA729271	0.220663738	0.022032202	0.220663738	stage_E14_vs_E10	ordinary_muscle_development		0	FALSE
ENSGALG00010027242	ENSGALG00010027242	PRJNA729271	0.45405476	0.022043207	0.45405476	stage_E14_vs_E10	ordinary_muscle_development		0	FALSE
ENSGALG00010005279	CCDC181	PRJNA729271	0.737945253	0.02204417	0.737945253	stage_E14_vs_E10	ordinary_muscle_development		0	FALSE
ENSGALG00010002088	OZFL1	PRJNA729271	-0.240538856	0.022072958	0.240538856	stage_E14_vs_E10	ordinary_muscle_development		0	FALSE
ENSGALG00010007874	ENSGALG00010007874	PRJNA729271	-1.414879509	0.022075475	1.414879509	stage_E14_vs_E10	ordinary_muscle_development	D_vs_E10;stage_1W_vs_E10;stage_3W_vs_E10;stage	1	TRUE
ENSGALG00010003619	HNF4G	PRJNA729271	-1.308469841	0.022088174	1.308469841	stage_E14_vs_E10	ordinary_muscle_development	D_vs_E10;stage_1W_vs_E10;stage_3W_vs_E10;stage	1	TRUE
ENSGALG00010005888	IMPG2	PRJNA729271	0.546880962	0.022160181	0.546880962	stage_E14_vs_E10	ordinary_muscle_development		0	FALSE
ENSGALG00010014952	C21orf58	PRJNA729271	-1.703632364	0.022208004	1.703632364	stage_E14_vs_E10	ordinary_muscle_development	D_vs_E10;stage_1W_vs_E10;stage_3W_vs_E10;stage	1	TRUE
ENSGALG00010027379	OPTC	PRJNA729271	-1.64166763	0.02232234	1.64166763	stage_E14_vs_E10	ordinary_muscle_development	D_vs_E10;stage_1W_vs_E10;stage_3W_vs_E10;stage	1	TRUE
ENSGALG00010019473	OXTR	PRJNA729271	-1.224053014	0.022356979	1.224053014	stage_E14_vs_E10	ordinary_muscle_development	D_vs_E10;stage_1W_vs_E10;stage_3W_vs_E10;stage	1	TRUE
ENSGALG00010000175	ENSGALG00010000175	PRJNA729271	-2.692741754	0.022610527	2.692741754	stage_E14_vs_E10	ordinary_muscle_development	D_vs_E10;stage_1W_vs_E10;stage_3W_vs_E10;stage	1	TRUE
ENSGALG00010028081	UBQLN4	PRJNA729271	-0.397575068	0.022662967	0.397575068	stage_E14_vs_E10	ordinary_muscle_development		0	FALSE
ENSGALG00010025452	TRIM33	PRJNA729271	0.303643532	0.02280697	0.303643532	stage_E14_vs_E10	ordinary_muscle_development		0	FALSE
ENSGALG00010010968	RRP7A	PRJNA729271	0.585206173	0.022870251	0.585206173	stage_E14_vs_E10	ordinary_muscle_development		0	FALSE
ENSGALG00010001878	XK	PRJNA729271	-0.41049341	0.02298196	0.41049341	stage_E14_vs_E10	ordinary_muscle_development		0	FALSE
ENSGALG00010016111	EPB41	PRJNA729271	-0.243644512	0.023323274	0.243644512	stage_E14_vs_E10	ordinary_muscle_development		0	FALSE
ENSGALG00010001129	TM4SF4	PRJNA729271	-2.353869076	0.023346258	2.353869076	stage_E14_vs_E10	ordinary_muscle_development	D_vs_E10;stage_1W_vs_E10;stage_3W_vs_E10;stage	1	TRUE
ENSGALG00010009602	TSTD2	PRJNA729271	-0.198636542	0.023356968	0.198636542	stage_E14_vs_E10	ordinary_muscle_development		0	FALSE
ENSGALG00010016177	ENSGALG00010016177	PRJNA729271	3.6466032	0.023380724	3.6466032	stage_E14_vs_E10	ordinary_muscle_development	D_vs_E10;stage_1W_vs_E10;stage_3W_vs_E10;stage	1	TRUE
ENSGALG00010000326	ENSGALG00010000326	PRJNA729271	4.228233594	0.023392909	4.228233594	stage_E14_vs_E10	ordinary_muscle_development	D_vs_E10;stage_1W_vs_E10;stage_3W_vs_E10;stage	1	TRUE
ENSGALG00010011925	RRP1B	PRJNA729271	-1.060283011	0.023399564	1.060283011	stage_E14_vs_E10	ordinary_muscle_development	D_vs_E10;stage_1W_vs_E10;stage_3W_vs_E10;stage	1	TRUE
ENSGALG00010013097	ENSGALG00010013097	PRJNA729271	-1.111116617	0.023561232	1.111116617	stage_E14_vs_E10	ordinary_muscle_development	D_vs_E10;stage_1W_vs_E10;stage_3W_vs_E10;stage	1	TRUE
ENSGALG00010016653	EPHA2	PRJNA729271	-0.533721787	0.02386249	0.533721787	stage_E14_vs_E10	ordinary_muscle_development		0	FALSE
ENSGALG00010023999	DGCR14	PRJNA729271	0.231535321	0.023877187	0.231535321	stage_E14_vs_E10	ordinary_muscle_development		0	FALSE
ENSGALG00010003018	MOGAT2	PRJNA729271	-2.644395531	0.023882242	2.644395531	stage_E14_vs_E10	ordinary_muscle_development	D_vs_E10;stage_1W_vs_E10;stage_3W_vs_E10;stage	1	TRUE
ENSGALG00010028887	SF3B4	PRJNA729271	-0.415878436	0.02398002	0.415878436	stage_E14_vs_E10	ordinary_muscle_development		0	FALSE
ENSGALG00010017256	CARHSP1	PRJNA729271	0.303762551	0.02407929	0.303762551	stage_E14_vs_E10	ordinary_muscle_development		0	FALSE
ENSGALG00010011181	ENSGALG00010011181	PRJNA729271	-0.686898319	0.024254103	0.686898319	stage_E14_vs_E10	ordinary_muscle_development		0	FALSE
ENSGALG00010013065	FKTN	PRJNA729271	-0.245549864	0.024389465	0.245549864	stage_E14_vs_E10	ordinary_muscle_development		0	FALSE
ENSGALG00010011218	ENSGALG00010011218	PRJNA729271	-1.439780953	0.024414849	1.439780953	stage_E14_vs_E10	ordinary_muscle_development	D_vs_E10;stage_1W_vs_E10;stage_3W_vs_E10;stage	1	TRUE
ENSGALG00010010317	ENSGALG00010010317	PRJNA729271	-0.255255634	0.024504788	0.255255634	stage_E14_vs_E10	ordinary_muscle_development		0	FALSE
ENSGALG00010001239	ENSGALG00010001239	PRJNA729271	0.580494994	0.024514285	0.580494994	stage_E14_vs_E10	ordinary_muscle_development		0	FALSE
ENSGALG00010022065	PABPC1L	PRJNA729271	0.589865104	0.024597642	0.589865104	stage_E14_vs_E10	ordinary_muscle_development		0	FALSE
ENSGALG00010016854	TAF1A	PRJNA729271	-0.250649676	0.024727808	0.250649676	stage_E14_vs_E10	ordinary_muscle_development		0	FALSE
ENSGALG00010007795	ZNF236	PRJNA729271	0.274985665	0.024767357	0.274985665	stage_E14_vs_E10	ordinary_muscle_development		0	FALSE
ENSGALG00010005242	CCDC80	PRJNA729271	0.5627141	0.024785834	0.5627141	stage_E14_vs_E10	ordinary_muscle_development		0	FALSE
ENSGALG00010022534	CFAP57	PRJNA729271	-1.32309299	0.024852765	1.32309299	stage_E14_vs_E10	ordinary_muscle_development	D_vs_E10;stage_1W_vs_E10;stage_3W_vs_E10;stage	1	TRUE
ENSGALG00010029171	ENSGALG00010029171	PRJNA729271	0.343791704	0.024924019	0.343791704	stage_E14_vs_E10	ordinary_muscle_development		0	FALSE
ENSGALG00010002442	ENSGALG00010002442	PRJNA729271	1.100900308	0.024976135	1.100900308	stage_E14_vs_E10	ordinary_muscle_development	D_vs_E10;stage_1W_vs_E10;stage_3W_vs_E10;stage	1	TRUE
ENSGALG00010013409	DAB2	PRJNA729271	-0.362333351	0.025078413	0.362333351	stage_E14_vs_E10	ordinary_muscle_development		0	FALSE
ENSGALG00010008821	ENSGALG00010008821	PRJNA729271	0.324780997	0.025091563	0.324780997	stage_E14_vs_E10	ordinary_muscle_development		0	FALSE
ENSGALG00010001906	SKAP2	PRJNA729271	0.306326481	0.025155811	0.306326481	stage_E14_vs_E10	ordinary_muscle_development		0	FALSE
ENSGALG00010021897	ENSGALG00010021897	PRJNA729271	-0.522986652	0.025191727	0.522986652	stage_E14_vs_E10	ordinary_muscle_development		0	FALSE
ENSGALG00010015970	THPO	PRJNA729271	0.886741644	0.025410869	0.886741644	stage_E14_vs_E10	ordinary_muscle_development		0	FALSE
ENSGALG00010024782	HOXD12	PRJNA729271	-5.540697025	0.025600236	5.540697025	stage_E14_vs_E10	ordinary_muscle_development	D_vs_E10;stage_1W_vs_E10;stage_3W_vs_E10;stage	1	TRUE
ENSGALG00010028866	HORMAD1	PRJNA729271	-1.172881313	0.025741829	1.172881313	stage_E14_vs_E10	ordinary_muscle_development	D_vs_E10;stage_1W_vs_E10;stage_3W_vs_E10;stage	1	TRUE
ENSGALG00010015769	TMEM50A	PRJNA729271	0.238741565	0.025814892	0.238741565	stage_E14_vs_E10	ordinary_muscle_development		0	FALSE
ENSGALG00010019346	POU3F4	PRJNA729271	-1.363884923	0.025989896	1.363884923	stage_E14_vs_E10	ordinary_muscle_development	D_vs_E10;stage_1W_vs_E10;stage_3W_vs_E10;stage	1	TRUE
ENSGALG00010006232	ZNF654	PRJNA729271	-0.207595832	0.026014139	0.207595832	stage_E14_vs_E10	ordinary_muscle_development		0	FALSE
ENSGALG00010029238	CAMKK1	PRJNA729271	0.56649256	0.026483815	0.56649256	stage_E14_vs_E10	ordinary_muscle_development		0	FALSE
ENSGALG00010022296	CIAPIN1	PRJNA729271	-0.232679659	0.026552388	0.232679659	stage_E14_vs_E10	ordinary_muscle_development		0	FALSE

ENSGALG00010020032	CHST12	PRJNA729271	-0.288061978	0.026608306	0.288061978	stage_E14_vs_E10	ordinary_muscle_development		0	FALSE
ENSGALG00010021271	ENSGALG00010021271	PRJNA729271	-1.098856877	0.026652813	1.098856877	stage_E14_vs_E10	ordinary_muscle_development	D_vs_E10;stage_1W_vs_E10;stage_3W_vs_E10;stage	1	TRUE
ENSGALG00010012711	ELL2	PRJNA729271	-0.296657779	0.0268879	0.296657779	stage_E14_vs_E10	ordinary_muscle_development		0	FALSE
ENSGALG00010021013	FAP	PRJNA729271	0.744206942	0.026893049	0.744206942	stage_E14_vs_E10	ordinary_muscle_development		0	FALSE
ENSGALG00010004383	ENSGALG00010004383	PRJNA729271	-1.260223344	0.027021181	1.260223344	stage_E14_vs_E10	ordinary_muscle_development	D_vs_E10;stage_1W_vs_E10;stage_3W_vs_E10;stage	1	TRUE
ENSGALG00010022748	ENSGALG00010022748	PRJNA729271	-0.806704534	0.02706831	0.806704534	stage_E14_vs_E10	ordinary_muscle_development		0	FALSE
ENSGALG00010016534	TRIT1	PRJNA729271	-0.33887865	0.027069176	0.33887865	stage_E14_vs_E10	ordinary_muscle_development		0	FALSE
ENSGALG00010026700	C8B	PRJNA729271	-2.467132842	0.027106209	2.467132842	stage_E14_vs_E10	ordinary_muscle_development	D_vs_E10;stage_1W_vs_E10;stage_3W_vs_E10;stage	1	TRUE
ENSGALG00010004868	NPAT	PRJNA729271	-0.284607831	0.027113654	0.284607831	stage_E14_vs_E10	ordinary_muscle_development		0	FALSE
ENSGALG00010007369	ENSGALG00010007369	PRJNA729271	0.341874815	0.027119425	0.341874815	stage_E14_vs_E10	ordinary_muscle_development		0	FALSE
ENSGALG00010005354	M1AP	PRJNA729271	-0.831843917	0.027121202	0.831843917	stage_E14_vs_E10	ordinary_muscle_development		0	FALSE
ENSGALG00010018285	BBIP1	PRJNA729271	-0.446013477	0.02722753	0.446013477	stage_E14_vs_E10	ordinary_muscle_development		0	FALSE
ENSGALG00010007857	ENSGALG00010007857	PRJNA729271	-0.80473066	0.027269178	0.80473066	stage_E14_vs_E10	ordinary_muscle_development		0	FALSE
ENSGALG00010006121	ENSGALG00010006121	PRJNA729271	-0.756662433	0.027273974	0.756662433	stage_E14_vs_E10	ordinary_muscle_development		0	FALSE
ENSGALG00010007049	ENSGALG00010007049	PRJNA729271	0.806788147	0.027293461	0.806788147	stage_E14_vs_E10	ordinary_muscle_development		0	FALSE
ENSGALG00010013453	PUM1	PRJNA729271	-0.175192286	0.027445271	0.175192286	stage_E14_vs_E10	ordinary_muscle_development		0	FALSE
ENSGALG00010025890	SCG2	PRJNA729271	1.180485905	0.027549307	1.180485905	stage_E14_vs_E10	ordinary_muscle_development	D_vs_E10;stage_1W_vs_E10;stage_3W_vs_E10;stage	1	TRUE
ENSGALG00010028017	ENSGALG00010028017	PRJNA729271	-3.091091952	0.027762528	3.091091952	stage_E14_vs_E10	ordinary_muscle_development	D_vs_E10;stage_1W_vs_E10;stage_3W_vs_E10;stage	1	TRUE
ENSGALG00010008969	PPP1R37	PRJNA729271	0.431886487	0.027967983	0.431886487	stage_E14_vs_E10	ordinary_muscle_development		0	FALSE
ENSGALG00010018908	ENSGALG00010018908	PRJNA729271	-0.568730319	0.027993857	0.568730319	stage_E14_vs_E10	ordinary_muscle_development		0	FALSE
ENSGALG00010008196	PDCD6	PRJNA729271	0.337461393	0.02813523	0.337461393	stage_E14_vs_E10	ordinary_muscle_development		0	FALSE
ENSGALG00010023258	ENSGALG00010023258	PRJNA729271	-0.910182872	0.028198095	0.910182872	stage_E14_vs_E10	ordinary_muscle_development		0	FALSE
ENSGALG00010012765	PHF5A	PRJNA729271	-0.253195808	0.028217041	0.253195808	stage_E14_vs_E10	ordinary_muscle_development		0	FALSE
ENSGALG00010008983	ENSGALG00010008983	PRJNA729271	0.760083176	0.02837728	0.760083176	stage_E14_vs_E10	ordinary_muscle_development		0	FALSE
ENSGALG00010010955	GTF3C3	PRJNA729271	0.166087692	0.028490551	0.166087692	stage_E14_vs_E10	ordinary_muscle_development		0	FALSE
ENSGALG00010016945	BDH2	PRJNA729271	0.33410135	0.028503521	0.33410135	stage_E14_vs_E10	ordinary_muscle_development		0	FALSE
ENSGALG00010000051	GDPGP1	PRJNA729271	0.43900997	0.028657545	0.43900997	stage_E14_vs_E10	ordinary_muscle_development		0	FALSE
ENSGALG00010017465	TMEM234	PRJNA729271	-0.473870762	0.029163576	0.473870762	stage_E14_vs_E10	ordinary_muscle_development		0	FALSE
ENSGALG00010015169	ENSGALG00010015169	PRJNA729271	1.232047769	0.029283176	1.232047769	stage_E14_vs_E10	ordinary_muscle_development	D_vs_E10;stage_1W_vs_E10;stage_3W_vs_E10;stage	1	TRUE
ENSGALG00010021455	CFAP58	PRJNA729271	-1.047338194	0.029405625	1.047338194	stage_E14_vs_E10	ordinary_muscle_development	D_vs_E10;stage_1W_vs_E10;stage_3W_vs_E10;stage	1	TRUE
ENSGALG00010019910	ENSGALG00010019910	PRJNA729271	0.565393798	0.029442974	0.565393798	stage_E14_vs_E10	ordinary_muscle_development		0	FALSE
ENSGALG00010016450	ENSGALG00010016450	PRJNA729271	-0.651119075	0.029609919	0.651119075	stage_E14_vs_E10	ordinary_muscle_development		0	FALSE
ENSGALG00010027250	ENSGALG00010027250	PRJNA729271	-0.395027291	0.029920367	0.395027291	stage_E14_vs_E10	ordinary_muscle_development		0	FALSE
ENSGALG00010028823	GFI1B	PRJNA729271	0.715841522	0.029977378	0.715841522	stage_E14_vs_E10	ordinary_muscle_development		0	FALSE
ENSGALG00010028035	ENSGALG00010028035	PRJNA729271	1.71590815	0.030087993	1.71590815	stage_E14_vs_E10	ordinary_muscle_development	D_vs_E10;stage_1W_vs_E10;stage_3W_vs_E10;stage	1	TRUE
ENSGALG00010009026	RAPGEF2	PRJNA729271	-0.311450879	0.030160884	0.311450879	stage_E14_vs_E10	ordinary_muscle_development		0	FALSE
ENSGALG00010012067	ENSGALG00010012067	PRJNA729271	0.1894206	0.030271026	0.1894206	stage_E14_vs_E10	ordinary_muscle_development		0	FALSE
ENSGALG00010018327	SNAP25	PRJNA729271	-1.266154687	0.030316734	1.266154687	stage_E14_vs_E10	ordinary_muscle_development	D_vs_E10;stage_1W_vs_E10;stage_3W_vs_E10;stage	1	TRUE
ENSGALG00010018736	EEF2KMT	PRJNA729271	0.297668164	0.030705887	0.297668164	stage_E14_vs_E10	ordinary_muscle_development		0	FALSE
ENSGALG00010003263	ENSGALG00010003263	PRJNA729271	1.724642152	0.030718783	1.724642152	stage_E14_vs_E10	ordinary_muscle_development	D_vs_E10;stage_1W_vs_E10;stage_3W_vs_E10;stage	1	TRUE
ENSGALG00010029780	TEX14	PRJNA729271	0.585888055	0.030751014	0.585888055	stage_E14_vs_E10	ordinary_muscle_development		0	FALSE
ENSGALG00010013823	GUF1	PRJNA729271	0.254208405	0.031028363	0.254208405	stage_E14_vs_E10	ordinary_muscle_development		0	FALSE
ENSGALG00010004717	ALKBH8	PRJNA729271	-0.431376553	0.031034239	0.431376553	stage_E14_vs_E10	ordinary_muscle_development		0	FALSE
ENSGALG00010000619	ENSGALG00010000619	PRJNA729271	0.686513564	0.031045233	0.686513564	stage_E14_vs_E10	ordinary_muscle_development		0	FALSE
ENSGALG00010024408	ENSGALG00010024408	PRJNA729271	-3.150688583	0.031145622	3.150688583	stage_E14_vs_E10	ordinary_muscle_development	D_vs_E10;stage_1W_vs_E10;stage_3W_vs_E10;stage	1	TRUE
ENSGALG00010006071	TEX30	PRJNA729271	-0.550721544	0.031511025	0.550721544	stage_E14_vs_E10	ordinary_muscle_development		0	FALSE
ENSGALG00010016113	DTWD1	PRJNA729271	0.333761866	0.031712425	0.333761866	stage_E14_vs_E10	ordinary_muscle_development		0	FALSE
ENSGALG00010022553	PRPF6	PRJNA729271	-0.391377432	0.031921032	0.391377432	stage_E14_vs_E10	ordinary_muscle_development		0	FALSE
ENSGALG00010029598	SUPT4H1	PRJNA729271	-0.338062255	0.03196674	0.338062255	stage_E14_vs_E10	ordinary_muscle_development		0	FALSE
ENSGALG00010014851	CALHM4	PRJNA729271	0.87722371	0.032145293	0.87722371	stage_E14_vs_E10	ordinary_muscle_development		0	FALSE
ENSGALG00010029546	WSB1	PRJNA729271	0.375841894	0.032227301	0.375841894	stage_E14_vs_E10	ordinary_muscle_development		0	FALSE
ENSGALG00010022799	DCAF17	PRJNA729271	-0.348252551	0.032342096	0.348252551	stage_E14_vs_E10	ordinary_muscle_development		0	FALSE
ENSGALG00010014998	NALF1	PRJNA729271	-1.263819484	0.032350702	1.263819484	stage_E14_vs_E10	ordinary_muscle_development	D_vs_E10;stage_1W_vs_E10;stage_3W_vs_E10;stage	1	TRUE
ENSGALG00010024101	CNEP1R1	PRJNA729271	0.20123159	0.032446076	0.20123159	stage_E14_vs_E10	ordinary_muscle_development		0	FALSE
ENSGALG00010016925	PHACTR4	PRJNA729271	-0.312641498	0.032459301	0.312641498	stage_E14_vs_E10	ordinary_muscle_development		0	FALSE
ENSGALG00010027705	S100A11	PRJNA729271	-0.56339074	0.032492711	0.56339074	stage_E14_vs_E10	ordinary_muscle_development		0	FALSE
ENSGALG00010020813	NPY4R	PRJNA729271	1.217367401	0.03255575	1.217367401	stage_E14_vs_E10	ordinary_muscle_development	D_vs_E10;stage_1W_vs_E10;stage_3W_vs_E10;stage	1	TRUE
ENSGALG00010011588	COLGALT2	PRJNA729271	-0.450596432	0.032951318	0.450596432	stage_E14_vs_E10	ordinary_muscle_development		0	FALSE
ENSGALG00010010748	SF3B1	PRJNA729271	-0.160802668	0.032962561	0.160802668	stage_E14_vs_E10	ordinary_muscle_development		0	FALSE
ENSGALG00010012154	CWC27	PRJNA729271	-0.255856665	0.033068481	0.255856665	stage_E14_vs_E10	ordinary_muscle_development		0	FALSE
ENSGALG00010026480	USP49	PRJNA729271	-0.479391628	0.033344123	0.479391628	stage_E14_vs_E10	ordinary_muscle_development		0	FALSE
ENSGALG00010026259	MED15	PRJNA729271	-0.199790043	0.033517847	0.199790043	stage_E14_vs_E10	ordinary_muscle_development		0	FALSE
ENSGALG00010002472	CDC37	PRJNA729271	-0.484567237	0.033712221	0.484567237	stage_E14_vs_E10	ordinary_muscle_development		0	FALSE
ENSGALG00010001436	ENSGALG00010001436	PRJNA729271	-3.488586652	0.03374748	3.488586652	stage_E14_vs_E10	ordinary_muscle_development	D_vs_E10;stage_1W_vs_E10;stage_3W_vs_E10;stage	1	TRUE
ENSGALG00010021206	HSD17B1	PRJNA729271	0.90616931	0.033757944	0.90616931	stage_E14_vs_E10	ordinary_muscle_development		0	FALSE
ENSGALG00010027380	GPX1	PRJNA729271	-0.206815788	0.033852515	0.206815788	stage_E14_vs_E10	ordinary_muscle_development		0	FALSE
ENSGALG00010029746	TRPV1	PRJNA729271	0.710805615	0.03402715	0.710805615	stage_E14_vs_E10	ordinary_muscle_development		0	FALSE
ENSGALG00010025706	ENSGALG00010025706	PRJNA729271	7.155736898	0.034082087	7.155736898	stage_E14_vs_E10	ordinary_muscle_development	D_vs_E10;stage_1W_vs_E10;stage_3W_vs_E10;stage	1	TRUE
ENSGALG00010017612	CLDN11	PRJNA729271	-0.989756239	0.034107608	0.989756239	stage_E14_vs_E10	ordinary_muscle_development		0	FALSE
ENSGALG00010017493	YPEL5	PRJNA729271	0.325749698	0.034113946	0.325749698	stage_E14_vs_E10	ordinary_muscle_development		0	FALSE
ENSGALG00010020845	RAB35	PRJNA729271	0.206720818	0.034281261	0.206720818	stage_E14_vs_E10	ordinary_muscle_development		0	FALSE
ENSGALG00010011416	ENSGALG00010011416	PRJNA729271	0.962157324	0.034317279	0.962157324	stage_E14_vs_E10	ordinary_muscle_development		0	FALSE
ENSGALG00010015038	PCSK5	PRJNA729271	-0.458906941	0.034378211	0.458906941	stage_E14_vs_E10	ordinary_muscle_development		0	FALSE
ENSGALG00010023062	STX2	PRJNA729271	-0.232672653	0.034489431	0.232672653	stage_E14_vs_E10	ordinary_muscle_development		0	FALSE
ENSGALG00010020302	HAAO	PRJNA729271	3.183588685	0.034597162	3.183588685	stage_E14_vs_E10	ordinary_muscle_development	D_vs_E10;stage_1W_vs_E10;stage_3W_vs_E10;stage	1	TRUE
ENSGALG00010003420	ENSGALG00010003420	PRJNA729271	1.417367343	0.034660969	1.417367343	stage_E14_vs_E10	ordinary_muscle_development	D_vs_E10;stage_1W_vs_E10;stage_3W_vs_E10;stage	1	TRUE
ENSGALG00010005851	SLC15A1	PRJNA729271	-2.56424225	0.034984913	2.56424225	stage_E14_vs_E10	ordinary_muscle_development	D_vs_E10;stage_1W_vs_E10;stage_3W_vs_E10;stage	1	TRUE
ENSGALG00010014697	ENSGALG00010014697	PRJNA729271	1.468720818	0.035106372	1.468720818	stage_E14_vs_E10	ordinary_muscle_development	D_vs_E10;stage_1W_vs_E10;stage_3W_vs_E10;stage	1	TRUE
ENSGALG00010019577	MXD1	PRJNA729271	-0.436681166	0.035156786	0.436681166	stage_E14_vs_E10	ordinary_muscle_development		0	FALSE
ENSGALG00010018537	ENSGALG00010018537	PRJNA729271	-1.606114413	0.035160007	1.606114413	stage_E14_vs_E10	ordinary_muscle_development	D_vs_E10;stage_1W_vs_E10;stage_3W_vs_E10;stage	1	TRUE
ENSGALG00010001779	ENSGALG00010001779	PRJNA729271	1.219777302	0.035298068	1.219777302	stage_E14_vs_E10	ordinary_muscle_development	D_vs_E10;stage_1W_vs_E10;stage_3W_vs_E10;stage	1	TRUE
ENSGALG00010017783	STAR	PRJNA729271	0.836170064	0.035450321	0.836170064	stage_E14_vs_E10	ordinary_muscle_development		0	FALSE
ENSGALG00010010179	CDHR2	PRJNA729271	1.821355018	0.035644444	1.821355018	stage_E14_vs_E10	ordinary_muscle_development	D_vs_E10;stage_1W_vs_E10;stage_3W_vs_E10;stage	1	TRUE
ENSGALG00010029299	PRKRIP1	PRJNA729271	-0.203100332	0.035665498	0.203100332	stage_E14_vs_E10	ordinary_muscle_development		0	FALSE
ENSGALG00010002328	ENSGALG00010002328	PRJNA729271	0.553341391	0.035682773	0.553341391	stage_E14_vs_E10	ordinary_muscle_development		0	FALSE
ENSGALG00010029179	PIGW	PRJNA729271	0.330022212	0.035831582	0.330022212	stage_E14_vs_E10	ordinary_muscle_development		0	FALSE
ENSGALG00010003374	SYCE2	PRJNA729271	-0.909969981	0.035850128	0.909969981	stage_E14_vs_E10	ordinary_muscle_development		0	FALSE
ENSGALG00010023431	BCAR1	PRJNA729271	-0.363173983	0.035850128	0.363173983	stage_E14_vs_E10	ordinary_muscle_development		0	FALSE
ENSGALG00010013646	CLDN1	PRJNA729271	0.546052414	0.035885639	0.546052414	stage_E14_vs_E10	ordinary_muscle_development		0	FALSE
ENSGALG00010012091	ENSGALG00010012091	PRJNA729271	-0.98315525	0.035966757	0.98315525	stage_E14_vs_E10	ordinary_muscle_development		0	FALSE
ENSGALG00010006670	ANKRD16	PRJNA729271	0.400150808	0.036111567	0.400150808	stage_E14_vs_E10	ordinary_muscle_development		0	FALSE
ENSGALG00010020283	FBXO42	PRJNA729271	-0.507989173	0.036148733	0.507989173	stage_E14_vs_E10	ordinary_muscle_development		0	FALSE
ENSGALG00010004739	ENSGALG00010004739	PRJNA729271	1.561142916	0.036163376	1.561142916	stage_E14_vs_E10	ordinary_muscle_development	D_vs_E10;stage_1W_vs_E10;stage_3W_vs_E10;stage	1	TRUE
ENSGALG00010029610	ERN1	PRJNA729271	-0.182348252	0.036177212	0.182348252	stage_E14_vs_E10	ordinary_muscle_development		0	FALSE
ENSGALG00010020680	CFAP221	PRJNA729271	-1.396931394	0.036203002	1.396931394	stage_E14_vs_E10	ordinary_muscle_development	D_vs_E10;stage_1W_vs_E10;stage_3W_vs_E10;stage	1	TRUE
ENSGALG00010004086	UTP3	PRJNA729271	-0.436916238	0.036410802	0.436916238	stage_E14_vs_E10	ordinary_muscle_development		0	FALSE
ENSGALG00010004328	ENSGALG00010004328	PRJNA729271	-1.552745005	0.03642628	1.552745005	stage_E14_vs_E10	ordinary_muscle_development	D_vs_E10;stage_1W_vs_E10;stage_3W_vs_E10;stage	1	TRUE
ENSGALG00010024368	DPAGT1	PRJNA729271	0.17391983	0.036699726	0.17391983	stage_E14_vs_E10	ordinary_muscle_development		0	FALSE
ENSGALG00010007324	ENSGALG00010007324	PRJNA729271	-0.320772373	0.036752291	0.320772373	stage_E14_vs_E10	ordinary_muscle_development		0	FALSE
ENSGALG00010012629	NUS1	PRJNA729271	-0.245266769	0.036825745	0.245266769	stage_E14_vs_E10	ordinary_muscle_development		0	FALSE
ENSGALG00010017083	ENSGALG00010017083	PRJNA729271	0.911974665	0.037246149	0.911974665	stage_E14_vs_E10	ordinary_muscle_development		0	FALSE
ENSGALG00010004109	AGMO	PRJNA729271	-1.197381714	0.037248007	1.197381714	stage_E14_vs_E10	ordinary_muscle_development	D_vs_E10;stage_1W_vs_E10;stage_3W_vs_E10;stage	1	TRUE
ENSGALG00010009665	DGKQ	PRJNA729271	0.373376698	0.037398162	0.373376698	stage_E14_vs_E10	ordinary_muscle_development		0	FALSE
ENSGALG00010016842	NPY7R	PRJNA729271	-0.616040905	0.037447901	0.616040905	stage_E14_vs_E10	ordinary_muscle_development		0	FALSE
ENSGALG00010012393	ENSGALG00010012393	PRJNA729271	-3.60919464	0.037462469	3.60919464	stage_E14_vs_E10	ordinary_muscle_development	D_vs_E10;stage_1W_vs_E10;stage_3W_vs_E10;stage	1	TRUE
ENSGALG00010020524	ENSGALG00010020524	PRJNA729271	0.954691341	0.037714055	0.954691341	stage_E14_vs_E10	ordinary_muscle_development		0	FALSE
ENSGALG00010017136	ENSGALG00010017136	PRJNA729271	-0.868298614	0.037828539	0.868298614	stage_E14_vs_E10	ordinary_muscle_development		0	FALSE
ENSGALG00010012991	MFSD9	PRJNA729271	0.222198824	0.037947188	0.222198824	stage_E14_vs_E10	ordinary_muscle_development		0	FALSE
ENSGALG00010028577	CERS2	PRJNA729271	0.291193478	0.038022824	0.291193478	stage_E14_vs_E10	ordinary_muscle_development		0	FALSE
ENSGALG00010004057	ENSGALG00010004057	PRJNA729271	-0.955395839	0.038644741	0.955395839	stage_E14_vs_E10	ordinary_muscle_development		0	FALSE
ENSGALG00010019744	KBP	PRJNA729271	-1.1221547	0.038661264	1.1221547	stage_E14_vs_E10	ordinary_muscle_development	D_vs_E10;stage_1W_vs_E10;stage_3W_vs_E10;stage	1	TRUE
ENSGALG00010000630	ENSGALG00010000630	PRJNA729271	0.794288198	0.038813536	0.794288198	stage_E14_vs_E10	ordinary_muscle_development		0	FALSE
ENSGALG00010013236	MON2	PRJNA729271	0.183885148	0.038962624	0.183885148	stage_E14_vs_E10	ordinary_muscle_development		0	FALSE
ENSGALG00010001173	IFT80	PRJNA729271	-0.247336962	0.039274395	0.247336962	stage_E14_vs_E10	ordinary_muscle_development		0	FALSE
ENSGALG00010017384	ARID4A	PRJNA729271	-0.201645841	0.039487949	0.201645841	stage_E14_vs_E10	ordinary_muscle_development		0	FALSE
ENSGALG00010011439	ENSGALG00010011439	PRJNA729271	-0.72321982	0.03963433	0.72321982	stage_E14_vs_E10	ordinary_muscle_development		0	FALSE
ENSGALG00010013143	ENSGALG00010013143	PRJNA729271	0.887805981	0.039754428	0.887805981	stage_E14_vs_E10	ordinary_muscle_development		0	FALSE
ENSGALG00010018518	ENSGALG00010018518	PRJNA729271	-2.271126844	0.040227086	2.271126844	stage_E14_vs_E10	ordinary_muscle_development	D_vs_E10;stage_1W_vs_E10;stage_3W_vs_E10;stage	1	TRUE
ENSGALG00010007004	ENSGALG00010007004	PRJNA729271	1.259770789	0.040240989	1.259770789	stage_E14_vs_E10	ordinary_muscle_development	D_vs_E10;stage_1W_vs_E10;stage_3W_vs_E10;stage	1	TRUE
ENSGALG00010004375	ENSGALG00010004375	PRJNA729271	-0.5080689	0.040382753	0.5080689	stage_E14_vs_E10	ordinary_muscle_development		0	FALSE
ENSGALG00010018993	ENSGALG00010018993	PRJNA729271	1.032164842	0.040537292	1.032164842	stage_E14_vs_E10	ordinary_muscle_development	D_vs_E10;stage_1W_vs_E10;stage_3W_vs_E10;stage	1	TRUE
ENSGALG00010004323	TRDMT1	PRJNA729271	-0.345443585	0.040602883	0.345443585	stage_E14_vs_E10	ordinary_muscle_development		0	FALSE
ENSGALG00010017719	ENSGALG00010017719	PRJNA729271	3.80405474	0.040694053	3.80405474	stage_E14_vs_E10	ordinary_muscle_development	D_vs_E10;stage_1W_vs_E10;stage_3W_vs_E10;stage	1	TRUE
ENSGALG00010028503	NDOR1	PRJNA729271	-0.274395509	0.040831493	0.274395509	stage_E14_vs_E10	ordinary_muscle_development		0	FALSE
ENSGALG00010013224	ATP6V0A4	PRJNA729271	1.504734441	0.04098772	1.504734441	stage_E14_vs_E10	ordinary_muscle_development	D_vs_E10;stage_1W_vs_E10;stage_3W_vs_E10;stage	1	TRUE
ENSGALG00010005981	AREL1	PRJNA729271	0.229233848	0.041239124	0.229233848	stage_E14_vs_E10	ordinary_muscle_development		0	FALSE
ENSGALG00010023201	ZNF280D	PRJNA729271	-0.276440074	0.041416802	0.276440074	stage_E14_vs_E10	ordinary_muscle_development		0	FALSE
ENSGALG00010029465	ENSGALG00010029465	PRJNA729271	-5.339064745	0.041900409	5.339064745	stage_E14_vs_E10	ordinary_muscle_development	D_vs_E10;stage_1W_vs_E10;stage_3W_vs_E10;stage	1	TRUE
ENSGALG00010005287	ENSGALG00010005287	PRJNA729271	-0.923985596	0.041971051	0.923985596	stage_E14_vs_E10	ordinary_muscle_development		0	FALSE
ENSGALG00010023101	CLCA2	PRJNA729271	2.222213307	0.042054695	2.222213307	stage_E14_vs_E10	ordinary_muscle_development	D_vs_E10;stage_1W_vs_E10;stage_3W_vs_E10;stage	1	TRUE
ENSGALG00010029198	PPM1D	PRJNA729271	0.37340208	0.042117155	0.37340208	stage_E14_vs_E10	ordinary_muscle_development		0	FALSE
ENSGALG00010002784	TRIM27.2	PRJNA729271	-1.372720669	0.042368546	1.372720669	stage_E14_vs_E10	ordinary_muscle_development	D_vs_E10;stage_1W_vs_E10;stage_3W_vs_E10;stage	1	TRUE
ENSGALG00010008438	SYBU	PRJNA729271	0.560286476	0.042411977	0.560286476	stage_E14_vs_E10	ordinary_muscle_development		0	FALSE
ENSGALG00010000613	ENSGALG00010000613	PRJNA729271	0.406796496	0.042466022	0.406796496	stage_E14_vs_E10	ordinary_muscle_development		0	FALSE
ENSGALG00010024561	ROBO3	PRJNA729271	-1.195231578	0.042697442	1.195231578	stage_E14_vs_E10	ordinary_muscle_development	D_vs_E10;stage_1W_vs_E10;stage_3W_vs_E10;stage	1	TRUE
ENSGALG00010017495	NT5M	PRJNA729271	0.286154744	0.042738632	0.286154744	stage_E14_vs_E10	ordinary_muscle_development		0	FALSE
ENSGALG00010021441	ENSGALG00010021441	PRJNA729271	-0.707177369	0.042749532	0.707177369	stage_E14_vs_E10	ordinary_muscle_development		0	FALSE
ENSGALG00010009020	ARHGAP35	PRJNA729271	-0.395418351	0.042871221	0.395418351	stage_E14_vs_E10	ordinary_muscle_development		0	FALSE
ENSGALG00010015436	SLC30A9	PRJNA729271	-0.210325549	0.043643204	0.210325549	stage_E14_vs_E10	ordinary_muscle_development		0	FALSE
ENSGALG00010016611	DNALI1	PRJNA729271	-0.453431917	0.04371417	0.453431917	stage_E14_vs_E10	ordinary_muscle_development		0	FALSE
ENSGALG00010003765	ENSGALG00010003765	PRJNA729271	1.888212509	0.043838574	1.888212509	stage_E14_vs_E10	ordinary_muscle_development	D_vs_E10;stage_1W_vs_E10;stage_3W_vs_E10;stage	1	TRUE
ENSGALG00010019205	ENSGALG00010019205	PRJNA729271	-0.577509422	0.043874702	0.577509422	stage_E14_vs_E10	ordinary_muscle_development		0	FALSE
ENSGALG00010012090	HELB	PRJNA729271	0.472343728	0.043915183	0.472343728	stage_E14_vs_E10	ordinary_muscle_development		0	FALSE
ENSGALG00010021636	C1orf159	PRJNA729271	0.416973577	0.044353855	0.416973577	stage_E14_vs_E10	ordinary_muscle_development		0	FALSE
ENSGALG00010011715	SLCO1B3	PRJNA729271	3.761244807	0.044633085	3.761244807	stage_E14_vs_E10	ordinary_muscle_development	D_vs_E10;stage_1W_vs_E10;stage_3W_vs_E10;stage	1	TRUE
ENSGALG00010014902	AASDH	PRJNA729271	0.273739942	0.044699818	0.273739942	stage_E14_vs_E10	ordinary_muscle_development		0	FALSE
ENSGALG00010027740	MARCHF2	PRJNA729271	-0.308729227	0.044919533	0.308729227	stage_E14_vs_E10	ordinary_muscle_development		0	FALSE
ENSGALG00010005645	FUCA2	PRJNA729271	0.33175323	0.044955141	0.33175323	stage_E14_vs_E10	ordinary_muscle_development		0	FALSE
ENSGALG00010028466	MAMDC4	PRJNA729271	1.342321898	0.04508925	1.342321898	stage_E14_vs_E10	ordinary_muscle_development	D_vs_E10;stage_1W_vs_E10;stage_3W_vs_E10;stage	1	TRUE
ENSGALG00010012878	GDNF	PRJNA729271	0.661776489	0.045092954	0.661776489	stage_E14_vs_E10	ordinary_muscle_development		0	FALSE
ENSGALG00010006054	ENSGALG00010006054	PRJNA729271	4.559795454	0.045141122	4.559795454	stage_E14_vs_E10	ordinary_muscle_development	D_vs_E10;stage_1W_vs_E10;stage_3W_vs_E10;stage	1	TRUE
ENSGALG00010020128	KIF6	PRJNA729271	-1.090578662	0.045157951	1.090578662	stage_E14_vs_E10	ordinary_muscle_development	D_vs_E10;stage_1W_vs_E10;stage_3W_vs_E10;stage	1	TRUE
ENSGALG00010023194	MRC2	PRJNA729271	-0.426350821	0.045162446	0.426350821	stage_E14_vs_E10	ordinary_muscle_development		0	FALSE
ENSGALG00010001070	C3orf33	PRJNA729271	-0.388234308	0.045210735	0.388234308	stage_E14_vs_E10	ordinary_muscle_development		0	FALSE
ENSGALG00010020468	ENSGALG00010020468	PRJNA729271	-1.199859361	0.045296353	1.199859361	stage_E14_vs_E10	ordinary_muscle_development	D_vs_E10;stage_1W_vs_E10;stage_3W_vs_E10;stage	1	TRUE
ENSGALG00010023012	ENSGALG00010023012	PRJNA729271	1.065694475	0.045403288	1.065694475	stage_E14_vs_E10	ordinary_muscle_development	D_vs_E10;stage_1W_vs_E10;stage_3W_vs_E10;stage	1	TRUE
ENSGALG00010008051	ENSGALG00010008051	PRJNA729271	-0.166351883	0.045413446	0.166351883	stage_E14_vs_E10	ordinary_muscle_development		0	FALSE
ENSGALG00010013388	SOWAHC	PRJNA729271	0.44406952	0.045427344	0.44406952	stage_E14_vs_E10	ordinary_muscle_development		0	FALSE
ENSGALG00010001367	ENSGALG00010001367	PRJNA729271	0.757561667	0.04561489	0.757561667	stage_E14_vs_E10	ordinary_muscle_development		0	FALSE
ENSGALG00010020525	ENSGALG00010020525	PRJNA729271	1.554114362	0.045846465	1.554114362	stage_E14_vs_E10	ordinary_muscle_development	D_vs_E10;stage_1W_vs_E10;stage_3W_vs_E10;stage	1	TRUE
ENSGALG00010001873	ENSGALG00010001873	PRJNA729271	-0.487603523	0.045930037	0.487603523	stage_E14_vs_E10	ordinary_muscle_development		0	FALSE
ENSGALG00010018206	KIZ	PRJNA729271	-0.269977192	0.04600084	0.269977192	stage_E14_vs_E10	ordinary_muscle_development		0	FALSE
ENSGALG00010012894	CLIC6	PRJNA729271	-1.475628335	0.046120249	1.475628335	stage_E14_vs_E10	ordinary_muscle_development	D_vs_E10;stage_1W_vs_E10;stage_3W_vs_E10;stage	1	TRUE
ENSGALG00010001158	FRMPD1	PRJNA729271	-0.803354955	0.046220901	0.803354955	stage_E14_vs_E10	ordinary_muscle_development		0	FALSE
ENSGALG00010005892	ENSGALG00010005892	PRJNA729271	1.753912551	0.046231689	1.753912551	stage_E14_vs_E10	ordinary_muscle_development	D_vs_E10;stage_1W_vs_E10;stage_3W_vs_E10;stage	1	TRUE
ENSGALG00010029667	TMEM104	PRJNA729271	-0.197685318	0.046625728	0.197685318	stage_E14_vs_E10	ordinary_muscle_development		0	FALSE
ENSGALG00010011891	HAL	PRJNA729271	1.098279849	0.046658662	1.098279849	stage_E14_vs_E10	ordinary_muscle_development	D_vs_E10;stage_1W_vs_E10;stage_3W_vs_E10;stage	1	TRUE
ENSGALG00010024898	NABP2	PRJNA729271	-0.657105783	0.047184598	0.657105783	stage_E14_vs_E10	ordinary_muscle_development		0	FALSE
ENSGALG00010027968	FKBP15	PRJNA729271	0.202019534	0.04735128	0.202019534	stage_E14_vs_E10	ordinary_muscle_development		0	FALSE
ENSGALG00010011523	PIGO	PRJNA729271	-0.261193958	0.047913259	0.261193958	stage_E14_vs_E10	ordinary_muscle_development		0	FALSE
ENSGALG00010020633	HOGA1	PRJNA729271	-0.774552967	0.048175893	0.774552967	stage_E14_vs_E10	ordinary_muscle_development		0	FALSE
ENSGALG00010009311	BNIP1	PRJNA729271	-0.234473381	0.048790914	0.234473381	stage_E14_vs_E10	ordinary_muscle_development		0	FALSE
ENSGALG00010024984	SHISAL2A	PRJNA729271	-1.187023464	0.049321843	1.187023464	stage_E14_vs_E10	ordinary_muscle_development	D_vs_E10;stage_1W_vs_E10;stage_3W_vs_E10;stage	1	TRUE
ENSGALG00010028045	ENSGALG00010028045	PRJNA729271	-1.401952581	0.049533044	1.401952581	stage_E14_vs_E10	ordinary_muscle_development	D_vs_E10;stage_1W_vs_E10;stage_3W_vs_E10;stage	1	TRUE
ENSGALG00010021628	REC114	PRJNA729271	-1.37873675	0.049641583	1.37873675	stage_E14_vs_E10	ordinary_muscle_development	D_vs_E10;stage_1W_vs_E10;stage_3W_vs_E10;stage	1	TRUE

ENSGALG00010014970	SLC51A	PRJNA729271	1.04474305	0.049811952	1.04474305	stage_E14_vs_E10	ordinary_muscle_development	D_vs_E10;stage_1W_vs_E10;stage_3W_vs_E10;stage	1	TRUE
ENSGALG00010002150	EIF3A	PRJNA729271	0.276662737	0.049925152	0.276662737	stage_E14_vs_E10	ordinary_muscle_development		0	FALSE
ENSGALG00010023519	DGKG	PRJNA729271	-0.503599086	0.050073304	0.503599086	stage_E14_vs_E10	ordinary_muscle_development		0	FALSE
ENSGALG00010003121	ARMC1	PRJNA729271	0.169367596	0.050077541	0.169367596	stage_E14_vs_E10	ordinary_muscle_development		0	FALSE
ENSGALG00010005830	USP12	PRJNA729271	-0.397493428	0.050368727	0.397493428	stage_E14_vs_E10	ordinary_muscle_development		0	FALSE
ENSGALG00010014047	HM13	PRJNA729271	0.343174848	0.050594385	0.343174848	stage_E14_vs_E10	ordinary_muscle_development		0	FALSE
ENSGALG00010030109	FBF1	PRJNA729271	0.189034818	0.050820785	0.189034818	stage_E14_vs_E10	ordinary_muscle_development		0	FALSE
ENSGALG00010003401	ENSGALG00010003401	PRJNA729271	-3.664787263	0.050920107	3.664787263	stage_E14_vs_E10	ordinary_muscle_development		0	FALSE
ENSGALG00010018363	TCTE1	PRJNA729271	-0.858882792	0.050945065	0.858882792	stage_E14_vs_E10	ordinary_muscle_development		0	FALSE
ENSGALG00010028560	ENSGALG00010028560	PRJNA729271	7.686309626	0.051041277	7.686309626	stage_E14_vs_E10	ordinary_muscle_development		0	FALSE
ENSGALG00010023903	TBKBP1	PRJNA729271	-0.870542364	0.051157634	0.870542364	stage_E14_vs_E10	ordinary_muscle_development		0	FALSE
ENSGALG00010023271	PATL2	PRJNA729271	0.844654116	0.051168754	0.844654116	stage_E14_vs_E10	ordinary_muscle_development		0	FALSE
ENSGALG00010023222	ENSGALG00010023222	PRJNA729271	-1.27216781	0.051389186	1.27216781	stage_E14_vs_E10	ordinary_muscle_development		0	FALSE
ENSGALG00010028515	ENSGALG00010028515	PRJNA729271	7.401223472	0.051573887	7.401223472	stage_E14_vs_E10	ordinary_muscle_development		0	FALSE
ENSGALG00010009025	UTP23	PRJNA729271	-0.206782172	0.051795902	0.206782172	stage_E14_vs_E10	ordinary_muscle_development		0	FALSE
ENSGALG00010007963	LDAH	PRJNA729271	0.184846957	0.052125484	0.184846957	stage_E14_vs_E10	ordinary_muscle_development		0	FALSE
ENSGALG00010020820	VWA5B1	PRJNA729271	-1.089789394	0.052151466	1.089789394	stage_E14_vs_E10	ordinary_muscle_development		0	FALSE
ENSGALG00010015062	ENSGALG00010015062	PRJNA729271	0.470201955	0.052542676	0.470201955	stage_E14_vs_E10	ordinary_muscle_development		0	FALSE
ENSGALG00010011109	ENSGALG00010011109	PRJNA729271	-2.590299052	0.052635946	2.590299052	stage_E14_vs_E10	ordinary_muscle_development		0	FALSE
ENSGALG00010009153	SECISBP2	PRJNA729271	0.253123767	0.052669698	0.253123767	stage_E14_vs_E10	ordinary_muscle_development		0	FALSE
ENSGALG00010005023	POGLUT3	PRJNA729271	0.367523871	0.052774229	0.367523871	stage_E14_vs_E10	ordinary_muscle_development		0	FALSE
ENSGALG00010011101	ENSGALG00010011101	PRJNA729271	-1.740865156	0.052806064	1.740865156	stage_E14_vs_E10	ordinary_muscle_development		0	FALSE
ENSGALG00010021277	ENSGALG00010021277	PRJNA729271	-0.513387374	0.052826671	0.513387374	stage_E14_vs_E10	ordinary_muscle_development		0	FALSE
ENSGALG00010000060	HYPK	PRJNA729271	0.494086296	0.052834309	0.494086296	stage_E14_vs_E10	ordinary_muscle_development		0	FALSE
ENSGALG00010022800	KLHL10	PRJNA729271	-0.986563314	0.053178416	0.986563314	stage_E14_vs_E10	ordinary_muscle_development		0	FALSE
ENSGALG00010020313	FRA10AC1	PRJNA729271	0.239921273	0.053222312	0.239921273	stage_E14_vs_E10	ordinary_muscle_development		0	FALSE
ENSGALG00010012781	SPIN1L	PRJNA729271	-6.736984209	0.053750896	6.736984209	stage_E14_vs_E10	ordinary_muscle_development		0	FALSE
ENSGALG00010003278	ENSGALG00010003278	PRJNA729271	1.011194927	0.054218194	1.011194927	stage_E14_vs_E10	ordinary_muscle_development		0	FALSE
ENSGALG00010012945	CLINT1	PRJNA729271	0.13898401	0.054255922	0.13898401	stage_E14_vs_E10	ordinary_muscle_development		0	FALSE
ENSGALG00010021667	TBX15	PRJNA729271	0.385834361	0.055390309	0.385834361	stage_E14_vs_E10	ordinary_muscle_development		0	FALSE
ENSGALG00010004214	REPS1	PRJNA729271	-0.245833164	0.055899176	0.245833164	stage_E14_vs_E10	ordinary_muscle_development		0	FALSE
ENSGALG00010013587	SLC26A1	PRJNA729271	-1.066965914	0.05610852	1.066965914	stage_E14_vs_E10	ordinary_muscle_development		0	FALSE
ENSGALG00010012353	PIGG	PRJNA729271	0.334790475	0.05665697	0.334790475	stage_E14_vs_E10	ordinary_muscle_development		0	FALSE
ENSGALG00010029490	ENSGALG00010029490	PRJNA729271	-1.286868611	0.056813491	1.286868611	stage_E14_vs_E10	ordinary_muscle_development		0	FALSE
ENSGALG00010008440	ENSGALG00010008440	PRJNA729271	0.624058025	0.057390706	0.624058025	stage_E14_vs_E10	ordinary_muscle_development		0	FALSE
ENSGALG00010026173	CDK12	PRJNA729271	0.323833244	0.057531327	0.323833244	stage_E14_vs_E10	ordinary_muscle_development		0	FALSE
ENSGALG00010007976	ENSGALG00010007976	PRJNA729271	0.590391575	0.057745321	0.590391575	stage_E14_vs_E10	ordinary_muscle_development		0	FALSE
ENSGALG00010008417	IGFBP1	PRJNA729271	-2.116349747	0.058177796	2.116349747	stage_E14_vs_E10	ordinary_muscle_development		0	FALSE
ENSGALG00010029620	ENSGALG00010029620	PRJNA729271	0.798385532	0.058267364	0.798385532	stage_E14_vs_E10	ordinary_muscle_development		0	FALSE
ENSGALG00010008307	ENSGALG00010008307	PRJNA729271	-1.476991733	0.058654727	1.476991733	stage_E14_vs_E10	ordinary_muscle_development		0	FALSE
ENSGALG00010027838	ENSGALG00010027838	PRJNA729271	-1.471599684	0.058737381	1.471599684	stage_E14_vs_E10	ordinary_muscle_development		0	FALSE
ENSGALG00010011636	GRIK1	PRJNA729271	-0.873976578	0.058737381	0.873976578	stage_E14_vs_E10	ordinary_muscle_development		0	FALSE
ENSGALG00010020428	SEMA4C	PRJNA729271	-0.301343014	0.058858718	0.301343014	stage_E14_vs_E10	ordinary_muscle_development		0	FALSE
ENSGALG00010011876	AMDHD1	PRJNA729271	-0.927789271	0.059519878	0.927789271	stage_E14_vs_E10	ordinary_muscle_development		0	FALSE
ENSGALG00010010680	ENSGALG00010010680	PRJNA729271	-1.33739837	0.059705434	1.33739837	stage_E14_vs_E10	ordinary_muscle_development		0	FALSE
ENSGALG00010009993	ENSGALG00010009993	PRJNA729271	-0.743233764	0.0597395	0.743233764	stage_E14_vs_E10	ordinary_muscle_development		0	FALSE
ENSGALG00010021621	ZNF518A	PRJNA729271	0.278981972	0.059787135	0.278981972	stage_E14_vs_E10	ordinary_muscle_development		0	FALSE
ENSGALG00010017688	SPDYA	PRJNA729271	-1.200410237	0.059857632	1.200410237	stage_E14_vs_E10	ordinary_muscle_development		0	FALSE
ENSGALG00010006064	DMTF1	PRJNA729271	0.161965674	0.060251345	0.161965674	stage_E14_vs_E10	ordinary_muscle_development		0	FALSE
ENSGALG00010015565	ENSGALG00010015565	PRJNA729271	-0.900907915	0.060539237	0.900907915	stage_E14_vs_E10	ordinary_muscle_development		0	FALSE
ENSGALG00010005849	SNX25	PRJNA729271	-0.21130582	0.060841832	0.21130582	stage_E14_vs_E10	ordinary_muscle_development		0	FALSE
ENSGALG00010003351	ENSGALG00010003351	PRJNA729271	2.08489098	0.06116899	2.08489098	stage_E14_vs_E10	ordinary_muscle_development		0	FALSE
ENSGALG00010023248	PRDM10	PRJNA729271	-0.246354573	0.061402232	0.246354573	stage_E14_vs_E10	ordinary_muscle_development		0	FALSE
ENSGALG00010001608	ENSGALG00010001608	PRJNA729271	-1.028522399	0.061493056	1.028522399	stage_E14_vs_E10	ordinary_muscle_development		0	FALSE
ENSGALG00010014965	ABHD13	PRJNA729271	-0.195448864	0.061765078	0.195448864	stage_E14_vs_E10	ordinary_muscle_development		0	FALSE
ENSGALG00010009719	YIPF3	PRJNA729271	-0.164029193	0.061815024	0.164029193	stage_E14_vs_E10	ordinary_muscle_development		0	FALSE
ENSGALG00010014696	SPINK5	PRJNA729271	1.238604032	0.06230752	1.238604032	stage_E14_vs_E10	ordinary_muscle_development		0	FALSE
ENSGALG00010007165	ENSGALG00010007165	PRJNA729271	0.643586877	0.063361962	0.643586877	stage_E14_vs_E10	ordinary_muscle_development		0	FALSE
ENSGALG00010021476	ALKBH2	PRJNA729271	0.234366078	0.064292559	0.234366078	stage_E14_vs_E10	ordinary_muscle_development		0	FALSE
ENSGALG00010009520	SPINK4	PRJNA729271	-0.789710557	0.064332159	0.789710557	stage_E14_vs_E10	ordinary_muscle_development		0	FALSE
ENSGALG00010003892	OPRK1	PRJNA729271	-5.065881662	0.064340476	5.065881662	stage_E14_vs_E10	ordinary_muscle_development		0	FALSE
ENSGALG00010003245	ENSGALG00010003245	PRJNA729271	-0.926905381	0.06472866	0.926905381	stage_E14_vs_E10	ordinary_muscle_development		0	FALSE
ENSGALG00010023866	AMBRA1	PRJNA729271	0.094947277	0.064895918	0.094947277	stage_E14_vs_E10	ordinary_muscle_development		0	FALSE
ENSGALG00010016468	TTC39B	PRJNA729271	0.328793432	0.06511869	0.328793432	stage_E14_vs_E10	ordinary_muscle_development		0	FALSE
ENSGALG00010018019	NAPB	PRJNA729271	0.686340125	0.065190737	0.686340125	stage_E14_vs_E10	ordinary_muscle_development		0	FALSE
ENSGALG00010006086	ENSGALG00010006086	PRJNA729271	-0.619682016	0.06571344	0.619682016	stage_E14_vs_E10	ordinary_muscle_development		0	FALSE
ENSGALG00010019872	VTI1A	PRJNA729271	0.230444467	0.066058055	0.230444467	stage_E14_vs_E10	ordinary_muscle_development		0	FALSE
ENSGALG00010020723	RPS24	PRJNA729271	0.377856559	0.06609803	0.377856559	stage_E14_vs_E10	ordinary_muscle_development		0	FALSE
ENSGALG00010013133	TXNDC15	PRJNA729271	-0.234647919	0.06626732	0.234647919	stage_E14_vs_E10	ordinary_muscle_development		0	FALSE
ENSGALG00010008005	ANKRD42	PRJNA729271	-0.705206514	0.066349098	0.705206514	stage_E14_vs_E10	ordinary_muscle_development		0	FALSE
ENSGALG00010009155	PAK4	PRJNA729271	-0.429005896	0.066490569	0.429005896	stage_E14_vs_E10	ordinary_muscle_development		0	FALSE
ENSGALG00010000804	ENSGALG00010000804	PRJNA729271	-1.008467834	0.066849874	1.008467834	stage_E14_vs_E10	ordinary_muscle_development		0	FALSE
ENSGALG00010001553	CNGA4	PRJNA729271	0.789701241	0.067065055	0.789701241	stage_E14_vs_E10	ordinary_muscle_development		0	FALSE
ENSGALG00010002985	ENSGALG00010002985	PRJNA729271	0.411460377	0.067987566	0.411460377	stage_E14_vs_E10	ordinary_muscle_development		0	FALSE
ENSGALG00010007217	MSH3	PRJNA729271	-0.303032497	0.068210226	0.303032497	stage_E14_vs_E10	ordinary_muscle_development		0	FALSE
ENSGALG00010022592	CCDC65	PRJNA729271	-0.885541157	0.068580573	0.885541157	stage_E14_vs_E10	ordinary_muscle_development		0	FALSE
ENSGALG00010007425	ARSA	PRJNA729271	-0.260978256	0.068774674	0.260978256	stage_E14_vs_E10	ordinary_muscle_development		0	FALSE
ENSGALG00010005337	DCTN1	PRJNA729271	0.295493942	0.068937744	0.295493942	stage_E14_vs_E10	ordinary_muscle_development		0	FALSE
ENSGALG00010017638	GFRA2	PRJNA729271	0.494854711	0.069032491	0.494854711	stage_E14_vs_E10	ordinary_muscle_development		0	FALSE
ENSGALG00010021059	ENSGALG00010021059	PRJNA729271	-2.63357695	0.069413791	2.63357695	stage_E14_vs_E10	ordinary_muscle_development		0	FALSE
ENSGALG00010027310	ARHGEF2	PRJNA729271	-0.250731219	0.06962206	0.250731219	stage_E14_vs_E10	ordinary_muscle_development		0	FALSE
ENSGALG00010012536	FBXO4	PRJNA729271	0.257475658	0.069912163	0.257475658	stage_E14_vs_E10	ordinary_muscle_development		0	FALSE
ENSGALG00010029303	SLC26A11	PRJNA729271	-0.317426102	0.069983554	0.317426102	stage_E14_vs_E10	ordinary_muscle_development		0	FALSE
ENSGALG00010021951	PSKH1	PRJNA729271	0.275673576	0.070125071	0.275673576	stage_E14_vs_E10	ordinary_muscle_development		0	FALSE
ENSGALG00010023711	ENSGALG00010023711	PRJNA729271	-0.685831702	0.070165946	0.685831702	stage_E14_vs_E10	ordinary_muscle_development		0	FALSE
ENSGALG00010021600	FMN1	PRJNA729271	-0.253251368	0.070373369	0.253251368	stage_E14_vs_E10	ordinary_muscle_development		0	FALSE
ENSGALG00010017675	ENSGALG00010017675	PRJNA729271	0.899222249	0.070815287	0.899222249	stage_E14_vs_E10	ordinary_muscle_development		0	FALSE
ENSGALG00010024125	ENSGALG00010024125	PRJNA729271	-3.239277991	0.0708305	3.239277991	stage_E14_vs_E10	ordinary_muscle_development		0	FALSE
ENSGALG00010015173	ENS-1	PRJNA729271	-1.463009518	0.071700526	1.463009518	stage_E14_vs_E10	ordinary_muscle_development		0	FALSE
ENSGALG00010025170	COMMD9	PRJNA729271	-0.191163819	0.071832644	0.191163819	stage_E14_vs_E10	ordinary_muscle_development		0	FALSE
ENSGALG00010014041	SKIV2L2	PRJNA729271	0.186558286	0.072102997	0.186558286	stage_E14_vs_E10	ordinary_muscle_development		0	FALSE
ENSGALG00010011382	ENSGALG00010011382	PRJNA729271	0.379511309	0.072418558	0.379511309	stage_E14_vs_E10	ordinary_muscle_development		0	FALSE
ENSGALG00010002908	S1PR2	PRJNA729271	-0.346064115	0.073033311	0.346064115	stage_E14_vs_E10	ordinary_muscle_development		0	FALSE
ENSGALG00010003136	NUP98	PRJNA729271	0.169794988	0.073723458	0.169794988	stage_E14_vs_E10	ordinary_muscle_development		0	FALSE
ENSGALG00010024264	AMER3	PRJNA729271	-0.575216317	0.073786021	0.575216317	stage_E14_vs_E10	ordinary_muscle_development		0	FALSE
ENSGALG00010002181	FIS1	PRJNA729271	0.497708335	0.073982847	0.497708335	stage_E14_vs_E10	ordinary_muscle_development		0	FALSE
ENSGALG00010002579	MATN3	PRJNA729271	-1.08544178	0.074097046	1.08544178	stage_E14_vs_E10	ordinary_muscle_development		0	FALSE
ENSGALG00010023226	ENSGALG00010023226	PRJNA729271	0.394958898	0.074241071	0.394958898	stage_E14_vs_E10	ordinary_muscle_development		0	FALSE
ENSGALG00010015261	IQCG	PRJNA729271	-0.88487374	0.074302282	0.88487374	stage_E14_vs_E10	ordinary_muscle_development		0	FALSE
ENSGALG00010024290	SOST	PRJNA729271	-0.976995435	0.074368648	0.976995435	stage_E14_vs_E10	ordinary_muscle_development		0	FALSE
ENSGALG00010025103	GSK3B	PRJNA729271	0.190322434	0.074942813	0.190322434	stage_E14_vs_E10	ordinary_muscle_development		0	FALSE
ENSGALG00010011442	ENSGALG00010011442	PRJNA729271	-3.991143992	0.075051249	3.991143992	stage_E14_vs_E10	ordinary_muscle_development		0	FALSE
ENSGALG00010017484	IQCC	PRJNA729271	0.257808762	0.075070859	0.257808762	stage_E14_vs_E10	ordinary_muscle_development		0	FALSE
ENSGALG00010017552	LARS2	PRJNA729271	-0.200114646	0.075368995	0.200114646	stage_E14_vs_E10	ordinary_muscle_development		0	FALSE
ENSGALG00010018006	DCUN1D3	PRJNA729271	-0.182004472	0.075788725	0.182004472	stage_E14_vs_E10	ordinary_muscle_development		0	FALSE
ENSGALG00010029219	TLCD1	PRJNA729271	0.379404144	0.076164084	0.379404144	stage_E14_vs_E10	ordinary_muscle_development		0	FALSE
ENSGALG00010018240	SOUL	PRJNA729271	-1.378491285	0.076871979	1.378491285	stage_E14_vs_E10	ordinary_muscle_development		0	FALSE
ENSGALG00010012892	ORMDL1	PRJNA729271	0.301788676	0.077663122	0.301788676	stage_E14_vs_E10	ordinary_muscle_development		0	FALSE
ENSGALG00010011042	SLC13A4	PRJNA729271	0.95025892	0.078289784	0.95025892	stage_E14_vs_E10	ordinary_muscle_development		0	FALSE
ENSGALG00010025877	ENSGALG00010025877	PRJNA729271	6.976983342	0.079149051	6.976983342	stage_E14_vs_E10	ordinary_muscle_development		0	FALSE
ENSGALG00010020618	ENSGALG00010020618	PRJNA729271	-0.616675012	0.079518114	0.616675012	stage_E14_vs_E10	ordinary_muscle_development		0	FALSE
ENSGALG00010001552	TUBGCP6	PRJNA729271	0.200188959	0.079704158	0.200188959	stage_E14_vs_E10	ordinary_muscle_development		0	FALSE
ENSGALG00010024342	HNF4A	PRJNA729271	2.355317798	0.080115664	2.355317798	stage_E14_vs_E10	ordinary_muscle_development		0	FALSE
ENSGALG00010025531	ENSGALG00010025531	PRJNA729271	0.146017187	0.080648168	0.146017187	stage_E14_vs_E10	ordinary_muscle_development		0	FALSE
ENSGALG00010011945	ENSGALG00010011945	PRJNA729271	-1.054522807	0.081037182	1.054522807	stage_E14_vs_E10	ordinary_muscle_development		0	FALSE
ENSGALG00010017447	CAPN13	PRJNA729271	-1.04881195	0.081301135	1.04881195	stage_E14_vs_E10	ordinary_muscle_development		0	FALSE
ENSGALG00010021922	MRPL51	PRJNA729271	-0.25477937	0.081329706	0.25477937	stage_E14_vs_E10	ordinary_muscle_development		0	FALSE
ENSGALG00010000239	ENSGALG00010000239	PRJNA729271	-8.750700671	0.081631732	8.750700671	stage_E14_vs_E10	ordinary_muscle_development		0	FALSE
ENSGALG00010012118	ENSGALG00010012118	PRJNA729271	-1.470939787	0.08258086	1.470939787	stage_E14_vs_E10	ordinary_muscle_development		0	FALSE
ENSGALG00010029224	FAM222B	PRJNA729271	-0.300260852	0.082729894	0.300260852	stage_E14_vs_E10	ordinary_muscle_development		0	FALSE
ENSGALG00010022441	FICD	PRJNA729271	0.21793551	0.082985445	0.21793551	stage_E14_vs_E10	ordinary_muscle_development		0	FALSE
ENSGALG00010014624	GPRC6A	PRJNA729271	0.753280083	0.083105387	0.753280083	stage_E14_vs_E10	ordinary_muscle_development		0	FALSE
ENSGALG00010023166	KDM2A	PRJNA729271	-0.208467819	0.083192609	0.208467819	stage_E14_vs_E10	ordinary_muscle_development		0	FALSE
ENSGALG00010017736	ENSGALG00010017736	PRJNA729271	7.886246683	0.08329002	7.886246683	stage_E14_vs_E10	ordinary_muscle_development		0	FALSE
ENSGALG00010019215	ENSGALG00010019215	PRJNA729271	-0.187405993	0.08480343	0.187405993	stage_E14_vs_E10	ordinary_muscle_development		0	FALSE
ENSGALG00010002023	CLCN4	PRJNA729271	-0.489699211	0.084821858	0.489699211	stage_E14_vs_E10	ordinary_muscle_development		0	FALSE
ENSGALG00010019185	ANKDD1A	PRJNA729271	-0.412763578	0.085022736	0.412763578	stage_E14_vs_E10	ordinary_muscle_development		0	FALSE
ENSGALG00010027479	HCN2	PRJNA729271	-0.502750506	0.086543383	0.502750506	stage_E14_vs_E10	ordinary_muscle_development		0	FALSE
ENSGALG00010008533	ENSGALG00010008533	PRJNA729271	-1.205182567	0.086557833	1.205182567	stage_E14_vs_E10	ordinary_muscle_development		0	FALSE
ENSGALG00010008297	ENSGALG00010008297	PRJNA729271	-0.446924429	0.088054684	0.446924429	stage_E14_vs_E10	ordinary_muscle_development		0	FALSE
ENSGALG00010022345	USP33	PRJNA729271	-0.244176134	0.088157176	0.244176134	stage_E14_vs_E10	ordinary_muscle_development		0	FALSE
ENSGALG00010000262	HNRNPKL	PRJNA729271	-6.357779544	0.088459196	6.357779544	stage_E14_vs_E10	ordinary_muscle_development		0	FALSE
ENSGALG00010000577	ENSGALG00010000577	PRJNA729271	-0.38689716	0.088648258	0.38689716	stage_E14_vs_E10	ordinary_muscle_development		0	FALSE
ENSGALG00010010417	OTOF	PRJNA729271	1.152968165	0.089127925	1.152968165	stage_E14_vs_E10	ordinary_muscle_development		0	FALSE
ENSGALG00010013168	VPS26C	PRJNA729271	-0.247846888	0.089832036	0.247846888	stage_E14_vs_E10	ordinary_muscle_development		0	FALSE
ENSGALG00010008430	ENSGALG00010008430	PRJNA729271	-0.83255461	0.090485056	0.83255461	stage_E14_vs_E10	ordinary_muscle_development		0	FALSE
ENSGALG00010028152	ENSGALG00010028152	PRJNA729271	-0.590357267	0.090910858	0.590357267	stage_E14_vs_E10	ordinary_muscle_development		0	FALSE
ENSGALG00010011930	TSPO	PRJNA729271	0.269580785	0.091748309	0.269580785	stage_E14_vs_E10	ordinary_muscle_development		0	FALSE
ENSGALG00010022012	ENSGALG00010022012	PRJNA729271	5.694742442	0.092065839	5.694742442	stage_E14_vs_E10	ordinary_muscle_development		0	FALSE
ENSGALG00010017369	CAPN14	PRJNA729271	-1.053616317	0.092484773	1.053616317	stage_E14_vs_E10	ordinary_muscle_development		0	FALSE
ENSGALG00010008411	ENSGALG00010008411	PRJNA729271	0.708680379	0.09272806	0.708680379	stage_E14_vs_E10	ordinary_muscle_development		0	FALSE
ENSGALG00010028478	PEA15	PRJNA729271	0.294021595	0.093342412	0.294021595	stage_E14_vs_E10	ordinary_muscle_development		0	FALSE
ENSGALG00010019318	NOG2	PRJNA729271	0.490827256	0.093546561	0.490827256	stage_E14_vs_E10	ordinary_muscle_development		0	FALSE
ENSGALG00010008306	ENSGALG00010008306	PRJNA729271	0.514974001	0.093956766	0.514974001	stage_E14_vs_E10	ordinary_muscle_development		0	FALSE
ENSGALG00010003304	ENSGALG00010003304	PRJNA729271	-1.365114558	0.094454377	1.365114558	stage_E14_vs_E10	ordinary_muscle_development		0	FALSE
ENSGALG00010002098	ENSGALG00010002098	PRJNA729271	0.75687604	0.095199056	0.75687604	stage_E14_vs_E10	ordinary_muscle_development		0	FALSE
ENSGALG00010013109	ENSGALG00010013109	PRJNA729271	2.588726817	0.095496437	2.588726817	stage_E14_vs_E10	ordinary_muscle_development		0	FALSE
ENSGALG00010003324	ENSGALG00010003324	PRJNA729271	-1.031203908	0.096741238	1.031203908	stage_E14_vs_E10	ordinary_muscle_development		0	FALSE
ENSGALG00010012959	TBC1D32	PRJNA729271	-0.28383539	0.097384336	0.28383539	stage_E14_vs_E10	ordinary_muscle_development		0	FALSE
ENSGALG00010029676	FADS6	PRJNA729271	-2.021298499	0.097496924	2.021298499	stage_E14_vs_E10	ordinary_muscle_development		0	FALSE
ENSGALG00010004326	ENSGALG00010004326	PRJNA729271	-1.093173433	0.09763976	1.093173433	stage_E14_vs_E10	ordinary_muscle_development		0	FALSE
ENSGALG00010027917	PPARG	PRJNA729271	0.758356162	0.097713103	0.758356162	stage_E14_vs_E10	ordinary_muscle_development		0	FALSE
ENSGALG00010013889	ENSGALG00010013889	PRJNA729271	0.61755046	0.097713103	0.61755046	stage_E14_vs_E10	ordinary_muscle_development		0	FALSE
ENSGALG00010016060	ENSGALG00010016060	PRJNA729271	2.453651375	0.097820385	2.453651375	stage_E14_vs_E10	ordinary_muscle_development		0	FALSE
ENSGALG00010028138	ENSGALG00010028138	PRJNA729271	-0.97776967	0.09888011	0.97776967	stage_E14_vs_E10	ordinary_muscle_development		0	FALSE
ENSGALG00010013202	ENSGALG00010013202	PRJNA729271	-1.300466852	0.098974689	1.300466852	stage_E14_vs_E10	ordinary_muscle_development		0	FALSE
ENSGALG00010000382	ENSGALG00010000382	PRJNA729271	-1.019235257	0.099087589	1.019235257	stage_E14_vs_E10	ordinary_muscle_development		0	FALSE
ENSGALG00010001755	CUZD1	PRJNA729271	-2.360811199	0.09935174	2.360811199	stage_E14_vs_E10	ordinary_muscle_development		0	FALSE
ENSGALG00010007002	EXOC4	PRJNA729271	-0.208755145	0.100174239	0.208755145	stage_E14_vs_E10	ordinary_muscle_development		0	FALSE
ENSGALG00010011161	ENSGALG00010011161	PRJNA729271	-0.164358781	0.101556033	0.164358781	stage_E14_vs_E10	ordinary_muscle_development		0	FALSE
ENSGALG00010022833	HRH3	PRJNA729271	-0.973447763	0.101945321	0.973447763	stage_E14_vs_E10	ordinary_muscle_development		0	FALSE
ENSGALG00010022388	CYP2W1	PRJNA729271	-0.771555783	0.101945321	0.771555783	stage_E14_vs_E10	ordinary_muscle_development		0	FALSE
ENSGALG00010011873	RFX3	PRJNA729271	-0.357283893	0.102055806	0.357283893	stage_E14_vs_E10	ordinary_muscle_development		0	FALSE
ENSGALG00010001186	ENSGALG00010001186	PRJNA729271	-0.708045414	0.102781254	0.708045414	stage_E14_vs_E10	ordinary_muscle_development		0	FALSE
ENSGALG00010017381	CA6	PRJNA729271	-0.855560853	0.103207759	0.855560853	stage_E14_vs_E10	ordinary_muscle_development		0	FALSE
ENSGALG00010018071	GPAT4	PRJNA729271	0.129238096	0.104667592	0.129238096	stage_E14_vs_E10	ordinary_muscle_development		0	FALSE
ENSGALG00010027121	ELF3	PRJNA729271	-1.7646492	0.107135289	1.7646492	stage_E14_vs_E10	ordinary_muscle_development		0	FALSE
ENSGALG00010028689	CFAP45	PRJNA729271	0.904718617	0.108107682	0.904718617	stage_E14_vs_E10	ordinary_muscle_development		0	FALSE
ENSGALG00010003639	ENSGALG00010003639	PRJNA729271	-1.197883373	0.108677996	1.197883373	stage_E14_vs_E10	ordinary_muscle_development		0	FALSE
ENSGALG00010010900	SLC34A2	PRJNA729271	-1.532617369	0.11006609	1.532617369	stage_E14_vs_E10	ordinary_muscle_development		0	FALSE
ENSGALG00010027703	NCSTN	PRJNA729271	-0.177583652	0.110115976	0.177583652	stage_E14_vs_E10	ordinary_muscle_development		0	FALSE
ENSGALG00010016620	ENSGALG00010016620	PRJNA729271	0.20544955	0.110150894	0.20544955	stage_E14_vs_E10	ordinary_muscle_development		0	FALSE
ENSGALG00010003915	MIOS	PRJNA729271	-0.131400731	0.110307844	0.131400731	stage_E14_vs_E10	ordinary_muscle_development		0	FALSE
ENSGALG00010001134	ENSGALG00010001134	PRJNA729271	-1.22835017	0.110882661	1.22835017	stage_E14_vs_E10	ordinary_muscle_development		0	FALSE
ENSGALG00010004975	SNW1	PRJNA729271	0.1182781	0.110891933	0.1182781	stage_E14_vs_E10	ordinary_muscle_development		0	FALSE

ENSGALG00010020730	RRP12	PRJNA729271	-0.801360428	0.112016306	0.801360428	stage_E14_vs_E10	ordinary_muscle_development	0	FALSE
ENSGALG00010002461	TOMT	PRJNA729271	0.543059323	0.112861302	0.543059323	stage_E14_vs_E10	ordinary_muscle_development	0	FALSE
ENSGALG00010016989	RHOG2	PRJNA729271	0.156326703	0.11316832	0.156326703	stage_E14_vs_E10	ordinary_muscle_development	0	FALSE
ENSGALG00010013163	ENSGALG00010013163	PRJNA729271	-0.817304748	0.114407973	0.817304748	stage_E14_vs_E10	ordinary_muscle_development	0	FALSE
ENSGALG00010012497	PLCXD3	PRJNA729271	-0.756556799	0.115087005	0.756556799	stage_E14_vs_E10	ordinary_muscle_development	0	FALSE
ENSGALG00010000176	ENSGALG00010000176	PRJNA729271	-0.772329995	0.116377414	0.772329995	stage_E14_vs_E10	ordinary_muscle_development	0	FALSE
ENSGALG00010016344	SMIM14	PRJNA729271	0.186389221	0.117173942	0.186389221	stage_E14_vs_E10	ordinary_muscle_development	0	FALSE
ENSGALG00010013404	ENSGALG00010013404	PRJNA729271	0.382884257	0.11798503	0.382884257	stage_E14_vs_E10	ordinary_muscle_development	0	FALSE
ENSGALG00010015838	IFNLR1	PRJNA729271	-2.182703027	0.118853817	2.182703027	stage_E14_vs_E10	ordinary_muscle_development	0	FALSE
ENSGALG00010007988	TARBP1	PRJNA729271	-0.205564035	0.118912106	0.205564035	stage_E14_vs_E10	ordinary_muscle_development	0	FALSE
ENSGALG00010000184	ENSGALG00010000184	PRJNA729271	5.788418408	0.119309708	5.788418408	stage_E14_vs_E10	ordinary_muscle_development	0	FALSE
ENSGALG00010019047	GHRL	PRJNA729271	-0.744721213	0.120403558	0.744721213	stage_E14_vs_E10	ordinary_muscle_development	0	FALSE
ENSGALG00010028534	ENSGALG00010028534	PRJNA729271	7.833582425	0.12044416	7.833582425	stage_E14_vs_E10	ordinary_muscle_development	0	FALSE
ENSGALG00010008897	NSMCE2	PRJNA729271	-0.274159356	0.120936278	0.274159356	stage_E14_vs_E10	ordinary_muscle_development	0	FALSE
ENSGALG00010017824	YAE1	PRJNA729271	-0.214029479	0.121848037	0.214029479	stage_E14_vs_E10	ordinary_muscle_development	0	FALSE
ENSGALG00010022557	WNT1	PRJNA729271	-0.690639524	0.122730045	0.690639524	stage_E14_vs_E10	ordinary_muscle_development	0	FALSE
ENSGALG00010009276	ENSGALG00010009276	PRJNA729271	-0.556102062	0.123561427	0.556102062	stage_E14_vs_E10	ordinary_muscle_development	0	FALSE
ENSGALG00010022211	ENSGALG00010022211	PRJNA729271	0.817299245	0.125304163	0.817299245	stage_E14_vs_E10	ordinary_muscle_development	0	FALSE
ENSGALG00010009772	ENSGALG00010009772	PRJNA729271	-0.965550942	0.125477503	0.965550942	stage_E14_vs_E10	ordinary_muscle_development	0	FALSE
ENSGALG00010017361	METTL22	PRJNA729271	-0.237523121	0.125514552	0.237523121	stage_E14_vs_E10	ordinary_muscle_development	0	FALSE
ENSGALG00010021612	BCS1L	PRJNA729271	-0.218508312	0.125765006	0.218508312	stage_E14_vs_E10	ordinary_muscle_development	0	FALSE
ENSGALG00010023008	CDHR5	PRJNA729271	-1.644120401	0.12636379	1.644120401	stage_E14_vs_E10	ordinary_muscle_development	0	FALSE
ENSGALG00010014002	ZDHHC18	PRJNA729271	-0.467160128	0.128805056	0.467160128	stage_E14_vs_E10	ordinary_muscle_development	0	FALSE
ENSGALG00010027952	CIB3	PRJNA729271	0.743146719	0.129131721	0.743146719	stage_E14_vs_E10	ordinary_muscle_development	0	FALSE
ENSGALG00010002827	SBSPON	PRJNA729271	0.539834276	0.129778339	0.539834276	stage_E14_vs_E10	ordinary_muscle_development	0	FALSE
ENSGALG00010027087	RAB7B	PRJNA729271	0.53284925	0.130194426	0.53284925	stage_E14_vs_E10	ordinary_muscle_development	0	FALSE
ENSGALG00010011867	ENSGALG00010011867	PRJNA729271	0.442535325	0.13181329	0.442535325	stage_E14_vs_E10	ordinary_muscle_development	0	FALSE
ENSGALG00010013248	ENSGALG00010013248	PRJNA729271	-1.103608915	0.13283082	1.103608915	stage_E14_vs_E10	ordinary_muscle_development	0	FALSE
ENSGALG00010000529	STIP1	PRJNA729271	0.305805389	0.134267587	0.305805389	stage_E14_vs_E10	ordinary_muscle_development	0	FALSE
ENSGALG00010026889	ENSGALG00010026889	PRJNA729271	-0.45622597	0.135306761	0.45622597	stage_E14_vs_E10	ordinary_muscle_development	0	FALSE
ENSGALG00010003827	RRH	PRJNA729271	-1.243582982	0.135688995	1.243582982	stage_E14_vs_E10	ordinary_muscle_development	0	FALSE
ENSGALG00010028603	SH3GLB2	PRJNA729271	-0.534315022	0.137955636	0.534315022	stage_E14_vs_E10	ordinary_muscle_development	0	FALSE
ENSGALG00010000434	TP53	PRJNA729271	0.346830307	0.138386128	0.346830307	stage_E14_vs_E10	ordinary_muscle_development	0	FALSE
ENSGALG00010015626	XKR7	PRJNA729271	-0.920874799	0.138993732	0.920874799	stage_E14_vs_E10	ordinary_muscle_development	0	FALSE
ENSGALG00010020768	DNAH8	PRJNA729271	-1.194013165	0.139396623	1.194013165	stage_E14_vs_E10	ordinary_muscle_development	0	FALSE
ENSGALG00010027225	CREB3L3	PRJNA729271	-1.18462935	0.142644769	1.18462935	stage_E14_vs_E10	ordinary_muscle_development	0	FALSE
ENSGALG00010006524	TAB2	PRJNA729271	-0.131218704	0.143519953	0.131218704	stage_E14_vs_E10	ordinary_muscle_development	0	FALSE
ENSGALG00010017148	MOK	PRJNA729271	-0.826554397	0.144264649	0.826554397	stage_E14_vs_E10	ordinary_muscle_development	0	FALSE
ENSGALG00010019899	ENSGALG00010019899	PRJNA729271	-0.527324598	0.144838936	0.527324598	stage_E14_vs_E10	ordinary_muscle_development	0	FALSE
ENSGALG00010012433	ENSGALG00010012433	PRJNA729271	0.908950372	0.145262041	0.908950372	stage_E14_vs_E10	ordinary_muscle_development	0	FALSE
ENSGALG00010006408	ENSGALG00010006408	PRJNA729271	-1.065479836	0.148087052	1.065479836	stage_E14_vs_E10	ordinary_muscle_development	0	FALSE
ENSGALG00010002567	COA4	PRJNA729271	0.356586026	0.148545326	0.356586026	stage_E14_vs_E10	ordinary_muscle_development	0	FALSE
ENSGALG00010018274	ZNF711	PRJNA729271	-0.148760461	0.150994645	0.148760461	stage_E14_vs_E10	ordinary_muscle_development	0	FALSE
ENSGALG00010029462	ENSGALG00010029462	PRJNA729271	2.829745256	0.153117027	2.829745256	stage_E14_vs_E10	ordinary_muscle_development	0	FALSE
ENSGALG00010015115	CLDN2	PRJNA729271	-0.546895017	0.153541252	0.546895017	stage_E14_vs_E10	ordinary_muscle_development	0	FALSE
ENSGALG00010008696	PFN4	PRJNA729271	-0.327947383	0.154449026	0.327947383	stage_E14_vs_E10	ordinary_muscle_development	0	FALSE
ENSGALG00010007933	CEP126	PRJNA729271	-0.792602653	0.155307172	0.792602653	stage_E14_vs_E10	ordinary_muscle_development	0	FALSE
ENSGALG00010010978	SERHL	PRJNA729271	-0.207519286	0.155416407	0.207519286	stage_E14_vs_E10	ordinary_muscle_development	0	FALSE
ENSGALG00010013347	ENSGALG00010013347	PRJNA729271	0.52862498	0.155452405	0.52862498	stage_E14_vs_E10	ordinary_muscle_development	0	FALSE
ENSGALG00010015314	GMEB1	PRJNA729271	-0.109869597	0.157053126	0.109869597	stage_E14_vs_E10	ordinary_muscle_development	0	FALSE
ENSGALG00010027236	PEX11G	PRJNA729271	0.422425708	0.157191763	0.422425708	stage_E14_vs_E10	ordinary_muscle_development	0	FALSE
ENSGALG00010012211	ENSGALG00010012211	PRJNA729271	-6.220358547	0.158573307	6.220358547	stage_E14_vs_E10	ordinary_muscle_development	0	FALSE
ENSGALG00010029603	RHBDL3	PRJNA729271	-0.665862632	0.161972076	0.665862632	stage_E14_vs_E10	ordinary_muscle_development	0	FALSE
ENSGALG00010000622	ENSGALG00010000622	PRJNA729271	-0.697163798	0.165367357	0.697163798	stage_E14_vs_E10	ordinary_muscle_development	0	FALSE
ENSGALG00010029497	ENSGALG00010029497	PRJNA729271	-0.891647679	0.165394143	0.891647679	stage_E14_vs_E10	ordinary_muscle_development	0	FALSE
ENSGALG00010026249	YPEL4	PRJNA729271	-0.925177938	0.165838194	0.925177938	stage_E14_vs_E10	ordinary_muscle_development	0	FALSE
ENSGALG00010025794	ENSGALG00010025794	PRJNA729271	6.643372129	0.166506742	6.643372129	stage_E14_vs_E10	ordinary_muscle_development	0	FALSE
ENSGALG00010023019	CABP1	PRJNA729271	0.483575989	0.167933643	0.483575989	stage_E14_vs_E10	ordinary_muscle_development	0	FALSE
ENSGALG00010028471	TPGS1	PRJNA729271	0.254490215	0.167933643	0.254490215	stage_E14_vs_E10	ordinary_muscle_development	0	FALSE
ENSGALG00010010369	ENSGALG00010010369	PRJNA729271	-0.395479009	0.168570936	0.395479009	stage_E14_vs_E10	ordinary_muscle_development	0	FALSE
ENSGALG00010025782	ENSGALG00010025782	PRJNA729271	7.399160195	0.169195994	7.399160195	stage_E14_vs_E10	ordinary_muscle_development	0	FALSE
ENSGALG00010022542	ENSGALG00010022542	PRJNA729271	1.090992097	0.169511693	1.090992097	stage_E14_vs_E10	ordinary_muscle_development	0	FALSE
ENSGALG00010020212	JMJD7	PRJNA729271	0.202680928	0.171129815	0.202680928	stage_E14_vs_E10	ordinary_muscle_development	0	FALSE
ENSGALG00010029445	SPHK1	PRJNA729271	-0.20312113	0.17525467	0.20312113	stage_E14_vs_E10	ordinary_muscle_development	0	FALSE
ENSGALG00010002339	ZNFY4	PRJNA729271	-0.456339852	0.175688762	0.456339852	stage_E14_vs_E10	ordinary_muscle_development	0	FALSE
ENSGALG00010013751	FUT10	PRJNA729271	-0.274596894	0.177797518	0.274596894	stage_E14_vs_E10	ordinary_muscle_development	0	FALSE
ENSGALG00010000632	ENSGALG00010000632	PRJNA729271	-0.624037673	0.178373409	0.624037673	stage_E14_vs_E10	ordinary_muscle_development	0	FALSE
ENSGALG00010014789	F7	PRJNA729271	-1.414958449	0.178476796	1.414958449	stage_E14_vs_E10	ordinary_muscle_development	0	FALSE
ENSGALG00010028453	ENSGALG00010028453	PRJNA729271	0.135035795	0.181696692	0.135035795	stage_E14_vs_E10	ordinary_muscle_development	0	FALSE
ENSGALG00010001842	GPR26	PRJNA729271	-0.819870889	0.181705446	0.819870889	stage_E14_vs_E10	ordinary_muscle_development	0	FALSE
ENSGALG00010028662	FCRL4	PRJNA729271	0.324123918	0.182656568	0.324123918	stage_E14_vs_E10	ordinary_muscle_development	0	FALSE
ENSGALG00010002955	BLEC1	PRJNA729271	-0.797188078	0.182957167	0.797188078	stage_E14_vs_E10	ordinary_muscle_development	0	FALSE
ENSGALG00010028575	SETDB1	PRJNA729271	-0.1692746	0.183181018	0.1692746	stage_E14_vs_E10	ordinary_muscle_development	0	FALSE
ENSGALG00010011459	ENSGALG00010011459	PRJNA729271	0.39282327	0.184055694	0.39282327	stage_E14_vs_E10	ordinary_muscle_development	0	FALSE
ENSGALG00010000471	ENSGALG00010000471	PRJNA729271	-0.289847294	0.185351454	0.289847294	stage_E14_vs_E10	ordinary_muscle_development	0	FALSE
ENSGALG00010002951	ENSGALG00010002951	PRJNA729271	-6.129514798	0.186802953	6.129514798	stage_E14_vs_E10	ordinary_muscle_development	0	FALSE
ENSGALG00010029413	B3GNTL1	PRJNA729271	0.316700077	0.188424803	0.316700077	stage_E14_vs_E10	ordinary_muscle_development	0	FALSE
ENSGALG00010007014	ENSGALG00010007014	PRJNA729271	-0.756395622	0.188776063	0.756395622	stage_E14_vs_E10	ordinary_muscle_development	0	FALSE
ENSGALG00010013508	TUBE1	PRJNA729271	-0.301855848	0.191485628	0.301855848	stage_E14_vs_E10	ordinary_muscle_development	0	FALSE
ENSGALG00010023813	CELA1	PRJNA729271	7.331689439	0.191653864	7.331689439	stage_E14_vs_E10	ordinary_muscle_development	0	FALSE
ENSGALG00010028023	ENSGALG00010028023	PRJNA729271	1.238836334	0.193846001	1.238836334	stage_E14_vs_E10	ordinary_muscle_development	0	FALSE
ENSGALG00010011430	ENSGALG00010011430	PRJNA729271	-0.469327741	0.197978418	0.469327741	stage_E14_vs_E10	ordinary_muscle_development	0	FALSE
ENSGALG00010013159	ENSGALG00010013159	PRJNA729271	-2.147094417	0.199836261	2.147094417	stage_E14_vs_E10	ordinary_muscle_development	0	FALSE
ENSGALG00010008253	ENSGALG00010008253	PRJNA729271	0.954352265	0.199907092	0.954352265	stage_E14_vs_E10	ordinary_muscle_development	0	FALSE
ENSGALG00010024703	HSF4	PRJNA729271	-0.851549858	0.201415668	0.851549858	stage_E14_vs_E10	ordinary_muscle_development	0	FALSE
ENSGALG00010024599	ENSGALG00010024599	PRJNA729271	0.287738827	0.202850946	0.287738827	stage_E14_vs_E10	ordinary_muscle_development	0	FALSE
ENSGALG00010010898	PHF24	PRJNA729271	-5.303862226	0.20559968	5.303862226	stage_E14_vs_E10	ordinary_muscle_development	0	FALSE
ENSGALG00010028712	FBXW2	PRJNA729271	-0.076502993	0.208231947	0.076502993	stage_E14_vs_E10	ordinary_muscle_development	0	FALSE
ENSGALG00010018872	KCTD9	PRJNA729271	0.134888563	0.211769494	0.134888563	stage_E14_vs_E10	ordinary_muscle_development	0	FALSE
ENSGALG00010019004	ECE2	PRJNA729271	-0.266360183	0.212520542	0.266360183	stage_E14_vs_E10	ordinary_muscle_development	0	FALSE
ENSGALG00010003303	MTHFD2L	PRJNA729271	-0.209859567	0.215882802	0.209859567	stage_E14_vs_E10	ordinary_muscle_development	0	FALSE
ENSGALG00010025563	TSPEAR	PRJNA729271	0.337810846	0.216189668	0.337810846	stage_E14_vs_E10	ordinary_muscle_development	0	FALSE
ENSGALG00010019174	ENSGALG00010019174	PRJNA729271	-0.690540456	0.21798761	0.690540456	stage_E14_vs_E10	ordinary_muscle_development	0	FALSE
ENSGALG00010020356	STX3	PRJNA729271	-0.529552676	0.218381345	0.529552676	stage_E14_vs_E10	ordinary_muscle_development	0	FALSE
ENSGALG00010024086	GABARAPL1	PRJNA729271	0.287942216	0.218965657	0.287942216	stage_E14_vs_E10	ordinary_muscle_development	0	FALSE
ENSGALG00010012294	ENSGALG00010012294	PRJNA729271	0.643005681	0.221922343	0.643005681	stage_E14_vs_E10	ordinary_muscle_development	0	FALSE
ENSGALG00010011882	ENSGALG00010011882	PRJNA729271	0.257008525	0.222762738	0.257008525	stage_E14_vs_E10	ordinary_muscle_development	0	FALSE
ENSGALG00010023615	PRSS3	PRJNA729271	6.622492485	0.22381398	6.622492485	stage_E14_vs_E10	ordinary_muscle_development	0	FALSE
ENSGALG00010012418	ENSGALG00010012418	PRJNA729271	1.013178678	0.225277232	1.013178678	stage_E14_vs_E10	ordinary_muscle_development	0	FALSE
ENSGALG00010016241	TOPAZ1	PRJNA729271	1.031627178	0.231133289	1.031627178	stage_E14_vs_E10	ordinary_muscle_development	0	FALSE
ENSGALG00010015807	ENSGALG00010015807	PRJNA729271	1.246137364	0.231591172	1.246137364	stage_E14_vs_E10	ordinary_muscle_development	0	FALSE
ENSGALG00010029533	MLXIPL	PRJNA729271	-1.062305717	0.232858688	1.062305717	stage_E14_vs_E10	ordinary_muscle_development	0	FALSE
ENSGALG00010004381	ENSGALG00010004381	PRJNA729271	-0.619693092	0.23672814	0.619693092	stage_E14_vs_E10	ordinary_muscle_development	0	FALSE
ENSGALG00010021853	ENSGALG00010021853	PRJNA729271	0.72265437	0.238649743	0.72265437	stage_E14_vs_E10	ordinary_muscle_development	0	FALSE
ENSGALG00010020955	FHAD1	PRJNA729271	-1.228085329	0.242531621	1.228085329	stage_E14_vs_E10	ordinary_muscle_development	0	FALSE
ENSGALG00010022269	CNTD1	PRJNA729271	-0.502545086	0.24404179	0.502545086	stage_E14_vs_E10	ordinary_muscle_development	0	FALSE
ENSGALG00010008328	ENSGALG00010008328	PRJNA729271	-0.613257116	0.248394973	0.613257116	stage_E14_vs_E10	ordinary_muscle_development	0	FALSE
ENSGALG00010002173	GCM1	PRJNA729271	-1.140351732	0.251718998	1.140351732	stage_E14_vs_E10	ordinary_muscle_development	0	FALSE
ENSGALG00010010661	CPEB2	PRJNA729271	-0.246280834	0.265276548	0.246280834	stage_E14_vs_E10	ordinary_muscle_development	0	FALSE
ENSGALG00010012342	ENSGALG00010012342	PRJNA729271	2.024216284	0.266416798	2.024216284	stage_E14_vs_E10	ordinary_muscle_development	0	FALSE
ENSGALG00010011120	ENSGALG00010011120	PRJNA729271	-3.704326776	0.267770275	3.704326776	stage_E14_vs_E10	ordinary_muscle_development	0	FALSE
ENSGALG00010001921	ENSGALG00010001921	PRJNA729271	-0.346236139	0.276100139	0.346236139	stage_E14_vs_E10	ordinary_muscle_development	0	FALSE
ENSGALG00010012791	CFAP418	PRJNA729271	-0.25516472	0.27721489	0.25516472	stage_E14_vs_E10	ordinary_muscle_development	0	FALSE
ENSGALG00010023426	KCNJ1	PRJNA729271	-0.573552581	0.277422882	0.573552581	stage_E14_vs_E10	ordinary_muscle_development	0	FALSE
ENSGALG00010012888	ENSGALG00010012888	PRJNA729271	-4.359032383	0.279202357	4.359032383	stage_E14_vs_E10	ordinary_muscle_development	0	FALSE
ENSGALG00010029464	ENSGALG00010029464	PRJNA729271	0.590809732	0.304484778	0.590809732	stage_E14_vs_E10	ordinary_muscle_development	0	FALSE
ENSGALG00010018880	TMEM17	PRJNA729271	-0.286311272	0.305016415	0.286311272	stage_E14_vs_E10	ordinary_muscle_development	0	FALSE
ENSGALG00010000188	ENSGALG00010000188	PRJNA729271	1.469011036	0.305851988	1.469011036	stage_E14_vs_E10	ordinary_muscle_development	0	FALSE
ENSGALG00010008529	GPR83	PRJNA729271	-0.515633643	0.306865985	0.515633643	stage_E14_vs_E10	ordinary_muscle_development	0	FALSE
ENSGALG00010011555	ENSGALG00010011555	PRJNA729271	-4.793529315	0.308880364	4.793529315	stage_E14_vs_E10	ordinary_muscle_development	0	FALSE
ENSGALG00010029474	ENSGALG00010029474	PRJNA729271	-1.200293891	0.311301464	1.200293891	stage_E14_vs_E10	ordinary_muscle_development	0	FALSE
ENSGALG00010008668	ENSGALG00010008668	PRJNA729271	-0.368608178	0.31843653	0.368608178	stage_E14_vs_E10	ordinary_muscle_development	0	FALSE
ENSGALG00010007010	ENSGALG00010007010	PRJNA729271	1.086733356	0.32078982	1.086733356	stage_E14_vs_E10	ordinary_muscle_development	0	FALSE
ENSGALG00010011964	ENSGALG00010011964	PRJNA729271	-4.783956721	0.321059959	4.783956721	stage_E14_vs_E10	ordinary_muscle_development	0	FALSE
ENSGALG00010002238	ENSGALG00010002238	PRJNA729271	0.40851691	0.326743128	0.40851691	stage_E14_vs_E10	ordinary_muscle_development	0	FALSE
ENSGALG00010018055	PEX5L	PRJNA729271	-0.525674442	0.336838373	0.525674442	stage_E14_vs_E10	ordinary_muscle_development	0	FALSE
ENSGALG00010000308	ENSGALG00010000308	PRJNA729271	0.344850044	0.347629299	0.344850044	stage_E14_vs_E10	ordinary_muscle_development	0	FALSE
ENSGALG00010024889	SLC2A4RG	PRJNA729271	-0.50864792	0.350700103	0.50864792	stage_E14_vs_E10	ordinary_muscle_development	0	FALSE
ENSGALG00010010352	MALT1	PRJNA729271	-0.112355697	0.359012593	0.112355697	stage_E14_vs_E10	ordinary_muscle_development	0	FALSE
ENSGALG00010003743	ARL9	PRJNA729271	-0.183806008	0.359601628	0.183806008	stage_E14_vs_E10	ordinary_muscle_development	0	FALSE
ENSGALG00010020334	CABP4	PRJNA729271	0.477284751	0.362484991	0.477284751	stage_E14_vs_E10	ordinary_muscle_development	0	FALSE
ENSGALG00010010395	GRP	PRJNA729271	-0.80968939	0.401133605	0.80968939	stage_E14_vs_E10	ordinary_muscle_development	0	FALSE
ENSGALG00010027595	GNAT1	PRJNA729271	0.387436758	0.403436056	0.387436758	stage_E14_vs_E10	ordinary_muscle_development	0	FALSE
ENSGALG00010013244	TRIM24	PRJNA729271	-0.109044071	0.424230993	0.109044071	stage_E14_vs_E10	ordinary_muscle_development	0	FALSE
ENSGALG00010012047	LMAN2	PRJNA729271	0.082219967	0.425942587	0.082219967	stage_E14_vs_E10	ordinary_muscle_development	0	FALSE
ENSGALG00010023986	SYCE3	PRJNA729271	-0.537065368	0.429790146	0.537065368	stage_E14_vs_E10	ordinary_muscle_development	0	FALSE
ENSGALG00010013554	ENSGALG00010013554	PRJNA729271	-4.708307854	0.438202327	4.708307854	stage_E14_vs_E10	ordinary_muscle_development	0	FALSE
ENSGALG00010029463	ENSGALG00010029463	PRJNA729271	-0.374963806	0.438372373	0.374963806	stage_E14_vs_E10	ordinary_muscle_development	0	FALSE
ENSGALG00010013134	ENSGALG00010013134	PRJNA729271	-4.645837164	0.441882811	4.645837164	stage_E14_vs_E10	ordinary_muscle_development	0	FALSE
ENSGALG00010003767	ENSGALG00010003767	PRJNA729271	-0.342689583	0.447115189	0.342689583	stage_E14_vs_E10	ordinary_muscle_development	0	FALSE
ENSGALG00010026539	ENSGALG00010026539	PRJNA729271	-0.31205719	0.455624655	0.31205719	stage_E14_vs_E10	ordinary_muscle_development	0	FALSE
ENSGALG00010029962	ENSGALG00010029962	PRJNA729271	-0.617502122	0.459089269	0.617502122	stage_E14_vs_E10	ordinary_muscle_development	0	FALSE
ENSGALG00010012323	SMAD7B	PRJNA729271	3.092418259	0.464905573	3.092418259	stage_E14_vs_E10	ordinary_muscle_development	0	FALSE
ENSGALG00010000172	ENSGALG00010000172	PRJNA729271	-1.037560678	0.47349254	1.037560678	stage_E14_vs_E10	ordinary_muscle_development	0	FALSE
ENSGALG00010003346	ENSGALG00010003346	PRJNA729271	4.064437297	0.501688578	4.064437297	stage_E14_vs_E10	ordinary_muscle_development	0	FALSE
ENSGALG00010013565	ENSGALG00010013565	PRJNA729271	4.077731775	0.55244964	4.077731775	stage_E14_vs_E10	ordinary_muscle_development	0	FALSE
ENSGALG00010011978	ENSGALG00010011978	PRJNA729271	3.124678358	0.603513842	3.124678358	stage_E14_vs_E10	ordinary_muscle_development	0	FALSE
