# Supplementary material for: Cross-cohort transcriptomic meta-analysis identifies conserved melanogenesis signatures and developmental background in black-boned chicken breast muscle
Source: Anim Biosci. 2026 Jun 24;39(10):260430. doi: 10.5713/ab.260430 (PMC13662945; doi:10.5713/ab.260430)
Supplement: Supplementary file 17 [file ab-260430-Supplementary-9.pdf]

Supplement 9. Sensitivity analysis of PRJNA729271 background filtering using alternative absolute log2 fold-change cutoffs while retaining adjusted p-value <0.05.

absolute log2FC cutoff	background flag rule	background flagged conserved genes	total conserved genes	fraction flagged	canonical anchors flagged	interpretation
0	PRJNA729271 adjusted p-value < 0.05 and maximum absolute log2 fold change >= cutoff	203	227	0.894273128	PMEL; TYRP1; DCT; TYR	All four canonical melanogenesis anchors remained background-flagged at this cutoff.
0.5	PRJNA729271 adjusted p-value < 0.05 and maximum absolute log2 fold change >= cutoff	194	227	0.854625551	PMEL; TYRP1; DCT; TYR	All four canonical melanogenesis anchors remained background-flagged at this cutoff.
1	PRJNA729271 adjusted p-value < 0.05 and maximum absolute log2 fold change >= cutoff	156	227	0.68722467	PMEL; TYRP1; DCT; TYR	All four canonical melanogenesis anchors remained background-flagged at this cutoff.
1.5	PRJNA729271 adjusted p-value < 0.05 and maximum absolute log2 fold change >= cutoff	125	227	0.550660793	PMEL; TYRP1; DCT; TYR	All four canonical melanogenesis anchors remained background-flagged at this cutoff.
2	PRJNA729271 adjusted p-value < 0.05 and maximum absolute log2 fold change >= cutoff	103	227	0.453744493	PMEL; TYRP1; DCT; TYR	All four canonical melanogenesis anchors remained background-flagged at this cutoff.
